# Supplementary material for: Genome-Wide Identification and Expression Pattern of the GRAS Gene Family in Pitaya (Selenicereus undatus L.)
Source: Biology (Basel). 2022 Dec 21;12(1):11. doi: 10.3390/biology12010011 (PMC9854919; doi:10.3390/biology12010011)
Supplement: Supplementary file 1 [file biology-12-00011-s001.zip › Supplementary file S5/HU06G00568.1_plantcare.html]

Content-Type: text/html; charset=ISO-8859-1


PlantCARE


Webmaster Firefox specific output  
To save the result:
click on the frame with the right mouse button and save the source code as a text file with extension .html  
REFERENCE:PlantCARE: a database of plant cis-acting regulatory elements and a portal to tools for in silico analysis of promoter sequences.  
Lescot, M., Déhais, P., Moreau, Y., De Moor, B., Rouzé ,P.,and Rombauts, S.  
Nucleic Acids Res., Database issue(2002), 30(1):325-327.   


---

>HU06G00568.1   
+ -Up\_Stream \_Len000CCCTGG GTCATTAAAA AAAAAGAAAG AAAGAAGATT GCGCTAAAAG TAGATTTTTA   
  
  
+ TCAGATTCAA GCTGCAGGTG ATAACCAAAT TGTCATTAAA GCAATGCAAG CGCATATCAA TACCCTTTTG   
  
  
+ GGAGATAGCT CCGACTGTTG AAGACATTAG GAGCATGATC TCCAACTGTG AATTTGTTTC ATTTACTCAT   
  
  
+ ATTCATATCT ACCATGTGGA TAATATGACT GCAGACTAGA TGGCCAAATT TGAATGCACA CTTATAACTT   
  
  
+ CAACTCTTTC TACTTTTTTC TTCCCCACCT TATCAGGAAT TTCTTTTAAT TGTCGTGGGA GACAACTTGG   
  
  
+ GTAGAACTCT TGCGAAAAGG GCAACTTAAC GTTTTGGTAC TTGCATTTTC CAAAAAAACA AAAAGGCTAA   
  
  
+ ACAATTCTCC GCATGCTGAC TTCACGGTAG ATATGATGTA TTTTTTAACA AAAGACATCT TCTGAATGAA   
  
  
+ ACGCTTAATA TCTTGGACCA TACTAGAAAT TAAATTGCCA CGGTGCATGA ATTTCACCTC GAACTGCTTG   
  
  
+ TATAAAGATC TTGTTATCAC CTTCAGTAAT CAGATACTTG TAACCAAATT GAATGACCAC AAAGACCACA   
  
  
+ TTCCTCACAA TCGTTGCCTC TAGAATAAGA ATGAATGTTT CTTCAAGGTT GAAAGCCCTT GCTAATGCGA   
  
  
+ ACCTGCCTAT ATGCCTAACT GGCTATATAT ATGTGCGTAG AACAGCATGA CATGAGTTAA TAATATAGCA   
  
  
+ GCTAAAGGGA ACTCTAATGT ACACATGATT GTAATTCACC TGCCTCGCTA GCTTACGAGC ATGACTCTTG   
  
  
+ ACTATTATTG TTTTAATTGA TCTTGAATTT TCATAGATTT TACTCCATTG TTTACTCATT TTGTTATAAT   
  
  
+ TAACCACCTG ATTTTAAACT TTAAAATACA CAAAGATTCA TCTAATATAA TTTTTTAGAT AAAGTTATGC   
  
  
+ ATACATATAT ACAAATTAAA TTATTTACGA TCTAATCATT TCCCGTTATA TATTTCACAC TAAAATTTAG   
  
  
+ AAGAGACAAT CTATTAAAAT TACATCACAT GTTTTGGGGG GGGGGGGATA TCAGATATGC ATGACATTCC   
  
  
+ TAATCCAATA ATGCAATGAA ACCCACCGAA TAGTTGAAAT GATTTGTTCT ATCCATGAAC TCACCAAGTC   
  
  
+ ACAATCCATC ACACTCGAAG GATATGCTTT TCTTTTTGAA GGGAATCTTG CTTAATCAAA CCTATAAACT   
  
  
+ TTTAAAAGCA ACAGACAAAA AAGTGATTAT AATGGTAGTG GTAGGATGAA GGCAGCTTTC AGAAAAGCGA   
  
  
+ TTGAATTTTT ATTGTATCCC ATGAAAACCC ATTGAATTTT TCTTTCTTTC TTTGTTTTTG TTACCTGGGT   
  
  
+ TTATGCAAAG GACGGCTGTT GTATTTAATA ATAACAAAAA CATAGGGCTT TGCGGATTGC TAATGCTGGG   
  
  
+ GATGGCATCA GTGCTCGAAT CTGTGTACGC TGTCATAGGA ACCCACTTGC TTCTGCTCAT TTATTTCTGT   
  
  
+ CTCTAGATCC CCTCCTCTCT CTCTCTCTCT CTCTAAGGTG TCTGCTCGTC TGTTCATATT CTCCTGAGGT   
  
  
+ TCTTCTTCAT CTATCTTGTT CTGGGTGTGT GCTGAGGTAC TTCTTTTCTT TATAAATTTT TCATCTTTTG   
  
  
+ TTGTTTTGCT TGGTGTTTAT GATTTTAGTT TTTGTCTTTA AGACGAGATT TGCTGTTAAT AGCAAGATTT   
  
  
+ CTGAACTAGT ATTGATCCTT TTTGTGGTAT ATTTGTTGAA TTTGATTAAT TTGTGTATTG TGTTTAGCTT   
  
  
+ AAGTAACTCG TTGTGATCTA ATTGATGCTC GGGTTTTGAC AGTTTGATTA GGGTTTTCGG TACTGGAGTT   
  
  
+ AAAGTTTTTT TATTTTTTAT AGGAGGAGTT AAAGGTAATA ATAAGAATTG ATGTTTGCTG ATTTTTCCTC   
  
  
+ TTTTCTTTTT TGGTTTCGAT TTGGTGGGCA GTTATCTGTT TATGGTTTTT GCAAATGGAT CCACAATTAG   
  
  
+ AGGAATTATA TGGACCTTTA CATCGAATCA AGTTCAATGA TCAAAGGGTG CCAATTTTAC CAAGTCGCAG   
  
  
+ TGTTGTTACT CCGATGAAAC TCCAAGATTC CAATTTGAAT CCAAGTGTTC CAAATCCAAG TGTTGTGAAT   
  
  
+ CCTCCTCCGT TGGTTCCCCC AAACCCAAAT CTTAATTTAG TTGTGGCATC TCAATATTCT GACATTGAGA   
  
  
+ CAGCCCTGAA CGAGGATTGT GATTTTAGTG ATGTTGTTCT TAAGTATATT AATCAACTTC TTATGGAAGA   
  
  
+ GGATGGGGAA GAGAAACTCC ATACTGATCA TGAACCTTCA GTCGTTGAGG CGGCAGAGAA GTCATTGTAT   
  
  
+ GAGGCGCTCG GGCAGAGATA TCCTCCTTCC GGAAACCGAA ATCAGTTGCC AGATGTTGAG CATGACGGCT   
  
  
+ TGACTGGGAG CAGTGTTAGG GCTCATAGTG GTGCAAGCAG TGTTAGGGCT CATAGTGACG CAAGTGGTGG   
  
  
+ TAGTGGCCTG ACAGGATATG GTTGGTATGG TGATCCTTAT AATTGGAGTC CTCAAAATGT CGTGAATTTT   
  
  
+ ACCATTTCCT CCTCGAAACC CTTATCGTGC AGCTCATCAG ATAGCTCGGG CAGATGGGTT AGTAAGTGGA   
  
  
+ AGTCAAATTC TGATTCACAG CTCAGCTCGG CCATTAATGC AAGTGGTTTA GTGGATGGGC CAGGGGACTC   
  
  
+ TCCTGTGAGT GCTCTTAGTG TGTCTGAAAT ATTCAATGAC AGTCAGTCAA TGTTGCAGTT TCAGAAAGGA   
  
  
+ TTTGAGGAAG CGAATAAATT TCTTCCAAAG AGTTCTTTGT ACAAGGGTTT TGCCAACACG GGATTGCCTT   
  
  
+ ATCAGAAGGC AAACAATAGT GCCCAAGATT TGTTGGTCAA TGTAGAGGAT GTTACTAGGG GAAAGAAGCA   
  
  
+ TCGTTATCCC GAAGAATTGC AGTCAGAAGA AGGGAGGATA AATAAGCAAT CAGCTGTTTC CCTGGCAGCT   
  
  
+ GATGAGGCAG TTGTTAGGTC TGAAATGTTC GATAGGGTAC TGCTTTGTAG TCGGGGAAAA CATGATGCTG   
  
  
+ CTCTCCGGGA AGCTTTACAG ACTGAACTAA ATAAGAGTCT GCGAAATGCC CCAGTTAAGG GGTCTAATAG   
  
  
+ TGGGAAAGGC CGTGGTAAGA AAGCGGGAAA GAAGAGGGAT GTAGTAGATT TAAGATCTCT CTTAACCCTA   
  
  
+ TGTGCACAAG CAGTTGCATC GAATGATCAC AGGAGTGCAA ATGACCTGCT TAGGCAAATT AGACAGCATT   
  
  
+ CTTCTCCTAG TGGGGATGGT AACCAAAGAA TGGCACATTA TTTTGCGGAT GGTCTTGAGG CACGCCTTGC   
  
  
+ TGGTGTAGGA ACTCCTATAT ATAACTGTCT TGTAACGGGT CCGGCATCGG CTGTAGATAT CTTGAGAGCT   
  
  
+ TACCACATGT TTCTTGCCAC ATGCCCATTT AAGAAAATGG GAAATTTCTT CTCTAATAGA ACGATTATGG   
  
  
+ CTGTGGCAGA GAATGCAACA TGCCTTCATA TAATTGATCT CGGTATTGTC TATGGTTTCC AATGGCCTTG   
  
  
+ TCTAATTCAG CGACTTTCAT CTAGGCCTGG TGGCCCCCCC AAACTTCGAA TAACCGGAGT AGATCTTCCA   
  
  
+ CAACCTGGGT TCCGACCAGC CAAAAGAGTT GAGGAGACAG GGCGTCGCTT GAAGAACTAT GCAGAGTCAT   
  
  
+ TTAATGTGCC CTTTGAGTTC AATGCTATAG CAAAGAAGTG GGAAACACTT ACCATTGAAG ATCTCAGGAT   
  
  
+ CAATAGCGAT GAGTTGCTTG TTGTCACCTG TATGTTTAGG TTTAAACATA TACCTGAGGA AACAGTGACC   
  
  
+ GTGGATTGCC CTAGGGATAC TGTTCTTAAC CTGATTGGGC GCATAAACCC AGCTGTTTTC ATACAAGGCA   
  
  
+ CTGTTAACGG GGCTTTCAAT TCTCCCTTTT TCATAGCTCG ATTTCGAGAG GCTCTATTTC ACTTCTCCAC   
  
  
+ TCTGTTTGAT ATGCTAGAGG CCAACCTGCC AAGGGACAAT AAGGAGAGGA TGCTAATTGA GAGAGAGATA   
  
  
+ TTTGGGAGGC AGGCAATGAA TGTGATTGCT TGTGAGGGTT TAGAGAGGAT AGAAAGGCCA GAGACGTACA   
  
  
+ AGCAGTGGCA AGTCCGAAAT GAAAGGGCAG GGTTTAGGCA GCTGCCTTTA GATCGCCAGA TTCTGGAAAT   
  
  
+ GGCTAAAAAG AGGGTGAAAT CTGTGTATAA CAAAGATTTC TCCATTGATG AAGACGGGCA CTGGTTGTTG   
  
  
+ CTGGGATGGA AGGGCAGAAT TGTGTACACA CTCACTACTT GGAAGCCTGC GGAGTA  

- -Up\_Stream \_Len000GGGACC CAGTAATTTT TTTTTCTTTC TTTCTTCTAA CGCGATTTTC ATCTAAAAAT   
  
  
- AGTCTAAGTT CGACGTCCAC TATTGGTTTA ACAGTAATTT CGTTACGTTC GCGTATAGTT ATGGGAAAAC   
  
  
- CCTCTATCGA GGCTGACAAC TTCTGTAATC CTCGTACTAG AGGTTGACAC TTAAACAAAG TAAATGAGTA   
  
  
- TAAGTATAGA TGGTACACCT ATTATACTGA CGTCTGATCT ACCGGTTTAA ACTTACGTGT GAATATTGAA   
  
  
- GTTGAGAAAG ATGAAAAAAG AAGGGGTGGA ATAGTCCTTA AAGAAAATTA ACAGCACCCT CTGTTGAACC   
  
  
- CATCTTGAGA ACGCTTTTCC CGTTGAATTG CAAAACCATG AACGTAAAAG GTTTTTTTGT TTTTCCGATT   
  
  
- TGTTAAGAGG CGTACGACTG AAGTGCCATC TATACTACAT AAAAAATTGT TTTCTGTAGA AGACTTACTT   
  
  
- TGCGAATTAT AGAACCTGGT ATGATCTTTA ATTTAACGGT GCCACGTACT TAAAGTGGAG CTTGACGAAC   
  
  
- ATATTTCTAG AACAATAGTG GAAGTCATTA GTCTATGAAC ATTGGTTTAA CTTACTGGTG TTTCTGGTGT   
  
  
- AAGGAGTGTT AGCAACGGAG ATCTTATTCT TACTTACAAA GAAGTTCCAA CTTTCGGGAA CGATTACGCT   
  
  
- TGGACGGATA TACGGATTGA CCGATATATA TACACGCATC TTGTCGTACT GTACTCAATT ATTATATCGT   
  
  
- CGATTTCCCT TGAGATTACA TGTGTACTAA CATTAAGTGG ACGGAGCGAT CGAATGCTCG TACTGAGAAC   
  
  
- TGATAATAAC AAAATTAACT AGAACTTAAA AGTATCTAAA ATGAGGTAAC AAATGAGTAA AACAATATTA   
  
  
- ATTGGTGGAC TAAAATTTGA AATTTTATGT GTTTCTAAGT AGATTATATT AAAAAATCTA TTTCAATACG   
  
  
- TATGTATATA TGTTTAATTT AATAAATGCT AGATTAGTAA AGGGCAATAT ATAAAGTGTG ATTTTAAATC   
  
  
- TTCTCTGTTA GATAATTTTA ATGTAGTGTA CAAAACCCCC CCCCCCCTAT AGTCTATACG TACTGTAAGG   
  
  
- ATTAGGTTAT TACGTTACTT TGGGTGGCTT ATCAACTTTA CTAAACAAGA TAGGTACTTG AGTGGTTCAG   
  
  
- TGTTAGGTAG TGTGAGCTTC CTATACGAAA AGAAAAACTT CCCTTAGAAC GAATTAGTTT GGATATTTGA   
  
  
- AAATTTTCGT TGTCTGTTTT TTCACTAATA TTACCATCAC CATCCTACTT CCGTCGAAAG TCTTTTCGCT   
  
  
- AACTTAAAAA TAACATAGGG TACTTTTGGG TAACTTAAAA AGAAAGAAAG AAACAAAAAC AATGGACCCA   
  
  
- AATACGTTTC CTGCCGACAA CATAAATTAT TATTGTTTTT GTATCCCGAA ACGCCTAACG ATTACGACCC   
  
  
- CTACCGTAGT CACGAGCTTA GACACATGCG ACAGTATCCT TGGGTGAACG AAGACGAGTA AATAAAGACA   
  
  
- GAGATCTAGG GGAGGAGAGA GAGAGAGAGA GAGATTCCAC AGACGAGCAG ACAAGTATAA GAGGACTCCA   
  
  
- AGAAGAAGTA GATAGAACAA GACCCACACA CGACTCCATG AAGAAAAGAA ATATTTAAAA AGTAGAAAAC   
  
  
- AACAAAACGA ACCACAAATA CTAAAATCAA AAACAGAAAT TCTGCTCTAA ACGACAATTA TCGTTCTAAA   
  
  
- GACTTGATCA TAACTAGGAA AAACACCATA TAAACAACTT AAACTAATTA AACACATAAC ACAAATCGAA   
  
  
- TTCATTGAGC AACACTAGAT TAACTACGAG CCCAAAACTG TCAAACTAAT CCCAAAAGCC ATGACCTCAA   
  
  
- TTTCAAAAAA ATAAAAAATA TCCTCCTCAA TTTCCATTAT TATTCTTAAC TACAAACGAC TAAAAAGGAG   
  
  
- AAAAGAAAAA ACCAAAGCTA AACCACCCGT CAATAGACAA ATACCAAAAA CGTTTACCTA GGTGTTAATC   
  
  
- TCCTTAATAT ACCTGGAAAT GTAGCTTAGT TCAAGTTACT AGTTTCCCAC GGTTAAAATG GTTCAGCGTC   
  
  
- ACAACAATGA GGCTACTTTG AGGTTCTAAG GTTAAACTTA GGTTCACAAG GTTTAGGTTC ACAACACTTA   
  
  
- GGAGGAGGCA ACCAAGGGGG TTTGGGTTTA GAATTAAATC AACACCGTAG AGTTATAAGA CTGTAACTCT   
  
  
- GTCGGGACTT GCTCCTAACA CTAAAATCAC TACAACAAGA ATTCATATAA TTAGTTGAAG AATACCTTCT   
  
  
- CCTACCCCTT CTCTTTGAGG TATGACTAGT ACTTGGAAGT CAGCAACTCC GCCGTCTCTT CAGTAACATA   
  
  
- CTCCGCGAGC CCGTCTCTAT AGGAGGAAGG CCTTTGGCTT TAGTCAACGG TCTACAACTC GTACTGCCGA   
  
  
- ACTGACCCTC GTCACAATCC CGAGTATCAC CACGTTCGTC ACAATCCCGA GTATCACTGC GTTCACCACC   
  
  
- ATCACCGGAC TGTCCTATAC CAACCATACC ACTAGGAATA TTAACCTCAG GAGTTTTACA GCACTTAAAA   
  
  
- TGGTAAAGGA GGAGCTTTGG GAATAGCACG TCGAGTAGTC TATCGAGCCC GTCTACCCAA TCATTCACCT   
  
  
- TCAGTTTAAG ACTAAGTGTC GAGTCGAGCC GGTAATTACG TTCACCAAAT CACCTACCCG GTCCCCTGAG   
  
  
- AGGACACTCA CGAGAATCAC ACAGACTTTA TAAGTTACTG TCAGTCAGTT ACAACGTCAA AGTCTTTCCT   
  
  
- AAACTCCTTC GCTTATTTAA AGAAGGTTTC TCAAGAAACA TGTTCCCAAA ACGGTTGTGC CCTAACGGAA   
  
  
- TAGTCTTCCG TTTGTTATCA CGGGTTCTAA ACAACCAGTT ACATCTCCTA CAATGATCCC CTTTCTTCGT   
  
  
- AGCAATAGGG CTTCTTAACG TCAGTCTTCT TCCCTCCTAT TTATTCGTTA GTCGACAAAG GGACCGTCGA   
  
  
- CTACTCCGTC AACAATCCAG ACTTTACAAG CTATCCCATG ACGAAACATC AGCCCCTTTT GTACTACGAC   
  
  
- GAGAGGCCCT TCGAAATGTC TGACTTGATT TATTCTCAGA CGCTTTACGG GGTCAATTCC CCAGATTATC   
  
  
- ACCCTTTCCG GCACCATTCT TTCGCCCTTT CTTCTCCCTA CATCATCTAA ATTCTAGAGA GAATTGGGAT   
  
  
- ACACGTGTTC GTCAACGTAG CTTACTAGTG TCCTCACGTT TACTGGACGA ATCCGTTTAA TCTGTCGTAA   
  
  
- GAAGAGGATC ACCCCTACCA TTGGTTTCTT ACCGTGTAAT AAAACGCCTA CCAGAACTCC GTGCGGAACG   
  
  
- ACCACATCCT TGAGGATATA TATTGACAGA ACATTGCCCA GGCCGTAGCC GACATCTATA GAACTCTCGA   
  
  
- ATGGTGTACA AAGAACGGTG TACGGGTAAA TTCTTTTACC CTTTAAAGAA GAGATTATCT TGCTAATACC   
  
  
- GACACCGTCT CTTACGTTGT ACGGAAGTAT ATTAACTAGA GCCATAACAG ATACCAAAGG TTACCGGAAC   
  
  
- AGATTAAGTC GCTGAAAGTA GATCCGGACC ACCGGGGGGG TTTGAAGCTT ATTGGCCTCA TCTAGAAGGT   
  
  
- GTTGGACCCA AGGCTGGTCG GTTTTCTCAA CTCCTCTGTC CCGCAGCGAA CTTCTTGATA CGTCTCAGTA   
  
  
- AATTACACGG GAAACTCAAG TTACGATATC GTTTCTTCAC CCTTTGTGAA TGGTAACTTC TAGAGTCCTA   
  
  
- GTTATCGCTA CTCAACGAAC AACAGTGGAC ATACAAATCC AAATTTGTAT ATGGACTCCT TTGTCACTGG   
  
  
- CACCTAACGG GATCCCTATG ACAAGAATTG GACTAACCCG CGTATTTGGG TCGACAAAAG TATGTTCCGT   
  
  
- GACAATTGCC CCGAAAGTTA AGAGGGAAAA AGTATCGAGC TAAAGCTCTC CGAGATAAAG TGAAGAGGTG   
  
  
- AGACAAACTA TACGATCTCC GGTTGGACGG TTCCCTGTTA TTCCTCTCCT ACGATTAACT CTCTCTCTAT   
  
  
- AAACCCTCCG TCCGTTACTT ACACTAACGA ACACTCCCAA ATCTCTCCTA TCTTTCCGGT CTCTGCATGT   
  
  
- TCGTCACCGT TCAGGCTTTA CTTTCCCGTC CCAAATCCGT CGACGGAAAT CTAGCGGTCT AAGACCTTTA   
  
  
- CCGATTTTTC TCCCACTTTA GACACATATT GTTTCTAAAG AGGTAACTAC TTCTGCCCGT GACCAACAAC   
  
  
- GACCCTACCT TCCCGTCTTA ACACATGTGT GAGTGATGAA CCTTCGGACG CCTCAT

  
  
Motifs Found  

+   

| Site Name | Organism | Position | Strand | Matrix score. | sequence | function |
| --- | --- | --- | --- | --- | --- | --- |
|  | organism | 3085 | + | 4 | motif\_sequence | short\_function |
|  | organism | 3039 | + | 4 | motif\_sequence | short\_function |
|  | organism | 2310 | - | 4 | motif\_sequence | short\_function |
|  | organism | 744 | - | 4 | motif\_sequence | short\_function |
|  | organism | 3510 | - | 4 | motif\_sequence | short\_function |
|  | organism | 481 | + | 4 | motif\_sequence | short\_function |
|  | organism | 3198 | - | 4 | motif\_sequence | short\_function |
|  | organism | 3224 | + | 4 | motif\_sequence | short\_function |
|  | organism | 2367 | - | 4 | motif\_sequence | short\_function |
|  | organism | 791 | + | 4 | motif\_sequence | short\_function |
|  | organism | 3771 | - | 4 | motif\_sequence | short\_function |
|  | organism | 1054 | - | 4 | motif\_sequence | short\_function |
|  | organism | 2644 | - | 4 | motif\_sequence | short\_function |
|  | organism | 2916 | - | 4 | motif\_sequence | short\_function |
|  | organism | 145 | - | 4 | motif\_sequence | short\_function |
|  | organism | 3185 | - | 4 | motif\_sequence | short\_function |
|  | organism | 735 | + | 4 | motif\_sequence | short\_function |
|  | organism | 3874 | + | 4 | motif\_sequence | short\_function |
|  | organism | 3327 | - | 4 | motif\_sequence | short\_function |
|  | organism | 3637 | + | 4 | motif\_sequence | short\_function |
|  | organism | 1594 | + | 4 | motif\_sequence | short\_function |
|  | organism | 64 | - | 4 | motif\_sequence | short\_function |
|  | organism | 3417 | - | 4 | motif\_sequence | short\_function |
|  | organism | 2322 | - | 4 | motif\_sequence | short\_function |
|  | organism | 221 | + | 4 | motif\_sequence | short\_function |
|  | organism | 3633 | - | 4 | motif\_sequence | short\_function |
|  | organism | 451 | - | 4 | motif\_sequence | short\_function |
|  | organism | 3705 | - | 4 | motif\_sequence | short\_function |
|  | organism | 3718 | + | 4 | motif\_sequence | short\_function |
|  | organism | 2396 | - | 4 | motif\_sequence | short\_function |
|  | organism | 4037 | - | 4 | motif\_sequence | short\_function |
|  | organism | 48 | - | 4 | motif\_sequence | short\_function |
|  | organism | 182 | + | 4 | motif\_sequence | short\_function |
|  | organism | 2732 | + | 4 | motif\_sequence | short\_function |

>HU06G00568.1   
+ -Up\_Stream \_Len000CCCTGG GTCATTAAAA AAAAAGAAAG AAAGAAGATT GCGCTAAAAG TAGATTTTTA   
  
  
+ TCAGATTCAA GCTGCAGGTG ATAACCAAAT TGTCATTAAA GCAATGCAAG CGCATATCAA TACCCTTTTG   
  
  
+ GGAGATAGCT CCGACTGTTG AAGACATTAG GAGCATGATC TCCAACTGTG AATTTGTTTC ATTTACTCAT   
  
  
+ ATTCATATCT ACCATGTGGA TAATATGACT GCAGACTAGA TGGCCAAATT TGAATGCACA CTTATAACTT   
  
  
+ CAACTCTTTC TACTTTTTTC TTCCCCACCT TATCAGGAAT TTCTTTTAAT TGTCGTGGGA GACAACTTGG   
  
  
+ GTAGAACTCT TGCGAAAAGG GCAACTTAAC GTTTTGGTAC TTGCATTTTC CAAAAAAACA AAAAGGCTAA   
  
  
+ ACAATTCTCC GCATGCTGAC TTCACGGTAG ATATGATGTA TTTTTTAACA AAAGACATCT TCTGAATGAA   
  
  
+ ACGCTTAATA TCTTGGACCA TACTAGAAAT TAAATTGCCA CGGTGCATGA ATTTCACCTC GAACTGCTTG   
  
  
+ TATAAAGATC TTGTTATCAC CTTCAGTAAT CAGATACTTG TAACCAAATT GAATGACCAC AAAGACCACA   
  
  
+ TTCCTCACAA TCGTTGCCTC TAGAATAAGA ATGAATGTTT CTTCAAGGTT GAAAGCCCTT GCTAATGCGA   
  
  
+ ACCTGCCTAT ATGCCTAACT GGCTATATAT ATGTGCGTAG AACAGCATGA CATGAGTTAA TAATATAGCA   
  
  
+ GCTAAAGGGA ACTCTAATGT ACACATGATT GTAATTCACC TGCCTCGCTA GCTTACGAGC ATGACTCTTG   
  
  
+ ACTATTATTG TTTTAATTGA TCTTGAATTT TCATAGATTT TACTCCATTG TTTACTCATT TTGTTATAAT   
  
  
+ TAACCACCTG ATTTTAAACT TTAAAATACA CAAAGATTCA TCTAATATAA TTTTTTAGAT AAAGTTATGC   
  
  
+ ATACATATAT ACAAATTAAA TTATTTACGA TCTAATCATT TCCCGTTATA TATTTCACAC TAAAATTTAG   
  
  
+ AAGAGACAAT CTATTAAAAT TACATCACAT GTTTTGGGGG GGGGGGGATA TCAGATATGC ATGACATTCC   
  
  
+ TAATCCAATA ATGCAATGAA ACCCACCGAA TAGTTGAAAT GATTTGTTCT ATCCATGAAC TCACCAAGTC   
  
  
+ ACAATCCATC ACACTCGAAG GATATGCTTT TCTTTTTGAA GGGAATCTTG CTTAATCAAA CCTATAAACT   
  
  
+ TTTAAAAGCA ACAGACAAAA AAGTGATTAT AATGGTAGTG GTAGGATGAA GGCAGCTTTC AGAAAAGCGA   
  
  
+ TTGAATTTTT ATTGTATCCC ATGAAAACCC ATTGAATTTT TCTTTCTTTC TTTGTTTTTG TTACCTGGGT   
  
  
+ TTATGCAAAG GACGGCTGTT GTATTTAATA ATAACAAAAA CATAGGGCTT TGCGGATTGC TAATGCTGGG   
  
  
+ GATGGCATCA GTGCTCGAAT CTGTGTACGC TGTCATAGGA ACCCACTTGC TTCTGCTCAT TTATTTCTGT   
  
  
+ CTCTAGATCC CCTCCTCTCT CTCTCTCTCT CTCTAAGGTG TCTGCTCGTC TGTTCATATT CTCCTGAGGT   
  
  
+ TCTTCTTCAT CTATCTTGTT CTGGGTGTGT GCTGAGGTAC TTCTTTTCTT TATAAATTTT TCATCTTTTG   
  
  
+ TTGTTTTGCT TGGTGTTTAT GATTTTAGTT TTTGTCTTTA AGACGAGATT TGCTGTTAAT AGCAAGATTT   
  
  
+ CTGAACTAGT ATTGATCCTT TTTGTGGTAT ATTTGTTGAA TTTGATTAAT TTGTGTATTG TGTTTAGCTT   
  
  
+ AAGTAACTCG TTGTGATCTA ATTGATGCTC GGGTTTTGAC AGTTTGATTA GGGTTTTCGG TACTGGAGTT   
  
  
+ AAAGTTTTTT TATTTTTTAT AGGAGGAGTT AAAGGTAATA ATAAGAATTG ATGTTTGCTG ATTTTTCCTC   
  
  
+ TTTTCTTTTT TGGTTTCGAT TTGGTGGGCA GTTATCTGTT TATGGTTTTT GCAAATGGAT CCACAATTAG   
  
  
+ AGGAATTATA TGGACCTTTA CATCGAATCA AGTTCAATGA TCAAAGGGTG CCAATTTTAC CAAGTCGCAG   
  
  
+ TGTTGTTACT CCGATGAAAC TCCAAGATTC CAATTTGAAT CCAAGTGTTC CAAATCCAAG TGTTGTGAAT   
  
  
+ CCTCCTCCGT TGGTTCCCCC AAACCCAAAT CTTAATTTAG TTGTGGCATC TCAATATTCT GACATTGAGA   
  
  
+ CAGCCCTGAA CGAGGATTGT GATTTTAGTG ATGTTGTTCT TAAGTATATT AATCAACTTC TTATGGAAGA   
  
  
+ GGATGGGGAA GAGAAACTCC ATACTGATCA TGAACCTTCA GTCGTTGAGG CGGCAGAGAA GTCATTGTAT   
  
  
+ GAGGCGCTCG GGCAGAGATA TCCTCCTTCC GGAAACCGAA ATCAGTTGCC AGATGTTGAG CATGACGGCT   
  
  
+ TGACTGGGAG CAGTGTTAGG GCTCATAGTG GTGCAAGCAG TGTTAGGGCT CATAGTGACG CAAGTGGTGG   
  
  
+ TAGTGGCCTG ACAGGATATG GTTGGTATGG TGATCCTTAT AATTGGAGTC CTCAAAATGT CGTGAATTTT   
  
  
+ ACCATTTCCT CCTCGAAACC CTTATCGTGC AGCTCATCAG ATAGCTCGGG CAGATGGGTT AGTAAGTGGA   
  
  
+ AGTCAAATTC TGATTCACAG CTCAGCTCGG CCATTAATGC AAGTGGTTTA GTGGATGGGC CAGGGGACTC   
  
  
+ TCCTGTGAGT GCTCTTAGTG TGTCTGAAAT ATTCAATGAC AGTCAGTCAA TGTTGCAGTT TCAGAAAGGA   
  
  
+ TTTGAGGAAG CGAATAAATT TCTTCCAAAG AGTTCTTTGT ACAAGGGTTT TGCCAACACG GGATTGCCTT   
  
  
+ ATCAGAAGGC AAACAATAGT GCCCAAGATT TGTTGGTCAA TGTAGAGGAT GTTACTAGGG GAAAGAAGCA   
  
  
+ TCGTTATCCC GAAGAATTGC AGTCAGAAGA AGGGAGGATA AATAAGCAAT CAGCTGTTTC CCTGGCAGCT   
  
  
+ GATGAGGCAG TTGTTAGGTC TGAAATGTTC GATAGGGTAC TGCTTTGTAG TCGGGGAAAA CATGATGCTG   
  
  
+ CTCTCCGGGA AGCTTTACAG ACTGAACTAA ATAAGAGTCT GCGAAATGCC CCAGTTAAGG GGTCTAATAG   
  
  
+ TGGGAAAGGC CGTGGTAAGA AAGCGGGAAA GAAGAGGGAT GTAGTAGATT TAAGATCTCT CTTAACCCTA   
  
  
+ TGTGCACAAG CAGTTGCATC GAATGATCAC AGGAGTGCAA ATGACCTGCT TAGGCAAATT AGACAGCATT   
  
  
+ CTTCTCCTAG TGGGGATGGT AACCAAAGAA TGGCACATTA TTTTGCGGAT GGTCTTGAGG CACGCCTTGC   
  
  
+ TGGTGTAGGA ACTCCTATAT ATAACTGTCT TGTAACGGGT CCGGCATCGG CTGTAGATAT CTTGAGAGCT   
  
  
+ TACCACATGT TTCTTGCCAC ATGCCCATTT AAGAAAATGG GAAATTTCTT CTCTAATAGA ACGATTATGG   
  
  
+ CTGTGGCAGA GAATGCAACA TGCCTTCATA TAATTGATCT CGGTATTGTC TATGGTTTCC AATGGCCTTG   
  
  
+ TCTAATTCAG CGACTTTCAT CTAGGCCTGG TGGCCCCCCC AAACTTCGAA TAACCGGAGT AGATCTTCCA   
  
  
+ CAACCTGGGT TCCGACCAGC CAAAAGAGTT GAGGAGACAG GGCGTCGCTT GAAGAACTAT GCAGAGTCAT   
  
  
+ TTAATGTGCC CTTTGAGTTC AATGCTATAG CAAAGAAGTG GGAAACACTT ACCATTGAAG ATCTCAGGAT   
  
  
+ CAATAGCGAT GAGTTGCTTG TTGTCACCTG TATGTTTAGG TTTAAACATA TACCTGAGGA AACAGTGACC   
  
  
+ GTGGATTGCC CTAGGGATAC TGTTCTTAAC CTGATTGGGC GCATAAACCC AGCTGTTTTC ATACAAGGCA   
  
  
+ CTGTTAACGG GGCTTTCAAT TCTCCCTTTT TCATAGCTCG ATTTCGAGAG GCTCTATTTC ACTTCTCCAC   
  
  
+ TCTGTTTGAT ATGCTAGAGG CCAACCTGCC AAGGGACAAT AAGGAGAGGA TGCTAATTGA GAGAGAGATA   
  
  
+ TTTGGGAGGC AGGCAATGAA TGTGATTGCT TGTGAGGGTT TAGAGAGGAT AGAAAGGCCA GAGACGTACA   
  
  
+ AGCAGTGGCA AGTCCGAAAT GAAAGGGCAG GGTTTAGGCA GCTGCCTTTA GATCGCCAGA TTCTGGAAAT   
  
  
+ GGCTAAAAAG AGGGTGAAAT CTGTGTATAA CAAAGATTTC TCCATTGATG AAGACGGGCA CTGGTTGTTG   
  
  
+ CTGGGATGGA AGGGCAGAAT TGTGTACACA CTCACTACTT GGAAGCCTGC GGAGTA  

- -Up\_Stream \_Len000GGGACC CAGTAATTTT TTTTTCTTTC TTTCTTCTAA CGCGATTTTC ATCTAAAAAT   
  
  
- AGTCTAAGTT CGACGTCCAC TATTGGTTTA ACAGTAATTT CGTTACGTTC GCGTATAGTT ATGGGAAAAC   
  
  
- CCTCTATCGA GGCTGACAAC TTCTGTAATC CTCGTACTAG AGGTTGACAC TTAAACAAAG TAAATGAGTA   
  
  
- TAAGTATAGA TGGTACACCT ATTATACTGA CGTCTGATCT ACCGGTTTAA ACTTACGTGT GAATATTGAA   
  
  
- GTTGAGAAAG ATGAAAAAAG AAGGGGTGGA ATAGTCCTTA AAGAAAATTA ACAGCACCCT CTGTTGAACC   
  
  
- CATCTTGAGA ACGCTTTTCC CGTTGAATTG CAAAACCATG AACGTAAAAG GTTTTTTTGT TTTTCCGATT   
  
  
- TGTTAAGAGG CGTACGACTG AAGTGCCATC TATACTACAT AAAAAATTGT TTTCTGTAGA AGACTTACTT   
  
  
- TGCGAATTAT AGAACCTGGT ATGATCTTTA ATTTAACGGT GCCACGTACT TAAAGTGGAG CTTGACGAAC   
  
  
- ATATTTCTAG AACAATAGTG GAAGTCATTA GTCTATGAAC ATTGGTTTAA CTTACTGGTG TTTCTGGTGT   
  
  
- AAGGAGTGTT AGCAACGGAG ATCTTATTCT TACTTACAAA GAAGTTCCAA CTTTCGGGAA CGATTACGCT   
  
  
- TGGACGGATA TACGGATTGA CCGATATATA TACACGCATC TTGTCGTACT GTACTCAATT ATTATATCGT   
  
  
- CGATTTCCCT TGAGATTACA TGTGTACTAA CATTAAGTGG ACGGAGCGAT CGAATGCTCG TACTGAGAAC   
  
  
- TGATAATAAC AAAATTAACT AGAACTTAAA AGTATCTAAA ATGAGGTAAC AAATGAGTAA AACAATATTA   
  
  
- ATTGGTGGAC TAAAATTTGA AATTTTATGT GTTTCTAAGT AGATTATATT AAAAAATCTA TTTCAATACG   
  
  
- TATGTATATA TGTTTAATTT AATAAATGCT AGATTAGTAA AGGGCAATAT ATAAAGTGTG ATTTTAAATC   
  
  
- TTCTCTGTTA GATAATTTTA ATGTAGTGTA CAAAACCCCC CCCCCCCTAT AGTCTATACG TACTGTAAGG   
  
  
- ATTAGGTTAT TACGTTACTT TGGGTGGCTT ATCAACTTTA CTAAACAAGA TAGGTACTTG AGTGGTTCAG   
  
  
- TGTTAGGTAG TGTGAGCTTC CTATACGAAA AGAAAAACTT CCCTTAGAAC GAATTAGTTT GGATATTTGA   
  
  
- AAATTTTCGT TGTCTGTTTT TTCACTAATA TTACCATCAC CATCCTACTT CCGTCGAAAG TCTTTTCGCT   
  
  
- AACTTAAAAA TAACATAGGG TACTTTTGGG TAACTTAAAA AGAAAGAAAG AAACAAAAAC AATGGACCCA   
  
  
- AATACGTTTC CTGCCGACAA CATAAATTAT TATTGTTTTT GTATCCCGAA ACGCCTAACG ATTACGACCC   
  
  
- CTACCGTAGT CACGAGCTTA GACACATGCG ACAGTATCCT TGGGTGAACG AAGACGAGTA AATAAAGACA   
  
  
- GAGATCTAGG GGAGGAGAGA GAGAGAGAGA GAGATTCCAC AGACGAGCAG ACAAGTATAA GAGGACTCCA   
  
  
- AGAAGAAGTA GATAGAACAA GACCCACACA CGACTCCATG AAGAAAAGAA ATATTTAAAA AGTAGAAAAC   
  
  
- AACAAAACGA ACCACAAATA CTAAAATCAA AAACAGAAAT TCTGCTCTAA ACGACAATTA TCGTTCTAAA   
  
  
- GACTTGATCA TAACTAGGAA AAACACCATA TAAACAACTT AAACTAATTA AACACATAAC ACAAATCGAA   
  
  
- TTCATTGAGC AACACTAGAT TAACTACGAG CCCAAAACTG TCAAACTAAT CCCAAAAGCC ATGACCTCAA   
  
  
- TTTCAAAAAA ATAAAAAATA TCCTCCTCAA TTTCCATTAT TATTCTTAAC TACAAACGAC TAAAAAGGAG   
  
  
- AAAAGAAAAA ACCAAAGCTA AACCACCCGT CAATAGACAA ATACCAAAAA CGTTTACCTA GGTGTTAATC   
  
  
- TCCTTAATAT ACCTGGAAAT GTAGCTTAGT TCAAGTTACT AGTTTCCCAC GGTTAAAATG GTTCAGCGTC   
  
  
- ACAACAATGA GGCTACTTTG AGGTTCTAAG GTTAAACTTA GGTTCACAAG GTTTAGGTTC ACAACACTTA   
  
  
- GGAGGAGGCA ACCAAGGGGG TTTGGGTTTA GAATTAAATC AACACCGTAG AGTTATAAGA CTGTAACTCT   
  
  
- GTCGGGACTT GCTCCTAACA CTAAAATCAC TACAACAAGA ATTCATATAA TTAGTTGAAG AATACCTTCT   
  
  
- CCTACCCCTT CTCTTTGAGG TATGACTAGT ACTTGGAAGT CAGCAACTCC GCCGTCTCTT CAGTAACATA   
  
  
- CTCCGCGAGC CCGTCTCTAT AGGAGGAAGG CCTTTGGCTT TAGTCAACGG TCTACAACTC GTACTGCCGA   
  
  
- ACTGACCCTC GTCACAATCC CGAGTATCAC CACGTTCGTC ACAATCCCGA GTATCACTGC GTTCACCACC   
  
  
- ATCACCGGAC TGTCCTATAC CAACCATACC ACTAGGAATA TTAACCTCAG GAGTTTTACA GCACTTAAAA   
  
  
- TGGTAAAGGA GGAGCTTTGG GAATAGCACG TCGAGTAGTC TATCGAGCCC GTCTACCCAA TCATTCACCT   
  
  
- TCAGTTTAAG ACTAAGTGTC GAGTCGAGCC GGTAATTACG TTCACCAAAT CACCTACCCG GTCCCCTGAG   
  
  
- AGGACACTCA CGAGAATCAC ACAGACTTTA TAAGTTACTG TCAGTCAGTT ACAACGTCAA AGTCTTTCCT   
  
  
- AAACTCCTTC GCTTATTTAA AGAAGGTTTC TCAAGAAACA TGTTCCCAAA ACGGTTGTGC CCTAACGGAA   
  
  
- TAGTCTTCCG TTTGTTATCA CGGGTTCTAA ACAACCAGTT ACATCTCCTA CAATGATCCC CTTTCTTCGT   
  
  
- AGCAATAGGG CTTCTTAACG TCAGTCTTCT TCCCTCCTAT TTATTCGTTA GTCGACAAAG GGACCGTCGA   
  
  
- CTACTCCGTC AACAATCCAG ACTTTACAAG CTATCCCATG ACGAAACATC AGCCCCTTTT GTACTACGAC   
  
  
- GAGAGGCCCT TCGAAATGTC TGACTTGATT TATTCTCAGA CGCTTTACGG GGTCAATTCC CCAGATTATC   
  
  
- ACCCTTTCCG GCACCATTCT TTCGCCCTTT CTTCTCCCTA CATCATCTAA ATTCTAGAGA GAATTGGGAT   
  
  
- ACACGTGTTC GTCAACGTAG CTTACTAGTG TCCTCACGTT TACTGGACGA ATCCGTTTAA TCTGTCGTAA   
  
  
- GAAGAGGATC ACCCCTACCA TTGGTTTCTT ACCGTGTAAT AAAACGCCTA CCAGAACTCC GTGCGGAACG   
  
  
- ACCACATCCT TGAGGATATA TATTGACAGA ACATTGCCCA GGCCGTAGCC GACATCTATA GAACTCTCGA   
  
  
- ATGGTGTACA AAGAACGGTG TACGGGTAAA TTCTTTTACC CTTTAAAGAA GAGATTATCT TGCTAATACC   
  
  
- GACACCGTCT CTTACGTTGT ACGGAAGTAT ATTAACTAGA GCCATAACAG ATACCAAAGG TTACCGGAAC   
  
  
- AGATTAAGTC GCTGAAAGTA GATCCGGACC ACCGGGGGGG TTTGAAGCTT ATTGGCCTCA TCTAGAAGGT   
  
  
- GTTGGACCCA AGGCTGGTCG GTTTTCTCAA CTCCTCTGTC CCGCAGCGAA CTTCTTGATA CGTCTCAGTA   
  
  
- AATTACACGG GAAACTCAAG TTACGATATC GTTTCTTCAC CCTTTGTGAA TGGTAACTTC TAGAGTCCTA   
  
  
- GTTATCGCTA CTCAACGAAC AACAGTGGAC ATACAAATCC AAATTTGTAT ATGGACTCCT TTGTCACTGG   
  
  
- CACCTAACGG GATCCCTATG ACAAGAATTG GACTAACCCG CGTATTTGGG TCGACAAAAG TATGTTCCGT   
  
  
- GACAATTGCC CCGAAAGTTA AGAGGGAAAA AGTATCGAGC TAAAGCTCTC CGAGATAAAG TGAAGAGGTG   
  
  
- AGACAAACTA TACGATCTCC GGTTGGACGG TTCCCTGTTA TTCCTCTCCT ACGATTAACT CTCTCTCTAT   
  
  
- AAACCCTCCG TCCGTTACTT ACACTAACGA ACACTCCCAA ATCTCTCCTA TCTTTCCGGT CTCTGCATGT   
  
  
- TCGTCACCGT TCAGGCTTTA CTTTCCCGTC CCAAATCCGT CGACGGAAAT CTAGCGGTCT AAGACCTTTA   
  
  
- CCGATTTTTC TCCCACTTTA GACACATATT GTTTCTAAAG AGGTAACTAC TTCTGCCCGT GACCAACAAC   
  
  
- GACCCTACCT TCCCGTCTTA ACACATGTGT GAGTGATGAA CCTTCGGACG CCTCAT

+     A-box

| Site Name | Organism | Position | Strand | Matrix score. | sequence | function |
| --- | --- | --- | --- | --- | --- | --- |
| A-box | Petroselinum crispum | 1414 | - | 6 | CCGTCC | cis-acting regulatory element |

>HU06G00568.1   
+ -Up\_Stream \_Len000CCCTGG GTCATTAAAA AAAAAGAAAG AAAGAAGATT GCGCTAAAAG TAGATTTTTA   
  
  
+ TCAGATTCAA GCTGCAGGTG ATAACCAAAT TGTCATTAAA GCAATGCAAG CGCATATCAA TACCCTTTTG   
  
  
+ GGAGATAGCT CCGACTGTTG AAGACATTAG GAGCATGATC TCCAACTGTG AATTTGTTTC ATTTACTCAT   
  
  
+ ATTCATATCT ACCATGTGGA TAATATGACT GCAGACTAGA TGGCCAAATT TGAATGCACA CTTATAACTT   
  
  
+ CAACTCTTTC TACTTTTTTC TTCCCCACCT TATCAGGAAT TTCTTTTAAT TGTCGTGGGA GACAACTTGG   
  
  
+ GTAGAACTCT TGCGAAAAGG GCAACTTAAC GTTTTGGTAC TTGCATTTTC CAAAAAAACA AAAAGGCTAA   
  
  
+ ACAATTCTCC GCATGCTGAC TTCACGGTAG ATATGATGTA TTTTTTAACA AAAGACATCT TCTGAATGAA   
  
  
+ ACGCTTAATA TCTTGGACCA TACTAGAAAT TAAATTGCCA CGGTGCATGA ATTTCACCTC GAACTGCTTG   
  
  
+ TATAAAGATC TTGTTATCAC CTTCAGTAAT CAGATACTTG TAACCAAATT GAATGACCAC AAAGACCACA   
  
  
+ TTCCTCACAA TCGTTGCCTC TAGAATAAGA ATGAATGTTT CTTCAAGGTT GAAAGCCCTT GCTAATGCGA   
  
  
+ ACCTGCCTAT ATGCCTAACT GGCTATATAT ATGTGCGTAG AACAGCATGA CATGAGTTAA TAATATAGCA   
  
  
+ GCTAAAGGGA ACTCTAATGT ACACATGATT GTAATTCACC TGCCTCGCTA GCTTACGAGC ATGACTCTTG   
  
  
+ ACTATTATTG TTTTAATTGA TCTTGAATTT TCATAGATTT TACTCCATTG TTTACTCATT TTGTTATAAT   
  
  
+ TAACCACCTG ATTTTAAACT TTAAAATACA CAAAGATTCA TCTAATATAA TTTTTTAGAT AAAGTTATGC   
  
  
+ ATACATATAT ACAAATTAAA TTATTTACGA TCTAATCATT TCCCGTTATA TATTTCACAC TAAAATTTAG   
  
  
+ AAGAGACAAT CTATTAAAAT TACATCACAT GTTTTGGGGG GGGGGGGATA TCAGATATGC ATGACATTCC   
  
  
+ TAATCCAATA ATGCAATGAA ACCCACCGAA TAGTTGAAAT GATTTGTTCT ATCCATGAAC TCACCAAGTC   
  
  
+ ACAATCCATC ACACTCGAAG GATATGCTTT TCTTTTTGAA GGGAATCTTG CTTAATCAAA CCTATAAACT   
  
  
+ TTTAAAAGCA ACAGACAAAA AAGTGATTAT AATGGTAGTG GTAGGATGAA GGCAGCTTTC AGAAAAGCGA   
  
  
+ TTGAATTTTT ATTGTATCCC ATGAAAACCC ATTGAATTTT TCTTTCTTTC TTTGTTTTTG TTACCTGGGT   
  
  
+ TTATGCAAAG GACGGCTGTT GTATTTAATA ATAACAAAAA CATAGGGCTT TGCGGATTGC TAATGCTGGG   
  
  
+ GATGGCATCA GTGCTCGAAT CTGTGTACGC TGTCATAGGA ACCCACTTGC TTCTGCTCAT TTATTTCTGT   
  
  
+ CTCTAGATCC CCTCCTCTCT CTCTCTCTCT CTCTAAGGTG TCTGCTCGTC TGTTCATATT CTCCTGAGGT   
  
  
+ TCTTCTTCAT CTATCTTGTT CTGGGTGTGT GCTGAGGTAC TTCTTTTCTT TATAAATTTT TCATCTTTTG   
  
  
+ TTGTTTTGCT TGGTGTTTAT GATTTTAGTT TTTGTCTTTA AGACGAGATT TGCTGTTAAT AGCAAGATTT   
  
  
+ CTGAACTAGT ATTGATCCTT TTTGTGGTAT ATTTGTTGAA TTTGATTAAT TTGTGTATTG TGTTTAGCTT   
  
  
+ AAGTAACTCG TTGTGATCTA ATTGATGCTC GGGTTTTGAC AGTTTGATTA GGGTTTTCGG TACTGGAGTT   
  
  
+ AAAGTTTTTT TATTTTTTAT AGGAGGAGTT AAAGGTAATA ATAAGAATTG ATGTTTGCTG ATTTTTCCTC   
  
  
+ TTTTCTTTTT TGGTTTCGAT TTGGTGGGCA GTTATCTGTT TATGGTTTTT GCAAATGGAT CCACAATTAG   
  
  
+ AGGAATTATA TGGACCTTTA CATCGAATCA AGTTCAATGA TCAAAGGGTG CCAATTTTAC CAAGTCGCAG   
  
  
+ TGTTGTTACT CCGATGAAAC TCCAAGATTC CAATTTGAAT CCAAGTGTTC CAAATCCAAG TGTTGTGAAT   
  
  
+ CCTCCTCCGT TGGTTCCCCC AAACCCAAAT CTTAATTTAG TTGTGGCATC TCAATATTCT GACATTGAGA   
  
  
+ CAGCCCTGAA CGAGGATTGT GATTTTAGTG ATGTTGTTCT TAAGTATATT AATCAACTTC TTATGGAAGA   
  
  
+ GGATGGGGAA GAGAAACTCC ATACTGATCA TGAACCTTCA GTCGTTGAGG CGGCAGAGAA GTCATTGTAT   
  
  
+ GAGGCGCTCG GGCAGAGATA TCCTCCTTCC GGAAACCGAA ATCAGTTGCC AGATGTTGAG CATGACGGCT   
  
  
+ TGACTGGGAG CAGTGTTAGG GCTCATAGTG GTGCAAGCAG TGTTAGGGCT CATAGTGACG CAAGTGGTGG   
  
  
+ TAGTGGCCTG ACAGGATATG GTTGGTATGG TGATCCTTAT AATTGGAGTC CTCAAAATGT CGTGAATTTT   
  
  
+ ACCATTTCCT CCTCGAAACC CTTATCGTGC AGCTCATCAG ATAGCTCGGG CAGATGGGTT AGTAAGTGGA   
  
  
+ AGTCAAATTC TGATTCACAG CTCAGCTCGG CCATTAATGC AAGTGGTTTA GTGGATGGGC CAGGGGACTC   
  
  
+ TCCTGTGAGT GCTCTTAGTG TGTCTGAAAT ATTCAATGAC AGTCAGTCAA TGTTGCAGTT TCAGAAAGGA   
  
  
+ TTTGAGGAAG CGAATAAATT TCTTCCAAAG AGTTCTTTGT ACAAGGGTTT TGCCAACACG GGATTGCCTT   
  
  
+ ATCAGAAGGC AAACAATAGT GCCCAAGATT TGTTGGTCAA TGTAGAGGAT GTTACTAGGG GAAAGAAGCA   
  
  
+ TCGTTATCCC GAAGAATTGC AGTCAGAAGA AGGGAGGATA AATAAGCAAT CAGCTGTTTC CCTGGCAGCT   
  
  
+ GATGAGGCAG TTGTTAGGTC TGAAATGTTC GATAGGGTAC TGCTTTGTAG TCGGGGAAAA CATGATGCTG   
  
  
+ CTCTCCGGGA AGCTTTACAG ACTGAACTAA ATAAGAGTCT GCGAAATGCC CCAGTTAAGG GGTCTAATAG   
  
  
+ TGGGAAAGGC CGTGGTAAGA AAGCGGGAAA GAAGAGGGAT GTAGTAGATT TAAGATCTCT CTTAACCCTA   
  
  
+ TGTGCACAAG CAGTTGCATC GAATGATCAC AGGAGTGCAA ATGACCTGCT TAGGCAAATT AGACAGCATT   
  
  
+ CTTCTCCTAG TGGGGATGGT AACCAAAGAA TGGCACATTA TTTTGCGGAT GGTCTTGAGG CACGCCTTGC   
  
  
+ TGGTGTAGGA ACTCCTATAT ATAACTGTCT TGTAACGGGT CCGGCATCGG CTGTAGATAT CTTGAGAGCT   
  
  
+ TACCACATGT TTCTTGCCAC ATGCCCATTT AAGAAAATGG GAAATTTCTT CTCTAATAGA ACGATTATGG   
  
  
+ CTGTGGCAGA GAATGCAACA TGCCTTCATA TAATTGATCT CGGTATTGTC TATGGTTTCC AATGGCCTTG   
  
  
+ TCTAATTCAG CGACTTTCAT CTAGGCCTGG TGGCCCCCCC AAACTTCGAA TAACCGGAGT AGATCTTCCA   
  
  
+ CAACCTGGGT TCCGACCAGC CAAAAGAGTT GAGGAGACAG GGCGTCGCTT GAAGAACTAT GCAGAGTCAT   
  
  
+ TTAATGTGCC CTTTGAGTTC AATGCTATAG CAAAGAAGTG GGAAACACTT ACCATTGAAG ATCTCAGGAT   
  
  
+ CAATAGCGAT GAGTTGCTTG TTGTCACCTG TATGTTTAGG TTTAAACATA TACCTGAGGA AACAGTGACC   
  
  
+ GTGGATTGCC CTAGGGATAC TGTTCTTAAC CTGATTGGGC GCATAAACCC AGCTGTTTTC ATACAAGGCA   
  
  
+ CTGTTAACGG GGCTTTCAAT TCTCCCTTTT TCATAGCTCG ATTTCGAGAG GCTCTATTTC ACTTCTCCAC   
  
  
+ TCTGTTTGAT ATGCTAGAGG CCAACCTGCC AAGGGACAAT AAGGAGAGGA TGCTAATTGA GAGAGAGATA   
  
  
+ TTTGGGAGGC AGGCAATGAA TGTGATTGCT TGTGAGGGTT TAGAGAGGAT AGAAAGGCCA GAGACGTACA   
  
  
+ AGCAGTGGCA AGTCCGAAAT GAAAGGGCAG GGTTTAGGCA GCTGCCTTTA GATCGCCAGA TTCTGGAAAT   
  
  
+ GGCTAAAAAG AGGGTGAAAT CTGTGTATAA CAAAGATTTC TCCATTGATG AAGACGGGCA CTGGTTGTTG   
  
  
+ CTGGGATGGA AGGGCAGAAT TGTGTACACA CTCACTACTT GGAAGCCTGC GGAGTA  

- -Up\_Stream \_Len000GGGACC CAGTAATTTT TTTTTCTTTC TTTCTTCTAA CGCGATTTTC ATCTAAAAAT   
  
  
- AGTCTAAGTT CGACGTCCAC TATTGGTTTA ACAGTAATTT CGTTACGTTC GCGTATAGTT ATGGGAAAAC   
  
  
- CCTCTATCGA GGCTGACAAC TTCTGTAATC CTCGTACTAG AGGTTGACAC TTAAACAAAG TAAATGAGTA   
  
  
- TAAGTATAGA TGGTACACCT ATTATACTGA CGTCTGATCT ACCGGTTTAA ACTTACGTGT GAATATTGAA   
  
  
- GTTGAGAAAG ATGAAAAAAG AAGGGGTGGA ATAGTCCTTA AAGAAAATTA ACAGCACCCT CTGTTGAACC   
  
  
- CATCTTGAGA ACGCTTTTCC CGTTGAATTG CAAAACCATG AACGTAAAAG GTTTTTTTGT TTTTCCGATT   
  
  
- TGTTAAGAGG CGTACGACTG AAGTGCCATC TATACTACAT AAAAAATTGT TTTCTGTAGA AGACTTACTT   
  
  
- TGCGAATTAT AGAACCTGGT ATGATCTTTA ATTTAACGGT GCCACGTACT TAAAGTGGAG CTTGACGAAC   
  
  
- ATATTTCTAG AACAATAGTG GAAGTCATTA GTCTATGAAC ATTGGTTTAA CTTACTGGTG TTTCTGGTGT   
  
  
- AAGGAGTGTT AGCAACGGAG ATCTTATTCT TACTTACAAA GAAGTTCCAA CTTTCGGGAA CGATTACGCT   
  
  
- TGGACGGATA TACGGATTGA CCGATATATA TACACGCATC TTGTCGTACT GTACTCAATT ATTATATCGT   
  
  
- CGATTTCCCT TGAGATTACA TGTGTACTAA CATTAAGTGG ACGGAGCGAT CGAATGCTCG TACTGAGAAC   
  
  
- TGATAATAAC AAAATTAACT AGAACTTAAA AGTATCTAAA ATGAGGTAAC AAATGAGTAA AACAATATTA   
  
  
- ATTGGTGGAC TAAAATTTGA AATTTTATGT GTTTCTAAGT AGATTATATT AAAAAATCTA TTTCAATACG   
  
  
- TATGTATATA TGTTTAATTT AATAAATGCT AGATTAGTAA AGGGCAATAT ATAAAGTGTG ATTTTAAATC   
  
  
- TTCTCTGTTA GATAATTTTA ATGTAGTGTA CAAAACCCCC CCCCCCCTAT AGTCTATACG TACTGTAAGG   
  
  
- ATTAGGTTAT TACGTTACTT TGGGTGGCTT ATCAACTTTA CTAAACAAGA TAGGTACTTG AGTGGTTCAG   
  
  
- TGTTAGGTAG TGTGAGCTTC CTATACGAAA AGAAAAACTT CCCTTAGAAC GAATTAGTTT GGATATTTGA   
  
  
- AAATTTTCGT TGTCTGTTTT TTCACTAATA TTACCATCAC CATCCTACTT CCGTCGAAAG TCTTTTCGCT   
  
  
- AACTTAAAAA TAACATAGGG TACTTTTGGG TAACTTAAAA AGAAAGAAAG AAACAAAAAC AATGGACCCA   
  
  
- AATACGTTTC CTGCCGACAA CATAAATTAT TATTGTTTTT GTATCCCGAA ACGCCTAACG ATTACGACCC   
  
  
- CTACCGTAGT CACGAGCTTA GACACATGCG ACAGTATCCT TGGGTGAACG AAGACGAGTA AATAAAGACA   
  
  
- GAGATCTAGG GGAGGAGAGA GAGAGAGAGA GAGATTCCAC AGACGAGCAG ACAAGTATAA GAGGACTCCA   
  
  
- AGAAGAAGTA GATAGAACAA GACCCACACA CGACTCCATG AAGAAAAGAA ATATTTAAAA AGTAGAAAAC   
  
  
- AACAAAACGA ACCACAAATA CTAAAATCAA AAACAGAAAT TCTGCTCTAA ACGACAATTA TCGTTCTAAA   
  
  
- GACTTGATCA TAACTAGGAA AAACACCATA TAAACAACTT AAACTAATTA AACACATAAC ACAAATCGAA   
  
  
- TTCATTGAGC AACACTAGAT TAACTACGAG CCCAAAACTG TCAAACTAAT CCCAAAAGCC ATGACCTCAA   
  
  
- TTTCAAAAAA ATAAAAAATA TCCTCCTCAA TTTCCATTAT TATTCTTAAC TACAAACGAC TAAAAAGGAG   
  
  
- AAAAGAAAAA ACCAAAGCTA AACCACCCGT CAATAGACAA ATACCAAAAA CGTTTACCTA GGTGTTAATC   
  
  
- TCCTTAATAT ACCTGGAAAT GTAGCTTAGT TCAAGTTACT AGTTTCCCAC GGTTAAAATG GTTCAGCGTC   
  
  
- ACAACAATGA GGCTACTTTG AGGTTCTAAG GTTAAACTTA GGTTCACAAG GTTTAGGTTC ACAACACTTA   
  
  
- GGAGGAGGCA ACCAAGGGGG TTTGGGTTTA GAATTAAATC AACACCGTAG AGTTATAAGA CTGTAACTCT   
  
  
- GTCGGGACTT GCTCCTAACA CTAAAATCAC TACAACAAGA ATTCATATAA TTAGTTGAAG AATACCTTCT   
  
  
- CCTACCCCTT CTCTTTGAGG TATGACTAGT ACTTGGAAGT CAGCAACTCC GCCGTCTCTT CAGTAACATA   
  
  
- CTCCGCGAGC CCGTCTCTAT AGGAGGAAGG CCTTTGGCTT TAGTCAACGG TCTACAACTC GTACTGCCGA   
  
  
- ACTGACCCTC GTCACAATCC CGAGTATCAC CACGTTCGTC ACAATCCCGA GTATCACTGC GTTCACCACC   
  
  
- ATCACCGGAC TGTCCTATAC CAACCATACC ACTAGGAATA TTAACCTCAG GAGTTTTACA GCACTTAAAA   
  
  
- TGGTAAAGGA GGAGCTTTGG GAATAGCACG TCGAGTAGTC TATCGAGCCC GTCTACCCAA TCATTCACCT   
  
  
- TCAGTTTAAG ACTAAGTGTC GAGTCGAGCC GGTAATTACG TTCACCAAAT CACCTACCCG GTCCCCTGAG   
  
  
- AGGACACTCA CGAGAATCAC ACAGACTTTA TAAGTTACTG TCAGTCAGTT ACAACGTCAA AGTCTTTCCT   
  
  
- AAACTCCTTC GCTTATTTAA AGAAGGTTTC TCAAGAAACA TGTTCCCAAA ACGGTTGTGC CCTAACGGAA   
  
  
- TAGTCTTCCG TTTGTTATCA CGGGTTCTAA ACAACCAGTT ACATCTCCTA CAATGATCCC CTTTCTTCGT   
  
  
- AGCAATAGGG CTTCTTAACG TCAGTCTTCT TCCCTCCTAT TTATTCGTTA GTCGACAAAG GGACCGTCGA   
  
  
- CTACTCCGTC AACAATCCAG ACTTTACAAG CTATCCCATG ACGAAACATC AGCCCCTTTT GTACTACGAC   
  
  
- GAGAGGCCCT TCGAAATGTC TGACTTGATT TATTCTCAGA CGCTTTACGG GGTCAATTCC CCAGATTATC   
  
  
- ACCCTTTCCG GCACCATTCT TTCGCCCTTT CTTCTCCCTA CATCATCTAA ATTCTAGAGA GAATTGGGAT   
  
  
- ACACGTGTTC GTCAACGTAG CTTACTAGTG TCCTCACGTT TACTGGACGA ATCCGTTTAA TCTGTCGTAA   
  
  
- GAAGAGGATC ACCCCTACCA TTGGTTTCTT ACCGTGTAAT AAAACGCCTA CCAGAACTCC GTGCGGAACG   
  
  
- ACCACATCCT TGAGGATATA TATTGACAGA ACATTGCCCA GGCCGTAGCC GACATCTATA GAACTCTCGA   
  
  
- ATGGTGTACA AAGAACGGTG TACGGGTAAA TTCTTTTACC CTTTAAAGAA GAGATTATCT TGCTAATACC   
  
  
- GACACCGTCT CTTACGTTGT ACGGAAGTAT ATTAACTAGA GCCATAACAG ATACCAAAGG TTACCGGAAC   
  
  
- AGATTAAGTC GCTGAAAGTA GATCCGGACC ACCGGGGGGG TTTGAAGCTT ATTGGCCTCA TCTAGAAGGT   
  
  
- GTTGGACCCA AGGCTGGTCG GTTTTCTCAA CTCCTCTGTC CCGCAGCGAA CTTCTTGATA CGTCTCAGTA   
  
  
- AATTACACGG GAAACTCAAG TTACGATATC GTTTCTTCAC CCTTTGTGAA TGGTAACTTC TAGAGTCCTA   
  
  
- GTTATCGCTA CTCAACGAAC AACAGTGGAC ATACAAATCC AAATTTGTAT ATGGACTCCT TTGTCACTGG   
  
  
- CACCTAACGG GATCCCTATG ACAAGAATTG GACTAACCCG CGTATTTGGG TCGACAAAAG TATGTTCCGT   
  
  
- GACAATTGCC CCGAAAGTTA AGAGGGAAAA AGTATCGAGC TAAAGCTCTC CGAGATAAAG TGAAGAGGTG   
  
  
- AGACAAACTA TACGATCTCC GGTTGGACGG TTCCCTGTTA TTCCTCTCCT ACGATTAACT CTCTCTCTAT   
  
  
- AAACCCTCCG TCCGTTACTT ACACTAACGA ACACTCCCAA ATCTCTCCTA TCTTTCCGGT CTCTGCATGT   
  
  
- TCGTCACCGT TCAGGCTTTA CTTTCCCGTC CCAAATCCGT CGACGGAAAT CTAGCGGTCT AAGACCTTTA   
  
  
- CCGATTTTTC TCCCACTTTA GACACATATT GTTTCTAAAG AGGTAACTAC TTCTGCCCGT GACCAACAAC   
  
  
- GACCCTACCT TCCCGTCTTA ACACATGTGT GAGTGATGAA CCTTCGGACG CCTCAT

+     AAGAA-motif

| Site Name | Organism | Position | Strand | Matrix score. | sequence | function |
| --- | --- | --- | --- | --- | --- | --- |
| AAGAA-motif | Avena sativa | 1374 | - | 7 | GAAAGAA |  |
| AAGAA-motif | Avena sativa | 2935 | + | 7 | GAAAGAA |  |
| AAGAA-motif | Avena sativa | 1378 | - | 7 | GAAAGAA |  |
| AAGAA-motif | Avena sativa | 3181 | + | 7 | GAAAGAA |  |
| AAGAA-motif | Avena sativa | 40 | + | 7 | GAAAGAA |  |
| AAGAA-motif | Avena sativa | 44 | + | 7 | GAAAGAA |  |

>HU06G00568.1   
+ -Up\_Stream \_Len000CCCTGG GTCATTAAAA AAAAAGAAAG AAAGAAGATT GCGCTAAAAG TAGATTTTTA   
  
  
+ TCAGATTCAA GCTGCAGGTG ATAACCAAAT TGTCATTAAA GCAATGCAAG CGCATATCAA TACCCTTTTG   
  
  
+ GGAGATAGCT CCGACTGTTG AAGACATTAG GAGCATGATC TCCAACTGTG AATTTGTTTC ATTTACTCAT   
  
  
+ ATTCATATCT ACCATGTGGA TAATATGACT GCAGACTAGA TGGCCAAATT TGAATGCACA CTTATAACTT   
  
  
+ CAACTCTTTC TACTTTTTTC TTCCCCACCT TATCAGGAAT TTCTTTTAAT TGTCGTGGGA GACAACTTGG   
  
  
+ GTAGAACTCT TGCGAAAAGG GCAACTTAAC GTTTTGGTAC TTGCATTTTC CAAAAAAACA AAAAGGCTAA   
  
  
+ ACAATTCTCC GCATGCTGAC TTCACGGTAG ATATGATGTA TTTTTTAACA AAAGACATCT TCTGAATGAA   
  
  
+ ACGCTTAATA TCTTGGACCA TACTAGAAAT TAAATTGCCA CGGTGCATGA ATTTCACCTC GAACTGCTTG   
  
  
+ TATAAAGATC TTGTTATCAC CTTCAGTAAT CAGATACTTG TAACCAAATT GAATGACCAC AAAGACCACA   
  
  
+ TTCCTCACAA TCGTTGCCTC TAGAATAAGA ATGAATGTTT CTTCAAGGTT GAAAGCCCTT GCTAATGCGA   
  
  
+ ACCTGCCTAT ATGCCTAACT GGCTATATAT ATGTGCGTAG AACAGCATGA CATGAGTTAA TAATATAGCA   
  
  
+ GCTAAAGGGA ACTCTAATGT ACACATGATT GTAATTCACC TGCCTCGCTA GCTTACGAGC ATGACTCTTG   
  
  
+ ACTATTATTG TTTTAATTGA TCTTGAATTT TCATAGATTT TACTCCATTG TTTACTCATT TTGTTATAAT   
  
  
+ TAACCACCTG ATTTTAAACT TTAAAATACA CAAAGATTCA TCTAATATAA TTTTTTAGAT AAAGTTATGC   
  
  
+ ATACATATAT ACAAATTAAA TTATTTACGA TCTAATCATT TCCCGTTATA TATTTCACAC TAAAATTTAG   
  
  
+ AAGAGACAAT CTATTAAAAT TACATCACAT GTTTTGGGGG GGGGGGGATA TCAGATATGC ATGACATTCC   
  
  
+ TAATCCAATA ATGCAATGAA ACCCACCGAA TAGTTGAAAT GATTTGTTCT ATCCATGAAC TCACCAAGTC   
  
  
+ ACAATCCATC ACACTCGAAG GATATGCTTT TCTTTTTGAA GGGAATCTTG CTTAATCAAA CCTATAAACT   
  
  
+ TTTAAAAGCA ACAGACAAAA AAGTGATTAT AATGGTAGTG GTAGGATGAA GGCAGCTTTC AGAAAAGCGA   
  
  
+ TTGAATTTTT ATTGTATCCC ATGAAAACCC ATTGAATTTT TCTTTCTTTC TTTGTTTTTG TTACCTGGGT   
  
  
+ TTATGCAAAG GACGGCTGTT GTATTTAATA ATAACAAAAA CATAGGGCTT TGCGGATTGC TAATGCTGGG   
  
  
+ GATGGCATCA GTGCTCGAAT CTGTGTACGC TGTCATAGGA ACCCACTTGC TTCTGCTCAT TTATTTCTGT   
  
  
+ CTCTAGATCC CCTCCTCTCT CTCTCTCTCT CTCTAAGGTG TCTGCTCGTC TGTTCATATT CTCCTGAGGT   
  
  
+ TCTTCTTCAT CTATCTTGTT CTGGGTGTGT GCTGAGGTAC TTCTTTTCTT TATAAATTTT TCATCTTTTG   
  
  
+ TTGTTTTGCT TGGTGTTTAT GATTTTAGTT TTTGTCTTTA AGACGAGATT TGCTGTTAAT AGCAAGATTT   
  
  
+ CTGAACTAGT ATTGATCCTT TTTGTGGTAT ATTTGTTGAA TTTGATTAAT TTGTGTATTG TGTTTAGCTT   
  
  
+ AAGTAACTCG TTGTGATCTA ATTGATGCTC GGGTTTTGAC AGTTTGATTA GGGTTTTCGG TACTGGAGTT   
  
  
+ AAAGTTTTTT TATTTTTTAT AGGAGGAGTT AAAGGTAATA ATAAGAATTG ATGTTTGCTG ATTTTTCCTC   
  
  
+ TTTTCTTTTT TGGTTTCGAT TTGGTGGGCA GTTATCTGTT TATGGTTTTT GCAAATGGAT CCACAATTAG   
  
  
+ AGGAATTATA TGGACCTTTA CATCGAATCA AGTTCAATGA TCAAAGGGTG CCAATTTTAC CAAGTCGCAG   
  
  
+ TGTTGTTACT CCGATGAAAC TCCAAGATTC CAATTTGAAT CCAAGTGTTC CAAATCCAAG TGTTGTGAAT   
  
  
+ CCTCCTCCGT TGGTTCCCCC AAACCCAAAT CTTAATTTAG TTGTGGCATC TCAATATTCT GACATTGAGA   
  
  
+ CAGCCCTGAA CGAGGATTGT GATTTTAGTG ATGTTGTTCT TAAGTATATT AATCAACTTC TTATGGAAGA   
  
  
+ GGATGGGGAA GAGAAACTCC ATACTGATCA TGAACCTTCA GTCGTTGAGG CGGCAGAGAA GTCATTGTAT   
  
  
+ GAGGCGCTCG GGCAGAGATA TCCTCCTTCC GGAAACCGAA ATCAGTTGCC AGATGTTGAG CATGACGGCT   
  
  
+ TGACTGGGAG CAGTGTTAGG GCTCATAGTG GTGCAAGCAG TGTTAGGGCT CATAGTGACG CAAGTGGTGG   
  
  
+ TAGTGGCCTG ACAGGATATG GTTGGTATGG TGATCCTTAT AATTGGAGTC CTCAAAATGT CGTGAATTTT   
  
  
+ ACCATTTCCT CCTCGAAACC CTTATCGTGC AGCTCATCAG ATAGCTCGGG CAGATGGGTT AGTAAGTGGA   
  
  
+ AGTCAAATTC TGATTCACAG CTCAGCTCGG CCATTAATGC AAGTGGTTTA GTGGATGGGC CAGGGGACTC   
  
  
+ TCCTGTGAGT GCTCTTAGTG TGTCTGAAAT ATTCAATGAC AGTCAGTCAA TGTTGCAGTT TCAGAAAGGA   
  
  
+ TTTGAGGAAG CGAATAAATT TCTTCCAAAG AGTTCTTTGT ACAAGGGTTT TGCCAACACG GGATTGCCTT   
  
  
+ ATCAGAAGGC AAACAATAGT GCCCAAGATT TGTTGGTCAA TGTAGAGGAT GTTACTAGGG GAAAGAAGCA   
  
  
+ TCGTTATCCC GAAGAATTGC AGTCAGAAGA AGGGAGGATA AATAAGCAAT CAGCTGTTTC CCTGGCAGCT   
  
  
+ GATGAGGCAG TTGTTAGGTC TGAAATGTTC GATAGGGTAC TGCTTTGTAG TCGGGGAAAA CATGATGCTG   
  
  
+ CTCTCCGGGA AGCTTTACAG ACTGAACTAA ATAAGAGTCT GCGAAATGCC CCAGTTAAGG GGTCTAATAG   
  
  
+ TGGGAAAGGC CGTGGTAAGA AAGCGGGAAA GAAGAGGGAT GTAGTAGATT TAAGATCTCT CTTAACCCTA   
  
  
+ TGTGCACAAG CAGTTGCATC GAATGATCAC AGGAGTGCAA ATGACCTGCT TAGGCAAATT AGACAGCATT   
  
  
+ CTTCTCCTAG TGGGGATGGT AACCAAAGAA TGGCACATTA TTTTGCGGAT GGTCTTGAGG CACGCCTTGC   
  
  
+ TGGTGTAGGA ACTCCTATAT ATAACTGTCT TGTAACGGGT CCGGCATCGG CTGTAGATAT CTTGAGAGCT   
  
  
+ TACCACATGT TTCTTGCCAC ATGCCCATTT AAGAAAATGG GAAATTTCTT CTCTAATAGA ACGATTATGG   
  
  
+ CTGTGGCAGA GAATGCAACA TGCCTTCATA TAATTGATCT CGGTATTGTC TATGGTTTCC AATGGCCTTG   
  
  
+ TCTAATTCAG CGACTTTCAT CTAGGCCTGG TGGCCCCCCC AAACTTCGAA TAACCGGAGT AGATCTTCCA   
  
  
+ CAACCTGGGT TCCGACCAGC CAAAAGAGTT GAGGAGACAG GGCGTCGCTT GAAGAACTAT GCAGAGTCAT   
  
  
+ TTAATGTGCC CTTTGAGTTC AATGCTATAG CAAAGAAGTG GGAAACACTT ACCATTGAAG ATCTCAGGAT   
  
  
+ CAATAGCGAT GAGTTGCTTG TTGTCACCTG TATGTTTAGG TTTAAACATA TACCTGAGGA AACAGTGACC   
  
  
+ GTGGATTGCC CTAGGGATAC TGTTCTTAAC CTGATTGGGC GCATAAACCC AGCTGTTTTC ATACAAGGCA   
  
  
+ CTGTTAACGG GGCTTTCAAT TCTCCCTTTT TCATAGCTCG ATTTCGAGAG GCTCTATTTC ACTTCTCCAC   
  
  
+ TCTGTTTGAT ATGCTAGAGG CCAACCTGCC AAGGGACAAT AAGGAGAGGA TGCTAATTGA GAGAGAGATA   
  
  
+ TTTGGGAGGC AGGCAATGAA TGTGATTGCT TGTGAGGGTT TAGAGAGGAT AGAAAGGCCA GAGACGTACA   
  
  
+ AGCAGTGGCA AGTCCGAAAT GAAAGGGCAG GGTTTAGGCA GCTGCCTTTA GATCGCCAGA TTCTGGAAAT   
  
  
+ GGCTAAAAAG AGGGTGAAAT CTGTGTATAA CAAAGATTTC TCCATTGATG AAGACGGGCA CTGGTTGTTG   
  
  
+ CTGGGATGGA AGGGCAGAAT TGTGTACACA CTCACTACTT GGAAGCCTGC GGAGTA  

- -Up\_Stream \_Len000GGGACC CAGTAATTTT TTTTTCTTTC TTTCTTCTAA CGCGATTTTC ATCTAAAAAT   
  
  
- AGTCTAAGTT CGACGTCCAC TATTGGTTTA ACAGTAATTT CGTTACGTTC GCGTATAGTT ATGGGAAAAC   
  
  
- CCTCTATCGA GGCTGACAAC TTCTGTAATC CTCGTACTAG AGGTTGACAC TTAAACAAAG TAAATGAGTA   
  
  
- TAAGTATAGA TGGTACACCT ATTATACTGA CGTCTGATCT ACCGGTTTAA ACTTACGTGT GAATATTGAA   
  
  
- GTTGAGAAAG ATGAAAAAAG AAGGGGTGGA ATAGTCCTTA AAGAAAATTA ACAGCACCCT CTGTTGAACC   
  
  
- CATCTTGAGA ACGCTTTTCC CGTTGAATTG CAAAACCATG AACGTAAAAG GTTTTTTTGT TTTTCCGATT   
  
  
- TGTTAAGAGG CGTACGACTG AAGTGCCATC TATACTACAT AAAAAATTGT TTTCTGTAGA AGACTTACTT   
  
  
- TGCGAATTAT AGAACCTGGT ATGATCTTTA ATTTAACGGT GCCACGTACT TAAAGTGGAG CTTGACGAAC   
  
  
- ATATTTCTAG AACAATAGTG GAAGTCATTA GTCTATGAAC ATTGGTTTAA CTTACTGGTG TTTCTGGTGT   
  
  
- AAGGAGTGTT AGCAACGGAG ATCTTATTCT TACTTACAAA GAAGTTCCAA CTTTCGGGAA CGATTACGCT   
  
  
- TGGACGGATA TACGGATTGA CCGATATATA TACACGCATC TTGTCGTACT GTACTCAATT ATTATATCGT   
  
  
- CGATTTCCCT TGAGATTACA TGTGTACTAA CATTAAGTGG ACGGAGCGAT CGAATGCTCG TACTGAGAAC   
  
  
- TGATAATAAC AAAATTAACT AGAACTTAAA AGTATCTAAA ATGAGGTAAC AAATGAGTAA AACAATATTA   
  
  
- ATTGGTGGAC TAAAATTTGA AATTTTATGT GTTTCTAAGT AGATTATATT AAAAAATCTA TTTCAATACG   
  
  
- TATGTATATA TGTTTAATTT AATAAATGCT AGATTAGTAA AGGGCAATAT ATAAAGTGTG ATTTTAAATC   
  
  
- TTCTCTGTTA GATAATTTTA ATGTAGTGTA CAAAACCCCC CCCCCCCTAT AGTCTATACG TACTGTAAGG   
  
  
- ATTAGGTTAT TACGTTACTT TGGGTGGCTT ATCAACTTTA CTAAACAAGA TAGGTACTTG AGTGGTTCAG   
  
  
- TGTTAGGTAG TGTGAGCTTC CTATACGAAA AGAAAAACTT CCCTTAGAAC GAATTAGTTT GGATATTTGA   
  
  
- AAATTTTCGT TGTCTGTTTT TTCACTAATA TTACCATCAC CATCCTACTT CCGTCGAAAG TCTTTTCGCT   
  
  
- AACTTAAAAA TAACATAGGG TACTTTTGGG TAACTTAAAA AGAAAGAAAG AAACAAAAAC AATGGACCCA   
  
  
- AATACGTTTC CTGCCGACAA CATAAATTAT TATTGTTTTT GTATCCCGAA ACGCCTAACG ATTACGACCC   
  
  
- CTACCGTAGT CACGAGCTTA GACACATGCG ACAGTATCCT TGGGTGAACG AAGACGAGTA AATAAAGACA   
  
  
- GAGATCTAGG GGAGGAGAGA GAGAGAGAGA GAGATTCCAC AGACGAGCAG ACAAGTATAA GAGGACTCCA   
  
  
- AGAAGAAGTA GATAGAACAA GACCCACACA CGACTCCATG AAGAAAAGAA ATATTTAAAA AGTAGAAAAC   
  
  
- AACAAAACGA ACCACAAATA CTAAAATCAA AAACAGAAAT TCTGCTCTAA ACGACAATTA TCGTTCTAAA   
  
  
- GACTTGATCA TAACTAGGAA AAACACCATA TAAACAACTT AAACTAATTA AACACATAAC ACAAATCGAA   
  
  
- TTCATTGAGC AACACTAGAT TAACTACGAG CCCAAAACTG TCAAACTAAT CCCAAAAGCC ATGACCTCAA   
  
  
- TTTCAAAAAA ATAAAAAATA TCCTCCTCAA TTTCCATTAT TATTCTTAAC TACAAACGAC TAAAAAGGAG   
  
  
- AAAAGAAAAA ACCAAAGCTA AACCACCCGT CAATAGACAA ATACCAAAAA CGTTTACCTA GGTGTTAATC   
  
  
- TCCTTAATAT ACCTGGAAAT GTAGCTTAGT TCAAGTTACT AGTTTCCCAC GGTTAAAATG GTTCAGCGTC   
  
  
- ACAACAATGA GGCTACTTTG AGGTTCTAAG GTTAAACTTA GGTTCACAAG GTTTAGGTTC ACAACACTTA   
  
  
- GGAGGAGGCA ACCAAGGGGG TTTGGGTTTA GAATTAAATC AACACCGTAG AGTTATAAGA CTGTAACTCT   
  
  
- GTCGGGACTT GCTCCTAACA CTAAAATCAC TACAACAAGA ATTCATATAA TTAGTTGAAG AATACCTTCT   
  
  
- CCTACCCCTT CTCTTTGAGG TATGACTAGT ACTTGGAAGT CAGCAACTCC GCCGTCTCTT CAGTAACATA   
  
  
- CTCCGCGAGC CCGTCTCTAT AGGAGGAAGG CCTTTGGCTT TAGTCAACGG TCTACAACTC GTACTGCCGA   
  
  
- ACTGACCCTC GTCACAATCC CGAGTATCAC CACGTTCGTC ACAATCCCGA GTATCACTGC GTTCACCACC   
  
  
- ATCACCGGAC TGTCCTATAC CAACCATACC ACTAGGAATA TTAACCTCAG GAGTTTTACA GCACTTAAAA   
  
  
- TGGTAAAGGA GGAGCTTTGG GAATAGCACG TCGAGTAGTC TATCGAGCCC GTCTACCCAA TCATTCACCT   
  
  
- TCAGTTTAAG ACTAAGTGTC GAGTCGAGCC GGTAATTACG TTCACCAAAT CACCTACCCG GTCCCCTGAG   
  
  
- AGGACACTCA CGAGAATCAC ACAGACTTTA TAAGTTACTG TCAGTCAGTT ACAACGTCAA AGTCTTTCCT   
  
  
- AAACTCCTTC GCTTATTTAA AGAAGGTTTC TCAAGAAACA TGTTCCCAAA ACGGTTGTGC CCTAACGGAA   
  
  
- TAGTCTTCCG TTTGTTATCA CGGGTTCTAA ACAACCAGTT ACATCTCCTA CAATGATCCC CTTTCTTCGT   
  
  
- AGCAATAGGG CTTCTTAACG TCAGTCTTCT TCCCTCCTAT TTATTCGTTA GTCGACAAAG GGACCGTCGA   
  
  
- CTACTCCGTC AACAATCCAG ACTTTACAAG CTATCCCATG ACGAAACATC AGCCCCTTTT GTACTACGAC   
  
  
- GAGAGGCCCT TCGAAATGTC TGACTTGATT TATTCTCAGA CGCTTTACGG GGTCAATTCC CCAGATTATC   
  
  
- ACCCTTTCCG GCACCATTCT TTCGCCCTTT CTTCTCCCTA CATCATCTAA ATTCTAGAGA GAATTGGGAT   
  
  
- ACACGTGTTC GTCAACGTAG CTTACTAGTG TCCTCACGTT TACTGGACGA ATCCGTTTAA TCTGTCGTAA   
  
  
- GAAGAGGATC ACCCCTACCA TTGGTTTCTT ACCGTGTAAT AAAACGCCTA CCAGAACTCC GTGCGGAACG   
  
  
- ACCACATCCT TGAGGATATA TATTGACAGA ACATTGCCCA GGCCGTAGCC GACATCTATA GAACTCTCGA   
  
  
- ATGGTGTACA AAGAACGGTG TACGGGTAAA TTCTTTTACC CTTTAAAGAA GAGATTATCT TGCTAATACC   
  
  
- GACACCGTCT CTTACGTTGT ACGGAAGTAT ATTAACTAGA GCCATAACAG ATACCAAAGG TTACCGGAAC   
  
  
- AGATTAAGTC GCTGAAAGTA GATCCGGACC ACCGGGGGGG TTTGAAGCTT ATTGGCCTCA TCTAGAAGGT   
  
  
- GTTGGACCCA AGGCTGGTCG GTTTTCTCAA CTCCTCTGTC CCGCAGCGAA CTTCTTGATA CGTCTCAGTA   
  
  
- AATTACACGG GAAACTCAAG TTACGATATC GTTTCTTCAC CCTTTGTGAA TGGTAACTTC TAGAGTCCTA   
  
  
- GTTATCGCTA CTCAACGAAC AACAGTGGAC ATACAAATCC AAATTTGTAT ATGGACTCCT TTGTCACTGG   
  
  
- CACCTAACGG GATCCCTATG ACAAGAATTG GACTAACCCG CGTATTTGGG TCGACAAAAG TATGTTCCGT   
  
  
- GACAATTGCC CCGAAAGTTA AGAGGGAAAA AGTATCGAGC TAAAGCTCTC CGAGATAAAG TGAAGAGGTG   
  
  
- AGACAAACTA TACGATCTCC GGTTGGACGG TTCCCTGTTA TTCCTCTCCT ACGATTAACT CTCTCTCTAT   
  
  
- AAACCCTCCG TCCGTTACTT ACACTAACGA ACACTCCCAA ATCTCTCCTA TCTTTCCGGT CTCTGCATGT   
  
  
- TCGTCACCGT TCAGGCTTTA CTTTCCCGTC CCAAATCCGT CGACGGAAAT CTAGCGGTCT AAGACCTTTA   
  
  
- CCGATTTTTC TCCCACTTTA GACACATATT GTTTCTAAAG AGGTAACTAC TTCTGCCCGT GACCAACAAC   
  
  
- GACCCTACCT TCCCGTCTTA ACACATGTGT GAGTGATGAA CCTTCGGACG CCTCAT

+     ARE

| Site Name | Organism | Position | Strand | Matrix score. | sequence | function |
| --- | --- | --- | --- | --- | --- | --- |
| ARE | Zea mays | 1975 | - | 6 | AAACCA | cis-acting regulatory element essential for the anaerobic induction |
| ARE | Zea mays | 2007 | - | 6 | AAACCA | cis-acting regulatory element essential for the anaerobic induction |
| ARE | Zea mays | 2708 | - | 6 | AAACCA | cis-acting regulatory element essential for the anaerobic induction |
| ARE | Zea mays | 3557 | - | 6 | AAACCA | cis-acting regulatory element essential for the anaerobic induction |

>HU06G00568.1   
+ -Up\_Stream \_Len000CCCTGG GTCATTAAAA AAAAAGAAAG AAAGAAGATT GCGCTAAAAG TAGATTTTTA   
  
  
+ TCAGATTCAA GCTGCAGGTG ATAACCAAAT TGTCATTAAA GCAATGCAAG CGCATATCAA TACCCTTTTG   
  
  
+ GGAGATAGCT CCGACTGTTG AAGACATTAG GAGCATGATC TCCAACTGTG AATTTGTTTC ATTTACTCAT   
  
  
+ ATTCATATCT ACCATGTGGA TAATATGACT GCAGACTAGA TGGCCAAATT TGAATGCACA CTTATAACTT   
  
  
+ CAACTCTTTC TACTTTTTTC TTCCCCACCT TATCAGGAAT TTCTTTTAAT TGTCGTGGGA GACAACTTGG   
  
  
+ GTAGAACTCT TGCGAAAAGG GCAACTTAAC GTTTTGGTAC TTGCATTTTC CAAAAAAACA AAAAGGCTAA   
  
  
+ ACAATTCTCC GCATGCTGAC TTCACGGTAG ATATGATGTA TTTTTTAACA AAAGACATCT TCTGAATGAA   
  
  
+ ACGCTTAATA TCTTGGACCA TACTAGAAAT TAAATTGCCA CGGTGCATGA ATTTCACCTC GAACTGCTTG   
  
  
+ TATAAAGATC TTGTTATCAC CTTCAGTAAT CAGATACTTG TAACCAAATT GAATGACCAC AAAGACCACA   
  
  
+ TTCCTCACAA TCGTTGCCTC TAGAATAAGA ATGAATGTTT CTTCAAGGTT GAAAGCCCTT GCTAATGCGA   
  
  
+ ACCTGCCTAT ATGCCTAACT GGCTATATAT ATGTGCGTAG AACAGCATGA CATGAGTTAA TAATATAGCA   
  
  
+ GCTAAAGGGA ACTCTAATGT ACACATGATT GTAATTCACC TGCCTCGCTA GCTTACGAGC ATGACTCTTG   
  
  
+ ACTATTATTG TTTTAATTGA TCTTGAATTT TCATAGATTT TACTCCATTG TTTACTCATT TTGTTATAAT   
  
  
+ TAACCACCTG ATTTTAAACT TTAAAATACA CAAAGATTCA TCTAATATAA TTTTTTAGAT AAAGTTATGC   
  
  
+ ATACATATAT ACAAATTAAA TTATTTACGA TCTAATCATT TCCCGTTATA TATTTCACAC TAAAATTTAG   
  
  
+ AAGAGACAAT CTATTAAAAT TACATCACAT GTTTTGGGGG GGGGGGGATA TCAGATATGC ATGACATTCC   
  
  
+ TAATCCAATA ATGCAATGAA ACCCACCGAA TAGTTGAAAT GATTTGTTCT ATCCATGAAC TCACCAAGTC   
  
  
+ ACAATCCATC ACACTCGAAG GATATGCTTT TCTTTTTGAA GGGAATCTTG CTTAATCAAA CCTATAAACT   
  
  
+ TTTAAAAGCA ACAGACAAAA AAGTGATTAT AATGGTAGTG GTAGGATGAA GGCAGCTTTC AGAAAAGCGA   
  
  
+ TTGAATTTTT ATTGTATCCC ATGAAAACCC ATTGAATTTT TCTTTCTTTC TTTGTTTTTG TTACCTGGGT   
  
  
+ TTATGCAAAG GACGGCTGTT GTATTTAATA ATAACAAAAA CATAGGGCTT TGCGGATTGC TAATGCTGGG   
  
  
+ GATGGCATCA GTGCTCGAAT CTGTGTACGC TGTCATAGGA ACCCACTTGC TTCTGCTCAT TTATTTCTGT   
  
  
+ CTCTAGATCC CCTCCTCTCT CTCTCTCTCT CTCTAAGGTG TCTGCTCGTC TGTTCATATT CTCCTGAGGT   
  
  
+ TCTTCTTCAT CTATCTTGTT CTGGGTGTGT GCTGAGGTAC TTCTTTTCTT TATAAATTTT TCATCTTTTG   
  
  
+ TTGTTTTGCT TGGTGTTTAT GATTTTAGTT TTTGTCTTTA AGACGAGATT TGCTGTTAAT AGCAAGATTT   
  
  
+ CTGAACTAGT ATTGATCCTT TTTGTGGTAT ATTTGTTGAA TTTGATTAAT TTGTGTATTG TGTTTAGCTT   
  
  
+ AAGTAACTCG TTGTGATCTA ATTGATGCTC GGGTTTTGAC AGTTTGATTA GGGTTTTCGG TACTGGAGTT   
  
  
+ AAAGTTTTTT TATTTTTTAT AGGAGGAGTT AAAGGTAATA ATAAGAATTG ATGTTTGCTG ATTTTTCCTC   
  
  
+ TTTTCTTTTT TGGTTTCGAT TTGGTGGGCA GTTATCTGTT TATGGTTTTT GCAAATGGAT CCACAATTAG   
  
  
+ AGGAATTATA TGGACCTTTA CATCGAATCA AGTTCAATGA TCAAAGGGTG CCAATTTTAC CAAGTCGCAG   
  
  
+ TGTTGTTACT CCGATGAAAC TCCAAGATTC CAATTTGAAT CCAAGTGTTC CAAATCCAAG TGTTGTGAAT   
  
  
+ CCTCCTCCGT TGGTTCCCCC AAACCCAAAT CTTAATTTAG TTGTGGCATC TCAATATTCT GACATTGAGA   
  
  
+ CAGCCCTGAA CGAGGATTGT GATTTTAGTG ATGTTGTTCT TAAGTATATT AATCAACTTC TTATGGAAGA   
  
  
+ GGATGGGGAA GAGAAACTCC ATACTGATCA TGAACCTTCA GTCGTTGAGG CGGCAGAGAA GTCATTGTAT   
  
  
+ GAGGCGCTCG GGCAGAGATA TCCTCCTTCC GGAAACCGAA ATCAGTTGCC AGATGTTGAG CATGACGGCT   
  
  
+ TGACTGGGAG CAGTGTTAGG GCTCATAGTG GTGCAAGCAG TGTTAGGGCT CATAGTGACG CAAGTGGTGG   
  
  
+ TAGTGGCCTG ACAGGATATG GTTGGTATGG TGATCCTTAT AATTGGAGTC CTCAAAATGT CGTGAATTTT   
  
  
+ ACCATTTCCT CCTCGAAACC CTTATCGTGC AGCTCATCAG ATAGCTCGGG CAGATGGGTT AGTAAGTGGA   
  
  
+ AGTCAAATTC TGATTCACAG CTCAGCTCGG CCATTAATGC AAGTGGTTTA GTGGATGGGC CAGGGGACTC   
  
  
+ TCCTGTGAGT GCTCTTAGTG TGTCTGAAAT ATTCAATGAC AGTCAGTCAA TGTTGCAGTT TCAGAAAGGA   
  
  
+ TTTGAGGAAG CGAATAAATT TCTTCCAAAG AGTTCTTTGT ACAAGGGTTT TGCCAACACG GGATTGCCTT   
  
  
+ ATCAGAAGGC AAACAATAGT GCCCAAGATT TGTTGGTCAA TGTAGAGGAT GTTACTAGGG GAAAGAAGCA   
  
  
+ TCGTTATCCC GAAGAATTGC AGTCAGAAGA AGGGAGGATA AATAAGCAAT CAGCTGTTTC CCTGGCAGCT   
  
  
+ GATGAGGCAG TTGTTAGGTC TGAAATGTTC GATAGGGTAC TGCTTTGTAG TCGGGGAAAA CATGATGCTG   
  
  
+ CTCTCCGGGA AGCTTTACAG ACTGAACTAA ATAAGAGTCT GCGAAATGCC CCAGTTAAGG GGTCTAATAG   
  
  
+ TGGGAAAGGC CGTGGTAAGA AAGCGGGAAA GAAGAGGGAT GTAGTAGATT TAAGATCTCT CTTAACCCTA   
  
  
+ TGTGCACAAG CAGTTGCATC GAATGATCAC AGGAGTGCAA ATGACCTGCT TAGGCAAATT AGACAGCATT   
  
  
+ CTTCTCCTAG TGGGGATGGT AACCAAAGAA TGGCACATTA TTTTGCGGAT GGTCTTGAGG CACGCCTTGC   
  
  
+ TGGTGTAGGA ACTCCTATAT ATAACTGTCT TGTAACGGGT CCGGCATCGG CTGTAGATAT CTTGAGAGCT   
  
  
+ TACCACATGT TTCTTGCCAC ATGCCCATTT AAGAAAATGG GAAATTTCTT CTCTAATAGA ACGATTATGG   
  
  
+ CTGTGGCAGA GAATGCAACA TGCCTTCATA TAATTGATCT CGGTATTGTC TATGGTTTCC AATGGCCTTG   
  
  
+ TCTAATTCAG CGACTTTCAT CTAGGCCTGG TGGCCCCCCC AAACTTCGAA TAACCGGAGT AGATCTTCCA   
  
  
+ CAACCTGGGT TCCGACCAGC CAAAAGAGTT GAGGAGACAG GGCGTCGCTT GAAGAACTAT GCAGAGTCAT   
  
  
+ TTAATGTGCC CTTTGAGTTC AATGCTATAG CAAAGAAGTG GGAAACACTT ACCATTGAAG ATCTCAGGAT   
  
  
+ CAATAGCGAT GAGTTGCTTG TTGTCACCTG TATGTTTAGG TTTAAACATA TACCTGAGGA AACAGTGACC   
  
  
+ GTGGATTGCC CTAGGGATAC TGTTCTTAAC CTGATTGGGC GCATAAACCC AGCTGTTTTC ATACAAGGCA   
  
  
+ CTGTTAACGG GGCTTTCAAT TCTCCCTTTT TCATAGCTCG ATTTCGAGAG GCTCTATTTC ACTTCTCCAC   
  
  
+ TCTGTTTGAT ATGCTAGAGG CCAACCTGCC AAGGGACAAT AAGGAGAGGA TGCTAATTGA GAGAGAGATA   
  
  
+ TTTGGGAGGC AGGCAATGAA TGTGATTGCT TGTGAGGGTT TAGAGAGGAT AGAAAGGCCA GAGACGTACA   
  
  
+ AGCAGTGGCA AGTCCGAAAT GAAAGGGCAG GGTTTAGGCA GCTGCCTTTA GATCGCCAGA TTCTGGAAAT   
  
  
+ GGCTAAAAAG AGGGTGAAAT CTGTGTATAA CAAAGATTTC TCCATTGATG AAGACGGGCA CTGGTTGTTG   
  
  
+ CTGGGATGGA AGGGCAGAAT TGTGTACACA CTCACTACTT GGAAGCCTGC GGAGTA  

- -Up\_Stream \_Len000GGGACC CAGTAATTTT TTTTTCTTTC TTTCTTCTAA CGCGATTTTC ATCTAAAAAT   
  
  
- AGTCTAAGTT CGACGTCCAC TATTGGTTTA ACAGTAATTT CGTTACGTTC GCGTATAGTT ATGGGAAAAC   
  
  
- CCTCTATCGA GGCTGACAAC TTCTGTAATC CTCGTACTAG AGGTTGACAC TTAAACAAAG TAAATGAGTA   
  
  
- TAAGTATAGA TGGTACACCT ATTATACTGA CGTCTGATCT ACCGGTTTAA ACTTACGTGT GAATATTGAA   
  
  
- GTTGAGAAAG ATGAAAAAAG AAGGGGTGGA ATAGTCCTTA AAGAAAATTA ACAGCACCCT CTGTTGAACC   
  
  
- CATCTTGAGA ACGCTTTTCC CGTTGAATTG CAAAACCATG AACGTAAAAG GTTTTTTTGT TTTTCCGATT   
  
  
- TGTTAAGAGG CGTACGACTG AAGTGCCATC TATACTACAT AAAAAATTGT TTTCTGTAGA AGACTTACTT   
  
  
- TGCGAATTAT AGAACCTGGT ATGATCTTTA ATTTAACGGT GCCACGTACT TAAAGTGGAG CTTGACGAAC   
  
  
- ATATTTCTAG AACAATAGTG GAAGTCATTA GTCTATGAAC ATTGGTTTAA CTTACTGGTG TTTCTGGTGT   
  
  
- AAGGAGTGTT AGCAACGGAG ATCTTATTCT TACTTACAAA GAAGTTCCAA CTTTCGGGAA CGATTACGCT   
  
  
- TGGACGGATA TACGGATTGA CCGATATATA TACACGCATC TTGTCGTACT GTACTCAATT ATTATATCGT   
  
  
- CGATTTCCCT TGAGATTACA TGTGTACTAA CATTAAGTGG ACGGAGCGAT CGAATGCTCG TACTGAGAAC   
  
  
- TGATAATAAC AAAATTAACT AGAACTTAAA AGTATCTAAA ATGAGGTAAC AAATGAGTAA AACAATATTA   
  
  
- ATTGGTGGAC TAAAATTTGA AATTTTATGT GTTTCTAAGT AGATTATATT AAAAAATCTA TTTCAATACG   
  
  
- TATGTATATA TGTTTAATTT AATAAATGCT AGATTAGTAA AGGGCAATAT ATAAAGTGTG ATTTTAAATC   
  
  
- TTCTCTGTTA GATAATTTTA ATGTAGTGTA CAAAACCCCC CCCCCCCTAT AGTCTATACG TACTGTAAGG   
  
  
- ATTAGGTTAT TACGTTACTT TGGGTGGCTT ATCAACTTTA CTAAACAAGA TAGGTACTTG AGTGGTTCAG   
  
  
- TGTTAGGTAG TGTGAGCTTC CTATACGAAA AGAAAAACTT CCCTTAGAAC GAATTAGTTT GGATATTTGA   
  
  
- AAATTTTCGT TGTCTGTTTT TTCACTAATA TTACCATCAC CATCCTACTT CCGTCGAAAG TCTTTTCGCT   
  
  
- AACTTAAAAA TAACATAGGG TACTTTTGGG TAACTTAAAA AGAAAGAAAG AAACAAAAAC AATGGACCCA   
  
  
- AATACGTTTC CTGCCGACAA CATAAATTAT TATTGTTTTT GTATCCCGAA ACGCCTAACG ATTACGACCC   
  
  
- CTACCGTAGT CACGAGCTTA GACACATGCG ACAGTATCCT TGGGTGAACG AAGACGAGTA AATAAAGACA   
  
  
- GAGATCTAGG GGAGGAGAGA GAGAGAGAGA GAGATTCCAC AGACGAGCAG ACAAGTATAA GAGGACTCCA   
  
  
- AGAAGAAGTA GATAGAACAA GACCCACACA CGACTCCATG AAGAAAAGAA ATATTTAAAA AGTAGAAAAC   
  
  
- AACAAAACGA ACCACAAATA CTAAAATCAA AAACAGAAAT TCTGCTCTAA ACGACAATTA TCGTTCTAAA   
  
  
- GACTTGATCA TAACTAGGAA AAACACCATA TAAACAACTT AAACTAATTA AACACATAAC ACAAATCGAA   
  
  
- TTCATTGAGC AACACTAGAT TAACTACGAG CCCAAAACTG TCAAACTAAT CCCAAAAGCC ATGACCTCAA   
  
  
- TTTCAAAAAA ATAAAAAATA TCCTCCTCAA TTTCCATTAT TATTCTTAAC TACAAACGAC TAAAAAGGAG   
  
  
- AAAAGAAAAA ACCAAAGCTA AACCACCCGT CAATAGACAA ATACCAAAAA CGTTTACCTA GGTGTTAATC   
  
  
- TCCTTAATAT ACCTGGAAAT GTAGCTTAGT TCAAGTTACT AGTTTCCCAC GGTTAAAATG GTTCAGCGTC   
  
  
- ACAACAATGA GGCTACTTTG AGGTTCTAAG GTTAAACTTA GGTTCACAAG GTTTAGGTTC ACAACACTTA   
  
  
- GGAGGAGGCA ACCAAGGGGG TTTGGGTTTA GAATTAAATC AACACCGTAG AGTTATAAGA CTGTAACTCT   
  
  
- GTCGGGACTT GCTCCTAACA CTAAAATCAC TACAACAAGA ATTCATATAA TTAGTTGAAG AATACCTTCT   
  
  
- CCTACCCCTT CTCTTTGAGG TATGACTAGT ACTTGGAAGT CAGCAACTCC GCCGTCTCTT CAGTAACATA   
  
  
- CTCCGCGAGC CCGTCTCTAT AGGAGGAAGG CCTTTGGCTT TAGTCAACGG TCTACAACTC GTACTGCCGA   
  
  
- ACTGACCCTC GTCACAATCC CGAGTATCAC CACGTTCGTC ACAATCCCGA GTATCACTGC GTTCACCACC   
  
  
- ATCACCGGAC TGTCCTATAC CAACCATACC ACTAGGAATA TTAACCTCAG GAGTTTTACA GCACTTAAAA   
  
  
- TGGTAAAGGA GGAGCTTTGG GAATAGCACG TCGAGTAGTC TATCGAGCCC GTCTACCCAA TCATTCACCT   
  
  
- TCAGTTTAAG ACTAAGTGTC GAGTCGAGCC GGTAATTACG TTCACCAAAT CACCTACCCG GTCCCCTGAG   
  
  
- AGGACACTCA CGAGAATCAC ACAGACTTTA TAAGTTACTG TCAGTCAGTT ACAACGTCAA AGTCTTTCCT   
  
  
- AAACTCCTTC GCTTATTTAA AGAAGGTTTC TCAAGAAACA TGTTCCCAAA ACGGTTGTGC CCTAACGGAA   
  
  
- TAGTCTTCCG TTTGTTATCA CGGGTTCTAA ACAACCAGTT ACATCTCCTA CAATGATCCC CTTTCTTCGT   
  
  
- AGCAATAGGG CTTCTTAACG TCAGTCTTCT TCCCTCCTAT TTATTCGTTA GTCGACAAAG GGACCGTCGA   
  
  
- CTACTCCGTC AACAATCCAG ACTTTACAAG CTATCCCATG ACGAAACATC AGCCCCTTTT GTACTACGAC   
  
  
- GAGAGGCCCT TCGAAATGTC TGACTTGATT TATTCTCAGA CGCTTTACGG GGTCAATTCC CCAGATTATC   
  
  
- ACCCTTTCCG GCACCATTCT TTCGCCCTTT CTTCTCCCTA CATCATCTAA ATTCTAGAGA GAATTGGGAT   
  
  
- ACACGTGTTC GTCAACGTAG CTTACTAGTG TCCTCACGTT TACTGGACGA ATCCGTTTAA TCTGTCGTAA   
  
  
- GAAGAGGATC ACCCCTACCA TTGGTTTCTT ACCGTGTAAT AAAACGCCTA CCAGAACTCC GTGCGGAACG   
  
  
- ACCACATCCT TGAGGATATA TATTGACAGA ACATTGCCCA GGCCGTAGCC GACATCTATA GAACTCTCGA   
  
  
- ATGGTGTACA AAGAACGGTG TACGGGTAAA TTCTTTTACC CTTTAAAGAA GAGATTATCT TGCTAATACC   
  
  
- GACACCGTCT CTTACGTTGT ACGGAAGTAT ATTAACTAGA GCCATAACAG ATACCAAAGG TTACCGGAAC   
  
  
- AGATTAAGTC GCTGAAAGTA GATCCGGACC ACCGGGGGGG TTTGAAGCTT ATTGGCCTCA TCTAGAAGGT   
  
  
- GTTGGACCCA AGGCTGGTCG GTTTTCTCAA CTCCTCTGTC CCGCAGCGAA CTTCTTGATA CGTCTCAGTA   
  
  
- AATTACACGG GAAACTCAAG TTACGATATC GTTTCTTCAC CCTTTGTGAA TGGTAACTTC TAGAGTCCTA   
  
  
- GTTATCGCTA CTCAACGAAC AACAGTGGAC ATACAAATCC AAATTTGTAT ATGGACTCCT TTGTCACTGG   
  
  
- CACCTAACGG GATCCCTATG ACAAGAATTG GACTAACCCG CGTATTTGGG TCGACAAAAG TATGTTCCGT   
  
  
- GACAATTGCC CCGAAAGTTA AGAGGGAAAA AGTATCGAGC TAAAGCTCTC CGAGATAAAG TGAAGAGGTG   
  
  
- AGACAAACTA TACGATCTCC GGTTGGACGG TTCCCTGTTA TTCCTCTCCT ACGATTAACT CTCTCTCTAT   
  
  
- AAACCCTCCG TCCGTTACTT ACACTAACGA ACACTCCCAA ATCTCTCCTA TCTTTCCGGT CTCTGCATGT   
  
  
- TCGTCACCGT TCAGGCTTTA CTTTCCCGTC CCAAATCCGT CGACGGAAAT CTAGCGGTCT AAGACCTTTA   
  
  
- CCGATTTTTC TCCCACTTTA GACACATATT GTTTCTAAAG AGGTAACTAC TTCTGCCCGT GACCAACAAC   
  
  
- GACCCTACCT TCCCGTCTTA ACACATGTGT GAGTGATGAA CCTTCGGACG CCTCAT

+     AT~TATA-box

| Site Name | Organism | Position | Strand | Matrix score. | sequence | function |
| --- | --- | --- | --- | --- | --- | --- |
| AT~TATA-box | Arabidopsis thaliana | 730 | + | 6 | TATATA |  |
| AT~TATA-box | Arabidopsis thaliana | 3380 | - | 6 | TATATA |  |
| AT~TATA-box | Arabidopsis thaliana | 728 | + | 6 | TATATA |  |
| AT~TATA-box | Arabidopsis thaliana | 3382 | - | 6 | TATATA |  |
| AT~TATA-box | Arabidopsis thaliana | 990 | + | 6 | TATATA |  |
| AT~TATA-box | Arabidopsis thaliana | 1031 | + | 6 | TATATA |  |

>HU06G00568.1   
+ -Up\_Stream \_Len000CCCTGG GTCATTAAAA AAAAAGAAAG AAAGAAGATT GCGCTAAAAG TAGATTTTTA   
  
  
+ TCAGATTCAA GCTGCAGGTG ATAACCAAAT TGTCATTAAA GCAATGCAAG CGCATATCAA TACCCTTTTG   
  
  
+ GGAGATAGCT CCGACTGTTG AAGACATTAG GAGCATGATC TCCAACTGTG AATTTGTTTC ATTTACTCAT   
  
  
+ ATTCATATCT ACCATGTGGA TAATATGACT GCAGACTAGA TGGCCAAATT TGAATGCACA CTTATAACTT   
  
  
+ CAACTCTTTC TACTTTTTTC TTCCCCACCT TATCAGGAAT TTCTTTTAAT TGTCGTGGGA GACAACTTGG   
  
  
+ GTAGAACTCT TGCGAAAAGG GCAACTTAAC GTTTTGGTAC TTGCATTTTC CAAAAAAACA AAAAGGCTAA   
  
  
+ ACAATTCTCC GCATGCTGAC TTCACGGTAG ATATGATGTA TTTTTTAACA AAAGACATCT TCTGAATGAA   
  
  
+ ACGCTTAATA TCTTGGACCA TACTAGAAAT TAAATTGCCA CGGTGCATGA ATTTCACCTC GAACTGCTTG   
  
  
+ TATAAAGATC TTGTTATCAC CTTCAGTAAT CAGATACTTG TAACCAAATT GAATGACCAC AAAGACCACA   
  
  
+ TTCCTCACAA TCGTTGCCTC TAGAATAAGA ATGAATGTTT CTTCAAGGTT GAAAGCCCTT GCTAATGCGA   
  
  
+ ACCTGCCTAT ATGCCTAACT GGCTATATAT ATGTGCGTAG AACAGCATGA CATGAGTTAA TAATATAGCA   
  
  
+ GCTAAAGGGA ACTCTAATGT ACACATGATT GTAATTCACC TGCCTCGCTA GCTTACGAGC ATGACTCTTG   
  
  
+ ACTATTATTG TTTTAATTGA TCTTGAATTT TCATAGATTT TACTCCATTG TTTACTCATT TTGTTATAAT   
  
  
+ TAACCACCTG ATTTTAAACT TTAAAATACA CAAAGATTCA TCTAATATAA TTTTTTAGAT AAAGTTATGC   
  
  
+ ATACATATAT ACAAATTAAA TTATTTACGA TCTAATCATT TCCCGTTATA TATTTCACAC TAAAATTTAG   
  
  
+ AAGAGACAAT CTATTAAAAT TACATCACAT GTTTTGGGGG GGGGGGGATA TCAGATATGC ATGACATTCC   
  
  
+ TAATCCAATA ATGCAATGAA ACCCACCGAA TAGTTGAAAT GATTTGTTCT ATCCATGAAC TCACCAAGTC   
  
  
+ ACAATCCATC ACACTCGAAG GATATGCTTT TCTTTTTGAA GGGAATCTTG CTTAATCAAA CCTATAAACT   
  
  
+ TTTAAAAGCA ACAGACAAAA AAGTGATTAT AATGGTAGTG GTAGGATGAA GGCAGCTTTC AGAAAAGCGA   
  
  
+ TTGAATTTTT ATTGTATCCC ATGAAAACCC ATTGAATTTT TCTTTCTTTC TTTGTTTTTG TTACCTGGGT   
  
  
+ TTATGCAAAG GACGGCTGTT GTATTTAATA ATAACAAAAA CATAGGGCTT TGCGGATTGC TAATGCTGGG   
  
  
+ GATGGCATCA GTGCTCGAAT CTGTGTACGC TGTCATAGGA ACCCACTTGC TTCTGCTCAT TTATTTCTGT   
  
  
+ CTCTAGATCC CCTCCTCTCT CTCTCTCTCT CTCTAAGGTG TCTGCTCGTC TGTTCATATT CTCCTGAGGT   
  
  
+ TCTTCTTCAT CTATCTTGTT CTGGGTGTGT GCTGAGGTAC TTCTTTTCTT TATAAATTTT TCATCTTTTG   
  
  
+ TTGTTTTGCT TGGTGTTTAT GATTTTAGTT TTTGTCTTTA AGACGAGATT TGCTGTTAAT AGCAAGATTT   
  
  
+ CTGAACTAGT ATTGATCCTT TTTGTGGTAT ATTTGTTGAA TTTGATTAAT TTGTGTATTG TGTTTAGCTT   
  
  
+ AAGTAACTCG TTGTGATCTA ATTGATGCTC GGGTTTTGAC AGTTTGATTA GGGTTTTCGG TACTGGAGTT   
  
  
+ AAAGTTTTTT TATTTTTTAT AGGAGGAGTT AAAGGTAATA ATAAGAATTG ATGTTTGCTG ATTTTTCCTC   
  
  
+ TTTTCTTTTT TGGTTTCGAT TTGGTGGGCA GTTATCTGTT TATGGTTTTT GCAAATGGAT CCACAATTAG   
  
  
+ AGGAATTATA TGGACCTTTA CATCGAATCA AGTTCAATGA TCAAAGGGTG CCAATTTTAC CAAGTCGCAG   
  
  
+ TGTTGTTACT CCGATGAAAC TCCAAGATTC CAATTTGAAT CCAAGTGTTC CAAATCCAAG TGTTGTGAAT   
  
  
+ CCTCCTCCGT TGGTTCCCCC AAACCCAAAT CTTAATTTAG TTGTGGCATC TCAATATTCT GACATTGAGA   
  
  
+ CAGCCCTGAA CGAGGATTGT GATTTTAGTG ATGTTGTTCT TAAGTATATT AATCAACTTC TTATGGAAGA   
  
  
+ GGATGGGGAA GAGAAACTCC ATACTGATCA TGAACCTTCA GTCGTTGAGG CGGCAGAGAA GTCATTGTAT   
  
  
+ GAGGCGCTCG GGCAGAGATA TCCTCCTTCC GGAAACCGAA ATCAGTTGCC AGATGTTGAG CATGACGGCT   
  
  
+ TGACTGGGAG CAGTGTTAGG GCTCATAGTG GTGCAAGCAG TGTTAGGGCT CATAGTGACG CAAGTGGTGG   
  
  
+ TAGTGGCCTG ACAGGATATG GTTGGTATGG TGATCCTTAT AATTGGAGTC CTCAAAATGT CGTGAATTTT   
  
  
+ ACCATTTCCT CCTCGAAACC CTTATCGTGC AGCTCATCAG ATAGCTCGGG CAGATGGGTT AGTAAGTGGA   
  
  
+ AGTCAAATTC TGATTCACAG CTCAGCTCGG CCATTAATGC AAGTGGTTTA GTGGATGGGC CAGGGGACTC   
  
  
+ TCCTGTGAGT GCTCTTAGTG TGTCTGAAAT ATTCAATGAC AGTCAGTCAA TGTTGCAGTT TCAGAAAGGA   
  
  
+ TTTGAGGAAG CGAATAAATT TCTTCCAAAG AGTTCTTTGT ACAAGGGTTT TGCCAACACG GGATTGCCTT   
  
  
+ ATCAGAAGGC AAACAATAGT GCCCAAGATT TGTTGGTCAA TGTAGAGGAT GTTACTAGGG GAAAGAAGCA   
  
  
+ TCGTTATCCC GAAGAATTGC AGTCAGAAGA AGGGAGGATA AATAAGCAAT CAGCTGTTTC CCTGGCAGCT   
  
  
+ GATGAGGCAG TTGTTAGGTC TGAAATGTTC GATAGGGTAC TGCTTTGTAG TCGGGGAAAA CATGATGCTG   
  
  
+ CTCTCCGGGA AGCTTTACAG ACTGAACTAA ATAAGAGTCT GCGAAATGCC CCAGTTAAGG GGTCTAATAG   
  
  
+ TGGGAAAGGC CGTGGTAAGA AAGCGGGAAA GAAGAGGGAT GTAGTAGATT TAAGATCTCT CTTAACCCTA   
  
  
+ TGTGCACAAG CAGTTGCATC GAATGATCAC AGGAGTGCAA ATGACCTGCT TAGGCAAATT AGACAGCATT   
  
  
+ CTTCTCCTAG TGGGGATGGT AACCAAAGAA TGGCACATTA TTTTGCGGAT GGTCTTGAGG CACGCCTTGC   
  
  
+ TGGTGTAGGA ACTCCTATAT ATAACTGTCT TGTAACGGGT CCGGCATCGG CTGTAGATAT CTTGAGAGCT   
  
  
+ TACCACATGT TTCTTGCCAC ATGCCCATTT AAGAAAATGG GAAATTTCTT CTCTAATAGA ACGATTATGG   
  
  
+ CTGTGGCAGA GAATGCAACA TGCCTTCATA TAATTGATCT CGGTATTGTC TATGGTTTCC AATGGCCTTG   
  
  
+ TCTAATTCAG CGACTTTCAT CTAGGCCTGG TGGCCCCCCC AAACTTCGAA TAACCGGAGT AGATCTTCCA   
  
  
+ CAACCTGGGT TCCGACCAGC CAAAAGAGTT GAGGAGACAG GGCGTCGCTT GAAGAACTAT GCAGAGTCAT   
  
  
+ TTAATGTGCC CTTTGAGTTC AATGCTATAG CAAAGAAGTG GGAAACACTT ACCATTGAAG ATCTCAGGAT   
  
  
+ CAATAGCGAT GAGTTGCTTG TTGTCACCTG TATGTTTAGG TTTAAACATA TACCTGAGGA AACAGTGACC   
  
  
+ GTGGATTGCC CTAGGGATAC TGTTCTTAAC CTGATTGGGC GCATAAACCC AGCTGTTTTC ATACAAGGCA   
  
  
+ CTGTTAACGG GGCTTTCAAT TCTCCCTTTT TCATAGCTCG ATTTCGAGAG GCTCTATTTC ACTTCTCCAC   
  
  
+ TCTGTTTGAT ATGCTAGAGG CCAACCTGCC AAGGGACAAT AAGGAGAGGA TGCTAATTGA GAGAGAGATA   
  
  
+ TTTGGGAGGC AGGCAATGAA TGTGATTGCT TGTGAGGGTT TAGAGAGGAT AGAAAGGCCA GAGACGTACA   
  
  
+ AGCAGTGGCA AGTCCGAAAT GAAAGGGCAG GGTTTAGGCA GCTGCCTTTA GATCGCCAGA TTCTGGAAAT   
  
  
+ GGCTAAAAAG AGGGTGAAAT CTGTGTATAA CAAAGATTTC TCCATTGATG AAGACGGGCA CTGGTTGTTG   
  
  
+ CTGGGATGGA AGGGCAGAAT TGTGTACACA CTCACTACTT GGAAGCCTGC GGAGTA  

- -Up\_Stream \_Len000GGGACC CAGTAATTTT TTTTTCTTTC TTTCTTCTAA CGCGATTTTC ATCTAAAAAT   
  
  
- AGTCTAAGTT CGACGTCCAC TATTGGTTTA ACAGTAATTT CGTTACGTTC GCGTATAGTT ATGGGAAAAC   
  
  
- CCTCTATCGA GGCTGACAAC TTCTGTAATC CTCGTACTAG AGGTTGACAC TTAAACAAAG TAAATGAGTA   
  
  
- TAAGTATAGA TGGTACACCT ATTATACTGA CGTCTGATCT ACCGGTTTAA ACTTACGTGT GAATATTGAA   
  
  
- GTTGAGAAAG ATGAAAAAAG AAGGGGTGGA ATAGTCCTTA AAGAAAATTA ACAGCACCCT CTGTTGAACC   
  
  
- CATCTTGAGA ACGCTTTTCC CGTTGAATTG CAAAACCATG AACGTAAAAG GTTTTTTTGT TTTTCCGATT   
  
  
- TGTTAAGAGG CGTACGACTG AAGTGCCATC TATACTACAT AAAAAATTGT TTTCTGTAGA AGACTTACTT   
  
  
- TGCGAATTAT AGAACCTGGT ATGATCTTTA ATTTAACGGT GCCACGTACT TAAAGTGGAG CTTGACGAAC   
  
  
- ATATTTCTAG AACAATAGTG GAAGTCATTA GTCTATGAAC ATTGGTTTAA CTTACTGGTG TTTCTGGTGT   
  
  
- AAGGAGTGTT AGCAACGGAG ATCTTATTCT TACTTACAAA GAAGTTCCAA CTTTCGGGAA CGATTACGCT   
  
  
- TGGACGGATA TACGGATTGA CCGATATATA TACACGCATC TTGTCGTACT GTACTCAATT ATTATATCGT   
  
  
- CGATTTCCCT TGAGATTACA TGTGTACTAA CATTAAGTGG ACGGAGCGAT CGAATGCTCG TACTGAGAAC   
  
  
- TGATAATAAC AAAATTAACT AGAACTTAAA AGTATCTAAA ATGAGGTAAC AAATGAGTAA AACAATATTA   
  
  
- ATTGGTGGAC TAAAATTTGA AATTTTATGT GTTTCTAAGT AGATTATATT AAAAAATCTA TTTCAATACG   
  
  
- TATGTATATA TGTTTAATTT AATAAATGCT AGATTAGTAA AGGGCAATAT ATAAAGTGTG ATTTTAAATC   
  
  
- TTCTCTGTTA GATAATTTTA ATGTAGTGTA CAAAACCCCC CCCCCCCTAT AGTCTATACG TACTGTAAGG   
  
  
- ATTAGGTTAT TACGTTACTT TGGGTGGCTT ATCAACTTTA CTAAACAAGA TAGGTACTTG AGTGGTTCAG   
  
  
- TGTTAGGTAG TGTGAGCTTC CTATACGAAA AGAAAAACTT CCCTTAGAAC GAATTAGTTT GGATATTTGA   
  
  
- AAATTTTCGT TGTCTGTTTT TTCACTAATA TTACCATCAC CATCCTACTT CCGTCGAAAG TCTTTTCGCT   
  
  
- AACTTAAAAA TAACATAGGG TACTTTTGGG TAACTTAAAA AGAAAGAAAG AAACAAAAAC AATGGACCCA   
  
  
- AATACGTTTC CTGCCGACAA CATAAATTAT TATTGTTTTT GTATCCCGAA ACGCCTAACG ATTACGACCC   
  
  
- CTACCGTAGT CACGAGCTTA GACACATGCG ACAGTATCCT TGGGTGAACG AAGACGAGTA AATAAAGACA   
  
  
- GAGATCTAGG GGAGGAGAGA GAGAGAGAGA GAGATTCCAC AGACGAGCAG ACAAGTATAA GAGGACTCCA   
  
  
- AGAAGAAGTA GATAGAACAA GACCCACACA CGACTCCATG AAGAAAAGAA ATATTTAAAA AGTAGAAAAC   
  
  
- AACAAAACGA ACCACAAATA CTAAAATCAA AAACAGAAAT TCTGCTCTAA ACGACAATTA TCGTTCTAAA   
  
  
- GACTTGATCA TAACTAGGAA AAACACCATA TAAACAACTT AAACTAATTA AACACATAAC ACAAATCGAA   
  
  
- TTCATTGAGC AACACTAGAT TAACTACGAG CCCAAAACTG TCAAACTAAT CCCAAAAGCC ATGACCTCAA   
  
  
- TTTCAAAAAA ATAAAAAATA TCCTCCTCAA TTTCCATTAT TATTCTTAAC TACAAACGAC TAAAAAGGAG   
  
  
- AAAAGAAAAA ACCAAAGCTA AACCACCCGT CAATAGACAA ATACCAAAAA CGTTTACCTA GGTGTTAATC   
  
  
- TCCTTAATAT ACCTGGAAAT GTAGCTTAGT TCAAGTTACT AGTTTCCCAC GGTTAAAATG GTTCAGCGTC   
  
  
- ACAACAATGA GGCTACTTTG AGGTTCTAAG GTTAAACTTA GGTTCACAAG GTTTAGGTTC ACAACACTTA   
  
  
- GGAGGAGGCA ACCAAGGGGG TTTGGGTTTA GAATTAAATC AACACCGTAG AGTTATAAGA CTGTAACTCT   
  
  
- GTCGGGACTT GCTCCTAACA CTAAAATCAC TACAACAAGA ATTCATATAA TTAGTTGAAG AATACCTTCT   
  
  
- CCTACCCCTT CTCTTTGAGG TATGACTAGT ACTTGGAAGT CAGCAACTCC GCCGTCTCTT CAGTAACATA   
  
  
- CTCCGCGAGC CCGTCTCTAT AGGAGGAAGG CCTTTGGCTT TAGTCAACGG TCTACAACTC GTACTGCCGA   
  
  
- ACTGACCCTC GTCACAATCC CGAGTATCAC CACGTTCGTC ACAATCCCGA GTATCACTGC GTTCACCACC   
  
  
- ATCACCGGAC TGTCCTATAC CAACCATACC ACTAGGAATA TTAACCTCAG GAGTTTTACA GCACTTAAAA   
  
  
- TGGTAAAGGA GGAGCTTTGG GAATAGCACG TCGAGTAGTC TATCGAGCCC GTCTACCCAA TCATTCACCT   
  
  
- TCAGTTTAAG ACTAAGTGTC GAGTCGAGCC GGTAATTACG TTCACCAAAT CACCTACCCG GTCCCCTGAG   
  
  
- AGGACACTCA CGAGAATCAC ACAGACTTTA TAAGTTACTG TCAGTCAGTT ACAACGTCAA AGTCTTTCCT   
  
  
- AAACTCCTTC GCTTATTTAA AGAAGGTTTC TCAAGAAACA TGTTCCCAAA ACGGTTGTGC CCTAACGGAA   
  
  
- TAGTCTTCCG TTTGTTATCA CGGGTTCTAA ACAACCAGTT ACATCTCCTA CAATGATCCC CTTTCTTCGT   
  
  
- AGCAATAGGG CTTCTTAACG TCAGTCTTCT TCCCTCCTAT TTATTCGTTA GTCGACAAAG GGACCGTCGA   
  
  
- CTACTCCGTC AACAATCCAG ACTTTACAAG CTATCCCATG ACGAAACATC AGCCCCTTTT GTACTACGAC   
  
  
- GAGAGGCCCT TCGAAATGTC TGACTTGATT TATTCTCAGA CGCTTTACGG GGTCAATTCC CCAGATTATC   
  
  
- ACCCTTTCCG GCACCATTCT TTCGCCCTTT CTTCTCCCTA CATCATCTAA ATTCTAGAGA GAATTGGGAT   
  
  
- ACACGTGTTC GTCAACGTAG CTTACTAGTG TCCTCACGTT TACTGGACGA ATCCGTTTAA TCTGTCGTAA   
  
  
- GAAGAGGATC ACCCCTACCA TTGGTTTCTT ACCGTGTAAT AAAACGCCTA CCAGAACTCC GTGCGGAACG   
  
  
- ACCACATCCT TGAGGATATA TATTGACAGA ACATTGCCCA GGCCGTAGCC GACATCTATA GAACTCTCGA   
  
  
- ATGGTGTACA AAGAACGGTG TACGGGTAAA TTCTTTTACC CTTTAAAGAA GAGATTATCT TGCTAATACC   
  
  
- GACACCGTCT CTTACGTTGT ACGGAAGTAT ATTAACTAGA GCCATAACAG ATACCAAAGG TTACCGGAAC   
  
  
- AGATTAAGTC GCTGAAAGTA GATCCGGACC ACCGGGGGGG TTTGAAGCTT ATTGGCCTCA TCTAGAAGGT   
  
  
- GTTGGACCCA AGGCTGGTCG GTTTTCTCAA CTCCTCTGTC CCGCAGCGAA CTTCTTGATA CGTCTCAGTA   
  
  
- AATTACACGG GAAACTCAAG TTACGATATC GTTTCTTCAC CCTTTGTGAA TGGTAACTTC TAGAGTCCTA   
  
  
- GTTATCGCTA CTCAACGAAC AACAGTGGAC ATACAAATCC AAATTTGTAT ATGGACTCCT TTGTCACTGG   
  
  
- CACCTAACGG GATCCCTATG ACAAGAATTG GACTAACCCG CGTATTTGGG TCGACAAAAG TATGTTCCGT   
  
  
- GACAATTGCC CCGAAAGTTA AGAGGGAAAA AGTATCGAGC TAAAGCTCTC CGAGATAAAG TGAAGAGGTG   
  
  
- AGACAAACTA TACGATCTCC GGTTGGACGG TTCCCTGTTA TTCCTCTCCT ACGATTAACT CTCTCTCTAT   
  
  
- AAACCCTCCG TCCGTTACTT ACACTAACGA ACACTCCCAA ATCTCTCCTA TCTTTCCGGT CTCTGCATGT   
  
  
- TCGTCACCGT TCAGGCTTTA CTTTCCCGTC CCAAATCCGT CGACGGAAAT CTAGCGGTCT AAGACCTTTA   
  
  
- CCGATTTTTC TCCCACTTTA GACACATATT GTTTCTAAAG AGGTAACTAC TTCTGCCCGT GACCAACAAC   
  
  
- GACCCTACCT TCCCGTCTTA ACACATGTGT GAGTGATGAA CCTTCGGACG CCTCAT

+     AuxRR-core

| Site Name | Organism | Position | Strand | Matrix score. | sequence | function |
| --- | --- | --- | --- | --- | --- | --- |
| AuxRR-core | Nicotiana tabacum | 2044 | - | 7 | GGTCCAT | cis-acting regulatory element involved in auxin responsiveness |

>HU06G00568.1   
+ -Up\_Stream \_Len000CCCTGG GTCATTAAAA AAAAAGAAAG AAAGAAGATT GCGCTAAAAG TAGATTTTTA   
  
  
+ TCAGATTCAA GCTGCAGGTG ATAACCAAAT TGTCATTAAA GCAATGCAAG CGCATATCAA TACCCTTTTG   
  
  
+ GGAGATAGCT CCGACTGTTG AAGACATTAG GAGCATGATC TCCAACTGTG AATTTGTTTC ATTTACTCAT   
  
  
+ ATTCATATCT ACCATGTGGA TAATATGACT GCAGACTAGA TGGCCAAATT TGAATGCACA CTTATAACTT   
  
  
+ CAACTCTTTC TACTTTTTTC TTCCCCACCT TATCAGGAAT TTCTTTTAAT TGTCGTGGGA GACAACTTGG   
  
  
+ GTAGAACTCT TGCGAAAAGG GCAACTTAAC GTTTTGGTAC TTGCATTTTC CAAAAAAACA AAAAGGCTAA   
  
  
+ ACAATTCTCC GCATGCTGAC TTCACGGTAG ATATGATGTA TTTTTTAACA AAAGACATCT TCTGAATGAA   
  
  
+ ACGCTTAATA TCTTGGACCA TACTAGAAAT TAAATTGCCA CGGTGCATGA ATTTCACCTC GAACTGCTTG   
  
  
+ TATAAAGATC TTGTTATCAC CTTCAGTAAT CAGATACTTG TAACCAAATT GAATGACCAC AAAGACCACA   
  
  
+ TTCCTCACAA TCGTTGCCTC TAGAATAAGA ATGAATGTTT CTTCAAGGTT GAAAGCCCTT GCTAATGCGA   
  
  
+ ACCTGCCTAT ATGCCTAACT GGCTATATAT ATGTGCGTAG AACAGCATGA CATGAGTTAA TAATATAGCA   
  
  
+ GCTAAAGGGA ACTCTAATGT ACACATGATT GTAATTCACC TGCCTCGCTA GCTTACGAGC ATGACTCTTG   
  
  
+ ACTATTATTG TTTTAATTGA TCTTGAATTT TCATAGATTT TACTCCATTG TTTACTCATT TTGTTATAAT   
  
  
+ TAACCACCTG ATTTTAAACT TTAAAATACA CAAAGATTCA TCTAATATAA TTTTTTAGAT AAAGTTATGC   
  
  
+ ATACATATAT ACAAATTAAA TTATTTACGA TCTAATCATT TCCCGTTATA TATTTCACAC TAAAATTTAG   
  
  
+ AAGAGACAAT CTATTAAAAT TACATCACAT GTTTTGGGGG GGGGGGGATA TCAGATATGC ATGACATTCC   
  
  
+ TAATCCAATA ATGCAATGAA ACCCACCGAA TAGTTGAAAT GATTTGTTCT ATCCATGAAC TCACCAAGTC   
  
  
+ ACAATCCATC ACACTCGAAG GATATGCTTT TCTTTTTGAA GGGAATCTTG CTTAATCAAA CCTATAAACT   
  
  
+ TTTAAAAGCA ACAGACAAAA AAGTGATTAT AATGGTAGTG GTAGGATGAA GGCAGCTTTC AGAAAAGCGA   
  
  
+ TTGAATTTTT ATTGTATCCC ATGAAAACCC ATTGAATTTT TCTTTCTTTC TTTGTTTTTG TTACCTGGGT   
  
  
+ TTATGCAAAG GACGGCTGTT GTATTTAATA ATAACAAAAA CATAGGGCTT TGCGGATTGC TAATGCTGGG   
  
  
+ GATGGCATCA GTGCTCGAAT CTGTGTACGC TGTCATAGGA ACCCACTTGC TTCTGCTCAT TTATTTCTGT   
  
  
+ CTCTAGATCC CCTCCTCTCT CTCTCTCTCT CTCTAAGGTG TCTGCTCGTC TGTTCATATT CTCCTGAGGT   
  
  
+ TCTTCTTCAT CTATCTTGTT CTGGGTGTGT GCTGAGGTAC TTCTTTTCTT TATAAATTTT TCATCTTTTG   
  
  
+ TTGTTTTGCT TGGTGTTTAT GATTTTAGTT TTTGTCTTTA AGACGAGATT TGCTGTTAAT AGCAAGATTT   
  
  
+ CTGAACTAGT ATTGATCCTT TTTGTGGTAT ATTTGTTGAA TTTGATTAAT TTGTGTATTG TGTTTAGCTT   
  
  
+ AAGTAACTCG TTGTGATCTA ATTGATGCTC GGGTTTTGAC AGTTTGATTA GGGTTTTCGG TACTGGAGTT   
  
  
+ AAAGTTTTTT TATTTTTTAT AGGAGGAGTT AAAGGTAATA ATAAGAATTG ATGTTTGCTG ATTTTTCCTC   
  
  
+ TTTTCTTTTT TGGTTTCGAT TTGGTGGGCA GTTATCTGTT TATGGTTTTT GCAAATGGAT CCACAATTAG   
  
  
+ AGGAATTATA TGGACCTTTA CATCGAATCA AGTTCAATGA TCAAAGGGTG CCAATTTTAC CAAGTCGCAG   
  
  
+ TGTTGTTACT CCGATGAAAC TCCAAGATTC CAATTTGAAT CCAAGTGTTC CAAATCCAAG TGTTGTGAAT   
  
  
+ CCTCCTCCGT TGGTTCCCCC AAACCCAAAT CTTAATTTAG TTGTGGCATC TCAATATTCT GACATTGAGA   
  
  
+ CAGCCCTGAA CGAGGATTGT GATTTTAGTG ATGTTGTTCT TAAGTATATT AATCAACTTC TTATGGAAGA   
  
  
+ GGATGGGGAA GAGAAACTCC ATACTGATCA TGAACCTTCA GTCGTTGAGG CGGCAGAGAA GTCATTGTAT   
  
  
+ GAGGCGCTCG GGCAGAGATA TCCTCCTTCC GGAAACCGAA ATCAGTTGCC AGATGTTGAG CATGACGGCT   
  
  
+ TGACTGGGAG CAGTGTTAGG GCTCATAGTG GTGCAAGCAG TGTTAGGGCT CATAGTGACG CAAGTGGTGG   
  
  
+ TAGTGGCCTG ACAGGATATG GTTGGTATGG TGATCCTTAT AATTGGAGTC CTCAAAATGT CGTGAATTTT   
  
  
+ ACCATTTCCT CCTCGAAACC CTTATCGTGC AGCTCATCAG ATAGCTCGGG CAGATGGGTT AGTAAGTGGA   
  
  
+ AGTCAAATTC TGATTCACAG CTCAGCTCGG CCATTAATGC AAGTGGTTTA GTGGATGGGC CAGGGGACTC   
  
  
+ TCCTGTGAGT GCTCTTAGTG TGTCTGAAAT ATTCAATGAC AGTCAGTCAA TGTTGCAGTT TCAGAAAGGA   
  
  
+ TTTGAGGAAG CGAATAAATT TCTTCCAAAG AGTTCTTTGT ACAAGGGTTT TGCCAACACG GGATTGCCTT   
  
  
+ ATCAGAAGGC AAACAATAGT GCCCAAGATT TGTTGGTCAA TGTAGAGGAT GTTACTAGGG GAAAGAAGCA   
  
  
+ TCGTTATCCC GAAGAATTGC AGTCAGAAGA AGGGAGGATA AATAAGCAAT CAGCTGTTTC CCTGGCAGCT   
  
  
+ GATGAGGCAG TTGTTAGGTC TGAAATGTTC GATAGGGTAC TGCTTTGTAG TCGGGGAAAA CATGATGCTG   
  
  
+ CTCTCCGGGA AGCTTTACAG ACTGAACTAA ATAAGAGTCT GCGAAATGCC CCAGTTAAGG GGTCTAATAG   
  
  
+ TGGGAAAGGC CGTGGTAAGA AAGCGGGAAA GAAGAGGGAT GTAGTAGATT TAAGATCTCT CTTAACCCTA   
  
  
+ TGTGCACAAG CAGTTGCATC GAATGATCAC AGGAGTGCAA ATGACCTGCT TAGGCAAATT AGACAGCATT   
  
  
+ CTTCTCCTAG TGGGGATGGT AACCAAAGAA TGGCACATTA TTTTGCGGAT GGTCTTGAGG CACGCCTTGC   
  
  
+ TGGTGTAGGA ACTCCTATAT ATAACTGTCT TGTAACGGGT CCGGCATCGG CTGTAGATAT CTTGAGAGCT   
  
  
+ TACCACATGT TTCTTGCCAC ATGCCCATTT AAGAAAATGG GAAATTTCTT CTCTAATAGA ACGATTATGG   
  
  
+ CTGTGGCAGA GAATGCAACA TGCCTTCATA TAATTGATCT CGGTATTGTC TATGGTTTCC AATGGCCTTG   
  
  
+ TCTAATTCAG CGACTTTCAT CTAGGCCTGG TGGCCCCCCC AAACTTCGAA TAACCGGAGT AGATCTTCCA   
  
  
+ CAACCTGGGT TCCGACCAGC CAAAAGAGTT GAGGAGACAG GGCGTCGCTT GAAGAACTAT GCAGAGTCAT   
  
  
+ TTAATGTGCC CTTTGAGTTC AATGCTATAG CAAAGAAGTG GGAAACACTT ACCATTGAAG ATCTCAGGAT   
  
  
+ CAATAGCGAT GAGTTGCTTG TTGTCACCTG TATGTTTAGG TTTAAACATA TACCTGAGGA AACAGTGACC   
  
  
+ GTGGATTGCC CTAGGGATAC TGTTCTTAAC CTGATTGGGC GCATAAACCC AGCTGTTTTC ATACAAGGCA   
  
  
+ CTGTTAACGG GGCTTTCAAT TCTCCCTTTT TCATAGCTCG ATTTCGAGAG GCTCTATTTC ACTTCTCCAC   
  
  
+ TCTGTTTGAT ATGCTAGAGG CCAACCTGCC AAGGGACAAT AAGGAGAGGA TGCTAATTGA GAGAGAGATA   
  
  
+ TTTGGGAGGC AGGCAATGAA TGTGATTGCT TGTGAGGGTT TAGAGAGGAT AGAAAGGCCA GAGACGTACA   
  
  
+ AGCAGTGGCA AGTCCGAAAT GAAAGGGCAG GGTTTAGGCA GCTGCCTTTA GATCGCCAGA TTCTGGAAAT   
  
  
+ GGCTAAAAAG AGGGTGAAAT CTGTGTATAA CAAAGATTTC TCCATTGATG AAGACGGGCA CTGGTTGTTG   
  
  
+ CTGGGATGGA AGGGCAGAAT TGTGTACACA CTCACTACTT GGAAGCCTGC GGAGTA  

- -Up\_Stream \_Len000GGGACC CAGTAATTTT TTTTTCTTTC TTTCTTCTAA CGCGATTTTC ATCTAAAAAT   
  
  
- AGTCTAAGTT CGACGTCCAC TATTGGTTTA ACAGTAATTT CGTTACGTTC GCGTATAGTT ATGGGAAAAC   
  
  
- CCTCTATCGA GGCTGACAAC TTCTGTAATC CTCGTACTAG AGGTTGACAC TTAAACAAAG TAAATGAGTA   
  
  
- TAAGTATAGA TGGTACACCT ATTATACTGA CGTCTGATCT ACCGGTTTAA ACTTACGTGT GAATATTGAA   
  
  
- GTTGAGAAAG ATGAAAAAAG AAGGGGTGGA ATAGTCCTTA AAGAAAATTA ACAGCACCCT CTGTTGAACC   
  
  
- CATCTTGAGA ACGCTTTTCC CGTTGAATTG CAAAACCATG AACGTAAAAG GTTTTTTTGT TTTTCCGATT   
  
  
- TGTTAAGAGG CGTACGACTG AAGTGCCATC TATACTACAT AAAAAATTGT TTTCTGTAGA AGACTTACTT   
  
  
- TGCGAATTAT AGAACCTGGT ATGATCTTTA ATTTAACGGT GCCACGTACT TAAAGTGGAG CTTGACGAAC   
  
  
- ATATTTCTAG AACAATAGTG GAAGTCATTA GTCTATGAAC ATTGGTTTAA CTTACTGGTG TTTCTGGTGT   
  
  
- AAGGAGTGTT AGCAACGGAG ATCTTATTCT TACTTACAAA GAAGTTCCAA CTTTCGGGAA CGATTACGCT   
  
  
- TGGACGGATA TACGGATTGA CCGATATATA TACACGCATC TTGTCGTACT GTACTCAATT ATTATATCGT   
  
  
- CGATTTCCCT TGAGATTACA TGTGTACTAA CATTAAGTGG ACGGAGCGAT CGAATGCTCG TACTGAGAAC   
  
  
- TGATAATAAC AAAATTAACT AGAACTTAAA AGTATCTAAA ATGAGGTAAC AAATGAGTAA AACAATATTA   
  
  
- ATTGGTGGAC TAAAATTTGA AATTTTATGT GTTTCTAAGT AGATTATATT AAAAAATCTA TTTCAATACG   
  
  
- TATGTATATA TGTTTAATTT AATAAATGCT AGATTAGTAA AGGGCAATAT ATAAAGTGTG ATTTTAAATC   
  
  
- TTCTCTGTTA GATAATTTTA ATGTAGTGTA CAAAACCCCC CCCCCCCTAT AGTCTATACG TACTGTAAGG   
  
  
- ATTAGGTTAT TACGTTACTT TGGGTGGCTT ATCAACTTTA CTAAACAAGA TAGGTACTTG AGTGGTTCAG   
  
  
- TGTTAGGTAG TGTGAGCTTC CTATACGAAA AGAAAAACTT CCCTTAGAAC GAATTAGTTT GGATATTTGA   
  
  
- AAATTTTCGT TGTCTGTTTT TTCACTAATA TTACCATCAC CATCCTACTT CCGTCGAAAG TCTTTTCGCT   
  
  
- AACTTAAAAA TAACATAGGG TACTTTTGGG TAACTTAAAA AGAAAGAAAG AAACAAAAAC AATGGACCCA   
  
  
- AATACGTTTC CTGCCGACAA CATAAATTAT TATTGTTTTT GTATCCCGAA ACGCCTAACG ATTACGACCC   
  
  
- CTACCGTAGT CACGAGCTTA GACACATGCG ACAGTATCCT TGGGTGAACG AAGACGAGTA AATAAAGACA   
  
  
- GAGATCTAGG GGAGGAGAGA GAGAGAGAGA GAGATTCCAC AGACGAGCAG ACAAGTATAA GAGGACTCCA   
  
  
- AGAAGAAGTA GATAGAACAA GACCCACACA CGACTCCATG AAGAAAAGAA ATATTTAAAA AGTAGAAAAC   
  
  
- AACAAAACGA ACCACAAATA CTAAAATCAA AAACAGAAAT TCTGCTCTAA ACGACAATTA TCGTTCTAAA   
  
  
- GACTTGATCA TAACTAGGAA AAACACCATA TAAACAACTT AAACTAATTA AACACATAAC ACAAATCGAA   
  
  
- TTCATTGAGC AACACTAGAT TAACTACGAG CCCAAAACTG TCAAACTAAT CCCAAAAGCC ATGACCTCAA   
  
  
- TTTCAAAAAA ATAAAAAATA TCCTCCTCAA TTTCCATTAT TATTCTTAAC TACAAACGAC TAAAAAGGAG   
  
  
- AAAAGAAAAA ACCAAAGCTA AACCACCCGT CAATAGACAA ATACCAAAAA CGTTTACCTA GGTGTTAATC   
  
  
- TCCTTAATAT ACCTGGAAAT GTAGCTTAGT TCAAGTTACT AGTTTCCCAC GGTTAAAATG GTTCAGCGTC   
  
  
- ACAACAATGA GGCTACTTTG AGGTTCTAAG GTTAAACTTA GGTTCACAAG GTTTAGGTTC ACAACACTTA   
  
  
- GGAGGAGGCA ACCAAGGGGG TTTGGGTTTA GAATTAAATC AACACCGTAG AGTTATAAGA CTGTAACTCT   
  
  
- GTCGGGACTT GCTCCTAACA CTAAAATCAC TACAACAAGA ATTCATATAA TTAGTTGAAG AATACCTTCT   
  
  
- CCTACCCCTT CTCTTTGAGG TATGACTAGT ACTTGGAAGT CAGCAACTCC GCCGTCTCTT CAGTAACATA   
  
  
- CTCCGCGAGC CCGTCTCTAT AGGAGGAAGG CCTTTGGCTT TAGTCAACGG TCTACAACTC GTACTGCCGA   
  
  
- ACTGACCCTC GTCACAATCC CGAGTATCAC CACGTTCGTC ACAATCCCGA GTATCACTGC GTTCACCACC   
  
  
- ATCACCGGAC TGTCCTATAC CAACCATACC ACTAGGAATA TTAACCTCAG GAGTTTTACA GCACTTAAAA   
  
  
- TGGTAAAGGA GGAGCTTTGG GAATAGCACG TCGAGTAGTC TATCGAGCCC GTCTACCCAA TCATTCACCT   
  
  
- TCAGTTTAAG ACTAAGTGTC GAGTCGAGCC GGTAATTACG TTCACCAAAT CACCTACCCG GTCCCCTGAG   
  
  
- AGGACACTCA CGAGAATCAC ACAGACTTTA TAAGTTACTG TCAGTCAGTT ACAACGTCAA AGTCTTTCCT   
  
  
- AAACTCCTTC GCTTATTTAA AGAAGGTTTC TCAAGAAACA TGTTCCCAAA ACGGTTGTGC CCTAACGGAA   
  
  
- TAGTCTTCCG TTTGTTATCA CGGGTTCTAA ACAACCAGTT ACATCTCCTA CAATGATCCC CTTTCTTCGT   
  
  
- AGCAATAGGG CTTCTTAACG TCAGTCTTCT TCCCTCCTAT TTATTCGTTA GTCGACAAAG GGACCGTCGA   
  
  
- CTACTCCGTC AACAATCCAG ACTTTACAAG CTATCCCATG ACGAAACATC AGCCCCTTTT GTACTACGAC   
  
  
- GAGAGGCCCT TCGAAATGTC TGACTTGATT TATTCTCAGA CGCTTTACGG GGTCAATTCC CCAGATTATC   
  
  
- ACCCTTTCCG GCACCATTCT TTCGCCCTTT CTTCTCCCTA CATCATCTAA ATTCTAGAGA GAATTGGGAT   
  
  
- ACACGTGTTC GTCAACGTAG CTTACTAGTG TCCTCACGTT TACTGGACGA ATCCGTTTAA TCTGTCGTAA   
  
  
- GAAGAGGATC ACCCCTACCA TTGGTTTCTT ACCGTGTAAT AAAACGCCTA CCAGAACTCC GTGCGGAACG   
  
  
- ACCACATCCT TGAGGATATA TATTGACAGA ACATTGCCCA GGCCGTAGCC GACATCTATA GAACTCTCGA   
  
  
- ATGGTGTACA AAGAACGGTG TACGGGTAAA TTCTTTTACC CTTTAAAGAA GAGATTATCT TGCTAATACC   
  
  
- GACACCGTCT CTTACGTTGT ACGGAAGTAT ATTAACTAGA GCCATAACAG ATACCAAAGG TTACCGGAAC   
  
  
- AGATTAAGTC GCTGAAAGTA GATCCGGACC ACCGGGGGGG TTTGAAGCTT ATTGGCCTCA TCTAGAAGGT   
  
  
- GTTGGACCCA AGGCTGGTCG GTTTTCTCAA CTCCTCTGTC CCGCAGCGAA CTTCTTGATA CGTCTCAGTA   
  
  
- AATTACACGG GAAACTCAAG TTACGATATC GTTTCTTCAC CCTTTGTGAA TGGTAACTTC TAGAGTCCTA   
  
  
- GTTATCGCTA CTCAACGAAC AACAGTGGAC ATACAAATCC AAATTTGTAT ATGGACTCCT TTGTCACTGG   
  
  
- CACCTAACGG GATCCCTATG ACAAGAATTG GACTAACCCG CGTATTTGGG TCGACAAAAG TATGTTCCGT   
  
  
- GACAATTGCC CCGAAAGTTA AGAGGGAAAA AGTATCGAGC TAAAGCTCTC CGAGATAAAG TGAAGAGGTG   
  
  
- AGACAAACTA TACGATCTCC GGTTGGACGG TTCCCTGTTA TTCCTCTCCT ACGATTAACT CTCTCTCTAT   
  
  
- AAACCCTCCG TCCGTTACTT ACACTAACGA ACACTCCCAA ATCTCTCCTA TCTTTCCGGT CTCTGCATGT   
  
  
- TCGTCACCGT TCAGGCTTTA CTTTCCCGTC CCAAATCCGT CGACGGAAAT CTAGCGGTCT AAGACCTTTA   
  
  
- CCGATTTTTC TCCCACTTTA GACACATATT GTTTCTAAAG AGGTAACTAC TTCTGCCCGT GACCAACAAC   
  
  
- GACCCTACCT TCCCGTCTTA ACACATGTGT GAGTGATGAA CCTTCGGACG CCTCAT

+     Box 4

| Site Name | Organism | Position | Strand | Matrix score. | sequence | function |
| --- | --- | --- | --- | --- | --- | --- |
| Box 4 | Petroselinum crispum | 2697 | - | 6 | ATTAAT | part of a conserved DNA module involved in light responsiveness |
| Box 4 | Petroselinum crispum | 2292 | - | 6 | ATTAAT | part of a conserved DNA module involved in light responsiveness |
| Box 4 | Petroselinum crispum | 1799 | + | 6 | ATTAAT | part of a conserved DNA module involved in light responsiveness |

>HU06G00568.1   
+ -Up\_Stream \_Len000CCCTGG GTCATTAAAA AAAAAGAAAG AAAGAAGATT GCGCTAAAAG TAGATTTTTA   
  
  
+ TCAGATTCAA GCTGCAGGTG ATAACCAAAT TGTCATTAAA GCAATGCAAG CGCATATCAA TACCCTTTTG   
  
  
+ GGAGATAGCT CCGACTGTTG AAGACATTAG GAGCATGATC TCCAACTGTG AATTTGTTTC ATTTACTCAT   
  
  
+ ATTCATATCT ACCATGTGGA TAATATGACT GCAGACTAGA TGGCCAAATT TGAATGCACA CTTATAACTT   
  
  
+ CAACTCTTTC TACTTTTTTC TTCCCCACCT TATCAGGAAT TTCTTTTAAT TGTCGTGGGA GACAACTTGG   
  
  
+ GTAGAACTCT TGCGAAAAGG GCAACTTAAC GTTTTGGTAC TTGCATTTTC CAAAAAAACA AAAAGGCTAA   
  
  
+ ACAATTCTCC GCATGCTGAC TTCACGGTAG ATATGATGTA TTTTTTAACA AAAGACATCT TCTGAATGAA   
  
  
+ ACGCTTAATA TCTTGGACCA TACTAGAAAT TAAATTGCCA CGGTGCATGA ATTTCACCTC GAACTGCTTG   
  
  
+ TATAAAGATC TTGTTATCAC CTTCAGTAAT CAGATACTTG TAACCAAATT GAATGACCAC AAAGACCACA   
  
  
+ TTCCTCACAA TCGTTGCCTC TAGAATAAGA ATGAATGTTT CTTCAAGGTT GAAAGCCCTT GCTAATGCGA   
  
  
+ ACCTGCCTAT ATGCCTAACT GGCTATATAT ATGTGCGTAG AACAGCATGA CATGAGTTAA TAATATAGCA   
  
  
+ GCTAAAGGGA ACTCTAATGT ACACATGATT GTAATTCACC TGCCTCGCTA GCTTACGAGC ATGACTCTTG   
  
  
+ ACTATTATTG TTTTAATTGA TCTTGAATTT TCATAGATTT TACTCCATTG TTTACTCATT TTGTTATAAT   
  
  
+ TAACCACCTG ATTTTAAACT TTAAAATACA CAAAGATTCA TCTAATATAA TTTTTTAGAT AAAGTTATGC   
  
  
+ ATACATATAT ACAAATTAAA TTATTTACGA TCTAATCATT TCCCGTTATA TATTTCACAC TAAAATTTAG   
  
  
+ AAGAGACAAT CTATTAAAAT TACATCACAT GTTTTGGGGG GGGGGGGATA TCAGATATGC ATGACATTCC   
  
  
+ TAATCCAATA ATGCAATGAA ACCCACCGAA TAGTTGAAAT GATTTGTTCT ATCCATGAAC TCACCAAGTC   
  
  
+ ACAATCCATC ACACTCGAAG GATATGCTTT TCTTTTTGAA GGGAATCTTG CTTAATCAAA CCTATAAACT   
  
  
+ TTTAAAAGCA ACAGACAAAA AAGTGATTAT AATGGTAGTG GTAGGATGAA GGCAGCTTTC AGAAAAGCGA   
  
  
+ TTGAATTTTT ATTGTATCCC ATGAAAACCC ATTGAATTTT TCTTTCTTTC TTTGTTTTTG TTACCTGGGT   
  
  
+ TTATGCAAAG GACGGCTGTT GTATTTAATA ATAACAAAAA CATAGGGCTT TGCGGATTGC TAATGCTGGG   
  
  
+ GATGGCATCA GTGCTCGAAT CTGTGTACGC TGTCATAGGA ACCCACTTGC TTCTGCTCAT TTATTTCTGT   
  
  
+ CTCTAGATCC CCTCCTCTCT CTCTCTCTCT CTCTAAGGTG TCTGCTCGTC TGTTCATATT CTCCTGAGGT   
  
  
+ TCTTCTTCAT CTATCTTGTT CTGGGTGTGT GCTGAGGTAC TTCTTTTCTT TATAAATTTT TCATCTTTTG   
  
  
+ TTGTTTTGCT TGGTGTTTAT GATTTTAGTT TTTGTCTTTA AGACGAGATT TGCTGTTAAT AGCAAGATTT   
  
  
+ CTGAACTAGT ATTGATCCTT TTTGTGGTAT ATTTGTTGAA TTTGATTAAT TTGTGTATTG TGTTTAGCTT   
  
  
+ AAGTAACTCG TTGTGATCTA ATTGATGCTC GGGTTTTGAC AGTTTGATTA GGGTTTTCGG TACTGGAGTT   
  
  
+ AAAGTTTTTT TATTTTTTAT AGGAGGAGTT AAAGGTAATA ATAAGAATTG ATGTTTGCTG ATTTTTCCTC   
  
  
+ TTTTCTTTTT TGGTTTCGAT TTGGTGGGCA GTTATCTGTT TATGGTTTTT GCAAATGGAT CCACAATTAG   
  
  
+ AGGAATTATA TGGACCTTTA CATCGAATCA AGTTCAATGA TCAAAGGGTG CCAATTTTAC CAAGTCGCAG   
  
  
+ TGTTGTTACT CCGATGAAAC TCCAAGATTC CAATTTGAAT CCAAGTGTTC CAAATCCAAG TGTTGTGAAT   
  
  
+ CCTCCTCCGT TGGTTCCCCC AAACCCAAAT CTTAATTTAG TTGTGGCATC TCAATATTCT GACATTGAGA   
  
  
+ CAGCCCTGAA CGAGGATTGT GATTTTAGTG ATGTTGTTCT TAAGTATATT AATCAACTTC TTATGGAAGA   
  
  
+ GGATGGGGAA GAGAAACTCC ATACTGATCA TGAACCTTCA GTCGTTGAGG CGGCAGAGAA GTCATTGTAT   
  
  
+ GAGGCGCTCG GGCAGAGATA TCCTCCTTCC GGAAACCGAA ATCAGTTGCC AGATGTTGAG CATGACGGCT   
  
  
+ TGACTGGGAG CAGTGTTAGG GCTCATAGTG GTGCAAGCAG TGTTAGGGCT CATAGTGACG CAAGTGGTGG   
  
  
+ TAGTGGCCTG ACAGGATATG GTTGGTATGG TGATCCTTAT AATTGGAGTC CTCAAAATGT CGTGAATTTT   
  
  
+ ACCATTTCCT CCTCGAAACC CTTATCGTGC AGCTCATCAG ATAGCTCGGG CAGATGGGTT AGTAAGTGGA   
  
  
+ AGTCAAATTC TGATTCACAG CTCAGCTCGG CCATTAATGC AAGTGGTTTA GTGGATGGGC CAGGGGACTC   
  
  
+ TCCTGTGAGT GCTCTTAGTG TGTCTGAAAT ATTCAATGAC AGTCAGTCAA TGTTGCAGTT TCAGAAAGGA   
  
  
+ TTTGAGGAAG CGAATAAATT TCTTCCAAAG AGTTCTTTGT ACAAGGGTTT TGCCAACACG GGATTGCCTT   
  
  
+ ATCAGAAGGC AAACAATAGT GCCCAAGATT TGTTGGTCAA TGTAGAGGAT GTTACTAGGG GAAAGAAGCA   
  
  
+ TCGTTATCCC GAAGAATTGC AGTCAGAAGA AGGGAGGATA AATAAGCAAT CAGCTGTTTC CCTGGCAGCT   
  
  
+ GATGAGGCAG TTGTTAGGTC TGAAATGTTC GATAGGGTAC TGCTTTGTAG TCGGGGAAAA CATGATGCTG   
  
  
+ CTCTCCGGGA AGCTTTACAG ACTGAACTAA ATAAGAGTCT GCGAAATGCC CCAGTTAAGG GGTCTAATAG   
  
  
+ TGGGAAAGGC CGTGGTAAGA AAGCGGGAAA GAAGAGGGAT GTAGTAGATT TAAGATCTCT CTTAACCCTA   
  
  
+ TGTGCACAAG CAGTTGCATC GAATGATCAC AGGAGTGCAA ATGACCTGCT TAGGCAAATT AGACAGCATT   
  
  
+ CTTCTCCTAG TGGGGATGGT AACCAAAGAA TGGCACATTA TTTTGCGGAT GGTCTTGAGG CACGCCTTGC   
  
  
+ TGGTGTAGGA ACTCCTATAT ATAACTGTCT TGTAACGGGT CCGGCATCGG CTGTAGATAT CTTGAGAGCT   
  
  
+ TACCACATGT TTCTTGCCAC ATGCCCATTT AAGAAAATGG GAAATTTCTT CTCTAATAGA ACGATTATGG   
  
  
+ CTGTGGCAGA GAATGCAACA TGCCTTCATA TAATTGATCT CGGTATTGTC TATGGTTTCC AATGGCCTTG   
  
  
+ TCTAATTCAG CGACTTTCAT CTAGGCCTGG TGGCCCCCCC AAACTTCGAA TAACCGGAGT AGATCTTCCA   
  
  
+ CAACCTGGGT TCCGACCAGC CAAAAGAGTT GAGGAGACAG GGCGTCGCTT GAAGAACTAT GCAGAGTCAT   
  
  
+ TTAATGTGCC CTTTGAGTTC AATGCTATAG CAAAGAAGTG GGAAACACTT ACCATTGAAG ATCTCAGGAT   
  
  
+ CAATAGCGAT GAGTTGCTTG TTGTCACCTG TATGTTTAGG TTTAAACATA TACCTGAGGA AACAGTGACC   
  
  
+ GTGGATTGCC CTAGGGATAC TGTTCTTAAC CTGATTGGGC GCATAAACCC AGCTGTTTTC ATACAAGGCA   
  
  
+ CTGTTAACGG GGCTTTCAAT TCTCCCTTTT TCATAGCTCG ATTTCGAGAG GCTCTATTTC ACTTCTCCAC   
  
  
+ TCTGTTTGAT ATGCTAGAGG CCAACCTGCC AAGGGACAAT AAGGAGAGGA TGCTAATTGA GAGAGAGATA   
  
  
+ TTTGGGAGGC AGGCAATGAA TGTGATTGCT TGTGAGGGTT TAGAGAGGAT AGAAAGGCCA GAGACGTACA   
  
  
+ AGCAGTGGCA AGTCCGAAAT GAAAGGGCAG GGTTTAGGCA GCTGCCTTTA GATCGCCAGA TTCTGGAAAT   
  
  
+ GGCTAAAAAG AGGGTGAAAT CTGTGTATAA CAAAGATTTC TCCATTGATG AAGACGGGCA CTGGTTGTTG   
  
  
+ CTGGGATGGA AGGGCAGAAT TGTGTACACA CTCACTACTT GGAAGCCTGC GGAGTA  

- -Up\_Stream \_Len000GGGACC CAGTAATTTT TTTTTCTTTC TTTCTTCTAA CGCGATTTTC ATCTAAAAAT   
  
  
- AGTCTAAGTT CGACGTCCAC TATTGGTTTA ACAGTAATTT CGTTACGTTC GCGTATAGTT ATGGGAAAAC   
  
  
- CCTCTATCGA GGCTGACAAC TTCTGTAATC CTCGTACTAG AGGTTGACAC TTAAACAAAG TAAATGAGTA   
  
  
- TAAGTATAGA TGGTACACCT ATTATACTGA CGTCTGATCT ACCGGTTTAA ACTTACGTGT GAATATTGAA   
  
  
- GTTGAGAAAG ATGAAAAAAG AAGGGGTGGA ATAGTCCTTA AAGAAAATTA ACAGCACCCT CTGTTGAACC   
  
  
- CATCTTGAGA ACGCTTTTCC CGTTGAATTG CAAAACCATG AACGTAAAAG GTTTTTTTGT TTTTCCGATT   
  
  
- TGTTAAGAGG CGTACGACTG AAGTGCCATC TATACTACAT AAAAAATTGT TTTCTGTAGA AGACTTACTT   
  
  
- TGCGAATTAT AGAACCTGGT ATGATCTTTA ATTTAACGGT GCCACGTACT TAAAGTGGAG CTTGACGAAC   
  
  
- ATATTTCTAG AACAATAGTG GAAGTCATTA GTCTATGAAC ATTGGTTTAA CTTACTGGTG TTTCTGGTGT   
  
  
- AAGGAGTGTT AGCAACGGAG ATCTTATTCT TACTTACAAA GAAGTTCCAA CTTTCGGGAA CGATTACGCT   
  
  
- TGGACGGATA TACGGATTGA CCGATATATA TACACGCATC TTGTCGTACT GTACTCAATT ATTATATCGT   
  
  
- CGATTTCCCT TGAGATTACA TGTGTACTAA CATTAAGTGG ACGGAGCGAT CGAATGCTCG TACTGAGAAC   
  
  
- TGATAATAAC AAAATTAACT AGAACTTAAA AGTATCTAAA ATGAGGTAAC AAATGAGTAA AACAATATTA   
  
  
- ATTGGTGGAC TAAAATTTGA AATTTTATGT GTTTCTAAGT AGATTATATT AAAAAATCTA TTTCAATACG   
  
  
- TATGTATATA TGTTTAATTT AATAAATGCT AGATTAGTAA AGGGCAATAT ATAAAGTGTG ATTTTAAATC   
  
  
- TTCTCTGTTA GATAATTTTA ATGTAGTGTA CAAAACCCCC CCCCCCCTAT AGTCTATACG TACTGTAAGG   
  
  
- ATTAGGTTAT TACGTTACTT TGGGTGGCTT ATCAACTTTA CTAAACAAGA TAGGTACTTG AGTGGTTCAG   
  
  
- TGTTAGGTAG TGTGAGCTTC CTATACGAAA AGAAAAACTT CCCTTAGAAC GAATTAGTTT GGATATTTGA   
  
  
- AAATTTTCGT TGTCTGTTTT TTCACTAATA TTACCATCAC CATCCTACTT CCGTCGAAAG TCTTTTCGCT   
  
  
- AACTTAAAAA TAACATAGGG TACTTTTGGG TAACTTAAAA AGAAAGAAAG AAACAAAAAC AATGGACCCA   
  
  
- AATACGTTTC CTGCCGACAA CATAAATTAT TATTGTTTTT GTATCCCGAA ACGCCTAACG ATTACGACCC   
  
  
- CTACCGTAGT CACGAGCTTA GACACATGCG ACAGTATCCT TGGGTGAACG AAGACGAGTA AATAAAGACA   
  
  
- GAGATCTAGG GGAGGAGAGA GAGAGAGAGA GAGATTCCAC AGACGAGCAG ACAAGTATAA GAGGACTCCA   
  
  
- AGAAGAAGTA GATAGAACAA GACCCACACA CGACTCCATG AAGAAAAGAA ATATTTAAAA AGTAGAAAAC   
  
  
- AACAAAACGA ACCACAAATA CTAAAATCAA AAACAGAAAT TCTGCTCTAA ACGACAATTA TCGTTCTAAA   
  
  
- GACTTGATCA TAACTAGGAA AAACACCATA TAAACAACTT AAACTAATTA AACACATAAC ACAAATCGAA   
  
  
- TTCATTGAGC AACACTAGAT TAACTACGAG CCCAAAACTG TCAAACTAAT CCCAAAAGCC ATGACCTCAA   
  
  
- TTTCAAAAAA ATAAAAAATA TCCTCCTCAA TTTCCATTAT TATTCTTAAC TACAAACGAC TAAAAAGGAG   
  
  
- AAAAGAAAAA ACCAAAGCTA AACCACCCGT CAATAGACAA ATACCAAAAA CGTTTACCTA GGTGTTAATC   
  
  
- TCCTTAATAT ACCTGGAAAT GTAGCTTAGT TCAAGTTACT AGTTTCCCAC GGTTAAAATG GTTCAGCGTC   
  
  
- ACAACAATGA GGCTACTTTG AGGTTCTAAG GTTAAACTTA GGTTCACAAG GTTTAGGTTC ACAACACTTA   
  
  
- GGAGGAGGCA ACCAAGGGGG TTTGGGTTTA GAATTAAATC AACACCGTAG AGTTATAAGA CTGTAACTCT   
  
  
- GTCGGGACTT GCTCCTAACA CTAAAATCAC TACAACAAGA ATTCATATAA TTAGTTGAAG AATACCTTCT   
  
  
- CCTACCCCTT CTCTTTGAGG TATGACTAGT ACTTGGAAGT CAGCAACTCC GCCGTCTCTT CAGTAACATA   
  
  
- CTCCGCGAGC CCGTCTCTAT AGGAGGAAGG CCTTTGGCTT TAGTCAACGG TCTACAACTC GTACTGCCGA   
  
  
- ACTGACCCTC GTCACAATCC CGAGTATCAC CACGTTCGTC ACAATCCCGA GTATCACTGC GTTCACCACC   
  
  
- ATCACCGGAC TGTCCTATAC CAACCATACC ACTAGGAATA TTAACCTCAG GAGTTTTACA GCACTTAAAA   
  
  
- TGGTAAAGGA GGAGCTTTGG GAATAGCACG TCGAGTAGTC TATCGAGCCC GTCTACCCAA TCATTCACCT   
  
  
- TCAGTTTAAG ACTAAGTGTC GAGTCGAGCC GGTAATTACG TTCACCAAAT CACCTACCCG GTCCCCTGAG   
  
  
- AGGACACTCA CGAGAATCAC ACAGACTTTA TAAGTTACTG TCAGTCAGTT ACAACGTCAA AGTCTTTCCT   
  
  
- AAACTCCTTC GCTTATTTAA AGAAGGTTTC TCAAGAAACA TGTTCCCAAA ACGGTTGTGC CCTAACGGAA   
  
  
- TAGTCTTCCG TTTGTTATCA CGGGTTCTAA ACAACCAGTT ACATCTCCTA CAATGATCCC CTTTCTTCGT   
  
  
- AGCAATAGGG CTTCTTAACG TCAGTCTTCT TCCCTCCTAT TTATTCGTTA GTCGACAAAG GGACCGTCGA   
  
  
- CTACTCCGTC AACAATCCAG ACTTTACAAG CTATCCCATG ACGAAACATC AGCCCCTTTT GTACTACGAC   
  
  
- GAGAGGCCCT TCGAAATGTC TGACTTGATT TATTCTCAGA CGCTTTACGG GGTCAATTCC CCAGATTATC   
  
  
- ACCCTTTCCG GCACCATTCT TTCGCCCTTT CTTCTCCCTA CATCATCTAA ATTCTAGAGA GAATTGGGAT   
  
  
- ACACGTGTTC GTCAACGTAG CTTACTAGTG TCCTCACGTT TACTGGACGA ATCCGTTTAA TCTGTCGTAA   
  
  
- GAAGAGGATC ACCCCTACCA TTGGTTTCTT ACCGTGTAAT AAAACGCCTA CCAGAACTCC GTGCGGAACG   
  
  
- ACCACATCCT TGAGGATATA TATTGACAGA ACATTGCCCA GGCCGTAGCC GACATCTATA GAACTCTCGA   
  
  
- ATGGTGTACA AAGAACGGTG TACGGGTAAA TTCTTTTACC CTTTAAAGAA GAGATTATCT TGCTAATACC   
  
  
- GACACCGTCT CTTACGTTGT ACGGAAGTAT ATTAACTAGA GCCATAACAG ATACCAAAGG TTACCGGAAC   
  
  
- AGATTAAGTC GCTGAAAGTA GATCCGGACC ACCGGGGGGG TTTGAAGCTT ATTGGCCTCA TCTAGAAGGT   
  
  
- GTTGGACCCA AGGCTGGTCG GTTTTCTCAA CTCCTCTGTC CCGCAGCGAA CTTCTTGATA CGTCTCAGTA   
  
  
- AATTACACGG GAAACTCAAG TTACGATATC GTTTCTTCAC CCTTTGTGAA TGGTAACTTC TAGAGTCCTA   
  
  
- GTTATCGCTA CTCAACGAAC AACAGTGGAC ATACAAATCC AAATTTGTAT ATGGACTCCT TTGTCACTGG   
  
  
- CACCTAACGG GATCCCTATG ACAAGAATTG GACTAACCCG CGTATTTGGG TCGACAAAAG TATGTTCCGT   
  
  
- GACAATTGCC CCGAAAGTTA AGAGGGAAAA AGTATCGAGC TAAAGCTCTC CGAGATAAAG TGAAGAGGTG   
  
  
- AGACAAACTA TACGATCTCC GGTTGGACGG TTCCCTGTTA TTCCTCTCCT ACGATTAACT CTCTCTCTAT   
  
  
- AAACCCTCCG TCCGTTACTT ACACTAACGA ACACTCCCAA ATCTCTCCTA TCTTTCCGGT CTCTGCATGT   
  
  
- TCGTCACCGT TCAGGCTTTA CTTTCCCGTC CCAAATCCGT CGACGGAAAT CTAGCGGTCT AAGACCTTTA   
  
  
- CCGATTTTTC TCCCACTTTA GACACATATT GTTTCTAAAG AGGTAACTAC TTCTGCCCGT GACCAACAAC   
  
  
- GACCCTACCT TCCCGTCTTA ACACATGTGT GAGTGATGAA CCTTCGGACG CCTCAT

+     CAAT-box

| Site Name | Organism | Position | Strand | Matrix score. | sequence | function |
| --- | --- | --- | --- | --- | --- | --- |
| CAAT-box | Pisum sativum | 2200 | + | 5 | CAAAT | common cis-acting element in promoter and enhancer regions |
| CAAT-box | Pisum sativum | 2016 | + | 5 | CAAAT | common cis-acting element in promoter and enhancer regions |
| CAAT-box | Nicotiana glutinosa | 642 | + | 4 | CAAT |  |
| CAAT-box | Pisum sativum | 196 | - | 5 | CAAAT | common cis-acting element in promoter and enhancer regions |
| CAAT-box | Pisum sativum | 1803 | - | 5 | CAAAT | common cis-acting element in promoter and enhancer regions |
| CAAT-box | Pisum sativum | 1794 | - | 5 | CAAAT | common cis-acting element in promoter and enhancer regions |
| CAAT-box | Nicotiana glutinosa | 2888 | + | 4 | CAAT |  |
| CAAT-box | Nicotiana glutinosa | 2238 | - | 4 | CAAT |  |
| CAAT-box | Nicotiana glutinosa | 3859 | - | 4 | CAAT |  |
| CAAT-box | Nicotiana glutinosa | 52 | - | 4 | CAAT |  |
| CAAT-box | Nicotiana glutinosa | 132 | + | 4 | CAAT |  |
| CAAT-box | Arabidopsis thaliana | 2134 | + | 5 | CCAAT | common cis-acting element in promoter and enhancer regions |
| CAAT-box | Nicotiana glutinosa | 612 | - | 4 | CAAT |  |
| CAAT-box | Nicotiana glutinosa | 4078 | + | 4 | CAAT |  |
| CAAT-box | Nicotiana glutinosa | 1941 | - | 4 | CAAT |  |
| CAAT-box | Pisum sativum | 1983 | - | 5 | CAAAT | common cis-acting element in promoter and enhancer regions |
| CAAT-box | Nicotiana glutinosa | 2867 | - | 4 | CAAT |  |
| CAAT-box | Nicotiana glutinosa | 528 | - | 4 | CAAT |  |
| CAAT-box | Nicotiana glutinosa | 3768 | - | 4 | CAAT |  |
| CAAT-box | Nicotiana glutinosa | 2028 | + | 4 | CAAT |  |
| CAAT-box | Pisum sativum | 1785 | - | 5 | CAAAT | common cis-acting element in promoter and enhancer regions |
| CAAT-box | Nicotiana glutinosa | 3564 | + | 4 | CAAT |  |
| CAAT-box | Nicotiana glutinosa | 1811 | - | 4 | CAAT |  |
| CAAT-box | Pisum sativum | 2137 | - | 5 | CAAAT | common cis-acting element in promoter and enhancer regions |
| CAAT-box | Pisum sativum | 996 | + | 5 | CAAAT | common cis-acting element in promoter and enhancer regions |
| CAAT-box | Nicotiana glutinosa | 1196 | + | 4 | CAAT |  |
| CAAT-box | Nicotiana glutinosa | 103 | - | 4 | CAAT |  |
| CAAT-box | Nicotiana glutinosa | 2991 | + | 4 | CAAT |  |
| CAAT-box | Nicotiana glutinosa | 802 | - | 4 | CAAT |  |
| CAAT-box | Nicotiana glutinosa | 851 | - | 4 | CAAT |  |
| CAAT-box | Pisum sativum | 4064 | - | 5 | CAAAT | common cis-acting element in promoter and enhancer regions |
| CAAT-box | Nicotiana glutinosa | 1460 | - | 4 | CAAT |  |
| CAAT-box | Arabidopsis thaliana | 2566 | - | 5 | CCAAT | common cis-acting element in promoter and enhancer regions |
| CAAT-box | Nicotiana glutinosa | 2912 | + | 4 | CAAT |  |
| CAAT-box | Nicotiana glutinosa | 1061 | + | 4 | CAAT |  |
| CAAT-box | Nicotiana glutinosa | 1845 | - | 4 | CAAT |  |
| CAAT-box | Pisum sativum | 100 | + | 5 | CAAAT | common cis-acting element in promoter and enhancer regions |
| CAAT-box | Pisum sativum | 3262 | + | 5 | CAAAT | common cis-acting element in promoter and enhancer regions |
| CAAT-box | Nicotiana glutinosa | 3549 | - | 4 | CAAT |  |
| CAAT-box | Nicotiana glutinosa | 2960 | - | 4 | CAAT |  |
| CAAT-box | Nicotiana glutinosa | 1345 | - | 4 | CAAT |  |
| CAAT-box | Pisum sativum | 609 | + | 5 | CAAAT | common cis-acting element in promoter and enhancer regions |
| CAAT-box | Pisum sativum | 2155 | + | 5 | CAAAT | common cis-acting element in promoter and enhancer regions |
| CAAT-box | Nicotiana glutinosa | 2069 | + | 4 | CAAT |  |
| CAAT-box | Nicotiana glutinosa | 116 | + | 4 | CAAT |  |
| CAAT-box | Nicotiana glutinosa | 1130 | + | 4 | CAAT |  |
| CAAT-box | Nicotiana glutinosa | 4293 | - | 4 | CAAT |  |
| CAAT-box | Nicotiana glutinosa | 2768 | + | 4 | CAAT |  |
| CAAT-box | Nicotiana glutinosa | 4248 | - | 4 | CAAT |  |
| CAAT-box | Nicotiana glutinosa | 2260 | - | 4 | CAAT |  |
| CAAT-box | Nicotiana glutinosa | 4089 | - | 4 | CAAT |  |
| CAAT-box | Nicotiana glutinosa | 3941 | + | 4 | CAAT |  |
| CAAT-box | Arabidopsis thaliana | 3888 | - | 5 | CCAAT | common cis-acting element in promoter and enhancer regions |
| CAAT-box | Nicotiana glutinosa | 1138 | + | 4 | CAAT |  |
| CAAT-box | Nicotiana glutinosa | 1765 | - | 4 | CAAT |  |
| CAAT-box | Nicotiana glutinosa | 891 | - | 4 | CAAT |  |
| CAAT-box | Nicotiana glutinosa | 426 | + | 4 | CAAT |  |
| CAAT-box | Nicotiana glutinosa | 2782 | + | 4 | CAAT |  |
| CAAT-box | Pisum sativum | 2902 | - | 5 | CAAAT | common cis-acting element in promoter and enhancer regions |
| CAAT-box | Pisum sativum | 259 | + | 5 | CAAAT | common cis-acting element in promoter and enhancer regions |
| CAAT-box | Nicotiana glutinosa | 2226 | + | 4 | CAAT |  |
| CAAT-box | Arabidopsis thaliana | 2085 | + | 5 | CCAAT | common cis-acting element in promoter and enhancer regions |
| CAAT-box | Arabidopsis thaliana | 1129 | + | 5 | CCAAT | common cis-acting element in promoter and enhancer regions |
| CAAT-box | Nicotiana glutinosa | 333 | - | 4 | CAAT |  |
| CAAT-box | Nicotiana glutinosa | 1365 | - | 4 | CAAT |  |
| CAAT-box | Nicotiana glutinosa | 4050 | - | 4 | CAAT |  |
| CAAT-box | Nicotiana glutinosa | 860 | - | 4 | CAAT |  |
| CAAT-box | Pisum sativum | 1732 | - | 5 | CAAAT | common cis-acting element in promoter and enhancer regions |
| CAAT-box | Pisum sativum | 262 | - | 5 | CAAAT | common cis-acting element in promoter and enhancer regions |
| CAAT-box | Nicotiana glutinosa | 2086 | + | 4 | CAAT |  |
| CAAT-box | Petunia hybrida | 2855 | + | 7 | TGCCAAC | common cis-acting element in promoter and enhancer regions |
| CAAT-box | Pisum sativum | 1166 | - | 5 | CAAAT | common cis-acting element in promoter and enhancer regions |
| CAAT-box | Nicotiana glutinosa | 2135 | + | 4 | CAAT |  |
| CAAT-box | Nicotiana glutinosa | 1334 | - | 4 | CAAT |  |
| CAAT-box | Nicotiana glutinosa | 4031 | + | 4 | CAAT |  |
| CAAT-box | Nicotiana glutinosa | 3734 | + | 4 | CAAT |  |
| CAAT-box | Pisum sativum | 2804 | - | 5 | CAAAT | common cis-acting element in promoter and enhancer regions |
| CAAT-box | Pisum sativum | 2668 | + | 5 | CAAAT | common cis-acting element in promoter and enhancer regions |
| CAAT-box | Nicotiana glutinosa | 2378 | - | 4 | CAAT |  |
| CAAT-box | Arabidopsis thaliana | 3563 | + | 5 | CCAAT | common cis-acting element in promoter and enhancer regions |
| CAAT-box | Pisum sativum | 3279 | + | 5 | CAAAT | common cis-acting element in promoter and enhancer regions |
| CAAT-box | Nicotiana glutinosa | 3537 | - | 4 | CAAT |  |
| CAAT-box | Nicotiana glutinosa | 3785 | + | 4 | CAAT |  |

>HU06G00568.1   
+ -Up\_Stream \_Len000CCCTGG GTCATTAAAA AAAAAGAAAG AAAGAAGATT GCGCTAAAAG TAGATTTTTA   
  
  
+ TCAGATTCAA GCTGCAGGTG ATAACCAAAT TGTCATTAAA GCAATGCAAG CGCATATCAA TACCCTTTTG   
  
  
+ GGAGATAGCT CCGACTGTTG AAGACATTAG GAGCATGATC TCCAACTGTG AATTTGTTTC ATTTACTCAT   
  
  
+ ATTCATATCT ACCATGTGGA TAATATGACT GCAGACTAGA TGGCCAAATT TGAATGCACA CTTATAACTT   
  
  
+ CAACTCTTTC TACTTTTTTC TTCCCCACCT TATCAGGAAT TTCTTTTAAT TGTCGTGGGA GACAACTTGG   
  
  
+ GTAGAACTCT TGCGAAAAGG GCAACTTAAC GTTTTGGTAC TTGCATTTTC CAAAAAAACA AAAAGGCTAA   
  
  
+ ACAATTCTCC GCATGCTGAC TTCACGGTAG ATATGATGTA TTTTTTAACA AAAGACATCT TCTGAATGAA   
  
  
+ ACGCTTAATA TCTTGGACCA TACTAGAAAT TAAATTGCCA CGGTGCATGA ATTTCACCTC GAACTGCTTG   
  
  
+ TATAAAGATC TTGTTATCAC CTTCAGTAAT CAGATACTTG TAACCAAATT GAATGACCAC AAAGACCACA   
  
  
+ TTCCTCACAA TCGTTGCCTC TAGAATAAGA ATGAATGTTT CTTCAAGGTT GAAAGCCCTT GCTAATGCGA   
  
  
+ ACCTGCCTAT ATGCCTAACT GGCTATATAT ATGTGCGTAG AACAGCATGA CATGAGTTAA TAATATAGCA   
  
  
+ GCTAAAGGGA ACTCTAATGT ACACATGATT GTAATTCACC TGCCTCGCTA GCTTACGAGC ATGACTCTTG   
  
  
+ ACTATTATTG TTTTAATTGA TCTTGAATTT TCATAGATTT TACTCCATTG TTTACTCATT TTGTTATAAT   
  
  
+ TAACCACCTG ATTTTAAACT TTAAAATACA CAAAGATTCA TCTAATATAA TTTTTTAGAT AAAGTTATGC   
  
  
+ ATACATATAT ACAAATTAAA TTATTTACGA TCTAATCATT TCCCGTTATA TATTTCACAC TAAAATTTAG   
  
  
+ AAGAGACAAT CTATTAAAAT TACATCACAT GTTTTGGGGG GGGGGGGATA TCAGATATGC ATGACATTCC   
  
  
+ TAATCCAATA ATGCAATGAA ACCCACCGAA TAGTTGAAAT GATTTGTTCT ATCCATGAAC TCACCAAGTC   
  
  
+ ACAATCCATC ACACTCGAAG GATATGCTTT TCTTTTTGAA GGGAATCTTG CTTAATCAAA CCTATAAACT   
  
  
+ TTTAAAAGCA ACAGACAAAA AAGTGATTAT AATGGTAGTG GTAGGATGAA GGCAGCTTTC AGAAAAGCGA   
  
  
+ TTGAATTTTT ATTGTATCCC ATGAAAACCC ATTGAATTTT TCTTTCTTTC TTTGTTTTTG TTACCTGGGT   
  
  
+ TTATGCAAAG GACGGCTGTT GTATTTAATA ATAACAAAAA CATAGGGCTT TGCGGATTGC TAATGCTGGG   
  
  
+ GATGGCATCA GTGCTCGAAT CTGTGTACGC TGTCATAGGA ACCCACTTGC TTCTGCTCAT TTATTTCTGT   
  
  
+ CTCTAGATCC CCTCCTCTCT CTCTCTCTCT CTCTAAGGTG TCTGCTCGTC TGTTCATATT CTCCTGAGGT   
  
  
+ TCTTCTTCAT CTATCTTGTT CTGGGTGTGT GCTGAGGTAC TTCTTTTCTT TATAAATTTT TCATCTTTTG   
  
  
+ TTGTTTTGCT TGGTGTTTAT GATTTTAGTT TTTGTCTTTA AGACGAGATT TGCTGTTAAT AGCAAGATTT   
  
  
+ CTGAACTAGT ATTGATCCTT TTTGTGGTAT ATTTGTTGAA TTTGATTAAT TTGTGTATTG TGTTTAGCTT   
  
  
+ AAGTAACTCG TTGTGATCTA ATTGATGCTC GGGTTTTGAC AGTTTGATTA GGGTTTTCGG TACTGGAGTT   
  
  
+ AAAGTTTTTT TATTTTTTAT AGGAGGAGTT AAAGGTAATA ATAAGAATTG ATGTTTGCTG ATTTTTCCTC   
  
  
+ TTTTCTTTTT TGGTTTCGAT TTGGTGGGCA GTTATCTGTT TATGGTTTTT GCAAATGGAT CCACAATTAG   
  
  
+ AGGAATTATA TGGACCTTTA CATCGAATCA AGTTCAATGA TCAAAGGGTG CCAATTTTAC CAAGTCGCAG   
  
  
+ TGTTGTTACT CCGATGAAAC TCCAAGATTC CAATTTGAAT CCAAGTGTTC CAAATCCAAG TGTTGTGAAT   
  
  
+ CCTCCTCCGT TGGTTCCCCC AAACCCAAAT CTTAATTTAG TTGTGGCATC TCAATATTCT GACATTGAGA   
  
  
+ CAGCCCTGAA CGAGGATTGT GATTTTAGTG ATGTTGTTCT TAAGTATATT AATCAACTTC TTATGGAAGA   
  
  
+ GGATGGGGAA GAGAAACTCC ATACTGATCA TGAACCTTCA GTCGTTGAGG CGGCAGAGAA GTCATTGTAT   
  
  
+ GAGGCGCTCG GGCAGAGATA TCCTCCTTCC GGAAACCGAA ATCAGTTGCC AGATGTTGAG CATGACGGCT   
  
  
+ TGACTGGGAG CAGTGTTAGG GCTCATAGTG GTGCAAGCAG TGTTAGGGCT CATAGTGACG CAAGTGGTGG   
  
  
+ TAGTGGCCTG ACAGGATATG GTTGGTATGG TGATCCTTAT AATTGGAGTC CTCAAAATGT CGTGAATTTT   
  
  
+ ACCATTTCCT CCTCGAAACC CTTATCGTGC AGCTCATCAG ATAGCTCGGG CAGATGGGTT AGTAAGTGGA   
  
  
+ AGTCAAATTC TGATTCACAG CTCAGCTCGG CCATTAATGC AAGTGGTTTA GTGGATGGGC CAGGGGACTC   
  
  
+ TCCTGTGAGT GCTCTTAGTG TGTCTGAAAT ATTCAATGAC AGTCAGTCAA TGTTGCAGTT TCAGAAAGGA   
  
  
+ TTTGAGGAAG CGAATAAATT TCTTCCAAAG AGTTCTTTGT ACAAGGGTTT TGCCAACACG GGATTGCCTT   
  
  
+ ATCAGAAGGC AAACAATAGT GCCCAAGATT TGTTGGTCAA TGTAGAGGAT GTTACTAGGG GAAAGAAGCA   
  
  
+ TCGTTATCCC GAAGAATTGC AGTCAGAAGA AGGGAGGATA AATAAGCAAT CAGCTGTTTC CCTGGCAGCT   
  
  
+ GATGAGGCAG TTGTTAGGTC TGAAATGTTC GATAGGGTAC TGCTTTGTAG TCGGGGAAAA CATGATGCTG   
  
  
+ CTCTCCGGGA AGCTTTACAG ACTGAACTAA ATAAGAGTCT GCGAAATGCC CCAGTTAAGG GGTCTAATAG   
  
  
+ TGGGAAAGGC CGTGGTAAGA AAGCGGGAAA GAAGAGGGAT GTAGTAGATT TAAGATCTCT CTTAACCCTA   
  
  
+ TGTGCACAAG CAGTTGCATC GAATGATCAC AGGAGTGCAA ATGACCTGCT TAGGCAAATT AGACAGCATT   
  
  
+ CTTCTCCTAG TGGGGATGGT AACCAAAGAA TGGCACATTA TTTTGCGGAT GGTCTTGAGG CACGCCTTGC   
  
  
+ TGGTGTAGGA ACTCCTATAT ATAACTGTCT TGTAACGGGT CCGGCATCGG CTGTAGATAT CTTGAGAGCT   
  
  
+ TACCACATGT TTCTTGCCAC ATGCCCATTT AAGAAAATGG GAAATTTCTT CTCTAATAGA ACGATTATGG   
  
  
+ CTGTGGCAGA GAATGCAACA TGCCTTCATA TAATTGATCT CGGTATTGTC TATGGTTTCC AATGGCCTTG   
  
  
+ TCTAATTCAG CGACTTTCAT CTAGGCCTGG TGGCCCCCCC AAACTTCGAA TAACCGGAGT AGATCTTCCA   
  
  
+ CAACCTGGGT TCCGACCAGC CAAAAGAGTT GAGGAGACAG GGCGTCGCTT GAAGAACTAT GCAGAGTCAT   
  
  
+ TTAATGTGCC CTTTGAGTTC AATGCTATAG CAAAGAAGTG GGAAACACTT ACCATTGAAG ATCTCAGGAT   
  
  
+ CAATAGCGAT GAGTTGCTTG TTGTCACCTG TATGTTTAGG TTTAAACATA TACCTGAGGA AACAGTGACC   
  
  
+ GTGGATTGCC CTAGGGATAC TGTTCTTAAC CTGATTGGGC GCATAAACCC AGCTGTTTTC ATACAAGGCA   
  
  
+ CTGTTAACGG GGCTTTCAAT TCTCCCTTTT TCATAGCTCG ATTTCGAGAG GCTCTATTTC ACTTCTCCAC   
  
  
+ TCTGTTTGAT ATGCTAGAGG CCAACCTGCC AAGGGACAAT AAGGAGAGGA TGCTAATTGA GAGAGAGATA   
  
  
+ TTTGGGAGGC AGGCAATGAA TGTGATTGCT TGTGAGGGTT TAGAGAGGAT AGAAAGGCCA GAGACGTACA   
  
  
+ AGCAGTGGCA AGTCCGAAAT GAAAGGGCAG GGTTTAGGCA GCTGCCTTTA GATCGCCAGA TTCTGGAAAT   
  
  
+ GGCTAAAAAG AGGGTGAAAT CTGTGTATAA CAAAGATTTC TCCATTGATG AAGACGGGCA CTGGTTGTTG   
  
  
+ CTGGGATGGA AGGGCAGAAT TGTGTACACA CTCACTACTT GGAAGCCTGC GGAGTA  

- -Up\_Stream \_Len000GGGACC CAGTAATTTT TTTTTCTTTC TTTCTTCTAA CGCGATTTTC ATCTAAAAAT   
  
  
- AGTCTAAGTT CGACGTCCAC TATTGGTTTA ACAGTAATTT CGTTACGTTC GCGTATAGTT ATGGGAAAAC   
  
  
- CCTCTATCGA GGCTGACAAC TTCTGTAATC CTCGTACTAG AGGTTGACAC TTAAACAAAG TAAATGAGTA   
  
  
- TAAGTATAGA TGGTACACCT ATTATACTGA CGTCTGATCT ACCGGTTTAA ACTTACGTGT GAATATTGAA   
  
  
- GTTGAGAAAG ATGAAAAAAG AAGGGGTGGA ATAGTCCTTA AAGAAAATTA ACAGCACCCT CTGTTGAACC   
  
  
- CATCTTGAGA ACGCTTTTCC CGTTGAATTG CAAAACCATG AACGTAAAAG GTTTTTTTGT TTTTCCGATT   
  
  
- TGTTAAGAGG CGTACGACTG AAGTGCCATC TATACTACAT AAAAAATTGT TTTCTGTAGA AGACTTACTT   
  
  
- TGCGAATTAT AGAACCTGGT ATGATCTTTA ATTTAACGGT GCCACGTACT TAAAGTGGAG CTTGACGAAC   
  
  
- ATATTTCTAG AACAATAGTG GAAGTCATTA GTCTATGAAC ATTGGTTTAA CTTACTGGTG TTTCTGGTGT   
  
  
- AAGGAGTGTT AGCAACGGAG ATCTTATTCT TACTTACAAA GAAGTTCCAA CTTTCGGGAA CGATTACGCT   
  
  
- TGGACGGATA TACGGATTGA CCGATATATA TACACGCATC TTGTCGTACT GTACTCAATT ATTATATCGT   
  
  
- CGATTTCCCT TGAGATTACA TGTGTACTAA CATTAAGTGG ACGGAGCGAT CGAATGCTCG TACTGAGAAC   
  
  
- TGATAATAAC AAAATTAACT AGAACTTAAA AGTATCTAAA ATGAGGTAAC AAATGAGTAA AACAATATTA   
  
  
- ATTGGTGGAC TAAAATTTGA AATTTTATGT GTTTCTAAGT AGATTATATT AAAAAATCTA TTTCAATACG   
  
  
- TATGTATATA TGTTTAATTT AATAAATGCT AGATTAGTAA AGGGCAATAT ATAAAGTGTG ATTTTAAATC   
  
  
- TTCTCTGTTA GATAATTTTA ATGTAGTGTA CAAAACCCCC CCCCCCCTAT AGTCTATACG TACTGTAAGG   
  
  
- ATTAGGTTAT TACGTTACTT TGGGTGGCTT ATCAACTTTA CTAAACAAGA TAGGTACTTG AGTGGTTCAG   
  
  
- TGTTAGGTAG TGTGAGCTTC CTATACGAAA AGAAAAACTT CCCTTAGAAC GAATTAGTTT GGATATTTGA   
  
  
- AAATTTTCGT TGTCTGTTTT TTCACTAATA TTACCATCAC CATCCTACTT CCGTCGAAAG TCTTTTCGCT   
  
  
- AACTTAAAAA TAACATAGGG TACTTTTGGG TAACTTAAAA AGAAAGAAAG AAACAAAAAC AATGGACCCA   
  
  
- AATACGTTTC CTGCCGACAA CATAAATTAT TATTGTTTTT GTATCCCGAA ACGCCTAACG ATTACGACCC   
  
  
- CTACCGTAGT CACGAGCTTA GACACATGCG ACAGTATCCT TGGGTGAACG AAGACGAGTA AATAAAGACA   
  
  
- GAGATCTAGG GGAGGAGAGA GAGAGAGAGA GAGATTCCAC AGACGAGCAG ACAAGTATAA GAGGACTCCA   
  
  
- AGAAGAAGTA GATAGAACAA GACCCACACA CGACTCCATG AAGAAAAGAA ATATTTAAAA AGTAGAAAAC   
  
  
- AACAAAACGA ACCACAAATA CTAAAATCAA AAACAGAAAT TCTGCTCTAA ACGACAATTA TCGTTCTAAA   
  
  
- GACTTGATCA TAACTAGGAA AAACACCATA TAAACAACTT AAACTAATTA AACACATAAC ACAAATCGAA   
  
  
- TTCATTGAGC AACACTAGAT TAACTACGAG CCCAAAACTG TCAAACTAAT CCCAAAAGCC ATGACCTCAA   
  
  
- TTTCAAAAAA ATAAAAAATA TCCTCCTCAA TTTCCATTAT TATTCTTAAC TACAAACGAC TAAAAAGGAG   
  
  
- AAAAGAAAAA ACCAAAGCTA AACCACCCGT CAATAGACAA ATACCAAAAA CGTTTACCTA GGTGTTAATC   
  
  
- TCCTTAATAT ACCTGGAAAT GTAGCTTAGT TCAAGTTACT AGTTTCCCAC GGTTAAAATG GTTCAGCGTC   
  
  
- ACAACAATGA GGCTACTTTG AGGTTCTAAG GTTAAACTTA GGTTCACAAG GTTTAGGTTC ACAACACTTA   
  
  
- GGAGGAGGCA ACCAAGGGGG TTTGGGTTTA GAATTAAATC AACACCGTAG AGTTATAAGA CTGTAACTCT   
  
  
- GTCGGGACTT GCTCCTAACA CTAAAATCAC TACAACAAGA ATTCATATAA TTAGTTGAAG AATACCTTCT   
  
  
- CCTACCCCTT CTCTTTGAGG TATGACTAGT ACTTGGAAGT CAGCAACTCC GCCGTCTCTT CAGTAACATA   
  
  
- CTCCGCGAGC CCGTCTCTAT AGGAGGAAGG CCTTTGGCTT TAGTCAACGG TCTACAACTC GTACTGCCGA   
  
  
- ACTGACCCTC GTCACAATCC CGAGTATCAC CACGTTCGTC ACAATCCCGA GTATCACTGC GTTCACCACC   
  
  
- ATCACCGGAC TGTCCTATAC CAACCATACC ACTAGGAATA TTAACCTCAG GAGTTTTACA GCACTTAAAA   
  
  
- TGGTAAAGGA GGAGCTTTGG GAATAGCACG TCGAGTAGTC TATCGAGCCC GTCTACCCAA TCATTCACCT   
  
  
- TCAGTTTAAG ACTAAGTGTC GAGTCGAGCC GGTAATTACG TTCACCAAAT CACCTACCCG GTCCCCTGAG   
  
  
- AGGACACTCA CGAGAATCAC ACAGACTTTA TAAGTTACTG TCAGTCAGTT ACAACGTCAA AGTCTTTCCT   
  
  
- AAACTCCTTC GCTTATTTAA AGAAGGTTTC TCAAGAAACA TGTTCCCAAA ACGGTTGTGC CCTAACGGAA   
  
  
- TAGTCTTCCG TTTGTTATCA CGGGTTCTAA ACAACCAGTT ACATCTCCTA CAATGATCCC CTTTCTTCGT   
  
  
- AGCAATAGGG CTTCTTAACG TCAGTCTTCT TCCCTCCTAT TTATTCGTTA GTCGACAAAG GGACCGTCGA   
  
  
- CTACTCCGTC AACAATCCAG ACTTTACAAG CTATCCCATG ACGAAACATC AGCCCCTTTT GTACTACGAC   
  
  
- GAGAGGCCCT TCGAAATGTC TGACTTGATT TATTCTCAGA CGCTTTACGG GGTCAATTCC CCAGATTATC   
  
  
- ACCCTTTCCG GCACCATTCT TTCGCCCTTT CTTCTCCCTA CATCATCTAA ATTCTAGAGA GAATTGGGAT   
  
  
- ACACGTGTTC GTCAACGTAG CTTACTAGTG TCCTCACGTT TACTGGACGA ATCCGTTTAA TCTGTCGTAA   
  
  
- GAAGAGGATC ACCCCTACCA TTGGTTTCTT ACCGTGTAAT AAAACGCCTA CCAGAACTCC GTGCGGAACG   
  
  
- ACCACATCCT TGAGGATATA TATTGACAGA ACATTGCCCA GGCCGTAGCC GACATCTATA GAACTCTCGA   
  
  
- ATGGTGTACA AAGAACGGTG TACGGGTAAA TTCTTTTACC CTTTAAAGAA GAGATTATCT TGCTAATACC   
  
  
- GACACCGTCT CTTACGTTGT ACGGAAGTAT ATTAACTAGA GCCATAACAG ATACCAAAGG TTACCGGAAC   
  
  
- AGATTAAGTC GCTGAAAGTA GATCCGGACC ACCGGGGGGG TTTGAAGCTT ATTGGCCTCA TCTAGAAGGT   
  
  
- GTTGGACCCA AGGCTGGTCG GTTTTCTCAA CTCCTCTGTC CCGCAGCGAA CTTCTTGATA CGTCTCAGTA   
  
  
- AATTACACGG GAAACTCAAG TTACGATATC GTTTCTTCAC CCTTTGTGAA TGGTAACTTC TAGAGTCCTA   
  
  
- GTTATCGCTA CTCAACGAAC AACAGTGGAC ATACAAATCC AAATTTGTAT ATGGACTCCT TTGTCACTGG   
  
  
- CACCTAACGG GATCCCTATG ACAAGAATTG GACTAACCCG CGTATTTGGG TCGACAAAAG TATGTTCCGT   
  
  
- GACAATTGCC CCGAAAGTTA AGAGGGAAAA AGTATCGAGC TAAAGCTCTC CGAGATAAAG TGAAGAGGTG   
  
  
- AGACAAACTA TACGATCTCC GGTTGGACGG TTCCCTGTTA TTCCTCTCCT ACGATTAACT CTCTCTCTAT   
  
  
- AAACCCTCCG TCCGTTACTT ACACTAACGA ACACTCCCAA ATCTCTCCTA TCTTTCCGGT CTCTGCATGT   
  
  
- TCGTCACCGT TCAGGCTTTA CTTTCCCGTC CCAAATCCGT CGACGGAAAT CTAGCGGTCT AAGACCTTTA   
  
  
- CCGATTTTTC TCCCACTTTA GACACATATT GTTTCTAAAG AGGTAACTAC TTCTGCCCGT GACCAACAAC   
  
  
- GACCCTACCT TCCCGTCTTA ACACATGTGT GAGTGATGAA CCTTCGGACG CCTCAT

+     CAT-box

| Site Name | Organism | Position | Strand | Matrix score. | sequence | function |
| --- | --- | --- | --- | --- | --- | --- |
| CAT-box | Arabidopsis thaliana | 2526 | - | 6 | GCCACT | cis-acting regulatory element related to meristem expression |
| CAT-box | Arabidopsis thaliana | 4138 | - | 6 | GCCACT | cis-acting regulatory element related to meristem expression |

>HU06G00568.1   
+ -Up\_Stream \_Len000CCCTGG GTCATTAAAA AAAAAGAAAG AAAGAAGATT GCGCTAAAAG TAGATTTTTA   
  
  
+ TCAGATTCAA GCTGCAGGTG ATAACCAAAT TGTCATTAAA GCAATGCAAG CGCATATCAA TACCCTTTTG   
  
  
+ GGAGATAGCT CCGACTGTTG AAGACATTAG GAGCATGATC TCCAACTGTG AATTTGTTTC ATTTACTCAT   
  
  
+ ATTCATATCT ACCATGTGGA TAATATGACT GCAGACTAGA TGGCCAAATT TGAATGCACA CTTATAACTT   
  
  
+ CAACTCTTTC TACTTTTTTC TTCCCCACCT TATCAGGAAT TTCTTTTAAT TGTCGTGGGA GACAACTTGG   
  
  
+ GTAGAACTCT TGCGAAAAGG GCAACTTAAC GTTTTGGTAC TTGCATTTTC CAAAAAAACA AAAAGGCTAA   
  
  
+ ACAATTCTCC GCATGCTGAC TTCACGGTAG ATATGATGTA TTTTTTAACA AAAGACATCT TCTGAATGAA   
  
  
+ ACGCTTAATA TCTTGGACCA TACTAGAAAT TAAATTGCCA CGGTGCATGA ATTTCACCTC GAACTGCTTG   
  
  
+ TATAAAGATC TTGTTATCAC CTTCAGTAAT CAGATACTTG TAACCAAATT GAATGACCAC AAAGACCACA   
  
  
+ TTCCTCACAA TCGTTGCCTC TAGAATAAGA ATGAATGTTT CTTCAAGGTT GAAAGCCCTT GCTAATGCGA   
  
  
+ ACCTGCCTAT ATGCCTAACT GGCTATATAT ATGTGCGTAG AACAGCATGA CATGAGTTAA TAATATAGCA   
  
  
+ GCTAAAGGGA ACTCTAATGT ACACATGATT GTAATTCACC TGCCTCGCTA GCTTACGAGC ATGACTCTTG   
  
  
+ ACTATTATTG TTTTAATTGA TCTTGAATTT TCATAGATTT TACTCCATTG TTTACTCATT TTGTTATAAT   
  
  
+ TAACCACCTG ATTTTAAACT TTAAAATACA CAAAGATTCA TCTAATATAA TTTTTTAGAT AAAGTTATGC   
  
  
+ ATACATATAT ACAAATTAAA TTATTTACGA TCTAATCATT TCCCGTTATA TATTTCACAC TAAAATTTAG   
  
  
+ AAGAGACAAT CTATTAAAAT TACATCACAT GTTTTGGGGG GGGGGGGATA TCAGATATGC ATGACATTCC   
  
  
+ TAATCCAATA ATGCAATGAA ACCCACCGAA TAGTTGAAAT GATTTGTTCT ATCCATGAAC TCACCAAGTC   
  
  
+ ACAATCCATC ACACTCGAAG GATATGCTTT TCTTTTTGAA GGGAATCTTG CTTAATCAAA CCTATAAACT   
  
  
+ TTTAAAAGCA ACAGACAAAA AAGTGATTAT AATGGTAGTG GTAGGATGAA GGCAGCTTTC AGAAAAGCGA   
  
  
+ TTGAATTTTT ATTGTATCCC ATGAAAACCC ATTGAATTTT TCTTTCTTTC TTTGTTTTTG TTACCTGGGT   
  
  
+ TTATGCAAAG GACGGCTGTT GTATTTAATA ATAACAAAAA CATAGGGCTT TGCGGATTGC TAATGCTGGG   
  
  
+ GATGGCATCA GTGCTCGAAT CTGTGTACGC TGTCATAGGA ACCCACTTGC TTCTGCTCAT TTATTTCTGT   
  
  
+ CTCTAGATCC CCTCCTCTCT CTCTCTCTCT CTCTAAGGTG TCTGCTCGTC TGTTCATATT CTCCTGAGGT   
  
  
+ TCTTCTTCAT CTATCTTGTT CTGGGTGTGT GCTGAGGTAC TTCTTTTCTT TATAAATTTT TCATCTTTTG   
  
  
+ TTGTTTTGCT TGGTGTTTAT GATTTTAGTT TTTGTCTTTA AGACGAGATT TGCTGTTAAT AGCAAGATTT   
  
  
+ CTGAACTAGT ATTGATCCTT TTTGTGGTAT ATTTGTTGAA TTTGATTAAT TTGTGTATTG TGTTTAGCTT   
  
  
+ AAGTAACTCG TTGTGATCTA ATTGATGCTC GGGTTTTGAC AGTTTGATTA GGGTTTTCGG TACTGGAGTT   
  
  
+ AAAGTTTTTT TATTTTTTAT AGGAGGAGTT AAAGGTAATA ATAAGAATTG ATGTTTGCTG ATTTTTCCTC   
  
  
+ TTTTCTTTTT TGGTTTCGAT TTGGTGGGCA GTTATCTGTT TATGGTTTTT GCAAATGGAT CCACAATTAG   
  
  
+ AGGAATTATA TGGACCTTTA CATCGAATCA AGTTCAATGA TCAAAGGGTG CCAATTTTAC CAAGTCGCAG   
  
  
+ TGTTGTTACT CCGATGAAAC TCCAAGATTC CAATTTGAAT CCAAGTGTTC CAAATCCAAG TGTTGTGAAT   
  
  
+ CCTCCTCCGT TGGTTCCCCC AAACCCAAAT CTTAATTTAG TTGTGGCATC TCAATATTCT GACATTGAGA   
  
  
+ CAGCCCTGAA CGAGGATTGT GATTTTAGTG ATGTTGTTCT TAAGTATATT AATCAACTTC TTATGGAAGA   
  
  
+ GGATGGGGAA GAGAAACTCC ATACTGATCA TGAACCTTCA GTCGTTGAGG CGGCAGAGAA GTCATTGTAT   
  
  
+ GAGGCGCTCG GGCAGAGATA TCCTCCTTCC GGAAACCGAA ATCAGTTGCC AGATGTTGAG CATGACGGCT   
  
  
+ TGACTGGGAG CAGTGTTAGG GCTCATAGTG GTGCAAGCAG TGTTAGGGCT CATAGTGACG CAAGTGGTGG   
  
  
+ TAGTGGCCTG ACAGGATATG GTTGGTATGG TGATCCTTAT AATTGGAGTC CTCAAAATGT CGTGAATTTT   
  
  
+ ACCATTTCCT CCTCGAAACC CTTATCGTGC AGCTCATCAG ATAGCTCGGG CAGATGGGTT AGTAAGTGGA   
  
  
+ AGTCAAATTC TGATTCACAG CTCAGCTCGG CCATTAATGC AAGTGGTTTA GTGGATGGGC CAGGGGACTC   
  
  
+ TCCTGTGAGT GCTCTTAGTG TGTCTGAAAT ATTCAATGAC AGTCAGTCAA TGTTGCAGTT TCAGAAAGGA   
  
  
+ TTTGAGGAAG CGAATAAATT TCTTCCAAAG AGTTCTTTGT ACAAGGGTTT TGCCAACACG GGATTGCCTT   
  
  
+ ATCAGAAGGC AAACAATAGT GCCCAAGATT TGTTGGTCAA TGTAGAGGAT GTTACTAGGG GAAAGAAGCA   
  
  
+ TCGTTATCCC GAAGAATTGC AGTCAGAAGA AGGGAGGATA AATAAGCAAT CAGCTGTTTC CCTGGCAGCT   
  
  
+ GATGAGGCAG TTGTTAGGTC TGAAATGTTC GATAGGGTAC TGCTTTGTAG TCGGGGAAAA CATGATGCTG   
  
  
+ CTCTCCGGGA AGCTTTACAG ACTGAACTAA ATAAGAGTCT GCGAAATGCC CCAGTTAAGG GGTCTAATAG   
  
  
+ TGGGAAAGGC CGTGGTAAGA AAGCGGGAAA GAAGAGGGAT GTAGTAGATT TAAGATCTCT CTTAACCCTA   
  
  
+ TGTGCACAAG CAGTTGCATC GAATGATCAC AGGAGTGCAA ATGACCTGCT TAGGCAAATT AGACAGCATT   
  
  
+ CTTCTCCTAG TGGGGATGGT AACCAAAGAA TGGCACATTA TTTTGCGGAT GGTCTTGAGG CACGCCTTGC   
  
  
+ TGGTGTAGGA ACTCCTATAT ATAACTGTCT TGTAACGGGT CCGGCATCGG CTGTAGATAT CTTGAGAGCT   
  
  
+ TACCACATGT TTCTTGCCAC ATGCCCATTT AAGAAAATGG GAAATTTCTT CTCTAATAGA ACGATTATGG   
  
  
+ CTGTGGCAGA GAATGCAACA TGCCTTCATA TAATTGATCT CGGTATTGTC TATGGTTTCC AATGGCCTTG   
  
  
+ TCTAATTCAG CGACTTTCAT CTAGGCCTGG TGGCCCCCCC AAACTTCGAA TAACCGGAGT AGATCTTCCA   
  
  
+ CAACCTGGGT TCCGACCAGC CAAAAGAGTT GAGGAGACAG GGCGTCGCTT GAAGAACTAT GCAGAGTCAT   
  
  
+ TTAATGTGCC CTTTGAGTTC AATGCTATAG CAAAGAAGTG GGAAACACTT ACCATTGAAG ATCTCAGGAT   
  
  
+ CAATAGCGAT GAGTTGCTTG TTGTCACCTG TATGTTTAGG TTTAAACATA TACCTGAGGA AACAGTGACC   
  
  
+ GTGGATTGCC CTAGGGATAC TGTTCTTAAC CTGATTGGGC GCATAAACCC AGCTGTTTTC ATACAAGGCA   
  
  
+ CTGTTAACGG GGCTTTCAAT TCTCCCTTTT TCATAGCTCG ATTTCGAGAG GCTCTATTTC ACTTCTCCAC   
  
  
+ TCTGTTTGAT ATGCTAGAGG CCAACCTGCC AAGGGACAAT AAGGAGAGGA TGCTAATTGA GAGAGAGATA   
  
  
+ TTTGGGAGGC AGGCAATGAA TGTGATTGCT TGTGAGGGTT TAGAGAGGAT AGAAAGGCCA GAGACGTACA   
  
  
+ AGCAGTGGCA AGTCCGAAAT GAAAGGGCAG GGTTTAGGCA GCTGCCTTTA GATCGCCAGA TTCTGGAAAT   
  
  
+ GGCTAAAAAG AGGGTGAAAT CTGTGTATAA CAAAGATTTC TCCATTGATG AAGACGGGCA CTGGTTGTTG   
  
  
+ CTGGGATGGA AGGGCAGAAT TGTGTACACA CTCACTACTT GGAAGCCTGC GGAGTA  

- -Up\_Stream \_Len000GGGACC CAGTAATTTT TTTTTCTTTC TTTCTTCTAA CGCGATTTTC ATCTAAAAAT   
  
  
- AGTCTAAGTT CGACGTCCAC TATTGGTTTA ACAGTAATTT CGTTACGTTC GCGTATAGTT ATGGGAAAAC   
  
  
- CCTCTATCGA GGCTGACAAC TTCTGTAATC CTCGTACTAG AGGTTGACAC TTAAACAAAG TAAATGAGTA   
  
  
- TAAGTATAGA TGGTACACCT ATTATACTGA CGTCTGATCT ACCGGTTTAA ACTTACGTGT GAATATTGAA   
  
  
- GTTGAGAAAG ATGAAAAAAG AAGGGGTGGA ATAGTCCTTA AAGAAAATTA ACAGCACCCT CTGTTGAACC   
  
  
- CATCTTGAGA ACGCTTTTCC CGTTGAATTG CAAAACCATG AACGTAAAAG GTTTTTTTGT TTTTCCGATT   
  
  
- TGTTAAGAGG CGTACGACTG AAGTGCCATC TATACTACAT AAAAAATTGT TTTCTGTAGA AGACTTACTT   
  
  
- TGCGAATTAT AGAACCTGGT ATGATCTTTA ATTTAACGGT GCCACGTACT TAAAGTGGAG CTTGACGAAC   
  
  
- ATATTTCTAG AACAATAGTG GAAGTCATTA GTCTATGAAC ATTGGTTTAA CTTACTGGTG TTTCTGGTGT   
  
  
- AAGGAGTGTT AGCAACGGAG ATCTTATTCT TACTTACAAA GAAGTTCCAA CTTTCGGGAA CGATTACGCT   
  
  
- TGGACGGATA TACGGATTGA CCGATATATA TACACGCATC TTGTCGTACT GTACTCAATT ATTATATCGT   
  
  
- CGATTTCCCT TGAGATTACA TGTGTACTAA CATTAAGTGG ACGGAGCGAT CGAATGCTCG TACTGAGAAC   
  
  
- TGATAATAAC AAAATTAACT AGAACTTAAA AGTATCTAAA ATGAGGTAAC AAATGAGTAA AACAATATTA   
  
  
- ATTGGTGGAC TAAAATTTGA AATTTTATGT GTTTCTAAGT AGATTATATT AAAAAATCTA TTTCAATACG   
  
  
- TATGTATATA TGTTTAATTT AATAAATGCT AGATTAGTAA AGGGCAATAT ATAAAGTGTG ATTTTAAATC   
  
  
- TTCTCTGTTA GATAATTTTA ATGTAGTGTA CAAAACCCCC CCCCCCCTAT AGTCTATACG TACTGTAAGG   
  
  
- ATTAGGTTAT TACGTTACTT TGGGTGGCTT ATCAACTTTA CTAAACAAGA TAGGTACTTG AGTGGTTCAG   
  
  
- TGTTAGGTAG TGTGAGCTTC CTATACGAAA AGAAAAACTT CCCTTAGAAC GAATTAGTTT GGATATTTGA   
  
  
- AAATTTTCGT TGTCTGTTTT TTCACTAATA TTACCATCAC CATCCTACTT CCGTCGAAAG TCTTTTCGCT   
  
  
- AACTTAAAAA TAACATAGGG TACTTTTGGG TAACTTAAAA AGAAAGAAAG AAACAAAAAC AATGGACCCA   
  
  
- AATACGTTTC CTGCCGACAA CATAAATTAT TATTGTTTTT GTATCCCGAA ACGCCTAACG ATTACGACCC   
  
  
- CTACCGTAGT CACGAGCTTA GACACATGCG ACAGTATCCT TGGGTGAACG AAGACGAGTA AATAAAGACA   
  
  
- GAGATCTAGG GGAGGAGAGA GAGAGAGAGA GAGATTCCAC AGACGAGCAG ACAAGTATAA GAGGACTCCA   
  
  
- AGAAGAAGTA GATAGAACAA GACCCACACA CGACTCCATG AAGAAAAGAA ATATTTAAAA AGTAGAAAAC   
  
  
- AACAAAACGA ACCACAAATA CTAAAATCAA AAACAGAAAT TCTGCTCTAA ACGACAATTA TCGTTCTAAA   
  
  
- GACTTGATCA TAACTAGGAA AAACACCATA TAAACAACTT AAACTAATTA AACACATAAC ACAAATCGAA   
  
  
- TTCATTGAGC AACACTAGAT TAACTACGAG CCCAAAACTG TCAAACTAAT CCCAAAAGCC ATGACCTCAA   
  
  
- TTTCAAAAAA ATAAAAAATA TCCTCCTCAA TTTCCATTAT TATTCTTAAC TACAAACGAC TAAAAAGGAG   
  
  
- AAAAGAAAAA ACCAAAGCTA AACCACCCGT CAATAGACAA ATACCAAAAA CGTTTACCTA GGTGTTAATC   
  
  
- TCCTTAATAT ACCTGGAAAT GTAGCTTAGT TCAAGTTACT AGTTTCCCAC GGTTAAAATG GTTCAGCGTC   
  
  
- ACAACAATGA GGCTACTTTG AGGTTCTAAG GTTAAACTTA GGTTCACAAG GTTTAGGTTC ACAACACTTA   
  
  
- GGAGGAGGCA ACCAAGGGGG TTTGGGTTTA GAATTAAATC AACACCGTAG AGTTATAAGA CTGTAACTCT   
  
  
- GTCGGGACTT GCTCCTAACA CTAAAATCAC TACAACAAGA ATTCATATAA TTAGTTGAAG AATACCTTCT   
  
  
- CCTACCCCTT CTCTTTGAGG TATGACTAGT ACTTGGAAGT CAGCAACTCC GCCGTCTCTT CAGTAACATA   
  
  
- CTCCGCGAGC CCGTCTCTAT AGGAGGAAGG CCTTTGGCTT TAGTCAACGG TCTACAACTC GTACTGCCGA   
  
  
- ACTGACCCTC GTCACAATCC CGAGTATCAC CACGTTCGTC ACAATCCCGA GTATCACTGC GTTCACCACC   
  
  
- ATCACCGGAC TGTCCTATAC CAACCATACC ACTAGGAATA TTAACCTCAG GAGTTTTACA GCACTTAAAA   
  
  
- TGGTAAAGGA GGAGCTTTGG GAATAGCACG TCGAGTAGTC TATCGAGCCC GTCTACCCAA TCATTCACCT   
  
  
- TCAGTTTAAG ACTAAGTGTC GAGTCGAGCC GGTAATTACG TTCACCAAAT CACCTACCCG GTCCCCTGAG   
  
  
- AGGACACTCA CGAGAATCAC ACAGACTTTA TAAGTTACTG TCAGTCAGTT ACAACGTCAA AGTCTTTCCT   
  
  
- AAACTCCTTC GCTTATTTAA AGAAGGTTTC TCAAGAAACA TGTTCCCAAA ACGGTTGTGC CCTAACGGAA   
  
  
- TAGTCTTCCG TTTGTTATCA CGGGTTCTAA ACAACCAGTT ACATCTCCTA CAATGATCCC CTTTCTTCGT   
  
  
- AGCAATAGGG CTTCTTAACG TCAGTCTTCT TCCCTCCTAT TTATTCGTTA GTCGACAAAG GGACCGTCGA   
  
  
- CTACTCCGTC AACAATCCAG ACTTTACAAG CTATCCCATG ACGAAACATC AGCCCCTTTT GTACTACGAC   
  
  
- GAGAGGCCCT TCGAAATGTC TGACTTGATT TATTCTCAGA CGCTTTACGG GGTCAATTCC CCAGATTATC   
  
  
- ACCCTTTCCG GCACCATTCT TTCGCCCTTT CTTCTCCCTA CATCATCTAA ATTCTAGAGA GAATTGGGAT   
  
  
- ACACGTGTTC GTCAACGTAG CTTACTAGTG TCCTCACGTT TACTGGACGA ATCCGTTTAA TCTGTCGTAA   
  
  
- GAAGAGGATC ACCCCTACCA TTGGTTTCTT ACCGTGTAAT AAAACGCCTA CCAGAACTCC GTGCGGAACG   
  
  
- ACCACATCCT TGAGGATATA TATTGACAGA ACATTGCCCA GGCCGTAGCC GACATCTATA GAACTCTCGA   
  
  
- ATGGTGTACA AAGAACGGTG TACGGGTAAA TTCTTTTACC CTTTAAAGAA GAGATTATCT TGCTAATACC   
  
  
- GACACCGTCT CTTACGTTGT ACGGAAGTAT ATTAACTAGA GCCATAACAG ATACCAAAGG TTACCGGAAC   
  
  
- AGATTAAGTC GCTGAAAGTA GATCCGGACC ACCGGGGGGG TTTGAAGCTT ATTGGCCTCA TCTAGAAGGT   
  
  
- GTTGGACCCA AGGCTGGTCG GTTTTCTCAA CTCCTCTGTC CCGCAGCGAA CTTCTTGATA CGTCTCAGTA   
  
  
- AATTACACGG GAAACTCAAG TTACGATATC GTTTCTTCAC CCTTTGTGAA TGGTAACTTC TAGAGTCCTA   
  
  
- GTTATCGCTA CTCAACGAAC AACAGTGGAC ATACAAATCC AAATTTGTAT ATGGACTCCT TTGTCACTGG   
  
  
- CACCTAACGG GATCCCTATG ACAAGAATTG GACTAACCCG CGTATTTGGG TCGACAAAAG TATGTTCCGT   
  
  
- GACAATTGCC CCGAAAGTTA AGAGGGAAAA AGTATCGAGC TAAAGCTCTC CGAGATAAAG TGAAGAGGTG   
  
  
- AGACAAACTA TACGATCTCC GGTTGGACGG TTCCCTGTTA TTCCTCTCCT ACGATTAACT CTCTCTCTAT   
  
  
- AAACCCTCCG TCCGTTACTT ACACTAACGA ACACTCCCAA ATCTCTCCTA TCTTTCCGGT CTCTGCATGT   
  
  
- TCGTCACCGT TCAGGCTTTA CTTTCCCGTC CCAAATCCGT CGACGGAAAT CTAGCGGTCT AAGACCTTTA   
  
  
- CCGATTTTTC TCCCACTTTA GACACATATT GTTTCTAAAG AGGTAACTAC TTCTGCCCGT GACCAACAAC   
  
  
- GACCCTACCT TCCCGTCTTA ACACATGTGT GAGTGATGAA CCTTCGGACG CCTCAT

+     CCAAT-box

| Site Name | Organism | Position | Strand | Matrix score. | sequence | function |
| --- | --- | --- | --- | --- | --- | --- |
| CCAAT-box | Hordeum vulgare | 2181 | - | 6 | CAACGG | MYBHv1 binding site |

>HU06G00568.1   
+ -Up\_Stream \_Len000CCCTGG GTCATTAAAA AAAAAGAAAG AAAGAAGATT GCGCTAAAAG TAGATTTTTA   
  
  
+ TCAGATTCAA GCTGCAGGTG ATAACCAAAT TGTCATTAAA GCAATGCAAG CGCATATCAA TACCCTTTTG   
  
  
+ GGAGATAGCT CCGACTGTTG AAGACATTAG GAGCATGATC TCCAACTGTG AATTTGTTTC ATTTACTCAT   
  
  
+ ATTCATATCT ACCATGTGGA TAATATGACT GCAGACTAGA TGGCCAAATT TGAATGCACA CTTATAACTT   
  
  
+ CAACTCTTTC TACTTTTTTC TTCCCCACCT TATCAGGAAT TTCTTTTAAT TGTCGTGGGA GACAACTTGG   
  
  
+ GTAGAACTCT TGCGAAAAGG GCAACTTAAC GTTTTGGTAC TTGCATTTTC CAAAAAAACA AAAAGGCTAA   
  
  
+ ACAATTCTCC GCATGCTGAC TTCACGGTAG ATATGATGTA TTTTTTAACA AAAGACATCT TCTGAATGAA   
  
  
+ ACGCTTAATA TCTTGGACCA TACTAGAAAT TAAATTGCCA CGGTGCATGA ATTTCACCTC GAACTGCTTG   
  
  
+ TATAAAGATC TTGTTATCAC CTTCAGTAAT CAGATACTTG TAACCAAATT GAATGACCAC AAAGACCACA   
  
  
+ TTCCTCACAA TCGTTGCCTC TAGAATAAGA ATGAATGTTT CTTCAAGGTT GAAAGCCCTT GCTAATGCGA   
  
  
+ ACCTGCCTAT ATGCCTAACT GGCTATATAT ATGTGCGTAG AACAGCATGA CATGAGTTAA TAATATAGCA   
  
  
+ GCTAAAGGGA ACTCTAATGT ACACATGATT GTAATTCACC TGCCTCGCTA GCTTACGAGC ATGACTCTTG   
  
  
+ ACTATTATTG TTTTAATTGA TCTTGAATTT TCATAGATTT TACTCCATTG TTTACTCATT TTGTTATAAT   
  
  
+ TAACCACCTG ATTTTAAACT TTAAAATACA CAAAGATTCA TCTAATATAA TTTTTTAGAT AAAGTTATGC   
  
  
+ ATACATATAT ACAAATTAAA TTATTTACGA TCTAATCATT TCCCGTTATA TATTTCACAC TAAAATTTAG   
  
  
+ AAGAGACAAT CTATTAAAAT TACATCACAT GTTTTGGGGG GGGGGGGATA TCAGATATGC ATGACATTCC   
  
  
+ TAATCCAATA ATGCAATGAA ACCCACCGAA TAGTTGAAAT GATTTGTTCT ATCCATGAAC TCACCAAGTC   
  
  
+ ACAATCCATC ACACTCGAAG GATATGCTTT TCTTTTTGAA GGGAATCTTG CTTAATCAAA CCTATAAACT   
  
  
+ TTTAAAAGCA ACAGACAAAA AAGTGATTAT AATGGTAGTG GTAGGATGAA GGCAGCTTTC AGAAAAGCGA   
  
  
+ TTGAATTTTT ATTGTATCCC ATGAAAACCC ATTGAATTTT TCTTTCTTTC TTTGTTTTTG TTACCTGGGT   
  
  
+ TTATGCAAAG GACGGCTGTT GTATTTAATA ATAACAAAAA CATAGGGCTT TGCGGATTGC TAATGCTGGG   
  
  
+ GATGGCATCA GTGCTCGAAT CTGTGTACGC TGTCATAGGA ACCCACTTGC TTCTGCTCAT TTATTTCTGT   
  
  
+ CTCTAGATCC CCTCCTCTCT CTCTCTCTCT CTCTAAGGTG TCTGCTCGTC TGTTCATATT CTCCTGAGGT   
  
  
+ TCTTCTTCAT CTATCTTGTT CTGGGTGTGT GCTGAGGTAC TTCTTTTCTT TATAAATTTT TCATCTTTTG   
  
  
+ TTGTTTTGCT TGGTGTTTAT GATTTTAGTT TTTGTCTTTA AGACGAGATT TGCTGTTAAT AGCAAGATTT   
  
  
+ CTGAACTAGT ATTGATCCTT TTTGTGGTAT ATTTGTTGAA TTTGATTAAT TTGTGTATTG TGTTTAGCTT   
  
  
+ AAGTAACTCG TTGTGATCTA ATTGATGCTC GGGTTTTGAC AGTTTGATTA GGGTTTTCGG TACTGGAGTT   
  
  
+ AAAGTTTTTT TATTTTTTAT AGGAGGAGTT AAAGGTAATA ATAAGAATTG ATGTTTGCTG ATTTTTCCTC   
  
  
+ TTTTCTTTTT TGGTTTCGAT TTGGTGGGCA GTTATCTGTT TATGGTTTTT GCAAATGGAT CCACAATTAG   
  
  
+ AGGAATTATA TGGACCTTTA CATCGAATCA AGTTCAATGA TCAAAGGGTG CCAATTTTAC CAAGTCGCAG   
  
  
+ TGTTGTTACT CCGATGAAAC TCCAAGATTC CAATTTGAAT CCAAGTGTTC CAAATCCAAG TGTTGTGAAT   
  
  
+ CCTCCTCCGT TGGTTCCCCC AAACCCAAAT CTTAATTTAG TTGTGGCATC TCAATATTCT GACATTGAGA   
  
  
+ CAGCCCTGAA CGAGGATTGT GATTTTAGTG ATGTTGTTCT TAAGTATATT AATCAACTTC TTATGGAAGA   
  
  
+ GGATGGGGAA GAGAAACTCC ATACTGATCA TGAACCTTCA GTCGTTGAGG CGGCAGAGAA GTCATTGTAT   
  
  
+ GAGGCGCTCG GGCAGAGATA TCCTCCTTCC GGAAACCGAA ATCAGTTGCC AGATGTTGAG CATGACGGCT   
  
  
+ TGACTGGGAG CAGTGTTAGG GCTCATAGTG GTGCAAGCAG TGTTAGGGCT CATAGTGACG CAAGTGGTGG   
  
  
+ TAGTGGCCTG ACAGGATATG GTTGGTATGG TGATCCTTAT AATTGGAGTC CTCAAAATGT CGTGAATTTT   
  
  
+ ACCATTTCCT CCTCGAAACC CTTATCGTGC AGCTCATCAG ATAGCTCGGG CAGATGGGTT AGTAAGTGGA   
  
  
+ AGTCAAATTC TGATTCACAG CTCAGCTCGG CCATTAATGC AAGTGGTTTA GTGGATGGGC CAGGGGACTC   
  
  
+ TCCTGTGAGT GCTCTTAGTG TGTCTGAAAT ATTCAATGAC AGTCAGTCAA TGTTGCAGTT TCAGAAAGGA   
  
  
+ TTTGAGGAAG CGAATAAATT TCTTCCAAAG AGTTCTTTGT ACAAGGGTTT TGCCAACACG GGATTGCCTT   
  
  
+ ATCAGAAGGC AAACAATAGT GCCCAAGATT TGTTGGTCAA TGTAGAGGAT GTTACTAGGG GAAAGAAGCA   
  
  
+ TCGTTATCCC GAAGAATTGC AGTCAGAAGA AGGGAGGATA AATAAGCAAT CAGCTGTTTC CCTGGCAGCT   
  
  
+ GATGAGGCAG TTGTTAGGTC TGAAATGTTC GATAGGGTAC TGCTTTGTAG TCGGGGAAAA CATGATGCTG   
  
  
+ CTCTCCGGGA AGCTTTACAG ACTGAACTAA ATAAGAGTCT GCGAAATGCC CCAGTTAAGG GGTCTAATAG   
  
  
+ TGGGAAAGGC CGTGGTAAGA AAGCGGGAAA GAAGAGGGAT GTAGTAGATT TAAGATCTCT CTTAACCCTA   
  
  
+ TGTGCACAAG CAGTTGCATC GAATGATCAC AGGAGTGCAA ATGACCTGCT TAGGCAAATT AGACAGCATT   
  
  
+ CTTCTCCTAG TGGGGATGGT AACCAAAGAA TGGCACATTA TTTTGCGGAT GGTCTTGAGG CACGCCTTGC   
  
  
+ TGGTGTAGGA ACTCCTATAT ATAACTGTCT TGTAACGGGT CCGGCATCGG CTGTAGATAT CTTGAGAGCT   
  
  
+ TACCACATGT TTCTTGCCAC ATGCCCATTT AAGAAAATGG GAAATTTCTT CTCTAATAGA ACGATTATGG   
  
  
+ CTGTGGCAGA GAATGCAACA TGCCTTCATA TAATTGATCT CGGTATTGTC TATGGTTTCC AATGGCCTTG   
  
  
+ TCTAATTCAG CGACTTTCAT CTAGGCCTGG TGGCCCCCCC AAACTTCGAA TAACCGGAGT AGATCTTCCA   
  
  
+ CAACCTGGGT TCCGACCAGC CAAAAGAGTT GAGGAGACAG GGCGTCGCTT GAAGAACTAT GCAGAGTCAT   
  
  
+ TTAATGTGCC CTTTGAGTTC AATGCTATAG CAAAGAAGTG GGAAACACTT ACCATTGAAG ATCTCAGGAT   
  
  
+ CAATAGCGAT GAGTTGCTTG TTGTCACCTG TATGTTTAGG TTTAAACATA TACCTGAGGA AACAGTGACC   
  
  
+ GTGGATTGCC CTAGGGATAC TGTTCTTAAC CTGATTGGGC GCATAAACCC AGCTGTTTTC ATACAAGGCA   
  
  
+ CTGTTAACGG GGCTTTCAAT TCTCCCTTTT TCATAGCTCG ATTTCGAGAG GCTCTATTTC ACTTCTCCAC   
  
  
+ TCTGTTTGAT ATGCTAGAGG CCAACCTGCC AAGGGACAAT AAGGAGAGGA TGCTAATTGA GAGAGAGATA   
  
  
+ TTTGGGAGGC AGGCAATGAA TGTGATTGCT TGTGAGGGTT TAGAGAGGAT AGAAAGGCCA GAGACGTACA   
  
  
+ AGCAGTGGCA AGTCCGAAAT GAAAGGGCAG GGTTTAGGCA GCTGCCTTTA GATCGCCAGA TTCTGGAAAT   
  
  
+ GGCTAAAAAG AGGGTGAAAT CTGTGTATAA CAAAGATTTC TCCATTGATG AAGACGGGCA CTGGTTGTTG   
  
  
+ CTGGGATGGA AGGGCAGAAT TGTGTACACA CTCACTACTT GGAAGCCTGC GGAGTA  

- -Up\_Stream \_Len000GGGACC CAGTAATTTT TTTTTCTTTC TTTCTTCTAA CGCGATTTTC ATCTAAAAAT   
  
  
- AGTCTAAGTT CGACGTCCAC TATTGGTTTA ACAGTAATTT CGTTACGTTC GCGTATAGTT ATGGGAAAAC   
  
  
- CCTCTATCGA GGCTGACAAC TTCTGTAATC CTCGTACTAG AGGTTGACAC TTAAACAAAG TAAATGAGTA   
  
  
- TAAGTATAGA TGGTACACCT ATTATACTGA CGTCTGATCT ACCGGTTTAA ACTTACGTGT GAATATTGAA   
  
  
- GTTGAGAAAG ATGAAAAAAG AAGGGGTGGA ATAGTCCTTA AAGAAAATTA ACAGCACCCT CTGTTGAACC   
  
  
- CATCTTGAGA ACGCTTTTCC CGTTGAATTG CAAAACCATG AACGTAAAAG GTTTTTTTGT TTTTCCGATT   
  
  
- TGTTAAGAGG CGTACGACTG AAGTGCCATC TATACTACAT AAAAAATTGT TTTCTGTAGA AGACTTACTT   
  
  
- TGCGAATTAT AGAACCTGGT ATGATCTTTA ATTTAACGGT GCCACGTACT TAAAGTGGAG CTTGACGAAC   
  
  
- ATATTTCTAG AACAATAGTG GAAGTCATTA GTCTATGAAC ATTGGTTTAA CTTACTGGTG TTTCTGGTGT   
  
  
- AAGGAGTGTT AGCAACGGAG ATCTTATTCT TACTTACAAA GAAGTTCCAA CTTTCGGGAA CGATTACGCT   
  
  
- TGGACGGATA TACGGATTGA CCGATATATA TACACGCATC TTGTCGTACT GTACTCAATT ATTATATCGT   
  
  
- CGATTTCCCT TGAGATTACA TGTGTACTAA CATTAAGTGG ACGGAGCGAT CGAATGCTCG TACTGAGAAC   
  
  
- TGATAATAAC AAAATTAACT AGAACTTAAA AGTATCTAAA ATGAGGTAAC AAATGAGTAA AACAATATTA   
  
  
- ATTGGTGGAC TAAAATTTGA AATTTTATGT GTTTCTAAGT AGATTATATT AAAAAATCTA TTTCAATACG   
  
  
- TATGTATATA TGTTTAATTT AATAAATGCT AGATTAGTAA AGGGCAATAT ATAAAGTGTG ATTTTAAATC   
  
  
- TTCTCTGTTA GATAATTTTA ATGTAGTGTA CAAAACCCCC CCCCCCCTAT AGTCTATACG TACTGTAAGG   
  
  
- ATTAGGTTAT TACGTTACTT TGGGTGGCTT ATCAACTTTA CTAAACAAGA TAGGTACTTG AGTGGTTCAG   
  
  
- TGTTAGGTAG TGTGAGCTTC CTATACGAAA AGAAAAACTT CCCTTAGAAC GAATTAGTTT GGATATTTGA   
  
  
- AAATTTTCGT TGTCTGTTTT TTCACTAATA TTACCATCAC CATCCTACTT CCGTCGAAAG TCTTTTCGCT   
  
  
- AACTTAAAAA TAACATAGGG TACTTTTGGG TAACTTAAAA AGAAAGAAAG AAACAAAAAC AATGGACCCA   
  
  
- AATACGTTTC CTGCCGACAA CATAAATTAT TATTGTTTTT GTATCCCGAA ACGCCTAACG ATTACGACCC   
  
  
- CTACCGTAGT CACGAGCTTA GACACATGCG ACAGTATCCT TGGGTGAACG AAGACGAGTA AATAAAGACA   
  
  
- GAGATCTAGG GGAGGAGAGA GAGAGAGAGA GAGATTCCAC AGACGAGCAG ACAAGTATAA GAGGACTCCA   
  
  
- AGAAGAAGTA GATAGAACAA GACCCACACA CGACTCCATG AAGAAAAGAA ATATTTAAAA AGTAGAAAAC   
  
  
- AACAAAACGA ACCACAAATA CTAAAATCAA AAACAGAAAT TCTGCTCTAA ACGACAATTA TCGTTCTAAA   
  
  
- GACTTGATCA TAACTAGGAA AAACACCATA TAAACAACTT AAACTAATTA AACACATAAC ACAAATCGAA   
  
  
- TTCATTGAGC AACACTAGAT TAACTACGAG CCCAAAACTG TCAAACTAAT CCCAAAAGCC ATGACCTCAA   
  
  
- TTTCAAAAAA ATAAAAAATA TCCTCCTCAA TTTCCATTAT TATTCTTAAC TACAAACGAC TAAAAAGGAG   
  
  
- AAAAGAAAAA ACCAAAGCTA AACCACCCGT CAATAGACAA ATACCAAAAA CGTTTACCTA GGTGTTAATC   
  
  
- TCCTTAATAT ACCTGGAAAT GTAGCTTAGT TCAAGTTACT AGTTTCCCAC GGTTAAAATG GTTCAGCGTC   
  
  
- ACAACAATGA GGCTACTTTG AGGTTCTAAG GTTAAACTTA GGTTCACAAG GTTTAGGTTC ACAACACTTA   
  
  
- GGAGGAGGCA ACCAAGGGGG TTTGGGTTTA GAATTAAATC AACACCGTAG AGTTATAAGA CTGTAACTCT   
  
  
- GTCGGGACTT GCTCCTAACA CTAAAATCAC TACAACAAGA ATTCATATAA TTAGTTGAAG AATACCTTCT   
  
  
- CCTACCCCTT CTCTTTGAGG TATGACTAGT ACTTGGAAGT CAGCAACTCC GCCGTCTCTT CAGTAACATA   
  
  
- CTCCGCGAGC CCGTCTCTAT AGGAGGAAGG CCTTTGGCTT TAGTCAACGG TCTACAACTC GTACTGCCGA   
  
  
- ACTGACCCTC GTCACAATCC CGAGTATCAC CACGTTCGTC ACAATCCCGA GTATCACTGC GTTCACCACC   
  
  
- ATCACCGGAC TGTCCTATAC CAACCATACC ACTAGGAATA TTAACCTCAG GAGTTTTACA GCACTTAAAA   
  
  
- TGGTAAAGGA GGAGCTTTGG GAATAGCACG TCGAGTAGTC TATCGAGCCC GTCTACCCAA TCATTCACCT   
  
  
- TCAGTTTAAG ACTAAGTGTC GAGTCGAGCC GGTAATTACG TTCACCAAAT CACCTACCCG GTCCCCTGAG   
  
  
- AGGACACTCA CGAGAATCAC ACAGACTTTA TAAGTTACTG TCAGTCAGTT ACAACGTCAA AGTCTTTCCT   
  
  
- AAACTCCTTC GCTTATTTAA AGAAGGTTTC TCAAGAAACA TGTTCCCAAA ACGGTTGTGC CCTAACGGAA   
  
  
- TAGTCTTCCG TTTGTTATCA CGGGTTCTAA ACAACCAGTT ACATCTCCTA CAATGATCCC CTTTCTTCGT   
  
  
- AGCAATAGGG CTTCTTAACG TCAGTCTTCT TCCCTCCTAT TTATTCGTTA GTCGACAAAG GGACCGTCGA   
  
  
- CTACTCCGTC AACAATCCAG ACTTTACAAG CTATCCCATG ACGAAACATC AGCCCCTTTT GTACTACGAC   
  
  
- GAGAGGCCCT TCGAAATGTC TGACTTGATT TATTCTCAGA CGCTTTACGG GGTCAATTCC CCAGATTATC   
  
  
- ACCCTTTCCG GCACCATTCT TTCGCCCTTT CTTCTCCCTA CATCATCTAA ATTCTAGAGA GAATTGGGAT   
  
  
- ACACGTGTTC GTCAACGTAG CTTACTAGTG TCCTCACGTT TACTGGACGA ATCCGTTTAA TCTGTCGTAA   
  
  
- GAAGAGGATC ACCCCTACCA TTGGTTTCTT ACCGTGTAAT AAAACGCCTA CCAGAACTCC GTGCGGAACG   
  
  
- ACCACATCCT TGAGGATATA TATTGACAGA ACATTGCCCA GGCCGTAGCC GACATCTATA GAACTCTCGA   
  
  
- ATGGTGTACA AAGAACGGTG TACGGGTAAA TTCTTTTACC CTTTAAAGAA GAGATTATCT TGCTAATACC   
  
  
- GACACCGTCT CTTACGTTGT ACGGAAGTAT ATTAACTAGA GCCATAACAG ATACCAAAGG TTACCGGAAC   
  
  
- AGATTAAGTC GCTGAAAGTA GATCCGGACC ACCGGGGGGG TTTGAAGCTT ATTGGCCTCA TCTAGAAGGT   
  
  
- GTTGGACCCA AGGCTGGTCG GTTTTCTCAA CTCCTCTGTC CCGCAGCGAA CTTCTTGATA CGTCTCAGTA   
  
  
- AATTACACGG GAAACTCAAG TTACGATATC GTTTCTTCAC CCTTTGTGAA TGGTAACTTC TAGAGTCCTA   
  
  
- GTTATCGCTA CTCAACGAAC AACAGTGGAC ATACAAATCC AAATTTGTAT ATGGACTCCT TTGTCACTGG   
  
  
- CACCTAACGG GATCCCTATG ACAAGAATTG GACTAACCCG CGTATTTGGG TCGACAAAAG TATGTTCCGT   
  
  
- GACAATTGCC CCGAAAGTTA AGAGGGAAAA AGTATCGAGC TAAAGCTCTC CGAGATAAAG TGAAGAGGTG   
  
  
- AGACAAACTA TACGATCTCC GGTTGGACGG TTCCCTGTTA TTCCTCTCCT ACGATTAACT CTCTCTCTAT   
  
  
- AAACCCTCCG TCCGTTACTT ACACTAACGA ACACTCCCAA ATCTCTCCTA TCTTTCCGGT CTCTGCATGT   
  
  
- TCGTCACCGT TCAGGCTTTA CTTTCCCGTC CCAAATCCGT CGACGGAAAT CTAGCGGTCT AAGACCTTTA   
  
  
- CCGATTTTTC TCCCACTTTA GACACATATT GTTTCTAAAG AGGTAACTAC TTCTGCCCGT GACCAACAAC   
  
  
- GACCCTACCT TCCCGTCTTA ACACATGTGT GAGTGATGAA CCTTCGGACG CCTCAT

+     CCGTCC motif

| Site Name | Organism | Position | Strand | Matrix score. | sequence | function |
| --- | --- | --- | --- | --- | --- | --- |
| CCGTCC motif | Nicotiana tabacum | 1414 | - | 6 | CCGTCC |  |

>HU06G00568.1   
+ -Up\_Stream \_Len000CCCTGG GTCATTAAAA AAAAAGAAAG AAAGAAGATT GCGCTAAAAG TAGATTTTTA   
  
  
+ TCAGATTCAA GCTGCAGGTG ATAACCAAAT TGTCATTAAA GCAATGCAAG CGCATATCAA TACCCTTTTG   
  
  
+ GGAGATAGCT CCGACTGTTG AAGACATTAG GAGCATGATC TCCAACTGTG AATTTGTTTC ATTTACTCAT   
  
  
+ ATTCATATCT ACCATGTGGA TAATATGACT GCAGACTAGA TGGCCAAATT TGAATGCACA CTTATAACTT   
  
  
+ CAACTCTTTC TACTTTTTTC TTCCCCACCT TATCAGGAAT TTCTTTTAAT TGTCGTGGGA GACAACTTGG   
  
  
+ GTAGAACTCT TGCGAAAAGG GCAACTTAAC GTTTTGGTAC TTGCATTTTC CAAAAAAACA AAAAGGCTAA   
  
  
+ ACAATTCTCC GCATGCTGAC TTCACGGTAG ATATGATGTA TTTTTTAACA AAAGACATCT TCTGAATGAA   
  
  
+ ACGCTTAATA TCTTGGACCA TACTAGAAAT TAAATTGCCA CGGTGCATGA ATTTCACCTC GAACTGCTTG   
  
  
+ TATAAAGATC TTGTTATCAC CTTCAGTAAT CAGATACTTG TAACCAAATT GAATGACCAC AAAGACCACA   
  
  
+ TTCCTCACAA TCGTTGCCTC TAGAATAAGA ATGAATGTTT CTTCAAGGTT GAAAGCCCTT GCTAATGCGA   
  
  
+ ACCTGCCTAT ATGCCTAACT GGCTATATAT ATGTGCGTAG AACAGCATGA CATGAGTTAA TAATATAGCA   
  
  
+ GCTAAAGGGA ACTCTAATGT ACACATGATT GTAATTCACC TGCCTCGCTA GCTTACGAGC ATGACTCTTG   
  
  
+ ACTATTATTG TTTTAATTGA TCTTGAATTT TCATAGATTT TACTCCATTG TTTACTCATT TTGTTATAAT   
  
  
+ TAACCACCTG ATTTTAAACT TTAAAATACA CAAAGATTCA TCTAATATAA TTTTTTAGAT AAAGTTATGC   
  
  
+ ATACATATAT ACAAATTAAA TTATTTACGA TCTAATCATT TCCCGTTATA TATTTCACAC TAAAATTTAG   
  
  
+ AAGAGACAAT CTATTAAAAT TACATCACAT GTTTTGGGGG GGGGGGGATA TCAGATATGC ATGACATTCC   
  
  
+ TAATCCAATA ATGCAATGAA ACCCACCGAA TAGTTGAAAT GATTTGTTCT ATCCATGAAC TCACCAAGTC   
  
  
+ ACAATCCATC ACACTCGAAG GATATGCTTT TCTTTTTGAA GGGAATCTTG CTTAATCAAA CCTATAAACT   
  
  
+ TTTAAAAGCA ACAGACAAAA AAGTGATTAT AATGGTAGTG GTAGGATGAA GGCAGCTTTC AGAAAAGCGA   
  
  
+ TTGAATTTTT ATTGTATCCC ATGAAAACCC ATTGAATTTT TCTTTCTTTC TTTGTTTTTG TTACCTGGGT   
  
  
+ TTATGCAAAG GACGGCTGTT GTATTTAATA ATAACAAAAA CATAGGGCTT TGCGGATTGC TAATGCTGGG   
  
  
+ GATGGCATCA GTGCTCGAAT CTGTGTACGC TGTCATAGGA ACCCACTTGC TTCTGCTCAT TTATTTCTGT   
  
  
+ CTCTAGATCC CCTCCTCTCT CTCTCTCTCT CTCTAAGGTG TCTGCTCGTC TGTTCATATT CTCCTGAGGT   
  
  
+ TCTTCTTCAT CTATCTTGTT CTGGGTGTGT GCTGAGGTAC TTCTTTTCTT TATAAATTTT TCATCTTTTG   
  
  
+ TTGTTTTGCT TGGTGTTTAT GATTTTAGTT TTTGTCTTTA AGACGAGATT TGCTGTTAAT AGCAAGATTT   
  
  
+ CTGAACTAGT ATTGATCCTT TTTGTGGTAT ATTTGTTGAA TTTGATTAAT TTGTGTATTG TGTTTAGCTT   
  
  
+ AAGTAACTCG TTGTGATCTA ATTGATGCTC GGGTTTTGAC AGTTTGATTA GGGTTTTCGG TACTGGAGTT   
  
  
+ AAAGTTTTTT TATTTTTTAT AGGAGGAGTT AAAGGTAATA ATAAGAATTG ATGTTTGCTG ATTTTTCCTC   
  
  
+ TTTTCTTTTT TGGTTTCGAT TTGGTGGGCA GTTATCTGTT TATGGTTTTT GCAAATGGAT CCACAATTAG   
  
  
+ AGGAATTATA TGGACCTTTA CATCGAATCA AGTTCAATGA TCAAAGGGTG CCAATTTTAC CAAGTCGCAG   
  
  
+ TGTTGTTACT CCGATGAAAC TCCAAGATTC CAATTTGAAT CCAAGTGTTC CAAATCCAAG TGTTGTGAAT   
  
  
+ CCTCCTCCGT TGGTTCCCCC AAACCCAAAT CTTAATTTAG TTGTGGCATC TCAATATTCT GACATTGAGA   
  
  
+ CAGCCCTGAA CGAGGATTGT GATTTTAGTG ATGTTGTTCT TAAGTATATT AATCAACTTC TTATGGAAGA   
  
  
+ GGATGGGGAA GAGAAACTCC ATACTGATCA TGAACCTTCA GTCGTTGAGG CGGCAGAGAA GTCATTGTAT   
  
  
+ GAGGCGCTCG GGCAGAGATA TCCTCCTTCC GGAAACCGAA ATCAGTTGCC AGATGTTGAG CATGACGGCT   
  
  
+ TGACTGGGAG CAGTGTTAGG GCTCATAGTG GTGCAAGCAG TGTTAGGGCT CATAGTGACG CAAGTGGTGG   
  
  
+ TAGTGGCCTG ACAGGATATG GTTGGTATGG TGATCCTTAT AATTGGAGTC CTCAAAATGT CGTGAATTTT   
  
  
+ ACCATTTCCT CCTCGAAACC CTTATCGTGC AGCTCATCAG ATAGCTCGGG CAGATGGGTT AGTAAGTGGA   
  
  
+ AGTCAAATTC TGATTCACAG CTCAGCTCGG CCATTAATGC AAGTGGTTTA GTGGATGGGC CAGGGGACTC   
  
  
+ TCCTGTGAGT GCTCTTAGTG TGTCTGAAAT ATTCAATGAC AGTCAGTCAA TGTTGCAGTT TCAGAAAGGA   
  
  
+ TTTGAGGAAG CGAATAAATT TCTTCCAAAG AGTTCTTTGT ACAAGGGTTT TGCCAACACG GGATTGCCTT   
  
  
+ ATCAGAAGGC AAACAATAGT GCCCAAGATT TGTTGGTCAA TGTAGAGGAT GTTACTAGGG GAAAGAAGCA   
  
  
+ TCGTTATCCC GAAGAATTGC AGTCAGAAGA AGGGAGGATA AATAAGCAAT CAGCTGTTTC CCTGGCAGCT   
  
  
+ GATGAGGCAG TTGTTAGGTC TGAAATGTTC GATAGGGTAC TGCTTTGTAG TCGGGGAAAA CATGATGCTG   
  
  
+ CTCTCCGGGA AGCTTTACAG ACTGAACTAA ATAAGAGTCT GCGAAATGCC CCAGTTAAGG GGTCTAATAG   
  
  
+ TGGGAAAGGC CGTGGTAAGA AAGCGGGAAA GAAGAGGGAT GTAGTAGATT TAAGATCTCT CTTAACCCTA   
  
  
+ TGTGCACAAG CAGTTGCATC GAATGATCAC AGGAGTGCAA ATGACCTGCT TAGGCAAATT AGACAGCATT   
  
  
+ CTTCTCCTAG TGGGGATGGT AACCAAAGAA TGGCACATTA TTTTGCGGAT GGTCTTGAGG CACGCCTTGC   
  
  
+ TGGTGTAGGA ACTCCTATAT ATAACTGTCT TGTAACGGGT CCGGCATCGG CTGTAGATAT CTTGAGAGCT   
  
  
+ TACCACATGT TTCTTGCCAC ATGCCCATTT AAGAAAATGG GAAATTTCTT CTCTAATAGA ACGATTATGG   
  
  
+ CTGTGGCAGA GAATGCAACA TGCCTTCATA TAATTGATCT CGGTATTGTC TATGGTTTCC AATGGCCTTG   
  
  
+ TCTAATTCAG CGACTTTCAT CTAGGCCTGG TGGCCCCCCC AAACTTCGAA TAACCGGAGT AGATCTTCCA   
  
  
+ CAACCTGGGT TCCGACCAGC CAAAAGAGTT GAGGAGACAG GGCGTCGCTT GAAGAACTAT GCAGAGTCAT   
  
  
+ TTAATGTGCC CTTTGAGTTC AATGCTATAG CAAAGAAGTG GGAAACACTT ACCATTGAAG ATCTCAGGAT   
  
  
+ CAATAGCGAT GAGTTGCTTG TTGTCACCTG TATGTTTAGG TTTAAACATA TACCTGAGGA AACAGTGACC   
  
  
+ GTGGATTGCC CTAGGGATAC TGTTCTTAAC CTGATTGGGC GCATAAACCC AGCTGTTTTC ATACAAGGCA   
  
  
+ CTGTTAACGG GGCTTTCAAT TCTCCCTTTT TCATAGCTCG ATTTCGAGAG GCTCTATTTC ACTTCTCCAC   
  
  
+ TCTGTTTGAT ATGCTAGAGG CCAACCTGCC AAGGGACAAT AAGGAGAGGA TGCTAATTGA GAGAGAGATA   
  
  
+ TTTGGGAGGC AGGCAATGAA TGTGATTGCT TGTGAGGGTT TAGAGAGGAT AGAAAGGCCA GAGACGTACA   
  
  
+ AGCAGTGGCA AGTCCGAAAT GAAAGGGCAG GGTTTAGGCA GCTGCCTTTA GATCGCCAGA TTCTGGAAAT   
  
  
+ GGCTAAAAAG AGGGTGAAAT CTGTGTATAA CAAAGATTTC TCCATTGATG AAGACGGGCA CTGGTTGTTG   
  
  
+ CTGGGATGGA AGGGCAGAAT TGTGTACACA CTCACTACTT GGAAGCCTGC GGAGTA  

- -Up\_Stream \_Len000GGGACC CAGTAATTTT TTTTTCTTTC TTTCTTCTAA CGCGATTTTC ATCTAAAAAT   
  
  
- AGTCTAAGTT CGACGTCCAC TATTGGTTTA ACAGTAATTT CGTTACGTTC GCGTATAGTT ATGGGAAAAC   
  
  
- CCTCTATCGA GGCTGACAAC TTCTGTAATC CTCGTACTAG AGGTTGACAC TTAAACAAAG TAAATGAGTA   
  
  
- TAAGTATAGA TGGTACACCT ATTATACTGA CGTCTGATCT ACCGGTTTAA ACTTACGTGT GAATATTGAA   
  
  
- GTTGAGAAAG ATGAAAAAAG AAGGGGTGGA ATAGTCCTTA AAGAAAATTA ACAGCACCCT CTGTTGAACC   
  
  
- CATCTTGAGA ACGCTTTTCC CGTTGAATTG CAAAACCATG AACGTAAAAG GTTTTTTTGT TTTTCCGATT   
  
  
- TGTTAAGAGG CGTACGACTG AAGTGCCATC TATACTACAT AAAAAATTGT TTTCTGTAGA AGACTTACTT   
  
  
- TGCGAATTAT AGAACCTGGT ATGATCTTTA ATTTAACGGT GCCACGTACT TAAAGTGGAG CTTGACGAAC   
  
  
- ATATTTCTAG AACAATAGTG GAAGTCATTA GTCTATGAAC ATTGGTTTAA CTTACTGGTG TTTCTGGTGT   
  
  
- AAGGAGTGTT AGCAACGGAG ATCTTATTCT TACTTACAAA GAAGTTCCAA CTTTCGGGAA CGATTACGCT   
  
  
- TGGACGGATA TACGGATTGA CCGATATATA TACACGCATC TTGTCGTACT GTACTCAATT ATTATATCGT   
  
  
- CGATTTCCCT TGAGATTACA TGTGTACTAA CATTAAGTGG ACGGAGCGAT CGAATGCTCG TACTGAGAAC   
  
  
- TGATAATAAC AAAATTAACT AGAACTTAAA AGTATCTAAA ATGAGGTAAC AAATGAGTAA AACAATATTA   
  
  
- ATTGGTGGAC TAAAATTTGA AATTTTATGT GTTTCTAAGT AGATTATATT AAAAAATCTA TTTCAATACG   
  
  
- TATGTATATA TGTTTAATTT AATAAATGCT AGATTAGTAA AGGGCAATAT ATAAAGTGTG ATTTTAAATC   
  
  
- TTCTCTGTTA GATAATTTTA ATGTAGTGTA CAAAACCCCC CCCCCCCTAT AGTCTATACG TACTGTAAGG   
  
  
- ATTAGGTTAT TACGTTACTT TGGGTGGCTT ATCAACTTTA CTAAACAAGA TAGGTACTTG AGTGGTTCAG   
  
  
- TGTTAGGTAG TGTGAGCTTC CTATACGAAA AGAAAAACTT CCCTTAGAAC GAATTAGTTT GGATATTTGA   
  
  
- AAATTTTCGT TGTCTGTTTT TTCACTAATA TTACCATCAC CATCCTACTT CCGTCGAAAG TCTTTTCGCT   
  
  
- AACTTAAAAA TAACATAGGG TACTTTTGGG TAACTTAAAA AGAAAGAAAG AAACAAAAAC AATGGACCCA   
  
  
- AATACGTTTC CTGCCGACAA CATAAATTAT TATTGTTTTT GTATCCCGAA ACGCCTAACG ATTACGACCC   
  
  
- CTACCGTAGT CACGAGCTTA GACACATGCG ACAGTATCCT TGGGTGAACG AAGACGAGTA AATAAAGACA   
  
  
- GAGATCTAGG GGAGGAGAGA GAGAGAGAGA GAGATTCCAC AGACGAGCAG ACAAGTATAA GAGGACTCCA   
  
  
- AGAAGAAGTA GATAGAACAA GACCCACACA CGACTCCATG AAGAAAAGAA ATATTTAAAA AGTAGAAAAC   
  
  
- AACAAAACGA ACCACAAATA CTAAAATCAA AAACAGAAAT TCTGCTCTAA ACGACAATTA TCGTTCTAAA   
  
  
- GACTTGATCA TAACTAGGAA AAACACCATA TAAACAACTT AAACTAATTA AACACATAAC ACAAATCGAA   
  
  
- TTCATTGAGC AACACTAGAT TAACTACGAG CCCAAAACTG TCAAACTAAT CCCAAAAGCC ATGACCTCAA   
  
  
- TTTCAAAAAA ATAAAAAATA TCCTCCTCAA TTTCCATTAT TATTCTTAAC TACAAACGAC TAAAAAGGAG   
  
  
- AAAAGAAAAA ACCAAAGCTA AACCACCCGT CAATAGACAA ATACCAAAAA CGTTTACCTA GGTGTTAATC   
  
  
- TCCTTAATAT ACCTGGAAAT GTAGCTTAGT TCAAGTTACT AGTTTCCCAC GGTTAAAATG GTTCAGCGTC   
  
  
- ACAACAATGA GGCTACTTTG AGGTTCTAAG GTTAAACTTA GGTTCACAAG GTTTAGGTTC ACAACACTTA   
  
  
- GGAGGAGGCA ACCAAGGGGG TTTGGGTTTA GAATTAAATC AACACCGTAG AGTTATAAGA CTGTAACTCT   
  
  
- GTCGGGACTT GCTCCTAACA CTAAAATCAC TACAACAAGA ATTCATATAA TTAGTTGAAG AATACCTTCT   
  
  
- CCTACCCCTT CTCTTTGAGG TATGACTAGT ACTTGGAAGT CAGCAACTCC GCCGTCTCTT CAGTAACATA   
  
  
- CTCCGCGAGC CCGTCTCTAT AGGAGGAAGG CCTTTGGCTT TAGTCAACGG TCTACAACTC GTACTGCCGA   
  
  
- ACTGACCCTC GTCACAATCC CGAGTATCAC CACGTTCGTC ACAATCCCGA GTATCACTGC GTTCACCACC   
  
  
- ATCACCGGAC TGTCCTATAC CAACCATACC ACTAGGAATA TTAACCTCAG GAGTTTTACA GCACTTAAAA   
  
  
- TGGTAAAGGA GGAGCTTTGG GAATAGCACG TCGAGTAGTC TATCGAGCCC GTCTACCCAA TCATTCACCT   
  
  
- TCAGTTTAAG ACTAAGTGTC GAGTCGAGCC GGTAATTACG TTCACCAAAT CACCTACCCG GTCCCCTGAG   
  
  
- AGGACACTCA CGAGAATCAC ACAGACTTTA TAAGTTACTG TCAGTCAGTT ACAACGTCAA AGTCTTTCCT   
  
  
- AAACTCCTTC GCTTATTTAA AGAAGGTTTC TCAAGAAACA TGTTCCCAAA ACGGTTGTGC CCTAACGGAA   
  
  
- TAGTCTTCCG TTTGTTATCA CGGGTTCTAA ACAACCAGTT ACATCTCCTA CAATGATCCC CTTTCTTCGT   
  
  
- AGCAATAGGG CTTCTTAACG TCAGTCTTCT TCCCTCCTAT TTATTCGTTA GTCGACAAAG GGACCGTCGA   
  
  
- CTACTCCGTC AACAATCCAG ACTTTACAAG CTATCCCATG ACGAAACATC AGCCCCTTTT GTACTACGAC   
  
  
- GAGAGGCCCT TCGAAATGTC TGACTTGATT TATTCTCAGA CGCTTTACGG GGTCAATTCC CCAGATTATC   
  
  
- ACCCTTTCCG GCACCATTCT TTCGCCCTTT CTTCTCCCTA CATCATCTAA ATTCTAGAGA GAATTGGGAT   
  
  
- ACACGTGTTC GTCAACGTAG CTTACTAGTG TCCTCACGTT TACTGGACGA ATCCGTTTAA TCTGTCGTAA   
  
  
- GAAGAGGATC ACCCCTACCA TTGGTTTCTT ACCGTGTAAT AAAACGCCTA CCAGAACTCC GTGCGGAACG   
  
  
- ACCACATCCT TGAGGATATA TATTGACAGA ACATTGCCCA GGCCGTAGCC GACATCTATA GAACTCTCGA   
  
  
- ATGGTGTACA AAGAACGGTG TACGGGTAAA TTCTTTTACC CTTTAAAGAA GAGATTATCT TGCTAATACC   
  
  
- GACACCGTCT CTTACGTTGT ACGGAAGTAT ATTAACTAGA GCCATAACAG ATACCAAAGG TTACCGGAAC   
  
  
- AGATTAAGTC GCTGAAAGTA GATCCGGACC ACCGGGGGGG TTTGAAGCTT ATTGGCCTCA TCTAGAAGGT   
  
  
- GTTGGACCCA AGGCTGGTCG GTTTTCTCAA CTCCTCTGTC CCGCAGCGAA CTTCTTGATA CGTCTCAGTA   
  
  
- AATTACACGG GAAACTCAAG TTACGATATC GTTTCTTCAC CCTTTGTGAA TGGTAACTTC TAGAGTCCTA   
  
  
- GTTATCGCTA CTCAACGAAC AACAGTGGAC ATACAAATCC AAATTTGTAT ATGGACTCCT TTGTCACTGG   
  
  
- CACCTAACGG GATCCCTATG ACAAGAATTG GACTAACCCG CGTATTTGGG TCGACAAAAG TATGTTCCGT   
  
  
- GACAATTGCC CCGAAAGTTA AGAGGGAAAA AGTATCGAGC TAAAGCTCTC CGAGATAAAG TGAAGAGGTG   
  
  
- AGACAAACTA TACGATCTCC GGTTGGACGG TTCCCTGTTA TTCCTCTCCT ACGATTAACT CTCTCTCTAT   
  
  
- AAACCCTCCG TCCGTTACTT ACACTAACGA ACACTCCCAA ATCTCTCCTA TCTTTCCGGT CTCTGCATGT   
  
  
- TCGTCACCGT TCAGGCTTTA CTTTCCCGTC CCAAATCCGT CGACGGAAAT CTAGCGGTCT AAGACCTTTA   
  
  
- CCGATTTTTC TCCCACTTTA GACACATATT GTTTCTAAAG AGGTAACTAC TTCTGCCCGT GACCAACAAC   
  
  
- GACCCTACCT TCCCGTCTTA ACACATGTGT GAGTGATGAA CCTTCGGACG CCTCAT

+     CCGTCC-box

| Site Name | Organism | Position | Strand | Matrix score. | sequence | function |
| --- | --- | --- | --- | --- | --- | --- |
| CCGTCC-box | Petroselinum hortense | 1414 | - | 6 | CCGTCC |  |

>HU06G00568.1   
+ -Up\_Stream \_Len000CCCTGG GTCATTAAAA AAAAAGAAAG AAAGAAGATT GCGCTAAAAG TAGATTTTTA   
  
  
+ TCAGATTCAA GCTGCAGGTG ATAACCAAAT TGTCATTAAA GCAATGCAAG CGCATATCAA TACCCTTTTG   
  
  
+ GGAGATAGCT CCGACTGTTG AAGACATTAG GAGCATGATC TCCAACTGTG AATTTGTTTC ATTTACTCAT   
  
  
+ ATTCATATCT ACCATGTGGA TAATATGACT GCAGACTAGA TGGCCAAATT TGAATGCACA CTTATAACTT   
  
  
+ CAACTCTTTC TACTTTTTTC TTCCCCACCT TATCAGGAAT TTCTTTTAAT TGTCGTGGGA GACAACTTGG   
  
  
+ GTAGAACTCT TGCGAAAAGG GCAACTTAAC GTTTTGGTAC TTGCATTTTC CAAAAAAACA AAAAGGCTAA   
  
  
+ ACAATTCTCC GCATGCTGAC TTCACGGTAG ATATGATGTA TTTTTTAACA AAAGACATCT TCTGAATGAA   
  
  
+ ACGCTTAATA TCTTGGACCA TACTAGAAAT TAAATTGCCA CGGTGCATGA ATTTCACCTC GAACTGCTTG   
  
  
+ TATAAAGATC TTGTTATCAC CTTCAGTAAT CAGATACTTG TAACCAAATT GAATGACCAC AAAGACCACA   
  
  
+ TTCCTCACAA TCGTTGCCTC TAGAATAAGA ATGAATGTTT CTTCAAGGTT GAAAGCCCTT GCTAATGCGA   
  
  
+ ACCTGCCTAT ATGCCTAACT GGCTATATAT ATGTGCGTAG AACAGCATGA CATGAGTTAA TAATATAGCA   
  
  
+ GCTAAAGGGA ACTCTAATGT ACACATGATT GTAATTCACC TGCCTCGCTA GCTTACGAGC ATGACTCTTG   
  
  
+ ACTATTATTG TTTTAATTGA TCTTGAATTT TCATAGATTT TACTCCATTG TTTACTCATT TTGTTATAAT   
  
  
+ TAACCACCTG ATTTTAAACT TTAAAATACA CAAAGATTCA TCTAATATAA TTTTTTAGAT AAAGTTATGC   
  
  
+ ATACATATAT ACAAATTAAA TTATTTACGA TCTAATCATT TCCCGTTATA TATTTCACAC TAAAATTTAG   
  
  
+ AAGAGACAAT CTATTAAAAT TACATCACAT GTTTTGGGGG GGGGGGGATA TCAGATATGC ATGACATTCC   
  
  
+ TAATCCAATA ATGCAATGAA ACCCACCGAA TAGTTGAAAT GATTTGTTCT ATCCATGAAC TCACCAAGTC   
  
  
+ ACAATCCATC ACACTCGAAG GATATGCTTT TCTTTTTGAA GGGAATCTTG CTTAATCAAA CCTATAAACT   
  
  
+ TTTAAAAGCA ACAGACAAAA AAGTGATTAT AATGGTAGTG GTAGGATGAA GGCAGCTTTC AGAAAAGCGA   
  
  
+ TTGAATTTTT ATTGTATCCC ATGAAAACCC ATTGAATTTT TCTTTCTTTC TTTGTTTTTG TTACCTGGGT   
  
  
+ TTATGCAAAG GACGGCTGTT GTATTTAATA ATAACAAAAA CATAGGGCTT TGCGGATTGC TAATGCTGGG   
  
  
+ GATGGCATCA GTGCTCGAAT CTGTGTACGC TGTCATAGGA ACCCACTTGC TTCTGCTCAT TTATTTCTGT   
  
  
+ CTCTAGATCC CCTCCTCTCT CTCTCTCTCT CTCTAAGGTG TCTGCTCGTC TGTTCATATT CTCCTGAGGT   
  
  
+ TCTTCTTCAT CTATCTTGTT CTGGGTGTGT GCTGAGGTAC TTCTTTTCTT TATAAATTTT TCATCTTTTG   
  
  
+ TTGTTTTGCT TGGTGTTTAT GATTTTAGTT TTTGTCTTTA AGACGAGATT TGCTGTTAAT AGCAAGATTT   
  
  
+ CTGAACTAGT ATTGATCCTT TTTGTGGTAT ATTTGTTGAA TTTGATTAAT TTGTGTATTG TGTTTAGCTT   
  
  
+ AAGTAACTCG TTGTGATCTA ATTGATGCTC GGGTTTTGAC AGTTTGATTA GGGTTTTCGG TACTGGAGTT   
  
  
+ AAAGTTTTTT TATTTTTTAT AGGAGGAGTT AAAGGTAATA ATAAGAATTG ATGTTTGCTG ATTTTTCCTC   
  
  
+ TTTTCTTTTT TGGTTTCGAT TTGGTGGGCA GTTATCTGTT TATGGTTTTT GCAAATGGAT CCACAATTAG   
  
  
+ AGGAATTATA TGGACCTTTA CATCGAATCA AGTTCAATGA TCAAAGGGTG CCAATTTTAC CAAGTCGCAG   
  
  
+ TGTTGTTACT CCGATGAAAC TCCAAGATTC CAATTTGAAT CCAAGTGTTC CAAATCCAAG TGTTGTGAAT   
  
  
+ CCTCCTCCGT TGGTTCCCCC AAACCCAAAT CTTAATTTAG TTGTGGCATC TCAATATTCT GACATTGAGA   
  
  
+ CAGCCCTGAA CGAGGATTGT GATTTTAGTG ATGTTGTTCT TAAGTATATT AATCAACTTC TTATGGAAGA   
  
  
+ GGATGGGGAA GAGAAACTCC ATACTGATCA TGAACCTTCA GTCGTTGAGG CGGCAGAGAA GTCATTGTAT   
  
  
+ GAGGCGCTCG GGCAGAGATA TCCTCCTTCC GGAAACCGAA ATCAGTTGCC AGATGTTGAG CATGACGGCT   
  
  
+ TGACTGGGAG CAGTGTTAGG GCTCATAGTG GTGCAAGCAG TGTTAGGGCT CATAGTGACG CAAGTGGTGG   
  
  
+ TAGTGGCCTG ACAGGATATG GTTGGTATGG TGATCCTTAT AATTGGAGTC CTCAAAATGT CGTGAATTTT   
  
  
+ ACCATTTCCT CCTCGAAACC CTTATCGTGC AGCTCATCAG ATAGCTCGGG CAGATGGGTT AGTAAGTGGA   
  
  
+ AGTCAAATTC TGATTCACAG CTCAGCTCGG CCATTAATGC AAGTGGTTTA GTGGATGGGC CAGGGGACTC   
  
  
+ TCCTGTGAGT GCTCTTAGTG TGTCTGAAAT ATTCAATGAC AGTCAGTCAA TGTTGCAGTT TCAGAAAGGA   
  
  
+ TTTGAGGAAG CGAATAAATT TCTTCCAAAG AGTTCTTTGT ACAAGGGTTT TGCCAACACG GGATTGCCTT   
  
  
+ ATCAGAAGGC AAACAATAGT GCCCAAGATT TGTTGGTCAA TGTAGAGGAT GTTACTAGGG GAAAGAAGCA   
  
  
+ TCGTTATCCC GAAGAATTGC AGTCAGAAGA AGGGAGGATA AATAAGCAAT CAGCTGTTTC CCTGGCAGCT   
  
  
+ GATGAGGCAG TTGTTAGGTC TGAAATGTTC GATAGGGTAC TGCTTTGTAG TCGGGGAAAA CATGATGCTG   
  
  
+ CTCTCCGGGA AGCTTTACAG ACTGAACTAA ATAAGAGTCT GCGAAATGCC CCAGTTAAGG GGTCTAATAG   
  
  
+ TGGGAAAGGC CGTGGTAAGA AAGCGGGAAA GAAGAGGGAT GTAGTAGATT TAAGATCTCT CTTAACCCTA   
  
  
+ TGTGCACAAG CAGTTGCATC GAATGATCAC AGGAGTGCAA ATGACCTGCT TAGGCAAATT AGACAGCATT   
  
  
+ CTTCTCCTAG TGGGGATGGT AACCAAAGAA TGGCACATTA TTTTGCGGAT GGTCTTGAGG CACGCCTTGC   
  
  
+ TGGTGTAGGA ACTCCTATAT ATAACTGTCT TGTAACGGGT CCGGCATCGG CTGTAGATAT CTTGAGAGCT   
  
  
+ TACCACATGT TTCTTGCCAC ATGCCCATTT AAGAAAATGG GAAATTTCTT CTCTAATAGA ACGATTATGG   
  
  
+ CTGTGGCAGA GAATGCAACA TGCCTTCATA TAATTGATCT CGGTATTGTC TATGGTTTCC AATGGCCTTG   
  
  
+ TCTAATTCAG CGACTTTCAT CTAGGCCTGG TGGCCCCCCC AAACTTCGAA TAACCGGAGT AGATCTTCCA   
  
  
+ CAACCTGGGT TCCGACCAGC CAAAAGAGTT GAGGAGACAG GGCGTCGCTT GAAGAACTAT GCAGAGTCAT   
  
  
+ TTAATGTGCC CTTTGAGTTC AATGCTATAG CAAAGAAGTG GGAAACACTT ACCATTGAAG ATCTCAGGAT   
  
  
+ CAATAGCGAT GAGTTGCTTG TTGTCACCTG TATGTTTAGG TTTAAACATA TACCTGAGGA AACAGTGACC   
  
  
+ GTGGATTGCC CTAGGGATAC TGTTCTTAAC CTGATTGGGC GCATAAACCC AGCTGTTTTC ATACAAGGCA   
  
  
+ CTGTTAACGG GGCTTTCAAT TCTCCCTTTT TCATAGCTCG ATTTCGAGAG GCTCTATTTC ACTTCTCCAC   
  
  
+ TCTGTTTGAT ATGCTAGAGG CCAACCTGCC AAGGGACAAT AAGGAGAGGA TGCTAATTGA GAGAGAGATA   
  
  
+ TTTGGGAGGC AGGCAATGAA TGTGATTGCT TGTGAGGGTT TAGAGAGGAT AGAAAGGCCA GAGACGTACA   
  
  
+ AGCAGTGGCA AGTCCGAAAT GAAAGGGCAG GGTTTAGGCA GCTGCCTTTA GATCGCCAGA TTCTGGAAAT   
  
  
+ GGCTAAAAAG AGGGTGAAAT CTGTGTATAA CAAAGATTTC TCCATTGATG AAGACGGGCA CTGGTTGTTG   
  
  
+ CTGGGATGGA AGGGCAGAAT TGTGTACACA CTCACTACTT GGAAGCCTGC GGAGTA  

- -Up\_Stream \_Len000GGGACC CAGTAATTTT TTTTTCTTTC TTTCTTCTAA CGCGATTTTC ATCTAAAAAT   
  
  
- AGTCTAAGTT CGACGTCCAC TATTGGTTTA ACAGTAATTT CGTTACGTTC GCGTATAGTT ATGGGAAAAC   
  
  
- CCTCTATCGA GGCTGACAAC TTCTGTAATC CTCGTACTAG AGGTTGACAC TTAAACAAAG TAAATGAGTA   
  
  
- TAAGTATAGA TGGTACACCT ATTATACTGA CGTCTGATCT ACCGGTTTAA ACTTACGTGT GAATATTGAA   
  
  
- GTTGAGAAAG ATGAAAAAAG AAGGGGTGGA ATAGTCCTTA AAGAAAATTA ACAGCACCCT CTGTTGAACC   
  
  
- CATCTTGAGA ACGCTTTTCC CGTTGAATTG CAAAACCATG AACGTAAAAG GTTTTTTTGT TTTTCCGATT   
  
  
- TGTTAAGAGG CGTACGACTG AAGTGCCATC TATACTACAT AAAAAATTGT TTTCTGTAGA AGACTTACTT   
  
  
- TGCGAATTAT AGAACCTGGT ATGATCTTTA ATTTAACGGT GCCACGTACT TAAAGTGGAG CTTGACGAAC   
  
  
- ATATTTCTAG AACAATAGTG GAAGTCATTA GTCTATGAAC ATTGGTTTAA CTTACTGGTG TTTCTGGTGT   
  
  
- AAGGAGTGTT AGCAACGGAG ATCTTATTCT TACTTACAAA GAAGTTCCAA CTTTCGGGAA CGATTACGCT   
  
  
- TGGACGGATA TACGGATTGA CCGATATATA TACACGCATC TTGTCGTACT GTACTCAATT ATTATATCGT   
  
  
- CGATTTCCCT TGAGATTACA TGTGTACTAA CATTAAGTGG ACGGAGCGAT CGAATGCTCG TACTGAGAAC   
  
  
- TGATAATAAC AAAATTAACT AGAACTTAAA AGTATCTAAA ATGAGGTAAC AAATGAGTAA AACAATATTA   
  
  
- ATTGGTGGAC TAAAATTTGA AATTTTATGT GTTTCTAAGT AGATTATATT AAAAAATCTA TTTCAATACG   
  
  
- TATGTATATA TGTTTAATTT AATAAATGCT AGATTAGTAA AGGGCAATAT ATAAAGTGTG ATTTTAAATC   
  
  
- TTCTCTGTTA GATAATTTTA ATGTAGTGTA CAAAACCCCC CCCCCCCTAT AGTCTATACG TACTGTAAGG   
  
  
- ATTAGGTTAT TACGTTACTT TGGGTGGCTT ATCAACTTTA CTAAACAAGA TAGGTACTTG AGTGGTTCAG   
  
  
- TGTTAGGTAG TGTGAGCTTC CTATACGAAA AGAAAAACTT CCCTTAGAAC GAATTAGTTT GGATATTTGA   
  
  
- AAATTTTCGT TGTCTGTTTT TTCACTAATA TTACCATCAC CATCCTACTT CCGTCGAAAG TCTTTTCGCT   
  
  
- AACTTAAAAA TAACATAGGG TACTTTTGGG TAACTTAAAA AGAAAGAAAG AAACAAAAAC AATGGACCCA   
  
  
- AATACGTTTC CTGCCGACAA CATAAATTAT TATTGTTTTT GTATCCCGAA ACGCCTAACG ATTACGACCC   
  
  
- CTACCGTAGT CACGAGCTTA GACACATGCG ACAGTATCCT TGGGTGAACG AAGACGAGTA AATAAAGACA   
  
  
- GAGATCTAGG GGAGGAGAGA GAGAGAGAGA GAGATTCCAC AGACGAGCAG ACAAGTATAA GAGGACTCCA   
  
  
- AGAAGAAGTA GATAGAACAA GACCCACACA CGACTCCATG AAGAAAAGAA ATATTTAAAA AGTAGAAAAC   
  
  
- AACAAAACGA ACCACAAATA CTAAAATCAA AAACAGAAAT TCTGCTCTAA ACGACAATTA TCGTTCTAAA   
  
  
- GACTTGATCA TAACTAGGAA AAACACCATA TAAACAACTT AAACTAATTA AACACATAAC ACAAATCGAA   
  
  
- TTCATTGAGC AACACTAGAT TAACTACGAG CCCAAAACTG TCAAACTAAT CCCAAAAGCC ATGACCTCAA   
  
  
- TTTCAAAAAA ATAAAAAATA TCCTCCTCAA TTTCCATTAT TATTCTTAAC TACAAACGAC TAAAAAGGAG   
  
  
- AAAAGAAAAA ACCAAAGCTA AACCACCCGT CAATAGACAA ATACCAAAAA CGTTTACCTA GGTGTTAATC   
  
  
- TCCTTAATAT ACCTGGAAAT GTAGCTTAGT TCAAGTTACT AGTTTCCCAC GGTTAAAATG GTTCAGCGTC   
  
  
- ACAACAATGA GGCTACTTTG AGGTTCTAAG GTTAAACTTA GGTTCACAAG GTTTAGGTTC ACAACACTTA   
  
  
- GGAGGAGGCA ACCAAGGGGG TTTGGGTTTA GAATTAAATC AACACCGTAG AGTTATAAGA CTGTAACTCT   
  
  
- GTCGGGACTT GCTCCTAACA CTAAAATCAC TACAACAAGA ATTCATATAA TTAGTTGAAG AATACCTTCT   
  
  
- CCTACCCCTT CTCTTTGAGG TATGACTAGT ACTTGGAAGT CAGCAACTCC GCCGTCTCTT CAGTAACATA   
  
  
- CTCCGCGAGC CCGTCTCTAT AGGAGGAAGG CCTTTGGCTT TAGTCAACGG TCTACAACTC GTACTGCCGA   
  
  
- ACTGACCCTC GTCACAATCC CGAGTATCAC CACGTTCGTC ACAATCCCGA GTATCACTGC GTTCACCACC   
  
  
- ATCACCGGAC TGTCCTATAC CAACCATACC ACTAGGAATA TTAACCTCAG GAGTTTTACA GCACTTAAAA   
  
  
- TGGTAAAGGA GGAGCTTTGG GAATAGCACG TCGAGTAGTC TATCGAGCCC GTCTACCCAA TCATTCACCT   
  
  
- TCAGTTTAAG ACTAAGTGTC GAGTCGAGCC GGTAATTACG TTCACCAAAT CACCTACCCG GTCCCCTGAG   
  
  
- AGGACACTCA CGAGAATCAC ACAGACTTTA TAAGTTACTG TCAGTCAGTT ACAACGTCAA AGTCTTTCCT   
  
  
- AAACTCCTTC GCTTATTTAA AGAAGGTTTC TCAAGAAACA TGTTCCCAAA ACGGTTGTGC CCTAACGGAA   
  
  
- TAGTCTTCCG TTTGTTATCA CGGGTTCTAA ACAACCAGTT ACATCTCCTA CAATGATCCC CTTTCTTCGT   
  
  
- AGCAATAGGG CTTCTTAACG TCAGTCTTCT TCCCTCCTAT TTATTCGTTA GTCGACAAAG GGACCGTCGA   
  
  
- CTACTCCGTC AACAATCCAG ACTTTACAAG CTATCCCATG ACGAAACATC AGCCCCTTTT GTACTACGAC   
  
  
- GAGAGGCCCT TCGAAATGTC TGACTTGATT TATTCTCAGA CGCTTTACGG GGTCAATTCC CCAGATTATC   
  
  
- ACCCTTTCCG GCACCATTCT TTCGCCCTTT CTTCTCCCTA CATCATCTAA ATTCTAGAGA GAATTGGGAT   
  
  
- ACACGTGTTC GTCAACGTAG CTTACTAGTG TCCTCACGTT TACTGGACGA ATCCGTTTAA TCTGTCGTAA   
  
  
- GAAGAGGATC ACCCCTACCA TTGGTTTCTT ACCGTGTAAT AAAACGCCTA CCAGAACTCC GTGCGGAACG   
  
  
- ACCACATCCT TGAGGATATA TATTGACAGA ACATTGCCCA GGCCGTAGCC GACATCTATA GAACTCTCGA   
  
  
- ATGGTGTACA AAGAACGGTG TACGGGTAAA TTCTTTTACC CTTTAAAGAA GAGATTATCT TGCTAATACC   
  
  
- GACACCGTCT CTTACGTTGT ACGGAAGTAT ATTAACTAGA GCCATAACAG ATACCAAAGG TTACCGGAAC   
  
  
- AGATTAAGTC GCTGAAAGTA GATCCGGACC ACCGGGGGGG TTTGAAGCTT ATTGGCCTCA TCTAGAAGGT   
  
  
- GTTGGACCCA AGGCTGGTCG GTTTTCTCAA CTCCTCTGTC CCGCAGCGAA CTTCTTGATA CGTCTCAGTA   
  
  
- AATTACACGG GAAACTCAAG TTACGATATC GTTTCTTCAC CCTTTGTGAA TGGTAACTTC TAGAGTCCTA   
  
  
- GTTATCGCTA CTCAACGAAC AACAGTGGAC ATACAAATCC AAATTTGTAT ATGGACTCCT TTGTCACTGG   
  
  
- CACCTAACGG GATCCCTATG ACAAGAATTG GACTAACCCG CGTATTTGGG TCGACAAAAG TATGTTCCGT   
  
  
- GACAATTGCC CCGAAAGTTA AGAGGGAAAA AGTATCGAGC TAAAGCTCTC CGAGATAAAG TGAAGAGGTG   
  
  
- AGACAAACTA TACGATCTCC GGTTGGACGG TTCCCTGTTA TTCCTCTCCT ACGATTAACT CTCTCTCTAT   
  
  
- AAACCCTCCG TCCGTTACTT ACACTAACGA ACACTCCCAA ATCTCTCCTA TCTTTCCGGT CTCTGCATGT   
  
  
- TCGTCACCGT TCAGGCTTTA CTTTCCCGTC CCAAATCCGT CGACGGAAAT CTAGCGGTCT AAGACCTTTA   
  
  
- CCGATTTTTC TCCCACTTTA GACACATATT GTTTCTAAAG AGGTAACTAC TTCTGCCCGT GACCAACAAC   
  
  
- GACCCTACCT TCCCGTCTTA ACACATGTGT GAGTGATGAA CCTTCGGACG CCTCAT

+     CGTCA-motif

| Site Name | Organism | Position | Strand | Matrix score. | sequence | function |
| --- | --- | --- | --- | --- | --- | --- |
| CGTCA-motif | Hordeum vulgare | 2510 | - | 5 | CGTCA | cis-acting regulatory element involved in the MeJA-responsiveness |
| CGTCA-motif | Hordeum vulgare | 2447 | - | 5 | CGTCA | cis-acting regulatory element involved in the MeJA-responsiveness |

>HU06G00568.1   
+ -Up\_Stream \_Len000CCCTGG GTCATTAAAA AAAAAGAAAG AAAGAAGATT GCGCTAAAAG TAGATTTTTA   
  
  
+ TCAGATTCAA GCTGCAGGTG ATAACCAAAT TGTCATTAAA GCAATGCAAG CGCATATCAA TACCCTTTTG   
  
  
+ GGAGATAGCT CCGACTGTTG AAGACATTAG GAGCATGATC TCCAACTGTG AATTTGTTTC ATTTACTCAT   
  
  
+ ATTCATATCT ACCATGTGGA TAATATGACT GCAGACTAGA TGGCCAAATT TGAATGCACA CTTATAACTT   
  
  
+ CAACTCTTTC TACTTTTTTC TTCCCCACCT TATCAGGAAT TTCTTTTAAT TGTCGTGGGA GACAACTTGG   
  
  
+ GTAGAACTCT TGCGAAAAGG GCAACTTAAC GTTTTGGTAC TTGCATTTTC CAAAAAAACA AAAAGGCTAA   
  
  
+ ACAATTCTCC GCATGCTGAC TTCACGGTAG ATATGATGTA TTTTTTAACA AAAGACATCT TCTGAATGAA   
  
  
+ ACGCTTAATA TCTTGGACCA TACTAGAAAT TAAATTGCCA CGGTGCATGA ATTTCACCTC GAACTGCTTG   
  
  
+ TATAAAGATC TTGTTATCAC CTTCAGTAAT CAGATACTTG TAACCAAATT GAATGACCAC AAAGACCACA   
  
  
+ TTCCTCACAA TCGTTGCCTC TAGAATAAGA ATGAATGTTT CTTCAAGGTT GAAAGCCCTT GCTAATGCGA   
  
  
+ ACCTGCCTAT ATGCCTAACT GGCTATATAT ATGTGCGTAG AACAGCATGA CATGAGTTAA TAATATAGCA   
  
  
+ GCTAAAGGGA ACTCTAATGT ACACATGATT GTAATTCACC TGCCTCGCTA GCTTACGAGC ATGACTCTTG   
  
  
+ ACTATTATTG TTTTAATTGA TCTTGAATTT TCATAGATTT TACTCCATTG TTTACTCATT TTGTTATAAT   
  
  
+ TAACCACCTG ATTTTAAACT TTAAAATACA CAAAGATTCA TCTAATATAA TTTTTTAGAT AAAGTTATGC   
  
  
+ ATACATATAT ACAAATTAAA TTATTTACGA TCTAATCATT TCCCGTTATA TATTTCACAC TAAAATTTAG   
  
  
+ AAGAGACAAT CTATTAAAAT TACATCACAT GTTTTGGGGG GGGGGGGATA TCAGATATGC ATGACATTCC   
  
  
+ TAATCCAATA ATGCAATGAA ACCCACCGAA TAGTTGAAAT GATTTGTTCT ATCCATGAAC TCACCAAGTC   
  
  
+ ACAATCCATC ACACTCGAAG GATATGCTTT TCTTTTTGAA GGGAATCTTG CTTAATCAAA CCTATAAACT   
  
  
+ TTTAAAAGCA ACAGACAAAA AAGTGATTAT AATGGTAGTG GTAGGATGAA GGCAGCTTTC AGAAAAGCGA   
  
  
+ TTGAATTTTT ATTGTATCCC ATGAAAACCC ATTGAATTTT TCTTTCTTTC TTTGTTTTTG TTACCTGGGT   
  
  
+ TTATGCAAAG GACGGCTGTT GTATTTAATA ATAACAAAAA CATAGGGCTT TGCGGATTGC TAATGCTGGG   
  
  
+ GATGGCATCA GTGCTCGAAT CTGTGTACGC TGTCATAGGA ACCCACTTGC TTCTGCTCAT TTATTTCTGT   
  
  
+ CTCTAGATCC CCTCCTCTCT CTCTCTCTCT CTCTAAGGTG TCTGCTCGTC TGTTCATATT CTCCTGAGGT   
  
  
+ TCTTCTTCAT CTATCTTGTT CTGGGTGTGT GCTGAGGTAC TTCTTTTCTT TATAAATTTT TCATCTTTTG   
  
  
+ TTGTTTTGCT TGGTGTTTAT GATTTTAGTT TTTGTCTTTA AGACGAGATT TGCTGTTAAT AGCAAGATTT   
  
  
+ CTGAACTAGT ATTGATCCTT TTTGTGGTAT ATTTGTTGAA TTTGATTAAT TTGTGTATTG TGTTTAGCTT   
  
  
+ AAGTAACTCG TTGTGATCTA ATTGATGCTC GGGTTTTGAC AGTTTGATTA GGGTTTTCGG TACTGGAGTT   
  
  
+ AAAGTTTTTT TATTTTTTAT AGGAGGAGTT AAAGGTAATA ATAAGAATTG ATGTTTGCTG ATTTTTCCTC   
  
  
+ TTTTCTTTTT TGGTTTCGAT TTGGTGGGCA GTTATCTGTT TATGGTTTTT GCAAATGGAT CCACAATTAG   
  
  
+ AGGAATTATA TGGACCTTTA CATCGAATCA AGTTCAATGA TCAAAGGGTG CCAATTTTAC CAAGTCGCAG   
  
  
+ TGTTGTTACT CCGATGAAAC TCCAAGATTC CAATTTGAAT CCAAGTGTTC CAAATCCAAG TGTTGTGAAT   
  
  
+ CCTCCTCCGT TGGTTCCCCC AAACCCAAAT CTTAATTTAG TTGTGGCATC TCAATATTCT GACATTGAGA   
  
  
+ CAGCCCTGAA CGAGGATTGT GATTTTAGTG ATGTTGTTCT TAAGTATATT AATCAACTTC TTATGGAAGA   
  
  
+ GGATGGGGAA GAGAAACTCC ATACTGATCA TGAACCTTCA GTCGTTGAGG CGGCAGAGAA GTCATTGTAT   
  
  
+ GAGGCGCTCG GGCAGAGATA TCCTCCTTCC GGAAACCGAA ATCAGTTGCC AGATGTTGAG CATGACGGCT   
  
  
+ TGACTGGGAG CAGTGTTAGG GCTCATAGTG GTGCAAGCAG TGTTAGGGCT CATAGTGACG CAAGTGGTGG   
  
  
+ TAGTGGCCTG ACAGGATATG GTTGGTATGG TGATCCTTAT AATTGGAGTC CTCAAAATGT CGTGAATTTT   
  
  
+ ACCATTTCCT CCTCGAAACC CTTATCGTGC AGCTCATCAG ATAGCTCGGG CAGATGGGTT AGTAAGTGGA   
  
  
+ AGTCAAATTC TGATTCACAG CTCAGCTCGG CCATTAATGC AAGTGGTTTA GTGGATGGGC CAGGGGACTC   
  
  
+ TCCTGTGAGT GCTCTTAGTG TGTCTGAAAT ATTCAATGAC AGTCAGTCAA TGTTGCAGTT TCAGAAAGGA   
  
  
+ TTTGAGGAAG CGAATAAATT TCTTCCAAAG AGTTCTTTGT ACAAGGGTTT TGCCAACACG GGATTGCCTT   
  
  
+ ATCAGAAGGC AAACAATAGT GCCCAAGATT TGTTGGTCAA TGTAGAGGAT GTTACTAGGG GAAAGAAGCA   
  
  
+ TCGTTATCCC GAAGAATTGC AGTCAGAAGA AGGGAGGATA AATAAGCAAT CAGCTGTTTC CCTGGCAGCT   
  
  
+ GATGAGGCAG TTGTTAGGTC TGAAATGTTC GATAGGGTAC TGCTTTGTAG TCGGGGAAAA CATGATGCTG   
  
  
+ CTCTCCGGGA AGCTTTACAG ACTGAACTAA ATAAGAGTCT GCGAAATGCC CCAGTTAAGG GGTCTAATAG   
  
  
+ TGGGAAAGGC CGTGGTAAGA AAGCGGGAAA GAAGAGGGAT GTAGTAGATT TAAGATCTCT CTTAACCCTA   
  
  
+ TGTGCACAAG CAGTTGCATC GAATGATCAC AGGAGTGCAA ATGACCTGCT TAGGCAAATT AGACAGCATT   
  
  
+ CTTCTCCTAG TGGGGATGGT AACCAAAGAA TGGCACATTA TTTTGCGGAT GGTCTTGAGG CACGCCTTGC   
  
  
+ TGGTGTAGGA ACTCCTATAT ATAACTGTCT TGTAACGGGT CCGGCATCGG CTGTAGATAT CTTGAGAGCT   
  
  
+ TACCACATGT TTCTTGCCAC ATGCCCATTT AAGAAAATGG GAAATTTCTT CTCTAATAGA ACGATTATGG   
  
  
+ CTGTGGCAGA GAATGCAACA TGCCTTCATA TAATTGATCT CGGTATTGTC TATGGTTTCC AATGGCCTTG   
  
  
+ TCTAATTCAG CGACTTTCAT CTAGGCCTGG TGGCCCCCCC AAACTTCGAA TAACCGGAGT AGATCTTCCA   
  
  
+ CAACCTGGGT TCCGACCAGC CAAAAGAGTT GAGGAGACAG GGCGTCGCTT GAAGAACTAT GCAGAGTCAT   
  
  
+ TTAATGTGCC CTTTGAGTTC AATGCTATAG CAAAGAAGTG GGAAACACTT ACCATTGAAG ATCTCAGGAT   
  
  
+ CAATAGCGAT GAGTTGCTTG TTGTCACCTG TATGTTTAGG TTTAAACATA TACCTGAGGA AACAGTGACC   
  
  
+ GTGGATTGCC CTAGGGATAC TGTTCTTAAC CTGATTGGGC GCATAAACCC AGCTGTTTTC ATACAAGGCA   
  
  
+ CTGTTAACGG GGCTTTCAAT TCTCCCTTTT TCATAGCTCG ATTTCGAGAG GCTCTATTTC ACTTCTCCAC   
  
  
+ TCTGTTTGAT ATGCTAGAGG CCAACCTGCC AAGGGACAAT AAGGAGAGGA TGCTAATTGA GAGAGAGATA   
  
  
+ TTTGGGAGGC AGGCAATGAA TGTGATTGCT TGTGAGGGTT TAGAGAGGAT AGAAAGGCCA GAGACGTACA   
  
  
+ AGCAGTGGCA AGTCCGAAAT GAAAGGGCAG GGTTTAGGCA GCTGCCTTTA GATCGCCAGA TTCTGGAAAT   
  
  
+ GGCTAAAAAG AGGGTGAAAT CTGTGTATAA CAAAGATTTC TCCATTGATG AAGACGGGCA CTGGTTGTTG   
  
  
+ CTGGGATGGA AGGGCAGAAT TGTGTACACA CTCACTACTT GGAAGCCTGC GGAGTA  

- -Up\_Stream \_Len000GGGACC CAGTAATTTT TTTTTCTTTC TTTCTTCTAA CGCGATTTTC ATCTAAAAAT   
  
  
- AGTCTAAGTT CGACGTCCAC TATTGGTTTA ACAGTAATTT CGTTACGTTC GCGTATAGTT ATGGGAAAAC   
  
  
- CCTCTATCGA GGCTGACAAC TTCTGTAATC CTCGTACTAG AGGTTGACAC TTAAACAAAG TAAATGAGTA   
  
  
- TAAGTATAGA TGGTACACCT ATTATACTGA CGTCTGATCT ACCGGTTTAA ACTTACGTGT GAATATTGAA   
  
  
- GTTGAGAAAG ATGAAAAAAG AAGGGGTGGA ATAGTCCTTA AAGAAAATTA ACAGCACCCT CTGTTGAACC   
  
  
- CATCTTGAGA ACGCTTTTCC CGTTGAATTG CAAAACCATG AACGTAAAAG GTTTTTTTGT TTTTCCGATT   
  
  
- TGTTAAGAGG CGTACGACTG AAGTGCCATC TATACTACAT AAAAAATTGT TTTCTGTAGA AGACTTACTT   
  
  
- TGCGAATTAT AGAACCTGGT ATGATCTTTA ATTTAACGGT GCCACGTACT TAAAGTGGAG CTTGACGAAC   
  
  
- ATATTTCTAG AACAATAGTG GAAGTCATTA GTCTATGAAC ATTGGTTTAA CTTACTGGTG TTTCTGGTGT   
  
  
- AAGGAGTGTT AGCAACGGAG ATCTTATTCT TACTTACAAA GAAGTTCCAA CTTTCGGGAA CGATTACGCT   
  
  
- TGGACGGATA TACGGATTGA CCGATATATA TACACGCATC TTGTCGTACT GTACTCAATT ATTATATCGT   
  
  
- CGATTTCCCT TGAGATTACA TGTGTACTAA CATTAAGTGG ACGGAGCGAT CGAATGCTCG TACTGAGAAC   
  
  
- TGATAATAAC AAAATTAACT AGAACTTAAA AGTATCTAAA ATGAGGTAAC AAATGAGTAA AACAATATTA   
  
  
- ATTGGTGGAC TAAAATTTGA AATTTTATGT GTTTCTAAGT AGATTATATT AAAAAATCTA TTTCAATACG   
  
  
- TATGTATATA TGTTTAATTT AATAAATGCT AGATTAGTAA AGGGCAATAT ATAAAGTGTG ATTTTAAATC   
  
  
- TTCTCTGTTA GATAATTTTA ATGTAGTGTA CAAAACCCCC CCCCCCCTAT AGTCTATACG TACTGTAAGG   
  
  
- ATTAGGTTAT TACGTTACTT TGGGTGGCTT ATCAACTTTA CTAAACAAGA TAGGTACTTG AGTGGTTCAG   
  
  
- TGTTAGGTAG TGTGAGCTTC CTATACGAAA AGAAAAACTT CCCTTAGAAC GAATTAGTTT GGATATTTGA   
  
  
- AAATTTTCGT TGTCTGTTTT TTCACTAATA TTACCATCAC CATCCTACTT CCGTCGAAAG TCTTTTCGCT   
  
  
- AACTTAAAAA TAACATAGGG TACTTTTGGG TAACTTAAAA AGAAAGAAAG AAACAAAAAC AATGGACCCA   
  
  
- AATACGTTTC CTGCCGACAA CATAAATTAT TATTGTTTTT GTATCCCGAA ACGCCTAACG ATTACGACCC   
  
  
- CTACCGTAGT CACGAGCTTA GACACATGCG ACAGTATCCT TGGGTGAACG AAGACGAGTA AATAAAGACA   
  
  
- GAGATCTAGG GGAGGAGAGA GAGAGAGAGA GAGATTCCAC AGACGAGCAG ACAAGTATAA GAGGACTCCA   
  
  
- AGAAGAAGTA GATAGAACAA GACCCACACA CGACTCCATG AAGAAAAGAA ATATTTAAAA AGTAGAAAAC   
  
  
- AACAAAACGA ACCACAAATA CTAAAATCAA AAACAGAAAT TCTGCTCTAA ACGACAATTA TCGTTCTAAA   
  
  
- GACTTGATCA TAACTAGGAA AAACACCATA TAAACAACTT AAACTAATTA AACACATAAC ACAAATCGAA   
  
  
- TTCATTGAGC AACACTAGAT TAACTACGAG CCCAAAACTG TCAAACTAAT CCCAAAAGCC ATGACCTCAA   
  
  
- TTTCAAAAAA ATAAAAAATA TCCTCCTCAA TTTCCATTAT TATTCTTAAC TACAAACGAC TAAAAAGGAG   
  
  
- AAAAGAAAAA ACCAAAGCTA AACCACCCGT CAATAGACAA ATACCAAAAA CGTTTACCTA GGTGTTAATC   
  
  
- TCCTTAATAT ACCTGGAAAT GTAGCTTAGT TCAAGTTACT AGTTTCCCAC GGTTAAAATG GTTCAGCGTC   
  
  
- ACAACAATGA GGCTACTTTG AGGTTCTAAG GTTAAACTTA GGTTCACAAG GTTTAGGTTC ACAACACTTA   
  
  
- GGAGGAGGCA ACCAAGGGGG TTTGGGTTTA GAATTAAATC AACACCGTAG AGTTATAAGA CTGTAACTCT   
  
  
- GTCGGGACTT GCTCCTAACA CTAAAATCAC TACAACAAGA ATTCATATAA TTAGTTGAAG AATACCTTCT   
  
  
- CCTACCCCTT CTCTTTGAGG TATGACTAGT ACTTGGAAGT CAGCAACTCC GCCGTCTCTT CAGTAACATA   
  
  
- CTCCGCGAGC CCGTCTCTAT AGGAGGAAGG CCTTTGGCTT TAGTCAACGG TCTACAACTC GTACTGCCGA   
  
  
- ACTGACCCTC GTCACAATCC CGAGTATCAC CACGTTCGTC ACAATCCCGA GTATCACTGC GTTCACCACC   
  
  
- ATCACCGGAC TGTCCTATAC CAACCATACC ACTAGGAATA TTAACCTCAG GAGTTTTACA GCACTTAAAA   
  
  
- TGGTAAAGGA GGAGCTTTGG GAATAGCACG TCGAGTAGTC TATCGAGCCC GTCTACCCAA TCATTCACCT   
  
  
- TCAGTTTAAG ACTAAGTGTC GAGTCGAGCC GGTAATTACG TTCACCAAAT CACCTACCCG GTCCCCTGAG   
  
  
- AGGACACTCA CGAGAATCAC ACAGACTTTA TAAGTTACTG TCAGTCAGTT ACAACGTCAA AGTCTTTCCT   
  
  
- AAACTCCTTC GCTTATTTAA AGAAGGTTTC TCAAGAAACA TGTTCCCAAA ACGGTTGTGC CCTAACGGAA   
  
  
- TAGTCTTCCG TTTGTTATCA CGGGTTCTAA ACAACCAGTT ACATCTCCTA CAATGATCCC CTTTCTTCGT   
  
  
- AGCAATAGGG CTTCTTAACG TCAGTCTTCT TCCCTCCTAT TTATTCGTTA GTCGACAAAG GGACCGTCGA   
  
  
- CTACTCCGTC AACAATCCAG ACTTTACAAG CTATCCCATG ACGAAACATC AGCCCCTTTT GTACTACGAC   
  
  
- GAGAGGCCCT TCGAAATGTC TGACTTGATT TATTCTCAGA CGCTTTACGG GGTCAATTCC CCAGATTATC   
  
  
- ACCCTTTCCG GCACCATTCT TTCGCCCTTT CTTCTCCCTA CATCATCTAA ATTCTAGAGA GAATTGGGAT   
  
  
- ACACGTGTTC GTCAACGTAG CTTACTAGTG TCCTCACGTT TACTGGACGA ATCCGTTTAA TCTGTCGTAA   
  
  
- GAAGAGGATC ACCCCTACCA TTGGTTTCTT ACCGTGTAAT AAAACGCCTA CCAGAACTCC GTGCGGAACG   
  
  
- ACCACATCCT TGAGGATATA TATTGACAGA ACATTGCCCA GGCCGTAGCC GACATCTATA GAACTCTCGA   
  
  
- ATGGTGTACA AAGAACGGTG TACGGGTAAA TTCTTTTACC CTTTAAAGAA GAGATTATCT TGCTAATACC   
  
  
- GACACCGTCT CTTACGTTGT ACGGAAGTAT ATTAACTAGA GCCATAACAG ATACCAAAGG TTACCGGAAC   
  
  
- AGATTAAGTC GCTGAAAGTA GATCCGGACC ACCGGGGGGG TTTGAAGCTT ATTGGCCTCA TCTAGAAGGT   
  
  
- GTTGGACCCA AGGCTGGTCG GTTTTCTCAA CTCCTCTGTC CCGCAGCGAA CTTCTTGATA CGTCTCAGTA   
  
  
- AATTACACGG GAAACTCAAG TTACGATATC GTTTCTTCAC CCTTTGTGAA TGGTAACTTC TAGAGTCCTA   
  
  
- GTTATCGCTA CTCAACGAAC AACAGTGGAC ATACAAATCC AAATTTGTAT ATGGACTCCT TTGTCACTGG   
  
  
- CACCTAACGG GATCCCTATG ACAAGAATTG GACTAACCCG CGTATTTGGG TCGACAAAAG TATGTTCCGT   
  
  
- GACAATTGCC CCGAAAGTTA AGAGGGAAAA AGTATCGAGC TAAAGCTCTC CGAGATAAAG TGAAGAGGTG   
  
  
- AGACAAACTA TACGATCTCC GGTTGGACGG TTCCCTGTTA TTCCTCTCCT ACGATTAACT CTCTCTCTAT   
  
  
- AAACCCTCCG TCCGTTACTT ACACTAACGA ACACTCCCAA ATCTCTCCTA TCTTTCCGGT CTCTGCATGT   
  
  
- TCGTCACCGT TCAGGCTTTA CTTTCCCGTC CCAAATCCGT CGACGGAAAT CTAGCGGTCT AAGACCTTTA   
  
  
- CCGATTTTTC TCCCACTTTA GACACATATT GTTTCTAAAG AGGTAACTAC TTCTGCCCGT GACCAACAAC   
  
  
- GACCCTACCT TCCCGTCTTA ACACATGTGT GAGTGATGAA CCTTCGGACG CCTCAT

+     CTAG-motif

| Site Name | Organism | Position | Strand | Matrix score. | sequence | function |
| --- | --- | --- | --- | --- | --- | --- |
| CTAG-motif | Avena sativa | 3296 | - | 9 | ACTAGCAGAA |  |

>HU06G00568.1   
+ -Up\_Stream \_Len000CCCTGG GTCATTAAAA AAAAAGAAAG AAAGAAGATT GCGCTAAAAG TAGATTTTTA   
  
  
+ TCAGATTCAA GCTGCAGGTG ATAACCAAAT TGTCATTAAA GCAATGCAAG CGCATATCAA TACCCTTTTG   
  
  
+ GGAGATAGCT CCGACTGTTG AAGACATTAG GAGCATGATC TCCAACTGTG AATTTGTTTC ATTTACTCAT   
  
  
+ ATTCATATCT ACCATGTGGA TAATATGACT GCAGACTAGA TGGCCAAATT TGAATGCACA CTTATAACTT   
  
  
+ CAACTCTTTC TACTTTTTTC TTCCCCACCT TATCAGGAAT TTCTTTTAAT TGTCGTGGGA GACAACTTGG   
  
  
+ GTAGAACTCT TGCGAAAAGG GCAACTTAAC GTTTTGGTAC TTGCATTTTC CAAAAAAACA AAAAGGCTAA   
  
  
+ ACAATTCTCC GCATGCTGAC TTCACGGTAG ATATGATGTA TTTTTTAACA AAAGACATCT TCTGAATGAA   
  
  
+ ACGCTTAATA TCTTGGACCA TACTAGAAAT TAAATTGCCA CGGTGCATGA ATTTCACCTC GAACTGCTTG   
  
  
+ TATAAAGATC TTGTTATCAC CTTCAGTAAT CAGATACTTG TAACCAAATT GAATGACCAC AAAGACCACA   
  
  
+ TTCCTCACAA TCGTTGCCTC TAGAATAAGA ATGAATGTTT CTTCAAGGTT GAAAGCCCTT GCTAATGCGA   
  
  
+ ACCTGCCTAT ATGCCTAACT GGCTATATAT ATGTGCGTAG AACAGCATGA CATGAGTTAA TAATATAGCA   
  
  
+ GCTAAAGGGA ACTCTAATGT ACACATGATT GTAATTCACC TGCCTCGCTA GCTTACGAGC ATGACTCTTG   
  
  
+ ACTATTATTG TTTTAATTGA TCTTGAATTT TCATAGATTT TACTCCATTG TTTACTCATT TTGTTATAAT   
  
  
+ TAACCACCTG ATTTTAAACT TTAAAATACA CAAAGATTCA TCTAATATAA TTTTTTAGAT AAAGTTATGC   
  
  
+ ATACATATAT ACAAATTAAA TTATTTACGA TCTAATCATT TCCCGTTATA TATTTCACAC TAAAATTTAG   
  
  
+ AAGAGACAAT CTATTAAAAT TACATCACAT GTTTTGGGGG GGGGGGGATA TCAGATATGC ATGACATTCC   
  
  
+ TAATCCAATA ATGCAATGAA ACCCACCGAA TAGTTGAAAT GATTTGTTCT ATCCATGAAC TCACCAAGTC   
  
  
+ ACAATCCATC ACACTCGAAG GATATGCTTT TCTTTTTGAA GGGAATCTTG CTTAATCAAA CCTATAAACT   
  
  
+ TTTAAAAGCA ACAGACAAAA AAGTGATTAT AATGGTAGTG GTAGGATGAA GGCAGCTTTC AGAAAAGCGA   
  
  
+ TTGAATTTTT ATTGTATCCC ATGAAAACCC ATTGAATTTT TCTTTCTTTC TTTGTTTTTG TTACCTGGGT   
  
  
+ TTATGCAAAG GACGGCTGTT GTATTTAATA ATAACAAAAA CATAGGGCTT TGCGGATTGC TAATGCTGGG   
  
  
+ GATGGCATCA GTGCTCGAAT CTGTGTACGC TGTCATAGGA ACCCACTTGC TTCTGCTCAT TTATTTCTGT   
  
  
+ CTCTAGATCC CCTCCTCTCT CTCTCTCTCT CTCTAAGGTG TCTGCTCGTC TGTTCATATT CTCCTGAGGT   
  
  
+ TCTTCTTCAT CTATCTTGTT CTGGGTGTGT GCTGAGGTAC TTCTTTTCTT TATAAATTTT TCATCTTTTG   
  
  
+ TTGTTTTGCT TGGTGTTTAT GATTTTAGTT TTTGTCTTTA AGACGAGATT TGCTGTTAAT AGCAAGATTT   
  
  
+ CTGAACTAGT ATTGATCCTT TTTGTGGTAT ATTTGTTGAA TTTGATTAAT TTGTGTATTG TGTTTAGCTT   
  
  
+ AAGTAACTCG TTGTGATCTA ATTGATGCTC GGGTTTTGAC AGTTTGATTA GGGTTTTCGG TACTGGAGTT   
  
  
+ AAAGTTTTTT TATTTTTTAT AGGAGGAGTT AAAGGTAATA ATAAGAATTG ATGTTTGCTG ATTTTTCCTC   
  
  
+ TTTTCTTTTT TGGTTTCGAT TTGGTGGGCA GTTATCTGTT TATGGTTTTT GCAAATGGAT CCACAATTAG   
  
  
+ AGGAATTATA TGGACCTTTA CATCGAATCA AGTTCAATGA TCAAAGGGTG CCAATTTTAC CAAGTCGCAG   
  
  
+ TGTTGTTACT CCGATGAAAC TCCAAGATTC CAATTTGAAT CCAAGTGTTC CAAATCCAAG TGTTGTGAAT   
  
  
+ CCTCCTCCGT TGGTTCCCCC AAACCCAAAT CTTAATTTAG TTGTGGCATC TCAATATTCT GACATTGAGA   
  
  
+ CAGCCCTGAA CGAGGATTGT GATTTTAGTG ATGTTGTTCT TAAGTATATT AATCAACTTC TTATGGAAGA   
  
  
+ GGATGGGGAA GAGAAACTCC ATACTGATCA TGAACCTTCA GTCGTTGAGG CGGCAGAGAA GTCATTGTAT   
  
  
+ GAGGCGCTCG GGCAGAGATA TCCTCCTTCC GGAAACCGAA ATCAGTTGCC AGATGTTGAG CATGACGGCT   
  
  
+ TGACTGGGAG CAGTGTTAGG GCTCATAGTG GTGCAAGCAG TGTTAGGGCT CATAGTGACG CAAGTGGTGG   
  
  
+ TAGTGGCCTG ACAGGATATG GTTGGTATGG TGATCCTTAT AATTGGAGTC CTCAAAATGT CGTGAATTTT   
  
  
+ ACCATTTCCT CCTCGAAACC CTTATCGTGC AGCTCATCAG ATAGCTCGGG CAGATGGGTT AGTAAGTGGA   
  
  
+ AGTCAAATTC TGATTCACAG CTCAGCTCGG CCATTAATGC AAGTGGTTTA GTGGATGGGC CAGGGGACTC   
  
  
+ TCCTGTGAGT GCTCTTAGTG TGTCTGAAAT ATTCAATGAC AGTCAGTCAA TGTTGCAGTT TCAGAAAGGA   
  
  
+ TTTGAGGAAG CGAATAAATT TCTTCCAAAG AGTTCTTTGT ACAAGGGTTT TGCCAACACG GGATTGCCTT   
  
  
+ ATCAGAAGGC AAACAATAGT GCCCAAGATT TGTTGGTCAA TGTAGAGGAT GTTACTAGGG GAAAGAAGCA   
  
  
+ TCGTTATCCC GAAGAATTGC AGTCAGAAGA AGGGAGGATA AATAAGCAAT CAGCTGTTTC CCTGGCAGCT   
  
  
+ GATGAGGCAG TTGTTAGGTC TGAAATGTTC GATAGGGTAC TGCTTTGTAG TCGGGGAAAA CATGATGCTG   
  
  
+ CTCTCCGGGA AGCTTTACAG ACTGAACTAA ATAAGAGTCT GCGAAATGCC CCAGTTAAGG GGTCTAATAG   
  
  
+ TGGGAAAGGC CGTGGTAAGA AAGCGGGAAA GAAGAGGGAT GTAGTAGATT TAAGATCTCT CTTAACCCTA   
  
  
+ TGTGCACAAG CAGTTGCATC GAATGATCAC AGGAGTGCAA ATGACCTGCT TAGGCAAATT AGACAGCATT   
  
  
+ CTTCTCCTAG TGGGGATGGT AACCAAAGAA TGGCACATTA TTTTGCGGAT GGTCTTGAGG CACGCCTTGC   
  
  
+ TGGTGTAGGA ACTCCTATAT ATAACTGTCT TGTAACGGGT CCGGCATCGG CTGTAGATAT CTTGAGAGCT   
  
  
+ TACCACATGT TTCTTGCCAC ATGCCCATTT AAGAAAATGG GAAATTTCTT CTCTAATAGA ACGATTATGG   
  
  
+ CTGTGGCAGA GAATGCAACA TGCCTTCATA TAATTGATCT CGGTATTGTC TATGGTTTCC AATGGCCTTG   
  
  
+ TCTAATTCAG CGACTTTCAT CTAGGCCTGG TGGCCCCCCC AAACTTCGAA TAACCGGAGT AGATCTTCCA   
  
  
+ CAACCTGGGT TCCGACCAGC CAAAAGAGTT GAGGAGACAG GGCGTCGCTT GAAGAACTAT GCAGAGTCAT   
  
  
+ TTAATGTGCC CTTTGAGTTC AATGCTATAG CAAAGAAGTG GGAAACACTT ACCATTGAAG ATCTCAGGAT   
  
  
+ CAATAGCGAT GAGTTGCTTG TTGTCACCTG TATGTTTAGG TTTAAACATA TACCTGAGGA AACAGTGACC   
  
  
+ GTGGATTGCC CTAGGGATAC TGTTCTTAAC CTGATTGGGC GCATAAACCC AGCTGTTTTC ATACAAGGCA   
  
  
+ CTGTTAACGG GGCTTTCAAT TCTCCCTTTT TCATAGCTCG ATTTCGAGAG GCTCTATTTC ACTTCTCCAC   
  
  
+ TCTGTTTGAT ATGCTAGAGG CCAACCTGCC AAGGGACAAT AAGGAGAGGA TGCTAATTGA GAGAGAGATA   
  
  
+ TTTGGGAGGC AGGCAATGAA TGTGATTGCT TGTGAGGGTT TAGAGAGGAT AGAAAGGCCA GAGACGTACA   
  
  
+ AGCAGTGGCA AGTCCGAAAT GAAAGGGCAG GGTTTAGGCA GCTGCCTTTA GATCGCCAGA TTCTGGAAAT   
  
  
+ GGCTAAAAAG AGGGTGAAAT CTGTGTATAA CAAAGATTTC TCCATTGATG AAGACGGGCA CTGGTTGTTG   
  
  
+ CTGGGATGGA AGGGCAGAAT TGTGTACACA CTCACTACTT GGAAGCCTGC GGAGTA  

- -Up\_Stream \_Len000GGGACC CAGTAATTTT TTTTTCTTTC TTTCTTCTAA CGCGATTTTC ATCTAAAAAT   
  
  
- AGTCTAAGTT CGACGTCCAC TATTGGTTTA ACAGTAATTT CGTTACGTTC GCGTATAGTT ATGGGAAAAC   
  
  
- CCTCTATCGA GGCTGACAAC TTCTGTAATC CTCGTACTAG AGGTTGACAC TTAAACAAAG TAAATGAGTA   
  
  
- TAAGTATAGA TGGTACACCT ATTATACTGA CGTCTGATCT ACCGGTTTAA ACTTACGTGT GAATATTGAA   
  
  
- GTTGAGAAAG ATGAAAAAAG AAGGGGTGGA ATAGTCCTTA AAGAAAATTA ACAGCACCCT CTGTTGAACC   
  
  
- CATCTTGAGA ACGCTTTTCC CGTTGAATTG CAAAACCATG AACGTAAAAG GTTTTTTTGT TTTTCCGATT   
  
  
- TGTTAAGAGG CGTACGACTG AAGTGCCATC TATACTACAT AAAAAATTGT TTTCTGTAGA AGACTTACTT   
  
  
- TGCGAATTAT AGAACCTGGT ATGATCTTTA ATTTAACGGT GCCACGTACT TAAAGTGGAG CTTGACGAAC   
  
  
- ATATTTCTAG AACAATAGTG GAAGTCATTA GTCTATGAAC ATTGGTTTAA CTTACTGGTG TTTCTGGTGT   
  
  
- AAGGAGTGTT AGCAACGGAG ATCTTATTCT TACTTACAAA GAAGTTCCAA CTTTCGGGAA CGATTACGCT   
  
  
- TGGACGGATA TACGGATTGA CCGATATATA TACACGCATC TTGTCGTACT GTACTCAATT ATTATATCGT   
  
  
- CGATTTCCCT TGAGATTACA TGTGTACTAA CATTAAGTGG ACGGAGCGAT CGAATGCTCG TACTGAGAAC   
  
  
- TGATAATAAC AAAATTAACT AGAACTTAAA AGTATCTAAA ATGAGGTAAC AAATGAGTAA AACAATATTA   
  
  
- ATTGGTGGAC TAAAATTTGA AATTTTATGT GTTTCTAAGT AGATTATATT AAAAAATCTA TTTCAATACG   
  
  
- TATGTATATA TGTTTAATTT AATAAATGCT AGATTAGTAA AGGGCAATAT ATAAAGTGTG ATTTTAAATC   
  
  
- TTCTCTGTTA GATAATTTTA ATGTAGTGTA CAAAACCCCC CCCCCCCTAT AGTCTATACG TACTGTAAGG   
  
  
- ATTAGGTTAT TACGTTACTT TGGGTGGCTT ATCAACTTTA CTAAACAAGA TAGGTACTTG AGTGGTTCAG   
  
  
- TGTTAGGTAG TGTGAGCTTC CTATACGAAA AGAAAAACTT CCCTTAGAAC GAATTAGTTT GGATATTTGA   
  
  
- AAATTTTCGT TGTCTGTTTT TTCACTAATA TTACCATCAC CATCCTACTT CCGTCGAAAG TCTTTTCGCT   
  
  
- AACTTAAAAA TAACATAGGG TACTTTTGGG TAACTTAAAA AGAAAGAAAG AAACAAAAAC AATGGACCCA   
  
  
- AATACGTTTC CTGCCGACAA CATAAATTAT TATTGTTTTT GTATCCCGAA ACGCCTAACG ATTACGACCC   
  
  
- CTACCGTAGT CACGAGCTTA GACACATGCG ACAGTATCCT TGGGTGAACG AAGACGAGTA AATAAAGACA   
  
  
- GAGATCTAGG GGAGGAGAGA GAGAGAGAGA GAGATTCCAC AGACGAGCAG ACAAGTATAA GAGGACTCCA   
  
  
- AGAAGAAGTA GATAGAACAA GACCCACACA CGACTCCATG AAGAAAAGAA ATATTTAAAA AGTAGAAAAC   
  
  
- AACAAAACGA ACCACAAATA CTAAAATCAA AAACAGAAAT TCTGCTCTAA ACGACAATTA TCGTTCTAAA   
  
  
- GACTTGATCA TAACTAGGAA AAACACCATA TAAACAACTT AAACTAATTA AACACATAAC ACAAATCGAA   
  
  
- TTCATTGAGC AACACTAGAT TAACTACGAG CCCAAAACTG TCAAACTAAT CCCAAAAGCC ATGACCTCAA   
  
  
- TTTCAAAAAA ATAAAAAATA TCCTCCTCAA TTTCCATTAT TATTCTTAAC TACAAACGAC TAAAAAGGAG   
  
  
- AAAAGAAAAA ACCAAAGCTA AACCACCCGT CAATAGACAA ATACCAAAAA CGTTTACCTA GGTGTTAATC   
  
  
- TCCTTAATAT ACCTGGAAAT GTAGCTTAGT TCAAGTTACT AGTTTCCCAC GGTTAAAATG GTTCAGCGTC   
  
  
- ACAACAATGA GGCTACTTTG AGGTTCTAAG GTTAAACTTA GGTTCACAAG GTTTAGGTTC ACAACACTTA   
  
  
- GGAGGAGGCA ACCAAGGGGG TTTGGGTTTA GAATTAAATC AACACCGTAG AGTTATAAGA CTGTAACTCT   
  
  
- GTCGGGACTT GCTCCTAACA CTAAAATCAC TACAACAAGA ATTCATATAA TTAGTTGAAG AATACCTTCT   
  
  
- CCTACCCCTT CTCTTTGAGG TATGACTAGT ACTTGGAAGT CAGCAACTCC GCCGTCTCTT CAGTAACATA   
  
  
- CTCCGCGAGC CCGTCTCTAT AGGAGGAAGG CCTTTGGCTT TAGTCAACGG TCTACAACTC GTACTGCCGA   
  
  
- ACTGACCCTC GTCACAATCC CGAGTATCAC CACGTTCGTC ACAATCCCGA GTATCACTGC GTTCACCACC   
  
  
- ATCACCGGAC TGTCCTATAC CAACCATACC ACTAGGAATA TTAACCTCAG GAGTTTTACA GCACTTAAAA   
  
  
- TGGTAAAGGA GGAGCTTTGG GAATAGCACG TCGAGTAGTC TATCGAGCCC GTCTACCCAA TCATTCACCT   
  
  
- TCAGTTTAAG ACTAAGTGTC GAGTCGAGCC GGTAATTACG TTCACCAAAT CACCTACCCG GTCCCCTGAG   
  
  
- AGGACACTCA CGAGAATCAC ACAGACTTTA TAAGTTACTG TCAGTCAGTT ACAACGTCAA AGTCTTTCCT   
  
  
- AAACTCCTTC GCTTATTTAA AGAAGGTTTC TCAAGAAACA TGTTCCCAAA ACGGTTGTGC CCTAACGGAA   
  
  
- TAGTCTTCCG TTTGTTATCA CGGGTTCTAA ACAACCAGTT ACATCTCCTA CAATGATCCC CTTTCTTCGT   
  
  
- AGCAATAGGG CTTCTTAACG TCAGTCTTCT TCCCTCCTAT TTATTCGTTA GTCGACAAAG GGACCGTCGA   
  
  
- CTACTCCGTC AACAATCCAG ACTTTACAAG CTATCCCATG ACGAAACATC AGCCCCTTTT GTACTACGAC   
  
  
- GAGAGGCCCT TCGAAATGTC TGACTTGATT TATTCTCAGA CGCTTTACGG GGTCAATTCC CCAGATTATC   
  
  
- ACCCTTTCCG GCACCATTCT TTCGCCCTTT CTTCTCCCTA CATCATCTAA ATTCTAGAGA GAATTGGGAT   
  
  
- ACACGTGTTC GTCAACGTAG CTTACTAGTG TCCTCACGTT TACTGGACGA ATCCGTTTAA TCTGTCGTAA   
  
  
- GAAGAGGATC ACCCCTACCA TTGGTTTCTT ACCGTGTAAT AAAACGCCTA CCAGAACTCC GTGCGGAACG   
  
  
- ACCACATCCT TGAGGATATA TATTGACAGA ACATTGCCCA GGCCGTAGCC GACATCTATA GAACTCTCGA   
  
  
- ATGGTGTACA AAGAACGGTG TACGGGTAAA TTCTTTTACC CTTTAAAGAA GAGATTATCT TGCTAATACC   
  
  
- GACACCGTCT CTTACGTTGT ACGGAAGTAT ATTAACTAGA GCCATAACAG ATACCAAAGG TTACCGGAAC   
  
  
- AGATTAAGTC GCTGAAAGTA GATCCGGACC ACCGGGGGGG TTTGAAGCTT ATTGGCCTCA TCTAGAAGGT   
  
  
- GTTGGACCCA AGGCTGGTCG GTTTTCTCAA CTCCTCTGTC CCGCAGCGAA CTTCTTGATA CGTCTCAGTA   
  
  
- AATTACACGG GAAACTCAAG TTACGATATC GTTTCTTCAC CCTTTGTGAA TGGTAACTTC TAGAGTCCTA   
  
  
- GTTATCGCTA CTCAACGAAC AACAGTGGAC ATACAAATCC AAATTTGTAT ATGGACTCCT TTGTCACTGG   
  
  
- CACCTAACGG GATCCCTATG ACAAGAATTG GACTAACCCG CGTATTTGGG TCGACAAAAG TATGTTCCGT   
  
  
- GACAATTGCC CCGAAAGTTA AGAGGGAAAA AGTATCGAGC TAAAGCTCTC CGAGATAAAG TGAAGAGGTG   
  
  
- AGACAAACTA TACGATCTCC GGTTGGACGG TTCCCTGTTA TTCCTCTCCT ACGATTAACT CTCTCTCTAT   
  
  
- AAACCCTCCG TCCGTTACTT ACACTAACGA ACACTCCCAA ATCTCTCCTA TCTTTCCGGT CTCTGCATGT   
  
  
- TCGTCACCGT TCAGGCTTTA CTTTCCCGTC CCAAATCCGT CGACGGAAAT CTAGCGGTCT AAGACCTTTA   
  
  
- CCGATTTTTC TCCCACTTTA GACACATATT GTTTCTAAAG AGGTAACTAC TTCTGCCCGT GACCAACAAC   
  
  
- GACCCTACCT TCCCGTCTTA ACACATGTGT GAGTGATGAA CCTTCGGACG CCTCAT

+     DRE1

| Site Name | Organism | Position | Strand | Matrix score. | sequence | function |
| --- | --- | --- | --- | --- | --- | --- |
| DRE1 | Zea mays | 3542 | - | 7 | ACCGAGA |  |

>HU06G00568.1   
+ -Up\_Stream \_Len000CCCTGG GTCATTAAAA AAAAAGAAAG AAAGAAGATT GCGCTAAAAG TAGATTTTTA   
  
  
+ TCAGATTCAA GCTGCAGGTG ATAACCAAAT TGTCATTAAA GCAATGCAAG CGCATATCAA TACCCTTTTG   
  
  
+ GGAGATAGCT CCGACTGTTG AAGACATTAG GAGCATGATC TCCAACTGTG AATTTGTTTC ATTTACTCAT   
  
  
+ ATTCATATCT ACCATGTGGA TAATATGACT GCAGACTAGA TGGCCAAATT TGAATGCACA CTTATAACTT   
  
  
+ CAACTCTTTC TACTTTTTTC TTCCCCACCT TATCAGGAAT TTCTTTTAAT TGTCGTGGGA GACAACTTGG   
  
  
+ GTAGAACTCT TGCGAAAAGG GCAACTTAAC GTTTTGGTAC TTGCATTTTC CAAAAAAACA AAAAGGCTAA   
  
  
+ ACAATTCTCC GCATGCTGAC TTCACGGTAG ATATGATGTA TTTTTTAACA AAAGACATCT TCTGAATGAA   
  
  
+ ACGCTTAATA TCTTGGACCA TACTAGAAAT TAAATTGCCA CGGTGCATGA ATTTCACCTC GAACTGCTTG   
  
  
+ TATAAAGATC TTGTTATCAC CTTCAGTAAT CAGATACTTG TAACCAAATT GAATGACCAC AAAGACCACA   
  
  
+ TTCCTCACAA TCGTTGCCTC TAGAATAAGA ATGAATGTTT CTTCAAGGTT GAAAGCCCTT GCTAATGCGA   
  
  
+ ACCTGCCTAT ATGCCTAACT GGCTATATAT ATGTGCGTAG AACAGCATGA CATGAGTTAA TAATATAGCA   
  
  
+ GCTAAAGGGA ACTCTAATGT ACACATGATT GTAATTCACC TGCCTCGCTA GCTTACGAGC ATGACTCTTG   
  
  
+ ACTATTATTG TTTTAATTGA TCTTGAATTT TCATAGATTT TACTCCATTG TTTACTCATT TTGTTATAAT   
  
  
+ TAACCACCTG ATTTTAAACT TTAAAATACA CAAAGATTCA TCTAATATAA TTTTTTAGAT AAAGTTATGC   
  
  
+ ATACATATAT ACAAATTAAA TTATTTACGA TCTAATCATT TCCCGTTATA TATTTCACAC TAAAATTTAG   
  
  
+ AAGAGACAAT CTATTAAAAT TACATCACAT GTTTTGGGGG GGGGGGGATA TCAGATATGC ATGACATTCC   
  
  
+ TAATCCAATA ATGCAATGAA ACCCACCGAA TAGTTGAAAT GATTTGTTCT ATCCATGAAC TCACCAAGTC   
  
  
+ ACAATCCATC ACACTCGAAG GATATGCTTT TCTTTTTGAA GGGAATCTTG CTTAATCAAA CCTATAAACT   
  
  
+ TTTAAAAGCA ACAGACAAAA AAGTGATTAT AATGGTAGTG GTAGGATGAA GGCAGCTTTC AGAAAAGCGA   
  
  
+ TTGAATTTTT ATTGTATCCC ATGAAAACCC ATTGAATTTT TCTTTCTTTC TTTGTTTTTG TTACCTGGGT   
  
  
+ TTATGCAAAG GACGGCTGTT GTATTTAATA ATAACAAAAA CATAGGGCTT TGCGGATTGC TAATGCTGGG   
  
  
+ GATGGCATCA GTGCTCGAAT CTGTGTACGC TGTCATAGGA ACCCACTTGC TTCTGCTCAT TTATTTCTGT   
  
  
+ CTCTAGATCC CCTCCTCTCT CTCTCTCTCT CTCTAAGGTG TCTGCTCGTC TGTTCATATT CTCCTGAGGT   
  
  
+ TCTTCTTCAT CTATCTTGTT CTGGGTGTGT GCTGAGGTAC TTCTTTTCTT TATAAATTTT TCATCTTTTG   
  
  
+ TTGTTTTGCT TGGTGTTTAT GATTTTAGTT TTTGTCTTTA AGACGAGATT TGCTGTTAAT AGCAAGATTT   
  
  
+ CTGAACTAGT ATTGATCCTT TTTGTGGTAT ATTTGTTGAA TTTGATTAAT TTGTGTATTG TGTTTAGCTT   
  
  
+ AAGTAACTCG TTGTGATCTA ATTGATGCTC GGGTTTTGAC AGTTTGATTA GGGTTTTCGG TACTGGAGTT   
  
  
+ AAAGTTTTTT TATTTTTTAT AGGAGGAGTT AAAGGTAATA ATAAGAATTG ATGTTTGCTG ATTTTTCCTC   
  
  
+ TTTTCTTTTT TGGTTTCGAT TTGGTGGGCA GTTATCTGTT TATGGTTTTT GCAAATGGAT CCACAATTAG   
  
  
+ AGGAATTATA TGGACCTTTA CATCGAATCA AGTTCAATGA TCAAAGGGTG CCAATTTTAC CAAGTCGCAG   
  
  
+ TGTTGTTACT CCGATGAAAC TCCAAGATTC CAATTTGAAT CCAAGTGTTC CAAATCCAAG TGTTGTGAAT   
  
  
+ CCTCCTCCGT TGGTTCCCCC AAACCCAAAT CTTAATTTAG TTGTGGCATC TCAATATTCT GACATTGAGA   
  
  
+ CAGCCCTGAA CGAGGATTGT GATTTTAGTG ATGTTGTTCT TAAGTATATT AATCAACTTC TTATGGAAGA   
  
  
+ GGATGGGGAA GAGAAACTCC ATACTGATCA TGAACCTTCA GTCGTTGAGG CGGCAGAGAA GTCATTGTAT   
  
  
+ GAGGCGCTCG GGCAGAGATA TCCTCCTTCC GGAAACCGAA ATCAGTTGCC AGATGTTGAG CATGACGGCT   
  
  
+ TGACTGGGAG CAGTGTTAGG GCTCATAGTG GTGCAAGCAG TGTTAGGGCT CATAGTGACG CAAGTGGTGG   
  
  
+ TAGTGGCCTG ACAGGATATG GTTGGTATGG TGATCCTTAT AATTGGAGTC CTCAAAATGT CGTGAATTTT   
  
  
+ ACCATTTCCT CCTCGAAACC CTTATCGTGC AGCTCATCAG ATAGCTCGGG CAGATGGGTT AGTAAGTGGA   
  
  
+ AGTCAAATTC TGATTCACAG CTCAGCTCGG CCATTAATGC AAGTGGTTTA GTGGATGGGC CAGGGGACTC   
  
  
+ TCCTGTGAGT GCTCTTAGTG TGTCTGAAAT ATTCAATGAC AGTCAGTCAA TGTTGCAGTT TCAGAAAGGA   
  
  
+ TTTGAGGAAG CGAATAAATT TCTTCCAAAG AGTTCTTTGT ACAAGGGTTT TGCCAACACG GGATTGCCTT   
  
  
+ ATCAGAAGGC AAACAATAGT GCCCAAGATT TGTTGGTCAA TGTAGAGGAT GTTACTAGGG GAAAGAAGCA   
  
  
+ TCGTTATCCC GAAGAATTGC AGTCAGAAGA AGGGAGGATA AATAAGCAAT CAGCTGTTTC CCTGGCAGCT   
  
  
+ GATGAGGCAG TTGTTAGGTC TGAAATGTTC GATAGGGTAC TGCTTTGTAG TCGGGGAAAA CATGATGCTG   
  
  
+ CTCTCCGGGA AGCTTTACAG ACTGAACTAA ATAAGAGTCT GCGAAATGCC CCAGTTAAGG GGTCTAATAG   
  
  
+ TGGGAAAGGC CGTGGTAAGA AAGCGGGAAA GAAGAGGGAT GTAGTAGATT TAAGATCTCT CTTAACCCTA   
  
  
+ TGTGCACAAG CAGTTGCATC GAATGATCAC AGGAGTGCAA ATGACCTGCT TAGGCAAATT AGACAGCATT   
  
  
+ CTTCTCCTAG TGGGGATGGT AACCAAAGAA TGGCACATTA TTTTGCGGAT GGTCTTGAGG CACGCCTTGC   
  
  
+ TGGTGTAGGA ACTCCTATAT ATAACTGTCT TGTAACGGGT CCGGCATCGG CTGTAGATAT CTTGAGAGCT   
  
  
+ TACCACATGT TTCTTGCCAC ATGCCCATTT AAGAAAATGG GAAATTTCTT CTCTAATAGA ACGATTATGG   
  
  
+ CTGTGGCAGA GAATGCAACA TGCCTTCATA TAATTGATCT CGGTATTGTC TATGGTTTCC AATGGCCTTG   
  
  
+ TCTAATTCAG CGACTTTCAT CTAGGCCTGG TGGCCCCCCC AAACTTCGAA TAACCGGAGT AGATCTTCCA   
  
  
+ CAACCTGGGT TCCGACCAGC CAAAAGAGTT GAGGAGACAG GGCGTCGCTT GAAGAACTAT GCAGAGTCAT   
  
  
+ TTAATGTGCC CTTTGAGTTC AATGCTATAG CAAAGAAGTG GGAAACACTT ACCATTGAAG ATCTCAGGAT   
  
  
+ CAATAGCGAT GAGTTGCTTG TTGTCACCTG TATGTTTAGG TTTAAACATA TACCTGAGGA AACAGTGACC   
  
  
+ GTGGATTGCC CTAGGGATAC TGTTCTTAAC CTGATTGGGC GCATAAACCC AGCTGTTTTC ATACAAGGCA   
  
  
+ CTGTTAACGG GGCTTTCAAT TCTCCCTTTT TCATAGCTCG ATTTCGAGAG GCTCTATTTC ACTTCTCCAC   
  
  
+ TCTGTTTGAT ATGCTAGAGG CCAACCTGCC AAGGGACAAT AAGGAGAGGA TGCTAATTGA GAGAGAGATA   
  
  
+ TTTGGGAGGC AGGCAATGAA TGTGATTGCT TGTGAGGGTT TAGAGAGGAT AGAAAGGCCA GAGACGTACA   
  
  
+ AGCAGTGGCA AGTCCGAAAT GAAAGGGCAG GGTTTAGGCA GCTGCCTTTA GATCGCCAGA TTCTGGAAAT   
  
  
+ GGCTAAAAAG AGGGTGAAAT CTGTGTATAA CAAAGATTTC TCCATTGATG AAGACGGGCA CTGGTTGTTG   
  
  
+ CTGGGATGGA AGGGCAGAAT TGTGTACACA CTCACTACTT GGAAGCCTGC GGAGTA  

- -Up\_Stream \_Len000GGGACC CAGTAATTTT TTTTTCTTTC TTTCTTCTAA CGCGATTTTC ATCTAAAAAT   
  
  
- AGTCTAAGTT CGACGTCCAC TATTGGTTTA ACAGTAATTT CGTTACGTTC GCGTATAGTT ATGGGAAAAC   
  
  
- CCTCTATCGA GGCTGACAAC TTCTGTAATC CTCGTACTAG AGGTTGACAC TTAAACAAAG TAAATGAGTA   
  
  
- TAAGTATAGA TGGTACACCT ATTATACTGA CGTCTGATCT ACCGGTTTAA ACTTACGTGT GAATATTGAA   
  
  
- GTTGAGAAAG ATGAAAAAAG AAGGGGTGGA ATAGTCCTTA AAGAAAATTA ACAGCACCCT CTGTTGAACC   
  
  
- CATCTTGAGA ACGCTTTTCC CGTTGAATTG CAAAACCATG AACGTAAAAG GTTTTTTTGT TTTTCCGATT   
  
  
- TGTTAAGAGG CGTACGACTG AAGTGCCATC TATACTACAT AAAAAATTGT TTTCTGTAGA AGACTTACTT   
  
  
- TGCGAATTAT AGAACCTGGT ATGATCTTTA ATTTAACGGT GCCACGTACT TAAAGTGGAG CTTGACGAAC   
  
  
- ATATTTCTAG AACAATAGTG GAAGTCATTA GTCTATGAAC ATTGGTTTAA CTTACTGGTG TTTCTGGTGT   
  
  
- AAGGAGTGTT AGCAACGGAG ATCTTATTCT TACTTACAAA GAAGTTCCAA CTTTCGGGAA CGATTACGCT   
  
  
- TGGACGGATA TACGGATTGA CCGATATATA TACACGCATC TTGTCGTACT GTACTCAATT ATTATATCGT   
  
  
- CGATTTCCCT TGAGATTACA TGTGTACTAA CATTAAGTGG ACGGAGCGAT CGAATGCTCG TACTGAGAAC   
  
  
- TGATAATAAC AAAATTAACT AGAACTTAAA AGTATCTAAA ATGAGGTAAC AAATGAGTAA AACAATATTA   
  
  
- ATTGGTGGAC TAAAATTTGA AATTTTATGT GTTTCTAAGT AGATTATATT AAAAAATCTA TTTCAATACG   
  
  
- TATGTATATA TGTTTAATTT AATAAATGCT AGATTAGTAA AGGGCAATAT ATAAAGTGTG ATTTTAAATC   
  
  
- TTCTCTGTTA GATAATTTTA ATGTAGTGTA CAAAACCCCC CCCCCCCTAT AGTCTATACG TACTGTAAGG   
  
  
- ATTAGGTTAT TACGTTACTT TGGGTGGCTT ATCAACTTTA CTAAACAAGA TAGGTACTTG AGTGGTTCAG   
  
  
- TGTTAGGTAG TGTGAGCTTC CTATACGAAA AGAAAAACTT CCCTTAGAAC GAATTAGTTT GGATATTTGA   
  
  
- AAATTTTCGT TGTCTGTTTT TTCACTAATA TTACCATCAC CATCCTACTT CCGTCGAAAG TCTTTTCGCT   
  
  
- AACTTAAAAA TAACATAGGG TACTTTTGGG TAACTTAAAA AGAAAGAAAG AAACAAAAAC AATGGACCCA   
  
  
- AATACGTTTC CTGCCGACAA CATAAATTAT TATTGTTTTT GTATCCCGAA ACGCCTAACG ATTACGACCC   
  
  
- CTACCGTAGT CACGAGCTTA GACACATGCG ACAGTATCCT TGGGTGAACG AAGACGAGTA AATAAAGACA   
  
  
- GAGATCTAGG GGAGGAGAGA GAGAGAGAGA GAGATTCCAC AGACGAGCAG ACAAGTATAA GAGGACTCCA   
  
  
- AGAAGAAGTA GATAGAACAA GACCCACACA CGACTCCATG AAGAAAAGAA ATATTTAAAA AGTAGAAAAC   
  
  
- AACAAAACGA ACCACAAATA CTAAAATCAA AAACAGAAAT TCTGCTCTAA ACGACAATTA TCGTTCTAAA   
  
  
- GACTTGATCA TAACTAGGAA AAACACCATA TAAACAACTT AAACTAATTA AACACATAAC ACAAATCGAA   
  
  
- TTCATTGAGC AACACTAGAT TAACTACGAG CCCAAAACTG TCAAACTAAT CCCAAAAGCC ATGACCTCAA   
  
  
- TTTCAAAAAA ATAAAAAATA TCCTCCTCAA TTTCCATTAT TATTCTTAAC TACAAACGAC TAAAAAGGAG   
  
  
- AAAAGAAAAA ACCAAAGCTA AACCACCCGT CAATAGACAA ATACCAAAAA CGTTTACCTA GGTGTTAATC   
  
  
- TCCTTAATAT ACCTGGAAAT GTAGCTTAGT TCAAGTTACT AGTTTCCCAC GGTTAAAATG GTTCAGCGTC   
  
  
- ACAACAATGA GGCTACTTTG AGGTTCTAAG GTTAAACTTA GGTTCACAAG GTTTAGGTTC ACAACACTTA   
  
  
- GGAGGAGGCA ACCAAGGGGG TTTGGGTTTA GAATTAAATC AACACCGTAG AGTTATAAGA CTGTAACTCT   
  
  
- GTCGGGACTT GCTCCTAACA CTAAAATCAC TACAACAAGA ATTCATATAA TTAGTTGAAG AATACCTTCT   
  
  
- CCTACCCCTT CTCTTTGAGG TATGACTAGT ACTTGGAAGT CAGCAACTCC GCCGTCTCTT CAGTAACATA   
  
  
- CTCCGCGAGC CCGTCTCTAT AGGAGGAAGG CCTTTGGCTT TAGTCAACGG TCTACAACTC GTACTGCCGA   
  
  
- ACTGACCCTC GTCACAATCC CGAGTATCAC CACGTTCGTC ACAATCCCGA GTATCACTGC GTTCACCACC   
  
  
- ATCACCGGAC TGTCCTATAC CAACCATACC ACTAGGAATA TTAACCTCAG GAGTTTTACA GCACTTAAAA   
  
  
- TGGTAAAGGA GGAGCTTTGG GAATAGCACG TCGAGTAGTC TATCGAGCCC GTCTACCCAA TCATTCACCT   
  
  
- TCAGTTTAAG ACTAAGTGTC GAGTCGAGCC GGTAATTACG TTCACCAAAT CACCTACCCG GTCCCCTGAG   
  
  
- AGGACACTCA CGAGAATCAC ACAGACTTTA TAAGTTACTG TCAGTCAGTT ACAACGTCAA AGTCTTTCCT   
  
  
- AAACTCCTTC GCTTATTTAA AGAAGGTTTC TCAAGAAACA TGTTCCCAAA ACGGTTGTGC CCTAACGGAA   
  
  
- TAGTCTTCCG TTTGTTATCA CGGGTTCTAA ACAACCAGTT ACATCTCCTA CAATGATCCC CTTTCTTCGT   
  
  
- AGCAATAGGG CTTCTTAACG TCAGTCTTCT TCCCTCCTAT TTATTCGTTA GTCGACAAAG GGACCGTCGA   
  
  
- CTACTCCGTC AACAATCCAG ACTTTACAAG CTATCCCATG ACGAAACATC AGCCCCTTTT GTACTACGAC   
  
  
- GAGAGGCCCT TCGAAATGTC TGACTTGATT TATTCTCAGA CGCTTTACGG GGTCAATTCC CCAGATTATC   
  
  
- ACCCTTTCCG GCACCATTCT TTCGCCCTTT CTTCTCCCTA CATCATCTAA ATTCTAGAGA GAATTGGGAT   
  
  
- ACACGTGTTC GTCAACGTAG CTTACTAGTG TCCTCACGTT TACTGGACGA ATCCGTTTAA TCTGTCGTAA   
  
  
- GAAGAGGATC ACCCCTACCA TTGGTTTCTT ACCGTGTAAT AAAACGCCTA CCAGAACTCC GTGCGGAACG   
  
  
- ACCACATCCT TGAGGATATA TATTGACAGA ACATTGCCCA GGCCGTAGCC GACATCTATA GAACTCTCGA   
  
  
- ATGGTGTACA AAGAACGGTG TACGGGTAAA TTCTTTTACC CTTTAAAGAA GAGATTATCT TGCTAATACC   
  
  
- GACACCGTCT CTTACGTTGT ACGGAAGTAT ATTAACTAGA GCCATAACAG ATACCAAAGG TTACCGGAAC   
  
  
- AGATTAAGTC GCTGAAAGTA GATCCGGACC ACCGGGGGGG TTTGAAGCTT ATTGGCCTCA TCTAGAAGGT   
  
  
- GTTGGACCCA AGGCTGGTCG GTTTTCTCAA CTCCTCTGTC CCGCAGCGAA CTTCTTGATA CGTCTCAGTA   
  
  
- AATTACACGG GAAACTCAAG TTACGATATC GTTTCTTCAC CCTTTGTGAA TGGTAACTTC TAGAGTCCTA   
  
  
- GTTATCGCTA CTCAACGAAC AACAGTGGAC ATACAAATCC AAATTTGTAT ATGGACTCCT TTGTCACTGG   
  
  
- CACCTAACGG GATCCCTATG ACAAGAATTG GACTAACCCG CGTATTTGGG TCGACAAAAG TATGTTCCGT   
  
  
- GACAATTGCC CCGAAAGTTA AGAGGGAAAA AGTATCGAGC TAAAGCTCTC CGAGATAAAG TGAAGAGGTG   
  
  
- AGACAAACTA TACGATCTCC GGTTGGACGG TTCCCTGTTA TTCCTCTCCT ACGATTAACT CTCTCTCTAT   
  
  
- AAACCCTCCG TCCGTTACTT ACACTAACGA ACACTCCCAA ATCTCTCCTA TCTTTCCGGT CTCTGCATGT   
  
  
- TCGTCACCGT TCAGGCTTTA CTTTCCCGTC CCAAATCCGT CGACGGAAAT CTAGCGGTCT AAGACCTTTA   
  
  
- CCGATTTTTC TCCCACTTTA GACACATATT GTTTCTAAAG AGGTAACTAC TTCTGCCCGT GACCAACAAC   
  
  
- GACCCTACCT TCCCGTCTTA ACACATGTGT GAGTGATGAA CCTTCGGACG CCTCAT

+     ERE

| Site Name | Organism | Position | Strand | Matrix score. | sequence | function |
| --- | --- | --- | --- | --- | --- | --- |
| ERE | Nicotiana glutinos | 934 | - | 8 | ATTTTAAA |  |
| ERE | Nicotiana glutinos | 925 | + | 8 | ATTTTAAA |  |

>HU06G00568.1   
+ -Up\_Stream \_Len000CCCTGG GTCATTAAAA AAAAAGAAAG AAAGAAGATT GCGCTAAAAG TAGATTTTTA   
  
  
+ TCAGATTCAA GCTGCAGGTG ATAACCAAAT TGTCATTAAA GCAATGCAAG CGCATATCAA TACCCTTTTG   
  
  
+ GGAGATAGCT CCGACTGTTG AAGACATTAG GAGCATGATC TCCAACTGTG AATTTGTTTC ATTTACTCAT   
  
  
+ ATTCATATCT ACCATGTGGA TAATATGACT GCAGACTAGA TGGCCAAATT TGAATGCACA CTTATAACTT   
  
  
+ CAACTCTTTC TACTTTTTTC TTCCCCACCT TATCAGGAAT TTCTTTTAAT TGTCGTGGGA GACAACTTGG   
  
  
+ GTAGAACTCT TGCGAAAAGG GCAACTTAAC GTTTTGGTAC TTGCATTTTC CAAAAAAACA AAAAGGCTAA   
  
  
+ ACAATTCTCC GCATGCTGAC TTCACGGTAG ATATGATGTA TTTTTTAACA AAAGACATCT TCTGAATGAA   
  
  
+ ACGCTTAATA TCTTGGACCA TACTAGAAAT TAAATTGCCA CGGTGCATGA ATTTCACCTC GAACTGCTTG   
  
  
+ TATAAAGATC TTGTTATCAC CTTCAGTAAT CAGATACTTG TAACCAAATT GAATGACCAC AAAGACCACA   
  
  
+ TTCCTCACAA TCGTTGCCTC TAGAATAAGA ATGAATGTTT CTTCAAGGTT GAAAGCCCTT GCTAATGCGA   
  
  
+ ACCTGCCTAT ATGCCTAACT GGCTATATAT ATGTGCGTAG AACAGCATGA CATGAGTTAA TAATATAGCA   
  
  
+ GCTAAAGGGA ACTCTAATGT ACACATGATT GTAATTCACC TGCCTCGCTA GCTTACGAGC ATGACTCTTG   
  
  
+ ACTATTATTG TTTTAATTGA TCTTGAATTT TCATAGATTT TACTCCATTG TTTACTCATT TTGTTATAAT   
  
  
+ TAACCACCTG ATTTTAAACT TTAAAATACA CAAAGATTCA TCTAATATAA TTTTTTAGAT AAAGTTATGC   
  
  
+ ATACATATAT ACAAATTAAA TTATTTACGA TCTAATCATT TCCCGTTATA TATTTCACAC TAAAATTTAG   
  
  
+ AAGAGACAAT CTATTAAAAT TACATCACAT GTTTTGGGGG GGGGGGGATA TCAGATATGC ATGACATTCC   
  
  
+ TAATCCAATA ATGCAATGAA ACCCACCGAA TAGTTGAAAT GATTTGTTCT ATCCATGAAC TCACCAAGTC   
  
  
+ ACAATCCATC ACACTCGAAG GATATGCTTT TCTTTTTGAA GGGAATCTTG CTTAATCAAA CCTATAAACT   
  
  
+ TTTAAAAGCA ACAGACAAAA AAGTGATTAT AATGGTAGTG GTAGGATGAA GGCAGCTTTC AGAAAAGCGA   
  
  
+ TTGAATTTTT ATTGTATCCC ATGAAAACCC ATTGAATTTT TCTTTCTTTC TTTGTTTTTG TTACCTGGGT   
  
  
+ TTATGCAAAG GACGGCTGTT GTATTTAATA ATAACAAAAA CATAGGGCTT TGCGGATTGC TAATGCTGGG   
  
  
+ GATGGCATCA GTGCTCGAAT CTGTGTACGC TGTCATAGGA ACCCACTTGC TTCTGCTCAT TTATTTCTGT   
  
  
+ CTCTAGATCC CCTCCTCTCT CTCTCTCTCT CTCTAAGGTG TCTGCTCGTC TGTTCATATT CTCCTGAGGT   
  
  
+ TCTTCTTCAT CTATCTTGTT CTGGGTGTGT GCTGAGGTAC TTCTTTTCTT TATAAATTTT TCATCTTTTG   
  
  
+ TTGTTTTGCT TGGTGTTTAT GATTTTAGTT TTTGTCTTTA AGACGAGATT TGCTGTTAAT AGCAAGATTT   
  
  
+ CTGAACTAGT ATTGATCCTT TTTGTGGTAT ATTTGTTGAA TTTGATTAAT TTGTGTATTG TGTTTAGCTT   
  
  
+ AAGTAACTCG TTGTGATCTA ATTGATGCTC GGGTTTTGAC AGTTTGATTA GGGTTTTCGG TACTGGAGTT   
  
  
+ AAAGTTTTTT TATTTTTTAT AGGAGGAGTT AAAGGTAATA ATAAGAATTG ATGTTTGCTG ATTTTTCCTC   
  
  
+ TTTTCTTTTT TGGTTTCGAT TTGGTGGGCA GTTATCTGTT TATGGTTTTT GCAAATGGAT CCACAATTAG   
  
  
+ AGGAATTATA TGGACCTTTA CATCGAATCA AGTTCAATGA TCAAAGGGTG CCAATTTTAC CAAGTCGCAG   
  
  
+ TGTTGTTACT CCGATGAAAC TCCAAGATTC CAATTTGAAT CCAAGTGTTC CAAATCCAAG TGTTGTGAAT   
  
  
+ CCTCCTCCGT TGGTTCCCCC AAACCCAAAT CTTAATTTAG TTGTGGCATC TCAATATTCT GACATTGAGA   
  
  
+ CAGCCCTGAA CGAGGATTGT GATTTTAGTG ATGTTGTTCT TAAGTATATT AATCAACTTC TTATGGAAGA   
  
  
+ GGATGGGGAA GAGAAACTCC ATACTGATCA TGAACCTTCA GTCGTTGAGG CGGCAGAGAA GTCATTGTAT   
  
  
+ GAGGCGCTCG GGCAGAGATA TCCTCCTTCC GGAAACCGAA ATCAGTTGCC AGATGTTGAG CATGACGGCT   
  
  
+ TGACTGGGAG CAGTGTTAGG GCTCATAGTG GTGCAAGCAG TGTTAGGGCT CATAGTGACG CAAGTGGTGG   
  
  
+ TAGTGGCCTG ACAGGATATG GTTGGTATGG TGATCCTTAT AATTGGAGTC CTCAAAATGT CGTGAATTTT   
  
  
+ ACCATTTCCT CCTCGAAACC CTTATCGTGC AGCTCATCAG ATAGCTCGGG CAGATGGGTT AGTAAGTGGA   
  
  
+ AGTCAAATTC TGATTCACAG CTCAGCTCGG CCATTAATGC AAGTGGTTTA GTGGATGGGC CAGGGGACTC   
  
  
+ TCCTGTGAGT GCTCTTAGTG TGTCTGAAAT ATTCAATGAC AGTCAGTCAA TGTTGCAGTT TCAGAAAGGA   
  
  
+ TTTGAGGAAG CGAATAAATT TCTTCCAAAG AGTTCTTTGT ACAAGGGTTT TGCCAACACG GGATTGCCTT   
  
  
+ ATCAGAAGGC AAACAATAGT GCCCAAGATT TGTTGGTCAA TGTAGAGGAT GTTACTAGGG GAAAGAAGCA   
  
  
+ TCGTTATCCC GAAGAATTGC AGTCAGAAGA AGGGAGGATA AATAAGCAAT CAGCTGTTTC CCTGGCAGCT   
  
  
+ GATGAGGCAG TTGTTAGGTC TGAAATGTTC GATAGGGTAC TGCTTTGTAG TCGGGGAAAA CATGATGCTG   
  
  
+ CTCTCCGGGA AGCTTTACAG ACTGAACTAA ATAAGAGTCT GCGAAATGCC CCAGTTAAGG GGTCTAATAG   
  
  
+ TGGGAAAGGC CGTGGTAAGA AAGCGGGAAA GAAGAGGGAT GTAGTAGATT TAAGATCTCT CTTAACCCTA   
  
  
+ TGTGCACAAG CAGTTGCATC GAATGATCAC AGGAGTGCAA ATGACCTGCT TAGGCAAATT AGACAGCATT   
  
  
+ CTTCTCCTAG TGGGGATGGT AACCAAAGAA TGGCACATTA TTTTGCGGAT GGTCTTGAGG CACGCCTTGC   
  
  
+ TGGTGTAGGA ACTCCTATAT ATAACTGTCT TGTAACGGGT CCGGCATCGG CTGTAGATAT CTTGAGAGCT   
  
  
+ TACCACATGT TTCTTGCCAC ATGCCCATTT AAGAAAATGG GAAATTTCTT CTCTAATAGA ACGATTATGG   
  
  
+ CTGTGGCAGA GAATGCAACA TGCCTTCATA TAATTGATCT CGGTATTGTC TATGGTTTCC AATGGCCTTG   
  
  
+ TCTAATTCAG CGACTTTCAT CTAGGCCTGG TGGCCCCCCC AAACTTCGAA TAACCGGAGT AGATCTTCCA   
  
  
+ CAACCTGGGT TCCGACCAGC CAAAAGAGTT GAGGAGACAG GGCGTCGCTT GAAGAACTAT GCAGAGTCAT   
  
  
+ TTAATGTGCC CTTTGAGTTC AATGCTATAG CAAAGAAGTG GGAAACACTT ACCATTGAAG ATCTCAGGAT   
  
  
+ CAATAGCGAT GAGTTGCTTG TTGTCACCTG TATGTTTAGG TTTAAACATA TACCTGAGGA AACAGTGACC   
  
  
+ GTGGATTGCC CTAGGGATAC TGTTCTTAAC CTGATTGGGC GCATAAACCC AGCTGTTTTC ATACAAGGCA   
  
  
+ CTGTTAACGG GGCTTTCAAT TCTCCCTTTT TCATAGCTCG ATTTCGAGAG GCTCTATTTC ACTTCTCCAC   
  
  
+ TCTGTTTGAT ATGCTAGAGG CCAACCTGCC AAGGGACAAT AAGGAGAGGA TGCTAATTGA GAGAGAGATA   
  
  
+ TTTGGGAGGC AGGCAATGAA TGTGATTGCT TGTGAGGGTT TAGAGAGGAT AGAAAGGCCA GAGACGTACA   
  
  
+ AGCAGTGGCA AGTCCGAAAT GAAAGGGCAG GGTTTAGGCA GCTGCCTTTA GATCGCCAGA TTCTGGAAAT   
  
  
+ GGCTAAAAAG AGGGTGAAAT CTGTGTATAA CAAAGATTTC TCCATTGATG AAGACGGGCA CTGGTTGTTG   
  
  
+ CTGGGATGGA AGGGCAGAAT TGTGTACACA CTCACTACTT GGAAGCCTGC GGAGTA  

- -Up\_Stream \_Len000GGGACC CAGTAATTTT TTTTTCTTTC TTTCTTCTAA CGCGATTTTC ATCTAAAAAT   
  
  
- AGTCTAAGTT CGACGTCCAC TATTGGTTTA ACAGTAATTT CGTTACGTTC GCGTATAGTT ATGGGAAAAC   
  
  
- CCTCTATCGA GGCTGACAAC TTCTGTAATC CTCGTACTAG AGGTTGACAC TTAAACAAAG TAAATGAGTA   
  
  
- TAAGTATAGA TGGTACACCT ATTATACTGA CGTCTGATCT ACCGGTTTAA ACTTACGTGT GAATATTGAA   
  
  
- GTTGAGAAAG ATGAAAAAAG AAGGGGTGGA ATAGTCCTTA AAGAAAATTA ACAGCACCCT CTGTTGAACC   
  
  
- CATCTTGAGA ACGCTTTTCC CGTTGAATTG CAAAACCATG AACGTAAAAG GTTTTTTTGT TTTTCCGATT   
  
  
- TGTTAAGAGG CGTACGACTG AAGTGCCATC TATACTACAT AAAAAATTGT TTTCTGTAGA AGACTTACTT   
  
  
- TGCGAATTAT AGAACCTGGT ATGATCTTTA ATTTAACGGT GCCACGTACT TAAAGTGGAG CTTGACGAAC   
  
  
- ATATTTCTAG AACAATAGTG GAAGTCATTA GTCTATGAAC ATTGGTTTAA CTTACTGGTG TTTCTGGTGT   
  
  
- AAGGAGTGTT AGCAACGGAG ATCTTATTCT TACTTACAAA GAAGTTCCAA CTTTCGGGAA CGATTACGCT   
  
  
- TGGACGGATA TACGGATTGA CCGATATATA TACACGCATC TTGTCGTACT GTACTCAATT ATTATATCGT   
  
  
- CGATTTCCCT TGAGATTACA TGTGTACTAA CATTAAGTGG ACGGAGCGAT CGAATGCTCG TACTGAGAAC   
  
  
- TGATAATAAC AAAATTAACT AGAACTTAAA AGTATCTAAA ATGAGGTAAC AAATGAGTAA AACAATATTA   
  
  
- ATTGGTGGAC TAAAATTTGA AATTTTATGT GTTTCTAAGT AGATTATATT AAAAAATCTA TTTCAATACG   
  
  
- TATGTATATA TGTTTAATTT AATAAATGCT AGATTAGTAA AGGGCAATAT ATAAAGTGTG ATTTTAAATC   
  
  
- TTCTCTGTTA GATAATTTTA ATGTAGTGTA CAAAACCCCC CCCCCCCTAT AGTCTATACG TACTGTAAGG   
  
  
- ATTAGGTTAT TACGTTACTT TGGGTGGCTT ATCAACTTTA CTAAACAAGA TAGGTACTTG AGTGGTTCAG   
  
  
- TGTTAGGTAG TGTGAGCTTC CTATACGAAA AGAAAAACTT CCCTTAGAAC GAATTAGTTT GGATATTTGA   
  
  
- AAATTTTCGT TGTCTGTTTT TTCACTAATA TTACCATCAC CATCCTACTT CCGTCGAAAG TCTTTTCGCT   
  
  
- AACTTAAAAA TAACATAGGG TACTTTTGGG TAACTTAAAA AGAAAGAAAG AAACAAAAAC AATGGACCCA   
  
  
- AATACGTTTC CTGCCGACAA CATAAATTAT TATTGTTTTT GTATCCCGAA ACGCCTAACG ATTACGACCC   
  
  
- CTACCGTAGT CACGAGCTTA GACACATGCG ACAGTATCCT TGGGTGAACG AAGACGAGTA AATAAAGACA   
  
  
- GAGATCTAGG GGAGGAGAGA GAGAGAGAGA GAGATTCCAC AGACGAGCAG ACAAGTATAA GAGGACTCCA   
  
  
- AGAAGAAGTA GATAGAACAA GACCCACACA CGACTCCATG AAGAAAAGAA ATATTTAAAA AGTAGAAAAC   
  
  
- AACAAAACGA ACCACAAATA CTAAAATCAA AAACAGAAAT TCTGCTCTAA ACGACAATTA TCGTTCTAAA   
  
  
- GACTTGATCA TAACTAGGAA AAACACCATA TAAACAACTT AAACTAATTA AACACATAAC ACAAATCGAA   
  
  
- TTCATTGAGC AACACTAGAT TAACTACGAG CCCAAAACTG TCAAACTAAT CCCAAAAGCC ATGACCTCAA   
  
  
- TTTCAAAAAA ATAAAAAATA TCCTCCTCAA TTTCCATTAT TATTCTTAAC TACAAACGAC TAAAAAGGAG   
  
  
- AAAAGAAAAA ACCAAAGCTA AACCACCCGT CAATAGACAA ATACCAAAAA CGTTTACCTA GGTGTTAATC   
  
  
- TCCTTAATAT ACCTGGAAAT GTAGCTTAGT TCAAGTTACT AGTTTCCCAC GGTTAAAATG GTTCAGCGTC   
  
  
- ACAACAATGA GGCTACTTTG AGGTTCTAAG GTTAAACTTA GGTTCACAAG GTTTAGGTTC ACAACACTTA   
  
  
- GGAGGAGGCA ACCAAGGGGG TTTGGGTTTA GAATTAAATC AACACCGTAG AGTTATAAGA CTGTAACTCT   
  
  
- GTCGGGACTT GCTCCTAACA CTAAAATCAC TACAACAAGA ATTCATATAA TTAGTTGAAG AATACCTTCT   
  
  
- CCTACCCCTT CTCTTTGAGG TATGACTAGT ACTTGGAAGT CAGCAACTCC GCCGTCTCTT CAGTAACATA   
  
  
- CTCCGCGAGC CCGTCTCTAT AGGAGGAAGG CCTTTGGCTT TAGTCAACGG TCTACAACTC GTACTGCCGA   
  
  
- ACTGACCCTC GTCACAATCC CGAGTATCAC CACGTTCGTC ACAATCCCGA GTATCACTGC GTTCACCACC   
  
  
- ATCACCGGAC TGTCCTATAC CAACCATACC ACTAGGAATA TTAACCTCAG GAGTTTTACA GCACTTAAAA   
  
  
- TGGTAAAGGA GGAGCTTTGG GAATAGCACG TCGAGTAGTC TATCGAGCCC GTCTACCCAA TCATTCACCT   
  
  
- TCAGTTTAAG ACTAAGTGTC GAGTCGAGCC GGTAATTACG TTCACCAAAT CACCTACCCG GTCCCCTGAG   
  
  
- AGGACACTCA CGAGAATCAC ACAGACTTTA TAAGTTACTG TCAGTCAGTT ACAACGTCAA AGTCTTTCCT   
  
  
- AAACTCCTTC GCTTATTTAA AGAAGGTTTC TCAAGAAACA TGTTCCCAAA ACGGTTGTGC CCTAACGGAA   
  
  
- TAGTCTTCCG TTTGTTATCA CGGGTTCTAA ACAACCAGTT ACATCTCCTA CAATGATCCC CTTTCTTCGT   
  
  
- AGCAATAGGG CTTCTTAACG TCAGTCTTCT TCCCTCCTAT TTATTCGTTA GTCGACAAAG GGACCGTCGA   
  
  
- CTACTCCGTC AACAATCCAG ACTTTACAAG CTATCCCATG ACGAAACATC AGCCCCTTTT GTACTACGAC   
  
  
- GAGAGGCCCT TCGAAATGTC TGACTTGATT TATTCTCAGA CGCTTTACGG GGTCAATTCC CCAGATTATC   
  
  
- ACCCTTTCCG GCACCATTCT TTCGCCCTTT CTTCTCCCTA CATCATCTAA ATTCTAGAGA GAATTGGGAT   
  
  
- ACACGTGTTC GTCAACGTAG CTTACTAGTG TCCTCACGTT TACTGGACGA ATCCGTTTAA TCTGTCGTAA   
  
  
- GAAGAGGATC ACCCCTACCA TTGGTTTCTT ACCGTGTAAT AAAACGCCTA CCAGAACTCC GTGCGGAACG   
  
  
- ACCACATCCT TGAGGATATA TATTGACAGA ACATTGCCCA GGCCGTAGCC GACATCTATA GAACTCTCGA   
  
  
- ATGGTGTACA AAGAACGGTG TACGGGTAAA TTCTTTTACC CTTTAAAGAA GAGATTATCT TGCTAATACC   
  
  
- GACACCGTCT CTTACGTTGT ACGGAAGTAT ATTAACTAGA GCCATAACAG ATACCAAAGG TTACCGGAAC   
  
  
- AGATTAAGTC GCTGAAAGTA GATCCGGACC ACCGGGGGGG TTTGAAGCTT ATTGGCCTCA TCTAGAAGGT   
  
  
- GTTGGACCCA AGGCTGGTCG GTTTTCTCAA CTCCTCTGTC CCGCAGCGAA CTTCTTGATA CGTCTCAGTA   
  
  
- AATTACACGG GAAACTCAAG TTACGATATC GTTTCTTCAC CCTTTGTGAA TGGTAACTTC TAGAGTCCTA   
  
  
- GTTATCGCTA CTCAACGAAC AACAGTGGAC ATACAAATCC AAATTTGTAT ATGGACTCCT TTGTCACTGG   
  
  
- CACCTAACGG GATCCCTATG ACAAGAATTG GACTAACCCG CGTATTTGGG TCGACAAAAG TATGTTCCGT   
  
  
- GACAATTGCC CCGAAAGTTA AGAGGGAAAA AGTATCGAGC TAAAGCTCTC CGAGATAAAG TGAAGAGGTG   
  
  
- AGACAAACTA TACGATCTCC GGTTGGACGG TTCCCTGTTA TTCCTCTCCT ACGATTAACT CTCTCTCTAT   
  
  
- AAACCCTCCG TCCGTTACTT ACACTAACGA ACACTCCCAA ATCTCTCCTA TCTTTCCGGT CTCTGCATGT   
  
  
- TCGTCACCGT TCAGGCTTTA CTTTCCCGTC CCAAATCCGT CGACGGAAAT CTAGCGGTCT AAGACCTTTA   
  
  
- CCGATTTTTC TCCCACTTTA GACACATATT GTTTCTAAAG AGGTAACTAC TTCTGCCCGT GACCAACAAC   
  
  
- GACCCTACCT TCCCGTCTTA ACACATGTGT GAGTGATGAA CCTTCGGACG CCTCAT

+     G-Box

| Site Name | Organism | Position | Strand | Matrix score. | sequence | function |
| --- | --- | --- | --- | --- | --- | --- |
| G-Box | Triticum aestivum | 224 | - | 10 | TCCACATGGCA | cis-acting regulatory element involved in light responsiveness |

>HU06G00568.1   
+ -Up\_Stream \_Len000CCCTGG GTCATTAAAA AAAAAGAAAG AAAGAAGATT GCGCTAAAAG TAGATTTTTA   
  
  
+ TCAGATTCAA GCTGCAGGTG ATAACCAAAT TGTCATTAAA GCAATGCAAG CGCATATCAA TACCCTTTTG   
  
  
+ GGAGATAGCT CCGACTGTTG AAGACATTAG GAGCATGATC TCCAACTGTG AATTTGTTTC ATTTACTCAT   
  
  
+ ATTCATATCT ACCATGTGGA TAATATGACT GCAGACTAGA TGGCCAAATT TGAATGCACA CTTATAACTT   
  
  
+ CAACTCTTTC TACTTTTTTC TTCCCCACCT TATCAGGAAT TTCTTTTAAT TGTCGTGGGA GACAACTTGG   
  
  
+ GTAGAACTCT TGCGAAAAGG GCAACTTAAC GTTTTGGTAC TTGCATTTTC CAAAAAAACA AAAAGGCTAA   
  
  
+ ACAATTCTCC GCATGCTGAC TTCACGGTAG ATATGATGTA TTTTTTAACA AAAGACATCT TCTGAATGAA   
  
  
+ ACGCTTAATA TCTTGGACCA TACTAGAAAT TAAATTGCCA CGGTGCATGA ATTTCACCTC GAACTGCTTG   
  
  
+ TATAAAGATC TTGTTATCAC CTTCAGTAAT CAGATACTTG TAACCAAATT GAATGACCAC AAAGACCACA   
  
  
+ TTCCTCACAA TCGTTGCCTC TAGAATAAGA ATGAATGTTT CTTCAAGGTT GAAAGCCCTT GCTAATGCGA   
  
  
+ ACCTGCCTAT ATGCCTAACT GGCTATATAT ATGTGCGTAG AACAGCATGA CATGAGTTAA TAATATAGCA   
  
  
+ GCTAAAGGGA ACTCTAATGT ACACATGATT GTAATTCACC TGCCTCGCTA GCTTACGAGC ATGACTCTTG   
  
  
+ ACTATTATTG TTTTAATTGA TCTTGAATTT TCATAGATTT TACTCCATTG TTTACTCATT TTGTTATAAT   
  
  
+ TAACCACCTG ATTTTAAACT TTAAAATACA CAAAGATTCA TCTAATATAA TTTTTTAGAT AAAGTTATGC   
  
  
+ ATACATATAT ACAAATTAAA TTATTTACGA TCTAATCATT TCCCGTTATA TATTTCACAC TAAAATTTAG   
  
  
+ AAGAGACAAT CTATTAAAAT TACATCACAT GTTTTGGGGG GGGGGGGATA TCAGATATGC ATGACATTCC   
  
  
+ TAATCCAATA ATGCAATGAA ACCCACCGAA TAGTTGAAAT GATTTGTTCT ATCCATGAAC TCACCAAGTC   
  
  
+ ACAATCCATC ACACTCGAAG GATATGCTTT TCTTTTTGAA GGGAATCTTG CTTAATCAAA CCTATAAACT   
  
  
+ TTTAAAAGCA ACAGACAAAA AAGTGATTAT AATGGTAGTG GTAGGATGAA GGCAGCTTTC AGAAAAGCGA   
  
  
+ TTGAATTTTT ATTGTATCCC ATGAAAACCC ATTGAATTTT TCTTTCTTTC TTTGTTTTTG TTACCTGGGT   
  
  
+ TTATGCAAAG GACGGCTGTT GTATTTAATA ATAACAAAAA CATAGGGCTT TGCGGATTGC TAATGCTGGG   
  
  
+ GATGGCATCA GTGCTCGAAT CTGTGTACGC TGTCATAGGA ACCCACTTGC TTCTGCTCAT TTATTTCTGT   
  
  
+ CTCTAGATCC CCTCCTCTCT CTCTCTCTCT CTCTAAGGTG TCTGCTCGTC TGTTCATATT CTCCTGAGGT   
  
  
+ TCTTCTTCAT CTATCTTGTT CTGGGTGTGT GCTGAGGTAC TTCTTTTCTT TATAAATTTT TCATCTTTTG   
  
  
+ TTGTTTTGCT TGGTGTTTAT GATTTTAGTT TTTGTCTTTA AGACGAGATT TGCTGTTAAT AGCAAGATTT   
  
  
+ CTGAACTAGT ATTGATCCTT TTTGTGGTAT ATTTGTTGAA TTTGATTAAT TTGTGTATTG TGTTTAGCTT   
  
  
+ AAGTAACTCG TTGTGATCTA ATTGATGCTC GGGTTTTGAC AGTTTGATTA GGGTTTTCGG TACTGGAGTT   
  
  
+ AAAGTTTTTT TATTTTTTAT AGGAGGAGTT AAAGGTAATA ATAAGAATTG ATGTTTGCTG ATTTTTCCTC   
  
  
+ TTTTCTTTTT TGGTTTCGAT TTGGTGGGCA GTTATCTGTT TATGGTTTTT GCAAATGGAT CCACAATTAG   
  
  
+ AGGAATTATA TGGACCTTTA CATCGAATCA AGTTCAATGA TCAAAGGGTG CCAATTTTAC CAAGTCGCAG   
  
  
+ TGTTGTTACT CCGATGAAAC TCCAAGATTC CAATTTGAAT CCAAGTGTTC CAAATCCAAG TGTTGTGAAT   
  
  
+ CCTCCTCCGT TGGTTCCCCC AAACCCAAAT CTTAATTTAG TTGTGGCATC TCAATATTCT GACATTGAGA   
  
  
+ CAGCCCTGAA CGAGGATTGT GATTTTAGTG ATGTTGTTCT TAAGTATATT AATCAACTTC TTATGGAAGA   
  
  
+ GGATGGGGAA GAGAAACTCC ATACTGATCA TGAACCTTCA GTCGTTGAGG CGGCAGAGAA GTCATTGTAT   
  
  
+ GAGGCGCTCG GGCAGAGATA TCCTCCTTCC GGAAACCGAA ATCAGTTGCC AGATGTTGAG CATGACGGCT   
  
  
+ TGACTGGGAG CAGTGTTAGG GCTCATAGTG GTGCAAGCAG TGTTAGGGCT CATAGTGACG CAAGTGGTGG   
  
  
+ TAGTGGCCTG ACAGGATATG GTTGGTATGG TGATCCTTAT AATTGGAGTC CTCAAAATGT CGTGAATTTT   
  
  
+ ACCATTTCCT CCTCGAAACC CTTATCGTGC AGCTCATCAG ATAGCTCGGG CAGATGGGTT AGTAAGTGGA   
  
  
+ AGTCAAATTC TGATTCACAG CTCAGCTCGG CCATTAATGC AAGTGGTTTA GTGGATGGGC CAGGGGACTC   
  
  
+ TCCTGTGAGT GCTCTTAGTG TGTCTGAAAT ATTCAATGAC AGTCAGTCAA TGTTGCAGTT TCAGAAAGGA   
  
  
+ TTTGAGGAAG CGAATAAATT TCTTCCAAAG AGTTCTTTGT ACAAGGGTTT TGCCAACACG GGATTGCCTT   
  
  
+ ATCAGAAGGC AAACAATAGT GCCCAAGATT TGTTGGTCAA TGTAGAGGAT GTTACTAGGG GAAAGAAGCA   
  
  
+ TCGTTATCCC GAAGAATTGC AGTCAGAAGA AGGGAGGATA AATAAGCAAT CAGCTGTTTC CCTGGCAGCT   
  
  
+ GATGAGGCAG TTGTTAGGTC TGAAATGTTC GATAGGGTAC TGCTTTGTAG TCGGGGAAAA CATGATGCTG   
  
  
+ CTCTCCGGGA AGCTTTACAG ACTGAACTAA ATAAGAGTCT GCGAAATGCC CCAGTTAAGG GGTCTAATAG   
  
  
+ TGGGAAAGGC CGTGGTAAGA AAGCGGGAAA GAAGAGGGAT GTAGTAGATT TAAGATCTCT CTTAACCCTA   
  
  
+ TGTGCACAAG CAGTTGCATC GAATGATCAC AGGAGTGCAA ATGACCTGCT TAGGCAAATT AGACAGCATT   
  
  
+ CTTCTCCTAG TGGGGATGGT AACCAAAGAA TGGCACATTA TTTTGCGGAT GGTCTTGAGG CACGCCTTGC   
  
  
+ TGGTGTAGGA ACTCCTATAT ATAACTGTCT TGTAACGGGT CCGGCATCGG CTGTAGATAT CTTGAGAGCT   
  
  
+ TACCACATGT TTCTTGCCAC ATGCCCATTT AAGAAAATGG GAAATTTCTT CTCTAATAGA ACGATTATGG   
  
  
+ CTGTGGCAGA GAATGCAACA TGCCTTCATA TAATTGATCT CGGTATTGTC TATGGTTTCC AATGGCCTTG   
  
  
+ TCTAATTCAG CGACTTTCAT CTAGGCCTGG TGGCCCCCCC AAACTTCGAA TAACCGGAGT AGATCTTCCA   
  
  
+ CAACCTGGGT TCCGACCAGC CAAAAGAGTT GAGGAGACAG GGCGTCGCTT GAAGAACTAT GCAGAGTCAT   
  
  
+ TTAATGTGCC CTTTGAGTTC AATGCTATAG CAAAGAAGTG GGAAACACTT ACCATTGAAG ATCTCAGGAT   
  
  
+ CAATAGCGAT GAGTTGCTTG TTGTCACCTG TATGTTTAGG TTTAAACATA TACCTGAGGA AACAGTGACC   
  
  
+ GTGGATTGCC CTAGGGATAC TGTTCTTAAC CTGATTGGGC GCATAAACCC AGCTGTTTTC ATACAAGGCA   
  
  
+ CTGTTAACGG GGCTTTCAAT TCTCCCTTTT TCATAGCTCG ATTTCGAGAG GCTCTATTTC ACTTCTCCAC   
  
  
+ TCTGTTTGAT ATGCTAGAGG CCAACCTGCC AAGGGACAAT AAGGAGAGGA TGCTAATTGA GAGAGAGATA   
  
  
+ TTTGGGAGGC AGGCAATGAA TGTGATTGCT TGTGAGGGTT TAGAGAGGAT AGAAAGGCCA GAGACGTACA   
  
  
+ AGCAGTGGCA AGTCCGAAAT GAAAGGGCAG GGTTTAGGCA GCTGCCTTTA GATCGCCAGA TTCTGGAAAT   
  
  
+ GGCTAAAAAG AGGGTGAAAT CTGTGTATAA CAAAGATTTC TCCATTGATG AAGACGGGCA CTGGTTGTTG   
  
  
+ CTGGGATGGA AGGGCAGAAT TGTGTACACA CTCACTACTT GGAAGCCTGC GGAGTA  

- -Up\_Stream \_Len000GGGACC CAGTAATTTT TTTTTCTTTC TTTCTTCTAA CGCGATTTTC ATCTAAAAAT   
  
  
- AGTCTAAGTT CGACGTCCAC TATTGGTTTA ACAGTAATTT CGTTACGTTC GCGTATAGTT ATGGGAAAAC   
  
  
- CCTCTATCGA GGCTGACAAC TTCTGTAATC CTCGTACTAG AGGTTGACAC TTAAACAAAG TAAATGAGTA   
  
  
- TAAGTATAGA TGGTACACCT ATTATACTGA CGTCTGATCT ACCGGTTTAA ACTTACGTGT GAATATTGAA   
  
  
- GTTGAGAAAG ATGAAAAAAG AAGGGGTGGA ATAGTCCTTA AAGAAAATTA ACAGCACCCT CTGTTGAACC   
  
  
- CATCTTGAGA ACGCTTTTCC CGTTGAATTG CAAAACCATG AACGTAAAAG GTTTTTTTGT TTTTCCGATT   
  
  
- TGTTAAGAGG CGTACGACTG AAGTGCCATC TATACTACAT AAAAAATTGT TTTCTGTAGA AGACTTACTT   
  
  
- TGCGAATTAT AGAACCTGGT ATGATCTTTA ATTTAACGGT GCCACGTACT TAAAGTGGAG CTTGACGAAC   
  
  
- ATATTTCTAG AACAATAGTG GAAGTCATTA GTCTATGAAC ATTGGTTTAA CTTACTGGTG TTTCTGGTGT   
  
  
- AAGGAGTGTT AGCAACGGAG ATCTTATTCT TACTTACAAA GAAGTTCCAA CTTTCGGGAA CGATTACGCT   
  
  
- TGGACGGATA TACGGATTGA CCGATATATA TACACGCATC TTGTCGTACT GTACTCAATT ATTATATCGT   
  
  
- CGATTTCCCT TGAGATTACA TGTGTACTAA CATTAAGTGG ACGGAGCGAT CGAATGCTCG TACTGAGAAC   
  
  
- TGATAATAAC AAAATTAACT AGAACTTAAA AGTATCTAAA ATGAGGTAAC AAATGAGTAA AACAATATTA   
  
  
- ATTGGTGGAC TAAAATTTGA AATTTTATGT GTTTCTAAGT AGATTATATT AAAAAATCTA TTTCAATACG   
  
  
- TATGTATATA TGTTTAATTT AATAAATGCT AGATTAGTAA AGGGCAATAT ATAAAGTGTG ATTTTAAATC   
  
  
- TTCTCTGTTA GATAATTTTA ATGTAGTGTA CAAAACCCCC CCCCCCCTAT AGTCTATACG TACTGTAAGG   
  
  
- ATTAGGTTAT TACGTTACTT TGGGTGGCTT ATCAACTTTA CTAAACAAGA TAGGTACTTG AGTGGTTCAG   
  
  
- TGTTAGGTAG TGTGAGCTTC CTATACGAAA AGAAAAACTT CCCTTAGAAC GAATTAGTTT GGATATTTGA   
  
  
- AAATTTTCGT TGTCTGTTTT TTCACTAATA TTACCATCAC CATCCTACTT CCGTCGAAAG TCTTTTCGCT   
  
  
- AACTTAAAAA TAACATAGGG TACTTTTGGG TAACTTAAAA AGAAAGAAAG AAACAAAAAC AATGGACCCA   
  
  
- AATACGTTTC CTGCCGACAA CATAAATTAT TATTGTTTTT GTATCCCGAA ACGCCTAACG ATTACGACCC   
  
  
- CTACCGTAGT CACGAGCTTA GACACATGCG ACAGTATCCT TGGGTGAACG AAGACGAGTA AATAAAGACA   
  
  
- GAGATCTAGG GGAGGAGAGA GAGAGAGAGA GAGATTCCAC AGACGAGCAG ACAAGTATAA GAGGACTCCA   
  
  
- AGAAGAAGTA GATAGAACAA GACCCACACA CGACTCCATG AAGAAAAGAA ATATTTAAAA AGTAGAAAAC   
  
  
- AACAAAACGA ACCACAAATA CTAAAATCAA AAACAGAAAT TCTGCTCTAA ACGACAATTA TCGTTCTAAA   
  
  
- GACTTGATCA TAACTAGGAA AAACACCATA TAAACAACTT AAACTAATTA AACACATAAC ACAAATCGAA   
  
  
- TTCATTGAGC AACACTAGAT TAACTACGAG CCCAAAACTG TCAAACTAAT CCCAAAAGCC ATGACCTCAA   
  
  
- TTTCAAAAAA ATAAAAAATA TCCTCCTCAA TTTCCATTAT TATTCTTAAC TACAAACGAC TAAAAAGGAG   
  
  
- AAAAGAAAAA ACCAAAGCTA AACCACCCGT CAATAGACAA ATACCAAAAA CGTTTACCTA GGTGTTAATC   
  
  
- TCCTTAATAT ACCTGGAAAT GTAGCTTAGT TCAAGTTACT AGTTTCCCAC GGTTAAAATG GTTCAGCGTC   
  
  
- ACAACAATGA GGCTACTTTG AGGTTCTAAG GTTAAACTTA GGTTCACAAG GTTTAGGTTC ACAACACTTA   
  
  
- GGAGGAGGCA ACCAAGGGGG TTTGGGTTTA GAATTAAATC AACACCGTAG AGTTATAAGA CTGTAACTCT   
  
  
- GTCGGGACTT GCTCCTAACA CTAAAATCAC TACAACAAGA ATTCATATAA TTAGTTGAAG AATACCTTCT   
  
  
- CCTACCCCTT CTCTTTGAGG TATGACTAGT ACTTGGAAGT CAGCAACTCC GCCGTCTCTT CAGTAACATA   
  
  
- CTCCGCGAGC CCGTCTCTAT AGGAGGAAGG CCTTTGGCTT TAGTCAACGG TCTACAACTC GTACTGCCGA   
  
  
- ACTGACCCTC GTCACAATCC CGAGTATCAC CACGTTCGTC ACAATCCCGA GTATCACTGC GTTCACCACC   
  
  
- ATCACCGGAC TGTCCTATAC CAACCATACC ACTAGGAATA TTAACCTCAG GAGTTTTACA GCACTTAAAA   
  
  
- TGGTAAAGGA GGAGCTTTGG GAATAGCACG TCGAGTAGTC TATCGAGCCC GTCTACCCAA TCATTCACCT   
  
  
- TCAGTTTAAG ACTAAGTGTC GAGTCGAGCC GGTAATTACG TTCACCAAAT CACCTACCCG GTCCCCTGAG   
  
  
- AGGACACTCA CGAGAATCAC ACAGACTTTA TAAGTTACTG TCAGTCAGTT ACAACGTCAA AGTCTTTCCT   
  
  
- AAACTCCTTC GCTTATTTAA AGAAGGTTTC TCAAGAAACA TGTTCCCAAA ACGGTTGTGC CCTAACGGAA   
  
  
- TAGTCTTCCG TTTGTTATCA CGGGTTCTAA ACAACCAGTT ACATCTCCTA CAATGATCCC CTTTCTTCGT   
  
  
- AGCAATAGGG CTTCTTAACG TCAGTCTTCT TCCCTCCTAT TTATTCGTTA GTCGACAAAG GGACCGTCGA   
  
  
- CTACTCCGTC AACAATCCAG ACTTTACAAG CTATCCCATG ACGAAACATC AGCCCCTTTT GTACTACGAC   
  
  
- GAGAGGCCCT TCGAAATGTC TGACTTGATT TATTCTCAGA CGCTTTACGG GGTCAATTCC CCAGATTATC   
  
  
- ACCCTTTCCG GCACCATTCT TTCGCCCTTT CTTCTCCCTA CATCATCTAA ATTCTAGAGA GAATTGGGAT   
  
  
- ACACGTGTTC GTCAACGTAG CTTACTAGTG TCCTCACGTT TACTGGACGA ATCCGTTTAA TCTGTCGTAA   
  
  
- GAAGAGGATC ACCCCTACCA TTGGTTTCTT ACCGTGTAAT AAAACGCCTA CCAGAACTCC GTGCGGAACG   
  
  
- ACCACATCCT TGAGGATATA TATTGACAGA ACATTGCCCA GGCCGTAGCC GACATCTATA GAACTCTCGA   
  
  
- ATGGTGTACA AAGAACGGTG TACGGGTAAA TTCTTTTACC CTTTAAAGAA GAGATTATCT TGCTAATACC   
  
  
- GACACCGTCT CTTACGTTGT ACGGAAGTAT ATTAACTAGA GCCATAACAG ATACCAAAGG TTACCGGAAC   
  
  
- AGATTAAGTC GCTGAAAGTA GATCCGGACC ACCGGGGGGG TTTGAAGCTT ATTGGCCTCA TCTAGAAGGT   
  
  
- GTTGGACCCA AGGCTGGTCG GTTTTCTCAA CTCCTCTGTC CCGCAGCGAA CTTCTTGATA CGTCTCAGTA   
  
  
- AATTACACGG GAAACTCAAG TTACGATATC GTTTCTTCAC CCTTTGTGAA TGGTAACTTC TAGAGTCCTA   
  
  
- GTTATCGCTA CTCAACGAAC AACAGTGGAC ATACAAATCC AAATTTGTAT ATGGACTCCT TTGTCACTGG   
  
  
- CACCTAACGG GATCCCTATG ACAAGAATTG GACTAACCCG CGTATTTGGG TCGACAAAAG TATGTTCCGT   
  
  
- GACAATTGCC CCGAAAGTTA AGAGGGAAAA AGTATCGAGC TAAAGCTCTC CGAGATAAAG TGAAGAGGTG   
  
  
- AGACAAACTA TACGATCTCC GGTTGGACGG TTCCCTGTTA TTCCTCTCCT ACGATTAACT CTCTCTCTAT   
  
  
- AAACCCTCCG TCCGTTACTT ACACTAACGA ACACTCCCAA ATCTCTCCTA TCTTTCCGGT CTCTGCATGT   
  
  
- TCGTCACCGT TCAGGCTTTA CTTTCCCGTC CCAAATCCGT CGACGGAAAT CTAGCGGTCT AAGACCTTTA   
  
  
- CCGATTTTTC TCCCACTTTA GACACATATT GTTTCTAAAG AGGTAACTAC TTCTGCCCGT GACCAACAAC   
  
  
- GACCCTACCT TCCCGTCTTA ACACATGTGT GAGTGATGAA CCTTCGGACG CCTCAT

+     G-box

| Site Name | Organism | Position | Strand | Matrix score. | sequence | function |
| --- | --- | --- | --- | --- | --- | --- |
| G-box | Zea mays | 2583 | - | 6 | CACGAC | cis-acting regulatory element involved in light responsiveness |
| G-box | Zea mays | 336 | - | 6 | CACGAC | cis-acting regulatory element involved in light responsiveness |

>HU06G00568.1   
+ -Up\_Stream \_Len000CCCTGG GTCATTAAAA AAAAAGAAAG AAAGAAGATT GCGCTAAAAG TAGATTTTTA   
  
  
+ TCAGATTCAA GCTGCAGGTG ATAACCAAAT TGTCATTAAA GCAATGCAAG CGCATATCAA TACCCTTTTG   
  
  
+ GGAGATAGCT CCGACTGTTG AAGACATTAG GAGCATGATC TCCAACTGTG AATTTGTTTC ATTTACTCAT   
  
  
+ ATTCATATCT ACCATGTGGA TAATATGACT GCAGACTAGA TGGCCAAATT TGAATGCACA CTTATAACTT   
  
  
+ CAACTCTTTC TACTTTTTTC TTCCCCACCT TATCAGGAAT TTCTTTTAAT TGTCGTGGGA GACAACTTGG   
  
  
+ GTAGAACTCT TGCGAAAAGG GCAACTTAAC GTTTTGGTAC TTGCATTTTC CAAAAAAACA AAAAGGCTAA   
  
  
+ ACAATTCTCC GCATGCTGAC TTCACGGTAG ATATGATGTA TTTTTTAACA AAAGACATCT TCTGAATGAA   
  
  
+ ACGCTTAATA TCTTGGACCA TACTAGAAAT TAAATTGCCA CGGTGCATGA ATTTCACCTC GAACTGCTTG   
  
  
+ TATAAAGATC TTGTTATCAC CTTCAGTAAT CAGATACTTG TAACCAAATT GAATGACCAC AAAGACCACA   
  
  
+ TTCCTCACAA TCGTTGCCTC TAGAATAAGA ATGAATGTTT CTTCAAGGTT GAAAGCCCTT GCTAATGCGA   
  
  
+ ACCTGCCTAT ATGCCTAACT GGCTATATAT ATGTGCGTAG AACAGCATGA CATGAGTTAA TAATATAGCA   
  
  
+ GCTAAAGGGA ACTCTAATGT ACACATGATT GTAATTCACC TGCCTCGCTA GCTTACGAGC ATGACTCTTG   
  
  
+ ACTATTATTG TTTTAATTGA TCTTGAATTT TCATAGATTT TACTCCATTG TTTACTCATT TTGTTATAAT   
  
  
+ TAACCACCTG ATTTTAAACT TTAAAATACA CAAAGATTCA TCTAATATAA TTTTTTAGAT AAAGTTATGC   
  
  
+ ATACATATAT ACAAATTAAA TTATTTACGA TCTAATCATT TCCCGTTATA TATTTCACAC TAAAATTTAG   
  
  
+ AAGAGACAAT CTATTAAAAT TACATCACAT GTTTTGGGGG GGGGGGGATA TCAGATATGC ATGACATTCC   
  
  
+ TAATCCAATA ATGCAATGAA ACCCACCGAA TAGTTGAAAT GATTTGTTCT ATCCATGAAC TCACCAAGTC   
  
  
+ ACAATCCATC ACACTCGAAG GATATGCTTT TCTTTTTGAA GGGAATCTTG CTTAATCAAA CCTATAAACT   
  
  
+ TTTAAAAGCA ACAGACAAAA AAGTGATTAT AATGGTAGTG GTAGGATGAA GGCAGCTTTC AGAAAAGCGA   
  
  
+ TTGAATTTTT ATTGTATCCC ATGAAAACCC ATTGAATTTT TCTTTCTTTC TTTGTTTTTG TTACCTGGGT   
  
  
+ TTATGCAAAG GACGGCTGTT GTATTTAATA ATAACAAAAA CATAGGGCTT TGCGGATTGC TAATGCTGGG   
  
  
+ GATGGCATCA GTGCTCGAAT CTGTGTACGC TGTCATAGGA ACCCACTTGC TTCTGCTCAT TTATTTCTGT   
  
  
+ CTCTAGATCC CCTCCTCTCT CTCTCTCTCT CTCTAAGGTG TCTGCTCGTC TGTTCATATT CTCCTGAGGT   
  
  
+ TCTTCTTCAT CTATCTTGTT CTGGGTGTGT GCTGAGGTAC TTCTTTTCTT TATAAATTTT TCATCTTTTG   
  
  
+ TTGTTTTGCT TGGTGTTTAT GATTTTAGTT TTTGTCTTTA AGACGAGATT TGCTGTTAAT AGCAAGATTT   
  
  
+ CTGAACTAGT ATTGATCCTT TTTGTGGTAT ATTTGTTGAA TTTGATTAAT TTGTGTATTG TGTTTAGCTT   
  
  
+ AAGTAACTCG TTGTGATCTA ATTGATGCTC GGGTTTTGAC AGTTTGATTA GGGTTTTCGG TACTGGAGTT   
  
  
+ AAAGTTTTTT TATTTTTTAT AGGAGGAGTT AAAGGTAATA ATAAGAATTG ATGTTTGCTG ATTTTTCCTC   
  
  
+ TTTTCTTTTT TGGTTTCGAT TTGGTGGGCA GTTATCTGTT TATGGTTTTT GCAAATGGAT CCACAATTAG   
  
  
+ AGGAATTATA TGGACCTTTA CATCGAATCA AGTTCAATGA TCAAAGGGTG CCAATTTTAC CAAGTCGCAG   
  
  
+ TGTTGTTACT CCGATGAAAC TCCAAGATTC CAATTTGAAT CCAAGTGTTC CAAATCCAAG TGTTGTGAAT   
  
  
+ CCTCCTCCGT TGGTTCCCCC AAACCCAAAT CTTAATTTAG TTGTGGCATC TCAATATTCT GACATTGAGA   
  
  
+ CAGCCCTGAA CGAGGATTGT GATTTTAGTG ATGTTGTTCT TAAGTATATT AATCAACTTC TTATGGAAGA   
  
  
+ GGATGGGGAA GAGAAACTCC ATACTGATCA TGAACCTTCA GTCGTTGAGG CGGCAGAGAA GTCATTGTAT   
  
  
+ GAGGCGCTCG GGCAGAGATA TCCTCCTTCC GGAAACCGAA ATCAGTTGCC AGATGTTGAG CATGACGGCT   
  
  
+ TGACTGGGAG CAGTGTTAGG GCTCATAGTG GTGCAAGCAG TGTTAGGGCT CATAGTGACG CAAGTGGTGG   
  
  
+ TAGTGGCCTG ACAGGATATG GTTGGTATGG TGATCCTTAT AATTGGAGTC CTCAAAATGT CGTGAATTTT   
  
  
+ ACCATTTCCT CCTCGAAACC CTTATCGTGC AGCTCATCAG ATAGCTCGGG CAGATGGGTT AGTAAGTGGA   
  
  
+ AGTCAAATTC TGATTCACAG CTCAGCTCGG CCATTAATGC AAGTGGTTTA GTGGATGGGC CAGGGGACTC   
  
  
+ TCCTGTGAGT GCTCTTAGTG TGTCTGAAAT ATTCAATGAC AGTCAGTCAA TGTTGCAGTT TCAGAAAGGA   
  
  
+ TTTGAGGAAG CGAATAAATT TCTTCCAAAG AGTTCTTTGT ACAAGGGTTT TGCCAACACG GGATTGCCTT   
  
  
+ ATCAGAAGGC AAACAATAGT GCCCAAGATT TGTTGGTCAA TGTAGAGGAT GTTACTAGGG GAAAGAAGCA   
  
  
+ TCGTTATCCC GAAGAATTGC AGTCAGAAGA AGGGAGGATA AATAAGCAAT CAGCTGTTTC CCTGGCAGCT   
  
  
+ GATGAGGCAG TTGTTAGGTC TGAAATGTTC GATAGGGTAC TGCTTTGTAG TCGGGGAAAA CATGATGCTG   
  
  
+ CTCTCCGGGA AGCTTTACAG ACTGAACTAA ATAAGAGTCT GCGAAATGCC CCAGTTAAGG GGTCTAATAG   
  
  
+ TGGGAAAGGC CGTGGTAAGA AAGCGGGAAA GAAGAGGGAT GTAGTAGATT TAAGATCTCT CTTAACCCTA   
  
  
+ TGTGCACAAG CAGTTGCATC GAATGATCAC AGGAGTGCAA ATGACCTGCT TAGGCAAATT AGACAGCATT   
  
  
+ CTTCTCCTAG TGGGGATGGT AACCAAAGAA TGGCACATTA TTTTGCGGAT GGTCTTGAGG CACGCCTTGC   
  
  
+ TGGTGTAGGA ACTCCTATAT ATAACTGTCT TGTAACGGGT CCGGCATCGG CTGTAGATAT CTTGAGAGCT   
  
  
+ TACCACATGT TTCTTGCCAC ATGCCCATTT AAGAAAATGG GAAATTTCTT CTCTAATAGA ACGATTATGG   
  
  
+ CTGTGGCAGA GAATGCAACA TGCCTTCATA TAATTGATCT CGGTATTGTC TATGGTTTCC AATGGCCTTG   
  
  
+ TCTAATTCAG CGACTTTCAT CTAGGCCTGG TGGCCCCCCC AAACTTCGAA TAACCGGAGT AGATCTTCCA   
  
  
+ CAACCTGGGT TCCGACCAGC CAAAAGAGTT GAGGAGACAG GGCGTCGCTT GAAGAACTAT GCAGAGTCAT   
  
  
+ TTAATGTGCC CTTTGAGTTC AATGCTATAG CAAAGAAGTG GGAAACACTT ACCATTGAAG ATCTCAGGAT   
  
  
+ CAATAGCGAT GAGTTGCTTG TTGTCACCTG TATGTTTAGG TTTAAACATA TACCTGAGGA AACAGTGACC   
  
  
+ GTGGATTGCC CTAGGGATAC TGTTCTTAAC CTGATTGGGC GCATAAACCC AGCTGTTTTC ATACAAGGCA   
  
  
+ CTGTTAACGG GGCTTTCAAT TCTCCCTTTT TCATAGCTCG ATTTCGAGAG GCTCTATTTC ACTTCTCCAC   
  
  
+ TCTGTTTGAT ATGCTAGAGG CCAACCTGCC AAGGGACAAT AAGGAGAGGA TGCTAATTGA GAGAGAGATA   
  
  
+ TTTGGGAGGC AGGCAATGAA TGTGATTGCT TGTGAGGGTT TAGAGAGGAT AGAAAGGCCA GAGACGTACA   
  
  
+ AGCAGTGGCA AGTCCGAAAT GAAAGGGCAG GGTTTAGGCA GCTGCCTTTA GATCGCCAGA TTCTGGAAAT   
  
  
+ GGCTAAAAAG AGGGTGAAAT CTGTGTATAA CAAAGATTTC TCCATTGATG AAGACGGGCA CTGGTTGTTG   
  
  
+ CTGGGATGGA AGGGCAGAAT TGTGTACACA CTCACTACTT GGAAGCCTGC GGAGTA  

- -Up\_Stream \_Len000GGGACC CAGTAATTTT TTTTTCTTTC TTTCTTCTAA CGCGATTTTC ATCTAAAAAT   
  
  
- AGTCTAAGTT CGACGTCCAC TATTGGTTTA ACAGTAATTT CGTTACGTTC GCGTATAGTT ATGGGAAAAC   
  
  
- CCTCTATCGA GGCTGACAAC TTCTGTAATC CTCGTACTAG AGGTTGACAC TTAAACAAAG TAAATGAGTA   
  
  
- TAAGTATAGA TGGTACACCT ATTATACTGA CGTCTGATCT ACCGGTTTAA ACTTACGTGT GAATATTGAA   
  
  
- GTTGAGAAAG ATGAAAAAAG AAGGGGTGGA ATAGTCCTTA AAGAAAATTA ACAGCACCCT CTGTTGAACC   
  
  
- CATCTTGAGA ACGCTTTTCC CGTTGAATTG CAAAACCATG AACGTAAAAG GTTTTTTTGT TTTTCCGATT   
  
  
- TGTTAAGAGG CGTACGACTG AAGTGCCATC TATACTACAT AAAAAATTGT TTTCTGTAGA AGACTTACTT   
  
  
- TGCGAATTAT AGAACCTGGT ATGATCTTTA ATTTAACGGT GCCACGTACT TAAAGTGGAG CTTGACGAAC   
  
  
- ATATTTCTAG AACAATAGTG GAAGTCATTA GTCTATGAAC ATTGGTTTAA CTTACTGGTG TTTCTGGTGT   
  
  
- AAGGAGTGTT AGCAACGGAG ATCTTATTCT TACTTACAAA GAAGTTCCAA CTTTCGGGAA CGATTACGCT   
  
  
- TGGACGGATA TACGGATTGA CCGATATATA TACACGCATC TTGTCGTACT GTACTCAATT ATTATATCGT   
  
  
- CGATTTCCCT TGAGATTACA TGTGTACTAA CATTAAGTGG ACGGAGCGAT CGAATGCTCG TACTGAGAAC   
  
  
- TGATAATAAC AAAATTAACT AGAACTTAAA AGTATCTAAA ATGAGGTAAC AAATGAGTAA AACAATATTA   
  
  
- ATTGGTGGAC TAAAATTTGA AATTTTATGT GTTTCTAAGT AGATTATATT AAAAAATCTA TTTCAATACG   
  
  
- TATGTATATA TGTTTAATTT AATAAATGCT AGATTAGTAA AGGGCAATAT ATAAAGTGTG ATTTTAAATC   
  
  
- TTCTCTGTTA GATAATTTTA ATGTAGTGTA CAAAACCCCC CCCCCCCTAT AGTCTATACG TACTGTAAGG   
  
  
- ATTAGGTTAT TACGTTACTT TGGGTGGCTT ATCAACTTTA CTAAACAAGA TAGGTACTTG AGTGGTTCAG   
  
  
- TGTTAGGTAG TGTGAGCTTC CTATACGAAA AGAAAAACTT CCCTTAGAAC GAATTAGTTT GGATATTTGA   
  
  
- AAATTTTCGT TGTCTGTTTT TTCACTAATA TTACCATCAC CATCCTACTT CCGTCGAAAG TCTTTTCGCT   
  
  
- AACTTAAAAA TAACATAGGG TACTTTTGGG TAACTTAAAA AGAAAGAAAG AAACAAAAAC AATGGACCCA   
  
  
- AATACGTTTC CTGCCGACAA CATAAATTAT TATTGTTTTT GTATCCCGAA ACGCCTAACG ATTACGACCC   
  
  
- CTACCGTAGT CACGAGCTTA GACACATGCG ACAGTATCCT TGGGTGAACG AAGACGAGTA AATAAAGACA   
  
  
- GAGATCTAGG GGAGGAGAGA GAGAGAGAGA GAGATTCCAC AGACGAGCAG ACAAGTATAA GAGGACTCCA   
  
  
- AGAAGAAGTA GATAGAACAA GACCCACACA CGACTCCATG AAGAAAAGAA ATATTTAAAA AGTAGAAAAC   
  
  
- AACAAAACGA ACCACAAATA CTAAAATCAA AAACAGAAAT TCTGCTCTAA ACGACAATTA TCGTTCTAAA   
  
  
- GACTTGATCA TAACTAGGAA AAACACCATA TAAACAACTT AAACTAATTA AACACATAAC ACAAATCGAA   
  
  
- TTCATTGAGC AACACTAGAT TAACTACGAG CCCAAAACTG TCAAACTAAT CCCAAAAGCC ATGACCTCAA   
  
  
- TTTCAAAAAA ATAAAAAATA TCCTCCTCAA TTTCCATTAT TATTCTTAAC TACAAACGAC TAAAAAGGAG   
  
  
- AAAAGAAAAA ACCAAAGCTA AACCACCCGT CAATAGACAA ATACCAAAAA CGTTTACCTA GGTGTTAATC   
  
  
- TCCTTAATAT ACCTGGAAAT GTAGCTTAGT TCAAGTTACT AGTTTCCCAC GGTTAAAATG GTTCAGCGTC   
  
  
- ACAACAATGA GGCTACTTTG AGGTTCTAAG GTTAAACTTA GGTTCACAAG GTTTAGGTTC ACAACACTTA   
  
  
- GGAGGAGGCA ACCAAGGGGG TTTGGGTTTA GAATTAAATC AACACCGTAG AGTTATAAGA CTGTAACTCT   
  
  
- GTCGGGACTT GCTCCTAACA CTAAAATCAC TACAACAAGA ATTCATATAA TTAGTTGAAG AATACCTTCT   
  
  
- CCTACCCCTT CTCTTTGAGG TATGACTAGT ACTTGGAAGT CAGCAACTCC GCCGTCTCTT CAGTAACATA   
  
  
- CTCCGCGAGC CCGTCTCTAT AGGAGGAAGG CCTTTGGCTT TAGTCAACGG TCTACAACTC GTACTGCCGA   
  
  
- ACTGACCCTC GTCACAATCC CGAGTATCAC CACGTTCGTC ACAATCCCGA GTATCACTGC GTTCACCACC   
  
  
- ATCACCGGAC TGTCCTATAC CAACCATACC ACTAGGAATA TTAACCTCAG GAGTTTTACA GCACTTAAAA   
  
  
- TGGTAAAGGA GGAGCTTTGG GAATAGCACG TCGAGTAGTC TATCGAGCCC GTCTACCCAA TCATTCACCT   
  
  
- TCAGTTTAAG ACTAAGTGTC GAGTCGAGCC GGTAATTACG TTCACCAAAT CACCTACCCG GTCCCCTGAG   
  
  
- AGGACACTCA CGAGAATCAC ACAGACTTTA TAAGTTACTG TCAGTCAGTT ACAACGTCAA AGTCTTTCCT   
  
  
- AAACTCCTTC GCTTATTTAA AGAAGGTTTC TCAAGAAACA TGTTCCCAAA ACGGTTGTGC CCTAACGGAA   
  
  
- TAGTCTTCCG TTTGTTATCA CGGGTTCTAA ACAACCAGTT ACATCTCCTA CAATGATCCC CTTTCTTCGT   
  
  
- AGCAATAGGG CTTCTTAACG TCAGTCTTCT TCCCTCCTAT TTATTCGTTA GTCGACAAAG GGACCGTCGA   
  
  
- CTACTCCGTC AACAATCCAG ACTTTACAAG CTATCCCATG ACGAAACATC AGCCCCTTTT GTACTACGAC   
  
  
- GAGAGGCCCT TCGAAATGTC TGACTTGATT TATTCTCAGA CGCTTTACGG GGTCAATTCC CCAGATTATC   
  
  
- ACCCTTTCCG GCACCATTCT TTCGCCCTTT CTTCTCCCTA CATCATCTAA ATTCTAGAGA GAATTGGGAT   
  
  
- ACACGTGTTC GTCAACGTAG CTTACTAGTG TCCTCACGTT TACTGGACGA ATCCGTTTAA TCTGTCGTAA   
  
  
- GAAGAGGATC ACCCCTACCA TTGGTTTCTT ACCGTGTAAT AAAACGCCTA CCAGAACTCC GTGCGGAACG   
  
  
- ACCACATCCT TGAGGATATA TATTGACAGA ACATTGCCCA GGCCGTAGCC GACATCTATA GAACTCTCGA   
  
  
- ATGGTGTACA AAGAACGGTG TACGGGTAAA TTCTTTTACC CTTTAAAGAA GAGATTATCT TGCTAATACC   
  
  
- GACACCGTCT CTTACGTTGT ACGGAAGTAT ATTAACTAGA GCCATAACAG ATACCAAAGG TTACCGGAAC   
  
  
- AGATTAAGTC GCTGAAAGTA GATCCGGACC ACCGGGGGGG TTTGAAGCTT ATTGGCCTCA TCTAGAAGGT   
  
  
- GTTGGACCCA AGGCTGGTCG GTTTTCTCAA CTCCTCTGTC CCGCAGCGAA CTTCTTGATA CGTCTCAGTA   
  
  
- AATTACACGG GAAACTCAAG TTACGATATC GTTTCTTCAC CCTTTGTGAA TGGTAACTTC TAGAGTCCTA   
  
  
- GTTATCGCTA CTCAACGAAC AACAGTGGAC ATACAAATCC AAATTTGTAT ATGGACTCCT TTGTCACTGG   
  
  
- CACCTAACGG GATCCCTATG ACAAGAATTG GACTAACCCG CGTATTTGGG TCGACAAAAG TATGTTCCGT   
  
  
- GACAATTGCC CCGAAAGTTA AGAGGGAAAA AGTATCGAGC TAAAGCTCTC CGAGATAAAG TGAAGAGGTG   
  
  
- AGACAAACTA TACGATCTCC GGTTGGACGG TTCCCTGTTA TTCCTCTCCT ACGATTAACT CTCTCTCTAT   
  
  
- AAACCCTCCG TCCGTTACTT ACACTAACGA ACACTCCCAA ATCTCTCCTA TCTTTCCGGT CTCTGCATGT   
  
  
- TCGTCACCGT TCAGGCTTTA CTTTCCCGTC CCAAATCCGT CGACGGAAAT CTAGCGGTCT AAGACCTTTA   
  
  
- CCGATTTTTC TCCCACTTTA GACACATATT GTTTCTAAAG AGGTAACTAC TTCTGCCCGT GACCAACAAC   
  
  
- GACCCTACCT TCCCGTCTTA ACACATGTGT GAGTGATGAA CCTTCGGACG CCTCAT

+     GARE-motif

| Site Name | Organism | Position | Strand | Matrix score. | sequence | function |
| --- | --- | --- | --- | --- | --- | --- |
| GARE-motif | Brassica oleracea | 1273 | - | 7 | TCTGTTG | gibberellin-responsive element |

>HU06G00568.1   
+ -Up\_Stream \_Len000CCCTGG GTCATTAAAA AAAAAGAAAG AAAGAAGATT GCGCTAAAAG TAGATTTTTA   
  
  
+ TCAGATTCAA GCTGCAGGTG ATAACCAAAT TGTCATTAAA GCAATGCAAG CGCATATCAA TACCCTTTTG   
  
  
+ GGAGATAGCT CCGACTGTTG AAGACATTAG GAGCATGATC TCCAACTGTG AATTTGTTTC ATTTACTCAT   
  
  
+ ATTCATATCT ACCATGTGGA TAATATGACT GCAGACTAGA TGGCCAAATT TGAATGCACA CTTATAACTT   
  
  
+ CAACTCTTTC TACTTTTTTC TTCCCCACCT TATCAGGAAT TTCTTTTAAT TGTCGTGGGA GACAACTTGG   
  
  
+ GTAGAACTCT TGCGAAAAGG GCAACTTAAC GTTTTGGTAC TTGCATTTTC CAAAAAAACA AAAAGGCTAA   
  
  
+ ACAATTCTCC GCATGCTGAC TTCACGGTAG ATATGATGTA TTTTTTAACA AAAGACATCT TCTGAATGAA   
  
  
+ ACGCTTAATA TCTTGGACCA TACTAGAAAT TAAATTGCCA CGGTGCATGA ATTTCACCTC GAACTGCTTG   
  
  
+ TATAAAGATC TTGTTATCAC CTTCAGTAAT CAGATACTTG TAACCAAATT GAATGACCAC AAAGACCACA   
  
  
+ TTCCTCACAA TCGTTGCCTC TAGAATAAGA ATGAATGTTT CTTCAAGGTT GAAAGCCCTT GCTAATGCGA   
  
  
+ ACCTGCCTAT ATGCCTAACT GGCTATATAT ATGTGCGTAG AACAGCATGA CATGAGTTAA TAATATAGCA   
  
  
+ GCTAAAGGGA ACTCTAATGT ACACATGATT GTAATTCACC TGCCTCGCTA GCTTACGAGC ATGACTCTTG   
  
  
+ ACTATTATTG TTTTAATTGA TCTTGAATTT TCATAGATTT TACTCCATTG TTTACTCATT TTGTTATAAT   
  
  
+ TAACCACCTG ATTTTAAACT TTAAAATACA CAAAGATTCA TCTAATATAA TTTTTTAGAT AAAGTTATGC   
  
  
+ ATACATATAT ACAAATTAAA TTATTTACGA TCTAATCATT TCCCGTTATA TATTTCACAC TAAAATTTAG   
  
  
+ AAGAGACAAT CTATTAAAAT TACATCACAT GTTTTGGGGG GGGGGGGATA TCAGATATGC ATGACATTCC   
  
  
+ TAATCCAATA ATGCAATGAA ACCCACCGAA TAGTTGAAAT GATTTGTTCT ATCCATGAAC TCACCAAGTC   
  
  
+ ACAATCCATC ACACTCGAAG GATATGCTTT TCTTTTTGAA GGGAATCTTG CTTAATCAAA CCTATAAACT   
  
  
+ TTTAAAAGCA ACAGACAAAA AAGTGATTAT AATGGTAGTG GTAGGATGAA GGCAGCTTTC AGAAAAGCGA   
  
  
+ TTGAATTTTT ATTGTATCCC ATGAAAACCC ATTGAATTTT TCTTTCTTTC TTTGTTTTTG TTACCTGGGT   
  
  
+ TTATGCAAAG GACGGCTGTT GTATTTAATA ATAACAAAAA CATAGGGCTT TGCGGATTGC TAATGCTGGG   
  
  
+ GATGGCATCA GTGCTCGAAT CTGTGTACGC TGTCATAGGA ACCCACTTGC TTCTGCTCAT TTATTTCTGT   
  
  
+ CTCTAGATCC CCTCCTCTCT CTCTCTCTCT CTCTAAGGTG TCTGCTCGTC TGTTCATATT CTCCTGAGGT   
  
  
+ TCTTCTTCAT CTATCTTGTT CTGGGTGTGT GCTGAGGTAC TTCTTTTCTT TATAAATTTT TCATCTTTTG   
  
  
+ TTGTTTTGCT TGGTGTTTAT GATTTTAGTT TTTGTCTTTA AGACGAGATT TGCTGTTAAT AGCAAGATTT   
  
  
+ CTGAACTAGT ATTGATCCTT TTTGTGGTAT ATTTGTTGAA TTTGATTAAT TTGTGTATTG TGTTTAGCTT   
  
  
+ AAGTAACTCG TTGTGATCTA ATTGATGCTC GGGTTTTGAC AGTTTGATTA GGGTTTTCGG TACTGGAGTT   
  
  
+ AAAGTTTTTT TATTTTTTAT AGGAGGAGTT AAAGGTAATA ATAAGAATTG ATGTTTGCTG ATTTTTCCTC   
  
  
+ TTTTCTTTTT TGGTTTCGAT TTGGTGGGCA GTTATCTGTT TATGGTTTTT GCAAATGGAT CCACAATTAG   
  
  
+ AGGAATTATA TGGACCTTTA CATCGAATCA AGTTCAATGA TCAAAGGGTG CCAATTTTAC CAAGTCGCAG   
  
  
+ TGTTGTTACT CCGATGAAAC TCCAAGATTC CAATTTGAAT CCAAGTGTTC CAAATCCAAG TGTTGTGAAT   
  
  
+ CCTCCTCCGT TGGTTCCCCC AAACCCAAAT CTTAATTTAG TTGTGGCATC TCAATATTCT GACATTGAGA   
  
  
+ CAGCCCTGAA CGAGGATTGT GATTTTAGTG ATGTTGTTCT TAAGTATATT AATCAACTTC TTATGGAAGA   
  
  
+ GGATGGGGAA GAGAAACTCC ATACTGATCA TGAACCTTCA GTCGTTGAGG CGGCAGAGAA GTCATTGTAT   
  
  
+ GAGGCGCTCG GGCAGAGATA TCCTCCTTCC GGAAACCGAA ATCAGTTGCC AGATGTTGAG CATGACGGCT   
  
  
+ TGACTGGGAG CAGTGTTAGG GCTCATAGTG GTGCAAGCAG TGTTAGGGCT CATAGTGACG CAAGTGGTGG   
  
  
+ TAGTGGCCTG ACAGGATATG GTTGGTATGG TGATCCTTAT AATTGGAGTC CTCAAAATGT CGTGAATTTT   
  
  
+ ACCATTTCCT CCTCGAAACC CTTATCGTGC AGCTCATCAG ATAGCTCGGG CAGATGGGTT AGTAAGTGGA   
  
  
+ AGTCAAATTC TGATTCACAG CTCAGCTCGG CCATTAATGC AAGTGGTTTA GTGGATGGGC CAGGGGACTC   
  
  
+ TCCTGTGAGT GCTCTTAGTG TGTCTGAAAT ATTCAATGAC AGTCAGTCAA TGTTGCAGTT TCAGAAAGGA   
  
  
+ TTTGAGGAAG CGAATAAATT TCTTCCAAAG AGTTCTTTGT ACAAGGGTTT TGCCAACACG GGATTGCCTT   
  
  
+ ATCAGAAGGC AAACAATAGT GCCCAAGATT TGTTGGTCAA TGTAGAGGAT GTTACTAGGG GAAAGAAGCA   
  
  
+ TCGTTATCCC GAAGAATTGC AGTCAGAAGA AGGGAGGATA AATAAGCAAT CAGCTGTTTC CCTGGCAGCT   
  
  
+ GATGAGGCAG TTGTTAGGTC TGAAATGTTC GATAGGGTAC TGCTTTGTAG TCGGGGAAAA CATGATGCTG   
  
  
+ CTCTCCGGGA AGCTTTACAG ACTGAACTAA ATAAGAGTCT GCGAAATGCC CCAGTTAAGG GGTCTAATAG   
  
  
+ TGGGAAAGGC CGTGGTAAGA AAGCGGGAAA GAAGAGGGAT GTAGTAGATT TAAGATCTCT CTTAACCCTA   
  
  
+ TGTGCACAAG CAGTTGCATC GAATGATCAC AGGAGTGCAA ATGACCTGCT TAGGCAAATT AGACAGCATT   
  
  
+ CTTCTCCTAG TGGGGATGGT AACCAAAGAA TGGCACATTA TTTTGCGGAT GGTCTTGAGG CACGCCTTGC   
  
  
+ TGGTGTAGGA ACTCCTATAT ATAACTGTCT TGTAACGGGT CCGGCATCGG CTGTAGATAT CTTGAGAGCT   
  
  
+ TACCACATGT TTCTTGCCAC ATGCCCATTT AAGAAAATGG GAAATTTCTT CTCTAATAGA ACGATTATGG   
  
  
+ CTGTGGCAGA GAATGCAACA TGCCTTCATA TAATTGATCT CGGTATTGTC TATGGTTTCC AATGGCCTTG   
  
  
+ TCTAATTCAG CGACTTTCAT CTAGGCCTGG TGGCCCCCCC AAACTTCGAA TAACCGGAGT AGATCTTCCA   
  
  
+ CAACCTGGGT TCCGACCAGC CAAAAGAGTT GAGGAGACAG GGCGTCGCTT GAAGAACTAT GCAGAGTCAT   
  
  
+ TTAATGTGCC CTTTGAGTTC AATGCTATAG CAAAGAAGTG GGAAACACTT ACCATTGAAG ATCTCAGGAT   
  
  
+ CAATAGCGAT GAGTTGCTTG TTGTCACCTG TATGTTTAGG TTTAAACATA TACCTGAGGA AACAGTGACC   
  
  
+ GTGGATTGCC CTAGGGATAC TGTTCTTAAC CTGATTGGGC GCATAAACCC AGCTGTTTTC ATACAAGGCA   
  
  
+ CTGTTAACGG GGCTTTCAAT TCTCCCTTTT TCATAGCTCG ATTTCGAGAG GCTCTATTTC ACTTCTCCAC   
  
  
+ TCTGTTTGAT ATGCTAGAGG CCAACCTGCC AAGGGACAAT AAGGAGAGGA TGCTAATTGA GAGAGAGATA   
  
  
+ TTTGGGAGGC AGGCAATGAA TGTGATTGCT TGTGAGGGTT TAGAGAGGAT AGAAAGGCCA GAGACGTACA   
  
  
+ AGCAGTGGCA AGTCCGAAAT GAAAGGGCAG GGTTTAGGCA GCTGCCTTTA GATCGCCAGA TTCTGGAAAT   
  
  
+ GGCTAAAAAG AGGGTGAAAT CTGTGTATAA CAAAGATTTC TCCATTGATG AAGACGGGCA CTGGTTGTTG   
  
  
+ CTGGGATGGA AGGGCAGAAT TGTGTACACA CTCACTACTT GGAAGCCTGC GGAGTA  

- -Up\_Stream \_Len000GGGACC CAGTAATTTT TTTTTCTTTC TTTCTTCTAA CGCGATTTTC ATCTAAAAAT   
  
  
- AGTCTAAGTT CGACGTCCAC TATTGGTTTA ACAGTAATTT CGTTACGTTC GCGTATAGTT ATGGGAAAAC   
  
  
- CCTCTATCGA GGCTGACAAC TTCTGTAATC CTCGTACTAG AGGTTGACAC TTAAACAAAG TAAATGAGTA   
  
  
- TAAGTATAGA TGGTACACCT ATTATACTGA CGTCTGATCT ACCGGTTTAA ACTTACGTGT GAATATTGAA   
  
  
- GTTGAGAAAG ATGAAAAAAG AAGGGGTGGA ATAGTCCTTA AAGAAAATTA ACAGCACCCT CTGTTGAACC   
  
  
- CATCTTGAGA ACGCTTTTCC CGTTGAATTG CAAAACCATG AACGTAAAAG GTTTTTTTGT TTTTCCGATT   
  
  
- TGTTAAGAGG CGTACGACTG AAGTGCCATC TATACTACAT AAAAAATTGT TTTCTGTAGA AGACTTACTT   
  
  
- TGCGAATTAT AGAACCTGGT ATGATCTTTA ATTTAACGGT GCCACGTACT TAAAGTGGAG CTTGACGAAC   
  
  
- ATATTTCTAG AACAATAGTG GAAGTCATTA GTCTATGAAC ATTGGTTTAA CTTACTGGTG TTTCTGGTGT   
  
  
- AAGGAGTGTT AGCAACGGAG ATCTTATTCT TACTTACAAA GAAGTTCCAA CTTTCGGGAA CGATTACGCT   
  
  
- TGGACGGATA TACGGATTGA CCGATATATA TACACGCATC TTGTCGTACT GTACTCAATT ATTATATCGT   
  
  
- CGATTTCCCT TGAGATTACA TGTGTACTAA CATTAAGTGG ACGGAGCGAT CGAATGCTCG TACTGAGAAC   
  
  
- TGATAATAAC AAAATTAACT AGAACTTAAA AGTATCTAAA ATGAGGTAAC AAATGAGTAA AACAATATTA   
  
  
- ATTGGTGGAC TAAAATTTGA AATTTTATGT GTTTCTAAGT AGATTATATT AAAAAATCTA TTTCAATACG   
  
  
- TATGTATATA TGTTTAATTT AATAAATGCT AGATTAGTAA AGGGCAATAT ATAAAGTGTG ATTTTAAATC   
  
  
- TTCTCTGTTA GATAATTTTA ATGTAGTGTA CAAAACCCCC CCCCCCCTAT AGTCTATACG TACTGTAAGG   
  
  
- ATTAGGTTAT TACGTTACTT TGGGTGGCTT ATCAACTTTA CTAAACAAGA TAGGTACTTG AGTGGTTCAG   
  
  
- TGTTAGGTAG TGTGAGCTTC CTATACGAAA AGAAAAACTT CCCTTAGAAC GAATTAGTTT GGATATTTGA   
  
  
- AAATTTTCGT TGTCTGTTTT TTCACTAATA TTACCATCAC CATCCTACTT CCGTCGAAAG TCTTTTCGCT   
  
  
- AACTTAAAAA TAACATAGGG TACTTTTGGG TAACTTAAAA AGAAAGAAAG AAACAAAAAC AATGGACCCA   
  
  
- AATACGTTTC CTGCCGACAA CATAAATTAT TATTGTTTTT GTATCCCGAA ACGCCTAACG ATTACGACCC   
  
  
- CTACCGTAGT CACGAGCTTA GACACATGCG ACAGTATCCT TGGGTGAACG AAGACGAGTA AATAAAGACA   
  
  
- GAGATCTAGG GGAGGAGAGA GAGAGAGAGA GAGATTCCAC AGACGAGCAG ACAAGTATAA GAGGACTCCA   
  
  
- AGAAGAAGTA GATAGAACAA GACCCACACA CGACTCCATG AAGAAAAGAA ATATTTAAAA AGTAGAAAAC   
  
  
- AACAAAACGA ACCACAAATA CTAAAATCAA AAACAGAAAT TCTGCTCTAA ACGACAATTA TCGTTCTAAA   
  
  
- GACTTGATCA TAACTAGGAA AAACACCATA TAAACAACTT AAACTAATTA AACACATAAC ACAAATCGAA   
  
  
- TTCATTGAGC AACACTAGAT TAACTACGAG CCCAAAACTG TCAAACTAAT CCCAAAAGCC ATGACCTCAA   
  
  
- TTTCAAAAAA ATAAAAAATA TCCTCCTCAA TTTCCATTAT TATTCTTAAC TACAAACGAC TAAAAAGGAG   
  
  
- AAAAGAAAAA ACCAAAGCTA AACCACCCGT CAATAGACAA ATACCAAAAA CGTTTACCTA GGTGTTAATC   
  
  
- TCCTTAATAT ACCTGGAAAT GTAGCTTAGT TCAAGTTACT AGTTTCCCAC GGTTAAAATG GTTCAGCGTC   
  
  
- ACAACAATGA GGCTACTTTG AGGTTCTAAG GTTAAACTTA GGTTCACAAG GTTTAGGTTC ACAACACTTA   
  
  
- GGAGGAGGCA ACCAAGGGGG TTTGGGTTTA GAATTAAATC AACACCGTAG AGTTATAAGA CTGTAACTCT   
  
  
- GTCGGGACTT GCTCCTAACA CTAAAATCAC TACAACAAGA ATTCATATAA TTAGTTGAAG AATACCTTCT   
  
  
- CCTACCCCTT CTCTTTGAGG TATGACTAGT ACTTGGAAGT CAGCAACTCC GCCGTCTCTT CAGTAACATA   
  
  
- CTCCGCGAGC CCGTCTCTAT AGGAGGAAGG CCTTTGGCTT TAGTCAACGG TCTACAACTC GTACTGCCGA   
  
  
- ACTGACCCTC GTCACAATCC CGAGTATCAC CACGTTCGTC ACAATCCCGA GTATCACTGC GTTCACCACC   
  
  
- ATCACCGGAC TGTCCTATAC CAACCATACC ACTAGGAATA TTAACCTCAG GAGTTTTACA GCACTTAAAA   
  
  
- TGGTAAAGGA GGAGCTTTGG GAATAGCACG TCGAGTAGTC TATCGAGCCC GTCTACCCAA TCATTCACCT   
  
  
- TCAGTTTAAG ACTAAGTGTC GAGTCGAGCC GGTAATTACG TTCACCAAAT CACCTACCCG GTCCCCTGAG   
  
  
- AGGACACTCA CGAGAATCAC ACAGACTTTA TAAGTTACTG TCAGTCAGTT ACAACGTCAA AGTCTTTCCT   
  
  
- AAACTCCTTC GCTTATTTAA AGAAGGTTTC TCAAGAAACA TGTTCCCAAA ACGGTTGTGC CCTAACGGAA   
  
  
- TAGTCTTCCG TTTGTTATCA CGGGTTCTAA ACAACCAGTT ACATCTCCTA CAATGATCCC CTTTCTTCGT   
  
  
- AGCAATAGGG CTTCTTAACG TCAGTCTTCT TCCCTCCTAT TTATTCGTTA GTCGACAAAG GGACCGTCGA   
  
  
- CTACTCCGTC AACAATCCAG ACTTTACAAG CTATCCCATG ACGAAACATC AGCCCCTTTT GTACTACGAC   
  
  
- GAGAGGCCCT TCGAAATGTC TGACTTGATT TATTCTCAGA CGCTTTACGG GGTCAATTCC CCAGATTATC   
  
  
- ACCCTTTCCG GCACCATTCT TTCGCCCTTT CTTCTCCCTA CATCATCTAA ATTCTAGAGA GAATTGGGAT   
  
  
- ACACGTGTTC GTCAACGTAG CTTACTAGTG TCCTCACGTT TACTGGACGA ATCCGTTTAA TCTGTCGTAA   
  
  
- GAAGAGGATC ACCCCTACCA TTGGTTTCTT ACCGTGTAAT AAAACGCCTA CCAGAACTCC GTGCGGAACG   
  
  
- ACCACATCCT TGAGGATATA TATTGACAGA ACATTGCCCA GGCCGTAGCC GACATCTATA GAACTCTCGA   
  
  
- ATGGTGTACA AAGAACGGTG TACGGGTAAA TTCTTTTACC CTTTAAAGAA GAGATTATCT TGCTAATACC   
  
  
- GACACCGTCT CTTACGTTGT ACGGAAGTAT ATTAACTAGA GCCATAACAG ATACCAAAGG TTACCGGAAC   
  
  
- AGATTAAGTC GCTGAAAGTA GATCCGGACC ACCGGGGGGG TTTGAAGCTT ATTGGCCTCA TCTAGAAGGT   
  
  
- GTTGGACCCA AGGCTGGTCG GTTTTCTCAA CTCCTCTGTC CCGCAGCGAA CTTCTTGATA CGTCTCAGTA   
  
  
- AATTACACGG GAAACTCAAG TTACGATATC GTTTCTTCAC CCTTTGTGAA TGGTAACTTC TAGAGTCCTA   
  
  
- GTTATCGCTA CTCAACGAAC AACAGTGGAC ATACAAATCC AAATTTGTAT ATGGACTCCT TTGTCACTGG   
  
  
- CACCTAACGG GATCCCTATG ACAAGAATTG GACTAACCCG CGTATTTGGG TCGACAAAAG TATGTTCCGT   
  
  
- GACAATTGCC CCGAAAGTTA AGAGGGAAAA AGTATCGAGC TAAAGCTCTC CGAGATAAAG TGAAGAGGTG   
  
  
- AGACAAACTA TACGATCTCC GGTTGGACGG TTCCCTGTTA TTCCTCTCCT ACGATTAACT CTCTCTCTAT   
  
  
- AAACCCTCCG TCCGTTACTT ACACTAACGA ACACTCCCAA ATCTCTCCTA TCTTTCCGGT CTCTGCATGT   
  
  
- TCGTCACCGT TCAGGCTTTA CTTTCCCGTC CCAAATCCGT CGACGGAAAT CTAGCGGTCT AAGACCTTTA   
  
  
- CCGATTTTTC TCCCACTTTA GACACATATT GTTTCTAAAG AGGTAACTAC TTCTGCCCGT GACCAACAAC   
  
  
- GACCCTACCT TCCCGTCTTA ACACATGTGT GAGTGATGAA CCTTCGGACG CCTCAT

+     GATA-motif

| Site Name | Organism | Position | Strand | Matrix score. | sequence | function |
| --- | --- | --- | --- | --- | --- | --- |
| GATA-motif | Pisum sativum | 3045 | + | 7 | GATAGGG | part of a light responsive element |

>HU06G00568.1   
+ -Up\_Stream \_Len000CCCTGG GTCATTAAAA AAAAAGAAAG AAAGAAGATT GCGCTAAAAG TAGATTTTTA   
  
  
+ TCAGATTCAA GCTGCAGGTG ATAACCAAAT TGTCATTAAA GCAATGCAAG CGCATATCAA TACCCTTTTG   
  
  
+ GGAGATAGCT CCGACTGTTG AAGACATTAG GAGCATGATC TCCAACTGTG AATTTGTTTC ATTTACTCAT   
  
  
+ ATTCATATCT ACCATGTGGA TAATATGACT GCAGACTAGA TGGCCAAATT TGAATGCACA CTTATAACTT   
  
  
+ CAACTCTTTC TACTTTTTTC TTCCCCACCT TATCAGGAAT TTCTTTTAAT TGTCGTGGGA GACAACTTGG   
  
  
+ GTAGAACTCT TGCGAAAAGG GCAACTTAAC GTTTTGGTAC TTGCATTTTC CAAAAAAACA AAAAGGCTAA   
  
  
+ ACAATTCTCC GCATGCTGAC TTCACGGTAG ATATGATGTA TTTTTTAACA AAAGACATCT TCTGAATGAA   
  
  
+ ACGCTTAATA TCTTGGACCA TACTAGAAAT TAAATTGCCA CGGTGCATGA ATTTCACCTC GAACTGCTTG   
  
  
+ TATAAAGATC TTGTTATCAC CTTCAGTAAT CAGATACTTG TAACCAAATT GAATGACCAC AAAGACCACA   
  
  
+ TTCCTCACAA TCGTTGCCTC TAGAATAAGA ATGAATGTTT CTTCAAGGTT GAAAGCCCTT GCTAATGCGA   
  
  
+ ACCTGCCTAT ATGCCTAACT GGCTATATAT ATGTGCGTAG AACAGCATGA CATGAGTTAA TAATATAGCA   
  
  
+ GCTAAAGGGA ACTCTAATGT ACACATGATT GTAATTCACC TGCCTCGCTA GCTTACGAGC ATGACTCTTG   
  
  
+ ACTATTATTG TTTTAATTGA TCTTGAATTT TCATAGATTT TACTCCATTG TTTACTCATT TTGTTATAAT   
  
  
+ TAACCACCTG ATTTTAAACT TTAAAATACA CAAAGATTCA TCTAATATAA TTTTTTAGAT AAAGTTATGC   
  
  
+ ATACATATAT ACAAATTAAA TTATTTACGA TCTAATCATT TCCCGTTATA TATTTCACAC TAAAATTTAG   
  
  
+ AAGAGACAAT CTATTAAAAT TACATCACAT GTTTTGGGGG GGGGGGGATA TCAGATATGC ATGACATTCC   
  
  
+ TAATCCAATA ATGCAATGAA ACCCACCGAA TAGTTGAAAT GATTTGTTCT ATCCATGAAC TCACCAAGTC   
  
  
+ ACAATCCATC ACACTCGAAG GATATGCTTT TCTTTTTGAA GGGAATCTTG CTTAATCAAA CCTATAAACT   
  
  
+ TTTAAAAGCA ACAGACAAAA AAGTGATTAT AATGGTAGTG GTAGGATGAA GGCAGCTTTC AGAAAAGCGA   
  
  
+ TTGAATTTTT ATTGTATCCC ATGAAAACCC ATTGAATTTT TCTTTCTTTC TTTGTTTTTG TTACCTGGGT   
  
  
+ TTATGCAAAG GACGGCTGTT GTATTTAATA ATAACAAAAA CATAGGGCTT TGCGGATTGC TAATGCTGGG   
  
  
+ GATGGCATCA GTGCTCGAAT CTGTGTACGC TGTCATAGGA ACCCACTTGC TTCTGCTCAT TTATTTCTGT   
  
  
+ CTCTAGATCC CCTCCTCTCT CTCTCTCTCT CTCTAAGGTG TCTGCTCGTC TGTTCATATT CTCCTGAGGT   
  
  
+ TCTTCTTCAT CTATCTTGTT CTGGGTGTGT GCTGAGGTAC TTCTTTTCTT TATAAATTTT TCATCTTTTG   
  
  
+ TTGTTTTGCT TGGTGTTTAT GATTTTAGTT TTTGTCTTTA AGACGAGATT TGCTGTTAAT AGCAAGATTT   
  
  
+ CTGAACTAGT ATTGATCCTT TTTGTGGTAT ATTTGTTGAA TTTGATTAAT TTGTGTATTG TGTTTAGCTT   
  
  
+ AAGTAACTCG TTGTGATCTA ATTGATGCTC GGGTTTTGAC AGTTTGATTA GGGTTTTCGG TACTGGAGTT   
  
  
+ AAAGTTTTTT TATTTTTTAT AGGAGGAGTT AAAGGTAATA ATAAGAATTG ATGTTTGCTG ATTTTTCCTC   
  
  
+ TTTTCTTTTT TGGTTTCGAT TTGGTGGGCA GTTATCTGTT TATGGTTTTT GCAAATGGAT CCACAATTAG   
  
  
+ AGGAATTATA TGGACCTTTA CATCGAATCA AGTTCAATGA TCAAAGGGTG CCAATTTTAC CAAGTCGCAG   
  
  
+ TGTTGTTACT CCGATGAAAC TCCAAGATTC CAATTTGAAT CCAAGTGTTC CAAATCCAAG TGTTGTGAAT   
  
  
+ CCTCCTCCGT TGGTTCCCCC AAACCCAAAT CTTAATTTAG TTGTGGCATC TCAATATTCT GACATTGAGA   
  
  
+ CAGCCCTGAA CGAGGATTGT GATTTTAGTG ATGTTGTTCT TAAGTATATT AATCAACTTC TTATGGAAGA   
  
  
+ GGATGGGGAA GAGAAACTCC ATACTGATCA TGAACCTTCA GTCGTTGAGG CGGCAGAGAA GTCATTGTAT   
  
  
+ GAGGCGCTCG GGCAGAGATA TCCTCCTTCC GGAAACCGAA ATCAGTTGCC AGATGTTGAG CATGACGGCT   
  
  
+ TGACTGGGAG CAGTGTTAGG GCTCATAGTG GTGCAAGCAG TGTTAGGGCT CATAGTGACG CAAGTGGTGG   
  
  
+ TAGTGGCCTG ACAGGATATG GTTGGTATGG TGATCCTTAT AATTGGAGTC CTCAAAATGT CGTGAATTTT   
  
  
+ ACCATTTCCT CCTCGAAACC CTTATCGTGC AGCTCATCAG ATAGCTCGGG CAGATGGGTT AGTAAGTGGA   
  
  
+ AGTCAAATTC TGATTCACAG CTCAGCTCGG CCATTAATGC AAGTGGTTTA GTGGATGGGC CAGGGGACTC   
  
  
+ TCCTGTGAGT GCTCTTAGTG TGTCTGAAAT ATTCAATGAC AGTCAGTCAA TGTTGCAGTT TCAGAAAGGA   
  
  
+ TTTGAGGAAG CGAATAAATT TCTTCCAAAG AGTTCTTTGT ACAAGGGTTT TGCCAACACG GGATTGCCTT   
  
  
+ ATCAGAAGGC AAACAATAGT GCCCAAGATT TGTTGGTCAA TGTAGAGGAT GTTACTAGGG GAAAGAAGCA   
  
  
+ TCGTTATCCC GAAGAATTGC AGTCAGAAGA AGGGAGGATA AATAAGCAAT CAGCTGTTTC CCTGGCAGCT   
  
  
+ GATGAGGCAG TTGTTAGGTC TGAAATGTTC GATAGGGTAC TGCTTTGTAG TCGGGGAAAA CATGATGCTG   
  
  
+ CTCTCCGGGA AGCTTTACAG ACTGAACTAA ATAAGAGTCT GCGAAATGCC CCAGTTAAGG GGTCTAATAG   
  
  
+ TGGGAAAGGC CGTGGTAAGA AAGCGGGAAA GAAGAGGGAT GTAGTAGATT TAAGATCTCT CTTAACCCTA   
  
  
+ TGTGCACAAG CAGTTGCATC GAATGATCAC AGGAGTGCAA ATGACCTGCT TAGGCAAATT AGACAGCATT   
  
  
+ CTTCTCCTAG TGGGGATGGT AACCAAAGAA TGGCACATTA TTTTGCGGAT GGTCTTGAGG CACGCCTTGC   
  
  
+ TGGTGTAGGA ACTCCTATAT ATAACTGTCT TGTAACGGGT CCGGCATCGG CTGTAGATAT CTTGAGAGCT   
  
  
+ TACCACATGT TTCTTGCCAC ATGCCCATTT AAGAAAATGG GAAATTTCTT CTCTAATAGA ACGATTATGG   
  
  
+ CTGTGGCAGA GAATGCAACA TGCCTTCATA TAATTGATCT CGGTATTGTC TATGGTTTCC AATGGCCTTG   
  
  
+ TCTAATTCAG CGACTTTCAT CTAGGCCTGG TGGCCCCCCC AAACTTCGAA TAACCGGAGT AGATCTTCCA   
  
  
+ CAACCTGGGT TCCGACCAGC CAAAAGAGTT GAGGAGACAG GGCGTCGCTT GAAGAACTAT GCAGAGTCAT   
  
  
+ TTAATGTGCC CTTTGAGTTC AATGCTATAG CAAAGAAGTG GGAAACACTT ACCATTGAAG ATCTCAGGAT   
  
  
+ CAATAGCGAT GAGTTGCTTG TTGTCACCTG TATGTTTAGG TTTAAACATA TACCTGAGGA AACAGTGACC   
  
  
+ GTGGATTGCC CTAGGGATAC TGTTCTTAAC CTGATTGGGC GCATAAACCC AGCTGTTTTC ATACAAGGCA   
  
  
+ CTGTTAACGG GGCTTTCAAT TCTCCCTTTT TCATAGCTCG ATTTCGAGAG GCTCTATTTC ACTTCTCCAC   
  
  
+ TCTGTTTGAT ATGCTAGAGG CCAACCTGCC AAGGGACAAT AAGGAGAGGA TGCTAATTGA GAGAGAGATA   
  
  
+ TTTGGGAGGC AGGCAATGAA TGTGATTGCT TGTGAGGGTT TAGAGAGGAT AGAAAGGCCA GAGACGTACA   
  
  
+ AGCAGTGGCA AGTCCGAAAT GAAAGGGCAG GGTTTAGGCA GCTGCCTTTA GATCGCCAGA TTCTGGAAAT   
  
  
+ GGCTAAAAAG AGGGTGAAAT CTGTGTATAA CAAAGATTTC TCCATTGATG AAGACGGGCA CTGGTTGTTG   
  
  
+ CTGGGATGGA AGGGCAGAAT TGTGTACACA CTCACTACTT GGAAGCCTGC GGAGTA  

- -Up\_Stream \_Len000GGGACC CAGTAATTTT TTTTTCTTTC TTTCTTCTAA CGCGATTTTC ATCTAAAAAT   
  
  
- AGTCTAAGTT CGACGTCCAC TATTGGTTTA ACAGTAATTT CGTTACGTTC GCGTATAGTT ATGGGAAAAC   
  
  
- CCTCTATCGA GGCTGACAAC TTCTGTAATC CTCGTACTAG AGGTTGACAC TTAAACAAAG TAAATGAGTA   
  
  
- TAAGTATAGA TGGTACACCT ATTATACTGA CGTCTGATCT ACCGGTTTAA ACTTACGTGT GAATATTGAA   
  
  
- GTTGAGAAAG ATGAAAAAAG AAGGGGTGGA ATAGTCCTTA AAGAAAATTA ACAGCACCCT CTGTTGAACC   
  
  
- CATCTTGAGA ACGCTTTTCC CGTTGAATTG CAAAACCATG AACGTAAAAG GTTTTTTTGT TTTTCCGATT   
  
  
- TGTTAAGAGG CGTACGACTG AAGTGCCATC TATACTACAT AAAAAATTGT TTTCTGTAGA AGACTTACTT   
  
  
- TGCGAATTAT AGAACCTGGT ATGATCTTTA ATTTAACGGT GCCACGTACT TAAAGTGGAG CTTGACGAAC   
  
  
- ATATTTCTAG AACAATAGTG GAAGTCATTA GTCTATGAAC ATTGGTTTAA CTTACTGGTG TTTCTGGTGT   
  
  
- AAGGAGTGTT AGCAACGGAG ATCTTATTCT TACTTACAAA GAAGTTCCAA CTTTCGGGAA CGATTACGCT   
  
  
- TGGACGGATA TACGGATTGA CCGATATATA TACACGCATC TTGTCGTACT GTACTCAATT ATTATATCGT   
  
  
- CGATTTCCCT TGAGATTACA TGTGTACTAA CATTAAGTGG ACGGAGCGAT CGAATGCTCG TACTGAGAAC   
  
  
- TGATAATAAC AAAATTAACT AGAACTTAAA AGTATCTAAA ATGAGGTAAC AAATGAGTAA AACAATATTA   
  
  
- ATTGGTGGAC TAAAATTTGA AATTTTATGT GTTTCTAAGT AGATTATATT AAAAAATCTA TTTCAATACG   
  
  
- TATGTATATA TGTTTAATTT AATAAATGCT AGATTAGTAA AGGGCAATAT ATAAAGTGTG ATTTTAAATC   
  
  
- TTCTCTGTTA GATAATTTTA ATGTAGTGTA CAAAACCCCC CCCCCCCTAT AGTCTATACG TACTGTAAGG   
  
  
- ATTAGGTTAT TACGTTACTT TGGGTGGCTT ATCAACTTTA CTAAACAAGA TAGGTACTTG AGTGGTTCAG   
  
  
- TGTTAGGTAG TGTGAGCTTC CTATACGAAA AGAAAAACTT CCCTTAGAAC GAATTAGTTT GGATATTTGA   
  
  
- AAATTTTCGT TGTCTGTTTT TTCACTAATA TTACCATCAC CATCCTACTT CCGTCGAAAG TCTTTTCGCT   
  
  
- AACTTAAAAA TAACATAGGG TACTTTTGGG TAACTTAAAA AGAAAGAAAG AAACAAAAAC AATGGACCCA   
  
  
- AATACGTTTC CTGCCGACAA CATAAATTAT TATTGTTTTT GTATCCCGAA ACGCCTAACG ATTACGACCC   
  
  
- CTACCGTAGT CACGAGCTTA GACACATGCG ACAGTATCCT TGGGTGAACG AAGACGAGTA AATAAAGACA   
  
  
- GAGATCTAGG GGAGGAGAGA GAGAGAGAGA GAGATTCCAC AGACGAGCAG ACAAGTATAA GAGGACTCCA   
  
  
- AGAAGAAGTA GATAGAACAA GACCCACACA CGACTCCATG AAGAAAAGAA ATATTTAAAA AGTAGAAAAC   
  
  
- AACAAAACGA ACCACAAATA CTAAAATCAA AAACAGAAAT TCTGCTCTAA ACGACAATTA TCGTTCTAAA   
  
  
- GACTTGATCA TAACTAGGAA AAACACCATA TAAACAACTT AAACTAATTA AACACATAAC ACAAATCGAA   
  
  
- TTCATTGAGC AACACTAGAT TAACTACGAG CCCAAAACTG TCAAACTAAT CCCAAAAGCC ATGACCTCAA   
  
  
- TTTCAAAAAA ATAAAAAATA TCCTCCTCAA TTTCCATTAT TATTCTTAAC TACAAACGAC TAAAAAGGAG   
  
  
- AAAAGAAAAA ACCAAAGCTA AACCACCCGT CAATAGACAA ATACCAAAAA CGTTTACCTA GGTGTTAATC   
  
  
- TCCTTAATAT ACCTGGAAAT GTAGCTTAGT TCAAGTTACT AGTTTCCCAC GGTTAAAATG GTTCAGCGTC   
  
  
- ACAACAATGA GGCTACTTTG AGGTTCTAAG GTTAAACTTA GGTTCACAAG GTTTAGGTTC ACAACACTTA   
  
  
- GGAGGAGGCA ACCAAGGGGG TTTGGGTTTA GAATTAAATC AACACCGTAG AGTTATAAGA CTGTAACTCT   
  
  
- GTCGGGACTT GCTCCTAACA CTAAAATCAC TACAACAAGA ATTCATATAA TTAGTTGAAG AATACCTTCT   
  
  
- CCTACCCCTT CTCTTTGAGG TATGACTAGT ACTTGGAAGT CAGCAACTCC GCCGTCTCTT CAGTAACATA   
  
  
- CTCCGCGAGC CCGTCTCTAT AGGAGGAAGG CCTTTGGCTT TAGTCAACGG TCTACAACTC GTACTGCCGA   
  
  
- ACTGACCCTC GTCACAATCC CGAGTATCAC CACGTTCGTC ACAATCCCGA GTATCACTGC GTTCACCACC   
  
  
- ATCACCGGAC TGTCCTATAC CAACCATACC ACTAGGAATA TTAACCTCAG GAGTTTTACA GCACTTAAAA   
  
  
- TGGTAAAGGA GGAGCTTTGG GAATAGCACG TCGAGTAGTC TATCGAGCCC GTCTACCCAA TCATTCACCT   
  
  
- TCAGTTTAAG ACTAAGTGTC GAGTCGAGCC GGTAATTACG TTCACCAAAT CACCTACCCG GTCCCCTGAG   
  
  
- AGGACACTCA CGAGAATCAC ACAGACTTTA TAAGTTACTG TCAGTCAGTT ACAACGTCAA AGTCTTTCCT   
  
  
- AAACTCCTTC GCTTATTTAA AGAAGGTTTC TCAAGAAACA TGTTCCCAAA ACGGTTGTGC CCTAACGGAA   
  
  
- TAGTCTTCCG TTTGTTATCA CGGGTTCTAA ACAACCAGTT ACATCTCCTA CAATGATCCC CTTTCTTCGT   
  
  
- AGCAATAGGG CTTCTTAACG TCAGTCTTCT TCCCTCCTAT TTATTCGTTA GTCGACAAAG GGACCGTCGA   
  
  
- CTACTCCGTC AACAATCCAG ACTTTACAAG CTATCCCATG ACGAAACATC AGCCCCTTTT GTACTACGAC   
  
  
- GAGAGGCCCT TCGAAATGTC TGACTTGATT TATTCTCAGA CGCTTTACGG GGTCAATTCC CCAGATTATC   
  
  
- ACCCTTTCCG GCACCATTCT TTCGCCCTTT CTTCTCCCTA CATCATCTAA ATTCTAGAGA GAATTGGGAT   
  
  
- ACACGTGTTC GTCAACGTAG CTTACTAGTG TCCTCACGTT TACTGGACGA ATCCGTTTAA TCTGTCGTAA   
  
  
- GAAGAGGATC ACCCCTACCA TTGGTTTCTT ACCGTGTAAT AAAACGCCTA CCAGAACTCC GTGCGGAACG   
  
  
- ACCACATCCT TGAGGATATA TATTGACAGA ACATTGCCCA GGCCGTAGCC GACATCTATA GAACTCTCGA   
  
  
- ATGGTGTACA AAGAACGGTG TACGGGTAAA TTCTTTTACC CTTTAAAGAA GAGATTATCT TGCTAATACC   
  
  
- GACACCGTCT CTTACGTTGT ACGGAAGTAT ATTAACTAGA GCCATAACAG ATACCAAAGG TTACCGGAAC   
  
  
- AGATTAAGTC GCTGAAAGTA GATCCGGACC ACCGGGGGGG TTTGAAGCTT ATTGGCCTCA TCTAGAAGGT   
  
  
- GTTGGACCCA AGGCTGGTCG GTTTTCTCAA CTCCTCTGTC CCGCAGCGAA CTTCTTGATA CGTCTCAGTA   
  
  
- AATTACACGG GAAACTCAAG TTACGATATC GTTTCTTCAC CCTTTGTGAA TGGTAACTTC TAGAGTCCTA   
  
  
- GTTATCGCTA CTCAACGAAC AACAGTGGAC ATACAAATCC AAATTTGTAT ATGGACTCCT TTGTCACTGG   
  
  
- CACCTAACGG GATCCCTATG ACAAGAATTG GACTAACCCG CGTATTTGGG TCGACAAAAG TATGTTCCGT   
  
  
- GACAATTGCC CCGAAAGTTA AGAGGGAAAA AGTATCGAGC TAAAGCTCTC CGAGATAAAG TGAAGAGGTG   
  
  
- AGACAAACTA TACGATCTCC GGTTGGACGG TTCCCTGTTA TTCCTCTCCT ACGATTAACT CTCTCTCTAT   
  
  
- AAACCCTCCG TCCGTTACTT ACACTAACGA ACACTCCCAA ATCTCTCCTA TCTTTCCGGT CTCTGCATGT   
  
  
- TCGTCACCGT TCAGGCTTTA CTTTCCCGTC CCAAATCCGT CGACGGAAAT CTAGCGGTCT AAGACCTTTA   
  
  
- CCGATTTTTC TCCCACTTTA GACACATATT GTTTCTAAAG AGGTAACTAC TTCTGCCCGT GACCAACAAC   
  
  
- GACCCTACCT TCCCGTCTTA ACACATGTGT GAGTGATGAA CCTTCGGACG CCTCAT

+     GT1-motif

| Site Name | Organism | Position | Strand | Matrix score. | sequence | function |
| --- | --- | --- | --- | --- | --- | --- |
| GT1-motif | Avena sativa | 913 | - | 7 | GGTTAAT | light responsive element |
| GT1-motif | Arabidopsis thaliana | 3216 | - | 6 | GGTTAA | light responsive element |
| GT1-motif | Arabidopsis thaliana | 914 | - | 6 | GGTTAA | light responsive element |
| GT1-motif | Arabidopsis thaliana | 3880 | - | 6 | GGTTAA | light responsive element |

>HU06G00568.1   
+ -Up\_Stream \_Len000CCCTGG GTCATTAAAA AAAAAGAAAG AAAGAAGATT GCGCTAAAAG TAGATTTTTA   
  
  
+ TCAGATTCAA GCTGCAGGTG ATAACCAAAT TGTCATTAAA GCAATGCAAG CGCATATCAA TACCCTTTTG   
  
  
+ GGAGATAGCT CCGACTGTTG AAGACATTAG GAGCATGATC TCCAACTGTG AATTTGTTTC ATTTACTCAT   
  
  
+ ATTCATATCT ACCATGTGGA TAATATGACT GCAGACTAGA TGGCCAAATT TGAATGCACA CTTATAACTT   
  
  
+ CAACTCTTTC TACTTTTTTC TTCCCCACCT TATCAGGAAT TTCTTTTAAT TGTCGTGGGA GACAACTTGG   
  
  
+ GTAGAACTCT TGCGAAAAGG GCAACTTAAC GTTTTGGTAC TTGCATTTTC CAAAAAAACA AAAAGGCTAA   
  
  
+ ACAATTCTCC GCATGCTGAC TTCACGGTAG ATATGATGTA TTTTTTAACA AAAGACATCT TCTGAATGAA   
  
  
+ ACGCTTAATA TCTTGGACCA TACTAGAAAT TAAATTGCCA CGGTGCATGA ATTTCACCTC GAACTGCTTG   
  
  
+ TATAAAGATC TTGTTATCAC CTTCAGTAAT CAGATACTTG TAACCAAATT GAATGACCAC AAAGACCACA   
  
  
+ TTCCTCACAA TCGTTGCCTC TAGAATAAGA ATGAATGTTT CTTCAAGGTT GAAAGCCCTT GCTAATGCGA   
  
  
+ ACCTGCCTAT ATGCCTAACT GGCTATATAT ATGTGCGTAG AACAGCATGA CATGAGTTAA TAATATAGCA   
  
  
+ GCTAAAGGGA ACTCTAATGT ACACATGATT GTAATTCACC TGCCTCGCTA GCTTACGAGC ATGACTCTTG   
  
  
+ ACTATTATTG TTTTAATTGA TCTTGAATTT TCATAGATTT TACTCCATTG TTTACTCATT TTGTTATAAT   
  
  
+ TAACCACCTG ATTTTAAACT TTAAAATACA CAAAGATTCA TCTAATATAA TTTTTTAGAT AAAGTTATGC   
  
  
+ ATACATATAT ACAAATTAAA TTATTTACGA TCTAATCATT TCCCGTTATA TATTTCACAC TAAAATTTAG   
  
  
+ AAGAGACAAT CTATTAAAAT TACATCACAT GTTTTGGGGG GGGGGGGATA TCAGATATGC ATGACATTCC   
  
  
+ TAATCCAATA ATGCAATGAA ACCCACCGAA TAGTTGAAAT GATTTGTTCT ATCCATGAAC TCACCAAGTC   
  
  
+ ACAATCCATC ACACTCGAAG GATATGCTTT TCTTTTTGAA GGGAATCTTG CTTAATCAAA CCTATAAACT   
  
  
+ TTTAAAAGCA ACAGACAAAA AAGTGATTAT AATGGTAGTG GTAGGATGAA GGCAGCTTTC AGAAAAGCGA   
  
  
+ TTGAATTTTT ATTGTATCCC ATGAAAACCC ATTGAATTTT TCTTTCTTTC TTTGTTTTTG TTACCTGGGT   
  
  
+ TTATGCAAAG GACGGCTGTT GTATTTAATA ATAACAAAAA CATAGGGCTT TGCGGATTGC TAATGCTGGG   
  
  
+ GATGGCATCA GTGCTCGAAT CTGTGTACGC TGTCATAGGA ACCCACTTGC TTCTGCTCAT TTATTTCTGT   
  
  
+ CTCTAGATCC CCTCCTCTCT CTCTCTCTCT CTCTAAGGTG TCTGCTCGTC TGTTCATATT CTCCTGAGGT   
  
  
+ TCTTCTTCAT CTATCTTGTT CTGGGTGTGT GCTGAGGTAC TTCTTTTCTT TATAAATTTT TCATCTTTTG   
  
  
+ TTGTTTTGCT TGGTGTTTAT GATTTTAGTT TTTGTCTTTA AGACGAGATT TGCTGTTAAT AGCAAGATTT   
  
  
+ CTGAACTAGT ATTGATCCTT TTTGTGGTAT ATTTGTTGAA TTTGATTAAT TTGTGTATTG TGTTTAGCTT   
  
  
+ AAGTAACTCG TTGTGATCTA ATTGATGCTC GGGTTTTGAC AGTTTGATTA GGGTTTTCGG TACTGGAGTT   
  
  
+ AAAGTTTTTT TATTTTTTAT AGGAGGAGTT AAAGGTAATA ATAAGAATTG ATGTTTGCTG ATTTTTCCTC   
  
  
+ TTTTCTTTTT TGGTTTCGAT TTGGTGGGCA GTTATCTGTT TATGGTTTTT GCAAATGGAT CCACAATTAG   
  
  
+ AGGAATTATA TGGACCTTTA CATCGAATCA AGTTCAATGA TCAAAGGGTG CCAATTTTAC CAAGTCGCAG   
  
  
+ TGTTGTTACT CCGATGAAAC TCCAAGATTC CAATTTGAAT CCAAGTGTTC CAAATCCAAG TGTTGTGAAT   
  
  
+ CCTCCTCCGT TGGTTCCCCC AAACCCAAAT CTTAATTTAG TTGTGGCATC TCAATATTCT GACATTGAGA   
  
  
+ CAGCCCTGAA CGAGGATTGT GATTTTAGTG ATGTTGTTCT TAAGTATATT AATCAACTTC TTATGGAAGA   
  
  
+ GGATGGGGAA GAGAAACTCC ATACTGATCA TGAACCTTCA GTCGTTGAGG CGGCAGAGAA GTCATTGTAT   
  
  
+ GAGGCGCTCG GGCAGAGATA TCCTCCTTCC GGAAACCGAA ATCAGTTGCC AGATGTTGAG CATGACGGCT   
  
  
+ TGACTGGGAG CAGTGTTAGG GCTCATAGTG GTGCAAGCAG TGTTAGGGCT CATAGTGACG CAAGTGGTGG   
  
  
+ TAGTGGCCTG ACAGGATATG GTTGGTATGG TGATCCTTAT AATTGGAGTC CTCAAAATGT CGTGAATTTT   
  
  
+ ACCATTTCCT CCTCGAAACC CTTATCGTGC AGCTCATCAG ATAGCTCGGG CAGATGGGTT AGTAAGTGGA   
  
  
+ AGTCAAATTC TGATTCACAG CTCAGCTCGG CCATTAATGC AAGTGGTTTA GTGGATGGGC CAGGGGACTC   
  
  
+ TCCTGTGAGT GCTCTTAGTG TGTCTGAAAT ATTCAATGAC AGTCAGTCAA TGTTGCAGTT TCAGAAAGGA   
  
  
+ TTTGAGGAAG CGAATAAATT TCTTCCAAAG AGTTCTTTGT ACAAGGGTTT TGCCAACACG GGATTGCCTT   
  
  
+ ATCAGAAGGC AAACAATAGT GCCCAAGATT TGTTGGTCAA TGTAGAGGAT GTTACTAGGG GAAAGAAGCA   
  
  
+ TCGTTATCCC GAAGAATTGC AGTCAGAAGA AGGGAGGATA AATAAGCAAT CAGCTGTTTC CCTGGCAGCT   
  
  
+ GATGAGGCAG TTGTTAGGTC TGAAATGTTC GATAGGGTAC TGCTTTGTAG TCGGGGAAAA CATGATGCTG   
  
  
+ CTCTCCGGGA AGCTTTACAG ACTGAACTAA ATAAGAGTCT GCGAAATGCC CCAGTTAAGG GGTCTAATAG   
  
  
+ TGGGAAAGGC CGTGGTAAGA AAGCGGGAAA GAAGAGGGAT GTAGTAGATT TAAGATCTCT CTTAACCCTA   
  
  
+ TGTGCACAAG CAGTTGCATC GAATGATCAC AGGAGTGCAA ATGACCTGCT TAGGCAAATT AGACAGCATT   
  
  
+ CTTCTCCTAG TGGGGATGGT AACCAAAGAA TGGCACATTA TTTTGCGGAT GGTCTTGAGG CACGCCTTGC   
  
  
+ TGGTGTAGGA ACTCCTATAT ATAACTGTCT TGTAACGGGT CCGGCATCGG CTGTAGATAT CTTGAGAGCT   
  
  
+ TACCACATGT TTCTTGCCAC ATGCCCATTT AAGAAAATGG GAAATTTCTT CTCTAATAGA ACGATTATGG   
  
  
+ CTGTGGCAGA GAATGCAACA TGCCTTCATA TAATTGATCT CGGTATTGTC TATGGTTTCC AATGGCCTTG   
  
  
+ TCTAATTCAG CGACTTTCAT CTAGGCCTGG TGGCCCCCCC AAACTTCGAA TAACCGGAGT AGATCTTCCA   
  
  
+ CAACCTGGGT TCCGACCAGC CAAAAGAGTT GAGGAGACAG GGCGTCGCTT GAAGAACTAT GCAGAGTCAT   
  
  
+ TTAATGTGCC CTTTGAGTTC AATGCTATAG CAAAGAAGTG GGAAACACTT ACCATTGAAG ATCTCAGGAT   
  
  
+ CAATAGCGAT GAGTTGCTTG TTGTCACCTG TATGTTTAGG TTTAAACATA TACCTGAGGA AACAGTGACC   
  
  
+ GTGGATTGCC CTAGGGATAC TGTTCTTAAC CTGATTGGGC GCATAAACCC AGCTGTTTTC ATACAAGGCA   
  
  
+ CTGTTAACGG GGCTTTCAAT TCTCCCTTTT TCATAGCTCG ATTTCGAGAG GCTCTATTTC ACTTCTCCAC   
  
  
+ TCTGTTTGAT ATGCTAGAGG CCAACCTGCC AAGGGACAAT AAGGAGAGGA TGCTAATTGA GAGAGAGATA   
  
  
+ TTTGGGAGGC AGGCAATGAA TGTGATTGCT TGTGAGGGTT TAGAGAGGAT AGAAAGGCCA GAGACGTACA   
  
  
+ AGCAGTGGCA AGTCCGAAAT GAAAGGGCAG GGTTTAGGCA GCTGCCTTTA GATCGCCAGA TTCTGGAAAT   
  
  
+ GGCTAAAAAG AGGGTGAAAT CTGTGTATAA CAAAGATTTC TCCATTGATG AAGACGGGCA CTGGTTGTTG   
  
  
+ CTGGGATGGA AGGGCAGAAT TGTGTACACA CTCACTACTT GGAAGCCTGC GGAGTA  

- -Up\_Stream \_Len000GGGACC CAGTAATTTT TTTTTCTTTC TTTCTTCTAA CGCGATTTTC ATCTAAAAAT   
  
  
- AGTCTAAGTT CGACGTCCAC TATTGGTTTA ACAGTAATTT CGTTACGTTC GCGTATAGTT ATGGGAAAAC   
  
  
- CCTCTATCGA GGCTGACAAC TTCTGTAATC CTCGTACTAG AGGTTGACAC TTAAACAAAG TAAATGAGTA   
  
  
- TAAGTATAGA TGGTACACCT ATTATACTGA CGTCTGATCT ACCGGTTTAA ACTTACGTGT GAATATTGAA   
  
  
- GTTGAGAAAG ATGAAAAAAG AAGGGGTGGA ATAGTCCTTA AAGAAAATTA ACAGCACCCT CTGTTGAACC   
  
  
- CATCTTGAGA ACGCTTTTCC CGTTGAATTG CAAAACCATG AACGTAAAAG GTTTTTTTGT TTTTCCGATT   
  
  
- TGTTAAGAGG CGTACGACTG AAGTGCCATC TATACTACAT AAAAAATTGT TTTCTGTAGA AGACTTACTT   
  
  
- TGCGAATTAT AGAACCTGGT ATGATCTTTA ATTTAACGGT GCCACGTACT TAAAGTGGAG CTTGACGAAC   
  
  
- ATATTTCTAG AACAATAGTG GAAGTCATTA GTCTATGAAC ATTGGTTTAA CTTACTGGTG TTTCTGGTGT   
  
  
- AAGGAGTGTT AGCAACGGAG ATCTTATTCT TACTTACAAA GAAGTTCCAA CTTTCGGGAA CGATTACGCT   
  
  
- TGGACGGATA TACGGATTGA CCGATATATA TACACGCATC TTGTCGTACT GTACTCAATT ATTATATCGT   
  
  
- CGATTTCCCT TGAGATTACA TGTGTACTAA CATTAAGTGG ACGGAGCGAT CGAATGCTCG TACTGAGAAC   
  
  
- TGATAATAAC AAAATTAACT AGAACTTAAA AGTATCTAAA ATGAGGTAAC AAATGAGTAA AACAATATTA   
  
  
- ATTGGTGGAC TAAAATTTGA AATTTTATGT GTTTCTAAGT AGATTATATT AAAAAATCTA TTTCAATACG   
  
  
- TATGTATATA TGTTTAATTT AATAAATGCT AGATTAGTAA AGGGCAATAT ATAAAGTGTG ATTTTAAATC   
  
  
- TTCTCTGTTA GATAATTTTA ATGTAGTGTA CAAAACCCCC CCCCCCCTAT AGTCTATACG TACTGTAAGG   
  
  
- ATTAGGTTAT TACGTTACTT TGGGTGGCTT ATCAACTTTA CTAAACAAGA TAGGTACTTG AGTGGTTCAG   
  
  
- TGTTAGGTAG TGTGAGCTTC CTATACGAAA AGAAAAACTT CCCTTAGAAC GAATTAGTTT GGATATTTGA   
  
  
- AAATTTTCGT TGTCTGTTTT TTCACTAATA TTACCATCAC CATCCTACTT CCGTCGAAAG TCTTTTCGCT   
  
  
- AACTTAAAAA TAACATAGGG TACTTTTGGG TAACTTAAAA AGAAAGAAAG AAACAAAAAC AATGGACCCA   
  
  
- AATACGTTTC CTGCCGACAA CATAAATTAT TATTGTTTTT GTATCCCGAA ACGCCTAACG ATTACGACCC   
  
  
- CTACCGTAGT CACGAGCTTA GACACATGCG ACAGTATCCT TGGGTGAACG AAGACGAGTA AATAAAGACA   
  
  
- GAGATCTAGG GGAGGAGAGA GAGAGAGAGA GAGATTCCAC AGACGAGCAG ACAAGTATAA GAGGACTCCA   
  
  
- AGAAGAAGTA GATAGAACAA GACCCACACA CGACTCCATG AAGAAAAGAA ATATTTAAAA AGTAGAAAAC   
  
  
- AACAAAACGA ACCACAAATA CTAAAATCAA AAACAGAAAT TCTGCTCTAA ACGACAATTA TCGTTCTAAA   
  
  
- GACTTGATCA TAACTAGGAA AAACACCATA TAAACAACTT AAACTAATTA AACACATAAC ACAAATCGAA   
  
  
- TTCATTGAGC AACACTAGAT TAACTACGAG CCCAAAACTG TCAAACTAAT CCCAAAAGCC ATGACCTCAA   
  
  
- TTTCAAAAAA ATAAAAAATA TCCTCCTCAA TTTCCATTAT TATTCTTAAC TACAAACGAC TAAAAAGGAG   
  
  
- AAAAGAAAAA ACCAAAGCTA AACCACCCGT CAATAGACAA ATACCAAAAA CGTTTACCTA GGTGTTAATC   
  
  
- TCCTTAATAT ACCTGGAAAT GTAGCTTAGT TCAAGTTACT AGTTTCCCAC GGTTAAAATG GTTCAGCGTC   
  
  
- ACAACAATGA GGCTACTTTG AGGTTCTAAG GTTAAACTTA GGTTCACAAG GTTTAGGTTC ACAACACTTA   
  
  
- GGAGGAGGCA ACCAAGGGGG TTTGGGTTTA GAATTAAATC AACACCGTAG AGTTATAAGA CTGTAACTCT   
  
  
- GTCGGGACTT GCTCCTAACA CTAAAATCAC TACAACAAGA ATTCATATAA TTAGTTGAAG AATACCTTCT   
  
  
- CCTACCCCTT CTCTTTGAGG TATGACTAGT ACTTGGAAGT CAGCAACTCC GCCGTCTCTT CAGTAACATA   
  
  
- CTCCGCGAGC CCGTCTCTAT AGGAGGAAGG CCTTTGGCTT TAGTCAACGG TCTACAACTC GTACTGCCGA   
  
  
- ACTGACCCTC GTCACAATCC CGAGTATCAC CACGTTCGTC ACAATCCCGA GTATCACTGC GTTCACCACC   
  
  
- ATCACCGGAC TGTCCTATAC CAACCATACC ACTAGGAATA TTAACCTCAG GAGTTTTACA GCACTTAAAA   
  
  
- TGGTAAAGGA GGAGCTTTGG GAATAGCACG TCGAGTAGTC TATCGAGCCC GTCTACCCAA TCATTCACCT   
  
  
- TCAGTTTAAG ACTAAGTGTC GAGTCGAGCC GGTAATTACG TTCACCAAAT CACCTACCCG GTCCCCTGAG   
  
  
- AGGACACTCA CGAGAATCAC ACAGACTTTA TAAGTTACTG TCAGTCAGTT ACAACGTCAA AGTCTTTCCT   
  
  
- AAACTCCTTC GCTTATTTAA AGAAGGTTTC TCAAGAAACA TGTTCCCAAA ACGGTTGTGC CCTAACGGAA   
  
  
- TAGTCTTCCG TTTGTTATCA CGGGTTCTAA ACAACCAGTT ACATCTCCTA CAATGATCCC CTTTCTTCGT   
  
  
- AGCAATAGGG CTTCTTAACG TCAGTCTTCT TCCCTCCTAT TTATTCGTTA GTCGACAAAG GGACCGTCGA   
  
  
- CTACTCCGTC AACAATCCAG ACTTTACAAG CTATCCCATG ACGAAACATC AGCCCCTTTT GTACTACGAC   
  
  
- GAGAGGCCCT TCGAAATGTC TGACTTGATT TATTCTCAGA CGCTTTACGG GGTCAATTCC CCAGATTATC   
  
  
- ACCCTTTCCG GCACCATTCT TTCGCCCTTT CTTCTCCCTA CATCATCTAA ATTCTAGAGA GAATTGGGAT   
  
  
- ACACGTGTTC GTCAACGTAG CTTACTAGTG TCCTCACGTT TACTGGACGA ATCCGTTTAA TCTGTCGTAA   
  
  
- GAAGAGGATC ACCCCTACCA TTGGTTTCTT ACCGTGTAAT AAAACGCCTA CCAGAACTCC GTGCGGAACG   
  
  
- ACCACATCCT TGAGGATATA TATTGACAGA ACATTGCCCA GGCCGTAGCC GACATCTATA GAACTCTCGA   
  
  
- ATGGTGTACA AAGAACGGTG TACGGGTAAA TTCTTTTACC CTTTAAAGAA GAGATTATCT TGCTAATACC   
  
  
- GACACCGTCT CTTACGTTGT ACGGAAGTAT ATTAACTAGA GCCATAACAG ATACCAAAGG TTACCGGAAC   
  
  
- AGATTAAGTC GCTGAAAGTA GATCCGGACC ACCGGGGGGG TTTGAAGCTT ATTGGCCTCA TCTAGAAGGT   
  
  
- GTTGGACCCA AGGCTGGTCG GTTTTCTCAA CTCCTCTGTC CCGCAGCGAA CTTCTTGATA CGTCTCAGTA   
  
  
- AATTACACGG GAAACTCAAG TTACGATATC GTTTCTTCAC CCTTTGTGAA TGGTAACTTC TAGAGTCCTA   
  
  
- GTTATCGCTA CTCAACGAAC AACAGTGGAC ATACAAATCC AAATTTGTAT ATGGACTCCT TTGTCACTGG   
  
  
- CACCTAACGG GATCCCTATG ACAAGAATTG GACTAACCCG CGTATTTGGG TCGACAAAAG TATGTTCCGT   
  
  
- GACAATTGCC CCGAAAGTTA AGAGGGAAAA AGTATCGAGC TAAAGCTCTC CGAGATAAAG TGAAGAGGTG   
  
  
- AGACAAACTA TACGATCTCC GGTTGGACGG TTCCCTGTTA TTCCTCTCCT ACGATTAACT CTCTCTCTAT   
  
  
- AAACCCTCCG TCCGTTACTT ACACTAACGA ACACTCCCAA ATCTCTCCTA TCTTTCCGGT CTCTGCATGT   
  
  
- TCGTCACCGT TCAGGCTTTA CTTTCCCGTC CCAAATCCGT CGACGGAAAT CTAGCGGTCT AAGACCTTTA   
  
  
- CCGATTTTTC TCCCACTTTA GACACATATT GTTTCTAAAG AGGTAACTAC TTCTGCCCGT GACCAACAAC   
  
  
- GACCCTACCT TCCCGTCTTA ACACATGTGT GAGTGATGAA CCTTCGGACG CCTCAT

+     I-box

| Site Name | Organism | Position | Strand | Matrix score. | sequence | function |
| --- | --- | --- | --- | --- | --- | --- |
| I-box | Zea mays | 310 | - | 9 | gGATAAGGTG | part of a light responsive element |
| I-box | Arabidopsis thaliana | 2612 | - | 9 | GATAAGGGT | part of a light responsive element |

>HU06G00568.1   
+ -Up\_Stream \_Len000CCCTGG GTCATTAAAA AAAAAGAAAG AAAGAAGATT GCGCTAAAAG TAGATTTTTA   
  
  
+ TCAGATTCAA GCTGCAGGTG ATAACCAAAT TGTCATTAAA GCAATGCAAG CGCATATCAA TACCCTTTTG   
  
  
+ GGAGATAGCT CCGACTGTTG AAGACATTAG GAGCATGATC TCCAACTGTG AATTTGTTTC ATTTACTCAT   
  
  
+ ATTCATATCT ACCATGTGGA TAATATGACT GCAGACTAGA TGGCCAAATT TGAATGCACA CTTATAACTT   
  
  
+ CAACTCTTTC TACTTTTTTC TTCCCCACCT TATCAGGAAT TTCTTTTAAT TGTCGTGGGA GACAACTTGG   
  
  
+ GTAGAACTCT TGCGAAAAGG GCAACTTAAC GTTTTGGTAC TTGCATTTTC CAAAAAAACA AAAAGGCTAA   
  
  
+ ACAATTCTCC GCATGCTGAC TTCACGGTAG ATATGATGTA TTTTTTAACA AAAGACATCT TCTGAATGAA   
  
  
+ ACGCTTAATA TCTTGGACCA TACTAGAAAT TAAATTGCCA CGGTGCATGA ATTTCACCTC GAACTGCTTG   
  
  
+ TATAAAGATC TTGTTATCAC CTTCAGTAAT CAGATACTTG TAACCAAATT GAATGACCAC AAAGACCACA   
  
  
+ TTCCTCACAA TCGTTGCCTC TAGAATAAGA ATGAATGTTT CTTCAAGGTT GAAAGCCCTT GCTAATGCGA   
  
  
+ ACCTGCCTAT ATGCCTAACT GGCTATATAT ATGTGCGTAG AACAGCATGA CATGAGTTAA TAATATAGCA   
  
  
+ GCTAAAGGGA ACTCTAATGT ACACATGATT GTAATTCACC TGCCTCGCTA GCTTACGAGC ATGACTCTTG   
  
  
+ ACTATTATTG TTTTAATTGA TCTTGAATTT TCATAGATTT TACTCCATTG TTTACTCATT TTGTTATAAT   
  
  
+ TAACCACCTG ATTTTAAACT TTAAAATACA CAAAGATTCA TCTAATATAA TTTTTTAGAT AAAGTTATGC   
  
  
+ ATACATATAT ACAAATTAAA TTATTTACGA TCTAATCATT TCCCGTTATA TATTTCACAC TAAAATTTAG   
  
  
+ AAGAGACAAT CTATTAAAAT TACATCACAT GTTTTGGGGG GGGGGGGATA TCAGATATGC ATGACATTCC   
  
  
+ TAATCCAATA ATGCAATGAA ACCCACCGAA TAGTTGAAAT GATTTGTTCT ATCCATGAAC TCACCAAGTC   
  
  
+ ACAATCCATC ACACTCGAAG GATATGCTTT TCTTTTTGAA GGGAATCTTG CTTAATCAAA CCTATAAACT   
  
  
+ TTTAAAAGCA ACAGACAAAA AAGTGATTAT AATGGTAGTG GTAGGATGAA GGCAGCTTTC AGAAAAGCGA   
  
  
+ TTGAATTTTT ATTGTATCCC ATGAAAACCC ATTGAATTTT TCTTTCTTTC TTTGTTTTTG TTACCTGGGT   
  
  
+ TTATGCAAAG GACGGCTGTT GTATTTAATA ATAACAAAAA CATAGGGCTT TGCGGATTGC TAATGCTGGG   
  
  
+ GATGGCATCA GTGCTCGAAT CTGTGTACGC TGTCATAGGA ACCCACTTGC TTCTGCTCAT TTATTTCTGT   
  
  
+ CTCTAGATCC CCTCCTCTCT CTCTCTCTCT CTCTAAGGTG TCTGCTCGTC TGTTCATATT CTCCTGAGGT   
  
  
+ TCTTCTTCAT CTATCTTGTT CTGGGTGTGT GCTGAGGTAC TTCTTTTCTT TATAAATTTT TCATCTTTTG   
  
  
+ TTGTTTTGCT TGGTGTTTAT GATTTTAGTT TTTGTCTTTA AGACGAGATT TGCTGTTAAT AGCAAGATTT   
  
  
+ CTGAACTAGT ATTGATCCTT TTTGTGGTAT ATTTGTTGAA TTTGATTAAT TTGTGTATTG TGTTTAGCTT   
  
  
+ AAGTAACTCG TTGTGATCTA ATTGATGCTC GGGTTTTGAC AGTTTGATTA GGGTTTTCGG TACTGGAGTT   
  
  
+ AAAGTTTTTT TATTTTTTAT AGGAGGAGTT AAAGGTAATA ATAAGAATTG ATGTTTGCTG ATTTTTCCTC   
  
  
+ TTTTCTTTTT TGGTTTCGAT TTGGTGGGCA GTTATCTGTT TATGGTTTTT GCAAATGGAT CCACAATTAG   
  
  
+ AGGAATTATA TGGACCTTTA CATCGAATCA AGTTCAATGA TCAAAGGGTG CCAATTTTAC CAAGTCGCAG   
  
  
+ TGTTGTTACT CCGATGAAAC TCCAAGATTC CAATTTGAAT CCAAGTGTTC CAAATCCAAG TGTTGTGAAT   
  
  
+ CCTCCTCCGT TGGTTCCCCC AAACCCAAAT CTTAATTTAG TTGTGGCATC TCAATATTCT GACATTGAGA   
  
  
+ CAGCCCTGAA CGAGGATTGT GATTTTAGTG ATGTTGTTCT TAAGTATATT AATCAACTTC TTATGGAAGA   
  
  
+ GGATGGGGAA GAGAAACTCC ATACTGATCA TGAACCTTCA GTCGTTGAGG CGGCAGAGAA GTCATTGTAT   
  
  
+ GAGGCGCTCG GGCAGAGATA TCCTCCTTCC GGAAACCGAA ATCAGTTGCC AGATGTTGAG CATGACGGCT   
  
  
+ TGACTGGGAG CAGTGTTAGG GCTCATAGTG GTGCAAGCAG TGTTAGGGCT CATAGTGACG CAAGTGGTGG   
  
  
+ TAGTGGCCTG ACAGGATATG GTTGGTATGG TGATCCTTAT AATTGGAGTC CTCAAAATGT CGTGAATTTT   
  
  
+ ACCATTTCCT CCTCGAAACC CTTATCGTGC AGCTCATCAG ATAGCTCGGG CAGATGGGTT AGTAAGTGGA   
  
  
+ AGTCAAATTC TGATTCACAG CTCAGCTCGG CCATTAATGC AAGTGGTTTA GTGGATGGGC CAGGGGACTC   
  
  
+ TCCTGTGAGT GCTCTTAGTG TGTCTGAAAT ATTCAATGAC AGTCAGTCAA TGTTGCAGTT TCAGAAAGGA   
  
  
+ TTTGAGGAAG CGAATAAATT TCTTCCAAAG AGTTCTTTGT ACAAGGGTTT TGCCAACACG GGATTGCCTT   
  
  
+ ATCAGAAGGC AAACAATAGT GCCCAAGATT TGTTGGTCAA TGTAGAGGAT GTTACTAGGG GAAAGAAGCA   
  
  
+ TCGTTATCCC GAAGAATTGC AGTCAGAAGA AGGGAGGATA AATAAGCAAT CAGCTGTTTC CCTGGCAGCT   
  
  
+ GATGAGGCAG TTGTTAGGTC TGAAATGTTC GATAGGGTAC TGCTTTGTAG TCGGGGAAAA CATGATGCTG   
  
  
+ CTCTCCGGGA AGCTTTACAG ACTGAACTAA ATAAGAGTCT GCGAAATGCC CCAGTTAAGG GGTCTAATAG   
  
  
+ TGGGAAAGGC CGTGGTAAGA AAGCGGGAAA GAAGAGGGAT GTAGTAGATT TAAGATCTCT CTTAACCCTA   
  
  
+ TGTGCACAAG CAGTTGCATC GAATGATCAC AGGAGTGCAA ATGACCTGCT TAGGCAAATT AGACAGCATT   
  
  
+ CTTCTCCTAG TGGGGATGGT AACCAAAGAA TGGCACATTA TTTTGCGGAT GGTCTTGAGG CACGCCTTGC   
  
  
+ TGGTGTAGGA ACTCCTATAT ATAACTGTCT TGTAACGGGT CCGGCATCGG CTGTAGATAT CTTGAGAGCT   
  
  
+ TACCACATGT TTCTTGCCAC ATGCCCATTT AAGAAAATGG GAAATTTCTT CTCTAATAGA ACGATTATGG   
  
  
+ CTGTGGCAGA GAATGCAACA TGCCTTCATA TAATTGATCT CGGTATTGTC TATGGTTTCC AATGGCCTTG   
  
  
+ TCTAATTCAG CGACTTTCAT CTAGGCCTGG TGGCCCCCCC AAACTTCGAA TAACCGGAGT AGATCTTCCA   
  
  
+ CAACCTGGGT TCCGACCAGC CAAAAGAGTT GAGGAGACAG GGCGTCGCTT GAAGAACTAT GCAGAGTCAT   
  
  
+ TTAATGTGCC CTTTGAGTTC AATGCTATAG CAAAGAAGTG GGAAACACTT ACCATTGAAG ATCTCAGGAT   
  
  
+ CAATAGCGAT GAGTTGCTTG TTGTCACCTG TATGTTTAGG TTTAAACATA TACCTGAGGA AACAGTGACC   
  
  
+ GTGGATTGCC CTAGGGATAC TGTTCTTAAC CTGATTGGGC GCATAAACCC AGCTGTTTTC ATACAAGGCA   
  
  
+ CTGTTAACGG GGCTTTCAAT TCTCCCTTTT TCATAGCTCG ATTTCGAGAG GCTCTATTTC ACTTCTCCAC   
  
  
+ TCTGTTTGAT ATGCTAGAGG CCAACCTGCC AAGGGACAAT AAGGAGAGGA TGCTAATTGA GAGAGAGATA   
  
  
+ TTTGGGAGGC AGGCAATGAA TGTGATTGCT TGTGAGGGTT TAGAGAGGAT AGAAAGGCCA GAGACGTACA   
  
  
+ AGCAGTGGCA AGTCCGAAAT GAAAGGGCAG GGTTTAGGCA GCTGCCTTTA GATCGCCAGA TTCTGGAAAT   
  
  
+ GGCTAAAAAG AGGGTGAAAT CTGTGTATAA CAAAGATTTC TCCATTGATG AAGACGGGCA CTGGTTGTTG   
  
  
+ CTGGGATGGA AGGGCAGAAT TGTGTACACA CTCACTACTT GGAAGCCTGC GGAGTA  

- -Up\_Stream \_Len000GGGACC CAGTAATTTT TTTTTCTTTC TTTCTTCTAA CGCGATTTTC ATCTAAAAAT   
  
  
- AGTCTAAGTT CGACGTCCAC TATTGGTTTA ACAGTAATTT CGTTACGTTC GCGTATAGTT ATGGGAAAAC   
  
  
- CCTCTATCGA GGCTGACAAC TTCTGTAATC CTCGTACTAG AGGTTGACAC TTAAACAAAG TAAATGAGTA   
  
  
- TAAGTATAGA TGGTACACCT ATTATACTGA CGTCTGATCT ACCGGTTTAA ACTTACGTGT GAATATTGAA   
  
  
- GTTGAGAAAG ATGAAAAAAG AAGGGGTGGA ATAGTCCTTA AAGAAAATTA ACAGCACCCT CTGTTGAACC   
  
  
- CATCTTGAGA ACGCTTTTCC CGTTGAATTG CAAAACCATG AACGTAAAAG GTTTTTTTGT TTTTCCGATT   
  
  
- TGTTAAGAGG CGTACGACTG AAGTGCCATC TATACTACAT AAAAAATTGT TTTCTGTAGA AGACTTACTT   
  
  
- TGCGAATTAT AGAACCTGGT ATGATCTTTA ATTTAACGGT GCCACGTACT TAAAGTGGAG CTTGACGAAC   
  
  
- ATATTTCTAG AACAATAGTG GAAGTCATTA GTCTATGAAC ATTGGTTTAA CTTACTGGTG TTTCTGGTGT   
  
  
- AAGGAGTGTT AGCAACGGAG ATCTTATTCT TACTTACAAA GAAGTTCCAA CTTTCGGGAA CGATTACGCT   
  
  
- TGGACGGATA TACGGATTGA CCGATATATA TACACGCATC TTGTCGTACT GTACTCAATT ATTATATCGT   
  
  
- CGATTTCCCT TGAGATTACA TGTGTACTAA CATTAAGTGG ACGGAGCGAT CGAATGCTCG TACTGAGAAC   
  
  
- TGATAATAAC AAAATTAACT AGAACTTAAA AGTATCTAAA ATGAGGTAAC AAATGAGTAA AACAATATTA   
  
  
- ATTGGTGGAC TAAAATTTGA AATTTTATGT GTTTCTAAGT AGATTATATT AAAAAATCTA TTTCAATACG   
  
  
- TATGTATATA TGTTTAATTT AATAAATGCT AGATTAGTAA AGGGCAATAT ATAAAGTGTG ATTTTAAATC   
  
  
- TTCTCTGTTA GATAATTTTA ATGTAGTGTA CAAAACCCCC CCCCCCCTAT AGTCTATACG TACTGTAAGG   
  
  
- ATTAGGTTAT TACGTTACTT TGGGTGGCTT ATCAACTTTA CTAAACAAGA TAGGTACTTG AGTGGTTCAG   
  
  
- TGTTAGGTAG TGTGAGCTTC CTATACGAAA AGAAAAACTT CCCTTAGAAC GAATTAGTTT GGATATTTGA   
  
  
- AAATTTTCGT TGTCTGTTTT TTCACTAATA TTACCATCAC CATCCTACTT CCGTCGAAAG TCTTTTCGCT   
  
  
- AACTTAAAAA TAACATAGGG TACTTTTGGG TAACTTAAAA AGAAAGAAAG AAACAAAAAC AATGGACCCA   
  
  
- AATACGTTTC CTGCCGACAA CATAAATTAT TATTGTTTTT GTATCCCGAA ACGCCTAACG ATTACGACCC   
  
  
- CTACCGTAGT CACGAGCTTA GACACATGCG ACAGTATCCT TGGGTGAACG AAGACGAGTA AATAAAGACA   
  
  
- GAGATCTAGG GGAGGAGAGA GAGAGAGAGA GAGATTCCAC AGACGAGCAG ACAAGTATAA GAGGACTCCA   
  
  
- AGAAGAAGTA GATAGAACAA GACCCACACA CGACTCCATG AAGAAAAGAA ATATTTAAAA AGTAGAAAAC   
  
  
- AACAAAACGA ACCACAAATA CTAAAATCAA AAACAGAAAT TCTGCTCTAA ACGACAATTA TCGTTCTAAA   
  
  
- GACTTGATCA TAACTAGGAA AAACACCATA TAAACAACTT AAACTAATTA AACACATAAC ACAAATCGAA   
  
  
- TTCATTGAGC AACACTAGAT TAACTACGAG CCCAAAACTG TCAAACTAAT CCCAAAAGCC ATGACCTCAA   
  
  
- TTTCAAAAAA ATAAAAAATA TCCTCCTCAA TTTCCATTAT TATTCTTAAC TACAAACGAC TAAAAAGGAG   
  
  
- AAAAGAAAAA ACCAAAGCTA AACCACCCGT CAATAGACAA ATACCAAAAA CGTTTACCTA GGTGTTAATC   
  
  
- TCCTTAATAT ACCTGGAAAT GTAGCTTAGT TCAAGTTACT AGTTTCCCAC GGTTAAAATG GTTCAGCGTC   
  
  
- ACAACAATGA GGCTACTTTG AGGTTCTAAG GTTAAACTTA GGTTCACAAG GTTTAGGTTC ACAACACTTA   
  
  
- GGAGGAGGCA ACCAAGGGGG TTTGGGTTTA GAATTAAATC AACACCGTAG AGTTATAAGA CTGTAACTCT   
  
  
- GTCGGGACTT GCTCCTAACA CTAAAATCAC TACAACAAGA ATTCATATAA TTAGTTGAAG AATACCTTCT   
  
  
- CCTACCCCTT CTCTTTGAGG TATGACTAGT ACTTGGAAGT CAGCAACTCC GCCGTCTCTT CAGTAACATA   
  
  
- CTCCGCGAGC CCGTCTCTAT AGGAGGAAGG CCTTTGGCTT TAGTCAACGG TCTACAACTC GTACTGCCGA   
  
  
- ACTGACCCTC GTCACAATCC CGAGTATCAC CACGTTCGTC ACAATCCCGA GTATCACTGC GTTCACCACC   
  
  
- ATCACCGGAC TGTCCTATAC CAACCATACC ACTAGGAATA TTAACCTCAG GAGTTTTACA GCACTTAAAA   
  
  
- TGGTAAAGGA GGAGCTTTGG GAATAGCACG TCGAGTAGTC TATCGAGCCC GTCTACCCAA TCATTCACCT   
  
  
- TCAGTTTAAG ACTAAGTGTC GAGTCGAGCC GGTAATTACG TTCACCAAAT CACCTACCCG GTCCCCTGAG   
  
  
- AGGACACTCA CGAGAATCAC ACAGACTTTA TAAGTTACTG TCAGTCAGTT ACAACGTCAA AGTCTTTCCT   
  
  
- AAACTCCTTC GCTTATTTAA AGAAGGTTTC TCAAGAAACA TGTTCCCAAA ACGGTTGTGC CCTAACGGAA   
  
  
- TAGTCTTCCG TTTGTTATCA CGGGTTCTAA ACAACCAGTT ACATCTCCTA CAATGATCCC CTTTCTTCGT   
  
  
- AGCAATAGGG CTTCTTAACG TCAGTCTTCT TCCCTCCTAT TTATTCGTTA GTCGACAAAG GGACCGTCGA   
  
  
- CTACTCCGTC AACAATCCAG ACTTTACAAG CTATCCCATG ACGAAACATC AGCCCCTTTT GTACTACGAC   
  
  
- GAGAGGCCCT TCGAAATGTC TGACTTGATT TATTCTCAGA CGCTTTACGG GGTCAATTCC CCAGATTATC   
  
  
- ACCCTTTCCG GCACCATTCT TTCGCCCTTT CTTCTCCCTA CATCATCTAA ATTCTAGAGA GAATTGGGAT   
  
  
- ACACGTGTTC GTCAACGTAG CTTACTAGTG TCCTCACGTT TACTGGACGA ATCCGTTTAA TCTGTCGTAA   
  
  
- GAAGAGGATC ACCCCTACCA TTGGTTTCTT ACCGTGTAAT AAAACGCCTA CCAGAACTCC GTGCGGAACG   
  
  
- ACCACATCCT TGAGGATATA TATTGACAGA ACATTGCCCA GGCCGTAGCC GACATCTATA GAACTCTCGA   
  
  
- ATGGTGTACA AAGAACGGTG TACGGGTAAA TTCTTTTACC CTTTAAAGAA GAGATTATCT TGCTAATACC   
  
  
- GACACCGTCT CTTACGTTGT ACGGAAGTAT ATTAACTAGA GCCATAACAG ATACCAAAGG TTACCGGAAC   
  
  
- AGATTAAGTC GCTGAAAGTA GATCCGGACC ACCGGGGGGG TTTGAAGCTT ATTGGCCTCA TCTAGAAGGT   
  
  
- GTTGGACCCA AGGCTGGTCG GTTTTCTCAA CTCCTCTGTC CCGCAGCGAA CTTCTTGATA CGTCTCAGTA   
  
  
- AATTACACGG GAAACTCAAG TTACGATATC GTTTCTTCAC CCTTTGTGAA TGGTAACTTC TAGAGTCCTA   
  
  
- GTTATCGCTA CTCAACGAAC AACAGTGGAC ATACAAATCC AAATTTGTAT ATGGACTCCT TTGTCACTGG   
  
  
- CACCTAACGG GATCCCTATG ACAAGAATTG GACTAACCCG CGTATTTGGG TCGACAAAAG TATGTTCCGT   
  
  
- GACAATTGCC CCGAAAGTTA AGAGGGAAAA AGTATCGAGC TAAAGCTCTC CGAGATAAAG TGAAGAGGTG   
  
  
- AGACAAACTA TACGATCTCC GGTTGGACGG TTCCCTGTTA TTCCTCTCCT ACGATTAACT CTCTCTCTAT   
  
  
- AAACCCTCCG TCCGTTACTT ACACTAACGA ACACTCCCAA ATCTCTCCTA TCTTTCCGGT CTCTGCATGT   
  
  
- TCGTCACCGT TCAGGCTTTA CTTTCCCGTC CCAAATCCGT CGACGGAAAT CTAGCGGTCT AAGACCTTTA   
  
  
- CCGATTTTTC TCCCACTTTA GACACATATT GTTTCTAAAG AGGTAACTAC TTCTGCCCGT GACCAACAAC   
  
  
- GACCCTACCT TCCCGTCTTA ACACATGTGT GAGTGATGAA CCTTCGGACG CCTCAT

+     LTR

| Site Name | Organism | Position | Strand | Matrix score. | sequence | function |
| --- | --- | --- | --- | --- | --- | --- |
| LTR | Hordeum vulgare | 2420 | + | 6 | CCGAAA | cis-acting element involved in low-temperature responsiveness |
| LTR | Hordeum vulgare | 4148 | + | 6 | CCGAAA | cis-acting element involved in low-temperature responsiveness |
| LTR | Hordeum vulgare | 1879 | - | 6 | CCGAAA | cis-acting element involved in low-temperature responsiveness |

>HU06G00568.1   
+ -Up\_Stream \_Len000CCCTGG GTCATTAAAA AAAAAGAAAG AAAGAAGATT GCGCTAAAAG TAGATTTTTA   
  
  
+ TCAGATTCAA GCTGCAGGTG ATAACCAAAT TGTCATTAAA GCAATGCAAG CGCATATCAA TACCCTTTTG   
  
  
+ GGAGATAGCT CCGACTGTTG AAGACATTAG GAGCATGATC TCCAACTGTG AATTTGTTTC ATTTACTCAT   
  
  
+ ATTCATATCT ACCATGTGGA TAATATGACT GCAGACTAGA TGGCCAAATT TGAATGCACA CTTATAACTT   
  
  
+ CAACTCTTTC TACTTTTTTC TTCCCCACCT TATCAGGAAT TTCTTTTAAT TGTCGTGGGA GACAACTTGG   
  
  
+ GTAGAACTCT TGCGAAAAGG GCAACTTAAC GTTTTGGTAC TTGCATTTTC CAAAAAAACA AAAAGGCTAA   
  
  
+ ACAATTCTCC GCATGCTGAC TTCACGGTAG ATATGATGTA TTTTTTAACA AAAGACATCT TCTGAATGAA   
  
  
+ ACGCTTAATA TCTTGGACCA TACTAGAAAT TAAATTGCCA CGGTGCATGA ATTTCACCTC GAACTGCTTG   
  
  
+ TATAAAGATC TTGTTATCAC CTTCAGTAAT CAGATACTTG TAACCAAATT GAATGACCAC AAAGACCACA   
  
  
+ TTCCTCACAA TCGTTGCCTC TAGAATAAGA ATGAATGTTT CTTCAAGGTT GAAAGCCCTT GCTAATGCGA   
  
  
+ ACCTGCCTAT ATGCCTAACT GGCTATATAT ATGTGCGTAG AACAGCATGA CATGAGTTAA TAATATAGCA   
  
  
+ GCTAAAGGGA ACTCTAATGT ACACATGATT GTAATTCACC TGCCTCGCTA GCTTACGAGC ATGACTCTTG   
  
  
+ ACTATTATTG TTTTAATTGA TCTTGAATTT TCATAGATTT TACTCCATTG TTTACTCATT TTGTTATAAT   
  
  
+ TAACCACCTG ATTTTAAACT TTAAAATACA CAAAGATTCA TCTAATATAA TTTTTTAGAT AAAGTTATGC   
  
  
+ ATACATATAT ACAAATTAAA TTATTTACGA TCTAATCATT TCCCGTTATA TATTTCACAC TAAAATTTAG   
  
  
+ AAGAGACAAT CTATTAAAAT TACATCACAT GTTTTGGGGG GGGGGGGATA TCAGATATGC ATGACATTCC   
  
  
+ TAATCCAATA ATGCAATGAA ACCCACCGAA TAGTTGAAAT GATTTGTTCT ATCCATGAAC TCACCAAGTC   
  
  
+ ACAATCCATC ACACTCGAAG GATATGCTTT TCTTTTTGAA GGGAATCTTG CTTAATCAAA CCTATAAACT   
  
  
+ TTTAAAAGCA ACAGACAAAA AAGTGATTAT AATGGTAGTG GTAGGATGAA GGCAGCTTTC AGAAAAGCGA   
  
  
+ TTGAATTTTT ATTGTATCCC ATGAAAACCC ATTGAATTTT TCTTTCTTTC TTTGTTTTTG TTACCTGGGT   
  
  
+ TTATGCAAAG GACGGCTGTT GTATTTAATA ATAACAAAAA CATAGGGCTT TGCGGATTGC TAATGCTGGG   
  
  
+ GATGGCATCA GTGCTCGAAT CTGTGTACGC TGTCATAGGA ACCCACTTGC TTCTGCTCAT TTATTTCTGT   
  
  
+ CTCTAGATCC CCTCCTCTCT CTCTCTCTCT CTCTAAGGTG TCTGCTCGTC TGTTCATATT CTCCTGAGGT   
  
  
+ TCTTCTTCAT CTATCTTGTT CTGGGTGTGT GCTGAGGTAC TTCTTTTCTT TATAAATTTT TCATCTTTTG   
  
  
+ TTGTTTTGCT TGGTGTTTAT GATTTTAGTT TTTGTCTTTA AGACGAGATT TGCTGTTAAT AGCAAGATTT   
  
  
+ CTGAACTAGT ATTGATCCTT TTTGTGGTAT ATTTGTTGAA TTTGATTAAT TTGTGTATTG TGTTTAGCTT   
  
  
+ AAGTAACTCG TTGTGATCTA ATTGATGCTC GGGTTTTGAC AGTTTGATTA GGGTTTTCGG TACTGGAGTT   
  
  
+ AAAGTTTTTT TATTTTTTAT AGGAGGAGTT AAAGGTAATA ATAAGAATTG ATGTTTGCTG ATTTTTCCTC   
  
  
+ TTTTCTTTTT TGGTTTCGAT TTGGTGGGCA GTTATCTGTT TATGGTTTTT GCAAATGGAT CCACAATTAG   
  
  
+ AGGAATTATA TGGACCTTTA CATCGAATCA AGTTCAATGA TCAAAGGGTG CCAATTTTAC CAAGTCGCAG   
  
  
+ TGTTGTTACT CCGATGAAAC TCCAAGATTC CAATTTGAAT CCAAGTGTTC CAAATCCAAG TGTTGTGAAT   
  
  
+ CCTCCTCCGT TGGTTCCCCC AAACCCAAAT CTTAATTTAG TTGTGGCATC TCAATATTCT GACATTGAGA   
  
  
+ CAGCCCTGAA CGAGGATTGT GATTTTAGTG ATGTTGTTCT TAAGTATATT AATCAACTTC TTATGGAAGA   
  
  
+ GGATGGGGAA GAGAAACTCC ATACTGATCA TGAACCTTCA GTCGTTGAGG CGGCAGAGAA GTCATTGTAT   
  
  
+ GAGGCGCTCG GGCAGAGATA TCCTCCTTCC GGAAACCGAA ATCAGTTGCC AGATGTTGAG CATGACGGCT   
  
  
+ TGACTGGGAG CAGTGTTAGG GCTCATAGTG GTGCAAGCAG TGTTAGGGCT CATAGTGACG CAAGTGGTGG   
  
  
+ TAGTGGCCTG ACAGGATATG GTTGGTATGG TGATCCTTAT AATTGGAGTC CTCAAAATGT CGTGAATTTT   
  
  
+ ACCATTTCCT CCTCGAAACC CTTATCGTGC AGCTCATCAG ATAGCTCGGG CAGATGGGTT AGTAAGTGGA   
  
  
+ AGTCAAATTC TGATTCACAG CTCAGCTCGG CCATTAATGC AAGTGGTTTA GTGGATGGGC CAGGGGACTC   
  
  
+ TCCTGTGAGT GCTCTTAGTG TGTCTGAAAT ATTCAATGAC AGTCAGTCAA TGTTGCAGTT TCAGAAAGGA   
  
  
+ TTTGAGGAAG CGAATAAATT TCTTCCAAAG AGTTCTTTGT ACAAGGGTTT TGCCAACACG GGATTGCCTT   
  
  
+ ATCAGAAGGC AAACAATAGT GCCCAAGATT TGTTGGTCAA TGTAGAGGAT GTTACTAGGG GAAAGAAGCA   
  
  
+ TCGTTATCCC GAAGAATTGC AGTCAGAAGA AGGGAGGATA AATAAGCAAT CAGCTGTTTC CCTGGCAGCT   
  
  
+ GATGAGGCAG TTGTTAGGTC TGAAATGTTC GATAGGGTAC TGCTTTGTAG TCGGGGAAAA CATGATGCTG   
  
  
+ CTCTCCGGGA AGCTTTACAG ACTGAACTAA ATAAGAGTCT GCGAAATGCC CCAGTTAAGG GGTCTAATAG   
  
  
+ TGGGAAAGGC CGTGGTAAGA AAGCGGGAAA GAAGAGGGAT GTAGTAGATT TAAGATCTCT CTTAACCCTA   
  
  
+ TGTGCACAAG CAGTTGCATC GAATGATCAC AGGAGTGCAA ATGACCTGCT TAGGCAAATT AGACAGCATT   
  
  
+ CTTCTCCTAG TGGGGATGGT AACCAAAGAA TGGCACATTA TTTTGCGGAT GGTCTTGAGG CACGCCTTGC   
  
  
+ TGGTGTAGGA ACTCCTATAT ATAACTGTCT TGTAACGGGT CCGGCATCGG CTGTAGATAT CTTGAGAGCT   
  
  
+ TACCACATGT TTCTTGCCAC ATGCCCATTT AAGAAAATGG GAAATTTCTT CTCTAATAGA ACGATTATGG   
  
  
+ CTGTGGCAGA GAATGCAACA TGCCTTCATA TAATTGATCT CGGTATTGTC TATGGTTTCC AATGGCCTTG   
  
  
+ TCTAATTCAG CGACTTTCAT CTAGGCCTGG TGGCCCCCCC AAACTTCGAA TAACCGGAGT AGATCTTCCA   
  
  
+ CAACCTGGGT TCCGACCAGC CAAAAGAGTT GAGGAGACAG GGCGTCGCTT GAAGAACTAT GCAGAGTCAT   
  
  
+ TTAATGTGCC CTTTGAGTTC AATGCTATAG CAAAGAAGTG GGAAACACTT ACCATTGAAG ATCTCAGGAT   
  
  
+ CAATAGCGAT GAGTTGCTTG TTGTCACCTG TATGTTTAGG TTTAAACATA TACCTGAGGA AACAGTGACC   
  
  
+ GTGGATTGCC CTAGGGATAC TGTTCTTAAC CTGATTGGGC GCATAAACCC AGCTGTTTTC ATACAAGGCA   
  
  
+ CTGTTAACGG GGCTTTCAAT TCTCCCTTTT TCATAGCTCG ATTTCGAGAG GCTCTATTTC ACTTCTCCAC   
  
  
+ TCTGTTTGAT ATGCTAGAGG CCAACCTGCC AAGGGACAAT AAGGAGAGGA TGCTAATTGA GAGAGAGATA   
  
  
+ TTTGGGAGGC AGGCAATGAA TGTGATTGCT TGTGAGGGTT TAGAGAGGAT AGAAAGGCCA GAGACGTACA   
  
  
+ AGCAGTGGCA AGTCCGAAAT GAAAGGGCAG GGTTTAGGCA GCTGCCTTTA GATCGCCAGA TTCTGGAAAT   
  
  
+ GGCTAAAAAG AGGGTGAAAT CTGTGTATAA CAAAGATTTC TCCATTGATG AAGACGGGCA CTGGTTGTTG   
  
  
+ CTGGGATGGA AGGGCAGAAT TGTGTACACA CTCACTACTT GGAAGCCTGC GGAGTA  

- -Up\_Stream \_Len000GGGACC CAGTAATTTT TTTTTCTTTC TTTCTTCTAA CGCGATTTTC ATCTAAAAAT   
  
  
- AGTCTAAGTT CGACGTCCAC TATTGGTTTA ACAGTAATTT CGTTACGTTC GCGTATAGTT ATGGGAAAAC   
  
  
- CCTCTATCGA GGCTGACAAC TTCTGTAATC CTCGTACTAG AGGTTGACAC TTAAACAAAG TAAATGAGTA   
  
  
- TAAGTATAGA TGGTACACCT ATTATACTGA CGTCTGATCT ACCGGTTTAA ACTTACGTGT GAATATTGAA   
  
  
- GTTGAGAAAG ATGAAAAAAG AAGGGGTGGA ATAGTCCTTA AAGAAAATTA ACAGCACCCT CTGTTGAACC   
  
  
- CATCTTGAGA ACGCTTTTCC CGTTGAATTG CAAAACCATG AACGTAAAAG GTTTTTTTGT TTTTCCGATT   
  
  
- TGTTAAGAGG CGTACGACTG AAGTGCCATC TATACTACAT AAAAAATTGT TTTCTGTAGA AGACTTACTT   
  
  
- TGCGAATTAT AGAACCTGGT ATGATCTTTA ATTTAACGGT GCCACGTACT TAAAGTGGAG CTTGACGAAC   
  
  
- ATATTTCTAG AACAATAGTG GAAGTCATTA GTCTATGAAC ATTGGTTTAA CTTACTGGTG TTTCTGGTGT   
  
  
- AAGGAGTGTT AGCAACGGAG ATCTTATTCT TACTTACAAA GAAGTTCCAA CTTTCGGGAA CGATTACGCT   
  
  
- TGGACGGATA TACGGATTGA CCGATATATA TACACGCATC TTGTCGTACT GTACTCAATT ATTATATCGT   
  
  
- CGATTTCCCT TGAGATTACA TGTGTACTAA CATTAAGTGG ACGGAGCGAT CGAATGCTCG TACTGAGAAC   
  
  
- TGATAATAAC AAAATTAACT AGAACTTAAA AGTATCTAAA ATGAGGTAAC AAATGAGTAA AACAATATTA   
  
  
- ATTGGTGGAC TAAAATTTGA AATTTTATGT GTTTCTAAGT AGATTATATT AAAAAATCTA TTTCAATACG   
  
  
- TATGTATATA TGTTTAATTT AATAAATGCT AGATTAGTAA AGGGCAATAT ATAAAGTGTG ATTTTAAATC   
  
  
- TTCTCTGTTA GATAATTTTA ATGTAGTGTA CAAAACCCCC CCCCCCCTAT AGTCTATACG TACTGTAAGG   
  
  
- ATTAGGTTAT TACGTTACTT TGGGTGGCTT ATCAACTTTA CTAAACAAGA TAGGTACTTG AGTGGTTCAG   
  
  
- TGTTAGGTAG TGTGAGCTTC CTATACGAAA AGAAAAACTT CCCTTAGAAC GAATTAGTTT GGATATTTGA   
  
  
- AAATTTTCGT TGTCTGTTTT TTCACTAATA TTACCATCAC CATCCTACTT CCGTCGAAAG TCTTTTCGCT   
  
  
- AACTTAAAAA TAACATAGGG TACTTTTGGG TAACTTAAAA AGAAAGAAAG AAACAAAAAC AATGGACCCA   
  
  
- AATACGTTTC CTGCCGACAA CATAAATTAT TATTGTTTTT GTATCCCGAA ACGCCTAACG ATTACGACCC   
  
  
- CTACCGTAGT CACGAGCTTA GACACATGCG ACAGTATCCT TGGGTGAACG AAGACGAGTA AATAAAGACA   
  
  
- GAGATCTAGG GGAGGAGAGA GAGAGAGAGA GAGATTCCAC AGACGAGCAG ACAAGTATAA GAGGACTCCA   
  
  
- AGAAGAAGTA GATAGAACAA GACCCACACA CGACTCCATG AAGAAAAGAA ATATTTAAAA AGTAGAAAAC   
  
  
- AACAAAACGA ACCACAAATA CTAAAATCAA AAACAGAAAT TCTGCTCTAA ACGACAATTA TCGTTCTAAA   
  
  
- GACTTGATCA TAACTAGGAA AAACACCATA TAAACAACTT AAACTAATTA AACACATAAC ACAAATCGAA   
  
  
- TTCATTGAGC AACACTAGAT TAACTACGAG CCCAAAACTG TCAAACTAAT CCCAAAAGCC ATGACCTCAA   
  
  
- TTTCAAAAAA ATAAAAAATA TCCTCCTCAA TTTCCATTAT TATTCTTAAC TACAAACGAC TAAAAAGGAG   
  
  
- AAAAGAAAAA ACCAAAGCTA AACCACCCGT CAATAGACAA ATACCAAAAA CGTTTACCTA GGTGTTAATC   
  
  
- TCCTTAATAT ACCTGGAAAT GTAGCTTAGT TCAAGTTACT AGTTTCCCAC GGTTAAAATG GTTCAGCGTC   
  
  
- ACAACAATGA GGCTACTTTG AGGTTCTAAG GTTAAACTTA GGTTCACAAG GTTTAGGTTC ACAACACTTA   
  
  
- GGAGGAGGCA ACCAAGGGGG TTTGGGTTTA GAATTAAATC AACACCGTAG AGTTATAAGA CTGTAACTCT   
  
  
- GTCGGGACTT GCTCCTAACA CTAAAATCAC TACAACAAGA ATTCATATAA TTAGTTGAAG AATACCTTCT   
  
  
- CCTACCCCTT CTCTTTGAGG TATGACTAGT ACTTGGAAGT CAGCAACTCC GCCGTCTCTT CAGTAACATA   
  
  
- CTCCGCGAGC CCGTCTCTAT AGGAGGAAGG CCTTTGGCTT TAGTCAACGG TCTACAACTC GTACTGCCGA   
  
  
- ACTGACCCTC GTCACAATCC CGAGTATCAC CACGTTCGTC ACAATCCCGA GTATCACTGC GTTCACCACC   
  
  
- ATCACCGGAC TGTCCTATAC CAACCATACC ACTAGGAATA TTAACCTCAG GAGTTTTACA GCACTTAAAA   
  
  
- TGGTAAAGGA GGAGCTTTGG GAATAGCACG TCGAGTAGTC TATCGAGCCC GTCTACCCAA TCATTCACCT   
  
  
- TCAGTTTAAG ACTAAGTGTC GAGTCGAGCC GGTAATTACG TTCACCAAAT CACCTACCCG GTCCCCTGAG   
  
  
- AGGACACTCA CGAGAATCAC ACAGACTTTA TAAGTTACTG TCAGTCAGTT ACAACGTCAA AGTCTTTCCT   
  
  
- AAACTCCTTC GCTTATTTAA AGAAGGTTTC TCAAGAAACA TGTTCCCAAA ACGGTTGTGC CCTAACGGAA   
  
  
- TAGTCTTCCG TTTGTTATCA CGGGTTCTAA ACAACCAGTT ACATCTCCTA CAATGATCCC CTTTCTTCGT   
  
  
- AGCAATAGGG CTTCTTAACG TCAGTCTTCT TCCCTCCTAT TTATTCGTTA GTCGACAAAG GGACCGTCGA   
  
  
- CTACTCCGTC AACAATCCAG ACTTTACAAG CTATCCCATG ACGAAACATC AGCCCCTTTT GTACTACGAC   
  
  
- GAGAGGCCCT TCGAAATGTC TGACTTGATT TATTCTCAGA CGCTTTACGG GGTCAATTCC CCAGATTATC   
  
  
- ACCCTTTCCG GCACCATTCT TTCGCCCTTT CTTCTCCCTA CATCATCTAA ATTCTAGAGA GAATTGGGAT   
  
  
- ACACGTGTTC GTCAACGTAG CTTACTAGTG TCCTCACGTT TACTGGACGA ATCCGTTTAA TCTGTCGTAA   
  
  
- GAAGAGGATC ACCCCTACCA TTGGTTTCTT ACCGTGTAAT AAAACGCCTA CCAGAACTCC GTGCGGAACG   
  
  
- ACCACATCCT TGAGGATATA TATTGACAGA ACATTGCCCA GGCCGTAGCC GACATCTATA GAACTCTCGA   
  
  
- ATGGTGTACA AAGAACGGTG TACGGGTAAA TTCTTTTACC CTTTAAAGAA GAGATTATCT TGCTAATACC   
  
  
- GACACCGTCT CTTACGTTGT ACGGAAGTAT ATTAACTAGA GCCATAACAG ATACCAAAGG TTACCGGAAC   
  
  
- AGATTAAGTC GCTGAAAGTA GATCCGGACC ACCGGGGGGG TTTGAAGCTT ATTGGCCTCA TCTAGAAGGT   
  
  
- GTTGGACCCA AGGCTGGTCG GTTTTCTCAA CTCCTCTGTC CCGCAGCGAA CTTCTTGATA CGTCTCAGTA   
  
  
- AATTACACGG GAAACTCAAG TTACGATATC GTTTCTTCAC CCTTTGTGAA TGGTAACTTC TAGAGTCCTA   
  
  
- GTTATCGCTA CTCAACGAAC AACAGTGGAC ATACAAATCC AAATTTGTAT ATGGACTCCT TTGTCACTGG   
  
  
- CACCTAACGG GATCCCTATG ACAAGAATTG GACTAACCCG CGTATTTGGG TCGACAAAAG TATGTTCCGT   
  
  
- GACAATTGCC CCGAAAGTTA AGAGGGAAAA AGTATCGAGC TAAAGCTCTC CGAGATAAAG TGAAGAGGTG   
  
  
- AGACAAACTA TACGATCTCC GGTTGGACGG TTCCCTGTTA TTCCTCTCCT ACGATTAACT CTCTCTCTAT   
  
  
- AAACCCTCCG TCCGTTACTT ACACTAACGA ACACTCCCAA ATCTCTCCTA TCTTTCCGGT CTCTGCATGT   
  
  
- TCGTCACCGT TCAGGCTTTA CTTTCCCGTC CCAAATCCGT CGACGGAAAT CTAGCGGTCT AAGACCTTTA   
  
  
- CCGATTTTTC TCCCACTTTA GACACATATT GTTTCTAAAG AGGTAACTAC TTCTGCCCGT GACCAACAAC   
  
  
- GACCCTACCT TCCCGTCTTA ACACATGTGT GAGTGATGAA CCTTCGGACG CCTCAT

+     MBS

| Site Name | Organism | Position | Strand | Matrix score. | sequence | function |
| --- | --- | --- | --- | --- | --- | --- |
| MBS | Arabidopsis thaliana | 187 | + | 6 | CAACTG | MYB binding site involved in drought-inducibility |
| MBS | Arabidopsis thaliana | 3022 | - | 6 | CAACTG | MYB binding site involved in drought-inducibility |
| MBS | Arabidopsis thaliana | 2427 | - | 6 | CAACTG | MYB binding site involved in drought-inducibility |
| MBS | Arabidopsis thaliana | 3235 | - | 6 | CAACTG | MYB binding site involved in drought-inducibility |

>HU06G00568.1   
+ -Up\_Stream \_Len000CCCTGG GTCATTAAAA AAAAAGAAAG AAAGAAGATT GCGCTAAAAG TAGATTTTTA   
  
  
+ TCAGATTCAA GCTGCAGGTG ATAACCAAAT TGTCATTAAA GCAATGCAAG CGCATATCAA TACCCTTTTG   
  
  
+ GGAGATAGCT CCGACTGTTG AAGACATTAG GAGCATGATC TCCAACTGTG AATTTGTTTC ATTTACTCAT   
  
  
+ ATTCATATCT ACCATGTGGA TAATATGACT GCAGACTAGA TGGCCAAATT TGAATGCACA CTTATAACTT   
  
  
+ CAACTCTTTC TACTTTTTTC TTCCCCACCT TATCAGGAAT TTCTTTTAAT TGTCGTGGGA GACAACTTGG   
  
  
+ GTAGAACTCT TGCGAAAAGG GCAACTTAAC GTTTTGGTAC TTGCATTTTC CAAAAAAACA AAAAGGCTAA   
  
  
+ ACAATTCTCC GCATGCTGAC TTCACGGTAG ATATGATGTA TTTTTTAACA AAAGACATCT TCTGAATGAA   
  
  
+ ACGCTTAATA TCTTGGACCA TACTAGAAAT TAAATTGCCA CGGTGCATGA ATTTCACCTC GAACTGCTTG   
  
  
+ TATAAAGATC TTGTTATCAC CTTCAGTAAT CAGATACTTG TAACCAAATT GAATGACCAC AAAGACCACA   
  
  
+ TTCCTCACAA TCGTTGCCTC TAGAATAAGA ATGAATGTTT CTTCAAGGTT GAAAGCCCTT GCTAATGCGA   
  
  
+ ACCTGCCTAT ATGCCTAACT GGCTATATAT ATGTGCGTAG AACAGCATGA CATGAGTTAA TAATATAGCA   
  
  
+ GCTAAAGGGA ACTCTAATGT ACACATGATT GTAATTCACC TGCCTCGCTA GCTTACGAGC ATGACTCTTG   
  
  
+ ACTATTATTG TTTTAATTGA TCTTGAATTT TCATAGATTT TACTCCATTG TTTACTCATT TTGTTATAAT   
  
  
+ TAACCACCTG ATTTTAAACT TTAAAATACA CAAAGATTCA TCTAATATAA TTTTTTAGAT AAAGTTATGC   
  
  
+ ATACATATAT ACAAATTAAA TTATTTACGA TCTAATCATT TCCCGTTATA TATTTCACAC TAAAATTTAG   
  
  
+ AAGAGACAAT CTATTAAAAT TACATCACAT GTTTTGGGGG GGGGGGGATA TCAGATATGC ATGACATTCC   
  
  
+ TAATCCAATA ATGCAATGAA ACCCACCGAA TAGTTGAAAT GATTTGTTCT ATCCATGAAC TCACCAAGTC   
  
  
+ ACAATCCATC ACACTCGAAG GATATGCTTT TCTTTTTGAA GGGAATCTTG CTTAATCAAA CCTATAAACT   
  
  
+ TTTAAAAGCA ACAGACAAAA AAGTGATTAT AATGGTAGTG GTAGGATGAA GGCAGCTTTC AGAAAAGCGA   
  
  
+ TTGAATTTTT ATTGTATCCC ATGAAAACCC ATTGAATTTT TCTTTCTTTC TTTGTTTTTG TTACCTGGGT   
  
  
+ TTATGCAAAG GACGGCTGTT GTATTTAATA ATAACAAAAA CATAGGGCTT TGCGGATTGC TAATGCTGGG   
  
  
+ GATGGCATCA GTGCTCGAAT CTGTGTACGC TGTCATAGGA ACCCACTTGC TTCTGCTCAT TTATTTCTGT   
  
  
+ CTCTAGATCC CCTCCTCTCT CTCTCTCTCT CTCTAAGGTG TCTGCTCGTC TGTTCATATT CTCCTGAGGT   
  
  
+ TCTTCTTCAT CTATCTTGTT CTGGGTGTGT GCTGAGGTAC TTCTTTTCTT TATAAATTTT TCATCTTTTG   
  
  
+ TTGTTTTGCT TGGTGTTTAT GATTTTAGTT TTTGTCTTTA AGACGAGATT TGCTGTTAAT AGCAAGATTT   
  
  
+ CTGAACTAGT ATTGATCCTT TTTGTGGTAT ATTTGTTGAA TTTGATTAAT TTGTGTATTG TGTTTAGCTT   
  
  
+ AAGTAACTCG TTGTGATCTA ATTGATGCTC GGGTTTTGAC AGTTTGATTA GGGTTTTCGG TACTGGAGTT   
  
  
+ AAAGTTTTTT TATTTTTTAT AGGAGGAGTT AAAGGTAATA ATAAGAATTG ATGTTTGCTG ATTTTTCCTC   
  
  
+ TTTTCTTTTT TGGTTTCGAT TTGGTGGGCA GTTATCTGTT TATGGTTTTT GCAAATGGAT CCACAATTAG   
  
  
+ AGGAATTATA TGGACCTTTA CATCGAATCA AGTTCAATGA TCAAAGGGTG CCAATTTTAC CAAGTCGCAG   
  
  
+ TGTTGTTACT CCGATGAAAC TCCAAGATTC CAATTTGAAT CCAAGTGTTC CAAATCCAAG TGTTGTGAAT   
  
  
+ CCTCCTCCGT TGGTTCCCCC AAACCCAAAT CTTAATTTAG TTGTGGCATC TCAATATTCT GACATTGAGA   
  
  
+ CAGCCCTGAA CGAGGATTGT GATTTTAGTG ATGTTGTTCT TAAGTATATT AATCAACTTC TTATGGAAGA   
  
  
+ GGATGGGGAA GAGAAACTCC ATACTGATCA TGAACCTTCA GTCGTTGAGG CGGCAGAGAA GTCATTGTAT   
  
  
+ GAGGCGCTCG GGCAGAGATA TCCTCCTTCC GGAAACCGAA ATCAGTTGCC AGATGTTGAG CATGACGGCT   
  
  
+ TGACTGGGAG CAGTGTTAGG GCTCATAGTG GTGCAAGCAG TGTTAGGGCT CATAGTGACG CAAGTGGTGG   
  
  
+ TAGTGGCCTG ACAGGATATG GTTGGTATGG TGATCCTTAT AATTGGAGTC CTCAAAATGT CGTGAATTTT   
  
  
+ ACCATTTCCT CCTCGAAACC CTTATCGTGC AGCTCATCAG ATAGCTCGGG CAGATGGGTT AGTAAGTGGA   
  
  
+ AGTCAAATTC TGATTCACAG CTCAGCTCGG CCATTAATGC AAGTGGTTTA GTGGATGGGC CAGGGGACTC   
  
  
+ TCCTGTGAGT GCTCTTAGTG TGTCTGAAAT ATTCAATGAC AGTCAGTCAA TGTTGCAGTT TCAGAAAGGA   
  
  
+ TTTGAGGAAG CGAATAAATT TCTTCCAAAG AGTTCTTTGT ACAAGGGTTT TGCCAACACG GGATTGCCTT   
  
  
+ ATCAGAAGGC AAACAATAGT GCCCAAGATT TGTTGGTCAA TGTAGAGGAT GTTACTAGGG GAAAGAAGCA   
  
  
+ TCGTTATCCC GAAGAATTGC AGTCAGAAGA AGGGAGGATA AATAAGCAAT CAGCTGTTTC CCTGGCAGCT   
  
  
+ GATGAGGCAG TTGTTAGGTC TGAAATGTTC GATAGGGTAC TGCTTTGTAG TCGGGGAAAA CATGATGCTG   
  
  
+ CTCTCCGGGA AGCTTTACAG ACTGAACTAA ATAAGAGTCT GCGAAATGCC CCAGTTAAGG GGTCTAATAG   
  
  
+ TGGGAAAGGC CGTGGTAAGA AAGCGGGAAA GAAGAGGGAT GTAGTAGATT TAAGATCTCT CTTAACCCTA   
  
  
+ TGTGCACAAG CAGTTGCATC GAATGATCAC AGGAGTGCAA ATGACCTGCT TAGGCAAATT AGACAGCATT   
  
  
+ CTTCTCCTAG TGGGGATGGT AACCAAAGAA TGGCACATTA TTTTGCGGAT GGTCTTGAGG CACGCCTTGC   
  
  
+ TGGTGTAGGA ACTCCTATAT ATAACTGTCT TGTAACGGGT CCGGCATCGG CTGTAGATAT CTTGAGAGCT   
  
  
+ TACCACATGT TTCTTGCCAC ATGCCCATTT AAGAAAATGG GAAATTTCTT CTCTAATAGA ACGATTATGG   
  
  
+ CTGTGGCAGA GAATGCAACA TGCCTTCATA TAATTGATCT CGGTATTGTC TATGGTTTCC AATGGCCTTG   
  
  
+ TCTAATTCAG CGACTTTCAT CTAGGCCTGG TGGCCCCCCC AAACTTCGAA TAACCGGAGT AGATCTTCCA   
  
  
+ CAACCTGGGT TCCGACCAGC CAAAAGAGTT GAGGAGACAG GGCGTCGCTT GAAGAACTAT GCAGAGTCAT   
  
  
+ TTAATGTGCC CTTTGAGTTC AATGCTATAG CAAAGAAGTG GGAAACACTT ACCATTGAAG ATCTCAGGAT   
  
  
+ CAATAGCGAT GAGTTGCTTG TTGTCACCTG TATGTTTAGG TTTAAACATA TACCTGAGGA AACAGTGACC   
  
  
+ GTGGATTGCC CTAGGGATAC TGTTCTTAAC CTGATTGGGC GCATAAACCC AGCTGTTTTC ATACAAGGCA   
  
  
+ CTGTTAACGG GGCTTTCAAT TCTCCCTTTT TCATAGCTCG ATTTCGAGAG GCTCTATTTC ACTTCTCCAC   
  
  
+ TCTGTTTGAT ATGCTAGAGG CCAACCTGCC AAGGGACAAT AAGGAGAGGA TGCTAATTGA GAGAGAGATA   
  
  
+ TTTGGGAGGC AGGCAATGAA TGTGATTGCT TGTGAGGGTT TAGAGAGGAT AGAAAGGCCA GAGACGTACA   
  
  
+ AGCAGTGGCA AGTCCGAAAT GAAAGGGCAG GGTTTAGGCA GCTGCCTTTA GATCGCCAGA TTCTGGAAAT   
  
  
+ GGCTAAAAAG AGGGTGAAAT CTGTGTATAA CAAAGATTTC TCCATTGATG AAGACGGGCA CTGGTTGTTG   
  
  
+ CTGGGATGGA AGGGCAGAAT TGTGTACACA CTCACTACTT GGAAGCCTGC GGAGTA  

- -Up\_Stream \_Len000GGGACC CAGTAATTTT TTTTTCTTTC TTTCTTCTAA CGCGATTTTC ATCTAAAAAT   
  
  
- AGTCTAAGTT CGACGTCCAC TATTGGTTTA ACAGTAATTT CGTTACGTTC GCGTATAGTT ATGGGAAAAC   
  
  
- CCTCTATCGA GGCTGACAAC TTCTGTAATC CTCGTACTAG AGGTTGACAC TTAAACAAAG TAAATGAGTA   
  
  
- TAAGTATAGA TGGTACACCT ATTATACTGA CGTCTGATCT ACCGGTTTAA ACTTACGTGT GAATATTGAA   
  
  
- GTTGAGAAAG ATGAAAAAAG AAGGGGTGGA ATAGTCCTTA AAGAAAATTA ACAGCACCCT CTGTTGAACC   
  
  
- CATCTTGAGA ACGCTTTTCC CGTTGAATTG CAAAACCATG AACGTAAAAG GTTTTTTTGT TTTTCCGATT   
  
  
- TGTTAAGAGG CGTACGACTG AAGTGCCATC TATACTACAT AAAAAATTGT TTTCTGTAGA AGACTTACTT   
  
  
- TGCGAATTAT AGAACCTGGT ATGATCTTTA ATTTAACGGT GCCACGTACT TAAAGTGGAG CTTGACGAAC   
  
  
- ATATTTCTAG AACAATAGTG GAAGTCATTA GTCTATGAAC ATTGGTTTAA CTTACTGGTG TTTCTGGTGT   
  
  
- AAGGAGTGTT AGCAACGGAG ATCTTATTCT TACTTACAAA GAAGTTCCAA CTTTCGGGAA CGATTACGCT   
  
  
- TGGACGGATA TACGGATTGA CCGATATATA TACACGCATC TTGTCGTACT GTACTCAATT ATTATATCGT   
  
  
- CGATTTCCCT TGAGATTACA TGTGTACTAA CATTAAGTGG ACGGAGCGAT CGAATGCTCG TACTGAGAAC   
  
  
- TGATAATAAC AAAATTAACT AGAACTTAAA AGTATCTAAA ATGAGGTAAC AAATGAGTAA AACAATATTA   
  
  
- ATTGGTGGAC TAAAATTTGA AATTTTATGT GTTTCTAAGT AGATTATATT AAAAAATCTA TTTCAATACG   
  
  
- TATGTATATA TGTTTAATTT AATAAATGCT AGATTAGTAA AGGGCAATAT ATAAAGTGTG ATTTTAAATC   
  
  
- TTCTCTGTTA GATAATTTTA ATGTAGTGTA CAAAACCCCC CCCCCCCTAT AGTCTATACG TACTGTAAGG   
  
  
- ATTAGGTTAT TACGTTACTT TGGGTGGCTT ATCAACTTTA CTAAACAAGA TAGGTACTTG AGTGGTTCAG   
  
  
- TGTTAGGTAG TGTGAGCTTC CTATACGAAA AGAAAAACTT CCCTTAGAAC GAATTAGTTT GGATATTTGA   
  
  
- AAATTTTCGT TGTCTGTTTT TTCACTAATA TTACCATCAC CATCCTACTT CCGTCGAAAG TCTTTTCGCT   
  
  
- AACTTAAAAA TAACATAGGG TACTTTTGGG TAACTTAAAA AGAAAGAAAG AAACAAAAAC AATGGACCCA   
  
  
- AATACGTTTC CTGCCGACAA CATAAATTAT TATTGTTTTT GTATCCCGAA ACGCCTAACG ATTACGACCC   
  
  
- CTACCGTAGT CACGAGCTTA GACACATGCG ACAGTATCCT TGGGTGAACG AAGACGAGTA AATAAAGACA   
  
  
- GAGATCTAGG GGAGGAGAGA GAGAGAGAGA GAGATTCCAC AGACGAGCAG ACAAGTATAA GAGGACTCCA   
  
  
- AGAAGAAGTA GATAGAACAA GACCCACACA CGACTCCATG AAGAAAAGAA ATATTTAAAA AGTAGAAAAC   
  
  
- AACAAAACGA ACCACAAATA CTAAAATCAA AAACAGAAAT TCTGCTCTAA ACGACAATTA TCGTTCTAAA   
  
  
- GACTTGATCA TAACTAGGAA AAACACCATA TAAACAACTT AAACTAATTA AACACATAAC ACAAATCGAA   
  
  
- TTCATTGAGC AACACTAGAT TAACTACGAG CCCAAAACTG TCAAACTAAT CCCAAAAGCC ATGACCTCAA   
  
  
- TTTCAAAAAA ATAAAAAATA TCCTCCTCAA TTTCCATTAT TATTCTTAAC TACAAACGAC TAAAAAGGAG   
  
  
- AAAAGAAAAA ACCAAAGCTA AACCACCCGT CAATAGACAA ATACCAAAAA CGTTTACCTA GGTGTTAATC   
  
  
- TCCTTAATAT ACCTGGAAAT GTAGCTTAGT TCAAGTTACT AGTTTCCCAC GGTTAAAATG GTTCAGCGTC   
  
  
- ACAACAATGA GGCTACTTTG AGGTTCTAAG GTTAAACTTA GGTTCACAAG GTTTAGGTTC ACAACACTTA   
  
  
- GGAGGAGGCA ACCAAGGGGG TTTGGGTTTA GAATTAAATC AACACCGTAG AGTTATAAGA CTGTAACTCT   
  
  
- GTCGGGACTT GCTCCTAACA CTAAAATCAC TACAACAAGA ATTCATATAA TTAGTTGAAG AATACCTTCT   
  
  
- CCTACCCCTT CTCTTTGAGG TATGACTAGT ACTTGGAAGT CAGCAACTCC GCCGTCTCTT CAGTAACATA   
  
  
- CTCCGCGAGC CCGTCTCTAT AGGAGGAAGG CCTTTGGCTT TAGTCAACGG TCTACAACTC GTACTGCCGA   
  
  
- ACTGACCCTC GTCACAATCC CGAGTATCAC CACGTTCGTC ACAATCCCGA GTATCACTGC GTTCACCACC   
  
  
- ATCACCGGAC TGTCCTATAC CAACCATACC ACTAGGAATA TTAACCTCAG GAGTTTTACA GCACTTAAAA   
  
  
- TGGTAAAGGA GGAGCTTTGG GAATAGCACG TCGAGTAGTC TATCGAGCCC GTCTACCCAA TCATTCACCT   
  
  
- TCAGTTTAAG ACTAAGTGTC GAGTCGAGCC GGTAATTACG TTCACCAAAT CACCTACCCG GTCCCCTGAG   
  
  
- AGGACACTCA CGAGAATCAC ACAGACTTTA TAAGTTACTG TCAGTCAGTT ACAACGTCAA AGTCTTTCCT   
  
  
- AAACTCCTTC GCTTATTTAA AGAAGGTTTC TCAAGAAACA TGTTCCCAAA ACGGTTGTGC CCTAACGGAA   
  
  
- TAGTCTTCCG TTTGTTATCA CGGGTTCTAA ACAACCAGTT ACATCTCCTA CAATGATCCC CTTTCTTCGT   
  
  
- AGCAATAGGG CTTCTTAACG TCAGTCTTCT TCCCTCCTAT TTATTCGTTA GTCGACAAAG GGACCGTCGA   
  
  
- CTACTCCGTC AACAATCCAG ACTTTACAAG CTATCCCATG ACGAAACATC AGCCCCTTTT GTACTACGAC   
  
  
- GAGAGGCCCT TCGAAATGTC TGACTTGATT TATTCTCAGA CGCTTTACGG GGTCAATTCC CCAGATTATC   
  
  
- ACCCTTTCCG GCACCATTCT TTCGCCCTTT CTTCTCCCTA CATCATCTAA ATTCTAGAGA GAATTGGGAT   
  
  
- ACACGTGTTC GTCAACGTAG CTTACTAGTG TCCTCACGTT TACTGGACGA ATCCGTTTAA TCTGTCGTAA   
  
  
- GAAGAGGATC ACCCCTACCA TTGGTTTCTT ACCGTGTAAT AAAACGCCTA CCAGAACTCC GTGCGGAACG   
  
  
- ACCACATCCT TGAGGATATA TATTGACAGA ACATTGCCCA GGCCGTAGCC GACATCTATA GAACTCTCGA   
  
  
- ATGGTGTACA AAGAACGGTG TACGGGTAAA TTCTTTTACC CTTTAAAGAA GAGATTATCT TGCTAATACC   
  
  
- GACACCGTCT CTTACGTTGT ACGGAAGTAT ATTAACTAGA GCCATAACAG ATACCAAAGG TTACCGGAAC   
  
  
- AGATTAAGTC GCTGAAAGTA GATCCGGACC ACCGGGGGGG TTTGAAGCTT ATTGGCCTCA TCTAGAAGGT   
  
  
- GTTGGACCCA AGGCTGGTCG GTTTTCTCAA CTCCTCTGTC CCGCAGCGAA CTTCTTGATA CGTCTCAGTA   
  
  
- AATTACACGG GAAACTCAAG TTACGATATC GTTTCTTCAC CCTTTGTGAA TGGTAACTTC TAGAGTCCTA   
  
  
- GTTATCGCTA CTCAACGAAC AACAGTGGAC ATACAAATCC AAATTTGTAT ATGGACTCCT TTGTCACTGG   
  
  
- CACCTAACGG GATCCCTATG ACAAGAATTG GACTAACCCG CGTATTTGGG TCGACAAAAG TATGTTCCGT   
  
  
- GACAATTGCC CCGAAAGTTA AGAGGGAAAA AGTATCGAGC TAAAGCTCTC CGAGATAAAG TGAAGAGGTG   
  
  
- AGACAAACTA TACGATCTCC GGTTGGACGG TTCCCTGTTA TTCCTCTCCT ACGATTAACT CTCTCTCTAT   
  
  
- AAACCCTCCG TCCGTTACTT ACACTAACGA ACACTCCCAA ATCTCTCCTA TCTTTCCGGT CTCTGCATGT   
  
  
- TCGTCACCGT TCAGGCTTTA CTTTCCCGTC CCAAATCCGT CGACGGAAAT CTAGCGGTCT AAGACCTTTA   
  
  
- CCGATTTTTC TCCCACTTTA GACACATATT GTTTCTAAAG AGGTAACTAC TTCTGCCCGT GACCAACAAC   
  
  
- GACCCTACCT TCCCGTCTTA ACACATGTGT GAGTGATGAA CCTTCGGACG CCTCAT

+     MRE

| Site Name | Organism | Position | Strand | Matrix score. | sequence | function |
| --- | --- | --- | --- | --- | --- | --- |
| MRE | Petroselinum crispum | 3820 | - | 7 | AACCTAA | MYB binding site involved in light responsiveness |

>HU06G00568.1   
+ -Up\_Stream \_Len000CCCTGG GTCATTAAAA AAAAAGAAAG AAAGAAGATT GCGCTAAAAG TAGATTTTTA   
  
  
+ TCAGATTCAA GCTGCAGGTG ATAACCAAAT TGTCATTAAA GCAATGCAAG CGCATATCAA TACCCTTTTG   
  
  
+ GGAGATAGCT CCGACTGTTG AAGACATTAG GAGCATGATC TCCAACTGTG AATTTGTTTC ATTTACTCAT   
  
  
+ ATTCATATCT ACCATGTGGA TAATATGACT GCAGACTAGA TGGCCAAATT TGAATGCACA CTTATAACTT   
  
  
+ CAACTCTTTC TACTTTTTTC TTCCCCACCT TATCAGGAAT TTCTTTTAAT TGTCGTGGGA GACAACTTGG   
  
  
+ GTAGAACTCT TGCGAAAAGG GCAACTTAAC GTTTTGGTAC TTGCATTTTC CAAAAAAACA AAAAGGCTAA   
  
  
+ ACAATTCTCC GCATGCTGAC TTCACGGTAG ATATGATGTA TTTTTTAACA AAAGACATCT TCTGAATGAA   
  
  
+ ACGCTTAATA TCTTGGACCA TACTAGAAAT TAAATTGCCA CGGTGCATGA ATTTCACCTC GAACTGCTTG   
  
  
+ TATAAAGATC TTGTTATCAC CTTCAGTAAT CAGATACTTG TAACCAAATT GAATGACCAC AAAGACCACA   
  
  
+ TTCCTCACAA TCGTTGCCTC TAGAATAAGA ATGAATGTTT CTTCAAGGTT GAAAGCCCTT GCTAATGCGA   
  
  
+ ACCTGCCTAT ATGCCTAACT GGCTATATAT ATGTGCGTAG AACAGCATGA CATGAGTTAA TAATATAGCA   
  
  
+ GCTAAAGGGA ACTCTAATGT ACACATGATT GTAATTCACC TGCCTCGCTA GCTTACGAGC ATGACTCTTG   
  
  
+ ACTATTATTG TTTTAATTGA TCTTGAATTT TCATAGATTT TACTCCATTG TTTACTCATT TTGTTATAAT   
  
  
+ TAACCACCTG ATTTTAAACT TTAAAATACA CAAAGATTCA TCTAATATAA TTTTTTAGAT AAAGTTATGC   
  
  
+ ATACATATAT ACAAATTAAA TTATTTACGA TCTAATCATT TCCCGTTATA TATTTCACAC TAAAATTTAG   
  
  
+ AAGAGACAAT CTATTAAAAT TACATCACAT GTTTTGGGGG GGGGGGGATA TCAGATATGC ATGACATTCC   
  
  
+ TAATCCAATA ATGCAATGAA ACCCACCGAA TAGTTGAAAT GATTTGTTCT ATCCATGAAC TCACCAAGTC   
  
  
+ ACAATCCATC ACACTCGAAG GATATGCTTT TCTTTTTGAA GGGAATCTTG CTTAATCAAA CCTATAAACT   
  
  
+ TTTAAAAGCA ACAGACAAAA AAGTGATTAT AATGGTAGTG GTAGGATGAA GGCAGCTTTC AGAAAAGCGA   
  
  
+ TTGAATTTTT ATTGTATCCC ATGAAAACCC ATTGAATTTT TCTTTCTTTC TTTGTTTTTG TTACCTGGGT   
  
  
+ TTATGCAAAG GACGGCTGTT GTATTTAATA ATAACAAAAA CATAGGGCTT TGCGGATTGC TAATGCTGGG   
  
  
+ GATGGCATCA GTGCTCGAAT CTGTGTACGC TGTCATAGGA ACCCACTTGC TTCTGCTCAT TTATTTCTGT   
  
  
+ CTCTAGATCC CCTCCTCTCT CTCTCTCTCT CTCTAAGGTG TCTGCTCGTC TGTTCATATT CTCCTGAGGT   
  
  
+ TCTTCTTCAT CTATCTTGTT CTGGGTGTGT GCTGAGGTAC TTCTTTTCTT TATAAATTTT TCATCTTTTG   
  
  
+ TTGTTTTGCT TGGTGTTTAT GATTTTAGTT TTTGTCTTTA AGACGAGATT TGCTGTTAAT AGCAAGATTT   
  
  
+ CTGAACTAGT ATTGATCCTT TTTGTGGTAT ATTTGTTGAA TTTGATTAAT TTGTGTATTG TGTTTAGCTT   
  
  
+ AAGTAACTCG TTGTGATCTA ATTGATGCTC GGGTTTTGAC AGTTTGATTA GGGTTTTCGG TACTGGAGTT   
  
  
+ AAAGTTTTTT TATTTTTTAT AGGAGGAGTT AAAGGTAATA ATAAGAATTG ATGTTTGCTG ATTTTTCCTC   
  
  
+ TTTTCTTTTT TGGTTTCGAT TTGGTGGGCA GTTATCTGTT TATGGTTTTT GCAAATGGAT CCACAATTAG   
  
  
+ AGGAATTATA TGGACCTTTA CATCGAATCA AGTTCAATGA TCAAAGGGTG CCAATTTTAC CAAGTCGCAG   
  
  
+ TGTTGTTACT CCGATGAAAC TCCAAGATTC CAATTTGAAT CCAAGTGTTC CAAATCCAAG TGTTGTGAAT   
  
  
+ CCTCCTCCGT TGGTTCCCCC AAACCCAAAT CTTAATTTAG TTGTGGCATC TCAATATTCT GACATTGAGA   
  
  
+ CAGCCCTGAA CGAGGATTGT GATTTTAGTG ATGTTGTTCT TAAGTATATT AATCAACTTC TTATGGAAGA   
  
  
+ GGATGGGGAA GAGAAACTCC ATACTGATCA TGAACCTTCA GTCGTTGAGG CGGCAGAGAA GTCATTGTAT   
  
  
+ GAGGCGCTCG GGCAGAGATA TCCTCCTTCC GGAAACCGAA ATCAGTTGCC AGATGTTGAG CATGACGGCT   
  
  
+ TGACTGGGAG CAGTGTTAGG GCTCATAGTG GTGCAAGCAG TGTTAGGGCT CATAGTGACG CAAGTGGTGG   
  
  
+ TAGTGGCCTG ACAGGATATG GTTGGTATGG TGATCCTTAT AATTGGAGTC CTCAAAATGT CGTGAATTTT   
  
  
+ ACCATTTCCT CCTCGAAACC CTTATCGTGC AGCTCATCAG ATAGCTCGGG CAGATGGGTT AGTAAGTGGA   
  
  
+ AGTCAAATTC TGATTCACAG CTCAGCTCGG CCATTAATGC AAGTGGTTTA GTGGATGGGC CAGGGGACTC   
  
  
+ TCCTGTGAGT GCTCTTAGTG TGTCTGAAAT ATTCAATGAC AGTCAGTCAA TGTTGCAGTT TCAGAAAGGA   
  
  
+ TTTGAGGAAG CGAATAAATT TCTTCCAAAG AGTTCTTTGT ACAAGGGTTT TGCCAACACG GGATTGCCTT   
  
  
+ ATCAGAAGGC AAACAATAGT GCCCAAGATT TGTTGGTCAA TGTAGAGGAT GTTACTAGGG GAAAGAAGCA   
  
  
+ TCGTTATCCC GAAGAATTGC AGTCAGAAGA AGGGAGGATA AATAAGCAAT CAGCTGTTTC CCTGGCAGCT   
  
  
+ GATGAGGCAG TTGTTAGGTC TGAAATGTTC GATAGGGTAC TGCTTTGTAG TCGGGGAAAA CATGATGCTG   
  
  
+ CTCTCCGGGA AGCTTTACAG ACTGAACTAA ATAAGAGTCT GCGAAATGCC CCAGTTAAGG GGTCTAATAG   
  
  
+ TGGGAAAGGC CGTGGTAAGA AAGCGGGAAA GAAGAGGGAT GTAGTAGATT TAAGATCTCT CTTAACCCTA   
  
  
+ TGTGCACAAG CAGTTGCATC GAATGATCAC AGGAGTGCAA ATGACCTGCT TAGGCAAATT AGACAGCATT   
  
  
+ CTTCTCCTAG TGGGGATGGT AACCAAAGAA TGGCACATTA TTTTGCGGAT GGTCTTGAGG CACGCCTTGC   
  
  
+ TGGTGTAGGA ACTCCTATAT ATAACTGTCT TGTAACGGGT CCGGCATCGG CTGTAGATAT CTTGAGAGCT   
  
  
+ TACCACATGT TTCTTGCCAC ATGCCCATTT AAGAAAATGG GAAATTTCTT CTCTAATAGA ACGATTATGG   
  
  
+ CTGTGGCAGA GAATGCAACA TGCCTTCATA TAATTGATCT CGGTATTGTC TATGGTTTCC AATGGCCTTG   
  
  
+ TCTAATTCAG CGACTTTCAT CTAGGCCTGG TGGCCCCCCC AAACTTCGAA TAACCGGAGT AGATCTTCCA   
  
  
+ CAACCTGGGT TCCGACCAGC CAAAAGAGTT GAGGAGACAG GGCGTCGCTT GAAGAACTAT GCAGAGTCAT   
  
  
+ TTAATGTGCC CTTTGAGTTC AATGCTATAG CAAAGAAGTG GGAAACACTT ACCATTGAAG ATCTCAGGAT   
  
  
+ CAATAGCGAT GAGTTGCTTG TTGTCACCTG TATGTTTAGG TTTAAACATA TACCTGAGGA AACAGTGACC   
  
  
+ GTGGATTGCC CTAGGGATAC TGTTCTTAAC CTGATTGGGC GCATAAACCC AGCTGTTTTC ATACAAGGCA   
  
  
+ CTGTTAACGG GGCTTTCAAT TCTCCCTTTT TCATAGCTCG ATTTCGAGAG GCTCTATTTC ACTTCTCCAC   
  
  
+ TCTGTTTGAT ATGCTAGAGG CCAACCTGCC AAGGGACAAT AAGGAGAGGA TGCTAATTGA GAGAGAGATA   
  
  
+ TTTGGGAGGC AGGCAATGAA TGTGATTGCT TGTGAGGGTT TAGAGAGGAT AGAAAGGCCA GAGACGTACA   
  
  
+ AGCAGTGGCA AGTCCGAAAT GAAAGGGCAG GGTTTAGGCA GCTGCCTTTA GATCGCCAGA TTCTGGAAAT   
  
  
+ GGCTAAAAAG AGGGTGAAAT CTGTGTATAA CAAAGATTTC TCCATTGATG AAGACGGGCA CTGGTTGTTG   
  
  
+ CTGGGATGGA AGGGCAGAAT TGTGTACACA CTCACTACTT GGAAGCCTGC GGAGTA  

- -Up\_Stream \_Len000GGGACC CAGTAATTTT TTTTTCTTTC TTTCTTCTAA CGCGATTTTC ATCTAAAAAT   
  
  
- AGTCTAAGTT CGACGTCCAC TATTGGTTTA ACAGTAATTT CGTTACGTTC GCGTATAGTT ATGGGAAAAC   
  
  
- CCTCTATCGA GGCTGACAAC TTCTGTAATC CTCGTACTAG AGGTTGACAC TTAAACAAAG TAAATGAGTA   
  
  
- TAAGTATAGA TGGTACACCT ATTATACTGA CGTCTGATCT ACCGGTTTAA ACTTACGTGT GAATATTGAA   
  
  
- GTTGAGAAAG ATGAAAAAAG AAGGGGTGGA ATAGTCCTTA AAGAAAATTA ACAGCACCCT CTGTTGAACC   
  
  
- CATCTTGAGA ACGCTTTTCC CGTTGAATTG CAAAACCATG AACGTAAAAG GTTTTTTTGT TTTTCCGATT   
  
  
- TGTTAAGAGG CGTACGACTG AAGTGCCATC TATACTACAT AAAAAATTGT TTTCTGTAGA AGACTTACTT   
  
  
- TGCGAATTAT AGAACCTGGT ATGATCTTTA ATTTAACGGT GCCACGTACT TAAAGTGGAG CTTGACGAAC   
  
  
- ATATTTCTAG AACAATAGTG GAAGTCATTA GTCTATGAAC ATTGGTTTAA CTTACTGGTG TTTCTGGTGT   
  
  
- AAGGAGTGTT AGCAACGGAG ATCTTATTCT TACTTACAAA GAAGTTCCAA CTTTCGGGAA CGATTACGCT   
  
  
- TGGACGGATA TACGGATTGA CCGATATATA TACACGCATC TTGTCGTACT GTACTCAATT ATTATATCGT   
  
  
- CGATTTCCCT TGAGATTACA TGTGTACTAA CATTAAGTGG ACGGAGCGAT CGAATGCTCG TACTGAGAAC   
  
  
- TGATAATAAC AAAATTAACT AGAACTTAAA AGTATCTAAA ATGAGGTAAC AAATGAGTAA AACAATATTA   
  
  
- ATTGGTGGAC TAAAATTTGA AATTTTATGT GTTTCTAAGT AGATTATATT AAAAAATCTA TTTCAATACG   
  
  
- TATGTATATA TGTTTAATTT AATAAATGCT AGATTAGTAA AGGGCAATAT ATAAAGTGTG ATTTTAAATC   
  
  
- TTCTCTGTTA GATAATTTTA ATGTAGTGTA CAAAACCCCC CCCCCCCTAT AGTCTATACG TACTGTAAGG   
  
  
- ATTAGGTTAT TACGTTACTT TGGGTGGCTT ATCAACTTTA CTAAACAAGA TAGGTACTTG AGTGGTTCAG   
  
  
- TGTTAGGTAG TGTGAGCTTC CTATACGAAA AGAAAAACTT CCCTTAGAAC GAATTAGTTT GGATATTTGA   
  
  
- AAATTTTCGT TGTCTGTTTT TTCACTAATA TTACCATCAC CATCCTACTT CCGTCGAAAG TCTTTTCGCT   
  
  
- AACTTAAAAA TAACATAGGG TACTTTTGGG TAACTTAAAA AGAAAGAAAG AAACAAAAAC AATGGACCCA   
  
  
- AATACGTTTC CTGCCGACAA CATAAATTAT TATTGTTTTT GTATCCCGAA ACGCCTAACG ATTACGACCC   
  
  
- CTACCGTAGT CACGAGCTTA GACACATGCG ACAGTATCCT TGGGTGAACG AAGACGAGTA AATAAAGACA   
  
  
- GAGATCTAGG GGAGGAGAGA GAGAGAGAGA GAGATTCCAC AGACGAGCAG ACAAGTATAA GAGGACTCCA   
  
  
- AGAAGAAGTA GATAGAACAA GACCCACACA CGACTCCATG AAGAAAAGAA ATATTTAAAA AGTAGAAAAC   
  
  
- AACAAAACGA ACCACAAATA CTAAAATCAA AAACAGAAAT TCTGCTCTAA ACGACAATTA TCGTTCTAAA   
  
  
- GACTTGATCA TAACTAGGAA AAACACCATA TAAACAACTT AAACTAATTA AACACATAAC ACAAATCGAA   
  
  
- TTCATTGAGC AACACTAGAT TAACTACGAG CCCAAAACTG TCAAACTAAT CCCAAAAGCC ATGACCTCAA   
  
  
- TTTCAAAAAA ATAAAAAATA TCCTCCTCAA TTTCCATTAT TATTCTTAAC TACAAACGAC TAAAAAGGAG   
  
  
- AAAAGAAAAA ACCAAAGCTA AACCACCCGT CAATAGACAA ATACCAAAAA CGTTTACCTA GGTGTTAATC   
  
  
- TCCTTAATAT ACCTGGAAAT GTAGCTTAGT TCAAGTTACT AGTTTCCCAC GGTTAAAATG GTTCAGCGTC   
  
  
- ACAACAATGA GGCTACTTTG AGGTTCTAAG GTTAAACTTA GGTTCACAAG GTTTAGGTTC ACAACACTTA   
  
  
- GGAGGAGGCA ACCAAGGGGG TTTGGGTTTA GAATTAAATC AACACCGTAG AGTTATAAGA CTGTAACTCT   
  
  
- GTCGGGACTT GCTCCTAACA CTAAAATCAC TACAACAAGA ATTCATATAA TTAGTTGAAG AATACCTTCT   
  
  
- CCTACCCCTT CTCTTTGAGG TATGACTAGT ACTTGGAAGT CAGCAACTCC GCCGTCTCTT CAGTAACATA   
  
  
- CTCCGCGAGC CCGTCTCTAT AGGAGGAAGG CCTTTGGCTT TAGTCAACGG TCTACAACTC GTACTGCCGA   
  
  
- ACTGACCCTC GTCACAATCC CGAGTATCAC CACGTTCGTC ACAATCCCGA GTATCACTGC GTTCACCACC   
  
  
- ATCACCGGAC TGTCCTATAC CAACCATACC ACTAGGAATA TTAACCTCAG GAGTTTTACA GCACTTAAAA   
  
  
- TGGTAAAGGA GGAGCTTTGG GAATAGCACG TCGAGTAGTC TATCGAGCCC GTCTACCCAA TCATTCACCT   
  
  
- TCAGTTTAAG ACTAAGTGTC GAGTCGAGCC GGTAATTACG TTCACCAAAT CACCTACCCG GTCCCCTGAG   
  
  
- AGGACACTCA CGAGAATCAC ACAGACTTTA TAAGTTACTG TCAGTCAGTT ACAACGTCAA AGTCTTTCCT   
  
  
- AAACTCCTTC GCTTATTTAA AGAAGGTTTC TCAAGAAACA TGTTCCCAAA ACGGTTGTGC CCTAACGGAA   
  
  
- TAGTCTTCCG TTTGTTATCA CGGGTTCTAA ACAACCAGTT ACATCTCCTA CAATGATCCC CTTTCTTCGT   
  
  
- AGCAATAGGG CTTCTTAACG TCAGTCTTCT TCCCTCCTAT TTATTCGTTA GTCGACAAAG GGACCGTCGA   
  
  
- CTACTCCGTC AACAATCCAG ACTTTACAAG CTATCCCATG ACGAAACATC AGCCCCTTTT GTACTACGAC   
  
  
- GAGAGGCCCT TCGAAATGTC TGACTTGATT TATTCTCAGA CGCTTTACGG GGTCAATTCC CCAGATTATC   
  
  
- ACCCTTTCCG GCACCATTCT TTCGCCCTTT CTTCTCCCTA CATCATCTAA ATTCTAGAGA GAATTGGGAT   
  
  
- ACACGTGTTC GTCAACGTAG CTTACTAGTG TCCTCACGTT TACTGGACGA ATCCGTTTAA TCTGTCGTAA   
  
  
- GAAGAGGATC ACCCCTACCA TTGGTTTCTT ACCGTGTAAT AAAACGCCTA CCAGAACTCC GTGCGGAACG   
  
  
- ACCACATCCT TGAGGATATA TATTGACAGA ACATTGCCCA GGCCGTAGCC GACATCTATA GAACTCTCGA   
  
  
- ATGGTGTACA AAGAACGGTG TACGGGTAAA TTCTTTTACC CTTTAAAGAA GAGATTATCT TGCTAATACC   
  
  
- GACACCGTCT CTTACGTTGT ACGGAAGTAT ATTAACTAGA GCCATAACAG ATACCAAAGG TTACCGGAAC   
  
  
- AGATTAAGTC GCTGAAAGTA GATCCGGACC ACCGGGGGGG TTTGAAGCTT ATTGGCCTCA TCTAGAAGGT   
  
  
- GTTGGACCCA AGGCTGGTCG GTTTTCTCAA CTCCTCTGTC CCGCAGCGAA CTTCTTGATA CGTCTCAGTA   
  
  
- AATTACACGG GAAACTCAAG TTACGATATC GTTTCTTCAC CCTTTGTGAA TGGTAACTTC TAGAGTCCTA   
  
  
- GTTATCGCTA CTCAACGAAC AACAGTGGAC ATACAAATCC AAATTTGTAT ATGGACTCCT TTGTCACTGG   
  
  
- CACCTAACGG GATCCCTATG ACAAGAATTG GACTAACCCG CGTATTTGGG TCGACAAAAG TATGTTCCGT   
  
  
- GACAATTGCC CCGAAAGTTA AGAGGGAAAA AGTATCGAGC TAAAGCTCTC CGAGATAAAG TGAAGAGGTG   
  
  
- AGACAAACTA TACGATCTCC GGTTGGACGG TTCCCTGTTA TTCCTCTCCT ACGATTAACT CTCTCTCTAT   
  
  
- AAACCCTCCG TCCGTTACTT ACACTAACGA ACACTCCCAA ATCTCTCCTA TCTTTCCGGT CTCTGCATGT   
  
  
- TCGTCACCGT TCAGGCTTTA CTTTCCCGTC CCAAATCCGT CGACGGAAAT CTAGCGGTCT AAGACCTTTA   
  
  
- CCGATTTTTC TCCCACTTTA GACACATATT GTTTCTAAAG AGGTAACTAC TTCTGCCCGT GACCAACAAC   
  
  
- GACCCTACCT TCCCGTCTTA ACACATGTGT GAGTGATGAA CCTTCGGACG CCTCAT

+     MYB

| Site Name | Organism | Position | Strand | Matrix score. | sequence | function |
| --- | --- | --- | --- | --- | --- | --- |
| MYB | Arabidopsis thaliana | 96 | + | 6 | TAACCA |  |
| MYB | Arabidopsis thaliana | 2543 | - | 6 | CAACCA |  |
| MYB | Arabidopsis thaliana | 915 | + | 6 | TAACCA |  |
| MYB | Arabidopsis thaliana | 4266 | - | 6 | CAACCA |  |
| MYB | Arabidopsis thaliana | 159 | - | 6 | CAACAG |  |
| MYB | Arabidopsis thaliana | 3314 | + | 6 | TAACCA |  |
| MYB | Arabidopsis thaliana | 605 | + | 6 | TAACCA |  |
| MYB | Arabidopsis thaliana | 1273 | + | 6 | CAACAG |  |
| MYB | Arabidopsis thaliana | 1420 | - | 6 | CAACAG |  |

>HU06G00568.1   
+ -Up\_Stream \_Len000CCCTGG GTCATTAAAA AAAAAGAAAG AAAGAAGATT GCGCTAAAAG TAGATTTTTA   
  
  
+ TCAGATTCAA GCTGCAGGTG ATAACCAAAT TGTCATTAAA GCAATGCAAG CGCATATCAA TACCCTTTTG   
  
  
+ GGAGATAGCT CCGACTGTTG AAGACATTAG GAGCATGATC TCCAACTGTG AATTTGTTTC ATTTACTCAT   
  
  
+ ATTCATATCT ACCATGTGGA TAATATGACT GCAGACTAGA TGGCCAAATT TGAATGCACA CTTATAACTT   
  
  
+ CAACTCTTTC TACTTTTTTC TTCCCCACCT TATCAGGAAT TTCTTTTAAT TGTCGTGGGA GACAACTTGG   
  
  
+ GTAGAACTCT TGCGAAAAGG GCAACTTAAC GTTTTGGTAC TTGCATTTTC CAAAAAAACA AAAAGGCTAA   
  
  
+ ACAATTCTCC GCATGCTGAC TTCACGGTAG ATATGATGTA TTTTTTAACA AAAGACATCT TCTGAATGAA   
  
  
+ ACGCTTAATA TCTTGGACCA TACTAGAAAT TAAATTGCCA CGGTGCATGA ATTTCACCTC GAACTGCTTG   
  
  
+ TATAAAGATC TTGTTATCAC CTTCAGTAAT CAGATACTTG TAACCAAATT GAATGACCAC AAAGACCACA   
  
  
+ TTCCTCACAA TCGTTGCCTC TAGAATAAGA ATGAATGTTT CTTCAAGGTT GAAAGCCCTT GCTAATGCGA   
  
  
+ ACCTGCCTAT ATGCCTAACT GGCTATATAT ATGTGCGTAG AACAGCATGA CATGAGTTAA TAATATAGCA   
  
  
+ GCTAAAGGGA ACTCTAATGT ACACATGATT GTAATTCACC TGCCTCGCTA GCTTACGAGC ATGACTCTTG   
  
  
+ ACTATTATTG TTTTAATTGA TCTTGAATTT TCATAGATTT TACTCCATTG TTTACTCATT TTGTTATAAT   
  
  
+ TAACCACCTG ATTTTAAACT TTAAAATACA CAAAGATTCA TCTAATATAA TTTTTTAGAT AAAGTTATGC   
  
  
+ ATACATATAT ACAAATTAAA TTATTTACGA TCTAATCATT TCCCGTTATA TATTTCACAC TAAAATTTAG   
  
  
+ AAGAGACAAT CTATTAAAAT TACATCACAT GTTTTGGGGG GGGGGGGATA TCAGATATGC ATGACATTCC   
  
  
+ TAATCCAATA ATGCAATGAA ACCCACCGAA TAGTTGAAAT GATTTGTTCT ATCCATGAAC TCACCAAGTC   
  
  
+ ACAATCCATC ACACTCGAAG GATATGCTTT TCTTTTTGAA GGGAATCTTG CTTAATCAAA CCTATAAACT   
  
  
+ TTTAAAAGCA ACAGACAAAA AAGTGATTAT AATGGTAGTG GTAGGATGAA GGCAGCTTTC AGAAAAGCGA   
  
  
+ TTGAATTTTT ATTGTATCCC ATGAAAACCC ATTGAATTTT TCTTTCTTTC TTTGTTTTTG TTACCTGGGT   
  
  
+ TTATGCAAAG GACGGCTGTT GTATTTAATA ATAACAAAAA CATAGGGCTT TGCGGATTGC TAATGCTGGG   
  
  
+ GATGGCATCA GTGCTCGAAT CTGTGTACGC TGTCATAGGA ACCCACTTGC TTCTGCTCAT TTATTTCTGT   
  
  
+ CTCTAGATCC CCTCCTCTCT CTCTCTCTCT CTCTAAGGTG TCTGCTCGTC TGTTCATATT CTCCTGAGGT   
  
  
+ TCTTCTTCAT CTATCTTGTT CTGGGTGTGT GCTGAGGTAC TTCTTTTCTT TATAAATTTT TCATCTTTTG   
  
  
+ TTGTTTTGCT TGGTGTTTAT GATTTTAGTT TTTGTCTTTA AGACGAGATT TGCTGTTAAT AGCAAGATTT   
  
  
+ CTGAACTAGT ATTGATCCTT TTTGTGGTAT ATTTGTTGAA TTTGATTAAT TTGTGTATTG TGTTTAGCTT   
  
  
+ AAGTAACTCG TTGTGATCTA ATTGATGCTC GGGTTTTGAC AGTTTGATTA GGGTTTTCGG TACTGGAGTT   
  
  
+ AAAGTTTTTT TATTTTTTAT AGGAGGAGTT AAAGGTAATA ATAAGAATTG ATGTTTGCTG ATTTTTCCTC   
  
  
+ TTTTCTTTTT TGGTTTCGAT TTGGTGGGCA GTTATCTGTT TATGGTTTTT GCAAATGGAT CCACAATTAG   
  
  
+ AGGAATTATA TGGACCTTTA CATCGAATCA AGTTCAATGA TCAAAGGGTG CCAATTTTAC CAAGTCGCAG   
  
  
+ TGTTGTTACT CCGATGAAAC TCCAAGATTC CAATTTGAAT CCAAGTGTTC CAAATCCAAG TGTTGTGAAT   
  
  
+ CCTCCTCCGT TGGTTCCCCC AAACCCAAAT CTTAATTTAG TTGTGGCATC TCAATATTCT GACATTGAGA   
  
  
+ CAGCCCTGAA CGAGGATTGT GATTTTAGTG ATGTTGTTCT TAAGTATATT AATCAACTTC TTATGGAAGA   
  
  
+ GGATGGGGAA GAGAAACTCC ATACTGATCA TGAACCTTCA GTCGTTGAGG CGGCAGAGAA GTCATTGTAT   
  
  
+ GAGGCGCTCG GGCAGAGATA TCCTCCTTCC GGAAACCGAA ATCAGTTGCC AGATGTTGAG CATGACGGCT   
  
  
+ TGACTGGGAG CAGTGTTAGG GCTCATAGTG GTGCAAGCAG TGTTAGGGCT CATAGTGACG CAAGTGGTGG   
  
  
+ TAGTGGCCTG ACAGGATATG GTTGGTATGG TGATCCTTAT AATTGGAGTC CTCAAAATGT CGTGAATTTT   
  
  
+ ACCATTTCCT CCTCGAAACC CTTATCGTGC AGCTCATCAG ATAGCTCGGG CAGATGGGTT AGTAAGTGGA   
  
  
+ AGTCAAATTC TGATTCACAG CTCAGCTCGG CCATTAATGC AAGTGGTTTA GTGGATGGGC CAGGGGACTC   
  
  
+ TCCTGTGAGT GCTCTTAGTG TGTCTGAAAT ATTCAATGAC AGTCAGTCAA TGTTGCAGTT TCAGAAAGGA   
  
  
+ TTTGAGGAAG CGAATAAATT TCTTCCAAAG AGTTCTTTGT ACAAGGGTTT TGCCAACACG GGATTGCCTT   
  
  
+ ATCAGAAGGC AAACAATAGT GCCCAAGATT TGTTGGTCAA TGTAGAGGAT GTTACTAGGG GAAAGAAGCA   
  
  
+ TCGTTATCCC GAAGAATTGC AGTCAGAAGA AGGGAGGATA AATAAGCAAT CAGCTGTTTC CCTGGCAGCT   
  
  
+ GATGAGGCAG TTGTTAGGTC TGAAATGTTC GATAGGGTAC TGCTTTGTAG TCGGGGAAAA CATGATGCTG   
  
  
+ CTCTCCGGGA AGCTTTACAG ACTGAACTAA ATAAGAGTCT GCGAAATGCC CCAGTTAAGG GGTCTAATAG   
  
  
+ TGGGAAAGGC CGTGGTAAGA AAGCGGGAAA GAAGAGGGAT GTAGTAGATT TAAGATCTCT CTTAACCCTA   
  
  
+ TGTGCACAAG CAGTTGCATC GAATGATCAC AGGAGTGCAA ATGACCTGCT TAGGCAAATT AGACAGCATT   
  
  
+ CTTCTCCTAG TGGGGATGGT AACCAAAGAA TGGCACATTA TTTTGCGGAT GGTCTTGAGG CACGCCTTGC   
  
  
+ TGGTGTAGGA ACTCCTATAT ATAACTGTCT TGTAACGGGT CCGGCATCGG CTGTAGATAT CTTGAGAGCT   
  
  
+ TACCACATGT TTCTTGCCAC ATGCCCATTT AAGAAAATGG GAAATTTCTT CTCTAATAGA ACGATTATGG   
  
  
+ CTGTGGCAGA GAATGCAACA TGCCTTCATA TAATTGATCT CGGTATTGTC TATGGTTTCC AATGGCCTTG   
  
  
+ TCTAATTCAG CGACTTTCAT CTAGGCCTGG TGGCCCCCCC AAACTTCGAA TAACCGGAGT AGATCTTCCA   
  
  
+ CAACCTGGGT TCCGACCAGC CAAAAGAGTT GAGGAGACAG GGCGTCGCTT GAAGAACTAT GCAGAGTCAT   
  
  
+ TTAATGTGCC CTTTGAGTTC AATGCTATAG CAAAGAAGTG GGAAACACTT ACCATTGAAG ATCTCAGGAT   
  
  
+ CAATAGCGAT GAGTTGCTTG TTGTCACCTG TATGTTTAGG TTTAAACATA TACCTGAGGA AACAGTGACC   
  
  
+ GTGGATTGCC CTAGGGATAC TGTTCTTAAC CTGATTGGGC GCATAAACCC AGCTGTTTTC ATACAAGGCA   
  
  
+ CTGTTAACGG GGCTTTCAAT TCTCCCTTTT TCATAGCTCG ATTTCGAGAG GCTCTATTTC ACTTCTCCAC   
  
  
+ TCTGTTTGAT ATGCTAGAGG CCAACCTGCC AAGGGACAAT AAGGAGAGGA TGCTAATTGA GAGAGAGATA   
  
  
+ TTTGGGAGGC AGGCAATGAA TGTGATTGCT TGTGAGGGTT TAGAGAGGAT AGAAAGGCCA GAGACGTACA   
  
  
+ AGCAGTGGCA AGTCCGAAAT GAAAGGGCAG GGTTTAGGCA GCTGCCTTTA GATCGCCAGA TTCTGGAAAT   
  
  
+ GGCTAAAAAG AGGGTGAAAT CTGTGTATAA CAAAGATTTC TCCATTGATG AAGACGGGCA CTGGTTGTTG   
  
  
+ CTGGGATGGA AGGGCAGAAT TGTGTACACA CTCACTACTT GGAAGCCTGC GGAGTA  

- -Up\_Stream \_Len000GGGACC CAGTAATTTT TTTTTCTTTC TTTCTTCTAA CGCGATTTTC ATCTAAAAAT   
  
  
- AGTCTAAGTT CGACGTCCAC TATTGGTTTA ACAGTAATTT CGTTACGTTC GCGTATAGTT ATGGGAAAAC   
  
  
- CCTCTATCGA GGCTGACAAC TTCTGTAATC CTCGTACTAG AGGTTGACAC TTAAACAAAG TAAATGAGTA   
  
  
- TAAGTATAGA TGGTACACCT ATTATACTGA CGTCTGATCT ACCGGTTTAA ACTTACGTGT GAATATTGAA   
  
  
- GTTGAGAAAG ATGAAAAAAG AAGGGGTGGA ATAGTCCTTA AAGAAAATTA ACAGCACCCT CTGTTGAACC   
  
  
- CATCTTGAGA ACGCTTTTCC CGTTGAATTG CAAAACCATG AACGTAAAAG GTTTTTTTGT TTTTCCGATT   
  
  
- TGTTAAGAGG CGTACGACTG AAGTGCCATC TATACTACAT AAAAAATTGT TTTCTGTAGA AGACTTACTT   
  
  
- TGCGAATTAT AGAACCTGGT ATGATCTTTA ATTTAACGGT GCCACGTACT TAAAGTGGAG CTTGACGAAC   
  
  
- ATATTTCTAG AACAATAGTG GAAGTCATTA GTCTATGAAC ATTGGTTTAA CTTACTGGTG TTTCTGGTGT   
  
  
- AAGGAGTGTT AGCAACGGAG ATCTTATTCT TACTTACAAA GAAGTTCCAA CTTTCGGGAA CGATTACGCT   
  
  
- TGGACGGATA TACGGATTGA CCGATATATA TACACGCATC TTGTCGTACT GTACTCAATT ATTATATCGT   
  
  
- CGATTTCCCT TGAGATTACA TGTGTACTAA CATTAAGTGG ACGGAGCGAT CGAATGCTCG TACTGAGAAC   
  
  
- TGATAATAAC AAAATTAACT AGAACTTAAA AGTATCTAAA ATGAGGTAAC AAATGAGTAA AACAATATTA   
  
  
- ATTGGTGGAC TAAAATTTGA AATTTTATGT GTTTCTAAGT AGATTATATT AAAAAATCTA TTTCAATACG   
  
  
- TATGTATATA TGTTTAATTT AATAAATGCT AGATTAGTAA AGGGCAATAT ATAAAGTGTG ATTTTAAATC   
  
  
- TTCTCTGTTA GATAATTTTA ATGTAGTGTA CAAAACCCCC CCCCCCCTAT AGTCTATACG TACTGTAAGG   
  
  
- ATTAGGTTAT TACGTTACTT TGGGTGGCTT ATCAACTTTA CTAAACAAGA TAGGTACTTG AGTGGTTCAG   
  
  
- TGTTAGGTAG TGTGAGCTTC CTATACGAAA AGAAAAACTT CCCTTAGAAC GAATTAGTTT GGATATTTGA   
  
  
- AAATTTTCGT TGTCTGTTTT TTCACTAATA TTACCATCAC CATCCTACTT CCGTCGAAAG TCTTTTCGCT   
  
  
- AACTTAAAAA TAACATAGGG TACTTTTGGG TAACTTAAAA AGAAAGAAAG AAACAAAAAC AATGGACCCA   
  
  
- AATACGTTTC CTGCCGACAA CATAAATTAT TATTGTTTTT GTATCCCGAA ACGCCTAACG ATTACGACCC   
  
  
- CTACCGTAGT CACGAGCTTA GACACATGCG ACAGTATCCT TGGGTGAACG AAGACGAGTA AATAAAGACA   
  
  
- GAGATCTAGG GGAGGAGAGA GAGAGAGAGA GAGATTCCAC AGACGAGCAG ACAAGTATAA GAGGACTCCA   
  
  
- AGAAGAAGTA GATAGAACAA GACCCACACA CGACTCCATG AAGAAAAGAA ATATTTAAAA AGTAGAAAAC   
  
  
- AACAAAACGA ACCACAAATA CTAAAATCAA AAACAGAAAT TCTGCTCTAA ACGACAATTA TCGTTCTAAA   
  
  
- GACTTGATCA TAACTAGGAA AAACACCATA TAAACAACTT AAACTAATTA AACACATAAC ACAAATCGAA   
  
  
- TTCATTGAGC AACACTAGAT TAACTACGAG CCCAAAACTG TCAAACTAAT CCCAAAAGCC ATGACCTCAA   
  
  
- TTTCAAAAAA ATAAAAAATA TCCTCCTCAA TTTCCATTAT TATTCTTAAC TACAAACGAC TAAAAAGGAG   
  
  
- AAAAGAAAAA ACCAAAGCTA AACCACCCGT CAATAGACAA ATACCAAAAA CGTTTACCTA GGTGTTAATC   
  
  
- TCCTTAATAT ACCTGGAAAT GTAGCTTAGT TCAAGTTACT AGTTTCCCAC GGTTAAAATG GTTCAGCGTC   
  
  
- ACAACAATGA GGCTACTTTG AGGTTCTAAG GTTAAACTTA GGTTCACAAG GTTTAGGTTC ACAACACTTA   
  
  
- GGAGGAGGCA ACCAAGGGGG TTTGGGTTTA GAATTAAATC AACACCGTAG AGTTATAAGA CTGTAACTCT   
  
  
- GTCGGGACTT GCTCCTAACA CTAAAATCAC TACAACAAGA ATTCATATAA TTAGTTGAAG AATACCTTCT   
  
  
- CCTACCCCTT CTCTTTGAGG TATGACTAGT ACTTGGAAGT CAGCAACTCC GCCGTCTCTT CAGTAACATA   
  
  
- CTCCGCGAGC CCGTCTCTAT AGGAGGAAGG CCTTTGGCTT TAGTCAACGG TCTACAACTC GTACTGCCGA   
  
  
- ACTGACCCTC GTCACAATCC CGAGTATCAC CACGTTCGTC ACAATCCCGA GTATCACTGC GTTCACCACC   
  
  
- ATCACCGGAC TGTCCTATAC CAACCATACC ACTAGGAATA TTAACCTCAG GAGTTTTACA GCACTTAAAA   
  
  
- TGGTAAAGGA GGAGCTTTGG GAATAGCACG TCGAGTAGTC TATCGAGCCC GTCTACCCAA TCATTCACCT   
  
  
- TCAGTTTAAG ACTAAGTGTC GAGTCGAGCC GGTAATTACG TTCACCAAAT CACCTACCCG GTCCCCTGAG   
  
  
- AGGACACTCA CGAGAATCAC ACAGACTTTA TAAGTTACTG TCAGTCAGTT ACAACGTCAA AGTCTTTCCT   
  
  
- AAACTCCTTC GCTTATTTAA AGAAGGTTTC TCAAGAAACA TGTTCCCAAA ACGGTTGTGC CCTAACGGAA   
  
  
- TAGTCTTCCG TTTGTTATCA CGGGTTCTAA ACAACCAGTT ACATCTCCTA CAATGATCCC CTTTCTTCGT   
  
  
- AGCAATAGGG CTTCTTAACG TCAGTCTTCT TCCCTCCTAT TTATTCGTTA GTCGACAAAG GGACCGTCGA   
  
  
- CTACTCCGTC AACAATCCAG ACTTTACAAG CTATCCCATG ACGAAACATC AGCCCCTTTT GTACTACGAC   
  
  
- GAGAGGCCCT TCGAAATGTC TGACTTGATT TATTCTCAGA CGCTTTACGG GGTCAATTCC CCAGATTATC   
  
  
- ACCCTTTCCG GCACCATTCT TTCGCCCTTT CTTCTCCCTA CATCATCTAA ATTCTAGAGA GAATTGGGAT   
  
  
- ACACGTGTTC GTCAACGTAG CTTACTAGTG TCCTCACGTT TACTGGACGA ATCCGTTTAA TCTGTCGTAA   
  
  
- GAAGAGGATC ACCCCTACCA TTGGTTTCTT ACCGTGTAAT AAAACGCCTA CCAGAACTCC GTGCGGAACG   
  
  
- ACCACATCCT TGAGGATATA TATTGACAGA ACATTGCCCA GGCCGTAGCC GACATCTATA GAACTCTCGA   
  
  
- ATGGTGTACA AAGAACGGTG TACGGGTAAA TTCTTTTACC CTTTAAAGAA GAGATTATCT TGCTAATACC   
  
  
- GACACCGTCT CTTACGTTGT ACGGAAGTAT ATTAACTAGA GCCATAACAG ATACCAAAGG TTACCGGAAC   
  
  
- AGATTAAGTC GCTGAAAGTA GATCCGGACC ACCGGGGGGG TTTGAAGCTT ATTGGCCTCA TCTAGAAGGT   
  
  
- GTTGGACCCA AGGCTGGTCG GTTTTCTCAA CTCCTCTGTC CCGCAGCGAA CTTCTTGATA CGTCTCAGTA   
  
  
- AATTACACGG GAAACTCAAG TTACGATATC GTTTCTTCAC CCTTTGTGAA TGGTAACTTC TAGAGTCCTA   
  
  
- GTTATCGCTA CTCAACGAAC AACAGTGGAC ATACAAATCC AAATTTGTAT ATGGACTCCT TTGTCACTGG   
  
  
- CACCTAACGG GATCCCTATG ACAAGAATTG GACTAACCCG CGTATTTGGG TCGACAAAAG TATGTTCCGT   
  
  
- GACAATTGCC CCGAAAGTTA AGAGGGAAAA AGTATCGAGC TAAAGCTCTC CGAGATAAAG TGAAGAGGTG   
  
  
- AGACAAACTA TACGATCTCC GGTTGGACGG TTCCCTGTTA TTCCTCTCCT ACGATTAACT CTCTCTCTAT   
  
  
- AAACCCTCCG TCCGTTACTT ACACTAACGA ACACTCCCAA ATCTCTCCTA TCTTTCCGGT CTCTGCATGT   
  
  
- TCGTCACCGT TCAGGCTTTA CTTTCCCGTC CCAAATCCGT CGACGGAAAT CTAGCGGTCT AAGACCTTTA   
  
  
- CCGATTTTTC TCCCACTTTA GACACATATT GTTTCTAAAG AGGTAACTAC TTCTGCCCGT GACCAACAAC   
  
  
- GACCCTACCT TCCCGTCTTA ACACATGTGT GAGTGATGAA CCTTCGGACG CCTCAT

+     MYB recognition site

| Site Name | Organism | Position | Strand | Matrix score. | sequence | function |
| --- | --- | --- | --- | --- | --- | --- |
| MYB recognition site | Arabidopsis thaliana | 2181 | + | 6 | CCGTTG |  |

>HU06G00568.1   
+ -Up\_Stream \_Len000CCCTGG GTCATTAAAA AAAAAGAAAG AAAGAAGATT GCGCTAAAAG TAGATTTTTA   
  
  
+ TCAGATTCAA GCTGCAGGTG ATAACCAAAT TGTCATTAAA GCAATGCAAG CGCATATCAA TACCCTTTTG   
  
  
+ GGAGATAGCT CCGACTGTTG AAGACATTAG GAGCATGATC TCCAACTGTG AATTTGTTTC ATTTACTCAT   
  
  
+ ATTCATATCT ACCATGTGGA TAATATGACT GCAGACTAGA TGGCCAAATT TGAATGCACA CTTATAACTT   
  
  
+ CAACTCTTTC TACTTTTTTC TTCCCCACCT TATCAGGAAT TTCTTTTAAT TGTCGTGGGA GACAACTTGG   
  
  
+ GTAGAACTCT TGCGAAAAGG GCAACTTAAC GTTTTGGTAC TTGCATTTTC CAAAAAAACA AAAAGGCTAA   
  
  
+ ACAATTCTCC GCATGCTGAC TTCACGGTAG ATATGATGTA TTTTTTAACA AAAGACATCT TCTGAATGAA   
  
  
+ ACGCTTAATA TCTTGGACCA TACTAGAAAT TAAATTGCCA CGGTGCATGA ATTTCACCTC GAACTGCTTG   
  
  
+ TATAAAGATC TTGTTATCAC CTTCAGTAAT CAGATACTTG TAACCAAATT GAATGACCAC AAAGACCACA   
  
  
+ TTCCTCACAA TCGTTGCCTC TAGAATAAGA ATGAATGTTT CTTCAAGGTT GAAAGCCCTT GCTAATGCGA   
  
  
+ ACCTGCCTAT ATGCCTAACT GGCTATATAT ATGTGCGTAG AACAGCATGA CATGAGTTAA TAATATAGCA   
  
  
+ GCTAAAGGGA ACTCTAATGT ACACATGATT GTAATTCACC TGCCTCGCTA GCTTACGAGC ATGACTCTTG   
  
  
+ ACTATTATTG TTTTAATTGA TCTTGAATTT TCATAGATTT TACTCCATTG TTTACTCATT TTGTTATAAT   
  
  
+ TAACCACCTG ATTTTAAACT TTAAAATACA CAAAGATTCA TCTAATATAA TTTTTTAGAT AAAGTTATGC   
  
  
+ ATACATATAT ACAAATTAAA TTATTTACGA TCTAATCATT TCCCGTTATA TATTTCACAC TAAAATTTAG   
  
  
+ AAGAGACAAT CTATTAAAAT TACATCACAT GTTTTGGGGG GGGGGGGATA TCAGATATGC ATGACATTCC   
  
  
+ TAATCCAATA ATGCAATGAA ACCCACCGAA TAGTTGAAAT GATTTGTTCT ATCCATGAAC TCACCAAGTC   
  
  
+ ACAATCCATC ACACTCGAAG GATATGCTTT TCTTTTTGAA GGGAATCTTG CTTAATCAAA CCTATAAACT   
  
  
+ TTTAAAAGCA ACAGACAAAA AAGTGATTAT AATGGTAGTG GTAGGATGAA GGCAGCTTTC AGAAAAGCGA   
  
  
+ TTGAATTTTT ATTGTATCCC ATGAAAACCC ATTGAATTTT TCTTTCTTTC TTTGTTTTTG TTACCTGGGT   
  
  
+ TTATGCAAAG GACGGCTGTT GTATTTAATA ATAACAAAAA CATAGGGCTT TGCGGATTGC TAATGCTGGG   
  
  
+ GATGGCATCA GTGCTCGAAT CTGTGTACGC TGTCATAGGA ACCCACTTGC TTCTGCTCAT TTATTTCTGT   
  
  
+ CTCTAGATCC CCTCCTCTCT CTCTCTCTCT CTCTAAGGTG TCTGCTCGTC TGTTCATATT CTCCTGAGGT   
  
  
+ TCTTCTTCAT CTATCTTGTT CTGGGTGTGT GCTGAGGTAC TTCTTTTCTT TATAAATTTT TCATCTTTTG   
  
  
+ TTGTTTTGCT TGGTGTTTAT GATTTTAGTT TTTGTCTTTA AGACGAGATT TGCTGTTAAT AGCAAGATTT   
  
  
+ CTGAACTAGT ATTGATCCTT TTTGTGGTAT ATTTGTTGAA TTTGATTAAT TTGTGTATTG TGTTTAGCTT   
  
  
+ AAGTAACTCG TTGTGATCTA ATTGATGCTC GGGTTTTGAC AGTTTGATTA GGGTTTTCGG TACTGGAGTT   
  
  
+ AAAGTTTTTT TATTTTTTAT AGGAGGAGTT AAAGGTAATA ATAAGAATTG ATGTTTGCTG ATTTTTCCTC   
  
  
+ TTTTCTTTTT TGGTTTCGAT TTGGTGGGCA GTTATCTGTT TATGGTTTTT GCAAATGGAT CCACAATTAG   
  
  
+ AGGAATTATA TGGACCTTTA CATCGAATCA AGTTCAATGA TCAAAGGGTG CCAATTTTAC CAAGTCGCAG   
  
  
+ TGTTGTTACT CCGATGAAAC TCCAAGATTC CAATTTGAAT CCAAGTGTTC CAAATCCAAG TGTTGTGAAT   
  
  
+ CCTCCTCCGT TGGTTCCCCC AAACCCAAAT CTTAATTTAG TTGTGGCATC TCAATATTCT GACATTGAGA   
  
  
+ CAGCCCTGAA CGAGGATTGT GATTTTAGTG ATGTTGTTCT TAAGTATATT AATCAACTTC TTATGGAAGA   
  
  
+ GGATGGGGAA GAGAAACTCC ATACTGATCA TGAACCTTCA GTCGTTGAGG CGGCAGAGAA GTCATTGTAT   
  
  
+ GAGGCGCTCG GGCAGAGATA TCCTCCTTCC GGAAACCGAA ATCAGTTGCC AGATGTTGAG CATGACGGCT   
  
  
+ TGACTGGGAG CAGTGTTAGG GCTCATAGTG GTGCAAGCAG TGTTAGGGCT CATAGTGACG CAAGTGGTGG   
  
  
+ TAGTGGCCTG ACAGGATATG GTTGGTATGG TGATCCTTAT AATTGGAGTC CTCAAAATGT CGTGAATTTT   
  
  
+ ACCATTTCCT CCTCGAAACC CTTATCGTGC AGCTCATCAG ATAGCTCGGG CAGATGGGTT AGTAAGTGGA   
  
  
+ AGTCAAATTC TGATTCACAG CTCAGCTCGG CCATTAATGC AAGTGGTTTA GTGGATGGGC CAGGGGACTC   
  
  
+ TCCTGTGAGT GCTCTTAGTG TGTCTGAAAT ATTCAATGAC AGTCAGTCAA TGTTGCAGTT TCAGAAAGGA   
  
  
+ TTTGAGGAAG CGAATAAATT TCTTCCAAAG AGTTCTTTGT ACAAGGGTTT TGCCAACACG GGATTGCCTT   
  
  
+ ATCAGAAGGC AAACAATAGT GCCCAAGATT TGTTGGTCAA TGTAGAGGAT GTTACTAGGG GAAAGAAGCA   
  
  
+ TCGTTATCCC GAAGAATTGC AGTCAGAAGA AGGGAGGATA AATAAGCAAT CAGCTGTTTC CCTGGCAGCT   
  
  
+ GATGAGGCAG TTGTTAGGTC TGAAATGTTC GATAGGGTAC TGCTTTGTAG TCGGGGAAAA CATGATGCTG   
  
  
+ CTCTCCGGGA AGCTTTACAG ACTGAACTAA ATAAGAGTCT GCGAAATGCC CCAGTTAAGG GGTCTAATAG   
  
  
+ TGGGAAAGGC CGTGGTAAGA AAGCGGGAAA GAAGAGGGAT GTAGTAGATT TAAGATCTCT CTTAACCCTA   
  
  
+ TGTGCACAAG CAGTTGCATC GAATGATCAC AGGAGTGCAA ATGACCTGCT TAGGCAAATT AGACAGCATT   
  
  
+ CTTCTCCTAG TGGGGATGGT AACCAAAGAA TGGCACATTA TTTTGCGGAT GGTCTTGAGG CACGCCTTGC   
  
  
+ TGGTGTAGGA ACTCCTATAT ATAACTGTCT TGTAACGGGT CCGGCATCGG CTGTAGATAT CTTGAGAGCT   
  
  
+ TACCACATGT TTCTTGCCAC ATGCCCATTT AAGAAAATGG GAAATTTCTT CTCTAATAGA ACGATTATGG   
  
  
+ CTGTGGCAGA GAATGCAACA TGCCTTCATA TAATTGATCT CGGTATTGTC TATGGTTTCC AATGGCCTTG   
  
  
+ TCTAATTCAG CGACTTTCAT CTAGGCCTGG TGGCCCCCCC AAACTTCGAA TAACCGGAGT AGATCTTCCA   
  
  
+ CAACCTGGGT TCCGACCAGC CAAAAGAGTT GAGGAGACAG GGCGTCGCTT GAAGAACTAT GCAGAGTCAT   
  
  
+ TTAATGTGCC CTTTGAGTTC AATGCTATAG CAAAGAAGTG GGAAACACTT ACCATTGAAG ATCTCAGGAT   
  
  
+ CAATAGCGAT GAGTTGCTTG TTGTCACCTG TATGTTTAGG TTTAAACATA TACCTGAGGA AACAGTGACC   
  
  
+ GTGGATTGCC CTAGGGATAC TGTTCTTAAC CTGATTGGGC GCATAAACCC AGCTGTTTTC ATACAAGGCA   
  
  
+ CTGTTAACGG GGCTTTCAAT TCTCCCTTTT TCATAGCTCG ATTTCGAGAG GCTCTATTTC ACTTCTCCAC   
  
  
+ TCTGTTTGAT ATGCTAGAGG CCAACCTGCC AAGGGACAAT AAGGAGAGGA TGCTAATTGA GAGAGAGATA   
  
  
+ TTTGGGAGGC AGGCAATGAA TGTGATTGCT TGTGAGGGTT TAGAGAGGAT AGAAAGGCCA GAGACGTACA   
  
  
+ AGCAGTGGCA AGTCCGAAAT GAAAGGGCAG GGTTTAGGCA GCTGCCTTTA GATCGCCAGA TTCTGGAAAT   
  
  
+ GGCTAAAAAG AGGGTGAAAT CTGTGTATAA CAAAGATTTC TCCATTGATG AAGACGGGCA CTGGTTGTTG   
  
  
+ CTGGGATGGA AGGGCAGAAT TGTGTACACA CTCACTACTT GGAAGCCTGC GGAGTA  

- -Up\_Stream \_Len000GGGACC CAGTAATTTT TTTTTCTTTC TTTCTTCTAA CGCGATTTTC ATCTAAAAAT   
  
  
- AGTCTAAGTT CGACGTCCAC TATTGGTTTA ACAGTAATTT CGTTACGTTC GCGTATAGTT ATGGGAAAAC   
  
  
- CCTCTATCGA GGCTGACAAC TTCTGTAATC CTCGTACTAG AGGTTGACAC TTAAACAAAG TAAATGAGTA   
  
  
- TAAGTATAGA TGGTACACCT ATTATACTGA CGTCTGATCT ACCGGTTTAA ACTTACGTGT GAATATTGAA   
  
  
- GTTGAGAAAG ATGAAAAAAG AAGGGGTGGA ATAGTCCTTA AAGAAAATTA ACAGCACCCT CTGTTGAACC   
  
  
- CATCTTGAGA ACGCTTTTCC CGTTGAATTG CAAAACCATG AACGTAAAAG GTTTTTTTGT TTTTCCGATT   
  
  
- TGTTAAGAGG CGTACGACTG AAGTGCCATC TATACTACAT AAAAAATTGT TTTCTGTAGA AGACTTACTT   
  
  
- TGCGAATTAT AGAACCTGGT ATGATCTTTA ATTTAACGGT GCCACGTACT TAAAGTGGAG CTTGACGAAC   
  
  
- ATATTTCTAG AACAATAGTG GAAGTCATTA GTCTATGAAC ATTGGTTTAA CTTACTGGTG TTTCTGGTGT   
  
  
- AAGGAGTGTT AGCAACGGAG ATCTTATTCT TACTTACAAA GAAGTTCCAA CTTTCGGGAA CGATTACGCT   
  
  
- TGGACGGATA TACGGATTGA CCGATATATA TACACGCATC TTGTCGTACT GTACTCAATT ATTATATCGT   
  
  
- CGATTTCCCT TGAGATTACA TGTGTACTAA CATTAAGTGG ACGGAGCGAT CGAATGCTCG TACTGAGAAC   
  
  
- TGATAATAAC AAAATTAACT AGAACTTAAA AGTATCTAAA ATGAGGTAAC AAATGAGTAA AACAATATTA   
  
  
- ATTGGTGGAC TAAAATTTGA AATTTTATGT GTTTCTAAGT AGATTATATT AAAAAATCTA TTTCAATACG   
  
  
- TATGTATATA TGTTTAATTT AATAAATGCT AGATTAGTAA AGGGCAATAT ATAAAGTGTG ATTTTAAATC   
  
  
- TTCTCTGTTA GATAATTTTA ATGTAGTGTA CAAAACCCCC CCCCCCCTAT AGTCTATACG TACTGTAAGG   
  
  
- ATTAGGTTAT TACGTTACTT TGGGTGGCTT ATCAACTTTA CTAAACAAGA TAGGTACTTG AGTGGTTCAG   
  
  
- TGTTAGGTAG TGTGAGCTTC CTATACGAAA AGAAAAACTT CCCTTAGAAC GAATTAGTTT GGATATTTGA   
  
  
- AAATTTTCGT TGTCTGTTTT TTCACTAATA TTACCATCAC CATCCTACTT CCGTCGAAAG TCTTTTCGCT   
  
  
- AACTTAAAAA TAACATAGGG TACTTTTGGG TAACTTAAAA AGAAAGAAAG AAACAAAAAC AATGGACCCA   
  
  
- AATACGTTTC CTGCCGACAA CATAAATTAT TATTGTTTTT GTATCCCGAA ACGCCTAACG ATTACGACCC   
  
  
- CTACCGTAGT CACGAGCTTA GACACATGCG ACAGTATCCT TGGGTGAACG AAGACGAGTA AATAAAGACA   
  
  
- GAGATCTAGG GGAGGAGAGA GAGAGAGAGA GAGATTCCAC AGACGAGCAG ACAAGTATAA GAGGACTCCA   
  
  
- AGAAGAAGTA GATAGAACAA GACCCACACA CGACTCCATG AAGAAAAGAA ATATTTAAAA AGTAGAAAAC   
  
  
- AACAAAACGA ACCACAAATA CTAAAATCAA AAACAGAAAT TCTGCTCTAA ACGACAATTA TCGTTCTAAA   
  
  
- GACTTGATCA TAACTAGGAA AAACACCATA TAAACAACTT AAACTAATTA AACACATAAC ACAAATCGAA   
  
  
- TTCATTGAGC AACACTAGAT TAACTACGAG CCCAAAACTG TCAAACTAAT CCCAAAAGCC ATGACCTCAA   
  
  
- TTTCAAAAAA ATAAAAAATA TCCTCCTCAA TTTCCATTAT TATTCTTAAC TACAAACGAC TAAAAAGGAG   
  
  
- AAAAGAAAAA ACCAAAGCTA AACCACCCGT CAATAGACAA ATACCAAAAA CGTTTACCTA GGTGTTAATC   
  
  
- TCCTTAATAT ACCTGGAAAT GTAGCTTAGT TCAAGTTACT AGTTTCCCAC GGTTAAAATG GTTCAGCGTC   
  
  
- ACAACAATGA GGCTACTTTG AGGTTCTAAG GTTAAACTTA GGTTCACAAG GTTTAGGTTC ACAACACTTA   
  
  
- GGAGGAGGCA ACCAAGGGGG TTTGGGTTTA GAATTAAATC AACACCGTAG AGTTATAAGA CTGTAACTCT   
  
  
- GTCGGGACTT GCTCCTAACA CTAAAATCAC TACAACAAGA ATTCATATAA TTAGTTGAAG AATACCTTCT   
  
  
- CCTACCCCTT CTCTTTGAGG TATGACTAGT ACTTGGAAGT CAGCAACTCC GCCGTCTCTT CAGTAACATA   
  
  
- CTCCGCGAGC CCGTCTCTAT AGGAGGAAGG CCTTTGGCTT TAGTCAACGG TCTACAACTC GTACTGCCGA   
  
  
- ACTGACCCTC GTCACAATCC CGAGTATCAC CACGTTCGTC ACAATCCCGA GTATCACTGC GTTCACCACC   
  
  
- ATCACCGGAC TGTCCTATAC CAACCATACC ACTAGGAATA TTAACCTCAG GAGTTTTACA GCACTTAAAA   
  
  
- TGGTAAAGGA GGAGCTTTGG GAATAGCACG TCGAGTAGTC TATCGAGCCC GTCTACCCAA TCATTCACCT   
  
  
- TCAGTTTAAG ACTAAGTGTC GAGTCGAGCC GGTAATTACG TTCACCAAAT CACCTACCCG GTCCCCTGAG   
  
  
- AGGACACTCA CGAGAATCAC ACAGACTTTA TAAGTTACTG TCAGTCAGTT ACAACGTCAA AGTCTTTCCT   
  
  
- AAACTCCTTC GCTTATTTAA AGAAGGTTTC TCAAGAAACA TGTTCCCAAA ACGGTTGTGC CCTAACGGAA   
  
  
- TAGTCTTCCG TTTGTTATCA CGGGTTCTAA ACAACCAGTT ACATCTCCTA CAATGATCCC CTTTCTTCGT   
  
  
- AGCAATAGGG CTTCTTAACG TCAGTCTTCT TCCCTCCTAT TTATTCGTTA GTCGACAAAG GGACCGTCGA   
  
  
- CTACTCCGTC AACAATCCAG ACTTTACAAG CTATCCCATG ACGAAACATC AGCCCCTTTT GTACTACGAC   
  
  
- GAGAGGCCCT TCGAAATGTC TGACTTGATT TATTCTCAGA CGCTTTACGG GGTCAATTCC CCAGATTATC   
  
  
- ACCCTTTCCG GCACCATTCT TTCGCCCTTT CTTCTCCCTA CATCATCTAA ATTCTAGAGA GAATTGGGAT   
  
  
- ACACGTGTTC GTCAACGTAG CTTACTAGTG TCCTCACGTT TACTGGACGA ATCCGTTTAA TCTGTCGTAA   
  
  
- GAAGAGGATC ACCCCTACCA TTGGTTTCTT ACCGTGTAAT AAAACGCCTA CCAGAACTCC GTGCGGAACG   
  
  
- ACCACATCCT TGAGGATATA TATTGACAGA ACATTGCCCA GGCCGTAGCC GACATCTATA GAACTCTCGA   
  
  
- ATGGTGTACA AAGAACGGTG TACGGGTAAA TTCTTTTACC CTTTAAAGAA GAGATTATCT TGCTAATACC   
  
  
- GACACCGTCT CTTACGTTGT ACGGAAGTAT ATTAACTAGA GCCATAACAG ATACCAAAGG TTACCGGAAC   
  
  
- AGATTAAGTC GCTGAAAGTA GATCCGGACC ACCGGGGGGG TTTGAAGCTT ATTGGCCTCA TCTAGAAGGT   
  
  
- GTTGGACCCA AGGCTGGTCG GTTTTCTCAA CTCCTCTGTC CCGCAGCGAA CTTCTTGATA CGTCTCAGTA   
  
  
- AATTACACGG GAAACTCAAG TTACGATATC GTTTCTTCAC CCTTTGTGAA TGGTAACTTC TAGAGTCCTA   
  
  
- GTTATCGCTA CTCAACGAAC AACAGTGGAC ATACAAATCC AAATTTGTAT ATGGACTCCT TTGTCACTGG   
  
  
- CACCTAACGG GATCCCTATG ACAAGAATTG GACTAACCCG CGTATTTGGG TCGACAAAAG TATGTTCCGT   
  
  
- GACAATTGCC CCGAAAGTTA AGAGGGAAAA AGTATCGAGC TAAAGCTCTC CGAGATAAAG TGAAGAGGTG   
  
  
- AGACAAACTA TACGATCTCC GGTTGGACGG TTCCCTGTTA TTCCTCTCCT ACGATTAACT CTCTCTCTAT   
  
  
- AAACCCTCCG TCCGTTACTT ACACTAACGA ACACTCCCAA ATCTCTCCTA TCTTTCCGGT CTCTGCATGT   
  
  
- TCGTCACCGT TCAGGCTTTA CTTTCCCGTC CCAAATCCGT CGACGGAAAT CTAGCGGTCT AAGACCTTTA   
  
  
- CCGATTTTTC TCCCACTTTA GACACATATT GTTTCTAAAG AGGTAACTAC TTCTGCCCGT GACCAACAAC   
  
  
- GACCCTACCT TCCCGTCTTA ACACATGTGT GAGTGATGAA CCTTCGGACG CCTCAT

+     MYB-like sequence

| Site Name | Organism | Position | Strand | Matrix score. | sequence | function |
| --- | --- | --- | --- | --- | --- | --- |
| MYB-like sequence | Arabidopsis thaliana | 3314 | + | 6 | TAACCA |  |
| MYB-like sequence | Arabidopsis thaliana | 915 | + | 6 | TAACCA |  |
| MYB-like sequence | Arabidopsis thaliana | 96 | + | 6 | TAACCA |  |
| MYB-like sequence | Arabidopsis thaliana | 605 | + | 6 | TAACCA |  |

>HU06G00568.1   
+ -Up\_Stream \_Len000CCCTGG GTCATTAAAA AAAAAGAAAG AAAGAAGATT GCGCTAAAAG TAGATTTTTA   
  
  
+ TCAGATTCAA GCTGCAGGTG ATAACCAAAT TGTCATTAAA GCAATGCAAG CGCATATCAA TACCCTTTTG   
  
  
+ GGAGATAGCT CCGACTGTTG AAGACATTAG GAGCATGATC TCCAACTGTG AATTTGTTTC ATTTACTCAT   
  
  
+ ATTCATATCT ACCATGTGGA TAATATGACT GCAGACTAGA TGGCCAAATT TGAATGCACA CTTATAACTT   
  
  
+ CAACTCTTTC TACTTTTTTC TTCCCCACCT TATCAGGAAT TTCTTTTAAT TGTCGTGGGA GACAACTTGG   
  
  
+ GTAGAACTCT TGCGAAAAGG GCAACTTAAC GTTTTGGTAC TTGCATTTTC CAAAAAAACA AAAAGGCTAA   
  
  
+ ACAATTCTCC GCATGCTGAC TTCACGGTAG ATATGATGTA TTTTTTAACA AAAGACATCT TCTGAATGAA   
  
  
+ ACGCTTAATA TCTTGGACCA TACTAGAAAT TAAATTGCCA CGGTGCATGA ATTTCACCTC GAACTGCTTG   
  
  
+ TATAAAGATC TTGTTATCAC CTTCAGTAAT CAGATACTTG TAACCAAATT GAATGACCAC AAAGACCACA   
  
  
+ TTCCTCACAA TCGTTGCCTC TAGAATAAGA ATGAATGTTT CTTCAAGGTT GAAAGCCCTT GCTAATGCGA   
  
  
+ ACCTGCCTAT ATGCCTAACT GGCTATATAT ATGTGCGTAG AACAGCATGA CATGAGTTAA TAATATAGCA   
  
  
+ GCTAAAGGGA ACTCTAATGT ACACATGATT GTAATTCACC TGCCTCGCTA GCTTACGAGC ATGACTCTTG   
  
  
+ ACTATTATTG TTTTAATTGA TCTTGAATTT TCATAGATTT TACTCCATTG TTTACTCATT TTGTTATAAT   
  
  
+ TAACCACCTG ATTTTAAACT TTAAAATACA CAAAGATTCA TCTAATATAA TTTTTTAGAT AAAGTTATGC   
  
  
+ ATACATATAT ACAAATTAAA TTATTTACGA TCTAATCATT TCCCGTTATA TATTTCACAC TAAAATTTAG   
  
  
+ AAGAGACAAT CTATTAAAAT TACATCACAT GTTTTGGGGG GGGGGGGATA TCAGATATGC ATGACATTCC   
  
  
+ TAATCCAATA ATGCAATGAA ACCCACCGAA TAGTTGAAAT GATTTGTTCT ATCCATGAAC TCACCAAGTC   
  
  
+ ACAATCCATC ACACTCGAAG GATATGCTTT TCTTTTTGAA GGGAATCTTG CTTAATCAAA CCTATAAACT   
  
  
+ TTTAAAAGCA ACAGACAAAA AAGTGATTAT AATGGTAGTG GTAGGATGAA GGCAGCTTTC AGAAAAGCGA   
  
  
+ TTGAATTTTT ATTGTATCCC ATGAAAACCC ATTGAATTTT TCTTTCTTTC TTTGTTTTTG TTACCTGGGT   
  
  
+ TTATGCAAAG GACGGCTGTT GTATTTAATA ATAACAAAAA CATAGGGCTT TGCGGATTGC TAATGCTGGG   
  
  
+ GATGGCATCA GTGCTCGAAT CTGTGTACGC TGTCATAGGA ACCCACTTGC TTCTGCTCAT TTATTTCTGT   
  
  
+ CTCTAGATCC CCTCCTCTCT CTCTCTCTCT CTCTAAGGTG TCTGCTCGTC TGTTCATATT CTCCTGAGGT   
  
  
+ TCTTCTTCAT CTATCTTGTT CTGGGTGTGT GCTGAGGTAC TTCTTTTCTT TATAAATTTT TCATCTTTTG   
  
  
+ TTGTTTTGCT TGGTGTTTAT GATTTTAGTT TTTGTCTTTA AGACGAGATT TGCTGTTAAT AGCAAGATTT   
  
  
+ CTGAACTAGT ATTGATCCTT TTTGTGGTAT ATTTGTTGAA TTTGATTAAT TTGTGTATTG TGTTTAGCTT   
  
  
+ AAGTAACTCG TTGTGATCTA ATTGATGCTC GGGTTTTGAC AGTTTGATTA GGGTTTTCGG TACTGGAGTT   
  
  
+ AAAGTTTTTT TATTTTTTAT AGGAGGAGTT AAAGGTAATA ATAAGAATTG ATGTTTGCTG ATTTTTCCTC   
  
  
+ TTTTCTTTTT TGGTTTCGAT TTGGTGGGCA GTTATCTGTT TATGGTTTTT GCAAATGGAT CCACAATTAG   
  
  
+ AGGAATTATA TGGACCTTTA CATCGAATCA AGTTCAATGA TCAAAGGGTG CCAATTTTAC CAAGTCGCAG   
  
  
+ TGTTGTTACT CCGATGAAAC TCCAAGATTC CAATTTGAAT CCAAGTGTTC CAAATCCAAG TGTTGTGAAT   
  
  
+ CCTCCTCCGT TGGTTCCCCC AAACCCAAAT CTTAATTTAG TTGTGGCATC TCAATATTCT GACATTGAGA   
  
  
+ CAGCCCTGAA CGAGGATTGT GATTTTAGTG ATGTTGTTCT TAAGTATATT AATCAACTTC TTATGGAAGA   
  
  
+ GGATGGGGAA GAGAAACTCC ATACTGATCA TGAACCTTCA GTCGTTGAGG CGGCAGAGAA GTCATTGTAT   
  
  
+ GAGGCGCTCG GGCAGAGATA TCCTCCTTCC GGAAACCGAA ATCAGTTGCC AGATGTTGAG CATGACGGCT   
  
  
+ TGACTGGGAG CAGTGTTAGG GCTCATAGTG GTGCAAGCAG TGTTAGGGCT CATAGTGACG CAAGTGGTGG   
  
  
+ TAGTGGCCTG ACAGGATATG GTTGGTATGG TGATCCTTAT AATTGGAGTC CTCAAAATGT CGTGAATTTT   
  
  
+ ACCATTTCCT CCTCGAAACC CTTATCGTGC AGCTCATCAG ATAGCTCGGG CAGATGGGTT AGTAAGTGGA   
  
  
+ AGTCAAATTC TGATTCACAG CTCAGCTCGG CCATTAATGC AAGTGGTTTA GTGGATGGGC CAGGGGACTC   
  
  
+ TCCTGTGAGT GCTCTTAGTG TGTCTGAAAT ATTCAATGAC AGTCAGTCAA TGTTGCAGTT TCAGAAAGGA   
  
  
+ TTTGAGGAAG CGAATAAATT TCTTCCAAAG AGTTCTTTGT ACAAGGGTTT TGCCAACACG GGATTGCCTT   
  
  
+ ATCAGAAGGC AAACAATAGT GCCCAAGATT TGTTGGTCAA TGTAGAGGAT GTTACTAGGG GAAAGAAGCA   
  
  
+ TCGTTATCCC GAAGAATTGC AGTCAGAAGA AGGGAGGATA AATAAGCAAT CAGCTGTTTC CCTGGCAGCT   
  
  
+ GATGAGGCAG TTGTTAGGTC TGAAATGTTC GATAGGGTAC TGCTTTGTAG TCGGGGAAAA CATGATGCTG   
  
  
+ CTCTCCGGGA AGCTTTACAG ACTGAACTAA ATAAGAGTCT GCGAAATGCC CCAGTTAAGG GGTCTAATAG   
  
  
+ TGGGAAAGGC CGTGGTAAGA AAGCGGGAAA GAAGAGGGAT GTAGTAGATT TAAGATCTCT CTTAACCCTA   
  
  
+ TGTGCACAAG CAGTTGCATC GAATGATCAC AGGAGTGCAA ATGACCTGCT TAGGCAAATT AGACAGCATT   
  
  
+ CTTCTCCTAG TGGGGATGGT AACCAAAGAA TGGCACATTA TTTTGCGGAT GGTCTTGAGG CACGCCTTGC   
  
  
+ TGGTGTAGGA ACTCCTATAT ATAACTGTCT TGTAACGGGT CCGGCATCGG CTGTAGATAT CTTGAGAGCT   
  
  
+ TACCACATGT TTCTTGCCAC ATGCCCATTT AAGAAAATGG GAAATTTCTT CTCTAATAGA ACGATTATGG   
  
  
+ CTGTGGCAGA GAATGCAACA TGCCTTCATA TAATTGATCT CGGTATTGTC TATGGTTTCC AATGGCCTTG   
  
  
+ TCTAATTCAG CGACTTTCAT CTAGGCCTGG TGGCCCCCCC AAACTTCGAA TAACCGGAGT AGATCTTCCA   
  
  
+ CAACCTGGGT TCCGACCAGC CAAAAGAGTT GAGGAGACAG GGCGTCGCTT GAAGAACTAT GCAGAGTCAT   
  
  
+ TTAATGTGCC CTTTGAGTTC AATGCTATAG CAAAGAAGTG GGAAACACTT ACCATTGAAG ATCTCAGGAT   
  
  
+ CAATAGCGAT GAGTTGCTTG TTGTCACCTG TATGTTTAGG TTTAAACATA TACCTGAGGA AACAGTGACC   
  
  
+ GTGGATTGCC CTAGGGATAC TGTTCTTAAC CTGATTGGGC GCATAAACCC AGCTGTTTTC ATACAAGGCA   
  
  
+ CTGTTAACGG GGCTTTCAAT TCTCCCTTTT TCATAGCTCG ATTTCGAGAG GCTCTATTTC ACTTCTCCAC   
  
  
+ TCTGTTTGAT ATGCTAGAGG CCAACCTGCC AAGGGACAAT AAGGAGAGGA TGCTAATTGA GAGAGAGATA   
  
  
+ TTTGGGAGGC AGGCAATGAA TGTGATTGCT TGTGAGGGTT TAGAGAGGAT AGAAAGGCCA GAGACGTACA   
  
  
+ AGCAGTGGCA AGTCCGAAAT GAAAGGGCAG GGTTTAGGCA GCTGCCTTTA GATCGCCAGA TTCTGGAAAT   
  
  
+ GGCTAAAAAG AGGGTGAAAT CTGTGTATAA CAAAGATTTC TCCATTGATG AAGACGGGCA CTGGTTGTTG   
  
  
+ CTGGGATGGA AGGGCAGAAT TGTGTACACA CTCACTACTT GGAAGCCTGC GGAGTA  

- -Up\_Stream \_Len000GGGACC CAGTAATTTT TTTTTCTTTC TTTCTTCTAA CGCGATTTTC ATCTAAAAAT   
  
  
- AGTCTAAGTT CGACGTCCAC TATTGGTTTA ACAGTAATTT CGTTACGTTC GCGTATAGTT ATGGGAAAAC   
  
  
- CCTCTATCGA GGCTGACAAC TTCTGTAATC CTCGTACTAG AGGTTGACAC TTAAACAAAG TAAATGAGTA   
  
  
- TAAGTATAGA TGGTACACCT ATTATACTGA CGTCTGATCT ACCGGTTTAA ACTTACGTGT GAATATTGAA   
  
  
- GTTGAGAAAG ATGAAAAAAG AAGGGGTGGA ATAGTCCTTA AAGAAAATTA ACAGCACCCT CTGTTGAACC   
  
  
- CATCTTGAGA ACGCTTTTCC CGTTGAATTG CAAAACCATG AACGTAAAAG GTTTTTTTGT TTTTCCGATT   
  
  
- TGTTAAGAGG CGTACGACTG AAGTGCCATC TATACTACAT AAAAAATTGT TTTCTGTAGA AGACTTACTT   
  
  
- TGCGAATTAT AGAACCTGGT ATGATCTTTA ATTTAACGGT GCCACGTACT TAAAGTGGAG CTTGACGAAC   
  
  
- ATATTTCTAG AACAATAGTG GAAGTCATTA GTCTATGAAC ATTGGTTTAA CTTACTGGTG TTTCTGGTGT   
  
  
- AAGGAGTGTT AGCAACGGAG ATCTTATTCT TACTTACAAA GAAGTTCCAA CTTTCGGGAA CGATTACGCT   
  
  
- TGGACGGATA TACGGATTGA CCGATATATA TACACGCATC TTGTCGTACT GTACTCAATT ATTATATCGT   
  
  
- CGATTTCCCT TGAGATTACA TGTGTACTAA CATTAAGTGG ACGGAGCGAT CGAATGCTCG TACTGAGAAC   
  
  
- TGATAATAAC AAAATTAACT AGAACTTAAA AGTATCTAAA ATGAGGTAAC AAATGAGTAA AACAATATTA   
  
  
- ATTGGTGGAC TAAAATTTGA AATTTTATGT GTTTCTAAGT AGATTATATT AAAAAATCTA TTTCAATACG   
  
  
- TATGTATATA TGTTTAATTT AATAAATGCT AGATTAGTAA AGGGCAATAT ATAAAGTGTG ATTTTAAATC   
  
  
- TTCTCTGTTA GATAATTTTA ATGTAGTGTA CAAAACCCCC CCCCCCCTAT AGTCTATACG TACTGTAAGG   
  
  
- ATTAGGTTAT TACGTTACTT TGGGTGGCTT ATCAACTTTA CTAAACAAGA TAGGTACTTG AGTGGTTCAG   
  
  
- TGTTAGGTAG TGTGAGCTTC CTATACGAAA AGAAAAACTT CCCTTAGAAC GAATTAGTTT GGATATTTGA   
  
  
- AAATTTTCGT TGTCTGTTTT TTCACTAATA TTACCATCAC CATCCTACTT CCGTCGAAAG TCTTTTCGCT   
  
  
- AACTTAAAAA TAACATAGGG TACTTTTGGG TAACTTAAAA AGAAAGAAAG AAACAAAAAC AATGGACCCA   
  
  
- AATACGTTTC CTGCCGACAA CATAAATTAT TATTGTTTTT GTATCCCGAA ACGCCTAACG ATTACGACCC   
  
  
- CTACCGTAGT CACGAGCTTA GACACATGCG ACAGTATCCT TGGGTGAACG AAGACGAGTA AATAAAGACA   
  
  
- GAGATCTAGG GGAGGAGAGA GAGAGAGAGA GAGATTCCAC AGACGAGCAG ACAAGTATAA GAGGACTCCA   
  
  
- AGAAGAAGTA GATAGAACAA GACCCACACA CGACTCCATG AAGAAAAGAA ATATTTAAAA AGTAGAAAAC   
  
  
- AACAAAACGA ACCACAAATA CTAAAATCAA AAACAGAAAT TCTGCTCTAA ACGACAATTA TCGTTCTAAA   
  
  
- GACTTGATCA TAACTAGGAA AAACACCATA TAAACAACTT AAACTAATTA AACACATAAC ACAAATCGAA   
  
  
- TTCATTGAGC AACACTAGAT TAACTACGAG CCCAAAACTG TCAAACTAAT CCCAAAAGCC ATGACCTCAA   
  
  
- TTTCAAAAAA ATAAAAAATA TCCTCCTCAA TTTCCATTAT TATTCTTAAC TACAAACGAC TAAAAAGGAG   
  
  
- AAAAGAAAAA ACCAAAGCTA AACCACCCGT CAATAGACAA ATACCAAAAA CGTTTACCTA GGTGTTAATC   
  
  
- TCCTTAATAT ACCTGGAAAT GTAGCTTAGT TCAAGTTACT AGTTTCCCAC GGTTAAAATG GTTCAGCGTC   
  
  
- ACAACAATGA GGCTACTTTG AGGTTCTAAG GTTAAACTTA GGTTCACAAG GTTTAGGTTC ACAACACTTA   
  
  
- GGAGGAGGCA ACCAAGGGGG TTTGGGTTTA GAATTAAATC AACACCGTAG AGTTATAAGA CTGTAACTCT   
  
  
- GTCGGGACTT GCTCCTAACA CTAAAATCAC TACAACAAGA ATTCATATAA TTAGTTGAAG AATACCTTCT   
  
  
- CCTACCCCTT CTCTTTGAGG TATGACTAGT ACTTGGAAGT CAGCAACTCC GCCGTCTCTT CAGTAACATA   
  
  
- CTCCGCGAGC CCGTCTCTAT AGGAGGAAGG CCTTTGGCTT TAGTCAACGG TCTACAACTC GTACTGCCGA   
  
  
- ACTGACCCTC GTCACAATCC CGAGTATCAC CACGTTCGTC ACAATCCCGA GTATCACTGC GTTCACCACC   
  
  
- ATCACCGGAC TGTCCTATAC CAACCATACC ACTAGGAATA TTAACCTCAG GAGTTTTACA GCACTTAAAA   
  
  
- TGGTAAAGGA GGAGCTTTGG GAATAGCACG TCGAGTAGTC TATCGAGCCC GTCTACCCAA TCATTCACCT   
  
  
- TCAGTTTAAG ACTAAGTGTC GAGTCGAGCC GGTAATTACG TTCACCAAAT CACCTACCCG GTCCCCTGAG   
  
  
- AGGACACTCA CGAGAATCAC ACAGACTTTA TAAGTTACTG TCAGTCAGTT ACAACGTCAA AGTCTTTCCT   
  
  
- AAACTCCTTC GCTTATTTAA AGAAGGTTTC TCAAGAAACA TGTTCCCAAA ACGGTTGTGC CCTAACGGAA   
  
  
- TAGTCTTCCG TTTGTTATCA CGGGTTCTAA ACAACCAGTT ACATCTCCTA CAATGATCCC CTTTCTTCGT   
  
  
- AGCAATAGGG CTTCTTAACG TCAGTCTTCT TCCCTCCTAT TTATTCGTTA GTCGACAAAG GGACCGTCGA   
  
  
- CTACTCCGTC AACAATCCAG ACTTTACAAG CTATCCCATG ACGAAACATC AGCCCCTTTT GTACTACGAC   
  
  
- GAGAGGCCCT TCGAAATGTC TGACTTGATT TATTCTCAGA CGCTTTACGG GGTCAATTCC CCAGATTATC   
  
  
- ACCCTTTCCG GCACCATTCT TTCGCCCTTT CTTCTCCCTA CATCATCTAA ATTCTAGAGA GAATTGGGAT   
  
  
- ACACGTGTTC GTCAACGTAG CTTACTAGTG TCCTCACGTT TACTGGACGA ATCCGTTTAA TCTGTCGTAA   
  
  
- GAAGAGGATC ACCCCTACCA TTGGTTTCTT ACCGTGTAAT AAAACGCCTA CCAGAACTCC GTGCGGAACG   
  
  
- ACCACATCCT TGAGGATATA TATTGACAGA ACATTGCCCA GGCCGTAGCC GACATCTATA GAACTCTCGA   
  
  
- ATGGTGTACA AAGAACGGTG TACGGGTAAA TTCTTTTACC CTTTAAAGAA GAGATTATCT TGCTAATACC   
  
  
- GACACCGTCT CTTACGTTGT ACGGAAGTAT ATTAACTAGA GCCATAACAG ATACCAAAGG TTACCGGAAC   
  
  
- AGATTAAGTC GCTGAAAGTA GATCCGGACC ACCGGGGGGG TTTGAAGCTT ATTGGCCTCA TCTAGAAGGT   
  
  
- GTTGGACCCA AGGCTGGTCG GTTTTCTCAA CTCCTCTGTC CCGCAGCGAA CTTCTTGATA CGTCTCAGTA   
  
  
- AATTACACGG GAAACTCAAG TTACGATATC GTTTCTTCAC CCTTTGTGAA TGGTAACTTC TAGAGTCCTA   
  
  
- GTTATCGCTA CTCAACGAAC AACAGTGGAC ATACAAATCC AAATTTGTAT ATGGACTCCT TTGTCACTGG   
  
  
- CACCTAACGG GATCCCTATG ACAAGAATTG GACTAACCCG CGTATTTGGG TCGACAAAAG TATGTTCCGT   
  
  
- GACAATTGCC CCGAAAGTTA AGAGGGAAAA AGTATCGAGC TAAAGCTCTC CGAGATAAAG TGAAGAGGTG   
  
  
- AGACAAACTA TACGATCTCC GGTTGGACGG TTCCCTGTTA TTCCTCTCCT ACGATTAACT CTCTCTCTAT   
  
  
- AAACCCTCCG TCCGTTACTT ACACTAACGA ACACTCCCAA ATCTCTCCTA TCTTTCCGGT CTCTGCATGT   
  
  
- TCGTCACCGT TCAGGCTTTA CTTTCCCGTC CCAAATCCGT CGACGGAAAT CTAGCGGTCT AAGACCTTTA   
  
  
- CCGATTTTTC TCCCACTTTA GACACATATT GTTTCTAAAG AGGTAACTAC TTCTGCCCGT GACCAACAAC   
  
  
- GACCCTACCT TCCCGTCTTA ACACATGTGT GAGTGATGAA CCTTCGGACG CCTCAT

+     MYC

| Site Name | Organism | Position | Strand | Matrix score. | sequence | function |
| --- | --- | --- | --- | --- | --- | --- |
| MYC | Arabidopsis thaliana | 2016 | - | 6 | CATTTG |  |
| MYC | Arabidopsis thaliana | 3438 | - | 6 | CATGTG |  |
| MYC | Arabidopsis thaliana | 1080 | - | 6 | CATGTG |  |
| MYC | Arabidopsis thaliana | 3452 | - | 6 | CATGTG |  |
| MYC | Arabidopsis thaliana | 3262 | - | 6 | CATTTG |  |
| MYC | Arabidopsis thaliana | 227 | + | 6 | CATGTG |  |
| MYC | Arabidopsis thaliana | 796 | - | 6 | CATGTG |  |

>HU06G00568.1   
+ -Up\_Stream \_Len000CCCTGG GTCATTAAAA AAAAAGAAAG AAAGAAGATT GCGCTAAAAG TAGATTTTTA   
  
  
+ TCAGATTCAA GCTGCAGGTG ATAACCAAAT TGTCATTAAA GCAATGCAAG CGCATATCAA TACCCTTTTG   
  
  
+ GGAGATAGCT CCGACTGTTG AAGACATTAG GAGCATGATC TCCAACTGTG AATTTGTTTC ATTTACTCAT   
  
  
+ ATTCATATCT ACCATGTGGA TAATATGACT GCAGACTAGA TGGCCAAATT TGAATGCACA CTTATAACTT   
  
  
+ CAACTCTTTC TACTTTTTTC TTCCCCACCT TATCAGGAAT TTCTTTTAAT TGTCGTGGGA GACAACTTGG   
  
  
+ GTAGAACTCT TGCGAAAAGG GCAACTTAAC GTTTTGGTAC TTGCATTTTC CAAAAAAACA AAAAGGCTAA   
  
  
+ ACAATTCTCC GCATGCTGAC TTCACGGTAG ATATGATGTA TTTTTTAACA AAAGACATCT TCTGAATGAA   
  
  
+ ACGCTTAATA TCTTGGACCA TACTAGAAAT TAAATTGCCA CGGTGCATGA ATTTCACCTC GAACTGCTTG   
  
  
+ TATAAAGATC TTGTTATCAC CTTCAGTAAT CAGATACTTG TAACCAAATT GAATGACCAC AAAGACCACA   
  
  
+ TTCCTCACAA TCGTTGCCTC TAGAATAAGA ATGAATGTTT CTTCAAGGTT GAAAGCCCTT GCTAATGCGA   
  
  
+ ACCTGCCTAT ATGCCTAACT GGCTATATAT ATGTGCGTAG AACAGCATGA CATGAGTTAA TAATATAGCA   
  
  
+ GCTAAAGGGA ACTCTAATGT ACACATGATT GTAATTCACC TGCCTCGCTA GCTTACGAGC ATGACTCTTG   
  
  
+ ACTATTATTG TTTTAATTGA TCTTGAATTT TCATAGATTT TACTCCATTG TTTACTCATT TTGTTATAAT   
  
  
+ TAACCACCTG ATTTTAAACT TTAAAATACA CAAAGATTCA TCTAATATAA TTTTTTAGAT AAAGTTATGC   
  
  
+ ATACATATAT ACAAATTAAA TTATTTACGA TCTAATCATT TCCCGTTATA TATTTCACAC TAAAATTTAG   
  
  
+ AAGAGACAAT CTATTAAAAT TACATCACAT GTTTTGGGGG GGGGGGGATA TCAGATATGC ATGACATTCC   
  
  
+ TAATCCAATA ATGCAATGAA ACCCACCGAA TAGTTGAAAT GATTTGTTCT ATCCATGAAC TCACCAAGTC   
  
  
+ ACAATCCATC ACACTCGAAG GATATGCTTT TCTTTTTGAA GGGAATCTTG CTTAATCAAA CCTATAAACT   
  
  
+ TTTAAAAGCA ACAGACAAAA AAGTGATTAT AATGGTAGTG GTAGGATGAA GGCAGCTTTC AGAAAAGCGA   
  
  
+ TTGAATTTTT ATTGTATCCC ATGAAAACCC ATTGAATTTT TCTTTCTTTC TTTGTTTTTG TTACCTGGGT   
  
  
+ TTATGCAAAG GACGGCTGTT GTATTTAATA ATAACAAAAA CATAGGGCTT TGCGGATTGC TAATGCTGGG   
  
  
+ GATGGCATCA GTGCTCGAAT CTGTGTACGC TGTCATAGGA ACCCACTTGC TTCTGCTCAT TTATTTCTGT   
  
  
+ CTCTAGATCC CCTCCTCTCT CTCTCTCTCT CTCTAAGGTG TCTGCTCGTC TGTTCATATT CTCCTGAGGT   
  
  
+ TCTTCTTCAT CTATCTTGTT CTGGGTGTGT GCTGAGGTAC TTCTTTTCTT TATAAATTTT TCATCTTTTG   
  
  
+ TTGTTTTGCT TGGTGTTTAT GATTTTAGTT TTTGTCTTTA AGACGAGATT TGCTGTTAAT AGCAAGATTT   
  
  
+ CTGAACTAGT ATTGATCCTT TTTGTGGTAT ATTTGTTGAA TTTGATTAAT TTGTGTATTG TGTTTAGCTT   
  
  
+ AAGTAACTCG TTGTGATCTA ATTGATGCTC GGGTTTTGAC AGTTTGATTA GGGTTTTCGG TACTGGAGTT   
  
  
+ AAAGTTTTTT TATTTTTTAT AGGAGGAGTT AAAGGTAATA ATAAGAATTG ATGTTTGCTG ATTTTTCCTC   
  
  
+ TTTTCTTTTT TGGTTTCGAT TTGGTGGGCA GTTATCTGTT TATGGTTTTT GCAAATGGAT CCACAATTAG   
  
  
+ AGGAATTATA TGGACCTTTA CATCGAATCA AGTTCAATGA TCAAAGGGTG CCAATTTTAC CAAGTCGCAG   
  
  
+ TGTTGTTACT CCGATGAAAC TCCAAGATTC CAATTTGAAT CCAAGTGTTC CAAATCCAAG TGTTGTGAAT   
  
  
+ CCTCCTCCGT TGGTTCCCCC AAACCCAAAT CTTAATTTAG TTGTGGCATC TCAATATTCT GACATTGAGA   
  
  
+ CAGCCCTGAA CGAGGATTGT GATTTTAGTG ATGTTGTTCT TAAGTATATT AATCAACTTC TTATGGAAGA   
  
  
+ GGATGGGGAA GAGAAACTCC ATACTGATCA TGAACCTTCA GTCGTTGAGG CGGCAGAGAA GTCATTGTAT   
  
  
+ GAGGCGCTCG GGCAGAGATA TCCTCCTTCC GGAAACCGAA ATCAGTTGCC AGATGTTGAG CATGACGGCT   
  
  
+ TGACTGGGAG CAGTGTTAGG GCTCATAGTG GTGCAAGCAG TGTTAGGGCT CATAGTGACG CAAGTGGTGG   
  
  
+ TAGTGGCCTG ACAGGATATG GTTGGTATGG TGATCCTTAT AATTGGAGTC CTCAAAATGT CGTGAATTTT   
  
  
+ ACCATTTCCT CCTCGAAACC CTTATCGTGC AGCTCATCAG ATAGCTCGGG CAGATGGGTT AGTAAGTGGA   
  
  
+ AGTCAAATTC TGATTCACAG CTCAGCTCGG CCATTAATGC AAGTGGTTTA GTGGATGGGC CAGGGGACTC   
  
  
+ TCCTGTGAGT GCTCTTAGTG TGTCTGAAAT ATTCAATGAC AGTCAGTCAA TGTTGCAGTT TCAGAAAGGA   
  
  
+ TTTGAGGAAG CGAATAAATT TCTTCCAAAG AGTTCTTTGT ACAAGGGTTT TGCCAACACG GGATTGCCTT   
  
  
+ ATCAGAAGGC AAACAATAGT GCCCAAGATT TGTTGGTCAA TGTAGAGGAT GTTACTAGGG GAAAGAAGCA   
  
  
+ TCGTTATCCC GAAGAATTGC AGTCAGAAGA AGGGAGGATA AATAAGCAAT CAGCTGTTTC CCTGGCAGCT   
  
  
+ GATGAGGCAG TTGTTAGGTC TGAAATGTTC GATAGGGTAC TGCTTTGTAG TCGGGGAAAA CATGATGCTG   
  
  
+ CTCTCCGGGA AGCTTTACAG ACTGAACTAA ATAAGAGTCT GCGAAATGCC CCAGTTAAGG GGTCTAATAG   
  
  
+ TGGGAAAGGC CGTGGTAAGA AAGCGGGAAA GAAGAGGGAT GTAGTAGATT TAAGATCTCT CTTAACCCTA   
  
  
+ TGTGCACAAG CAGTTGCATC GAATGATCAC AGGAGTGCAA ATGACCTGCT TAGGCAAATT AGACAGCATT   
  
  
+ CTTCTCCTAG TGGGGATGGT AACCAAAGAA TGGCACATTA TTTTGCGGAT GGTCTTGAGG CACGCCTTGC   
  
  
+ TGGTGTAGGA ACTCCTATAT ATAACTGTCT TGTAACGGGT CCGGCATCGG CTGTAGATAT CTTGAGAGCT   
  
  
+ TACCACATGT TTCTTGCCAC ATGCCCATTT AAGAAAATGG GAAATTTCTT CTCTAATAGA ACGATTATGG   
  
  
+ CTGTGGCAGA GAATGCAACA TGCCTTCATA TAATTGATCT CGGTATTGTC TATGGTTTCC AATGGCCTTG   
  
  
+ TCTAATTCAG CGACTTTCAT CTAGGCCTGG TGGCCCCCCC AAACTTCGAA TAACCGGAGT AGATCTTCCA   
  
  
+ CAACCTGGGT TCCGACCAGC CAAAAGAGTT GAGGAGACAG GGCGTCGCTT GAAGAACTAT GCAGAGTCAT   
  
  
+ TTAATGTGCC CTTTGAGTTC AATGCTATAG CAAAGAAGTG GGAAACACTT ACCATTGAAG ATCTCAGGAT   
  
  
+ CAATAGCGAT GAGTTGCTTG TTGTCACCTG TATGTTTAGG TTTAAACATA TACCTGAGGA AACAGTGACC   
  
  
+ GTGGATTGCC CTAGGGATAC TGTTCTTAAC CTGATTGGGC GCATAAACCC AGCTGTTTTC ATACAAGGCA   
  
  
+ CTGTTAACGG GGCTTTCAAT TCTCCCTTTT TCATAGCTCG ATTTCGAGAG GCTCTATTTC ACTTCTCCAC   
  
  
+ TCTGTTTGAT ATGCTAGAGG CCAACCTGCC AAGGGACAAT AAGGAGAGGA TGCTAATTGA GAGAGAGATA   
  
  
+ TTTGGGAGGC AGGCAATGAA TGTGATTGCT TGTGAGGGTT TAGAGAGGAT AGAAAGGCCA GAGACGTACA   
  
  
+ AGCAGTGGCA AGTCCGAAAT GAAAGGGCAG GGTTTAGGCA GCTGCCTTTA GATCGCCAGA TTCTGGAAAT   
  
  
+ GGCTAAAAAG AGGGTGAAAT CTGTGTATAA CAAAGATTTC TCCATTGATG AAGACGGGCA CTGGTTGTTG   
  
  
+ CTGGGATGGA AGGGCAGAAT TGTGTACACA CTCACTACTT GGAAGCCTGC GGAGTA  

- -Up\_Stream \_Len000GGGACC CAGTAATTTT TTTTTCTTTC TTTCTTCTAA CGCGATTTTC ATCTAAAAAT   
  
  
- AGTCTAAGTT CGACGTCCAC TATTGGTTTA ACAGTAATTT CGTTACGTTC GCGTATAGTT ATGGGAAAAC   
  
  
- CCTCTATCGA GGCTGACAAC TTCTGTAATC CTCGTACTAG AGGTTGACAC TTAAACAAAG TAAATGAGTA   
  
  
- TAAGTATAGA TGGTACACCT ATTATACTGA CGTCTGATCT ACCGGTTTAA ACTTACGTGT GAATATTGAA   
  
  
- GTTGAGAAAG ATGAAAAAAG AAGGGGTGGA ATAGTCCTTA AAGAAAATTA ACAGCACCCT CTGTTGAACC   
  
  
- CATCTTGAGA ACGCTTTTCC CGTTGAATTG CAAAACCATG AACGTAAAAG GTTTTTTTGT TTTTCCGATT   
  
  
- TGTTAAGAGG CGTACGACTG AAGTGCCATC TATACTACAT AAAAAATTGT TTTCTGTAGA AGACTTACTT   
  
  
- TGCGAATTAT AGAACCTGGT ATGATCTTTA ATTTAACGGT GCCACGTACT TAAAGTGGAG CTTGACGAAC   
  
  
- ATATTTCTAG AACAATAGTG GAAGTCATTA GTCTATGAAC ATTGGTTTAA CTTACTGGTG TTTCTGGTGT   
  
  
- AAGGAGTGTT AGCAACGGAG ATCTTATTCT TACTTACAAA GAAGTTCCAA CTTTCGGGAA CGATTACGCT   
  
  
- TGGACGGATA TACGGATTGA CCGATATATA TACACGCATC TTGTCGTACT GTACTCAATT ATTATATCGT   
  
  
- CGATTTCCCT TGAGATTACA TGTGTACTAA CATTAAGTGG ACGGAGCGAT CGAATGCTCG TACTGAGAAC   
  
  
- TGATAATAAC AAAATTAACT AGAACTTAAA AGTATCTAAA ATGAGGTAAC AAATGAGTAA AACAATATTA   
  
  
- ATTGGTGGAC TAAAATTTGA AATTTTATGT GTTTCTAAGT AGATTATATT AAAAAATCTA TTTCAATACG   
  
  
- TATGTATATA TGTTTAATTT AATAAATGCT AGATTAGTAA AGGGCAATAT ATAAAGTGTG ATTTTAAATC   
  
  
- TTCTCTGTTA GATAATTTTA ATGTAGTGTA CAAAACCCCC CCCCCCCTAT AGTCTATACG TACTGTAAGG   
  
  
- ATTAGGTTAT TACGTTACTT TGGGTGGCTT ATCAACTTTA CTAAACAAGA TAGGTACTTG AGTGGTTCAG   
  
  
- TGTTAGGTAG TGTGAGCTTC CTATACGAAA AGAAAAACTT CCCTTAGAAC GAATTAGTTT GGATATTTGA   
  
  
- AAATTTTCGT TGTCTGTTTT TTCACTAATA TTACCATCAC CATCCTACTT CCGTCGAAAG TCTTTTCGCT   
  
  
- AACTTAAAAA TAACATAGGG TACTTTTGGG TAACTTAAAA AGAAAGAAAG AAACAAAAAC AATGGACCCA   
  
  
- AATACGTTTC CTGCCGACAA CATAAATTAT TATTGTTTTT GTATCCCGAA ACGCCTAACG ATTACGACCC   
  
  
- CTACCGTAGT CACGAGCTTA GACACATGCG ACAGTATCCT TGGGTGAACG AAGACGAGTA AATAAAGACA   
  
  
- GAGATCTAGG GGAGGAGAGA GAGAGAGAGA GAGATTCCAC AGACGAGCAG ACAAGTATAA GAGGACTCCA   
  
  
- AGAAGAAGTA GATAGAACAA GACCCACACA CGACTCCATG AAGAAAAGAA ATATTTAAAA AGTAGAAAAC   
  
  
- AACAAAACGA ACCACAAATA CTAAAATCAA AAACAGAAAT TCTGCTCTAA ACGACAATTA TCGTTCTAAA   
  
  
- GACTTGATCA TAACTAGGAA AAACACCATA TAAACAACTT AAACTAATTA AACACATAAC ACAAATCGAA   
  
  
- TTCATTGAGC AACACTAGAT TAACTACGAG CCCAAAACTG TCAAACTAAT CCCAAAAGCC ATGACCTCAA   
  
  
- TTTCAAAAAA ATAAAAAATA TCCTCCTCAA TTTCCATTAT TATTCTTAAC TACAAACGAC TAAAAAGGAG   
  
  
- AAAAGAAAAA ACCAAAGCTA AACCACCCGT CAATAGACAA ATACCAAAAA CGTTTACCTA GGTGTTAATC   
  
  
- TCCTTAATAT ACCTGGAAAT GTAGCTTAGT TCAAGTTACT AGTTTCCCAC GGTTAAAATG GTTCAGCGTC   
  
  
- ACAACAATGA GGCTACTTTG AGGTTCTAAG GTTAAACTTA GGTTCACAAG GTTTAGGTTC ACAACACTTA   
  
  
- GGAGGAGGCA ACCAAGGGGG TTTGGGTTTA GAATTAAATC AACACCGTAG AGTTATAAGA CTGTAACTCT   
  
  
- GTCGGGACTT GCTCCTAACA CTAAAATCAC TACAACAAGA ATTCATATAA TTAGTTGAAG AATACCTTCT   
  
  
- CCTACCCCTT CTCTTTGAGG TATGACTAGT ACTTGGAAGT CAGCAACTCC GCCGTCTCTT CAGTAACATA   
  
  
- CTCCGCGAGC CCGTCTCTAT AGGAGGAAGG CCTTTGGCTT TAGTCAACGG TCTACAACTC GTACTGCCGA   
  
  
- ACTGACCCTC GTCACAATCC CGAGTATCAC CACGTTCGTC ACAATCCCGA GTATCACTGC GTTCACCACC   
  
  
- ATCACCGGAC TGTCCTATAC CAACCATACC ACTAGGAATA TTAACCTCAG GAGTTTTACA GCACTTAAAA   
  
  
- TGGTAAAGGA GGAGCTTTGG GAATAGCACG TCGAGTAGTC TATCGAGCCC GTCTACCCAA TCATTCACCT   
  
  
- TCAGTTTAAG ACTAAGTGTC GAGTCGAGCC GGTAATTACG TTCACCAAAT CACCTACCCG GTCCCCTGAG   
  
  
- AGGACACTCA CGAGAATCAC ACAGACTTTA TAAGTTACTG TCAGTCAGTT ACAACGTCAA AGTCTTTCCT   
  
  
- AAACTCCTTC GCTTATTTAA AGAAGGTTTC TCAAGAAACA TGTTCCCAAA ACGGTTGTGC CCTAACGGAA   
  
  
- TAGTCTTCCG TTTGTTATCA CGGGTTCTAA ACAACCAGTT ACATCTCCTA CAATGATCCC CTTTCTTCGT   
  
  
- AGCAATAGGG CTTCTTAACG TCAGTCTTCT TCCCTCCTAT TTATTCGTTA GTCGACAAAG GGACCGTCGA   
  
  
- CTACTCCGTC AACAATCCAG ACTTTACAAG CTATCCCATG ACGAAACATC AGCCCCTTTT GTACTACGAC   
  
  
- GAGAGGCCCT TCGAAATGTC TGACTTGATT TATTCTCAGA CGCTTTACGG GGTCAATTCC CCAGATTATC   
  
  
- ACCCTTTCCG GCACCATTCT TTCGCCCTTT CTTCTCCCTA CATCATCTAA ATTCTAGAGA GAATTGGGAT   
  
  
- ACACGTGTTC GTCAACGTAG CTTACTAGTG TCCTCACGTT TACTGGACGA ATCCGTTTAA TCTGTCGTAA   
  
  
- GAAGAGGATC ACCCCTACCA TTGGTTTCTT ACCGTGTAAT AAAACGCCTA CCAGAACTCC GTGCGGAACG   
  
  
- ACCACATCCT TGAGGATATA TATTGACAGA ACATTGCCCA GGCCGTAGCC GACATCTATA GAACTCTCGA   
  
  
- ATGGTGTACA AAGAACGGTG TACGGGTAAA TTCTTTTACC CTTTAAAGAA GAGATTATCT TGCTAATACC   
  
  
- GACACCGTCT CTTACGTTGT ACGGAAGTAT ATTAACTAGA GCCATAACAG ATACCAAAGG TTACCGGAAC   
  
  
- AGATTAAGTC GCTGAAAGTA GATCCGGACC ACCGGGGGGG TTTGAAGCTT ATTGGCCTCA TCTAGAAGGT   
  
  
- GTTGGACCCA AGGCTGGTCG GTTTTCTCAA CTCCTCTGTC CCGCAGCGAA CTTCTTGATA CGTCTCAGTA   
  
  
- AATTACACGG GAAACTCAAG TTACGATATC GTTTCTTCAC CCTTTGTGAA TGGTAACTTC TAGAGTCCTA   
  
  
- GTTATCGCTA CTCAACGAAC AACAGTGGAC ATACAAATCC AAATTTGTAT ATGGACTCCT TTGTCACTGG   
  
  
- CACCTAACGG GATCCCTATG ACAAGAATTG GACTAACCCG CGTATTTGGG TCGACAAAAG TATGTTCCGT   
  
  
- GACAATTGCC CCGAAAGTTA AGAGGGAAAA AGTATCGAGC TAAAGCTCTC CGAGATAAAG TGAAGAGGTG   
  
  
- AGACAAACTA TACGATCTCC GGTTGGACGG TTCCCTGTTA TTCCTCTCCT ACGATTAACT CTCTCTCTAT   
  
  
- AAACCCTCCG TCCGTTACTT ACACTAACGA ACACTCCCAA ATCTCTCCTA TCTTTCCGGT CTCTGCATGT   
  
  
- TCGTCACCGT TCAGGCTTTA CTTTCCCGTC CCAAATCCGT CGACGGAAAT CTAGCGGTCT AAGACCTTTA   
  
  
- CCGATTTTTC TCCCACTTTA GACACATATT GTTTCTAAAG AGGTAACTAC TTCTGCCCGT GACCAACAAC   
  
  
- GACCCTACCT TCCCGTCTTA ACACATGTGT GAGTGATGAA CCTTCGGACG CCTCAT

+     Myb

| Site Name | Organism | Position | Strand | Matrix score. | sequence | function |
| --- | --- | --- | --- | --- | --- | --- |
| Myb | Arabidopsis thaliana | 3022 | - | 6 | CAACTG |  |
| Myb | Arabidopsis thaliana | 2427 | - | 6 | CAACTG |  |
| Myb | Arabidopsis thaliana | 3136 | - | 6 | TAACTG |  |
| Myb | Arabidopsis thaliana | 3235 | - | 6 | CAACTG |  |
| Myb | Arabidopsis thaliana | 720 | + | 6 | TAACTG |  |
| Myb | Arabidopsis thaliana | 1993 | - | 6 | TAACTG |  |
| Myb | Arabidopsis thaliana | 3386 | + | 6 | TAACTG |  |
| Myb | Arabidopsis thaliana | 187 | + | 6 | CAACTG |  |

>HU06G00568.1   
+ -Up\_Stream \_Len000CCCTGG GTCATTAAAA AAAAAGAAAG AAAGAAGATT GCGCTAAAAG TAGATTTTTA   
  
  
+ TCAGATTCAA GCTGCAGGTG ATAACCAAAT TGTCATTAAA GCAATGCAAG CGCATATCAA TACCCTTTTG   
  
  
+ GGAGATAGCT CCGACTGTTG AAGACATTAG GAGCATGATC TCCAACTGTG AATTTGTTTC ATTTACTCAT   
  
  
+ ATTCATATCT ACCATGTGGA TAATATGACT GCAGACTAGA TGGCCAAATT TGAATGCACA CTTATAACTT   
  
  
+ CAACTCTTTC TACTTTTTTC TTCCCCACCT TATCAGGAAT TTCTTTTAAT TGTCGTGGGA GACAACTTGG   
  
  
+ GTAGAACTCT TGCGAAAAGG GCAACTTAAC GTTTTGGTAC TTGCATTTTC CAAAAAAACA AAAAGGCTAA   
  
  
+ ACAATTCTCC GCATGCTGAC TTCACGGTAG ATATGATGTA TTTTTTAACA AAAGACATCT TCTGAATGAA   
  
  
+ ACGCTTAATA TCTTGGACCA TACTAGAAAT TAAATTGCCA CGGTGCATGA ATTTCACCTC GAACTGCTTG   
  
  
+ TATAAAGATC TTGTTATCAC CTTCAGTAAT CAGATACTTG TAACCAAATT GAATGACCAC AAAGACCACA   
  
  
+ TTCCTCACAA TCGTTGCCTC TAGAATAAGA ATGAATGTTT CTTCAAGGTT GAAAGCCCTT GCTAATGCGA   
  
  
+ ACCTGCCTAT ATGCCTAACT GGCTATATAT ATGTGCGTAG AACAGCATGA CATGAGTTAA TAATATAGCA   
  
  
+ GCTAAAGGGA ACTCTAATGT ACACATGATT GTAATTCACC TGCCTCGCTA GCTTACGAGC ATGACTCTTG   
  
  
+ ACTATTATTG TTTTAATTGA TCTTGAATTT TCATAGATTT TACTCCATTG TTTACTCATT TTGTTATAAT   
  
  
+ TAACCACCTG ATTTTAAACT TTAAAATACA CAAAGATTCA TCTAATATAA TTTTTTAGAT AAAGTTATGC   
  
  
+ ATACATATAT ACAAATTAAA TTATTTACGA TCTAATCATT TCCCGTTATA TATTTCACAC TAAAATTTAG   
  
  
+ AAGAGACAAT CTATTAAAAT TACATCACAT GTTTTGGGGG GGGGGGGATA TCAGATATGC ATGACATTCC   
  
  
+ TAATCCAATA ATGCAATGAA ACCCACCGAA TAGTTGAAAT GATTTGTTCT ATCCATGAAC TCACCAAGTC   
  
  
+ ACAATCCATC ACACTCGAAG GATATGCTTT TCTTTTTGAA GGGAATCTTG CTTAATCAAA CCTATAAACT   
  
  
+ TTTAAAAGCA ACAGACAAAA AAGTGATTAT AATGGTAGTG GTAGGATGAA GGCAGCTTTC AGAAAAGCGA   
  
  
+ TTGAATTTTT ATTGTATCCC ATGAAAACCC ATTGAATTTT TCTTTCTTTC TTTGTTTTTG TTACCTGGGT   
  
  
+ TTATGCAAAG GACGGCTGTT GTATTTAATA ATAACAAAAA CATAGGGCTT TGCGGATTGC TAATGCTGGG   
  
  
+ GATGGCATCA GTGCTCGAAT CTGTGTACGC TGTCATAGGA ACCCACTTGC TTCTGCTCAT TTATTTCTGT   
  
  
+ CTCTAGATCC CCTCCTCTCT CTCTCTCTCT CTCTAAGGTG TCTGCTCGTC TGTTCATATT CTCCTGAGGT   
  
  
+ TCTTCTTCAT CTATCTTGTT CTGGGTGTGT GCTGAGGTAC TTCTTTTCTT TATAAATTTT TCATCTTTTG   
  
  
+ TTGTTTTGCT TGGTGTTTAT GATTTTAGTT TTTGTCTTTA AGACGAGATT TGCTGTTAAT AGCAAGATTT   
  
  
+ CTGAACTAGT ATTGATCCTT TTTGTGGTAT ATTTGTTGAA TTTGATTAAT TTGTGTATTG TGTTTAGCTT   
  
  
+ AAGTAACTCG TTGTGATCTA ATTGATGCTC GGGTTTTGAC AGTTTGATTA GGGTTTTCGG TACTGGAGTT   
  
  
+ AAAGTTTTTT TATTTTTTAT AGGAGGAGTT AAAGGTAATA ATAAGAATTG ATGTTTGCTG ATTTTTCCTC   
  
  
+ TTTTCTTTTT TGGTTTCGAT TTGGTGGGCA GTTATCTGTT TATGGTTTTT GCAAATGGAT CCACAATTAG   
  
  
+ AGGAATTATA TGGACCTTTA CATCGAATCA AGTTCAATGA TCAAAGGGTG CCAATTTTAC CAAGTCGCAG   
  
  
+ TGTTGTTACT CCGATGAAAC TCCAAGATTC CAATTTGAAT CCAAGTGTTC CAAATCCAAG TGTTGTGAAT   
  
  
+ CCTCCTCCGT TGGTTCCCCC AAACCCAAAT CTTAATTTAG TTGTGGCATC TCAATATTCT GACATTGAGA   
  
  
+ CAGCCCTGAA CGAGGATTGT GATTTTAGTG ATGTTGTTCT TAAGTATATT AATCAACTTC TTATGGAAGA   
  
  
+ GGATGGGGAA GAGAAACTCC ATACTGATCA TGAACCTTCA GTCGTTGAGG CGGCAGAGAA GTCATTGTAT   
  
  
+ GAGGCGCTCG GGCAGAGATA TCCTCCTTCC GGAAACCGAA ATCAGTTGCC AGATGTTGAG CATGACGGCT   
  
  
+ TGACTGGGAG CAGTGTTAGG GCTCATAGTG GTGCAAGCAG TGTTAGGGCT CATAGTGACG CAAGTGGTGG   
  
  
+ TAGTGGCCTG ACAGGATATG GTTGGTATGG TGATCCTTAT AATTGGAGTC CTCAAAATGT CGTGAATTTT   
  
  
+ ACCATTTCCT CCTCGAAACC CTTATCGTGC AGCTCATCAG ATAGCTCGGG CAGATGGGTT AGTAAGTGGA   
  
  
+ AGTCAAATTC TGATTCACAG CTCAGCTCGG CCATTAATGC AAGTGGTTTA GTGGATGGGC CAGGGGACTC   
  
  
+ TCCTGTGAGT GCTCTTAGTG TGTCTGAAAT ATTCAATGAC AGTCAGTCAA TGTTGCAGTT TCAGAAAGGA   
  
  
+ TTTGAGGAAG CGAATAAATT TCTTCCAAAG AGTTCTTTGT ACAAGGGTTT TGCCAACACG GGATTGCCTT   
  
  
+ ATCAGAAGGC AAACAATAGT GCCCAAGATT TGTTGGTCAA TGTAGAGGAT GTTACTAGGG GAAAGAAGCA   
  
  
+ TCGTTATCCC GAAGAATTGC AGTCAGAAGA AGGGAGGATA AATAAGCAAT CAGCTGTTTC CCTGGCAGCT   
  
  
+ GATGAGGCAG TTGTTAGGTC TGAAATGTTC GATAGGGTAC TGCTTTGTAG TCGGGGAAAA CATGATGCTG   
  
  
+ CTCTCCGGGA AGCTTTACAG ACTGAACTAA ATAAGAGTCT GCGAAATGCC CCAGTTAAGG GGTCTAATAG   
  
  
+ TGGGAAAGGC CGTGGTAAGA AAGCGGGAAA GAAGAGGGAT GTAGTAGATT TAAGATCTCT CTTAACCCTA   
  
  
+ TGTGCACAAG CAGTTGCATC GAATGATCAC AGGAGTGCAA ATGACCTGCT TAGGCAAATT AGACAGCATT   
  
  
+ CTTCTCCTAG TGGGGATGGT AACCAAAGAA TGGCACATTA TTTTGCGGAT GGTCTTGAGG CACGCCTTGC   
  
  
+ TGGTGTAGGA ACTCCTATAT ATAACTGTCT TGTAACGGGT CCGGCATCGG CTGTAGATAT CTTGAGAGCT   
  
  
+ TACCACATGT TTCTTGCCAC ATGCCCATTT AAGAAAATGG GAAATTTCTT CTCTAATAGA ACGATTATGG   
  
  
+ CTGTGGCAGA GAATGCAACA TGCCTTCATA TAATTGATCT CGGTATTGTC TATGGTTTCC AATGGCCTTG   
  
  
+ TCTAATTCAG CGACTTTCAT CTAGGCCTGG TGGCCCCCCC AAACTTCGAA TAACCGGAGT AGATCTTCCA   
  
  
+ CAACCTGGGT TCCGACCAGC CAAAAGAGTT GAGGAGACAG GGCGTCGCTT GAAGAACTAT GCAGAGTCAT   
  
  
+ TTAATGTGCC CTTTGAGTTC AATGCTATAG CAAAGAAGTG GGAAACACTT ACCATTGAAG ATCTCAGGAT   
  
  
+ CAATAGCGAT GAGTTGCTTG TTGTCACCTG TATGTTTAGG TTTAAACATA TACCTGAGGA AACAGTGACC   
  
  
+ GTGGATTGCC CTAGGGATAC TGTTCTTAAC CTGATTGGGC GCATAAACCC AGCTGTTTTC ATACAAGGCA   
  
  
+ CTGTTAACGG GGCTTTCAAT TCTCCCTTTT TCATAGCTCG ATTTCGAGAG GCTCTATTTC ACTTCTCCAC   
  
  
+ TCTGTTTGAT ATGCTAGAGG CCAACCTGCC AAGGGACAAT AAGGAGAGGA TGCTAATTGA GAGAGAGATA   
  
  
+ TTTGGGAGGC AGGCAATGAA TGTGATTGCT TGTGAGGGTT TAGAGAGGAT AGAAAGGCCA GAGACGTACA   
  
  
+ AGCAGTGGCA AGTCCGAAAT GAAAGGGCAG GGTTTAGGCA GCTGCCTTTA GATCGCCAGA TTCTGGAAAT   
  
  
+ GGCTAAAAAG AGGGTGAAAT CTGTGTATAA CAAAGATTTC TCCATTGATG AAGACGGGCA CTGGTTGTTG   
  
  
+ CTGGGATGGA AGGGCAGAAT TGTGTACACA CTCACTACTT GGAAGCCTGC GGAGTA  

- -Up\_Stream \_Len000GGGACC CAGTAATTTT TTTTTCTTTC TTTCTTCTAA CGCGATTTTC ATCTAAAAAT   
  
  
- AGTCTAAGTT CGACGTCCAC TATTGGTTTA ACAGTAATTT CGTTACGTTC GCGTATAGTT ATGGGAAAAC   
  
  
- CCTCTATCGA GGCTGACAAC TTCTGTAATC CTCGTACTAG AGGTTGACAC TTAAACAAAG TAAATGAGTA   
  
  
- TAAGTATAGA TGGTACACCT ATTATACTGA CGTCTGATCT ACCGGTTTAA ACTTACGTGT GAATATTGAA   
  
  
- GTTGAGAAAG ATGAAAAAAG AAGGGGTGGA ATAGTCCTTA AAGAAAATTA ACAGCACCCT CTGTTGAACC   
  
  
- CATCTTGAGA ACGCTTTTCC CGTTGAATTG CAAAACCATG AACGTAAAAG GTTTTTTTGT TTTTCCGATT   
  
  
- TGTTAAGAGG CGTACGACTG AAGTGCCATC TATACTACAT AAAAAATTGT TTTCTGTAGA AGACTTACTT   
  
  
- TGCGAATTAT AGAACCTGGT ATGATCTTTA ATTTAACGGT GCCACGTACT TAAAGTGGAG CTTGACGAAC   
  
  
- ATATTTCTAG AACAATAGTG GAAGTCATTA GTCTATGAAC ATTGGTTTAA CTTACTGGTG TTTCTGGTGT   
  
  
- AAGGAGTGTT AGCAACGGAG ATCTTATTCT TACTTACAAA GAAGTTCCAA CTTTCGGGAA CGATTACGCT   
  
  
- TGGACGGATA TACGGATTGA CCGATATATA TACACGCATC TTGTCGTACT GTACTCAATT ATTATATCGT   
  
  
- CGATTTCCCT TGAGATTACA TGTGTACTAA CATTAAGTGG ACGGAGCGAT CGAATGCTCG TACTGAGAAC   
  
  
- TGATAATAAC AAAATTAACT AGAACTTAAA AGTATCTAAA ATGAGGTAAC AAATGAGTAA AACAATATTA   
  
  
- ATTGGTGGAC TAAAATTTGA AATTTTATGT GTTTCTAAGT AGATTATATT AAAAAATCTA TTTCAATACG   
  
  
- TATGTATATA TGTTTAATTT AATAAATGCT AGATTAGTAA AGGGCAATAT ATAAAGTGTG ATTTTAAATC   
  
  
- TTCTCTGTTA GATAATTTTA ATGTAGTGTA CAAAACCCCC CCCCCCCTAT AGTCTATACG TACTGTAAGG   
  
  
- ATTAGGTTAT TACGTTACTT TGGGTGGCTT ATCAACTTTA CTAAACAAGA TAGGTACTTG AGTGGTTCAG   
  
  
- TGTTAGGTAG TGTGAGCTTC CTATACGAAA AGAAAAACTT CCCTTAGAAC GAATTAGTTT GGATATTTGA   
  
  
- AAATTTTCGT TGTCTGTTTT TTCACTAATA TTACCATCAC CATCCTACTT CCGTCGAAAG TCTTTTCGCT   
  
  
- AACTTAAAAA TAACATAGGG TACTTTTGGG TAACTTAAAA AGAAAGAAAG AAACAAAAAC AATGGACCCA   
  
  
- AATACGTTTC CTGCCGACAA CATAAATTAT TATTGTTTTT GTATCCCGAA ACGCCTAACG ATTACGACCC   
  
  
- CTACCGTAGT CACGAGCTTA GACACATGCG ACAGTATCCT TGGGTGAACG AAGACGAGTA AATAAAGACA   
  
  
- GAGATCTAGG GGAGGAGAGA GAGAGAGAGA GAGATTCCAC AGACGAGCAG ACAAGTATAA GAGGACTCCA   
  
  
- AGAAGAAGTA GATAGAACAA GACCCACACA CGACTCCATG AAGAAAAGAA ATATTTAAAA AGTAGAAAAC   
  
  
- AACAAAACGA ACCACAAATA CTAAAATCAA AAACAGAAAT TCTGCTCTAA ACGACAATTA TCGTTCTAAA   
  
  
- GACTTGATCA TAACTAGGAA AAACACCATA TAAACAACTT AAACTAATTA AACACATAAC ACAAATCGAA   
  
  
- TTCATTGAGC AACACTAGAT TAACTACGAG CCCAAAACTG TCAAACTAAT CCCAAAAGCC ATGACCTCAA   
  
  
- TTTCAAAAAA ATAAAAAATA TCCTCCTCAA TTTCCATTAT TATTCTTAAC TACAAACGAC TAAAAAGGAG   
  
  
- AAAAGAAAAA ACCAAAGCTA AACCACCCGT CAATAGACAA ATACCAAAAA CGTTTACCTA GGTGTTAATC   
  
  
- TCCTTAATAT ACCTGGAAAT GTAGCTTAGT TCAAGTTACT AGTTTCCCAC GGTTAAAATG GTTCAGCGTC   
  
  
- ACAACAATGA GGCTACTTTG AGGTTCTAAG GTTAAACTTA GGTTCACAAG GTTTAGGTTC ACAACACTTA   
  
  
- GGAGGAGGCA ACCAAGGGGG TTTGGGTTTA GAATTAAATC AACACCGTAG AGTTATAAGA CTGTAACTCT   
  
  
- GTCGGGACTT GCTCCTAACA CTAAAATCAC TACAACAAGA ATTCATATAA TTAGTTGAAG AATACCTTCT   
  
  
- CCTACCCCTT CTCTTTGAGG TATGACTAGT ACTTGGAAGT CAGCAACTCC GCCGTCTCTT CAGTAACATA   
  
  
- CTCCGCGAGC CCGTCTCTAT AGGAGGAAGG CCTTTGGCTT TAGTCAACGG TCTACAACTC GTACTGCCGA   
  
  
- ACTGACCCTC GTCACAATCC CGAGTATCAC CACGTTCGTC ACAATCCCGA GTATCACTGC GTTCACCACC   
  
  
- ATCACCGGAC TGTCCTATAC CAACCATACC ACTAGGAATA TTAACCTCAG GAGTTTTACA GCACTTAAAA   
  
  
- TGGTAAAGGA GGAGCTTTGG GAATAGCACG TCGAGTAGTC TATCGAGCCC GTCTACCCAA TCATTCACCT   
  
  
- TCAGTTTAAG ACTAAGTGTC GAGTCGAGCC GGTAATTACG TTCACCAAAT CACCTACCCG GTCCCCTGAG   
  
  
- AGGACACTCA CGAGAATCAC ACAGACTTTA TAAGTTACTG TCAGTCAGTT ACAACGTCAA AGTCTTTCCT   
  
  
- AAACTCCTTC GCTTATTTAA AGAAGGTTTC TCAAGAAACA TGTTCCCAAA ACGGTTGTGC CCTAACGGAA   
  
  
- TAGTCTTCCG TTTGTTATCA CGGGTTCTAA ACAACCAGTT ACATCTCCTA CAATGATCCC CTTTCTTCGT   
  
  
- AGCAATAGGG CTTCTTAACG TCAGTCTTCT TCCCTCCTAT TTATTCGTTA GTCGACAAAG GGACCGTCGA   
  
  
- CTACTCCGTC AACAATCCAG ACTTTACAAG CTATCCCATG ACGAAACATC AGCCCCTTTT GTACTACGAC   
  
  
- GAGAGGCCCT TCGAAATGTC TGACTTGATT TATTCTCAGA CGCTTTACGG GGTCAATTCC CCAGATTATC   
  
  
- ACCCTTTCCG GCACCATTCT TTCGCCCTTT CTTCTCCCTA CATCATCTAA ATTCTAGAGA GAATTGGGAT   
  
  
- ACACGTGTTC GTCAACGTAG CTTACTAGTG TCCTCACGTT TACTGGACGA ATCCGTTTAA TCTGTCGTAA   
  
  
- GAAGAGGATC ACCCCTACCA TTGGTTTCTT ACCGTGTAAT AAAACGCCTA CCAGAACTCC GTGCGGAACG   
  
  
- ACCACATCCT TGAGGATATA TATTGACAGA ACATTGCCCA GGCCGTAGCC GACATCTATA GAACTCTCGA   
  
  
- ATGGTGTACA AAGAACGGTG TACGGGTAAA TTCTTTTACC CTTTAAAGAA GAGATTATCT TGCTAATACC   
  
  
- GACACCGTCT CTTACGTTGT ACGGAAGTAT ATTAACTAGA GCCATAACAG ATACCAAAGG TTACCGGAAC   
  
  
- AGATTAAGTC GCTGAAAGTA GATCCGGACC ACCGGGGGGG TTTGAAGCTT ATTGGCCTCA TCTAGAAGGT   
  
  
- GTTGGACCCA AGGCTGGTCG GTTTTCTCAA CTCCTCTGTC CCGCAGCGAA CTTCTTGATA CGTCTCAGTA   
  
  
- AATTACACGG GAAACTCAAG TTACGATATC GTTTCTTCAC CCTTTGTGAA TGGTAACTTC TAGAGTCCTA   
  
  
- GTTATCGCTA CTCAACGAAC AACAGTGGAC ATACAAATCC AAATTTGTAT ATGGACTCCT TTGTCACTGG   
  
  
- CACCTAACGG GATCCCTATG ACAAGAATTG GACTAACCCG CGTATTTGGG TCGACAAAAG TATGTTCCGT   
  
  
- GACAATTGCC CCGAAAGTTA AGAGGGAAAA AGTATCGAGC TAAAGCTCTC CGAGATAAAG TGAAGAGGTG   
  
  
- AGACAAACTA TACGATCTCC GGTTGGACGG TTCCCTGTTA TTCCTCTCCT ACGATTAACT CTCTCTCTAT   
  
  
- AAACCCTCCG TCCGTTACTT ACACTAACGA ACACTCCCAA ATCTCTCCTA TCTTTCCGGT CTCTGCATGT   
  
  
- TCGTCACCGT TCAGGCTTTA CTTTCCCGTC CCAAATCCGT CGACGGAAAT CTAGCGGTCT AAGACCTTTA   
  
  
- CCGATTTTTC TCCCACTTTA GACACATATT GTTTCTAAAG AGGTAACTAC TTCTGCCCGT GACCAACAAC   
  
  
- GACCCTACCT TCCCGTCTTA ACACATGTGT GAGTGATGAA CCTTCGGACG CCTCAT

+     Myb-binding site

| Site Name | Organism | Position | Strand | Matrix score. | sequence | function |
| --- | --- | --- | --- | --- | --- | --- |
| Myb-binding site | Nicotiana tabacum | 1273 | + | 6 | CAACAG |  |
| Myb-binding site | Nicotiana tabacum | 159 | - | 6 | CAACAG |  |
| Myb-binding site | Nicotiana tabacum | 1420 | - | 6 | CAACAG |  |

>HU06G00568.1   
+ -Up\_Stream \_Len000CCCTGG GTCATTAAAA AAAAAGAAAG AAAGAAGATT GCGCTAAAAG TAGATTTTTA   
  
  
+ TCAGATTCAA GCTGCAGGTG ATAACCAAAT TGTCATTAAA GCAATGCAAG CGCATATCAA TACCCTTTTG   
  
  
+ GGAGATAGCT CCGACTGTTG AAGACATTAG GAGCATGATC TCCAACTGTG AATTTGTTTC ATTTACTCAT   
  
  
+ ATTCATATCT ACCATGTGGA TAATATGACT GCAGACTAGA TGGCCAAATT TGAATGCACA CTTATAACTT   
  
  
+ CAACTCTTTC TACTTTTTTC TTCCCCACCT TATCAGGAAT TTCTTTTAAT TGTCGTGGGA GACAACTTGG   
  
  
+ GTAGAACTCT TGCGAAAAGG GCAACTTAAC GTTTTGGTAC TTGCATTTTC CAAAAAAACA AAAAGGCTAA   
  
  
+ ACAATTCTCC GCATGCTGAC TTCACGGTAG ATATGATGTA TTTTTTAACA AAAGACATCT TCTGAATGAA   
  
  
+ ACGCTTAATA TCTTGGACCA TACTAGAAAT TAAATTGCCA CGGTGCATGA ATTTCACCTC GAACTGCTTG   
  
  
+ TATAAAGATC TTGTTATCAC CTTCAGTAAT CAGATACTTG TAACCAAATT GAATGACCAC AAAGACCACA   
  
  
+ TTCCTCACAA TCGTTGCCTC TAGAATAAGA ATGAATGTTT CTTCAAGGTT GAAAGCCCTT GCTAATGCGA   
  
  
+ ACCTGCCTAT ATGCCTAACT GGCTATATAT ATGTGCGTAG AACAGCATGA CATGAGTTAA TAATATAGCA   
  
  
+ GCTAAAGGGA ACTCTAATGT ACACATGATT GTAATTCACC TGCCTCGCTA GCTTACGAGC ATGACTCTTG   
  
  
+ ACTATTATTG TTTTAATTGA TCTTGAATTT TCATAGATTT TACTCCATTG TTTACTCATT TTGTTATAAT   
  
  
+ TAACCACCTG ATTTTAAACT TTAAAATACA CAAAGATTCA TCTAATATAA TTTTTTAGAT AAAGTTATGC   
  
  
+ ATACATATAT ACAAATTAAA TTATTTACGA TCTAATCATT TCCCGTTATA TATTTCACAC TAAAATTTAG   
  
  
+ AAGAGACAAT CTATTAAAAT TACATCACAT GTTTTGGGGG GGGGGGGATA TCAGATATGC ATGACATTCC   
  
  
+ TAATCCAATA ATGCAATGAA ACCCACCGAA TAGTTGAAAT GATTTGTTCT ATCCATGAAC TCACCAAGTC   
  
  
+ ACAATCCATC ACACTCGAAG GATATGCTTT TCTTTTTGAA GGGAATCTTG CTTAATCAAA CCTATAAACT   
  
  
+ TTTAAAAGCA ACAGACAAAA AAGTGATTAT AATGGTAGTG GTAGGATGAA GGCAGCTTTC AGAAAAGCGA   
  
  
+ TTGAATTTTT ATTGTATCCC ATGAAAACCC ATTGAATTTT TCTTTCTTTC TTTGTTTTTG TTACCTGGGT   
  
  
+ TTATGCAAAG GACGGCTGTT GTATTTAATA ATAACAAAAA CATAGGGCTT TGCGGATTGC TAATGCTGGG   
  
  
+ GATGGCATCA GTGCTCGAAT CTGTGTACGC TGTCATAGGA ACCCACTTGC TTCTGCTCAT TTATTTCTGT   
  
  
+ CTCTAGATCC CCTCCTCTCT CTCTCTCTCT CTCTAAGGTG TCTGCTCGTC TGTTCATATT CTCCTGAGGT   
  
  
+ TCTTCTTCAT CTATCTTGTT CTGGGTGTGT GCTGAGGTAC TTCTTTTCTT TATAAATTTT TCATCTTTTG   
  
  
+ TTGTTTTGCT TGGTGTTTAT GATTTTAGTT TTTGTCTTTA AGACGAGATT TGCTGTTAAT AGCAAGATTT   
  
  
+ CTGAACTAGT ATTGATCCTT TTTGTGGTAT ATTTGTTGAA TTTGATTAAT TTGTGTATTG TGTTTAGCTT   
  
  
+ AAGTAACTCG TTGTGATCTA ATTGATGCTC GGGTTTTGAC AGTTTGATTA GGGTTTTCGG TACTGGAGTT   
  
  
+ AAAGTTTTTT TATTTTTTAT AGGAGGAGTT AAAGGTAATA ATAAGAATTG ATGTTTGCTG ATTTTTCCTC   
  
  
+ TTTTCTTTTT TGGTTTCGAT TTGGTGGGCA GTTATCTGTT TATGGTTTTT GCAAATGGAT CCACAATTAG   
  
  
+ AGGAATTATA TGGACCTTTA CATCGAATCA AGTTCAATGA TCAAAGGGTG CCAATTTTAC CAAGTCGCAG   
  
  
+ TGTTGTTACT CCGATGAAAC TCCAAGATTC CAATTTGAAT CCAAGTGTTC CAAATCCAAG TGTTGTGAAT   
  
  
+ CCTCCTCCGT TGGTTCCCCC AAACCCAAAT CTTAATTTAG TTGTGGCATC TCAATATTCT GACATTGAGA   
  
  
+ CAGCCCTGAA CGAGGATTGT GATTTTAGTG ATGTTGTTCT TAAGTATATT AATCAACTTC TTATGGAAGA   
  
  
+ GGATGGGGAA GAGAAACTCC ATACTGATCA TGAACCTTCA GTCGTTGAGG CGGCAGAGAA GTCATTGTAT   
  
  
+ GAGGCGCTCG GGCAGAGATA TCCTCCTTCC GGAAACCGAA ATCAGTTGCC AGATGTTGAG CATGACGGCT   
  
  
+ TGACTGGGAG CAGTGTTAGG GCTCATAGTG GTGCAAGCAG TGTTAGGGCT CATAGTGACG CAAGTGGTGG   
  
  
+ TAGTGGCCTG ACAGGATATG GTTGGTATGG TGATCCTTAT AATTGGAGTC CTCAAAATGT CGTGAATTTT   
  
  
+ ACCATTTCCT CCTCGAAACC CTTATCGTGC AGCTCATCAG ATAGCTCGGG CAGATGGGTT AGTAAGTGGA   
  
  
+ AGTCAAATTC TGATTCACAG CTCAGCTCGG CCATTAATGC AAGTGGTTTA GTGGATGGGC CAGGGGACTC   
  
  
+ TCCTGTGAGT GCTCTTAGTG TGTCTGAAAT ATTCAATGAC AGTCAGTCAA TGTTGCAGTT TCAGAAAGGA   
  
  
+ TTTGAGGAAG CGAATAAATT TCTTCCAAAG AGTTCTTTGT ACAAGGGTTT TGCCAACACG GGATTGCCTT   
  
  
+ ATCAGAAGGC AAACAATAGT GCCCAAGATT TGTTGGTCAA TGTAGAGGAT GTTACTAGGG GAAAGAAGCA   
  
  
+ TCGTTATCCC GAAGAATTGC AGTCAGAAGA AGGGAGGATA AATAAGCAAT CAGCTGTTTC CCTGGCAGCT   
  
  
+ GATGAGGCAG TTGTTAGGTC TGAAATGTTC GATAGGGTAC TGCTTTGTAG TCGGGGAAAA CATGATGCTG   
  
  
+ CTCTCCGGGA AGCTTTACAG ACTGAACTAA ATAAGAGTCT GCGAAATGCC CCAGTTAAGG GGTCTAATAG   
  
  
+ TGGGAAAGGC CGTGGTAAGA AAGCGGGAAA GAAGAGGGAT GTAGTAGATT TAAGATCTCT CTTAACCCTA   
  
  
+ TGTGCACAAG CAGTTGCATC GAATGATCAC AGGAGTGCAA ATGACCTGCT TAGGCAAATT AGACAGCATT   
  
  
+ CTTCTCCTAG TGGGGATGGT AACCAAAGAA TGGCACATTA TTTTGCGGAT GGTCTTGAGG CACGCCTTGC   
  
  
+ TGGTGTAGGA ACTCCTATAT ATAACTGTCT TGTAACGGGT CCGGCATCGG CTGTAGATAT CTTGAGAGCT   
  
  
+ TACCACATGT TTCTTGCCAC ATGCCCATTT AAGAAAATGG GAAATTTCTT CTCTAATAGA ACGATTATGG   
  
  
+ CTGTGGCAGA GAATGCAACA TGCCTTCATA TAATTGATCT CGGTATTGTC TATGGTTTCC AATGGCCTTG   
  
  
+ TCTAATTCAG CGACTTTCAT CTAGGCCTGG TGGCCCCCCC AAACTTCGAA TAACCGGAGT AGATCTTCCA   
  
  
+ CAACCTGGGT TCCGACCAGC CAAAAGAGTT GAGGAGACAG GGCGTCGCTT GAAGAACTAT GCAGAGTCAT   
  
  
+ TTAATGTGCC CTTTGAGTTC AATGCTATAG CAAAGAAGTG GGAAACACTT ACCATTGAAG ATCTCAGGAT   
  
  
+ CAATAGCGAT GAGTTGCTTG TTGTCACCTG TATGTTTAGG TTTAAACATA TACCTGAGGA AACAGTGACC   
  
  
+ GTGGATTGCC CTAGGGATAC TGTTCTTAAC CTGATTGGGC GCATAAACCC AGCTGTTTTC ATACAAGGCA   
  
  
+ CTGTTAACGG GGCTTTCAAT TCTCCCTTTT TCATAGCTCG ATTTCGAGAG GCTCTATTTC ACTTCTCCAC   
  
  
+ TCTGTTTGAT ATGCTAGAGG CCAACCTGCC AAGGGACAAT AAGGAGAGGA TGCTAATTGA GAGAGAGATA   
  
  
+ TTTGGGAGGC AGGCAATGAA TGTGATTGCT TGTGAGGGTT TAGAGAGGAT AGAAAGGCCA GAGACGTACA   
  
  
+ AGCAGTGGCA AGTCCGAAAT GAAAGGGCAG GGTTTAGGCA GCTGCCTTTA GATCGCCAGA TTCTGGAAAT   
  
  
+ GGCTAAAAAG AGGGTGAAAT CTGTGTATAA CAAAGATTTC TCCATTGATG AAGACGGGCA CTGGTTGTTG   
  
  
+ CTGGGATGGA AGGGCAGAAT TGTGTACACA CTCACTACTT GGAAGCCTGC GGAGTA  

- -Up\_Stream \_Len000GGGACC CAGTAATTTT TTTTTCTTTC TTTCTTCTAA CGCGATTTTC ATCTAAAAAT   
  
  
- AGTCTAAGTT CGACGTCCAC TATTGGTTTA ACAGTAATTT CGTTACGTTC GCGTATAGTT ATGGGAAAAC   
  
  
- CCTCTATCGA GGCTGACAAC TTCTGTAATC CTCGTACTAG AGGTTGACAC TTAAACAAAG TAAATGAGTA   
  
  
- TAAGTATAGA TGGTACACCT ATTATACTGA CGTCTGATCT ACCGGTTTAA ACTTACGTGT GAATATTGAA   
  
  
- GTTGAGAAAG ATGAAAAAAG AAGGGGTGGA ATAGTCCTTA AAGAAAATTA ACAGCACCCT CTGTTGAACC   
  
  
- CATCTTGAGA ACGCTTTTCC CGTTGAATTG CAAAACCATG AACGTAAAAG GTTTTTTTGT TTTTCCGATT   
  
  
- TGTTAAGAGG CGTACGACTG AAGTGCCATC TATACTACAT AAAAAATTGT TTTCTGTAGA AGACTTACTT   
  
  
- TGCGAATTAT AGAACCTGGT ATGATCTTTA ATTTAACGGT GCCACGTACT TAAAGTGGAG CTTGACGAAC   
  
  
- ATATTTCTAG AACAATAGTG GAAGTCATTA GTCTATGAAC ATTGGTTTAA CTTACTGGTG TTTCTGGTGT   
  
  
- AAGGAGTGTT AGCAACGGAG ATCTTATTCT TACTTACAAA GAAGTTCCAA CTTTCGGGAA CGATTACGCT   
  
  
- TGGACGGATA TACGGATTGA CCGATATATA TACACGCATC TTGTCGTACT GTACTCAATT ATTATATCGT   
  
  
- CGATTTCCCT TGAGATTACA TGTGTACTAA CATTAAGTGG ACGGAGCGAT CGAATGCTCG TACTGAGAAC   
  
  
- TGATAATAAC AAAATTAACT AGAACTTAAA AGTATCTAAA ATGAGGTAAC AAATGAGTAA AACAATATTA   
  
  
- ATTGGTGGAC TAAAATTTGA AATTTTATGT GTTTCTAAGT AGATTATATT AAAAAATCTA TTTCAATACG   
  
  
- TATGTATATA TGTTTAATTT AATAAATGCT AGATTAGTAA AGGGCAATAT ATAAAGTGTG ATTTTAAATC   
  
  
- TTCTCTGTTA GATAATTTTA ATGTAGTGTA CAAAACCCCC CCCCCCCTAT AGTCTATACG TACTGTAAGG   
  
  
- ATTAGGTTAT TACGTTACTT TGGGTGGCTT ATCAACTTTA CTAAACAAGA TAGGTACTTG AGTGGTTCAG   
  
  
- TGTTAGGTAG TGTGAGCTTC CTATACGAAA AGAAAAACTT CCCTTAGAAC GAATTAGTTT GGATATTTGA   
  
  
- AAATTTTCGT TGTCTGTTTT TTCACTAATA TTACCATCAC CATCCTACTT CCGTCGAAAG TCTTTTCGCT   
  
  
- AACTTAAAAA TAACATAGGG TACTTTTGGG TAACTTAAAA AGAAAGAAAG AAACAAAAAC AATGGACCCA   
  
  
- AATACGTTTC CTGCCGACAA CATAAATTAT TATTGTTTTT GTATCCCGAA ACGCCTAACG ATTACGACCC   
  
  
- CTACCGTAGT CACGAGCTTA GACACATGCG ACAGTATCCT TGGGTGAACG AAGACGAGTA AATAAAGACA   
  
  
- GAGATCTAGG GGAGGAGAGA GAGAGAGAGA GAGATTCCAC AGACGAGCAG ACAAGTATAA GAGGACTCCA   
  
  
- AGAAGAAGTA GATAGAACAA GACCCACACA CGACTCCATG AAGAAAAGAA ATATTTAAAA AGTAGAAAAC   
  
  
- AACAAAACGA ACCACAAATA CTAAAATCAA AAACAGAAAT TCTGCTCTAA ACGACAATTA TCGTTCTAAA   
  
  
- GACTTGATCA TAACTAGGAA AAACACCATA TAAACAACTT AAACTAATTA AACACATAAC ACAAATCGAA   
  
  
- TTCATTGAGC AACACTAGAT TAACTACGAG CCCAAAACTG TCAAACTAAT CCCAAAAGCC ATGACCTCAA   
  
  
- TTTCAAAAAA ATAAAAAATA TCCTCCTCAA TTTCCATTAT TATTCTTAAC TACAAACGAC TAAAAAGGAG   
  
  
- AAAAGAAAAA ACCAAAGCTA AACCACCCGT CAATAGACAA ATACCAAAAA CGTTTACCTA GGTGTTAATC   
  
  
- TCCTTAATAT ACCTGGAAAT GTAGCTTAGT TCAAGTTACT AGTTTCCCAC GGTTAAAATG GTTCAGCGTC   
  
  
- ACAACAATGA GGCTACTTTG AGGTTCTAAG GTTAAACTTA GGTTCACAAG GTTTAGGTTC ACAACACTTA   
  
  
- GGAGGAGGCA ACCAAGGGGG TTTGGGTTTA GAATTAAATC AACACCGTAG AGTTATAAGA CTGTAACTCT   
  
  
- GTCGGGACTT GCTCCTAACA CTAAAATCAC TACAACAAGA ATTCATATAA TTAGTTGAAG AATACCTTCT   
  
  
- CCTACCCCTT CTCTTTGAGG TATGACTAGT ACTTGGAAGT CAGCAACTCC GCCGTCTCTT CAGTAACATA   
  
  
- CTCCGCGAGC CCGTCTCTAT AGGAGGAAGG CCTTTGGCTT TAGTCAACGG TCTACAACTC GTACTGCCGA   
  
  
- ACTGACCCTC GTCACAATCC CGAGTATCAC CACGTTCGTC ACAATCCCGA GTATCACTGC GTTCACCACC   
  
  
- ATCACCGGAC TGTCCTATAC CAACCATACC ACTAGGAATA TTAACCTCAG GAGTTTTACA GCACTTAAAA   
  
  
- TGGTAAAGGA GGAGCTTTGG GAATAGCACG TCGAGTAGTC TATCGAGCCC GTCTACCCAA TCATTCACCT   
  
  
- TCAGTTTAAG ACTAAGTGTC GAGTCGAGCC GGTAATTACG TTCACCAAAT CACCTACCCG GTCCCCTGAG   
  
  
- AGGACACTCA CGAGAATCAC ACAGACTTTA TAAGTTACTG TCAGTCAGTT ACAACGTCAA AGTCTTTCCT   
  
  
- AAACTCCTTC GCTTATTTAA AGAAGGTTTC TCAAGAAACA TGTTCCCAAA ACGGTTGTGC CCTAACGGAA   
  
  
- TAGTCTTCCG TTTGTTATCA CGGGTTCTAA ACAACCAGTT ACATCTCCTA CAATGATCCC CTTTCTTCGT   
  
  
- AGCAATAGGG CTTCTTAACG TCAGTCTTCT TCCCTCCTAT TTATTCGTTA GTCGACAAAG GGACCGTCGA   
  
  
- CTACTCCGTC AACAATCCAG ACTTTACAAG CTATCCCATG ACGAAACATC AGCCCCTTTT GTACTACGAC   
  
  
- GAGAGGCCCT TCGAAATGTC TGACTTGATT TATTCTCAGA CGCTTTACGG GGTCAATTCC CCAGATTATC   
  
  
- ACCCTTTCCG GCACCATTCT TTCGCCCTTT CTTCTCCCTA CATCATCTAA ATTCTAGAGA GAATTGGGAT   
  
  
- ACACGTGTTC GTCAACGTAG CTTACTAGTG TCCTCACGTT TACTGGACGA ATCCGTTTAA TCTGTCGTAA   
  
  
- GAAGAGGATC ACCCCTACCA TTGGTTTCTT ACCGTGTAAT AAAACGCCTA CCAGAACTCC GTGCGGAACG   
  
  
- ACCACATCCT TGAGGATATA TATTGACAGA ACATTGCCCA GGCCGTAGCC GACATCTATA GAACTCTCGA   
  
  
- ATGGTGTACA AAGAACGGTG TACGGGTAAA TTCTTTTACC CTTTAAAGAA GAGATTATCT TGCTAATACC   
  
  
- GACACCGTCT CTTACGTTGT ACGGAAGTAT ATTAACTAGA GCCATAACAG ATACCAAAGG TTACCGGAAC   
  
  
- AGATTAAGTC GCTGAAAGTA GATCCGGACC ACCGGGGGGG TTTGAAGCTT ATTGGCCTCA TCTAGAAGGT   
  
  
- GTTGGACCCA AGGCTGGTCG GTTTTCTCAA CTCCTCTGTC CCGCAGCGAA CTTCTTGATA CGTCTCAGTA   
  
  
- AATTACACGG GAAACTCAAG TTACGATATC GTTTCTTCAC CCTTTGTGAA TGGTAACTTC TAGAGTCCTA   
  
  
- GTTATCGCTA CTCAACGAAC AACAGTGGAC ATACAAATCC AAATTTGTAT ATGGACTCCT TTGTCACTGG   
  
  
- CACCTAACGG GATCCCTATG ACAAGAATTG GACTAACCCG CGTATTTGGG TCGACAAAAG TATGTTCCGT   
  
  
- GACAATTGCC CCGAAAGTTA AGAGGGAAAA AGTATCGAGC TAAAGCTCTC CGAGATAAAG TGAAGAGGTG   
  
  
- AGACAAACTA TACGATCTCC GGTTGGACGG TTCCCTGTTA TTCCTCTCCT ACGATTAACT CTCTCTCTAT   
  
  
- AAACCCTCCG TCCGTTACTT ACACTAACGA ACACTCCCAA ATCTCTCCTA TCTTTCCGGT CTCTGCATGT   
  
  
- TCGTCACCGT TCAGGCTTTA CTTTCCCGTC CCAAATCCGT CGACGGAAAT CTAGCGGTCT AAGACCTTTA   
  
  
- CCGATTTTTC TCCCACTTTA GACACATATT GTTTCTAAAG AGGTAACTAC TTCTGCCCGT GACCAACAAC   
  
  
- GACCCTACCT TCCCGTCTTA ACACATGTGT GAGTGATGAA CCTTCGGACG CCTCAT

+     Myc

| Site Name | Organism | Position | Strand | Matrix score. | sequence | function |
| --- | --- | --- | --- | --- | --- | --- |
| Myc | Arabidopsis thaliana | 3212 | + | 7 | TCTCTTA |  |

>HU06G00568.1   
+ -Up\_Stream \_Len000CCCTGG GTCATTAAAA AAAAAGAAAG AAAGAAGATT GCGCTAAAAG TAGATTTTTA   
  
  
+ TCAGATTCAA GCTGCAGGTG ATAACCAAAT TGTCATTAAA GCAATGCAAG CGCATATCAA TACCCTTTTG   
  
  
+ GGAGATAGCT CCGACTGTTG AAGACATTAG GAGCATGATC TCCAACTGTG AATTTGTTTC ATTTACTCAT   
  
  
+ ATTCATATCT ACCATGTGGA TAATATGACT GCAGACTAGA TGGCCAAATT TGAATGCACA CTTATAACTT   
  
  
+ CAACTCTTTC TACTTTTTTC TTCCCCACCT TATCAGGAAT TTCTTTTAAT TGTCGTGGGA GACAACTTGG   
  
  
+ GTAGAACTCT TGCGAAAAGG GCAACTTAAC GTTTTGGTAC TTGCATTTTC CAAAAAAACA AAAAGGCTAA   
  
  
+ ACAATTCTCC GCATGCTGAC TTCACGGTAG ATATGATGTA TTTTTTAACA AAAGACATCT TCTGAATGAA   
  
  
+ ACGCTTAATA TCTTGGACCA TACTAGAAAT TAAATTGCCA CGGTGCATGA ATTTCACCTC GAACTGCTTG   
  
  
+ TATAAAGATC TTGTTATCAC CTTCAGTAAT CAGATACTTG TAACCAAATT GAATGACCAC AAAGACCACA   
  
  
+ TTCCTCACAA TCGTTGCCTC TAGAATAAGA ATGAATGTTT CTTCAAGGTT GAAAGCCCTT GCTAATGCGA   
  
  
+ ACCTGCCTAT ATGCCTAACT GGCTATATAT ATGTGCGTAG AACAGCATGA CATGAGTTAA TAATATAGCA   
  
  
+ GCTAAAGGGA ACTCTAATGT ACACATGATT GTAATTCACC TGCCTCGCTA GCTTACGAGC ATGACTCTTG   
  
  
+ ACTATTATTG TTTTAATTGA TCTTGAATTT TCATAGATTT TACTCCATTG TTTACTCATT TTGTTATAAT   
  
  
+ TAACCACCTG ATTTTAAACT TTAAAATACA CAAAGATTCA TCTAATATAA TTTTTTAGAT AAAGTTATGC   
  
  
+ ATACATATAT ACAAATTAAA TTATTTACGA TCTAATCATT TCCCGTTATA TATTTCACAC TAAAATTTAG   
  
  
+ AAGAGACAAT CTATTAAAAT TACATCACAT GTTTTGGGGG GGGGGGGATA TCAGATATGC ATGACATTCC   
  
  
+ TAATCCAATA ATGCAATGAA ACCCACCGAA TAGTTGAAAT GATTTGTTCT ATCCATGAAC TCACCAAGTC   
  
  
+ ACAATCCATC ACACTCGAAG GATATGCTTT TCTTTTTGAA GGGAATCTTG CTTAATCAAA CCTATAAACT   
  
  
+ TTTAAAAGCA ACAGACAAAA AAGTGATTAT AATGGTAGTG GTAGGATGAA GGCAGCTTTC AGAAAAGCGA   
  
  
+ TTGAATTTTT ATTGTATCCC ATGAAAACCC ATTGAATTTT TCTTTCTTTC TTTGTTTTTG TTACCTGGGT   
  
  
+ TTATGCAAAG GACGGCTGTT GTATTTAATA ATAACAAAAA CATAGGGCTT TGCGGATTGC TAATGCTGGG   
  
  
+ GATGGCATCA GTGCTCGAAT CTGTGTACGC TGTCATAGGA ACCCACTTGC TTCTGCTCAT TTATTTCTGT   
  
  
+ CTCTAGATCC CCTCCTCTCT CTCTCTCTCT CTCTAAGGTG TCTGCTCGTC TGTTCATATT CTCCTGAGGT   
  
  
+ TCTTCTTCAT CTATCTTGTT CTGGGTGTGT GCTGAGGTAC TTCTTTTCTT TATAAATTTT TCATCTTTTG   
  
  
+ TTGTTTTGCT TGGTGTTTAT GATTTTAGTT TTTGTCTTTA AGACGAGATT TGCTGTTAAT AGCAAGATTT   
  
  
+ CTGAACTAGT ATTGATCCTT TTTGTGGTAT ATTTGTTGAA TTTGATTAAT TTGTGTATTG TGTTTAGCTT   
  
  
+ AAGTAACTCG TTGTGATCTA ATTGATGCTC GGGTTTTGAC AGTTTGATTA GGGTTTTCGG TACTGGAGTT   
  
  
+ AAAGTTTTTT TATTTTTTAT AGGAGGAGTT AAAGGTAATA ATAAGAATTG ATGTTTGCTG ATTTTTCCTC   
  
  
+ TTTTCTTTTT TGGTTTCGAT TTGGTGGGCA GTTATCTGTT TATGGTTTTT GCAAATGGAT CCACAATTAG   
  
  
+ AGGAATTATA TGGACCTTTA CATCGAATCA AGTTCAATGA TCAAAGGGTG CCAATTTTAC CAAGTCGCAG   
  
  
+ TGTTGTTACT CCGATGAAAC TCCAAGATTC CAATTTGAAT CCAAGTGTTC CAAATCCAAG TGTTGTGAAT   
  
  
+ CCTCCTCCGT TGGTTCCCCC AAACCCAAAT CTTAATTTAG TTGTGGCATC TCAATATTCT GACATTGAGA   
  
  
+ CAGCCCTGAA CGAGGATTGT GATTTTAGTG ATGTTGTTCT TAAGTATATT AATCAACTTC TTATGGAAGA   
  
  
+ GGATGGGGAA GAGAAACTCC ATACTGATCA TGAACCTTCA GTCGTTGAGG CGGCAGAGAA GTCATTGTAT   
  
  
+ GAGGCGCTCG GGCAGAGATA TCCTCCTTCC GGAAACCGAA ATCAGTTGCC AGATGTTGAG CATGACGGCT   
  
  
+ TGACTGGGAG CAGTGTTAGG GCTCATAGTG GTGCAAGCAG TGTTAGGGCT CATAGTGACG CAAGTGGTGG   
  
  
+ TAGTGGCCTG ACAGGATATG GTTGGTATGG TGATCCTTAT AATTGGAGTC CTCAAAATGT CGTGAATTTT   
  
  
+ ACCATTTCCT CCTCGAAACC CTTATCGTGC AGCTCATCAG ATAGCTCGGG CAGATGGGTT AGTAAGTGGA   
  
  
+ AGTCAAATTC TGATTCACAG CTCAGCTCGG CCATTAATGC AAGTGGTTTA GTGGATGGGC CAGGGGACTC   
  
  
+ TCCTGTGAGT GCTCTTAGTG TGTCTGAAAT ATTCAATGAC AGTCAGTCAA TGTTGCAGTT TCAGAAAGGA   
  
  
+ TTTGAGGAAG CGAATAAATT TCTTCCAAAG AGTTCTTTGT ACAAGGGTTT TGCCAACACG GGATTGCCTT   
  
  
+ ATCAGAAGGC AAACAATAGT GCCCAAGATT TGTTGGTCAA TGTAGAGGAT GTTACTAGGG GAAAGAAGCA   
  
  
+ TCGTTATCCC GAAGAATTGC AGTCAGAAGA AGGGAGGATA AATAAGCAAT CAGCTGTTTC CCTGGCAGCT   
  
  
+ GATGAGGCAG TTGTTAGGTC TGAAATGTTC GATAGGGTAC TGCTTTGTAG TCGGGGAAAA CATGATGCTG   
  
  
+ CTCTCCGGGA AGCTTTACAG ACTGAACTAA ATAAGAGTCT GCGAAATGCC CCAGTTAAGG GGTCTAATAG   
  
  
+ TGGGAAAGGC CGTGGTAAGA AAGCGGGAAA GAAGAGGGAT GTAGTAGATT TAAGATCTCT CTTAACCCTA   
  
  
+ TGTGCACAAG CAGTTGCATC GAATGATCAC AGGAGTGCAA ATGACCTGCT TAGGCAAATT AGACAGCATT   
  
  
+ CTTCTCCTAG TGGGGATGGT AACCAAAGAA TGGCACATTA TTTTGCGGAT GGTCTTGAGG CACGCCTTGC   
  
  
+ TGGTGTAGGA ACTCCTATAT ATAACTGTCT TGTAACGGGT CCGGCATCGG CTGTAGATAT CTTGAGAGCT   
  
  
+ TACCACATGT TTCTTGCCAC ATGCCCATTT AAGAAAATGG GAAATTTCTT CTCTAATAGA ACGATTATGG   
  
  
+ CTGTGGCAGA GAATGCAACA TGCCTTCATA TAATTGATCT CGGTATTGTC TATGGTTTCC AATGGCCTTG   
  
  
+ TCTAATTCAG CGACTTTCAT CTAGGCCTGG TGGCCCCCCC AAACTTCGAA TAACCGGAGT AGATCTTCCA   
  
  
+ CAACCTGGGT TCCGACCAGC CAAAAGAGTT GAGGAGACAG GGCGTCGCTT GAAGAACTAT GCAGAGTCAT   
  
  
+ TTAATGTGCC CTTTGAGTTC AATGCTATAG CAAAGAAGTG GGAAACACTT ACCATTGAAG ATCTCAGGAT   
  
  
+ CAATAGCGAT GAGTTGCTTG TTGTCACCTG TATGTTTAGG TTTAAACATA TACCTGAGGA AACAGTGACC   
  
  
+ GTGGATTGCC CTAGGGATAC TGTTCTTAAC CTGATTGGGC GCATAAACCC AGCTGTTTTC ATACAAGGCA   
  
  
+ CTGTTAACGG GGCTTTCAAT TCTCCCTTTT TCATAGCTCG ATTTCGAGAG GCTCTATTTC ACTTCTCCAC   
  
  
+ TCTGTTTGAT ATGCTAGAGG CCAACCTGCC AAGGGACAAT AAGGAGAGGA TGCTAATTGA GAGAGAGATA   
  
  
+ TTTGGGAGGC AGGCAATGAA TGTGATTGCT TGTGAGGGTT TAGAGAGGAT AGAAAGGCCA GAGACGTACA   
  
  
+ AGCAGTGGCA AGTCCGAAAT GAAAGGGCAG GGTTTAGGCA GCTGCCTTTA GATCGCCAGA TTCTGGAAAT   
  
  
+ GGCTAAAAAG AGGGTGAAAT CTGTGTATAA CAAAGATTTC TCCATTGATG AAGACGGGCA CTGGTTGTTG   
  
  
+ CTGGGATGGA AGGGCAGAAT TGTGTACACA CTCACTACTT GGAAGCCTGC GGAGTA  

- -Up\_Stream \_Len000GGGACC CAGTAATTTT TTTTTCTTTC TTTCTTCTAA CGCGATTTTC ATCTAAAAAT   
  
  
- AGTCTAAGTT CGACGTCCAC TATTGGTTTA ACAGTAATTT CGTTACGTTC GCGTATAGTT ATGGGAAAAC   
  
  
- CCTCTATCGA GGCTGACAAC TTCTGTAATC CTCGTACTAG AGGTTGACAC TTAAACAAAG TAAATGAGTA   
  
  
- TAAGTATAGA TGGTACACCT ATTATACTGA CGTCTGATCT ACCGGTTTAA ACTTACGTGT GAATATTGAA   
  
  
- GTTGAGAAAG ATGAAAAAAG AAGGGGTGGA ATAGTCCTTA AAGAAAATTA ACAGCACCCT CTGTTGAACC   
  
  
- CATCTTGAGA ACGCTTTTCC CGTTGAATTG CAAAACCATG AACGTAAAAG GTTTTTTTGT TTTTCCGATT   
  
  
- TGTTAAGAGG CGTACGACTG AAGTGCCATC TATACTACAT AAAAAATTGT TTTCTGTAGA AGACTTACTT   
  
  
- TGCGAATTAT AGAACCTGGT ATGATCTTTA ATTTAACGGT GCCACGTACT TAAAGTGGAG CTTGACGAAC   
  
  
- ATATTTCTAG AACAATAGTG GAAGTCATTA GTCTATGAAC ATTGGTTTAA CTTACTGGTG TTTCTGGTGT   
  
  
- AAGGAGTGTT AGCAACGGAG ATCTTATTCT TACTTACAAA GAAGTTCCAA CTTTCGGGAA CGATTACGCT   
  
  
- TGGACGGATA TACGGATTGA CCGATATATA TACACGCATC TTGTCGTACT GTACTCAATT ATTATATCGT   
  
  
- CGATTTCCCT TGAGATTACA TGTGTACTAA CATTAAGTGG ACGGAGCGAT CGAATGCTCG TACTGAGAAC   
  
  
- TGATAATAAC AAAATTAACT AGAACTTAAA AGTATCTAAA ATGAGGTAAC AAATGAGTAA AACAATATTA   
  
  
- ATTGGTGGAC TAAAATTTGA AATTTTATGT GTTTCTAAGT AGATTATATT AAAAAATCTA TTTCAATACG   
  
  
- TATGTATATA TGTTTAATTT AATAAATGCT AGATTAGTAA AGGGCAATAT ATAAAGTGTG ATTTTAAATC   
  
  
- TTCTCTGTTA GATAATTTTA ATGTAGTGTA CAAAACCCCC CCCCCCCTAT AGTCTATACG TACTGTAAGG   
  
  
- ATTAGGTTAT TACGTTACTT TGGGTGGCTT ATCAACTTTA CTAAACAAGA TAGGTACTTG AGTGGTTCAG   
  
  
- TGTTAGGTAG TGTGAGCTTC CTATACGAAA AGAAAAACTT CCCTTAGAAC GAATTAGTTT GGATATTTGA   
  
  
- AAATTTTCGT TGTCTGTTTT TTCACTAATA TTACCATCAC CATCCTACTT CCGTCGAAAG TCTTTTCGCT   
  
  
- AACTTAAAAA TAACATAGGG TACTTTTGGG TAACTTAAAA AGAAAGAAAG AAACAAAAAC AATGGACCCA   
  
  
- AATACGTTTC CTGCCGACAA CATAAATTAT TATTGTTTTT GTATCCCGAA ACGCCTAACG ATTACGACCC   
  
  
- CTACCGTAGT CACGAGCTTA GACACATGCG ACAGTATCCT TGGGTGAACG AAGACGAGTA AATAAAGACA   
  
  
- GAGATCTAGG GGAGGAGAGA GAGAGAGAGA GAGATTCCAC AGACGAGCAG ACAAGTATAA GAGGACTCCA   
  
  
- AGAAGAAGTA GATAGAACAA GACCCACACA CGACTCCATG AAGAAAAGAA ATATTTAAAA AGTAGAAAAC   
  
  
- AACAAAACGA ACCACAAATA CTAAAATCAA AAACAGAAAT TCTGCTCTAA ACGACAATTA TCGTTCTAAA   
  
  
- GACTTGATCA TAACTAGGAA AAACACCATA TAAACAACTT AAACTAATTA AACACATAAC ACAAATCGAA   
  
  
- TTCATTGAGC AACACTAGAT TAACTACGAG CCCAAAACTG TCAAACTAAT CCCAAAAGCC ATGACCTCAA   
  
  
- TTTCAAAAAA ATAAAAAATA TCCTCCTCAA TTTCCATTAT TATTCTTAAC TACAAACGAC TAAAAAGGAG   
  
  
- AAAAGAAAAA ACCAAAGCTA AACCACCCGT CAATAGACAA ATACCAAAAA CGTTTACCTA GGTGTTAATC   
  
  
- TCCTTAATAT ACCTGGAAAT GTAGCTTAGT TCAAGTTACT AGTTTCCCAC GGTTAAAATG GTTCAGCGTC   
  
  
- ACAACAATGA GGCTACTTTG AGGTTCTAAG GTTAAACTTA GGTTCACAAG GTTTAGGTTC ACAACACTTA   
  
  
- GGAGGAGGCA ACCAAGGGGG TTTGGGTTTA GAATTAAATC AACACCGTAG AGTTATAAGA CTGTAACTCT   
  
  
- GTCGGGACTT GCTCCTAACA CTAAAATCAC TACAACAAGA ATTCATATAA TTAGTTGAAG AATACCTTCT   
  
  
- CCTACCCCTT CTCTTTGAGG TATGACTAGT ACTTGGAAGT CAGCAACTCC GCCGTCTCTT CAGTAACATA   
  
  
- CTCCGCGAGC CCGTCTCTAT AGGAGGAAGG CCTTTGGCTT TAGTCAACGG TCTACAACTC GTACTGCCGA   
  
  
- ACTGACCCTC GTCACAATCC CGAGTATCAC CACGTTCGTC ACAATCCCGA GTATCACTGC GTTCACCACC   
  
  
- ATCACCGGAC TGTCCTATAC CAACCATACC ACTAGGAATA TTAACCTCAG GAGTTTTACA GCACTTAAAA   
  
  
- TGGTAAAGGA GGAGCTTTGG GAATAGCACG TCGAGTAGTC TATCGAGCCC GTCTACCCAA TCATTCACCT   
  
  
- TCAGTTTAAG ACTAAGTGTC GAGTCGAGCC GGTAATTACG TTCACCAAAT CACCTACCCG GTCCCCTGAG   
  
  
- AGGACACTCA CGAGAATCAC ACAGACTTTA TAAGTTACTG TCAGTCAGTT ACAACGTCAA AGTCTTTCCT   
  
  
- AAACTCCTTC GCTTATTTAA AGAAGGTTTC TCAAGAAACA TGTTCCCAAA ACGGTTGTGC CCTAACGGAA   
  
  
- TAGTCTTCCG TTTGTTATCA CGGGTTCTAA ACAACCAGTT ACATCTCCTA CAATGATCCC CTTTCTTCGT   
  
  
- AGCAATAGGG CTTCTTAACG TCAGTCTTCT TCCCTCCTAT TTATTCGTTA GTCGACAAAG GGACCGTCGA   
  
  
- CTACTCCGTC AACAATCCAG ACTTTACAAG CTATCCCATG ACGAAACATC AGCCCCTTTT GTACTACGAC   
  
  
- GAGAGGCCCT TCGAAATGTC TGACTTGATT TATTCTCAGA CGCTTTACGG GGTCAATTCC CCAGATTATC   
  
  
- ACCCTTTCCG GCACCATTCT TTCGCCCTTT CTTCTCCCTA CATCATCTAA ATTCTAGAGA GAATTGGGAT   
  
  
- ACACGTGTTC GTCAACGTAG CTTACTAGTG TCCTCACGTT TACTGGACGA ATCCGTTTAA TCTGTCGTAA   
  
  
- GAAGAGGATC ACCCCTACCA TTGGTTTCTT ACCGTGTAAT AAAACGCCTA CCAGAACTCC GTGCGGAACG   
  
  
- ACCACATCCT TGAGGATATA TATTGACAGA ACATTGCCCA GGCCGTAGCC GACATCTATA GAACTCTCGA   
  
  
- ATGGTGTACA AAGAACGGTG TACGGGTAAA TTCTTTTACC CTTTAAAGAA GAGATTATCT TGCTAATACC   
  
  
- GACACCGTCT CTTACGTTGT ACGGAAGTAT ATTAACTAGA GCCATAACAG ATACCAAAGG TTACCGGAAC   
  
  
- AGATTAAGTC GCTGAAAGTA GATCCGGACC ACCGGGGGGG TTTGAAGCTT ATTGGCCTCA TCTAGAAGGT   
  
  
- GTTGGACCCA AGGCTGGTCG GTTTTCTCAA CTCCTCTGTC CCGCAGCGAA CTTCTTGATA CGTCTCAGTA   
  
  
- AATTACACGG GAAACTCAAG TTACGATATC GTTTCTTCAC CCTTTGTGAA TGGTAACTTC TAGAGTCCTA   
  
  
- GTTATCGCTA CTCAACGAAC AACAGTGGAC ATACAAATCC AAATTTGTAT ATGGACTCCT TTGTCACTGG   
  
  
- CACCTAACGG GATCCCTATG ACAAGAATTG GACTAACCCG CGTATTTGGG TCGACAAAAG TATGTTCCGT   
  
  
- GACAATTGCC CCGAAAGTTA AGAGGGAAAA AGTATCGAGC TAAAGCTCTC CGAGATAAAG TGAAGAGGTG   
  
  
- AGACAAACTA TACGATCTCC GGTTGGACGG TTCCCTGTTA TTCCTCTCCT ACGATTAACT CTCTCTCTAT   
  
  
- AAACCCTCCG TCCGTTACTT ACACTAACGA ACACTCCCAA ATCTCTCCTA TCTTTCCGGT CTCTGCATGT   
  
  
- TCGTCACCGT TCAGGCTTTA CTTTCCCGTC CCAAATCCGT CGACGGAAAT CTAGCGGTCT AAGACCTTTA   
  
  
- CCGATTTTTC TCCCACTTTA GACACATATT GTTTCTAAAG AGGTAACTAC TTCTGCCCGT GACCAACAAC   
  
  
- GACCCTACCT TCCCGTCTTA ACACATGTGT GAGTGATGAA CCTTCGGACG CCTCAT

+     P-box

| Site Name | Organism | Position | Strand | Matrix score. | sequence | function |
| --- | --- | --- | --- | --- | --- | --- |
| P-box | Oryza sativa | 138 | + | 7 | CCTTTTG | gibberellin-responsive element |

>HU06G00568.1   
+ -Up\_Stream \_Len000CCCTGG GTCATTAAAA AAAAAGAAAG AAAGAAGATT GCGCTAAAAG TAGATTTTTA   
  
  
+ TCAGATTCAA GCTGCAGGTG ATAACCAAAT TGTCATTAAA GCAATGCAAG CGCATATCAA TACCCTTTTG   
  
  
+ GGAGATAGCT CCGACTGTTG AAGACATTAG GAGCATGATC TCCAACTGTG AATTTGTTTC ATTTACTCAT   
  
  
+ ATTCATATCT ACCATGTGGA TAATATGACT GCAGACTAGA TGGCCAAATT TGAATGCACA CTTATAACTT   
  
  
+ CAACTCTTTC TACTTTTTTC TTCCCCACCT TATCAGGAAT TTCTTTTAAT TGTCGTGGGA GACAACTTGG   
  
  
+ GTAGAACTCT TGCGAAAAGG GCAACTTAAC GTTTTGGTAC TTGCATTTTC CAAAAAAACA AAAAGGCTAA   
  
  
+ ACAATTCTCC GCATGCTGAC TTCACGGTAG ATATGATGTA TTTTTTAACA AAAGACATCT TCTGAATGAA   
  
  
+ ACGCTTAATA TCTTGGACCA TACTAGAAAT TAAATTGCCA CGGTGCATGA ATTTCACCTC GAACTGCTTG   
  
  
+ TATAAAGATC TTGTTATCAC CTTCAGTAAT CAGATACTTG TAACCAAATT GAATGACCAC AAAGACCACA   
  
  
+ TTCCTCACAA TCGTTGCCTC TAGAATAAGA ATGAATGTTT CTTCAAGGTT GAAAGCCCTT GCTAATGCGA   
  
  
+ ACCTGCCTAT ATGCCTAACT GGCTATATAT ATGTGCGTAG AACAGCATGA CATGAGTTAA TAATATAGCA   
  
  
+ GCTAAAGGGA ACTCTAATGT ACACATGATT GTAATTCACC TGCCTCGCTA GCTTACGAGC ATGACTCTTG   
  
  
+ ACTATTATTG TTTTAATTGA TCTTGAATTT TCATAGATTT TACTCCATTG TTTACTCATT TTGTTATAAT   
  
  
+ TAACCACCTG ATTTTAAACT TTAAAATACA CAAAGATTCA TCTAATATAA TTTTTTAGAT AAAGTTATGC   
  
  
+ ATACATATAT ACAAATTAAA TTATTTACGA TCTAATCATT TCCCGTTATA TATTTCACAC TAAAATTTAG   
  
  
+ AAGAGACAAT CTATTAAAAT TACATCACAT GTTTTGGGGG GGGGGGGATA TCAGATATGC ATGACATTCC   
  
  
+ TAATCCAATA ATGCAATGAA ACCCACCGAA TAGTTGAAAT GATTTGTTCT ATCCATGAAC TCACCAAGTC   
  
  
+ ACAATCCATC ACACTCGAAG GATATGCTTT TCTTTTTGAA GGGAATCTTG CTTAATCAAA CCTATAAACT   
  
  
+ TTTAAAAGCA ACAGACAAAA AAGTGATTAT AATGGTAGTG GTAGGATGAA GGCAGCTTTC AGAAAAGCGA   
  
  
+ TTGAATTTTT ATTGTATCCC ATGAAAACCC ATTGAATTTT TCTTTCTTTC TTTGTTTTTG TTACCTGGGT   
  
  
+ TTATGCAAAG GACGGCTGTT GTATTTAATA ATAACAAAAA CATAGGGCTT TGCGGATTGC TAATGCTGGG   
  
  
+ GATGGCATCA GTGCTCGAAT CTGTGTACGC TGTCATAGGA ACCCACTTGC TTCTGCTCAT TTATTTCTGT   
  
  
+ CTCTAGATCC CCTCCTCTCT CTCTCTCTCT CTCTAAGGTG TCTGCTCGTC TGTTCATATT CTCCTGAGGT   
  
  
+ TCTTCTTCAT CTATCTTGTT CTGGGTGTGT GCTGAGGTAC TTCTTTTCTT TATAAATTTT TCATCTTTTG   
  
  
+ TTGTTTTGCT TGGTGTTTAT GATTTTAGTT TTTGTCTTTA AGACGAGATT TGCTGTTAAT AGCAAGATTT   
  
  
+ CTGAACTAGT ATTGATCCTT TTTGTGGTAT ATTTGTTGAA TTTGATTAAT TTGTGTATTG TGTTTAGCTT   
  
  
+ AAGTAACTCG TTGTGATCTA ATTGATGCTC GGGTTTTGAC AGTTTGATTA GGGTTTTCGG TACTGGAGTT   
  
  
+ AAAGTTTTTT TATTTTTTAT AGGAGGAGTT AAAGGTAATA ATAAGAATTG ATGTTTGCTG ATTTTTCCTC   
  
  
+ TTTTCTTTTT TGGTTTCGAT TTGGTGGGCA GTTATCTGTT TATGGTTTTT GCAAATGGAT CCACAATTAG   
  
  
+ AGGAATTATA TGGACCTTTA CATCGAATCA AGTTCAATGA TCAAAGGGTG CCAATTTTAC CAAGTCGCAG   
  
  
+ TGTTGTTACT CCGATGAAAC TCCAAGATTC CAATTTGAAT CCAAGTGTTC CAAATCCAAG TGTTGTGAAT   
  
  
+ CCTCCTCCGT TGGTTCCCCC AAACCCAAAT CTTAATTTAG TTGTGGCATC TCAATATTCT GACATTGAGA   
  
  
+ CAGCCCTGAA CGAGGATTGT GATTTTAGTG ATGTTGTTCT TAAGTATATT AATCAACTTC TTATGGAAGA   
  
  
+ GGATGGGGAA GAGAAACTCC ATACTGATCA TGAACCTTCA GTCGTTGAGG CGGCAGAGAA GTCATTGTAT   
  
  
+ GAGGCGCTCG GGCAGAGATA TCCTCCTTCC GGAAACCGAA ATCAGTTGCC AGATGTTGAG CATGACGGCT   
  
  
+ TGACTGGGAG CAGTGTTAGG GCTCATAGTG GTGCAAGCAG TGTTAGGGCT CATAGTGACG CAAGTGGTGG   
  
  
+ TAGTGGCCTG ACAGGATATG GTTGGTATGG TGATCCTTAT AATTGGAGTC CTCAAAATGT CGTGAATTTT   
  
  
+ ACCATTTCCT CCTCGAAACC CTTATCGTGC AGCTCATCAG ATAGCTCGGG CAGATGGGTT AGTAAGTGGA   
  
  
+ AGTCAAATTC TGATTCACAG CTCAGCTCGG CCATTAATGC AAGTGGTTTA GTGGATGGGC CAGGGGACTC   
  
  
+ TCCTGTGAGT GCTCTTAGTG TGTCTGAAAT ATTCAATGAC AGTCAGTCAA TGTTGCAGTT TCAGAAAGGA   
  
  
+ TTTGAGGAAG CGAATAAATT TCTTCCAAAG AGTTCTTTGT ACAAGGGTTT TGCCAACACG GGATTGCCTT   
  
  
+ ATCAGAAGGC AAACAATAGT GCCCAAGATT TGTTGGTCAA TGTAGAGGAT GTTACTAGGG GAAAGAAGCA   
  
  
+ TCGTTATCCC GAAGAATTGC AGTCAGAAGA AGGGAGGATA AATAAGCAAT CAGCTGTTTC CCTGGCAGCT   
  
  
+ GATGAGGCAG TTGTTAGGTC TGAAATGTTC GATAGGGTAC TGCTTTGTAG TCGGGGAAAA CATGATGCTG   
  
  
+ CTCTCCGGGA AGCTTTACAG ACTGAACTAA ATAAGAGTCT GCGAAATGCC CCAGTTAAGG GGTCTAATAG   
  
  
+ TGGGAAAGGC CGTGGTAAGA AAGCGGGAAA GAAGAGGGAT GTAGTAGATT TAAGATCTCT CTTAACCCTA   
  
  
+ TGTGCACAAG CAGTTGCATC GAATGATCAC AGGAGTGCAA ATGACCTGCT TAGGCAAATT AGACAGCATT   
  
  
+ CTTCTCCTAG TGGGGATGGT AACCAAAGAA TGGCACATTA TTTTGCGGAT GGTCTTGAGG CACGCCTTGC   
  
  
+ TGGTGTAGGA ACTCCTATAT ATAACTGTCT TGTAACGGGT CCGGCATCGG CTGTAGATAT CTTGAGAGCT   
  
  
+ TACCACATGT TTCTTGCCAC ATGCCCATTT AAGAAAATGG GAAATTTCTT CTCTAATAGA ACGATTATGG   
  
  
+ CTGTGGCAGA GAATGCAACA TGCCTTCATA TAATTGATCT CGGTATTGTC TATGGTTTCC AATGGCCTTG   
  
  
+ TCTAATTCAG CGACTTTCAT CTAGGCCTGG TGGCCCCCCC AAACTTCGAA TAACCGGAGT AGATCTTCCA   
  
  
+ CAACCTGGGT TCCGACCAGC CAAAAGAGTT GAGGAGACAG GGCGTCGCTT GAAGAACTAT GCAGAGTCAT   
  
  
+ TTAATGTGCC CTTTGAGTTC AATGCTATAG CAAAGAAGTG GGAAACACTT ACCATTGAAG ATCTCAGGAT   
  
  
+ CAATAGCGAT GAGTTGCTTG TTGTCACCTG TATGTTTAGG TTTAAACATA TACCTGAGGA AACAGTGACC   
  
  
+ GTGGATTGCC CTAGGGATAC TGTTCTTAAC CTGATTGGGC GCATAAACCC AGCTGTTTTC ATACAAGGCA   
  
  
+ CTGTTAACGG GGCTTTCAAT TCTCCCTTTT TCATAGCTCG ATTTCGAGAG GCTCTATTTC ACTTCTCCAC   
  
  
+ TCTGTTTGAT ATGCTAGAGG CCAACCTGCC AAGGGACAAT AAGGAGAGGA TGCTAATTGA GAGAGAGATA   
  
  
+ TTTGGGAGGC AGGCAATGAA TGTGATTGCT TGTGAGGGTT TAGAGAGGAT AGAAAGGCCA GAGACGTACA   
  
  
+ AGCAGTGGCA AGTCCGAAAT GAAAGGGCAG GGTTTAGGCA GCTGCCTTTA GATCGCCAGA TTCTGGAAAT   
  
  
+ GGCTAAAAAG AGGGTGAAAT CTGTGTATAA CAAAGATTTC TCCATTGATG AAGACGGGCA CTGGTTGTTG   
  
  
+ CTGGGATGGA AGGGCAGAAT TGTGTACACA CTCACTACTT GGAAGCCTGC GGAGTA  

- -Up\_Stream \_Len000GGGACC CAGTAATTTT TTTTTCTTTC TTTCTTCTAA CGCGATTTTC ATCTAAAAAT   
  
  
- AGTCTAAGTT CGACGTCCAC TATTGGTTTA ACAGTAATTT CGTTACGTTC GCGTATAGTT ATGGGAAAAC   
  
  
- CCTCTATCGA GGCTGACAAC TTCTGTAATC CTCGTACTAG AGGTTGACAC TTAAACAAAG TAAATGAGTA   
  
  
- TAAGTATAGA TGGTACACCT ATTATACTGA CGTCTGATCT ACCGGTTTAA ACTTACGTGT GAATATTGAA   
  
  
- GTTGAGAAAG ATGAAAAAAG AAGGGGTGGA ATAGTCCTTA AAGAAAATTA ACAGCACCCT CTGTTGAACC   
  
  
- CATCTTGAGA ACGCTTTTCC CGTTGAATTG CAAAACCATG AACGTAAAAG GTTTTTTTGT TTTTCCGATT   
  
  
- TGTTAAGAGG CGTACGACTG AAGTGCCATC TATACTACAT AAAAAATTGT TTTCTGTAGA AGACTTACTT   
  
  
- TGCGAATTAT AGAACCTGGT ATGATCTTTA ATTTAACGGT GCCACGTACT TAAAGTGGAG CTTGACGAAC   
  
  
- ATATTTCTAG AACAATAGTG GAAGTCATTA GTCTATGAAC ATTGGTTTAA CTTACTGGTG TTTCTGGTGT   
  
  
- AAGGAGTGTT AGCAACGGAG ATCTTATTCT TACTTACAAA GAAGTTCCAA CTTTCGGGAA CGATTACGCT   
  
  
- TGGACGGATA TACGGATTGA CCGATATATA TACACGCATC TTGTCGTACT GTACTCAATT ATTATATCGT   
  
  
- CGATTTCCCT TGAGATTACA TGTGTACTAA CATTAAGTGG ACGGAGCGAT CGAATGCTCG TACTGAGAAC   
  
  
- TGATAATAAC AAAATTAACT AGAACTTAAA AGTATCTAAA ATGAGGTAAC AAATGAGTAA AACAATATTA   
  
  
- ATTGGTGGAC TAAAATTTGA AATTTTATGT GTTTCTAAGT AGATTATATT AAAAAATCTA TTTCAATACG   
  
  
- TATGTATATA TGTTTAATTT AATAAATGCT AGATTAGTAA AGGGCAATAT ATAAAGTGTG ATTTTAAATC   
  
  
- TTCTCTGTTA GATAATTTTA ATGTAGTGTA CAAAACCCCC CCCCCCCTAT AGTCTATACG TACTGTAAGG   
  
  
- ATTAGGTTAT TACGTTACTT TGGGTGGCTT ATCAACTTTA CTAAACAAGA TAGGTACTTG AGTGGTTCAG   
  
  
- TGTTAGGTAG TGTGAGCTTC CTATACGAAA AGAAAAACTT CCCTTAGAAC GAATTAGTTT GGATATTTGA   
  
  
- AAATTTTCGT TGTCTGTTTT TTCACTAATA TTACCATCAC CATCCTACTT CCGTCGAAAG TCTTTTCGCT   
  
  
- AACTTAAAAA TAACATAGGG TACTTTTGGG TAACTTAAAA AGAAAGAAAG AAACAAAAAC AATGGACCCA   
  
  
- AATACGTTTC CTGCCGACAA CATAAATTAT TATTGTTTTT GTATCCCGAA ACGCCTAACG ATTACGACCC   
  
  
- CTACCGTAGT CACGAGCTTA GACACATGCG ACAGTATCCT TGGGTGAACG AAGACGAGTA AATAAAGACA   
  
  
- GAGATCTAGG GGAGGAGAGA GAGAGAGAGA GAGATTCCAC AGACGAGCAG ACAAGTATAA GAGGACTCCA   
  
  
- AGAAGAAGTA GATAGAACAA GACCCACACA CGACTCCATG AAGAAAAGAA ATATTTAAAA AGTAGAAAAC   
  
  
- AACAAAACGA ACCACAAATA CTAAAATCAA AAACAGAAAT TCTGCTCTAA ACGACAATTA TCGTTCTAAA   
  
  
- GACTTGATCA TAACTAGGAA AAACACCATA TAAACAACTT AAACTAATTA AACACATAAC ACAAATCGAA   
  
  
- TTCATTGAGC AACACTAGAT TAACTACGAG CCCAAAACTG TCAAACTAAT CCCAAAAGCC ATGACCTCAA   
  
  
- TTTCAAAAAA ATAAAAAATA TCCTCCTCAA TTTCCATTAT TATTCTTAAC TACAAACGAC TAAAAAGGAG   
  
  
- AAAAGAAAAA ACCAAAGCTA AACCACCCGT CAATAGACAA ATACCAAAAA CGTTTACCTA GGTGTTAATC   
  
  
- TCCTTAATAT ACCTGGAAAT GTAGCTTAGT TCAAGTTACT AGTTTCCCAC GGTTAAAATG GTTCAGCGTC   
  
  
- ACAACAATGA GGCTACTTTG AGGTTCTAAG GTTAAACTTA GGTTCACAAG GTTTAGGTTC ACAACACTTA   
  
  
- GGAGGAGGCA ACCAAGGGGG TTTGGGTTTA GAATTAAATC AACACCGTAG AGTTATAAGA CTGTAACTCT   
  
  
- GTCGGGACTT GCTCCTAACA CTAAAATCAC TACAACAAGA ATTCATATAA TTAGTTGAAG AATACCTTCT   
  
  
- CCTACCCCTT CTCTTTGAGG TATGACTAGT ACTTGGAAGT CAGCAACTCC GCCGTCTCTT CAGTAACATA   
  
  
- CTCCGCGAGC CCGTCTCTAT AGGAGGAAGG CCTTTGGCTT TAGTCAACGG TCTACAACTC GTACTGCCGA   
  
  
- ACTGACCCTC GTCACAATCC CGAGTATCAC CACGTTCGTC ACAATCCCGA GTATCACTGC GTTCACCACC   
  
  
- ATCACCGGAC TGTCCTATAC CAACCATACC ACTAGGAATA TTAACCTCAG GAGTTTTACA GCACTTAAAA   
  
  
- TGGTAAAGGA GGAGCTTTGG GAATAGCACG TCGAGTAGTC TATCGAGCCC GTCTACCCAA TCATTCACCT   
  
  
- TCAGTTTAAG ACTAAGTGTC GAGTCGAGCC GGTAATTACG TTCACCAAAT CACCTACCCG GTCCCCTGAG   
  
  
- AGGACACTCA CGAGAATCAC ACAGACTTTA TAAGTTACTG TCAGTCAGTT ACAACGTCAA AGTCTTTCCT   
  
  
- AAACTCCTTC GCTTATTTAA AGAAGGTTTC TCAAGAAACA TGTTCCCAAA ACGGTTGTGC CCTAACGGAA   
  
  
- TAGTCTTCCG TTTGTTATCA CGGGTTCTAA ACAACCAGTT ACATCTCCTA CAATGATCCC CTTTCTTCGT   
  
  
- AGCAATAGGG CTTCTTAACG TCAGTCTTCT TCCCTCCTAT TTATTCGTTA GTCGACAAAG GGACCGTCGA   
  
  
- CTACTCCGTC AACAATCCAG ACTTTACAAG CTATCCCATG ACGAAACATC AGCCCCTTTT GTACTACGAC   
  
  
- GAGAGGCCCT TCGAAATGTC TGACTTGATT TATTCTCAGA CGCTTTACGG GGTCAATTCC CCAGATTATC   
  
  
- ACCCTTTCCG GCACCATTCT TTCGCCCTTT CTTCTCCCTA CATCATCTAA ATTCTAGAGA GAATTGGGAT   
  
  
- ACACGTGTTC GTCAACGTAG CTTACTAGTG TCCTCACGTT TACTGGACGA ATCCGTTTAA TCTGTCGTAA   
  
  
- GAAGAGGATC ACCCCTACCA TTGGTTTCTT ACCGTGTAAT AAAACGCCTA CCAGAACTCC GTGCGGAACG   
  
  
- ACCACATCCT TGAGGATATA TATTGACAGA ACATTGCCCA GGCCGTAGCC GACATCTATA GAACTCTCGA   
  
  
- ATGGTGTACA AAGAACGGTG TACGGGTAAA TTCTTTTACC CTTTAAAGAA GAGATTATCT TGCTAATACC   
  
  
- GACACCGTCT CTTACGTTGT ACGGAAGTAT ATTAACTAGA GCCATAACAG ATACCAAAGG TTACCGGAAC   
  
  
- AGATTAAGTC GCTGAAAGTA GATCCGGACC ACCGGGGGGG TTTGAAGCTT ATTGGCCTCA TCTAGAAGGT   
  
  
- GTTGGACCCA AGGCTGGTCG GTTTTCTCAA CTCCTCTGTC CCGCAGCGAA CTTCTTGATA CGTCTCAGTA   
  
  
- AATTACACGG GAAACTCAAG TTACGATATC GTTTCTTCAC CCTTTGTGAA TGGTAACTTC TAGAGTCCTA   
  
  
- GTTATCGCTA CTCAACGAAC AACAGTGGAC ATACAAATCC AAATTTGTAT ATGGACTCCT TTGTCACTGG   
  
  
- CACCTAACGG GATCCCTATG ACAAGAATTG GACTAACCCG CGTATTTGGG TCGACAAAAG TATGTTCCGT   
  
  
- GACAATTGCC CCGAAAGTTA AGAGGGAAAA AGTATCGAGC TAAAGCTCTC CGAGATAAAG TGAAGAGGTG   
  
  
- AGACAAACTA TACGATCTCC GGTTGGACGG TTCCCTGTTA TTCCTCTCCT ACGATTAACT CTCTCTCTAT   
  
  
- AAACCCTCCG TCCGTTACTT ACACTAACGA ACACTCCCAA ATCTCTCCTA TCTTTCCGGT CTCTGCATGT   
  
  
- TCGTCACCGT TCAGGCTTTA CTTTCCCGTC CCAAATCCGT CGACGGAAAT CTAGCGGTCT AAGACCTTTA   
  
  
- CCGATTTTTC TCCCACTTTA GACACATATT GTTTCTAAAG AGGTAACTAC TTCTGCCCGT GACCAACAAC   
  
  
- GACCCTACCT TCCCGTCTTA ACACATGTGT GAGTGATGAA CCTTCGGACG CCTCAT

+     STRE

| Site Name | Organism | Position | Strand | Matrix score. | sequence | function |
| --- | --- | --- | --- | --- | --- | --- |
| STRE | Arabidopsis thaliana | 2931 | + | 5 | AGGGG |  |
| STRE | Arabidopsis thaliana | 3142 | + | 5 | AGGGG |  |
| STRE | Arabidopsis thaliana | 2726 | + | 5 | AGGGG |  |
| STRE | Arabidopsis thaliana | 1553 | - | 5 | AGGGG |  |

>HU06G00568.1   
+ -Up\_Stream \_Len000CCCTGG GTCATTAAAA AAAAAGAAAG AAAGAAGATT GCGCTAAAAG TAGATTTTTA   
  
  
+ TCAGATTCAA GCTGCAGGTG ATAACCAAAT TGTCATTAAA GCAATGCAAG CGCATATCAA TACCCTTTTG   
  
  
+ GGAGATAGCT CCGACTGTTG AAGACATTAG GAGCATGATC TCCAACTGTG AATTTGTTTC ATTTACTCAT   
  
  
+ ATTCATATCT ACCATGTGGA TAATATGACT GCAGACTAGA TGGCCAAATT TGAATGCACA CTTATAACTT   
  
  
+ CAACTCTTTC TACTTTTTTC TTCCCCACCT TATCAGGAAT TTCTTTTAAT TGTCGTGGGA GACAACTTGG   
  
  
+ GTAGAACTCT TGCGAAAAGG GCAACTTAAC GTTTTGGTAC TTGCATTTTC CAAAAAAACA AAAAGGCTAA   
  
  
+ ACAATTCTCC GCATGCTGAC TTCACGGTAG ATATGATGTA TTTTTTAACA AAAGACATCT TCTGAATGAA   
  
  
+ ACGCTTAATA TCTTGGACCA TACTAGAAAT TAAATTGCCA CGGTGCATGA ATTTCACCTC GAACTGCTTG   
  
  
+ TATAAAGATC TTGTTATCAC CTTCAGTAAT CAGATACTTG TAACCAAATT GAATGACCAC AAAGACCACA   
  
  
+ TTCCTCACAA TCGTTGCCTC TAGAATAAGA ATGAATGTTT CTTCAAGGTT GAAAGCCCTT GCTAATGCGA   
  
  
+ ACCTGCCTAT ATGCCTAACT GGCTATATAT ATGTGCGTAG AACAGCATGA CATGAGTTAA TAATATAGCA   
  
  
+ GCTAAAGGGA ACTCTAATGT ACACATGATT GTAATTCACC TGCCTCGCTA GCTTACGAGC ATGACTCTTG   
  
  
+ ACTATTATTG TTTTAATTGA TCTTGAATTT TCATAGATTT TACTCCATTG TTTACTCATT TTGTTATAAT   
  
  
+ TAACCACCTG ATTTTAAACT TTAAAATACA CAAAGATTCA TCTAATATAA TTTTTTAGAT AAAGTTATGC   
  
  
+ ATACATATAT ACAAATTAAA TTATTTACGA TCTAATCATT TCCCGTTATA TATTTCACAC TAAAATTTAG   
  
  
+ AAGAGACAAT CTATTAAAAT TACATCACAT GTTTTGGGGG GGGGGGGATA TCAGATATGC ATGACATTCC   
  
  
+ TAATCCAATA ATGCAATGAA ACCCACCGAA TAGTTGAAAT GATTTGTTCT ATCCATGAAC TCACCAAGTC   
  
  
+ ACAATCCATC ACACTCGAAG GATATGCTTT TCTTTTTGAA GGGAATCTTG CTTAATCAAA CCTATAAACT   
  
  
+ TTTAAAAGCA ACAGACAAAA AAGTGATTAT AATGGTAGTG GTAGGATGAA GGCAGCTTTC AGAAAAGCGA   
  
  
+ TTGAATTTTT ATTGTATCCC ATGAAAACCC ATTGAATTTT TCTTTCTTTC TTTGTTTTTG TTACCTGGGT   
  
  
+ TTATGCAAAG GACGGCTGTT GTATTTAATA ATAACAAAAA CATAGGGCTT TGCGGATTGC TAATGCTGGG   
  
  
+ GATGGCATCA GTGCTCGAAT CTGTGTACGC TGTCATAGGA ACCCACTTGC TTCTGCTCAT TTATTTCTGT   
  
  
+ CTCTAGATCC CCTCCTCTCT CTCTCTCTCT CTCTAAGGTG TCTGCTCGTC TGTTCATATT CTCCTGAGGT   
  
  
+ TCTTCTTCAT CTATCTTGTT CTGGGTGTGT GCTGAGGTAC TTCTTTTCTT TATAAATTTT TCATCTTTTG   
  
  
+ TTGTTTTGCT TGGTGTTTAT GATTTTAGTT TTTGTCTTTA AGACGAGATT TGCTGTTAAT AGCAAGATTT   
  
  
+ CTGAACTAGT ATTGATCCTT TTTGTGGTAT ATTTGTTGAA TTTGATTAAT TTGTGTATTG TGTTTAGCTT   
  
  
+ AAGTAACTCG TTGTGATCTA ATTGATGCTC GGGTTTTGAC AGTTTGATTA GGGTTTTCGG TACTGGAGTT   
  
  
+ AAAGTTTTTT TATTTTTTAT AGGAGGAGTT AAAGGTAATA ATAAGAATTG ATGTTTGCTG ATTTTTCCTC   
  
  
+ TTTTCTTTTT TGGTTTCGAT TTGGTGGGCA GTTATCTGTT TATGGTTTTT GCAAATGGAT CCACAATTAG   
  
  
+ AGGAATTATA TGGACCTTTA CATCGAATCA AGTTCAATGA TCAAAGGGTG CCAATTTTAC CAAGTCGCAG   
  
  
+ TGTTGTTACT CCGATGAAAC TCCAAGATTC CAATTTGAAT CCAAGTGTTC CAAATCCAAG TGTTGTGAAT   
  
  
+ CCTCCTCCGT TGGTTCCCCC AAACCCAAAT CTTAATTTAG TTGTGGCATC TCAATATTCT GACATTGAGA   
  
  
+ CAGCCCTGAA CGAGGATTGT GATTTTAGTG ATGTTGTTCT TAAGTATATT AATCAACTTC TTATGGAAGA   
  
  
+ GGATGGGGAA GAGAAACTCC ATACTGATCA TGAACCTTCA GTCGTTGAGG CGGCAGAGAA GTCATTGTAT   
  
  
+ GAGGCGCTCG GGCAGAGATA TCCTCCTTCC GGAAACCGAA ATCAGTTGCC AGATGTTGAG CATGACGGCT   
  
  
+ TGACTGGGAG CAGTGTTAGG GCTCATAGTG GTGCAAGCAG TGTTAGGGCT CATAGTGACG CAAGTGGTGG   
  
  
+ TAGTGGCCTG ACAGGATATG GTTGGTATGG TGATCCTTAT AATTGGAGTC CTCAAAATGT CGTGAATTTT   
  
  
+ ACCATTTCCT CCTCGAAACC CTTATCGTGC AGCTCATCAG ATAGCTCGGG CAGATGGGTT AGTAAGTGGA   
  
  
+ AGTCAAATTC TGATTCACAG CTCAGCTCGG CCATTAATGC AAGTGGTTTA GTGGATGGGC CAGGGGACTC   
  
  
+ TCCTGTGAGT GCTCTTAGTG TGTCTGAAAT ATTCAATGAC AGTCAGTCAA TGTTGCAGTT TCAGAAAGGA   
  
  
+ TTTGAGGAAG CGAATAAATT TCTTCCAAAG AGTTCTTTGT ACAAGGGTTT TGCCAACACG GGATTGCCTT   
  
  
+ ATCAGAAGGC AAACAATAGT GCCCAAGATT TGTTGGTCAA TGTAGAGGAT GTTACTAGGG GAAAGAAGCA   
  
  
+ TCGTTATCCC GAAGAATTGC AGTCAGAAGA AGGGAGGATA AATAAGCAAT CAGCTGTTTC CCTGGCAGCT   
  
  
+ GATGAGGCAG TTGTTAGGTC TGAAATGTTC GATAGGGTAC TGCTTTGTAG TCGGGGAAAA CATGATGCTG   
  
  
+ CTCTCCGGGA AGCTTTACAG ACTGAACTAA ATAAGAGTCT GCGAAATGCC CCAGTTAAGG GGTCTAATAG   
  
  
+ TGGGAAAGGC CGTGGTAAGA AAGCGGGAAA GAAGAGGGAT GTAGTAGATT TAAGATCTCT CTTAACCCTA   
  
  
+ TGTGCACAAG CAGTTGCATC GAATGATCAC AGGAGTGCAA ATGACCTGCT TAGGCAAATT AGACAGCATT   
  
  
+ CTTCTCCTAG TGGGGATGGT AACCAAAGAA TGGCACATTA TTTTGCGGAT GGTCTTGAGG CACGCCTTGC   
  
  
+ TGGTGTAGGA ACTCCTATAT ATAACTGTCT TGTAACGGGT CCGGCATCGG CTGTAGATAT CTTGAGAGCT   
  
  
+ TACCACATGT TTCTTGCCAC ATGCCCATTT AAGAAAATGG GAAATTTCTT CTCTAATAGA ACGATTATGG   
  
  
+ CTGTGGCAGA GAATGCAACA TGCCTTCATA TAATTGATCT CGGTATTGTC TATGGTTTCC AATGGCCTTG   
  
  
+ TCTAATTCAG CGACTTTCAT CTAGGCCTGG TGGCCCCCCC AAACTTCGAA TAACCGGAGT AGATCTTCCA   
  
  
+ CAACCTGGGT TCCGACCAGC CAAAAGAGTT GAGGAGACAG GGCGTCGCTT GAAGAACTAT GCAGAGTCAT   
  
  
+ TTAATGTGCC CTTTGAGTTC AATGCTATAG CAAAGAAGTG GGAAACACTT ACCATTGAAG ATCTCAGGAT   
  
  
+ CAATAGCGAT GAGTTGCTTG TTGTCACCTG TATGTTTAGG TTTAAACATA TACCTGAGGA AACAGTGACC   
  
  
+ GTGGATTGCC CTAGGGATAC TGTTCTTAAC CTGATTGGGC GCATAAACCC AGCTGTTTTC ATACAAGGCA   
  
  
+ CTGTTAACGG GGCTTTCAAT TCTCCCTTTT TCATAGCTCG ATTTCGAGAG GCTCTATTTC ACTTCTCCAC   
  
  
+ TCTGTTTGAT ATGCTAGAGG CCAACCTGCC AAGGGACAAT AAGGAGAGGA TGCTAATTGA GAGAGAGATA   
  
  
+ TTTGGGAGGC AGGCAATGAA TGTGATTGCT TGTGAGGGTT TAGAGAGGAT AGAAAGGCCA GAGACGTACA   
  
  
+ AGCAGTGGCA AGTCCGAAAT GAAAGGGCAG GGTTTAGGCA GCTGCCTTTA GATCGCCAGA TTCTGGAAAT   
  
  
+ GGCTAAAAAG AGGGTGAAAT CTGTGTATAA CAAAGATTTC TCCATTGATG AAGACGGGCA CTGGTTGTTG   
  
  
+ CTGGGATGGA AGGGCAGAAT TGTGTACACA CTCACTACTT GGAAGCCTGC GGAGTA  

- -Up\_Stream \_Len000GGGACC CAGTAATTTT TTTTTCTTTC TTTCTTCTAA CGCGATTTTC ATCTAAAAAT   
  
  
- AGTCTAAGTT CGACGTCCAC TATTGGTTTA ACAGTAATTT CGTTACGTTC GCGTATAGTT ATGGGAAAAC   
  
  
- CCTCTATCGA GGCTGACAAC TTCTGTAATC CTCGTACTAG AGGTTGACAC TTAAACAAAG TAAATGAGTA   
  
  
- TAAGTATAGA TGGTACACCT ATTATACTGA CGTCTGATCT ACCGGTTTAA ACTTACGTGT GAATATTGAA   
  
  
- GTTGAGAAAG ATGAAAAAAG AAGGGGTGGA ATAGTCCTTA AAGAAAATTA ACAGCACCCT CTGTTGAACC   
  
  
- CATCTTGAGA ACGCTTTTCC CGTTGAATTG CAAAACCATG AACGTAAAAG GTTTTTTTGT TTTTCCGATT   
  
  
- TGTTAAGAGG CGTACGACTG AAGTGCCATC TATACTACAT AAAAAATTGT TTTCTGTAGA AGACTTACTT   
  
  
- TGCGAATTAT AGAACCTGGT ATGATCTTTA ATTTAACGGT GCCACGTACT TAAAGTGGAG CTTGACGAAC   
  
  
- ATATTTCTAG AACAATAGTG GAAGTCATTA GTCTATGAAC ATTGGTTTAA CTTACTGGTG TTTCTGGTGT   
  
  
- AAGGAGTGTT AGCAACGGAG ATCTTATTCT TACTTACAAA GAAGTTCCAA CTTTCGGGAA CGATTACGCT   
  
  
- TGGACGGATA TACGGATTGA CCGATATATA TACACGCATC TTGTCGTACT GTACTCAATT ATTATATCGT   
  
  
- CGATTTCCCT TGAGATTACA TGTGTACTAA CATTAAGTGG ACGGAGCGAT CGAATGCTCG TACTGAGAAC   
  
  
- TGATAATAAC AAAATTAACT AGAACTTAAA AGTATCTAAA ATGAGGTAAC AAATGAGTAA AACAATATTA   
  
  
- ATTGGTGGAC TAAAATTTGA AATTTTATGT GTTTCTAAGT AGATTATATT AAAAAATCTA TTTCAATACG   
  
  
- TATGTATATA TGTTTAATTT AATAAATGCT AGATTAGTAA AGGGCAATAT ATAAAGTGTG ATTTTAAATC   
  
  
- TTCTCTGTTA GATAATTTTA ATGTAGTGTA CAAAACCCCC CCCCCCCTAT AGTCTATACG TACTGTAAGG   
  
  
- ATTAGGTTAT TACGTTACTT TGGGTGGCTT ATCAACTTTA CTAAACAAGA TAGGTACTTG AGTGGTTCAG   
  
  
- TGTTAGGTAG TGTGAGCTTC CTATACGAAA AGAAAAACTT CCCTTAGAAC GAATTAGTTT GGATATTTGA   
  
  
- AAATTTTCGT TGTCTGTTTT TTCACTAATA TTACCATCAC CATCCTACTT CCGTCGAAAG TCTTTTCGCT   
  
  
- AACTTAAAAA TAACATAGGG TACTTTTGGG TAACTTAAAA AGAAAGAAAG AAACAAAAAC AATGGACCCA   
  
  
- AATACGTTTC CTGCCGACAA CATAAATTAT TATTGTTTTT GTATCCCGAA ACGCCTAACG ATTACGACCC   
  
  
- CTACCGTAGT CACGAGCTTA GACACATGCG ACAGTATCCT TGGGTGAACG AAGACGAGTA AATAAAGACA   
  
  
- GAGATCTAGG GGAGGAGAGA GAGAGAGAGA GAGATTCCAC AGACGAGCAG ACAAGTATAA GAGGACTCCA   
  
  
- AGAAGAAGTA GATAGAACAA GACCCACACA CGACTCCATG AAGAAAAGAA ATATTTAAAA AGTAGAAAAC   
  
  
- AACAAAACGA ACCACAAATA CTAAAATCAA AAACAGAAAT TCTGCTCTAA ACGACAATTA TCGTTCTAAA   
  
  
- GACTTGATCA TAACTAGGAA AAACACCATA TAAACAACTT AAACTAATTA AACACATAAC ACAAATCGAA   
  
  
- TTCATTGAGC AACACTAGAT TAACTACGAG CCCAAAACTG TCAAACTAAT CCCAAAAGCC ATGACCTCAA   
  
  
- TTTCAAAAAA ATAAAAAATA TCCTCCTCAA TTTCCATTAT TATTCTTAAC TACAAACGAC TAAAAAGGAG   
  
  
- AAAAGAAAAA ACCAAAGCTA AACCACCCGT CAATAGACAA ATACCAAAAA CGTTTACCTA GGTGTTAATC   
  
  
- TCCTTAATAT ACCTGGAAAT GTAGCTTAGT TCAAGTTACT AGTTTCCCAC GGTTAAAATG GTTCAGCGTC   
  
  
- ACAACAATGA GGCTACTTTG AGGTTCTAAG GTTAAACTTA GGTTCACAAG GTTTAGGTTC ACAACACTTA   
  
  
- GGAGGAGGCA ACCAAGGGGG TTTGGGTTTA GAATTAAATC AACACCGTAG AGTTATAAGA CTGTAACTCT   
  
  
- GTCGGGACTT GCTCCTAACA CTAAAATCAC TACAACAAGA ATTCATATAA TTAGTTGAAG AATACCTTCT
[truncated: 223,177 more chars]
