# Supplementary material for: Genome-Wide Identification and Expression Pattern of the GRAS Gene Family in Pitaya (Selenicereus undatus L.)
Source: Biology (Basel). 2022 Dec 21;12(1):11. doi: 10.3390/biology12010011 (PMC9854919; doi:10.3390/biology12010011)
Supplement: Supplementary file 1 [file biology-12-00011-s001.zip › Supplementary file S5/HU06G02537.1_plantcare.html]

Content-Type: text/html; charset=ISO-8859-1


PlantCARE


Webmaster Firefox specific output  
To save the result:
click on the frame with the right mouse button and save the source code as a text file with extension .html  
REFERENCE:PlantCARE: a database of plant cis-acting regulatory elements and a portal to tools for in silico analysis of promoter sequences.  
Lescot, M., Déhais, P., Moreau, Y., De Moor, B., Rouzé ,P.,and Rombauts, S.  
Nucleic Acids Res., Database issue(2002), 30(1):325-327.   


---

>HU06G02537.1   
+ +Up\_Stream \_Len000CCTCGG TGTGGGATTC CGGGTTGATA AATAGGGACC AGCGAAATGT TCAAATAATT   
  
  
+ GGTGAAGGAG TCTCTGTTTG AATGCCTACT AATGCTTTTT TTTTTTTTTT GAATAATTTG TCAGAAAGTT   
  
  
+ AGGCTGCATT GGAAGGGGCG GCCTACGAAG GCTTTTCAGC AAATAAATAA CCAGCTTTCA TTTTGAAATT   
  
  
+ TTTTTTAAAA AATGAAACTC ATTTCACTTA AAAATTTACC ATAATATATA TATATATATA TATATATATA   
  
  
+ TATATATATA TATTTGATTT AACGTTAAGC AAACTTTTAA CTTATGTATC ATTTTACGAC CGTAAAAAAA   
  
  
+ AAAGAAATAT AACACAATAT GTAATAGTAT TTAAGTTTTT GATAAATAAT ATTTTAACAT AATTTGTGTG   
  
  
+ TAGTATGAAT TTAAATCTTA GTACTAATAT AATTATGTAT ACTTGGCTAT AAGATCTCAA TATTAGTATT   
  
  
+ TTATGATTCA ATGTAATGAT GATGCAACTG TGTGTAATTA GAAAGCCTTT GATTGCAGAT ATCTATCTTA   
  
  
+ TTGATACACA TCTACCAATT GAGCTTTTTC AAGATTTGAA GACGGTAGTC AAGTATCTTT GGGAGATTAC   
  
  
+ GTTAGAATTA CTTCATGATA AATTAGACAT ATTTTATACT TCTAATCTTA AATAAGTCTC CATCCTTTCA   
  
  
+ TCATATTTTA AAGAAATGTA CAAGTAAATG TCAATAGTTT GAAACGTTCC TTATCATAAA AGTCATACAT   
  
  
+ ATGTAGGACA TTTTCAAGTT TATGTAAACC ATATGATATA GTTCAAGATA ATTGTCAAAC AAGTTATGAA   
  
  
+ ATCTAAAGGT GTTTGAACGT AATTTCAAGC TAATCTTAGC AAGTTATAGT ATTATTTAAA CTACGACTAG   
  
  
+ ACACAACATT TTGTTCATTT ATATAACACT TAGAATGCAA AAAAATCGGT ATTACTAATA CTGTATCAAG   
  
  
+ AATTGAAGTG TTATGCATTA TACTGATAAG GGATAATATT TAAATTACTT TACTGTTATA GAAGCCAAAA   
  
  
+ ATCTAAACTT TCATACAGAC GTCGGACTAA ATTAGAAAAT TCTTTAACTT AATAGAAAGG ATGACATAAA   
  
  
+ TAAAAATAAA AACCTAAATA GCATAATAAC CATTCTACAC AAAAGTCAAT CTTCTAATAG AAAAAAGCCA   
  
  
+ TAAGTATTCA CTTATTTACC ATTTTATTCC TAATCTACTG TCTGTCTATT CACACCATAA TTCATGAACC   
  
  
+ AATGTGGAGT TTCTGTTTCA TTTCATGGGG TGATTTATGT CTGACAATGA TCCAACAAAC TGAGCCTCAC   
  
  
+ GCGCGGAACA CGAGAGTGGC TGTACTTATG GTGTGAAAGA GCTCTACAAG GTGAGATTGA GGAGGCTACG   
  
  
+ CGTTGGAGTT ATCACCACCG TTGGATTTGT TTTCTTTGGA GCCACGTTAT CACTCTTCAG ACGCCGATCC   
  
  
+ AACGGCCCTT ACTTCCATCT AGTCCCCACT CTCCAACTCT ACGCAATCAA CGCCTTCCGC ATTTAAATAT   
  
  
+ TTCCTTCTCT ATCGCCTACT TTCTCTGTCT CACAATTTTT GTCCGTATTT TGAATTTTCT CAAAATTATC   
  
  
+ ATATAATTTA TTTGCCTTCA ACTAAAGTAT TTATTAAATT AGTTAATGTA TTTAAATTGA ACTAAAAAAG   
  
  
+ AGAAAAAGAA AGAAAACAAC CCCACGAAAG CAAAACGACA GAGGCCAACT TCTCTCTCTA GAGTGTAGAG   
  
  
+ CCCCATCTCG GTTATAATTA TGAGAATCAA TTCCTTCTCA CAAGAGAAAC TCTCGCTCTC TCTCTCTCTC   
  
  
+ CATAAGACAT CACACCAATT GTATGAATTT TCTCTCTCCT GATTTCTGTT TTCCTTCTTG TTGTCCCCTT   
  
  
+ CTAATGCGTT AGTGCGGCCA AAACCACCAT AAACCATCTC TCTTTCAAGA AAGCAGCAAC AACAAAGAGA   
  
  
+ GACAGAGAGA GAGAGAGGGG GAGAAAGATC ACATCTTTTG GGTTTCCGGC GAATATGACG TCTGGGTTTT   
  
  
+ CGGGCGATTT CTTCTACGGC GGTGGAGGCG GTGGCGGAGG AGGTGTCCGG CCTTCTATCG GCGGTGTACA   
  
  
+ AGGCGGGGTT GTCCCATATG GGTTCCGGCA ACAACAACAA CAACAGCAAC AGCAGCAGCA GCAGCAACAG   
  
  
+ CTACATCTAC AGCTACAACA ACAGCGGGGG ATTTTCTCGG CGGATCAGAT CAGCGCTCAA CGAAGGGCGG   
  
  
+ ATTTGTTGAT GGGTAAGCGT TCTTTCGCTG ATTTTCAATC TCAACAACAG CAAATCAGTC TTTTGCAGCA   
  
  
+ GCAGCAGCAG CAACAACAAC AACAGCCCTT TGCGCTCTTG CAAAAACAAC AGCAGTTTCA ACAACAATTA   
  
  
+ CAACAACAAC AGCCACTTTC TCTCCTCCTT CAACAACAGT ACTTGATGAG ATCCGTAAAA CAGAGAACCA   
  
  
+ ACAATTTCGC AGCATCTCCG ATTTCTCCTC TTTCTTCTTC CATTGATCTT TCTTCCGCTA ATAATTCCCG   
  
  
+ GTTCGGTTTG CCGGTTTACC AACAACAACT CCGGTCTCAA CAACAACCAC CTATCTCTCT CCTCGCCCAC   
  
  
+ AACAACAACC AATTCAACCT CTCAAATAAT AACAATTCTT CTTTAATCCA AAACTCCACC AACACTCTTC   
  
  
+ TTGCTCCCGC AGCCAGCAAT AACACCCTAA TGAAAAATTC GGGCTTTTCC TCATCTGGGT TTTCTAATTC   
  
  
+ GGGTCCAAAC CTGGTCCAAC CGAAGCAGGA AATCGGGTAC TCGAATATGG GTACCAATAT GGGTATGAAT   
  
  
+ CTGGTCCAGC CCAAACCCGA CCCTGAGGAT CACGGAATAA TGAACACCCT TCAAGAATTG GAGAAGGAGC   
  
  
+ TTTTGAATGA CGATGAGGAA AACCAGCCGG AAAACGACGC CGTTTCAGTC ATCACCAACA GCGAGTGGTC   
  
  
+ CGAGACCTTC CAGAATCTGA TGAGTCCCGA CCCGAGTTCG AGCCCGAATG CCACCAACAC CCACCCGGGT   
  
  
+ TCGGGTTCTG CTAGTACCAG CGGCAGCAAC AACACTACAA CCACCCCTGC AAACAACAAT AACATGATAT   
  
  
+ CTACCTCGCC GACGTCGTCC ACGTCATCCT GCTGCTCCAC GTCGGCATCG GCGTCTCCGC CGGCGGCGGT   
  
  
+ GGCGACCCCG CAGGTACCAG CCAAGCAGCT GATCTCGGAA GCAGCGGCGG CGATTTCGGA CGGAAAGATT   
  
  
+ GAATCCGCCA TGGAAGTGAT GAACCGGCTG TCCCAGGTTG CCAATTCTAG GGGGAGCTCG GAACAAAAGC   
  
  
+ TGGCTTTTTA CATGGGCCAC GCGCTTCGGT CGCGCGTGAG TCCCACCGAA CACGCTCCCC CTATTGTGGA   
  
  
+ GCTTTTCCAA AAGGAGCATA TGGCGGCCAT GTATTCATTG TACGAGCTCT CTCATTGTTT CAAATTTGGT   
  
  
+ CTTTTGGCTG CCAATTTGGT GATGTTGGAG GCCATTACTT CCGCCCTTCA TGAGGGTCTG AAAATTCATG   
  
  
+ TTGTGGATTT TGATATTGGG CATGGTGGTC AGTACATCAA CCTCCTACGC GCCTTGGGGG AGCGTAGGGC   
  
  
+ TCAGTCGGGG AGCGCGGTGT CGAATGAGGT GAGGATCACT GTGGTTGAGA CCGAGGTCGG TAATGGTGGT   
  
  
+ GCGGCGGGTG CGGAGGCTGT TAAGGATGGC CTGGTGAAAT TGGCTGAGAA GGTGGGGATT AGTTTGAAGT   
  
  
+ TTGAGGCTTT GACTCGGAAG ATCTCCGAGT TAACCCGGGA GTCACTGGGG TGTGAGGAGG GGGAGGCCGT   
  
  
+ GGTGGTGAAT TTTGCGTTCA AACTTTACCG GGTACCGGAT GAGAGCGTGT CGATGGAGAA TCTGAGGGAC   
  
  
+ GAGCTCCTGC GCCGGGTGAA GGCGCTAAGG CCACGCGCCG TCACATTGGT GGAGCAGGAC TTAAATGCGA   
  
  
+ ATACGGCACC GTTCATGACG CGCGTGAGTG GCGCTTGTGA GTACTATAGA GCATTGTTCG ACTCGCTCGA   
  
  
+ CTCGATGGTG CCGAGGGATA ACCCGGACCG GGTCCGGGTC GAGGAGGCCT TGGGGCGGAA GATGGCAAAC   
  
  
+ TCGGTGGCTT GCGAGGGCCG GGAACGGGTT GAGCGGGCGG AGGTGTTTGG GAAGTGGCGG GCGAGGATGA   
  
  
+ GTATGGCAGG GTTCGAGTTG AAGGCACTGA GTCAGAGTGT GGCCGAGTCA ATGCGAACCA AACTCAGCTC   
  
  
+ GGTTCTGGGC GGAAAGCCGG GTTTCACGGT CAAGGAGGAG AGCGGTGGGG TGGGGTTCGG GTGGATGGGA   
  
  
+ CGAACTCTCA CCGTCGCATC GGCCTGGCGT TA  

- +Up\_Stream \_Len000GGAGCC ACACCCTAAG GCCCAACTAT TTATCCCTGG TCGCTTTACA AGTTTATTAA   
  
  
- CCACTTCCTC AGAGACAAAC TTACGGATGA TTACGAAAAA AAAAAAAAAA CTTATTAAAC AGTCTTTCAA   
  
  
- TCCGACGTAA CCTTCCCCGC CGGATGCTTC CGAAAAGTCG TTTATTTATT GGTCGAAAGT AAAACTTTAA   
  
  
- AAAAAATTTT TTACTTTGAG TAAAGTGAAT TTTTAAATGG TATTATATAT ATATATATAT ATATATATAT   
  
  
- ATATATATAT ATAAACTAAA TTGCAATTCG TTTGAAAATT GAATACATAG TAAAATGCTG GCATTTTTTT   
  
  
- TTTCTTTATA TTGTGTTATA CATTATCATA AATTCAAAAA CTATTTATTA TAAAATTGTA TTAAACACAC   
  
  
- ATCATACTTA AATTTAGAAT CATGATTATA TTAATACATA TGAACCGATA TTCTAGAGTT ATAATCATAA   
  
  
- AATACTAAGT TACATTACTA CTACGTTGAC ACACATTAAT CTTTCGGAAA CTAACGTCTA TAGATAGAAT   
  
  
- AACTATGTGT AGATGGTTAA CTCGAAAAAG TTCTAAACTT CTGCCATCAG TTCATAGAAA CCCTCTAATG   
  
  
- CAATCTTAAT GAAGTACTAT TTAATCTGTA TAAAATATGA AGATTAGAAT TTATTCAGAG GTAGGAAAGT   
  
  
- AGTATAAAAT TTCTTTACAT GTTCATTTAC AGTTATCAAA CTTTGCAAGG AATAGTATTT TCAGTATGTA   
  
  
- TACATCCTGT AAAAGTTCAA ATACATTTGG TATACTATAT CAAGTTCTAT TAACAGTTTG TTCAATACTT   
  
  
- TAGATTTCCA CAAACTTGCA TTAAAGTTCG ATTAGAATCG TTCAATATCA TAATAAATTT GATGCTGATC   
  
  
- TGTGTTGTAA AACAAGTAAA TATATTGTGA ATCTTACGTT TTTTTAGCCA TAATGATTAT GACATAGTTC   
  
  
- TTAACTTCAC AATACGTAAT ATGACTATTC CCTATTATAA ATTTAATGAA ATGACAATAT CTTCGGTTTT   
  
  
- TAGATTTGAA AGTATGTCTG CAGCCTGATT TAATCTTTTA AGAAATTGAA TTATCTTTCC TACTGTATTT   
  
  
- ATTTTTATTT TTGGATTTAT CGTATTATTG GTAAGATGTG TTTTCAGTTA GAAGATTATC TTTTTTCGGT   
  
  
- ATTCATAAGT GAATAAATGG TAAAATAAGG ATTAGATGAC AGACAGATAA GTGTGGTATT AAGTACTTGG   
  
  
- TTACACCTCA AAGACAAAGT AAAGTACCCC ACTAAATACA GACTGTTACT AGGTTGTTTG ACTCGGAGTG   
  
  
- CGCGCCTTGT GCTCTCACCG ACATGAATAC CACACTTTCT CGAGATGTTC CACTCTAACT CCTCCGATGC   
  
  
- GCAACCTCAA TAGTGGTGGC AACCTAAACA AAAGAAACCT CGGTGCAATA GTGAGAAGTC TGCGGCTAGG   
  
  
- TTGCCGGGAA TGAAGGTAGA TCAGGGGTGA GAGGTTGAGA TGCGTTAGTT GCGGAAGGCG TAAATTTATA   
  
  
- AAGGAAGAGA TAGCGGATGA AAGAGACAGA GTGTTAAAAA CAGGCATAAA ACTTAAAAGA GTTTTAATAG   
  
  
- TATATTAAAT AAACGGAAGT TGATTTCATA AATAATTTAA TCAATTACAT AAATTTAACT TGATTTTTTC   
  
  
- TCTTTTTCTT TCTTTTGTTG GGGTGCTTTC GTTTTGCTGT CTCCGGTTGA AGAGAGAGAT CTCACATCTC   
  
  
- GGGGTAGAGC CAATATTAAT ACTCTTAGTT AAGGAAGAGT GTTCTCTTTG AGAGCGAGAG AGAGAGAGAG   
  
  
- GTATTCTGTA GTGTGGTTAA CATACTTAAA AGAGAGAGGA CTAAAGACAA AAGGAAGAAC AACAGGGGAA   
  
  
- GATTACGCAA TCACGCCGGT TTTGGTGGTA TTTGGTAGAG AGAAAGTTCT TTCGTCGTTG TTGTTTCTCT   
  
  
- CTGTCTCTCT CTCTCTCCCC CTCTTTCTAG TGTAGAAAAC CCAAAGGCCG CTTATACTGC AGACCCAAAA   
  
  
- GCCCGCTAAA GAAGATGCCG CCACCTCCGC CACCGCCTCC TCCACAGGCC GGAAGATAGC CGCCACATGT   
  
  
- TCCGCCCCAA CAGGGTATAC CCAAGGCCGT TGTTGTTGTT GTTGTCGTTG TCGTCGTCGT CGTCGTTGTC   
  
  
- GATGTAGATG TCGATGTTGT TGTCGCCCCC TAAAAGAGCC GCCTAGTCTA GTCGCGAGTT GCTTCCCGCC   
  
  
- TAAACAACTA CCCATTCGCA AGAAAGCGAC TAAAAGTTAG AGTTGTTGTC GTTTAGTCAG AAAACGTCGT   
  
  
- CGTCGTCGTC GTTGTTGTTG TTGTCGGGAA ACGCGAGAAC GTTTTTGTTG TCGTCAAAGT TGTTGTTAAT   
  
  
- GTTGTTGTTG TCGGTGAAAG AGAGGAGGAA GTTGTTGTCA TGAACTACTC TAGGCATTTT GTCTCTTGGT   
  
  
- TGTTAAAGCG TCGTAGAGGC TAAAGAGGAG AAAGAAGAAG GTAACTAGAA AGAAGGCGAT TATTAAGGGC   
  
  
- CAAGCCAAAC GGCCAAATGG TTGTTGTTGA GGCCAGAGTT GTTGTTGGTG GATAGAGAGA GGAGCGGGTG   
  
  
- TTGTTGTTGG TTAAGTTGGA GAGTTTATTA TTGTTAAGAA GAAATTAGGT TTTGAGGTGG TTGTGAGAAG   
  
  
- AACGAGGGCG TCGGTCGTTA TTGTGGGATT ACTTTTTAAG CCCGAAAAGG AGTAGACCCA AAAGATTAAG   
  
  
- CCCAGGTTTG GACCAGGTTG GCTTCGTCCT TTAGCCCATG AGCTTATACC CATGGTTATA CCCATACTTA   
  
  
- GACCAGGTCG GGTTTGGGCT GGGACTCCTA GTGCCTTATT ACTTGTGGGA AGTTCTTAAC CTCTTCCTCG   
  
  
- AAAACTTACT GCTACTCCTT TTGGTCGGCC TTTTGCTGCG GCAAAGTCAG TAGTGGTTGT CGCTCACCAG   
  
  
- GCTCTGGAAG GTCTTAGACT ACTCAGGGCT GGGCTCAAGC TCGGGCTTAC GGTGGTTGTG GGTGGGCCCA   
  
  
- AGCCCAAGAC GATCATGGTC GCCGTCGTTG TTGTGATGTT GGTGGGGACG TTTGTTGTTA TTGTACTATA   
  
  
- GATGGAGCGG CTGCAGCAGG TGCAGTAGGA CGACGAGGTG CAGCCGTAGC CGCAGAGGCG GCCGCCGCCA   
  
  
- CCGCTGGGGC GTCCATGGTC GGTTCGTCGA CTAGAGCCTT CGTCGCCGCC GCTAAAGCCT GCCTTTCTAA   
  
  
- CTTAGGCGGT ACCTTCACTA CTTGGCCGAC AGGGTCCAAC GGTTAAGATC CCCCTCGAGC CTTGTTTTCG   
  
  
- ACCGAAAAAT GTACCCGGTG CGCGAAGCCA GCGCGCACTC AGGGTGGCTT GTGCGAGGGG GATAACACCT   
  
  
- CGAAAAGGTT TTCCTCGTAT ACCGCCGGTA CATAAGTAAC ATGCTCGAGA GAGTAACAAA GTTTAAACCA   
  
  
- GAAAACCGAC GGTTAAACCA CTACAACCTC CGGTAATGAA GGCGGGAAGT ACTCCCAGAC TTTTAAGTAC   
  
  
- AACACCTAAA ACTATAACCC GTACCACCAG TCATGTAGTT GGAGGATGCG CGGAACCCCC TCGCATCCCG   
  
  
- AGTCAGCCCC TCGCGCCACA GCTTACTCCA CTCCTAGTGA CACCAACTCT GGCTCCAGCC ATTACCACCA   
  
  
- CGCCGCCCAC GCCTCCGACA ATTCCTACCG GACCACTTTA ACCGACTCTT CCACCCCTAA TCAAACTTCA   
  
  
- AACTCCGAAA CTGAGCCTTC TAGAGGCTCA ATTGGGCCCT CAGTGACCCC ACACTCCTCC CCCTCCGGCA   
  
  
- CCACCACTTA AAACGCAAGT TTGAAATGGC CCATGGCCTA CTCTCGCACA GCTACCTCTT AGACTCCCTG   
  
  
- CTCGAGGACG CGGCCCACTT CCGCGATTCC GGTGCGCGGC AGTGTAACCA CCTCGTCCTG AATTTACGCT   
  
  
- TATGCCGTGG CAAGTACTGC GCGCACTCAC CGCGAACACT CATGATATCT CGTAACAAGC TGAGCGAGCT   
  
  
- GAGCTACCAC GGCTCCCTAT TGGGCCTGGC CCAGGCCCAG CTCCTCCGGA ACCCCGCCTT CTACCGTTTG   
  
  
- AGCCACCGAA CGCTCCCGGC CCTTGCCCAA CTCGCCCGCC TCCACAAACC CTTCACCGCC CGCTCCTACT   
  
  
- CATACCGTCC CAAGCTCAAC TTCCGTGACT CAGTCTCACA CCGGCTCAGT TACGCTTGGT TTGAGTCGAG   
  
  
- CCAAGACCCG CCTTTCGGCC CAAAGTGCCA GTTCCTCCTC TCGCCACCCC ACCCCAAGCC CACCTACCCT   
  
  
- GCTTGAGAGT GGCAGCGTAG CCGGACCGCA AT

  
  
Motifs Found  

+   

| Site Name | Organism | Position | Strand | Matrix score. | sequence | function |
| --- | --- | --- | --- | --- | --- | --- |
|  | organism | 3731 | - | 4 | motif\_sequence | short\_function |
|  | organism | 4241 | - | 4 | motif\_sequence | short\_function |
|  | organism | 2581 | + | 4 | motif\_sequence | short\_function |
|  | organism | 1511 | + | 4 | motif\_sequence | short\_function |
|  | organism | 1749 | - | 4 | motif\_sequence | short\_function |
|  | organism | 3083 | + | 4 | motif\_sequence | short\_function |
|  | organism | 2659 | + | 4 | motif\_sequence | short\_function |
|  | organism | 1457 | + | 4 | motif\_sequence | short\_function |
|  | organism | 3735 | + | 4 | motif\_sequence | short\_function |
|  | organism | 1173 | + | 4 | motif\_sequence | short\_function |
|  | organism | 1503 | + | 4 | motif\_sequence | short\_function |
|  | organism | 1858 | + | 4 | motif\_sequence | short\_function |
|  | organism | 1227 | + | 4 | motif\_sequence | short\_function |
|  | organism | 720 | + | 4 | motif\_sequence | short\_function |
|  | organism | 780 | - | 4 | motif\_sequence | short\_function |
|  | organism | 574 | + | 4 | motif\_sequence | short\_function |
|  | organism | 4052 | - | 4 | motif\_sequence | short\_function |
|  | organism | 1820 | + | 4 | motif\_sequence | short\_function |
|  | organism | 2179 | + | 4 | motif\_sequence | short\_function |
|  | organism | 61 | + | 4 | motif\_sequence | short\_function |
|  | organism | 549 | - | 4 | motif\_sequence | short\_function |
|  | organism | 3252 | + | 4 | motif\_sequence | short\_function |
|  | organism | 3536 | - | 4 | motif\_sequence | short\_function |
|  | organism | 1354 | + | 4 | motif\_sequence | short\_function |
|  | organism | 626 | - | 4 | motif\_sequence | short\_function |
|  | organism | 2404 | + | 4 | motif\_sequence | short\_function |
|  | organism | 1376 | + | 4 | motif\_sequence | short\_function |
|  | organism | 2468 | + | 4 | motif\_sequence | short\_function |

>HU06G02537.1   
+ +Up\_Stream \_Len000CCTCGG TGTGGGATTC CGGGTTGATA AATAGGGACC AGCGAAATGT TCAAATAATT   
  
  
+ GGTGAAGGAG TCTCTGTTTG AATGCCTACT AATGCTTTTT TTTTTTTTTT GAATAATTTG TCAGAAAGTT   
  
  
+ AGGCTGCATT GGAAGGGGCG GCCTACGAAG GCTTTTCAGC AAATAAATAA CCAGCTTTCA TTTTGAAATT   
  
  
+ TTTTTTAAAA AATGAAACTC ATTTCACTTA AAAATTTACC ATAATATATA TATATATATA TATATATATA   
  
  
+ TATATATATA TATTTGATTT AACGTTAAGC AAACTTTTAA CTTATGTATC ATTTTACGAC CGTAAAAAAA   
  
  
+ AAAGAAATAT AACACAATAT GTAATAGTAT TTAAGTTTTT GATAAATAAT ATTTTAACAT AATTTGTGTG   
  
  
+ TAGTATGAAT TTAAATCTTA GTACTAATAT AATTATGTAT ACTTGGCTAT AAGATCTCAA TATTAGTATT   
  
  
+ TTATGATTCA ATGTAATGAT GATGCAACTG TGTGTAATTA GAAAGCCTTT GATTGCAGAT ATCTATCTTA   
  
  
+ TTGATACACA TCTACCAATT GAGCTTTTTC AAGATTTGAA GACGGTAGTC AAGTATCTTT GGGAGATTAC   
  
  
+ GTTAGAATTA CTTCATGATA AATTAGACAT ATTTTATACT TCTAATCTTA AATAAGTCTC CATCCTTTCA   
  
  
+ TCATATTTTA AAGAAATGTA CAAGTAAATG TCAATAGTTT GAAACGTTCC TTATCATAAA AGTCATACAT   
  
  
+ ATGTAGGACA TTTTCAAGTT TATGTAAACC ATATGATATA GTTCAAGATA ATTGTCAAAC AAGTTATGAA   
  
  
+ ATCTAAAGGT GTTTGAACGT AATTTCAAGC TAATCTTAGC AAGTTATAGT ATTATTTAAA CTACGACTAG   
  
  
+ ACACAACATT TTGTTCATTT ATATAACACT TAGAATGCAA AAAAATCGGT ATTACTAATA CTGTATCAAG   
  
  
+ AATTGAAGTG TTATGCATTA TACTGATAAG GGATAATATT TAAATTACTT TACTGTTATA GAAGCCAAAA   
  
  
+ ATCTAAACTT TCATACAGAC GTCGGACTAA ATTAGAAAAT TCTTTAACTT AATAGAAAGG ATGACATAAA   
  
  
+ TAAAAATAAA AACCTAAATA GCATAATAAC CATTCTACAC AAAAGTCAAT CTTCTAATAG AAAAAAGCCA   
  
  
+ TAAGTATTCA CTTATTTACC ATTTTATTCC TAATCTACTG TCTGTCTATT CACACCATAA TTCATGAACC   
  
  
+ AATGTGGAGT TTCTGTTTCA TTTCATGGGG TGATTTATGT CTGACAATGA TCCAACAAAC TGAGCCTCAC   
  
  
+ GCGCGGAACA CGAGAGTGGC TGTACTTATG GTGTGAAAGA GCTCTACAAG GTGAGATTGA GGAGGCTACG   
  
  
+ CGTTGGAGTT ATCACCACCG TTGGATTTGT TTTCTTTGGA GCCACGTTAT CACTCTTCAG ACGCCGATCC   
  
  
+ AACGGCCCTT ACTTCCATCT AGTCCCCACT CTCCAACTCT ACGCAATCAA CGCCTTCCGC ATTTAAATAT   
  
  
+ TTCCTTCTCT ATCGCCTACT TTCTCTGTCT CACAATTTTT GTCCGTATTT TGAATTTTCT CAAAATTATC   
  
  
+ ATATAATTTA TTTGCCTTCA ACTAAAGTAT TTATTAAATT AGTTAATGTA TTTAAATTGA ACTAAAAAAG   
  
  
+ AGAAAAAGAA AGAAAACAAC CCCACGAAAG CAAAACGACA GAGGCCAACT TCTCTCTCTA GAGTGTAGAG   
  
  
+ CCCCATCTCG GTTATAATTA TGAGAATCAA TTCCTTCTCA CAAGAGAAAC TCTCGCTCTC TCTCTCTCTC   
  
  
+ CATAAGACAT CACACCAATT GTATGAATTT TCTCTCTCCT GATTTCTGTT TTCCTTCTTG TTGTCCCCTT   
  
  
+ CTAATGCGTT AGTGCGGCCA AAACCACCAT AAACCATCTC TCTTTCAAGA AAGCAGCAAC AACAAAGAGA   
  
  
+ GACAGAGAGA GAGAGAGGGG GAGAAAGATC ACATCTTTTG GGTTTCCGGC GAATATGACG TCTGGGTTTT   
  
  
+ CGGGCGATTT CTTCTACGGC GGTGGAGGCG GTGGCGGAGG AGGTGTCCGG CCTTCTATCG GCGGTGTACA   
  
  
+ AGGCGGGGTT GTCCCATATG GGTTCCGGCA ACAACAACAA CAACAGCAAC AGCAGCAGCA GCAGCAACAG   
  
  
+ CTACATCTAC AGCTACAACA ACAGCGGGGG ATTTTCTCGG CGGATCAGAT CAGCGCTCAA CGAAGGGCGG   
  
  
+ ATTTGTTGAT GGGTAAGCGT TCTTTCGCTG ATTTTCAATC TCAACAACAG CAAATCAGTC TTTTGCAGCA   
  
  
+ GCAGCAGCAG CAACAACAAC AACAGCCCTT TGCGCTCTTG CAAAAACAAC AGCAGTTTCA ACAACAATTA   
  
  
+ CAACAACAAC AGCCACTTTC TCTCCTCCTT CAACAACAGT ACTTGATGAG ATCCGTAAAA CAGAGAACCA   
  
  
+ ACAATTTCGC AGCATCTCCG ATTTCTCCTC TTTCTTCTTC CATTGATCTT TCTTCCGCTA ATAATTCCCG   
  
  
+ GTTCGGTTTG CCGGTTTACC AACAACAACT CCGGTCTCAA CAACAACCAC CTATCTCTCT CCTCGCCCAC   
  
  
+ AACAACAACC AATTCAACCT CTCAAATAAT AACAATTCTT CTTTAATCCA AAACTCCACC AACACTCTTC   
  
  
+ TTGCTCCCGC AGCCAGCAAT AACACCCTAA TGAAAAATTC GGGCTTTTCC TCATCTGGGT TTTCTAATTC   
  
  
+ GGGTCCAAAC CTGGTCCAAC CGAAGCAGGA AATCGGGTAC TCGAATATGG GTACCAATAT GGGTATGAAT   
  
  
+ CTGGTCCAGC CCAAACCCGA CCCTGAGGAT CACGGAATAA TGAACACCCT TCAAGAATTG GAGAAGGAGC   
  
  
+ TTTTGAATGA CGATGAGGAA AACCAGCCGG AAAACGACGC CGTTTCAGTC ATCACCAACA GCGAGTGGTC   
  
  
+ CGAGACCTTC CAGAATCTGA TGAGTCCCGA CCCGAGTTCG AGCCCGAATG CCACCAACAC CCACCCGGGT   
  
  
+ TCGGGTTCTG CTAGTACCAG CGGCAGCAAC AACACTACAA CCACCCCTGC AAACAACAAT AACATGATAT   
  
  
+ CTACCTCGCC GACGTCGTCC ACGTCATCCT GCTGCTCCAC GTCGGCATCG GCGTCTCCGC CGGCGGCGGT   
  
  
+ GGCGACCCCG CAGGTACCAG CCAAGCAGCT GATCTCGGAA GCAGCGGCGG CGATTTCGGA CGGAAAGATT   
  
  
+ GAATCCGCCA TGGAAGTGAT GAACCGGCTG TCCCAGGTTG CCAATTCTAG GGGGAGCTCG GAACAAAAGC   
  
  
+ TGGCTTTTTA CATGGGCCAC GCGCTTCGGT CGCGCGTGAG TCCCACCGAA CACGCTCCCC CTATTGTGGA   
  
  
+ GCTTTTCCAA AAGGAGCATA TGGCGGCCAT GTATTCATTG TACGAGCTCT CTCATTGTTT CAAATTTGGT   
  
  
+ CTTTTGGCTG CCAATTTGGT GATGTTGGAG GCCATTACTT CCGCCCTTCA TGAGGGTCTG AAAATTCATG   
  
  
+ TTGTGGATTT TGATATTGGG CATGGTGGTC AGTACATCAA CCTCCTACGC GCCTTGGGGG AGCGTAGGGC   
  
  
+ TCAGTCGGGG AGCGCGGTGT CGAATGAGGT GAGGATCACT GTGGTTGAGA CCGAGGTCGG TAATGGTGGT   
  
  
+ GCGGCGGGTG CGGAGGCTGT TAAGGATGGC CTGGTGAAAT TGGCTGAGAA GGTGGGGATT AGTTTGAAGT   
  
  
+ TTGAGGCTTT GACTCGGAAG ATCTCCGAGT TAACCCGGGA GTCACTGGGG TGTGAGGAGG GGGAGGCCGT   
  
  
+ GGTGGTGAAT TTTGCGTTCA AACTTTACCG GGTACCGGAT GAGAGCGTGT CGATGGAGAA TCTGAGGGAC   
  
  
+ GAGCTCCTGC GCCGGGTGAA GGCGCTAAGG CCACGCGCCG TCACATTGGT GGAGCAGGAC TTAAATGCGA   
  
  
+ ATACGGCACC GTTCATGACG CGCGTGAGTG GCGCTTGTGA GTACTATAGA GCATTGTTCG ACTCGCTCGA   
  
  
+ CTCGATGGTG CCGAGGGATA ACCCGGACCG GGTCCGGGTC GAGGAGGCCT TGGGGCGGAA GATGGCAAAC   
  
  
+ TCGGTGGCTT GCGAGGGCCG GGAACGGGTT GAGCGGGCGG AGGTGTTTGG GAAGTGGCGG GCGAGGATGA   
  
  
+ GTATGGCAGG GTTCGAGTTG AAGGCACTGA GTCAGAGTGT GGCCGAGTCA ATGCGAACCA AACTCAGCTC   
  
  
+ GGTTCTGGGC GGAAAGCCGG GTTTCACGGT CAAGGAGGAG AGCGGTGGGG TGGGGTTCGG GTGGATGGGA   
  
  
+ CGAACTCTCA CCGTCGCATC GGCCTGGCGT TA  

- +Up\_Stream \_Len000GGAGCC ACACCCTAAG GCCCAACTAT TTATCCCTGG TCGCTTTACA AGTTTATTAA   
  
  
- CCACTTCCTC AGAGACAAAC TTACGGATGA TTACGAAAAA AAAAAAAAAA CTTATTAAAC AGTCTTTCAA   
  
  
- TCCGACGTAA CCTTCCCCGC CGGATGCTTC CGAAAAGTCG TTTATTTATT GGTCGAAAGT AAAACTTTAA   
  
  
- AAAAAATTTT TTACTTTGAG TAAAGTGAAT TTTTAAATGG TATTATATAT ATATATATAT ATATATATAT   
  
  
- ATATATATAT ATAAACTAAA TTGCAATTCG TTTGAAAATT GAATACATAG TAAAATGCTG GCATTTTTTT   
  
  
- TTTCTTTATA TTGTGTTATA CATTATCATA AATTCAAAAA CTATTTATTA TAAAATTGTA TTAAACACAC   
  
  
- ATCATACTTA AATTTAGAAT CATGATTATA TTAATACATA TGAACCGATA TTCTAGAGTT ATAATCATAA   
  
  
- AATACTAAGT TACATTACTA CTACGTTGAC ACACATTAAT CTTTCGGAAA CTAACGTCTA TAGATAGAAT   
  
  
- AACTATGTGT AGATGGTTAA CTCGAAAAAG TTCTAAACTT CTGCCATCAG TTCATAGAAA CCCTCTAATG   
  
  
- CAATCTTAAT GAAGTACTAT TTAATCTGTA TAAAATATGA AGATTAGAAT TTATTCAGAG GTAGGAAAGT   
  
  
- AGTATAAAAT TTCTTTACAT GTTCATTTAC AGTTATCAAA CTTTGCAAGG AATAGTATTT TCAGTATGTA   
  
  
- TACATCCTGT AAAAGTTCAA ATACATTTGG TATACTATAT CAAGTTCTAT TAACAGTTTG TTCAATACTT   
  
  
- TAGATTTCCA CAAACTTGCA TTAAAGTTCG ATTAGAATCG TTCAATATCA TAATAAATTT GATGCTGATC   
  
  
- TGTGTTGTAA AACAAGTAAA TATATTGTGA ATCTTACGTT TTTTTAGCCA TAATGATTAT GACATAGTTC   
  
  
- TTAACTTCAC AATACGTAAT ATGACTATTC CCTATTATAA ATTTAATGAA ATGACAATAT CTTCGGTTTT   
  
  
- TAGATTTGAA AGTATGTCTG CAGCCTGATT TAATCTTTTA AGAAATTGAA TTATCTTTCC TACTGTATTT   
  
  
- ATTTTTATTT TTGGATTTAT CGTATTATTG GTAAGATGTG TTTTCAGTTA GAAGATTATC TTTTTTCGGT   
  
  
- ATTCATAAGT GAATAAATGG TAAAATAAGG ATTAGATGAC AGACAGATAA GTGTGGTATT AAGTACTTGG   
  
  
- TTACACCTCA AAGACAAAGT AAAGTACCCC ACTAAATACA GACTGTTACT AGGTTGTTTG ACTCGGAGTG   
  
  
- CGCGCCTTGT GCTCTCACCG ACATGAATAC CACACTTTCT CGAGATGTTC CACTCTAACT CCTCCGATGC   
  
  
- GCAACCTCAA TAGTGGTGGC AACCTAAACA AAAGAAACCT CGGTGCAATA GTGAGAAGTC TGCGGCTAGG   
  
  
- TTGCCGGGAA TGAAGGTAGA TCAGGGGTGA GAGGTTGAGA TGCGTTAGTT GCGGAAGGCG TAAATTTATA   
  
  
- AAGGAAGAGA TAGCGGATGA AAGAGACAGA GTGTTAAAAA CAGGCATAAA ACTTAAAAGA GTTTTAATAG   
  
  
- TATATTAAAT AAACGGAAGT TGATTTCATA AATAATTTAA TCAATTACAT AAATTTAACT TGATTTTTTC   
  
  
- TCTTTTTCTT TCTTTTGTTG GGGTGCTTTC GTTTTGCTGT CTCCGGTTGA AGAGAGAGAT CTCACATCTC   
  
  
- GGGGTAGAGC CAATATTAAT ACTCTTAGTT AAGGAAGAGT GTTCTCTTTG AGAGCGAGAG AGAGAGAGAG   
  
  
- GTATTCTGTA GTGTGGTTAA CATACTTAAA AGAGAGAGGA CTAAAGACAA AAGGAAGAAC AACAGGGGAA   
  
  
- GATTACGCAA TCACGCCGGT TTTGGTGGTA TTTGGTAGAG AGAAAGTTCT TTCGTCGTTG TTGTTTCTCT   
  
  
- CTGTCTCTCT CTCTCTCCCC CTCTTTCTAG TGTAGAAAAC CCAAAGGCCG CTTATACTGC AGACCCAAAA   
  
  
- GCCCGCTAAA GAAGATGCCG CCACCTCCGC CACCGCCTCC TCCACAGGCC GGAAGATAGC CGCCACATGT   
  
  
- TCCGCCCCAA CAGGGTATAC CCAAGGCCGT TGTTGTTGTT GTTGTCGTTG TCGTCGTCGT CGTCGTTGTC   
  
  
- GATGTAGATG TCGATGTTGT TGTCGCCCCC TAAAAGAGCC GCCTAGTCTA GTCGCGAGTT GCTTCCCGCC   
  
  
- TAAACAACTA CCCATTCGCA AGAAAGCGAC TAAAAGTTAG AGTTGTTGTC GTTTAGTCAG AAAACGTCGT   
  
  
- CGTCGTCGTC GTTGTTGTTG TTGTCGGGAA ACGCGAGAAC GTTTTTGTTG TCGTCAAAGT TGTTGTTAAT   
  
  
- GTTGTTGTTG TCGGTGAAAG AGAGGAGGAA GTTGTTGTCA TGAACTACTC TAGGCATTTT GTCTCTTGGT   
  
  
- TGTTAAAGCG TCGTAGAGGC TAAAGAGGAG AAAGAAGAAG GTAACTAGAA AGAAGGCGAT TATTAAGGGC   
  
  
- CAAGCCAAAC GGCCAAATGG TTGTTGTTGA GGCCAGAGTT GTTGTTGGTG GATAGAGAGA GGAGCGGGTG   
  
  
- TTGTTGTTGG TTAAGTTGGA GAGTTTATTA TTGTTAAGAA GAAATTAGGT TTTGAGGTGG TTGTGAGAAG   
  
  
- AACGAGGGCG TCGGTCGTTA TTGTGGGATT ACTTTTTAAG CCCGAAAAGG AGTAGACCCA AAAGATTAAG   
  
  
- CCCAGGTTTG GACCAGGTTG GCTTCGTCCT TTAGCCCATG AGCTTATACC CATGGTTATA CCCATACTTA   
  
  
- GACCAGGTCG GGTTTGGGCT GGGACTCCTA GTGCCTTATT ACTTGTGGGA AGTTCTTAAC CTCTTCCTCG   
  
  
- AAAACTTACT GCTACTCCTT TTGGTCGGCC TTTTGCTGCG GCAAAGTCAG TAGTGGTTGT CGCTCACCAG   
  
  
- GCTCTGGAAG GTCTTAGACT ACTCAGGGCT GGGCTCAAGC TCGGGCTTAC GGTGGTTGTG GGTGGGCCCA   
  
  
- AGCCCAAGAC GATCATGGTC GCCGTCGTTG TTGTGATGTT GGTGGGGACG TTTGTTGTTA TTGTACTATA   
  
  
- GATGGAGCGG CTGCAGCAGG TGCAGTAGGA CGACGAGGTG CAGCCGTAGC CGCAGAGGCG GCCGCCGCCA   
  
  
- CCGCTGGGGC GTCCATGGTC GGTTCGTCGA CTAGAGCCTT CGTCGCCGCC GCTAAAGCCT GCCTTTCTAA   
  
  
- CTTAGGCGGT ACCTTCACTA CTTGGCCGAC AGGGTCCAAC GGTTAAGATC CCCCTCGAGC CTTGTTTTCG   
  
  
- ACCGAAAAAT GTACCCGGTG CGCGAAGCCA GCGCGCACTC AGGGTGGCTT GTGCGAGGGG GATAACACCT   
  
  
- CGAAAAGGTT TTCCTCGTAT ACCGCCGGTA CATAAGTAAC ATGCTCGAGA GAGTAACAAA GTTTAAACCA   
  
  
- GAAAACCGAC GGTTAAACCA CTACAACCTC CGGTAATGAA GGCGGGAAGT ACTCCCAGAC TTTTAAGTAC   
  
  
- AACACCTAAA ACTATAACCC GTACCACCAG TCATGTAGTT GGAGGATGCG CGGAACCCCC TCGCATCCCG   
  
  
- AGTCAGCCCC TCGCGCCACA GCTTACTCCA CTCCTAGTGA CACCAACTCT GGCTCCAGCC ATTACCACCA   
  
  
- CGCCGCCCAC GCCTCCGACA ATTCCTACCG GACCACTTTA ACCGACTCTT CCACCCCTAA TCAAACTTCA   
  
  
- AACTCCGAAA CTGAGCCTTC TAGAGGCTCA ATTGGGCCCT CAGTGACCCC ACACTCCTCC CCCTCCGGCA   
  
  
- CCACCACTTA AAACGCAAGT TTGAAATGGC CCATGGCCTA CTCTCGCACA GCTACCTCTT AGACTCCCTG   
  
  
- CTCGAGGACG CGGCCCACTT CCGCGATTCC GGTGCGCGGC AGTGTAACCA CCTCGTCCTG AATTTACGCT   
  
  
- TATGCCGTGG CAAGTACTGC GCGCACTCAC CGCGAACACT CATGATATCT CGTAACAAGC TGAGCGAGCT   
  
  
- GAGCTACCAC GGCTCCCTAT TGGGCCTGGC CCAGGCCCAG CTCCTCCGGA ACCCCGCCTT CTACCGTTTG   
  
  
- AGCCACCGAA CGCTCCCGGC CCTTGCCCAA CTCGCCCGCC TCCACAAACC CTTCACCGCC CGCTCCTACT   
  
  
- CATACCGTCC CAAGCTCAAC TTCCGTGACT CAGTCTCACA CCGGCTCAGT TACGCTTGGT TTGAGTCGAG   
  
  
- CCAAGACCCG CCTTTCGGCC CAAAGTGCCA GTTCCTCCTC TCGCCACCCC ACCCCAAGCC CACCTACCCT   
  
  
- GCTTGAGAGT GGCAGCGTAG CCGGACCGCA AT

+     A-box

| Site Name | Organism | Position | Strand | Matrix score. | sequence | function |
| --- | --- | --- | --- | --- | --- | --- |
| A-box | Petroselinum crispum | 3212 | - | 6 | CCGTCC | cis-acting regulatory element |

>HU06G02537.1   
+ +Up\_Stream \_Len000CCTCGG TGTGGGATTC CGGGTTGATA AATAGGGACC AGCGAAATGT TCAAATAATT   
  
  
+ GGTGAAGGAG TCTCTGTTTG AATGCCTACT AATGCTTTTT TTTTTTTTTT GAATAATTTG TCAGAAAGTT   
  
  
+ AGGCTGCATT GGAAGGGGCG GCCTACGAAG GCTTTTCAGC AAATAAATAA CCAGCTTTCA TTTTGAAATT   
  
  
+ TTTTTTAAAA AATGAAACTC ATTTCACTTA AAAATTTACC ATAATATATA TATATATATA TATATATATA   
  
  
+ TATATATATA TATTTGATTT AACGTTAAGC AAACTTTTAA CTTATGTATC ATTTTACGAC CGTAAAAAAA   
  
  
+ AAAGAAATAT AACACAATAT GTAATAGTAT TTAAGTTTTT GATAAATAAT ATTTTAACAT AATTTGTGTG   
  
  
+ TAGTATGAAT TTAAATCTTA GTACTAATAT AATTATGTAT ACTTGGCTAT AAGATCTCAA TATTAGTATT   
  
  
+ TTATGATTCA ATGTAATGAT GATGCAACTG TGTGTAATTA GAAAGCCTTT GATTGCAGAT ATCTATCTTA   
  
  
+ TTGATACACA TCTACCAATT GAGCTTTTTC AAGATTTGAA GACGGTAGTC AAGTATCTTT GGGAGATTAC   
  
  
+ GTTAGAATTA CTTCATGATA AATTAGACAT ATTTTATACT TCTAATCTTA AATAAGTCTC CATCCTTTCA   
  
  
+ TCATATTTTA AAGAAATGTA CAAGTAAATG TCAATAGTTT GAAACGTTCC TTATCATAAA AGTCATACAT   
  
  
+ ATGTAGGACA TTTTCAAGTT TATGTAAACC ATATGATATA GTTCAAGATA ATTGTCAAAC AAGTTATGAA   
  
  
+ ATCTAAAGGT GTTTGAACGT AATTTCAAGC TAATCTTAGC AAGTTATAGT ATTATTTAAA CTACGACTAG   
  
  
+ ACACAACATT TTGTTCATTT ATATAACACT TAGAATGCAA AAAAATCGGT ATTACTAATA CTGTATCAAG   
  
  
+ AATTGAAGTG TTATGCATTA TACTGATAAG GGATAATATT TAAATTACTT TACTGTTATA GAAGCCAAAA   
  
  
+ ATCTAAACTT TCATACAGAC GTCGGACTAA ATTAGAAAAT TCTTTAACTT AATAGAAAGG ATGACATAAA   
  
  
+ TAAAAATAAA AACCTAAATA GCATAATAAC CATTCTACAC AAAAGTCAAT CTTCTAATAG AAAAAAGCCA   
  
  
+ TAAGTATTCA CTTATTTACC ATTTTATTCC TAATCTACTG TCTGTCTATT CACACCATAA TTCATGAACC   
  
  
+ AATGTGGAGT TTCTGTTTCA TTTCATGGGG TGATTTATGT CTGACAATGA TCCAACAAAC TGAGCCTCAC   
  
  
+ GCGCGGAACA CGAGAGTGGC TGTACTTATG GTGTGAAAGA GCTCTACAAG GTGAGATTGA GGAGGCTACG   
  
  
+ CGTTGGAGTT ATCACCACCG TTGGATTTGT TTTCTTTGGA GCCACGTTAT CACTCTTCAG ACGCCGATCC   
  
  
+ AACGGCCCTT ACTTCCATCT AGTCCCCACT CTCCAACTCT ACGCAATCAA CGCCTTCCGC ATTTAAATAT   
  
  
+ TTCCTTCTCT ATCGCCTACT TTCTCTGTCT CACAATTTTT GTCCGTATTT TGAATTTTCT CAAAATTATC   
  
  
+ ATATAATTTA TTTGCCTTCA ACTAAAGTAT TTATTAAATT AGTTAATGTA TTTAAATTGA ACTAAAAAAG   
  
  
+ AGAAAAAGAA AGAAAACAAC CCCACGAAAG CAAAACGACA GAGGCCAACT TCTCTCTCTA GAGTGTAGAG   
  
  
+ CCCCATCTCG GTTATAATTA TGAGAATCAA TTCCTTCTCA CAAGAGAAAC TCTCGCTCTC TCTCTCTCTC   
  
  
+ CATAAGACAT CACACCAATT GTATGAATTT TCTCTCTCCT GATTTCTGTT TTCCTTCTTG TTGTCCCCTT   
  
  
+ CTAATGCGTT AGTGCGGCCA AAACCACCAT AAACCATCTC TCTTTCAAGA AAGCAGCAAC AACAAAGAGA   
  
  
+ GACAGAGAGA GAGAGAGGGG GAGAAAGATC ACATCTTTTG GGTTTCCGGC GAATATGACG TCTGGGTTTT   
  
  
+ CGGGCGATTT CTTCTACGGC GGTGGAGGCG GTGGCGGAGG AGGTGTCCGG CCTTCTATCG GCGGTGTACA   
  
  
+ AGGCGGGGTT GTCCCATATG GGTTCCGGCA ACAACAACAA CAACAGCAAC AGCAGCAGCA GCAGCAACAG   
  
  
+ CTACATCTAC AGCTACAACA ACAGCGGGGG ATTTTCTCGG CGGATCAGAT CAGCGCTCAA CGAAGGGCGG   
  
  
+ ATTTGTTGAT GGGTAAGCGT TCTTTCGCTG ATTTTCAATC TCAACAACAG CAAATCAGTC TTTTGCAGCA   
  
  
+ GCAGCAGCAG CAACAACAAC AACAGCCCTT TGCGCTCTTG CAAAAACAAC AGCAGTTTCA ACAACAATTA   
  
  
+ CAACAACAAC AGCCACTTTC TCTCCTCCTT CAACAACAGT ACTTGATGAG ATCCGTAAAA CAGAGAACCA   
  
  
+ ACAATTTCGC AGCATCTCCG ATTTCTCCTC TTTCTTCTTC CATTGATCTT TCTTCCGCTA ATAATTCCCG   
  
  
+ GTTCGGTTTG CCGGTTTACC AACAACAACT CCGGTCTCAA CAACAACCAC CTATCTCTCT CCTCGCCCAC   
  
  
+ AACAACAACC AATTCAACCT CTCAAATAAT AACAATTCTT CTTTAATCCA AAACTCCACC AACACTCTTC   
  
  
+ TTGCTCCCGC AGCCAGCAAT AACACCCTAA TGAAAAATTC GGGCTTTTCC TCATCTGGGT TTTCTAATTC   
  
  
+ GGGTCCAAAC CTGGTCCAAC CGAAGCAGGA AATCGGGTAC TCGAATATGG GTACCAATAT GGGTATGAAT   
  
  
+ CTGGTCCAGC CCAAACCCGA CCCTGAGGAT CACGGAATAA TGAACACCCT TCAAGAATTG GAGAAGGAGC   
  
  
+ TTTTGAATGA CGATGAGGAA AACCAGCCGG AAAACGACGC CGTTTCAGTC ATCACCAACA GCGAGTGGTC   
  
  
+ CGAGACCTTC CAGAATCTGA TGAGTCCCGA CCCGAGTTCG AGCCCGAATG CCACCAACAC CCACCCGGGT   
  
  
+ TCGGGTTCTG CTAGTACCAG CGGCAGCAAC AACACTACAA CCACCCCTGC AAACAACAAT AACATGATAT   
  
  
+ CTACCTCGCC GACGTCGTCC ACGTCATCCT GCTGCTCCAC GTCGGCATCG GCGTCTCCGC CGGCGGCGGT   
  
  
+ GGCGACCCCG CAGGTACCAG CCAAGCAGCT GATCTCGGAA GCAGCGGCGG CGATTTCGGA CGGAAAGATT   
  
  
+ GAATCCGCCA TGGAAGTGAT GAACCGGCTG TCCCAGGTTG CCAATTCTAG GGGGAGCTCG GAACAAAAGC   
  
  
+ TGGCTTTTTA CATGGGCCAC GCGCTTCGGT CGCGCGTGAG TCCCACCGAA CACGCTCCCC CTATTGTGGA   
  
  
+ GCTTTTCCAA AAGGAGCATA TGGCGGCCAT GTATTCATTG TACGAGCTCT CTCATTGTTT CAAATTTGGT   
  
  
+ CTTTTGGCTG CCAATTTGGT GATGTTGGAG GCCATTACTT CCGCCCTTCA TGAGGGTCTG AAAATTCATG   
  
  
+ TTGTGGATTT TGATATTGGG CATGGTGGTC AGTACATCAA CCTCCTACGC GCCTTGGGGG AGCGTAGGGC   
  
  
+ TCAGTCGGGG AGCGCGGTGT CGAATGAGGT GAGGATCACT GTGGTTGAGA CCGAGGTCGG TAATGGTGGT   
  
  
+ GCGGCGGGTG CGGAGGCTGT TAAGGATGGC CTGGTGAAAT TGGCTGAGAA GGTGGGGATT AGTTTGAAGT   
  
  
+ TTGAGGCTTT GACTCGGAAG ATCTCCGAGT TAACCCGGGA GTCACTGGGG TGTGAGGAGG GGGAGGCCGT   
  
  
+ GGTGGTGAAT TTTGCGTTCA AACTTTACCG GGTACCGGAT GAGAGCGTGT CGATGGAGAA TCTGAGGGAC   
  
  
+ GAGCTCCTGC GCCGGGTGAA GGCGCTAAGG CCACGCGCCG TCACATTGGT GGAGCAGGAC TTAAATGCGA   
  
  
+ ATACGGCACC GTTCATGACG CGCGTGAGTG GCGCTTGTGA GTACTATAGA GCATTGTTCG ACTCGCTCGA   
  
  
+ CTCGATGGTG CCGAGGGATA ACCCGGACCG GGTCCGGGTC GAGGAGGCCT TGGGGCGGAA GATGGCAAAC   
  
  
+ TCGGTGGCTT GCGAGGGCCG GGAACGGGTT GAGCGGGCGG AGGTGTTTGG GAAGTGGCGG GCGAGGATGA   
  
  
+ GTATGGCAGG GTTCGAGTTG AAGGCACTGA GTCAGAGTGT GGCCGAGTCA ATGCGAACCA AACTCAGCTC   
  
  
+ GGTTCTGGGC GGAAAGCCGG GTTTCACGGT CAAGGAGGAG AGCGGTGGGG TGGGGTTCGG GTGGATGGGA   
  
  
+ CGAACTCTCA CCGTCGCATC GGCCTGGCGT TA  

- +Up\_Stream \_Len000GGAGCC ACACCCTAAG GCCCAACTAT TTATCCCTGG TCGCTTTACA AGTTTATTAA   
  
  
- CCACTTCCTC AGAGACAAAC TTACGGATGA TTACGAAAAA AAAAAAAAAA CTTATTAAAC AGTCTTTCAA   
  
  
- TCCGACGTAA CCTTCCCCGC CGGATGCTTC CGAAAAGTCG TTTATTTATT GGTCGAAAGT AAAACTTTAA   
  
  
- AAAAAATTTT TTACTTTGAG TAAAGTGAAT TTTTAAATGG TATTATATAT ATATATATAT ATATATATAT   
  
  
- ATATATATAT ATAAACTAAA TTGCAATTCG TTTGAAAATT GAATACATAG TAAAATGCTG GCATTTTTTT   
  
  
- TTTCTTTATA TTGTGTTATA CATTATCATA AATTCAAAAA CTATTTATTA TAAAATTGTA TTAAACACAC   
  
  
- ATCATACTTA AATTTAGAAT CATGATTATA TTAATACATA TGAACCGATA TTCTAGAGTT ATAATCATAA   
  
  
- AATACTAAGT TACATTACTA CTACGTTGAC ACACATTAAT CTTTCGGAAA CTAACGTCTA TAGATAGAAT   
  
  
- AACTATGTGT AGATGGTTAA CTCGAAAAAG TTCTAAACTT CTGCCATCAG TTCATAGAAA CCCTCTAATG   
  
  
- CAATCTTAAT GAAGTACTAT TTAATCTGTA TAAAATATGA AGATTAGAAT TTATTCAGAG GTAGGAAAGT   
  
  
- AGTATAAAAT TTCTTTACAT GTTCATTTAC AGTTATCAAA CTTTGCAAGG AATAGTATTT TCAGTATGTA   
  
  
- TACATCCTGT AAAAGTTCAA ATACATTTGG TATACTATAT CAAGTTCTAT TAACAGTTTG TTCAATACTT   
  
  
- TAGATTTCCA CAAACTTGCA TTAAAGTTCG ATTAGAATCG TTCAATATCA TAATAAATTT GATGCTGATC   
  
  
- TGTGTTGTAA AACAAGTAAA TATATTGTGA ATCTTACGTT TTTTTAGCCA TAATGATTAT GACATAGTTC   
  
  
- TTAACTTCAC AATACGTAAT ATGACTATTC CCTATTATAA ATTTAATGAA ATGACAATAT CTTCGGTTTT   
  
  
- TAGATTTGAA AGTATGTCTG CAGCCTGATT TAATCTTTTA AGAAATTGAA TTATCTTTCC TACTGTATTT   
  
  
- ATTTTTATTT TTGGATTTAT CGTATTATTG GTAAGATGTG TTTTCAGTTA GAAGATTATC TTTTTTCGGT   
  
  
- ATTCATAAGT GAATAAATGG TAAAATAAGG ATTAGATGAC AGACAGATAA GTGTGGTATT AAGTACTTGG   
  
  
- TTACACCTCA AAGACAAAGT AAAGTACCCC ACTAAATACA GACTGTTACT AGGTTGTTTG ACTCGGAGTG   
  
  
- CGCGCCTTGT GCTCTCACCG ACATGAATAC CACACTTTCT CGAGATGTTC CACTCTAACT CCTCCGATGC   
  
  
- GCAACCTCAA TAGTGGTGGC AACCTAAACA AAAGAAACCT CGGTGCAATA GTGAGAAGTC TGCGGCTAGG   
  
  
- TTGCCGGGAA TGAAGGTAGA TCAGGGGTGA GAGGTTGAGA TGCGTTAGTT GCGGAAGGCG TAAATTTATA   
  
  
- AAGGAAGAGA TAGCGGATGA AAGAGACAGA GTGTTAAAAA CAGGCATAAA ACTTAAAAGA GTTTTAATAG   
  
  
- TATATTAAAT AAACGGAAGT TGATTTCATA AATAATTTAA TCAATTACAT AAATTTAACT TGATTTTTTC   
  
  
- TCTTTTTCTT TCTTTTGTTG GGGTGCTTTC GTTTTGCTGT CTCCGGTTGA AGAGAGAGAT CTCACATCTC   
  
  
- GGGGTAGAGC CAATATTAAT ACTCTTAGTT AAGGAAGAGT GTTCTCTTTG AGAGCGAGAG AGAGAGAGAG   
  
  
- GTATTCTGTA GTGTGGTTAA CATACTTAAA AGAGAGAGGA CTAAAGACAA AAGGAAGAAC AACAGGGGAA   
  
  
- GATTACGCAA TCACGCCGGT TTTGGTGGTA TTTGGTAGAG AGAAAGTTCT TTCGTCGTTG TTGTTTCTCT   
  
  
- CTGTCTCTCT CTCTCTCCCC CTCTTTCTAG TGTAGAAAAC CCAAAGGCCG CTTATACTGC AGACCCAAAA   
  
  
- GCCCGCTAAA GAAGATGCCG CCACCTCCGC CACCGCCTCC TCCACAGGCC GGAAGATAGC CGCCACATGT   
  
  
- TCCGCCCCAA CAGGGTATAC CCAAGGCCGT TGTTGTTGTT GTTGTCGTTG TCGTCGTCGT CGTCGTTGTC   
  
  
- GATGTAGATG TCGATGTTGT TGTCGCCCCC TAAAAGAGCC GCCTAGTCTA GTCGCGAGTT GCTTCCCGCC   
  
  
- TAAACAACTA CCCATTCGCA AGAAAGCGAC TAAAAGTTAG AGTTGTTGTC GTTTAGTCAG AAAACGTCGT   
  
  
- CGTCGTCGTC GTTGTTGTTG TTGTCGGGAA ACGCGAGAAC GTTTTTGTTG TCGTCAAAGT TGTTGTTAAT   
  
  
- GTTGTTGTTG TCGGTGAAAG AGAGGAGGAA GTTGTTGTCA TGAACTACTC TAGGCATTTT GTCTCTTGGT   
  
  
- TGTTAAAGCG TCGTAGAGGC TAAAGAGGAG AAAGAAGAAG GTAACTAGAA AGAAGGCGAT TATTAAGGGC   
  
  
- CAAGCCAAAC GGCCAAATGG TTGTTGTTGA GGCCAGAGTT GTTGTTGGTG GATAGAGAGA GGAGCGGGTG   
  
  
- TTGTTGTTGG TTAAGTTGGA GAGTTTATTA TTGTTAAGAA GAAATTAGGT TTTGAGGTGG TTGTGAGAAG   
  
  
- AACGAGGGCG TCGGTCGTTA TTGTGGGATT ACTTTTTAAG CCCGAAAAGG AGTAGACCCA AAAGATTAAG   
  
  
- CCCAGGTTTG GACCAGGTTG GCTTCGTCCT TTAGCCCATG AGCTTATACC CATGGTTATA CCCATACTTA   
  
  
- GACCAGGTCG GGTTTGGGCT GGGACTCCTA GTGCCTTATT ACTTGTGGGA AGTTCTTAAC CTCTTCCTCG   
  
  
- AAAACTTACT GCTACTCCTT TTGGTCGGCC TTTTGCTGCG GCAAAGTCAG TAGTGGTTGT CGCTCACCAG   
  
  
- GCTCTGGAAG GTCTTAGACT ACTCAGGGCT GGGCTCAAGC TCGGGCTTAC GGTGGTTGTG GGTGGGCCCA   
  
  
- AGCCCAAGAC GATCATGGTC GCCGTCGTTG TTGTGATGTT GGTGGGGACG TTTGTTGTTA TTGTACTATA   
  
  
- GATGGAGCGG CTGCAGCAGG TGCAGTAGGA CGACGAGGTG CAGCCGTAGC CGCAGAGGCG GCCGCCGCCA   
  
  
- CCGCTGGGGC GTCCATGGTC GGTTCGTCGA CTAGAGCCTT CGTCGCCGCC GCTAAAGCCT GCCTTTCTAA   
  
  
- CTTAGGCGGT ACCTTCACTA CTTGGCCGAC AGGGTCCAAC GGTTAAGATC CCCCTCGAGC CTTGTTTTCG   
  
  
- ACCGAAAAAT GTACCCGGTG CGCGAAGCCA GCGCGCACTC AGGGTGGCTT GTGCGAGGGG GATAACACCT   
  
  
- CGAAAAGGTT TTCCTCGTAT ACCGCCGGTA CATAAGTAAC ATGCTCGAGA GAGTAACAAA GTTTAAACCA   
  
  
- GAAAACCGAC GGTTAAACCA CTACAACCTC CGGTAATGAA GGCGGGAAGT ACTCCCAGAC TTTTAAGTAC   
  
  
- AACACCTAAA ACTATAACCC GTACCACCAG TCATGTAGTT GGAGGATGCG CGGAACCCCC TCGCATCCCG   
  
  
- AGTCAGCCCC TCGCGCCACA GCTTACTCCA CTCCTAGTGA CACCAACTCT GGCTCCAGCC ATTACCACCA   
  
  
- CGCCGCCCAC GCCTCCGACA ATTCCTACCG GACCACTTTA ACCGACTCTT CCACCCCTAA TCAAACTTCA   
  
  
- AACTCCGAAA CTGAGCCTTC TAGAGGCTCA ATTGGGCCCT CAGTGACCCC ACACTCCTCC CCCTCCGGCA   
  
  
- CCACCACTTA AAACGCAAGT TTGAAATGGC CCATGGCCTA CTCTCGCACA GCTACCTCTT AGACTCCCTG   
  
  
- CTCGAGGACG CGGCCCACTT CCGCGATTCC GGTGCGCGGC AGTGTAACCA CCTCGTCCTG AATTTACGCT   
  
  
- TATGCCGTGG CAAGTACTGC GCGCACTCAC CGCGAACACT CATGATATCT CGTAACAAGC TGAGCGAGCT   
  
  
- GAGCTACCAC GGCTCCCTAT TGGGCCTGGC CCAGGCCCAG CTCCTCCGGA ACCCCGCCTT CTACCGTTTG   
  
  
- AGCCACCGAA CGCTCCCGGC CCTTGCCCAA CTCGCCCGCC TCCACAAACC CTTCACCGCC CGCTCCTACT   
  
  
- CATACCGTCC CAAGCTCAAC TTCCGTGACT CAGTCTCACA CCGGCTCAGT TACGCTTGGT TTGAGTCGAG   
  
  
- CCAAGACCCG CCTTTCGGCC CAAAGTGCCA GTTCCTCCTC TCGCCACCCC ACCCCAAGCC CACCTACCCT   
  
  
- GCTTGAGAGT GGCAGCGTAG CCGGACCGCA AT

+     AAGAA-motif

| Site Name | Organism | Position | Strand | Matrix score. | sequence | function |
| --- | --- | --- | --- | --- | --- | --- |
| AAGAA-motif | Avena sativa | 1692 | + | 7 | GAAAGAA |  |
| AAGAA-motif | Avena sativa | 2264 | - | 7 | GAAAGAA |  |

>HU06G02537.1   
+ +Up\_Stream \_Len000CCTCGG TGTGGGATTC CGGGTTGATA AATAGGGACC AGCGAAATGT TCAAATAATT   
  
  
+ GGTGAAGGAG TCTCTGTTTG AATGCCTACT AATGCTTTTT TTTTTTTTTT GAATAATTTG TCAGAAAGTT   
  
  
+ AGGCTGCATT GGAAGGGGCG GCCTACGAAG GCTTTTCAGC AAATAAATAA CCAGCTTTCA TTTTGAAATT   
  
  
+ TTTTTTAAAA AATGAAACTC ATTTCACTTA AAAATTTACC ATAATATATA TATATATATA TATATATATA   
  
  
+ TATATATATA TATTTGATTT AACGTTAAGC AAACTTTTAA CTTATGTATC ATTTTACGAC CGTAAAAAAA   
  
  
+ AAAGAAATAT AACACAATAT GTAATAGTAT TTAAGTTTTT GATAAATAAT ATTTTAACAT AATTTGTGTG   
  
  
+ TAGTATGAAT TTAAATCTTA GTACTAATAT AATTATGTAT ACTTGGCTAT AAGATCTCAA TATTAGTATT   
  
  
+ TTATGATTCA ATGTAATGAT GATGCAACTG TGTGTAATTA GAAAGCCTTT GATTGCAGAT ATCTATCTTA   
  
  
+ TTGATACACA TCTACCAATT GAGCTTTTTC AAGATTTGAA GACGGTAGTC AAGTATCTTT GGGAGATTAC   
  
  
+ GTTAGAATTA CTTCATGATA AATTAGACAT ATTTTATACT TCTAATCTTA AATAAGTCTC CATCCTTTCA   
  
  
+ TCATATTTTA AAGAAATGTA CAAGTAAATG TCAATAGTTT GAAACGTTCC TTATCATAAA AGTCATACAT   
  
  
+ ATGTAGGACA TTTTCAAGTT TATGTAAACC ATATGATATA GTTCAAGATA ATTGTCAAAC AAGTTATGAA   
  
  
+ ATCTAAAGGT GTTTGAACGT AATTTCAAGC TAATCTTAGC AAGTTATAGT ATTATTTAAA CTACGACTAG   
  
  
+ ACACAACATT TTGTTCATTT ATATAACACT TAGAATGCAA AAAAATCGGT ATTACTAATA CTGTATCAAG   
  
  
+ AATTGAAGTG TTATGCATTA TACTGATAAG GGATAATATT TAAATTACTT TACTGTTATA GAAGCCAAAA   
  
  
+ ATCTAAACTT TCATACAGAC GTCGGACTAA ATTAGAAAAT TCTTTAACTT AATAGAAAGG ATGACATAAA   
  
  
+ TAAAAATAAA AACCTAAATA GCATAATAAC CATTCTACAC AAAAGTCAAT CTTCTAATAG AAAAAAGCCA   
  
  
+ TAAGTATTCA CTTATTTACC ATTTTATTCC TAATCTACTG TCTGTCTATT CACACCATAA TTCATGAACC   
  
  
+ AATGTGGAGT TTCTGTTTCA TTTCATGGGG TGATTTATGT CTGACAATGA TCCAACAAAC TGAGCCTCAC   
  
  
+ GCGCGGAACA CGAGAGTGGC TGTACTTATG GTGTGAAAGA GCTCTACAAG GTGAGATTGA GGAGGCTACG   
  
  
+ CGTTGGAGTT ATCACCACCG TTGGATTTGT TTTCTTTGGA GCCACGTTAT CACTCTTCAG ACGCCGATCC   
  
  
+ AACGGCCCTT ACTTCCATCT AGTCCCCACT CTCCAACTCT ACGCAATCAA CGCCTTCCGC ATTTAAATAT   
  
  
+ TTCCTTCTCT ATCGCCTACT TTCTCTGTCT CACAATTTTT GTCCGTATTT TGAATTTTCT CAAAATTATC   
  
  
+ ATATAATTTA TTTGCCTTCA ACTAAAGTAT TTATTAAATT AGTTAATGTA TTTAAATTGA ACTAAAAAAG   
  
  
+ AGAAAAAGAA AGAAAACAAC CCCACGAAAG CAAAACGACA GAGGCCAACT TCTCTCTCTA GAGTGTAGAG   
  
  
+ CCCCATCTCG GTTATAATTA TGAGAATCAA TTCCTTCTCA CAAGAGAAAC TCTCGCTCTC TCTCTCTCTC   
  
  
+ CATAAGACAT CACACCAATT GTATGAATTT TCTCTCTCCT GATTTCTGTT TTCCTTCTTG TTGTCCCCTT   
  
  
+ CTAATGCGTT AGTGCGGCCA AAACCACCAT AAACCATCTC TCTTTCAAGA AAGCAGCAAC AACAAAGAGA   
  
  
+ GACAGAGAGA GAGAGAGGGG GAGAAAGATC ACATCTTTTG GGTTTCCGGC GAATATGACG TCTGGGTTTT   
  
  
+ CGGGCGATTT CTTCTACGGC GGTGGAGGCG GTGGCGGAGG AGGTGTCCGG CCTTCTATCG GCGGTGTACA   
  
  
+ AGGCGGGGTT GTCCCATATG GGTTCCGGCA ACAACAACAA CAACAGCAAC AGCAGCAGCA GCAGCAACAG   
  
  
+ CTACATCTAC AGCTACAACA ACAGCGGGGG ATTTTCTCGG CGGATCAGAT CAGCGCTCAA CGAAGGGCGG   
  
  
+ ATTTGTTGAT GGGTAAGCGT TCTTTCGCTG ATTTTCAATC TCAACAACAG CAAATCAGTC TTTTGCAGCA   
  
  
+ GCAGCAGCAG CAACAACAAC AACAGCCCTT TGCGCTCTTG CAAAAACAAC AGCAGTTTCA ACAACAATTA   
  
  
+ CAACAACAAC AGCCACTTTC TCTCCTCCTT CAACAACAGT ACTTGATGAG ATCCGTAAAA CAGAGAACCA   
  
  
+ ACAATTTCGC AGCATCTCCG ATTTCTCCTC TTTCTTCTTC CATTGATCTT TCTTCCGCTA ATAATTCCCG   
  
  
+ GTTCGGTTTG CCGGTTTACC AACAACAACT CCGGTCTCAA CAACAACCAC CTATCTCTCT CCTCGCCCAC   
  
  
+ AACAACAACC AATTCAACCT CTCAAATAAT AACAATTCTT CTTTAATCCA AAACTCCACC AACACTCTTC   
  
  
+ TTGCTCCCGC AGCCAGCAAT AACACCCTAA TGAAAAATTC GGGCTTTTCC TCATCTGGGT TTTCTAATTC   
  
  
+ GGGTCCAAAC CTGGTCCAAC CGAAGCAGGA AATCGGGTAC TCGAATATGG GTACCAATAT GGGTATGAAT   
  
  
+ CTGGTCCAGC CCAAACCCGA CCCTGAGGAT CACGGAATAA TGAACACCCT TCAAGAATTG GAGAAGGAGC   
  
  
+ TTTTGAATGA CGATGAGGAA AACCAGCCGG AAAACGACGC CGTTTCAGTC ATCACCAACA GCGAGTGGTC   
  
  
+ CGAGACCTTC CAGAATCTGA TGAGTCCCGA CCCGAGTTCG AGCCCGAATG CCACCAACAC CCACCCGGGT   
  
  
+ TCGGGTTCTG CTAGTACCAG CGGCAGCAAC AACACTACAA CCACCCCTGC AAACAACAAT AACATGATAT   
  
  
+ CTACCTCGCC GACGTCGTCC ACGTCATCCT GCTGCTCCAC GTCGGCATCG GCGTCTCCGC CGGCGGCGGT   
  
  
+ GGCGACCCCG CAGGTACCAG CCAAGCAGCT GATCTCGGAA GCAGCGGCGG CGATTTCGGA CGGAAAGATT   
  
  
+ GAATCCGCCA TGGAAGTGAT GAACCGGCTG TCCCAGGTTG CCAATTCTAG GGGGAGCTCG GAACAAAAGC   
  
  
+ TGGCTTTTTA CATGGGCCAC GCGCTTCGGT CGCGCGTGAG TCCCACCGAA CACGCTCCCC CTATTGTGGA   
  
  
+ GCTTTTCCAA AAGGAGCATA TGGCGGCCAT GTATTCATTG TACGAGCTCT CTCATTGTTT CAAATTTGGT   
  
  
+ CTTTTGGCTG CCAATTTGGT GATGTTGGAG GCCATTACTT CCGCCCTTCA TGAGGGTCTG AAAATTCATG   
  
  
+ TTGTGGATTT TGATATTGGG CATGGTGGTC AGTACATCAA CCTCCTACGC GCCTTGGGGG AGCGTAGGGC   
  
  
+ TCAGTCGGGG AGCGCGGTGT CGAATGAGGT GAGGATCACT GTGGTTGAGA CCGAGGTCGG TAATGGTGGT   
  
  
+ GCGGCGGGTG CGGAGGCTGT TAAGGATGGC CTGGTGAAAT TGGCTGAGAA GGTGGGGATT AGTTTGAAGT   
  
  
+ TTGAGGCTTT GACTCGGAAG ATCTCCGAGT TAACCCGGGA GTCACTGGGG TGTGAGGAGG GGGAGGCCGT   
  
  
+ GGTGGTGAAT TTTGCGTTCA AACTTTACCG GGTACCGGAT GAGAGCGTGT CGATGGAGAA TCTGAGGGAC   
  
  
+ GAGCTCCTGC GCCGGGTGAA GGCGCTAAGG CCACGCGCCG TCACATTGGT GGAGCAGGAC TTAAATGCGA   
  
  
+ ATACGGCACC GTTCATGACG CGCGTGAGTG GCGCTTGTGA GTACTATAGA GCATTGTTCG ACTCGCTCGA   
  
  
+ CTCGATGGTG CCGAGGGATA ACCCGGACCG GGTCCGGGTC GAGGAGGCCT TGGGGCGGAA GATGGCAAAC   
  
  
+ TCGGTGGCTT GCGAGGGCCG GGAACGGGTT GAGCGGGCGG AGGTGTTTGG GAAGTGGCGG GCGAGGATGA   
  
  
+ GTATGGCAGG GTTCGAGTTG AAGGCACTGA GTCAGAGTGT GGCCGAGTCA ATGCGAACCA AACTCAGCTC   
  
  
+ GGTTCTGGGC GGAAAGCCGG GTTTCACGGT CAAGGAGGAG AGCGGTGGGG TGGGGTTCGG GTGGATGGGA   
  
  
+ CGAACTCTCA CCGTCGCATC GGCCTGGCGT TA  

- +Up\_Stream \_Len000GGAGCC ACACCCTAAG GCCCAACTAT TTATCCCTGG TCGCTTTACA AGTTTATTAA   
  
  
- CCACTTCCTC AGAGACAAAC TTACGGATGA TTACGAAAAA AAAAAAAAAA CTTATTAAAC AGTCTTTCAA   
  
  
- TCCGACGTAA CCTTCCCCGC CGGATGCTTC CGAAAAGTCG TTTATTTATT GGTCGAAAGT AAAACTTTAA   
  
  
- AAAAAATTTT TTACTTTGAG TAAAGTGAAT TTTTAAATGG TATTATATAT ATATATATAT ATATATATAT   
  
  
- ATATATATAT ATAAACTAAA TTGCAATTCG TTTGAAAATT GAATACATAG TAAAATGCTG GCATTTTTTT   
  
  
- TTTCTTTATA TTGTGTTATA CATTATCATA AATTCAAAAA CTATTTATTA TAAAATTGTA TTAAACACAC   
  
  
- ATCATACTTA AATTTAGAAT CATGATTATA TTAATACATA TGAACCGATA TTCTAGAGTT ATAATCATAA   
  
  
- AATACTAAGT TACATTACTA CTACGTTGAC ACACATTAAT CTTTCGGAAA CTAACGTCTA TAGATAGAAT   
  
  
- AACTATGTGT AGATGGTTAA CTCGAAAAAG TTCTAAACTT CTGCCATCAG TTCATAGAAA CCCTCTAATG   
  
  
- CAATCTTAAT GAAGTACTAT TTAATCTGTA TAAAATATGA AGATTAGAAT TTATTCAGAG GTAGGAAAGT   
  
  
- AGTATAAAAT TTCTTTACAT GTTCATTTAC AGTTATCAAA CTTTGCAAGG AATAGTATTT TCAGTATGTA   
  
  
- TACATCCTGT AAAAGTTCAA ATACATTTGG TATACTATAT CAAGTTCTAT TAACAGTTTG TTCAATACTT   
  
  
- TAGATTTCCA CAAACTTGCA TTAAAGTTCG ATTAGAATCG TTCAATATCA TAATAAATTT GATGCTGATC   
  
  
- TGTGTTGTAA AACAAGTAAA TATATTGTGA ATCTTACGTT TTTTTAGCCA TAATGATTAT GACATAGTTC   
  
  
- TTAACTTCAC AATACGTAAT ATGACTATTC CCTATTATAA ATTTAATGAA ATGACAATAT CTTCGGTTTT   
  
  
- TAGATTTGAA AGTATGTCTG CAGCCTGATT TAATCTTTTA AGAAATTGAA TTATCTTTCC TACTGTATTT   
  
  
- ATTTTTATTT TTGGATTTAT CGTATTATTG GTAAGATGTG TTTTCAGTTA GAAGATTATC TTTTTTCGGT   
  
  
- ATTCATAAGT GAATAAATGG TAAAATAAGG ATTAGATGAC AGACAGATAA GTGTGGTATT AAGTACTTGG   
  
  
- TTACACCTCA AAGACAAAGT AAAGTACCCC ACTAAATACA GACTGTTACT AGGTTGTTTG ACTCGGAGTG   
  
  
- CGCGCCTTGT GCTCTCACCG ACATGAATAC CACACTTTCT CGAGATGTTC CACTCTAACT CCTCCGATGC   
  
  
- GCAACCTCAA TAGTGGTGGC AACCTAAACA AAAGAAACCT CGGTGCAATA GTGAGAAGTC TGCGGCTAGG   
  
  
- TTGCCGGGAA TGAAGGTAGA TCAGGGGTGA GAGGTTGAGA TGCGTTAGTT GCGGAAGGCG TAAATTTATA   
  
  
- AAGGAAGAGA TAGCGGATGA AAGAGACAGA GTGTTAAAAA CAGGCATAAA ACTTAAAAGA GTTTTAATAG   
  
  
- TATATTAAAT AAACGGAAGT TGATTTCATA AATAATTTAA TCAATTACAT AAATTTAACT TGATTTTTTC   
  
  
- TCTTTTTCTT TCTTTTGTTG GGGTGCTTTC GTTTTGCTGT CTCCGGTTGA AGAGAGAGAT CTCACATCTC   
  
  
- GGGGTAGAGC CAATATTAAT ACTCTTAGTT AAGGAAGAGT GTTCTCTTTG AGAGCGAGAG AGAGAGAGAG   
  
  
- GTATTCTGTA GTGTGGTTAA CATACTTAAA AGAGAGAGGA CTAAAGACAA AAGGAAGAAC AACAGGGGAA   
  
  
- GATTACGCAA TCACGCCGGT TTTGGTGGTA TTTGGTAGAG AGAAAGTTCT TTCGTCGTTG TTGTTTCTCT   
  
  
- CTGTCTCTCT CTCTCTCCCC CTCTTTCTAG TGTAGAAAAC CCAAAGGCCG CTTATACTGC AGACCCAAAA   
  
  
- GCCCGCTAAA GAAGATGCCG CCACCTCCGC CACCGCCTCC TCCACAGGCC GGAAGATAGC CGCCACATGT   
  
  
- TCCGCCCCAA CAGGGTATAC CCAAGGCCGT TGTTGTTGTT GTTGTCGTTG TCGTCGTCGT CGTCGTTGTC   
  
  
- GATGTAGATG TCGATGTTGT TGTCGCCCCC TAAAAGAGCC GCCTAGTCTA GTCGCGAGTT GCTTCCCGCC   
  
  
- TAAACAACTA CCCATTCGCA AGAAAGCGAC TAAAAGTTAG AGTTGTTGTC GTTTAGTCAG AAAACGTCGT   
  
  
- CGTCGTCGTC GTTGTTGTTG TTGTCGGGAA ACGCGAGAAC GTTTTTGTTG TCGTCAAAGT TGTTGTTAAT   
  
  
- GTTGTTGTTG TCGGTGAAAG AGAGGAGGAA GTTGTTGTCA TGAACTACTC TAGGCATTTT GTCTCTTGGT   
  
  
- TGTTAAAGCG TCGTAGAGGC TAAAGAGGAG AAAGAAGAAG GTAACTAGAA AGAAGGCGAT TATTAAGGGC   
  
  
- CAAGCCAAAC GGCCAAATGG TTGTTGTTGA GGCCAGAGTT GTTGTTGGTG GATAGAGAGA GGAGCGGGTG   
  
  
- TTGTTGTTGG TTAAGTTGGA GAGTTTATTA TTGTTAAGAA GAAATTAGGT TTTGAGGTGG TTGTGAGAAG   
  
  
- AACGAGGGCG TCGGTCGTTA TTGTGGGATT ACTTTTTAAG CCCGAAAAGG AGTAGACCCA AAAGATTAAG   
  
  
- CCCAGGTTTG GACCAGGTTG GCTTCGTCCT TTAGCCCATG AGCTTATACC CATGGTTATA CCCATACTTA   
  
  
- GACCAGGTCG GGTTTGGGCT GGGACTCCTA GTGCCTTATT ACTTGTGGGA AGTTCTTAAC CTCTTCCTCG   
  
  
- AAAACTTACT GCTACTCCTT TTGGTCGGCC TTTTGCTGCG GCAAAGTCAG TAGTGGTTGT CGCTCACCAG   
  
  
- GCTCTGGAAG GTCTTAGACT ACTCAGGGCT GGGCTCAAGC TCGGGCTTAC GGTGGTTGTG GGTGGGCCCA   
  
  
- AGCCCAAGAC GATCATGGTC GCCGTCGTTG TTGTGATGTT GGTGGGGACG TTTGTTGTTA TTGTACTATA   
  
  
- GATGGAGCGG CTGCAGCAGG TGCAGTAGGA CGACGAGGTG CAGCCGTAGC CGCAGAGGCG GCCGCCGCCA   
  
  
- CCGCTGGGGC GTCCATGGTC GGTTCGTCGA CTAGAGCCTT CGTCGCCGCC GCTAAAGCCT GCCTTTCTAA   
  
  
- CTTAGGCGGT ACCTTCACTA CTTGGCCGAC AGGGTCCAAC GGTTAAGATC CCCCTCGAGC CTTGTTTTCG   
  
  
- ACCGAAAAAT GTACCCGGTG CGCGAAGCCA GCGCGCACTC AGGGTGGCTT GTGCGAGGGG GATAACACCT   
  
  
- CGAAAAGGTT TTCCTCGTAT ACCGCCGGTA CATAAGTAAC ATGCTCGAGA GAGTAACAAA GTTTAAACCA   
  
  
- GAAAACCGAC GGTTAAACCA CTACAACCTC CGGTAATGAA GGCGGGAAGT ACTCCCAGAC TTTTAAGTAC   
  
  
- AACACCTAAA ACTATAACCC GTACCACCAG TCATGTAGTT GGAGGATGCG CGGAACCCCC TCGCATCCCG   
  
  
- AGTCAGCCCC TCGCGCCACA GCTTACTCCA CTCCTAGTGA CACCAACTCT GGCTCCAGCC ATTACCACCA   
  
  
- CGCCGCCCAC GCCTCCGACA ATTCCTACCG GACCACTTTA ACCGACTCTT CCACCCCTAA TCAAACTTCA   
  
  
- AACTCCGAAA CTGAGCCTTC TAGAGGCTCA ATTGGGCCCT CAGTGACCCC ACACTCCTCC CCCTCCGGCA   
  
  
- CCACCACTTA AAACGCAAGT TTGAAATGGC CCATGGCCTA CTCTCGCACA GCTACCTCTT AGACTCCCTG   
  
  
- CTCGAGGACG CGGCCCACTT CCGCGATTCC GGTGCGCGGC AGTGTAACCA CCTCGTCCTG AATTTACGCT   
  
  
- TATGCCGTGG CAAGTACTGC GCGCACTCAC CGCGAACACT CATGATATCT CGTAACAAGC TGAGCGAGCT   
  
  
- GAGCTACCAC GGCTCCCTAT TGGGCCTGGC CCAGGCCCAG CTCCTCCGGA ACCCCGCCTT CTACCGTTTG   
  
  
- AGCCACCGAA CGCTCCCGGC CCTTGCCCAA CTCGCCCGCC TCCACAAACC CTTCACCGCC CGCTCCTACT   
  
  
- CATACCGTCC CAAGCTCAAC TTCCGTGACT CAGTCTCACA CCGGCTCAGT TACGCTTGGT TTGAGTCGAG   
  
  
- CCAAGACCCG CCTTTCGGCC CAAAGTGCCA GTTCCTCCTC TCGCCACCCC ACCCCAAGCC CACCTACCCT   
  
  
- GCTTGAGAGT GGCAGCGTAG CCGGACCGCA AT

+     ABRE

| Site Name | Organism | Position | Strand | Matrix score. | sequence | function |
| --- | --- | --- | --- | --- | --- | --- |
| ABRE | Arabidopsis thaliana | 342 | - | 7 | TACGGTC | cis-acting element involved in the abscisic acid responsiveness |
| ABRE | Arabidopsis thaliana | 4014 | + | 7 | AACCCGG | cis-acting element involved in the abscisic acid responsiveness |
| ABRE | Arabidopsis thaliana | 34 | - | 7 | AACCCGG | cis-acting element involved in the abscisic acid responsiveness |
| ABRE | Arabidopsis thaliana | 3122 | - | 5 | ACGTG | cis-acting element involved in the abscisic acid responsiveness |
| ABRE | Arabidopsis thaliana | 3104 | - | 5 | ACGTG | cis-acting element involved in the abscisic acid responsiveness |
| ABRE | Arabidopsis thaliana | 3746 | + | 7 | AACCCGG | cis-acting element involved in the abscisic acid responsiveness |
| ABRE | Arabidopsis thaliana | 4221 | - | 7 | AACCCGG | cis-acting element involved in the abscisic acid responsiveness |
| ABRE | Arabidopsis thaliana | 3009 | - | 7 | AACCCGG | cis-acting element involved in the abscisic acid responsiveness |
| ABRE | Arabidopsis thaliana | 1447 | - | 5 | ACGTG | cis-acting element involved in the abscisic acid responsiveness |
| ABRE | Oryza sativa | 3884 | - | 9 | GCCGCGTGGC | cis-acting element involved in the abscisic acid responsiveness |

>HU06G02537.1   
+ +Up\_Stream \_Len000CCTCGG TGTGGGATTC CGGGTTGATA AATAGGGACC AGCGAAATGT TCAAATAATT   
  
  
+ GGTGAAGGAG TCTCTGTTTG AATGCCTACT AATGCTTTTT TTTTTTTTTT GAATAATTTG TCAGAAAGTT   
  
  
+ AGGCTGCATT GGAAGGGGCG GCCTACGAAG GCTTTTCAGC AAATAAATAA CCAGCTTTCA TTTTGAAATT   
  
  
+ TTTTTTAAAA AATGAAACTC ATTTCACTTA AAAATTTACC ATAATATATA TATATATATA TATATATATA   
  
  
+ TATATATATA TATTTGATTT AACGTTAAGC AAACTTTTAA CTTATGTATC ATTTTACGAC CGTAAAAAAA   
  
  
+ AAAGAAATAT AACACAATAT GTAATAGTAT TTAAGTTTTT GATAAATAAT ATTTTAACAT AATTTGTGTG   
  
  
+ TAGTATGAAT TTAAATCTTA GTACTAATAT AATTATGTAT ACTTGGCTAT AAGATCTCAA TATTAGTATT   
  
  
+ TTATGATTCA ATGTAATGAT GATGCAACTG TGTGTAATTA GAAAGCCTTT GATTGCAGAT ATCTATCTTA   
  
  
+ TTGATACACA TCTACCAATT GAGCTTTTTC AAGATTTGAA GACGGTAGTC AAGTATCTTT GGGAGATTAC   
  
  
+ GTTAGAATTA CTTCATGATA AATTAGACAT ATTTTATACT TCTAATCTTA AATAAGTCTC CATCCTTTCA   
  
  
+ TCATATTTTA AAGAAATGTA CAAGTAAATG TCAATAGTTT GAAACGTTCC TTATCATAAA AGTCATACAT   
  
  
+ ATGTAGGACA TTTTCAAGTT TATGTAAACC ATATGATATA GTTCAAGATA ATTGTCAAAC AAGTTATGAA   
  
  
+ ATCTAAAGGT GTTTGAACGT AATTTCAAGC TAATCTTAGC AAGTTATAGT ATTATTTAAA CTACGACTAG   
  
  
+ ACACAACATT TTGTTCATTT ATATAACACT TAGAATGCAA AAAAATCGGT ATTACTAATA CTGTATCAAG   
  
  
+ AATTGAAGTG TTATGCATTA TACTGATAAG GGATAATATT TAAATTACTT TACTGTTATA GAAGCCAAAA   
  
  
+ ATCTAAACTT TCATACAGAC GTCGGACTAA ATTAGAAAAT TCTTTAACTT AATAGAAAGG ATGACATAAA   
  
  
+ TAAAAATAAA AACCTAAATA GCATAATAAC CATTCTACAC AAAAGTCAAT CTTCTAATAG AAAAAAGCCA   
  
  
+ TAAGTATTCA CTTATTTACC ATTTTATTCC TAATCTACTG TCTGTCTATT CACACCATAA TTCATGAACC   
  
  
+ AATGTGGAGT TTCTGTTTCA TTTCATGGGG TGATTTATGT CTGACAATGA TCCAACAAAC TGAGCCTCAC   
  
  
+ GCGCGGAACA CGAGAGTGGC TGTACTTATG GTGTGAAAGA GCTCTACAAG GTGAGATTGA GGAGGCTACG   
  
  
+ CGTTGGAGTT ATCACCACCG TTGGATTTGT TTTCTTTGGA GCCACGTTAT CACTCTTCAG ACGCCGATCC   
  
  
+ AACGGCCCTT ACTTCCATCT AGTCCCCACT CTCCAACTCT ACGCAATCAA CGCCTTCCGC ATTTAAATAT   
  
  
+ TTCCTTCTCT ATCGCCTACT TTCTCTGTCT CACAATTTTT GTCCGTATTT TGAATTTTCT CAAAATTATC   
  
  
+ ATATAATTTA TTTGCCTTCA ACTAAAGTAT TTATTAAATT AGTTAATGTA TTTAAATTGA ACTAAAAAAG   
  
  
+ AGAAAAAGAA AGAAAACAAC CCCACGAAAG CAAAACGACA GAGGCCAACT TCTCTCTCTA GAGTGTAGAG   
  
  
+ CCCCATCTCG GTTATAATTA TGAGAATCAA TTCCTTCTCA CAAGAGAAAC TCTCGCTCTC TCTCTCTCTC   
  
  
+ CATAAGACAT CACACCAATT GTATGAATTT TCTCTCTCCT GATTTCTGTT TTCCTTCTTG TTGTCCCCTT   
  
  
+ CTAATGCGTT AGTGCGGCCA AAACCACCAT AAACCATCTC TCTTTCAAGA AAGCAGCAAC AACAAAGAGA   
  
  
+ GACAGAGAGA GAGAGAGGGG GAGAAAGATC ACATCTTTTG GGTTTCCGGC GAATATGACG TCTGGGTTTT   
  
  
+ CGGGCGATTT CTTCTACGGC GGTGGAGGCG GTGGCGGAGG AGGTGTCCGG CCTTCTATCG GCGGTGTACA   
  
  
+ AGGCGGGGTT GTCCCATATG GGTTCCGGCA ACAACAACAA CAACAGCAAC AGCAGCAGCA GCAGCAACAG   
  
  
+ CTACATCTAC AGCTACAACA ACAGCGGGGG ATTTTCTCGG CGGATCAGAT CAGCGCTCAA CGAAGGGCGG   
  
  
+ ATTTGTTGAT GGGTAAGCGT TCTTTCGCTG ATTTTCAATC TCAACAACAG CAAATCAGTC TTTTGCAGCA   
  
  
+ GCAGCAGCAG CAACAACAAC AACAGCCCTT TGCGCTCTTG CAAAAACAAC AGCAGTTTCA ACAACAATTA   
  
  
+ CAACAACAAC AGCCACTTTC TCTCCTCCTT CAACAACAGT ACTTGATGAG ATCCGTAAAA CAGAGAACCA   
  
  
+ ACAATTTCGC AGCATCTCCG ATTTCTCCTC TTTCTTCTTC CATTGATCTT TCTTCCGCTA ATAATTCCCG   
  
  
+ GTTCGGTTTG CCGGTTTACC AACAACAACT CCGGTCTCAA CAACAACCAC CTATCTCTCT CCTCGCCCAC   
  
  
+ AACAACAACC AATTCAACCT CTCAAATAAT AACAATTCTT CTTTAATCCA AAACTCCACC AACACTCTTC   
  
  
+ TTGCTCCCGC AGCCAGCAAT AACACCCTAA TGAAAAATTC GGGCTTTTCC TCATCTGGGT TTTCTAATTC   
  
  
+ GGGTCCAAAC CTGGTCCAAC CGAAGCAGGA AATCGGGTAC TCGAATATGG GTACCAATAT GGGTATGAAT   
  
  
+ CTGGTCCAGC CCAAACCCGA CCCTGAGGAT CACGGAATAA TGAACACCCT TCAAGAATTG GAGAAGGAGC   
  
  
+ TTTTGAATGA CGATGAGGAA AACCAGCCGG AAAACGACGC CGTTTCAGTC ATCACCAACA GCGAGTGGTC   
  
  
+ CGAGACCTTC CAGAATCTGA TGAGTCCCGA CCCGAGTTCG AGCCCGAATG CCACCAACAC CCACCCGGGT   
  
  
+ TCGGGTTCTG CTAGTACCAG CGGCAGCAAC AACACTACAA CCACCCCTGC AAACAACAAT AACATGATAT   
  
  
+ CTACCTCGCC GACGTCGTCC ACGTCATCCT GCTGCTCCAC GTCGGCATCG GCGTCTCCGC CGGCGGCGGT   
  
  
+ GGCGACCCCG CAGGTACCAG CCAAGCAGCT GATCTCGGAA GCAGCGGCGG CGATTTCGGA CGGAAAGATT   
  
  
+ GAATCCGCCA TGGAAGTGAT GAACCGGCTG TCCCAGGTTG CCAATTCTAG GGGGAGCTCG GAACAAAAGC   
  
  
+ TGGCTTTTTA CATGGGCCAC GCGCTTCGGT CGCGCGTGAG TCCCACCGAA CACGCTCCCC CTATTGTGGA   
  
  
+ GCTTTTCCAA AAGGAGCATA TGGCGGCCAT GTATTCATTG TACGAGCTCT CTCATTGTTT CAAATTTGGT   
  
  
+ CTTTTGGCTG CCAATTTGGT GATGTTGGAG GCCATTACTT CCGCCCTTCA TGAGGGTCTG AAAATTCATG   
  
  
+ TTGTGGATTT TGATATTGGG CATGGTGGTC AGTACATCAA CCTCCTACGC GCCTTGGGGG AGCGTAGGGC   
  
  
+ TCAGTCGGGG AGCGCGGTGT CGAATGAGGT GAGGATCACT GTGGTTGAGA CCGAGGTCGG TAATGGTGGT   
  
  
+ GCGGCGGGTG CGGAGGCTGT TAAGGATGGC CTGGTGAAAT TGGCTGAGAA GGTGGGGATT AGTTTGAAGT   
  
  
+ TTGAGGCTTT GACTCGGAAG ATCTCCGAGT TAACCCGGGA GTCACTGGGG TGTGAGGAGG GGGAGGCCGT   
  
  
+ GGTGGTGAAT TTTGCGTTCA AACTTTACCG GGTACCGGAT GAGAGCGTGT CGATGGAGAA TCTGAGGGAC   
  
  
+ GAGCTCCTGC GCCGGGTGAA GGCGCTAAGG CCACGCGCCG TCACATTGGT GGAGCAGGAC TTAAATGCGA   
  
  
+ ATACGGCACC GTTCATGACG CGCGTGAGTG GCGCTTGTGA GTACTATAGA GCATTGTTCG ACTCGCTCGA   
  
  
+ CTCGATGGTG CCGAGGGATA ACCCGGACCG GGTCCGGGTC GAGGAGGCCT TGGGGCGGAA GATGGCAAAC   
  
  
+ TCGGTGGCTT GCGAGGGCCG GGAACGGGTT GAGCGGGCGG AGGTGTTTGG GAAGTGGCGG GCGAGGATGA   
  
  
+ GTATGGCAGG GTTCGAGTTG AAGGCACTGA GTCAGAGTGT GGCCGAGTCA ATGCGAACCA AACTCAGCTC   
  
  
+ GGTTCTGGGC GGAAAGCCGG GTTTCACGGT CAAGGAGGAG AGCGGTGGGG TGGGGTTCGG GTGGATGGGA   
  
  
+ CGAACTCTCA CCGTCGCATC GGCCTGGCGT TA  

- +Up\_Stream \_Len000GGAGCC ACACCCTAAG GCCCAACTAT TTATCCCTGG TCGCTTTACA AGTTTATTAA   
  
  
- CCACTTCCTC AGAGACAAAC TTACGGATGA TTACGAAAAA AAAAAAAAAA CTTATTAAAC AGTCTTTCAA   
  
  
- TCCGACGTAA CCTTCCCCGC CGGATGCTTC CGAAAAGTCG TTTATTTATT GGTCGAAAGT AAAACTTTAA   
  
  
- AAAAAATTTT TTACTTTGAG TAAAGTGAAT TTTTAAATGG TATTATATAT ATATATATAT ATATATATAT   
  
  
- ATATATATAT ATAAACTAAA TTGCAATTCG TTTGAAAATT GAATACATAG TAAAATGCTG GCATTTTTTT   
  
  
- TTTCTTTATA TTGTGTTATA CATTATCATA AATTCAAAAA CTATTTATTA TAAAATTGTA TTAAACACAC   
  
  
- ATCATACTTA AATTTAGAAT CATGATTATA TTAATACATA TGAACCGATA TTCTAGAGTT ATAATCATAA   
  
  
- AATACTAAGT TACATTACTA CTACGTTGAC ACACATTAAT CTTTCGGAAA CTAACGTCTA TAGATAGAAT   
  
  
- AACTATGTGT AGATGGTTAA CTCGAAAAAG TTCTAAACTT CTGCCATCAG TTCATAGAAA CCCTCTAATG   
  
  
- CAATCTTAAT GAAGTACTAT TTAATCTGTA TAAAATATGA AGATTAGAAT TTATTCAGAG GTAGGAAAGT   
  
  
- AGTATAAAAT TTCTTTACAT GTTCATTTAC AGTTATCAAA CTTTGCAAGG AATAGTATTT TCAGTATGTA   
  
  
- TACATCCTGT AAAAGTTCAA ATACATTTGG TATACTATAT CAAGTTCTAT TAACAGTTTG TTCAATACTT   
  
  
- TAGATTTCCA CAAACTTGCA TTAAAGTTCG ATTAGAATCG TTCAATATCA TAATAAATTT GATGCTGATC   
  
  
- TGTGTTGTAA AACAAGTAAA TATATTGTGA ATCTTACGTT TTTTTAGCCA TAATGATTAT GACATAGTTC   
  
  
- TTAACTTCAC AATACGTAAT ATGACTATTC CCTATTATAA ATTTAATGAA ATGACAATAT CTTCGGTTTT   
  
  
- TAGATTTGAA AGTATGTCTG CAGCCTGATT TAATCTTTTA AGAAATTGAA TTATCTTTCC TACTGTATTT   
  
  
- ATTTTTATTT TTGGATTTAT CGTATTATTG GTAAGATGTG TTTTCAGTTA GAAGATTATC TTTTTTCGGT   
  
  
- ATTCATAAGT GAATAAATGG TAAAATAAGG ATTAGATGAC AGACAGATAA GTGTGGTATT AAGTACTTGG   
  
  
- TTACACCTCA AAGACAAAGT AAAGTACCCC ACTAAATACA GACTGTTACT AGGTTGTTTG ACTCGGAGTG   
  
  
- CGCGCCTTGT GCTCTCACCG ACATGAATAC CACACTTTCT CGAGATGTTC CACTCTAACT CCTCCGATGC   
  
  
- GCAACCTCAA TAGTGGTGGC AACCTAAACA AAAGAAACCT CGGTGCAATA GTGAGAAGTC TGCGGCTAGG   
  
  
- TTGCCGGGAA TGAAGGTAGA TCAGGGGTGA GAGGTTGAGA TGCGTTAGTT GCGGAAGGCG TAAATTTATA   
  
  
- AAGGAAGAGA TAGCGGATGA AAGAGACAGA GTGTTAAAAA CAGGCATAAA ACTTAAAAGA GTTTTAATAG   
  
  
- TATATTAAAT AAACGGAAGT TGATTTCATA AATAATTTAA TCAATTACAT AAATTTAACT TGATTTTTTC   
  
  
- TCTTTTTCTT TCTTTTGTTG GGGTGCTTTC GTTTTGCTGT CTCCGGTTGA AGAGAGAGAT CTCACATCTC   
  
  
- GGGGTAGAGC CAATATTAAT ACTCTTAGTT AAGGAAGAGT GTTCTCTTTG AGAGCGAGAG AGAGAGAGAG   
  
  
- GTATTCTGTA GTGTGGTTAA CATACTTAAA AGAGAGAGGA CTAAAGACAA AAGGAAGAAC AACAGGGGAA   
  
  
- GATTACGCAA TCACGCCGGT TTTGGTGGTA TTTGGTAGAG AGAAAGTTCT TTCGTCGTTG TTGTTTCTCT   
  
  
- CTGTCTCTCT CTCTCTCCCC CTCTTTCTAG TGTAGAAAAC CCAAAGGCCG CTTATACTGC AGACCCAAAA   
  
  
- GCCCGCTAAA GAAGATGCCG CCACCTCCGC CACCGCCTCC TCCACAGGCC GGAAGATAGC CGCCACATGT   
  
  
- TCCGCCCCAA CAGGGTATAC CCAAGGCCGT TGTTGTTGTT GTTGTCGTTG TCGTCGTCGT CGTCGTTGTC   
  
  
- GATGTAGATG TCGATGTTGT TGTCGCCCCC TAAAAGAGCC GCCTAGTCTA GTCGCGAGTT GCTTCCCGCC   
  
  
- TAAACAACTA CCCATTCGCA AGAAAGCGAC TAAAAGTTAG AGTTGTTGTC GTTTAGTCAG AAAACGTCGT   
  
  
- CGTCGTCGTC GTTGTTGTTG TTGTCGGGAA ACGCGAGAAC GTTTTTGTTG TCGTCAAAGT TGTTGTTAAT   
  
  
- GTTGTTGTTG TCGGTGAAAG AGAGGAGGAA GTTGTTGTCA TGAACTACTC TAGGCATTTT GTCTCTTGGT   
  
  
- TGTTAAAGCG TCGTAGAGGC TAAAGAGGAG AAAGAAGAAG GTAACTAGAA AGAAGGCGAT TATTAAGGGC   
  
  
- CAAGCCAAAC GGCCAAATGG TTGTTGTTGA GGCCAGAGTT GTTGTTGGTG GATAGAGAGA GGAGCGGGTG   
  
  
- TTGTTGTTGG TTAAGTTGGA GAGTTTATTA TTGTTAAGAA GAAATTAGGT TTTGAGGTGG TTGTGAGAAG   
  
  
- AACGAGGGCG TCGGTCGTTA TTGTGGGATT ACTTTTTAAG CCCGAAAAGG AGTAGACCCA AAAGATTAAG   
  
  
- CCCAGGTTTG GACCAGGTTG GCTTCGTCCT TTAGCCCATG AGCTTATACC CATGGTTATA CCCATACTTA   
  
  
- GACCAGGTCG GGTTTGGGCT GGGACTCCTA GTGCCTTATT ACTTGTGGGA AGTTCTTAAC CTCTTCCTCG   
  
  
- AAAACTTACT GCTACTCCTT TTGGTCGGCC TTTTGCTGCG GCAAAGTCAG TAGTGGTTGT CGCTCACCAG   
  
  
- GCTCTGGAAG GTCTTAGACT ACTCAGGGCT GGGCTCAAGC TCGGGCTTAC GGTGGTTGTG GGTGGGCCCA   
  
  
- AGCCCAAGAC GATCATGGTC GCCGTCGTTG TTGTGATGTT GGTGGGGACG TTTGTTGTTA TTGTACTATA   
  
  
- GATGGAGCGG CTGCAGCAGG TGCAGTAGGA CGACGAGGTG CAGCCGTAGC CGCAGAGGCG GCCGCCGCCA   
  
  
- CCGCTGGGGC GTCCATGGTC GGTTCGTCGA CTAGAGCCTT CGTCGCCGCC GCTAAAGCCT GCCTTTCTAA   
  
  
- CTTAGGCGGT ACCTTCACTA CTTGGCCGAC AGGGTCCAAC GGTTAAGATC CCCCTCGAGC CTTGTTTTCG   
  
  
- ACCGAAAAAT GTACCCGGTG CGCGAAGCCA GCGCGCACTC AGGGTGGCTT GTGCGAGGGG GATAACACCT   
  
  
- CGAAAAGGTT TTCCTCGTAT ACCGCCGGTA CATAAGTAAC ATGCTCGAGA GAGTAACAAA GTTTAAACCA   
  
  
- GAAAACCGAC GGTTAAACCA CTACAACCTC CGGTAATGAA GGCGGGAAGT ACTCCCAGAC TTTTAAGTAC   
  
  
- AACACCTAAA ACTATAACCC GTACCACCAG TCATGTAGTT GGAGGATGCG CGGAACCCCC TCGCATCCCG   
  
  
- AGTCAGCCCC TCGCGCCACA GCTTACTCCA CTCCTAGTGA CACCAACTCT GGCTCCAGCC ATTACCACCA   
  
  
- CGCCGCCCAC GCCTCCGACA ATTCCTACCG GACCACTTTA ACCGACTCTT CCACCCCTAA TCAAACTTCA   
  
  
- AACTCCGAAA CTGAGCCTTC TAGAGGCTCA ATTGGGCCCT CAGTGACCCC ACACTCCTCC CCCTCCGGCA   
  
  
- CCACCACTTA AAACGCAAGT TTGAAATGGC CCATGGCCTA CTCTCGCACA GCTACCTCTT AGACTCCCTG   
  
  
- CTCGAGGACG CGGCCCACTT CCGCGATTCC GGTGCGCGGC AGTGTAACCA CCTCGTCCTG AATTTACGCT   
  
  
- TATGCCGTGG CAAGTACTGC GCGCACTCAC CGCGAACACT CATGATATCT CGTAACAAGC TGAGCGAGCT   
  
  
- GAGCTACCAC GGCTCCCTAT TGGGCCTGGC CCAGGCCCAG CTCCTCCGGA ACCCCGCCTT CTACCGTTTG   
  
  
- AGCCACCGAA CGCTCCCGGC CCTTGCCCAA CTCGCCCGCC TCCACAAACC CTTCACCGCC CGCTCCTACT   
  
  
- CATACCGTCC CAAGCTCAAC TTCCGTGACT CAGTCTCACA CCGGCTCAGT TACGCTTGGT TTGAGTCGAG   
  
  
- CCAAGACCCG CCTTTCGGCC CAAAGTGCCA GTTCCTCCTC TCGCCACCCC ACCCCAAGCC CACCTACCCT   
  
  
- GCTTGAGAGT GGCAGCGTAG CCGGACCGCA AT

+     AC-II

| Site Name | Organism | Position | Strand | Matrix score. | sequence | function |
| --- | --- | --- | --- | --- | --- | --- |
| AC-II | Phaseolus vulgaris | 2995 | + | 11 | CCACCAACCCCC |  |

>HU06G02537.1   
+ +Up\_Stream \_Len000CCTCGG TGTGGGATTC CGGGTTGATA AATAGGGACC AGCGAAATGT TCAAATAATT   
  
  
+ GGTGAAGGAG TCTCTGTTTG AATGCCTACT AATGCTTTTT TTTTTTTTTT GAATAATTTG TCAGAAAGTT   
  
  
+ AGGCTGCATT GGAAGGGGCG GCCTACGAAG GCTTTTCAGC AAATAAATAA CCAGCTTTCA TTTTGAAATT   
  
  
+ TTTTTTAAAA AATGAAACTC ATTTCACTTA AAAATTTACC ATAATATATA TATATATATA TATATATATA   
  
  
+ TATATATATA TATTTGATTT AACGTTAAGC AAACTTTTAA CTTATGTATC ATTTTACGAC CGTAAAAAAA   
  
  
+ AAAGAAATAT AACACAATAT GTAATAGTAT TTAAGTTTTT GATAAATAAT ATTTTAACAT AATTTGTGTG   
  
  
+ TAGTATGAAT TTAAATCTTA GTACTAATAT AATTATGTAT ACTTGGCTAT AAGATCTCAA TATTAGTATT   
  
  
+ TTATGATTCA ATGTAATGAT GATGCAACTG TGTGTAATTA GAAAGCCTTT GATTGCAGAT ATCTATCTTA   
  
  
+ TTGATACACA TCTACCAATT GAGCTTTTTC AAGATTTGAA GACGGTAGTC AAGTATCTTT GGGAGATTAC   
  
  
+ GTTAGAATTA CTTCATGATA AATTAGACAT ATTTTATACT TCTAATCTTA AATAAGTCTC CATCCTTTCA   
  
  
+ TCATATTTTA AAGAAATGTA CAAGTAAATG TCAATAGTTT GAAACGTTCC TTATCATAAA AGTCATACAT   
  
  
+ ATGTAGGACA TTTTCAAGTT TATGTAAACC ATATGATATA GTTCAAGATA ATTGTCAAAC AAGTTATGAA   
  
  
+ ATCTAAAGGT GTTTGAACGT AATTTCAAGC TAATCTTAGC AAGTTATAGT ATTATTTAAA CTACGACTAG   
  
  
+ ACACAACATT TTGTTCATTT ATATAACACT TAGAATGCAA AAAAATCGGT ATTACTAATA CTGTATCAAG   
  
  
+ AATTGAAGTG TTATGCATTA TACTGATAAG GGATAATATT TAAATTACTT TACTGTTATA GAAGCCAAAA   
  
  
+ ATCTAAACTT TCATACAGAC GTCGGACTAA ATTAGAAAAT TCTTTAACTT AATAGAAAGG ATGACATAAA   
  
  
+ TAAAAATAAA AACCTAAATA GCATAATAAC CATTCTACAC AAAAGTCAAT CTTCTAATAG AAAAAAGCCA   
  
  
+ TAAGTATTCA CTTATTTACC ATTTTATTCC TAATCTACTG TCTGTCTATT CACACCATAA TTCATGAACC   
  
  
+ AATGTGGAGT TTCTGTTTCA TTTCATGGGG TGATTTATGT CTGACAATGA TCCAACAAAC TGAGCCTCAC   
  
  
+ GCGCGGAACA CGAGAGTGGC TGTACTTATG GTGTGAAAGA GCTCTACAAG GTGAGATTGA GGAGGCTACG   
  
  
+ CGTTGGAGTT ATCACCACCG TTGGATTTGT TTTCTTTGGA GCCACGTTAT CACTCTTCAG ACGCCGATCC   
  
  
+ AACGGCCCTT ACTTCCATCT AGTCCCCACT CTCCAACTCT ACGCAATCAA CGCCTTCCGC ATTTAAATAT   
  
  
+ TTCCTTCTCT ATCGCCTACT TTCTCTGTCT CACAATTTTT GTCCGTATTT TGAATTTTCT CAAAATTATC   
  
  
+ ATATAATTTA TTTGCCTTCA ACTAAAGTAT TTATTAAATT AGTTAATGTA TTTAAATTGA ACTAAAAAAG   
  
  
+ AGAAAAAGAA AGAAAACAAC CCCACGAAAG CAAAACGACA GAGGCCAACT TCTCTCTCTA GAGTGTAGAG   
  
  
+ CCCCATCTCG GTTATAATTA TGAGAATCAA TTCCTTCTCA CAAGAGAAAC TCTCGCTCTC TCTCTCTCTC   
  
  
+ CATAAGACAT CACACCAATT GTATGAATTT TCTCTCTCCT GATTTCTGTT TTCCTTCTTG TTGTCCCCTT   
  
  
+ CTAATGCGTT AGTGCGGCCA AAACCACCAT AAACCATCTC TCTTTCAAGA AAGCAGCAAC AACAAAGAGA   
  
  
+ GACAGAGAGA GAGAGAGGGG GAGAAAGATC ACATCTTTTG GGTTTCCGGC GAATATGACG TCTGGGTTTT   
  
  
+ CGGGCGATTT CTTCTACGGC GGTGGAGGCG GTGGCGGAGG AGGTGTCCGG CCTTCTATCG GCGGTGTACA   
  
  
+ AGGCGGGGTT GTCCCATATG GGTTCCGGCA ACAACAACAA CAACAGCAAC AGCAGCAGCA GCAGCAACAG   
  
  
+ CTACATCTAC AGCTACAACA ACAGCGGGGG ATTTTCTCGG CGGATCAGAT CAGCGCTCAA CGAAGGGCGG   
  
  
+ ATTTGTTGAT GGGTAAGCGT TCTTTCGCTG ATTTTCAATC TCAACAACAG CAAATCAGTC TTTTGCAGCA   
  
  
+ GCAGCAGCAG CAACAACAAC AACAGCCCTT TGCGCTCTTG CAAAAACAAC AGCAGTTTCA ACAACAATTA   
  
  
+ CAACAACAAC AGCCACTTTC TCTCCTCCTT CAACAACAGT ACTTGATGAG ATCCGTAAAA CAGAGAACCA   
  
  
+ ACAATTTCGC AGCATCTCCG ATTTCTCCTC TTTCTTCTTC CATTGATCTT TCTTCCGCTA ATAATTCCCG   
  
  
+ GTTCGGTTTG CCGGTTTACC AACAACAACT CCGGTCTCAA CAACAACCAC CTATCTCTCT CCTCGCCCAC   
  
  
+ AACAACAACC AATTCAACCT CTCAAATAAT AACAATTCTT CTTTAATCCA AAACTCCACC AACACTCTTC   
  
  
+ TTGCTCCCGC AGCCAGCAAT AACACCCTAA TGAAAAATTC GGGCTTTTCC TCATCTGGGT TTTCTAATTC   
  
  
+ GGGTCCAAAC CTGGTCCAAC CGAAGCAGGA AATCGGGTAC TCGAATATGG GTACCAATAT GGGTATGAAT   
  
  
+ CTGGTCCAGC CCAAACCCGA CCCTGAGGAT CACGGAATAA TGAACACCCT TCAAGAATTG GAGAAGGAGC   
  
  
+ TTTTGAATGA CGATGAGGAA AACCAGCCGG AAAACGACGC CGTTTCAGTC ATCACCAACA GCGAGTGGTC   
  
  
+ CGAGACCTTC CAGAATCTGA TGAGTCCCGA CCCGAGTTCG AGCCCGAATG CCACCAACAC CCACCCGGGT   
  
  
+ TCGGGTTCTG CTAGTACCAG CGGCAGCAAC AACACTACAA CCACCCCTGC AAACAACAAT AACATGATAT   
  
  
+ CTACCTCGCC GACGTCGTCC ACGTCATCCT GCTGCTCCAC GTCGGCATCG GCGTCTCCGC CGGCGGCGGT   
  
  
+ GGCGACCCCG CAGGTACCAG CCAAGCAGCT GATCTCGGAA GCAGCGGCGG CGATTTCGGA CGGAAAGATT   
  
  
+ GAATCCGCCA TGGAAGTGAT GAACCGGCTG TCCCAGGTTG CCAATTCTAG GGGGAGCTCG GAACAAAAGC   
  
  
+ TGGCTTTTTA CATGGGCCAC GCGCTTCGGT CGCGCGTGAG TCCCACCGAA CACGCTCCCC CTATTGTGGA   
  
  
+ GCTTTTCCAA AAGGAGCATA TGGCGGCCAT GTATTCATTG TACGAGCTCT CTCATTGTTT CAAATTTGGT   
  
  
+ CTTTTGGCTG CCAATTTGGT GATGTTGGAG GCCATTACTT CCGCCCTTCA TGAGGGTCTG AAAATTCATG   
  
  
+ TTGTGGATTT TGATATTGGG CATGGTGGTC AGTACATCAA CCTCCTACGC GCCTTGGGGG AGCGTAGGGC   
  
  
+ TCAGTCGGGG AGCGCGGTGT CGAATGAGGT GAGGATCACT GTGGTTGAGA CCGAGGTCGG TAATGGTGGT   
  
  
+ GCGGCGGGTG CGGAGGCTGT TAAGGATGGC CTGGTGAAAT TGGCTGAGAA GGTGGGGATT AGTTTGAAGT   
  
  
+ TTGAGGCTTT GACTCGGAAG ATCTCCGAGT TAACCCGGGA GTCACTGGGG TGTGAGGAGG GGGAGGCCGT   
  
  
+ GGTGGTGAAT TTTGCGTTCA AACTTTACCG GGTACCGGAT GAGAGCGTGT CGATGGAGAA TCTGAGGGAC   
  
  
+ GAGCTCCTGC GCCGGGTGAA GGCGCTAAGG CCACGCGCCG TCACATTGGT GGAGCAGGAC TTAAATGCGA   
  
  
+ ATACGGCACC GTTCATGACG CGCGTGAGTG GCGCTTGTGA GTACTATAGA GCATTGTTCG ACTCGCTCGA   
  
  
+ CTCGATGGTG CCGAGGGATA ACCCGGACCG GGTCCGGGTC GAGGAGGCCT TGGGGCGGAA GATGGCAAAC   
  
  
+ TCGGTGGCTT GCGAGGGCCG GGAACGGGTT GAGCGGGCGG AGGTGTTTGG GAAGTGGCGG GCGAGGATGA   
  
  
+ GTATGGCAGG GTTCGAGTTG AAGGCACTGA GTCAGAGTGT GGCCGAGTCA ATGCGAACCA AACTCAGCTC   
  
  
+ GGTTCTGGGC GGAAAGCCGG GTTTCACGGT CAAGGAGGAG AGCGGTGGGG TGGGGTTCGG GTGGATGGGA   
  
  
+ CGAACTCTCA CCGTCGCATC GGCCTGGCGT TA  

- +Up\_Stream \_Len000GGAGCC ACACCCTAAG GCCCAACTAT TTATCCCTGG TCGCTTTACA AGTTTATTAA   
  
  
- CCACTTCCTC AGAGACAAAC TTACGGATGA TTACGAAAAA AAAAAAAAAA CTTATTAAAC AGTCTTTCAA   
  
  
- TCCGACGTAA CCTTCCCCGC CGGATGCTTC CGAAAAGTCG TTTATTTATT GGTCGAAAGT AAAACTTTAA   
  
  
- AAAAAATTTT TTACTTTGAG TAAAGTGAAT TTTTAAATGG TATTATATAT ATATATATAT ATATATATAT   
  
  
- ATATATATAT ATAAACTAAA TTGCAATTCG TTTGAAAATT GAATACATAG TAAAATGCTG GCATTTTTTT   
  
  
- TTTCTTTATA TTGTGTTATA CATTATCATA AATTCAAAAA CTATTTATTA TAAAATTGTA TTAAACACAC   
  
  
- ATCATACTTA AATTTAGAAT CATGATTATA TTAATACATA TGAACCGATA TTCTAGAGTT ATAATCATAA   
  
  
- AATACTAAGT TACATTACTA CTACGTTGAC ACACATTAAT CTTTCGGAAA CTAACGTCTA TAGATAGAAT   
  
  
- AACTATGTGT AGATGGTTAA CTCGAAAAAG TTCTAAACTT CTGCCATCAG TTCATAGAAA CCCTCTAATG   
  
  
- CAATCTTAAT GAAGTACTAT TTAATCTGTA TAAAATATGA AGATTAGAAT TTATTCAGAG GTAGGAAAGT   
  
  
- AGTATAAAAT TTCTTTACAT GTTCATTTAC AGTTATCAAA CTTTGCAAGG AATAGTATTT TCAGTATGTA   
  
  
- TACATCCTGT AAAAGTTCAA ATACATTTGG TATACTATAT CAAGTTCTAT TAACAGTTTG TTCAATACTT   
  
  
- TAGATTTCCA CAAACTTGCA TTAAAGTTCG ATTAGAATCG TTCAATATCA TAATAAATTT GATGCTGATC   
  
  
- TGTGTTGTAA AACAAGTAAA TATATTGTGA ATCTTACGTT TTTTTAGCCA TAATGATTAT GACATAGTTC   
  
  
- TTAACTTCAC AATACGTAAT ATGACTATTC CCTATTATAA ATTTAATGAA ATGACAATAT CTTCGGTTTT   
  
  
- TAGATTTGAA AGTATGTCTG CAGCCTGATT TAATCTTTTA AGAAATTGAA TTATCTTTCC TACTGTATTT   
  
  
- ATTTTTATTT TTGGATTTAT CGTATTATTG GTAAGATGTG TTTTCAGTTA GAAGATTATC TTTTTTCGGT   
  
  
- ATTCATAAGT GAATAAATGG TAAAATAAGG ATTAGATGAC AGACAGATAA GTGTGGTATT AAGTACTTGG   
  
  
- TTACACCTCA AAGACAAAGT AAAGTACCCC ACTAAATACA GACTGTTACT AGGTTGTTTG ACTCGGAGTG   
  
  
- CGCGCCTTGT GCTCTCACCG ACATGAATAC CACACTTTCT CGAGATGTTC CACTCTAACT CCTCCGATGC   
  
  
- GCAACCTCAA TAGTGGTGGC AACCTAAACA AAAGAAACCT CGGTGCAATA GTGAGAAGTC TGCGGCTAGG   
  
  
- TTGCCGGGAA TGAAGGTAGA TCAGGGGTGA GAGGTTGAGA TGCGTTAGTT GCGGAAGGCG TAAATTTATA   
  
  
- AAGGAAGAGA TAGCGGATGA AAGAGACAGA GTGTTAAAAA CAGGCATAAA ACTTAAAAGA GTTTTAATAG   
  
  
- TATATTAAAT AAACGGAAGT TGATTTCATA AATAATTTAA TCAATTACAT AAATTTAACT TGATTTTTTC   
  
  
- TCTTTTTCTT TCTTTTGTTG GGGTGCTTTC GTTTTGCTGT CTCCGGTTGA AGAGAGAGAT CTCACATCTC   
  
  
- GGGGTAGAGC CAATATTAAT ACTCTTAGTT AAGGAAGAGT GTTCTCTTTG AGAGCGAGAG AGAGAGAGAG   
  
  
- GTATTCTGTA GTGTGGTTAA CATACTTAAA AGAGAGAGGA CTAAAGACAA AAGGAAGAAC AACAGGGGAA   
  
  
- GATTACGCAA TCACGCCGGT TTTGGTGGTA TTTGGTAGAG AGAAAGTTCT TTCGTCGTTG TTGTTTCTCT   
  
  
- CTGTCTCTCT CTCTCTCCCC CTCTTTCTAG TGTAGAAAAC CCAAAGGCCG CTTATACTGC AGACCCAAAA   
  
  
- GCCCGCTAAA GAAGATGCCG CCACCTCCGC CACCGCCTCC TCCACAGGCC GGAAGATAGC CGCCACATGT   
  
  
- TCCGCCCCAA CAGGGTATAC CCAAGGCCGT TGTTGTTGTT GTTGTCGTTG TCGTCGTCGT CGTCGTTGTC   
  
  
- GATGTAGATG TCGATGTTGT TGTCGCCCCC TAAAAGAGCC GCCTAGTCTA GTCGCGAGTT GCTTCCCGCC   
  
  
- TAAACAACTA CCCATTCGCA AGAAAGCGAC TAAAAGTTAG AGTTGTTGTC GTTTAGTCAG AAAACGTCGT   
  
  
- CGTCGTCGTC GTTGTTGTTG TTGTCGGGAA ACGCGAGAAC GTTTTTGTTG TCGTCAAAGT TGTTGTTAAT   
  
  
- GTTGTTGTTG TCGGTGAAAG AGAGGAGGAA GTTGTTGTCA TGAACTACTC TAGGCATTTT GTCTCTTGGT   
  
  
- TGTTAAAGCG TCGTAGAGGC TAAAGAGGAG AAAGAAGAAG GTAACTAGAA AGAAGGCGAT TATTAAGGGC   
  
  
- CAAGCCAAAC GGCCAAATGG TTGTTGTTGA GGCCAGAGTT GTTGTTGGTG GATAGAGAGA GGAGCGGGTG   
  
  
- TTGTTGTTGG TTAAGTTGGA GAGTTTATTA TTGTTAAGAA GAAATTAGGT TTTGAGGTGG TTGTGAGAAG   
  
  
- AACGAGGGCG TCGGTCGTTA TTGTGGGATT ACTTTTTAAG CCCGAAAAGG AGTAGACCCA AAAGATTAAG   
  
  
- CCCAGGTTTG GACCAGGTTG GCTTCGTCCT TTAGCCCATG AGCTTATACC CATGGTTATA CCCATACTTA   
  
  
- GACCAGGTCG GGTTTGGGCT GGGACTCCTA GTGCCTTATT ACTTGTGGGA AGTTCTTAAC CTCTTCCTCG   
  
  
- AAAACTTACT GCTACTCCTT TTGGTCGGCC TTTTGCTGCG GCAAAGTCAG TAGTGGTTGT CGCTCACCAG   
  
  
- GCTCTGGAAG GTCTTAGACT ACTCAGGGCT GGGCTCAAGC TCGGGCTTAC GGTGGTTGTG GGTGGGCCCA   
  
  
- AGCCCAAGAC GATCATGGTC GCCGTCGTTG TTGTGATGTT GGTGGGGACG TTTGTTGTTA TTGTACTATA   
  
  
- GATGGAGCGG CTGCAGCAGG TGCAGTAGGA CGACGAGGTG CAGCCGTAGC CGCAGAGGCG GCCGCCGCCA   
  
  
- CCGCTGGGGC GTCCATGGTC GGTTCGTCGA CTAGAGCCTT CGTCGCCGCC GCTAAAGCCT GCCTTTCTAA   
  
  
- CTTAGGCGGT ACCTTCACTA CTTGGCCGAC AGGGTCCAAC GGTTAAGATC CCCCTCGAGC CTTGTTTTCG   
  
  
- ACCGAAAAAT GTACCCGGTG CGCGAAGCCA GCGCGCACTC AGGGTGGCTT GTGCGAGGGG GATAACACCT   
  
  
- CGAAAAGGTT TTCCTCGTAT ACCGCCGGTA CATAAGTAAC ATGCTCGAGA GAGTAACAAA GTTTAAACCA   
  
  
- GAAAACCGAC GGTTAAACCA CTACAACCTC CGGTAATGAA GGCGGGAAGT ACTCCCAGAC TTTTAAGTAC   
  
  
- AACACCTAAA ACTATAACCC GTACCACCAG TCATGTAGTT GGAGGATGCG CGGAACCCCC TCGCATCCCG   
  
  
- AGTCAGCCCC TCGCGCCACA GCTTACTCCA CTCCTAGTGA CACCAACTCT GGCTCCAGCC ATTACCACCA   
  
  
- CGCCGCCCAC GCCTCCGACA ATTCCTACCG GACCACTTTA ACCGACTCTT CCACCCCTAA TCAAACTTCA   
  
  
- AACTCCGAAA CTGAGCCTTC TAGAGGCTCA ATTGGGCCCT CAGTGACCCC ACACTCCTCC CCCTCCGGCA   
  
  
- CCACCACTTA AAACGCAAGT TTGAAATGGC CCATGGCCTA CTCTCGCACA GCTACCTCTT AGACTCCCTG   
  
  
- CTCGAGGACG CGGCCCACTT CCGCGATTCC GGTGCGCGGC AGTGTAACCA CCTCGTCCTG AATTTACGCT   
  
  
- TATGCCGTGG CAAGTACTGC GCGCACTCAC CGCGAACACT CATGATATCT CGTAACAAGC TGAGCGAGCT   
  
  
- GAGCTACCAC GGCTCCCTAT TGGGCCTGGC CCAGGCCCAG CTCCTCCGGA ACCCCGCCTT CTACCGTTTG   
  
  
- AGCCACCGAA CGCTCCCGGC CCTTGCCCAA CTCGCCCGCC TCCACAAACC CTTCACCGCC CGCTCCTACT   
  
  
- CATACCGTCC CAAGCTCAAC TTCCGTGACT CAGTCTCACA CCGGCTCAGT TACGCTTGGT TTGAGTCGAG   
  
  
- CCAAGACCCG CCTTTCGGCC CAAAGTGCCA GTTCCTCCTC TCGCCACCCC ACCCCAAGCC CACCTACCCT   
  
  
- GCTTGAGAGT GGCAGCGTAG CCGGACCGCA AT

+     ACE

| Site Name | Organism | Position | Strand | Matrix score. | sequence | function |
| --- | --- | --- | --- | --- | --- | --- |
| ACE | Petroselinum crispum | 1636 | + | 9 | CTAACGTATT | cis-acting element involved in light responsiveness |
| ACE | Petroselinum crispum | 630 | - | 9 | CTAACGTATT | cis-acting element involved in light responsiveness |
| ACE | Petroselinum crispum | 1897 | - | 9 | CTAACGTATT | cis-acting element involved in light responsiveness |

>HU06G02537.1   
+ +Up\_Stream \_Len000CCTCGG TGTGGGATTC CGGGTTGATA AATAGGGACC AGCGAAATGT TCAAATAATT   
  
  
+ GGTGAAGGAG TCTCTGTTTG AATGCCTACT AATGCTTTTT TTTTTTTTTT GAATAATTTG TCAGAAAGTT   
  
  
+ AGGCTGCATT GGAAGGGGCG GCCTACGAAG GCTTTTCAGC AAATAAATAA CCAGCTTTCA TTTTGAAATT   
  
  
+ TTTTTTAAAA AATGAAACTC ATTTCACTTA AAAATTTACC ATAATATATA TATATATATA TATATATATA   
  
  
+ TATATATATA TATTTGATTT AACGTTAAGC AAACTTTTAA CTTATGTATC ATTTTACGAC CGTAAAAAAA   
  
  
+ AAAGAAATAT AACACAATAT GTAATAGTAT TTAAGTTTTT GATAAATAAT ATTTTAACAT AATTTGTGTG   
  
  
+ TAGTATGAAT TTAAATCTTA GTACTAATAT AATTATGTAT ACTTGGCTAT AAGATCTCAA TATTAGTATT   
  
  
+ TTATGATTCA ATGTAATGAT GATGCAACTG TGTGTAATTA GAAAGCCTTT GATTGCAGAT ATCTATCTTA   
  
  
+ TTGATACACA TCTACCAATT GAGCTTTTTC AAGATTTGAA GACGGTAGTC AAGTATCTTT GGGAGATTAC   
  
  
+ GTTAGAATTA CTTCATGATA AATTAGACAT ATTTTATACT TCTAATCTTA AATAAGTCTC CATCCTTTCA   
  
  
+ TCATATTTTA AAGAAATGTA CAAGTAAATG TCAATAGTTT GAAACGTTCC TTATCATAAA AGTCATACAT   
  
  
+ ATGTAGGACA TTTTCAAGTT TATGTAAACC ATATGATATA GTTCAAGATA ATTGTCAAAC AAGTTATGAA   
  
  
+ ATCTAAAGGT GTTTGAACGT AATTTCAAGC TAATCTTAGC AAGTTATAGT ATTATTTAAA CTACGACTAG   
  
  
+ ACACAACATT TTGTTCATTT ATATAACACT TAGAATGCAA AAAAATCGGT ATTACTAATA CTGTATCAAG   
  
  
+ AATTGAAGTG TTATGCATTA TACTGATAAG GGATAATATT TAAATTACTT TACTGTTATA GAAGCCAAAA   
  
  
+ ATCTAAACTT TCATACAGAC GTCGGACTAA ATTAGAAAAT TCTTTAACTT AATAGAAAGG ATGACATAAA   
  
  
+ TAAAAATAAA AACCTAAATA GCATAATAAC CATTCTACAC AAAAGTCAAT CTTCTAATAG AAAAAAGCCA   
  
  
+ TAAGTATTCA CTTATTTACC ATTTTATTCC TAATCTACTG TCTGTCTATT CACACCATAA TTCATGAACC   
  
  
+ AATGTGGAGT TTCTGTTTCA TTTCATGGGG TGATTTATGT CTGACAATGA TCCAACAAAC TGAGCCTCAC   
  
  
+ GCGCGGAACA CGAGAGTGGC TGTACTTATG GTGTGAAAGA GCTCTACAAG GTGAGATTGA GGAGGCTACG   
  
  
+ CGTTGGAGTT ATCACCACCG TTGGATTTGT TTTCTTTGGA GCCACGTTAT CACTCTTCAG ACGCCGATCC   
  
  
+ AACGGCCCTT ACTTCCATCT AGTCCCCACT CTCCAACTCT ACGCAATCAA CGCCTTCCGC ATTTAAATAT   
  
  
+ TTCCTTCTCT ATCGCCTACT TTCTCTGTCT CACAATTTTT GTCCGTATTT TGAATTTTCT CAAAATTATC   
  
  
+ ATATAATTTA TTTGCCTTCA ACTAAAGTAT TTATTAAATT AGTTAATGTA TTTAAATTGA ACTAAAAAAG   
  
  
+ AGAAAAAGAA AGAAAACAAC CCCACGAAAG CAAAACGACA GAGGCCAACT TCTCTCTCTA GAGTGTAGAG   
  
  
+ CCCCATCTCG GTTATAATTA TGAGAATCAA TTCCTTCTCA CAAGAGAAAC TCTCGCTCTC TCTCTCTCTC   
  
  
+ CATAAGACAT CACACCAATT GTATGAATTT TCTCTCTCCT GATTTCTGTT TTCCTTCTTG TTGTCCCCTT   
  
  
+ CTAATGCGTT AGTGCGGCCA AAACCACCAT AAACCATCTC TCTTTCAAGA AAGCAGCAAC AACAAAGAGA   
  
  
+ GACAGAGAGA GAGAGAGGGG GAGAAAGATC ACATCTTTTG GGTTTCCGGC GAATATGACG TCTGGGTTTT   
  
  
+ CGGGCGATTT CTTCTACGGC GGTGGAGGCG GTGGCGGAGG AGGTGTCCGG CCTTCTATCG GCGGTGTACA   
  
  
+ AGGCGGGGTT GTCCCATATG GGTTCCGGCA ACAACAACAA CAACAGCAAC AGCAGCAGCA GCAGCAACAG   
  
  
+ CTACATCTAC AGCTACAACA ACAGCGGGGG ATTTTCTCGG CGGATCAGAT CAGCGCTCAA CGAAGGGCGG   
  
  
+ ATTTGTTGAT GGGTAAGCGT TCTTTCGCTG ATTTTCAATC TCAACAACAG CAAATCAGTC TTTTGCAGCA   
  
  
+ GCAGCAGCAG CAACAACAAC AACAGCCCTT TGCGCTCTTG CAAAAACAAC AGCAGTTTCA ACAACAATTA   
  
  
+ CAACAACAAC AGCCACTTTC TCTCCTCCTT CAACAACAGT ACTTGATGAG ATCCGTAAAA CAGAGAACCA   
  
  
+ ACAATTTCGC AGCATCTCCG ATTTCTCCTC TTTCTTCTTC CATTGATCTT TCTTCCGCTA ATAATTCCCG   
  
  
+ GTTCGGTTTG CCGGTTTACC AACAACAACT CCGGTCTCAA CAACAACCAC CTATCTCTCT CCTCGCCCAC   
  
  
+ AACAACAACC AATTCAACCT CTCAAATAAT AACAATTCTT CTTTAATCCA AAACTCCACC AACACTCTTC   
  
  
+ TTGCTCCCGC AGCCAGCAAT AACACCCTAA TGAAAAATTC GGGCTTTTCC TCATCTGGGT TTTCTAATTC   
  
  
+ GGGTCCAAAC CTGGTCCAAC CGAAGCAGGA AATCGGGTAC TCGAATATGG GTACCAATAT GGGTATGAAT   
  
  
+ CTGGTCCAGC CCAAACCCGA CCCTGAGGAT CACGGAATAA TGAACACCCT TCAAGAATTG GAGAAGGAGC   
  
  
+ TTTTGAATGA CGATGAGGAA AACCAGCCGG AAAACGACGC CGTTTCAGTC ATCACCAACA GCGAGTGGTC   
  
  
+ CGAGACCTTC CAGAATCTGA TGAGTCCCGA CCCGAGTTCG AGCCCGAATG CCACCAACAC CCACCCGGGT   
  
  
+ TCGGGTTCTG CTAGTACCAG CGGCAGCAAC AACACTACAA CCACCCCTGC AAACAACAAT AACATGATAT   
  
  
+ CTACCTCGCC GACGTCGTCC ACGTCATCCT GCTGCTCCAC GTCGGCATCG GCGTCTCCGC CGGCGGCGGT   
  
  
+ GGCGACCCCG CAGGTACCAG CCAAGCAGCT GATCTCGGAA GCAGCGGCGG CGATTTCGGA CGGAAAGATT   
  
  
+ GAATCCGCCA TGGAAGTGAT GAACCGGCTG TCCCAGGTTG CCAATTCTAG GGGGAGCTCG GAACAAAAGC   
  
  
+ TGGCTTTTTA CATGGGCCAC GCGCTTCGGT CGCGCGTGAG TCCCACCGAA CACGCTCCCC CTATTGTGGA   
  
  
+ GCTTTTCCAA AAGGAGCATA TGGCGGCCAT GTATTCATTG TACGAGCTCT CTCATTGTTT CAAATTTGGT   
  
  
+ CTTTTGGCTG CCAATTTGGT GATGTTGGAG GCCATTACTT CCGCCCTTCA TGAGGGTCTG AAAATTCATG   
  
  
+ TTGTGGATTT TGATATTGGG CATGGTGGTC AGTACATCAA CCTCCTACGC GCCTTGGGGG AGCGTAGGGC   
  
  
+ TCAGTCGGGG AGCGCGGTGT CGAATGAGGT GAGGATCACT GTGGTTGAGA CCGAGGTCGG TAATGGTGGT   
  
  
+ GCGGCGGGTG CGGAGGCTGT TAAGGATGGC CTGGTGAAAT TGGCTGAGAA GGTGGGGATT AGTTTGAAGT   
  
  
+ TTGAGGCTTT GACTCGGAAG ATCTCCGAGT TAACCCGGGA GTCACTGGGG TGTGAGGAGG GGGAGGCCGT   
  
  
+ GGTGGTGAAT TTTGCGTTCA AACTTTACCG GGTACCGGAT GAGAGCGTGT CGATGGAGAA TCTGAGGGAC   
  
  
+ GAGCTCCTGC GCCGGGTGAA GGCGCTAAGG CCACGCGCCG TCACATTGGT GGAGCAGGAC TTAAATGCGA   
  
  
+ ATACGGCACC GTTCATGACG CGCGTGAGTG GCGCTTGTGA GTACTATAGA GCATTGTTCG ACTCGCTCGA   
  
  
+ CTCGATGGTG CCGAGGGATA ACCCGGACCG GGTCCGGGTC GAGGAGGCCT TGGGGCGGAA GATGGCAAAC   
  
  
+ TCGGTGGCTT GCGAGGGCCG GGAACGGGTT GAGCGGGCGG AGGTGTTTGG GAAGTGGCGG GCGAGGATGA   
  
  
+ GTATGGCAGG GTTCGAGTTG AAGGCACTGA GTCAGAGTGT GGCCGAGTCA ATGCGAACCA AACTCAGCTC   
  
  
+ GGTTCTGGGC GGAAAGCCGG GTTTCACGGT CAAGGAGGAG AGCGGTGGGG TGGGGTTCGG GTGGATGGGA   
  
  
+ CGAACTCTCA CCGTCGCATC GGCCTGGCGT TA  

- +Up\_Stream \_Len000GGAGCC ACACCCTAAG GCCCAACTAT TTATCCCTGG TCGCTTTACA AGTTTATTAA   
  
  
- CCACTTCCTC AGAGACAAAC TTACGGATGA TTACGAAAAA AAAAAAAAAA CTTATTAAAC AGTCTTTCAA   
  
  
- TCCGACGTAA CCTTCCCCGC CGGATGCTTC CGAAAAGTCG TTTATTTATT GGTCGAAAGT AAAACTTTAA   
  
  
- AAAAAATTTT TTACTTTGAG TAAAGTGAAT TTTTAAATGG TATTATATAT ATATATATAT ATATATATAT   
  
  
- ATATATATAT ATAAACTAAA TTGCAATTCG TTTGAAAATT GAATACATAG TAAAATGCTG GCATTTTTTT   
  
  
- TTTCTTTATA TTGTGTTATA CATTATCATA AATTCAAAAA CTATTTATTA TAAAATTGTA TTAAACACAC   
  
  
- ATCATACTTA AATTTAGAAT CATGATTATA TTAATACATA TGAACCGATA TTCTAGAGTT ATAATCATAA   
  
  
- AATACTAAGT TACATTACTA CTACGTTGAC ACACATTAAT CTTTCGGAAA CTAACGTCTA TAGATAGAAT   
  
  
- AACTATGTGT AGATGGTTAA CTCGAAAAAG TTCTAAACTT CTGCCATCAG TTCATAGAAA CCCTCTAATG   
  
  
- CAATCTTAAT GAAGTACTAT TTAATCTGTA TAAAATATGA AGATTAGAAT TTATTCAGAG GTAGGAAAGT   
  
  
- AGTATAAAAT TTCTTTACAT GTTCATTTAC AGTTATCAAA CTTTGCAAGG AATAGTATTT TCAGTATGTA   
  
  
- TACATCCTGT AAAAGTTCAA ATACATTTGG TATACTATAT CAAGTTCTAT TAACAGTTTG TTCAATACTT   
  
  
- TAGATTTCCA CAAACTTGCA TTAAAGTTCG ATTAGAATCG TTCAATATCA TAATAAATTT GATGCTGATC   
  
  
- TGTGTTGTAA AACAAGTAAA TATATTGTGA ATCTTACGTT TTTTTAGCCA TAATGATTAT GACATAGTTC   
  
  
- TTAACTTCAC AATACGTAAT ATGACTATTC CCTATTATAA ATTTAATGAA ATGACAATAT CTTCGGTTTT   
  
  
- TAGATTTGAA AGTATGTCTG CAGCCTGATT TAATCTTTTA AGAAATTGAA TTATCTTTCC TACTGTATTT   
  
  
- ATTTTTATTT TTGGATTTAT CGTATTATTG GTAAGATGTG TTTTCAGTTA GAAGATTATC TTTTTTCGGT   
  
  
- ATTCATAAGT GAATAAATGG TAAAATAAGG ATTAGATGAC AGACAGATAA GTGTGGTATT AAGTACTTGG   
  
  
- TTACACCTCA AAGACAAAGT AAAGTACCCC ACTAAATACA GACTGTTACT AGGTTGTTTG ACTCGGAGTG   
  
  
- CGCGCCTTGT GCTCTCACCG ACATGAATAC CACACTTTCT CGAGATGTTC CACTCTAACT CCTCCGATGC   
  
  
- GCAACCTCAA TAGTGGTGGC AACCTAAACA AAAGAAACCT CGGTGCAATA GTGAGAAGTC TGCGGCTAGG   
  
  
- TTGCCGGGAA TGAAGGTAGA TCAGGGGTGA GAGGTTGAGA TGCGTTAGTT GCGGAAGGCG TAAATTTATA   
  
  
- AAGGAAGAGA TAGCGGATGA AAGAGACAGA GTGTTAAAAA CAGGCATAAA ACTTAAAAGA GTTTTAATAG   
  
  
- TATATTAAAT AAACGGAAGT TGATTTCATA AATAATTTAA TCAATTACAT AAATTTAACT TGATTTTTTC   
  
  
- TCTTTTTCTT TCTTTTGTTG GGGTGCTTTC GTTTTGCTGT CTCCGGTTGA AGAGAGAGAT CTCACATCTC   
  
  
- GGGGTAGAGC CAATATTAAT ACTCTTAGTT AAGGAAGAGT GTTCTCTTTG AGAGCGAGAG AGAGAGAGAG   
  
  
- GTATTCTGTA GTGTGGTTAA CATACTTAAA AGAGAGAGGA CTAAAGACAA AAGGAAGAAC AACAGGGGAA   
  
  
- GATTACGCAA TCACGCCGGT TTTGGTGGTA TTTGGTAGAG AGAAAGTTCT TTCGTCGTTG TTGTTTCTCT   
  
  
- CTGTCTCTCT CTCTCTCCCC CTCTTTCTAG TGTAGAAAAC CCAAAGGCCG CTTATACTGC AGACCCAAAA   
  
  
- GCCCGCTAAA GAAGATGCCG CCACCTCCGC CACCGCCTCC TCCACAGGCC GGAAGATAGC CGCCACATGT   
  
  
- TCCGCCCCAA CAGGGTATAC CCAAGGCCGT TGTTGTTGTT GTTGTCGTTG TCGTCGTCGT CGTCGTTGTC   
  
  
- GATGTAGATG TCGATGTTGT TGTCGCCCCC TAAAAGAGCC GCCTAGTCTA GTCGCGAGTT GCTTCCCGCC   
  
  
- TAAACAACTA CCCATTCGCA AGAAAGCGAC TAAAAGTTAG AGTTGTTGTC GTTTAGTCAG AAAACGTCGT   
  
  
- CGTCGTCGTC GTTGTTGTTG TTGTCGGGAA ACGCGAGAAC GTTTTTGTTG TCGTCAAAGT TGTTGTTAAT   
  
  
- GTTGTTGTTG TCGGTGAAAG AGAGGAGGAA GTTGTTGTCA TGAACTACTC TAGGCATTTT GTCTCTTGGT   
  
  
- TGTTAAAGCG TCGTAGAGGC TAAAGAGGAG AAAGAAGAAG GTAACTAGAA AGAAGGCGAT TATTAAGGGC   
  
  
- CAAGCCAAAC GGCCAAATGG TTGTTGTTGA GGCCAGAGTT GTTGTTGGTG GATAGAGAGA GGAGCGGGTG   
  
  
- TTGTTGTTGG TTAAGTTGGA GAGTTTATTA TTGTTAAGAA GAAATTAGGT TTTGAGGTGG TTGTGAGAAG   
  
  
- AACGAGGGCG TCGGTCGTTA TTGTGGGATT ACTTTTTAAG CCCGAAAAGG AGTAGACCCA AAAGATTAAG   
  
  
- CCCAGGTTTG GACCAGGTTG GCTTCGTCCT TTAGCCCATG AGCTTATACC CATGGTTATA CCCATACTTA   
  
  
- GACCAGGTCG GGTTTGGGCT GGGACTCCTA GTGCCTTATT ACTTGTGGGA AGTTCTTAAC CTCTTCCTCG   
  
  
- AAAACTTACT GCTACTCCTT TTGGTCGGCC TTTTGCTGCG GCAAAGTCAG TAGTGGTTGT CGCTCACCAG   
  
  
- GCTCTGGAAG GTCTTAGACT ACTCAGGGCT GGGCTCAAGC TCGGGCTTAC GGTGGTTGTG GGTGGGCCCA   
  
  
- AGCCCAAGAC GATCATGGTC GCCGTCGTTG TTGTGATGTT GGTGGGGACG TTTGTTGTTA TTGTACTATA   
  
  
- GATGGAGCGG CTGCAGCAGG TGCAGTAGGA CGACGAGGTG CAGCCGTAGC CGCAGAGGCG GCCGCCGCCA   
  
  
- CCGCTGGGGC GTCCATGGTC GGTTCGTCGA CTAGAGCCTT CGTCGCCGCC GCTAAAGCCT GCCTTTCTAA   
  
  
- CTTAGGCGGT ACCTTCACTA CTTGGCCGAC AGGGTCCAAC GGTTAAGATC CCCCTCGAGC CTTGTTTTCG   
  
  
- ACCGAAAAAT GTACCCGGTG CGCGAAGCCA GCGCGCACTC AGGGTGGCTT GTGCGAGGGG GATAACACCT   
  
  
- CGAAAAGGTT TTCCTCGTAT ACCGCCGGTA CATAAGTAAC ATGCTCGAGA GAGTAACAAA GTTTAAACCA   
  
  
- GAAAACCGAC GGTTAAACCA CTACAACCTC CGGTAATGAA GGCGGGAAGT ACTCCCAGAC TTTTAAGTAC   
  
  
- AACACCTAAA ACTATAACCC GTACCACCAG TCATGTAGTT GGAGGATGCG CGGAACCCCC TCGCATCCCG   
  
  
- AGTCAGCCCC TCGCGCCACA GCTTACTCCA CTCCTAGTGA CACCAACTCT GGCTCCAGCC ATTACCACCA   
  
  
- CGCCGCCCAC GCCTCCGACA ATTCCTACCG GACCACTTTA ACCGACTCTT CCACCCCTAA TCAAACTTCA   
  
  
- AACTCCGAAA CTGAGCCTTC TAGAGGCTCA ATTGGGCCCT CAGTGACCCC ACACTCCTCC CCCTCCGGCA   
  
  
- CCACCACTTA AAACGCAAGT TTGAAATGGC CCATGGCCTA CTCTCGCACA GCTACCTCTT AGACTCCCTG   
  
  
- CTCGAGGACG CGGCCCACTT CCGCGATTCC GGTGCGCGGC AGTGTAACCA CCTCGTCCTG AATTTACGCT   
  
  
- TATGCCGTGG CAAGTACTGC GCGCACTCAC CGCGAACACT CATGATATCT CGTAACAAGC TGAGCGAGCT   
  
  
- GAGCTACCAC GGCTCCCTAT TGGGCCTGGC CCAGGCCCAG CTCCTCCGGA ACCCCGCCTT CTACCGTTTG   
  
  
- AGCCACCGAA CGCTCCCGGC CCTTGCCCAA CTCGCCCGCC TCCACAAACC CTTCACCGCC CGCTCCTACT   
  
  
- CATACCGTCC CAAGCTCAAC TTCCGTGACT CAGTCTCACA CCGGCTCAGT TACGCTTGGT TTGAGTCGAG   
  
  
- CCAAGACCCG CCTTTCGGCC CAAAGTGCCA GTTCCTCCTC TCGCCACCCC ACCCCAAGCC CACCTACCCT   
  
  
- GCTTGAGAGT GGCAGCGTAG CCGGACCGCA AT

+     ACTCATCCT sequence

| Site Name | Organism | Position | Strand | Matrix score. | sequence | function |
| --- | --- | --- | --- | --- | --- | --- |
| ACTCATCCT sequence | Arabidopsis thaliana | 4128 | - | 9 | ACTCATCCT |  |

>HU06G02537.1   
+ +Up\_Stream \_Len000CCTCGG TGTGGGATTC CGGGTTGATA AATAGGGACC AGCGAAATGT TCAAATAATT   
  
  
+ GGTGAAGGAG TCTCTGTTTG AATGCCTACT AATGCTTTTT TTTTTTTTTT GAATAATTTG TCAGAAAGTT   
  
  
+ AGGCTGCATT GGAAGGGGCG GCCTACGAAG GCTTTTCAGC AAATAAATAA CCAGCTTTCA TTTTGAAATT   
  
  
+ TTTTTTAAAA AATGAAACTC ATTTCACTTA AAAATTTACC ATAATATATA TATATATATA TATATATATA   
  
  
+ TATATATATA TATTTGATTT AACGTTAAGC AAACTTTTAA CTTATGTATC ATTTTACGAC CGTAAAAAAA   
  
  
+ AAAGAAATAT AACACAATAT GTAATAGTAT TTAAGTTTTT GATAAATAAT ATTTTAACAT AATTTGTGTG   
  
  
+ TAGTATGAAT TTAAATCTTA GTACTAATAT AATTATGTAT ACTTGGCTAT AAGATCTCAA TATTAGTATT   
  
  
+ TTATGATTCA ATGTAATGAT GATGCAACTG TGTGTAATTA GAAAGCCTTT GATTGCAGAT ATCTATCTTA   
  
  
+ TTGATACACA TCTACCAATT GAGCTTTTTC AAGATTTGAA GACGGTAGTC AAGTATCTTT GGGAGATTAC   
  
  
+ GTTAGAATTA CTTCATGATA AATTAGACAT ATTTTATACT TCTAATCTTA AATAAGTCTC CATCCTTTCA   
  
  
+ TCATATTTTA AAGAAATGTA CAAGTAAATG TCAATAGTTT GAAACGTTCC TTATCATAAA AGTCATACAT   
  
  
+ ATGTAGGACA TTTTCAAGTT TATGTAAACC ATATGATATA GTTCAAGATA ATTGTCAAAC AAGTTATGAA   
  
  
+ ATCTAAAGGT GTTTGAACGT AATTTCAAGC TAATCTTAGC AAGTTATAGT ATTATTTAAA CTACGACTAG   
  
  
+ ACACAACATT TTGTTCATTT ATATAACACT TAGAATGCAA AAAAATCGGT ATTACTAATA CTGTATCAAG   
  
  
+ AATTGAAGTG TTATGCATTA TACTGATAAG GGATAATATT TAAATTACTT TACTGTTATA GAAGCCAAAA   
  
  
+ ATCTAAACTT TCATACAGAC GTCGGACTAA ATTAGAAAAT TCTTTAACTT AATAGAAAGG ATGACATAAA   
  
  
+ TAAAAATAAA AACCTAAATA GCATAATAAC CATTCTACAC AAAAGTCAAT CTTCTAATAG AAAAAAGCCA   
  
  
+ TAAGTATTCA CTTATTTACC ATTTTATTCC TAATCTACTG TCTGTCTATT CACACCATAA TTCATGAACC   
  
  
+ AATGTGGAGT TTCTGTTTCA TTTCATGGGG TGATTTATGT CTGACAATGA TCCAACAAAC TGAGCCTCAC   
  
  
+ GCGCGGAACA CGAGAGTGGC TGTACTTATG GTGTGAAAGA GCTCTACAAG GTGAGATTGA GGAGGCTACG   
  
  
+ CGTTGGAGTT ATCACCACCG TTGGATTTGT TTTCTTTGGA GCCACGTTAT CACTCTTCAG ACGCCGATCC   
  
  
+ AACGGCCCTT ACTTCCATCT AGTCCCCACT CTCCAACTCT ACGCAATCAA CGCCTTCCGC ATTTAAATAT   
  
  
+ TTCCTTCTCT ATCGCCTACT TTCTCTGTCT CACAATTTTT GTCCGTATTT TGAATTTTCT CAAAATTATC   
  
  
+ ATATAATTTA TTTGCCTTCA ACTAAAGTAT TTATTAAATT AGTTAATGTA TTTAAATTGA ACTAAAAAAG   
  
  
+ AGAAAAAGAA AGAAAACAAC CCCACGAAAG CAAAACGACA GAGGCCAACT TCTCTCTCTA GAGTGTAGAG   
  
  
+ CCCCATCTCG GTTATAATTA TGAGAATCAA TTCCTTCTCA CAAGAGAAAC TCTCGCTCTC TCTCTCTCTC   
  
  
+ CATAAGACAT CACACCAATT GTATGAATTT TCTCTCTCCT GATTTCTGTT TTCCTTCTTG TTGTCCCCTT   
  
  
+ CTAATGCGTT AGTGCGGCCA AAACCACCAT AAACCATCTC TCTTTCAAGA AAGCAGCAAC AACAAAGAGA   
  
  
+ GACAGAGAGA GAGAGAGGGG GAGAAAGATC ACATCTTTTG GGTTTCCGGC GAATATGACG TCTGGGTTTT   
  
  
+ CGGGCGATTT CTTCTACGGC GGTGGAGGCG GTGGCGGAGG AGGTGTCCGG CCTTCTATCG GCGGTGTACA   
  
  
+ AGGCGGGGTT GTCCCATATG GGTTCCGGCA ACAACAACAA CAACAGCAAC AGCAGCAGCA GCAGCAACAG   
  
  
+ CTACATCTAC AGCTACAACA ACAGCGGGGG ATTTTCTCGG CGGATCAGAT CAGCGCTCAA CGAAGGGCGG   
  
  
+ ATTTGTTGAT GGGTAAGCGT TCTTTCGCTG ATTTTCAATC TCAACAACAG CAAATCAGTC TTTTGCAGCA   
  
  
+ GCAGCAGCAG CAACAACAAC AACAGCCCTT TGCGCTCTTG CAAAAACAAC AGCAGTTTCA ACAACAATTA   
  
  
+ CAACAACAAC AGCCACTTTC TCTCCTCCTT CAACAACAGT ACTTGATGAG ATCCGTAAAA CAGAGAACCA   
  
  
+ ACAATTTCGC AGCATCTCCG ATTTCTCCTC TTTCTTCTTC CATTGATCTT TCTTCCGCTA ATAATTCCCG   
  
  
+ GTTCGGTTTG CCGGTTTACC AACAACAACT CCGGTCTCAA CAACAACCAC CTATCTCTCT CCTCGCCCAC   
  
  
+ AACAACAACC AATTCAACCT CTCAAATAAT AACAATTCTT CTTTAATCCA AAACTCCACC AACACTCTTC   
  
  
+ TTGCTCCCGC AGCCAGCAAT AACACCCTAA TGAAAAATTC GGGCTTTTCC TCATCTGGGT TTTCTAATTC   
  
  
+ GGGTCCAAAC CTGGTCCAAC CGAAGCAGGA AATCGGGTAC TCGAATATGG GTACCAATAT GGGTATGAAT   
  
  
+ CTGGTCCAGC CCAAACCCGA CCCTGAGGAT CACGGAATAA TGAACACCCT TCAAGAATTG GAGAAGGAGC   
  
  
+ TTTTGAATGA CGATGAGGAA AACCAGCCGG AAAACGACGC CGTTTCAGTC ATCACCAACA GCGAGTGGTC   
  
  
+ CGAGACCTTC CAGAATCTGA TGAGTCCCGA CCCGAGTTCG AGCCCGAATG CCACCAACAC CCACCCGGGT   
  
  
+ TCGGGTTCTG CTAGTACCAG CGGCAGCAAC AACACTACAA CCACCCCTGC AAACAACAAT AACATGATAT   
  
  
+ CTACCTCGCC GACGTCGTCC ACGTCATCCT GCTGCTCCAC GTCGGCATCG GCGTCTCCGC CGGCGGCGGT   
  
  
+ GGCGACCCCG CAGGTACCAG CCAAGCAGCT GATCTCGGAA GCAGCGGCGG CGATTTCGGA CGGAAAGATT   
  
  
+ GAATCCGCCA TGGAAGTGAT GAACCGGCTG TCCCAGGTTG CCAATTCTAG GGGGAGCTCG GAACAAAAGC   
  
  
+ TGGCTTTTTA CATGGGCCAC GCGCTTCGGT CGCGCGTGAG TCCCACCGAA CACGCTCCCC CTATTGTGGA   
  
  
+ GCTTTTCCAA AAGGAGCATA TGGCGGCCAT GTATTCATTG TACGAGCTCT CTCATTGTTT CAAATTTGGT   
  
  
+ CTTTTGGCTG CCAATTTGGT GATGTTGGAG GCCATTACTT CCGCCCTTCA TGAGGGTCTG AAAATTCATG   
  
  
+ TTGTGGATTT TGATATTGGG CATGGTGGTC AGTACATCAA CCTCCTACGC GCCTTGGGGG AGCGTAGGGC   
  
  
+ TCAGTCGGGG AGCGCGGTGT CGAATGAGGT GAGGATCACT GTGGTTGAGA CCGAGGTCGG TAATGGTGGT   
  
  
+ GCGGCGGGTG CGGAGGCTGT TAAGGATGGC CTGGTGAAAT TGGCTGAGAA GGTGGGGATT AGTTTGAAGT   
  
  
+ TTGAGGCTTT GACTCGGAAG ATCTCCGAGT TAACCCGGGA GTCACTGGGG TGTGAGGAGG GGGAGGCCGT   
  
  
+ GGTGGTGAAT TTTGCGTTCA AACTTTACCG GGTACCGGAT GAGAGCGTGT CGATGGAGAA TCTGAGGGAC   
  
  
+ GAGCTCCTGC GCCGGGTGAA GGCGCTAAGG CCACGCGCCG TCACATTGGT GGAGCAGGAC TTAAATGCGA   
  
  
+ ATACGGCACC GTTCATGACG CGCGTGAGTG GCGCTTGTGA GTACTATAGA GCATTGTTCG ACTCGCTCGA   
  
  
+ CTCGATGGTG CCGAGGGATA ACCCGGACCG GGTCCGGGTC GAGGAGGCCT TGGGGCGGAA GATGGCAAAC   
  
  
+ TCGGTGGCTT GCGAGGGCCG GGAACGGGTT GAGCGGGCGG AGGTGTTTGG GAAGTGGCGG GCGAGGATGA   
  
  
+ GTATGGCAGG GTTCGAGTTG AAGGCACTGA GTCAGAGTGT GGCCGAGTCA ATGCGAACCA AACTCAGCTC   
  
  
+ GGTTCTGGGC GGAAAGCCGG GTTTCACGGT CAAGGAGGAG AGCGGTGGGG TGGGGTTCGG GTGGATGGGA   
  
  
+ CGAACTCTCA CCGTCGCATC GGCCTGGCGT TA  

- +Up\_Stream \_Len000GGAGCC ACACCCTAAG GCCCAACTAT TTATCCCTGG TCGCTTTACA AGTTTATTAA   
  
  
- CCACTTCCTC AGAGACAAAC TTACGGATGA TTACGAAAAA AAAAAAAAAA CTTATTAAAC AGTCTTTCAA   
  
  
- TCCGACGTAA CCTTCCCCGC CGGATGCTTC CGAAAAGTCG TTTATTTATT GGTCGAAAGT AAAACTTTAA   
  
  
- AAAAAATTTT TTACTTTGAG TAAAGTGAAT TTTTAAATGG TATTATATAT ATATATATAT ATATATATAT   
  
  
- ATATATATAT ATAAACTAAA TTGCAATTCG TTTGAAAATT GAATACATAG TAAAATGCTG GCATTTTTTT   
  
  
- TTTCTTTATA TTGTGTTATA CATTATCATA AATTCAAAAA CTATTTATTA TAAAATTGTA TTAAACACAC   
  
  
- ATCATACTTA AATTTAGAAT CATGATTATA TTAATACATA TGAACCGATA TTCTAGAGTT ATAATCATAA   
  
  
- AATACTAAGT TACATTACTA CTACGTTGAC ACACATTAAT CTTTCGGAAA CTAACGTCTA TAGATAGAAT   
  
  
- AACTATGTGT AGATGGTTAA CTCGAAAAAG TTCTAAACTT CTGCCATCAG TTCATAGAAA CCCTCTAATG   
  
  
- CAATCTTAAT GAAGTACTAT TTAATCTGTA TAAAATATGA AGATTAGAAT TTATTCAGAG GTAGGAAAGT   
  
  
- AGTATAAAAT TTCTTTACAT GTTCATTTAC AGTTATCAAA CTTTGCAAGG AATAGTATTT TCAGTATGTA   
  
  
- TACATCCTGT AAAAGTTCAA ATACATTTGG TATACTATAT CAAGTTCTAT TAACAGTTTG TTCAATACTT   
  
  
- TAGATTTCCA CAAACTTGCA TTAAAGTTCG ATTAGAATCG TTCAATATCA TAATAAATTT GATGCTGATC   
  
  
- TGTGTTGTAA AACAAGTAAA TATATTGTGA ATCTTACGTT TTTTTAGCCA TAATGATTAT GACATAGTTC   
  
  
- TTAACTTCAC AATACGTAAT ATGACTATTC CCTATTATAA ATTTAATGAA ATGACAATAT CTTCGGTTTT   
  
  
- TAGATTTGAA AGTATGTCTG CAGCCTGATT TAATCTTTTA AGAAATTGAA TTATCTTTCC TACTGTATTT   
  
  
- ATTTTTATTT TTGGATTTAT CGTATTATTG GTAAGATGTG TTTTCAGTTA GAAGATTATC TTTTTTCGGT   
  
  
- ATTCATAAGT GAATAAATGG TAAAATAAGG ATTAGATGAC AGACAGATAA GTGTGGTATT AAGTACTTGG   
  
  
- TTACACCTCA AAGACAAAGT AAAGTACCCC ACTAAATACA GACTGTTACT AGGTTGTTTG ACTCGGAGTG   
  
  
- CGCGCCTTGT GCTCTCACCG ACATGAATAC CACACTTTCT CGAGATGTTC CACTCTAACT CCTCCGATGC   
  
  
- GCAACCTCAA TAGTGGTGGC AACCTAAACA AAAGAAACCT CGGTGCAATA GTGAGAAGTC TGCGGCTAGG   
  
  
- TTGCCGGGAA TGAAGGTAGA TCAGGGGTGA GAGGTTGAGA TGCGTTAGTT GCGGAAGGCG TAAATTTATA   
  
  
- AAGGAAGAGA TAGCGGATGA AAGAGACAGA GTGTTAAAAA CAGGCATAAA ACTTAAAAGA GTTTTAATAG   
  
  
- TATATTAAAT AAACGGAAGT TGATTTCATA AATAATTTAA TCAATTACAT AAATTTAACT TGATTTTTTC   
  
  
- TCTTTTTCTT TCTTTTGTTG GGGTGCTTTC GTTTTGCTGT CTCCGGTTGA AGAGAGAGAT CTCACATCTC   
  
  
- GGGGTAGAGC CAATATTAAT ACTCTTAGTT AAGGAAGAGT GTTCTCTTTG AGAGCGAGAG AGAGAGAGAG   
  
  
- GTATTCTGTA GTGTGGTTAA CATACTTAAA AGAGAGAGGA CTAAAGACAA AAGGAAGAAC AACAGGGGAA   
  
  
- GATTACGCAA TCACGCCGGT TTTGGTGGTA TTTGGTAGAG AGAAAGTTCT TTCGTCGTTG TTGTTTCTCT   
  
  
- CTGTCTCTCT CTCTCTCCCC CTCTTTCTAG TGTAGAAAAC CCAAAGGCCG CTTATACTGC AGACCCAAAA   
  
  
- GCCCGCTAAA GAAGATGCCG CCACCTCCGC CACCGCCTCC TCCACAGGCC GGAAGATAGC CGCCACATGT   
  
  
- TCCGCCCCAA CAGGGTATAC CCAAGGCCGT TGTTGTTGTT GTTGTCGTTG TCGTCGTCGT CGTCGTTGTC   
  
  
- GATGTAGATG TCGATGTTGT TGTCGCCCCC TAAAAGAGCC GCCTAGTCTA GTCGCGAGTT GCTTCCCGCC   
  
  
- TAAACAACTA CCCATTCGCA AGAAAGCGAC TAAAAGTTAG AGTTGTTGTC GTTTAGTCAG AAAACGTCGT   
  
  
- CGTCGTCGTC GTTGTTGTTG TTGTCGGGAA ACGCGAGAAC GTTTTTGTTG TCGTCAAAGT TGTTGTTAAT   
  
  
- GTTGTTGTTG TCGGTGAAAG AGAGGAGGAA GTTGTTGTCA TGAACTACTC TAGGCATTTT GTCTCTTGGT   
  
  
- TGTTAAAGCG TCGTAGAGGC TAAAGAGGAG AAAGAAGAAG GTAACTAGAA AGAAGGCGAT TATTAAGGGC   
  
  
- CAAGCCAAAC GGCCAAATGG TTGTTGTTGA GGCCAGAGTT GTTGTTGGTG GATAGAGAGA GGAGCGGGTG   
  
  
- TTGTTGTTGG TTAAGTTGGA GAGTTTATTA TTGTTAAGAA GAAATTAGGT TTTGAGGTGG TTGTGAGAAG   
  
  
- AACGAGGGCG TCGGTCGTTA TTGTGGGATT ACTTTTTAAG CCCGAAAAGG AGTAGACCCA AAAGATTAAG   
  
  
- CCCAGGTTTG GACCAGGTTG GCTTCGTCCT TTAGCCCATG AGCTTATACC CATGGTTATA CCCATACTTA   
  
  
- GACCAGGTCG GGTTTGGGCT GGGACTCCTA GTGCCTTATT ACTTGTGGGA AGTTCTTAAC CTCTTCCTCG   
  
  
- AAAACTTACT GCTACTCCTT TTGGTCGGCC TTTTGCTGCG GCAAAGTCAG TAGTGGTTGT CGCTCACCAG   
  
  
- GCTCTGGAAG GTCTTAGACT ACTCAGGGCT GGGCTCAAGC TCGGGCTTAC GGTGGTTGTG GGTGGGCCCA   
  
  
- AGCCCAAGAC GATCATGGTC GCCGTCGTTG TTGTGATGTT GGTGGGGACG TTTGTTGTTA TTGTACTATA   
  
  
- GATGGAGCGG CTGCAGCAGG TGCAGTAGGA CGACGAGGTG CAGCCGTAGC CGCAGAGGCG GCCGCCGCCA   
  
  
- CCGCTGGGGC GTCCATGGTC GGTTCGTCGA CTAGAGCCTT CGTCGCCGCC GCTAAAGCCT GCCTTTCTAA   
  
  
- CTTAGGCGGT ACCTTCACTA CTTGGCCGAC AGGGTCCAAC GGTTAAGATC CCCCTCGAGC CTTGTTTTCG   
  
  
- ACCGAAAAAT GTACCCGGTG CGCGAAGCCA GCGCGCACTC AGGGTGGCTT GTGCGAGGGG GATAACACCT   
  
  
- CGAAAAGGTT TTCCTCGTAT ACCGCCGGTA CATAAGTAAC ATGCTCGAGA GAGTAACAAA GTTTAAACCA   
  
  
- GAAAACCGAC GGTTAAACCA CTACAACCTC CGGTAATGAA GGCGGGAAGT ACTCCCAGAC TTTTAAGTAC   
  
  
- AACACCTAAA ACTATAACCC GTACCACCAG TCATGTAGTT GGAGGATGCG CGGAACCCCC TCGCATCCCG   
  
  
- AGTCAGCCCC TCGCGCCACA GCTTACTCCA CTCCTAGTGA CACCAACTCT GGCTCCAGCC ATTACCACCA   
  
  
- CGCCGCCCAC GCCTCCGACA ATTCCTACCG GACCACTTTA ACCGACTCTT CCACCCCTAA TCAAACTTCA   
  
  
- AACTCCGAAA CTGAGCCTTC TAGAGGCTCA ATTGGGCCCT CAGTGACCCC ACACTCCTCC CCCTCCGGCA   
  
  
- CCACCACTTA AAACGCAAGT TTGAAATGGC CCATGGCCTA CTCTCGCACA GCTACCTCTT AGACTCCCTG   
  
  
- CTCGAGGACG CGGCCCACTT CCGCGATTCC GGTGCGCGGC AGTGTAACCA CCTCGTCCTG AATTTACGCT   
  
  
- TATGCCGTGG CAAGTACTGC GCGCACTCAC CGCGAACACT CATGATATCT CGTAACAAGC TGAGCGAGCT   
  
  
- GAGCTACCAC GGCTCCCTAT TGGGCCTGGC CCAGGCCCAG CTCCTCCGGA ACCCCGCCTT CTACCGTTTG   
  
  
- AGCCACCGAA CGCTCCCGGC CCTTGCCCAA CTCGCCCGCC TCCACAAACC CTTCACCGCC CGCTCCTACT   
  
  
- CATACCGTCC CAAGCTCAAC TTCCGTGACT CAGTCTCACA CCGGCTCAGT TACGCTTGGT TTGAGTCGAG   
  
  
- CCAAGACCCG CCTTTCGGCC CAAAGTGCCA GTTCCTCCTC TCGCCACCCC ACCCCAAGCC CACCTACCCT   
  
  
- GCTTGAGAGT GGCAGCGTAG CCGGACCGCA AT

+     ARE

| Site Name | Organism | Position | Strand | Matrix score. | sequence | function |
| --- | --- | --- | --- | --- | --- | --- |
| ARE | Zea mays | 1925 | + | 6 | AAACCA | cis-acting regulatory element essential for the anaerobic induction |
| ARE | Zea mays | 1915 | + | 6 | AAACCA | cis-acting regulatory element essential for the anaerobic induction |
| ARE | Zea mays | 800 | + | 6 | AAACCA | cis-acting regulatory element essential for the anaerobic induction |
| ARE | Zea mays | 2894 | + | 6 | AAACCA | cis-acting regulatory element essential for the anaerobic induction |

>HU06G02537.1   
+ +Up\_Stream \_Len000CCTCGG TGTGGGATTC CGGGTTGATA AATAGGGACC AGCGAAATGT TCAAATAATT   
  
  
+ GGTGAAGGAG TCTCTGTTTG AATGCCTACT AATGCTTTTT TTTTTTTTTT GAATAATTTG TCAGAAAGTT   
  
  
+ AGGCTGCATT GGAAGGGGCG GCCTACGAAG GCTTTTCAGC AAATAAATAA CCAGCTTTCA TTTTGAAATT   
  
  
+ TTTTTTAAAA AATGAAACTC ATTTCACTTA AAAATTTACC ATAATATATA TATATATATA TATATATATA   
  
  
+ TATATATATA TATTTGATTT AACGTTAAGC AAACTTTTAA CTTATGTATC ATTTTACGAC CGTAAAAAAA   
  
  
+ AAAGAAATAT AACACAATAT GTAATAGTAT TTAAGTTTTT GATAAATAAT ATTTTAACAT AATTTGTGTG   
  
  
+ TAGTATGAAT TTAAATCTTA GTACTAATAT AATTATGTAT ACTTGGCTAT AAGATCTCAA TATTAGTATT   
  
  
+ TTATGATTCA ATGTAATGAT GATGCAACTG TGTGTAATTA GAAAGCCTTT GATTGCAGAT ATCTATCTTA   
  
  
+ TTGATACACA TCTACCAATT GAGCTTTTTC AAGATTTGAA GACGGTAGTC AAGTATCTTT GGGAGATTAC   
  
  
+ GTTAGAATTA CTTCATGATA AATTAGACAT ATTTTATACT TCTAATCTTA AATAAGTCTC CATCCTTTCA   
  
  
+ TCATATTTTA AAGAAATGTA CAAGTAAATG TCAATAGTTT GAAACGTTCC TTATCATAAA AGTCATACAT   
  
  
+ ATGTAGGACA TTTTCAAGTT TATGTAAACC ATATGATATA GTTCAAGATA ATTGTCAAAC AAGTTATGAA   
  
  
+ ATCTAAAGGT GTTTGAACGT AATTTCAAGC TAATCTTAGC AAGTTATAGT ATTATTTAAA CTACGACTAG   
  
  
+ ACACAACATT TTGTTCATTT ATATAACACT TAGAATGCAA AAAAATCGGT ATTACTAATA CTGTATCAAG   
  
  
+ AATTGAAGTG TTATGCATTA TACTGATAAG GGATAATATT TAAATTACTT TACTGTTATA GAAGCCAAAA   
  
  
+ ATCTAAACTT TCATACAGAC GTCGGACTAA ATTAGAAAAT TCTTTAACTT AATAGAAAGG ATGACATAAA   
  
  
+ TAAAAATAAA AACCTAAATA GCATAATAAC CATTCTACAC AAAAGTCAAT CTTCTAATAG AAAAAAGCCA   
  
  
+ TAAGTATTCA CTTATTTACC ATTTTATTCC TAATCTACTG TCTGTCTATT CACACCATAA TTCATGAACC   
  
  
+ AATGTGGAGT TTCTGTTTCA TTTCATGGGG TGATTTATGT CTGACAATGA TCCAACAAAC TGAGCCTCAC   
  
  
+ GCGCGGAACA CGAGAGTGGC TGTACTTATG GTGTGAAAGA GCTCTACAAG GTGAGATTGA GGAGGCTACG   
  
  
+ CGTTGGAGTT ATCACCACCG TTGGATTTGT TTTCTTTGGA GCCACGTTAT CACTCTTCAG ACGCCGATCC   
  
  
+ AACGGCCCTT ACTTCCATCT AGTCCCCACT CTCCAACTCT ACGCAATCAA CGCCTTCCGC ATTTAAATAT   
  
  
+ TTCCTTCTCT ATCGCCTACT TTCTCTGTCT CACAATTTTT GTCCGTATTT TGAATTTTCT CAAAATTATC   
  
  
+ ATATAATTTA TTTGCCTTCA ACTAAAGTAT TTATTAAATT AGTTAATGTA TTTAAATTGA ACTAAAAAAG   
  
  
+ AGAAAAAGAA AGAAAACAAC CCCACGAAAG CAAAACGACA GAGGCCAACT TCTCTCTCTA GAGTGTAGAG   
  
  
+ CCCCATCTCG GTTATAATTA TGAGAATCAA TTCCTTCTCA CAAGAGAAAC TCTCGCTCTC TCTCTCTCTC   
  
  
+ CATAAGACAT CACACCAATT GTATGAATTT TCTCTCTCCT GATTTCTGTT TTCCTTCTTG TTGTCCCCTT   
  
  
+ CTAATGCGTT AGTGCGGCCA AAACCACCAT AAACCATCTC TCTTTCAAGA AAGCAGCAAC AACAAAGAGA   
  
  
+ GACAGAGAGA GAGAGAGGGG GAGAAAGATC ACATCTTTTG GGTTTCCGGC GAATATGACG TCTGGGTTTT   
  
  
+ CGGGCGATTT CTTCTACGGC GGTGGAGGCG GTGGCGGAGG AGGTGTCCGG CCTTCTATCG GCGGTGTACA   
  
  
+ AGGCGGGGTT GTCCCATATG GGTTCCGGCA ACAACAACAA CAACAGCAAC AGCAGCAGCA GCAGCAACAG   
  
  
+ CTACATCTAC AGCTACAACA ACAGCGGGGG ATTTTCTCGG CGGATCAGAT CAGCGCTCAA CGAAGGGCGG   
  
  
+ ATTTGTTGAT GGGTAAGCGT TCTTTCGCTG ATTTTCAATC TCAACAACAG CAAATCAGTC TTTTGCAGCA   
  
  
+ GCAGCAGCAG CAACAACAAC AACAGCCCTT TGCGCTCTTG CAAAAACAAC AGCAGTTTCA ACAACAATTA   
  
  
+ CAACAACAAC AGCCACTTTC TCTCCTCCTT CAACAACAGT ACTTGATGAG ATCCGTAAAA CAGAGAACCA   
  
  
+ ACAATTTCGC AGCATCTCCG ATTTCTCCTC TTTCTTCTTC CATTGATCTT TCTTCCGCTA ATAATTCCCG   
  
  
+ GTTCGGTTTG CCGGTTTACC AACAACAACT CCGGTCTCAA CAACAACCAC CTATCTCTCT CCTCGCCCAC   
  
  
+ AACAACAACC AATTCAACCT CTCAAATAAT AACAATTCTT CTTTAATCCA AAACTCCACC AACACTCTTC   
  
  
+ TTGCTCCCGC AGCCAGCAAT AACACCCTAA TGAAAAATTC GGGCTTTTCC TCATCTGGGT TTTCTAATTC   
  
  
+ GGGTCCAAAC CTGGTCCAAC CGAAGCAGGA AATCGGGTAC TCGAATATGG GTACCAATAT GGGTATGAAT   
  
  
+ CTGGTCCAGC CCAAACCCGA CCCTGAGGAT CACGGAATAA TGAACACCCT TCAAGAATTG GAGAAGGAGC   
  
  
+ TTTTGAATGA CGATGAGGAA AACCAGCCGG AAAACGACGC CGTTTCAGTC ATCACCAACA GCGAGTGGTC   
  
  
+ CGAGACCTTC CAGAATCTGA TGAGTCCCGA CCCGAGTTCG AGCCCGAATG CCACCAACAC CCACCCGGGT   
  
  
+ TCGGGTTCTG CTAGTACCAG CGGCAGCAAC AACACTACAA CCACCCCTGC AAACAACAAT AACATGATAT   
  
  
+ CTACCTCGCC GACGTCGTCC ACGTCATCCT GCTGCTCCAC GTCGGCATCG GCGTCTCCGC CGGCGGCGGT   
  
  
+ GGCGACCCCG CAGGTACCAG CCAAGCAGCT GATCTCGGAA GCAGCGGCGG CGATTTCGGA CGGAAAGATT   
  
  
+ GAATCCGCCA TGGAAGTGAT GAACCGGCTG TCCCAGGTTG CCAATTCTAG GGGGAGCTCG GAACAAAAGC   
  
  
+ TGGCTTTTTA CATGGGCCAC GCGCTTCGGT CGCGCGTGAG TCCCACCGAA CACGCTCCCC CTATTGTGGA   
  
  
+ GCTTTTCCAA AAGGAGCATA TGGCGGCCAT GTATTCATTG TACGAGCTCT CTCATTGTTT CAAATTTGGT   
  
  
+ CTTTTGGCTG CCAATTTGGT GATGTTGGAG GCCATTACTT CCGCCCTTCA TGAGGGTCTG AAAATTCATG   
  
  
+ TTGTGGATTT TGATATTGGG CATGGTGGTC AGTACATCAA CCTCCTACGC GCCTTGGGGG AGCGTAGGGC   
  
  
+ TCAGTCGGGG AGCGCGGTGT CGAATGAGGT GAGGATCACT GTGGTTGAGA CCGAGGTCGG TAATGGTGGT   
  
  
+ GCGGCGGGTG CGGAGGCTGT TAAGGATGGC CTGGTGAAAT TGGCTGAGAA GGTGGGGATT AGTTTGAAGT   
  
  
+ TTGAGGCTTT GACTCGGAAG ATCTCCGAGT TAACCCGGGA GTCACTGGGG TGTGAGGAGG GGGAGGCCGT   
  
  
+ GGTGGTGAAT TTTGCGTTCA AACTTTACCG GGTACCGGAT GAGAGCGTGT CGATGGAGAA TCTGAGGGAC   
  
  
+ GAGCTCCTGC GCCGGGTGAA GGCGCTAAGG CCACGCGCCG TCACATTGGT GGAGCAGGAC TTAAATGCGA   
  
  
+ ATACGGCACC GTTCATGACG CGCGTGAGTG GCGCTTGTGA GTACTATAGA GCATTGTTCG ACTCGCTCGA   
  
  
+ CTCGATGGTG CCGAGGGATA ACCCGGACCG GGTCCGGGTC GAGGAGGCCT TGGGGCGGAA GATGGCAAAC   
  
  
+ TCGGTGGCTT GCGAGGGCCG GGAACGGGTT GAGCGGGCGG AGGTGTTTGG GAAGTGGCGG GCGAGGATGA   
  
  
+ GTATGGCAGG GTTCGAGTTG AAGGCACTGA GTCAGAGTGT GGCCGAGTCA ATGCGAACCA AACTCAGCTC   
  
  
+ GGTTCTGGGC GGAAAGCCGG GTTTCACGGT CAAGGAGGAG AGCGGTGGGG TGGGGTTCGG GTGGATGGGA   
  
  
+ CGAACTCTCA CCGTCGCATC GGCCTGGCGT TA  

- +Up\_Stream \_Len000GGAGCC ACACCCTAAG GCCCAACTAT TTATCCCTGG TCGCTTTACA AGTTTATTAA   
  
  
- CCACTTCCTC AGAGACAAAC TTACGGATGA TTACGAAAAA AAAAAAAAAA CTTATTAAAC AGTCTTTCAA   
  
  
- TCCGACGTAA CCTTCCCCGC CGGATGCTTC CGAAAAGTCG TTTATTTATT GGTCGAAAGT AAAACTTTAA   
  
  
- AAAAAATTTT TTACTTTGAG TAAAGTGAAT TTTTAAATGG TATTATATAT ATATATATAT ATATATATAT   
  
  
- ATATATATAT ATAAACTAAA TTGCAATTCG TTTGAAAATT GAATACATAG TAAAATGCTG GCATTTTTTT   
  
  
- TTTCTTTATA TTGTGTTATA CATTATCATA AATTCAAAAA CTATTTATTA TAAAATTGTA TTAAACACAC   
  
  
- ATCATACTTA AATTTAGAAT CATGATTATA TTAATACATA TGAACCGATA TTCTAGAGTT ATAATCATAA   
  
  
- AATACTAAGT TACATTACTA CTACGTTGAC ACACATTAAT CTTTCGGAAA CTAACGTCTA TAGATAGAAT   
  
  
- AACTATGTGT AGATGGTTAA CTCGAAAAAG TTCTAAACTT CTGCCATCAG TTCATAGAAA CCCTCTAATG   
  
  
- CAATCTTAAT GAAGTACTAT TTAATCTGTA TAAAATATGA AGATTAGAAT TTATTCAGAG GTAGGAAAGT   
  
  
- AGTATAAAAT TTCTTTACAT GTTCATTTAC AGTTATCAAA CTTTGCAAGG AATAGTATTT TCAGTATGTA   
  
  
- TACATCCTGT AAAAGTTCAA ATACATTTGG TATACTATAT CAAGTTCTAT TAACAGTTTG TTCAATACTT   
  
  
- TAGATTTCCA CAAACTTGCA TTAAAGTTCG ATTAGAATCG TTCAATATCA TAATAAATTT GATGCTGATC   
  
  
- TGTGTTGTAA AACAAGTAAA TATATTGTGA ATCTTACGTT TTTTTAGCCA TAATGATTAT GACATAGTTC   
  
  
- TTAACTTCAC AATACGTAAT ATGACTATTC CCTATTATAA ATTTAATGAA ATGACAATAT CTTCGGTTTT   
  
  
- TAGATTTGAA AGTATGTCTG CAGCCTGATT TAATCTTTTA AGAAATTGAA TTATCTTTCC TACTGTATTT   
  
  
- ATTTTTATTT TTGGATTTAT CGTATTATTG GTAAGATGTG TTTTCAGTTA GAAGATTATC TTTTTTCGGT   
  
  
- ATTCATAAGT GAATAAATGG TAAAATAAGG ATTAGATGAC AGACAGATAA GTGTGGTATT AAGTACTTGG   
  
  
- TTACACCTCA AAGACAAAGT AAAGTACCCC ACTAAATACA GACTGTTACT AGGTTGTTTG ACTCGGAGTG   
  
  
- CGCGCCTTGT GCTCTCACCG ACATGAATAC CACACTTTCT CGAGATGTTC CACTCTAACT CCTCCGATGC   
  
  
- GCAACCTCAA TAGTGGTGGC AACCTAAACA AAAGAAACCT CGGTGCAATA GTGAGAAGTC TGCGGCTAGG   
  
  
- TTGCCGGGAA TGAAGGTAGA TCAGGGGTGA GAGGTTGAGA TGCGTTAGTT GCGGAAGGCG TAAATTTATA   
  
  
- AAGGAAGAGA TAGCGGATGA AAGAGACAGA GTGTTAAAAA CAGGCATAAA ACTTAAAAGA GTTTTAATAG   
  
  
- TATATTAAAT AAACGGAAGT TGATTTCATA AATAATTTAA TCAATTACAT AAATTTAACT TGATTTTTTC   
  
  
- TCTTTTTCTT TCTTTTGTTG GGGTGCTTTC GTTTTGCTGT CTCCGGTTGA AGAGAGAGAT CTCACATCTC   
  
  
- GGGGTAGAGC CAATATTAAT ACTCTTAGTT AAGGAAGAGT GTTCTCTTTG AGAGCGAGAG AGAGAGAGAG   
  
  
- GTATTCTGTA GTGTGGTTAA CATACTTAAA AGAGAGAGGA CTAAAGACAA AAGGAAGAAC AACAGGGGAA   
  
  
- GATTACGCAA TCACGCCGGT TTTGGTGGTA TTTGGTAGAG AGAAAGTTCT TTCGTCGTTG TTGTTTCTCT   
  
  
- CTGTCTCTCT CTCTCTCCCC CTCTTTCTAG TGTAGAAAAC CCAAAGGCCG CTTATACTGC AGACCCAAAA   
  
  
- GCCCGCTAAA GAAGATGCCG CCACCTCCGC CACCGCCTCC TCCACAGGCC GGAAGATAGC CGCCACATGT   
  
  
- TCCGCCCCAA CAGGGTATAC CCAAGGCCGT TGTTGTTGTT GTTGTCGTTG TCGTCGTCGT CGTCGTTGTC   
  
  
- GATGTAGATG TCGATGTTGT TGTCGCCCCC TAAAAGAGCC GCCTAGTCTA GTCGCGAGTT GCTTCCCGCC   
  
  
- TAAACAACTA CCCATTCGCA AGAAAGCGAC TAAAAGTTAG AGTTGTTGTC GTTTAGTCAG AAAACGTCGT   
  
  
- CGTCGTCGTC GTTGTTGTTG TTGTCGGGAA ACGCGAGAAC GTTTTTGTTG TCGTCAAAGT TGTTGTTAAT   
  
  
- GTTGTTGTTG TCGGTGAAAG AGAGGAGGAA GTTGTTGTCA TGAACTACTC TAGGCATTTT GTCTCTTGGT   
  
  
- TGTTAAAGCG TCGTAGAGGC TAAAGAGGAG AAAGAAGAAG GTAACTAGAA AGAAGGCGAT TATTAAGGGC   
  
  
- CAAGCCAAAC GGCCAAATGG TTGTTGTTGA GGCCAGAGTT GTTGTTGGTG GATAGAGAGA GGAGCGGGTG   
  
  
- TTGTTGTTGG TTAAGTTGGA GAGTTTATTA TTGTTAAGAA GAAATTAGGT TTTGAGGTGG TTGTGAGAAG   
  
  
- AACGAGGGCG TCGGTCGTTA TTGTGGGATT ACTTTTTAAG CCCGAAAAGG AGTAGACCCA AAAGATTAAG   
  
  
- CCCAGGTTTG GACCAGGTTG GCTTCGTCCT TTAGCCCATG AGCTTATACC CATGGTTATA CCCATACTTA   
  
  
- GACCAGGTCG GGTTTGGGCT GGGACTCCTA GTGCCTTATT ACTTGTGGGA AGTTCTTAAC CTCTTCCTCG   
  
  
- AAAACTTACT GCTACTCCTT TTGGTCGGCC TTTTGCTGCG GCAAAGTCAG TAGTGGTTGT CGCTCACCAG   
  
  
- GCTCTGGAAG GTCTTAGACT ACTCAGGGCT GGGCTCAAGC TCGGGCTTAC GGTGGTTGTG GGTGGGCCCA   
  
  
- AGCCCAAGAC GATCATGGTC GCCGTCGTTG TTGTGATGTT GGTGGGGACG TTTGTTGTTA TTGTACTATA   
  
  
- GATGGAGCGG CTGCAGCAGG TGCAGTAGGA CGACGAGGTG CAGCCGTAGC CGCAGAGGCG GCCGCCGCCA   
  
  
- CCGCTGGGGC GTCCATGGTC GGTTCGTCGA CTAGAGCCTT CGTCGCCGCC GCTAAAGCCT GCCTTTCTAA   
  
  
- CTTAGGCGGT ACCTTCACTA CTTGGCCGAC AGGGTCCAAC GGTTAAGATC CCCCTCGAGC CTTGTTTTCG   
  
  
- ACCGAAAAAT GTACCCGGTG CGCGAAGCCA GCGCGCACTC AGGGTGGCTT GTGCGAGGGG GATAACACCT   
  
  
- CGAAAAGGTT TTCCTCGTAT ACCGCCGGTA CATAAGTAAC ATGCTCGAGA GAGTAACAAA GTTTAAACCA   
  
  
- GAAAACCGAC GGTTAAACCA CTACAACCTC CGGTAATGAA GGCGGGAAGT ACTCCCAGAC TTTTAAGTAC   
  
  
- AACACCTAAA ACTATAACCC GTACCACCAG TCATGTAGTT GGAGGATGCG CGGAACCCCC TCGCATCCCG   
  
  
- AGTCAGCCCC TCGCGCCACA GCTTACTCCA CTCCTAGTGA CACCAACTCT GGCTCCAGCC ATTACCACCA   
  
  
- CGCCGCCCAC GCCTCCGACA ATTCCTACCG GACCACTTTA ACCGACTCTT CCACCCCTAA TCAAACTTCA   
  
  
- AACTCCGAAA CTGAGCCTTC TAGAGGCTCA ATTGGGCCCT CAGTGACCCC ACACTCCTCC CCCTCCGGCA   
  
  
- CCACCACTTA AAACGCAAGT TTGAAATGGC CCATGGCCTA CTCTCGCACA GCTACCTCTT AGACTCCCTG   
  
  
- CTCGAGGACG CGGCCCACTT CCGCGATTCC GGTGCGCGGC AGTGTAACCA CCTCGTCCTG AATTTACGCT   
  
  
- TATGCCGTGG CAAGTACTGC GCGCACTCAC CGCGAACACT CATGATATCT CGTAACAAGC TGAGCGAGCT   
  
  
- GAGCTACCAC GGCTCCCTAT TGGGCCTGGC CCAGGCCCAG CTCCTCCGGA ACCCCGCCTT CTACCGTTTG   
  
  
- AGCCACCGAA CGCTCCCGGC CCTTGCCCAA CTCGCCCGCC TCCACAAACC CTTCACCGCC CGCTCCTACT   
  
  
- CATACCGTCC CAAGCTCAAC TTCCGTGACT CAGTCTCACA CCGGCTCAGT TACGCTTGGT TTGAGTCGAG   
  
  
- CCAAGACCCG CCTTTCGGCC CAAAGTGCCA GTTCCTCCTC TCGCCACCCC ACCCCAAGCC CACCTACCCT   
  
  
- GCTTGAGAGT GGCAGCGTAG CCGGACCGCA AT

+     AT-rich sequence

| Site Name | Organism | Position | Strand | Matrix score. | sequence | function |
| --- | --- | --- | --- | --- | --- | --- |
| AT-rich sequence | Pisum sativum | 489 | - | 9 | TAAAATACT | element for maximal elicitor-mediated activation (2copies) |

>HU06G02537.1   
+ +Up\_Stream \_Len000CCTCGG TGTGGGATTC CGGGTTGATA AATAGGGACC AGCGAAATGT TCAAATAATT   
  
  
+ GGTGAAGGAG TCTCTGTTTG AATGCCTACT AATGCTTTTT TTTTTTTTTT GAATAATTTG TCAGAAAGTT   
  
  
+ AGGCTGCATT GGAAGGGGCG GCCTACGAAG GCTTTTCAGC AAATAAATAA CCAGCTTTCA TTTTGAAATT   
  
  
+ TTTTTTAAAA AATGAAACTC ATTTCACTTA AAAATTTACC ATAATATATA TATATATATA TATATATATA   
  
  
+ TATATATATA TATTTGATTT AACGTTAAGC AAACTTTTAA CTTATGTATC ATTTTACGAC CGTAAAAAAA   
  
  
+ AAAGAAATAT AACACAATAT GTAATAGTAT TTAAGTTTTT GATAAATAAT ATTTTAACAT AATTTGTGTG   
  
  
+ TAGTATGAAT TTAAATCTTA GTACTAATAT AATTATGTAT ACTTGGCTAT AAGATCTCAA TATTAGTATT   
  
  
+ TTATGATTCA ATGTAATGAT GATGCAACTG TGTGTAATTA GAAAGCCTTT GATTGCAGAT ATCTATCTTA   
  
  
+ TTGATACACA TCTACCAATT GAGCTTTTTC AAGATTTGAA GACGGTAGTC AAGTATCTTT GGGAGATTAC   
  
  
+ GTTAGAATTA CTTCATGATA AATTAGACAT ATTTTATACT TCTAATCTTA AATAAGTCTC CATCCTTTCA   
  
  
+ TCATATTTTA AAGAAATGTA CAAGTAAATG TCAATAGTTT GAAACGTTCC TTATCATAAA AGTCATACAT   
  
  
+ ATGTAGGACA TTTTCAAGTT TATGTAAACC ATATGATATA GTTCAAGATA ATTGTCAAAC AAGTTATGAA   
  
  
+ ATCTAAAGGT GTTTGAACGT AATTTCAAGC TAATCTTAGC AAGTTATAGT ATTATTTAAA CTACGACTAG   
  
  
+ ACACAACATT TTGTTCATTT ATATAACACT TAGAATGCAA AAAAATCGGT ATTACTAATA CTGTATCAAG   
  
  
+ AATTGAAGTG TTATGCATTA TACTGATAAG GGATAATATT TAAATTACTT TACTGTTATA GAAGCCAAAA   
  
  
+ ATCTAAACTT TCATACAGAC GTCGGACTAA ATTAGAAAAT TCTTTAACTT AATAGAAAGG ATGACATAAA   
  
  
+ TAAAAATAAA AACCTAAATA GCATAATAAC CATTCTACAC AAAAGTCAAT CTTCTAATAG AAAAAAGCCA   
  
  
+ TAAGTATTCA CTTATTTACC ATTTTATTCC TAATCTACTG TCTGTCTATT CACACCATAA TTCATGAACC   
  
  
+ AATGTGGAGT TTCTGTTTCA TTTCATGGGG TGATTTATGT CTGACAATGA TCCAACAAAC TGAGCCTCAC   
  
  
+ GCGCGGAACA CGAGAGTGGC TGTACTTATG GTGTGAAAGA GCTCTACAAG GTGAGATTGA GGAGGCTACG   
  
  
+ CGTTGGAGTT ATCACCACCG TTGGATTTGT TTTCTTTGGA GCCACGTTAT CACTCTTCAG ACGCCGATCC   
  
  
+ AACGGCCCTT ACTTCCATCT AGTCCCCACT CTCCAACTCT ACGCAATCAA CGCCTTCCGC ATTTAAATAT   
  
  
+ TTCCTTCTCT ATCGCCTACT TTCTCTGTCT CACAATTTTT GTCCGTATTT TGAATTTTCT CAAAATTATC   
  
  
+ ATATAATTTA TTTGCCTTCA ACTAAAGTAT TTATTAAATT AGTTAATGTA TTTAAATTGA ACTAAAAAAG   
  
  
+ AGAAAAAGAA AGAAAACAAC CCCACGAAAG CAAAACGACA GAGGCCAACT TCTCTCTCTA GAGTGTAGAG   
  
  
+ CCCCATCTCG GTTATAATTA TGAGAATCAA TTCCTTCTCA CAAGAGAAAC TCTCGCTCTC TCTCTCTCTC   
  
  
+ CATAAGACAT CACACCAATT GTATGAATTT TCTCTCTCCT GATTTCTGTT TTCCTTCTTG TTGTCCCCTT   
  
  
+ CTAATGCGTT AGTGCGGCCA AAACCACCAT AAACCATCTC TCTTTCAAGA AAGCAGCAAC AACAAAGAGA   
  
  
+ GACAGAGAGA GAGAGAGGGG GAGAAAGATC ACATCTTTTG GGTTTCCGGC GAATATGACG TCTGGGTTTT   
  
  
+ CGGGCGATTT CTTCTACGGC GGTGGAGGCG GTGGCGGAGG AGGTGTCCGG CCTTCTATCG GCGGTGTACA   
  
  
+ AGGCGGGGTT GTCCCATATG GGTTCCGGCA ACAACAACAA CAACAGCAAC AGCAGCAGCA GCAGCAACAG   
  
  
+ CTACATCTAC AGCTACAACA ACAGCGGGGG ATTTTCTCGG CGGATCAGAT CAGCGCTCAA CGAAGGGCGG   
  
  
+ ATTTGTTGAT GGGTAAGCGT TCTTTCGCTG ATTTTCAATC TCAACAACAG CAAATCAGTC TTTTGCAGCA   
  
  
+ GCAGCAGCAG CAACAACAAC AACAGCCCTT TGCGCTCTTG CAAAAACAAC AGCAGTTTCA ACAACAATTA   
  
  
+ CAACAACAAC AGCCACTTTC TCTCCTCCTT CAACAACAGT ACTTGATGAG ATCCGTAAAA CAGAGAACCA   
  
  
+ ACAATTTCGC AGCATCTCCG ATTTCTCCTC TTTCTTCTTC CATTGATCTT TCTTCCGCTA ATAATTCCCG   
  
  
+ GTTCGGTTTG CCGGTTTACC AACAACAACT CCGGTCTCAA CAACAACCAC CTATCTCTCT CCTCGCCCAC   
  
  
+ AACAACAACC AATTCAACCT CTCAAATAAT AACAATTCTT CTTTAATCCA AAACTCCACC AACACTCTTC   
  
  
+ TTGCTCCCGC AGCCAGCAAT AACACCCTAA TGAAAAATTC GGGCTTTTCC TCATCTGGGT TTTCTAATTC   
  
  
+ GGGTCCAAAC CTGGTCCAAC CGAAGCAGGA AATCGGGTAC TCGAATATGG GTACCAATAT GGGTATGAAT   
  
  
+ CTGGTCCAGC CCAAACCCGA CCCTGAGGAT CACGGAATAA TGAACACCCT TCAAGAATTG GAGAAGGAGC   
  
  
+ TTTTGAATGA CGATGAGGAA AACCAGCCGG AAAACGACGC CGTTTCAGTC ATCACCAACA GCGAGTGGTC   
  
  
+ CGAGACCTTC CAGAATCTGA TGAGTCCCGA CCCGAGTTCG AGCCCGAATG CCACCAACAC CCACCCGGGT   
  
  
+ TCGGGTTCTG CTAGTACCAG CGGCAGCAAC AACACTACAA CCACCCCTGC AAACAACAAT AACATGATAT   
  
  
+ CTACCTCGCC GACGTCGTCC ACGTCATCCT GCTGCTCCAC GTCGGCATCG GCGTCTCCGC CGGCGGCGGT   
  
  
+ GGCGACCCCG CAGGTACCAG CCAAGCAGCT GATCTCGGAA GCAGCGGCGG CGATTTCGGA CGGAAAGATT   
  
  
+ GAATCCGCCA TGGAAGTGAT GAACCGGCTG TCCCAGGTTG CCAATTCTAG GGGGAGCTCG GAACAAAAGC   
  
  
+ TGGCTTTTTA CATGGGCCAC GCGCTTCGGT CGCGCGTGAG TCCCACCGAA CACGCTCCCC CTATTGTGGA   
  
  
+ GCTTTTCCAA AAGGAGCATA TGGCGGCCAT GTATTCATTG TACGAGCTCT CTCATTGTTT CAAATTTGGT   
  
  
+ CTTTTGGCTG CCAATTTGGT GATGTTGGAG GCCATTACTT CCGCCCTTCA TGAGGGTCTG AAAATTCATG   
  
  
+ TTGTGGATTT TGATATTGGG CATGGTGGTC AGTACATCAA CCTCCTACGC GCCTTGGGGG AGCGTAGGGC   
  
  
+ TCAGTCGGGG AGCGCGGTGT CGAATGAGGT GAGGATCACT GTGGTTGAGA CCGAGGTCGG TAATGGTGGT   
  
  
+ GCGGCGGGTG CGGAGGCTGT TAAGGATGGC CTGGTGAAAT TGGCTGAGAA GGTGGGGATT AGTTTGAAGT   
  
  
+ TTGAGGCTTT GACTCGGAAG ATCTCCGAGT TAACCCGGGA GTCACTGGGG TGTGAGGAGG GGGAGGCCGT   
  
  
+ GGTGGTGAAT TTTGCGTTCA AACTTTACCG GGTACCGGAT GAGAGCGTGT CGATGGAGAA TCTGAGGGAC   
  
  
+ GAGCTCCTGC GCCGGGTGAA GGCGCTAAGG CCACGCGCCG TCACATTGGT GGAGCAGGAC TTAAATGCGA   
  
  
+ ATACGGCACC GTTCATGACG CGCGTGAGTG GCGCTTGTGA GTACTATAGA GCATTGTTCG ACTCGCTCGA   
  
  
+ CTCGATGGTG CCGAGGGATA ACCCGGACCG GGTCCGGGTC GAGGAGGCCT TGGGGCGGAA GATGGCAAAC   
  
  
+ TCGGTGGCTT GCGAGGGCCG GGAACGGGTT GAGCGGGCGG AGGTGTTTGG GAAGTGGCGG GCGAGGATGA   
  
  
+ GTATGGCAGG GTTCGAGTTG AAGGCACTGA GTCAGAGTGT GGCCGAGTCA ATGCGAACCA AACTCAGCTC   
  
  
+ GGTTCTGGGC GGAAAGCCGG GTTTCACGGT CAAGGAGGAG AGCGGTGGGG TGGGGTTCGG GTGGATGGGA   
  
  
+ CGAACTCTCA CCGTCGCATC GGCCTGGCGT TA  

- +Up\_Stream \_Len000GGAGCC ACACCCTAAG GCCCAACTAT TTATCCCTGG TCGCTTTACA AGTTTATTAA   
  
  
- CCACTTCCTC AGAGACAAAC TTACGGATGA TTACGAAAAA AAAAAAAAAA CTTATTAAAC AGTCTTTCAA   
  
  
- TCCGACGTAA CCTTCCCCGC CGGATGCTTC CGAAAAGTCG TTTATTTATT GGTCGAAAGT AAAACTTTAA   
  
  
- AAAAAATTTT TTACTTTGAG TAAAGTGAAT TTTTAAATGG TATTATATAT ATATATATAT ATATATATAT   
  
  
- ATATATATAT ATAAACTAAA TTGCAATTCG TTTGAAAATT GAATACATAG TAAAATGCTG GCATTTTTTT   
  
  
- TTTCTTTATA TTGTGTTATA CATTATCATA AATTCAAAAA CTATTTATTA TAAAATTGTA TTAAACACAC   
  
  
- ATCATACTTA AATTTAGAAT CATGATTATA TTAATACATA TGAACCGATA TTCTAGAGTT ATAATCATAA   
  
  
- AATACTAAGT TACATTACTA CTACGTTGAC ACACATTAAT CTTTCGGAAA CTAACGTCTA TAGATAGAAT   
  
  
- AACTATGTGT AGATGGTTAA CTCGAAAAAG TTCTAAACTT CTGCCATCAG TTCATAGAAA CCCTCTAATG   
  
  
- CAATCTTAAT GAAGTACTAT TTAATCTGTA TAAAATATGA AGATTAGAAT TTATTCAGAG GTAGGAAAGT   
  
  
- AGTATAAAAT TTCTTTACAT GTTCATTTAC AGTTATCAAA CTTTGCAAGG AATAGTATTT TCAGTATGTA   
  
  
- TACATCCTGT AAAAGTTCAA ATACATTTGG TATACTATAT CAAGTTCTAT TAACAGTTTG TTCAATACTT   
  
  
- TAGATTTCCA CAAACTTGCA TTAAAGTTCG ATTAGAATCG TTCAATATCA TAATAAATTT GATGCTGATC   
  
  
- TGTGTTGTAA AACAAGTAAA TATATTGTGA ATCTTACGTT TTTTTAGCCA TAATGATTAT GACATAGTTC   
  
  
- TTAACTTCAC AATACGTAAT ATGACTATTC CCTATTATAA ATTTAATGAA ATGACAATAT CTTCGGTTTT   
  
  
- TAGATTTGAA AGTATGTCTG CAGCCTGATT TAATCTTTTA AGAAATTGAA TTATCTTTCC TACTGTATTT   
  
  
- ATTTTTATTT TTGGATTTAT CGTATTATTG GTAAGATGTG TTTTCAGTTA GAAGATTATC TTTTTTCGGT   
  
  
- ATTCATAAGT GAATAAATGG TAAAATAAGG ATTAGATGAC AGACAGATAA GTGTGGTATT AAGTACTTGG   
  
  
- TTACACCTCA AAGACAAAGT AAAGTACCCC ACTAAATACA GACTGTTACT AGGTTGTTTG ACTCGGAGTG   
  
  
- CGCGCCTTGT GCTCTCACCG ACATGAATAC CACACTTTCT CGAGATGTTC CACTCTAACT CCTCCGATGC   
  
  
- GCAACCTCAA TAGTGGTGGC AACCTAAACA AAAGAAACCT CGGTGCAATA GTGAGAAGTC TGCGGCTAGG   
  
  
- TTGCCGGGAA TGAAGGTAGA TCAGGGGTGA GAGGTTGAGA TGCGTTAGTT GCGGAAGGCG TAAATTTATA   
  
  
- AAGGAAGAGA TAGCGGATGA AAGAGACAGA GTGTTAAAAA CAGGCATAAA ACTTAAAAGA GTTTTAATAG   
  
  
- TATATTAAAT AAACGGAAGT TGATTTCATA AATAATTTAA TCAATTACAT AAATTTAACT TGATTTTTTC   
  
  
- TCTTTTTCTT TCTTTTGTTG GGGTGCTTTC GTTTTGCTGT CTCCGGTTGA AGAGAGAGAT CTCACATCTC   
  
  
- GGGGTAGAGC CAATATTAAT ACTCTTAGTT AAGGAAGAGT GTTCTCTTTG AGAGCGAGAG AGAGAGAGAG   
  
  
- GTATTCTGTA GTGTGGTTAA CATACTTAAA AGAGAGAGGA CTAAAGACAA AAGGAAGAAC AACAGGGGAA   
  
  
- GATTACGCAA TCACGCCGGT TTTGGTGGTA TTTGGTAGAG AGAAAGTTCT TTCGTCGTTG TTGTTTCTCT   
  
  
- CTGTCTCTCT CTCTCTCCCC CTCTTTCTAG TGTAGAAAAC CCAAAGGCCG CTTATACTGC AGACCCAAAA   
  
  
- GCCCGCTAAA GAAGATGCCG CCACCTCCGC CACCGCCTCC TCCACAGGCC GGAAGATAGC CGCCACATGT   
  
  
- TCCGCCCCAA CAGGGTATAC CCAAGGCCGT TGTTGTTGTT GTTGTCGTTG TCGTCGTCGT CGTCGTTGTC   
  
  
- GATGTAGATG TCGATGTTGT TGTCGCCCCC TAAAAGAGCC GCCTAGTCTA GTCGCGAGTT GCTTCCCGCC   
  
  
- TAAACAACTA CCCATTCGCA AGAAAGCGAC TAAAAGTTAG AGTTGTTGTC GTTTAGTCAG AAAACGTCGT   
  
  
- CGTCGTCGTC GTTGTTGTTG TTGTCGGGAA ACGCGAGAAC GTTTTTGTTG TCGTCAAAGT TGTTGTTAAT   
  
  
- GTTGTTGTTG TCGGTGAAAG AGAGGAGGAA GTTGTTGTCA TGAACTACTC TAGGCATTTT GTCTCTTGGT   
  
  
- TGTTAAAGCG TCGTAGAGGC TAAAGAGGAG AAAGAAGAAG GTAACTAGAA AGAAGGCGAT TATTAAGGGC   
  
  
- CAAGCCAAAC GGCCAAATGG TTGTTGTTGA GGCCAGAGTT GTTGTTGGTG GATAGAGAGA GGAGCGGGTG   
  
  
- TTGTTGTTGG TTAAGTTGGA GAGTTTATTA TTGTTAAGAA GAAATTAGGT TTTGAGGTGG TTGTGAGAAG   
  
  
- AACGAGGGCG TCGGTCGTTA TTGTGGGATT ACTTTTTAAG CCCGAAAAGG AGTAGACCCA AAAGATTAAG   
  
  
- CCCAGGTTTG GACCAGGTTG GCTTCGTCCT TTAGCCCATG AGCTTATACC CATGGTTATA CCCATACTTA   
  
  
- GACCAGGTCG GGTTTGGGCT GGGACTCCTA GTGCCTTATT ACTTGTGGGA AGTTCTTAAC CTCTTCCTCG   
  
  
- AAAACTTACT GCTACTCCTT TTGGTCGGCC TTTTGCTGCG GCAAAGTCAG TAGTGGTTGT CGCTCACCAG   
  
  
- GCTCTGGAAG GTCTTAGACT ACTCAGGGCT GGGCTCAAGC TCGGGCTTAC GGTGGTTGTG GGTGGGCCCA   
  
  
- AGCCCAAGAC GATCATGGTC GCCGTCGTTG TTGTGATGTT GGTGGGGACG TTTGTTGTTA TTGTACTATA   
  
  
- GATGGAGCGG CTGCAGCAGG TGCAGTAGGA CGACGAGGTG CAGCCGTAGC CGCAGAGGCG GCCGCCGCCA   
  
  
- CCGCTGGGGC GTCCATGGTC GGTTCGTCGA CTAGAGCCTT CGTCGCCGCC GCTAAAGCCT GCCTTTCTAA   
  
  
- CTTAGGCGGT ACCTTCACTA CTTGGCCGAC AGGGTCCAAC GGTTAAGATC CCCCTCGAGC CTTGTTTTCG   
  
  
- ACCGAAAAAT GTACCCGGTG CGCGAAGCCA GCGCGCACTC AGGGTGGCTT GTGCGAGGGG GATAACACCT   
  
  
- CGAAAAGGTT TTCCTCGTAT ACCGCCGGTA CATAAGTAAC ATGCTCGAGA GAGTAACAAA GTTTAAACCA   
  
  
- GAAAACCGAC GGTTAAACCA CTACAACCTC CGGTAATGAA GGCGGGAAGT ACTCCCAGAC TTTTAAGTAC   
  
  
- AACACCTAAA ACTATAACCC GTACCACCAG TCATGTAGTT GGAGGATGCG CGGAACCCCC TCGCATCCCG   
  
  
- AGTCAGCCCC TCGCGCCACA GCTTACTCCA CTCCTAGTGA CACCAACTCT GGCTCCAGCC ATTACCACCA   
  
  
- CGCCGCCCAC GCCTCCGACA ATTCCTACCG GACCACTTTA ACCGACTCTT CCACCCCTAA TCAAACTTCA   
  
  
- AACTCCGAAA CTGAGCCTTC TAGAGGCTCA ATTGGGCCCT CAGTGACCCC ACACTCCTCC CCCTCCGGCA   
  
  
- CCACCACTTA AAACGCAAGT TTGAAATGGC CCATGGCCTA CTCTCGCACA GCTACCTCTT AGACTCCCTG   
  
  
- CTCGAGGACG CGGCCCACTT CCGCGATTCC GGTGCGCGGC AGTGTAACCA CCTCGTCCTG AATTTACGCT   
  
  
- TATGCCGTGG CAAGTACTGC GCGCACTCAC CGCGAACACT CATGATATCT CGTAACAAGC TGAGCGAGCT   
  
  
- GAGCTACCAC GGCTCCCTAT TGGGCCTGGC CCAGGCCCAG CTCCTCCGGA ACCCCGCCTT CTACCGTTTG   
  
  
- AGCCACCGAA CGCTCCCGGC CCTTGCCCAA CTCGCCCGCC TCCACAAACC CTTCACCGCC CGCTCCTACT   
  
  
- CATACCGTCC CAAGCTCAAC TTCCGTGACT CAGTCTCACA CCGGCTCAGT TACGCTTGGT TTGAGTCGAG   
  
  
- CCAAGACCCG CCTTTCGGCC CAAAGTGCCA GTTCCTCCTC TCGCCACCCC ACCCCAAGCC CACCTACCCT   
  
  
- GCTTGAGAGT GGCAGCGTAG CCGGACCGCA AT

+     ATCT-motif

| Site Name | Organism | Position | Strand | Matrix score. | sequence | function |
| --- | --- | --- | --- | --- | --- | --- |
| ATCT-motif | Pisum sativum | 3700 | - | 9 | AATCTAATCC | part of a conserved DNA module involved in light responsiveness |

>HU06G02537.1   
+ +Up\_Stream \_Len000CCTCGG TGTGGGATTC CGGGTTGATA AATAGGGACC AGCGAAATGT TCAAATAATT   
  
  
+ GGTGAAGGAG TCTCTGTTTG AATGCCTACT AATGCTTTTT TTTTTTTTTT GAATAATTTG TCAGAAAGTT   
  
  
+ AGGCTGCATT GGAAGGGGCG GCCTACGAAG GCTTTTCAGC AAATAAATAA CCAGCTTTCA TTTTGAAATT   
  
  
+ TTTTTTAAAA AATGAAACTC ATTTCACTTA AAAATTTACC ATAATATATA TATATATATA TATATATATA   
  
  
+ TATATATATA TATTTGATTT AACGTTAAGC AAACTTTTAA CTTATGTATC ATTTTACGAC CGTAAAAAAA   
  
  
+ AAAGAAATAT AACACAATAT GTAATAGTAT TTAAGTTTTT GATAAATAAT ATTTTAACAT AATTTGTGTG   
  
  
+ TAGTATGAAT TTAAATCTTA GTACTAATAT AATTATGTAT ACTTGGCTAT AAGATCTCAA TATTAGTATT   
  
  
+ TTATGATTCA ATGTAATGAT GATGCAACTG TGTGTAATTA GAAAGCCTTT GATTGCAGAT ATCTATCTTA   
  
  
+ TTGATACACA TCTACCAATT GAGCTTTTTC AAGATTTGAA GACGGTAGTC AAGTATCTTT GGGAGATTAC   
  
  
+ GTTAGAATTA CTTCATGATA AATTAGACAT ATTTTATACT TCTAATCTTA AATAAGTCTC CATCCTTTCA   
  
  
+ TCATATTTTA AAGAAATGTA CAAGTAAATG TCAATAGTTT GAAACGTTCC TTATCATAAA AGTCATACAT   
  
  
+ ATGTAGGACA TTTTCAAGTT TATGTAAACC ATATGATATA GTTCAAGATA ATTGTCAAAC AAGTTATGAA   
  
  
+ ATCTAAAGGT GTTTGAACGT AATTTCAAGC TAATCTTAGC AAGTTATAGT ATTATTTAAA CTACGACTAG   
  
  
+ ACACAACATT TTGTTCATTT ATATAACACT TAGAATGCAA AAAAATCGGT ATTACTAATA CTGTATCAAG   
  
  
+ AATTGAAGTG TTATGCATTA TACTGATAAG GGATAATATT TAAATTACTT TACTGTTATA GAAGCCAAAA   
  
  
+ ATCTAAACTT TCATACAGAC GTCGGACTAA ATTAGAAAAT TCTTTAACTT AATAGAAAGG ATGACATAAA   
  
  
+ TAAAAATAAA AACCTAAATA GCATAATAAC CATTCTACAC AAAAGTCAAT CTTCTAATAG AAAAAAGCCA   
  
  
+ TAAGTATTCA CTTATTTACC ATTTTATTCC TAATCTACTG TCTGTCTATT CACACCATAA TTCATGAACC   
  
  
+ AATGTGGAGT TTCTGTTTCA TTTCATGGGG TGATTTATGT CTGACAATGA TCCAACAAAC TGAGCCTCAC   
  
  
+ GCGCGGAACA CGAGAGTGGC TGTACTTATG GTGTGAAAGA GCTCTACAAG GTGAGATTGA GGAGGCTACG   
  
  
+ CGTTGGAGTT ATCACCACCG TTGGATTTGT TTTCTTTGGA GCCACGTTAT CACTCTTCAG ACGCCGATCC   
  
  
+ AACGGCCCTT ACTTCCATCT AGTCCCCACT CTCCAACTCT ACGCAATCAA CGCCTTCCGC ATTTAAATAT   
  
  
+ TTCCTTCTCT ATCGCCTACT TTCTCTGTCT CACAATTTTT GTCCGTATTT TGAATTTTCT CAAAATTATC   
  
  
+ ATATAATTTA TTTGCCTTCA ACTAAAGTAT TTATTAAATT AGTTAATGTA TTTAAATTGA ACTAAAAAAG   
  
  
+ AGAAAAAGAA AGAAAACAAC CCCACGAAAG CAAAACGACA GAGGCCAACT TCTCTCTCTA GAGTGTAGAG   
  
  
+ CCCCATCTCG GTTATAATTA TGAGAATCAA TTCCTTCTCA CAAGAGAAAC TCTCGCTCTC TCTCTCTCTC   
  
  
+ CATAAGACAT CACACCAATT GTATGAATTT TCTCTCTCCT GATTTCTGTT TTCCTTCTTG TTGTCCCCTT   
  
  
+ CTAATGCGTT AGTGCGGCCA AAACCACCAT AAACCATCTC TCTTTCAAGA AAGCAGCAAC AACAAAGAGA   
  
  
+ GACAGAGAGA GAGAGAGGGG GAGAAAGATC ACATCTTTTG GGTTTCCGGC GAATATGACG TCTGGGTTTT   
  
  
+ CGGGCGATTT CTTCTACGGC GGTGGAGGCG GTGGCGGAGG AGGTGTCCGG CCTTCTATCG GCGGTGTACA   
  
  
+ AGGCGGGGTT GTCCCATATG GGTTCCGGCA ACAACAACAA CAACAGCAAC AGCAGCAGCA GCAGCAACAG   
  
  
+ CTACATCTAC AGCTACAACA ACAGCGGGGG ATTTTCTCGG CGGATCAGAT CAGCGCTCAA CGAAGGGCGG   
  
  
+ ATTTGTTGAT GGGTAAGCGT TCTTTCGCTG ATTTTCAATC TCAACAACAG CAAATCAGTC TTTTGCAGCA   
  
  
+ GCAGCAGCAG CAACAACAAC AACAGCCCTT TGCGCTCTTG CAAAAACAAC AGCAGTTTCA ACAACAATTA   
  
  
+ CAACAACAAC AGCCACTTTC TCTCCTCCTT CAACAACAGT ACTTGATGAG ATCCGTAAAA CAGAGAACCA   
  
  
+ ACAATTTCGC AGCATCTCCG ATTTCTCCTC TTTCTTCTTC CATTGATCTT TCTTCCGCTA ATAATTCCCG   
  
  
+ GTTCGGTTTG CCGGTTTACC AACAACAACT CCGGTCTCAA CAACAACCAC CTATCTCTCT CCTCGCCCAC   
  
  
+ AACAACAACC AATTCAACCT CTCAAATAAT AACAATTCTT CTTTAATCCA AAACTCCACC AACACTCTTC   
  
  
+ TTGCTCCCGC AGCCAGCAAT AACACCCTAA TGAAAAATTC GGGCTTTTCC TCATCTGGGT TTTCTAATTC   
  
  
+ GGGTCCAAAC CTGGTCCAAC CGAAGCAGGA AATCGGGTAC TCGAATATGG GTACCAATAT GGGTATGAAT   
  
  
+ CTGGTCCAGC CCAAACCCGA CCCTGAGGAT CACGGAATAA TGAACACCCT TCAAGAATTG GAGAAGGAGC   
  
  
+ TTTTGAATGA CGATGAGGAA AACCAGCCGG AAAACGACGC CGTTTCAGTC ATCACCAACA GCGAGTGGTC   
  
  
+ CGAGACCTTC CAGAATCTGA TGAGTCCCGA CCCGAGTTCG AGCCCGAATG CCACCAACAC CCACCCGGGT   
  
  
+ TCGGGTTCTG CTAGTACCAG CGGCAGCAAC AACACTACAA CCACCCCTGC AAACAACAAT AACATGATAT   
  
  
+ CTACCTCGCC GACGTCGTCC ACGTCATCCT GCTGCTCCAC GTCGGCATCG GCGTCTCCGC CGGCGGCGGT   
  
  
+ GGCGACCCCG CAGGTACCAG CCAAGCAGCT GATCTCGGAA GCAGCGGCGG CGATTTCGGA CGGAAAGATT   
  
  
+ GAATCCGCCA TGGAAGTGAT GAACCGGCTG TCCCAGGTTG CCAATTCTAG GGGGAGCTCG GAACAAAAGC   
  
  
+ TGGCTTTTTA CATGGGCCAC GCGCTTCGGT CGCGCGTGAG TCCCACCGAA CACGCTCCCC CTATTGTGGA   
  
  
+ GCTTTTCCAA AAGGAGCATA TGGCGGCCAT GTATTCATTG TACGAGCTCT CTCATTGTTT CAAATTTGGT   
  
  
+ CTTTTGGCTG CCAATTTGGT GATGTTGGAG GCCATTACTT CCGCCCTTCA TGAGGGTCTG AAAATTCATG   
  
  
+ TTGTGGATTT TGATATTGGG CATGGTGGTC AGTACATCAA CCTCCTACGC GCCTTGGGGG AGCGTAGGGC   
  
  
+ TCAGTCGGGG AGCGCGGTGT CGAATGAGGT GAGGATCACT GTGGTTGAGA CCGAGGTCGG TAATGGTGGT   
  
  
+ GCGGCGGGTG CGGAGGCTGT TAAGGATGGC CTGGTGAAAT TGGCTGAGAA GGTGGGGATT AGTTTGAAGT   
  
  
+ TTGAGGCTTT GACTCGGAAG ATCTCCGAGT TAACCCGGGA GTCACTGGGG TGTGAGGAGG GGGAGGCCGT   
  
  
+ GGTGGTGAAT TTTGCGTTCA AACTTTACCG GGTACCGGAT GAGAGCGTGT CGATGGAGAA TCTGAGGGAC   
  
  
+ GAGCTCCTGC GCCGGGTGAA GGCGCTAAGG CCACGCGCCG TCACATTGGT GGAGCAGGAC TTAAATGCGA   
  
  
+ ATACGGCACC GTTCATGACG CGCGTGAGTG GCGCTTGTGA GTACTATAGA GCATTGTTCG ACTCGCTCGA   
  
  
+ CTCGATGGTG CCGAGGGATA ACCCGGACCG GGTCCGGGTC GAGGAGGCCT TGGGGCGGAA GATGGCAAAC   
  
  
+ TCGGTGGCTT GCGAGGGCCG GGAACGGGTT GAGCGGGCGG AGGTGTTTGG GAAGTGGCGG GCGAGGATGA   
  
  
+ GTATGGCAGG GTTCGAGTTG AAGGCACTGA GTCAGAGTGT GGCCGAGTCA ATGCGAACCA AACTCAGCTC   
  
  
+ GGTTCTGGGC GGAAAGCCGG GTTTCACGGT CAAGGAGGAG AGCGGTGGGG TGGGGTTCGG GTGGATGGGA   
  
  
+ CGAACTCTCA CCGTCGCATC GGCCTGGCGT TA  

- +Up\_Stream \_Len000GGAGCC ACACCCTAAG GCCCAACTAT TTATCCCTGG TCGCTTTACA AGTTTATTAA   
  
  
- CCACTTCCTC AGAGACAAAC TTACGGATGA TTACGAAAAA AAAAAAAAAA CTTATTAAAC AGTCTTTCAA   
  
  
- TCCGACGTAA CCTTCCCCGC CGGATGCTTC CGAAAAGTCG TTTATTTATT GGTCGAAAGT AAAACTTTAA   
  
  
- AAAAAATTTT TTACTTTGAG TAAAGTGAAT TTTTAAATGG TATTATATAT ATATATATAT ATATATATAT   
  
  
- ATATATATAT ATAAACTAAA TTGCAATTCG TTTGAAAATT GAATACATAG TAAAATGCTG GCATTTTTTT   
  
  
- TTTCTTTATA TTGTGTTATA CATTATCATA AATTCAAAAA CTATTTATTA TAAAATTGTA TTAAACACAC   
  
  
- ATCATACTTA AATTTAGAAT CATGATTATA TTAATACATA TGAACCGATA TTCTAGAGTT ATAATCATAA   
  
  
- AATACTAAGT TACATTACTA CTACGTTGAC ACACATTAAT CTTTCGGAAA CTAACGTCTA TAGATAGAAT   
  
  
- AACTATGTGT AGATGGTTAA CTCGAAAAAG TTCTAAACTT CTGCCATCAG TTCATAGAAA CCCTCTAATG   
  
  
- CAATCTTAAT GAAGTACTAT TTAATCTGTA TAAAATATGA AGATTAGAAT TTATTCAGAG GTAGGAAAGT   
  
  
- AGTATAAAAT TTCTTTACAT GTTCATTTAC AGTTATCAAA CTTTGCAAGG AATAGTATTT TCAGTATGTA   
  
  
- TACATCCTGT AAAAGTTCAA ATACATTTGG TATACTATAT CAAGTTCTAT TAACAGTTTG TTCAATACTT   
  
  
- TAGATTTCCA CAAACTTGCA TTAAAGTTCG ATTAGAATCG TTCAATATCA TAATAAATTT GATGCTGATC   
  
  
- TGTGTTGTAA AACAAGTAAA TATATTGTGA ATCTTACGTT TTTTTAGCCA TAATGATTAT GACATAGTTC   
  
  
- TTAACTTCAC AATACGTAAT ATGACTATTC CCTATTATAA ATTTAATGAA ATGACAATAT CTTCGGTTTT   
  
  
- TAGATTTGAA AGTATGTCTG CAGCCTGATT TAATCTTTTA AGAAATTGAA TTATCTTTCC TACTGTATTT   
  
  
- ATTTTTATTT TTGGATTTAT CGTATTATTG GTAAGATGTG TTTTCAGTTA GAAGATTATC TTTTTTCGGT   
  
  
- ATTCATAAGT GAATAAATGG TAAAATAAGG ATTAGATGAC AGACAGATAA GTGTGGTATT AAGTACTTGG   
  
  
- TTACACCTCA AAGACAAAGT AAAGTACCCC ACTAAATACA GACTGTTACT AGGTTGTTTG ACTCGGAGTG   
  
  
- CGCGCCTTGT GCTCTCACCG ACATGAATAC CACACTTTCT CGAGATGTTC CACTCTAACT CCTCCGATGC   
  
  
- GCAACCTCAA TAGTGGTGGC AACCTAAACA AAAGAAACCT CGGTGCAATA GTGAGAAGTC TGCGGCTAGG   
  
  
- TTGCCGGGAA TGAAGGTAGA TCAGGGGTGA GAGGTTGAGA TGCGTTAGTT GCGGAAGGCG TAAATTTATA   
  
  
- AAGGAAGAGA TAGCGGATGA AAGAGACAGA GTGTTAAAAA CAGGCATAAA ACTTAAAAGA GTTTTAATAG   
  
  
- TATATTAAAT AAACGGAAGT TGATTTCATA AATAATTTAA TCAATTACAT AAATTTAACT TGATTTTTTC   
  
  
- TCTTTTTCTT TCTTTTGTTG GGGTGCTTTC GTTTTGCTGT CTCCGGTTGA AGAGAGAGAT CTCACATCTC   
  
  
- GGGGTAGAGC CAATATTAAT ACTCTTAGTT AAGGAAGAGT GTTCTCTTTG AGAGCGAGAG AGAGAGAGAG   
  
  
- GTATTCTGTA GTGTGGTTAA CATACTTAAA AGAGAGAGGA CTAAAGACAA AAGGAAGAAC AACAGGGGAA   
  
  
- GATTACGCAA TCACGCCGGT TTTGGTGGTA TTTGGTAGAG AGAAAGTTCT TTCGTCGTTG TTGTTTCTCT   
  
  
- CTGTCTCTCT CTCTCTCCCC CTCTTTCTAG TGTAGAAAAC CCAAAGGCCG CTTATACTGC AGACCCAAAA   
  
  
- GCCCGCTAAA GAAGATGCCG CCACCTCCGC CACCGCCTCC TCCACAGGCC GGAAGATAGC CGCCACATGT   
  
  
- TCCGCCCCAA CAGGGTATAC CCAAGGCCGT TGTTGTTGTT GTTGTCGTTG TCGTCGTCGT CGTCGTTGTC   
  
  
- GATGTAGATG TCGATGTTGT TGTCGCCCCC TAAAAGAGCC GCCTAGTCTA GTCGCGAGTT GCTTCCCGCC   
  
  
- TAAACAACTA CCCATTCGCA AGAAAGCGAC TAAAAGTTAG AGTTGTTGTC GTTTAGTCAG AAAACGTCGT   
  
  
- CGTCGTCGTC GTTGTTGTTG TTGTCGGGAA ACGCGAGAAC GTTTTTGTTG TCGTCAAAGT TGTTGTTAAT   
  
  
- GTTGTTGTTG TCGGTGAAAG AGAGGAGGAA GTTGTTGTCA TGAACTACTC TAGGCATTTT GTCTCTTGGT   
  
  
- TGTTAAAGCG TCGTAGAGGC TAAAGAGGAG AAAGAAGAAG GTAACTAGAA AGAAGGCGAT TATTAAGGGC   
  
  
- CAAGCCAAAC GGCCAAATGG TTGTTGTTGA GGCCAGAGTT GTTGTTGGTG GATAGAGAGA GGAGCGGGTG   
  
  
- TTGTTGTTGG TTAAGTTGGA GAGTTTATTA TTGTTAAGAA GAAATTAGGT TTTGAGGTGG TTGTGAGAAG   
  
  
- AACGAGGGCG TCGGTCGTTA TTGTGGGATT ACTTTTTAAG CCCGAAAAGG AGTAGACCCA AAAGATTAAG   
  
  
- CCCAGGTTTG GACCAGGTTG GCTTCGTCCT TTAGCCCATG AGCTTATACC CATGGTTATA CCCATACTTA   
  
  
- GACCAGGTCG GGTTTGGGCT GGGACTCCTA GTGCCTTATT ACTTGTGGGA AGTTCTTAAC CTCTTCCTCG   
  
  
- AAAACTTACT GCTACTCCTT TTGGTCGGCC TTTTGCTGCG GCAAAGTCAG TAGTGGTTGT CGCTCACCAG   
  
  
- GCTCTGGAAG GTCTTAGACT ACTCAGGGCT GGGCTCAAGC TCGGGCTTAC GGTGGTTGTG GGTGGGCCCA   
  
  
- AGCCCAAGAC GATCATGGTC GCCGTCGTTG TTGTGATGTT GGTGGGGACG TTTGTTGTTA TTGTACTATA   
  
  
- GATGGAGCGG CTGCAGCAGG TGCAGTAGGA CGACGAGGTG CAGCCGTAGC CGCAGAGGCG GCCGCCGCCA   
  
  
- CCGCTGGGGC GTCCATGGTC GGTTCGTCGA CTAGAGCCTT CGTCGCCGCC GCTAAAGCCT GCCTTTCTAA   
  
  
- CTTAGGCGGT ACCTTCACTA CTTGGCCGAC AGGGTCCAAC GGTTAAGATC CCCCTCGAGC CTTGTTTTCG   
  
  
- ACCGAAAAAT GTACCCGGTG CGCGAAGCCA GCGCGCACTC AGGGTGGCTT GTGCGAGGGG GATAACACCT   
  
  
- CGAAAAGGTT TTCCTCGTAT ACCGCCGGTA CATAAGTAAC ATGCTCGAGA GAGTAACAAA GTTTAAACCA   
  
  
- GAAAACCGAC GGTTAAACCA CTACAACCTC CGGTAATGAA GGCGGGAAGT ACTCCCAGAC TTTTAAGTAC   
  
  
- AACACCTAAA ACTATAACCC GTACCACCAG TCATGTAGTT GGAGGATGCG CGGAACCCCC TCGCATCCCG   
  
  
- AGTCAGCCCC TCGCGCCACA GCTTACTCCA CTCCTAGTGA CACCAACTCT GGCTCCAGCC ATTACCACCA   
  
  
- CGCCGCCCAC GCCTCCGACA ATTCCTACCG GACCACTTTA ACCGACTCTT CCACCCCTAA TCAAACTTCA   
  
  
- AACTCCGAAA CTGAGCCTTC TAGAGGCTCA ATTGGGCCCT CAGTGACCCC ACACTCCTCC CCCTCCGGCA   
  
  
- CCACCACTTA AAACGCAAGT TTGAAATGGC CCATGGCCTA CTCTCGCACA GCTACCTCTT AGACTCCCTG   
  
  
- CTCGAGGACG CGGCCCACTT CCGCGATTCC GGTGCGCGGC AGTGTAACCA CCTCGTCCTG AATTTACGCT   
  
  
- TATGCCGTGG CAAGTACTGC GCGCACTCAC CGCGAACACT CATGATATCT CGTAACAAGC TGAGCGAGCT   
  
  
- GAGCTACCAC GGCTCCCTAT TGGGCCTGGC CCAGGCCCAG CTCCTCCGGA ACCCCGCCTT CTACCGTTTG   
  
  
- AGCCACCGAA CGCTCCCGGC CCTTGCCCAA CTCGCCCGCC TCCACAAACC CTTCACCGCC CGCTCCTACT   
  
  
- CATACCGTCC CAAGCTCAAC TTCCGTGACT CAGTCTCACA CCGGCTCAGT TACGCTTGGT TTGAGTCGAG   
  
  
- CCAAGACCCG CCTTTCGGCC CAAAGTGCCA GTTCCTCCTC TCGCCACCCC ACCCCAAGCC CACCTACCCT   
  
  
- GCTTGAGAGT GGCAGCGTAG CCGGACCGCA AT

+     AT~TATA-box

| Site Name | Organism | Position | Strand | Matrix score. | sequence | function |
| --- | --- | --- | --- | --- | --- | --- |
| AT~TATA-box | Arabidopsis thaliana | 283 | + | 6 | TATATA |  |
| AT~TATA-box | Arabidopsis thaliana | 287 | + | 6 | TATATA |  |
| AT~TATA-box | Arabidopsis thaliana | 265 | + | 6 | TATATA |  |
| AT~TATA-box | Arabidopsis thaliana | 259 | + | 6 | TATATA |  |
| AT~TATA-box | Arabidopsis thaliana | 291 | + | 6 | TATATA |  |
| AT~TATA-box | Arabidopsis thaliana | 277 | + | 6 | TATATA |  |
| AT~TATA-box | Arabidopsis thaliana | 285 | + | 6 | TATATA |  |
| AT~TATA-box | Arabidopsis thaliana | 261 | + | 6 | TATATA |  |
| AT~TATA-box | Arabidopsis thaliana | 267 | + | 6 | TATATA |  |
| AT~TATA-box | Arabidopsis thaliana | 934 | + | 6 | TATATA |  |
| AT~TATA-box | Arabidopsis thaliana | 273 | + | 6 | TATATA |  |
| AT~TATA-box | Arabidopsis thaliana | 271 | + | 6 | TATATA |  |
| AT~TATA-box | Arabidopsis thaliana | 289 | + | 6 | TATATA |  |
| AT~TATA-box | Arabidopsis thaliana | 263 | + | 6 | TATATA |  |
| AT~TATA-box | Arabidopsis thaliana | 269 | + | 6 | TATATA |  |
| AT~TATA-box | Arabidopsis thaliana | 279 | + | 6 | TATATA |  |
| AT~TATA-box | Arabidopsis thaliana | 275 | + | 6 | TATATA |  |
| AT~TATA-box | Arabidopsis thaliana | 932 | - | 8 | TATATAAA |  |
| AT~TATA-box | Arabidopsis thaliana | 281 | + | 6 | TATATA |  |

>HU06G02537.1   
+ +Up\_Stream \_Len000CCTCGG TGTGGGATTC CGGGTTGATA AATAGGGACC AGCGAAATGT TCAAATAATT   
  
  
+ GGTGAAGGAG TCTCTGTTTG AATGCCTACT AATGCTTTTT TTTTTTTTTT GAATAATTTG TCAGAAAGTT   
  
  
+ AGGCTGCATT GGAAGGGGCG GCCTACGAAG GCTTTTCAGC AAATAAATAA CCAGCTTTCA TTTTGAAATT   
  
  
+ TTTTTTAAAA AATGAAACTC ATTTCACTTA AAAATTTACC ATAATATATA TATATATATA TATATATATA   
  
  
+ TATATATATA TATTTGATTT AACGTTAAGC AAACTTTTAA CTTATGTATC ATTTTACGAC CGTAAAAAAA   
  
  
+ AAAGAAATAT AACACAATAT GTAATAGTAT TTAAGTTTTT GATAAATAAT ATTTTAACAT AATTTGTGTG   
  
  
+ TAGTATGAAT TTAAATCTTA GTACTAATAT AATTATGTAT ACTTGGCTAT AAGATCTCAA TATTAGTATT   
  
  
+ TTATGATTCA ATGTAATGAT GATGCAACTG TGTGTAATTA GAAAGCCTTT GATTGCAGAT ATCTATCTTA   
  
  
+ TTGATACACA TCTACCAATT GAGCTTTTTC AAGATTTGAA GACGGTAGTC AAGTATCTTT GGGAGATTAC   
  
  
+ GTTAGAATTA CTTCATGATA AATTAGACAT ATTTTATACT TCTAATCTTA AATAAGTCTC CATCCTTTCA   
  
  
+ TCATATTTTA AAGAAATGTA CAAGTAAATG TCAATAGTTT GAAACGTTCC TTATCATAAA AGTCATACAT   
  
  
+ ATGTAGGACA TTTTCAAGTT TATGTAAACC ATATGATATA GTTCAAGATA ATTGTCAAAC AAGTTATGAA   
  
  
+ ATCTAAAGGT GTTTGAACGT AATTTCAAGC TAATCTTAGC AAGTTATAGT ATTATTTAAA CTACGACTAG   
  
  
+ ACACAACATT TTGTTCATTT ATATAACACT TAGAATGCAA AAAAATCGGT ATTACTAATA CTGTATCAAG   
  
  
+ AATTGAAGTG TTATGCATTA TACTGATAAG GGATAATATT TAAATTACTT TACTGTTATA GAAGCCAAAA   
  
  
+ ATCTAAACTT TCATACAGAC GTCGGACTAA ATTAGAAAAT TCTTTAACTT AATAGAAAGG ATGACATAAA   
  
  
+ TAAAAATAAA AACCTAAATA GCATAATAAC CATTCTACAC AAAAGTCAAT CTTCTAATAG AAAAAAGCCA   
  
  
+ TAAGTATTCA CTTATTTACC ATTTTATTCC TAATCTACTG TCTGTCTATT CACACCATAA TTCATGAACC   
  
  
+ AATGTGGAGT TTCTGTTTCA TTTCATGGGG TGATTTATGT CTGACAATGA TCCAACAAAC TGAGCCTCAC   
  
  
+ GCGCGGAACA CGAGAGTGGC TGTACTTATG GTGTGAAAGA GCTCTACAAG GTGAGATTGA GGAGGCTACG   
  
  
+ CGTTGGAGTT ATCACCACCG TTGGATTTGT TTTCTTTGGA GCCACGTTAT CACTCTTCAG ACGCCGATCC   
  
  
+ AACGGCCCTT ACTTCCATCT AGTCCCCACT CTCCAACTCT ACGCAATCAA CGCCTTCCGC ATTTAAATAT   
  
  
+ TTCCTTCTCT ATCGCCTACT TTCTCTGTCT CACAATTTTT GTCCGTATTT TGAATTTTCT CAAAATTATC   
  
  
+ ATATAATTTA TTTGCCTTCA ACTAAAGTAT TTATTAAATT AGTTAATGTA TTTAAATTGA ACTAAAAAAG   
  
  
+ AGAAAAAGAA AGAAAACAAC CCCACGAAAG CAAAACGACA GAGGCCAACT TCTCTCTCTA GAGTGTAGAG   
  
  
+ CCCCATCTCG GTTATAATTA TGAGAATCAA TTCCTTCTCA CAAGAGAAAC TCTCGCTCTC TCTCTCTCTC   
  
  
+ CATAAGACAT CACACCAATT GTATGAATTT TCTCTCTCCT GATTTCTGTT TTCCTTCTTG TTGTCCCCTT   
  
  
+ CTAATGCGTT AGTGCGGCCA AAACCACCAT AAACCATCTC TCTTTCAAGA AAGCAGCAAC AACAAAGAGA   
  
  
+ GACAGAGAGA GAGAGAGGGG GAGAAAGATC ACATCTTTTG GGTTTCCGGC GAATATGACG TCTGGGTTTT   
  
  
+ CGGGCGATTT CTTCTACGGC GGTGGAGGCG GTGGCGGAGG AGGTGTCCGG CCTTCTATCG GCGGTGTACA   
  
  
+ AGGCGGGGTT GTCCCATATG GGTTCCGGCA ACAACAACAA CAACAGCAAC AGCAGCAGCA GCAGCAACAG   
  
  
+ CTACATCTAC AGCTACAACA ACAGCGGGGG ATTTTCTCGG CGGATCAGAT CAGCGCTCAA CGAAGGGCGG   
  
  
+ ATTTGTTGAT GGGTAAGCGT TCTTTCGCTG ATTTTCAATC TCAACAACAG CAAATCAGTC TTTTGCAGCA   
  
  
+ GCAGCAGCAG CAACAACAAC AACAGCCCTT TGCGCTCTTG CAAAAACAAC AGCAGTTTCA ACAACAATTA   
  
  
+ CAACAACAAC AGCCACTTTC TCTCCTCCTT CAACAACAGT ACTTGATGAG ATCCGTAAAA CAGAGAACCA   
  
  
+ ACAATTTCGC AGCATCTCCG ATTTCTCCTC TTTCTTCTTC CATTGATCTT TCTTCCGCTA ATAATTCCCG   
  
  
+ GTTCGGTTTG CCGGTTTACC AACAACAACT CCGGTCTCAA CAACAACCAC CTATCTCTCT CCTCGCCCAC   
  
  
+ AACAACAACC AATTCAACCT CTCAAATAAT AACAATTCTT CTTTAATCCA AAACTCCACC AACACTCTTC   
  
  
+ TTGCTCCCGC AGCCAGCAAT AACACCCTAA TGAAAAATTC GGGCTTTTCC TCATCTGGGT TTTCTAATTC   
  
  
+ GGGTCCAAAC CTGGTCCAAC CGAAGCAGGA AATCGGGTAC TCGAATATGG GTACCAATAT GGGTATGAAT   
  
  
+ CTGGTCCAGC CCAAACCCGA CCCTGAGGAT CACGGAATAA TGAACACCCT TCAAGAATTG GAGAAGGAGC   
  
  
+ TTTTGAATGA CGATGAGGAA AACCAGCCGG AAAACGACGC CGTTTCAGTC ATCACCAACA GCGAGTGGTC   
  
  
+ CGAGACCTTC CAGAATCTGA TGAGTCCCGA CCCGAGTTCG AGCCCGAATG CCACCAACAC CCACCCGGGT   
  
  
+ TCGGGTTCTG CTAGTACCAG CGGCAGCAAC AACACTACAA CCACCCCTGC AAACAACAAT AACATGATAT   
  
  
+ CTACCTCGCC GACGTCGTCC ACGTCATCCT GCTGCTCCAC GTCGGCATCG GCGTCTCCGC CGGCGGCGGT   
  
  
+ GGCGACCCCG CAGGTACCAG CCAAGCAGCT GATCTCGGAA GCAGCGGCGG CGATTTCGGA CGGAAAGATT   
  
  
+ GAATCCGCCA TGGAAGTGAT GAACCGGCTG TCCCAGGTTG CCAATTCTAG GGGGAGCTCG GAACAAAAGC   
  
  
+ TGGCTTTTTA CATGGGCCAC GCGCTTCGGT CGCGCGTGAG TCCCACCGAA CACGCTCCCC CTATTGTGGA   
  
  
+ GCTTTTCCAA AAGGAGCATA TGGCGGCCAT GTATTCATTG TACGAGCTCT CTCATTGTTT CAAATTTGGT   
  
  
+ CTTTTGGCTG CCAATTTGGT GATGTTGGAG GCCATTACTT CCGCCCTTCA TGAGGGTCTG AAAATTCATG   
  
  
+ TTGTGGATTT TGATATTGGG CATGGTGGTC AGTACATCAA CCTCCTACGC GCCTTGGGGG AGCGTAGGGC   
  
  
+ TCAGTCGGGG AGCGCGGTGT CGAATGAGGT GAGGATCACT GTGGTTGAGA CCGAGGTCGG TAATGGTGGT   
  
  
+ GCGGCGGGTG CGGAGGCTGT TAAGGATGGC CTGGTGAAAT TGGCTGAGAA GGTGGGGATT AGTTTGAAGT   
  
  
+ TTGAGGCTTT GACTCGGAAG ATCTCCGAGT TAACCCGGGA GTCACTGGGG TGTGAGGAGG GGGAGGCCGT   
  
  
+ GGTGGTGAAT TTTGCGTTCA AACTTTACCG GGTACCGGAT GAGAGCGTGT CGATGGAGAA TCTGAGGGAC   
  
  
+ GAGCTCCTGC GCCGGGTGAA GGCGCTAAGG CCACGCGCCG TCACATTGGT GGAGCAGGAC TTAAATGCGA   
  
  
+ ATACGGCACC GTTCATGACG CGCGTGAGTG GCGCTTGTGA GTACTATAGA GCATTGTTCG ACTCGCTCGA   
  
  
+ CTCGATGGTG CCGAGGGATA ACCCGGACCG GGTCCGGGTC GAGGAGGCCT TGGGGCGGAA GATGGCAAAC   
  
  
+ TCGGTGGCTT GCGAGGGCCG GGAACGGGTT GAGCGGGCGG AGGTGTTTGG GAAGTGGCGG GCGAGGATGA   
  
  
+ GTATGGCAGG GTTCGAGTTG AAGGCACTGA GTCAGAGTGT GGCCGAGTCA ATGCGAACCA AACTCAGCTC   
  
  
+ GGTTCTGGGC GGAAAGCCGG GTTTCACGGT CAAGGAGGAG AGCGGTGGGG TGGGGTTCGG GTGGATGGGA   
  
  
+ CGAACTCTCA CCGTCGCATC GGCCTGGCGT TA  

- +Up\_Stream \_Len000GGAGCC ACACCCTAAG GCCCAACTAT TTATCCCTGG TCGCTTTACA AGTTTATTAA   
  
  
- CCACTTCCTC AGAGACAAAC TTACGGATGA TTACGAAAAA AAAAAAAAAA CTTATTAAAC AGTCTTTCAA   
  
  
- TCCGACGTAA CCTTCCCCGC CGGATGCTTC CGAAAAGTCG TTTATTTATT GGTCGAAAGT AAAACTTTAA   
  
  
- AAAAAATTTT TTACTTTGAG TAAAGTGAAT TTTTAAATGG TATTATATAT ATATATATAT ATATATATAT   
  
  
- ATATATATAT ATAAACTAAA TTGCAATTCG TTTGAAAATT GAATACATAG TAAAATGCTG GCATTTTTTT   
  
  
- TTTCTTTATA TTGTGTTATA CATTATCATA AATTCAAAAA CTATTTATTA TAAAATTGTA TTAAACACAC   
  
  
- ATCATACTTA AATTTAGAAT CATGATTATA TTAATACATA TGAACCGATA TTCTAGAGTT ATAATCATAA   
  
  
- AATACTAAGT TACATTACTA CTACGTTGAC ACACATTAAT CTTTCGGAAA CTAACGTCTA TAGATAGAAT   
  
  
- AACTATGTGT AGATGGTTAA CTCGAAAAAG TTCTAAACTT CTGCCATCAG TTCATAGAAA CCCTCTAATG   
  
  
- CAATCTTAAT GAAGTACTAT TTAATCTGTA TAAAATATGA AGATTAGAAT TTATTCAGAG GTAGGAAAGT   
  
  
- AGTATAAAAT TTCTTTACAT GTTCATTTAC AGTTATCAAA CTTTGCAAGG AATAGTATTT TCAGTATGTA   
  
  
- TACATCCTGT AAAAGTTCAA ATACATTTGG TATACTATAT CAAGTTCTAT TAACAGTTTG TTCAATACTT   
  
  
- TAGATTTCCA CAAACTTGCA TTAAAGTTCG ATTAGAATCG TTCAATATCA TAATAAATTT GATGCTGATC   
  
  
- TGTGTTGTAA AACAAGTAAA TATATTGTGA ATCTTACGTT TTTTTAGCCA TAATGATTAT GACATAGTTC   
  
  
- TTAACTTCAC AATACGTAAT ATGACTATTC CCTATTATAA ATTTAATGAA ATGACAATAT CTTCGGTTTT   
  
  
- TAGATTTGAA AGTATGTCTG CAGCCTGATT TAATCTTTTA AGAAATTGAA TTATCTTTCC TACTGTATTT   
  
  
- ATTTTTATTT TTGGATTTAT CGTATTATTG GTAAGATGTG TTTTCAGTTA GAAGATTATC TTTTTTCGGT   
  
  
- ATTCATAAGT GAATAAATGG TAAAATAAGG ATTAGATGAC AGACAGATAA GTGTGGTATT AAGTACTTGG   
  
  
- TTACACCTCA AAGACAAAGT AAAGTACCCC ACTAAATACA GACTGTTACT AGGTTGTTTG ACTCGGAGTG   
  
  
- CGCGCCTTGT GCTCTCACCG ACATGAATAC CACACTTTCT CGAGATGTTC CACTCTAACT CCTCCGATGC   
  
  
- GCAACCTCAA TAGTGGTGGC AACCTAAACA AAAGAAACCT CGGTGCAATA GTGAGAAGTC TGCGGCTAGG   
  
  
- TTGCCGGGAA TGAAGGTAGA TCAGGGGTGA GAGGTTGAGA TGCGTTAGTT GCGGAAGGCG TAAATTTATA   
  
  
- AAGGAAGAGA TAGCGGATGA AAGAGACAGA GTGTTAAAAA CAGGCATAAA ACTTAAAAGA GTTTTAATAG   
  
  
- TATATTAAAT AAACGGAAGT TGATTTCATA AATAATTTAA TCAATTACAT AAATTTAACT TGATTTTTTC   
  
  
- TCTTTTTCTT TCTTTTGTTG GGGTGCTTTC GTTTTGCTGT CTCCGGTTGA AGAGAGAGAT CTCACATCTC   
  
  
- GGGGTAGAGC CAATATTAAT ACTCTTAGTT AAGGAAGAGT GTTCTCTTTG AGAGCGAGAG AGAGAGAGAG   
  
  
- GTATTCTGTA GTGTGGTTAA CATACTTAAA AGAGAGAGGA CTAAAGACAA AAGGAAGAAC AACAGGGGAA   
  
  
- GATTACGCAA TCACGCCGGT TTTGGTGGTA TTTGGTAGAG AGAAAGTTCT TTCGTCGTTG TTGTTTCTCT   
  
  
- CTGTCTCTCT CTCTCTCCCC CTCTTTCTAG TGTAGAAAAC CCAAAGGCCG CTTATACTGC AGACCCAAAA   
  
  
- GCCCGCTAAA GAAGATGCCG CCACCTCCGC CACCGCCTCC TCCACAGGCC GGAAGATAGC CGCCACATGT   
  
  
- TCCGCCCCAA CAGGGTATAC CCAAGGCCGT TGTTGTTGTT GTTGTCGTTG TCGTCGTCGT CGTCGTTGTC   
  
  
- GATGTAGATG TCGATGTTGT TGTCGCCCCC TAAAAGAGCC GCCTAGTCTA GTCGCGAGTT GCTTCCCGCC   
  
  
- TAAACAACTA CCCATTCGCA AGAAAGCGAC TAAAAGTTAG AGTTGTTGTC GTTTAGTCAG AAAACGTCGT   
  
  
- CGTCGTCGTC GTTGTTGTTG TTGTCGGGAA ACGCGAGAAC GTTTTTGTTG TCGTCAAAGT TGTTGTTAAT   
  
  
- GTTGTTGTTG TCGGTGAAAG AGAGGAGGAA GTTGTTGTCA TGAACTACTC TAGGCATTTT GTCTCTTGGT   
  
  
- TGTTAAAGCG TCGTAGAGGC TAAAGAGGAG AAAGAAGAAG GTAACTAGAA AGAAGGCGAT TATTAAGGGC   
  
  
- CAAGCCAAAC GGCCAAATGG TTGTTGTTGA GGCCAGAGTT GTTGTTGGTG GATAGAGAGA GGAGCGGGTG   
  
  
- TTGTTGTTGG TTAAGTTGGA GAGTTTATTA TTGTTAAGAA GAAATTAGGT TTTGAGGTGG TTGTGAGAAG   
  
  
- AACGAGGGCG TCGGTCGTTA TTGTGGGATT ACTTTTTAAG CCCGAAAAGG AGTAGACCCA AAAGATTAAG   
  
  
- CCCAGGTTTG GACCAGGTTG GCTTCGTCCT TTAGCCCATG AGCTTATACC CATGGTTATA CCCATACTTA   
  
  
- GACCAGGTCG GGTTTGGGCT GGGACTCCTA GTGCCTTATT ACTTGTGGGA AGTTCTTAAC CTCTTCCTCG   
  
  
- AAAACTTACT GCTACTCCTT TTGGTCGGCC TTTTGCTGCG GCAAAGTCAG TAGTGGTTGT CGCTCACCAG   
  
  
- GCTCTGGAAG GTCTTAGACT ACTCAGGGCT GGGCTCAAGC TCGGGCTTAC GGTGGTTGTG GGTGGGCCCA   
  
  
- AGCCCAAGAC GATCATGGTC GCCGTCGTTG TTGTGATGTT GGTGGGGACG TTTGTTGTTA TTGTACTATA   
  
  
- GATGGAGCGG CTGCAGCAGG TGCAGTAGGA CGACGAGGTG CAGCCGTAGC CGCAGAGGCG GCCGCCGCCA   
  
  
- CCGCTGGGGC GTCCATGGTC GGTTCGTCGA CTAGAGCCTT CGTCGCCGCC GCTAAAGCCT GCCTTTCTAA   
  
  
- CTTAGGCGGT ACCTTCACTA CTTGGCCGAC AGGGTCCAAC GGTTAAGATC CCCCTCGAGC CTTGTTTTCG   
  
  
- ACCGAAAAAT GTACCCGGTG CGCGAAGCCA GCGCGCACTC AGGGTGGCTT GTGCGAGGGG GATAACACCT   
  
  
- CGAAAAGGTT TTCCTCGTAT ACCGCCGGTA CATAAGTAAC ATGCTCGAGA GAGTAACAAA GTTTAAACCA   
  
  
- GAAAACCGAC GGTTAAACCA CTACAACCTC CGGTAATGAA GGCGGGAAGT ACTCCCAGAC TTTTAAGTAC   
  
  
- AACACCTAAA ACTATAACCC GTACCACCAG TCATGTAGTT GGAGGATGCG CGGAACCCCC TCGCATCCCG   
  
  
- AGTCAGCCCC TCGCGCCACA GCTTACTCCA CTCCTAGTGA CACCAACTCT GGCTCCAGCC ATTACCACCA   
  
  
- CGCCGCCCAC GCCTCCGACA ATTCCTACCG GACCACTTTA ACCGACTCTT CCACCCCTAA TCAAACTTCA   
  
  
- AACTCCGAAA CTGAGCCTTC TAGAGGCTCA ATTGGGCCCT CAGTGACCCC ACACTCCTCC CCCTCCGGCA   
  
  
- CCACCACTTA AAACGCAAGT TTGAAATGGC CCATGGCCTA CTCTCGCACA GCTACCTCTT AGACTCCCTG   
  
  
- CTCGAGGACG CGGCCCACTT CCGCGATTCC GGTGCGCGGC AGTGTAACCA CCTCGTCCTG AATTTACGCT   
  
  
- TATGCCGTGG CAAGTACTGC GCGCACTCAC CGCGAACACT CATGATATCT CGTAACAAGC TGAGCGAGCT   
  
  
- GAGCTACCAC GGCTCCCTAT TGGGCCTGGC CCAGGCCCAG CTCCTCCGGA ACCCCGCCTT CTACCGTTTG   
  
  
- AGCCACCGAA CGCTCCCGGC CCTTGCCCAA CTCGCCCGCC TCCACAAACC CTTCACCGCC CGCTCCTACT   
  
  
- CATACCGTCC CAAGCTCAAC TTCCGTGACT CAGTCTCACA CCGGCTCAGT TACGCTTGGT TTGAGTCGAG   
  
  
- CCAAGACCCG CCTTTCGGCC CAAAGTGCCA GTTCCTCCTC TCGCCACCCC ACCCCAAGCC CACCTACCCT   
  
  
- GCTTGAGAGT GGCAGCGTAG CCGGACCGCA AT

+     AuxRE

| Site Name | Organism | Position | Strand | Matrix score. | sequence | function |
| --- | --- | --- | --- | --- | --- | --- |
| AuxRE | Glycine max | 561 | - | 11 | TGTCTCAATAAG | part of an auxin-responsive element |

>HU06G02537.1   
+ +Up\_Stream \_Len000CCTCGG TGTGGGATTC CGGGTTGATA AATAGGGACC AGCGAAATGT TCAAATAATT   
  
  
+ GGTGAAGGAG TCTCTGTTTG AATGCCTACT AATGCTTTTT TTTTTTTTTT GAATAATTTG TCAGAAAGTT   
  
  
+ AGGCTGCATT GGAAGGGGCG GCCTACGAAG GCTTTTCAGC AAATAAATAA CCAGCTTTCA TTTTGAAATT   
  
  
+ TTTTTTAAAA AATGAAACTC ATTTCACTTA AAAATTTACC ATAATATATA TATATATATA TATATATATA   
  
  
+ TATATATATA TATTTGATTT AACGTTAAGC AAACTTTTAA CTTATGTATC ATTTTACGAC CGTAAAAAAA   
  
  
+ AAAGAAATAT AACACAATAT GTAATAGTAT TTAAGTTTTT GATAAATAAT ATTTTAACAT AATTTGTGTG   
  
  
+ TAGTATGAAT TTAAATCTTA GTACTAATAT AATTATGTAT ACTTGGCTAT AAGATCTCAA TATTAGTATT   
  
  
+ TTATGATTCA ATGTAATGAT GATGCAACTG TGTGTAATTA GAAAGCCTTT GATTGCAGAT ATCTATCTTA   
  
  
+ TTGATACACA TCTACCAATT GAGCTTTTTC AAGATTTGAA GACGGTAGTC AAGTATCTTT GGGAGATTAC   
  
  
+ GTTAGAATTA CTTCATGATA AATTAGACAT ATTTTATACT TCTAATCTTA AATAAGTCTC CATCCTTTCA   
  
  
+ TCATATTTTA AAGAAATGTA CAAGTAAATG TCAATAGTTT GAAACGTTCC TTATCATAAA AGTCATACAT   
  
  
+ ATGTAGGACA TTTTCAAGTT TATGTAAACC ATATGATATA GTTCAAGATA ATTGTCAAAC AAGTTATGAA   
  
  
+ ATCTAAAGGT GTTTGAACGT AATTTCAAGC TAATCTTAGC AAGTTATAGT ATTATTTAAA CTACGACTAG   
  
  
+ ACACAACATT TTGTTCATTT ATATAACACT TAGAATGCAA AAAAATCGGT ATTACTAATA CTGTATCAAG   
  
  
+ AATTGAAGTG TTATGCATTA TACTGATAAG GGATAATATT TAAATTACTT TACTGTTATA GAAGCCAAAA   
  
  
+ ATCTAAACTT TCATACAGAC GTCGGACTAA ATTAGAAAAT TCTTTAACTT AATAGAAAGG ATGACATAAA   
  
  
+ TAAAAATAAA AACCTAAATA GCATAATAAC CATTCTACAC AAAAGTCAAT CTTCTAATAG AAAAAAGCCA   
  
  
+ TAAGTATTCA CTTATTTACC ATTTTATTCC TAATCTACTG TCTGTCTATT CACACCATAA TTCATGAACC   
  
  
+ AATGTGGAGT TTCTGTTTCA TTTCATGGGG TGATTTATGT CTGACAATGA TCCAACAAAC TGAGCCTCAC   
  
  
+ GCGCGGAACA CGAGAGTGGC TGTACTTATG GTGTGAAAGA GCTCTACAAG GTGAGATTGA GGAGGCTACG   
  
  
+ CGTTGGAGTT ATCACCACCG TTGGATTTGT TTTCTTTGGA GCCACGTTAT CACTCTTCAG ACGCCGATCC   
  
  
+ AACGGCCCTT ACTTCCATCT AGTCCCCACT CTCCAACTCT ACGCAATCAA CGCCTTCCGC ATTTAAATAT   
  
  
+ TTCCTTCTCT ATCGCCTACT TTCTCTGTCT CACAATTTTT GTCCGTATTT TGAATTTTCT CAAAATTATC   
  
  
+ ATATAATTTA TTTGCCTTCA ACTAAAGTAT TTATTAAATT AGTTAATGTA TTTAAATTGA ACTAAAAAAG   
  
  
+ AGAAAAAGAA AGAAAACAAC CCCACGAAAG CAAAACGACA GAGGCCAACT TCTCTCTCTA GAGTGTAGAG   
  
  
+ CCCCATCTCG GTTATAATTA TGAGAATCAA TTCCTTCTCA CAAGAGAAAC TCTCGCTCTC TCTCTCTCTC   
  
  
+ CATAAGACAT CACACCAATT GTATGAATTT TCTCTCTCCT GATTTCTGTT TTCCTTCTTG TTGTCCCCTT   
  
  
+ CTAATGCGTT AGTGCGGCCA AAACCACCAT AAACCATCTC TCTTTCAAGA AAGCAGCAAC AACAAAGAGA   
  
  
+ GACAGAGAGA GAGAGAGGGG GAGAAAGATC ACATCTTTTG GGTTTCCGGC GAATATGACG TCTGGGTTTT   
  
  
+ CGGGCGATTT CTTCTACGGC GGTGGAGGCG GTGGCGGAGG AGGTGTCCGG CCTTCTATCG GCGGTGTACA   
  
  
+ AGGCGGGGTT GTCCCATATG GGTTCCGGCA ACAACAACAA CAACAGCAAC AGCAGCAGCA GCAGCAACAG   
  
  
+ CTACATCTAC AGCTACAACA ACAGCGGGGG ATTTTCTCGG CGGATCAGAT CAGCGCTCAA CGAAGGGCGG   
  
  
+ ATTTGTTGAT GGGTAAGCGT TCTTTCGCTG ATTTTCAATC TCAACAACAG CAAATCAGTC TTTTGCAGCA   
  
  
+ GCAGCAGCAG CAACAACAAC AACAGCCCTT TGCGCTCTTG CAAAAACAAC AGCAGTTTCA ACAACAATTA   
  
  
+ CAACAACAAC AGCCACTTTC TCTCCTCCTT CAACAACAGT ACTTGATGAG ATCCGTAAAA CAGAGAACCA   
  
  
+ ACAATTTCGC AGCATCTCCG ATTTCTCCTC TTTCTTCTTC CATTGATCTT TCTTCCGCTA ATAATTCCCG   
  
  
+ GTTCGGTTTG CCGGTTTACC AACAACAACT CCGGTCTCAA CAACAACCAC CTATCTCTCT CCTCGCCCAC   
  
  
+ AACAACAACC AATTCAACCT CTCAAATAAT AACAATTCTT CTTTAATCCA AAACTCCACC AACACTCTTC   
  
  
+ TTGCTCCCGC AGCCAGCAAT AACACCCTAA TGAAAAATTC GGGCTTTTCC TCATCTGGGT TTTCTAATTC   
  
  
+ GGGTCCAAAC CTGGTCCAAC CGAAGCAGGA AATCGGGTAC TCGAATATGG GTACCAATAT GGGTATGAAT   
  
  
+ CTGGTCCAGC CCAAACCCGA CCCTGAGGAT CACGGAATAA TGAACACCCT TCAAGAATTG GAGAAGGAGC   
  
  
+ TTTTGAATGA CGATGAGGAA AACCAGCCGG AAAACGACGC CGTTTCAGTC ATCACCAACA GCGAGTGGTC   
  
  
+ CGAGACCTTC CAGAATCTGA TGAGTCCCGA CCCGAGTTCG AGCCCGAATG CCACCAACAC CCACCCGGGT   
  
  
+ TCGGGTTCTG CTAGTACCAG CGGCAGCAAC AACACTACAA CCACCCCTGC AAACAACAAT AACATGATAT   
  
  
+ CTACCTCGCC GACGTCGTCC ACGTCATCCT GCTGCTCCAC GTCGGCATCG GCGTCTCCGC CGGCGGCGGT   
  
  
+ GGCGACCCCG CAGGTACCAG CCAAGCAGCT GATCTCGGAA GCAGCGGCGG CGATTTCGGA CGGAAAGATT   
  
  
+ GAATCCGCCA TGGAAGTGAT GAACCGGCTG TCCCAGGTTG CCAATTCTAG GGGGAGCTCG GAACAAAAGC   
  
  
+ TGGCTTTTTA CATGGGCCAC GCGCTTCGGT CGCGCGTGAG TCCCACCGAA CACGCTCCCC CTATTGTGGA   
  
  
+ GCTTTTCCAA AAGGAGCATA TGGCGGCCAT GTATTCATTG TACGAGCTCT CTCATTGTTT CAAATTTGGT   
  
  
+ CTTTTGGCTG CCAATTTGGT GATGTTGGAG GCCATTACTT CCGCCCTTCA TGAGGGTCTG AAAATTCATG   
  
  
+ TTGTGGATTT TGATATTGGG CATGGTGGTC AGTACATCAA CCTCCTACGC GCCTTGGGGG AGCGTAGGGC   
  
  
+ TCAGTCGGGG AGCGCGGTGT CGAATGAGGT GAGGATCACT GTGGTTGAGA CCGAGGTCGG TAATGGTGGT   
  
  
+ GCGGCGGGTG CGGAGGCTGT TAAGGATGGC CTGGTGAAAT TGGCTGAGAA GGTGGGGATT AGTTTGAAGT   
  
  
+ TTGAGGCTTT GACTCGGAAG ATCTCCGAGT TAACCCGGGA GTCACTGGGG TGTGAGGAGG GGGAGGCCGT   
  
  
+ GGTGGTGAAT TTTGCGTTCA AACTTTACCG GGTACCGGAT GAGAGCGTGT CGATGGAGAA TCTGAGGGAC   
  
  
+ GAGCTCCTGC GCCGGGTGAA GGCGCTAAGG CCACGCGCCG TCACATTGGT GGAGCAGGAC TTAAATGCGA   
  
  
+ ATACGGCACC GTTCATGACG CGCGTGAGTG GCGCTTGTGA GTACTATAGA GCATTGTTCG ACTCGCTCGA   
  
  
+ CTCGATGGTG CCGAGGGATA ACCCGGACCG GGTCCGGGTC GAGGAGGCCT TGGGGCGGAA GATGGCAAAC   
  
  
+ TCGGTGGCTT GCGAGGGCCG GGAACGGGTT GAGCGGGCGG AGGTGTTTGG GAAGTGGCGG GCGAGGATGA   
  
  
+ GTATGGCAGG GTTCGAGTTG AAGGCACTGA GTCAGAGTGT GGCCGAGTCA ATGCGAACCA AACTCAGCTC   
  
  
+ GGTTCTGGGC GGAAAGCCGG GTTTCACGGT CAAGGAGGAG AGCGGTGGGG TGGGGTTCGG GTGGATGGGA   
  
  
+ CGAACTCTCA CCGTCGCATC GGCCTGGCGT TA  

- +Up\_Stream \_Len000GGAGCC ACACCCTAAG GCCCAACTAT TTATCCCTGG TCGCTTTACA AGTTTATTAA   
  
  
- CCACTTCCTC AGAGACAAAC TTACGGATGA TTACGAAAAA AAAAAAAAAA CTTATTAAAC AGTCTTTCAA   
  
  
- TCCGACGTAA CCTTCCCCGC CGGATGCTTC CGAAAAGTCG TTTATTTATT GGTCGAAAGT AAAACTTTAA   
  
  
- AAAAAATTTT TTACTTTGAG TAAAGTGAAT TTTTAAATGG TATTATATAT ATATATATAT ATATATATAT   
  
  
- ATATATATAT ATAAACTAAA TTGCAATTCG TTTGAAAATT GAATACATAG TAAAATGCTG GCATTTTTTT   
  
  
- TTTCTTTATA TTGTGTTATA CATTATCATA AATTCAAAAA CTATTTATTA TAAAATTGTA TTAAACACAC   
  
  
- ATCATACTTA AATTTAGAAT CATGATTATA TTAATACATA TGAACCGATA TTCTAGAGTT ATAATCATAA   
  
  
- AATACTAAGT TACATTACTA CTACGTTGAC ACACATTAAT CTTTCGGAAA CTAACGTCTA TAGATAGAAT   
  
  
- AACTATGTGT AGATGGTTAA CTCGAAAAAG TTCTAAACTT CTGCCATCAG TTCATAGAAA CCCTCTAATG   
  
  
- CAATCTTAAT GAAGTACTAT TTAATCTGTA TAAAATATGA AGATTAGAAT TTATTCAGAG GTAGGAAAGT   
  
  
- AGTATAAAAT TTCTTTACAT GTTCATTTAC AGTTATCAAA CTTTGCAAGG AATAGTATTT TCAGTATGTA   
  
  
- TACATCCTGT AAAAGTTCAA ATACATTTGG TATACTATAT CAAGTTCTAT TAACAGTTTG TTCAATACTT   
  
  
- TAGATTTCCA CAAACTTGCA TTAAAGTTCG ATTAGAATCG TTCAATATCA TAATAAATTT GATGCTGATC   
  
  
- TGTGTTGTAA AACAAGTAAA TATATTGTGA ATCTTACGTT TTTTTAGCCA TAATGATTAT GACATAGTTC   
  
  
- TTAACTTCAC AATACGTAAT ATGACTATTC CCTATTATAA ATTTAATGAA ATGACAATAT CTTCGGTTTT   
  
  
- TAGATTTGAA AGTATGTCTG CAGCCTGATT TAATCTTTTA AGAAATTGAA TTATCTTTCC TACTGTATTT   
  
  
- ATTTTTATTT TTGGATTTAT CGTATTATTG GTAAGATGTG TTTTCAGTTA GAAGATTATC TTTTTTCGGT   
  
  
- ATTCATAAGT GAATAAATGG TAAAATAAGG ATTAGATGAC AGACAGATAA GTGTGGTATT AAGTACTTGG   
  
  
- TTACACCTCA AAGACAAAGT AAAGTACCCC ACTAAATACA GACTGTTACT AGGTTGTTTG ACTCGGAGTG   
  
  
- CGCGCCTTGT GCTCTCACCG ACATGAATAC CACACTTTCT CGAGATGTTC CACTCTAACT CCTCCGATGC   
  
  
- GCAACCTCAA TAGTGGTGGC AACCTAAACA AAAGAAACCT CGGTGCAATA GTGAGAAGTC TGCGGCTAGG   
  
  
- TTGCCGGGAA TGAAGGTAGA TCAGGGGTGA GAGGTTGAGA TGCGTTAGTT GCGGAAGGCG TAAATTTATA   
  
  
- AAGGAAGAGA TAGCGGATGA AAGAGACAGA GTGTTAAAAA CAGGCATAAA ACTTAAAAGA GTTTTAATAG   
  
  
- TATATTAAAT AAACGGAAGT TGATTTCATA AATAATTTAA TCAATTACAT AAATTTAACT TGATTTTTTC   
  
  
- TCTTTTTCTT TCTTTTGTTG GGGTGCTTTC GTTTTGCTGT CTCCGGTTGA AGAGAGAGAT CTCACATCTC   
  
  
- GGGGTAGAGC CAATATTAAT ACTCTTAGTT AAGGAAGAGT GTTCTCTTTG AGAGCGAGAG AGAGAGAGAG   
  
  
- GTATTCTGTA GTGTGGTTAA CATACTTAAA AGAGAGAGGA CTAAAGACAA AAGGAAGAAC AACAGGGGAA   
  
  
- GATTACGCAA TCACGCCGGT TTTGGTGGTA TTTGGTAGAG AGAAAGTTCT TTCGTCGTTG TTGTTTCTCT   
  
  
- CTGTCTCTCT CTCTCTCCCC CTCTTTCTAG TGTAGAAAAC CCAAAGGCCG CTTATACTGC AGACCCAAAA   
  
  
- GCCCGCTAAA GAAGATGCCG CCACCTCCGC CACCGCCTCC TCCACAGGCC GGAAGATAGC CGCCACATGT   
  
  
- TCCGCCCCAA CAGGGTATAC CCAAGGCCGT TGTTGTTGTT GTTGTCGTTG TCGTCGTCGT CGTCGTTGTC   
  
  
- GATGTAGATG TCGATGTTGT TGTCGCCCCC TAAAAGAGCC GCCTAGTCTA GTCGCGAGTT GCTTCCCGCC   
  
  
- TAAACAACTA CCCATTCGCA AGAAAGCGAC TAAAAGTTAG AGTTGTTGTC GTTTAGTCAG AAAACGTCGT   
  
  
- CGTCGTCGTC GTTGTTGTTG TTGTCGGGAA ACGCGAGAAC GTTTTTGTTG TCGTCAAAGT TGTTGTTAAT   
  
  
- GTTGTTGTTG TCGGTGAAAG AGAGGAGGAA GTTGTTGTCA TGAACTACTC TAGGCATTTT GTCTCTTGGT   
  
  
- TGTTAAAGCG TCGTAGAGGC TAAAGAGGAG AAAGAAGAAG GTAACTAGAA AGAAGGCGAT TATTAAGGGC   
  
  
- CAAGCCAAAC GGCCAAATGG TTGTTGTTGA GGCCAGAGTT GTTGTTGGTG GATAGAGAGA GGAGCGGGTG   
  
  
- TTGTTGTTGG TTAAGTTGGA GAGTTTATTA TTGTTAAGAA GAAATTAGGT TTTGAGGTGG TTGTGAGAAG   
  
  
- AACGAGGGCG TCGGTCGTTA TTGTGGGATT ACTTTTTAAG CCCGAAAAGG AGTAGACCCA AAAGATTAAG   
  
  
- CCCAGGTTTG GACCAGGTTG GCTTCGTCCT TTAGCCCATG AGCTTATACC CATGGTTATA CCCATACTTA   
  
  
- GACCAGGTCG GGTTTGGGCT GGGACTCCTA GTGCCTTATT ACTTGTGGGA AGTTCTTAAC CTCTTCCTCG   
  
  
- AAAACTTACT GCTACTCCTT TTGGTCGGCC TTTTGCTGCG GCAAAGTCAG TAGTGGTTGT CGCTCACCAG   
  
  
- GCTCTGGAAG GTCTTAGACT ACTCAGGGCT GGGCTCAAGC TCGGGCTTAC GGTGGTTGTG GGTGGGCCCA   
  
  
- AGCCCAAGAC GATCATGGTC GCCGTCGTTG TTGTGATGTT GGTGGGGACG TTTGTTGTTA TTGTACTATA   
  
  
- GATGGAGCGG CTGCAGCAGG TGCAGTAGGA CGACGAGGTG CAGCCGTAGC CGCAGAGGCG GCCGCCGCCA   
  
  
- CCGCTGGGGC GTCCATGGTC GGTTCGTCGA CTAGAGCCTT CGTCGCCGCC GCTAAAGCCT GCCTTTCTAA   
  
  
- CTTAGGCGGT ACCTTCACTA CTTGGCCGAC AGGGTCCAAC GGTTAAGATC CCCCTCGAGC CTTGTTTTCG   
  
  
- ACCGAAAAAT GTACCCGGTG CGCGAAGCCA GCGCGCACTC AGGGTGGCTT GTGCGAGGGG GATAACACCT   
  
  
- CGAAAAGGTT TTCCTCGTAT ACCGCCGGTA CATAAGTAAC ATGCTCGAGA GAGTAACAAA GTTTAAACCA   
  
  
- GAAAACCGAC GGTTAAACCA CTACAACCTC CGGTAATGAA GGCGGGAAGT ACTCCCAGAC TTTTAAGTAC   
  
  
- AACACCTAAA ACTATAACCC GTACCACCAG TCATGTAGTT GGAGGATGCG CGGAACCCCC TCGCATCCCG   
  
  
- AGTCAGCCCC TCGCGCCACA GCTTACTCCA CTCCTAGTGA CACCAACTCT GGCTCCAGCC ATTACCACCA   
  
  
- CGCCGCCCAC GCCTCCGACA ATTCCTACCG GACCACTTTA ACCGACTCTT CCACCCCTAA TCAAACTTCA   
  
  
- AACTCCGAAA CTGAGCCTTC TAGAGGCTCA ATTGGGCCCT CAGTGACCCC ACACTCCTCC CCCTCCGGCA   
  
  
- CCACCACTTA AAACGCAAGT TTGAAATGGC CCATGGCCTA CTCTCGCACA GCTACCTCTT AGACTCCCTG   
  
  
- CTCGAGGACG CGGCCCACTT CCGCGATTCC GGTGCGCGGC AGTGTAACCA CCTCGTCCTG AATTTACGCT   
  
  
- TATGCCGTGG CAAGTACTGC GCGCACTCAC CGCGAACACT CATGATATCT CGTAACAAGC TGAGCGAGCT   
  
  
- GAGCTACCAC GGCTCCCTAT TGGGCCTGGC CCAGGCCCAG CTCCTCCGGA ACCCCGCCTT CTACCGTTTG   
  
  
- AGCCACCGAA CGCTCCCGGC CCTTGCCCAA CTCGCCCGCC TCCACAAACC CTTCACCGCC CGCTCCTACT   
  
  
- CATACCGTCC CAAGCTCAAC TTCCGTGACT CAGTCTCACA CCGGCTCAGT TACGCTTGGT TTGAGTCGAG   
  
  
- CCAAGACCCG CCTTTCGGCC CAAAGTGCCA GTTCCTCCTC TCGCCACCCC ACCCCAAGCC CACCTACCCT   
  
  
- GCTTGAGAGT GGCAGCGTAG CCGGACCGCA AT

+     CAAT-box

| Site Name | Organism | Position | Strand | Matrix score. | sequence | function |
| --- | --- | --- | --- | --- | --- | --- |
| CAAT-box | Nicotiana glutinosa | 3446 | + | 4 | CAAT |  |
| CAAT-box | Pisum sativum | 3425 | + | 5 | CAAAT | common cis-acting element in promoter and enhancer regions |
| CAAT-box | Nicotiana glutinosa | 3401 | - | 4 | CAAT |  |
| CAAT-box | Arabidopsis thaliana | 3445 | + | 5 | CCAAT | common cis-acting element in promoter and enhancer regions |
| CAAT-box | Nicotiana glutinosa | 3357 | - | 4 | CAAT |  |
| CAAT-box | Arabidopsis thaliana | 2861 | - | 5 | CCAAT | common cis-acting element in promoter and enhancer regions |
| CAAT-box | Nicotiana glutinosa | 1577 | + | 4 | CAAT |  |
| CAAT-box | Arabidopsis thaliana | 2603 | + | 5 | CCAAT | common cis-acting element in promoter and enhancer regions |
| CAAT-box | Nicotiana glutinosa | 1518 | + | 4 | CAAT |  |
| CAAT-box | Nicotiana glutinosa | 482 | + | 4 | CAAT |  |
| CAAT-box | Pisum sativum | 1429 | - | 5 | CAAAT | common cis-acting element in promoter and enhancer regions |
| CAAT-box | Nicotiana glutinosa | 1840 | + | 4 | CAAT |  |
| CAAT-box | Nicotiana glutinosa | 1390 | - | 4 | CAAT |  |
| CAAT-box | Pisum sativum | 66 | + | 5 | CAAAT | common cis-acting element in promoter and enhancer regions |
| CAAT-box | Arabidopsis thaliana | 3899 | - | 5 | CCAAT | common cis-acting element in promoter and enhancer regions |
| CAAT-box | Pisum sativum | 416 | - | 5 | CAAAT | common cis-acting element in promoter and enhancer regions |
| CAAT-box | Arabidopsis thaliana | 72 | - | 5 | CCAAT | common cis-acting element in promoter and enhancer regions |
| CAAT-box | Nicotiana glutinosa | 2627 | + | 4 | CAAT |  |
| CAAT-box | Nicotiana glutinosa | 2681 | + | 4 | CAAT |  |
| CAAT-box | Nicotiana glutinosa | 564 | - | 4 | CAAT |  |
| CAAT-box | Nicotiana glutinosa | 2604 | + | 4 | CAAT |  |
| CAAT-box | Nicotiana glutinosa | 736 | + | 4 | CAAT |  |
| CAAT-box | Nicotiana glutinosa | 546 | - | 4 | CAAT |  |
| CAAT-box | Nicotiana glutinosa | 3222 | - | 4 | CAAT |  |
| CAAT-box | Arabidopsis thaliana | 3683 | - | 5 | CCAAT | common cis-acting element in promoter and enhancer regions |
| CAAT-box | Pisum sativum | 1624 | - | 5 | CAAAT | common cis-acting element in promoter and enhancer regions |
| CAAT-box | Pisum sativum | 598 | - | 5 | CAAAT | common cis-acting element in promoter and enhancer regions |
| CAAT-box | Arabidopsis thaliana | 3265 | + | 5 | CCAAT | common cis-acting element in promoter and enhancer regions |
| CAAT-box | Nicotiana glutinosa | 986 | - | 4 | CAAT |  |
| CAAT-box | Pisum sativum | 2617 | + | 5 | CAAAT | common cis-acting element in promoter and enhancer regions |
| CAAT-box | Nicotiana glutinosa | 3977 | - | 4 | CAAT |  |
| CAAT-box | Nicotiana glutinosa | 2496 | - | 4 | CAAT |  |
| CAAT-box | Nicotiana glutinosa | 2280 | + | 4 | CAAT |  |
| CAAT-box | Pisum sativum | 184 | + | 5 | CAAAT | common cis-acting element in promoter and enhancer regions |
| CAAT-box | Pisum sativum | 130 | - | 5 | CAAAT | common cis-acting element in promoter and enhancer regions |
| CAAT-box | Arabidopsis thaliana | 1839 | + | 5 | CCAAT | common cis-acting element in promoter and enhancer regions |
| CAAT-box | Pisum sativum | 2295 | + | 5 | CAAAT | common cis-acting element in promoter and enhancer regions |
| CAAT-box | Pisum sativum | 296 | - | 5 | CAAAT | common cis-acting element in promoter and enhancer regions |
| CAAT-box | Arabidopsis thaliana | 1263 | + | 5 | CCAAT | common cis-acting element in promoter and enhancer regions |
| CAAT-box | Nicotiana glutinosa | 2379 | + | 4 | CAAT |  |
| CAAT-box | Nicotiana glutinosa | 503 | + | 4 | CAAT |  |
| CAAT-box | Nicotiana glutinosa | 582 | - | 4 | CAAT |  |
| CAAT-box | Nicotiana glutinosa | 3418 | - | 4 | CAAT |  |
| CAAT-box | Arabidopsis thaliana | 2788 | + | 5 | CCAAT | common cis-acting element in promoter and enhancer regions |
| CAAT-box | Nicotiana glutinosa | 3266 | + | 4 | CAAT |  |
| CAAT-box | Nicotiana glutinosa | 4183 | + | 4 | CAAT |  |
| CAAT-box | Nicotiana glutinosa | 1670 | - | 4 | CAAT |  |
| CAAT-box | Nicotiana glutinosa | 1842 | - | 4 | CAAT |  |
| CAAT-box | Arabidopsis thaliana | 579 | + | 5 | CCAAT | common cis-acting element in promoter and enhancer regions |
| CAAT-box | Nicotiana glutinosa | 2789 | + | 4 | CAAT |  |
| CAAT-box | Nicotiana glutinosa | 825 | - | 4 | CAAT |  |
| CAAT-box | Pisum sativum | 3428 | - | 5 | CAAAT | common cis-acting element in promoter and enhancer regions |
| CAAT-box | Arabidopsis thaliana | 152 | - | 5 | CCAAT | common cis-acting element in promoter and enhancer regions |
| CAAT-box | Nicotiana glutinosa | 1782 | + | 4 | CAAT |  |
| CAAT-box | Nicotiana glutinosa | 2456 | + | 4 | CAAT |  |
| CAAT-box | Pisum sativum | 3448 | - | 5 | CAAAT | common cis-acting element in promoter and enhancer regions |
| CAAT-box | Nicotiana glutinosa | 580 | + | 4 | CAAT |  |
| CAAT-box | Nicotiana glutinosa | 1264 | + | 4 | CAAT |  |
| CAAT-box | Nicotiana glutinosa | 1171 | + | 4 | CAAT |  |
| CAAT-box | Nicotiana glutinosa | 3071 | + | 4 | CAAT |  |
| CAAT-box | Pisum sativum | 2245 | - | 5 | CAAAT | common cis-acting element in promoter and enhancer regions |
| CAAT-box | Nicotiana glutinosa | 369 | + | 4 | CAAT |  |
| CAAT-box | Nicotiana glutinosa | 1309 | + | 4 | CAAT |  |
| CAAT-box | Arabidopsis thaliana | 3519 | - | 5 | CCAAT | common cis-acting element in promoter and enhancer regions |

>HU06G02537.1   
+ +Up\_Stream \_Len000CCTCGG TGTGGGATTC CGGGTTGATA AATAGGGACC AGCGAAATGT TCAAATAATT   
  
  
+ GGTGAAGGAG TCTCTGTTTG AATGCCTACT AATGCTTTTT TTTTTTTTTT GAATAATTTG TCAGAAAGTT   
  
  
+ AGGCTGCATT GGAAGGGGCG GCCTACGAAG GCTTTTCAGC AAATAAATAA CCAGCTTTCA TTTTGAAATT   
  
  
+ TTTTTTAAAA AATGAAACTC ATTTCACTTA AAAATTTACC ATAATATATA TATATATATA TATATATATA   
  
  
+ TATATATATA TATTTGATTT AACGTTAAGC AAACTTTTAA CTTATGTATC ATTTTACGAC CGTAAAAAAA   
  
  
+ AAAGAAATAT AACACAATAT GTAATAGTAT TTAAGTTTTT GATAAATAAT ATTTTAACAT AATTTGTGTG   
  
  
+ TAGTATGAAT TTAAATCTTA GTACTAATAT AATTATGTAT ACTTGGCTAT AAGATCTCAA TATTAGTATT   
  
  
+ TTATGATTCA ATGTAATGAT GATGCAACTG TGTGTAATTA GAAAGCCTTT GATTGCAGAT ATCTATCTTA   
  
  
+ TTGATACACA TCTACCAATT GAGCTTTTTC AAGATTTGAA GACGGTAGTC AAGTATCTTT GGGAGATTAC   
  
  
+ GTTAGAATTA CTTCATGATA AATTAGACAT ATTTTATACT TCTAATCTTA AATAAGTCTC CATCCTTTCA   
  
  
+ TCATATTTTA AAGAAATGTA CAAGTAAATG TCAATAGTTT GAAACGTTCC TTATCATAAA AGTCATACAT   
  
  
+ ATGTAGGACA TTTTCAAGTT TATGTAAACC ATATGATATA GTTCAAGATA ATTGTCAAAC AAGTTATGAA   
  
  
+ ATCTAAAGGT GTTTGAACGT AATTTCAAGC TAATCTTAGC AAGTTATAGT ATTATTTAAA CTACGACTAG   
  
  
+ ACACAACATT TTGTTCATTT ATATAACACT TAGAATGCAA AAAAATCGGT ATTACTAATA CTGTATCAAG   
  
  
+ AATTGAAGTG TTATGCATTA TACTGATAAG GGATAATATT TAAATTACTT TACTGTTATA GAAGCCAAAA   
  
  
+ ATCTAAACTT TCATACAGAC GTCGGACTAA ATTAGAAAAT TCTTTAACTT AATAGAAAGG ATGACATAAA   
  
  
+ TAAAAATAAA AACCTAAATA GCATAATAAC CATTCTACAC AAAAGTCAAT CTTCTAATAG AAAAAAGCCA   
  
  
+ TAAGTATTCA CTTATTTACC ATTTTATTCC TAATCTACTG TCTGTCTATT CACACCATAA TTCATGAACC   
  
  
+ AATGTGGAGT TTCTGTTTCA TTTCATGGGG TGATTTATGT CTGACAATGA TCCAACAAAC TGAGCCTCAC   
  
  
+ GCGCGGAACA CGAGAGTGGC TGTACTTATG GTGTGAAAGA GCTCTACAAG GTGAGATTGA GGAGGCTACG   
  
  
+ CGTTGGAGTT ATCACCACCG TTGGATTTGT TTTCTTTGGA GCCACGTTAT CACTCTTCAG ACGCCGATCC   
  
  
+ AACGGCCCTT ACTTCCATCT AGTCCCCACT CTCCAACTCT ACGCAATCAA CGCCTTCCGC ATTTAAATAT   
  
  
+ TTCCTTCTCT ATCGCCTACT TTCTCTGTCT CACAATTTTT GTCCGTATTT TGAATTTTCT CAAAATTATC   
  
  
+ ATATAATTTA TTTGCCTTCA ACTAAAGTAT TTATTAAATT AGTTAATGTA TTTAAATTGA ACTAAAAAAG   
  
  
+ AGAAAAAGAA AGAAAACAAC CCCACGAAAG CAAAACGACA GAGGCCAACT TCTCTCTCTA GAGTGTAGAG   
  
  
+ CCCCATCTCG GTTATAATTA TGAGAATCAA TTCCTTCTCA CAAGAGAAAC TCTCGCTCTC TCTCTCTCTC   
  
  
+ CATAAGACAT CACACCAATT GTATGAATTT TCTCTCTCCT GATTTCTGTT TTCCTTCTTG TTGTCCCCTT   
  
  
+ CTAATGCGTT AGTGCGGCCA AAACCACCAT AAACCATCTC TCTTTCAAGA AAGCAGCAAC AACAAAGAGA   
  
  
+ GACAGAGAGA GAGAGAGGGG GAGAAAGATC ACATCTTTTG GGTTTCCGGC GAATATGACG TCTGGGTTTT   
  
  
+ CGGGCGATTT CTTCTACGGC GGTGGAGGCG GTGGCGGAGG AGGTGTCCGG CCTTCTATCG GCGGTGTACA   
  
  
+ AGGCGGGGTT GTCCCATATG GGTTCCGGCA ACAACAACAA CAACAGCAAC AGCAGCAGCA GCAGCAACAG   
  
  
+ CTACATCTAC AGCTACAACA ACAGCGGGGG ATTTTCTCGG CGGATCAGAT CAGCGCTCAA CGAAGGGCGG   
  
  
+ ATTTGTTGAT GGGTAAGCGT TCTTTCGCTG ATTTTCAATC TCAACAACAG CAAATCAGTC TTTTGCAGCA   
  
  
+ GCAGCAGCAG CAACAACAAC AACAGCCCTT TGCGCTCTTG CAAAAACAAC AGCAGTTTCA ACAACAATTA   
  
  
+ CAACAACAAC AGCCACTTTC TCTCCTCCTT CAACAACAGT ACTTGATGAG ATCCGTAAAA CAGAGAACCA   
  
  
+ ACAATTTCGC AGCATCTCCG ATTTCTCCTC TTTCTTCTTC CATTGATCTT TCTTCCGCTA ATAATTCCCG   
  
  
+ GTTCGGTTTG CCGGTTTACC AACAACAACT CCGGTCTCAA CAACAACCAC CTATCTCTCT CCTCGCCCAC   
  
  
+ AACAACAACC AATTCAACCT CTCAAATAAT AACAATTCTT CTTTAATCCA AAACTCCACC AACACTCTTC   
  
  
+ TTGCTCCCGC AGCCAGCAAT AACACCCTAA TGAAAAATTC GGGCTTTTCC TCATCTGGGT TTTCTAATTC   
  
  
+ GGGTCCAAAC CTGGTCCAAC CGAAGCAGGA AATCGGGTAC TCGAATATGG GTACCAATAT GGGTATGAAT   
  
  
+ CTGGTCCAGC CCAAACCCGA CCCTGAGGAT CACGGAATAA TGAACACCCT TCAAGAATTG GAGAAGGAGC   
  
  
+ TTTTGAATGA CGATGAGGAA AACCAGCCGG AAAACGACGC CGTTTCAGTC ATCACCAACA GCGAGTGGTC   
  
  
+ CGAGACCTTC CAGAATCTGA TGAGTCCCGA CCCGAGTTCG AGCCCGAATG CCACCAACAC CCACCCGGGT   
  
  
+ TCGGGTTCTG CTAGTACCAG CGGCAGCAAC AACACTACAA CCACCCCTGC AAACAACAAT AACATGATAT   
  
  
+ CTACCTCGCC GACGTCGTCC ACGTCATCCT GCTGCTCCAC GTCGGCATCG GCGTCTCCGC CGGCGGCGGT   
  
  
+ GGCGACCCCG CAGGTACCAG CCAAGCAGCT GATCTCGGAA GCAGCGGCGG CGATTTCGGA CGGAAAGATT   
  
  
+ GAATCCGCCA TGGAAGTGAT GAACCGGCTG TCCCAGGTTG CCAATTCTAG GGGGAGCTCG GAACAAAAGC   
  
  
+ TGGCTTTTTA CATGGGCCAC GCGCTTCGGT CGCGCGTGAG TCCCACCGAA CACGCTCCCC CTATTGTGGA   
  
  
+ GCTTTTCCAA AAGGAGCATA TGGCGGCCAT GTATTCATTG TACGAGCTCT CTCATTGTTT CAAATTTGGT   
  
  
+ CTTTTGGCTG CCAATTTGGT GATGTTGGAG GCCATTACTT CCGCCCTTCA TGAGGGTCTG AAAATTCATG   
  
  
+ TTGTGGATTT TGATATTGGG CATGGTGGTC AGTACATCAA CCTCCTACGC GCCTTGGGGG AGCGTAGGGC   
  
  
+ TCAGTCGGGG AGCGCGGTGT CGAATGAGGT GAGGATCACT GTGGTTGAGA CCGAGGTCGG TAATGGTGGT   
  
  
+ GCGGCGGGTG CGGAGGCTGT TAAGGATGGC CTGGTGAAAT TGGCTGAGAA GGTGGGGATT AGTTTGAAGT   
  
  
+ TTGAGGCTTT GACTCGGAAG ATCTCCGAGT TAACCCGGGA GTCACTGGGG TGTGAGGAGG GGGAGGCCGT   
  
  
+ GGTGGTGAAT TTTGCGTTCA AACTTTACCG GGTACCGGAT GAGAGCGTGT CGATGGAGAA TCTGAGGGAC   
  
  
+ GAGCTCCTGC GCCGGGTGAA GGCGCTAAGG CCACGCGCCG TCACATTGGT GGAGCAGGAC TTAAATGCGA   
  
  
+ ATACGGCACC GTTCATGACG CGCGTGAGTG GCGCTTGTGA GTACTATAGA GCATTGTTCG ACTCGCTCGA   
  
  
+ CTCGATGGTG CCGAGGGATA ACCCGGACCG GGTCCGGGTC GAGGAGGCCT TGGGGCGGAA GATGGCAAAC   
  
  
+ TCGGTGGCTT GCGAGGGCCG GGAACGGGTT GAGCGGGCGG AGGTGTTTGG GAAGTGGCGG GCGAGGATGA   
  
  
+ GTATGGCAGG GTTCGAGTTG AAGGCACTGA GTCAGAGTGT GGCCGAGTCA ATGCGAACCA AACTCAGCTC   
  
  
+ GGTTCTGGGC GGAAAGCCGG GTTTCACGGT CAAGGAGGAG AGCGGTGGGG TGGGGTTCGG GTGGATGGGA   
  
  
+ CGAACTCTCA CCGTCGCATC GGCCTGGCGT TA  

- +Up\_Stream \_Len000GGAGCC ACACCCTAAG GCCCAACTAT TTATCCCTGG TCGCTTTACA AGTTTATTAA   
  
  
- CCACTTCCTC AGAGACAAAC TTACGGATGA TTACGAAAAA AAAAAAAAAA CTTATTAAAC AGTCTTTCAA   
  
  
- TCCGACGTAA CCTTCCCCGC CGGATGCTTC CGAAAAGTCG TTTATTTATT GGTCGAAAGT AAAACTTTAA   
  
  
- AAAAAATTTT TTACTTTGAG TAAAGTGAAT TTTTAAATGG TATTATATAT ATATATATAT ATATATATAT   
  
  
- ATATATATAT ATAAACTAAA TTGCAATTCG TTTGAAAATT GAATACATAG TAAAATGCTG GCATTTTTTT   
  
  
- TTTCTTTATA TTGTGTTATA CATTATCATA AATTCAAAAA CTATTTATTA TAAAATTGTA TTAAACACAC   
  
  
- ATCATACTTA AATTTAGAAT CATGATTATA TTAATACATA TGAACCGATA TTCTAGAGTT ATAATCATAA   
  
  
- AATACTAAGT TACATTACTA CTACGTTGAC ACACATTAAT CTTTCGGAAA CTAACGTCTA TAGATAGAAT   
  
  
- AACTATGTGT AGATGGTTAA CTCGAAAAAG TTCTAAACTT CTGCCATCAG TTCATAGAAA CCCTCTAATG   
  
  
- CAATCTTAAT GAAGTACTAT TTAATCTGTA TAAAATATGA AGATTAGAAT TTATTCAGAG GTAGGAAAGT   
  
  
- AGTATAAAAT TTCTTTACAT GTTCATTTAC AGTTATCAAA CTTTGCAAGG AATAGTATTT TCAGTATGTA   
  
  
- TACATCCTGT AAAAGTTCAA ATACATTTGG TATACTATAT CAAGTTCTAT TAACAGTTTG TTCAATACTT   
  
  
- TAGATTTCCA CAAACTTGCA TTAAAGTTCG ATTAGAATCG TTCAATATCA TAATAAATTT GATGCTGATC   
  
  
- TGTGTTGTAA AACAAGTAAA TATATTGTGA ATCTTACGTT TTTTTAGCCA TAATGATTAT GACATAGTTC   
  
  
- TTAACTTCAC AATACGTAAT ATGACTATTC CCTATTATAA ATTTAATGAA ATGACAATAT CTTCGGTTTT   
  
  
- TAGATTTGAA AGTATGTCTG CAGCCTGATT TAATCTTTTA AGAAATTGAA TTATCTTTCC TACTGTATTT   
  
  
- ATTTTTATTT TTGGATTTAT CGTATTATTG GTAAGATGTG TTTTCAGTTA GAAGATTATC TTTTTTCGGT   
  
  
- ATTCATAAGT GAATAAATGG TAAAATAAGG ATTAGATGAC AGACAGATAA GTGTGGTATT AAGTACTTGG   
  
  
- TTACACCTCA AAGACAAAGT AAAGTACCCC ACTAAATACA GACTGTTACT AGGTTGTTTG ACTCGGAGTG   
  
  
- CGCGCCTTGT GCTCTCACCG ACATGAATAC CACACTTTCT CGAGATGTTC CACTCTAACT CCTCCGATGC   
  
  
- GCAACCTCAA TAGTGGTGGC AACCTAAACA AAAGAAACCT CGGTGCAATA GTGAGAAGTC TGCGGCTAGG   
  
  
- TTGCCGGGAA TGAAGGTAGA TCAGGGGTGA GAGGTTGAGA TGCGTTAGTT GCGGAAGGCG TAAATTTATA   
  
  
- AAGGAAGAGA TAGCGGATGA AAGAGACAGA GTGTTAAAAA CAGGCATAAA ACTTAAAAGA GTTTTAATAG   
  
  
- TATATTAAAT AAACGGAAGT TGATTTCATA AATAATTTAA TCAATTACAT AAATTTAACT TGATTTTTTC   
  
  
- TCTTTTTCTT TCTTTTGTTG GGGTGCTTTC GTTTTGCTGT CTCCGGTTGA AGAGAGAGAT CTCACATCTC   
  
  
- GGGGTAGAGC CAATATTAAT ACTCTTAGTT AAGGAAGAGT GTTCTCTTTG AGAGCGAGAG AGAGAGAGAG   
  
  
- GTATTCTGTA GTGTGGTTAA CATACTTAAA AGAGAGAGGA CTAAAGACAA AAGGAAGAAC AACAGGGGAA   
  
  
- GATTACGCAA TCACGCCGGT TTTGGTGGTA TTTGGTAGAG AGAAAGTTCT TTCGTCGTTG TTGTTTCTCT   
  
  
- CTGTCTCTCT CTCTCTCCCC CTCTTTCTAG TGTAGAAAAC CCAAAGGCCG CTTATACTGC AGACCCAAAA   
  
  
- GCCCGCTAAA GAAGATGCCG CCACCTCCGC CACCGCCTCC TCCACAGGCC GGAAGATAGC CGCCACATGT   
  
  
- TCCGCCCCAA CAGGGTATAC CCAAGGCCGT TGTTGTTGTT GTTGTCGTTG TCGTCGTCGT CGTCGTTGTC   
  
  
- GATGTAGATG TCGATGTTGT TGTCGCCCCC TAAAAGAGCC GCCTAGTCTA GTCGCGAGTT GCTTCCCGCC   
  
  
- TAAACAACTA CCCATTCGCA AGAAAGCGAC TAAAAGTTAG AGTTGTTGTC GTTTAGTCAG AAAACGTCGT   
  
  
- CGTCGTCGTC GTTGTTGTTG TTGTCGGGAA ACGCGAGAAC GTTTTTGTTG TCGTCAAAGT TGTTGTTAAT   
  
  
- GTTGTTGTTG TCGGTGAAAG AGAGGAGGAA GTTGTTGTCA TGAACTACTC TAGGCATTTT GTCTCTTGGT   
  
  
- TGTTAAAGCG TCGTAGAGGC TAAAGAGGAG AAAGAAGAAG GTAACTAGAA AGAAGGCGAT TATTAAGGGC   
  
  
- CAAGCCAAAC GGCCAAATGG TTGTTGTTGA GGCCAGAGTT GTTGTTGGTG GATAGAGAGA GGAGCGGGTG   
  
  
- TTGTTGTTGG TTAAGTTGGA GAGTTTATTA TTGTTAAGAA GAAATTAGGT TTTGAGGTGG TTGTGAGAAG   
  
  
- AACGAGGGCG TCGGTCGTTA TTGTGGGATT ACTTTTTAAG CCCGAAAAGG AGTAGACCCA AAAGATTAAG   
  
  
- CCCAGGTTTG GACCAGGTTG GCTTCGTCCT TTAGCCCATG AGCTTATACC CATGGTTATA CCCATACTTA   
  
  
- GACCAGGTCG GGTTTGGGCT GGGACTCCTA GTGCCTTATT ACTTGTGGGA AGTTCTTAAC CTCTTCCTCG   
  
  
- AAAACTTACT GCTACTCCTT TTGGTCGGCC TTTTGCTGCG GCAAAGTCAG TAGTGGTTGT CGCTCACCAG   
  
  
- GCTCTGGAAG GTCTTAGACT ACTCAGGGCT GGGCTCAAGC TCGGGCTTAC GGTGGTTGTG GGTGGGCCCA   
  
  
- AGCCCAAGAC GATCATGGTC GCCGTCGTTG TTGTGATGTT GGTGGGGACG TTTGTTGTTA TTGTACTATA   
  
  
- GATGGAGCGG CTGCAGCAGG TGCAGTAGGA CGACGAGGTG CAGCCGTAGC CGCAGAGGCG GCCGCCGCCA   
  
  
- CCGCTGGGGC GTCCATGGTC GGTTCGTCGA CTAGAGCCTT CGTCGCCGCC GCTAAAGCCT GCCTTTCTAA   
  
  
- CTTAGGCGGT ACCTTCACTA CTTGGCCGAC AGGGTCCAAC GGTTAAGATC CCCCTCGAGC CTTGTTTTCG   
  
  
- ACCGAAAAAT GTACCCGGTG CGCGAAGCCA GCGCGCACTC AGGGTGGCTT GTGCGAGGGG GATAACACCT   
  
  
- CGAAAAGGTT TTCCTCGTAT ACCGCCGGTA CATAAGTAAC ATGCTCGAGA GAGTAACAAA GTTTAAACCA   
  
  
- GAAAACCGAC GGTTAAACCA CTACAACCTC CGGTAATGAA GGCGGGAAGT ACTCCCAGAC TTTTAAGTAC   
  
  
- AACACCTAAA ACTATAACCC GTACCACCAG TCATGTAGTT GGAGGATGCG CGGAACCCCC TCGCATCCCG   
  
  
- AGTCAGCCCC TCGCGCCACA GCTTACTCCA CTCCTAGTGA CACCAACTCT GGCTCCAGCC ATTACCACCA   
  
  
- CGCCGCCCAC GCCTCCGACA ATTCCTACCG GACCACTTTA ACCGACTCTT CCACCCCTAA TCAAACTTCA   
  
  
- AACTCCGAAA CTGAGCCTTC TAGAGGCTCA ATTGGGCCCT CAGTGACCCC ACACTCCTCC CCCTCCGGCA   
  
  
- CCACCACTTA AAACGCAAGT TTGAAATGGC CCATGGCCTA CTCTCGCACA GCTACCTCTT AGACTCCCTG   
  
  
- CTCGAGGACG CGGCCCACTT CCGCGATTCC GGTGCGCGGC AGTGTAACCA CCTCGTCCTG AATTTACGCT   
  
  
- TATGCCGTGG CAAGTACTGC GCGCACTCAC CGCGAACACT CATGATATCT CGTAACAAGC TGAGCGAGCT   
  
  
- GAGCTACCAC GGCTCCCTAT TGGGCCTGGC CCAGGCCCAG CTCCTCCGGA ACCCCGCCTT CTACCGTTTG   
  
  
- AGCCACCGAA CGCTCCCGGC CCTTGCCCAA CTCGCCCGCC TCCACAAACC CTTCACCGCC CGCTCCTACT   
  
  
- CATACCGTCC CAAGCTCAAC TTCCGTGACT CAGTCTCACA CCGGCTCAGT TACGCTTGGT TTGAGTCGAG   
  
  
- CCAAGACCCG CCTTTCGGCC CAAAGTGCCA GTTCCTCCTC TCGCCACCCC ACCCCAAGCC CACCTACCCT   
  
  
- GCTTGAGAGT GGCAGCGTAG CCGGACCGCA AT

+     CAT-box

| Site Name | Organism | Position | Strand | Matrix score. | sequence | function |
| --- | --- | --- | --- | --- | --- | --- |
| CAT-box | Arabidopsis thaliana | 4117 | - | 6 | GCCACT | cis-acting regulatory element related to meristem expression |
| CAT-box | Arabidopsis thaliana | 3951 | - | 6 | GCCACT | cis-acting regulatory element related to meristem expression |
| CAT-box | Arabidopsis thaliana | 1349 | - | 6 | GCCACT | cis-acting regulatory element related to meristem expression |
| CAT-box | Arabidopsis thaliana | 2396 | + | 6 | GCCACT | cis-acting regulatory element related to meristem expression |

>HU06G02537.1   
+ +Up\_Stream \_Len000CCTCGG TGTGGGATTC CGGGTTGATA AATAGGGACC AGCGAAATGT TCAAATAATT   
  
  
+ GGTGAAGGAG TCTCTGTTTG AATGCCTACT AATGCTTTTT TTTTTTTTTT GAATAATTTG TCAGAAAGTT   
  
  
+ AGGCTGCATT GGAAGGGGCG GCCTACGAAG GCTTTTCAGC AAATAAATAA CCAGCTTTCA TTTTGAAATT   
  
  
+ TTTTTTAAAA AATGAAACTC ATTTCACTTA AAAATTTACC ATAATATATA TATATATATA TATATATATA   
  
  
+ TATATATATA TATTTGATTT AACGTTAAGC AAACTTTTAA CTTATGTATC ATTTTACGAC CGTAAAAAAA   
  
  
+ AAAGAAATAT AACACAATAT GTAATAGTAT TTAAGTTTTT GATAAATAAT ATTTTAACAT AATTTGTGTG   
  
  
+ TAGTATGAAT TTAAATCTTA GTACTAATAT AATTATGTAT ACTTGGCTAT AAGATCTCAA TATTAGTATT   
  
  
+ TTATGATTCA ATGTAATGAT GATGCAACTG TGTGTAATTA GAAAGCCTTT GATTGCAGAT ATCTATCTTA   
  
  
+ TTGATACACA TCTACCAATT GAGCTTTTTC AAGATTTGAA GACGGTAGTC AAGTATCTTT GGGAGATTAC   
  
  
+ GTTAGAATTA CTTCATGATA AATTAGACAT ATTTTATACT TCTAATCTTA AATAAGTCTC CATCCTTTCA   
  
  
+ TCATATTTTA AAGAAATGTA CAAGTAAATG TCAATAGTTT GAAACGTTCC TTATCATAAA AGTCATACAT   
  
  
+ ATGTAGGACA TTTTCAAGTT TATGTAAACC ATATGATATA GTTCAAGATA ATTGTCAAAC AAGTTATGAA   
  
  
+ ATCTAAAGGT GTTTGAACGT AATTTCAAGC TAATCTTAGC AAGTTATAGT ATTATTTAAA CTACGACTAG   
  
  
+ ACACAACATT TTGTTCATTT ATATAACACT TAGAATGCAA AAAAATCGGT ATTACTAATA CTGTATCAAG   
  
  
+ AATTGAAGTG TTATGCATTA TACTGATAAG GGATAATATT TAAATTACTT TACTGTTATA GAAGCCAAAA   
  
  
+ ATCTAAACTT TCATACAGAC GTCGGACTAA ATTAGAAAAT TCTTTAACTT AATAGAAAGG ATGACATAAA   
  
  
+ TAAAAATAAA AACCTAAATA GCATAATAAC CATTCTACAC AAAAGTCAAT CTTCTAATAG AAAAAAGCCA   
  
  
+ TAAGTATTCA CTTATTTACC ATTTTATTCC TAATCTACTG TCTGTCTATT CACACCATAA TTCATGAACC   
  
  
+ AATGTGGAGT TTCTGTTTCA TTTCATGGGG TGATTTATGT CTGACAATGA TCCAACAAAC TGAGCCTCAC   
  
  
+ GCGCGGAACA CGAGAGTGGC TGTACTTATG GTGTGAAAGA GCTCTACAAG GTGAGATTGA GGAGGCTACG   
  
  
+ CGTTGGAGTT ATCACCACCG TTGGATTTGT TTTCTTTGGA GCCACGTTAT CACTCTTCAG ACGCCGATCC   
  
  
+ AACGGCCCTT ACTTCCATCT AGTCCCCACT CTCCAACTCT ACGCAATCAA CGCCTTCCGC ATTTAAATAT   
  
  
+ TTCCTTCTCT ATCGCCTACT TTCTCTGTCT CACAATTTTT GTCCGTATTT TGAATTTTCT CAAAATTATC   
  
  
+ ATATAATTTA TTTGCCTTCA ACTAAAGTAT TTATTAAATT AGTTAATGTA TTTAAATTGA ACTAAAAAAG   
  
  
+ AGAAAAAGAA AGAAAACAAC CCCACGAAAG CAAAACGACA GAGGCCAACT TCTCTCTCTA GAGTGTAGAG   
  
  
+ CCCCATCTCG GTTATAATTA TGAGAATCAA TTCCTTCTCA CAAGAGAAAC TCTCGCTCTC TCTCTCTCTC   
  
  
+ CATAAGACAT CACACCAATT GTATGAATTT TCTCTCTCCT GATTTCTGTT TTCCTTCTTG TTGTCCCCTT   
  
  
+ CTAATGCGTT AGTGCGGCCA AAACCACCAT AAACCATCTC TCTTTCAAGA AAGCAGCAAC AACAAAGAGA   
  
  
+ GACAGAGAGA GAGAGAGGGG GAGAAAGATC ACATCTTTTG GGTTTCCGGC GAATATGACG TCTGGGTTTT   
  
  
+ CGGGCGATTT CTTCTACGGC GGTGGAGGCG GTGGCGGAGG AGGTGTCCGG CCTTCTATCG GCGGTGTACA   
  
  
+ AGGCGGGGTT GTCCCATATG GGTTCCGGCA ACAACAACAA CAACAGCAAC AGCAGCAGCA GCAGCAACAG   
  
  
+ CTACATCTAC AGCTACAACA ACAGCGGGGG ATTTTCTCGG CGGATCAGAT CAGCGCTCAA CGAAGGGCGG   
  
  
+ ATTTGTTGAT GGGTAAGCGT TCTTTCGCTG ATTTTCAATC TCAACAACAG CAAATCAGTC TTTTGCAGCA   
  
  
+ GCAGCAGCAG CAACAACAAC AACAGCCCTT TGCGCTCTTG CAAAAACAAC AGCAGTTTCA ACAACAATTA   
  
  
+ CAACAACAAC AGCCACTTTC TCTCCTCCTT CAACAACAGT ACTTGATGAG ATCCGTAAAA CAGAGAACCA   
  
  
+ ACAATTTCGC AGCATCTCCG ATTTCTCCTC TTTCTTCTTC CATTGATCTT TCTTCCGCTA ATAATTCCCG   
  
  
+ GTTCGGTTTG CCGGTTTACC AACAACAACT CCGGTCTCAA CAACAACCAC CTATCTCTCT CCTCGCCCAC   
  
  
+ AACAACAACC AATTCAACCT CTCAAATAAT AACAATTCTT CTTTAATCCA AAACTCCACC AACACTCTTC   
  
  
+ TTGCTCCCGC AGCCAGCAAT AACACCCTAA TGAAAAATTC GGGCTTTTCC TCATCTGGGT TTTCTAATTC   
  
  
+ GGGTCCAAAC CTGGTCCAAC CGAAGCAGGA AATCGGGTAC TCGAATATGG GTACCAATAT GGGTATGAAT   
  
  
+ CTGGTCCAGC CCAAACCCGA CCCTGAGGAT CACGGAATAA TGAACACCCT TCAAGAATTG GAGAAGGAGC   
  
  
+ TTTTGAATGA CGATGAGGAA AACCAGCCGG AAAACGACGC CGTTTCAGTC ATCACCAACA GCGAGTGGTC   
  
  
+ CGAGACCTTC CAGAATCTGA TGAGTCCCGA CCCGAGTTCG AGCCCGAATG CCACCAACAC CCACCCGGGT   
  
  
+ TCGGGTTCTG CTAGTACCAG CGGCAGCAAC AACACTACAA CCACCCCTGC AAACAACAAT AACATGATAT   
  
  
+ CTACCTCGCC GACGTCGTCC ACGTCATCCT GCTGCTCCAC GTCGGCATCG GCGTCTCCGC CGGCGGCGGT   
  
  
+ GGCGACCCCG CAGGTACCAG CCAAGCAGCT GATCTCGGAA GCAGCGGCGG CGATTTCGGA CGGAAAGATT   
  
  
+ GAATCCGCCA TGGAAGTGAT GAACCGGCTG TCCCAGGTTG CCAATTCTAG GGGGAGCTCG GAACAAAAGC   
  
  
+ TGGCTTTTTA CATGGGCCAC GCGCTTCGGT CGCGCGTGAG TCCCACCGAA CACGCTCCCC CTATTGTGGA   
  
  
+ GCTTTTCCAA AAGGAGCATA TGGCGGCCAT GTATTCATTG TACGAGCTCT CTCATTGTTT CAAATTTGGT   
  
  
+ CTTTTGGCTG CCAATTTGGT GATGTTGGAG GCCATTACTT CCGCCCTTCA TGAGGGTCTG AAAATTCATG   
  
  
+ TTGTGGATTT TGATATTGGG CATGGTGGTC AGTACATCAA CCTCCTACGC GCCTTGGGGG AGCGTAGGGC   
  
  
+ TCAGTCGGGG AGCGCGGTGT CGAATGAGGT GAGGATCACT GTGGTTGAGA CCGAGGTCGG TAATGGTGGT   
  
  
+ GCGGCGGGTG CGGAGGCTGT TAAGGATGGC CTGGTGAAAT TGGCTGAGAA GGTGGGGATT AGTTTGAAGT   
  
  
+ TTGAGGCTTT GACTCGGAAG ATCTCCGAGT TAACCCGGGA GTCACTGGGG TGTGAGGAGG GGGAGGCCGT   
  
  
+ GGTGGTGAAT TTTGCGTTCA AACTTTACCG GGTACCGGAT GAGAGCGTGT CGATGGAGAA TCTGAGGGAC   
  
  
+ GAGCTCCTGC GCCGGGTGAA GGCGCTAAGG CCACGCGCCG TCACATTGGT GGAGCAGGAC TTAAATGCGA   
  
  
+ ATACGGCACC GTTCATGACG CGCGTGAGTG GCGCTTGTGA GTACTATAGA GCATTGTTCG ACTCGCTCGA   
  
  
+ CTCGATGGTG CCGAGGGATA ACCCGGACCG GGTCCGGGTC GAGGAGGCCT TGGGGCGGAA GATGGCAAAC   
  
  
+ TCGGTGGCTT GCGAGGGCCG GGAACGGGTT GAGCGGGCGG AGGTGTTTGG GAAGTGGCGG GCGAGGATGA   
  
  
+ GTATGGCAGG GTTCGAGTTG AAGGCACTGA GTCAGAGTGT GGCCGAGTCA ATGCGAACCA AACTCAGCTC   
  
  
+ GGTTCTGGGC GGAAAGCCGG GTTTCACGGT CAAGGAGGAG AGCGGTGGGG TGGGGTTCGG GTGGATGGGA   
  
  
+ CGAACTCTCA CCGTCGCATC GGCCTGGCGT TA  

- +Up\_Stream \_Len000GGAGCC ACACCCTAAG GCCCAACTAT TTATCCCTGG TCGCTTTACA AGTTTATTAA   
  
  
- CCACTTCCTC AGAGACAAAC TTACGGATGA TTACGAAAAA AAAAAAAAAA CTTATTAAAC AGTCTTTCAA   
  
  
- TCCGACGTAA CCTTCCCCGC CGGATGCTTC CGAAAAGTCG TTTATTTATT GGTCGAAAGT AAAACTTTAA   
  
  
- AAAAAATTTT TTACTTTGAG TAAAGTGAAT TTTTAAATGG TATTATATAT ATATATATAT ATATATATAT   
  
  
- ATATATATAT ATAAACTAAA TTGCAATTCG TTTGAAAATT GAATACATAG TAAAATGCTG GCATTTTTTT   
  
  
- TTTCTTTATA TTGTGTTATA CATTATCATA AATTCAAAAA CTATTTATTA TAAAATTGTA TTAAACACAC   
  
  
- ATCATACTTA AATTTAGAAT CATGATTATA TTAATACATA TGAACCGATA TTCTAGAGTT ATAATCATAA   
  
  
- AATACTAAGT TACATTACTA CTACGTTGAC ACACATTAAT CTTTCGGAAA CTAACGTCTA TAGATAGAAT   
  
  
- AACTATGTGT AGATGGTTAA CTCGAAAAAG TTCTAAACTT CTGCCATCAG TTCATAGAAA CCCTCTAATG   
  
  
- CAATCTTAAT GAAGTACTAT TTAATCTGTA TAAAATATGA AGATTAGAAT TTATTCAGAG GTAGGAAAGT   
  
  
- AGTATAAAAT TTCTTTACAT GTTCATTTAC AGTTATCAAA CTTTGCAAGG AATAGTATTT TCAGTATGTA   
  
  
- TACATCCTGT AAAAGTTCAA ATACATTTGG TATACTATAT CAAGTTCTAT TAACAGTTTG TTCAATACTT   
  
  
- TAGATTTCCA CAAACTTGCA TTAAAGTTCG ATTAGAATCG TTCAATATCA TAATAAATTT GATGCTGATC   
  
  
- TGTGTTGTAA AACAAGTAAA TATATTGTGA ATCTTACGTT TTTTTAGCCA TAATGATTAT GACATAGTTC   
  
  
- TTAACTTCAC AATACGTAAT ATGACTATTC CCTATTATAA ATTTAATGAA ATGACAATAT CTTCGGTTTT   
  
  
- TAGATTTGAA AGTATGTCTG CAGCCTGATT TAATCTTTTA AGAAATTGAA TTATCTTTCC TACTGTATTT   
  
  
- ATTTTTATTT TTGGATTTAT CGTATTATTG GTAAGATGTG TTTTCAGTTA GAAGATTATC TTTTTTCGGT   
  
  
- ATTCATAAGT GAATAAATGG TAAAATAAGG ATTAGATGAC AGACAGATAA GTGTGGTATT AAGTACTTGG   
  
  
- TTACACCTCA AAGACAAAGT AAAGTACCCC ACTAAATACA GACTGTTACT AGGTTGTTTG ACTCGGAGTG   
  
  
- CGCGCCTTGT GCTCTCACCG ACATGAATAC CACACTTTCT CGAGATGTTC CACTCTAACT CCTCCGATGC   
  
  
- GCAACCTCAA TAGTGGTGGC AACCTAAACA AAAGAAACCT CGGTGCAATA GTGAGAAGTC TGCGGCTAGG   
  
  
- TTGCCGGGAA TGAAGGTAGA TCAGGGGTGA GAGGTTGAGA TGCGTTAGTT GCGGAAGGCG TAAATTTATA   
  
  
- AAGGAAGAGA TAGCGGATGA AAGAGACAGA GTGTTAAAAA CAGGCATAAA ACTTAAAAGA GTTTTAATAG   
  
  
- TATATTAAAT AAACGGAAGT TGATTTCATA AATAATTTAA TCAATTACAT AAATTTAACT TGATTTTTTC   
  
  
- TCTTTTTCTT TCTTTTGTTG GGGTGCTTTC GTTTTGCTGT CTCCGGTTGA AGAGAGAGAT CTCACATCTC   
  
  
- GGGGTAGAGC CAATATTAAT ACTCTTAGTT AAGGAAGAGT GTTCTCTTTG AGAGCGAGAG AGAGAGAGAG   
  
  
- GTATTCTGTA GTGTGGTTAA CATACTTAAA AGAGAGAGGA CTAAAGACAA AAGGAAGAAC AACAGGGGAA   
  
  
- GATTACGCAA TCACGCCGGT TTTGGTGGTA TTTGGTAGAG AGAAAGTTCT TTCGTCGTTG TTGTTTCTCT   
  
  
- CTGTCTCTCT CTCTCTCCCC CTCTTTCTAG TGTAGAAAAC CCAAAGGCCG CTTATACTGC AGACCCAAAA   
  
  
- GCCCGCTAAA GAAGATGCCG CCACCTCCGC CACCGCCTCC TCCACAGGCC GGAAGATAGC CGCCACATGT   
  
  
- TCCGCCCCAA CAGGGTATAC CCAAGGCCGT TGTTGTTGTT GTTGTCGTTG TCGTCGTCGT CGTCGTTGTC   
  
  
- GATGTAGATG TCGATGTTGT TGTCGCCCCC TAAAAGAGCC GCCTAGTCTA GTCGCGAGTT GCTTCCCGCC   
  
  
- TAAACAACTA CCCATTCGCA AGAAAGCGAC TAAAAGTTAG AGTTGTTGTC GTTTAGTCAG AAAACGTCGT   
  
  
- CGTCGTCGTC GTTGTTGTTG TTGTCGGGAA ACGCGAGAAC GTTTTTGTTG TCGTCAAAGT TGTTGTTAAT   
  
  
- GTTGTTGTTG TCGGTGAAAG AGAGGAGGAA GTTGTTGTCA TGAACTACTC TAGGCATTTT GTCTCTTGGT   
  
  
- TGTTAAAGCG TCGTAGAGGC TAAAGAGGAG AAAGAAGAAG GTAACTAGAA AGAAGGCGAT TATTAAGGGC   
  
  
- CAAGCCAAAC GGCCAAATGG TTGTTGTTGA GGCCAGAGTT GTTGTTGGTG GATAGAGAGA GGAGCGGGTG   
  
  
- TTGTTGTTGG TTAAGTTGGA GAGTTTATTA TTGTTAAGAA GAAATTAGGT TTTGAGGTGG TTGTGAGAAG   
  
  
- AACGAGGGCG TCGGTCGTTA TTGTGGGATT ACTTTTTAAG CCCGAAAAGG AGTAGACCCA AAAGATTAAG   
  
  
- CCCAGGTTTG GACCAGGTTG GCTTCGTCCT TTAGCCCATG AGCTTATACC CATGGTTATA CCCATACTTA   
  
  
- GACCAGGTCG GGTTTGGGCT GGGACTCCTA GTGCCTTATT ACTTGTGGGA AGTTCTTAAC CTCTTCCTCG   
  
  
- AAAACTTACT GCTACTCCTT TTGGTCGGCC TTTTGCTGCG GCAAAGTCAG TAGTGGTTGT CGCTCACCAG   
  
  
- GCTCTGGAAG GTCTTAGACT ACTCAGGGCT GGGCTCAAGC TCGGGCTTAC GGTGGTTGTG GGTGGGCCCA   
  
  
- AGCCCAAGAC GATCATGGTC GCCGTCGTTG TTGTGATGTT GGTGGGGACG TTTGTTGTTA TTGTACTATA   
  
  
- GATGGAGCGG CTGCAGCAGG TGCAGTAGGA CGACGAGGTG CAGCCGTAGC CGCAGAGGCG GCCGCCGCCA   
  
  
- CCGCTGGGGC GTCCATGGTC GGTTCGTCGA CTAGAGCCTT CGTCGCCGCC GCTAAAGCCT GCCTTTCTAA   
  
  
- CTTAGGCGGT ACCTTCACTA CTTGGCCGAC AGGGTCCAAC GGTTAAGATC CCCCTCGAGC CTTGTTTTCG   
  
  
- ACCGAAAAAT GTACCCGGTG CGCGAAGCCA GCGCGCACTC AGGGTGGCTT GTGCGAGGGG GATAACACCT   
  
  
- CGAAAAGGTT TTCCTCGTAT ACCGCCGGTA CATAAGTAAC ATGCTCGAGA GAGTAACAAA GTTTAAACCA   
  
  
- GAAAACCGAC GGTTAAACCA CTACAACCTC CGGTAATGAA GGCGGGAAGT ACTCCCAGAC TTTTAAGTAC   
  
  
- AACACCTAAA ACTATAACCC GTACCACCAG TCATGTAGTT GGAGGATGCG CGGAACCCCC TCGCATCCCG   
  
  
- AGTCAGCCCC TCGCGCCACA GCTTACTCCA CTCCTAGTGA CACCAACTCT GGCTCCAGCC ATTACCACCA   
  
  
- CGCCGCCCAC GCCTCCGACA ATTCCTACCG GACCACTTTA ACCGACTCTT CCACCCCTAA TCAAACTTCA   
  
  
- AACTCCGAAA CTGAGCCTTC TAGAGGCTCA ATTGGGCCCT CAGTGACCCC ACACTCCTCC CCCTCCGGCA   
  
  
- CCACCACTTA AAACGCAAGT TTGAAATGGC CCATGGCCTA CTCTCGCACA GCTACCTCTT AGACTCCCTG   
  
  
- CTCGAGGACG CGGCCCACTT CCGCGATTCC GGTGCGCGGC AGTGTAACCA CCTCGTCCTG AATTTACGCT   
  
  
- TATGCCGTGG CAAGTACTGC GCGCACTCAC CGCGAACACT CATGATATCT CGTAACAAGC TGAGCGAGCT   
  
  
- GAGCTACCAC GGCTCCCTAT TGGGCCTGGC CCAGGCCCAG CTCCTCCGGA ACCCCGCCTT CTACCGTTTG   
  
  
- AGCCACCGAA CGCTCCCGGC CCTTGCCCAA CTCGCCCGCC TCCACAAACC CTTCACCGCC CGCTCCTACT   
  
  
- CATACCGTCC CAAGCTCAAC TTCCGTGACT CAGTCTCACA CCGGCTCAGT TACGCTTGGT TTGAGTCGAG   
  
  
- CCAAGACCCG CCTTTCGGCC CAAAGTGCCA GTTCCTCCTC TCGCCACCCC ACCCCAAGCC CACCTACCCT   
  
  
- GCTTGAGAGT GGCAGCGTAG CCGGACCGCA AT

+     CCAAT-box

| Site Name | Organism | Position | Strand | Matrix score. | sequence | function |
| --- | --- | --- | --- | --- | --- | --- |
| CCAAT-box | Hordeum vulgare | 1422 | - | 6 | CAACGG | MYBHv1 binding site |
| CCAAT-box | Hordeum vulgare | 1474 | + | 6 | CAACGG | MYBHv1 binding site |

>HU06G02537.1   
+ +Up\_Stream \_Len000CCTCGG TGTGGGATTC CGGGTTGATA AATAGGGACC AGCGAAATGT TCAAATAATT   
  
  
+ GGTGAAGGAG TCTCTGTTTG AATGCCTACT AATGCTTTTT TTTTTTTTTT GAATAATTTG TCAGAAAGTT   
  
  
+ AGGCTGCATT GGAAGGGGCG GCCTACGAAG GCTTTTCAGC AAATAAATAA CCAGCTTTCA TTTTGAAATT   
  
  
+ TTTTTTAAAA AATGAAACTC ATTTCACTTA AAAATTTACC ATAATATATA TATATATATA TATATATATA   
  
  
+ TATATATATA TATTTGATTT AACGTTAAGC AAACTTTTAA CTTATGTATC ATTTTACGAC CGTAAAAAAA   
  
  
+ AAAGAAATAT AACACAATAT GTAATAGTAT TTAAGTTTTT GATAAATAAT ATTTTAACAT AATTTGTGTG   
  
  
+ TAGTATGAAT TTAAATCTTA GTACTAATAT AATTATGTAT ACTTGGCTAT AAGATCTCAA TATTAGTATT   
  
  
+ TTATGATTCA ATGTAATGAT GATGCAACTG TGTGTAATTA GAAAGCCTTT GATTGCAGAT ATCTATCTTA   
  
  
+ TTGATACACA TCTACCAATT GAGCTTTTTC AAGATTTGAA GACGGTAGTC AAGTATCTTT GGGAGATTAC   
  
  
+ GTTAGAATTA CTTCATGATA AATTAGACAT ATTTTATACT TCTAATCTTA AATAAGTCTC CATCCTTTCA   
  
  
+ TCATATTTTA AAGAAATGTA CAAGTAAATG TCAATAGTTT GAAACGTTCC TTATCATAAA AGTCATACAT   
  
  
+ ATGTAGGACA TTTTCAAGTT TATGTAAACC ATATGATATA GTTCAAGATA ATTGTCAAAC AAGTTATGAA   
  
  
+ ATCTAAAGGT GTTTGAACGT AATTTCAAGC TAATCTTAGC AAGTTATAGT ATTATTTAAA CTACGACTAG   
  
  
+ ACACAACATT TTGTTCATTT ATATAACACT TAGAATGCAA AAAAATCGGT ATTACTAATA CTGTATCAAG   
  
  
+ AATTGAAGTG TTATGCATTA TACTGATAAG GGATAATATT TAAATTACTT TACTGTTATA GAAGCCAAAA   
  
  
+ ATCTAAACTT TCATACAGAC GTCGGACTAA ATTAGAAAAT TCTTTAACTT AATAGAAAGG ATGACATAAA   
  
  
+ TAAAAATAAA AACCTAAATA GCATAATAAC CATTCTACAC AAAAGTCAAT CTTCTAATAG AAAAAAGCCA   
  
  
+ TAAGTATTCA CTTATTTACC ATTTTATTCC TAATCTACTG TCTGTCTATT CACACCATAA TTCATGAACC   
  
  
+ AATGTGGAGT TTCTGTTTCA TTTCATGGGG TGATTTATGT CTGACAATGA TCCAACAAAC TGAGCCTCAC   
  
  
+ GCGCGGAACA CGAGAGTGGC TGTACTTATG GTGTGAAAGA GCTCTACAAG GTGAGATTGA GGAGGCTACG   
  
  
+ CGTTGGAGTT ATCACCACCG TTGGATTTGT TTTCTTTGGA GCCACGTTAT CACTCTTCAG ACGCCGATCC   
  
  
+ AACGGCCCTT ACTTCCATCT AGTCCCCACT CTCCAACTCT ACGCAATCAA CGCCTTCCGC ATTTAAATAT   
  
  
+ TTCCTTCTCT ATCGCCTACT TTCTCTGTCT CACAATTTTT GTCCGTATTT TGAATTTTCT CAAAATTATC   
  
  
+ ATATAATTTA TTTGCCTTCA ACTAAAGTAT TTATTAAATT AGTTAATGTA TTTAAATTGA ACTAAAAAAG   
  
  
+ AGAAAAAGAA AGAAAACAAC CCCACGAAAG CAAAACGACA GAGGCCAACT TCTCTCTCTA GAGTGTAGAG   
  
  
+ CCCCATCTCG GTTATAATTA TGAGAATCAA TTCCTTCTCA CAAGAGAAAC TCTCGCTCTC TCTCTCTCTC   
  
  
+ CATAAGACAT CACACCAATT GTATGAATTT TCTCTCTCCT GATTTCTGTT TTCCTTCTTG TTGTCCCCTT   
  
  
+ CTAATGCGTT AGTGCGGCCA AAACCACCAT AAACCATCTC TCTTTCAAGA AAGCAGCAAC AACAAAGAGA   
  
  
+ GACAGAGAGA GAGAGAGGGG GAGAAAGATC ACATCTTTTG GGTTTCCGGC GAATATGACG TCTGGGTTTT   
  
  
+ CGGGCGATTT CTTCTACGGC GGTGGAGGCG GTGGCGGAGG AGGTGTCCGG CCTTCTATCG GCGGTGTACA   
  
  
+ AGGCGGGGTT GTCCCATATG GGTTCCGGCA ACAACAACAA CAACAGCAAC AGCAGCAGCA GCAGCAACAG   
  
  
+ CTACATCTAC AGCTACAACA ACAGCGGGGG ATTTTCTCGG CGGATCAGAT CAGCGCTCAA CGAAGGGCGG   
  
  
+ ATTTGTTGAT GGGTAAGCGT TCTTTCGCTG ATTTTCAATC TCAACAACAG CAAATCAGTC TTTTGCAGCA   
  
  
+ GCAGCAGCAG CAACAACAAC AACAGCCCTT TGCGCTCTTG CAAAAACAAC AGCAGTTTCA ACAACAATTA   
  
  
+ CAACAACAAC AGCCACTTTC TCTCCTCCTT CAACAACAGT ACTTGATGAG ATCCGTAAAA CAGAGAACCA   
  
  
+ ACAATTTCGC AGCATCTCCG ATTTCTCCTC TTTCTTCTTC CATTGATCTT TCTTCCGCTA ATAATTCCCG   
  
  
+ GTTCGGTTTG CCGGTTTACC AACAACAACT CCGGTCTCAA CAACAACCAC CTATCTCTCT CCTCGCCCAC   
  
  
+ AACAACAACC AATTCAACCT CTCAAATAAT AACAATTCTT CTTTAATCCA AAACTCCACC AACACTCTTC   
  
  
+ TTGCTCCCGC AGCCAGCAAT AACACCCTAA TGAAAAATTC GGGCTTTTCC TCATCTGGGT TTTCTAATTC   
  
  
+ GGGTCCAAAC CTGGTCCAAC CGAAGCAGGA AATCGGGTAC TCGAATATGG GTACCAATAT GGGTATGAAT   
  
  
+ CTGGTCCAGC CCAAACCCGA CCCTGAGGAT CACGGAATAA TGAACACCCT TCAAGAATTG GAGAAGGAGC   
  
  
+ TTTTGAATGA CGATGAGGAA AACCAGCCGG AAAACGACGC CGTTTCAGTC ATCACCAACA GCGAGTGGTC   
  
  
+ CGAGACCTTC CAGAATCTGA TGAGTCCCGA CCCGAGTTCG AGCCCGAATG CCACCAACAC CCACCCGGGT   
  
  
+ TCGGGTTCTG CTAGTACCAG CGGCAGCAAC AACACTACAA CCACCCCTGC AAACAACAAT AACATGATAT   
  
  
+ CTACCTCGCC GACGTCGTCC ACGTCATCCT GCTGCTCCAC GTCGGCATCG GCGTCTCCGC CGGCGGCGGT   
  
  
+ GGCGACCCCG CAGGTACCAG CCAAGCAGCT GATCTCGGAA GCAGCGGCGG CGATTTCGGA CGGAAAGATT   
  
  
+ GAATCCGCCA TGGAAGTGAT GAACCGGCTG TCCCAGGTTG CCAATTCTAG GGGGAGCTCG GAACAAAAGC   
  
  
+ TGGCTTTTTA CATGGGCCAC GCGCTTCGGT CGCGCGTGAG TCCCACCGAA CACGCTCCCC CTATTGTGGA   
  
  
+ GCTTTTCCAA AAGGAGCATA TGGCGGCCAT GTATTCATTG TACGAGCTCT CTCATTGTTT CAAATTTGGT   
  
  
+ CTTTTGGCTG CCAATTTGGT GATGTTGGAG GCCATTACTT CCGCCCTTCA TGAGGGTCTG AAAATTCATG   
  
  
+ TTGTGGATTT TGATATTGGG CATGGTGGTC AGTACATCAA CCTCCTACGC GCCTTGGGGG AGCGTAGGGC   
  
  
+ TCAGTCGGGG AGCGCGGTGT CGAATGAGGT GAGGATCACT GTGGTTGAGA CCGAGGTCGG TAATGGTGGT   
  
  
+ GCGGCGGGTG CGGAGGCTGT TAAGGATGGC CTGGTGAAAT TGGCTGAGAA GGTGGGGATT AGTTTGAAGT   
  
  
+ TTGAGGCTTT GACTCGGAAG ATCTCCGAGT TAACCCGGGA GTCACTGGGG TGTGAGGAGG GGGAGGCCGT   
  
  
+ GGTGGTGAAT TTTGCGTTCA AACTTTACCG GGTACCGGAT GAGAGCGTGT CGATGGAGAA TCTGAGGGAC   
  
  
+ GAGCTCCTGC GCCGGGTGAA GGCGCTAAGG CCACGCGCCG TCACATTGGT GGAGCAGGAC TTAAATGCGA   
  
  
+ ATACGGCACC GTTCATGACG CGCGTGAGTG GCGCTTGTGA GTACTATAGA GCATTGTTCG ACTCGCTCGA   
  
  
+ CTCGATGGTG CCGAGGGATA ACCCGGACCG GGTCCGGGTC GAGGAGGCCT TGGGGCGGAA GATGGCAAAC   
  
  
+ TCGGTGGCTT GCGAGGGCCG GGAACGGGTT GAGCGGGCGG AGGTGTTTGG GAAGTGGCGG GCGAGGATGA   
  
  
+ GTATGGCAGG GTTCGAGTTG AAGGCACTGA GTCAGAGTGT GGCCGAGTCA ATGCGAACCA AACTCAGCTC   
  
  
+ GGTTCTGGGC GGAAAGCCGG GTTTCACGGT CAAGGAGGAG AGCGGTGGGG TGGGGTTCGG GTGGATGGGA   
  
  
+ CGAACTCTCA CCGTCGCATC GGCCTGGCGT TA  

- +Up\_Stream \_Len000GGAGCC ACACCCTAAG GCCCAACTAT TTATCCCTGG TCGCTTTACA AGTTTATTAA   
  
  
- CCACTTCCTC AGAGACAAAC TTACGGATGA TTACGAAAAA AAAAAAAAAA CTTATTAAAC AGTCTTTCAA   
  
  
- TCCGACGTAA CCTTCCCCGC CGGATGCTTC CGAAAAGTCG TTTATTTATT GGTCGAAAGT AAAACTTTAA   
  
  
- AAAAAATTTT TTACTTTGAG TAAAGTGAAT TTTTAAATGG TATTATATAT ATATATATAT ATATATATAT   
  
  
- ATATATATAT ATAAACTAAA TTGCAATTCG TTTGAAAATT GAATACATAG TAAAATGCTG GCATTTTTTT   
  
  
- TTTCTTTATA TTGTGTTATA CATTATCATA AATTCAAAAA CTATTTATTA TAAAATTGTA TTAAACACAC   
  
  
- ATCATACTTA AATTTAGAAT CATGATTATA TTAATACATA TGAACCGATA TTCTAGAGTT ATAATCATAA   
  
  
- AATACTAAGT TACATTACTA CTACGTTGAC ACACATTAAT CTTTCGGAAA CTAACGTCTA TAGATAGAAT   
  
  
- AACTATGTGT AGATGGTTAA CTCGAAAAAG TTCTAAACTT CTGCCATCAG TTCATAGAAA CCCTCTAATG   
  
  
- CAATCTTAAT GAAGTACTAT TTAATCTGTA TAAAATATGA AGATTAGAAT TTATTCAGAG GTAGGAAAGT   
  
  
- AGTATAAAAT TTCTTTACAT GTTCATTTAC AGTTATCAAA CTTTGCAAGG AATAGTATTT TCAGTATGTA   
  
  
- TACATCCTGT AAAAGTTCAA ATACATTTGG TATACTATAT CAAGTTCTAT TAACAGTTTG TTCAATACTT   
  
  
- TAGATTTCCA CAAACTTGCA TTAAAGTTCG ATTAGAATCG TTCAATATCA TAATAAATTT GATGCTGATC   
  
  
- TGTGTTGTAA AACAAGTAAA TATATTGTGA ATCTTACGTT TTTTTAGCCA TAATGATTAT GACATAGTTC   
  
  
- TTAACTTCAC AATACGTAAT ATGACTATTC CCTATTATAA ATTTAATGAA ATGACAATAT CTTCGGTTTT   
  
  
- TAGATTTGAA AGTATGTCTG CAGCCTGATT TAATCTTTTA AGAAATTGAA TTATCTTTCC TACTGTATTT   
  
  
- ATTTTTATTT TTGGATTTAT CGTATTATTG GTAAGATGTG TTTTCAGTTA GAAGATTATC TTTTTTCGGT   
  
  
- ATTCATAAGT GAATAAATGG TAAAATAAGG ATTAGATGAC AGACAGATAA GTGTGGTATT AAGTACTTGG   
  
  
- TTACACCTCA AAGACAAAGT AAAGTACCCC ACTAAATACA GACTGTTACT AGGTTGTTTG ACTCGGAGTG   
  
  
- CGCGCCTTGT GCTCTCACCG ACATGAATAC CACACTTTCT CGAGATGTTC CACTCTAACT CCTCCGATGC   
  
  
- GCAACCTCAA TAGTGGTGGC AACCTAAACA AAAGAAACCT CGGTGCAATA GTGAGAAGTC TGCGGCTAGG   
  
  
- TTGCCGGGAA TGAAGGTAGA TCAGGGGTGA GAGGTTGAGA TGCGTTAGTT GCGGAAGGCG TAAATTTATA   
  
  
- AAGGAAGAGA TAGCGGATGA AAGAGACAGA GTGTTAAAAA CAGGCATAAA ACTTAAAAGA GTTTTAATAG   
  
  
- TATATTAAAT AAACGGAAGT TGATTTCATA AATAATTTAA TCAATTACAT AAATTTAACT TGATTTTTTC   
  
  
- TCTTTTTCTT TCTTTTGTTG GGGTGCTTTC GTTTTGCTGT CTCCGGTTGA AGAGAGAGAT CTCACATCTC   
  
  
- GGGGTAGAGC CAATATTAAT ACTCTTAGTT AAGGAAGAGT GTTCTCTTTG AGAGCGAGAG AGAGAGAGAG   
  
  
- GTATTCTGTA GTGTGGTTAA CATACTTAAA AGAGAGAGGA CTAAAGACAA AAGGAAGAAC AACAGGGGAA   
  
  
- GATTACGCAA TCACGCCGGT TTTGGTGGTA TTTGGTAGAG AGAAAGTTCT TTCGTCGTTG TTGTTTCTCT   
  
  
- CTGTCTCTCT CTCTCTCCCC CTCTTTCTAG TGTAGAAAAC CCAAAGGCCG CTTATACTGC AGACCCAAAA   
  
  
- GCCCGCTAAA GAAGATGCCG CCACCTCCGC CACCGCCTCC TCCACAGGCC GGAAGATAGC CGCCACATGT   
  
  
- TCCGCCCCAA CAGGGTATAC CCAAGGCCGT TGTTGTTGTT GTTGTCGTTG TCGTCGTCGT CGTCGTTGTC   
  
  
- GATGTAGATG TCGATGTTGT TGTCGCCCCC TAAAAGAGCC GCCTAGTCTA GTCGCGAGTT GCTTCCCGCC   
  
  
- TAAACAACTA CCCATTCGCA AGAAAGCGAC TAAAAGTTAG AGTTGTTGTC GTTTAGTCAG AAAACGTCGT   
  
  
- CGTCGTCGTC GTTGTTGTTG TTGTCGGGAA ACGCGAGAAC GTTTTTGTTG TCGTCAAAGT TGTTGTTAAT   
  
  
- GTTGTTGTTG TCGGTGAAAG AGAGGAGGAA GTTGTTGTCA TGAACTACTC TAGGCATTTT GTCTCTTGGT   
  
  
- TGTTAAAGCG TCGTAGAGGC TAAAGAGGAG AAAGAAGAAG GTAACTAGAA AGAAGGCGAT TATTAAGGGC   
  
  
- CAAGCCAAAC GGCCAAATGG TTGTTGTTGA GGCCAGAGTT GTTGTTGGTG GATAGAGAGA GGAGCGGGTG   
  
  
- TTGTTGTTGG TTAAGTTGGA GAGTTTATTA TTGTTAAGAA GAAATTAGGT TTTGAGGTGG TTGTGAGAAG   
  
  
- AACGAGGGCG TCGGTCGTTA TTGTGGGATT ACTTTTTAAG CCCGAAAAGG AGTAGACCCA AAAGATTAAG   
  
  
- CCCAGGTTTG GACCAGGTTG GCTTCGTCCT TTAGCCCATG AGCTTATACC CATGGTTATA CCCATACTTA   
  
  
- GACCAGGTCG GGTTTGGGCT GGGACTCCTA GTGCCTTATT ACTTGTGGGA AGTTCTTAAC CTCTTCCTCG   
  
  
- AAAACTTACT GCTACTCCTT TTGGTCGGCC TTTTGCTGCG GCAAAGTCAG TAGTGGTTGT CGCTCACCAG   
  
  
- GCTCTGGAAG GTCTTAGACT ACTCAGGGCT GGGCTCAAGC TCGGGCTTAC GGTGGTTGTG GGTGGGCCCA   
  
  
- AGCCCAAGAC GATCATGGTC GCCGTCGTTG TTGTGATGTT GGTGGGGACG TTTGTTGTTA TTGTACTATA   
  
  
- GATGGAGCGG CTGCAGCAGG TGCAGTAGGA CGACGAGGTG CAGCCGTAGC CGCAGAGGCG GCCGCCGCCA   
  
  
- CCGCTGGGGC GTCCATGGTC GGTTCGTCGA CTAGAGCCTT CGTCGCCGCC GCTAAAGCCT GCCTTTCTAA   
  
  
- CTTAGGCGGT ACCTTCACTA CTTGGCCGAC AGGGTCCAAC GGTTAAGATC CCCCTCGAGC CTTGTTTTCG   
  
  
- ACCGAAAAAT GTACCCGGTG CGCGAAGCCA GCGCGCACTC AGGGTGGCTT GTGCGAGGGG GATAACACCT   
  
  
- CGAAAAGGTT TTCCTCGTAT ACCGCCGGTA CATAAGTAAC ATGCTCGAGA GAGTAACAAA GTTTAAACCA   
  
  
- GAAAACCGAC GGTTAAACCA CTACAACCTC CGGTAATGAA GGCGGGAAGT ACTCCCAGAC TTTTAAGTAC   
  
  
- AACACCTAAA ACTATAACCC GTACCACCAG TCATGTAGTT GGAGGATGCG CGGAACCCCC TCGCATCCCG   
  
  
- AGTCAGCCCC TCGCGCCACA GCTTACTCCA CTCCTAGTGA CACCAACTCT GGCTCCAGCC ATTACCACCA   
  
  
- CGCCGCCCAC GCCTCCGACA ATTCCTACCG GACCACTTTA ACCGACTCTT CCACCCCTAA TCAAACTTCA   
  
  
- AACTCCGAAA CTGAGCCTTC TAGAGGCTCA ATTGGGCCCT CAGTGACCCC ACACTCCTCC CCCTCCGGCA   
  
  
- CCACCACTTA AAACGCAAGT TTGAAATGGC CCATGGCCTA CTCTCGCACA GCTACCTCTT AGACTCCCTG   
  
  
- CTCGAGGACG CGGCCCACTT CCGCGATTCC GGTGCGCGGC AGTGTAACCA CCTCGTCCTG AATTTACGCT   
  
  
- TATGCCGTGG CAAGTACTGC GCGCACTCAC CGCGAACACT CATGATATCT CGTAACAAGC TGAGCGAGCT   
  
  
- GAGCTACCAC GGCTCCCTAT TGGGCCTGGC CCAGGCCCAG CTCCTCCGGA ACCCCGCCTT CTACCGTTTG   
  
  
- AGCCACCGAA CGCTCCCGGC CCTTGCCCAA CTCGCCCGCC TCCACAAACC CTTCACCGCC CGCTCCTACT   
  
  
- CATACCGTCC CAAGCTCAAC TTCCGTGACT CAGTCTCACA CCGGCTCAGT TACGCTTGGT TTGAGTCGAG   
  
  
- CCAAGACCCG CCTTTCGGCC CAAAGTGCCA GTTCCTCCTC TCGCCACCCC ACCCCAAGCC CACCTACCCT   
  
  
- GCTTGAGAGT GGCAGCGTAG CCGGACCGCA AT

+     CCGTCC motif

| Site Name | Organism | Position | Strand | Matrix score. | sequence | function |
| --- | --- | --- | --- | --- | --- | --- |
| CCGTCC motif | Nicotiana tabacum | 3212 | - | 6 | CCGTCC |  |

>HU06G02537.1   
+ +Up\_Stream \_Len000CCTCGG TGTGGGATTC CGGGTTGATA AATAGGGACC AGCGAAATGT TCAAATAATT   
  
  
+ GGTGAAGGAG TCTCTGTTTG AATGCCTACT AATGCTTTTT TTTTTTTTTT GAATAATTTG TCAGAAAGTT   
  
  
+ AGGCTGCATT GGAAGGGGCG GCCTACGAAG GCTTTTCAGC AAATAAATAA CCAGCTTTCA TTTTGAAATT   
  
  
+ TTTTTTAAAA AATGAAACTC ATTTCACTTA AAAATTTACC ATAATATATA TATATATATA TATATATATA   
  
  
+ TATATATATA TATTTGATTT AACGTTAAGC AAACTTTTAA CTTATGTATC ATTTTACGAC CGTAAAAAAA   
  
  
+ AAAGAAATAT AACACAATAT GTAATAGTAT TTAAGTTTTT GATAAATAAT ATTTTAACAT AATTTGTGTG   
  
  
+ TAGTATGAAT TTAAATCTTA GTACTAATAT AATTATGTAT ACTTGGCTAT AAGATCTCAA TATTAGTATT   
  
  
+ TTATGATTCA ATGTAATGAT GATGCAACTG TGTGTAATTA GAAAGCCTTT GATTGCAGAT ATCTATCTTA   
  
  
+ TTGATACACA TCTACCAATT GAGCTTTTTC AAGATTTGAA GACGGTAGTC AAGTATCTTT GGGAGATTAC   
  
  
+ GTTAGAATTA CTTCATGATA AATTAGACAT ATTTTATACT TCTAATCTTA AATAAGTCTC CATCCTTTCA   
  
  
+ TCATATTTTA AAGAAATGTA CAAGTAAATG TCAATAGTTT GAAACGTTCC TTATCATAAA AGTCATACAT   
  
  
+ ATGTAGGACA TTTTCAAGTT TATGTAAACC ATATGATATA GTTCAAGATA ATTGTCAAAC AAGTTATGAA   
  
  
+ ATCTAAAGGT GTTTGAACGT AATTTCAAGC TAATCTTAGC AAGTTATAGT ATTATTTAAA CTACGACTAG   
  
  
+ ACACAACATT TTGTTCATTT ATATAACACT TAGAATGCAA AAAAATCGGT ATTACTAATA CTGTATCAAG   
  
  
+ AATTGAAGTG TTATGCATTA TACTGATAAG GGATAATATT TAAATTACTT TACTGTTATA GAAGCCAAAA   
  
  
+ ATCTAAACTT TCATACAGAC GTCGGACTAA ATTAGAAAAT TCTTTAACTT AATAGAAAGG ATGACATAAA   
  
  
+ TAAAAATAAA AACCTAAATA GCATAATAAC CATTCTACAC AAAAGTCAAT CTTCTAATAG AAAAAAGCCA   
  
  
+ TAAGTATTCA CTTATTTACC ATTTTATTCC TAATCTACTG TCTGTCTATT CACACCATAA TTCATGAACC   
  
  
+ AATGTGGAGT TTCTGTTTCA TTTCATGGGG TGATTTATGT CTGACAATGA TCCAACAAAC TGAGCCTCAC   
  
  
+ GCGCGGAACA CGAGAGTGGC TGTACTTATG GTGTGAAAGA GCTCTACAAG GTGAGATTGA GGAGGCTACG   
  
  
+ CGTTGGAGTT ATCACCACCG TTGGATTTGT TTTCTTTGGA GCCACGTTAT CACTCTTCAG ACGCCGATCC   
  
  
+ AACGGCCCTT ACTTCCATCT AGTCCCCACT CTCCAACTCT ACGCAATCAA CGCCTTCCGC ATTTAAATAT   
  
  
+ TTCCTTCTCT ATCGCCTACT TTCTCTGTCT CACAATTTTT GTCCGTATTT TGAATTTTCT CAAAATTATC   
  
  
+ ATATAATTTA TTTGCCTTCA ACTAAAGTAT TTATTAAATT AGTTAATGTA TTTAAATTGA ACTAAAAAAG   
  
  
+ AGAAAAAGAA AGAAAACAAC CCCACGAAAG CAAAACGACA GAGGCCAACT TCTCTCTCTA GAGTGTAGAG   
  
  
+ CCCCATCTCG GTTATAATTA TGAGAATCAA TTCCTTCTCA CAAGAGAAAC TCTCGCTCTC TCTCTCTCTC   
  
  
+ CATAAGACAT CACACCAATT GTATGAATTT TCTCTCTCCT GATTTCTGTT TTCCTTCTTG TTGTCCCCTT   
  
  
+ CTAATGCGTT AGTGCGGCCA AAACCACCAT AAACCATCTC TCTTTCAAGA AAGCAGCAAC AACAAAGAGA   
  
  
+ GACAGAGAGA GAGAGAGGGG GAGAAAGATC ACATCTTTTG GGTTTCCGGC GAATATGACG TCTGGGTTTT   
  
  
+ CGGGCGATTT CTTCTACGGC GGTGGAGGCG GTGGCGGAGG AGGTGTCCGG CCTTCTATCG GCGGTGTACA   
  
  
+ AGGCGGGGTT GTCCCATATG GGTTCCGGCA ACAACAACAA CAACAGCAAC AGCAGCAGCA GCAGCAACAG   
  
  
+ CTACATCTAC AGCTACAACA ACAGCGGGGG ATTTTCTCGG CGGATCAGAT CAGCGCTCAA CGAAGGGCGG   
  
  
+ ATTTGTTGAT GGGTAAGCGT TCTTTCGCTG ATTTTCAATC TCAACAACAG CAAATCAGTC TTTTGCAGCA   
  
  
+ GCAGCAGCAG CAACAACAAC AACAGCCCTT TGCGCTCTTG CAAAAACAAC AGCAGTTTCA ACAACAATTA   
  
  
+ CAACAACAAC AGCCACTTTC TCTCCTCCTT CAACAACAGT ACTTGATGAG ATCCGTAAAA CAGAGAACCA   
  
  
+ ACAATTTCGC AGCATCTCCG ATTTCTCCTC TTTCTTCTTC CATTGATCTT TCTTCCGCTA ATAATTCCCG   
  
  
+ GTTCGGTTTG CCGGTTTACC AACAACAACT CCGGTCTCAA CAACAACCAC CTATCTCTCT CCTCGCCCAC   
  
  
+ AACAACAACC AATTCAACCT CTCAAATAAT AACAATTCTT CTTTAATCCA AAACTCCACC AACACTCTTC   
  
  
+ TTGCTCCCGC AGCCAGCAAT AACACCCTAA TGAAAAATTC GGGCTTTTCC TCATCTGGGT TTTCTAATTC   
  
  
+ GGGTCCAAAC CTGGTCCAAC CGAAGCAGGA AATCGGGTAC TCGAATATGG GTACCAATAT GGGTATGAAT   
  
  
+ CTGGTCCAGC CCAAACCCGA CCCTGAGGAT CACGGAATAA TGAACACCCT TCAAGAATTG GAGAAGGAGC   
  
  
+ TTTTGAATGA CGATGAGGAA AACCAGCCGG AAAACGACGC CGTTTCAGTC ATCACCAACA GCGAGTGGTC   
  
  
+ CGAGACCTTC CAGAATCTGA TGAGTCCCGA CCCGAGTTCG AGCCCGAATG CCACCAACAC CCACCCGGGT   
  
  
+ TCGGGTTCTG CTAGTACCAG CGGCAGCAAC AACACTACAA CCACCCCTGC AAACAACAAT AACATGATAT   
  
  
+ CTACCTCGCC GACGTCGTCC ACGTCATCCT GCTGCTCCAC GTCGGCATCG GCGTCTCCGC CGGCGGCGGT   
  
  
+ GGCGACCCCG CAGGTACCAG CCAAGCAGCT GATCTCGGAA GCAGCGGCGG CGATTTCGGA CGGAAAGATT   
  
  
+ GAATCCGCCA TGGAAGTGAT GAACCGGCTG TCCCAGGTTG CCAATTCTAG GGGGAGCTCG GAACAAAAGC   
  
  
+ TGGCTTTTTA CATGGGCCAC GCGCTTCGGT CGCGCGTGAG TCCCACCGAA CACGCTCCCC CTATTGTGGA   
  
  
+ GCTTTTCCAA AAGGAGCATA TGGCGGCCAT GTATTCATTG TACGAGCTCT CTCATTGTTT CAAATTTGGT   
  
  
+ CTTTTGGCTG CCAATTTGGT GATGTTGGAG GCCATTACTT CCGCCCTTCA TGAGGGTCTG AAAATTCATG   
  
  
+ TTGTGGATTT TGATATTGGG CATGGTGGTC AGTACATCAA CCTCCTACGC GCCTTGGGGG AGCGTAGGGC   
  
  
+ TCAGTCGGGG AGCGCGGTGT CGAATGAGGT GAGGATCACT GTGGTTGAGA CCGAGGTCGG TAATGGTGGT   
  
  
+ GCGGCGGGTG CGGAGGCTGT TAAGGATGGC CTGGTGAAAT TGGCTGAGAA GGTGGGGATT AGTTTGAAGT   
  
  
+ TTGAGGCTTT GACTCGGAAG ATCTCCGAGT TAACCCGGGA GTCACTGGGG TGTGAGGAGG GGGAGGCCGT   
  
  
+ GGTGGTGAAT TTTGCGTTCA AACTTTACCG GGTACCGGAT GAGAGCGTGT CGATGGAGAA TCTGAGGGAC   
  
  
+ GAGCTCCTGC GCCGGGTGAA GGCGCTAAGG CCACGCGCCG TCACATTGGT GGAGCAGGAC TTAAATGCGA   
  
  
+ ATACGGCACC GTTCATGACG CGCGTGAGTG GCGCTTGTGA GTACTATAGA GCATTGTTCG ACTCGCTCGA   
  
  
+ CTCGATGGTG CCGAGGGATA ACCCGGACCG GGTCCGGGTC GAGGAGGCCT TGGGGCGGAA GATGGCAAAC   
  
  
+ TCGGTGGCTT GCGAGGGCCG GGAACGGGTT GAGCGGGCGG AGGTGTTTGG GAAGTGGCGG GCGAGGATGA   
  
  
+ GTATGGCAGG GTTCGAGTTG AAGGCACTGA GTCAGAGTGT GGCCGAGTCA ATGCGAACCA AACTCAGCTC   
  
  
+ GGTTCTGGGC GGAAAGCCGG GTTTCACGGT CAAGGAGGAG AGCGGTGGGG TGGGGTTCGG GTGGATGGGA   
  
  
+ CGAACTCTCA CCGTCGCATC GGCCTGGCGT TA  

- +Up\_Stream \_Len000GGAGCC ACACCCTAAG GCCCAACTAT TTATCCCTGG TCGCTTTACA AGTTTATTAA   
  
  
- CCACTTCCTC AGAGACAAAC TTACGGATGA TTACGAAAAA AAAAAAAAAA CTTATTAAAC AGTCTTTCAA   
  
  
- TCCGACGTAA CCTTCCCCGC CGGATGCTTC CGAAAAGTCG TTTATTTATT GGTCGAAAGT AAAACTTTAA   
  
  
- AAAAAATTTT TTACTTTGAG TAAAGTGAAT TTTTAAATGG TATTATATAT ATATATATAT ATATATATAT   
  
  
- ATATATATAT ATAAACTAAA TTGCAATTCG TTTGAAAATT GAATACATAG TAAAATGCTG GCATTTTTTT   
  
  
- TTTCTTTATA TTGTGTTATA CATTATCATA AATTCAAAAA CTATTTATTA TAAAATTGTA TTAAACACAC   
  
  
- ATCATACTTA AATTTAGAAT CATGATTATA TTAATACATA TGAACCGATA TTCTAGAGTT ATAATCATAA   
  
  
- AATACTAAGT TACATTACTA CTACGTTGAC ACACATTAAT CTTTCGGAAA CTAACGTCTA TAGATAGAAT   
  
  
- AACTATGTGT AGATGGTTAA CTCGAAAAAG TTCTAAACTT CTGCCATCAG TTCATAGAAA CCCTCTAATG   
  
  
- CAATCTTAAT GAAGTACTAT TTAATCTGTA TAAAATATGA AGATTAGAAT TTATTCAGAG GTAGGAAAGT   
  
  
- AGTATAAAAT TTCTTTACAT GTTCATTTAC AGTTATCAAA CTTTGCAAGG AATAGTATTT TCAGTATGTA   
  
  
- TACATCCTGT AAAAGTTCAA ATACATTTGG TATACTATAT CAAGTTCTAT TAACAGTTTG TTCAATACTT   
  
  
- TAGATTTCCA CAAACTTGCA TTAAAGTTCG ATTAGAATCG TTCAATATCA TAATAAATTT GATGCTGATC   
  
  
- TGTGTTGTAA AACAAGTAAA TATATTGTGA ATCTTACGTT TTTTTAGCCA TAATGATTAT GACATAGTTC   
  
  
- TTAACTTCAC AATACGTAAT ATGACTATTC CCTATTATAA ATTTAATGAA ATGACAATAT CTTCGGTTTT   
  
  
- TAGATTTGAA AGTATGTCTG CAGCCTGATT TAATCTTTTA AGAAATTGAA TTATCTTTCC TACTGTATTT   
  
  
- ATTTTTATTT TTGGATTTAT CGTATTATTG GTAAGATGTG TTTTCAGTTA GAAGATTATC TTTTTTCGGT   
  
  
- ATTCATAAGT GAATAAATGG TAAAATAAGG ATTAGATGAC AGACAGATAA GTGTGGTATT AAGTACTTGG   
  
  
- TTACACCTCA AAGACAAAGT AAAGTACCCC ACTAAATACA GACTGTTACT AGGTTGTTTG ACTCGGAGTG   
  
  
- CGCGCCTTGT GCTCTCACCG ACATGAATAC CACACTTTCT CGAGATGTTC CACTCTAACT CCTCCGATGC   
  
  
- GCAACCTCAA TAGTGGTGGC AACCTAAACA AAAGAAACCT CGGTGCAATA GTGAGAAGTC TGCGGCTAGG   
  
  
- TTGCCGGGAA TGAAGGTAGA TCAGGGGTGA GAGGTTGAGA TGCGTTAGTT GCGGAAGGCG TAAATTTATA   
  
  
- AAGGAAGAGA TAGCGGATGA AAGAGACAGA GTGTTAAAAA CAGGCATAAA ACTTAAAAGA GTTTTAATAG   
  
  
- TATATTAAAT AAACGGAAGT TGATTTCATA AATAATTTAA TCAATTACAT AAATTTAACT TGATTTTTTC   
  
  
- TCTTTTTCTT TCTTTTGTTG GGGTGCTTTC GTTTTGCTGT CTCCGGTTGA AGAGAGAGAT CTCACATCTC   
  
  
- GGGGTAGAGC CAATATTAAT ACTCTTAGTT AAGGAAGAGT GTTCTCTTTG AGAGCGAGAG AGAGAGAGAG   
  
  
- GTATTCTGTA GTGTGGTTAA CATACTTAAA AGAGAGAGGA CTAAAGACAA AAGGAAGAAC AACAGGGGAA   
  
  
- GATTACGCAA TCACGCCGGT TTTGGTGGTA TTTGGTAGAG AGAAAGTTCT TTCGTCGTTG TTGTTTCTCT   
  
  
- CTGTCTCTCT CTCTCTCCCC CTCTTTCTAG TGTAGAAAAC CCAAAGGCCG CTTATACTGC AGACCCAAAA   
  
  
- GCCCGCTAAA GAAGATGCCG CCACCTCCGC CACCGCCTCC TCCACAGGCC GGAAGATAGC CGCCACATGT   
  
  
- TCCGCCCCAA CAGGGTATAC CCAAGGCCGT TGTTGTTGTT GTTGTCGTTG TCGTCGTCGT CGTCGTTGTC   
  
  
- GATGTAGATG TCGATGTTGT TGTCGCCCCC TAAAAGAGCC GCCTAGTCTA GTCGCGAGTT GCTTCCCGCC   
  
  
- TAAACAACTA CCCATTCGCA AGAAAGCGAC TAAAAGTTAG AGTTGTTGTC GTTTAGTCAG AAAACGTCGT   
  
  
- CGTCGTCGTC GTTGTTGTTG TTGTCGGGAA ACGCGAGAAC GTTTTTGTTG TCGTCAAAGT TGTTGTTAAT   
  
  
- GTTGTTGTTG TCGGTGAAAG AGAGGAGGAA GTTGTTGTCA TGAACTACTC TAGGCATTTT GTCTCTTGGT   
  
  
- TGTTAAAGCG TCGTAGAGGC TAAAGAGGAG AAAGAAGAAG GTAACTAGAA AGAAGGCGAT TATTAAGGGC   
  
  
- CAAGCCAAAC GGCCAAATGG TTGTTGTTGA GGCCAGAGTT GTTGTTGGTG GATAGAGAGA GGAGCGGGTG   
  
  
- TTGTTGTTGG TTAAGTTGGA GAGTTTATTA TTGTTAAGAA GAAATTAGGT TTTGAGGTGG TTGTGAGAAG   
  
  
- AACGAGGGCG TCGGTCGTTA TTGTGGGATT ACTTTTTAAG CCCGAAAAGG AGTAGACCCA AAAGATTAAG   
  
  
- CCCAGGTTTG GACCAGGTTG GCTTCGTCCT TTAGCCCATG AGCTTATACC CATGGTTATA CCCATACTTA   
  
  
- GACCAGGTCG GGTTTGGGCT GGGACTCCTA GTGCCTTATT ACTTGTGGGA AGTTCTTAAC CTCTTCCTCG   
  
  
- AAAACTTACT GCTACTCCTT TTGGTCGGCC TTTTGCTGCG GCAAAGTCAG TAGTGGTTGT CGCTCACCAG   
  
  
- GCTCTGGAAG GTCTTAGACT ACTCAGGGCT GGGCTCAAGC TCGGGCTTAC GGTGGTTGTG GGTGGGCCCA   
  
  
- AGCCCAAGAC GATCATGGTC GCCGTCGTTG TTGTGATGTT GGTGGGGACG TTTGTTGTTA TTGTACTATA   
  
  
- GATGGAGCGG CTGCAGCAGG TGCAGTAGGA CGACGAGGTG CAGCCGTAGC CGCAGAGGCG GCCGCCGCCA   
  
  
- CCGCTGGGGC GTCCATGGTC GGTTCGTCGA CTAGAGCCTT CGTCGCCGCC GCTAAAGCCT GCCTTTCTAA   
  
  
- CTTAGGCGGT ACCTTCACTA CTTGGCCGAC AGGGTCCAAC GGTTAAGATC CCCCTCGAGC CTTGTTTTCG   
  
  
- ACCGAAAAAT GTACCCGGTG CGCGAAGCCA GCGCGCACTC AGGGTGGCTT GTGCGAGGGG GATAACACCT   
  
  
- CGAAAAGGTT TTCCTCGTAT ACCGCCGGTA CATAAGTAAC ATGCTCGAGA GAGTAACAAA GTTTAAACCA   
  
  
- GAAAACCGAC GGTTAAACCA CTACAACCTC CGGTAATGAA GGCGGGAAGT ACTCCCAGAC TTTTAAGTAC   
  
  
- AACACCTAAA ACTATAACCC GTACCACCAG TCATGTAGTT GGAGGATGCG CGGAACCCCC TCGCATCCCG   
  
  
- AGTCAGCCCC TCGCGCCACA GCTTACTCCA CTCCTAGTGA CACCAACTCT GGCTCCAGCC ATTACCACCA   
  
  
- CGCCGCCCAC GCCTCCGACA ATTCCTACCG GACCACTTTA ACCGACTCTT CCACCCCTAA TCAAACTTCA   
  
  
- AACTCCGAAA CTGAGCCTTC TAGAGGCTCA ATTGGGCCCT CAGTGACCCC ACACTCCTCC CCCTCCGGCA   
  
  
- CCACCACTTA AAACGCAAGT TTGAAATGGC CCATGGCCTA CTCTCGCACA GCTACCTCTT AGACTCCCTG   
  
  
- CTCGAGGACG CGGCCCACTT CCGCGATTCC GGTGCGCGGC AGTGTAACCA CCTCGTCCTG AATTTACGCT   
  
  
- TATGCCGTGG CAAGTACTGC GCGCACTCAC CGCGAACACT CATGATATCT CGTAACAAGC TGAGCGAGCT   
  
  
- GAGCTACCAC GGCTCCCTAT TGGGCCTGGC CCAGGCCCAG CTCCTCCGGA ACCCCGCCTT CTACCGTTTG   
  
  
- AGCCACCGAA CGCTCCCGGC CCTTGCCCAA CTCGCCCGCC TCCACAAACC CTTCACCGCC CGCTCCTACT   
  
  
- CATACCGTCC CAAGCTCAAC TTCCGTGACT CAGTCTCACA CCGGCTCAGT TACGCTTGGT TTGAGTCGAG   
  
  
- CCAAGACCCG CCTTTCGGCC CAAAGTGCCA GTTCCTCCTC TCGCCACCCC ACCCCAAGCC CACCTACCCT   
  
  
- GCTTGAGAGT GGCAGCGTAG CCGGACCGCA AT

+     CCGTCC-box

| Site Name | Organism | Position | Strand | Matrix score. | sequence | function |
| --- | --- | --- | --- | --- | --- | --- |
| CCGTCC-box | Petroselinum hortense | 3212 | - | 6 | CCGTCC |  |

>HU06G02537.1   
+ +Up\_Stream \_Len000CCTCGG TGTGGGATTC CGGGTTGATA AATAGGGACC AGCGAAATGT TCAAATAATT   
  
  
+ GGTGAAGGAG TCTCTGTTTG AATGCCTACT AATGCTTTTT TTTTTTTTTT GAATAATTTG TCAGAAAGTT   
  
  
+ AGGCTGCATT GGAAGGGGCG GCCTACGAAG GCTTTTCAGC AAATAAATAA CCAGCTTTCA TTTTGAAATT   
  
  
+ TTTTTTAAAA AATGAAACTC ATTTCACTTA AAAATTTACC ATAATATATA TATATATATA TATATATATA   
  
  
+ TATATATATA TATTTGATTT AACGTTAAGC AAACTTTTAA CTTATGTATC ATTTTACGAC CGTAAAAAAA   
  
  
+ AAAGAAATAT AACACAATAT GTAATAGTAT TTAAGTTTTT GATAAATAAT ATTTTAACAT AATTTGTGTG   
  
  
+ TAGTATGAAT TTAAATCTTA GTACTAATAT AATTATGTAT ACTTGGCTAT AAGATCTCAA TATTAGTATT   
  
  
+ TTATGATTCA ATGTAATGAT GATGCAACTG TGTGTAATTA GAAAGCCTTT GATTGCAGAT ATCTATCTTA   
  
  
+ TTGATACACA TCTACCAATT GAGCTTTTTC AAGATTTGAA GACGGTAGTC AAGTATCTTT GGGAGATTAC   
  
  
+ GTTAGAATTA CTTCATGATA AATTAGACAT ATTTTATACT TCTAATCTTA AATAAGTCTC CATCCTTTCA   
  
  
+ TCATATTTTA AAGAAATGTA CAAGTAAATG TCAATAGTTT GAAACGTTCC TTATCATAAA AGTCATACAT   
  
  
+ ATGTAGGACA TTTTCAAGTT TATGTAAACC ATATGATATA GTTCAAGATA ATTGTCAAAC AAGTTATGAA   
  
  
+ ATCTAAAGGT GTTTGAACGT AATTTCAAGC TAATCTTAGC AAGTTATAGT ATTATTTAAA CTACGACTAG   
  
  
+ ACACAACATT TTGTTCATTT ATATAACACT TAGAATGCAA AAAAATCGGT ATTACTAATA CTGTATCAAG   
  
  
+ AATTGAAGTG TTATGCATTA TACTGATAAG GGATAATATT TAAATTACTT TACTGTTATA GAAGCCAAAA   
  
  
+ ATCTAAACTT TCATACAGAC GTCGGACTAA ATTAGAAAAT TCTTTAACTT AATAGAAAGG ATGACATAAA   
  
  
+ TAAAAATAAA AACCTAAATA GCATAATAAC CATTCTACAC AAAAGTCAAT CTTCTAATAG AAAAAAGCCA   
  
  
+ TAAGTATTCA CTTATTTACC ATTTTATTCC TAATCTACTG TCTGTCTATT CACACCATAA TTCATGAACC   
  
  
+ AATGTGGAGT TTCTGTTTCA TTTCATGGGG TGATTTATGT CTGACAATGA TCCAACAAAC TGAGCCTCAC   
  
  
+ GCGCGGAACA CGAGAGTGGC TGTACTTATG GTGTGAAAGA GCTCTACAAG GTGAGATTGA GGAGGCTACG   
  
  
+ CGTTGGAGTT ATCACCACCG TTGGATTTGT TTTCTTTGGA GCCACGTTAT CACTCTTCAG ACGCCGATCC   
  
  
+ AACGGCCCTT ACTTCCATCT AGTCCCCACT CTCCAACTCT ACGCAATCAA CGCCTTCCGC ATTTAAATAT   
  
  
+ TTCCTTCTCT ATCGCCTACT TTCTCTGTCT CACAATTTTT GTCCGTATTT TGAATTTTCT CAAAATTATC   
  
  
+ ATATAATTTA TTTGCCTTCA ACTAAAGTAT TTATTAAATT AGTTAATGTA TTTAAATTGA ACTAAAAAAG   
  
  
+ AGAAAAAGAA AGAAAACAAC CCCACGAAAG CAAAACGACA GAGGCCAACT TCTCTCTCTA GAGTGTAGAG   
  
  
+ CCCCATCTCG GTTATAATTA TGAGAATCAA TTCCTTCTCA CAAGAGAAAC TCTCGCTCTC TCTCTCTCTC   
  
  
+ CATAAGACAT CACACCAATT GTATGAATTT TCTCTCTCCT GATTTCTGTT TTCCTTCTTG TTGTCCCCTT   
  
  
+ CTAATGCGTT AGTGCGGCCA AAACCACCAT AAACCATCTC TCTTTCAAGA AAGCAGCAAC AACAAAGAGA   
  
  
+ GACAGAGAGA GAGAGAGGGG GAGAAAGATC ACATCTTTTG GGTTTCCGGC GAATATGACG TCTGGGTTTT   
  
  
+ CGGGCGATTT CTTCTACGGC GGTGGAGGCG GTGGCGGAGG AGGTGTCCGG CCTTCTATCG GCGGTGTACA   
  
  
+ AGGCGGGGTT GTCCCATATG GGTTCCGGCA ACAACAACAA CAACAGCAAC AGCAGCAGCA GCAGCAACAG   
  
  
+ CTACATCTAC AGCTACAACA ACAGCGGGGG ATTTTCTCGG CGGATCAGAT CAGCGCTCAA CGAAGGGCGG   
  
  
+ ATTTGTTGAT GGGTAAGCGT TCTTTCGCTG ATTTTCAATC TCAACAACAG CAAATCAGTC TTTTGCAGCA   
  
  
+ GCAGCAGCAG CAACAACAAC AACAGCCCTT TGCGCTCTTG CAAAAACAAC AGCAGTTTCA ACAACAATTA   
  
  
+ CAACAACAAC AGCCACTTTC TCTCCTCCTT CAACAACAGT ACTTGATGAG ATCCGTAAAA CAGAGAACCA   
  
  
+ ACAATTTCGC AGCATCTCCG ATTTCTCCTC TTTCTTCTTC CATTGATCTT TCTTCCGCTA ATAATTCCCG   
  
  
+ GTTCGGTTTG CCGGTTTACC AACAACAACT CCGGTCTCAA CAACAACCAC CTATCTCTCT CCTCGCCCAC   
  
  
+ AACAACAACC AATTCAACCT CTCAAATAAT AACAATTCTT CTTTAATCCA AAACTCCACC AACACTCTTC   
  
  
+ TTGCTCCCGC AGCCAGCAAT AACACCCTAA TGAAAAATTC GGGCTTTTCC TCATCTGGGT TTTCTAATTC   
  
  
+ GGGTCCAAAC CTGGTCCAAC CGAAGCAGGA AATCGGGTAC TCGAATATGG GTACCAATAT GGGTATGAAT   
  
  
+ CTGGTCCAGC CCAAACCCGA CCCTGAGGAT CACGGAATAA TGAACACCCT TCAAGAATTG GAGAAGGAGC   
  
  
+ TTTTGAATGA CGATGAGGAA AACCAGCCGG AAAACGACGC CGTTTCAGTC ATCACCAACA GCGAGTGGTC   
  
  
+ CGAGACCTTC CAGAATCTGA TGAGTCCCGA CCCGAGTTCG AGCCCGAATG CCACCAACAC CCACCCGGGT   
  
  
+ TCGGGTTCTG CTAGTACCAG CGGCAGCAAC AACACTACAA CCACCCCTGC AAACAACAAT AACATGATAT   
  
  
+ CTACCTCGCC GACGTCGTCC ACGTCATCCT GCTGCTCCAC GTCGGCATCG GCGTCTCCGC CGGCGGCGGT   
  
  
+ GGCGACCCCG CAGGTACCAG CCAAGCAGCT GATCTCGGAA GCAGCGGCGG CGATTTCGGA CGGAAAGATT   
  
  
+ GAATCCGCCA TGGAAGTGAT GAACCGGCTG TCCCAGGTTG CCAATTCTAG GGGGAGCTCG GAACAAAAGC   
  
  
+ TGGCTTTTTA CATGGGCCAC GCGCTTCGGT CGCGCGTGAG TCCCACCGAA CACGCTCCCC CTATTGTGGA   
  
  
+ GCTTTTCCAA AAGGAGCATA TGGCGGCCAT GTATTCATTG TACGAGCTCT CTCATTGTTT CAAATTTGGT   
  
  
+ CTTTTGGCTG CCAATTTGGT GATGTTGGAG GCCATTACTT CCGCCCTTCA TGAGGGTCTG AAAATTCATG   
  
  
+ TTGTGGATTT TGATATTGGG CATGGTGGTC AGTACATCAA CCTCCTACGC GCCTTGGGGG AGCGTAGGGC   
  
  
+ TCAGTCGGGG AGCGCGGTGT CGAATGAGGT GAGGATCACT GTGGTTGAGA CCGAGGTCGG TAATGGTGGT   
  
  
+ GCGGCGGGTG CGGAGGCTGT TAAGGATGGC CTGGTGAAAT TGGCTGAGAA GGTGGGGATT AGTTTGAAGT   
  
  
+ TTGAGGCTTT GACTCGGAAG ATCTCCGAGT TAACCCGGGA GTCACTGGGG TGTGAGGAGG GGGAGGCCGT   
  
  
+ GGTGGTGAAT TTTGCGTTCA AACTTTACCG GGTACCGGAT GAGAGCGTGT CGATGGAGAA TCTGAGGGAC   
  
  
+ GAGCTCCTGC GCCGGGTGAA GGCGCTAAGG CCACGCGCCG TCACATTGGT GGAGCAGGAC TTAAATGCGA   
  
  
+ ATACGGCACC GTTCATGACG CGCGTGAGTG GCGCTTGTGA GTACTATAGA GCATTGTTCG ACTCGCTCGA   
  
  
+ CTCGATGGTG CCGAGGGATA ACCCGGACCG GGTCCGGGTC GAGGAGGCCT TGGGGCGGAA GATGGCAAAC   
  
  
+ TCGGTGGCTT GCGAGGGCCG GGAACGGGTT GAGCGGGCGG AGGTGTTTGG GAAGTGGCGG GCGAGGATGA   
  
  
+ GTATGGCAGG GTTCGAGTTG AAGGCACTGA GTCAGAGTGT GGCCGAGTCA ATGCGAACCA AACTCAGCTC   
  
  
+ GGTTCTGGGC GGAAAGCCGG GTTTCACGGT CAAGGAGGAG AGCGGTGGGG TGGGGTTCGG GTGGATGGGA   
  
  
+ CGAACTCTCA CCGTCGCATC GGCCTGGCGT TA  

- +Up\_Stream \_Len000GGAGCC ACACCCTAAG GCCCAACTAT TTATCCCTGG TCGCTTTACA AGTTTATTAA   
  
  
- CCACTTCCTC AGAGACAAAC TTACGGATGA TTACGAAAAA AAAAAAAAAA CTTATTAAAC AGTCTTTCAA   
  
  
- TCCGACGTAA CCTTCCCCGC CGGATGCTTC CGAAAAGTCG TTTATTTATT GGTCGAAAGT AAAACTTTAA   
  
  
- AAAAAATTTT TTACTTTGAG TAAAGTGAAT TTTTAAATGG TATTATATAT ATATATATAT ATATATATAT   
  
  
- ATATATATAT ATAAACTAAA TTGCAATTCG TTTGAAAATT GAATACATAG TAAAATGCTG GCATTTTTTT   
  
  
- TTTCTTTATA TTGTGTTATA CATTATCATA AATTCAAAAA CTATTTATTA TAAAATTGTA TTAAACACAC   
  
  
- ATCATACTTA AATTTAGAAT CATGATTATA TTAATACATA TGAACCGATA TTCTAGAGTT ATAATCATAA   
  
  
- AATACTAAGT TACATTACTA CTACGTTGAC ACACATTAAT CTTTCGGAAA CTAACGTCTA TAGATAGAAT   
  
  
- AACTATGTGT AGATGGTTAA CTCGAAAAAG TTCTAAACTT CTGCCATCAG TTCATAGAAA CCCTCTAATG   
  
  
- CAATCTTAAT GAAGTACTAT TTAATCTGTA TAAAATATGA AGATTAGAAT TTATTCAGAG GTAGGAAAGT   
  
  
- AGTATAAAAT TTCTTTACAT GTTCATTTAC AGTTATCAAA CTTTGCAAGG AATAGTATTT TCAGTATGTA   
  
  
- TACATCCTGT AAAAGTTCAA ATACATTTGG TATACTATAT CAAGTTCTAT TAACAGTTTG TTCAATACTT   
  
  
- TAGATTTCCA CAAACTTGCA TTAAAGTTCG ATTAGAATCG TTCAATATCA TAATAAATTT GATGCTGATC   
  
  
- TGTGTTGTAA AACAAGTAAA TATATTGTGA ATCTTACGTT TTTTTAGCCA TAATGATTAT GACATAGTTC   
  
  
- TTAACTTCAC AATACGTAAT ATGACTATTC CCTATTATAA ATTTAATGAA ATGACAATAT CTTCGGTTTT   
  
  
- TAGATTTGAA AGTATGTCTG CAGCCTGATT TAATCTTTTA AGAAATTGAA TTATCTTTCC TACTGTATTT   
  
  
- ATTTTTATTT TTGGATTTAT CGTATTATTG GTAAGATGTG TTTTCAGTTA GAAGATTATC TTTTTTCGGT   
  
  
- ATTCATAAGT GAATAAATGG TAAAATAAGG ATTAGATGAC AGACAGATAA GTGTGGTATT AAGTACTTGG   
  
  
- TTACACCTCA AAGACAAAGT AAAGTACCCC ACTAAATACA GACTGTTACT AGGTTGTTTG ACTCGGAGTG   
  
  
- CGCGCCTTGT GCTCTCACCG ACATGAATAC CACACTTTCT CGAGATGTTC CACTCTAACT CCTCCGATGC   
  
  
- GCAACCTCAA TAGTGGTGGC AACCTAAACA AAAGAAACCT CGGTGCAATA GTGAGAAGTC TGCGGCTAGG   
  
  
- TTGCCGGGAA TGAAGGTAGA TCAGGGGTGA GAGGTTGAGA TGCGTTAGTT GCGGAAGGCG TAAATTTATA   
  
  
- AAGGAAGAGA TAGCGGATGA AAGAGACAGA GTGTTAAAAA CAGGCATAAA ACTTAAAAGA GTTTTAATAG   
  
  
- TATATTAAAT AAACGGAAGT TGATTTCATA AATAATTTAA TCAATTACAT AAATTTAACT TGATTTTTTC   
  
  
- TCTTTTTCTT TCTTTTGTTG GGGTGCTTTC GTTTTGCTGT CTCCGGTTGA AGAGAGAGAT CTCACATCTC   
  
  
- GGGGTAGAGC CAATATTAAT ACTCTTAGTT AAGGAAGAGT GTTCTCTTTG AGAGCGAGAG AGAGAGAGAG   
  
  
- GTATTCTGTA GTGTGGTTAA CATACTTAAA AGAGAGAGGA CTAAAGACAA AAGGAAGAAC AACAGGGGAA   
  
  
- GATTACGCAA TCACGCCGGT TTTGGTGGTA TTTGGTAGAG AGAAAGTTCT TTCGTCGTTG TTGTTTCTCT   
  
  
- CTGTCTCTCT CTCTCTCCCC CTCTTTCTAG TGTAGAAAAC CCAAAGGCCG CTTATACTGC AGACCCAAAA   
  
  
- GCCCGCTAAA GAAGATGCCG CCACCTCCGC CACCGCCTCC TCCACAGGCC GGAAGATAGC CGCCACATGT   
  
  
- TCCGCCCCAA CAGGGTATAC CCAAGGCCGT TGTTGTTGTT GTTGTCGTTG TCGTCGTCGT CGTCGTTGTC   
  
  
- GATGTAGATG TCGATGTTGT TGTCGCCCCC TAAAAGAGCC GCCTAGTCTA GTCGCGAGTT GCTTCCCGCC   
  
  
- TAAACAACTA CCCATTCGCA AGAAAGCGAC TAAAAGTTAG AGTTGTTGTC GTTTAGTCAG AAAACGTCGT   
  
  
- CGTCGTCGTC GTTGTTGTTG TTGTCGGGAA ACGCGAGAAC GTTTTTGTTG TCGTCAAAGT TGTTGTTAAT   
  
  
- GTTGTTGTTG TCGGTGAAAG AGAGGAGGAA GTTGTTGTCA TGAACTACTC TAGGCATTTT GTCTCTTGGT   
  
  
- TGTTAAAGCG TCGTAGAGGC TAAAGAGGAG AAAGAAGAAG GTAACTAGAA AGAAGGCGAT TATTAAGGGC   
  
  
- CAAGCCAAAC GGCCAAATGG TTGTTGTTGA GGCCAGAGTT GTTGTTGGTG GATAGAGAGA GGAGCGGGTG   
  
  
- TTGTTGTTGG TTAAGTTGGA GAGTTTATTA TTGTTAAGAA GAAATTAGGT TTTGAGGTGG TTGTGAGAAG   
  
  
- AACGAGGGCG TCGGTCGTTA TTGTGGGATT ACTTTTTAAG CCCGAAAAGG AGTAGACCCA AAAGATTAAG   
  
  
- CCCAGGTTTG GACCAGGTTG GCTTCGTCCT TTAGCCCATG AGCTTATACC CATGGTTATA CCCATACTTA   
  
  
- GACCAGGTCG GGTTTGGGCT GGGACTCCTA GTGCCTTATT ACTTGTGGGA AGTTCTTAAC CTCTTCCTCG   
  
  
- AAAACTTACT GCTACTCCTT TTGGTCGGCC TTTTGCTGCG GCAAAGTCAG TAGTGGTTGT CGCTCACCAG   
  
  
- GCTCTGGAAG GTCTTAGACT ACTCAGGGCT GGGCTCAAGC TCGGGCTTAC GGTGGTTGTG GGTGGGCCCA   
  
  
- AGCCCAAGAC GATCATGGTC GCCGTCGTTG TTGTGATGTT GGTGGGGACG TTTGTTGTTA TTGTACTATA   
  
  
- GATGGAGCGG CTGCAGCAGG TGCAGTAGGA CGACGAGGTG CAGCCGTAGC CGCAGAGGCG GCCGCCGCCA   
  
  
- CCGCTGGGGC GTCCATGGTC GGTTCGTCGA CTAGAGCCTT CGTCGCCGCC GCTAAAGCCT GCCTTTCTAA   
  
  
- CTTAGGCGGT ACCTTCACTA CTTGGCCGAC AGGGTCCAAC GGTTAAGATC CCCCTCGAGC CTTGTTTTCG   
  
  
- ACCGAAAAAT GTACCCGGTG CGCGAAGCCA GCGCGCACTC AGGGTGGCTT GTGCGAGGGG GATAACACCT   
  
  
- CGAAAAGGTT TTCCTCGTAT ACCGCCGGTA CATAAGTAAC ATGCTCGAGA GAGTAACAAA GTTTAAACCA   
  
  
- GAAAACCGAC GGTTAAACCA CTACAACCTC CGGTAATGAA GGCGGGAAGT ACTCCCAGAC TTTTAAGTAC   
  
  
- AACACCTAAA ACTATAACCC GTACCACCAG TCATGTAGTT GGAGGATGCG CGGAACCCCC TCGCATCCCG   
  
  
- AGTCAGCCCC TCGCGCCACA GCTTACTCCA CTCCTAGTGA CACCAACTCT GGCTCCAGCC ATTACCACCA   
  
  
- CGCCGCCCAC GCCTCCGACA ATTCCTACCG GACCACTTTA ACCGACTCTT CCACCCCTAA TCAAACTTCA   
  
  
- AACTCCGAAA CTGAGCCTTC TAGAGGCTCA ATTGGGCCCT CAGTGACCCC ACACTCCTCC CCCTCCGGCA   
  
  
- CCACCACTTA AAACGCAAGT TTGAAATGGC CCATGGCCTA CTCTCGCACA GCTACCTCTT AGACTCCCTG   
  
  
- CTCGAGGACG CGGCCCACTT CCGCGATTCC GGTGCGCGGC AGTGTAACCA CCTCGTCCTG AATTTACGCT   
  
  
- TATGCCGTGG CAAGTACTGC GCGCACTCAC CGCGAACACT CATGATATCT CGTAACAAGC TGAGCGAGCT   
  
  
- GAGCTACCAC GGCTCCCTAT TGGGCCTGGC CCAGGCCCAG CTCCTCCGGA ACCCCGCCTT CTACCGTTTG   
  
  
- AGCCACCGAA CGCTCCCGGC CCTTGCCCAA CTCGCCCGCC TCCACAAACC CTTCACCGCC CGCTCCTACT   
  
  
- CATACCGTCC CAAGCTCAAC TTCCGTGACT CAGTCTCACA CCGGCTCAGT TACGCTTGGT TTGAGTCGAG   
  
  
- CCAAGACCCG CCTTTCGGCC CAAAGTGCCA GTTCCTCCTC TCGCCACCCC ACCCCAAGCC CACCTACCCT   
  
  
- GCTTGAGAGT GGCAGCGTAG CCGGACCGCA AT

+     CGTCA-motif

| Site Name | Organism | Position | Strand | Matrix score. | sequence | function |
| --- | --- | --- | --- | --- | --- | --- |
| CGTCA-motif | Hordeum vulgare | 3940 | - | 5 | CGTCA | cis-acting regulatory element involved in the MeJA-responsiveness |
| CGTCA-motif | Hordeum vulgare | 3893 | + | 5 | CGTCA | cis-acting regulatory element involved in the MeJA-responsiveness |
| CGTCA-motif | Hordeum vulgare | 3106 | + | 5 | CGTCA | cis-acting regulatory element involved in the MeJA-responsiveness |
| CGTCA-motif | Hordeum vulgare | 2882 | - | 5 | CGTCA | cis-acting regulatory element involved in the MeJA-responsiveness |
| CGTCA-motif | Hordeum vulgare | 2020 | - | 5 | CGTCA | cis-acting regulatory element involved in the MeJA-responsiveness |

>HU06G02537.1   
+ +Up\_Stream \_Len000CCTCGG TGTGGGATTC CGGGTTGATA AATAGGGACC AGCGAAATGT TCAAATAATT   
  
  
+ GGTGAAGGAG TCTCTGTTTG AATGCCTACT AATGCTTTTT TTTTTTTTTT GAATAATTTG TCAGAAAGTT   
  
  
+ AGGCTGCATT GGAAGGGGCG GCCTACGAAG GCTTTTCAGC AAATAAATAA CCAGCTTTCA TTTTGAAATT   
  
  
+ TTTTTTAAAA AATGAAACTC ATTTCACTTA AAAATTTACC ATAATATATA TATATATATA TATATATATA   
  
  
+ TATATATATA TATTTGATTT AACGTTAAGC AAACTTTTAA CTTATGTATC ATTTTACGAC CGTAAAAAAA   
  
  
+ AAAGAAATAT AACACAATAT GTAATAGTAT TTAAGTTTTT GATAAATAAT ATTTTAACAT AATTTGTGTG   
  
  
+ TAGTATGAAT TTAAATCTTA GTACTAATAT AATTATGTAT ACTTGGCTAT AAGATCTCAA TATTAGTATT   
  
  
+ TTATGATTCA ATGTAATGAT GATGCAACTG TGTGTAATTA GAAAGCCTTT GATTGCAGAT ATCTATCTTA   
  
  
+ TTGATACACA TCTACCAATT GAGCTTTTTC AAGATTTGAA GACGGTAGTC AAGTATCTTT GGGAGATTAC   
  
  
+ GTTAGAATTA CTTCATGATA AATTAGACAT ATTTTATACT TCTAATCTTA AATAAGTCTC CATCCTTTCA   
  
  
+ TCATATTTTA AAGAAATGTA CAAGTAAATG TCAATAGTTT GAAACGTTCC TTATCATAAA AGTCATACAT   
  
  
+ ATGTAGGACA TTTTCAAGTT TATGTAAACC ATATGATATA GTTCAAGATA ATTGTCAAAC AAGTTATGAA   
  
  
+ ATCTAAAGGT GTTTGAACGT AATTTCAAGC TAATCTTAGC AAGTTATAGT ATTATTTAAA CTACGACTAG   
  
  
+ ACACAACATT TTGTTCATTT ATATAACACT TAGAATGCAA AAAAATCGGT ATTACTAATA CTGTATCAAG   
  
  
+ AATTGAAGTG TTATGCATTA TACTGATAAG GGATAATATT TAAATTACTT TACTGTTATA GAAGCCAAAA   
  
  
+ ATCTAAACTT TCATACAGAC GTCGGACTAA ATTAGAAAAT TCTTTAACTT AATAGAAAGG ATGACATAAA   
  
  
+ TAAAAATAAA AACCTAAATA GCATAATAAC CATTCTACAC AAAAGTCAAT CTTCTAATAG AAAAAAGCCA   
  
  
+ TAAGTATTCA CTTATTTACC ATTTTATTCC TAATCTACTG TCTGTCTATT CACACCATAA TTCATGAACC   
  
  
+ AATGTGGAGT TTCTGTTTCA TTTCATGGGG TGATTTATGT CTGACAATGA TCCAACAAAC TGAGCCTCAC   
  
  
+ GCGCGGAACA CGAGAGTGGC TGTACTTATG GTGTGAAAGA GCTCTACAAG GTGAGATTGA GGAGGCTACG   
  
  
+ CGTTGGAGTT ATCACCACCG TTGGATTTGT TTTCTTTGGA GCCACGTTAT CACTCTTCAG ACGCCGATCC   
  
  
+ AACGGCCCTT ACTTCCATCT AGTCCCCACT CTCCAACTCT ACGCAATCAA CGCCTTCCGC ATTTAAATAT   
  
  
+ TTCCTTCTCT ATCGCCTACT TTCTCTGTCT CACAATTTTT GTCCGTATTT TGAATTTTCT CAAAATTATC   
  
  
+ ATATAATTTA TTTGCCTTCA ACTAAAGTAT TTATTAAATT AGTTAATGTA TTTAAATTGA ACTAAAAAAG   
  
  
+ AGAAAAAGAA AGAAAACAAC CCCACGAAAG CAAAACGACA GAGGCCAACT TCTCTCTCTA GAGTGTAGAG   
  
  
+ CCCCATCTCG GTTATAATTA TGAGAATCAA TTCCTTCTCA CAAGAGAAAC TCTCGCTCTC TCTCTCTCTC   
  
  
+ CATAAGACAT CACACCAATT GTATGAATTT TCTCTCTCCT GATTTCTGTT TTCCTTCTTG TTGTCCCCTT   
  
  
+ CTAATGCGTT AGTGCGGCCA AAACCACCAT AAACCATCTC TCTTTCAAGA AAGCAGCAAC AACAAAGAGA   
  
  
+ GACAGAGAGA GAGAGAGGGG GAGAAAGATC ACATCTTTTG GGTTTCCGGC GAATATGACG TCTGGGTTTT   
  
  
+ CGGGCGATTT CTTCTACGGC GGTGGAGGCG GTGGCGGAGG AGGTGTCCGG CCTTCTATCG GCGGTGTACA   
  
  
+ AGGCGGGGTT GTCCCATATG GGTTCCGGCA ACAACAACAA CAACAGCAAC AGCAGCAGCA GCAGCAACAG   
  
  
+ CTACATCTAC AGCTACAACA ACAGCGGGGG ATTTTCTCGG CGGATCAGAT CAGCGCTCAA CGAAGGGCGG   
  
  
+ ATTTGTTGAT GGGTAAGCGT TCTTTCGCTG ATTTTCAATC TCAACAACAG CAAATCAGTC TTTTGCAGCA   
  
  
+ GCAGCAGCAG CAACAACAAC AACAGCCCTT TGCGCTCTTG CAAAAACAAC AGCAGTTTCA ACAACAATTA   
  
  
+ CAACAACAAC AGCCACTTTC TCTCCTCCTT CAACAACAGT ACTTGATGAG ATCCGTAAAA CAGAGAACCA   
  
  
+ ACAATTTCGC AGCATCTCCG ATTTCTCCTC TTTCTTCTTC CATTGATCTT TCTTCCGCTA ATAATTCCCG   
  
  
+ GTTCGGTTTG CCGGTTTACC AACAACAACT CCGGTCTCAA CAACAACCAC CTATCTCTCT CCTCGCCCAC   
  
  
+ AACAACAACC AATTCAACCT CTCAAATAAT AACAATTCTT CTTTAATCCA AAACTCCACC AACACTCTTC   
  
  
+ TTGCTCCCGC AGCCAGCAAT AACACCCTAA TGAAAAATTC GGGCTTTTCC TCATCTGGGT TTTCTAATTC   
  
  
+ GGGTCCAAAC CTGGTCCAAC CGAAGCAGGA AATCGGGTAC TCGAATATGG GTACCAATAT GGGTATGAAT   
  
  
+ CTGGTCCAGC CCAAACCCGA CCCTGAGGAT CACGGAATAA TGAACACCCT TCAAGAATTG GAGAAGGAGC   
  
  
+ TTTTGAATGA CGATGAGGAA AACCAGCCGG AAAACGACGC CGTTTCAGTC ATCACCAACA GCGAGTGGTC   
  
  
+ CGAGACCTTC CAGAATCTGA TGAGTCCCGA CCCGAGTTCG AGCCCGAATG CCACCAACAC CCACCCGGGT   
  
  
+ TCGGGTTCTG CTAGTACCAG CGGCAGCAAC AACACTACAA CCACCCCTGC AAACAACAAT AACATGATAT   
  
  
+ CTACCTCGCC GACGTCGTCC ACGTCATCCT GCTGCTCCAC GTCGGCATCG GCGTCTCCGC CGGCGGCGGT   
  
  
+ GGCGACCCCG CAGGTACCAG CCAAGCAGCT GATCTCGGAA GCAGCGGCGG CGATTTCGGA CGGAAAGATT   
  
  
+ GAATCCGCCA TGGAAGTGAT GAACCGGCTG TCCCAGGTTG CCAATTCTAG GGGGAGCTCG GAACAAAAGC   
  
  
+ TGGCTTTTTA CATGGGCCAC GCGCTTCGGT CGCGCGTGAG TCCCACCGAA CACGCTCCCC CTATTGTGGA   
  
  
+ GCTTTTCCAA AAGGAGCATA TGGCGGCCAT GTATTCATTG TACGAGCTCT CTCATTGTTT CAAATTTGGT   
  
  
+ CTTTTGGCTG CCAATTTGGT GATGTTGGAG GCCATTACTT CCGCCCTTCA TGAGGGTCTG AAAATTCATG   
  
  
+ TTGTGGATTT TGATATTGGG CATGGTGGTC AGTACATCAA CCTCCTACGC GCCTTGGGGG AGCGTAGGGC   
  
  
+ TCAGTCGGGG AGCGCGGTGT CGAATGAGGT GAGGATCACT GTGGTTGAGA CCGAGGTCGG TAATGGTGGT   
  
  
+ GCGGCGGGTG CGGAGGCTGT TAAGGATGGC CTGGTGAAAT TGGCTGAGAA GGTGGGGATT AGTTTGAAGT   
  
  
+ TTGAGGCTTT GACTCGGAAG ATCTCCGAGT TAACCCGGGA GTCACTGGGG TGTGAGGAGG GGGAGGCCGT   
  
  
+ GGTGGTGAAT TTTGCGTTCA AACTTTACCG GGTACCGGAT GAGAGCGTGT CGATGGAGAA TCTGAGGGAC   
  
  
+ GAGCTCCTGC GCCGGGTGAA GGCGCTAAGG CCACGCGCCG TCACATTGGT GGAGCAGGAC TTAAATGCGA   
  
  
+ ATACGGCACC GTTCATGACG CGCGTGAGTG GCGCTTGTGA GTACTATAGA GCATTGTTCG ACTCGCTCGA   
  
  
+ CTCGATGGTG CCGAGGGATA ACCCGGACCG GGTCCGGGTC GAGGAGGCCT TGGGGCGGAA GATGGCAAAC   
  
  
+ TCGGTGGCTT GCGAGGGCCG GGAACGGGTT GAGCGGGCGG AGGTGTTTGG GAAGTGGCGG GCGAGGATGA   
  
  
+ GTATGGCAGG GTTCGAGTTG AAGGCACTGA GTCAGAGTGT GGCCGAGTCA ATGCGAACCA AACTCAGCTC   
  
  
+ GGTTCTGGGC GGAAAGCCGG GTTTCACGGT CAAGGAGGAG AGCGGTGGGG TGGGGTTCGG GTGGATGGGA   
  
  
+ CGAACTCTCA CCGTCGCATC GGCCTGGCGT TA  

- +Up\_Stream \_Len000GGAGCC ACACCCTAAG GCCCAACTAT TTATCCCTGG TCGCTTTACA AGTTTATTAA   
  
  
- CCACTTCCTC AGAGACAAAC TTACGGATGA TTACGAAAAA AAAAAAAAAA CTTATTAAAC AGTCTTTCAA   
  
  
- TCCGACGTAA CCTTCCCCGC CGGATGCTTC CGAAAAGTCG TTTATTTATT GGTCGAAAGT AAAACTTTAA   
  
  
- AAAAAATTTT TTACTTTGAG TAAAGTGAAT TTTTAAATGG TATTATATAT ATATATATAT ATATATATAT   
  
  
- ATATATATAT ATAAACTAAA TTGCAATTCG TTTGAAAATT GAATACATAG TAAAATGCTG GCATTTTTTT   
  
  
- TTTCTTTATA TTGTGTTATA CATTATCATA AATTCAAAAA CTATTTATTA TAAAATTGTA TTAAACACAC   
  
  
- ATCATACTTA AATTTAGAAT CATGATTATA TTAATACATA TGAACCGATA TTCTAGAGTT ATAATCATAA   
  
  
- AATACTAAGT TACATTACTA CTACGTTGAC ACACATTAAT CTTTCGGAAA CTAACGTCTA TAGATAGAAT   
  
  
- AACTATGTGT AGATGGTTAA CTCGAAAAAG TTCTAAACTT CTGCCATCAG TTCATAGAAA CCCTCTAATG   
  
  
- CAATCTTAAT GAAGTACTAT TTAATCTGTA TAAAATATGA AGATTAGAAT TTATTCAGAG GTAGGAAAGT   
  
  
- AGTATAAAAT TTCTTTACAT GTTCATTTAC AGTTATCAAA CTTTGCAAGG AATAGTATTT TCAGTATGTA   
  
  
- TACATCCTGT AAAAGTTCAA ATACATTTGG TATACTATAT CAAGTTCTAT TAACAGTTTG TTCAATACTT   
  
  
- TAGATTTCCA CAAACTTGCA TTAAAGTTCG ATTAGAATCG TTCAATATCA TAATAAATTT GATGCTGATC   
  
  
- TGTGTTGTAA AACAAGTAAA TATATTGTGA ATCTTACGTT TTTTTAGCCA TAATGATTAT GACATAGTTC   
  
  
- TTAACTTCAC AATACGTAAT ATGACTATTC CCTATTATAA ATTTAATGAA ATGACAATAT CTTCGGTTTT   
  
  
- TAGATTTGAA AGTATGTCTG CAGCCTGATT TAATCTTTTA AGAAATTGAA TTATCTTTCC TACTGTATTT   
  
  
- ATTTTTATTT TTGGATTTAT CGTATTATTG GTAAGATGTG TTTTCAGTTA GAAGATTATC TTTTTTCGGT   
  
  
- ATTCATAAGT GAATAAATGG TAAAATAAGG ATTAGATGAC AGACAGATAA GTGTGGTATT AAGTACTTGG   
  
  
- TTACACCTCA AAGACAAAGT AAAGTACCCC ACTAAATACA GACTGTTACT AGGTTGTTTG ACTCGGAGTG   
  
  
- CGCGCCTTGT GCTCTCACCG ACATGAATAC CACACTTTCT CGAGATGTTC CACTCTAACT CCTCCGATGC   
  
  
- GCAACCTCAA TAGTGGTGGC AACCTAAACA AAAGAAACCT CGGTGCAATA GTGAGAAGTC TGCGGCTAGG   
  
  
- TTGCCGGGAA TGAAGGTAGA TCAGGGGTGA GAGGTTGAGA TGCGTTAGTT GCGGAAGGCG TAAATTTATA   
  
  
- AAGGAAGAGA TAGCGGATGA AAGAGACAGA GTGTTAAAAA CAGGCATAAA ACTTAAAAGA GTTTTAATAG   
  
  
- TATATTAAAT AAACGGAAGT TGATTTCATA AATAATTTAA TCAATTACAT AAATTTAACT TGATTTTTTC   
  
  
- TCTTTTTCTT TCTTTTGTTG GGGTGCTTTC GTTTTGCTGT CTCCGGTTGA AGAGAGAGAT CTCACATCTC   
  
  
- GGGGTAGAGC CAATATTAAT ACTCTTAGTT AAGGAAGAGT GTTCTCTTTG AGAGCGAGAG AGAGAGAGAG   
  
  
- GTATTCTGTA GTGTGGTTAA CATACTTAAA AGAGAGAGGA CTAAAGACAA AAGGAAGAAC AACAGGGGAA   
  
  
- GATTACGCAA TCACGCCGGT TTTGGTGGTA TTTGGTAGAG AGAAAGTTCT TTCGTCGTTG TTGTTTCTCT   
  
  
- CTGTCTCTCT CTCTCTCCCC CTCTTTCTAG TGTAGAAAAC CCAAAGGCCG CTTATACTGC AGACCCAAAA   
  
  
- GCCCGCTAAA GAAGATGCCG CCACCTCCGC CACCGCCTCC TCCACAGGCC GGAAGATAGC CGCCACATGT   
  
  
- TCCGCCCCAA CAGGGTATAC CCAAGGCCGT TGTTGTTGTT GTTGTCGTTG TCGTCGTCGT CGTCGTTGTC   
  
  
- GATGTAGATG TCGATGTTGT TGTCGCCCCC TAAAAGAGCC GCCTAGTCTA GTCGCGAGTT GCTTCCCGCC   
  
  
- TAAACAACTA CCCATTCGCA AGAAAGCGAC TAAAAGTTAG AGTTGTTGTC GTTTAGTCAG AAAACGTCGT   
  
  
- CGTCGTCGTC GTTGTTGTTG TTGTCGGGAA ACGCGAGAAC GTTTTTGTTG TCGTCAAAGT TGTTGTTAAT   
  
  
- GTTGTTGTTG TCGGTGAAAG AGAGGAGGAA GTTGTTGTCA TGAACTACTC TAGGCATTTT GTCTCTTGGT   
  
  
- TGTTAAAGCG TCGTAGAGGC TAAAGAGGAG AAAGAAGAAG GTAACTAGAA AGAAGGCGAT TATTAAGGGC   
  
  
- CAAGCCAAAC GGCCAAATGG TTGTTGTTGA GGCCAGAGTT GTTGTTGGTG GATAGAGAGA GGAGCGGGTG   
  
  
- TTGTTGTTGG TTAAGTTGGA GAGTTTATTA TTGTTAAGAA GAAATTAGGT TTTGAGGTGG TTGTGAGAAG   
  
  
- AACGAGGGCG TCGGTCGTTA TTGTGGGATT ACTTTTTAAG CCCGAAAAGG AGTAGACCCA AAAGATTAAG   
  
  
- CCCAGGTTTG GACCAGGTTG GCTTCGTCCT TTAGCCCATG AGCTTATACC CATGGTTATA CCCATACTTA   
  
  
- GACCAGGTCG GGTTTGGGCT GGGACTCCTA GTGCCTTATT ACTTGTGGGA AGTTCTTAAC CTCTTCCTCG   
  
  
- AAAACTTACT GCTACTCCTT TTGGTCGGCC TTTTGCTGCG GCAAAGTCAG TAGTGGTTGT CGCTCACCAG   
  
  
- GCTCTGGAAG GTCTTAGACT ACTCAGGGCT GGGCTCAAGC TCGGGCTTAC GGTGGTTGTG GGTGGGCCCA   
  
  
- AGCCCAAGAC GATCATGGTC GCCGTCGTTG TTGTGATGTT GGTGGGGACG TTTGTTGTTA TTGTACTATA   
  
  
- GATGGAGCGG CTGCAGCAGG TGCAGTAGGA CGACGAGGTG CAGCCGTAGC CGCAGAGGCG GCCGCCGCCA   
  
  
- CCGCTGGGGC GTCCATGGTC GGTTCGTCGA CTAGAGCCTT CGTCGCCGCC GCTAAAGCCT GCCTTTCTAA   
  
  
- CTTAGGCGGT ACCTTCACTA CTTGGCCGAC AGGGTCCAAC GGTTAAGATC CCCCTCGAGC CTTGTTTTCG   
  
  
- ACCGAAAAAT GTACCCGGTG CGCGAAGCCA GCGCGCACTC AGGGTGGCTT GTGCGAGGGG GATAACACCT   
  
  
- CGAAAAGGTT TTCCTCGTAT ACCGCCGGTA CATAAGTAAC ATGCTCGAGA GAGTAACAAA GTTTAAACCA   
  
  
- GAAAACCGAC GGTTAAACCA CTACAACCTC CGGTAATGAA GGCGGGAAGT ACTCCCAGAC TTTTAAGTAC   
  
  
- AACACCTAAA ACTATAACCC GTACCACCAG TCATGTAGTT GGAGGATGCG CGGAACCCCC TCGCATCCCG   
  
  
- AGTCAGCCCC TCGCGCCACA GCTTACTCCA CTCCTAGTGA CACCAACTCT GGCTCCAGCC ATTACCACCA   
  
  
- CGCCGCCCAC GCCTCCGACA ATTCCTACCG GACCACTTTA ACCGACTCTT CCACCCCTAA TCAAACTTCA   
  
  
- AACTCCGAAA CTGAGCCTTC TAGAGGCTCA ATTGGGCCCT CAGTGACCCC ACACTCCTCC CCCTCCGGCA   
  
  
- CCACCACTTA AAACGCAAGT TTGAAATGGC CCATGGCCTA CTCTCGCACA GCTACCTCTT AGACTCCCTG   
  
  
- CTCGAGGACG CGGCCCACTT CCGCGATTCC GGTGCGCGGC AGTGTAACCA CCTCGTCCTG AATTTACGCT   
  
  
- TATGCCGTGG CAAGTACTGC GCGCACTCAC CGCGAACACT CATGATATCT CGTAACAAGC TGAGCGAGCT   
  
  
- GAGCTACCAC GGCTCCCTAT TGGGCCTGGC CCAGGCCCAG CTCCTCCGGA ACCCCGCCTT CTACCGTTTG   
  
  
- AGCCACCGAA CGCTCCCGGC CCTTGCCCAA CTCGCCCGCC TCCACAAACC CTTCACCGCC CGCTCCTACT   
  
  
- CATACCGTCC CAAGCTCAAC TTCCGTGACT CAGTCTCACA CCGGCTCAGT TACGCTTGGT TTGAGTCGAG   
  
  
- CCAAGACCCG CCTTTCGGCC CAAAGTGCCA GTTCCTCCTC TCGCCACCCC ACCCCAAGCC CACCTACCCT   
  
  
- GCTTGAGAGT GGCAGCGTAG CCGGACCGCA AT

+     CTAG-motif

| Site Name | Organism | Position | Strand | Matrix score. | sequence | function |
| --- | --- | --- | --- | --- | --- | --- |
| CTAG-motif | Avena sativa | 3020 | - | 10 | ACTAGCAGAA |  |

>HU06G02537.1   
+ +Up\_Stream \_Len000CCTCGG TGTGGGATTC CGGGTTGATA AATAGGGACC AGCGAAATGT TCAAATAATT   
  
  
+ GGTGAAGGAG TCTCTGTTTG AATGCCTACT AATGCTTTTT TTTTTTTTTT GAATAATTTG TCAGAAAGTT   
  
  
+ AGGCTGCATT GGAAGGGGCG GCCTACGAAG GCTTTTCAGC AAATAAATAA CCAGCTTTCA TTTTGAAATT   
  
  
+ TTTTTTAAAA AATGAAACTC ATTTCACTTA AAAATTTACC ATAATATATA TATATATATA TATATATATA   
  
  
+ TATATATATA TATTTGATTT AACGTTAAGC AAACTTTTAA CTTATGTATC ATTTTACGAC CGTAAAAAAA   
  
  
+ AAAGAAATAT AACACAATAT GTAATAGTAT TTAAGTTTTT GATAAATAAT ATTTTAACAT AATTTGTGTG   
  
  
+ TAGTATGAAT TTAAATCTTA GTACTAATAT AATTATGTAT ACTTGGCTAT AAGATCTCAA TATTAGTATT   
  
  
+ TTATGATTCA ATGTAATGAT GATGCAACTG TGTGTAATTA GAAAGCCTTT GATTGCAGAT ATCTATCTTA   
  
  
+ TTGATACACA TCTACCAATT GAGCTTTTTC AAGATTTGAA GACGGTAGTC AAGTATCTTT GGGAGATTAC   
  
  
+ GTTAGAATTA CTTCATGATA AATTAGACAT ATTTTATACT TCTAATCTTA AATAAGTCTC CATCCTTTCA   
  
  
+ TCATATTTTA AAGAAATGTA CAAGTAAATG TCAATAGTTT GAAACGTTCC TTATCATAAA AGTCATACAT   
  
  
+ ATGTAGGACA TTTTCAAGTT TATGTAAACC ATATGATATA GTTCAAGATA ATTGTCAAAC AAGTTATGAA   
  
  
+ ATCTAAAGGT GTTTGAACGT AATTTCAAGC TAATCTTAGC AAGTTATAGT ATTATTTAAA CTACGACTAG   
  
  
+ ACACAACATT TTGTTCATTT ATATAACACT TAGAATGCAA AAAAATCGGT ATTACTAATA CTGTATCAAG   
  
  
+ AATTGAAGTG TTATGCATTA TACTGATAAG GGATAATATT TAAATTACTT TACTGTTATA GAAGCCAAAA   
  
  
+ ATCTAAACTT TCATACAGAC GTCGGACTAA ATTAGAAAAT TCTTTAACTT AATAGAAAGG ATGACATAAA   
  
  
+ TAAAAATAAA AACCTAAATA GCATAATAAC CATTCTACAC AAAAGTCAAT CTTCTAATAG AAAAAAGCCA   
  
  
+ TAAGTATTCA CTTATTTACC ATTTTATTCC TAATCTACTG TCTGTCTATT CACACCATAA TTCATGAACC   
  
  
+ AATGTGGAGT TTCTGTTTCA TTTCATGGGG TGATTTATGT CTGACAATGA TCCAACAAAC TGAGCCTCAC   
  
  
+ GCGCGGAACA CGAGAGTGGC TGTACTTATG GTGTGAAAGA GCTCTACAAG GTGAGATTGA GGAGGCTACG   
  
  
+ CGTTGGAGTT ATCACCACCG TTGGATTTGT TTTCTTTGGA GCCACGTTAT CACTCTTCAG ACGCCGATCC   
  
  
+ AACGGCCCTT ACTTCCATCT AGTCCCCACT CTCCAACTCT ACGCAATCAA CGCCTTCCGC ATTTAAATAT   
  
  
+ TTCCTTCTCT ATCGCCTACT TTCTCTGTCT CACAATTTTT GTCCGTATTT TGAATTTTCT CAAAATTATC   
  
  
+ ATATAATTTA TTTGCCTTCA ACTAAAGTAT TTATTAAATT AGTTAATGTA TTTAAATTGA ACTAAAAAAG   
  
  
+ AGAAAAAGAA AGAAAACAAC CCCACGAAAG CAAAACGACA GAGGCCAACT TCTCTCTCTA GAGTGTAGAG   
  
  
+ CCCCATCTCG GTTATAATTA TGAGAATCAA TTCCTTCTCA CAAGAGAAAC TCTCGCTCTC TCTCTCTCTC   
  
  
+ CATAAGACAT CACACCAATT GTATGAATTT TCTCTCTCCT GATTTCTGTT TTCCTTCTTG TTGTCCCCTT   
  
  
+ CTAATGCGTT AGTGCGGCCA AAACCACCAT AAACCATCTC TCTTTCAAGA AAGCAGCAAC AACAAAGAGA   
  
  
+ GACAGAGAGA GAGAGAGGGG GAGAAAGATC ACATCTTTTG GGTTTCCGGC GAATATGACG TCTGGGTTTT   
  
  
+ CGGGCGATTT CTTCTACGGC GGTGGAGGCG GTGGCGGAGG AGGTGTCCGG CCTTCTATCG GCGGTGTACA   
  
  
+ AGGCGGGGTT GTCCCATATG GGTTCCGGCA ACAACAACAA CAACAGCAAC AGCAGCAGCA GCAGCAACAG   
  
  
+ CTACATCTAC AGCTACAACA ACAGCGGGGG ATTTTCTCGG CGGATCAGAT CAGCGCTCAA CGAAGGGCGG   
  
  
+ ATTTGTTGAT GGGTAAGCGT TCTTTCGCTG ATTTTCAATC TCAACAACAG CAAATCAGTC TTTTGCAGCA   
  
  
+ GCAGCAGCAG CAACAACAAC AACAGCCCTT TGCGCTCTTG CAAAAACAAC AGCAGTTTCA ACAACAATTA   
  
  
+ CAACAACAAC AGCCACTTTC TCTCCTCCTT CAACAACAGT ACTTGATGAG ATCCGTAAAA CAGAGAACCA   
  
  
+ ACAATTTCGC AGCATCTCCG ATTTCTCCTC TTTCTTCTTC CATTGATCTT TCTTCCGCTA ATAATTCCCG   
  
  
+ GTTCGGTTTG CCGGTTTACC AACAACAACT CCGGTCTCAA CAACAACCAC CTATCTCTCT CCTCGCCCAC   
  
  
+ AACAACAACC AATTCAACCT CTCAAATAAT AACAATTCTT CTTTAATCCA AAACTCCACC AACACTCTTC   
  
  
+ TTGCTCCCGC AGCCAGCAAT AACACCCTAA TGAAAAATTC GGGCTTTTCC TCATCTGGGT TTTCTAATTC   
  
  
+ GGGTCCAAAC CTGGTCCAAC CGAAGCAGGA AATCGGGTAC TCGAATATGG GTACCAATAT GGGTATGAAT   
  
  
+ CTGGTCCAGC CCAAACCCGA CCCTGAGGAT CACGGAATAA TGAACACCCT TCAAGAATTG GAGAAGGAGC   
  
  
+ TTTTGAATGA CGATGAGGAA AACCAGCCGG AAAACGACGC CGTTTCAGTC ATCACCAACA GCGAGTGGTC   
  
  
+ CGAGACCTTC CAGAATCTGA TGAGTCCCGA CCCGAGTTCG AGCCCGAATG CCACCAACAC CCACCCGGGT   
  
  
+ TCGGGTTCTG CTAGTACCAG CGGCAGCAAC AACACTACAA CCACCCCTGC AAACAACAAT AACATGATAT   
  
  
+ CTACCTCGCC GACGTCGTCC ACGTCATCCT GCTGCTCCAC GTCGGCATCG GCGTCTCCGC CGGCGGCGGT   
  
  
+ GGCGACCCCG CAGGTACCAG CCAAGCAGCT GATCTCGGAA GCAGCGGCGG CGATTTCGGA CGGAAAGATT   
  
  
+ GAATCCGCCA TGGAAGTGAT GAACCGGCTG TCCCAGGTTG CCAATTCTAG GGGGAGCTCG GAACAAAAGC   
  
  
+ TGGCTTTTTA CATGGGCCAC GCGCTTCGGT CGCGCGTGAG TCCCACCGAA CACGCTCCCC CTATTGTGGA   
  
  
+ GCTTTTCCAA AAGGAGCATA TGGCGGCCAT GTATTCATTG TACGAGCTCT CTCATTGTTT CAAATTTGGT   
  
  
+ CTTTTGGCTG CCAATTTGGT GATGTTGGAG GCCATTACTT CCGCCCTTCA TGAGGGTCTG AAAATTCATG   
  
  
+ TTGTGGATTT TGATATTGGG CATGGTGGTC AGTACATCAA CCTCCTACGC GCCTTGGGGG AGCGTAGGGC   
  
  
+ TCAGTCGGGG AGCGCGGTGT CGAATGAGGT GAGGATCACT GTGGTTGAGA CCGAGGTCGG TAATGGTGGT   
  
  
+ GCGGCGGGTG CGGAGGCTGT TAAGGATGGC CTGGTGAAAT TGGCTGAGAA GGTGGGGATT AGTTTGAAGT   
  
  
+ TTGAGGCTTT GACTCGGAAG ATCTCCGAGT TAACCCGGGA GTCACTGGGG TGTGAGGAGG GGGAGGCCGT   
  
  
+ GGTGGTGAAT TTTGCGTTCA AACTTTACCG GGTACCGGAT GAGAGCGTGT CGATGGAGAA TCTGAGGGAC   
  
  
+ GAGCTCCTGC GCCGGGTGAA GGCGCTAAGG CCACGCGCCG TCACATTGGT GGAGCAGGAC TTAAATGCGA   
  
  
+ ATACGGCACC GTTCATGACG CGCGTGAGTG GCGCTTGTGA GTACTATAGA GCATTGTTCG ACTCGCTCGA   
  
  
+ CTCGATGGTG CCGAGGGATA ACCCGGACCG GGTCCGGGTC GAGGAGGCCT TGGGGCGGAA GATGGCAAAC   
  
  
+ TCGGTGGCTT GCGAGGGCCG GGAACGGGTT GAGCGGGCGG AGGTGTTTGG GAAGTGGCGG GCGAGGATGA   
  
  
+ GTATGGCAGG GTTCGAGTTG AAGGCACTGA GTCAGAGTGT GGCCGAGTCA ATGCGAACCA AACTCAGCTC   
  
  
+ GGTTCTGGGC GGAAAGCCGG GTTTCACGGT CAAGGAGGAG AGCGGTGGGG TGGGGTTCGG GTGGATGGGA   
  
  
+ CGAACTCTCA CCGTCGCATC GGCCTGGCGT TA  

- +Up\_Stream \_Len000GGAGCC ACACCCTAAG GCCCAACTAT TTATCCCTGG TCGCTTTACA AGTTTATTAA   
  
  
- CCACTTCCTC AGAGACAAAC TTACGGATGA TTACGAAAAA AAAAAAAAAA CTTATTAAAC AGTCTTTCAA   
  
  
- TCCGACGTAA CCTTCCCCGC CGGATGCTTC CGAAAAGTCG TTTATTTATT GGTCGAAAGT AAAACTTTAA   
  
  
- AAAAAATTTT TTACTTTGAG TAAAGTGAAT TTTTAAATGG TATTATATAT ATATATATAT ATATATATAT   
  
  
- ATATATATAT ATAAACTAAA TTGCAATTCG TTTGAAAATT GAATACATAG TAAAATGCTG GCATTTTTTT   
  
  
- TTTCTTTATA TTGTGTTATA CATTATCATA AATTCAAAAA CTATTTATTA TAAAATTGTA TTAAACACAC   
  
  
- ATCATACTTA AATTTAGAAT CATGATTATA TTAATACATA TGAACCGATA TTCTAGAGTT ATAATCATAA   
  
  
- AATACTAAGT TACATTACTA CTACGTTGAC ACACATTAAT CTTTCGGAAA CTAACGTCTA TAGATAGAAT   
  
  
- AACTATGTGT AGATGGTTAA CTCGAAAAAG TTCTAAACTT CTGCCATCAG TTCATAGAAA CCCTCTAATG   
  
  
- CAATCTTAAT GAAGTACTAT TTAATCTGTA TAAAATATGA AGATTAGAAT TTATTCAGAG GTAGGAAAGT   
  
  
- AGTATAAAAT TTCTTTACAT GTTCATTTAC AGTTATCAAA CTTTGCAAGG AATAGTATTT TCAGTATGTA   
  
  
- TACATCCTGT AAAAGTTCAA ATACATTTGG TATACTATAT CAAGTTCTAT TAACAGTTTG TTCAATACTT   
  
  
- TAGATTTCCA CAAACTTGCA TTAAAGTTCG ATTAGAATCG TTCAATATCA TAATAAATTT GATGCTGATC   
  
  
- TGTGTTGTAA AACAAGTAAA TATATTGTGA ATCTTACGTT TTTTTAGCCA TAATGATTAT GACATAGTTC   
  
  
- TTAACTTCAC AATACGTAAT ATGACTATTC CCTATTATAA ATTTAATGAA ATGACAATAT CTTCGGTTTT   
  
  
- TAGATTTGAA AGTATGTCTG CAGCCTGATT TAATCTTTTA AGAAATTGAA TTATCTTTCC TACTGTATTT   
  
  
- ATTTTTATTT TTGGATTTAT CGTATTATTG GTAAGATGTG TTTTCAGTTA GAAGATTATC TTTTTTCGGT   
  
  
- ATTCATAAGT GAATAAATGG TAAAATAAGG ATTAGATGAC AGACAGATAA GTGTGGTATT AAGTACTTGG   
  
  
- TTACACCTCA AAGACAAAGT AAAGTACCCC ACTAAATACA GACTGTTACT AGGTTGTTTG ACTCGGAGTG   
  
  
- CGCGCCTTGT GCTCTCACCG ACATGAATAC CACACTTTCT CGAGATGTTC CACTCTAACT CCTCCGATGC   
  
  
- GCAACCTCAA TAGTGGTGGC AACCTAAACA AAAGAAACCT CGGTGCAATA GTGAGAAGTC TGCGGCTAGG   
  
  
- TTGCCGGGAA TGAAGGTAGA TCAGGGGTGA GAGGTTGAGA TGCGTTAGTT GCGGAAGGCG TAAATTTATA   
  
  
- AAGGAAGAGA TAGCGGATGA AAGAGACAGA GTGTTAAAAA CAGGCATAAA ACTTAAAAGA GTTTTAATAG   
  
  
- TATATTAAAT AAACGGAAGT TGATTTCATA AATAATTTAA TCAATTACAT AAATTTAACT TGATTTTTTC   
  
  
- TCTTTTTCTT TCTTTTGTTG GGGTGCTTTC GTTTTGCTGT CTCCGGTTGA AGAGAGAGAT CTCACATCTC   
  
  
- GGGGTAGAGC CAATATTAAT ACTCTTAGTT AAGGAAGAGT GTTCTCTTTG AGAGCGAGAG AGAGAGAGAG   
  
  
- GTATTCTGTA GTGTGGTTAA CATACTTAAA AGAGAGAGGA CTAAAGACAA AAGGAAGAAC AACAGGGGAA   
  
  
- GATTACGCAA TCACGCCGGT TTTGGTGGTA TTTGGTAGAG AGAAAGTTCT TTCGTCGTTG TTGTTTCTCT   
  
  
- CTGTCTCTCT CTCTCTCCCC CTCTTTCTAG TGTAGAAAAC CCAAAGGCCG CTTATACTGC AGACCCAAAA   
  
  
- GCCCGCTAAA GAAGATGCCG CCACCTCCGC CACCGCCTCC TCCACAGGCC GGAAGATAGC CGCCACATGT   
  
  
- TCCGCCCCAA CAGGGTATAC CCAAGGCCGT TGTTGTTGTT GTTGTCGTTG TCGTCGTCGT CGTCGTTGTC   
  
  
- GATGTAGATG TCGATGTTGT TGTCGCCCCC TAAAAGAGCC GCCTAGTCTA GTCGCGAGTT GCTTCCCGCC   
  
  
- TAAACAACTA CCCATTCGCA AGAAAGCGAC TAAAAGTTAG AGTTGTTGTC GTTTAGTCAG AAAACGTCGT   
  
  
- CGTCGTCGTC GTTGTTGTTG TTGTCGGGAA ACGCGAGAAC GTTTTTGTTG TCGTCAAAGT TGTTGTTAAT   
  
  
- GTTGTTGTTG TCGGTGAAAG AGAGGAGGAA GTTGTTGTCA TGAACTACTC TAGGCATTTT GTCTCTTGGT   
  
  
- TGTTAAAGCG TCGTAGAGGC TAAAGAGGAG AAAGAAGAAG GTAACTAGAA AGAAGGCGAT TATTAAGGGC   
  
  
- CAAGCCAAAC GGCCAAATGG TTGTTGTTGA GGCCAGAGTT GTTGTTGGTG GATAGAGAGA GGAGCGGGTG   
  
  
- TTGTTGTTGG TTAAGTTGGA GAGTTTATTA TTGTTAAGAA GAAATTAGGT TTTGAGGTGG TTGTGAGAAG   
  
  
- AACGAGGGCG TCGGTCGTTA TTGTGGGATT ACTTTTTAAG CCCGAAAAGG AGTAGACCCA AAAGATTAAG   
  
  
- CCCAGGTTTG GACCAGGTTG GCTTCGTCCT TTAGCCCATG AGCTTATACC CATGGTTATA CCCATACTTA   
  
  
- GACCAGGTCG GGTTTGGGCT GGGACTCCTA GTGCCTTATT ACTTGTGGGA AGTTCTTAAC CTCTTCCTCG   
  
  
- AAAACTTACT GCTACTCCTT TTGGTCGGCC TTTTGCTGCG GCAAAGTCAG TAGTGGTTGT CGCTCACCAG   
  
  
- GCTCTGGAAG GTCTTAGACT ACTCAGGGCT GGGCTCAAGC TCGGGCTTAC GGTGGTTGTG GGTGGGCCCA   
  
  
- AGCCCAAGAC GATCATGGTC GCCGTCGTTG TTGTGATGTT GGTGGGGACG TTTGTTGTTA TTGTACTATA   
  
  
- GATGGAGCGG CTGCAGCAGG TGCAGTAGGA CGACGAGGTG CAGCCGTAGC CGCAGAGGCG GCCGCCGCCA   
  
  
- CCGCTGGGGC GTCCATGGTC GGTTCGTCGA CTAGAGCCTT CGTCGCCGCC GCTAAAGCCT GCCTTTCTAA   
  
  
- CTTAGGCGGT ACCTTCACTA CTTGGCCGAC AGGGTCCAAC GGTTAAGATC CCCCTCGAGC CTTGTTTTCG   
  
  
- ACCGAAAAAT GTACCCGGTG CGCGAAGCCA GCGCGCACTC AGGGTGGCTT GTGCGAGGGG GATAACACCT   
  
  
- CGAAAAGGTT TTCCTCGTAT ACCGCCGGTA CATAAGTAAC ATGCTCGAGA GAGTAACAAA GTTTAAACCA   
  
  
- GAAAACCGAC GGTTAAACCA CTACAACCTC CGGTAATGAA GGCGGGAAGT ACTCCCAGAC TTTTAAGTAC   
  
  
- AACACCTAAA ACTATAACCC GTACCACCAG TCATGTAGTT GGAGGATGCG CGGAACCCCC TCGCATCCCG   
  
  
- AGTCAGCCCC TCGCGCCACA GCTTACTCCA CTCCTAGTGA CACCAACTCT GGCTCCAGCC ATTACCACCA   
  
  
- CGCCGCCCAC GCCTCCGACA ATTCCTACCG GACCACTTTA ACCGACTCTT CCACCCCTAA TCAAACTTCA   
  
  
- AACTCCGAAA CTGAGCCTTC TAGAGGCTCA ATTGGGCCCT CAGTGACCCC ACACTCCTCC CCCTCCGGCA   
  
  
- CCACCACTTA AAACGCAAGT TTGAAATGGC CCATGGCCTA CTCTCGCACA GCTACCTCTT AGACTCCCTG   
  
  
- CTCGAGGACG CGGCCCACTT CCGCGATTCC GGTGCGCGGC AGTGTAACCA CCTCGTCCTG AATTTACGCT   
  
  
- TATGCCGTGG CAAGTACTGC GCGCACTCAC CGCGAACACT CATGATATCT CGTAACAAGC TGAGCGAGCT   
  
  
- GAGCTACCAC GGCTCCCTAT TGGGCCTGGC CCAGGCCCAG CTCCTCCGGA ACCCCGCCTT CTACCGTTTG   
  
  
- AGCCACCGAA CGCTCCCGGC CCTTGCCCAA CTCGCCCGCC TCCACAAACC CTTCACCGCC CGCTCCTACT   
  
  
- CATACCGTCC CAAGCTCAAC TTCCGTGACT CAGTCTCACA CCGGCTCAGT TACGCTTGGT TTGAGTCGAG   
  
  
- CCAAGACCCG CCTTTCGGCC CAAAGTGCCA GTTCCTCCTC TCGCCACCCC ACCCCAAGCC CACCTACCCT   
  
  
- GCTTGAGAGT GGCAGCGTAG CCGGACCGCA AT

+     DRE core

| Site Name | Organism | Position | Strand | Matrix score. | sequence | function |
| --- | --- | --- | --- | --- | --- | --- |
| DRE core | Arabidopsis thaliana | 3125 | - | 6 | GCCGAC |  |
| DRE core | Arabidopsis thaliana | 3092 | + | 6 | GCCGAC |  |

>HU06G02537.1   
+ +Up\_Stream \_Len000CCTCGG TGTGGGATTC CGGGTTGATA AATAGGGACC AGCGAAATGT TCAAATAATT   
  
  
+ GGTGAAGGAG TCTCTGTTTG AATGCCTACT AATGCTTTTT TTTTTTTTTT GAATAATTTG TCAGAAAGTT   
  
  
+ AGGCTGCATT GGAAGGGGCG GCCTACGAAG GCTTTTCAGC AAATAAATAA CCAGCTTTCA TTTTGAAATT   
  
  
+ TTTTTTAAAA AATGAAACTC ATTTCACTTA AAAATTTACC ATAATATATA TATATATATA TATATATATA   
  
  
+ TATATATATA TATTTGATTT AACGTTAAGC AAACTTTTAA CTTATGTATC ATTTTACGAC CGTAAAAAAA   
  
  
+ AAAGAAATAT AACACAATAT GTAATAGTAT TTAAGTTTTT GATAAATAAT ATTTTAACAT AATTTGTGTG   
  
  
+ TAGTATGAAT TTAAATCTTA GTACTAATAT AATTATGTAT ACTTGGCTAT AAGATCTCAA TATTAGTATT   
  
  
+ TTATGATTCA ATGTAATGAT GATGCAACTG TGTGTAATTA GAAAGCCTTT GATTGCAGAT ATCTATCTTA   
  
  
+ TTGATACACA TCTACCAATT GAGCTTTTTC AAGATTTGAA GACGGTAGTC AAGTATCTTT GGGAGATTAC   
  
  
+ GTTAGAATTA CTTCATGATA AATTAGACAT ATTTTATACT TCTAATCTTA AATAAGTCTC CATCCTTTCA   
  
  
+ TCATATTTTA AAGAAATGTA CAAGTAAATG TCAATAGTTT GAAACGTTCC TTATCATAAA AGTCATACAT   
  
  
+ ATGTAGGACA TTTTCAAGTT TATGTAAACC ATATGATATA GTTCAAGATA ATTGTCAAAC AAGTTATGAA   
  
  
+ ATCTAAAGGT GTTTGAACGT AATTTCAAGC TAATCTTAGC AAGTTATAGT ATTATTTAAA CTACGACTAG   
  
  
+ ACACAACATT TTGTTCATTT ATATAACACT TAGAATGCAA AAAAATCGGT ATTACTAATA CTGTATCAAG   
  
  
+ AATTGAAGTG TTATGCATTA TACTGATAAG GGATAATATT TAAATTACTT TACTGTTATA GAAGCCAAAA   
  
  
+ ATCTAAACTT TCATACAGAC GTCGGACTAA ATTAGAAAAT TCTTTAACTT AATAGAAAGG ATGACATAAA   
  
  
+ TAAAAATAAA AACCTAAATA GCATAATAAC CATTCTACAC AAAAGTCAAT CTTCTAATAG AAAAAAGCCA   
  
  
+ TAAGTATTCA CTTATTTACC ATTTTATTCC TAATCTACTG TCTGTCTATT CACACCATAA TTCATGAACC   
  
  
+ AATGTGGAGT TTCTGTTTCA TTTCATGGGG TGATTTATGT CTGACAATGA TCCAACAAAC TGAGCCTCAC   
  
  
+ GCGCGGAACA CGAGAGTGGC TGTACTTATG GTGTGAAAGA GCTCTACAAG GTGAGATTGA GGAGGCTACG   
  
  
+ CGTTGGAGTT ATCACCACCG TTGGATTTGT TTTCTTTGGA GCCACGTTAT CACTCTTCAG ACGCCGATCC   
  
  
+ AACGGCCCTT ACTTCCATCT AGTCCCCACT CTCCAACTCT ACGCAATCAA CGCCTTCCGC ATTTAAATAT   
  
  
+ TTCCTTCTCT ATCGCCTACT TTCTCTGTCT CACAATTTTT GTCCGTATTT TGAATTTTCT CAAAATTATC   
  
  
+ ATATAATTTA TTTGCCTTCA ACTAAAGTAT TTATTAAATT AGTTAATGTA TTTAAATTGA ACTAAAAAAG   
  
  
+ AGAAAAAGAA AGAAAACAAC CCCACGAAAG CAAAACGACA GAGGCCAACT TCTCTCTCTA GAGTGTAGAG   
  
  
+ CCCCATCTCG GTTATAATTA TGAGAATCAA TTCCTTCTCA CAAGAGAAAC TCTCGCTCTC TCTCTCTCTC   
  
  
+ CATAAGACAT CACACCAATT GTATGAATTT TCTCTCTCCT GATTTCTGTT TTCCTTCTTG TTGTCCCCTT   
  
  
+ CTAATGCGTT AGTGCGGCCA AAACCACCAT AAACCATCTC TCTTTCAAGA AAGCAGCAAC AACAAAGAGA   
  
  
+ GACAGAGAGA GAGAGAGGGG GAGAAAGATC ACATCTTTTG GGTTTCCGGC GAATATGACG TCTGGGTTTT   
  
  
+ CGGGCGATTT CTTCTACGGC GGTGGAGGCG GTGGCGGAGG AGGTGTCCGG CCTTCTATCG GCGGTGTACA   
  
  
+ AGGCGGGGTT GTCCCATATG GGTTCCGGCA ACAACAACAA CAACAGCAAC AGCAGCAGCA GCAGCAACAG   
  
  
+ CTACATCTAC AGCTACAACA ACAGCGGGGG ATTTTCTCGG CGGATCAGAT CAGCGCTCAA CGAAGGGCGG   
  
  
+ ATTTGTTGAT GGGTAAGCGT TCTTTCGCTG ATTTTCAATC TCAACAACAG CAAATCAGTC TTTTGCAGCA   
  
  
+ GCAGCAGCAG CAACAACAAC AACAGCCCTT TGCGCTCTTG CAAAAACAAC AGCAGTTTCA ACAACAATTA   
  
  
+ CAACAACAAC AGCCACTTTC TCTCCTCCTT CAACAACAGT ACTTGATGAG ATCCGTAAAA CAGAGAACCA   
  
  
+ ACAATTTCGC AGCATCTCCG ATTTCTCCTC TTTCTTCTTC CATTGATCTT TCTTCCGCTA ATAATTCCCG   
  
  
+ GTTCGGTTTG CCGGTTTACC AACAACAACT CCGGTCTCAA CAACAACCAC CTATCTCTCT CCTCGCCCAC   
  
  
+ AACAACAACC AATTCAACCT CTCAAATAAT AACAATTCTT CTTTAATCCA AAACTCCACC AACACTCTTC   
  
  
+ TTGCTCCCGC AGCCAGCAAT AACACCCTAA TGAAAAATTC GGGCTTTTCC TCATCTGGGT TTTCTAATTC   
  
  
+ GGGTCCAAAC CTGGTCCAAC CGAAGCAGGA AATCGGGTAC TCGAATATGG GTACCAATAT GGGTATGAAT   
  
  
+ CTGGTCCAGC CCAAACCCGA CCCTGAGGAT CACGGAATAA TGAACACCCT TCAAGAATTG GAGAAGGAGC   
  
  
+ TTTTGAATGA CGATGAGGAA AACCAGCCGG AAAACGACGC CGTTTCAGTC ATCACCAACA GCGAGTGGTC   
  
  
+ CGAGACCTTC CAGAATCTGA TGAGTCCCGA CCCGAGTTCG AGCCCGAATG CCACCAACAC CCACCCGGGT   
  
  
+ TCGGGTTCTG CTAGTACCAG CGGCAGCAAC AACACTACAA CCACCCCTGC AAACAACAAT AACATGATAT   
  
  
+ CTACCTCGCC GACGTCGTCC ACGTCATCCT GCTGCTCCAC GTCGGCATCG GCGTCTCCGC CGGCGGCGGT   
  
  
+ GGCGACCCCG CAGGTACCAG CCAAGCAGCT GATCTCGGAA GCAGCGGCGG CGATTTCGGA CGGAAAGATT   
  
  
+ GAATCCGCCA TGGAAGTGAT GAACCGGCTG TCCCAGGTTG CCAATTCTAG GGGGAGCTCG GAACAAAAGC   
  
  
+ TGGCTTTTTA CATGGGCCAC GCGCTTCGGT CGCGCGTGAG TCCCACCGAA CACGCTCCCC CTATTGTGGA   
  
  
+ GCTTTTCCAA AAGGAGCATA TGGCGGCCAT GTATTCATTG TACGAGCTCT CTCATTGTTT CAAATTTGGT   
  
  
+ CTTTTGGCTG CCAATTTGGT GATGTTGGAG GCCATTACTT CCGCCCTTCA TGAGGGTCTG AAAATTCATG   
  
  
+ TTGTGGATTT TGATATTGGG CATGGTGGTC AGTACATCAA CCTCCTACGC GCCTTGGGGG AGCGTAGGGC   
  
  
+ TCAGTCGGGG AGCGCGGTGT CGAATGAGGT GAGGATCACT GTGGTTGAGA CCGAGGTCGG TAATGGTGGT   
  
  
+ GCGGCGGGTG CGGAGGCTGT TAAGGATGGC CTGGTGAAAT TGGCTGAGAA GGTGGGGATT AGTTTGAAGT   
  
  
+ TTGAGGCTTT GACTCGGAAG ATCTCCGAGT TAACCCGGGA GTCACTGGGG TGTGAGGAGG GGGAGGCCGT   
  
  
+ GGTGGTGAAT TTTGCGTTCA AACTTTACCG GGTACCGGAT GAGAGCGTGT CGATGGAGAA TCTGAGGGAC   
  
  
+ GAGCTCCTGC GCCGGGTGAA GGCGCTAAGG CCACGCGCCG TCACATTGGT GGAGCAGGAC TTAAATGCGA   
  
  
+ ATACGGCACC GTTCATGACG CGCGTGAGTG GCGCTTGTGA GTACTATAGA GCATTGTTCG ACTCGCTCGA   
  
  
+ CTCGATGGTG CCGAGGGATA ACCCGGACCG GGTCCGGGTC GAGGAGGCCT TGGGGCGGAA GATGGCAAAC   
  
  
+ TCGGTGGCTT GCGAGGGCCG GGAACGGGTT GAGCGGGCGG AGGTGTTTGG GAAGTGGCGG GCGAGGATGA   
  
  
+ GTATGGCAGG GTTCGAGTTG AAGGCACTGA GTCAGAGTGT GGCCGAGTCA ATGCGAACCA AACTCAGCTC   
  
  
+ GGTTCTGGGC GGAAAGCCGG GTTTCACGGT CAAGGAGGAG AGCGGTGGGG TGGGGTTCGG GTGGATGGGA   
  
  
+ CGAACTCTCA CCGTCGCATC GGCCTGGCGT TA  

- +Up\_Stream \_Len000GGAGCC ACACCCTAAG GCCCAACTAT TTATCCCTGG TCGCTTTACA AGTTTATTAA   
  
  
- CCACTTCCTC AGAGACAAAC TTACGGATGA TTACGAAAAA AAAAAAAAAA CTTATTAAAC AGTCTTTCAA   
  
  
- TCCGACGTAA CCTTCCCCGC CGGATGCTTC CGAAAAGTCG TTTATTTATT GGTCGAAAGT AAAACTTTAA   
  
  
- AAAAAATTTT TTACTTTGAG TAAAGTGAAT TTTTAAATGG TATTATATAT ATATATATAT ATATATATAT   
  
  
- ATATATATAT ATAAACTAAA TTGCAATTCG TTTGAAAATT GAATACATAG TAAAATGCTG GCATTTTTTT   
  
  
- TTTCTTTATA TTGTGTTATA CATTATCATA AATTCAAAAA CTATTTATTA TAAAATTGTA TTAAACACAC   
  
  
- ATCATACTTA AATTTAGAAT CATGATTATA TTAATACATA TGAACCGATA TTCTAGAGTT ATAATCATAA   
  
  
- AATACTAAGT TACATTACTA CTACGTTGAC ACACATTAAT CTTTCGGAAA CTAACGTCTA TAGATAGAAT   
  
  
- AACTATGTGT AGATGGTTAA CTCGAAAAAG TTCTAAACTT CTGCCATCAG TTCATAGAAA CCCTCTAATG   
  
  
- CAATCTTAAT GAAGTACTAT TTAATCTGTA TAAAATATGA AGATTAGAAT TTATTCAGAG GTAGGAAAGT   
  
  
- AGTATAAAAT TTCTTTACAT GTTCATTTAC AGTTATCAAA CTTTGCAAGG AATAGTATTT TCAGTATGTA   
  
  
- TACATCCTGT AAAAGTTCAA ATACATTTGG TATACTATAT CAAGTTCTAT TAACAGTTTG TTCAATACTT   
  
  
- TAGATTTCCA CAAACTTGCA TTAAAGTTCG ATTAGAATCG TTCAATATCA TAATAAATTT GATGCTGATC   
  
  
- TGTGTTGTAA AACAAGTAAA TATATTGTGA ATCTTACGTT TTTTTAGCCA TAATGATTAT GACATAGTTC   
  
  
- TTAACTTCAC AATACGTAAT ATGACTATTC CCTATTATAA ATTTAATGAA ATGACAATAT CTTCGGTTTT   
  
  
- TAGATTTGAA AGTATGTCTG CAGCCTGATT TAATCTTTTA AGAAATTGAA TTATCTTTCC TACTGTATTT   
  
  
- ATTTTTATTT TTGGATTTAT CGTATTATTG GTAAGATGTG TTTTCAGTTA GAAGATTATC TTTTTTCGGT   
  
  
- ATTCATAAGT GAATAAATGG TAAAATAAGG ATTAGATGAC AGACAGATAA GTGTGGTATT AAGTACTTGG   
  
  
- TTACACCTCA AAGACAAAGT AAAGTACCCC ACTAAATACA GACTGTTACT AGGTTGTTTG ACTCGGAGTG   
  
  
- CGCGCCTTGT GCTCTCACCG ACATGAATAC CACACTTTCT CGAGATGTTC CACTCTAACT CCTCCGATGC   
  
  
- GCAACCTCAA TAGTGGTGGC AACCTAAACA AAAGAAACCT CGGTGCAATA GTGAGAAGTC TGCGGCTAGG   
  
  
- TTGCCGGGAA TGAAGGTAGA TCAGGGGTGA GAGGTTGAGA TGCGTTAGTT GCGGAAGGCG TAAATTTATA   
  
  
- AAGGAAGAGA TAGCGGATGA AAGAGACAGA GTGTTAAAAA CAGGCATAAA ACTTAAAAGA GTTTTAATAG   
  
  
- TATATTAAAT AAACGGAAGT TGATTTCATA AATAATTTAA TCAATTACAT AAATTTAACT TGATTTTTTC   
  
  
- TCTTTTTCTT TCTTTTGTTG GGGTGCTTTC GTTTTGCTGT CTCCGGTTGA AGAGAGAGAT CTCACATCTC   
  
  
- GGGGTAGAGC CAATATTAAT ACTCTTAGTT AAGGAAGAGT GTTCTCTTTG AGAGCGAGAG AGAGAGAGAG   
  
  
- GTATTCTGTA GTGTGGTTAA CATACTTAAA AGAGAGAGGA CTAAAGACAA AAGGAAGAAC AACAGGGGAA   
  
  
- GATTACGCAA TCACGCCGGT TTTGGTGGTA TTTGGTAGAG AGAAAGTTCT TTCGTCGTTG TTGTTTCTCT   
  
  
- CTGTCTCTCT CTCTCTCCCC CTCTTTCTAG TGTAGAAAAC CCAAAGGCCG CTTATACTGC AGACCCAAAA   
  
  
- GCCCGCTAAA GAAGATGCCG CCACCTCCGC CACCGCCTCC TCCACAGGCC GGAAGATAGC CGCCACATGT   
  
  
- TCCGCCCCAA CAGGGTATAC CCAAGGCCGT TGTTGTTGTT GTTGTCGTTG TCGTCGTCGT CGTCGTTGTC   
  
  
- GATGTAGATG TCGATGTTGT TGTCGCCCCC TAAAAGAGCC GCCTAGTCTA GTCGCGAGTT GCTTCCCGCC   
  
  
- TAAACAACTA CCCATTCGCA AGAAAGCGAC TAAAAGTTAG AGTTGTTGTC GTTTAGTCAG AAAACGTCGT   
  
  
- CGTCGTCGTC GTTGTTGTTG TTGTCGGGAA ACGCGAGAAC GTTTTTGTTG TCGTCAAAGT TGTTGTTAAT   
  
  
- GTTGTTGTTG TCGGTGAAAG AGAGGAGGAA GTTGTTGTCA TGAACTACTC TAGGCATTTT GTCTCTTGGT   
  
  
- TGTTAAAGCG TCGTAGAGGC TAAAGAGGAG AAAGAAGAAG GTAACTAGAA AGAAGGCGAT TATTAAGGGC   
  
  
- CAAGCCAAAC GGCCAAATGG TTGTTGTTGA GGCCAGAGTT GTTGTTGGTG GATAGAGAGA GGAGCGGGTG   
  
  
- TTGTTGTTGG TTAAGTTGGA GAGTTTATTA TTGTTAAGAA GAAATTAGGT TTTGAGGTGG TTGTGAGAAG   
  
  
- AACGAGGGCG TCGGTCGTTA TTGTGGGATT ACTTTTTAAG CCCGAAAAGG AGTAGACCCA AAAGATTAAG   
  
  
- CCCAGGTTTG GACCAGGTTG GCTTCGTCCT TTAGCCCATG AGCTTATACC CATGGTTATA CCCATACTTA   
  
  
- GACCAGGTCG GGTTTGGGCT GGGACTCCTA GTGCCTTATT ACTTGTGGGA AGTTCTTAAC CTCTTCCTCG   
  
  
- AAAACTTACT GCTACTCCTT TTGGTCGGCC TTTTGCTGCG GCAAAGTCAG TAGTGGTTGT CGCTCACCAG   
  
  
- GCTCTGGAAG GTCTTAGACT ACTCAGGGCT GGGCTCAAGC TCGGGCTTAC GGTGGTTGTG GGTGGGCCCA   
  
  
- AGCCCAAGAC GATCATGGTC GCCGTCGTTG TTGTGATGTT GGTGGGGACG TTTGTTGTTA TTGTACTATA   
  
  
- GATGGAGCGG CTGCAGCAGG TGCAGTAGGA CGACGAGGTG CAGCCGTAGC CGCAGAGGCG GCCGCCGCCA   
  
  
- CCGCTGGGGC GTCCATGGTC GGTTCGTCGA CTAGAGCCTT CGTCGCCGCC GCTAAAGCCT GCCTTTCTAA   
  
  
- CTTAGGCGGT ACCTTCACTA CTTGGCCGAC AGGGTCCAAC GGTTAAGATC CCCCTCGAGC CTTGTTTTCG   
  
  
- ACCGAAAAAT GTACCCGGTG CGCGAAGCCA GCGCGCACTC AGGGTGGCTT GTGCGAGGGG GATAACACCT   
  
  
- CGAAAAGGTT TTCCTCGTAT ACCGCCGGTA CATAAGTAAC ATGCTCGAGA GAGTAACAAA GTTTAAACCA   
  
  
- GAAAACCGAC GGTTAAACCA CTACAACCTC CGGTAATGAA GGCGGGAAGT ACTCCCAGAC TTTTAAGTAC   
  
  
- AACACCTAAA ACTATAACCC GTACCACCAG TCATGTAGTT GGAGGATGCG CGGAACCCCC TCGCATCCCG   
  
  
- AGTCAGCCCC TCGCGCCACA GCTTACTCCA CTCCTAGTGA CACCAACTCT GGCTCCAGCC ATTACCACCA   
  
  
- CGCCGCCCAC GCCTCCGACA ATTCCTACCG GACCACTTTA ACCGACTCTT CCACCCCTAA TCAAACTTCA   
  
  
- AACTCCGAAA CTGAGCCTTC TAGAGGCTCA ATTGGGCCCT CAGTGACCCC ACACTCCTCC CCCTCCGGCA   
  
  
- CCACCACTTA AAACGCAAGT TTGAAATGGC CCATGGCCTA CTCTCGCACA GCTACCTCTT AGACTCCCTG   
  
  
- CTCGAGGACG CGGCCCACTT CCGCGATTCC GGTGCGCGGC AGTGTAACCA CCTCGTCCTG AATTTACGCT   
  
  
- TATGCCGTGG CAAGTACTGC GCGCACTCAC CGCGAACACT CATGATATCT CGTAACAAGC TGAGCGAGCT   
  
  
- GAGCTACCAC GGCTCCCTAT TGGGCCTGGC CCAGGCCCAG CTCCTCCGGA ACCCCGCCTT CTACCGTTTG   
  
  
- AGCCACCGAA CGCTCCCGGC CCTTGCCCAA CTCGCCCGCC TCCACAAACC CTTCACCGCC CGCTCCTACT   
  
  
- CATACCGTCC CAAGCTCAAC TTCCGTGACT CAGTCTCACA CCGGCTCAGT TACGCTTGGT TTGAGTCGAG   
  
  
- CCAAGACCCG CCTTTCGGCC CAAAGTGCCA GTTCCTCCTC TCGCCACCCC ACCCCAAGCC CACCTACCCT   
  
  
- GCTTGAGAGT GGCAGCGTAG CCGGACCGCA AT

+     DRE1

| Site Name | Organism | Position | Strand | Matrix score. | sequence | function |
| --- | --- | --- | --- | --- | --- | --- |
| DRE1 | Zea mays | 1760 | - | 7 | ACCGAGA |  |

>HU06G02537.1   
+ +Up\_Stream \_Len000CCTCGG TGTGGGATTC CGGGTTGATA AATAGGGACC AGCGAAATGT TCAAATAATT   
  
  
+ GGTGAAGGAG TCTCTGTTTG AATGCCTACT AATGCTTTTT TTTTTTTTTT GAATAATTTG TCAGAAAGTT   
  
  
+ AGGCTGCATT GGAAGGGGCG GCCTACGAAG GCTTTTCAGC AAATAAATAA CCAGCTTTCA TTTTGAAATT   
  
  
+ TTTTTTAAAA AATGAAACTC ATTTCACTTA AAAATTTACC ATAATATATA TATATATATA TATATATATA   
  
  
+ TATATATATA TATTTGATTT AACGTTAAGC AAACTTTTAA CTTATGTATC ATTTTACGAC CGTAAAAAAA   
  
  
+ AAAGAAATAT AACACAATAT GTAATAGTAT TTAAGTTTTT GATAAATAAT ATTTTAACAT AATTTGTGTG   
  
  
+ TAGTATGAAT TTAAATCTTA GTACTAATAT AATTATGTAT ACTTGGCTAT AAGATCTCAA TATTAGTATT   
  
  
+ TTATGATTCA ATGTAATGAT GATGCAACTG TGTGTAATTA GAAAGCCTTT GATTGCAGAT ATCTATCTTA   
  
  
+ TTGATACACA TCTACCAATT GAGCTTTTTC AAGATTTGAA GACGGTAGTC AAGTATCTTT GGGAGATTAC   
  
  
+ GTTAGAATTA CTTCATGATA AATTAGACAT ATTTTATACT TCTAATCTTA AATAAGTCTC CATCCTTTCA   
  
  
+ TCATATTTTA AAGAAATGTA CAAGTAAATG TCAATAGTTT GAAACGTTCC TTATCATAAA AGTCATACAT   
  
  
+ ATGTAGGACA TTTTCAAGTT TATGTAAACC ATATGATATA GTTCAAGATA ATTGTCAAAC AAGTTATGAA   
  
  
+ ATCTAAAGGT GTTTGAACGT AATTTCAAGC TAATCTTAGC AAGTTATAGT ATTATTTAAA CTACGACTAG   
  
  
+ ACACAACATT TTGTTCATTT ATATAACACT TAGAATGCAA AAAAATCGGT ATTACTAATA CTGTATCAAG   
  
  
+ AATTGAAGTG TTATGCATTA TACTGATAAG GGATAATATT TAAATTACTT TACTGTTATA GAAGCCAAAA   
  
  
+ ATCTAAACTT TCATACAGAC GTCGGACTAA ATTAGAAAAT TCTTTAACTT AATAGAAAGG ATGACATAAA   
  
  
+ TAAAAATAAA AACCTAAATA GCATAATAAC CATTCTACAC AAAAGTCAAT CTTCTAATAG AAAAAAGCCA   
  
  
+ TAAGTATTCA CTTATTTACC ATTTTATTCC TAATCTACTG TCTGTCTATT CACACCATAA TTCATGAACC   
  
  
+ AATGTGGAGT TTCTGTTTCA TTTCATGGGG TGATTTATGT CTGACAATGA TCCAACAAAC TGAGCCTCAC   
  
  
+ GCGCGGAACA CGAGAGTGGC TGTACTTATG GTGTGAAAGA GCTCTACAAG GTGAGATTGA GGAGGCTACG   
  
  
+ CGTTGGAGTT ATCACCACCG TTGGATTTGT TTTCTTTGGA GCCACGTTAT CACTCTTCAG ACGCCGATCC   
  
  
+ AACGGCCCTT ACTTCCATCT AGTCCCCACT CTCCAACTCT ACGCAATCAA CGCCTTCCGC ATTTAAATAT   
  
  
+ TTCCTTCTCT ATCGCCTACT TTCTCTGTCT CACAATTTTT GTCCGTATTT TGAATTTTCT CAAAATTATC   
  
  
+ ATATAATTTA TTTGCCTTCA ACTAAAGTAT TTATTAAATT AGTTAATGTA TTTAAATTGA ACTAAAAAAG   
  
  
+ AGAAAAAGAA AGAAAACAAC CCCACGAAAG CAAAACGACA GAGGCCAACT TCTCTCTCTA GAGTGTAGAG   
  
  
+ CCCCATCTCG GTTATAATTA TGAGAATCAA TTCCTTCTCA CAAGAGAAAC TCTCGCTCTC TCTCTCTCTC   
  
  
+ CATAAGACAT CACACCAATT GTATGAATTT TCTCTCTCCT GATTTCTGTT TTCCTTCTTG TTGTCCCCTT   
  
  
+ CTAATGCGTT AGTGCGGCCA AAACCACCAT AAACCATCTC TCTTTCAAGA AAGCAGCAAC AACAAAGAGA   
  
  
+ GACAGAGAGA GAGAGAGGGG GAGAAAGATC ACATCTTTTG GGTTTCCGGC GAATATGACG TCTGGGTTTT   
  
  
+ CGGGCGATTT CTTCTACGGC GGTGGAGGCG GTGGCGGAGG AGGTGTCCGG CCTTCTATCG GCGGTGTACA   
  
  
+ AGGCGGGGTT GTCCCATATG GGTTCCGGCA ACAACAACAA CAACAGCAAC AGCAGCAGCA GCAGCAACAG   
  
  
+ CTACATCTAC AGCTACAACA ACAGCGGGGG ATTTTCTCGG CGGATCAGAT CAGCGCTCAA CGAAGGGCGG   
  
  
+ ATTTGTTGAT GGGTAAGCGT TCTTTCGCTG ATTTTCAATC TCAACAACAG CAAATCAGTC TTTTGCAGCA   
  
  
+ GCAGCAGCAG CAACAACAAC AACAGCCCTT TGCGCTCTTG CAAAAACAAC AGCAGTTTCA ACAACAATTA   
  
  
+ CAACAACAAC AGCCACTTTC TCTCCTCCTT CAACAACAGT ACTTGATGAG ATCCGTAAAA CAGAGAACCA   
  
  
+ ACAATTTCGC AGCATCTCCG ATTTCTCCTC TTTCTTCTTC CATTGATCTT TCTTCCGCTA ATAATTCCCG   
  
  
+ GTTCGGTTTG CCGGTTTACC AACAACAACT CCGGTCTCAA CAACAACCAC CTATCTCTCT CCTCGCCCAC   
  
  
+ AACAACAACC AATTCAACCT CTCAAATAAT AACAATTCTT CTTTAATCCA AAACTCCACC AACACTCTTC   
  
  
+ TTGCTCCCGC AGCCAGCAAT AACACCCTAA TGAAAAATTC GGGCTTTTCC TCATCTGGGT TTTCTAATTC   
  
  
+ GGGTCCAAAC CTGGTCCAAC CGAAGCAGGA AATCGGGTAC TCGAATATGG GTACCAATAT GGGTATGAAT   
  
  
+ CTGGTCCAGC CCAAACCCGA CCCTGAGGAT CACGGAATAA TGAACACCCT TCAAGAATTG GAGAAGGAGC   
  
  
+ TTTTGAATGA CGATGAGGAA AACCAGCCGG AAAACGACGC CGTTTCAGTC ATCACCAACA GCGAGTGGTC   
  
  
+ CGAGACCTTC CAGAATCTGA TGAGTCCCGA CCCGAGTTCG AGCCCGAATG CCACCAACAC CCACCCGGGT   
  
  
+ TCGGGTTCTG CTAGTACCAG CGGCAGCAAC AACACTACAA CCACCCCTGC AAACAACAAT AACATGATAT   
  
  
+ CTACCTCGCC GACGTCGTCC ACGTCATCCT GCTGCTCCAC GTCGGCATCG GCGTCTCCGC CGGCGGCGGT   
  
  
+ GGCGACCCCG CAGGTACCAG CCAAGCAGCT GATCTCGGAA GCAGCGGCGG CGATTTCGGA CGGAAAGATT   
  
  
+ GAATCCGCCA TGGAAGTGAT GAACCGGCTG TCCCAGGTTG CCAATTCTAG GGGGAGCTCG GAACAAAAGC   
  
  
+ TGGCTTTTTA CATGGGCCAC GCGCTTCGGT CGCGCGTGAG TCCCACCGAA CACGCTCCCC CTATTGTGGA   
  
  
+ GCTTTTCCAA AAGGAGCATA TGGCGGCCAT GTATTCATTG TACGAGCTCT CTCATTGTTT CAAATTTGGT   
  
  
+ CTTTTGGCTG CCAATTTGGT GATGTTGGAG GCCATTACTT CCGCCCTTCA TGAGGGTCTG AAAATTCATG   
  
  
+ TTGTGGATTT TGATATTGGG CATGGTGGTC AGTACATCAA CCTCCTACGC GCCTTGGGGG AGCGTAGGGC   
  
  
+ TCAGTCGGGG AGCGCGGTGT CGAATGAGGT GAGGATCACT GTGGTTGAGA CCGAGGTCGG TAATGGTGGT   
  
  
+ GCGGCGGGTG CGGAGGCTGT TAAGGATGGC CTGGTGAAAT TGGCTGAGAA GGTGGGGATT AGTTTGAAGT   
  
  
+ TTGAGGCTTT GACTCGGAAG ATCTCCGAGT TAACCCGGGA GTCACTGGGG TGTGAGGAGG GGGAGGCCGT   
  
  
+ GGTGGTGAAT TTTGCGTTCA AACTTTACCG GGTACCGGAT GAGAGCGTGT CGATGGAGAA TCTGAGGGAC   
  
  
+ GAGCTCCTGC GCCGGGTGAA GGCGCTAAGG CCACGCGCCG TCACATTGGT GGAGCAGGAC TTAAATGCGA   
  
  
+ ATACGGCACC GTTCATGACG CGCGTGAGTG GCGCTTGTGA GTACTATAGA GCATTGTTCG ACTCGCTCGA   
  
  
+ CTCGATGGTG CCGAGGGATA ACCCGGACCG GGTCCGGGTC GAGGAGGCCT TGGGGCGGAA GATGGCAAAC   
  
  
+ TCGGTGGCTT GCGAGGGCCG GGAACGGGTT GAGCGGGCGG AGGTGTTTGG GAAGTGGCGG GCGAGGATGA   
  
  
+ GTATGGCAGG GTTCGAGTTG AAGGCACTGA GTCAGAGTGT GGCCGAGTCA ATGCGAACCA AACTCAGCTC   
  
  
+ GGTTCTGGGC GGAAAGCCGG GTTTCACGGT CAAGGAGGAG AGCGGTGGGG TGGGGTTCGG GTGGATGGGA   
  
  
+ CGAACTCTCA CCGTCGCATC GGCCTGGCGT TA  

- +Up\_Stream \_Len000GGAGCC ACACCCTAAG GCCCAACTAT TTATCCCTGG TCGCTTTACA AGTTTATTAA   
  
  
- CCACTTCCTC AGAGACAAAC TTACGGATGA TTACGAAAAA AAAAAAAAAA CTTATTAAAC AGTCTTTCAA   
  
  
- TCCGACGTAA CCTTCCCCGC CGGATGCTTC CGAAAAGTCG TTTATTTATT GGTCGAAAGT AAAACTTTAA   
  
  
- AAAAAATTTT TTACTTTGAG TAAAGTGAAT TTTTAAATGG TATTATATAT ATATATATAT ATATATATAT   
  
  
- ATATATATAT ATAAACTAAA TTGCAATTCG TTTGAAAATT GAATACATAG TAAAATGCTG GCATTTTTTT   
  
  
- TTTCTTTATA TTGTGTTATA CATTATCATA AATTCAAAAA CTATTTATTA TAAAATTGTA TTAAACACAC   
  
  
- ATCATACTTA AATTTAGAAT CATGATTATA TTAATACATA TGAACCGATA TTCTAGAGTT ATAATCATAA   
  
  
- AATACTAAGT TACATTACTA CTACGTTGAC ACACATTAAT CTTTCGGAAA CTAACGTCTA TAGATAGAAT   
  
  
- AACTATGTGT AGATGGTTAA CTCGAAAAAG TTCTAAACTT CTGCCATCAG TTCATAGAAA CCCTCTAATG   
  
  
- CAATCTTAAT GAAGTACTAT TTAATCTGTA TAAAATATGA AGATTAGAAT TTATTCAGAG GTAGGAAAGT   
  
  
- AGTATAAAAT TTCTTTACAT GTTCATTTAC AGTTATCAAA CTTTGCAAGG AATAGTATTT TCAGTATGTA   
  
  
- TACATCCTGT AAAAGTTCAA ATACATTTGG TATACTATAT CAAGTTCTAT TAACAGTTTG TTCAATACTT   
  
  
- TAGATTTCCA CAAACTTGCA TTAAAGTTCG ATTAGAATCG TTCAATATCA TAATAAATTT GATGCTGATC   
  
  
- TGTGTTGTAA AACAAGTAAA TATATTGTGA ATCTTACGTT TTTTTAGCCA TAATGATTAT GACATAGTTC   
  
  
- TTAACTTCAC AATACGTAAT ATGACTATTC CCTATTATAA ATTTAATGAA ATGACAATAT CTTCGGTTTT   
  
  
- TAGATTTGAA AGTATGTCTG CAGCCTGATT TAATCTTTTA AGAAATTGAA TTATCTTTCC TACTGTATTT   
  
  
- ATTTTTATTT TTGGATTTAT CGTATTATTG GTAAGATGTG TTTTCAGTTA GAAGATTATC TTTTTTCGGT   
  
  
- ATTCATAAGT GAATAAATGG TAAAATAAGG ATTAGATGAC AGACAGATAA GTGTGGTATT AAGTACTTGG   
  
  
- TTACACCTCA AAGACAAAGT AAAGTACCCC ACTAAATACA GACTGTTACT AGGTTGTTTG ACTCGGAGTG   
  
  
- CGCGCCTTGT GCTCTCACCG ACATGAATAC CACACTTTCT CGAGATGTTC CACTCTAACT CCTCCGATGC   
  
  
- GCAACCTCAA TAGTGGTGGC AACCTAAACA AAAGAAACCT CGGTGCAATA GTGAGAAGTC TGCGGCTAGG   
  
  
- TTGCCGGGAA TGAAGGTAGA TCAGGGGTGA GAGGTTGAGA TGCGTTAGTT GCGGAAGGCG TAAATTTATA   
  
  
- AAGGAAGAGA TAGCGGATGA AAGAGACAGA GTGTTAAAAA CAGGCATAAA ACTTAAAAGA GTTTTAATAG   
  
  
- TATATTAAAT AAACGGAAGT TGATTTCATA AATAATTTAA TCAATTACAT AAATTTAACT TGATTTTTTC   
  
  
- TCTTTTTCTT TCTTTTGTTG GGGTGCTTTC GTTTTGCTGT CTCCGGTTGA AGAGAGAGAT CTCACATCTC   
  
  
- GGGGTAGAGC CAATATTAAT ACTCTTAGTT AAGGAAGAGT GTTCTCTTTG AGAGCGAGAG AGAGAGAGAG   
  
  
- GTATTCTGTA GTGTGGTTAA CATACTTAAA AGAGAGAGGA CTAAAGACAA AAGGAAGAAC AACAGGGGAA   
  
  
- GATTACGCAA TCACGCCGGT TTTGGTGGTA TTTGGTAGAG AGAAAGTTCT TTCGTCGTTG TTGTTTCTCT   
  
  
- CTGTCTCTCT CTCTCTCCCC CTCTTTCTAG TGTAGAAAAC CCAAAGGCCG CTTATACTGC AGACCCAAAA   
  
  
- GCCCGCTAAA GAAGATGCCG CCACCTCCGC CACCGCCTCC TCCACAGGCC GGAAGATAGC CGCCACATGT   
  
  
- TCCGCCCCAA CAGGGTATAC CCAAGGCCGT TGTTGTTGTT GTTGTCGTTG TCGTCGTCGT CGTCGTTGTC   
  
  
- GATGTAGATG TCGATGTTGT TGTCGCCCCC TAAAAGAGCC GCCTAGTCTA GTCGCGAGTT GCTTCCCGCC   
  
  
- TAAACAACTA CCCATTCGCA AGAAAGCGAC TAAAAGTTAG AGTTGTTGTC GTTTAGTCAG AAAACGTCGT   
  
  
- CGTCGTCGTC GTTGTTGTTG TTGTCGGGAA ACGCGAGAAC GTTTTTGTTG TCGTCAAAGT TGTTGTTAAT   
  
  
- GTTGTTGTTG TCGGTGAAAG AGAGGAGGAA GTTGTTGTCA TGAACTACTC TAGGCATTTT GTCTCTTGGT   
  
  
- TGTTAAAGCG TCGTAGAGGC TAAAGAGGAG AAAGAAGAAG GTAACTAGAA AGAAGGCGAT TATTAAGGGC   
  
  
- CAAGCCAAAC GGCCAAATGG TTGTTGTTGA GGCCAGAGTT GTTGTTGGTG GATAGAGAGA GGAGCGGGTG   
  
  
- TTGTTGTTGG TTAAGTTGGA GAGTTTATTA TTGTTAAGAA GAAATTAGGT TTTGAGGTGG TTGTGAGAAG   
  
  
- AACGAGGGCG TCGGTCGTTA TTGTGGGATT ACTTTTTAAG CCCGAAAAGG AGTAGACCCA AAAGATTAAG   
  
  
- CCCAGGTTTG GACCAGGTTG GCTTCGTCCT TTAGCCCATG AGCTTATACC CATGGTTATA CCCATACTTA   
  
  
- GACCAGGTCG GGTTTGGGCT GGGACTCCTA GTGCCTTATT ACTTGTGGGA AGTTCTTAAC CTCTTCCTCG   
  
  
- AAAACTTACT GCTACTCCTT TTGGTCGGCC TTTTGCTGCG GCAAAGTCAG TAGTGGTTGT CGCTCACCAG   
  
  
- GCTCTGGAAG GTCTTAGACT ACTCAGGGCT GGGCTCAAGC TCGGGCTTAC GGTGGTTGTG GGTGGGCCCA   
  
  
- AGCCCAAGAC GATCATGGTC GCCGTCGTTG TTGTGATGTT GGTGGGGACG TTTGTTGTTA TTGTACTATA   
  
  
- GATGGAGCGG CTGCAGCAGG TGCAGTAGGA CGACGAGGTG CAGCCGTAGC CGCAGAGGCG GCCGCCGCCA   
  
  
- CCGCTGGGGC GTCCATGGTC GGTTCGTCGA CTAGAGCCTT CGTCGCCGCC GCTAAAGCCT GCCTTTCTAA   
  
  
- CTTAGGCGGT ACCTTCACTA CTTGGCCGAC AGGGTCCAAC GGTTAAGATC CCCCTCGAGC CTTGTTTTCG   
  
  
- ACCGAAAAAT GTACCCGGTG CGCGAAGCCA GCGCGCACTC AGGGTGGCTT GTGCGAGGGG GATAACACCT   
  
  
- CGAAAAGGTT TTCCTCGTAT ACCGCCGGTA CATAAGTAAC ATGCTCGAGA GAGTAACAAA GTTTAAACCA   
  
  
- GAAAACCGAC GGTTAAACCA CTACAACCTC CGGTAATGAA GGCGGGAAGT ACTCCCAGAC TTTTAAGTAC   
  
  
- AACACCTAAA ACTATAACCC GTACCACCAG TCATGTAGTT GGAGGATGCG CGGAACCCCC TCGCATCCCG   
  
  
- AGTCAGCCCC TCGCGCCACA GCTTACTCCA CTCCTAGTGA CACCAACTCT GGCTCCAGCC ATTACCACCA   
  
  
- CGCCGCCCAC GCCTCCGACA ATTCCTACCG GACCACTTTA ACCGACTCTT CCACCCCTAA TCAAACTTCA   
  
  
- AACTCCGAAA CTGAGCCTTC TAGAGGCTCA ATTGGGCCCT CAGTGACCCC ACACTCCTCC CCCTCCGGCA   
  
  
- CCACCACTTA AAACGCAAGT TTGAAATGGC CCATGGCCTA CTCTCGCACA GCTACCTCTT AGACTCCCTG   
  
  
- CTCGAGGACG CGGCCCACTT CCGCGATTCC GGTGCGCGGC AGTGTAACCA CCTCGTCCTG AATTTACGCT   
  
  
- TATGCCGTGG CAAGTACTGC GCGCACTCAC CGCGAACACT CATGATATCT CGTAACAAGC TGAGCGAGCT   
  
  
- GAGCTACCAC GGCTCCCTAT TGGGCCTGGC CCAGGCCCAG CTCCTCCGGA ACCCCGCCTT CTACCGTTTG   
  
  
- AGCCACCGAA CGCTCCCGGC CCTTGCCCAA CTCGCCCGCC TCCACAAACC CTTCACCGCC CGCTCCTACT   
  
  
- CATACCGTCC CAAGCTCAAC TTCCGTGACT CAGTCTCACA CCGGCTCAGT TACGCTTGGT TTGAGTCGAG   
  
  
- CCAAGACCCG CCTTTCGGCC CAAAGTGCCA GTTCCTCCTC TCGCCACCCC ACCCCAAGCC CACCTACCCT   
  
  
- GCTTGAGAGT GGCAGCGTAG CCGGACCGCA AT

+     ERE

| Site Name | Organism | Position | Strand | Matrix score. | sequence | function |
| --- | --- | --- | --- | --- | --- | --- |
| ERE | Nicotiana glutinos | 839 | - | 8 | ATTTCATA |  |
| ERE | Nicotiana glutinos | 709 | + | 8 | ATTTTAAA |  |

>HU06G02537.1   
+ +Up\_Stream \_Len000CCTCGG TGTGGGATTC CGGGTTGATA AATAGGGACC AGCGAAATGT TCAAATAATT   
  
  
+ GGTGAAGGAG TCTCTGTTTG AATGCCTACT AATGCTTTTT TTTTTTTTTT GAATAATTTG TCAGAAAGTT   
  
  
+ AGGCTGCATT GGAAGGGGCG GCCTACGAAG GCTTTTCAGC AAATAAATAA CCAGCTTTCA TTTTGAAATT   
  
  
+ TTTTTTAAAA AATGAAACTC ATTTCACTTA AAAATTTACC ATAATATATA TATATATATA TATATATATA   
  
  
+ TATATATATA TATTTGATTT AACGTTAAGC AAACTTTTAA CTTATGTATC ATTTTACGAC CGTAAAAAAA   
  
  
+ AAAGAAATAT AACACAATAT GTAATAGTAT TTAAGTTTTT GATAAATAAT ATTTTAACAT AATTTGTGTG   
  
  
+ TAGTATGAAT TTAAATCTTA GTACTAATAT AATTATGTAT ACTTGGCTAT AAGATCTCAA TATTAGTATT   
  
  
+ TTATGATTCA ATGTAATGAT GATGCAACTG TGTGTAATTA GAAAGCCTTT GATTGCAGAT ATCTATCTTA   
  
  
+ TTGATACACA TCTACCAATT GAGCTTTTTC AAGATTTGAA GACGGTAGTC AAGTATCTTT GGGAGATTAC   
  
  
+ GTTAGAATTA CTTCATGATA AATTAGACAT ATTTTATACT TCTAATCTTA AATAAGTCTC CATCCTTTCA   
  
  
+ TCATATTTTA AAGAAATGTA CAAGTAAATG TCAATAGTTT GAAACGTTCC TTATCATAAA AGTCATACAT   
  
  
+ ATGTAGGACA TTTTCAAGTT TATGTAAACC ATATGATATA GTTCAAGATA ATTGTCAAAC AAGTTATGAA   
  
  
+ ATCTAAAGGT GTTTGAACGT AATTTCAAGC TAATCTTAGC AAGTTATAGT ATTATTTAAA CTACGACTAG   
  
  
+ ACACAACATT TTGTTCATTT ATATAACACT TAGAATGCAA AAAAATCGGT ATTACTAATA CTGTATCAAG   
  
  
+ AATTGAAGTG TTATGCATTA TACTGATAAG GGATAATATT TAAATTACTT TACTGTTATA GAAGCCAAAA   
  
  
+ ATCTAAACTT TCATACAGAC GTCGGACTAA ATTAGAAAAT TCTTTAACTT AATAGAAAGG ATGACATAAA   
  
  
+ TAAAAATAAA AACCTAAATA GCATAATAAC CATTCTACAC AAAAGTCAAT CTTCTAATAG AAAAAAGCCA   
  
  
+ TAAGTATTCA CTTATTTACC ATTTTATTCC TAATCTACTG TCTGTCTATT CACACCATAA TTCATGAACC   
  
  
+ AATGTGGAGT TTCTGTTTCA TTTCATGGGG TGATTTATGT CTGACAATGA TCCAACAAAC TGAGCCTCAC   
  
  
+ GCGCGGAACA CGAGAGTGGC TGTACTTATG GTGTGAAAGA GCTCTACAAG GTGAGATTGA GGAGGCTACG   
  
  
+ CGTTGGAGTT ATCACCACCG TTGGATTTGT TTTCTTTGGA GCCACGTTAT CACTCTTCAG ACGCCGATCC   
  
  
+ AACGGCCCTT ACTTCCATCT AGTCCCCACT CTCCAACTCT ACGCAATCAA CGCCTTCCGC ATTTAAATAT   
  
  
+ TTCCTTCTCT ATCGCCTACT TTCTCTGTCT CACAATTTTT GTCCGTATTT TGAATTTTCT CAAAATTATC   
  
  
+ ATATAATTTA TTTGCCTTCA ACTAAAGTAT TTATTAAATT AGTTAATGTA TTTAAATTGA ACTAAAAAAG   
  
  
+ AGAAAAAGAA AGAAAACAAC CCCACGAAAG CAAAACGACA GAGGCCAACT TCTCTCTCTA GAGTGTAGAG   
  
  
+ CCCCATCTCG GTTATAATTA TGAGAATCAA TTCCTTCTCA CAAGAGAAAC TCTCGCTCTC TCTCTCTCTC   
  
  
+ CATAAGACAT CACACCAATT GTATGAATTT TCTCTCTCCT GATTTCTGTT TTCCTTCTTG TTGTCCCCTT   
  
  
+ CTAATGCGTT AGTGCGGCCA AAACCACCAT AAACCATCTC TCTTTCAAGA AAGCAGCAAC AACAAAGAGA   
  
  
+ GACAGAGAGA GAGAGAGGGG GAGAAAGATC ACATCTTTTG GGTTTCCGGC GAATATGACG TCTGGGTTTT   
  
  
+ CGGGCGATTT CTTCTACGGC GGTGGAGGCG GTGGCGGAGG AGGTGTCCGG CCTTCTATCG GCGGTGTACA   
  
  
+ AGGCGGGGTT GTCCCATATG GGTTCCGGCA ACAACAACAA CAACAGCAAC AGCAGCAGCA GCAGCAACAG   
  
  
+ CTACATCTAC AGCTACAACA ACAGCGGGGG ATTTTCTCGG CGGATCAGAT CAGCGCTCAA CGAAGGGCGG   
  
  
+ ATTTGTTGAT GGGTAAGCGT TCTTTCGCTG ATTTTCAATC TCAACAACAG CAAATCAGTC TTTTGCAGCA   
  
  
+ GCAGCAGCAG CAACAACAAC AACAGCCCTT TGCGCTCTTG CAAAAACAAC AGCAGTTTCA ACAACAATTA   
  
  
+ CAACAACAAC AGCCACTTTC TCTCCTCCTT CAACAACAGT ACTTGATGAG ATCCGTAAAA CAGAGAACCA   
  
  
+ ACAATTTCGC AGCATCTCCG ATTTCTCCTC TTTCTTCTTC CATTGATCTT TCTTCCGCTA ATAATTCCCG   
  
  
+ GTTCGGTTTG CCGGTTTACC AACAACAACT CCGGTCTCAA CAACAACCAC CTATCTCTCT CCTCGCCCAC   
  
  
+ AACAACAACC AATTCAACCT CTCAAATAAT AACAATTCTT CTTTAATCCA AAACTCCACC AACACTCTTC   
  
  
+ TTGCTCCCGC AGCCAGCAAT AACACCCTAA TGAAAAATTC GGGCTTTTCC TCATCTGGGT TTTCTAATTC   
  
  
+ GGGTCCAAAC CTGGTCCAAC CGAAGCAGGA AATCGGGTAC TCGAATATGG GTACCAATAT GGGTATGAAT   
  
  
+ CTGGTCCAGC CCAAACCCGA CCCTGAGGAT CACGGAATAA TGAACACCCT TCAAGAATTG GAGAAGGAGC   
  
  
+ TTTTGAATGA CGATGAGGAA AACCAGCCGG AAAACGACGC CGTTTCAGTC ATCACCAACA GCGAGTGGTC   
  
  
+ CGAGACCTTC CAGAATCTGA TGAGTCCCGA CCCGAGTTCG AGCCCGAATG CCACCAACAC CCACCCGGGT   
  
  
+ TCGGGTTCTG CTAGTACCAG CGGCAGCAAC AACACTACAA CCACCCCTGC AAACAACAAT AACATGATAT   
  
  
+ CTACCTCGCC GACGTCGTCC ACGTCATCCT GCTGCTCCAC GTCGGCATCG GCGTCTCCGC CGGCGGCGGT   
  
  
+ GGCGACCCCG CAGGTACCAG CCAAGCAGCT GATCTCGGAA GCAGCGGCGG CGATTTCGGA CGGAAAGATT   
  
  
+ GAATCCGCCA TGGAAGTGAT GAACCGGCTG TCCCAGGTTG CCAATTCTAG GGGGAGCTCG GAACAAAAGC   
  
  
+ TGGCTTTTTA CATGGGCCAC GCGCTTCGGT CGCGCGTGAG TCCCACCGAA CACGCTCCCC CTATTGTGGA   
  
  
+ GCTTTTCCAA AAGGAGCATA TGGCGGCCAT GTATTCATTG TACGAGCTCT CTCATTGTTT CAAATTTGGT   
  
  
+ CTTTTGGCTG CCAATTTGGT GATGTTGGAG GCCATTACTT CCGCCCTTCA TGAGGGTCTG AAAATTCATG   
  
  
+ TTGTGGATTT TGATATTGGG CATGGTGGTC AGTACATCAA CCTCCTACGC GCCTTGGGGG AGCGTAGGGC   
  
  
+ TCAGTCGGGG AGCGCGGTGT CGAATGAGGT GAGGATCACT GTGGTTGAGA CCGAGGTCGG TAATGGTGGT   
  
  
+ GCGGCGGGTG CGGAGGCTGT TAAGGATGGC CTGGTGAAAT TGGCTGAGAA GGTGGGGATT AGTTTGAAGT   
  
  
+ TTGAGGCTTT GACTCGGAAG ATCTCCGAGT TAACCCGGGA GTCACTGGGG TGTGAGGAGG GGGAGGCCGT   
  
  
+ GGTGGTGAAT TTTGCGTTCA AACTTTACCG GGTACCGGAT GAGAGCGTGT CGATGGAGAA TCTGAGGGAC   
  
  
+ GAGCTCCTGC GCCGGGTGAA GGCGCTAAGG CCACGCGCCG TCACATTGGT GGAGCAGGAC TTAAATGCGA   
  
  
+ ATACGGCACC GTTCATGACG CGCGTGAGTG GCGCTTGTGA GTACTATAGA GCATTGTTCG ACTCGCTCGA   
  
  
+ CTCGATGGTG CCGAGGGATA ACCCGGACCG GGTCCGGGTC GAGGAGGCCT TGGGGCGGAA GATGGCAAAC   
  
  
+ TCGGTGGCTT GCGAGGGCCG GGAACGGGTT GAGCGGGCGG AGGTGTTTGG GAAGTGGCGG GCGAGGATGA   
  
  
+ GTATGGCAGG GTTCGAGTTG AAGGCACTGA GTCAGAGTGT GGCCGAGTCA ATGCGAACCA AACTCAGCTC   
  
  
+ GGTTCTGGGC GGAAAGCCGG GTTTCACGGT CAAGGAGGAG AGCGGTGGGG TGGGGTTCGG GTGGATGGGA   
  
  
+ CGAACTCTCA CCGTCGCATC GGCCTGGCGT TA  

- +Up\_Stream \_Len000GGAGCC ACACCCTAAG GCCCAACTAT TTATCCCTGG TCGCTTTACA AGTTTATTAA   
  
  
- CCACTTCCTC AGAGACAAAC TTACGGATGA TTACGAAAAA AAAAAAAAAA CTTATTAAAC AGTCTTTCAA   
  
  
- TCCGACGTAA CCTTCCCCGC CGGATGCTTC CGAAAAGTCG TTTATTTATT GGTCGAAAGT AAAACTTTAA   
  
  
- AAAAAATTTT TTACTTTGAG TAAAGTGAAT TTTTAAATGG TATTATATAT ATATATATAT ATATATATAT   
  
  
- ATATATATAT ATAAACTAAA TTGCAATTCG TTTGAAAATT GAATACATAG TAAAATGCTG GCATTTTTTT   
  
  
- TTTCTTTATA TTGTGTTATA CATTATCATA AATTCAAAAA CTATTTATTA TAAAATTGTA TTAAACACAC   
  
  
- ATCATACTTA AATTTAGAAT CATGATTATA TTAATACATA TGAACCGATA TTCTAGAGTT ATAATCATAA   
  
  
- AATACTAAGT TACATTACTA CTACGTTGAC ACACATTAAT CTTTCGGAAA CTAACGTCTA TAGATAGAAT   
  
  
- AACTATGTGT AGATGGTTAA CTCGAAAAAG TTCTAAACTT CTGCCATCAG TTCATAGAAA CCCTCTAATG   
  
  
- CAATCTTAAT GAAGTACTAT TTAATCTGTA TAAAATATGA AGATTAGAAT TTATTCAGAG GTAGGAAAGT   
  
  
- AGTATAAAAT TTCTTTACAT GTTCATTTAC AGTTATCAAA CTTTGCAAGG AATAGTATTT TCAGTATGTA   
  
  
- TACATCCTGT AAAAGTTCAA ATACATTTGG TATACTATAT CAAGTTCTAT TAACAGTTTG TTCAATACTT   
  
  
- TAGATTTCCA CAAACTTGCA TTAAAGTTCG ATTAGAATCG TTCAATATCA TAATAAATTT GATGCTGATC   
  
  
- TGTGTTGTAA AACAAGTAAA TATATTGTGA ATCTTACGTT TTTTTAGCCA TAATGATTAT GACATAGTTC   
  
  
- TTAACTTCAC AATACGTAAT ATGACTATTC CCTATTATAA ATTTAATGAA ATGACAATAT CTTCGGTTTT   
  
  
- TAGATTTGAA AGTATGTCTG CAGCCTGATT TAATCTTTTA AGAAATTGAA TTATCTTTCC TACTGTATTT   
  
  
- ATTTTTATTT TTGGATTTAT CGTATTATTG GTAAGATGTG TTTTCAGTTA GAAGATTATC TTTTTTCGGT   
  
  
- ATTCATAAGT GAATAAATGG TAAAATAAGG ATTAGATGAC AGACAGATAA GTGTGGTATT AAGTACTTGG   
  
  
- TTACACCTCA AAGACAAAGT AAAGTACCCC ACTAAATACA GACTGTTACT AGGTTGTTTG ACTCGGAGTG   
  
  
- CGCGCCTTGT GCTCTCACCG ACATGAATAC CACACTTTCT CGAGATGTTC CACTCTAACT CCTCCGATGC   
  
  
- GCAACCTCAA TAGTGGTGGC AACCTAAACA AAAGAAACCT CGGTGCAATA GTGAGAAGTC TGCGGCTAGG   
  
  
- TTGCCGGGAA TGAAGGTAGA TCAGGGGTGA GAGGTTGAGA TGCGTTAGTT GCGGAAGGCG TAAATTTATA   
  
  
- AAGGAAGAGA TAGCGGATGA AAGAGACAGA GTGTTAAAAA CAGGCATAAA ACTTAAAAGA GTTTTAATAG   
  
  
- TATATTAAAT AAACGGAAGT TGATTTCATA AATAATTTAA TCAATTACAT AAATTTAACT TGATTTTTTC   
  
  
- TCTTTTTCTT TCTTTTGTTG GGGTGCTTTC GTTTTGCTGT CTCCGGTTGA AGAGAGAGAT CTCACATCTC   
  
  
- GGGGTAGAGC CAATATTAAT ACTCTTAGTT AAGGAAGAGT GTTCTCTTTG AGAGCGAGAG AGAGAGAGAG   
  
  
- GTATTCTGTA GTGTGGTTAA CATACTTAAA AGAGAGAGGA CTAAAGACAA AAGGAAGAAC AACAGGGGAA   
  
  
- GATTACGCAA TCACGCCGGT TTTGGTGGTA TTTGGTAGAG AGAAAGTTCT TTCGTCGTTG TTGTTTCTCT   
  
  
- CTGTCTCTCT CTCTCTCCCC CTCTTTCTAG TGTAGAAAAC CCAAAGGCCG CTTATACTGC AGACCCAAAA   
  
  
- GCCCGCTAAA GAAGATGCCG CCACCTCCGC CACCGCCTCC TCCACAGGCC GGAAGATAGC CGCCACATGT   
  
  
- TCCGCCCCAA CAGGGTATAC CCAAGGCCGT TGTTGTTGTT GTTGTCGTTG TCGTCGTCGT CGTCGTTGTC   
  
  
- GATGTAGATG TCGATGTTGT TGTCGCCCCC TAAAAGAGCC GCCTAGTCTA GTCGCGAGTT GCTTCCCGCC   
  
  
- TAAACAACTA CCCATTCGCA AGAAAGCGAC TAAAAGTTAG AGTTGTTGTC GTTTAGTCAG AAAACGTCGT   
  
  
- CGTCGTCGTC GTTGTTGTTG TTGTCGGGAA ACGCGAGAAC GTTTTTGTTG TCGTCAAAGT TGTTGTTAAT   
  
  
- GTTGTTGTTG TCGGTGAAAG AGAGGAGGAA GTTGTTGTCA TGAACTACTC TAGGCATTTT GTCTCTTGGT   
  
  
- TGTTAAAGCG TCGTAGAGGC TAAAGAGGAG AAAGAAGAAG GTAACTAGAA AGAAGGCGAT TATTAAGGGC   
  
  
- CAAGCCAAAC GGCCAAATGG TTGTTGTTGA GGCCAGAGTT GTTGTTGGTG GATAGAGAGA GGAGCGGGTG   
  
  
- TTGTTGTTGG TTAAGTTGGA GAGTTTATTA TTGTTAAGAA GAAATTAGGT TTTGAGGTGG TTGTGAGAAG   
  
  
- AACGAGGGCG TCGGTCGTTA TTGTGGGATT ACTTTTTAAG CCCGAAAAGG AGTAGACCCA AAAGATTAAG   
  
  
- CCCAGGTTTG GACCAGGTTG GCTTCGTCCT TTAGCCCATG AGCTTATACC CATGGTTATA CCCATACTTA   
  
  
- GACCAGGTCG GGTTTGGGCT GGGACTCCTA GTGCCTTATT ACTTGTGGGA AGTTCTTAAC CTCTTCCTCG   
  
  
- AAAACTTACT GCTACTCCTT TTGGTCGGCC TTTTGCTGCG GCAAAGTCAG TAGTGGTTGT CGCTCACCAG   
  
  
- GCTCTGGAAG GTCTTAGACT ACTCAGGGCT GGGCTCAAGC TCGGGCTTAC GGTGGTTGTG GGTGGGCCCA   
  
  
- AGCCCAAGAC GATCATGGTC GCCGTCGTTG TTGTGATGTT GGTGGGGACG TTTGTTGTTA TTGTACTATA   
  
  
- GATGGAGCGG CTGCAGCAGG TGCAGTAGGA CGACGAGGTG CAGCCGTAGC CGCAGAGGCG GCCGCCGCCA   
  
  
- CCGCTGGGGC GTCCATGGTC GGTTCGTCGA CTAGAGCCTT CGTCGCCGCC GCTAAAGCCT GCCTTTCTAA   
  
  
- CTTAGGCGGT ACCTTCACTA CTTGGCCGAC AGGGTCCAAC GGTTAAGATC CCCCTCGAGC CTTGTTTTCG   
  
  
- ACCGAAAAAT GTACCCGGTG CGCGAAGCCA GCGCGCACTC AGGGTGGCTT GTGCGAGGGG GATAACACCT   
  
  
- CGAAAAGGTT TTCCTCGTAT ACCGCCGGTA CATAAGTAAC ATGCTCGAGA GAGTAACAAA GTTTAAACCA   
  
  
- GAAAACCGAC GGTTAAACCA CTACAACCTC CGGTAATGAA GGCGGGAAGT ACTCCCAGAC TTTTAAGTAC   
  
  
- AACACCTAAA ACTATAACCC GTACCACCAG TCATGTAGTT GGAGGATGCG CGGAACCCCC TCGCATCCCG   
  
  
- AGTCAGCCCC TCGCGCCACA GCTTACTCCA CTCCTAGTGA CACCAACTCT GGCTCCAGCC ATTACCACCA   
  
  
- CGCCGCCCAC GCCTCCGACA ATTCCTACCG GACCACTTTA ACCGACTCTT CCACCCCTAA TCAAACTTCA   
  
  
- AACTCCGAAA CTGAGCCTTC TAGAGGCTCA ATTGGGCCCT CAGTGACCCC ACACTCCTCC CCCTCCGGCA   
  
  
- CCACCACTTA AAACGCAAGT TTGAAATGGC CCATGGCCTA CTCTCGCACA GCTACCTCTT AGACTCCCTG   
  
  
- CTCGAGGACG CGGCCCACTT CCGCGATTCC GGTGCGCGGC AGTGTAACCA CCTCGTCCTG AATTTACGCT   
  
  
- TATGCCGTGG CAAGTACTGC GCGCACTCAC CGCGAACACT CATGATATCT CGTAACAAGC TGAGCGAGCT   
  
  
- GAGCTACCAC GGCTCCCTAT TGGGCCTGGC CCAGGCCCAG CTCCTCCGGA ACCCCGCCTT CTACCGTTTG   
  
  
- AGCCACCGAA CGCTCCCGGC CCTTGCCCAA CTCGCCCGCC TCCACAAACC CTTCACCGCC CGCTCCTACT   
  
  
- CATACCGTCC CAAGCTCAAC TTCCGTGACT CAGTCTCACA CCGGCTCAGT TACGCTTGGT TTGAGTCGAG   
  
  
- CCAAGACCCG CCTTTCGGCC CAAAGTGCCA GTTCCTCCTC TCGCCACCCC ACCCCAAGCC CACCTACCCT   
  
  
- GCTTGAGAGT GGCAGCGTAG CCGGACCGCA AT

+     G-Box

| Site Name | Organism | Position | Strand | Matrix score. | sequence | function |
| --- | --- | --- | --- | --- | --- | --- |
| G-Box | Pisum sativum | 1447 | + | 6 | CACGTT | cis-acting regulatory element involved in light responsiveness |

>HU06G02537.1   
+ +Up\_Stream \_Len000CCTCGG TGTGGGATTC CGGGTTGATA AATAGGGACC AGCGAAATGT TCAAATAATT   
  
  
+ GGTGAAGGAG TCTCTGTTTG AATGCCTACT AATGCTTTTT TTTTTTTTTT GAATAATTTG TCAGAAAGTT   
  
  
+ AGGCTGCATT GGAAGGGGCG GCCTACGAAG GCTTTTCAGC AAATAAATAA CCAGCTTTCA TTTTGAAATT   
  
  
+ TTTTTTAAAA AATGAAACTC ATTTCACTTA AAAATTTACC ATAATATATA TATATATATA TATATATATA   
  
  
+ TATATATATA TATTTGATTT AACGTTAAGC AAACTTTTAA CTTATGTATC ATTTTACGAC CGTAAAAAAA   
  
  
+ AAAGAAATAT AACACAATAT GTAATAGTAT TTAAGTTTTT GATAAATAAT ATTTTAACAT AATTTGTGTG   
  
  
+ TAGTATGAAT TTAAATCTTA GTACTAATAT AATTATGTAT ACTTGGCTAT AAGATCTCAA TATTAGTATT   
  
  
+ TTATGATTCA ATGTAATGAT GATGCAACTG TGTGTAATTA GAAAGCCTTT GATTGCAGAT ATCTATCTTA   
  
  
+ TTGATACACA TCTACCAATT GAGCTTTTTC AAGATTTGAA GACGGTAGTC AAGTATCTTT GGGAGATTAC   
  
  
+ GTTAGAATTA CTTCATGATA AATTAGACAT ATTTTATACT TCTAATCTTA AATAAGTCTC CATCCTTTCA   
  
  
+ TCATATTTTA AAGAAATGTA CAAGTAAATG TCAATAGTTT GAAACGTTCC TTATCATAAA AGTCATACAT   
  
  
+ ATGTAGGACA TTTTCAAGTT TATGTAAACC ATATGATATA GTTCAAGATA ATTGTCAAAC AAGTTATGAA   
  
  
+ ATCTAAAGGT GTTTGAACGT AATTTCAAGC TAATCTTAGC AAGTTATAGT ATTATTTAAA CTACGACTAG   
  
  
+ ACACAACATT TTGTTCATTT ATATAACACT TAGAATGCAA AAAAATCGGT ATTACTAATA CTGTATCAAG   
  
  
+ AATTGAAGTG TTATGCATTA TACTGATAAG GGATAATATT TAAATTACTT TACTGTTATA GAAGCCAAAA   
  
  
+ ATCTAAACTT TCATACAGAC GTCGGACTAA ATTAGAAAAT TCTTTAACTT AATAGAAAGG ATGACATAAA   
  
  
+ TAAAAATAAA AACCTAAATA GCATAATAAC CATTCTACAC AAAAGTCAAT CTTCTAATAG AAAAAAGCCA   
  
  
+ TAAGTATTCA CTTATTTACC ATTTTATTCC TAATCTACTG TCTGTCTATT CACACCATAA TTCATGAACC   
  
  
+ AATGTGGAGT TTCTGTTTCA TTTCATGGGG TGATTTATGT CTGACAATGA TCCAACAAAC TGAGCCTCAC   
  
  
+ GCGCGGAACA CGAGAGTGGC TGTACTTATG GTGTGAAAGA GCTCTACAAG GTGAGATTGA GGAGGCTACG   
  
  
+ CGTTGGAGTT ATCACCACCG TTGGATTTGT TTTCTTTGGA GCCACGTTAT CACTCTTCAG ACGCCGATCC   
  
  
+ AACGGCCCTT ACTTCCATCT AGTCCCCACT CTCCAACTCT ACGCAATCAA CGCCTTCCGC ATTTAAATAT   
  
  
+ TTCCTTCTCT ATCGCCTACT TTCTCTGTCT CACAATTTTT GTCCGTATTT TGAATTTTCT CAAAATTATC   
  
  
+ ATATAATTTA TTTGCCTTCA ACTAAAGTAT TTATTAAATT AGTTAATGTA TTTAAATTGA ACTAAAAAAG   
  
  
+ AGAAAAAGAA AGAAAACAAC CCCACGAAAG CAAAACGACA GAGGCCAACT TCTCTCTCTA GAGTGTAGAG   
  
  
+ CCCCATCTCG GTTATAATTA TGAGAATCAA TTCCTTCTCA CAAGAGAAAC TCTCGCTCTC TCTCTCTCTC   
  
  
+ CATAAGACAT CACACCAATT GTATGAATTT TCTCTCTCCT GATTTCTGTT TTCCTTCTTG TTGTCCCCTT   
  
  
+ CTAATGCGTT AGTGCGGCCA AAACCACCAT AAACCATCTC TCTTTCAAGA AAGCAGCAAC AACAAAGAGA   
  
  
+ GACAGAGAGA GAGAGAGGGG GAGAAAGATC ACATCTTTTG GGTTTCCGGC GAATATGACG TCTGGGTTTT   
  
  
+ CGGGCGATTT CTTCTACGGC GGTGGAGGCG GTGGCGGAGG AGGTGTCCGG CCTTCTATCG GCGGTGTACA   
  
  
+ AGGCGGGGTT GTCCCATATG GGTTCCGGCA ACAACAACAA CAACAGCAAC AGCAGCAGCA GCAGCAACAG   
  
  
+ CTACATCTAC AGCTACAACA ACAGCGGGGG ATTTTCTCGG CGGATCAGAT CAGCGCTCAA CGAAGGGCGG   
  
  
+ ATTTGTTGAT GGGTAAGCGT TCTTTCGCTG ATTTTCAATC TCAACAACAG CAAATCAGTC TTTTGCAGCA   
  
  
+ GCAGCAGCAG CAACAACAAC AACAGCCCTT TGCGCTCTTG CAAAAACAAC AGCAGTTTCA ACAACAATTA   
  
  
+ CAACAACAAC AGCCACTTTC TCTCCTCCTT CAACAACAGT ACTTGATGAG ATCCGTAAAA CAGAGAACCA   
  
  
+ ACAATTTCGC AGCATCTCCG ATTTCTCCTC TTTCTTCTTC CATTGATCTT TCTTCCGCTA ATAATTCCCG   
  
  
+ GTTCGGTTTG CCGGTTTACC AACAACAACT CCGGTCTCAA CAACAACCAC CTATCTCTCT CCTCGCCCAC   
  
  
+ AACAACAACC AATTCAACCT CTCAAATAAT AACAATTCTT CTTTAATCCA AAACTCCACC AACACTCTTC   
  
  
+ TTGCTCCCGC AGCCAGCAAT AACACCCTAA TGAAAAATTC GGGCTTTTCC TCATCTGGGT TTTCTAATTC   
  
  
+ GGGTCCAAAC CTGGTCCAAC CGAAGCAGGA AATCGGGTAC TCGAATATGG GTACCAATAT GGGTATGAAT   
  
  
+ CTGGTCCAGC CCAAACCCGA CCCTGAGGAT CACGGAATAA TGAACACCCT TCAAGAATTG GAGAAGGAGC   
  
  
+ TTTTGAATGA CGATGAGGAA AACCAGCCGG AAAACGACGC CGTTTCAGTC ATCACCAACA GCGAGTGGTC   
  
  
+ CGAGACCTTC CAGAATCTGA TGAGTCCCGA CCCGAGTTCG AGCCCGAATG CCACCAACAC CCACCCGGGT   
  
  
+ TCGGGTTCTG CTAGTACCAG CGGCAGCAAC AACACTACAA CCACCCCTGC AAACAACAAT AACATGATAT   
  
  
+ CTACCTCGCC GACGTCGTCC ACGTCATCCT GCTGCTCCAC GTCGGCATCG GCGTCTCCGC CGGCGGCGGT   
  
  
+ GGCGACCCCG CAGGTACCAG CCAAGCAGCT GATCTCGGAA GCAGCGGCGG CGATTTCGGA CGGAAAGATT   
  
  
+ GAATCCGCCA TGGAAGTGAT GAACCGGCTG TCCCAGGTTG CCAATTCTAG GGGGAGCTCG GAACAAAAGC   
  
  
+ TGGCTTTTTA CATGGGCCAC GCGCTTCGGT CGCGCGTGAG TCCCACCGAA CACGCTCCCC CTATTGTGGA   
  
  
+ GCTTTTCCAA AAGGAGCATA TGGCGGCCAT GTATTCATTG TACGAGCTCT CTCATTGTTT CAAATTTGGT   
  
  
+ CTTTTGGCTG CCAATTTGGT GATGTTGGAG GCCATTACTT CCGCCCTTCA TGAGGGTCTG AAAATTCATG   
  
  
+ TTGTGGATTT TGATATTGGG CATGGTGGTC AGTACATCAA CCTCCTACGC GCCTTGGGGG AGCGTAGGGC   
  
  
+ TCAGTCGGGG AGCGCGGTGT CGAATGAGGT GAGGATCACT GTGGTTGAGA CCGAGGTCGG TAATGGTGGT   
  
  
+ GCGGCGGGTG CGGAGGCTGT TAAGGATGGC CTGGTGAAAT TGGCTGAGAA GGTGGGGATT AGTTTGAAGT   
  
  
+ TTGAGGCTTT GACTCGGAAG ATCTCCGAGT TAACCCGGGA GTCACTGGGG TGTGAGGAGG GGGAGGCCGT   
  
  
+ GGTGGTGAAT TTTGCGTTCA AACTTTACCG GGTACCGGAT GAGAGCGTGT CGATGGAGAA TCTGAGGGAC   
  
  
+ GAGCTCCTGC GCCGGGTGAA GGCGCTAAGG CCACGCGCCG TCACATTGGT GGAGCAGGAC TTAAATGCGA   
  
  
+ ATACGGCACC GTTCATGACG CGCGTGAGTG GCGCTTGTGA GTACTATAGA GCATTGTTCG ACTCGCTCGA   
  
  
+ CTCGATGGTG CCGAGGGATA ACCCGGACCG GGTCCGGGTC GAGGAGGCCT TGGGGCGGAA GATGGCAAAC   
  
  
+ TCGGTGGCTT GCGAGGGCCG GGAACGGGTT GAGCGGGCGG AGGTGTTTGG GAAGTGGCGG GCGAGGATGA   
  
  
+ GTATGGCAGG GTTCGAGTTG AAGGCACTGA GTCAGAGTGT GGCCGAGTCA ATGCGAACCA AACTCAGCTC   
  
  
+ GGTTCTGGGC GGAAAGCCGG GTTTCACGGT CAAGGAGGAG AGCGGTGGGG TGGGGTTCGG GTGGATGGGA   
  
  
+ CGAACTCTCA CCGTCGCATC GGCCTGGCGT TA  

- +Up\_Stream \_Len000GGAGCC ACACCCTAAG GCCCAACTAT TTATCCCTGG TCGCTTTACA AGTTTATTAA   
  
  
- CCACTTCCTC AGAGACAAAC TTACGGATGA TTACGAAAAA AAAAAAAAAA CTTATTAAAC AGTCTTTCAA   
  
  
- TCCGACGTAA CCTTCCCCGC CGGATGCTTC CGAAAAGTCG TTTATTTATT GGTCGAAAGT AAAACTTTAA   
  
  
- AAAAAATTTT TTACTTTGAG TAAAGTGAAT TTTTAAATGG TATTATATAT ATATATATAT ATATATATAT   
  
  
- ATATATATAT ATAAACTAAA TTGCAATTCG TTTGAAAATT GAATACATAG TAAAATGCTG GCATTTTTTT   
  
  
- TTTCTTTATA TTGTGTTATA CATTATCATA AATTCAAAAA CTATTTATTA TAAAATTGTA TTAAACACAC   
  
  
- ATCATACTTA AATTTAGAAT CATGATTATA TTAATACATA TGAACCGATA TTCTAGAGTT ATAATCATAA   
  
  
- AATACTAAGT TACATTACTA CTACGTTGAC ACACATTAAT CTTTCGGAAA CTAACGTCTA TAGATAGAAT   
  
  
- AACTATGTGT AGATGGTTAA CTCGAAAAAG TTCTAAACTT CTGCCATCAG TTCATAGAAA CCCTCTAATG   
  
  
- CAATCTTAAT GAAGTACTAT TTAATCTGTA TAAAATATGA AGATTAGAAT TTATTCAGAG GTAGGAAAGT   
  
  
- AGTATAAAAT TTCTTTACAT GTTCATTTAC AGTTATCAAA CTTTGCAAGG AATAGTATTT TCAGTATGTA   
  
  
- TACATCCTGT AAAAGTTCAA ATACATTTGG TATACTATAT CAAGTTCTAT TAACAGTTTG TTCAATACTT   
  
  
- TAGATTTCCA CAAACTTGCA TTAAAGTTCG ATTAGAATCG TTCAATATCA TAATAAATTT GATGCTGATC   
  
  
- TGTGTTGTAA AACAAGTAAA TATATTGTGA ATCTTACGTT TTTTTAGCCA TAATGATTAT GACATAGTTC   
  
  
- TTAACTTCAC AATACGTAAT ATGACTATTC CCTATTATAA ATTTAATGAA ATGACAATAT CTTCGGTTTT   
  
  
- TAGATTTGAA AGTATGTCTG CAGCCTGATT TAATCTTTTA AGAAATTGAA TTATCTTTCC TACTGTATTT   
  
  
- ATTTTTATTT TTGGATTTAT CGTATTATTG GTAAGATGTG TTTTCAGTTA GAAGATTATC TTTTTTCGGT   
  
  
- ATTCATAAGT GAATAAATGG TAAAATAAGG ATTAGATGAC AGACAGATAA GTGTGGTATT AAGTACTTGG   
  
  
- TTACACCTCA AAGACAAAGT AAAGTACCCC ACTAAATACA GACTGTTACT AGGTTGTTTG ACTCGGAGTG   
  
  
- CGCGCCTTGT GCTCTCACCG ACATGAATAC CACACTTTCT CGAGATGTTC CACTCTAACT CCTCCGATGC   
  
  
- GCAACCTCAA TAGTGGTGGC AACCTAAACA AAAGAAACCT CGGTGCAATA GTGAGAAGTC TGCGGCTAGG   
  
  
- TTGCCGGGAA TGAAGGTAGA TCAGGGGTGA GAGGTTGAGA TGCGTTAGTT GCGGAAGGCG TAAATTTATA   
  
  
- AAGGAAGAGA TAGCGGATGA AAGAGACAGA GTGTTAAAAA CAGGCATAAA ACTTAAAAGA GTTTTAATAG   
  
  
- TATATTAAAT AAACGGAAGT TGATTTCATA AATAATTTAA TCAATTACAT AAATTTAACT TGATTTTTTC   
  
  
- TCTTTTTCTT TCTTTTGTTG GGGTGCTTTC GTTTTGCTGT CTCCGGTTGA AGAGAGAGAT CTCACATCTC   
  
  
- GGGGTAGAGC CAATATTAAT ACTCTTAGTT AAGGAAGAGT GTTCTCTTTG AGAGCGAGAG AGAGAGAGAG   
  
  
- GTATTCTGTA GTGTGGTTAA CATACTTAAA AGAGAGAGGA CTAAAGACAA AAGGAAGAAC AACAGGGGAA   
  
  
- GATTACGCAA TCACGCCGGT TTTGGTGGTA TTTGGTAGAG AGAAAGTTCT TTCGTCGTTG TTGTTTCTCT   
  
  
- CTGTCTCTCT CTCTCTCCCC CTCTTTCTAG TGTAGAAAAC CCAAAGGCCG CTTATACTGC AGACCCAAAA   
  
  
- GCCCGCTAAA GAAGATGCCG CCACCTCCGC CACCGCCTCC TCCACAGGCC GGAAGATAGC CGCCACATGT   
  
  
- TCCGCCCCAA CAGGGTATAC CCAAGGCCGT TGTTGTTGTT GTTGTCGTTG TCGTCGTCGT CGTCGTTGTC   
  
  
- GATGTAGATG TCGATGTTGT TGTCGCCCCC TAAAAGAGCC GCCTAGTCTA GTCGCGAGTT GCTTCCCGCC   
  
  
- TAAACAACTA CCCATTCGCA AGAAAGCGAC TAAAAGTTAG AGTTGTTGTC GTTTAGTCAG AAAACGTCGT   
  
  
- CGTCGTCGTC GTTGTTGTTG TTGTCGGGAA ACGCGAGAAC GTTTTTGTTG TCGTCAAAGT TGTTGTTAAT   
  
  
- GTTGTTGTTG TCGGTGAAAG AGAGGAGGAA GTTGTTGTCA TGAACTACTC TAGGCATTTT GTCTCTTGGT   
  
  
- TGTTAAAGCG TCGTAGAGGC TAAAGAGGAG AAAGAAGAAG GTAACTAGAA AGAAGGCGAT TATTAAGGGC   
  
  
- CAAGCCAAAC GGCCAAATGG TTGTTGTTGA GGCCAGAGTT GTTGTTGGTG GATAGAGAGA GGAGCGGGTG   
  
  
- TTGTTGTTGG TTAAGTTGGA GAGTTTATTA TTGTTAAGAA GAAATTAGGT TTTGAGGTGG TTGTGAGAAG   
  
  
- AACGAGGGCG TCGGTCGTTA TTGTGGGATT ACTTTTTAAG CCCGAAAAGG AGTAGACCCA AAAGATTAAG   
  
  
- CCCAGGTTTG GACCAGGTTG GCTTCGTCCT TTAGCCCATG AGCTTATACC CATGGTTATA CCCATACTTA   
  
  
- GACCAGGTCG GGTTTGGGCT GGGACTCCTA GTGCCTTATT ACTTGTGGGA AGTTCTTAAC CTCTTCCTCG   
  
  
- AAAACTTACT GCTACTCCTT TTGGTCGGCC TTTTGCTGCG GCAAAGTCAG TAGTGGTTGT CGCTCACCAG   
  
  
- GCTCTGGAAG GTCTTAGACT ACTCAGGGCT GGGCTCAAGC TCGGGCTTAC GGTGGTTGTG GGTGGGCCCA   
  
  
- AGCCCAAGAC GATCATGGTC GCCGTCGTTG TTGTGATGTT GGTGGGGACG TTTGTTGTTA TTGTACTATA   
  
  
- GATGGAGCGG CTGCAGCAGG TGCAGTAGGA CGACGAGGTG CAGCCGTAGC CGCAGAGGCG GCCGCCGCCA   
  
  
- CCGCTGGGGC GTCCATGGTC GGTTCGTCGA CTAGAGCCTT CGTCGCCGCC GCTAAAGCCT GCCTTTCTAA   
  
  
- CTTAGGCGGT ACCTTCACTA CTTGGCCGAC AGGGTCCAAC GGTTAAGATC CCCCTCGAGC CTTGTTTTCG   
  
  
- ACCGAAAAAT GTACCCGGTG CGCGAAGCCA GCGCGCACTC AGGGTGGCTT GTGCGAGGGG GATAACACCT   
  
  
- CGAAAAGGTT TTCCTCGTAT ACCGCCGGTA CATAAGTAAC ATGCTCGAGA GAGTAACAAA GTTTAAACCA   
  
  
- GAAAACCGAC GGTTAAACCA CTACAACCTC CGGTAATGAA GGCGGGAAGT ACTCCCAGAC TTTTAAGTAC   
  
  
- AACACCTAAA ACTATAACCC GTACCACCAG TCATGTAGTT GGAGGATGCG CGGAACCCCC TCGCATCCCG   
  
  
- AGTCAGCCCC TCGCGCCACA GCTTACTCCA CTCCTAGTGA CACCAACTCT GGCTCCAGCC ATTACCACCA   
  
  
- CGCCGCCCAC GCCTCCGACA ATTCCTACCG GACCACTTTA ACCGACTCTT CCACCCCTAA TCAAACTTCA   
  
  
- AACTCCGAAA CTGAGCCTTC TAGAGGCTCA ATTGGGCCCT CAGTGACCCC ACACTCCTCC CCCTCCGGCA   
  
  
- CCACCACTTA AAACGCAAGT TTGAAATGGC CCATGGCCTA CTCTCGCACA GCTACCTCTT AGACTCCCTG   
  
  
- CTCGAGGACG CGGCCCACTT CCGCGATTCC GGTGCGCGGC AGTGTAACCA CCTCGTCCTG AATTTACGCT   
  
  
- TATGCCGTGG CAAGTACTGC GCGCACTCAC CGCGAACACT CATGATATCT CGTAACAAGC TGAGCGAGCT   
  
  
- GAGCTACCAC GGCTCCCTAT TGGGCCTGGC CCAGGCCCAG CTCCTCCGGA ACCCCGCCTT CTACCGTTTG   
  
  
- AGCCACCGAA CGCTCCCGGC CCTTGCCCAA CTCGCCCGCC TCCACAAACC CTTCACCGCC CGCTCCTACT   
  
  
- CATACCGTCC CAAGCTCAAC TTCCGTGACT CAGTCTCACA CCGGCTCAGT TACGCTTGGT TTGAGTCGAG   
  
  
- CCAAGACCCG CCTTTCGGCC CAAAGTGCCA GTTCCTCCTC TCGCCACCCC ACCCCAAGCC CACCTACCCT   
  
  
- GCTTGAGAGT GGCAGCGTAG CCGGACCGCA AT

+     G-box

| Site Name | Organism | Position | Strand | Matrix score. | sequence | function |
| --- | --- | --- | --- | --- | --- | --- |
| G-box | Zea mays | 3122 | + | 6 | CACGTC | cis-acting regulatory element involved in light responsiveness |
| G-box | Zea mays | 3104 | + | 6 | CACGTC | cis-acting regulatory element involved in light responsiveness |
| G-box | Brassica oleracea | 938 | + | 9 | TAACACGTAG | cis-acting regulatory element involved in light responsiveness |

>HU06G02537.1   
+ +Up\_Stream \_Len000CCTCGG TGTGGGATTC CGGGTTGATA AATAGGGACC AGCGAAATGT TCAAATAATT   
  
  
+ GGTGAAGGAG TCTCTGTTTG AATGCCTACT AATGCTTTTT TTTTTTTTTT GAATAATTTG TCAGAAAGTT   
  
  
+ AGGCTGCATT GGAAGGGGCG GCCTACGAAG GCTTTTCAGC AAATAAATAA CCAGCTTTCA TTTTGAAATT   
  
  
+ TTTTTTAAAA AATGAAACTC ATTTCACTTA AAAATTTACC ATAATATATA TATATATATA TATATATATA   
  
  
+ TATATATATA TATTTGATTT AACGTTAAGC AAACTTTTAA CTTATGTATC ATTTTACGAC CGTAAAAAAA   
  
  
+ AAAGAAATAT AACACAATAT GTAATAGTAT TTAAGTTTTT GATAAATAAT ATTTTAACAT AATTTGTGTG   
  
  
+ TAGTATGAAT TTAAATCTTA GTACTAATAT AATTATGTAT ACTTGGCTAT AAGATCTCAA TATTAGTATT   
  
  
+ TTATGATTCA ATGTAATGAT GATGCAACTG TGTGTAATTA GAAAGCCTTT GATTGCAGAT ATCTATCTTA   
  
  
+ TTGATACACA TCTACCAATT GAGCTTTTTC AAGATTTGAA GACGGTAGTC AAGTATCTTT GGGAGATTAC   
  
  
+ GTTAGAATTA CTTCATGATA AATTAGACAT ATTTTATACT TCTAATCTTA AATAAGTCTC CATCCTTTCA   
  
  
+ TCATATTTTA AAGAAATGTA CAAGTAAATG TCAATAGTTT GAAACGTTCC TTATCATAAA AGTCATACAT   
  
  
+ ATGTAGGACA TTTTCAAGTT TATGTAAACC ATATGATATA GTTCAAGATA ATTGTCAAAC AAGTTATGAA   
  
  
+ ATCTAAAGGT GTTTGAACGT AATTTCAAGC TAATCTTAGC AAGTTATAGT ATTATTTAAA CTACGACTAG   
  
  
+ ACACAACATT TTGTTCATTT ATATAACACT TAGAATGCAA AAAAATCGGT ATTACTAATA CTGTATCAAG   
  
  
+ AATTGAAGTG TTATGCATTA TACTGATAAG GGATAATATT TAAATTACTT TACTGTTATA GAAGCCAAAA   
  
  
+ ATCTAAACTT TCATACAGAC GTCGGACTAA ATTAGAAAAT TCTTTAACTT AATAGAAAGG ATGACATAAA   
  
  
+ TAAAAATAAA AACCTAAATA GCATAATAAC CATTCTACAC AAAAGTCAAT CTTCTAATAG AAAAAAGCCA   
  
  
+ TAAGTATTCA CTTATTTACC ATTTTATTCC TAATCTACTG TCTGTCTATT CACACCATAA TTCATGAACC   
  
  
+ AATGTGGAGT TTCTGTTTCA TTTCATGGGG TGATTTATGT CTGACAATGA TCCAACAAAC TGAGCCTCAC   
  
  
+ GCGCGGAACA CGAGAGTGGC TGTACTTATG GTGTGAAAGA GCTCTACAAG GTGAGATTGA GGAGGCTACG   
  
  
+ CGTTGGAGTT ATCACCACCG TTGGATTTGT TTTCTTTGGA GCCACGTTAT CACTCTTCAG ACGCCGATCC   
  
  
+ AACGGCCCTT ACTTCCATCT AGTCCCCACT CTCCAACTCT ACGCAATCAA CGCCTTCCGC ATTTAAATAT   
  
  
+ TTCCTTCTCT ATCGCCTACT TTCTCTGTCT CACAATTTTT GTCCGTATTT TGAATTTTCT CAAAATTATC   
  
  
+ ATATAATTTA TTTGCCTTCA ACTAAAGTAT TTATTAAATT AGTTAATGTA TTTAAATTGA ACTAAAAAAG   
  
  
+ AGAAAAAGAA AGAAAACAAC CCCACGAAAG CAAAACGACA GAGGCCAACT TCTCTCTCTA GAGTGTAGAG   
  
  
+ CCCCATCTCG GTTATAATTA TGAGAATCAA TTCCTTCTCA CAAGAGAAAC TCTCGCTCTC TCTCTCTCTC   
  
  
+ CATAAGACAT CACACCAATT GTATGAATTT TCTCTCTCCT GATTTCTGTT TTCCTTCTTG TTGTCCCCTT   
  
  
+ CTAATGCGTT AGTGCGGCCA AAACCACCAT AAACCATCTC TCTTTCAAGA AAGCAGCAAC AACAAAGAGA   
  
  
+ GACAGAGAGA GAGAGAGGGG GAGAAAGATC ACATCTTTTG GGTTTCCGGC GAATATGACG TCTGGGTTTT   
  
  
+ CGGGCGATTT CTTCTACGGC GGTGGAGGCG GTGGCGGAGG AGGTGTCCGG CCTTCTATCG GCGGTGTACA   
  
  
+ AGGCGGGGTT GTCCCATATG GGTTCCGGCA ACAACAACAA CAACAGCAAC AGCAGCAGCA GCAGCAACAG   
  
  
+ CTACATCTAC AGCTACAACA ACAGCGGGGG ATTTTCTCGG CGGATCAGAT CAGCGCTCAA CGAAGGGCGG   
  
  
+ ATTTGTTGAT GGGTAAGCGT TCTTTCGCTG ATTTTCAATC TCAACAACAG CAAATCAGTC TTTTGCAGCA   
  
  
+ GCAGCAGCAG CAACAACAAC AACAGCCCTT TGCGCTCTTG CAAAAACAAC AGCAGTTTCA ACAACAATTA   
  
  
+ CAACAACAAC AGCCACTTTC TCTCCTCCTT CAACAACAGT ACTTGATGAG ATCCGTAAAA CAGAGAACCA   
  
  
+ ACAATTTCGC AGCATCTCCG ATTTCTCCTC TTTCTTCTTC CATTGATCTT TCTTCCGCTA ATAATTCCCG   
  
  
+ GTTCGGTTTG CCGGTTTACC AACAACAACT CCGGTCTCAA CAACAACCAC CTATCTCTCT CCTCGCCCAC   
  
  
+ AACAACAACC AATTCAACCT CTCAAATAAT AACAATTCTT CTTTAATCCA AAACTCCACC AACACTCTTC   
  
  
+ TTGCTCCCGC AGCCAGCAAT AACACCCTAA TGAAAAATTC GGGCTTTTCC TCATCTGGGT TTTCTAATTC   
  
  
+ GGGTCCAAAC CTGGTCCAAC CGAAGCAGGA AATCGGGTAC TCGAATATGG GTACCAATAT GGGTATGAAT   
  
  
+ CTGGTCCAGC CCAAACCCGA CCCTGAGGAT CACGGAATAA TGAACACCCT TCAAGAATTG GAGAAGGAGC   
  
  
+ TTTTGAATGA CGATGAGGAA AACCAGCCGG AAAACGACGC CGTTTCAGTC ATCACCAACA GCGAGTGGTC   
  
  
+ CGAGACCTTC CAGAATCTGA TGAGTCCCGA CCCGAGTTCG AGCCCGAATG CCACCAACAC CCACCCGGGT   
  
  
+ TCGGGTTCTG CTAGTACCAG CGGCAGCAAC AACACTACAA CCACCCCTGC AAACAACAAT AACATGATAT   
  
  
+ CTACCTCGCC GACGTCGTCC ACGTCATCCT GCTGCTCCAC GTCGGCATCG GCGTCTCCGC CGGCGGCGGT   
  
  
+ GGCGACCCCG CAGGTACCAG CCAAGCAGCT GATCTCGGAA GCAGCGGCGG CGATTTCGGA CGGAAAGATT   
  
  
+ GAATCCGCCA TGGAAGTGAT GAACCGGCTG TCCCAGGTTG CCAATTCTAG GGGGAGCTCG GAACAAAAGC   
  
  
+ TGGCTTTTTA CATGGGCCAC GCGCTTCGGT CGCGCGTGAG TCCCACCGAA CACGCTCCCC CTATTGTGGA   
  
  
+ GCTTTTCCAA AAGGAGCATA TGGCGGCCAT GTATTCATTG TACGAGCTCT CTCATTGTTT CAAATTTGGT   
  
  
+ CTTTTGGCTG CCAATTTGGT GATGTTGGAG GCCATTACTT CCGCCCTTCA TGAGGGTCTG AAAATTCATG   
  
  
+ TTGTGGATTT TGATATTGGG CATGGTGGTC AGTACATCAA CCTCCTACGC GCCTTGGGGG AGCGTAGGGC   
  
  
+ TCAGTCGGGG AGCGCGGTGT CGAATGAGGT GAGGATCACT GTGGTTGAGA CCGAGGTCGG TAATGGTGGT   
  
  
+ GCGGCGGGTG CGGAGGCTGT TAAGGATGGC CTGGTGAAAT TGGCTGAGAA GGTGGGGATT AGTTTGAAGT   
  
  
+ TTGAGGCTTT GACTCGGAAG ATCTCCGAGT TAACCCGGGA GTCACTGGGG TGTGAGGAGG GGGAGGCCGT   
  
  
+ GGTGGTGAAT TTTGCGTTCA AACTTTACCG GGTACCGGAT GAGAGCGTGT CGATGGAGAA TCTGAGGGAC   
  
  
+ GAGCTCCTGC GCCGGGTGAA GGCGCTAAGG CCACGCGCCG TCACATTGGT GGAGCAGGAC TTAAATGCGA   
  
  
+ ATACGGCACC GTTCATGACG CGCGTGAGTG GCGCTTGTGA GTACTATAGA GCATTGTTCG ACTCGCTCGA   
  
  
+ CTCGATGGTG CCGAGGGATA ACCCGGACCG GGTCCGGGTC GAGGAGGCCT TGGGGCGGAA GATGGCAAAC   
  
  
+ TCGGTGGCTT GCGAGGGCCG GGAACGGGTT GAGCGGGCGG AGGTGTTTGG GAAGTGGCGG GCGAGGATGA   
  
  
+ GTATGGCAGG GTTCGAGTTG AAGGCACTGA GTCAGAGTGT GGCCGAGTCA ATGCGAACCA AACTCAGCTC   
  
  
+ GGTTCTGGGC GGAAAGCCGG GTTTCACGGT CAAGGAGGAG AGCGGTGGGG TGGGGTTCGG GTGGATGGGA   
  
  
+ CGAACTCTCA CCGTCGCATC GGCCTGGCGT TA  

- +Up\_Stream \_Len000GGAGCC ACACCCTAAG GCCCAACTAT TTATCCCTGG TCGCTTTACA AGTTTATTAA   
  
  
- CCACTTCCTC AGAGACAAAC TTACGGATGA TTACGAAAAA AAAAAAAAAA CTTATTAAAC AGTCTTTCAA   
  
  
- TCCGACGTAA CCTTCCCCGC CGGATGCTTC CGAAAAGTCG TTTATTTATT GGTCGAAAGT AAAACTTTAA   
  
  
- AAAAAATTTT TTACTTTGAG TAAAGTGAAT TTTTAAATGG TATTATATAT ATATATATAT ATATATATAT   
  
  
- ATATATATAT ATAAACTAAA TTGCAATTCG TTTGAAAATT GAATACATAG TAAAATGCTG GCATTTTTTT   
  
  
- TTTCTTTATA TTGTGTTATA CATTATCATA AATTCAAAAA CTATTTATTA TAAAATTGTA TTAAACACAC   
  
  
- ATCATACTTA AATTTAGAAT CATGATTATA TTAATACATA TGAACCGATA TTCTAGAGTT ATAATCATAA   
  
  
- AATACTAAGT TACATTACTA CTACGTTGAC ACACATTAAT CTTTCGGAAA CTAACGTCTA TAGATAGAAT   
  
  
- AACTATGTGT AGATGGTTAA CTCGAAAAAG TTCTAAACTT CTGCCATCAG TTCATAGAAA CCCTCTAATG   
  
  
- CAATCTTAAT GAAGTACTAT TTAATCTGTA TAAAATATGA AGATTAGAAT TTATTCAGAG GTAGGAAAGT   
  
  
- AGTATAAAAT TTCTTTACAT GTTCATTTAC AGTTATCAAA CTTTGCAAGG AATAGTATTT TCAGTATGTA   
  
  
- TACATCCTGT AAAAGTTCAA ATACATTTGG TATACTATAT CAAGTTCTAT TAACAGTTTG TTCAATACTT   
  
  
- TAGATTTCCA CAAACTTGCA TTAAAGTTCG ATTAGAATCG TTCAATATCA TAATAAATTT GATGCTGATC   
  
  
- TGTGTTGTAA AACAAGTAAA TATATTGTGA ATCTTACGTT TTTTTAGCCA TAATGATTAT GACATAGTTC   
  
  
- TTAACTTCAC AATACGTAAT ATGACTATTC CCTATTATAA ATTTAATGAA ATGACAATAT CTTCGGTTTT   
  
  
- TAGATTTGAA AGTATGTCTG CAGCCTGATT TAATCTTTTA AGAAATTGAA TTATCTTTCC TACTGTATTT   
  
  
- ATTTTTATTT TTGGATTTAT CGTATTATTG GTAAGATGTG TTTTCAGTTA GAAGATTATC TTTTTTCGGT   
  
  
- ATTCATAAGT GAATAAATGG TAAAATAAGG ATTAGATGAC AGACAGATAA GTGTGGTATT AAGTACTTGG   
  
  
- TTACACCTCA AAGACAAAGT AAAGTACCCC ACTAAATACA GACTGTTACT AGGTTGTTTG ACTCGGAGTG   
  
  
- CGCGCCTTGT GCTCTCACCG ACATGAATAC CACACTTTCT CGAGATGTTC CACTCTAACT CCTCCGATGC   
  
  
- GCAACCTCAA TAGTGGTGGC AACCTAAACA AAAGAAACCT CGGTGCAATA GTGAGAAGTC TGCGGCTAGG   
  
  
- TTGCCGGGAA TGAAGGTAGA TCAGGGGTGA GAGGTTGAGA TGCGTTAGTT GCGGAAGGCG TAAATTTATA   
  
  
- AAGGAAGAGA TAGCGGATGA AAGAGACAGA GTGTTAAAAA CAGGCATAAA ACTTAAAAGA GTTTTAATAG   
  
  
- TATATTAAAT AAACGGAAGT TGATTTCATA AATAATTTAA TCAATTACAT AAATTTAACT TGATTTTTTC   
  
  
- TCTTTTTCTT TCTTTTGTTG GGGTGCTTTC GTTTTGCTGT CTCCGGTTGA AGAGAGAGAT CTCACATCTC   
  
  
- GGGGTAGAGC CAATATTAAT ACTCTTAGTT AAGGAAGAGT GTTCTCTTTG AGAGCGAGAG AGAGAGAGAG   
  
  
- GTATTCTGTA GTGTGGTTAA CATACTTAAA AGAGAGAGGA CTAAAGACAA AAGGAAGAAC AACAGGGGAA   
  
  
- GATTACGCAA TCACGCCGGT TTTGGTGGTA TTTGGTAGAG AGAAAGTTCT TTCGTCGTTG TTGTTTCTCT   
  
  
- CTGTCTCTCT CTCTCTCCCC CTCTTTCTAG TGTAGAAAAC CCAAAGGCCG CTTATACTGC AGACCCAAAA   
  
  
- GCCCGCTAAA GAAGATGCCG CCACCTCCGC CACCGCCTCC TCCACAGGCC GGAAGATAGC CGCCACATGT   
  
  
- TCCGCCCCAA CAGGGTATAC CCAAGGCCGT TGTTGTTGTT GTTGTCGTTG TCGTCGTCGT CGTCGTTGTC   
  
  
- GATGTAGATG TCGATGTTGT TGTCGCCCCC TAAAAGAGCC GCCTAGTCTA GTCGCGAGTT GCTTCCCGCC   
  
  
- TAAACAACTA CCCATTCGCA AGAAAGCGAC TAAAAGTTAG AGTTGTTGTC GTTTAGTCAG AAAACGTCGT   
  
  
- CGTCGTCGTC GTTGTTGTTG TTGTCGGGAA ACGCGAGAAC GTTTTTGTTG TCGTCAAAGT TGTTGTTAAT   
  
  
- GTTGTTGTTG TCGGTGAAAG AGAGGAGGAA GTTGTTGTCA TGAACTACTC TAGGCATTTT GTCTCTTGGT   
  
  
- TGTTAAAGCG TCGTAGAGGC TAAAGAGGAG AAAGAAGAAG GTAACTAGAA AGAAGGCGAT TATTAAGGGC   
  
  
- CAAGCCAAAC GGCCAAATGG TTGTTGTTGA GGCCAGAGTT GTTGTTGGTG GATAGAGAGA GGAGCGGGTG   
  
  
- TTGTTGTTGG TTAAGTTGGA GAGTTTATTA TTGTTAAGAA GAAATTAGGT TTTGAGGTGG TTGTGAGAAG   
  
  
- AACGAGGGCG TCGGTCGTTA TTGTGGGATT ACTTTTTAAG CCCGAAAAGG AGTAGACCCA AAAGATTAAG   
  
  
- CCCAGGTTTG GACCAGGTTG GCTTCGTCCT TTAGCCCATG AGCTTATACC CATGGTTATA CCCATACTTA   
  
  
- GACCAGGTCG GGTTTGGGCT GGGACTCCTA GTGCCTTATT ACTTGTGGGA AGTTCTTAAC CTCTTCCTCG   
  
  
- AAAACTTACT GCTACTCCTT TTGGTCGGCC TTTTGCTGCG GCAAAGTCAG TAGTGGTTGT CGCTCACCAG   
  
  
- GCTCTGGAAG GTCTTAGACT ACTCAGGGCT GGGCTCAAGC TCGGGCTTAC GGTGGTTGTG GGTGGGCCCA   
  
  
- AGCCCAAGAC GATCATGGTC GCCGTCGTTG TTGTGATGTT GGTGGGGACG TTTGTTGTTA TTGTACTATA   
  
  
- GATGGAGCGG CTGCAGCAGG TGCAGTAGGA CGACGAGGTG CAGCCGTAGC CGCAGAGGCG GCCGCCGCCA   
  
  
- CCGCTGGGGC GTCCATGGTC GGTTCGTCGA CTAGAGCCTT CGTCGCCGCC GCTAAAGCCT GCCTTTCTAA   
  
  
- CTTAGGCGGT ACCTTCACTA CTTGGCCGAC AGGGTCCAAC GGTTAAGATC CCCCTCGAGC CTTGTTTTCG   
  
  
- ACCGAAAAAT GTACCCGGTG CGCGAAGCCA GCGCGCACTC AGGGTGGCTT GTGCGAGGGG GATAACACCT   
  
  
- CGAAAAGGTT TTCCTCGTAT ACCGCCGGTA CATAAGTAAC ATGCTCGAGA GAGTAACAAA GTTTAAACCA   
  
  
- GAAAACCGAC GGTTAAACCA CTACAACCTC CGGTAATGAA GGCGGGAAGT ACTCCCAGAC TTTTAAGTAC   
  
  
- AACACCTAAA ACTATAACCC GTACCACCAG TCATGTAGTT GGAGGATGCG CGGAACCCCC TCGCATCCCG   
  
  
- AGTCAGCCCC TCGCGCCACA GCTTACTCCA CTCCTAGTGA CACCAACTCT GGCTCCAGCC ATTACCACCA   
  
  
- CGCCGCCCAC GCCTCCGACA ATTCCTACCG GACCACTTTA ACCGACTCTT CCACCCCTAA TCAAACTTCA   
  
  
- AACTCCGAAA CTGAGCCTTC TAGAGGCTCA ATTGGGCCCT CAGTGACCCC ACACTCCTCC CCCTCCGGCA   
  
  
- CCACCACTTA AAACGCAAGT TTGAAATGGC CCATGGCCTA CTCTCGCACA GCTACCTCTT AGACTCCCTG   
  
  
- CTCGAGGACG CGGCCCACTT CCGCGATTCC GGTGCGCGGC AGTGTAACCA CCTCGTCCTG AATTTACGCT   
  
  
- TATGCCGTGG CAAGTACTGC GCGCACTCAC CGCGAACACT CATGATATCT CGTAACAAGC TGAGCGAGCT   
  
  
- GAGCTACCAC GGCTCCCTAT TGGGCCTGGC CCAGGCCCAG CTCCTCCGGA ACCCCGCCTT CTACCGTTTG   
  
  
- AGCCACCGAA CGCTCCCGGC CCTTGCCCAA CTCGCCCGCC TCCACAAACC CTTCACCGCC CGCTCCTACT   
  
  
- CATACCGTCC CAAGCTCAAC TTCCGTGACT CAGTCTCACA CCGGCTCAGT TACGCTTGGT TTGAGTCGAG   
  
  
- CCAAGACCCG CCTTTCGGCC CAAAGTGCCA GTTCCTCCTC TCGCCACCCC ACCCCAAGCC CACCTACCCT   
  
  
- GCTTGAGAGT GGCAGCGTAG CCGGACCGCA AT

+     GC-motif

| Site Name | Organism | Position | Strand | Matrix score. | sequence | function |
| --- | --- | --- | --- | --- | --- | --- |
| GC-motif | Zea mays | 2199 | - | 6 | CCCCCG | enhancer-like element involved in anoxic specific inducibility |

>HU06G02537.1   
+ +Up\_Stream \_Len000CCTCGG TGTGGGATTC CGGGTTGATA AATAGGGACC AGCGAAATGT TCAAATAATT   
  
  
+ GGTGAAGGAG TCTCTGTTTG AATGCCTACT AATGCTTTTT TTTTTTTTTT GAATAATTTG TCAGAAAGTT   
  
  
+ AGGCTGCATT GGAAGGGGCG GCCTACGAAG GCTTTTCAGC AAATAAATAA CCAGCTTTCA TTTTGAAATT   
  
  
+ TTTTTTAAAA AATGAAACTC ATTTCACTTA AAAATTTACC ATAATATATA TATATATATA TATATATATA   
  
  
+ TATATATATA TATTTGATTT AACGTTAAGC AAACTTTTAA CTTATGTATC ATTTTACGAC CGTAAAAAAA   
  
  
+ AAAGAAATAT AACACAATAT GTAATAGTAT TTAAGTTTTT GATAAATAAT ATTTTAACAT AATTTGTGTG   
  
  
+ TAGTATGAAT TTAAATCTTA GTACTAATAT AATTATGTAT ACTTGGCTAT AAGATCTCAA TATTAGTATT   
  
  
+ TTATGATTCA ATGTAATGAT GATGCAACTG TGTGTAATTA GAAAGCCTTT GATTGCAGAT ATCTATCTTA   
  
  
+ TTGATACACA TCTACCAATT GAGCTTTTTC AAGATTTGAA GACGGTAGTC AAGTATCTTT GGGAGATTAC   
  
  
+ GTTAGAATTA CTTCATGATA AATTAGACAT ATTTTATACT TCTAATCTTA AATAAGTCTC CATCCTTTCA   
  
  
+ TCATATTTTA AAGAAATGTA CAAGTAAATG TCAATAGTTT GAAACGTTCC TTATCATAAA AGTCATACAT   
  
  
+ ATGTAGGACA TTTTCAAGTT TATGTAAACC ATATGATATA GTTCAAGATA ATTGTCAAAC AAGTTATGAA   
  
  
+ ATCTAAAGGT GTTTGAACGT AATTTCAAGC TAATCTTAGC AAGTTATAGT ATTATTTAAA CTACGACTAG   
  
  
+ ACACAACATT TTGTTCATTT ATATAACACT TAGAATGCAA AAAAATCGGT ATTACTAATA CTGTATCAAG   
  
  
+ AATTGAAGTG TTATGCATTA TACTGATAAG GGATAATATT TAAATTACTT TACTGTTATA GAAGCCAAAA   
  
  
+ ATCTAAACTT TCATACAGAC GTCGGACTAA ATTAGAAAAT TCTTTAACTT AATAGAAAGG ATGACATAAA   
  
  
+ TAAAAATAAA AACCTAAATA GCATAATAAC CATTCTACAC AAAAGTCAAT CTTCTAATAG AAAAAAGCCA   
  
  
+ TAAGTATTCA CTTATTTACC ATTTTATTCC TAATCTACTG TCTGTCTATT CACACCATAA TTCATGAACC   
  
  
+ AATGTGGAGT TTCTGTTTCA TTTCATGGGG TGATTTATGT CTGACAATGA TCCAACAAAC TGAGCCTCAC   
  
  
+ GCGCGGAACA CGAGAGTGGC TGTACTTATG GTGTGAAAGA GCTCTACAAG GTGAGATTGA GGAGGCTACG   
  
  
+ CGTTGGAGTT ATCACCACCG TTGGATTTGT TTTCTTTGGA GCCACGTTAT CACTCTTCAG ACGCCGATCC   
  
  
+ AACGGCCCTT ACTTCCATCT AGTCCCCACT CTCCAACTCT ACGCAATCAA CGCCTTCCGC ATTTAAATAT   
  
  
+ TTCCTTCTCT ATCGCCTACT TTCTCTGTCT CACAATTTTT GTCCGTATTT TGAATTTTCT CAAAATTATC   
  
  
+ ATATAATTTA TTTGCCTTCA ACTAAAGTAT TTATTAAATT AGTTAATGTA TTTAAATTGA ACTAAAAAAG   
  
  
+ AGAAAAAGAA AGAAAACAAC CCCACGAAAG CAAAACGACA GAGGCCAACT TCTCTCTCTA GAGTGTAGAG   
  
  
+ CCCCATCTCG GTTATAATTA TGAGAATCAA TTCCTTCTCA CAAGAGAAAC TCTCGCTCTC TCTCTCTCTC   
  
  
+ CATAAGACAT CACACCAATT GTATGAATTT TCTCTCTCCT GATTTCTGTT TTCCTTCTTG TTGTCCCCTT   
  
  
+ CTAATGCGTT AGTGCGGCCA AAACCACCAT AAACCATCTC TCTTTCAAGA AAGCAGCAAC AACAAAGAGA   
  
  
+ GACAGAGAGA GAGAGAGGGG GAGAAAGATC ACATCTTTTG GGTTTCCGGC GAATATGACG TCTGGGTTTT   
  
  
+ CGGGCGATTT CTTCTACGGC GGTGGAGGCG GTGGCGGAGG AGGTGTCCGG CCTTCTATCG GCGGTGTACA   
  
  
+ AGGCGGGGTT GTCCCATATG GGTTCCGGCA ACAACAACAA CAACAGCAAC AGCAGCAGCA GCAGCAACAG   
  
  
+ CTACATCTAC AGCTACAACA ACAGCGGGGG ATTTTCTCGG CGGATCAGAT CAGCGCTCAA CGAAGGGCGG   
  
  
+ ATTTGTTGAT GGGTAAGCGT TCTTTCGCTG ATTTTCAATC TCAACAACAG CAAATCAGTC TTTTGCAGCA   
  
  
+ GCAGCAGCAG CAACAACAAC AACAGCCCTT TGCGCTCTTG CAAAAACAAC AGCAGTTTCA ACAACAATTA   
  
  
+ CAACAACAAC AGCCACTTTC TCTCCTCCTT CAACAACAGT ACTTGATGAG ATCCGTAAAA CAGAGAACCA   
  
  
+ ACAATTTCGC AGCATCTCCG ATTTCTCCTC TTTCTTCTTC CATTGATCTT TCTTCCGCTA ATAATTCCCG   
  
  
+ GTTCGGTTTG CCGGTTTACC AACAACAACT CCGGTCTCAA CAACAACCAC CTATCTCTCT CCTCGCCCAC   
  
  
+ AACAACAACC AATTCAACCT CTCAAATAAT AACAATTCTT CTTTAATCCA AAACTCCACC AACACTCTTC   
  
  
+ TTGCTCCCGC AGCCAGCAAT AACACCCTAA TGAAAAATTC GGGCTTTTCC TCATCTGGGT TTTCTAATTC   
  
  
+ GGGTCCAAAC CTGGTCCAAC CGAAGCAGGA AATCGGGTAC TCGAATATGG GTACCAATAT GGGTATGAAT   
  
  
+ CTGGTCCAGC CCAAACCCGA CCCTGAGGAT CACGGAATAA TGAACACCCT TCAAGAATTG GAGAAGGAGC   
  
  
+ TTTTGAATGA CGATGAGGAA AACCAGCCGG AAAACGACGC CGTTTCAGTC ATCACCAACA GCGAGTGGTC   
  
  
+ CGAGACCTTC CAGAATCTGA TGAGTCCCGA CCCGAGTTCG AGCCCGAATG CCACCAACAC CCACCCGGGT   
  
  
+ TCGGGTTCTG CTAGTACCAG CGGCAGCAAC AACACTACAA CCACCCCTGC AAACAACAAT AACATGATAT   
  
  
+ CTACCTCGCC GACGTCGTCC ACGTCATCCT GCTGCTCCAC GTCGGCATCG GCGTCTCCGC CGGCGGCGGT   
  
  
+ GGCGACCCCG CAGGTACCAG CCAAGCAGCT GATCTCGGAA GCAGCGGCGG CGATTTCGGA CGGAAAGATT   
  
  
+ GAATCCGCCA TGGAAGTGAT GAACCGGCTG TCCCAGGTTG CCAATTCTAG GGGGAGCTCG GAACAAAAGC   
  
  
+ TGGCTTTTTA CATGGGCCAC GCGCTTCGGT CGCGCGTGAG TCCCACCGAA CACGCTCCCC CTATTGTGGA   
  
  
+ GCTTTTCCAA AAGGAGCATA TGGCGGCCAT GTATTCATTG TACGAGCTCT CTCATTGTTT CAAATTTGGT   
  
  
+ CTTTTGGCTG CCAATTTGGT GATGTTGGAG GCCATTACTT CCGCCCTTCA TGAGGGTCTG AAAATTCATG   
  
  
+ TTGTGGATTT TGATATTGGG CATGGTGGTC AGTACATCAA CCTCCTACGC GCCTTGGGGG AGCGTAGGGC   
  
  
+ TCAGTCGGGG AGCGCGGTGT CGAATGAGGT GAGGATCACT GTGGTTGAGA CCGAGGTCGG TAATGGTGGT   
  
  
+ GCGGCGGGTG CGGAGGCTGT TAAGGATGGC CTGGTGAAAT TGGCTGAGAA GGTGGGGATT AGTTTGAAGT   
  
  
+ TTGAGGCTTT GACTCGGAAG ATCTCCGAGT TAACCCGGGA GTCACTGGGG TGTGAGGAGG GGGAGGCCGT   
  
  
+ GGTGGTGAAT TTTGCGTTCA AACTTTACCG GGTACCGGAT GAGAGCGTGT CGATGGAGAA TCTGAGGGAC   
  
  
+ GAGCTCCTGC GCCGGGTGAA GGCGCTAAGG CCACGCGCCG TCACATTGGT GGAGCAGGAC TTAAATGCGA   
  
  
+ ATACGGCACC GTTCATGACG CGCGTGAGTG GCGCTTGTGA GTACTATAGA GCATTGTTCG ACTCGCTCGA   
  
  
+ CTCGATGGTG CCGAGGGATA ACCCGGACCG GGTCCGGGTC GAGGAGGCCT TGGGGCGGAA GATGGCAAAC   
  
  
+ TCGGTGGCTT GCGAGGGCCG GGAACGGGTT GAGCGGGCGG AGGTGTTTGG GAAGTGGCGG GCGAGGATGA   
  
  
+ GTATGGCAGG GTTCGAGTTG AAGGCACTGA GTCAGAGTGT GGCCGAGTCA ATGCGAACCA AACTCAGCTC   
  
  
+ GGTTCTGGGC GGAAAGCCGG GTTTCACGGT CAAGGAGGAG AGCGGTGGGG TGGGGTTCGG GTGGATGGGA   
  
  
+ CGAACTCTCA CCGTCGCATC GGCCTGGCGT TA  

- +Up\_Stream \_Len000GGAGCC ACACCCTAAG GCCCAACTAT TTATCCCTGG TCGCTTTACA AGTTTATTAA   
  
  
- CCACTTCCTC AGAGACAAAC TTACGGATGA TTACGAAAAA AAAAAAAAAA CTTATTAAAC AGTCTTTCAA   
  
  
- TCCGACGTAA CCTTCCCCGC CGGATGCTTC CGAAAAGTCG TTTATTTATT GGTCGAAAGT AAAACTTTAA   
  
  
- AAAAAATTTT TTACTTTGAG TAAAGTGAAT TTTTAAATGG TATTATATAT ATATATATAT ATATATATAT   
  
  
- ATATATATAT ATAAACTAAA TTGCAATTCG TTTGAAAATT GAATACATAG TAAAATGCTG GCATTTTTTT   
  
  
- TTTCTTTATA TTGTGTTATA CATTATCATA AATTCAAAAA CTATTTATTA TAAAATTGTA TTAAACACAC   
  
  
- ATCATACTTA AATTTAGAAT CATGATTATA TTAATACATA TGAACCGATA TTCTAGAGTT ATAATCATAA   
  
  
- AATACTAAGT TACATTACTA CTACGTTGAC ACACATTAAT CTTTCGGAAA CTAACGTCTA TAGATAGAAT   
  
  
- AACTATGTGT AGATGGTTAA CTCGAAAAAG TTCTAAACTT CTGCCATCAG TTCATAGAAA CCCTCTAATG   
  
  
- CAATCTTAAT GAAGTACTAT TTAATCTGTA TAAAATATGA AGATTAGAAT TTATTCAGAG GTAGGAAAGT   
  
  
- AGTATAAAAT TTCTTTACAT GTTCATTTAC AGTTATCAAA CTTTGCAAGG AATAGTATTT TCAGTATGTA   
  
  
- TACATCCTGT AAAAGTTCAA ATACATTTGG TATACTATAT CAAGTTCTAT TAACAGTTTG TTCAATACTT   
  
  
- TAGATTTCCA CAAACTTGCA TTAAAGTTCG ATTAGAATCG TTCAATATCA TAATAAATTT GATGCTGATC   
  
  
- TGTGTTGTAA AACAAGTAAA TATATTGTGA ATCTTACGTT TTTTTAGCCA TAATGATTAT GACATAGTTC   
  
  
- TTAACTTCAC AATACGTAAT ATGACTATTC CCTATTATAA ATTTAATGAA ATGACAATAT CTTCGGTTTT   
  
  
- TAGATTTGAA AGTATGTCTG CAGCCTGATT TAATCTTTTA AGAAATTGAA TTATCTTTCC TACTGTATTT   
  
  
- ATTTTTATTT TTGGATTTAT CGTATTATTG GTAAGATGTG TTTTCAGTTA GAAGATTATC TTTTTTCGGT   
  
  
- ATTCATAAGT GAATAAATGG TAAAATAAGG ATTAGATGAC AGACAGATAA GTGTGGTATT AAGTACTTGG   
  
  
- TTACACCTCA AAGACAAAGT AAAGTACCCC ACTAAATACA GACTGTTACT AGGTTGTTTG ACTCGGAGTG   
  
  
- CGCGCCTTGT GCTCTCACCG ACATGAATAC CACACTTTCT CGAGATGTTC CACTCTAACT CCTCCGATGC   
  
  
- GCAACCTCAA TAGTGGTGGC AACCTAAACA AAAGAAACCT CGGTGCAATA GTGAGAAGTC TGCGGCTAGG   
  
  
- TTGCCGGGAA TGAAGGTAGA TCAGGGGTGA GAGGTTGAGA TGCGTTAGTT GCGGAAGGCG TAAATTTATA   
  
  
- AAGGAAGAGA TAGCGGATGA AAGAGACAGA GTGTTAAAAA CAGGCATAAA ACTTAAAAGA GTTTTAATAG   
  
  
- TATATTAAAT AAACGGAAGT TGATTTCATA AATAATTTAA TCAATTACAT AAATTTAACT TGATTTTTTC   
  
  
- TCTTTTTCTT TCTTTTGTTG GGGTGCTTTC GTTTTGCTGT CTCCGGTTGA AGAGAGAGAT CTCACATCTC   
  
  
- GGGGTAGAGC CAATATTAAT ACTCTTAGTT AAGGAAGAGT GTTCTCTTTG AGAGCGAGAG AGAGAGAGAG   
  
  
- GTATTCTGTA GTGTGGTTAA CATACTTAAA AGAGAGAGGA CTAAAGACAA AAGGAAGAAC AACAGGGGAA   
  
  
- GATTACGCAA TCACGCCGGT TTTGGTGGTA TTTGGTAGAG AGAAAGTTCT TTCGTCGTTG TTGTTTCTCT   
  
  
- CTGTCTCTCT CTCTCTCCCC CTCTTTCTAG TGTAGAAAAC CCAAAGGCCG CTTATACTGC AGACCCAAAA   
  
  
- GCCCGCTAAA GAAGATGCCG CCACCTCCGC CACCGCCTCC TCCACAGGCC GGAAGATAGC CGCCACATGT   
  
  
- TCCGCCCCAA CAGGGTATAC CCAAGGCCGT TGTTGTTGTT GTTGTCGTTG TCGTCGTCGT CGTCGTTGTC   
  
  
- GATGTAGATG TCGATGTTGT TGTCGCCCCC TAAAAGAGCC GCCTAGTCTA GTCGCGAGTT GCTTCCCGCC   
  
  
- TAAACAACTA CCCATTCGCA AGAAAGCGAC TAAAAGTTAG AGTTGTTGTC GTTTAGTCAG AAAACGTCGT   
  
  
- CGTCGTCGTC GTTGTTGTTG TTGTCGGGAA ACGCGAGAAC GTTTTTGTTG TCGTCAAAGT TGTTGTTAAT   
  
  
- GTTGTTGTTG TCGGTGAAAG AGAGGAGGAA GTTGTTGTCA TGAACTACTC TAGGCATTTT GTCTCTTGGT   
  
  
- TGTTAAAGCG TCGTAGAGGC TAAAGAGGAG AAAGAAGAAG GTAACTAGAA AGAAGGCGAT TATTAAGGGC   
  
  
- CAAGCCAAAC GGCCAAATGG TTGTTGTTGA GGCCAGAGTT GTTGTTGGTG GATAGAGAGA GGAGCGGGTG   
  
  
- TTGTTGTTGG TTAAGTTGGA GAGTTTATTA TTGTTAAGAA GAAATTAGGT TTTGAGGTGG TTGTGAGAAG   
  
  
- AACGAGGGCG TCGGTCGTTA TTGTGGGATT ACTTTTTAAG CCCGAAAAGG AGTAGACCCA AAAGATTAAG   
  
  
- CCCAGGTTTG GACCAGGTTG GCTTCGTCCT TTAGCCCATG AGCTTATACC CATGGTTATA CCCATACTTA   
  
  
- GACCAGGTCG GGTTTGGGCT GGGACTCCTA GTGCCTTATT ACTTGTGGGA AGTTCTTAAC CTCTTCCTCG   
  
  
- AAAACTTACT GCTACTCCTT TTGGTCGGCC TTTTGCTGCG GCAAAGTCAG TAGTGGTTGT CGCTCACCAG   
  
  
- GCTCTGGAAG GTCTTAGACT ACTCAGGGCT GGGCTCAAGC TCGGGCTTAC GGTGGTTGTG GGTGGGCCCA   
  
  
- AGCCCAAGAC GATCATGGTC GCCGTCGTTG TTGTGATGTT GGTGGGGACG TTTGTTGTTA TTGTACTATA   
  
  
- GATGGAGCGG CTGCAGCAGG TGCAGTAGGA CGACGAGGTG CAGCCGTAGC CGCAGAGGCG GCCGCCGCCA   
  
  
- CCGCTGGGGC GTCCATGGTC GGTTCGTCGA CTAGAGCCTT CGTCGCCGCC GCTAAAGCCT GCCTTTCTAA   
  
  
- CTTAGGCGGT ACCTTCACTA CTTGGCCGAC AGGGTCCAAC GGTTAAGATC CCCCTCGAGC CTTGTTTTCG   
  
  
- ACCGAAAAAT GTACCCGGTG CGCGAAGCCA GCGCGCACTC AGGGTGGCTT GTGCGAGGGG GATAACACCT   
  
  
- CGAAAAGGTT TTCCTCGTAT ACCGCCGGTA CATAAGTAAC ATGCTCGAGA GAGTAACAAA GTTTAAACCA   
  
  
- GAAAACCGAC GGTTAAACCA CTACAACCTC CGGTAATGAA GGCGGGAAGT ACTCCCAGAC TTTTAAGTAC   
  
  
- AACACCTAAA ACTATAACCC GTACCACCAG TCATGTAGTT GGAGGATGCG CGGAACCCCC TCGCATCCCG   
  
  
- AGTCAGCCCC TCGCGCCACA GCTTACTCCA CTCCTAGTGA CACCAACTCT GGCTCCAGCC ATTACCACCA   
  
  
- CGCCGCCCAC GCCTCCGACA ATTCCTACCG GACCACTTTA ACCGACTCTT CCACCCCTAA TCAAACTTCA   
  
  
- AACTCCGAAA CTGAGCCTTC TAGAGGCTCA ATTGGGCCCT CAGTGACCCC ACACTCCTCC CCCTCCGGCA   
  
  
- CCACCACTTA AAACGCAAGT TTGAAATGGC CCATGGCCTA CTCTCGCACA GCTACCTCTT AGACTCCCTG   
  
  
- CTCGAGGACG CGGCCCACTT CCGCGATTCC GGTGCGCGGC AGTGTAACCA CCTCGTCCTG AATTTACGCT   
  
  
- TATGCCGTGG CAAGTACTGC GCGCACTCAC CGCGAACACT CATGATATCT CGTAACAAGC TGAGCGAGCT   
  
  
- GAGCTACCAC GGCTCCCTAT TGGGCCTGGC CCAGGCCCAG CTCCTCCGGA ACCCCGCCTT CTACCGTTTG   
  
  
- AGCCACCGAA CGCTCCCGGC CCTTGCCCAA CTCGCCCGCC TCCACAAACC CTTCACCGCC CGCTCCTACT   
  
  
- CATACCGTCC CAAGCTCAAC TTCCGTGACT CAGTCTCACA CCGGCTCAGT TACGCTTGGT TTGAGTCGAG   
  
  
- CCAAGACCCG CCTTTCGGCC CAAAGTGCCA GTTCCTCCTC TCGCCACCCC ACCCCAAGCC CACCTACCCT   
  
  
- GCTTGAGAGT GGCAGCGTAG CCGGACCGCA AT

+     GCN4\_motif

| Site Name | Organism | Position | Strand | Matrix score. | sequence | function |
| --- | --- | --- | --- | --- | --- | --- |
| GCN4\_motif | Oryza sativa | 4162 | + | 7 | TGAGTCA | cis-regulatory element involved in endosperm expression |

>HU06G02537.1   
+ +Up\_Stream \_Len000CCTCGG TGTGGGATTC CGGGTTGATA AATAGGGACC AGCGAAATGT TCAAATAATT   
  
  
+ GGTGAAGGAG TCTCTGTTTG AATGCCTACT AATGCTTTTT TTTTTTTTTT GAATAATTTG TCAGAAAGTT   
  
  
+ AGGCTGCATT GGAAGGGGCG GCCTACGAAG GCTTTTCAGC AAATAAATAA CCAGCTTTCA TTTTGAAATT   
  
  
+ TTTTTTAAAA AATGAAACTC ATTTCACTTA AAAATTTACC ATAATATATA TATATATATA TATATATATA   
  
  
+ TATATATATA TATTTGATTT AACGTTAAGC AAACTTTTAA CTTATGTATC ATTTTACGAC CGTAAAAAAA   
  
  
+ AAAGAAATAT AACACAATAT GTAATAGTAT TTAAGTTTTT GATAAATAAT ATTTTAACAT AATTTGTGTG   
  
  
+ TAGTATGAAT TTAAATCTTA GTACTAATAT AATTATGTAT ACTTGGCTAT AAGATCTCAA TATTAGTATT   
  
  
+ TTATGATTCA ATGTAATGAT GATGCAACTG TGTGTAATTA GAAAGCCTTT GATTGCAGAT ATCTATCTTA   
  
  
+ TTGATACACA TCTACCAATT GAGCTTTTTC AAGATTTGAA GACGGTAGTC AAGTATCTTT GGGAGATTAC   
  
  
+ GTTAGAATTA CTTCATGATA AATTAGACAT ATTTTATACT TCTAATCTTA AATAAGTCTC CATCCTTTCA   
  
  
+ TCATATTTTA AAGAAATGTA CAAGTAAATG TCAATAGTTT GAAACGTTCC TTATCATAAA AGTCATACAT   
  
  
+ ATGTAGGACA TTTTCAAGTT TATGTAAACC ATATGATATA GTTCAAGATA ATTGTCAAAC AAGTTATGAA   
  
  
+ ATCTAAAGGT GTTTGAACGT AATTTCAAGC TAATCTTAGC AAGTTATAGT ATTATTTAAA CTACGACTAG   
  
  
+ ACACAACATT TTGTTCATTT ATATAACACT TAGAATGCAA AAAAATCGGT ATTACTAATA CTGTATCAAG   
  
  
+ AATTGAAGTG TTATGCATTA TACTGATAAG GGATAATATT TAAATTACTT TACTGTTATA GAAGCCAAAA   
  
  
+ ATCTAAACTT TCATACAGAC GTCGGACTAA ATTAGAAAAT TCTTTAACTT AATAGAAAGG ATGACATAAA   
  
  
+ TAAAAATAAA AACCTAAATA GCATAATAAC CATTCTACAC AAAAGTCAAT CTTCTAATAG AAAAAAGCCA   
  
  
+ TAAGTATTCA CTTATTTACC ATTTTATTCC TAATCTACTG TCTGTCTATT CACACCATAA TTCATGAACC   
  
  
+ AATGTGGAGT TTCTGTTTCA TTTCATGGGG TGATTTATGT CTGACAATGA TCCAACAAAC TGAGCCTCAC   
  
  
+ GCGCGGAACA CGAGAGTGGC TGTACTTATG GTGTGAAAGA GCTCTACAAG GTGAGATTGA GGAGGCTACG   
  
  
+ CGTTGGAGTT ATCACCACCG TTGGATTTGT TTTCTTTGGA GCCACGTTAT CACTCTTCAG ACGCCGATCC   
  
  
+ AACGGCCCTT ACTTCCATCT AGTCCCCACT CTCCAACTCT ACGCAATCAA CGCCTTCCGC ATTTAAATAT   
  
  
+ TTCCTTCTCT ATCGCCTACT TTCTCTGTCT CACAATTTTT GTCCGTATTT TGAATTTTCT CAAAATTATC   
  
  
+ ATATAATTTA TTTGCCTTCA ACTAAAGTAT TTATTAAATT AGTTAATGTA TTTAAATTGA ACTAAAAAAG   
  
  
+ AGAAAAAGAA AGAAAACAAC CCCACGAAAG CAAAACGACA GAGGCCAACT TCTCTCTCTA GAGTGTAGAG   
  
  
+ CCCCATCTCG GTTATAATTA TGAGAATCAA TTCCTTCTCA CAAGAGAAAC TCTCGCTCTC TCTCTCTCTC   
  
  
+ CATAAGACAT CACACCAATT GTATGAATTT TCTCTCTCCT GATTTCTGTT TTCCTTCTTG TTGTCCCCTT   
  
  
+ CTAATGCGTT AGTGCGGCCA AAACCACCAT AAACCATCTC TCTTTCAAGA AAGCAGCAAC AACAAAGAGA   
  
  
+ GACAGAGAGA GAGAGAGGGG GAGAAAGATC ACATCTTTTG GGTTTCCGGC GAATATGACG TCTGGGTTTT   
  
  
+ CGGGCGATTT CTTCTACGGC GGTGGAGGCG GTGGCGGAGG AGGTGTCCGG CCTTCTATCG GCGGTGTACA   
  
  
+ AGGCGGGGTT GTCCCATATG GGTTCCGGCA ACAACAACAA CAACAGCAAC AGCAGCAGCA GCAGCAACAG   
  
  
+ CTACATCTAC AGCTACAACA ACAGCGGGGG ATTTTCTCGG CGGATCAGAT CAGCGCTCAA CGAAGGGCGG   
  
  
+ ATTTGTTGAT GGGTAAGCGT TCTTTCGCTG ATTTTCAATC TCAACAACAG CAAATCAGTC TTTTGCAGCA   
  
  
+ GCAGCAGCAG CAACAACAAC AACAGCCCTT TGCGCTCTTG CAAAAACAAC AGCAGTTTCA ACAACAATTA   
  
  
+ CAACAACAAC AGCCACTTTC TCTCCTCCTT CAACAACAGT ACTTGATGAG ATCCGTAAAA CAGAGAACCA   
  
  
+ ACAATTTCGC AGCATCTCCG ATTTCTCCTC TTTCTTCTTC CATTGATCTT TCTTCCGCTA ATAATTCCCG   
  
  
+ GTTCGGTTTG CCGGTTTACC AACAACAACT CCGGTCTCAA CAACAACCAC CTATCTCTCT CCTCGCCCAC   
  
  
+ AACAACAACC AATTCAACCT CTCAAATAAT AACAATTCTT CTTTAATCCA AAACTCCACC AACACTCTTC   
  
  
+ TTGCTCCCGC AGCCAGCAAT AACACCCTAA TGAAAAATTC GGGCTTTTCC TCATCTGGGT TTTCTAATTC   
  
  
+ GGGTCCAAAC CTGGTCCAAC CGAAGCAGGA AATCGGGTAC TCGAATATGG GTACCAATAT GGGTATGAAT   
  
  
+ CTGGTCCAGC CCAAACCCGA CCCTGAGGAT CACGGAATAA TGAACACCCT TCAAGAATTG GAGAAGGAGC   
  
  
+ TTTTGAATGA CGATGAGGAA AACCAGCCGG AAAACGACGC CGTTTCAGTC ATCACCAACA GCGAGTGGTC   
  
  
+ CGAGACCTTC CAGAATCTGA TGAGTCCCGA CCCGAGTTCG AGCCCGAATG CCACCAACAC CCACCCGGGT   
  
  
+ TCGGGTTCTG CTAGTACCAG CGGCAGCAAC AACACTACAA CCACCCCTGC AAACAACAAT AACATGATAT   
  
  
+ CTACCTCGCC GACGTCGTCC ACGTCATCCT GCTGCTCCAC GTCGGCATCG GCGTCTCCGC CGGCGGCGGT   
  
  
+ GGCGACCCCG CAGGTACCAG CCAAGCAGCT GATCTCGGAA GCAGCGGCGG CGATTTCGGA CGGAAAGATT   
  
  
+ GAATCCGCCA TGGAAGTGAT GAACCGGCTG TCCCAGGTTG CCAATTCTAG GGGGAGCTCG GAACAAAAGC   
  
  
+ TGGCTTTTTA CATGGGCCAC GCGCTTCGGT CGCGCGTGAG TCCCACCGAA CACGCTCCCC CTATTGTGGA   
  
  
+ GCTTTTCCAA AAGGAGCATA TGGCGGCCAT GTATTCATTG TACGAGCTCT CTCATTGTTT CAAATTTGGT   
  
  
+ CTTTTGGCTG CCAATTTGGT GATGTTGGAG GCCATTACTT CCGCCCTTCA TGAGGGTCTG AAAATTCATG   
  
  
+ TTGTGGATTT TGATATTGGG CATGGTGGTC AGTACATCAA CCTCCTACGC GCCTTGGGGG AGCGTAGGGC   
  
  
+ TCAGTCGGGG AGCGCGGTGT CGAATGAGGT GAGGATCACT GTGGTTGAGA CCGAGGTCGG TAATGGTGGT   
  
  
+ GCGGCGGGTG CGGAGGCTGT TAAGGATGGC CTGGTGAAAT TGGCTGAGAA GGTGGGGATT AGTTTGAAGT   
  
  
+ TTGAGGCTTT GACTCGGAAG ATCTCCGAGT TAACCCGGGA GTCACTGGGG TGTGAGGAGG GGGAGGCCGT   
  
  
+ GGTGGTGAAT TTTGCGTTCA AACTTTACCG GGTACCGGAT GAGAGCGTGT CGATGGAGAA TCTGAGGGAC   
  
  
+ GAGCTCCTGC GCCGGGTGAA GGCGCTAAGG CCACGCGCCG TCACATTGGT GGAGCAGGAC TTAAATGCGA   
  
  
+ ATACGGCACC GTTCATGACG CGCGTGAGTG GCGCTTGTGA GTACTATAGA GCATTGTTCG ACTCGCTCGA   
  
  
+ CTCGATGGTG CCGAGGGATA ACCCGGACCG GGTCCGGGTC GAGGAGGCCT TGGGGCGGAA GATGGCAAAC   
  
  
+ TCGGTGGCTT GCGAGGGCCG GGAACGGGTT GAGCGGGCGG AGGTGTTTGG GAAGTGGCGG GCGAGGATGA   
  
  
+ GTATGGCAGG GTTCGAGTTG AAGGCACTGA GTCAGAGTGT GGCCGAGTCA ATGCGAACCA AACTCAGCTC   
  
  
+ GGTTCTGGGC GGAAAGCCGG GTTTCACGGT CAAGGAGGAG AGCGGTGGGG TGGGGTTCGG GTGGATGGGA   
  
  
+ CGAACTCTCA CCGTCGCATC GGCCTGGCGT TA  

- +Up\_Stream \_Len000GGAGCC ACACCCTAAG GCCCAACTAT TTATCCCTGG TCGCTTTACA AGTTTATTAA   
  
  
- CCACTTCCTC AGAGACAAAC TTACGGATGA TTACGAAAAA AAAAAAAAAA CTTATTAAAC AGTCTTTCAA   
  
  
- TCCGACGTAA CCTTCCCCGC CGGATGCTTC CGAAAAGTCG TTTATTTATT GGTCGAAAGT AAAACTTTAA   
  
  
- AAAAAATTTT TTACTTTGAG TAAAGTGAAT TTTTAAATGG TATTATATAT ATATATATAT ATATATATAT   
  
  
- ATATATATAT ATAAACTAAA TTGCAATTCG TTTGAAAATT GAATACATAG TAAAATGCTG GCATTTTTTT   
  
  
- TTTCTTTATA TTGTGTTATA CATTATCATA AATTCAAAAA CTATTTATTA TAAAATTGTA TTAAACACAC   
  
  
- ATCATACTTA AATTTAGAAT CATGATTATA TTAATACATA TGAACCGATA TTCTAGAGTT ATAATCATAA   
  
  
- AATACTAAGT TACATTACTA CTACGTTGAC ACACATTAAT CTTTCGGAAA CTAACGTCTA TAGATAGAAT   
  
  
- AACTATGTGT AGATGGTTAA CTCGAAAAAG TTCTAAACTT CTGCCATCAG TTCATAGAAA CCCTCTAATG   
  
  
- CAATCTTAAT GAAGTACTAT TTAATCTGTA TAAAATATGA AGATTAGAAT TTATTCAGAG GTAGGAAAGT   
  
  
- AGTATAAAAT TTCTTTACAT GTTCATTTAC AGTTATCAAA CTTTGCAAGG AATAGTATTT TCAGTATGTA   
  
  
- TACATCCTGT AAAAGTTCAA ATACATTTGG TATACTATAT CAAGTTCTAT TAACAGTTTG TTCAATACTT   
  
  
- TAGATTTCCA CAAACTTGCA TTAAAGTTCG ATTAGAATCG TTCAATATCA TAATAAATTT GATGCTGATC   
  
  
- TGTGTTGTAA AACAAGTAAA TATATTGTGA ATCTTACGTT TTTTTAGCCA TAATGATTAT GACATAGTTC   
  
  
- TTAACTTCAC AATACGTAAT ATGACTATTC CCTATTATAA ATTTAATGAA ATGACAATAT CTTCGGTTTT   
  
  
- TAGATTTGAA AGTATGTCTG CAGCCTGATT TAATCTTTTA AGAAATTGAA TTATCTTTCC TACTGTATTT   
  
  
- ATTTTTATTT TTGGATTTAT CGTATTATTG GTAAGATGTG TTTTCAGTTA GAAGATTATC TTTTTTCGGT   
  
  
- ATTCATAAGT GAATAAATGG TAAAATAAGG ATTAGATGAC AGACAGATAA GTGTGGTATT AAGTACTTGG   
  
  
- TTACACCTCA AAGACAAAGT AAAGTACCCC ACTAAATACA GACTGTTACT AGGTTGTTTG ACTCGGAGTG   
  
  
- CGCGCCTTGT GCTCTCACCG ACATGAATAC CACACTTTCT CGAGATGTTC CACTCTAACT CCTCCGATGC   
  
  
- GCAACCTCAA TAGTGGTGGC AACCTAAACA AAAGAAACCT CGGTGCAATA GTGAGAAGTC TGCGGCTAGG   
  
  
- TTGCCGGGAA TGAAGGTAGA TCAGGGGTGA GAGGTTGAGA TGCGTTAGTT GCGGAAGGCG TAAATTTATA   
  
  
- AAGGAAGAGA TAGCGGATGA AAGAGACAGA GTGTTAAAAA CAGGCATAAA ACTTAAAAGA GTTTTAATAG   
  
  
- TATATTAAAT AAACGGAAGT TGATTTCATA AATAATTTAA TCAATTACAT AAATTTAACT TGATTTTTTC   
  
  
- TCTTTTTCTT TCTTTTGTTG GGGTGCTTTC GTTTTGCTGT CTCCGGTTGA AGAGAGAGAT CTCACATCTC   
  
  
- GGGGTAGAGC CAATATTAAT ACTCTTAGTT AAGGAAGAGT GTTCTCTTTG AGAGCGAGAG AGAGAGAGAG   
  
  
- GTATTCTGTA GTGTGGTTAA CATACTTAAA AGAGAGAGGA CTAAAGACAA AAGGAAGAAC AACAGGGGAA   
  
  
- GATTACGCAA TCACGCCGGT TTTGGTGGTA TTTGGTAGAG AGAAAGTTCT TTCGTCGTTG TTGTTTCTCT   
  
  
- CTGTCTCTCT CTCTCTCCCC CTCTTTCTAG TGTAGAAAAC CCAAAGGCCG CTTATACTGC AGACCCAAAA   
  
  
- GCCCGCTAAA GAAGATGCCG CCACCTCCGC CACCGCCTCC TCCACAGGCC GGAAGATAGC CGCCACATGT   
  
  
- TCCGCCCCAA CAGGGTATAC CCAAGGCCGT TGTTGTTGTT GTTGTCGTTG TCGTCGTCGT CGTCGTTGTC   
  
  
- GATGTAGATG TCGATGTTGT TGTCGCCCCC TAAAAGAGCC GCCTAGTCTA GTCGCGAGTT GCTTCCCGCC   
  
  
- TAAACAACTA CCCATTCGCA AGAAAGCGAC TAAAAGTTAG AGTTGTTGTC GTTTAGTCAG AAAACGTCGT   
  
  
- CGTCGTCGTC GTTGTTGTTG TTGTCGGGAA ACGCGAGAAC GTTTTTGTTG TCGTCAAAGT TGTTGTTAAT   
  
  
- GTTGTTGTTG TCGGTGAAAG AGAGGAGGAA GTTGTTGTCA TGAACTACTC TAGGCATTTT GTCTCTTGGT   
  
  
- TGTTAAAGCG TCGTAGAGGC TAAAGAGGAG AAAGAAGAAG GTAACTAGAA AGAAGGCGAT TATTAAGGGC   
  
  
- CAAGCCAAAC GGCCAAATGG TTGTTGTTGA GGCCAGAGTT GTTGTTGGTG GATAGAGAGA GGAGCGGGTG   
  
  
- TTGTTGTTGG TTAAGTTGGA GAGTTTATTA TTGTTAAGAA GAAATTAGGT TTTGAGGTGG TTGTGAGAAG   
  
  
- AACGAGGGCG TCGGTCGTTA TTGTGGGATT ACTTTTTAAG CCCGAAAAGG AGTAGACCCA AAAGATTAAG   
  
  
- CCCAGGTTTG GACCAGGTTG GCTTCGTCCT TTAGCCCATG AGCTTATACC CATGGTTATA CCCATACTTA   
  
  
- GACCAGGTCG GGTTTGGGCT GGGACTCCTA GTGCCTTATT ACTTGTGGGA AGTTCTTAAC CTCTTCCTCG   
  
  
- AAAACTTACT GCTACTCCTT TTGGTCGGCC TTTTGCTGCG GCAAAGTCAG TAGTGGTTGT CGCTCACCAG   
  
  
- GCTCTGGAAG GTCTTAGACT ACTCAGGGCT GGGCTCAAGC TCGGGCTTAC GGTGGTTGTG GGTGGGCCCA   
  
  
- AGCCCAAGAC GATCATGGTC GCCGTCGTTG TTGTGATGTT GGTGGGGACG TTTGTTGTTA TTGTACTATA   
  
  
- GATGGAGCGG CTGCAGCAGG TGCAGTAGGA CGACGAGGTG CAGCCGTAGC CGCAGAGGCG GCCGCCGCCA   
  
  
- CCGCTGGGGC GTCCATGGTC GGTTCGTCGA CTAGAGCCTT CGTCGCCGCC GCTAAAGCCT GCCTTTCTAA   
  
  
- CTTAGGCGGT ACCTTCACTA CTTGGCCGAC AGGGTCCAAC GGTTAAGATC CCCCTCGAGC CTTGTTTTCG   
  
  
- ACCGAAAAAT GTACCCGGTG CGCGAAGCCA GCGCGCACTC AGGGTGGCTT GTGCGAGGGG GATAACACCT   
  
  
- CGAAAAGGTT TTCCTCGTAT ACCGCCGGTA CATAAGTAAC ATGCTCGAGA GAGTAACAAA GTTTAAACCA   
  
  
- GAAAACCGAC GGTTAAACCA CTACAACCTC CGGTAATGAA GGCGGGAAGT ACTCCCAGAC TTTTAAGTAC   
  
  
- AACACCTAAA ACTATAACCC GTACCACCAG TCATGTAGTT GGAGGATGCG CGGAACCCCC TCGCATCCCG   
  
  
- AGTCAGCCCC TCGCGCCACA GCTTACTCCA CTCCTAGTGA CACCAACTCT GGCTCCAGCC ATTACCACCA   
  
  
- CGCCGCCCAC GCCTCCGACA ATTCCTACCG GACCACTTTA ACCGACTCTT CCACCCCTAA TCAAACTTCA   
  
  
- AACTCCGAAA CTGAGCCTTC TAGAGGCTCA ATTGGGCCCT CAGTGACCCC ACACTCCTCC CCCTCCGGCA   
  
  
- CCACCACTTA AAACGCAAGT TTGAAATGGC CCATGGCCTA CTCTCGCACA GCTACCTCTT AGACTCCCTG   
  
  
- CTCGAGGACG CGGCCCACTT CCGCGATTCC GGTGCGCGGC AGTGTAACCA CCTCGTCCTG AATTTACGCT   
  
  
- TATGCCGTGG CAAGTACTGC GCGCACTCAC CGCGAACACT CATGATATCT CGTAACAAGC TGAGCGAGCT   
  
  
- GAGCTACCAC GGCTCCCTAT TGGGCCTGGC CCAGGCCCAG CTCCTCCGGA ACCCCGCCTT CTACCGTTTG   
  
  
- AGCCACCGAA CGCTCCCGGC CCTTGCCCAA CTCGCCCGCC TCCACAAACC CTTCACCGCC CGCTCCTACT   
  
  
- CATACCGTCC CAAGCTCAAC TTCCGTGACT CAGTCTCACA CCGGCTCAGT TACGCTTGGT TTGAGTCGAG   
  
  
- CCAAGACCCG CCTTTCGGCC CAAAGTGCCA GTTCCTCCTC TCGCCACCCC ACCCCAAGCC CACCTACCCT   
  
  
- GCTTGAGAGT GGCAGCGTAG CCGGACCGCA AT

+     GT1-motif

| Site Name | Organism | Position | Strand | Matrix score. | sequence | function |
| --- | --- | --- | --- | --- | --- | --- |
| GT1-motif | Arabidopsis thaliana | 3744 | - | 6 | GGTTAA | light responsive element |

>HU06G02537.1   
+ +Up\_Stream \_Len000CCTCGG TGTGGGATTC CGGGTTGATA AATAGGGACC AGCGAAATGT TCAAATAATT   
  
  
+ GGTGAAGGAG TCTCTGTTTG AATGCCTACT AATGCTTTTT TTTTTTTTTT GAATAATTTG TCAGAAAGTT   
  
  
+ AGGCTGCATT GGAAGGGGCG GCCTACGAAG GCTTTTCAGC AAATAAATAA CCAGCTTTCA TTTTGAAATT   
  
  
+ TTTTTTAAAA AATGAAACTC ATTTCACTTA AAAATTTACC ATAATATATA TATATATATA TATATATATA   
  
  
+ TATATATATA TATTTGATTT AACGTTAAGC AAACTTTTAA CTTATGTATC ATTTTACGAC CGTAAAAAAA   
  
  
+ AAAGAAATAT AACACAATAT GTAATAGTAT TTAAGTTTTT GATAAATAAT ATTTTAACAT AATTTGTGTG   
  
  
+ TAGTATGAAT TTAAATCTTA GTACTAATAT AATTATGTAT ACTTGGCTAT AAGATCTCAA TATTAGTATT   
  
  
+ TTATGATTCA ATGTAATGAT GATGCAACTG TGTGTAATTA GAAAGCCTTT GATTGCAGAT ATCTATCTTA   
  
  
+ TTGATACACA TCTACCAATT GAGCTTTTTC AAGATTTGAA GACGGTAGTC AAGTATCTTT GGGAGATTAC   
  
  
+ GTTAGAATTA CTTCATGATA AATTAGACAT ATTTTATACT TCTAATCTTA AATAAGTCTC CATCCTTTCA   
  
  
+ TCATATTTTA AAGAAATGTA CAAGTAAATG TCAATAGTTT GAAACGTTCC TTATCATAAA AGTCATACAT   
  
  
+ ATGTAGGACA TTTTCAAGTT TATGTAAACC ATATGATATA GTTCAAGATA ATTGTCAAAC AAGTTATGAA   
  
  
+ ATCTAAAGGT GTTTGAACGT AATTTCAAGC TAATCTTAGC AAGTTATAGT ATTATTTAAA CTACGACTAG   
  
  
+ ACACAACATT TTGTTCATTT ATATAACACT TAGAATGCAA AAAAATCGGT ATTACTAATA CTGTATCAAG   
  
  
+ AATTGAAGTG TTATGCATTA TACTGATAAG GGATAATATT TAAATTACTT TACTGTTATA GAAGCCAAAA   
  
  
+ ATCTAAACTT TCATACAGAC GTCGGACTAA ATTAGAAAAT TCTTTAACTT AATAGAAAGG ATGACATAAA   
  
  
+ TAAAAATAAA AACCTAAATA GCATAATAAC CATTCTACAC AAAAGTCAAT CTTCTAATAG AAAAAAGCCA   
  
  
+ TAAGTATTCA CTTATTTACC ATTTTATTCC TAATCTACTG TCTGTCTATT CACACCATAA TTCATGAACC   
  
  
+ AATGTGGAGT TTCTGTTTCA TTTCATGGGG TGATTTATGT CTGACAATGA TCCAACAAAC TGAGCCTCAC   
  
  
+ GCGCGGAACA CGAGAGTGGC TGTACTTATG GTGTGAAAGA GCTCTACAAG GTGAGATTGA GGAGGCTACG   
  
  
+ CGTTGGAGTT ATCACCACCG TTGGATTTGT TTTCTTTGGA GCCACGTTAT CACTCTTCAG ACGCCGATCC   
  
  
+ AACGGCCCTT ACTTCCATCT AGTCCCCACT CTCCAACTCT ACGCAATCAA CGCCTTCCGC ATTTAAATAT   
  
  
+ TTCCTTCTCT ATCGCCTACT TTCTCTGTCT CACAATTTTT GTCCGTATTT TGAATTTTCT CAAAATTATC   
  
  
+ ATATAATTTA TTTGCCTTCA ACTAAAGTAT TTATTAAATT AGTTAATGTA TTTAAATTGA ACTAAAAAAG   
  
  
+ AGAAAAAGAA AGAAAACAAC CCCACGAAAG CAAAACGACA GAGGCCAACT TCTCTCTCTA GAGTGTAGAG   
  
  
+ CCCCATCTCG GTTATAATTA TGAGAATCAA TTCCTTCTCA CAAGAGAAAC TCTCGCTCTC TCTCTCTCTC   
  
  
+ CATAAGACAT CACACCAATT GTATGAATTT TCTCTCTCCT GATTTCTGTT TTCCTTCTTG TTGTCCCCTT   
  
  
+ CTAATGCGTT AGTGCGGCCA AAACCACCAT AAACCATCTC TCTTTCAAGA AAGCAGCAAC AACAAAGAGA   
  
  
+ GACAGAGAGA GAGAGAGGGG GAGAAAGATC ACATCTTTTG GGTTTCCGGC GAATATGACG TCTGGGTTTT   
  
  
+ CGGGCGATTT CTTCTACGGC GGTGGAGGCG GTGGCGGAGG AGGTGTCCGG CCTTCTATCG GCGGTGTACA   
  
  
+ AGGCGGGGTT GTCCCATATG GGTTCCGGCA ACAACAACAA CAACAGCAAC AGCAGCAGCA GCAGCAACAG   
  
  
+ CTACATCTAC AGCTACAACA ACAGCGGGGG ATTTTCTCGG CGGATCAGAT CAGCGCTCAA CGAAGGGCGG   
  
  
+ ATTTGTTGAT GGGTAAGCGT TCTTTCGCTG ATTTTCAATC TCAACAACAG CAAATCAGTC TTTTGCAGCA   
  
  
+ GCAGCAGCAG CAACAACAAC AACAGCCCTT TGCGCTCTTG CAAAAACAAC AGCAGTTTCA ACAACAATTA   
  
  
+ CAACAACAAC AGCCACTTTC TCTCCTCCTT CAACAACAGT ACTTGATGAG ATCCGTAAAA CAGAGAACCA   
  
  
+ ACAATTTCGC AGCATCTCCG ATTTCTCCTC TTTCTTCTTC CATTGATCTT TCTTCCGCTA ATAATTCCCG   
  
  
+ GTTCGGTTTG CCGGTTTACC AACAACAACT CCGGTCTCAA CAACAACCAC CTATCTCTCT CCTCGCCCAC   
  
  
+ AACAACAACC AATTCAACCT CTCAAATAAT AACAATTCTT CTTTAATCCA AAACTCCACC AACACTCTTC   
  
  
+ TTGCTCCCGC AGCCAGCAAT AACACCCTAA TGAAAAATTC GGGCTTTTCC TCATCTGGGT TTTCTAATTC   
  
  
+ GGGTCCAAAC CTGGTCCAAC CGAAGCAGGA AATCGGGTAC TCGAATATGG GTACCAATAT GGGTATGAAT   
  
  
+ CTGGTCCAGC CCAAACCCGA CCCTGAGGAT CACGGAATAA TGAACACCCT TCAAGAATTG GAGAAGGAGC   
  
  
+ TTTTGAATGA CGATGAGGAA AACCAGCCGG AAAACGACGC CGTTTCAGTC ATCACCAACA GCGAGTGGTC   
  
  
+ CGAGACCTTC CAGAATCTGA TGAGTCCCGA CCCGAGTTCG AGCCCGAATG CCACCAACAC CCACCCGGGT   
  
  
+ TCGGGTTCTG CTAGTACCAG CGGCAGCAAC AACACTACAA CCACCCCTGC AAACAACAAT AACATGATAT   
  
  
+ CTACCTCGCC GACGTCGTCC ACGTCATCCT GCTGCTCCAC GTCGGCATCG GCGTCTCCGC CGGCGGCGGT   
  
  
+ GGCGACCCCG CAGGTACCAG CCAAGCAGCT GATCTCGGAA GCAGCGGCGG CGATTTCGGA CGGAAAGATT   
  
  
+ GAATCCGCCA TGGAAGTGAT GAACCGGCTG TCCCAGGTTG CCAATTCTAG GGGGAGCTCG GAACAAAAGC   
  
  
+ TGGCTTTTTA CATGGGCCAC GCGCTTCGGT CGCGCGTGAG TCCCACCGAA CACGCTCCCC CTATTGTGGA   
  
  
+ GCTTTTCCAA AAGGAGCATA TGGCGGCCAT GTATTCATTG TACGAGCTCT CTCATTGTTT CAAATTTGGT   
  
  
+ CTTTTGGCTG CCAATTTGGT GATGTTGGAG GCCATTACTT CCGCCCTTCA TGAGGGTCTG AAAATTCATG   
  
  
+ TTGTGGATTT TGATATTGGG CATGGTGGTC AGTACATCAA CCTCCTACGC GCCTTGGGGG AGCGTAGGGC   
  
  
+ TCAGTCGGGG AGCGCGGTGT CGAATGAGGT GAGGATCACT GTGGTTGAGA CCGAGGTCGG TAATGGTGGT   
  
  
+ GCGGCGGGTG CGGAGGCTGT TAAGGATGGC CTGGTGAAAT TGGCTGAGAA GGTGGGGATT AGTTTGAAGT   
  
  
+ TTGAGGCTTT GACTCGGAAG ATCTCCGAGT TAACCCGGGA GTCACTGGGG TGTGAGGAGG GGGAGGCCGT   
  
  
+ GGTGGTGAAT TTTGCGTTCA AACTTTACCG GGTACCGGAT GAGAGCGTGT CGATGGAGAA TCTGAGGGAC   
  
  
+ GAGCTCCTGC GCCGGGTGAA GGCGCTAAGG CCACGCGCCG TCACATTGGT GGAGCAGGAC TTAAATGCGA   
  
  
+ ATACGGCACC GTTCATGACG CGCGTGAGTG GCGCTTGTGA GTACTATAGA GCATTGTTCG ACTCGCTCGA   
  
  
+ CTCGATGGTG CCGAGGGATA ACCCGGACCG GGTCCGGGTC GAGGAGGCCT TGGGGCGGAA GATGGCAAAC   
  
  
+ TCGGTGGCTT GCGAGGGCCG GGAACGGGTT GAGCGGGCGG AGGTGTTTGG GAAGTGGCGG GCGAGGATGA   
  
  
+ GTATGGCAGG GTTCGAGTTG AAGGCACTGA GTCAGAGTGT GGCCGAGTCA ATGCGAACCA AACTCAGCTC   
  
  
+ GGTTCTGGGC GGAAAGCCGG GTTTCACGGT CAAGGAGGAG AGCGGTGGGG TGGGGTTCGG GTGGATGGGA   
  
  
+ CGAACTCTCA CCGTCGCATC GGCCTGGCGT TA  

- +Up\_Stream \_Len000GGAGCC ACACCCTAAG GCCCAACTAT TTATCCCTGG TCGCTTTACA AGTTTATTAA   
  
  
- CCACTTCCTC AGAGACAAAC TTACGGATGA TTACGAAAAA AAAAAAAAAA CTTATTAAAC AGTCTTTCAA   
  
  
- TCCGACGTAA CCTTCCCCGC CGGATGCTTC CGAAAAGTCG TTTATTTATT GGTCGAAAGT AAAACTTTAA   
  
  
- AAAAAATTTT TTACTTTGAG TAAAGTGAAT TTTTAAATGG TATTATATAT ATATATATAT ATATATATAT   
  
  
- ATATATATAT ATAAACTAAA TTGCAATTCG TTTGAAAATT GAATACATAG TAAAATGCTG GCATTTTTTT   
  
  
- TTTCTTTATA TTGTGTTATA CATTATCATA AATTCAAAAA CTATTTATTA TAAAATTGTA TTAAACACAC   
  
  
- ATCATACTTA AATTTAGAAT CATGATTATA TTAATACATA TGAACCGATA TTCTAGAGTT ATAATCATAA   
  
  
- AATACTAAGT TACATTACTA CTACGTTGAC ACACATTAAT CTTTCGGAAA CTAACGTCTA TAGATAGAAT   
  
  
- AACTATGTGT AGATGGTTAA CTCGAAAAAG TTCTAAACTT CTGCCATCAG TTCATAGAAA CCCTCTAATG   
  
  
- CAATCTTAAT GAAGTACTAT TTAATCTGTA TAAAATATGA AGATTAGAAT TTATTCAGAG GTAGGAAAGT   
  
  
- AGTATAAAAT TTCTTTACAT GTTCATTTAC AGTTATCAAA CTTTGCAAGG AATAGTATTT TCAGTATGTA   
  
  
- TACATCCTGT AAAAGTTCAA ATACATTTGG TATACTATAT CAAGTTCTAT TAACAGTTTG TTCAATACTT   
  
  
- TAGATTTCCA CAAACTTGCA TTAAAGTTCG ATTAGAATCG TTCAATATCA TAATAAATTT GATGCTGATC   
  
  
- TGTGTTGTAA AACAAGTAAA TATATTGTGA ATCTTACGTT TTTTTAGCCA TAATGATTAT GACATAGTTC   
  
  
- TTAACTTCAC AATACGTAAT ATGACTATTC CCTATTATAA ATTTAATGAA ATGACAATAT CTTCGGTTTT   
  
  
- TAGATTTGAA AGTATGTCTG CAGCCTGATT TAATCTTTTA AGAAATTGAA TTATCTTTCC TACTGTATTT   
  
  
- ATTTTTATTT TTGGATTTAT CGTATTATTG GTAAGATGTG TTTTCAGTTA GAAGATTATC TTTTTTCGGT   
  
  
- ATTCATAAGT GAATAAATGG TAAAATAAGG ATTAGATGAC AGACAGATAA GTGTGGTATT AAGTACTTGG   
  
  
- TTACACCTCA AAGACAAAGT AAAGTACCCC ACTAAATACA GACTGTTACT AGGTTGTTTG ACTCGGAGTG   
  
  
- CGCGCCTTGT GCTCTCACCG ACATGAATAC CACACTTTCT CGAGATGTTC CACTCTAACT CCTCCGATGC   
  
  
- GCAACCTCAA TAGTGGTGGC AACCTAAACA AAAGAAACCT CGGTGCAATA GTGAGAAGTC TGCGGCTAGG   
  
  
- TTGCCGGGAA TGAAGGTAGA TCAGGGGTGA GAGGTTGAGA TGCGTTAGTT GCGGAAGGCG TAAATTTATA   
  
  
- AAGGAAGAGA TAGCGGATGA AAGAGACAGA GTGTTAAAAA CAGGCATAAA ACTTAAAAGA GTTTTAATAG   
  
  
- TATATTAAAT AAACGGAAGT TGATTTCATA AATAATTTAA TCAATTACAT AAATTTAACT TGATTTTTTC   
  
  
- TCTTTTTCTT TCTTTTGTTG GGGTGCTTTC GTTTTGCTGT CTCCGGTTGA AGAGAGAGAT CTCACATCTC   
  
  
- GGGGTAGAGC CAATATTAAT ACTCTTAGTT AAGGAAGAGT GTTCTCTTTG AGAGCGAGAG AGAGAGAGAG   
  
  
- GTATTCTGTA GTGTGGTTAA CATACTTAAA AGAGAGAGGA CTAAAGACAA AAGGAAGAAC AACAGGGGAA   
  
  
- GATTACGCAA TCACGCCGGT TTTGGTGGTA TTTGGTAGAG AGAAAGTTCT TTCGTCGTTG TTGTTTCTCT   
  
  
- CTGTCTCTCT CTCTCTCCCC CTCTTTCTAG TGTAGAAAAC CCAAAGGCCG CTTATACTGC AGACCCAAAA   
  
  
- GCCCGCTAAA GAAGATGCCG CCACCTCCGC CACCGCCTCC TCCACAGGCC GGAAGATAGC CGCCACATGT   
  
  
- TCCGCCCCAA CAGGGTATAC CCAAGGCCGT TGTTGTTGTT GTTGTCGTTG TCGTCGTCGT CGTCGTTGTC   
  
  
- GATGTAGATG TCGATGTTGT TGTCGCCCCC TAAAAGAGCC GCCTAGTCTA GTCGCGAGTT GCTTCCCGCC   
  
  
- TAAACAACTA CCCATTCGCA AGAAAGCGAC TAAAAGTTAG AGTTGTTGTC GTTTAGTCAG AAAACGTCGT   
  
  
- CGTCGTCGTC GTTGTTGTTG TTGTCGGGAA ACGCGAGAAC GTTTTTGTTG TCGTCAAAGT TGTTGTTAAT   
  
  
- GTTGTTGTTG TCGGTGAAAG AGAGGAGGAA GTTGTTGTCA TGAACTACTC TAGGCATTTT GTCTCTTGGT   
  
  
- TGTTAAAGCG TCGTAGAGGC TAAAGAGGAG AAAGAAGAAG GTAACTAGAA AGAAGGCGAT TATTAAGGGC   
  
  
- CAAGCCAAAC GGCCAAATGG TTGTTGTTGA GGCCAGAGTT GTTGTTGGTG GATAGAGAGA GGAGCGGGTG   
  
  
- TTGTTGTTGG TTAAGTTGGA GAGTTTATTA TTGTTAAGAA GAAATTAGGT TTTGAGGTGG TTGTGAGAAG   
  
  
- AACGAGGGCG TCGGTCGTTA TTGTGGGATT ACTTTTTAAG CCCGAAAAGG AGTAGACCCA AAAGATTAAG   
  
  
- CCCAGGTTTG GACCAGGTTG GCTTCGTCCT TTAGCCCATG AGCTTATACC CATGGTTATA CCCATACTTA   
  
  
- GACCAGGTCG GGTTTGGGCT GGGACTCCTA GTGCCTTATT ACTTGTGGGA AGTTCTTAAC CTCTTCCTCG   
  
  
- AAAACTTACT GCTACTCCTT TTGGTCGGCC TTTTGCTGCG GCAAAGTCAG TAGTGGTTGT CGCTCACCAG   
  
  
- GCTCTGGAAG GTCTTAGACT ACTCAGGGCT GGGCTCAAGC TCGGGCTTAC GGTGGTTGTG GGTGGGCCCA   
  
  
- AGCCCAAGAC GATCATGGTC GCCGTCGTTG TTGTGATGTT GGTGGGGACG TTTGTTGTTA TTGTACTATA   
  
  
- GATGGAGCGG CTGCAGCAGG TGCAGTAGGA CGACGAGGTG CAGCCGTAGC CGCAGAGGCG GCCGCCGCCA   
  
  
- CCGCTGGGGC GTCCATGGTC GGTTCGTCGA CTAGAGCCTT CGTCGCCGCC GCTAAAGCCT GCCTTTCTAA   
  
  
- CTTAGGCGGT ACCTTCACTA CTTGGCCGAC AGGGTCCAAC GGTTAAGATC CCCCTCGAGC CTTGTTTTCG   
  
  
- ACCGAAAAAT GTACCCGGTG CGCGAAGCCA GCGCGCACTC AGGGTGGCTT GTGCGAGGGG GATAACACCT   
  
  
- CGAAAAGGTT TTCCTCGTAT ACCGCCGGTA CATAAGTAAC ATGCTCGAGA GAGTAACAAA GTTTAAACCA   
  
  
- GAAAACCGAC GGTTAAACCA CTACAACCTC CGGTAATGAA GGCGGGAAGT ACTCCCAGAC TTTTAAGTAC   
  
  
- AACACCTAAA ACTATAACCC GTACCACCAG TCATGTAGTT GGAGGATGCG CGGAACCCCC TCGCATCCCG   
  
  
- AGTCAGCCCC TCGCGCCACA GCTTACTCCA CTCCTAGTGA CACCAACTCT GGCTCCAGCC ATTACCACCA   
  
  
- CGCCGCCCAC GCCTCCGACA ATTCCTACCG GACCACTTTA ACCGACTCTT CCACCCCTAA TCAAACTTCA   
  
  
- AACTCCGAAA CTGAGCCTTC TAGAGGCTCA ATTGGGCCCT CAGTGACCCC ACACTCCTCC CCCTCCGGCA   
  
  
- CCACCACTTA AAACGCAAGT TTGAAATGGC CCATGGCCTA CTCTCGCACA GCTACCTCTT AGACTCCCTG   
  
  
- CTCGAGGACG CGGCCCACTT CCGCGATTCC GGTGCGCGGC AGTGTAACCA CCTCGTCCTG AATTTACGCT   
  
  
- TATGCCGTGG CAAGTACTGC GCGCACTCAC CGCGAACACT CATGATATCT CGTAACAAGC TGAGCGAGCT   
  
  
- GAGCTACCAC GGCTCCCTAT TGGGCCTGGC CCAGGCCCAG CTCCTCCGGA ACCCCGCCTT CTACCGTTTG   
  
  
- AGCCACCGAA CGCTCCCGGC CCTTGCCCAA CTCGCCCGCC TCCACAAACC CTTCACCGCC CGCTCCTACT   
  
  
- CATACCGTCC CAAGCTCAAC TTCCGTGACT CAGTCTCACA CCGGCTCAGT TACGCTTGGT TTGAGTCGAG   
  
  
- CCAAGACCCG CCTTTCGGCC CAAAGTGCCA GTTCCTCCTC TCGCCACCCC ACCCCAAGCC CACCTACCCT   
  
  
- GCTTGAGAGT GGCAGCGTAG CCGGACCGCA AT

+     GTGGC-motif

| Site Name | Organism | Position | Strand | Matrix score. | sequence | function |
| --- | --- | --- | --- | --- | --- | --- |
| GTGGC-motif | Hordeum vulgare | 4167 | + | 10 | CAGCGTGTGGC | part of a light responsive element |

>HU06G02537.1   
+ +Up\_Stream \_Len000CCTCGG TGTGGGATTC CGGGTTGATA AATAGGGACC AGCGAAATGT TCAAATAATT   
  
  
+ GGTGAAGGAG TCTCTGTTTG AATGCCTACT AATGCTTTTT TTTTTTTTTT GAATAATTTG TCAGAAAGTT   
  
  
+ AGGCTGCATT GGAAGGGGCG GCCTACGAAG GCTTTTCAGC AAATAAATAA CCAGCTTTCA TTTTGAAATT   
  
  
+ TTTTTTAAAA AATGAAACTC ATTTCACTTA AAAATTTACC ATAATATATA TATATATATA TATATATATA   
  
  
+ TATATATATA TATTTGATTT AACGTTAAGC AAACTTTTAA CTTATGTATC ATTTTACGAC CGTAAAAAAA   
  
  
+ AAAGAAATAT AACACAATAT GTAATAGTAT TTAAGTTTTT GATAAATAAT ATTTTAACAT AATTTGTGTG   
  
  
+ TAGTATGAAT TTAAATCTTA GTACTAATAT AATTATGTAT ACTTGGCTAT AAGATCTCAA TATTAGTATT   
  
  
+ TTATGATTCA ATGTAATGAT GATGCAACTG TGTGTAATTA GAAAGCCTTT GATTGCAGAT ATCTATCTTA   
  
  
+ TTGATACACA TCTACCAATT GAGCTTTTTC AAGATTTGAA GACGGTAGTC AAGTATCTTT GGGAGATTAC   
  
  
+ GTTAGAATTA CTTCATGATA AATTAGACAT ATTTTATACT TCTAATCTTA AATAAGTCTC CATCCTTTCA   
  
  
+ TCATATTTTA AAGAAATGTA CAAGTAAATG TCAATAGTTT GAAACGTTCC TTATCATAAA AGTCATACAT   
  
  
+ ATGTAGGACA TTTTCAAGTT TATGTAAACC ATATGATATA GTTCAAGATA ATTGTCAAAC AAGTTATGAA   
  
  
+ ATCTAAAGGT GTTTGAACGT AATTTCAAGC TAATCTTAGC AAGTTATAGT ATTATTTAAA CTACGACTAG   
  
  
+ ACACAACATT TTGTTCATTT ATATAACACT TAGAATGCAA AAAAATCGGT ATTACTAATA CTGTATCAAG   
  
  
+ AATTGAAGTG TTATGCATTA TACTGATAAG GGATAATATT TAAATTACTT TACTGTTATA GAAGCCAAAA   
  
  
+ ATCTAAACTT TCATACAGAC GTCGGACTAA ATTAGAAAAT TCTTTAACTT AATAGAAAGG ATGACATAAA   
  
  
+ TAAAAATAAA AACCTAAATA GCATAATAAC CATTCTACAC AAAAGTCAAT CTTCTAATAG AAAAAAGCCA   
  
  
+ TAAGTATTCA CTTATTTACC ATTTTATTCC TAATCTACTG TCTGTCTATT CACACCATAA TTCATGAACC   
  
  
+ AATGTGGAGT TTCTGTTTCA TTTCATGGGG TGATTTATGT CTGACAATGA TCCAACAAAC TGAGCCTCAC   
  
  
+ GCGCGGAACA CGAGAGTGGC TGTACTTATG GTGTGAAAGA GCTCTACAAG GTGAGATTGA GGAGGCTACG   
  
  
+ CGTTGGAGTT ATCACCACCG TTGGATTTGT TTTCTTTGGA GCCACGTTAT CACTCTTCAG ACGCCGATCC   
  
  
+ AACGGCCCTT ACTTCCATCT AGTCCCCACT CTCCAACTCT ACGCAATCAA CGCCTTCCGC ATTTAAATAT   
  
  
+ TTCCTTCTCT ATCGCCTACT TTCTCTGTCT CACAATTTTT GTCCGTATTT TGAATTTTCT CAAAATTATC   
  
  
+ ATATAATTTA TTTGCCTTCA ACTAAAGTAT TTATTAAATT AGTTAATGTA TTTAAATTGA ACTAAAAAAG   
  
  
+ AGAAAAAGAA AGAAAACAAC CCCACGAAAG CAAAACGACA GAGGCCAACT TCTCTCTCTA GAGTGTAGAG   
  
  
+ CCCCATCTCG GTTATAATTA TGAGAATCAA TTCCTTCTCA CAAGAGAAAC TCTCGCTCTC TCTCTCTCTC   
  
  
+ CATAAGACAT CACACCAATT GTATGAATTT TCTCTCTCCT GATTTCTGTT TTCCTTCTTG TTGTCCCCTT   
  
  
+ CTAATGCGTT AGTGCGGCCA AAACCACCAT AAACCATCTC TCTTTCAAGA AAGCAGCAAC AACAAAGAGA   
  
  
+ GACAGAGAGA GAGAGAGGGG GAGAAAGATC ACATCTTTTG GGTTTCCGGC GAATATGACG TCTGGGTTTT   
  
  
+ CGGGCGATTT CTTCTACGGC GGTGGAGGCG GTGGCGGAGG AGGTGTCCGG CCTTCTATCG GCGGTGTACA   
  
  
+ AGGCGGGGTT GTCCCATATG GGTTCCGGCA ACAACAACAA CAACAGCAAC AGCAGCAGCA GCAGCAACAG   
  
  
+ CTACATCTAC AGCTACAACA ACAGCGGGGG ATTTTCTCGG CGGATCAGAT CAGCGCTCAA CGAAGGGCGG   
  
  
+ ATTTGTTGAT GGGTAAGCGT TCTTTCGCTG ATTTTCAATC TCAACAACAG CAAATCAGTC TTTTGCAGCA   
  
  
+ GCAGCAGCAG CAACAACAAC AACAGCCCTT TGCGCTCTTG CAAAAACAAC AGCAGTTTCA ACAACAATTA   
  
  
+ CAACAACAAC AGCCACTTTC TCTCCTCCTT CAACAACAGT ACTTGATGAG ATCCGTAAAA CAGAGAACCA   
  
  
+ ACAATTTCGC AGCATCTCCG ATTTCTCCTC TTTCTTCTTC CATTGATCTT TCTTCCGCTA ATAATTCCCG   
  
  
+ GTTCGGTTTG CCGGTTTACC AACAACAACT CCGGTCTCAA CAACAACCAC CTATCTCTCT CCTCGCCCAC   
  
  
+ AACAACAACC AATTCAACCT CTCAAATAAT AACAATTCTT CTTTAATCCA AAACTCCACC AACACTCTTC   
  
  
+ TTGCTCCCGC AGCCAGCAAT AACACCCTAA TGAAAAATTC GGGCTTTTCC TCATCTGGGT TTTCTAATTC   
  
  
+ GGGTCCAAAC CTGGTCCAAC CGAAGCAGGA AATCGGGTAC TCGAATATGG GTACCAATAT GGGTATGAAT   
  
  
+ CTGGTCCAGC CCAAACCCGA CCCTGAGGAT CACGGAATAA TGAACACCCT TCAAGAATTG GAGAAGGAGC   
  
  
+ TTTTGAATGA CGATGAGGAA AACCAGCCGG AAAACGACGC CGTTTCAGTC ATCACCAACA GCGAGTGGTC   
  
  
+ CGAGACCTTC CAGAATCTGA TGAGTCCCGA CCCGAGTTCG AGCCCGAATG CCACCAACAC CCACCCGGGT   
  
  
+ TCGGGTTCTG CTAGTACCAG CGGCAGCAAC AACACTACAA CCACCCCTGC AAACAACAAT AACATGATAT   
  
  
+ CTACCTCGCC GACGTCGTCC ACGTCATCCT GCTGCTCCAC GTCGGCATCG GCGTCTCCGC CGGCGGCGGT   
  
  
+ GGCGACCCCG CAGGTACCAG CCAAGCAGCT GATCTCGGAA GCAGCGGCGG CGATTTCGGA CGGAAAGATT   
  
  
+ GAATCCGCCA TGGAAGTGAT GAACCGGCTG TCCCAGGTTG CCAATTCTAG GGGGAGCTCG GAACAAAAGC   
  
  
+ TGGCTTTTTA CATGGGCCAC GCGCTTCGGT CGCGCGTGAG TCCCACCGAA CACGCTCCCC CTATTGTGGA   
  
  
+ GCTTTTCCAA AAGGAGCATA TGGCGGCCAT GTATTCATTG TACGAGCTCT CTCATTGTTT CAAATTTGGT   
  
  
+ CTTTTGGCTG CCAATTTGGT GATGTTGGAG GCCATTACTT CCGCCCTTCA TGAGGGTCTG AAAATTCATG   
  
  
+ TTGTGGATTT TGATATTGGG CATGGTGGTC AGTACATCAA CCTCCTACGC GCCTTGGGGG AGCGTAGGGC   
  
  
+ TCAGTCGGGG AGCGCGGTGT CGAATGAGGT GAGGATCACT GTGGTTGAGA CCGAGGTCGG TAATGGTGGT   
  
  
+ GCGGCGGGTG CGGAGGCTGT TAAGGATGGC CTGGTGAAAT TGGCTGAGAA GGTGGGGATT AGTTTGAAGT   
  
  
+ TTGAGGCTTT GACTCGGAAG ATCTCCGAGT TAACCCGGGA GTCACTGGGG TGTGAGGAGG GGGAGGCCGT   
  
  
+ GGTGGTGAAT TTTGCGTTCA AACTTTACCG GGTACCGGAT GAGAGCGTGT CGATGGAGAA TCTGAGGGAC   
  
  
+ GAGCTCCTGC GCCGGGTGAA GGCGCTAAGG CCACGCGCCG TCACATTGGT GGAGCAGGAC TTAAATGCGA   
  
  
+ ATACGGCACC GTTCATGACG CGCGTGAGTG GCGCTTGTGA GTACTATAGA GCATTGTTCG ACTCGCTCGA   
  
  
+ CTCGATGGTG CCGAGGGATA ACCCGGACCG GGTCCGGGTC GAGGAGGCCT TGGGGCGGAA GATGGCAAAC   
  
  
+ TCGGTGGCTT GCGAGGGCCG GGAACGGGTT GAGCGGGCGG AGGTGTTTGG GAAGTGGCGG GCGAGGATGA   
  
  
+ GTATGGCAGG GTTCGAGTTG AAGGCACTGA GTCAGAGTGT GGCCGAGTCA ATGCGAACCA AACTCAGCTC   
  
  
+ GGTTCTGGGC GGAAAGCCGG GTTTCACGGT CAAGGAGGAG AGCGGTGGGG TGGGGTTCGG GTGGATGGGA   
  
  
+ CGAACTCTCA CCGTCGCATC GGCCTGGCGT TA  

- +Up\_Stream \_Len000GGAGCC ACACCCTAAG GCCCAACTAT TTATCCCTGG TCGCTTTACA AGTTTATTAA   
  
  
- CCACTTCCTC AGAGACAAAC TTACGGATGA TTACGAAAAA AAAAAAAAAA CTTATTAAAC AGTCTTTCAA   
  
  
- TCCGACGTAA CCTTCCCCGC CGGATGCTTC CGAAAAGTCG TTTATTTATT GGTCGAAAGT AAAACTTTAA   
  
  
- AAAAAATTTT TTACTTTGAG TAAAGTGAAT TTTTAAATGG TATTATATAT ATATATATAT ATATATATAT   
  
  
- ATATATATAT ATAAACTAAA TTGCAATTCG TTTGAAAATT GAATACATAG TAAAATGCTG GCATTTTTTT   
  
  
- TTTCTTTATA TTGTGTTATA CATTATCATA AATTCAAAAA CTATTTATTA TAAAATTGTA TTAAACACAC   
  
  
- ATCATACTTA AATTTAGAAT CATGATTATA TTAATACATA TGAACCGATA TTCTAGAGTT ATAATCATAA   
  
  
- AATACTAAGT TACATTACTA CTACGTTGAC ACACATTAAT CTTTCGGAAA CTAACGTCTA TAGATAGAAT   
  
  
- AACTATGTGT AGATGGTTAA CTCGAAAAAG TTCTAAACTT CTGCCATCAG TTCATAGAAA CCCTCTAATG   
  
  
- CAATCTTAAT GAAGTACTAT TTAATCTGTA TAAAATATGA AGATTAGAAT TTATTCAGAG GTAGGAAAGT   
  
  
- AGTATAAAAT TTCTTTACAT GTTCATTTAC AGTTATCAAA CTTTGCAAGG AATAGTATTT TCAGTATGTA   
  
  
- TACATCCTGT AAAAGTTCAA ATACATTTGG TATACTATAT CAAGTTCTAT TAACAGTTTG TTCAATACTT   
  
  
- TAGATTTCCA CAAACTTGCA TTAAAGTTCG ATTAGAATCG TTCAATATCA TAATAAATTT GATGCTGATC   
  
  
- TGTGTTGTAA AACAAGTAAA TATATTGTGA ATCTTACGTT TTTTTAGCCA TAATGATTAT GACATAGTTC   
  
  
- TTAACTTCAC AATACGTAAT ATGACTATTC CCTATTATAA ATTTAATGAA ATGACAATAT CTTCGGTTTT   
  
  
- TAGATTTGAA AGTATGTCTG CAGCCTGATT TAATCTTTTA AGAAATTGAA TTATCTTTCC TACTGTATTT   
  
  
- ATTTTTATTT TTGGATTTAT CGTATTATTG GTAAGATGTG TTTTCAGTTA GAAGATTATC TTTTTTCGGT   
  
  
- ATTCATAAGT GAATAAATGG TAAAATAAGG ATTAGATGAC AGACAGATAA GTGTGGTATT AAGTACTTGG   
  
  
- TTACACCTCA AAGACAAAGT AAAGTACCCC ACTAAATACA GACTGTTACT AGGTTGTTTG ACTCGGAGTG   
  
  
- CGCGCCTTGT GCTCTCACCG ACATGAATAC CACACTTTCT CGAGATGTTC CACTCTAACT CCTCCGATGC   
  
  
- GCAACCTCAA TAGTGGTGGC AACCTAAACA AAAGAAACCT CGGTGCAATA GTGAGAAGTC TGCGGCTAGG   
  
  
- TTGCCGGGAA TGAAGGTAGA TCAGGGGTGA GAGGTTGAGA TGCGTTAGTT GCGGAAGGCG TAAATTTATA   
  
  
- AAGGAAGAGA TAGCGGATGA AAGAGACAGA GTGTTAAAAA CAGGCATAAA ACTTAAAAGA GTTTTAATAG   
  
  
- TATATTAAAT AAACGGAAGT TGATTTCATA AATAATTTAA TCAATTACAT AAATTTAACT TGATTTTTTC   
  
  
- TCTTTTTCTT TCTTTTGTTG GGGTGCTTTC GTTTTGCTGT CTCCGGTTGA AGAGAGAGAT CTCACATCTC   
  
  
- GGGGTAGAGC CAATATTAAT ACTCTTAGTT AAGGAAGAGT GTTCTCTTTG AGAGCGAGAG AGAGAGAGAG   
  
  
- GTATTCTGTA GTGTGGTTAA CATACTTAAA AGAGAGAGGA CTAAAGACAA AAGGAAGAAC AACAGGGGAA   
  
  
- GATTACGCAA TCACGCCGGT TTTGGTGGTA TTTGGTAGAG AGAAAGTTCT TTCGTCGTTG TTGTTTCTCT   
  
  
- CTGTCTCTCT CTCTCTCCCC CTCTTTCTAG TGTAGAAAAC CCAAAGGCCG CTTATACTGC AGACCCAAAA   
  
  
- GCCCGCTAAA GAAGATGCCG CCACCTCCGC CACCGCCTCC TCCACAGGCC GGAAGATAGC CGCCACATGT   
  
  
- TCCGCCCCAA CAGGGTATAC CCAAGGCCGT TGTTGTTGTT GTTGTCGTTG TCGTCGTCGT CGTCGTTGTC   
  
  
- GATGTAGATG TCGATGTTGT TGTCGCCCCC TAAAAGAGCC GCCTAGTCTA GTCGCGAGTT GCTTCCCGCC   
  
  
- TAAACAACTA CCCATTCGCA AGAAAGCGAC TAAAAGTTAG AGTTGTTGTC GTTTAGTCAG AAAACGTCGT   
  
  
- CGTCGTCGTC GTTGTTGTTG TTGTCGGGAA ACGCGAGAAC GTTTTTGTTG TCGTCAAAGT TGTTGTTAAT   
  
  
- GTTGTTGTTG TCGGTGAAAG AGAGGAGGAA GTTGTTGTCA TGAACTACTC TAGGCATTTT GTCTCTTGGT   
  
  
- TGTTAAAGCG TCGTAGAGGC TAAAGAGGAG AAAGAAGAAG GTAACTAGAA AGAAGGCGAT TATTAAGGGC   
  
  
- CAAGCCAAAC GGCCAAATGG TTGTTGTTGA GGCCAGAGTT GTTGTTGGTG GATAGAGAGA GGAGCGGGTG   
  
  
- TTGTTGTTGG TTAAGTTGGA GAGTTTATTA TTGTTAAGAA GAAATTAGGT TTTGAGGTGG TTGTGAGAAG   
  
  
- AACGAGGGCG TCGGTCGTTA TTGTGGGATT ACTTTTTAAG CCCGAAAAGG AGTAGACCCA AAAGATTAAG   
  
  
- CCCAGGTTTG GACCAGGTTG GCTTCGTCCT TTAGCCCATG AGCTTATACC CATGGTTATA CCCATACTTA   
  
  
- GACCAGGTCG GGTTTGGGCT GGGACTCCTA GTGCCTTATT ACTTGTGGGA AGTTCTTAAC CTCTTCCTCG   
  
  
- AAAACTTACT GCTACTCCTT TTGGTCGGCC TTTTGCTGCG GCAAAGTCAG TAGTGGTTGT CGCTCACCAG   
  
  
- GCTCTGGAAG GTCTTAGACT ACTCAGGGCT GGGCTCAAGC TCGGGCTTAC GGTGGTTGTG GGTGGGCCCA   
  
  
- AGCCCAAGAC GATCATGGTC GCCGTCGTTG TTGTGATGTT GGTGGGGACG TTTGTTGTTA TTGTACTATA   
  
  
- GATGGAGCGG CTGCAGCAGG TGCAGTAGGA CGACGAGGTG CAGCCGTAGC CGCAGAGGCG GCCGCCGCCA   
  
  
- CCGCTGGGGC GTCCATGGTC GGTTCGTCGA CTAGAGCCTT CGTCGCCGCC GCTAAAGCCT GCCTTTCTAA   
  
  
- CTTAGGCGGT ACCTTCACTA CTTGGCCGAC AGGGTCCAAC GGTTAAGATC CCCCTCGAGC CTTGTTTTCG   
  
  
- ACCGAAAAAT GTACCCGGTG CGCGAAGCCA GCGCGCACTC AGGGTGGCTT GTGCGAGGGG GATAACACCT   
  
  
- CGAAAAGGTT TTCCTCGTAT ACCGCCGGTA CATAAGTAAC ATGCTCGAGA GAGTAACAAA GTTTAAACCA   
  
  
- GAAAACCGAC GGTTAAACCA CTACAACCTC CGGTAATGAA GGCGGGAAGT ACTCCCAGAC TTTTAAGTAC   
  
  
- AACACCTAAA ACTATAACCC GTACCACCAG TCATGTAGTT GGAGGATGCG CGGAACCCCC TCGCATCCCG   
  
  
- AGTCAGCCCC TCGCGCCACA GCTTACTCCA CTCCTAGTGA CACCAACTCT GGCTCCAGCC ATTACCACCA   
  
  
- CGCCGCCCAC GCCTCCGACA ATTCCTACCG GACCACTTTA ACCGACTCTT CCACCCCTAA TCAAACTTCA   
  
  
- AACTCCGAAA CTGAGCCTTC TAGAGGCTCA ATTGGGCCCT CAGTGACCCC ACACTCCTCC CCCTCCGGCA   
  
  
- CCACCACTTA AAACGCAAGT TTGAAATGGC CCATGGCCTA CTCTCGCACA GCTACCTCTT AGACTCCCTG   
  
  
- CTCGAGGACG CGGCCCACTT CCGCGATTCC GGTGCGCGGC AGTGTAACCA CCTCGTCCTG AATTTACGCT   
  
  
- TATGCCGTGG CAAGTACTGC GCGCACTCAC CGCGAACACT CATGATATCT CGTAACAAGC TGAGCGAGCT   
  
  
- GAGCTACCAC GGCTCCCTAT TGGGCCTGGC CCAGGCCCAG CTCCTCCGGA ACCCCGCCTT CTACCGTTTG   
  
  
- AGCCACCGAA CGCTCCCGGC CCTTGCCCAA CTCGCCCGCC TCCACAAACC CTTCACCGCC CGCTCCTACT   
  
  
- CATACCGTCC CAAGCTCAAC TTCCGTGACT CAGTCTCACA CCGGCTCAGT TACGCTTGGT TTGAGTCGAG   
  
  
- CCAAGACCCG CCTTTCGGCC CAAAGTGCCA GTTCCTCCTC TCGCCACCCC ACCCCAAGCC CACCTACCCT   
  
  
- GCTTGAGAGT GGCAGCGTAG CCGGACCGCA AT

+     LTR

| Site Name | Organism | Position | Strand | Matrix score. | sequence | function |
| --- | --- | --- | --- | --- | --- | --- |
| LTR | Hordeum vulgare | 2032 | - | 6 | CCGAAA | cis-acting element involved in low-temperature responsiveness |
| LTR | Hordeum vulgare | 3208 | - | 6 | CCGAAA | cis-acting element involved in low-temperature responsiveness |

>HU06G02537.1   
+ +Up\_Stream \_Len000CCTCGG TGTGGGATTC CGGGTTGATA AATAGGGACC AGCGAAATGT TCAAATAATT   
  
  
+ GGTGAAGGAG TCTCTGTTTG AATGCCTACT AATGCTTTTT TTTTTTTTTT GAATAATTTG TCAGAAAGTT   
  
  
+ AGGCTGCATT GGAAGGGGCG GCCTACGAAG GCTTTTCAGC AAATAAATAA CCAGCTTTCA TTTTGAAATT   
  
  
+ TTTTTTAAAA AATGAAACTC ATTTCACTTA AAAATTTACC ATAATATATA TATATATATA TATATATATA   
  
  
+ TATATATATA TATTTGATTT AACGTTAAGC AAACTTTTAA CTTATGTATC ATTTTACGAC CGTAAAAAAA   
  
  
+ AAAGAAATAT AACACAATAT GTAATAGTAT TTAAGTTTTT GATAAATAAT ATTTTAACAT AATTTGTGTG   
  
  
+ TAGTATGAAT TTAAATCTTA GTACTAATAT AATTATGTAT ACTTGGCTAT AAGATCTCAA TATTAGTATT   
  
  
+ TTATGATTCA ATGTAATGAT GATGCAACTG TGTGTAATTA GAAAGCCTTT GATTGCAGAT ATCTATCTTA   
  
  
+ TTGATACACA TCTACCAATT GAGCTTTTTC AAGATTTGAA GACGGTAGTC AAGTATCTTT GGGAGATTAC   
  
  
+ GTTAGAATTA CTTCATGATA AATTAGACAT ATTTTATACT TCTAATCTTA AATAAGTCTC CATCCTTTCA   
  
  
+ TCATATTTTA AAGAAATGTA CAAGTAAATG TCAATAGTTT GAAACGTTCC TTATCATAAA AGTCATACAT   
  
  
+ ATGTAGGACA TTTTCAAGTT TATGTAAACC ATATGATATA GTTCAAGATA ATTGTCAAAC AAGTTATGAA   
  
  
+ ATCTAAAGGT GTTTGAACGT AATTTCAAGC TAATCTTAGC AAGTTATAGT ATTATTTAAA CTACGACTAG   
  
  
+ ACACAACATT TTGTTCATTT ATATAACACT TAGAATGCAA AAAAATCGGT ATTACTAATA CTGTATCAAG   
  
  
+ AATTGAAGTG TTATGCATTA TACTGATAAG GGATAATATT TAAATTACTT TACTGTTATA GAAGCCAAAA   
  
  
+ ATCTAAACTT TCATACAGAC GTCGGACTAA ATTAGAAAAT TCTTTAACTT AATAGAAAGG ATGACATAAA   
  
  
+ TAAAAATAAA AACCTAAATA GCATAATAAC CATTCTACAC AAAAGTCAAT CTTCTAATAG AAAAAAGCCA   
  
  
+ TAAGTATTCA CTTATTTACC ATTTTATTCC TAATCTACTG TCTGTCTATT CACACCATAA TTCATGAACC   
  
  
+ AATGTGGAGT TTCTGTTTCA TTTCATGGGG TGATTTATGT CTGACAATGA TCCAACAAAC TGAGCCTCAC   
  
  
+ GCGCGGAACA CGAGAGTGGC TGTACTTATG GTGTGAAAGA GCTCTACAAG GTGAGATTGA GGAGGCTACG   
  
  
+ CGTTGGAGTT ATCACCACCG TTGGATTTGT TTTCTTTGGA GCCACGTTAT CACTCTTCAG ACGCCGATCC   
  
  
+ AACGGCCCTT ACTTCCATCT AGTCCCCACT CTCCAACTCT ACGCAATCAA CGCCTTCCGC ATTTAAATAT   
  
  
+ TTCCTTCTCT ATCGCCTACT TTCTCTGTCT CACAATTTTT GTCCGTATTT TGAATTTTCT CAAAATTATC   
  
  
+ ATATAATTTA TTTGCCTTCA ACTAAAGTAT TTATTAAATT AGTTAATGTA TTTAAATTGA ACTAAAAAAG   
  
  
+ AGAAAAAGAA AGAAAACAAC CCCACGAAAG CAAAACGACA GAGGCCAACT TCTCTCTCTA GAGTGTAGAG   
  
  
+ CCCCATCTCG GTTATAATTA TGAGAATCAA TTCCTTCTCA CAAGAGAAAC TCTCGCTCTC TCTCTCTCTC   
  
  
+ CATAAGACAT CACACCAATT GTATGAATTT TCTCTCTCCT GATTTCTGTT TTCCTTCTTG TTGTCCCCTT   
  
  
+ CTAATGCGTT AGTGCGGCCA AAACCACCAT AAACCATCTC TCTTTCAAGA AAGCAGCAAC AACAAAGAGA   
  
  
+ GACAGAGAGA GAGAGAGGGG GAGAAAGATC ACATCTTTTG GGTTTCCGGC GAATATGACG TCTGGGTTTT   
  
  
+ CGGGCGATTT CTTCTACGGC GGTGGAGGCG GTGGCGGAGG AGGTGTCCGG CCTTCTATCG GCGGTGTACA   
  
  
+ AGGCGGGGTT GTCCCATATG GGTTCCGGCA ACAACAACAA CAACAGCAAC AGCAGCAGCA GCAGCAACAG   
  
  
+ CTACATCTAC AGCTACAACA ACAGCGGGGG ATTTTCTCGG CGGATCAGAT CAGCGCTCAA CGAAGGGCGG   
  
  
+ ATTTGTTGAT GGGTAAGCGT TCTTTCGCTG ATTTTCAATC TCAACAACAG CAAATCAGTC TTTTGCAGCA   
  
  
+ GCAGCAGCAG CAACAACAAC AACAGCCCTT TGCGCTCTTG CAAAAACAAC AGCAGTTTCA ACAACAATTA   
  
  
+ CAACAACAAC AGCCACTTTC TCTCCTCCTT CAACAACAGT ACTTGATGAG ATCCGTAAAA CAGAGAACCA   
  
  
+ ACAATTTCGC AGCATCTCCG ATTTCTCCTC TTTCTTCTTC CATTGATCTT TCTTCCGCTA ATAATTCCCG   
  
  
+ GTTCGGTTTG CCGGTTTACC AACAACAACT CCGGTCTCAA CAACAACCAC CTATCTCTCT CCTCGCCCAC   
  
  
+ AACAACAACC AATTCAACCT CTCAAATAAT AACAATTCTT CTTTAATCCA AAACTCCACC AACACTCTTC   
  
  
+ TTGCTCCCGC AGCCAGCAAT AACACCCTAA TGAAAAATTC GGGCTTTTCC TCATCTGGGT TTTCTAATTC   
  
  
+ GGGTCCAAAC CTGGTCCAAC CGAAGCAGGA AATCGGGTAC TCGAATATGG GTACCAATAT GGGTATGAAT   
  
  
+ CTGGTCCAGC CCAAACCCGA CCCTGAGGAT CACGGAATAA TGAACACCCT TCAAGAATTG GAGAAGGAGC   
  
  
+ TTTTGAATGA CGATGAGGAA AACCAGCCGG AAAACGACGC CGTTTCAGTC ATCACCAACA GCGAGTGGTC   
  
  
+ CGAGACCTTC CAGAATCTGA TGAGTCCCGA CCCGAGTTCG AGCCCGAATG CCACCAACAC CCACCCGGGT   
  
  
+ TCGGGTTCTG CTAGTACCAG CGGCAGCAAC AACACTACAA CCACCCCTGC AAACAACAAT AACATGATAT   
  
  
+ CTACCTCGCC GACGTCGTCC ACGTCATCCT GCTGCTCCAC GTCGGCATCG GCGTCTCCGC CGGCGGCGGT   
  
  
+ GGCGACCCCG CAGGTACCAG CCAAGCAGCT GATCTCGGAA GCAGCGGCGG CGATTTCGGA CGGAAAGATT   
  
  
+ GAATCCGCCA TGGAAGTGAT GAACCGGCTG TCCCAGGTTG CCAATTCTAG GGGGAGCTCG GAACAAAAGC   
  
  
+ TGGCTTTTTA CATGGGCCAC GCGCTTCGGT CGCGCGTGAG TCCCACCGAA CACGCTCCCC CTATTGTGGA   
  
  
+ GCTTTTCCAA AAGGAGCATA TGGCGGCCAT GTATTCATTG TACGAGCTCT CTCATTGTTT CAAATTTGGT   
  
  
+ CTTTTGGCTG CCAATTTGGT GATGTTGGAG GCCATTACTT CCGCCCTTCA TGAGGGTCTG AAAATTCATG   
  
  
+ TTGTGGATTT TGATATTGGG CATGGTGGTC AGTACATCAA CCTCCTACGC GCCTTGGGGG AGCGTAGGGC   
  
  
+ TCAGTCGGGG AGCGCGGTGT CGAATGAGGT GAGGATCACT GTGGTTGAGA CCGAGGTCGG TAATGGTGGT   
  
  
+ GCGGCGGGTG CGGAGGCTGT TAAGGATGGC CTGGTGAAAT TGGCTGAGAA GGTGGGGATT AGTTTGAAGT   
  
  
+ TTGAGGCTTT GACTCGGAAG ATCTCCGAGT TAACCCGGGA GTCACTGGGG TGTGAGGAGG GGGAGGCCGT   
  
  
+ GGTGGTGAAT TTTGCGTTCA AACTTTACCG GGTACCGGAT GAGAGCGTGT CGATGGAGAA TCTGAGGGAC   
  
  
+ GAGCTCCTGC GCCGGGTGAA GGCGCTAAGG CCACGCGCCG TCACATTGGT GGAGCAGGAC TTAAATGCGA   
  
  
+ ATACGGCACC GTTCATGACG CGCGTGAGTG GCGCTTGTGA GTACTATAGA GCATTGTTCG ACTCGCTCGA   
  
  
+ CTCGATGGTG CCGAGGGATA ACCCGGACCG GGTCCGGGTC GAGGAGGCCT TGGGGCGGAA GATGGCAAAC   
  
  
+ TCGGTGGCTT GCGAGGGCCG GGAACGGGTT GAGCGGGCGG AGGTGTTTGG GAAGTGGCGG GCGAGGATGA   
  
  
+ GTATGGCAGG GTTCGAGTTG AAGGCACTGA GTCAGAGTGT GGCCGAGTCA ATGCGAACCA AACTCAGCTC   
  
  
+ GGTTCTGGGC GGAAAGCCGG GTTTCACGGT CAAGGAGGAG AGCGGTGGGG TGGGGTTCGG GTGGATGGGA   
  
  
+ CGAACTCTCA CCGTCGCATC GGCCTGGCGT TA  

- +Up\_Stream \_Len000GGAGCC ACACCCTAAG GCCCAACTAT TTATCCCTGG TCGCTTTACA AGTTTATTAA   
  
  
- CCACTTCCTC AGAGACAAAC TTACGGATGA TTACGAAAAA AAAAAAAAAA CTTATTAAAC AGTCTTTCAA   
  
  
- TCCGACGTAA CCTTCCCCGC CGGATGCTTC CGAAAAGTCG TTTATTTATT GGTCGAAAGT AAAACTTTAA   
  
  
- AAAAAATTTT TTACTTTGAG TAAAGTGAAT TTTTAAATGG TATTATATAT ATATATATAT ATATATATAT   
  
  
- ATATATATAT ATAAACTAAA TTGCAATTCG TTTGAAAATT GAATACATAG TAAAATGCTG GCATTTTTTT   
  
  
- TTTCTTTATA TTGTGTTATA CATTATCATA AATTCAAAAA CTATTTATTA TAAAATTGTA TTAAACACAC   
  
  
- ATCATACTTA AATTTAGAAT CATGATTATA TTAATACATA TGAACCGATA TTCTAGAGTT ATAATCATAA   
  
  
- AATACTAAGT TACATTACTA CTACGTTGAC ACACATTAAT CTTTCGGAAA CTAACGTCTA TAGATAGAAT   
  
  
- AACTATGTGT AGATGGTTAA CTCGAAAAAG TTCTAAACTT CTGCCATCAG TTCATAGAAA CCCTCTAATG   
  
  
- CAATCTTAAT GAAGTACTAT TTAATCTGTA TAAAATATGA AGATTAGAAT TTATTCAGAG GTAGGAAAGT   
  
  
- AGTATAAAAT TTCTTTACAT GTTCATTTAC AGTTATCAAA CTTTGCAAGG AATAGTATTT TCAGTATGTA   
  
  
- TACATCCTGT AAAAGTTCAA ATACATTTGG TATACTATAT CAAGTTCTAT TAACAGTTTG TTCAATACTT   
  
  
- TAGATTTCCA CAAACTTGCA TTAAAGTTCG ATTAGAATCG TTCAATATCA TAATAAATTT GATGCTGATC   
  
  
- TGTGTTGTAA AACAAGTAAA TATATTGTGA ATCTTACGTT TTTTTAGCCA TAATGATTAT GACATAGTTC   
  
  
- TTAACTTCAC AATACGTAAT ATGACTATTC CCTATTATAA ATTTAATGAA ATGACAATAT CTTCGGTTTT   
  
  
- TAGATTTGAA AGTATGTCTG CAGCCTGATT TAATCTTTTA AGAAATTGAA TTATCTTTCC TACTGTATTT   
  
  
- ATTTTTATTT TTGGATTTAT CGTATTATTG GTAAGATGTG TTTTCAGTTA GAAGATTATC TTTTTTCGGT   
  
  
- ATTCATAAGT GAATAAATGG TAAAATAAGG ATTAGATGAC AGACAGATAA GTGTGGTATT AAGTACTTGG   
  
  
- TTACACCTCA AAGACAAAGT AAAGTACCCC ACTAAATACA GACTGTTACT AGGTTGTTTG ACTCGGAGTG   
  
  
- CGCGCCTTGT GCTCTCACCG ACATGAATAC CACACTTTCT CGAGATGTTC CACTCTAACT CCTCCGATGC   
  
  
- GCAACCTCAA TAGTGGTGGC AACCTAAACA AAAGAAACCT CGGTGCAATA GTGAGAAGTC TGCGGCTAGG   
  
  
- TTGCCGGGAA TGAAGGTAGA TCAGGGGTGA GAGGTTGAGA TGCGTTAGTT GCGGAAGGCG TAAATTTATA   
  
  
- AAGGAAGAGA TAGCGGATGA AAGAGACAGA GTGTTAAAAA CAGGCATAAA ACTTAAAAGA GTTTTAATAG   
  
  
- TATATTAAAT AAACGGAAGT TGATTTCATA AATAATTTAA TCAATTACAT AAATTTAACT TGATTTTTTC   
  
  
- TCTTTTTCTT TCTTTTGTTG GGGTGCTTTC GTTTTGCTGT CTCCGGTTGA AGAGAGAGAT CTCACATCTC   
  
  
- GGGGTAGAGC CAATATTAAT ACTCTTAGTT AAGGAAGAGT GTTCTCTTTG AGAGCGAGAG AGAGAGAGAG   
  
  
- GTATTCTGTA GTGTGGTTAA CATACTTAAA AGAGAGAGGA CTAAAGACAA AAGGAAGAAC AACAGGGGAA   
  
  
- GATTACGCAA TCACGCCGGT TTTGGTGGTA TTTGGTAGAG AGAAAGTTCT TTCGTCGTTG TTGTTTCTCT   
  
  
- CTGTCTCTCT CTCTCTCCCC CTCTTTCTAG TGTAGAAAAC CCAAAGGCCG CTTATACTGC AGACCCAAAA   
  
  
- GCCCGCTAAA GAAGATGCCG CCACCTCCGC CACCGCCTCC TCCACAGGCC GGAAGATAGC CGCCACATGT   
  
  
- TCCGCCCCAA CAGGGTATAC CCAAGGCCGT TGTTGTTGTT GTTGTCGTTG TCGTCGTCGT CGTCGTTGTC   
  
  
- GATGTAGATG TCGATGTTGT TGTCGCCCCC TAAAAGAGCC GCCTAGTCTA GTCGCGAGTT GCTTCCCGCC   
  
  
- TAAACAACTA CCCATTCGCA AGAAAGCGAC TAAAAGTTAG AGTTGTTGTC GTTTAGTCAG AAAACGTCGT   
  
  
- CGTCGTCGTC GTTGTTGTTG TTGTCGGGAA ACGCGAGAAC GTTTTTGTTG TCGTCAAAGT TGTTGTTAAT   
  
  
- GTTGTTGTTG TCGGTGAAAG AGAGGAGGAA GTTGTTGTCA TGAACTACTC TAGGCATTTT GTCTCTTGGT   
  
  
- TGTTAAAGCG TCGTAGAGGC TAAAGAGGAG AAAGAAGAAG GTAACTAGAA AGAAGGCGAT TATTAAGGGC   
  
  
- CAAGCCAAAC GGCCAAATGG TTGTTGTTGA GGCCAGAGTT GTTGTTGGTG GATAGAGAGA GGAGCGGGTG   
  
  
- TTGTTGTTGG TTAAGTTGGA GAGTTTATTA TTGTTAAGAA GAAATTAGGT TTTGAGGTGG TTGTGAGAAG   
  
  
- AACGAGGGCG TCGGTCGTTA TTGTGGGATT ACTTTTTAAG CCCGAAAAGG AGTAGACCCA AAAGATTAAG   
  
  
- CCCAGGTTTG GACCAGGTTG GCTTCGTCCT TTAGCCCATG AGCTTATACC CATGGTTATA CCCATACTTA   
  
  
- GACCAGGTCG GGTTTGGGCT GGGACTCCTA GTGCCTTATT ACTTGTGGGA AGTTCTTAAC CTCTTCCTCG   
  
  
- AAAACTTACT GCTACTCCTT TTGGTCGGCC TTTTGCTGCG GCAAAGTCAG TAGTGGTTGT CGCTCACCAG   
  
  
- GCTCTGGAAG GTCTTAGACT ACTCAGGGCT GGGCTCAAGC TCGGGCTTAC GGTGGTTGTG GGTGGGCCCA   
  
  
- AGCCCAAGAC GATCATGGTC GCCGTCGTTG TTGTGATGTT GGTGGGGACG TTTGTTGTTA TTGTACTATA   
  
  
- GATGGAGCGG CTGCAGCAGG TGCAGTAGGA CGACGAGGTG CAGCCGTAGC CGCAGAGGCG GCCGCCGCCA   
  
  
- CCGCTGGGGC GTCCATGGTC GGTTCGTCGA CTAGAGCCTT CGTCGCCGCC GCTAAAGCCT GCCTTTCTAA   
  
  
- CTTAGGCGGT ACCTTCACTA CTTGGCCGAC AGGGTCCAAC GGTTAAGATC CCCCTCGAGC CTTGTTTTCG   
  
  
- ACCGAAAAAT GTACCCGGTG CGCGAAGCCA GCGCGCACTC AGGGTGGCTT GTGCGAGGGG GATAACACCT   
  
  
- CGAAAAGGTT TTCCTCGTAT ACCGCCGGTA CATAAGTAAC ATGCTCGAGA GAGTAACAAA GTTTAAACCA   
  
  
- GAAAACCGAC GGTTAAACCA CTACAACCTC CGGTAATGAA GGCGGGAAGT ACTCCCAGAC TTTTAAGTAC   
  
  
- AACACCTAAA ACTATAACCC GTACCACCAG TCATGTAGTT GGAGGATGCG CGGAACCCCC TCGCATCCCG   
  
  
- AGTCAGCCCC TCGCGCCACA GCTTACTCCA CTCCTAGTGA CACCAACTCT GGCTCCAGCC ATTACCACCA   
  
  
- CGCCGCCCAC GCCTCCGACA ATTCCTACCG GACCACTTTA ACCGACTCTT CCACCCCTAA TCAAACTTCA   
  
  
- AACTCCGAAA CTGAGCCTTC TAGAGGCTCA ATTGGGCCCT CAGTGACCCC ACACTCCTCC CCCTCCGGCA   
  
  
- CCACCACTTA AAACGCAAGT TTGAAATGGC CCATGGCCTA CTCTCGCACA GCTACCTCTT AGACTCCCTG   
  
  
- CTCGAGGACG CGGCCCACTT CCGCGATTCC GGTGCGCGGC AGTGTAACCA CCTCGTCCTG AATTTACGCT   
  
  
- TATGCCGTGG CAAGTACTGC GCGCACTCAC CGCGAACACT CATGATATCT CGTAACAAGC TGAGCGAGCT   
  
  
- GAGCTACCAC GGCTCCCTAT TGGGCCTGGC CCAGGCCCAG CTCCTCCGGA ACCCCGCCTT CTACCGTTTG   
  
  
- AGCCACCGAA CGCTCCCGGC CCTTGCCCAA CTCGCCCGCC TCCACAAACC CTTCACCGCC CGCTCCTACT   
  
  
- CATACCGTCC CAAGCTCAAC TTCCGTGACT CAGTCTCACA CCGGCTCAGT TACGCTTGGT TTGAGTCGAG   
  
  
- CCAAGACCCG CCTTTCGGCC CAAAGTGCCA GTTCCTCCTC TCGCCACCCC ACCCCAAGCC CACCTACCCT   
  
  
- GCTTGAGAGT GGCAGCGTAG CCGGACCGCA AT

+     MBS

| Site Name | Organism | Position | Strand | Matrix score. | sequence | function |
| --- | --- | --- | --- | --- | --- | --- |
| MBS | Arabidopsis thaliana | 519 | + | 6 | CAACTG | MYB binding site involved in drought-inducibility |

>HU06G02537.1   
+ +Up\_Stream \_Len000CCTCGG TGTGGGATTC CGGGTTGATA AATAGGGACC AGCGAAATGT TCAAATAATT   
  
  
+ GGTGAAGGAG TCTCTGTTTG AATGCCTACT AATGCTTTTT TTTTTTTTTT GAATAATTTG TCAGAAAGTT   
  
  
+ AGGCTGCATT GGAAGGGGCG GCCTACGAAG GCTTTTCAGC AAATAAATAA CCAGCTTTCA TTTTGAAATT   
  
  
+ TTTTTTAAAA AATGAAACTC ATTTCACTTA AAAATTTACC ATAATATATA TATATATATA TATATATATA   
  
  
+ TATATATATA TATTTGATTT AACGTTAAGC AAACTTTTAA CTTATGTATC ATTTTACGAC CGTAAAAAAA   
  
  
+ AAAGAAATAT AACACAATAT GTAATAGTAT TTAAGTTTTT GATAAATAAT ATTTTAACAT AATTTGTGTG   
  
  
+ TAGTATGAAT TTAAATCTTA GTACTAATAT AATTATGTAT ACTTGGCTAT AAGATCTCAA TATTAGTATT   
  
  
+ TTATGATTCA ATGTAATGAT GATGCAACTG TGTGTAATTA GAAAGCCTTT GATTGCAGAT ATCTATCTTA   
  
  
+ TTGATACACA TCTACCAATT GAGCTTTTTC AAGATTTGAA GACGGTAGTC AAGTATCTTT GGGAGATTAC   
  
  
+ GTTAGAATTA CTTCATGATA AATTAGACAT ATTTTATACT TCTAATCTTA AATAAGTCTC CATCCTTTCA   
  
  
+ TCATATTTTA AAGAAATGTA CAAGTAAATG TCAATAGTTT GAAACGTTCC TTATCATAAA AGTCATACAT   
  
  
+ ATGTAGGACA TTTTCAAGTT TATGTAAACC ATATGATATA GTTCAAGATA ATTGTCAAAC AAGTTATGAA   
  
  
+ ATCTAAAGGT GTTTGAACGT AATTTCAAGC TAATCTTAGC AAGTTATAGT ATTATTTAAA CTACGACTAG   
  
  
+ ACACAACATT TTGTTCATTT ATATAACACT TAGAATGCAA AAAAATCGGT ATTACTAATA CTGTATCAAG   
  
  
+ AATTGAAGTG TTATGCATTA TACTGATAAG GGATAATATT TAAATTACTT TACTGTTATA GAAGCCAAAA   
  
  
+ ATCTAAACTT TCATACAGAC GTCGGACTAA ATTAGAAAAT TCTTTAACTT AATAGAAAGG ATGACATAAA   
  
  
+ TAAAAATAAA AACCTAAATA GCATAATAAC CATTCTACAC AAAAGTCAAT CTTCTAATAG AAAAAAGCCA   
  
  
+ TAAGTATTCA CTTATTTACC ATTTTATTCC TAATCTACTG TCTGTCTATT CACACCATAA TTCATGAACC   
  
  
+ AATGTGGAGT TTCTGTTTCA TTTCATGGGG TGATTTATGT CTGACAATGA TCCAACAAAC TGAGCCTCAC   
  
  
+ GCGCGGAACA CGAGAGTGGC TGTACTTATG GTGTGAAAGA GCTCTACAAG GTGAGATTGA GGAGGCTACG   
  
  
+ CGTTGGAGTT ATCACCACCG TTGGATTTGT TTTCTTTGGA GCCACGTTAT CACTCTTCAG ACGCCGATCC   
  
  
+ AACGGCCCTT ACTTCCATCT AGTCCCCACT CTCCAACTCT ACGCAATCAA CGCCTTCCGC ATTTAAATAT   
  
  
+ TTCCTTCTCT ATCGCCTACT TTCTCTGTCT CACAATTTTT GTCCGTATTT TGAATTTTCT CAAAATTATC   
  
  
+ ATATAATTTA TTTGCCTTCA ACTAAAGTAT TTATTAAATT AGTTAATGTA TTTAAATTGA ACTAAAAAAG   
  
  
+ AGAAAAAGAA AGAAAACAAC CCCACGAAAG CAAAACGACA GAGGCCAACT TCTCTCTCTA GAGTGTAGAG   
  
  
+ CCCCATCTCG GTTATAATTA TGAGAATCAA TTCCTTCTCA CAAGAGAAAC TCTCGCTCTC TCTCTCTCTC   
  
  
+ CATAAGACAT CACACCAATT GTATGAATTT TCTCTCTCCT GATTTCTGTT TTCCTTCTTG TTGTCCCCTT   
  
  
+ CTAATGCGTT AGTGCGGCCA AAACCACCAT AAACCATCTC TCTTTCAAGA AAGCAGCAAC AACAAAGAGA   
  
  
+ GACAGAGAGA GAGAGAGGGG GAGAAAGATC ACATCTTTTG GGTTTCCGGC GAATATGACG TCTGGGTTTT   
  
  
+ CGGGCGATTT CTTCTACGGC GGTGGAGGCG GTGGCGGAGG AGGTGTCCGG CCTTCTATCG GCGGTGTACA   
  
  
+ AGGCGGGGTT GTCCCATATG GGTTCCGGCA ACAACAACAA CAACAGCAAC AGCAGCAGCA GCAGCAACAG   
  
  
+ CTACATCTAC AGCTACAACA ACAGCGGGGG ATTTTCTCGG CGGATCAGAT CAGCGCTCAA CGAAGGGCGG   
  
  
+ ATTTGTTGAT GGGTAAGCGT TCTTTCGCTG ATTTTCAATC TCAACAACAG CAAATCAGTC TTTTGCAGCA   
  
  
+ GCAGCAGCAG CAACAACAAC AACAGCCCTT TGCGCTCTTG CAAAAACAAC AGCAGTTTCA ACAACAATTA   
  
  
+ CAACAACAAC AGCCACTTTC TCTCCTCCTT CAACAACAGT ACTTGATGAG ATCCGTAAAA CAGAGAACCA   
  
  
+ ACAATTTCGC AGCATCTCCG ATTTCTCCTC TTTCTTCTTC CATTGATCTT TCTTCCGCTA ATAATTCCCG   
  
  
+ GTTCGGTTTG CCGGTTTACC AACAACAACT CCGGTCTCAA CAACAACCAC CTATCTCTCT CCTCGCCCAC   
  
  
+ AACAACAACC AATTCAACCT CTCAAATAAT AACAATTCTT CTTTAATCCA AAACTCCACC AACACTCTTC   
  
  
+ TTGCTCCCGC AGCCAGCAAT AACACCCTAA TGAAAAATTC GGGCTTTTCC TCATCTGGGT TTTCTAATTC   
  
  
+ GGGTCCAAAC CTGGTCCAAC CGAAGCAGGA AATCGGGTAC TCGAATATGG GTACCAATAT GGGTATGAAT   
  
  
+ CTGGTCCAGC CCAAACCCGA CCCTGAGGAT CACGGAATAA TGAACACCCT TCAAGAATTG GAGAAGGAGC   
  
  
+ TTTTGAATGA CGATGAGGAA AACCAGCCGG AAAACGACGC CGTTTCAGTC ATCACCAACA GCGAGTGGTC   
  
  
+ CGAGACCTTC CAGAATCTGA TGAGTCCCGA CCCGAGTTCG AGCCCGAATG CCACCAACAC CCACCCGGGT   
  
  
+ TCGGGTTCTG CTAGTACCAG CGGCAGCAAC AACACTACAA CCACCCCTGC AAACAACAAT AACATGATAT   
  
  
+ CTACCTCGCC GACGTCGTCC ACGTCATCCT GCTGCTCCAC GTCGGCATCG GCGTCTCCGC CGGCGGCGGT   
  
  
+ GGCGACCCCG CAGGTACCAG CCAAGCAGCT GATCTCGGAA GCAGCGGCGG CGATTTCGGA CGGAAAGATT   
  
  
+ GAATCCGCCA TGGAAGTGAT GAACCGGCTG TCCCAGGTTG CCAATTCTAG GGGGAGCTCG GAACAAAAGC   
  
  
+ TGGCTTTTTA CATGGGCCAC GCGCTTCGGT CGCGCGTGAG TCCCACCGAA CACGCTCCCC CTATTGTGGA   
  
  
+ GCTTTTCCAA AAGGAGCATA TGGCGGCCAT GTATTCATTG TACGAGCTCT CTCATTGTTT CAAATTTGGT   
  
  
+ CTTTTGGCTG CCAATTTGGT GATGTTGGAG GCCATTACTT CCGCCCTTCA TGAGGGTCTG AAAATTCATG   
  
  
+ TTGTGGATTT TGATATTGGG CATGGTGGTC AGTACATCAA CCTCCTACGC GCCTTGGGGG AGCGTAGGGC   
  
  
+ TCAGTCGGGG AGCGCGGTGT CGAATGAGGT GAGGATCACT GTGGTTGAGA CCGAGGTCGG TAATGGTGGT   
  
  
+ GCGGCGGGTG CGGAGGCTGT TAAGGATGGC CTGGTGAAAT TGGCTGAGAA GGTGGGGATT AGTTTGAAGT   
  
  
+ TTGAGGCTTT GACTCGGAAG ATCTCCGAGT TAACCCGGGA GTCACTGGGG TGTGAGGAGG GGGAGGCCGT   
  
  
+ GGTGGTGAAT TTTGCGTTCA AACTTTACCG GGTACCGGAT GAGAGCGTGT CGATGGAGAA TCTGAGGGAC   
  
  
+ GAGCTCCTGC GCCGGGTGAA GGCGCTAAGG CCACGCGCCG TCACATTGGT GGAGCAGGAC TTAAATGCGA   
  
  
+ ATACGGCACC GTTCATGACG CGCGTGAGTG GCGCTTGTGA GTACTATAGA GCATTGTTCG ACTCGCTCGA   
  
  
+ CTCGATGGTG CCGAGGGATA ACCCGGACCG GGTCCGGGTC GAGGAGGCCT TGGGGCGGAA GATGGCAAAC   
  
  
+ TCGGTGGCTT GCGAGGGCCG GGAACGGGTT GAGCGGGCGG AGGTGTTTGG GAAGTGGCGG GCGAGGATGA   
  
  
+ GTATGGCAGG GTTCGAGTTG AAGGCACTGA GTCAGAGTGT GGCCGAGTCA ATGCGAACCA AACTCAGCTC   
  
  
+ GGTTCTGGGC GGAAAGCCGG GTTTCACGGT CAAGGAGGAG AGCGGTGGGG TGGGGTTCGG GTGGATGGGA   
  
  
+ CGAACTCTCA CCGTCGCATC GGCCTGGCGT TA  

- +Up\_Stream \_Len000GGAGCC ACACCCTAAG GCCCAACTAT TTATCCCTGG TCGCTTTACA AGTTTATTAA   
  
  
- CCACTTCCTC AGAGACAAAC TTACGGATGA TTACGAAAAA AAAAAAAAAA CTTATTAAAC AGTCTTTCAA   
  
  
- TCCGACGTAA CCTTCCCCGC CGGATGCTTC CGAAAAGTCG TTTATTTATT GGTCGAAAGT AAAACTTTAA   
  
  
- AAAAAATTTT TTACTTTGAG TAAAGTGAAT TTTTAAATGG TATTATATAT ATATATATAT ATATATATAT   
  
  
- ATATATATAT ATAAACTAAA TTGCAATTCG TTTGAAAATT GAATACATAG TAAAATGCTG GCATTTTTTT   
  
  
- TTTCTTTATA TTGTGTTATA CATTATCATA AATTCAAAAA CTATTTATTA TAAAATTGTA TTAAACACAC   
  
  
- ATCATACTTA AATTTAGAAT CATGATTATA TTAATACATA TGAACCGATA TTCTAGAGTT ATAATCATAA   
  
  
- AATACTAAGT TACATTACTA CTACGTTGAC ACACATTAAT CTTTCGGAAA CTAACGTCTA TAGATAGAAT   
  
  
- AACTATGTGT AGATGGTTAA CTCGAAAAAG TTCTAAACTT CTGCCATCAG TTCATAGAAA CCCTCTAATG   
  
  
- CAATCTTAAT GAAGTACTAT TTAATCTGTA TAAAATATGA AGATTAGAAT TTATTCAGAG GTAGGAAAGT   
  
  
- AGTATAAAAT TTCTTTACAT GTTCATTTAC AGTTATCAAA CTTTGCAAGG AATAGTATTT TCAGTATGTA   
  
  
- TACATCCTGT AAAAGTTCAA ATACATTTGG TATACTATAT CAAGTTCTAT TAACAGTTTG TTCAATACTT   
  
  
- TAGATTTCCA CAAACTTGCA TTAAAGTTCG ATTAGAATCG TTCAATATCA TAATAAATTT GATGCTGATC   
  
  
- TGTGTTGTAA AACAAGTAAA TATATTGTGA ATCTTACGTT TTTTTAGCCA TAATGATTAT GACATAGTTC   
  
  
- TTAACTTCAC AATACGTAAT ATGACTATTC CCTATTATAA ATTTAATGAA ATGACAATAT CTTCGGTTTT   
  
  
- TAGATTTGAA AGTATGTCTG CAGCCTGATT TAATCTTTTA AGAAATTGAA TTATCTTTCC TACTGTATTT   
  
  
- ATTTTTATTT TTGGATTTAT CGTATTATTG GTAAGATGTG TTTTCAGTTA GAAGATTATC TTTTTTCGGT   
  
  
- ATTCATAAGT GAATAAATGG TAAAATAAGG ATTAGATGAC AGACAGATAA GTGTGGTATT AAGTACTTGG   
  
  
- TTACACCTCA AAGACAAAGT AAAGTACCCC ACTAAATACA GACTGTTACT AGGTTGTTTG ACTCGGAGTG   
  
  
- CGCGCCTTGT GCTCTCACCG ACATGAATAC CACACTTTCT CGAGATGTTC CACTCTAACT CCTCCGATGC   
  
  
- GCAACCTCAA TAGTGGTGGC AACCTAAACA AAAGAAACCT CGGTGCAATA GTGAGAAGTC TGCGGCTAGG   
  
  
- TTGCCGGGAA TGAAGGTAGA TCAGGGGTGA GAGGTTGAGA TGCGTTAGTT GCGGAAGGCG TAAATTTATA   
  
  
- AAGGAAGAGA TAGCGGATGA AAGAGACAGA GTGTTAAAAA CAGGCATAAA ACTTAAAAGA GTTTTAATAG   
  
  
- TATATTAAAT AAACGGAAGT TGATTTCATA AATAATTTAA TCAATTACAT AAATTTAACT TGATTTTTTC   
  
  
- TCTTTTTCTT TCTTTTGTTG GGGTGCTTTC GTTTTGCTGT CTCCGGTTGA AGAGAGAGAT CTCACATCTC   
  
  
- GGGGTAGAGC CAATATTAAT ACTCTTAGTT AAGGAAGAGT GTTCTCTTTG AGAGCGAGAG AGAGAGAGAG   
  
  
- GTATTCTGTA GTGTGGTTAA CATACTTAAA AGAGAGAGGA CTAAAGACAA AAGGAAGAAC AACAGGGGAA   
  
  
- GATTACGCAA TCACGCCGGT TTTGGTGGTA TTTGGTAGAG AGAAAGTTCT TTCGTCGTTG TTGTTTCTCT   
  
  
- CTGTCTCTCT CTCTCTCCCC CTCTTTCTAG TGTAGAAAAC CCAAAGGCCG CTTATACTGC AGACCCAAAA   
  
  
- GCCCGCTAAA GAAGATGCCG CCACCTCCGC CACCGCCTCC TCCACAGGCC GGAAGATAGC CGCCACATGT   
  
  
- TCCGCCCCAA CAGGGTATAC CCAAGGCCGT TGTTGTTGTT GTTGTCGTTG TCGTCGTCGT CGTCGTTGTC   
  
  
- GATGTAGATG TCGATGTTGT TGTCGCCCCC TAAAAGAGCC GCCTAGTCTA GTCGCGAGTT GCTTCCCGCC   
  
  
- TAAACAACTA CCCATTCGCA AGAAAGCGAC TAAAAGTTAG AGTTGTTGTC GTTTAGTCAG AAAACGTCGT   
  
  
- CGTCGTCGTC GTTGTTGTTG TTGTCGGGAA ACGCGAGAAC GTTTTTGTTG TCGTCAAAGT TGTTGTTAAT   
  
  
- GTTGTTGTTG TCGGTGAAAG AGAGGAGGAA GTTGTTGTCA TGAACTACTC TAGGCATTTT GTCTCTTGGT   
  
  
- TGTTAAAGCG TCGTAGAGGC TAAAGAGGAG AAAGAAGAAG GTAACTAGAA AGAAGGCGAT TATTAAGGGC   
  
  
- CAAGCCAAAC GGCCAAATGG TTGTTGTTGA GGCCAGAGTT GTTGTTGGTG GATAGAGAGA GGAGCGGGTG   
  
  
- TTGTTGTTGG TTAAGTTGGA GAGTTTATTA TTGTTAAGAA GAAATTAGGT TTTGAGGTGG TTGTGAGAAG   
  
  
- AACGAGGGCG TCGGTCGTTA TTGTGGGATT ACTTTTTAAG CCCGAAAAGG AGTAGACCCA AAAGATTAAG   
  
  
- CCCAGGTTTG GACCAGGTTG GCTTCGTCCT TTAGCCCATG AGCTTATACC CATGGTTATA CCCATACTTA   
  
  
- GACCAGGTCG GGTTTGGGCT GGGACTCCTA GTGCCTTATT ACTTGTGGGA AGTTCTTAAC CTCTTCCTCG   
  
  
- AAAACTTACT GCTACTCCTT TTGGTCGGCC TTTTGCTGCG GCAAAGTCAG TAGTGGTTGT CGCTCACCAG   
  
  
- GCTCTGGAAG GTCTTAGACT ACTCAGGGCT GGGCTCAAGC TCGGGCTTAC GGTGGTTGTG GGTGGGCCCA   
  
  
- AGCCCAAGAC GATCATGGTC GCCGTCGTTG TTGTGATGTT GGTGGGGACG TTTGTTGTTA TTGTACTATA   
  
  
- GATGGAGCGG CTGCAGCAGG TGCAGTAGGA CGACGAGGTG CAGCCGTAGC CGCAGAGGCG GCCGCCGCCA   
  
  
- CCGCTGGGGC GTCCATGGTC GGTTCGTCGA CTAGAGCCTT CGTCGCCGCC GCTAAAGCCT GCCTTTCTAA   
  
  
- CTTAGGCGGT ACCTTCACTA CTTGGCCGAC AGGGTCCAAC GGTTAAGATC CCCCTCGAGC CTTGTTTTCG   
  
  
- ACCGAAAAAT GTACCCGGTG CGCGAAGCCA GCGCGCACTC AGGGTGGCTT GTGCGAGGGG GATAACACCT   
  
  
- CGAAAAGGTT TTCCTCGTAT ACCGCCGGTA CATAAGTAAC ATGCTCGAGA GAGTAACAAA GTTTAAACCA   
  
  
- GAAAACCGAC GGTTAAACCA CTACAACCTC CGGTAATGAA GGCGGGAAGT ACTCCCAGAC TTTTAAGTAC   
  
  
- AACACCTAAA ACTATAACCC GTACCACCAG TCATGTAGTT GGAGGATGCG CGGAACCCCC TCGCATCCCG   
  
  
- AGTCAGCCCC TCGCGCCACA GCTTACTCCA CTCCTAGTGA CACCAACTCT GGCTCCAGCC ATTACCACCA   
  
  
- CGCCGCCCAC GCCTCCGACA ATTCCTACCG GACCACTTTA ACCGACTCTT CCACCCCTAA TCAAACTTCA   
  
  
- AACTCCGAAA CTGAGCCTTC TAGAGGCTCA ATTGGGCCCT CAGTGACCCC ACACTCCTCC CCCTCCGGCA   
  
  
- CCACCACTTA AAACGCAAGT TTGAAATGGC CCATGGCCTA CTCTCGCACA GCTACCTCTT AGACTCCCTG   
  
  
- CTCGAGGACG CGGCCCACTT CCGCGATTCC GGTGCGCGGC AGTGTAACCA CCTCGTCCTG AATTTACGCT   
  
  
- TATGCCGTGG CAAGTACTGC GCGCACTCAC CGCGAACACT CATGATATCT CGTAACAAGC TGAGCGAGCT   
  
  
- GAGCTACCAC GGCTCCCTAT TGGGCCTGGC CCAGGCCCAG CTCCTCCGGA ACCCCGCCTT CTACCGTTTG   
  
  
- AGCCACCGAA CGCTCCCGGC CCTTGCCCAA CTCGCCCGCC TCCACAAACC CTTCACCGCC CGCTCCTACT   
  
  
- CATACCGTCC CAAGCTCAAC TTCCGTGACT CAGTCTCACA CCGGCTCAGT TACGCTTGGT TTGAGTCGAG   
  
  
- CCAAGACCCG CCTTTCGGCC CAAAGTGCCA GTTCCTCCTC TCGCCACCCC ACCCCAAGCC CACCTACCCT   
  
  
- GCTTGAGAGT GGCAGCGTAG CCGGACCGCA AT

+     MRE

| Site Name | Organism | Position | Strand | Matrix score. | sequence | function |
| --- | --- | --- | --- | --- | --- | --- |
| MRE | Petroselinum crispum | 1135 | + | 7 | AACCTAA | MYB binding site involved in light responsiveness |

>HU06G02537.1   
+ +Up\_Stream \_Len000CCTCGG TGTGGGATTC CGGGTTGATA AATAGGGACC AGCGAAATGT TCAAATAATT   
  
  
+ GGTGAAGGAG TCTCTGTTTG AATGCCTACT AATGCTTTTT TTTTTTTTTT GAATAATTTG TCAGAAAGTT   
  
  
+ AGGCTGCATT GGAAGGGGCG GCCTACGAAG GCTTTTCAGC AAATAAATAA CCAGCTTTCA TTTTGAAATT   
  
  
+ TTTTTTAAAA AATGAAACTC ATTTCACTTA AAAATTTACC ATAATATATA TATATATATA TATATATATA   
  
  
+ TATATATATA TATTTGATTT AACGTTAAGC AAACTTTTAA CTTATGTATC ATTTTACGAC CGTAAAAAAA   
  
  
+ AAAGAAATAT AACACAATAT GTAATAGTAT TTAAGTTTTT GATAAATAAT ATTTTAACAT AATTTGTGTG   
  
  
+ TAGTATGAAT TTAAATCTTA GTACTAATAT AATTATGTAT ACTTGGCTAT AAGATCTCAA TATTAGTATT   
  
  
+ TTATGATTCA ATGTAATGAT GATGCAACTG TGTGTAATTA GAAAGCCTTT GATTGCAGAT ATCTATCTTA   
  
  
+ TTGATACACA TCTACCAATT GAGCTTTTTC AAGATTTGAA GACGGTAGTC AAGTATCTTT GGGAGATTAC   
  
  
+ GTTAGAATTA CTTCATGATA AATTAGACAT ATTTTATACT TCTAATCTTA AATAAGTCTC CATCCTTTCA   
  
  
+ TCATATTTTA AAGAAATGTA CAAGTAAATG TCAATAGTTT GAAACGTTCC TTATCATAAA AGTCATACAT   
  
  
+ ATGTAGGACA TTTTCAAGTT TATGTAAACC ATATGATATA GTTCAAGATA ATTGTCAAAC AAGTTATGAA   
  
  
+ ATCTAAAGGT GTTTGAACGT AATTTCAAGC TAATCTTAGC AAGTTATAGT ATTATTTAAA CTACGACTAG   
  
  
+ ACACAACATT TTGTTCATTT ATATAACACT TAGAATGCAA AAAAATCGGT ATTACTAATA CTGTATCAAG   
  
  
+ AATTGAAGTG TTATGCATTA TACTGATAAG GGATAATATT TAAATTACTT TACTGTTATA GAAGCCAAAA   
  
  
+ ATCTAAACTT TCATACAGAC GTCGGACTAA ATTAGAAAAT TCTTTAACTT AATAGAAAGG ATGACATAAA   
  
  
+ TAAAAATAAA AACCTAAATA GCATAATAAC CATTCTACAC AAAAGTCAAT CTTCTAATAG AAAAAAGCCA   
  
  
+ TAAGTATTCA CTTATTTACC ATTTTATTCC TAATCTACTG TCTGTCTATT CACACCATAA TTCATGAACC   
  
  
+ AATGTGGAGT TTCTGTTTCA TTTCATGGGG TGATTTATGT CTGACAATGA TCCAACAAAC TGAGCCTCAC   
  
  
+ GCGCGGAACA CGAGAGTGGC TGTACTTATG GTGTGAAAGA GCTCTACAAG GTGAGATTGA GGAGGCTACG   
  
  
+ CGTTGGAGTT ATCACCACCG TTGGATTTGT TTTCTTTGGA GCCACGTTAT CACTCTTCAG ACGCCGATCC   
  
  
+ AACGGCCCTT ACTTCCATCT AGTCCCCACT CTCCAACTCT ACGCAATCAA CGCCTTCCGC ATTTAAATAT   
  
  
+ TTCCTTCTCT ATCGCCTACT TTCTCTGTCT CACAATTTTT GTCCGTATTT TGAATTTTCT CAAAATTATC   
  
  
+ ATATAATTTA TTTGCCTTCA ACTAAAGTAT TTATTAAATT AGTTAATGTA TTTAAATTGA ACTAAAAAAG   
  
  
+ AGAAAAAGAA AGAAAACAAC CCCACGAAAG CAAAACGACA GAGGCCAACT TCTCTCTCTA GAGTGTAGAG   
  
  
+ CCCCATCTCG GTTATAATTA TGAGAATCAA TTCCTTCTCA CAAGAGAAAC TCTCGCTCTC TCTCTCTCTC   
  
  
+ CATAAGACAT CACACCAATT GTATGAATTT TCTCTCTCCT GATTTCTGTT TTCCTTCTTG TTGTCCCCTT   
  
  
+ CTAATGCGTT AGTGCGGCCA AAACCACCAT AAACCATCTC TCTTTCAAGA AAGCAGCAAC AACAAAGAGA   
  
  
+ GACAGAGAGA GAGAGAGGGG GAGAAAGATC ACATCTTTTG GGTTTCCGGC GAATATGACG TCTGGGTTTT   
  
  
+ CGGGCGATTT CTTCTACGGC GGTGGAGGCG GTGGCGGAGG AGGTGTCCGG CCTTCTATCG GCGGTGTACA   
  
  
+ AGGCGGGGTT GTCCCATATG GGTTCCGGCA ACAACAACAA CAACAGCAAC AGCAGCAGCA GCAGCAACAG   
  
  
+ CTACATCTAC AGCTACAACA ACAGCGGGGG ATTTTCTCGG CGGATCAGAT CAGCGCTCAA CGAAGGGCGG   
  
  
+ ATTTGTTGAT GGGTAAGCGT TCTTTCGCTG ATTTTCAATC TCAACAACAG CAAATCAGTC TTTTGCAGCA   
  
  
+ GCAGCAGCAG CAACAACAAC AACAGCCCTT TGCGCTCTTG CAAAAACAAC AGCAGTTTCA ACAACAATTA   
  
  
+ CAACAACAAC AGCCACTTTC TCTCCTCCTT CAACAACAGT ACTTGATGAG ATCCGTAAAA CAGAGAACCA   
  
  
+ ACAATTTCGC AGCATCTCCG ATTTCTCCTC TTTCTTCTTC CATTGATCTT TCTTCCGCTA ATAATTCCCG   
  
  
+ GTTCGGTTTG CCGGTTTACC AACAACAACT CCGGTCTCAA CAACAACCAC CTATCTCTCT CCTCGCCCAC   
  
  
+ AACAACAACC AATTCAACCT CTCAAATAAT AACAATTCTT CTTTAATCCA AAACTCCACC AACACTCTTC   
  
  
+ TTGCTCCCGC AGCCAGCAAT AACACCCTAA TGAAAAATTC GGGCTTTTCC TCATCTGGGT TTTCTAATTC   
  
  
+ GGGTCCAAAC CTGGTCCAAC CGAAGCAGGA AATCGGGTAC TCGAATATGG GTACCAATAT GGGTATGAAT   
  
  
+ CTGGTCCAGC CCAAACCCGA CCCTGAGGAT CACGGAATAA TGAACACCCT TCAAGAATTG GAGAAGGAGC   
  
  
+ TTTTGAATGA CGATGAGGAA AACCAGCCGG AAAACGACGC CGTTTCAGTC ATCACCAACA GCGAGTGGTC   
  
  
+ CGAGACCTTC CAGAATCTGA TGAGTCCCGA CCCGAGTTCG AGCCCGAATG CCACCAACAC CCACCCGGGT   
  
  
+ TCGGGTTCTG CTAGTACCAG CGGCAGCAAC AACACTACAA CCACCCCTGC AAACAACAAT AACATGATAT   
  
  
+ CTACCTCGCC GACGTCGTCC ACGTCATCCT GCTGCTCCAC GTCGGCATCG GCGTCTCCGC CGGCGGCGGT   
  
  
+ GGCGACCCCG CAGGTACCAG CCAAGCAGCT GATCTCGGAA GCAGCGGCGG CGATTTCGGA CGGAAAGATT   
  
  
+ GAATCCGCCA TGGAAGTGAT GAACCGGCTG TCCCAGGTTG CCAATTCTAG GGGGAGCTCG GAACAAAAGC   
  
  
+ TGGCTTTTTA CATGGGCCAC GCGCTTCGGT CGCGCGTGAG TCCCACCGAA CACGCTCCCC CTATTGTGGA   
  
  
+ GCTTTTCCAA AAGGAGCATA TGGCGGCCAT GTATTCATTG TACGAGCTCT CTCATTGTTT CAAATTTGGT   
  
  
+ CTTTTGGCTG CCAATTTGGT GATGTTGGAG GCCATTACTT CCGCCCTTCA TGAGGGTCTG AAAATTCATG   
  
  
+ TTGTGGATTT TGATATTGGG CATGGTGGTC AGTACATCAA CCTCCTACGC GCCTTGGGGG AGCGTAGGGC   
  
  
+ TCAGTCGGGG AGCGCGGTGT CGAATGAGGT GAGGATCACT GTGGTTGAGA CCGAGGTCGG TAATGGTGGT   
  
  
+ GCGGCGGGTG CGGAGGCTGT TAAGGATGGC CTGGTGAAAT TGGCTGAGAA GGTGGGGATT AGTTTGAAGT   
  
  
+ TTGAGGCTTT GACTCGGAAG ATCTCCGAGT TAACCCGGGA GTCACTGGGG TGTGAGGAGG GGGAGGCCGT   
  
  
+ GGTGGTGAAT TTTGCGTTCA AACTTTACCG GGTACCGGAT GAGAGCGTGT CGATGGAGAA TCTGAGGGAC   
  
  
+ GAGCTCCTGC GCCGGGTGAA GGCGCTAAGG CCACGCGCCG TCACATTGGT GGAGCAGGAC TTAAATGCGA   
  
  
+ ATACGGCACC GTTCATGACG CGCGTGAGTG GCGCTTGTGA GTACTATAGA GCATTGTTCG ACTCGCTCGA   
  
  
+ CTCGATGGTG CCGAGGGATA ACCCGGACCG GGTCCGGGTC GAGGAGGCCT TGGGGCGGAA GATGGCAAAC   
  
  
+ TCGGTGGCTT GCGAGGGCCG GGAACGGGTT GAGCGGGCGG AGGTGTTTGG GAAGTGGCGG GCGAGGATGA   
  
  
+ GTATGGCAGG GTTCGAGTTG AAGGCACTGA GTCAGAGTGT GGCCGAGTCA ATGCGAACCA AACTCAGCTC   
  
  
+ GGTTCTGGGC GGAAAGCCGG GTTTCACGGT CAAGGAGGAG AGCGGTGGGG TGGGGTTCGG GTGGATGGGA   
  
  
+ CGAACTCTCA CCGTCGCATC GGCCTGGCGT TA  

- +Up\_Stream \_Len000GGAGCC ACACCCTAAG GCCCAACTAT TTATCCCTGG TCGCTTTACA AGTTTATTAA   
  
  
- CCACTTCCTC AGAGACAAAC TTACGGATGA TTACGAAAAA AAAAAAAAAA CTTATTAAAC AGTCTTTCAA   
  
  
- TCCGACGTAA CCTTCCCCGC CGGATGCTTC CGAAAAGTCG TTTATTTATT GGTCGAAAGT AAAACTTTAA   
  
  
- AAAAAATTTT TTACTTTGAG TAAAGTGAAT TTTTAAATGG TATTATATAT ATATATATAT ATATATATAT   
  
  
- ATATATATAT ATAAACTAAA TTGCAATTCG TTTGAAAATT GAATACATAG TAAAATGCTG GCATTTTTTT   
  
  
- TTTCTTTATA TTGTGTTATA CATTATCATA AATTCAAAAA CTATTTATTA TAAAATTGTA TTAAACACAC   
  
  
- ATCATACTTA AATTTAGAAT CATGATTATA TTAATACATA TGAACCGATA TTCTAGAGTT ATAATCATAA   
  
  
- AATACTAAGT TACATTACTA CTACGTTGAC ACACATTAAT CTTTCGGAAA CTAACGTCTA TAGATAGAAT   
  
  
- AACTATGTGT AGATGGTTAA CTCGAAAAAG TTCTAAACTT CTGCCATCAG TTCATAGAAA CCCTCTAATG   
  
  
- CAATCTTAAT GAAGTACTAT TTAATCTGTA TAAAATATGA AGATTAGAAT TTATTCAGAG GTAGGAAAGT   
  
  
- AGTATAAAAT TTCTTTACAT GTTCATTTAC AGTTATCAAA CTTTGCAAGG AATAGTATTT TCAGTATGTA   
  
  
- TACATCCTGT AAAAGTTCAA ATACATTTGG TATACTATAT CAAGTTCTAT TAACAGTTTG TTCAATACTT   
  
  
- TAGATTTCCA CAAACTTGCA TTAAAGTTCG ATTAGAATCG TTCAATATCA TAATAAATTT GATGCTGATC   
  
  
- TGTGTTGTAA AACAAGTAAA TATATTGTGA ATCTTACGTT TTTTTAGCCA TAATGATTAT GACATAGTTC   
  
  
- TTAACTTCAC AATACGTAAT ATGACTATTC CCTATTATAA ATTTAATGAA ATGACAATAT CTTCGGTTTT   
  
  
- TAGATTTGAA AGTATGTCTG CAGCCTGATT TAATCTTTTA AGAAATTGAA TTATCTTTCC TACTGTATTT   
  
  
- ATTTTTATTT TTGGATTTAT CGTATTATTG GTAAGATGTG TTTTCAGTTA GAAGATTATC TTTTTTCGGT   
  
  
- ATTCATAAGT GAATAAATGG TAAAATAAGG ATTAGATGAC AGACAGATAA GTGTGGTATT AAGTACTTGG   
  
  
- TTACACCTCA AAGACAAAGT AAAGTACCCC ACTAAATACA GACTGTTACT AGGTTGTTTG ACTCGGAGTG   
  
  
- CGCGCCTTGT GCTCTCACCG ACATGAATAC CACACTTTCT CGAGATGTTC CACTCTAACT CCTCCGATGC   
  
  
- GCAACCTCAA TAGTGGTGGC AACCTAAACA AAAGAAACCT CGGTGCAATA GTGAGAAGTC TGCGGCTAGG   
  
  
- TTGCCGGGAA TGAAGGTAGA TCAGGGGTGA GAGGTTGAGA TGCGTTAGTT GCGGAAGGCG TAAATTTATA   
  
  
- AAGGAAGAGA TAGCGGATGA AAGAGACAGA GTGTTAAAAA CAGGCATAAA ACTTAAAAGA GTTTTAATAG   
  
  
- TATATTAAAT AAACGGAAGT TGATTTCATA AATAATTTAA TCAATTACAT AAATTTAACT TGATTTTTTC   
  
  
- TCTTTTTCTT TCTTTTGTTG GGGTGCTTTC GTTTTGCTGT CTCCGGTTGA AGAGAGAGAT CTCACATCTC   
  
  
- GGGGTAGAGC CAATATTAAT ACTCTTAGTT AAGGAAGAGT GTTCTCTTTG AGAGCGAGAG AGAGAGAGAG   
  
  
- GTATTCTGTA GTGTGGTTAA CATACTTAAA AGAGAGAGGA CTAAAGACAA AAGGAAGAAC AACAGGGGAA   
  
  
- GATTACGCAA TCACGCCGGT TTTGGTGGTA TTTGGTAGAG AGAAAGTTCT TTCGTCGTTG TTGTTTCTCT   
  
  
- CTGTCTCTCT CTCTCTCCCC CTCTTTCTAG TGTAGAAAAC CCAAAGGCCG CTTATACTGC AGACCCAAAA   
  
  
- GCCCGCTAAA GAAGATGCCG CCACCTCCGC CACCGCCTCC TCCACAGGCC GGAAGATAGC CGCCACATGT   
  
  
- TCCGCCCCAA CAGGGTATAC CCAAGGCCGT TGTTGTTGTT GTTGTCGTTG TCGTCGTCGT CGTCGTTGTC   
  
  
- GATGTAGATG TCGATGTTGT TGTCGCCCCC TAAAAGAGCC GCCTAGTCTA GTCGCGAGTT GCTTCCCGCC   
  
  
- TAAACAACTA CCCATTCGCA AGAAAGCGAC TAAAAGTTAG AGTTGTTGTC GTTTAGTCAG AAAACGTCGT   
  
  
- CGTCGTCGTC GTTGTTGTTG TTGTCGGGAA ACGCGAGAAC GTTTTTGTTG TCGTCAAAGT TGTTGTTAAT   
  
  
- GTTGTTGTTG TCGGTGAAAG AGAGGAGGAA GTTGTTGTCA TGAACTACTC TAGGCATTTT GTCTCTTGGT   
  
  
- TGTTAAAGCG TCGTAGAGGC TAAAGAGGAG AAAGAAGAAG GTAACTAGAA AGAAGGCGAT TATTAAGGGC   
  
  
- CAAGCCAAAC GGCCAAATGG TTGTTGTTGA GGCCAGAGTT GTTGTTGGTG GATAGAGAGA GGAGCGGGTG   
  
  
- TTGTTGTTGG TTAAGTTGGA GAGTTTATTA TTGTTAAGAA GAAATTAGGT TTTGAGGTGG TTGTGAGAAG   
  
  
- AACGAGGGCG TCGGTCGTTA TTGTGGGATT ACTTTTTAAG CCCGAAAAGG AGTAGACCCA AAAGATTAAG   
  
  
- CCCAGGTTTG GACCAGGTTG GCTTCGTCCT TTAGCCCATG AGCTTATACC CATGGTTATA CCCATACTTA   
  
  
- GACCAGGTCG GGTTTGGGCT GGGACTCCTA GTGCCTTATT ACTTGTGGGA AGTTCTTAAC CTCTTCCTCG   
  
  
- AAAACTTACT GCTACTCCTT TTGGTCGGCC TTTTGCTGCG GCAAAGTCAG TAGTGGTTGT CGCTCACCAG   
  
  
- GCTCTGGAAG GTCTTAGACT ACTCAGGGCT GGGCTCAAGC TCGGGCTTAC GGTGGTTGTG GGTGGGCCCA   
  
  
- AGCCCAAGAC GATCATGGTC GCCGTCGTTG TTGTGATGTT GGTGGGGACG TTTGTTGTTA TTGTACTATA   
  
  
- GATGGAGCGG CTGCAGCAGG TGCAGTAGGA CGACGAGGTG CAGCCGTAGC CGCAGAGGCG GCCGCCGCCA   
  
  
- CCGCTGGGGC GTCCATGGTC GGTTCGTCGA CTAGAGCCTT CGTCGCCGCC GCTAAAGCCT GCCTTTCTAA   
  
  
- CTTAGGCGGT ACCTTCACTA CTTGGCCGAC AGGGTCCAAC GGTTAAGATC CCCCTCGAGC CTTGTTTTCG   
  
  
- ACCGAAAAAT GTACCCGGTG CGCGAAGCCA GCGCGCACTC AGGGTGGCTT GTGCGAGGGG GATAACACCT   
  
  
- CGAAAAGGTT TTCCTCGTAT ACCGCCGGTA CATAAGTAAC ATGCTCGAGA GAGTAACAAA GTTTAAACCA   
  
  
- GAAAACCGAC GGTTAAACCA CTACAACCTC CGGTAATGAA GGCGGGAAGT ACTCCCAGAC TTTTAAGTAC   
  
  
- AACACCTAAA ACTATAACCC GTACCACCAG TCATGTAGTT GGAGGATGCG CGGAACCCCC TCGCATCCCG   
  
  
- AGTCAGCCCC TCGCGCCACA GCTTACTCCA CTCCTAGTGA CACCAACTCT GGCTCCAGCC ATTACCACCA   
  
  
- CGCCGCCCAC GCCTCCGACA ATTCCTACCG GACCACTTTA ACCGACTCTT CCACCCCTAA TCAAACTTCA   
  
  
- AACTCCGAAA CTGAGCCTTC TAGAGGCTCA ATTGGGCCCT CAGTGACCCC ACACTCCTCC CCCTCCGGCA   
  
  
- CCACCACTTA AAACGCAAGT TTGAAATGGC CCATGGCCTA CTCTCGCACA GCTACCTCTT AGACTCCCTG   
  
  
- CTCGAGGACG CGGCCCACTT CCGCGATTCC GGTGCGCGGC AGTGTAACCA CCTCGTCCTG AATTTACGCT   
  
  
- TATGCCGTGG CAAGTACTGC GCGCACTCAC CGCGAACACT CATGATATCT CGTAACAAGC TGAGCGAGCT   
  
  
- GAGCTACCAC GGCTCCCTAT TGGGCCTGGC CCAGGCCCAG CTCCTCCGGA ACCCCGCCTT CTACCGTTTG   
  
  
- AGCCACCGAA CGCTCCCGGC CCTTGCCCAA CTCGCCCGCC TCCACAAACC CTTCACCGCC CGCTCCTACT   
  
  
- CATACCGTCC CAAGCTCAAC TTCCGTGACT CAGTCTCACA CCGGCTCAGT TACGCTTGGT TTGAGTCGAG   
  
  
- CCAAGACCCG CCTTTCGGCC CAAAGTGCCA GTTCCTCCTC TCGCCACCCC ACCCCAAGCC CACCTACCCT   
  
  
- GCTTGAGAGT GGCAGCGTAG CCGGACCGCA AT

+     MSA-like

| Site Name | Organism | Position | Strand | Matrix score. | sequence | function |
| --- | --- | --- | --- | --- | --- | --- |
| MSA-like | Catharanthus roseus | 1421 | - | 9 | TCCAACGGT | cis-acting element involved in cell cycle regulation |

>HU06G02537.1   
+ +Up\_Stream \_Len000CCTCGG TGTGGGATTC CGGGTTGATA AATAGGGACC AGCGAAATGT TCAAATAATT   
  
  
+ GGTGAAGGAG TCTCTGTTTG AATGCCTACT AATGCTTTTT TTTTTTTTTT GAATAATTTG TCAGAAAGTT   
  
  
+ AGGCTGCATT GGAAGGGGCG GCCTACGAAG GCTTTTCAGC AAATAAATAA CCAGCTTTCA TTTTGAAATT   
  
  
+ TTTTTTAAAA AATGAAACTC ATTTCACTTA AAAATTTACC ATAATATATA TATATATATA TATATATATA   
  
  
+ TATATATATA TATTTGATTT AACGTTAAGC AAACTTTTAA CTTATGTATC ATTTTACGAC CGTAAAAAAA   
  
  
+ AAAGAAATAT AACACAATAT GTAATAGTAT TTAAGTTTTT GATAAATAAT ATTTTAACAT AATTTGTGTG   
  
  
+ TAGTATGAAT TTAAATCTTA GTACTAATAT AATTATGTAT ACTTGGCTAT AAGATCTCAA TATTAGTATT   
  
  
+ TTATGATTCA ATGTAATGAT GATGCAACTG TGTGTAATTA GAAAGCCTTT GATTGCAGAT ATCTATCTTA   
  
  
+ TTGATACACA TCTACCAATT GAGCTTTTTC AAGATTTGAA GACGGTAGTC AAGTATCTTT GGGAGATTAC   
  
  
+ GTTAGAATTA CTTCATGATA AATTAGACAT ATTTTATACT TCTAATCTTA AATAAGTCTC CATCCTTTCA   
  
  
+ TCATATTTTA AAGAAATGTA CAAGTAAATG TCAATAGTTT GAAACGTTCC TTATCATAAA AGTCATACAT   
  
  
+ ATGTAGGACA TTTTCAAGTT TATGTAAACC ATATGATATA GTTCAAGATA ATTGTCAAAC AAGTTATGAA   
  
  
+ ATCTAAAGGT GTTTGAACGT AATTTCAAGC TAATCTTAGC AAGTTATAGT ATTATTTAAA CTACGACTAG   
  
  
+ ACACAACATT TTGTTCATTT ATATAACACT TAGAATGCAA AAAAATCGGT ATTACTAATA CTGTATCAAG   
  
  
+ AATTGAAGTG TTATGCATTA TACTGATAAG GGATAATATT TAAATTACTT TACTGTTATA GAAGCCAAAA   
  
  
+ ATCTAAACTT TCATACAGAC GTCGGACTAA ATTAGAAAAT TCTTTAACTT AATAGAAAGG ATGACATAAA   
  
  
+ TAAAAATAAA AACCTAAATA GCATAATAAC CATTCTACAC AAAAGTCAAT CTTCTAATAG AAAAAAGCCA   
  
  
+ TAAGTATTCA CTTATTTACC ATTTTATTCC TAATCTACTG TCTGTCTATT CACACCATAA TTCATGAACC   
  
  
+ AATGTGGAGT TTCTGTTTCA TTTCATGGGG TGATTTATGT CTGACAATGA TCCAACAAAC TGAGCCTCAC   
  
  
+ GCGCGGAACA CGAGAGTGGC TGTACTTATG GTGTGAAAGA GCTCTACAAG GTGAGATTGA GGAGGCTACG   
  
  
+ CGTTGGAGTT ATCACCACCG TTGGATTTGT TTTCTTTGGA GCCACGTTAT CACTCTTCAG ACGCCGATCC   
  
  
+ AACGGCCCTT ACTTCCATCT AGTCCCCACT CTCCAACTCT ACGCAATCAA CGCCTTCCGC ATTTAAATAT   
  
  
+ TTCCTTCTCT ATCGCCTACT TTCTCTGTCT CACAATTTTT GTCCGTATTT TGAATTTTCT CAAAATTATC   
  
  
+ ATATAATTTA TTTGCCTTCA ACTAAAGTAT TTATTAAATT AGTTAATGTA TTTAAATTGA ACTAAAAAAG   
  
  
+ AGAAAAAGAA AGAAAACAAC CCCACGAAAG CAAAACGACA GAGGCCAACT TCTCTCTCTA GAGTGTAGAG   
  
  
+ CCCCATCTCG GTTATAATTA TGAGAATCAA TTCCTTCTCA CAAGAGAAAC TCTCGCTCTC TCTCTCTCTC   
  
  
+ CATAAGACAT CACACCAATT GTATGAATTT TCTCTCTCCT GATTTCTGTT TTCCTTCTTG TTGTCCCCTT   
  
  
+ CTAATGCGTT AGTGCGGCCA AAACCACCAT AAACCATCTC TCTTTCAAGA AAGCAGCAAC AACAAAGAGA   
  
  
+ GACAGAGAGA GAGAGAGGGG GAGAAAGATC ACATCTTTTG GGTTTCCGGC GAATATGACG TCTGGGTTTT   
  
  
+ CGGGCGATTT CTTCTACGGC GGTGGAGGCG GTGGCGGAGG AGGTGTCCGG CCTTCTATCG GCGGTGTACA   
  
  
+ AGGCGGGGTT GTCCCATATG GGTTCCGGCA ACAACAACAA CAACAGCAAC AGCAGCAGCA GCAGCAACAG   
  
  
+ CTACATCTAC AGCTACAACA ACAGCGGGGG ATTTTCTCGG CGGATCAGAT CAGCGCTCAA CGAAGGGCGG   
  
  
+ ATTTGTTGAT GGGTAAGCGT TCTTTCGCTG ATTTTCAATC TCAACAACAG CAAATCAGTC TTTTGCAGCA   
  
  
+ GCAGCAGCAG CAACAACAAC AACAGCCCTT TGCGCTCTTG CAAAAACAAC AGCAGTTTCA ACAACAATTA   
  
  
+ CAACAACAAC AGCCACTTTC TCTCCTCCTT CAACAACAGT ACTTGATGAG ATCCGTAAAA CAGAGAACCA   
  
  
+ ACAATTTCGC AGCATCTCCG ATTTCTCCTC TTTCTTCTTC CATTGATCTT TCTTCCGCTA ATAATTCCCG   
  
  
+ GTTCGGTTTG CCGGTTTACC AACAACAACT CCGGTCTCAA CAACAACCAC CTATCTCTCT CCTCGCCCAC   
  
  
+ AACAACAACC AATTCAACCT CTCAAATAAT AACAATTCTT CTTTAATCCA AAACTCCACC AACACTCTTC   
  
  
+ TTGCTCCCGC AGCCAGCAAT AACACCCTAA TGAAAAATTC GGGCTTTTCC TCATCTGGGT TTTCTAATTC   
  
  
+ GGGTCCAAAC CTGGTCCAAC CGAAGCAGGA AATCGGGTAC TCGAATATGG GTACCAATAT GGGTATGAAT   
  
  
+ CTGGTCCAGC CCAAACCCGA CCCTGAGGAT CACGGAATAA TGAACACCCT TCAAGAATTG GAGAAGGAGC   
  
  
+ TTTTGAATGA CGATGAGGAA AACCAGCCGG AAAACGACGC CGTTTCAGTC ATCACCAACA GCGAGTGGTC   
  
  
+ CGAGACCTTC CAGAATCTGA TGAGTCCCGA CCCGAGTTCG AGCCCGAATG CCACCAACAC CCACCCGGGT   
  
  
+ TCGGGTTCTG CTAGTACCAG CGGCAGCAAC AACACTACAA CCACCCCTGC AAACAACAAT AACATGATAT   
  
  
+ CTACCTCGCC GACGTCGTCC ACGTCATCCT GCTGCTCCAC GTCGGCATCG GCGTCTCCGC CGGCGGCGGT   
  
  
+ GGCGACCCCG CAGGTACCAG CCAAGCAGCT GATCTCGGAA GCAGCGGCGG CGATTTCGGA CGGAAAGATT   
  
  
+ GAATCCGCCA TGGAAGTGAT GAACCGGCTG TCCCAGGTTG CCAATTCTAG GGGGAGCTCG GAACAAAAGC   
  
  
+ TGGCTTTTTA CATGGGCCAC GCGCTTCGGT CGCGCGTGAG TCCCACCGAA CACGCTCCCC CTATTGTGGA   
  
  
+ GCTTTTCCAA AAGGAGCATA TGGCGGCCAT GTATTCATTG TACGAGCTCT CTCATTGTTT CAAATTTGGT   
  
  
+ CTTTTGGCTG CCAATTTGGT GATGTTGGAG GCCATTACTT CCGCCCTTCA TGAGGGTCTG AAAATTCATG   
  
  
+ TTGTGGATTT TGATATTGGG CATGGTGGTC AGTACATCAA CCTCCTACGC GCCTTGGGGG AGCGTAGGGC   
  
  
+ TCAGTCGGGG AGCGCGGTGT CGAATGAGGT GAGGATCACT GTGGTTGAGA CCGAGGTCGG TAATGGTGGT   
  
  
+ GCGGCGGGTG CGGAGGCTGT TAAGGATGGC CTGGTGAAAT TGGCTGAGAA GGTGGGGATT AGTTTGAAGT   
  
  
+ TTGAGGCTTT GACTCGGAAG ATCTCCGAGT TAACCCGGGA GTCACTGGGG TGTGAGGAGG GGGAGGCCGT   
  
  
+ GGTGGTGAAT TTTGCGTTCA AACTTTACCG GGTACCGGAT GAGAGCGTGT CGATGGAGAA TCTGAGGGAC   
  
  
+ GAGCTCCTGC GCCGGGTGAA GGCGCTAAGG CCACGCGCCG TCACATTGGT GGAGCAGGAC TTAAATGCGA   
  
  
+ ATACGGCACC GTTCATGACG CGCGTGAGTG GCGCTTGTGA GTACTATAGA GCATTGTTCG ACTCGCTCGA   
  
  
+ CTCGATGGTG CCGAGGGATA ACCCGGACCG GGTCCGGGTC GAGGAGGCCT TGGGGCGGAA GATGGCAAAC   
  
  
+ TCGGTGGCTT GCGAGGGCCG GGAACGGGTT GAGCGGGCGG AGGTGTTTGG GAAGTGGCGG GCGAGGATGA   
  
  
+ GTATGGCAGG GTTCGAGTTG AAGGCACTGA GTCAGAGTGT GGCCGAGTCA ATGCGAACCA AACTCAGCTC   
  
  
+ GGTTCTGGGC GGAAAGCCGG GTTTCACGGT CAAGGAGGAG AGCGGTGGGG TGGGGTTCGG GTGGATGGGA   
  
  
+ CGAACTCTCA CCGTCGCATC GGCCTGGCGT TA  

- +Up\_Stream \_Len000GGAGCC ACACCCTAAG GCCCAACTAT TTATCCCTGG TCGCTTTACA AGTTTATTAA   
  
  
- CCACTTCCTC AGAGACAAAC TTACGGATGA TTACGAAAAA AAAAAAAAAA CTTATTAAAC AGTCTTTCAA   
  
  
- TCCGACGTAA CCTTCCCCGC CGGATGCTTC CGAAAAGTCG TTTATTTATT GGTCGAAAGT AAAACTTTAA   
  
  
- AAAAAATTTT TTACTTTGAG TAAAGTGAAT TTTTAAATGG TATTATATAT ATATATATAT ATATATATAT   
  
  
- ATATATATAT ATAAACTAAA TTGCAATTCG TTTGAAAATT GAATACATAG TAAAATGCTG GCATTTTTTT   
  
  
- TTTCTTTATA TTGTGTTATA CATTATCATA AATTCAAAAA CTATTTATTA TAAAATTGTA TTAAACACAC   
  
  
- ATCATACTTA AATTTAGAAT CATGATTATA TTAATACATA TGAACCGATA TTCTAGAGTT ATAATCATAA   
  
  
- AATACTAAGT TACATTACTA CTACGTTGAC ACACATTAAT CTTTCGGAAA CTAACGTCTA TAGATAGAAT   
  
  
- AACTATGTGT AGATGGTTAA CTCGAAAAAG TTCTAAACTT CTGCCATCAG TTCATAGAAA CCCTCTAATG   
  
  
- CAATCTTAAT GAAGTACTAT TTAATCTGTA TAAAATATGA AGATTAGAAT TTATTCAGAG GTAGGAAAGT   
  
  
- AGTATAAAAT TTCTTTACAT GTTCATTTAC AGTTATCAAA CTTTGCAAGG AATAGTATTT TCAGTATGTA   
  
  
- TACATCCTGT AAAAGTTCAA ATACATTTGG TATACTATAT CAAGTTCTAT TAACAGTTTG TTCAATACTT   
  
  
- TAGATTTCCA CAAACTTGCA TTAAAGTTCG ATTAGAATCG TTCAATATCA TAATAAATTT GATGCTGATC   
  
  
- TGTGTTGTAA AACAAGTAAA TATATTGTGA ATCTTACGTT TTTTTAGCCA TAATGATTAT GACATAGTTC   
  
  
- TTAACTTCAC AATACGTAAT ATGACTATTC CCTATTATAA ATTTAATGAA ATGACAATAT CTTCGGTTTT   
  
  
- TAGATTTGAA AGTATGTCTG CAGCCTGATT TAATCTTTTA AGAAATTGAA TTATCTTTCC TACTGTATTT   
  
  
- ATTTTTATTT TTGGATTTAT CGTATTATTG GTAAGATGTG TTTTCAGTTA GAAGATTATC TTTTTTCGGT   
  
  
- ATTCATAAGT GAATAAATGG TAAAATAAGG ATTAGATGAC AGACAGATAA GTGTGGTATT AAGTACTTGG   
  
  
- TTACACCTCA AAGACAAAGT AAAGTACCCC ACTAAATACA GACTGTTACT AGGTTGTTTG ACTCGGAGTG   
  
  
- CGCGCCTTGT GCTCTCACCG ACATGAATAC CACACTTTCT CGAGATGTTC CACTCTAACT CCTCCGATGC   
  
  
- GCAACCTCAA TAGTGGTGGC AACCTAAACA AAAGAAACCT CGGTGCAATA GTGAGAAGTC TGCGGCTAGG   
  
  
- TTGCCGGGAA TGAAGGTAGA TCAGGGGTGA GAGGTTGAGA TGCGTTAGTT GCGGAAGGCG TAAATTTATA   
  
  
- AAGGAAGAGA TAGCGGATGA AAGAGACAGA GTGTTAAAAA CAGGCATAAA ACTTAAAAGA GTTTTAATAG   
  
  
- TATATTAAAT AAACGGAAGT TGATTTCATA AATAATTTAA TCAATTACAT AAATTTAACT TGATTTTTTC   
  
  
- TCTTTTTCTT TCTTTTGTTG GGGTGCTTTC GTTTTGCTGT CTCCGGTTGA AGAGAGAGAT CTCACATCTC   
  
  
- GGGGTAGAGC CAATATTAAT ACTCTTAGTT AAGGAAGAGT GTTCTCTTTG AGAGCGAGAG AGAGAGAGAG   
  
  
- GTATTCTGTA GTGTGGTTAA CATACTTAAA AGAGAGAGGA CTAAAGACAA AAGGAAGAAC AACAGGGGAA   
  
  
- GATTACGCAA TCACGCCGGT TTTGGTGGTA TTTGGTAGAG AGAAAGTTCT TTCGTCGTTG TTGTTTCTCT   
  
  
- CTGTCTCTCT CTCTCTCCCC CTCTTTCTAG TGTAGAAAAC CCAAAGGCCG CTTATACTGC AGACCCAAAA   
  
  
- GCCCGCTAAA GAAGATGCCG CCACCTCCGC CACCGCCTCC TCCACAGGCC GGAAGATAGC CGCCACATGT   
  
  
- TCCGCCCCAA CAGGGTATAC CCAAGGCCGT TGTTGTTGTT GTTGTCGTTG TCGTCGTCGT CGTCGTTGTC   
  
  
- GATGTAGATG TCGATGTTGT TGTCGCCCCC TAAAAGAGCC GCCTAGTCTA GTCGCGAGTT GCTTCCCGCC   
  
  
- TAAACAACTA CCCATTCGCA AGAAAGCGAC TAAAAGTTAG AGTTGTTGTC GTTTAGTCAG AAAACGTCGT   
  
  
- CGTCGTCGTC GTTGTTGTTG TTGTCGGGAA ACGCGAGAAC GTTTTTGTTG TCGTCAAAGT TGTTGTTAAT   
  
  
- GTTGTTGTTG TCGGTGAAAG AGAGGAGGAA GTTGTTGTCA TGAACTACTC TAGGCATTTT GTCTCTTGGT   
  
  
- TGTTAAAGCG TCGTAGAGGC TAAAGAGGAG AAAGAAGAAG GTAACTAGAA AGAAGGCGAT TATTAAGGGC   
  
  
- CAAGCCAAAC GGCCAAATGG TTGTTGTTGA GGCCAGAGTT GTTGTTGGTG GATAGAGAGA GGAGCGGGTG   
  
  
- TTGTTGTTGG TTAAGTTGGA GAGTTTATTA TTGTTAAGAA GAAATTAGGT TTTGAGGTGG TTGTGAGAAG   
  
  
- AACGAGGGCG TCGGTCGTTA TTGTGGGATT ACTTTTTAAG CCCGAAAAGG AGTAGACCCA AAAGATTAAG   
  
  
- CCCAGGTTTG GACCAGGTTG GCTTCGTCCT TTAGCCCATG AGCTTATACC CATGGTTATA CCCATACTTA   
  
  
- GACCAGGTCG GGTTTGGGCT GGGACTCCTA GTGCCTTATT ACTTGTGGGA AGTTCTTAAC CTCTTCCTCG   
  
  
- AAAACTTACT GCTACTCCTT TTGGTCGGCC TTTTGCTGCG GCAAAGTCAG TAGTGGTTGT CGCTCACCAG   
  
  
- GCTCTGGAAG GTCTTAGACT ACTCAGGGCT GGGCTCAAGC TCGGGCTTAC GGTGGTTGTG GGTGGGCCCA   
  
  
- AGCCCAAGAC GATCATGGTC GCCGTCGTTG TTGTGATGTT GGTGGGGACG TTTGTTGTTA TTGTACTATA   
  
  
- GATGGAGCGG CTGCAGCAGG TGCAGTAGGA CGACGAGGTG CAGCCGTAGC CGCAGAGGCG GCCGCCGCCA   
  
  
- CCGCTGGGGC GTCCATGGTC GGTTCGTCGA CTAGAGCCTT CGTCGCCGCC GCTAAAGCCT GCCTTTCTAA   
  
  
- CTTAGGCGGT ACCTTCACTA CTTGGCCGAC AGGGTCCAAC GGTTAAGATC CCCCTCGAGC CTTGTTTTCG   
  
  
- ACCGAAAAAT GTACCCGGTG CGCGAAGCCA GCGCGCACTC AGGGTGGCTT GTGCGAGGGG GATAACACCT   
  
  
- CGAAAAGGTT TTCCTCGTAT ACCGCCGGTA CATAAGTAAC ATGCTCGAGA GAGTAACAAA GTTTAAACCA   
  
  
- GAAAACCGAC GGTTAAACCA CTACAACCTC CGGTAATGAA GGCGGGAAGT ACTCCCAGAC TTTTAAGTAC   
  
  
- AACACCTAAA ACTATAACCC GTACCACCAG TCATGTAGTT GGAGGATGCG CGGAACCCCC TCGCATCCCG   
  
  
- AGTCAGCCCC TCGCGCCACA GCTTACTCCA CTCCTAGTGA CACCAACTCT GGCTCCAGCC ATTACCACCA   
  
  
- CGCCGCCCAC GCCTCCGACA ATTCCTACCG GACCACTTTA ACCGACTCTT CCACCCCTAA TCAAACTTCA   
  
  
- AACTCCGAAA CTGAGCCTTC TAGAGGCTCA ATTGGGCCCT CAGTGACCCC ACACTCCTCC CCCTCCGGCA   
  
  
- CCACCACTTA AAACGCAAGT TTGAAATGGC CCATGGCCTA CTCTCGCACA GCTACCTCTT AGACTCCCTG   
  
  
- CTCGAGGACG CGGCCCACTT CCGCGATTCC GGTGCGCGGC AGTGTAACCA CCTCGTCCTG AATTTACGCT   
  
  
- TATGCCGTGG CAAGTACTGC GCGCACTCAC CGCGAACACT CATGATATCT CGTAACAAGC TGAGCGAGCT   
  
  
- GAGCTACCAC GGCTCCCTAT TGGGCCTGGC CCAGGCCCAG CTCCTCCGGA ACCCCGCCTT CTACCGTTTG   
  
  
- AGCCACCGAA CGCTCCCGGC CCTTGCCCAA CTCGCCCGCC TCCACAAACC CTTCACCGCC CGCTCCTACT   
  
  
- CATACCGTCC CAAGCTCAAC TTCCGTGACT CAGTCTCACA CCGGCTCAGT TACGCTTGGT TTGAGTCGAG   
  
  
- CCAAGACCCG CCTTTCGGCC CAAAGTGCCA GTTCCTCCTC TCGCCACCCC ACCCCAAGCC CACCTACCCT   
  
  
- GCTTGAGAGT GGCAGCGTAG CCGGACCGCA AT

+     MYB

| Site Name | Organism | Position | Strand | Matrix score. | sequence | function |
| --- | --- | --- | --- | --- | --- | --- |
| MYB | Arabidopsis thaliana | 3616 | - | 6 | CAACCA |  |
| MYB | Arabidopsis thaliana | 2568 | + | 6 | CAACCA |  |
| MYB | Arabidopsis thaliana | 2361 | + | 6 | CAACAG |  |
| MYB | Arabidopsis thaliana | 2289 | + | 6 | CAACAG |  |
| MYB | Arabidopsis thaliana | 2151 | + | 6 | CAACAG |  |
| MYB | Arabidopsis thaliana | 2145 | + | 6 | CAACAG |  |
| MYB | Arabidopsis thaliana | 3052 | + | 6 | CAACCA |  |
| MYB | Arabidopsis thaliana | 2930 | + | 6 | CAACAG |  |
| MYB | Arabidopsis thaliana | 2600 | + | 6 | CAACCA |  |
| MYB | Arabidopsis thaliana | 1151 | + | 6 | TAACCA |  |
| MYB | Arabidopsis thaliana | 2391 | + | 6 | CAACAG |  |
| MYB | Arabidopsis thaliana | 2418 | + | 6 | CAACAG |  |
| MYB | Arabidopsis thaliana | 2169 | + | 6 | CAACAG |  |
| MYB | Arabidopsis thaliana | 2193 | + | 6 | CAACAG |  |
| MYB | Arabidopsis thaliana | 192 | + | 6 | TAACCA |  |
| MYB | Arabidopsis thaliana | 2334 | + | 6 | CAACAG |  |

>HU06G02537.1   
+ +Up\_Stream \_Len000CCTCGG TGTGGGATTC CGGGTTGATA AATAGGGACC AGCGAAATGT TCAAATAATT   
  
  
+ GGTGAAGGAG TCTCTGTTTG AATGCCTACT AATGCTTTTT TTTTTTTTTT GAATAATTTG TCAGAAAGTT   
  
  
+ AGGCTGCATT GGAAGGGGCG GCCTACGAAG GCTTTTCAGC AAATAAATAA CCAGCTTTCA TTTTGAAATT   
  
  
+ TTTTTTAAAA AATGAAACTC ATTTCACTTA AAAATTTACC ATAATATATA TATATATATA TATATATATA   
  
  
+ TATATATATA TATTTGATTT AACGTTAAGC AAACTTTTAA CTTATGTATC ATTTTACGAC CGTAAAAAAA   
  
  
+ AAAGAAATAT AACACAATAT GTAATAGTAT TTAAGTTTTT GATAAATAAT ATTTTAACAT AATTTGTGTG   
  
  
+ TAGTATGAAT TTAAATCTTA GTACTAATAT AATTATGTAT ACTTGGCTAT AAGATCTCAA TATTAGTATT   
  
  
+ TTATGATTCA ATGTAATGAT GATGCAACTG TGTGTAATTA GAAAGCCTTT GATTGCAGAT ATCTATCTTA   
  
  
+ TTGATACACA TCTACCAATT GAGCTTTTTC AAGATTTGAA GACGGTAGTC AAGTATCTTT GGGAGATTAC   
  
  
+ GTTAGAATTA CTTCATGATA AATTAGACAT ATTTTATACT TCTAATCTTA AATAAGTCTC CATCCTTTCA   
  
  
+ TCATATTTTA AAGAAATGTA CAAGTAAATG TCAATAGTTT GAAACGTTCC TTATCATAAA AGTCATACAT   
  
  
+ ATGTAGGACA TTTTCAAGTT TATGTAAACC ATATGATATA GTTCAAGATA ATTGTCAAAC AAGTTATGAA   
  
  
+ ATCTAAAGGT GTTTGAACGT AATTTCAAGC TAATCTTAGC AAGTTATAGT ATTATTTAAA CTACGACTAG   
  
  
+ ACACAACATT TTGTTCATTT ATATAACACT TAGAATGCAA AAAAATCGGT ATTACTAATA CTGTATCAAG   
  
  
+ AATTGAAGTG TTATGCATTA TACTGATAAG GGATAATATT TAAATTACTT TACTGTTATA GAAGCCAAAA   
  
  
+ ATCTAAACTT TCATACAGAC GTCGGACTAA ATTAGAAAAT TCTTTAACTT AATAGAAAGG ATGACATAAA   
  
  
+ TAAAAATAAA AACCTAAATA GCATAATAAC CATTCTACAC AAAAGTCAAT CTTCTAATAG AAAAAAGCCA   
  
  
+ TAAGTATTCA CTTATTTACC ATTTTATTCC TAATCTACTG TCTGTCTATT CACACCATAA TTCATGAACC   
  
  
+ AATGTGGAGT TTCTGTTTCA TTTCATGGGG TGATTTATGT CTGACAATGA TCCAACAAAC TGAGCCTCAC   
  
  
+ GCGCGGAACA CGAGAGTGGC TGTACTTATG GTGTGAAAGA GCTCTACAAG GTGAGATTGA GGAGGCTACG   
  
  
+ CGTTGGAGTT ATCACCACCG TTGGATTTGT TTTCTTTGGA GCCACGTTAT CACTCTTCAG ACGCCGATCC   
  
  
+ AACGGCCCTT ACTTCCATCT AGTCCCCACT CTCCAACTCT ACGCAATCAA CGCCTTCCGC ATTTAAATAT   
  
  
+ TTCCTTCTCT ATCGCCTACT TTCTCTGTCT CACAATTTTT GTCCGTATTT TGAATTTTCT CAAAATTATC   
  
  
+ ATATAATTTA TTTGCCTTCA ACTAAAGTAT TTATTAAATT AGTTAATGTA TTTAAATTGA ACTAAAAAAG   
  
  
+ AGAAAAAGAA AGAAAACAAC CCCACGAAAG CAAAACGACA GAGGCCAACT TCTCTCTCTA GAGTGTAGAG   
  
  
+ CCCCATCTCG GTTATAATTA TGAGAATCAA TTCCTTCTCA CAAGAGAAAC TCTCGCTCTC TCTCTCTCTC   
  
  
+ CATAAGACAT CACACCAATT GTATGAATTT TCTCTCTCCT GATTTCTGTT TTCCTTCTTG TTGTCCCCTT   
  
  
+ CTAATGCGTT AGTGCGGCCA AAACCACCAT AAACCATCTC TCTTTCAAGA AAGCAGCAAC AACAAAGAGA   
  
  
+ GACAGAGAGA GAGAGAGGGG GAGAAAGATC ACATCTTTTG GGTTTCCGGC GAATATGACG TCTGGGTTTT   
  
  
+ CGGGCGATTT CTTCTACGGC GGTGGAGGCG GTGGCGGAGG AGGTGTCCGG CCTTCTATCG GCGGTGTACA   
  
  
+ AGGCGGGGTT GTCCCATATG GGTTCCGGCA ACAACAACAA CAACAGCAAC AGCAGCAGCA GCAGCAACAG   
  
  
+ CTACATCTAC AGCTACAACA ACAGCGGGGG ATTTTCTCGG CGGATCAGAT CAGCGCTCAA CGAAGGGCGG   
  
  
+ ATTTGTTGAT GGGTAAGCGT TCTTTCGCTG ATTTTCAATC TCAACAACAG CAAATCAGTC TTTTGCAGCA   
  
  
+ GCAGCAGCAG CAACAACAAC AACAGCCCTT TGCGCTCTTG CAAAAACAAC AGCAGTTTCA ACAACAATTA   
  
  
+ CAACAACAAC AGCCACTTTC TCTCCTCCTT CAACAACAGT ACTTGATGAG ATCCGTAAAA CAGAGAACCA   
  
  
+ ACAATTTCGC AGCATCTCCG ATTTCTCCTC TTTCTTCTTC CATTGATCTT TCTTCCGCTA ATAATTCCCG   
  
  
+ GTTCGGTTTG CCGGTTTACC AACAACAACT CCGGTCTCAA CAACAACCAC CTATCTCTCT CCTCGCCCAC   
  
  
+ AACAACAACC AATTCAACCT CTCAAATAAT AACAATTCTT CTTTAATCCA AAACTCCACC AACACTCTTC   
  
  
+ TTGCTCCCGC AGCCAGCAAT AACACCCTAA TGAAAAATTC GGGCTTTTCC TCATCTGGGT TTTCTAATTC   
  
  
+ GGGTCCAAAC CTGGTCCAAC CGAAGCAGGA AATCGGGTAC TCGAATATGG GTACCAATAT GGGTATGAAT   
  
  
+ CTGGTCCAGC CCAAACCCGA CCCTGAGGAT CACGGAATAA TGAACACCCT TCAAGAATTG GAGAAGGAGC   
  
  
+ TTTTGAATGA CGATGAGGAA AACCAGCCGG AAAACGACGC CGTTTCAGTC ATCACCAACA GCGAGTGGTC   
  
  
+ CGAGACCTTC CAGAATCTGA TGAGTCCCGA CCCGAGTTCG AGCCCGAATG CCACCAACAC CCACCCGGGT   
  
  
+ TCGGGTTCTG CTAGTACCAG CGGCAGCAAC AACACTACAA CCACCCCTGC AAACAACAAT AACATGATAT   
  
  
+ CTACCTCGCC GACGTCGTCC ACGTCATCCT GCTGCTCCAC GTCGGCATCG GCGTCTCCGC CGGCGGCGGT   
  
  
+ GGCGACCCCG CAGGTACCAG CCAAGCAGCT GATCTCGGAA GCAGCGGCGG CGATTTCGGA CGGAAAGATT   
  
  
+ GAATCCGCCA TGGAAGTGAT GAACCGGCTG TCCCAGGTTG CCAATTCTAG GGGGAGCTCG GAACAAAAGC   
  
  
+ TGGCTTTTTA CATGGGCCAC GCGCTTCGGT CGCGCGTGAG TCCCACCGAA CACGCTCCCC CTATTGTGGA   
  
  
+ GCTTTTCCAA AAGGAGCATA TGGCGGCCAT GTATTCATTG TACGAGCTCT CTCATTGTTT CAAATTTGGT   
  
  
+ CTTTTGGCTG CCAATTTGGT GATGTTGGAG GCCATTACTT CCGCCCTTCA TGAGGGTCTG AAAATTCATG   
  
  
+ TTGTGGATTT TGATATTGGG CATGGTGGTC AGTACATCAA CCTCCTACGC GCCTTGGGGG AGCGTAGGGC   
  
  
+ TCAGTCGGGG AGCGCGGTGT CGAATGAGGT GAGGATCACT GTGGTTGAGA CCGAGGTCGG TAATGGTGGT   
  
  
+ GCGGCGGGTG CGGAGGCTGT TAAGGATGGC CTGGTGAAAT TGGCTGAGAA GGTGGGGATT AGTTTGAAGT   
  
  
+ TTGAGGCTTT GACTCGGAAG ATCTCCGAGT TAACCCGGGA GTCACTGGGG TGTGAGGAGG GGGAGGCCGT   
  
  
+ GGTGGTGAAT TTTGCGTTCA AACTTTACCG GGTACCGGAT GAGAGCGTGT CGATGGAGAA TCTGAGGGAC   
  
  
+ GAGCTCCTGC GCCGGGTGAA GGCGCTAAGG CCACGCGCCG TCACATTGGT GGAGCAGGAC TTAAATGCGA   
  
  
+ ATACGGCACC GTTCATGACG CGCGTGAGTG GCGCTTGTGA GTACTATAGA GCATTGTTCG ACTCGCTCGA   
  
  
+ CTCGATGGTG CCGAGGGATA ACCCGGACCG GGTCCGGGTC GAGGAGGCCT TGGGGCGGAA GATGGCAAAC   
  
  
+ TCGGTGGCTT GCGAGGGCCG GGAACGGGTT GAGCGGGCGG AGGTGTTTGG GAAGTGGCGG GCGAGGATGA   
  
  
+ GTATGGCAGG GTTCGAGTTG AAGGCACTGA GTCAGAGTGT GGCCGAGTCA ATGCGAACCA AACTCAGCTC   
  
  
+ GGTTCTGGGC GGAAAGCCGG GTTTCACGGT CAAGGAGGAG AGCGGTGGGG TGGGGTTCGG GTGGATGGGA   
  
  
+ CGAACTCTCA CCGTCGCATC GGCCTGGCGT TA  

- +Up\_Stream \_Len000GGAGCC ACACCCTAAG GCCCAACTAT TTATCCCTGG TCGCTTTACA AGTTTATTAA   
  
  
- CCACTTCCTC AGAGACAAAC TTACGGATGA TTACGAAAAA AAAAAAAAAA CTTATTAAAC AGTCTTTCAA   
  
  
- TCCGACGTAA CCTTCCCCGC CGGATGCTTC CGAAAAGTCG TTTATTTATT GGTCGAAAGT AAAACTTTAA   
  
  
- AAAAAATTTT TTACTTTGAG TAAAGTGAAT TTTTAAATGG TATTATATAT ATATATATAT ATATATATAT   
  
  
- ATATATATAT ATAAACTAAA TTGCAATTCG TTTGAAAATT GAATACATAG TAAAATGCTG GCATTTTTTT   
  
  
- TTTCTTTATA TTGTGTTATA CATTATCATA AATTCAAAAA CTATTTATTA TAAAATTGTA TTAAACACAC   
  
  
- ATCATACTTA AATTTAGAAT CATGATTATA TTAATACATA TGAACCGATA TTCTAGAGTT ATAATCATAA   
  
  
- AATACTAAGT TACATTACTA CTACGTTGAC ACACATTAAT CTTTCGGAAA CTAACGTCTA TAGATAGAAT   
  
  
- AACTATGTGT AGATGGTTAA CTCGAAAAAG TTCTAAACTT CTGCCATCAG TTCATAGAAA CCCTCTAATG   
  
  
- CAATCTTAAT GAAGTACTAT TTAATCTGTA TAAAATATGA AGATTAGAAT TTATTCAGAG GTAGGAAAGT   
  
  
- AGTATAAAAT TTCTTTACAT GTTCATTTAC AGTTATCAAA CTTTGCAAGG AATAGTATTT TCAGTATGTA   
  
  
- TACATCCTGT AAAAGTTCAA ATACATTTGG TATACTATAT CAAGTTCTAT TAACAGTTTG TTCAATACTT   
  
  
- TAGATTTCCA CAAACTTGCA TTAAAGTTCG ATTAGAATCG TTCAATATCA TAATAAATTT GATGCTGATC   
  
  
- TGTGTTGTAA AACAAGTAAA TATATTGTGA ATCTTACGTT TTTTTAGCCA TAATGATTAT GACATAGTTC   
  
  
- TTAACTTCAC AATACGTAAT ATGACTATTC CCTATTATAA ATTTAATGAA ATGACAATAT CTTCGGTTTT   
  
  
- TAGATTTGAA AGTATGTCTG CAGCCTGATT TAATCTTTTA AGAAATTGAA TTATCTTTCC TACTGTATTT   
  
  
- ATTTTTATTT TTGGATTTAT CGTATTATTG GTAAGATGTG TTTTCAGTTA GAAGATTATC TTTTTTCGGT   
  
  
- ATTCATAAGT GAATAAATGG TAAAATAAGG ATTAGATGAC AGACAGATAA GTGTGGTATT AAGTACTTGG   
  
  
- TTACACCTCA AAGACAAAGT AAAGTACCCC ACTAAATACA GACTGTTACT AGGTTGTTTG ACTCGGAGTG   
  
  
- CGCGCCTTGT GCTCTCACCG ACATGAATAC CACACTTTCT CGAGATGTTC CACTCTAACT CCTCCGATGC   
  
  
- GCAACCTCAA TAGTGGTGGC AACCTAAACA AAAGAAACCT CGGTGCAATA GTGAGAAGTC TGCGGCTAGG   
  
  
- TTGCCGGGAA TGAAGGTAGA TCAGGGGTGA GAGGTTGAGA TGCGTTAGTT GCGGAAGGCG TAAATTTATA   
  
  
- AAGGAAGAGA TAGCGGATGA AAGAGACAGA GTGTTAAAAA CAGGCATAAA ACTTAAAAGA GTTTTAATAG   
  
  
- TATATTAAAT AAACGGAAGT TGATTTCATA AATAATTTAA TCAATTACAT AAATTTAACT TGATTTTTTC   
  
  
- TCTTTTTCTT TCTTTTGTTG GGGTGCTTTC GTTTTGCTGT CTCCGGTTGA AGAGAGAGAT CTCACATCTC   
  
  
- GGGGTAGAGC CAATATTAAT ACTCTTAGTT AAGGAAGAGT GTTCTCTTTG AGAGCGAGAG AGAGAGAGAG   
  
  
- GTATTCTGTA GTGTGGTTAA CATACTTAAA AGAGAGAGGA CTAAAGACAA AAGGAAGAAC AACAGGGGAA   
  
  
- GATTACGCAA TCACGCCGGT TTTGGTGGTA TTTGGTAGAG AGAAAGTTCT TTCGTCGTTG TTGTTTCTCT   
  
  
- CTGTCTCTCT CTCTCTCCCC CTCTTTCTAG TGTAGAAAAC CCAAAGGCCG CTTATACTGC AGACCCAAAA   
  
  
- GCCCGCTAAA GAAGATGCCG CCACCTCCGC CACCGCCTCC TCCACAGGCC GGAAGATAGC CGCCACATGT   
  
  
- TCCGCCCCAA CAGGGTATAC CCAAGGCCGT TGTTGTTGTT GTTGTCGTTG TCGTCGTCGT CGTCGTTGTC   
  
  
- GATGTAGATG TCGATGTTGT TGTCGCCCCC TAAAAGAGCC GCCTAGTCTA GTCGCGAGTT GCTTCCCGCC   
  
  
- TAAACAACTA CCCATTCGCA AGAAAGCGAC TAAAAGTTAG AGTTGTTGTC GTTTAGTCAG AAAACGTCGT   
  
  
- CGTCGTCGTC GTTGTTGTTG TTGTCGGGAA ACGCGAGAAC GTTTTTGTTG TCGTCAAAGT TGTTGTTAAT   
  
  
- GTTGTTGTTG TCGGTGAAAG AGAGGAGGAA GTTGTTGTCA TGAACTACTC TAGGCATTTT GTCTCTTGGT   
  
  
- TGTTAAAGCG TCGTAGAGGC TAAAGAGGAG AAAGAAGAAG GTAACTAGAA AGAAGGCGAT TATTAAGGGC   
  
  
- CAAGCCAAAC GGCCAAATGG TTGTTGTTGA GGCCAGAGTT GTTGTTGGTG GATAGAGAGA GGAGCGGGTG   
  
  
- TTGTTGTTGG TTAAGTTGGA GAGTTTATTA TTGTTAAGAA GAAATTAGGT TTTGAGGTGG TTGTGAGAAG   
  
  
- AACGAGGGCG TCGGTCGTTA TTGTGGGATT ACTTTTTAAG CCCGAAAAGG AGTAGACCCA AAAGATTAAG   
  
  
- CCCAGGTTTG GACCAGGTTG GCTTCGTCCT TTAGCCCATG AGCTTATACC CATGGTTATA CCCATACTTA   
  
  
- GACCAGGTCG GGTTTGGGCT GGGACTCCTA GTGCCTTATT ACTTGTGGGA AGTTCTTAAC CTCTTCCTCG   
  
  
- AAAACTTACT GCTACTCCTT TTGGTCGGCC TTTTGCTGCG GCAAAGTCAG TAGTGGTTGT CGCTCACCAG   
  
  
- GCTCTGGAAG GTCTTAGACT ACTCAGGGCT GGGCTCAAGC TCGGGCTTAC GGTGGTTGTG GGTGGGCCCA   
  
  
- AGCCCAAGAC GATCATGGTC GCCGTCGTTG TTGTGATGTT GGTGGGGACG TTTGTTGTTA TTGTACTATA   
  
  
- GATGGAGCGG CTGCAGCAGG TGCAGTAGGA CGACGAGGTG CAGCCGTAGC CGCAGAGGCG GCCGCCGCCA   
  
  
- CCGCTGGGGC GTCCATGGTC GGTTCGTCGA CTAGAGCCTT CGTCGCCGCC GCTAAAGCCT GCCTTTCTAA   
  
  
- CTTAGGCGGT ACCTTCACTA CTTGGCCGAC AGGGTCCAAC GGTTAAGATC CCCCTCGAGC CTTGTTTTCG   
  
  
- ACCGAAAAAT GTACCCGGTG CGCGAAGCCA GCGCGCACTC AGGGTGGCTT GTGCGAGGGG GATAACACCT   
  
  
- CGAAAAGGTT TTCCTCGTAT ACCGCCGGTA CATAAGTAAC ATGCTCGAGA GAGTAACAAA GTTTAAACCA   
  
  
- GAAAACCGAC GGTTAAACCA CTACAACCTC CGGTAATGAA GGCGGGAAGT ACTCCCAGAC TTTTAAGTAC   
  
  
- AACACCTAAA ACTATAACCC GTACCACCAG TCATGTAGTT GGAGGATGCG CGGAACCCCC TCGCATCCCG   
  
  
- AGTCAGCCCC TCGCGCCACA GCTTACTCCA CTCCTAGTGA CACCAACTCT GGCTCCAGCC ATTACCACCA   
  
  
- CGCCGCCCAC GCCTCCGACA ATTCCTACCG GACCACTTTA ACCGACTCTT CCACCCCTAA TCAAACTTCA   
  
  
- AACTCCGAAA CTGAGCCTTC TAGAGGCTCA ATTGGGCCCT CAGTGACCCC ACACTCCTCC CCCTCCGGCA   
  
  
- CCACCACTTA AAACGCAAGT TTGAAATGGC CCATGGCCTA CTCTCGCACA GCTACCTCTT AGACTCCCTG   
  
  
- CTCGAGGACG CGGCCCACTT CCGCGATTCC GGTGCGCGGC AGTGTAACCA CCTCGTCCTG AATTTACGCT   
  
  
- TATGCCGTGG CAAGTACTGC GCGCACTCAC CGCGAACACT CATGATATCT CGTAACAAGC TGAGCGAGCT   
  
  
- GAGCTACCAC GGCTCCCTAT TGGGCCTGGC CCAGGCCCAG CTCCTCCGGA ACCCCGCCTT CTACCGTTTG   
  
  
- AGCCACCGAA CGCTCCCGGC CCTTGCCCAA CTCGCCCGCC TCCACAAACC CTTCACCGCC CGCTCCTACT   
  
  
- CATACCGTCC CAAGCTCAAC TTCCGTGACT CAGTCTCACA CCGGCTCAGT TACGCTTGGT TTGAGTCGAG   
  
  
- CCAAGACCCG CCTTTCGGCC CAAAGTGCCA GTTCCTCCTC TCGCCACCCC ACCCCAAGCC CACCTACCCT   
  
  
- GCTTGAGAGT GGCAGCGTAG CCGGACCGCA AT

+     MYB recognition site

| Site Name | Organism | Position | Strand | Matrix score. | sequence | function |
| --- | --- | --- | --- | --- | --- | --- |
| MYB recognition site | Arabidopsis thaliana | 1422 | + | 6 | CCGTTG |  |
| MYB recognition site | Arabidopsis thaliana | 1474 | - | 6 | CCGTTG |  |

>HU06G02537.1   
+ +Up\_Stream \_Len000CCTCGG TGTGGGATTC CGGGTTGATA AATAGGGACC AGCGAAATGT TCAAATAATT   
  
  
+ GGTGAAGGAG TCTCTGTTTG AATGCCTACT AATGCTTTTT TTTTTTTTTT GAATAATTTG TCAGAAAGTT   
  
  
+ AGGCTGCATT GGAAGGGGCG GCCTACGAAG GCTTTTCAGC AAATAAATAA CCAGCTTTCA TTTTGAAATT   
  
  
+ TTTTTTAAAA AATGAAACTC ATTTCACTTA AAAATTTACC ATAATATATA TATATATATA TATATATATA   
  
  
+ TATATATATA TATTTGATTT AACGTTAAGC AAACTTTTAA CTTATGTATC ATTTTACGAC CGTAAAAAAA   
  
  
+ AAAGAAATAT AACACAATAT GTAATAGTAT TTAAGTTTTT GATAAATAAT ATTTTAACAT AATTTGTGTG   
  
  
+ TAGTATGAAT TTAAATCTTA GTACTAATAT AATTATGTAT ACTTGGCTAT AAGATCTCAA TATTAGTATT   
  
  
+ TTATGATTCA ATGTAATGAT GATGCAACTG TGTGTAATTA GAAAGCCTTT GATTGCAGAT ATCTATCTTA   
  
  
+ TTGATACACA TCTACCAATT GAGCTTTTTC AAGATTTGAA GACGGTAGTC AAGTATCTTT GGGAGATTAC   
  
  
+ GTTAGAATTA CTTCATGATA AATTAGACAT ATTTTATACT TCTAATCTTA AATAAGTCTC CATCCTTTCA   
  
  
+ TCATATTTTA AAGAAATGTA CAAGTAAATG TCAATAGTTT GAAACGTTCC TTATCATAAA AGTCATACAT   
  
  
+ ATGTAGGACA TTTTCAAGTT TATGTAAACC ATATGATATA GTTCAAGATA ATTGTCAAAC AAGTTATGAA   
  
  
+ ATCTAAAGGT GTTTGAACGT AATTTCAAGC TAATCTTAGC AAGTTATAGT ATTATTTAAA CTACGACTAG   
  
  
+ ACACAACATT TTGTTCATTT ATATAACACT TAGAATGCAA AAAAATCGGT ATTACTAATA CTGTATCAAG   
  
  
+ AATTGAAGTG TTATGCATTA TACTGATAAG GGATAATATT TAAATTACTT TACTGTTATA GAAGCCAAAA   
  
  
+ ATCTAAACTT TCATACAGAC GTCGGACTAA ATTAGAAAAT TCTTTAACTT AATAGAAAGG ATGACATAAA   
  
  
+ TAAAAATAAA AACCTAAATA GCATAATAAC CATTCTACAC AAAAGTCAAT CTTCTAATAG AAAAAAGCCA   
  
  
+ TAAGTATTCA CTTATTTACC ATTTTATTCC TAATCTACTG TCTGTCTATT CACACCATAA TTCATGAACC   
  
  
+ AATGTGGAGT TTCTGTTTCA TTTCATGGGG TGATTTATGT CTGACAATGA TCCAACAAAC TGAGCCTCAC   
  
  
+ GCGCGGAACA CGAGAGTGGC TGTACTTATG GTGTGAAAGA GCTCTACAAG GTGAGATTGA GGAGGCTACG   
  
  
+ CGTTGGAGTT ATCACCACCG TTGGATTTGT TTTCTTTGGA GCCACGTTAT CACTCTTCAG ACGCCGATCC   
  
  
+ AACGGCCCTT ACTTCCATCT AGTCCCCACT CTCCAACTCT ACGCAATCAA CGCCTTCCGC ATTTAAATAT   
  
  
+ TTCCTTCTCT ATCGCCTACT TTCTCTGTCT CACAATTTTT GTCCGTATTT TGAATTTTCT CAAAATTATC   
  
  
+ ATATAATTTA TTTGCCTTCA ACTAAAGTAT TTATTAAATT AGTTAATGTA TTTAAATTGA ACTAAAAAAG   
  
  
+ AGAAAAAGAA AGAAAACAAC CCCACGAAAG CAAAACGACA GAGGCCAACT TCTCTCTCTA GAGTGTAGAG   
  
  
+ CCCCATCTCG GTTATAATTA TGAGAATCAA TTCCTTCTCA CAAGAGAAAC TCTCGCTCTC TCTCTCTCTC   
  
  
+ CATAAGACAT CACACCAATT GTATGAATTT TCTCTCTCCT GATTTCTGTT TTCCTTCTTG TTGTCCCCTT   
  
  
+ CTAATGCGTT AGTGCGGCCA AAACCACCAT AAACCATCTC TCTTTCAAGA AAGCAGCAAC AACAAAGAGA   
  
  
+ GACAGAGAGA GAGAGAGGGG GAGAAAGATC ACATCTTTTG GGTTTCCGGC GAATATGACG TCTGGGTTTT   
  
  
+ CGGGCGATTT CTTCTACGGC GGTGGAGGCG GTGGCGGAGG AGGTGTCCGG CCTTCTATCG GCGGTGTACA   
  
  
+ AGGCGGGGTT GTCCCATATG GGTTCCGGCA ACAACAACAA CAACAGCAAC AGCAGCAGCA GCAGCAACAG   
  
  
+ CTACATCTAC AGCTACAACA ACAGCGGGGG ATTTTCTCGG CGGATCAGAT CAGCGCTCAA CGAAGGGCGG   
  
  
+ ATTTGTTGAT GGGTAAGCGT TCTTTCGCTG ATTTTCAATC TCAACAACAG CAAATCAGTC TTTTGCAGCA   
  
  
+ GCAGCAGCAG CAACAACAAC AACAGCCCTT TGCGCTCTTG CAAAAACAAC AGCAGTTTCA ACAACAATTA   
  
  
+ CAACAACAAC AGCCACTTTC TCTCCTCCTT CAACAACAGT ACTTGATGAG ATCCGTAAAA CAGAGAACCA   
  
  
+ ACAATTTCGC AGCATCTCCG ATTTCTCCTC TTTCTTCTTC CATTGATCTT TCTTCCGCTA ATAATTCCCG   
  
  
+ GTTCGGTTTG CCGGTTTACC AACAACAACT CCGGTCTCAA CAACAACCAC CTATCTCTCT CCTCGCCCAC   
  
  
+ AACAACAACC AATTCAACCT CTCAAATAAT AACAATTCTT CTTTAATCCA AAACTCCACC AACACTCTTC   
  
  
+ TTGCTCCCGC AGCCAGCAAT AACACCCTAA TGAAAAATTC GGGCTTTTCC TCATCTGGGT TTTCTAATTC   
  
  
+ GGGTCCAAAC CTGGTCCAAC CGAAGCAGGA AATCGGGTAC TCGAATATGG GTACCAATAT GGGTATGAAT   
  
  
+ CTGGTCCAGC CCAAACCCGA CCCTGAGGAT CACGGAATAA TGAACACCCT TCAAGAATTG GAGAAGGAGC   
  
  
+ TTTTGAATGA CGATGAGGAA AACCAGCCGG AAAACGACGC CGTTTCAGTC ATCACCAACA GCGAGTGGTC   
  
  
+ CGAGACCTTC CAGAATCTGA TGAGTCCCGA CCCGAGTTCG AGCCCGAATG CCACCAACAC CCACCCGGGT   
  
  
+ TCGGGTTCTG CTAGTACCAG CGGCAGCAAC AACACTACAA CCACCCCTGC AAACAACAAT AACATGATAT   
  
  
+ CTACCTCGCC GACGTCGTCC ACGTCATCCT GCTGCTCCAC GTCGGCATCG GCGTCTCCGC CGGCGGCGGT   
  
  
+ GGCGACCCCG CAGGTACCAG CCAAGCAGCT GATCTCGGAA GCAGCGGCGG CGATTTCGGA CGGAAAGATT   
  
  
+ GAATCCGCCA TGGAAGTGAT GAACCGGCTG TCCCAGGTTG CCAATTCTAG GGGGAGCTCG GAACAAAAGC   
  
  
+ TGGCTTTTTA CATGGGCCAC GCGCTTCGGT CGCGCGTGAG TCCCACCGAA CACGCTCCCC CTATTGTGGA   
  
  
+ GCTTTTCCAA AAGGAGCATA TGGCGGCCAT GTATTCATTG TACGAGCTCT CTCATTGTTT CAAATTTGGT   
  
  
+ CTTTTGGCTG CCAATTTGGT GATGTTGGAG GCCATTACTT CCGCCCTTCA TGAGGGTCTG AAAATTCATG   
  
  
+ TTGTGGATTT TGATATTGGG CATGGTGGTC AGTACATCAA CCTCCTACGC GCCTTGGGGG AGCGTAGGGC   
  
  
+ TCAGTCGGGG AGCGCGGTGT CGAATGAGGT GAGGATCACT GTGGTTGAGA CCGAGGTCGG TAATGGTGGT   
  
  
+ GCGGCGGGTG CGGAGGCTGT TAAGGATGGC CTGGTGAAAT TGGCTGAGAA GGTGGGGATT AGTTTGAAGT   
  
  
+ TTGAGGCTTT GACTCGGAAG ATCTCCGAGT TAACCCGGGA GTCACTGGGG TGTGAGGAGG GGGAGGCCGT   
  
  
+ GGTGGTGAAT TTTGCGTTCA AACTTTACCG GGTACCGGAT GAGAGCGTGT CGATGGAGAA TCTGAGGGAC   
  
  
+ GAGCTCCTGC GCCGGGTGAA GGCGCTAAGG CCACGCGCCG TCACATTGGT GGAGCAGGAC TTAAATGCGA   
  
  
+ ATACGGCACC GTTCATGACG CGCGTGAGTG GCGCTTGTGA GTACTATAGA GCATTGTTCG ACTCGCTCGA   
  
  
+ CTCGATGGTG CCGAGGGATA ACCCGGACCG GGTCCGGGTC GAGGAGGCCT TGGGGCGGAA GATGGCAAAC   
  
  
+ TCGGTGGCTT GCGAGGGCCG GGAACGGGTT GAGCGGGCGG AGGTGTTTGG GAAGTGGCGG GCGAGGATGA   
  
  
+ GTATGGCAGG GTTCGAGTTG AAGGCACTGA GTCAGAGTGT GGCCGAGTCA ATGCGAACCA AACTCAGCTC   
  
  
+ GGTTCTGGGC GGAAAGCCGG GTTTCACGGT CAAGGAGGAG AGCGGTGGGG TGGGGTTCGG GTGGATGGGA   
  
  
+ CGAACTCTCA CCGTCGCATC GGCCTGGCGT TA  

- +Up\_Stream \_Len000GGAGCC ACACCCTAAG GCCCAACTAT TTATCCCTGG TCGCTTTACA AGTTTATTAA   
  
  
- CCACTTCCTC AGAGACAAAC TTACGGATGA TTACGAAAAA AAAAAAAAAA CTTATTAAAC AGTCTTTCAA   
  
  
- TCCGACGTAA CCTTCCCCGC CGGATGCTTC CGAAAAGTCG TTTATTTATT GGTCGAAAGT AAAACTTTAA   
  
  
- AAAAAATTTT TTACTTTGAG TAAAGTGAAT TTTTAAATGG TATTATATAT ATATATATAT ATATATATAT   
  
  
- ATATATATAT ATAAACTAAA TTGCAATTCG TTTGAAAATT GAATACATAG TAAAATGCTG GCATTTTTTT   
  
  
- TTTCTTTATA TTGTGTTATA CATTATCATA AATTCAAAAA CTATTTATTA TAAAATTGTA TTAAACACAC   
  
  
- ATCATACTTA AATTTAGAAT CATGATTATA TTAATACATA TGAACCGATA TTCTAGAGTT ATAATCATAA   
  
  
- AATACTAAGT TACATTACTA CTACGTTGAC ACACATTAAT CTTTCGGAAA CTAACGTCTA TAGATAGAAT   
  
  
- AACTATGTGT AGATGGTTAA CTCGAAAAAG TTCTAAACTT CTGCCATCAG TTCATAGAAA CCCTCTAATG   
  
  
- CAATCTTAAT GAAGTACTAT TTAATCTGTA TAAAATATGA AGATTAGAAT TTATTCAGAG GTAGGAAAGT   
  
  
- AGTATAAAAT TTCTTTACAT GTTCATTTAC AGTTATCAAA CTTTGCAAGG AATAGTATTT TCAGTATGTA   
  
  
- TACATCCTGT AAAAGTTCAA ATACATTTGG TATACTATAT CAAGTTCTAT TAACAGTTTG TTCAATACTT   
  
  
- TAGATTTCCA CAAACTTGCA TTAAAGTTCG ATTAGAATCG TTCAATATCA TAATAAATTT GATGCTGATC   
  
  
- TGTGTTGTAA AACAAGTAAA TATATTGTGA ATCTTACGTT TTTTTAGCCA TAATGATTAT GACATAGTTC   
  
  
- TTAACTTCAC AATACGTAAT ATGACTATTC CCTATTATAA ATTTAATGAA ATGACAATAT CTTCGGTTTT   
  
  
- TAGATTTGAA AGTATGTCTG CAGCCTGATT TAATCTTTTA AGAAATTGAA TTATCTTTCC TACTGTATTT   
  
  
- ATTTTTATTT TTGGATTTAT CGTATTATTG GTAAGATGTG TTTTCAGTTA GAAGATTATC TTTTTTCGGT   
  
  
- ATTCATAAGT GAATAAATGG TAAAATAAGG ATTAGATGAC AGACAGATAA GTGTGGTATT AAGTACTTGG   
  
  
- TTACACCTCA AAGACAAAGT AAAGTACCCC ACTAAATACA GACTGTTACT AGGTTGTTTG ACTCGGAGTG   
  
  
- CGCGCCTTGT GCTCTCACCG ACATGAATAC CACACTTTCT CGAGATGTTC CACTCTAACT CCTCCGATGC   
  
  
- GCAACCTCAA TAGTGGTGGC AACCTAAACA AAAGAAACCT CGGTGCAATA GTGAGAAGTC TGCGGCTAGG   
  
  
- TTGCCGGGAA TGAAGGTAGA TCAGGGGTGA GAGGTTGAGA TGCGTTAGTT GCGGAAGGCG TAAATTTATA   
  
  
- AAGGAAGAGA TAGCGGATGA AAGAGACAGA GTGTTAAAAA CAGGCATAAA ACTTAAAAGA GTTTTAATAG   
  
  
- TATATTAAAT AAACGGAAGT TGATTTCATA AATAATTTAA TCAATTACAT AAATTTAACT TGATTTTTTC   
  
  
- TCTTTTTCTT TCTTTTGTTG GGGTGCTTTC GTTTTGCTGT CTCCGGTTGA AGAGAGAGAT CTCACATCTC   
  
  
- GGGGTAGAGC CAATATTAAT ACTCTTAGTT AAGGAAGAGT GTTCTCTTTG AGAGCGAGAG AGAGAGAGAG   
  
  
- GTATTCTGTA GTGTGGTTAA CATACTTAAA AGAGAGAGGA CTAAAGACAA AAGGAAGAAC AACAGGGGAA   
  
  
- GATTACGCAA TCACGCCGGT TTTGGTGGTA TTTGGTAGAG AGAAAGTTCT TTCGTCGTTG TTGTTTCTCT   
  
  
- CTGTCTCTCT CTCTCTCCCC CTCTTTCTAG TGTAGAAAAC CCAAAGGCCG CTTATACTGC AGACCCAAAA   
  
  
- GCCCGCTAAA GAAGATGCCG CCACCTCCGC CACCGCCTCC TCCACAGGCC GGAAGATAGC CGCCACATGT   
  
  
- TCCGCCCCAA CAGGGTATAC CCAAGGCCGT TGTTGTTGTT GTTGTCGTTG TCGTCGTCGT CGTCGTTGTC   
  
  
- GATGTAGATG TCGATGTTGT TGTCGCCCCC TAAAAGAGCC GCCTAGTCTA GTCGCGAGTT GCTTCCCGCC   
  
  
- TAAACAACTA CCCATTCGCA AGAAAGCGAC TAAAAGTTAG AGTTGTTGTC GTTTAGTCAG AAAACGTCGT   
  
  
- CGTCGTCGTC GTTGTTGTTG TTGTCGGGAA ACGCGAGAAC GTTTTTGTTG TCGTCAAAGT TGTTGTTAAT   
  
  
- GTTGTTGTTG TCGGTGAAAG AGAGGAGGAA GTTGTTGTCA TGAACTACTC TAGGCATTTT GTCTCTTGGT   
  
  
- TGTTAAAGCG TCGTAGAGGC TAAAGAGGAG AAAGAAGAAG GTAACTAGAA AGAAGGCGAT TATTAAGGGC   
  
  
- CAAGCCAAAC GGCCAAATGG TTGTTGTTGA GGCCAGAGTT GTTGTTGGTG GATAGAGAGA GGAGCGGGTG   
  
  
- TTGTTGTTGG TTAAGTTGGA GAGTTTATTA TTGTTAAGAA GAAATTAGGT TTTGAGGTGG TTGTGAGAAG   
  
  
- AACGAGGGCG TCGGTCGTTA TTGTGGGATT ACTTTTTAAG CCCGAAAAGG AGTAGACCCA AAAGATTAAG   
  
  
- CCCAGGTTTG GACCAGGTTG GCTTCGTCCT TTAGCCCATG AGCTTATACC CATGGTTATA CCCATACTTA   
  
  
- GACCAGGTCG GGTTTGGGCT GGGACTCCTA GTGCCTTATT ACTTGTGGGA AGTTCTTAAC CTCTTCCTCG   
  
  
- AAAACTTACT GCTACTCCTT TTGGTCGGCC TTTTGCTGCG GCAAAGTCAG TAGTGGTTGT CGCTCACCAG   
  
  
- GCTCTGGAAG GTCTTAGACT ACTCAGGGCT GGGCTCAAGC TCGGGCTTAC GGTGGTTGTG GGTGGGCCCA   
  
  
- AGCCCAAGAC GATCATGGTC GCCGTCGTTG TTGTGATGTT GGTGGGGACG TTTGTTGTTA TTGTACTATA   
  
  
- GATGGAGCGG CTGCAGCAGG TGCAGTAGGA CGACGAGGTG CAGCCGTAGC CGCAGAGGCG GCCGCCGCCA   
  
  
- CCGCTGGGGC GTCCATGGTC GGTTCGTCGA CTAGAGCCTT CGTCGCCGCC GCTAAAGCCT GCCTTTCTAA   
  
  
- CTTAGGCGGT ACCTTCACTA CTTGGCCGAC AGGGTCCAAC GGTTAAGATC CCCCTCGAGC CTTGTTTTCG   
  
  
- ACCGAAAAAT GTACCCGGTG CGCGAAGCCA GCGCGCACTC AGGGTGGCTT GTGCGAGGGG GATAACACCT   
  
  
- CGAAAAGGTT TTCCTCGTAT ACCGCCGGTA CATAAGTAAC ATGCTCGAGA GAGTAACAAA GTTTAAACCA   
  
  
- GAAAACCGAC GGTTAAACCA CTACAACCTC CGGTAATGAA GGCGGGAAGT ACTCCCAGAC TTTTAAGTAC   
  
  
- AACACCTAAA ACTATAACCC GTACCACCAG TCATGTAGTT GGAGGATGCG CGGAACCCCC TCGCATCCCG   
  
  
- AGTCAGCCCC TCGCGCCACA GCTTACTCCA CTCCTAGTGA CACCAACTCT GGCTCCAGCC ATTACCACCA   
  
  
- CGCCGCCCAC GCCTCCGACA ATTCCTACCG GACCACTTTA ACCGACTCTT CCACCCCTAA TCAAACTTCA   
  
  
- AACTCCGAAA CTGAGCCTTC TAGAGGCTCA ATTGGGCCCT CAGTGACCCC ACACTCCTCC CCCTCCGGCA   
  
  
- CCACCACTTA AAACGCAAGT TTGAAATGGC CCATGGCCTA CTCTCGCACA GCTACCTCTT AGACTCCCTG   
  
  
- CTCGAGGACG CGGCCCACTT CCGCGATTCC GGTGCGCGGC AGTGTAACCA CCTCGTCCTG AATTTACGCT   
  
  
- TATGCCGTGG CAAGTACTGC GCGCACTCAC CGCGAACACT CATGATATCT CGTAACAAGC TGAGCGAGCT
[truncated: 246,598 more chars]
