# Supplementary material for: Genome-Wide Identification and Expression Pattern of the GRAS Gene Family in Pitaya (Selenicereus undatus L.)
Source: Biology (Basel). 2022 Dec 21;12(1):11. doi: 10.3390/biology12010011 (PMC9854919; doi:10.3390/biology12010011)
Supplement: Supplementary file 1 [file biology-12-00011-s001.zip › Supplementary file S5/HU07G00272.1_plantcare.html]

Content-Type: text/html; charset=ISO-8859-1


PlantCARE


Webmaster Firefox specific output  
To save the result:
click on the frame with the right mouse button and save the source code as a text file with extension .html  
REFERENCE:PlantCARE: a database of plant cis-acting regulatory elements and a portal to tools for in silico analysis of promoter sequences.  
Lescot, M., Déhais, P., Moreau, Y., De Moor, B., Rouzé ,P.,and Rombauts, S.  
Nucleic Acids Res., Database issue(2002), 30(1):325-327.   


---

>HU07G00272.1   
+ -Up\_Stream \_Len000ACCAAC GATTCTTTAC TGAATCAGCT ACATTTTTGG ATTGTGTTAA GGTATCTTTT   
  
  
+ AGATCTTCAG GAGGAGGGGG CTGAAGTAGC TTGGTTACTT TTGCGCTTAT CAACTGTGGA AGAATATTAT   
  
  
+ TGAGACTAGT GTAGAAGAAT TAGTAGTTTC CTCCACATTG GAAAAAAACC ATGAAATTAA CGCTTTAGGC   
  
  
+ CCTCAGAGTA CCAAGTGTCA TCGTAAATGA TTGAAATTTC AAGTCTTAGC TGAGTCATGA AGTCCTGATT   
  
  
+ TCCCATGACA AATTGATGTT GTTGTCTAAA GCTTGGCGGG TGATTTGAAA GAAGATTTAG ATCAGGCTGA   
  
  
+ CAATACACCA AATCTGGGAG CAAGGAACTG GATTTTTCTG TGTCTAGAAA AGCTTTCCGC TTTCTGAACA   
  
  
+ ATTGAGAAAA CCATGCATAG GAAATTTCTT GATATTTAGA TCATAGCAGA CTTAACAATC TGGCTTTACC   
  
  
+ GAACTGTCCT CTACTTCTAA GTTGTGAAGG TGTGAAAGAT AAACCATGTC AAACTTAGTG TCCAACAGAA   
  
  
+ ACGCATCTCT ACTTCTCCAG GCAAAGTTGT ACTTCAGATC TTCTACACCT TTTCTAGGCA GGACTTTGGG   
  
  
+ ATATAGAGTT TGGCCTGGGT GAAAAGTAGG AAGTGGGGAA GTAGAAAATG ATGAAAGAAG GCACCACTTG   
  
  
+ GGTCCAGATA CATAGGCCTA CGCACGAGAA CCTTTGCATG GTCTAGATAC ATGACTGTAT ACTTCTTCCT   
  
  
+ TTCCTTTACC TGTTGGAGAT TATCCTAGGA TTTTACATGG CACCAGCTGG ATTTGTTAAT GACATAAACA   
  
  
+ TATCTTTAAT TACAGTTGCT TATGGTATAC ATTTAAACTA TTCGATAGTT GACAGCTGAT GTCCTTATCC   
  
  
+ TTGTGCAGCT TATTCCTCGA TAGAATAATC TTTGGACATT TCTGTCTAGG TGATAAAGGG CAAAAACTAA   
  
  
+ GATACAAGAA ATAGAACATG TCCTCCATTT ACAAGACGTA TCTGTTTTTG GTTTTGGTCA ATGATCCCTG   
  
  
+ TTGTCCTCAA GCATTAACAC AGAATGATCA TTATTGACTT GGACTGAAAG GTTTTGGGAA CTCCATAGTA   
  
  
+ TGCCTTTTCA ATAAAATCAA AAGCAAATTT GCTGTTCACT GATAACTGCA GTTGTGTTGC TTTGCTGTTT   
  
  
+ CCATACCAGA CTCGTAACCA AGCTGATAGC AAATGTGCAA AGGTACAGTA TAATTAACGA TGTTTTCATA   
  
  
+ TTGCTACGAG TTCTGAAACA TGATATAAGA AAACACCAGA ACCCAAATAC CTACATAGGT CCTAGCCCTG   
  
  
+ GAATGGCTAT TTTAAATATC CAAATGGACT TACAGAATTC GCAGAGTTGT TTGGTTTGTT TAACAATCAT   
  
  
+ TGAATTGTTA GCTATGAGAA TCTGCGGGAC AATCGTAATC TTTGGCGATT ACTTCGATTG GCATACTGAT   
  
  
+ GTTTTTTTAT TTGCGTATCT AAGCATGGTG GTGTACCTTT GATTTGTGTG TCTAGGTAGT GTGTGTAGAT   
  
  
+ AAATATCATA GAACTCAGAC GTTGGTAGGG TTATGTTGAG CACTTTCTCT TGCTGTTTGT CCTTTGTGAT   
  
  
+ GGCACCTCTT GCTTTTCCGA ACCACCAAAA GTCTCATTGA CAAAACTCTT CAAACATTGC AAACTGCCCC   
  
  
+ CTTAAATATG TTTTATTAAA AAAAATGGAA GAGCTCAAAC TGTTGAGTGA GCCTAGATTC CAAGATTTAG   
  
  
+ AAACAGTAAA CTTGATAGAT ATATTTATAT GAACTATTGT TAGATGCAAC ACCCCCAACT TGAAACACAA   
  
  
+ AAGAGAAATT AAAAACAAAA AGGTTTTGCA CTATTTATAT ATCCTGTGTT CTTTGTGCTA GTCTTATTTA   
  
  
+ ATGTTAATTG GTAAGCATTA TTTGTTGGAT TATTCCACTG CACTTTCTCT TACTAACTTC AGCATTTCAT   
  
  
+ TCCTTGTTCT TCAGCTTTTG AACTTCTTTA GCCTATTTTG TTGTCTTTAT GGTTATGGAA GCCATCCAGA   
  
  
+ AGAAGAATGA TGAACTTCTG AGTCTTAGCT TGGCCATTGT CGGTCACTCT GATCGCACTG AGAAAAAACT   
  
  
+ GAAGAGGCGG ATTGATGTCT CCGATCCTCT GATCACTTCA GGTGAAGGCT GCGAAGGGAA GATAATCAGG   
  
  
+ CTCCTCCAAG AGAGACAGAA CTTGTTGAAC ATCAAGCAAA GAGGAAAAGG CGTCATTCAA GAAGGGAAGG   
  
  
+ GTCTTCATCT TATCCATCTG CTCCTTGTAT CTGCCACCTT AATCAACGAA AACAACATCA GTTCAGCTGT   
  
  
+ TGATAATCTT ATTGAGCTCT TCCAATATGT CTCCGAAAGT GGTGATTCAG GACAAAGGGT TGCTGCTTAC   
  
  
+ TTTGCGGATG GGTTAACAGC AAGGATCTTA ACTCAGAGGT CTCCATTTTA TCGCCTGATA CTGGGCAAAC   
  
  
+ CAGCACCTGC TGAAGAGTTT TCAGCTTTTA CTCACTTATA TAGAGCCTCT CCATTCTATC AGTTTGCTCA   
  
  
+ TTTCACAGCC AATCAGGCAA TTGTAGAAGC ATTTGAGAGG GAGGAAGAGA GTAACAACTG GGCTTTGCAT   
  
  
+ GTAATTGATT TGGACATCTT GCATGGTTTC CAGTGGCCTT CTCTTATTCA GTCTCTCTCC GAAGAGGCTA   
  
  
+ CTTGTTCAAA CCGTTCATTG TCTCTTCAAA TTACAGGCTT CGGAAGAAGC TTAGAAGAGC TCATTGAAAC   
  
  
+ AGAAGCTCGG CTGGTAAGTT TCTCTAAGAG CTTTCAGAAT ATCAACTTTG AATTCCACGG GTTTCTGAGA   
  
  
+ GGCTCGAAAC TCAGGAACCT AAGGAGGGAG AATGAAACAG TAGTTGTAAA TCTCGTTTTC CACCTCAGTA   
  
  
+ CTTTGAAAGA TACCGTACAG ATTTCTGACA CCTTGACTTC TGTACATTCA CTAAACCCCT CTATTGTGGT   
  
  
+ ATTAGTCGAG AGAGAAGGAA GTCGAAACAG ATGTGGGTTC CTCTCAAGTT ATGTAGATGC TCTGCATTAT   
  
  
+ TATGCTGCAA TGTTTGACTC TTTAGATGAT TGCCTCCCAC TTGAAAGTCC TGAGAGGCTG AGCATAGAGA   
  
  
+ AGAACCATCT TGGAAAAGAG ATCAAAGAAG CCATAGGTTG TGAGAAGGAT GAAACAAACT ATCTGAAGTT   
  
  
+ TGAGATGCTG GAGACTTGGA GAGGGAGGAT GGAGAGTCAT GGATTTTCAG GTATGAAGCT AAGTTCCAGG   
  
  
+ GCAACTATAC AAGCAAAGCT GCTTTTAAAA ATGGGAAGCC ATTATCATAC CTATTTGGAA GAAGACTGTG   
  
  
+ GAGGTGGTGG GTTCAGAGTT TGTGAACGAG ATGATGGAAT GGCTATCTCT CTTGGCTGGC AAGGTAGGTT   
  
  
+ CCTCGCAACT GCCTCGGTAT GGCGTTCTGT GTG  

- -Up\_Stream \_Len000TGGTTG CTAAGAAATG ACTTAGTCGA TGTAAAAACC TAACACAATT CCATAGAAAA   
  
  
- TCTAGAAGTC CTCCTCCCCC GACTTCATCG AACCAATGAA AACGCGAATA GTTGACACCT TCTTATAATA   
  
  
- ACTCTGATCA CATCTTCTTA ATCATCAAAG GAGGTGTAAC CTTTTTTTGG TACTTTAATT GCGAAATCCG   
  
  
- GGAGTCTCAT GGTTCACAGT AGCATTTACT AACTTTAAAG TTCAGAATCG ACTCAGTACT TCAGGACTAA   
  
  
- AGGGTACTGT TTAACTACAA CAACAGATTT CGAACCGCCC ACTAAACTTT CTTCTAAATC TAGTCCGACT   
  
  
- GTTATGTGGT TTAGACCCTC GTTCCTTGAC CTAAAAAGAC ACAGATCTTT TCGAAAGGCG AAAGACTTGT   
  
  
- TAACTCTTTT GGTACGTATC CTTTAAAGAA CTATAAATCT AGTATCGTCT GAATTGTTAG ACCGAAATGG   
  
  
- CTTGACAGGA GATGAAGATT CAACACTTCC ACACTTTCTA TTTGGTACAG TTTGAATCAC AGGTTGTCTT   
  
  
- TGCGTAGAGA TGAAGAGGTC CGTTTCAACA TGAAGTCTAG AAGATGTGGA AAAGATCCGT CCTGAAACCC   
  
  
- TATATCTCAA ACCGGACCCA CTTTTCATCC TTCACCCCTT CATCTTTTAC TACTTTCTTC CGTGGTGAAC   
  
  
- CCAGGTCTAT GTATCCGGAT GCGTGCTCTT GGAAACGTAC CAGATCTATG TACTGACATA TGAAGAAGGA   
  
  
- AAGGAAATGG ACAACCTCTA ATAGGATCCT AAAATGTACC GTGGTCGACC TAAACAATTA CTGTATTTGT   
  
  
- ATAGAAATTA ATGTCAACGA ATACCATATG TAAATTTGAT AAGCTATCAA CTGTCGACTA CAGGAATAGG   
  
  
- AACACGTCGA ATAAGGAGCT ATCTTATTAG AAACCTGTAA AGACAGATCC ACTATTTCCC GTTTTTGATT   
  
  
- CTATGTTCTT TATCTTGTAC AGGAGGTAAA TGTTCTGCAT AGACAAAAAC CAAAACCAGT TACTAGGGAC   
  
  
- AACAGGAGTT CGTAATTGTG TCTTACTAGT AATAACTGAA CCTGACTTTC CAAAACCCTT GAGGTATCAT   
  
  
- ACGGAAAAGT TATTTTAGTT TTCGTTTAAA CGACAAGTGA CTATTGACGT CAACACAACG AAACGACAAA   
  
  
- GGTATGGTCT GAGCATTGGT TCGACTATCG TTTACACGTT TCCATGTCAT ATTAATTGCT ACAAAAGTAT   
  
  
- AACGATGCTC AAGACTTTGT ACTATATTCT TTTGTGGTCT TGGGTTTATG GATGTATCCA GGATCGGGAC   
  
  
- CTTACCGATA AAATTTATAG GTTTACCTGA ATGTCTTAAG CGTCTCAACA AACCAAACAA ATTGTTAGTA   
  
  
- ACTTAACAAT CGATACTCTT AGACGCCCTG TTAGCATTAG AAACCGCTAA TGAAGCTAAC CGTATGACTA   
  
  
- CAAAAAAATA AACGCATAGA TTCGTACCAC CACATGGAAA CTAAACACAC AGATCCATCA CACACATCTA   
  
  
- TTTATAGTAT CTTGAGTCTG CAACCATCCC AATACAACTC GTGAAAGAGA ACGACAAACA GGAAACACTA   
  
  
- CCGTGGAGAA CGAAAAGGCT TGGTGGTTTT CAGAGTAACT GTTTTGAGAA GTTTGTAACG TTTGACGGGG   
  
  
- GAATTTATAC AAAATAATTT TTTTTACCTT CTCGAGTTTG ACAACTCACT CGGATCTAAG GTTCTAAATC   
  
  
- TTTGTCATTT GAACTATCTA TATAAATATA CTTGATAACA ATCTACGTTG TGGGGGTTGA ACTTTGTGTT   
  
  
- TTCTCTTTAA TTTTTGTTTT TCCAAAACGT GATAAATATA TAGGACACAA GAAACACGAT CAGAATAAAT   
  
  
- TACAATTAAC CATTCGTAAT AAACAACCTA ATAAGGTGAC GTGAAAGAGA ATGATTGAAG TCGTAAAGTA   
  
  
- AGGAACAAGA AGTCGAAAAC TTGAAGAAAT CGGATAAAAC AACAGAAATA CCAATACCTT CGGTAGGTCT   
  
  
- TCTTCTTACT ACTTGAAGAC TCAGAATCGA ACCGGTAACA GCCAGTGAGA CTAGCGTGAC TCTTTTTTGA   
  
  
- CTTCTCCGCC TAACTACAGA GGCTAGGAGA CTAGTGAAGT CCACTTCCGA CGCTTCCCTT CTATTAGTCC   
  
  
- GAGGAGGTTC TCTCTGTCTT GAACAACTTG TAGTTCGTTT CTCCTTTTCC GCAGTAAGTT CTTCCCTTCC   
  
  
- CAGAAGTAGA ATAGGTAGAC GAGGAACATA GACGGTGGAA TTAGTTGCTT TTGTTGTAGT CAAGTCGACA   
  
  
- ACTATTAGAA TAACTCGAGA AGGTTATACA GAGGCTTTCA CCACTAAGTC CTGTTTCCCA ACGACGAATG   
  
  
- AAACGCCTAC CCAATTGTCG TTCCTAGAAT TGAGTCTCCA GAGGTAAAAT AGCGGACTAT GACCCGTTTG   
  
  
- GTCGTGGACG ACTTCTCAAA AGTCGAAAAT GAGTGAATAT ATCTCGGAGA GGTAAGATAG TCAAACGAGT   
  
  
- AAAGTGTCGG TTAGTCCGTT AACATCTTCG TAAACTCTCC CTCCTTCTCT CATTGTTGAC CCGAAACGTA   
  
  
- CATTAACTAA ACCTGTAGAA CGTACCAAAG GTCACCGGAA GAGAATAAGT CAGAGAGAGG CTTCTCCGAT   
  
  
- GAACAAGTTT GGCAAGTAAC AGAGAAGTTT AATGTCCGAA GCCTTCTTCG AATCTTCTCG AGTAACTTTG   
  
  
- TCTTCGAGCC GACCATTCAA AGAGATTCTC GAAAGTCTTA TAGTTGAAAC TTAAGGTGCC CAAAGACTCT   
  
  
- CCGAGCTTTG AGTCCTTGGA TTCCTCCCTC TTACTTTGTC ATCAACATTT AGAGCAAAAG GTGGAGTCAT   
  
  
- GAAACTTTCT ATGGCATGTC TAAAGACTGT GGAACTGAAG ACATGTAAGT GATTTGGGGA GATAACACCA   
  
  
- TAATCAGCTC TCTCTTCCTT CAGCTTTGTC TACACCCAAG GAGAGTTCAA TACATCTACG AGACGTAATA   
  
  
- ATACGACGTT ACAAACTGAG AAATCTACTA ACGGAGGGTG AACTTTCAGG ACTCTCCGAC TCGTATCTCT   
  
  
- TCTTGGTAGA ACCTTTTCTC TAGTTTCTTC GGTATCCAAC ACTCTTCCTA CTTTGTTTGA TAGACTTCAA   
  
  
- ACTCTACGAC CTCTGAACCT CTCCCTCCTA CCTCTCAGTA CCTAAAAGTC CATACTTCGA TTCAAGGTCC   
  
  
- CGTTGATATG TTCGTTTCGA CGAAAATTTT TACCCTTCGG TAATAGTATG GATAAACCTT CTTCTGACAC   
  
  
- CTCCACCACC CAAGTCTCAA ACACTTGCTC TACTACCTTA CCGATAGAGA GAACCGACCG TTCCATCCAA   
  
  
- GGAGCGTTGA CGGAGCCATA CCGCAAGACA CAC

  
  
Motifs Found  

+   

| Site Name | Organism | Position | Strand | Matrix score. | sequence | function |
| --- | --- | --- | --- | --- | --- | --- |
|  | organism | 2273 | + | 4 | motif\_sequence | short\_function |
|  | organism | 2260 | + | 4 | motif\_sequence | short\_function |
|  | organism | 2105 | - | 4 | motif\_sequence | short\_function |
|  | organism | 2162 | - | 4 | motif\_sequence | short\_function |
|  | organism | 2606 | - | 4 | motif\_sequence | short\_function |
|  | organism | 3172 | - | 4 | motif\_sequence | short\_function |
|  | organism | 3004 | + | 4 | motif\_sequence | short\_function |
|  | organism | 3185 | - | 4 | motif\_sequence | short\_function |
|  | organism | 2889 | - | 4 | motif\_sequence | short\_function |
|  | organism | 2718 | - | 4 | motif\_sequence | short\_function |
|  | organism | 2649 | + | 4 | motif\_sequence | short\_function |
|  | organism | 998 | - | 4 | motif\_sequence | short\_function |
|  | organism | 903 | + | 4 | motif\_sequence | short\_function |
|  | organism | 2997 | - | 4 | motif\_sequence | short\_function |
|  | organism | 2916 | - | 4 | motif\_sequence | short\_function |
|  | organism | 2568 | - | 4 | motif\_sequence | short\_function |
|  | organism | 948 | - | 4 | motif\_sequence | short\_function |
|  | organism | 2686 | + | 4 | motif\_sequence | short\_function |
|  | organism | 789 | - | 4 | motif\_sequence | short\_function |
|  | organism | 2331 | + | 4 | motif\_sequence | short\_function |
|  | organism | 503 | + | 4 | motif\_sequence | short\_function |
|  | organism | 498 | + | 4 | motif\_sequence | short\_function |
|  | organism | 335 | - | 4 | motif\_sequence | short\_function |
|  | organism | 2201 | - | 4 | motif\_sequence | short\_function |
|  | organism | 2655 | - | 4 | motif\_sequence | short\_function |
|  | organism | 1002 | + | 4 | motif\_sequence | short\_function |
|  | organism | 1375 | - | 4 | motif\_sequence | short\_function |
|  | organism | 77 | + | 4 | motif\_sequence | short\_function |
|  | organism | 2466 | - | 4 | motif\_sequence | short\_function |
|  | organism | 571 | + | 4 | motif\_sequence | short\_function |
|  | organism | 2501 | + | 4 | motif\_sequence | short\_function |
|  | organism | 2914 | + | 4 | motif\_sequence | short\_function |
|  | organism | 1660 | + | 4 | motif\_sequence | short\_function |
|  | organism | 1227 | + | 4 | motif\_sequence | short\_function |
|  | organism | 1237 | - | 4 | motif\_sequence | short\_function |
|  | organism | 602 | + | 4 | motif\_sequence | short\_function |
|  | organism | 1712 | - | 4 | motif\_sequence | short\_function |
|  | organism | 1424 | + | 4 | motif\_sequence | short\_function |
|  | organism | 1539 | - | 4 | motif\_sequence | short\_function |
|  | organism | 1156 | + | 4 | motif\_sequence | short\_function |

>HU07G00272.1   
+ -Up\_Stream \_Len000ACCAAC GATTCTTTAC TGAATCAGCT ACATTTTTGG ATTGTGTTAA GGTATCTTTT   
  
  
+ AGATCTTCAG GAGGAGGGGG CTGAAGTAGC TTGGTTACTT TTGCGCTTAT CAACTGTGGA AGAATATTAT   
  
  
+ TGAGACTAGT GTAGAAGAAT TAGTAGTTTC CTCCACATTG GAAAAAAACC ATGAAATTAA CGCTTTAGGC   
  
  
+ CCTCAGAGTA CCAAGTGTCA TCGTAAATGA TTGAAATTTC AAGTCTTAGC TGAGTCATGA AGTCCTGATT   
  
  
+ TCCCATGACA AATTGATGTT GTTGTCTAAA GCTTGGCGGG TGATTTGAAA GAAGATTTAG ATCAGGCTGA   
  
  
+ CAATACACCA AATCTGGGAG CAAGGAACTG GATTTTTCTG TGTCTAGAAA AGCTTTCCGC TTTCTGAACA   
  
  
+ ATTGAGAAAA CCATGCATAG GAAATTTCTT GATATTTAGA TCATAGCAGA CTTAACAATC TGGCTTTACC   
  
  
+ GAACTGTCCT CTACTTCTAA GTTGTGAAGG TGTGAAAGAT AAACCATGTC AAACTTAGTG TCCAACAGAA   
  
  
+ ACGCATCTCT ACTTCTCCAG GCAAAGTTGT ACTTCAGATC TTCTACACCT TTTCTAGGCA GGACTTTGGG   
  
  
+ ATATAGAGTT TGGCCTGGGT GAAAAGTAGG AAGTGGGGAA GTAGAAAATG ATGAAAGAAG GCACCACTTG   
  
  
+ GGTCCAGATA CATAGGCCTA CGCACGAGAA CCTTTGCATG GTCTAGATAC ATGACTGTAT ACTTCTTCCT   
  
  
+ TTCCTTTACC TGTTGGAGAT TATCCTAGGA TTTTACATGG CACCAGCTGG ATTTGTTAAT GACATAAACA   
  
  
+ TATCTTTAAT TACAGTTGCT TATGGTATAC ATTTAAACTA TTCGATAGTT GACAGCTGAT GTCCTTATCC   
  
  
+ TTGTGCAGCT TATTCCTCGA TAGAATAATC TTTGGACATT TCTGTCTAGG TGATAAAGGG CAAAAACTAA   
  
  
+ GATACAAGAA ATAGAACATG TCCTCCATTT ACAAGACGTA TCTGTTTTTG GTTTTGGTCA ATGATCCCTG   
  
  
+ TTGTCCTCAA GCATTAACAC AGAATGATCA TTATTGACTT GGACTGAAAG GTTTTGGGAA CTCCATAGTA   
  
  
+ TGCCTTTTCA ATAAAATCAA AAGCAAATTT GCTGTTCACT GATAACTGCA GTTGTGTTGC TTTGCTGTTT   
  
  
+ CCATACCAGA CTCGTAACCA AGCTGATAGC AAATGTGCAA AGGTACAGTA TAATTAACGA TGTTTTCATA   
  
  
+ TTGCTACGAG TTCTGAAACA TGATATAAGA AAACACCAGA ACCCAAATAC CTACATAGGT CCTAGCCCTG   
  
  
+ GAATGGCTAT TTTAAATATC CAAATGGACT TACAGAATTC GCAGAGTTGT TTGGTTTGTT TAACAATCAT   
  
  
+ TGAATTGTTA GCTATGAGAA TCTGCGGGAC AATCGTAATC TTTGGCGATT ACTTCGATTG GCATACTGAT   
  
  
+ GTTTTTTTAT TTGCGTATCT AAGCATGGTG GTGTACCTTT GATTTGTGTG TCTAGGTAGT GTGTGTAGAT   
  
  
+ AAATATCATA GAACTCAGAC GTTGGTAGGG TTATGTTGAG CACTTTCTCT TGCTGTTTGT CCTTTGTGAT   
  
  
+ GGCACCTCTT GCTTTTCCGA ACCACCAAAA GTCTCATTGA CAAAACTCTT CAAACATTGC AAACTGCCCC   
  
  
+ CTTAAATATG TTTTATTAAA AAAAATGGAA GAGCTCAAAC TGTTGAGTGA GCCTAGATTC CAAGATTTAG   
  
  
+ AAACAGTAAA CTTGATAGAT ATATTTATAT GAACTATTGT TAGATGCAAC ACCCCCAACT TGAAACACAA   
  
  
+ AAGAGAAATT AAAAACAAAA AGGTTTTGCA CTATTTATAT ATCCTGTGTT CTTTGTGCTA GTCTTATTTA   
  
  
+ ATGTTAATTG GTAAGCATTA TTTGTTGGAT TATTCCACTG CACTTTCTCT TACTAACTTC AGCATTTCAT   
  
  
+ TCCTTGTTCT TCAGCTTTTG AACTTCTTTA GCCTATTTTG TTGTCTTTAT GGTTATGGAA GCCATCCAGA   
  
  
+ AGAAGAATGA TGAACTTCTG AGTCTTAGCT TGGCCATTGT CGGTCACTCT GATCGCACTG AGAAAAAACT   
  
  
+ GAAGAGGCGG ATTGATGTCT CCGATCCTCT GATCACTTCA GGTGAAGGCT GCGAAGGGAA GATAATCAGG   
  
  
+ CTCCTCCAAG AGAGACAGAA CTTGTTGAAC ATCAAGCAAA GAGGAAAAGG CGTCATTCAA GAAGGGAAGG   
  
  
+ GTCTTCATCT TATCCATCTG CTCCTTGTAT CTGCCACCTT AATCAACGAA AACAACATCA GTTCAGCTGT   
  
  
+ TGATAATCTT ATTGAGCTCT TCCAATATGT CTCCGAAAGT GGTGATTCAG GACAAAGGGT TGCTGCTTAC   
  
  
+ TTTGCGGATG GGTTAACAGC AAGGATCTTA ACTCAGAGGT CTCCATTTTA TCGCCTGATA CTGGGCAAAC   
  
  
+ CAGCACCTGC TGAAGAGTTT TCAGCTTTTA CTCACTTATA TAGAGCCTCT CCATTCTATC AGTTTGCTCA   
  
  
+ TTTCACAGCC AATCAGGCAA TTGTAGAAGC ATTTGAGAGG GAGGAAGAGA GTAACAACTG GGCTTTGCAT   
  
  
+ GTAATTGATT TGGACATCTT GCATGGTTTC CAGTGGCCTT CTCTTATTCA GTCTCTCTCC GAAGAGGCTA   
  
  
+ CTTGTTCAAA CCGTTCATTG TCTCTTCAAA TTACAGGCTT CGGAAGAAGC TTAGAAGAGC TCATTGAAAC   
  
  
+ AGAAGCTCGG CTGGTAAGTT TCTCTAAGAG CTTTCAGAAT ATCAACTTTG AATTCCACGG GTTTCTGAGA   
  
  
+ GGCTCGAAAC TCAGGAACCT AAGGAGGGAG AATGAAACAG TAGTTGTAAA TCTCGTTTTC CACCTCAGTA   
  
  
+ CTTTGAAAGA TACCGTACAG ATTTCTGACA CCTTGACTTC TGTACATTCA CTAAACCCCT CTATTGTGGT   
  
  
+ ATTAGTCGAG AGAGAAGGAA GTCGAAACAG ATGTGGGTTC CTCTCAAGTT ATGTAGATGC TCTGCATTAT   
  
  
+ TATGCTGCAA TGTTTGACTC TTTAGATGAT TGCCTCCCAC TTGAAAGTCC TGAGAGGCTG AGCATAGAGA   
  
  
+ AGAACCATCT TGGAAAAGAG ATCAAAGAAG CCATAGGTTG TGAGAAGGAT GAAACAAACT ATCTGAAGTT   
  
  
+ TGAGATGCTG GAGACTTGGA GAGGGAGGAT GGAGAGTCAT GGATTTTCAG GTATGAAGCT AAGTTCCAGG   
  
  
+ GCAACTATAC AAGCAAAGCT GCTTTTAAAA ATGGGAAGCC ATTATCATAC CTATTTGGAA GAAGACTGTG   
  
  
+ GAGGTGGTGG GTTCAGAGTT TGTGAACGAG ATGATGGAAT GGCTATCTCT CTTGGCTGGC AAGGTAGGTT   
  
  
+ CCTCGCAACT GCCTCGGTAT GGCGTTCTGT GTG  

- -Up\_Stream \_Len000TGGTTG CTAAGAAATG ACTTAGTCGA TGTAAAAACC TAACACAATT CCATAGAAAA   
  
  
- TCTAGAAGTC CTCCTCCCCC GACTTCATCG AACCAATGAA AACGCGAATA GTTGACACCT TCTTATAATA   
  
  
- ACTCTGATCA CATCTTCTTA ATCATCAAAG GAGGTGTAAC CTTTTTTTGG TACTTTAATT GCGAAATCCG   
  
  
- GGAGTCTCAT GGTTCACAGT AGCATTTACT AACTTTAAAG TTCAGAATCG ACTCAGTACT TCAGGACTAA   
  
  
- AGGGTACTGT TTAACTACAA CAACAGATTT CGAACCGCCC ACTAAACTTT CTTCTAAATC TAGTCCGACT   
  
  
- GTTATGTGGT TTAGACCCTC GTTCCTTGAC CTAAAAAGAC ACAGATCTTT TCGAAAGGCG AAAGACTTGT   
  
  
- TAACTCTTTT GGTACGTATC CTTTAAAGAA CTATAAATCT AGTATCGTCT GAATTGTTAG ACCGAAATGG   
  
  
- CTTGACAGGA GATGAAGATT CAACACTTCC ACACTTTCTA TTTGGTACAG TTTGAATCAC AGGTTGTCTT   
  
  
- TGCGTAGAGA TGAAGAGGTC CGTTTCAACA TGAAGTCTAG AAGATGTGGA AAAGATCCGT CCTGAAACCC   
  
  
- TATATCTCAA ACCGGACCCA CTTTTCATCC TTCACCCCTT CATCTTTTAC TACTTTCTTC CGTGGTGAAC   
  
  
- CCAGGTCTAT GTATCCGGAT GCGTGCTCTT GGAAACGTAC CAGATCTATG TACTGACATA TGAAGAAGGA   
  
  
- AAGGAAATGG ACAACCTCTA ATAGGATCCT AAAATGTACC GTGGTCGACC TAAACAATTA CTGTATTTGT   
  
  
- ATAGAAATTA ATGTCAACGA ATACCATATG TAAATTTGAT AAGCTATCAA CTGTCGACTA CAGGAATAGG   
  
  
- AACACGTCGA ATAAGGAGCT ATCTTATTAG AAACCTGTAA AGACAGATCC ACTATTTCCC GTTTTTGATT   
  
  
- CTATGTTCTT TATCTTGTAC AGGAGGTAAA TGTTCTGCAT AGACAAAAAC CAAAACCAGT TACTAGGGAC   
  
  
- AACAGGAGTT CGTAATTGTG TCTTACTAGT AATAACTGAA CCTGACTTTC CAAAACCCTT GAGGTATCAT   
  
  
- ACGGAAAAGT TATTTTAGTT TTCGTTTAAA CGACAAGTGA CTATTGACGT CAACACAACG AAACGACAAA   
  
  
- GGTATGGTCT GAGCATTGGT TCGACTATCG TTTACACGTT TCCATGTCAT ATTAATTGCT ACAAAAGTAT   
  
  
- AACGATGCTC AAGACTTTGT ACTATATTCT TTTGTGGTCT TGGGTTTATG GATGTATCCA GGATCGGGAC   
  
  
- CTTACCGATA AAATTTATAG GTTTACCTGA ATGTCTTAAG CGTCTCAACA AACCAAACAA ATTGTTAGTA   
  
  
- ACTTAACAAT CGATACTCTT AGACGCCCTG TTAGCATTAG AAACCGCTAA TGAAGCTAAC CGTATGACTA   
  
  
- CAAAAAAATA AACGCATAGA TTCGTACCAC CACATGGAAA CTAAACACAC AGATCCATCA CACACATCTA   
  
  
- TTTATAGTAT CTTGAGTCTG CAACCATCCC AATACAACTC GTGAAAGAGA ACGACAAACA GGAAACACTA   
  
  
- CCGTGGAGAA CGAAAAGGCT TGGTGGTTTT CAGAGTAACT GTTTTGAGAA GTTTGTAACG TTTGACGGGG   
  
  
- GAATTTATAC AAAATAATTT TTTTTACCTT CTCGAGTTTG ACAACTCACT CGGATCTAAG GTTCTAAATC   
  
  
- TTTGTCATTT GAACTATCTA TATAAATATA CTTGATAACA ATCTACGTTG TGGGGGTTGA ACTTTGTGTT   
  
  
- TTCTCTTTAA TTTTTGTTTT TCCAAAACGT GATAAATATA TAGGACACAA GAAACACGAT CAGAATAAAT   
  
  
- TACAATTAAC CATTCGTAAT AAACAACCTA ATAAGGTGAC GTGAAAGAGA ATGATTGAAG TCGTAAAGTA   
  
  
- AGGAACAAGA AGTCGAAAAC TTGAAGAAAT CGGATAAAAC AACAGAAATA CCAATACCTT CGGTAGGTCT   
  
  
- TCTTCTTACT ACTTGAAGAC TCAGAATCGA ACCGGTAACA GCCAGTGAGA CTAGCGTGAC TCTTTTTTGA   
  
  
- CTTCTCCGCC TAACTACAGA GGCTAGGAGA CTAGTGAAGT CCACTTCCGA CGCTTCCCTT CTATTAGTCC   
  
  
- GAGGAGGTTC TCTCTGTCTT GAACAACTTG TAGTTCGTTT CTCCTTTTCC GCAGTAAGTT CTTCCCTTCC   
  
  
- CAGAAGTAGA ATAGGTAGAC GAGGAACATA GACGGTGGAA TTAGTTGCTT TTGTTGTAGT CAAGTCGACA   
  
  
- ACTATTAGAA TAACTCGAGA AGGTTATACA GAGGCTTTCA CCACTAAGTC CTGTTTCCCA ACGACGAATG   
  
  
- AAACGCCTAC CCAATTGTCG TTCCTAGAAT TGAGTCTCCA GAGGTAAAAT AGCGGACTAT GACCCGTTTG   
  
  
- GTCGTGGACG ACTTCTCAAA AGTCGAAAAT GAGTGAATAT ATCTCGGAGA GGTAAGATAG TCAAACGAGT   
  
  
- AAAGTGTCGG TTAGTCCGTT AACATCTTCG TAAACTCTCC CTCCTTCTCT CATTGTTGAC CCGAAACGTA   
  
  
- CATTAACTAA ACCTGTAGAA CGTACCAAAG GTCACCGGAA GAGAATAAGT CAGAGAGAGG CTTCTCCGAT   
  
  
- GAACAAGTTT GGCAAGTAAC AGAGAAGTTT AATGTCCGAA GCCTTCTTCG AATCTTCTCG AGTAACTTTG   
  
  
- TCTTCGAGCC GACCATTCAA AGAGATTCTC GAAAGTCTTA TAGTTGAAAC TTAAGGTGCC CAAAGACTCT   
  
  
- CCGAGCTTTG AGTCCTTGGA TTCCTCCCTC TTACTTTGTC ATCAACATTT AGAGCAAAAG GTGGAGTCAT   
  
  
- GAAACTTTCT ATGGCATGTC TAAAGACTGT GGAACTGAAG ACATGTAAGT GATTTGGGGA GATAACACCA   
  
  
- TAATCAGCTC TCTCTTCCTT CAGCTTTGTC TACACCCAAG GAGAGTTCAA TACATCTACG AGACGTAATA   
  
  
- ATACGACGTT ACAAACTGAG AAATCTACTA ACGGAGGGTG AACTTTCAGG ACTCTCCGAC TCGTATCTCT   
  
  
- TCTTGGTAGA ACCTTTTCTC TAGTTTCTTC GGTATCCAAC ACTCTTCCTA CTTTGTTTGA TAGACTTCAA   
  
  
- ACTCTACGAC CTCTGAACCT CTCCCTCCTA CCTCTCAGTA CCTAAAAGTC CATACTTCGA TTCAAGGTCC   
  
  
- CGTTGATATG TTCGTTTCGA CGAAAATTTT TACCCTTCGG TAATAGTATG GATAAACCTT CTTCTGACAC   
  
  
- CTCCACCACC CAAGTCTCAA ACACTTGCTC TACTACCTTA CCGATAGAGA GAACCGACCG TTCCATCCAA   
  
  
- GGAGCGTTGA CGGAGCCATA CCGCAAGACA CAC

+     AAGAA-motif

| Site Name | Organism | Position | Strand | Matrix score. | sequence | function |
| --- | --- | --- | --- | --- | --- | --- |
| AAGAA-motif | Avena sativa | 775 | - | 9 | gGTAAAGAAA |  |
| AAGAA-motif | Avena sativa | 687 | + | 7 | GAAAGAA |  |
| AAGAA-motif | Avena sativa | 331 | + | 7 | GAAAGAA |  |

>HU07G00272.1   
+ -Up\_Stream \_Len000ACCAAC GATTCTTTAC TGAATCAGCT ACATTTTTGG ATTGTGTTAA GGTATCTTTT   
  
  
+ AGATCTTCAG GAGGAGGGGG CTGAAGTAGC TTGGTTACTT TTGCGCTTAT CAACTGTGGA AGAATATTAT   
  
  
+ TGAGACTAGT GTAGAAGAAT TAGTAGTTTC CTCCACATTG GAAAAAAACC ATGAAATTAA CGCTTTAGGC   
  
  
+ CCTCAGAGTA CCAAGTGTCA TCGTAAATGA TTGAAATTTC AAGTCTTAGC TGAGTCATGA AGTCCTGATT   
  
  
+ TCCCATGACA AATTGATGTT GTTGTCTAAA GCTTGGCGGG TGATTTGAAA GAAGATTTAG ATCAGGCTGA   
  
  
+ CAATACACCA AATCTGGGAG CAAGGAACTG GATTTTTCTG TGTCTAGAAA AGCTTTCCGC TTTCTGAACA   
  
  
+ ATTGAGAAAA CCATGCATAG GAAATTTCTT GATATTTAGA TCATAGCAGA CTTAACAATC TGGCTTTACC   
  
  
+ GAACTGTCCT CTACTTCTAA GTTGTGAAGG TGTGAAAGAT AAACCATGTC AAACTTAGTG TCCAACAGAA   
  
  
+ ACGCATCTCT ACTTCTCCAG GCAAAGTTGT ACTTCAGATC TTCTACACCT TTTCTAGGCA GGACTTTGGG   
  
  
+ ATATAGAGTT TGGCCTGGGT GAAAAGTAGG AAGTGGGGAA GTAGAAAATG ATGAAAGAAG GCACCACTTG   
  
  
+ GGTCCAGATA CATAGGCCTA CGCACGAGAA CCTTTGCATG GTCTAGATAC ATGACTGTAT ACTTCTTCCT   
  
  
+ TTCCTTTACC TGTTGGAGAT TATCCTAGGA TTTTACATGG CACCAGCTGG ATTTGTTAAT GACATAAACA   
  
  
+ TATCTTTAAT TACAGTTGCT TATGGTATAC ATTTAAACTA TTCGATAGTT GACAGCTGAT GTCCTTATCC   
  
  
+ TTGTGCAGCT TATTCCTCGA TAGAATAATC TTTGGACATT TCTGTCTAGG TGATAAAGGG CAAAAACTAA   
  
  
+ GATACAAGAA ATAGAACATG TCCTCCATTT ACAAGACGTA TCTGTTTTTG GTTTTGGTCA ATGATCCCTG   
  
  
+ TTGTCCTCAA GCATTAACAC AGAATGATCA TTATTGACTT GGACTGAAAG GTTTTGGGAA CTCCATAGTA   
  
  
+ TGCCTTTTCA ATAAAATCAA AAGCAAATTT GCTGTTCACT GATAACTGCA GTTGTGTTGC TTTGCTGTTT   
  
  
+ CCATACCAGA CTCGTAACCA AGCTGATAGC AAATGTGCAA AGGTACAGTA TAATTAACGA TGTTTTCATA   
  
  
+ TTGCTACGAG TTCTGAAACA TGATATAAGA AAACACCAGA ACCCAAATAC CTACATAGGT CCTAGCCCTG   
  
  
+ GAATGGCTAT TTTAAATATC CAAATGGACT TACAGAATTC GCAGAGTTGT TTGGTTTGTT TAACAATCAT   
  
  
+ TGAATTGTTA GCTATGAGAA TCTGCGGGAC AATCGTAATC TTTGGCGATT ACTTCGATTG GCATACTGAT   
  
  
+ GTTTTTTTAT TTGCGTATCT AAGCATGGTG GTGTACCTTT GATTTGTGTG TCTAGGTAGT GTGTGTAGAT   
  
  
+ AAATATCATA GAACTCAGAC GTTGGTAGGG TTATGTTGAG CACTTTCTCT TGCTGTTTGT CCTTTGTGAT   
  
  
+ GGCACCTCTT GCTTTTCCGA ACCACCAAAA GTCTCATTGA CAAAACTCTT CAAACATTGC AAACTGCCCC   
  
  
+ CTTAAATATG TTTTATTAAA AAAAATGGAA GAGCTCAAAC TGTTGAGTGA GCCTAGATTC CAAGATTTAG   
  
  
+ AAACAGTAAA CTTGATAGAT ATATTTATAT GAACTATTGT TAGATGCAAC ACCCCCAACT TGAAACACAA   
  
  
+ AAGAGAAATT AAAAACAAAA AGGTTTTGCA CTATTTATAT ATCCTGTGTT CTTTGTGCTA GTCTTATTTA   
  
  
+ ATGTTAATTG GTAAGCATTA TTTGTTGGAT TATTCCACTG CACTTTCTCT TACTAACTTC AGCATTTCAT   
  
  
+ TCCTTGTTCT TCAGCTTTTG AACTTCTTTA GCCTATTTTG TTGTCTTTAT GGTTATGGAA GCCATCCAGA   
  
  
+ AGAAGAATGA TGAACTTCTG AGTCTTAGCT TGGCCATTGT CGGTCACTCT GATCGCACTG AGAAAAAACT   
  
  
+ GAAGAGGCGG ATTGATGTCT CCGATCCTCT GATCACTTCA GGTGAAGGCT GCGAAGGGAA GATAATCAGG   
  
  
+ CTCCTCCAAG AGAGACAGAA CTTGTTGAAC ATCAAGCAAA GAGGAAAAGG CGTCATTCAA GAAGGGAAGG   
  
  
+ GTCTTCATCT TATCCATCTG CTCCTTGTAT CTGCCACCTT AATCAACGAA AACAACATCA GTTCAGCTGT   
  
  
+ TGATAATCTT ATTGAGCTCT TCCAATATGT CTCCGAAAGT GGTGATTCAG GACAAAGGGT TGCTGCTTAC   
  
  
+ TTTGCGGATG GGTTAACAGC AAGGATCTTA ACTCAGAGGT CTCCATTTTA TCGCCTGATA CTGGGCAAAC   
  
  
+ CAGCACCTGC TGAAGAGTTT TCAGCTTTTA CTCACTTATA TAGAGCCTCT CCATTCTATC AGTTTGCTCA   
  
  
+ TTTCACAGCC AATCAGGCAA TTGTAGAAGC ATTTGAGAGG GAGGAAGAGA GTAACAACTG GGCTTTGCAT   
  
  
+ GTAATTGATT TGGACATCTT GCATGGTTTC CAGTGGCCTT CTCTTATTCA GTCTCTCTCC GAAGAGGCTA   
  
  
+ CTTGTTCAAA CCGTTCATTG TCTCTTCAAA TTACAGGCTT CGGAAGAAGC TTAGAAGAGC TCATTGAAAC   
  
  
+ AGAAGCTCGG CTGGTAAGTT TCTCTAAGAG CTTTCAGAAT ATCAACTTTG AATTCCACGG GTTTCTGAGA   
  
  
+ GGCTCGAAAC TCAGGAACCT AAGGAGGGAG AATGAAACAG TAGTTGTAAA TCTCGTTTTC CACCTCAGTA   
  
  
+ CTTTGAAAGA TACCGTACAG ATTTCTGACA CCTTGACTTC TGTACATTCA CTAAACCCCT CTATTGTGGT   
  
  
+ ATTAGTCGAG AGAGAAGGAA GTCGAAACAG ATGTGGGTTC CTCTCAAGTT ATGTAGATGC TCTGCATTAT   
  
  
+ TATGCTGCAA TGTTTGACTC TTTAGATGAT TGCCTCCCAC TTGAAAGTCC TGAGAGGCTG AGCATAGAGA   
  
  
+ AGAACCATCT TGGAAAAGAG ATCAAAGAAG CCATAGGTTG TGAGAAGGAT GAAACAAACT ATCTGAAGTT   
  
  
+ TGAGATGCTG GAGACTTGGA GAGGGAGGAT GGAGAGTCAT GGATTTTCAG GTATGAAGCT AAGTTCCAGG   
  
  
+ GCAACTATAC AAGCAAAGCT GCTTTTAAAA ATGGGAAGCC ATTATCATAC CTATTTGGAA GAAGACTGTG   
  
  
+ GAGGTGGTGG GTTCAGAGTT TGTGAACGAG ATGATGGAAT GGCTATCTCT CTTGGCTGGC AAGGTAGGTT   
  
  
+ CCTCGCAACT GCCTCGGTAT GGCGTTCTGT GTG  

- -Up\_Stream \_Len000TGGTTG CTAAGAAATG ACTTAGTCGA TGTAAAAACC TAACACAATT CCATAGAAAA   
  
  
- TCTAGAAGTC CTCCTCCCCC GACTTCATCG AACCAATGAA AACGCGAATA GTTGACACCT TCTTATAATA   
  
  
- ACTCTGATCA CATCTTCTTA ATCATCAAAG GAGGTGTAAC CTTTTTTTGG TACTTTAATT GCGAAATCCG   
  
  
- GGAGTCTCAT GGTTCACAGT AGCATTTACT AACTTTAAAG TTCAGAATCG ACTCAGTACT TCAGGACTAA   
  
  
- AGGGTACTGT TTAACTACAA CAACAGATTT CGAACCGCCC ACTAAACTTT CTTCTAAATC TAGTCCGACT   
  
  
- GTTATGTGGT TTAGACCCTC GTTCCTTGAC CTAAAAAGAC ACAGATCTTT TCGAAAGGCG AAAGACTTGT   
  
  
- TAACTCTTTT GGTACGTATC CTTTAAAGAA CTATAAATCT AGTATCGTCT GAATTGTTAG ACCGAAATGG   
  
  
- CTTGACAGGA GATGAAGATT CAACACTTCC ACACTTTCTA TTTGGTACAG TTTGAATCAC AGGTTGTCTT   
  
  
- TGCGTAGAGA TGAAGAGGTC CGTTTCAACA TGAAGTCTAG AAGATGTGGA AAAGATCCGT CCTGAAACCC   
  
  
- TATATCTCAA ACCGGACCCA CTTTTCATCC TTCACCCCTT CATCTTTTAC TACTTTCTTC CGTGGTGAAC   
  
  
- CCAGGTCTAT GTATCCGGAT GCGTGCTCTT GGAAACGTAC CAGATCTATG TACTGACATA TGAAGAAGGA   
  
  
- AAGGAAATGG ACAACCTCTA ATAGGATCCT AAAATGTACC GTGGTCGACC TAAACAATTA CTGTATTTGT   
  
  
- ATAGAAATTA ATGTCAACGA ATACCATATG TAAATTTGAT AAGCTATCAA CTGTCGACTA CAGGAATAGG   
  
  
- AACACGTCGA ATAAGGAGCT ATCTTATTAG AAACCTGTAA AGACAGATCC ACTATTTCCC GTTTTTGATT   
  
  
- CTATGTTCTT TATCTTGTAC AGGAGGTAAA TGTTCTGCAT AGACAAAAAC CAAAACCAGT TACTAGGGAC   
  
  
- AACAGGAGTT CGTAATTGTG TCTTACTAGT AATAACTGAA CCTGACTTTC CAAAACCCTT GAGGTATCAT   
  
  
- ACGGAAAAGT TATTTTAGTT TTCGTTTAAA CGACAAGTGA CTATTGACGT CAACACAACG AAACGACAAA   
  
  
- GGTATGGTCT GAGCATTGGT TCGACTATCG TTTACACGTT TCCATGTCAT ATTAATTGCT ACAAAAGTAT   
  
  
- AACGATGCTC AAGACTTTGT ACTATATTCT TTTGTGGTCT TGGGTTTATG GATGTATCCA GGATCGGGAC   
  
  
- CTTACCGATA AAATTTATAG GTTTACCTGA ATGTCTTAAG CGTCTCAACA AACCAAACAA ATTGTTAGTA   
  
  
- ACTTAACAAT CGATACTCTT AGACGCCCTG TTAGCATTAG AAACCGCTAA TGAAGCTAAC CGTATGACTA   
  
  
- CAAAAAAATA AACGCATAGA TTCGTACCAC CACATGGAAA CTAAACACAC AGATCCATCA CACACATCTA   
  
  
- TTTATAGTAT CTTGAGTCTG CAACCATCCC AATACAACTC GTGAAAGAGA ACGACAAACA GGAAACACTA   
  
  
- CCGTGGAGAA CGAAAAGGCT TGGTGGTTTT CAGAGTAACT GTTTTGAGAA GTTTGTAACG TTTGACGGGG   
  
  
- GAATTTATAC AAAATAATTT TTTTTACCTT CTCGAGTTTG ACAACTCACT CGGATCTAAG GTTCTAAATC   
  
  
- TTTGTCATTT GAACTATCTA TATAAATATA CTTGATAACA ATCTACGTTG TGGGGGTTGA ACTTTGTGTT   
  
  
- TTCTCTTTAA TTTTTGTTTT TCCAAAACGT GATAAATATA TAGGACACAA GAAACACGAT CAGAATAAAT   
  
  
- TACAATTAAC CATTCGTAAT AAACAACCTA ATAAGGTGAC GTGAAAGAGA ATGATTGAAG TCGTAAAGTA   
  
  
- AGGAACAAGA AGTCGAAAAC TTGAAGAAAT CGGATAAAAC AACAGAAATA CCAATACCTT CGGTAGGTCT   
  
  
- TCTTCTTACT ACTTGAAGAC TCAGAATCGA ACCGGTAACA GCCAGTGAGA CTAGCGTGAC TCTTTTTTGA   
  
  
- CTTCTCCGCC TAACTACAGA GGCTAGGAGA CTAGTGAAGT CCACTTCCGA CGCTTCCCTT CTATTAGTCC   
  
  
- GAGGAGGTTC TCTCTGTCTT GAACAACTTG TAGTTCGTTT CTCCTTTTCC GCAGTAAGTT CTTCCCTTCC   
  
  
- CAGAAGTAGA ATAGGTAGAC GAGGAACATA GACGGTGGAA TTAGTTGCTT TTGTTGTAGT CAAGTCGACA   
  
  
- ACTATTAGAA TAACTCGAGA AGGTTATACA GAGGCTTTCA CCACTAAGTC CTGTTTCCCA ACGACGAATG   
  
  
- AAACGCCTAC CCAATTGTCG TTCCTAGAAT TGAGTCTCCA GAGGTAAAAT AGCGGACTAT GACCCGTTTG   
  
  
- GTCGTGGACG ACTTCTCAAA AGTCGAAAAT GAGTGAATAT ATCTCGGAGA GGTAAGATAG TCAAACGAGT   
  
  
- AAAGTGTCGG TTAGTCCGTT AACATCTTCG TAAACTCTCC CTCCTTCTCT CATTGTTGAC CCGAAACGTA   
  
  
- CATTAACTAA ACCTGTAGAA CGTACCAAAG GTCACCGGAA GAGAATAAGT CAGAGAGAGG CTTCTCCGAT   
  
  
- GAACAAGTTT GGCAAGTAAC AGAGAAGTTT AATGTCCGAA GCCTTCTTCG AATCTTCTCG AGTAACTTTG   
  
  
- TCTTCGAGCC GACCATTCAA AGAGATTCTC GAAAGTCTTA TAGTTGAAAC TTAAGGTGCC CAAAGACTCT   
  
  
- CCGAGCTTTG AGTCCTTGGA TTCCTCCCTC TTACTTTGTC ATCAACATTT AGAGCAAAAG GTGGAGTCAT   
  
  
- GAAACTTTCT ATGGCATGTC TAAAGACTGT GGAACTGAAG ACATGTAAGT GATTTGGGGA GATAACACCA   
  
  
- TAATCAGCTC TCTCTTCCTT CAGCTTTGTC TACACCCAAG GAGAGTTCAA TACATCTACG AGACGTAATA   
  
  
- ATACGACGTT ACAAACTGAG AAATCTACTA ACGGAGGGTG AACTTTCAGG ACTCTCCGAC TCGTATCTCT   
  
  
- TCTTGGTAGA ACCTTTTCTC TAGTTTCTTC GGTATCCAAC ACTCTTCCTA CTTTGTTTGA TAGACTTCAA   
  
  
- ACTCTACGAC CTCTGAACCT CTCCCTCCTA CCTCTCAGTA CCTAAAAGTC CATACTTCGA TTCAAGGTCC   
  
  
- CGTTGATATG TTCGTTTCGA CGAAAATTTT TACCCTTCGG TAATAGTATG GATAAACCTT CTTCTGACAC   
  
  
- CTCCACCACC CAAGTCTCAA ACACTTGCTC TACTACCTTA CCGATAGAGA GAACCGACCG TTCCATCCAA   
  
  
- GGAGCGTTGA CGGAGCCATA CCGCAAGACA CAC

+     AE-box

| Site Name | Organism | Position | Strand | Matrix score. | sequence | function |
| --- | --- | --- | --- | --- | --- | --- |
| AE-box | Arabidopsis thaliana | 2750 | - | 8 | AGAAACTT | part of a module for light response |

>HU07G00272.1   
+ -Up\_Stream \_Len000ACCAAC GATTCTTTAC TGAATCAGCT ACATTTTTGG ATTGTGTTAA GGTATCTTTT   
  
  
+ AGATCTTCAG GAGGAGGGGG CTGAAGTAGC TTGGTTACTT TTGCGCTTAT CAACTGTGGA AGAATATTAT   
  
  
+ TGAGACTAGT GTAGAAGAAT TAGTAGTTTC CTCCACATTG GAAAAAAACC ATGAAATTAA CGCTTTAGGC   
  
  
+ CCTCAGAGTA CCAAGTGTCA TCGTAAATGA TTGAAATTTC AAGTCTTAGC TGAGTCATGA AGTCCTGATT   
  
  
+ TCCCATGACA AATTGATGTT GTTGTCTAAA GCTTGGCGGG TGATTTGAAA GAAGATTTAG ATCAGGCTGA   
  
  
+ CAATACACCA AATCTGGGAG CAAGGAACTG GATTTTTCTG TGTCTAGAAA AGCTTTCCGC TTTCTGAACA   
  
  
+ ATTGAGAAAA CCATGCATAG GAAATTTCTT GATATTTAGA TCATAGCAGA CTTAACAATC TGGCTTTACC   
  
  
+ GAACTGTCCT CTACTTCTAA GTTGTGAAGG TGTGAAAGAT AAACCATGTC AAACTTAGTG TCCAACAGAA   
  
  
+ ACGCATCTCT ACTTCTCCAG GCAAAGTTGT ACTTCAGATC TTCTACACCT TTTCTAGGCA GGACTTTGGG   
  
  
+ ATATAGAGTT TGGCCTGGGT GAAAAGTAGG AAGTGGGGAA GTAGAAAATG ATGAAAGAAG GCACCACTTG   
  
  
+ GGTCCAGATA CATAGGCCTA CGCACGAGAA CCTTTGCATG GTCTAGATAC ATGACTGTAT ACTTCTTCCT   
  
  
+ TTCCTTTACC TGTTGGAGAT TATCCTAGGA TTTTACATGG CACCAGCTGG ATTTGTTAAT GACATAAACA   
  
  
+ TATCTTTAAT TACAGTTGCT TATGGTATAC ATTTAAACTA TTCGATAGTT GACAGCTGAT GTCCTTATCC   
  
  
+ TTGTGCAGCT TATTCCTCGA TAGAATAATC TTTGGACATT TCTGTCTAGG TGATAAAGGG CAAAAACTAA   
  
  
+ GATACAAGAA ATAGAACATG TCCTCCATTT ACAAGACGTA TCTGTTTTTG GTTTTGGTCA ATGATCCCTG   
  
  
+ TTGTCCTCAA GCATTAACAC AGAATGATCA TTATTGACTT GGACTGAAAG GTTTTGGGAA CTCCATAGTA   
  
  
+ TGCCTTTTCA ATAAAATCAA AAGCAAATTT GCTGTTCACT GATAACTGCA GTTGTGTTGC TTTGCTGTTT   
  
  
+ CCATACCAGA CTCGTAACCA AGCTGATAGC AAATGTGCAA AGGTACAGTA TAATTAACGA TGTTTTCATA   
  
  
+ TTGCTACGAG TTCTGAAACA TGATATAAGA AAACACCAGA ACCCAAATAC CTACATAGGT CCTAGCCCTG   
  
  
+ GAATGGCTAT TTTAAATATC CAAATGGACT TACAGAATTC GCAGAGTTGT TTGGTTTGTT TAACAATCAT   
  
  
+ TGAATTGTTA GCTATGAGAA TCTGCGGGAC AATCGTAATC TTTGGCGATT ACTTCGATTG GCATACTGAT   
  
  
+ GTTTTTTTAT TTGCGTATCT AAGCATGGTG GTGTACCTTT GATTTGTGTG TCTAGGTAGT GTGTGTAGAT   
  
  
+ AAATATCATA GAACTCAGAC GTTGGTAGGG TTATGTTGAG CACTTTCTCT TGCTGTTTGT CCTTTGTGAT   
  
  
+ GGCACCTCTT GCTTTTCCGA ACCACCAAAA GTCTCATTGA CAAAACTCTT CAAACATTGC AAACTGCCCC   
  
  
+ CTTAAATATG TTTTATTAAA AAAAATGGAA GAGCTCAAAC TGTTGAGTGA GCCTAGATTC CAAGATTTAG   
  
  
+ AAACAGTAAA CTTGATAGAT ATATTTATAT GAACTATTGT TAGATGCAAC ACCCCCAACT TGAAACACAA   
  
  
+ AAGAGAAATT AAAAACAAAA AGGTTTTGCA CTATTTATAT ATCCTGTGTT CTTTGTGCTA GTCTTATTTA   
  
  
+ ATGTTAATTG GTAAGCATTA TTTGTTGGAT TATTCCACTG CACTTTCTCT TACTAACTTC AGCATTTCAT   
  
  
+ TCCTTGTTCT TCAGCTTTTG AACTTCTTTA GCCTATTTTG TTGTCTTTAT GGTTATGGAA GCCATCCAGA   
  
  
+ AGAAGAATGA TGAACTTCTG AGTCTTAGCT TGGCCATTGT CGGTCACTCT GATCGCACTG AGAAAAAACT   
  
  
+ GAAGAGGCGG ATTGATGTCT CCGATCCTCT GATCACTTCA GGTGAAGGCT GCGAAGGGAA GATAATCAGG   
  
  
+ CTCCTCCAAG AGAGACAGAA CTTGTTGAAC ATCAAGCAAA GAGGAAAAGG CGTCATTCAA GAAGGGAAGG   
  
  
+ GTCTTCATCT TATCCATCTG CTCCTTGTAT CTGCCACCTT AATCAACGAA AACAACATCA GTTCAGCTGT   
  
  
+ TGATAATCTT ATTGAGCTCT TCCAATATGT CTCCGAAAGT GGTGATTCAG GACAAAGGGT TGCTGCTTAC   
  
  
+ TTTGCGGATG GGTTAACAGC AAGGATCTTA ACTCAGAGGT CTCCATTTTA TCGCCTGATA CTGGGCAAAC   
  
  
+ CAGCACCTGC TGAAGAGTTT TCAGCTTTTA CTCACTTATA TAGAGCCTCT CCATTCTATC AGTTTGCTCA   
  
  
+ TTTCACAGCC AATCAGGCAA TTGTAGAAGC ATTTGAGAGG GAGGAAGAGA GTAACAACTG GGCTTTGCAT   
  
  
+ GTAATTGATT TGGACATCTT GCATGGTTTC CAGTGGCCTT CTCTTATTCA GTCTCTCTCC GAAGAGGCTA   
  
  
+ CTTGTTCAAA CCGTTCATTG TCTCTTCAAA TTACAGGCTT CGGAAGAAGC TTAGAAGAGC TCATTGAAAC   
  
  
+ AGAAGCTCGG CTGGTAAGTT TCTCTAAGAG CTTTCAGAAT ATCAACTTTG AATTCCACGG GTTTCTGAGA   
  
  
+ GGCTCGAAAC TCAGGAACCT AAGGAGGGAG AATGAAACAG TAGTTGTAAA TCTCGTTTTC CACCTCAGTA   
  
  
+ CTTTGAAAGA TACCGTACAG ATTTCTGACA CCTTGACTTC TGTACATTCA CTAAACCCCT CTATTGTGGT   
  
  
+ ATTAGTCGAG AGAGAAGGAA GTCGAAACAG ATGTGGGTTC CTCTCAAGTT ATGTAGATGC TCTGCATTAT   
  
  
+ TATGCTGCAA TGTTTGACTC TTTAGATGAT TGCCTCCCAC TTGAAAGTCC TGAGAGGCTG AGCATAGAGA   
  
  
+ AGAACCATCT TGGAAAAGAG ATCAAAGAAG CCATAGGTTG TGAGAAGGAT GAAACAAACT ATCTGAAGTT   
  
  
+ TGAGATGCTG GAGACTTGGA GAGGGAGGAT GGAGAGTCAT GGATTTTCAG GTATGAAGCT AAGTTCCAGG   
  
  
+ GCAACTATAC AAGCAAAGCT GCTTTTAAAA ATGGGAAGCC ATTATCATAC CTATTTGGAA GAAGACTGTG   
  
  
+ GAGGTGGTGG GTTCAGAGTT TGTGAACGAG ATGATGGAAT GGCTATCTCT CTTGGCTGGC AAGGTAGGTT   
  
  
+ CCTCGCAACT GCCTCGGTAT GGCGTTCTGT GTG  

- -Up\_Stream \_Len000TGGTTG CTAAGAAATG ACTTAGTCGA TGTAAAAACC TAACACAATT CCATAGAAAA   
  
  
- TCTAGAAGTC CTCCTCCCCC GACTTCATCG AACCAATGAA AACGCGAATA GTTGACACCT TCTTATAATA   
  
  
- ACTCTGATCA CATCTTCTTA ATCATCAAAG GAGGTGTAAC CTTTTTTTGG TACTTTAATT GCGAAATCCG   
  
  
- GGAGTCTCAT GGTTCACAGT AGCATTTACT AACTTTAAAG TTCAGAATCG ACTCAGTACT TCAGGACTAA   
  
  
- AGGGTACTGT TTAACTACAA CAACAGATTT CGAACCGCCC ACTAAACTTT CTTCTAAATC TAGTCCGACT   
  
  
- GTTATGTGGT TTAGACCCTC GTTCCTTGAC CTAAAAAGAC ACAGATCTTT TCGAAAGGCG AAAGACTTGT   
  
  
- TAACTCTTTT GGTACGTATC CTTTAAAGAA CTATAAATCT AGTATCGTCT GAATTGTTAG ACCGAAATGG   
  
  
- CTTGACAGGA GATGAAGATT CAACACTTCC ACACTTTCTA TTTGGTACAG TTTGAATCAC AGGTTGTCTT   
  
  
- TGCGTAGAGA TGAAGAGGTC CGTTTCAACA TGAAGTCTAG AAGATGTGGA AAAGATCCGT CCTGAAACCC   
  
  
- TATATCTCAA ACCGGACCCA CTTTTCATCC TTCACCCCTT CATCTTTTAC TACTTTCTTC CGTGGTGAAC   
  
  
- CCAGGTCTAT GTATCCGGAT GCGTGCTCTT GGAAACGTAC CAGATCTATG TACTGACATA TGAAGAAGGA   
  
  
- AAGGAAATGG ACAACCTCTA ATAGGATCCT AAAATGTACC GTGGTCGACC TAAACAATTA CTGTATTTGT   
  
  
- ATAGAAATTA ATGTCAACGA ATACCATATG TAAATTTGAT AAGCTATCAA CTGTCGACTA CAGGAATAGG   
  
  
- AACACGTCGA ATAAGGAGCT ATCTTATTAG AAACCTGTAA AGACAGATCC ACTATTTCCC GTTTTTGATT   
  
  
- CTATGTTCTT TATCTTGTAC AGGAGGTAAA TGTTCTGCAT AGACAAAAAC CAAAACCAGT TACTAGGGAC   
  
  
- AACAGGAGTT CGTAATTGTG TCTTACTAGT AATAACTGAA CCTGACTTTC CAAAACCCTT GAGGTATCAT   
  
  
- ACGGAAAAGT TATTTTAGTT TTCGTTTAAA CGACAAGTGA CTATTGACGT CAACACAACG AAACGACAAA   
  
  
- GGTATGGTCT GAGCATTGGT TCGACTATCG TTTACACGTT TCCATGTCAT ATTAATTGCT ACAAAAGTAT   
  
  
- AACGATGCTC AAGACTTTGT ACTATATTCT TTTGTGGTCT TGGGTTTATG GATGTATCCA GGATCGGGAC   
  
  
- CTTACCGATA AAATTTATAG GTTTACCTGA ATGTCTTAAG CGTCTCAACA AACCAAACAA ATTGTTAGTA   
  
  
- ACTTAACAAT CGATACTCTT AGACGCCCTG TTAGCATTAG AAACCGCTAA TGAAGCTAAC CGTATGACTA   
  
  
- CAAAAAAATA AACGCATAGA TTCGTACCAC CACATGGAAA CTAAACACAC AGATCCATCA CACACATCTA   
  
  
- TTTATAGTAT CTTGAGTCTG CAACCATCCC AATACAACTC GTGAAAGAGA ACGACAAACA GGAAACACTA   
  
  
- CCGTGGAGAA CGAAAAGGCT TGGTGGTTTT CAGAGTAACT GTTTTGAGAA GTTTGTAACG TTTGACGGGG   
  
  
- GAATTTATAC AAAATAATTT TTTTTACCTT CTCGAGTTTG ACAACTCACT CGGATCTAAG GTTCTAAATC   
  
  
- TTTGTCATTT GAACTATCTA TATAAATATA CTTGATAACA ATCTACGTTG TGGGGGTTGA ACTTTGTGTT   
  
  
- TTCTCTTTAA TTTTTGTTTT TCCAAAACGT GATAAATATA TAGGACACAA GAAACACGAT CAGAATAAAT   
  
  
- TACAATTAAC CATTCGTAAT AAACAACCTA ATAAGGTGAC GTGAAAGAGA ATGATTGAAG TCGTAAAGTA   
  
  
- AGGAACAAGA AGTCGAAAAC TTGAAGAAAT CGGATAAAAC AACAGAAATA CCAATACCTT CGGTAGGTCT   
  
  
- TCTTCTTACT ACTTGAAGAC TCAGAATCGA ACCGGTAACA GCCAGTGAGA CTAGCGTGAC TCTTTTTTGA   
  
  
- CTTCTCCGCC TAACTACAGA GGCTAGGAGA CTAGTGAAGT CCACTTCCGA CGCTTCCCTT CTATTAGTCC   
  
  
- GAGGAGGTTC TCTCTGTCTT GAACAACTTG TAGTTCGTTT CTCCTTTTCC GCAGTAAGTT CTTCCCTTCC   
  
  
- CAGAAGTAGA ATAGGTAGAC GAGGAACATA GACGGTGGAA TTAGTTGCTT TTGTTGTAGT CAAGTCGACA   
  
  
- ACTATTAGAA TAACTCGAGA AGGTTATACA GAGGCTTTCA CCACTAAGTC CTGTTTCCCA ACGACGAATG   
  
  
- AAACGCCTAC CCAATTGTCG TTCCTAGAAT TGAGTCTCCA GAGGTAAAAT AGCGGACTAT GACCCGTTTG   
  
  
- GTCGTGGACG ACTTCTCAAA AGTCGAAAAT GAGTGAATAT ATCTCGGAGA GGTAAGATAG TCAAACGAGT   
  
  
- AAAGTGTCGG TTAGTCCGTT AACATCTTCG TAAACTCTCC CTCCTTCTCT CATTGTTGAC CCGAAACGTA   
  
  
- CATTAACTAA ACCTGTAGAA CGTACCAAAG GTCACCGGAA GAGAATAAGT CAGAGAGAGG CTTCTCCGAT   
  
  
- GAACAAGTTT GGCAAGTAAC AGAGAAGTTT AATGTCCGAA GCCTTCTTCG AATCTTCTCG AGTAACTTTG   
  
  
- TCTTCGAGCC GACCATTCAA AGAGATTCTC GAAAGTCTTA TAGTTGAAAC TTAAGGTGCC CAAAGACTCT   
  
  
- CCGAGCTTTG AGTCCTTGGA TTCCTCCCTC TTACTTTGTC ATCAACATTT AGAGCAAAAG GTGGAGTCAT   
  
  
- GAAACTTTCT ATGGCATGTC TAAAGACTGT GGAACTGAAG ACATGTAAGT GATTTGGGGA GATAACACCA   
  
  
- TAATCAGCTC TCTCTTCCTT CAGCTTTGTC TACACCCAAG GAGAGTTCAA TACATCTACG AGACGTAATA   
  
  
- ATACGACGTT ACAAACTGAG AAATCTACTA ACGGAGGGTG AACTTTCAGG ACTCTCCGAC TCGTATCTCT   
  
  
- TCTTGGTAGA ACCTTTTCTC TAGTTTCTTC GGTATCCAAC ACTCTTCCTA CTTTGTTTGA TAGACTTCAA   
  
  
- ACTCTACGAC CTCTGAACCT CTCCCTCCTA CCTCTCAGTA CCTAAAAGTC CATACTTCGA TTCAAGGTCC   
  
  
- CGTTGATATG TTCGTTTCGA CGAAAATTTT TACCCTTCGG TAATAGTATG GATAAACCTT CTTCTGACAC   
  
  
- CTCCACCACC CAAGTCTCAA ACACTTGCTC TACTACCTTA CCGATAGAGA GAACCGACCG TTCCATCCAA   
  
  
- GGAGCGTTGA CGGAGCCATA CCGCAAGACA CAC

+     ARE

| Site Name | Organism | Position | Strand | Matrix score. | sequence | function |
| --- | --- | --- | --- | --- | --- | --- |
| ARE | Zea mays | 1033 | - | 6 | AAACCA | cis-acting regulatory element essential for the anaerobic induction |
| ARE | Zea mays | 432 | + | 6 | AAACCA | cis-acting regulatory element essential for the anaerobic induction |
| ARE | Zea mays | 2451 | + | 6 | AAACCA | cis-acting regulatory element essential for the anaerobic induction |
| ARE | Zea mays | 190 | + | 6 | AAACCA | cis-acting regulatory element essential for the anaerobic induction |
| ARE | Zea mays | 2618 | - | 6 | AAACCA | cis-acting regulatory element essential for the anaerobic induction |
| ARE | Zea mays | 535 | + | 6 | AAACCA | cis-acting regulatory element essential for the anaerobic induction |
| ARE | Zea mays | 1386 | - | 6 | AAACCA | cis-acting regulatory element essential for the anaerobic induction |

>HU07G00272.1   
+ -Up\_Stream \_Len000ACCAAC GATTCTTTAC TGAATCAGCT ACATTTTTGG ATTGTGTTAA GGTATCTTTT   
  
  
+ AGATCTTCAG GAGGAGGGGG CTGAAGTAGC TTGGTTACTT TTGCGCTTAT CAACTGTGGA AGAATATTAT   
  
  
+ TGAGACTAGT GTAGAAGAAT TAGTAGTTTC CTCCACATTG GAAAAAAACC ATGAAATTAA CGCTTTAGGC   
  
  
+ CCTCAGAGTA CCAAGTGTCA TCGTAAATGA TTGAAATTTC AAGTCTTAGC TGAGTCATGA AGTCCTGATT   
  
  
+ TCCCATGACA AATTGATGTT GTTGTCTAAA GCTTGGCGGG TGATTTGAAA GAAGATTTAG ATCAGGCTGA   
  
  
+ CAATACACCA AATCTGGGAG CAAGGAACTG GATTTTTCTG TGTCTAGAAA AGCTTTCCGC TTTCTGAACA   
  
  
+ ATTGAGAAAA CCATGCATAG GAAATTTCTT GATATTTAGA TCATAGCAGA CTTAACAATC TGGCTTTACC   
  
  
+ GAACTGTCCT CTACTTCTAA GTTGTGAAGG TGTGAAAGAT AAACCATGTC AAACTTAGTG TCCAACAGAA   
  
  
+ ACGCATCTCT ACTTCTCCAG GCAAAGTTGT ACTTCAGATC TTCTACACCT TTTCTAGGCA GGACTTTGGG   
  
  
+ ATATAGAGTT TGGCCTGGGT GAAAAGTAGG AAGTGGGGAA GTAGAAAATG ATGAAAGAAG GCACCACTTG   
  
  
+ GGTCCAGATA CATAGGCCTA CGCACGAGAA CCTTTGCATG GTCTAGATAC ATGACTGTAT ACTTCTTCCT   
  
  
+ TTCCTTTACC TGTTGGAGAT TATCCTAGGA TTTTACATGG CACCAGCTGG ATTTGTTAAT GACATAAACA   
  
  
+ TATCTTTAAT TACAGTTGCT TATGGTATAC ATTTAAACTA TTCGATAGTT GACAGCTGAT GTCCTTATCC   
  
  
+ TTGTGCAGCT TATTCCTCGA TAGAATAATC TTTGGACATT TCTGTCTAGG TGATAAAGGG CAAAAACTAA   
  
  
+ GATACAAGAA ATAGAACATG TCCTCCATTT ACAAGACGTA TCTGTTTTTG GTTTTGGTCA ATGATCCCTG   
  
  
+ TTGTCCTCAA GCATTAACAC AGAATGATCA TTATTGACTT GGACTGAAAG GTTTTGGGAA CTCCATAGTA   
  
  
+ TGCCTTTTCA ATAAAATCAA AAGCAAATTT GCTGTTCACT GATAACTGCA GTTGTGTTGC TTTGCTGTTT   
  
  
+ CCATACCAGA CTCGTAACCA AGCTGATAGC AAATGTGCAA AGGTACAGTA TAATTAACGA TGTTTTCATA   
  
  
+ TTGCTACGAG TTCTGAAACA TGATATAAGA AAACACCAGA ACCCAAATAC CTACATAGGT CCTAGCCCTG   
  
  
+ GAATGGCTAT TTTAAATATC CAAATGGACT TACAGAATTC GCAGAGTTGT TTGGTTTGTT TAACAATCAT   
  
  
+ TGAATTGTTA GCTATGAGAA TCTGCGGGAC AATCGTAATC TTTGGCGATT ACTTCGATTG GCATACTGAT   
  
  
+ GTTTTTTTAT TTGCGTATCT AAGCATGGTG GTGTACCTTT GATTTGTGTG TCTAGGTAGT GTGTGTAGAT   
  
  
+ AAATATCATA GAACTCAGAC GTTGGTAGGG TTATGTTGAG CACTTTCTCT TGCTGTTTGT CCTTTGTGAT   
  
  
+ GGCACCTCTT GCTTTTCCGA ACCACCAAAA GTCTCATTGA CAAAACTCTT CAAACATTGC AAACTGCCCC   
  
  
+ CTTAAATATG TTTTATTAAA AAAAATGGAA GAGCTCAAAC TGTTGAGTGA GCCTAGATTC CAAGATTTAG   
  
  
+ AAACAGTAAA CTTGATAGAT ATATTTATAT GAACTATTGT TAGATGCAAC ACCCCCAACT TGAAACACAA   
  
  
+ AAGAGAAATT AAAAACAAAA AGGTTTTGCA CTATTTATAT ATCCTGTGTT CTTTGTGCTA GTCTTATTTA   
  
  
+ ATGTTAATTG GTAAGCATTA TTTGTTGGAT TATTCCACTG CACTTTCTCT TACTAACTTC AGCATTTCAT   
  
  
+ TCCTTGTTCT TCAGCTTTTG AACTTCTTTA GCCTATTTTG TTGTCTTTAT GGTTATGGAA GCCATCCAGA   
  
  
+ AGAAGAATGA TGAACTTCTG AGTCTTAGCT TGGCCATTGT CGGTCACTCT GATCGCACTG AGAAAAAACT   
  
  
+ GAAGAGGCGG ATTGATGTCT CCGATCCTCT GATCACTTCA GGTGAAGGCT GCGAAGGGAA GATAATCAGG   
  
  
+ CTCCTCCAAG AGAGACAGAA CTTGTTGAAC ATCAAGCAAA GAGGAAAAGG CGTCATTCAA GAAGGGAAGG   
  
  
+ GTCTTCATCT TATCCATCTG CTCCTTGTAT CTGCCACCTT AATCAACGAA AACAACATCA GTTCAGCTGT   
  
  
+ TGATAATCTT ATTGAGCTCT TCCAATATGT CTCCGAAAGT GGTGATTCAG GACAAAGGGT TGCTGCTTAC   
  
  
+ TTTGCGGATG GGTTAACAGC AAGGATCTTA ACTCAGAGGT CTCCATTTTA TCGCCTGATA CTGGGCAAAC   
  
  
+ CAGCACCTGC TGAAGAGTTT TCAGCTTTTA CTCACTTATA TAGAGCCTCT CCATTCTATC AGTTTGCTCA   
  
  
+ TTTCACAGCC AATCAGGCAA TTGTAGAAGC ATTTGAGAGG GAGGAAGAGA GTAACAACTG GGCTTTGCAT   
  
  
+ GTAATTGATT TGGACATCTT GCATGGTTTC CAGTGGCCTT CTCTTATTCA GTCTCTCTCC GAAGAGGCTA   
  
  
+ CTTGTTCAAA CCGTTCATTG TCTCTTCAAA TTACAGGCTT CGGAAGAAGC TTAGAAGAGC TCATTGAAAC   
  
  
+ AGAAGCTCGG CTGGTAAGTT TCTCTAAGAG CTTTCAGAAT ATCAACTTTG AATTCCACGG GTTTCTGAGA   
  
  
+ GGCTCGAAAC TCAGGAACCT AAGGAGGGAG AATGAAACAG TAGTTGTAAA TCTCGTTTTC CACCTCAGTA   
  
  
+ CTTTGAAAGA TACCGTACAG ATTTCTGACA CCTTGACTTC TGTACATTCA CTAAACCCCT CTATTGTGGT   
  
  
+ ATTAGTCGAG AGAGAAGGAA GTCGAAACAG ATGTGGGTTC CTCTCAAGTT ATGTAGATGC TCTGCATTAT   
  
  
+ TATGCTGCAA TGTTTGACTC TTTAGATGAT TGCCTCCCAC TTGAAAGTCC TGAGAGGCTG AGCATAGAGA   
  
  
+ AGAACCATCT TGGAAAAGAG ATCAAAGAAG CCATAGGTTG TGAGAAGGAT GAAACAAACT ATCTGAAGTT   
  
  
+ TGAGATGCTG GAGACTTGGA GAGGGAGGAT GGAGAGTCAT GGATTTTCAG GTATGAAGCT AAGTTCCAGG   
  
  
+ GCAACTATAC AAGCAAAGCT GCTTTTAAAA ATGGGAAGCC ATTATCATAC CTATTTGGAA GAAGACTGTG   
  
  
+ GAGGTGGTGG GTTCAGAGTT TGTGAACGAG ATGATGGAAT GGCTATCTCT CTTGGCTGGC AAGGTAGGTT   
  
  
+ CCTCGCAACT GCCTCGGTAT GGCGTTCTGT GTG  

- -Up\_Stream \_Len000TGGTTG CTAAGAAATG ACTTAGTCGA TGTAAAAACC TAACACAATT CCATAGAAAA   
  
  
- TCTAGAAGTC CTCCTCCCCC GACTTCATCG AACCAATGAA AACGCGAATA GTTGACACCT TCTTATAATA   
  
  
- ACTCTGATCA CATCTTCTTA ATCATCAAAG GAGGTGTAAC CTTTTTTTGG TACTTTAATT GCGAAATCCG   
  
  
- GGAGTCTCAT GGTTCACAGT AGCATTTACT AACTTTAAAG TTCAGAATCG ACTCAGTACT TCAGGACTAA   
  
  
- AGGGTACTGT TTAACTACAA CAACAGATTT CGAACCGCCC ACTAAACTTT CTTCTAAATC TAGTCCGACT   
  
  
- GTTATGTGGT TTAGACCCTC GTTCCTTGAC CTAAAAAGAC ACAGATCTTT TCGAAAGGCG AAAGACTTGT   
  
  
- TAACTCTTTT GGTACGTATC CTTTAAAGAA CTATAAATCT AGTATCGTCT GAATTGTTAG ACCGAAATGG   
  
  
- CTTGACAGGA GATGAAGATT CAACACTTCC ACACTTTCTA TTTGGTACAG TTTGAATCAC AGGTTGTCTT   
  
  
- TGCGTAGAGA TGAAGAGGTC CGTTTCAACA TGAAGTCTAG AAGATGTGGA AAAGATCCGT CCTGAAACCC   
  
  
- TATATCTCAA ACCGGACCCA CTTTTCATCC TTCACCCCTT CATCTTTTAC TACTTTCTTC CGTGGTGAAC   
  
  
- CCAGGTCTAT GTATCCGGAT GCGTGCTCTT GGAAACGTAC CAGATCTATG TACTGACATA TGAAGAAGGA   
  
  
- AAGGAAATGG ACAACCTCTA ATAGGATCCT AAAATGTACC GTGGTCGACC TAAACAATTA CTGTATTTGT   
  
  
- ATAGAAATTA ATGTCAACGA ATACCATATG TAAATTTGAT AAGCTATCAA CTGTCGACTA CAGGAATAGG   
  
  
- AACACGTCGA ATAAGGAGCT ATCTTATTAG AAACCTGTAA AGACAGATCC ACTATTTCCC GTTTTTGATT   
  
  
- CTATGTTCTT TATCTTGTAC AGGAGGTAAA TGTTCTGCAT AGACAAAAAC CAAAACCAGT TACTAGGGAC   
  
  
- AACAGGAGTT CGTAATTGTG TCTTACTAGT AATAACTGAA CCTGACTTTC CAAAACCCTT GAGGTATCAT   
  
  
- ACGGAAAAGT TATTTTAGTT TTCGTTTAAA CGACAAGTGA CTATTGACGT CAACACAACG AAACGACAAA   
  
  
- GGTATGGTCT GAGCATTGGT TCGACTATCG TTTACACGTT TCCATGTCAT ATTAATTGCT ACAAAAGTAT   
  
  
- AACGATGCTC AAGACTTTGT ACTATATTCT TTTGTGGTCT TGGGTTTATG GATGTATCCA GGATCGGGAC   
  
  
- CTTACCGATA AAATTTATAG GTTTACCTGA ATGTCTTAAG CGTCTCAACA AACCAAACAA ATTGTTAGTA   
  
  
- ACTTAACAAT CGATACTCTT AGACGCCCTG TTAGCATTAG AAACCGCTAA TGAAGCTAAC CGTATGACTA   
  
  
- CAAAAAAATA AACGCATAGA TTCGTACCAC CACATGGAAA CTAAACACAC AGATCCATCA CACACATCTA   
  
  
- TTTATAGTAT CTTGAGTCTG CAACCATCCC AATACAACTC GTGAAAGAGA ACGACAAACA GGAAACACTA   
  
  
- CCGTGGAGAA CGAAAAGGCT TGGTGGTTTT CAGAGTAACT GTTTTGAGAA GTTTGTAACG TTTGACGGGG   
  
  
- GAATTTATAC AAAATAATTT TTTTTACCTT CTCGAGTTTG ACAACTCACT CGGATCTAAG GTTCTAAATC   
  
  
- TTTGTCATTT GAACTATCTA TATAAATATA CTTGATAACA ATCTACGTTG TGGGGGTTGA ACTTTGTGTT   
  
  
- TTCTCTTTAA TTTTTGTTTT TCCAAAACGT GATAAATATA TAGGACACAA GAAACACGAT CAGAATAAAT   
  
  
- TACAATTAAC CATTCGTAAT AAACAACCTA ATAAGGTGAC GTGAAAGAGA ATGATTGAAG TCGTAAAGTA   
  
  
- AGGAACAAGA AGTCGAAAAC TTGAAGAAAT CGGATAAAAC AACAGAAATA CCAATACCTT CGGTAGGTCT   
  
  
- TCTTCTTACT ACTTGAAGAC TCAGAATCGA ACCGGTAACA GCCAGTGAGA CTAGCGTGAC TCTTTTTTGA   
  
  
- CTTCTCCGCC TAACTACAGA GGCTAGGAGA CTAGTGAAGT CCACTTCCGA CGCTTCCCTT CTATTAGTCC   
  
  
- GAGGAGGTTC TCTCTGTCTT GAACAACTTG TAGTTCGTTT CTCCTTTTCC GCAGTAAGTT CTTCCCTTCC   
  
  
- CAGAAGTAGA ATAGGTAGAC GAGGAACATA GACGGTGGAA TTAGTTGCTT TTGTTGTAGT CAAGTCGACA   
  
  
- ACTATTAGAA TAACTCGAGA AGGTTATACA GAGGCTTTCA CCACTAAGTC CTGTTTCCCA ACGACGAATG   
  
  
- AAACGCCTAC CCAATTGTCG TTCCTAGAAT TGAGTCTCCA GAGGTAAAAT AGCGGACTAT GACCCGTTTG   
  
  
- GTCGTGGACG ACTTCTCAAA AGTCGAAAAT GAGTGAATAT ATCTCGGAGA GGTAAGATAG TCAAACGAGT   
  
  
- AAAGTGTCGG TTAGTCCGTT AACATCTTCG TAAACTCTCC CTCCTTCTCT CATTGTTGAC CCGAAACGTA   
  
  
- CATTAACTAA ACCTGTAGAA CGTACCAAAG GTCACCGGAA GAGAATAAGT CAGAGAGAGG CTTCTCCGAT   
  
  
- GAACAAGTTT GGCAAGTAAC AGAGAAGTTT AATGTCCGAA GCCTTCTTCG AATCTTCTCG AGTAACTTTG   
  
  
- TCTTCGAGCC GACCATTCAA AGAGATTCTC GAAAGTCTTA TAGTTGAAAC TTAAGGTGCC CAAAGACTCT   
  
  
- CCGAGCTTTG AGTCCTTGGA TTCCTCCCTC TTACTTTGTC ATCAACATTT AGAGCAAAAG GTGGAGTCAT   
  
  
- GAAACTTTCT ATGGCATGTC TAAAGACTGT GGAACTGAAG ACATGTAAGT GATTTGGGGA GATAACACCA   
  
  
- TAATCAGCTC TCTCTTCCTT CAGCTTTGTC TACACCCAAG GAGAGTTCAA TACATCTACG AGACGTAATA   
  
  
- ATACGACGTT ACAAACTGAG AAATCTACTA ACGGAGGGTG AACTTTCAGG ACTCTCCGAC TCGTATCTCT   
  
  
- TCTTGGTAGA ACCTTTTCTC TAGTTTCTTC GGTATCCAAC ACTCTTCCTA CTTTGTTTGA TAGACTTCAA   
  
  
- ACTCTACGAC CTCTGAACCT CTCCCTCCTA CCTCTCAGTA CCTAAAAGTC CATACTTCGA TTCAAGGTCC   
  
  
- CGTTGATATG TTCGTTTCGA CGAAAATTTT TACCCTTCGG TAATAGTATG GATAAACCTT CTTCTGACAC   
  
  
- CTCCACCACC CAAGTCTCAA ACACTTGCTC TACTACCTTA CCGATAGAGA GAACCGACCG TTCCATCCAA   
  
  
- GGAGCGTTGA CGGAGCCATA CCGCAAGACA CAC

+     AT~TATA-box

| Site Name | Organism | Position | Strand | Matrix score. | sequence | function |
| --- | --- | --- | --- | --- | --- | --- |
| AT~TATA-box | Arabidopsis thaliana | 2491 | - | 6 | TATATA |  |
| AT~TATA-box | Arabidopsis thaliana | 1860 | - | 6 | TATATA |  |
| AT~TATA-box | Arabidopsis thaliana | 1858 | - | 8 | TATATAAA |  |

>HU07G00272.1   
+ -Up\_Stream \_Len000ACCAAC GATTCTTTAC TGAATCAGCT ACATTTTTGG ATTGTGTTAA GGTATCTTTT   
  
  
+ AGATCTTCAG GAGGAGGGGG CTGAAGTAGC TTGGTTACTT TTGCGCTTAT CAACTGTGGA AGAATATTAT   
  
  
+ TGAGACTAGT GTAGAAGAAT TAGTAGTTTC CTCCACATTG GAAAAAAACC ATGAAATTAA CGCTTTAGGC   
  
  
+ CCTCAGAGTA CCAAGTGTCA TCGTAAATGA TTGAAATTTC AAGTCTTAGC TGAGTCATGA AGTCCTGATT   
  
  
+ TCCCATGACA AATTGATGTT GTTGTCTAAA GCTTGGCGGG TGATTTGAAA GAAGATTTAG ATCAGGCTGA   
  
  
+ CAATACACCA AATCTGGGAG CAAGGAACTG GATTTTTCTG TGTCTAGAAA AGCTTTCCGC TTTCTGAACA   
  
  
+ ATTGAGAAAA CCATGCATAG GAAATTTCTT GATATTTAGA TCATAGCAGA CTTAACAATC TGGCTTTACC   
  
  
+ GAACTGTCCT CTACTTCTAA GTTGTGAAGG TGTGAAAGAT AAACCATGTC AAACTTAGTG TCCAACAGAA   
  
  
+ ACGCATCTCT ACTTCTCCAG GCAAAGTTGT ACTTCAGATC TTCTACACCT TTTCTAGGCA GGACTTTGGG   
  
  
+ ATATAGAGTT TGGCCTGGGT GAAAAGTAGG AAGTGGGGAA GTAGAAAATG ATGAAAGAAG GCACCACTTG   
  
  
+ GGTCCAGATA CATAGGCCTA CGCACGAGAA CCTTTGCATG GTCTAGATAC ATGACTGTAT ACTTCTTCCT   
  
  
+ TTCCTTTACC TGTTGGAGAT TATCCTAGGA TTTTACATGG CACCAGCTGG ATTTGTTAAT GACATAAACA   
  
  
+ TATCTTTAAT TACAGTTGCT TATGGTATAC ATTTAAACTA TTCGATAGTT GACAGCTGAT GTCCTTATCC   
  
  
+ TTGTGCAGCT TATTCCTCGA TAGAATAATC TTTGGACATT TCTGTCTAGG TGATAAAGGG CAAAAACTAA   
  
  
+ GATACAAGAA ATAGAACATG TCCTCCATTT ACAAGACGTA TCTGTTTTTG GTTTTGGTCA ATGATCCCTG   
  
  
+ TTGTCCTCAA GCATTAACAC AGAATGATCA TTATTGACTT GGACTGAAAG GTTTTGGGAA CTCCATAGTA   
  
  
+ TGCCTTTTCA ATAAAATCAA AAGCAAATTT GCTGTTCACT GATAACTGCA GTTGTGTTGC TTTGCTGTTT   
  
  
+ CCATACCAGA CTCGTAACCA AGCTGATAGC AAATGTGCAA AGGTACAGTA TAATTAACGA TGTTTTCATA   
  
  
+ TTGCTACGAG TTCTGAAACA TGATATAAGA AAACACCAGA ACCCAAATAC CTACATAGGT CCTAGCCCTG   
  
  
+ GAATGGCTAT TTTAAATATC CAAATGGACT TACAGAATTC GCAGAGTTGT TTGGTTTGTT TAACAATCAT   
  
  
+ TGAATTGTTA GCTATGAGAA TCTGCGGGAC AATCGTAATC TTTGGCGATT ACTTCGATTG GCATACTGAT   
  
  
+ GTTTTTTTAT TTGCGTATCT AAGCATGGTG GTGTACCTTT GATTTGTGTG TCTAGGTAGT GTGTGTAGAT   
  
  
+ AAATATCATA GAACTCAGAC GTTGGTAGGG TTATGTTGAG CACTTTCTCT TGCTGTTTGT CCTTTGTGAT   
  
  
+ GGCACCTCTT GCTTTTCCGA ACCACCAAAA GTCTCATTGA CAAAACTCTT CAAACATTGC AAACTGCCCC   
  
  
+ CTTAAATATG TTTTATTAAA AAAAATGGAA GAGCTCAAAC TGTTGAGTGA GCCTAGATTC CAAGATTTAG   
  
  
+ AAACAGTAAA CTTGATAGAT ATATTTATAT GAACTATTGT TAGATGCAAC ACCCCCAACT TGAAACACAA   
  
  
+ AAGAGAAATT AAAAACAAAA AGGTTTTGCA CTATTTATAT ATCCTGTGTT CTTTGTGCTA GTCTTATTTA   
  
  
+ ATGTTAATTG GTAAGCATTA TTTGTTGGAT TATTCCACTG CACTTTCTCT TACTAACTTC AGCATTTCAT   
  
  
+ TCCTTGTTCT TCAGCTTTTG AACTTCTTTA GCCTATTTTG TTGTCTTTAT GGTTATGGAA GCCATCCAGA   
  
  
+ AGAAGAATGA TGAACTTCTG AGTCTTAGCT TGGCCATTGT CGGTCACTCT GATCGCACTG AGAAAAAACT   
  
  
+ GAAGAGGCGG ATTGATGTCT CCGATCCTCT GATCACTTCA GGTGAAGGCT GCGAAGGGAA GATAATCAGG   
  
  
+ CTCCTCCAAG AGAGACAGAA CTTGTTGAAC ATCAAGCAAA GAGGAAAAGG CGTCATTCAA GAAGGGAAGG   
  
  
+ GTCTTCATCT TATCCATCTG CTCCTTGTAT CTGCCACCTT AATCAACGAA AACAACATCA GTTCAGCTGT   
  
  
+ TGATAATCTT ATTGAGCTCT TCCAATATGT CTCCGAAAGT GGTGATTCAG GACAAAGGGT TGCTGCTTAC   
  
  
+ TTTGCGGATG GGTTAACAGC AAGGATCTTA ACTCAGAGGT CTCCATTTTA TCGCCTGATA CTGGGCAAAC   
  
  
+ CAGCACCTGC TGAAGAGTTT TCAGCTTTTA CTCACTTATA TAGAGCCTCT CCATTCTATC AGTTTGCTCA   
  
  
+ TTTCACAGCC AATCAGGCAA TTGTAGAAGC ATTTGAGAGG GAGGAAGAGA GTAACAACTG GGCTTTGCAT   
  
  
+ GTAATTGATT TGGACATCTT GCATGGTTTC CAGTGGCCTT CTCTTATTCA GTCTCTCTCC GAAGAGGCTA   
  
  
+ CTTGTTCAAA CCGTTCATTG TCTCTTCAAA TTACAGGCTT CGGAAGAAGC TTAGAAGAGC TCATTGAAAC   
  
  
+ AGAAGCTCGG CTGGTAAGTT TCTCTAAGAG CTTTCAGAAT ATCAACTTTG AATTCCACGG GTTTCTGAGA   
  
  
+ GGCTCGAAAC TCAGGAACCT AAGGAGGGAG AATGAAACAG TAGTTGTAAA TCTCGTTTTC CACCTCAGTA   
  
  
+ CTTTGAAAGA TACCGTACAG ATTTCTGACA CCTTGACTTC TGTACATTCA CTAAACCCCT CTATTGTGGT   
  
  
+ ATTAGTCGAG AGAGAAGGAA GTCGAAACAG ATGTGGGTTC CTCTCAAGTT ATGTAGATGC TCTGCATTAT   
  
  
+ TATGCTGCAA TGTTTGACTC TTTAGATGAT TGCCTCCCAC TTGAAAGTCC TGAGAGGCTG AGCATAGAGA   
  
  
+ AGAACCATCT TGGAAAAGAG ATCAAAGAAG CCATAGGTTG TGAGAAGGAT GAAACAAACT ATCTGAAGTT   
  
  
+ TGAGATGCTG GAGACTTGGA GAGGGAGGAT GGAGAGTCAT GGATTTTCAG GTATGAAGCT AAGTTCCAGG   
  
  
+ GCAACTATAC AAGCAAAGCT GCTTTTAAAA ATGGGAAGCC ATTATCATAC CTATTTGGAA GAAGACTGTG   
  
  
+ GAGGTGGTGG GTTCAGAGTT TGTGAACGAG ATGATGGAAT GGCTATCTCT CTTGGCTGGC AAGGTAGGTT   
  
  
+ CCTCGCAACT GCCTCGGTAT GGCGTTCTGT GTG  

- -Up\_Stream \_Len000TGGTTG CTAAGAAATG ACTTAGTCGA TGTAAAAACC TAACACAATT CCATAGAAAA   
  
  
- TCTAGAAGTC CTCCTCCCCC GACTTCATCG AACCAATGAA AACGCGAATA GTTGACACCT TCTTATAATA   
  
  
- ACTCTGATCA CATCTTCTTA ATCATCAAAG GAGGTGTAAC CTTTTTTTGG TACTTTAATT GCGAAATCCG   
  
  
- GGAGTCTCAT GGTTCACAGT AGCATTTACT AACTTTAAAG TTCAGAATCG ACTCAGTACT TCAGGACTAA   
  
  
- AGGGTACTGT TTAACTACAA CAACAGATTT CGAACCGCCC ACTAAACTTT CTTCTAAATC TAGTCCGACT   
  
  
- GTTATGTGGT TTAGACCCTC GTTCCTTGAC CTAAAAAGAC ACAGATCTTT TCGAAAGGCG AAAGACTTGT   
  
  
- TAACTCTTTT GGTACGTATC CTTTAAAGAA CTATAAATCT AGTATCGTCT GAATTGTTAG ACCGAAATGG   
  
  
- CTTGACAGGA GATGAAGATT CAACACTTCC ACACTTTCTA TTTGGTACAG TTTGAATCAC AGGTTGTCTT   
  
  
- TGCGTAGAGA TGAAGAGGTC CGTTTCAACA TGAAGTCTAG AAGATGTGGA AAAGATCCGT CCTGAAACCC   
  
  
- TATATCTCAA ACCGGACCCA CTTTTCATCC TTCACCCCTT CATCTTTTAC TACTTTCTTC CGTGGTGAAC   
  
  
- CCAGGTCTAT GTATCCGGAT GCGTGCTCTT GGAAACGTAC CAGATCTATG TACTGACATA TGAAGAAGGA   
  
  
- AAGGAAATGG ACAACCTCTA ATAGGATCCT AAAATGTACC GTGGTCGACC TAAACAATTA CTGTATTTGT   
  
  
- ATAGAAATTA ATGTCAACGA ATACCATATG TAAATTTGAT AAGCTATCAA CTGTCGACTA CAGGAATAGG   
  
  
- AACACGTCGA ATAAGGAGCT ATCTTATTAG AAACCTGTAA AGACAGATCC ACTATTTCCC GTTTTTGATT   
  
  
- CTATGTTCTT TATCTTGTAC AGGAGGTAAA TGTTCTGCAT AGACAAAAAC CAAAACCAGT TACTAGGGAC   
  
  
- AACAGGAGTT CGTAATTGTG TCTTACTAGT AATAACTGAA CCTGACTTTC CAAAACCCTT GAGGTATCAT   
  
  
- ACGGAAAAGT TATTTTAGTT TTCGTTTAAA CGACAAGTGA CTATTGACGT CAACACAACG AAACGACAAA   
  
  
- GGTATGGTCT GAGCATTGGT TCGACTATCG TTTACACGTT TCCATGTCAT ATTAATTGCT ACAAAAGTAT   
  
  
- AACGATGCTC AAGACTTTGT ACTATATTCT TTTGTGGTCT TGGGTTTATG GATGTATCCA GGATCGGGAC   
  
  
- CTTACCGATA AAATTTATAG GTTTACCTGA ATGTCTTAAG CGTCTCAACA AACCAAACAA ATTGTTAGTA   
  
  
- ACTTAACAAT CGATACTCTT AGACGCCCTG TTAGCATTAG AAACCGCTAA TGAAGCTAAC CGTATGACTA   
  
  
- CAAAAAAATA AACGCATAGA TTCGTACCAC CACATGGAAA CTAAACACAC AGATCCATCA CACACATCTA   
  
  
- TTTATAGTAT CTTGAGTCTG CAACCATCCC AATACAACTC GTGAAAGAGA ACGACAAACA GGAAACACTA   
  
  
- CCGTGGAGAA CGAAAAGGCT TGGTGGTTTT CAGAGTAACT GTTTTGAGAA GTTTGTAACG TTTGACGGGG   
  
  
- GAATTTATAC AAAATAATTT TTTTTACCTT CTCGAGTTTG ACAACTCACT CGGATCTAAG GTTCTAAATC   
  
  
- TTTGTCATTT GAACTATCTA TATAAATATA CTTGATAACA ATCTACGTTG TGGGGGTTGA ACTTTGTGTT   
  
  
- TTCTCTTTAA TTTTTGTTTT TCCAAAACGT GATAAATATA TAGGACACAA GAAACACGAT CAGAATAAAT   
  
  
- TACAATTAAC CATTCGTAAT AAACAACCTA ATAAGGTGAC GTGAAAGAGA ATGATTGAAG TCGTAAAGTA   
  
  
- AGGAACAAGA AGTCGAAAAC TTGAAGAAAT CGGATAAAAC AACAGAAATA CCAATACCTT CGGTAGGTCT   
  
  
- TCTTCTTACT ACTTGAAGAC TCAGAATCGA ACCGGTAACA GCCAGTGAGA CTAGCGTGAC TCTTTTTTGA   
  
  
- CTTCTCCGCC TAACTACAGA GGCTAGGAGA CTAGTGAAGT CCACTTCCGA CGCTTCCCTT CTATTAGTCC   
  
  
- GAGGAGGTTC TCTCTGTCTT GAACAACTTG TAGTTCGTTT CTCCTTTTCC GCAGTAAGTT CTTCCCTTCC   
  
  
- CAGAAGTAGA ATAGGTAGAC GAGGAACATA GACGGTGGAA TTAGTTGCTT TTGTTGTAGT CAAGTCGACA   
  
  
- ACTATTAGAA TAACTCGAGA AGGTTATACA GAGGCTTTCA CCACTAAGTC CTGTTTCCCA ACGACGAATG   
  
  
- AAACGCCTAC CCAATTGTCG TTCCTAGAAT TGAGTCTCCA GAGGTAAAAT AGCGGACTAT GACCCGTTTG   
  
  
- GTCGTGGACG ACTTCTCAAA AGTCGAAAAT GAGTGAATAT ATCTCGGAGA GGTAAGATAG TCAAACGAGT   
  
  
- AAAGTGTCGG TTAGTCCGTT AACATCTTCG TAAACTCTCC CTCCTTCTCT CATTGTTGAC CCGAAACGTA   
  
  
- CATTAACTAA ACCTGTAGAA CGTACCAAAG GTCACCGGAA GAGAATAAGT CAGAGAGAGG CTTCTCCGAT   
  
  
- GAACAAGTTT GGCAAGTAAC AGAGAAGTTT AATGTCCGAA GCCTTCTTCG AATCTTCTCG AGTAACTTTG   
  
  
- TCTTCGAGCC GACCATTCAA AGAGATTCTC GAAAGTCTTA TAGTTGAAAC TTAAGGTGCC CAAAGACTCT   
  
  
- CCGAGCTTTG AGTCCTTGGA TTCCTCCCTC TTACTTTGTC ATCAACATTT AGAGCAAAAG GTGGAGTCAT   
  
  
- GAAACTTTCT ATGGCATGTC TAAAGACTGT GGAACTGAAG ACATGTAAGT GATTTGGGGA GATAACACCA   
  
  
- TAATCAGCTC TCTCTTCCTT CAGCTTTGTC TACACCCAAG GAGAGTTCAA TACATCTACG AGACGTAATA   
  
  
- ATACGACGTT ACAAACTGAG AAATCTACTA ACGGAGGGTG AACTTTCAGG ACTCTCCGAC TCGTATCTCT   
  
  
- TCTTGGTAGA ACCTTTTCTC TAGTTTCTTC GGTATCCAAC ACTCTTCCTA CTTTGTTTGA TAGACTTCAA   
  
  
- ACTCTACGAC CTCTGAACCT CTCCCTCCTA CCTCTCAGTA CCTAAAAGTC CATACTTCGA TTCAAGGTCC   
  
  
- CGTTGATATG TTCGTTTCGA CGAAAATTTT TACCCTTCGG TAATAGTATG GATAAACCTT CTTCTGACAC   
  
  
- CTCCACCACC CAAGTCTCAA ACACTTGCTC TACTACCTTA CCGATAGAGA GAACCGACCG TTCCATCCAA   
  
  
- GGAGCGTTGA CGGAGCCATA CCGCAAGACA CAC

+     CAAT-box

| Site Name | Organism | Position | Strand | Matrix score. | sequence | function |
| --- | --- | --- | --- | --- | --- | --- |
| CAAT-box | Nicotiana glutinosa | 3043 | - | 4 | CAAT |  |
| CAAT-box | Nicotiana glutinosa | 2937 | - | 4 | CAAT |  |
| CAAT-box | Nicotiana glutinosa | 2544 | - | 4 | CAAT |  |
| CAAT-box | Pisum sativum | 2691 | + | 5 | CAAAT | common cis-acting element in promoter and enhancer regions |
| CAAT-box | Nicotiana glutinosa | 2681 | - | 4 | CAAT |  |
| CAAT-box | Nicotiana glutinosa | 2534 | + | 4 | CAAT |  |
| CAAT-box | Nicotiana glutinosa | 1264 | - | 4 | CAAT |  |
| CAAT-box | Nicotiana glutinosa | 2598 | - | 4 | CAAT |  |
| CAAT-box | Nicotiana glutinosa | 2727 | - | 4 | CAAT |  |
| CAAT-box | Nicotiana glutinosa | 2325 | - | 4 | CAAT |  |
| CAAT-box | Nicotiana glutinosa | 2070 | - | 4 | CAAT |  |
| CAAT-box | Nicotiana glutinosa | 355 | + | 4 | CAAT |  |
| CAAT-box | Nicotiana glutinosa | 143 | - | 4 | CAAT |  |
| CAAT-box | Pisum sativum | 327 | - | 5 | CAAAT | common cis-acting element in promoter and enhancer regions |
| CAAT-box | Nicotiana glutinosa | 296 | - | 4 | CAAT |  |
| CAAT-box | Nicotiana glutinosa | 1043 | + | 4 | CAAT |  |
| CAAT-box | Pisum sativum | 2602 | - | 5 | CAAAT | common cis-acting element in promoter and enhancer regions |
| CAAT-box | Nicotiana glutinosa | 480 | + | 4 | CAAT |  |
| CAAT-box | Nicotiana glutinosa | 425 | - | 4 | CAAT |  |
| CAAT-box | Pisum sativum | 1516 | - | 5 | CAAAT | common cis-acting element in promoter and enhancer regions |
| CAAT-box | Nicotiana glutinosa | 2542 | + | 4 | CAAT |  |
| CAAT-box | Pisum sativum | 293 | + | 5 | CAAAT | common cis-acting element in promoter and enhancer regions |
| CAAT-box | Nicotiana glutinosa | 55 | - | 4 | CAAT |  |
| CAAT-box | Arabidopsis thaliana | 181 | - | 5 | CCAAT | common cis-acting element in promoter and enhancer regions |
| CAAT-box | Arabidopsis thaliana | 2336 | + | 5 | CCAAT | common cis-acting element in promoter and enhancer regions |
| CAAT-box | Nicotiana glutinosa | 1790 | - | 4 | CAAT |  |
| CAAT-box | Nicotiana glutinosa | 1133 | + | 4 | CAAT |  |
| CAAT-box | Arabidopsis thaliana | 1461 | - | 5 | CCAAT | common cis-acting element in promoter and enhancer regions |
| CAAT-box | Nicotiana glutinosa | 1434 | + | 4 | CAAT |  |
| CAAT-box | Nicotiana glutinosa | 244 | - | 4 | CAAT |  |
| CAAT-box | Pisum sativum | 3277 | - | 5 | CAAAT | common cis-acting element in promoter and enhancer regions |
| CAAT-box | Pisum sativum | 825 | - | 5 | CAAAT | common cis-acting element in promoter and enhancer regions |
| CAAT-box | Pisum sativum | 1151 | - | 5 | CAAAT | common cis-acting element in promoter and enhancer regions |
| CAAT-box | Nicotiana glutinosa | 1670 | - | 4 | CAAT |  |
| CAAT-box | Nicotiana glutinosa | 1650 | - | 4 | CAAT |  |
| CAAT-box | Nicotiana glutinosa | 3022 | + | 4 | CAAT |  |
| CAAT-box | Nicotiana glutinosa | 423 | + | 4 | CAAT |  |
| CAAT-box | Pisum sativum | 363 | + | 5 | CAAAT | common cis-acting element in promoter and enhancer regions |
| CAAT-box | Pisum sativum | 1308 | + | 5 | CAAAT | common cis-acting element in promoter and enhancer regions |
| CAAT-box | Nicotiana glutinosa | 1398 | + | 4 | CAAT |  |
| CAAT-box | Nicotiana glutinosa | 2337 | + | 4 | CAAT |  |
| CAAT-box | Nicotiana glutinosa | 1087 | - | 4 | CAAT |  |
| CAAT-box | Arabidopsis thaliana | 2533 | + | 5 | CCAAT | common cis-acting element in promoter and enhancer regions |
| CAAT-box | Pisum sativum | 1483 | - | 5 | CAAAT | common cis-acting element in promoter and enhancer regions |
| CAAT-box | Pisum sativum | 2555 | - | 5 | CAAAT | common cis-acting element in promoter and enhancer regions |
| CAAT-box | Nicotiana glutinosa | 1403 | - | 4 | CAAT |  |
| CAAT-box | Arabidopsis thaliana | 1901 | - | 5 | CCAAT | common cis-acting element in promoter and enhancer regions |
| CAAT-box | Pisum sativum | 1224 | + | 5 | CAAAT | common cis-acting element in promoter and enhancer regions |
| CAAT-box | Nicotiana glutinosa | 2115 | - | 4 | CAAT |  |
| CAAT-box | Pisum sativum | 1148 | + | 5 | CAAAT | common cis-acting element in promoter and enhancer regions |
| CAAT-box | Pisum sativum | 1914 | - | 5 | CAAAT | common cis-acting element in promoter and enhancer regions |
| CAAT-box | Nicotiana glutinosa | 1408 | - | 4 | CAAT |  |
| CAAT-box | Pisum sativum | 1355 | + | 5 | CAAAT | common cis-acting element in promoter and enhancer regions |

>HU07G00272.1   
+ -Up\_Stream \_Len000ACCAAC GATTCTTTAC TGAATCAGCT ACATTTTTGG ATTGTGTTAA GGTATCTTTT   
  
  
+ AGATCTTCAG GAGGAGGGGG CTGAAGTAGC TTGGTTACTT TTGCGCTTAT CAACTGTGGA AGAATATTAT   
  
  
+ TGAGACTAGT GTAGAAGAAT TAGTAGTTTC CTCCACATTG GAAAAAAACC ATGAAATTAA CGCTTTAGGC   
  
  
+ CCTCAGAGTA CCAAGTGTCA TCGTAAATGA TTGAAATTTC AAGTCTTAGC TGAGTCATGA AGTCCTGATT   
  
  
+ TCCCATGACA AATTGATGTT GTTGTCTAAA GCTTGGCGGG TGATTTGAAA GAAGATTTAG ATCAGGCTGA   
  
  
+ CAATACACCA AATCTGGGAG CAAGGAACTG GATTTTTCTG TGTCTAGAAA AGCTTTCCGC TTTCTGAACA   
  
  
+ ATTGAGAAAA CCATGCATAG GAAATTTCTT GATATTTAGA TCATAGCAGA CTTAACAATC TGGCTTTACC   
  
  
+ GAACTGTCCT CTACTTCTAA GTTGTGAAGG TGTGAAAGAT AAACCATGTC AAACTTAGTG TCCAACAGAA   
  
  
+ ACGCATCTCT ACTTCTCCAG GCAAAGTTGT ACTTCAGATC TTCTACACCT TTTCTAGGCA GGACTTTGGG   
  
  
+ ATATAGAGTT TGGCCTGGGT GAAAAGTAGG AAGTGGGGAA GTAGAAAATG ATGAAAGAAG GCACCACTTG   
  
  
+ GGTCCAGATA CATAGGCCTA CGCACGAGAA CCTTTGCATG GTCTAGATAC ATGACTGTAT ACTTCTTCCT   
  
  
+ TTCCTTTACC TGTTGGAGAT TATCCTAGGA TTTTACATGG CACCAGCTGG ATTTGTTAAT GACATAAACA   
  
  
+ TATCTTTAAT TACAGTTGCT TATGGTATAC ATTTAAACTA TTCGATAGTT GACAGCTGAT GTCCTTATCC   
  
  
+ TTGTGCAGCT TATTCCTCGA TAGAATAATC TTTGGACATT TCTGTCTAGG TGATAAAGGG CAAAAACTAA   
  
  
+ GATACAAGAA ATAGAACATG TCCTCCATTT ACAAGACGTA TCTGTTTTTG GTTTTGGTCA ATGATCCCTG   
  
  
+ TTGTCCTCAA GCATTAACAC AGAATGATCA TTATTGACTT GGACTGAAAG GTTTTGGGAA CTCCATAGTA   
  
  
+ TGCCTTTTCA ATAAAATCAA AAGCAAATTT GCTGTTCACT GATAACTGCA GTTGTGTTGC TTTGCTGTTT   
  
  
+ CCATACCAGA CTCGTAACCA AGCTGATAGC AAATGTGCAA AGGTACAGTA TAATTAACGA TGTTTTCATA   
  
  
+ TTGCTACGAG TTCTGAAACA TGATATAAGA AAACACCAGA ACCCAAATAC CTACATAGGT CCTAGCCCTG   
  
  
+ GAATGGCTAT TTTAAATATC CAAATGGACT TACAGAATTC GCAGAGTTGT TTGGTTTGTT TAACAATCAT   
  
  
+ TGAATTGTTA GCTATGAGAA TCTGCGGGAC AATCGTAATC TTTGGCGATT ACTTCGATTG GCATACTGAT   
  
  
+ GTTTTTTTAT TTGCGTATCT AAGCATGGTG GTGTACCTTT GATTTGTGTG TCTAGGTAGT GTGTGTAGAT   
  
  
+ AAATATCATA GAACTCAGAC GTTGGTAGGG TTATGTTGAG CACTTTCTCT TGCTGTTTGT CCTTTGTGAT   
  
  
+ GGCACCTCTT GCTTTTCCGA ACCACCAAAA GTCTCATTGA CAAAACTCTT CAAACATTGC AAACTGCCCC   
  
  
+ CTTAAATATG TTTTATTAAA AAAAATGGAA GAGCTCAAAC TGTTGAGTGA GCCTAGATTC CAAGATTTAG   
  
  
+ AAACAGTAAA CTTGATAGAT ATATTTATAT GAACTATTGT TAGATGCAAC ACCCCCAACT TGAAACACAA   
  
  
+ AAGAGAAATT AAAAACAAAA AGGTTTTGCA CTATTTATAT ATCCTGTGTT CTTTGTGCTA GTCTTATTTA   
  
  
+ ATGTTAATTG GTAAGCATTA TTTGTTGGAT TATTCCACTG CACTTTCTCT TACTAACTTC AGCATTTCAT   
  
  
+ TCCTTGTTCT TCAGCTTTTG AACTTCTTTA GCCTATTTTG TTGTCTTTAT GGTTATGGAA GCCATCCAGA   
  
  
+ AGAAGAATGA TGAACTTCTG AGTCTTAGCT TGGCCATTGT CGGTCACTCT GATCGCACTG AGAAAAAACT   
  
  
+ GAAGAGGCGG ATTGATGTCT CCGATCCTCT GATCACTTCA GGTGAAGGCT GCGAAGGGAA GATAATCAGG   
  
  
+ CTCCTCCAAG AGAGACAGAA CTTGTTGAAC ATCAAGCAAA GAGGAAAAGG CGTCATTCAA GAAGGGAAGG   
  
  
+ GTCTTCATCT TATCCATCTG CTCCTTGTAT CTGCCACCTT AATCAACGAA AACAACATCA GTTCAGCTGT   
  
  
+ TGATAATCTT ATTGAGCTCT TCCAATATGT CTCCGAAAGT GGTGATTCAG GACAAAGGGT TGCTGCTTAC   
  
  
+ TTTGCGGATG GGTTAACAGC AAGGATCTTA ACTCAGAGGT CTCCATTTTA TCGCCTGATA CTGGGCAAAC   
  
  
+ CAGCACCTGC TGAAGAGTTT TCAGCTTTTA CTCACTTATA TAGAGCCTCT CCATTCTATC AGTTTGCTCA   
  
  
+ TTTCACAGCC AATCAGGCAA TTGTAGAAGC ATTTGAGAGG GAGGAAGAGA GTAACAACTG GGCTTTGCAT   
  
  
+ GTAATTGATT TGGACATCTT GCATGGTTTC CAGTGGCCTT CTCTTATTCA GTCTCTCTCC GAAGAGGCTA   
  
  
+ CTTGTTCAAA CCGTTCATTG TCTCTTCAAA TTACAGGCTT CGGAAGAAGC TTAGAAGAGC TCATTGAAAC   
  
  
+ AGAAGCTCGG CTGGTAAGTT TCTCTAAGAG CTTTCAGAAT ATCAACTTTG AATTCCACGG GTTTCTGAGA   
  
  
+ GGCTCGAAAC TCAGGAACCT AAGGAGGGAG AATGAAACAG TAGTTGTAAA TCTCGTTTTC CACCTCAGTA   
  
  
+ CTTTGAAAGA TACCGTACAG ATTTCTGACA CCTTGACTTC TGTACATTCA CTAAACCCCT CTATTGTGGT   
  
  
+ ATTAGTCGAG AGAGAAGGAA GTCGAAACAG ATGTGGGTTC CTCTCAAGTT ATGTAGATGC TCTGCATTAT   
  
  
+ TATGCTGCAA TGTTTGACTC TTTAGATGAT TGCCTCCCAC TTGAAAGTCC TGAGAGGCTG AGCATAGAGA   
  
  
+ AGAACCATCT TGGAAAAGAG ATCAAAGAAG CCATAGGTTG TGAGAAGGAT GAAACAAACT ATCTGAAGTT   
  
  
+ TGAGATGCTG GAGACTTGGA GAGGGAGGAT GGAGAGTCAT GGATTTTCAG GTATGAAGCT AAGTTCCAGG   
  
  
+ GCAACTATAC AAGCAAAGCT GCTTTTAAAA ATGGGAAGCC ATTATCATAC CTATTTGGAA GAAGACTGTG   
  
  
+ GAGGTGGTGG GTTCAGAGTT TGTGAACGAG ATGATGGAAT GGCTATCTCT CTTGGCTGGC AAGGTAGGTT   
  
  
+ CCTCGCAACT GCCTCGGTAT GGCGTTCTGT GTG  

- -Up\_Stream \_Len000TGGTTG CTAAGAAATG ACTTAGTCGA TGTAAAAACC TAACACAATT CCATAGAAAA   
  
  
- TCTAGAAGTC CTCCTCCCCC GACTTCATCG AACCAATGAA AACGCGAATA GTTGACACCT TCTTATAATA   
  
  
- ACTCTGATCA CATCTTCTTA ATCATCAAAG GAGGTGTAAC CTTTTTTTGG TACTTTAATT GCGAAATCCG   
  
  
- GGAGTCTCAT GGTTCACAGT AGCATTTACT AACTTTAAAG TTCAGAATCG ACTCAGTACT TCAGGACTAA   
  
  
- AGGGTACTGT TTAACTACAA CAACAGATTT CGAACCGCCC ACTAAACTTT CTTCTAAATC TAGTCCGACT   
  
  
- GTTATGTGGT TTAGACCCTC GTTCCTTGAC CTAAAAAGAC ACAGATCTTT TCGAAAGGCG AAAGACTTGT   
  
  
- TAACTCTTTT GGTACGTATC CTTTAAAGAA CTATAAATCT AGTATCGTCT GAATTGTTAG ACCGAAATGG   
  
  
- CTTGACAGGA GATGAAGATT CAACACTTCC ACACTTTCTA TTTGGTACAG TTTGAATCAC AGGTTGTCTT   
  
  
- TGCGTAGAGA TGAAGAGGTC CGTTTCAACA TGAAGTCTAG AAGATGTGGA AAAGATCCGT CCTGAAACCC   
  
  
- TATATCTCAA ACCGGACCCA CTTTTCATCC TTCACCCCTT CATCTTTTAC TACTTTCTTC CGTGGTGAAC   
  
  
- CCAGGTCTAT GTATCCGGAT GCGTGCTCTT GGAAACGTAC CAGATCTATG TACTGACATA TGAAGAAGGA   
  
  
- AAGGAAATGG ACAACCTCTA ATAGGATCCT AAAATGTACC GTGGTCGACC TAAACAATTA CTGTATTTGT   
  
  
- ATAGAAATTA ATGTCAACGA ATACCATATG TAAATTTGAT AAGCTATCAA CTGTCGACTA CAGGAATAGG   
  
  
- AACACGTCGA ATAAGGAGCT ATCTTATTAG AAACCTGTAA AGACAGATCC ACTATTTCCC GTTTTTGATT   
  
  
- CTATGTTCTT TATCTTGTAC AGGAGGTAAA TGTTCTGCAT AGACAAAAAC CAAAACCAGT TACTAGGGAC   
  
  
- AACAGGAGTT CGTAATTGTG TCTTACTAGT AATAACTGAA CCTGACTTTC CAAAACCCTT GAGGTATCAT   
  
  
- ACGGAAAAGT TATTTTAGTT TTCGTTTAAA CGACAAGTGA CTATTGACGT CAACACAACG AAACGACAAA   
  
  
- GGTATGGTCT GAGCATTGGT TCGACTATCG TTTACACGTT TCCATGTCAT ATTAATTGCT ACAAAAGTAT   
  
  
- AACGATGCTC AAGACTTTGT ACTATATTCT TTTGTGGTCT TGGGTTTATG GATGTATCCA GGATCGGGAC   
  
  
- CTTACCGATA AAATTTATAG GTTTACCTGA ATGTCTTAAG CGTCTCAACA AACCAAACAA ATTGTTAGTA   
  
  
- ACTTAACAAT CGATACTCTT AGACGCCCTG TTAGCATTAG AAACCGCTAA TGAAGCTAAC CGTATGACTA   
  
  
- CAAAAAAATA AACGCATAGA TTCGTACCAC CACATGGAAA CTAAACACAC AGATCCATCA CACACATCTA   
  
  
- TTTATAGTAT CTTGAGTCTG CAACCATCCC AATACAACTC GTGAAAGAGA ACGACAAACA GGAAACACTA   
  
  
- CCGTGGAGAA CGAAAAGGCT TGGTGGTTTT CAGAGTAACT GTTTTGAGAA GTTTGTAACG TTTGACGGGG   
  
  
- GAATTTATAC AAAATAATTT TTTTTACCTT CTCGAGTTTG ACAACTCACT CGGATCTAAG GTTCTAAATC   
  
  
- TTTGTCATTT GAACTATCTA TATAAATATA CTTGATAACA ATCTACGTTG TGGGGGTTGA ACTTTGTGTT   
  
  
- TTCTCTTTAA TTTTTGTTTT TCCAAAACGT GATAAATATA TAGGACACAA GAAACACGAT CAGAATAAAT   
  
  
- TACAATTAAC CATTCGTAAT AAACAACCTA ATAAGGTGAC GTGAAAGAGA ATGATTGAAG TCGTAAAGTA   
  
  
- AGGAACAAGA AGTCGAAAAC TTGAAGAAAT CGGATAAAAC AACAGAAATA CCAATACCTT CGGTAGGTCT   
  
  
- TCTTCTTACT ACTTGAAGAC TCAGAATCGA ACCGGTAACA GCCAGTGAGA CTAGCGTGAC TCTTTTTTGA   
  
  
- CTTCTCCGCC TAACTACAGA GGCTAGGAGA CTAGTGAAGT CCACTTCCGA CGCTTCCCTT CTATTAGTCC   
  
  
- GAGGAGGTTC TCTCTGTCTT GAACAACTTG TAGTTCGTTT CTCCTTTTCC GCAGTAAGTT CTTCCCTTCC   
  
  
- CAGAAGTAGA ATAGGTAGAC GAGGAACATA GACGGTGGAA TTAGTTGCTT TTGTTGTAGT CAAGTCGACA   
  
  
- ACTATTAGAA TAACTCGAGA AGGTTATACA GAGGCTTTCA CCACTAAGTC CTGTTTCCCA ACGACGAATG   
  
  
- AAACGCCTAC CCAATTGTCG TTCCTAGAAT TGAGTCTCCA GAGGTAAAAT AGCGGACTAT GACCCGTTTG   
  
  
- GTCGTGGACG ACTTCTCAAA AGTCGAAAAT GAGTGAATAT ATCTCGGAGA GGTAAGATAG TCAAACGAGT   
  
  
- AAAGTGTCGG TTAGTCCGTT AACATCTTCG TAAACTCTCC CTCCTTCTCT CATTGTTGAC CCGAAACGTA   
  
  
- CATTAACTAA ACCTGTAGAA CGTACCAAAG GTCACCGGAA GAGAATAAGT CAGAGAGAGG CTTCTCCGAT   
  
  
- GAACAAGTTT GGCAAGTAAC AGAGAAGTTT AATGTCCGAA GCCTTCTTCG AATCTTCTCG AGTAACTTTG   
  
  
- TCTTCGAGCC GACCATTCAA AGAGATTCTC GAAAGTCTTA TAGTTGAAAC TTAAGGTGCC CAAAGACTCT   
  
  
- CCGAGCTTTG AGTCCTTGGA TTCCTCCCTC TTACTTTGTC ATCAACATTT AGAGCAAAAG GTGGAGTCAT   
  
  
- GAAACTTTCT ATGGCATGTC TAAAGACTGT GGAACTGAAG ACATGTAAGT GATTTGGGGA GATAACACCA   
  
  
- TAATCAGCTC TCTCTTCCTT CAGCTTTGTC TACACCCAAG GAGAGTTCAA TACATCTACG AGACGTAATA   
  
  
- ATACGACGTT ACAAACTGAG AAATCTACTA ACGGAGGGTG AACTTTCAGG ACTCTCCGAC TCGTATCTCT   
  
  
- TCTTGGTAGA ACCTTTTCTC TAGTTTCTTC GGTATCCAAC ACTCTTCCTA CTTTGTTTGA TAGACTTCAA   
  
  
- ACTCTACGAC CTCTGAACCT CTCCCTCCTA CCTCTCAGTA CCTAAAAGTC CATACTTCGA TTCAAGGTCC   
  
  
- CGTTGATATG TTCGTTTCGA CGAAAATTTT TACCCTTCGG TAATAGTATG GATAAACCTT CTTCTGACAC   
  
  
- CTCCACCACC CAAGTCTCAA ACACTTGCTC TACTACCTTA CCGATAGAGA GAACCGACCG TTCCATCCAA   
  
  
- GGAGCGTTGA CGGAGCCATA CCGCAAGACA CAC

+     CAT-box

| Site Name | Organism | Position | Strand | Matrix score. | sequence | function |
| --- | --- | --- | --- | --- | --- | --- |
| CAT-box | Arabidopsis thaliana | 2626 | - | 6 | GCCACT | cis-acting regulatory element related to meristem expression |

>HU07G00272.1   
+ -Up\_Stream \_Len000ACCAAC GATTCTTTAC TGAATCAGCT ACATTTTTGG ATTGTGTTAA GGTATCTTTT   
  
  
+ AGATCTTCAG GAGGAGGGGG CTGAAGTAGC TTGGTTACTT TTGCGCTTAT CAACTGTGGA AGAATATTAT   
  
  
+ TGAGACTAGT GTAGAAGAAT TAGTAGTTTC CTCCACATTG GAAAAAAACC ATGAAATTAA CGCTTTAGGC   
  
  
+ CCTCAGAGTA CCAAGTGTCA TCGTAAATGA TTGAAATTTC AAGTCTTAGC TGAGTCATGA AGTCCTGATT   
  
  
+ TCCCATGACA AATTGATGTT GTTGTCTAAA GCTTGGCGGG TGATTTGAAA GAAGATTTAG ATCAGGCTGA   
  
  
+ CAATACACCA AATCTGGGAG CAAGGAACTG GATTTTTCTG TGTCTAGAAA AGCTTTCCGC TTTCTGAACA   
  
  
+ ATTGAGAAAA CCATGCATAG GAAATTTCTT GATATTTAGA TCATAGCAGA CTTAACAATC TGGCTTTACC   
  
  
+ GAACTGTCCT CTACTTCTAA GTTGTGAAGG TGTGAAAGAT AAACCATGTC AAACTTAGTG TCCAACAGAA   
  
  
+ ACGCATCTCT ACTTCTCCAG GCAAAGTTGT ACTTCAGATC TTCTACACCT TTTCTAGGCA GGACTTTGGG   
  
  
+ ATATAGAGTT TGGCCTGGGT GAAAAGTAGG AAGTGGGGAA GTAGAAAATG ATGAAAGAAG GCACCACTTG   
  
  
+ GGTCCAGATA CATAGGCCTA CGCACGAGAA CCTTTGCATG GTCTAGATAC ATGACTGTAT ACTTCTTCCT   
  
  
+ TTCCTTTACC TGTTGGAGAT TATCCTAGGA TTTTACATGG CACCAGCTGG ATTTGTTAAT GACATAAACA   
  
  
+ TATCTTTAAT TACAGTTGCT TATGGTATAC ATTTAAACTA TTCGATAGTT GACAGCTGAT GTCCTTATCC   
  
  
+ TTGTGCAGCT TATTCCTCGA TAGAATAATC TTTGGACATT TCTGTCTAGG TGATAAAGGG CAAAAACTAA   
  
  
+ GATACAAGAA ATAGAACATG TCCTCCATTT ACAAGACGTA TCTGTTTTTG GTTTTGGTCA ATGATCCCTG   
  
  
+ TTGTCCTCAA GCATTAACAC AGAATGATCA TTATTGACTT GGACTGAAAG GTTTTGGGAA CTCCATAGTA   
  
  
+ TGCCTTTTCA ATAAAATCAA AAGCAAATTT GCTGTTCACT GATAACTGCA GTTGTGTTGC TTTGCTGTTT   
  
  
+ CCATACCAGA CTCGTAACCA AGCTGATAGC AAATGTGCAA AGGTACAGTA TAATTAACGA TGTTTTCATA   
  
  
+ TTGCTACGAG TTCTGAAACA TGATATAAGA AAACACCAGA ACCCAAATAC CTACATAGGT CCTAGCCCTG   
  
  
+ GAATGGCTAT TTTAAATATC CAAATGGACT TACAGAATTC GCAGAGTTGT TTGGTTTGTT TAACAATCAT   
  
  
+ TGAATTGTTA GCTATGAGAA TCTGCGGGAC AATCGTAATC TTTGGCGATT ACTTCGATTG GCATACTGAT   
  
  
+ GTTTTTTTAT TTGCGTATCT AAGCATGGTG GTGTACCTTT GATTTGTGTG TCTAGGTAGT GTGTGTAGAT   
  
  
+ AAATATCATA GAACTCAGAC GTTGGTAGGG TTATGTTGAG CACTTTCTCT TGCTGTTTGT CCTTTGTGAT   
  
  
+ GGCACCTCTT GCTTTTCCGA ACCACCAAAA GTCTCATTGA CAAAACTCTT CAAACATTGC AAACTGCCCC   
  
  
+ CTTAAATATG TTTTATTAAA AAAAATGGAA GAGCTCAAAC TGTTGAGTGA GCCTAGATTC CAAGATTTAG   
  
  
+ AAACAGTAAA CTTGATAGAT ATATTTATAT GAACTATTGT TAGATGCAAC ACCCCCAACT TGAAACACAA   
  
  
+ AAGAGAAATT AAAAACAAAA AGGTTTTGCA CTATTTATAT ATCCTGTGTT CTTTGTGCTA GTCTTATTTA   
  
  
+ ATGTTAATTG GTAAGCATTA TTTGTTGGAT TATTCCACTG CACTTTCTCT TACTAACTTC AGCATTTCAT   
  
  
+ TCCTTGTTCT TCAGCTTTTG AACTTCTTTA GCCTATTTTG TTGTCTTTAT GGTTATGGAA GCCATCCAGA   
  
  
+ AGAAGAATGA TGAACTTCTG AGTCTTAGCT TGGCCATTGT CGGTCACTCT GATCGCACTG AGAAAAAACT   
  
  
+ GAAGAGGCGG ATTGATGTCT CCGATCCTCT GATCACTTCA GGTGAAGGCT GCGAAGGGAA GATAATCAGG   
  
  
+ CTCCTCCAAG AGAGACAGAA CTTGTTGAAC ATCAAGCAAA GAGGAAAAGG CGTCATTCAA GAAGGGAAGG   
  
  
+ GTCTTCATCT TATCCATCTG CTCCTTGTAT CTGCCACCTT AATCAACGAA AACAACATCA GTTCAGCTGT   
  
  
+ TGATAATCTT ATTGAGCTCT TCCAATATGT CTCCGAAAGT GGTGATTCAG GACAAAGGGT TGCTGCTTAC   
  
  
+ TTTGCGGATG GGTTAACAGC AAGGATCTTA ACTCAGAGGT CTCCATTTTA TCGCCTGATA CTGGGCAAAC   
  
  
+ CAGCACCTGC TGAAGAGTTT TCAGCTTTTA CTCACTTATA TAGAGCCTCT CCATTCTATC AGTTTGCTCA   
  
  
+ TTTCACAGCC AATCAGGCAA TTGTAGAAGC ATTTGAGAGG GAGGAAGAGA GTAACAACTG GGCTTTGCAT   
  
  
+ GTAATTGATT TGGACATCTT GCATGGTTTC CAGTGGCCTT CTCTTATTCA GTCTCTCTCC GAAGAGGCTA   
  
  
+ CTTGTTCAAA CCGTTCATTG TCTCTTCAAA TTACAGGCTT CGGAAGAAGC TTAGAAGAGC TCATTGAAAC   
  
  
+ AGAAGCTCGG CTGGTAAGTT TCTCTAAGAG CTTTCAGAAT ATCAACTTTG AATTCCACGG GTTTCTGAGA   
  
  
+ GGCTCGAAAC TCAGGAACCT AAGGAGGGAG AATGAAACAG TAGTTGTAAA TCTCGTTTTC CACCTCAGTA   
  
  
+ CTTTGAAAGA TACCGTACAG ATTTCTGACA CCTTGACTTC TGTACATTCA CTAAACCCCT CTATTGTGGT   
  
  
+ ATTAGTCGAG AGAGAAGGAA GTCGAAACAG ATGTGGGTTC CTCTCAAGTT ATGTAGATGC TCTGCATTAT   
  
  
+ TATGCTGCAA TGTTTGACTC TTTAGATGAT TGCCTCCCAC TTGAAAGTCC TGAGAGGCTG AGCATAGAGA   
  
  
+ AGAACCATCT TGGAAAAGAG ATCAAAGAAG CCATAGGTTG TGAGAAGGAT GAAACAAACT ATCTGAAGTT   
  
  
+ TGAGATGCTG GAGACTTGGA GAGGGAGGAT GGAGAGTCAT GGATTTTCAG GTATGAAGCT AAGTTCCAGG   
  
  
+ GCAACTATAC AAGCAAAGCT GCTTTTAAAA ATGGGAAGCC ATTATCATAC CTATTTGGAA GAAGACTGTG   
  
  
+ GAGGTGGTGG GTTCAGAGTT TGTGAACGAG ATGATGGAAT GGCTATCTCT CTTGGCTGGC AAGGTAGGTT   
  
  
+ CCTCGCAACT GCCTCGGTAT GGCGTTCTGT GTG  

- -Up\_Stream \_Len000TGGTTG CTAAGAAATG ACTTAGTCGA TGTAAAAACC TAACACAATT CCATAGAAAA   
  
  
- TCTAGAAGTC CTCCTCCCCC GACTTCATCG AACCAATGAA AACGCGAATA GTTGACACCT TCTTATAATA   
  
  
- ACTCTGATCA CATCTTCTTA ATCATCAAAG GAGGTGTAAC CTTTTTTTGG TACTTTAATT GCGAAATCCG   
  
  
- GGAGTCTCAT GGTTCACAGT AGCATTTACT AACTTTAAAG TTCAGAATCG ACTCAGTACT TCAGGACTAA   
  
  
- AGGGTACTGT TTAACTACAA CAACAGATTT CGAACCGCCC ACTAAACTTT CTTCTAAATC TAGTCCGACT   
  
  
- GTTATGTGGT TTAGACCCTC GTTCCTTGAC CTAAAAAGAC ACAGATCTTT TCGAAAGGCG AAAGACTTGT   
  
  
- TAACTCTTTT GGTACGTATC CTTTAAAGAA CTATAAATCT AGTATCGTCT GAATTGTTAG ACCGAAATGG   
  
  
- CTTGACAGGA GATGAAGATT CAACACTTCC ACACTTTCTA TTTGGTACAG TTTGAATCAC AGGTTGTCTT   
  
  
- TGCGTAGAGA TGAAGAGGTC CGTTTCAACA TGAAGTCTAG AAGATGTGGA AAAGATCCGT CCTGAAACCC   
  
  
- TATATCTCAA ACCGGACCCA CTTTTCATCC TTCACCCCTT CATCTTTTAC TACTTTCTTC CGTGGTGAAC   
  
  
- CCAGGTCTAT GTATCCGGAT GCGTGCTCTT GGAAACGTAC CAGATCTATG TACTGACATA TGAAGAAGGA   
  
  
- AAGGAAATGG ACAACCTCTA ATAGGATCCT AAAATGTACC GTGGTCGACC TAAACAATTA CTGTATTTGT   
  
  
- ATAGAAATTA ATGTCAACGA ATACCATATG TAAATTTGAT AAGCTATCAA CTGTCGACTA CAGGAATAGG   
  
  
- AACACGTCGA ATAAGGAGCT ATCTTATTAG AAACCTGTAA AGACAGATCC ACTATTTCCC GTTTTTGATT   
  
  
- CTATGTTCTT TATCTTGTAC AGGAGGTAAA TGTTCTGCAT AGACAAAAAC CAAAACCAGT TACTAGGGAC   
  
  
- AACAGGAGTT CGTAATTGTG TCTTACTAGT AATAACTGAA CCTGACTTTC CAAAACCCTT GAGGTATCAT   
  
  
- ACGGAAAAGT TATTTTAGTT TTCGTTTAAA CGACAAGTGA CTATTGACGT CAACACAACG AAACGACAAA   
  
  
- GGTATGGTCT GAGCATTGGT TCGACTATCG TTTACACGTT TCCATGTCAT ATTAATTGCT ACAAAAGTAT   
  
  
- AACGATGCTC AAGACTTTGT ACTATATTCT TTTGTGGTCT TGGGTTTATG GATGTATCCA GGATCGGGAC   
  
  
- CTTACCGATA AAATTTATAG GTTTACCTGA ATGTCTTAAG CGTCTCAACA AACCAAACAA ATTGTTAGTA   
  
  
- ACTTAACAAT CGATACTCTT AGACGCCCTG TTAGCATTAG AAACCGCTAA TGAAGCTAAC CGTATGACTA   
  
  
- CAAAAAAATA AACGCATAGA TTCGTACCAC CACATGGAAA CTAAACACAC AGATCCATCA CACACATCTA   
  
  
- TTTATAGTAT CTTGAGTCTG CAACCATCCC AATACAACTC GTGAAAGAGA ACGACAAACA GGAAACACTA   
  
  
- CCGTGGAGAA CGAAAAGGCT TGGTGGTTTT CAGAGTAACT GTTTTGAGAA GTTTGTAACG TTTGACGGGG   
  
  
- GAATTTATAC AAAATAATTT TTTTTACCTT CTCGAGTTTG ACAACTCACT CGGATCTAAG GTTCTAAATC   
  
  
- TTTGTCATTT GAACTATCTA TATAAATATA CTTGATAACA ATCTACGTTG TGGGGGTTGA ACTTTGTGTT   
  
  
- TTCTCTTTAA TTTTTGTTTT TCCAAAACGT GATAAATATA TAGGACACAA GAAACACGAT CAGAATAAAT   
  
  
- TACAATTAAC CATTCGTAAT AAACAACCTA ATAAGGTGAC GTGAAAGAGA ATGATTGAAG TCGTAAAGTA   
  
  
- AGGAACAAGA AGTCGAAAAC TTGAAGAAAT CGGATAAAAC AACAGAAATA CCAATACCTT CGGTAGGTCT   
  
  
- TCTTCTTACT ACTTGAAGAC TCAGAATCGA ACCGGTAACA GCCAGTGAGA CTAGCGTGAC TCTTTTTTGA   
  
  
- CTTCTCCGCC TAACTACAGA GGCTAGGAGA CTAGTGAAGT CCACTTCCGA CGCTTCCCTT CTATTAGTCC   
  
  
- GAGGAGGTTC TCTCTGTCTT GAACAACTTG TAGTTCGTTT CTCCTTTTCC GCAGTAAGTT CTTCCCTTCC   
  
  
- CAGAAGTAGA ATAGGTAGAC GAGGAACATA GACGGTGGAA TTAGTTGCTT TTGTTGTAGT CAAGTCGACA   
  
  
- ACTATTAGAA TAACTCGAGA AGGTTATACA GAGGCTTTCA CCACTAAGTC CTGTTTCCCA ACGACGAATG   
  
  
- AAACGCCTAC CCAATTGTCG TTCCTAGAAT TGAGTCTCCA GAGGTAAAAT AGCGGACTAT GACCCGTTTG   
  
  
- GTCGTGGACG ACTTCTCAAA AGTCGAAAAT GAGTGAATAT ATCTCGGAGA GGTAAGATAG TCAAACGAGT   
  
  
- AAAGTGTCGG TTAGTCCGTT AACATCTTCG TAAACTCTCC CTCCTTCTCT CATTGTTGAC CCGAAACGTA   
  
  
- CATTAACTAA ACCTGTAGAA CGTACCAAAG GTCACCGGAA GAGAATAAGT CAGAGAGAGG CTTCTCCGAT   
  
  
- GAACAAGTTT GGCAAGTAAC AGAGAAGTTT AATGTCCGAA GCCTTCTTCG AATCTTCTCG AGTAACTTTG   
  
  
- TCTTCGAGCC GACCATTCAA AGAGATTCTC GAAAGTCTTA TAGTTGAAAC TTAAGGTGCC CAAAGACTCT   
  
  
- CCGAGCTTTG AGTCCTTGGA TTCCTCCCTC TTACTTTGTC ATCAACATTT AGAGCAAAAG GTGGAGTCAT   
  
  
- GAAACTTTCT ATGGCATGTC TAAAGACTGT GGAACTGAAG ACATGTAAGT GATTTGGGGA GATAACACCA   
  
  
- TAATCAGCTC TCTCTTCCTT CAGCTTTGTC TACACCCAAG GAGAGTTCAA TACATCTACG AGACGTAATA   
  
  
- ATACGACGTT ACAAACTGAG AAATCTACTA ACGGAGGGTG AACTTTCAGG ACTCTCCGAC TCGTATCTCT   
  
  
- TCTTGGTAGA ACCTTTTCTC TAGTTTCTTC GGTATCCAAC ACTCTTCCTA CTTTGTTTGA TAGACTTCAA   
  
  
- ACTCTACGAC CTCTGAACCT CTCCCTCCTA CCTCTCAGTA CCTAAAAGTC CATACTTCGA TTCAAGGTCC   
  
  
- CGTTGATATG TTCGTTTCGA CGAAAATTTT TACCCTTCGG TAATAGTATG GATAAACCTT CTTCTGACAC   
  
  
- CTCCACCACC CAAGTCTCAA ACACTTGCTC TACTACCTTA CCGATAGAGA GAACCGACCG TTCCATCCAA   
  
  
- GGAGCGTTGA CGGAGCCATA CCGCAAGACA CAC

+     CGTCA-motif

| Site Name | Organism | Position | Strand | Matrix score. | sequence | function |
| --- | --- | --- | --- | --- | --- | --- |
| CGTCA-motif | Hordeum vulgare | 2225 | + | 5 | CGTCA | cis-acting regulatory element involved in the MeJA-responsiveness |

>HU07G00272.1   
+ -Up\_Stream \_Len000ACCAAC GATTCTTTAC TGAATCAGCT ACATTTTTGG ATTGTGTTAA GGTATCTTTT   
  
  
+ AGATCTTCAG GAGGAGGGGG CTGAAGTAGC TTGGTTACTT TTGCGCTTAT CAACTGTGGA AGAATATTAT   
  
  
+ TGAGACTAGT GTAGAAGAAT TAGTAGTTTC CTCCACATTG GAAAAAAACC ATGAAATTAA CGCTTTAGGC   
  
  
+ CCTCAGAGTA CCAAGTGTCA TCGTAAATGA TTGAAATTTC AAGTCTTAGC TGAGTCATGA AGTCCTGATT   
  
  
+ TCCCATGACA AATTGATGTT GTTGTCTAAA GCTTGGCGGG TGATTTGAAA GAAGATTTAG ATCAGGCTGA   
  
  
+ CAATACACCA AATCTGGGAG CAAGGAACTG GATTTTTCTG TGTCTAGAAA AGCTTTCCGC TTTCTGAACA   
  
  
+ ATTGAGAAAA CCATGCATAG GAAATTTCTT GATATTTAGA TCATAGCAGA CTTAACAATC TGGCTTTACC   
  
  
+ GAACTGTCCT CTACTTCTAA GTTGTGAAGG TGTGAAAGAT AAACCATGTC AAACTTAGTG TCCAACAGAA   
  
  
+ ACGCATCTCT ACTTCTCCAG GCAAAGTTGT ACTTCAGATC TTCTACACCT TTTCTAGGCA GGACTTTGGG   
  
  
+ ATATAGAGTT TGGCCTGGGT GAAAAGTAGG AAGTGGGGAA GTAGAAAATG ATGAAAGAAG GCACCACTTG   
  
  
+ GGTCCAGATA CATAGGCCTA CGCACGAGAA CCTTTGCATG GTCTAGATAC ATGACTGTAT ACTTCTTCCT   
  
  
+ TTCCTTTACC TGTTGGAGAT TATCCTAGGA TTTTACATGG CACCAGCTGG ATTTGTTAAT GACATAAACA   
  
  
+ TATCTTTAAT TACAGTTGCT TATGGTATAC ATTTAAACTA TTCGATAGTT GACAGCTGAT GTCCTTATCC   
  
  
+ TTGTGCAGCT TATTCCTCGA TAGAATAATC TTTGGACATT TCTGTCTAGG TGATAAAGGG CAAAAACTAA   
  
  
+ GATACAAGAA ATAGAACATG TCCTCCATTT ACAAGACGTA TCTGTTTTTG GTTTTGGTCA ATGATCCCTG   
  
  
+ TTGTCCTCAA GCATTAACAC AGAATGATCA TTATTGACTT GGACTGAAAG GTTTTGGGAA CTCCATAGTA   
  
  
+ TGCCTTTTCA ATAAAATCAA AAGCAAATTT GCTGTTCACT GATAACTGCA GTTGTGTTGC TTTGCTGTTT   
  
  
+ CCATACCAGA CTCGTAACCA AGCTGATAGC AAATGTGCAA AGGTACAGTA TAATTAACGA TGTTTTCATA   
  
  
+ TTGCTACGAG TTCTGAAACA TGATATAAGA AAACACCAGA ACCCAAATAC CTACATAGGT CCTAGCCCTG   
  
  
+ GAATGGCTAT TTTAAATATC CAAATGGACT TACAGAATTC GCAGAGTTGT TTGGTTTGTT TAACAATCAT   
  
  
+ TGAATTGTTA GCTATGAGAA TCTGCGGGAC AATCGTAATC TTTGGCGATT ACTTCGATTG GCATACTGAT   
  
  
+ GTTTTTTTAT TTGCGTATCT AAGCATGGTG GTGTACCTTT GATTTGTGTG TCTAGGTAGT GTGTGTAGAT   
  
  
+ AAATATCATA GAACTCAGAC GTTGGTAGGG TTATGTTGAG CACTTTCTCT TGCTGTTTGT CCTTTGTGAT   
  
  
+ GGCACCTCTT GCTTTTCCGA ACCACCAAAA GTCTCATTGA CAAAACTCTT CAAACATTGC AAACTGCCCC   
  
  
+ CTTAAATATG TTTTATTAAA AAAAATGGAA GAGCTCAAAC TGTTGAGTGA GCCTAGATTC CAAGATTTAG   
  
  
+ AAACAGTAAA CTTGATAGAT ATATTTATAT GAACTATTGT TAGATGCAAC ACCCCCAACT TGAAACACAA   
  
  
+ AAGAGAAATT AAAAACAAAA AGGTTTTGCA CTATTTATAT ATCCTGTGTT CTTTGTGCTA GTCTTATTTA   
  
  
+ ATGTTAATTG GTAAGCATTA TTTGTTGGAT TATTCCACTG CACTTTCTCT TACTAACTTC AGCATTTCAT   
  
  
+ TCCTTGTTCT TCAGCTTTTG AACTTCTTTA GCCTATTTTG TTGTCTTTAT GGTTATGGAA GCCATCCAGA   
  
  
+ AGAAGAATGA TGAACTTCTG AGTCTTAGCT TGGCCATTGT CGGTCACTCT GATCGCACTG AGAAAAAACT   
  
  
+ GAAGAGGCGG ATTGATGTCT CCGATCCTCT GATCACTTCA GGTGAAGGCT GCGAAGGGAA GATAATCAGG   
  
  
+ CTCCTCCAAG AGAGACAGAA CTTGTTGAAC ATCAAGCAAA GAGGAAAAGG CGTCATTCAA GAAGGGAAGG   
  
  
+ GTCTTCATCT TATCCATCTG CTCCTTGTAT CTGCCACCTT AATCAACGAA AACAACATCA GTTCAGCTGT   
  
  
+ TGATAATCTT ATTGAGCTCT TCCAATATGT CTCCGAAAGT GGTGATTCAG GACAAAGGGT TGCTGCTTAC   
  
  
+ TTTGCGGATG GGTTAACAGC AAGGATCTTA ACTCAGAGGT CTCCATTTTA TCGCCTGATA CTGGGCAAAC   
  
  
+ CAGCACCTGC TGAAGAGTTT TCAGCTTTTA CTCACTTATA TAGAGCCTCT CCATTCTATC AGTTTGCTCA   
  
  
+ TTTCACAGCC AATCAGGCAA TTGTAGAAGC ATTTGAGAGG GAGGAAGAGA GTAACAACTG GGCTTTGCAT   
  
  
+ GTAATTGATT TGGACATCTT GCATGGTTTC CAGTGGCCTT CTCTTATTCA GTCTCTCTCC GAAGAGGCTA   
  
  
+ CTTGTTCAAA CCGTTCATTG TCTCTTCAAA TTACAGGCTT CGGAAGAAGC TTAGAAGAGC TCATTGAAAC   
  
  
+ AGAAGCTCGG CTGGTAAGTT TCTCTAAGAG CTTTCAGAAT ATCAACTTTG AATTCCACGG GTTTCTGAGA   
  
  
+ GGCTCGAAAC TCAGGAACCT AAGGAGGGAG AATGAAACAG TAGTTGTAAA TCTCGTTTTC CACCTCAGTA   
  
  
+ CTTTGAAAGA TACCGTACAG ATTTCTGACA CCTTGACTTC TGTACATTCA CTAAACCCCT CTATTGTGGT   
  
  
+ ATTAGTCGAG AGAGAAGGAA GTCGAAACAG ATGTGGGTTC CTCTCAAGTT ATGTAGATGC TCTGCATTAT   
  
  
+ TATGCTGCAA TGTTTGACTC TTTAGATGAT TGCCTCCCAC TTGAAAGTCC TGAGAGGCTG AGCATAGAGA   
  
  
+ AGAACCATCT TGGAAAAGAG ATCAAAGAAG CCATAGGTTG TGAGAAGGAT GAAACAAACT ATCTGAAGTT   
  
  
+ TGAGATGCTG GAGACTTGGA GAGGGAGGAT GGAGAGTCAT GGATTTTCAG GTATGAAGCT AAGTTCCAGG   
  
  
+ GCAACTATAC AAGCAAAGCT GCTTTTAAAA ATGGGAAGCC ATTATCATAC CTATTTGGAA GAAGACTGTG   
  
  
+ GAGGTGGTGG GTTCAGAGTT TGTGAACGAG ATGATGGAAT GGCTATCTCT CTTGGCTGGC AAGGTAGGTT   
  
  
+ CCTCGCAACT GCCTCGGTAT GGCGTTCTGT GTG  

- -Up\_Stream \_Len000TGGTTG CTAAGAAATG ACTTAGTCGA TGTAAAAACC TAACACAATT CCATAGAAAA   
  
  
- TCTAGAAGTC CTCCTCCCCC GACTTCATCG AACCAATGAA AACGCGAATA GTTGACACCT TCTTATAATA   
  
  
- ACTCTGATCA CATCTTCTTA ATCATCAAAG GAGGTGTAAC CTTTTTTTGG TACTTTAATT GCGAAATCCG   
  
  
- GGAGTCTCAT GGTTCACAGT AGCATTTACT AACTTTAAAG TTCAGAATCG ACTCAGTACT TCAGGACTAA   
  
  
- AGGGTACTGT TTAACTACAA CAACAGATTT CGAACCGCCC ACTAAACTTT CTTCTAAATC TAGTCCGACT   
  
  
- GTTATGTGGT TTAGACCCTC GTTCCTTGAC CTAAAAAGAC ACAGATCTTT TCGAAAGGCG AAAGACTTGT   
  
  
- TAACTCTTTT GGTACGTATC CTTTAAAGAA CTATAAATCT AGTATCGTCT GAATTGTTAG ACCGAAATGG   
  
  
- CTTGACAGGA GATGAAGATT CAACACTTCC ACACTTTCTA TTTGGTACAG TTTGAATCAC AGGTTGTCTT   
  
  
- TGCGTAGAGA TGAAGAGGTC CGTTTCAACA TGAAGTCTAG AAGATGTGGA AAAGATCCGT CCTGAAACCC   
  
  
- TATATCTCAA ACCGGACCCA CTTTTCATCC TTCACCCCTT CATCTTTTAC TACTTTCTTC CGTGGTGAAC   
  
  
- CCAGGTCTAT GTATCCGGAT GCGTGCTCTT GGAAACGTAC CAGATCTATG TACTGACATA TGAAGAAGGA   
  
  
- AAGGAAATGG ACAACCTCTA ATAGGATCCT AAAATGTACC GTGGTCGACC TAAACAATTA CTGTATTTGT   
  
  
- ATAGAAATTA ATGTCAACGA ATACCATATG TAAATTTGAT AAGCTATCAA CTGTCGACTA CAGGAATAGG   
  
  
- AACACGTCGA ATAAGGAGCT ATCTTATTAG AAACCTGTAA AGACAGATCC ACTATTTCCC GTTTTTGATT   
  
  
- CTATGTTCTT TATCTTGTAC AGGAGGTAAA TGTTCTGCAT AGACAAAAAC CAAAACCAGT TACTAGGGAC   
  
  
- AACAGGAGTT CGTAATTGTG TCTTACTAGT AATAACTGAA CCTGACTTTC CAAAACCCTT GAGGTATCAT   
  
  
- ACGGAAAAGT TATTTTAGTT TTCGTTTAAA CGACAAGTGA CTATTGACGT CAACACAACG AAACGACAAA   
  
  
- GGTATGGTCT GAGCATTGGT TCGACTATCG TTTACACGTT TCCATGTCAT ATTAATTGCT ACAAAAGTAT   
  
  
- AACGATGCTC AAGACTTTGT ACTATATTCT TTTGTGGTCT TGGGTTTATG GATGTATCCA GGATCGGGAC   
  
  
- CTTACCGATA AAATTTATAG GTTTACCTGA ATGTCTTAAG CGTCTCAACA AACCAAACAA ATTGTTAGTA   
  
  
- ACTTAACAAT CGATACTCTT AGACGCCCTG TTAGCATTAG AAACCGCTAA TGAAGCTAAC CGTATGACTA   
  
  
- CAAAAAAATA AACGCATAGA TTCGTACCAC CACATGGAAA CTAAACACAC AGATCCATCA CACACATCTA   
  
  
- TTTATAGTAT CTTGAGTCTG CAACCATCCC AATACAACTC GTGAAAGAGA ACGACAAACA GGAAACACTA   
  
  
- CCGTGGAGAA CGAAAAGGCT TGGTGGTTTT CAGAGTAACT GTTTTGAGAA GTTTGTAACG TTTGACGGGG   
  
  
- GAATTTATAC AAAATAATTT TTTTTACCTT CTCGAGTTTG ACAACTCACT CGGATCTAAG GTTCTAAATC   
  
  
- TTTGTCATTT GAACTATCTA TATAAATATA CTTGATAACA ATCTACGTTG TGGGGGTTGA ACTTTGTGTT   
  
  
- TTCTCTTTAA TTTTTGTTTT TCCAAAACGT GATAAATATA TAGGACACAA GAAACACGAT CAGAATAAAT   
  
  
- TACAATTAAC CATTCGTAAT AAACAACCTA ATAAGGTGAC GTGAAAGAGA ATGATTGAAG TCGTAAAGTA   
  
  
- AGGAACAAGA AGTCGAAAAC TTGAAGAAAT CGGATAAAAC AACAGAAATA CCAATACCTT CGGTAGGTCT   
  
  
- TCTTCTTACT ACTTGAAGAC TCAGAATCGA ACCGGTAACA GCCAGTGAGA CTAGCGTGAC TCTTTTTTGA   
  
  
- CTTCTCCGCC TAACTACAGA GGCTAGGAGA CTAGTGAAGT CCACTTCCGA CGCTTCCCTT CTATTAGTCC   
  
  
- GAGGAGGTTC TCTCTGTCTT GAACAACTTG TAGTTCGTTT CTCCTTTTCC GCAGTAAGTT CTTCCCTTCC   
  
  
- CAGAAGTAGA ATAGGTAGAC GAGGAACATA GACGGTGGAA TTAGTTGCTT TTGTTGTAGT CAAGTCGACA   
  
  
- ACTATTAGAA TAACTCGAGA AGGTTATACA GAGGCTTTCA CCACTAAGTC CTGTTTCCCA ACGACGAATG   
  
  
- AAACGCCTAC CCAATTGTCG TTCCTAGAAT TGAGTCTCCA GAGGTAAAAT AGCGGACTAT GACCCGTTTG   
  
  
- GTCGTGGACG ACTTCTCAAA AGTCGAAAAT GAGTGAATAT ATCTCGGAGA GGTAAGATAG TCAAACGAGT   
  
  
- AAAGTGTCGG TTAGTCCGTT AACATCTTCG TAAACTCTCC CTCCTTCTCT CATTGTTGAC CCGAAACGTA   
  
  
- CATTAACTAA ACCTGTAGAA CGTACCAAAG GTCACCGGAA GAGAATAAGT CAGAGAGAGG CTTCTCCGAT   
  
  
- GAACAAGTTT GGCAAGTAAC AGAGAAGTTT AATGTCCGAA GCCTTCTTCG AATCTTCTCG AGTAACTTTG   
  
  
- TCTTCGAGCC GACCATTCAA AGAGATTCTC GAAAGTCTTA TAGTTGAAAC TTAAGGTGCC CAAAGACTCT   
  
  
- CCGAGCTTTG AGTCCTTGGA TTCCTCCCTC TTACTTTGTC ATCAACATTT AGAGCAAAAG GTGGAGTCAT   
  
  
- GAAACTTTCT ATGGCATGTC TAAAGACTGT GGAACTGAAG ACATGTAAGT GATTTGGGGA GATAACACCA   
  
  
- TAATCAGCTC TCTCTTCCTT CAGCTTTGTC TACACCCAAG GAGAGTTCAA TACATCTACG AGACGTAATA   
  
  
- ATACGACGTT ACAAACTGAG AAATCTACTA ACGGAGGGTG AACTTTCAGG ACTCTCCGAC TCGTATCTCT   
  
  
- TCTTGGTAGA ACCTTTTCTC TAGTTTCTTC GGTATCCAAC ACTCTTCCTA CTTTGTTTGA TAGACTTCAA   
  
  
- ACTCTACGAC CTCTGAACCT CTCCCTCCTA CCTCTCAGTA CCTAAAAGTC CATACTTCGA TTCAAGGTCC   
  
  
- CGTTGATATG TTCGTTTCGA CGAAAATTTT TACCCTTCGG TAATAGTATG GATAAACCTT CTTCTGACAC   
  
  
- CTCCACCACC CAAGTCTCAA ACACTTGCTC TACTACCTTA CCGATAGAGA GAACCGACCG TTCCATCCAA   
  
  
- GGAGCGTTGA CGGAGCCATA CCGCAAGACA CAC

+     CTAG-motif

| Site Name | Organism | Position | Strand | Matrix score. | sequence | function |
| --- | --- | --- | --- | --- | --- | --- |
| CTAG-motif | Avena sativa | 1877 | - | 9 | ACTAGCAGAA |  |

>HU07G00272.1   
+ -Up\_Stream \_Len000ACCAAC GATTCTTTAC TGAATCAGCT ACATTTTTGG ATTGTGTTAA GGTATCTTTT   
  
  
+ AGATCTTCAG GAGGAGGGGG CTGAAGTAGC TTGGTTACTT TTGCGCTTAT CAACTGTGGA AGAATATTAT   
  
  
+ TGAGACTAGT GTAGAAGAAT TAGTAGTTTC CTCCACATTG GAAAAAAACC ATGAAATTAA CGCTTTAGGC   
  
  
+ CCTCAGAGTA CCAAGTGTCA TCGTAAATGA TTGAAATTTC AAGTCTTAGC TGAGTCATGA AGTCCTGATT   
  
  
+ TCCCATGACA AATTGATGTT GTTGTCTAAA GCTTGGCGGG TGATTTGAAA GAAGATTTAG ATCAGGCTGA   
  
  
+ CAATACACCA AATCTGGGAG CAAGGAACTG GATTTTTCTG TGTCTAGAAA AGCTTTCCGC TTTCTGAACA   
  
  
+ ATTGAGAAAA CCATGCATAG GAAATTTCTT GATATTTAGA TCATAGCAGA CTTAACAATC TGGCTTTACC   
  
  
+ GAACTGTCCT CTACTTCTAA GTTGTGAAGG TGTGAAAGAT AAACCATGTC AAACTTAGTG TCCAACAGAA   
  
  
+ ACGCATCTCT ACTTCTCCAG GCAAAGTTGT ACTTCAGATC TTCTACACCT TTTCTAGGCA GGACTTTGGG   
  
  
+ ATATAGAGTT TGGCCTGGGT GAAAAGTAGG AAGTGGGGAA GTAGAAAATG ATGAAAGAAG GCACCACTTG   
  
  
+ GGTCCAGATA CATAGGCCTA CGCACGAGAA CCTTTGCATG GTCTAGATAC ATGACTGTAT ACTTCTTCCT   
  
  
+ TTCCTTTACC TGTTGGAGAT TATCCTAGGA TTTTACATGG CACCAGCTGG ATTTGTTAAT GACATAAACA   
  
  
+ TATCTTTAAT TACAGTTGCT TATGGTATAC ATTTAAACTA TTCGATAGTT GACAGCTGAT GTCCTTATCC   
  
  
+ TTGTGCAGCT TATTCCTCGA TAGAATAATC TTTGGACATT TCTGTCTAGG TGATAAAGGG CAAAAACTAA   
  
  
+ GATACAAGAA ATAGAACATG TCCTCCATTT ACAAGACGTA TCTGTTTTTG GTTTTGGTCA ATGATCCCTG   
  
  
+ TTGTCCTCAA GCATTAACAC AGAATGATCA TTATTGACTT GGACTGAAAG GTTTTGGGAA CTCCATAGTA   
  
  
+ TGCCTTTTCA ATAAAATCAA AAGCAAATTT GCTGTTCACT GATAACTGCA GTTGTGTTGC TTTGCTGTTT   
  
  
+ CCATACCAGA CTCGTAACCA AGCTGATAGC AAATGTGCAA AGGTACAGTA TAATTAACGA TGTTTTCATA   
  
  
+ TTGCTACGAG TTCTGAAACA TGATATAAGA AAACACCAGA ACCCAAATAC CTACATAGGT CCTAGCCCTG   
  
  
+ GAATGGCTAT TTTAAATATC CAAATGGACT TACAGAATTC GCAGAGTTGT TTGGTTTGTT TAACAATCAT   
  
  
+ TGAATTGTTA GCTATGAGAA TCTGCGGGAC AATCGTAATC TTTGGCGATT ACTTCGATTG GCATACTGAT   
  
  
+ GTTTTTTTAT TTGCGTATCT AAGCATGGTG GTGTACCTTT GATTTGTGTG TCTAGGTAGT GTGTGTAGAT   
  
  
+ AAATATCATA GAACTCAGAC GTTGGTAGGG TTATGTTGAG CACTTTCTCT TGCTGTTTGT CCTTTGTGAT   
  
  
+ GGCACCTCTT GCTTTTCCGA ACCACCAAAA GTCTCATTGA CAAAACTCTT CAAACATTGC AAACTGCCCC   
  
  
+ CTTAAATATG TTTTATTAAA AAAAATGGAA GAGCTCAAAC TGTTGAGTGA GCCTAGATTC CAAGATTTAG   
  
  
+ AAACAGTAAA CTTGATAGAT ATATTTATAT GAACTATTGT TAGATGCAAC ACCCCCAACT TGAAACACAA   
  
  
+ AAGAGAAATT AAAAACAAAA AGGTTTTGCA CTATTTATAT ATCCTGTGTT CTTTGTGCTA GTCTTATTTA   
  
  
+ ATGTTAATTG GTAAGCATTA TTTGTTGGAT TATTCCACTG CACTTTCTCT TACTAACTTC AGCATTTCAT   
  
  
+ TCCTTGTTCT TCAGCTTTTG AACTTCTTTA GCCTATTTTG TTGTCTTTAT GGTTATGGAA GCCATCCAGA   
  
  
+ AGAAGAATGA TGAACTTCTG AGTCTTAGCT TGGCCATTGT CGGTCACTCT GATCGCACTG AGAAAAAACT   
  
  
+ GAAGAGGCGG ATTGATGTCT CCGATCCTCT GATCACTTCA GGTGAAGGCT GCGAAGGGAA GATAATCAGG   
  
  
+ CTCCTCCAAG AGAGACAGAA CTTGTTGAAC ATCAAGCAAA GAGGAAAAGG CGTCATTCAA GAAGGGAAGG   
  
  
+ GTCTTCATCT TATCCATCTG CTCCTTGTAT CTGCCACCTT AATCAACGAA AACAACATCA GTTCAGCTGT   
  
  
+ TGATAATCTT ATTGAGCTCT TCCAATATGT CTCCGAAAGT GGTGATTCAG GACAAAGGGT TGCTGCTTAC   
  
  
+ TTTGCGGATG GGTTAACAGC AAGGATCTTA ACTCAGAGGT CTCCATTTTA TCGCCTGATA CTGGGCAAAC   
  
  
+ CAGCACCTGC TGAAGAGTTT TCAGCTTTTA CTCACTTATA TAGAGCCTCT CCATTCTATC AGTTTGCTCA   
  
  
+ TTTCACAGCC AATCAGGCAA TTGTAGAAGC ATTTGAGAGG GAGGAAGAGA GTAACAACTG GGCTTTGCAT   
  
  
+ GTAATTGATT TGGACATCTT GCATGGTTTC CAGTGGCCTT CTCTTATTCA GTCTCTCTCC GAAGAGGCTA   
  
  
+ CTTGTTCAAA CCGTTCATTG TCTCTTCAAA TTACAGGCTT CGGAAGAAGC TTAGAAGAGC TCATTGAAAC   
  
  
+ AGAAGCTCGG CTGGTAAGTT TCTCTAAGAG CTTTCAGAAT ATCAACTTTG AATTCCACGG GTTTCTGAGA   
  
  
+ GGCTCGAAAC TCAGGAACCT AAGGAGGGAG AATGAAACAG TAGTTGTAAA TCTCGTTTTC CACCTCAGTA   
  
  
+ CTTTGAAAGA TACCGTACAG ATTTCTGACA CCTTGACTTC TGTACATTCA CTAAACCCCT CTATTGTGGT   
  
  
+ ATTAGTCGAG AGAGAAGGAA GTCGAAACAG ATGTGGGTTC CTCTCAAGTT ATGTAGATGC TCTGCATTAT   
  
  
+ TATGCTGCAA TGTTTGACTC TTTAGATGAT TGCCTCCCAC TTGAAAGTCC TGAGAGGCTG AGCATAGAGA   
  
  
+ AGAACCATCT TGGAAAAGAG ATCAAAGAAG CCATAGGTTG TGAGAAGGAT GAAACAAACT ATCTGAAGTT   
  
  
+ TGAGATGCTG GAGACTTGGA GAGGGAGGAT GGAGAGTCAT GGATTTTCAG GTATGAAGCT AAGTTCCAGG   
  
  
+ GCAACTATAC AAGCAAAGCT GCTTTTAAAA ATGGGAAGCC ATTATCATAC CTATTTGGAA GAAGACTGTG   
  
  
+ GAGGTGGTGG GTTCAGAGTT TGTGAACGAG ATGATGGAAT GGCTATCTCT CTTGGCTGGC AAGGTAGGTT   
  
  
+ CCTCGCAACT GCCTCGGTAT GGCGTTCTGT GTG  

- -Up\_Stream \_Len000TGGTTG CTAAGAAATG ACTTAGTCGA TGTAAAAACC TAACACAATT CCATAGAAAA   
  
  
- TCTAGAAGTC CTCCTCCCCC GACTTCATCG AACCAATGAA AACGCGAATA GTTGACACCT TCTTATAATA   
  
  
- ACTCTGATCA CATCTTCTTA ATCATCAAAG GAGGTGTAAC CTTTTTTTGG TACTTTAATT GCGAAATCCG   
  
  
- GGAGTCTCAT GGTTCACAGT AGCATTTACT AACTTTAAAG TTCAGAATCG ACTCAGTACT TCAGGACTAA   
  
  
- AGGGTACTGT TTAACTACAA CAACAGATTT CGAACCGCCC ACTAAACTTT CTTCTAAATC TAGTCCGACT   
  
  
- GTTATGTGGT TTAGACCCTC GTTCCTTGAC CTAAAAAGAC ACAGATCTTT TCGAAAGGCG AAAGACTTGT   
  
  
- TAACTCTTTT GGTACGTATC CTTTAAAGAA CTATAAATCT AGTATCGTCT GAATTGTTAG ACCGAAATGG   
  
  
- CTTGACAGGA GATGAAGATT CAACACTTCC ACACTTTCTA TTTGGTACAG TTTGAATCAC AGGTTGTCTT   
  
  
- TGCGTAGAGA TGAAGAGGTC CGTTTCAACA TGAAGTCTAG AAGATGTGGA AAAGATCCGT CCTGAAACCC   
  
  
- TATATCTCAA ACCGGACCCA CTTTTCATCC TTCACCCCTT CATCTTTTAC TACTTTCTTC CGTGGTGAAC   
  
  
- CCAGGTCTAT GTATCCGGAT GCGTGCTCTT GGAAACGTAC CAGATCTATG TACTGACATA TGAAGAAGGA   
  
  
- AAGGAAATGG ACAACCTCTA ATAGGATCCT AAAATGTACC GTGGTCGACC TAAACAATTA CTGTATTTGT   
  
  
- ATAGAAATTA ATGTCAACGA ATACCATATG TAAATTTGAT AAGCTATCAA CTGTCGACTA CAGGAATAGG   
  
  
- AACACGTCGA ATAAGGAGCT ATCTTATTAG AAACCTGTAA AGACAGATCC ACTATTTCCC GTTTTTGATT   
  
  
- CTATGTTCTT TATCTTGTAC AGGAGGTAAA TGTTCTGCAT AGACAAAAAC CAAAACCAGT TACTAGGGAC   
  
  
- AACAGGAGTT CGTAATTGTG TCTTACTAGT AATAACTGAA CCTGACTTTC CAAAACCCTT GAGGTATCAT   
  
  
- ACGGAAAAGT TATTTTAGTT TTCGTTTAAA CGACAAGTGA CTATTGACGT CAACACAACG AAACGACAAA   
  
  
- GGTATGGTCT GAGCATTGGT TCGACTATCG TTTACACGTT TCCATGTCAT ATTAATTGCT ACAAAAGTAT   
  
  
- AACGATGCTC AAGACTTTGT ACTATATTCT TTTGTGGTCT TGGGTTTATG GATGTATCCA GGATCGGGAC   
  
  
- CTTACCGATA AAATTTATAG GTTTACCTGA ATGTCTTAAG CGTCTCAACA AACCAAACAA ATTGTTAGTA   
  
  
- ACTTAACAAT CGATACTCTT AGACGCCCTG TTAGCATTAG AAACCGCTAA TGAAGCTAAC CGTATGACTA   
  
  
- CAAAAAAATA AACGCATAGA TTCGTACCAC CACATGGAAA CTAAACACAC AGATCCATCA CACACATCTA   
  
  
- TTTATAGTAT CTTGAGTCTG CAACCATCCC AATACAACTC GTGAAAGAGA ACGACAAACA GGAAACACTA   
  
  
- CCGTGGAGAA CGAAAAGGCT TGGTGGTTTT CAGAGTAACT GTTTTGAGAA GTTTGTAACG TTTGACGGGG   
  
  
- GAATTTATAC AAAATAATTT TTTTTACCTT CTCGAGTTTG ACAACTCACT CGGATCTAAG GTTCTAAATC   
  
  
- TTTGTCATTT GAACTATCTA TATAAATATA CTTGATAACA ATCTACGTTG TGGGGGTTGA ACTTTGTGTT   
  
  
- TTCTCTTTAA TTTTTGTTTT TCCAAAACGT GATAAATATA TAGGACACAA GAAACACGAT CAGAATAAAT   
  
  
- TACAATTAAC CATTCGTAAT AAACAACCTA ATAAGGTGAC GTGAAAGAGA ATGATTGAAG TCGTAAAGTA   
  
  
- AGGAACAAGA AGTCGAAAAC TTGAAGAAAT CGGATAAAAC AACAGAAATA CCAATACCTT CGGTAGGTCT   
  
  
- TCTTCTTACT ACTTGAAGAC TCAGAATCGA ACCGGTAACA GCCAGTGAGA CTAGCGTGAC TCTTTTTTGA   
  
  
- CTTCTCCGCC TAACTACAGA GGCTAGGAGA CTAGTGAAGT CCACTTCCGA CGCTTCCCTT CTATTAGTCC   
  
  
- GAGGAGGTTC TCTCTGTCTT GAACAACTTG TAGTTCGTTT CTCCTTTTCC GCAGTAAGTT CTTCCCTTCC   
  
  
- CAGAAGTAGA ATAGGTAGAC GAGGAACATA GACGGTGGAA TTAGTTGCTT TTGTTGTAGT CAAGTCGACA   
  
  
- ACTATTAGAA TAACTCGAGA AGGTTATACA GAGGCTTTCA CCACTAAGTC CTGTTTCCCA ACGACGAATG   
  
  
- AAACGCCTAC CCAATTGTCG TTCCTAGAAT TGAGTCTCCA GAGGTAAAAT AGCGGACTAT GACCCGTTTG   
  
  
- GTCGTGGACG ACTTCTCAAA AGTCGAAAAT GAGTGAATAT ATCTCGGAGA GGTAAGATAG TCAAACGAGT   
  
  
- AAAGTGTCGG TTAGTCCGTT AACATCTTCG TAAACTCTCC CTCCTTCTCT CATTGTTGAC CCGAAACGTA   
  
  
- CATTAACTAA ACCTGTAGAA CGTACCAAAG GTCACCGGAA GAGAATAAGT CAGAGAGAGG CTTCTCCGAT   
  
  
- GAACAAGTTT GGCAAGTAAC AGAGAAGTTT AATGTCCGAA GCCTTCTTCG AATCTTCTCG AGTAACTTTG   
  
  
- TCTTCGAGCC GACCATTCAA AGAGATTCTC GAAAGTCTTA TAGTTGAAAC TTAAGGTGCC CAAAGACTCT   
  
  
- CCGAGCTTTG AGTCCTTGGA TTCCTCCCTC TTACTTTGTC ATCAACATTT AGAGCAAAAG GTGGAGTCAT   
  
  
- GAAACTTTCT ATGGCATGTC TAAAGACTGT GGAACTGAAG ACATGTAAGT GATTTGGGGA GATAACACCA   
  
  
- TAATCAGCTC TCTCTTCCTT CAGCTTTGTC TACACCCAAG GAGAGTTCAA TACATCTACG AGACGTAATA   
  
  
- ATACGACGTT ACAAACTGAG AAATCTACTA ACGGAGGGTG AACTTTCAGG ACTCTCCGAC TCGTATCTCT   
  
  
- TCTTGGTAGA ACCTTTTCTC TAGTTTCTTC GGTATCCAAC ACTCTTCCTA CTTTGTTTGA TAGACTTCAA   
  
  
- ACTCTACGAC CTCTGAACCT CTCCCTCCTA CCTCTCAGTA CCTAAAAGTC CATACTTCGA TTCAAGGTCC   
  
  
- CGTTGATATG TTCGTTTCGA CGAAAATTTT TACCCTTCGG TAATAGTATG GATAAACCTT CTTCTGACAC   
  
  
- CTCCACCACC CAAGTCTCAA ACACTTGCTC TACTACCTTA CCGATAGAGA GAACCGACCG TTCCATCCAA   
  
  
- GGAGCGTTGA CGGAGCCATA CCGCAAGACA CAC

+     ERE

| Site Name | Organism | Position | Strand | Matrix score. | sequence | function |
| --- | --- | --- | --- | --- | --- | --- |
| ERE | Nicotiana glutinos | 1343 | + | 8 | ATTTTAAA |  |

>HU07G00272.1   
+ -Up\_Stream \_Len000ACCAAC GATTCTTTAC TGAATCAGCT ACATTTTTGG ATTGTGTTAA GGTATCTTTT   
  
  
+ AGATCTTCAG GAGGAGGGGG CTGAAGTAGC TTGGTTACTT TTGCGCTTAT CAACTGTGGA AGAATATTAT   
  
  
+ TGAGACTAGT GTAGAAGAAT TAGTAGTTTC CTCCACATTG GAAAAAAACC ATGAAATTAA CGCTTTAGGC   
  
  
+ CCTCAGAGTA CCAAGTGTCA TCGTAAATGA TTGAAATTTC AAGTCTTAGC TGAGTCATGA AGTCCTGATT   
  
  
+ TCCCATGACA AATTGATGTT GTTGTCTAAA GCTTGGCGGG TGATTTGAAA GAAGATTTAG ATCAGGCTGA   
  
  
+ CAATACACCA AATCTGGGAG CAAGGAACTG GATTTTTCTG TGTCTAGAAA AGCTTTCCGC TTTCTGAACA   
  
  
+ ATTGAGAAAA CCATGCATAG GAAATTTCTT GATATTTAGA TCATAGCAGA CTTAACAATC TGGCTTTACC   
  
  
+ GAACTGTCCT CTACTTCTAA GTTGTGAAGG TGTGAAAGAT AAACCATGTC AAACTTAGTG TCCAACAGAA   
  
  
+ ACGCATCTCT ACTTCTCCAG GCAAAGTTGT ACTTCAGATC TTCTACACCT TTTCTAGGCA GGACTTTGGG   
  
  
+ ATATAGAGTT TGGCCTGGGT GAAAAGTAGG AAGTGGGGAA GTAGAAAATG ATGAAAGAAG GCACCACTTG   
  
  
+ GGTCCAGATA CATAGGCCTA CGCACGAGAA CCTTTGCATG GTCTAGATAC ATGACTGTAT ACTTCTTCCT   
  
  
+ TTCCTTTACC TGTTGGAGAT TATCCTAGGA TTTTACATGG CACCAGCTGG ATTTGTTAAT GACATAAACA   
  
  
+ TATCTTTAAT TACAGTTGCT TATGGTATAC ATTTAAACTA TTCGATAGTT GACAGCTGAT GTCCTTATCC   
  
  
+ TTGTGCAGCT TATTCCTCGA TAGAATAATC TTTGGACATT TCTGTCTAGG TGATAAAGGG CAAAAACTAA   
  
  
+ GATACAAGAA ATAGAACATG TCCTCCATTT ACAAGACGTA TCTGTTTTTG GTTTTGGTCA ATGATCCCTG   
  
  
+ TTGTCCTCAA GCATTAACAC AGAATGATCA TTATTGACTT GGACTGAAAG GTTTTGGGAA CTCCATAGTA   
  
  
+ TGCCTTTTCA ATAAAATCAA AAGCAAATTT GCTGTTCACT GATAACTGCA GTTGTGTTGC TTTGCTGTTT   
  
  
+ CCATACCAGA CTCGTAACCA AGCTGATAGC AAATGTGCAA AGGTACAGTA TAATTAACGA TGTTTTCATA   
  
  
+ TTGCTACGAG TTCTGAAACA TGATATAAGA AAACACCAGA ACCCAAATAC CTACATAGGT CCTAGCCCTG   
  
  
+ GAATGGCTAT TTTAAATATC CAAATGGACT TACAGAATTC GCAGAGTTGT TTGGTTTGTT TAACAATCAT   
  
  
+ TGAATTGTTA GCTATGAGAA TCTGCGGGAC AATCGTAATC TTTGGCGATT ACTTCGATTG GCATACTGAT   
  
  
+ GTTTTTTTAT TTGCGTATCT AAGCATGGTG GTGTACCTTT GATTTGTGTG TCTAGGTAGT GTGTGTAGAT   
  
  
+ AAATATCATA GAACTCAGAC GTTGGTAGGG TTATGTTGAG CACTTTCTCT TGCTGTTTGT CCTTTGTGAT   
  
  
+ GGCACCTCTT GCTTTTCCGA ACCACCAAAA GTCTCATTGA CAAAACTCTT CAAACATTGC AAACTGCCCC   
  
  
+ CTTAAATATG TTTTATTAAA AAAAATGGAA GAGCTCAAAC TGTTGAGTGA GCCTAGATTC CAAGATTTAG   
  
  
+ AAACAGTAAA CTTGATAGAT ATATTTATAT GAACTATTGT TAGATGCAAC ACCCCCAACT TGAAACACAA   
  
  
+ AAGAGAAATT AAAAACAAAA AGGTTTTGCA CTATTTATAT ATCCTGTGTT CTTTGTGCTA GTCTTATTTA   
  
  
+ ATGTTAATTG GTAAGCATTA TTTGTTGGAT TATTCCACTG CACTTTCTCT TACTAACTTC AGCATTTCAT   
  
  
+ TCCTTGTTCT TCAGCTTTTG AACTTCTTTA GCCTATTTTG TTGTCTTTAT GGTTATGGAA GCCATCCAGA   
  
  
+ AGAAGAATGA TGAACTTCTG AGTCTTAGCT TGGCCATTGT CGGTCACTCT GATCGCACTG AGAAAAAACT   
  
  
+ GAAGAGGCGG ATTGATGTCT CCGATCCTCT GATCACTTCA GGTGAAGGCT GCGAAGGGAA GATAATCAGG   
  
  
+ CTCCTCCAAG AGAGACAGAA CTTGTTGAAC ATCAAGCAAA GAGGAAAAGG CGTCATTCAA GAAGGGAAGG   
  
  
+ GTCTTCATCT TATCCATCTG CTCCTTGTAT CTGCCACCTT AATCAACGAA AACAACATCA GTTCAGCTGT   
  
  
+ TGATAATCTT ATTGAGCTCT TCCAATATGT CTCCGAAAGT GGTGATTCAG GACAAAGGGT TGCTGCTTAC   
  
  
+ TTTGCGGATG GGTTAACAGC AAGGATCTTA ACTCAGAGGT CTCCATTTTA TCGCCTGATA CTGGGCAAAC   
  
  
+ CAGCACCTGC TGAAGAGTTT TCAGCTTTTA CTCACTTATA TAGAGCCTCT CCATTCTATC AGTTTGCTCA   
  
  
+ TTTCACAGCC AATCAGGCAA TTGTAGAAGC ATTTGAGAGG GAGGAAGAGA GTAACAACTG GGCTTTGCAT   
  
  
+ GTAATTGATT TGGACATCTT GCATGGTTTC CAGTGGCCTT CTCTTATTCA GTCTCTCTCC GAAGAGGCTA   
  
  
+ CTTGTTCAAA CCGTTCATTG TCTCTTCAAA TTACAGGCTT CGGAAGAAGC TTAGAAGAGC TCATTGAAAC   
  
  
+ AGAAGCTCGG CTGGTAAGTT TCTCTAAGAG CTTTCAGAAT ATCAACTTTG AATTCCACGG GTTTCTGAGA   
  
  
+ GGCTCGAAAC TCAGGAACCT AAGGAGGGAG AATGAAACAG TAGTTGTAAA TCTCGTTTTC CACCTCAGTA   
  
  
+ CTTTGAAAGA TACCGTACAG ATTTCTGACA CCTTGACTTC TGTACATTCA CTAAACCCCT CTATTGTGGT   
  
  
+ ATTAGTCGAG AGAGAAGGAA GTCGAAACAG ATGTGGGTTC CTCTCAAGTT ATGTAGATGC TCTGCATTAT   
  
  
+ TATGCTGCAA TGTTTGACTC TTTAGATGAT TGCCTCCCAC TTGAAAGTCC TGAGAGGCTG AGCATAGAGA   
  
  
+ AGAACCATCT TGGAAAAGAG ATCAAAGAAG CCATAGGTTG TGAGAAGGAT GAAACAAACT ATCTGAAGTT   
  
  
+ TGAGATGCTG GAGACTTGGA GAGGGAGGAT GGAGAGTCAT GGATTTTCAG GTATGAAGCT AAGTTCCAGG   
  
  
+ GCAACTATAC AAGCAAAGCT GCTTTTAAAA ATGGGAAGCC ATTATCATAC CTATTTGGAA GAAGACTGTG   
  
  
+ GAGGTGGTGG GTTCAGAGTT TGTGAACGAG ATGATGGAAT GGCTATCTCT CTTGGCTGGC AAGGTAGGTT   
  
  
+ CCTCGCAACT GCCTCGGTAT GGCGTTCTGT GTG  

- -Up\_Stream \_Len000TGGTTG CTAAGAAATG ACTTAGTCGA TGTAAAAACC TAACACAATT CCATAGAAAA   
  
  
- TCTAGAAGTC CTCCTCCCCC GACTTCATCG AACCAATGAA AACGCGAATA GTTGACACCT TCTTATAATA   
  
  
- ACTCTGATCA CATCTTCTTA ATCATCAAAG GAGGTGTAAC CTTTTTTTGG TACTTTAATT GCGAAATCCG   
  
  
- GGAGTCTCAT GGTTCACAGT AGCATTTACT AACTTTAAAG TTCAGAATCG ACTCAGTACT TCAGGACTAA   
  
  
- AGGGTACTGT TTAACTACAA CAACAGATTT CGAACCGCCC ACTAAACTTT CTTCTAAATC TAGTCCGACT   
  
  
- GTTATGTGGT TTAGACCCTC GTTCCTTGAC CTAAAAAGAC ACAGATCTTT TCGAAAGGCG AAAGACTTGT   
  
  
- TAACTCTTTT GGTACGTATC CTTTAAAGAA CTATAAATCT AGTATCGTCT GAATTGTTAG ACCGAAATGG   
  
  
- CTTGACAGGA GATGAAGATT CAACACTTCC ACACTTTCTA TTTGGTACAG TTTGAATCAC AGGTTGTCTT   
  
  
- TGCGTAGAGA TGAAGAGGTC CGTTTCAACA TGAAGTCTAG AAGATGTGGA AAAGATCCGT CCTGAAACCC   
  
  
- TATATCTCAA ACCGGACCCA CTTTTCATCC TTCACCCCTT CATCTTTTAC TACTTTCTTC CGTGGTGAAC   
  
  
- CCAGGTCTAT GTATCCGGAT GCGTGCTCTT GGAAACGTAC CAGATCTATG TACTGACATA TGAAGAAGGA   
  
  
- AAGGAAATGG ACAACCTCTA ATAGGATCCT AAAATGTACC GTGGTCGACC TAAACAATTA CTGTATTTGT   
  
  
- ATAGAAATTA ATGTCAACGA ATACCATATG TAAATTTGAT AAGCTATCAA CTGTCGACTA CAGGAATAGG   
  
  
- AACACGTCGA ATAAGGAGCT ATCTTATTAG AAACCTGTAA AGACAGATCC ACTATTTCCC GTTTTTGATT   
  
  
- CTATGTTCTT TATCTTGTAC AGGAGGTAAA TGTTCTGCAT AGACAAAAAC CAAAACCAGT TACTAGGGAC   
  
  
- AACAGGAGTT CGTAATTGTG TCTTACTAGT AATAACTGAA CCTGACTTTC CAAAACCCTT GAGGTATCAT   
  
  
- ACGGAAAAGT TATTTTAGTT TTCGTTTAAA CGACAAGTGA CTATTGACGT CAACACAACG AAACGACAAA   
  
  
- GGTATGGTCT GAGCATTGGT TCGACTATCG TTTACACGTT TCCATGTCAT ATTAATTGCT ACAAAAGTAT   
  
  
- AACGATGCTC AAGACTTTGT ACTATATTCT TTTGTGGTCT TGGGTTTATG GATGTATCCA GGATCGGGAC   
  
  
- CTTACCGATA AAATTTATAG GTTTACCTGA ATGTCTTAAG CGTCTCAACA AACCAAACAA ATTGTTAGTA   
  
  
- ACTTAACAAT CGATACTCTT AGACGCCCTG TTAGCATTAG AAACCGCTAA TGAAGCTAAC CGTATGACTA   
  
  
- CAAAAAAATA AACGCATAGA TTCGTACCAC CACATGGAAA CTAAACACAC AGATCCATCA CACACATCTA   
  
  
- TTTATAGTAT CTTGAGTCTG CAACCATCCC AATACAACTC GTGAAAGAGA ACGACAAACA GGAAACACTA   
  
  
- CCGTGGAGAA CGAAAAGGCT TGGTGGTTTT CAGAGTAACT GTTTTGAGAA GTTTGTAACG TTTGACGGGG   
  
  
- GAATTTATAC AAAATAATTT TTTTTACCTT CTCGAGTTTG ACAACTCACT CGGATCTAAG GTTCTAAATC   
  
  
- TTTGTCATTT GAACTATCTA TATAAATATA CTTGATAACA ATCTACGTTG TGGGGGTTGA ACTTTGTGTT   
  
  
- TTCTCTTTAA TTTTTGTTTT TCCAAAACGT GATAAATATA TAGGACACAA GAAACACGAT CAGAATAAAT   
  
  
- TACAATTAAC CATTCGTAAT AAACAACCTA ATAAGGTGAC GTGAAAGAGA ATGATTGAAG TCGTAAAGTA   
  
  
- AGGAACAAGA AGTCGAAAAC TTGAAGAAAT CGGATAAAAC AACAGAAATA CCAATACCTT CGGTAGGTCT   
  
  
- TCTTCTTACT ACTTGAAGAC TCAGAATCGA ACCGGTAACA GCCAGTGAGA CTAGCGTGAC TCTTTTTTGA   
  
  
- CTTCTCCGCC TAACTACAGA GGCTAGGAGA CTAGTGAAGT CCACTTCCGA CGCTTCCCTT CTATTAGTCC   
  
  
- GAGGAGGTTC TCTCTGTCTT GAACAACTTG TAGTTCGTTT CTCCTTTTCC GCAGTAAGTT CTTCCCTTCC   
  
  
- CAGAAGTAGA ATAGGTAGAC GAGGAACATA GACGGTGGAA TTAGTTGCTT TTGTTGTAGT CAAGTCGACA   
  
  
- ACTATTAGAA TAACTCGAGA AGGTTATACA GAGGCTTTCA CCACTAAGTC CTGTTTCCCA ACGACGAATG   
  
  
- AAACGCCTAC CCAATTGTCG TTCCTAGAAT TGAGTCTCCA GAGGTAAAAT AGCGGACTAT GACCCGTTTG   
  
  
- GTCGTGGACG ACTTCTCAAA AGTCGAAAAT GAGTGAATAT ATCTCGGAGA GGTAAGATAG TCAAACGAGT   
  
  
- AAAGTGTCGG TTAGTCCGTT AACATCTTCG TAAACTCTCC CTCCTTCTCT CATTGTTGAC CCGAAACGTA   
  
  
- CATTAACTAA ACCTGTAGAA CGTACCAAAG GTCACCGGAA GAGAATAAGT CAGAGAGAGG CTTCTCCGAT   
  
  
- GAACAAGTTT GGCAAGTAAC AGAGAAGTTT AATGTCCGAA GCCTTCTTCG AATCTTCTCG AGTAACTTTG   
  
  
- TCTTCGAGCC GACCATTCAA AGAGATTCTC GAAAGTCTTA TAGTTGAAAC TTAAGGTGCC CAAAGACTCT   
  
  
- CCGAGCTTTG AGTCCTTGGA TTCCTCCCTC TTACTTTGTC ATCAACATTT AGAGCAAAAG GTGGAGTCAT   
  
  
- GAAACTTTCT ATGGCATGTC TAAAGACTGT GGAACTGAAG ACATGTAAGT GATTTGGGGA GATAACACCA   
  
  
- TAATCAGCTC TCTCTTCCTT CAGCTTTGTC TACACCCAAG GAGAGTTCAA TACATCTACG AGACGTAATA   
  
  
- ATACGACGTT ACAAACTGAG AAATCTACTA ACGGAGGGTG AACTTTCAGG ACTCTCCGAC TCGTATCTCT   
  
  
- TCTTGGTAGA ACCTTTTCTC TAGTTTCTTC GGTATCCAAC ACTCTTCCTA CTTTGTTTGA TAGACTTCAA   
  
  
- ACTCTACGAC CTCTGAACCT CTCCCTCCTA CCTCTCAGTA CCTAAAAGTC CATACTTCGA TTCAAGGTCC   
  
  
- CGTTGATATG TTCGTTTCGA CGAAAATTTT TACCCTTCGG TAATAGTATG GATAAACCTT CTTCTGACAC   
  
  
- CTCCACCACC CAAGTCTCAA ACACTTGCTC TACTACCTTA CCGATAGAGA GAACCGACCG TTCCATCCAA   
  
  
- GGAGCGTTGA CGGAGCCATA CCGCAAGACA CAC

+     GARE-motif

| Site Name | Organism | Position | Strand | Matrix score. | sequence | function |
| --- | --- | --- | --- | --- | --- | --- |
| GARE-motif | Brassica oleracea | 557 | - | 7 | TCTGTTG | gibberellin-responsive element |

>HU07G00272.1   
+ -Up\_Stream \_Len000ACCAAC GATTCTTTAC TGAATCAGCT ACATTTTTGG ATTGTGTTAA GGTATCTTTT   
  
  
+ AGATCTTCAG GAGGAGGGGG CTGAAGTAGC TTGGTTACTT TTGCGCTTAT CAACTGTGGA AGAATATTAT   
  
  
+ TGAGACTAGT GTAGAAGAAT TAGTAGTTTC CTCCACATTG GAAAAAAACC ATGAAATTAA CGCTTTAGGC   
  
  
+ CCTCAGAGTA CCAAGTGTCA TCGTAAATGA TTGAAATTTC AAGTCTTAGC TGAGTCATGA AGTCCTGATT   
  
  
+ TCCCATGACA AATTGATGTT GTTGTCTAAA GCTTGGCGGG TGATTTGAAA GAAGATTTAG ATCAGGCTGA   
  
  
+ CAATACACCA AATCTGGGAG CAAGGAACTG GATTTTTCTG TGTCTAGAAA AGCTTTCCGC TTTCTGAACA   
  
  
+ ATTGAGAAAA CCATGCATAG GAAATTTCTT GATATTTAGA TCATAGCAGA CTTAACAATC TGGCTTTACC   
  
  
+ GAACTGTCCT CTACTTCTAA GTTGTGAAGG TGTGAAAGAT AAACCATGTC AAACTTAGTG TCCAACAGAA   
  
  
+ ACGCATCTCT ACTTCTCCAG GCAAAGTTGT ACTTCAGATC TTCTACACCT TTTCTAGGCA GGACTTTGGG   
  
  
+ ATATAGAGTT TGGCCTGGGT GAAAAGTAGG AAGTGGGGAA GTAGAAAATG ATGAAAGAAG GCACCACTTG   
  
  
+ GGTCCAGATA CATAGGCCTA CGCACGAGAA CCTTTGCATG GTCTAGATAC ATGACTGTAT ACTTCTTCCT   
  
  
+ TTCCTTTACC TGTTGGAGAT TATCCTAGGA TTTTACATGG CACCAGCTGG ATTTGTTAAT GACATAAACA   
  
  
+ TATCTTTAAT TACAGTTGCT TATGGTATAC ATTTAAACTA TTCGATAGTT GACAGCTGAT GTCCTTATCC   
  
  
+ TTGTGCAGCT TATTCCTCGA TAGAATAATC TTTGGACATT TCTGTCTAGG TGATAAAGGG CAAAAACTAA   
  
  
+ GATACAAGAA ATAGAACATG TCCTCCATTT ACAAGACGTA TCTGTTTTTG GTTTTGGTCA ATGATCCCTG   
  
  
+ TTGTCCTCAA GCATTAACAC AGAATGATCA TTATTGACTT GGACTGAAAG GTTTTGGGAA CTCCATAGTA   
  
  
+ TGCCTTTTCA ATAAAATCAA AAGCAAATTT GCTGTTCACT GATAACTGCA GTTGTGTTGC TTTGCTGTTT   
  
  
+ CCATACCAGA CTCGTAACCA AGCTGATAGC AAATGTGCAA AGGTACAGTA TAATTAACGA TGTTTTCATA   
  
  
+ TTGCTACGAG TTCTGAAACA TGATATAAGA AAACACCAGA ACCCAAATAC CTACATAGGT CCTAGCCCTG   
  
  
+ GAATGGCTAT TTTAAATATC CAAATGGACT TACAGAATTC GCAGAGTTGT TTGGTTTGTT TAACAATCAT   
  
  
+ TGAATTGTTA GCTATGAGAA TCTGCGGGAC AATCGTAATC TTTGGCGATT ACTTCGATTG GCATACTGAT   
  
  
+ GTTTTTTTAT TTGCGTATCT AAGCATGGTG GTGTACCTTT GATTTGTGTG TCTAGGTAGT GTGTGTAGAT   
  
  
+ AAATATCATA GAACTCAGAC GTTGGTAGGG TTATGTTGAG CACTTTCTCT TGCTGTTTGT CCTTTGTGAT   
  
  
+ GGCACCTCTT GCTTTTCCGA ACCACCAAAA GTCTCATTGA CAAAACTCTT CAAACATTGC AAACTGCCCC   
  
  
+ CTTAAATATG TTTTATTAAA AAAAATGGAA GAGCTCAAAC TGTTGAGTGA GCCTAGATTC CAAGATTTAG   
  
  
+ AAACAGTAAA CTTGATAGAT ATATTTATAT GAACTATTGT TAGATGCAAC ACCCCCAACT TGAAACACAA   
  
  
+ AAGAGAAATT AAAAACAAAA AGGTTTTGCA CTATTTATAT ATCCTGTGTT CTTTGTGCTA GTCTTATTTA   
  
  
+ ATGTTAATTG GTAAGCATTA TTTGTTGGAT TATTCCACTG CACTTTCTCT TACTAACTTC AGCATTTCAT   
  
  
+ TCCTTGTTCT TCAGCTTTTG AACTTCTTTA GCCTATTTTG TTGTCTTTAT GGTTATGGAA GCCATCCAGA   
  
  
+ AGAAGAATGA TGAACTTCTG AGTCTTAGCT TGGCCATTGT CGGTCACTCT GATCGCACTG AGAAAAAACT   
  
  
+ GAAGAGGCGG ATTGATGTCT CCGATCCTCT GATCACTTCA GGTGAAGGCT GCGAAGGGAA GATAATCAGG   
  
  
+ CTCCTCCAAG AGAGACAGAA CTTGTTGAAC ATCAAGCAAA GAGGAAAAGG CGTCATTCAA GAAGGGAAGG   
  
  
+ GTCTTCATCT TATCCATCTG CTCCTTGTAT CTGCCACCTT AATCAACGAA AACAACATCA GTTCAGCTGT   
  
  
+ TGATAATCTT ATTGAGCTCT TCCAATATGT CTCCGAAAGT GGTGATTCAG GACAAAGGGT TGCTGCTTAC   
  
  
+ TTTGCGGATG GGTTAACAGC AAGGATCTTA ACTCAGAGGT CTCCATTTTA TCGCCTGATA CTGGGCAAAC   
  
  
+ CAGCACCTGC TGAAGAGTTT TCAGCTTTTA CTCACTTATA TAGAGCCTCT CCATTCTATC AGTTTGCTCA   
  
  
+ TTTCACAGCC AATCAGGCAA TTGTAGAAGC ATTTGAGAGG GAGGAAGAGA GTAACAACTG GGCTTTGCAT   
  
  
+ GTAATTGATT TGGACATCTT GCATGGTTTC CAGTGGCCTT CTCTTATTCA GTCTCTCTCC GAAGAGGCTA   
  
  
+ CTTGTTCAAA CCGTTCATTG TCTCTTCAAA TTACAGGCTT CGGAAGAAGC TTAGAAGAGC TCATTGAAAC   
  
  
+ AGAAGCTCGG CTGGTAAGTT TCTCTAAGAG CTTTCAGAAT ATCAACTTTG AATTCCACGG GTTTCTGAGA   
  
  
+ GGCTCGAAAC TCAGGAACCT AAGGAGGGAG AATGAAACAG TAGTTGTAAA TCTCGTTTTC CACCTCAGTA   
  
  
+ CTTTGAAAGA TACCGTACAG ATTTCTGACA CCTTGACTTC TGTACATTCA CTAAACCCCT CTATTGTGGT   
  
  
+ ATTAGTCGAG AGAGAAGGAA GTCGAAACAG ATGTGGGTTC CTCTCAAGTT ATGTAGATGC TCTGCATTAT   
  
  
+ TATGCTGCAA TGTTTGACTC TTTAGATGAT TGCCTCCCAC TTGAAAGTCC TGAGAGGCTG AGCATAGAGA   
  
  
+ AGAACCATCT TGGAAAAGAG ATCAAAGAAG CCATAGGTTG TGAGAAGGAT GAAACAAACT ATCTGAAGTT   
  
  
+ TGAGATGCTG GAGACTTGGA GAGGGAGGAT GGAGAGTCAT GGATTTTCAG GTATGAAGCT AAGTTCCAGG   
  
  
+ GCAACTATAC AAGCAAAGCT GCTTTTAAAA ATGGGAAGCC ATTATCATAC CTATTTGGAA GAAGACTGTG   
  
  
+ GAGGTGGTGG GTTCAGAGTT TGTGAACGAG ATGATGGAAT GGCTATCTCT CTTGGCTGGC AAGGTAGGTT   
  
  
+ CCTCGCAACT GCCTCGGTAT GGCGTTCTGT GTG  

- -Up\_Stream \_Len000TGGTTG CTAAGAAATG ACTTAGTCGA TGTAAAAACC TAACACAATT CCATAGAAAA   
  
  
- TCTAGAAGTC CTCCTCCCCC GACTTCATCG AACCAATGAA AACGCGAATA GTTGACACCT TCTTATAATA   
  
  
- ACTCTGATCA CATCTTCTTA ATCATCAAAG GAGGTGTAAC CTTTTTTTGG TACTTTAATT GCGAAATCCG   
  
  
- GGAGTCTCAT GGTTCACAGT AGCATTTACT AACTTTAAAG TTCAGAATCG ACTCAGTACT TCAGGACTAA   
  
  
- AGGGTACTGT TTAACTACAA CAACAGATTT CGAACCGCCC ACTAAACTTT CTTCTAAATC TAGTCCGACT   
  
  
- GTTATGTGGT TTAGACCCTC GTTCCTTGAC CTAAAAAGAC ACAGATCTTT TCGAAAGGCG AAAGACTTGT   
  
  
- TAACTCTTTT GGTACGTATC CTTTAAAGAA CTATAAATCT AGTATCGTCT GAATTGTTAG ACCGAAATGG   
  
  
- CTTGACAGGA GATGAAGATT CAACACTTCC ACACTTTCTA TTTGGTACAG TTTGAATCAC AGGTTGTCTT   
  
  
- TGCGTAGAGA TGAAGAGGTC CGTTTCAACA TGAAGTCTAG AAGATGTGGA AAAGATCCGT CCTGAAACCC   
  
  
- TATATCTCAA ACCGGACCCA CTTTTCATCC TTCACCCCTT CATCTTTTAC TACTTTCTTC CGTGGTGAAC   
  
  
- CCAGGTCTAT GTATCCGGAT GCGTGCTCTT GGAAACGTAC CAGATCTATG TACTGACATA TGAAGAAGGA   
  
  
- AAGGAAATGG ACAACCTCTA ATAGGATCCT AAAATGTACC GTGGTCGACC TAAACAATTA CTGTATTTGT   
  
  
- ATAGAAATTA ATGTCAACGA ATACCATATG TAAATTTGAT AAGCTATCAA CTGTCGACTA CAGGAATAGG   
  
  
- AACACGTCGA ATAAGGAGCT ATCTTATTAG AAACCTGTAA AGACAGATCC ACTATTTCCC GTTTTTGATT   
  
  
- CTATGTTCTT TATCTTGTAC AGGAGGTAAA TGTTCTGCAT AGACAAAAAC CAAAACCAGT TACTAGGGAC   
  
  
- AACAGGAGTT CGTAATTGTG TCTTACTAGT AATAACTGAA CCTGACTTTC CAAAACCCTT GAGGTATCAT   
  
  
- ACGGAAAAGT TATTTTAGTT TTCGTTTAAA CGACAAGTGA CTATTGACGT CAACACAACG AAACGACAAA   
  
  
- GGTATGGTCT GAGCATTGGT TCGACTATCG TTTACACGTT TCCATGTCAT ATTAATTGCT ACAAAAGTAT   
  
  
- AACGATGCTC AAGACTTTGT ACTATATTCT TTTGTGGTCT TGGGTTTATG GATGTATCCA GGATCGGGAC   
  
  
- CTTACCGATA AAATTTATAG GTTTACCTGA ATGTCTTAAG CGTCTCAACA AACCAAACAA ATTGTTAGTA   
  
  
- ACTTAACAAT CGATACTCTT AGACGCCCTG TTAGCATTAG AAACCGCTAA TGAAGCTAAC CGTATGACTA   
  
  
- CAAAAAAATA AACGCATAGA TTCGTACCAC CACATGGAAA CTAAACACAC AGATCCATCA CACACATCTA   
  
  
- TTTATAGTAT CTTGAGTCTG CAACCATCCC AATACAACTC GTGAAAGAGA ACGACAAACA GGAAACACTA   
  
  
- CCGTGGAGAA CGAAAAGGCT TGGTGGTTTT CAGAGTAACT GTTTTGAGAA GTTTGTAACG TTTGACGGGG   
  
  
- GAATTTATAC AAAATAATTT TTTTTACCTT CTCGAGTTTG ACAACTCACT CGGATCTAAG GTTCTAAATC   
  
  
- TTTGTCATTT GAACTATCTA TATAAATATA CTTGATAACA ATCTACGTTG TGGGGGTTGA ACTTTGTGTT   
  
  
- TTCTCTTTAA TTTTTGTTTT TCCAAAACGT GATAAATATA TAGGACACAA GAAACACGAT CAGAATAAAT   
  
  
- TACAATTAAC CATTCGTAAT AAACAACCTA ATAAGGTGAC GTGAAAGAGA ATGATTGAAG TCGTAAAGTA   
  
  
- AGGAACAAGA AGTCGAAAAC TTGAAGAAAT CGGATAAAAC AACAGAAATA CCAATACCTT CGGTAGGTCT   
  
  
- TCTTCTTACT ACTTGAAGAC TCAGAATCGA ACCGGTAACA GCCAGTGAGA CTAGCGTGAC TCTTTTTTGA   
  
  
- CTTCTCCGCC TAACTACAGA GGCTAGGAGA CTAGTGAAGT CCACTTCCGA CGCTTCCCTT CTATTAGTCC   
  
  
- GAGGAGGTTC TCTCTGTCTT GAACAACTTG TAGTTCGTTT CTCCTTTTCC GCAGTAAGTT CTTCCCTTCC   
  
  
- CAGAAGTAGA ATAGGTAGAC GAGGAACATA GACGGTGGAA TTAGTTGCTT TTGTTGTAGT CAAGTCGACA   
  
  
- ACTATTAGAA TAACTCGAGA AGGTTATACA GAGGCTTTCA CCACTAAGTC CTGTTTCCCA ACGACGAATG   
  
  
- AAACGCCTAC CCAATTGTCG TTCCTAGAAT TGAGTCTCCA GAGGTAAAAT AGCGGACTAT GACCCGTTTG   
  
  
- GTCGTGGACG ACTTCTCAAA AGTCGAAAAT GAGTGAATAT ATCTCGGAGA GGTAAGATAG TCAAACGAGT   
  
  
- AAAGTGTCGG TTAGTCCGTT AACATCTTCG TAAACTCTCC CTCCTTCTCT CATTGTTGAC CCGAAACGTA   
  
  
- CATTAACTAA ACCTGTAGAA CGTACCAAAG GTCACCGGAA GAGAATAAGT CAGAGAGAGG CTTCTCCGAT   
  
  
- GAACAAGTTT GGCAAGTAAC AGAGAAGTTT AATGTCCGAA GCCTTCTTCG AATCTTCTCG AGTAACTTTG   
  
  
- TCTTCGAGCC GACCATTCAA AGAGATTCTC GAAAGTCTTA TAGTTGAAAC TTAAGGTGCC CAAAGACTCT   
  
  
- CCGAGCTTTG AGTCCTTGGA TTCCTCCCTC TTACTTTGTC ATCAACATTT AGAGCAAAAG GTGGAGTCAT   
  
  
- GAAACTTTCT ATGGCATGTC TAAAGACTGT GGAACTGAAG ACATGTAAGT GATTTGGGGA GATAACACCA   
  
  
- TAATCAGCTC TCTCTTCCTT CAGCTTTGTC TACACCCAAG GAGAGTTCAA TACATCTACG AGACGTAATA   
  
  
- ATACGACGTT ACAAACTGAG AAATCTACTA ACGGAGGGTG AACTTTCAGG ACTCTCCGAC TCGTATCTCT   
  
  
- TCTTGGTAGA ACCTTTTCTC TAGTTTCTTC GGTATCCAAC ACTCTTCCTA CTTTGTTTGA TAGACTTCAA   
  
  
- ACTCTACGAC CTCTGAACCT CTCCCTCCTA CCTCTCAGTA CCTAAAAGTC CATACTTCGA TTCAAGGTCC   
  
  
- CGTTGATATG TTCGTTTCGA CGAAAATTTT TACCCTTCGG TAATAGTATG GATAAACCTT CTTCTGACAC   
  
  
- CTCCACCACC CAAGTCTCAA ACACTTGCTC TACTACCTTA CCGATAGAGA GAACCGACCG TTCCATCCAA   
  
  
- GGAGCGTTGA CGGAGCCATA CCGCAAGACA CAC

+     GATA-motif

| Site Name | Organism | Position | Strand | Matrix score. | sequence | function |
| --- | --- | --- | --- | --- | --- | --- |
| GATA-motif | Solanum tuberosum | 907 | - | 10 | AAGGATAAGG | part of a light responsive element |

>HU07G00272.1   
+ -Up\_Stream \_Len000ACCAAC GATTCTTTAC TGAATCAGCT ACATTTTTGG ATTGTGTTAA GGTATCTTTT   
  
  
+ AGATCTTCAG GAGGAGGGGG CTGAAGTAGC TTGGTTACTT TTGCGCTTAT CAACTGTGGA AGAATATTAT   
  
  
+ TGAGACTAGT GTAGAAGAAT TAGTAGTTTC CTCCACATTG GAAAAAAACC ATGAAATTAA CGCTTTAGGC   
  
  
+ CCTCAGAGTA CCAAGTGTCA TCGTAAATGA TTGAAATTTC AAGTCTTAGC TGAGTCATGA AGTCCTGATT   
  
  
+ TCCCATGACA AATTGATGTT GTTGTCTAAA GCTTGGCGGG TGATTTGAAA GAAGATTTAG ATCAGGCTGA   
  
  
+ CAATACACCA AATCTGGGAG CAAGGAACTG GATTTTTCTG TGTCTAGAAA AGCTTTCCGC TTTCTGAACA   
  
  
+ ATTGAGAAAA CCATGCATAG GAAATTTCTT GATATTTAGA TCATAGCAGA CTTAACAATC TGGCTTTACC   
  
  
+ GAACTGTCCT CTACTTCTAA GTTGTGAAGG TGTGAAAGAT AAACCATGTC AAACTTAGTG TCCAACAGAA   
  
  
+ ACGCATCTCT ACTTCTCCAG GCAAAGTTGT ACTTCAGATC TTCTACACCT TTTCTAGGCA GGACTTTGGG   
  
  
+ ATATAGAGTT TGGCCTGGGT GAAAAGTAGG AAGTGGGGAA GTAGAAAATG ATGAAAGAAG GCACCACTTG   
  
  
+ GGTCCAGATA CATAGGCCTA CGCACGAGAA CCTTTGCATG GTCTAGATAC ATGACTGTAT ACTTCTTCCT   
  
  
+ TTCCTTTACC TGTTGGAGAT TATCCTAGGA TTTTACATGG CACCAGCTGG ATTTGTTAAT GACATAAACA   
  
  
+ TATCTTTAAT TACAGTTGCT TATGGTATAC ATTTAAACTA TTCGATAGTT GACAGCTGAT GTCCTTATCC   
  
  
+ TTGTGCAGCT TATTCCTCGA TAGAATAATC TTTGGACATT TCTGTCTAGG TGATAAAGGG CAAAAACTAA   
  
  
+ GATACAAGAA ATAGAACATG TCCTCCATTT ACAAGACGTA TCTGTTTTTG GTTTTGGTCA ATGATCCCTG   
  
  
+ TTGTCCTCAA GCATTAACAC AGAATGATCA TTATTGACTT GGACTGAAAG GTTTTGGGAA CTCCATAGTA   
  
  
+ TGCCTTTTCA ATAAAATCAA AAGCAAATTT GCTGTTCACT GATAACTGCA GTTGTGTTGC TTTGCTGTTT   
  
  
+ CCATACCAGA CTCGTAACCA AGCTGATAGC AAATGTGCAA AGGTACAGTA TAATTAACGA TGTTTTCATA   
  
  
+ TTGCTACGAG TTCTGAAACA TGATATAAGA AAACACCAGA ACCCAAATAC CTACATAGGT CCTAGCCCTG   
  
  
+ GAATGGCTAT TTTAAATATC CAAATGGACT TACAGAATTC GCAGAGTTGT TTGGTTTGTT TAACAATCAT   
  
  
+ TGAATTGTTA GCTATGAGAA TCTGCGGGAC AATCGTAATC TTTGGCGATT ACTTCGATTG GCATACTGAT   
  
  
+ GTTTTTTTAT TTGCGTATCT AAGCATGGTG GTGTACCTTT GATTTGTGTG TCTAGGTAGT GTGTGTAGAT   
  
  
+ AAATATCATA GAACTCAGAC GTTGGTAGGG TTATGTTGAG CACTTTCTCT TGCTGTTTGT CCTTTGTGAT   
  
  
+ GGCACCTCTT GCTTTTCCGA ACCACCAAAA GTCTCATTGA CAAAACTCTT CAAACATTGC AAACTGCCCC   
  
  
+ CTTAAATATG TTTTATTAAA AAAAATGGAA GAGCTCAAAC TGTTGAGTGA GCCTAGATTC CAAGATTTAG   
  
  
+ AAACAGTAAA CTTGATAGAT ATATTTATAT GAACTATTGT TAGATGCAAC ACCCCCAACT TGAAACACAA   
  
  
+ AAGAGAAATT AAAAACAAAA AGGTTTTGCA CTATTTATAT ATCCTGTGTT CTTTGTGCTA GTCTTATTTA   
  
  
+ ATGTTAATTG GTAAGCATTA TTTGTTGGAT TATTCCACTG CACTTTCTCT TACTAACTTC AGCATTTCAT   
  
  
+ TCCTTGTTCT TCAGCTTTTG AACTTCTTTA GCCTATTTTG TTGTCTTTAT GGTTATGGAA GCCATCCAGA   
  
  
+ AGAAGAATGA TGAACTTCTG AGTCTTAGCT TGGCCATTGT CGGTCACTCT GATCGCACTG AGAAAAAACT   
  
  
+ GAAGAGGCGG ATTGATGTCT CCGATCCTCT GATCACTTCA GGTGAAGGCT GCGAAGGGAA GATAATCAGG   
  
  
+ CTCCTCCAAG AGAGACAGAA CTTGTTGAAC ATCAAGCAAA GAGGAAAAGG CGTCATTCAA GAAGGGAAGG   
  
  
+ GTCTTCATCT TATCCATCTG CTCCTTGTAT CTGCCACCTT AATCAACGAA AACAACATCA GTTCAGCTGT   
  
  
+ TGATAATCTT ATTGAGCTCT TCCAATATGT CTCCGAAAGT GGTGATTCAG GACAAAGGGT TGCTGCTTAC   
  
  
+ TTTGCGGATG GGTTAACAGC AAGGATCTTA ACTCAGAGGT CTCCATTTTA TCGCCTGATA CTGGGCAAAC   
  
  
+ CAGCACCTGC TGAAGAGTTT TCAGCTTTTA CTCACTTATA TAGAGCCTCT CCATTCTATC AGTTTGCTCA   
  
  
+ TTTCACAGCC AATCAGGCAA TTGTAGAAGC ATTTGAGAGG GAGGAAGAGA GTAACAACTG GGCTTTGCAT   
  
  
+ GTAATTGATT TGGACATCTT GCATGGTTTC CAGTGGCCTT CTCTTATTCA GTCTCTCTCC GAAGAGGCTA   
  
  
+ CTTGTTCAAA CCGTTCATTG TCTCTTCAAA TTACAGGCTT CGGAAGAAGC TTAGAAGAGC TCATTGAAAC   
  
  
+ AGAAGCTCGG CTGGTAAGTT TCTCTAAGAG CTTTCAGAAT ATCAACTTTG AATTCCACGG GTTTCTGAGA   
  
  
+ GGCTCGAAAC TCAGGAACCT AAGGAGGGAG AATGAAACAG TAGTTGTAAA TCTCGTTTTC CACCTCAGTA   
  
  
+ CTTTGAAAGA TACCGTACAG ATTTCTGACA CCTTGACTTC TGTACATTCA CTAAACCCCT CTATTGTGGT   
  
  
+ ATTAGTCGAG AGAGAAGGAA GTCGAAACAG ATGTGGGTTC CTCTCAAGTT ATGTAGATGC TCTGCATTAT   
  
  
+ TATGCTGCAA TGTTTGACTC TTTAGATGAT TGCCTCCCAC TTGAAAGTCC TGAGAGGCTG AGCATAGAGA   
  
  
+ AGAACCATCT TGGAAAAGAG ATCAAAGAAG CCATAGGTTG TGAGAAGGAT GAAACAAACT ATCTGAAGTT   
  
  
+ TGAGATGCTG GAGACTTGGA GAGGGAGGAT GGAGAGTCAT GGATTTTCAG GTATGAAGCT AAGTTCCAGG   
  
  
+ GCAACTATAC AAGCAAAGCT GCTTTTAAAA ATGGGAAGCC ATTATCATAC CTATTTGGAA GAAGACTGTG   
  
  
+ GAGGTGGTGG GTTCAGAGTT TGTGAACGAG ATGATGGAAT GGCTATCTCT CTTGGCTGGC AAGGTAGGTT   
  
  
+ CCTCGCAACT GCCTCGGTAT GGCGTTCTGT GTG  

- -Up\_Stream \_Len000TGGTTG CTAAGAAATG ACTTAGTCGA TGTAAAAACC TAACACAATT CCATAGAAAA   
  
  
- TCTAGAAGTC CTCCTCCCCC GACTTCATCG AACCAATGAA AACGCGAATA GTTGACACCT TCTTATAATA   
  
  
- ACTCTGATCA CATCTTCTTA ATCATCAAAG GAGGTGTAAC CTTTTTTTGG TACTTTAATT GCGAAATCCG   
  
  
- GGAGTCTCAT GGTTCACAGT AGCATTTACT AACTTTAAAG TTCAGAATCG ACTCAGTACT TCAGGACTAA   
  
  
- AGGGTACTGT TTAACTACAA CAACAGATTT CGAACCGCCC ACTAAACTTT CTTCTAAATC TAGTCCGACT   
  
  
- GTTATGTGGT TTAGACCCTC GTTCCTTGAC CTAAAAAGAC ACAGATCTTT TCGAAAGGCG AAAGACTTGT   
  
  
- TAACTCTTTT GGTACGTATC CTTTAAAGAA CTATAAATCT AGTATCGTCT GAATTGTTAG ACCGAAATGG   
  
  
- CTTGACAGGA GATGAAGATT CAACACTTCC ACACTTTCTA TTTGGTACAG TTTGAATCAC AGGTTGTCTT   
  
  
- TGCGTAGAGA TGAAGAGGTC CGTTTCAACA TGAAGTCTAG AAGATGTGGA AAAGATCCGT CCTGAAACCC   
  
  
- TATATCTCAA ACCGGACCCA CTTTTCATCC TTCACCCCTT CATCTTTTAC TACTTTCTTC CGTGGTGAAC   
  
  
- CCAGGTCTAT GTATCCGGAT GCGTGCTCTT GGAAACGTAC CAGATCTATG TACTGACATA TGAAGAAGGA   
  
  
- AAGGAAATGG ACAACCTCTA ATAGGATCCT AAAATGTACC GTGGTCGACC TAAACAATTA CTGTATTTGT   
  
  
- ATAGAAATTA ATGTCAACGA ATACCATATG TAAATTTGAT AAGCTATCAA CTGTCGACTA CAGGAATAGG   
  
  
- AACACGTCGA ATAAGGAGCT ATCTTATTAG AAACCTGTAA AGACAGATCC ACTATTTCCC GTTTTTGATT   
  
  
- CTATGTTCTT TATCTTGTAC AGGAGGTAAA TGTTCTGCAT AGACAAAAAC CAAAACCAGT TACTAGGGAC   
  
  
- AACAGGAGTT CGTAATTGTG TCTTACTAGT AATAACTGAA CCTGACTTTC CAAAACCCTT GAGGTATCAT   
  
  
- ACGGAAAAGT TATTTTAGTT TTCGTTTAAA CGACAAGTGA CTATTGACGT CAACACAACG AAACGACAAA   
  
  
- GGTATGGTCT GAGCATTGGT TCGACTATCG TTTACACGTT TCCATGTCAT ATTAATTGCT ACAAAAGTAT   
  
  
- AACGATGCTC AAGACTTTGT ACTATATTCT TTTGTGGTCT TGGGTTTATG GATGTATCCA GGATCGGGAC   
  
  
- CTTACCGATA AAATTTATAG GTTTACCTGA ATGTCTTAAG CGTCTCAACA AACCAAACAA ATTGTTAGTA   
  
  
- ACTTAACAAT CGATACTCTT AGACGCCCTG TTAGCATTAG AAACCGCTAA TGAAGCTAAC CGTATGACTA   
  
  
- CAAAAAAATA AACGCATAGA TTCGTACCAC CACATGGAAA CTAAACACAC AGATCCATCA CACACATCTA   
  
  
- TTTATAGTAT CTTGAGTCTG CAACCATCCC AATACAACTC GTGAAAGAGA ACGACAAACA GGAAACACTA   
  
  
- CCGTGGAGAA CGAAAAGGCT TGGTGGTTTT CAGAGTAACT GTTTTGAGAA GTTTGTAACG TTTGACGGGG   
  
  
- GAATTTATAC AAAATAATTT TTTTTACCTT CTCGAGTTTG ACAACTCACT CGGATCTAAG GTTCTAAATC   
  
  
- TTTGTCATTT GAACTATCTA TATAAATATA CTTGATAACA ATCTACGTTG TGGGGGTTGA ACTTTGTGTT   
  
  
- TTCTCTTTAA TTTTTGTTTT TCCAAAACGT GATAAATATA TAGGACACAA GAAACACGAT CAGAATAAAT   
  
  
- TACAATTAAC CATTCGTAAT AAACAACCTA ATAAGGTGAC GTGAAAGAGA ATGATTGAAG TCGTAAAGTA   
  
  
- AGGAACAAGA AGTCGAAAAC TTGAAGAAAT CGGATAAAAC AACAGAAATA CCAATACCTT CGGTAGGTCT   
  
  
- TCTTCTTACT ACTTGAAGAC TCAGAATCGA ACCGGTAACA GCCAGTGAGA CTAGCGTGAC TCTTTTTTGA   
  
  
- CTTCTCCGCC TAACTACAGA GGCTAGGAGA CTAGTGAAGT CCACTTCCGA CGCTTCCCTT CTATTAGTCC   
  
  
- GAGGAGGTTC TCTCTGTCTT GAACAACTTG TAGTTCGTTT CTCCTTTTCC GCAGTAAGTT CTTCCCTTCC   
  
  
- CAGAAGTAGA ATAGGTAGAC GAGGAACATA GACGGTGGAA TTAGTTGCTT TTGTTGTAGT CAAGTCGACA   
  
  
- ACTATTAGAA TAACTCGAGA AGGTTATACA GAGGCTTTCA CCACTAAGTC CTGTTTCCCA ACGACGAATG   
  
  
- AAACGCCTAC CCAATTGTCG TTCCTAGAAT TGAGTCTCCA GAGGTAAAAT AGCGGACTAT GACCCGTTTG   
  
  
- GTCGTGGACG ACTTCTCAAA AGTCGAAAAT GAGTGAATAT ATCTCGGAGA GGTAAGATAG TCAAACGAGT   
  
  
- AAAGTGTCGG TTAGTCCGTT AACATCTTCG TAAACTCTCC CTCCTTCTCT CATTGTTGAC CCGAAACGTA   
  
  
- CATTAACTAA ACCTGTAGAA CGTACCAAAG GTCACCGGAA GAGAATAAGT CAGAGAGAGG CTTCTCCGAT   
  
  
- GAACAAGTTT GGCAAGTAAC AGAGAAGTTT AATGTCCGAA GCCTTCTTCG AATCTTCTCG AGTAACTTTG   
  
  
- TCTTCGAGCC GACCATTCAA AGAGATTCTC GAAAGTCTTA TAGTTGAAAC TTAAGGTGCC CAAAGACTCT   
  
  
- CCGAGCTTTG AGTCCTTGGA TTCCTCCCTC TTACTTTGTC ATCAACATTT AGAGCAAAAG GTGGAGTCAT   
  
  
- GAAACTTTCT ATGGCATGTC TAAAGACTGT GGAACTGAAG ACATGTAAGT GATTTGGGGA GATAACACCA   
  
  
- TAATCAGCTC TCTCTTCCTT CAGCTTTGTC TACACCCAAG GAGAGTTCAA TACATCTACG AGACGTAATA   
  
  
- ATACGACGTT ACAAACTGAG AAATCTACTA ACGGAGGGTG AACTTTCAGG ACTCTCCGAC TCGTATCTCT   
  
  
- TCTTGGTAGA ACCTTTTCTC TAGTTTCTTC GGTATCCAAC ACTCTTCCTA CTTTGTTTGA TAGACTTCAA   
  
  
- ACTCTACGAC CTCTGAACCT CTCCCTCCTA CCTCTCAGTA CCTAAAAGTC CATACTTCGA TTCAAGGTCC   
  
  
- CGTTGATATG TTCGTTTCGA CGAAAATTTT TACCCTTCGG TAATAGTATG GATAAACCTT CTTCTGACAC   
  
  
- CTCCACCACC CAAGTCTCAA ACACTTGCTC TACTACCTTA CCGATAGAGA GAACCGACCG TTCCATCCAA   
  
  
- GGAGCGTTGA CGGAGCCATA CCGCAAGACA CAC

+     GCN4\_motif

| Site Name | Organism | Position | Strand | Matrix score. | sequence | function |
| --- | --- | --- | --- | --- | --- | --- |
| GCN4\_motif | Oryza sativa | 265 | + | 7 | TGAGTCA | cis-regulatory element involved in endosperm expression |

>HU07G00272.1   
+ -Up\_Stream \_Len000ACCAAC GATTCTTTAC TGAATCAGCT ACATTTTTGG ATTGTGTTAA GGTATCTTTT   
  
  
+ AGATCTTCAG GAGGAGGGGG CTGAAGTAGC TTGGTTACTT TTGCGCTTAT CAACTGTGGA AGAATATTAT   
  
  
+ TGAGACTAGT GTAGAAGAAT TAGTAGTTTC CTCCACATTG GAAAAAAACC ATGAAATTAA CGCTTTAGGC   
  
  
+ CCTCAGAGTA CCAAGTGTCA TCGTAAATGA TTGAAATTTC AAGTCTTAGC TGAGTCATGA AGTCCTGATT   
  
  
+ TCCCATGACA AATTGATGTT GTTGTCTAAA GCTTGGCGGG TGATTTGAAA GAAGATTTAG ATCAGGCTGA   
  
  
+ CAATACACCA AATCTGGGAG CAAGGAACTG GATTTTTCTG TGTCTAGAAA AGCTTTCCGC TTTCTGAACA   
  
  
+ ATTGAGAAAA CCATGCATAG GAAATTTCTT GATATTTAGA TCATAGCAGA CTTAACAATC TGGCTTTACC   
  
  
+ GAACTGTCCT CTACTTCTAA GTTGTGAAGG TGTGAAAGAT AAACCATGTC AAACTTAGTG TCCAACAGAA   
  
  
+ ACGCATCTCT ACTTCTCCAG GCAAAGTTGT ACTTCAGATC TTCTACACCT TTTCTAGGCA GGACTTTGGG   
  
  
+ ATATAGAGTT TGGCCTGGGT GAAAAGTAGG AAGTGGGGAA GTAGAAAATG ATGAAAGAAG GCACCACTTG   
  
  
+ GGTCCAGATA CATAGGCCTA CGCACGAGAA CCTTTGCATG GTCTAGATAC ATGACTGTAT ACTTCTTCCT   
  
  
+ TTCCTTTACC TGTTGGAGAT TATCCTAGGA TTTTACATGG CACCAGCTGG ATTTGTTAAT GACATAAACA   
  
  
+ TATCTTTAAT TACAGTTGCT TATGGTATAC ATTTAAACTA TTCGATAGTT GACAGCTGAT GTCCTTATCC   
  
  
+ TTGTGCAGCT TATTCCTCGA TAGAATAATC TTTGGACATT TCTGTCTAGG TGATAAAGGG CAAAAACTAA   
  
  
+ GATACAAGAA ATAGAACATG TCCTCCATTT ACAAGACGTA TCTGTTTTTG GTTTTGGTCA ATGATCCCTG   
  
  
+ TTGTCCTCAA GCATTAACAC AGAATGATCA TTATTGACTT GGACTGAAAG GTTTTGGGAA CTCCATAGTA   
  
  
+ TGCCTTTTCA ATAAAATCAA AAGCAAATTT GCTGTTCACT GATAACTGCA GTTGTGTTGC TTTGCTGTTT   
  
  
+ CCATACCAGA CTCGTAACCA AGCTGATAGC AAATGTGCAA AGGTACAGTA TAATTAACGA TGTTTTCATA   
  
  
+ TTGCTACGAG TTCTGAAACA TGATATAAGA AAACACCAGA ACCCAAATAC CTACATAGGT CCTAGCCCTG   
  
  
+ GAATGGCTAT TTTAAATATC CAAATGGACT TACAGAATTC GCAGAGTTGT TTGGTTTGTT TAACAATCAT   
  
  
+ TGAATTGTTA GCTATGAGAA TCTGCGGGAC AATCGTAATC TTTGGCGATT ACTTCGATTG GCATACTGAT   
  
  
+ GTTTTTTTAT TTGCGTATCT AAGCATGGTG GTGTACCTTT GATTTGTGTG TCTAGGTAGT GTGTGTAGAT   
  
  
+ AAATATCATA GAACTCAGAC GTTGGTAGGG TTATGTTGAG CACTTTCTCT TGCTGTTTGT CCTTTGTGAT   
  
  
+ GGCACCTCTT GCTTTTCCGA ACCACCAAAA GTCTCATTGA CAAAACTCTT CAAACATTGC AAACTGCCCC   
  
  
+ CTTAAATATG TTTTATTAAA AAAAATGGAA GAGCTCAAAC TGTTGAGTGA GCCTAGATTC CAAGATTTAG   
  
  
+ AAACAGTAAA CTTGATAGAT ATATTTATAT GAACTATTGT TAGATGCAAC ACCCCCAACT TGAAACACAA   
  
  
+ AAGAGAAATT AAAAACAAAA AGGTTTTGCA CTATTTATAT ATCCTGTGTT CTTTGTGCTA GTCTTATTTA   
  
  
+ ATGTTAATTG GTAAGCATTA TTTGTTGGAT TATTCCACTG CACTTTCTCT TACTAACTTC AGCATTTCAT   
  
  
+ TCCTTGTTCT TCAGCTTTTG AACTTCTTTA GCCTATTTTG TTGTCTTTAT GGTTATGGAA GCCATCCAGA   
  
  
+ AGAAGAATGA TGAACTTCTG AGTCTTAGCT TGGCCATTGT CGGTCACTCT GATCGCACTG AGAAAAAACT   
  
  
+ GAAGAGGCGG ATTGATGTCT CCGATCCTCT GATCACTTCA GGTGAAGGCT GCGAAGGGAA GATAATCAGG   
  
  
+ CTCCTCCAAG AGAGACAGAA CTTGTTGAAC ATCAAGCAAA GAGGAAAAGG CGTCATTCAA GAAGGGAAGG   
  
  
+ GTCTTCATCT TATCCATCTG CTCCTTGTAT CTGCCACCTT AATCAACGAA AACAACATCA GTTCAGCTGT   
  
  
+ TGATAATCTT ATTGAGCTCT TCCAATATGT CTCCGAAAGT GGTGATTCAG GACAAAGGGT TGCTGCTTAC   
  
  
+ TTTGCGGATG GGTTAACAGC AAGGATCTTA ACTCAGAGGT CTCCATTTTA TCGCCTGATA CTGGGCAAAC   
  
  
+ CAGCACCTGC TGAAGAGTTT TCAGCTTTTA CTCACTTATA TAGAGCCTCT CCATTCTATC AGTTTGCTCA   
  
  
+ TTTCACAGCC AATCAGGCAA TTGTAGAAGC ATTTGAGAGG GAGGAAGAGA GTAACAACTG GGCTTTGCAT   
  
  
+ GTAATTGATT TGGACATCTT GCATGGTTTC CAGTGGCCTT CTCTTATTCA GTCTCTCTCC GAAGAGGCTA   
  
  
+ CTTGTTCAAA CCGTTCATTG TCTCTTCAAA TTACAGGCTT CGGAAGAAGC TTAGAAGAGC TCATTGAAAC   
  
  
+ AGAAGCTCGG CTGGTAAGTT TCTCTAAGAG CTTTCAGAAT ATCAACTTTG AATTCCACGG GTTTCTGAGA   
  
  
+ GGCTCGAAAC TCAGGAACCT AAGGAGGGAG AATGAAACAG TAGTTGTAAA TCTCGTTTTC CACCTCAGTA   
  
  
+ CTTTGAAAGA TACCGTACAG ATTTCTGACA CCTTGACTTC TGTACATTCA CTAAACCCCT CTATTGTGGT   
  
  
+ ATTAGTCGAG AGAGAAGGAA GTCGAAACAG ATGTGGGTTC CTCTCAAGTT ATGTAGATGC TCTGCATTAT   
  
  
+ TATGCTGCAA TGTTTGACTC TTTAGATGAT TGCCTCCCAC TTGAAAGTCC TGAGAGGCTG AGCATAGAGA   
  
  
+ AGAACCATCT TGGAAAAGAG ATCAAAGAAG CCATAGGTTG TGAGAAGGAT GAAACAAACT ATCTGAAGTT   
  
  
+ TGAGATGCTG GAGACTTGGA GAGGGAGGAT GGAGAGTCAT GGATTTTCAG GTATGAAGCT AAGTTCCAGG   
  
  
+ GCAACTATAC AAGCAAAGCT GCTTTTAAAA ATGGGAAGCC ATTATCATAC CTATTTGGAA GAAGACTGTG   
  
  
+ GAGGTGGTGG GTTCAGAGTT TGTGAACGAG ATGATGGAAT GGCTATCTCT CTTGGCTGGC AAGGTAGGTT   
  
  
+ CCTCGCAACT GCCTCGGTAT GGCGTTCTGT GTG  

- -Up\_Stream \_Len000TGGTTG CTAAGAAATG ACTTAGTCGA TGTAAAAACC TAACACAATT CCATAGAAAA   
  
  
- TCTAGAAGTC CTCCTCCCCC GACTTCATCG AACCAATGAA AACGCGAATA GTTGACACCT TCTTATAATA   
  
  
- ACTCTGATCA CATCTTCTTA ATCATCAAAG GAGGTGTAAC CTTTTTTTGG TACTTTAATT GCGAAATCCG   
  
  
- GGAGTCTCAT GGTTCACAGT AGCATTTACT AACTTTAAAG TTCAGAATCG ACTCAGTACT TCAGGACTAA   
  
  
- AGGGTACTGT TTAACTACAA CAACAGATTT CGAACCGCCC ACTAAACTTT CTTCTAAATC TAGTCCGACT   
  
  
- GTTATGTGGT TTAGACCCTC GTTCCTTGAC CTAAAAAGAC ACAGATCTTT TCGAAAGGCG AAAGACTTGT   
  
  
- TAACTCTTTT GGTACGTATC CTTTAAAGAA CTATAAATCT AGTATCGTCT GAATTGTTAG ACCGAAATGG   
  
  
- CTTGACAGGA GATGAAGATT CAACACTTCC ACACTTTCTA TTTGGTACAG TTTGAATCAC AGGTTGTCTT   
  
  
- TGCGTAGAGA TGAAGAGGTC CGTTTCAACA TGAAGTCTAG AAGATGTGGA AAAGATCCGT CCTGAAACCC   
  
  
- TATATCTCAA ACCGGACCCA CTTTTCATCC TTCACCCCTT CATCTTTTAC TACTTTCTTC CGTGGTGAAC   
  
  
- CCAGGTCTAT GTATCCGGAT GCGTGCTCTT GGAAACGTAC CAGATCTATG TACTGACATA TGAAGAAGGA   
  
  
- AAGGAAATGG ACAACCTCTA ATAGGATCCT AAAATGTACC GTGGTCGACC TAAACAATTA CTGTATTTGT   
  
  
- ATAGAAATTA ATGTCAACGA ATACCATATG TAAATTTGAT AAGCTATCAA CTGTCGACTA CAGGAATAGG   
  
  
- AACACGTCGA ATAAGGAGCT ATCTTATTAG AAACCTGTAA AGACAGATCC ACTATTTCCC GTTTTTGATT   
  
  
- CTATGTTCTT TATCTTGTAC AGGAGGTAAA TGTTCTGCAT AGACAAAAAC CAAAACCAGT TACTAGGGAC   
  
  
- AACAGGAGTT CGTAATTGTG TCTTACTAGT AATAACTGAA CCTGACTTTC CAAAACCCTT GAGGTATCAT   
  
  
- ACGGAAAAGT TATTTTAGTT TTCGTTTAAA CGACAAGTGA CTATTGACGT CAACACAACG AAACGACAAA   
  
  
- GGTATGGTCT GAGCATTGGT TCGACTATCG TTTACACGTT TCCATGTCAT ATTAATTGCT ACAAAAGTAT   
  
  
- AACGATGCTC AAGACTTTGT ACTATATTCT TTTGTGGTCT TGGGTTTATG GATGTATCCA GGATCGGGAC   
  
  
- CTTACCGATA AAATTTATAG GTTTACCTGA ATGTCTTAAG CGTCTCAACA AACCAAACAA ATTGTTAGTA   
  
  
- ACTTAACAAT CGATACTCTT AGACGCCCTG TTAGCATTAG AAACCGCTAA TGAAGCTAAC CGTATGACTA   
  
  
- CAAAAAAATA AACGCATAGA TTCGTACCAC CACATGGAAA CTAAACACAC AGATCCATCA CACACATCTA   
  
  
- TTTATAGTAT CTTGAGTCTG CAACCATCCC AATACAACTC GTGAAAGAGA ACGACAAACA GGAAACACTA   
  
  
- CCGTGGAGAA CGAAAAGGCT TGGTGGTTTT CAGAGTAACT GTTTTGAGAA GTTTGTAACG TTTGACGGGG   
  
  
- GAATTTATAC AAAATAATTT TTTTTACCTT CTCGAGTTTG ACAACTCACT CGGATCTAAG GTTCTAAATC   
  
  
- TTTGTCATTT GAACTATCTA TATAAATATA CTTGATAACA ATCTACGTTG TGGGGGTTGA ACTTTGTGTT   
  
  
- TTCTCTTTAA TTTTTGTTTT TCCAAAACGT GATAAATATA TAGGACACAA GAAACACGAT CAGAATAAAT   
  
  
- TACAATTAAC CATTCGTAAT AAACAACCTA ATAAGGTGAC GTGAAAGAGA ATGATTGAAG TCGTAAAGTA   
  
  
- AGGAACAAGA AGTCGAAAAC TTGAAGAAAT CGGATAAAAC AACAGAAATA CCAATACCTT CGGTAGGTCT   
  
  
- TCTTCTTACT ACTTGAAGAC TCAGAATCGA ACCGGTAACA GCCAGTGAGA CTAGCGTGAC TCTTTTTTGA   
  
  
- CTTCTCCGCC TAACTACAGA GGCTAGGAGA CTAGTGAAGT CCACTTCCGA CGCTTCCCTT CTATTAGTCC   
  
  
- GAGGAGGTTC TCTCTGTCTT GAACAACTTG TAGTTCGTTT CTCCTTTTCC GCAGTAAGTT CTTCCCTTCC   
  
  
- CAGAAGTAGA ATAGGTAGAC GAGGAACATA GACGGTGGAA TTAGTTGCTT TTGTTGTAGT CAAGTCGACA   
  
  
- ACTATTAGAA TAACTCGAGA AGGTTATACA GAGGCTTTCA CCACTAAGTC CTGTTTCCCA ACGACGAATG   
  
  
- AAACGCCTAC CCAATTGTCG TTCCTAGAAT TGAGTCTCCA GAGGTAAAAT AGCGGACTAT GACCCGTTTG   
  
  
- GTCGTGGACG ACTTCTCAAA AGTCGAAAAT GAGTGAATAT ATCTCGGAGA GGTAAGATAG TCAAACGAGT   
  
  
- AAAGTGTCGG TTAGTCCGTT AACATCTTCG TAAACTCTCC CTCCTTCTCT CATTGTTGAC CCGAAACGTA   
  
  
- CATTAACTAA ACCTGTAGAA CGTACCAAAG GTCACCGGAA GAGAATAAGT CAGAGAGAGG CTTCTCCGAT   
  
  
- GAACAAGTTT GGCAAGTAAC AGAGAAGTTT AATGTCCGAA GCCTTCTTCG AATCTTCTCG AGTAACTTTG   
  
  
- TCTTCGAGCC GACCATTCAA AGAGATTCTC GAAAGTCTTA TAGTTGAAAC TTAAGGTGCC CAAAGACTCT   
  
  
- CCGAGCTTTG AGTCCTTGGA TTCCTCCCTC TTACTTTGTC ATCAACATTT AGAGCAAAAG GTGGAGTCAT   
  
  
- GAAACTTTCT ATGGCATGTC TAAAGACTGT GGAACTGAAG ACATGTAAGT GATTTGGGGA GATAACACCA   
  
  
- TAATCAGCTC TCTCTTCCTT CAGCTTTGTC TACACCCAAG GAGAGTTCAA TACATCTACG AGACGTAATA   
  
  
- ATACGACGTT ACAAACTGAG AAATCTACTA ACGGAGGGTG AACTTTCAGG ACTCTCCGAC TCGTATCTCT   
  
  
- TCTTGGTAGA ACCTTTTCTC TAGTTTCTTC GGTATCCAAC ACTCTTCCTA CTTTGTTTGA TAGACTTCAA   
  
  
- ACTCTACGAC CTCTGAACCT CTCCCTCCTA CCTCTCAGTA CCTAAAAGTC CATACTTCGA TTCAAGGTCC   
  
  
- CGTTGATATG TTCGTTTCGA CGAAAATTTT TACCCTTCGG TAATAGTATG GATAAACCTT CTTCTGACAC   
  
  
- CTCCACCACC CAAGTCTCAA ACACTTGCTC TACTACCTTA CCGATAGAGA GAACCGACCG TTCCATCCAA   
  
  
- GGAGCGTTGA CGGAGCCATA CCGCAAGACA CAC

+     GT1-motif

| Site Name | Organism | Position | Strand | Matrix score. | sequence | function |
| --- | --- | --- | --- | --- | --- | --- |
| GT1-motif | Arabidopsis thaliana | 2395 | + | 6 | GGTTAA | light responsive element |

>HU07G00272.1   
+ -Up\_Stream \_Len000ACCAAC GATTCTTTAC TGAATCAGCT ACATTTTTGG ATTGTGTTAA GGTATCTTTT   
  
  
+ AGATCTTCAG GAGGAGGGGG CTGAAGTAGC TTGGTTACTT TTGCGCTTAT CAACTGTGGA AGAATATTAT   
  
  
+ TGAGACTAGT GTAGAAGAAT TAGTAGTTTC CTCCACATTG GAAAAAAACC ATGAAATTAA CGCTTTAGGC   
  
  
+ CCTCAGAGTA CCAAGTGTCA TCGTAAATGA TTGAAATTTC AAGTCTTAGC TGAGTCATGA AGTCCTGATT   
  
  
+ TCCCATGACA AATTGATGTT GTTGTCTAAA GCTTGGCGGG TGATTTGAAA GAAGATTTAG ATCAGGCTGA   
  
  
+ CAATACACCA AATCTGGGAG CAAGGAACTG GATTTTTCTG TGTCTAGAAA AGCTTTCCGC TTTCTGAACA   
  
  
+ ATTGAGAAAA CCATGCATAG GAAATTTCTT GATATTTAGA TCATAGCAGA CTTAACAATC TGGCTTTACC   
  
  
+ GAACTGTCCT CTACTTCTAA GTTGTGAAGG TGTGAAAGAT AAACCATGTC AAACTTAGTG TCCAACAGAA   
  
  
+ ACGCATCTCT ACTTCTCCAG GCAAAGTTGT ACTTCAGATC TTCTACACCT TTTCTAGGCA GGACTTTGGG   
  
  
+ ATATAGAGTT TGGCCTGGGT GAAAAGTAGG AAGTGGGGAA GTAGAAAATG ATGAAAGAAG GCACCACTTG   
  
  
+ GGTCCAGATA CATAGGCCTA CGCACGAGAA CCTTTGCATG GTCTAGATAC ATGACTGTAT ACTTCTTCCT   
  
  
+ TTCCTTTACC TGTTGGAGAT TATCCTAGGA TTTTACATGG CACCAGCTGG ATTTGTTAAT GACATAAACA   
  
  
+ TATCTTTAAT TACAGTTGCT TATGGTATAC ATTTAAACTA TTCGATAGTT GACAGCTGAT GTCCTTATCC   
  
  
+ TTGTGCAGCT TATTCCTCGA TAGAATAATC TTTGGACATT TCTGTCTAGG TGATAAAGGG CAAAAACTAA   
  
  
+ GATACAAGAA ATAGAACATG TCCTCCATTT ACAAGACGTA TCTGTTTTTG GTTTTGGTCA ATGATCCCTG   
  
  
+ TTGTCCTCAA GCATTAACAC AGAATGATCA TTATTGACTT GGACTGAAAG GTTTTGGGAA CTCCATAGTA   
  
  
+ TGCCTTTTCA ATAAAATCAA AAGCAAATTT GCTGTTCACT GATAACTGCA GTTGTGTTGC TTTGCTGTTT   
  
  
+ CCATACCAGA CTCGTAACCA AGCTGATAGC AAATGTGCAA AGGTACAGTA TAATTAACGA TGTTTTCATA   
  
  
+ TTGCTACGAG TTCTGAAACA TGATATAAGA AAACACCAGA ACCCAAATAC CTACATAGGT CCTAGCCCTG   
  
  
+ GAATGGCTAT TTTAAATATC CAAATGGACT TACAGAATTC GCAGAGTTGT TTGGTTTGTT TAACAATCAT   
  
  
+ TGAATTGTTA GCTATGAGAA TCTGCGGGAC AATCGTAATC TTTGGCGATT ACTTCGATTG GCATACTGAT   
  
  
+ GTTTTTTTAT TTGCGTATCT AAGCATGGTG GTGTACCTTT GATTTGTGTG TCTAGGTAGT GTGTGTAGAT   
  
  
+ AAATATCATA GAACTCAGAC GTTGGTAGGG TTATGTTGAG CACTTTCTCT TGCTGTTTGT CCTTTGTGAT   
  
  
+ GGCACCTCTT GCTTTTCCGA ACCACCAAAA GTCTCATTGA CAAAACTCTT CAAACATTGC AAACTGCCCC   
  
  
+ CTTAAATATG TTTTATTAAA AAAAATGGAA GAGCTCAAAC TGTTGAGTGA GCCTAGATTC CAAGATTTAG   
  
  
+ AAACAGTAAA CTTGATAGAT ATATTTATAT GAACTATTGT TAGATGCAAC ACCCCCAACT TGAAACACAA   
  
  
+ AAGAGAAATT AAAAACAAAA AGGTTTTGCA CTATTTATAT ATCCTGTGTT CTTTGTGCTA GTCTTATTTA   
  
  
+ ATGTTAATTG GTAAGCATTA TTTGTTGGAT TATTCCACTG CACTTTCTCT TACTAACTTC AGCATTTCAT   
  
  
+ TCCTTGTTCT TCAGCTTTTG AACTTCTTTA GCCTATTTTG TTGTCTTTAT GGTTATGGAA GCCATCCAGA   
  
  
+ AGAAGAATGA TGAACTTCTG AGTCTTAGCT TGGCCATTGT CGGTCACTCT GATCGCACTG AGAAAAAACT   
  
  
+ GAAGAGGCGG ATTGATGTCT CCGATCCTCT GATCACTTCA GGTGAAGGCT GCGAAGGGAA GATAATCAGG   
  
  
+ CTCCTCCAAG AGAGACAGAA CTTGTTGAAC ATCAAGCAAA GAGGAAAAGG CGTCATTCAA GAAGGGAAGG   
  
  
+ GTCTTCATCT TATCCATCTG CTCCTTGTAT CTGCCACCTT AATCAACGAA AACAACATCA GTTCAGCTGT   
  
  
+ TGATAATCTT ATTGAGCTCT TCCAATATGT CTCCGAAAGT GGTGATTCAG GACAAAGGGT TGCTGCTTAC   
  
  
+ TTTGCGGATG GGTTAACAGC AAGGATCTTA ACTCAGAGGT CTCCATTTTA TCGCCTGATA CTGGGCAAAC   
  
  
+ CAGCACCTGC TGAAGAGTTT TCAGCTTTTA CTCACTTATA TAGAGCCTCT CCATTCTATC AGTTTGCTCA   
  
  
+ TTTCACAGCC AATCAGGCAA TTGTAGAAGC ATTTGAGAGG GAGGAAGAGA GTAACAACTG GGCTTTGCAT   
  
  
+ GTAATTGATT TGGACATCTT GCATGGTTTC CAGTGGCCTT CTCTTATTCA GTCTCTCTCC GAAGAGGCTA   
  
  
+ CTTGTTCAAA CCGTTCATTG TCTCTTCAAA TTACAGGCTT CGGAAGAAGC TTAGAAGAGC TCATTGAAAC   
  
  
+ AGAAGCTCGG CTGGTAAGTT TCTCTAAGAG CTTTCAGAAT ATCAACTTTG AATTCCACGG GTTTCTGAGA   
  
  
+ GGCTCGAAAC TCAGGAACCT AAGGAGGGAG AATGAAACAG TAGTTGTAAA TCTCGTTTTC CACCTCAGTA   
  
  
+ CTTTGAAAGA TACCGTACAG ATTTCTGACA CCTTGACTTC TGTACATTCA CTAAACCCCT CTATTGTGGT   
  
  
+ ATTAGTCGAG AGAGAAGGAA GTCGAAACAG ATGTGGGTTC CTCTCAAGTT ATGTAGATGC TCTGCATTAT   
  
  
+ TATGCTGCAA TGTTTGACTC TTTAGATGAT TGCCTCCCAC TTGAAAGTCC TGAGAGGCTG AGCATAGAGA   
  
  
+ AGAACCATCT TGGAAAAGAG ATCAAAGAAG CCATAGGTTG TGAGAAGGAT GAAACAAACT ATCTGAAGTT   
  
  
+ TGAGATGCTG GAGACTTGGA GAGGGAGGAT GGAGAGTCAT GGATTTTCAG GTATGAAGCT AAGTTCCAGG   
  
  
+ GCAACTATAC AAGCAAAGCT GCTTTTAAAA ATGGGAAGCC ATTATCATAC CTATTTGGAA GAAGACTGTG   
  
  
+ GAGGTGGTGG GTTCAGAGTT TGTGAACGAG ATGATGGAAT GGCTATCTCT CTTGGCTGGC AAGGTAGGTT   
  
  
+ CCTCGCAACT GCCTCGGTAT GGCGTTCTGT GTG  

- -Up\_Stream \_Len000TGGTTG CTAAGAAATG ACTTAGTCGA TGTAAAAACC TAACACAATT CCATAGAAAA   
  
  
- TCTAGAAGTC CTCCTCCCCC GACTTCATCG AACCAATGAA AACGCGAATA GTTGACACCT TCTTATAATA   
  
  
- ACTCTGATCA CATCTTCTTA ATCATCAAAG GAGGTGTAAC CTTTTTTTGG TACTTTAATT GCGAAATCCG   
  
  
- GGAGTCTCAT GGTTCACAGT AGCATTTACT AACTTTAAAG TTCAGAATCG ACTCAGTACT TCAGGACTAA   
  
  
- AGGGTACTGT TTAACTACAA CAACAGATTT CGAACCGCCC ACTAAACTTT CTTCTAAATC TAGTCCGACT   
  
  
- GTTATGTGGT TTAGACCCTC GTTCCTTGAC CTAAAAAGAC ACAGATCTTT TCGAAAGGCG AAAGACTTGT   
  
  
- TAACTCTTTT GGTACGTATC CTTTAAAGAA CTATAAATCT AGTATCGTCT GAATTGTTAG ACCGAAATGG   
  
  
- CTTGACAGGA GATGAAGATT CAACACTTCC ACACTTTCTA TTTGGTACAG TTTGAATCAC AGGTTGTCTT   
  
  
- TGCGTAGAGA TGAAGAGGTC CGTTTCAACA TGAAGTCTAG AAGATGTGGA AAAGATCCGT CCTGAAACCC   
  
  
- TATATCTCAA ACCGGACCCA CTTTTCATCC TTCACCCCTT CATCTTTTAC TACTTTCTTC CGTGGTGAAC   
  
  
- CCAGGTCTAT GTATCCGGAT GCGTGCTCTT GGAAACGTAC CAGATCTATG TACTGACATA TGAAGAAGGA   
  
  
- AAGGAAATGG ACAACCTCTA ATAGGATCCT AAAATGTACC GTGGTCGACC TAAACAATTA CTGTATTTGT   
  
  
- ATAGAAATTA ATGTCAACGA ATACCATATG TAAATTTGAT AAGCTATCAA CTGTCGACTA CAGGAATAGG   
  
  
- AACACGTCGA ATAAGGAGCT ATCTTATTAG AAACCTGTAA AGACAGATCC ACTATTTCCC GTTTTTGATT   
  
  
- CTATGTTCTT TATCTTGTAC AGGAGGTAAA TGTTCTGCAT AGACAAAAAC CAAAACCAGT TACTAGGGAC   
  
  
- AACAGGAGTT CGTAATTGTG TCTTACTAGT AATAACTGAA CCTGACTTTC CAAAACCCTT GAGGTATCAT   
  
  
- ACGGAAAAGT TATTTTAGTT TTCGTTTAAA CGACAAGTGA CTATTGACGT CAACACAACG AAACGACAAA   
  
  
- GGTATGGTCT GAGCATTGGT TCGACTATCG TTTACACGTT TCCATGTCAT ATTAATTGCT ACAAAAGTAT   
  
  
- AACGATGCTC AAGACTTTGT ACTATATTCT TTTGTGGTCT TGGGTTTATG GATGTATCCA GGATCGGGAC   
  
  
- CTTACCGATA AAATTTATAG GTTTACCTGA ATGTCTTAAG CGTCTCAACA AACCAAACAA ATTGTTAGTA   
  
  
- ACTTAACAAT CGATACTCTT AGACGCCCTG TTAGCATTAG AAACCGCTAA TGAAGCTAAC CGTATGACTA   
  
  
- CAAAAAAATA AACGCATAGA TTCGTACCAC CACATGGAAA CTAAACACAC AGATCCATCA CACACATCTA   
  
  
- TTTATAGTAT CTTGAGTCTG CAACCATCCC AATACAACTC GTGAAAGAGA ACGACAAACA GGAAACACTA   
  
  
- CCGTGGAGAA CGAAAAGGCT TGGTGGTTTT CAGAGTAACT GTTTTGAGAA GTTTGTAACG TTTGACGGGG   
  
  
- GAATTTATAC AAAATAATTT TTTTTACCTT CTCGAGTTTG ACAACTCACT CGGATCTAAG GTTCTAAATC   
  
  
- TTTGTCATTT GAACTATCTA TATAAATATA CTTGATAACA ATCTACGTTG TGGGGGTTGA ACTTTGTGTT   
  
  
- TTCTCTTTAA TTTTTGTTTT TCCAAAACGT GATAAATATA TAGGACACAA GAAACACGAT CAGAATAAAT   
  
  
- TACAATTAAC CATTCGTAAT AAACAACCTA ATAAGGTGAC GTGAAAGAGA ATGATTGAAG TCGTAAAGTA   
  
  
- AGGAACAAGA AGTCGAAAAC TTGAAGAAAT CGGATAAAAC AACAGAAATA CCAATACCTT CGGTAGGTCT   
  
  
- TCTTCTTACT ACTTGAAGAC TCAGAATCGA ACCGGTAACA GCCAGTGAGA CTAGCGTGAC TCTTTTTTGA   
  
  
- CTTCTCCGCC TAACTACAGA GGCTAGGAGA CTAGTGAAGT CCACTTCCGA CGCTTCCCTT CTATTAGTCC   
  
  
- GAGGAGGTTC TCTCTGTCTT GAACAACTTG TAGTTCGTTT CTCCTTTTCC GCAGTAAGTT CTTCCCTTCC   
  
  
- CAGAAGTAGA ATAGGTAGAC GAGGAACATA GACGGTGGAA TTAGTTGCTT TTGTTGTAGT CAAGTCGACA   
  
  
- ACTATTAGAA TAACTCGAGA AGGTTATACA GAGGCTTTCA CCACTAAGTC CTGTTTCCCA ACGACGAATG   
  
  
- AAACGCCTAC CCAATTGTCG TTCCTAGAAT TGAGTCTCCA GAGGTAAAAT AGCGGACTAT GACCCGTTTG   
  
  
- GTCGTGGACG ACTTCTCAAA AGTCGAAAAT GAGTGAATAT ATCTCGGAGA GGTAAGATAG TCAAACGAGT   
  
  
- AAAGTGTCGG TTAGTCCGTT AACATCTTCG TAAACTCTCC CTCCTTCTCT CATTGTTGAC CCGAAACGTA   
  
  
- CATTAACTAA ACCTGTAGAA CGTACCAAAG GTCACCGGAA GAGAATAAGT CAGAGAGAGG CTTCTCCGAT   
  
  
- GAACAAGTTT GGCAAGTAAC AGAGAAGTTT AATGTCCGAA GCCTTCTTCG AATCTTCTCG AGTAACTTTG   
  
  
- TCTTCGAGCC GACCATTCAA AGAGATTCTC GAAAGTCTTA TAGTTGAAAC TTAAGGTGCC CAAAGACTCT   
  
  
- CCGAGCTTTG AGTCCTTGGA TTCCTCCCTC TTACTTTGTC ATCAACATTT AGAGCAAAAG GTGGAGTCAT   
  
  
- GAAACTTTCT ATGGCATGTC TAAAGACTGT GGAACTGAAG ACATGTAAGT GATTTGGGGA GATAACACCA   
  
  
- TAATCAGCTC TCTCTTCCTT CAGCTTTGTC TACACCCAAG GAGAGTTCAA TACATCTACG AGACGTAATA   
  
  
- ATACGACGTT ACAAACTGAG AAATCTACTA ACGGAGGGTG AACTTTCAGG ACTCTCCGAC TCGTATCTCT   
  
  
- TCTTGGTAGA ACCTTTTCTC TAGTTTCTTC GGTATCCAAC ACTCTTCCTA CTTTGTTTGA TAGACTTCAA   
  
  
- ACTCTACGAC CTCTGAACCT CTCCCTCCTA CCTCTCAGTA CCTAAAAGTC CATACTTCGA TTCAAGGTCC   
  
  
- CGTTGATATG TTCGTTTCGA CGAAAATTTT TACCCTTCGG TAATAGTATG GATAAACCTT CTTCTGACAC   
  
  
- CTCCACCACC CAAGTCTCAA ACACTTGCTC TACTACCTTA CCGATAGAGA GAACCGACCG TTCCATCCAA   
  
  
- GGAGCGTTGA CGGAGCCATA CCGCAAGACA CAC

+     HD-Zip 1

| Site Name | Organism | Position | Strand | Matrix score. | sequence | function |
| --- | --- | --- | --- | --- | --- | --- |
| HD-Zip 1 | Arabidopsis thaliana | 1398 | + | 8 | CAAT(A/T)ATTG | element involved in differentiation of the palisade mesophyll cells |

>HU07G00272.1   
+ -Up\_Stream \_Len000ACCAAC GATTCTTTAC TGAATCAGCT ACATTTTTGG ATTGTGTTAA GGTATCTTTT   
  
  
+ AGATCTTCAG GAGGAGGGGG CTGAAGTAGC TTGGTTACTT TTGCGCTTAT CAACTGTGGA AGAATATTAT   
  
  
+ TGAGACTAGT GTAGAAGAAT TAGTAGTTTC CTCCACATTG GAAAAAAACC ATGAAATTAA CGCTTTAGGC   
  
  
+ CCTCAGAGTA CCAAGTGTCA TCGTAAATGA TTGAAATTTC AAGTCTTAGC TGAGTCATGA AGTCCTGATT   
  
  
+ TCCCATGACA AATTGATGTT GTTGTCTAAA GCTTGGCGGG TGATTTGAAA GAAGATTTAG ATCAGGCTGA   
  
  
+ CAATACACCA AATCTGGGAG CAAGGAACTG GATTTTTCTG TGTCTAGAAA AGCTTTCCGC TTTCTGAACA   
  
  
+ ATTGAGAAAA CCATGCATAG GAAATTTCTT GATATTTAGA TCATAGCAGA CTTAACAATC TGGCTTTACC   
  
  
+ GAACTGTCCT CTACTTCTAA GTTGTGAAGG TGTGAAAGAT AAACCATGTC AAACTTAGTG TCCAACAGAA   
  
  
+ ACGCATCTCT ACTTCTCCAG GCAAAGTTGT ACTTCAGATC TTCTACACCT TTTCTAGGCA GGACTTTGGG   
  
  
+ ATATAGAGTT TGGCCTGGGT GAAAAGTAGG AAGTGGGGAA GTAGAAAATG ATGAAAGAAG GCACCACTTG   
  
  
+ GGTCCAGATA CATAGGCCTA CGCACGAGAA CCTTTGCATG GTCTAGATAC ATGACTGTAT ACTTCTTCCT   
  
  
+ TTCCTTTACC TGTTGGAGAT TATCCTAGGA TTTTACATGG CACCAGCTGG ATTTGTTAAT GACATAAACA   
  
  
+ TATCTTTAAT TACAGTTGCT TATGGTATAC ATTTAAACTA TTCGATAGTT GACAGCTGAT GTCCTTATCC   
  
  
+ TTGTGCAGCT TATTCCTCGA TAGAATAATC TTTGGACATT TCTGTCTAGG TGATAAAGGG CAAAAACTAA   
  
  
+ GATACAAGAA ATAGAACATG TCCTCCATTT ACAAGACGTA TCTGTTTTTG GTTTTGGTCA ATGATCCCTG   
  
  
+ TTGTCCTCAA GCATTAACAC AGAATGATCA TTATTGACTT GGACTGAAAG GTTTTGGGAA CTCCATAGTA   
  
  
+ TGCCTTTTCA ATAAAATCAA AAGCAAATTT GCTGTTCACT GATAACTGCA GTTGTGTTGC TTTGCTGTTT   
  
  
+ CCATACCAGA CTCGTAACCA AGCTGATAGC AAATGTGCAA AGGTACAGTA TAATTAACGA TGTTTTCATA   
  
  
+ TTGCTACGAG TTCTGAAACA TGATATAAGA AAACACCAGA ACCCAAATAC CTACATAGGT CCTAGCCCTG   
  
  
+ GAATGGCTAT TTTAAATATC CAAATGGACT TACAGAATTC GCAGAGTTGT TTGGTTTGTT TAACAATCAT   
  
  
+ TGAATTGTTA GCTATGAGAA TCTGCGGGAC AATCGTAATC TTTGGCGATT ACTTCGATTG GCATACTGAT   
  
  
+ GTTTTTTTAT TTGCGTATCT AAGCATGGTG GTGTACCTTT GATTTGTGTG TCTAGGTAGT GTGTGTAGAT   
  
  
+ AAATATCATA GAACTCAGAC GTTGGTAGGG TTATGTTGAG CACTTTCTCT TGCTGTTTGT CCTTTGTGAT   
  
  
+ GGCACCTCTT GCTTTTCCGA ACCACCAAAA GTCTCATTGA CAAAACTCTT CAAACATTGC AAACTGCCCC   
  
  
+ CTTAAATATG TTTTATTAAA AAAAATGGAA GAGCTCAAAC TGTTGAGTGA GCCTAGATTC CAAGATTTAG   
  
  
+ AAACAGTAAA CTTGATAGAT ATATTTATAT GAACTATTGT TAGATGCAAC ACCCCCAACT TGAAACACAA   
  
  
+ AAGAGAAATT AAAAACAAAA AGGTTTTGCA CTATTTATAT ATCCTGTGTT CTTTGTGCTA GTCTTATTTA   
  
  
+ ATGTTAATTG GTAAGCATTA TTTGTTGGAT TATTCCACTG CACTTTCTCT TACTAACTTC AGCATTTCAT   
  
  
+ TCCTTGTTCT TCAGCTTTTG AACTTCTTTA GCCTATTTTG TTGTCTTTAT GGTTATGGAA GCCATCCAGA   
  
  
+ AGAAGAATGA TGAACTTCTG AGTCTTAGCT TGGCCATTGT CGGTCACTCT GATCGCACTG AGAAAAAACT   
  
  
+ GAAGAGGCGG ATTGATGTCT CCGATCCTCT GATCACTTCA GGTGAAGGCT GCGAAGGGAA GATAATCAGG   
  
  
+ CTCCTCCAAG AGAGACAGAA CTTGTTGAAC ATCAAGCAAA GAGGAAAAGG CGTCATTCAA GAAGGGAAGG   
  
  
+ GTCTTCATCT TATCCATCTG CTCCTTGTAT CTGCCACCTT AATCAACGAA AACAACATCA GTTCAGCTGT   
  
  
+ TGATAATCTT ATTGAGCTCT TCCAATATGT CTCCGAAAGT GGTGATTCAG GACAAAGGGT TGCTGCTTAC   
  
  
+ TTTGCGGATG GGTTAACAGC AAGGATCTTA ACTCAGAGGT CTCCATTTTA TCGCCTGATA CTGGGCAAAC   
  
  
+ CAGCACCTGC TGAAGAGTTT TCAGCTTTTA CTCACTTATA TAGAGCCTCT CCATTCTATC AGTTTGCTCA   
  
  
+ TTTCACAGCC AATCAGGCAA TTGTAGAAGC ATTTGAGAGG GAGGAAGAGA GTAACAACTG GGCTTTGCAT   
  
  
+ GTAATTGATT TGGACATCTT GCATGGTTTC CAGTGGCCTT CTCTTATTCA GTCTCTCTCC GAAGAGGCTA   
  
  
+ CTTGTTCAAA CCGTTCATTG TCTCTTCAAA TTACAGGCTT CGGAAGAAGC TTAGAAGAGC TCATTGAAAC   
  
  
+ AGAAGCTCGG CTGGTAAGTT TCTCTAAGAG CTTTCAGAAT ATCAACTTTG AATTCCACGG GTTTCTGAGA   
  
  
+ GGCTCGAAAC TCAGGAACCT AAGGAGGGAG AATGAAACAG TAGTTGTAAA TCTCGTTTTC CACCTCAGTA   
  
  
+ CTTTGAAAGA TACCGTACAG ATTTCTGACA CCTTGACTTC TGTACATTCA CTAAACCCCT CTATTGTGGT   
  
  
+ ATTAGTCGAG AGAGAAGGAA GTCGAAACAG ATGTGGGTTC CTCTCAAGTT ATGTAGATGC TCTGCATTAT   
  
  
+ TATGCTGCAA TGTTTGACTC TTTAGATGAT TGCCTCCCAC TTGAAAGTCC TGAGAGGCTG AGCATAGAGA   
  
  
+ AGAACCATCT TGGAAAAGAG ATCAAAGAAG CCATAGGTTG TGAGAAGGAT GAAACAAACT ATCTGAAGTT   
  
  
+ TGAGATGCTG GAGACTTGGA GAGGGAGGAT GGAGAGTCAT GGATTTTCAG GTATGAAGCT AAGTTCCAGG   
  
  
+ GCAACTATAC AAGCAAAGCT GCTTTTAAAA ATGGGAAGCC ATTATCATAC CTATTTGGAA GAAGACTGTG   
  
  
+ GAGGTGGTGG GTTCAGAGTT TGTGAACGAG ATGATGGAAT GGCTATCTCT CTTGGCTGGC AAGGTAGGTT   
  
  
+ CCTCGCAACT GCCTCGGTAT GGCGTTCTGT GTG  

- -Up\_Stream \_Len000TGGTTG CTAAGAAATG ACTTAGTCGA TGTAAAAACC TAACACAATT CCATAGAAAA   
  
  
- TCTAGAAGTC CTCCTCCCCC GACTTCATCG AACCAATGAA AACGCGAATA GTTGACACCT TCTTATAATA   
  
  
- ACTCTGATCA CATCTTCTTA ATCATCAAAG GAGGTGTAAC CTTTTTTTGG TACTTTAATT GCGAAATCCG   
  
  
- GGAGTCTCAT GGTTCACAGT AGCATTTACT AACTTTAAAG TTCAGAATCG ACTCAGTACT TCAGGACTAA   
  
  
- AGGGTACTGT TTAACTACAA CAACAGATTT CGAACCGCCC ACTAAACTTT CTTCTAAATC TAGTCCGACT   
  
  
- GTTATGTGGT TTAGACCCTC GTTCCTTGAC CTAAAAAGAC ACAGATCTTT TCGAAAGGCG AAAGACTTGT   
  
  
- TAACTCTTTT GGTACGTATC CTTTAAAGAA CTATAAATCT AGTATCGTCT GAATTGTTAG ACCGAAATGG   
  
  
- CTTGACAGGA GATGAAGATT CAACACTTCC ACACTTTCTA TTTGGTACAG TTTGAATCAC AGGTTGTCTT   
  
  
- TGCGTAGAGA TGAAGAGGTC CGTTTCAACA TGAAGTCTAG AAGATGTGGA AAAGATCCGT CCTGAAACCC   
  
  
- TATATCTCAA ACCGGACCCA CTTTTCATCC TTCACCCCTT CATCTTTTAC TACTTTCTTC CGTGGTGAAC   
  
  
- CCAGGTCTAT GTATCCGGAT GCGTGCTCTT GGAAACGTAC CAGATCTATG TACTGACATA TGAAGAAGGA   
  
  
- AAGGAAATGG ACAACCTCTA ATAGGATCCT AAAATGTACC GTGGTCGACC TAAACAATTA CTGTATTTGT   
  
  
- ATAGAAATTA ATGTCAACGA ATACCATATG TAAATTTGAT AAGCTATCAA CTGTCGACTA CAGGAATAGG   
  
  
- AACACGTCGA ATAAGGAGCT ATCTTATTAG AAACCTGTAA AGACAGATCC ACTATTTCCC GTTTTTGATT   
  
  
- CTATGTTCTT TATCTTGTAC AGGAGGTAAA TGTTCTGCAT AGACAAAAAC CAAAACCAGT TACTAGGGAC   
  
  
- AACAGGAGTT CGTAATTGTG TCTTACTAGT AATAACTGAA CCTGACTTTC CAAAACCCTT GAGGTATCAT   
  
  
- ACGGAAAAGT TATTTTAGTT TTCGTTTAAA CGACAAGTGA CTATTGACGT CAACACAACG AAACGACAAA   
  
  
- GGTATGGTCT GAGCATTGGT TCGACTATCG TTTACACGTT TCCATGTCAT ATTAATTGCT ACAAAAGTAT   
  
  
- AACGATGCTC AAGACTTTGT ACTATATTCT TTTGTGGTCT TGGGTTTATG GATGTATCCA GGATCGGGAC   
  
  
- CTTACCGATA AAATTTATAG GTTTACCTGA ATGTCTTAAG CGTCTCAACA AACCAAACAA ATTGTTAGTA   
  
  
- ACTTAACAAT CGATACTCTT AGACGCCCTG TTAGCATTAG AAACCGCTAA TGAAGCTAAC CGTATGACTA   
  
  
- CAAAAAAATA AACGCATAGA TTCGTACCAC CACATGGAAA CTAAACACAC AGATCCATCA CACACATCTA   
  
  
- TTTATAGTAT CTTGAGTCTG CAACCATCCC AATACAACTC GTGAAAGAGA ACGACAAACA GGAAACACTA   
  
  
- CCGTGGAGAA CGAAAAGGCT TGGTGGTTTT CAGAGTAACT GTTTTGAGAA GTTTGTAACG TTTGACGGGG   
  
  
- GAATTTATAC AAAATAATTT TTTTTACCTT CTCGAGTTTG ACAACTCACT CGGATCTAAG GTTCTAAATC   
  
  
- TTTGTCATTT GAACTATCTA TATAAATATA CTTGATAACA ATCTACGTTG TGGGGGTTGA ACTTTGTGTT   
  
  
- TTCTCTTTAA TTTTTGTTTT TCCAAAACGT GATAAATATA TAGGACACAA GAAACACGAT CAGAATAAAT   
  
  
- TACAATTAAC CATTCGTAAT AAACAACCTA ATAAGGTGAC GTGAAAGAGA ATGATTGAAG TCGTAAAGTA   
  
  
- AGGAACAAGA AGTCGAAAAC TTGAAGAAAT CGGATAAAAC AACAGAAATA CCAATACCTT CGGTAGGTCT   
  
  
- TCTTCTTACT ACTTGAAGAC TCAGAATCGA ACCGGTAACA GCCAGTGAGA CTAGCGTGAC TCTTTTTTGA   
  
  
- CTTCTCCGCC TAACTACAGA GGCTAGGAGA CTAGTGAAGT CCACTTCCGA CGCTTCCCTT CTATTAGTCC   
  
  
- GAGGAGGTTC TCTCTGTCTT GAACAACTTG TAGTTCGTTT CTCCTTTTCC GCAGTAAGTT CTTCCCTTCC   
  
  
- CAGAAGTAGA ATAGGTAGAC GAGGAACATA GACGGTGGAA TTAGTTGCTT TTGTTGTAGT CAAGTCGACA   
  
  
- ACTATTAGAA TAACTCGAGA AGGTTATACA GAGGCTTTCA CCACTAAGTC CTGTTTCCCA ACGACGAATG   
  
  
- AAACGCCTAC CCAATTGTCG TTCCTAGAAT TGAGTCTCCA GAGGTAAAAT AGCGGACTAT GACCCGTTTG   
  
  
- GTCGTGGACG ACTTCTCAAA AGTCGAAAAT GAGTGAATAT ATCTCGGAGA GGTAAGATAG TCAAACGAGT   
  
  
- AAAGTGTCGG TTAGTCCGTT AACATCTTCG TAAACTCTCC CTCCTTCTCT CATTGTTGAC CCGAAACGTA   
  
  
- CATTAACTAA ACCTGTAGAA CGTACCAAAG GTCACCGGAA GAGAATAAGT CAGAGAGAGG CTTCTCCGAT   
  
  
- GAACAAGTTT GGCAAGTAAC AGAGAAGTTT AATGTCCGAA GCCTTCTTCG AATCTTCTCG AGTAACTTTG   
  
  
- TCTTCGAGCC GACCATTCAA AGAGATTCTC GAAAGTCTTA TAGTTGAAAC TTAAGGTGCC CAAAGACTCT   
  
  
- CCGAGCTTTG AGTCCTTGGA TTCCTCCCTC TTACTTTGTC ATCAACATTT AGAGCAAAAG GTGGAGTCAT   
  
  
- GAAACTTTCT ATGGCATGTC TAAAGACTGT GGAACTGAAG ACATGTAAGT GATTTGGGGA GATAACACCA   
  
  
- TAATCAGCTC TCTCTTCCTT CAGCTTTGTC TACACCCAAG GAGAGTTCAA TACATCTACG AGACGTAATA   
  
  
- ATACGACGTT ACAAACTGAG AAATCTACTA ACGGAGGGTG AACTTTCAGG ACTCTCCGAC TCGTATCTCT   
  
  
- TCTTGGTAGA ACCTTTTCTC TAGTTTCTTC GGTATCCAAC ACTCTTCCTA CTTTGTTTGA TAGACTTCAA   
  
  
- ACTCTACGAC CTCTGAACCT CTCCCTCCTA CCTCTCAGTA CCTAAAAGTC CATACTTCGA TTCAAGGTCC   
  
  
- CGTTGATATG TTCGTTTCGA CGAAAATTTT TACCCTTCGG TAATAGTATG GATAAACCTT CTTCTGACAC   
  
  
- CTCCACCACC CAAGTCTCAA ACACTTGCTC TACTACCTTA CCGATAGAGA GAACCGACCG TTCCATCCAA   
  
  
- GGAGCGTTGA CGGAGCCATA CCGCAAGACA CAC

+     I-box

| Site Name | Organism | Position | Strand | Matrix score. | sequence | function |
| --- | --- | --- | --- | --- | --- | --- |
| I-box | Arabidopsis thaliana | 907 | + | 9 | ccttatcct | part of a light responsive element |
| I-box | Zea mays | 2250 | - | 9 | gGATAAGGTG | part of a light responsive element |

>HU07G00272.1   
+ -Up\_Stream \_Len000ACCAAC GATTCTTTAC TGAATCAGCT ACATTTTTGG ATTGTGTTAA GGTATCTTTT   
  
  
+ AGATCTTCAG GAGGAGGGGG CTGAAGTAGC TTGGTTACTT TTGCGCTTAT CAACTGTGGA AGAATATTAT   
  
  
+ TGAGACTAGT GTAGAAGAAT TAGTAGTTTC CTCCACATTG GAAAAAAACC ATGAAATTAA CGCTTTAGGC   
  
  
+ CCTCAGAGTA CCAAGTGTCA TCGTAAATGA TTGAAATTTC AAGTCTTAGC TGAGTCATGA AGTCCTGATT   
  
  
+ TCCCATGACA AATTGATGTT GTTGTCTAAA GCTTGGCGGG TGATTTGAAA GAAGATTTAG ATCAGGCTGA   
  
  
+ CAATACACCA AATCTGGGAG CAAGGAACTG GATTTTTCTG TGTCTAGAAA AGCTTTCCGC TTTCTGAACA   
  
  
+ ATTGAGAAAA CCATGCATAG GAAATTTCTT GATATTTAGA TCATAGCAGA CTTAACAATC TGGCTTTACC   
  
  
+ GAACTGTCCT CTACTTCTAA GTTGTGAAGG TGTGAAAGAT AAACCATGTC AAACTTAGTG TCCAACAGAA   
  
  
+ ACGCATCTCT ACTTCTCCAG GCAAAGTTGT ACTTCAGATC TTCTACACCT TTTCTAGGCA GGACTTTGGG   
  
  
+ ATATAGAGTT TGGCCTGGGT GAAAAGTAGG AAGTGGGGAA GTAGAAAATG ATGAAAGAAG GCACCACTTG   
  
  
+ GGTCCAGATA CATAGGCCTA CGCACGAGAA CCTTTGCATG GTCTAGATAC ATGACTGTAT ACTTCTTCCT   
  
  
+ TTCCTTTACC TGTTGGAGAT TATCCTAGGA TTTTACATGG CACCAGCTGG ATTTGTTAAT GACATAAACA   
  
  
+ TATCTTTAAT TACAGTTGCT TATGGTATAC ATTTAAACTA TTCGATAGTT GACAGCTGAT GTCCTTATCC   
  
  
+ TTGTGCAGCT TATTCCTCGA TAGAATAATC TTTGGACATT TCTGTCTAGG TGATAAAGGG CAAAAACTAA   
  
  
+ GATACAAGAA ATAGAACATG TCCTCCATTT ACAAGACGTA TCTGTTTTTG GTTTTGGTCA ATGATCCCTG   
  
  
+ TTGTCCTCAA GCATTAACAC AGAATGATCA TTATTGACTT GGACTGAAAG GTTTTGGGAA CTCCATAGTA   
  
  
+ TGCCTTTTCA ATAAAATCAA AAGCAAATTT GCTGTTCACT GATAACTGCA GTTGTGTTGC TTTGCTGTTT   
  
  
+ CCATACCAGA CTCGTAACCA AGCTGATAGC AAATGTGCAA AGGTACAGTA TAATTAACGA TGTTTTCATA   
  
  
+ TTGCTACGAG TTCTGAAACA TGATATAAGA AAACACCAGA ACCCAAATAC CTACATAGGT CCTAGCCCTG   
  
  
+ GAATGGCTAT TTTAAATATC CAAATGGACT TACAGAATTC GCAGAGTTGT TTGGTTTGTT TAACAATCAT   
  
  
+ TGAATTGTTA GCTATGAGAA TCTGCGGGAC AATCGTAATC TTTGGCGATT ACTTCGATTG GCATACTGAT   
  
  
+ GTTTTTTTAT TTGCGTATCT AAGCATGGTG GTGTACCTTT GATTTGTGTG TCTAGGTAGT GTGTGTAGAT   
  
  
+ AAATATCATA GAACTCAGAC GTTGGTAGGG TTATGTTGAG CACTTTCTCT TGCTGTTTGT CCTTTGTGAT   
  
  
+ GGCACCTCTT GCTTTTCCGA ACCACCAAAA GTCTCATTGA CAAAACTCTT CAAACATTGC AAACTGCCCC   
  
  
+ CTTAAATATG TTTTATTAAA AAAAATGGAA GAGCTCAAAC TGTTGAGTGA GCCTAGATTC CAAGATTTAG   
  
  
+ AAACAGTAAA CTTGATAGAT ATATTTATAT GAACTATTGT TAGATGCAAC ACCCCCAACT TGAAACACAA   
  
  
+ AAGAGAAATT AAAAACAAAA AGGTTTTGCA CTATTTATAT ATCCTGTGTT CTTTGTGCTA GTCTTATTTA   
  
  
+ ATGTTAATTG GTAAGCATTA TTTGTTGGAT TATTCCACTG CACTTTCTCT TACTAACTTC AGCATTTCAT   
  
  
+ TCCTTGTTCT TCAGCTTTTG AACTTCTTTA GCCTATTTTG TTGTCTTTAT GGTTATGGAA GCCATCCAGA   
  
  
+ AGAAGAATGA TGAACTTCTG AGTCTTAGCT TGGCCATTGT CGGTCACTCT GATCGCACTG AGAAAAAACT   
  
  
+ GAAGAGGCGG ATTGATGTCT CCGATCCTCT GATCACTTCA GGTGAAGGCT GCGAAGGGAA GATAATCAGG   
  
  
+ CTCCTCCAAG AGAGACAGAA CTTGTTGAAC ATCAAGCAAA GAGGAAAAGG CGTCATTCAA GAAGGGAAGG   
  
  
+ GTCTTCATCT TATCCATCTG CTCCTTGTAT CTGCCACCTT AATCAACGAA AACAACATCA GTTCAGCTGT   
  
  
+ TGATAATCTT ATTGAGCTCT TCCAATATGT CTCCGAAAGT GGTGATTCAG GACAAAGGGT TGCTGCTTAC   
  
  
+ TTTGCGGATG GGTTAACAGC AAGGATCTTA ACTCAGAGGT CTCCATTTTA TCGCCTGATA CTGGGCAAAC   
  
  
+ CAGCACCTGC TGAAGAGTTT TCAGCTTTTA CTCACTTATA TAGAGCCTCT CCATTCTATC AGTTTGCTCA   
  
  
+ TTTCACAGCC AATCAGGCAA TTGTAGAAGC ATTTGAGAGG GAGGAAGAGA GTAACAACTG GGCTTTGCAT   
  
  
+ GTAATTGATT TGGACATCTT GCATGGTTTC CAGTGGCCTT CTCTTATTCA GTCTCTCTCC GAAGAGGCTA   
  
  
+ CTTGTTCAAA CCGTTCATTG TCTCTTCAAA TTACAGGCTT CGGAAGAAGC TTAGAAGAGC TCATTGAAAC   
  
  
+ AGAAGCTCGG CTGGTAAGTT TCTCTAAGAG CTTTCAGAAT ATCAACTTTG AATTCCACGG GTTTCTGAGA   
  
  
+ GGCTCGAAAC TCAGGAACCT AAGGAGGGAG AATGAAACAG TAGTTGTAAA TCTCGTTTTC CACCTCAGTA   
  
  
+ CTTTGAAAGA TACCGTACAG ATTTCTGACA CCTTGACTTC TGTACATTCA CTAAACCCCT CTATTGTGGT   
  
  
+ ATTAGTCGAG AGAGAAGGAA GTCGAAACAG ATGTGGGTTC CTCTCAAGTT ATGTAGATGC TCTGCATTAT   
  
  
+ TATGCTGCAA TGTTTGACTC TTTAGATGAT TGCCTCCCAC TTGAAAGTCC TGAGAGGCTG AGCATAGAGA   
  
  
+ AGAACCATCT TGGAAAAGAG ATCAAAGAAG CCATAGGTTG TGAGAAGGAT GAAACAAACT ATCTGAAGTT   
  
  
+ TGAGATGCTG GAGACTTGGA GAGGGAGGAT GGAGAGTCAT GGATTTTCAG GTATGAAGCT AAGTTCCAGG   
  
  
+ GCAACTATAC AAGCAAAGCT GCTTTTAAAA ATGGGAAGCC ATTATCATAC CTATTTGGAA GAAGACTGTG   
  
  
+ GAGGTGGTGG GTTCAGAGTT TGTGAACGAG ATGATGGAAT GGCTATCTCT CTTGGCTGGC AAGGTAGGTT   
  
  
+ CCTCGCAACT GCCTCGGTAT GGCGTTCTGT GTG  

- -Up\_Stream \_Len000TGGTTG CTAAGAAATG ACTTAGTCGA TGTAAAAACC TAACACAATT CCATAGAAAA   
  
  
- TCTAGAAGTC CTCCTCCCCC GACTTCATCG AACCAATGAA AACGCGAATA GTTGACACCT TCTTATAATA   
  
  
- ACTCTGATCA CATCTTCTTA ATCATCAAAG GAGGTGTAAC CTTTTTTTGG TACTTTAATT GCGAAATCCG   
  
  
- GGAGTCTCAT GGTTCACAGT AGCATTTACT AACTTTAAAG TTCAGAATCG ACTCAGTACT TCAGGACTAA   
  
  
- AGGGTACTGT TTAACTACAA CAACAGATTT CGAACCGCCC ACTAAACTTT CTTCTAAATC TAGTCCGACT   
  
  
- GTTATGTGGT TTAGACCCTC GTTCCTTGAC CTAAAAAGAC ACAGATCTTT TCGAAAGGCG AAAGACTTGT   
  
  
- TAACTCTTTT GGTACGTATC CTTTAAAGAA CTATAAATCT AGTATCGTCT GAATTGTTAG ACCGAAATGG   
  
  
- CTTGACAGGA GATGAAGATT CAACACTTCC ACACTTTCTA TTTGGTACAG TTTGAATCAC AGGTTGTCTT   
  
  
- TGCGTAGAGA TGAAGAGGTC CGTTTCAACA TGAAGTCTAG AAGATGTGGA AAAGATCCGT CCTGAAACCC   
  
  
- TATATCTCAA ACCGGACCCA CTTTTCATCC TTCACCCCTT CATCTTTTAC TACTTTCTTC CGTGGTGAAC   
  
  
- CCAGGTCTAT GTATCCGGAT GCGTGCTCTT GGAAACGTAC CAGATCTATG TACTGACATA TGAAGAAGGA   
  
  
- AAGGAAATGG ACAACCTCTA ATAGGATCCT AAAATGTACC GTGGTCGACC TAAACAATTA CTGTATTTGT   
  
  
- ATAGAAATTA ATGTCAACGA ATACCATATG TAAATTTGAT AAGCTATCAA CTGTCGACTA CAGGAATAGG   
  
  
- AACACGTCGA ATAAGGAGCT ATCTTATTAG AAACCTGTAA AGACAGATCC ACTATTTCCC GTTTTTGATT   
  
  
- CTATGTTCTT TATCTTGTAC AGGAGGTAAA TGTTCTGCAT AGACAAAAAC CAAAACCAGT TACTAGGGAC   
  
  
- AACAGGAGTT CGTAATTGTG TCTTACTAGT AATAACTGAA CCTGACTTTC CAAAACCCTT GAGGTATCAT   
  
  
- ACGGAAAAGT TATTTTAGTT TTCGTTTAAA CGACAAGTGA CTATTGACGT CAACACAACG AAACGACAAA   
  
  
- GGTATGGTCT GAGCATTGGT TCGACTATCG TTTACACGTT TCCATGTCAT ATTAATTGCT ACAAAAGTAT   
  
  
- AACGATGCTC AAGACTTTGT ACTATATTCT TTTGTGGTCT TGGGTTTATG GATGTATCCA GGATCGGGAC   
  
  
- CTTACCGATA AAATTTATAG GTTTACCTGA ATGTCTTAAG CGTCTCAACA AACCAAACAA ATTGTTAGTA   
  
  
- ACTTAACAAT CGATACTCTT AGACGCCCTG TTAGCATTAG AAACCGCTAA TGAAGCTAAC CGTATGACTA   
  
  
- CAAAAAAATA AACGCATAGA TTCGTACCAC CACATGGAAA CTAAACACAC AGATCCATCA CACACATCTA   
  
  
- TTTATAGTAT CTTGAGTCTG CAACCATCCC AATACAACTC GTGAAAGAGA ACGACAAACA GGAAACACTA   
  
  
- CCGTGGAGAA CGAAAAGGCT TGGTGGTTTT CAGAGTAACT GTTTTGAGAA GTTTGTAACG TTTGACGGGG   
  
  
- GAATTTATAC AAAATAATTT TTTTTACCTT CTCGAGTTTG ACAACTCACT CGGATCTAAG GTTCTAAATC   
  
  
- TTTGTCATTT GAACTATCTA TATAAATATA CTTGATAACA ATCTACGTTG TGGGGGTTGA ACTTTGTGTT   
  
  
- TTCTCTTTAA TTTTTGTTTT TCCAAAACGT GATAAATATA TAGGACACAA GAAACACGAT CAGAATAAAT   
  
  
- TACAATTAAC CATTCGTAAT AAACAACCTA ATAAGGTGAC GTGAAAGAGA ATGATTGAAG TCGTAAAGTA   
  
  
- AGGAACAAGA AGTCGAAAAC TTGAAGAAAT CGGATAAAAC AACAGAAATA CCAATACCTT CGGTAGGTCT   
  
  
- TCTTCTTACT ACTTGAAGAC TCAGAATCGA ACCGGTAACA GCCAGTGAGA CTAGCGTGAC TCTTTTTTGA   
  
  
- CTTCTCCGCC TAACTACAGA GGCTAGGAGA CTAGTGAAGT CCACTTCCGA CGCTTCCCTT CTATTAGTCC   
  
  
- GAGGAGGTTC TCTCTGTCTT GAACAACTTG TAGTTCGTTT CTCCTTTTCC GCAGTAAGTT CTTCCCTTCC   
  
  
- CAGAAGTAGA ATAGGTAGAC GAGGAACATA GACGGTGGAA TTAGTTGCTT TTGTTGTAGT CAAGTCGACA   
  
  
- ACTATTAGAA TAACTCGAGA AGGTTATACA GAGGCTTTCA CCACTAAGTC CTGTTTCCCA ACGACGAATG   
  
  
- AAACGCCTAC CCAATTGTCG TTCCTAGAAT TGAGTCTCCA GAGGTAAAAT AGCGGACTAT GACCCGTTTG   
  
  
- GTCGTGGACG ACTTCTCAAA AGTCGAAAAT GAGTGAATAT ATCTCGGAGA GGTAAGATAG TCAAACGAGT   
  
  
- AAAGTGTCGG TTAGTCCGTT AACATCTTCG TAAACTCTCC CTCCTTCTCT CATTGTTGAC CCGAAACGTA   
  
  
- CATTAACTAA ACCTGTAGAA CGTACCAAAG GTCACCGGAA GAGAATAAGT CAGAGAGAGG CTTCTCCGAT   
  
  
- GAACAAGTTT GGCAAGTAAC AGAGAAGTTT AATGTCCGAA GCCTTCTTCG AATCTTCTCG AGTAACTTTG   
  
  
- TCTTCGAGCC GACCATTCAA AGAGATTCTC GAAAGTCTTA TAGTTGAAAC TTAAGGTGCC CAAAGACTCT   
  
  
- CCGAGCTTTG AGTCCTTGGA TTCCTCCCTC TTACTTTGTC ATCAACATTT AGAGCAAAAG GTGGAGTCAT   
  
  
- GAAACTTTCT ATGGCATGTC TAAAGACTGT GGAACTGAAG ACATGTAAGT GATTTGGGGA GATAACACCA   
  
  
- TAATCAGCTC TCTCTTCCTT CAGCTTTGTC TACACCCAAG GAGAGTTCAA TACATCTACG AGACGTAATA   
  
  
- ATACGACGTT ACAAACTGAG AAATCTACTA ACGGAGGGTG AACTTTCAGG ACTCTCCGAC TCGTATCTCT   
  
  
- TCTTGGTAGA ACCTTTTCTC TAGTTTCTTC GGTATCCAAC ACTCTTCCTA CTTTGTTTGA TAGACTTCAA   
  
  
- ACTCTACGAC CTCTGAACCT CTCCCTCCTA CCTCTCAGTA CCTAAAAGTC CATACTTCGA TTCAAGGTCC   
  
  
- CGTTGATATG TTCGTTTCGA CGAAAATTTT TACCCTTCGG TAATAGTATG GATAAACCTT CTTCTGACAC   
  
  
- CTCCACCACC CAAGTCTCAA ACACTTGCTC TACTACCTTA CCGATAGAGA GAACCGACCG TTCCATCCAA   
  
  
- GGAGCGTTGA CGGAGCCATA CCGCAAGACA CAC

+     LAMP-element

| Site Name | Organism | Position | Strand | Matrix score. | sequence | function |
| --- | --- | --- | --- | --- | --- | --- |
| LAMP-element | Pisum sativum | 965 | - | 8 | CTTTATCA | part of a light responsive element |

>HU07G00272.1   
+ -Up\_Stream \_Len000ACCAAC GATTCTTTAC TGAATCAGCT ACATTTTTGG ATTGTGTTAA GGTATCTTTT   
  
  
+ AGATCTTCAG GAGGAGGGGG CTGAAGTAGC TTGGTTACTT TTGCGCTTAT CAACTGTGGA AGAATATTAT   
  
  
+ TGAGACTAGT GTAGAAGAAT TAGTAGTTTC CTCCACATTG GAAAAAAACC ATGAAATTAA CGCTTTAGGC   
  
  
+ CCTCAGAGTA CCAAGTGTCA TCGTAAATGA TTGAAATTTC AAGTCTTAGC TGAGTCATGA AGTCCTGATT   
  
  
+ TCCCATGACA AATTGATGTT GTTGTCTAAA GCTTGGCGGG TGATTTGAAA GAAGATTTAG ATCAGGCTGA   
  
  
+ CAATACACCA AATCTGGGAG CAAGGAACTG GATTTTTCTG TGTCTAGAAA AGCTTTCCGC TTTCTGAACA   
  
  
+ ATTGAGAAAA CCATGCATAG GAAATTTCTT GATATTTAGA TCATAGCAGA CTTAACAATC TGGCTTTACC   
  
  
+ GAACTGTCCT CTACTTCTAA GTTGTGAAGG TGTGAAAGAT AAACCATGTC AAACTTAGTG TCCAACAGAA   
  
  
+ ACGCATCTCT ACTTCTCCAG GCAAAGTTGT ACTTCAGATC TTCTACACCT TTTCTAGGCA GGACTTTGGG   
  
  
+ ATATAGAGTT TGGCCTGGGT GAAAAGTAGG AAGTGGGGAA GTAGAAAATG ATGAAAGAAG GCACCACTTG   
  
  
+ GGTCCAGATA CATAGGCCTA CGCACGAGAA CCTTTGCATG GTCTAGATAC ATGACTGTAT ACTTCTTCCT   
  
  
+ TTCCTTTACC TGTTGGAGAT TATCCTAGGA TTTTACATGG CACCAGCTGG ATTTGTTAAT GACATAAACA   
  
  
+ TATCTTTAAT TACAGTTGCT TATGGTATAC ATTTAAACTA TTCGATAGTT GACAGCTGAT GTCCTTATCC   
  
  
+ TTGTGCAGCT TATTCCTCGA TAGAATAATC TTTGGACATT TCTGTCTAGG TGATAAAGGG CAAAAACTAA   
  
  
+ GATACAAGAA ATAGAACATG TCCTCCATTT ACAAGACGTA TCTGTTTTTG GTTTTGGTCA ATGATCCCTG   
  
  
+ TTGTCCTCAA GCATTAACAC AGAATGATCA TTATTGACTT GGACTGAAAG GTTTTGGGAA CTCCATAGTA   
  
  
+ TGCCTTTTCA ATAAAATCAA AAGCAAATTT GCTGTTCACT GATAACTGCA GTTGTGTTGC TTTGCTGTTT   
  
  
+ CCATACCAGA CTCGTAACCA AGCTGATAGC AAATGTGCAA AGGTACAGTA TAATTAACGA TGTTTTCATA   
  
  
+ TTGCTACGAG TTCTGAAACA TGATATAAGA AAACACCAGA ACCCAAATAC CTACATAGGT CCTAGCCCTG   
  
  
+ GAATGGCTAT TTTAAATATC CAAATGGACT TACAGAATTC GCAGAGTTGT TTGGTTTGTT TAACAATCAT   
  
  
+ TGAATTGTTA GCTATGAGAA TCTGCGGGAC AATCGTAATC TTTGGCGATT ACTTCGATTG GCATACTGAT   
  
  
+ GTTTTTTTAT TTGCGTATCT AAGCATGGTG GTGTACCTTT GATTTGTGTG TCTAGGTAGT GTGTGTAGAT   
  
  
+ AAATATCATA GAACTCAGAC GTTGGTAGGG TTATGTTGAG CACTTTCTCT TGCTGTTTGT CCTTTGTGAT   
  
  
+ GGCACCTCTT GCTTTTCCGA ACCACCAAAA GTCTCATTGA CAAAACTCTT CAAACATTGC AAACTGCCCC   
  
  
+ CTTAAATATG TTTTATTAAA AAAAATGGAA GAGCTCAAAC TGTTGAGTGA GCCTAGATTC CAAGATTTAG   
  
  
+ AAACAGTAAA CTTGATAGAT ATATTTATAT GAACTATTGT TAGATGCAAC ACCCCCAACT TGAAACACAA   
  
  
+ AAGAGAAATT AAAAACAAAA AGGTTTTGCA CTATTTATAT ATCCTGTGTT CTTTGTGCTA GTCTTATTTA   
  
  
+ ATGTTAATTG GTAAGCATTA TTTGTTGGAT TATTCCACTG CACTTTCTCT TACTAACTTC AGCATTTCAT   
  
  
+ TCCTTGTTCT TCAGCTTTTG AACTTCTTTA GCCTATTTTG TTGTCTTTAT GGTTATGGAA GCCATCCAGA   
  
  
+ AGAAGAATGA TGAACTTCTG AGTCTTAGCT TGGCCATTGT CGGTCACTCT GATCGCACTG AGAAAAAACT   
  
  
+ GAAGAGGCGG ATTGATGTCT CCGATCCTCT GATCACTTCA GGTGAAGGCT GCGAAGGGAA GATAATCAGG   
  
  
+ CTCCTCCAAG AGAGACAGAA CTTGTTGAAC ATCAAGCAAA GAGGAAAAGG CGTCATTCAA GAAGGGAAGG   
  
  
+ GTCTTCATCT TATCCATCTG CTCCTTGTAT CTGCCACCTT AATCAACGAA AACAACATCA GTTCAGCTGT   
  
  
+ TGATAATCTT ATTGAGCTCT TCCAATATGT CTCCGAAAGT GGTGATTCAG GACAAAGGGT TGCTGCTTAC   
  
  
+ TTTGCGGATG GGTTAACAGC AAGGATCTTA ACTCAGAGGT CTCCATTTTA TCGCCTGATA CTGGGCAAAC   
  
  
+ CAGCACCTGC TGAAGAGTTT TCAGCTTTTA CTCACTTATA TAGAGCCTCT CCATTCTATC AGTTTGCTCA   
  
  
+ TTTCACAGCC AATCAGGCAA TTGTAGAAGC ATTTGAGAGG GAGGAAGAGA GTAACAACTG GGCTTTGCAT   
  
  
+ GTAATTGATT TGGACATCTT GCATGGTTTC CAGTGGCCTT CTCTTATTCA GTCTCTCTCC GAAGAGGCTA   
  
  
+ CTTGTTCAAA CCGTTCATTG TCTCTTCAAA TTACAGGCTT CGGAAGAAGC TTAGAAGAGC TCATTGAAAC   
  
  
+ AGAAGCTCGG CTGGTAAGTT TCTCTAAGAG CTTTCAGAAT ATCAACTTTG AATTCCACGG GTTTCTGAGA   
  
  
+ GGCTCGAAAC TCAGGAACCT AAGGAGGGAG AATGAAACAG TAGTTGTAAA TCTCGTTTTC CACCTCAGTA   
  
  
+ CTTTGAAAGA TACCGTACAG ATTTCTGACA CCTTGACTTC TGTACATTCA CTAAACCCCT CTATTGTGGT   
  
  
+ ATTAGTCGAG AGAGAAGGAA GTCGAAACAG ATGTGGGTTC CTCTCAAGTT ATGTAGATGC TCTGCATTAT   
  
  
+ TATGCTGCAA TGTTTGACTC TTTAGATGAT TGCCTCCCAC TTGAAAGTCC TGAGAGGCTG AGCATAGAGA   
  
  
+ AGAACCATCT TGGAAAAGAG ATCAAAGAAG CCATAGGTTG TGAGAAGGAT GAAACAAACT ATCTGAAGTT   
  
  
+ TGAGATGCTG GAGACTTGGA GAGGGAGGAT GGAGAGTCAT GGATTTTCAG GTATGAAGCT AAGTTCCAGG   
  
  
+ GCAACTATAC AAGCAAAGCT GCTTTTAAAA ATGGGAAGCC ATTATCATAC CTATTTGGAA GAAGACTGTG   
  
  
+ GAGGTGGTGG GTTCAGAGTT TGTGAACGAG ATGATGGAAT GGCTATCTCT CTTGGCTGGC AAGGTAGGTT   
  
  
+ CCTCGCAACT GCCTCGGTAT GGCGTTCTGT GTG  

- -Up\_Stream \_Len000TGGTTG CTAAGAAATG ACTTAGTCGA TGTAAAAACC TAACACAATT CCATAGAAAA   
  
  
- TCTAGAAGTC CTCCTCCCCC GACTTCATCG AACCAATGAA AACGCGAATA GTTGACACCT TCTTATAATA   
  
  
- ACTCTGATCA CATCTTCTTA ATCATCAAAG GAGGTGTAAC CTTTTTTTGG TACTTTAATT GCGAAATCCG   
  
  
- GGAGTCTCAT GGTTCACAGT AGCATTTACT AACTTTAAAG TTCAGAATCG ACTCAGTACT TCAGGACTAA   
  
  
- AGGGTACTGT TTAACTACAA CAACAGATTT CGAACCGCCC ACTAAACTTT CTTCTAAATC TAGTCCGACT   
  
  
- GTTATGTGGT TTAGACCCTC GTTCCTTGAC CTAAAAAGAC ACAGATCTTT TCGAAAGGCG AAAGACTTGT   
  
  
- TAACTCTTTT GGTACGTATC CTTTAAAGAA CTATAAATCT AGTATCGTCT GAATTGTTAG ACCGAAATGG   
  
  
- CTTGACAGGA GATGAAGATT CAACACTTCC ACACTTTCTA TTTGGTACAG TTTGAATCAC AGGTTGTCTT   
  
  
- TGCGTAGAGA TGAAGAGGTC CGTTTCAACA TGAAGTCTAG AAGATGTGGA AAAGATCCGT CCTGAAACCC   
  
  
- TATATCTCAA ACCGGACCCA CTTTTCATCC TTCACCCCTT CATCTTTTAC TACTTTCTTC CGTGGTGAAC   
  
  
- CCAGGTCTAT GTATCCGGAT GCGTGCTCTT GGAAACGTAC CAGATCTATG TACTGACATA TGAAGAAGGA   
  
  
- AAGGAAATGG ACAACCTCTA ATAGGATCCT AAAATGTACC GTGGTCGACC TAAACAATTA CTGTATTTGT   
  
  
- ATAGAAATTA ATGTCAACGA ATACCATATG TAAATTTGAT AAGCTATCAA CTGTCGACTA CAGGAATAGG   
  
  
- AACACGTCGA ATAAGGAGCT ATCTTATTAG AAACCTGTAA AGACAGATCC ACTATTTCCC GTTTTTGATT   
  
  
- CTATGTTCTT TATCTTGTAC AGGAGGTAAA TGTTCTGCAT AGACAAAAAC CAAAACCAGT TACTAGGGAC   
  
  
- AACAGGAGTT CGTAATTGTG TCTTACTAGT AATAACTGAA CCTGACTTTC CAAAACCCTT GAGGTATCAT   
  
  
- ACGGAAAAGT TATTTTAGTT TTCGTTTAAA CGACAAGTGA CTATTGACGT CAACACAACG AAACGACAAA   
  
  
- GGTATGGTCT GAGCATTGGT TCGACTATCG TTTACACGTT TCCATGTCAT ATTAATTGCT ACAAAAGTAT   
  
  
- AACGATGCTC AAGACTTTGT ACTATATTCT TTTGTGGTCT TGGGTTTATG GATGTATCCA GGATCGGGAC   
  
  
- CTTACCGATA AAATTTATAG GTTTACCTGA ATGTCTTAAG CGTCTCAACA AACCAAACAA ATTGTTAGTA   
  
  
- ACTTAACAAT CGATACTCTT AGACGCCCTG TTAGCATTAG AAACCGCTAA TGAAGCTAAC CGTATGACTA   
  
  
- CAAAAAAATA AACGCATAGA TTCGTACCAC CACATGGAAA CTAAACACAC AGATCCATCA CACACATCTA   
  
  
- TTTATAGTAT CTTGAGTCTG CAACCATCCC AATACAACTC GTGAAAGAGA ACGACAAACA GGAAACACTA   
  
  
- CCGTGGAGAA CGAAAAGGCT TGGTGGTTTT CAGAGTAACT GTTTTGAGAA GTTTGTAACG TTTGACGGGG   
  
  
- GAATTTATAC AAAATAATTT TTTTTACCTT CTCGAGTTTG ACAACTCACT CGGATCTAAG GTTCTAAATC   
  
  
- TTTGTCATTT GAACTATCTA TATAAATATA CTTGATAACA ATCTACGTTG TGGGGGTTGA ACTTTGTGTT   
  
  
- TTCTCTTTAA TTTTTGTTTT TCCAAAACGT GATAAATATA TAGGACACAA GAAACACGAT CAGAATAAAT   
  
  
- TACAATTAAC CATTCGTAAT AAACAACCTA ATAAGGTGAC GTGAAAGAGA ATGATTGAAG TCGTAAAGTA   
  
  
- AGGAACAAGA AGTCGAAAAC TTGAAGAAAT CGGATAAAAC AACAGAAATA CCAATACCTT CGGTAGGTCT   
  
  
- TCTTCTTACT ACTTGAAGAC TCAGAATCGA ACCGGTAACA GCCAGTGAGA CTAGCGTGAC TCTTTTTTGA   
  
  
- CTTCTCCGCC TAACTACAGA GGCTAGGAGA CTAGTGAAGT CCACTTCCGA CGCTTCCCTT CTATTAGTCC   
  
  
- GAGGAGGTTC TCTCTGTCTT GAACAACTTG TAGTTCGTTT CTCCTTTTCC GCAGTAAGTT CTTCCCTTCC   
  
  
- CAGAAGTAGA ATAGGTAGAC GAGGAACATA GACGGTGGAA TTAGTTGCTT TTGTTGTAGT CAAGTCGACA   
  
  
- ACTATTAGAA TAACTCGAGA AGGTTATACA GAGGCTTTCA CCACTAAGTC CTGTTTCCCA ACGACGAATG   
  
  
- AAACGCCTAC CCAATTGTCG TTCCTAGAAT TGAGTCTCCA GAGGTAAAAT AGCGGACTAT GACCCGTTTG   
  
  
- GTCGTGGACG ACTTCTCAAA AGTCGAAAAT GAGTGAATAT ATCTCGGAGA GGTAAGATAG TCAAACGAGT   
  
  
- AAAGTGTCGG TTAGTCCGTT AACATCTTCG TAAACTCTCC CTCCTTCTCT CATTGTTGAC CCGAAACGTA   
  
  
- CATTAACTAA ACCTGTAGAA CGTACCAAAG GTCACCGGAA GAGAATAAGT CAGAGAGAGG CTTCTCCGAT   
  
  
- GAACAAGTTT GGCAAGTAAC AGAGAAGTTT AATGTCCGAA GCCTTCTTCG AATCTTCTCG AGTAACTTTG   
  
  
- TCTTCGAGCC GACCATTCAA AGAGATTCTC GAAAGTCTTA TAGTTGAAAC TTAAGGTGCC CAAAGACTCT   
  
  
- CCGAGCTTTG AGTCCTTGGA TTCCTCCCTC TTACTTTGTC ATCAACATTT AGAGCAAAAG GTGGAGTCAT   
  
  
- GAAACTTTCT ATGGCATGTC TAAAGACTGT GGAACTGAAG ACATGTAAGT GATTTGGGGA GATAACACCA   
  
  
- TAATCAGCTC TCTCTTCCTT CAGCTTTGTC TACACCCAAG GAGAGTTCAA TACATCTACG AGACGTAATA   
  
  
- ATACGACGTT ACAAACTGAG AAATCTACTA ACGGAGGGTG AACTTTCAGG ACTCTCCGAC TCGTATCTCT   
  
  
- TCTTGGTAGA ACCTTTTCTC TAGTTTCTTC GGTATCCAAC ACTCTTCCTA CTTTGTTTGA TAGACTTCAA   
  
  
- ACTCTACGAC CTCTGAACCT CTCCCTCCTA CCTCTCAGTA CCTAAAAGTC CATACTTCGA TTCAAGGTCC   
  
  
- CGTTGATATG TTCGTTTCGA CGAAAATTTT TACCCTTCGG TAATAGTATG GATAAACCTT CTTCTGACAC   
  
  
- CTCCACCACC CAAGTCTCAA ACACTTGCTC TACTACCTTA CCGATAGAGA GAACCGACCG TTCCATCCAA   
  
  
- GGAGCGTTGA CGGAGCCATA CCGCAAGACA CAC

+     LTR

| Site Name | Organism | Position | Strand | Matrix score. | sequence | function |
| --- | --- | --- | --- | --- | --- | --- |
| LTR | Hordeum vulgare | 2347 | + | 6 | CCGAAA | cis-acting element involved in low-temperature responsiveness |

>HU07G00272.1   
+ -Up\_Stream \_Len000ACCAAC GATTCTTTAC TGAATCAGCT ACATTTTTGG ATTGTGTTAA GGTATCTTTT   
  
  
+ AGATCTTCAG GAGGAGGGGG CTGAAGTAGC TTGGTTACTT TTGCGCTTAT CAACTGTGGA AGAATATTAT   
  
  
+ TGAGACTAGT GTAGAAGAAT TAGTAGTTTC CTCCACATTG GAAAAAAACC ATGAAATTAA CGCTTTAGGC   
  
  
+ CCTCAGAGTA CCAAGTGTCA TCGTAAATGA TTGAAATTTC AAGTCTTAGC TGAGTCATGA AGTCCTGATT   
  
  
+ TCCCATGACA AATTGATGTT GTTGTCTAAA GCTTGGCGGG TGATTTGAAA GAAGATTTAG ATCAGGCTGA   
  
  
+ CAATACACCA AATCTGGGAG CAAGGAACTG GATTTTTCTG TGTCTAGAAA AGCTTTCCGC TTTCTGAACA   
  
  
+ ATTGAGAAAA CCATGCATAG GAAATTTCTT GATATTTAGA TCATAGCAGA CTTAACAATC TGGCTTTACC   
  
  
+ GAACTGTCCT CTACTTCTAA GTTGTGAAGG TGTGAAAGAT AAACCATGTC AAACTTAGTG TCCAACAGAA   
  
  
+ ACGCATCTCT ACTTCTCCAG GCAAAGTTGT ACTTCAGATC TTCTACACCT TTTCTAGGCA GGACTTTGGG   
  
  
+ ATATAGAGTT TGGCCTGGGT GAAAAGTAGG AAGTGGGGAA GTAGAAAATG ATGAAAGAAG GCACCACTTG   
  
  
+ GGTCCAGATA CATAGGCCTA CGCACGAGAA CCTTTGCATG GTCTAGATAC ATGACTGTAT ACTTCTTCCT   
  
  
+ TTCCTTTACC TGTTGGAGAT TATCCTAGGA TTTTACATGG CACCAGCTGG ATTTGTTAAT GACATAAACA   
  
  
+ TATCTTTAAT TACAGTTGCT TATGGTATAC ATTTAAACTA TTCGATAGTT GACAGCTGAT GTCCTTATCC   
  
  
+ TTGTGCAGCT TATTCCTCGA TAGAATAATC TTTGGACATT TCTGTCTAGG TGATAAAGGG CAAAAACTAA   
  
  
+ GATACAAGAA ATAGAACATG TCCTCCATTT ACAAGACGTA TCTGTTTTTG GTTTTGGTCA ATGATCCCTG   
  
  
+ TTGTCCTCAA GCATTAACAC AGAATGATCA TTATTGACTT GGACTGAAAG GTTTTGGGAA CTCCATAGTA   
  
  
+ TGCCTTTTCA ATAAAATCAA AAGCAAATTT GCTGTTCACT GATAACTGCA GTTGTGTTGC TTTGCTGTTT   
  
  
+ CCATACCAGA CTCGTAACCA AGCTGATAGC AAATGTGCAA AGGTACAGTA TAATTAACGA TGTTTTCATA   
  
  
+ TTGCTACGAG TTCTGAAACA TGATATAAGA AAACACCAGA ACCCAAATAC CTACATAGGT CCTAGCCCTG   
  
  
+ GAATGGCTAT TTTAAATATC CAAATGGACT TACAGAATTC GCAGAGTTGT TTGGTTTGTT TAACAATCAT   
  
  
+ TGAATTGTTA GCTATGAGAA TCTGCGGGAC AATCGTAATC TTTGGCGATT ACTTCGATTG GCATACTGAT   
  
  
+ GTTTTTTTAT TTGCGTATCT AAGCATGGTG GTGTACCTTT GATTTGTGTG TCTAGGTAGT GTGTGTAGAT   
  
  
+ AAATATCATA GAACTCAGAC GTTGGTAGGG TTATGTTGAG CACTTTCTCT TGCTGTTTGT CCTTTGTGAT   
  
  
+ GGCACCTCTT GCTTTTCCGA ACCACCAAAA GTCTCATTGA CAAAACTCTT CAAACATTGC AAACTGCCCC   
  
  
+ CTTAAATATG TTTTATTAAA AAAAATGGAA GAGCTCAAAC TGTTGAGTGA GCCTAGATTC CAAGATTTAG   
  
  
+ AAACAGTAAA CTTGATAGAT ATATTTATAT GAACTATTGT TAGATGCAAC ACCCCCAACT TGAAACACAA   
  
  
+ AAGAGAAATT AAAAACAAAA AGGTTTTGCA CTATTTATAT ATCCTGTGTT CTTTGTGCTA GTCTTATTTA   
  
  
+ ATGTTAATTG GTAAGCATTA TTTGTTGGAT TATTCCACTG CACTTTCTCT TACTAACTTC AGCATTTCAT   
  
  
+ TCCTTGTTCT TCAGCTTTTG AACTTCTTTA GCCTATTTTG TTGTCTTTAT GGTTATGGAA GCCATCCAGA   
  
  
+ AGAAGAATGA TGAACTTCTG AGTCTTAGCT TGGCCATTGT CGGTCACTCT GATCGCACTG AGAAAAAACT   
  
  
+ GAAGAGGCGG ATTGATGTCT CCGATCCTCT GATCACTTCA GGTGAAGGCT GCGAAGGGAA GATAATCAGG   
  
  
+ CTCCTCCAAG AGAGACAGAA CTTGTTGAAC ATCAAGCAAA GAGGAAAAGG CGTCATTCAA GAAGGGAAGG   
  
  
+ GTCTTCATCT TATCCATCTG CTCCTTGTAT CTGCCACCTT AATCAACGAA AACAACATCA GTTCAGCTGT   
  
  
+ TGATAATCTT ATTGAGCTCT TCCAATATGT CTCCGAAAGT GGTGATTCAG GACAAAGGGT TGCTGCTTAC   
  
  
+ TTTGCGGATG GGTTAACAGC AAGGATCTTA ACTCAGAGGT CTCCATTTTA TCGCCTGATA CTGGGCAAAC   
  
  
+ CAGCACCTGC TGAAGAGTTT TCAGCTTTTA CTCACTTATA TAGAGCCTCT CCATTCTATC AGTTTGCTCA   
  
  
+ TTTCACAGCC AATCAGGCAA TTGTAGAAGC ATTTGAGAGG GAGGAAGAGA GTAACAACTG GGCTTTGCAT   
  
  
+ GTAATTGATT TGGACATCTT GCATGGTTTC CAGTGGCCTT CTCTTATTCA GTCTCTCTCC GAAGAGGCTA   
  
  
+ CTTGTTCAAA CCGTTCATTG TCTCTTCAAA TTACAGGCTT CGGAAGAAGC TTAGAAGAGC TCATTGAAAC   
  
  
+ AGAAGCTCGG CTGGTAAGTT TCTCTAAGAG CTTTCAGAAT ATCAACTTTG AATTCCACGG GTTTCTGAGA   
  
  
+ GGCTCGAAAC TCAGGAACCT AAGGAGGGAG AATGAAACAG TAGTTGTAAA TCTCGTTTTC CACCTCAGTA   
  
  
+ CTTTGAAAGA TACCGTACAG ATTTCTGACA CCTTGACTTC TGTACATTCA CTAAACCCCT CTATTGTGGT   
  
  
+ ATTAGTCGAG AGAGAAGGAA GTCGAAACAG ATGTGGGTTC CTCTCAAGTT ATGTAGATGC TCTGCATTAT   
  
  
+ TATGCTGCAA TGTTTGACTC TTTAGATGAT TGCCTCCCAC TTGAAAGTCC TGAGAGGCTG AGCATAGAGA   
  
  
+ AGAACCATCT TGGAAAAGAG ATCAAAGAAG CCATAGGTTG TGAGAAGGAT GAAACAAACT ATCTGAAGTT   
  
  
+ TGAGATGCTG GAGACTTGGA GAGGGAGGAT GGAGAGTCAT GGATTTTCAG GTATGAAGCT AAGTTCCAGG   
  
  
+ GCAACTATAC AAGCAAAGCT GCTTTTAAAA ATGGGAAGCC ATTATCATAC CTATTTGGAA GAAGACTGTG   
  
  
+ GAGGTGGTGG GTTCAGAGTT TGTGAACGAG ATGATGGAAT GGCTATCTCT CTTGGCTGGC AAGGTAGGTT   
  
  
+ CCTCGCAACT GCCTCGGTAT GGCGTTCTGT GTG  

- -Up\_Stream \_Len000TGGTTG CTAAGAAATG ACTTAGTCGA TGTAAAAACC TAACACAATT CCATAGAAAA   
  
  
- TCTAGAAGTC CTCCTCCCCC GACTTCATCG AACCAATGAA AACGCGAATA GTTGACACCT TCTTATAATA   
  
  
- ACTCTGATCA CATCTTCTTA ATCATCAAAG GAGGTGTAAC CTTTTTTTGG TACTTTAATT GCGAAATCCG   
  
  
- GGAGTCTCAT GGTTCACAGT AGCATTTACT AACTTTAAAG TTCAGAATCG ACTCAGTACT TCAGGACTAA   
  
  
- AGGGTACTGT TTAACTACAA CAACAGATTT CGAACCGCCC ACTAAACTTT CTTCTAAATC TAGTCCGACT   
  
  
- GTTATGTGGT TTAGACCCTC GTTCCTTGAC CTAAAAAGAC ACAGATCTTT TCGAAAGGCG AAAGACTTGT   
  
  
- TAACTCTTTT GGTACGTATC CTTTAAAGAA CTATAAATCT AGTATCGTCT GAATTGTTAG ACCGAAATGG   
  
  
- CTTGACAGGA GATGAAGATT CAACACTTCC ACACTTTCTA TTTGGTACAG TTTGAATCAC AGGTTGTCTT   
  
  
- TGCGTAGAGA TGAAGAGGTC CGTTTCAACA TGAAGTCTAG AAGATGTGGA AAAGATCCGT CCTGAAACCC   
  
  
- TATATCTCAA ACCGGACCCA CTTTTCATCC TTCACCCCTT CATCTTTTAC TACTTTCTTC CGTGGTGAAC   
  
  
- CCAGGTCTAT GTATCCGGAT GCGTGCTCTT GGAAACGTAC CAGATCTATG TACTGACATA TGAAGAAGGA   
  
  
- AAGGAAATGG ACAACCTCTA ATAGGATCCT AAAATGTACC GTGGTCGACC TAAACAATTA CTGTATTTGT   
  
  
- ATAGAAATTA ATGTCAACGA ATACCATATG TAAATTTGAT AAGCTATCAA CTGTCGACTA CAGGAATAGG   
  
  
- AACACGTCGA ATAAGGAGCT ATCTTATTAG AAACCTGTAA AGACAGATCC ACTATTTCCC GTTTTTGATT   
  
  
- CTATGTTCTT TATCTTGTAC AGGAGGTAAA TGTTCTGCAT AGACAAAAAC CAAAACCAGT TACTAGGGAC   
  
  
- AACAGGAGTT CGTAATTGTG TCTTACTAGT AATAACTGAA CCTGACTTTC CAAAACCCTT GAGGTATCAT   
  
  
- ACGGAAAAGT TATTTTAGTT TTCGTTTAAA CGACAAGTGA CTATTGACGT CAACACAACG AAACGACAAA   
  
  
- GGTATGGTCT GAGCATTGGT TCGACTATCG TTTACACGTT TCCATGTCAT ATTAATTGCT ACAAAAGTAT   
  
  
- AACGATGCTC AAGACTTTGT ACTATATTCT TTTGTGGTCT TGGGTTTATG GATGTATCCA GGATCGGGAC   
  
  
- CTTACCGATA AAATTTATAG GTTTACCTGA ATGTCTTAAG CGTCTCAACA AACCAAACAA ATTGTTAGTA   
  
  
- ACTTAACAAT CGATACTCTT AGACGCCCTG TTAGCATTAG AAACCGCTAA TGAAGCTAAC CGTATGACTA   
  
  
- CAAAAAAATA AACGCATAGA TTCGTACCAC CACATGGAAA CTAAACACAC AGATCCATCA CACACATCTA   
  
  
- TTTATAGTAT CTTGAGTCTG CAACCATCCC AATACAACTC GTGAAAGAGA ACGACAAACA GGAAACACTA   
  
  
- CCGTGGAGAA CGAAAAGGCT TGGTGGTTTT CAGAGTAACT GTTTTGAGAA GTTTGTAACG TTTGACGGGG   
  
  
- GAATTTATAC AAAATAATTT TTTTTACCTT CTCGAGTTTG ACAACTCACT CGGATCTAAG GTTCTAAATC   
  
  
- TTTGTCATTT GAACTATCTA TATAAATATA CTTGATAACA ATCTACGTTG TGGGGGTTGA ACTTTGTGTT   
  
  
- TTCTCTTTAA TTTTTGTTTT TCCAAAACGT GATAAATATA TAGGACACAA GAAACACGAT CAGAATAAAT   
  
  
- TACAATTAAC CATTCGTAAT AAACAACCTA ATAAGGTGAC GTGAAAGAGA ATGATTGAAG TCGTAAAGTA   
  
  
- AGGAACAAGA AGTCGAAAAC TTGAAGAAAT CGGATAAAAC AACAGAAATA CCAATACCTT CGGTAGGTCT   
  
  
- TCTTCTTACT ACTTGAAGAC TCAGAATCGA ACCGGTAACA GCCAGTGAGA CTAGCGTGAC TCTTTTTTGA   
  
  
- CTTCTCCGCC TAACTACAGA GGCTAGGAGA CTAGTGAAGT CCACTTCCGA CGCTTCCCTT CTATTAGTCC   
  
  
- GAGGAGGTTC TCTCTGTCTT GAACAACTTG TAGTTCGTTT CTCCTTTTCC GCAGTAAGTT CTTCCCTTCC   
  
  
- CAGAAGTAGA ATAGGTAGAC GAGGAACATA GACGGTGGAA TTAGTTGCTT TTGTTGTAGT CAAGTCGACA   
  
  
- ACTATTAGAA TAACTCGAGA AGGTTATACA GAGGCTTTCA CCACTAAGTC CTGTTTCCCA ACGACGAATG   
  
  
- AAACGCCTAC CCAATTGTCG TTCCTAGAAT TGAGTCTCCA GAGGTAAAAT AGCGGACTAT GACCCGTTTG   
  
  
- GTCGTGGACG ACTTCTCAAA AGTCGAAAAT GAGTGAATAT ATCTCGGAGA GGTAAGATAG TCAAACGAGT   
  
  
- AAAGTGTCGG TTAGTCCGTT AACATCTTCG TAAACTCTCC CTCCTTCTCT CATTGTTGAC CCGAAACGTA   
  
  
- CATTAACTAA ACCTGTAGAA CGTACCAAAG GTCACCGGAA GAGAATAAGT CAGAGAGAGG CTTCTCCGAT   
  
  
- GAACAAGTTT GGCAAGTAAC AGAGAAGTTT AATGTCCGAA GCCTTCTTCG AATCTTCTCG AGTAACTTTG   
  
  
- TCTTCGAGCC GACCATTCAA AGAGATTCTC GAAAGTCTTA TAGTTGAAAC TTAAGGTGCC CAAAGACTCT   
  
  
- CCGAGCTTTG AGTCCTTGGA TTCCTCCCTC TTACTTTGTC ATCAACATTT AGAGCAAAAG GTGGAGTCAT   
  
  
- GAAACTTTCT ATGGCATGTC TAAAGACTGT GGAACTGAAG ACATGTAAGT GATTTGGGGA GATAACACCA   
  
  
- TAATCAGCTC TCTCTTCCTT CAGCTTTGTC TACACCCAAG GAGAGTTCAA TACATCTACG AGACGTAATA   
  
  
- ATACGACGTT ACAAACTGAG AAATCTACTA ACGGAGGGTG AACTTTCAGG ACTCTCCGAC TCGTATCTCT   
  
  
- TCTTGGTAGA ACCTTTTCTC TAGTTTCTTC GGTATCCAAC ACTCTTCCTA CTTTGTTTGA TAGACTTCAA   
  
  
- ACTCTACGAC CTCTGAACCT CTCCCTCCTA CCTCTCAGTA CCTAAAAGTC CATACTTCGA TTCAAGGTCC   
  
  
- CGTTGATATG TTCGTTTCGA CGAAAATTTT TACCCTTCGG TAATAGTATG GATAAACCTT CTTCTGACAC   
  
  
- CTCCACCACC CAAGTCTCAA ACACTTGCTC TACTACCTTA CCGATAGAGA GAACCGACCG TTCCATCCAA   
  
  
- GGAGCGTTGA CGGAGCCATA CCGCAAGACA CAC

+     MBS

| Site Name | Organism | Position | Strand | Matrix score. | sequence | function |
| --- | --- | --- | --- | --- | --- | --- |
| MBS | Arabidopsis thaliana | 1173 | - | 6 | CAACTG | MYB binding site involved in drought-inducibility |
| MBS | Arabidopsis thaliana | 125 | + | 6 | CAACTG | MYB binding site involved in drought-inducibility |
| MBS | Arabidopsis thaliana | 857 | - | 6 | CAACTG | MYB binding site involved in drought-inducibility |
| MBS | Arabidopsis thaliana | 2579 | + | 6 | CAACTG | MYB binding site involved in drought-inducibility |
| MBS | Arabidopsis thaliana | 3370 | + | 6 | CAACTG | MYB binding site involved in drought-inducibility |

>HU07G00272.1   
+ -Up\_Stream \_Len000ACCAAC GATTCTTTAC TGAATCAGCT ACATTTTTGG ATTGTGTTAA GGTATCTTTT   
  
  
+ AGATCTTCAG GAGGAGGGGG CTGAAGTAGC TTGGTTACTT TTGCGCTTAT CAACTGTGGA AGAATATTAT   
  
  
+ TGAGACTAGT GTAGAAGAAT TAGTAGTTTC CTCCACATTG GAAAAAAACC ATGAAATTAA CGCTTTAGGC   
  
  
+ CCTCAGAGTA CCAAGTGTCA TCGTAAATGA TTGAAATTTC AAGTCTTAGC TGAGTCATGA AGTCCTGATT   
  
  
+ TCCCATGACA AATTGATGTT GTTGTCTAAA GCTTGGCGGG TGATTTGAAA GAAGATTTAG ATCAGGCTGA   
  
  
+ CAATACACCA AATCTGGGAG CAAGGAACTG GATTTTTCTG TGTCTAGAAA AGCTTTCCGC TTTCTGAACA   
  
  
+ ATTGAGAAAA CCATGCATAG GAAATTTCTT GATATTTAGA TCATAGCAGA CTTAACAATC TGGCTTTACC   
  
  
+ GAACTGTCCT CTACTTCTAA GTTGTGAAGG TGTGAAAGAT AAACCATGTC AAACTTAGTG TCCAACAGAA   
  
  
+ ACGCATCTCT ACTTCTCCAG GCAAAGTTGT ACTTCAGATC TTCTACACCT TTTCTAGGCA GGACTTTGGG   
  
  
+ ATATAGAGTT TGGCCTGGGT GAAAAGTAGG AAGTGGGGAA GTAGAAAATG ATGAAAGAAG GCACCACTTG   
  
  
+ GGTCCAGATA CATAGGCCTA CGCACGAGAA CCTTTGCATG GTCTAGATAC ATGACTGTAT ACTTCTTCCT   
  
  
+ TTCCTTTACC TGTTGGAGAT TATCCTAGGA TTTTACATGG CACCAGCTGG ATTTGTTAAT GACATAAACA   
  
  
+ TATCTTTAAT TACAGTTGCT TATGGTATAC ATTTAAACTA TTCGATAGTT GACAGCTGAT GTCCTTATCC   
  
  
+ TTGTGCAGCT TATTCCTCGA TAGAATAATC TTTGGACATT TCTGTCTAGG TGATAAAGGG CAAAAACTAA   
  
  
+ GATACAAGAA ATAGAACATG TCCTCCATTT ACAAGACGTA TCTGTTTTTG GTTTTGGTCA ATGATCCCTG   
  
  
+ TTGTCCTCAA GCATTAACAC AGAATGATCA TTATTGACTT GGACTGAAAG GTTTTGGGAA CTCCATAGTA   
  
  
+ TGCCTTTTCA ATAAAATCAA AAGCAAATTT GCTGTTCACT GATAACTGCA GTTGTGTTGC TTTGCTGTTT   
  
  
+ CCATACCAGA CTCGTAACCA AGCTGATAGC AAATGTGCAA AGGTACAGTA TAATTAACGA TGTTTTCATA   
  
  
+ TTGCTACGAG TTCTGAAACA TGATATAAGA AAACACCAGA ACCCAAATAC CTACATAGGT CCTAGCCCTG   
  
  
+ GAATGGCTAT TTTAAATATC CAAATGGACT TACAGAATTC GCAGAGTTGT TTGGTTTGTT TAACAATCAT   
  
  
+ TGAATTGTTA GCTATGAGAA TCTGCGGGAC AATCGTAATC TTTGGCGATT ACTTCGATTG GCATACTGAT   
  
  
+ GTTTTTTTAT TTGCGTATCT AAGCATGGTG GTGTACCTTT GATTTGTGTG TCTAGGTAGT GTGTGTAGAT   
  
  
+ AAATATCATA GAACTCAGAC GTTGGTAGGG TTATGTTGAG CACTTTCTCT TGCTGTTTGT CCTTTGTGAT   
  
  
+ GGCACCTCTT GCTTTTCCGA ACCACCAAAA GTCTCATTGA CAAAACTCTT CAAACATTGC AAACTGCCCC   
  
  
+ CTTAAATATG TTTTATTAAA AAAAATGGAA GAGCTCAAAC TGTTGAGTGA GCCTAGATTC CAAGATTTAG   
  
  
+ AAACAGTAAA CTTGATAGAT ATATTTATAT GAACTATTGT TAGATGCAAC ACCCCCAACT TGAAACACAA   
  
  
+ AAGAGAAATT AAAAACAAAA AGGTTTTGCA CTATTTATAT ATCCTGTGTT CTTTGTGCTA GTCTTATTTA   
  
  
+ ATGTTAATTG GTAAGCATTA TTTGTTGGAT TATTCCACTG CACTTTCTCT TACTAACTTC AGCATTTCAT   
  
  
+ TCCTTGTTCT TCAGCTTTTG AACTTCTTTA GCCTATTTTG TTGTCTTTAT GGTTATGGAA GCCATCCAGA   
  
  
+ AGAAGAATGA TGAACTTCTG AGTCTTAGCT TGGCCATTGT CGGTCACTCT GATCGCACTG AGAAAAAACT   
  
  
+ GAAGAGGCGG ATTGATGTCT CCGATCCTCT GATCACTTCA GGTGAAGGCT GCGAAGGGAA GATAATCAGG   
  
  
+ CTCCTCCAAG AGAGACAGAA CTTGTTGAAC ATCAAGCAAA GAGGAAAAGG CGTCATTCAA GAAGGGAAGG   
  
  
+ GTCTTCATCT TATCCATCTG CTCCTTGTAT CTGCCACCTT AATCAACGAA AACAACATCA GTTCAGCTGT   
  
  
+ TGATAATCTT ATTGAGCTCT TCCAATATGT CTCCGAAAGT GGTGATTCAG GACAAAGGGT TGCTGCTTAC   
  
  
+ TTTGCGGATG GGTTAACAGC AAGGATCTTA ACTCAGAGGT CTCCATTTTA TCGCCTGATA CTGGGCAAAC   
  
  
+ CAGCACCTGC TGAAGAGTTT TCAGCTTTTA CTCACTTATA TAGAGCCTCT CCATTCTATC AGTTTGCTCA   
  
  
+ TTTCACAGCC AATCAGGCAA TTGTAGAAGC ATTTGAGAGG GAGGAAGAGA GTAACAACTG GGCTTTGCAT   
  
  
+ GTAATTGATT TGGACATCTT GCATGGTTTC CAGTGGCCTT CTCTTATTCA GTCTCTCTCC GAAGAGGCTA   
  
  
+ CTTGTTCAAA CCGTTCATTG TCTCTTCAAA TTACAGGCTT CGGAAGAAGC TTAGAAGAGC TCATTGAAAC   
  
  
+ AGAAGCTCGG CTGGTAAGTT TCTCTAAGAG CTTTCAGAAT ATCAACTTTG AATTCCACGG GTTTCTGAGA   
  
  
+ GGCTCGAAAC TCAGGAACCT AAGGAGGGAG AATGAAACAG TAGTTGTAAA TCTCGTTTTC CACCTCAGTA   
  
  
+ CTTTGAAAGA TACCGTACAG ATTTCTGACA CCTTGACTTC TGTACATTCA CTAAACCCCT CTATTGTGGT   
  
  
+ ATTAGTCGAG AGAGAAGGAA GTCGAAACAG ATGTGGGTTC CTCTCAAGTT ATGTAGATGC TCTGCATTAT   
  
  
+ TATGCTGCAA TGTTTGACTC TTTAGATGAT TGCCTCCCAC TTGAAAGTCC TGAGAGGCTG AGCATAGAGA   
  
  
+ AGAACCATCT TGGAAAAGAG ATCAAAGAAG CCATAGGTTG TGAGAAGGAT GAAACAAACT ATCTGAAGTT   
  
  
+ TGAGATGCTG GAGACTTGGA GAGGGAGGAT GGAGAGTCAT GGATTTTCAG GTATGAAGCT AAGTTCCAGG   
  
  
+ GCAACTATAC AAGCAAAGCT GCTTTTAAAA ATGGGAAGCC ATTATCATAC CTATTTGGAA GAAGACTGTG   
  
  
+ GAGGTGGTGG GTTCAGAGTT TGTGAACGAG ATGATGGAAT GGCTATCTCT CTTGGCTGGC AAGGTAGGTT   
  
  
+ CCTCGCAACT GCCTCGGTAT GGCGTTCTGT GTG  

- -Up\_Stream \_Len000TGGTTG CTAAGAAATG ACTTAGTCGA TGTAAAAACC TAACACAATT CCATAGAAAA   
  
  
- TCTAGAAGTC CTCCTCCCCC GACTTCATCG AACCAATGAA AACGCGAATA GTTGACACCT TCTTATAATA   
  
  
- ACTCTGATCA CATCTTCTTA ATCATCAAAG GAGGTGTAAC CTTTTTTTGG TACTTTAATT GCGAAATCCG   
  
  
- GGAGTCTCAT GGTTCACAGT AGCATTTACT AACTTTAAAG TTCAGAATCG ACTCAGTACT TCAGGACTAA   
  
  
- AGGGTACTGT TTAACTACAA CAACAGATTT CGAACCGCCC ACTAAACTTT CTTCTAAATC TAGTCCGACT   
  
  
- GTTATGTGGT TTAGACCCTC GTTCCTTGAC CTAAAAAGAC ACAGATCTTT TCGAAAGGCG AAAGACTTGT   
  
  
- TAACTCTTTT GGTACGTATC CTTTAAAGAA CTATAAATCT AGTATCGTCT GAATTGTTAG ACCGAAATGG   
  
  
- CTTGACAGGA GATGAAGATT CAACACTTCC ACACTTTCTA TTTGGTACAG TTTGAATCAC AGGTTGTCTT   
  
  
- TGCGTAGAGA TGAAGAGGTC CGTTTCAACA TGAAGTCTAG AAGATGTGGA AAAGATCCGT CCTGAAACCC   
  
  
- TATATCTCAA ACCGGACCCA CTTTTCATCC TTCACCCCTT CATCTTTTAC TACTTTCTTC CGTGGTGAAC   
  
  
- CCAGGTCTAT GTATCCGGAT GCGTGCTCTT GGAAACGTAC CAGATCTATG TACTGACATA TGAAGAAGGA   
  
  
- AAGGAAATGG ACAACCTCTA ATAGGATCCT AAAATGTACC GTGGTCGACC TAAACAATTA CTGTATTTGT   
  
  
- ATAGAAATTA ATGTCAACGA ATACCATATG TAAATTTGAT AAGCTATCAA CTGTCGACTA CAGGAATAGG   
  
  
- AACACGTCGA ATAAGGAGCT ATCTTATTAG AAACCTGTAA AGACAGATCC ACTATTTCCC GTTTTTGATT   
  
  
- CTATGTTCTT TATCTTGTAC AGGAGGTAAA TGTTCTGCAT AGACAAAAAC CAAAACCAGT TACTAGGGAC   
  
  
- AACAGGAGTT CGTAATTGTG TCTTACTAGT AATAACTGAA CCTGACTTTC CAAAACCCTT GAGGTATCAT   
  
  
- ACGGAAAAGT TATTTTAGTT TTCGTTTAAA CGACAAGTGA CTATTGACGT CAACACAACG AAACGACAAA   
  
  
- GGTATGGTCT GAGCATTGGT TCGACTATCG TTTACACGTT TCCATGTCAT ATTAATTGCT ACAAAAGTAT   
  
  
- AACGATGCTC AAGACTTTGT ACTATATTCT TTTGTGGTCT TGGGTTTATG GATGTATCCA GGATCGGGAC   
  
  
- CTTACCGATA AAATTTATAG GTTTACCTGA ATGTCTTAAG CGTCTCAACA AACCAAACAA ATTGTTAGTA   
  
  
- ACTTAACAAT CGATACTCTT AGACGCCCTG TTAGCATTAG AAACCGCTAA TGAAGCTAAC CGTATGACTA   
  
  
- CAAAAAAATA AACGCATAGA TTCGTACCAC CACATGGAAA CTAAACACAC AGATCCATCA CACACATCTA   
  
  
- TTTATAGTAT CTTGAGTCTG CAACCATCCC AATACAACTC GTGAAAGAGA ACGACAAACA GGAAACACTA   
  
  
- CCGTGGAGAA CGAAAAGGCT TGGTGGTTTT CAGAGTAACT GTTTTGAGAA GTTTGTAACG TTTGACGGGG   
  
  
- GAATTTATAC AAAATAATTT TTTTTACCTT CTCGAGTTTG ACAACTCACT CGGATCTAAG GTTCTAAATC   
  
  
- TTTGTCATTT GAACTATCTA TATAAATATA CTTGATAACA ATCTACGTTG TGGGGGTTGA ACTTTGTGTT   
  
  
- TTCTCTTTAA TTTTTGTTTT TCCAAAACGT GATAAATATA TAGGACACAA GAAACACGAT CAGAATAAAT   
  
  
- TACAATTAAC CATTCGTAAT AAACAACCTA ATAAGGTGAC GTGAAAGAGA ATGATTGAAG TCGTAAAGTA   
  
  
- AGGAACAAGA AGTCGAAAAC TTGAAGAAAT CGGATAAAAC AACAGAAATA CCAATACCTT CGGTAGGTCT   
  
  
- TCTTCTTACT ACTTGAAGAC TCAGAATCGA ACCGGTAACA GCCAGTGAGA CTAGCGTGAC TCTTTTTTGA   
  
  
- CTTCTCCGCC TAACTACAGA GGCTAGGAGA CTAGTGAAGT CCACTTCCGA CGCTTCCCTT CTATTAGTCC   
  
  
- GAGGAGGTTC TCTCTGTCTT GAACAACTTG TAGTTCGTTT CTCCTTTTCC GCAGTAAGTT CTTCCCTTCC   
  
  
- CAGAAGTAGA ATAGGTAGAC GAGGAACATA GACGGTGGAA TTAGTTGCTT TTGTTGTAGT CAAGTCGACA   
  
  
- ACTATTAGAA TAACTCGAGA AGGTTATACA GAGGCTTTCA CCACTAAGTC CTGTTTCCCA ACGACGAATG   
  
  
- AAACGCCTAC CCAATTGTCG TTCCTAGAAT TGAGTCTCCA GAGGTAAAAT AGCGGACTAT GACCCGTTTG   
  
  
- GTCGTGGACG ACTTCTCAAA AGTCGAAAAT GAGTGAATAT ATCTCGGAGA GGTAAGATAG TCAAACGAGT   
  
  
- AAAGTGTCGG TTAGTCCGTT AACATCTTCG TAAACTCTCC CTCCTTCTCT CATTGTTGAC CCGAAACGTA   
  
  
- CATTAACTAA ACCTGTAGAA CGTACCAAAG GTCACCGGAA GAGAATAAGT CAGAGAGAGG CTTCTCCGAT   
  
  
- GAACAAGTTT GGCAAGTAAC AGAGAAGTTT AATGTCCGAA GCCTTCTTCG AATCTTCTCG AGTAACTTTG   
  
  
- TCTTCGAGCC GACCATTCAA AGAGATTCTC GAAAGTCTTA TAGTTGAAAC TTAAGGTGCC CAAAGACTCT   
  
  
- CCGAGCTTTG AGTCCTTGGA TTCCTCCCTC TTACTTTGTC ATCAACATTT AGAGCAAAAG GTGGAGTCAT   
  
  
- GAAACTTTCT ATGGCATGTC TAAAGACTGT GGAACTGAAG ACATGTAAGT GATTTGGGGA GATAACACCA   
  
  
- TAATCAGCTC TCTCTTCCTT CAGCTTTGTC TACACCCAAG GAGAGTTCAA TACATCTACG AGACGTAATA   
  
  
- ATACGACGTT ACAAACTGAG AAATCTACTA ACGGAGGGTG AACTTTCAGG ACTCTCCGAC TCGTATCTCT   
  
  
- TCTTGGTAGA ACCTTTTCTC TAGTTTCTTC GGTATCCAAC ACTCTTCCTA CTTTGTTTGA TAGACTTCAA   
  
  
- ACTCTACGAC CTCTGAACCT CTCCCTCCTA CCTCTCAGTA CCTAAAAGTC CATACTTCGA TTCAAGGTCC   
  
  
- CGTTGATATG TTCGTTTCGA CGAAAATTTT TACCCTTCGG TAATAGTATG GATAAACCTT CTTCTGACAC   
  
  
- CTCCACCACC CAAGTCTCAA ACACTTGCTC TACTACCTTA CCGATAGAGA GAACCGACCG TTCCATCCAA   
  
  
- GGAGCGTTGA CGGAGCCATA CCGCAAGACA CAC

+     MRE

| Site Name | Organism | Position | Strand | Matrix score. | sequence | function |
| --- | --- | --- | --- | --- | --- | --- |
| MRE | Petroselinum crispum | 2820 | + | 7 | AACCTAA | MYB binding site involved in light responsiveness |

>HU07G00272.1   
+ -Up\_Stream \_Len000ACCAAC GATTCTTTAC TGAATCAGCT ACATTTTTGG ATTGTGTTAA GGTATCTTTT   
  
  
+ AGATCTTCAG GAGGAGGGGG CTGAAGTAGC TTGGTTACTT TTGCGCTTAT CAACTGTGGA AGAATATTAT   
  
  
+ TGAGACTAGT GTAGAAGAAT TAGTAGTTTC CTCCACATTG GAAAAAAACC ATGAAATTAA CGCTTTAGGC   
  
  
+ CCTCAGAGTA CCAAGTGTCA TCGTAAATGA TTGAAATTTC AAGTCTTAGC TGAGTCATGA AGTCCTGATT   
  
  
+ TCCCATGACA AATTGATGTT GTTGTCTAAA GCTTGGCGGG TGATTTGAAA GAAGATTTAG ATCAGGCTGA   
  
  
+ CAATACACCA AATCTGGGAG CAAGGAACTG GATTTTTCTG TGTCTAGAAA AGCTTTCCGC TTTCTGAACA   
  
  
+ ATTGAGAAAA CCATGCATAG GAAATTTCTT GATATTTAGA TCATAGCAGA CTTAACAATC TGGCTTTACC   
  
  
+ GAACTGTCCT CTACTTCTAA GTTGTGAAGG TGTGAAAGAT AAACCATGTC AAACTTAGTG TCCAACAGAA   
  
  
+ ACGCATCTCT ACTTCTCCAG GCAAAGTTGT ACTTCAGATC TTCTACACCT TTTCTAGGCA GGACTTTGGG   
  
  
+ ATATAGAGTT TGGCCTGGGT GAAAAGTAGG AAGTGGGGAA GTAGAAAATG ATGAAAGAAG GCACCACTTG   
  
  
+ GGTCCAGATA CATAGGCCTA CGCACGAGAA CCTTTGCATG GTCTAGATAC ATGACTGTAT ACTTCTTCCT   
  
  
+ TTCCTTTACC TGTTGGAGAT TATCCTAGGA TTTTACATGG CACCAGCTGG ATTTGTTAAT GACATAAACA   
  
  
+ TATCTTTAAT TACAGTTGCT TATGGTATAC ATTTAAACTA TTCGATAGTT GACAGCTGAT GTCCTTATCC   
  
  
+ TTGTGCAGCT TATTCCTCGA TAGAATAATC TTTGGACATT TCTGTCTAGG TGATAAAGGG CAAAAACTAA   
  
  
+ GATACAAGAA ATAGAACATG TCCTCCATTT ACAAGACGTA TCTGTTTTTG GTTTTGGTCA ATGATCCCTG   
  
  
+ TTGTCCTCAA GCATTAACAC AGAATGATCA TTATTGACTT GGACTGAAAG GTTTTGGGAA CTCCATAGTA   
  
  
+ TGCCTTTTCA ATAAAATCAA AAGCAAATTT GCTGTTCACT GATAACTGCA GTTGTGTTGC TTTGCTGTTT   
  
  
+ CCATACCAGA CTCGTAACCA AGCTGATAGC AAATGTGCAA AGGTACAGTA TAATTAACGA TGTTTTCATA   
  
  
+ TTGCTACGAG TTCTGAAACA TGATATAAGA AAACACCAGA ACCCAAATAC CTACATAGGT CCTAGCCCTG   
  
  
+ GAATGGCTAT TTTAAATATC CAAATGGACT TACAGAATTC GCAGAGTTGT TTGGTTTGTT TAACAATCAT   
  
  
+ TGAATTGTTA GCTATGAGAA TCTGCGGGAC AATCGTAATC TTTGGCGATT ACTTCGATTG GCATACTGAT   
  
  
+ GTTTTTTTAT TTGCGTATCT AAGCATGGTG GTGTACCTTT GATTTGTGTG TCTAGGTAGT GTGTGTAGAT   
  
  
+ AAATATCATA GAACTCAGAC GTTGGTAGGG TTATGTTGAG CACTTTCTCT TGCTGTTTGT CCTTTGTGAT   
  
  
+ GGCACCTCTT GCTTTTCCGA ACCACCAAAA GTCTCATTGA CAAAACTCTT CAAACATTGC AAACTGCCCC   
  
  
+ CTTAAATATG TTTTATTAAA AAAAATGGAA GAGCTCAAAC TGTTGAGTGA GCCTAGATTC CAAGATTTAG   
  
  
+ AAACAGTAAA CTTGATAGAT ATATTTATAT GAACTATTGT TAGATGCAAC ACCCCCAACT TGAAACACAA   
  
  
+ AAGAGAAATT AAAAACAAAA AGGTTTTGCA CTATTTATAT ATCCTGTGTT CTTTGTGCTA GTCTTATTTA   
  
  
+ ATGTTAATTG GTAAGCATTA TTTGTTGGAT TATTCCACTG CACTTTCTCT TACTAACTTC AGCATTTCAT   
  
  
+ TCCTTGTTCT TCAGCTTTTG AACTTCTTTA GCCTATTTTG TTGTCTTTAT GGTTATGGAA GCCATCCAGA   
  
  
+ AGAAGAATGA TGAACTTCTG AGTCTTAGCT TGGCCATTGT CGGTCACTCT GATCGCACTG AGAAAAAACT   
  
  
+ GAAGAGGCGG ATTGATGTCT CCGATCCTCT GATCACTTCA GGTGAAGGCT GCGAAGGGAA GATAATCAGG   
  
  
+ CTCCTCCAAG AGAGACAGAA CTTGTTGAAC ATCAAGCAAA GAGGAAAAGG CGTCATTCAA GAAGGGAAGG   
  
  
+ GTCTTCATCT TATCCATCTG CTCCTTGTAT CTGCCACCTT AATCAACGAA AACAACATCA GTTCAGCTGT   
  
  
+ TGATAATCTT ATTGAGCTCT TCCAATATGT CTCCGAAAGT GGTGATTCAG GACAAAGGGT TGCTGCTTAC   
  
  
+ TTTGCGGATG GGTTAACAGC AAGGATCTTA ACTCAGAGGT CTCCATTTTA TCGCCTGATA CTGGGCAAAC   
  
  
+ CAGCACCTGC TGAAGAGTTT TCAGCTTTTA CTCACTTATA TAGAGCCTCT CCATTCTATC AGTTTGCTCA   
  
  
+ TTTCACAGCC AATCAGGCAA TTGTAGAAGC ATTTGAGAGG GAGGAAGAGA GTAACAACTG GGCTTTGCAT   
  
  
+ GTAATTGATT TGGACATCTT GCATGGTTTC CAGTGGCCTT CTCTTATTCA GTCTCTCTCC GAAGAGGCTA   
  
  
+ CTTGTTCAAA CCGTTCATTG TCTCTTCAAA TTACAGGCTT CGGAAGAAGC TTAGAAGAGC TCATTGAAAC   
  
  
+ AGAAGCTCGG CTGGTAAGTT TCTCTAAGAG CTTTCAGAAT ATCAACTTTG AATTCCACGG GTTTCTGAGA   
  
  
+ GGCTCGAAAC TCAGGAACCT AAGGAGGGAG AATGAAACAG TAGTTGTAAA TCTCGTTTTC CACCTCAGTA   
  
  
+ CTTTGAAAGA TACCGTACAG ATTTCTGACA CCTTGACTTC TGTACATTCA CTAAACCCCT CTATTGTGGT   
  
  
+ ATTAGTCGAG AGAGAAGGAA GTCGAAACAG ATGTGGGTTC CTCTCAAGTT ATGTAGATGC TCTGCATTAT   
  
  
+ TATGCTGCAA TGTTTGACTC TTTAGATGAT TGCCTCCCAC TTGAAAGTCC TGAGAGGCTG AGCATAGAGA   
  
  
+ AGAACCATCT TGGAAAAGAG ATCAAAGAAG CCATAGGTTG TGAGAAGGAT GAAACAAACT ATCTGAAGTT   
  
  
+ TGAGATGCTG GAGACTTGGA GAGGGAGGAT GGAGAGTCAT GGATTTTCAG GTATGAAGCT AAGTTCCAGG   
  
  
+ GCAACTATAC AAGCAAAGCT GCTTTTAAAA ATGGGAAGCC ATTATCATAC CTATTTGGAA GAAGACTGTG   
  
  
+ GAGGTGGTGG GTTCAGAGTT TGTGAACGAG ATGATGGAAT GGCTATCTCT CTTGGCTGGC AAGGTAGGTT   
  
  
+ CCTCGCAACT GCCTCGGTAT GGCGTTCTGT GTG  

- -Up\_Stream \_Len000TGGTTG CTAAGAAATG ACTTAGTCGA TGTAAAAACC TAACACAATT CCATAGAAAA   
  
  
- TCTAGAAGTC CTCCTCCCCC GACTTCATCG AACCAATGAA AACGCGAATA GTTGACACCT TCTTATAATA   
  
  
- ACTCTGATCA CATCTTCTTA ATCATCAAAG GAGGTGTAAC CTTTTTTTGG TACTTTAATT GCGAAATCCG   
  
  
- GGAGTCTCAT GGTTCACAGT AGCATTTACT AACTTTAAAG TTCAGAATCG ACTCAGTACT TCAGGACTAA   
  
  
- AGGGTACTGT TTAACTACAA CAACAGATTT CGAACCGCCC ACTAAACTTT CTTCTAAATC TAGTCCGACT   
  
  
- GTTATGTGGT TTAGACCCTC GTTCCTTGAC CTAAAAAGAC ACAGATCTTT TCGAAAGGCG AAAGACTTGT   
  
  
- TAACTCTTTT GGTACGTATC CTTTAAAGAA CTATAAATCT AGTATCGTCT GAATTGTTAG ACCGAAATGG   
  
  
- CTTGACAGGA GATGAAGATT CAACACTTCC ACACTTTCTA TTTGGTACAG TTTGAATCAC AGGTTGTCTT   
  
  
- TGCGTAGAGA TGAAGAGGTC CGTTTCAACA TGAAGTCTAG AAGATGTGGA AAAGATCCGT CCTGAAACCC   
  
  
- TATATCTCAA ACCGGACCCA CTTTTCATCC TTCACCCCTT CATCTTTTAC TACTTTCTTC CGTGGTGAAC   
  
  
- CCAGGTCTAT GTATCCGGAT GCGTGCTCTT GGAAACGTAC CAGATCTATG TACTGACATA TGAAGAAGGA   
  
  
- AAGGAAATGG ACAACCTCTA ATAGGATCCT AAAATGTACC GTGGTCGACC TAAACAATTA CTGTATTTGT   
  
  
- ATAGAAATTA ATGTCAACGA ATACCATATG TAAATTTGAT AAGCTATCAA CTGTCGACTA CAGGAATAGG   
  
  
- AACACGTCGA ATAAGGAGCT ATCTTATTAG AAACCTGTAA AGACAGATCC ACTATTTCCC GTTTTTGATT   
  
  
- CTATGTTCTT TATCTTGTAC AGGAGGTAAA TGTTCTGCAT AGACAAAAAC CAAAACCAGT TACTAGGGAC   
  
  
- AACAGGAGTT CGTAATTGTG TCTTACTAGT AATAACTGAA CCTGACTTTC CAAAACCCTT GAGGTATCAT   
  
  
- ACGGAAAAGT TATTTTAGTT TTCGTTTAAA CGACAAGTGA CTATTGACGT CAACACAACG AAACGACAAA   
  
  
- GGTATGGTCT GAGCATTGGT TCGACTATCG TTTACACGTT TCCATGTCAT ATTAATTGCT ACAAAAGTAT   
  
  
- AACGATGCTC AAGACTTTGT ACTATATTCT TTTGTGGTCT TGGGTTTATG GATGTATCCA GGATCGGGAC   
  
  
- CTTACCGATA AAATTTATAG GTTTACCTGA ATGTCTTAAG CGTCTCAACA AACCAAACAA ATTGTTAGTA   
  
  
- ACTTAACAAT CGATACTCTT AGACGCCCTG TTAGCATTAG AAACCGCTAA TGAAGCTAAC CGTATGACTA   
  
  
- CAAAAAAATA AACGCATAGA TTCGTACCAC CACATGGAAA CTAAACACAC AGATCCATCA CACACATCTA   
  
  
- TTTATAGTAT CTTGAGTCTG CAACCATCCC AATACAACTC GTGAAAGAGA ACGACAAACA GGAAACACTA   
  
  
- CCGTGGAGAA CGAAAAGGCT TGGTGGTTTT CAGAGTAACT GTTTTGAGAA GTTTGTAACG TTTGACGGGG   
  
  
- GAATTTATAC AAAATAATTT TTTTTACCTT CTCGAGTTTG ACAACTCACT CGGATCTAAG GTTCTAAATC   
  
  
- TTTGTCATTT GAACTATCTA TATAAATATA CTTGATAACA ATCTACGTTG TGGGGGTTGA ACTTTGTGTT   
  
  
- TTCTCTTTAA TTTTTGTTTT TCCAAAACGT GATAAATATA TAGGACACAA GAAACACGAT CAGAATAAAT   
  
  
- TACAATTAAC CATTCGTAAT AAACAACCTA ATAAGGTGAC GTGAAAGAGA ATGATTGAAG TCGTAAAGTA   
  
  
- AGGAACAAGA AGTCGAAAAC TTGAAGAAAT CGGATAAAAC AACAGAAATA CCAATACCTT CGGTAGGTCT   
  
  
- TCTTCTTACT ACTTGAAGAC TCAGAATCGA ACCGGTAACA GCCAGTGAGA CTAGCGTGAC TCTTTTTTGA   
  
  
- CTTCTCCGCC TAACTACAGA GGCTAGGAGA CTAGTGAAGT CCACTTCCGA CGCTTCCCTT CTATTAGTCC   
  
  
- GAGGAGGTTC TCTCTGTCTT GAACAACTTG TAGTTCGTTT CTCCTTTTCC GCAGTAAGTT CTTCCCTTCC   
  
  
- CAGAAGTAGA ATAGGTAGAC GAGGAACATA GACGGTGGAA TTAGTTGCTT TTGTTGTAGT CAAGTCGACA   
  
  
- ACTATTAGAA TAACTCGAGA AGGTTATACA GAGGCTTTCA CCACTAAGTC CTGTTTCCCA ACGACGAATG   
  
  
- AAACGCCTAC CCAATTGTCG TTCCTAGAAT TGAGTCTCCA GAGGTAAAAT AGCGGACTAT GACCCGTTTG   
  
  
- GTCGTGGACG ACTTCTCAAA AGTCGAAAAT GAGTGAATAT ATCTCGGAGA GGTAAGATAG TCAAACGAGT   
  
  
- AAAGTGTCGG TTAGTCCGTT AACATCTTCG TAAACTCTCC CTCCTTCTCT CATTGTTGAC CCGAAACGTA   
  
  
- CATTAACTAA ACCTGTAGAA CGTACCAAAG GTCACCGGAA GAGAATAAGT CAGAGAGAGG CTTCTCCGAT   
  
  
- GAACAAGTTT GGCAAGTAAC AGAGAAGTTT AATGTCCGAA GCCTTCTTCG AATCTTCTCG AGTAACTTTG   
  
  
- TCTTCGAGCC GACCATTCAA AGAGATTCTC GAAAGTCTTA TAGTTGAAAC TTAAGGTGCC CAAAGACTCT   
  
  
- CCGAGCTTTG AGTCCTTGGA TTCCTCCCTC TTACTTTGTC ATCAACATTT AGAGCAAAAG GTGGAGTCAT   
  
  
- GAAACTTTCT ATGGCATGTC TAAAGACTGT GGAACTGAAG ACATGTAAGT GATTTGGGGA GATAACACCA   
  
  
- TAATCAGCTC TCTCTTCCTT CAGCTTTGTC TACACCCAAG GAGAGTTCAA TACATCTACG AGACGTAATA   
  
  
- ATACGACGTT ACAAACTGAG AAATCTACTA ACGGAGGGTG AACTTTCAGG ACTCTCCGAC TCGTATCTCT   
  
  
- TCTTGGTAGA ACCTTTTCTC TAGTTTCTTC GGTATCCAAC ACTCTTCCTA CTTTGTTTGA TAGACTTCAA   
  
  
- ACTCTACGAC CTCTGAACCT CTCCCTCCTA CCTCTCAGTA CCTAAAAGTC CATACTTCGA TTCAAGGTCC   
  
  
- CGTTGATATG TTCGTTTCGA CGAAAATTTT TACCCTTCGG TAATAGTATG GATAAACCTT CTTCTGACAC   
  
  
- CTCCACCACC CAAGTCTCAA ACACTTGCTC TACTACCTTA CCGATAGAGA GAACCGACCG TTCCATCCAA   
  
  
- GGAGCGTTGA CGGAGCCATA CCGCAAGACA CAC

+     MYB

| Site Name | Organism | Position | Strand | Matrix score. | sequence | function |
| --- | --- | --- | --- | --- | --- | --- |
| MYB | Arabidopsis thaliana | 2014 | - | 6 | TAACCA |  |
| MYB | Arabidopsis thaliana | 557 | + | 6 | CAACAG |  |
| MYB | Arabidopsis thaliana | 106 | - | 6 | TAACCA |  |
| MYB | Arabidopsis thaliana | 2311 | - | 6 | CAACAG |  |
| MYB | Arabidopsis thaliana | 1052 | - | 6 | CAACAG |  |
| MYB | Arabidopsis thaliana | 1209 | + | 6 | TAACCA |  |
| MYB | Arabidopsis thaliana | 1724 | - | 6 | CAACAG |  |
| MYB | Arabidopsis thaliana | 784 | - | 6 | CAACAG |  |

>HU07G00272.1   
+ -Up\_Stream \_Len000ACCAAC GATTCTTTAC TGAATCAGCT ACATTTTTGG ATTGTGTTAA GGTATCTTTT   
  
  
+ AGATCTTCAG GAGGAGGGGG CTGAAGTAGC TTGGTTACTT TTGCGCTTAT CAACTGTGGA AGAATATTAT   
  
  
+ TGAGACTAGT GTAGAAGAAT TAGTAGTTTC CTCCACATTG GAAAAAAACC ATGAAATTAA CGCTTTAGGC   
  
  
+ CCTCAGAGTA CCAAGTGTCA TCGTAAATGA TTGAAATTTC AAGTCTTAGC TGAGTCATGA AGTCCTGATT   
  
  
+ TCCCATGACA AATTGATGTT GTTGTCTAAA GCTTGGCGGG TGATTTGAAA GAAGATTTAG ATCAGGCTGA   
  
  
+ CAATACACCA AATCTGGGAG CAAGGAACTG GATTTTTCTG TGTCTAGAAA AGCTTTCCGC TTTCTGAACA   
  
  
+ ATTGAGAAAA CCATGCATAG GAAATTTCTT GATATTTAGA TCATAGCAGA CTTAACAATC TGGCTTTACC   
  
  
+ GAACTGTCCT CTACTTCTAA GTTGTGAAGG TGTGAAAGAT AAACCATGTC AAACTTAGTG TCCAACAGAA   
  
  
+ ACGCATCTCT ACTTCTCCAG GCAAAGTTGT ACTTCAGATC TTCTACACCT TTTCTAGGCA GGACTTTGGG   
  
  
+ ATATAGAGTT TGGCCTGGGT GAAAAGTAGG AAGTGGGGAA GTAGAAAATG ATGAAAGAAG GCACCACTTG   
  
  
+ GGTCCAGATA CATAGGCCTA CGCACGAGAA CCTTTGCATG GTCTAGATAC ATGACTGTAT ACTTCTTCCT   
  
  
+ TTCCTTTACC TGTTGGAGAT TATCCTAGGA TTTTACATGG CACCAGCTGG ATTTGTTAAT GACATAAACA   
  
  
+ TATCTTTAAT TACAGTTGCT TATGGTATAC ATTTAAACTA TTCGATAGTT GACAGCTGAT GTCCTTATCC   
  
  
+ TTGTGCAGCT TATTCCTCGA TAGAATAATC TTTGGACATT TCTGTCTAGG TGATAAAGGG CAAAAACTAA   
  
  
+ GATACAAGAA ATAGAACATG TCCTCCATTT ACAAGACGTA TCTGTTTTTG GTTTTGGTCA ATGATCCCTG   
  
  
+ TTGTCCTCAA GCATTAACAC AGAATGATCA TTATTGACTT GGACTGAAAG GTTTTGGGAA CTCCATAGTA   
  
  
+ TGCCTTTTCA ATAAAATCAA AAGCAAATTT GCTGTTCACT GATAACTGCA GTTGTGTTGC TTTGCTGTTT   
  
  
+ CCATACCAGA CTCGTAACCA AGCTGATAGC AAATGTGCAA AGGTACAGTA TAATTAACGA TGTTTTCATA   
  
  
+ TTGCTACGAG TTCTGAAACA TGATATAAGA AAACACCAGA ACCCAAATAC CTACATAGGT CCTAGCCCTG   
  
  
+ GAATGGCTAT TTTAAATATC CAAATGGACT TACAGAATTC GCAGAGTTGT TTGGTTTGTT TAACAATCAT   
  
  
+ TGAATTGTTA GCTATGAGAA TCTGCGGGAC AATCGTAATC TTTGGCGATT ACTTCGATTG GCATACTGAT   
  
  
+ GTTTTTTTAT TTGCGTATCT AAGCATGGTG GTGTACCTTT GATTTGTGTG TCTAGGTAGT GTGTGTAGAT   
  
  
+ AAATATCATA GAACTCAGAC GTTGGTAGGG TTATGTTGAG CACTTTCTCT TGCTGTTTGT CCTTTGTGAT   
  
  
+ GGCACCTCTT GCTTTTCCGA ACCACCAAAA GTCTCATTGA CAAAACTCTT CAAACATTGC AAACTGCCCC   
  
  
+ CTTAAATATG TTTTATTAAA AAAAATGGAA GAGCTCAAAC TGTTGAGTGA GCCTAGATTC CAAGATTTAG   
  
  
+ AAACAGTAAA CTTGATAGAT ATATTTATAT GAACTATTGT TAGATGCAAC ACCCCCAACT TGAAACACAA   
  
  
+ AAGAGAAATT AAAAACAAAA AGGTTTTGCA CTATTTATAT ATCCTGTGTT CTTTGTGCTA GTCTTATTTA   
  
  
+ ATGTTAATTG GTAAGCATTA TTTGTTGGAT TATTCCACTG CACTTTCTCT TACTAACTTC AGCATTTCAT   
  
  
+ TCCTTGTTCT TCAGCTTTTG AACTTCTTTA GCCTATTTTG TTGTCTTTAT GGTTATGGAA GCCATCCAGA   
  
  
+ AGAAGAATGA TGAACTTCTG AGTCTTAGCT TGGCCATTGT CGGTCACTCT GATCGCACTG AGAAAAAACT   
  
  
+ GAAGAGGCGG ATTGATGTCT CCGATCCTCT GATCACTTCA GGTGAAGGCT GCGAAGGGAA GATAATCAGG   
  
  
+ CTCCTCCAAG AGAGACAGAA CTTGTTGAAC ATCAAGCAAA GAGGAAAAGG CGTCATTCAA GAAGGGAAGG   
  
  
+ GTCTTCATCT TATCCATCTG CTCCTTGTAT CTGCCACCTT AATCAACGAA AACAACATCA GTTCAGCTGT   
  
  
+ TGATAATCTT ATTGAGCTCT TCCAATATGT CTCCGAAAGT GGTGATTCAG GACAAAGGGT TGCTGCTTAC   
  
  
+ TTTGCGGATG GGTTAACAGC AAGGATCTTA ACTCAGAGGT CTCCATTTTA TCGCCTGATA CTGGGCAAAC   
  
  
+ CAGCACCTGC TGAAGAGTTT TCAGCTTTTA CTCACTTATA TAGAGCCTCT CCATTCTATC AGTTTGCTCA   
  
  
+ TTTCACAGCC AATCAGGCAA TTGTAGAAGC ATTTGAGAGG GAGGAAGAGA GTAACAACTG GGCTTTGCAT   
  
  
+ GTAATTGATT TGGACATCTT GCATGGTTTC CAGTGGCCTT CTCTTATTCA GTCTCTCTCC GAAGAGGCTA   
  
  
+ CTTGTTCAAA CCGTTCATTG TCTCTTCAAA TTACAGGCTT CGGAAGAAGC TTAGAAGAGC TCATTGAAAC   
  
  
+ AGAAGCTCGG CTGGTAAGTT TCTCTAAGAG CTTTCAGAAT ATCAACTTTG AATTCCACGG GTTTCTGAGA   
  
  
+ GGCTCGAAAC TCAGGAACCT AAGGAGGGAG AATGAAACAG TAGTTGTAAA TCTCGTTTTC CACCTCAGTA   
  
  
+ CTTTGAAAGA TACCGTACAG ATTTCTGACA CCTTGACTTC TGTACATTCA CTAAACCCCT CTATTGTGGT   
  
  
+ ATTAGTCGAG AGAGAAGGAA GTCGAAACAG ATGTGGGTTC CTCTCAAGTT ATGTAGATGC TCTGCATTAT   
  
  
+ TATGCTGCAA TGTTTGACTC TTTAGATGAT TGCCTCCCAC TTGAAAGTCC TGAGAGGCTG AGCATAGAGA   
  
  
+ AGAACCATCT TGGAAAAGAG ATCAAAGAAG CCATAGGTTG TGAGAAGGAT GAAACAAACT ATCTGAAGTT   
  
  
+ TGAGATGCTG GAGACTTGGA GAGGGAGGAT GGAGAGTCAT GGATTTTCAG GTATGAAGCT AAGTTCCAGG   
  
  
+ GCAACTATAC AAGCAAAGCT GCTTTTAAAA ATGGGAAGCC ATTATCATAC CTATTTGGAA GAAGACTGTG   
  
  
+ GAGGTGGTGG GTTCAGAGTT TGTGAACGAG ATGATGGAAT GGCTATCTCT CTTGGCTGGC AAGGTAGGTT   
  
  
+ CCTCGCAACT GCCTCGGTAT GGCGTTCTGT GTG  

- -Up\_Stream \_Len000TGGTTG CTAAGAAATG ACTTAGTCGA TGTAAAAACC TAACACAATT CCATAGAAAA   
  
  
- TCTAGAAGTC CTCCTCCCCC GACTTCATCG AACCAATGAA AACGCGAATA GTTGACACCT TCTTATAATA   
  
  
- ACTCTGATCA CATCTTCTTA ATCATCAAAG GAGGTGTAAC CTTTTTTTGG TACTTTAATT GCGAAATCCG   
  
  
- GGAGTCTCAT GGTTCACAGT AGCATTTACT AACTTTAAAG TTCAGAATCG ACTCAGTACT TCAGGACTAA   
  
  
- AGGGTACTGT TTAACTACAA CAACAGATTT CGAACCGCCC ACTAAACTTT CTTCTAAATC TAGTCCGACT   
  
  
- GTTATGTGGT TTAGACCCTC GTTCCTTGAC CTAAAAAGAC ACAGATCTTT TCGAAAGGCG AAAGACTTGT   
  
  
- TAACTCTTTT GGTACGTATC CTTTAAAGAA CTATAAATCT AGTATCGTCT GAATTGTTAG ACCGAAATGG   
  
  
- CTTGACAGGA GATGAAGATT CAACACTTCC ACACTTTCTA TTTGGTACAG TTTGAATCAC AGGTTGTCTT   
  
  
- TGCGTAGAGA TGAAGAGGTC CGTTTCAACA TGAAGTCTAG AAGATGTGGA AAAGATCCGT CCTGAAACCC   
  
  
- TATATCTCAA ACCGGACCCA CTTTTCATCC TTCACCCCTT CATCTTTTAC TACTTTCTTC CGTGGTGAAC   
  
  
- CCAGGTCTAT GTATCCGGAT GCGTGCTCTT GGAAACGTAC CAGATCTATG TACTGACATA TGAAGAAGGA   
  
  
- AAGGAAATGG ACAACCTCTA ATAGGATCCT AAAATGTACC GTGGTCGACC TAAACAATTA CTGTATTTGT   
  
  
- ATAGAAATTA ATGTCAACGA ATACCATATG TAAATTTGAT AAGCTATCAA CTGTCGACTA CAGGAATAGG   
  
  
- AACACGTCGA ATAAGGAGCT ATCTTATTAG AAACCTGTAA AGACAGATCC ACTATTTCCC GTTTTTGATT   
  
  
- CTATGTTCTT TATCTTGTAC AGGAGGTAAA TGTTCTGCAT AGACAAAAAC CAAAACCAGT TACTAGGGAC   
  
  
- AACAGGAGTT CGTAATTGTG TCTTACTAGT AATAACTGAA CCTGACTTTC CAAAACCCTT GAGGTATCAT   
  
  
- ACGGAAAAGT TATTTTAGTT TTCGTTTAAA CGACAAGTGA CTATTGACGT CAACACAACG AAACGACAAA   
  
  
- GGTATGGTCT GAGCATTGGT TCGACTATCG TTTACACGTT TCCATGTCAT ATTAATTGCT ACAAAAGTAT   
  
  
- AACGATGCTC AAGACTTTGT ACTATATTCT TTTGTGGTCT TGGGTTTATG GATGTATCCA GGATCGGGAC   
  
  
- CTTACCGATA AAATTTATAG GTTTACCTGA ATGTCTTAAG CGTCTCAACA AACCAAACAA ATTGTTAGTA   
  
  
- ACTTAACAAT CGATACTCTT AGACGCCCTG TTAGCATTAG AAACCGCTAA TGAAGCTAAC CGTATGACTA   
  
  
- CAAAAAAATA AACGCATAGA TTCGTACCAC CACATGGAAA CTAAACACAC AGATCCATCA CACACATCTA   
  
  
- TTTATAGTAT CTTGAGTCTG CAACCATCCC AATACAACTC GTGAAAGAGA ACGACAAACA GGAAACACTA   
  
  
- CCGTGGAGAA CGAAAAGGCT TGGTGGTTTT CAGAGTAACT GTTTTGAGAA GTTTGTAACG TTTGACGGGG   
  
  
- GAATTTATAC AAAATAATTT TTTTTACCTT CTCGAGTTTG ACAACTCACT CGGATCTAAG GTTCTAAATC   
  
  
- TTTGTCATTT GAACTATCTA TATAAATATA CTTGATAACA ATCTACGTTG TGGGGGTTGA ACTTTGTGTT   
  
  
- TTCTCTTTAA TTTTTGTTTT TCCAAAACGT GATAAATATA TAGGACACAA GAAACACGAT CAGAATAAAT   
  
  
- TACAATTAAC CATTCGTAAT AAACAACCTA ATAAGGTGAC GTGAAAGAGA ATGATTGAAG TCGTAAAGTA   
  
  
- AGGAACAAGA AGTCGAAAAC TTGAAGAAAT CGGATAAAAC AACAGAAATA CCAATACCTT CGGTAGGTCT   
  
  
- TCTTCTTACT ACTTGAAGAC TCAGAATCGA ACCGGTAACA GCCAGTGAGA CTAGCGTGAC TCTTTTTTGA   
  
  
- CTTCTCCGCC TAACTACAGA GGCTAGGAGA CTAGTGAAGT CCACTTCCGA CGCTTCCCTT CTATTAGTCC   
  
  
- GAGGAGGTTC TCTCTGTCTT GAACAACTTG TAGTTCGTTT CTCCTTTTCC GCAGTAAGTT CTTCCCTTCC   
  
  
- CAGAAGTAGA ATAGGTAGAC GAGGAACATA GACGGTGGAA TTAGTTGCTT TTGTTGTAGT CAAGTCGACA   
  
  
- ACTATTAGAA TAACTCGAGA AGGTTATACA GAGGCTTTCA CCACTAAGTC CTGTTTCCCA ACGACGAATG   
  
  
- AAACGCCTAC CCAATTGTCG TTCCTAGAAT TGAGTCTCCA GAGGTAAAAT AGCGGACTAT GACCCGTTTG   
  
  
- GTCGTGGACG ACTTCTCAAA AGTCGAAAAT GAGTGAATAT ATCTCGGAGA GGTAAGATAG TCAAACGAGT   
  
  
- AAAGTGTCGG TTAGTCCGTT AACATCTTCG TAAACTCTCC CTCCTTCTCT CATTGTTGAC CCGAAACGTA   
  
  
- CATTAACTAA ACCTGTAGAA CGTACCAAAG GTCACCGGAA GAGAATAAGT CAGAGAGAGG CTTCTCCGAT   
  
  
- GAACAAGTTT GGCAAGTAAC AGAGAAGTTT AATGTCCGAA GCCTTCTTCG AATCTTCTCG AGTAACTTTG   
  
  
- TCTTCGAGCC GACCATTCAA AGAGATTCTC GAAAGTCTTA TAGTTGAAAC TTAAGGTGCC CAAAGACTCT   
  
  
- CCGAGCTTTG AGTCCTTGGA TTCCTCCCTC TTACTTTGTC ATCAACATTT AGAGCAAAAG GTGGAGTCAT   
  
  
- GAAACTTTCT ATGGCATGTC TAAAGACTGT GGAACTGAAG ACATGTAAGT GATTTGGGGA GATAACACCA   
  
  
- TAATCAGCTC TCTCTTCCTT CAGCTTTGTC TACACCCAAG GAGAGTTCAA TACATCTACG AGACGTAATA   
  
  
- ATACGACGTT ACAAACTGAG AAATCTACTA ACGGAGGGTG AACTTTCAGG ACTCTCCGAC TCGTATCTCT   
  
  
- TCTTGGTAGA ACCTTTTCTC TAGTTTCTTC GGTATCCAAC ACTCTTCCTA CTTTGTTTGA TAGACTTCAA   
  
  
- ACTCTACGAC CTCTGAACCT CTCCCTCCTA CCTCTCAGTA CCTAAAAGTC CATACTTCGA TTCAAGGTCC   
  
  
- CGTTGATATG TTCGTTTCGA CGAAAATTTT TACCCTTCGG TAATAGTATG GATAAACCTT CTTCTGACAC   
  
  
- CTCCACCACC CAAGTCTCAA ACACTTGCTC TACTACCTTA CCGATAGAGA GAACCGACCG TTCCATCCAA   
  
  
- GGAGCGTTGA CGGAGCCATA CCGCAAGACA CAC

+     MYB-like sequence

| Site Name | Organism | Position | Strand | Matrix score. | sequence | function |
| --- | --- | --- | --- | --- | --- | --- |
| MYB-like sequence | Arabidopsis thaliana | 2014 | - | 6 | TAACCA |  |
| MYB-like sequence | Arabidopsis thaliana | 106 | - | 6 | TAACCA |  |
| MYB-like sequence | Arabidopsis thaliana | 1209 | + | 6 | TAACCA |  |

>HU07G00272.1   
+ -Up\_Stream \_Len000ACCAAC GATTCTTTAC TGAATCAGCT ACATTTTTGG ATTGTGTTAA GGTATCTTTT   
  
  
+ AGATCTTCAG GAGGAGGGGG CTGAAGTAGC TTGGTTACTT TTGCGCTTAT CAACTGTGGA AGAATATTAT   
  
  
+ TGAGACTAGT GTAGAAGAAT TAGTAGTTTC CTCCACATTG GAAAAAAACC ATGAAATTAA CGCTTTAGGC   
  
  
+ CCTCAGAGTA CCAAGTGTCA TCGTAAATGA TTGAAATTTC AAGTCTTAGC TGAGTCATGA AGTCCTGATT   
  
  
+ TCCCATGACA AATTGATGTT GTTGTCTAAA GCTTGGCGGG TGATTTGAAA GAAGATTTAG ATCAGGCTGA   
  
  
+ CAATACACCA AATCTGGGAG CAAGGAACTG GATTTTTCTG TGTCTAGAAA AGCTTTCCGC TTTCTGAACA   
  
  
+ ATTGAGAAAA CCATGCATAG GAAATTTCTT GATATTTAGA TCATAGCAGA CTTAACAATC TGGCTTTACC   
  
  
+ GAACTGTCCT CTACTTCTAA GTTGTGAAGG TGTGAAAGAT AAACCATGTC AAACTTAGTG TCCAACAGAA   
  
  
+ ACGCATCTCT ACTTCTCCAG GCAAAGTTGT ACTTCAGATC TTCTACACCT TTTCTAGGCA GGACTTTGGG   
  
  
+ ATATAGAGTT TGGCCTGGGT GAAAAGTAGG AAGTGGGGAA GTAGAAAATG ATGAAAGAAG GCACCACTTG   
  
  
+ GGTCCAGATA CATAGGCCTA CGCACGAGAA CCTTTGCATG GTCTAGATAC ATGACTGTAT ACTTCTTCCT   
  
  
+ TTCCTTTACC TGTTGGAGAT TATCCTAGGA TTTTACATGG CACCAGCTGG ATTTGTTAAT GACATAAACA   
  
  
+ TATCTTTAAT TACAGTTGCT TATGGTATAC ATTTAAACTA TTCGATAGTT GACAGCTGAT GTCCTTATCC   
  
  
+ TTGTGCAGCT TATTCCTCGA TAGAATAATC TTTGGACATT TCTGTCTAGG TGATAAAGGG CAAAAACTAA   
  
  
+ GATACAAGAA ATAGAACATG TCCTCCATTT ACAAGACGTA TCTGTTTTTG GTTTTGGTCA ATGATCCCTG   
  
  
+ TTGTCCTCAA GCATTAACAC AGAATGATCA TTATTGACTT GGACTGAAAG GTTTTGGGAA CTCCATAGTA   
  
  
+ TGCCTTTTCA ATAAAATCAA AAGCAAATTT GCTGTTCACT GATAACTGCA GTTGTGTTGC TTTGCTGTTT   
  
  
+ CCATACCAGA CTCGTAACCA AGCTGATAGC AAATGTGCAA AGGTACAGTA TAATTAACGA TGTTTTCATA   
  
  
+ TTGCTACGAG TTCTGAAACA TGATATAAGA AAACACCAGA ACCCAAATAC CTACATAGGT CCTAGCCCTG   
  
  
+ GAATGGCTAT TTTAAATATC CAAATGGACT TACAGAATTC GCAGAGTTGT TTGGTTTGTT TAACAATCAT   
  
  
+ TGAATTGTTA GCTATGAGAA TCTGCGGGAC AATCGTAATC TTTGGCGATT ACTTCGATTG GCATACTGAT   
  
  
+ GTTTTTTTAT TTGCGTATCT AAGCATGGTG GTGTACCTTT GATTTGTGTG TCTAGGTAGT GTGTGTAGAT   
  
  
+ AAATATCATA GAACTCAGAC GTTGGTAGGG TTATGTTGAG CACTTTCTCT TGCTGTTTGT CCTTTGTGAT   
  
  
+ GGCACCTCTT GCTTTTCCGA ACCACCAAAA GTCTCATTGA CAAAACTCTT CAAACATTGC AAACTGCCCC   
  
  
+ CTTAAATATG TTTTATTAAA AAAAATGGAA GAGCTCAAAC TGTTGAGTGA GCCTAGATTC CAAGATTTAG   
  
  
+ AAACAGTAAA CTTGATAGAT ATATTTATAT GAACTATTGT TAGATGCAAC ACCCCCAACT TGAAACACAA   
  
  
+ AAGAGAAATT AAAAACAAAA AGGTTTTGCA CTATTTATAT ATCCTGTGTT CTTTGTGCTA GTCTTATTTA   
  
  
+ ATGTTAATTG GTAAGCATTA TTTGTTGGAT TATTCCACTG CACTTTCTCT TACTAACTTC AGCATTTCAT   
  
  
+ TCCTTGTTCT TCAGCTTTTG AACTTCTTTA GCCTATTTTG TTGTCTTTAT GGTTATGGAA GCCATCCAGA   
  
  
+ AGAAGAATGA TGAACTTCTG AGTCTTAGCT TGGCCATTGT CGGTCACTCT GATCGCACTG AGAAAAAACT   
  
  
+ GAAGAGGCGG ATTGATGTCT CCGATCCTCT GATCACTTCA GGTGAAGGCT GCGAAGGGAA GATAATCAGG   
  
  
+ CTCCTCCAAG AGAGACAGAA CTTGTTGAAC ATCAAGCAAA GAGGAAAAGG CGTCATTCAA GAAGGGAAGG   
  
  
+ GTCTTCATCT TATCCATCTG CTCCTTGTAT CTGCCACCTT AATCAACGAA AACAACATCA GTTCAGCTGT   
  
  
+ TGATAATCTT ATTGAGCTCT TCCAATATGT CTCCGAAAGT GGTGATTCAG GACAAAGGGT TGCTGCTTAC   
  
  
+ TTTGCGGATG GGTTAACAGC AAGGATCTTA ACTCAGAGGT CTCCATTTTA TCGCCTGATA CTGGGCAAAC   
  
  
+ CAGCACCTGC TGAAGAGTTT TCAGCTTTTA CTCACTTATA TAGAGCCTCT CCATTCTATC AGTTTGCTCA   
  
  
+ TTTCACAGCC AATCAGGCAA TTGTAGAAGC ATTTGAGAGG GAGGAAGAGA GTAACAACTG GGCTTTGCAT   
  
  
+ GTAATTGATT TGGACATCTT GCATGGTTTC CAGTGGCCTT CTCTTATTCA GTCTCTCTCC GAAGAGGCTA   
  
  
+ CTTGTTCAAA CCGTTCATTG TCTCTTCAAA TTACAGGCTT CGGAAGAAGC TTAGAAGAGC TCATTGAAAC   
  
  
+ AGAAGCTCGG CTGGTAAGTT TCTCTAAGAG CTTTCAGAAT ATCAACTTTG AATTCCACGG GTTTCTGAGA   
  
  
+ GGCTCGAAAC TCAGGAACCT AAGGAGGGAG AATGAAACAG TAGTTGTAAA TCTCGTTTTC CACCTCAGTA   
  
  
+ CTTTGAAAGA TACCGTACAG ATTTCTGACA CCTTGACTTC TGTACATTCA CTAAACCCCT CTATTGTGGT   
  
  
+ ATTAGTCGAG AGAGAAGGAA GTCGAAACAG ATGTGGGTTC CTCTCAAGTT ATGTAGATGC TCTGCATTAT   
  
  
+ TATGCTGCAA TGTTTGACTC TTTAGATGAT TGCCTCCCAC TTGAAAGTCC TGAGAGGCTG AGCATAGAGA   
  
  
+ AGAACCATCT TGGAAAAGAG ATCAAAGAAG CCATAGGTTG TGAGAAGGAT GAAACAAACT ATCTGAAGTT   
  
  
+ TGAGATGCTG GAGACTTGGA GAGGGAGGAT GGAGAGTCAT GGATTTTCAG GTATGAAGCT AAGTTCCAGG   
  
  
+ GCAACTATAC AAGCAAAGCT GCTTTTAAAA ATGGGAAGCC ATTATCATAC CTATTTGGAA GAAGACTGTG   
  
  
+ GAGGTGGTGG GTTCAGAGTT TGTGAACGAG ATGATGGAAT GGCTATCTCT CTTGGCTGGC AAGGTAGGTT   
  
  
+ CCTCGCAACT GCCTCGGTAT GGCGTTCTGT GTG  

- -Up\_Stream \_Len000TGGTTG CTAAGAAATG ACTTAGTCGA TGTAAAAACC TAACACAATT CCATAGAAAA   
  
  
- TCTAGAAGTC CTCCTCCCCC GACTTCATCG AACCAATGAA AACGCGAATA GTTGACACCT TCTTATAATA   
  
  
- ACTCTGATCA CATCTTCTTA ATCATCAAAG GAGGTGTAAC CTTTTTTTGG TACTTTAATT GCGAAATCCG   
  
  
- GGAGTCTCAT GGTTCACAGT AGCATTTACT AACTTTAAAG TTCAGAATCG ACTCAGTACT TCAGGACTAA   
  
  
- AGGGTACTGT TTAACTACAA CAACAGATTT CGAACCGCCC ACTAAACTTT CTTCTAAATC TAGTCCGACT   
  
  
- GTTATGTGGT TTAGACCCTC GTTCCTTGAC CTAAAAAGAC ACAGATCTTT TCGAAAGGCG AAAGACTTGT   
  
  
- TAACTCTTTT GGTACGTATC CTTTAAAGAA CTATAAATCT AGTATCGTCT GAATTGTTAG ACCGAAATGG   
  
  
- CTTGACAGGA GATGAAGATT CAACACTTCC ACACTTTCTA TTTGGTACAG TTTGAATCAC AGGTTGTCTT   
  
  
- TGCGTAGAGA TGAAGAGGTC CGTTTCAACA TGAAGTCTAG AAGATGTGGA AAAGATCCGT CCTGAAACCC   
  
  
- TATATCTCAA ACCGGACCCA CTTTTCATCC TTCACCCCTT CATCTTTTAC TACTTTCTTC CGTGGTGAAC   
  
  
- CCAGGTCTAT GTATCCGGAT GCGTGCTCTT GGAAACGTAC CAGATCTATG TACTGACATA TGAAGAAGGA   
  
  
- AAGGAAATGG ACAACCTCTA ATAGGATCCT AAAATGTACC GTGGTCGACC TAAACAATTA CTGTATTTGT   
  
  
- ATAGAAATTA ATGTCAACGA ATACCATATG TAAATTTGAT AAGCTATCAA CTGTCGACTA CAGGAATAGG   
  
  
- AACACGTCGA ATAAGGAGCT ATCTTATTAG AAACCTGTAA AGACAGATCC ACTATTTCCC GTTTTTGATT   
  
  
- CTATGTTCTT TATCTTGTAC AGGAGGTAAA TGTTCTGCAT AGACAAAAAC CAAAACCAGT TACTAGGGAC   
  
  
- AACAGGAGTT CGTAATTGTG TCTTACTAGT AATAACTGAA CCTGACTTTC CAAAACCCTT GAGGTATCAT   
  
  
- ACGGAAAAGT TATTTTAGTT TTCGTTTAAA CGACAAGTGA CTATTGACGT CAACACAACG AAACGACAAA   
  
  
- GGTATGGTCT GAGCATTGGT TCGACTATCG TTTACACGTT TCCATGTCAT ATTAATTGCT ACAAAAGTAT   
  
  
- AACGATGCTC AAGACTTTGT ACTATATTCT TTTGTGGTCT TGGGTTTATG GATGTATCCA GGATCGGGAC   
  
  
- CTTACCGATA AAATTTATAG GTTTACCTGA ATGTCTTAAG CGTCTCAACA AACCAAACAA ATTGTTAGTA   
  
  
- ACTTAACAAT CGATACTCTT AGACGCCCTG TTAGCATTAG AAACCGCTAA TGAAGCTAAC CGTATGACTA   
  
  
- CAAAAAAATA AACGCATAGA TTCGTACCAC CACATGGAAA CTAAACACAC AGATCCATCA CACACATCTA   
  
  
- TTTATAGTAT CTTGAGTCTG CAACCATCCC AATACAACTC GTGAAAGAGA ACGACAAACA GGAAACACTA   
  
  
- CCGTGGAGAA CGAAAAGGCT TGGTGGTTTT CAGAGTAACT GTTTTGAGAA GTTTGTAACG TTTGACGGGG   
  
  
- GAATTTATAC AAAATAATTT TTTTTACCTT CTCGAGTTTG ACAACTCACT CGGATCTAAG GTTCTAAATC   
  
  
- TTTGTCATTT GAACTATCTA TATAAATATA CTTGATAACA ATCTACGTTG TGGGGGTTGA ACTTTGTGTT   
  
  
- TTCTCTTTAA TTTTTGTTTT TCCAAAACGT GATAAATATA TAGGACACAA GAAACACGAT CAGAATAAAT   
  
  
- TACAATTAAC CATTCGTAAT AAACAACCTA ATAAGGTGAC GTGAAAGAGA ATGATTGAAG TCGTAAAGTA   
  
  
- AGGAACAAGA AGTCGAAAAC TTGAAGAAAT CGGATAAAAC AACAGAAATA CCAATACCTT CGGTAGGTCT   
  
  
- TCTTCTTACT ACTTGAAGAC TCAGAATCGA ACCGGTAACA GCCAGTGAGA CTAGCGTGAC TCTTTTTTGA   
  
  
- CTTCTCCGCC TAACTACAGA GGCTAGGAGA CTAGTGAAGT CCACTTCCGA CGCTTCCCTT CTATTAGTCC   
  
  
- GAGGAGGTTC TCTCTGTCTT GAACAACTTG TAGTTCGTTT CTCCTTTTCC GCAGTAAGTT CTTCCCTTCC   
  
  
- CAGAAGTAGA ATAGGTAGAC GAGGAACATA GACGGTGGAA TTAGTTGCTT TTGTTGTAGT CAAGTCGACA   
  
  
- ACTATTAGAA TAACTCGAGA AGGTTATACA GAGGCTTTCA CCACTAAGTC CTGTTTCCCA ACGACGAATG   
  
  
- AAACGCCTAC CCAATTGTCG TTCCTAGAAT TGAGTCTCCA GAGGTAAAAT AGCGGACTAT GACCCGTTTG   
  
  
- GTCGTGGACG ACTTCTCAAA AGTCGAAAAT GAGTGAATAT ATCTCGGAGA GGTAAGATAG TCAAACGAGT   
  
  
- AAAGTGTCGG TTAGTCCGTT AACATCTTCG TAAACTCTCC CTCCTTCTCT CATTGTTGAC CCGAAACGTA   
  
  
- CATTAACTAA ACCTGTAGAA CGTACCAAAG GTCACCGGAA GAGAATAAGT CAGAGAGAGG CTTCTCCGAT   
  
  
- GAACAAGTTT GGCAAGTAAC AGAGAAGTTT AATGTCCGAA GCCTTCTTCG AATCTTCTCG AGTAACTTTG   
  
  
- TCTTCGAGCC GACCATTCAA AGAGATTCTC GAAAGTCTTA TAGTTGAAAC TTAAGGTGCC CAAAGACTCT   
  
  
- CCGAGCTTTG AGTCCTTGGA TTCCTCCCTC TTACTTTGTC ATCAACATTT AGAGCAAAAG GTGGAGTCAT   
  
  
- GAAACTTTCT ATGGCATGTC TAAAGACTGT GGAACTGAAG ACATGTAAGT GATTTGGGGA GATAACACCA   
  
  
- TAATCAGCTC TCTCTTCCTT CAGCTTTGTC TACACCCAAG GAGAGTTCAA TACATCTACG AGACGTAATA   
  
  
- ATACGACGTT ACAAACTGAG AAATCTACTA ACGGAGGGTG AACTTTCAGG ACTCTCCGAC TCGTATCTCT   
  
  
- TCTTGGTAGA ACCTTTTCTC TAGTTTCTTC GGTATCCAAC ACTCTTCCTA CTTTGTTTGA TAGACTTCAA   
  
  
- ACTCTACGAC CTCTGAACCT CTCCCTCCTA CCTCTCAGTA CCTAAAAGTC CATACTTCGA TTCAAGGTCC   
  
  
- CGTTGATATG TTCGTTTCGA CGAAAATTTT TACCCTTCGG TAATAGTATG GATAAACCTT CTTCTGACAC   
  
  
- CTCCACCACC CAAGTCTCAA ACACTTGCTC TACTACCTTA CCGATAGAGA GAACCGACCG TTCCATCCAA   
  
  
- GGAGCGTTGA CGGAGCCATA CCGCAAGACA CAC

+     MYC

| Site Name | Organism | Position | Strand | Matrix score. | sequence | function |
| --- | --- | --- | --- | --- | --- | --- |
| MYC | Arabidopsis thaliana | 2554 | + | 6 | CATTTG |  |
| MYC | Arabidopsis thaliana | 2542 | - | 6 | CAATTG |  |
| MYC | Arabidopsis thaliana | 423 | + | 6 | CAATTG |  |
| MYC | Arabidopsis thaliana | 1224 | - | 6 | CATTTG |  |
| MYC | Arabidopsis thaliana | 1355 | - | 6 | CATTTG |  |

>HU07G00272.1   
+ -Up\_Stream \_Len000ACCAAC GATTCTTTAC TGAATCAGCT ACATTTTTGG ATTGTGTTAA GGTATCTTTT   
  
  
+ AGATCTTCAG GAGGAGGGGG CTGAAGTAGC TTGGTTACTT TTGCGCTTAT CAACTGTGGA AGAATATTAT   
  
  
+ TGAGACTAGT GTAGAAGAAT TAGTAGTTTC CTCCACATTG GAAAAAAACC ATGAAATTAA CGCTTTAGGC   
  
  
+ CCTCAGAGTA CCAAGTGTCA TCGTAAATGA TTGAAATTTC AAGTCTTAGC TGAGTCATGA AGTCCTGATT   
  
  
+ TCCCATGACA AATTGATGTT GTTGTCTAAA GCTTGGCGGG TGATTTGAAA GAAGATTTAG ATCAGGCTGA   
  
  
+ CAATACACCA AATCTGGGAG CAAGGAACTG GATTTTTCTG TGTCTAGAAA AGCTTTCCGC TTTCTGAACA   
  
  
+ ATTGAGAAAA CCATGCATAG GAAATTTCTT GATATTTAGA TCATAGCAGA CTTAACAATC TGGCTTTACC   
  
  
+ GAACTGTCCT CTACTTCTAA GTTGTGAAGG TGTGAAAGAT AAACCATGTC AAACTTAGTG TCCAACAGAA   
  
  
+ ACGCATCTCT ACTTCTCCAG GCAAAGTTGT ACTTCAGATC TTCTACACCT TTTCTAGGCA GGACTTTGGG   
  
  
+ ATATAGAGTT TGGCCTGGGT GAAAAGTAGG AAGTGGGGAA GTAGAAAATG ATGAAAGAAG GCACCACTTG   
  
  
+ GGTCCAGATA CATAGGCCTA CGCACGAGAA CCTTTGCATG GTCTAGATAC ATGACTGTAT ACTTCTTCCT   
  
  
+ TTCCTTTACC TGTTGGAGAT TATCCTAGGA TTTTACATGG CACCAGCTGG ATTTGTTAAT GACATAAACA   
  
  
+ TATCTTTAAT TACAGTTGCT TATGGTATAC ATTTAAACTA TTCGATAGTT GACAGCTGAT GTCCTTATCC   
  
  
+ TTGTGCAGCT TATTCCTCGA TAGAATAATC TTTGGACATT TCTGTCTAGG TGATAAAGGG CAAAAACTAA   
  
  
+ GATACAAGAA ATAGAACATG TCCTCCATTT ACAAGACGTA TCTGTTTTTG GTTTTGGTCA ATGATCCCTG   
  
  
+ TTGTCCTCAA GCATTAACAC AGAATGATCA TTATTGACTT GGACTGAAAG GTTTTGGGAA CTCCATAGTA   
  
  
+ TGCCTTTTCA ATAAAATCAA AAGCAAATTT GCTGTTCACT GATAACTGCA GTTGTGTTGC TTTGCTGTTT   
  
  
+ CCATACCAGA CTCGTAACCA AGCTGATAGC AAATGTGCAA AGGTACAGTA TAATTAACGA TGTTTTCATA   
  
  
+ TTGCTACGAG TTCTGAAACA TGATATAAGA AAACACCAGA ACCCAAATAC CTACATAGGT CCTAGCCCTG   
  
  
+ GAATGGCTAT TTTAAATATC CAAATGGACT TACAGAATTC GCAGAGTTGT TTGGTTTGTT TAACAATCAT   
  
  
+ TGAATTGTTA GCTATGAGAA TCTGCGGGAC AATCGTAATC TTTGGCGATT ACTTCGATTG GCATACTGAT   
  
  
+ GTTTTTTTAT TTGCGTATCT AAGCATGGTG GTGTACCTTT GATTTGTGTG TCTAGGTAGT GTGTGTAGAT   
  
  
+ AAATATCATA GAACTCAGAC GTTGGTAGGG TTATGTTGAG CACTTTCTCT TGCTGTTTGT CCTTTGTGAT   
  
  
+ GGCACCTCTT GCTTTTCCGA ACCACCAAAA GTCTCATTGA CAAAACTCTT CAAACATTGC AAACTGCCCC   
  
  
+ CTTAAATATG TTTTATTAAA AAAAATGGAA GAGCTCAAAC TGTTGAGTGA GCCTAGATTC CAAGATTTAG   
  
  
+ AAACAGTAAA CTTGATAGAT ATATTTATAT GAACTATTGT TAGATGCAAC ACCCCCAACT TGAAACACAA   
  
  
+ AAGAGAAATT AAAAACAAAA AGGTTTTGCA CTATTTATAT ATCCTGTGTT CTTTGTGCTA GTCTTATTTA   
  
  
+ ATGTTAATTG GTAAGCATTA TTTGTTGGAT TATTCCACTG CACTTTCTCT TACTAACTTC AGCATTTCAT   
  
  
+ TCCTTGTTCT TCAGCTTTTG AACTTCTTTA GCCTATTTTG TTGTCTTTAT GGTTATGGAA GCCATCCAGA   
  
  
+ AGAAGAATGA TGAACTTCTG AGTCTTAGCT TGGCCATTGT CGGTCACTCT GATCGCACTG AGAAAAAACT   
  
  
+ GAAGAGGCGG ATTGATGTCT CCGATCCTCT GATCACTTCA GGTGAAGGCT GCGAAGGGAA GATAATCAGG   
  
  
+ CTCCTCCAAG AGAGACAGAA CTTGTTGAAC ATCAAGCAAA GAGGAAAAGG CGTCATTCAA GAAGGGAAGG   
  
  
+ GTCTTCATCT TATCCATCTG CTCCTTGTAT CTGCCACCTT AATCAACGAA AACAACATCA GTTCAGCTGT   
  
  
+ TGATAATCTT ATTGAGCTCT TCCAATATGT CTCCGAAAGT GGTGATTCAG GACAAAGGGT TGCTGCTTAC   
  
  
+ TTTGCGGATG GGTTAACAGC AAGGATCTTA ACTCAGAGGT CTCCATTTTA TCGCCTGATA CTGGGCAAAC   
  
  
+ CAGCACCTGC TGAAGAGTTT TCAGCTTTTA CTCACTTATA TAGAGCCTCT CCATTCTATC AGTTTGCTCA   
  
  
+ TTTCACAGCC AATCAGGCAA TTGTAGAAGC ATTTGAGAGG GAGGAAGAGA GTAACAACTG GGCTTTGCAT   
  
  
+ GTAATTGATT TGGACATCTT GCATGGTTTC CAGTGGCCTT CTCTTATTCA GTCTCTCTCC GAAGAGGCTA   
  
  
+ CTTGTTCAAA CCGTTCATTG TCTCTTCAAA TTACAGGCTT CGGAAGAAGC TTAGAAGAGC TCATTGAAAC   
  
  
+ AGAAGCTCGG CTGGTAAGTT TCTCTAAGAG CTTTCAGAAT ATCAACTTTG AATTCCACGG GTTTCTGAGA   
  
  
+ GGCTCGAAAC TCAGGAACCT AAGGAGGGAG AATGAAACAG TAGTTGTAAA TCTCGTTTTC CACCTCAGTA   
  
  
+ CTTTGAAAGA TACCGTACAG ATTTCTGACA CCTTGACTTC TGTACATTCA CTAAACCCCT CTATTGTGGT   
  
  
+ ATTAGTCGAG AGAGAAGGAA GTCGAAACAG ATGTGGGTTC CTCTCAAGTT ATGTAGATGC TCTGCATTAT   
  
  
+ TATGCTGCAA TGTTTGACTC TTTAGATGAT TGCCTCCCAC TTGAAAGTCC TGAGAGGCTG AGCATAGAGA   
  
  
+ AGAACCATCT TGGAAAAGAG ATCAAAGAAG CCATAGGTTG TGAGAAGGAT GAAACAAACT ATCTGAAGTT   
  
  
+ TGAGATGCTG GAGACTTGGA GAGGGAGGAT GGAGAGTCAT GGATTTTCAG GTATGAAGCT AAGTTCCAGG   
  
  
+ GCAACTATAC AAGCAAAGCT GCTTTTAAAA ATGGGAAGCC ATTATCATAC CTATTTGGAA GAAGACTGTG   
  
  
+ GAGGTGGTGG GTTCAGAGTT TGTGAACGAG ATGATGGAAT GGCTATCTCT CTTGGCTGGC AAGGTAGGTT   
  
  
+ CCTCGCAACT GCCTCGGTAT GGCGTTCTGT GTG  

- -Up\_Stream \_Len000TGGTTG CTAAGAAATG ACTTAGTCGA TGTAAAAACC TAACACAATT CCATAGAAAA   
  
  
- TCTAGAAGTC CTCCTCCCCC GACTTCATCG AACCAATGAA AACGCGAATA GTTGACACCT TCTTATAATA   
  
  
- ACTCTGATCA CATCTTCTTA ATCATCAAAG GAGGTGTAAC CTTTTTTTGG TACTTTAATT GCGAAATCCG   
  
  
- GGAGTCTCAT GGTTCACAGT AGCATTTACT AACTTTAAAG TTCAGAATCG ACTCAGTACT TCAGGACTAA   
  
  
- AGGGTACTGT TTAACTACAA CAACAGATTT CGAACCGCCC ACTAAACTTT CTTCTAAATC TAGTCCGACT   
  
  
- GTTATGTGGT TTAGACCCTC GTTCCTTGAC CTAAAAAGAC ACAGATCTTT TCGAAAGGCG AAAGACTTGT   
  
  
- TAACTCTTTT GGTACGTATC CTTTAAAGAA CTATAAATCT AGTATCGTCT GAATTGTTAG ACCGAAATGG   
  
  
- CTTGACAGGA GATGAAGATT CAACACTTCC ACACTTTCTA TTTGGTACAG TTTGAATCAC AGGTTGTCTT   
  
  
- TGCGTAGAGA TGAAGAGGTC CGTTTCAACA TGAAGTCTAG AAGATGTGGA AAAGATCCGT CCTGAAACCC   
  
  
- TATATCTCAA ACCGGACCCA CTTTTCATCC TTCACCCCTT CATCTTTTAC TACTTTCTTC CGTGGTGAAC   
  
  
- CCAGGTCTAT GTATCCGGAT GCGTGCTCTT GGAAACGTAC CAGATCTATG TACTGACATA TGAAGAAGGA   
  
  
- AAGGAAATGG ACAACCTCTA ATAGGATCCT AAAATGTACC GTGGTCGACC TAAACAATTA CTGTATTTGT   
  
  
- ATAGAAATTA ATGTCAACGA ATACCATATG TAAATTTGAT AAGCTATCAA CTGTCGACTA CAGGAATAGG   
  
  
- AACACGTCGA ATAAGGAGCT ATCTTATTAG AAACCTGTAA AGACAGATCC ACTATTTCCC GTTTTTGATT   
  
  
- CTATGTTCTT TATCTTGTAC AGGAGGTAAA TGTTCTGCAT AGACAAAAAC CAAAACCAGT TACTAGGGAC   
  
  
- AACAGGAGTT CGTAATTGTG TCTTACTAGT AATAACTGAA CCTGACTTTC CAAAACCCTT GAGGTATCAT   
  
  
- ACGGAAAAGT TATTTTAGTT TTCGTTTAAA CGACAAGTGA CTATTGACGT CAACACAACG AAACGACAAA   
  
  
- GGTATGGTCT GAGCATTGGT TCGACTATCG TTTACACGTT TCCATGTCAT ATTAATTGCT ACAAAAGTAT   
  
  
- AACGATGCTC AAGACTTTGT ACTATATTCT TTTGTGGTCT TGGGTTTATG GATGTATCCA GGATCGGGAC   
  
  
- CTTACCGATA AAATTTATAG GTTTACCTGA ATGTCTTAAG CGTCTCAACA AACCAAACAA ATTGTTAGTA   
  
  
- ACTTAACAAT CGATACTCTT AGACGCCCTG TTAGCATTAG AAACCGCTAA TGAAGCTAAC CGTATGACTA   
  
  
- CAAAAAAATA AACGCATAGA TTCGTACCAC CACATGGAAA CTAAACACAC AGATCCATCA CACACATCTA   
  
  
- TTTATAGTAT CTTGAGTCTG CAACCATCCC AATACAACTC GTGAAAGAGA ACGACAAACA GGAAACACTA   
  
  
- CCGTGGAGAA CGAAAAGGCT TGGTGGTTTT CAGAGTAACT GTTTTGAGAA GTTTGTAACG TTTGACGGGG   
  
  
- GAATTTATAC AAAATAATTT TTTTTACCTT CTCGAGTTTG ACAACTCACT CGGATCTAAG GTTCTAAATC   
  
  
- TTTGTCATTT GAACTATCTA TATAAATATA CTTGATAACA ATCTACGTTG TGGGGGTTGA ACTTTGTGTT   
  
  
- TTCTCTTTAA TTTTTGTTTT TCCAAAACGT GATAAATATA TAGGACACAA GAAACACGAT CAGAATAAAT   
  
  
- TACAATTAAC CATTCGTAAT AAACAACCTA ATAAGGTGAC GTGAAAGAGA ATGATTGAAG TCGTAAAGTA   
  
  
- AGGAACAAGA AGTCGAAAAC TTGAAGAAAT CGGATAAAAC AACAGAAATA CCAATACCTT CGGTAGGTCT   
  
  
- TCTTCTTACT ACTTGAAGAC TCAGAATCGA ACCGGTAACA GCCAGTGAGA CTAGCGTGAC TCTTTTTTGA   
  
  
- CTTCTCCGCC TAACTACAGA GGCTAGGAGA CTAGTGAAGT CCACTTCCGA CGCTTCCCTT CTATTAGTCC   
  
  
- GAGGAGGTTC TCTCTGTCTT GAACAACTTG TAGTTCGTTT CTCCTTTTCC GCAGTAAGTT CTTCCCTTCC   
  
  
- CAGAAGTAGA ATAGGTAGAC GAGGAACATA GACGGTGGAA TTAGTTGCTT TTGTTGTAGT CAAGTCGACA   
  
  
- ACTATTAGAA TAACTCGAGA AGGTTATACA GAGGCTTTCA CCACTAAGTC CTGTTTCCCA ACGACGAATG   
  
  
- AAACGCCTAC CCAATTGTCG TTCCTAGAAT TGAGTCTCCA GAGGTAAAAT AGCGGACTAT GACCCGTTTG   
  
  
- GTCGTGGACG ACTTCTCAAA AGTCGAAAAT GAGTGAATAT ATCTCGGAGA GGTAAGATAG TCAAACGAGT   
  
  
- AAAGTGTCGG TTAGTCCGTT AACATCTTCG TAAACTCTCC CTCCTTCTCT CATTGTTGAC CCGAAACGTA   
  
  
- CATTAACTAA ACCTGTAGAA CGTACCAAAG GTCACCGGAA GAGAATAAGT CAGAGAGAGG CTTCTCCGAT   
  
  
- GAACAAGTTT GGCAAGTAAC AGAGAAGTTT AATGTCCGAA GCCTTCTTCG AATCTTCTCG AGTAACTTTG   
  
  
- TCTTCGAGCC GACCATTCAA AGAGATTCTC GAAAGTCTTA TAGTTGAAAC TTAAGGTGCC CAAAGACTCT   
  
  
- CCGAGCTTTG AGTCCTTGGA TTCCTCCCTC TTACTTTGTC ATCAACATTT AGAGCAAAAG GTGGAGTCAT   
  
  
- GAAACTTTCT ATGGCATGTC TAAAGACTGT GGAACTGAAG ACATGTAAGT GATTTGGGGA GATAACACCA   
  
  
- TAATCAGCTC TCTCTTCCTT CAGCTTTGTC TACACCCAAG GAGAGTTCAA TACATCTACG AGACGTAATA   
  
  
- ATACGACGTT ACAAACTGAG AAATCTACTA ACGGAGGGTG AACTTTCAGG ACTCTCCGAC TCGTATCTCT   
  
  
- TCTTGGTAGA ACCTTTTCTC TAGTTTCTTC GGTATCCAAC ACTCTTCCTA CTTTGTTTGA TAGACTTCAA   
  
  
- ACTCTACGAC CTCTGAACCT CTCCCTCCTA CCTCTCAGTA CCTAAAAGTC CATACTTCGA TTCAAGGTCC   
  
  
- CGTTGATATG TTCGTTTCGA CGAAAATTTT TACCCTTCGG TAATAGTATG GATAAACCTT CTTCTGACAC   
  
  
- CTCCACCACC CAAGTCTCAA ACACTTGCTC TACTACCTTA CCGATAGAGA GAACCGACCG TTCCATCCAA   
  
  
- GGAGCGTTGA CGGAGCCATA CCGCAAGACA CAC

+     Myb

| Site Name | Organism | Position | Strand | Matrix score. | sequence | function |
| --- | --- | --- | --- | --- | --- | --- |
| Myb | Arabidopsis thaliana | 857 | - | 6 | CAACTG |  |
| Myb | Arabidopsis thaliana | 2579 | + | 6 | CAACTG |  |
| Myb | Arabidopsis thaliana | 3370 | + | 6 | CAACTG |  |
| Myb | Arabidopsis thaliana | 1167 | + | 6 | TAACTG |  |
| Myb | Arabidopsis thaliana | 125 | + | 6 | CAACTG |  |
| Myb | Arabidopsis thaliana | 1173 | - | 6 | CAACTG |  |

>HU07G00272.1   
+ -Up\_Stream \_Len000ACCAAC GATTCTTTAC TGAATCAGCT ACATTTTTGG ATTGTGTTAA GGTATCTTTT   
  
  
+ AGATCTTCAG GAGGAGGGGG CTGAAGTAGC TTGGTTACTT TTGCGCTTAT CAACTGTGGA AGAATATTAT   
  
  
+ TGAGACTAGT GTAGAAGAAT TAGTAGTTTC CTCCACATTG GAAAAAAACC ATGAAATTAA CGCTTTAGGC   
  
  
+ CCTCAGAGTA CCAAGTGTCA TCGTAAATGA TTGAAATTTC AAGTCTTAGC TGAGTCATGA AGTCCTGATT   
  
  
+ TCCCATGACA AATTGATGTT GTTGTCTAAA GCTTGGCGGG TGATTTGAAA GAAGATTTAG ATCAGGCTGA   
  
  
+ CAATACACCA AATCTGGGAG CAAGGAACTG GATTTTTCTG TGTCTAGAAA AGCTTTCCGC TTTCTGAACA   
  
  
+ ATTGAGAAAA CCATGCATAG GAAATTTCTT GATATTTAGA TCATAGCAGA CTTAACAATC TGGCTTTACC   
  
  
+ GAACTGTCCT CTACTTCTAA GTTGTGAAGG TGTGAAAGAT AAACCATGTC AAACTTAGTG TCCAACAGAA   
  
  
+ ACGCATCTCT ACTTCTCCAG GCAAAGTTGT ACTTCAGATC TTCTACACCT TTTCTAGGCA GGACTTTGGG   
  
  
+ ATATAGAGTT TGGCCTGGGT GAAAAGTAGG AAGTGGGGAA GTAGAAAATG ATGAAAGAAG GCACCACTTG   
  
  
+ GGTCCAGATA CATAGGCCTA CGCACGAGAA CCTTTGCATG GTCTAGATAC ATGACTGTAT ACTTCTTCCT   
  
  
+ TTCCTTTACC TGTTGGAGAT TATCCTAGGA TTTTACATGG CACCAGCTGG ATTTGTTAAT GACATAAACA   
  
  
+ TATCTTTAAT TACAGTTGCT TATGGTATAC ATTTAAACTA TTCGATAGTT GACAGCTGAT GTCCTTATCC   
  
  
+ TTGTGCAGCT TATTCCTCGA TAGAATAATC TTTGGACATT TCTGTCTAGG TGATAAAGGG CAAAAACTAA   
  
  
+ GATACAAGAA ATAGAACATG TCCTCCATTT ACAAGACGTA TCTGTTTTTG GTTTTGGTCA ATGATCCCTG   
  
  
+ TTGTCCTCAA GCATTAACAC AGAATGATCA TTATTGACTT GGACTGAAAG GTTTTGGGAA CTCCATAGTA   
  
  
+ TGCCTTTTCA ATAAAATCAA AAGCAAATTT GCTGTTCACT GATAACTGCA GTTGTGTTGC TTTGCTGTTT   
  
  
+ CCATACCAGA CTCGTAACCA AGCTGATAGC AAATGTGCAA AGGTACAGTA TAATTAACGA TGTTTTCATA   
  
  
+ TTGCTACGAG TTCTGAAACA TGATATAAGA AAACACCAGA ACCCAAATAC CTACATAGGT CCTAGCCCTG   
  
  
+ GAATGGCTAT TTTAAATATC CAAATGGACT TACAGAATTC GCAGAGTTGT TTGGTTTGTT TAACAATCAT   
  
  
+ TGAATTGTTA GCTATGAGAA TCTGCGGGAC AATCGTAATC TTTGGCGATT ACTTCGATTG GCATACTGAT   
  
  
+ GTTTTTTTAT TTGCGTATCT AAGCATGGTG GTGTACCTTT GATTTGTGTG TCTAGGTAGT GTGTGTAGAT   
  
  
+ AAATATCATA GAACTCAGAC GTTGGTAGGG TTATGTTGAG CACTTTCTCT TGCTGTTTGT CCTTTGTGAT   
  
  
+ GGCACCTCTT GCTTTTCCGA ACCACCAAAA GTCTCATTGA CAAAACTCTT CAAACATTGC AAACTGCCCC   
  
  
+ CTTAAATATG TTTTATTAAA AAAAATGGAA GAGCTCAAAC TGTTGAGTGA GCCTAGATTC CAAGATTTAG   
  
  
+ AAACAGTAAA CTTGATAGAT ATATTTATAT GAACTATTGT TAGATGCAAC ACCCCCAACT TGAAACACAA   
  
  
+ AAGAGAAATT AAAAACAAAA AGGTTTTGCA CTATTTATAT ATCCTGTGTT CTTTGTGCTA GTCTTATTTA   
  
  
+ ATGTTAATTG GTAAGCATTA TTTGTTGGAT TATTCCACTG CACTTTCTCT TACTAACTTC AGCATTTCAT   
  
  
+ TCCTTGTTCT TCAGCTTTTG AACTTCTTTA GCCTATTTTG TTGTCTTTAT GGTTATGGAA GCCATCCAGA   
  
  
+ AGAAGAATGA TGAACTTCTG AGTCTTAGCT TGGCCATTGT CGGTCACTCT GATCGCACTG AGAAAAAACT   
  
  
+ GAAGAGGCGG ATTGATGTCT CCGATCCTCT GATCACTTCA GGTGAAGGCT GCGAAGGGAA GATAATCAGG   
  
  
+ CTCCTCCAAG AGAGACAGAA CTTGTTGAAC ATCAAGCAAA GAGGAAAAGG CGTCATTCAA GAAGGGAAGG   
  
  
+ GTCTTCATCT TATCCATCTG CTCCTTGTAT CTGCCACCTT AATCAACGAA AACAACATCA GTTCAGCTGT   
  
  
+ TGATAATCTT ATTGAGCTCT TCCAATATGT CTCCGAAAGT GGTGATTCAG GACAAAGGGT TGCTGCTTAC   
  
  
+ TTTGCGGATG GGTTAACAGC AAGGATCTTA ACTCAGAGGT CTCCATTTTA TCGCCTGATA CTGGGCAAAC   
  
  
+ CAGCACCTGC TGAAGAGTTT TCAGCTTTTA CTCACTTATA TAGAGCCTCT CCATTCTATC AGTTTGCTCA   
  
  
+ TTTCACAGCC AATCAGGCAA TTGTAGAAGC ATTTGAGAGG GAGGAAGAGA GTAACAACTG GGCTTTGCAT   
  
  
+ GTAATTGATT TGGACATCTT GCATGGTTTC CAGTGGCCTT CTCTTATTCA GTCTCTCTCC GAAGAGGCTA   
  
  
+ CTTGTTCAAA CCGTTCATTG TCTCTTCAAA TTACAGGCTT CGGAAGAAGC TTAGAAGAGC TCATTGAAAC   
  
  
+ AGAAGCTCGG CTGGTAAGTT TCTCTAAGAG CTTTCAGAAT ATCAACTTTG AATTCCACGG GTTTCTGAGA   
  
  
+ GGCTCGAAAC TCAGGAACCT AAGGAGGGAG AATGAAACAG TAGTTGTAAA TCTCGTTTTC CACCTCAGTA   
  
  
+ CTTTGAAAGA TACCGTACAG ATTTCTGACA CCTTGACTTC TGTACATTCA CTAAACCCCT CTATTGTGGT   
  
  
+ ATTAGTCGAG AGAGAAGGAA GTCGAAACAG ATGTGGGTTC CTCTCAAGTT ATGTAGATGC TCTGCATTAT   
  
  
+ TATGCTGCAA TGTTTGACTC TTTAGATGAT TGCCTCCCAC TTGAAAGTCC TGAGAGGCTG AGCATAGAGA   
  
  
+ AGAACCATCT TGGAAAAGAG ATCAAAGAAG CCATAGGTTG TGAGAAGGAT GAAACAAACT ATCTGAAGTT   
  
  
+ TGAGATGCTG GAGACTTGGA GAGGGAGGAT GGAGAGTCAT GGATTTTCAG GTATGAAGCT AAGTTCCAGG   
  
  
+ GCAACTATAC AAGCAAAGCT GCTTTTAAAA ATGGGAAGCC ATTATCATAC CTATTTGGAA GAAGACTGTG   
  
  
+ GAGGTGGTGG GTTCAGAGTT TGTGAACGAG ATGATGGAAT GGCTATCTCT CTTGGCTGGC AAGGTAGGTT   
  
  
+ CCTCGCAACT GCCTCGGTAT GGCGTTCTGT GTG  

- -Up\_Stream \_Len000TGGTTG CTAAGAAATG ACTTAGTCGA TGTAAAAACC TAACACAATT CCATAGAAAA   
  
  
- TCTAGAAGTC CTCCTCCCCC GACTTCATCG AACCAATGAA AACGCGAATA GTTGACACCT TCTTATAATA   
  
  
- ACTCTGATCA CATCTTCTTA ATCATCAAAG GAGGTGTAAC CTTTTTTTGG TACTTTAATT GCGAAATCCG   
  
  
- GGAGTCTCAT GGTTCACAGT AGCATTTACT AACTTTAAAG TTCAGAATCG ACTCAGTACT TCAGGACTAA   
  
  
- AGGGTACTGT TTAACTACAA CAACAGATTT CGAACCGCCC ACTAAACTTT CTTCTAAATC TAGTCCGACT   
  
  
- GTTATGTGGT TTAGACCCTC GTTCCTTGAC CTAAAAAGAC ACAGATCTTT TCGAAAGGCG AAAGACTTGT   
  
  
- TAACTCTTTT GGTACGTATC CTTTAAAGAA CTATAAATCT AGTATCGTCT GAATTGTTAG ACCGAAATGG   
  
  
- CTTGACAGGA GATGAAGATT CAACACTTCC ACACTTTCTA TTTGGTACAG TTTGAATCAC AGGTTGTCTT   
  
  
- TGCGTAGAGA TGAAGAGGTC CGTTTCAACA TGAAGTCTAG AAGATGTGGA AAAGATCCGT CCTGAAACCC   
  
  
- TATATCTCAA ACCGGACCCA CTTTTCATCC TTCACCCCTT CATCTTTTAC TACTTTCTTC CGTGGTGAAC   
  
  
- CCAGGTCTAT GTATCCGGAT GCGTGCTCTT GGAAACGTAC CAGATCTATG TACTGACATA TGAAGAAGGA   
  
  
- AAGGAAATGG ACAACCTCTA ATAGGATCCT AAAATGTACC GTGGTCGACC TAAACAATTA CTGTATTTGT   
  
  
- ATAGAAATTA ATGTCAACGA ATACCATATG TAAATTTGAT AAGCTATCAA CTGTCGACTA CAGGAATAGG   
  
  
- AACACGTCGA ATAAGGAGCT ATCTTATTAG AAACCTGTAA AGACAGATCC ACTATTTCCC GTTTTTGATT   
  
  
- CTATGTTCTT TATCTTGTAC AGGAGGTAAA TGTTCTGCAT AGACAAAAAC CAAAACCAGT TACTAGGGAC   
  
  
- AACAGGAGTT CGTAATTGTG TCTTACTAGT AATAACTGAA CCTGACTTTC CAAAACCCTT GAGGTATCAT   
  
  
- ACGGAAAAGT TATTTTAGTT TTCGTTTAAA CGACAAGTGA CTATTGACGT CAACACAACG AAACGACAAA   
  
  
- GGTATGGTCT GAGCATTGGT TCGACTATCG TTTACACGTT TCCATGTCAT ATTAATTGCT ACAAAAGTAT   
  
  
- AACGATGCTC AAGACTTTGT ACTATATTCT TTTGTGGTCT TGGGTTTATG GATGTATCCA GGATCGGGAC   
  
  
- CTTACCGATA AAATTTATAG GTTTACCTGA ATGTCTTAAG CGTCTCAACA AACCAAACAA ATTGTTAGTA   
  
  
- ACTTAACAAT CGATACTCTT AGACGCCCTG TTAGCATTAG AAACCGCTAA TGAAGCTAAC CGTATGACTA   
  
  
- CAAAAAAATA AACGCATAGA TTCGTACCAC CACATGGAAA CTAAACACAC AGATCCATCA CACACATCTA   
  
  
- TTTATAGTAT CTTGAGTCTG CAACCATCCC AATACAACTC GTGAAAGAGA ACGACAAACA GGAAACACTA   
  
  
- CCGTGGAGAA CGAAAAGGCT TGGTGGTTTT CAGAGTAACT GTTTTGAGAA GTTTGTAACG TTTGACGGGG   
  
  
- GAATTTATAC AAAATAATTT TTTTTACCTT CTCGAGTTTG ACAACTCACT CGGATCTAAG GTTCTAAATC   
  
  
- TTTGTCATTT GAACTATCTA TATAAATATA CTTGATAACA ATCTACGTTG TGGGGGTTGA ACTTTGTGTT   
  
  
- TTCTCTTTAA TTTTTGTTTT TCCAAAACGT GATAAATATA TAGGACACAA GAAACACGAT CAGAATAAAT   
  
  
- TACAATTAAC CATTCGTAAT AAACAACCTA ATAAGGTGAC GTGAAAGAGA ATGATTGAAG TCGTAAAGTA   
  
  
- AGGAACAAGA AGTCGAAAAC TTGAAGAAAT CGGATAAAAC AACAGAAATA CCAATACCTT CGGTAGGTCT   
  
  
- TCTTCTTACT ACTTGAAGAC TCAGAATCGA ACCGGTAACA GCCAGTGAGA CTAGCGTGAC TCTTTTTTGA   
  
  
- CTTCTCCGCC TAACTACAGA GGCTAGGAGA CTAGTGAAGT CCACTTCCGA CGCTTCCCTT CTATTAGTCC   
  
  
- GAGGAGGTTC TCTCTGTCTT GAACAACTTG TAGTTCGTTT CTCCTTTTCC GCAGTAAGTT CTTCCCTTCC   
  
  
- CAGAAGTAGA ATAGGTAGAC GAGGAACATA GACGGTGGAA TTAGTTGCTT TTGTTGTAGT CAAGTCGACA   
  
  
- ACTATTAGAA TAACTCGAGA AGGTTATACA GAGGCTTTCA CCACTAAGTC CTGTTTCCCA ACGACGAATG   
  
  
- AAACGCCTAC CCAATTGTCG TTCCTAGAAT TGAGTCTCCA GAGGTAAAAT AGCGGACTAT GACCCGTTTG   
  
  
- GTCGTGGACG ACTTCTCAAA AGTCGAAAAT GAGTGAATAT ATCTCGGAGA GGTAAGATAG TCAAACGAGT   
  
  
- AAAGTGTCGG TTAGTCCGTT AACATCTTCG TAAACTCTCC CTCCTTCTCT CATTGTTGAC CCGAAACGTA   
  
  
- CATTAACTAA ACCTGTAGAA CGTACCAAAG GTCACCGGAA GAGAATAAGT CAGAGAGAGG CTTCTCCGAT   
  
  
- GAACAAGTTT GGCAAGTAAC AGAGAAGTTT AATGTCCGAA GCCTTCTTCG AATCTTCTCG AGTAACTTTG   
  
  
- TCTTCGAGCC GACCATTCAA AGAGATTCTC GAAAGTCTTA TAGTTGAAAC TTAAGGTGCC CAAAGACTCT   
  
  
- CCGAGCTTTG AGTCCTTGGA TTCCTCCCTC TTACTTTGTC ATCAACATTT AGAGCAAAAG GTGGAGTCAT   
  
  
- GAAACTTTCT ATGGCATGTC TAAAGACTGT GGAACTGAAG ACATGTAAGT GATTTGGGGA GATAACACCA   
  
  
- TAATCAGCTC TCTCTTCCTT CAGCTTTGTC TACACCCAAG GAGAGTTCAA TACATCTACG AGACGTAATA   
  
  
- ATACGACGTT ACAAACTGAG AAATCTACTA ACGGAGGGTG AACTTTCAGG ACTCTCCGAC TCGTATCTCT   
  
  
- TCTTGGTAGA ACCTTTTCTC TAGTTTCTTC GGTATCCAAC ACTCTTCCTA CTTTGTTTGA TAGACTTCAA   
  
  
- ACTCTACGAC CTCTGAACCT CTCCCTCCTA CCTCTCAGTA CCTAAAAGTC CATACTTCGA TTCAAGGTCC   
  
  
- CGTTGATATG TTCGTTTCGA CGAAAATTTT TACCCTTCGG TAATAGTATG GATAAACCTT CTTCTGACAC   
  
  
- CTCCACCACC CAAGTCTCAA ACACTTGCTC TACTACCTTA CCGATAGAGA GAACCGACCG TTCCATCCAA   
  
  
- GGAGCGTTGA CGGAGCCATA CCGCAAGACA CAC

+     Myb-binding site

| Site Name | Organism | Position | Strand | Matrix score. | sequence | function |
| --- | --- | --- | --- | --- | --- | --- |
| Myb-binding site | Nicotiana tabacum | 784 | - | 6 | CAACAG |  |
| Myb-binding site | Nicotiana tabacum | 2311 | - | 6 | CAACAG |  |
| Myb-binding site | Nicotiana tabacum | 1052 | - | 6 | CAACAG |  |
| Myb-binding site | Nicotiana tabacum | 1724 | - | 6 | CAACAG |  |
| Myb-binding site | Nicotiana tabacum | 557 | + | 6 | CAACAG |  |

>HU07G00272.1   
+ -Up\_Stream \_Len000ACCAAC GATTCTTTAC TGAATCAGCT ACATTTTTGG ATTGTGTTAA GGTATCTTTT   
  
  
+ AGATCTTCAG GAGGAGGGGG CTGAAGTAGC TTGGTTACTT TTGCGCTTAT CAACTGTGGA AGAATATTAT   
  
  
+ TGAGACTAGT GTAGAAGAAT TAGTAGTTTC CTCCACATTG GAAAAAAACC ATGAAATTAA CGCTTTAGGC   
  
  
+ CCTCAGAGTA CCAAGTGTCA TCGTAAATGA TTGAAATTTC AAGTCTTAGC TGAGTCATGA AGTCCTGATT   
  
  
+ TCCCATGACA AATTGATGTT GTTGTCTAAA GCTTGGCGGG TGATTTGAAA GAAGATTTAG ATCAGGCTGA   
  
  
+ CAATACACCA AATCTGGGAG CAAGGAACTG GATTTTTCTG TGTCTAGAAA AGCTTTCCGC TTTCTGAACA   
  
  
+ ATTGAGAAAA CCATGCATAG GAAATTTCTT GATATTTAGA TCATAGCAGA CTTAACAATC TGGCTTTACC   
  
  
+ GAACTGTCCT CTACTTCTAA GTTGTGAAGG TGTGAAAGAT AAACCATGTC AAACTTAGTG TCCAACAGAA   
  
  
+ ACGCATCTCT ACTTCTCCAG GCAAAGTTGT ACTTCAGATC TTCTACACCT TTTCTAGGCA GGACTTTGGG   
  
  
+ ATATAGAGTT TGGCCTGGGT GAAAAGTAGG AAGTGGGGAA GTAGAAAATG ATGAAAGAAG GCACCACTTG   
  
  
+ GGTCCAGATA CATAGGCCTA CGCACGAGAA CCTTTGCATG GTCTAGATAC ATGACTGTAT ACTTCTTCCT   
  
  
+ TTCCTTTACC TGTTGGAGAT TATCCTAGGA TTTTACATGG CACCAGCTGG ATTTGTTAAT GACATAAACA   
  
  
+ TATCTTTAAT TACAGTTGCT TATGGTATAC ATTTAAACTA TTCGATAGTT GACAGCTGAT GTCCTTATCC   
  
  
+ TTGTGCAGCT TATTCCTCGA TAGAATAATC TTTGGACATT TCTGTCTAGG TGATAAAGGG CAAAAACTAA   
  
  
+ GATACAAGAA ATAGAACATG TCCTCCATTT ACAAGACGTA TCTGTTTTTG GTTTTGGTCA ATGATCCCTG   
  
  
+ TTGTCCTCAA GCATTAACAC AGAATGATCA TTATTGACTT GGACTGAAAG GTTTTGGGAA CTCCATAGTA   
  
  
+ TGCCTTTTCA ATAAAATCAA AAGCAAATTT GCTGTTCACT GATAACTGCA GTTGTGTTGC TTTGCTGTTT   
  
  
+ CCATACCAGA CTCGTAACCA AGCTGATAGC AAATGTGCAA AGGTACAGTA TAATTAACGA TGTTTTCATA   
  
  
+ TTGCTACGAG TTCTGAAACA TGATATAAGA AAACACCAGA ACCCAAATAC CTACATAGGT CCTAGCCCTG   
  
  
+ GAATGGCTAT TTTAAATATC CAAATGGACT TACAGAATTC GCAGAGTTGT TTGGTTTGTT TAACAATCAT   
  
  
+ TGAATTGTTA GCTATGAGAA TCTGCGGGAC AATCGTAATC TTTGGCGATT ACTTCGATTG GCATACTGAT   
  
  
+ GTTTTTTTAT TTGCGTATCT AAGCATGGTG GTGTACCTTT GATTTGTGTG TCTAGGTAGT GTGTGTAGAT   
  
  
+ AAATATCATA GAACTCAGAC GTTGGTAGGG TTATGTTGAG CACTTTCTCT TGCTGTTTGT CCTTTGTGAT   
  
  
+ GGCACCTCTT GCTTTTCCGA ACCACCAAAA GTCTCATTGA CAAAACTCTT CAAACATTGC AAACTGCCCC   
  
  
+ CTTAAATATG TTTTATTAAA AAAAATGGAA GAGCTCAAAC TGTTGAGTGA GCCTAGATTC CAAGATTTAG   
  
  
+ AAACAGTAAA CTTGATAGAT ATATTTATAT GAACTATTGT TAGATGCAAC ACCCCCAACT TGAAACACAA   
  
  
+ AAGAGAAATT AAAAACAAAA AGGTTTTGCA CTATTTATAT ATCCTGTGTT CTTTGTGCTA GTCTTATTTA   
  
  
+ ATGTTAATTG GTAAGCATTA TTTGTTGGAT TATTCCACTG CACTTTCTCT TACTAACTTC AGCATTTCAT   
  
  
+ TCCTTGTTCT TCAGCTTTTG AACTTCTTTA GCCTATTTTG TTGTCTTTAT GGTTATGGAA GCCATCCAGA   
  
  
+ AGAAGAATGA TGAACTTCTG AGTCTTAGCT TGGCCATTGT CGGTCACTCT GATCGCACTG AGAAAAAACT   
  
  
+ GAAGAGGCGG ATTGATGTCT CCGATCCTCT GATCACTTCA GGTGAAGGCT GCGAAGGGAA GATAATCAGG   
  
  
+ CTCCTCCAAG AGAGACAGAA CTTGTTGAAC ATCAAGCAAA GAGGAAAAGG CGTCATTCAA GAAGGGAAGG   
  
  
+ GTCTTCATCT TATCCATCTG CTCCTTGTAT CTGCCACCTT AATCAACGAA AACAACATCA GTTCAGCTGT   
  
  
+ TGATAATCTT ATTGAGCTCT TCCAATATGT CTCCGAAAGT GGTGATTCAG GACAAAGGGT TGCTGCTTAC   
  
  
+ TTTGCGGATG GGTTAACAGC AAGGATCTTA ACTCAGAGGT CTCCATTTTA TCGCCTGATA CTGGGCAAAC   
  
  
+ CAGCACCTGC TGAAGAGTTT TCAGCTTTTA CTCACTTATA TAGAGCCTCT CCATTCTATC AGTTTGCTCA   
  
  
+ TTTCACAGCC AATCAGGCAA TTGTAGAAGC ATTTGAGAGG GAGGAAGAGA GTAACAACTG GGCTTTGCAT   
  
  
+ GTAATTGATT TGGACATCTT GCATGGTTTC CAGTGGCCTT CTCTTATTCA GTCTCTCTCC GAAGAGGCTA   
  
  
+ CTTGTTCAAA CCGTTCATTG TCTCTTCAAA TTACAGGCTT CGGAAGAAGC TTAGAAGAGC TCATTGAAAC   
  
  
+ AGAAGCTCGG CTGGTAAGTT TCTCTAAGAG CTTTCAGAAT ATCAACTTTG AATTCCACGG GTTTCTGAGA   
  
  
+ GGCTCGAAAC TCAGGAACCT AAGGAGGGAG AATGAAACAG TAGTTGTAAA TCTCGTTTTC CACCTCAGTA   
  
  
+ CTTTGAAAGA TACCGTACAG ATTTCTGACA CCTTGACTTC TGTACATTCA CTAAACCCCT CTATTGTGGT   
  
  
+ ATTAGTCGAG AGAGAAGGAA GTCGAAACAG ATGTGGGTTC CTCTCAAGTT ATGTAGATGC TCTGCATTAT   
  
  
+ TATGCTGCAA TGTTTGACTC TTTAGATGAT TGCCTCCCAC TTGAAAGTCC TGAGAGGCTG AGCATAGAGA   
  
  
+ AGAACCATCT TGGAAAAGAG ATCAAAGAAG CCATAGGTTG TGAGAAGGAT GAAACAAACT ATCTGAAGTT   
  
  
+ TGAGATGCTG GAGACTTGGA GAGGGAGGAT GGAGAGTCAT GGATTTTCAG GTATGAAGCT AAGTTCCAGG   
  
  
+ GCAACTATAC AAGCAAAGCT GCTTTTAAAA ATGGGAAGCC ATTATCATAC CTATTTGGAA GAAGACTGTG   
  
  
+ GAGGTGGTGG GTTCAGAGTT TGTGAACGAG ATGATGGAAT GGCTATCTCT CTTGGCTGGC AAGGTAGGTT   
  
  
+ CCTCGCAACT GCCTCGGTAT GGCGTTCTGT GTG  

- -Up\_Stream \_Len000TGGTTG CTAAGAAATG ACTTAGTCGA TGTAAAAACC TAACACAATT CCATAGAAAA   
  
  
- TCTAGAAGTC CTCCTCCCCC GACTTCATCG AACCAATGAA AACGCGAATA GTTGACACCT TCTTATAATA   
  
  
- ACTCTGATCA CATCTTCTTA ATCATCAAAG GAGGTGTAAC CTTTTTTTGG TACTTTAATT GCGAAATCCG   
  
  
- GGAGTCTCAT GGTTCACAGT AGCATTTACT AACTTTAAAG TTCAGAATCG ACTCAGTACT TCAGGACTAA   
  
  
- AGGGTACTGT TTAACTACAA CAACAGATTT CGAACCGCCC ACTAAACTTT CTTCTAAATC TAGTCCGACT   
  
  
- GTTATGTGGT TTAGACCCTC GTTCCTTGAC CTAAAAAGAC ACAGATCTTT TCGAAAGGCG AAAGACTTGT   
  
  
- TAACTCTTTT GGTACGTATC CTTTAAAGAA CTATAAATCT AGTATCGTCT GAATTGTTAG ACCGAAATGG   
  
  
- CTTGACAGGA GATGAAGATT CAACACTTCC ACACTTTCTA TTTGGTACAG TTTGAATCAC AGGTTGTCTT   
  
  
- TGCGTAGAGA TGAAGAGGTC CGTTTCAACA TGAAGTCTAG AAGATGTGGA AAAGATCCGT CCTGAAACCC   
  
  
- TATATCTCAA ACCGGACCCA CTTTTCATCC TTCACCCCTT CATCTTTTAC TACTTTCTTC CGTGGTGAAC   
  
  
- CCAGGTCTAT GTATCCGGAT GCGTGCTCTT GGAAACGTAC CAGATCTATG TACTGACATA TGAAGAAGGA   
  
  
- AAGGAAATGG ACAACCTCTA ATAGGATCCT AAAATGTACC GTGGTCGACC TAAACAATTA CTGTATTTGT   
  
  
- ATAGAAATTA ATGTCAACGA ATACCATATG TAAATTTGAT AAGCTATCAA CTGTCGACTA CAGGAATAGG   
  
  
- AACACGTCGA ATAAGGAGCT ATCTTATTAG AAACCTGTAA AGACAGATCC ACTATTTCCC GTTTTTGATT   
  
  
- CTATGTTCTT TATCTTGTAC AGGAGGTAAA TGTTCTGCAT AGACAAAAAC CAAAACCAGT TACTAGGGAC   
  
  
- AACAGGAGTT CGTAATTGTG TCTTACTAGT AATAACTGAA CCTGACTTTC CAAAACCCTT GAGGTATCAT   
  
  
- ACGGAAAAGT TATTTTAGTT TTCGTTTAAA CGACAAGTGA CTATTGACGT CAACACAACG AAACGACAAA   
  
  
- GGTATGGTCT GAGCATTGGT TCGACTATCG TTTACACGTT TCCATGTCAT ATTAATTGCT ACAAAAGTAT   
  
  
- AACGATGCTC AAGACTTTGT ACTATATTCT TTTGTGGTCT TGGGTTTATG GATGTATCCA GGATCGGGAC   
  
  
- CTTACCGATA AAATTTATAG GTTTACCTGA ATGTCTTAAG CGTCTCAACA AACCAAACAA ATTGTTAGTA   
  
  
- ACTTAACAAT CGATACTCTT AGACGCCCTG TTAGCATTAG AAACCGCTAA TGAAGCTAAC CGTATGACTA   
  
  
- CAAAAAAATA AACGCATAGA TTCGTACCAC CACATGGAAA CTAAACACAC AGATCCATCA CACACATCTA   
  
  
- TTTATAGTAT CTTGAGTCTG CAACCATCCC AATACAACTC GTGAAAGAGA ACGACAAACA GGAAACACTA   
  
  
- CCGTGGAGAA CGAAAAGGCT TGGTGGTTTT CAGAGTAACT GTTTTGAGAA GTTTGTAACG TTTGACGGGG   
  
  
- GAATTTATAC AAAATAATTT TTTTTACCTT CTCGAGTTTG ACAACTCACT CGGATCTAAG GTTCTAAATC   
  
  
- TTTGTCATTT GAACTATCTA TATAAATATA CTTGATAACA ATCTACGTTG TGGGGGTTGA ACTTTGTGTT   
  
  
- TTCTCTTTAA TTTTTGTTTT TCCAAAACGT GATAAATATA TAGGACACAA GAAACACGAT CAGAATAAAT   
  
  
- TACAATTAAC CATTCGTAAT AAACAACCTA ATAAGGTGAC GTGAAAGAGA ATGATTGAAG TCGTAAAGTA   
  
  
- AGGAACAAGA AGTCGAAAAC TTGAAGAAAT CGGATAAAAC AACAGAAATA CCAATACCTT CGGTAGGTCT   
  
  
- TCTTCTTACT ACTTGAAGAC TCAGAATCGA ACCGGTAACA GCCAGTGAGA CTAGCGTGAC TCTTTTTTGA   
  
  
- CTTCTCCGCC TAACTACAGA GGCTAGGAGA CTAGTGAAGT CCACTTCCGA CGCTTCCCTT CTATTAGTCC   
  
  
- GAGGAGGTTC TCTCTGTCTT GAACAACTTG TAGTTCGTTT CTCCTTTTCC GCAGTAAGTT CTTCCCTTCC   
  
  
- CAGAAGTAGA ATAGGTAGAC GAGGAACATA GACGGTGGAA TTAGTTGCTT TTGTTGTAGT CAAGTCGACA   
  
  
- ACTATTAGAA TAACTCGAGA AGGTTATACA GAGGCTTTCA CCACTAAGTC CTGTTTCCCA ACGACGAATG   
  
  
- AAACGCCTAC CCAATTGTCG TTCCTAGAAT TGAGTCTCCA GAGGTAAAAT AGCGGACTAT GACCCGTTTG   
  
  
- GTCGTGGACG ACTTCTCAAA AGTCGAAAAT GAGTGAATAT ATCTCGGAGA GGTAAGATAG TCAAACGAGT   
  
  
- AAAGTGTCGG TTAGTCCGTT AACATCTTCG TAAACTCTCC CTCCTTCTCT CATTGTTGAC CCGAAACGTA   
  
  
- CATTAACTAA ACCTGTAGAA CGTACCAAAG GTCACCGGAA GAGAATAAGT CAGAGAGAGG CTTCTCCGAT   
  
  
- GAACAAGTTT GGCAAGTAAC AGAGAAGTTT AATGTCCGAA GCCTTCTTCG AATCTTCTCG AGTAACTTTG   
  
  
- TCTTCGAGCC GACCATTCAA AGAGATTCTC GAAAGTCTTA TAGTTGAAAC TTAAGGTGCC CAAAGACTCT   
  
  
- CCGAGCTTTG AGTCCTTGGA TTCCTCCCTC TTACTTTGTC ATCAACATTT AGAGCAAAAG GTGGAGTCAT   
  
  
- GAAACTTTCT ATGGCATGTC TAAAGACTGT GGAACTGAAG ACATGTAAGT GATTTGGGGA GATAACACCA   
  
  
- TAATCAGCTC TCTCTTCCTT CAGCTTTGTC TACACCCAAG GAGAGTTCAA TACATCTACG AGACGTAATA   
  
  
- ATACGACGTT ACAAACTGAG AAATCTACTA ACGGAGGGTG AACTTTCAGG ACTCTCCGAC TCGTATCTCT   
  
  
- TCTTGGTAGA ACCTTTTCTC TAGTTTCTTC GGTATCCAAC ACTCTTCCTA CTTTGTTTGA TAGACTTCAA   
  
  
- ACTCTACGAC CTCTGAACCT CTCCCTCCTA CCTCTCAGTA CCTAAAAGTC CATACTTCGA TTCAAGGTCC   
  
  
- CGTTGATATG TTCGTTTCGA CGAAAATTTT TACCCTTCGG TAATAGTATG GATAAACCTT CTTCTGACAC   
  
  
- CTCCACCACC CAAGTCTCAA ACACTTGCTC TACTACCTTA CCGATAGAGA GAACCGACCG TTCCATCCAA   
  
  
- GGAGCGTTGA CGGAGCCATA CCGCAAGACA CAC

+     Myc

| Site Name | Organism | Position | Strand | Matrix score. | sequence | function |
| --- | --- | --- | --- | --- | --- | --- |
| Myc | Arabidopsis thaliana | 1940 | + | 7 | TCTCTTA |  |
| Myc | Arabidopsis thaliana | 2634 | + | 7 | TCTCTTA |  |

>HU07G00272.1   
+ -Up\_Stream \_Len000ACCAAC GATTCTTTAC TGAATCAGCT ACATTTTTGG ATTGTGTTAA GGTATCTTTT   
  
  
+ AGATCTTCAG GAGGAGGGGG CTGAAGTAGC TTGGTTACTT TTGCGCTTAT CAACTGTGGA AGAATATTAT   
  
  
+ TGAGACTAGT GTAGAAGAAT TAGTAGTTTC CTCCACATTG GAAAAAAACC ATGAAATTAA CGCTTTAGGC   
  
  
+ CCTCAGAGTA CCAAGTGTCA TCGTAAATGA TTGAAATTTC AAGTCTTAGC TGAGTCATGA AGTCCTGATT   
  
  
+ TCCCATGACA AATTGATGTT GTTGTCTAAA GCTTGGCGGG TGATTTGAAA GAAGATTTAG ATCAGGCTGA   
  
  
+ CAATACACCA AATCTGGGAG CAAGGAACTG GATTTTTCTG TGTCTAGAAA AGCTTTCCGC TTTCTGAACA   
  
  
+ ATTGAGAAAA CCATGCATAG GAAATTTCTT GATATTTAGA TCATAGCAGA CTTAACAATC TGGCTTTACC   
  
  
+ GAACTGTCCT CTACTTCTAA GTTGTGAAGG TGTGAAAGAT AAACCATGTC AAACTTAGTG TCCAACAGAA   
  
  
+ ACGCATCTCT ACTTCTCCAG GCAAAGTTGT ACTTCAGATC TTCTACACCT TTTCTAGGCA GGACTTTGGG   
  
  
+ ATATAGAGTT TGGCCTGGGT GAAAAGTAGG AAGTGGGGAA GTAGAAAATG ATGAAAGAAG GCACCACTTG   
  
  
+ GGTCCAGATA CATAGGCCTA CGCACGAGAA CCTTTGCATG GTCTAGATAC ATGACTGTAT ACTTCTTCCT   
  
  
+ TTCCTTTACC TGTTGGAGAT TATCCTAGGA TTTTACATGG CACCAGCTGG ATTTGTTAAT GACATAAACA   
  
  
+ TATCTTTAAT TACAGTTGCT TATGGTATAC ATTTAAACTA TTCGATAGTT GACAGCTGAT GTCCTTATCC   
  
  
+ TTGTGCAGCT TATTCCTCGA TAGAATAATC TTTGGACATT TCTGTCTAGG TGATAAAGGG CAAAAACTAA   
  
  
+ GATACAAGAA ATAGAACATG TCCTCCATTT ACAAGACGTA TCTGTTTTTG GTTTTGGTCA ATGATCCCTG   
  
  
+ TTGTCCTCAA GCATTAACAC AGAATGATCA TTATTGACTT GGACTGAAAG GTTTTGGGAA CTCCATAGTA   
  
  
+ TGCCTTTTCA ATAAAATCAA AAGCAAATTT GCTGTTCACT GATAACTGCA GTTGTGTTGC TTTGCTGTTT   
  
  
+ CCATACCAGA CTCGTAACCA AGCTGATAGC AAATGTGCAA AGGTACAGTA TAATTAACGA TGTTTTCATA   
  
  
+ TTGCTACGAG TTCTGAAACA TGATATAAGA AAACACCAGA ACCCAAATAC CTACATAGGT CCTAGCCCTG   
  
  
+ GAATGGCTAT TTTAAATATC CAAATGGACT TACAGAATTC GCAGAGTTGT TTGGTTTGTT TAACAATCAT   
  
  
+ TGAATTGTTA GCTATGAGAA TCTGCGGGAC AATCGTAATC TTTGGCGATT ACTTCGATTG GCATACTGAT   
  
  
+ GTTTTTTTAT TTGCGTATCT AAGCATGGTG GTGTACCTTT GATTTGTGTG TCTAGGTAGT GTGTGTAGAT   
  
  
+ AAATATCATA GAACTCAGAC GTTGGTAGGG TTATGTTGAG CACTTTCTCT TGCTGTTTGT CCTTTGTGAT   
  
  
+ GGCACCTCTT GCTTTTCCGA ACCACCAAAA GTCTCATTGA CAAAACTCTT CAAACATTGC AAACTGCCCC   
  
  
+ CTTAAATATG TTTTATTAAA AAAAATGGAA GAGCTCAAAC TGTTGAGTGA GCCTAGATTC CAAGATTTAG   
  
  
+ AAACAGTAAA CTTGATAGAT ATATTTATAT GAACTATTGT TAGATGCAAC ACCCCCAACT TGAAACACAA   
  
  
+ AAGAGAAATT AAAAACAAAA AGGTTTTGCA CTATTTATAT ATCCTGTGTT CTTTGTGCTA GTCTTATTTA   
  
  
+ ATGTTAATTG GTAAGCATTA TTTGTTGGAT TATTCCACTG CACTTTCTCT TACTAACTTC AGCATTTCAT   
  
  
+ TCCTTGTTCT TCAGCTTTTG AACTTCTTTA GCCTATTTTG TTGTCTTTAT GGTTATGGAA GCCATCCAGA   
  
  
+ AGAAGAATGA TGAACTTCTG AGTCTTAGCT TGGCCATTGT CGGTCACTCT GATCGCACTG AGAAAAAACT   
  
  
+ GAAGAGGCGG ATTGATGTCT CCGATCCTCT GATCACTTCA GGTGAAGGCT GCGAAGGGAA GATAATCAGG   
  
  
+ CTCCTCCAAG AGAGACAGAA CTTGTTGAAC ATCAAGCAAA GAGGAAAAGG CGTCATTCAA GAAGGGAAGG   
  
  
+ GTCTTCATCT TATCCATCTG CTCCTTGTAT CTGCCACCTT AATCAACGAA AACAACATCA GTTCAGCTGT   
  
  
+ TGATAATCTT ATTGAGCTCT TCCAATATGT CTCCGAAAGT GGTGATTCAG GACAAAGGGT TGCTGCTTAC   
  
  
+ TTTGCGGATG GGTTAACAGC AAGGATCTTA ACTCAGAGGT CTCCATTTTA TCGCCTGATA CTGGGCAAAC   
  
  
+ CAGCACCTGC TGAAGAGTTT TCAGCTTTTA CTCACTTATA TAGAGCCTCT CCATTCTATC AGTTTGCTCA   
  
  
+ TTTCACAGCC AATCAGGCAA TTGTAGAAGC ATTTGAGAGG GAGGAAGAGA GTAACAACTG GGCTTTGCAT   
  
  
+ GTAATTGATT TGGACATCTT GCATGGTTTC CAGTGGCCTT CTCTTATTCA GTCTCTCTCC GAAGAGGCTA   
  
  
+ CTTGTTCAAA CCGTTCATTG TCTCTTCAAA TTACAGGCTT CGGAAGAAGC TTAGAAGAGC TCATTGAAAC   
  
  
+ AGAAGCTCGG CTGGTAAGTT TCTCTAAGAG CTTTCAGAAT ATCAACTTTG AATTCCACGG GTTTCTGAGA   
  
  
+ GGCTCGAAAC TCAGGAACCT AAGGAGGGAG AATGAAACAG TAGTTGTAAA TCTCGTTTTC CACCTCAGTA   
  
  
+ CTTTGAAAGA TACCGTACAG ATTTCTGACA CCTTGACTTC TGTACATTCA CTAAACCCCT CTATTGTGGT   
  
  
+ ATTAGTCGAG AGAGAAGGAA GTCGAAACAG ATGTGGGTTC CTCTCAAGTT ATGTAGATGC TCTGCATTAT   
  
  
+ TATGCTGCAA TGTTTGACTC TTTAGATGAT TGCCTCCCAC TTGAAAGTCC TGAGAGGCTG AGCATAGAGA   
  
  
+ AGAACCATCT TGGAAAAGAG ATCAAAGAAG CCATAGGTTG TGAGAAGGAT GAAACAAACT ATCTGAAGTT   
  
  
+ TGAGATGCTG GAGACTTGGA GAGGGAGGAT GGAGAGTCAT GGATTTTCAG GTATGAAGCT AAGTTCCAGG   
  
  
+ GCAACTATAC AAGCAAAGCT GCTTTTAAAA ATGGGAAGCC ATTATCATAC CTATTTGGAA GAAGACTGTG   
  
  
+ GAGGTGGTGG GTTCAGAGTT TGTGAACGAG ATGATGGAAT GGCTATCTCT CTTGGCTGGC AAGGTAGGTT   
  
  
+ CCTCGCAACT GCCTCGGTAT GGCGTTCTGT GTG  

- -Up\_Stream \_Len000TGGTTG CTAAGAAATG ACTTAGTCGA TGTAAAAACC TAACACAATT CCATAGAAAA   
  
  
- TCTAGAAGTC CTCCTCCCCC GACTTCATCG AACCAATGAA AACGCGAATA GTTGACACCT TCTTATAATA   
  
  
- ACTCTGATCA CATCTTCTTA ATCATCAAAG GAGGTGTAAC CTTTTTTTGG TACTTTAATT GCGAAATCCG   
  
  
- GGAGTCTCAT GGTTCACAGT AGCATTTACT AACTTTAAAG TTCAGAATCG ACTCAGTACT TCAGGACTAA   
  
  
- AGGGTACTGT TTAACTACAA CAACAGATTT CGAACCGCCC ACTAAACTTT CTTCTAAATC TAGTCCGACT   
  
  
- GTTATGTGGT TTAGACCCTC GTTCCTTGAC CTAAAAAGAC ACAGATCTTT TCGAAAGGCG AAAGACTTGT   
  
  
- TAACTCTTTT GGTACGTATC CTTTAAAGAA CTATAAATCT AGTATCGTCT GAATTGTTAG ACCGAAATGG   
  
  
- CTTGACAGGA GATGAAGATT CAACACTTCC ACACTTTCTA TTTGGTACAG TTTGAATCAC AGGTTGTCTT   
  
  
- TGCGTAGAGA TGAAGAGGTC CGTTTCAACA TGAAGTCTAG AAGATGTGGA AAAGATCCGT CCTGAAACCC   
  
  
- TATATCTCAA ACCGGACCCA CTTTTCATCC TTCACCCCTT CATCTTTTAC TACTTTCTTC CGTGGTGAAC   
  
  
- CCAGGTCTAT GTATCCGGAT GCGTGCTCTT GGAAACGTAC CAGATCTATG TACTGACATA TGAAGAAGGA   
  
  
- AAGGAAATGG ACAACCTCTA ATAGGATCCT AAAATGTACC GTGGTCGACC TAAACAATTA CTGTATTTGT   
  
  
- ATAGAAATTA ATGTCAACGA ATACCATATG TAAATTTGAT AAGCTATCAA CTGTCGACTA CAGGAATAGG   
  
  
- AACACGTCGA ATAAGGAGCT ATCTTATTAG AAACCTGTAA AGACAGATCC ACTATTTCCC GTTTTTGATT   
  
  
- CTATGTTCTT TATCTTGTAC AGGAGGTAAA TGTTCTGCAT AGACAAAAAC CAAAACCAGT TACTAGGGAC   
  
  
- AACAGGAGTT CGTAATTGTG TCTTACTAGT AATAACTGAA CCTGACTTTC CAAAACCCTT GAGGTATCAT   
  
  
- ACGGAAAAGT TATTTTAGTT TTCGTTTAAA CGACAAGTGA CTATTGACGT CAACACAACG AAACGACAAA   
  
  
- GGTATGGTCT GAGCATTGGT TCGACTATCG TTTACACGTT TCCATGTCAT ATTAATTGCT ACAAAAGTAT   
  
  
- AACGATGCTC AAGACTTTGT ACTATATTCT TTTGTGGTCT TGGGTTTATG GATGTATCCA GGATCGGGAC   
  
  
- CTTACCGATA AAATTTATAG GTTTACCTGA ATGTCTTAAG CGTCTCAACA AACCAAACAA ATTGTTAGTA   
  
  
- ACTTAACAAT CGATACTCTT AGACGCCCTG TTAGCATTAG AAACCGCTAA TGAAGCTAAC CGTATGACTA   
  
  
- CAAAAAAATA AACGCATAGA TTCGTACCAC CACATGGAAA CTAAACACAC AGATCCATCA CACACATCTA   
  
  
- TTTATAGTAT CTTGAGTCTG CAACCATCCC AATACAACTC GTGAAAGAGA ACGACAAACA GGAAACACTA   
  
  
- CCGTGGAGAA CGAAAAGGCT TGGTGGTTTT CAGAGTAACT GTTTTGAGAA GTTTGTAACG TTTGACGGGG   
  
  
- GAATTTATAC AAAATAATTT TTTTTACCTT CTCGAGTTTG ACAACTCACT CGGATCTAAG GTTCTAAATC   
  
  
- TTTGTCATTT GAACTATCTA TATAAATATA CTTGATAACA ATCTACGTTG TGGGGGTTGA ACTTTGTGTT   
  
  
- TTCTCTTTAA TTTTTGTTTT TCCAAAACGT GATAAATATA TAGGACACAA GAAACACGAT CAGAATAAAT   
  
  
- TACAATTAAC CATTCGTAAT AAACAACCTA ATAAGGTGAC GTGAAAGAGA ATGATTGAAG TCGTAAAGTA   
  
  
- AGGAACAAGA AGTCGAAAAC TTGAAGAAAT CGGATAAAAC AACAGAAATA CCAATACCTT CGGTAGGTCT   
  
  
- TCTTCTTACT ACTTGAAGAC TCAGAATCGA ACCGGTAACA GCCAGTGAGA CTAGCGTGAC TCTTTTTTGA   
  
  
- CTTCTCCGCC TAACTACAGA GGCTAGGAGA CTAGTGAAGT CCACTTCCGA CGCTTCCCTT CTATTAGTCC   
  
  
- GAGGAGGTTC TCTCTGTCTT GAACAACTTG TAGTTCGTTT CTCCTTTTCC GCAGTAAGTT CTTCCCTTCC   
  
  
- CAGAAGTAGA ATAGGTAGAC GAGGAACATA GACGGTGGAA TTAGTTGCTT TTGTTGTAGT CAAGTCGACA   
  
  
- ACTATTAGAA TAACTCGAGA AGGTTATACA GAGGCTTTCA CCACTAAGTC CTGTTTCCCA ACGACGAATG   
  
  
- AAACGCCTAC CCAATTGTCG TTCCTAGAAT TGAGTCTCCA GAGGTAAAAT AGCGGACTAT GACCCGTTTG   
  
  
- GTCGTGGACG ACTTCTCAAA AGTCGAAAAT GAGTGAATAT ATCTCGGAGA GGTAAGATAG TCAAACGAGT   
  
  
- AAAGTGTCGG TTAGTCCGTT AACATCTTCG TAAACTCTCC CTCCTTCTCT CATTGTTGAC CCGAAACGTA   
  
  
- CATTAACTAA ACCTGTAGAA CGTACCAAAG GTCACCGGAA GAGAATAAGT CAGAGAGAGG CTTCTCCGAT   
  
  
- GAACAAGTTT GGCAAGTAAC AGAGAAGTTT AATGTCCGAA GCCTTCTTCG AATCTTCTCG AGTAACTTTG   
  
  
- TCTTCGAGCC GACCATTCAA AGAGATTCTC GAAAGTCTTA TAGTTGAAAC TTAAGGTGCC CAAAGACTCT   
  
  
- CCGAGCTTTG AGTCCTTGGA TTCCTCCCTC TTACTTTGTC ATCAACATTT AGAGCAAAAG GTGGAGTCAT   
  
  
- GAAACTTTCT ATGGCATGTC TAAAGACTGT GGAACTGAAG ACATGTAAGT GATTTGGGGA GATAACACCA   
  
  
- TAATCAGCTC TCTCTTCCTT CAGCTTTGTC TACACCCAAG GAGAGTTCAA TACATCTACG AGACGTAATA   
  
  
- ATACGACGTT ACAAACTGAG AAATCTACTA ACGGAGGGTG AACTTTCAGG ACTCTCCGAC TCGTATCTCT   
  
  
- TCTTGGTAGA ACCTTTTCTC TAGTTTCTTC GGTATCCAAC ACTCTTCCTA CTTTGTTTGA TAGACTTCAA   
  
  
- ACTCTACGAC CTCTGAACCT CTCCCTCCTA CCTCTCAGTA CCTAAAAGTC CATACTTCGA TTCAAGGTCC   
  
  
- CGTTGATATG TTCGTTTCGA CGAAAATTTT TACCCTTCGG TAATAGTATG GATAAACCTT CTTCTGACAC   
  
  
- CTCCACCACC CAAGTCTCAA ACACTTGCTC TACTACCTTA CCGATAGAGA GAACCGACCG TTCCATCCAA   
  
  
- GGAGCGTTGA CGGAGCCATA CCGCAAGACA CAC

+     STRE

| Site Name | Organism | Position | Strand | Matrix score. | sequence | function |
| --- | --- | --- | --- | --- | --- | --- |
| STRE | Arabidopsis thaliana | 89 | + | 5 | AGGGG |  |
| STRE | Arabidopsis thaliana | 1682 | - | 5 | AGGGG |  |
| STRE | Arabidopsis thaliana | 2930 | - | 5 | AGGGG |  |

>HU07G00272.1   
+ -Up\_Stream \_Len000ACCAAC GATTCTTTAC TGAATCAGCT ACATTTTTGG ATTGTGTTAA GGTATCTTTT   
  
  
+ AGATCTTCAG GAGGAGGGGG CTGAAGTAGC TTGGTTACTT TTGCGCTTAT CAACTGTGGA AGAATATTAT   
  
  
+ TGAGACTAGT GTAGAAGAAT TAGTAGTTTC CTCCACATTG GAAAAAAACC ATGAAATTAA CGCTTTAGGC   
  
  
+ CCTCAGAGTA CCAAGTGTCA TCGTAAATGA TTGAAATTTC AAGTCTTAGC TGAGTCATGA AGTCCTGATT   
  
  
+ TCCCATGACA AATTGATGTT GTTGTCTAAA GCTTGGCGGG TGATTTGAAA GAAGATTTAG ATCAGGCTGA   
  
  
+ CAATACACCA AATCTGGGAG CAAGGAACTG GATTTTTCTG TGTCTAGAAA AGCTTTCCGC TTTCTGAACA   
  
  
+ ATTGAGAAAA CCATGCATAG GAAATTTCTT GATATTTAGA TCATAGCAGA CTTAACAATC TGGCTTTACC   
  
  
+ GAACTGTCCT CTACTTCTAA GTTGTGAAGG TGTGAAAGAT AAACCATGTC AAACTTAGTG TCCAACAGAA   
  
  
+ ACGCATCTCT ACTTCTCCAG GCAAAGTTGT ACTTCAGATC TTCTACACCT TTTCTAGGCA GGACTTTGGG   
  
  
+ ATATAGAGTT TGGCCTGGGT GAAAAGTAGG AAGTGGGGAA GTAGAAAATG ATGAAAGAAG GCACCACTTG   
  
  
+ GGTCCAGATA CATAGGCCTA CGCACGAGAA CCTTTGCATG GTCTAGATAC ATGACTGTAT ACTTCTTCCT   
  
  
+ TTCCTTTACC TGTTGGAGAT TATCCTAGGA TTTTACATGG CACCAGCTGG ATTTGTTAAT GACATAAACA   
  
  
+ TATCTTTAAT TACAGTTGCT TATGGTATAC ATTTAAACTA TTCGATAGTT GACAGCTGAT GTCCTTATCC   
  
  
+ TTGTGCAGCT TATTCCTCGA TAGAATAATC TTTGGACATT TCTGTCTAGG TGATAAAGGG CAAAAACTAA   
  
  
+ GATACAAGAA ATAGAACATG TCCTCCATTT ACAAGACGTA TCTGTTTTTG GTTTTGGTCA ATGATCCCTG   
  
  
+ TTGTCCTCAA GCATTAACAC AGAATGATCA TTATTGACTT GGACTGAAAG GTTTTGGGAA CTCCATAGTA   
  
  
+ TGCCTTTTCA ATAAAATCAA AAGCAAATTT GCTGTTCACT GATAACTGCA GTTGTGTTGC TTTGCTGTTT   
  
  
+ CCATACCAGA CTCGTAACCA AGCTGATAGC AAATGTGCAA AGGTACAGTA TAATTAACGA TGTTTTCATA   
  
  
+ TTGCTACGAG TTCTGAAACA TGATATAAGA AAACACCAGA ACCCAAATAC CTACATAGGT CCTAGCCCTG   
  
  
+ GAATGGCTAT TTTAAATATC CAAATGGACT TACAGAATTC GCAGAGTTGT TTGGTTTGTT TAACAATCAT   
  
  
+ TGAATTGTTA GCTATGAGAA TCTGCGGGAC AATCGTAATC TTTGGCGATT ACTTCGATTG GCATACTGAT   
  
  
+ GTTTTTTTAT TTGCGTATCT AAGCATGGTG GTGTACCTTT GATTTGTGTG TCTAGGTAGT GTGTGTAGAT   
  
  
+ AAATATCATA GAACTCAGAC GTTGGTAGGG TTATGTTGAG CACTTTCTCT TGCTGTTTGT CCTTTGTGAT   
  
  
+ GGCACCTCTT GCTTTTCCGA ACCACCAAAA GTCTCATTGA CAAAACTCTT CAAACATTGC AAACTGCCCC   
  
  
+ CTTAAATATG TTTTATTAAA AAAAATGGAA GAGCTCAAAC TGTTGAGTGA GCCTAGATTC CAAGATTTAG   
  
  
+ AAACAGTAAA CTTGATAGAT ATATTTATAT GAACTATTGT TAGATGCAAC ACCCCCAACT TGAAACACAA   
  
  
+ AAGAGAAATT AAAAACAAAA AGGTTTTGCA CTATTTATAT ATCCTGTGTT CTTTGTGCTA GTCTTATTTA   
  
  
+ ATGTTAATTG GTAAGCATTA TTTGTTGGAT TATTCCACTG CACTTTCTCT TACTAACTTC AGCATTTCAT   
  
  
+ TCCTTGTTCT TCAGCTTTTG AACTTCTTTA GCCTATTTTG TTGTCTTTAT GGTTATGGAA GCCATCCAGA   
  
  
+ AGAAGAATGA TGAACTTCTG AGTCTTAGCT TGGCCATTGT CGGTCACTCT GATCGCACTG AGAAAAAACT   
  
  
+ GAAGAGGCGG ATTGATGTCT CCGATCCTCT GATCACTTCA GGTGAAGGCT GCGAAGGGAA GATAATCAGG   
  
  
+ CTCCTCCAAG AGAGACAGAA CTTGTTGAAC ATCAAGCAAA GAGGAAAAGG CGTCATTCAA GAAGGGAAGG   
  
  
+ GTCTTCATCT TATCCATCTG CTCCTTGTAT CTGCCACCTT AATCAACGAA AACAACATCA GTTCAGCTGT   
  
  
+ TGATAATCTT ATTGAGCTCT TCCAATATGT CTCCGAAAGT GGTGATTCAG GACAAAGGGT TGCTGCTTAC   
  
  
+ TTTGCGGATG GGTTAACAGC AAGGATCTTA ACTCAGAGGT CTCCATTTTA TCGCCTGATA CTGGGCAAAC   
  
  
+ CAGCACCTGC TGAAGAGTTT TCAGCTTTTA CTCACTTATA TAGAGCCTCT CCATTCTATC AGTTTGCTCA   
  
  
+ TTTCACAGCC AATCAGGCAA TTGTAGAAGC ATTTGAGAGG GAGGAAGAGA GTAACAACTG GGCTTTGCAT   
  
  
+ GTAATTGATT TGGACATCTT GCATGGTTTC CAGTGGCCTT CTCTTATTCA GTCTCTCTCC GAAGAGGCTA   
  
  
+ CTTGTTCAAA CCGTTCATTG TCTCTTCAAA TTACAGGCTT CGGAAGAAGC TTAGAAGAGC TCATTGAAAC   
  
  
+ AGAAGCTCGG CTGGTAAGTT TCTCTAAGAG CTTTCAGAAT ATCAACTTTG AATTCCACGG GTTTCTGAGA   
  
  
+ GGCTCGAAAC TCAGGAACCT AAGGAGGGAG AATGAAACAG TAGTTGTAAA TCTCGTTTTC CACCTCAGTA   
  
  
+ CTTTGAAAGA TACCGTACAG ATTTCTGACA CCTTGACTTC TGTACATTCA CTAAACCCCT CTATTGTGGT   
  
  
+ ATTAGTCGAG AGAGAAGGAA GTCGAAACAG ATGTGGGTTC CTCTCAAGTT ATGTAGATGC TCTGCATTAT   
  
  
+ TATGCTGCAA TGTTTGACTC TTTAGATGAT TGCCTCCCAC TTGAAAGTCC TGAGAGGCTG AGCATAGAGA   
  
  
+ AGAACCATCT TGGAAAAGAG ATCAAAGAAG CCATAGGTTG TGAGAAGGAT GAAACAAACT ATCTGAAGTT   
  
  
+ TGAGATGCTG GAGACTTGGA GAGGGAGGAT GGAGAGTCAT GGATTTTCAG GTATGAAGCT AAGTTCCAGG   
  
  
+ GCAACTATAC AAGCAAAGCT GCTTTTAAAA ATGGGAAGCC ATTATCATAC CTATTTGGAA GAAGACTGTG   
  
  
+ GAGGTGGTGG GTTCAGAGTT TGTGAACGAG ATGATGGAAT GGCTATCTCT CTTGGCTGGC AAGGTAGGTT   
  
  
+ CCTCGCAACT GCCTCGGTAT GGCGTTCTGT GTG  

- -Up\_Stream \_Len000TGGTTG CTAAGAAATG ACTTAGTCGA TGTAAAAACC TAACACAATT CCATAGAAAA   
  
  
- TCTAGAAGTC CTCCTCCCCC GACTTCATCG AACCAATGAA AACGCGAATA GTTGACACCT TCTTATAATA   
  
  
- ACTCTGATCA CATCTTCTTA ATCATCAAAG GAGGTGTAAC CTTTTTTTGG TACTTTAATT GCGAAATCCG   
  
  
- GGAGTCTCAT GGTTCACAGT AGCATTTACT AACTTTAAAG TTCAGAATCG ACTCAGTACT TCAGGACTAA   
  
  
- AGGGTACTGT TTAACTACAA CAACAGATTT CGAACCGCCC ACTAAACTTT CTTCTAAATC TAGTCCGACT   
  
  
- GTTATGTGGT TTAGACCCTC GTTCCTTGAC CTAAAAAGAC ACAGATCTTT TCGAAAGGCG AAAGACTTGT   
  
  
- TAACTCTTTT GGTACGTATC CTTTAAAGAA CTATAAATCT AGTATCGTCT GAATTGTTAG ACCGAAATGG   
  
  
- CTTGACAGGA GATGAAGATT CAACACTTCC ACACTTTCTA TTTGGTACAG TTTGAATCAC AGGTTGTCTT   
  
  
- TGCGTAGAGA TGAAGAGGTC CGTTTCAACA TGAAGTCTAG AAGATGTGGA AAAGATCCGT CCTGAAACCC   
  
  
- TATATCTCAA ACCGGACCCA CTTTTCATCC TTCACCCCTT CATCTTTTAC TACTTTCTTC CGTGGTGAAC   
  
  
- CCAGGTCTAT GTATCCGGAT GCGTGCTCTT GGAAACGTAC CAGATCTATG TACTGACATA TGAAGAAGGA   
  
  
- AAGGAAATGG ACAACCTCTA ATAGGATCCT AAAATGTACC GTGGTCGACC TAAACAATTA CTGTATTTGT   
  
  
- ATAGAAATTA ATGTCAACGA ATACCATATG TAAATTTGAT AAGCTATCAA CTGTCGACTA CAGGAATAGG   
  
  
- AACACGTCGA ATAAGGAGCT ATCTTATTAG AAACCTGTAA AGACAGATCC ACTATTTCCC GTTTTTGATT   
  
  
- CTATGTTCTT TATCTTGTAC AGGAGGTAAA TGTTCTGCAT AGACAAAAAC CAAAACCAGT TACTAGGGAC   
  
  
- AACAGGAGTT CGTAATTGTG TCTTACTAGT AATAACTGAA CCTGACTTTC CAAAACCCTT GAGGTATCAT   
  
  
- ACGGAAAAGT TATTTTAGTT TTCGTTTAAA CGACAAGTGA CTATTGACGT CAACACAACG AAACGACAAA   
  
  
- GGTATGGTCT GAGCATTGGT TCGACTATCG TTTACACGTT TCCATGTCAT ATTAATTGCT ACAAAAGTAT   
  
  
- AACGATGCTC AAGACTTTGT ACTATATTCT TTTGTGGTCT TGGGTTTATG GATGTATCCA GGATCGGGAC   
  
  
- CTTACCGATA AAATTTATAG GTTTACCTGA ATGTCTTAAG CGTCTCAACA AACCAAACAA ATTGTTAGTA   
  
  
- ACTTAACAAT CGATACTCTT AGACGCCCTG TTAGCATTAG AAACCGCTAA TGAAGCTAAC CGTATGACTA   
  
  
- CAAAAAAATA AACGCATAGA TTCGTACCAC CACATGGAAA CTAAACACAC AGATCCATCA CACACATCTA   
  
  
- TTTATAGTAT CTTGAGTCTG CAACCATCCC AATACAACTC GTGAAAGAGA ACGACAAACA GGAAACACTA   
  
  
- CCGTGGAGAA CGAAAAGGCT TGGTGGTTTT CAGAGTAACT GTTTTGAGAA GTTTGTAACG TTTGACGGGG   
  
  
- GAATTTATAC AAAATAATTT TTTTTACCTT CTCGAGTTTG ACAACTCACT CGGATCTAAG GTTCTAAATC   
  
  
- TTTGTCATTT GAACTATCTA TATAAATATA CTTGATAACA ATCTACGTTG TGGGGGTTGA ACTTTGTGTT   
  
  
- TTCTCTTTAA TTTTTGTTTT TCCAAAACGT GATAAATATA TAGGACACAA GAAACACGAT CAGAATAAAT   
  
  
- TACAATTAAC CATTCGTAAT AAACAACCTA ATAAGGTGAC GTGAAAGAGA ATGATTGAAG TCGTAAAGTA   
  
  
- AGGAACAAGA AGTCGAAAAC TTGAAGAAAT CGGATAAAAC AACAGAAATA CCAATACCTT CGGTAGGTCT   
  
  
- TCTTCTTACT ACTTGAAGAC TCAGAATCGA ACCGGTAACA GCCAGTGAGA CTAGCGTGAC TCTTTTTTGA   
  
  
- CTTCTCCGCC TAACTACAGA GGCTAGGAGA CTAGTGAAGT CCACTTCCGA CGCTTCCCTT CTATTAGTCC   
  
  
- GAGGAGGTTC TCTCTGTCTT GAACAACTTG TAGTTCGTTT CTCCTTTTCC GCAGTAAGTT CTTCCCTTCC   
  
  
- CAGAAGTAGA ATAGGTAGAC GAGGAACATA GACGGTGGAA TTAGTTGCTT TTGTTGTAGT CAAGTCGACA   
  
  
- ACTATTAGAA TAACTCGAGA AGGTTATACA GAGGCTTTCA CCACTAAGTC CTGTTTCCCA ACGACGAATG   
  
  
- AAACGCCTAC CCAATTGTCG TTCCTAGAAT TGAGTCTCCA GAGGTAAAAT AGCGGACTAT GACCCGTTTG   
  
  
- GTCGTGGACG ACTTCTCAAA AGTCGAAAAT GAGTGAATAT ATCTCGGAGA GGTAAGATAG TCAAACGAGT   
  
  
- AAAGTGTCGG TTAGTCCGTT AACATCTTCG TAAACTCTCC CTCCTTCTCT CATTGTTGAC CCGAAACGTA   
  
  
- CATTAACTAA ACCTGTAGAA CGTACCAAAG GTCACCGGAA GAGAATAAGT CAGAGAGAGG CTTCTCCGAT   
  
  
- GAACAAGTTT GGCAAGTAAC AGAGAAGTTT AATGTCCGAA GCCTTCTTCG AATCTTCTCG AGTAACTTTG   
  
  
- TCTTCGAGCC GACCATTCAA AGAGATTCTC GAAAGTCTTA TAGTTGAAAC TTAAGGTGCC CAAAGACTCT   
  
  
- CCGAGCTTTG AGTCCTTGGA TTCCTCCCTC TTACTTTGTC ATCAACATTT AGAGCAAAAG GTGGAGTCAT   
  
  
- GAAACTTTCT ATGGCATGTC TAAAGACTGT GGAACTGAAG ACATGTAAGT GATTTGGGGA GATAACACCA   
  
  
- TAATCAGCTC TCTCTTCCTT CAGCTTTGTC TACACCCAAG GAGAGTTCAA TACATCTACG AGACGTAATA   
  
  
- ATACGACGTT ACAAACTGAG AAATCTACTA ACGGAGGGTG AACTTTCAGG ACTCTCCGAC TCGTATCTCT   
  
  
- TCTTGGTAGA ACCTTTTCTC TAGTTTCTTC GGTATCCAAC ACTCTTCCTA CTTTGTTTGA TAGACTTCAA   
  
  
- ACTCTACGAC CTCTGAACCT CTCCCTCCTA CCTCTCAGTA CCTAAAAGTC CATACTTCGA TTCAAGGTCC   
  
  
- CGTTGATATG TTCGTTTCGA CGAAAATTTT TACCCTTCGG TAATAGTATG GATAAACCTT CTTCTGACAC   
  
  
- CTCCACCACC CAAGTCTCAA ACACTTGCTC TACTACCTTA CCGATAGAGA GAACCGACCG TTCCATCCAA   
  
  
- GGAGCGTTGA CGGAGCCATA CCGCAAGACA CAC

+     TATA-box

| Site Name | Organism | Position | Strand | Matrix score. | sequence | function |
| --- | --- | --- | --- | --- | --- | --- |
| TATA-box | Arabidopsis thaliana | 1862 | - | 4 | TATA | core promoter element around -30 of transcription start |
| TATA-box | Arabidopsis thaliana | 2491 | - | 6 | TATATA | core promoter element around -30 of transcription start |
| TATA-box | Arabidopsis thaliana | 1779 | - | 5 | TATAA | core promoter element around -30 of transcription start |
| TATA-box | Arabidopsis thaliana | 1288 | + | 4 | TATA | core promoter element around -30 of transcription start |
| TATA-box | Arabidopsis thaliana | 1860 | - | 6 | TATATA | core promoter element around -30 of transcription start |
| TATA-box | Arabidopsis thaliana | 2490 | - | 7 | TATATAA | core promoter element around -30 of transcription start |
| TATA-box | Arabidopsis thaliana | 1859 | - | 7 | TATATAA | core promoter element around -30 of transcription start |
| TATA-box | Arabidopsis thaliana | 3230 | - | 4 | TATA | core promoter element around -30 of transcription start |
| TATA-box | Arabidopsis thaliana | 2493 | - | 4 | TATA | core promoter element around -30 of transcription start |
| TATA-box | Brassica juncea | 1857 | - | 7 | TATAAAT | core promoter element around -30 of transcription start |
| TATA-box | Arabidopsis thaliana | 1780 | - | 4 | TATA | core promoter element around -30 of transcription start |
| TATA-box | Arabidopsis thaliana | 1345 | - | 8 | TATTTAAA | core promoter element around -30 of transcription start |
| TATA-box | Brassica oleracea | 1287 | + | 6 | ATATAA | core promoter element around -30 of transcription start |
| TATA-box | Brassica napus | 1861 | - | 6 | ATATAT | core promoter element around -30 of transcription start |
| TATA-box | Arabidopsis thaliana | 1856 | - | 9 | taTATAAAtc | core promoter element around -30 of transcription start |
| TATA-box | Arabidopsis thaliana | 870 | + | 4 | TATA | core promoter element around -30 of transcription start |
| TATA-box | Arabidopsis thaliana | 762 | + | 4 | TATA | core promoter element around -30 of transcription start |
| TATA-box | Daucus carota | 1776 | - | 8 | TATAAATA | core promoter element around -30 of transcription start |
| TATA-box | Arabidopsis thaliana | 1774 | - | 4 | TATA | core promoter element around -30 of transcription start |
| TATA-box | Helianthus annuus | 760 | - | 6 | TATACA | core promoter element around -30 of transcription start |
| TATA-box | Helianthus annuus | 1858 | - | 6 | TATAAA | core promoter element around -30 of transcription start |
| TATA-box | Arabidopsis thaliana | 1243 | + | 4 | TATA | core promoter element around -30 of transcription start |
| TATA-box | Helianthus annuus | 1778 | - | 6 | TATAAA | core promoter element around -30 of transcription start |
| TATA-box | Brassica napus | 1773 | - | 6 | ATATAT | core promoter element around -30 of transcription start |
| TATA-box | Arabidopsis thaliana | 636 | + | 4 | TATA | core promoter element around -30 of transcription start |
| TATA-box | Brassica juncea | 1777 | - | 7 | TATAAAT | core promoter element around -30 of transcription start |

>HU07G00272.1   
+ -Up\_Stream \_Len000ACCAAC GATTCTTTAC TGAATCAGCT ACATTTTTGG ATTGTGTTAA GGTATCTTTT   
  
  
+ AGATCTTCAG GAGGAGGGGG CTGAAGTAGC TTGGTTACTT TTGCGCTTAT CAACTGTGGA AGAATATTAT   
  
  
+ TGAGACTAGT GTAGAAGAAT TAGTAGTTTC CTCCACATTG GAAAAAAACC ATGAAATTAA CGCTTTAGGC   
  
  
+ CCTCAGAGTA CCAAGTGTCA TCGTAAATGA TTGAAATTTC AAGTCTTAGC TGAGTCATGA AGTCCTGATT   
  
  
+ TCCCATGACA AATTGATGTT GTTGTCTAAA GCTTGGCGGG TGATTTGAAA GAAGATTTAG ATCAGGCTGA   
  
  
+ CAATACACCA AATCTGGGAG CAAGGAACTG GATTTTTCTG TGTCTAGAAA AGCTTTCCGC TTTCTGAACA   
  
  
+ ATTGAGAAAA CCATGCATAG GAAATTTCTT GATATTTAGA TCATAGCAGA CTTAACAATC TGGCTTTACC   
  
  
+ GAACTGTCCT CTACTTCTAA GTTGTGAAGG TGTGAAAGAT AAACCATGTC AAACTTAGTG TCCAACAGAA   
  
  
+ ACGCATCTCT ACTTCTCCAG GCAAAGTTGT ACTTCAGATC TTCTACACCT TTTCTAGGCA GGACTTTGGG   
  
  
+ ATATAGAGTT TGGCCTGGGT GAAAAGTAGG AAGTGGGGAA GTAGAAAATG ATGAAAGAAG GCACCACTTG   
  
  
+ GGTCCAGATA CATAGGCCTA CGCACGAGAA CCTTTGCATG GTCTAGATAC ATGACTGTAT ACTTCTTCCT   
  
  
+ TTCCTTTACC TGTTGGAGAT TATCCTAGGA TTTTACATGG CACCAGCTGG ATTTGTTAAT GACATAAACA   
  
  
+ TATCTTTAAT TACAGTTGCT TATGGTATAC ATTTAAACTA TTCGATAGTT GACAGCTGAT GTCCTTATCC   
  
  
+ TTGTGCAGCT TATTCCTCGA TAGAATAATC TTTGGACATT TCTGTCTAGG TGATAAAGGG CAAAAACTAA   
  
  
+ GATACAAGAA ATAGAACATG TCCTCCATTT ACAAGACGTA TCTGTTTTTG GTTTTGGTCA ATGATCCCTG   
  
  
+ TTGTCCTCAA GCATTAACAC AGAATGATCA TTATTGACTT GGACTGAAAG GTTTTGGGAA CTCCATAGTA   
  
  
+ TGCCTTTTCA ATAAAATCAA AAGCAAATTT GCTGTTCACT GATAACTGCA GTTGTGTTGC TTTGCTGTTT   
  
  
+ CCATACCAGA CTCGTAACCA AGCTGATAGC AAATGTGCAA AGGTACAGTA TAATTAACGA TGTTTTCATA   
  
  
+ TTGCTACGAG TTCTGAAACA TGATATAAGA AAACACCAGA ACCCAAATAC CTACATAGGT CCTAGCCCTG   
  
  
+ GAATGGCTAT TTTAAATATC CAAATGGACT TACAGAATTC GCAGAGTTGT TTGGTTTGTT TAACAATCAT   
  
  
+ TGAATTGTTA GCTATGAGAA TCTGCGGGAC AATCGTAATC TTTGGCGATT ACTTCGATTG GCATACTGAT   
  
  
+ GTTTTTTTAT TTGCGTATCT AAGCATGGTG GTGTACCTTT GATTTGTGTG TCTAGGTAGT GTGTGTAGAT   
  
  
+ AAATATCATA GAACTCAGAC GTTGGTAGGG TTATGTTGAG CACTTTCTCT TGCTGTTTGT CCTTTGTGAT   
  
  
+ GGCACCTCTT GCTTTTCCGA ACCACCAAAA GTCTCATTGA CAAAACTCTT CAAACATTGC AAACTGCCCC   
  
  
+ CTTAAATATG TTTTATTAAA AAAAATGGAA GAGCTCAAAC TGTTGAGTGA GCCTAGATTC CAAGATTTAG   
  
  
+ AAACAGTAAA CTTGATAGAT ATATTTATAT GAACTATTGT TAGATGCAAC ACCCCCAACT TGAAACACAA   
  
  
+ AAGAGAAATT AAAAACAAAA AGGTTTTGCA CTATTTATAT ATCCTGTGTT CTTTGTGCTA GTCTTATTTA   
  
  
+ ATGTTAATTG GTAAGCATTA TTTGTTGGAT TATTCCACTG CACTTTCTCT TACTAACTTC AGCATTTCAT   
  
  
+ TCCTTGTTCT TCAGCTTTTG AACTTCTTTA GCCTATTTTG TTGTCTTTAT GGTTATGGAA GCCATCCAGA   
  
  
+ AGAAGAATGA TGAACTTCTG AGTCTTAGCT TGGCCATTGT CGGTCACTCT GATCGCACTG AGAAAAAACT   
  
  
+ GAAGAGGCGG ATTGATGTCT CCGATCCTCT GATCACTTCA GGTGAAGGCT GCGAAGGGAA GATAATCAGG   
  
  
+ CTCCTCCAAG AGAGACAGAA CTTGTTGAAC ATCAAGCAAA GAGGAAAAGG CGTCATTCAA GAAGGGAAGG   
  
  
+ GTCTTCATCT TATCCATCTG CTCCTTGTAT CTGCCACCTT AATCAACGAA AACAACATCA GTTCAGCTGT   
  
  
+ TGATAATCTT ATTGAGCTCT TCCAATATGT CTCCGAAAGT GGTGATTCAG GACAAAGGGT TGCTGCTTAC   
  
  
+ TTTGCGGATG GGTTAACAGC AAGGATCTTA ACTCAGAGGT CTCCATTTTA TCGCCTGATA CTGGGCAAAC   
  
  
+ CAGCACCTGC TGAAGAGTTT TCAGCTTTTA CTCACTTATA TAGAGCCTCT CCATTCTATC AGTTTGCTCA   
  
  
+ TTTCACAGCC AATCAGGCAA TTGTAGAAGC ATTTGAGAGG GAGGAAGAGA GTAACAACTG GGCTTTGCAT   
  
  
+ GTAATTGATT TGGACATCTT GCATGGTTTC CAGTGGCCTT CTCTTATTCA GTCTCTCTCC GAAGAGGCTA   
  
  
+ CTTGTTCAAA CCGTTCATTG TCTCTTCAAA TTACAGGCTT CGGAAGAAGC TTAGAAGAGC TCATTGAAAC   
  
  
+ AGAAGCTCGG CTGGTAAGTT TCTCTAAGAG CTTTCAGAAT ATCAACTTTG AATTCCACGG GTTTCTGAGA   
  
  
+ GGCTCGAAAC TCAGGAACCT AAGGAGGGAG AATGAAACAG TAGTTGTAAA TCTCGTTTTC CACCTCAGTA   
  
  
+ CTTTGAAAGA TACCGTACAG ATTTCTGACA CCTTGACTTC TGTACATTCA CTAAACCCCT CTATTGTGGT   
  
  
+ ATTAGTCGAG AGAGAAGGAA GTCGAAACAG ATGTGGGTTC CTCTCAAGTT ATGTAGATGC TCTGCATTAT   
  
  
+ TATGCTGCAA TGTTTGACTC TTTAGATGAT TGCCTCCCAC TTGAAAGTCC TGAGAGGCTG AGCATAGAGA   
  
  
+ AGAACCATCT TGGAAAAGAG ATCAAAGAAG CCATAGGTTG TGAGAAGGAT GAAACAAACT ATCTGAAGTT   
  
  
+ TGAGATGCTG GAGACTTGGA GAGGGAGGAT GGAGAGTCAT GGATTTTCAG GTATGAAGCT AAGTTCCAGG   
  
  
+ GCAACTATAC AAGCAAAGCT GCTTTTAAAA ATGGGAAGCC ATTATCATAC CTATTTGGAA GAAGACTGTG   
  
  
+ GAGGTGGTGG GTTCAGAGTT TGTGAACGAG ATGATGGAAT GGCTATCTCT CTTGGCTGGC AAGGTAGGTT   
  
  
+ CCTCGCAACT GCCTCGGTAT GGCGTTCTGT GTG  

- -Up\_Stream \_Len000TGGTTG CTAAGAAATG ACTTAGTCGA TGTAAAAACC TAACACAATT CCATAGAAAA   
  
  
- TCTAGAAGTC CTCCTCCCCC GACTTCATCG AACCAATGAA AACGCGAATA GTTGACACCT TCTTATAATA   
  
  
- ACTCTGATCA CATCTTCTTA ATCATCAAAG GAGGTGTAAC CTTTTTTTGG TACTTTAATT GCGAAATCCG   
  
  
- GGAGTCTCAT GGTTCACAGT AGCATTTACT AACTTTAAAG TTCAGAATCG ACTCAGTACT TCAGGACTAA   
  
  
- AGGGTACTGT TTAACTACAA CAACAGATTT CGAACCGCCC ACTAAACTTT CTTCTAAATC TAGTCCGACT   
  
  
- GTTATGTGGT TTAGACCCTC GTTCCTTGAC CTAAAAAGAC ACAGATCTTT TCGAAAGGCG AAAGACTTGT   
  
  
- TAACTCTTTT GGTACGTATC CTTTAAAGAA CTATAAATCT AGTATCGTCT GAATTGTTAG ACCGAAATGG   
  
  
- CTTGACAGGA GATGAAGATT CAACACTTCC ACACTTTCTA TTTGGTACAG TTTGAATCAC AGGTTGTCTT   
  
  
- TGCGTAGAGA TGAAGAGGTC CGTTTCAACA TGAAGTCTAG AAGATGTGGA AAAGATCCGT CCTGAAACCC   
  
  
- TATATCTCAA ACCGGACCCA CTTTTCATCC TTCACCCCTT CATCTTTTAC TACTTTCTTC CGTGGTGAAC   
  
  
- CCAGGTCTAT GTATCCGGAT GCGTGCTCTT GGAAACGTAC CAGATCTATG TACTGACATA TGAAGAAGGA   
  
  
- AAGGAAATGG ACAACCTCTA ATAGGATCCT AAAATGTACC GTGGTCGACC TAAACAATTA CTGTATTTGT   
  
  
- ATAGAAATTA ATGTCAACGA ATACCATATG TAAATTTGAT AAGCTATCAA CTGTCGACTA CAGGAATAGG   
  
  
- AACACGTCGA ATAAGGAGCT ATCTTATTAG AAACCTGTAA AGACAGATCC ACTATTTCCC GTTTTTGATT   
  
  
- CTATGTTCTT TATCTTGTAC AGGAGGTAAA TGTTCTGCAT AGACAAAAAC CAAAACCAGT TACTAGGGAC   
  
  
- AACAGGAGTT CGTAATTGTG TCTTACTAGT AATAACTGAA CCTGACTTTC CAAAACCCTT GAGGTATCAT   
  
  
- ACGGAAAAGT TATTTTAGTT TTCGTTTAAA CGACAAGTGA CTATTGACGT CAACACAACG AAACGACAAA   
  
  
- GGTATGGTCT GAGCATTGGT TCGACTATCG TTTACACGTT TCCATGTCAT ATTAATTGCT ACAAAAGTAT   
  
  
- AACGATGCTC AAGACTTTGT ACTATATTCT TTTGTGGTCT TGGGTTTATG GATGTATCCA GGATCGGGAC   
  
  
- CTTACCGATA AAATTTATAG GTTTACCTGA ATGTCTTAAG CGTCTCAACA AACCAAACAA ATTGTTAGTA   
  
  
- ACTTAACAAT CGATACTCTT AGACGCCCTG TTAGCATTAG AAACCGCTAA TGAAGCTAAC CGTATGACTA   
  
  
- CAAAAAAATA AACGCATAGA TTCGTACCAC CACATGGAAA CTAAACACAC AGATCCATCA CACACATCTA   
  
  
- TTTATAGTAT CTTGAGTCTG CAACCATCCC AATACAACTC GTGAAAGAGA ACGACAAACA GGAAACACTA   
  
  
- CCGTGGAGAA CGAAAAGGCT TGGTGGTTTT CAGAGTAACT GTTTTGAGAA GTTTGTAACG TTTGACGGGG   
  
  
- GAATTTATAC AAAATAATTT TTTTTACCTT CTCGAGTTTG ACAACTCACT CGGATCTAAG GTTCTAAATC   
  
  
- TTTGTCATTT GAACTATCTA TATAAATATA CTTGATAACA ATCTACGTTG TGGGGGTTGA ACTTTGTGTT   
  
  
- TTCTCTTTAA TTTTTGTTTT TCCAAAACGT GATAAATATA TAGGACACAA GAAACACGAT CAGAATAAAT   
  
  
- TACAATTAAC CATTCGTAAT AAACAACCTA ATAAGGTGAC GTGAAAGAGA ATGATTGAAG TCGTAAAGTA   
  
  
- AGGAACAAGA AGTCGAAAAC TTGAAGAAAT CGGATAAAAC AACAGAAATA CCAATACCTT CGGTAGGTCT   
  
  
- TCTTCTTACT ACTTGAAGAC TCAGAATCGA ACCGGTAACA GCCAGTGAGA CTAGCGTGAC TCTTTTTTGA   
  
  
- CTTCTCCGCC TAACTACAGA GGCTAGGAGA CTAGTGAAGT CCACTTCCGA CGCTTCCCTT CTATTAGTCC   
  
  
- GAGGAGGTTC TCTCTGTCTT GAACAACTTG TAGTTCGTTT CTCCTTTTCC GCAGTAAGTT CTTCCCTTCC   
  
  
- CAGAAGTAGA ATAGGTAGAC GAGGAACATA GACGGTGGAA TTAGTTGCTT TTGTTGTAGT CAAGTCGACA   
  
  
- ACTATTAGAA TAACTCGAGA AGGTTATACA GAGGCTTTCA CCACTAAGTC CTGTTTCCCA ACGACGAATG   
  
  
- AAACGCCTAC CCAATTGTCG TTCCTAGAAT TGAGTCTCCA GAGGTAAAAT AGCGGACTAT GACCCGTTTG   
  
  
- GTCGTGGACG ACTTCTCAAA AGTCGAAAAT GAGTGAATAT ATCTCGGAGA GGTAAGATAG TCAAACGAGT   
  
  
- AAAGTGTCGG TTAGTCCGTT AACATCTTCG TAAACTCTCC CTCCTTCTCT CATTGTTGAC CCGAAACGTA   
  
  
- CATTAACTAA ACCTGTAGAA CGTACCAAAG GTCACCGGAA GAGAATAAGT CAGAGAGAGG CTTCTCCGAT   
  
  
- GAACAAGTTT GGCAAGTAAC AGAGAAGTTT AATGTCCGAA GCCTTCTTCG AATCTTCTCG AGTAACTTTG   
  
  
- TCTTCGAGCC GACCATTCAA AGAGATTCTC GAAAGTCTTA TAGTTGAAAC TTAAGGTGCC CAAAGACTCT   
  
  
- CCGAGCTTTG AGTCCTTGGA TTCCTCCCTC TTACTTTGTC ATCAACATTT AGAGCAAAAG GTGGAGTCAT   
  
  
- GAAACTTTCT ATGGCATGTC TAAAGACTGT GGAACTGAAG ACATGTAAGT GATTTGGGGA GATAACACCA   
  
  
- TAATCAGCTC TCTCTTCCTT CAGCTTTGTC TACACCCAAG GAGAGTTCAA TACATCTACG AGACGTAATA   
  
  
- ATACGACGTT ACAAACTGAG AAATCTACTA ACGGAGGGTG AACTTTCAGG ACTCTCCGAC TCGTATCTCT   
  
  
- TCTTGGTAGA ACCTTTTCTC TAGTTTCTTC GGTATCCAAC ACTCTTCCTA CTTTGTTTGA TAGACTTCAA   
  
  
- ACTCTACGAC CTCTGAACCT CTCCCTCCTA CCTCTCAGTA CCTAAAAGTC CATACTTCGA TTCAAGGTCC   
  
  
- CGTTGATATG TTCGTTTCGA CGAAAATTTT TACCCTTCGG TAATAGTATG GATAAACCTT CTTCTGACAC   
  
  
- CTCCACCACC CAAGTCTCAA ACACTTGCTC TACTACCTTA CCGATAGAGA GAACCGACCG TTCCATCCAA   
  
  
- GGAGCGTTGA CGGAGCCATA CCGCAAGACA CAC

+     TATC-box

| Site Name | Organism | Position | Strand | Matrix score. | sequence | function |
| --- | --- | --- | --- | --- | --- | --- |
| TATC-box | Oryza sativa | 631 | - | 7 | TATCCCA | cis-acting element involved in gibberellin-responsiveness |

>HU07G00272.1   
+ -Up\_Stream \_Len000ACCAAC GATTCTTTAC TGAATCAGCT ACATTTTTGG ATTGTGTTAA GGTATCTTTT   
  
  
+ AGATCTTCAG GAGGAGGGGG CTGAAGTAGC TTGGTTACTT TTGCGCTTAT CAACTGTGGA AGAATATTAT   
  
  
+ TGAGACTAGT GTAGAAGAAT TAGTAGTTTC CTCCACATTG GAAAAAAACC ATGAAATTAA CGCTTTAGGC   
  
  
+ CCTCAGAGTA CCAAGTGTCA TCGTAAATGA TTGAAATTTC AAGTCTTAGC TGAGTCATGA AGTCCTGATT   
  
  
+ TCCCATGACA AATTGATGTT GTTGTCTAAA GCTTGGCGGG TGATTTGAAA GAAGATTTAG ATCAGGCTGA   
  
  
+ CAATACACCA AATCTGGGAG CAAGGAACTG GATTTTTCTG TGTCTAGAAA AGCTTTCCGC TTTCTGAACA   
  
  
+ ATTGAGAAAA CCATGCATAG GAAATTTCTT GATATTTAGA TCATAGCAGA CTTAACAATC TGGCTTTACC   
  
  
+ GAACTGTCCT CTACTTCTAA GTTGTGAAGG TGTGAAAGAT AAACCATGTC AAACTTAGTG TCCAACAGAA   
  
  
+ ACGCATCTCT ACTTCTCCAG GCAAAGTTGT ACTTCAGATC TTCTACACCT TTTCTAGGCA GGACTTTGGG   
  
  
+ ATATAGAGTT TGGCCTGGGT GAAAAGTAGG AAGTGGGGAA GTAGAAAATG ATGAAAGAAG GCACCACTTG   
  
  
+ GGTCCAGATA CATAGGCCTA CGCACGAGAA CCTTTGCATG GTCTAGATAC ATGACTGTAT ACTTCTTCCT   
  
  
+ TTCCTTTACC TGTTGGAGAT TATCCTAGGA TTTTACATGG CACCAGCTGG ATTTGTTAAT GACATAAACA   
  
  
+ TATCTTTAAT TACAGTTGCT TATGGTATAC ATTTAAACTA TTCGATAGTT GACAGCTGAT GTCCTTATCC   
  
  
+ TTGTGCAGCT TATTCCTCGA TAGAATAATC TTTGGACATT TCTGTCTAGG TGATAAAGGG CAAAAACTAA   
  
  
+ GATACAAGAA ATAGAACATG TCCTCCATTT ACAAGACGTA TCTGTTTTTG GTTTTGGTCA ATGATCCCTG   
  
  
+ TTGTCCTCAA GCATTAACAC AGAATGATCA TTATTGACTT GGACTGAAAG GTTTTGGGAA CTCCATAGTA   
  
  
+ TGCCTTTTCA ATAAAATCAA AAGCAAATTT GCTGTTCACT GATAACTGCA GTTGTGTTGC TTTGCTGTTT   
  
  
+ CCATACCAGA CTCGTAACCA AGCTGATAGC AAATGTGCAA AGGTACAGTA TAATTAACGA TGTTTTCATA   
  
  
+ TTGCTACGAG TTCTGAAACA TGATATAAGA AAACACCAGA ACCCAAATAC CTACATAGGT CCTAGCCCTG   
  
  
+ GAATGGCTAT TTTAAATATC CAAATGGACT TACAGAATTC GCAGAGTTGT TTGGTTTGTT TAACAATCAT   
  
  
+ TGAATTGTTA GCTATGAGAA TCTGCGGGAC AATCGTAATC TTTGGCGATT ACTTCGATTG GCATACTGAT   
  
  
+ GTTTTTTTAT TTGCGTATCT AAGCATGGTG GTGTACCTTT GATTTGTGTG TCTAGGTAGT GTGTGTAGAT   
  
  
+ AAATATCATA GAACTCAGAC GTTGGTAGGG TTATGTTGAG CACTTTCTCT TGCTGTTTGT CCTTTGTGAT   
  
  
+ GGCACCTCTT GCTTTTCCGA ACCACCAAAA GTCTCATTGA CAAAACTCTT CAAACATTGC AAACTGCCCC   
  
  
+ CTTAAATATG TTTTATTAAA AAAAATGGAA GAGCTCAAAC TGTTGAGTGA GCCTAGATTC CAAGATTTAG   
  
  
+ AAACAGTAAA CTTGATAGAT ATATTTATAT GAACTATTGT TAGATGCAAC ACCCCCAACT TGAAACACAA   
  
  
+ AAGAGAAATT AAAAACAAAA AGGTTTTGCA CTATTTATAT ATCCTGTGTT CTTTGTGCTA GTCTTATTTA   
  
  
+ ATGTTAATTG GTAAGCATTA TTTGTTGGAT TATTCCACTG CACTTTCTCT TACTAACTTC AGCATTTCAT   
  
  
+ TCCTTGTTCT TCAGCTTTTG AACTTCTTTA GCCTATTTTG TTGTCTTTAT GGTTATGGAA GCCATCCAGA   
  
  
+ AGAAGAATGA TGAACTTCTG AGTCTTAGCT TGGCCATTGT CGGTCACTCT GATCGCACTG AGAAAAAACT   
  
  
+ GAAGAGGCGG ATTGATGTCT CCGATCCTCT GATCACTTCA GGTGAAGGCT GCGAAGGGAA GATAATCAGG   
  
  
+ CTCCTCCAAG AGAGACAGAA CTTGTTGAAC ATCAAGCAAA GAGGAAAAGG CGTCATTCAA GAAGGGAAGG   
  
  
+ GTCTTCATCT TATCCATCTG CTCCTTGTAT CTGCCACCTT AATCAACGAA AACAACATCA GTTCAGCTGT   
  
  
+ TGATAATCTT ATTGAGCTCT TCCAATATGT CTCCGAAAGT GGTGATTCAG GACAAAGGGT TGCTGCTTAC   
  
  
+ TTTGCGGATG GGTTAACAGC AAGGATCTTA ACTCAGAGGT CTCCATTTTA TCGCCTGATA CTGGGCAAAC   
  
  
+ CAGCACCTGC TGAAGAGTTT TCAGCTTTTA CTCACTTATA TAGAGCCTCT CCATTCTATC AGTTTGCTCA   
  
  
+ TTTCACAGCC AATCAGGCAA TTGTAGAAGC ATTTGAGAGG GAGGAAGAGA GTAACAACTG GGCTTTGCAT   
  
  
+ GTAATTGATT TGGACATCTT GCATGGTTTC CAGTGGCCTT CTCTTATTCA GTCTCTCTCC GAAGAGGCTA   
  
  
+ CTTGTTCAAA CCGTTCATTG TCTCTTCAAA TTACAGGCTT CGGAAGAAGC TTAGAAGAGC TCATTGAAAC   
  
  
+ AGAAGCTCGG CTGGTAAGTT TCTCTAAGAG CTTTCAGAAT ATCAACTTTG AATTCCACGG GTTTCTGAGA   
  
  
+ GGCTCGAAAC TCAGGAACCT AAGGAGGGAG AATGAAACAG TAGTTGTAAA TCTCGTTTTC CACCTCAGTA   
  
  
+ CTTTGAAAGA TACCGTACAG ATTTCTGACA CCTTGACTTC TGTACATTCA CTAAACCCCT CTATTGTGGT   
  
  
+ ATTAGTCGAG AGAGAAGGAA GTCGAAACAG ATGTGGGTTC CTCTCAAGTT ATGTAGATGC TCTGCATTAT   
  
  
+ TATGCTGCAA TGTTTGACTC TTTAGATGAT TGCCTCCCAC TTGAAAGTCC TGAGAGGCTG AGCATAGAGA   
  
  
+ AGAACCATCT TGGAAAAGAG ATCAAAGAAG CCATAGGTTG TGAGAAGGAT GAAACAAACT ATCTGAAGTT   
  
  
+ TGAGATGCTG GAGACTTGGA GAGGGAGGAT GGAGAGTCAT GGATTTTCAG GTATGAAGCT AAGTTCCAGG   
  
  
+ GCAACTATAC AAGCAAAGCT GCTTTTAAAA ATGGGAAGCC ATTATCATAC CTATTTGGAA GAAGACTGTG   
  
  
+ GAGGTGGTGG GTTCAGAGTT TGTGAACGAG ATGATGGAAT GGCTATCTCT CTTGGCTGGC AAGGTAGGTT   
  
  
+ CCTCGCAACT GCCTCGGTAT GGCGTTCTGT GTG  

- -Up\_Stream \_Len000TGGTTG CTAAGAAATG ACTTAGTCGA TGTAAAAACC TAACACAATT CCATAGAAAA   
  
  
- TCTAGAAGTC CTCCTCCCCC GACTTCATCG AACCAATGAA AACGCGAATA GTTGACACCT TCTTATAATA   
  
  
- ACTCTGATCA CATCTTCTTA ATCATCAAAG GAGGTGTAAC CTTTTTTTGG TACTTTAATT GCGAAATCCG   
  
  
- GGAGTCTCAT GGTTCACAGT AGCATTTACT AACTTTAAAG TTCAGAATCG ACTCAGTACT TCAGGACTAA   
  
  
- AGGGTACTGT TTAACTACAA CAACAGATTT CGAACCGCCC ACTAAACTTT CTTCTAAATC TAGTCCGACT   
  
  
- GTTATGTGGT TTAGACCCTC GTTCCTTGAC CTAAAAAGAC ACAGATCTTT TCGAAAGGCG AAAGACTTGT   
  
  
- TAACTCTTTT GGTACGTATC CTTTAAAGAA CTATAAATCT AGTATCGTCT GAATTGTTAG ACCGAAATGG   
  
  
- CTTGACAGGA GATGAAGATT CAACACTTCC ACACTTTCTA TTTGGTACAG TTTGAATCAC AGGTTGTCTT   
  
  
- TGCGTAGAGA TGAAGAGGTC CGTTTCAACA TGAAGTCTAG AAGATGTGGA AAAGATCCGT CCTGAAACCC   
  
  
- TATATCTCAA ACCGGACCCA CTTTTCATCC TTCACCCCTT CATCTTTTAC TACTTTCTTC CGTGGTGAAC   
  
  
- CCAGGTCTAT GTATCCGGAT GCGTGCTCTT GGAAACGTAC CAGATCTATG TACTGACATA TGAAGAAGGA   
  
  
- AAGGAAATGG ACAACCTCTA ATAGGATCCT AAAATGTACC GTGGTCGACC TAAACAATTA CTGTATTTGT   
  
  
- ATAGAAATTA ATGTCAACGA ATACCATATG TAAATTTGAT AAGCTATCAA CTGTCGACTA CAGGAATAGG   
  
  
- AACACGTCGA ATAAGGAGCT ATCTTATTAG AAACCTGTAA AGACAGATCC ACTATTTCCC GTTTTTGATT   
  
  
- CTATGTTCTT TATCTTGTAC AGGAGGTAAA TGTTCTGCAT AGACAAAAAC CAAAACCAGT TACTAGGGAC   
  
  
- AACAGGAGTT CGTAATTGTG TCTTACTAGT AATAACTGAA CCTGACTTTC CAAAACCCTT GAGGTATCAT   
  
  
- ACGGAAAAGT TATTTTAGTT TTCGTTTAAA CGACAAGTGA CTATTGACGT CAACACAACG AAACGACAAA   
  
  
- GGTATGGTCT GAGCATTGGT TCGACTATCG TTTACACGTT TCCATGTCAT ATTAATTGCT ACAAAAGTAT   
  
  
- AACGATGCTC AAGACTTTGT ACTATATTCT TTTGTGGTCT TGGGTTTATG GATGTATCCA GGATCGGGAC   
  
  
- CTTACCGATA AAATTTATAG GTTTACCTGA ATGTCTTAAG CGTCTCAACA AACCAAACAA ATTGTTAGTA   
  
  
- ACTTAACAAT CGATACTCTT AGACGCCCTG TTAGCATTAG AAACCGCTAA TGAAGCTAAC CGTATGACTA   
  
  
- CAAAAAAATA AACGCATAGA TTCGTACCAC CACATGGAAA CTAAACACAC AGATCCATCA CACACATCTA   
  
  
- TTTATAGTAT CTTGAGTCTG CAACCATCCC AATACAACTC GTGAAAGAGA ACGACAAACA GGAAACACTA   
  
  
- CCGTGGAGAA CGAAAAGGCT TGGTGGTTTT CAGAGTAACT GTTTTGAGAA GTTTGTAACG TTTGACGGGG   
  
  
- GAATTTATAC AAAATAATTT TTTTTACCTT CTCGAGTTTG ACAACTCACT CGGATCTAAG GTTCTAAATC   
  
  
- TTTGTCATTT GAACTATCTA TATAAATATA CTTGATAACA ATCTACGTTG TGGGGGTTGA ACTTTGTGTT   
  
  
- TTCTCTTTAA TTTTTGTTTT TCCAAAACGT GATAAATATA TAGGACACAA GAAACACGAT CAGAATAAAT   
  
  
- TACAATTAAC CATTCGTAAT AAACAACCTA ATAAGGTGAC GTGAAAGAGA ATGATTGAAG TCGTAAAGTA   
  
  
- AGGAACAAGA AGTCGAAAAC TTGAAGAAAT CGGATAAAAC AACAGAAATA CCAATACCTT CGGTAGGTCT   
  
  
- TCTTCTTACT ACTTGAAGAC TCAGAATCGA ACCGGTAACA GCCAGTGAGA CTAGCGTGAC TCTTTTTTGA   
  
  
- CTTCTCCGCC TAACTACAGA GGCTAGGAGA CTAGTGAAGT CCACTTCCGA CGCTTCCCTT CTATTAGTCC   
  
  
- GAGGAGGTTC TCTCTGTCTT GAACAACTTG TAGTTCGTTT CTCCTTTTCC GCAGTAAGTT CTTCCCTTCC   
  
  
- CAGAAGTAGA ATAGGTAGAC GAGGAACATA GACGGTGGAA TTAGTTGCTT TTGTTGTAGT CAAGTCGACA   
  
  
- ACTATTAGAA TAACTCGAGA AGGTTATACA GAGGCTTTCA CCACTAAGTC CTGTTTCCCA ACGACGAATG   
  
  
- AAACGCCTAC CCAATTGTCG TTCCTAGAAT TGAGTCTCCA GAGGTAAAAT AGCGGACTAT GACCCGTTTG   
  
  
- GTCGTGGACG ACTTCTCAAA AGTCGAAAAT GAGTGAATAT ATCTCGGAGA GGTAAGATAG TCAAACGAGT   
  
  
- AAAGTGTCGG TTAGTCCGTT AACATCTTCG TAAACTCTCC CTCCTTCTCT CATTGTTGAC CCGAAACGTA   
  
  
- CATTAACTAA ACCTGTAGAA CGTACCAAAG GTCACCGGAA GAGAATAAGT CAGAGAGAGG CTTCTCCGAT   
  
  
- GAACAAGTTT GGCAAGTAAC AGAGAAGTTT AATGTCCGAA GCCTTCTTCG AATCTTCTCG AGTAACTTTG   
  
  
- TCTTCGAGCC GACCATTCAA AGAGATTCTC GAAAGTCTTA TAGTTGAAAC TTAAGGTGCC CAAAGACTCT   
  
  
- CCGAGCTTTG AGTCCTTGGA TTCCTCCCTC TTACTTTGTC ATCAACATTT AGAGCAAAAG GTGGAGTCAT   
  
  
- GAAACTTTCT ATGGCATGTC TAAAGACTGT GGAACTGAAG ACATGTAAGT GATTTGGGGA GATAACACCA   
  
  
- TAATCAGCTC TCTCTTCCTT CAGCTTTGTC TACACCCAAG GAGAGTTCAA TACATCTACG AGACGTAATA   
  
  
- ATACGACGTT ACAAACTGAG AAATCTACTA ACGGAGGGTG AACTTTCAGG ACTCTCCGAC TCGTATCTCT   
  
  
- TCTTGGTAGA ACCTTTTCTC TAGTTTCTTC GGTATCCAAC ACTCTTCCTA CTTTGTTTGA TAGACTTCAA   
  
  
- ACTCTACGAC CTCTGAACCT CTCCCTCCTA CCTCTCAGTA CCTAAAAGTC CATACTTCGA TTCAAGGTCC   
  
  
- CGTTGATATG TTCGTTTCGA CGAAAATTTT TACCCTTCGG TAATAGTATG GATAAACCTT CTTCTGACAC   
  
  
- CTCCACCACC CAAGTCTCAA ACACTTGCTC TACTACCTTA CCGATAGAGA GAACCGACCG TTCCATCCAA   
  
  
- GGAGCGTTGA CGGAGCCATA CCGCAAGACA CAC

+     TC-rich repeats

| Site Name | Organism | Position | Strand | Matrix score. | sequence | function |
| --- | --- | --- | --- | --- | --- | --- |
| TC-rich repeats | Nicotiana tabacum | 1289 | - | 9 | GTTTTCTTAC | cis-acting element involved in defense and stress responsiveness |

>HU07G00272.1   
+ -Up\_Stream \_Len000ACCAAC GATTCTTTAC TGAATCAGCT ACATTTTTGG ATTGTGTTAA GGTATCTTTT   
  
  
+ AGATCTTCAG GAGGAGGGGG CTGAAGTAGC TTGGTTACTT TTGCGCTTAT CAACTGTGGA AGAATATTAT   
  
  
+ TGAGACTAGT GTAGAAGAAT TAGTAGTTTC CTCCACATTG GAAAAAAACC ATGAAATTAA CGCTTTAGGC   
  
  
+ CCTCAGAGTA CCAAGTGTCA TCGTAAATGA TTGAAATTTC AAGTCTTAGC TGAGTCATGA AGTCCTGATT   
  
  
+ TCCCATGACA AATTGATGTT GTTGTCTAAA GCTTGGCGGG TGATTTGAAA GAAGATTTAG ATCAGGCTGA   
  
  
+ CAATACACCA AATCTGGGAG CAAGGAACTG GATTTTTCTG TGTCTAGAAA AGCTTTCCGC TTTCTGAACA   
  
  
+ ATTGAGAAAA CCATGCATAG GAAATTTCTT GATATTTAGA TCATAGCAGA CTTAACAATC TGGCTTTACC   
  
  
+ GAACTGTCCT CTACTTCTAA GTTGTGAAGG TGTGAAAGAT AAACCATGTC AAACTTAGTG TCCAACAGAA   
  
  
+ ACGCATCTCT ACTTCTCCAG GCAAAGTTGT ACTTCAGATC TTCTACACCT TTTCTAGGCA GGACTTTGGG   
  
  
+ ATATAGAGTT TGGCCTGGGT GAAAAGTAGG AAGTGGGGAA GTAGAAAATG ATGAAAGAAG GCACCACTTG   
  
  
+ GGTCCAGATA CATAGGCCTA CGCACGAGAA CCTTTGCATG GTCTAGATAC ATGACTGTAT ACTTCTTCCT   
  
  
+ TTCCTTTACC TGTTGGAGAT TATCCTAGGA TTTTACATGG CACCAGCTGG ATTTGTTAAT GACATAAACA   
  
  
+ TATCTTTAAT TACAGTTGCT TATGGTATAC ATTTAAACTA TTCGATAGTT GACAGCTGAT GTCCTTATCC   
  
  
+ TTGTGCAGCT TATTCCTCGA TAGAATAATC TTTGGACATT TCTGTCTAGG TGATAAAGGG CAAAAACTAA   
  
  
+ GATACAAGAA ATAGAACATG TCCTCCATTT ACAAGACGTA TCTGTTTTTG GTTTTGGTCA ATGATCCCTG   
  
  
+ TTGTCCTCAA GCATTAACAC AGAATGATCA TTATTGACTT GGACTGAAAG GTTTTGGGAA CTCCATAGTA   
  
  
+ TGCCTTTTCA ATAAAATCAA AAGCAAATTT GCTGTTCACT GATAACTGCA GTTGTGTTGC TTTGCTGTTT   
  
  
+ CCATACCAGA CTCGTAACCA AGCTGATAGC AAATGTGCAA AGGTACAGTA TAATTAACGA TGTTTTCATA   
  
  
+ TTGCTACGAG TTCTGAAACA TGATATAAGA AAACACCAGA ACCCAAATAC CTACATAGGT CCTAGCCCTG   
  
  
+ GAATGGCTAT TTTAAATATC CAAATGGACT TACAGAATTC GCAGAGTTGT TTGGTTTGTT TAACAATCAT   
  
  
+ TGAATTGTTA GCTATGAGAA TCTGCGGGAC AATCGTAATC TTTGGCGATT ACTTCGATTG GCATACTGAT   
  
  
+ GTTTTTTTAT TTGCGTATCT AAGCATGGTG GTGTACCTTT GATTTGTGTG TCTAGGTAGT GTGTGTAGAT   
  
  
+ AAATATCATA GAACTCAGAC GTTGGTAGGG TTATGTTGAG CACTTTCTCT TGCTGTTTGT CCTTTGTGAT   
  
  
+ GGCACCTCTT GCTTTTCCGA ACCACCAAAA GTCTCATTGA CAAAACTCTT CAAACATTGC AAACTGCCCC   
  
  
+ CTTAAATATG TTTTATTAAA AAAAATGGAA GAGCTCAAAC TGTTGAGTGA GCCTAGATTC CAAGATTTAG   
  
  
+ AAACAGTAAA CTTGATAGAT ATATTTATAT GAACTATTGT TAGATGCAAC ACCCCCAACT TGAAACACAA   
  
  
+ AAGAGAAATT AAAAACAAAA AGGTTTTGCA CTATTTATAT ATCCTGTGTT CTTTGTGCTA GTCTTATTTA   
  
  
+ ATGTTAATTG GTAAGCATTA TTTGTTGGAT TATTCCACTG CACTTTCTCT TACTAACTTC AGCATTTCAT   
  
  
+ TCCTTGTTCT TCAGCTTTTG AACTTCTTTA GCCTATTTTG TTGTCTTTAT GGTTATGGAA GCCATCCAGA   
  
  
+ AGAAGAATGA TGAACTTCTG AGTCTTAGCT TGGCCATTGT CGGTCACTCT GATCGCACTG AGAAAAAACT   
  
  
+ GAAGAGGCGG ATTGATGTCT CCGATCCTCT GATCACTTCA GGTGAAGGCT GCGAAGGGAA GATAATCAGG   
  
  
+ CTCCTCCAAG AGAGACAGAA CTTGTTGAAC ATCAAGCAAA GAGGAAAAGG CGTCATTCAA GAAGGGAAGG   
  
  
+ GTCTTCATCT TATCCATCTG CTCCTTGTAT CTGCCACCTT AATCAACGAA AACAACATCA GTTCAGCTGT   
  
  
+ TGATAATCTT ATTGAGCTCT TCCAATATGT CTCCGAAAGT GGTGATTCAG GACAAAGGGT TGCTGCTTAC   
  
  
+ TTTGCGGATG GGTTAACAGC AAGGATCTTA ACTCAGAGGT CTCCATTTTA TCGCCTGATA CTGGGCAAAC   
  
  
+ CAGCACCTGC TGAAGAGTTT TCAGCTTTTA CTCACTTATA TAGAGCCTCT CCATTCTATC AGTTTGCTCA   
  
  
+ TTTCACAGCC AATCAGGCAA TTGTAGAAGC ATTTGAGAGG GAGGAAGAGA GTAACAACTG GGCTTTGCAT   
  
  
+ GTAATTGATT TGGACATCTT GCATGGTTTC CAGTGGCCTT CTCTTATTCA GTCTCTCTCC GAAGAGGCTA   
  
  
+ CTTGTTCAAA CCGTTCATTG TCTCTTCAAA TTACAGGCTT CGGAAGAAGC TTAGAAGAGC TCATTGAAAC   
  
  
+ AGAAGCTCGG CTGGTAAGTT TCTCTAAGAG CTTTCAGAAT ATCAACTTTG AATTCCACGG GTTTCTGAGA   
  
  
+ GGCTCGAAAC TCAGGAACCT AAGGAGGGAG AATGAAACAG TAGTTGTAAA TCTCGTTTTC CACCTCAGTA   
  
  
+ CTTTGAAAGA TACCGTACAG ATTTCTGACA CCTTGACTTC TGTACATTCA CTAAACCCCT CTATTGTGGT   
  
  
+ ATTAGTCGAG AGAGAAGGAA GTCGAAACAG ATGTGGGTTC CTCTCAAGTT ATGTAGATGC TCTGCATTAT   
  
  
+ TATGCTGCAA TGTTTGACTC TTTAGATGAT TGCCTCCCAC TTGAAAGTCC TGAGAGGCTG AGCATAGAGA   
  
  
+ AGAACCATCT TGGAAAAGAG ATCAAAGAAG CCATAGGTTG TGAGAAGGAT GAAACAAACT ATCTGAAGTT   
  
  
+ TGAGATGCTG GAGACTTGGA GAGGGAGGAT GGAGAGTCAT GGATTTTCAG GTATGAAGCT AAGTTCCAGG   
  
  
+ GCAACTATAC AAGCAAAGCT GCTTTTAAAA ATGGGAAGCC ATTATCATAC CTATTTGGAA GAAGACTGTG   
  
  
+ GAGGTGGTGG GTTCAGAGTT TGTGAACGAG ATGATGGAAT GGCTATCTCT CTTGGCTGGC AAGGTAGGTT   
  
  
+ CCTCGCAACT GCCTCGGTAT GGCGTTCTGT GTG  

- -Up\_Stream \_Len000TGGTTG CTAAGAAATG ACTTAGTCGA TGTAAAAACC TAACACAATT CCATAGAAAA   
  
  
- TCTAGAAGTC CTCCTCCCCC GACTTCATCG AACCAATGAA AACGCGAATA GTTGACACCT TCTTATAATA   
  
  
- ACTCTGATCA CATCTTCTTA ATCATCAAAG GAGGTGTAAC CTTTTTTTGG TACTTTAATT GCGAAATCCG   
  
  
- GGAGTCTCAT GGTTCACAGT AGCATTTACT AACTTTAAAG TTCAGAATCG ACTCAGTACT TCAGGACTAA   
  
  
- AGGGTACTGT TTAACTACAA CAACAGATTT CGAACCGCCC ACTAAACTTT CTTCTAAATC TAGTCCGACT   
  
  
- GTTATGTGGT TTAGACCCTC GTTCCTTGAC CTAAAAAGAC ACAGATCTTT TCGAAAGGCG AAAGACTTGT   
  
  
- TAACTCTTTT GGTACGTATC CTTTAAAGAA CTATAAATCT AGTATCGTCT GAATTGTTAG ACCGAAATGG   
  
  
- CTTGACAGGA GATGAAGATT CAACACTTCC ACACTTTCTA TTTGGTACAG TTTGAATCAC AGGTTGTCTT   
  
  
- TGCGTAGAGA TGAAGAGGTC CGTTTCAACA TGAAGTCTAG AAGATGTGGA AAAGATCCGT CCTGAAACCC   
  
  
- TATATCTCAA ACCGGACCCA CTTTTCATCC TTCACCCCTT CATCTTTTAC TACTTTCTTC CGTGGTGAAC   
  
  
- CCAGGTCTAT GTATCCGGAT GCGTGCTCTT GGAAACGTAC CAGATCTATG TACTGACATA TGAAGAAGGA   
  
  
- AAGGAAATGG ACAACCTCTA ATAGGATCCT AAAATGTACC GTGGTCGACC TAAACAATTA CTGTATTTGT   
  
  
- ATAGAAATTA ATGTCAACGA ATACCATATG TAAATTTGAT AAGCTATCAA CTGTCGACTA CAGGAATAGG   
  
  
- AACACGTCGA ATAAGGAGCT ATCTTATTAG AAACCTGTAA AGACAGATCC ACTATTTCCC GTTTTTGATT   
  
  
- CTATGTTCTT TATCTTGTAC AGGAGGTAAA TGTTCTGCAT AGACAAAAAC CAAAACCAGT TACTAGGGAC   
  
  
- AACAGGAGTT CGTAATTGTG TCTTACTAGT AATAACTGAA CCTGACTTTC CAAAACCCTT GAGGTATCAT   
  
  
- ACGGAAAAGT TATTTTAGTT TTCGTTTAAA CGACAAGTGA CTATTGACGT CAACACAACG AAACGACAAA   
  
  
- GGTATGGTCT GAGCATTGGT TCGACTATCG TTTACACGTT TCCATGTCAT ATTAATTGCT ACAAAAGTAT   
  
  
- AACGATGCTC AAGACTTTGT ACTATATTCT TTTGTGGTCT TGGGTTTATG GATGTATCCA GGATCGGGAC   
  
  
- CTTACCGATA AAATTTATAG GTTTACCTGA ATGTCTTAAG CGTCTCAACA AACCAAACAA ATTGTTAGTA   
  
  
- ACTTAACAAT CGATACTCTT AGACGCCCTG TTAGCATTAG AAACCGCTAA TGAAGCTAAC CGTATGACTA   
  
  
- CAAAAAAATA AACGCATAGA TTCGTACCAC CACATGGAAA CTAAACACAC AGATCCATCA CACACATCTA   
  
  
- TTTATAGTAT CTTGAGTCTG CAACCATCCC AATACAACTC GTGAAAGAGA ACGACAAACA GGAAACACTA   
  
  
- CCGTGGAGAA CGAAAAGGCT TGGTGGTTTT CAGAGTAACT GTTTTGAGAA GTTTGTAACG TTTGACGGGG   
  
  
- GAATTTATAC AAAATAATTT TTTTTACCTT CTCGAGTTTG ACAACTCACT CGGATCTAAG GTTCTAAATC   
  
  
- TTTGTCATTT GAACTATCTA TATAAATATA CTTGATAACA ATCTACGTTG TGGGGGTTGA ACTTTGTGTT   
  
  
- TTCTCTTTAA TTTTTGTTTT TCCAAAACGT GATAAATATA TAGGACACAA GAAACACGAT CAGAATAAAT   
  
  
- TACAATTAAC CATTCGTAAT AAACAACCTA ATAAGGTGAC GTGAAAGAGA ATGATTGAAG TCGTAAAGTA   
  
  
- AGGAACAAGA AGTCGAAAAC TTGAAGAAAT CGGATAAAAC AACAGAAATA CCAATACCTT CGGTAGGTCT   
  
  
- TCTTCTTACT ACTTGAAGAC TCAGAATCGA ACCGGTAACA GCCAGTGAGA CTAGCGTGAC TCTTTTTTGA   
  
  
- CTTCTCCGCC TAACTACAGA GGCTAGGAGA CTAGTGAAGT CCACTTCCGA CGCTTCCCTT CTATTAGTCC   
  
  
- GAGGAGGTTC TCTCTGTCTT GAACAACTTG TAGTTCGTTT CTCCTTTTCC GCAGTAAGTT CTTCCCTTCC   
  
  
- CAGAAGTAGA ATAGGTAGAC GAGGAACATA GACGGTGGAA TTAGTTGCTT TTGTTGTAGT CAAGTCGACA   
  
  
- ACTATTAGAA TAACTCGAGA AGGTTATACA GAGGCTTTCA CCACTAAGTC CTGTTTCCCA ACGACGAATG   
  
  
- AAACGCCTAC CCAATTGTCG TTCCTAGAAT TGAGTCTCCA GAGGTAAAAT AGCGGACTAT GACCCGTTTG   
  
  
- GTCGTGGACG ACTTCTCAAA AGTCGAAAAT GAGTGAATAT ATCTCGGAGA GGTAAGATAG TCAAACGAGT   
  
  
- AAAGTGTCGG TTAGTCCGTT AACATCTTCG TAAACTCTCC CTCCTTCTCT CATTGTTGAC CCGAAACGTA   
  
  
- CATTAACTAA ACCTGTAGAA CGTACCAAAG GTCACCGGAA GAGAATAAGT CAGAGAGAGG CTTCTCCGAT   
  
  
- GAACAAGTTT GGCAAGTAAC AGAGAAGTTT AATGTCCGAA GCCTTCTTCG AATCTTCTCG AGTAACTTTG   
  
  
- TCTTCGAGCC GACCATTCAA AGAGATTCTC GAAAGTCTTA TAGTTGAAAC TTAAGGTGCC CAAAGACTCT   
  
  
- CCGAGCTTTG AGTCCTTGGA TTCCTCCCTC TTACTTTGTC ATCAACATTT AGAGCAAAAG GTGGAGTCAT   
  
  
- GAAACTTTCT ATGGCATGTC TAAAGACTGT GGAACTGAAG ACATGTAAGT GATTTGGGGA GATAACACCA   
  
  
- TAATCAGCTC TCTCTTCCTT CAGCTTTGTC TACACCCAAG GAGAGTTCAA TACATCTACG AGACGTAATA   
  
  
- ATACGACGTT ACAAACTGAG AAATCTACTA ACGGAGGGTG AACTTTCAGG ACTCTCCGAC TCGTATCTCT   
  
  
- TCTTGGTAGA ACCTTTTCTC TAGTTTCTTC GGTATCCAAC ACTCTTCCTA CTTTGTTTGA TAGACTTCAA   
  
  
- ACTCTACGAC CTCTGAACCT CTCCCTCCTA CCTCTCAGTA CCTAAAAGTC CATACTTCGA TTCAAGGTCC   
  
  
- CGTTGATATG TTCGTTTCGA CGAAAATTTT TACCCTTCGG TAATAGTATG GATAAACCTT CTTCTGACAC   
  
  
- CTCCACCACC CAAGTCTCAA ACACTTGCTC TACTACCTTA CCGATAGAGA GAACCGACCG TTCCATCCAA   
  
  
- GGAGCGTTGA CGGAGCCATA CCGCAAGACA CAC

+     TCA-element

| Site Name | Organism | Position | Strand | Matrix score. | sequence | function |
| --- | --- | --- | --- | --- | --- | --- |
| TCA-element | Nicotiana tabacum | 1703 | - | 9 | CCATCTTTTT | cis-acting element involved in salicylic acid responsiveness |
| TCA-element | Brassica oleracea | 2652 | + | 9 | TCAGAAGAGG | cis-acting element involved in salicylic acid responsiveness |

>HU07G00272.1   
+ -Up\_Stream \_Len000ACCAAC GATTCTTTAC TGAATCAGCT ACATTTTTGG ATTGTGTTAA GGTATCTTTT   
  
  
+ AGATCTTCAG GAGGAGGGGG CTGAAGTAGC TTGGTTACTT TTGCGCTTAT CAACTGTGGA AGAATATTAT   
  
  
+ TGAGACTAGT GTAGAAGAAT TAGTAGTTTC CTCCACATTG GAAAAAAACC ATGAAATTAA CGCTTTAGGC   
  
  
+ CCTCAGAGTA CCAAGTGTCA TCGTAAATGA TTGAAATTTC AAGTCTTAGC TGAGTCATGA AGTCCTGATT   
  
  
+ TCCCATGACA AATTGATGTT GTTGTCTAAA GCTTGGCGGG TGATTTGAAA GAAGATTTAG ATCAGGCTGA   
  
  
+ CAATACACCA AATCTGGGAG CAAGGAACTG GATTTTTCTG TGTCTAGAAA AGCTTTCCGC TTTCTGAACA   
  
  
+ ATTGAGAAAA CCATGCATAG GAAATTTCTT GATATTTAGA TCATAGCAGA CTTAACAATC TGGCTTTACC   
  
  
+ GAACTGTCCT CTACTTCTAA GTTGTGAAGG TGTGAAAGAT AAACCATGTC AAACTTAGTG TCCAACAGAA   
  
  
+ ACGCATCTCT ACTTCTCCAG GCAAAGTTGT ACTTCAGATC TTCTACACCT TTTCTAGGCA GGACTTTGGG   
  
  
+ ATATAGAGTT TGGCCTGGGT GAAAAGTAGG AAGTGGGGAA GTAGAAAATG ATGAAAGAAG GCACCACTTG   
  
  
+ GGTCCAGATA CATAGGCCTA CGCACGAGAA CCTTTGCATG GTCTAGATAC ATGACTGTAT ACTTCTTCCT   
  
  
+ TTCCTTTACC TGTTGGAGAT TATCCTAGGA TTTTACATGG CACCAGCTGG ATTTGTTAAT GACATAAACA   
  
  
+ TATCTTTAAT TACAGTTGCT TATGGTATAC ATTTAAACTA TTCGATAGTT GACAGCTGAT GTCCTTATCC   
  
  
+ TTGTGCAGCT TATTCCTCGA TAGAATAATC TTTGGACATT TCTGTCTAGG TGATAAAGGG CAAAAACTAA   
  
  
+ GATACAAGAA ATAGAACATG TCCTCCATTT ACAAGACGTA TCTGTTTTTG GTTTTGGTCA ATGATCCCTG   
  
  
+ TTGTCCTCAA GCATTAACAC AGAATGATCA TTATTGACTT GGACTGAAAG GTTTTGGGAA CTCCATAGTA   
  
  
+ TGCCTTTTCA ATAAAATCAA AAGCAAATTT GCTGTTCACT GATAACTGCA GTTGTGTTGC TTTGCTGTTT   
  
  
+ CCATACCAGA CTCGTAACCA AGCTGATAGC AAATGTGCAA AGGTACAGTA TAATTAACGA TGTTTTCATA   
  
  
+ TTGCTACGAG TTCTGAAACA TGATATAAGA AAACACCAGA ACCCAAATAC CTACATAGGT CCTAGCCCTG   
  
  
+ GAATGGCTAT TTTAAATATC CAAATGGACT TACAGAATTC GCAGAGTTGT TTGGTTTGTT TAACAATCAT   
  
  
+ TGAATTGTTA GCTATGAGAA TCTGCGGGAC AATCGTAATC TTTGGCGATT ACTTCGATTG GCATACTGAT   
  
  
+ GTTTTTTTAT TTGCGTATCT AAGCATGGTG GTGTACCTTT GATTTGTGTG TCTAGGTAGT GTGTGTAGAT   
  
  
+ AAATATCATA GAACTCAGAC GTTGGTAGGG TTATGTTGAG CACTTTCTCT TGCTGTTTGT CCTTTGTGAT   
  
  
+ GGCACCTCTT GCTTTTCCGA ACCACCAAAA GTCTCATTGA CAAAACTCTT CAAACATTGC AAACTGCCCC   
  
  
+ CTTAAATATG TTTTATTAAA AAAAATGGAA GAGCTCAAAC TGTTGAGTGA GCCTAGATTC CAAGATTTAG   
  
  
+ AAACAGTAAA CTTGATAGAT ATATTTATAT GAACTATTGT TAGATGCAAC ACCCCCAACT TGAAACACAA   
  
  
+ AAGAGAAATT AAAAACAAAA AGGTTTTGCA CTATTTATAT ATCCTGTGTT CTTTGTGCTA GTCTTATTTA   
  
  
+ ATGTTAATTG GTAAGCATTA TTTGTTGGAT TATTCCACTG CACTTTCTCT TACTAACTTC AGCATTTCAT   
  
  
+ TCCTTGTTCT TCAGCTTTTG AACTTCTTTA GCCTATTTTG TTGTCTTTAT GGTTATGGAA GCCATCCAGA   
  
  
+ AGAAGAATGA TGAACTTCTG AGTCTTAGCT TGGCCATTGT CGGTCACTCT GATCGCACTG AGAAAAAACT   
  
  
+ GAAGAGGCGG ATTGATGTCT CCGATCCTCT GATCACTTCA GGTGAAGGCT GCGAAGGGAA GATAATCAGG   
  
  
+ CTCCTCCAAG AGAGACAGAA CTTGTTGAAC ATCAAGCAAA GAGGAAAAGG CGTCATTCAA GAAGGGAAGG   
  
  
+ GTCTTCATCT TATCCATCTG CTCCTTGTAT CTGCCACCTT AATCAACGAA AACAACATCA GTTCAGCTGT   
  
  
+ TGATAATCTT ATTGAGCTCT TCCAATATGT CTCCGAAAGT GGTGATTCAG GACAAAGGGT TGCTGCTTAC   
  
  
+ TTTGCGGATG GGTTAACAGC AAGGATCTTA ACTCAGAGGT CTCCATTTTA TCGCCTGATA CTGGGCAAAC   
  
  
+ CAGCACCTGC TGAAGAGTTT TCAGCTTTTA CTCACTTATA TAGAGCCTCT CCATTCTATC AGTTTGCTCA   
  
  
+ TTTCACAGCC AATCAGGCAA TTGTAGAAGC ATTTGAGAGG GAGGAAGAGA GTAACAACTG GGCTTTGCAT   
  
  
+ GTAATTGATT TGGACATCTT GCATGGTTTC CAGTGGCCTT CTCTTATTCA GTCTCTCTCC GAAGAGGCTA   
  
  
+ CTTGTTCAAA CCGTTCATTG TCTCTTCAAA TTACAGGCTT CGGAAGAAGC TTAGAAGAGC TCATTGAAAC   
  
  
+ AGAAGCTCGG CTGGTAAGTT TCTCTAAGAG CTTTCAGAAT ATCAACTTTG AATTCCACGG GTTTCTGAGA   
  
  
+ GGCTCGAAAC TCAGGAACCT AAGGAGGGAG AATGAAACAG TAGTTGTAAA TCTCGTTTTC CACCTCAGTA   
  
  
+ CTTTGAAAGA TACCGTACAG ATTTCTGACA CCTTGACTTC TGTACATTCA CTAAACCCCT CTATTGTGGT   
  
  
+ ATTAGTCGAG AGAGAAGGAA GTCGAAACAG ATGTGGGTTC CTCTCAAGTT ATGTAGATGC TCTGCATTAT   
  
  
+ TATGCTGCAA TGTTTGACTC TTTAGATGAT TGCCTCCCAC TTGAAAGTCC TGAGAGGCTG AGCATAGAGA   
  
  
+ AGAACCATCT TGGAAAAGAG ATCAAAGAAG CCATAGGTTG TGAGAAGGAT GAAACAAACT ATCTGAAGTT   
  
  
+ TGAGATGCTG GAGACTTGGA GAGGGAGGAT GGAGAGTCAT GGATTTTCAG GTATGAAGCT AAGTTCCAGG   
  
  
+ GCAACTATAC AAGCAAAGCT GCTTTTAAAA ATGGGAAGCC ATTATCATAC CTATTTGGAA GAAGACTGTG   
  
  
+ GAGGTGGTGG GTTCAGAGTT TGTGAACGAG ATGATGGAAT GGCTATCTCT CTTGGCTGGC AAGGTAGGTT   
  
  
+ CCTCGCAACT GCCTCGGTAT GGCGTTCTGT GTG  

- -Up\_Stream \_Len000TGGTTG CTAAGAAATG ACTTAGTCGA TGTAAAAACC TAACACAATT CCATAGAAAA   
  
  
- TCTAGAAGTC CTCCTCCCCC GACTTCATCG AACCAATGAA AACGCGAATA GTTGACACCT TCTTATAATA   
  
  
- ACTCTGATCA CATCTTCTTA ATCATCAAAG GAGGTGTAAC CTTTTTTTGG TACTTTAATT GCGAAATCCG   
  
  
- GGAGTCTCAT GGTTCACAGT AGCATTTACT AACTTTAAAG TTCAGAATCG ACTCAGTACT TCAGGACTAA   
  
  
- AGGGTACTGT TTAACTACAA CAACAGATTT CGAACCGCCC ACTAAACTTT CTTCTAAATC TAGTCCGACT   
  
  
- GTTATGTGGT TTAGACCCTC GTTCCTTGAC CTAAAAAGAC ACAGATCTTT TCGAAAGGCG AAAGACTTGT   
  
  
- TAACTCTTTT GGTACGTATC CTTTAAAGAA CTATAAATCT AGTATCGTCT GAATTGTTAG ACCGAAATGG   
  
  
- CTTGACAGGA GATGAAGATT CAACACTTCC ACACTTTCTA TTTGGTACAG TTTGAATCAC AGGTTGTCTT   
  
  
- TGCGTAGAGA TGAAGAGGTC CGTTTCAACA TGAAGTCTAG AAGATGTGGA AAAGATCCGT CCTGAAACCC   
  
  
- TATATCTCAA ACCGGACCCA CTTTTCATCC TTCACCCCTT CATCTTTTAC TACTTTCTTC CGTGGTGAAC   
  
  
- CCAGGTCTAT GTATCCGGAT GCGTGCTCTT GGAAACGTAC CAGATCTATG TACTGACATA TGAAGAAGGA   
  
  
- AAGGAAATGG ACAACCTCTA ATAGGATCCT AAAATGTACC GTGGTCGACC TAAACAATTA CTGTATTTGT   
  
  
- ATAGAAATTA ATGTCAACGA ATACCATATG TAAATTTGAT AAGCTATCAA CTGTCGACTA CAGGAATAGG   
  
  
- AACACGTCGA ATAAGGAGCT ATCTTATTAG AAACCTGTAA AGACAGATCC ACTATTTCCC GTTTTTGATT   
  
  
- CTATGTTCTT TATCTTGTAC AGGAGGTAAA TGTTCTGCAT AGACAAAAAC CAAAACCAGT TACTAGGGAC   
  
  
- AACAGGAGTT CGTAATTGTG TCTTACTAGT AATAACTGAA CCTGACTTTC CAAAACCCTT GAGGTATCAT   
  
  
- ACGGAAAAGT TATTTTAGTT TTCGTTTAAA CGACAAGTGA CTATTGACGT CAACACAACG AAACGACAAA   
  
  
- GGTATGGTCT GAGCATTGGT TCGACTATCG TTTACACGTT TCCATGTCAT ATTAATTGCT ACAAAAGTAT   
  
  
- AACGATGCTC AAGACTTTGT ACTATATTCT TTTGTGGTCT TGGGTTTATG GATGTATCCA GGATCGGGAC   
  
  
- CTTACCGATA AAATTTATAG GTTTACCTGA ATGTCTTAAG CGTCTCAACA AACCAAACAA ATTGTTAGTA   
  
  
- ACTTAACAAT CGATACTCTT AGACGCCCTG TTAGCATTAG AAACCGCTAA TGAAGCTAAC CGTATGACTA   
  
  
- CAAAAAAATA AACGCATAGA TTCGTACCAC CACATGGAAA CTAAACACAC AGATCCATCA CACACATCTA   
  
  
- TTTATAGTAT CTTGAGTCTG CAACCATCCC AATACAACTC GTGAAAGAGA ACGACAAACA GGAAACACTA   
  
  
- CCGTGGAGAA CGAAAAGGCT TGGTGGTTTT CAGAGTAACT GTTTTGAGAA GTTTGTAACG TTTGACGGGG   
  
  
- GAATTTATAC AAAATAATTT TTTTTACCTT CTCGAGTTTG ACAACTCACT CGGATCTAAG GTTCTAAATC   
  
  
- TTTGTCATTT GAACTATCTA TATAAATATA CTTGATAACA ATCTACGTTG TGGGGGTTGA ACTTTGTGTT   
  
  
- TTCTCTTTAA TTTTTGTTTT TCCAAAACGT GATAAATATA TAGGACACAA GAAACACGAT CAGAATAAAT   
  
  
- TACAATTAAC CATTCGTAAT AAACAACCTA ATAAGGTGAC GTGAAAGAGA ATGATTGAAG TCGTAAAGTA   
  
  
- AGGAACAAGA AGTCGAAAAC TTGAAGAAAT CGGATAAAAC AACAGAAATA CCAATACCTT CGGTAGGTCT   
  
  
- TCTTCTTACT ACTTGAAGAC TCAGAATCGA ACCGGTAACA GCCAGTGAGA CTAGCGTGAC TCTTTTTTGA   
  
  
- CTTCTCCGCC TAACTACAGA GGCTAGGAGA CTAGTGAAGT CCACTTCCGA CGCTTCCCTT CTATTAGTCC   
  
  
- GAGGAGGTTC TCTCTGTCTT GAACAACTTG TAGTTCGTTT CTCCTTTTCC GCAGTAAGTT CTTCCCTTCC   
  
  
- CAGAAGTAGA ATAGGTAGAC GAGGAACATA GACGGTGGAA TTAGTTGCTT TTGTTGTAGT CAAGTCGACA   
  
  
- ACTATTAGAA TAACTCGAGA AGGTTATACA GAGGCTTTCA CCACTAAGTC CTGTTTCCCA ACGACGAATG   
  
  
- AAACGCCTAC CCAATTGTCG TTCCTAGAAT TGAGTCTCCA GAGGTAAAAT AGCGGACTAT GACCCGTTTG   
  
  
- GTCGTGGACG ACTTCTCAAA AGTCGAAAAT GAGTGAATAT ATCTCGGAGA GGTAAGATAG TCAAACGAGT   
  
  
- AAAGTGTCGG TTAGTCCGTT AACATCTTCG TAAACTCTCC CTCCTTCTCT CATTGTTGAC CCGAAACGTA   
  
  
- CATTAACTAA ACCTGTAGAA CGTACCAAAG GTCACCGGAA GAGAATAAGT CAGAGAGAGG CTTCTCCGAT   
  
  
- GAACAAGTTT GGCAAGTAAC AGAGAAGTTT AATGTCCGAA GCCTTCTTCG AATCTTCTCG AGTAACTTTG   
  
  
- TCTTCGAGCC GACCATTCAA AGAGATTCTC GAAAGTCTTA TAGTTGAAAC TTAAGGTGCC CAAAGACTCT   
  
  
- CCGAGCTTTG AGTCCTTGGA TTCCTCCCTC TTACTTTGTC ATCAACATTT AGAGCAAAAG GTGGAGTCAT   
  
  
- GAAACTTTCT ATGGCATGTC TAAAGACTGT GGAACTGAAG ACATGTAAGT GATTTGGGGA GATAACACCA   
  
  
- TAATCAGCTC TCTCTTCCTT CAGCTTTGTC TACACCCAAG GAGAGTTCAA TACATCTACG AGACGTAATA   
  
  
- ATACGACGTT ACAAACTGAG AAATCTACTA ACGGAGGGTG AACTTTCAGG ACTCTCCGAC TCGTATCTCT   
  
  
- TCTTGGTAGA ACCTTTTCTC TAGTTTCTTC GGTATCCAAC ACTCTTCCTA CTTTGTTTGA TAGACTTCAA   
  
  
- ACTCTACGAC CTCTGAACCT CTCCCTCCTA CCTCTCAGTA CCTAAAAGTC CATACTTCGA TTCAAGGTCC   
  
  
- CGTTGATATG TTCGTTTCGA CGAAAATTTT TACCCTTCGG TAATAGTATG GATAAACCTT CTTCTGACAC   
  
  
- CTCCACCACC CAAGTCTCAA ACACTTGCTC TACTACCTTA CCGATAGAGA GAACCGACCG TTCCATCCAA   
  
  
- GGAGCGTTGA CGGAGCCATA CCGCAAGACA CAC

+     TCCC-motif

| Site Name | Organism | Position | Strand | Matrix score. | sequence | function |
| --- | --- | --- | --- | --- | --- | --- |
| TCCC-motif | Spinacia oleracea | 2829 | - | 7 | TCTCCCT | part of a light responsive element |

>HU07G00272.1   
+ -Up\_Stream \_Len000ACCAAC GATTCTTTAC TGAATCAGCT ACATTTTTGG ATTGTGTTAA GGTATCTTTT   
  
  
+ AGATCTTCAG GAGGAGGGGG CTGAAGTAGC TTGGTTACTT TTGCGCTTAT CAACTGTGGA AGAATATTAT   
  
  
+ TGAGACTAGT GTAGAAGAAT TAGTAGTTTC CTCCACATTG GAAAAAAACC ATGAAATTAA CGCTTTAGGC   
  
  
+ CCTCAGAGTA CCAAGTGTCA TCGTAAATGA TTGAAATTTC AAGTCTTAGC TGAGTCATGA AGTCCTGATT   
  
  
+ TCCCATGACA AATTGATGTT GTTGTCTAAA GCTTGGCGGG TGATTTGAAA GAAGATTTAG ATCAGGCTGA   
  
  
+ CAATACACCA AATCTGGGAG CAAGGAACTG GATTTTTCTG TGTCTAGAAA AGCTTTCCGC TTTCTGAACA   
  
  
+ ATTGAGAAAA CCATGCATAG GAAATTTCTT GATATTTAGA TCATAGCAGA CTTAACAATC TGGCTTTACC   
  
  
+ GAACTGTCCT CTACTTCTAA GTTGTGAAGG TGTGAAAGAT AAACCATGTC AAACTTAGTG TCCAACAGAA   
  
  
+ ACGCATCTCT ACTTCTCCAG GCAAAGTTGT ACTTCAGATC TTCTACACCT TTTCTAGGCA GGACTTTGGG   
  
  
+ ATATAGAGTT TGGCCTGGGT GAAAAGTAGG AAGTGGGGAA GTAGAAAATG ATGAAAGAAG GCACCACTTG   
  
  
+ GGTCCAGATA CATAGGCCTA CGCACGAGAA CCTTTGCATG GTCTAGATAC ATGACTGTAT ACTTCTTCCT   
  
  
+ TTCCTTTACC TGTTGGAGAT TATCCTAGGA TTTTACATGG CACCAGCTGG ATTTGTTAAT GACATAAACA   
  
  
+ TATCTTTAAT TACAGTTGCT TATGGTATAC ATTTAAACTA TTCGATAGTT GACAGCTGAT GTCCTTATCC   
  
  
+ TTGTGCAGCT TATTCCTCGA TAGAATAATC TTTGGACATT TCTGTCTAGG TGATAAAGGG CAAAAACTAA   
  
  
+ GATACAAGAA ATAGAACATG TCCTCCATTT ACAAGACGTA TCTGTTTTTG GTTTTGGTCA ATGATCCCTG   
  
  
+ TTGTCCTCAA GCATTAACAC AGAATGATCA TTATTGACTT GGACTGAAAG GTTTTGGGAA CTCCATAGTA   
  
  
+ TGCCTTTTCA ATAAAATCAA AAGCAAATTT GCTGTTCACT GATAACTGCA GTTGTGTTGC TTTGCTGTTT   
  
  
+ CCATACCAGA CTCGTAACCA AGCTGATAGC AAATGTGCAA AGGTACAGTA TAATTAACGA TGTTTTCATA   
  
  
+ TTGCTACGAG TTCTGAAACA TGATATAAGA AAACACCAGA ACCCAAATAC CTACATAGGT CCTAGCCCTG   
  
  
+ GAATGGCTAT TTTAAATATC CAAATGGACT TACAGAATTC GCAGAGTTGT TTGGTTTGTT TAACAATCAT   
  
  
+ TGAATTGTTA GCTATGAGAA TCTGCGGGAC AATCGTAATC TTTGGCGATT ACTTCGATTG GCATACTGAT   
  
  
+ GTTTTTTTAT TTGCGTATCT AAGCATGGTG GTGTACCTTT GATTTGTGTG TCTAGGTAGT GTGTGTAGAT   
  
  
+ AAATATCATA GAACTCAGAC GTTGGTAGGG TTATGTTGAG CACTTTCTCT TGCTGTTTGT CCTTTGTGAT   
  
  
+ GGCACCTCTT GCTTTTCCGA ACCACCAAAA GTCTCATTGA CAAAACTCTT CAAACATTGC AAACTGCCCC   
  
  
+ CTTAAATATG TTTTATTAAA AAAAATGGAA GAGCTCAAAC TGTTGAGTGA GCCTAGATTC CAAGATTTAG   
  
  
+ AAACAGTAAA CTTGATAGAT ATATTTATAT GAACTATTGT TAGATGCAAC ACCCCCAACT TGAAACACAA   
  
  
+ AAGAGAAATT AAAAACAAAA AGGTTTTGCA CTATTTATAT ATCCTGTGTT CTTTGTGCTA GTCTTATTTA   
  
  
+ ATGTTAATTG GTAAGCATTA TTTGTTGGAT TATTCCACTG CACTTTCTCT TACTAACTTC AGCATTTCAT   
  
  
+ TCCTTGTTCT TCAGCTTTTG AACTTCTTTA GCCTATTTTG TTGTCTTTAT GGTTATGGAA GCCATCCAGA   
  
  
+ AGAAGAATGA TGAACTTCTG AGTCTTAGCT TGGCCATTGT CGGTCACTCT GATCGCACTG AGAAAAAACT   
  
  
+ GAAGAGGCGG ATTGATGTCT CCGATCCTCT GATCACTTCA GGTGAAGGCT GCGAAGGGAA GATAATCAGG   
  
  
+ CTCCTCCAAG AGAGACAGAA CTTGTTGAAC ATCAAGCAAA GAGGAAAAGG CGTCATTCAA GAAGGGAAGG   
  
  
+ GTCTTCATCT TATCCATCTG CTCCTTGTAT CTGCCACCTT AATCAACGAA AACAACATCA GTTCAGCTGT   
  
  
+ TGATAATCTT ATTGAGCTCT TCCAATATGT CTCCGAAAGT GGTGATTCAG GACAAAGGGT TGCTGCTTAC   
  
  
+ TTTGCGGATG GGTTAACAGC AAGGATCTTA ACTCAGAGGT CTCCATTTTA TCGCCTGATA CTGGGCAAAC   
  
  
+ CAGCACCTGC TGAAGAGTTT TCAGCTTTTA CTCACTTATA TAGAGCCTCT CCATTCTATC AGTTTGCTCA   
  
  
+ TTTCACAGCC AATCAGGCAA TTGTAGAAGC ATTTGAGAGG GAGGAAGAGA GTAACAACTG GGCTTTGCAT   
  
  
+ GTAATTGATT TGGACATCTT GCATGGTTTC CAGTGGCCTT CTCTTATTCA GTCTCTCTCC GAAGAGGCTA   
  
  
+ CTTGTTCAAA CCGTTCATTG TCTCTTCAAA TTACAGGCTT CGGAAGAAGC TTAGAAGAGC TCATTGAAAC   
  
  
+ AGAAGCTCGG CTGGTAAGTT TCTCTAAGAG CTTTCAGAAT ATCAACTTTG AATTCCACGG GTTTCTGAGA   
  
  
+ GGCTCGAAAC TCAGGAACCT AAGGAGGGAG AATGAAACAG TAGTTGTAAA TCTCGTTTTC CACCTCAGTA   
  
  
+ CTTTGAAAGA TACCGTACAG ATTTCTGACA CCTTGACTTC TGTACATTCA CTAAACCCCT CTATTGTGGT   
  
  
+ ATTAGTCGAG AGAGAAGGAA GTCGAAACAG ATGTGGGTTC CTCTCAAGTT ATGTAGATGC TCTGCATTAT   
  
  
+ TATGCTGCAA TGTTTGACTC TTTAGATGAT TGCCTCCCAC TTGAAAGTCC TGAGAGGCTG AGCATAGAGA   
  
  
+ AGAACCATCT TGGAAAAGAG ATCAAAGAAG CCATAGGTTG TGAGAAGGAT GAAACAAACT ATCTGAAGTT   
  
  
+ TGAGATGCTG GAGACTTGGA GAGGGAGGAT GGAGAGTCAT GGATTTTCAG GTATGAAGCT AAGTTCCAGG   
  
  
+ GCAACTATAC AAGCAAAGCT GCTTTTAAAA ATGGGAAGCC ATTATCATAC CTATTTGGAA GAAGACTGTG   
  
  
+ GAGGTGGTGG GTTCAGAGTT TGTGAACGAG ATGATGGAAT GGCTATCTCT CTTGGCTGGC AAGGTAGGTT   
  
  
+ CCTCGCAACT GCCTCGGTAT GGCGTTCTGT GTG  

- -Up\_Stream \_Len000TGGTTG CTAAGAAATG ACTTAGTCGA TGTAAAAACC TAACACAATT CCATAGAAAA   
  
  
- TCTAGAAGTC CTCCTCCCCC GACTTCATCG AACCAATGAA AACGCGAATA GTTGACACCT TCTTATAATA   
  
  
- ACTCTGATCA CATCTTCTTA ATCATCAAAG GAGGTGTAAC CTTTTTTTGG TACTTTAATT GCGAAATCCG   
  
  
- GGAGTCTCAT GGTTCACAGT AGCATTTACT AACTTTAAAG TTCAGAATCG ACTCAGTACT TCAGGACTAA   
  
  
- AGGGTACTGT TTAACTACAA CAACAGATTT CGAACCGCCC ACTAAACTTT CTTCTAAATC TAGTCCGACT   
  
  
- GTTATGTGGT TTAGACCCTC GTTCCTTGAC CTAAAAAGAC ACAGATCTTT TCGAAAGGCG AAAGACTTGT   
  
  
- TAACTCTTTT GGTACGTATC CTTTAAAGAA CTATAAATCT AGTATCGTCT GAATTGTTAG ACCGAAATGG   
  
  
- CTTGACAGGA GATGAAGATT CAACACTTCC ACACTTTCTA TTTGGTACAG TTTGAATCAC AGGTTGTCTT   
  
  
- TGCGTAGAGA TGAAGAGGTC CGTTTCAACA TGAAGTCTAG AAGATGTGGA AAAGATCCGT CCTGAAACCC   
  
  
- TATATCTCAA ACCGGACCCA CTTTTCATCC TTCACCCCTT CATCTTTTAC TACTTTCTTC CGTGGTGAAC   
  
  
- CCAGGTCTAT GTATCCGGAT GCGTGCTCTT GGAAACGTAC CAGATCTATG TACTGACATA TGAAGAAGGA   
  
  
- AAGGAAATGG ACAACCTCTA ATAGGATCCT AAAATGTACC GTGGTCGACC TAAACAATTA CTGTATTTGT   
  
  
- ATAGAAATTA ATGTCAACGA ATACCATATG TAAATTTGAT AAGCTATCAA CTGTCGACTA CAGGAATAGG   
  
  
- AACACGTCGA ATAAGGAGCT ATCTTATTAG AAACCTGTAA AGACAGATCC ACTATTTCCC GTTTTTGATT   
  
  
- CTATGTTCTT TATCTTGTAC AGGAGGTAAA TGTTCTGCAT AGACAAAAAC CAAAACCAGT TACTAGGGAC   
  
  
- AACAGGAGTT CGTAATTGTG TCTTACTAGT AATAACTGAA CCTGACTTTC CAAAACCCTT GAGGTATCAT   
  
  
- ACGGAAAAGT TATTTTAGTT TTCGTTTAAA CGACAAGTGA CTATTGACGT CAACACAACG AAACGACAAA   
  
  
- GGTATGGTCT GAGCATTGGT TCGACTATCG TTTACACGTT TCCATGTCAT ATTAATTGCT ACAAAAGTAT   
  
  
- AACGATGCTC AAGACTTTGT ACTATATTCT TTTGTGGTCT TGGGTTTATG GATGTATCCA GGATCGGGAC   
  
  
- CTTACCGATA AAATTTATAG GTTTACCTGA ATGTCTTAAG CGTCTCAACA AACCAAACAA ATTGTTAGTA   
  
  
- ACTTAACAAT CGATACTCTT AGACGCCCTG TTAGCATTAG AAACCGCTAA TGAAGCTAAC CGTATGACTA   
  
  
- CAAAAAAATA AACGCATAGA TTCGTACCAC CACATGGAAA CTAAACACAC AGATCCATCA CACACATCTA   
  
  
- TTTATAGTAT CTTGAGTCTG CAACCATCCC AATACAACTC GTGAAAGAGA ACGACAAACA GGAAACACTA   
  
  
- CCGTGGAGAA CGAAAAGGCT TGGTGGTTTT CAGAGTAACT GTTTTGAGAA GTTTGTAACG TTTGACGGGG   
  
  
- GAATTTATAC AAAATAATTT TTTTTACCTT CTCGAGTTTG ACAACTCACT CGGATCTAAG GTTCTAAATC   
  
  
- TTTGTCATTT GAACTATCTA TATAAATATA CTTGATAACA ATCTACGTTG TGGGGGTTGA ACTTTGTGTT   
  
  
- TTCTCTTTAA TTTTTGTTTT TCCAAAACGT GATAAATATA TAGGACACAA GAAACACGAT CAGAATAAAT   
  
  
- TACAATTAAC CATTCGTAAT AAACAACCTA ATAAGGTGAC GTGAAAGAGA ATGATTGAAG TCGTAAAGTA   
  
  
- AGGAACAAGA AGTCGAAAAC TTGAAGAAAT CGGATAAAAC AACAGAAATA CCAATACCTT CGGTAGGTCT   
  
  
- TCTTCTTACT ACTTGAAGAC TCAGAATCGA ACCGGTAACA GCCAGTGAGA CTAGCGTGAC TCTTTTTTGA   
  
  
- CTTCTCCGCC TAACTACAGA GGCTAGGAGA CTAGTGAAGT CCACTTCCGA CGCTTCCCTT CTATTAGTCC   
  
  
- GAGGAGGTTC TCTCTGTCTT GAACAACTTG TAGTTCGTTT CTCCTTTTCC GCAGTAAGTT CTTCCCTTCC   
  
  
- CAGAAGTAGA ATAGGTAGAC GAGGAACATA GACGGTGGAA TTAGTTGCTT TTGTTGTAGT CAAGTCGACA   
  
  
- ACTATTAGAA TAACTCGAGA AGGTTATACA GAGGCTTTCA CCACTAAGTC CTGTTTCCCA ACGACGAATG   
  
  
- AAACGCCTAC CCAATTGTCG TTCCTAGAAT TGAGTCTCCA GAGGTAAAAT AGCGGACTAT GACCCGTTTG   
  
  
- GTCGTGGACG ACTTCTCAAA AGTCGAAAAT GAGTGAATAT ATCTCGGAGA GGTAAGATAG TCAAACGAGT   
  
  
- AAAGTGTCGG TTAGTCCGTT AACATCTTCG TAAACTCTCC CTCCTTCTCT CATTGTTGAC CCGAAACGTA   
  
  
- CATTAACTAA ACCTGTAGAA CGTACCAAAG GTCACCGGAA GAGAATAAGT CAGAGAGAGG CTTCTCCGAT   
  
  
- GAACAAGTTT GGCAAGTAAC AGAGAAGTTT AATGTCCGAA GCCTTCTTCG AATCTTCTCG AGTAACTTTG   
  
  
- TCTTCGAGCC GACCATTCAA AGAGATTCTC GAAAGTCTTA TAGTTGAAAC TTAAGGTGCC CAAAGACTCT   
  
  
- CCGAGCTTTG AGTCCTTGGA TTCCTCCCTC TTACTTTGTC ATCAACATTT AGAGCAAAAG GTGGAGTCAT   
  
  
- GAAACTTTCT ATGGCATGTC TAAAGACTGT GGAACTGAAG ACATGTAAGT GATTTGGGGA GATAACACCA   
  
  
- TAATCAGCTC TCTCTTCCTT CAGCTTTGTC TACACCCAAG GAGAGTTCAA TACATCTACG AGACGTAATA   
  
  
- ATACGACGTT ACAAACTGAG AAATCTACTA ACGGAGGGTG AACTTTCAGG ACTCTCCGAC TCGTATCTCT   
  
  
- TCTTGGTAGA ACCTTTTCTC TAGTTTCTTC GGTATCCAAC ACTCTTCCTA CTTTGTTTGA TAGACTTCAA   
  
  
- ACTCTACGAC CTCTGAACCT CTCCCTCCTA CCTCTCAGTA CCTAAAAGTC CATACTTCGA TTCAAGGTCC   
  
  
- CGTTGATATG TTCGTTTCGA CGAAAATTTT TACCCTTCGG TAATAGTATG GATAAACCTT CTTCTGACAC   
  
  
- CTCCACCACC CAAGTCTCAA ACACTTGCTC TACTACCTTA CCGATAGAGA GAACCGACCG TTCCATCCAA   
  
  
- GGAGCGTTGA CGGAGCCATA CCGCAAGACA CAC

+     TCT-motif

| Site Name | Organism | Position | Strand | Matrix score. | sequence | function |
| --- | --- | --- | --- | --- | --- | --- |
| TCT-motif | Arabidopsis thaliana | 1942 | + | 6 | TCTTAC | part of a light responsive element |

>HU07G00272.1   
+ -Up\_Stream \_Len000ACCAAC GATTCTTTAC TGAATCAGCT ACATTTTTGG ATTGTGTTAA GGTATCTTTT   
  
  
+ AGATCTTCAG GAGGAGGGGG CTGAAGTAGC TTGGTTACTT TTGCGCTTAT CAACTGTGGA AGAATATTAT   
  
  
+ TGAGACTAGT GTAGAAGAAT TAGTAGTTTC CTCCACATTG GAAAAAAACC ATGAAATTAA CGCTTTAGGC   
  
  
+ CCTCAGAGTA CCAAGTGTCA TCGTAAATGA TTGAAATTTC AAGTCTTAGC TGAGTCATGA AGTCCTGATT   
  
  
+ TCCCATGACA AATTGATGTT GTTGTCTAAA GCTTGGCGGG TGATTTGAAA GAAGATTTAG ATCAGGCTGA   
  
  
+ CAATACACCA AATCTGGGAG CAAGGAACTG GATTTTTCTG TGTCTAGAAA AGCTTTCCGC TTTCTGAACA   
  
  
+ ATTGAGAAAA CCATGCATAG GAAATTTCTT GATATTTAGA TCATAGCAGA CTTAACAATC TGGCTTTACC   
  
  
+ GAACTGTCCT CTACTTCTAA GTTGTGAAGG TGTGAAAGAT AAACCATGTC AAACTTAGTG TCCAACAGAA   
  
  
+ ACGCATCTCT ACTTCTCCAG GCAAAGTTGT ACTTCAGATC TTCTACACCT TTTCTAGGCA GGACTTTGGG   
  
  
+ ATATAGAGTT TGGCCTGGGT GAAAAGTAGG AAGTGGGGAA GTAGAAAATG ATGAAAGAAG GCACCACTTG   
  
  
+ GGTCCAGATA CATAGGCCTA CGCACGAGAA CCTTTGCATG GTCTAGATAC ATGACTGTAT ACTTCTTCCT   
  
  
+ TTCCTTTACC TGTTGGAGAT TATCCTAGGA TTTTACATGG CACCAGCTGG ATTTGTTAAT GACATAAACA   
  
  
+ TATCTTTAAT TACAGTTGCT TATGGTATAC ATTTAAACTA TTCGATAGTT GACAGCTGAT GTCCTTATCC   
  
  
+ TTGTGCAGCT TATTCCTCGA TAGAATAATC TTTGGACATT TCTGTCTAGG TGATAAAGGG CAAAAACTAA   
  
  
+ GATACAAGAA ATAGAACATG TCCTCCATTT ACAAGACGTA TCTGTTTTTG GTTTTGGTCA ATGATCCCTG   
  
  
+ TTGTCCTCAA GCATTAACAC AGAATGATCA TTATTGACTT GGACTGAAAG GTTTTGGGAA CTCCATAGTA   
  
  
+ TGCCTTTTCA ATAAAATCAA AAGCAAATTT GCTGTTCACT GATAACTGCA GTTGTGTTGC TTTGCTGTTT   
  
  
+ CCATACCAGA CTCGTAACCA AGCTGATAGC AAATGTGCAA AGGTACAGTA TAATTAACGA TGTTTTCATA   
  
  
+ TTGCTACGAG TTCTGAAACA TGATATAAGA AAACACCAGA ACCCAAATAC CTACATAGGT CCTAGCCCTG   
  
  
+ GAATGGCTAT TTTAAATATC CAAATGGACT TACAGAATTC GCAGAGTTGT TTGGTTTGTT TAACAATCAT   
  
  
+ TGAATTGTTA GCTATGAGAA TCTGCGGGAC AATCGTAATC TTTGGCGATT ACTTCGATTG GCATACTGAT   
  
  
+ GTTTTTTTAT TTGCGTATCT AAGCATGGTG GTGTACCTTT GATTTGTGTG TCTAGGTAGT GTGTGTAGAT   
  
  
+ AAATATCATA GAACTCAGAC GTTGGTAGGG TTATGTTGAG CACTTTCTCT TGCTGTTTGT CCTTTGTGAT   
  
  
+ GGCACCTCTT GCTTTTCCGA ACCACCAAAA GTCTCATTGA CAAAACTCTT CAAACATTGC AAACTGCCCC   
  
  
+ CTTAAATATG TTTTATTAAA AAAAATGGAA GAGCTCAAAC TGTTGAGTGA GCCTAGATTC CAAGATTTAG   
  
  
+ AAACAGTAAA CTTGATAGAT ATATTTATAT GAACTATTGT TAGATGCAAC ACCCCCAACT TGAAACACAA   
  
  
+ AAGAGAAATT AAAAACAAAA AGGTTTTGCA CTATTTATAT ATCCTGTGTT CTTTGTGCTA GTCTTATTTA   
  
  
+ ATGTTAATTG GTAAGCATTA TTTGTTGGAT TATTCCACTG CACTTTCTCT TACTAACTTC AGCATTTCAT   
  
  
+ TCCTTGTTCT TCAGCTTTTG AACTTCTTTA GCCTATTTTG TTGTCTTTAT GGTTATGGAA GCCATCCAGA   
  
  
+ AGAAGAATGA TGAACTTCTG AGTCTTAGCT TGGCCATTGT CGGTCACTCT GATCGCACTG AGAAAAAACT   
  
  
+ GAAGAGGCGG ATTGATGTCT CCGATCCTCT GATCACTTCA GGTGAAGGCT GCGAAGGGAA GATAATCAGG   
  
  
+ CTCCTCCAAG AGAGACAGAA CTTGTTGAAC ATCAAGCAAA GAGGAAAAGG CGTCATTCAA GAAGGGAAGG   
  
  
+ GTCTTCATCT TATCCATCTG CTCCTTGTAT CTGCCACCTT AATCAACGAA AACAACATCA GTTCAGCTGT   
  
  
+ TGATAATCTT ATTGAGCTCT TCCAATATGT CTCCGAAAGT GGTGATTCAG GACAAAGGGT TGCTGCTTAC   
  
  
+ TTTGCGGATG GGTTAACAGC AAGGATCTTA ACTCAGAGGT CTCCATTTTA TCGCCTGATA CTGGGCAAAC   
  
  
+ CAGCACCTGC TGAAGAGTTT TCAGCTTTTA CTCACTTATA TAGAGCCTCT CCATTCTATC AGTTTGCTCA   
  
  
+ TTTCACAGCC AATCAGGCAA TTGTAGAAGC ATTTGAGAGG GAGGAAGAGA GTAACAACTG GGCTTTGCAT   
  
  
+ GTAATTGATT TGGACATCTT GCATGGTTTC CAGTGGCCTT CTCTTATTCA GTCTCTCTCC GAAGAGGCTA   
  
  
+ CTTGTTCAAA CCGTTCATTG TCTCTTCAAA TTACAGGCTT CGGAAGAAGC TTAGAAGAGC TCATTGAAAC   
  
  
+ AGAAGCTCGG CTGGTAAGTT TCTCTAAGAG CTTTCAGAAT ATCAACTTTG AATTCCACGG GTTTCTGAGA   
  
  
+ GGCTCGAAAC TCAGGAACCT AAGGAGGGAG AATGAAACAG TAGTTGTAAA TCTCGTTTTC CACCTCAGTA   
  
  
+ CTTTGAAAGA TACCGTACAG ATTTCTGACA CCTTGACTTC TGTACATTCA CTAAACCCCT CTATTGTGGT   
  
  
+ ATTAGTCGAG AGAGAAGGAA GTCGAAACAG ATGTGGGTTC CTCTCAAGTT ATGTAGATGC TCTGCATTAT   
  
  
+ TATGCTGCAA TGTTTGACTC TTTAGATGAT TGCCTCCCAC TTGAAAGTCC TGAGAGGCTG AGCATAGAGA   
  
  
+ AGAACCATCT TGGAAAAGAG ATCAAAGAAG CCATAGGTTG TGAGAAGGAT GAAACAAACT ATCTGAAGTT   
  
  
+ TGAGATGCTG GAGACTTGGA GAGGGAGGAT GGAGAGTCAT GGATTTTCAG GTATGAAGCT AAGTTCCAGG   
  
  
+ GCAACTATAC AAGCAAAGCT GCTTTTAAAA ATGGGAAGCC ATTATCATAC CTATTTGGAA GAAGACTGTG   
  
  
+ GAGGTGGTGG GTTCAGAGTT TGTGAACGAG ATGATGGAAT GGCTATCTCT CTTGGCTGGC AAGGTAGGTT   
  
  
+ CCTCGCAACT GCCTCGGTAT GGCGTTCTGT GTG  

- -Up\_Stream \_Len000TGGTTG CTAAGAAATG ACTTAGTCGA TGTAAAAACC TAACACAATT CCATAGAAAA   
  
  
- TCTAGAAGTC CTCCTCCCCC GACTTCATCG AACCAATGAA AACGCGAATA GTTGACACCT TCTTATAATA   
  
  
- ACTCTGATCA CATCTTCTTA ATCATCAAAG GAGGTGTAAC CTTTTTTTGG TACTTTAATT GCGAAATCCG   
  
  
- GGAGTCTCAT GGTTCACAGT AGCATTTACT AACTTTAAAG TTCAGAATCG ACTCAGTACT TCAGGACTAA   
  
  
- AGGGTACTGT TTAACTACAA CAACAGATTT CGAACCGCCC ACTAAACTTT CTTCTAAATC TAGTCCGACT   
  
  
- GTTATGTGGT TTAGACCCTC GTTCCTTGAC CTAAAAAGAC ACAGATCTTT TCGAAAGGCG AAAGACTTGT   
  
  
- TAACTCTTTT GGTACGTATC CTTTAAAGAA CTATAAATCT AGTATCGTCT GAATTGTTAG ACCGAAATGG   
  
  
- CTTGACAGGA GATGAAGATT CAACACTTCC ACACTTTCTA TTTGGTACAG TTTGAATCAC AGGTTGTCTT   
  
  
- TGCGTAGAGA TGAAGAGGTC CGTTTCAACA TGAAGTCTAG AAGATGTGGA AAAGATCCGT CCTGAAACCC   
  
  
- TATATCTCAA ACCGGACCCA CTTTTCATCC TTCACCCCTT CATCTTTTAC TACTTTCTTC CGTGGTGAAC   
  
  
- CCAGGTCTAT GTATCCGGAT GCGTGCTCTT GGAAACGTAC CAGATCTATG TACTGACATA TGAAGAAGGA   
  
  
- AAGGAAATGG ACAACCTCTA ATAGGATCCT AAAATGTACC GTGGTCGACC TAAACAATTA CTGTATTTGT   
  
  
- ATAGAAATTA ATGTCAACGA ATACCATATG TAAATTTGAT AAGCTATCAA CTGTCGACTA CAGGAATAGG   
  
  
- AACACGTCGA ATAAGGAGCT ATCTTATTAG AAACCTGTAA AGACAGATCC ACTATTTCCC GTTTTTGATT   
  
  
- CTATGTTCTT TATCTTGTAC AGGAGGTAAA TGTTCTGCAT AGACAAAAAC CAAAACCAGT TACTAGGGAC   
  
  
- AACAGGAGTT CGTAATTGTG TCTTACTAGT AATAACTGAA CCTGACTTTC CAAAACCCTT GAGGTATCAT   
  
  
- ACGGAAAAGT TATTTTAGTT TTCGTTTAAA CGACAAGTGA CTATTGACGT CAACACAACG AAACGACAAA   
  
  
- GGTATGGTCT GAGCATTGGT TCGACTATCG TTTACACGTT TCCATGTCAT ATTAATTGCT ACAAAAGTAT   
  
  
- AACGATGCTC AAGACTTTGT ACTATATTCT TTTGTGGTCT TGGGTTTATG GATGTATCCA GGATCGGGAC   
  
  
- CTTACCGATA AAATTTATAG GTTTACCTGA ATGTCTTAAG CGTCTCAACA AACCAAACAA ATTGTTAGTA   
  
  
- ACTTAACAAT CGATACTCTT AGACGCCCTG TTAGCATTAG AAACCGCTAA TGAAGCTAAC CGTATGACTA   
  
  
- CAAAAAAATA AACGCATAGA TTCGTACCAC CACATGGAAA CTAAACACAC AGATCCATCA CACACATCTA   
  
  
- TTTATAGTAT CTTGAGTCTG CAACCATCCC AATACAACTC GTGAAAGAGA ACGACAAACA GGAAACACTA   
  
  
- CCGTGGAGAA CGAAAAGGCT TGGTGGTTTT CAGAGTAACT GTTTTGAGAA GTTTGTAACG TTTGACGGGG   
  
  
- GAATTTATAC AAAATAATTT TTTTTACCTT CTCGAGTTTG ACAACTCACT CGGATCTAAG GTTCTAAATC   
  
  
- TTTGTCATTT GAACTATCTA TATAAATATA CTTGATAACA ATCTACGTTG TGGGGGTTGA ACTTTGTGTT   
  
  
- TTCTCTTTAA TTTTTGTTTT TCCAAAACGT GATAAATATA TAGGACACAA GAAACACGAT CAGAATAAAT   
  
  
- TACAATTAAC CATTCGTAAT AAACAACCTA ATAAGGTGAC GTGAAAGAGA ATGATTGAAG TCGTAAAGTA   
  
  
- AGGAACAAGA AGTCGAAAAC TTGAAGAAAT CGGATAAAAC AACAGAAATA CCAATACCTT CGGTAGGTCT   
  
  
- TCTTCTTACT ACTTGAAGAC TCAGAATCGA ACCGGTAACA GCCAGTGAGA CTAGCGTGAC TCTTTTTTGA   
  
  
- CTTCTCCGCC TAACTACAGA GGCTAGGAGA CTAGTGAAGT CCACTTCCGA CGCTTCCCTT CTATTAGTCC   
  
  
- GAGGAGGTTC TCTCTGTCTT GAACAACTTG TAGTTCGTTT CTCCTTTTCC GCAGTAAGTT CTTCCCTTCC   
  
  
- CAGAAGTAGA ATAGGTAGAC GAGGAACATA GACGGTGGAA TTAGTTGCTT TTGTTGTAGT CAAGTCGACA   
  
  
- ACTATTAGAA TAACTCGAGA AGGTTATACA GAGGCTTTCA CCACTAAGTC CTGTTTCCCA ACGACGAATG   
  
  
- AAACGCCTAC CCAATTGTCG TTCCTAGAAT TGAGTCTCCA GAGGTAAAAT AGCGGACTAT GACCCGTTTG   
  
  
- GTCGTGGACG ACTTCTCAAA AGTCGAAAAT GAGTGAATAT ATCTCGGAGA GGTAAGATAG TCAAACGAGT   
  
  
- AAAGTGTCGG TTAGTCCGTT AACATCTTCG TAAACTCTCC CTCCTTCTCT CATTGTTGAC CCGAAACGTA   
  
  
- CATTAACTAA ACCTGTAGAA CGTACCAAAG GTCACCGGAA GAGAATAAGT CAGAGAGAGG CTTCTCCGAT   
  
  
- GAACAAGTTT GGCAAGTAAC AGAGAAGTTT AATGTCCGAA GCCTTCTTCG AATCTTCTCG AGTAACTTTG   
  
  
- TCTTCGAGCC GACCATTCAA AGAGATTCTC GAAAGTCTTA TAGTTGAAAC TTAAGGTGCC CAAAGACTCT   
  
  
- CCGAGCTTTG AGTCCTTGGA TTCCTCCCTC TTACTTTGTC ATCAACATTT AGAGCAAAAG GTGGAGTCAT   
  
  
- GAAACTTTCT ATGGCATGTC TAAAGACTGT GGAACTGAAG ACATGTAAGT GATTTGGGGA GATAACACCA   
  
  
- TAATCAGCTC TCTCTTCCTT CAGCTTTGTC TACACCCAAG GAGAGTTCAA TACATCTACG AGACGTAATA   
  
  
- ATACGACGTT ACAAACTGAG AAATCTACTA ACGGAGGGTG AACTTTCAGG ACTCTCCGAC TCGTATCTCT   
  
  
- TCTTGGTAGA ACCTTTTCTC TAGTTTCTTC GGTATCCAAC ACTCTTCCTA CTTTGTTTGA TAGACTTCAA   
  
  
- ACTCTACGAC CTCTGAACCT CTCCCTCCTA CCTCTCAGTA CCTAAAAGTC CATACTTCGA TTCAAGGTCC   
  
  
- CGTTGATATG TTCGTTTCGA CGAAAATTTT TACCCTTCGG TAATAGTATG GATAAACCTT CTTCTGACAC   
  
  
- CTCCACCACC CAAGTCTCAA ACACTTGCTC TACTACCTTA CCGATAGAGA GAACCGACCG TTCCATCCAA   
  
  
- GGAGCGTTGA CGGAGCCATA CCGCAAGACA CAC

+     TGACG-motif

| Site Name | Organism | Position | Strand | Matrix score. | sequence | function |
| --- | --- | --- | --- | --- | --- | --- |
| TGACG-motif | Hordeum vulgare | 2225 | - | 5 | TGACG | cis-acting regulatory element involved in the MeJA-responsiveness |

>HU07G00272.1   
+ -Up\_Stream \_Len000ACCAAC GATTCTTTAC TGAATCAGCT ACATTTTTGG ATTGTGTTAA GGTATCTTTT   
  
  
+ AGATCTTCAG GAGGAGGGGG CTGAAGTAGC TTGGTTACTT TTGCGCTTAT CAACTGTGGA AGAATATTAT   
  
  
+ TGAGACTAGT GTAGAAGAAT TAGTAGTTTC CTCCACATTG GAAAAAAACC ATGAAATTAA CGCTTTAGGC   
  
  
+ CCTCAGAGTA CCAAGTGTCA TCGTAAATGA TTGAAATTTC AAGTCTTAGC TGAGTCATGA AGTCCTGATT   
  
  
+ TCCCATGACA AATTGATGTT GTTGTCTAAA GCTTGGCGGG TGATTTGAAA GAAGATTTAG ATCAGGCTGA   
  
  
+ CAATACACCA AATCTGGGAG CAAGGAACTG GATTTTTCTG TGTCTAGAAA AGCTTTCCGC TTTCTGAACA   
  
  
+ ATTGAGAAAA CCATGCATAG GAAATTTCTT GATATTTAGA TCATAGCAGA CTTAACAATC TGGCTTTACC   
  
  
+ GAACTGTCCT CTACTTCTAA GTTGTGAAGG TGTGAAAGAT AAACCATGTC AAACTTAGTG TCCAACAGAA   
  
  
+ ACGCATCTCT ACTTCTCCAG GCAAAGTTGT ACTTCAGATC TTCTACACCT TTTCTAGGCA GGACTTTGGG   
  
  
+ ATATAGAGTT TGGCCTGGGT GAAAAGTAGG AAGTGGGGAA GTAGAAAATG ATGAAAGAAG GCACCACTTG   
  
  
+ GGTCCAGATA CATAGGCCTA CGCACGAGAA CCTTTGCATG GTCTAGATAC ATGACTGTAT ACTTCTTCCT   
  
  
+ TTCCTTTACC TGTTGGAGAT TATCCTAGGA TTTTACATGG CACCAGCTGG ATTTGTTAAT GACATAAACA   
  
  
+ TATCTTTAAT TACAGTTGCT TATGGTATAC ATTTAAACTA TTCGATAGTT GACAGCTGAT GTCCTTATCC   
  
  
+ TTGTGCAGCT TATTCCTCGA TAGAATAATC TTTGGACATT TCTGTCTAGG TGATAAAGGG CAAAAACTAA   
  
  
+ GATACAAGAA ATAGAACATG TCCTCCATTT ACAAGACGTA TCTGTTTTTG GTTTTGGTCA ATGATCCCTG   
  
  
+ TTGTCCTCAA GCATTAACAC AGAATGATCA TTATTGACTT GGACTGAAAG GTTTTGGGAA CTCCATAGTA   
  
  
+ TGCCTTTTCA ATAAAATCAA AAGCAAATTT GCTGTTCACT GATAACTGCA GTTGTGTTGC TTTGCTGTTT   
  
  
+ CCATACCAGA CTCGTAACCA AGCTGATAGC AAATGTGCAA AGGTACAGTA TAATTAACGA TGTTTTCATA   
  
  
+ TTGCTACGAG TTCTGAAACA TGATATAAGA AAACACCAGA ACCCAAATAC CTACATAGGT CCTAGCCCTG   
  
  
+ GAATGGCTAT TTTAAATATC CAAATGGACT TACAGAATTC GCAGAGTTGT TTGGTTTGTT TAACAATCAT   
  
  
+ TGAATTGTTA GCTATGAGAA TCTGCGGGAC AATCGTAATC TTTGGCGATT ACTTCGATTG GCATACTGAT   
  
  
+ GTTTTTTTAT TTGCGTATCT AAGCATGGTG GTGTACCTTT GATTTGTGTG TCTAGGTAGT GTGTGTAGAT   
  
  
+ AAATATCATA GAACTCAGAC GTTGGTAGGG TTATGTTGAG CACTTTCTCT TGCTGTTTGT CCTTTGTGAT   
  
  
+ GGCACCTCTT GCTTTTCCGA ACCACCAAAA GTCTCATTGA CAAAACTCTT CAAACATTGC AAACTGCCCC   
  
  
+ CTTAAATATG TTTTATTAAA AAAAATGGAA GAGCTCAAAC TGTTGAGTGA GCCTAGATTC CAAGATTTAG   
  
  
+ AAACAGTAAA CTTGATAGAT ATATTTATAT GAACTATTGT TAGATGCAAC ACCCCCAACT TGAAACACAA   
  
  
+ AAGAGAAATT AAAAACAAAA AGGTTTTGCA CTATTTATAT ATCCTGTGTT CTTTGTGCTA GTCTTATTTA   
  
  
+ ATGTTAATTG GTAAGCATTA TTTGTTGGAT TATTCCACTG CACTTTCTCT TACTAACTTC AGCATTTCAT   
  
  
+ TCCTTGTTCT TCAGCTTTTG AACTTCTTTA GCCTATTTTG TTGTCTTTAT GGTTATGGAA GCCATCCAGA   
  
  
+ AGAAGAATGA TGAACTTCTG AGTCTTAGCT TGGCCATTGT CGGTCACTCT GATCGCACTG AGAAAAAACT   
  
  
+ GAAGAGGCGG ATTGATGTCT CCGATCCTCT GATCACTTCA GGTGAAGGCT GCGAAGGGAA GATAATCAGG   
  
  
+ CTCCTCCAAG AGAGACAGAA CTTGTTGAAC ATCAAGCAAA GAGGAAAAGG CGTCATTCAA GAAGGGAAGG   
  
  
+ GTCTTCATCT TATCCATCTG CTCCTTGTAT CTGCCACCTT AATCAACGAA AACAACATCA GTTCAGCTGT   
  
  
+ TGATAATCTT ATTGAGCTCT TCCAATATGT CTCCGAAAGT GGTGATTCAG GACAAAGGGT TGCTGCTTAC   
  
  
+ TTTGCGGATG GGTTAACAGC AAGGATCTTA ACTCAGAGGT CTCCATTTTA TCGCCTGATA CTGGGCAAAC   
  
  
+ CAGCACCTGC TGAAGAGTTT TCAGCTTTTA CTCACTTATA TAGAGCCTCT CCATTCTATC AGTTTGCTCA   
  
  
+ TTTCACAGCC AATCAGGCAA TTGTAGAAGC ATTTGAGAGG GAGGAAGAGA GTAACAACTG GGCTTTGCAT   
  
  
+ GTAATTGATT TGGACATCTT GCATGGTTTC CAGTGGCCTT CTCTTATTCA GTCTCTCTCC GAAGAGGCTA   
  
  
+ CTTGTTCAAA CCGTTCATTG TCTCTTCAAA TTACAGGCTT CGGAAGAAGC TTAGAAGAGC TCATTGAAAC   
  
  
+ AGAAGCTCGG CTGGTAAGTT TCTCTAAGAG CTTTCAGAAT ATCAACTTTG AATTCCACGG GTTTCTGAGA   
  
  
+ GGCTCGAAAC TCAGGAACCT AAGGAGGGAG AATGAAACAG TAGTTGTAAA TCTCGTTTTC CACCTCAGTA   
  
  
+ CTTTGAAAGA TACCGTACAG ATTTCTGACA CCTTGACTTC TGTACATTCA CTAAACCCCT CTATTGTGGT   
  
  
+ ATTAGTCGAG AGAGAAGGAA GTCGAAACAG ATGTGGGTTC CTCTCAAGTT ATGTAGATGC TCTGCATTAT   
  
  
+ TATGCTGCAA TGTTTGACTC TTTAGATGAT TGCCTCCCAC TTGAAAGTCC TGAGAGGCTG AGCATAGAGA   
  
  
+ AGAACCATCT TGGAAAAGAG ATCAAAGAAG CCATAGGTTG TGAGAAGGAT GAAACAAACT ATCTGAAGTT   
  
  
+ TGAGATGCTG GAGACTTGGA GAGGGAGGAT GGAGAGTCAT GGATTTTCAG GTATGAAGCT AAGTTCCAGG   
  
  
+ GCAACTATAC AAGCAAAGCT GCTTTTAAAA ATGGGAAGCC ATTATCATAC CTATTTGGAA GAAGACTGTG   
  
  
+ GAGGTGGTGG GTTCAGAGTT TGTGAACGAG ATGATGGAAT GGCTATCTCT CTTGGCTGGC AAGGTAGGTT   
  
  
+ CCTCGCAACT GCCTCGGTAT GGCGTTCTGT GTG  

- -Up\_Stream \_Len000TGGTTG CTAAGAAATG ACTTAGTCGA TGTAAAAACC TAACACAATT CCATAGAAAA   
  
  
- TCTAGAAGTC CTCCTCCCCC GACTTCATCG AACCAATGAA AACGCGAATA GTTGACACCT TCTTATAATA   
  
  
- ACTCTGATCA CATCTTCTTA ATCATCAAAG GAGGTGTAAC CTTTTTTTGG TACTTTAATT GCGAAATCCG   
  
  
- GGAGTCTCAT GGTTCACAGT AGCATTTACT AACTTTAAAG TTCAGAATCG ACTCAGTACT TCAGGACTAA   
  
  
- AGGGTACTGT TTAACTACAA CAACAGATTT CGAACCGCCC ACTAAACTTT CTTCTAAATC TAGTCCGACT   
  
  
- GTTATGTGGT TTAGACCCTC GTTCCTTGAC CTAAAAAGAC ACAGATCTTT TCGAAAGGCG AAAGACTTGT   
  
  
- TAACTCTTTT GGTACGTATC CTTTAAAGAA CTATAAATCT AGTATCGTCT GAATTGTTAG ACCGAAATGG   
  
  
- CTTGACAGGA GATGAAGATT CAACACTTCC ACACTTTCTA TTTGGTACAG TTTGAATCAC AGGTTGTCTT   
  
  
- TGCGTAGAGA TGAAGAGGTC CGTTTCAACA TGAAGTCTAG AAGATGTGGA AAAGATCCGT CCTGAAACCC   
  
  
- TATATCTCAA ACCGGACCCA CTTTTCATCC TTCACCCCTT CATCTTTTAC TACTTTCTTC CGTGGTGAAC   
  
  
- CCAGGTCTAT GTATCCGGAT GCGTGCTCTT GGAAACGTAC CAGATCTATG TACTGACATA TGAAGAAGGA   
  
  
- AAGGAAATGG ACAACCTCTA ATAGGATCCT AAAATGTACC GTGGTCGACC TAAACAATTA CTGTATTTGT   
  
  
- ATAGAAATTA ATGTCAACGA ATACCATATG TAAATTTGAT AAGCTATCAA CTGTCGACTA CAGGAATAGG   
  
  
- AACACGTCGA ATAAGGAGCT ATCTTATTAG AAACCTGTAA AGACAGATCC ACTATTTCCC GTTTTTGATT   
  
  
- CTATGTTCTT TATCTTGTAC AGGAGGTAAA TGTTCTGCAT AGACAAAAAC CAAAACCAGT TACTAGGGAC   
  
  
- AACAGGAGTT CGTAATTGTG TCTTACTAGT AATAACTGAA CCTGACTTTC CAAAACCCTT GAGGTATCAT   
  
  
- ACGGAAAAGT TATTTTAGTT TTCGTTTAAA CGACAAGTGA CTATTGACGT CAACACAACG AAACGACAAA   
  
  
- GGTATGGTCT GAGCATTGGT TCGACTATCG TTTACACGTT TCCATGTCAT ATTAATTGCT ACAAAAGTAT   
  
  
- AACGATGCTC AAGACTTTGT ACTATATTCT TTTGTGGTCT TGGGTTTATG GATGTATCCA GGATCGGGAC   
  
  
- CTTACCGATA AAATTTATAG GTTTACCTGA ATGTCTTAAG CGTCTCAACA AACCAAACAA ATTGTTAGTA   
  
  
- ACTTAACAAT CGATACTCTT AGACGCCCTG TTAGCATTAG AAACCGCTAA TGAAGCTAAC CGTATGACTA   
  
  
- CAAAAAAATA AACGCATAGA TTCGTACCAC CACATGGAAA CTAAACACAC AGATCCATCA CACACATCTA   
  
  
- TTTATAGTAT CTTGAGTCTG CAACCATCCC AATACAACTC GTGAAAGAGA ACGACAAACA GGAAACACTA   
  
  
- CCGTGGAGAA CGAAAAGGCT TGGTGGTTTT CAGAGTAACT GTTTTGAGAA GTTTGTAACG TTTGACGGGG   
  
  
- GAATTTATAC AAAATAATTT TTTTTACCTT CTCGAGTTTG ACAACTCACT CGGATCTAAG GTTCTAAATC   
  
  
- TTTGTCATTT GAACTATCTA TATAAATATA CTTGATAACA ATCTACGTTG TGGGGGTTGA ACTTTGTGTT   
  
  
- TTCTCTTTAA TTTTTGTTTT TCCAAAACGT GATAAATATA TAGGACACAA GAAACACGAT CAGAATAAAT   
  
  
- TACAATTAAC CATTCGTAAT AAACAACCTA ATAAGGTGAC GTGAAAGAGA ATGATTGAAG TCGTAAAGTA   
  
  
- AGGAACAAGA AGTCGAAAAC TTGAAGAAAT CGGATAAAAC AACAGAAATA CCAATACCTT CGGTAGGTCT   
  
  
- TCTTCTTACT ACTTGAAGAC TCAGAATCGA ACCGGTAACA GCCAGTGAGA CTAGCGTGAC TCTTTTTTGA   
  
  
- CTTCTCCGCC TAACTACAGA GGCTAGGAGA CTAGTGAAGT CCACTTCCGA CGCTTCCCTT CTATTAGTCC   
  
  
- GAGGAGGTTC TCTCTGTCTT GAACAACTTG TAGTTCGTTT CTCCTTTTCC GCAGTAAGTT CTTCCCTTCC   
  
  
- CAGAAGTAGA ATAGGTAGAC GAGGAACATA GACGGTGGAA TTAGTTGCTT TTGTTGTAGT CAAGTCGACA   
  
  
- ACTATTAGAA TAACTCGAGA AGGTTATACA GAGGCTTTCA CCACTAAGTC CTGTTTCCCA ACGACGAATG   
  
  
- AAACGCCTAC CCAATTGTCG TTCCTAGAAT TGAGTCTCCA GAGGTAAAAT AGCGGACTAT GACCCGTTTG   
  
  
- GTCGTGGACG ACTTCTCAAA AGTCGAAAAT GAGTGAATAT ATCTCGGAGA GGTAAGATAG TCAAACGAGT   
  
  
- AAAGTGTCGG TTAGTCCGTT AACATCTTCG TAAACTCTCC CTCCTTCTCT CATTGTTGAC CCGAAACGTA   
  
  
- CATTAACTAA ACCTGTAGAA CGTACCAAAG GTCACCGGAA GAGAATAAGT CAGAGAGAGG CTTCTCCGAT   
  
  
- GAACAAGTTT GGCAAGTAAC AGAGAAGTTT AATGTCCGAA GCCTTCTTCG AATCTTCTCG AGTAACTTTG   
  
  
- TCTTCGAGCC GACCATTCAA AGAGATTCTC GAAAGTCTTA TAGTTGAAAC TTAAGGTGCC CAAAGACTCT   
  
  
- CCGAGCTTTG AGTCCTTGGA TTCCTCCCTC TTACTTTGTC ATCAACATTT AGAGCAAAAG GTGGAGTCAT   
  
  
- GAAACTTTCT ATGGCATGTC TAAAGACTGT GGAACTGAAG ACATGTAAGT GATTTGGGGA GATAACACCA   
  
  
- TAATCAGCTC TCTCTTCCTT CAGCTTTGTC TACACCCAAG GAGAGTTCAA TACATCTACG AGACGTAATA   
  
  
- ATACGACGTT ACAAACTGAG AAATCTACTA ACGGAGGGTG AACTTTCAGG ACTCTCCGAC TCGTATCTCT   
  
  
- TCTTGGTAGA ACCTTTTCTC TAGTTTCTTC GGTATCCAAC ACTCTTCCTA CTTTGTTTGA TAGACTTCAA   
  
  
- ACTCTACGAC CTCTGAACCT CTCCCTCCTA CCTCTCAGTA CCTAAAAGTC CATACTTCGA TTCAAGGTCC   
  
  
- CGTTGATATG TTCGTTTCGA CGAAAATTTT TACCCTTCGG TAATAGTATG GATAAACCTT CTTCTGACAC   
  
  
- CTCCACCACC CAAGTCTCAA ACACTTGCTC TACTACCTTA CCGATAGAGA GAACCGACCG TTCCATCCAA   
  
  
- GGAGCGTTGA CGGAGCCATA CCGCAAGACA CAC

+     Unnamed\_\_1

| Site Name | Organism | Position | Strand | Matrix score. | sequence | function |
| --- | --- | --- | --- | --- | --- | --- |
| Unnamed\_\_1 | Zea mays | 2789 | - | 5 | CGTGG |  |

>HU07G00272.1   
+ -Up\_Stream \_Len000ACCAAC GATTCTTTAC TGAATCAGCT ACATTTTTGG ATTGTGTTAA GGTATCTTTT   
  
  
+ AGATCTTCAG GAGGAGGGGG CTGAAGTAGC TTGGTTACTT TTGCGCTTAT CAACTGTGGA AGAATATTAT   
  
  
+ TGAGACTAGT GTAGAAGAAT TAGTAGTTTC CTCCACATTG GAAAAAAACC ATGAAATTAA CGCTTTAGGC   
  
  
+ CCTCAGAGTA CCAAGTGTCA TCGTAAATGA TTGAAATTTC AAGTCTTAGC TGAGTCATGA AGTCCTGATT   
  
  
+ TCCCATGACA AATTGATGTT GTTGTCTAAA GCTTGGCGGG TGATTTGAAA GAAGATTTAG ATCAGGCTGA   
  
  
+ CAATACACCA AATCTGGGAG CAAGGAACTG GATTTTTCTG TGTCTAGAAA AGCTTTCCGC TTTCTGAACA   
  
  
+ ATTGAGAAAA CCATGCATAG GAAATTTCTT GATATTTAGA TCATAGCAGA CTTAACAATC TGGCTTTACC   
  
  
+ GAACTGTCCT CTACTTCTAA GTTGTGAAGG TGTGAAAGAT AAACCATGTC AAACTTAGTG TCCAACAGAA   
  
  
+ ACGCATCTCT ACTTCTCCAG GCAAAGTTGT ACTTCAGATC TTCTACACCT TTTCTAGGCA GGACTTTGGG   
  
  
+ ATATAGAGTT TGGCCTGGGT GAAAAGTAGG AAGTGGGGAA GTAGAAAATG ATGAAAGAAG GCACCACTTG   
  
  
+ GGTCCAGATA CATAGGCCTA CGCACGAGAA CCTTTGCATG GTCTAGATAC ATGACTGTAT ACTTCTTCCT   
  
  
+ TTCCTTTACC TGTTGGAGAT TATCCTAGGA TTTTACATGG CACCAGCTGG ATTTGTTAAT GACATAAACA   
  
  
+ TATCTTTAAT TACAGTTGCT TATGGTATAC ATTTAAACTA TTCGATAGTT GACAGCTGAT GTCCTTATCC   
  
  
+ TTGTGCAGCT TATTCCTCGA TAGAATAATC TTTGGACATT TCTGTCTAGG TGATAAAGGG CAAAAACTAA   
  
  
+ GATACAAGAA ATAGAACATG TCCTCCATTT ACAAGACGTA TCTGTTTTTG GTTTTGGTCA ATGATCCCTG   
  
  
+ TTGTCCTCAA GCATTAACAC AGAATGATCA TTATTGACTT GGACTGAAAG GTTTTGGGAA CTCCATAGTA   
  
  
+ TGCCTTTTCA ATAAAATCAA AAGCAAATTT GCTGTTCACT GATAACTGCA GTTGTGTTGC TTTGCTGTTT   
  
  
+ CCATACCAGA CTCGTAACCA AGCTGATAGC AAATGTGCAA AGGTACAGTA TAATTAACGA TGTTTTCATA   
  
  
+ TTGCTACGAG TTCTGAAACA TGATATAAGA AAACACCAGA ACCCAAATAC CTACATAGGT CCTAGCCCTG   
  
  
+ GAATGGCTAT TTTAAATATC CAAATGGACT TACAGAATTC GCAGAGTTGT TTGGTTTGTT TAACAATCAT   
  
  
+ TGAATTGTTA GCTATGAGAA TCTGCGGGAC AATCGTAATC TTTGGCGATT ACTTCGATTG GCATACTGAT   
  
  
+ GTTTTTTTAT TTGCGTATCT AAGCATGGTG GTGTACCTTT GATTTGTGTG TCTAGGTAGT GTGTGTAGAT   
  
  
+ AAATATCATA GAACTCAGAC GTTGGTAGGG TTATGTTGAG CACTTTCTCT TGCTGTTTGT CCTTTGTGAT   
  
  
+ GGCACCTCTT GCTTTTCCGA ACCACCAAAA GTCTCATTGA CAAAACTCTT CAAACATTGC AAACTGCCCC   
  
  
+ CTTAAATATG TTTTATTAAA AAAAATGGAA GAGCTCAAAC TGTTGAGTGA GCCTAGATTC CAAGATTTAG   
  
  
+ AAACAGTAAA CTTGATAGAT ATATTTATAT GAACTATTGT TAGATGCAAC ACCCCCAACT TGAAACACAA   
  
  
+ AAGAGAAATT AAAAACAAAA AGGTTTTGCA CTATTTATAT ATCCTGTGTT CTTTGTGCTA GTCTTATTTA   
  
  
+ ATGTTAATTG GTAAGCATTA TTTGTTGGAT TATTCCACTG CACTTTCTCT TACTAACTTC AGCATTTCAT   
  
  
+ TCCTTGTTCT TCAGCTTTTG AACTTCTTTA GCCTATTTTG TTGTCTTTAT GGTTATGGAA GCCATCCAGA   
  
  
+ AGAAGAATGA TGAACTTCTG AGTCTTAGCT TGGCCATTGT CGGTCACTCT GATCGCACTG AGAAAAAACT   
  
  
+ GAAGAGGCGG ATTGATGTCT CCGATCCTCT GATCACTTCA GGTGAAGGCT GCGAAGGGAA GATAATCAGG   
  
  
+ CTCCTCCAAG AGAGACAGAA CTTGTTGAAC ATCAAGCAAA GAGGAAAAGG CGTCATTCAA GAAGGGAAGG   
  
  
+ GTCTTCATCT TATCCATCTG CTCCTTGTAT CTGCCACCTT AATCAACGAA AACAACATCA GTTCAGCTGT   
  
  
+ TGATAATCTT ATTGAGCTCT TCCAATATGT CTCCGAAAGT GGTGATTCAG GACAAAGGGT TGCTGCTTAC   
  
  
+ TTTGCGGATG GGTTAACAGC AAGGATCTTA ACTCAGAGGT CTCCATTTTA TCGCCTGATA CTGGGCAAAC   
  
  
+ CAGCACCTGC TGAAGAGTTT TCAGCTTTTA CTCACTTATA TAGAGCCTCT CCATTCTATC AGTTTGCTCA   
  
  
+ TTTCACAGCC AATCAGGCAA TTGTAGAAGC ATTTGAGAGG GAGGAAGAGA GTAACAACTG GGCTTTGCAT   
  
  
+ GTAATTGATT TGGACATCTT GCATGGTTTC CAGTGGCCTT CTCTTATTCA GTCTCTCTCC GAAGAGGCTA   
  
  
+ CTTGTTCAAA CCGTTCATTG TCTCTTCAAA TTACAGGCTT CGGAAGAAGC TTAGAAGAGC TCATTGAAAC   
  
  
+ AGAAGCTCGG CTGGTAAGTT TCTCTAAGAG CTTTCAGAAT ATCAACTTTG AATTCCACGG GTTTCTGAGA   
  
  
+ GGCTCGAAAC TCAGGAACCT AAGGAGGGAG AATGAAACAG TAGTTGTAAA TCTCGTTTTC CACCTCAGTA   
  
  
+ CTTTGAAAGA TACCGTACAG ATTTCTGACA CCTTGACTTC TGTACATTCA CTAAACCCCT CTATTGTGGT   
  
  
+ ATTAGTCGAG AGAGAAGGAA GTCGAAACAG ATGTGGGTTC CTCTCAAGTT ATGTAGATGC TCTGCATTAT   
  
  
+ TATGCTGCAA TGTTTGACTC TTTAGATGAT TGCCTCCCAC TTGAAAGTCC TGAGAGGCTG AGCATAGAGA   
  
  
+ AGAACCATCT TGGAAAAGAG ATCAAAGAAG CCATAGGTTG TGAGAAGGAT GAAACAAACT ATCTGAAGTT   
  
  
+ TGAGATGCTG GAGACTTGGA GAGGGAGGAT GGAGAGTCAT GGATTTTCAG GTATGAAGCT AAGTTCCAGG   
  
  
+ GCAACTATAC AAGCAAAGCT GCTTTTAAAA ATGGGAAGCC ATTATCATAC CTATTTGGAA GAAGACTGTG   
  
  
+ GAGGTGGTGG GTTCAGAGTT TGTGAACGAG ATGATGGAAT GGCTATCTCT CTTGGCTGGC AAGGTAGGTT   
  
  
+ CCTCGCAACT GCCTCGGTAT GGCGTTCTGT GTG  

- -Up\_Stream \_Len000TGGTTG CTAAGAAATG ACTTAGTCGA TGTAAAAACC TAACACAATT CCATAGAAAA   
  
  
- TCTAGAAGTC CTCCTCCCCC GACTTCATCG AACCAATGAA AACGCGAATA GTTGACACCT TCTTATAATA   
  
  
- ACTCTGATCA CATCTTCTTA ATCATCAAAG GAGGTGTAAC CTTTTTTTGG TACTTTAATT GCGAAATCCG   
  
  
- GGAGTCTCAT GGTTCACAGT AGCATTTACT AACTTTAAAG TTCAGAATCG ACTCAGTACT TCAGGACTAA   
  
  
- AGGGTACTGT TTAACTACAA CAACAGATTT CGAACCGCCC ACTAAACTTT CTTCTAAATC TAGTCCGACT   
  
  
- GTTATGTGGT TTAGACCCTC GTTCCTTGAC CTAAAAAGAC ACAGATCTTT TCGAAAGGCG AAAGACTTGT   
  
  
- TAACTCTTTT GGTACGTATC CTTTAAAGAA CTATAAATCT AGTATCGTCT GAATTGTTAG ACCGAAATGG   
  
  
- CTTGACAGGA GATGAAGATT CAACACTTCC ACACTTTCTA TTTGGTACAG TTTGAATCAC AGGTTGTCTT   
  
  
- TGCGTAGAGA TGAAGAGGTC CGTTTCAACA TGAAGTCTAG AAGATGTGGA AAAGATCCGT CCTGAAACCC   
  
  
- TATATCTCAA ACCGGACCCA CTTTTCATCC TTCACCCCTT CATCTTTTAC TACTTTCTTC CGTGGTGAAC   
  
  
- CCAGGTCTAT GTATCCGGAT GCGTGCTCTT GGAAACGTAC CAGATCTATG TACTGACATA TGAAGAAGGA   
  
  
- AAGGAAATGG ACAACCTCTA ATAGGATCCT AAAATGTACC GTGGTCGACC TAAACAATTA CTGTATTTGT   
  
  
- ATAGAAATTA ATGTCAACGA ATACCATATG TAAATTTGAT AAGCTATCAA CTGTCGACTA CAGGAATAGG   
  
  
- AACACGTCGA ATAAGGAGCT ATCTTATTAG AAACCTGTAA AGACAGATCC ACTATTTCCC GTTTTTGATT   
  
  
- CTATGTTCTT TATCTTGTAC AGGAGGTAAA TGTTCTGCAT AGACAAAAAC CAAAACCAGT TACTAGGGAC   
  
  
- AACAGGAGTT CGTAATTGTG TCTTACTAGT AATAACTGAA CCTGACTTTC CAAAACCCTT GAGGTATCAT   
  
  
- ACGGAAAAGT TATTTTAGTT TTCGTTTAAA CGACAAGTGA CTATTGACGT CAACACAACG AAACGACAAA   
  
  
- GGTATGGTCT GAGCATTGGT TCGACTATCG TTTACACGTT TCCATGTCAT ATTAATTGCT ACAAAAGTAT   
  
  
- AACGATGCTC AAGACTTTGT ACTATATTCT TTTGTGGTCT TGGGTTTATG GATGTATCCA GGATCGGGAC   
  
  
- CTTACCGATA AAATTTATAG GTTTACCTGA ATGTCTTAAG CGTCTCAACA AACCAAACAA ATTGTTAGTA   
  
  
- ACTTAACAAT CGATACTCTT AGACGCCCTG TTAGCATTAG AAACCGCTAA TGAAGCTAAC CGTATGACTA   
  
  
- CAAAAAAATA AACGCATAGA TTCGTACCAC CACATGGAAA CTAAACACAC AGATCCATCA CACACATCTA   
  
  
- TTTATAGTAT CTTGAGTCTG CAACCATCCC AATACAACTC GTGAAAGAGA ACGACAAACA GGAAACACTA   
  
  
- CCGTGGAGAA CGAAAAGGCT TGGTGGTTTT CAGAGTAACT GTTTTGAGAA GTTTGTAACG TTTGACGGGG   
  
  
- GAATTTATAC AAAATAATTT TTTTTACCTT CTCGAGTTTG ACAACTCACT CGGATCTAAG GTTCTAAATC   
  
  
- TTTGTCATTT GAACTATCTA TATAAATATA CTTGATAACA ATCTACGTTG TGGGGGTTGA ACTTTGTGTT   
  
  
- TTCTCTTTAA TTTTTGTTTT TCCAAAACGT GATAAATATA TAGGACACAA GAAACACGAT CAGAATAAAT   
  
  
- TACAATTAAC CATTCGTAAT AAACAACCTA ATAAGGTGAC GTGAAAGAGA ATGATTGAAG TCGTAAAGTA   
  
  
- AGGAACAAGA AGTCGAAAAC TTGAAGAAAT CGGATAAAAC AACAGAAATA CCAATACCTT CGGTAGGTCT   
  
  
- TCTTCTTACT ACTTGAAGAC TCAGAATCGA ACCGGTAACA GCCAGTGAGA CTAGCGTGAC TCTTTTTTGA   
  
  
- CTTCTCCGCC TAACTACAGA GGCTAGGAGA CTAGTGAAGT CCACTTCCGA CGCTTCCCTT CTATTAGTCC   
  
  
- GAGGAGGTTC TCTCTGTCTT GAACAACTTG TAGTTCGTTT CTCCTTTTCC GCAGTAAGTT CTTCCCTTCC   
  
  
- CAGAAGTAGA ATAGGTAGAC GAGGAACATA GACGGTGGAA TTAGTTGCTT TTGTTGTAGT CAAGTCGACA   
  
  
- ACTATTAGAA TAACTCGAGA AGGTTATACA GAGGCTTTCA CCACTAAGTC CTGTTTCCCA ACGACGAATG   
  
  
- AAACGCCTAC CCAATTGTCG TTCCTAGAAT TGAGTCTCCA GAGGTAAAAT AGCGGACTAT GACCCGTTTG   
  
  
- GTCGTGGACG ACTTCTCAAA AGTCGAAAAT GAGTGAATAT ATCTCGGAGA GGTAAGATAG TCAAACGAGT   
  
  
- AAAGTGTCGG TTAGTCCGTT AACATCTTCG TAAACTCTCC CTCCTTCTCT CATTGTTGAC CCGAAACGTA   
  
  
- CATTAACTAA ACCTGTAGAA CGTACCAAAG GTCACCGGAA GAGAATAAGT CAGAGAGAGG CTTCTCCGAT   
  
  
- GAACAAGTTT GGCAAGTAAC AGAGAAGTTT AATGTCCGAA GCCTTCTTCG AATCTTCTCG AGTAACTTTG   
  
  
- TCTTCGAGCC GACCATTCAA AGAGATTCTC GAAAGTCTTA TAGTTGAAAC TTAAGGTGCC CAAAGACTCT   
  
  
- CCGAGCTTTG AGTCCTTGGA TTCCTCCCTC TTACTTTGTC ATCAACATTT AGAGCAAAAG GTGGAGTCAT   
  
  
- GAAACTTTCT ATGGCATGTC TAAAGACTGT GGAACTGAAG ACATGTAAGT GATTTGGGGA GATAACACCA   
  
  
- TAATCAGCTC TCTCTTCCTT CAGCTTTGTC TACACCCAAG GAGAGTTCAA TACATCTACG AGACGTAATA   
  
  
- ATACGACGTT ACAAACTGAG AAATCTACTA ACGGAGGGTG AACTTTCAGG ACTCTCCGAC TCGTATCTCT   
  
  
- TCTTGGTAGA ACCTTTTCTC TAGTTTCTTC GGTATCCAAC ACTCTTCCTA CTTTGTTTGA TAGACTTCAA   
  
  
- ACTCTACGAC CTCTGAACCT CTCCCTCCTA CCTCTCAGTA CCTAAAAGTC CATACTTCGA TTCAAGGTCC   
  
  
- CGTTGATATG TTCGTTTCGA CGAAAATTTT TACCCTTCGG TAATAGTATG GATAAACCTT CTTCTGACAC   
  
  
- CTCCACCACC CAAGTCTCAA ACACTTGCTC TACTACCTTA CCGATAGAGA GAACCGACCG TTCCATCCAA   
  
  
- GGAGCGTTGA CGGAGCCATA CCGCAAGACA CAC

+     Unnamed\_\_4

| Site Name | Organism | Position | Strand | Matrix score. | sequence | function |
| --- | --- | --- | --- | --- | --- | --- |
| Unnamed\_\_4 | Petroselinum hortense | 3294 | - | 4 | CTCC |  |
| Unnamed\_\_4 | Petroselinum hortense | 371 | - | 4 | CTCC |  |
| Unnamed\_\_4 | Petroselinum hortense | 3185 | - | 4 | CTCC |  |
| Unnamed\_\_4 | Petroselinum hortense | 84 | - | 4 | CTCC |  |
| Unnamed\_\_4 | Petroselinum hortense | 1115 | + | 4 | CTCC |  |
| Unnamed\_\_4 | Petroselinum hortense | 2345 | + | 4 | CTCC |  |
| Unnamed\_\_4 | Petroselinum hortense | 2178 | + | 4 | CTCC |  |
| Unnamed\_\_4 | Petroselinum hortense | 87 | - | 4 | CTCC |  |
| Unnamed\_\_4 | Petroselinum hortense | 1007 | + | 4 | CTCC |  |
| Unnamed\_\_4 | Petroselinum hortense | 3178 | - | 4 | CTCC |  |
| Unnamed\_\_4 | Petroselinum hortense | 2265 | + | 4 | CTCC |  |
| Unnamed\_\_4 | Petroselinum hortense | 2831 | - | 4 | CTCC |  |
| Unnamed\_\_4 | Petroselinum hortense | 2503 | + | 4 | CTCC |  |
| Unnamed\_\_4 | Petroselinum hortense | 2123 | + | 4 | CTCC |  |
| Unnamed\_\_4 | Petroselinum hortense | 2425 | + | 4 | CTCC |  |
| Unnamed\_\_4 | Petroselinum hortense | 3172 | - | 4 | CTCC |  |
| Unnamed\_\_4 | Petroselinum hortense | 175 | + | 4 | CTCC |  |
| Unnamed\_\_4 | Petroselinum hortense | 2564 | - | 4 | CTCC |  |
| Unnamed\_\_4 | Petroselinum hortense | 3164 | - | 4 | CTCC |  |
| Unnamed\_\_4 | Petroselinum hortense | 789 | - | 4 | CTCC |  |
| Unnamed\_\_4 | Petroselinum hortense | 2175 | + | 4 | CTCC |  |
| Unnamed\_\_4 | Petroselinum hortense | 3048 | + | 4 | CTCC |  |
| Unnamed\_\_4 | Petroselinum hortense | 579 | + | 4 | CTCC |  |
| Unnamed\_\_4 | Petroselinum hortense | 2827 | - | 4 | CTCC |  |
| Unnamed\_\_4 | Petroselinum hortense | 2651 | + | 4 | CTCC |  |

>HU07G00272.1   
+ -Up\_Stream \_Len000ACCAAC GATTCTTTAC TGAATCAGCT ACATTTTTGG ATTGTGTTAA GGTATCTTTT   
  
  
+ AGATCTTCAG GAGGAGGGGG CTGAAGTAGC TTGGTTACTT TTGCGCTTAT CAACTGTGGA AGAATATTAT   
  
  
+ TGAGACTAGT GTAGAAGAAT TAGTAGTTTC CTCCACATTG GAAAAAAACC ATGAAATTAA CGCTTTAGGC   
  
  
+ CCTCAGAGTA CCAAGTGTCA TCGTAAATGA TTGAAATTTC AAGTCTTAGC TGAGTCATGA AGTCCTGATT   
  
  
+ TCCCATGACA AATTGATGTT GTTGTCTAAA GCTTGGCGGG TGATTTGAAA GAAGATTTAG ATCAGGCTGA   
  
  
+ CAATACACCA AATCTGGGAG CAAGGAACTG GATTTTTCTG TGTCTAGAAA AGCTTTCCGC TTTCTGAACA   
  
  
+ ATTGAGAAAA CCATGCATAG GAAATTTCTT GATATTTAGA TCATAGCAGA CTTAACAATC TGGCTTTACC   
  
  
+ GAACTGTCCT CTACTTCTAA GTTGTGAAGG TGTGAAAGAT AAACCATGTC AAACTTAGTG TCCAACAGAA   
  
  
+ ACGCATCTCT ACTTCTCCAG GCAAAGTTGT ACTTCAGATC TTCTACACCT TTTCTAGGCA GGACTTTGGG   
  
  
+ ATATAGAGTT TGGCCTGGGT GAAAAGTAGG AAGTGGGGAA GTAGAAAATG ATGAAAGAAG GCACCACTTG   
  
  
+ GGTCCAGATA CATAGGCCTA CGCACGAGAA CCTTTGCATG GTCTAGATAC ATGACTGTAT ACTTCTTCCT   
  
  
+ TTCCTTTACC TGTTGGAGAT TATCCTAGGA TTTTACATGG CACCAGCTGG ATTTGTTAAT GACATAAACA   
  
  
+ TATCTTTAAT TACAGTTGCT TATGGTATAC ATTTAAACTA TTCGATAGTT GACAGCTGAT GTCCTTATCC   
  
  
+ TTGTGCAGCT TATTCCTCGA TAGAATAATC TTTGGACATT TCTGTCTAGG TGATAAAGGG CAAAAACTAA   
  
  
+ GATACAAGAA ATAGAACATG TCCTCCATTT ACAAGACGTA TCTGTTTTTG GTTTTGGTCA ATGATCCCTG   
  
  
+ TTGTCCTCAA GCATTAACAC AGAATGATCA TTATTGACTT GGACTGAAAG GTTTTGGGAA CTCCATAGTA   
  
  
+ TGCCTTTTCA ATAAAATCAA AAGCAAATTT GCTGTTCACT GATAACTGCA GTTGTGTTGC TTTGCTGTTT   
  
  
+ CCATACCAGA CTCGTAACCA AGCTGATAGC AAATGTGCAA AGGTACAGTA TAATTAACGA TGTTTTCATA   
  
  
+ TTGCTACGAG TTCTGAAACA TGATATAAGA AAACACCAGA ACCCAAATAC CTACATAGGT CCTAGCCCTG   
  
  
+ GAATGGCTAT TTTAAATATC CAAATGGACT TACAGAATTC GCAGAGTTGT TTGGTTTGTT TAACAATCAT   
  
  
+ TGAATTGTTA GCTATGAGAA TCTGCGGGAC AATCGTAATC TTTGGCGATT ACTTCGATTG GCATACTGAT   
  
  
+ GTTTTTTTAT TTGCGTATCT AAGCATGGTG GTGTACCTTT GATTTGTGTG TCTAGGTAGT GTGTGTAGAT   
  
  
+ AAATATCATA GAACTCAGAC GTTGGTAGGG TTATGTTGAG CACTTTCTCT TGCTGTTTGT CCTTTGTGAT   
  
  
+ GGCACCTCTT GCTTTTCCGA ACCACCAAAA GTCTCATTGA CAAAACTCTT CAAACATTGC AAACTGCCCC   
  
  
+ CTTAAATATG TTTTATTAAA AAAAATGGAA GAGCTCAAAC TGTTGAGTGA GCCTAGATTC CAAGATTTAG   
  
  
+ AAACAGTAAA CTTGATAGAT ATATTTATAT GAACTATTGT TAGATGCAAC ACCCCCAACT TGAAACACAA   
  
  
+ AAGAGAAATT AAAAACAAAA AGGTTTTGCA CTATTTATAT ATCCTGTGTT CTTTGTGCTA GTCTTATTTA   
  
  
+ ATGTTAATTG GTAAGCATTA TTTGTTGGAT TATTCCACTG CACTTTCTCT TACTAACTTC AGCATTTCAT   
  
  
+ TCCTTGTTCT TCAGCTTTTG AACTTCTTTA GCCTATTTTG TTGTCTTTAT GGTTATGGAA GCCATCCAGA   
  
  
+ AGAAGAATGA TGAACTTCTG AGTCTTAGCT TGGCCATTGT CGGTCACTCT GATCGCACTG AGAAAAAACT   
  
  
+ GAAGAGGCGG ATTGATGTCT CCGATCCTCT GATCACTTCA GGTGAAGGCT GCGAAGGGAA GATAATCAGG   
  
  
+ CTCCTCCAAG AGAGACAGAA CTTGTTGAAC ATCAAGCAAA GAGGAAAAGG CGTCATTCAA GAAGGGAAGG   
  
  
+ GTCTTCATCT TATCCATCTG CTCCTTGTAT CTGCCACCTT AATCAACGAA AACAACATCA GTTCAGCTGT   
  
  
+ TGATAATCTT ATTGAGCTCT TCCAATATGT CTCCGAAAGT GGTGATTCAG GACAAAGGGT TGCTGCTTAC   
  
  
+ TTTGCGGATG GGTTAACAGC AAGGATCTTA ACTCAGAGGT CTCCATTTTA TCGCCTGATA CTGGGCAAAC   
  
  
+ CAGCACCTGC TGAAGAGTTT TCAGCTTTTA CTCACTTATA TAGAGCCTCT CCATTCTATC AGTTTGCTCA   
  
  
+ TTTCACAGCC AATCAGGCAA TTGTAGAAGC ATTTGAGAGG GAGGAAGAGA GTAACAACTG GGCTTTGCAT   
  
  
+ GTAATTGATT TGGACATCTT GCATGGTTTC CAGTGGCCTT CTCTTATTCA GTCTCTCTCC GAAGAGGCTA   
  
  
+ CTTGTTCAAA CCGTTCATTG TCTCTTCAAA TTACAGGCTT CGGAAGAAGC TTAGAAGAGC TCATTGAAAC   
  
  
+ AGAAGCTCGG CTGGTAAGTT TCTCTAAGAG CTTTCAGAAT ATCAACTTTG AATTCCACGG GTTTCTGAGA   
  
  
+ GGCTCGAAAC TCAGGAACCT AAGGAGGGAG AATGAAACAG TAGTTGTAAA TCTCGTTTTC CACCTCAGTA   
  
  
+ CTTTGAAAGA TACCGTACAG ATTTCTGACA CCTTGACTTC TGTACATTCA CTAAACCCCT CTATTGTGGT   
  
  
+ ATTAGTCGAG AGAGAAGGAA GTCGAAACAG ATGTGGGTTC CTCTCAAGTT ATGTAGATGC TCTGCATTAT   
  
  
+ TATGCTGCAA TGTTTGACTC TTTAGATGAT TGCCTCCCAC TTGAAAGTCC TGAGAGGCTG AGCATAGAGA   
  
  
+ AGAACCATCT TGGAAAAGAG ATCAAAGAAG CCATAGGTTG TGAGAAGGAT GAAACAAACT ATCTGAAGTT   
  
  
+ TGAGATGCTG GAGACTTGGA GAGGGAGGAT GGAGAGTCAT GGATTTTCAG GTATGAAGCT AAGTTCCAGG   
  
  
+ GCAACTATAC AAGCAAAGCT GCTTTTAAAA ATGGGAAGCC ATTATCATAC CTATTTGGAA GAAGACTGTG   
  
  
+ GAGGTGGTGG GTTCAGAGTT TGTGAACGAG ATGATGGAAT GGCTATCTCT CTTGGCTGGC AAGGTAGGTT   
  
  
+ CCTCGCAACT GCCTCGGTAT GGCGTTCTGT GTG  

- -Up\_Stream \_Len000TGGTTG CTAAGAAATG ACTTAGTCGA TGTAAAAACC TAACACAATT CCATAGAAAA   
  
  
- TCTAGAAGTC CTCCTCCCCC GACTTCATCG AACCAATGAA AACGCGAATA GTTGACACCT TCTTATAATA   
  
  
- ACTCTGATCA CATCTTCTTA ATCATCAAAG GAGGTGTAAC CTTTTTTTGG TACTTTAATT GCGAAATCCG   
  
  
- GGAGTCTCAT GGTTCACAGT AGCATTTACT AACTTTAAAG TTCAGAATCG ACTCAGTACT TCAGGACTAA   
  
  
- AGGGTACTGT TTAACTACAA CAACAGATTT CGAACCGCCC ACTAAACTTT CTTCTAAATC TAGTCCGACT   
  
  
- GTTATGTGGT TTAGACCCTC GTTCCTTGAC CTAAAAAGAC ACAGATCTTT TCGAAAGGCG AAAGACTTGT   
  
  
- TAACTCTTTT GGTACGTATC CTTTAAAGAA CTATAAATCT AGTATCGTCT GAATTGTTAG ACCGAAATGG   
  
  
- CTTGACAGGA GATGAAGATT CAACACTTCC ACACTTTCTA TTTGGTACAG TTTGAATCAC AGGTTGTCTT   
  
  
- TGCGTAGAGA TGAAGAGGTC CGTTTCAACA TGAAGTCTAG AAGATGTGGA AAAGATCCGT CCTGAAACCC   
  
  
- TATATCTCAA ACCGGACCCA CTTTTCATCC TTCACCCCTT CATCTTTTAC TACTTTCTTC CGTGGTGAAC   
  
  
- CCAGGTCTAT GTATCCGGAT GCGTGCTCTT GGAAACGTAC CAGATCTATG TACTGACATA TGAAGAAGGA   
  
  
- AAGGAAATGG ACAACCTCTA ATAGGATCCT AAAATGTACC GTGGTCGACC TAAACAATTA CTGTATTTGT   
  
  
- ATAGAAATTA ATGTCAACGA ATACCATATG TAAATTTGAT AAGCTATCAA CTGTCGACTA CAGGAATAGG   
  
  
- AACACGTCGA ATAAGGAGCT ATCTTATTAG AAACCTGTAA AGACAGATCC ACTATTTCCC GTTTTTGATT   
  
  
- CTATGTTCTT TATCTTGTAC AGGAGGTAAA TGTTCTGCAT AGACAAAAAC CAAAACCAGT TACTAGGGAC   
  
  
- AACAGGAGTT CGTAATTGTG TCTTACTAGT AATAACTGAA CCTGACTTTC CAAAACCCTT GAGGTATCAT   
  
  
- ACGGAAAAGT TATTTTAGTT TTCGTTTAAA CGACAAGTGA CTATTGACGT CAACACAACG AAACGACAAA   
  
  
- GGTATGGTCT GAGCATTGGT TCGACTATCG TTTACACGTT TCCATGTCAT ATTAATTGCT ACAAAAGTAT   
  
  
- AACGATGCTC AAGACTTTGT ACTATATTCT TTTGTGGTCT TGGGTTTATG GATGTATCCA GGATCGGGAC   
  
  
- CTTACCGATA AAATTTATAG GTTTACCTGA ATGTCTTAAG CGTCTCAACA AACCAAACAA ATTGTTAGTA   
  
  
- ACTTAACAAT CGATACTCTT AGACGCCCTG TTAGCATTAG AAACCGCTAA TGAAGCTAAC CGTATGACTA   
  
  
- CAAAAAAATA AACGCATAGA TTCGTACCAC CACATGGAAA CTAAACACAC AGATCCATCA CACACATCTA   
  
  
- TTTATAGTAT CTTGAGTCTG CAACCATCCC AATACAACTC GTGAAAGAGA ACGACAAACA GGAAACACTA   
  
  
- CCGTGGAGAA CGAAAAGGCT TGGTGGTTTT CAGAGTAACT GTTTTGAGAA GTTTGTAACG TTTGACGGGG   
  
  
- GAATTTATAC AAAATAATTT TTTTTACCTT CTCGAGTTTG ACAACTCACT CGGATCTAAG GTTCTAAATC   
  
  
- TTTGTCATTT GAACTATCTA TATAAATATA CTTGATAACA ATCTACGTTG TGGGGGTTGA ACTTTGTGTT   
  
  
- TTCTCTTTAA TTTTTGTTTT TCCAAAACGT GATAAATATA TAGGACACAA GAAACACGAT CAGAATAAAT   
  
  
- TACAATTAAC CATTCGTAAT AAACAACCTA ATAAGGTGAC GTGAAAGAGA ATGATTGAAG TCGTAAAGTA   
  
  
- AGGAACAAGA AGTCGAAAAC TTGAAGAAAT CGGATAAAAC AACAGAAATA CCAATACCTT CGGTAGGTCT   
  
  
- TCTTCTTACT ACTTGAAGAC TCAGAATCGA ACCGGTAACA GCCAGTGAGA CTAGCGTGAC TCTTTTTTGA   
  
  
- CTTCTCCGCC TAACTACAGA GGCTAGGAGA CTAGTGAAGT CCACTTCCGA CGCTTCCCTT CTATTAGTCC   
  
  
- GAGGAGGTTC TCTCTGTCTT GAACAACTTG TAGTTCGTTT CTCCTTTTCC GCAGTAAGTT CTTCCCTTCC   
  
  
- CAGAAGTAGA ATAGGTAGAC GAGGAACATA GACGGTGGAA TTAGTTGCTT TTGTTGTAGT CAAGTCGACA   
  
  
- ACTATTAGAA TAACTCGAGA AGGTTATACA GAGGCTTTCA CCACTAAGTC CTGTTTCCCA ACGACGAATG   
  
  
- AAACGCCTAC CCAATTGTCG TTCCTAGAAT TGAGTCTCCA GAGGTAAAAT AGCGGACTAT GACCCGTTTG   
  
  
- GTCGTGGACG ACTTCTCAAA AGTCGAAAAT GAGTGAATAT ATCTCGGAGA GGTAAGATAG TCAAACGAGT   
  
  
- AAAGTGTCGG TTAGTCCGTT AACATCTTCG TAAACTCTCC CTCCTTCTCT CATTGTTGAC CCGAAACGTA   
  
  
- CATTAACTAA ACCTGTAGAA CGTACCAAAG GTCACCGGAA GAGAATAAGT CAGAGAGAGG CTTCTCCGAT   
  
  
- GAACAAGTTT GGCAAGTAAC AGAGAAGTTT AATGTCCGAA GCCTTCTTCG AATCTTCTCG AGTAACTTTG   
  
  
- TCTTCGAGCC GACCATTCAA AGAGATTCTC GAAAGTCTTA TAGTTGAAAC TTAAGGTGCC CAAAGACTCT   
  
  
- CCGAGCTTTG AGTCCTTGGA TTCCTCCCTC TTACTTTGTC ATCAACATTT AGAGCAAAAG GTGGAGTCAT   
  
  
- GAAACTTTCT ATGGCATGTC TAAAGACTGT GGAACTGAAG ACATGTAAGT GATTTGGGGA GATAACACCA   
  
  
- TAATCAGCTC TCTCTTCCTT CAGCTTTGTC TACACCCAAG GAGAGTTCAA TACATCTACG AGACGTAATA   
  
  
- ATACGACGTT ACAAACTGAG AAATCTACTA ACGGAGGGTG AACTTTCAGG ACTCTCCGAC TCGTATCTCT   
  
  
- TCTTGGTAGA ACCTTTTCTC TAGTTTCTTC GGTATCCAAC ACTCTTCCTA CTTTGTTTGA TAGACTTCAA   
  
  
- ACTCTACGAC CTCTGAACCT CTCCCTCCTA CCTCTCAGTA CCTAAAAGTC CATACTTCGA TTCAAGGTCC   
  
  
- CGTTGATATG TTCGTTTCGA CGAAAATTTT TACCCTTCGG TAATAGTATG GATAAACCTT CTTCTGACAC   
  
  
- CTCCACCACC CAAGTCTCAA ACACTTGCTC TACTACCTTA CCGATAGAGA GAACCGACCG TTCCATCCAA   
  
  
- GGAGCGTTGA CGGAGCCATA CCGCAAGACA CAC

+     Unnamed\_\_6

| Site Name | Organism | Position | Strand | Matrix score. | sequence | function |
| --- | --- | --- | --- | --- | --- | --- |
| Unnamed\_\_6 | Zea mays | 1773 | - | 10 | taTAAATATct |  |

>HU07G00272.1   
+ -Up\_Stream \_Len000ACCAAC GATTCTTTAC TGAATCAGCT ACATTTTTGG ATTGTGTTAA GGTATCTTTT   
  
  
+ AGATCTTCAG GAGGAGGGGG CTGAAGTAGC TTGGTTACTT TTGCGCTTAT CAACTGTGGA AGAATATTAT   
  
  
+ TGAGACTAGT GTAGAAGAAT TAGTAGTTTC CTCCACATTG GAAAAAAACC ATGAAATTAA CGCTTTAGGC   
  
  
+ CCTCAGAGTA CCAAGTGTCA TCGTAAATGA TTGAAATTTC AAGTCTTAGC TGAGTCATGA AGTCCTGATT   
  
  
+ TCCCATGACA AATTGATGTT GTTGTCTAAA GCTTGGCGGG TGATTTGAAA GAAGATTTAG ATCAGGCTGA   
  
  
+ CAATACACCA AATCTGGGAG CAAGGAACTG GATTTTTCTG TGTCTAGAAA AGCTTTCCGC TTTCTGAACA   
  
  
+ ATTGAGAAAA CCATGCATAG GAAATTTCTT GATATTTAGA TCATAGCAGA CTTAACAATC TGGCTTTACC   
  
  
+ GAACTGTCCT CTACTTCTAA GTTGTGAAGG TGTGAAAGAT AAACCATGTC AAACTTAGTG TCCAACAGAA   
  
  
+ ACGCATCTCT ACTTCTCCAG GCAAAGTTGT ACTTCAGATC TTCTACACCT TTTCTAGGCA GGACTTTGGG   
  
  
+ ATATAGAGTT TGGCCTGGGT GAAAAGTAGG AAGTGGGGAA GTAGAAAATG ATGAAAGAAG GCACCACTTG   
  
  
+ GGTCCAGATA CATAGGCCTA CGCACGAGAA CCTTTGCATG GTCTAGATAC ATGACTGTAT ACTTCTTCCT   
  
  
+ TTCCTTTACC TGTTGGAGAT TATCCTAGGA TTTTACATGG CACCAGCTGG ATTTGTTAAT GACATAAACA   
  
  
+ TATCTTTAAT TACAGTTGCT TATGGTATAC ATTTAAACTA TTCGATAGTT GACAGCTGAT GTCCTTATCC   
  
  
+ TTGTGCAGCT TATTCCTCGA TAGAATAATC TTTGGACATT TCTGTCTAGG TGATAAAGGG CAAAAACTAA   
  
  
+ GATACAAGAA ATAGAACATG TCCTCCATTT ACAAGACGTA TCTGTTTTTG GTTTTGGTCA ATGATCCCTG   
  
  
+ TTGTCCTCAA GCATTAACAC AGAATGATCA TTATTGACTT GGACTGAAAG GTTTTGGGAA CTCCATAGTA   
  
  
+ TGCCTTTTCA ATAAAATCAA AAGCAAATTT GCTGTTCACT GATAACTGCA GTTGTGTTGC TTTGCTGTTT   
  
  
+ CCATACCAGA CTCGTAACCA AGCTGATAGC AAATGTGCAA AGGTACAGTA TAATTAACGA TGTTTTCATA   
  
  
+ TTGCTACGAG TTCTGAAACA TGATATAAGA AAACACCAGA ACCCAAATAC CTACATAGGT CCTAGCCCTG   
  
  
+ GAATGGCTAT TTTAAATATC CAAATGGACT TACAGAATTC GCAGAGTTGT TTGGTTTGTT TAACAATCAT   
  
  
+ TGAATTGTTA GCTATGAGAA TCTGCGGGAC AATCGTAATC TTTGGCGATT ACTTCGATTG GCATACTGAT   
  
  
+ GTTTTTTTAT TTGCGTATCT AAGCATGGTG GTGTACCTTT GATTTGTGTG TCTAGGTAGT GTGTGTAGAT   
  
  
+ AAATATCATA GAACTCAGAC GTTGGTAGGG TTATGTTGAG CACTTTCTCT TGCTGTTTGT CCTTTGTGAT   
  
  
+ GGCACCTCTT GCTTTTCCGA ACCACCAAAA GTCTCATTGA CAAAACTCTT CAAACATTGC AAACTGCCCC   
  
  
+ CTTAAATATG TTTTATTAAA AAAAATGGAA GAGCTCAAAC TGTTGAGTGA GCCTAGATTC CAAGATTTAG   
  
  
+ AAACAGTAAA CTTGATAGAT ATATTTATAT GAACTATTGT TAGATGCAAC ACCCCCAACT TGAAACACAA   
  
  
+ AAGAGAAATT AAAAACAAAA AGGTTTTGCA CTATTTATAT ATCCTGTGTT CTTTGTGCTA GTCTTATTTA   
  
  
+ ATGTTAATTG GTAAGCATTA TTTGTTGGAT TATTCCACTG CACTTTCTCT TACTAACTTC AGCATTTCAT   
  
  
+ TCCTTGTTCT TCAGCTTTTG AACTTCTTTA GCCTATTTTG TTGTCTTTAT GGTTATGGAA GCCATCCAGA   
  
  
+ AGAAGAATGA TGAACTTCTG AGTCTTAGCT TGGCCATTGT CGGTCACTCT GATCGCACTG AGAAAAAACT   
  
  
+ GAAGAGGCGG ATTGATGTCT CCGATCCTCT GATCACTTCA GGTGAAGGCT GCGAAGGGAA GATAATCAGG   
  
  
+ CTCCTCCAAG AGAGACAGAA CTTGTTGAAC ATCAAGCAAA GAGGAAAAGG CGTCATTCAA GAAGGGAAGG   
  
  
+ GTCTTCATCT TATCCATCTG CTCCTTGTAT CTGCCACCTT AATCAACGAA AACAACATCA GTTCAGCTGT   
  
  
+ TGATAATCTT ATTGAGCTCT TCCAATATGT CTCCGAAAGT GGTGATTCAG GACAAAGGGT TGCTGCTTAC   
  
  
+ TTTGCGGATG GGTTAACAGC AAGGATCTTA ACTCAGAGGT CTCCATTTTA TCGCCTGATA CTGGGCAAAC   
  
  
+ CAGCACCTGC TGAAGAGTTT TCAGCTTTTA CTCACTTATA TAGAGCCTCT CCATTCTATC AGTTTGCTCA   
  
  
+ TTTCACAGCC AATCAGGCAA TTGTAGAAGC ATTTGAGAGG GAGGAAGAGA GTAACAACTG GGCTTTGCAT   
  
  
+ GTAATTGATT TGGACATCTT GCATGGTTTC CAGTGGCCTT CTCTTATTCA GTCTCTCTCC GAAGAGGCTA   
  
  
+ CTTGTTCAAA CCGTTCATTG TCTCTTCAAA TTACAGGCTT CGGAAGAAGC TTAGAAGAGC TCATTGAAAC   
  
  
+ AGAAGCTCGG CTGGTAAGTT TCTCTAAGAG CTTTCAGAAT ATCAACTTTG AATTCCACGG GTTTCTGAGA   
  
  
+ GGCTCGAAAC TCAGGAACCT AAGGAGGGAG AATGAAACAG TAGTTGTAAA TCTCGTTTTC CACCTCAGTA   
  
  
+ CTTTGAAAGA TACCGTACAG ATTTCTGACA CCTTGACTTC TGTACATTCA CTAAACCCCT CTATTGTGGT   
  
  
+ ATTAGTCGAG AGAGAAGGAA GTCGAAACAG ATGTGGGTTC CTCTCAAGTT ATGTAGATGC TCTGCATTAT   
  
  
+ TATGCTGCAA TGTTTGACTC TTTAGATGAT TGCCTCCCAC TTGAAAGTCC TGAGAGGCTG AGCATAGAGA   
  
  
+ AGAACCATCT TGGAAAAGAG ATCAAAGAAG CCATAGGTTG TGAGAAGGAT GAAACAAACT ATCTGAAGTT   
  
  
+ TGAGATGCTG GAGACTTGGA GAGGGAGGAT GGAGAGTCAT GGATTTTCAG GTATGAAGCT AAGTTCCAGG   
  
  
+ GCAACTATAC AAGCAAAGCT GCTTTTAAAA ATGGGAAGCC ATTATCATAC CTATTTGGAA GAAGACTGTG   
  
  
+ GAGGTGGTGG GTTCAGAGTT TGTGAACGAG ATGATGGAAT GGCTATCTCT CTTGGCTGGC AAGGTAGGTT   
  
  
+ CCTCGCAACT GCCTCGGTAT GGCGTTCTGT GTG  

- -Up\_Stream \_Len000TGGTTG CTAAGAAATG ACTTAGTCGA TGTAAAAACC TAACACAATT CCATAGAAAA   
  
  
- TCTAGAAGTC CTCCTCCCCC GACTTCATCG AACCAATGAA AACGCGAATA GTTGACACCT TCTTATAATA   
  
  
- ACTCTGATCA CATCTTCTTA ATCATCAAAG GAGGTGTAAC CTTTTTTTGG TACTTTAATT GCGAAATCCG   
  
  
- GGAGTCTCAT GGTTCACAGT AGCATTTACT AACTTTAAAG TTCAGAATCG ACTCAGTACT TCAGGACTAA   
  
  
- AGGGTACTGT TTAACTACAA CAACAGATTT CGAACCGCCC ACTAAACTTT CTTCTAAATC TAGTCCGACT   
  
  
- GTTATGTGGT TTAGACCCTC GTTCCTTGAC CTAAAAAGAC ACAGATCTTT TCGAAAGGCG AAAGACTTGT   
  
  
- TAACTCTTTT GGTACGTATC CTTTAAAGAA CTATAAATCT AGTATCGTCT GAATTGTTAG ACCGAAATGG   
  
  
- CTTGACAGGA GATGAAGATT CAACACTTCC ACACTTTCTA TTTGGTACAG TTTGAATCAC AGGTTGTCTT   
  
  
- TGCGTAGAGA TGAAGAGGTC CGTTTCAACA TGAAGTCTAG AAGATGTGGA AAAGATCCGT CCTGAAACCC   
  
  
- TATATCTCAA ACCGGACCCA CTTTTCATCC TTCACCCCTT CATCTTTTAC TACTTTCTTC CGTGGTGAAC   
  
  
- CCAGGTCTAT GTATCCGGAT GCGTGCTCTT GGAAACGTAC CAGATCTATG TACTGACATA TGAAGAAGGA   
  
  
- AAGGAAATGG ACAACCTCTA ATAGGATCCT AAAATGTACC GTGGTCGACC TAAACAATTA CTGTATTTGT   
  
  
- ATAGAAATTA ATGTCAACGA ATACCATATG TAAATTTGAT AAGCTATCAA CTGTCGACTA CAGGAATAGG   
  
  
- AACACGTCGA ATAAGGAGCT ATCTTATTAG AAACCTGTAA AGACAGATCC ACTATTTCCC GTTTTTGATT   
  
  
- CTATGTTCTT TATCTTGTAC AGGAGGTAAA TGTTCTGCAT AGACAAAAAC CAAAACCAGT TACTAGGGAC   
  
  
- AACAGGAGTT CGTAATTGTG TCTTACTAGT AATAACTGAA CCTGACTTTC CAAAACCCTT GAGGTATCAT   
  
  
- ACGGAAAAGT TATTTTAGTT TTCGTTTAAA CGACAAGTGA CTATTGACGT CAACACAACG AAACGACAAA   
  
  
- GGTATGGTCT GAGCATTGGT TCGACTATCG TTTACACGTT TCCATGTCAT ATTAATTGCT ACAAAAGTAT   
  
  
- AACGATGCTC AAGACTTTGT ACTATATTCT TTTGTGGTCT TGGGTTTATG GATGTATCCA GGATCGGGAC   
  
  
- CTTACCGATA AAATTTATAG GTTTACCTGA ATGTCTTAAG CGTCTCAACA AACCAAACAA ATTGTTAGTA   
  
  
- ACTTAACAAT CGATACTCTT AGACGCCCTG TTAGCATTAG AAACCGCTAA TGAAGCTAAC CGTATGACTA   
  
  
- CAAAAAAATA AACGCATAGA TTCGTACCAC CACATGGAAA CTAAACACAC AGATCCATCA CACACATCTA   
  
  
- TTTATAGTAT CTTGAGTCTG CAACCATCCC AATACAACTC GTGAAAGAGA ACGACAAACA GGAAACACTA   
  
  
- CCGTGGAGAA CGAAAAGGCT TGGTGGTTTT CAGAGTAACT GTTTTGAGAA GTTTGTAACG TTTGACGGGG   
  
  
- GAATTTATAC AAAATAATTT TTTTTACCTT CTCGAGTTTG ACAACTCACT CGGATCTAAG GTTCTAAATC   
  
  
- TTTGTCATTT GAACTATCTA TATAAATATA CTTGATAACA ATCTACGTTG TGGGGGTTGA ACTTTGTGTT   
  
  
- TTCTCTTTAA TTTTTGTTTT TCCAAAACGT GATAAATATA TAGGACACAA GAAACACGAT CAGAATAAAT   
  
  
- TACAATTAAC CATTCGTAAT AAACAACCTA ATAAGGTGAC GTGAAAGAGA ATGATTGAAG TCGTAAAGTA   
  
  
- AGGAACAAGA AGTCGAAAAC TTGAAGAAAT CGGATAAAAC AACAGAAATA CCAATACCTT CGGTAGGTCT   
  
  
- TCTTCTTACT ACTTGAAGAC TCAGAATCGA ACCGGTAACA GCCAGTGAGA CTAGCGTGAC TCTTTTTTGA   
  
  
- CTTCTCCGCC TAACTACAGA GGCTAGGAGA CTAGTGAAGT CCACTTCCGA CGCTTCCCTT CTATTAGTCC   
  
  
- GAGGAGGTTC TCTCTGTCTT GAACAACTTG TAGTTCGTTT CTCCTTTTCC GCAGTAAGTT CTTCCCTTCC   
  
  
- CAGAAGTAGA ATAGGTAGAC GAGGAACATA GACGGTGGAA TTAGTTGCTT TTGTTGTAGT CAAGTCGACA   
  
  
- ACTATTAGAA TAACTCGAGA AGGTTATACA GAGGCTTTCA CCACTAAGTC CTGTTTCCCA ACGACGAATG   
  
  
- AAACGCCTAC CCAATTGTCG TTCCTAGAAT TGAGTCTCCA GAGGTAAAAT AGCGGACTAT GACCCGTTTG   
  
  
- GTCGTGGACG ACTTCTCAAA AGTCGAAAAT GAGTGAATAT ATCTCGGAGA GGTAAGATAG TCAAACGAGT   
  
  
- AAAGTGTCGG TTAGTCCGTT AACATCTTCG TAAACTCTCC CTCCTTCTCT CATTGTTGAC CCGAAACGTA   
  
  
- CATTAACTAA ACCTGTAGAA CGTACCAAAG GTCACCGGAA GAGAATAAGT CAGAGAGAGG CTTCTCCGAT   
  
  
- GAACAAGTTT GGCAAGTAAC AGAGAAGTTT AATGTCCGAA GCCTTCTTCG AATCTTCTCG AGTAACTTTG   
  
  
- TCTTCGAGCC GACCATTCAA AGAGATTCTC GAAAGTCTTA TAGTTGAAAC TTAAGGTGCC CAAAGACTCT   
  
  
- CCGAGCTTTG AGTCCTTGGA TTCCTCCCTC TTACTTTGTC ATCAACATTT AGAGCAAAAG GTGGAGTCAT   
  
  
- GAAACTTTCT ATGGCATGTC TAAAGACTGT GGAACTGAAG ACATGTAAGT GATTTGGGGA GATAACACCA   
  
  
- TAATCAGCTC TCTCTTCCTT CAGCTTTGTC TACACCCAAG GAGAGTTCAA TACATCTACG AGACGTAATA   
  
  
- ATACGACGTT ACAAACTGAG AAATCTACTA ACGGAGGGTG AACTTTCAGG ACTCTCCGAC TCGTATCTCT   
  
  
- TCTTGGTAGA ACCTTTTCTC TAGTTTCTTC GGTATCCAAC ACTCTTCCTA CTTTGTTTGA TAGACTTCAA   
  
  
- ACTCTACGAC CTCTGAACCT CTCCCTCCTA CCTCTCAGTA CCTAAAAGTC CATACTTCGA TTCAAGGTCC   
  
  
- CGTTGATATG TTCGTTTCGA CGAAAATTTT TACCCTTCGG TAATAGTATG GATAAACCTT CTTCTGACAC   
  
  
- CTCCACCACC CAAGTCTCAA ACACTTGCTC TACTACCTTA CCGATAGAGA GAACCGACCG TTCCATCCAA   
  
  
- GGAGCGTTGA CGGAGCCATA CCGCAAGACA CAC

+     W box

| Site Name | Organism | Position | Strand | Matrix score. | sequence | function |
| --- | --- | --- | --- | --- | --- | --- |
| W box | Arabidopsis thaliana | 1040 | - | 6 | TTGACC |  |

>HU07G00272.1   
+ -Up\_Stream \_Len000ACCAAC GATTCTTTAC TGAATCAGCT ACATTTTTGG ATTGTGTTAA GGTATCTTTT   
  
  
+ AGATCTTCAG GAGGAGGGGG CTGAAGTAGC TTGGTTACTT TTGCGCTTAT CAACTGTGGA AGAATATTAT   
  
  
+ TGAGACTAGT GTAGAAGAAT TAGTAGTTTC CTCCACATTG GAAAAAAACC ATGAAATTAA CGCTTTAGGC   
  
  
+ CCTCAGAGTA CCAAGTGTCA TCGTAAATGA TTGAAATTTC AAGTCTTAGC TGAGTCATGA AGTCCTGATT   
  
  
+ TCCCATGACA AATTGATGTT GTTGTCTAAA GCTTGGCGGG TGATTTGAAA GAAGATTTAG ATCAGGCTGA   
  
  
+ CAATACACCA AATCTGGGAG CAAGGAACTG GATTTTTCTG TGTCTAGAAA AGCTTTCCGC TTTCTGAACA   
  
  
+ ATTGAGAAAA CCATGCATAG GAAATTTCTT GATATTTAGA TCATAGCAGA CTTAACAATC TGGCTTTACC   
  
  
+ GAACTGTCCT CTACTTCTAA GTTGTGAAGG TGTGAAAGAT AAACCATGTC AAACTTAGTG TCCAACAGAA   
  
  
+ ACGCATCTCT ACTTCTCCAG GCAAAGTTGT ACTTCAGATC TTCTACACCT TTTCTAGGCA GGACTTTGGG   
  
  
+ ATATAGAGTT TGGCCTGGGT GAAAAGTAGG AAGTGGGGAA GTAGAAAATG ATGAAAGAAG GCACCACTTG   
  
  
+ GGTCCAGATA CATAGGCCTA CGCACGAGAA CCTTTGCATG GTCTAGATAC ATGACTGTAT ACTTCTTCCT   
  
  
+ TTCCTTTACC TGTTGGAGAT TATCCTAGGA TTTTACATGG CACCAGCTGG ATTTGTTAAT GACATAAACA   
  
  
+ TATCTTTAAT TACAGTTGCT TATGGTATAC ATTTAAACTA TTCGATAGTT GACAGCTGAT GTCCTTATCC   
  
  
+ TTGTGCAGCT TATTCCTCGA TAGAATAATC TTTGGACATT TCTGTCTAGG TGATAAAGGG CAAAAACTAA   
  
  
+ GATACAAGAA ATAGAACATG TCCTCCATTT ACAAGACGTA TCTGTTTTTG GTTTTGGTCA ATGATCCCTG   
  
  
+ TTGTCCTCAA GCATTAACAC AGAATGATCA TTATTGACTT GGACTGAAAG GTTTTGGGAA CTCCATAGTA   
  
  
+ TGCCTTTTCA ATAAAATCAA AAGCAAATTT GCTGTTCACT GATAACTGCA GTTGTGTTGC TTTGCTGTTT   
  
  
+ CCATACCAGA CTCGTAACCA AGCTGATAGC AAATGTGCAA AGGTACAGTA TAATTAACGA TGTTTTCATA   
  
  
+ TTGCTACGAG TTCTGAAACA TGATATAAGA AAACACCAGA ACCCAAATAC CTACATAGGT CCTAGCCCTG   
  
  
+ GAATGGCTAT TTTAAATATC CAAATGGACT TACAGAATTC GCAGAGTTGT TTGGTTTGTT TAACAATCAT   
  
  
+ TGAATTGTTA GCTATGAGAA TCTGCGGGAC AATCGTAATC TTTGGCGATT ACTTCGATTG GCATACTGAT   
  
  
+ GTTTTTTTAT TTGCGTATCT AAGCATGGTG GTGTACCTTT GATTTGTGTG TCTAGGTAGT GTGTGTAGAT   
  
  
+ AAATATCATA GAACTCAGAC GTTGGTAGGG TTATGTTGAG CACTTTCTCT TGCTGTTTGT CCTTTGTGAT   
  
  
+ GGCACCTCTT GCTTTTCCGA ACCACCAAAA GTCTCATTGA CAAAACTCTT CAAACATTGC AAACTGCCCC   
  
  
+ CTTAAATATG TTTTATTAAA AAAAATGGAA GAGCTCAAAC TGTTGAGTGA GCCTAGATTC CAAGATTTAG   
  
  
+ AAACAGTAAA CTTGATAGAT ATATTTATAT GAACTATTGT TAGATGCAAC ACCCCCAACT TGAAACACAA   
  
  
+ AAGAGAAATT AAAAACAAAA AGGTTTTGCA CTATTTATAT ATCCTGTGTT CTTTGTGCTA GTCTTATTTA   
  
  
+ ATGTTAATTG GTAAGCATTA TTTGTTGGAT TATTCCACTG CACTTTCTCT TACTAACTTC AGCATTTCAT   
  
  
+ TCCTTGTTCT TCAGCTTTTG AACTTCTTTA GCCTATTTTG TTGTCTTTAT GGTTATGGAA GCCATCCAGA   
  
  
+ AGAAGAATGA TGAACTTCTG AGTCTTAGCT TGGCCATTGT CGGTCACTCT GATCGCACTG AGAAAAAACT   
  
  
+ GAAGAGGCGG ATTGATGTCT CCGATCCTCT GATCACTTCA GGTGAAGGCT GCGAAGGGAA GATAATCAGG   
  
  
+ CTCCTCCAAG AGAGACAGAA CTTGTTGAAC ATCAAGCAAA GAGGAAAAGG CGTCATTCAA GAAGGGAAGG   
  
  
+ GTCTTCATCT TATCCATCTG CTCCTTGTAT CTGCCACCTT AATCAACGAA AACAACATCA GTTCAGCTGT   
  
  
+ TGATAATCTT ATTGAGCTCT TCCAATATGT CTCCGAAAGT GGTGATTCAG GACAAAGGGT TGCTGCTTAC   
  
  
+ TTTGCGGATG GGTTAACAGC AAGGATCTTA ACTCAGAGGT CTCCATTTTA TCGCCTGATA CTGGGCAAAC   
  
  
+ CAGCACCTGC TGAAGAGTTT TCAGCTTTTA CTCACTTATA TAGAGCCTCT CCATTCTATC AGTTTGCTCA   
  
  
+ TTTCACAGCC AATCAGGCAA TTGTAGAAGC ATTTGAGAGG GAGGAAGAGA GTAACAACTG GGCTTTGCAT   
  
  
+ GTAATTGATT TGGACATCTT GCATGGTTTC CAGTGGCCTT CTCTTATTCA GTCTCTCTCC GAAGAGGCTA   
  
  
+ CTTGTTCAAA CCGTTCATTG TCTCTTCAAA TTACAGGCTT CGGAAGAAGC TTAGAAGAGC TCATTGAAAC   
  
  
+ AGAAGCTCGG CTGGTAAGTT TCTCTAAGAG CTTTCAGAAT ATCAACTTTG AATTCCACGG GTTTCTGAGA   
  
  
+ GGCTCGAAAC TCAGGAACCT AAGGAGGGAG AATGAAACAG TAGTTGTAAA TCTCGTTTTC CACCTCAGTA   
  
  
+ CTTTGAAAGA TACCGTACAG ATTTCTGACA CCTTGACTTC TGTACATTCA CTAAACCCCT CTATTGTGGT   
  
  
+ ATTAGTCGAG AGAGAAGGAA GTCGAAACAG ATGTGGGTTC CTCTCAAGTT ATGTAGATGC TCTGCATTAT   
  
  
+ TATGCTGCAA TGTTTGACTC TTTAGATGAT TGCCTCCCAC TTGAAAGTCC TGAGAGGCTG AGCATAGAGA   
  
  
+ AGAACCATCT TGGAAAAGAG ATCAAAGAAG CCATAGGTTG TGAGAAGGAT GAAACAAACT ATCTGAAGTT   
  
  
+ TGAGATGCTG GAGACTTGGA GAGGGAGGAT GGAGAGTCAT GGATTTTCAG GTATGAAGCT AAGTTCCAGG   
  
  
+ GCAACTATAC AAGCAAAGCT GCTTTTAAAA ATGGGAAGCC ATTATCATAC CTATTTGGAA GAAGACTGTG   
  
  
+ GAGGTGGTGG GTTCAGAGTT TGTGAACGAG ATGATGGAAT GGCTATCTCT CTTGGCTGGC AAGGTAGGTT   
  
  
+ CCTCGCAACT GCCTCGGTAT GGCGTTCTGT GTG  

- -Up\_Stream \_Len000TGGTTG CTAAGAAATG ACTTAGTCGA TGTAAAAACC TAACACAATT CCATAGAAAA   
  
  
- TCTAGAAGTC CTCCTCCCCC GACTTCATCG AACCAATGAA AACGCGAATA GTTGACACCT TCTTATAATA   
  
  
- ACTCTGATCA CATCTTCTTA ATCATCAAAG GAGGTGTAAC CTTTTTTTGG TACTTTAATT GCGAAATCCG   
  
  
- GGAGTCTCAT GGTTCACAGT AGCATTTACT AACTTTAAAG TTCAGAATCG ACTCAGTACT TCAGGACTAA   
  
  
- AGGGTACTGT TTAACTACAA CAACAGATTT CGAACCGCCC ACTAAACTTT CTTCTAAATC TAGTCCGACT   
  
  
- GTTATGTGGT TTAGACCCTC GTTCCTTGAC CTAAAAAGAC ACAGATCTTT TCGAAAGGCG AAAGACTTGT   
  
  
- TAACTCTTTT GGTACGTATC CTTTAAAGAA CTATAAATCT AGTATCGTCT GAATTGTTAG ACCGAAATGG   
  
  
- CTTGACAGGA GATGAAGATT CAACACTTCC ACACTTTCTA TTTGGTACAG TTTGAATCAC AGGTTGTCTT   
  
  
- TGCGTAGAGA TGAAGAGGTC CGTTTCAACA TGAAGTCTAG AAGATGTGGA AAAGATCCGT CCTGAAACCC   
  
  
- TATATCTCAA ACCGGACCCA CTTTTCATCC TTCACCCCTT CATCTTTTAC TACTTTCTTC CGTGGTGAAC   
  
  
- CCAGGTCTAT GTATCCGGAT GCGTGCTCTT GGAAACGTAC CAGATCTATG TACTGACATA TGAAGAAGGA   
  
  
- AAGGAAATGG ACAACCTCTA ATAGGATCCT AAAATGTACC GTGGTCGACC TAAACAATTA CTGTATTTGT   
  
  
- ATAGAAATTA ATGTCAACGA ATACCATATG TAAATTTGAT AAGCTATCAA CTGTCGACTA CAGGAATAGG   
  
  
- AACACGTCGA ATAAGGAGCT ATCTTATTAG AAACCTGTAA AGACAGATCC ACTATTTCCC GTTTTTGATT   
  
  
- CTATGTTCTT TATCTTGTAC AGGAGGTAAA TGTTCTGCAT AGACAAAAAC CAAAACCAGT TACTAGGGAC   
  
  
- AACAGGAGTT CGTAATTGTG TCTTACTAGT AATAACTGAA CCTGACTTTC CAAAACCCTT GAGGTATCAT   
  
  
- ACGGAAAAGT TATTTTAGTT TTCGTTTAAA CGACAAGTGA CTATTGACGT CAACACAACG AAACGACAAA   
  
  
- GGTATGGTCT GAGCATTGGT TCGACTATCG TTTACACGTT TCCATGTCAT ATTAATTGCT ACAAAAGTAT   
  
  
- AACGATGCTC AAGACTTTGT ACTATATTCT TTTGTGGTCT TGGGTTTATG GATGTATCCA GGATCGGGAC   
  
  
- CTTACCGATA AAATTTATAG GTTTACCTGA ATGTCTTAAG CGTCTCAACA AACCAAACAA ATTGTTAGTA   
  
  
- ACTTAACAAT CGATACTCTT AGACGCCCTG TTAGCATTAG AAACCGCTAA TGAAGCTAAC CGTATGACTA   
  
  
- CAAAAAAATA AACGCATAGA TTCGTACCAC CACATGGAAA CTAAACACAC AGATCCATCA CACACATCTA   
  
  
- TTTATAGTAT CTTGAGTCTG CAACCATCCC AATACAACTC GTGAAAGAGA ACGACAAACA GGAAACACTA   
  
  
- CCGTGGAGAA CGAAAAGGCT TGGTGGTTTT CAGAGTAACT GTTTTGAGAA GTTTGTAACG TTTGACGGGG   
  
  
- GAATTTATAC AAAATAATTT TTTTTACCTT CTCGAGTTTG ACAACTCACT CGGATCTAAG GTTCTAAATC   
  
  
- TTTGTCATTT GAACTATCTA TATAAATATA CTTGATAACA ATCTACGTTG TGGGGGTTGA ACTTTGTGTT   
  
  
- TTCTCTTTAA TTTTTGTTTT TCCAAAACGT GATAAATATA TAGGACACAA GAAACACGAT CAGAATAAAT   
  
  
- TACAATTAAC CATTCGTAAT AAACAACCTA ATAAGGTGAC GTGAAAGAGA ATGATTGAAG TCGTAAAGTA   
  
  
- AGGAACAAGA AGTCGAAAAC TTGAAGAAAT CGGATAAAAC AACAGAAATA CCAATACCTT CGGTAGGTCT   
  
  
- TCTTCTTACT ACTTGAAGAC TCAGAATCGA ACCGGTAACA GCCAGTGAGA CTAGCGTGAC TCTTTTTTGA   
  
  
- CTTCTCCGCC TAACTACAGA GGCTAGGAGA CTAGTGAAGT CCACTTCCGA CGCTTCCCTT CTATTAGTCC   
  
  
- GAGGAGGTTC TCTCTGTCTT GAACAACTTG TAGTTCGTTT CTCCTTTTCC GCAGTAAGTT CTTCCCTTCC   
  
  
- CAGAAGTAGA ATAGGTAGAC GAGGAACATA GACGGTGGAA TTAGTTGCTT TTGTTGTAGT CAAGTCGACA   
  
  
- ACTATTAGAA TAACTCGAGA AGGTTATACA GAGGCTTTCA CCACTAAGTC CTGTTTCCCA ACGACGAATG   
  
  
- AAACGCCTAC CCAATTGTCG TTCCTAGAAT TGAGTCTCCA GAGGTAAAAT AGCGGACTAT GACCCGTTTG   
  
  
- GTCGTGGACG ACTTCTCAAA AGTCGAAAAT GAGTGAATAT ATCTCGGAGA GGTAAGATAG TCAAACGAGT   
  
  
- AAAGTGTCGG TTAGTCCGTT AACATCTTCG TAAACTCTCC CTCCTTCTCT CATTGTTGAC CCGAAACGTA   
  
  
- CATTAACTAA ACCTGTAGAA CGTACCAAAG GTCACCGGAA GAGAATAAGT CAGAGAGAGG CTTCTCCGAT   
  
  
- GAACAAGTTT GGCAAGTAAC AGAGAAGTTT AATGTCCGAA GCCTTCTTCG AATCTTCTCG AGTAACTTTG   
  
  
- TCTTCGAGCC GACCATTCAA AGAGATTCTC GAAAGTCTTA TAGTTGAAAC TTAAGGTGCC CAAAGACTCT   
  
  
- CCGAGCTTTG AGTCCTTGGA TTCCTCCCTC TTACTTTGTC ATCAACATTT AGAGCAAAAG GTGGAGTCAT   
  
  
- GAAACTTTCT ATGGCATGTC TAAAGACTGT GGAACTGAAG ACATGTAAGT GATTTGGGGA GATAACACCA   
  
  
- TAATCAGCTC TCTCTTCCTT CAGCTTTGTC TACACCCAAG GAGAGTTCAA TACATCTACG AGACGTAATA   
  
  
- ATACGACGTT ACAAACTGAG AAATCTACTA ACGGAGGGTG AACTTTCAGG ACTCTCCGAC TCGTATCTCT   
  
  
- TCTTGGTAGA ACCTTTTCTC TAGTTTCTTC GGTATCCAAC ACTCTTCCTA CTTTGTTTGA TAGACTTCAA   
  
  
- ACTCTACGAC CTCTGAACCT CTCCCTCCTA CCTCTCAGTA CCTAAAAGTC CATACTTCGA TTCAAGGTCC   
  
  
- CGTTGATATG TTCGTTTCGA CGAAAATTTT TACCCTTCGG TAATAGTATG GATAAACCTT CTTCTGACAC   
  
  
- CTCCACCACC CAAGTCTCAA ACACTTGCTC TACTACCTTA CCGATAGAGA GAACCGACCG TTCCATCCAA   
  
  
- GGAGCGTTGA CGGAGCCATA CCGCAAGACA CAC

+     WRE3

| Site Name | Organism | Position | Strand | Matrix score. | sequence | function |
| --- | --- | --- | --- | --- | --- | --- |
| WRE3 | Pisum sativum | 3296 | - | 6 | CCACCT |  |
| WRE3 | Pisum sativum | 2278 | + | 6 | CCACCT |  |
| WRE3 | Pisum sativum | 2864 | + | 6 | CCACCT |  |

>HU07G00272.1   
+ -Up\_Stream \_Len000ACCAAC GATTCTTTAC TGAATCAGCT ACATTTTTGG ATTGTGTTAA GGTATCTTTT   
  
  
+ AGATCTTCAG GAGGAGGGGG CTGAAGTAGC TTGGTTACTT TTGCGCTTAT CAACTGTGGA AGAATATTAT   
  
  
+ TGAGACTAGT GTAGAAGAAT TAGTAGTTTC CTCCACATTG GAAAAAAACC ATGAAATTAA CGCTTTAGGC   
  
  
+ CCTCAGAGTA CCAAGTGTCA TCGTAAATGA TTGAAATTTC AAGTCTTAGC TGAGTCATGA AGTCCTGATT   
  
  
+ TCCCATGACA AATTGATGTT GTTGTCTAAA GCTTGGCGGG TGATTTGAAA GAAGATTTAG ATCAGGCTGA   
  
  
+ CAATACACCA AATCTGGGAG CAAGGAACTG GATTTTTCTG TGTCTAGAAA AGCTTTCCGC TTTCTGAACA   
  
  
+ ATTGAGAAAA CCATGCATAG GAAATTTCTT GATATTTAGA TCATAGCAGA CTTAACAATC TGGCTTTACC   
  
  
+ GAACTGTCCT CTACTTCTAA GTTGTGAAGG TGTGAAAGAT AAACCATGTC AAACTTAGTG TCCAACAGAA   
  
  
+ ACGCATCTCT ACTTCTCCAG GCAAAGTTGT ACTTCAGATC TTCTACACCT TTTCTAGGCA GGACTTTGGG   
  
  
+ ATATAGAGTT TGGCCTGGGT GAAAAGTAGG AAGTGGGGAA GTAGAAAATG ATGAAAGAAG GCACCACTTG   
  
  
+ GGTCCAGATA CATAGGCCTA CGCACGAGAA CCTTTGCATG GTCTAGATAC ATGACTGTAT ACTTCTTCCT   
  
  
+ TTCCTTTACC TGTTGGAGAT TATCCTAGGA TTTTACATGG CACCAGCTGG ATTTGTTAAT GACATAAACA   
  
  
+ TATCTTTAAT TACAGTTGCT TATGGTATAC ATTTAAACTA TTCGATAGTT GACAGCTGAT GTCCTTATCC   
  
  
+ TTGTGCAGCT TATTCCTCGA TAGAATAATC TTTGGACATT TCTGTCTAGG TGATAAAGGG CAAAAACTAA   
  
  
+ GATACAAGAA ATAGAACATG TCCTCCATTT ACAAGACGTA TCTGTTTTTG GTTTTGGTCA ATGATCCCTG   
  
  
+ TTGTCCTCAA GCATTAACAC AGAATGATCA TTATTGACTT GGACTGAAAG GTTTTGGGAA CTCCATAGTA   
  
  
+ TGCCTTTTCA ATAAAATCAA AAGCAAATTT GCTGTTCACT GATAACTGCA GTTGTGTTGC TTTGCTGTTT   
  
  
+ CCATACCAGA CTCGTAACCA AGCTGATAGC AAATGTGCAA AGGTACAGTA TAATTAACGA TGTTTTCATA   
  
  
+ TTGCTACGAG TTCTGAAACA TGATATAAGA AAACACCAGA ACCCAAATAC CTACATAGGT CCTAGCCCTG   
  
  
+ GAATGGCTAT TTTAAATATC CAAATGGACT TACAGAATTC GCAGAGTTGT TTGGTTTGTT TAACAATCAT   
  
  
+ TGAATTGTTA GCTATGAGAA TCTGCGGGAC AATCGTAATC TTTGGCGATT ACTTCGATTG GCATACTGAT   
  
  
+ GTTTTTTTAT TTGCGTATCT AAGCATGGTG GTGTACCTTT GATTTGTGTG TCTAGGTAGT GTGTGTAGAT   
  
  
+ AAATATCATA GAACTCAGAC GTTGGTAGGG TTATGTTGAG CACTTTCTCT TGCTGTTTGT CCTTTGTGAT   
  
  
+ GGCACCTCTT GCTTTTCCGA ACCACCAAAA GTCTCATTGA CAAAACTCTT CAAACATTGC AAACTGCCCC   
  
  
+ CTTAAATATG TTTTATTAAA AAAAATGGAA GAGCTCAAAC TGTTGAGTGA GCCTAGATTC CAAGATTTAG   
  
  
+ AAACAGTAAA CTTGATAGAT ATATTTATAT GAACTATTGT TAGATGCAAC ACCCCCAACT TGAAACACAA   
  
  
+ AAGAGAAATT AAAAACAAAA AGGTTTTGCA CTATTTATAT ATCCTGTGTT CTTTGTGCTA GTCTTATTTA   
  
  
+ ATGTTAATTG GTAAGCATTA TTTGTTGGAT TATTCCACTG CACTTTCTCT TACTAACTTC AGCATTTCAT   
  
  
+ TCCTTGTTCT TCAGCTTTTG AACTTCTTTA GCCTATTTTG TTGTCTTTAT GGTTATGGAA GCCATCCAGA   
  
  
+ AGAAGAATGA TGAACTTCTG AGTCTTAGCT TGGCCATTGT CGGTCACTCT GATCGCACTG AGAAAAAACT   
  
  
+ GAAGAGGCGG ATTGATGTCT CCGATCCTCT GATCACTTCA GGTGAAGGCT GCGAAGGGAA GATAATCAGG   
  
  
+ CTCCTCCAAG AGAGACAGAA CTTGTTGAAC ATCAAGCAAA GAGGAAAAGG CGTCATTCAA GAAGGGAAGG   
  
  
+ GTCTTCATCT TATCCATCTG CTCCTTGTAT CTGCCACCTT AATCAACGAA AACAACATCA GTTCAGCTGT   
  
  
+ TGATAATCTT ATTGAGCTCT TCCAATATGT CTCCGAAAGT GGTGATTCAG GACAAAGGGT TGCTGCTTAC   
  
  
+ TTTGCGGATG GGTTAACAGC AAGGATCTTA ACTCAGAGGT CTCCATTTTA TCGCCTGATA CTGGGCAAAC   
  
  
+ CAGCACCTGC TGAAGAGTTT TCAGCTTTTA CTCACTTATA TAGAGCCTCT CCATTCTATC AGTTTGCTCA   
  
  
+ TTTCACAGCC AATCAGGCAA TTGTAGAAGC ATTTGAGAGG GAGGAAGAGA GTAACAACTG GGCTTTGCAT   
  
  
+ GTAATTGATT TGGACATCTT GCATGGTTTC CAGTGGCCTT CTCTTATTCA GTCTCTCTCC GAAGAGGCTA   
  
  
+ CTTGTTCAAA CCGTTCATTG TCTCTTCAAA TTACAGGCTT CGGAAGAAGC TTAGAAGAGC TCATTGAAAC   
  
  
+ AGAAGCTCGG CTGGTAAGTT TCTCTAAGAG CTTTCAGAAT ATCAACTTTG AATTCCACGG GTTTCTGAGA   
  
  
+ GGCTCGAAAC TCAGGAACCT AAGGAGGGAG AATGAAACAG TAGTTGTAAA TCTCGTTTTC CACCTCAGTA   
  
  
+ CTTTGAAAGA TACCGTACAG ATTTCTGACA CCTTGACTTC TGTACATTCA CTAAACCCCT CTATTGTGGT   
  
  
+ ATTAGTCGAG AGAGAAGGAA GTCGAAACAG ATGTGGGTTC CTCTCAAGTT ATGTAGATGC TCTGCATTAT   
  
  
+ TATGCTGCAA TGTTTGACTC TTTAGATGAT TGCCTCCCAC TTGAAAGTCC TGAGAGGCTG AGCATAGAGA   
  
  
+ AGAACCATCT TGGAAAAGAG ATCAAAGAAG CCATAGGTTG TGAGAAGGAT GAAACAAACT ATCTGAAGTT   
  
  
+ TGAGATGCTG GAGACTTGGA GAGGGAGGAT GGAGAGTCAT GGATTTTCAG GTATGAAGCT AAGTTCCAGG   
  
  
+ GCAACTATAC AAGCAAAGCT GCTTTTAAAA ATGGGAAGCC ATTATCATAC CTATTTGGAA GAAGACTGTG   
  
  
+ GAGGTGGTGG GTTCAGAGTT TGTGAACGAG ATGATGGAAT GGCTATCTCT CTTGGCTGGC AAGGTAGGTT   
  
  
+ CCTCGCAACT GCCTCGGTAT GGCGTTCTGT GTG  

- -Up\_Stream \_Len000TGGTTG CTAAGAAATG ACTTAGTCGA TGTAAAAACC TAACACAATT CCATAGAAAA   
  
  
- TCTAGAAGTC CTCCTCCCCC GACTTCATCG AACCAATGAA AACGCGAATA GTTGACACCT TCTTATAATA   
  
  
- ACTCTGATCA CATCTTCTTA ATCATCAAAG GAGGTGTAAC CTTTTTTTGG TACTTTAATT GCGAAATCCG   
  
  
- GGAGTCTCAT GGTTCACAGT AGCATTTACT AACTTTAAAG TTCAGAATCG ACTCAGTACT TCAGGACTAA   
  
  
- AGGGTACTGT TTAACTACAA CAACAGATTT CGAACCGCCC ACTAAACTTT CTTCTAAATC TAGTCCGACT   
  
  
- GTTATGTGGT TTAGACCCTC GTTCCTTGAC CTAAAAAGAC ACAGATCTTT TCGAAAGGCG AAAGACTTGT   
  
  
- TAACTCTTTT GGTACGTATC CTTTAAAGAA CTATAAATCT AGTATCGTCT GAATTGTTAG ACCGAAATGG   
  
  
- CTTGACAGGA GATGAAGATT CAACACTTCC ACACTTTCTA TTTGGTACAG TTTGAATCAC AGGTTGTCTT   
  
  
- TGCGTAGAGA TGAAGAGGTC CGTTTCAACA TGAAGTCTAG AAGATGTGGA AAAGATCCGT CCTGAAACCC   
  
  
- TATATCTCAA ACCGGACCCA CTTTTCATCC TTCACCCCTT CATCTTTTAC TACTTTCTTC CGTGGTGAAC   
  
  
- CCAGGTCTAT GTATCCGGAT GCGTGCTCTT GGAAACGTAC CAGATCTATG TACTGACATA TGAAGAAGGA   
  
  
- AAGGAAATGG ACAACCTCTA ATAGGATCCT AAAATGTACC GTGGTCGACC TAAACAATTA CTGTATTTGT   
  
  
- ATAGAAATTA ATGTCAACGA ATACCATATG TAAATTTGAT AAGCTATCAA CTGTCGACTA CAGGAATAGG   
  
  
- AACACGTCGA ATAAGGAGCT ATCTTATTAG AAACCTGTAA AGACAGATCC ACTATTTCCC GTTTTTGATT   
  
  
- CTATGTTCTT TATCTTGTAC AGGAGGTAAA TGTTCTGCAT AGACAAAAAC CAAAACCAGT TACTAGGGAC   
  
  
- AACAGGAGTT CGTAATTGTG TCTTACTAGT AATAACTGAA CCTGACTTTC CAAAACCCTT GAGGTATCAT   
  
  
- ACGGAAAAGT TATTTTAGTT TTCGTTTAAA CGACAAGTGA CTATTGACGT CAACACAACG AAACGACAAA   
  
  
- GGTATGGTCT GAGCATTGGT TCGACTATCG TTTACACGTT TCCATGTCAT ATTAATTGCT ACAAAAGTAT   
  
  
- AACGATGCTC AAGACTTTGT ACTATATTCT TTTGTGGTCT TGGGTTTATG GATGTATCCA GGATCGGGAC   
  
  
- CTTACCGATA AAATTTATAG GTTTACCTGA ATGTCTTAAG CGTCTCAACA AACCAAACAA ATTGTTAGTA   
  
  
- ACTTAACAAT CGATACTCTT AGACGCCCTG TTAGCATTAG AAACCGCTAA TGAAGCTAAC CGTATGACTA   
  
  
- CAAAAAAATA AACGCATAGA TTCGTACCAC CACATGGAAA CTAAACACAC AGATCCATCA CACACATCTA   
  
  
- TTTATAGTAT CTTGAGTCTG CAACCATCCC AATACAACTC GTGAAAGAGA ACGACAAACA GGAAACACTA   
  
  
- CCGTGGAGAA CGAAAAGGCT TGGTGGTTTT CAGAGTAACT GTTTTGAGAA GTTTGTAACG TTTGACGGGG   
  
  
- GAATTTATAC AAAATAATTT TTTTTACCTT CTCGAGTTTG ACAACTCACT CGGATCTAAG GTTCTAAATC   
  
  
- TTTGTCATTT GAACTATCTA TATAAATATA CTTGATAACA ATCTACGTTG TGGGGGTTGA ACTTTGTGTT   
  
  
- TTCTCTTTAA TTTTTGTTTT TCCAAAACGT GATAAATATA TAGGACACAA GAAACACGAT CAGAATAAAT   
  
  
- TACAATTAAC CATTCGTAAT AAACAACCTA ATAAGGTGAC GTGAAAGAGA ATGATTGAAG TCGTAAAGTA   
  
  
- AGGAACAAGA AGTCGAAAAC TTGAAGAAAT CGGATAAAAC AACAGAAATA CCAATACCTT CGGTAGGTCT   
  
  
- TCTTCTTACT ACTTGAAGAC TCAGAATCGA ACCGGTAACA GCCAGTGAGA CTAGCGTGAC TCTTTTTTGA   
  
  
- CTTCTCCGCC TAACTACAGA GGCTAGGAGA CTAGTGAAGT CCACTTCCGA CGCTTCCCTT CTATTAGTCC   
  
  
- GAGGAGGTTC TCTCTGTCTT GAACAACTTG TAGTTCGTTT CTCCTTTTCC GCAGTAAGTT CTTCCCTTCC   
  
  
- CAGAAGTAGA ATAGGTAGAC GAGGAACATA GACGGTGGAA TTAGTTGCTT TTGTTGTAGT CAAGTCGACA   
  
  
- ACTATTAGAA TAACTCGAGA AGGTTATACA GAGGCTTTCA CCACTAAGTC CTGTTTCCCA ACGACGAATG   
  
  
- AAACGCCTAC CCAATTGTCG TTCCTAGAAT TGAGTCTCCA GAGGTAAAAT AGCGGACTAT GACCCGTTTG   
  
  
- GTCGTGGACG ACTTCTCAAA AGTCGAAAAT GAGTGAATAT ATCTCGGAGA GGTAAGATAG TCAAACGAGT   
  
  
- AAAGTGTCGG TTAGTCCGTT AACATCTTCG TAAACTCTCC CTCCTTCTCT CATTGTTGAC CCGAAACGTA   
  
  
- CATTAACTAA ACCTGTAGAA CGTACCAAAG GTCACCGGAA GAGAATAAGT CAGAGAGAGG CTTCTCCGAT   
  
  
- GAACAAGTTT GGCAAGTAAC AGAGAAGTTT AATGTCCGAA GCCTTCTTCG AATCTTCTCG AGTAACTTTG   
  
  
- TCTTCGAGCC GACCATTCAA AGAGATTCTC GAAAGTCTTA TAGTTGAAAC TTAAGGTGCC CAAAGACTCT   
  
  
- CCGAGCTTTG AGTCCTTGGA TTCCTCCCTC TTACTTTGTC ATCAACATTT AGAGCAAAAG GTGGAGTCAT   
  
  
- GAAACTTTCT ATGGCATGTC TAAAGACTGT GGAACTGAAG ACATGTAAGT GATTTGGGGA GATAACACCA   
  
  
- TAATCAGCTC TCTCTTCCTT CAGCTTTGTC TACACCCAAG GAGAGTTCAA TACATCTACG AGACGTAATA   
  
  
- ATACGACGTT ACAAACTGAG AAATCTACTA ACGGAGGGTG AACTTTCAGG ACTCTCCGAC TCGTATCTCT   
  
  
- TCTTGGTAGA ACCTTTTCTC TAGTTTCTTC GGTATCCAAC ACTCTTCCTA CTTTGTTTGA TAGACTTCAA   
  
  
- ACTCTACGAC CTCTGAACCT CTCCCTCCTA CCTCTCAGTA CCTAAAAGTC CATACTTCGA TTCAAGGTCC   
  
  
- CGTTGATATG TTCGTTTCGA CGAAAATTTT TACCCTTCGG TAATAGTATG GATAAACCTT CTTCTGACAC   
  
  
- CTCCACCACC CAAGTCTCAA ACACTTGCTC TACTACCTTA CCGATAGAGA GAACCGACCG TTCCATCCAA   
  
  
- GGAGCGTTGA CGGAGCCATA CCGCAAGACA CAC

+     WUN-motif

| Site Name | Organism | Position | Strand | Matrix score. | sequence | function |
| --- | --- | --- | --- | --- | --- | --- |
| WUN-motif | Nicotiana glutinosa | 2593 | - | 9 | CAATTACAT |  |
| WUN-motif | Brassica oleracea | 443 | - | 9 | AAATTTCCT | wound-responsive element |
| WUN-motif | Nicotiana glutinosa | 446 | + | 9 | AAATTTCTT |  |

>HU07G00272.1   
+ -Up\_Stream \_Len000ACCAAC GATTCTTTAC TGAATCAGCT ACATTTTTGG ATTGTGTTAA GGTATCTTTT   
  
  
+ AGATCTTCAG GAGGAGGGGG CTGAAGTAGC TTGGTTACTT TTGCGCTTAT CAACTGTGGA AGAATATTAT   
  
  
+ TGAGACTAGT GTAGAAGAAT TAGTAGTTTC CTCCACATTG GAAAAAAACC ATGAAATTAA CGCTTTAGGC   
  
  
+ CCTCAGAGTA CCAAGTGTCA TCGTAAATGA TTGAAATTTC AAGTCTTAGC TGAGTCATGA AGTCCTGATT   
  
  
+ TCCCATGACA AATTGATGTT GTTGTCTAAA GCTTGGCGGG TGATTTGAAA GAAGATTTAG ATCAGGCTGA   
  
  
+ CAATACACCA AATCTGGGAG CAAGGAACTG GATTTTTCTG TGTCTAGAAA AGCTTTCCGC TTTCTGAACA   
  
  
+ ATTGAGAAAA CCATGCATAG GAAATTTCTT GATATTTAGA TCATAGCAGA CTTAACAATC TGGCTTTACC   
  
  
+ GAACTGTCCT CTACTTCTAA GTTGTGAAGG TGTGAAAGAT AAACCATGTC AAACTTAGTG TCCAACAGAA   
  
  
+ ACGCATCTCT ACTTCTCCAG GCAAAGTTGT ACTTCAGATC TTCTACACCT TTTCTAGGCA GGACTTTGGG   
  
  
+ ATATAGAGTT TGGCCTGGGT GAAAAGTAGG AAGTGGGGAA GTAGAAAATG ATGAAAGAAG GCACCACTTG   
  
  
+ GGTCCAGATA CATAGGCCTA CGCACGAGAA CCTTTGCATG GTCTAGATAC ATGACTGTAT ACTTCTTCCT   
  
  
+ TTCCTTTACC TGTTGGAGAT TATCCTAGGA TTTTACATGG CACCAGCTGG ATTTGTTAAT GACATAAACA   
  
  
+ TATCTTTAAT TACAGTTGCT TATGGTATAC ATTTAAACTA TTCGATAGTT GACAGCTGAT GTCCTTATCC   
  
  
+ TTGTGCAGCT TATTCCTCGA TAGAATAATC TTTGGACATT TCTGTCTAGG TGATAAAGGG CAAAAACTAA   
  
  
+ GATACAAGAA ATAGAACATG TCCTCCATTT ACAAGACGTA TCTGTTTTTG GTTTTGGTCA ATGATCCCTG   
  
  
+ TTGTCCTCAA GCATTAACAC AGAATGATCA TTATTGACTT GGACTGAAAG GTTTTGGGAA CTCCATAGTA   
  
  
+ TGCCTTTTCA ATAAAATCAA AAGCAAATTT GCTGTTCACT GATAACTGCA GTTGTGTTGC TTTGCTGTTT   
  
  
+ CCATACCAGA CTCGTAACCA AGCTGATAGC AAATGTGCAA AGGTACAGTA TAATTAACGA TGTTTTCATA   
  
  
+ TTGCTACGAG TTCTGAAACA TGATATAAGA AAACACCAGA ACCCAAATAC CTACATAGGT CCTAGCCCTG   
  
  
+ GAATGGCTAT TTTAAATATC CAAATGGACT TACAGAATTC GCAGAGTTGT TTGGTTTGTT TAACAATCAT   
  
  
+ TGAATTGTTA GCTATGAGAA TCTGCGGGAC AATCGTAATC TTTGGCGATT ACTTCGATTG GCATACTGAT   
  
  
+ GTTTTTTTAT TTGCGTATCT AAGCATGGTG GTGTACCTTT GATTTGTGTG TCTAGGTAGT GTGTGTAGAT   
  
  
+ AAATATCATA GAACTCAGAC GTTGGTAGGG TTATGTTGAG CACTTTCTCT TGCTGTTTGT CCTTTGTGAT   
  
  
+ GGCACCTCTT GCTTTTCCGA ACCACCAAAA GTCTCATTGA CAAAACTCTT CAAACATTGC AAACTGCCCC   
  
  
+ CTTAAATATG TTTTATTAAA AAAAATGGAA GAGCTCAAAC TGTTGAGTGA GCCTAGATTC CAAGATTTAG   
  
  
+ AAACAGTAAA CTTGATAGAT ATATTTATAT GAACTATTGT TAGATGCAAC ACCCCCAACT TGAAACACAA   
  
  
+ AAGAGAAATT AAAAACAAAA AGGTTTTGCA CTATTTATAT ATCCTGTGTT CTTTGTGCTA GTCTTATTTA   
  
  
+ ATGTTAATTG GTAAGCATTA TTTGTTGGAT TATTCCACTG CACTTTCTCT TACTAACTTC AGCATTTCAT   
  
  
+ TCCTTGTTCT TCAGCTTTTG AACTTCTTTA GCCTATTTTG TTGTCTTTAT GGTTATGGAA GCCATCCAGA   
  
  
+ AGAAGAATGA TGAACTTCTG AGTCTTAGCT TGGCCATTGT CGGTCACTCT GATCGCACTG AGAAAAAACT   
  
  
+ GAAGAGGCGG ATTGATGTCT CCGATCCTCT GATCACTTCA GGTGAAGGCT GCGAAGGGAA GATAATCAGG   
  
  
+ CTCCTCCAAG AGAGACAGAA CTTGTTGAAC ATCAAGCAAA GAGGAAAAGG CGTCATTCAA GAAGGGAAGG   
  
  
+ GTCTTCATCT TATCCATCTG CTCCTTGTAT CTGCCACCTT AATCAACGAA AACAACATCA GTTCAGCTGT   
  
  
+ TGATAATCTT ATTGAGCTCT TCCAATATGT CTCCGAAAGT GGTGATTCAG GACAAAGGGT TGCTGCTTAC   
  
  
+ TTTGCGGATG GGTTAACAGC AAGGATCTTA ACTCAGAGGT CTCCATTTTA TCGCCTGATA CTGGGCAAAC   
  
  
+ CAGCACCTGC TGAAGAGTTT TCAGCTTTTA CTCACTTATA TAGAGCCTCT CCATTCTATC AGTTTGCTCA   
  
  
+ TTTCACAGCC AATCAGGCAA TTGTAGAAGC ATTTGAGAGG GAGGAAGAGA GTAACAACTG GGCTTTGCAT   
  
  
+ GTAATTGATT TGGACATCTT GCATGGTTTC CAGTGGCCTT CTCTTATTCA GTCTCTCTCC GAAGAGGCTA   
  
  
+ CTTGTTCAAA CCGTTCATTG TCTCTTCAAA TTACAGGCTT CGGAAGAAGC TTAGAAGAGC TCATTGAAAC   
  
  
+ AGAAGCTCGG CTGGTAAGTT TCTCTAAGAG CTTTCAGAAT ATCAACTTTG AATTCCACGG GTTTCTGAGA   
  
  
+ GGCTCGAAAC TCAGGAACCT AAGGAGGGAG AATGAAACAG TAGTTGTAAA TCTCGTTTTC CACCTCAGTA   
  
  
+ CTTTGAAAGA TACCGTACAG ATTTCTGACA CCTTGACTTC TGTACATTCA CTAAACCCCT CTATTGTGGT   
  
  
+ ATTAGTCGAG AGAGAAGGAA GTCGAAACAG ATGTGGGTTC CTCTCAAGTT ATGTAGATGC TCTGCATTAT   
  
  
+ TATGCTGCAA TGTTTGACTC TTTAGATGAT TGCCTCCCAC TTGAAAGTCC TGAGAGGCTG AGCATAGAGA   
  
  
+ AGAACCATCT TGGAAAAGAG ATCAAAGAAG CCATAGGTTG TGAGAAGGAT GAAACAAACT ATCTGAAGTT   
  
  
+ TGAGATGCTG GAGACTTGGA GAGGGAGGAT GGAGAGTCAT GGATTTTCAG GTATGAAGCT AAGTTCCAGG   
  
  
+ GCAACTATAC AAGCAAAGCT GCTTTTAAAA ATGGGAAGCC ATTATCATAC CTATTTGGAA GAAGACTGTG   
  
  
+ GAGGTGGTGG GTTCAGAGTT TGTGAACGAG ATGATGGAAT GGCTATCTCT CTTGGCTGGC AAGGTAGGTT   
  
  
+ CCTCGCAACT GCCTCGGTAT GGCGTTCTGT GTG  

- -Up\_Stream \_Len000TGGTTG CTAAGAAATG ACTTAGTCGA TGTAAAAACC TAACACAATT CCATAGAAAA   
  
  
- TCTAGAAGTC CTCCTCCCCC GACTTCATCG AACCAATGAA AACGCGAATA GTTGACACCT TCTTATAATA   
  
  
- ACTCTGATCA CATCTTCTTA ATCATCAAAG GAGGTGTAAC CTTTTTTTGG TACTTTAATT GCGAAATCCG   
  
  
- GGAGTCTCAT GGTTCACAGT AGCATTTACT AACTTTAAAG TTCAGAATCG ACTCAGTACT TCAGGACTAA   
  
  
- AGGGTACTGT TTAACTACAA CAACAGATTT CGAACCGCCC ACTAAACTTT CTTCTAAATC TAGTCCGACT   
  
  
- GTTATGTGGT TTAGACCCTC GTTCCTTGAC CTAAAAAGAC ACAGATCTTT TCGAAAGGCG AAAGACTTGT   
  
  
- TAACTCTTTT GGTACGTATC CTTTAAAGAA CTATAAATCT AGTATCGTCT GAATTGTTAG ACCGAAATGG   
  
  
- CTTGACAGGA GATGAAGATT CAACACTTCC ACACTTTCTA TTTGGTACAG TTTGAATCAC AGGTTGTCTT   
  
  
- TGCGTAGAGA TGAAGAGGTC CGTTTCAACA TGAAGTCTAG AAGATGTGGA AAAGATCCGT CCTGAAACCC   
  
  
- TATATCTCAA ACCGGACCCA CTTTTCATCC TTCACCCCTT CATCTTTTAC TACTTTCTTC CGTGGTGAAC   
  
  
- CCAGGTCTAT GTATCCGGAT GCGTGCTCTT GGAAACGTAC CAGATCTATG TACTGACATA TGAAGAAGGA   
  
  
- AAGGAAATGG ACAACCTCTA ATAGGATCCT AAAATGTACC GTGGTCGACC TAAACAATTA CTGTATTTGT   
  
  
- ATAGAAATTA ATGTCAACGA ATACCATATG TAAATTTGAT AAGCTATCAA CTGTCGACTA CAGGAATAGG   
  
  
- AACACGTCGA ATAAGGAGCT ATCTTATTAG AAACCTGTAA AGACAGATCC ACTATTTCCC GTTTTTGATT   
  
  
- CTATGTTCTT TATCTTGTAC AGGAGGTAAA TGTTCTGCAT AGACAAAAAC CAAAACCAGT TACTAGGGAC   
  
  
- AACAGGAGTT CGTAATTGTG TCTTACTAGT AATAACTGAA CCTGACTTTC CAAAACCCTT GAGGTATCAT   
  
  
- ACGGAAAAGT TATTTTAGTT TTCGTTTAAA CGACAAGTGA CTATTGACGT CAACACAACG AAACGACAAA   
  
  
- GGTATGGTCT GAGCATTGGT TCGACTATCG TTTACACGTT TCCATGTCAT ATTAATTGCT ACAAAAGTAT   
  
  
- AACGATGCTC AAGACTTTGT ACTATATTCT TTTGTGGTCT TGGGTTTATG GATGTATCCA GGATCGGGAC   
  
  
- CTTACCGATA AAATTTATAG GTTTACCTGA ATGTCTTAAG CGTCTCAACA AACCAAACAA ATTGTTAGTA   
  
  
- ACTTAACAAT CGATACTCTT AGACGCCCTG TTAGCATTAG AAACCGCTAA TGAAGCTAAC CGTATGACTA   
  
  
- CAAAAAAATA AACGCATAGA TTCGTACCAC CACATGGAAA CTAAACACAC AGATCCATCA CACACATCTA   
  
  
- TTTATAGTAT CTTGAGTCTG CAACCATCCC AATACAACTC GTGAAAGAGA ACGACAAACA GGAAACACTA   
  
  
- CCGTGGAGAA CGAAAAGGCT TGGTGGTTTT CAGAGTAACT GTTTTGAGAA GTTTGTAACG TTTGACGGGG   
  
  
- GAATTTATAC AAAATAATTT TTTTTACCTT CTCGAGTTTG ACAACTCACT CGGATCTAAG GTTCTAAATC   
  
  
- TTTGTCATTT GAACTATCTA TATAAATATA CTTGATAACA ATCTACGTTG TGGGGGTTGA ACTTTGTGTT   
  
  
- TTCTCTTTAA TTTTTGTTTT TCCAAAACGT GATAAATATA TAGGACACAA GAAACACGAT CAGAATAAAT   
  
  
- TACAATTAAC CATTCGTAAT AAACAACCTA ATAAGGTGAC GTGAAAGAGA ATGATTGAAG TCGTAAAGTA   
  
  
- AGGAACAAGA AGTCGAAAAC TTGAAGAAAT CGGATAAAAC AACAGAAATA CCAATACCTT CGGTAGGTCT   
  
  
- TCTTCTTACT ACTTGAAGAC TCAGAATCGA ACCGGTAACA GCCAGTGAGA CTAGCGTGAC TCTTTTTTGA   
  
  
- CTTCTCCGCC TAACTACAGA GGCTAGGAGA CTAGTGAAGT CCACTTCCGA CGCTTCCCTT CTATTAGTCC   
  
  
- GAGGAGGTTC TCTCTGTCTT GAACAACTTG TAGTTCGTTT CTCCTTTTCC GCAGTAAGTT CTTCCCTTCC   
  
  
- CAGAAGTAGA ATAGGTAGAC GAGGAACATA GACGGTGGAA TTAGTTGCTT TTGTTGTAGT CAAGTCGACA   
  
  
- ACTATTAGAA TAACTCGAGA AGGTTATACA GAGGCTTTCA CCACTAAGTC CTGTTTCCCA ACGACGAATG   
  
  
- AAACGCCTAC CCAATTGTCG TTCCTAGAAT TGAGTCTCCA GAGGTAAAAT AGCGGACTAT GACCCGTTTG   
  
  
- GTCGTGGACG ACTTCTCAAA AGTCGAAAAT GAGTGAATAT ATCTCGGAGA GGTAAGATAG TCAAACGAGT   
  
  
- AAAGTGTCGG TTAGTCCGTT AACATCTTCG TAAACTCTCC CTCCTTCTCT CATTGTTGAC CCGAAACGTA   
  
  
- CATTAACTAA ACCTGTAGAA CGTACCAAAG GTCACCGGAA GAGAATAAGT CAGAGAGAGG CTTCTCCGAT   
  
  
- GAACAAGTTT GGCAAGTAAC AGAGAAGTTT AATGTCCGAA GCCTTCTTCG AATCTTCTCG AGTAACTTTG   
  
  
- TCTTCGAGCC GACCATTCAA AGAGATTCTC GAAAGTCTTA TAGTTGAAAC TTAAGGTGCC CAAAGACTCT   
  
  
- CCGAGCTTTG AGTCCTTGGA TTCCTCCCTC TTACTTTGTC ATCAACATTT AGAGCAAAAG GTGGAGTCAT   
  
  
- GAAACTTTCT ATGGCATGTC TAAAGACTGT GGAACTGAAG ACATGTAAGT GATTTGGGGA GATAACACCA   
  
  
- TAATCAGCTC TCTCTTCCTT CAGCTTTGTC TACACCCAAG GAGAGTTCAA TACATCTACG AGACGTAATA   
  
  
- ATACGACGTT ACAAACTGAG AAATCTACTA ACGGAGGGTG AACTTTCAGG ACTCTCCGAC TCGTATCTCT   
  
  
- TCTTGGTAGA ACCTTTTCTC TAGTTTCTTC GGTATCCAAC ACTCTTCCTA CTTTGTTTGA TAGACTTCAA   
  
  
- ACTCTACGAC CTCTGAACCT CTCCCTCCTA CCTCTCAGTA CCTAAAAGTC CATACTTCGA TTCAAGGTCC   
  
  
- CGTTGATATG TTCGTTTCGA CGAAAATTTT TACCCTTCGG TAATAGTATG GATAAACCTT CTTCTGACAC   
  
  
- CTCCACCACC CAAGTCTCAA ACACTTGCTC TACTACCTTA CCGATAGAGA GAACCGACCG TTCCATCCAA   
  
  
- GGAGCGTTGA CGGAGCCATA CCGCAAGACA CAC

+     as-1

| Site Name | Organism | Position | Strand | Matrix score. | sequence | function |
| --- | --- | --- | --- | --- | --- | --- |
| as-1 | Arabidopsis thaliana | 2225 | - | 5 | TGACG |  |

>HU07G00272.1   
+ -Up\_Stream \_Len000ACCAAC GATTCTTTAC TGAATCAGCT ACATTTTTGG ATTGTGTTAA GGTATCTTTT   
  
  
+ AGATCTTCAG GAGGAGGGGG CTGAAGTAGC TTGGTTACTT TTGCGCTTAT CAACTGTGGA AGAATATTAT   
  
  
+ TGAGACTAGT GTAGAAGAAT TAGTAGTTTC CTCCACATTG GAAAAAAACC ATGAAATTAA CGCTTTAGGC   
  
  
+ CCTCAGAGTA CCAAGTGTCA TCGTAAATGA TTGAAATTTC AAGTCTTAGC TGAGTCATGA AGTCCTGATT   
  
  
+ TCCCATGACA AATTGATGTT GTTGTCTAAA GCTTGGCGGG TGATTTGAAA GAAGATTTAG ATCAGGCTGA   
  
  
+ CAATACACCA AATCTGGGAG CAAGGAACTG GATTTTTCTG TGTCTAGAAA AGCTTTCCGC TTTCTGAACA   
  
  
+ ATTGAGAAAA CCATGCATAG GAAATTTCTT GATATTTAGA TCATAGCAGA CTTAACAATC TGGCTTTACC   
  
  
+ GAACTGTCCT CTACTTCTAA GTTGTGAAGG TGTGAAAGAT AAACCATGTC AAACTTAGTG TCCAACAGAA   
  
  
+ ACGCATCTCT ACTTCTCCAG GCAAAGTTGT ACTTCAGATC TTCTACACCT TTTCTAGGCA GGACTTTGGG   
  
  
+ ATATAGAGTT TGGCCTGGGT GAAAAGTAGG AAGTGGGGAA GTAGAAAATG ATGAAAGAAG GCACCACTTG   
  
  
+ GGTCCAGATA CATAGGCCTA CGCACGAGAA CCTTTGCATG GTCTAGATAC ATGACTGTAT ACTTCTTCCT   
  
  
+ TTCCTTTACC TGTTGGAGAT TATCCTAGGA TTTTACATGG CACCAGCTGG ATTTGTTAAT GACATAAACA   
  
  
+ TATCTTTAAT TACAGTTGCT TATGGTATAC ATTTAAACTA TTCGATAGTT GACAGCTGAT GTCCTTATCC   
  
  
+ TTGTGCAGCT TATTCCTCGA TAGAATAATC TTTGGACATT TCTGTCTAGG TGATAAAGGG CAAAAACTAA   
  
  
+ GATACAAGAA ATAGAACATG TCCTCCATTT ACAAGACGTA TCTGTTTTTG GTTTTGGTCA ATGATCCCTG   
  
  
+ TTGTCCTCAA GCATTAACAC AGAATGATCA TTATTGACTT GGACTGAAAG GTTTTGGGAA CTCCATAGTA   
  
  
+ TGCCTTTTCA ATAAAATCAA AAGCAAATTT GCTGTTCACT GATAACTGCA GTTGTGTTGC TTTGCTGTTT   
  
  
+ CCATACCAGA CTCGTAACCA AGCTGATAGC AAATGTGCAA AGGTACAGTA TAATTAACGA TGTTTTCATA   
  
  
+ TTGCTACGAG TTCTGAAACA TGATATAAGA AAACACCAGA ACCCAAATAC CTACATAGGT CCTAGCCCTG   
  
  
+ GAATGGCTAT TTTAAATATC CAAATGGACT TACAGAATTC GCAGAGTTGT TTGGTTTGTT TAACAATCAT   
  
  
+ TGAATTGTTA GCTATGAGAA TCTGCGGGAC AATCGTAATC TTTGGCGATT ACTTCGATTG GCATACTGAT   
  
  
+ GTTTTTTTAT TTGCGTATCT AAGCATGGTG GTGTACCTTT GATTTGTGTG TCTAGGTAGT GTGTGTAGAT   
  
  
+ AAATATCATA GAACTCAGAC GTTGGTAGGG TTATGTTGAG CACTTTCTCT TGCTGTTTGT CCTTTGTGAT   
  
  
+ GGCACCTCTT GCTTTTCCGA ACCACCAAAA GTCTCATTGA CAAAACTCTT CAAACATTGC AAACTGCCCC   
  
  
+ CTTAAATATG TTTTATTAAA AAAAATGGAA GAGCTCAAAC TGTTGAGTGA GCCTAGATTC CAAGATTTAG   
  
  
+ AAACAGTAAA CTTGATAGAT ATATTTATAT GAACTATTGT TAGATGCAAC ACCCCCAACT TGAAACACAA   
  
  
+ AAGAGAAATT AAAAACAAAA AGGTTTTGCA CTATTTATAT ATCCTGTGTT CTTTGTGCTA GTCTTATTTA   
  
  
+ ATGTTAATTG GTAAGCATTA TTTGTTGGAT TATTCCACTG CACTTTCTCT TACTAACTTC AGCATTTCAT   
  
  
+ TCCTTGTTCT TCAGCTTTTG AACTTCTTTA GCCTATTTTG TTGTCTTTAT GGTTATGGAA GCCATCCAGA   
  
  
+ AGAAGAATGA TGAACTTCTG AGTCTTAGCT TGGCCATTGT CGGTCACTCT GATCGCACTG AGAAAAAACT   
  
  
+ GAAGAGGCGG ATTGATGTCT CCGATCCTCT GATCACTTCA GGTGAAGGCT GCGAAGGGAA GATAATCAGG   
  
  
+ CTCCTCCAAG AGAGACAGAA CTTGTTGAAC ATCAAGCAAA GAGGAAAAGG CGTCATTCAA GAAGGGAAGG   
  
  
+ GTCTTCATCT TATCCATCTG CTCCTTGTAT CTGCCACCTT AATCAACGAA AACAACATCA GTTCAGCTGT   
  
  
+ TGATAATCTT ATTGAGCTCT TCCAATATGT CTCCGAAAGT GGTGATTCAG GACAAAGGGT TGCTGCTTAC   
  
  
+ TTTGCGGATG GGTTAACAGC AAGGATCTTA ACTCAGAGGT CTCCATTTTA TCGCCTGATA CTGGGCAAAC   
  
  
+ CAGCACCTGC TGAAGAGTTT TCAGCTTTTA CTCACTTATA TAGAGCCTCT CCATTCTATC AGTTTGCTCA   
  
  
+ TTTCACAGCC AATCAGGCAA TTGTAGAAGC ATTTGAGAGG GAGGAAGAGA GTAACAACTG GGCTTTGCAT   
  
  
+ GTAATTGATT TGGACATCTT GCATGGTTTC CAGTGGCCTT CTCTTATTCA GTCTCTCTCC GAAGAGGCTA   
  
  
+ CTTGTTCAAA CCGTTCATTG TCTCTTCAAA TTACAGGCTT CGGAAGAAGC TTAGAAGAGC TCATTGAAAC   
  
  
+ AGAAGCTCGG CTGGTAAGTT TCTCTAAGAG CTTTCAGAAT ATCAACTTTG AATTCCACGG GTTTCTGAGA   
  
  
+ GGCTCGAAAC TCAGGAACCT AAGGAGGGAG AATGAAACAG TAGTTGTAAA TCTCGTTTTC CACCTCAGTA   
  
  
+ CTTTGAAAGA TACCGTACAG ATTTCTGACA CCTTGACTTC TGTACATTCA CTAAACCCCT CTATTGTGGT   
  
  
+ ATTAGTCGAG AGAGAAGGAA GTCGAAACAG ATGTGGGTTC CTCTCAAGTT ATGTAGATGC TCTGCATTAT   
  
  
+ TATGCTGCAA TGTTTGACTC TTTAGATGAT TGCCTCCCAC TTGAAAGTCC TGAGAGGCTG AGCATAGAGA   
  
  
+ AGAACCATCT TGGAAAAGAG ATCAAAGAAG CCATAGGTTG TGAGAAGGAT GAAACAAACT ATCTGAAGTT   
  
  
+ TGAGATGCTG GAGACTTGGA GAGGGAGGAT GGAGAGTCAT GGATTTTCAG GTATGAAGCT AAGTTCCAGG   
  
  
+ GCAACTATAC AAGCAAAGCT GCTTTTAAAA ATGGGAAGCC ATTATCATAC CTATTTGGAA GAAGACTGTG   
  
  
+ GAGGTGGTGG GTTCAGAGTT TGTGAACGAG ATGATGGAAT GGCTATCTCT CTTGGCTGGC AAGGTAGGTT   
  
  
+ CCTCGCAACT GCCTCGGTAT GGCGTTCTGT GTG  

- -Up\_Stream \_Len000TGGTTG CTAAGAAATG ACTTAGTCGA TGTAAAAACC TAACACAATT CCATAGAAAA   
  
  
- TCTAGAAGTC CTCCTCCCCC GACTTCATCG AACCAATGAA AACGCGAATA GTTGACACCT TCTTATAATA   
  
  
- ACTCTGATCA CATCTTCTTA ATCATCAAAG GAGGTGTAAC CTTTTTTTGG TACTTTAATT GCGAAATCCG   
  
  
- GGAGTCTCAT GGTTCACAGT AGCATTTACT AACTTTAAAG TTCAGAATCG ACTCAGTACT TCAGGACTAA   
  
  
- AGGGTACTGT TTAACTACAA CAACAGATTT CGAACCGCCC ACTAAACTTT CTTCTAAATC TAGTCCGACT   
  
  
- GTTATGTGGT TTAGACCCTC GTTCCTTGAC CTAAAAAGAC ACAGATCTTT TCGAAAGGCG AAAGACTTGT   
  
  
- TAACTCTTTT GGTACGTATC CTTTAAAGAA CTATAAATCT AGTATCGTCT GAATTGTTAG ACCGAAATGG   
  
  
- CTTGACAGGA GATGAAGATT CAACACTTCC ACACTTTCTA TTTGGTACAG TTTGAATCAC AGGTTGTCTT   
  
  
- TGCGTAGAGA TGAAGAGGTC CGTTTCAACA TGAAGTCTAG AAGATGTGGA AAAGATCCGT CCTGAAACCC   
  
  
- TATATCTCAA ACCGGACCCA CTTTTCATCC TTCACCCCTT CATCTTTTAC TACTTTCTTC CGTGGTGAAC   
  
  
- CCAGGTCTAT GTATCCGGAT GCGTGCTCTT GGAAACGTAC CAGATCTATG TACTGACATA TGAAGAAGGA   
  
  
- AAGGAAATGG ACAACCTCTA ATAGGATCCT AAAATGTACC GTGGTCGACC TAAACAATTA CTGTATTTGT   
  
  
- ATAGAAATTA ATGTCAACGA ATACCATATG TAAATTTGAT AAGCTATCAA CTGTCGACTA CAGGAATAGG   
  
  
- AACACGTCGA ATAAGGAGCT ATCTTATTAG AAACCTGTAA AGACAGATCC ACTATTTCCC GTTTTTGATT   
  
  
- CTATGTTCTT TATCTTGTAC AGGAGGTAAA TGTTCTGCAT AGACAAAAAC CAAAACCAGT TACTAGGGAC   
  
  
- AACAGGAGTT CGTAATTGTG TCTTACTAGT AATAACTGAA CCTGACTTTC CAAAACCCTT GAGGTATCAT   
  
  
- ACGGAAAAGT TATTTTAGTT TTCGTTTAAA CGACAAGTGA CTATTGACGT CAACACAACG AAACGACAAA   
  
  
- GGTATGGTCT GAGCATTGGT TCGACTATCG TTTACACGTT TCCATGTCAT ATTAATTGCT ACAAAAGTAT   
  
  
- AACGATGCTC AAGACTTTGT ACTATATTCT TTTGTGGTCT TGGGTTTATG GATGTATCCA GGATCGGGAC   
  
  
- CTTACCGATA AAATTTATAG GTTTACCTGA ATGTCTTAAG CGTCTCAACA AACCAAACAA ATTGTTAGTA   
  
  
- ACTTAACAAT CGATACTCTT AGACGCCCTG TTAGCATTAG AAACCGCTAA TGAAGCTAAC CGTATGACTA   
  
  
- CAAAAAAATA AACGCATAGA TTCGTACCAC CACATGGAAA CTAAACACAC AGATCCATCA CACACATCTA   
  
  
- TTTATAGTAT CTTGAGTCTG CAACCATCCC AATACAACTC GTGAAAGAGA ACGACAAACA GGAAACACTA   
  
  
- CCGTGGAGAA CGAAAAGGCT TGGTGGTTTT CAGAGTAACT GTTTTGAGAA GTTTGTAACG TTTGACGGGG   
  
  
- GAATTTATAC AAAATAATTT TTTTTACCTT CTCGAGTTTG ACAACTCACT CGGATCTAAG GTTCTAAATC   
  
  
- TTTGTCATTT GAACTATCTA TATAAATATA CTTGATAACA ATCTACGTTG TGGGGGTTGA ACTTTGTGTT   
  
  
- TTCTCTTTAA TTTTTGTTTT TCCAAAACGT GATAAATATA TAGGACACAA GAAACACGAT CAGAATAAAT   
  
  
- TACAATTAAC CATTCGTAAT AAACAACCTA ATAAGGTGAC GTGAAAGAGA ATGATTGAAG TCGTAAAGTA   
  
  
- AGGAACAAGA AGTCGAAAAC TTGAAGAAAT CGGATAAAAC AACAGAAATA CCAATACCTT CGGTAGGTCT   
  
  
- TCTTCTTACT ACTTGAAGAC TCAGAATCGA ACCGGTAACA GCCAGTGAGA CTAGCGTGAC TCTTTTTTGA   
  
  
- CTTCTCCGCC TAACTACAGA GGCTAGGAGA CTAGTGAAGT CCACTTCCGA CGCTTCCCTT CTATTAGTCC   
  
  
- GAGGAGGTTC TCTCTGTCTT GAACAACTTG TAGTTCGTTT CTCCTTTTCC GCAGTAAGTT CTTCCCTTCC   
  
  
- CAGAAGTAGA ATAGGTAGAC GAGGAACATA GACGGTGGAA TTAGTTGCTT TTGTTGTAGT CAAGTCGACA   
  
  
- ACTATTAGAA TAACTCGAGA AGGTTATACA GAGGCTTTCA CCACTAAGTC CTGTTTCCCA ACGACGAATG   
  
  
- AAACGCCTAC CCAATTGTCG TTCCTAGAAT TGAGTCTCCA GAGGTAAAAT AGCGGACTAT GACCCGTTTG   
  
  
- GTCGTGGACG ACTTCTCAAA AGTCGAAAAT GAGTGAATAT ATCTCGGAGA GGTAAGATAG TCAAACGAGT   
  
  
- AAAGTGTCGG TTAGTCCGTT AACATCTTCG TAAACTCTCC CTCCTTCTCT CATTGTTGAC CCGAAACGTA   
  
  
- CATTAACTAA ACCTGTAGAA CGTACCAAAG GTCACCGGAA GAGAATAAGT CAGAGAGAGG CTTCTCCGAT   
  
  
- GAACAAGTTT GGCAAGTAAC AGAGAAGTTT AATGTCCGAA GCCTTCTTCG AATCTTCTCG AGTAACTTTG   
  
  
- TCTTCGAGCC GACCATTCAA AGAGATTCTC GAAAGTCTTA TAGTTGAAAC TTAAGGTGCC CAAAGACTCT   
  
  
- CCGAGCTTTG AGTCCTTGGA TTCCTCCCTC TTACTTTGTC ATCAACATTT AGAGCAAAAG GTGGAGTCAT   
  
  
- GAAACTTTCT ATGGCATGTC TAAAGACTGT GGAACTGAAG ACATGTAAGT GATTTGGGGA GATAACACCA   
  
  
- TAATCAGCTC TCTCTTCCTT CAGCTTTGTC TACACCCAAG GAGAGTTCAA TACATCTACG AGACGTAATA   
  
  
- ATACGACGTT ACAAACTGAG AAATCTACTA ACGGAGGGTG AACTTTCAGG ACTCTCCGAC TCGTATCTCT   
  
  
- TCTTGGTAGA ACCTTTTCTC TAGTTTCTTC GGTATCCAAC ACTCTTCCTA CTTTGTTTGA TAGACTTCAA   
  
  
- ACTCTACGAC CTCTGAACCT CTCCCTCCTA CCTCTCAGTA CCTAAAAGTC CATACTTCGA TTCAAGGTCC   
  
  
- CGTTGATATG TTCGTTTCGA CGAAAATTTT TACCCTTCGG TAATAGTATG GATAAACCTT CTTCTGACAC   
  
  
- CTCCACCACC CAAGTCTCAA ACACTTGCTC TACTACCTTA CCGATAGAGA GAACCGACCG TTCCATCCAA   
  
  
- GGAGCGTTGA CGGAGCCATA CCGCAAGACA CAC
