# Supplementary material for: Genome-Wide Identification and Expression Pattern of the GRAS Gene Family in Pitaya (Selenicereus undatus L.)
Source: Biology (Basel). 2022 Dec 21;12(1):11. doi: 10.3390/biology12010011 (PMC9854919; doi:10.3390/biology12010011)
Supplement: Supplementary file 1 [file biology-12-00011-s001.zip › Supplementary file S5/HU07G02246.1_plantcare.html]

Content-Type: text/html; charset=ISO-8859-1


PlantCARE


Webmaster Firefox specific output  
To save the result:
click on the frame with the right mouse button and save the source code as a text file with extension .html  
REFERENCE:PlantCARE: a database of plant cis-acting regulatory elements and a portal to tools for in silico analysis of promoter sequences.  
Lescot, M., Déhais, P., Moreau, Y., De Moor, B., Rouzé ,P.,and Rombauts, S.  
Nucleic Acids Res., Database issue(2002), 30(1):325-327.   


---

>HU07G02246.1   
+ -Up\_Stream \_Len000ATTTGA TGTGTATGCT GACTACTTTT GATGATGATG ATCACAGTGA TGCATATTTA   
  
  
+ GAGCTGGTAA ATTGGTCATT CGATTCGAGT ACGGGTCGGG TCAAGTTCTG ATTAAGTGAC ATTTCGCGTC   
  
  
+ GTTTTGGTTT AGTTCGGGTC GGATCGATTT CGGGTTAAAT AATTTTTGGT GGAATACGCT TGTCATGCCA   
  
  
+ AACACAAGCA ACTTTGTTAA AAATTTCGAT TCGGGTCAGG TCAATTCAGG TTTGGGGTCC ACTTTCGAGT   
  
  
+ GAGCATATTT CGGATGTCGG GTCGCGTATG GGTCCGGGTC ATTCGGTTTG AATTTCGGAT CTTGGATCAA   
  
  
+ TTTTGCTAAG TATGTGCATA TTCAACCCTT GATGACATAG GAAACGCTTT GTCATCTCCA CCAATCAGAT   
  
  
+ ATCGTTATCT CTTTAATCAT CGCCATAATA GCATGAGTAA GCATGACCAT CTTTTTCAAA CATGTGATAC   
  
  
+ AGTTAAGGCC TGTTCTTTTC GTTAATAAAT CCCAATTTAT CAATTTTAAT ATATACTTTA ACGAATTTTA   
  
  
+ ACAAAAAATT TTAATTTCAA TCCGCTATAA CAAAAAAAAT ACCAACTTCA ACTTATTATA TAAAAAAAAT   
  
  
+ ATACAATTTT TTTTCAATTT TAGCCAACTT CAACAAAAAA AAAAAAAATT TAACAAATCT CAATCAACTT   
  
  
+ CACCCATTTC TCAACTTATA TCTCCTAATG AAAAGAACAG ATCCTTAGCA AGACTTCTCG ATTCATTTAA   
  
  
+ AAGGATAGTA TGGTAAATTC ATTTTATACA AGTGTCAAGT GTGAATATCT TTTTCACATC AGGTGATATA   
  
  
+ TTAGTAGGAC TTTTCGATTC ACTTATCTTC CTCTTTATGT TTTGTTAATG TGATAGTAAC GATAGGATTG   
  
  
+ CATGTATTTT TTCCAACTAT TCATTTACAA CTATCAATAT CTATTGATAA ATATGTTAAA ATAATTATAT   
  
  
+ GTTATTTAAA TTATTATTTG TTACTTAAGG TGTCGACTTT GTCGAAAGCG ACTTAATTCT AATTAAGATA   
  
  
+ GCGGCAGAGA AAGTTACACC TGATGACCAA GTTAGATCTG GTAAAACTAA CCATAGATCT GAAACCCAAA   
  
  
+ CATAATGATG TGTATTCATA CCCGACCTGA CATCTGAAAT AAGTTTACTC AAAAGTGGTT CCGAAACTTG   
  
  
+ AATTGACTCG AACCGAACCG TATTTGACTC GAAATTTTCT ACAAAGTTGC TTATTTTTGC ATGAAGACAT   
  
  
+ ATTGAGCCAA AAATTAGTTG ATTCGAAGTC AACTTAAACA AAAATTCATT CGACTCAAAC TGAAATATCA   
  
  
+ CTTAACTAAA ACTATGACTC AACTCAAACT TGATCTGATC CGTATTCAAA TCAAATACTC CATTTGCTAG   
  
  
+ GTCTAGAACC AGCCACGACC CCACATGGAC TATGCGGTTC AATGAAACGG CCAAATATAT CGCAATTTCT   
  
  
+ GTCTCCATCA ATCACACCCA TTTGCTACAC CCTGTTAATA TTTGACTCGA CCCTGTGAAT TGCCTTCGCC   
  
  
+ AGTTTTGTTT AATCTCTCTG GTTGCTTCCC ATCCAAGTCT GAATAGTATA GTTTGTACAG TGTGTACTAC   
  
  
+ TAGAAAGCAC GTTCCTTGAA TCTTCTTTCA AAGTTGTTTT TAAAGGGTTG AGTGATCCAT TACGACTTTT   
  
  
+ GATCTGGGTT CATCGCCTCC TCTCCTAATC CCTCACCCGG GTACCCTCAA TTCTCTATCG TCTCTGTTGC   
  
  
+ TCAGCTAATT CAAATTCCTT TAGTTGGTCA TCATTGAAAT CTGAAATCAG GGTGTTTTTC TTTTTATTAT   
  
  
+ TATCTGGCAG TTGGTTTGTT TATCTCTTGG TTTTTGGGGC TTATTTGGGT GCTGGTTGAT GTTATCCTGC   
  
  
+ TTGGGTTGAA TCTTCATCAG GTTTATTTTT GATTCATAGT TGAGGGTGAA AATTATTTCT CATACCCACC   
  
  
+ ACCTGTTTGT TCTTTTGTCT CTGAGAAAAA CCAAAAAGCT TTCTCTTTTT CCTGATGCAT CCTGTGCTCG   
  
  
+ TTGATCCTGA GTTAGTGAAC CATGCGTCCA AATTCAAGCC TGATTTGCTC TCAAACTTTG ATAAACAACA   
  
  
+ AGAATTTAGC AACGGGATCG AACAAGATGA CATCTTGCAA AACCCTGATC TGTGTTTTGA TTCAGAAACC   
  
  
+ CCTTCAGTTG ATCTAACTGA GAGTGTCATC AGTTCTGATT CTGTAGAAGT GCCTGATTTC TCAGATGCTT   
  
  
+ GCCTCAAGTT CATAAGTGAG ATTCTCCTAG AAGAGGATTT GGATGAAAGT CCTGCCTCTG CTCAAGAATT   
  
  
+ TAGGGCTCTC CAAGCCACTG AGAAGTCCCT GTATGATGCT CTTGGAGAGG GCTACTCTTT TTCATCTGAT   
  
  
+ AACAGCCCAT CATCATTAGG ACAGAGTATT GAGCACCAAA ATGAGAATTT TGAATTCAGT TCCGGTTATC   
  
  
+ CTGGAATTGA GGGCTATGTT AATGGTGATA TCACGTTCGA GTCCAACTGG ATGTTCAACC TAAGCCAATT   
  
  
+ GGATCCTGTT CTTACTTTGG ATGATATTTC TCAGCCCTTG TCTGCATCAA ACTCCCGATC ATCTGGGTCG   
  
  
+ AGCAATGGCT TTGATGATTC AGGGGATGGG GCAGCCACAT CTCCTGGCAG TACAGTTACA TCAACAGTCC   
  
  
+ CAGAGAAAAG GGTTGAATCT GTCAATCGCT CAAGGAGGAA GAAAAATCGT AAAAGGGATG AAGGTGGTCA   
  
  
+ TGAGGAAGGG AGGAGTAACA AGCAGCAAGC TTCATCCAAT GAAGATTATG TTGAGATGAA GGAGTTTGAT   
  
  
+ GATGTACTGC TCTGCAAAGA AGAGAAAGAT GTTATTGCAA ACTGCACCAA TGATTCCTCA CCCGTCGAAG   
  
  
+ CGAGTGATAA GTTGCAGAAG AAAGGGGGGA AGGGGAAAAC ATCGCGTGGG AAGAAGCAGA ATAGCACAAT   
  
  
+ AGAAGAGGTG GATCTGAGGA CTCTTCTCAC TCACTGTGCT CAAGCAATTT CAAATTTTGA TCTTAGGAGT   
  
  
+ GCAAATGAGC ATCTCAGGCA AATAAGGCAG CATTCTTCAC AATATGGTGA TAGCCTCCAG AGGCTTGCCC   
  
  
+ ATTATTTTGC TAATGGTCTT GAGGCTCGCA TAGCTGGCAC TGGTTCAACA ATCTCTGCTA ATGTTGTTGA   
  
  
+ TGCTCGAATC ACGTCATCTG ATTTCTTAAA GGCTTATAGG CTATATATGT CAGCCGTTCC TTTCAAAAGG   
  
  
+ ATGTTATATT TTCTCGCCAA CAAGACAATA CGGAAGTTGG CTGAGAAGGC AACCAGGATC CACATCATTG   
  
  
+ ATTTTGGAGT CTTGTTGGGT TTACAGTGGC CCTGTCTCAT ACAAAACCTC TCAAAGAGAC CCACTGGCCC   
  
  
+ GCCAAGCCTC CGTATTACTG GGATAGACTA TCCCCAGCAT GGTTTCCGGC CATCAGAAAG GGTTGAAGCG   
  
  
+ ACTGGCCGTC GGTTAGAAGG GTATTGCGAG AGATTTAATG TGCCGTTCAA GTATAAGGCC ATAGCTAAAA   
  
  
+ ATTGGGAGAG TATAAAGCTA GAGGATCTAG AAATTGATGA GAATGAGATG GTGTTTGTCA ATTGCATGCT   
  
  
+ TCGCTCTGGA ACACTGCTTG ATGAGACAGT GGTGGCAGAC AGTCCAAAGG ATGCTTTCTT AAGGTTAATC   
  
  
+ AGAGAGATAA ATCCCCGTCT TTTCATTCAT GGGACTATCA ATGGATCATT CAATGCTCCA TTCTTCATCA   
  
  
+ CTCGGTTCAG GGAGGCGCTC TTCCACTACT CTTCTTTATT TGATATCTTT GAAGCAACTA TGCCCCGTGA   
  
  
+ AGATCATGAA AGGCTCCTGG TTGAGAGTGA GATACAGGGC AAAGAAGCTT TGAATGTCAT AGCATGCGAA   
  
  
+ GGTGCCGAGA GGATTCAAAG GCCTGAAACA TACAAGCAAT GGCAAGCAAG GACAACAAGG GCCGGGTTTA   
  
  
+ GGCAGCTTCC ACTAGACCGG GAGCTTGTGA GCAGAGCAAA GGCTATGGTG AAAGCAAACT ACCATAAGGA   
  
  
+ TTTTGTGGTG GACGAGGACA GGCATTGGAT GCTACACGGT TGGAAAGGAA GAATCTTCTG TGCACTCTCT   
  
  
+ GTTTGGCAAC CCAACTG  

- -Up\_Stream \_Len000TAAACT ACACATACGA CTGATGAAAA CTACTACTAC TAGTGTCACT ACGTATAAAT   
  
  
- CTCGACCATT TAACCAGTAA GCTAAGCTCA TGCCCAGCCC AGTTCAAGAC TAATTCACTG TAAAGCGCAG   
  
  
- CAAAACCAAA TCAAGCCCAG CCTAGCTAAA GCCCAATTTA TTAAAAACCA CCTTATGCGA ACAGTACGGT   
  
  
- TTGTGTTCGT TGAAACAATT TTTAAAGCTA AGCCCAGTCC AGTTAAGTCC AAACCCCAGG TGAAAGCTCA   
  
  
- CTCGTATAAA GCCTACAGCC CAGCGCATAC CCAGGCCCAG TAAGCCAAAC TTAAAGCCTA GAACCTAGTT   
  
  
- AAAACGATTC ATACACGTAT AAGTTGGGAA CTACTGTATC CTTTGCGAAA CAGTAGAGGT GGTTAGTCTA   
  
  
- TAGCAATAGA GAAATTAGTA GCGGTATTAT CGTACTCATT CGTACTGGTA GAAAAAGTTT GTACACTATG   
  
  
- TCAATTCCGG ACAAGAAAAG CAATTATTTA GGGTTAAATA GTTAAAATTA TATATGAAAT TGCTTAAAAT   
  
  
- TGTTTTTTAA AATTAAAGTT AGGCGATATT GTTTTTTTTA TGGTTGAAGT TGAATAATAT ATTTTTTTTA   
  
  
- TATGTTAAAA AAAAGTTAAA ATCGGTTGAA GTTGTTTTTT TTTTTTTTAA ATTGTTTAGA GTTAGTTGAA   
  
  
- GTGGGTAAAG AGTTGAATAT AGAGGATTAC TTTTCTTGTC TAGGAATCGT TCTGAAGAGC TAAGTAAATT   
  
  
- TTCCTATCAT ACCATTTAAG TAAAATATGT TCACAGTTCA CACTTATAGA AAAAGTGTAG TCCACTATAT   
  
  
- AATCATCCTG AAAAGCTAAG TGAATAGAAG GAGAAATACA AAACAATTAC ACTATCATTG CTATCCTAAC   
  
  
- GTACATAAAA AAGGTTGATA AGTAAATGTT GATAGTTATA GATAACTATT TATACAATTT TATTAATATA   
  
  
- CAATAAATTT AATAATAAAC AATGAATTCC ACAGCTGAAA CAGCTTTCGC TGAATTAAGA TTAATTCTAT   
  
  
- CGCCGTCTCT TTCAATGTGG ACTACTGGTT CAATCTAGAC CATTTTGATT GGTATCTAGA CTTTGGGTTT   
  
  
- GTATTACTAC ACATAAGTAT GGGCTGGACT GTAGACTTTA TTCAAATGAG TTTTCACCAA GGCTTTGAAC   
  
  
- TTAACTGAGC TTGGCTTGGC ATAAACTGAG CTTTAAAAGA TGTTTCAACG AATAAAAACG TACTTCTGTA   
  
  
- TAACTCGGTT TTTAATCAAC TAAGCTTCAG TTGAATTTGT TTTTAAGTAA GCTGAGTTTG ACTTTATAGT   
  
  
- GAATTGATTT TGATACTGAG TTGAGTTTGA ACTAGACTAG GCATAAGTTT AGTTTATGAG GTAAACGATC   
  
  
- CAGATCTTGG TCGGTGCTGG GGTGTACCTG ATACGCCAAG TTACTTTGCC GGTTTATATA GCGTTAAAGA   
  
  
- CAGAGGTAGT TAGTGTGGGT AAACGATGTG GGACAATTAT AAACTGAGCT GGGACACTTA ACGGAAGCGG   
  
  
- TCAAAACAAA TTAGAGAGAC CAACGAAGGG TAGGTTCAGA CTTATCATAT CAAACATGTC ACACATGATG   
  
  
- ATCTTTCGTG CAAGGAACTT AGAAGAAAGT TTCAACAAAA ATTTCCCAAC TCACTAGGTA ATGCTGAAAA   
  
  
- CTAGACCCAA GTAGCGGAGG AGAGGATTAG GGAGTGGGCC CATGGGAGTT AAGAGATAGC AGAGACAACG   
  
  
- AGTCGATTAA GTTTAAGGAA ATCAACCAGT AGTAACTTTA GACTTTAGTC CCACAAAAAG AAAAATAATA   
  
  
- ATAGACCGTC AACCAAACAA ATAGAGAACC AAAAACCCCG AATAAACCCA CGACCAACTA CAATAGGACG   
  
  
- AACCCAACTT AGAAGTAGTC CAAATAAAAA CTAAGTATCA ACTCCCACTT TTAATAAAGA GTATGGGTGG   
  
  
- TGGACAAACA AGAAAACAGA GACTCTTTTT GGTTTTTCGA AAGAGAAAAA GGACTACGTA GGACACGAGC   
  
  
- AACTAGGACT CAATCACTTG GTACGCAGGT TTAAGTTCGG ACTAAACGAG AGTTTGAAAC TATTTGTTGT   
  
  
- TCTTAAATCG TTGCCCTAGC TTGTTCTACT GTAGAACGTT TTGGGACTAG ACACAAAACT AAGTCTTTGG   
  
  
- GGAAGTCAAC TAGATTGACT CTCACAGTAG TCAAGACTAA GACATCTTCA CGGACTAAAG AGTCTACGAA   
  
  
- CGGAGTTCAA GTATTCACTC TAAGAGGATC TTCTCCTAAA CCTACTTTCA GGACGGAGAC GAGTTCTTAA   
  
  
- ATCCCGAGAG GTTCGGTGAC TCTTCAGGGA CATACTACGA GAACCTCTCC CGATGAGAAA AAGTAGACTA   
  
  
- TTGTCGGGTA GTAGTAATCC TGTCTCATAA CTCGTGGTTT TACTCTTAAA ACTTAAGTCA AGGCCAATAG   
  
  
- GACCTTAACT CCCGATACAA TTACCACTAT AGTGCAAGCT CAGGTTGACC TACAAGTTGG ATTCGGTTAA   
  
  
- CCTAGGACAA GAATGAAACC TACTATAAAG AGTCGGGAAC AGACGTAGTT TGAGGGCTAG TAGACCCAGC   
  
  
- TCGTTACCGA AACTACTAAG TCCCCTACCC CGTCGGTGTA GAGGACCGTC ATGTCAATGT AGTTGTCAGG   
  
  
- GTCTCTTTTC CCAACTTAGA CAGTTAGCGA GTTCCTCCTT CTTTTTAGCA TTTTCCCTAC TTCCACCAGT   
  
  
- ACTCCTTCCC TCCTCATTGT TCGTCGTTCG AAGTAGGTTA CTTCTAATAC AACTCTACTT CCTCAAACTA   
  
  
- CTACATGACG AGACGTTTCT TCTCTTTCTA CAATAACGTT TGACGTGGTT ACTAAGGAGT GGGCAGCTTC   
  
  
- GCTCACTATT CAACGTCTTC TTTCCCCCCT TCCCCTTTTG TAGCGCACCC TTCTTCGTCT TATCGTGTTA   
  
  
- TCTTCTCCAC CTAGACTCCT GAGAAGAGTG AGTGACACGA GTTCGTTAAA GTTTAAAACT AGAATCCTCA   
  
  
- CGTTTACTCG TAGAGTCCGT TTATTCCGTC GTAAGAAGTG TTATACCACT ATCGGAGGTC TCCGAACGGG   
  
  
- TAATAAAACG ATTACCAGAA CTCCGAGCGT ATCGACCGTG ACCAAGTTGT TAGAGACGAT TACAACAACT   
  
  
- ACGAGCTTAG TGCAGTAGAC TAAAGAATTT CCGAATATCC GATATATACA GTCGGCAAGG AAAGTTTTCC   
  
  
- TACAATATAA AAGAGCGGTT GTTCTGTTAT GCCTTCAACC GACTCTTCCG TTGGTCCTAG GTGTAGTAAC   
  
  
- TAAAACCTCA GAACAACCCA AATGTCACCG GGACAGAGTA TGTTTTGGAG AGTTTCTCTG GGTGACCGGG   
  
  
- CGGTTCGGAG GCATAATGAC CCTATCTGAT AGGGGTCGTA CCAAAGGCCG GTAGTCTTTC CCAACTTCGC   
  
  
- TGACCGGCAG CCAATCTTCC CATAACGCTC TCTAAATTAC ACGGCAAGTT CATATTCCGG TATCGATTTT   
  
  
- TAACCCTCTC ATATTTCGAT CTCCTAGATC TTTAACTACT CTTACTCTAC CACAAACAGT TAACGTACGA   
  
  
- AGCGAGACCT TGTGACGAAC TACTCTGTCA CCACCGTCTG TCAGGTTTCC TACGAAAGAA TTCCAATTAG   
  
  
- TCTCTCTATT TAGGGGCAGA AAAGTAAGTA CCCTGATAGT TACCTAGTAA GTTACGAGGT AAGAAGTAGT   
  
  
- GAGCCAAGTC CCTCCGCGAG AAGGTGATGA GAAGAAATAA ACTATAGAAA CTTCGTTGAT ACGGGGCACT   
  
  
- TCTAGTACTT TCCGAGGACC AACTCTCACT CTATGTCCCG TTTCTTCGAA ACTTACAGTA TCGTACGCTT   
  
  
- CCACGGCTCT CCTAAGTTTC CGGACTTTGT ATGTTCGTTA CCGTTCGTTC CTGTTGTTCC CGGCCCAAAT   
  
  
- CCGTCGAAGG TGATCTGGCC CTCGAACACT CGTCTCGTTT CCGATACCAC TTTCGTTTGA TGGTATTCCT   
  
  
- AAAACACCAC CTGCTCCTGT CCGTAACCTA CGATGTGCCA ACCTTTCCTT CTTAGAAGAC ACGTGAGAGA   
  
  
- CAAACCGTTG GGTTGAC

  
  
Motifs Found  

+   

| Site Name | Organism | Position | Strand | Matrix score. | sequence | function |
| --- | --- | --- | --- | --- | --- | --- |
|  | organism | 2823 | - | 4 | motif\_sequence | short\_function |
|  | organism | 2946 | - | 4 | motif\_sequence | short\_function |
|  | organism | 2530 | + | 4 | motif\_sequence | short\_function |
|  | organism | 2320 | + | 4 | motif\_sequence | short\_function |
|  | organism | 2274 | - | 4 | motif\_sequence | short\_function |
|  | organism | 3955 | - | 4 | motif\_sequence | short\_function |
|  | organism | 2978 | + | 4 | motif\_sequence | short\_function |
|  | organism | 1634 | + | 4 | motif\_sequence | short\_function |
|  | organism | 3137 | + | 4 | motif\_sequence | short\_function |
|  | organism | 2300 | + | 4 | motif\_sequence | short\_function |
|  | organism | 3732 | + | 4 | motif\_sequence | short\_function |
|  | organism | 4047 | + | 4 | motif\_sequence | short\_function |
|  | organism | 2633 | + | 4 | motif\_sequence | short\_function |
|  | organism | 869 | + | 4 | motif\_sequence | short\_function |
|  | organism | 1058 | - | 4 | motif\_sequence | short\_function |
|  | organism | 2644 | - | 4 | motif\_sequence | short\_function |
|  | organism | 2403 | - | 4 | motif\_sequence | short\_function |
|  | organism | 3783 | - | 4 | motif\_sequence | short\_function |
|  | organism | 2965 | + | 4 | motif\_sequence | short\_function |
|  | organism | 408 | + | 4 | motif\_sequence | short\_function |
|  | organism | 1904 | + | 4 | motif\_sequence | short\_function |
|  | organism | 3509 | - | 4 | motif\_sequence | short\_function |
|  | organism | 1599 | - | 4 | motif\_sequence | short\_function |
|  | organism | 2775 | - | 4 | motif\_sequence | short\_function |
|  | organism | 2358 | - | 4 | motif\_sequence | short\_function |
|  | organism | 366 | + | 4 | motif\_sequence | short\_function |
|  | organism | 4010 | - | 4 | motif\_sequence | short\_function |
|  | organism | 2026 | + | 4 | motif\_sequence | short\_function |
|  | organism | 3743 | + | 4 | motif\_sequence | short\_function |
|  | organism | 3472 | + | 4 | motif\_sequence | short\_function |
|  | organism | 504 | + | 4 | motif\_sequence | short\_function |
|  | organism | 2505 | + | 4 | motif\_sequence | short\_function |
|  | organism | 2806 | + | 4 | motif\_sequence | short\_function |
|  | organism | 1704 | + | 4 | motif\_sequence | short\_function |
|  | organism | 2814 | + | 4 | motif\_sequence | short\_function |
|  | organism | 739 | - | 4 | motif\_sequence | short\_function |
|  | organism | 4052 | + | 4 | motif\_sequence | short\_function |
|  | organism | 724 | + | 4 | motif\_sequence | short\_function |

>HU07G02246.1   
+ -Up\_Stream \_Len000ATTTGA TGTGTATGCT GACTACTTTT GATGATGATG ATCACAGTGA TGCATATTTA   
  
  
+ GAGCTGGTAA ATTGGTCATT CGATTCGAGT ACGGGTCGGG TCAAGTTCTG ATTAAGTGAC ATTTCGCGTC   
  
  
+ GTTTTGGTTT AGTTCGGGTC GGATCGATTT CGGGTTAAAT AATTTTTGGT GGAATACGCT TGTCATGCCA   
  
  
+ AACACAAGCA ACTTTGTTAA AAATTTCGAT TCGGGTCAGG TCAATTCAGG TTTGGGGTCC ACTTTCGAGT   
  
  
+ GAGCATATTT CGGATGTCGG GTCGCGTATG GGTCCGGGTC ATTCGGTTTG AATTTCGGAT CTTGGATCAA   
  
  
+ TTTTGCTAAG TATGTGCATA TTCAACCCTT GATGACATAG GAAACGCTTT GTCATCTCCA CCAATCAGAT   
  
  
+ ATCGTTATCT CTTTAATCAT CGCCATAATA GCATGAGTAA GCATGACCAT CTTTTTCAAA CATGTGATAC   
  
  
+ AGTTAAGGCC TGTTCTTTTC GTTAATAAAT CCCAATTTAT CAATTTTAAT ATATACTTTA ACGAATTTTA   
  
  
+ ACAAAAAATT TTAATTTCAA TCCGCTATAA CAAAAAAAAT ACCAACTTCA ACTTATTATA TAAAAAAAAT   
  
  
+ ATACAATTTT TTTTCAATTT TAGCCAACTT CAACAAAAAA AAAAAAAATT TAACAAATCT CAATCAACTT   
  
  
+ CACCCATTTC TCAACTTATA TCTCCTAATG AAAAGAACAG ATCCTTAGCA AGACTTCTCG ATTCATTTAA   
  
  
+ AAGGATAGTA TGGTAAATTC ATTTTATACA AGTGTCAAGT GTGAATATCT TTTTCACATC AGGTGATATA   
  
  
+ TTAGTAGGAC TTTTCGATTC ACTTATCTTC CTCTTTATGT TTTGTTAATG TGATAGTAAC GATAGGATTG   
  
  
+ CATGTATTTT TTCCAACTAT TCATTTACAA CTATCAATAT CTATTGATAA ATATGTTAAA ATAATTATAT   
  
  
+ GTTATTTAAA TTATTATTTG TTACTTAAGG TGTCGACTTT GTCGAAAGCG ACTTAATTCT AATTAAGATA   
  
  
+ GCGGCAGAGA AAGTTACACC TGATGACCAA GTTAGATCTG GTAAAACTAA CCATAGATCT GAAACCCAAA   
  
  
+ CATAATGATG TGTATTCATA CCCGACCTGA CATCTGAAAT AAGTTTACTC AAAAGTGGTT CCGAAACTTG   
  
  
+ AATTGACTCG AACCGAACCG TATTTGACTC GAAATTTTCT ACAAAGTTGC TTATTTTTGC ATGAAGACAT   
  
  
+ ATTGAGCCAA AAATTAGTTG ATTCGAAGTC AACTTAAACA AAAATTCATT CGACTCAAAC TGAAATATCA   
  
  
+ CTTAACTAAA ACTATGACTC AACTCAAACT TGATCTGATC CGTATTCAAA TCAAATACTC CATTTGCTAG   
  
  
+ GTCTAGAACC AGCCACGACC CCACATGGAC TATGCGGTTC AATGAAACGG CCAAATATAT CGCAATTTCT   
  
  
+ GTCTCCATCA ATCACACCCA TTTGCTACAC CCTGTTAATA TTTGACTCGA CCCTGTGAAT TGCCTTCGCC   
  
  
+ AGTTTTGTTT AATCTCTCTG GTTGCTTCCC ATCCAAGTCT GAATAGTATA GTTTGTACAG TGTGTACTAC   
  
  
+ TAGAAAGCAC GTTCCTTGAA TCTTCTTTCA AAGTTGTTTT TAAAGGGTTG AGTGATCCAT TACGACTTTT   
  
  
+ GATCTGGGTT CATCGCCTCC TCTCCTAATC CCTCACCCGG GTACCCTCAA TTCTCTATCG TCTCTGTTGC   
  
  
+ TCAGCTAATT CAAATTCCTT TAGTTGGTCA TCATTGAAAT CTGAAATCAG GGTGTTTTTC TTTTTATTAT   
  
  
+ TATCTGGCAG TTGGTTTGTT TATCTCTTGG TTTTTGGGGC TTATTTGGGT GCTGGTTGAT GTTATCCTGC   
  
  
+ TTGGGTTGAA TCTTCATCAG GTTTATTTTT GATTCATAGT TGAGGGTGAA AATTATTTCT CATACCCACC   
  
  
+ ACCTGTTTGT TCTTTTGTCT CTGAGAAAAA CCAAAAAGCT TTCTCTTTTT CCTGATGCAT CCTGTGCTCG   
  
  
+ TTGATCCTGA GTTAGTGAAC CATGCGTCCA AATTCAAGCC TGATTTGCTC TCAAACTTTG ATAAACAACA   
  
  
+ AGAATTTAGC AACGGGATCG AACAAGATGA CATCTTGCAA AACCCTGATC TGTGTTTTGA TTCAGAAACC   
  
  
+ CCTTCAGTTG ATCTAACTGA GAGTGTCATC AGTTCTGATT CTGTAGAAGT GCCTGATTTC TCAGATGCTT   
  
  
+ GCCTCAAGTT CATAAGTGAG ATTCTCCTAG AAGAGGATTT GGATGAAAGT CCTGCCTCTG CTCAAGAATT   
  
  
+ TAGGGCTCTC CAAGCCACTG AGAAGTCCCT GTATGATGCT CTTGGAGAGG GCTACTCTTT TTCATCTGAT   
  
  
+ AACAGCCCAT CATCATTAGG ACAGAGTATT GAGCACCAAA ATGAGAATTT TGAATTCAGT TCCGGTTATC   
  
  
+ CTGGAATTGA GGGCTATGTT AATGGTGATA TCACGTTCGA GTCCAACTGG ATGTTCAACC TAAGCCAATT   
  
  
+ GGATCCTGTT CTTACTTTGG ATGATATTTC TCAGCCCTTG TCTGCATCAA ACTCCCGATC ATCTGGGTCG   
  
  
+ AGCAATGGCT TTGATGATTC AGGGGATGGG GCAGCCACAT CTCCTGGCAG TACAGTTACA TCAACAGTCC   
  
  
+ CAGAGAAAAG GGTTGAATCT GTCAATCGCT CAAGGAGGAA GAAAAATCGT AAAAGGGATG AAGGTGGTCA   
  
  
+ TGAGGAAGGG AGGAGTAACA AGCAGCAAGC TTCATCCAAT GAAGATTATG TTGAGATGAA GGAGTTTGAT   
  
  
+ GATGTACTGC TCTGCAAAGA AGAGAAAGAT GTTATTGCAA ACTGCACCAA TGATTCCTCA CCCGTCGAAG   
  
  
+ CGAGTGATAA GTTGCAGAAG AAAGGGGGGA AGGGGAAAAC ATCGCGTGGG AAGAAGCAGA ATAGCACAAT   
  
  
+ AGAAGAGGTG GATCTGAGGA CTCTTCTCAC TCACTGTGCT CAAGCAATTT CAAATTTTGA TCTTAGGAGT   
  
  
+ GCAAATGAGC ATCTCAGGCA AATAAGGCAG CATTCTTCAC AATATGGTGA TAGCCTCCAG AGGCTTGCCC   
  
  
+ ATTATTTTGC TAATGGTCTT GAGGCTCGCA TAGCTGGCAC TGGTTCAACA ATCTCTGCTA ATGTTGTTGA   
  
  
+ TGCTCGAATC ACGTCATCTG ATTTCTTAAA GGCTTATAGG CTATATATGT CAGCCGTTCC TTTCAAAAGG   
  
  
+ ATGTTATATT TTCTCGCCAA CAAGACAATA CGGAAGTTGG CTGAGAAGGC AACCAGGATC CACATCATTG   
  
  
+ ATTTTGGAGT CTTGTTGGGT TTACAGTGGC CCTGTCTCAT ACAAAACCTC TCAAAGAGAC CCACTGGCCC   
  
  
+ GCCAAGCCTC CGTATTACTG GGATAGACTA TCCCCAGCAT GGTTTCCGGC CATCAGAAAG GGTTGAAGCG   
  
  
+ ACTGGCCGTC GGTTAGAAGG GTATTGCGAG AGATTTAATG TGCCGTTCAA GTATAAGGCC ATAGCTAAAA   
  
  
+ ATTGGGAGAG TATAAAGCTA GAGGATCTAG AAATTGATGA GAATGAGATG GTGTTTGTCA ATTGCATGCT   
  
  
+ TCGCTCTGGA ACACTGCTTG ATGAGACAGT GGTGGCAGAC AGTCCAAAGG ATGCTTTCTT AAGGTTAATC   
  
  
+ AGAGAGATAA ATCCCCGTCT TTTCATTCAT GGGACTATCA ATGGATCATT CAATGCTCCA TTCTTCATCA   
  
  
+ CTCGGTTCAG GGAGGCGCTC TTCCACTACT CTTCTTTATT TGATATCTTT GAAGCAACTA TGCCCCGTGA   
  
  
+ AGATCATGAA AGGCTCCTGG TTGAGAGTGA GATACAGGGC AAAGAAGCTT TGAATGTCAT AGCATGCGAA   
  
  
+ GGTGCCGAGA GGATTCAAAG GCCTGAAACA TACAAGCAAT GGCAAGCAAG GACAACAAGG GCCGGGTTTA   
  
  
+ GGCAGCTTCC ACTAGACCGG GAGCTTGTGA GCAGAGCAAA GGCTATGGTG AAAGCAAACT ACCATAAGGA   
  
  
+ TTTTGTGGTG GACGAGGACA GGCATTGGAT GCTACACGGT TGGAAAGGAA GAATCTTCTG TGCACTCTCT   
  
  
+ GTTTGGCAAC CCAACTG  

- -Up\_Stream \_Len000TAAACT ACACATACGA CTGATGAAAA CTACTACTAC TAGTGTCACT ACGTATAAAT   
  
  
- CTCGACCATT TAACCAGTAA GCTAAGCTCA TGCCCAGCCC AGTTCAAGAC TAATTCACTG TAAAGCGCAG   
  
  
- CAAAACCAAA TCAAGCCCAG CCTAGCTAAA GCCCAATTTA TTAAAAACCA CCTTATGCGA ACAGTACGGT   
  
  
- TTGTGTTCGT TGAAACAATT TTTAAAGCTA AGCCCAGTCC AGTTAAGTCC AAACCCCAGG TGAAAGCTCA   
  
  
- CTCGTATAAA GCCTACAGCC CAGCGCATAC CCAGGCCCAG TAAGCCAAAC TTAAAGCCTA GAACCTAGTT   
  
  
- AAAACGATTC ATACACGTAT AAGTTGGGAA CTACTGTATC CTTTGCGAAA CAGTAGAGGT GGTTAGTCTA   
  
  
- TAGCAATAGA GAAATTAGTA GCGGTATTAT CGTACTCATT CGTACTGGTA GAAAAAGTTT GTACACTATG   
  
  
- TCAATTCCGG ACAAGAAAAG CAATTATTTA GGGTTAAATA GTTAAAATTA TATATGAAAT TGCTTAAAAT   
  
  
- TGTTTTTTAA AATTAAAGTT AGGCGATATT GTTTTTTTTA TGGTTGAAGT TGAATAATAT ATTTTTTTTA   
  
  
- TATGTTAAAA AAAAGTTAAA ATCGGTTGAA GTTGTTTTTT TTTTTTTTAA ATTGTTTAGA GTTAGTTGAA   
  
  
- GTGGGTAAAG AGTTGAATAT AGAGGATTAC TTTTCTTGTC TAGGAATCGT TCTGAAGAGC TAAGTAAATT   
  
  
- TTCCTATCAT ACCATTTAAG TAAAATATGT TCACAGTTCA CACTTATAGA AAAAGTGTAG TCCACTATAT   
  
  
- AATCATCCTG AAAAGCTAAG TGAATAGAAG GAGAAATACA AAACAATTAC ACTATCATTG CTATCCTAAC   
  
  
- GTACATAAAA AAGGTTGATA AGTAAATGTT GATAGTTATA GATAACTATT TATACAATTT TATTAATATA   
  
  
- CAATAAATTT AATAATAAAC AATGAATTCC ACAGCTGAAA CAGCTTTCGC TGAATTAAGA TTAATTCTAT   
  
  
- CGCCGTCTCT TTCAATGTGG ACTACTGGTT CAATCTAGAC CATTTTGATT GGTATCTAGA CTTTGGGTTT   
  
  
- GTATTACTAC ACATAAGTAT GGGCTGGACT GTAGACTTTA TTCAAATGAG TTTTCACCAA GGCTTTGAAC   
  
  
- TTAACTGAGC TTGGCTTGGC ATAAACTGAG CTTTAAAAGA TGTTTCAACG AATAAAAACG TACTTCTGTA   
  
  
- TAACTCGGTT TTTAATCAAC TAAGCTTCAG TTGAATTTGT TTTTAAGTAA GCTGAGTTTG ACTTTATAGT   
  
  
- GAATTGATTT TGATACTGAG TTGAGTTTGA ACTAGACTAG GCATAAGTTT AGTTTATGAG GTAAACGATC   
  
  
- CAGATCTTGG TCGGTGCTGG GGTGTACCTG ATACGCCAAG TTACTTTGCC GGTTTATATA GCGTTAAAGA   
  
  
- CAGAGGTAGT TAGTGTGGGT AAACGATGTG GGACAATTAT AAACTGAGCT GGGACACTTA ACGGAAGCGG   
  
  
- TCAAAACAAA TTAGAGAGAC CAACGAAGGG TAGGTTCAGA CTTATCATAT CAAACATGTC ACACATGATG   
  
  
- ATCTTTCGTG CAAGGAACTT AGAAGAAAGT TTCAACAAAA ATTTCCCAAC TCACTAGGTA ATGCTGAAAA   
  
  
- CTAGACCCAA GTAGCGGAGG AGAGGATTAG GGAGTGGGCC CATGGGAGTT AAGAGATAGC AGAGACAACG   
  
  
- AGTCGATTAA GTTTAAGGAA ATCAACCAGT AGTAACTTTA GACTTTAGTC CCACAAAAAG AAAAATAATA   
  
  
- ATAGACCGTC AACCAAACAA ATAGAGAACC AAAAACCCCG AATAAACCCA CGACCAACTA CAATAGGACG   
  
  
- AACCCAACTT AGAAGTAGTC CAAATAAAAA CTAAGTATCA ACTCCCACTT TTAATAAAGA GTATGGGTGG   
  
  
- TGGACAAACA AGAAAACAGA GACTCTTTTT GGTTTTTCGA AAGAGAAAAA GGACTACGTA GGACACGAGC   
  
  
- AACTAGGACT CAATCACTTG GTACGCAGGT TTAAGTTCGG ACTAAACGAG AGTTTGAAAC TATTTGTTGT   
  
  
- TCTTAAATCG TTGCCCTAGC TTGTTCTACT GTAGAACGTT TTGGGACTAG ACACAAAACT AAGTCTTTGG   
  
  
- GGAAGTCAAC TAGATTGACT CTCACAGTAG TCAAGACTAA GACATCTTCA CGGACTAAAG AGTCTACGAA   
  
  
- CGGAGTTCAA GTATTCACTC TAAGAGGATC TTCTCCTAAA CCTACTTTCA GGACGGAGAC GAGTTCTTAA   
  
  
- ATCCCGAGAG GTTCGGTGAC TCTTCAGGGA CATACTACGA GAACCTCTCC CGATGAGAAA AAGTAGACTA   
  
  
- TTGTCGGGTA GTAGTAATCC TGTCTCATAA CTCGTGGTTT TACTCTTAAA ACTTAAGTCA AGGCCAATAG   
  
  
- GACCTTAACT CCCGATACAA TTACCACTAT AGTGCAAGCT CAGGTTGACC TACAAGTTGG ATTCGGTTAA   
  
  
- CCTAGGACAA GAATGAAACC TACTATAAAG AGTCGGGAAC AGACGTAGTT TGAGGGCTAG TAGACCCAGC   
  
  
- TCGTTACCGA AACTACTAAG TCCCCTACCC CGTCGGTGTA GAGGACCGTC ATGTCAATGT AGTTGTCAGG   
  
  
- GTCTCTTTTC CCAACTTAGA CAGTTAGCGA GTTCCTCCTT CTTTTTAGCA TTTTCCCTAC TTCCACCAGT   
  
  
- ACTCCTTCCC TCCTCATTGT TCGTCGTTCG AAGTAGGTTA CTTCTAATAC AACTCTACTT CCTCAAACTA   
  
  
- CTACATGACG AGACGTTTCT TCTCTTTCTA CAATAACGTT TGACGTGGTT ACTAAGGAGT GGGCAGCTTC   
  
  
- GCTCACTATT CAACGTCTTC TTTCCCCCCT TCCCCTTTTG TAGCGCACCC TTCTTCGTCT TATCGTGTTA   
  
  
- TCTTCTCCAC CTAGACTCCT GAGAAGAGTG AGTGACACGA GTTCGTTAAA GTTTAAAACT AGAATCCTCA   
  
  
- CGTTTACTCG TAGAGTCCGT TTATTCCGTC GTAAGAAGTG TTATACCACT ATCGGAGGTC TCCGAACGGG   
  
  
- TAATAAAACG ATTACCAGAA CTCCGAGCGT ATCGACCGTG ACCAAGTTGT TAGAGACGAT TACAACAACT   
  
  
- ACGAGCTTAG TGCAGTAGAC TAAAGAATTT CCGAATATCC GATATATACA GTCGGCAAGG AAAGTTTTCC   
  
  
- TACAATATAA AAGAGCGGTT GTTCTGTTAT GCCTTCAACC GACTCTTCCG TTGGTCCTAG GTGTAGTAAC   
  
  
- TAAAACCTCA GAACAACCCA AATGTCACCG GGACAGAGTA TGTTTTGGAG AGTTTCTCTG GGTGACCGGG   
  
  
- CGGTTCGGAG GCATAATGAC CCTATCTGAT AGGGGTCGTA CCAAAGGCCG GTAGTCTTTC CCAACTTCGC   
  
  
- TGACCGGCAG CCAATCTTCC CATAACGCTC TCTAAATTAC ACGGCAAGTT CATATTCCGG TATCGATTTT   
  
  
- TAACCCTCTC ATATTTCGAT CTCCTAGATC TTTAACTACT CTTACTCTAC CACAAACAGT TAACGTACGA   
  
  
- AGCGAGACCT TGTGACGAAC TACTCTGTCA CCACCGTCTG TCAGGTTTCC TACGAAAGAA TTCCAATTAG   
  
  
- TCTCTCTATT TAGGGGCAGA AAAGTAAGTA CCCTGATAGT TACCTAGTAA GTTACGAGGT AAGAAGTAGT   
  
  
- GAGCCAAGTC CCTCCGCGAG AAGGTGATGA GAAGAAATAA ACTATAGAAA CTTCGTTGAT ACGGGGCACT   
  
  
- TCTAGTACTT TCCGAGGACC AACTCTCACT CTATGTCCCG TTTCTTCGAA ACTTACAGTA TCGTACGCTT   
  
  
- CCACGGCTCT CCTAAGTTTC CGGACTTTGT ATGTTCGTTA CCGTTCGTTC CTGTTGTTCC CGGCCCAAAT   
  
  
- CCGTCGAAGG TGATCTGGCC CTCGAACACT CGTCTCGTTT CCGATACCAC TTTCGTTTGA TGGTATTCCT   
  
  
- AAAACACCAC CTGCTCCTGT CCGTAACCTA CGATGTGCCA ACCTTTCCTT CTTAGAAGAC ACGTGAGAGA   
  
  
- CAAACCGTTG GGTTGAC

+     AAGAA-motif

| Site Name | Organism | Position | Strand | Matrix score. | sequence | function |
| --- | --- | --- | --- | --- | --- | --- |
| AAGAA-motif | Avena sativa | 1637 | - | 7 | GAAAGAA |  |

>HU07G02246.1   
+ -Up\_Stream \_Len000ATTTGA TGTGTATGCT GACTACTTTT GATGATGATG ATCACAGTGA TGCATATTTA   
  
  
+ GAGCTGGTAA ATTGGTCATT CGATTCGAGT ACGGGTCGGG TCAAGTTCTG ATTAAGTGAC ATTTCGCGTC   
  
  
+ GTTTTGGTTT AGTTCGGGTC GGATCGATTT CGGGTTAAAT AATTTTTGGT GGAATACGCT TGTCATGCCA   
  
  
+ AACACAAGCA ACTTTGTTAA AAATTTCGAT TCGGGTCAGG TCAATTCAGG TTTGGGGTCC ACTTTCGAGT   
  
  
+ GAGCATATTT CGGATGTCGG GTCGCGTATG GGTCCGGGTC ATTCGGTTTG AATTTCGGAT CTTGGATCAA   
  
  
+ TTTTGCTAAG TATGTGCATA TTCAACCCTT GATGACATAG GAAACGCTTT GTCATCTCCA CCAATCAGAT   
  
  
+ ATCGTTATCT CTTTAATCAT CGCCATAATA GCATGAGTAA GCATGACCAT CTTTTTCAAA CATGTGATAC   
  
  
+ AGTTAAGGCC TGTTCTTTTC GTTAATAAAT CCCAATTTAT CAATTTTAAT ATATACTTTA ACGAATTTTA   
  
  
+ ACAAAAAATT TTAATTTCAA TCCGCTATAA CAAAAAAAAT ACCAACTTCA ACTTATTATA TAAAAAAAAT   
  
  
+ ATACAATTTT TTTTCAATTT TAGCCAACTT CAACAAAAAA AAAAAAAATT TAACAAATCT CAATCAACTT   
  
  
+ CACCCATTTC TCAACTTATA TCTCCTAATG AAAAGAACAG ATCCTTAGCA AGACTTCTCG ATTCATTTAA   
  
  
+ AAGGATAGTA TGGTAAATTC ATTTTATACA AGTGTCAAGT GTGAATATCT TTTTCACATC AGGTGATATA   
  
  
+ TTAGTAGGAC TTTTCGATTC ACTTATCTTC CTCTTTATGT TTTGTTAATG TGATAGTAAC GATAGGATTG   
  
  
+ CATGTATTTT TTCCAACTAT TCATTTACAA CTATCAATAT CTATTGATAA ATATGTTAAA ATAATTATAT   
  
  
+ GTTATTTAAA TTATTATTTG TTACTTAAGG TGTCGACTTT GTCGAAAGCG ACTTAATTCT AATTAAGATA   
  
  
+ GCGGCAGAGA AAGTTACACC TGATGACCAA GTTAGATCTG GTAAAACTAA CCATAGATCT GAAACCCAAA   
  
  
+ CATAATGATG TGTATTCATA CCCGACCTGA CATCTGAAAT AAGTTTACTC AAAAGTGGTT CCGAAACTTG   
  
  
+ AATTGACTCG AACCGAACCG TATTTGACTC GAAATTTTCT ACAAAGTTGC TTATTTTTGC ATGAAGACAT   
  
  
+ ATTGAGCCAA AAATTAGTTG ATTCGAAGTC AACTTAAACA AAAATTCATT CGACTCAAAC TGAAATATCA   
  
  
+ CTTAACTAAA ACTATGACTC AACTCAAACT TGATCTGATC CGTATTCAAA TCAAATACTC CATTTGCTAG   
  
  
+ GTCTAGAACC AGCCACGACC CCACATGGAC TATGCGGTTC AATGAAACGG CCAAATATAT CGCAATTTCT   
  
  
+ GTCTCCATCA ATCACACCCA TTTGCTACAC CCTGTTAATA TTTGACTCGA CCCTGTGAAT TGCCTTCGCC   
  
  
+ AGTTTTGTTT AATCTCTCTG GTTGCTTCCC ATCCAAGTCT GAATAGTATA GTTTGTACAG TGTGTACTAC   
  
  
+ TAGAAAGCAC GTTCCTTGAA TCTTCTTTCA AAGTTGTTTT TAAAGGGTTG AGTGATCCAT TACGACTTTT   
  
  
+ GATCTGGGTT CATCGCCTCC TCTCCTAATC CCTCACCCGG GTACCCTCAA TTCTCTATCG TCTCTGTTGC   
  
  
+ TCAGCTAATT CAAATTCCTT TAGTTGGTCA TCATTGAAAT CTGAAATCAG GGTGTTTTTC TTTTTATTAT   
  
  
+ TATCTGGCAG TTGGTTTGTT TATCTCTTGG TTTTTGGGGC TTATTTGGGT GCTGGTTGAT GTTATCCTGC   
  
  
+ TTGGGTTGAA TCTTCATCAG GTTTATTTTT GATTCATAGT TGAGGGTGAA AATTATTTCT CATACCCACC   
  
  
+ ACCTGTTTGT TCTTTTGTCT CTGAGAAAAA CCAAAAAGCT TTCTCTTTTT CCTGATGCAT CCTGTGCTCG   
  
  
+ TTGATCCTGA GTTAGTGAAC CATGCGTCCA AATTCAAGCC TGATTTGCTC TCAAACTTTG ATAAACAACA   
  
  
+ AGAATTTAGC AACGGGATCG AACAAGATGA CATCTTGCAA AACCCTGATC TGTGTTTTGA TTCAGAAACC   
  
  
+ CCTTCAGTTG ATCTAACTGA GAGTGTCATC AGTTCTGATT CTGTAGAAGT GCCTGATTTC TCAGATGCTT   
  
  
+ GCCTCAAGTT CATAAGTGAG ATTCTCCTAG AAGAGGATTT GGATGAAAGT CCTGCCTCTG CTCAAGAATT   
  
  
+ TAGGGCTCTC CAAGCCACTG AGAAGTCCCT GTATGATGCT CTTGGAGAGG GCTACTCTTT TTCATCTGAT   
  
  
+ AACAGCCCAT CATCATTAGG ACAGAGTATT GAGCACCAAA ATGAGAATTT TGAATTCAGT TCCGGTTATC   
  
  
+ CTGGAATTGA GGGCTATGTT AATGGTGATA TCACGTTCGA GTCCAACTGG ATGTTCAACC TAAGCCAATT   
  
  
+ GGATCCTGTT CTTACTTTGG ATGATATTTC TCAGCCCTTG TCTGCATCAA ACTCCCGATC ATCTGGGTCG   
  
  
+ AGCAATGGCT TTGATGATTC AGGGGATGGG GCAGCCACAT CTCCTGGCAG TACAGTTACA TCAACAGTCC   
  
  
+ CAGAGAAAAG GGTTGAATCT GTCAATCGCT CAAGGAGGAA GAAAAATCGT AAAAGGGATG AAGGTGGTCA   
  
  
+ TGAGGAAGGG AGGAGTAACA AGCAGCAAGC TTCATCCAAT GAAGATTATG TTGAGATGAA GGAGTTTGAT   
  
  
+ GATGTACTGC TCTGCAAAGA AGAGAAAGAT GTTATTGCAA ACTGCACCAA TGATTCCTCA CCCGTCGAAG   
  
  
+ CGAGTGATAA GTTGCAGAAG AAAGGGGGGA AGGGGAAAAC ATCGCGTGGG AAGAAGCAGA ATAGCACAAT   
  
  
+ AGAAGAGGTG GATCTGAGGA CTCTTCTCAC TCACTGTGCT CAAGCAATTT CAAATTTTGA TCTTAGGAGT   
  
  
+ GCAAATGAGC ATCTCAGGCA AATAAGGCAG CATTCTTCAC AATATGGTGA TAGCCTCCAG AGGCTTGCCC   
  
  
+ ATTATTTTGC TAATGGTCTT GAGGCTCGCA TAGCTGGCAC TGGTTCAACA ATCTCTGCTA ATGTTGTTGA   
  
  
+ TGCTCGAATC ACGTCATCTG ATTTCTTAAA GGCTTATAGG CTATATATGT CAGCCGTTCC TTTCAAAAGG   
  
  
+ ATGTTATATT TTCTCGCCAA CAAGACAATA CGGAAGTTGG CTGAGAAGGC AACCAGGATC CACATCATTG   
  
  
+ ATTTTGGAGT CTTGTTGGGT TTACAGTGGC CCTGTCTCAT ACAAAACCTC TCAAAGAGAC CCACTGGCCC   
  
  
+ GCCAAGCCTC CGTATTACTG GGATAGACTA TCCCCAGCAT GGTTTCCGGC CATCAGAAAG GGTTGAAGCG   
  
  
+ ACTGGCCGTC GGTTAGAAGG GTATTGCGAG AGATTTAATG TGCCGTTCAA GTATAAGGCC ATAGCTAAAA   
  
  
+ ATTGGGAGAG TATAAAGCTA GAGGATCTAG AAATTGATGA GAATGAGATG GTGTTTGTCA ATTGCATGCT   
  
  
+ TCGCTCTGGA ACACTGCTTG ATGAGACAGT GGTGGCAGAC AGTCCAAAGG ATGCTTTCTT AAGGTTAATC   
  
  
+ AGAGAGATAA ATCCCCGTCT TTTCATTCAT GGGACTATCA ATGGATCATT CAATGCTCCA TTCTTCATCA   
  
  
+ CTCGGTTCAG GGAGGCGCTC TTCCACTACT CTTCTTTATT TGATATCTTT GAAGCAACTA TGCCCCGTGA   
  
  
+ AGATCATGAA AGGCTCCTGG TTGAGAGTGA GATACAGGGC AAAGAAGCTT TGAATGTCAT AGCATGCGAA   
  
  
+ GGTGCCGAGA GGATTCAAAG GCCTGAAACA TACAAGCAAT GGCAAGCAAG GACAACAAGG GCCGGGTTTA   
  
  
+ GGCAGCTTCC ACTAGACCGG GAGCTTGTGA GCAGAGCAAA GGCTATGGTG AAAGCAAACT ACCATAAGGA   
  
  
+ TTTTGTGGTG GACGAGGACA GGCATTGGAT GCTACACGGT TGGAAAGGAA GAATCTTCTG TGCACTCTCT   
  
  
+ GTTTGGCAAC CCAACTG  

- -Up\_Stream \_Len000TAAACT ACACATACGA CTGATGAAAA CTACTACTAC TAGTGTCACT ACGTATAAAT   
  
  
- CTCGACCATT TAACCAGTAA GCTAAGCTCA TGCCCAGCCC AGTTCAAGAC TAATTCACTG TAAAGCGCAG   
  
  
- CAAAACCAAA TCAAGCCCAG CCTAGCTAAA GCCCAATTTA TTAAAAACCA CCTTATGCGA ACAGTACGGT   
  
  
- TTGTGTTCGT TGAAACAATT TTTAAAGCTA AGCCCAGTCC AGTTAAGTCC AAACCCCAGG TGAAAGCTCA   
  
  
- CTCGTATAAA GCCTACAGCC CAGCGCATAC CCAGGCCCAG TAAGCCAAAC TTAAAGCCTA GAACCTAGTT   
  
  
- AAAACGATTC ATACACGTAT AAGTTGGGAA CTACTGTATC CTTTGCGAAA CAGTAGAGGT GGTTAGTCTA   
  
  
- TAGCAATAGA GAAATTAGTA GCGGTATTAT CGTACTCATT CGTACTGGTA GAAAAAGTTT GTACACTATG   
  
  
- TCAATTCCGG ACAAGAAAAG CAATTATTTA GGGTTAAATA GTTAAAATTA TATATGAAAT TGCTTAAAAT   
  
  
- TGTTTTTTAA AATTAAAGTT AGGCGATATT GTTTTTTTTA TGGTTGAAGT TGAATAATAT ATTTTTTTTA   
  
  
- TATGTTAAAA AAAAGTTAAA ATCGGTTGAA GTTGTTTTTT TTTTTTTTAA ATTGTTTAGA GTTAGTTGAA   
  
  
- GTGGGTAAAG AGTTGAATAT AGAGGATTAC TTTTCTTGTC TAGGAATCGT TCTGAAGAGC TAAGTAAATT   
  
  
- TTCCTATCAT ACCATTTAAG TAAAATATGT TCACAGTTCA CACTTATAGA AAAAGTGTAG TCCACTATAT   
  
  
- AATCATCCTG AAAAGCTAAG TGAATAGAAG GAGAAATACA AAACAATTAC ACTATCATTG CTATCCTAAC   
  
  
- GTACATAAAA AAGGTTGATA AGTAAATGTT GATAGTTATA GATAACTATT TATACAATTT TATTAATATA   
  
  
- CAATAAATTT AATAATAAAC AATGAATTCC ACAGCTGAAA CAGCTTTCGC TGAATTAAGA TTAATTCTAT   
  
  
- CGCCGTCTCT TTCAATGTGG ACTACTGGTT CAATCTAGAC CATTTTGATT GGTATCTAGA CTTTGGGTTT   
  
  
- GTATTACTAC ACATAAGTAT GGGCTGGACT GTAGACTTTA TTCAAATGAG TTTTCACCAA GGCTTTGAAC   
  
  
- TTAACTGAGC TTGGCTTGGC ATAAACTGAG CTTTAAAAGA TGTTTCAACG AATAAAAACG TACTTCTGTA   
  
  
- TAACTCGGTT TTTAATCAAC TAAGCTTCAG TTGAATTTGT TTTTAAGTAA GCTGAGTTTG ACTTTATAGT   
  
  
- GAATTGATTT TGATACTGAG TTGAGTTTGA ACTAGACTAG GCATAAGTTT AGTTTATGAG GTAAACGATC   
  
  
- CAGATCTTGG TCGGTGCTGG GGTGTACCTG ATACGCCAAG TTACTTTGCC GGTTTATATA GCGTTAAAGA   
  
  
- CAGAGGTAGT TAGTGTGGGT AAACGATGTG GGACAATTAT AAACTGAGCT GGGACACTTA ACGGAAGCGG   
  
  
- TCAAAACAAA TTAGAGAGAC CAACGAAGGG TAGGTTCAGA CTTATCATAT CAAACATGTC ACACATGATG   
  
  
- ATCTTTCGTG CAAGGAACTT AGAAGAAAGT TTCAACAAAA ATTTCCCAAC TCACTAGGTA ATGCTGAAAA   
  
  
- CTAGACCCAA GTAGCGGAGG AGAGGATTAG GGAGTGGGCC CATGGGAGTT AAGAGATAGC AGAGACAACG   
  
  
- AGTCGATTAA GTTTAAGGAA ATCAACCAGT AGTAACTTTA GACTTTAGTC CCACAAAAAG AAAAATAATA   
  
  
- ATAGACCGTC AACCAAACAA ATAGAGAACC AAAAACCCCG AATAAACCCA CGACCAACTA CAATAGGACG   
  
  
- AACCCAACTT AGAAGTAGTC CAAATAAAAA CTAAGTATCA ACTCCCACTT TTAATAAAGA GTATGGGTGG   
  
  
- TGGACAAACA AGAAAACAGA GACTCTTTTT GGTTTTTCGA AAGAGAAAAA GGACTACGTA GGACACGAGC   
  
  
- AACTAGGACT CAATCACTTG GTACGCAGGT TTAAGTTCGG ACTAAACGAG AGTTTGAAAC TATTTGTTGT   
  
  
- TCTTAAATCG TTGCCCTAGC TTGTTCTACT GTAGAACGTT TTGGGACTAG ACACAAAACT AAGTCTTTGG   
  
  
- GGAAGTCAAC TAGATTGACT CTCACAGTAG TCAAGACTAA GACATCTTCA CGGACTAAAG AGTCTACGAA   
  
  
- CGGAGTTCAA GTATTCACTC TAAGAGGATC TTCTCCTAAA CCTACTTTCA GGACGGAGAC GAGTTCTTAA   
  
  
- ATCCCGAGAG GTTCGGTGAC TCTTCAGGGA CATACTACGA GAACCTCTCC CGATGAGAAA AAGTAGACTA   
  
  
- TTGTCGGGTA GTAGTAATCC TGTCTCATAA CTCGTGGTTT TACTCTTAAA ACTTAAGTCA AGGCCAATAG   
  
  
- GACCTTAACT CCCGATACAA TTACCACTAT AGTGCAAGCT CAGGTTGACC TACAAGTTGG ATTCGGTTAA   
  
  
- CCTAGGACAA GAATGAAACC TACTATAAAG AGTCGGGAAC AGACGTAGTT TGAGGGCTAG TAGACCCAGC   
  
  
- TCGTTACCGA AACTACTAAG TCCCCTACCC CGTCGGTGTA GAGGACCGTC ATGTCAATGT AGTTGTCAGG   
  
  
- GTCTCTTTTC CCAACTTAGA CAGTTAGCGA GTTCCTCCTT CTTTTTAGCA TTTTCCCTAC TTCCACCAGT   
  
  
- ACTCCTTCCC TCCTCATTGT TCGTCGTTCG AAGTAGGTTA CTTCTAATAC AACTCTACTT CCTCAAACTA   
  
  
- CTACATGACG AGACGTTTCT TCTCTTTCTA CAATAACGTT TGACGTGGTT ACTAAGGAGT GGGCAGCTTC   
  
  
- GCTCACTATT CAACGTCTTC TTTCCCCCCT TCCCCTTTTG TAGCGCACCC TTCTTCGTCT TATCGTGTTA   
  
  
- TCTTCTCCAC CTAGACTCCT GAGAAGAGTG AGTGACACGA GTTCGTTAAA GTTTAAAACT AGAATCCTCA   
  
  
- CGTTTACTCG TAGAGTCCGT TTATTCCGTC GTAAGAAGTG TTATACCACT ATCGGAGGTC TCCGAACGGG   
  
  
- TAATAAAACG ATTACCAGAA CTCCGAGCGT ATCGACCGTG ACCAAGTTGT TAGAGACGAT TACAACAACT   
  
  
- ACGAGCTTAG TGCAGTAGAC TAAAGAATTT CCGAATATCC GATATATACA GTCGGCAAGG AAAGTTTTCC   
  
  
- TACAATATAA AAGAGCGGTT GTTCTGTTAT GCCTTCAACC GACTCTTCCG TTGGTCCTAG GTGTAGTAAC   
  
  
- TAAAACCTCA GAACAACCCA AATGTCACCG GGACAGAGTA TGTTTTGGAG AGTTTCTCTG GGTGACCGGG   
  
  
- CGGTTCGGAG GCATAATGAC CCTATCTGAT AGGGGTCGTA CCAAAGGCCG GTAGTCTTTC CCAACTTCGC   
  
  
- TGACCGGCAG CCAATCTTCC CATAACGCTC TCTAAATTAC ACGGCAAGTT CATATTCCGG TATCGATTTT   
  
  
- TAACCCTCTC ATATTTCGAT CTCCTAGATC TTTAACTACT CTTACTCTAC CACAAACAGT TAACGTACGA   
  
  
- AGCGAGACCT TGTGACGAAC TACTCTGTCA CCACCGTCTG TCAGGTTTCC TACGAAAGAA TTCCAATTAG   
  
  
- TCTCTCTATT TAGGGGCAGA AAAGTAAGTA CCCTGATAGT TACCTAGTAA GTTACGAGGT AAGAAGTAGT   
  
  
- GAGCCAAGTC CCTCCGCGAG AAGGTGATGA GAAGAAATAA ACTATAGAAA CTTCGTTGAT ACGGGGCACT   
  
  
- TCTAGTACTT TCCGAGGACC AACTCTCACT CTATGTCCCG TTTCTTCGAA ACTTACAGTA TCGTACGCTT   
  
  
- CCACGGCTCT CCTAAGTTTC CGGACTTTGT ATGTTCGTTA CCGTTCGTTC CTGTTGTTCC CGGCCCAAAT   
  
  
- CCGTCGAAGG TGATCTGGCC CTCGAACACT CGTCTCGTTT CCGATACCAC TTTCGTTTGA TGGTATTCCT   
  
  
- AAAACACCAC CTGCTCCTGT CCGTAACCTA CGATGTGCCA ACCTTTCCTT CTTAGAAGAC ACGTGAGAGA   
  
  
- CAAACCGTTG GGTTGAC

+     ABRE

| Site Name | Organism | Position | Strand | Matrix score. | sequence | function |
| --- | --- | --- | --- | --- | --- | --- |
| ABRE | Arabidopsis thaliana | 2486 | - | 5 | ACGTG | cis-acting element involved in the abscisic acid responsiveness |
| ABRE | Arabidopsis thaliana | 1622 | - | 5 | ACGTG | cis-acting element involved in the abscisic acid responsiveness |
| ABRE | Arabidopsis thaliana | 3916 | - | 7 | AACCCGG | cis-acting element involved in the abscisic acid responsiveness |
| ABRE | Arabidopsis thaliana | 3164 | - | 5 | ACGTG | cis-acting element involved in the abscisic acid responsiveness |

>HU07G02246.1   
+ -Up\_Stream \_Len000ATTTGA TGTGTATGCT GACTACTTTT GATGATGATG ATCACAGTGA TGCATATTTA   
  
  
+ GAGCTGGTAA ATTGGTCATT CGATTCGAGT ACGGGTCGGG TCAAGTTCTG ATTAAGTGAC ATTTCGCGTC   
  
  
+ GTTTTGGTTT AGTTCGGGTC GGATCGATTT CGGGTTAAAT AATTTTTGGT GGAATACGCT TGTCATGCCA   
  
  
+ AACACAAGCA ACTTTGTTAA AAATTTCGAT TCGGGTCAGG TCAATTCAGG TTTGGGGTCC ACTTTCGAGT   
  
  
+ GAGCATATTT CGGATGTCGG GTCGCGTATG GGTCCGGGTC ATTCGGTTTG AATTTCGGAT CTTGGATCAA   
  
  
+ TTTTGCTAAG TATGTGCATA TTCAACCCTT GATGACATAG GAAACGCTTT GTCATCTCCA CCAATCAGAT   
  
  
+ ATCGTTATCT CTTTAATCAT CGCCATAATA GCATGAGTAA GCATGACCAT CTTTTTCAAA CATGTGATAC   
  
  
+ AGTTAAGGCC TGTTCTTTTC GTTAATAAAT CCCAATTTAT CAATTTTAAT ATATACTTTA ACGAATTTTA   
  
  
+ ACAAAAAATT TTAATTTCAA TCCGCTATAA CAAAAAAAAT ACCAACTTCA ACTTATTATA TAAAAAAAAT   
  
  
+ ATACAATTTT TTTTCAATTT TAGCCAACTT CAACAAAAAA AAAAAAAATT TAACAAATCT CAATCAACTT   
  
  
+ CACCCATTTC TCAACTTATA TCTCCTAATG AAAAGAACAG ATCCTTAGCA AGACTTCTCG ATTCATTTAA   
  
  
+ AAGGATAGTA TGGTAAATTC ATTTTATACA AGTGTCAAGT GTGAATATCT TTTTCACATC AGGTGATATA   
  
  
+ TTAGTAGGAC TTTTCGATTC ACTTATCTTC CTCTTTATGT TTTGTTAATG TGATAGTAAC GATAGGATTG   
  
  
+ CATGTATTTT TTCCAACTAT TCATTTACAA CTATCAATAT CTATTGATAA ATATGTTAAA ATAATTATAT   
  
  
+ GTTATTTAAA TTATTATTTG TTACTTAAGG TGTCGACTTT GTCGAAAGCG ACTTAATTCT AATTAAGATA   
  
  
+ GCGGCAGAGA AAGTTACACC TGATGACCAA GTTAGATCTG GTAAAACTAA CCATAGATCT GAAACCCAAA   
  
  
+ CATAATGATG TGTATTCATA CCCGACCTGA CATCTGAAAT AAGTTTACTC AAAAGTGGTT CCGAAACTTG   
  
  
+ AATTGACTCG AACCGAACCG TATTTGACTC GAAATTTTCT ACAAAGTTGC TTATTTTTGC ATGAAGACAT   
  
  
+ ATTGAGCCAA AAATTAGTTG ATTCGAAGTC AACTTAAACA AAAATTCATT CGACTCAAAC TGAAATATCA   
  
  
+ CTTAACTAAA ACTATGACTC AACTCAAACT TGATCTGATC CGTATTCAAA TCAAATACTC CATTTGCTAG   
  
  
+ GTCTAGAACC AGCCACGACC CCACATGGAC TATGCGGTTC AATGAAACGG CCAAATATAT CGCAATTTCT   
  
  
+ GTCTCCATCA ATCACACCCA TTTGCTACAC CCTGTTAATA TTTGACTCGA CCCTGTGAAT TGCCTTCGCC   
  
  
+ AGTTTTGTTT AATCTCTCTG GTTGCTTCCC ATCCAAGTCT GAATAGTATA GTTTGTACAG TGTGTACTAC   
  
  
+ TAGAAAGCAC GTTCCTTGAA TCTTCTTTCA AAGTTGTTTT TAAAGGGTTG AGTGATCCAT TACGACTTTT   
  
  
+ GATCTGGGTT CATCGCCTCC TCTCCTAATC CCTCACCCGG GTACCCTCAA TTCTCTATCG TCTCTGTTGC   
  
  
+ TCAGCTAATT CAAATTCCTT TAGTTGGTCA TCATTGAAAT CTGAAATCAG GGTGTTTTTC TTTTTATTAT   
  
  
+ TATCTGGCAG TTGGTTTGTT TATCTCTTGG TTTTTGGGGC TTATTTGGGT GCTGGTTGAT GTTATCCTGC   
  
  
+ TTGGGTTGAA TCTTCATCAG GTTTATTTTT GATTCATAGT TGAGGGTGAA AATTATTTCT CATACCCACC   
  
  
+ ACCTGTTTGT TCTTTTGTCT CTGAGAAAAA CCAAAAAGCT TTCTCTTTTT CCTGATGCAT CCTGTGCTCG   
  
  
+ TTGATCCTGA GTTAGTGAAC CATGCGTCCA AATTCAAGCC TGATTTGCTC TCAAACTTTG ATAAACAACA   
  
  
+ AGAATTTAGC AACGGGATCG AACAAGATGA CATCTTGCAA AACCCTGATC TGTGTTTTGA TTCAGAAACC   
  
  
+ CCTTCAGTTG ATCTAACTGA GAGTGTCATC AGTTCTGATT CTGTAGAAGT GCCTGATTTC TCAGATGCTT   
  
  
+ GCCTCAAGTT CATAAGTGAG ATTCTCCTAG AAGAGGATTT GGATGAAAGT CCTGCCTCTG CTCAAGAATT   
  
  
+ TAGGGCTCTC CAAGCCACTG AGAAGTCCCT GTATGATGCT CTTGGAGAGG GCTACTCTTT TTCATCTGAT   
  
  
+ AACAGCCCAT CATCATTAGG ACAGAGTATT GAGCACCAAA ATGAGAATTT TGAATTCAGT TCCGGTTATC   
  
  
+ CTGGAATTGA GGGCTATGTT AATGGTGATA TCACGTTCGA GTCCAACTGG ATGTTCAACC TAAGCCAATT   
  
  
+ GGATCCTGTT CTTACTTTGG ATGATATTTC TCAGCCCTTG TCTGCATCAA ACTCCCGATC ATCTGGGTCG   
  
  
+ AGCAATGGCT TTGATGATTC AGGGGATGGG GCAGCCACAT CTCCTGGCAG TACAGTTACA TCAACAGTCC   
  
  
+ CAGAGAAAAG GGTTGAATCT GTCAATCGCT CAAGGAGGAA GAAAAATCGT AAAAGGGATG AAGGTGGTCA   
  
  
+ TGAGGAAGGG AGGAGTAACA AGCAGCAAGC TTCATCCAAT GAAGATTATG TTGAGATGAA GGAGTTTGAT   
  
  
+ GATGTACTGC TCTGCAAAGA AGAGAAAGAT GTTATTGCAA ACTGCACCAA TGATTCCTCA CCCGTCGAAG   
  
  
+ CGAGTGATAA GTTGCAGAAG AAAGGGGGGA AGGGGAAAAC ATCGCGTGGG AAGAAGCAGA ATAGCACAAT   
  
  
+ AGAAGAGGTG GATCTGAGGA CTCTTCTCAC TCACTGTGCT CAAGCAATTT CAAATTTTGA TCTTAGGAGT   
  
  
+ GCAAATGAGC ATCTCAGGCA AATAAGGCAG CATTCTTCAC AATATGGTGA TAGCCTCCAG AGGCTTGCCC   
  
  
+ ATTATTTTGC TAATGGTCTT GAGGCTCGCA TAGCTGGCAC TGGTTCAACA ATCTCTGCTA ATGTTGTTGA   
  
  
+ TGCTCGAATC ACGTCATCTG ATTTCTTAAA GGCTTATAGG CTATATATGT CAGCCGTTCC TTTCAAAAGG   
  
  
+ ATGTTATATT TTCTCGCCAA CAAGACAATA CGGAAGTTGG CTGAGAAGGC AACCAGGATC CACATCATTG   
  
  
+ ATTTTGGAGT CTTGTTGGGT TTACAGTGGC CCTGTCTCAT ACAAAACCTC TCAAAGAGAC CCACTGGCCC   
  
  
+ GCCAAGCCTC CGTATTACTG GGATAGACTA TCCCCAGCAT GGTTTCCGGC CATCAGAAAG GGTTGAAGCG   
  
  
+ ACTGGCCGTC GGTTAGAAGG GTATTGCGAG AGATTTAATG TGCCGTTCAA GTATAAGGCC ATAGCTAAAA   
  
  
+ ATTGGGAGAG TATAAAGCTA GAGGATCTAG AAATTGATGA GAATGAGATG GTGTTTGTCA ATTGCATGCT   
  
  
+ TCGCTCTGGA ACACTGCTTG ATGAGACAGT GGTGGCAGAC AGTCCAAAGG ATGCTTTCTT AAGGTTAATC   
  
  
+ AGAGAGATAA ATCCCCGTCT TTTCATTCAT GGGACTATCA ATGGATCATT CAATGCTCCA TTCTTCATCA   
  
  
+ CTCGGTTCAG GGAGGCGCTC TTCCACTACT CTTCTTTATT TGATATCTTT GAAGCAACTA TGCCCCGTGA   
  
  
+ AGATCATGAA AGGCTCCTGG TTGAGAGTGA GATACAGGGC AAAGAAGCTT TGAATGTCAT AGCATGCGAA   
  
  
+ GGTGCCGAGA GGATTCAAAG GCCTGAAACA TACAAGCAAT GGCAAGCAAG GACAACAAGG GCCGGGTTTA   
  
  
+ GGCAGCTTCC ACTAGACCGG GAGCTTGTGA GCAGAGCAAA GGCTATGGTG AAAGCAAACT ACCATAAGGA   
  
  
+ TTTTGTGGTG GACGAGGACA GGCATTGGAT GCTACACGGT TGGAAAGGAA GAATCTTCTG TGCACTCTCT   
  
  
+ GTTTGGCAAC CCAACTG  

- -Up\_Stream \_Len000TAAACT ACACATACGA CTGATGAAAA CTACTACTAC TAGTGTCACT ACGTATAAAT   
  
  
- CTCGACCATT TAACCAGTAA GCTAAGCTCA TGCCCAGCCC AGTTCAAGAC TAATTCACTG TAAAGCGCAG   
  
  
- CAAAACCAAA TCAAGCCCAG CCTAGCTAAA GCCCAATTTA TTAAAAACCA CCTTATGCGA ACAGTACGGT   
  
  
- TTGTGTTCGT TGAAACAATT TTTAAAGCTA AGCCCAGTCC AGTTAAGTCC AAACCCCAGG TGAAAGCTCA   
  
  
- CTCGTATAAA GCCTACAGCC CAGCGCATAC CCAGGCCCAG TAAGCCAAAC TTAAAGCCTA GAACCTAGTT   
  
  
- AAAACGATTC ATACACGTAT AAGTTGGGAA CTACTGTATC CTTTGCGAAA CAGTAGAGGT GGTTAGTCTA   
  
  
- TAGCAATAGA GAAATTAGTA GCGGTATTAT CGTACTCATT CGTACTGGTA GAAAAAGTTT GTACACTATG   
  
  
- TCAATTCCGG ACAAGAAAAG CAATTATTTA GGGTTAAATA GTTAAAATTA TATATGAAAT TGCTTAAAAT   
  
  
- TGTTTTTTAA AATTAAAGTT AGGCGATATT GTTTTTTTTA TGGTTGAAGT TGAATAATAT ATTTTTTTTA   
  
  
- TATGTTAAAA AAAAGTTAAA ATCGGTTGAA GTTGTTTTTT TTTTTTTTAA ATTGTTTAGA GTTAGTTGAA   
  
  
- GTGGGTAAAG AGTTGAATAT AGAGGATTAC TTTTCTTGTC TAGGAATCGT TCTGAAGAGC TAAGTAAATT   
  
  
- TTCCTATCAT ACCATTTAAG TAAAATATGT TCACAGTTCA CACTTATAGA AAAAGTGTAG TCCACTATAT   
  
  
- AATCATCCTG AAAAGCTAAG TGAATAGAAG GAGAAATACA AAACAATTAC ACTATCATTG CTATCCTAAC   
  
  
- GTACATAAAA AAGGTTGATA AGTAAATGTT GATAGTTATA GATAACTATT TATACAATTT TATTAATATA   
  
  
- CAATAAATTT AATAATAAAC AATGAATTCC ACAGCTGAAA CAGCTTTCGC TGAATTAAGA TTAATTCTAT   
  
  
- CGCCGTCTCT TTCAATGTGG ACTACTGGTT CAATCTAGAC CATTTTGATT GGTATCTAGA CTTTGGGTTT   
  
  
- GTATTACTAC ACATAAGTAT GGGCTGGACT GTAGACTTTA TTCAAATGAG TTTTCACCAA GGCTTTGAAC   
  
  
- TTAACTGAGC TTGGCTTGGC ATAAACTGAG CTTTAAAAGA TGTTTCAACG AATAAAAACG TACTTCTGTA   
  
  
- TAACTCGGTT TTTAATCAAC TAAGCTTCAG TTGAATTTGT TTTTAAGTAA GCTGAGTTTG ACTTTATAGT   
  
  
- GAATTGATTT TGATACTGAG TTGAGTTTGA ACTAGACTAG GCATAAGTTT AGTTTATGAG GTAAACGATC   
  
  
- CAGATCTTGG TCGGTGCTGG GGTGTACCTG ATACGCCAAG TTACTTTGCC GGTTTATATA GCGTTAAAGA   
  
  
- CAGAGGTAGT TAGTGTGGGT AAACGATGTG GGACAATTAT AAACTGAGCT GGGACACTTA ACGGAAGCGG   
  
  
- TCAAAACAAA TTAGAGAGAC CAACGAAGGG TAGGTTCAGA CTTATCATAT CAAACATGTC ACACATGATG   
  
  
- ATCTTTCGTG CAAGGAACTT AGAAGAAAGT TTCAACAAAA ATTTCCCAAC TCACTAGGTA ATGCTGAAAA   
  
  
- CTAGACCCAA GTAGCGGAGG AGAGGATTAG GGAGTGGGCC CATGGGAGTT AAGAGATAGC AGAGACAACG   
  
  
- AGTCGATTAA GTTTAAGGAA ATCAACCAGT AGTAACTTTA GACTTTAGTC CCACAAAAAG AAAAATAATA   
  
  
- ATAGACCGTC AACCAAACAA ATAGAGAACC AAAAACCCCG AATAAACCCA CGACCAACTA CAATAGGACG   
  
  
- AACCCAACTT AGAAGTAGTC CAAATAAAAA CTAAGTATCA ACTCCCACTT TTAATAAAGA GTATGGGTGG   
  
  
- TGGACAAACA AGAAAACAGA GACTCTTTTT GGTTTTTCGA AAGAGAAAAA GGACTACGTA GGACACGAGC   
  
  
- AACTAGGACT CAATCACTTG GTACGCAGGT TTAAGTTCGG ACTAAACGAG AGTTTGAAAC TATTTGTTGT   
  
  
- TCTTAAATCG TTGCCCTAGC TTGTTCTACT GTAGAACGTT TTGGGACTAG ACACAAAACT AAGTCTTTGG   
  
  
- GGAAGTCAAC TAGATTGACT CTCACAGTAG TCAAGACTAA GACATCTTCA CGGACTAAAG AGTCTACGAA   
  
  
- CGGAGTTCAA GTATTCACTC TAAGAGGATC TTCTCCTAAA CCTACTTTCA GGACGGAGAC GAGTTCTTAA   
  
  
- ATCCCGAGAG GTTCGGTGAC TCTTCAGGGA CATACTACGA GAACCTCTCC CGATGAGAAA AAGTAGACTA   
  
  
- TTGTCGGGTA GTAGTAATCC TGTCTCATAA CTCGTGGTTT TACTCTTAAA ACTTAAGTCA AGGCCAATAG   
  
  
- GACCTTAACT CCCGATACAA TTACCACTAT AGTGCAAGCT CAGGTTGACC TACAAGTTGG ATTCGGTTAA   
  
  
- CCTAGGACAA GAATGAAACC TACTATAAAG AGTCGGGAAC AGACGTAGTT TGAGGGCTAG TAGACCCAGC   
  
  
- TCGTTACCGA AACTACTAAG TCCCCTACCC CGTCGGTGTA GAGGACCGTC ATGTCAATGT AGTTGTCAGG   
  
  
- GTCTCTTTTC CCAACTTAGA CAGTTAGCGA GTTCCTCCTT CTTTTTAGCA TTTTCCCTAC TTCCACCAGT   
  
  
- ACTCCTTCCC TCCTCATTGT TCGTCGTTCG AAGTAGGTTA CTTCTAATAC AACTCTACTT CCTCAAACTA   
  
  
- CTACATGACG AGACGTTTCT TCTCTTTCTA CAATAACGTT TGACGTGGTT ACTAAGGAGT GGGCAGCTTC   
  
  
- GCTCACTATT CAACGTCTTC TTTCCCCCCT TCCCCTTTTG TAGCGCACCC TTCTTCGTCT TATCGTGTTA   
  
  
- TCTTCTCCAC CTAGACTCCT GAGAAGAGTG AGTGACACGA GTTCGTTAAA GTTTAAAACT AGAATCCTCA   
  
  
- CGTTTACTCG TAGAGTCCGT TTATTCCGTC GTAAGAAGTG TTATACCACT ATCGGAGGTC TCCGAACGGG   
  
  
- TAATAAAACG ATTACCAGAA CTCCGAGCGT ATCGACCGTG ACCAAGTTGT TAGAGACGAT TACAACAACT   
  
  
- ACGAGCTTAG TGCAGTAGAC TAAAGAATTT CCGAATATCC GATATATACA GTCGGCAAGG AAAGTTTTCC   
  
  
- TACAATATAA AAGAGCGGTT GTTCTGTTAT GCCTTCAACC GACTCTTCCG TTGGTCCTAG GTGTAGTAAC   
  
  
- TAAAACCTCA GAACAACCCA AATGTCACCG GGACAGAGTA TGTTTTGGAG AGTTTCTCTG GGTGACCGGG   
  
  
- CGGTTCGGAG GCATAATGAC CCTATCTGAT AGGGGTCGTA CCAAAGGCCG GTAGTCTTTC CCAACTTCGC   
  
  
- TGACCGGCAG CCAATCTTCC CATAACGCTC TCTAAATTAC ACGGCAAGTT CATATTCCGG TATCGATTTT   
  
  
- TAACCCTCTC ATATTTCGAT CTCCTAGATC TTTAACTACT CTTACTCTAC CACAAACAGT TAACGTACGA   
  
  
- AGCGAGACCT TGTGACGAAC TACTCTGTCA CCACCGTCTG TCAGGTTTCC TACGAAAGAA TTCCAATTAG   
  
  
- TCTCTCTATT TAGGGGCAGA AAAGTAAGTA CCCTGATAGT TACCTAGTAA GTTACGAGGT AAGAAGTAGT   
  
  
- GAGCCAAGTC CCTCCGCGAG AAGGTGATGA GAAGAAATAA ACTATAGAAA CTTCGTTGAT ACGGGGCACT   
  
  
- TCTAGTACTT TCCGAGGACC AACTCTCACT CTATGTCCCG TTTCTTCGAA ACTTACAGTA TCGTACGCTT   
  
  
- CCACGGCTCT CCTAAGTTTC CGGACTTTGT ATGTTCGTTA CCGTTCGTTC CTGTTGTTCC CGGCCCAAAT   
  
  
- CCGTCGAAGG TGATCTGGCC CTCGAACACT CGTCTCGTTT CCGATACCAC TTTCGTTTGA TGGTATTCCT   
  
  
- AAAACACCAC CTGCTCCTGT CCGTAACCTA CGATGTGCCA ACCTTTCCTT CTTAGAAGAC ACGTGAGAGA   
  
  
- CAAACCGTTG GGTTGAC

+     AC-I

| Site Name | Organism | Position | Strand | Matrix score. | sequence | function |
| --- | --- | --- | --- | --- | --- | --- |
| AC-I | Phaseolus vulgaris | 1954 | + | 8.5 | (T/C)C(T/C)(C/T)ACC(T/C)ACC |  |

>HU07G02246.1   
+ -Up\_Stream \_Len000ATTTGA TGTGTATGCT GACTACTTTT GATGATGATG ATCACAGTGA TGCATATTTA   
  
  
+ GAGCTGGTAA ATTGGTCATT CGATTCGAGT ACGGGTCGGG TCAAGTTCTG ATTAAGTGAC ATTTCGCGTC   
  
  
+ GTTTTGGTTT AGTTCGGGTC GGATCGATTT CGGGTTAAAT AATTTTTGGT GGAATACGCT TGTCATGCCA   
  
  
+ AACACAAGCA ACTTTGTTAA AAATTTCGAT TCGGGTCAGG TCAATTCAGG TTTGGGGTCC ACTTTCGAGT   
  
  
+ GAGCATATTT CGGATGTCGG GTCGCGTATG GGTCCGGGTC ATTCGGTTTG AATTTCGGAT CTTGGATCAA   
  
  
+ TTTTGCTAAG TATGTGCATA TTCAACCCTT GATGACATAG GAAACGCTTT GTCATCTCCA CCAATCAGAT   
  
  
+ ATCGTTATCT CTTTAATCAT CGCCATAATA GCATGAGTAA GCATGACCAT CTTTTTCAAA CATGTGATAC   
  
  
+ AGTTAAGGCC TGTTCTTTTC GTTAATAAAT CCCAATTTAT CAATTTTAAT ATATACTTTA ACGAATTTTA   
  
  
+ ACAAAAAATT TTAATTTCAA TCCGCTATAA CAAAAAAAAT ACCAACTTCA ACTTATTATA TAAAAAAAAT   
  
  
+ ATACAATTTT TTTTCAATTT TAGCCAACTT CAACAAAAAA AAAAAAAATT TAACAAATCT CAATCAACTT   
  
  
+ CACCCATTTC TCAACTTATA TCTCCTAATG AAAAGAACAG ATCCTTAGCA AGACTTCTCG ATTCATTTAA   
  
  
+ AAGGATAGTA TGGTAAATTC ATTTTATACA AGTGTCAAGT GTGAATATCT TTTTCACATC AGGTGATATA   
  
  
+ TTAGTAGGAC TTTTCGATTC ACTTATCTTC CTCTTTATGT TTTGTTAATG TGATAGTAAC GATAGGATTG   
  
  
+ CATGTATTTT TTCCAACTAT TCATTTACAA CTATCAATAT CTATTGATAA ATATGTTAAA ATAATTATAT   
  
  
+ GTTATTTAAA TTATTATTTG TTACTTAAGG TGTCGACTTT GTCGAAAGCG ACTTAATTCT AATTAAGATA   
  
  
+ GCGGCAGAGA AAGTTACACC TGATGACCAA GTTAGATCTG GTAAAACTAA CCATAGATCT GAAACCCAAA   
  
  
+ CATAATGATG TGTATTCATA CCCGACCTGA CATCTGAAAT AAGTTTACTC AAAAGTGGTT CCGAAACTTG   
  
  
+ AATTGACTCG AACCGAACCG TATTTGACTC GAAATTTTCT ACAAAGTTGC TTATTTTTGC ATGAAGACAT   
  
  
+ ATTGAGCCAA AAATTAGTTG ATTCGAAGTC AACTTAAACA AAAATTCATT CGACTCAAAC TGAAATATCA   
  
  
+ CTTAACTAAA ACTATGACTC AACTCAAACT TGATCTGATC CGTATTCAAA TCAAATACTC CATTTGCTAG   
  
  
+ GTCTAGAACC AGCCACGACC CCACATGGAC TATGCGGTTC AATGAAACGG CCAAATATAT CGCAATTTCT   
  
  
+ GTCTCCATCA ATCACACCCA TTTGCTACAC CCTGTTAATA TTTGACTCGA CCCTGTGAAT TGCCTTCGCC   
  
  
+ AGTTTTGTTT AATCTCTCTG GTTGCTTCCC ATCCAAGTCT GAATAGTATA GTTTGTACAG TGTGTACTAC   
  
  
+ TAGAAAGCAC GTTCCTTGAA TCTTCTTTCA AAGTTGTTTT TAAAGGGTTG AGTGATCCAT TACGACTTTT   
  
  
+ GATCTGGGTT CATCGCCTCC TCTCCTAATC CCTCACCCGG GTACCCTCAA TTCTCTATCG TCTCTGTTGC   
  
  
+ TCAGCTAATT CAAATTCCTT TAGTTGGTCA TCATTGAAAT CTGAAATCAG GGTGTTTTTC TTTTTATTAT   
  
  
+ TATCTGGCAG TTGGTTTGTT TATCTCTTGG TTTTTGGGGC TTATTTGGGT GCTGGTTGAT GTTATCCTGC   
  
  
+ TTGGGTTGAA TCTTCATCAG GTTTATTTTT GATTCATAGT TGAGGGTGAA AATTATTTCT CATACCCACC   
  
  
+ ACCTGTTTGT TCTTTTGTCT CTGAGAAAAA CCAAAAAGCT TTCTCTTTTT CCTGATGCAT CCTGTGCTCG   
  
  
+ TTGATCCTGA GTTAGTGAAC CATGCGTCCA AATTCAAGCC TGATTTGCTC TCAAACTTTG ATAAACAACA   
  
  
+ AGAATTTAGC AACGGGATCG AACAAGATGA CATCTTGCAA AACCCTGATC TGTGTTTTGA TTCAGAAACC   
  
  
+ CCTTCAGTTG ATCTAACTGA GAGTGTCATC AGTTCTGATT CTGTAGAAGT GCCTGATTTC TCAGATGCTT   
  
  
+ GCCTCAAGTT CATAAGTGAG ATTCTCCTAG AAGAGGATTT GGATGAAAGT CCTGCCTCTG CTCAAGAATT   
  
  
+ TAGGGCTCTC CAAGCCACTG AGAAGTCCCT GTATGATGCT CTTGGAGAGG GCTACTCTTT TTCATCTGAT   
  
  
+ AACAGCCCAT CATCATTAGG ACAGAGTATT GAGCACCAAA ATGAGAATTT TGAATTCAGT TCCGGTTATC   
  
  
+ CTGGAATTGA GGGCTATGTT AATGGTGATA TCACGTTCGA GTCCAACTGG ATGTTCAACC TAAGCCAATT   
  
  
+ GGATCCTGTT CTTACTTTGG ATGATATTTC TCAGCCCTTG TCTGCATCAA ACTCCCGATC ATCTGGGTCG   
  
  
+ AGCAATGGCT TTGATGATTC AGGGGATGGG GCAGCCACAT CTCCTGGCAG TACAGTTACA TCAACAGTCC   
  
  
+ CAGAGAAAAG GGTTGAATCT GTCAATCGCT CAAGGAGGAA GAAAAATCGT AAAAGGGATG AAGGTGGTCA   
  
  
+ TGAGGAAGGG AGGAGTAACA AGCAGCAAGC TTCATCCAAT GAAGATTATG TTGAGATGAA GGAGTTTGAT   
  
  
+ GATGTACTGC TCTGCAAAGA AGAGAAAGAT GTTATTGCAA ACTGCACCAA TGATTCCTCA CCCGTCGAAG   
  
  
+ CGAGTGATAA GTTGCAGAAG AAAGGGGGGA AGGGGAAAAC ATCGCGTGGG AAGAAGCAGA ATAGCACAAT   
  
  
+ AGAAGAGGTG GATCTGAGGA CTCTTCTCAC TCACTGTGCT CAAGCAATTT CAAATTTTGA TCTTAGGAGT   
  
  
+ GCAAATGAGC ATCTCAGGCA AATAAGGCAG CATTCTTCAC AATATGGTGA TAGCCTCCAG AGGCTTGCCC   
  
  
+ ATTATTTTGC TAATGGTCTT GAGGCTCGCA TAGCTGGCAC TGGTTCAACA ATCTCTGCTA ATGTTGTTGA   
  
  
+ TGCTCGAATC ACGTCATCTG ATTTCTTAAA GGCTTATAGG CTATATATGT CAGCCGTTCC TTTCAAAAGG   
  
  
+ ATGTTATATT TTCTCGCCAA CAAGACAATA CGGAAGTTGG CTGAGAAGGC AACCAGGATC CACATCATTG   
  
  
+ ATTTTGGAGT CTTGTTGGGT TTACAGTGGC CCTGTCTCAT ACAAAACCTC TCAAAGAGAC CCACTGGCCC   
  
  
+ GCCAAGCCTC CGTATTACTG GGATAGACTA TCCCCAGCAT GGTTTCCGGC CATCAGAAAG GGTTGAAGCG   
  
  
+ ACTGGCCGTC GGTTAGAAGG GTATTGCGAG AGATTTAATG TGCCGTTCAA GTATAAGGCC ATAGCTAAAA   
  
  
+ ATTGGGAGAG TATAAAGCTA GAGGATCTAG AAATTGATGA GAATGAGATG GTGTTTGTCA ATTGCATGCT   
  
  
+ TCGCTCTGGA ACACTGCTTG ATGAGACAGT GGTGGCAGAC AGTCCAAAGG ATGCTTTCTT AAGGTTAATC   
  
  
+ AGAGAGATAA ATCCCCGTCT TTTCATTCAT GGGACTATCA ATGGATCATT CAATGCTCCA TTCTTCATCA   
  
  
+ CTCGGTTCAG GGAGGCGCTC TTCCACTACT CTTCTTTATT TGATATCTTT GAAGCAACTA TGCCCCGTGA   
  
  
+ AGATCATGAA AGGCTCCTGG TTGAGAGTGA GATACAGGGC AAAGAAGCTT TGAATGTCAT AGCATGCGAA   
  
  
+ GGTGCCGAGA GGATTCAAAG GCCTGAAACA TACAAGCAAT GGCAAGCAAG GACAACAAGG GCCGGGTTTA   
  
  
+ GGCAGCTTCC ACTAGACCGG GAGCTTGTGA GCAGAGCAAA GGCTATGGTG AAAGCAAACT ACCATAAGGA   
  
  
+ TTTTGTGGTG GACGAGGACA GGCATTGGAT GCTACACGGT TGGAAAGGAA GAATCTTCTG TGCACTCTCT   
  
  
+ GTTTGGCAAC CCAACTG  

- -Up\_Stream \_Len000TAAACT ACACATACGA CTGATGAAAA CTACTACTAC TAGTGTCACT ACGTATAAAT   
  
  
- CTCGACCATT TAACCAGTAA GCTAAGCTCA TGCCCAGCCC AGTTCAAGAC TAATTCACTG TAAAGCGCAG   
  
  
- CAAAACCAAA TCAAGCCCAG CCTAGCTAAA GCCCAATTTA TTAAAAACCA CCTTATGCGA ACAGTACGGT   
  
  
- TTGTGTTCGT TGAAACAATT TTTAAAGCTA AGCCCAGTCC AGTTAAGTCC AAACCCCAGG TGAAAGCTCA   
  
  
- CTCGTATAAA GCCTACAGCC CAGCGCATAC CCAGGCCCAG TAAGCCAAAC TTAAAGCCTA GAACCTAGTT   
  
  
- AAAACGATTC ATACACGTAT AAGTTGGGAA CTACTGTATC CTTTGCGAAA CAGTAGAGGT GGTTAGTCTA   
  
  
- TAGCAATAGA GAAATTAGTA GCGGTATTAT CGTACTCATT CGTACTGGTA GAAAAAGTTT GTACACTATG   
  
  
- TCAATTCCGG ACAAGAAAAG CAATTATTTA GGGTTAAATA GTTAAAATTA TATATGAAAT TGCTTAAAAT   
  
  
- TGTTTTTTAA AATTAAAGTT AGGCGATATT GTTTTTTTTA TGGTTGAAGT TGAATAATAT ATTTTTTTTA   
  
  
- TATGTTAAAA AAAAGTTAAA ATCGGTTGAA GTTGTTTTTT TTTTTTTTAA ATTGTTTAGA GTTAGTTGAA   
  
  
- GTGGGTAAAG AGTTGAATAT AGAGGATTAC TTTTCTTGTC TAGGAATCGT TCTGAAGAGC TAAGTAAATT   
  
  
- TTCCTATCAT ACCATTTAAG TAAAATATGT TCACAGTTCA CACTTATAGA AAAAGTGTAG TCCACTATAT   
  
  
- AATCATCCTG AAAAGCTAAG TGAATAGAAG GAGAAATACA AAACAATTAC ACTATCATTG CTATCCTAAC   
  
  
- GTACATAAAA AAGGTTGATA AGTAAATGTT GATAGTTATA GATAACTATT TATACAATTT TATTAATATA   
  
  
- CAATAAATTT AATAATAAAC AATGAATTCC ACAGCTGAAA CAGCTTTCGC TGAATTAAGA TTAATTCTAT   
  
  
- CGCCGTCTCT TTCAATGTGG ACTACTGGTT CAATCTAGAC CATTTTGATT GGTATCTAGA CTTTGGGTTT   
  
  
- GTATTACTAC ACATAAGTAT GGGCTGGACT GTAGACTTTA TTCAAATGAG TTTTCACCAA GGCTTTGAAC   
  
  
- TTAACTGAGC TTGGCTTGGC ATAAACTGAG CTTTAAAAGA TGTTTCAACG AATAAAAACG TACTTCTGTA   
  
  
- TAACTCGGTT TTTAATCAAC TAAGCTTCAG TTGAATTTGT TTTTAAGTAA GCTGAGTTTG ACTTTATAGT   
  
  
- GAATTGATTT TGATACTGAG TTGAGTTTGA ACTAGACTAG GCATAAGTTT AGTTTATGAG GTAAACGATC   
  
  
- CAGATCTTGG TCGGTGCTGG GGTGTACCTG ATACGCCAAG TTACTTTGCC GGTTTATATA GCGTTAAAGA   
  
  
- CAGAGGTAGT TAGTGTGGGT AAACGATGTG GGACAATTAT AAACTGAGCT GGGACACTTA ACGGAAGCGG   
  
  
- TCAAAACAAA TTAGAGAGAC CAACGAAGGG TAGGTTCAGA CTTATCATAT CAAACATGTC ACACATGATG   
  
  
- ATCTTTCGTG CAAGGAACTT AGAAGAAAGT TTCAACAAAA ATTTCCCAAC TCACTAGGTA ATGCTGAAAA   
  
  
- CTAGACCCAA GTAGCGGAGG AGAGGATTAG GGAGTGGGCC CATGGGAGTT AAGAGATAGC AGAGACAACG   
  
  
- AGTCGATTAA GTTTAAGGAA ATCAACCAGT AGTAACTTTA GACTTTAGTC CCACAAAAAG AAAAATAATA   
  
  
- ATAGACCGTC AACCAAACAA ATAGAGAACC AAAAACCCCG AATAAACCCA CGACCAACTA CAATAGGACG   
  
  
- AACCCAACTT AGAAGTAGTC CAAATAAAAA CTAAGTATCA ACTCCCACTT TTAATAAAGA GTATGGGTGG   
  
  
- TGGACAAACA AGAAAACAGA GACTCTTTTT GGTTTTTCGA AAGAGAAAAA GGACTACGTA GGACACGAGC   
  
  
- AACTAGGACT CAATCACTTG GTACGCAGGT TTAAGTTCGG ACTAAACGAG AGTTTGAAAC TATTTGTTGT   
  
  
- TCTTAAATCG TTGCCCTAGC TTGTTCTACT GTAGAACGTT TTGGGACTAG ACACAAAACT AAGTCTTTGG   
  
  
- GGAAGTCAAC TAGATTGACT CTCACAGTAG TCAAGACTAA GACATCTTCA CGGACTAAAG AGTCTACGAA   
  
  
- CGGAGTTCAA GTATTCACTC TAAGAGGATC TTCTCCTAAA CCTACTTTCA GGACGGAGAC GAGTTCTTAA   
  
  
- ATCCCGAGAG GTTCGGTGAC TCTTCAGGGA CATACTACGA GAACCTCTCC CGATGAGAAA AAGTAGACTA   
  
  
- TTGTCGGGTA GTAGTAATCC TGTCTCATAA CTCGTGGTTT TACTCTTAAA ACTTAAGTCA AGGCCAATAG   
  
  
- GACCTTAACT CCCGATACAA TTACCACTAT AGTGCAAGCT CAGGTTGACC TACAAGTTGG ATTCGGTTAA   
  
  
- CCTAGGACAA GAATGAAACC TACTATAAAG AGTCGGGAAC AGACGTAGTT TGAGGGCTAG TAGACCCAGC   
  
  
- TCGTTACCGA AACTACTAAG TCCCCTACCC CGTCGGTGTA GAGGACCGTC ATGTCAATGT AGTTGTCAGG   
  
  
- GTCTCTTTTC CCAACTTAGA CAGTTAGCGA GTTCCTCCTT CTTTTTAGCA TTTTCCCTAC TTCCACCAGT   
  
  
- ACTCCTTCCC TCCTCATTGT TCGTCGTTCG AAGTAGGTTA CTTCTAATAC AACTCTACTT CCTCAAACTA   
  
  
- CTACATGACG AGACGTTTCT TCTCTTTCTA CAATAACGTT TGACGTGGTT ACTAAGGAGT GGGCAGCTTC   
  
  
- GCTCACTATT CAACGTCTTC TTTCCCCCCT TCCCCTTTTG TAGCGCACCC TTCTTCGTCT TATCGTGTTA   
  
  
- TCTTCTCCAC CTAGACTCCT GAGAAGAGTG AGTGACACGA GTTCGTTAAA GTTTAAAACT AGAATCCTCA   
  
  
- CGTTTACTCG TAGAGTCCGT TTATTCCGTC GTAAGAAGTG TTATACCACT ATCGGAGGTC TCCGAACGGG   
  
  
- TAATAAAACG ATTACCAGAA CTCCGAGCGT ATCGACCGTG ACCAAGTTGT TAGAGACGAT TACAACAACT   
  
  
- ACGAGCTTAG TGCAGTAGAC TAAAGAATTT CCGAATATCC GATATATACA GTCGGCAAGG AAAGTTTTCC   
  
  
- TACAATATAA AAGAGCGGTT GTTCTGTTAT GCCTTCAACC GACTCTTCCG TTGGTCCTAG GTGTAGTAAC   
  
  
- TAAAACCTCA GAACAACCCA AATGTCACCG GGACAGAGTA TGTTTTGGAG AGTTTCTCTG GGTGACCGGG   
  
  
- CGGTTCGGAG GCATAATGAC CCTATCTGAT AGGGGTCGTA CCAAAGGCCG GTAGTCTTTC CCAACTTCGC   
  
  
- TGACCGGCAG CCAATCTTCC CATAACGCTC TCTAAATTAC ACGGCAAGTT CATATTCCGG TATCGATTTT   
  
  
- TAACCCTCTC ATATTTCGAT CTCCTAGATC TTTAACTACT CTTACTCTAC CACAAACAGT TAACGTACGA   
  
  
- AGCGAGACCT TGTGACGAAC TACTCTGTCA CCACCGTCTG TCAGGTTTCC TACGAAAGAA TTCCAATTAG   
  
  
- TCTCTCTATT TAGGGGCAGA AAAGTAAGTA CCCTGATAGT TACCTAGTAA GTTACGAGGT AAGAAGTAGT   
  
  
- GAGCCAAGTC CCTCCGCGAG AAGGTGATGA GAAGAAATAA ACTATAGAAA CTTCGTTGAT ACGGGGCACT   
  
  
- TCTAGTACTT TCCGAGGACC AACTCTCACT CTATGTCCCG TTTCTTCGAA ACTTACAGTA TCGTACGCTT   
  
  
- CCACGGCTCT CCTAAGTTTC CGGACTTTGT ATGTTCGTTA CCGTTCGTTC CTGTTGTTCC CGGCCCAAAT   
  
  
- CCGTCGAAGG TGATCTGGCC CTCGAACACT CGTCTCGTTT CCGATACCAC TTTCGTTTGA TGGTATTCCT   
  
  
- AAAACACCAC CTGCTCCTGT CCGTAACCTA CGATGTGCCA ACCTTTCCTT CTTAGAAGAC ACGTGAGAGA   
  
  
- CAAACCGTTG GGTTGAC

+     AP-1

| Site Name | Organism | Position | Strand | Matrix score. | sequence | function |
| --- | --- | --- | --- | --- | --- | --- |
| AP-1 | Arabidopsis thaliana | 2042 | + | 8 | TGAGTTAG |  |

>HU07G02246.1   
+ -Up\_Stream \_Len000ATTTGA TGTGTATGCT GACTACTTTT GATGATGATG ATCACAGTGA TGCATATTTA   
  
  
+ GAGCTGGTAA ATTGGTCATT CGATTCGAGT ACGGGTCGGG TCAAGTTCTG ATTAAGTGAC ATTTCGCGTC   
  
  
+ GTTTTGGTTT AGTTCGGGTC GGATCGATTT CGGGTTAAAT AATTTTTGGT GGAATACGCT TGTCATGCCA   
  
  
+ AACACAAGCA ACTTTGTTAA AAATTTCGAT TCGGGTCAGG TCAATTCAGG TTTGGGGTCC ACTTTCGAGT   
  
  
+ GAGCATATTT CGGATGTCGG GTCGCGTATG GGTCCGGGTC ATTCGGTTTG AATTTCGGAT CTTGGATCAA   
  
  
+ TTTTGCTAAG TATGTGCATA TTCAACCCTT GATGACATAG GAAACGCTTT GTCATCTCCA CCAATCAGAT   
  
  
+ ATCGTTATCT CTTTAATCAT CGCCATAATA GCATGAGTAA GCATGACCAT CTTTTTCAAA CATGTGATAC   
  
  
+ AGTTAAGGCC TGTTCTTTTC GTTAATAAAT CCCAATTTAT CAATTTTAAT ATATACTTTA ACGAATTTTA   
  
  
+ ACAAAAAATT TTAATTTCAA TCCGCTATAA CAAAAAAAAT ACCAACTTCA ACTTATTATA TAAAAAAAAT   
  
  
+ ATACAATTTT TTTTCAATTT TAGCCAACTT CAACAAAAAA AAAAAAAATT TAACAAATCT CAATCAACTT   
  
  
+ CACCCATTTC TCAACTTATA TCTCCTAATG AAAAGAACAG ATCCTTAGCA AGACTTCTCG ATTCATTTAA   
  
  
+ AAGGATAGTA TGGTAAATTC ATTTTATACA AGTGTCAAGT GTGAATATCT TTTTCACATC AGGTGATATA   
  
  
+ TTAGTAGGAC TTTTCGATTC ACTTATCTTC CTCTTTATGT TTTGTTAATG TGATAGTAAC GATAGGATTG   
  
  
+ CATGTATTTT TTCCAACTAT TCATTTACAA CTATCAATAT CTATTGATAA ATATGTTAAA ATAATTATAT   
  
  
+ GTTATTTAAA TTATTATTTG TTACTTAAGG TGTCGACTTT GTCGAAAGCG ACTTAATTCT AATTAAGATA   
  
  
+ GCGGCAGAGA AAGTTACACC TGATGACCAA GTTAGATCTG GTAAAACTAA CCATAGATCT GAAACCCAAA   
  
  
+ CATAATGATG TGTATTCATA CCCGACCTGA CATCTGAAAT AAGTTTACTC AAAAGTGGTT CCGAAACTTG   
  
  
+ AATTGACTCG AACCGAACCG TATTTGACTC GAAATTTTCT ACAAAGTTGC TTATTTTTGC ATGAAGACAT   
  
  
+ ATTGAGCCAA AAATTAGTTG ATTCGAAGTC AACTTAAACA AAAATTCATT CGACTCAAAC TGAAATATCA   
  
  
+ CTTAACTAAA ACTATGACTC AACTCAAACT TGATCTGATC CGTATTCAAA TCAAATACTC CATTTGCTAG   
  
  
+ GTCTAGAACC AGCCACGACC CCACATGGAC TATGCGGTTC AATGAAACGG CCAAATATAT CGCAATTTCT   
  
  
+ GTCTCCATCA ATCACACCCA TTTGCTACAC CCTGTTAATA TTTGACTCGA CCCTGTGAAT TGCCTTCGCC   
  
  
+ AGTTTTGTTT AATCTCTCTG GTTGCTTCCC ATCCAAGTCT GAATAGTATA GTTTGTACAG TGTGTACTAC   
  
  
+ TAGAAAGCAC GTTCCTTGAA TCTTCTTTCA AAGTTGTTTT TAAAGGGTTG AGTGATCCAT TACGACTTTT   
  
  
+ GATCTGGGTT CATCGCCTCC TCTCCTAATC CCTCACCCGG GTACCCTCAA TTCTCTATCG TCTCTGTTGC   
  
  
+ TCAGCTAATT CAAATTCCTT TAGTTGGTCA TCATTGAAAT CTGAAATCAG GGTGTTTTTC TTTTTATTAT   
  
  
+ TATCTGGCAG TTGGTTTGTT TATCTCTTGG TTTTTGGGGC TTATTTGGGT GCTGGTTGAT GTTATCCTGC   
  
  
+ TTGGGTTGAA TCTTCATCAG GTTTATTTTT GATTCATAGT TGAGGGTGAA AATTATTTCT CATACCCACC   
  
  
+ ACCTGTTTGT TCTTTTGTCT CTGAGAAAAA CCAAAAAGCT TTCTCTTTTT CCTGATGCAT CCTGTGCTCG   
  
  
+ TTGATCCTGA GTTAGTGAAC CATGCGTCCA AATTCAAGCC TGATTTGCTC TCAAACTTTG ATAAACAACA   
  
  
+ AGAATTTAGC AACGGGATCG AACAAGATGA CATCTTGCAA AACCCTGATC TGTGTTTTGA TTCAGAAACC   
  
  
+ CCTTCAGTTG ATCTAACTGA GAGTGTCATC AGTTCTGATT CTGTAGAAGT GCCTGATTTC TCAGATGCTT   
  
  
+ GCCTCAAGTT CATAAGTGAG ATTCTCCTAG AAGAGGATTT GGATGAAAGT CCTGCCTCTG CTCAAGAATT   
  
  
+ TAGGGCTCTC CAAGCCACTG AGAAGTCCCT GTATGATGCT CTTGGAGAGG GCTACTCTTT TTCATCTGAT   
  
  
+ AACAGCCCAT CATCATTAGG ACAGAGTATT GAGCACCAAA ATGAGAATTT TGAATTCAGT TCCGGTTATC   
  
  
+ CTGGAATTGA GGGCTATGTT AATGGTGATA TCACGTTCGA GTCCAACTGG ATGTTCAACC TAAGCCAATT   
  
  
+ GGATCCTGTT CTTACTTTGG ATGATATTTC TCAGCCCTTG TCTGCATCAA ACTCCCGATC ATCTGGGTCG   
  
  
+ AGCAATGGCT TTGATGATTC AGGGGATGGG GCAGCCACAT CTCCTGGCAG TACAGTTACA TCAACAGTCC   
  
  
+ CAGAGAAAAG GGTTGAATCT GTCAATCGCT CAAGGAGGAA GAAAAATCGT AAAAGGGATG AAGGTGGTCA   
  
  
+ TGAGGAAGGG AGGAGTAACA AGCAGCAAGC TTCATCCAAT GAAGATTATG TTGAGATGAA GGAGTTTGAT   
  
  
+ GATGTACTGC TCTGCAAAGA AGAGAAAGAT GTTATTGCAA ACTGCACCAA TGATTCCTCA CCCGTCGAAG   
  
  
+ CGAGTGATAA GTTGCAGAAG AAAGGGGGGA AGGGGAAAAC ATCGCGTGGG AAGAAGCAGA ATAGCACAAT   
  
  
+ AGAAGAGGTG GATCTGAGGA CTCTTCTCAC TCACTGTGCT CAAGCAATTT CAAATTTTGA TCTTAGGAGT   
  
  
+ GCAAATGAGC ATCTCAGGCA AATAAGGCAG CATTCTTCAC AATATGGTGA TAGCCTCCAG AGGCTTGCCC   
  
  
+ ATTATTTTGC TAATGGTCTT GAGGCTCGCA TAGCTGGCAC TGGTTCAACA ATCTCTGCTA ATGTTGTTGA   
  
  
+ TGCTCGAATC ACGTCATCTG ATTTCTTAAA GGCTTATAGG CTATATATGT CAGCCGTTCC TTTCAAAAGG   
  
  
+ ATGTTATATT TTCTCGCCAA CAAGACAATA CGGAAGTTGG CTGAGAAGGC AACCAGGATC CACATCATTG   
  
  
+ ATTTTGGAGT CTTGTTGGGT TTACAGTGGC CCTGTCTCAT ACAAAACCTC TCAAAGAGAC CCACTGGCCC   
  
  
+ GCCAAGCCTC CGTATTACTG GGATAGACTA TCCCCAGCAT GGTTTCCGGC CATCAGAAAG GGTTGAAGCG   
  
  
+ ACTGGCCGTC GGTTAGAAGG GTATTGCGAG AGATTTAATG TGCCGTTCAA GTATAAGGCC ATAGCTAAAA   
  
  
+ ATTGGGAGAG TATAAAGCTA GAGGATCTAG AAATTGATGA GAATGAGATG GTGTTTGTCA ATTGCATGCT   
  
  
+ TCGCTCTGGA ACACTGCTTG ATGAGACAGT GGTGGCAGAC AGTCCAAAGG ATGCTTTCTT AAGGTTAATC   
  
  
+ AGAGAGATAA ATCCCCGTCT TTTCATTCAT GGGACTATCA ATGGATCATT CAATGCTCCA TTCTTCATCA   
  
  
+ CTCGGTTCAG GGAGGCGCTC TTCCACTACT CTTCTTTATT TGATATCTTT GAAGCAACTA TGCCCCGTGA   
  
  
+ AGATCATGAA AGGCTCCTGG TTGAGAGTGA GATACAGGGC AAAGAAGCTT TGAATGTCAT AGCATGCGAA   
  
  
+ GGTGCCGAGA GGATTCAAAG GCCTGAAACA TACAAGCAAT GGCAAGCAAG GACAACAAGG GCCGGGTTTA   
  
  
+ GGCAGCTTCC ACTAGACCGG GAGCTTGTGA GCAGAGCAAA GGCTATGGTG AAAGCAAACT ACCATAAGGA   
  
  
+ TTTTGTGGTG GACGAGGACA GGCATTGGAT GCTACACGGT TGGAAAGGAA GAATCTTCTG TGCACTCTCT   
  
  
+ GTTTGGCAAC CCAACTG  

- -Up\_Stream \_Len000TAAACT ACACATACGA CTGATGAAAA CTACTACTAC TAGTGTCACT ACGTATAAAT   
  
  
- CTCGACCATT TAACCAGTAA GCTAAGCTCA TGCCCAGCCC AGTTCAAGAC TAATTCACTG TAAAGCGCAG   
  
  
- CAAAACCAAA TCAAGCCCAG CCTAGCTAAA GCCCAATTTA TTAAAAACCA CCTTATGCGA ACAGTACGGT   
  
  
- TTGTGTTCGT TGAAACAATT TTTAAAGCTA AGCCCAGTCC AGTTAAGTCC AAACCCCAGG TGAAAGCTCA   
  
  
- CTCGTATAAA GCCTACAGCC CAGCGCATAC CCAGGCCCAG TAAGCCAAAC TTAAAGCCTA GAACCTAGTT   
  
  
- AAAACGATTC ATACACGTAT AAGTTGGGAA CTACTGTATC CTTTGCGAAA CAGTAGAGGT GGTTAGTCTA   
  
  
- TAGCAATAGA GAAATTAGTA GCGGTATTAT CGTACTCATT CGTACTGGTA GAAAAAGTTT GTACACTATG   
  
  
- TCAATTCCGG ACAAGAAAAG CAATTATTTA GGGTTAAATA GTTAAAATTA TATATGAAAT TGCTTAAAAT   
  
  
- TGTTTTTTAA AATTAAAGTT AGGCGATATT GTTTTTTTTA TGGTTGAAGT TGAATAATAT ATTTTTTTTA   
  
  
- TATGTTAAAA AAAAGTTAAA ATCGGTTGAA GTTGTTTTTT TTTTTTTTAA ATTGTTTAGA GTTAGTTGAA   
  
  
- GTGGGTAAAG AGTTGAATAT AGAGGATTAC TTTTCTTGTC TAGGAATCGT TCTGAAGAGC TAAGTAAATT   
  
  
- TTCCTATCAT ACCATTTAAG TAAAATATGT TCACAGTTCA CACTTATAGA AAAAGTGTAG TCCACTATAT   
  
  
- AATCATCCTG AAAAGCTAAG TGAATAGAAG GAGAAATACA AAACAATTAC ACTATCATTG CTATCCTAAC   
  
  
- GTACATAAAA AAGGTTGATA AGTAAATGTT GATAGTTATA GATAACTATT TATACAATTT TATTAATATA   
  
  
- CAATAAATTT AATAATAAAC AATGAATTCC ACAGCTGAAA CAGCTTTCGC TGAATTAAGA TTAATTCTAT   
  
  
- CGCCGTCTCT TTCAATGTGG ACTACTGGTT CAATCTAGAC CATTTTGATT GGTATCTAGA CTTTGGGTTT   
  
  
- GTATTACTAC ACATAAGTAT GGGCTGGACT GTAGACTTTA TTCAAATGAG TTTTCACCAA GGCTTTGAAC   
  
  
- TTAACTGAGC TTGGCTTGGC ATAAACTGAG CTTTAAAAGA TGTTTCAACG AATAAAAACG TACTTCTGTA   
  
  
- TAACTCGGTT TTTAATCAAC TAAGCTTCAG TTGAATTTGT TTTTAAGTAA GCTGAGTTTG ACTTTATAGT   
  
  
- GAATTGATTT TGATACTGAG TTGAGTTTGA ACTAGACTAG GCATAAGTTT AGTTTATGAG GTAAACGATC   
  
  
- CAGATCTTGG TCGGTGCTGG GGTGTACCTG ATACGCCAAG TTACTTTGCC GGTTTATATA GCGTTAAAGA   
  
  
- CAGAGGTAGT TAGTGTGGGT AAACGATGTG GGACAATTAT AAACTGAGCT GGGACACTTA ACGGAAGCGG   
  
  
- TCAAAACAAA TTAGAGAGAC CAACGAAGGG TAGGTTCAGA CTTATCATAT CAAACATGTC ACACATGATG   
  
  
- ATCTTTCGTG CAAGGAACTT AGAAGAAAGT TTCAACAAAA ATTTCCCAAC TCACTAGGTA ATGCTGAAAA   
  
  
- CTAGACCCAA GTAGCGGAGG AGAGGATTAG GGAGTGGGCC CATGGGAGTT AAGAGATAGC AGAGACAACG   
  
  
- AGTCGATTAA GTTTAAGGAA ATCAACCAGT AGTAACTTTA GACTTTAGTC CCACAAAAAG AAAAATAATA   
  
  
- ATAGACCGTC AACCAAACAA ATAGAGAACC AAAAACCCCG AATAAACCCA CGACCAACTA CAATAGGACG   
  
  
- AACCCAACTT AGAAGTAGTC CAAATAAAAA CTAAGTATCA ACTCCCACTT TTAATAAAGA GTATGGGTGG   
  
  
- TGGACAAACA AGAAAACAGA GACTCTTTTT GGTTTTTCGA AAGAGAAAAA GGACTACGTA GGACACGAGC   
  
  
- AACTAGGACT CAATCACTTG GTACGCAGGT TTAAGTTCGG ACTAAACGAG AGTTTGAAAC TATTTGTTGT   
  
  
- TCTTAAATCG TTGCCCTAGC TTGTTCTACT GTAGAACGTT TTGGGACTAG ACACAAAACT AAGTCTTTGG   
  
  
- GGAAGTCAAC TAGATTGACT CTCACAGTAG TCAAGACTAA GACATCTTCA CGGACTAAAG AGTCTACGAA   
  
  
- CGGAGTTCAA GTATTCACTC TAAGAGGATC TTCTCCTAAA CCTACTTTCA GGACGGAGAC GAGTTCTTAA   
  
  
- ATCCCGAGAG GTTCGGTGAC TCTTCAGGGA CATACTACGA GAACCTCTCC CGATGAGAAA AAGTAGACTA   
  
  
- TTGTCGGGTA GTAGTAATCC TGTCTCATAA CTCGTGGTTT TACTCTTAAA ACTTAAGTCA AGGCCAATAG   
  
  
- GACCTTAACT CCCGATACAA TTACCACTAT AGTGCAAGCT CAGGTTGACC TACAAGTTGG ATTCGGTTAA   
  
  
- CCTAGGACAA GAATGAAACC TACTATAAAG AGTCGGGAAC AGACGTAGTT TGAGGGCTAG TAGACCCAGC   
  
  
- TCGTTACCGA AACTACTAAG TCCCCTACCC CGTCGGTGTA GAGGACCGTC ATGTCAATGT AGTTGTCAGG   
  
  
- GTCTCTTTTC CCAACTTAGA CAGTTAGCGA GTTCCTCCTT CTTTTTAGCA TTTTCCCTAC TTCCACCAGT   
  
  
- ACTCCTTCCC TCCTCATTGT TCGTCGTTCG AAGTAGGTTA CTTCTAATAC AACTCTACTT CCTCAAACTA   
  
  
- CTACATGACG AGACGTTTCT TCTCTTTCTA CAATAACGTT TGACGTGGTT ACTAAGGAGT GGGCAGCTTC   
  
  
- GCTCACTATT CAACGTCTTC TTTCCCCCCT TCCCCTTTTG TAGCGCACCC TTCTTCGTCT TATCGTGTTA   
  
  
- TCTTCTCCAC CTAGACTCCT GAGAAGAGTG AGTGACACGA GTTCGTTAAA GTTTAAAACT AGAATCCTCA   
  
  
- CGTTTACTCG TAGAGTCCGT TTATTCCGTC GTAAGAAGTG TTATACCACT ATCGGAGGTC TCCGAACGGG   
  
  
- TAATAAAACG ATTACCAGAA CTCCGAGCGT ATCGACCGTG ACCAAGTTGT TAGAGACGAT TACAACAACT   
  
  
- ACGAGCTTAG TGCAGTAGAC TAAAGAATTT CCGAATATCC GATATATACA GTCGGCAAGG AAAGTTTTCC   
  
  
- TACAATATAA AAGAGCGGTT GTTCTGTTAT GCCTTCAACC GACTCTTCCG TTGGTCCTAG GTGTAGTAAC   
  
  
- TAAAACCTCA GAACAACCCA AATGTCACCG GGACAGAGTA TGTTTTGGAG AGTTTCTCTG GGTGACCGGG   
  
  
- CGGTTCGGAG GCATAATGAC CCTATCTGAT AGGGGTCGTA CCAAAGGCCG GTAGTCTTTC CCAACTTCGC   
  
  
- TGACCGGCAG CCAATCTTCC CATAACGCTC TCTAAATTAC ACGGCAAGTT CATATTCCGG TATCGATTTT   
  
  
- TAACCCTCTC ATATTTCGAT CTCCTAGATC TTTAACTACT CTTACTCTAC CACAAACAGT TAACGTACGA   
  
  
- AGCGAGACCT TGTGACGAAC TACTCTGTCA CCACCGTCTG TCAGGTTTCC TACGAAAGAA TTCCAATTAG   
  
  
- TCTCTCTATT TAGGGGCAGA AAAGTAAGTA CCCTGATAGT TACCTAGTAA GTTACGAGGT AAGAAGTAGT   
  
  
- GAGCCAAGTC CCTCCGCGAG AAGGTGATGA GAAGAAATAA ACTATAGAAA CTTCGTTGAT ACGGGGCACT   
  
  
- TCTAGTACTT TCCGAGGACC AACTCTCACT CTATGTCCCG TTTCTTCGAA ACTTACAGTA TCGTACGCTT   
  
  
- CCACGGCTCT CCTAAGTTTC CGGACTTTGT ATGTTCGTTA CCGTTCGTTC CTGTTGTTCC CGGCCCAAAT   
  
  
- CCGTCGAAGG TGATCTGGCC CTCGAACACT CGTCTCGTTT CCGATACCAC TTTCGTTTGA TGGTATTCCT   
  
  
- AAAACACCAC CTGCTCCTGT CCGTAACCTA CGATGTGCCA ACCTTTCCTT CTTAGAAGAC ACGTGAGAGA   
  
  
- CAAACCGTTG GGTTGAC

+     ARE

| Site Name | Organism | Position | Strand | Matrix score. | sequence | function |
| --- | --- | --- | --- | --- | --- | --- |
| ARE | Zea mays | 3404 | - | 6 | AAACCA | cis-acting regulatory element essential for the anaerobic induction |
| ARE | Zea mays | 1992 | + | 6 | AAACCA | cis-acting regulatory element essential for the anaerobic induction |
| ARE | Zea mays | 149 | - | 6 | AAACCA | cis-acting regulatory element essential for the anaerobic induction |
| ARE | Zea mays | 1852 | - | 6 | AAACCA | cis-acting regulatory element essential for the anaerobic induction |
| ARE | Zea mays | 1836 | - | 6 | AAACCA | cis-acting regulatory element essential for the anaerobic induction |

>HU07G02246.1   
+ -Up\_Stream \_Len000ATTTGA TGTGTATGCT GACTACTTTT GATGATGATG ATCACAGTGA TGCATATTTA   
  
  
+ GAGCTGGTAA ATTGGTCATT CGATTCGAGT ACGGGTCGGG TCAAGTTCTG ATTAAGTGAC ATTTCGCGTC   
  
  
+ GTTTTGGTTT AGTTCGGGTC GGATCGATTT CGGGTTAAAT AATTTTTGGT GGAATACGCT TGTCATGCCA   
  
  
+ AACACAAGCA ACTTTGTTAA AAATTTCGAT TCGGGTCAGG TCAATTCAGG TTTGGGGTCC ACTTTCGAGT   
  
  
+ GAGCATATTT CGGATGTCGG GTCGCGTATG GGTCCGGGTC ATTCGGTTTG AATTTCGGAT CTTGGATCAA   
  
  
+ TTTTGCTAAG TATGTGCATA TTCAACCCTT GATGACATAG GAAACGCTTT GTCATCTCCA CCAATCAGAT   
  
  
+ ATCGTTATCT CTTTAATCAT CGCCATAATA GCATGAGTAA GCATGACCAT CTTTTTCAAA CATGTGATAC   
  
  
+ AGTTAAGGCC TGTTCTTTTC GTTAATAAAT CCCAATTTAT CAATTTTAAT ATATACTTTA ACGAATTTTA   
  
  
+ ACAAAAAATT TTAATTTCAA TCCGCTATAA CAAAAAAAAT ACCAACTTCA ACTTATTATA TAAAAAAAAT   
  
  
+ ATACAATTTT TTTTCAATTT TAGCCAACTT CAACAAAAAA AAAAAAAATT TAACAAATCT CAATCAACTT   
  
  
+ CACCCATTTC TCAACTTATA TCTCCTAATG AAAAGAACAG ATCCTTAGCA AGACTTCTCG ATTCATTTAA   
  
  
+ AAGGATAGTA TGGTAAATTC ATTTTATACA AGTGTCAAGT GTGAATATCT TTTTCACATC AGGTGATATA   
  
  
+ TTAGTAGGAC TTTTCGATTC ACTTATCTTC CTCTTTATGT TTTGTTAATG TGATAGTAAC GATAGGATTG   
  
  
+ CATGTATTTT TTCCAACTAT TCATTTACAA CTATCAATAT CTATTGATAA ATATGTTAAA ATAATTATAT   
  
  
+ GTTATTTAAA TTATTATTTG TTACTTAAGG TGTCGACTTT GTCGAAAGCG ACTTAATTCT AATTAAGATA   
  
  
+ GCGGCAGAGA AAGTTACACC TGATGACCAA GTTAGATCTG GTAAAACTAA CCATAGATCT GAAACCCAAA   
  
  
+ CATAATGATG TGTATTCATA CCCGACCTGA CATCTGAAAT AAGTTTACTC AAAAGTGGTT CCGAAACTTG   
  
  
+ AATTGACTCG AACCGAACCG TATTTGACTC GAAATTTTCT ACAAAGTTGC TTATTTTTGC ATGAAGACAT   
  
  
+ ATTGAGCCAA AAATTAGTTG ATTCGAAGTC AACTTAAACA AAAATTCATT CGACTCAAAC TGAAATATCA   
  
  
+ CTTAACTAAA ACTATGACTC AACTCAAACT TGATCTGATC CGTATTCAAA TCAAATACTC CATTTGCTAG   
  
  
+ GTCTAGAACC AGCCACGACC CCACATGGAC TATGCGGTTC AATGAAACGG CCAAATATAT CGCAATTTCT   
  
  
+ GTCTCCATCA ATCACACCCA TTTGCTACAC CCTGTTAATA TTTGACTCGA CCCTGTGAAT TGCCTTCGCC   
  
  
+ AGTTTTGTTT AATCTCTCTG GTTGCTTCCC ATCCAAGTCT GAATAGTATA GTTTGTACAG TGTGTACTAC   
  
  
+ TAGAAAGCAC GTTCCTTGAA TCTTCTTTCA AAGTTGTTTT TAAAGGGTTG AGTGATCCAT TACGACTTTT   
  
  
+ GATCTGGGTT CATCGCCTCC TCTCCTAATC CCTCACCCGG GTACCCTCAA TTCTCTATCG TCTCTGTTGC   
  
  
+ TCAGCTAATT CAAATTCCTT TAGTTGGTCA TCATTGAAAT CTGAAATCAG GGTGTTTTTC TTTTTATTAT   
  
  
+ TATCTGGCAG TTGGTTTGTT TATCTCTTGG TTTTTGGGGC TTATTTGGGT GCTGGTTGAT GTTATCCTGC   
  
  
+ TTGGGTTGAA TCTTCATCAG GTTTATTTTT GATTCATAGT TGAGGGTGAA AATTATTTCT CATACCCACC   
  
  
+ ACCTGTTTGT TCTTTTGTCT CTGAGAAAAA CCAAAAAGCT TTCTCTTTTT CCTGATGCAT CCTGTGCTCG   
  
  
+ TTGATCCTGA GTTAGTGAAC CATGCGTCCA AATTCAAGCC TGATTTGCTC TCAAACTTTG ATAAACAACA   
  
  
+ AGAATTTAGC AACGGGATCG AACAAGATGA CATCTTGCAA AACCCTGATC TGTGTTTTGA TTCAGAAACC   
  
  
+ CCTTCAGTTG ATCTAACTGA GAGTGTCATC AGTTCTGATT CTGTAGAAGT GCCTGATTTC TCAGATGCTT   
  
  
+ GCCTCAAGTT CATAAGTGAG ATTCTCCTAG AAGAGGATTT GGATGAAAGT CCTGCCTCTG CTCAAGAATT   
  
  
+ TAGGGCTCTC CAAGCCACTG AGAAGTCCCT GTATGATGCT CTTGGAGAGG GCTACTCTTT TTCATCTGAT   
  
  
+ AACAGCCCAT CATCATTAGG ACAGAGTATT GAGCACCAAA ATGAGAATTT TGAATTCAGT TCCGGTTATC   
  
  
+ CTGGAATTGA GGGCTATGTT AATGGTGATA TCACGTTCGA GTCCAACTGG ATGTTCAACC TAAGCCAATT   
  
  
+ GGATCCTGTT CTTACTTTGG ATGATATTTC TCAGCCCTTG TCTGCATCAA ACTCCCGATC ATCTGGGTCG   
  
  
+ AGCAATGGCT TTGATGATTC AGGGGATGGG GCAGCCACAT CTCCTGGCAG TACAGTTACA TCAACAGTCC   
  
  
+ CAGAGAAAAG GGTTGAATCT GTCAATCGCT CAAGGAGGAA GAAAAATCGT AAAAGGGATG AAGGTGGTCA   
  
  
+ TGAGGAAGGG AGGAGTAACA AGCAGCAAGC TTCATCCAAT GAAGATTATG TTGAGATGAA GGAGTTTGAT   
  
  
+ GATGTACTGC TCTGCAAAGA AGAGAAAGAT GTTATTGCAA ACTGCACCAA TGATTCCTCA CCCGTCGAAG   
  
  
+ CGAGTGATAA GTTGCAGAAG AAAGGGGGGA AGGGGAAAAC ATCGCGTGGG AAGAAGCAGA ATAGCACAAT   
  
  
+ AGAAGAGGTG GATCTGAGGA CTCTTCTCAC TCACTGTGCT CAAGCAATTT CAAATTTTGA TCTTAGGAGT   
  
  
+ GCAAATGAGC ATCTCAGGCA AATAAGGCAG CATTCTTCAC AATATGGTGA TAGCCTCCAG AGGCTTGCCC   
  
  
+ ATTATTTTGC TAATGGTCTT GAGGCTCGCA TAGCTGGCAC TGGTTCAACA ATCTCTGCTA ATGTTGTTGA   
  
  
+ TGCTCGAATC ACGTCATCTG ATTTCTTAAA GGCTTATAGG CTATATATGT CAGCCGTTCC TTTCAAAAGG   
  
  
+ ATGTTATATT TTCTCGCCAA CAAGACAATA CGGAAGTTGG CTGAGAAGGC AACCAGGATC CACATCATTG   
  
  
+ ATTTTGGAGT CTTGTTGGGT TTACAGTGGC CCTGTCTCAT ACAAAACCTC TCAAAGAGAC CCACTGGCCC   
  
  
+ GCCAAGCCTC CGTATTACTG GGATAGACTA TCCCCAGCAT GGTTTCCGGC CATCAGAAAG GGTTGAAGCG   
  
  
+ ACTGGCCGTC GGTTAGAAGG GTATTGCGAG AGATTTAATG TGCCGTTCAA GTATAAGGCC ATAGCTAAAA   
  
  
+ ATTGGGAGAG TATAAAGCTA GAGGATCTAG AAATTGATGA GAATGAGATG GTGTTTGTCA ATTGCATGCT   
  
  
+ TCGCTCTGGA ACACTGCTTG ATGAGACAGT GGTGGCAGAC AGTCCAAAGG ATGCTTTCTT AAGGTTAATC   
  
  
+ AGAGAGATAA ATCCCCGTCT TTTCATTCAT GGGACTATCA ATGGATCATT CAATGCTCCA TTCTTCATCA   
  
  
+ CTCGGTTCAG GGAGGCGCTC TTCCACTACT CTTCTTTATT TGATATCTTT GAAGCAACTA TGCCCCGTGA   
  
  
+ AGATCATGAA AGGCTCCTGG TTGAGAGTGA GATACAGGGC AAAGAAGCTT TGAATGTCAT AGCATGCGAA   
  
  
+ GGTGCCGAGA GGATTCAAAG GCCTGAAACA TACAAGCAAT GGCAAGCAAG GACAACAAGG GCCGGGTTTA   
  
  
+ GGCAGCTTCC ACTAGACCGG GAGCTTGTGA GCAGAGCAAA GGCTATGGTG AAAGCAAACT ACCATAAGGA   
  
  
+ TTTTGTGGTG GACGAGGACA GGCATTGGAT GCTACACGGT TGGAAAGGAA GAATCTTCTG TGCACTCTCT   
  
  
+ GTTTGGCAAC CCAACTG  

- -Up\_Stream \_Len000TAAACT ACACATACGA CTGATGAAAA CTACTACTAC TAGTGTCACT ACGTATAAAT   
  
  
- CTCGACCATT TAACCAGTAA GCTAAGCTCA TGCCCAGCCC AGTTCAAGAC TAATTCACTG TAAAGCGCAG   
  
  
- CAAAACCAAA TCAAGCCCAG CCTAGCTAAA GCCCAATTTA TTAAAAACCA CCTTATGCGA ACAGTACGGT   
  
  
- TTGTGTTCGT TGAAACAATT TTTAAAGCTA AGCCCAGTCC AGTTAAGTCC AAACCCCAGG TGAAAGCTCA   
  
  
- CTCGTATAAA GCCTACAGCC CAGCGCATAC CCAGGCCCAG TAAGCCAAAC TTAAAGCCTA GAACCTAGTT   
  
  
- AAAACGATTC ATACACGTAT AAGTTGGGAA CTACTGTATC CTTTGCGAAA CAGTAGAGGT GGTTAGTCTA   
  
  
- TAGCAATAGA GAAATTAGTA GCGGTATTAT CGTACTCATT CGTACTGGTA GAAAAAGTTT GTACACTATG   
  
  
- TCAATTCCGG ACAAGAAAAG CAATTATTTA GGGTTAAATA GTTAAAATTA TATATGAAAT TGCTTAAAAT   
  
  
- TGTTTTTTAA AATTAAAGTT AGGCGATATT GTTTTTTTTA TGGTTGAAGT TGAATAATAT ATTTTTTTTA   
  
  
- TATGTTAAAA AAAAGTTAAA ATCGGTTGAA GTTGTTTTTT TTTTTTTTAA ATTGTTTAGA GTTAGTTGAA   
  
  
- GTGGGTAAAG AGTTGAATAT AGAGGATTAC TTTTCTTGTC TAGGAATCGT TCTGAAGAGC TAAGTAAATT   
  
  
- TTCCTATCAT ACCATTTAAG TAAAATATGT TCACAGTTCA CACTTATAGA AAAAGTGTAG TCCACTATAT   
  
  
- AATCATCCTG AAAAGCTAAG TGAATAGAAG GAGAAATACA AAACAATTAC ACTATCATTG CTATCCTAAC   
  
  
- GTACATAAAA AAGGTTGATA AGTAAATGTT GATAGTTATA GATAACTATT TATACAATTT TATTAATATA   
  
  
- CAATAAATTT AATAATAAAC AATGAATTCC ACAGCTGAAA CAGCTTTCGC TGAATTAAGA TTAATTCTAT   
  
  
- CGCCGTCTCT TTCAATGTGG ACTACTGGTT CAATCTAGAC CATTTTGATT GGTATCTAGA CTTTGGGTTT   
  
  
- GTATTACTAC ACATAAGTAT GGGCTGGACT GTAGACTTTA TTCAAATGAG TTTTCACCAA GGCTTTGAAC   
  
  
- TTAACTGAGC TTGGCTTGGC ATAAACTGAG CTTTAAAAGA TGTTTCAACG AATAAAAACG TACTTCTGTA   
  
  
- TAACTCGGTT TTTAATCAAC TAAGCTTCAG TTGAATTTGT TTTTAAGTAA GCTGAGTTTG ACTTTATAGT   
  
  
- GAATTGATTT TGATACTGAG TTGAGTTTGA ACTAGACTAG GCATAAGTTT AGTTTATGAG GTAAACGATC   
  
  
- CAGATCTTGG TCGGTGCTGG GGTGTACCTG ATACGCCAAG TTACTTTGCC GGTTTATATA GCGTTAAAGA   
  
  
- CAGAGGTAGT TAGTGTGGGT AAACGATGTG GGACAATTAT AAACTGAGCT GGGACACTTA ACGGAAGCGG   
  
  
- TCAAAACAAA TTAGAGAGAC CAACGAAGGG TAGGTTCAGA CTTATCATAT CAAACATGTC ACACATGATG   
  
  
- ATCTTTCGTG CAAGGAACTT AGAAGAAAGT TTCAACAAAA ATTTCCCAAC TCACTAGGTA ATGCTGAAAA   
  
  
- CTAGACCCAA GTAGCGGAGG AGAGGATTAG GGAGTGGGCC CATGGGAGTT AAGAGATAGC AGAGACAACG   
  
  
- AGTCGATTAA GTTTAAGGAA ATCAACCAGT AGTAACTTTA GACTTTAGTC CCACAAAAAG AAAAATAATA   
  
  
- ATAGACCGTC AACCAAACAA ATAGAGAACC AAAAACCCCG AATAAACCCA CGACCAACTA CAATAGGACG   
  
  
- AACCCAACTT AGAAGTAGTC CAAATAAAAA CTAAGTATCA ACTCCCACTT TTAATAAAGA GTATGGGTGG   
  
  
- TGGACAAACA AGAAAACAGA GACTCTTTTT GGTTTTTCGA AAGAGAAAAA GGACTACGTA GGACACGAGC   
  
  
- AACTAGGACT CAATCACTTG GTACGCAGGT TTAAGTTCGG ACTAAACGAG AGTTTGAAAC TATTTGTTGT   
  
  
- TCTTAAATCG TTGCCCTAGC TTGTTCTACT GTAGAACGTT TTGGGACTAG ACACAAAACT AAGTCTTTGG   
  
  
- GGAAGTCAAC TAGATTGACT CTCACAGTAG TCAAGACTAA GACATCTTCA CGGACTAAAG AGTCTACGAA   
  
  
- CGGAGTTCAA GTATTCACTC TAAGAGGATC TTCTCCTAAA CCTACTTTCA GGACGGAGAC GAGTTCTTAA   
  
  
- ATCCCGAGAG GTTCGGTGAC TCTTCAGGGA CATACTACGA GAACCTCTCC CGATGAGAAA AAGTAGACTA   
  
  
- TTGTCGGGTA GTAGTAATCC TGTCTCATAA CTCGTGGTTT TACTCTTAAA ACTTAAGTCA AGGCCAATAG   
  
  
- GACCTTAACT CCCGATACAA TTACCACTAT AGTGCAAGCT CAGGTTGACC TACAAGTTGG ATTCGGTTAA   
  
  
- CCTAGGACAA GAATGAAACC TACTATAAAG AGTCGGGAAC AGACGTAGTT TGAGGGCTAG TAGACCCAGC   
  
  
- TCGTTACCGA AACTACTAAG TCCCCTACCC CGTCGGTGTA GAGGACCGTC ATGTCAATGT AGTTGTCAGG   
  
  
- GTCTCTTTTC CCAACTTAGA CAGTTAGCGA GTTCCTCCTT CTTTTTAGCA TTTTCCCTAC TTCCACCAGT   
  
  
- ACTCCTTCCC TCCTCATTGT TCGTCGTTCG AAGTAGGTTA CTTCTAATAC AACTCTACTT CCTCAAACTA   
  
  
- CTACATGACG AGACGTTTCT TCTCTTTCTA CAATAACGTT TGACGTGGTT ACTAAGGAGT GGGCAGCTTC   
  
  
- GCTCACTATT CAACGTCTTC TTTCCCCCCT TCCCCTTTTG TAGCGCACCC TTCTTCGTCT TATCGTGTTA   
  
  
- TCTTCTCCAC CTAGACTCCT GAGAAGAGTG AGTGACACGA GTTCGTTAAA GTTTAAAACT AGAATCCTCA   
  
  
- CGTTTACTCG TAGAGTCCGT TTATTCCGTC GTAAGAAGTG TTATACCACT ATCGGAGGTC TCCGAACGGG   
  
  
- TAATAAAACG ATTACCAGAA CTCCGAGCGT ATCGACCGTG ACCAAGTTGT TAGAGACGAT TACAACAACT   
  
  
- ACGAGCTTAG TGCAGTAGAC TAAAGAATTT CCGAATATCC GATATATACA GTCGGCAAGG AAAGTTTTCC   
  
  
- TACAATATAA AAGAGCGGTT GTTCTGTTAT GCCTTCAACC GACTCTTCCG TTGGTCCTAG GTGTAGTAAC   
  
  
- TAAAACCTCA GAACAACCCA AATGTCACCG GGACAGAGTA TGTTTTGGAG AGTTTCTCTG GGTGACCGGG   
  
  
- CGGTTCGGAG GCATAATGAC CCTATCTGAT AGGGGTCGTA CCAAAGGCCG GTAGTCTTTC CCAACTTCGC   
  
  
- TGACCGGCAG CCAATCTTCC CATAACGCTC TCTAAATTAC ACGGCAAGTT CATATTCCGG TATCGATTTT   
  
  
- TAACCCTCTC ATATTTCGAT CTCCTAGATC TTTAACTACT CTTACTCTAC CACAAACAGT TAACGTACGA   
  
  
- AGCGAGACCT TGTGACGAAC TACTCTGTCA CCACCGTCTG TCAGGTTTCC TACGAAAGAA TTCCAATTAG   
  
  
- TCTCTCTATT TAGGGGCAGA AAAGTAAGTA CCCTGATAGT TACCTAGTAA GTTACGAGGT AAGAAGTAGT   
  
  
- GAGCCAAGTC CCTCCGCGAG AAGGTGATGA GAAGAAATAA ACTATAGAAA CTTCGTTGAT ACGGGGCACT   
  
  
- TCTAGTACTT TCCGAGGACC AACTCTCACT CTATGTCCCG TTTCTTCGAA ACTTACAGTA TCGTACGCTT   
  
  
- CCACGGCTCT CCTAAGTTTC CGGACTTTGT ATGTTCGTTA CCGTTCGTTC CTGTTGTTCC CGGCCCAAAT   
  
  
- CCGTCGAAGG TGATCTGGCC CTCGAACACT CGTCTCGTTT CCGATACCAC TTTCGTTTGA TGGTATTCCT   
  
  
- AAAACACCAC CTGCTCCTGT CCGTAACCTA CGATGTGCCA ACCTTTCCTT CTTAGAAGAC ACGTGAGAGA   
  
  
- CAAACCGTTG GGTTGAC

+     AT~TATA-box

| Site Name | Organism | Position | Strand | Matrix score. | sequence | function |
| --- | --- | --- | --- | --- | --- | --- |
| AT~TATA-box | Arabidopsis thaliana | 3196 | - | 6 | TATATA |  |
| AT~TATA-box | Arabidopsis thaliana | 621 | + | 6 | TATATA |  |
| AT~TATA-box | Arabidopsis thaliana | 544 | + | 6 | TATATA |  |

>HU07G02246.1   
+ -Up\_Stream \_Len000ATTTGA TGTGTATGCT GACTACTTTT GATGATGATG ATCACAGTGA TGCATATTTA   
  
  
+ GAGCTGGTAA ATTGGTCATT CGATTCGAGT ACGGGTCGGG TCAAGTTCTG ATTAAGTGAC ATTTCGCGTC   
  
  
+ GTTTTGGTTT AGTTCGGGTC GGATCGATTT CGGGTTAAAT AATTTTTGGT GGAATACGCT TGTCATGCCA   
  
  
+ AACACAAGCA ACTTTGTTAA AAATTTCGAT TCGGGTCAGG TCAATTCAGG TTTGGGGTCC ACTTTCGAGT   
  
  
+ GAGCATATTT CGGATGTCGG GTCGCGTATG GGTCCGGGTC ATTCGGTTTG AATTTCGGAT CTTGGATCAA   
  
  
+ TTTTGCTAAG TATGTGCATA TTCAACCCTT GATGACATAG GAAACGCTTT GTCATCTCCA CCAATCAGAT   
  
  
+ ATCGTTATCT CTTTAATCAT CGCCATAATA GCATGAGTAA GCATGACCAT CTTTTTCAAA CATGTGATAC   
  
  
+ AGTTAAGGCC TGTTCTTTTC GTTAATAAAT CCCAATTTAT CAATTTTAAT ATATACTTTA ACGAATTTTA   
  
  
+ ACAAAAAATT TTAATTTCAA TCCGCTATAA CAAAAAAAAT ACCAACTTCA ACTTATTATA TAAAAAAAAT   
  
  
+ ATACAATTTT TTTTCAATTT TAGCCAACTT CAACAAAAAA AAAAAAAATT TAACAAATCT CAATCAACTT   
  
  
+ CACCCATTTC TCAACTTATA TCTCCTAATG AAAAGAACAG ATCCTTAGCA AGACTTCTCG ATTCATTTAA   
  
  
+ AAGGATAGTA TGGTAAATTC ATTTTATACA AGTGTCAAGT GTGAATATCT TTTTCACATC AGGTGATATA   
  
  
+ TTAGTAGGAC TTTTCGATTC ACTTATCTTC CTCTTTATGT TTTGTTAATG TGATAGTAAC GATAGGATTG   
  
  
+ CATGTATTTT TTCCAACTAT TCATTTACAA CTATCAATAT CTATTGATAA ATATGTTAAA ATAATTATAT   
  
  
+ GTTATTTAAA TTATTATTTG TTACTTAAGG TGTCGACTTT GTCGAAAGCG ACTTAATTCT AATTAAGATA   
  
  
+ GCGGCAGAGA AAGTTACACC TGATGACCAA GTTAGATCTG GTAAAACTAA CCATAGATCT GAAACCCAAA   
  
  
+ CATAATGATG TGTATTCATA CCCGACCTGA CATCTGAAAT AAGTTTACTC AAAAGTGGTT CCGAAACTTG   
  
  
+ AATTGACTCG AACCGAACCG TATTTGACTC GAAATTTTCT ACAAAGTTGC TTATTTTTGC ATGAAGACAT   
  
  
+ ATTGAGCCAA AAATTAGTTG ATTCGAAGTC AACTTAAACA AAAATTCATT CGACTCAAAC TGAAATATCA   
  
  
+ CTTAACTAAA ACTATGACTC AACTCAAACT TGATCTGATC CGTATTCAAA TCAAATACTC CATTTGCTAG   
  
  
+ GTCTAGAACC AGCCACGACC CCACATGGAC TATGCGGTTC AATGAAACGG CCAAATATAT CGCAATTTCT   
  
  
+ GTCTCCATCA ATCACACCCA TTTGCTACAC CCTGTTAATA TTTGACTCGA CCCTGTGAAT TGCCTTCGCC   
  
  
+ AGTTTTGTTT AATCTCTCTG GTTGCTTCCC ATCCAAGTCT GAATAGTATA GTTTGTACAG TGTGTACTAC   
  
  
+ TAGAAAGCAC GTTCCTTGAA TCTTCTTTCA AAGTTGTTTT TAAAGGGTTG AGTGATCCAT TACGACTTTT   
  
  
+ GATCTGGGTT CATCGCCTCC TCTCCTAATC CCTCACCCGG GTACCCTCAA TTCTCTATCG TCTCTGTTGC   
  
  
+ TCAGCTAATT CAAATTCCTT TAGTTGGTCA TCATTGAAAT CTGAAATCAG GGTGTTTTTC TTTTTATTAT   
  
  
+ TATCTGGCAG TTGGTTTGTT TATCTCTTGG TTTTTGGGGC TTATTTGGGT GCTGGTTGAT GTTATCCTGC   
  
  
+ TTGGGTTGAA TCTTCATCAG GTTTATTTTT GATTCATAGT TGAGGGTGAA AATTATTTCT CATACCCACC   
  
  
+ ACCTGTTTGT TCTTTTGTCT CTGAGAAAAA CCAAAAAGCT TTCTCTTTTT CCTGATGCAT CCTGTGCTCG   
  
  
+ TTGATCCTGA GTTAGTGAAC CATGCGTCCA AATTCAAGCC TGATTTGCTC TCAAACTTTG ATAAACAACA   
  
  
+ AGAATTTAGC AACGGGATCG AACAAGATGA CATCTTGCAA AACCCTGATC TGTGTTTTGA TTCAGAAACC   
  
  
+ CCTTCAGTTG ATCTAACTGA GAGTGTCATC AGTTCTGATT CTGTAGAAGT GCCTGATTTC TCAGATGCTT   
  
  
+ GCCTCAAGTT CATAAGTGAG ATTCTCCTAG AAGAGGATTT GGATGAAAGT CCTGCCTCTG CTCAAGAATT   
  
  
+ TAGGGCTCTC CAAGCCACTG AGAAGTCCCT GTATGATGCT CTTGGAGAGG GCTACTCTTT TTCATCTGAT   
  
  
+ AACAGCCCAT CATCATTAGG ACAGAGTATT GAGCACCAAA ATGAGAATTT TGAATTCAGT TCCGGTTATC   
  
  
+ CTGGAATTGA GGGCTATGTT AATGGTGATA TCACGTTCGA GTCCAACTGG ATGTTCAACC TAAGCCAATT   
  
  
+ GGATCCTGTT CTTACTTTGG ATGATATTTC TCAGCCCTTG TCTGCATCAA ACTCCCGATC ATCTGGGTCG   
  
  
+ AGCAATGGCT TTGATGATTC AGGGGATGGG GCAGCCACAT CTCCTGGCAG TACAGTTACA TCAACAGTCC   
  
  
+ CAGAGAAAAG GGTTGAATCT GTCAATCGCT CAAGGAGGAA GAAAAATCGT AAAAGGGATG AAGGTGGTCA   
  
  
+ TGAGGAAGGG AGGAGTAACA AGCAGCAAGC TTCATCCAAT GAAGATTATG TTGAGATGAA GGAGTTTGAT   
  
  
+ GATGTACTGC TCTGCAAAGA AGAGAAAGAT GTTATTGCAA ACTGCACCAA TGATTCCTCA CCCGTCGAAG   
  
  
+ CGAGTGATAA GTTGCAGAAG AAAGGGGGGA AGGGGAAAAC ATCGCGTGGG AAGAAGCAGA ATAGCACAAT   
  
  
+ AGAAGAGGTG GATCTGAGGA CTCTTCTCAC TCACTGTGCT CAAGCAATTT CAAATTTTGA TCTTAGGAGT   
  
  
+ GCAAATGAGC ATCTCAGGCA AATAAGGCAG CATTCTTCAC AATATGGTGA TAGCCTCCAG AGGCTTGCCC   
  
  
+ ATTATTTTGC TAATGGTCTT GAGGCTCGCA TAGCTGGCAC TGGTTCAACA ATCTCTGCTA ATGTTGTTGA   
  
  
+ TGCTCGAATC ACGTCATCTG ATTTCTTAAA GGCTTATAGG CTATATATGT CAGCCGTTCC TTTCAAAAGG   
  
  
+ ATGTTATATT TTCTCGCCAA CAAGACAATA CGGAAGTTGG CTGAGAAGGC AACCAGGATC CACATCATTG   
  
  
+ ATTTTGGAGT CTTGTTGGGT TTACAGTGGC CCTGTCTCAT ACAAAACCTC TCAAAGAGAC CCACTGGCCC   
  
  
+ GCCAAGCCTC CGTATTACTG GGATAGACTA TCCCCAGCAT GGTTTCCGGC CATCAGAAAG GGTTGAAGCG   
  
  
+ ACTGGCCGTC GGTTAGAAGG GTATTGCGAG AGATTTAATG TGCCGTTCAA GTATAAGGCC ATAGCTAAAA   
  
  
+ ATTGGGAGAG TATAAAGCTA GAGGATCTAG AAATTGATGA GAATGAGATG GTGTTTGTCA ATTGCATGCT   
  
  
+ TCGCTCTGGA ACACTGCTTG ATGAGACAGT GGTGGCAGAC AGTCCAAAGG ATGCTTTCTT AAGGTTAATC   
  
  
+ AGAGAGATAA ATCCCCGTCT TTTCATTCAT GGGACTATCA ATGGATCATT CAATGCTCCA TTCTTCATCA   
  
  
+ CTCGGTTCAG GGAGGCGCTC TTCCACTACT CTTCTTTATT TGATATCTTT GAAGCAACTA TGCCCCGTGA   
  
  
+ AGATCATGAA AGGCTCCTGG TTGAGAGTGA GATACAGGGC AAAGAAGCTT TGAATGTCAT AGCATGCGAA   
  
  
+ GGTGCCGAGA GGATTCAAAG GCCTGAAACA TACAAGCAAT GGCAAGCAAG GACAACAAGG GCCGGGTTTA   
  
  
+ GGCAGCTTCC ACTAGACCGG GAGCTTGTGA GCAGAGCAAA GGCTATGGTG AAAGCAAACT ACCATAAGGA   
  
  
+ TTTTGTGGTG GACGAGGACA GGCATTGGAT GCTACACGGT TGGAAAGGAA GAATCTTCTG TGCACTCTCT   
  
  
+ GTTTGGCAAC CCAACTG  

- -Up\_Stream \_Len000TAAACT ACACATACGA CTGATGAAAA CTACTACTAC TAGTGTCACT ACGTATAAAT   
  
  
- CTCGACCATT TAACCAGTAA GCTAAGCTCA TGCCCAGCCC AGTTCAAGAC TAATTCACTG TAAAGCGCAG   
  
  
- CAAAACCAAA TCAAGCCCAG CCTAGCTAAA GCCCAATTTA TTAAAAACCA CCTTATGCGA ACAGTACGGT   
  
  
- TTGTGTTCGT TGAAACAATT TTTAAAGCTA AGCCCAGTCC AGTTAAGTCC AAACCCCAGG TGAAAGCTCA   
  
  
- CTCGTATAAA GCCTACAGCC CAGCGCATAC CCAGGCCCAG TAAGCCAAAC TTAAAGCCTA GAACCTAGTT   
  
  
- AAAACGATTC ATACACGTAT AAGTTGGGAA CTACTGTATC CTTTGCGAAA CAGTAGAGGT GGTTAGTCTA   
  
  
- TAGCAATAGA GAAATTAGTA GCGGTATTAT CGTACTCATT CGTACTGGTA GAAAAAGTTT GTACACTATG   
  
  
- TCAATTCCGG ACAAGAAAAG CAATTATTTA GGGTTAAATA GTTAAAATTA TATATGAAAT TGCTTAAAAT   
  
  
- TGTTTTTTAA AATTAAAGTT AGGCGATATT GTTTTTTTTA TGGTTGAAGT TGAATAATAT ATTTTTTTTA   
  
  
- TATGTTAAAA AAAAGTTAAA ATCGGTTGAA GTTGTTTTTT TTTTTTTTAA ATTGTTTAGA GTTAGTTGAA   
  
  
- GTGGGTAAAG AGTTGAATAT AGAGGATTAC TTTTCTTGTC TAGGAATCGT TCTGAAGAGC TAAGTAAATT   
  
  
- TTCCTATCAT ACCATTTAAG TAAAATATGT TCACAGTTCA CACTTATAGA AAAAGTGTAG TCCACTATAT   
  
  
- AATCATCCTG AAAAGCTAAG TGAATAGAAG GAGAAATACA AAACAATTAC ACTATCATTG CTATCCTAAC   
  
  
- GTACATAAAA AAGGTTGATA AGTAAATGTT GATAGTTATA GATAACTATT TATACAATTT TATTAATATA   
  
  
- CAATAAATTT AATAATAAAC AATGAATTCC ACAGCTGAAA CAGCTTTCGC TGAATTAAGA TTAATTCTAT   
  
  
- CGCCGTCTCT TTCAATGTGG ACTACTGGTT CAATCTAGAC CATTTTGATT GGTATCTAGA CTTTGGGTTT   
  
  
- GTATTACTAC ACATAAGTAT GGGCTGGACT GTAGACTTTA TTCAAATGAG TTTTCACCAA GGCTTTGAAC   
  
  
- TTAACTGAGC TTGGCTTGGC ATAAACTGAG CTTTAAAAGA TGTTTCAACG AATAAAAACG TACTTCTGTA   
  
  
- TAACTCGGTT TTTAATCAAC TAAGCTTCAG TTGAATTTGT TTTTAAGTAA GCTGAGTTTG ACTTTATAGT   
  
  
- GAATTGATTT TGATACTGAG TTGAGTTTGA ACTAGACTAG GCATAAGTTT AGTTTATGAG GTAAACGATC   
  
  
- CAGATCTTGG TCGGTGCTGG GGTGTACCTG ATACGCCAAG TTACTTTGCC GGTTTATATA GCGTTAAAGA   
  
  
- CAGAGGTAGT TAGTGTGGGT AAACGATGTG GGACAATTAT AAACTGAGCT GGGACACTTA ACGGAAGCGG   
  
  
- TCAAAACAAA TTAGAGAGAC CAACGAAGGG TAGGTTCAGA CTTATCATAT CAAACATGTC ACACATGATG   
  
  
- ATCTTTCGTG CAAGGAACTT AGAAGAAAGT TTCAACAAAA ATTTCCCAAC TCACTAGGTA ATGCTGAAAA   
  
  
- CTAGACCCAA GTAGCGGAGG AGAGGATTAG GGAGTGGGCC CATGGGAGTT AAGAGATAGC AGAGACAACG   
  
  
- AGTCGATTAA GTTTAAGGAA ATCAACCAGT AGTAACTTTA GACTTTAGTC CCACAAAAAG AAAAATAATA   
  
  
- ATAGACCGTC AACCAAACAA ATAGAGAACC AAAAACCCCG AATAAACCCA CGACCAACTA CAATAGGACG   
  
  
- AACCCAACTT AGAAGTAGTC CAAATAAAAA CTAAGTATCA ACTCCCACTT TTAATAAAGA GTATGGGTGG   
  
  
- TGGACAAACA AGAAAACAGA GACTCTTTTT GGTTTTTCGA AAGAGAAAAA GGACTACGTA GGACACGAGC   
  
  
- AACTAGGACT CAATCACTTG GTACGCAGGT TTAAGTTCGG ACTAAACGAG AGTTTGAAAC TATTTGTTGT   
  
  
- TCTTAAATCG TTGCCCTAGC TTGTTCTACT GTAGAACGTT TTGGGACTAG ACACAAAACT AAGTCTTTGG   
  
  
- GGAAGTCAAC TAGATTGACT CTCACAGTAG TCAAGACTAA GACATCTTCA CGGACTAAAG AGTCTACGAA   
  
  
- CGGAGTTCAA GTATTCACTC TAAGAGGATC TTCTCCTAAA CCTACTTTCA GGACGGAGAC GAGTTCTTAA   
  
  
- ATCCCGAGAG GTTCGGTGAC TCTTCAGGGA CATACTACGA GAACCTCTCC CGATGAGAAA AAGTAGACTA   
  
  
- TTGTCGGGTA GTAGTAATCC TGTCTCATAA CTCGTGGTTT TACTCTTAAA ACTTAAGTCA AGGCCAATAG   
  
  
- GACCTTAACT CCCGATACAA TTACCACTAT AGTGCAAGCT CAGGTTGACC TACAAGTTGG ATTCGGTTAA   
  
  
- CCTAGGACAA GAATGAAACC TACTATAAAG AGTCGGGAAC AGACGTAGTT TGAGGGCTAG TAGACCCAGC   
  
  
- TCGTTACCGA AACTACTAAG TCCCCTACCC CGTCGGTGTA GAGGACCGTC ATGTCAATGT AGTTGTCAGG   
  
  
- GTCTCTTTTC CCAACTTAGA CAGTTAGCGA GTTCCTCCTT CTTTTTAGCA TTTTCCCTAC TTCCACCAGT   
  
  
- ACTCCTTCCC TCCTCATTGT TCGTCGTTCG AAGTAGGTTA CTTCTAATAC AACTCTACTT CCTCAAACTA   
  
  
- CTACATGACG AGACGTTTCT TCTCTTTCTA CAATAACGTT TGACGTGGTT ACTAAGGAGT GGGCAGCTTC   
  
  
- GCTCACTATT CAACGTCTTC TTTCCCCCCT TCCCCTTTTG TAGCGCACCC TTCTTCGTCT TATCGTGTTA   
  
  
- TCTTCTCCAC CTAGACTCCT GAGAAGAGTG AGTGACACGA GTTCGTTAAA GTTTAAAACT AGAATCCTCA   
  
  
- CGTTTACTCG TAGAGTCCGT TTATTCCGTC GTAAGAAGTG TTATACCACT ATCGGAGGTC TCCGAACGGG   
  
  
- TAATAAAACG ATTACCAGAA CTCCGAGCGT ATCGACCGTG ACCAAGTTGT TAGAGACGAT TACAACAACT   
  
  
- ACGAGCTTAG TGCAGTAGAC TAAAGAATTT CCGAATATCC GATATATACA GTCGGCAAGG AAAGTTTTCC   
  
  
- TACAATATAA AAGAGCGGTT GTTCTGTTAT GCCTTCAACC GACTCTTCCG TTGGTCCTAG GTGTAGTAAC   
  
  
- TAAAACCTCA GAACAACCCA AATGTCACCG GGACAGAGTA TGTTTTGGAG AGTTTCTCTG GGTGACCGGG   
  
  
- CGGTTCGGAG GCATAATGAC CCTATCTGAT AGGGGTCGTA CCAAAGGCCG GTAGTCTTTC CCAACTTCGC   
  
  
- TGACCGGCAG CCAATCTTCC CATAACGCTC TCTAAATTAC ACGGCAAGTT CATATTCCGG TATCGATTTT   
  
  
- TAACCCTCTC ATATTTCGAT CTCCTAGATC TTTAACTACT CTTACTCTAC CACAAACAGT TAACGTACGA   
  
  
- AGCGAGACCT TGTGACGAAC TACTCTGTCA CCACCGTCTG TCAGGTTTCC TACGAAAGAA TTCCAATTAG   
  
  
- TCTCTCTATT TAGGGGCAGA AAAGTAAGTA CCCTGATAGT TACCTAGTAA GTTACGAGGT AAGAAGTAGT   
  
  
- GAGCCAAGTC CCTCCGCGAG AAGGTGATGA GAAGAAATAA ACTATAGAAA CTTCGTTGAT ACGGGGCACT   
  
  
- TCTAGTACTT TCCGAGGACC AACTCTCACT CTATGTCCCG TTTCTTCGAA ACTTACAGTA TCGTACGCTT   
  
  
- CCACGGCTCT CCTAAGTTTC CGGACTTTGT ATGTTCGTTA CCGTTCGTTC CTGTTGTTCC CGGCCCAAAT   
  
  
- CCGTCGAAGG TGATCTGGCC CTCGAACACT CGTCTCGTTT CCGATACCAC TTTCGTTTGA TGGTATTCCT   
  
  
- AAAACACCAC CTGCTCCTGT CCGTAACCTA CGATGTGCCA ACCTTTCCTT CTTAGAAGAC ACGTGAGAGA   
  
  
- CAAACCGTTG GGTTGAC

+     CAAT-box

| Site Name | Organism | Position | Strand | Matrix score. | sequence | function |
| --- | --- | --- | --- | --- | --- | --- |
| CAAT-box | Pisum sativum | 3752 | - | 5 | CAAAT | common cis-acting element in promoter and enhancer regions |
| CAAT-box | Nicotiana glutinosa | 3537 | - | 4 | CAAT |  |
| CAAT-box | Nicotiana glutinosa | 3891 | + | 4 | CAAT |  |
| CAAT-box | Arabidopsis thaliana | 3505 | - | 5 | CCAAT | common cis-acting element in promoter and enhancer regions |
| CAAT-box | Pisum sativum | 2995 | + | 5 | CAAAT | common cis-acting element in promoter and enhancer regions |
| CAAT-box | Nicotiana glutinosa | 2989 | + | 4 | CAAT |  |
| CAAT-box | Nicotiana glutinosa | 2838 | - | 4 | CAAT |  |
| CAAT-box | Arabidopsis thaliana | 2770 | + | 5 | CCAAT | common cis-acting element in promoter and enhancer regions |
| CAAT-box | Nicotiana glutinosa | 2597 | + | 4 | CAAT |  |
| CAAT-box | Arabidopsis thaliana | 3503 | - | 8 | CCCAATTT | common cis-acting element in promoter and enhancer regions |
| CAAT-box | Nicotiana glutinosa | 2941 | + | 4 | CAAT |  |
| CAAT-box | Pisum sativum | 3016 | + | 5 | CAAAT | common cis-acting element in promoter and enhancer regions |
| CAAT-box | Nicotiana glutinosa | 3054 | + | 4 | CAAT |  |
| CAAT-box | Nicotiana glutinosa | 2852 | + | 4 | CAAT |  |
| CAAT-box | Arabidopsis thaliana | 2522 | - | 5 | CCAAT | common cis-acting element in promoter and enhancer regions |
| CAAT-box | Arabidopsis thaliana | 4018 | - | 5 | CCAAT | common cis-acting element in promoter and enhancer regions |
| CAAT-box | Nicotiana glutinosa | 3683 | + | 4 | CAAT |  |
| CAAT-box | Nicotiana glutinosa | 3695 | + | 4 | CAAT |  |
| CAAT-box | Nicotiana glutinosa | 3565 | - | 4 | CAAT |  |
| CAAT-box | Nicotiana glutinosa | 3563 | + | 4 | CAAT |  |
| CAAT-box | Pisum sativum | 1765 | + | 5 | CAAAT | common cis-acting element in promoter and enhancer regions |
| CAAT-box | Nicotiana glutinosa | 1467 | + | 4 | CAAT |  |
| CAAT-box | Arabidopsis thaliana | 526 | + | 5 | CCAAT | common cis-acting element in promoter and enhancer regions |
| CAAT-box | Pisum sativum | 1867 | - | 5 | CAAAT | common cis-acting element in promoter and enhancer regions |
| CAAT-box | Nicotiana glutinosa | 416 | + | 4 | CAAT |  |
| CAAT-box | Pisum sativum | 1381 | + | 5 | CAAAT | common cis-acting element in promoter and enhancer regions |
| CAAT-box | Pisum sativum | 2077 | - | 5 | CAAAT | common cis-acting element in promoter and enhancer regions |
| CAAT-box | Pisum sativum | 1386 | + | 5 | CAAAT | common cis-acting element in promoter and enhancer regions |
| CAAT-box | Arabidopsis thaliana | 415 | + | 5 | CCAAT | common cis-acting element in promoter and enhancer regions |
| CAAT-box | Pisum sativum | 1514 | - | 5 | CAAAT | common cis-acting element in promoter and enhancer regions |
| CAAT-box | Pisum sativum | 1456 | + | 5 | CAAAT | common cis-acting element in promoter and enhancer regions |
| CAAT-box | Nicotiana glutinosa | 2771 | + | 4 | CAAT |  |
| CAAT-box | Nicotiana glutinosa | 2520 | + | 4 | CAAT |  |
| CAAT-box | Nicotiana glutinosa | 1483 | + | 4 | CAAT |  |
| CAAT-box | Nicotiana glutinosa | 535 | + | 4 | CAAT |  |
| CAAT-box | Nicotiana glutinosa | 3457 | - | 4 | CAAT |  |
| CAAT-box | Nicotiana glutinosa | 957 | - | 4 | CAAT |  |
| CAAT-box | Arabidopsis thaliana | 2851 | + | 5 | CCAAT | common cis-acting element in promoter and enhancer regions |
| CAAT-box | Nicotiana glutinosa | 638 | + | 4 | CAAT |  |
| CAAT-box | Nicotiana glutinosa | 3291 | - | 4 | CAAT |  |
| CAAT-box | Nicotiana glutinosa | 949 | + | 4 | CAAT |  |
| CAAT-box | Nicotiana glutinosa | 2687 | + | 4 | CAAT |  |
| CAAT-box | Nicotiana glutinosa | 695 | + | 4 | CAAT |  |
| CAAT-box | Nicotiana glutinosa | 527 | + | 4 | CAAT |  |
| CAAT-box | Arabidopsis thaliana | 525 | + | 8 | CCCAATTT | common cis-acting element in promoter and enhancer regions |
| CAAT-box | Nicotiana glutinosa | 3133 | + | 4 | CAAT |  |
| CAAT-box | Pisum sativum | 2281 | - | 5 | CAAAT | common cis-acting element in promoter and enhancer regions |
| CAAT-box | Pisum sativum | 1396 | - | 5 | CAAAT | common cis-acting element in promoter and enhancer regions |
| CAAT-box | Nicotiana glutinosa | 1444 | + | 4 | CAAT |  |
| CAAT-box | Nicotiana glutinosa | 3250 | + | 4 | CAAT |  |
| CAAT-box | Nicotiana glutinosa | 649 | + | 4 | CAAT |  |
| CAAT-box | Arabidopsis thaliana | 2519 | + | 5 | CCAAT | common cis-acting element in promoter and enhancer regions |
| CAAT-box | Nicotiana glutinosa | 2460 | - | 4 | CAAT |  |
| CAAT-box | Pisum sativum | 2063 | + | 5 | CAAAT | common cis-acting element in promoter and enhancer regions |
| CAAT-box | Nicotiana glutinosa | 256 | + | 4 | CAAT |  |
| CAAT-box | Nicotiana glutinosa | 2412 | - | 4 | CAAT |  |
| CAAT-box | Nicotiana glutinosa | 1732 | + | 4 | CAAT |  |
| CAAT-box | Nicotiana glutinosa | 582 | + | 4 | CAAT |  |
| CAAT-box | Nicotiana glutinosa | 352 | + | 4 | CAAT |  |
| CAAT-box | Arabidopsis thaliana | 85 | - | 5 | CCAAT | common cis-acting element in promoter and enhancer regions |
| CAAT-box | Pisum sativum | 3033 | + | 5 | CAAAT | common cis-acting element in promoter and enhancer regions |
| CAAT-box | Pisum sativum | 1000 | - | 5 | CAAAT | common cis-acting element in promoter and enhancer regions |
| CAAT-box | Pisum sativum | 1494 | - | 5 | CAAAT | common cis-acting element in promoter and enhancer regions |
| CAAT-box | Nicotiana glutinosa | 1196 | - | 4 | CAAT |  |
| CAAT-box | Nicotiana glutinosa | 911 | - | 4 | CAAT |  |
| CAAT-box | Pisum sativum | 19 | - | 5 | CAAAT | common cis-acting element in promoter and enhancer regions |
| CAAT-box | Pisum sativum | 688 | + | 5 | CAAAT | common cis-acting element in promoter and enhancer regions |
| CAAT-box | Nicotiana glutinosa | 1787 | - | 4 | CAAT |  |
| CAAT-box | Pisum sativum | 1216 | - | 5 | CAAAT | common cis-acting element in promoter and enhancer regions |
| CAAT-box | Nicotiana glutinosa | 1533 | - | 4 | CAAT |  |
| CAAT-box | Nicotiana glutinosa | 1265 | - | 4 | CAAT |  |

>HU07G02246.1   
+ -Up\_Stream \_Len000ATTTGA TGTGTATGCT GACTACTTTT GATGATGATG ATCACAGTGA TGCATATTTA   
  
  
+ GAGCTGGTAA ATTGGTCATT CGATTCGAGT ACGGGTCGGG TCAAGTTCTG ATTAAGTGAC ATTTCGCGTC   
  
  
+ GTTTTGGTTT AGTTCGGGTC GGATCGATTT CGGGTTAAAT AATTTTTGGT GGAATACGCT TGTCATGCCA   
  
  
+ AACACAAGCA ACTTTGTTAA AAATTTCGAT TCGGGTCAGG TCAATTCAGG TTTGGGGTCC ACTTTCGAGT   
  
  
+ GAGCATATTT CGGATGTCGG GTCGCGTATG GGTCCGGGTC ATTCGGTTTG AATTTCGGAT CTTGGATCAA   
  
  
+ TTTTGCTAAG TATGTGCATA TTCAACCCTT GATGACATAG GAAACGCTTT GTCATCTCCA CCAATCAGAT   
  
  
+ ATCGTTATCT CTTTAATCAT CGCCATAATA GCATGAGTAA GCATGACCAT CTTTTTCAAA CATGTGATAC   
  
  
+ AGTTAAGGCC TGTTCTTTTC GTTAATAAAT CCCAATTTAT CAATTTTAAT ATATACTTTA ACGAATTTTA   
  
  
+ ACAAAAAATT TTAATTTCAA TCCGCTATAA CAAAAAAAAT ACCAACTTCA ACTTATTATA TAAAAAAAAT   
  
  
+ ATACAATTTT TTTTCAATTT TAGCCAACTT CAACAAAAAA AAAAAAAATT TAACAAATCT CAATCAACTT   
  
  
+ CACCCATTTC TCAACTTATA TCTCCTAATG AAAAGAACAG ATCCTTAGCA AGACTTCTCG ATTCATTTAA   
  
  
+ AAGGATAGTA TGGTAAATTC ATTTTATACA AGTGTCAAGT GTGAATATCT TTTTCACATC AGGTGATATA   
  
  
+ TTAGTAGGAC TTTTCGATTC ACTTATCTTC CTCTTTATGT TTTGTTAATG TGATAGTAAC GATAGGATTG   
  
  
+ CATGTATTTT TTCCAACTAT TCATTTACAA CTATCAATAT CTATTGATAA ATATGTTAAA ATAATTATAT   
  
  
+ GTTATTTAAA TTATTATTTG TTACTTAAGG TGTCGACTTT GTCGAAAGCG ACTTAATTCT AATTAAGATA   
  
  
+ GCGGCAGAGA AAGTTACACC TGATGACCAA GTTAGATCTG GTAAAACTAA CCATAGATCT GAAACCCAAA   
  
  
+ CATAATGATG TGTATTCATA CCCGACCTGA CATCTGAAAT AAGTTTACTC AAAAGTGGTT CCGAAACTTG   
  
  
+ AATTGACTCG AACCGAACCG TATTTGACTC GAAATTTTCT ACAAAGTTGC TTATTTTTGC ATGAAGACAT   
  
  
+ ATTGAGCCAA AAATTAGTTG ATTCGAAGTC AACTTAAACA AAAATTCATT CGACTCAAAC TGAAATATCA   
  
  
+ CTTAACTAAA ACTATGACTC AACTCAAACT TGATCTGATC CGTATTCAAA TCAAATACTC CATTTGCTAG   
  
  
+ GTCTAGAACC AGCCACGACC CCACATGGAC TATGCGGTTC AATGAAACGG CCAAATATAT CGCAATTTCT   
  
  
+ GTCTCCATCA ATCACACCCA TTTGCTACAC CCTGTTAATA TTTGACTCGA CCCTGTGAAT TGCCTTCGCC   
  
  
+ AGTTTTGTTT AATCTCTCTG GTTGCTTCCC ATCCAAGTCT GAATAGTATA GTTTGTACAG TGTGTACTAC   
  
  
+ TAGAAAGCAC GTTCCTTGAA TCTTCTTTCA AAGTTGTTTT TAAAGGGTTG AGTGATCCAT TACGACTTTT   
  
  
+ GATCTGGGTT CATCGCCTCC TCTCCTAATC CCTCACCCGG GTACCCTCAA TTCTCTATCG TCTCTGTTGC   
  
  
+ TCAGCTAATT CAAATTCCTT TAGTTGGTCA TCATTGAAAT CTGAAATCAG GGTGTTTTTC TTTTTATTAT   
  
  
+ TATCTGGCAG TTGGTTTGTT TATCTCTTGG TTTTTGGGGC TTATTTGGGT GCTGGTTGAT GTTATCCTGC   
  
  
+ TTGGGTTGAA TCTTCATCAG GTTTATTTTT GATTCATAGT TGAGGGTGAA AATTATTTCT CATACCCACC   
  
  
+ ACCTGTTTGT TCTTTTGTCT CTGAGAAAAA CCAAAAAGCT TTCTCTTTTT CCTGATGCAT CCTGTGCTCG   
  
  
+ TTGATCCTGA GTTAGTGAAC CATGCGTCCA AATTCAAGCC TGATTTGCTC TCAAACTTTG ATAAACAACA   
  
  
+ AGAATTTAGC AACGGGATCG AACAAGATGA CATCTTGCAA AACCCTGATC TGTGTTTTGA TTCAGAAACC   
  
  
+ CCTTCAGTTG ATCTAACTGA GAGTGTCATC AGTTCTGATT CTGTAGAAGT GCCTGATTTC TCAGATGCTT   
  
  
+ GCCTCAAGTT CATAAGTGAG ATTCTCCTAG AAGAGGATTT GGATGAAAGT CCTGCCTCTG CTCAAGAATT   
  
  
+ TAGGGCTCTC CAAGCCACTG AGAAGTCCCT GTATGATGCT CTTGGAGAGG GCTACTCTTT TTCATCTGAT   
  
  
+ AACAGCCCAT CATCATTAGG ACAGAGTATT GAGCACCAAA ATGAGAATTT TGAATTCAGT TCCGGTTATC   
  
  
+ CTGGAATTGA GGGCTATGTT AATGGTGATA TCACGTTCGA GTCCAACTGG ATGTTCAACC TAAGCCAATT   
  
  
+ GGATCCTGTT CTTACTTTGG ATGATATTTC TCAGCCCTTG TCTGCATCAA ACTCCCGATC ATCTGGGTCG   
  
  
+ AGCAATGGCT TTGATGATTC AGGGGATGGG GCAGCCACAT CTCCTGGCAG TACAGTTACA TCAACAGTCC   
  
  
+ CAGAGAAAAG GGTTGAATCT GTCAATCGCT CAAGGAGGAA GAAAAATCGT AAAAGGGATG AAGGTGGTCA   
  
  
+ TGAGGAAGGG AGGAGTAACA AGCAGCAAGC TTCATCCAAT GAAGATTATG TTGAGATGAA GGAGTTTGAT   
  
  
+ GATGTACTGC TCTGCAAAGA AGAGAAAGAT GTTATTGCAA ACTGCACCAA TGATTCCTCA CCCGTCGAAG   
  
  
+ CGAGTGATAA GTTGCAGAAG AAAGGGGGGA AGGGGAAAAC ATCGCGTGGG AAGAAGCAGA ATAGCACAAT   
  
  
+ AGAAGAGGTG GATCTGAGGA CTCTTCTCAC TCACTGTGCT CAAGCAATTT CAAATTTTGA TCTTAGGAGT   
  
  
+ GCAAATGAGC ATCTCAGGCA AATAAGGCAG CATTCTTCAC AATATGGTGA TAGCCTCCAG AGGCTTGCCC   
  
  
+ ATTATTTTGC TAATGGTCTT GAGGCTCGCA TAGCTGGCAC TGGTTCAACA ATCTCTGCTA ATGTTGTTGA   
  
  
+ TGCTCGAATC ACGTCATCTG ATTTCTTAAA GGCTTATAGG CTATATATGT CAGCCGTTCC TTTCAAAAGG   
  
  
+ ATGTTATATT TTCTCGCCAA CAAGACAATA CGGAAGTTGG CTGAGAAGGC AACCAGGATC CACATCATTG   
  
  
+ ATTTTGGAGT CTTGTTGGGT TTACAGTGGC CCTGTCTCAT ACAAAACCTC TCAAAGAGAC CCACTGGCCC   
  
  
+ GCCAAGCCTC CGTATTACTG GGATAGACTA TCCCCAGCAT GGTTTCCGGC CATCAGAAAG GGTTGAAGCG   
  
  
+ ACTGGCCGTC GGTTAGAAGG GTATTGCGAG AGATTTAATG TGCCGTTCAA GTATAAGGCC ATAGCTAAAA   
  
  
+ ATTGGGAGAG TATAAAGCTA GAGGATCTAG AAATTGATGA GAATGAGATG GTGTTTGTCA ATTGCATGCT   
  
  
+ TCGCTCTGGA ACACTGCTTG ATGAGACAGT GGTGGCAGAC AGTCCAAAGG ATGCTTTCTT AAGGTTAATC   
  
  
+ AGAGAGATAA ATCCCCGTCT TTTCATTCAT GGGACTATCA ATGGATCATT CAATGCTCCA TTCTTCATCA   
  
  
+ CTCGGTTCAG GGAGGCGCTC TTCCACTACT CTTCTTTATT TGATATCTTT GAAGCAACTA TGCCCCGTGA   
  
  
+ AGATCATGAA AGGCTCCTGG TTGAGAGTGA GATACAGGGC AAAGAAGCTT TGAATGTCAT AGCATGCGAA   
  
  
+ GGTGCCGAGA GGATTCAAAG GCCTGAAACA TACAAGCAAT GGCAAGCAAG GACAACAAGG GCCGGGTTTA   
  
  
+ GGCAGCTTCC ACTAGACCGG GAGCTTGTGA GCAGAGCAAA GGCTATGGTG AAAGCAAACT ACCATAAGGA   
  
  
+ TTTTGTGGTG GACGAGGACA GGCATTGGAT GCTACACGGT TGGAAAGGAA GAATCTTCTG TGCACTCTCT   
  
  
+ GTTTGGCAAC CCAACTG  

- -Up\_Stream \_Len000TAAACT ACACATACGA CTGATGAAAA CTACTACTAC TAGTGTCACT ACGTATAAAT   
  
  
- CTCGACCATT TAACCAGTAA GCTAAGCTCA TGCCCAGCCC AGTTCAAGAC TAATTCACTG TAAAGCGCAG   
  
  
- CAAAACCAAA TCAAGCCCAG CCTAGCTAAA GCCCAATTTA TTAAAAACCA CCTTATGCGA ACAGTACGGT   
  
  
- TTGTGTTCGT TGAAACAATT TTTAAAGCTA AGCCCAGTCC AGTTAAGTCC AAACCCCAGG TGAAAGCTCA   
  
  
- CTCGTATAAA GCCTACAGCC CAGCGCATAC CCAGGCCCAG TAAGCCAAAC TTAAAGCCTA GAACCTAGTT   
  
  
- AAAACGATTC ATACACGTAT AAGTTGGGAA CTACTGTATC CTTTGCGAAA CAGTAGAGGT GGTTAGTCTA   
  
  
- TAGCAATAGA GAAATTAGTA GCGGTATTAT CGTACTCATT CGTACTGGTA GAAAAAGTTT GTACACTATG   
  
  
- TCAATTCCGG ACAAGAAAAG CAATTATTTA GGGTTAAATA GTTAAAATTA TATATGAAAT TGCTTAAAAT   
  
  
- TGTTTTTTAA AATTAAAGTT AGGCGATATT GTTTTTTTTA TGGTTGAAGT TGAATAATAT ATTTTTTTTA   
  
  
- TATGTTAAAA AAAAGTTAAA ATCGGTTGAA GTTGTTTTTT TTTTTTTTAA ATTGTTTAGA GTTAGTTGAA   
  
  
- GTGGGTAAAG AGTTGAATAT AGAGGATTAC TTTTCTTGTC TAGGAATCGT TCTGAAGAGC TAAGTAAATT   
  
  
- TTCCTATCAT ACCATTTAAG TAAAATATGT TCACAGTTCA CACTTATAGA AAAAGTGTAG TCCACTATAT   
  
  
- AATCATCCTG AAAAGCTAAG TGAATAGAAG GAGAAATACA AAACAATTAC ACTATCATTG CTATCCTAAC   
  
  
- GTACATAAAA AAGGTTGATA AGTAAATGTT GATAGTTATA GATAACTATT TATACAATTT TATTAATATA   
  
  
- CAATAAATTT AATAATAAAC AATGAATTCC ACAGCTGAAA CAGCTTTCGC TGAATTAAGA TTAATTCTAT   
  
  
- CGCCGTCTCT TTCAATGTGG ACTACTGGTT CAATCTAGAC CATTTTGATT GGTATCTAGA CTTTGGGTTT   
  
  
- GTATTACTAC ACATAAGTAT GGGCTGGACT GTAGACTTTA TTCAAATGAG TTTTCACCAA GGCTTTGAAC   
  
  
- TTAACTGAGC TTGGCTTGGC ATAAACTGAG CTTTAAAAGA TGTTTCAACG AATAAAAACG TACTTCTGTA   
  
  
- TAACTCGGTT TTTAATCAAC TAAGCTTCAG TTGAATTTGT TTTTAAGTAA GCTGAGTTTG ACTTTATAGT   
  
  
- GAATTGATTT TGATACTGAG TTGAGTTTGA ACTAGACTAG GCATAAGTTT AGTTTATGAG GTAAACGATC   
  
  
- CAGATCTTGG TCGGTGCTGG GGTGTACCTG ATACGCCAAG TTACTTTGCC GGTTTATATA GCGTTAAAGA   
  
  
- CAGAGGTAGT TAGTGTGGGT AAACGATGTG GGACAATTAT AAACTGAGCT GGGACACTTA ACGGAAGCGG   
  
  
- TCAAAACAAA TTAGAGAGAC CAACGAAGGG TAGGTTCAGA CTTATCATAT CAAACATGTC ACACATGATG   
  
  
- ATCTTTCGTG CAAGGAACTT AGAAGAAAGT TTCAACAAAA ATTTCCCAAC TCACTAGGTA ATGCTGAAAA   
  
  
- CTAGACCCAA GTAGCGGAGG AGAGGATTAG GGAGTGGGCC CATGGGAGTT AAGAGATAGC AGAGACAACG   
  
  
- AGTCGATTAA GTTTAAGGAA ATCAACCAGT AGTAACTTTA GACTTTAGTC CCACAAAAAG AAAAATAATA   
  
  
- ATAGACCGTC AACCAAACAA ATAGAGAACC AAAAACCCCG AATAAACCCA CGACCAACTA CAATAGGACG   
  
  
- AACCCAACTT AGAAGTAGTC CAAATAAAAA CTAAGTATCA ACTCCCACTT TTAATAAAGA GTATGGGTGG   
  
  
- TGGACAAACA AGAAAACAGA GACTCTTTTT GGTTTTTCGA AAGAGAAAAA GGACTACGTA GGACACGAGC   
  
  
- AACTAGGACT CAATCACTTG GTACGCAGGT TTAAGTTCGG ACTAAACGAG AGTTTGAAAC TATTTGTTGT   
  
  
- TCTTAAATCG TTGCCCTAGC TTGTTCTACT GTAGAACGTT TTGGGACTAG ACACAAAACT AAGTCTTTGG   
  
  
- GGAAGTCAAC TAGATTGACT CTCACAGTAG TCAAGACTAA GACATCTTCA CGGACTAAAG AGTCTACGAA   
  
  
- CGGAGTTCAA GTATTCACTC TAAGAGGATC TTCTCCTAAA CCTACTTTCA GGACGGAGAC GAGTTCTTAA   
  
  
- ATCCCGAGAG GTTCGGTGAC TCTTCAGGGA CATACTACGA GAACCTCTCC CGATGAGAAA AAGTAGACTA   
  
  
- TTGTCGGGTA GTAGTAATCC TGTCTCATAA CTCGTGGTTT TACTCTTAAA ACTTAAGTCA AGGCCAATAG   
  
  
- GACCTTAACT CCCGATACAA TTACCACTAT AGTGCAAGCT CAGGTTGACC TACAAGTTGG ATTCGGTTAA   
  
  
- CCTAGGACAA GAATGAAACC TACTATAAAG AGTCGGGAAC AGACGTAGTT TGAGGGCTAG TAGACCCAGC   
  
  
- TCGTTACCGA AACTACTAAG TCCCCTACCC CGTCGGTGTA GAGGACCGTC ATGTCAATGT AGTTGTCAGG   
  
  
- GTCTCTTTTC CCAACTTAGA CAGTTAGCGA GTTCCTCCTT CTTTTTAGCA TTTTCCCTAC TTCCACCAGT   
  
  
- ACTCCTTCCC TCCTCATTGT TCGTCGTTCG AAGTAGGTTA CTTCTAATAC AACTCTACTT CCTCAAACTA   
  
  
- CTACATGACG AGACGTTTCT TCTCTTTCTA CAATAACGTT TGACGTGGTT ACTAAGGAGT GGGCAGCTTC   
  
  
- GCTCACTATT CAACGTCTTC TTTCCCCCCT TCCCCTTTTG TAGCGCACCC TTCTTCGTCT TATCGTGTTA   
  
  
- TCTTCTCCAC CTAGACTCCT GAGAAGAGTG AGTGACACGA GTTCGTTAAA GTTTAAAACT AGAATCCTCA   
  
  
- CGTTTACTCG TAGAGTCCGT TTATTCCGTC GTAAGAAGTG TTATACCACT ATCGGAGGTC TCCGAACGGG   
  
  
- TAATAAAACG ATTACCAGAA CTCCGAGCGT ATCGACCGTG ACCAAGTTGT TAGAGACGAT TACAACAACT   
  
  
- ACGAGCTTAG TGCAGTAGAC TAAAGAATTT CCGAATATCC GATATATACA GTCGGCAAGG AAAGTTTTCC   
  
  
- TACAATATAA AAGAGCGGTT GTTCTGTTAT GCCTTCAACC GACTCTTCCG TTGGTCCTAG GTGTAGTAAC   
  
  
- TAAAACCTCA GAACAACCCA AATGTCACCG GGACAGAGTA TGTTTTGGAG AGTTTCTCTG GGTGACCGGG   
  
  
- CGGTTCGGAG GCATAATGAC CCTATCTGAT AGGGGTCGTA CCAAAGGCCG GTAGTCTTTC CCAACTTCGC   
  
  
- TGACCGGCAG CCAATCTTCC CATAACGCTC TCTAAATTAC ACGGCAAGTT CATATTCCGG TATCGATTTT   
  
  
- TAACCCTCTC ATATTTCGAT CTCCTAGATC TTTAACTACT CTTACTCTAC CACAAACAGT TAACGTACGA   
  
  
- AGCGAGACCT TGTGACGAAC TACTCTGTCA CCACCGTCTG TCAGGTTTCC TACGAAAGAA TTCCAATTAG   
  
  
- TCTCTCTATT TAGGGGCAGA AAAGTAAGTA CCCTGATAGT TACCTAGTAA GTTACGAGGT AAGAAGTAGT   
  
  
- GAGCCAAGTC CCTCCGCGAG AAGGTGATGA GAAGAAATAA ACTATAGAAA CTTCGTTGAT ACGGGGCACT   
  
  
- TCTAGTACTT TCCGAGGACC AACTCTCACT CTATGTCCCG TTTCTTCGAA ACTTACAGTA TCGTACGCTT   
  
  
- CCACGGCTCT CCTAAGTTTC CGGACTTTGT ATGTTCGTTA CCGTTCGTTC CTGTTGTTCC CGGCCCAAAT   
  
  
- CCGTCGAAGG TGATCTGGCC CTCGAACACT CGTCTCGTTT CCGATACCAC TTTCGTTTGA TGGTATTCCT   
  
  
- AAAACACCAC CTGCTCCTGT CCGTAACCTA CGATGTGCCA ACCTTTCCTT CTTAGAAGAC ACGTGAGAGA   
  
  
- CAAACCGTTG GGTTGAC

+     CAT-box

| Site Name | Organism | Position | Strand | Matrix score. | sequence | function |
| --- | --- | --- | --- | --- | --- | --- |
| CAT-box | Arabidopsis thaliana | 2328 | + | 6 | GCCACT | cis-acting regulatory element related to meristem expression |
| CAT-box | Arabidopsis thaliana | 3319 | - | 6 | GCCACT | cis-acting regulatory element related to meristem expression |

>HU07G02246.1   
+ -Up\_Stream \_Len000ATTTGA TGTGTATGCT GACTACTTTT GATGATGATG ATCACAGTGA TGCATATTTA   
  
  
+ GAGCTGGTAA ATTGGTCATT CGATTCGAGT ACGGGTCGGG TCAAGTTCTG ATTAAGTGAC ATTTCGCGTC   
  
  
+ GTTTTGGTTT AGTTCGGGTC GGATCGATTT CGGGTTAAAT AATTTTTGGT GGAATACGCT TGTCATGCCA   
  
  
+ AACACAAGCA ACTTTGTTAA AAATTTCGAT TCGGGTCAGG TCAATTCAGG TTTGGGGTCC ACTTTCGAGT   
  
  
+ GAGCATATTT CGGATGTCGG GTCGCGTATG GGTCCGGGTC ATTCGGTTTG AATTTCGGAT CTTGGATCAA   
  
  
+ TTTTGCTAAG TATGTGCATA TTCAACCCTT GATGACATAG GAAACGCTTT GTCATCTCCA CCAATCAGAT   
  
  
+ ATCGTTATCT CTTTAATCAT CGCCATAATA GCATGAGTAA GCATGACCAT CTTTTTCAAA CATGTGATAC   
  
  
+ AGTTAAGGCC TGTTCTTTTC GTTAATAAAT CCCAATTTAT CAATTTTAAT ATATACTTTA ACGAATTTTA   
  
  
+ ACAAAAAATT TTAATTTCAA TCCGCTATAA CAAAAAAAAT ACCAACTTCA ACTTATTATA TAAAAAAAAT   
  
  
+ ATACAATTTT TTTTCAATTT TAGCCAACTT CAACAAAAAA AAAAAAAATT TAACAAATCT CAATCAACTT   
  
  
+ CACCCATTTC TCAACTTATA TCTCCTAATG AAAAGAACAG ATCCTTAGCA AGACTTCTCG ATTCATTTAA   
  
  
+ AAGGATAGTA TGGTAAATTC ATTTTATACA AGTGTCAAGT GTGAATATCT TTTTCACATC AGGTGATATA   
  
  
+ TTAGTAGGAC TTTTCGATTC ACTTATCTTC CTCTTTATGT TTTGTTAATG TGATAGTAAC GATAGGATTG   
  
  
+ CATGTATTTT TTCCAACTAT TCATTTACAA CTATCAATAT CTATTGATAA ATATGTTAAA ATAATTATAT   
  
  
+ GTTATTTAAA TTATTATTTG TTACTTAAGG TGTCGACTTT GTCGAAAGCG ACTTAATTCT AATTAAGATA   
  
  
+ GCGGCAGAGA AAGTTACACC TGATGACCAA GTTAGATCTG GTAAAACTAA CCATAGATCT GAAACCCAAA   
  
  
+ CATAATGATG TGTATTCATA CCCGACCTGA CATCTGAAAT AAGTTTACTC AAAAGTGGTT CCGAAACTTG   
  
  
+ AATTGACTCG AACCGAACCG TATTTGACTC GAAATTTTCT ACAAAGTTGC TTATTTTTGC ATGAAGACAT   
  
  
+ ATTGAGCCAA AAATTAGTTG ATTCGAAGTC AACTTAAACA AAAATTCATT CGACTCAAAC TGAAATATCA   
  
  
+ CTTAACTAAA ACTATGACTC AACTCAAACT TGATCTGATC CGTATTCAAA TCAAATACTC CATTTGCTAG   
  
  
+ GTCTAGAACC AGCCACGACC CCACATGGAC TATGCGGTTC AATGAAACGG CCAAATATAT CGCAATTTCT   
  
  
+ GTCTCCATCA ATCACACCCA TTTGCTACAC CCTGTTAATA TTTGACTCGA CCCTGTGAAT TGCCTTCGCC   
  
  
+ AGTTTTGTTT AATCTCTCTG GTTGCTTCCC ATCCAAGTCT GAATAGTATA GTTTGTACAG TGTGTACTAC   
  
  
+ TAGAAAGCAC GTTCCTTGAA TCTTCTTTCA AAGTTGTTTT TAAAGGGTTG AGTGATCCAT TACGACTTTT   
  
  
+ GATCTGGGTT CATCGCCTCC TCTCCTAATC CCTCACCCGG GTACCCTCAA TTCTCTATCG TCTCTGTTGC   
  
  
+ TCAGCTAATT CAAATTCCTT TAGTTGGTCA TCATTGAAAT CTGAAATCAG GGTGTTTTTC TTTTTATTAT   
  
  
+ TATCTGGCAG TTGGTTTGTT TATCTCTTGG TTTTTGGGGC TTATTTGGGT GCTGGTTGAT GTTATCCTGC   
  
  
+ TTGGGTTGAA TCTTCATCAG GTTTATTTTT GATTCATAGT TGAGGGTGAA AATTATTTCT CATACCCACC   
  
  
+ ACCTGTTTGT TCTTTTGTCT CTGAGAAAAA CCAAAAAGCT TTCTCTTTTT CCTGATGCAT CCTGTGCTCG   
  
  
+ TTGATCCTGA GTTAGTGAAC CATGCGTCCA AATTCAAGCC TGATTTGCTC TCAAACTTTG ATAAACAACA   
  
  
+ AGAATTTAGC AACGGGATCG AACAAGATGA CATCTTGCAA AACCCTGATC TGTGTTTTGA TTCAGAAACC   
  
  
+ CCTTCAGTTG ATCTAACTGA GAGTGTCATC AGTTCTGATT CTGTAGAAGT GCCTGATTTC TCAGATGCTT   
  
  
+ GCCTCAAGTT CATAAGTGAG ATTCTCCTAG AAGAGGATTT GGATGAAAGT CCTGCCTCTG CTCAAGAATT   
  
  
+ TAGGGCTCTC CAAGCCACTG AGAAGTCCCT GTATGATGCT CTTGGAGAGG GCTACTCTTT TTCATCTGAT   
  
  
+ AACAGCCCAT CATCATTAGG ACAGAGTATT GAGCACCAAA ATGAGAATTT TGAATTCAGT TCCGGTTATC   
  
  
+ CTGGAATTGA GGGCTATGTT AATGGTGATA TCACGTTCGA GTCCAACTGG ATGTTCAACC TAAGCCAATT   
  
  
+ GGATCCTGTT CTTACTTTGG ATGATATTTC TCAGCCCTTG TCTGCATCAA ACTCCCGATC ATCTGGGTCG   
  
  
+ AGCAATGGCT TTGATGATTC AGGGGATGGG GCAGCCACAT CTCCTGGCAG TACAGTTACA TCAACAGTCC   
  
  
+ CAGAGAAAAG GGTTGAATCT GTCAATCGCT CAAGGAGGAA GAAAAATCGT AAAAGGGATG AAGGTGGTCA   
  
  
+ TGAGGAAGGG AGGAGTAACA AGCAGCAAGC TTCATCCAAT GAAGATTATG TTGAGATGAA GGAGTTTGAT   
  
  
+ GATGTACTGC TCTGCAAAGA AGAGAAAGAT GTTATTGCAA ACTGCACCAA TGATTCCTCA CCCGTCGAAG   
  
  
+ CGAGTGATAA GTTGCAGAAG AAAGGGGGGA AGGGGAAAAC ATCGCGTGGG AAGAAGCAGA ATAGCACAAT   
  
  
+ AGAAGAGGTG GATCTGAGGA CTCTTCTCAC TCACTGTGCT CAAGCAATTT CAAATTTTGA TCTTAGGAGT   
  
  
+ GCAAATGAGC ATCTCAGGCA AATAAGGCAG CATTCTTCAC AATATGGTGA TAGCCTCCAG AGGCTTGCCC   
  
  
+ ATTATTTTGC TAATGGTCTT GAGGCTCGCA TAGCTGGCAC TGGTTCAACA ATCTCTGCTA ATGTTGTTGA   
  
  
+ TGCTCGAATC ACGTCATCTG ATTTCTTAAA GGCTTATAGG CTATATATGT CAGCCGTTCC TTTCAAAAGG   
  
  
+ ATGTTATATT TTCTCGCCAA CAAGACAATA CGGAAGTTGG CTGAGAAGGC AACCAGGATC CACATCATTG   
  
  
+ ATTTTGGAGT CTTGTTGGGT TTACAGTGGC CCTGTCTCAT ACAAAACCTC TCAAAGAGAC CCACTGGCCC   
  
  
+ GCCAAGCCTC CGTATTACTG GGATAGACTA TCCCCAGCAT GGTTTCCGGC CATCAGAAAG GGTTGAAGCG   
  
  
+ ACTGGCCGTC GGTTAGAAGG GTATTGCGAG AGATTTAATG TGCCGTTCAA GTATAAGGCC ATAGCTAAAA   
  
  
+ ATTGGGAGAG TATAAAGCTA GAGGATCTAG AAATTGATGA GAATGAGATG GTGTTTGTCA ATTGCATGCT   
  
  
+ TCGCTCTGGA ACACTGCTTG ATGAGACAGT GGTGGCAGAC AGTCCAAAGG ATGCTTTCTT AAGGTTAATC   
  
  
+ AGAGAGATAA ATCCCCGTCT TTTCATTCAT GGGACTATCA ATGGATCATT CAATGCTCCA TTCTTCATCA   
  
  
+ CTCGGTTCAG GGAGGCGCTC TTCCACTACT CTTCTTTATT TGATATCTTT GAAGCAACTA TGCCCCGTGA   
  
  
+ AGATCATGAA AGGCTCCTGG TTGAGAGTGA GATACAGGGC AAAGAAGCTT TGAATGTCAT AGCATGCGAA   
  
  
+ GGTGCCGAGA GGATTCAAAG GCCTGAAACA TACAAGCAAT GGCAAGCAAG GACAACAAGG GCCGGGTTTA   
  
  
+ GGCAGCTTCC ACTAGACCGG GAGCTTGTGA GCAGAGCAAA GGCTATGGTG AAAGCAAACT ACCATAAGGA   
  
  
+ TTTTGTGGTG GACGAGGACA GGCATTGGAT GCTACACGGT TGGAAAGGAA GAATCTTCTG TGCACTCTCT   
  
  
+ GTTTGGCAAC CCAACTG  

- -Up\_Stream \_Len000TAAACT ACACATACGA CTGATGAAAA CTACTACTAC TAGTGTCACT ACGTATAAAT   
  
  
- CTCGACCATT TAACCAGTAA GCTAAGCTCA TGCCCAGCCC AGTTCAAGAC TAATTCACTG TAAAGCGCAG   
  
  
- CAAAACCAAA TCAAGCCCAG CCTAGCTAAA GCCCAATTTA TTAAAAACCA CCTTATGCGA ACAGTACGGT   
  
  
- TTGTGTTCGT TGAAACAATT TTTAAAGCTA AGCCCAGTCC AGTTAAGTCC AAACCCCAGG TGAAAGCTCA   
  
  
- CTCGTATAAA GCCTACAGCC CAGCGCATAC CCAGGCCCAG TAAGCCAAAC TTAAAGCCTA GAACCTAGTT   
  
  
- AAAACGATTC ATACACGTAT AAGTTGGGAA CTACTGTATC CTTTGCGAAA CAGTAGAGGT GGTTAGTCTA   
  
  
- TAGCAATAGA GAAATTAGTA GCGGTATTAT CGTACTCATT CGTACTGGTA GAAAAAGTTT GTACACTATG   
  
  
- TCAATTCCGG ACAAGAAAAG CAATTATTTA GGGTTAAATA GTTAAAATTA TATATGAAAT TGCTTAAAAT   
  
  
- TGTTTTTTAA AATTAAAGTT AGGCGATATT GTTTTTTTTA TGGTTGAAGT TGAATAATAT ATTTTTTTTA   
  
  
- TATGTTAAAA AAAAGTTAAA ATCGGTTGAA GTTGTTTTTT TTTTTTTTAA ATTGTTTAGA GTTAGTTGAA   
  
  
- GTGGGTAAAG AGTTGAATAT AGAGGATTAC TTTTCTTGTC TAGGAATCGT TCTGAAGAGC TAAGTAAATT   
  
  
- TTCCTATCAT ACCATTTAAG TAAAATATGT TCACAGTTCA CACTTATAGA AAAAGTGTAG TCCACTATAT   
  
  
- AATCATCCTG AAAAGCTAAG TGAATAGAAG GAGAAATACA AAACAATTAC ACTATCATTG CTATCCTAAC   
  
  
- GTACATAAAA AAGGTTGATA AGTAAATGTT GATAGTTATA GATAACTATT TATACAATTT TATTAATATA   
  
  
- CAATAAATTT AATAATAAAC AATGAATTCC ACAGCTGAAA CAGCTTTCGC TGAATTAAGA TTAATTCTAT   
  
  
- CGCCGTCTCT TTCAATGTGG ACTACTGGTT CAATCTAGAC CATTTTGATT GGTATCTAGA CTTTGGGTTT   
  
  
- GTATTACTAC ACATAAGTAT GGGCTGGACT GTAGACTTTA TTCAAATGAG TTTTCACCAA GGCTTTGAAC   
  
  
- TTAACTGAGC TTGGCTTGGC ATAAACTGAG CTTTAAAAGA TGTTTCAACG AATAAAAACG TACTTCTGTA   
  
  
- TAACTCGGTT TTTAATCAAC TAAGCTTCAG TTGAATTTGT TTTTAAGTAA GCTGAGTTTG ACTTTATAGT   
  
  
- GAATTGATTT TGATACTGAG TTGAGTTTGA ACTAGACTAG GCATAAGTTT AGTTTATGAG GTAAACGATC   
  
  
- CAGATCTTGG TCGGTGCTGG GGTGTACCTG ATACGCCAAG TTACTTTGCC GGTTTATATA GCGTTAAAGA   
  
  
- CAGAGGTAGT TAGTGTGGGT AAACGATGTG GGACAATTAT AAACTGAGCT GGGACACTTA ACGGAAGCGG   
  
  
- TCAAAACAAA TTAGAGAGAC CAACGAAGGG TAGGTTCAGA CTTATCATAT CAAACATGTC ACACATGATG   
  
  
- ATCTTTCGTG CAAGGAACTT AGAAGAAAGT TTCAACAAAA ATTTCCCAAC TCACTAGGTA ATGCTGAAAA   
  
  
- CTAGACCCAA GTAGCGGAGG AGAGGATTAG GGAGTGGGCC CATGGGAGTT AAGAGATAGC AGAGACAACG   
  
  
- AGTCGATTAA GTTTAAGGAA ATCAACCAGT AGTAACTTTA GACTTTAGTC CCACAAAAAG AAAAATAATA   
  
  
- ATAGACCGTC AACCAAACAA ATAGAGAACC AAAAACCCCG AATAAACCCA CGACCAACTA CAATAGGACG   
  
  
- AACCCAACTT AGAAGTAGTC CAAATAAAAA CTAAGTATCA ACTCCCACTT TTAATAAAGA GTATGGGTGG   
  
  
- TGGACAAACA AGAAAACAGA GACTCTTTTT GGTTTTTCGA AAGAGAAAAA GGACTACGTA GGACACGAGC   
  
  
- AACTAGGACT CAATCACTTG GTACGCAGGT TTAAGTTCGG ACTAAACGAG AGTTTGAAAC TATTTGTTGT   
  
  
- TCTTAAATCG TTGCCCTAGC TTGTTCTACT GTAGAACGTT TTGGGACTAG ACACAAAACT AAGTCTTTGG   
  
  
- GGAAGTCAAC TAGATTGACT CTCACAGTAG TCAAGACTAA GACATCTTCA CGGACTAAAG AGTCTACGAA   
  
  
- CGGAGTTCAA GTATTCACTC TAAGAGGATC TTCTCCTAAA CCTACTTTCA GGACGGAGAC GAGTTCTTAA   
  
  
- ATCCCGAGAG GTTCGGTGAC TCTTCAGGGA CATACTACGA GAACCTCTCC CGATGAGAAA AAGTAGACTA   
  
  
- TTGTCGGGTA GTAGTAATCC TGTCTCATAA CTCGTGGTTT TACTCTTAAA ACTTAAGTCA AGGCCAATAG   
  
  
- GACCTTAACT CCCGATACAA TTACCACTAT AGTGCAAGCT CAGGTTGACC TACAAGTTGG ATTCGGTTAA   
  
  
- CCTAGGACAA GAATGAAACC TACTATAAAG AGTCGGGAAC AGACGTAGTT TGAGGGCTAG TAGACCCAGC   
  
  
- TCGTTACCGA AACTACTAAG TCCCCTACCC CGTCGGTGTA GAGGACCGTC ATGTCAATGT AGTTGTCAGG   
  
  
- GTCTCTTTTC CCAACTTAGA CAGTTAGCGA GTTCCTCCTT CTTTTTAGCA TTTTCCCTAC TTCCACCAGT   
  
  
- ACTCCTTCCC TCCTCATTGT TCGTCGTTCG AAGTAGGTTA CTTCTAATAC AACTCTACTT CCTCAAACTA   
  
  
- CTACATGACG AGACGTTTCT TCTCTTTCTA CAATAACGTT TGACGTGGTT ACTAAGGAGT GGGCAGCTTC   
  
  
- GCTCACTATT CAACGTCTTC TTTCCCCCCT TCCCCTTTTG TAGCGCACCC TTCTTCGTCT TATCGTGTTA   
  
  
- TCTTCTCCAC CTAGACTCCT GAGAAGAGTG AGTGACACGA GTTCGTTAAA GTTTAAAACT AGAATCCTCA   
  
  
- CGTTTACTCG TAGAGTCCGT TTATTCCGTC GTAAGAAGTG TTATACCACT ATCGGAGGTC TCCGAACGGG   
  
  
- TAATAAAACG ATTACCAGAA CTCCGAGCGT ATCGACCGTG ACCAAGTTGT TAGAGACGAT TACAACAACT   
  
  
- ACGAGCTTAG TGCAGTAGAC TAAAGAATTT CCGAATATCC GATATATACA GTCGGCAAGG AAAGTTTTCC   
  
  
- TACAATATAA AAGAGCGGTT GTTCTGTTAT GCCTTCAACC GACTCTTCCG TTGGTCCTAG GTGTAGTAAC   
  
  
- TAAAACCTCA GAACAACCCA AATGTCACCG GGACAGAGTA TGTTTTGGAG AGTTTCTCTG GGTGACCGGG   
  
  
- CGGTTCGGAG GCATAATGAC CCTATCTGAT AGGGGTCGTA CCAAAGGCCG GTAGTCTTTC CCAACTTCGC   
  
  
- TGACCGGCAG CCAATCTTCC CATAACGCTC TCTAAATTAC ACGGCAAGTT CATATTCCGG TATCGATTTT   
  
  
- TAACCCTCTC ATATTTCGAT CTCCTAGATC TTTAACTACT CTTACTCTAC CACAAACAGT TAACGTACGA   
  
  
- AGCGAGACCT TGTGACGAAC TACTCTGTCA CCACCGTCTG TCAGGTTTCC TACGAAAGAA TTCCAATTAG   
  
  
- TCTCTCTATT TAGGGGCAGA AAAGTAAGTA CCCTGATAGT TACCTAGTAA GTTACGAGGT AAGAAGTAGT   
  
  
- GAGCCAAGTC CCTCCGCGAG AAGGTGATGA GAAGAAATAA ACTATAGAAA CTTCGTTGAT ACGGGGCACT   
  
  
- TCTAGTACTT TCCGAGGACC AACTCTCACT CTATGTCCCG TTTCTTCGAA ACTTACAGTA TCGTACGCTT   
  
  
- CCACGGCTCT CCTAAGTTTC CGGACTTTGT ATGTTCGTTA CCGTTCGTTC CTGTTGTTCC CGGCCCAAAT   
  
  
- CCGTCGAAGG TGATCTGGCC CTCGAACACT CGTCTCGTTT CCGATACCAC TTTCGTTTGA TGGTATTCCT   
  
  
- AAAACACCAC CTGCTCCTGT CCGTAACCTA CGATGTGCCA ACCTTTCCTT CTTAGAAGAC ACGTGAGAGA   
  
  
- CAAACCGTTG GGTTGAC

+     CCAAT-box

| Site Name | Organism | Position | Strand | Matrix score. | sequence | function |
| --- | --- | --- | --- | --- | --- | --- |
| CCAAT-box | Hordeum vulgare | 2114 | + | 6 | CAACGG | MYBHv1 binding site |

>HU07G02246.1   
+ -Up\_Stream \_Len000ATTTGA TGTGTATGCT GACTACTTTT GATGATGATG ATCACAGTGA TGCATATTTA   
  
  
+ GAGCTGGTAA ATTGGTCATT CGATTCGAGT ACGGGTCGGG TCAAGTTCTG ATTAAGTGAC ATTTCGCGTC   
  
  
+ GTTTTGGTTT AGTTCGGGTC GGATCGATTT CGGGTTAAAT AATTTTTGGT GGAATACGCT TGTCATGCCA   
  
  
+ AACACAAGCA ACTTTGTTAA AAATTTCGAT TCGGGTCAGG TCAATTCAGG TTTGGGGTCC ACTTTCGAGT   
  
  
+ GAGCATATTT CGGATGTCGG GTCGCGTATG GGTCCGGGTC ATTCGGTTTG AATTTCGGAT CTTGGATCAA   
  
  
+ TTTTGCTAAG TATGTGCATA TTCAACCCTT GATGACATAG GAAACGCTTT GTCATCTCCA CCAATCAGAT   
  
  
+ ATCGTTATCT CTTTAATCAT CGCCATAATA GCATGAGTAA GCATGACCAT CTTTTTCAAA CATGTGATAC   
  
  
+ AGTTAAGGCC TGTTCTTTTC GTTAATAAAT CCCAATTTAT CAATTTTAAT ATATACTTTA ACGAATTTTA   
  
  
+ ACAAAAAATT TTAATTTCAA TCCGCTATAA CAAAAAAAAT ACCAACTTCA ACTTATTATA TAAAAAAAAT   
  
  
+ ATACAATTTT TTTTCAATTT TAGCCAACTT CAACAAAAAA AAAAAAAATT TAACAAATCT CAATCAACTT   
  
  
+ CACCCATTTC TCAACTTATA TCTCCTAATG AAAAGAACAG ATCCTTAGCA AGACTTCTCG ATTCATTTAA   
  
  
+ AAGGATAGTA TGGTAAATTC ATTTTATACA AGTGTCAAGT GTGAATATCT TTTTCACATC AGGTGATATA   
  
  
+ TTAGTAGGAC TTTTCGATTC ACTTATCTTC CTCTTTATGT TTTGTTAATG TGATAGTAAC GATAGGATTG   
  
  
+ CATGTATTTT TTCCAACTAT TCATTTACAA CTATCAATAT CTATTGATAA ATATGTTAAA ATAATTATAT   
  
  
+ GTTATTTAAA TTATTATTTG TTACTTAAGG TGTCGACTTT GTCGAAAGCG ACTTAATTCT AATTAAGATA   
  
  
+ GCGGCAGAGA AAGTTACACC TGATGACCAA GTTAGATCTG GTAAAACTAA CCATAGATCT GAAACCCAAA   
  
  
+ CATAATGATG TGTATTCATA CCCGACCTGA CATCTGAAAT AAGTTTACTC AAAAGTGGTT CCGAAACTTG   
  
  
+ AATTGACTCG AACCGAACCG TATTTGACTC GAAATTTTCT ACAAAGTTGC TTATTTTTGC ATGAAGACAT   
  
  
+ ATTGAGCCAA AAATTAGTTG ATTCGAAGTC AACTTAAACA AAAATTCATT CGACTCAAAC TGAAATATCA   
  
  
+ CTTAACTAAA ACTATGACTC AACTCAAACT TGATCTGATC CGTATTCAAA TCAAATACTC CATTTGCTAG   
  
  
+ GTCTAGAACC AGCCACGACC CCACATGGAC TATGCGGTTC AATGAAACGG CCAAATATAT CGCAATTTCT   
  
  
+ GTCTCCATCA ATCACACCCA TTTGCTACAC CCTGTTAATA TTTGACTCGA CCCTGTGAAT TGCCTTCGCC   
  
  
+ AGTTTTGTTT AATCTCTCTG GTTGCTTCCC ATCCAAGTCT GAATAGTATA GTTTGTACAG TGTGTACTAC   
  
  
+ TAGAAAGCAC GTTCCTTGAA TCTTCTTTCA AAGTTGTTTT TAAAGGGTTG AGTGATCCAT TACGACTTTT   
  
  
+ GATCTGGGTT CATCGCCTCC TCTCCTAATC CCTCACCCGG GTACCCTCAA TTCTCTATCG TCTCTGTTGC   
  
  
+ TCAGCTAATT CAAATTCCTT TAGTTGGTCA TCATTGAAAT CTGAAATCAG GGTGTTTTTC TTTTTATTAT   
  
  
+ TATCTGGCAG TTGGTTTGTT TATCTCTTGG TTTTTGGGGC TTATTTGGGT GCTGGTTGAT GTTATCCTGC   
  
  
+ TTGGGTTGAA TCTTCATCAG GTTTATTTTT GATTCATAGT TGAGGGTGAA AATTATTTCT CATACCCACC   
  
  
+ ACCTGTTTGT TCTTTTGTCT CTGAGAAAAA CCAAAAAGCT TTCTCTTTTT CCTGATGCAT CCTGTGCTCG   
  
  
+ TTGATCCTGA GTTAGTGAAC CATGCGTCCA AATTCAAGCC TGATTTGCTC TCAAACTTTG ATAAACAACA   
  
  
+ AGAATTTAGC AACGGGATCG AACAAGATGA CATCTTGCAA AACCCTGATC TGTGTTTTGA TTCAGAAACC   
  
  
+ CCTTCAGTTG ATCTAACTGA GAGTGTCATC AGTTCTGATT CTGTAGAAGT GCCTGATTTC TCAGATGCTT   
  
  
+ GCCTCAAGTT CATAAGTGAG ATTCTCCTAG AAGAGGATTT GGATGAAAGT CCTGCCTCTG CTCAAGAATT   
  
  
+ TAGGGCTCTC CAAGCCACTG AGAAGTCCCT GTATGATGCT CTTGGAGAGG GCTACTCTTT TTCATCTGAT   
  
  
+ AACAGCCCAT CATCATTAGG ACAGAGTATT GAGCACCAAA ATGAGAATTT TGAATTCAGT TCCGGTTATC   
  
  
+ CTGGAATTGA GGGCTATGTT AATGGTGATA TCACGTTCGA GTCCAACTGG ATGTTCAACC TAAGCCAATT   
  
  
+ GGATCCTGTT CTTACTTTGG ATGATATTTC TCAGCCCTTG TCTGCATCAA ACTCCCGATC ATCTGGGTCG   
  
  
+ AGCAATGGCT TTGATGATTC AGGGGATGGG GCAGCCACAT CTCCTGGCAG TACAGTTACA TCAACAGTCC   
  
  
+ CAGAGAAAAG GGTTGAATCT GTCAATCGCT CAAGGAGGAA GAAAAATCGT AAAAGGGATG AAGGTGGTCA   
  
  
+ TGAGGAAGGG AGGAGTAACA AGCAGCAAGC TTCATCCAAT GAAGATTATG TTGAGATGAA GGAGTTTGAT   
  
  
+ GATGTACTGC TCTGCAAAGA AGAGAAAGAT GTTATTGCAA ACTGCACCAA TGATTCCTCA CCCGTCGAAG   
  
  
+ CGAGTGATAA GTTGCAGAAG AAAGGGGGGA AGGGGAAAAC ATCGCGTGGG AAGAAGCAGA ATAGCACAAT   
  
  
+ AGAAGAGGTG GATCTGAGGA CTCTTCTCAC TCACTGTGCT CAAGCAATTT CAAATTTTGA TCTTAGGAGT   
  
  
+ GCAAATGAGC ATCTCAGGCA AATAAGGCAG CATTCTTCAC AATATGGTGA TAGCCTCCAG AGGCTTGCCC   
  
  
+ ATTATTTTGC TAATGGTCTT GAGGCTCGCA TAGCTGGCAC TGGTTCAACA ATCTCTGCTA ATGTTGTTGA   
  
  
+ TGCTCGAATC ACGTCATCTG ATTTCTTAAA GGCTTATAGG CTATATATGT CAGCCGTTCC TTTCAAAAGG   
  
  
+ ATGTTATATT TTCTCGCCAA CAAGACAATA CGGAAGTTGG CTGAGAAGGC AACCAGGATC CACATCATTG   
  
  
+ ATTTTGGAGT CTTGTTGGGT TTACAGTGGC CCTGTCTCAT ACAAAACCTC TCAAAGAGAC CCACTGGCCC   
  
  
+ GCCAAGCCTC CGTATTACTG GGATAGACTA TCCCCAGCAT GGTTTCCGGC CATCAGAAAG GGTTGAAGCG   
  
  
+ ACTGGCCGTC GGTTAGAAGG GTATTGCGAG AGATTTAATG TGCCGTTCAA GTATAAGGCC ATAGCTAAAA   
  
  
+ ATTGGGAGAG TATAAAGCTA GAGGATCTAG AAATTGATGA GAATGAGATG GTGTTTGTCA ATTGCATGCT   
  
  
+ TCGCTCTGGA ACACTGCTTG ATGAGACAGT GGTGGCAGAC AGTCCAAAGG ATGCTTTCTT AAGGTTAATC   
  
  
+ AGAGAGATAA ATCCCCGTCT TTTCATTCAT GGGACTATCA ATGGATCATT CAATGCTCCA TTCTTCATCA   
  
  
+ CTCGGTTCAG GGAGGCGCTC TTCCACTACT CTTCTTTATT TGATATCTTT GAAGCAACTA TGCCCCGTGA   
  
  
+ AGATCATGAA AGGCTCCTGG TTGAGAGTGA GATACAGGGC AAAGAAGCTT TGAATGTCAT AGCATGCGAA   
  
  
+ GGTGCCGAGA GGATTCAAAG GCCTGAAACA TACAAGCAAT GGCAAGCAAG GACAACAAGG GCCGGGTTTA   
  
  
+ GGCAGCTTCC ACTAGACCGG GAGCTTGTGA GCAGAGCAAA GGCTATGGTG AAAGCAAACT ACCATAAGGA   
  
  
+ TTTTGTGGTG GACGAGGACA GGCATTGGAT GCTACACGGT TGGAAAGGAA GAATCTTCTG TGCACTCTCT   
  
  
+ GTTTGGCAAC CCAACTG  

- -Up\_Stream \_Len000TAAACT ACACATACGA CTGATGAAAA CTACTACTAC TAGTGTCACT ACGTATAAAT   
  
  
- CTCGACCATT TAACCAGTAA GCTAAGCTCA TGCCCAGCCC AGTTCAAGAC TAATTCACTG TAAAGCGCAG   
  
  
- CAAAACCAAA TCAAGCCCAG CCTAGCTAAA GCCCAATTTA TTAAAAACCA CCTTATGCGA ACAGTACGGT   
  
  
- TTGTGTTCGT TGAAACAATT TTTAAAGCTA AGCCCAGTCC AGTTAAGTCC AAACCCCAGG TGAAAGCTCA   
  
  
- CTCGTATAAA GCCTACAGCC CAGCGCATAC CCAGGCCCAG TAAGCCAAAC TTAAAGCCTA GAACCTAGTT   
  
  
- AAAACGATTC ATACACGTAT AAGTTGGGAA CTACTGTATC CTTTGCGAAA CAGTAGAGGT GGTTAGTCTA   
  
  
- TAGCAATAGA GAAATTAGTA GCGGTATTAT CGTACTCATT CGTACTGGTA GAAAAAGTTT GTACACTATG   
  
  
- TCAATTCCGG ACAAGAAAAG CAATTATTTA GGGTTAAATA GTTAAAATTA TATATGAAAT TGCTTAAAAT   
  
  
- TGTTTTTTAA AATTAAAGTT AGGCGATATT GTTTTTTTTA TGGTTGAAGT TGAATAATAT ATTTTTTTTA   
  
  
- TATGTTAAAA AAAAGTTAAA ATCGGTTGAA GTTGTTTTTT TTTTTTTTAA ATTGTTTAGA GTTAGTTGAA   
  
  
- GTGGGTAAAG AGTTGAATAT AGAGGATTAC TTTTCTTGTC TAGGAATCGT TCTGAAGAGC TAAGTAAATT   
  
  
- TTCCTATCAT ACCATTTAAG TAAAATATGT TCACAGTTCA CACTTATAGA AAAAGTGTAG TCCACTATAT   
  
  
- AATCATCCTG AAAAGCTAAG TGAATAGAAG GAGAAATACA AAACAATTAC ACTATCATTG CTATCCTAAC   
  
  
- GTACATAAAA AAGGTTGATA AGTAAATGTT GATAGTTATA GATAACTATT TATACAATTT TATTAATATA   
  
  
- CAATAAATTT AATAATAAAC AATGAATTCC ACAGCTGAAA CAGCTTTCGC TGAATTAAGA TTAATTCTAT   
  
  
- CGCCGTCTCT TTCAATGTGG ACTACTGGTT CAATCTAGAC CATTTTGATT GGTATCTAGA CTTTGGGTTT   
  
  
- GTATTACTAC ACATAAGTAT GGGCTGGACT GTAGACTTTA TTCAAATGAG TTTTCACCAA GGCTTTGAAC   
  
  
- TTAACTGAGC TTGGCTTGGC ATAAACTGAG CTTTAAAAGA TGTTTCAACG AATAAAAACG TACTTCTGTA   
  
  
- TAACTCGGTT TTTAATCAAC TAAGCTTCAG TTGAATTTGT TTTTAAGTAA GCTGAGTTTG ACTTTATAGT   
  
  
- GAATTGATTT TGATACTGAG TTGAGTTTGA ACTAGACTAG GCATAAGTTT AGTTTATGAG GTAAACGATC   
  
  
- CAGATCTTGG TCGGTGCTGG GGTGTACCTG ATACGCCAAG TTACTTTGCC GGTTTATATA GCGTTAAAGA   
  
  
- CAGAGGTAGT TAGTGTGGGT AAACGATGTG GGACAATTAT AAACTGAGCT GGGACACTTA ACGGAAGCGG   
  
  
- TCAAAACAAA TTAGAGAGAC CAACGAAGGG TAGGTTCAGA CTTATCATAT CAAACATGTC ACACATGATG   
  
  
- ATCTTTCGTG CAAGGAACTT AGAAGAAAGT TTCAACAAAA ATTTCCCAAC TCACTAGGTA ATGCTGAAAA   
  
  
- CTAGACCCAA GTAGCGGAGG AGAGGATTAG GGAGTGGGCC CATGGGAGTT AAGAGATAGC AGAGACAACG   
  
  
- AGTCGATTAA GTTTAAGGAA ATCAACCAGT AGTAACTTTA GACTTTAGTC CCACAAAAAG AAAAATAATA   
  
  
- ATAGACCGTC AACCAAACAA ATAGAGAACC AAAAACCCCG AATAAACCCA CGACCAACTA CAATAGGACG   
  
  
- AACCCAACTT AGAAGTAGTC CAAATAAAAA CTAAGTATCA ACTCCCACTT TTAATAAAGA GTATGGGTGG   
  
  
- TGGACAAACA AGAAAACAGA GACTCTTTTT GGTTTTTCGA AAGAGAAAAA GGACTACGTA GGACACGAGC   
  
  
- AACTAGGACT CAATCACTTG GTACGCAGGT TTAAGTTCGG ACTAAACGAG AGTTTGAAAC TATTTGTTGT   
  
  
- TCTTAAATCG TTGCCCTAGC TTGTTCTACT GTAGAACGTT TTGGGACTAG ACACAAAACT AAGTCTTTGG   
  
  
- GGAAGTCAAC TAGATTGACT CTCACAGTAG TCAAGACTAA GACATCTTCA CGGACTAAAG AGTCTACGAA   
  
  
- CGGAGTTCAA GTATTCACTC TAAGAGGATC TTCTCCTAAA CCTACTTTCA GGACGGAGAC GAGTTCTTAA   
  
  
- ATCCCGAGAG GTTCGGTGAC TCTTCAGGGA CATACTACGA GAACCTCTCC CGATGAGAAA AAGTAGACTA   
  
  
- TTGTCGGGTA GTAGTAATCC TGTCTCATAA CTCGTGGTTT TACTCTTAAA ACTTAAGTCA AGGCCAATAG   
  
  
- GACCTTAACT CCCGATACAA TTACCACTAT AGTGCAAGCT CAGGTTGACC TACAAGTTGG ATTCGGTTAA   
  
  
- CCTAGGACAA GAATGAAACC TACTATAAAG AGTCGGGAAC AGACGTAGTT TGAGGGCTAG TAGACCCAGC   
  
  
- TCGTTACCGA AACTACTAAG TCCCCTACCC CGTCGGTGTA GAGGACCGTC ATGTCAATGT AGTTGTCAGG   
  
  
- GTCTCTTTTC CCAACTTAGA CAGTTAGCGA GTTCCTCCTT CTTTTTAGCA TTTTCCCTAC TTCCACCAGT   
  
  
- ACTCCTTCCC TCCTCATTGT TCGTCGTTCG AAGTAGGTTA CTTCTAATAC AACTCTACTT CCTCAAACTA   
  
  
- CTACATGACG AGACGTTTCT TCTCTTTCTA CAATAACGTT TGACGTGGTT ACTAAGGAGT GGGCAGCTTC   
  
  
- GCTCACTATT CAACGTCTTC TTTCCCCCCT TCCCCTTTTG TAGCGCACCC TTCTTCGTCT TATCGTGTTA   
  
  
- TCTTCTCCAC CTAGACTCCT GAGAAGAGTG AGTGACACGA GTTCGTTAAA GTTTAAAACT AGAATCCTCA   
  
  
- CGTTTACTCG TAGAGTCCGT TTATTCCGTC GTAAGAAGTG TTATACCACT ATCGGAGGTC TCCGAACGGG   
  
  
- TAATAAAACG ATTACCAGAA CTCCGAGCGT ATCGACCGTG ACCAAGTTGT TAGAGACGAT TACAACAACT   
  
  
- ACGAGCTTAG TGCAGTAGAC TAAAGAATTT CCGAATATCC GATATATACA GTCGGCAAGG AAAGTTTTCC   
  
  
- TACAATATAA AAGAGCGGTT GTTCTGTTAT GCCTTCAACC GACTCTTCCG TTGGTCCTAG GTGTAGTAAC   
  
  
- TAAAACCTCA GAACAACCCA AATGTCACCG GGACAGAGTA TGTTTTGGAG AGTTTCTCTG GGTGACCGGG   
  
  
- CGGTTCGGAG GCATAATGAC CCTATCTGAT AGGGGTCGTA CCAAAGGCCG GTAGTCTTTC CCAACTTCGC   
  
  
- TGACCGGCAG CCAATCTTCC CATAACGCTC TCTAAATTAC ACGGCAAGTT CATATTCCGG TATCGATTTT   
  
  
- TAACCCTCTC ATATTTCGAT CTCCTAGATC TTTAACTACT CTTACTCTAC CACAAACAGT TAACGTACGA   
  
  
- AGCGAGACCT TGTGACGAAC TACTCTGTCA CCACCGTCTG TCAGGTTTCC TACGAAAGAA TTCCAATTAG   
  
  
- TCTCTCTATT TAGGGGCAGA AAAGTAAGTA CCCTGATAGT TACCTAGTAA GTTACGAGGT AAGAAGTAGT   
  
  
- GAGCCAAGTC CCTCCGCGAG AAGGTGATGA GAAGAAATAA ACTATAGAAA CTTCGTTGAT ACGGGGCACT   
  
  
- TCTAGTACTT TCCGAGGACC AACTCTCACT CTATGTCCCG TTTCTTCGAA ACTTACAGTA TCGTACGCTT   
  
  
- CCACGGCTCT CCTAAGTTTC CGGACTTTGT ATGTTCGTTA CCGTTCGTTC CTGTTGTTCC CGGCCCAAAT   
  
  
- CCGTCGAAGG TGATCTGGCC CTCGAACACT CGTCTCGTTT CCGATACCAC TTTCGTTTGA TGGTATTCCT   
  
  
- AAAACACCAC CTGCTCCTGT CCGTAACCTA CGATGTGCCA ACCTTTCCTT CTTAGAAGAC ACGTGAGAGA   
  
  
- CAAACCGTTG GGTTGAC

+     CGTCA-motif

| Site Name | Organism | Position | Strand | Matrix score. | sequence | function |
| --- | --- | --- | --- | --- | --- | --- |
| CGTCA-motif | Hordeum vulgare | 3166 | + | 5 | CGTCA | cis-acting regulatory element involved in the MeJA-responsiveness |

>HU07G02246.1   
+ -Up\_Stream \_Len000ATTTGA TGTGTATGCT GACTACTTTT GATGATGATG ATCACAGTGA TGCATATTTA   
  
  
+ GAGCTGGTAA ATTGGTCATT CGATTCGAGT ACGGGTCGGG TCAAGTTCTG ATTAAGTGAC ATTTCGCGTC   
  
  
+ GTTTTGGTTT AGTTCGGGTC GGATCGATTT CGGGTTAAAT AATTTTTGGT GGAATACGCT TGTCATGCCA   
  
  
+ AACACAAGCA ACTTTGTTAA AAATTTCGAT TCGGGTCAGG TCAATTCAGG TTTGGGGTCC ACTTTCGAGT   
  
  
+ GAGCATATTT CGGATGTCGG GTCGCGTATG GGTCCGGGTC ATTCGGTTTG AATTTCGGAT CTTGGATCAA   
  
  
+ TTTTGCTAAG TATGTGCATA TTCAACCCTT GATGACATAG GAAACGCTTT GTCATCTCCA CCAATCAGAT   
  
  
+ ATCGTTATCT CTTTAATCAT CGCCATAATA GCATGAGTAA GCATGACCAT CTTTTTCAAA CATGTGATAC   
  
  
+ AGTTAAGGCC TGTTCTTTTC GTTAATAAAT CCCAATTTAT CAATTTTAAT ATATACTTTA ACGAATTTTA   
  
  
+ ACAAAAAATT TTAATTTCAA TCCGCTATAA CAAAAAAAAT ACCAACTTCA ACTTATTATA TAAAAAAAAT   
  
  
+ ATACAATTTT TTTTCAATTT TAGCCAACTT CAACAAAAAA AAAAAAAATT TAACAAATCT CAATCAACTT   
  
  
+ CACCCATTTC TCAACTTATA TCTCCTAATG AAAAGAACAG ATCCTTAGCA AGACTTCTCG ATTCATTTAA   
  
  
+ AAGGATAGTA TGGTAAATTC ATTTTATACA AGTGTCAAGT GTGAATATCT TTTTCACATC AGGTGATATA   
  
  
+ TTAGTAGGAC TTTTCGATTC ACTTATCTTC CTCTTTATGT TTTGTTAATG TGATAGTAAC GATAGGATTG   
  
  
+ CATGTATTTT TTCCAACTAT TCATTTACAA CTATCAATAT CTATTGATAA ATATGTTAAA ATAATTATAT   
  
  
+ GTTATTTAAA TTATTATTTG TTACTTAAGG TGTCGACTTT GTCGAAAGCG ACTTAATTCT AATTAAGATA   
  
  
+ GCGGCAGAGA AAGTTACACC TGATGACCAA GTTAGATCTG GTAAAACTAA CCATAGATCT GAAACCCAAA   
  
  
+ CATAATGATG TGTATTCATA CCCGACCTGA CATCTGAAAT AAGTTTACTC AAAAGTGGTT CCGAAACTTG   
  
  
+ AATTGACTCG AACCGAACCG TATTTGACTC GAAATTTTCT ACAAAGTTGC TTATTTTTGC ATGAAGACAT   
  
  
+ ATTGAGCCAA AAATTAGTTG ATTCGAAGTC AACTTAAACA AAAATTCATT CGACTCAAAC TGAAATATCA   
  
  
+ CTTAACTAAA ACTATGACTC AACTCAAACT TGATCTGATC CGTATTCAAA TCAAATACTC CATTTGCTAG   
  
  
+ GTCTAGAACC AGCCACGACC CCACATGGAC TATGCGGTTC AATGAAACGG CCAAATATAT CGCAATTTCT   
  
  
+ GTCTCCATCA ATCACACCCA TTTGCTACAC CCTGTTAATA TTTGACTCGA CCCTGTGAAT TGCCTTCGCC   
  
  
+ AGTTTTGTTT AATCTCTCTG GTTGCTTCCC ATCCAAGTCT GAATAGTATA GTTTGTACAG TGTGTACTAC   
  
  
+ TAGAAAGCAC GTTCCTTGAA TCTTCTTTCA AAGTTGTTTT TAAAGGGTTG AGTGATCCAT TACGACTTTT   
  
  
+ GATCTGGGTT CATCGCCTCC TCTCCTAATC CCTCACCCGG GTACCCTCAA TTCTCTATCG TCTCTGTTGC   
  
  
+ TCAGCTAATT CAAATTCCTT TAGTTGGTCA TCATTGAAAT CTGAAATCAG GGTGTTTTTC TTTTTATTAT   
  
  
+ TATCTGGCAG TTGGTTTGTT TATCTCTTGG TTTTTGGGGC TTATTTGGGT GCTGGTTGAT GTTATCCTGC   
  
  
+ TTGGGTTGAA TCTTCATCAG GTTTATTTTT GATTCATAGT TGAGGGTGAA AATTATTTCT CATACCCACC   
  
  
+ ACCTGTTTGT TCTTTTGTCT CTGAGAAAAA CCAAAAAGCT TTCTCTTTTT CCTGATGCAT CCTGTGCTCG   
  
  
+ TTGATCCTGA GTTAGTGAAC CATGCGTCCA AATTCAAGCC TGATTTGCTC TCAAACTTTG ATAAACAACA   
  
  
+ AGAATTTAGC AACGGGATCG AACAAGATGA CATCTTGCAA AACCCTGATC TGTGTTTTGA TTCAGAAACC   
  
  
+ CCTTCAGTTG ATCTAACTGA GAGTGTCATC AGTTCTGATT CTGTAGAAGT GCCTGATTTC TCAGATGCTT   
  
  
+ GCCTCAAGTT CATAAGTGAG ATTCTCCTAG AAGAGGATTT GGATGAAAGT CCTGCCTCTG CTCAAGAATT   
  
  
+ TAGGGCTCTC CAAGCCACTG AGAAGTCCCT GTATGATGCT CTTGGAGAGG GCTACTCTTT TTCATCTGAT   
  
  
+ AACAGCCCAT CATCATTAGG ACAGAGTATT GAGCACCAAA ATGAGAATTT TGAATTCAGT TCCGGTTATC   
  
  
+ CTGGAATTGA GGGCTATGTT AATGGTGATA TCACGTTCGA GTCCAACTGG ATGTTCAACC TAAGCCAATT   
  
  
+ GGATCCTGTT CTTACTTTGG ATGATATTTC TCAGCCCTTG TCTGCATCAA ACTCCCGATC ATCTGGGTCG   
  
  
+ AGCAATGGCT TTGATGATTC AGGGGATGGG GCAGCCACAT CTCCTGGCAG TACAGTTACA TCAACAGTCC   
  
  
+ CAGAGAAAAG GGTTGAATCT GTCAATCGCT CAAGGAGGAA GAAAAATCGT AAAAGGGATG AAGGTGGTCA   
  
  
+ TGAGGAAGGG AGGAGTAACA AGCAGCAAGC TTCATCCAAT GAAGATTATG TTGAGATGAA GGAGTTTGAT   
  
  
+ GATGTACTGC TCTGCAAAGA AGAGAAAGAT GTTATTGCAA ACTGCACCAA TGATTCCTCA CCCGTCGAAG   
  
  
+ CGAGTGATAA GTTGCAGAAG AAAGGGGGGA AGGGGAAAAC ATCGCGTGGG AAGAAGCAGA ATAGCACAAT   
  
  
+ AGAAGAGGTG GATCTGAGGA CTCTTCTCAC TCACTGTGCT CAAGCAATTT CAAATTTTGA TCTTAGGAGT   
  
  
+ GCAAATGAGC ATCTCAGGCA AATAAGGCAG CATTCTTCAC AATATGGTGA TAGCCTCCAG AGGCTTGCCC   
  
  
+ ATTATTTTGC TAATGGTCTT GAGGCTCGCA TAGCTGGCAC TGGTTCAACA ATCTCTGCTA ATGTTGTTGA   
  
  
+ TGCTCGAATC ACGTCATCTG ATTTCTTAAA GGCTTATAGG CTATATATGT CAGCCGTTCC TTTCAAAAGG   
  
  
+ ATGTTATATT TTCTCGCCAA CAAGACAATA CGGAAGTTGG CTGAGAAGGC AACCAGGATC CACATCATTG   
  
  
+ ATTTTGGAGT CTTGTTGGGT TTACAGTGGC CCTGTCTCAT ACAAAACCTC TCAAAGAGAC CCACTGGCCC   
  
  
+ GCCAAGCCTC CGTATTACTG GGATAGACTA TCCCCAGCAT GGTTTCCGGC CATCAGAAAG GGTTGAAGCG   
  
  
+ ACTGGCCGTC GGTTAGAAGG GTATTGCGAG AGATTTAATG TGCCGTTCAA GTATAAGGCC ATAGCTAAAA   
  
  
+ ATTGGGAGAG TATAAAGCTA GAGGATCTAG AAATTGATGA GAATGAGATG GTGTTTGTCA ATTGCATGCT   
  
  
+ TCGCTCTGGA ACACTGCTTG ATGAGACAGT GGTGGCAGAC AGTCCAAAGG ATGCTTTCTT AAGGTTAATC   
  
  
+ AGAGAGATAA ATCCCCGTCT TTTCATTCAT GGGACTATCA ATGGATCATT CAATGCTCCA TTCTTCATCA   
  
  
+ CTCGGTTCAG GGAGGCGCTC TTCCACTACT CTTCTTTATT TGATATCTTT GAAGCAACTA TGCCCCGTGA   
  
  
+ AGATCATGAA AGGCTCCTGG TTGAGAGTGA GATACAGGGC AAAGAAGCTT TGAATGTCAT AGCATGCGAA   
  
  
+ GGTGCCGAGA GGATTCAAAG GCCTGAAACA TACAAGCAAT GGCAAGCAAG GACAACAAGG GCCGGGTTTA   
  
  
+ GGCAGCTTCC ACTAGACCGG GAGCTTGTGA GCAGAGCAAA GGCTATGGTG AAAGCAAACT ACCATAAGGA   
  
  
+ TTTTGTGGTG GACGAGGACA GGCATTGGAT GCTACACGGT TGGAAAGGAA GAATCTTCTG TGCACTCTCT   
  
  
+ GTTTGGCAAC CCAACTG  

- -Up\_Stream \_Len000TAAACT ACACATACGA CTGATGAAAA CTACTACTAC TAGTGTCACT ACGTATAAAT   
  
  
- CTCGACCATT TAACCAGTAA GCTAAGCTCA TGCCCAGCCC AGTTCAAGAC TAATTCACTG TAAAGCGCAG   
  
  
- CAAAACCAAA TCAAGCCCAG CCTAGCTAAA GCCCAATTTA TTAAAAACCA CCTTATGCGA ACAGTACGGT   
  
  
- TTGTGTTCGT TGAAACAATT TTTAAAGCTA AGCCCAGTCC AGTTAAGTCC AAACCCCAGG TGAAAGCTCA   
  
  
- CTCGTATAAA GCCTACAGCC CAGCGCATAC CCAGGCCCAG TAAGCCAAAC TTAAAGCCTA GAACCTAGTT   
  
  
- AAAACGATTC ATACACGTAT AAGTTGGGAA CTACTGTATC CTTTGCGAAA CAGTAGAGGT GGTTAGTCTA   
  
  
- TAGCAATAGA GAAATTAGTA GCGGTATTAT CGTACTCATT CGTACTGGTA GAAAAAGTTT GTACACTATG   
  
  
- TCAATTCCGG ACAAGAAAAG CAATTATTTA GGGTTAAATA GTTAAAATTA TATATGAAAT TGCTTAAAAT   
  
  
- TGTTTTTTAA AATTAAAGTT AGGCGATATT GTTTTTTTTA TGGTTGAAGT TGAATAATAT ATTTTTTTTA   
  
  
- TATGTTAAAA AAAAGTTAAA ATCGGTTGAA GTTGTTTTTT TTTTTTTTAA ATTGTTTAGA GTTAGTTGAA   
  
  
- GTGGGTAAAG AGTTGAATAT AGAGGATTAC TTTTCTTGTC TAGGAATCGT TCTGAAGAGC TAAGTAAATT   
  
  
- TTCCTATCAT ACCATTTAAG TAAAATATGT TCACAGTTCA CACTTATAGA AAAAGTGTAG TCCACTATAT   
  
  
- AATCATCCTG AAAAGCTAAG TGAATAGAAG GAGAAATACA AAACAATTAC ACTATCATTG CTATCCTAAC   
  
  
- GTACATAAAA AAGGTTGATA AGTAAATGTT GATAGTTATA GATAACTATT TATACAATTT TATTAATATA   
  
  
- CAATAAATTT AATAATAAAC AATGAATTCC ACAGCTGAAA CAGCTTTCGC TGAATTAAGA TTAATTCTAT   
  
  
- CGCCGTCTCT TTCAATGTGG ACTACTGGTT CAATCTAGAC CATTTTGATT GGTATCTAGA CTTTGGGTTT   
  
  
- GTATTACTAC ACATAAGTAT GGGCTGGACT GTAGACTTTA TTCAAATGAG TTTTCACCAA GGCTTTGAAC   
  
  
- TTAACTGAGC TTGGCTTGGC ATAAACTGAG CTTTAAAAGA TGTTTCAACG AATAAAAACG TACTTCTGTA   
  
  
- TAACTCGGTT TTTAATCAAC TAAGCTTCAG TTGAATTTGT TTTTAAGTAA GCTGAGTTTG ACTTTATAGT   
  
  
- GAATTGATTT TGATACTGAG TTGAGTTTGA ACTAGACTAG GCATAAGTTT AGTTTATGAG GTAAACGATC   
  
  
- CAGATCTTGG TCGGTGCTGG GGTGTACCTG ATACGCCAAG TTACTTTGCC GGTTTATATA GCGTTAAAGA   
  
  
- CAGAGGTAGT TAGTGTGGGT AAACGATGTG GGACAATTAT AAACTGAGCT GGGACACTTA ACGGAAGCGG   
  
  
- TCAAAACAAA TTAGAGAGAC CAACGAAGGG TAGGTTCAGA CTTATCATAT CAAACATGTC ACACATGATG   
  
  
- ATCTTTCGTG CAAGGAACTT AGAAGAAAGT TTCAACAAAA ATTTCCCAAC TCACTAGGTA ATGCTGAAAA   
  
  
- CTAGACCCAA GTAGCGGAGG AGAGGATTAG GGAGTGGGCC CATGGGAGTT AAGAGATAGC AGAGACAACG   
  
  
- AGTCGATTAA GTTTAAGGAA ATCAACCAGT AGTAACTTTA GACTTTAGTC CCACAAAAAG AAAAATAATA   
  
  
- ATAGACCGTC AACCAAACAA ATAGAGAACC AAAAACCCCG AATAAACCCA CGACCAACTA CAATAGGACG   
  
  
- AACCCAACTT AGAAGTAGTC CAAATAAAAA CTAAGTATCA ACTCCCACTT TTAATAAAGA GTATGGGTGG   
  
  
- TGGACAAACA AGAAAACAGA GACTCTTTTT GGTTTTTCGA AAGAGAAAAA GGACTACGTA GGACACGAGC   
  
  
- AACTAGGACT CAATCACTTG GTACGCAGGT TTAAGTTCGG ACTAAACGAG AGTTTGAAAC TATTTGTTGT   
  
  
- TCTTAAATCG TTGCCCTAGC TTGTTCTACT GTAGAACGTT TTGGGACTAG ACACAAAACT AAGTCTTTGG   
  
  
- GGAAGTCAAC TAGATTGACT CTCACAGTAG TCAAGACTAA GACATCTTCA CGGACTAAAG AGTCTACGAA   
  
  
- CGGAGTTCAA GTATTCACTC TAAGAGGATC TTCTCCTAAA CCTACTTTCA GGACGGAGAC GAGTTCTTAA   
  
  
- ATCCCGAGAG GTTCGGTGAC TCTTCAGGGA CATACTACGA GAACCTCTCC CGATGAGAAA AAGTAGACTA   
  
  
- TTGTCGGGTA GTAGTAATCC TGTCTCATAA CTCGTGGTTT TACTCTTAAA ACTTAAGTCA AGGCCAATAG   
  
  
- GACCTTAACT CCCGATACAA TTACCACTAT AGTGCAAGCT CAGGTTGACC TACAAGTTGG ATTCGGTTAA   
  
  
- CCTAGGACAA GAATGAAACC TACTATAAAG AGTCGGGAAC AGACGTAGTT TGAGGGCTAG TAGACCCAGC   
  
  
- TCGTTACCGA AACTACTAAG TCCCCTACCC CGTCGGTGTA GAGGACCGTC ATGTCAATGT AGTTGTCAGG   
  
  
- GTCTCTTTTC CCAACTTAGA CAGTTAGCGA GTTCCTCCTT CTTTTTAGCA TTTTCCCTAC TTCCACCAGT   
  
  
- ACTCCTTCCC TCCTCATTGT TCGTCGTTCG AAGTAGGTTA CTTCTAATAC AACTCTACTT CCTCAAACTA   
  
  
- CTACATGACG AGACGTTTCT TCTCTTTCTA CAATAACGTT TGACGTGGTT ACTAAGGAGT GGGCAGCTTC   
  
  
- GCTCACTATT CAACGTCTTC TTTCCCCCCT TCCCCTTTTG TAGCGCACCC TTCTTCGTCT TATCGTGTTA   
  
  
- TCTTCTCCAC CTAGACTCCT GAGAAGAGTG AGTGACACGA GTTCGTTAAA GTTTAAAACT AGAATCCTCA   
  
  
- CGTTTACTCG TAGAGTCCGT TTATTCCGTC GTAAGAAGTG TTATACCACT ATCGGAGGTC TCCGAACGGG   
  
  
- TAATAAAACG ATTACCAGAA CTCCGAGCGT ATCGACCGTG ACCAAGTTGT TAGAGACGAT TACAACAACT   
  
  
- ACGAGCTTAG TGCAGTAGAC TAAAGAATTT CCGAATATCC GATATATACA GTCGGCAAGG AAAGTTTTCC   
  
  
- TACAATATAA AAGAGCGGTT GTTCTGTTAT GCCTTCAACC GACTCTTCCG TTGGTCCTAG GTGTAGTAAC   
  
  
- TAAAACCTCA GAACAACCCA AATGTCACCG GGACAGAGTA TGTTTTGGAG AGTTTCTCTG GGTGACCGGG   
  
  
- CGGTTCGGAG GCATAATGAC CCTATCTGAT AGGGGTCGTA CCAAAGGCCG GTAGTCTTTC CCAACTTCGC   
  
  
- TGACCGGCAG CCAATCTTCC CATAACGCTC TCTAAATTAC ACGGCAAGTT CATATTCCGG TATCGATTTT   
  
  
- TAACCCTCTC ATATTTCGAT CTCCTAGATC TTTAACTACT CTTACTCTAC CACAAACAGT TAACGTACGA   
  
  
- AGCGAGACCT TGTGACGAAC TACTCTGTCA CCACCGTCTG TCAGGTTTCC TACGAAAGAA TTCCAATTAG   
  
  
- TCTCTCTATT TAGGGGCAGA AAAGTAAGTA CCCTGATAGT TACCTAGTAA GTTACGAGGT AAGAAGTAGT   
  
  
- GAGCCAAGTC CCTCCGCGAG AAGGTGATGA GAAGAAATAA ACTATAGAAA CTTCGTTGAT ACGGGGCACT   
  
  
- TCTAGTACTT TCCGAGGACC AACTCTCACT CTATGTCCCG TTTCTTCGAA ACTTACAGTA TCGTACGCTT   
  
  
- CCACGGCTCT CCTAAGTTTC CGGACTTTGT ATGTTCGTTA CCGTTCGTTC CTGTTGTTCC CGGCCCAAAT   
  
  
- CCGTCGAAGG TGATCTGGCC CTCGAACACT CGTCTCGTTT CCGATACCAC TTTCGTTTGA TGGTATTCCT   
  
  
- AAAACACCAC CTGCTCCTGT CCGTAACCTA CGATGTGCCA ACCTTTCCTT CTTAGAAGAC ACGTGAGAGA   
  
  
- CAAACCGTTG GGTTGAC

+     G-Box

| Site Name | Organism | Position | Strand | Matrix score. | sequence | function |
| --- | --- | --- | --- | --- | --- | --- |
| G-Box | Pisum sativum | 2486 | + | 6 | CACGTT | cis-acting regulatory element involved in light responsiveness |
| G-Box | Pisum sativum | 1622 | + | 6 | CACGTT | cis-acting regulatory element involved in light responsiveness |

>HU07G02246.1   
+ -Up\_Stream \_Len000ATTTGA TGTGTATGCT GACTACTTTT GATGATGATG ATCACAGTGA TGCATATTTA   
  
  
+ GAGCTGGTAA ATTGGTCATT CGATTCGAGT ACGGGTCGGG TCAAGTTCTG ATTAAGTGAC ATTTCGCGTC   
  
  
+ GTTTTGGTTT AGTTCGGGTC GGATCGATTT CGGGTTAAAT AATTTTTGGT GGAATACGCT TGTCATGCCA   
  
  
+ AACACAAGCA ACTTTGTTAA AAATTTCGAT TCGGGTCAGG TCAATTCAGG TTTGGGGTCC ACTTTCGAGT   
  
  
+ GAGCATATTT CGGATGTCGG GTCGCGTATG GGTCCGGGTC ATTCGGTTTG AATTTCGGAT CTTGGATCAA   
  
  
+ TTTTGCTAAG TATGTGCATA TTCAACCCTT GATGACATAG GAAACGCTTT GTCATCTCCA CCAATCAGAT   
  
  
+ ATCGTTATCT CTTTAATCAT CGCCATAATA GCATGAGTAA GCATGACCAT CTTTTTCAAA CATGTGATAC   
  
  
+ AGTTAAGGCC TGTTCTTTTC GTTAATAAAT CCCAATTTAT CAATTTTAAT ATATACTTTA ACGAATTTTA   
  
  
+ ACAAAAAATT TTAATTTCAA TCCGCTATAA CAAAAAAAAT ACCAACTTCA ACTTATTATA TAAAAAAAAT   
  
  
+ ATACAATTTT TTTTCAATTT TAGCCAACTT CAACAAAAAA AAAAAAAATT TAACAAATCT CAATCAACTT   
  
  
+ CACCCATTTC TCAACTTATA TCTCCTAATG AAAAGAACAG ATCCTTAGCA AGACTTCTCG ATTCATTTAA   
  
  
+ AAGGATAGTA TGGTAAATTC ATTTTATACA AGTGTCAAGT GTGAATATCT TTTTCACATC AGGTGATATA   
  
  
+ TTAGTAGGAC TTTTCGATTC ACTTATCTTC CTCTTTATGT TTTGTTAATG TGATAGTAAC GATAGGATTG   
  
  
+ CATGTATTTT TTCCAACTAT TCATTTACAA CTATCAATAT CTATTGATAA ATATGTTAAA ATAATTATAT   
  
  
+ GTTATTTAAA TTATTATTTG TTACTTAAGG TGTCGACTTT GTCGAAAGCG ACTTAATTCT AATTAAGATA   
  
  
+ GCGGCAGAGA AAGTTACACC TGATGACCAA GTTAGATCTG GTAAAACTAA CCATAGATCT GAAACCCAAA   
  
  
+ CATAATGATG TGTATTCATA CCCGACCTGA CATCTGAAAT AAGTTTACTC AAAAGTGGTT CCGAAACTTG   
  
  
+ AATTGACTCG AACCGAACCG TATTTGACTC GAAATTTTCT ACAAAGTTGC TTATTTTTGC ATGAAGACAT   
  
  
+ ATTGAGCCAA AAATTAGTTG ATTCGAAGTC AACTTAAACA AAAATTCATT CGACTCAAAC TGAAATATCA   
  
  
+ CTTAACTAAA ACTATGACTC AACTCAAACT TGATCTGATC CGTATTCAAA TCAAATACTC CATTTGCTAG   
  
  
+ GTCTAGAACC AGCCACGACC CCACATGGAC TATGCGGTTC AATGAAACGG CCAAATATAT CGCAATTTCT   
  
  
+ GTCTCCATCA ATCACACCCA TTTGCTACAC CCTGTTAATA TTTGACTCGA CCCTGTGAAT TGCCTTCGCC   
  
  
+ AGTTTTGTTT AATCTCTCTG GTTGCTTCCC ATCCAAGTCT GAATAGTATA GTTTGTACAG TGTGTACTAC   
  
  
+ TAGAAAGCAC GTTCCTTGAA TCTTCTTTCA AAGTTGTTTT TAAAGGGTTG AGTGATCCAT TACGACTTTT   
  
  
+ GATCTGGGTT CATCGCCTCC TCTCCTAATC CCTCACCCGG GTACCCTCAA TTCTCTATCG TCTCTGTTGC   
  
  
+ TCAGCTAATT CAAATTCCTT TAGTTGGTCA TCATTGAAAT CTGAAATCAG GGTGTTTTTC TTTTTATTAT   
  
  
+ TATCTGGCAG TTGGTTTGTT TATCTCTTGG TTTTTGGGGC TTATTTGGGT GCTGGTTGAT GTTATCCTGC   
  
  
+ TTGGGTTGAA TCTTCATCAG GTTTATTTTT GATTCATAGT TGAGGGTGAA AATTATTTCT CATACCCACC   
  
  
+ ACCTGTTTGT TCTTTTGTCT CTGAGAAAAA CCAAAAAGCT TTCTCTTTTT CCTGATGCAT CCTGTGCTCG   
  
  
+ TTGATCCTGA GTTAGTGAAC CATGCGTCCA AATTCAAGCC TGATTTGCTC TCAAACTTTG ATAAACAACA   
  
  
+ AGAATTTAGC AACGGGATCG AACAAGATGA CATCTTGCAA AACCCTGATC TGTGTTTTGA TTCAGAAACC   
  
  
+ CCTTCAGTTG ATCTAACTGA GAGTGTCATC AGTTCTGATT CTGTAGAAGT GCCTGATTTC TCAGATGCTT   
  
  
+ GCCTCAAGTT CATAAGTGAG ATTCTCCTAG AAGAGGATTT GGATGAAAGT CCTGCCTCTG CTCAAGAATT   
  
  
+ TAGGGCTCTC CAAGCCACTG AGAAGTCCCT GTATGATGCT CTTGGAGAGG GCTACTCTTT TTCATCTGAT   
  
  
+ AACAGCCCAT CATCATTAGG ACAGAGTATT GAGCACCAAA ATGAGAATTT TGAATTCAGT TCCGGTTATC   
  
  
+ CTGGAATTGA GGGCTATGTT AATGGTGATA TCACGTTCGA GTCCAACTGG ATGTTCAACC TAAGCCAATT   
  
  
+ GGATCCTGTT CTTACTTTGG ATGATATTTC TCAGCCCTTG TCTGCATCAA ACTCCCGATC ATCTGGGTCG   
  
  
+ AGCAATGGCT TTGATGATTC AGGGGATGGG GCAGCCACAT CTCCTGGCAG TACAGTTACA TCAACAGTCC   
  
  
+ CAGAGAAAAG GGTTGAATCT GTCAATCGCT CAAGGAGGAA GAAAAATCGT AAAAGGGATG AAGGTGGTCA   
  
  
+ TGAGGAAGGG AGGAGTAACA AGCAGCAAGC TTCATCCAAT GAAGATTATG TTGAGATGAA GGAGTTTGAT   
  
  
+ GATGTACTGC TCTGCAAAGA AGAGAAAGAT GTTATTGCAA ACTGCACCAA TGATTCCTCA CCCGTCGAAG   
  
  
+ CGAGTGATAA GTTGCAGAAG AAAGGGGGGA AGGGGAAAAC ATCGCGTGGG AAGAAGCAGA ATAGCACAAT   
  
  
+ AGAAGAGGTG GATCTGAGGA CTCTTCTCAC TCACTGTGCT CAAGCAATTT CAAATTTTGA TCTTAGGAGT   
  
  
+ GCAAATGAGC ATCTCAGGCA AATAAGGCAG CATTCTTCAC AATATGGTGA TAGCCTCCAG AGGCTTGCCC   
  
  
+ ATTATTTTGC TAATGGTCTT GAGGCTCGCA TAGCTGGCAC TGGTTCAACA ATCTCTGCTA ATGTTGTTGA   
  
  
+ TGCTCGAATC ACGTCATCTG ATTTCTTAAA GGCTTATAGG CTATATATGT CAGCCGTTCC TTTCAAAAGG   
  
  
+ ATGTTATATT TTCTCGCCAA CAAGACAATA CGGAAGTTGG CTGAGAAGGC AACCAGGATC CACATCATTG   
  
  
+ ATTTTGGAGT CTTGTTGGGT TTACAGTGGC CCTGTCTCAT ACAAAACCTC TCAAAGAGAC CCACTGGCCC   
  
  
+ GCCAAGCCTC CGTATTACTG GGATAGACTA TCCCCAGCAT GGTTTCCGGC CATCAGAAAG GGTTGAAGCG   
  
  
+ ACTGGCCGTC GGTTAGAAGG GTATTGCGAG AGATTTAATG TGCCGTTCAA GTATAAGGCC ATAGCTAAAA   
  
  
+ ATTGGGAGAG TATAAAGCTA GAGGATCTAG AAATTGATGA GAATGAGATG GTGTTTGTCA ATTGCATGCT   
  
  
+ TCGCTCTGGA ACACTGCTTG ATGAGACAGT GGTGGCAGAC AGTCCAAAGG ATGCTTTCTT AAGGTTAATC   
  
  
+ AGAGAGATAA ATCCCCGTCT TTTCATTCAT GGGACTATCA ATGGATCATT CAATGCTCCA TTCTTCATCA   
  
  
+ CTCGGTTCAG GGAGGCGCTC TTCCACTACT CTTCTTTATT TGATATCTTT GAAGCAACTA TGCCCCGTGA   
  
  
+ AGATCATGAA AGGCTCCTGG TTGAGAGTGA GATACAGGGC AAAGAAGCTT TGAATGTCAT AGCATGCGAA   
  
  
+ GGTGCCGAGA GGATTCAAAG GCCTGAAACA TACAAGCAAT GGCAAGCAAG GACAACAAGG GCCGGGTTTA   
  
  
+ GGCAGCTTCC ACTAGACCGG GAGCTTGTGA GCAGAGCAAA GGCTATGGTG AAAGCAAACT ACCATAAGGA   
  
  
+ TTTTGTGGTG GACGAGGACA GGCATTGGAT GCTACACGGT TGGAAAGGAA GAATCTTCTG TGCACTCTCT   
  
  
+ GTTTGGCAAC CCAACTG  

- -Up\_Stream \_Len000TAAACT ACACATACGA CTGATGAAAA CTACTACTAC TAGTGTCACT ACGTATAAAT   
  
  
- CTCGACCATT TAACCAGTAA GCTAAGCTCA TGCCCAGCCC AGTTCAAGAC TAATTCACTG TAAAGCGCAG   
  
  
- CAAAACCAAA TCAAGCCCAG CCTAGCTAAA GCCCAATTTA TTAAAAACCA CCTTATGCGA ACAGTACGGT   
  
  
- TTGTGTTCGT TGAAACAATT TTTAAAGCTA AGCCCAGTCC AGTTAAGTCC AAACCCCAGG TGAAAGCTCA   
  
  
- CTCGTATAAA GCCTACAGCC CAGCGCATAC CCAGGCCCAG TAAGCCAAAC TTAAAGCCTA GAACCTAGTT   
  
  
- AAAACGATTC ATACACGTAT AAGTTGGGAA CTACTGTATC CTTTGCGAAA CAGTAGAGGT GGTTAGTCTA   
  
  
- TAGCAATAGA GAAATTAGTA GCGGTATTAT CGTACTCATT CGTACTGGTA GAAAAAGTTT GTACACTATG   
  
  
- TCAATTCCGG ACAAGAAAAG CAATTATTTA GGGTTAAATA GTTAAAATTA TATATGAAAT TGCTTAAAAT   
  
  
- TGTTTTTTAA AATTAAAGTT AGGCGATATT GTTTTTTTTA TGGTTGAAGT TGAATAATAT ATTTTTTTTA   
  
  
- TATGTTAAAA AAAAGTTAAA ATCGGTTGAA GTTGTTTTTT TTTTTTTTAA ATTGTTTAGA GTTAGTTGAA   
  
  
- GTGGGTAAAG AGTTGAATAT AGAGGATTAC TTTTCTTGTC TAGGAATCGT TCTGAAGAGC TAAGTAAATT   
  
  
- TTCCTATCAT ACCATTTAAG TAAAATATGT TCACAGTTCA CACTTATAGA AAAAGTGTAG TCCACTATAT   
  
  
- AATCATCCTG AAAAGCTAAG TGAATAGAAG GAGAAATACA AAACAATTAC ACTATCATTG CTATCCTAAC   
  
  
- GTACATAAAA AAGGTTGATA AGTAAATGTT GATAGTTATA GATAACTATT TATACAATTT TATTAATATA   
  
  
- CAATAAATTT AATAATAAAC AATGAATTCC ACAGCTGAAA CAGCTTTCGC TGAATTAAGA TTAATTCTAT   
  
  
- CGCCGTCTCT TTCAATGTGG ACTACTGGTT CAATCTAGAC CATTTTGATT GGTATCTAGA CTTTGGGTTT   
  
  
- GTATTACTAC ACATAAGTAT GGGCTGGACT GTAGACTTTA TTCAAATGAG TTTTCACCAA GGCTTTGAAC   
  
  
- TTAACTGAGC TTGGCTTGGC ATAAACTGAG CTTTAAAAGA TGTTTCAACG AATAAAAACG TACTTCTGTA   
  
  
- TAACTCGGTT TTTAATCAAC TAAGCTTCAG TTGAATTTGT TTTTAAGTAA GCTGAGTTTG ACTTTATAGT   
  
  
- GAATTGATTT TGATACTGAG TTGAGTTTGA ACTAGACTAG GCATAAGTTT AGTTTATGAG GTAAACGATC   
  
  
- CAGATCTTGG TCGGTGCTGG GGTGTACCTG ATACGCCAAG TTACTTTGCC GGTTTATATA GCGTTAAAGA   
  
  
- CAGAGGTAGT TAGTGTGGGT AAACGATGTG GGACAATTAT AAACTGAGCT GGGACACTTA ACGGAAGCGG   
  
  
- TCAAAACAAA TTAGAGAGAC CAACGAAGGG TAGGTTCAGA CTTATCATAT CAAACATGTC ACACATGATG   
  
  
- ATCTTTCGTG CAAGGAACTT AGAAGAAAGT TTCAACAAAA ATTTCCCAAC TCACTAGGTA ATGCTGAAAA   
  
  
- CTAGACCCAA GTAGCGGAGG AGAGGATTAG GGAGTGGGCC CATGGGAGTT AAGAGATAGC AGAGACAACG   
  
  
- AGTCGATTAA GTTTAAGGAA ATCAACCAGT AGTAACTTTA GACTTTAGTC CCACAAAAAG AAAAATAATA   
  
  
- ATAGACCGTC AACCAAACAA ATAGAGAACC AAAAACCCCG AATAAACCCA CGACCAACTA CAATAGGACG   
  
  
- AACCCAACTT AGAAGTAGTC CAAATAAAAA CTAAGTATCA ACTCCCACTT TTAATAAAGA GTATGGGTGG   
  
  
- TGGACAAACA AGAAAACAGA GACTCTTTTT GGTTTTTCGA AAGAGAAAAA GGACTACGTA GGACACGAGC   
  
  
- AACTAGGACT CAATCACTTG GTACGCAGGT TTAAGTTCGG ACTAAACGAG AGTTTGAAAC TATTTGTTGT   
  
  
- TCTTAAATCG TTGCCCTAGC TTGTTCTACT GTAGAACGTT TTGGGACTAG ACACAAAACT AAGTCTTTGG   
  
  
- GGAAGTCAAC TAGATTGACT CTCACAGTAG TCAAGACTAA GACATCTTCA CGGACTAAAG AGTCTACGAA   
  
  
- CGGAGTTCAA GTATTCACTC TAAGAGGATC TTCTCCTAAA CCTACTTTCA GGACGGAGAC GAGTTCTTAA   
  
  
- ATCCCGAGAG GTTCGGTGAC TCTTCAGGGA CATACTACGA GAACCTCTCC CGATGAGAAA AAGTAGACTA   
  
  
- TTGTCGGGTA GTAGTAATCC TGTCTCATAA CTCGTGGTTT TACTCTTAAA ACTTAAGTCA AGGCCAATAG   
  
  
- GACCTTAACT CCCGATACAA TTACCACTAT AGTGCAAGCT CAGGTTGACC TACAAGTTGG ATTCGGTTAA   
  
  
- CCTAGGACAA GAATGAAACC TACTATAAAG AGTCGGGAAC AGACGTAGTT TGAGGGCTAG TAGACCCAGC   
  
  
- TCGTTACCGA AACTACTAAG TCCCCTACCC CGTCGGTGTA GAGGACCGTC ATGTCAATGT AGTTGTCAGG   
  
  
- GTCTCTTTTC CCAACTTAGA CAGTTAGCGA GTTCCTCCTT CTTTTTAGCA TTTTCCCTAC TTCCACCAGT   
  
  
- ACTCCTTCCC TCCTCATTGT TCGTCGTTCG AAGTAGGTTA CTTCTAATAC AACTCTACTT CCTCAAACTA   
  
  
- CTACATGACG AGACGTTTCT TCTCTTTCTA CAATAACGTT TGACGTGGTT ACTAAGGAGT GGGCAGCTTC   
  
  
- GCTCACTATT CAACGTCTTC TTTCCCCCCT TCCCCTTTTG TAGCGCACCC TTCTTCGTCT TATCGTGTTA   
  
  
- TCTTCTCCAC CTAGACTCCT GAGAAGAGTG AGTGACACGA GTTCGTTAAA GTTTAAAACT AGAATCCTCA   
  
  
- CGTTTACTCG TAGAGTCCGT TTATTCCGTC GTAAGAAGTG TTATACCACT ATCGGAGGTC TCCGAACGGG   
  
  
- TAATAAAACG ATTACCAGAA CTCCGAGCGT ATCGACCGTG ACCAAGTTGT TAGAGACGAT TACAACAACT   
  
  
- ACGAGCTTAG TGCAGTAGAC TAAAGAATTT CCGAATATCC GATATATACA GTCGGCAAGG AAAGTTTTCC   
  
  
- TACAATATAA AAGAGCGGTT GTTCTGTTAT GCCTTCAACC GACTCTTCCG TTGGTCCTAG GTGTAGTAAC   
  
  
- TAAAACCTCA GAACAACCCA AATGTCACCG GGACAGAGTA TGTTTTGGAG AGTTTCTCTG GGTGACCGGG   
  
  
- CGGTTCGGAG GCATAATGAC CCTATCTGAT AGGGGTCGTA CCAAAGGCCG GTAGTCTTTC CCAACTTCGC   
  
  
- TGACCGGCAG CCAATCTTCC CATAACGCTC TCTAAATTAC ACGGCAAGTT CATATTCCGG TATCGATTTT   
  
  
- TAACCCTCTC ATATTTCGAT CTCCTAGATC TTTAACTACT CTTACTCTAC CACAAACAGT TAACGTACGA   
  
  
- AGCGAGACCT TGTGACGAAC TACTCTGTCA CCACCGTCTG TCAGGTTTCC TACGAAAGAA TTCCAATTAG   
  
  
- TCTCTCTATT TAGGGGCAGA AAAGTAAGTA CCCTGATAGT TACCTAGTAA GTTACGAGGT AAGAAGTAGT   
  
  
- GAGCCAAGTC CCTCCGCGAG AAGGTGATGA GAAGAAATAA ACTATAGAAA CTTCGTTGAT ACGGGGCACT   
  
  
- TCTAGTACTT TCCGAGGACC AACTCTCACT CTATGTCCCG TTTCTTCGAA ACTTACAGTA TCGTACGCTT   
  
  
- CCACGGCTCT CCTAAGTTTC CGGACTTTGT ATGTTCGTTA CCGTTCGTTC CTGTTGTTCC CGGCCCAAAT   
  
  
- CCGTCGAAGG TGATCTGGCC CTCGAACACT CGTCTCGTTT CCGATACCAC TTTCGTTTGA TGGTATTCCT   
  
  
- AAAACACCAC CTGCTCCTGT CCGTAACCTA CGATGTGCCA ACCTTTCCTT CTTAGAAGAC ACGTGAGAGA   
  
  
- CAAACCGTTG GGTTGAC

+     G-box

| Site Name | Organism | Position | Strand | Matrix score. | sequence | function |
| --- | --- | --- | --- | --- | --- | --- |
| G-box | Zea mays | 1418 | + | 6 | CACGAC | cis-acting regulatory element involved in light responsiveness |
| G-box | Zea mays | 3164 | + | 6 | CACGTC | cis-acting regulatory element involved in light responsiveness |

>HU07G02246.1   
+ -Up\_Stream \_Len000ATTTGA TGTGTATGCT GACTACTTTT GATGATGATG ATCACAGTGA TGCATATTTA   
  
  
+ GAGCTGGTAA ATTGGTCATT CGATTCGAGT ACGGGTCGGG TCAAGTTCTG ATTAAGTGAC ATTTCGCGTC   
  
  
+ GTTTTGGTTT AGTTCGGGTC GGATCGATTT CGGGTTAAAT AATTTTTGGT GGAATACGCT TGTCATGCCA   
  
  
+ AACACAAGCA ACTTTGTTAA AAATTTCGAT TCGGGTCAGG TCAATTCAGG TTTGGGGTCC ACTTTCGAGT   
  
  
+ GAGCATATTT CGGATGTCGG GTCGCGTATG GGTCCGGGTC ATTCGGTTTG AATTTCGGAT CTTGGATCAA   
  
  
+ TTTTGCTAAG TATGTGCATA TTCAACCCTT GATGACATAG GAAACGCTTT GTCATCTCCA CCAATCAGAT   
  
  
+ ATCGTTATCT CTTTAATCAT CGCCATAATA GCATGAGTAA GCATGACCAT CTTTTTCAAA CATGTGATAC   
  
  
+ AGTTAAGGCC TGTTCTTTTC GTTAATAAAT CCCAATTTAT CAATTTTAAT ATATACTTTA ACGAATTTTA   
  
  
+ ACAAAAAATT TTAATTTCAA TCCGCTATAA CAAAAAAAAT ACCAACTTCA ACTTATTATA TAAAAAAAAT   
  
  
+ ATACAATTTT TTTTCAATTT TAGCCAACTT CAACAAAAAA AAAAAAAATT TAACAAATCT CAATCAACTT   
  
  
+ CACCCATTTC TCAACTTATA TCTCCTAATG AAAAGAACAG ATCCTTAGCA AGACTTCTCG ATTCATTTAA   
  
  
+ AAGGATAGTA TGGTAAATTC ATTTTATACA AGTGTCAAGT GTGAATATCT TTTTCACATC AGGTGATATA   
  
  
+ TTAGTAGGAC TTTTCGATTC ACTTATCTTC CTCTTTATGT TTTGTTAATG TGATAGTAAC GATAGGATTG   
  
  
+ CATGTATTTT TTCCAACTAT TCATTTACAA CTATCAATAT CTATTGATAA ATATGTTAAA ATAATTATAT   
  
  
+ GTTATTTAAA TTATTATTTG TTACTTAAGG TGTCGACTTT GTCGAAAGCG ACTTAATTCT AATTAAGATA   
  
  
+ GCGGCAGAGA AAGTTACACC TGATGACCAA GTTAGATCTG GTAAAACTAA CCATAGATCT GAAACCCAAA   
  
  
+ CATAATGATG TGTATTCATA CCCGACCTGA CATCTGAAAT AAGTTTACTC AAAAGTGGTT CCGAAACTTG   
  
  
+ AATTGACTCG AACCGAACCG TATTTGACTC GAAATTTTCT ACAAAGTTGC TTATTTTTGC ATGAAGACAT   
  
  
+ ATTGAGCCAA AAATTAGTTG ATTCGAAGTC AACTTAAACA AAAATTCATT CGACTCAAAC TGAAATATCA   
  
  
+ CTTAACTAAA ACTATGACTC AACTCAAACT TGATCTGATC CGTATTCAAA TCAAATACTC CATTTGCTAG   
  
  
+ GTCTAGAACC AGCCACGACC CCACATGGAC TATGCGGTTC AATGAAACGG CCAAATATAT CGCAATTTCT   
  
  
+ GTCTCCATCA ATCACACCCA TTTGCTACAC CCTGTTAATA TTTGACTCGA CCCTGTGAAT TGCCTTCGCC   
  
  
+ AGTTTTGTTT AATCTCTCTG GTTGCTTCCC ATCCAAGTCT GAATAGTATA GTTTGTACAG TGTGTACTAC   
  
  
+ TAGAAAGCAC GTTCCTTGAA TCTTCTTTCA AAGTTGTTTT TAAAGGGTTG AGTGATCCAT TACGACTTTT   
  
  
+ GATCTGGGTT CATCGCCTCC TCTCCTAATC CCTCACCCGG GTACCCTCAA TTCTCTATCG TCTCTGTTGC   
  
  
+ TCAGCTAATT CAAATTCCTT TAGTTGGTCA TCATTGAAAT CTGAAATCAG GGTGTTTTTC TTTTTATTAT   
  
  
+ TATCTGGCAG TTGGTTTGTT TATCTCTTGG TTTTTGGGGC TTATTTGGGT GCTGGTTGAT GTTATCCTGC   
  
  
+ TTGGGTTGAA TCTTCATCAG GTTTATTTTT GATTCATAGT TGAGGGTGAA AATTATTTCT CATACCCACC   
  
  
+ ACCTGTTTGT TCTTTTGTCT CTGAGAAAAA CCAAAAAGCT TTCTCTTTTT CCTGATGCAT CCTGTGCTCG   
  
  
+ TTGATCCTGA GTTAGTGAAC CATGCGTCCA AATTCAAGCC TGATTTGCTC TCAAACTTTG ATAAACAACA   
  
  
+ AGAATTTAGC AACGGGATCG AACAAGATGA CATCTTGCAA AACCCTGATC TGTGTTTTGA TTCAGAAACC   
  
  
+ CCTTCAGTTG ATCTAACTGA GAGTGTCATC AGTTCTGATT CTGTAGAAGT GCCTGATTTC TCAGATGCTT   
  
  
+ GCCTCAAGTT CATAAGTGAG ATTCTCCTAG AAGAGGATTT GGATGAAAGT CCTGCCTCTG CTCAAGAATT   
  
  
+ TAGGGCTCTC CAAGCCACTG AGAAGTCCCT GTATGATGCT CTTGGAGAGG GCTACTCTTT TTCATCTGAT   
  
  
+ AACAGCCCAT CATCATTAGG ACAGAGTATT GAGCACCAAA ATGAGAATTT TGAATTCAGT TCCGGTTATC   
  
  
+ CTGGAATTGA GGGCTATGTT AATGGTGATA TCACGTTCGA GTCCAACTGG ATGTTCAACC TAAGCCAATT   
  
  
+ GGATCCTGTT CTTACTTTGG ATGATATTTC TCAGCCCTTG TCTGCATCAA ACTCCCGATC ATCTGGGTCG   
  
  
+ AGCAATGGCT TTGATGATTC AGGGGATGGG GCAGCCACAT CTCCTGGCAG TACAGTTACA TCAACAGTCC   
  
  
+ CAGAGAAAAG GGTTGAATCT GTCAATCGCT CAAGGAGGAA GAAAAATCGT AAAAGGGATG AAGGTGGTCA   
  
  
+ TGAGGAAGGG AGGAGTAACA AGCAGCAAGC TTCATCCAAT GAAGATTATG TTGAGATGAA GGAGTTTGAT   
  
  
+ GATGTACTGC TCTGCAAAGA AGAGAAAGAT GTTATTGCAA ACTGCACCAA TGATTCCTCA CCCGTCGAAG   
  
  
+ CGAGTGATAA GTTGCAGAAG AAAGGGGGGA AGGGGAAAAC ATCGCGTGGG AAGAAGCAGA ATAGCACAAT   
  
  
+ AGAAGAGGTG GATCTGAGGA CTCTTCTCAC TCACTGTGCT CAAGCAATTT CAAATTTTGA TCTTAGGAGT   
  
  
+ GCAAATGAGC ATCTCAGGCA AATAAGGCAG CATTCTTCAC AATATGGTGA TAGCCTCCAG AGGCTTGCCC   
  
  
+ ATTATTTTGC TAATGGTCTT GAGGCTCGCA TAGCTGGCAC TGGTTCAACA ATCTCTGCTA ATGTTGTTGA   
  
  
+ TGCTCGAATC ACGTCATCTG ATTTCTTAAA GGCTTATAGG CTATATATGT CAGCCGTTCC TTTCAAAAGG   
  
  
+ ATGTTATATT TTCTCGCCAA CAAGACAATA CGGAAGTTGG CTGAGAAGGC AACCAGGATC CACATCATTG   
  
  
+ ATTTTGGAGT CTTGTTGGGT TTACAGTGGC CCTGTCTCAT ACAAAACCTC TCAAAGAGAC CCACTGGCCC   
  
  
+ GCCAAGCCTC CGTATTACTG GGATAGACTA TCCCCAGCAT GGTTTCCGGC CATCAGAAAG GGTTGAAGCG   
  
  
+ ACTGGCCGTC GGTTAGAAGG GTATTGCGAG AGATTTAATG TGCCGTTCAA GTATAAGGCC ATAGCTAAAA   
  
  
+ ATTGGGAGAG TATAAAGCTA GAGGATCTAG AAATTGATGA GAATGAGATG GTGTTTGTCA ATTGCATGCT   
  
  
+ TCGCTCTGGA ACACTGCTTG ATGAGACAGT GGTGGCAGAC AGTCCAAAGG ATGCTTTCTT AAGGTTAATC   
  
  
+ AGAGAGATAA ATCCCCGTCT TTTCATTCAT GGGACTATCA ATGGATCATT CAATGCTCCA TTCTTCATCA   
  
  
+ CTCGGTTCAG GGAGGCGCTC TTCCACTACT CTTCTTTATT TGATATCTTT GAAGCAACTA TGCCCCGTGA   
  
  
+ AGATCATGAA AGGCTCCTGG TTGAGAGTGA GATACAGGGC AAAGAAGCTT TGAATGTCAT AGCATGCGAA   
  
  
+ GGTGCCGAGA GGATTCAAAG GCCTGAAACA TACAAGCAAT GGCAAGCAAG GACAACAAGG GCCGGGTTTA   
  
  
+ GGCAGCTTCC ACTAGACCGG GAGCTTGTGA GCAGAGCAAA GGCTATGGTG AAAGCAAACT ACCATAAGGA   
  
  
+ TTTTGTGGTG GACGAGGACA GGCATTGGAT GCTACACGGT TGGAAAGGAA GAATCTTCTG TGCACTCTCT   
  
  
+ GTTTGGCAAC CCAACTG  

- -Up\_Stream \_Len000TAAACT ACACATACGA CTGATGAAAA CTACTACTAC TAGTGTCACT ACGTATAAAT   
  
  
- CTCGACCATT TAACCAGTAA GCTAAGCTCA TGCCCAGCCC AGTTCAAGAC TAATTCACTG TAAAGCGCAG   
  
  
- CAAAACCAAA TCAAGCCCAG CCTAGCTAAA GCCCAATTTA TTAAAAACCA CCTTATGCGA ACAGTACGGT   
  
  
- TTGTGTTCGT TGAAACAATT TTTAAAGCTA AGCCCAGTCC AGTTAAGTCC AAACCCCAGG TGAAAGCTCA   
  
  
- CTCGTATAAA GCCTACAGCC CAGCGCATAC CCAGGCCCAG TAAGCCAAAC TTAAAGCCTA GAACCTAGTT   
  
  
- AAAACGATTC ATACACGTAT AAGTTGGGAA CTACTGTATC CTTTGCGAAA CAGTAGAGGT GGTTAGTCTA   
  
  
- TAGCAATAGA GAAATTAGTA GCGGTATTAT CGTACTCATT CGTACTGGTA GAAAAAGTTT GTACACTATG   
  
  
- TCAATTCCGG ACAAGAAAAG CAATTATTTA GGGTTAAATA GTTAAAATTA TATATGAAAT TGCTTAAAAT   
  
  
- TGTTTTTTAA AATTAAAGTT AGGCGATATT GTTTTTTTTA TGGTTGAAGT TGAATAATAT ATTTTTTTTA   
  
  
- TATGTTAAAA AAAAGTTAAA ATCGGTTGAA GTTGTTTTTT TTTTTTTTAA ATTGTTTAGA GTTAGTTGAA   
  
  
- GTGGGTAAAG AGTTGAATAT AGAGGATTAC TTTTCTTGTC TAGGAATCGT TCTGAAGAGC TAAGTAAATT   
  
  
- TTCCTATCAT ACCATTTAAG TAAAATATGT TCACAGTTCA CACTTATAGA AAAAGTGTAG TCCACTATAT   
  
  
- AATCATCCTG AAAAGCTAAG TGAATAGAAG GAGAAATACA AAACAATTAC ACTATCATTG CTATCCTAAC   
  
  
- GTACATAAAA AAGGTTGATA AGTAAATGTT GATAGTTATA GATAACTATT TATACAATTT TATTAATATA   
  
  
- CAATAAATTT AATAATAAAC AATGAATTCC ACAGCTGAAA CAGCTTTCGC TGAATTAAGA TTAATTCTAT   
  
  
- CGCCGTCTCT TTCAATGTGG ACTACTGGTT CAATCTAGAC CATTTTGATT GGTATCTAGA CTTTGGGTTT   
  
  
- GTATTACTAC ACATAAGTAT GGGCTGGACT GTAGACTTTA TTCAAATGAG TTTTCACCAA GGCTTTGAAC   
  
  
- TTAACTGAGC TTGGCTTGGC ATAAACTGAG CTTTAAAAGA TGTTTCAACG AATAAAAACG TACTTCTGTA   
  
  
- TAACTCGGTT TTTAATCAAC TAAGCTTCAG TTGAATTTGT TTTTAAGTAA GCTGAGTTTG ACTTTATAGT   
  
  
- GAATTGATTT TGATACTGAG TTGAGTTTGA ACTAGACTAG GCATAAGTTT AGTTTATGAG GTAAACGATC   
  
  
- CAGATCTTGG TCGGTGCTGG GGTGTACCTG ATACGCCAAG TTACTTTGCC GGTTTATATA GCGTTAAAGA   
  
  
- CAGAGGTAGT TAGTGTGGGT AAACGATGTG GGACAATTAT AAACTGAGCT GGGACACTTA ACGGAAGCGG   
  
  
- TCAAAACAAA TTAGAGAGAC CAACGAAGGG TAGGTTCAGA CTTATCATAT CAAACATGTC ACACATGATG   
  
  
- ATCTTTCGTG CAAGGAACTT AGAAGAAAGT TTCAACAAAA ATTTCCCAAC TCACTAGGTA ATGCTGAAAA   
  
  
- CTAGACCCAA GTAGCGGAGG AGAGGATTAG GGAGTGGGCC CATGGGAGTT AAGAGATAGC AGAGACAACG   
  
  
- AGTCGATTAA GTTTAAGGAA ATCAACCAGT AGTAACTTTA GACTTTAGTC CCACAAAAAG AAAAATAATA   
  
  
- ATAGACCGTC AACCAAACAA ATAGAGAACC AAAAACCCCG AATAAACCCA CGACCAACTA CAATAGGACG   
  
  
- AACCCAACTT AGAAGTAGTC CAAATAAAAA CTAAGTATCA ACTCCCACTT TTAATAAAGA GTATGGGTGG   
  
  
- TGGACAAACA AGAAAACAGA GACTCTTTTT GGTTTTTCGA AAGAGAAAAA GGACTACGTA GGACACGAGC   
  
  
- AACTAGGACT CAATCACTTG GTACGCAGGT TTAAGTTCGG ACTAAACGAG AGTTTGAAAC TATTTGTTGT   
  
  
- TCTTAAATCG TTGCCCTAGC TTGTTCTACT GTAGAACGTT TTGGGACTAG ACACAAAACT AAGTCTTTGG   
  
  
- GGAAGTCAAC TAGATTGACT CTCACAGTAG TCAAGACTAA GACATCTTCA CGGACTAAAG AGTCTACGAA   
  
  
- CGGAGTTCAA GTATTCACTC TAAGAGGATC TTCTCCTAAA CCTACTTTCA GGACGGAGAC GAGTTCTTAA   
  
  
- ATCCCGAGAG GTTCGGTGAC TCTTCAGGGA CATACTACGA GAACCTCTCC CGATGAGAAA AAGTAGACTA   
  
  
- TTGTCGGGTA GTAGTAATCC TGTCTCATAA CTCGTGGTTT TACTCTTAAA ACTTAAGTCA AGGCCAATAG   
  
  
- GACCTTAACT CCCGATACAA TTACCACTAT AGTGCAAGCT CAGGTTGACC TACAAGTTGG ATTCGGTTAA   
  
  
- CCTAGGACAA GAATGAAACC TACTATAAAG AGTCGGGAAC AGACGTAGTT TGAGGGCTAG TAGACCCAGC   
  
  
- TCGTTACCGA AACTACTAAG TCCCCTACCC CGTCGGTGTA GAGGACCGTC ATGTCAATGT AGTTGTCAGG   
  
  
- GTCTCTTTTC CCAACTTAGA CAGTTAGCGA GTTCCTCCTT CTTTTTAGCA TTTTCCCTAC TTCCACCAGT   
  
  
- ACTCCTTCCC TCCTCATTGT TCGTCGTTCG AAGTAGGTTA CTTCTAATAC AACTCTACTT CCTCAAACTA   
  
  
- CTACATGACG AGACGTTTCT TCTCTTTCTA CAATAACGTT TGACGTGGTT ACTAAGGAGT GGGCAGCTTC   
  
  
- GCTCACTATT CAACGTCTTC TTTCCCCCCT TCCCCTTTTG TAGCGCACCC TTCTTCGTCT TATCGTGTTA   
  
  
- TCTTCTCCAC CTAGACTCCT GAGAAGAGTG AGTGACACGA GTTCGTTAAA GTTTAAAACT AGAATCCTCA   
  
  
- CGTTTACTCG TAGAGTCCGT TTATTCCGTC GTAAGAAGTG TTATACCACT ATCGGAGGTC TCCGAACGGG   
  
  
- TAATAAAACG ATTACCAGAA CTCCGAGCGT ATCGACCGTG ACCAAGTTGT TAGAGACGAT TACAACAACT   
  
  
- ACGAGCTTAG TGCAGTAGAC TAAAGAATTT CCGAATATCC GATATATACA GTCGGCAAGG AAAGTTTTCC   
  
  
- TACAATATAA AAGAGCGGTT GTTCTGTTAT GCCTTCAACC GACTCTTCCG TTGGTCCTAG GTGTAGTAAC   
  
  
- TAAAACCTCA GAACAACCCA AATGTCACCG GGACAGAGTA TGTTTTGGAG AGTTTCTCTG GGTGACCGGG   
  
  
- CGGTTCGGAG GCATAATGAC CCTATCTGAT AGGGGTCGTA CCAAAGGCCG GTAGTCTTTC CCAACTTCGC   
  
  
- TGACCGGCAG CCAATCTTCC CATAACGCTC TCTAAATTAC ACGGCAAGTT CATATTCCGG TATCGATTTT   
  
  
- TAACCCTCTC ATATTTCGAT CTCCTAGATC TTTAACTACT CTTACTCTAC CACAAACAGT TAACGTACGA   
  
  
- AGCGAGACCT TGTGACGAAC TACTCTGTCA CCACCGTCTG TCAGGTTTCC TACGAAAGAA TTCCAATTAG   
  
  
- TCTCTCTATT TAGGGGCAGA AAAGTAAGTA CCCTGATAGT TACCTAGTAA GTTACGAGGT AAGAAGTAGT   
  
  
- GAGCCAAGTC CCTCCGCGAG AAGGTGATGA GAAGAAATAA ACTATAGAAA CTTCGTTGAT ACGGGGCACT   
  
  
- TCTAGTACTT TCCGAGGACC AACTCTCACT CTATGTCCCG TTTCTTCGAA ACTTACAGTA TCGTACGCTT   
  
  
- CCACGGCTCT CCTAAGTTTC CGGACTTTGT ATGTTCGTTA CCGTTCGTTC CTGTTGTTCC CGGCCCAAAT   
  
  
- CCGTCGAAGG TGATCTGGCC CTCGAACACT CGTCTCGTTT CCGATACCAC TTTCGTTTGA TGGTATTCCT   
  
  
- AAAACACCAC CTGCTCCTGT CCGTAACCTA CGATGTGCCA ACCTTTCCTT CTTAGAAGAC ACGTGAGAGA   
  
  
- CAAACCGTTG GGTTGAC

+     GARE-motif

| Site Name | Organism | Position | Strand | Matrix score. | sequence | function |
| --- | --- | --- | --- | --- | --- | --- |
| GARE-motif | Brassica oleracea | 1747 | + | 7 | TCTGTTG | gibberellin-responsive element |

>HU07G02246.1   
+ -Up\_Stream \_Len000ATTTGA TGTGTATGCT GACTACTTTT GATGATGATG ATCACAGTGA TGCATATTTA   
  
  
+ GAGCTGGTAA ATTGGTCATT CGATTCGAGT ACGGGTCGGG TCAAGTTCTG ATTAAGTGAC ATTTCGCGTC   
  
  
+ GTTTTGGTTT AGTTCGGGTC GGATCGATTT CGGGTTAAAT AATTTTTGGT GGAATACGCT TGTCATGCCA   
  
  
+ AACACAAGCA ACTTTGTTAA AAATTTCGAT TCGGGTCAGG TCAATTCAGG TTTGGGGTCC ACTTTCGAGT   
  
  
+ GAGCATATTT CGGATGTCGG GTCGCGTATG GGTCCGGGTC ATTCGGTTTG AATTTCGGAT CTTGGATCAA   
  
  
+ TTTTGCTAAG TATGTGCATA TTCAACCCTT GATGACATAG GAAACGCTTT GTCATCTCCA CCAATCAGAT   
  
  
+ ATCGTTATCT CTTTAATCAT CGCCATAATA GCATGAGTAA GCATGACCAT CTTTTTCAAA CATGTGATAC   
  
  
+ AGTTAAGGCC TGTTCTTTTC GTTAATAAAT CCCAATTTAT CAATTTTAAT ATATACTTTA ACGAATTTTA   
  
  
+ ACAAAAAATT TTAATTTCAA TCCGCTATAA CAAAAAAAAT ACCAACTTCA ACTTATTATA TAAAAAAAAT   
  
  
+ ATACAATTTT TTTTCAATTT TAGCCAACTT CAACAAAAAA AAAAAAAATT TAACAAATCT CAATCAACTT   
  
  
+ CACCCATTTC TCAACTTATA TCTCCTAATG AAAAGAACAG ATCCTTAGCA AGACTTCTCG ATTCATTTAA   
  
  
+ AAGGATAGTA TGGTAAATTC ATTTTATACA AGTGTCAAGT GTGAATATCT TTTTCACATC AGGTGATATA   
  
  
+ TTAGTAGGAC TTTTCGATTC ACTTATCTTC CTCTTTATGT TTTGTTAATG TGATAGTAAC GATAGGATTG   
  
  
+ CATGTATTTT TTCCAACTAT TCATTTACAA CTATCAATAT CTATTGATAA ATATGTTAAA ATAATTATAT   
  
  
+ GTTATTTAAA TTATTATTTG TTACTTAAGG TGTCGACTTT GTCGAAAGCG ACTTAATTCT AATTAAGATA   
  
  
+ GCGGCAGAGA AAGTTACACC TGATGACCAA GTTAGATCTG GTAAAACTAA CCATAGATCT GAAACCCAAA   
  
  
+ CATAATGATG TGTATTCATA CCCGACCTGA CATCTGAAAT AAGTTTACTC AAAAGTGGTT CCGAAACTTG   
  
  
+ AATTGACTCG AACCGAACCG TATTTGACTC GAAATTTTCT ACAAAGTTGC TTATTTTTGC ATGAAGACAT   
  
  
+ ATTGAGCCAA AAATTAGTTG ATTCGAAGTC AACTTAAACA AAAATTCATT CGACTCAAAC TGAAATATCA   
  
  
+ CTTAACTAAA ACTATGACTC AACTCAAACT TGATCTGATC CGTATTCAAA TCAAATACTC CATTTGCTAG   
  
  
+ GTCTAGAACC AGCCACGACC CCACATGGAC TATGCGGTTC AATGAAACGG CCAAATATAT CGCAATTTCT   
  
  
+ GTCTCCATCA ATCACACCCA TTTGCTACAC CCTGTTAATA TTTGACTCGA CCCTGTGAAT TGCCTTCGCC   
  
  
+ AGTTTTGTTT AATCTCTCTG GTTGCTTCCC ATCCAAGTCT GAATAGTATA GTTTGTACAG TGTGTACTAC   
  
  
+ TAGAAAGCAC GTTCCTTGAA TCTTCTTTCA AAGTTGTTTT TAAAGGGTTG AGTGATCCAT TACGACTTTT   
  
  
+ GATCTGGGTT CATCGCCTCC TCTCCTAATC CCTCACCCGG GTACCCTCAA TTCTCTATCG TCTCTGTTGC   
  
  
+ TCAGCTAATT CAAATTCCTT TAGTTGGTCA TCATTGAAAT CTGAAATCAG GGTGTTTTTC TTTTTATTAT   
  
  
+ TATCTGGCAG TTGGTTTGTT TATCTCTTGG TTTTTGGGGC TTATTTGGGT GCTGGTTGAT GTTATCCTGC   
  
  
+ TTGGGTTGAA TCTTCATCAG GTTTATTTTT GATTCATAGT TGAGGGTGAA AATTATTTCT CATACCCACC   
  
  
+ ACCTGTTTGT TCTTTTGTCT CTGAGAAAAA CCAAAAAGCT TTCTCTTTTT CCTGATGCAT CCTGTGCTCG   
  
  
+ TTGATCCTGA GTTAGTGAAC CATGCGTCCA AATTCAAGCC TGATTTGCTC TCAAACTTTG ATAAACAACA   
  
  
+ AGAATTTAGC AACGGGATCG AACAAGATGA CATCTTGCAA AACCCTGATC TGTGTTTTGA TTCAGAAACC   
  
  
+ CCTTCAGTTG ATCTAACTGA GAGTGTCATC AGTTCTGATT CTGTAGAAGT GCCTGATTTC TCAGATGCTT   
  
  
+ GCCTCAAGTT CATAAGTGAG ATTCTCCTAG AAGAGGATTT GGATGAAAGT CCTGCCTCTG CTCAAGAATT   
  
  
+ TAGGGCTCTC CAAGCCACTG AGAAGTCCCT GTATGATGCT CTTGGAGAGG GCTACTCTTT TTCATCTGAT   
  
  
+ AACAGCCCAT CATCATTAGG ACAGAGTATT GAGCACCAAA ATGAGAATTT TGAATTCAGT TCCGGTTATC   
  
  
+ CTGGAATTGA GGGCTATGTT AATGGTGATA TCACGTTCGA GTCCAACTGG ATGTTCAACC TAAGCCAATT   
  
  
+ GGATCCTGTT CTTACTTTGG ATGATATTTC TCAGCCCTTG TCTGCATCAA ACTCCCGATC ATCTGGGTCG   
  
  
+ AGCAATGGCT TTGATGATTC AGGGGATGGG GCAGCCACAT CTCCTGGCAG TACAGTTACA TCAACAGTCC   
  
  
+ CAGAGAAAAG GGTTGAATCT GTCAATCGCT CAAGGAGGAA GAAAAATCGT AAAAGGGATG AAGGTGGTCA   
  
  
+ TGAGGAAGGG AGGAGTAACA AGCAGCAAGC TTCATCCAAT GAAGATTATG TTGAGATGAA GGAGTTTGAT   
  
  
+ GATGTACTGC TCTGCAAAGA AGAGAAAGAT GTTATTGCAA ACTGCACCAA TGATTCCTCA CCCGTCGAAG   
  
  
+ CGAGTGATAA GTTGCAGAAG AAAGGGGGGA AGGGGAAAAC ATCGCGTGGG AAGAAGCAGA ATAGCACAAT   
  
  
+ AGAAGAGGTG GATCTGAGGA CTCTTCTCAC TCACTGTGCT CAAGCAATTT CAAATTTTGA TCTTAGGAGT   
  
  
+ GCAAATGAGC ATCTCAGGCA AATAAGGCAG CATTCTTCAC AATATGGTGA TAGCCTCCAG AGGCTTGCCC   
  
  
+ ATTATTTTGC TAATGGTCTT GAGGCTCGCA TAGCTGGCAC TGGTTCAACA ATCTCTGCTA ATGTTGTTGA   
  
  
+ TGCTCGAATC ACGTCATCTG ATTTCTTAAA GGCTTATAGG CTATATATGT CAGCCGTTCC TTTCAAAAGG   
  
  
+ ATGTTATATT TTCTCGCCAA CAAGACAATA CGGAAGTTGG CTGAGAAGGC AACCAGGATC CACATCATTG   
  
  
+ ATTTTGGAGT CTTGTTGGGT TTACAGTGGC CCTGTCTCAT ACAAAACCTC TCAAAGAGAC CCACTGGCCC   
  
  
+ GCCAAGCCTC CGTATTACTG GGATAGACTA TCCCCAGCAT GGTTTCCGGC CATCAGAAAG GGTTGAAGCG   
  
  
+ ACTGGCCGTC GGTTAGAAGG GTATTGCGAG AGATTTAATG TGCCGTTCAA GTATAAGGCC ATAGCTAAAA   
  
  
+ ATTGGGAGAG TATAAAGCTA GAGGATCTAG AAATTGATGA GAATGAGATG GTGTTTGTCA ATTGCATGCT   
  
  
+ TCGCTCTGGA ACACTGCTTG ATGAGACAGT GGTGGCAGAC AGTCCAAAGG ATGCTTTCTT AAGGTTAATC   
  
  
+ AGAGAGATAA ATCCCCGTCT TTTCATTCAT GGGACTATCA ATGGATCATT CAATGCTCCA TTCTTCATCA   
  
  
+ CTCGGTTCAG GGAGGCGCTC TTCCACTACT CTTCTTTATT TGATATCTTT GAAGCAACTA TGCCCCGTGA   
  
  
+ AGATCATGAA AGGCTCCTGG TTGAGAGTGA GATACAGGGC AAAGAAGCTT TGAATGTCAT AGCATGCGAA   
  
  
+ GGTGCCGAGA GGATTCAAAG GCCTGAAACA TACAAGCAAT GGCAAGCAAG GACAACAAGG GCCGGGTTTA   
  
  
+ GGCAGCTTCC ACTAGACCGG GAGCTTGTGA GCAGAGCAAA GGCTATGGTG AAAGCAAACT ACCATAAGGA   
  
  
+ TTTTGTGGTG GACGAGGACA GGCATTGGAT GCTACACGGT TGGAAAGGAA GAATCTTCTG TGCACTCTCT   
  
  
+ GTTTGGCAAC CCAACTG  

- -Up\_Stream \_Len000TAAACT ACACATACGA CTGATGAAAA CTACTACTAC TAGTGTCACT ACGTATAAAT   
  
  
- CTCGACCATT TAACCAGTAA GCTAAGCTCA TGCCCAGCCC AGTTCAAGAC TAATTCACTG TAAAGCGCAG   
  
  
- CAAAACCAAA TCAAGCCCAG CCTAGCTAAA GCCCAATTTA TTAAAAACCA CCTTATGCGA ACAGTACGGT   
  
  
- TTGTGTTCGT TGAAACAATT TTTAAAGCTA AGCCCAGTCC AGTTAAGTCC AAACCCCAGG TGAAAGCTCA   
  
  
- CTCGTATAAA GCCTACAGCC CAGCGCATAC CCAGGCCCAG TAAGCCAAAC TTAAAGCCTA GAACCTAGTT   
  
  
- AAAACGATTC ATACACGTAT AAGTTGGGAA CTACTGTATC CTTTGCGAAA CAGTAGAGGT GGTTAGTCTA   
  
  
- TAGCAATAGA GAAATTAGTA GCGGTATTAT CGTACTCATT CGTACTGGTA GAAAAAGTTT GTACACTATG   
  
  
- TCAATTCCGG ACAAGAAAAG CAATTATTTA GGGTTAAATA GTTAAAATTA TATATGAAAT TGCTTAAAAT   
  
  
- TGTTTTTTAA AATTAAAGTT AGGCGATATT GTTTTTTTTA TGGTTGAAGT TGAATAATAT ATTTTTTTTA   
  
  
- TATGTTAAAA AAAAGTTAAA ATCGGTTGAA GTTGTTTTTT TTTTTTTTAA ATTGTTTAGA GTTAGTTGAA   
  
  
- GTGGGTAAAG AGTTGAATAT AGAGGATTAC TTTTCTTGTC TAGGAATCGT TCTGAAGAGC TAAGTAAATT   
  
  
- TTCCTATCAT ACCATTTAAG TAAAATATGT TCACAGTTCA CACTTATAGA AAAAGTGTAG TCCACTATAT   
  
  
- AATCATCCTG AAAAGCTAAG TGAATAGAAG GAGAAATACA AAACAATTAC ACTATCATTG CTATCCTAAC   
  
  
- GTACATAAAA AAGGTTGATA AGTAAATGTT GATAGTTATA GATAACTATT TATACAATTT TATTAATATA   
  
  
- CAATAAATTT AATAATAAAC AATGAATTCC ACAGCTGAAA CAGCTTTCGC TGAATTAAGA TTAATTCTAT   
  
  
- CGCCGTCTCT TTCAATGTGG ACTACTGGTT CAATCTAGAC CATTTTGATT GGTATCTAGA CTTTGGGTTT   
  
  
- GTATTACTAC ACATAAGTAT GGGCTGGACT GTAGACTTTA TTCAAATGAG TTTTCACCAA GGCTTTGAAC   
  
  
- TTAACTGAGC TTGGCTTGGC ATAAACTGAG CTTTAAAAGA TGTTTCAACG AATAAAAACG TACTTCTGTA   
  
  
- TAACTCGGTT TTTAATCAAC TAAGCTTCAG TTGAATTTGT TTTTAAGTAA GCTGAGTTTG ACTTTATAGT   
  
  
- GAATTGATTT TGATACTGAG TTGAGTTTGA ACTAGACTAG GCATAAGTTT AGTTTATGAG GTAAACGATC   
  
  
- CAGATCTTGG TCGGTGCTGG GGTGTACCTG ATACGCCAAG TTACTTTGCC GGTTTATATA GCGTTAAAGA   
  
  
- CAGAGGTAGT TAGTGTGGGT AAACGATGTG GGACAATTAT AAACTGAGCT GGGACACTTA ACGGAAGCGG   
  
  
- TCAAAACAAA TTAGAGAGAC CAACGAAGGG TAGGTTCAGA CTTATCATAT CAAACATGTC ACACATGATG   
  
  
- ATCTTTCGTG CAAGGAACTT AGAAGAAAGT TTCAACAAAA ATTTCCCAAC TCACTAGGTA ATGCTGAAAA   
  
  
- CTAGACCCAA GTAGCGGAGG AGAGGATTAG GGAGTGGGCC CATGGGAGTT AAGAGATAGC AGAGACAACG   
  
  
- AGTCGATTAA GTTTAAGGAA ATCAACCAGT AGTAACTTTA GACTTTAGTC CCACAAAAAG AAAAATAATA   
  
  
- ATAGACCGTC AACCAAACAA ATAGAGAACC AAAAACCCCG AATAAACCCA CGACCAACTA CAATAGGACG   
  
  
- AACCCAACTT AGAAGTAGTC CAAATAAAAA CTAAGTATCA ACTCCCACTT TTAATAAAGA GTATGGGTGG   
  
  
- TGGACAAACA AGAAAACAGA GACTCTTTTT GGTTTTTCGA AAGAGAAAAA GGACTACGTA GGACACGAGC   
  
  
- AACTAGGACT CAATCACTTG GTACGCAGGT TTAAGTTCGG ACTAAACGAG AGTTTGAAAC TATTTGTTGT   
  
  
- TCTTAAATCG TTGCCCTAGC TTGTTCTACT GTAGAACGTT TTGGGACTAG ACACAAAACT AAGTCTTTGG   
  
  
- GGAAGTCAAC TAGATTGACT CTCACAGTAG TCAAGACTAA GACATCTTCA CGGACTAAAG AGTCTACGAA   
  
  
- CGGAGTTCAA GTATTCACTC TAAGAGGATC TTCTCCTAAA CCTACTTTCA GGACGGAGAC GAGTTCTTAA   
  
  
- ATCCCGAGAG GTTCGGTGAC TCTTCAGGGA CATACTACGA GAACCTCTCC CGATGAGAAA AAGTAGACTA   
  
  
- TTGTCGGGTA GTAGTAATCC TGTCTCATAA CTCGTGGTTT TACTCTTAAA ACTTAAGTCA AGGCCAATAG   
  
  
- GACCTTAACT CCCGATACAA TTACCACTAT AGTGCAAGCT CAGGTTGACC TACAAGTTGG ATTCGGTTAA   
  
  
- CCTAGGACAA GAATGAAACC TACTATAAAG AGTCGGGAAC AGACGTAGTT TGAGGGCTAG TAGACCCAGC   
  
  
- TCGTTACCGA AACTACTAAG TCCCCTACCC CGTCGGTGTA GAGGACCGTC ATGTCAATGT AGTTGTCAGG   
  
  
- GTCTCTTTTC CCAACTTAGA CAGTTAGCGA GTTCCTCCTT CTTTTTAGCA TTTTCCCTAC TTCCACCAGT   
  
  
- ACTCCTTCCC TCCTCATTGT TCGTCGTTCG AAGTAGGTTA CTTCTAATAC AACTCTACTT CCTCAAACTA   
  
  
- CTACATGACG AGACGTTTCT TCTCTTTCTA CAATAACGTT TGACGTGGTT ACTAAGGAGT GGGCAGCTTC   
  
  
- GCTCACTATT CAACGTCTTC TTTCCCCCCT TCCCCTTTTG TAGCGCACCC TTCTTCGTCT TATCGTGTTA   
  
  
- TCTTCTCCAC CTAGACTCCT GAGAAGAGTG AGTGACACGA GTTCGTTAAA GTTTAAAACT AGAATCCTCA   
  
  
- CGTTTACTCG TAGAGTCCGT TTATTCCGTC GTAAGAAGTG TTATACCACT ATCGGAGGTC TCCGAACGGG   
  
  
- TAATAAAACG ATTACCAGAA CTCCGAGCGT ATCGACCGTG ACCAAGTTGT TAGAGACGAT TACAACAACT   
  
  
- ACGAGCTTAG TGCAGTAGAC TAAAGAATTT CCGAATATCC GATATATACA GTCGGCAAGG AAAGTTTTCC   
  
  
- TACAATATAA AAGAGCGGTT GTTCTGTTAT GCCTTCAACC GACTCTTCCG TTGGTCCTAG GTGTAGTAAC   
  
  
- TAAAACCTCA GAACAACCCA AATGTCACCG GGACAGAGTA TGTTTTGGAG AGTTTCTCTG GGTGACCGGG   
  
  
- CGGTTCGGAG GCATAATGAC CCTATCTGAT AGGGGTCGTA CCAAAGGCCG GTAGTCTTTC CCAACTTCGC   
  
  
- TGACCGGCAG CCAATCTTCC CATAACGCTC TCTAAATTAC ACGGCAAGTT CATATTCCGG TATCGATTTT   
  
  
- TAACCCTCTC ATATTTCGAT CTCCTAGATC TTTAACTACT CTTACTCTAC CACAAACAGT TAACGTACGA   
  
  
- AGCGAGACCT TGTGACGAAC TACTCTGTCA CCACCGTCTG TCAGGTTTCC TACGAAAGAA TTCCAATTAG   
  
  
- TCTCTCTATT TAGGGGCAGA AAAGTAAGTA CCCTGATAGT TACCTAGTAA GTTACGAGGT AAGAAGTAGT   
  
  
- GAGCCAAGTC CCTCCGCGAG AAGGTGATGA GAAGAAATAA ACTATAGAAA CTTCGTTGAT ACGGGGCACT   
  
  
- TCTAGTACTT TCCGAGGACC AACTCTCACT CTATGTCCCG TTTCTTCGAA ACTTACAGTA TCGTACGCTT   
  
  
- CCACGGCTCT CCTAAGTTTC CGGACTTTGT ATGTTCGTTA CCGTTCGTTC CTGTTGTTCC CGGCCCAAAT   
  
  
- CCGTCGAAGG TGATCTGGCC CTCGAACACT CGTCTCGTTT CCGATACCAC TTTCGTTTGA TGGTATTCCT   
  
  
- AAAACACCAC CTGCTCCTGT CCGTAACCTA CGATGTGCCA ACCTTTCCTT CTTAGAAGAC ACGTGAGAGA   
  
  
- CAAACCGTTG GGTTGAC

+     GATA-motif

| Site Name | Organism | Position | Strand | Matrix score. | sequence | function |
| --- | --- | --- | --- | --- | --- | --- |
| GATA-motif | Arabidopsis thaliana | 905 | + | 7 | GATAGGA | part of a light responsive element |
| GATA-motif | Solanum tuberosum | 3483 | + | 9 | AAGGATAAGG | part of a light responsive element |

>HU07G02246.1   
+ -Up\_Stream \_Len000ATTTGA TGTGTATGCT GACTACTTTT GATGATGATG ATCACAGTGA TGCATATTTA   
  
  
+ GAGCTGGTAA ATTGGTCATT CGATTCGAGT ACGGGTCGGG TCAAGTTCTG ATTAAGTGAC ATTTCGCGTC   
  
  
+ GTTTTGGTTT AGTTCGGGTC GGATCGATTT CGGGTTAAAT AATTTTTGGT GGAATACGCT TGTCATGCCA   
  
  
+ AACACAAGCA ACTTTGTTAA AAATTTCGAT TCGGGTCAGG TCAATTCAGG TTTGGGGTCC ACTTTCGAGT   
  
  
+ GAGCATATTT CGGATGTCGG GTCGCGTATG GGTCCGGGTC ATTCGGTTTG AATTTCGGAT CTTGGATCAA   
  
  
+ TTTTGCTAAG TATGTGCATA TTCAACCCTT GATGACATAG GAAACGCTTT GTCATCTCCA CCAATCAGAT   
  
  
+ ATCGTTATCT CTTTAATCAT CGCCATAATA GCATGAGTAA GCATGACCAT CTTTTTCAAA CATGTGATAC   
  
  
+ AGTTAAGGCC TGTTCTTTTC GTTAATAAAT CCCAATTTAT CAATTTTAAT ATATACTTTA ACGAATTTTA   
  
  
+ ACAAAAAATT TTAATTTCAA TCCGCTATAA CAAAAAAAAT ACCAACTTCA ACTTATTATA TAAAAAAAAT   
  
  
+ ATACAATTTT TTTTCAATTT TAGCCAACTT CAACAAAAAA AAAAAAAATT TAACAAATCT CAATCAACTT   
  
  
+ CACCCATTTC TCAACTTATA TCTCCTAATG AAAAGAACAG ATCCTTAGCA AGACTTCTCG ATTCATTTAA   
  
  
+ AAGGATAGTA TGGTAAATTC ATTTTATACA AGTGTCAAGT GTGAATATCT TTTTCACATC AGGTGATATA   
  
  
+ TTAGTAGGAC TTTTCGATTC ACTTATCTTC CTCTTTATGT TTTGTTAATG TGATAGTAAC GATAGGATTG   
  
  
+ CATGTATTTT TTCCAACTAT TCATTTACAA CTATCAATAT CTATTGATAA ATATGTTAAA ATAATTATAT   
  
  
+ GTTATTTAAA TTATTATTTG TTACTTAAGG TGTCGACTTT GTCGAAAGCG ACTTAATTCT AATTAAGATA   
  
  
+ GCGGCAGAGA AAGTTACACC TGATGACCAA GTTAGATCTG GTAAAACTAA CCATAGATCT GAAACCCAAA   
  
  
+ CATAATGATG TGTATTCATA CCCGACCTGA CATCTGAAAT AAGTTTACTC AAAAGTGGTT CCGAAACTTG   
  
  
+ AATTGACTCG AACCGAACCG TATTTGACTC GAAATTTTCT ACAAAGTTGC TTATTTTTGC ATGAAGACAT   
  
  
+ ATTGAGCCAA AAATTAGTTG ATTCGAAGTC AACTTAAACA AAAATTCATT CGACTCAAAC TGAAATATCA   
  
  
+ CTTAACTAAA ACTATGACTC AACTCAAACT TGATCTGATC CGTATTCAAA TCAAATACTC CATTTGCTAG   
  
  
+ GTCTAGAACC AGCCACGACC CCACATGGAC TATGCGGTTC AATGAAACGG CCAAATATAT CGCAATTTCT   
  
  
+ GTCTCCATCA ATCACACCCA TTTGCTACAC CCTGTTAATA TTTGACTCGA CCCTGTGAAT TGCCTTCGCC   
  
  
+ AGTTTTGTTT AATCTCTCTG GTTGCTTCCC ATCCAAGTCT GAATAGTATA GTTTGTACAG TGTGTACTAC   
  
  
+ TAGAAAGCAC GTTCCTTGAA TCTTCTTTCA AAGTTGTTTT TAAAGGGTTG AGTGATCCAT TACGACTTTT   
  
  
+ GATCTGGGTT CATCGCCTCC TCTCCTAATC CCTCACCCGG GTACCCTCAA TTCTCTATCG TCTCTGTTGC   
  
  
+ TCAGCTAATT CAAATTCCTT TAGTTGGTCA TCATTGAAAT CTGAAATCAG GGTGTTTTTC TTTTTATTAT   
  
  
+ TATCTGGCAG TTGGTTTGTT TATCTCTTGG TTTTTGGGGC TTATTTGGGT GCTGGTTGAT GTTATCCTGC   
  
  
+ TTGGGTTGAA TCTTCATCAG GTTTATTTTT GATTCATAGT TGAGGGTGAA AATTATTTCT CATACCCACC   
  
  
+ ACCTGTTTGT TCTTTTGTCT CTGAGAAAAA CCAAAAAGCT TTCTCTTTTT CCTGATGCAT CCTGTGCTCG   
  
  
+ TTGATCCTGA GTTAGTGAAC CATGCGTCCA AATTCAAGCC TGATTTGCTC TCAAACTTTG ATAAACAACA   
  
  
+ AGAATTTAGC AACGGGATCG AACAAGATGA CATCTTGCAA AACCCTGATC TGTGTTTTGA TTCAGAAACC   
  
  
+ CCTTCAGTTG ATCTAACTGA GAGTGTCATC AGTTCTGATT CTGTAGAAGT GCCTGATTTC TCAGATGCTT   
  
  
+ GCCTCAAGTT CATAAGTGAG ATTCTCCTAG AAGAGGATTT GGATGAAAGT CCTGCCTCTG CTCAAGAATT   
  
  
+ TAGGGCTCTC CAAGCCACTG AGAAGTCCCT GTATGATGCT CTTGGAGAGG GCTACTCTTT TTCATCTGAT   
  
  
+ AACAGCCCAT CATCATTAGG ACAGAGTATT GAGCACCAAA ATGAGAATTT TGAATTCAGT TCCGGTTATC   
  
  
+ CTGGAATTGA GGGCTATGTT AATGGTGATA TCACGTTCGA GTCCAACTGG ATGTTCAACC TAAGCCAATT   
  
  
+ GGATCCTGTT CTTACTTTGG ATGATATTTC TCAGCCCTTG TCTGCATCAA ACTCCCGATC ATCTGGGTCG   
  
  
+ AGCAATGGCT TTGATGATTC AGGGGATGGG GCAGCCACAT CTCCTGGCAG TACAGTTACA TCAACAGTCC   
  
  
+ CAGAGAAAAG GGTTGAATCT GTCAATCGCT CAAGGAGGAA GAAAAATCGT AAAAGGGATG AAGGTGGTCA   
  
  
+ TGAGGAAGGG AGGAGTAACA AGCAGCAAGC TTCATCCAAT GAAGATTATG TTGAGATGAA GGAGTTTGAT   
  
  
+ GATGTACTGC TCTGCAAAGA AGAGAAAGAT GTTATTGCAA ACTGCACCAA TGATTCCTCA CCCGTCGAAG   
  
  
+ CGAGTGATAA GTTGCAGAAG AAAGGGGGGA AGGGGAAAAC ATCGCGTGGG AAGAAGCAGA ATAGCACAAT   
  
  
+ AGAAGAGGTG GATCTGAGGA CTCTTCTCAC TCACTGTGCT CAAGCAATTT CAAATTTTGA TCTTAGGAGT   
  
  
+ GCAAATGAGC ATCTCAGGCA AATAAGGCAG CATTCTTCAC AATATGGTGA TAGCCTCCAG AGGCTTGCCC   
  
  
+ ATTATTTTGC TAATGGTCTT GAGGCTCGCA TAGCTGGCAC TGGTTCAACA ATCTCTGCTA ATGTTGTTGA   
  
  
+ TGCTCGAATC ACGTCATCTG ATTTCTTAAA GGCTTATAGG CTATATATGT CAGCCGTTCC TTTCAAAAGG   
  
  
+ ATGTTATATT TTCTCGCCAA CAAGACAATA CGGAAGTTGG CTGAGAAGGC AACCAGGATC CACATCATTG   
  
  
+ ATTTTGGAGT CTTGTTGGGT TTACAGTGGC CCTGTCTCAT ACAAAACCTC TCAAAGAGAC CCACTGGCCC   
  
  
+ GCCAAGCCTC CGTATTACTG GGATAGACTA TCCCCAGCAT GGTTTCCGGC CATCAGAAAG GGTTGAAGCG   
  
  
+ ACTGGCCGTC GGTTAGAAGG GTATTGCGAG AGATTTAATG TGCCGTTCAA GTATAAGGCC ATAGCTAAAA   
  
  
+ ATTGGGAGAG TATAAAGCTA GAGGATCTAG AAATTGATGA GAATGAGATG GTGTTTGTCA ATTGCATGCT   
  
  
+ TCGCTCTGGA ACACTGCTTG ATGAGACAGT GGTGGCAGAC AGTCCAAAGG ATGCTTTCTT AAGGTTAATC   
  
  
+ AGAGAGATAA ATCCCCGTCT TTTCATTCAT GGGACTATCA ATGGATCATT CAATGCTCCA TTCTTCATCA   
  
  
+ CTCGGTTCAG GGAGGCGCTC TTCCACTACT CTTCTTTATT TGATATCTTT GAAGCAACTA TGCCCCGTGA   
  
  
+ AGATCATGAA AGGCTCCTGG TTGAGAGTGA GATACAGGGC AAAGAAGCTT TGAATGTCAT AGCATGCGAA   
  
  
+ GGTGCCGAGA GGATTCAAAG GCCTGAAACA TACAAGCAAT GGCAAGCAAG GACAACAAGG GCCGGGTTTA   
  
  
+ GGCAGCTTCC ACTAGACCGG GAGCTTGTGA GCAGAGCAAA GGCTATGGTG AAAGCAAACT ACCATAAGGA   
  
  
+ TTTTGTGGTG GACGAGGACA GGCATTGGAT GCTACACGGT TGGAAAGGAA GAATCTTCTG TGCACTCTCT   
  
  
+ GTTTGGCAAC CCAACTG  

- -Up\_Stream \_Len000TAAACT ACACATACGA CTGATGAAAA CTACTACTAC TAGTGTCACT ACGTATAAAT   
  
  
- CTCGACCATT TAACCAGTAA GCTAAGCTCA TGCCCAGCCC AGTTCAAGAC TAATTCACTG TAAAGCGCAG   
  
  
- CAAAACCAAA TCAAGCCCAG CCTAGCTAAA GCCCAATTTA TTAAAAACCA CCTTATGCGA ACAGTACGGT   
  
  
- TTGTGTTCGT TGAAACAATT TTTAAAGCTA AGCCCAGTCC AGTTAAGTCC AAACCCCAGG TGAAAGCTCA   
  
  
- CTCGTATAAA GCCTACAGCC CAGCGCATAC CCAGGCCCAG TAAGCCAAAC TTAAAGCCTA GAACCTAGTT   
  
  
- AAAACGATTC ATACACGTAT AAGTTGGGAA CTACTGTATC CTTTGCGAAA CAGTAGAGGT GGTTAGTCTA   
  
  
- TAGCAATAGA GAAATTAGTA GCGGTATTAT CGTACTCATT CGTACTGGTA GAAAAAGTTT GTACACTATG   
  
  
- TCAATTCCGG ACAAGAAAAG CAATTATTTA GGGTTAAATA GTTAAAATTA TATATGAAAT TGCTTAAAAT   
  
  
- TGTTTTTTAA AATTAAAGTT AGGCGATATT GTTTTTTTTA TGGTTGAAGT TGAATAATAT ATTTTTTTTA   
  
  
- TATGTTAAAA AAAAGTTAAA ATCGGTTGAA GTTGTTTTTT TTTTTTTTAA ATTGTTTAGA GTTAGTTGAA   
  
  
- GTGGGTAAAG AGTTGAATAT AGAGGATTAC TTTTCTTGTC TAGGAATCGT TCTGAAGAGC TAAGTAAATT   
  
  
- TTCCTATCAT ACCATTTAAG TAAAATATGT TCACAGTTCA CACTTATAGA AAAAGTGTAG TCCACTATAT   
  
  
- AATCATCCTG AAAAGCTAAG TGAATAGAAG GAGAAATACA AAACAATTAC ACTATCATTG CTATCCTAAC   
  
  
- GTACATAAAA AAGGTTGATA AGTAAATGTT GATAGTTATA GATAACTATT TATACAATTT TATTAATATA   
  
  
- CAATAAATTT AATAATAAAC AATGAATTCC ACAGCTGAAA CAGCTTTCGC TGAATTAAGA TTAATTCTAT   
  
  
- CGCCGTCTCT TTCAATGTGG ACTACTGGTT CAATCTAGAC CATTTTGATT GGTATCTAGA CTTTGGGTTT   
  
  
- GTATTACTAC ACATAAGTAT GGGCTGGACT GTAGACTTTA TTCAAATGAG TTTTCACCAA GGCTTTGAAC   
  
  
- TTAACTGAGC TTGGCTTGGC ATAAACTGAG CTTTAAAAGA TGTTTCAACG AATAAAAACG TACTTCTGTA   
  
  
- TAACTCGGTT TTTAATCAAC TAAGCTTCAG TTGAATTTGT TTTTAAGTAA GCTGAGTTTG ACTTTATAGT   
  
  
- GAATTGATTT TGATACTGAG TTGAGTTTGA ACTAGACTAG GCATAAGTTT AGTTTATGAG GTAAACGATC   
  
  
- CAGATCTTGG TCGGTGCTGG GGTGTACCTG ATACGCCAAG TTACTTTGCC GGTTTATATA GCGTTAAAGA   
  
  
- CAGAGGTAGT TAGTGTGGGT AAACGATGTG GGACAATTAT AAACTGAGCT GGGACACTTA ACGGAAGCGG   
  
  
- TCAAAACAAA TTAGAGAGAC CAACGAAGGG TAGGTTCAGA CTTATCATAT CAAACATGTC ACACATGATG   
  
  
- ATCTTTCGTG CAAGGAACTT AGAAGAAAGT TTCAACAAAA ATTTCCCAAC TCACTAGGTA ATGCTGAAAA   
  
  
- CTAGACCCAA GTAGCGGAGG AGAGGATTAG GGAGTGGGCC CATGGGAGTT AAGAGATAGC AGAGACAACG   
  
  
- AGTCGATTAA GTTTAAGGAA ATCAACCAGT AGTAACTTTA GACTTTAGTC CCACAAAAAG AAAAATAATA   
  
  
- ATAGACCGTC AACCAAACAA ATAGAGAACC AAAAACCCCG AATAAACCCA CGACCAACTA CAATAGGACG   
  
  
- AACCCAACTT AGAAGTAGTC CAAATAAAAA CTAAGTATCA ACTCCCACTT TTAATAAAGA GTATGGGTGG   
  
  
- TGGACAAACA AGAAAACAGA GACTCTTTTT GGTTTTTCGA AAGAGAAAAA GGACTACGTA GGACACGAGC   
  
  
- AACTAGGACT CAATCACTTG GTACGCAGGT TTAAGTTCGG ACTAAACGAG AGTTTGAAAC TATTTGTTGT   
  
  
- TCTTAAATCG TTGCCCTAGC TTGTTCTACT GTAGAACGTT TTGGGACTAG ACACAAAACT AAGTCTTTGG   
  
  
- GGAAGTCAAC TAGATTGACT CTCACAGTAG TCAAGACTAA GACATCTTCA CGGACTAAAG AGTCTACGAA   
  
  
- CGGAGTTCAA GTATTCACTC TAAGAGGATC TTCTCCTAAA CCTACTTTCA GGACGGAGAC GAGTTCTTAA   
  
  
- ATCCCGAGAG GTTCGGTGAC TCTTCAGGGA CATACTACGA GAACCTCTCC CGATGAGAAA AAGTAGACTA   
  
  
- TTGTCGGGTA GTAGTAATCC TGTCTCATAA CTCGTGGTTT TACTCTTAAA ACTTAAGTCA AGGCCAATAG   
  
  
- GACCTTAACT CCCGATACAA TTACCACTAT AGTGCAAGCT CAGGTTGACC TACAAGTTGG ATTCGGTTAA   
  
  
- CCTAGGACAA GAATGAAACC TACTATAAAG AGTCGGGAAC AGACGTAGTT TGAGGGCTAG TAGACCCAGC   
  
  
- TCGTTACCGA AACTACTAAG TCCCCTACCC CGTCGGTGTA GAGGACCGTC ATGTCAATGT AGTTGTCAGG   
  
  
- GTCTCTTTTC CCAACTTAGA CAGTTAGCGA GTTCCTCCTT CTTTTTAGCA TTTTCCCTAC TTCCACCAGT   
  
  
- ACTCCTTCCC TCCTCATTGT TCGTCGTTCG AAGTAGGTTA CTTCTAATAC AACTCTACTT CCTCAAACTA   
  
  
- CTACATGACG AGACGTTTCT TCTCTTTCTA CAATAACGTT TGACGTGGTT ACTAAGGAGT GGGCAGCTTC   
  
  
- GCTCACTATT CAACGTCTTC TTTCCCCCCT TCCCCTTTTG TAGCGCACCC TTCTTCGTCT TATCGTGTTA   
  
  
- TCTTCTCCAC CTAGACTCCT GAGAAGAGTG AGTGACACGA GTTCGTTAAA GTTTAAAACT AGAATCCTCA   
  
  
- CGTTTACTCG TAGAGTCCGT TTATTCCGTC GTAAGAAGTG TTATACCACT ATCGGAGGTC TCCGAACGGG   
  
  
- TAATAAAACG ATTACCAGAA CTCCGAGCGT ATCGACCGTG ACCAAGTTGT TAGAGACGAT TACAACAACT   
  
  
- ACGAGCTTAG TGCAGTAGAC TAAAGAATTT CCGAATATCC GATATATACA GTCGGCAAGG AAAGTTTTCC   
  
  
- TACAATATAA AAGAGCGGTT GTTCTGTTAT GCCTTCAACC GACTCTTCCG TTGGTCCTAG GTGTAGTAAC   
  
  
- TAAAACCTCA GAACAACCCA AATGTCACCG GGACAGAGTA TGTTTTGGAG AGTTTCTCTG GGTGACCGGG   
  
  
- CGGTTCGGAG GCATAATGAC CCTATCTGAT AGGGGTCGTA CCAAAGGCCG GTAGTCTTTC CCAACTTCGC   
  
  
- TGACCGGCAG CCAATCTTCC CATAACGCTC TCTAAATTAC ACGGCAAGTT CATATTCCGG TATCGATTTT   
  
  
- TAACCCTCTC ATATTTCGAT CTCCTAGATC TTTAACTACT CTTACTCTAC CACAAACAGT TAACGTACGA   
  
  
- AGCGAGACCT TGTGACGAAC TACTCTGTCA CCACCGTCTG TCAGGTTTCC TACGAAAGAA TTCCAATTAG   
  
  
- TCTCTCTATT TAGGGGCAGA AAAGTAAGTA CCCTGATAGT TACCTAGTAA GTTACGAGGT AAGAAGTAGT   
  
  
- GAGCCAAGTC CCTCCGCGAG AAGGTGATGA GAAGAAATAA ACTATAGAAA CTTCGTTGAT ACGGGGCACT   
  
  
- TCTAGTACTT TCCGAGGACC AACTCTCACT CTATGTCCCG TTTCTTCGAA ACTTACAGTA TCGTACGCTT   
  
  
- CCACGGCTCT CCTAAGTTTC CGGACTTTGT ATGTTCGTTA CCGTTCGTTC CTGTTGTTCC CGGCCCAAAT   
  
  
- CCGTCGAAGG TGATCTGGCC CTCGAACACT CGTCTCGTTT CCGATACCAC TTTCGTTTGA TGGTATTCCT   
  
  
- AAAACACCAC CTGCTCCTGT CCGTAACCTA CGATGTGCCA ACCTTTCCTT CTTAGAAGAC ACGTGAGAGA   
  
  
- CAAACCGTTG GGTTGAC

+     GCN4\_motif

| Site Name | Organism | Position | Strand | Matrix score. | sequence | function |
| --- | --- | --- | --- | --- | --- | --- |
| GCN4\_motif | Oryza sativa | 1349 | - | 7 | TGAGTCA | cis-regulatory element involved in endosperm expression |

>HU07G02246.1   
+ -Up\_Stream \_Len000ATTTGA TGTGTATGCT GACTACTTTT GATGATGATG ATCACAGTGA TGCATATTTA   
  
  
+ GAGCTGGTAA ATTGGTCATT CGATTCGAGT ACGGGTCGGG TCAAGTTCTG ATTAAGTGAC ATTTCGCGTC   
  
  
+ GTTTTGGTTT AGTTCGGGTC GGATCGATTT CGGGTTAAAT AATTTTTGGT GGAATACGCT TGTCATGCCA   
  
  
+ AACACAAGCA ACTTTGTTAA AAATTTCGAT TCGGGTCAGG TCAATTCAGG TTTGGGGTCC ACTTTCGAGT   
  
  
+ GAGCATATTT CGGATGTCGG GTCGCGTATG GGTCCGGGTC ATTCGGTTTG AATTTCGGAT CTTGGATCAA   
  
  
+ TTTTGCTAAG TATGTGCATA TTCAACCCTT GATGACATAG GAAACGCTTT GTCATCTCCA CCAATCAGAT   
  
  
+ ATCGTTATCT CTTTAATCAT CGCCATAATA GCATGAGTAA GCATGACCAT CTTTTTCAAA CATGTGATAC   
  
  
+ AGTTAAGGCC TGTTCTTTTC GTTAATAAAT CCCAATTTAT CAATTTTAAT ATATACTTTA ACGAATTTTA   
  
  
+ ACAAAAAATT TTAATTTCAA TCCGCTATAA CAAAAAAAAT ACCAACTTCA ACTTATTATA TAAAAAAAAT   
  
  
+ ATACAATTTT TTTTCAATTT TAGCCAACTT CAACAAAAAA AAAAAAAATT TAACAAATCT CAATCAACTT   
  
  
+ CACCCATTTC TCAACTTATA TCTCCTAATG AAAAGAACAG ATCCTTAGCA AGACTTCTCG ATTCATTTAA   
  
  
+ AAGGATAGTA TGGTAAATTC ATTTTATACA AGTGTCAAGT GTGAATATCT TTTTCACATC AGGTGATATA   
  
  
+ TTAGTAGGAC TTTTCGATTC ACTTATCTTC CTCTTTATGT TTTGTTAATG TGATAGTAAC GATAGGATTG   
  
  
+ CATGTATTTT TTCCAACTAT TCATTTACAA CTATCAATAT CTATTGATAA ATATGTTAAA ATAATTATAT   
  
  
+ GTTATTTAAA TTATTATTTG TTACTTAAGG TGTCGACTTT GTCGAAAGCG ACTTAATTCT AATTAAGATA   
  
  
+ GCGGCAGAGA AAGTTACACC TGATGACCAA GTTAGATCTG GTAAAACTAA CCATAGATCT GAAACCCAAA   
  
  
+ CATAATGATG TGTATTCATA CCCGACCTGA CATCTGAAAT AAGTTTACTC AAAAGTGGTT CCGAAACTTG   
  
  
+ AATTGACTCG AACCGAACCG TATTTGACTC GAAATTTTCT ACAAAGTTGC TTATTTTTGC ATGAAGACAT   
  
  
+ ATTGAGCCAA AAATTAGTTG ATTCGAAGTC AACTTAAACA AAAATTCATT CGACTCAAAC TGAAATATCA   
  
  
+ CTTAACTAAA ACTATGACTC AACTCAAACT TGATCTGATC CGTATTCAAA TCAAATACTC CATTTGCTAG   
  
  
+ GTCTAGAACC AGCCACGACC CCACATGGAC TATGCGGTTC AATGAAACGG CCAAATATAT CGCAATTTCT   
  
  
+ GTCTCCATCA ATCACACCCA TTTGCTACAC CCTGTTAATA TTTGACTCGA CCCTGTGAAT TGCCTTCGCC   
  
  
+ AGTTTTGTTT AATCTCTCTG GTTGCTTCCC ATCCAAGTCT GAATAGTATA GTTTGTACAG TGTGTACTAC   
  
  
+ TAGAAAGCAC GTTCCTTGAA TCTTCTTTCA AAGTTGTTTT TAAAGGGTTG AGTGATCCAT TACGACTTTT   
  
  
+ GATCTGGGTT CATCGCCTCC TCTCCTAATC CCTCACCCGG GTACCCTCAA TTCTCTATCG TCTCTGTTGC   
  
  
+ TCAGCTAATT CAAATTCCTT TAGTTGGTCA TCATTGAAAT CTGAAATCAG GGTGTTTTTC TTTTTATTAT   
  
  
+ TATCTGGCAG TTGGTTTGTT TATCTCTTGG TTTTTGGGGC TTATTTGGGT GCTGGTTGAT GTTATCCTGC   
  
  
+ TTGGGTTGAA TCTTCATCAG GTTTATTTTT GATTCATAGT TGAGGGTGAA AATTATTTCT CATACCCACC   
  
  
+ ACCTGTTTGT TCTTTTGTCT CTGAGAAAAA CCAAAAAGCT TTCTCTTTTT CCTGATGCAT CCTGTGCTCG   
  
  
+ TTGATCCTGA GTTAGTGAAC CATGCGTCCA AATTCAAGCC TGATTTGCTC TCAAACTTTG ATAAACAACA   
  
  
+ AGAATTTAGC AACGGGATCG AACAAGATGA CATCTTGCAA AACCCTGATC TGTGTTTTGA TTCAGAAACC   
  
  
+ CCTTCAGTTG ATCTAACTGA GAGTGTCATC AGTTCTGATT CTGTAGAAGT GCCTGATTTC TCAGATGCTT   
  
  
+ GCCTCAAGTT CATAAGTGAG ATTCTCCTAG AAGAGGATTT GGATGAAAGT CCTGCCTCTG CTCAAGAATT   
  
  
+ TAGGGCTCTC CAAGCCACTG AGAAGTCCCT GTATGATGCT CTTGGAGAGG GCTACTCTTT TTCATCTGAT   
  
  
+ AACAGCCCAT CATCATTAGG ACAGAGTATT GAGCACCAAA ATGAGAATTT TGAATTCAGT TCCGGTTATC   
  
  
+ CTGGAATTGA GGGCTATGTT AATGGTGATA TCACGTTCGA GTCCAACTGG ATGTTCAACC TAAGCCAATT   
  
  
+ GGATCCTGTT CTTACTTTGG ATGATATTTC TCAGCCCTTG TCTGCATCAA ACTCCCGATC ATCTGGGTCG   
  
  
+ AGCAATGGCT TTGATGATTC AGGGGATGGG GCAGCCACAT CTCCTGGCAG TACAGTTACA TCAACAGTCC   
  
  
+ CAGAGAAAAG GGTTGAATCT GTCAATCGCT CAAGGAGGAA GAAAAATCGT AAAAGGGATG AAGGTGGTCA   
  
  
+ TGAGGAAGGG AGGAGTAACA AGCAGCAAGC TTCATCCAAT GAAGATTATG TTGAGATGAA GGAGTTTGAT   
  
  
+ GATGTACTGC TCTGCAAAGA AGAGAAAGAT GTTATTGCAA ACTGCACCAA TGATTCCTCA CCCGTCGAAG   
  
  
+ CGAGTGATAA GTTGCAGAAG AAAGGGGGGA AGGGGAAAAC ATCGCGTGGG AAGAAGCAGA ATAGCACAAT   
  
  
+ AGAAGAGGTG GATCTGAGGA CTCTTCTCAC TCACTGTGCT CAAGCAATTT CAAATTTTGA TCTTAGGAGT   
  
  
+ GCAAATGAGC ATCTCAGGCA AATAAGGCAG CATTCTTCAC AATATGGTGA TAGCCTCCAG AGGCTTGCCC   
  
  
+ ATTATTTTGC TAATGGTCTT GAGGCTCGCA TAGCTGGCAC TGGTTCAACA ATCTCTGCTA ATGTTGTTGA   
  
  
+ TGCTCGAATC ACGTCATCTG ATTTCTTAAA GGCTTATAGG CTATATATGT CAGCCGTTCC TTTCAAAAGG   
  
  
+ ATGTTATATT TTCTCGCCAA CAAGACAATA CGGAAGTTGG CTGAGAAGGC AACCAGGATC CACATCATTG   
  
  
+ ATTTTGGAGT CTTGTTGGGT TTACAGTGGC CCTGTCTCAT ACAAAACCTC TCAAAGAGAC CCACTGGCCC   
  
  
+ GCCAAGCCTC CGTATTACTG GGATAGACTA TCCCCAGCAT GGTTTCCGGC CATCAGAAAG GGTTGAAGCG   
  
  
+ ACTGGCCGTC GGTTAGAAGG GTATTGCGAG AGATTTAATG TGCCGTTCAA GTATAAGGCC ATAGCTAAAA   
  
  
+ ATTGGGAGAG TATAAAGCTA GAGGATCTAG AAATTGATGA GAATGAGATG GTGTTTGTCA ATTGCATGCT   
  
  
+ TCGCTCTGGA ACACTGCTTG ATGAGACAGT GGTGGCAGAC AGTCCAAAGG ATGCTTTCTT AAGGTTAATC   
  
  
+ AGAGAGATAA ATCCCCGTCT TTTCATTCAT GGGACTATCA ATGGATCATT CAATGCTCCA TTCTTCATCA   
  
  
+ CTCGGTTCAG GGAGGCGCTC TTCCACTACT CTTCTTTATT TGATATCTTT GAAGCAACTA TGCCCCGTGA   
  
  
+ AGATCATGAA AGGCTCCTGG TTGAGAGTGA GATACAGGGC AAAGAAGCTT TGAATGTCAT AGCATGCGAA   
  
  
+ GGTGCCGAGA GGATTCAAAG GCCTGAAACA TACAAGCAAT GGCAAGCAAG GACAACAAGG GCCGGGTTTA   
  
  
+ GGCAGCTTCC ACTAGACCGG GAGCTTGTGA GCAGAGCAAA GGCTATGGTG AAAGCAAACT ACCATAAGGA   
  
  
+ TTTTGTGGTG GACGAGGACA GGCATTGGAT GCTACACGGT TGGAAAGGAA GAATCTTCTG TGCACTCTCT   
  
  
+ GTTTGGCAAC CCAACTG  

- -Up\_Stream \_Len000TAAACT ACACATACGA CTGATGAAAA CTACTACTAC TAGTGTCACT ACGTATAAAT   
  
  
- CTCGACCATT TAACCAGTAA GCTAAGCTCA TGCCCAGCCC AGTTCAAGAC TAATTCACTG TAAAGCGCAG   
  
  
- CAAAACCAAA TCAAGCCCAG CCTAGCTAAA GCCCAATTTA TTAAAAACCA CCTTATGCGA ACAGTACGGT   
  
  
- TTGTGTTCGT TGAAACAATT TTTAAAGCTA AGCCCAGTCC AGTTAAGTCC AAACCCCAGG TGAAAGCTCA   
  
  
- CTCGTATAAA GCCTACAGCC CAGCGCATAC CCAGGCCCAG TAAGCCAAAC TTAAAGCCTA GAACCTAGTT   
  
  
- AAAACGATTC ATACACGTAT AAGTTGGGAA CTACTGTATC CTTTGCGAAA CAGTAGAGGT GGTTAGTCTA   
  
  
- TAGCAATAGA GAAATTAGTA GCGGTATTAT CGTACTCATT CGTACTGGTA GAAAAAGTTT GTACACTATG   
  
  
- TCAATTCCGG ACAAGAAAAG CAATTATTTA GGGTTAAATA GTTAAAATTA TATATGAAAT TGCTTAAAAT   
  
  
- TGTTTTTTAA AATTAAAGTT AGGCGATATT GTTTTTTTTA TGGTTGAAGT TGAATAATAT ATTTTTTTTA   
  
  
- TATGTTAAAA AAAAGTTAAA ATCGGTTGAA GTTGTTTTTT TTTTTTTTAA ATTGTTTAGA GTTAGTTGAA   
  
  
- GTGGGTAAAG AGTTGAATAT AGAGGATTAC TTTTCTTGTC TAGGAATCGT TCTGAAGAGC TAAGTAAATT   
  
  
- TTCCTATCAT ACCATTTAAG TAAAATATGT TCACAGTTCA CACTTATAGA AAAAGTGTAG TCCACTATAT   
  
  
- AATCATCCTG AAAAGCTAAG TGAATAGAAG GAGAAATACA AAACAATTAC ACTATCATTG CTATCCTAAC   
  
  
- GTACATAAAA AAGGTTGATA AGTAAATGTT GATAGTTATA GATAACTATT TATACAATTT TATTAATATA   
  
  
- CAATAAATTT AATAATAAAC AATGAATTCC ACAGCTGAAA CAGCTTTCGC TGAATTAAGA TTAATTCTAT   
  
  
- CGCCGTCTCT TTCAATGTGG ACTACTGGTT CAATCTAGAC CATTTTGATT GGTATCTAGA CTTTGGGTTT   
  
  
- GTATTACTAC ACATAAGTAT GGGCTGGACT GTAGACTTTA TTCAAATGAG TTTTCACCAA GGCTTTGAAC   
  
  
- TTAACTGAGC TTGGCTTGGC ATAAACTGAG CTTTAAAAGA TGTTTCAACG AATAAAAACG TACTTCTGTA   
  
  
- TAACTCGGTT TTTAATCAAC TAAGCTTCAG TTGAATTTGT TTTTAAGTAA GCTGAGTTTG ACTTTATAGT   
  
  
- GAATTGATTT TGATACTGAG TTGAGTTTGA ACTAGACTAG GCATAAGTTT AGTTTATGAG GTAAACGATC   
  
  
- CAGATCTTGG TCGGTGCTGG GGTGTACCTG ATACGCCAAG TTACTTTGCC GGTTTATATA GCGTTAAAGA   
  
  
- CAGAGGTAGT TAGTGTGGGT AAACGATGTG GGACAATTAT AAACTGAGCT GGGACACTTA ACGGAAGCGG   
  
  
- TCAAAACAAA TTAGAGAGAC CAACGAAGGG TAGGTTCAGA CTTATCATAT CAAACATGTC ACACATGATG   
  
  
- ATCTTTCGTG CAAGGAACTT AGAAGAAAGT TTCAACAAAA ATTTCCCAAC TCACTAGGTA ATGCTGAAAA   
  
  
- CTAGACCCAA GTAGCGGAGG AGAGGATTAG GGAGTGGGCC CATGGGAGTT AAGAGATAGC AGAGACAACG   
  
  
- AGTCGATTAA GTTTAAGGAA ATCAACCAGT AGTAACTTTA GACTTTAGTC CCACAAAAAG AAAAATAATA   
  
  
- ATAGACCGTC AACCAAACAA ATAGAGAACC AAAAACCCCG AATAAACCCA CGACCAACTA CAATAGGACG   
  
  
- AACCCAACTT AGAAGTAGTC CAAATAAAAA CTAAGTATCA ACTCCCACTT TTAATAAAGA GTATGGGTGG   
  
  
- TGGACAAACA AGAAAACAGA GACTCTTTTT GGTTTTTCGA AAGAGAAAAA GGACTACGTA GGACACGAGC   
  
  
- AACTAGGACT CAATCACTTG GTACGCAGGT TTAAGTTCGG ACTAAACGAG AGTTTGAAAC TATTTGTTGT   
  
  
- TCTTAAATCG TTGCCCTAGC TTGTTCTACT GTAGAACGTT TTGGGACTAG ACACAAAACT AAGTCTTTGG   
  
  
- GGAAGTCAAC TAGATTGACT CTCACAGTAG TCAAGACTAA GACATCTTCA CGGACTAAAG AGTCTACGAA   
  
  
- CGGAGTTCAA GTATTCACTC TAAGAGGATC TTCTCCTAAA CCTACTTTCA GGACGGAGAC GAGTTCTTAA   
  
  
- ATCCCGAGAG GTTCGGTGAC TCTTCAGGGA CATACTACGA GAACCTCTCC CGATGAGAAA AAGTAGACTA   
  
  
- TTGTCGGGTA GTAGTAATCC TGTCTCATAA CTCGTGGTTT TACTCTTAAA ACTTAAGTCA AGGCCAATAG   
  
  
- GACCTTAACT CCCGATACAA TTACCACTAT AGTGCAAGCT CAGGTTGACC TACAAGTTGG ATTCGGTTAA   
  
  
- CCTAGGACAA GAATGAAACC TACTATAAAG AGTCGGGAAC AGACGTAGTT TGAGGGCTAG TAGACCCAGC   
  
  
- TCGTTACCGA AACTACTAAG TCCCCTACCC CGTCGGTGTA GAGGACCGTC ATGTCAATGT AGTTGTCAGG   
  
  
- GTCTCTTTTC CCAACTTAGA CAGTTAGCGA GTTCCTCCTT CTTTTTAGCA TTTTCCCTAC TTCCACCAGT   
  
  
- ACTCCTTCCC TCCTCATTGT TCGTCGTTCG AAGTAGGTTA CTTCTAATAC AACTCTACTT CCTCAAACTA   
  
  
- CTACATGACG AGACGTTTCT TCTCTTTCTA CAATAACGTT TGACGTGGTT ACTAAGGAGT GGGCAGCTTC   
  
  
- GCTCACTATT CAACGTCTTC TTTCCCCCCT TCCCCTTTTG TAGCGCACCC TTCTTCGTCT TATCGTGTTA   
  
  
- TCTTCTCCAC CTAGACTCCT GAGAAGAGTG AGTGACACGA GTTCGTTAAA GTTTAAAACT AGAATCCTCA   
  
  
- CGTTTACTCG TAGAGTCCGT TTATTCCGTC GTAAGAAGTG TTATACCACT ATCGGAGGTC TCCGAACGGG   
  
  
- TAATAAAACG ATTACCAGAA CTCCGAGCGT ATCGACCGTG ACCAAGTTGT TAGAGACGAT TACAACAACT   
  
  
- ACGAGCTTAG TGCAGTAGAC TAAAGAATTT CCGAATATCC GATATATACA GTCGGCAAGG AAAGTTTTCC   
  
  
- TACAATATAA AAGAGCGGTT GTTCTGTTAT GCCTTCAACC GACTCTTCCG TTGGTCCTAG GTGTAGTAAC   
  
  
- TAAAACCTCA GAACAACCCA AATGTCACCG GGACAGAGTA TGTTTTGGAG AGTTTCTCTG GGTGACCGGG   
  
  
- CGGTTCGGAG GCATAATGAC CCTATCTGAT AGGGGTCGTA CCAAAGGCCG GTAGTCTTTC CCAACTTCGC   
  
  
- TGACCGGCAG CCAATCTTCC CATAACGCTC TCTAAATTAC ACGGCAAGTT CATATTCCGG TATCGATTTT   
  
  
- TAACCCTCTC ATATTTCGAT CTCCTAGATC TTTAACTACT CTTACTCTAC CACAAACAGT TAACGTACGA   
  
  
- AGCGAGACCT TGTGACGAAC TACTCTGTCA CCACCGTCTG TCAGGTTTCC TACGAAAGAA TTCCAATTAG   
  
  
- TCTCTCTATT TAGGGGCAGA AAAGTAAGTA CCCTGATAGT TACCTAGTAA GTTACGAGGT AAGAAGTAGT   
  
  
- GAGCCAAGTC CCTCCGCGAG AAGGTGATGA GAAGAAATAA ACTATAGAAA CTTCGTTGAT ACGGGGCACT   
  
  
- TCTAGTACTT TCCGAGGACC AACTCTCACT CTATGTCCCG TTTCTTCGAA ACTTACAGTA TCGTACGCTT   
  
  
- CCACGGCTCT CCTAAGTTTC CGGACTTTGT ATGTTCGTTA CCGTTCGTTC CTGTTGTTCC CGGCCCAAAT   
  
  
- CCGTCGAAGG TGATCTGGCC CTCGAACACT CGTCTCGTTT CCGATACCAC TTTCGTTTGA TGGTATTCCT   
  
  
- AAAACACCAC CTGCTCCTGT CCGTAACCTA CGATGTGCCA ACCTTTCCTT CTTAGAAGAC ACGTGAGAGA   
  
  
- CAAACCGTTG GGTTGAC

+     GGA-motif

| Site Name | Organism | Position | Strand | Matrix score. | sequence | function |
| --- | --- | --- | --- | --- | --- | --- |
| GGA-motif | Flaveria trinervia | 394 | + | 12 | GGAGACGCTTTGT | part of a light responsive element |

>HU07G02246.1   
+ -Up\_Stream \_Len000ATTTGA TGTGTATGCT GACTACTTTT GATGATGATG ATCACAGTGA TGCATATTTA   
  
  
+ GAGCTGGTAA ATTGGTCATT CGATTCGAGT ACGGGTCGGG TCAAGTTCTG ATTAAGTGAC ATTTCGCGTC   
  
  
+ GTTTTGGTTT AGTTCGGGTC GGATCGATTT CGGGTTAAAT AATTTTTGGT GGAATACGCT TGTCATGCCA   
  
  
+ AACACAAGCA ACTTTGTTAA AAATTTCGAT TCGGGTCAGG TCAATTCAGG TTTGGGGTCC ACTTTCGAGT   
  
  
+ GAGCATATTT CGGATGTCGG GTCGCGTATG GGTCCGGGTC ATTCGGTTTG AATTTCGGAT CTTGGATCAA   
  
  
+ TTTTGCTAAG TATGTGCATA TTCAACCCTT GATGACATAG GAAACGCTTT GTCATCTCCA CCAATCAGAT   
  
  
+ ATCGTTATCT CTTTAATCAT CGCCATAATA GCATGAGTAA GCATGACCAT CTTTTTCAAA CATGTGATAC   
  
  
+ AGTTAAGGCC TGTTCTTTTC GTTAATAAAT CCCAATTTAT CAATTTTAAT ATATACTTTA ACGAATTTTA   
  
  
+ ACAAAAAATT TTAATTTCAA TCCGCTATAA CAAAAAAAAT ACCAACTTCA ACTTATTATA TAAAAAAAAT   
  
  
+ ATACAATTTT TTTTCAATTT TAGCCAACTT CAACAAAAAA AAAAAAAATT TAACAAATCT CAATCAACTT   
  
  
+ CACCCATTTC TCAACTTATA TCTCCTAATG AAAAGAACAG ATCCTTAGCA AGACTTCTCG ATTCATTTAA   
  
  
+ AAGGATAGTA TGGTAAATTC ATTTTATACA AGTGTCAAGT GTGAATATCT TTTTCACATC AGGTGATATA   
  
  
+ TTAGTAGGAC TTTTCGATTC ACTTATCTTC CTCTTTATGT TTTGTTAATG TGATAGTAAC GATAGGATTG   
  
  
+ CATGTATTTT TTCCAACTAT TCATTTACAA CTATCAATAT CTATTGATAA ATATGTTAAA ATAATTATAT   
  
  
+ GTTATTTAAA TTATTATTTG TTACTTAAGG TGTCGACTTT GTCGAAAGCG ACTTAATTCT AATTAAGATA   
  
  
+ GCGGCAGAGA AAGTTACACC TGATGACCAA GTTAGATCTG GTAAAACTAA CCATAGATCT GAAACCCAAA   
  
  
+ CATAATGATG TGTATTCATA CCCGACCTGA CATCTGAAAT AAGTTTACTC AAAAGTGGTT CCGAAACTTG   
  
  
+ AATTGACTCG AACCGAACCG TATTTGACTC GAAATTTTCT ACAAAGTTGC TTATTTTTGC ATGAAGACAT   
  
  
+ ATTGAGCCAA AAATTAGTTG ATTCGAAGTC AACTTAAACA AAAATTCATT CGACTCAAAC TGAAATATCA   
  
  
+ CTTAACTAAA ACTATGACTC AACTCAAACT TGATCTGATC CGTATTCAAA TCAAATACTC CATTTGCTAG   
  
  
+ GTCTAGAACC AGCCACGACC CCACATGGAC TATGCGGTTC AATGAAACGG CCAAATATAT CGCAATTTCT   
  
  
+ GTCTCCATCA ATCACACCCA TTTGCTACAC CCTGTTAATA TTTGACTCGA CCCTGTGAAT TGCCTTCGCC   
  
  
+ AGTTTTGTTT AATCTCTCTG GTTGCTTCCC ATCCAAGTCT GAATAGTATA GTTTGTACAG TGTGTACTAC   
  
  
+ TAGAAAGCAC GTTCCTTGAA TCTTCTTTCA AAGTTGTTTT TAAAGGGTTG AGTGATCCAT TACGACTTTT   
  
  
+ GATCTGGGTT CATCGCCTCC TCTCCTAATC CCTCACCCGG GTACCCTCAA TTCTCTATCG TCTCTGTTGC   
  
  
+ TCAGCTAATT CAAATTCCTT TAGTTGGTCA TCATTGAAAT CTGAAATCAG GGTGTTTTTC TTTTTATTAT   
  
  
+ TATCTGGCAG TTGGTTTGTT TATCTCTTGG TTTTTGGGGC TTATTTGGGT GCTGGTTGAT GTTATCCTGC   
  
  
+ TTGGGTTGAA TCTTCATCAG GTTTATTTTT GATTCATAGT TGAGGGTGAA AATTATTTCT CATACCCACC   
  
  
+ ACCTGTTTGT TCTTTTGTCT CTGAGAAAAA CCAAAAAGCT TTCTCTTTTT CCTGATGCAT CCTGTGCTCG   
  
  
+ TTGATCCTGA GTTAGTGAAC CATGCGTCCA AATTCAAGCC TGATTTGCTC TCAAACTTTG ATAAACAACA   
  
  
+ AGAATTTAGC AACGGGATCG AACAAGATGA CATCTTGCAA AACCCTGATC TGTGTTTTGA TTCAGAAACC   
  
  
+ CCTTCAGTTG ATCTAACTGA GAGTGTCATC AGTTCTGATT CTGTAGAAGT GCCTGATTTC TCAGATGCTT   
  
  
+ GCCTCAAGTT CATAAGTGAG ATTCTCCTAG AAGAGGATTT GGATGAAAGT CCTGCCTCTG CTCAAGAATT   
  
  
+ TAGGGCTCTC CAAGCCACTG AGAAGTCCCT GTATGATGCT CTTGGAGAGG GCTACTCTTT TTCATCTGAT   
  
  
+ AACAGCCCAT CATCATTAGG ACAGAGTATT GAGCACCAAA ATGAGAATTT TGAATTCAGT TCCGGTTATC   
  
  
+ CTGGAATTGA GGGCTATGTT AATGGTGATA TCACGTTCGA GTCCAACTGG ATGTTCAACC TAAGCCAATT   
  
  
+ GGATCCTGTT CTTACTTTGG ATGATATTTC TCAGCCCTTG TCTGCATCAA ACTCCCGATC ATCTGGGTCG   
  
  
+ AGCAATGGCT TTGATGATTC AGGGGATGGG GCAGCCACAT CTCCTGGCAG TACAGTTACA TCAACAGTCC   
  
  
+ CAGAGAAAAG GGTTGAATCT GTCAATCGCT CAAGGAGGAA GAAAAATCGT AAAAGGGATG AAGGTGGTCA   
  
  
+ TGAGGAAGGG AGGAGTAACA AGCAGCAAGC TTCATCCAAT GAAGATTATG TTGAGATGAA GGAGTTTGAT   
  
  
+ GATGTACTGC TCTGCAAAGA AGAGAAAGAT GTTATTGCAA ACTGCACCAA TGATTCCTCA CCCGTCGAAG   
  
  
+ CGAGTGATAA GTTGCAGAAG AAAGGGGGGA AGGGGAAAAC ATCGCGTGGG AAGAAGCAGA ATAGCACAAT   
  
  
+ AGAAGAGGTG GATCTGAGGA CTCTTCTCAC TCACTGTGCT CAAGCAATTT CAAATTTTGA TCTTAGGAGT   
  
  
+ GCAAATGAGC ATCTCAGGCA AATAAGGCAG CATTCTTCAC AATATGGTGA TAGCCTCCAG AGGCTTGCCC   
  
  
+ ATTATTTTGC TAATGGTCTT GAGGCTCGCA TAGCTGGCAC TGGTTCAACA ATCTCTGCTA ATGTTGTTGA   
  
  
+ TGCTCGAATC ACGTCATCTG ATTTCTTAAA GGCTTATAGG CTATATATGT CAGCCGTTCC TTTCAAAAGG   
  
  
+ ATGTTATATT TTCTCGCCAA CAAGACAATA CGGAAGTTGG CTGAGAAGGC AACCAGGATC CACATCATTG   
  
  
+ ATTTTGGAGT CTTGTTGGGT TTACAGTGGC CCTGTCTCAT ACAAAACCTC TCAAAGAGAC CCACTGGCCC   
  
  
+ GCCAAGCCTC CGTATTACTG GGATAGACTA TCCCCAGCAT GGTTTCCGGC CATCAGAAAG GGTTGAAGCG   
  
  
+ ACTGGCCGTC GGTTAGAAGG GTATTGCGAG AGATTTAATG TGCCGTTCAA GTATAAGGCC ATAGCTAAAA   
  
  
+ ATTGGGAGAG TATAAAGCTA GAGGATCTAG AAATTGATGA GAATGAGATG GTGTTTGTCA ATTGCATGCT   
  
  
+ TCGCTCTGGA ACACTGCTTG ATGAGACAGT GGTGGCAGAC AGTCCAAAGG ATGCTTTCTT AAGGTTAATC   
  
  
+ AGAGAGATAA ATCCCCGTCT TTTCATTCAT GGGACTATCA ATGGATCATT CAATGCTCCA TTCTTCATCA   
  
  
+ CTCGGTTCAG GGAGGCGCTC TTCCACTACT CTTCTTTATT TGATATCTTT GAAGCAACTA TGCCCCGTGA   
  
  
+ AGATCATGAA AGGCTCCTGG TTGAGAGTGA GATACAGGGC AAAGAAGCTT TGAATGTCAT AGCATGCGAA   
  
  
+ GGTGCCGAGA GGATTCAAAG GCCTGAAACA TACAAGCAAT GGCAAGCAAG GACAACAAGG GCCGGGTTTA   
  
  
+ GGCAGCTTCC ACTAGACCGG GAGCTTGTGA GCAGAGCAAA GGCTATGGTG AAAGCAAACT ACCATAAGGA   
  
  
+ TTTTGTGGTG GACGAGGACA GGCATTGGAT GCTACACGGT TGGAAAGGAA GAATCTTCTG TGCACTCTCT   
  
  
+ GTTTGGCAAC CCAACTG  

- -Up\_Stream \_Len000TAAACT ACACATACGA CTGATGAAAA CTACTACTAC TAGTGTCACT ACGTATAAAT   
  
  
- CTCGACCATT TAACCAGTAA GCTAAGCTCA TGCCCAGCCC AGTTCAAGAC TAATTCACTG TAAAGCGCAG   
  
  
- CAAAACCAAA TCAAGCCCAG CCTAGCTAAA GCCCAATTTA TTAAAAACCA CCTTATGCGA ACAGTACGGT   
  
  
- TTGTGTTCGT TGAAACAATT TTTAAAGCTA AGCCCAGTCC AGTTAAGTCC AAACCCCAGG TGAAAGCTCA   
  
  
- CTCGTATAAA GCCTACAGCC CAGCGCATAC CCAGGCCCAG TAAGCCAAAC TTAAAGCCTA GAACCTAGTT   
  
  
- AAAACGATTC ATACACGTAT AAGTTGGGAA CTACTGTATC CTTTGCGAAA CAGTAGAGGT GGTTAGTCTA   
  
  
- TAGCAATAGA GAAATTAGTA GCGGTATTAT CGTACTCATT CGTACTGGTA GAAAAAGTTT GTACACTATG   
  
  
- TCAATTCCGG ACAAGAAAAG CAATTATTTA GGGTTAAATA GTTAAAATTA TATATGAAAT TGCTTAAAAT   
  
  
- TGTTTTTTAA AATTAAAGTT AGGCGATATT GTTTTTTTTA TGGTTGAAGT TGAATAATAT ATTTTTTTTA   
  
  
- TATGTTAAAA AAAAGTTAAA ATCGGTTGAA GTTGTTTTTT TTTTTTTTAA ATTGTTTAGA GTTAGTTGAA   
  
  
- GTGGGTAAAG AGTTGAATAT AGAGGATTAC TTTTCTTGTC TAGGAATCGT TCTGAAGAGC TAAGTAAATT   
  
  
- TTCCTATCAT ACCATTTAAG TAAAATATGT TCACAGTTCA CACTTATAGA AAAAGTGTAG TCCACTATAT   
  
  
- AATCATCCTG AAAAGCTAAG TGAATAGAAG GAGAAATACA AAACAATTAC ACTATCATTG CTATCCTAAC   
  
  
- GTACATAAAA AAGGTTGATA AGTAAATGTT GATAGTTATA GATAACTATT TATACAATTT TATTAATATA   
  
  
- CAATAAATTT AATAATAAAC AATGAATTCC ACAGCTGAAA CAGCTTTCGC TGAATTAAGA TTAATTCTAT   
  
  
- CGCCGTCTCT TTCAATGTGG ACTACTGGTT CAATCTAGAC CATTTTGATT GGTATCTAGA CTTTGGGTTT   
  
  
- GTATTACTAC ACATAAGTAT GGGCTGGACT GTAGACTTTA TTCAAATGAG TTTTCACCAA GGCTTTGAAC   
  
  
- TTAACTGAGC TTGGCTTGGC ATAAACTGAG CTTTAAAAGA TGTTTCAACG AATAAAAACG TACTTCTGTA   
  
  
- TAACTCGGTT TTTAATCAAC TAAGCTTCAG TTGAATTTGT TTTTAAGTAA GCTGAGTTTG ACTTTATAGT   
  
  
- GAATTGATTT TGATACTGAG TTGAGTTTGA ACTAGACTAG GCATAAGTTT AGTTTATGAG GTAAACGATC   
  
  
- CAGATCTTGG TCGGTGCTGG GGTGTACCTG ATACGCCAAG TTACTTTGCC GGTTTATATA GCGTTAAAGA   
  
  
- CAGAGGTAGT TAGTGTGGGT AAACGATGTG GGACAATTAT AAACTGAGCT GGGACACTTA ACGGAAGCGG   
  
  
- TCAAAACAAA TTAGAGAGAC CAACGAAGGG TAGGTTCAGA CTTATCATAT CAAACATGTC ACACATGATG   
  
  
- ATCTTTCGTG CAAGGAACTT AGAAGAAAGT TTCAACAAAA ATTTCCCAAC TCACTAGGTA ATGCTGAAAA   
  
  
- CTAGACCCAA GTAGCGGAGG AGAGGATTAG GGAGTGGGCC CATGGGAGTT AAGAGATAGC AGAGACAACG   
  
  
- AGTCGATTAA GTTTAAGGAA ATCAACCAGT AGTAACTTTA GACTTTAGTC CCACAAAAAG AAAAATAATA   
  
  
- ATAGACCGTC AACCAAACAA ATAGAGAACC AAAAACCCCG AATAAACCCA CGACCAACTA CAATAGGACG   
  
  
- AACCCAACTT AGAAGTAGTC CAAATAAAAA CTAAGTATCA ACTCCCACTT TTAATAAAGA GTATGGGTGG   
  
  
- TGGACAAACA AGAAAACAGA GACTCTTTTT GGTTTTTCGA AAGAGAAAAA GGACTACGTA GGACACGAGC   
  
  
- AACTAGGACT CAATCACTTG GTACGCAGGT TTAAGTTCGG ACTAAACGAG AGTTTGAAAC TATTTGTTGT   
  
  
- TCTTAAATCG TTGCCCTAGC TTGTTCTACT GTAGAACGTT TTGGGACTAG ACACAAAACT AAGTCTTTGG   
  
  
- GGAAGTCAAC TAGATTGACT CTCACAGTAG TCAAGACTAA GACATCTTCA CGGACTAAAG AGTCTACGAA   
  
  
- CGGAGTTCAA GTATTCACTC TAAGAGGATC TTCTCCTAAA CCTACTTTCA GGACGGAGAC GAGTTCTTAA   
  
  
- ATCCCGAGAG GTTCGGTGAC TCTTCAGGGA CATACTACGA GAACCTCTCC CGATGAGAAA AAGTAGACTA   
  
  
- TTGTCGGGTA GTAGTAATCC TGTCTCATAA CTCGTGGTTT TACTCTTAAA ACTTAAGTCA AGGCCAATAG   
  
  
- GACCTTAACT CCCGATACAA TTACCACTAT AGTGCAAGCT CAGGTTGACC TACAAGTTGG ATTCGGTTAA   
  
  
- CCTAGGACAA GAATGAAACC TACTATAAAG AGTCGGGAAC AGACGTAGTT TGAGGGCTAG TAGACCCAGC   
  
  
- TCGTTACCGA AACTACTAAG TCCCCTACCC CGTCGGTGTA GAGGACCGTC ATGTCAATGT AGTTGTCAGG   
  
  
- GTCTCTTTTC CCAACTTAGA CAGTTAGCGA GTTCCTCCTT CTTTTTAGCA TTTTCCCTAC TTCCACCAGT   
  
  
- ACTCCTTCCC TCCTCATTGT TCGTCGTTCG AAGTAGGTTA CTTCTAATAC AACTCTACTT CCTCAAACTA   
  
  
- CTACATGACG AGACGTTTCT TCTCTTTCTA CAATAACGTT TGACGTGGTT ACTAAGGAGT GGGCAGCTTC   
  
  
- GCTCACTATT CAACGTCTTC TTTCCCCCCT TCCCCTTTTG TAGCGCACCC TTCTTCGTCT TATCGTGTTA   
  
  
- TCTTCTCCAC CTAGACTCCT GAGAAGAGTG AGTGACACGA GTTCGTTAAA GTTTAAAACT AGAATCCTCA   
  
  
- CGTTTACTCG TAGAGTCCGT TTATTCCGTC GTAAGAAGTG TTATACCACT ATCGGAGGTC TCCGAACGGG   
  
  
- TAATAAAACG ATTACCAGAA CTCCGAGCGT ATCGACCGTG ACCAAGTTGT TAGAGACGAT TACAACAACT   
  
  
- ACGAGCTTAG TGCAGTAGAC TAAAGAATTT CCGAATATCC GATATATACA GTCGGCAAGG AAAGTTTTCC   
  
  
- TACAATATAA AAGAGCGGTT GTTCTGTTAT GCCTTCAACC GACTCTTCCG TTGGTCCTAG GTGTAGTAAC   
  
  
- TAAAACCTCA GAACAACCCA AATGTCACCG GGACAGAGTA TGTTTTGGAG AGTTTCTCTG GGTGACCGGG   
  
  
- CGGTTCGGAG GCATAATGAC CCTATCTGAT AGGGGTCGTA CCAAAGGCCG GTAGTCTTTC CCAACTTCGC   
  
  
- TGACCGGCAG CCAATCTTCC CATAACGCTC TCTAAATTAC ACGGCAAGTT CATATTCCGG TATCGATTTT   
  
  
- TAACCCTCTC ATATTTCGAT CTCCTAGATC TTTAACTACT CTTACTCTAC CACAAACAGT TAACGTACGA   
  
  
- AGCGAGACCT TGTGACGAAC TACTCTGTCA CCACCGTCTG TCAGGTTTCC TACGAAAGAA TTCCAATTAG   
  
  
- TCTCTCTATT TAGGGGCAGA AAAGTAAGTA CCCTGATAGT TACCTAGTAA GTTACGAGGT AAGAAGTAGT   
  
  
- GAGCCAAGTC CCTCCGCGAG AAGGTGATGA GAAGAAATAA ACTATAGAAA CTTCGTTGAT ACGGGGCACT   
  
  
- TCTAGTACTT TCCGAGGACC AACTCTCACT CTATGTCCCG TTTCTTCGAA ACTTACAGTA TCGTACGCTT   
  
  
- CCACGGCTCT CCTAAGTTTC CGGACTTTGT ATGTTCGTTA CCGTTCGTTC CTGTTGTTCC CGGCCCAAAT   
  
  
- CCGTCGAAGG TGATCTGGCC CTCGAACACT CGTCTCGTTT CCGATACCAC TTTCGTTTGA TGGTATTCCT   
  
  
- AAAACACCAC CTGCTCCTGT CCGTAACCTA CGATGTGCCA ACCTTTCCTT CTTAGAAGAC ACGTGAGAGA   
  
  
- CAAACCGTTG GGTTGAC

+     GT1-motif

| Site Name | Organism | Position | Strand | Matrix score. | sequence | function |
| --- | --- | --- | --- | --- | --- | --- |
| GT1-motif | Arabidopsis thaliana | 177 | + | 6 | GGTTAA | light responsive element |
| GT1-motif | Avena sativa | 3637 | + | 7 | GGTTAAT | light responsive element |

>HU07G02246.1   
+ -Up\_Stream \_Len000ATTTGA TGTGTATGCT GACTACTTTT GATGATGATG ATCACAGTGA TGCATATTTA   
  
  
+ GAGCTGGTAA ATTGGTCATT CGATTCGAGT ACGGGTCGGG TCAAGTTCTG ATTAAGTGAC ATTTCGCGTC   
  
  
+ GTTTTGGTTT AGTTCGGGTC GGATCGATTT CGGGTTAAAT AATTTTTGGT GGAATACGCT TGTCATGCCA   
  
  
+ AACACAAGCA ACTTTGTTAA AAATTTCGAT TCGGGTCAGG TCAATTCAGG TTTGGGGTCC ACTTTCGAGT   
  
  
+ GAGCATATTT CGGATGTCGG GTCGCGTATG GGTCCGGGTC ATTCGGTTTG AATTTCGGAT CTTGGATCAA   
  
  
+ TTTTGCTAAG TATGTGCATA TTCAACCCTT GATGACATAG GAAACGCTTT GTCATCTCCA CCAATCAGAT   
  
  
+ ATCGTTATCT CTTTAATCAT CGCCATAATA GCATGAGTAA GCATGACCAT CTTTTTCAAA CATGTGATAC   
  
  
+ AGTTAAGGCC TGTTCTTTTC GTTAATAAAT CCCAATTTAT CAATTTTAAT ATATACTTTA ACGAATTTTA   
  
  
+ ACAAAAAATT TTAATTTCAA TCCGCTATAA CAAAAAAAAT ACCAACTTCA ACTTATTATA TAAAAAAAAT   
  
  
+ ATACAATTTT TTTTCAATTT TAGCCAACTT CAACAAAAAA AAAAAAAATT TAACAAATCT CAATCAACTT   
  
  
+ CACCCATTTC TCAACTTATA TCTCCTAATG AAAAGAACAG ATCCTTAGCA AGACTTCTCG ATTCATTTAA   
  
  
+ AAGGATAGTA TGGTAAATTC ATTTTATACA AGTGTCAAGT GTGAATATCT TTTTCACATC AGGTGATATA   
  
  
+ TTAGTAGGAC TTTTCGATTC ACTTATCTTC CTCTTTATGT TTTGTTAATG TGATAGTAAC GATAGGATTG   
  
  
+ CATGTATTTT TTCCAACTAT TCATTTACAA CTATCAATAT CTATTGATAA ATATGTTAAA ATAATTATAT   
  
  
+ GTTATTTAAA TTATTATTTG TTACTTAAGG TGTCGACTTT GTCGAAAGCG ACTTAATTCT AATTAAGATA   
  
  
+ GCGGCAGAGA AAGTTACACC TGATGACCAA GTTAGATCTG GTAAAACTAA CCATAGATCT GAAACCCAAA   
  
  
+ CATAATGATG TGTATTCATA CCCGACCTGA CATCTGAAAT AAGTTTACTC AAAAGTGGTT CCGAAACTTG   
  
  
+ AATTGACTCG AACCGAACCG TATTTGACTC GAAATTTTCT ACAAAGTTGC TTATTTTTGC ATGAAGACAT   
  
  
+ ATTGAGCCAA AAATTAGTTG ATTCGAAGTC AACTTAAACA AAAATTCATT CGACTCAAAC TGAAATATCA   
  
  
+ CTTAACTAAA ACTATGACTC AACTCAAACT TGATCTGATC CGTATTCAAA TCAAATACTC CATTTGCTAG   
  
  
+ GTCTAGAACC AGCCACGACC CCACATGGAC TATGCGGTTC AATGAAACGG CCAAATATAT CGCAATTTCT   
  
  
+ GTCTCCATCA ATCACACCCA TTTGCTACAC CCTGTTAATA TTTGACTCGA CCCTGTGAAT TGCCTTCGCC   
  
  
+ AGTTTTGTTT AATCTCTCTG GTTGCTTCCC ATCCAAGTCT GAATAGTATA GTTTGTACAG TGTGTACTAC   
  
  
+ TAGAAAGCAC GTTCCTTGAA TCTTCTTTCA AAGTTGTTTT TAAAGGGTTG AGTGATCCAT TACGACTTTT   
  
  
+ GATCTGGGTT CATCGCCTCC TCTCCTAATC CCTCACCCGG GTACCCTCAA TTCTCTATCG TCTCTGTTGC   
  
  
+ TCAGCTAATT CAAATTCCTT TAGTTGGTCA TCATTGAAAT CTGAAATCAG GGTGTTTTTC TTTTTATTAT   
  
  
+ TATCTGGCAG TTGGTTTGTT TATCTCTTGG TTTTTGGGGC TTATTTGGGT GCTGGTTGAT GTTATCCTGC   
  
  
+ TTGGGTTGAA TCTTCATCAG GTTTATTTTT GATTCATAGT TGAGGGTGAA AATTATTTCT CATACCCACC   
  
  
+ ACCTGTTTGT TCTTTTGTCT CTGAGAAAAA CCAAAAAGCT TTCTCTTTTT CCTGATGCAT CCTGTGCTCG   
  
  
+ TTGATCCTGA GTTAGTGAAC CATGCGTCCA AATTCAAGCC TGATTTGCTC TCAAACTTTG ATAAACAACA   
  
  
+ AGAATTTAGC AACGGGATCG AACAAGATGA CATCTTGCAA AACCCTGATC TGTGTTTTGA TTCAGAAACC   
  
  
+ CCTTCAGTTG ATCTAACTGA GAGTGTCATC AGTTCTGATT CTGTAGAAGT GCCTGATTTC TCAGATGCTT   
  
  
+ GCCTCAAGTT CATAAGTGAG ATTCTCCTAG AAGAGGATTT GGATGAAAGT CCTGCCTCTG CTCAAGAATT   
  
  
+ TAGGGCTCTC CAAGCCACTG AGAAGTCCCT GTATGATGCT CTTGGAGAGG GCTACTCTTT TTCATCTGAT   
  
  
+ AACAGCCCAT CATCATTAGG ACAGAGTATT GAGCACCAAA ATGAGAATTT TGAATTCAGT TCCGGTTATC   
  
  
+ CTGGAATTGA GGGCTATGTT AATGGTGATA TCACGTTCGA GTCCAACTGG ATGTTCAACC TAAGCCAATT   
  
  
+ GGATCCTGTT CTTACTTTGG ATGATATTTC TCAGCCCTTG TCTGCATCAA ACTCCCGATC ATCTGGGTCG   
  
  
+ AGCAATGGCT TTGATGATTC AGGGGATGGG GCAGCCACAT CTCCTGGCAG TACAGTTACA TCAACAGTCC   
  
  
+ CAGAGAAAAG GGTTGAATCT GTCAATCGCT CAAGGAGGAA GAAAAATCGT AAAAGGGATG AAGGTGGTCA   
  
  
+ TGAGGAAGGG AGGAGTAACA AGCAGCAAGC TTCATCCAAT GAAGATTATG TTGAGATGAA GGAGTTTGAT   
  
  
+ GATGTACTGC TCTGCAAAGA AGAGAAAGAT GTTATTGCAA ACTGCACCAA TGATTCCTCA CCCGTCGAAG   
  
  
+ CGAGTGATAA GTTGCAGAAG AAAGGGGGGA AGGGGAAAAC ATCGCGTGGG AAGAAGCAGA ATAGCACAAT   
  
  
+ AGAAGAGGTG GATCTGAGGA CTCTTCTCAC TCACTGTGCT CAAGCAATTT CAAATTTTGA TCTTAGGAGT   
  
  
+ GCAAATGAGC ATCTCAGGCA AATAAGGCAG CATTCTTCAC AATATGGTGA TAGCCTCCAG AGGCTTGCCC   
  
  
+ ATTATTTTGC TAATGGTCTT GAGGCTCGCA TAGCTGGCAC TGGTTCAACA ATCTCTGCTA ATGTTGTTGA   
  
  
+ TGCTCGAATC ACGTCATCTG ATTTCTTAAA GGCTTATAGG CTATATATGT CAGCCGTTCC TTTCAAAAGG   
  
  
+ ATGTTATATT TTCTCGCCAA CAAGACAATA CGGAAGTTGG CTGAGAAGGC AACCAGGATC CACATCATTG   
  
  
+ ATTTTGGAGT CTTGTTGGGT TTACAGTGGC CCTGTCTCAT ACAAAACCTC TCAAAGAGAC CCACTGGCCC   
  
  
+ GCCAAGCCTC CGTATTACTG GGATAGACTA TCCCCAGCAT GGTTTCCGGC CATCAGAAAG GGTTGAAGCG   
  
  
+ ACTGGCCGTC GGTTAGAAGG GTATTGCGAG AGATTTAATG TGCCGTTCAA GTATAAGGCC ATAGCTAAAA   
  
  
+ ATTGGGAGAG TATAAAGCTA GAGGATCTAG AAATTGATGA GAATGAGATG GTGTTTGTCA ATTGCATGCT   
  
  
+ TCGCTCTGGA ACACTGCTTG ATGAGACAGT GGTGGCAGAC AGTCCAAAGG ATGCTTTCTT AAGGTTAATC   
  
  
+ AGAGAGATAA ATCCCCGTCT TTTCATTCAT GGGACTATCA ATGGATCATT CAATGCTCCA TTCTTCATCA   
  
  
+ CTCGGTTCAG GGAGGCGCTC TTCCACTACT CTTCTTTATT TGATATCTTT GAAGCAACTA TGCCCCGTGA   
  
  
+ AGATCATGAA AGGCTCCTGG TTGAGAGTGA GATACAGGGC AAAGAAGCTT TGAATGTCAT AGCATGCGAA   
  
  
+ GGTGCCGAGA GGATTCAAAG GCCTGAAACA TACAAGCAAT GGCAAGCAAG GACAACAAGG GCCGGGTTTA   
  
  
+ GGCAGCTTCC ACTAGACCGG GAGCTTGTGA GCAGAGCAAA GGCTATGGTG AAAGCAAACT ACCATAAGGA   
  
  
+ TTTTGTGGTG GACGAGGACA GGCATTGGAT GCTACACGGT TGGAAAGGAA GAATCTTCTG TGCACTCTCT   
  
  
+ GTTTGGCAAC CCAACTG  

- -Up\_Stream \_Len000TAAACT ACACATACGA CTGATGAAAA CTACTACTAC TAGTGTCACT ACGTATAAAT   
  
  
- CTCGACCATT TAACCAGTAA GCTAAGCTCA TGCCCAGCCC AGTTCAAGAC TAATTCACTG TAAAGCGCAG   
  
  
- CAAAACCAAA TCAAGCCCAG CCTAGCTAAA GCCCAATTTA TTAAAAACCA CCTTATGCGA ACAGTACGGT   
  
  
- TTGTGTTCGT TGAAACAATT TTTAAAGCTA AGCCCAGTCC AGTTAAGTCC AAACCCCAGG TGAAAGCTCA   
  
  
- CTCGTATAAA GCCTACAGCC CAGCGCATAC CCAGGCCCAG TAAGCCAAAC TTAAAGCCTA GAACCTAGTT   
  
  
- AAAACGATTC ATACACGTAT AAGTTGGGAA CTACTGTATC CTTTGCGAAA CAGTAGAGGT GGTTAGTCTA   
  
  
- TAGCAATAGA GAAATTAGTA GCGGTATTAT CGTACTCATT CGTACTGGTA GAAAAAGTTT GTACACTATG   
  
  
- TCAATTCCGG ACAAGAAAAG CAATTATTTA GGGTTAAATA GTTAAAATTA TATATGAAAT TGCTTAAAAT   
  
  
- TGTTTTTTAA AATTAAAGTT AGGCGATATT GTTTTTTTTA TGGTTGAAGT TGAATAATAT ATTTTTTTTA   
  
  
- TATGTTAAAA AAAAGTTAAA ATCGGTTGAA GTTGTTTTTT TTTTTTTTAA ATTGTTTAGA GTTAGTTGAA   
  
  
- GTGGGTAAAG AGTTGAATAT AGAGGATTAC TTTTCTTGTC TAGGAATCGT TCTGAAGAGC TAAGTAAATT   
  
  
- TTCCTATCAT ACCATTTAAG TAAAATATGT TCACAGTTCA CACTTATAGA AAAAGTGTAG TCCACTATAT   
  
  
- AATCATCCTG AAAAGCTAAG TGAATAGAAG GAGAAATACA AAACAATTAC ACTATCATTG CTATCCTAAC   
  
  
- GTACATAAAA AAGGTTGATA AGTAAATGTT GATAGTTATA GATAACTATT TATACAATTT TATTAATATA   
  
  
- CAATAAATTT AATAATAAAC AATGAATTCC ACAGCTGAAA CAGCTTTCGC TGAATTAAGA TTAATTCTAT   
  
  
- CGCCGTCTCT TTCAATGTGG ACTACTGGTT CAATCTAGAC CATTTTGATT GGTATCTAGA CTTTGGGTTT   
  
  
- GTATTACTAC ACATAAGTAT GGGCTGGACT GTAGACTTTA TTCAAATGAG TTTTCACCAA GGCTTTGAAC   
  
  
- TTAACTGAGC TTGGCTTGGC ATAAACTGAG CTTTAAAAGA TGTTTCAACG AATAAAAACG TACTTCTGTA   
  
  
- TAACTCGGTT TTTAATCAAC TAAGCTTCAG TTGAATTTGT TTTTAAGTAA GCTGAGTTTG ACTTTATAGT   
  
  
- GAATTGATTT TGATACTGAG TTGAGTTTGA ACTAGACTAG GCATAAGTTT AGTTTATGAG GTAAACGATC   
  
  
- CAGATCTTGG TCGGTGCTGG GGTGTACCTG ATACGCCAAG TTACTTTGCC GGTTTATATA GCGTTAAAGA   
  
  
- CAGAGGTAGT TAGTGTGGGT AAACGATGTG GGACAATTAT AAACTGAGCT GGGACACTTA ACGGAAGCGG   
  
  
- TCAAAACAAA TTAGAGAGAC CAACGAAGGG TAGGTTCAGA CTTATCATAT CAAACATGTC ACACATGATG   
  
  
- ATCTTTCGTG CAAGGAACTT AGAAGAAAGT TTCAACAAAA ATTTCCCAAC TCACTAGGTA ATGCTGAAAA   
  
  
- CTAGACCCAA GTAGCGGAGG AGAGGATTAG GGAGTGGGCC CATGGGAGTT AAGAGATAGC AGAGACAACG   
  
  
- AGTCGATTAA GTTTAAGGAA ATCAACCAGT AGTAACTTTA GACTTTAGTC CCACAAAAAG AAAAATAATA   
  
  
- ATAGACCGTC AACCAAACAA ATAGAGAACC AAAAACCCCG AATAAACCCA CGACCAACTA CAATAGGACG   
  
  
- AACCCAACTT AGAAGTAGTC CAAATAAAAA CTAAGTATCA ACTCCCACTT TTAATAAAGA GTATGGGTGG   
  
  
- TGGACAAACA AGAAAACAGA GACTCTTTTT GGTTTTTCGA AAGAGAAAAA GGACTACGTA GGACACGAGC   
  
  
- AACTAGGACT CAATCACTTG GTACGCAGGT TTAAGTTCGG ACTAAACGAG AGTTTGAAAC TATTTGTTGT   
  
  
- TCTTAAATCG TTGCCCTAGC TTGTTCTACT GTAGAACGTT TTGGGACTAG ACACAAAACT AAGTCTTTGG   
  
  
- GGAAGTCAAC TAGATTGACT CTCACAGTAG TCAAGACTAA GACATCTTCA CGGACTAAAG AGTCTACGAA   
  
  
- CGGAGTTCAA GTATTCACTC TAAGAGGATC TTCTCCTAAA CCTACTTTCA GGACGGAGAC GAGTTCTTAA   
  
  
- ATCCCGAGAG GTTCGGTGAC TCTTCAGGGA CATACTACGA GAACCTCTCC CGATGAGAAA AAGTAGACTA   
  
  
- TTGTCGGGTA GTAGTAATCC TGTCTCATAA CTCGTGGTTT TACTCTTAAA ACTTAAGTCA AGGCCAATAG   
  
  
- GACCTTAACT CCCGATACAA TTACCACTAT AGTGCAAGCT CAGGTTGACC TACAAGTTGG ATTCGGTTAA   
  
  
- CCTAGGACAA GAATGAAACC TACTATAAAG AGTCGGGAAC AGACGTAGTT TGAGGGCTAG TAGACCCAGC   
  
  
- TCGTTACCGA AACTACTAAG TCCCCTACCC CGTCGGTGTA GAGGACCGTC ATGTCAATGT AGTTGTCAGG   
  
  
- GTCTCTTTTC CCAACTTAGA CAGTTAGCGA GTTCCTCCTT CTTTTTAGCA TTTTCCCTAC TTCCACCAGT   
  
  
- ACTCCTTCCC TCCTCATTGT TCGTCGTTCG AAGTAGGTTA CTTCTAATAC AACTCTACTT CCTCAAACTA   
  
  
- CTACATGACG AGACGTTTCT TCTCTTTCTA CAATAACGTT TGACGTGGTT ACTAAGGAGT GGGCAGCTTC   
  
  
- GCTCACTATT CAACGTCTTC TTTCCCCCCT TCCCCTTTTG TAGCGCACCC TTCTTCGTCT TATCGTGTTA   
  
  
- TCTTCTCCAC CTAGACTCCT GAGAAGAGTG AGTGACACGA GTTCGTTAAA GTTTAAAACT AGAATCCTCA   
  
  
- CGTTTACTCG TAGAGTCCGT TTATTCCGTC GTAAGAAGTG TTATACCACT ATCGGAGGTC TCCGAACGGG   
  
  
- TAATAAAACG ATTACCAGAA CTCCGAGCGT ATCGACCGTG ACCAAGTTGT TAGAGACGAT TACAACAACT   
  
  
- ACGAGCTTAG TGCAGTAGAC TAAAGAATTT CCGAATATCC GATATATACA GTCGGCAAGG AAAGTTTTCC   
  
  
- TACAATATAA AAGAGCGGTT GTTCTGTTAT GCCTTCAACC GACTCTTCCG TTGGTCCTAG GTGTAGTAAC   
  
  
- TAAAACCTCA GAACAACCCA AATGTCACCG GGACAGAGTA TGTTTTGGAG AGTTTCTCTG GGTGACCGGG   
  
  
- CGGTTCGGAG GCATAATGAC CCTATCTGAT AGGGGTCGTA CCAAAGGCCG GTAGTCTTTC CCAACTTCGC   
  
  
- TGACCGGCAG CCAATCTTCC CATAACGCTC TCTAAATTAC ACGGCAAGTT CATATTCCGG TATCGATTTT   
  
  
- TAACCCTCTC ATATTTCGAT CTCCTAGATC TTTAACTACT CTTACTCTAC CACAAACAGT TAACGTACGA   
  
  
- AGCGAGACCT TGTGACGAAC TACTCTGTCA CCACCGTCTG TCAGGTTTCC TACGAAAGAA TTCCAATTAG   
  
  
- TCTCTCTATT TAGGGGCAGA AAAGTAAGTA CCCTGATAGT TACCTAGTAA GTTACGAGGT AAGAAGTAGT   
  
  
- GAGCCAAGTC CCTCCGCGAG AAGGTGATGA GAAGAAATAA ACTATAGAAA CTTCGTTGAT ACGGGGCACT   
  
  
- TCTAGTACTT TCCGAGGACC AACTCTCACT CTATGTCCCG TTTCTTCGAA ACTTACAGTA TCGTACGCTT   
  
  
- CCACGGCTCT CCTAAGTTTC CGGACTTTGT ATGTTCGTTA CCGTTCGTTC CTGTTGTTCC CGGCCCAAAT   
  
  
- CCGTCGAAGG TGATCTGGCC CTCGAACACT CGTCTCGTTT CCGATACCAC TTTCGTTTGA TGGTATTCCT   
  
  
- AAAACACCAC CTGCTCCTGT CCGTAACCTA CGATGTGCCA ACCTTTCCTT CTTAGAAGAC ACGTGAGAGA   
  
  
- CAAACCGTTG GGTTGAC

+     I-box

| Site Name | Organism | Position | Strand | Matrix score. | sequence | function |
| --- | --- | --- | --- | --- | --- | --- |
| I-box | Larix laricina | 3485 | + | 10 | GTATAAGGCC | part of a light responsive element |

>HU07G02246.1   
+ -Up\_Stream \_Len000ATTTGA TGTGTATGCT GACTACTTTT GATGATGATG ATCACAGTGA TGCATATTTA   
  
  
+ GAGCTGGTAA ATTGGTCATT CGATTCGAGT ACGGGTCGGG TCAAGTTCTG ATTAAGTGAC ATTTCGCGTC   
  
  
+ GTTTTGGTTT AGTTCGGGTC GGATCGATTT CGGGTTAAAT AATTTTTGGT GGAATACGCT TGTCATGCCA   
  
  
+ AACACAAGCA ACTTTGTTAA AAATTTCGAT TCGGGTCAGG TCAATTCAGG TTTGGGGTCC ACTTTCGAGT   
  
  
+ GAGCATATTT CGGATGTCGG GTCGCGTATG GGTCCGGGTC ATTCGGTTTG AATTTCGGAT CTTGGATCAA   
  
  
+ TTTTGCTAAG TATGTGCATA TTCAACCCTT GATGACATAG GAAACGCTTT GTCATCTCCA CCAATCAGAT   
  
  
+ ATCGTTATCT CTTTAATCAT CGCCATAATA GCATGAGTAA GCATGACCAT CTTTTTCAAA CATGTGATAC   
  
  
+ AGTTAAGGCC TGTTCTTTTC GTTAATAAAT CCCAATTTAT CAATTTTAAT ATATACTTTA ACGAATTTTA   
  
  
+ ACAAAAAATT TTAATTTCAA TCCGCTATAA CAAAAAAAAT ACCAACTTCA ACTTATTATA TAAAAAAAAT   
  
  
+ ATACAATTTT TTTTCAATTT TAGCCAACTT CAACAAAAAA AAAAAAAATT TAACAAATCT CAATCAACTT   
  
  
+ CACCCATTTC TCAACTTATA TCTCCTAATG AAAAGAACAG ATCCTTAGCA AGACTTCTCG ATTCATTTAA   
  
  
+ AAGGATAGTA TGGTAAATTC ATTTTATACA AGTGTCAAGT GTGAATATCT TTTTCACATC AGGTGATATA   
  
  
+ TTAGTAGGAC TTTTCGATTC ACTTATCTTC CTCTTTATGT TTTGTTAATG TGATAGTAAC GATAGGATTG   
  
  
+ CATGTATTTT TTCCAACTAT TCATTTACAA CTATCAATAT CTATTGATAA ATATGTTAAA ATAATTATAT   
  
  
+ GTTATTTAAA TTATTATTTG TTACTTAAGG TGTCGACTTT GTCGAAAGCG ACTTAATTCT AATTAAGATA   
  
  
+ GCGGCAGAGA AAGTTACACC TGATGACCAA GTTAGATCTG GTAAAACTAA CCATAGATCT GAAACCCAAA   
  
  
+ CATAATGATG TGTATTCATA CCCGACCTGA CATCTGAAAT AAGTTTACTC AAAAGTGGTT CCGAAACTTG   
  
  
+ AATTGACTCG AACCGAACCG TATTTGACTC GAAATTTTCT ACAAAGTTGC TTATTTTTGC ATGAAGACAT   
  
  
+ ATTGAGCCAA AAATTAGTTG ATTCGAAGTC AACTTAAACA AAAATTCATT CGACTCAAAC TGAAATATCA   
  
  
+ CTTAACTAAA ACTATGACTC AACTCAAACT TGATCTGATC CGTATTCAAA TCAAATACTC CATTTGCTAG   
  
  
+ GTCTAGAACC AGCCACGACC CCACATGGAC TATGCGGTTC AATGAAACGG CCAAATATAT CGCAATTTCT   
  
  
+ GTCTCCATCA ATCACACCCA TTTGCTACAC CCTGTTAATA TTTGACTCGA CCCTGTGAAT TGCCTTCGCC   
  
  
+ AGTTTTGTTT AATCTCTCTG GTTGCTTCCC ATCCAAGTCT GAATAGTATA GTTTGTACAG TGTGTACTAC   
  
  
+ TAGAAAGCAC GTTCCTTGAA TCTTCTTTCA AAGTTGTTTT TAAAGGGTTG AGTGATCCAT TACGACTTTT   
  
  
+ GATCTGGGTT CATCGCCTCC TCTCCTAATC CCTCACCCGG GTACCCTCAA TTCTCTATCG TCTCTGTTGC   
  
  
+ TCAGCTAATT CAAATTCCTT TAGTTGGTCA TCATTGAAAT CTGAAATCAG GGTGTTTTTC TTTTTATTAT   
  
  
+ TATCTGGCAG TTGGTTTGTT TATCTCTTGG TTTTTGGGGC TTATTTGGGT GCTGGTTGAT GTTATCCTGC   
  
  
+ TTGGGTTGAA TCTTCATCAG GTTTATTTTT GATTCATAGT TGAGGGTGAA AATTATTTCT CATACCCACC   
  
  
+ ACCTGTTTGT TCTTTTGTCT CTGAGAAAAA CCAAAAAGCT TTCTCTTTTT CCTGATGCAT CCTGTGCTCG   
  
  
+ TTGATCCTGA GTTAGTGAAC CATGCGTCCA AATTCAAGCC TGATTTGCTC TCAAACTTTG ATAAACAACA   
  
  
+ AGAATTTAGC AACGGGATCG AACAAGATGA CATCTTGCAA AACCCTGATC TGTGTTTTGA TTCAGAAACC   
  
  
+ CCTTCAGTTG ATCTAACTGA GAGTGTCATC AGTTCTGATT CTGTAGAAGT GCCTGATTTC TCAGATGCTT   
  
  
+ GCCTCAAGTT CATAAGTGAG ATTCTCCTAG AAGAGGATTT GGATGAAAGT CCTGCCTCTG CTCAAGAATT   
  
  
+ TAGGGCTCTC CAAGCCACTG AGAAGTCCCT GTATGATGCT CTTGGAGAGG GCTACTCTTT TTCATCTGAT   
  
  
+ AACAGCCCAT CATCATTAGG ACAGAGTATT GAGCACCAAA ATGAGAATTT TGAATTCAGT TCCGGTTATC   
  
  
+ CTGGAATTGA GGGCTATGTT AATGGTGATA TCACGTTCGA GTCCAACTGG ATGTTCAACC TAAGCCAATT   
  
  
+ GGATCCTGTT CTTACTTTGG ATGATATTTC TCAGCCCTTG TCTGCATCAA ACTCCCGATC ATCTGGGTCG   
  
  
+ AGCAATGGCT TTGATGATTC AGGGGATGGG GCAGCCACAT CTCCTGGCAG TACAGTTACA TCAACAGTCC   
  
  
+ CAGAGAAAAG GGTTGAATCT GTCAATCGCT CAAGGAGGAA GAAAAATCGT AAAAGGGATG AAGGTGGTCA   
  
  
+ TGAGGAAGGG AGGAGTAACA AGCAGCAAGC TTCATCCAAT GAAGATTATG TTGAGATGAA GGAGTTTGAT   
  
  
+ GATGTACTGC TCTGCAAAGA AGAGAAAGAT GTTATTGCAA ACTGCACCAA TGATTCCTCA CCCGTCGAAG   
  
  
+ CGAGTGATAA GTTGCAGAAG AAAGGGGGGA AGGGGAAAAC ATCGCGTGGG AAGAAGCAGA ATAGCACAAT   
  
  
+ AGAAGAGGTG GATCTGAGGA CTCTTCTCAC TCACTGTGCT CAAGCAATTT CAAATTTTGA TCTTAGGAGT   
  
  
+ GCAAATGAGC ATCTCAGGCA AATAAGGCAG CATTCTTCAC AATATGGTGA TAGCCTCCAG AGGCTTGCCC   
  
  
+ ATTATTTTGC TAATGGTCTT GAGGCTCGCA TAGCTGGCAC TGGTTCAACA ATCTCTGCTA ATGTTGTTGA   
  
  
+ TGCTCGAATC ACGTCATCTG ATTTCTTAAA GGCTTATAGG CTATATATGT CAGCCGTTCC TTTCAAAAGG   
  
  
+ ATGTTATATT TTCTCGCCAA CAAGACAATA CGGAAGTTGG CTGAGAAGGC AACCAGGATC CACATCATTG   
  
  
+ ATTTTGGAGT CTTGTTGGGT TTACAGTGGC CCTGTCTCAT ACAAAACCTC TCAAAGAGAC CCACTGGCCC   
  
  
+ GCCAAGCCTC CGTATTACTG GGATAGACTA TCCCCAGCAT GGTTTCCGGC CATCAGAAAG GGTTGAAGCG   
  
  
+ ACTGGCCGTC GGTTAGAAGG GTATTGCGAG AGATTTAATG TGCCGTTCAA GTATAAGGCC ATAGCTAAAA   
  
  
+ ATTGGGAGAG TATAAAGCTA GAGGATCTAG AAATTGATGA GAATGAGATG GTGTTTGTCA ATTGCATGCT   
  
  
+ TCGCTCTGGA ACACTGCTTG ATGAGACAGT GGTGGCAGAC AGTCCAAAGG ATGCTTTCTT AAGGTTAATC   
  
  
+ AGAGAGATAA ATCCCCGTCT TTTCATTCAT GGGACTATCA ATGGATCATT CAATGCTCCA TTCTTCATCA   
  
  
+ CTCGGTTCAG GGAGGCGCTC TTCCACTACT CTTCTTTATT TGATATCTTT GAAGCAACTA TGCCCCGTGA   
  
  
+ AGATCATGAA AGGCTCCTGG TTGAGAGTGA GATACAGGGC AAAGAAGCTT TGAATGTCAT AGCATGCGAA   
  
  
+ GGTGCCGAGA GGATTCAAAG GCCTGAAACA TACAAGCAAT GGCAAGCAAG GACAACAAGG GCCGGGTTTA   
  
  
+ GGCAGCTTCC ACTAGACCGG GAGCTTGTGA GCAGAGCAAA GGCTATGGTG AAAGCAAACT ACCATAAGGA   
  
  
+ TTTTGTGGTG GACGAGGACA GGCATTGGAT GCTACACGGT TGGAAAGGAA GAATCTTCTG TGCACTCTCT   
  
  
+ GTTTGGCAAC CCAACTG  

- -Up\_Stream \_Len000TAAACT ACACATACGA CTGATGAAAA CTACTACTAC TAGTGTCACT ACGTATAAAT   
  
  
- CTCGACCATT TAACCAGTAA GCTAAGCTCA TGCCCAGCCC AGTTCAAGAC TAATTCACTG TAAAGCGCAG   
  
  
- CAAAACCAAA TCAAGCCCAG CCTAGCTAAA GCCCAATTTA TTAAAAACCA CCTTATGCGA ACAGTACGGT   
  
  
- TTGTGTTCGT TGAAACAATT TTTAAAGCTA AGCCCAGTCC AGTTAAGTCC AAACCCCAGG TGAAAGCTCA   
  
  
- CTCGTATAAA GCCTACAGCC CAGCGCATAC CCAGGCCCAG TAAGCCAAAC TTAAAGCCTA GAACCTAGTT   
  
  
- AAAACGATTC ATACACGTAT AAGTTGGGAA CTACTGTATC CTTTGCGAAA CAGTAGAGGT GGTTAGTCTA   
  
  
- TAGCAATAGA GAAATTAGTA GCGGTATTAT CGTACTCATT CGTACTGGTA GAAAAAGTTT GTACACTATG   
  
  
- TCAATTCCGG ACAAGAAAAG CAATTATTTA GGGTTAAATA GTTAAAATTA TATATGAAAT TGCTTAAAAT   
  
  
- TGTTTTTTAA AATTAAAGTT AGGCGATATT GTTTTTTTTA TGGTTGAAGT TGAATAATAT ATTTTTTTTA   
  
  
- TATGTTAAAA AAAAGTTAAA ATCGGTTGAA GTTGTTTTTT TTTTTTTTAA ATTGTTTAGA GTTAGTTGAA   
  
  
- GTGGGTAAAG AGTTGAATAT AGAGGATTAC TTTTCTTGTC TAGGAATCGT TCTGAAGAGC TAAGTAAATT   
  
  
- TTCCTATCAT ACCATTTAAG TAAAATATGT TCACAGTTCA CACTTATAGA AAAAGTGTAG TCCACTATAT   
  
  
- AATCATCCTG AAAAGCTAAG TGAATAGAAG GAGAAATACA AAACAATTAC ACTATCATTG CTATCCTAAC   
  
  
- GTACATAAAA AAGGTTGATA AGTAAATGTT GATAGTTATA GATAACTATT TATACAATTT TATTAATATA   
  
  
- CAATAAATTT AATAATAAAC AATGAATTCC ACAGCTGAAA CAGCTTTCGC TGAATTAAGA TTAATTCTAT   
  
  
- CGCCGTCTCT TTCAATGTGG ACTACTGGTT CAATCTAGAC CATTTTGATT GGTATCTAGA CTTTGGGTTT   
  
  
- GTATTACTAC ACATAAGTAT GGGCTGGACT GTAGACTTTA TTCAAATGAG TTTTCACCAA GGCTTTGAAC   
  
  
- TTAACTGAGC TTGGCTTGGC ATAAACTGAG CTTTAAAAGA TGTTTCAACG AATAAAAACG TACTTCTGTA   
  
  
- TAACTCGGTT TTTAATCAAC TAAGCTTCAG TTGAATTTGT TTTTAAGTAA GCTGAGTTTG ACTTTATAGT   
  
  
- GAATTGATTT TGATACTGAG TTGAGTTTGA ACTAGACTAG GCATAAGTTT AGTTTATGAG GTAAACGATC   
  
  
- CAGATCTTGG TCGGTGCTGG GGTGTACCTG ATACGCCAAG TTACTTTGCC GGTTTATATA GCGTTAAAGA   
  
  
- CAGAGGTAGT TAGTGTGGGT AAACGATGTG GGACAATTAT AAACTGAGCT GGGACACTTA ACGGAAGCGG   
  
  
- TCAAAACAAA TTAGAGAGAC CAACGAAGGG TAGGTTCAGA CTTATCATAT CAAACATGTC ACACATGATG   
  
  
- ATCTTTCGTG CAAGGAACTT AGAAGAAAGT TTCAACAAAA ATTTCCCAAC TCACTAGGTA ATGCTGAAAA   
  
  
- CTAGACCCAA GTAGCGGAGG AGAGGATTAG GGAGTGGGCC CATGGGAGTT AAGAGATAGC AGAGACAACG   
  
  
- AGTCGATTAA GTTTAAGGAA ATCAACCAGT AGTAACTTTA GACTTTAGTC CCACAAAAAG AAAAATAATA   
  
  
- ATAGACCGTC AACCAAACAA ATAGAGAACC AAAAACCCCG AATAAACCCA CGACCAACTA CAATAGGACG   
  
  
- AACCCAACTT AGAAGTAGTC CAAATAAAAA CTAAGTATCA ACTCCCACTT TTAATAAAGA GTATGGGTGG   
  
  
- TGGACAAACA AGAAAACAGA GACTCTTTTT GGTTTTTCGA AAGAGAAAAA GGACTACGTA GGACACGAGC   
  
  
- AACTAGGACT CAATCACTTG GTACGCAGGT TTAAGTTCGG ACTAAACGAG AGTTTGAAAC TATTTGTTGT   
  
  
- TCTTAAATCG TTGCCCTAGC TTGTTCTACT GTAGAACGTT TTGGGACTAG ACACAAAACT AAGTCTTTGG   
  
  
- GGAAGTCAAC TAGATTGACT CTCACAGTAG TCAAGACTAA GACATCTTCA CGGACTAAAG AGTCTACGAA   
  
  
- CGGAGTTCAA GTATTCACTC TAAGAGGATC TTCTCCTAAA CCTACTTTCA GGACGGAGAC GAGTTCTTAA   
  
  
- ATCCCGAGAG GTTCGGTGAC TCTTCAGGGA CATACTACGA GAACCTCTCC CGATGAGAAA AAGTAGACTA   
  
  
- TTGTCGGGTA GTAGTAATCC TGTCTCATAA CTCGTGGTTT TACTCTTAAA ACTTAAGTCA AGGCCAATAG   
  
  
- GACCTTAACT CCCGATACAA TTACCACTAT AGTGCAAGCT CAGGTTGACC TACAAGTTGG ATTCGGTTAA   
  
  
- CCTAGGACAA GAATGAAACC TACTATAAAG AGTCGGGAAC AGACGTAGTT TGAGGGCTAG TAGACCCAGC   
  
  
- TCGTTACCGA AACTACTAAG TCCCCTACCC CGTCGGTGTA GAGGACCGTC ATGTCAATGT AGTTGTCAGG   
  
  
- GTCTCTTTTC CCAACTTAGA CAGTTAGCGA GTTCCTCCTT CTTTTTAGCA TTTTCCCTAC TTCCACCAGT   
  
  
- ACTCCTTCCC TCCTCATTGT TCGTCGTTCG AAGTAGGTTA CTTCTAATAC AACTCTACTT CCTCAAACTA   
  
  
- CTACATGACG AGACGTTTCT TCTCTTTCTA CAATAACGTT TGACGTGGTT ACTAAGGAGT GGGCAGCTTC   
  
  
- GCTCACTATT CAACGTCTTC TTTCCCCCCT TCCCCTTTTG TAGCGCACCC TTCTTCGTCT TATCGTGTTA   
  
  
- TCTTCTCCAC CTAGACTCCT GAGAAGAGTG AGTGACACGA GTTCGTTAAA GTTTAAAACT AGAATCCTCA   
  
  
- CGTTTACTCG TAGAGTCCGT TTATTCCGTC GTAAGAAGTG TTATACCACT ATCGGAGGTC TCCGAACGGG   
  
  
- TAATAAAACG ATTACCAGAA CTCCGAGCGT ATCGACCGTG ACCAAGTTGT TAGAGACGAT TACAACAACT   
  
  
- ACGAGCTTAG TGCAGTAGAC TAAAGAATTT CCGAATATCC GATATATACA GTCGGCAAGG AAAGTTTTCC   
  
  
- TACAATATAA AAGAGCGGTT GTTCTGTTAT GCCTTCAACC GACTCTTCCG TTGGTCCTAG GTGTAGTAAC   
  
  
- TAAAACCTCA GAACAACCCA AATGTCACCG GGACAGAGTA TGTTTTGGAG AGTTTCTCTG GGTGACCGGG   
  
  
- CGGTTCGGAG GCATAATGAC CCTATCTGAT AGGGGTCGTA CCAAAGGCCG GTAGTCTTTC CCAACTTCGC   
  
  
- TGACCGGCAG CCAATCTTCC CATAACGCTC TCTAAATTAC ACGGCAAGTT CATATTCCGG TATCGATTTT   
  
  
- TAACCCTCTC ATATTTCGAT CTCCTAGATC TTTAACTACT CTTACTCTAC CACAAACAGT TAACGTACGA   
  
  
- AGCGAGACCT TGTGACGAAC TACTCTGTCA CCACCGTCTG TCAGGTTTCC TACGAAAGAA TTCCAATTAG   
  
  
- TCTCTCTATT TAGGGGCAGA AAAGTAAGTA CCCTGATAGT TACCTAGTAA GTTACGAGGT AAGAAGTAGT   
  
  
- GAGCCAAGTC CCTCCGCGAG AAGGTGATGA GAAGAAATAA ACTATAGAAA CTTCGTTGAT ACGGGGCACT   
  
  
- TCTAGTACTT TCCGAGGACC AACTCTCACT CTATGTCCCG TTTCTTCGAA ACTTACAGTA TCGTACGCTT   
  
  
- CCACGGCTCT CCTAAGTTTC CGGACTTTGT ATGTTCGTTA CCGTTCGTTC CTGTTGTTCC CGGCCCAAAT   
  
  
- CCGTCGAAGG TGATCTGGCC CTCGAACACT CGTCTCGTTT CCGATACCAC TTTCGTTTGA TGGTATTCCT   
  
  
- AAAACACCAC CTGCTCCTGT CCGTAACCTA CGATGTGCCA ACCTTTCCTT CTTAGAAGAC ACGTGAGAGA   
  
  
- CAAACCGTTG GGTTGAC

+     LTR

| Site Name | Organism | Position | Strand | Matrix score. | sequence | function |
| --- | --- | --- | --- | --- | --- | --- |
| LTR | Hordeum vulgare | 292 | - | 6 | CCGAAA | cis-acting element involved in low-temperature responsiveness |
| LTR | Hordeum vulgare | 172 | - | 6 | CCGAAA | cis-acting element involved in low-temperature responsiveness |
| LTR | Hordeum vulgare | 337 | - | 6 | CCGAAA | cis-acting element involved in low-temperature responsiveness |
| LTR | Hordeum vulgare | 1185 | + | 6 | CCGAAA | cis-acting element involved in low-temperature responsiveness |

>HU07G02246.1   
+ -Up\_Stream \_Len000ATTTGA TGTGTATGCT GACTACTTTT GATGATGATG ATCACAGTGA TGCATATTTA   
  
  
+ GAGCTGGTAA ATTGGTCATT CGATTCGAGT ACGGGTCGGG TCAAGTTCTG ATTAAGTGAC ATTTCGCGTC   
  
  
+ GTTTTGGTTT AGTTCGGGTC GGATCGATTT CGGGTTAAAT AATTTTTGGT GGAATACGCT TGTCATGCCA   
  
  
+ AACACAAGCA ACTTTGTTAA AAATTTCGAT TCGGGTCAGG TCAATTCAGG TTTGGGGTCC ACTTTCGAGT   
  
  
+ GAGCATATTT CGGATGTCGG GTCGCGTATG GGTCCGGGTC ATTCGGTTTG AATTTCGGAT CTTGGATCAA   
  
  
+ TTTTGCTAAG TATGTGCATA TTCAACCCTT GATGACATAG GAAACGCTTT GTCATCTCCA CCAATCAGAT   
  
  
+ ATCGTTATCT CTTTAATCAT CGCCATAATA GCATGAGTAA GCATGACCAT CTTTTTCAAA CATGTGATAC   
  
  
+ AGTTAAGGCC TGTTCTTTTC GTTAATAAAT CCCAATTTAT CAATTTTAAT ATATACTTTA ACGAATTTTA   
  
  
+ ACAAAAAATT TTAATTTCAA TCCGCTATAA CAAAAAAAAT ACCAACTTCA ACTTATTATA TAAAAAAAAT   
  
  
+ ATACAATTTT TTTTCAATTT TAGCCAACTT CAACAAAAAA AAAAAAAATT TAACAAATCT CAATCAACTT   
  
  
+ CACCCATTTC TCAACTTATA TCTCCTAATG AAAAGAACAG ATCCTTAGCA AGACTTCTCG ATTCATTTAA   
  
  
+ AAGGATAGTA TGGTAAATTC ATTTTATACA AGTGTCAAGT GTGAATATCT TTTTCACATC AGGTGATATA   
  
  
+ TTAGTAGGAC TTTTCGATTC ACTTATCTTC CTCTTTATGT TTTGTTAATG TGATAGTAAC GATAGGATTG   
  
  
+ CATGTATTTT TTCCAACTAT TCATTTACAA CTATCAATAT CTATTGATAA ATATGTTAAA ATAATTATAT   
  
  
+ GTTATTTAAA TTATTATTTG TTACTTAAGG TGTCGACTTT GTCGAAAGCG ACTTAATTCT AATTAAGATA   
  
  
+ GCGGCAGAGA AAGTTACACC TGATGACCAA GTTAGATCTG GTAAAACTAA CCATAGATCT GAAACCCAAA   
  
  
+ CATAATGATG TGTATTCATA CCCGACCTGA CATCTGAAAT AAGTTTACTC AAAAGTGGTT CCGAAACTTG   
  
  
+ AATTGACTCG AACCGAACCG TATTTGACTC GAAATTTTCT ACAAAGTTGC TTATTTTTGC ATGAAGACAT   
  
  
+ ATTGAGCCAA AAATTAGTTG ATTCGAAGTC AACTTAAACA AAAATTCATT CGACTCAAAC TGAAATATCA   
  
  
+ CTTAACTAAA ACTATGACTC AACTCAAACT TGATCTGATC CGTATTCAAA TCAAATACTC CATTTGCTAG   
  
  
+ GTCTAGAACC AGCCACGACC CCACATGGAC TATGCGGTTC AATGAAACGG CCAAATATAT CGCAATTTCT   
  
  
+ GTCTCCATCA ATCACACCCA TTTGCTACAC CCTGTTAATA TTTGACTCGA CCCTGTGAAT TGCCTTCGCC   
  
  
+ AGTTTTGTTT AATCTCTCTG GTTGCTTCCC ATCCAAGTCT GAATAGTATA GTTTGTACAG TGTGTACTAC   
  
  
+ TAGAAAGCAC GTTCCTTGAA TCTTCTTTCA AAGTTGTTTT TAAAGGGTTG AGTGATCCAT TACGACTTTT   
  
  
+ GATCTGGGTT CATCGCCTCC TCTCCTAATC CCTCACCCGG GTACCCTCAA TTCTCTATCG TCTCTGTTGC   
  
  
+ TCAGCTAATT CAAATTCCTT TAGTTGGTCA TCATTGAAAT CTGAAATCAG GGTGTTTTTC TTTTTATTAT   
  
  
+ TATCTGGCAG TTGGTTTGTT TATCTCTTGG TTTTTGGGGC TTATTTGGGT GCTGGTTGAT GTTATCCTGC   
  
  
+ TTGGGTTGAA TCTTCATCAG GTTTATTTTT GATTCATAGT TGAGGGTGAA AATTATTTCT CATACCCACC   
  
  
+ ACCTGTTTGT TCTTTTGTCT CTGAGAAAAA CCAAAAAGCT TTCTCTTTTT CCTGATGCAT CCTGTGCTCG   
  
  
+ TTGATCCTGA GTTAGTGAAC CATGCGTCCA AATTCAAGCC TGATTTGCTC TCAAACTTTG ATAAACAACA   
  
  
+ AGAATTTAGC AACGGGATCG AACAAGATGA CATCTTGCAA AACCCTGATC TGTGTTTTGA TTCAGAAACC   
  
  
+ CCTTCAGTTG ATCTAACTGA GAGTGTCATC AGTTCTGATT CTGTAGAAGT GCCTGATTTC TCAGATGCTT   
  
  
+ GCCTCAAGTT CATAAGTGAG ATTCTCCTAG AAGAGGATTT GGATGAAAGT CCTGCCTCTG CTCAAGAATT   
  
  
+ TAGGGCTCTC CAAGCCACTG AGAAGTCCCT GTATGATGCT CTTGGAGAGG GCTACTCTTT TTCATCTGAT   
  
  
+ AACAGCCCAT CATCATTAGG ACAGAGTATT GAGCACCAAA ATGAGAATTT TGAATTCAGT TCCGGTTATC   
  
  
+ CTGGAATTGA GGGCTATGTT AATGGTGATA TCACGTTCGA GTCCAACTGG ATGTTCAACC TAAGCCAATT   
  
  
+ GGATCCTGTT CTTACTTTGG ATGATATTTC TCAGCCCTTG TCTGCATCAA ACTCCCGATC ATCTGGGTCG   
  
  
+ AGCAATGGCT TTGATGATTC AGGGGATGGG GCAGCCACAT CTCCTGGCAG TACAGTTACA TCAACAGTCC   
  
  
+ CAGAGAAAAG GGTTGAATCT GTCAATCGCT CAAGGAGGAA GAAAAATCGT AAAAGGGATG AAGGTGGTCA   
  
  
+ TGAGGAAGGG AGGAGTAACA AGCAGCAAGC TTCATCCAAT GAAGATTATG TTGAGATGAA GGAGTTTGAT   
  
  
+ GATGTACTGC TCTGCAAAGA AGAGAAAGAT GTTATTGCAA ACTGCACCAA TGATTCCTCA CCCGTCGAAG   
  
  
+ CGAGTGATAA GTTGCAGAAG AAAGGGGGGA AGGGGAAAAC ATCGCGTGGG AAGAAGCAGA ATAGCACAAT   
  
  
+ AGAAGAGGTG GATCTGAGGA CTCTTCTCAC TCACTGTGCT CAAGCAATTT CAAATTTTGA TCTTAGGAGT   
  
  
+ GCAAATGAGC ATCTCAGGCA AATAAGGCAG CATTCTTCAC AATATGGTGA TAGCCTCCAG AGGCTTGCCC   
  
  
+ ATTATTTTGC TAATGGTCTT GAGGCTCGCA TAGCTGGCAC TGGTTCAACA ATCTCTGCTA ATGTTGTTGA   
  
  
+ TGCTCGAATC ACGTCATCTG ATTTCTTAAA GGCTTATAGG CTATATATGT CAGCCGTTCC TTTCAAAAGG   
  
  
+ ATGTTATATT TTCTCGCCAA CAAGACAATA CGGAAGTTGG CTGAGAAGGC AACCAGGATC CACATCATTG   
  
  
+ ATTTTGGAGT CTTGTTGGGT TTACAGTGGC CCTGTCTCAT ACAAAACCTC TCAAAGAGAC CCACTGGCCC   
  
  
+ GCCAAGCCTC CGTATTACTG GGATAGACTA TCCCCAGCAT GGTTTCCGGC CATCAGAAAG GGTTGAAGCG   
  
  
+ ACTGGCCGTC GGTTAGAAGG GTATTGCGAG AGATTTAATG TGCCGTTCAA GTATAAGGCC ATAGCTAAAA   
  
  
+ ATTGGGAGAG TATAAAGCTA GAGGATCTAG AAATTGATGA GAATGAGATG GTGTTTGTCA ATTGCATGCT   
  
  
+ TCGCTCTGGA ACACTGCTTG ATGAGACAGT GGTGGCAGAC AGTCCAAAGG ATGCTTTCTT AAGGTTAATC   
  
  
+ AGAGAGATAA ATCCCCGTCT TTTCATTCAT GGGACTATCA ATGGATCATT CAATGCTCCA TTCTTCATCA   
  
  
+ CTCGGTTCAG GGAGGCGCTC TTCCACTACT CTTCTTTATT TGATATCTTT GAAGCAACTA TGCCCCGTGA   
  
  
+ AGATCATGAA AGGCTCCTGG TTGAGAGTGA GATACAGGGC AAAGAAGCTT TGAATGTCAT AGCATGCGAA   
  
  
+ GGTGCCGAGA GGATTCAAAG GCCTGAAACA TACAAGCAAT GGCAAGCAAG GACAACAAGG GCCGGGTTTA   
  
  
+ GGCAGCTTCC ACTAGACCGG GAGCTTGTGA GCAGAGCAAA GGCTATGGTG AAAGCAAACT ACCATAAGGA   
  
  
+ TTTTGTGGTG GACGAGGACA GGCATTGGAT GCTACACGGT TGGAAAGGAA GAATCTTCTG TGCACTCTCT   
  
  
+ GTTTGGCAAC CCAACTG  

- -Up\_Stream \_Len000TAAACT ACACATACGA CTGATGAAAA CTACTACTAC TAGTGTCACT ACGTATAAAT   
  
  
- CTCGACCATT TAACCAGTAA GCTAAGCTCA TGCCCAGCCC AGTTCAAGAC TAATTCACTG TAAAGCGCAG   
  
  
- CAAAACCAAA TCAAGCCCAG CCTAGCTAAA GCCCAATTTA TTAAAAACCA CCTTATGCGA ACAGTACGGT   
  
  
- TTGTGTTCGT TGAAACAATT TTTAAAGCTA AGCCCAGTCC AGTTAAGTCC AAACCCCAGG TGAAAGCTCA   
  
  
- CTCGTATAAA GCCTACAGCC CAGCGCATAC CCAGGCCCAG TAAGCCAAAC TTAAAGCCTA GAACCTAGTT   
  
  
- AAAACGATTC ATACACGTAT AAGTTGGGAA CTACTGTATC CTTTGCGAAA CAGTAGAGGT GGTTAGTCTA   
  
  
- TAGCAATAGA GAAATTAGTA GCGGTATTAT CGTACTCATT CGTACTGGTA GAAAAAGTTT GTACACTATG   
  
  
- TCAATTCCGG ACAAGAAAAG CAATTATTTA GGGTTAAATA GTTAAAATTA TATATGAAAT TGCTTAAAAT   
  
  
- TGTTTTTTAA AATTAAAGTT AGGCGATATT GTTTTTTTTA TGGTTGAAGT TGAATAATAT ATTTTTTTTA   
  
  
- TATGTTAAAA AAAAGTTAAA ATCGGTTGAA GTTGTTTTTT TTTTTTTTAA ATTGTTTAGA GTTAGTTGAA   
  
  
- GTGGGTAAAG AGTTGAATAT AGAGGATTAC TTTTCTTGTC TAGGAATCGT TCTGAAGAGC TAAGTAAATT   
  
  
- TTCCTATCAT ACCATTTAAG TAAAATATGT TCACAGTTCA CACTTATAGA AAAAGTGTAG TCCACTATAT   
  
  
- AATCATCCTG AAAAGCTAAG TGAATAGAAG GAGAAATACA AAACAATTAC ACTATCATTG CTATCCTAAC   
  
  
- GTACATAAAA AAGGTTGATA AGTAAATGTT GATAGTTATA GATAACTATT TATACAATTT TATTAATATA   
  
  
- CAATAAATTT AATAATAAAC AATGAATTCC ACAGCTGAAA CAGCTTTCGC TGAATTAAGA TTAATTCTAT   
  
  
- CGCCGTCTCT TTCAATGTGG ACTACTGGTT CAATCTAGAC CATTTTGATT GGTATCTAGA CTTTGGGTTT   
  
  
- GTATTACTAC ACATAAGTAT GGGCTGGACT GTAGACTTTA TTCAAATGAG TTTTCACCAA GGCTTTGAAC   
  
  
- TTAACTGAGC TTGGCTTGGC ATAAACTGAG CTTTAAAAGA TGTTTCAACG AATAAAAACG TACTTCTGTA   
  
  
- TAACTCGGTT TTTAATCAAC TAAGCTTCAG TTGAATTTGT TTTTAAGTAA GCTGAGTTTG ACTTTATAGT   
  
  
- GAATTGATTT TGATACTGAG TTGAGTTTGA ACTAGACTAG GCATAAGTTT AGTTTATGAG GTAAACGATC   
  
  
- CAGATCTTGG TCGGTGCTGG GGTGTACCTG ATACGCCAAG TTACTTTGCC GGTTTATATA GCGTTAAAGA   
  
  
- CAGAGGTAGT TAGTGTGGGT AAACGATGTG GGACAATTAT AAACTGAGCT GGGACACTTA ACGGAAGCGG   
  
  
- TCAAAACAAA TTAGAGAGAC CAACGAAGGG TAGGTTCAGA CTTATCATAT CAAACATGTC ACACATGATG   
  
  
- ATCTTTCGTG CAAGGAACTT AGAAGAAAGT TTCAACAAAA ATTTCCCAAC TCACTAGGTA ATGCTGAAAA   
  
  
- CTAGACCCAA GTAGCGGAGG AGAGGATTAG GGAGTGGGCC CATGGGAGTT AAGAGATAGC AGAGACAACG   
  
  
- AGTCGATTAA GTTTAAGGAA ATCAACCAGT AGTAACTTTA GACTTTAGTC CCACAAAAAG AAAAATAATA   
  
  
- ATAGACCGTC AACCAAACAA ATAGAGAACC AAAAACCCCG AATAAACCCA CGACCAACTA CAATAGGACG   
  
  
- AACCCAACTT AGAAGTAGTC CAAATAAAAA CTAAGTATCA ACTCCCACTT TTAATAAAGA GTATGGGTGG   
  
  
- TGGACAAACA AGAAAACAGA GACTCTTTTT GGTTTTTCGA AAGAGAAAAA GGACTACGTA GGACACGAGC   
  
  
- AACTAGGACT CAATCACTTG GTACGCAGGT TTAAGTTCGG ACTAAACGAG AGTTTGAAAC TATTTGTTGT   
  
  
- TCTTAAATCG TTGCCCTAGC TTGTTCTACT GTAGAACGTT TTGGGACTAG ACACAAAACT AAGTCTTTGG   
  
  
- GGAAGTCAAC TAGATTGACT CTCACAGTAG TCAAGACTAA GACATCTTCA CGGACTAAAG AGTCTACGAA   
  
  
- CGGAGTTCAA GTATTCACTC TAAGAGGATC TTCTCCTAAA CCTACTTTCA GGACGGAGAC GAGTTCTTAA   
  
  
- ATCCCGAGAG GTTCGGTGAC TCTTCAGGGA CATACTACGA GAACCTCTCC CGATGAGAAA AAGTAGACTA   
  
  
- TTGTCGGGTA GTAGTAATCC TGTCTCATAA CTCGTGGTTT TACTCTTAAA ACTTAAGTCA AGGCCAATAG   
  
  
- GACCTTAACT CCCGATACAA TTACCACTAT AGTGCAAGCT CAGGTTGACC TACAAGTTGG ATTCGGTTAA   
  
  
- CCTAGGACAA GAATGAAACC TACTATAAAG AGTCGGGAAC AGACGTAGTT TGAGGGCTAG TAGACCCAGC   
  
  
- TCGTTACCGA AACTACTAAG TCCCCTACCC CGTCGGTGTA GAGGACCGTC ATGTCAATGT AGTTGTCAGG   
  
  
- GTCTCTTTTC CCAACTTAGA CAGTTAGCGA GTTCCTCCTT CTTTTTAGCA TTTTCCCTAC TTCCACCAGT   
  
  
- ACTCCTTCCC TCCTCATTGT TCGTCGTTCG AAGTAGGTTA CTTCTAATAC AACTCTACTT CCTCAAACTA   
  
  
- CTACATGACG AGACGTTTCT TCTCTTTCTA CAATAACGTT TGACGTGGTT ACTAAGGAGT GGGCAGCTTC   
  
  
- GCTCACTATT CAACGTCTTC TTTCCCCCCT TCCCCTTTTG TAGCGCACCC TTCTTCGTCT TATCGTGTTA   
  
  
- TCTTCTCCAC CTAGACTCCT GAGAAGAGTG AGTGACACGA GTTCGTTAAA GTTTAAAACT AGAATCCTCA   
  
  
- CGTTTACTCG TAGAGTCCGT TTATTCCGTC GTAAGAAGTG TTATACCACT ATCGGAGGTC TCCGAACGGG   
  
  
- TAATAAAACG ATTACCAGAA CTCCGAGCGT ATCGACCGTG ACCAAGTTGT TAGAGACGAT TACAACAACT   
  
  
- ACGAGCTTAG TGCAGTAGAC TAAAGAATTT CCGAATATCC GATATATACA GTCGGCAAGG AAAGTTTTCC   
  
  
- TACAATATAA AAGAGCGGTT GTTCTGTTAT GCCTTCAACC GACTCTTCCG TTGGTCCTAG GTGTAGTAAC   
  
  
- TAAAACCTCA GAACAACCCA AATGTCACCG GGACAGAGTA TGTTTTGGAG AGTTTCTCTG GGTGACCGGG   
  
  
- CGGTTCGGAG GCATAATGAC CCTATCTGAT AGGGGTCGTA CCAAAGGCCG GTAGTCTTTC CCAACTTCGC   
  
  
- TGACCGGCAG CCAATCTTCC CATAACGCTC TCTAAATTAC ACGGCAAGTT CATATTCCGG TATCGATTTT   
  
  
- TAACCCTCTC ATATTTCGAT CTCCTAGATC TTTAACTACT CTTACTCTAC CACAAACAGT TAACGTACGA   
  
  
- AGCGAGACCT TGTGACGAAC TACTCTGTCA CCACCGTCTG TCAGGTTTCC TACGAAAGAA TTCCAATTAG   
  
  
- TCTCTCTATT TAGGGGCAGA AAAGTAAGTA CCCTGATAGT TACCTAGTAA GTTACGAGGT AAGAAGTAGT   
  
  
- GAGCCAAGTC CCTCCGCGAG AAGGTGATGA GAAGAAATAA ACTATAGAAA CTTCGTTGAT ACGGGGCACT   
  
  
- TCTAGTACTT TCCGAGGACC AACTCTCACT CTATGTCCCG TTTCTTCGAA ACTTACAGTA TCGTACGCTT   
  
  
- CCACGGCTCT CCTAAGTTTC CGGACTTTGT ATGTTCGTTA CCGTTCGTTC CTGTTGTTCC CGGCCCAAAT   
  
  
- CCGTCGAAGG TGATCTGGCC CTCGAACACT CGTCTCGTTT CCGATACCAC TTTCGTTTGA TGGTATTCCT   
  
  
- AAAACACCAC CTGCTCCTGT CCGTAACCTA CGATGTGCCA ACCTTTCCTT CTTAGAAGAC ACGTGAGAGA   
  
  
- CAAACCGTTG GGTTGAC

+     MBS

| Site Name | Organism | Position | Strand | Matrix score. | sequence | function |
| --- | --- | --- | --- | --- | --- | --- |
| MBS | Arabidopsis thaliana | 2498 | + | 6 | CAACTG | MYB binding site involved in drought-inducibility |
| MBS | Arabidopsis thaliana | 1832 | - | 6 | CAACTG | MYB binding site involved in drought-inducibility |
| MBS | Arabidopsis thaliana | 2179 | - | 6 | CAACTG | MYB binding site involved in drought-inducibility |
| MBS | Arabidopsis thaliana | 4076 | + | 6 | CAACTG | MYB binding site involved in drought-inducibility |

>HU07G02246.1   
+ -Up\_Stream \_Len000ATTTGA TGTGTATGCT GACTACTTTT GATGATGATG ATCACAGTGA TGCATATTTA   
  
  
+ GAGCTGGTAA ATTGGTCATT CGATTCGAGT ACGGGTCGGG TCAAGTTCTG ATTAAGTGAC ATTTCGCGTC   
  
  
+ GTTTTGGTTT AGTTCGGGTC GGATCGATTT CGGGTTAAAT AATTTTTGGT GGAATACGCT TGTCATGCCA   
  
  
+ AACACAAGCA ACTTTGTTAA AAATTTCGAT TCGGGTCAGG TCAATTCAGG TTTGGGGTCC ACTTTCGAGT   
  
  
+ GAGCATATTT CGGATGTCGG GTCGCGTATG GGTCCGGGTC ATTCGGTTTG AATTTCGGAT CTTGGATCAA   
  
  
+ TTTTGCTAAG TATGTGCATA TTCAACCCTT GATGACATAG GAAACGCTTT GTCATCTCCA CCAATCAGAT   
  
  
+ ATCGTTATCT CTTTAATCAT CGCCATAATA GCATGAGTAA GCATGACCAT CTTTTTCAAA CATGTGATAC   
  
  
+ AGTTAAGGCC TGTTCTTTTC GTTAATAAAT CCCAATTTAT CAATTTTAAT ATATACTTTA ACGAATTTTA   
  
  
+ ACAAAAAATT TTAATTTCAA TCCGCTATAA CAAAAAAAAT ACCAACTTCA ACTTATTATA TAAAAAAAAT   
  
  
+ ATACAATTTT TTTTCAATTT TAGCCAACTT CAACAAAAAA AAAAAAAATT TAACAAATCT CAATCAACTT   
  
  
+ CACCCATTTC TCAACTTATA TCTCCTAATG AAAAGAACAG ATCCTTAGCA AGACTTCTCG ATTCATTTAA   
  
  
+ AAGGATAGTA TGGTAAATTC ATTTTATACA AGTGTCAAGT GTGAATATCT TTTTCACATC AGGTGATATA   
  
  
+ TTAGTAGGAC TTTTCGATTC ACTTATCTTC CTCTTTATGT TTTGTTAATG TGATAGTAAC GATAGGATTG   
  
  
+ CATGTATTTT TTCCAACTAT TCATTTACAA CTATCAATAT CTATTGATAA ATATGTTAAA ATAATTATAT   
  
  
+ GTTATTTAAA TTATTATTTG TTACTTAAGG TGTCGACTTT GTCGAAAGCG ACTTAATTCT AATTAAGATA   
  
  
+ GCGGCAGAGA AAGTTACACC TGATGACCAA GTTAGATCTG GTAAAACTAA CCATAGATCT GAAACCCAAA   
  
  
+ CATAATGATG TGTATTCATA CCCGACCTGA CATCTGAAAT AAGTTTACTC AAAAGTGGTT CCGAAACTTG   
  
  
+ AATTGACTCG AACCGAACCG TATTTGACTC GAAATTTTCT ACAAAGTTGC TTATTTTTGC ATGAAGACAT   
  
  
+ ATTGAGCCAA AAATTAGTTG ATTCGAAGTC AACTTAAACA AAAATTCATT CGACTCAAAC TGAAATATCA   
  
  
+ CTTAACTAAA ACTATGACTC AACTCAAACT TGATCTGATC CGTATTCAAA TCAAATACTC CATTTGCTAG   
  
  
+ GTCTAGAACC AGCCACGACC CCACATGGAC TATGCGGTTC AATGAAACGG CCAAATATAT CGCAATTTCT   
  
  
+ GTCTCCATCA ATCACACCCA TTTGCTACAC CCTGTTAATA TTTGACTCGA CCCTGTGAAT TGCCTTCGCC   
  
  
+ AGTTTTGTTT AATCTCTCTG GTTGCTTCCC ATCCAAGTCT GAATAGTATA GTTTGTACAG TGTGTACTAC   
  
  
+ TAGAAAGCAC GTTCCTTGAA TCTTCTTTCA AAGTTGTTTT TAAAGGGTTG AGTGATCCAT TACGACTTTT   
  
  
+ GATCTGGGTT CATCGCCTCC TCTCCTAATC CCTCACCCGG GTACCCTCAA TTCTCTATCG TCTCTGTTGC   
  
  
+ TCAGCTAATT CAAATTCCTT TAGTTGGTCA TCATTGAAAT CTGAAATCAG GGTGTTTTTC TTTTTATTAT   
  
  
+ TATCTGGCAG TTGGTTTGTT TATCTCTTGG TTTTTGGGGC TTATTTGGGT GCTGGTTGAT GTTATCCTGC   
  
  
+ TTGGGTTGAA TCTTCATCAG GTTTATTTTT GATTCATAGT TGAGGGTGAA AATTATTTCT CATACCCACC   
  
  
+ ACCTGTTTGT TCTTTTGTCT CTGAGAAAAA CCAAAAAGCT TTCTCTTTTT CCTGATGCAT CCTGTGCTCG   
  
  
+ TTGATCCTGA GTTAGTGAAC CATGCGTCCA AATTCAAGCC TGATTTGCTC TCAAACTTTG ATAAACAACA   
  
  
+ AGAATTTAGC AACGGGATCG AACAAGATGA CATCTTGCAA AACCCTGATC TGTGTTTTGA TTCAGAAACC   
  
  
+ CCTTCAGTTG ATCTAACTGA GAGTGTCATC AGTTCTGATT CTGTAGAAGT GCCTGATTTC TCAGATGCTT   
  
  
+ GCCTCAAGTT CATAAGTGAG ATTCTCCTAG AAGAGGATTT GGATGAAAGT CCTGCCTCTG CTCAAGAATT   
  
  
+ TAGGGCTCTC CAAGCCACTG AGAAGTCCCT GTATGATGCT CTTGGAGAGG GCTACTCTTT TTCATCTGAT   
  
  
+ AACAGCCCAT CATCATTAGG ACAGAGTATT GAGCACCAAA ATGAGAATTT TGAATTCAGT TCCGGTTATC   
  
  
+ CTGGAATTGA GGGCTATGTT AATGGTGATA TCACGTTCGA GTCCAACTGG ATGTTCAACC TAAGCCAATT   
  
  
+ GGATCCTGTT CTTACTTTGG ATGATATTTC TCAGCCCTTG TCTGCATCAA ACTCCCGATC ATCTGGGTCG   
  
  
+ AGCAATGGCT TTGATGATTC AGGGGATGGG GCAGCCACAT CTCCTGGCAG TACAGTTACA TCAACAGTCC   
  
  
+ CAGAGAAAAG GGTTGAATCT GTCAATCGCT CAAGGAGGAA GAAAAATCGT AAAAGGGATG AAGGTGGTCA   
  
  
+ TGAGGAAGGG AGGAGTAACA AGCAGCAAGC TTCATCCAAT GAAGATTATG TTGAGATGAA GGAGTTTGAT   
  
  
+ GATGTACTGC TCTGCAAAGA AGAGAAAGAT GTTATTGCAA ACTGCACCAA TGATTCCTCA CCCGTCGAAG   
  
  
+ CGAGTGATAA GTTGCAGAAG AAAGGGGGGA AGGGGAAAAC ATCGCGTGGG AAGAAGCAGA ATAGCACAAT   
  
  
+ AGAAGAGGTG GATCTGAGGA CTCTTCTCAC TCACTGTGCT CAAGCAATTT CAAATTTTGA TCTTAGGAGT   
  
  
+ GCAAATGAGC ATCTCAGGCA AATAAGGCAG CATTCTTCAC AATATGGTGA TAGCCTCCAG AGGCTTGCCC   
  
  
+ ATTATTTTGC TAATGGTCTT GAGGCTCGCA TAGCTGGCAC TGGTTCAACA ATCTCTGCTA ATGTTGTTGA   
  
  
+ TGCTCGAATC ACGTCATCTG ATTTCTTAAA GGCTTATAGG CTATATATGT CAGCCGTTCC TTTCAAAAGG   
  
  
+ ATGTTATATT TTCTCGCCAA CAAGACAATA CGGAAGTTGG CTGAGAAGGC AACCAGGATC CACATCATTG   
  
  
+ ATTTTGGAGT CTTGTTGGGT TTACAGTGGC CCTGTCTCAT ACAAAACCTC TCAAAGAGAC CCACTGGCCC   
  
  
+ GCCAAGCCTC CGTATTACTG GGATAGACTA TCCCCAGCAT GGTTTCCGGC CATCAGAAAG GGTTGAAGCG   
  
  
+ ACTGGCCGTC GGTTAGAAGG GTATTGCGAG AGATTTAATG TGCCGTTCAA GTATAAGGCC ATAGCTAAAA   
  
  
+ ATTGGGAGAG TATAAAGCTA GAGGATCTAG AAATTGATGA GAATGAGATG GTGTTTGTCA ATTGCATGCT   
  
  
+ TCGCTCTGGA ACACTGCTTG ATGAGACAGT GGTGGCAGAC AGTCCAAAGG ATGCTTTCTT AAGGTTAATC   
  
  
+ AGAGAGATAA ATCCCCGTCT TTTCATTCAT GGGACTATCA ATGGATCATT CAATGCTCCA TTCTTCATCA   
  
  
+ CTCGGTTCAG GGAGGCGCTC TTCCACTACT CTTCTTTATT TGATATCTTT GAAGCAACTA TGCCCCGTGA   
  
  
+ AGATCATGAA AGGCTCCTGG TTGAGAGTGA GATACAGGGC AAAGAAGCTT TGAATGTCAT AGCATGCGAA   
  
  
+ GGTGCCGAGA GGATTCAAAG GCCTGAAACA TACAAGCAAT GGCAAGCAAG GACAACAAGG GCCGGGTTTA   
  
  
+ GGCAGCTTCC ACTAGACCGG GAGCTTGTGA GCAGAGCAAA GGCTATGGTG AAAGCAAACT ACCATAAGGA   
  
  
+ TTTTGTGGTG GACGAGGACA GGCATTGGAT GCTACACGGT TGGAAAGGAA GAATCTTCTG TGCACTCTCT   
  
  
+ GTTTGGCAAC CCAACTG  

- -Up\_Stream \_Len000TAAACT ACACATACGA CTGATGAAAA CTACTACTAC TAGTGTCACT ACGTATAAAT   
  
  
- CTCGACCATT TAACCAGTAA GCTAAGCTCA TGCCCAGCCC AGTTCAAGAC TAATTCACTG TAAAGCGCAG   
  
  
- CAAAACCAAA TCAAGCCCAG CCTAGCTAAA GCCCAATTTA TTAAAAACCA CCTTATGCGA ACAGTACGGT   
  
  
- TTGTGTTCGT TGAAACAATT TTTAAAGCTA AGCCCAGTCC AGTTAAGTCC AAACCCCAGG TGAAAGCTCA   
  
  
- CTCGTATAAA GCCTACAGCC CAGCGCATAC CCAGGCCCAG TAAGCCAAAC TTAAAGCCTA GAACCTAGTT   
  
  
- AAAACGATTC ATACACGTAT AAGTTGGGAA CTACTGTATC CTTTGCGAAA CAGTAGAGGT GGTTAGTCTA   
  
  
- TAGCAATAGA GAAATTAGTA GCGGTATTAT CGTACTCATT CGTACTGGTA GAAAAAGTTT GTACACTATG   
  
  
- TCAATTCCGG ACAAGAAAAG CAATTATTTA GGGTTAAATA GTTAAAATTA TATATGAAAT TGCTTAAAAT   
  
  
- TGTTTTTTAA AATTAAAGTT AGGCGATATT GTTTTTTTTA TGGTTGAAGT TGAATAATAT ATTTTTTTTA   
  
  
- TATGTTAAAA AAAAGTTAAA ATCGGTTGAA GTTGTTTTTT TTTTTTTTAA ATTGTTTAGA GTTAGTTGAA   
  
  
- GTGGGTAAAG AGTTGAATAT AGAGGATTAC TTTTCTTGTC TAGGAATCGT TCTGAAGAGC TAAGTAAATT   
  
  
- TTCCTATCAT ACCATTTAAG TAAAATATGT TCACAGTTCA CACTTATAGA AAAAGTGTAG TCCACTATAT   
  
  
- AATCATCCTG AAAAGCTAAG TGAATAGAAG GAGAAATACA AAACAATTAC ACTATCATTG CTATCCTAAC   
  
  
- GTACATAAAA AAGGTTGATA AGTAAATGTT GATAGTTATA GATAACTATT TATACAATTT TATTAATATA   
  
  
- CAATAAATTT AATAATAAAC AATGAATTCC ACAGCTGAAA CAGCTTTCGC TGAATTAAGA TTAATTCTAT   
  
  
- CGCCGTCTCT TTCAATGTGG ACTACTGGTT CAATCTAGAC CATTTTGATT GGTATCTAGA CTTTGGGTTT   
  
  
- GTATTACTAC ACATAAGTAT GGGCTGGACT GTAGACTTTA TTCAAATGAG TTTTCACCAA GGCTTTGAAC   
  
  
- TTAACTGAGC TTGGCTTGGC ATAAACTGAG CTTTAAAAGA TGTTTCAACG AATAAAAACG TACTTCTGTA   
  
  
- TAACTCGGTT TTTAATCAAC TAAGCTTCAG TTGAATTTGT TTTTAAGTAA GCTGAGTTTG ACTTTATAGT   
  
  
- GAATTGATTT TGATACTGAG TTGAGTTTGA ACTAGACTAG GCATAAGTTT AGTTTATGAG GTAAACGATC   
  
  
- CAGATCTTGG TCGGTGCTGG GGTGTACCTG ATACGCCAAG TTACTTTGCC GGTTTATATA GCGTTAAAGA   
  
  
- CAGAGGTAGT TAGTGTGGGT AAACGATGTG GGACAATTAT AAACTGAGCT GGGACACTTA ACGGAAGCGG   
  
  
- TCAAAACAAA TTAGAGAGAC CAACGAAGGG TAGGTTCAGA CTTATCATAT CAAACATGTC ACACATGATG   
  
  
- ATCTTTCGTG CAAGGAACTT AGAAGAAAGT TTCAACAAAA ATTTCCCAAC TCACTAGGTA ATGCTGAAAA   
  
  
- CTAGACCCAA GTAGCGGAGG AGAGGATTAG GGAGTGGGCC CATGGGAGTT AAGAGATAGC AGAGACAACG   
  
  
- AGTCGATTAA GTTTAAGGAA ATCAACCAGT AGTAACTTTA GACTTTAGTC CCACAAAAAG AAAAATAATA   
  
  
- ATAGACCGTC AACCAAACAA ATAGAGAACC AAAAACCCCG AATAAACCCA CGACCAACTA CAATAGGACG   
  
  
- AACCCAACTT AGAAGTAGTC CAAATAAAAA CTAAGTATCA ACTCCCACTT TTAATAAAGA GTATGGGTGG   
  
  
- TGGACAAACA AGAAAACAGA GACTCTTTTT GGTTTTTCGA AAGAGAAAAA GGACTACGTA GGACACGAGC   
  
  
- AACTAGGACT CAATCACTTG GTACGCAGGT TTAAGTTCGG ACTAAACGAG AGTTTGAAAC TATTTGTTGT   
  
  
- TCTTAAATCG TTGCCCTAGC TTGTTCTACT GTAGAACGTT TTGGGACTAG ACACAAAACT AAGTCTTTGG   
  
  
- GGAAGTCAAC TAGATTGACT CTCACAGTAG TCAAGACTAA GACATCTTCA CGGACTAAAG AGTCTACGAA   
  
  
- CGGAGTTCAA GTATTCACTC TAAGAGGATC TTCTCCTAAA CCTACTTTCA GGACGGAGAC GAGTTCTTAA   
  
  
- ATCCCGAGAG GTTCGGTGAC TCTTCAGGGA CATACTACGA GAACCTCTCC CGATGAGAAA AAGTAGACTA   
  
  
- TTGTCGGGTA GTAGTAATCC TGTCTCATAA CTCGTGGTTT TACTCTTAAA ACTTAAGTCA AGGCCAATAG   
  
  
- GACCTTAACT CCCGATACAA TTACCACTAT AGTGCAAGCT CAGGTTGACC TACAAGTTGG ATTCGGTTAA   
  
  
- CCTAGGACAA GAATGAAACC TACTATAAAG AGTCGGGAAC AGACGTAGTT TGAGGGCTAG TAGACCCAGC   
  
  
- TCGTTACCGA AACTACTAAG TCCCCTACCC CGTCGGTGTA GAGGACCGTC ATGTCAATGT AGTTGTCAGG   
  
  
- GTCTCTTTTC CCAACTTAGA CAGTTAGCGA GTTCCTCCTT CTTTTTAGCA TTTTCCCTAC TTCCACCAGT   
  
  
- ACTCCTTCCC TCCTCATTGT TCGTCGTTCG AAGTAGGTTA CTTCTAATAC AACTCTACTT CCTCAAACTA   
  
  
- CTACATGACG AGACGTTTCT TCTCTTTCTA CAATAACGTT TGACGTGGTT ACTAAGGAGT GGGCAGCTTC   
  
  
- GCTCACTATT CAACGTCTTC TTTCCCCCCT TCCCCTTTTG TAGCGCACCC TTCTTCGTCT TATCGTGTTA   
  
  
- TCTTCTCCAC CTAGACTCCT GAGAAGAGTG AGTGACACGA GTTCGTTAAA GTTTAAAACT AGAATCCTCA   
  
  
- CGTTTACTCG TAGAGTCCGT TTATTCCGTC GTAAGAAGTG TTATACCACT ATCGGAGGTC TCCGAACGGG   
  
  
- TAATAAAACG ATTACCAGAA CTCCGAGCGT ATCGACCGTG ACCAAGTTGT TAGAGACGAT TACAACAACT   
  
  
- ACGAGCTTAG TGCAGTAGAC TAAAGAATTT CCGAATATCC GATATATACA GTCGGCAAGG AAAGTTTTCC   
  
  
- TACAATATAA AAGAGCGGTT GTTCTGTTAT GCCTTCAACC GACTCTTCCG TTGGTCCTAG GTGTAGTAAC   
  
  
- TAAAACCTCA GAACAACCCA AATGTCACCG GGACAGAGTA TGTTTTGGAG AGTTTCTCTG GGTGACCGGG   
  
  
- CGGTTCGGAG GCATAATGAC CCTATCTGAT AGGGGTCGTA CCAAAGGCCG GTAGTCTTTC CCAACTTCGC   
  
  
- TGACCGGCAG CCAATCTTCC CATAACGCTC TCTAAATTAC ACGGCAAGTT CATATTCCGG TATCGATTTT   
  
  
- TAACCCTCTC ATATTTCGAT CTCCTAGATC TTTAACTACT CTTACTCTAC CACAAACAGT TAACGTACGA   
  
  
- AGCGAGACCT TGTGACGAAC TACTCTGTCA CCACCGTCTG TCAGGTTTCC TACGAAAGAA TTCCAATTAG   
  
  
- TCTCTCTATT TAGGGGCAGA AAAGTAAGTA CCCTGATAGT TACCTAGTAA GTTACGAGGT AAGAAGTAGT   
  
  
- GAGCCAAGTC CCTCCGCGAG AAGGTGATGA GAAGAAATAA ACTATAGAAA CTTCGTTGAT ACGGGGCACT   
  
  
- TCTAGTACTT TCCGAGGACC AACTCTCACT CTATGTCCCG TTTCTTCGAA ACTTACAGTA TCGTACGCTT   
  
  
- CCACGGCTCT CCTAAGTTTC CGGACTTTGT ATGTTCGTTA CCGTTCGTTC CTGTTGTTCC CGGCCCAAAT   
  
  
- CCGTCGAAGG TGATCTGGCC CTCGAACACT CGTCTCGTTT CCGATACCAC TTTCGTTTGA TGGTATTCCT   
  
  
- AAAACACCAC CTGCTCCTGT CCGTAACCTA CGATGTGCCA ACCTTTCCTT CTTAGAAGAC ACGTGAGAGA   
  
  
- CAAACCGTTG GGTTGAC

+     MRE

| Site Name | Organism | Position | Strand | Matrix score. | sequence | function |
| --- | --- | --- | --- | --- | --- | --- |
| MRE | Petroselinum crispum | 2511 | + | 7 | AACCTAA | MYB binding site involved in light responsiveness |

>HU07G02246.1   
+ -Up\_Stream \_Len000ATTTGA TGTGTATGCT GACTACTTTT GATGATGATG ATCACAGTGA TGCATATTTA   
  
  
+ GAGCTGGTAA ATTGGTCATT CGATTCGAGT ACGGGTCGGG TCAAGTTCTG ATTAAGTGAC ATTTCGCGTC   
  
  
+ GTTTTGGTTT AGTTCGGGTC GGATCGATTT CGGGTTAAAT AATTTTTGGT GGAATACGCT TGTCATGCCA   
  
  
+ AACACAAGCA ACTTTGTTAA AAATTTCGAT TCGGGTCAGG TCAATTCAGG TTTGGGGTCC ACTTTCGAGT   
  
  
+ GAGCATATTT CGGATGTCGG GTCGCGTATG GGTCCGGGTC ATTCGGTTTG AATTTCGGAT CTTGGATCAA   
  
  
+ TTTTGCTAAG TATGTGCATA TTCAACCCTT GATGACATAG GAAACGCTTT GTCATCTCCA CCAATCAGAT   
  
  
+ ATCGTTATCT CTTTAATCAT CGCCATAATA GCATGAGTAA GCATGACCAT CTTTTTCAAA CATGTGATAC   
  
  
+ AGTTAAGGCC TGTTCTTTTC GTTAATAAAT CCCAATTTAT CAATTTTAAT ATATACTTTA ACGAATTTTA   
  
  
+ ACAAAAAATT TTAATTTCAA TCCGCTATAA CAAAAAAAAT ACCAACTTCA ACTTATTATA TAAAAAAAAT   
  
  
+ ATACAATTTT TTTTCAATTT TAGCCAACTT CAACAAAAAA AAAAAAAATT TAACAAATCT CAATCAACTT   
  
  
+ CACCCATTTC TCAACTTATA TCTCCTAATG AAAAGAACAG ATCCTTAGCA AGACTTCTCG ATTCATTTAA   
  
  
+ AAGGATAGTA TGGTAAATTC ATTTTATACA AGTGTCAAGT GTGAATATCT TTTTCACATC AGGTGATATA   
  
  
+ TTAGTAGGAC TTTTCGATTC ACTTATCTTC CTCTTTATGT TTTGTTAATG TGATAGTAAC GATAGGATTG   
  
  
+ CATGTATTTT TTCCAACTAT TCATTTACAA CTATCAATAT CTATTGATAA ATATGTTAAA ATAATTATAT   
  
  
+ GTTATTTAAA TTATTATTTG TTACTTAAGG TGTCGACTTT GTCGAAAGCG ACTTAATTCT AATTAAGATA   
  
  
+ GCGGCAGAGA AAGTTACACC TGATGACCAA GTTAGATCTG GTAAAACTAA CCATAGATCT GAAACCCAAA   
  
  
+ CATAATGATG TGTATTCATA CCCGACCTGA CATCTGAAAT AAGTTTACTC AAAAGTGGTT CCGAAACTTG   
  
  
+ AATTGACTCG AACCGAACCG TATTTGACTC GAAATTTTCT ACAAAGTTGC TTATTTTTGC ATGAAGACAT   
  
  
+ ATTGAGCCAA AAATTAGTTG ATTCGAAGTC AACTTAAACA AAAATTCATT CGACTCAAAC TGAAATATCA   
  
  
+ CTTAACTAAA ACTATGACTC AACTCAAACT TGATCTGATC CGTATTCAAA TCAAATACTC CATTTGCTAG   
  
  
+ GTCTAGAACC AGCCACGACC CCACATGGAC TATGCGGTTC AATGAAACGG CCAAATATAT CGCAATTTCT   
  
  
+ GTCTCCATCA ATCACACCCA TTTGCTACAC CCTGTTAATA TTTGACTCGA CCCTGTGAAT TGCCTTCGCC   
  
  
+ AGTTTTGTTT AATCTCTCTG GTTGCTTCCC ATCCAAGTCT GAATAGTATA GTTTGTACAG TGTGTACTAC   
  
  
+ TAGAAAGCAC GTTCCTTGAA TCTTCTTTCA AAGTTGTTTT TAAAGGGTTG AGTGATCCAT TACGACTTTT   
  
  
+ GATCTGGGTT CATCGCCTCC TCTCCTAATC CCTCACCCGG GTACCCTCAA TTCTCTATCG TCTCTGTTGC   
  
  
+ TCAGCTAATT CAAATTCCTT TAGTTGGTCA TCATTGAAAT CTGAAATCAG GGTGTTTTTC TTTTTATTAT   
  
  
+ TATCTGGCAG TTGGTTTGTT TATCTCTTGG TTTTTGGGGC TTATTTGGGT GCTGGTTGAT GTTATCCTGC   
  
  
+ TTGGGTTGAA TCTTCATCAG GTTTATTTTT GATTCATAGT TGAGGGTGAA AATTATTTCT CATACCCACC   
  
  
+ ACCTGTTTGT TCTTTTGTCT CTGAGAAAAA CCAAAAAGCT TTCTCTTTTT CCTGATGCAT CCTGTGCTCG   
  
  
+ TTGATCCTGA GTTAGTGAAC CATGCGTCCA AATTCAAGCC TGATTTGCTC TCAAACTTTG ATAAACAACA   
  
  
+ AGAATTTAGC AACGGGATCG AACAAGATGA CATCTTGCAA AACCCTGATC TGTGTTTTGA TTCAGAAACC   
  
  
+ CCTTCAGTTG ATCTAACTGA GAGTGTCATC AGTTCTGATT CTGTAGAAGT GCCTGATTTC TCAGATGCTT   
  
  
+ GCCTCAAGTT CATAAGTGAG ATTCTCCTAG AAGAGGATTT GGATGAAAGT CCTGCCTCTG CTCAAGAATT   
  
  
+ TAGGGCTCTC CAAGCCACTG AGAAGTCCCT GTATGATGCT CTTGGAGAGG GCTACTCTTT TTCATCTGAT   
  
  
+ AACAGCCCAT CATCATTAGG ACAGAGTATT GAGCACCAAA ATGAGAATTT TGAATTCAGT TCCGGTTATC   
  
  
+ CTGGAATTGA GGGCTATGTT AATGGTGATA TCACGTTCGA GTCCAACTGG ATGTTCAACC TAAGCCAATT   
  
  
+ GGATCCTGTT CTTACTTTGG ATGATATTTC TCAGCCCTTG TCTGCATCAA ACTCCCGATC ATCTGGGTCG   
  
  
+ AGCAATGGCT TTGATGATTC AGGGGATGGG GCAGCCACAT CTCCTGGCAG TACAGTTACA TCAACAGTCC   
  
  
+ CAGAGAAAAG GGTTGAATCT GTCAATCGCT CAAGGAGGAA GAAAAATCGT AAAAGGGATG AAGGTGGTCA   
  
  
+ TGAGGAAGGG AGGAGTAACA AGCAGCAAGC TTCATCCAAT GAAGATTATG TTGAGATGAA GGAGTTTGAT   
  
  
+ GATGTACTGC TCTGCAAAGA AGAGAAAGAT GTTATTGCAA ACTGCACCAA TGATTCCTCA CCCGTCGAAG   
  
  
+ CGAGTGATAA GTTGCAGAAG AAAGGGGGGA AGGGGAAAAC ATCGCGTGGG AAGAAGCAGA ATAGCACAAT   
  
  
+ AGAAGAGGTG GATCTGAGGA CTCTTCTCAC TCACTGTGCT CAAGCAATTT CAAATTTTGA TCTTAGGAGT   
  
  
+ GCAAATGAGC ATCTCAGGCA AATAAGGCAG CATTCTTCAC AATATGGTGA TAGCCTCCAG AGGCTTGCCC   
  
  
+ ATTATTTTGC TAATGGTCTT GAGGCTCGCA TAGCTGGCAC TGGTTCAACA ATCTCTGCTA ATGTTGTTGA   
  
  
+ TGCTCGAATC ACGTCATCTG ATTTCTTAAA GGCTTATAGG CTATATATGT CAGCCGTTCC TTTCAAAAGG   
  
  
+ ATGTTATATT TTCTCGCCAA CAAGACAATA CGGAAGTTGG CTGAGAAGGC AACCAGGATC CACATCATTG   
  
  
+ ATTTTGGAGT CTTGTTGGGT TTACAGTGGC CCTGTCTCAT ACAAAACCTC TCAAAGAGAC CCACTGGCCC   
  
  
+ GCCAAGCCTC CGTATTACTG GGATAGACTA TCCCCAGCAT GGTTTCCGGC CATCAGAAAG GGTTGAAGCG   
  
  
+ ACTGGCCGTC GGTTAGAAGG GTATTGCGAG AGATTTAATG TGCCGTTCAA GTATAAGGCC ATAGCTAAAA   
  
  
+ ATTGGGAGAG TATAAAGCTA GAGGATCTAG AAATTGATGA GAATGAGATG GTGTTTGTCA ATTGCATGCT   
  
  
+ TCGCTCTGGA ACACTGCTTG ATGAGACAGT GGTGGCAGAC AGTCCAAAGG ATGCTTTCTT AAGGTTAATC   
  
  
+ AGAGAGATAA ATCCCCGTCT TTTCATTCAT GGGACTATCA ATGGATCATT CAATGCTCCA TTCTTCATCA   
  
  
+ CTCGGTTCAG GGAGGCGCTC TTCCACTACT CTTCTTTATT TGATATCTTT GAAGCAACTA TGCCCCGTGA   
  
  
+ AGATCATGAA AGGCTCCTGG TTGAGAGTGA GATACAGGGC AAAGAAGCTT TGAATGTCAT AGCATGCGAA   
  
  
+ GGTGCCGAGA GGATTCAAAG GCCTGAAACA TACAAGCAAT GGCAAGCAAG GACAACAAGG GCCGGGTTTA   
  
  
+ GGCAGCTTCC ACTAGACCGG GAGCTTGTGA GCAGAGCAAA GGCTATGGTG AAAGCAAACT ACCATAAGGA   
  
  
+ TTTTGTGGTG GACGAGGACA GGCATTGGAT GCTACACGGT TGGAAAGGAA GAATCTTCTG TGCACTCTCT   
  
  
+ GTTTGGCAAC CCAACTG  

- -Up\_Stream \_Len000TAAACT ACACATACGA CTGATGAAAA CTACTACTAC TAGTGTCACT ACGTATAAAT   
  
  
- CTCGACCATT TAACCAGTAA GCTAAGCTCA TGCCCAGCCC AGTTCAAGAC TAATTCACTG TAAAGCGCAG   
  
  
- CAAAACCAAA TCAAGCCCAG CCTAGCTAAA GCCCAATTTA TTAAAAACCA CCTTATGCGA ACAGTACGGT   
  
  
- TTGTGTTCGT TGAAACAATT TTTAAAGCTA AGCCCAGTCC AGTTAAGTCC AAACCCCAGG TGAAAGCTCA   
  
  
- CTCGTATAAA GCCTACAGCC CAGCGCATAC CCAGGCCCAG TAAGCCAAAC TTAAAGCCTA GAACCTAGTT   
  
  
- AAAACGATTC ATACACGTAT AAGTTGGGAA CTACTGTATC CTTTGCGAAA CAGTAGAGGT GGTTAGTCTA   
  
  
- TAGCAATAGA GAAATTAGTA GCGGTATTAT CGTACTCATT CGTACTGGTA GAAAAAGTTT GTACACTATG   
  
  
- TCAATTCCGG ACAAGAAAAG CAATTATTTA GGGTTAAATA GTTAAAATTA TATATGAAAT TGCTTAAAAT   
  
  
- TGTTTTTTAA AATTAAAGTT AGGCGATATT GTTTTTTTTA TGGTTGAAGT TGAATAATAT ATTTTTTTTA   
  
  
- TATGTTAAAA AAAAGTTAAA ATCGGTTGAA GTTGTTTTTT TTTTTTTTAA ATTGTTTAGA GTTAGTTGAA   
  
  
- GTGGGTAAAG AGTTGAATAT AGAGGATTAC TTTTCTTGTC TAGGAATCGT TCTGAAGAGC TAAGTAAATT   
  
  
- TTCCTATCAT ACCATTTAAG TAAAATATGT TCACAGTTCA CACTTATAGA AAAAGTGTAG TCCACTATAT   
  
  
- AATCATCCTG AAAAGCTAAG TGAATAGAAG GAGAAATACA AAACAATTAC ACTATCATTG CTATCCTAAC   
  
  
- GTACATAAAA AAGGTTGATA AGTAAATGTT GATAGTTATA GATAACTATT TATACAATTT TATTAATATA   
  
  
- CAATAAATTT AATAATAAAC AATGAATTCC ACAGCTGAAA CAGCTTTCGC TGAATTAAGA TTAATTCTAT   
  
  
- CGCCGTCTCT TTCAATGTGG ACTACTGGTT CAATCTAGAC CATTTTGATT GGTATCTAGA CTTTGGGTTT   
  
  
- GTATTACTAC ACATAAGTAT GGGCTGGACT GTAGACTTTA TTCAAATGAG TTTTCACCAA GGCTTTGAAC   
  
  
- TTAACTGAGC TTGGCTTGGC ATAAACTGAG CTTTAAAAGA TGTTTCAACG AATAAAAACG TACTTCTGTA   
  
  
- TAACTCGGTT TTTAATCAAC TAAGCTTCAG TTGAATTTGT TTTTAAGTAA GCTGAGTTTG ACTTTATAGT   
  
  
- GAATTGATTT TGATACTGAG TTGAGTTTGA ACTAGACTAG GCATAAGTTT AGTTTATGAG GTAAACGATC   
  
  
- CAGATCTTGG TCGGTGCTGG GGTGTACCTG ATACGCCAAG TTACTTTGCC GGTTTATATA GCGTTAAAGA   
  
  
- CAGAGGTAGT TAGTGTGGGT AAACGATGTG GGACAATTAT AAACTGAGCT GGGACACTTA ACGGAAGCGG   
  
  
- TCAAAACAAA TTAGAGAGAC CAACGAAGGG TAGGTTCAGA CTTATCATAT CAAACATGTC ACACATGATG   
  
  
- ATCTTTCGTG CAAGGAACTT AGAAGAAAGT TTCAACAAAA ATTTCCCAAC TCACTAGGTA ATGCTGAAAA   
  
  
- CTAGACCCAA GTAGCGGAGG AGAGGATTAG GGAGTGGGCC CATGGGAGTT AAGAGATAGC AGAGACAACG   
  
  
- AGTCGATTAA GTTTAAGGAA ATCAACCAGT AGTAACTTTA GACTTTAGTC CCACAAAAAG AAAAATAATA   
  
  
- ATAGACCGTC AACCAAACAA ATAGAGAACC AAAAACCCCG AATAAACCCA CGACCAACTA CAATAGGACG   
  
  
- AACCCAACTT AGAAGTAGTC CAAATAAAAA CTAAGTATCA ACTCCCACTT TTAATAAAGA GTATGGGTGG   
  
  
- TGGACAAACA AGAAAACAGA GACTCTTTTT GGTTTTTCGA AAGAGAAAAA GGACTACGTA GGACACGAGC   
  
  
- AACTAGGACT CAATCACTTG GTACGCAGGT TTAAGTTCGG ACTAAACGAG AGTTTGAAAC TATTTGTTGT   
  
  
- TCTTAAATCG TTGCCCTAGC TTGTTCTACT GTAGAACGTT TTGGGACTAG ACACAAAACT AAGTCTTTGG   
  
  
- GGAAGTCAAC TAGATTGACT CTCACAGTAG TCAAGACTAA GACATCTTCA CGGACTAAAG AGTCTACGAA   
  
  
- CGGAGTTCAA GTATTCACTC TAAGAGGATC TTCTCCTAAA CCTACTTTCA GGACGGAGAC GAGTTCTTAA   
  
  
- ATCCCGAGAG GTTCGGTGAC TCTTCAGGGA CATACTACGA GAACCTCTCC CGATGAGAAA AAGTAGACTA   
  
  
- TTGTCGGGTA GTAGTAATCC TGTCTCATAA CTCGTGGTTT TACTCTTAAA ACTTAAGTCA AGGCCAATAG   
  
  
- GACCTTAACT CCCGATACAA TTACCACTAT AGTGCAAGCT CAGGTTGACC TACAAGTTGG ATTCGGTTAA   
  
  
- CCTAGGACAA GAATGAAACC TACTATAAAG AGTCGGGAAC AGACGTAGTT TGAGGGCTAG TAGACCCAGC   
  
  
- TCGTTACCGA AACTACTAAG TCCCCTACCC CGTCGGTGTA GAGGACCGTC ATGTCAATGT AGTTGTCAGG   
  
  
- GTCTCTTTTC CCAACTTAGA CAGTTAGCGA GTTCCTCCTT CTTTTTAGCA TTTTCCCTAC TTCCACCAGT   
  
  
- ACTCCTTCCC TCCTCATTGT TCGTCGTTCG AAGTAGGTTA CTTCTAATAC AACTCTACTT CCTCAAACTA   
  
  
- CTACATGACG AGACGTTTCT TCTCTTTCTA CAATAACGTT TGACGTGGTT ACTAAGGAGT GGGCAGCTTC   
  
  
- GCTCACTATT CAACGTCTTC TTTCCCCCCT TCCCCTTTTG TAGCGCACCC TTCTTCGTCT TATCGTGTTA   
  
  
- TCTTCTCCAC CTAGACTCCT GAGAAGAGTG AGTGACACGA GTTCGTTAAA GTTTAAAACT AGAATCCTCA   
  
  
- CGTTTACTCG TAGAGTCCGT TTATTCCGTC GTAAGAAGTG TTATACCACT ATCGGAGGTC TCCGAACGGG   
  
  
- TAATAAAACG ATTACCAGAA CTCCGAGCGT ATCGACCGTG ACCAAGTTGT TAGAGACGAT TACAACAACT   
  
  
- ACGAGCTTAG TGCAGTAGAC TAAAGAATTT CCGAATATCC GATATATACA GTCGGCAAGG AAAGTTTTCC   
  
  
- TACAATATAA AAGAGCGGTT GTTCTGTTAT GCCTTCAACC GACTCTTCCG TTGGTCCTAG GTGTAGTAAC   
  
  
- TAAAACCTCA GAACAACCCA AATGTCACCG GGACAGAGTA TGTTTTGGAG AGTTTCTCTG GGTGACCGGG   
  
  
- CGGTTCGGAG GCATAATGAC CCTATCTGAT AGGGGTCGTA CCAAAGGCCG GTAGTCTTTC CCAACTTCGC   
  
  
- TGACCGGCAG CCAATCTTCC CATAACGCTC TCTAAATTAC ACGGCAAGTT CATATTCCGG TATCGATTTT   
  
  
- TAACCCTCTC ATATTTCGAT CTCCTAGATC TTTAACTACT CTTACTCTAC CACAAACAGT TAACGTACGA   
  
  
- AGCGAGACCT TGTGACGAAC TACTCTGTCA CCACCGTCTG TCAGGTTTCC TACGAAAGAA TTCCAATTAG   
  
  
- TCTCTCTATT TAGGGGCAGA AAAGTAAGTA CCCTGATAGT TACCTAGTAA GTTACGAGGT AAGAAGTAGT   
  
  
- GAGCCAAGTC CCTCCGCGAG AAGGTGATGA GAAGAAATAA ACTATAGAAA CTTCGTTGAT ACGGGGCACT   
  
  
- TCTAGTACTT TCCGAGGACC AACTCTCACT CTATGTCCCG TTTCTTCGAA ACTTACAGTA TCGTACGCTT   
  
  
- CCACGGCTCT CCTAAGTTTC CGGACTTTGT ATGTTCGTTA CCGTTCGTTC CTGTTGTTCC CGGCCCAAAT   
  
  
- CCGTCGAAGG TGATCTGGCC CTCGAACACT CGTCTCGTTT CCGATACCAC TTTCGTTTGA TGGTATTCCT   
  
  
- AAAACACCAC CTGCTCCTGT CCGTAACCTA CGATGTGCCA ACCTTTCCTT CTTAGAAGAC ACGTGAGAGA   
  
  
- CAAACCGTTG GGTTGAC

+     MYB

| Site Name | Organism | Position | Strand | Matrix score. | sequence | function |
| --- | --- | --- | --- | --- | --- | --- |
| MYB | Arabidopsis thaliana | 3802 | - | 6 | CAACCA |  |
| MYB | Arabidopsis thaliana | 1748 | - | 6 | CAACAG |  |
| MYB | Arabidopsis thaliana | 3274 | + | 6 | CAACCA |  |
| MYB | Arabidopsis thaliana | 1102 | + | 6 | TAACCA |  |
| MYB | Arabidopsis thaliana | 1563 | - | 6 | CAACCA |  |
| MYB | Arabidopsis thaliana | 2656 | + | 6 | CAACAG |  |
| MYB | Arabidopsis thaliana | 1877 | - | 6 | CAACCA |  |

>HU07G02246.1   
+ -Up\_Stream \_Len000ATTTGA TGTGTATGCT GACTACTTTT GATGATGATG ATCACAGTGA TGCATATTTA   
  
  
+ GAGCTGGTAA ATTGGTCATT CGATTCGAGT ACGGGTCGGG TCAAGTTCTG ATTAAGTGAC ATTTCGCGTC   
  
  
+ GTTTTGGTTT AGTTCGGGTC GGATCGATTT CGGGTTAAAT AATTTTTGGT GGAATACGCT TGTCATGCCA   
  
  
+ AACACAAGCA ACTTTGTTAA AAATTTCGAT TCGGGTCAGG TCAATTCAGG TTTGGGGTCC ACTTTCGAGT   
  
  
+ GAGCATATTT CGGATGTCGG GTCGCGTATG GGTCCGGGTC ATTCGGTTTG AATTTCGGAT CTTGGATCAA   
  
  
+ TTTTGCTAAG TATGTGCATA TTCAACCCTT GATGACATAG GAAACGCTTT GTCATCTCCA CCAATCAGAT   
  
  
+ ATCGTTATCT CTTTAATCAT CGCCATAATA GCATGAGTAA GCATGACCAT CTTTTTCAAA CATGTGATAC   
  
  
+ AGTTAAGGCC TGTTCTTTTC GTTAATAAAT CCCAATTTAT CAATTTTAAT ATATACTTTA ACGAATTTTA   
  
  
+ ACAAAAAATT TTAATTTCAA TCCGCTATAA CAAAAAAAAT ACCAACTTCA ACTTATTATA TAAAAAAAAT   
  
  
+ ATACAATTTT TTTTCAATTT TAGCCAACTT CAACAAAAAA AAAAAAAATT TAACAAATCT CAATCAACTT   
  
  
+ CACCCATTTC TCAACTTATA TCTCCTAATG AAAAGAACAG ATCCTTAGCA AGACTTCTCG ATTCATTTAA   
  
  
+ AAGGATAGTA TGGTAAATTC ATTTTATACA AGTGTCAAGT GTGAATATCT TTTTCACATC AGGTGATATA   
  
  
+ TTAGTAGGAC TTTTCGATTC ACTTATCTTC CTCTTTATGT TTTGTTAATG TGATAGTAAC GATAGGATTG   
  
  
+ CATGTATTTT TTCCAACTAT TCATTTACAA CTATCAATAT CTATTGATAA ATATGTTAAA ATAATTATAT   
  
  
+ GTTATTTAAA TTATTATTTG TTACTTAAGG TGTCGACTTT GTCGAAAGCG ACTTAATTCT AATTAAGATA   
  
  
+ GCGGCAGAGA AAGTTACACC TGATGACCAA GTTAGATCTG GTAAAACTAA CCATAGATCT GAAACCCAAA   
  
  
+ CATAATGATG TGTATTCATA CCCGACCTGA CATCTGAAAT AAGTTTACTC AAAAGTGGTT CCGAAACTTG   
  
  
+ AATTGACTCG AACCGAACCG TATTTGACTC GAAATTTTCT ACAAAGTTGC TTATTTTTGC ATGAAGACAT   
  
  
+ ATTGAGCCAA AAATTAGTTG ATTCGAAGTC AACTTAAACA AAAATTCATT CGACTCAAAC TGAAATATCA   
  
  
+ CTTAACTAAA ACTATGACTC AACTCAAACT TGATCTGATC CGTATTCAAA TCAAATACTC CATTTGCTAG   
  
  
+ GTCTAGAACC AGCCACGACC CCACATGGAC TATGCGGTTC AATGAAACGG CCAAATATAT CGCAATTTCT   
  
  
+ GTCTCCATCA ATCACACCCA TTTGCTACAC CCTGTTAATA TTTGACTCGA CCCTGTGAAT TGCCTTCGCC   
  
  
+ AGTTTTGTTT AATCTCTCTG GTTGCTTCCC ATCCAAGTCT GAATAGTATA GTTTGTACAG TGTGTACTAC   
  
  
+ TAGAAAGCAC GTTCCTTGAA TCTTCTTTCA AAGTTGTTTT TAAAGGGTTG AGTGATCCAT TACGACTTTT   
  
  
+ GATCTGGGTT CATCGCCTCC TCTCCTAATC CCTCACCCGG GTACCCTCAA TTCTCTATCG TCTCTGTTGC   
  
  
+ TCAGCTAATT CAAATTCCTT TAGTTGGTCA TCATTGAAAT CTGAAATCAG GGTGTTTTTC TTTTTATTAT   
  
  
+ TATCTGGCAG TTGGTTTGTT TATCTCTTGG TTTTTGGGGC TTATTTGGGT GCTGGTTGAT GTTATCCTGC   
  
  
+ TTGGGTTGAA TCTTCATCAG GTTTATTTTT GATTCATAGT TGAGGGTGAA AATTATTTCT CATACCCACC   
  
  
+ ACCTGTTTGT TCTTTTGTCT CTGAGAAAAA CCAAAAAGCT TTCTCTTTTT CCTGATGCAT CCTGTGCTCG   
  
  
+ TTGATCCTGA GTTAGTGAAC CATGCGTCCA AATTCAAGCC TGATTTGCTC TCAAACTTTG ATAAACAACA   
  
  
+ AGAATTTAGC AACGGGATCG AACAAGATGA CATCTTGCAA AACCCTGATC TGTGTTTTGA TTCAGAAACC   
  
  
+ CCTTCAGTTG ATCTAACTGA GAGTGTCATC AGTTCTGATT CTGTAGAAGT GCCTGATTTC TCAGATGCTT   
  
  
+ GCCTCAAGTT CATAAGTGAG ATTCTCCTAG AAGAGGATTT GGATGAAAGT CCTGCCTCTG CTCAAGAATT   
  
  
+ TAGGGCTCTC CAAGCCACTG AGAAGTCCCT GTATGATGCT CTTGGAGAGG GCTACTCTTT TTCATCTGAT   
  
  
+ AACAGCCCAT CATCATTAGG ACAGAGTATT GAGCACCAAA ATGAGAATTT TGAATTCAGT TCCGGTTATC   
  
  
+ CTGGAATTGA GGGCTATGTT AATGGTGATA TCACGTTCGA GTCCAACTGG ATGTTCAACC TAAGCCAATT   
  
  
+ GGATCCTGTT CTTACTTTGG ATGATATTTC TCAGCCCTTG TCTGCATCAA ACTCCCGATC ATCTGGGTCG   
  
  
+ AGCAATGGCT TTGATGATTC AGGGGATGGG GCAGCCACAT CTCCTGGCAG TACAGTTACA TCAACAGTCC   
  
  
+ CAGAGAAAAG GGTTGAATCT GTCAATCGCT CAAGGAGGAA GAAAAATCGT AAAAGGGATG AAGGTGGTCA   
  
  
+ TGAGGAAGGG AGGAGTAACA AGCAGCAAGC TTCATCCAAT GAAGATTATG TTGAGATGAA GGAGTTTGAT   
  
  
+ GATGTACTGC TCTGCAAAGA AGAGAAAGAT GTTATTGCAA ACTGCACCAA TGATTCCTCA CCCGTCGAAG   
  
  
+ CGAGTGATAA GTTGCAGAAG AAAGGGGGGA AGGGGAAAAC ATCGCGTGGG AAGAAGCAGA ATAGCACAAT   
  
  
+ AGAAGAGGTG GATCTGAGGA CTCTTCTCAC TCACTGTGCT CAAGCAATTT CAAATTTTGA TCTTAGGAGT   
  
  
+ GCAAATGAGC ATCTCAGGCA AATAAGGCAG CATTCTTCAC AATATGGTGA TAGCCTCCAG AGGCTTGCCC   
  
  
+ ATTATTTTGC TAATGGTCTT GAGGCTCGCA TAGCTGGCAC TGGTTCAACA ATCTCTGCTA ATGTTGTTGA   
  
  
+ TGCTCGAATC ACGTCATCTG ATTTCTTAAA GGCTTATAGG CTATATATGT CAGCCGTTCC TTTCAAAAGG   
  
  
+ ATGTTATATT TTCTCGCCAA CAAGACAATA CGGAAGTTGG CTGAGAAGGC AACCAGGATC CACATCATTG   
  
  
+ ATTTTGGAGT CTTGTTGGGT TTACAGTGGC CCTGTCTCAT ACAAAACCTC TCAAAGAGAC CCACTGGCCC   
  
  
+ GCCAAGCCTC CGTATTACTG GGATAGACTA TCCCCAGCAT GGTTTCCGGC CATCAGAAAG GGTTGAAGCG   
  
  
+ ACTGGCCGTC GGTTAGAAGG GTATTGCGAG AGATTTAATG TGCCGTTCAA GTATAAGGCC ATAGCTAAAA   
  
  
+ ATTGGGAGAG TATAAAGCTA GAGGATCTAG AAATTGATGA GAATGAGATG GTGTTTGTCA ATTGCATGCT   
  
  
+ TCGCTCTGGA ACACTGCTTG ATGAGACAGT GGTGGCAGAC AGTCCAAAGG ATGCTTTCTT AAGGTTAATC   
  
  
+ AGAGAGATAA ATCCCCGTCT TTTCATTCAT GGGACTATCA ATGGATCATT CAATGCTCCA TTCTTCATCA   
  
  
+ CTCGGTTCAG GGAGGCGCTC TTCCACTACT CTTCTTTATT TGATATCTTT GAAGCAACTA TGCCCCGTGA   
  
  
+ AGATCATGAA AGGCTCCTGG TTGAGAGTGA GATACAGGGC AAAGAAGCTT TGAATGTCAT AGCATGCGAA   
  
  
+ GGTGCCGAGA GGATTCAAAG GCCTGAAACA TACAAGCAAT GGCAAGCAAG GACAACAAGG GCCGGGTTTA   
  
  
+ GGCAGCTTCC ACTAGACCGG GAGCTTGTGA GCAGAGCAAA GGCTATGGTG AAAGCAAACT ACCATAAGGA   
  
  
+ TTTTGTGGTG GACGAGGACA GGCATTGGAT GCTACACGGT TGGAAAGGAA GAATCTTCTG TGCACTCTCT   
  
  
+ GTTTGGCAAC CCAACTG  

- -Up\_Stream \_Len000TAAACT ACACATACGA CTGATGAAAA CTACTACTAC TAGTGTCACT ACGTATAAAT   
  
  
- CTCGACCATT TAACCAGTAA GCTAAGCTCA TGCCCAGCCC AGTTCAAGAC TAATTCACTG TAAAGCGCAG   
  
  
- CAAAACCAAA TCAAGCCCAG CCTAGCTAAA GCCCAATTTA TTAAAAACCA CCTTATGCGA ACAGTACGGT   
  
  
- TTGTGTTCGT TGAAACAATT TTTAAAGCTA AGCCCAGTCC AGTTAAGTCC AAACCCCAGG TGAAAGCTCA   
  
  
- CTCGTATAAA GCCTACAGCC CAGCGCATAC CCAGGCCCAG TAAGCCAAAC TTAAAGCCTA GAACCTAGTT   
  
  
- AAAACGATTC ATACACGTAT AAGTTGGGAA CTACTGTATC CTTTGCGAAA CAGTAGAGGT GGTTAGTCTA   
  
  
- TAGCAATAGA GAAATTAGTA GCGGTATTAT CGTACTCATT CGTACTGGTA GAAAAAGTTT GTACACTATG   
  
  
- TCAATTCCGG ACAAGAAAAG CAATTATTTA GGGTTAAATA GTTAAAATTA TATATGAAAT TGCTTAAAAT   
  
  
- TGTTTTTTAA AATTAAAGTT AGGCGATATT GTTTTTTTTA TGGTTGAAGT TGAATAATAT ATTTTTTTTA   
  
  
- TATGTTAAAA AAAAGTTAAA ATCGGTTGAA GTTGTTTTTT TTTTTTTTAA ATTGTTTAGA GTTAGTTGAA   
  
  
- GTGGGTAAAG AGTTGAATAT AGAGGATTAC TTTTCTTGTC TAGGAATCGT TCTGAAGAGC TAAGTAAATT   
  
  
- TTCCTATCAT ACCATTTAAG TAAAATATGT TCACAGTTCA CACTTATAGA AAAAGTGTAG TCCACTATAT   
  
  
- AATCATCCTG AAAAGCTAAG TGAATAGAAG GAGAAATACA AAACAATTAC ACTATCATTG CTATCCTAAC   
  
  
- GTACATAAAA AAGGTTGATA AGTAAATGTT GATAGTTATA GATAACTATT TATACAATTT TATTAATATA   
  
  
- CAATAAATTT AATAATAAAC AATGAATTCC ACAGCTGAAA CAGCTTTCGC TGAATTAAGA TTAATTCTAT   
  
  
- CGCCGTCTCT TTCAATGTGG ACTACTGGTT CAATCTAGAC CATTTTGATT GGTATCTAGA CTTTGGGTTT   
  
  
- GTATTACTAC ACATAAGTAT GGGCTGGACT GTAGACTTTA TTCAAATGAG TTTTCACCAA GGCTTTGAAC   
  
  
- TTAACTGAGC TTGGCTTGGC ATAAACTGAG CTTTAAAAGA TGTTTCAACG AATAAAAACG TACTTCTGTA   
  
  
- TAACTCGGTT TTTAATCAAC TAAGCTTCAG TTGAATTTGT TTTTAAGTAA GCTGAGTTTG ACTTTATAGT   
  
  
- GAATTGATTT TGATACTGAG TTGAGTTTGA ACTAGACTAG GCATAAGTTT AGTTTATGAG GTAAACGATC   
  
  
- CAGATCTTGG TCGGTGCTGG GGTGTACCTG ATACGCCAAG TTACTTTGCC GGTTTATATA GCGTTAAAGA   
  
  
- CAGAGGTAGT TAGTGTGGGT AAACGATGTG GGACAATTAT AAACTGAGCT GGGACACTTA ACGGAAGCGG   
  
  
- TCAAAACAAA TTAGAGAGAC CAACGAAGGG TAGGTTCAGA CTTATCATAT CAAACATGTC ACACATGATG   
  
  
- ATCTTTCGTG CAAGGAACTT AGAAGAAAGT TTCAACAAAA ATTTCCCAAC TCACTAGGTA ATGCTGAAAA   
  
  
- CTAGACCCAA GTAGCGGAGG AGAGGATTAG GGAGTGGGCC CATGGGAGTT AAGAGATAGC AGAGACAACG   
  
  
- AGTCGATTAA GTTTAAGGAA ATCAACCAGT AGTAACTTTA GACTTTAGTC CCACAAAAAG AAAAATAATA   
  
  
- ATAGACCGTC AACCAAACAA ATAGAGAACC AAAAACCCCG AATAAACCCA CGACCAACTA CAATAGGACG   
  
  
- AACCCAACTT AGAAGTAGTC CAAATAAAAA CTAAGTATCA ACTCCCACTT TTAATAAAGA GTATGGGTGG   
  
  
- TGGACAAACA AGAAAACAGA GACTCTTTTT GGTTTTTCGA AAGAGAAAAA GGACTACGTA GGACACGAGC   
  
  
- AACTAGGACT CAATCACTTG GTACGCAGGT TTAAGTTCGG ACTAAACGAG AGTTTGAAAC TATTTGTTGT   
  
  
- TCTTAAATCG TTGCCCTAGC TTGTTCTACT GTAGAACGTT TTGGGACTAG ACACAAAACT AAGTCTTTGG   
  
  
- GGAAGTCAAC TAGATTGACT CTCACAGTAG TCAAGACTAA GACATCTTCA CGGACTAAAG AGTCTACGAA   
  
  
- CGGAGTTCAA GTATTCACTC TAAGAGGATC TTCTCCTAAA CCTACTTTCA GGACGGAGAC GAGTTCTTAA   
  
  
- ATCCCGAGAG GTTCGGTGAC TCTTCAGGGA CATACTACGA GAACCTCTCC CGATGAGAAA AAGTAGACTA   
  
  
- TTGTCGGGTA GTAGTAATCC TGTCTCATAA CTCGTGGTTT TACTCTTAAA ACTTAAGTCA AGGCCAATAG   
  
  
- GACCTTAACT CCCGATACAA TTACCACTAT AGTGCAAGCT CAGGTTGACC TACAAGTTGG ATTCGGTTAA   
  
  
- CCTAGGACAA GAATGAAACC TACTATAAAG AGTCGGGAAC AGACGTAGTT TGAGGGCTAG TAGACCCAGC   
  
  
- TCGTTACCGA AACTACTAAG TCCCCTACCC CGTCGGTGTA GAGGACCGTC ATGTCAATGT AGTTGTCAGG   
  
  
- GTCTCTTTTC CCAACTTAGA CAGTTAGCGA GTTCCTCCTT CTTTTTAGCA TTTTCCCTAC TTCCACCAGT   
  
  
- ACTCCTTCCC TCCTCATTGT TCGTCGTTCG AAGTAGGTTA CTTCTAATAC AACTCTACTT CCTCAAACTA   
  
  
- CTACATGACG AGACGTTTCT TCTCTTTCTA CAATAACGTT TGACGTGGTT ACTAAGGAGT GGGCAGCTTC   
  
  
- GCTCACTATT CAACGTCTTC TTTCCCCCCT TCCCCTTTTG TAGCGCACCC TTCTTCGTCT TATCGTGTTA   
  
  
- TCTTCTCCAC CTAGACTCCT GAGAAGAGTG AGTGACACGA GTTCGTTAAA GTTTAAAACT AGAATCCTCA   
  
  
- CGTTTACTCG TAGAGTCCGT TTATTCCGTC GTAAGAAGTG TTATACCACT ATCGGAGGTC TCCGAACGGG   
  
  
- TAATAAAACG ATTACCAGAA CTCCGAGCGT ATCGACCGTG ACCAAGTTGT TAGAGACGAT TACAACAACT   
  
  
- ACGAGCTTAG TGCAGTAGAC TAAAGAATTT CCGAATATCC GATATATACA GTCGGCAAGG AAAGTTTTCC   
  
  
- TACAATATAA AAGAGCGGTT GTTCTGTTAT GCCTTCAACC GACTCTTCCG TTGGTCCTAG GTGTAGTAAC   
  
  
- TAAAACCTCA GAACAACCCA AATGTCACCG GGACAGAGTA TGTTTTGGAG AGTTTCTCTG GGTGACCGGG   
  
  
- CGGTTCGGAG GCATAATGAC CCTATCTGAT AGGGGTCGTA CCAAAGGCCG GTAGTCTTTC CCAACTTCGC   
  
  
- TGACCGGCAG CCAATCTTCC CATAACGCTC TCTAAATTAC ACGGCAAGTT CATATTCCGG TATCGATTTT   
  
  
- TAACCCTCTC ATATTTCGAT CTCCTAGATC TTTAACTACT CTTACTCTAC CACAAACAGT TAACGTACGA   
  
  
- AGCGAGACCT TGTGACGAAC TACTCTGTCA CCACCGTCTG TCAGGTTTCC TACGAAAGAA TTCCAATTAG   
  
  
- TCTCTCTATT TAGGGGCAGA AAAGTAAGTA CCCTGATAGT TACCTAGTAA GTTACGAGGT AAGAAGTAGT   
  
  
- GAGCCAAGTC CCTCCGCGAG AAGGTGATGA GAAGAAATAA ACTATAGAAA CTTCGTTGAT ACGGGGCACT   
  
  
- TCTAGTACTT TCCGAGGACC AACTCTCACT CTATGTCCCG TTTCTTCGAA ACTTACAGTA TCGTACGCTT   
  
  
- CCACGGCTCT CCTAAGTTTC CGGACTTTGT ATGTTCGTTA CCGTTCGTTC CTGTTGTTCC CGGCCCAAAT   
  
  
- CCGTCGAAGG TGATCTGGCC CTCGAACACT CGTCTCGTTT CCGATACCAC TTTCGTTTGA TGGTATTCCT   
  
  
- AAAACACCAC CTGCTCCTGT CCGTAACCTA CGATGTGCCA ACCTTTCCTT CTTAGAAGAC ACGTGAGAGA   
  
  
- CAAACCGTTG GGTTGAC

+     MYB recognition site

| Site Name | Organism | Position | Strand | Matrix score. | sequence | function |
| --- | --- | --- | --- | --- | --- | --- |
| MYB recognition site | Arabidopsis thaliana | 2114 | - | 6 | CCGTTG |  |

>HU07G02246.1   
+ -Up\_Stream \_Len000ATTTGA TGTGTATGCT GACTACTTTT GATGATGATG ATCACAGTGA TGCATATTTA   
  
  
+ GAGCTGGTAA ATTGGTCATT CGATTCGAGT ACGGGTCGGG TCAAGTTCTG ATTAAGTGAC ATTTCGCGTC   
  
  
+ GTTTTGGTTT AGTTCGGGTC GGATCGATTT CGGGTTAAAT AATTTTTGGT GGAATACGCT TGTCATGCCA   
  
  
+ AACACAAGCA ACTTTGTTAA AAATTTCGAT TCGGGTCAGG TCAATTCAGG TTTGGGGTCC ACTTTCGAGT   
  
  
+ GAGCATATTT CGGATGTCGG GTCGCGTATG GGTCCGGGTC ATTCGGTTTG AATTTCGGAT CTTGGATCAA   
  
  
+ TTTTGCTAAG TATGTGCATA TTCAACCCTT GATGACATAG GAAACGCTTT GTCATCTCCA CCAATCAGAT   
  
  
+ ATCGTTATCT CTTTAATCAT CGCCATAATA GCATGAGTAA GCATGACCAT CTTTTTCAAA CATGTGATAC   
  
  
+ AGTTAAGGCC TGTTCTTTTC GTTAATAAAT CCCAATTTAT CAATTTTAAT ATATACTTTA ACGAATTTTA   
  
  
+ ACAAAAAATT TTAATTTCAA TCCGCTATAA CAAAAAAAAT ACCAACTTCA ACTTATTATA TAAAAAAAAT   
  
  
+ ATACAATTTT TTTTCAATTT TAGCCAACTT CAACAAAAAA AAAAAAAATT TAACAAATCT CAATCAACTT   
  
  
+ CACCCATTTC TCAACTTATA TCTCCTAATG AAAAGAACAG ATCCTTAGCA AGACTTCTCG ATTCATTTAA   
  
  
+ AAGGATAGTA TGGTAAATTC ATTTTATACA AGTGTCAAGT GTGAATATCT TTTTCACATC AGGTGATATA   
  
  
+ TTAGTAGGAC TTTTCGATTC ACTTATCTTC CTCTTTATGT TTTGTTAATG TGATAGTAAC GATAGGATTG   
  
  
+ CATGTATTTT TTCCAACTAT TCATTTACAA CTATCAATAT CTATTGATAA ATATGTTAAA ATAATTATAT   
  
  
+ GTTATTTAAA TTATTATTTG TTACTTAAGG TGTCGACTTT GTCGAAAGCG ACTTAATTCT AATTAAGATA   
  
  
+ GCGGCAGAGA AAGTTACACC TGATGACCAA GTTAGATCTG GTAAAACTAA CCATAGATCT GAAACCCAAA   
  
  
+ CATAATGATG TGTATTCATA CCCGACCTGA CATCTGAAAT AAGTTTACTC AAAAGTGGTT CCGAAACTTG   
  
  
+ AATTGACTCG AACCGAACCG TATTTGACTC GAAATTTTCT ACAAAGTTGC TTATTTTTGC ATGAAGACAT   
  
  
+ ATTGAGCCAA AAATTAGTTG ATTCGAAGTC AACTTAAACA AAAATTCATT CGACTCAAAC TGAAATATCA   
  
  
+ CTTAACTAAA ACTATGACTC AACTCAAACT TGATCTGATC CGTATTCAAA TCAAATACTC CATTTGCTAG   
  
  
+ GTCTAGAACC AGCCACGACC CCACATGGAC TATGCGGTTC AATGAAACGG CCAAATATAT CGCAATTTCT   
  
  
+ GTCTCCATCA ATCACACCCA TTTGCTACAC CCTGTTAATA TTTGACTCGA CCCTGTGAAT TGCCTTCGCC   
  
  
+ AGTTTTGTTT AATCTCTCTG GTTGCTTCCC ATCCAAGTCT GAATAGTATA GTTTGTACAG TGTGTACTAC   
  
  
+ TAGAAAGCAC GTTCCTTGAA TCTTCTTTCA AAGTTGTTTT TAAAGGGTTG AGTGATCCAT TACGACTTTT   
  
  
+ GATCTGGGTT CATCGCCTCC TCTCCTAATC CCTCACCCGG GTACCCTCAA TTCTCTATCG TCTCTGTTGC   
  
  
+ TCAGCTAATT CAAATTCCTT TAGTTGGTCA TCATTGAAAT CTGAAATCAG GGTGTTTTTC TTTTTATTAT   
  
  
+ TATCTGGCAG TTGGTTTGTT TATCTCTTGG TTTTTGGGGC TTATTTGGGT GCTGGTTGAT GTTATCCTGC   
  
  
+ TTGGGTTGAA TCTTCATCAG GTTTATTTTT GATTCATAGT TGAGGGTGAA AATTATTTCT CATACCCACC   
  
  
+ ACCTGTTTGT TCTTTTGTCT CTGAGAAAAA CCAAAAAGCT TTCTCTTTTT CCTGATGCAT CCTGTGCTCG   
  
  
+ TTGATCCTGA GTTAGTGAAC CATGCGTCCA AATTCAAGCC TGATTTGCTC TCAAACTTTG ATAAACAACA   
  
  
+ AGAATTTAGC AACGGGATCG AACAAGATGA CATCTTGCAA AACCCTGATC TGTGTTTTGA TTCAGAAACC   
  
  
+ CCTTCAGTTG ATCTAACTGA GAGTGTCATC AGTTCTGATT CTGTAGAAGT GCCTGATTTC TCAGATGCTT   
  
  
+ GCCTCAAGTT CATAAGTGAG ATTCTCCTAG AAGAGGATTT GGATGAAAGT CCTGCCTCTG CTCAAGAATT   
  
  
+ TAGGGCTCTC CAAGCCACTG AGAAGTCCCT GTATGATGCT CTTGGAGAGG GCTACTCTTT TTCATCTGAT   
  
  
+ AACAGCCCAT CATCATTAGG ACAGAGTATT GAGCACCAAA ATGAGAATTT TGAATTCAGT TCCGGTTATC   
  
  
+ CTGGAATTGA GGGCTATGTT AATGGTGATA TCACGTTCGA GTCCAACTGG ATGTTCAACC TAAGCCAATT   
  
  
+ GGATCCTGTT CTTACTTTGG ATGATATTTC TCAGCCCTTG TCTGCATCAA ACTCCCGATC ATCTGGGTCG   
  
  
+ AGCAATGGCT TTGATGATTC AGGGGATGGG GCAGCCACAT CTCCTGGCAG TACAGTTACA TCAACAGTCC   
  
  
+ CAGAGAAAAG GGTTGAATCT GTCAATCGCT CAAGGAGGAA GAAAAATCGT AAAAGGGATG AAGGTGGTCA   
  
  
+ TGAGGAAGGG AGGAGTAACA AGCAGCAAGC TTCATCCAAT GAAGATTATG TTGAGATGAA GGAGTTTGAT   
  
  
+ GATGTACTGC TCTGCAAAGA AGAGAAAGAT GTTATTGCAA ACTGCACCAA TGATTCCTCA CCCGTCGAAG   
  
  
+ CGAGTGATAA GTTGCAGAAG AAAGGGGGGA AGGGGAAAAC ATCGCGTGGG AAGAAGCAGA ATAGCACAAT   
  
  
+ AGAAGAGGTG GATCTGAGGA CTCTTCTCAC TCACTGTGCT CAAGCAATTT CAAATTTTGA TCTTAGGAGT   
  
  
+ GCAAATGAGC ATCTCAGGCA AATAAGGCAG CATTCTTCAC AATATGGTGA TAGCCTCCAG AGGCTTGCCC   
  
  
+ ATTATTTTGC TAATGGTCTT GAGGCTCGCA TAGCTGGCAC TGGTTCAACA ATCTCTGCTA ATGTTGTTGA   
  
  
+ TGCTCGAATC ACGTCATCTG ATTTCTTAAA GGCTTATAGG CTATATATGT CAGCCGTTCC TTTCAAAAGG   
  
  
+ ATGTTATATT TTCTCGCCAA CAAGACAATA CGGAAGTTGG CTGAGAAGGC AACCAGGATC CACATCATTG   
  
  
+ ATTTTGGAGT CTTGTTGGGT TTACAGTGGC CCTGTCTCAT ACAAAACCTC TCAAAGAGAC CCACTGGCCC   
  
  
+ GCCAAGCCTC CGTATTACTG GGATAGACTA TCCCCAGCAT GGTTTCCGGC CATCAGAAAG GGTTGAAGCG   
  
  
+ ACTGGCCGTC GGTTAGAAGG GTATTGCGAG AGATTTAATG TGCCGTTCAA GTATAAGGCC ATAGCTAAAA   
  
  
+ ATTGGGAGAG TATAAAGCTA GAGGATCTAG AAATTGATGA GAATGAGATG GTGTTTGTCA ATTGCATGCT   
  
  
+ TCGCTCTGGA ACACTGCTTG ATGAGACAGT GGTGGCAGAC AGTCCAAAGG ATGCTTTCTT AAGGTTAATC   
  
  
+ AGAGAGATAA ATCCCCGTCT TTTCATTCAT GGGACTATCA ATGGATCATT CAATGCTCCA TTCTTCATCA   
  
  
+ CTCGGTTCAG GGAGGCGCTC TTCCACTACT CTTCTTTATT TGATATCTTT GAAGCAACTA TGCCCCGTGA   
  
  
+ AGATCATGAA AGGCTCCTGG TTGAGAGTGA GATACAGGGC AAAGAAGCTT TGAATGTCAT AGCATGCGAA   
  
  
+ GGTGCCGAGA GGATTCAAAG GCCTGAAACA TACAAGCAAT GGCAAGCAAG GACAACAAGG GCCGGGTTTA   
  
  
+ GGCAGCTTCC ACTAGACCGG GAGCTTGTGA GCAGAGCAAA GGCTATGGTG AAAGCAAACT ACCATAAGGA   
  
  
+ TTTTGTGGTG GACGAGGACA GGCATTGGAT GCTACACGGT TGGAAAGGAA GAATCTTCTG TGCACTCTCT   
  
  
+ GTTTGGCAAC CCAACTG  

- -Up\_Stream \_Len000TAAACT ACACATACGA CTGATGAAAA CTACTACTAC TAGTGTCACT ACGTATAAAT   
  
  
- CTCGACCATT TAACCAGTAA GCTAAGCTCA TGCCCAGCCC AGTTCAAGAC TAATTCACTG TAAAGCGCAG   
  
  
- CAAAACCAAA TCAAGCCCAG CCTAGCTAAA GCCCAATTTA TTAAAAACCA CCTTATGCGA ACAGTACGGT   
  
  
- TTGTGTTCGT TGAAACAATT TTTAAAGCTA AGCCCAGTCC AGTTAAGTCC AAACCCCAGG TGAAAGCTCA   
  
  
- CTCGTATAAA GCCTACAGCC CAGCGCATAC CCAGGCCCAG TAAGCCAAAC TTAAAGCCTA GAACCTAGTT   
  
  
- AAAACGATTC ATACACGTAT AAGTTGGGAA CTACTGTATC CTTTGCGAAA CAGTAGAGGT GGTTAGTCTA   
  
  
- TAGCAATAGA GAAATTAGTA GCGGTATTAT CGTACTCATT CGTACTGGTA GAAAAAGTTT GTACACTATG   
  
  
- TCAATTCCGG ACAAGAAAAG CAATTATTTA GGGTTAAATA GTTAAAATTA TATATGAAAT TGCTTAAAAT   
  
  
- TGTTTTTTAA AATTAAAGTT AGGCGATATT GTTTTTTTTA TGGTTGAAGT TGAATAATAT ATTTTTTTTA   
  
  
- TATGTTAAAA AAAAGTTAAA ATCGGTTGAA GTTGTTTTTT TTTTTTTTAA ATTGTTTAGA GTTAGTTGAA   
  
  
- GTGGGTAAAG AGTTGAATAT AGAGGATTAC TTTTCTTGTC TAGGAATCGT TCTGAAGAGC TAAGTAAATT   
  
  
- TTCCTATCAT ACCATTTAAG TAAAATATGT TCACAGTTCA CACTTATAGA AAAAGTGTAG TCCACTATAT   
  
  
- AATCATCCTG AAAAGCTAAG TGAATAGAAG GAGAAATACA AAACAATTAC ACTATCATTG CTATCCTAAC   
  
  
- GTACATAAAA AAGGTTGATA AGTAAATGTT GATAGTTATA GATAACTATT TATACAATTT TATTAATATA   
  
  
- CAATAAATTT AATAATAAAC AATGAATTCC ACAGCTGAAA CAGCTTTCGC TGAATTAAGA TTAATTCTAT   
  
  
- CGCCGTCTCT TTCAATGTGG ACTACTGGTT CAATCTAGAC CATTTTGATT GGTATCTAGA CTTTGGGTTT   
  
  
- GTATTACTAC ACATAAGTAT GGGCTGGACT GTAGACTTTA TTCAAATGAG TTTTCACCAA GGCTTTGAAC   
  
  
- TTAACTGAGC TTGGCTTGGC ATAAACTGAG CTTTAAAAGA TGTTTCAACG AATAAAAACG TACTTCTGTA   
  
  
- TAACTCGGTT TTTAATCAAC TAAGCTTCAG TTGAATTTGT TTTTAAGTAA GCTGAGTTTG ACTTTATAGT   
  
  
- GAATTGATTT TGATACTGAG TTGAGTTTGA ACTAGACTAG GCATAAGTTT AGTTTATGAG GTAAACGATC   
  
  
- CAGATCTTGG TCGGTGCTGG GGTGTACCTG ATACGCCAAG TTACTTTGCC GGTTTATATA GCGTTAAAGA   
  
  
- CAGAGGTAGT TAGTGTGGGT AAACGATGTG GGACAATTAT AAACTGAGCT GGGACACTTA ACGGAAGCGG   
  
  
- TCAAAACAAA TTAGAGAGAC CAACGAAGGG TAGGTTCAGA CTTATCATAT CAAACATGTC ACACATGATG   
  
  
- ATCTTTCGTG CAAGGAACTT AGAAGAAAGT TTCAACAAAA ATTTCCCAAC TCACTAGGTA ATGCTGAAAA   
  
  
- CTAGACCCAA GTAGCGGAGG AGAGGATTAG GGAGTGGGCC CATGGGAGTT AAGAGATAGC AGAGACAACG   
  
  
- AGTCGATTAA GTTTAAGGAA ATCAACCAGT AGTAACTTTA GACTTTAGTC CCACAAAAAG AAAAATAATA   
  
  
- ATAGACCGTC AACCAAACAA ATAGAGAACC AAAAACCCCG AATAAACCCA CGACCAACTA CAATAGGACG   
  
  
- AACCCAACTT AGAAGTAGTC CAAATAAAAA CTAAGTATCA ACTCCCACTT TTAATAAAGA GTATGGGTGG   
  
  
- TGGACAAACA AGAAAACAGA GACTCTTTTT GGTTTTTCGA AAGAGAAAAA GGACTACGTA GGACACGAGC   
  
  
- AACTAGGACT CAATCACTTG GTACGCAGGT TTAAGTTCGG ACTAAACGAG AGTTTGAAAC TATTTGTTGT   
  
  
- TCTTAAATCG TTGCCCTAGC TTGTTCTACT GTAGAACGTT TTGGGACTAG ACACAAAACT AAGTCTTTGG   
  
  
- GGAAGTCAAC TAGATTGACT CTCACAGTAG TCAAGACTAA GACATCTTCA CGGACTAAAG AGTCTACGAA   
  
  
- CGGAGTTCAA GTATTCACTC TAAGAGGATC TTCTCCTAAA CCTACTTTCA GGACGGAGAC GAGTTCTTAA   
  
  
- ATCCCGAGAG GTTCGGTGAC TCTTCAGGGA CATACTACGA GAACCTCTCC CGATGAGAAA AAGTAGACTA   
  
  
- TTGTCGGGTA GTAGTAATCC TGTCTCATAA CTCGTGGTTT TACTCTTAAA ACTTAAGTCA AGGCCAATAG   
  
  
- GACCTTAACT CCCGATACAA TTACCACTAT AGTGCAAGCT CAGGTTGACC TACAAGTTGG ATTCGGTTAA   
  
  
- CCTAGGACAA GAATGAAACC TACTATAAAG AGTCGGGAAC AGACGTAGTT TGAGGGCTAG TAGACCCAGC   
  
  
- TCGTTACCGA AACTACTAAG TCCCCTACCC CGTCGGTGTA GAGGACCGTC ATGTCAATGT AGTTGTCAGG   
  
  
- GTCTCTTTTC CCAACTTAGA CAGTTAGCGA GTTCCTCCTT CTTTTTAGCA TTTTCCCTAC TTCCACCAGT   
  
  
- ACTCCTTCCC TCCTCATTGT TCGTCGTTCG AAGTAGGTTA CTTCTAATAC AACTCTACTT CCTCAAACTA   
  
  
- CTACATGACG AGACGTTTCT TCTCTTTCTA CAATAACGTT TGACGTGGTT ACTAAGGAGT GGGCAGCTTC   
  
  
- GCTCACTATT CAACGTCTTC TTTCCCCCCT TCCCCTTTTG TAGCGCACCC TTCTTCGTCT TATCGTGTTA   
  
  
- TCTTCTCCAC CTAGACTCCT GAGAAGAGTG AGTGACACGA GTTCGTTAAA GTTTAAAACT AGAATCCTCA   
  
  
- CGTTTACTCG TAGAGTCCGT TTATTCCGTC GTAAGAAGTG TTATACCACT ATCGGAGGTC TCCGAACGGG   
  
  
- TAATAAAACG ATTACCAGAA CTCCGAGCGT ATCGACCGTG ACCAAGTTGT TAGAGACGAT TACAACAACT   
  
  
- ACGAGCTTAG TGCAGTAGAC TAAAGAATTT CCGAATATCC GATATATACA GTCGGCAAGG AAAGTTTTCC   
  
  
- TACAATATAA AAGAGCGGTT GTTCTGTTAT GCCTTCAACC GACTCTTCCG TTGGTCCTAG GTGTAGTAAC   
  
  
- TAAAACCTCA GAACAACCCA AATGTCACCG GGACAGAGTA TGTTTTGGAG AGTTTCTCTG GGTGACCGGG   
  
  
- CGGTTCGGAG GCATAATGAC CCTATCTGAT AGGGGTCGTA CCAAAGGCCG GTAGTCTTTC CCAACTTCGC   
  
  
- TGACCGGCAG CCAATCTTCC CATAACGCTC TCTAAATTAC ACGGCAAGTT CATATTCCGG TATCGATTTT   
  
  
- TAACCCTCTC ATATTTCGAT CTCCTAGATC TTTAACTACT CTTACTCTAC CACAAACAGT TAACGTACGA   
  
  
- AGCGAGACCT TGTGACGAAC TACTCTGTCA CCACCGTCTG TCAGGTTTCC TACGAAAGAA TTCCAATTAG   
  
  
- TCTCTCTATT TAGGGGCAGA AAAGTAAGTA CCCTGATAGT TACCTAGTAA GTTACGAGGT AAGAAGTAGT   
  
  
- GAGCCAAGTC CCTCCGCGAG AAGGTGATGA GAAGAAATAA ACTATAGAAA CTTCGTTGAT ACGGGGCACT   
  
  
- TCTAGTACTT TCCGAGGACC AACTCTCACT CTATGTCCCG TTTCTTCGAA ACTTACAGTA TCGTACGCTT   
  
  
- CCACGGCTCT CCTAAGTTTC CGGACTTTGT ATGTTCGTTA CCGTTCGTTC CTGTTGTTCC CGGCCCAAAT   
  
  
- CCGTCGAAGG TGATCTGGCC CTCGAACACT CGTCTCGTTT CCGATACCAC TTTCGTTTGA TGGTATTCCT   
  
  
- AAAACACCAC CTGCTCCTGT CCGTAACCTA CGATGTGCCA ACCTTTCCTT CTTAGAAGAC ACGTGAGAGA   
  
  
- CAAACCGTTG GGTTGAC

+     MYB-like sequence

| Site Name | Organism | Position | Strand | Matrix score. | sequence | function |
| --- | --- | --- | --- | --- | --- | --- |
| MYB-like sequence | Arabidopsis thaliana | 1102 | + | 6 | TAACCA |  |

>HU07G02246.1   
+ -Up\_Stream \_Len000ATTTGA TGTGTATGCT GACTACTTTT GATGATGATG ATCACAGTGA TGCATATTTA   
  
  
+ GAGCTGGTAA ATTGGTCATT CGATTCGAGT ACGGGTCGGG TCAAGTTCTG ATTAAGTGAC ATTTCGCGTC   
  
  
+ GTTTTGGTTT AGTTCGGGTC GGATCGATTT CGGGTTAAAT AATTTTTGGT GGAATACGCT TGTCATGCCA   
  
  
+ AACACAAGCA ACTTTGTTAA AAATTTCGAT TCGGGTCAGG TCAATTCAGG TTTGGGGTCC ACTTTCGAGT   
  
  
+ GAGCATATTT CGGATGTCGG GTCGCGTATG GGTCCGGGTC ATTCGGTTTG AATTTCGGAT CTTGGATCAA   
  
  
+ TTTTGCTAAG TATGTGCATA TTCAACCCTT GATGACATAG GAAACGCTTT GTCATCTCCA CCAATCAGAT   
  
  
+ ATCGTTATCT CTTTAATCAT CGCCATAATA GCATGAGTAA GCATGACCAT CTTTTTCAAA CATGTGATAC   
  
  
+ AGTTAAGGCC TGTTCTTTTC GTTAATAAAT CCCAATTTAT CAATTTTAAT ATATACTTTA ACGAATTTTA   
  
  
+ ACAAAAAATT TTAATTTCAA TCCGCTATAA CAAAAAAAAT ACCAACTTCA ACTTATTATA TAAAAAAAAT   
  
  
+ ATACAATTTT TTTTCAATTT TAGCCAACTT CAACAAAAAA AAAAAAAATT TAACAAATCT CAATCAACTT   
  
  
+ CACCCATTTC TCAACTTATA TCTCCTAATG AAAAGAACAG ATCCTTAGCA AGACTTCTCG ATTCATTTAA   
  
  
+ AAGGATAGTA TGGTAAATTC ATTTTATACA AGTGTCAAGT GTGAATATCT TTTTCACATC AGGTGATATA   
  
  
+ TTAGTAGGAC TTTTCGATTC ACTTATCTTC CTCTTTATGT TTTGTTAATG TGATAGTAAC GATAGGATTG   
  
  
+ CATGTATTTT TTCCAACTAT TCATTTACAA CTATCAATAT CTATTGATAA ATATGTTAAA ATAATTATAT   
  
  
+ GTTATTTAAA TTATTATTTG TTACTTAAGG TGTCGACTTT GTCGAAAGCG ACTTAATTCT AATTAAGATA   
  
  
+ GCGGCAGAGA AAGTTACACC TGATGACCAA GTTAGATCTG GTAAAACTAA CCATAGATCT GAAACCCAAA   
  
  
+ CATAATGATG TGTATTCATA CCCGACCTGA CATCTGAAAT AAGTTTACTC AAAAGTGGTT CCGAAACTTG   
  
  
+ AATTGACTCG AACCGAACCG TATTTGACTC GAAATTTTCT ACAAAGTTGC TTATTTTTGC ATGAAGACAT   
  
  
+ ATTGAGCCAA AAATTAGTTG ATTCGAAGTC AACTTAAACA AAAATTCATT CGACTCAAAC TGAAATATCA   
  
  
+ CTTAACTAAA ACTATGACTC AACTCAAACT TGATCTGATC CGTATTCAAA TCAAATACTC CATTTGCTAG   
  
  
+ GTCTAGAACC AGCCACGACC CCACATGGAC TATGCGGTTC AATGAAACGG CCAAATATAT CGCAATTTCT   
  
  
+ GTCTCCATCA ATCACACCCA TTTGCTACAC CCTGTTAATA TTTGACTCGA CCCTGTGAAT TGCCTTCGCC   
  
  
+ AGTTTTGTTT AATCTCTCTG GTTGCTTCCC ATCCAAGTCT GAATAGTATA GTTTGTACAG TGTGTACTAC   
  
  
+ TAGAAAGCAC GTTCCTTGAA TCTTCTTTCA AAGTTGTTTT TAAAGGGTTG AGTGATCCAT TACGACTTTT   
  
  
+ GATCTGGGTT CATCGCCTCC TCTCCTAATC CCTCACCCGG GTACCCTCAA TTCTCTATCG TCTCTGTTGC   
  
  
+ TCAGCTAATT CAAATTCCTT TAGTTGGTCA TCATTGAAAT CTGAAATCAG GGTGTTTTTC TTTTTATTAT   
  
  
+ TATCTGGCAG TTGGTTTGTT TATCTCTTGG TTTTTGGGGC TTATTTGGGT GCTGGTTGAT GTTATCCTGC   
  
  
+ TTGGGTTGAA TCTTCATCAG GTTTATTTTT GATTCATAGT TGAGGGTGAA AATTATTTCT CATACCCACC   
  
  
+ ACCTGTTTGT TCTTTTGTCT CTGAGAAAAA CCAAAAAGCT TTCTCTTTTT CCTGATGCAT CCTGTGCTCG   
  
  
+ TTGATCCTGA GTTAGTGAAC CATGCGTCCA AATTCAAGCC TGATTTGCTC TCAAACTTTG ATAAACAACA   
  
  
+ AGAATTTAGC AACGGGATCG AACAAGATGA CATCTTGCAA AACCCTGATC TGTGTTTTGA TTCAGAAACC   
  
  
+ CCTTCAGTTG ATCTAACTGA GAGTGTCATC AGTTCTGATT CTGTAGAAGT GCCTGATTTC TCAGATGCTT   
  
  
+ GCCTCAAGTT CATAAGTGAG ATTCTCCTAG AAGAGGATTT GGATGAAAGT CCTGCCTCTG CTCAAGAATT   
  
  
+ TAGGGCTCTC CAAGCCACTG AGAAGTCCCT GTATGATGCT CTTGGAGAGG GCTACTCTTT TTCATCTGAT   
  
  
+ AACAGCCCAT CATCATTAGG ACAGAGTATT GAGCACCAAA ATGAGAATTT TGAATTCAGT TCCGGTTATC   
  
  
+ CTGGAATTGA GGGCTATGTT AATGGTGATA TCACGTTCGA GTCCAACTGG ATGTTCAACC TAAGCCAATT   
  
  
+ GGATCCTGTT CTTACTTTGG ATGATATTTC TCAGCCCTTG TCTGCATCAA ACTCCCGATC ATCTGGGTCG   
  
  
+ AGCAATGGCT TTGATGATTC AGGGGATGGG GCAGCCACAT CTCCTGGCAG TACAGTTACA TCAACAGTCC   
  
  
+ CAGAGAAAAG GGTTGAATCT GTCAATCGCT CAAGGAGGAA GAAAAATCGT AAAAGGGATG AAGGTGGTCA   
  
  
+ TGAGGAAGGG AGGAGTAACA AGCAGCAAGC TTCATCCAAT GAAGATTATG TTGAGATGAA GGAGTTTGAT   
  
  
+ GATGTACTGC TCTGCAAAGA AGAGAAAGAT GTTATTGCAA ACTGCACCAA TGATTCCTCA CCCGTCGAAG   
  
  
+ CGAGTGATAA GTTGCAGAAG AAAGGGGGGA AGGGGAAAAC ATCGCGTGGG AAGAAGCAGA ATAGCACAAT   
  
  
+ AGAAGAGGTG GATCTGAGGA CTCTTCTCAC TCACTGTGCT CAAGCAATTT CAAATTTTGA TCTTAGGAGT   
  
  
+ GCAAATGAGC ATCTCAGGCA AATAAGGCAG CATTCTTCAC AATATGGTGA TAGCCTCCAG AGGCTTGCCC   
  
  
+ ATTATTTTGC TAATGGTCTT GAGGCTCGCA TAGCTGGCAC TGGTTCAACA ATCTCTGCTA ATGTTGTTGA   
  
  
+ TGCTCGAATC ACGTCATCTG ATTTCTTAAA GGCTTATAGG CTATATATGT CAGCCGTTCC TTTCAAAAGG   
  
  
+ ATGTTATATT TTCTCGCCAA CAAGACAATA CGGAAGTTGG CTGAGAAGGC AACCAGGATC CACATCATTG   
  
  
+ ATTTTGGAGT CTTGTTGGGT TTACAGTGGC CCTGTCTCAT ACAAAACCTC TCAAAGAGAC CCACTGGCCC   
  
  
+ GCCAAGCCTC CGTATTACTG GGATAGACTA TCCCCAGCAT GGTTTCCGGC CATCAGAAAG GGTTGAAGCG   
  
  
+ ACTGGCCGTC GGTTAGAAGG GTATTGCGAG AGATTTAATG TGCCGTTCAA GTATAAGGCC ATAGCTAAAA   
  
  
+ ATTGGGAGAG TATAAAGCTA GAGGATCTAG AAATTGATGA GAATGAGATG GTGTTTGTCA ATTGCATGCT   
  
  
+ TCGCTCTGGA ACACTGCTTG ATGAGACAGT GGTGGCAGAC AGTCCAAAGG ATGCTTTCTT AAGGTTAATC   
  
  
+ AGAGAGATAA ATCCCCGTCT TTTCATTCAT GGGACTATCA ATGGATCATT CAATGCTCCA TTCTTCATCA   
  
  
+ CTCGGTTCAG GGAGGCGCTC TTCCACTACT CTTCTTTATT TGATATCTTT GAAGCAACTA TGCCCCGTGA   
  
  
+ AGATCATGAA AGGCTCCTGG TTGAGAGTGA GATACAGGGC AAAGAAGCTT TGAATGTCAT AGCATGCGAA   
  
  
+ GGTGCCGAGA GGATTCAAAG GCCTGAAACA TACAAGCAAT GGCAAGCAAG GACAACAAGG GCCGGGTTTA   
  
  
+ GGCAGCTTCC ACTAGACCGG GAGCTTGTGA GCAGAGCAAA GGCTATGGTG AAAGCAAACT ACCATAAGGA   
  
  
+ TTTTGTGGTG GACGAGGACA GGCATTGGAT GCTACACGGT TGGAAAGGAA GAATCTTCTG TGCACTCTCT   
  
  
+ GTTTGGCAAC CCAACTG  

- -Up\_Stream \_Len000TAAACT ACACATACGA CTGATGAAAA CTACTACTAC TAGTGTCACT ACGTATAAAT   
  
  
- CTCGACCATT TAACCAGTAA GCTAAGCTCA TGCCCAGCCC AGTTCAAGAC TAATTCACTG TAAAGCGCAG   
  
  
- CAAAACCAAA TCAAGCCCAG CCTAGCTAAA GCCCAATTTA TTAAAAACCA CCTTATGCGA ACAGTACGGT   
  
  
- TTGTGTTCGT TGAAACAATT TTTAAAGCTA AGCCCAGTCC AGTTAAGTCC AAACCCCAGG TGAAAGCTCA   
  
  
- CTCGTATAAA GCCTACAGCC CAGCGCATAC CCAGGCCCAG TAAGCCAAAC TTAAAGCCTA GAACCTAGTT   
  
  
- AAAACGATTC ATACACGTAT AAGTTGGGAA CTACTGTATC CTTTGCGAAA CAGTAGAGGT GGTTAGTCTA   
  
  
- TAGCAATAGA GAAATTAGTA GCGGTATTAT CGTACTCATT CGTACTGGTA GAAAAAGTTT GTACACTATG   
  
  
- TCAATTCCGG ACAAGAAAAG CAATTATTTA GGGTTAAATA GTTAAAATTA TATATGAAAT TGCTTAAAAT   
  
  
- TGTTTTTTAA AATTAAAGTT AGGCGATATT GTTTTTTTTA TGGTTGAAGT TGAATAATAT ATTTTTTTTA   
  
  
- TATGTTAAAA AAAAGTTAAA ATCGGTTGAA GTTGTTTTTT TTTTTTTTAA ATTGTTTAGA GTTAGTTGAA   
  
  
- GTGGGTAAAG AGTTGAATAT AGAGGATTAC TTTTCTTGTC TAGGAATCGT TCTGAAGAGC TAAGTAAATT   
  
  
- TTCCTATCAT ACCATTTAAG TAAAATATGT TCACAGTTCA CACTTATAGA AAAAGTGTAG TCCACTATAT   
  
  
- AATCATCCTG AAAAGCTAAG TGAATAGAAG GAGAAATACA AAACAATTAC ACTATCATTG CTATCCTAAC   
  
  
- GTACATAAAA AAGGTTGATA AGTAAATGTT GATAGTTATA GATAACTATT TATACAATTT TATTAATATA   
  
  
- CAATAAATTT AATAATAAAC AATGAATTCC ACAGCTGAAA CAGCTTTCGC TGAATTAAGA TTAATTCTAT   
  
  
- CGCCGTCTCT TTCAATGTGG ACTACTGGTT CAATCTAGAC CATTTTGATT GGTATCTAGA CTTTGGGTTT   
  
  
- GTATTACTAC ACATAAGTAT GGGCTGGACT GTAGACTTTA TTCAAATGAG TTTTCACCAA GGCTTTGAAC   
  
  
- TTAACTGAGC TTGGCTTGGC ATAAACTGAG CTTTAAAAGA TGTTTCAACG AATAAAAACG TACTTCTGTA   
  
  
- TAACTCGGTT TTTAATCAAC TAAGCTTCAG TTGAATTTGT TTTTAAGTAA GCTGAGTTTG ACTTTATAGT   
  
  
- GAATTGATTT TGATACTGAG TTGAGTTTGA ACTAGACTAG GCATAAGTTT AGTTTATGAG GTAAACGATC   
  
  
- CAGATCTTGG TCGGTGCTGG GGTGTACCTG ATACGCCAAG TTACTTTGCC GGTTTATATA GCGTTAAAGA   
  
  
- CAGAGGTAGT TAGTGTGGGT AAACGATGTG GGACAATTAT AAACTGAGCT GGGACACTTA ACGGAAGCGG   
  
  
- TCAAAACAAA TTAGAGAGAC CAACGAAGGG TAGGTTCAGA CTTATCATAT CAAACATGTC ACACATGATG   
  
  
- ATCTTTCGTG CAAGGAACTT AGAAGAAAGT TTCAACAAAA ATTTCCCAAC TCACTAGGTA ATGCTGAAAA   
  
  
- CTAGACCCAA GTAGCGGAGG AGAGGATTAG GGAGTGGGCC CATGGGAGTT AAGAGATAGC AGAGACAACG   
  
  
- AGTCGATTAA GTTTAAGGAA ATCAACCAGT AGTAACTTTA GACTTTAGTC CCACAAAAAG AAAAATAATA   
  
  
- ATAGACCGTC AACCAAACAA ATAGAGAACC AAAAACCCCG AATAAACCCA CGACCAACTA CAATAGGACG   
  
  
- AACCCAACTT AGAAGTAGTC CAAATAAAAA CTAAGTATCA ACTCCCACTT TTAATAAAGA GTATGGGTGG   
  
  
- TGGACAAACA AGAAAACAGA GACTCTTTTT GGTTTTTCGA AAGAGAAAAA GGACTACGTA GGACACGAGC   
  
  
- AACTAGGACT CAATCACTTG GTACGCAGGT TTAAGTTCGG ACTAAACGAG AGTTTGAAAC TATTTGTTGT   
  
  
- TCTTAAATCG TTGCCCTAGC TTGTTCTACT GTAGAACGTT TTGGGACTAG ACACAAAACT AAGTCTTTGG   
  
  
- GGAAGTCAAC TAGATTGACT CTCACAGTAG TCAAGACTAA GACATCTTCA CGGACTAAAG AGTCTACGAA   
  
  
- CGGAGTTCAA GTATTCACTC TAAGAGGATC TTCTCCTAAA CCTACTTTCA GGACGGAGAC GAGTTCTTAA   
  
  
- ATCCCGAGAG GTTCGGTGAC TCTTCAGGGA CATACTACGA GAACCTCTCC CGATGAGAAA AAGTAGACTA   
  
  
- TTGTCGGGTA GTAGTAATCC TGTCTCATAA CTCGTGGTTT TACTCTTAAA ACTTAAGTCA AGGCCAATAG   
  
  
- GACCTTAACT CCCGATACAA TTACCACTAT AGTGCAAGCT CAGGTTGACC TACAAGTTGG ATTCGGTTAA   
  
  
- CCTAGGACAA GAATGAAACC TACTATAAAG AGTCGGGAAC AGACGTAGTT TGAGGGCTAG TAGACCCAGC   
  
  
- TCGTTACCGA AACTACTAAG TCCCCTACCC CGTCGGTGTA GAGGACCGTC ATGTCAATGT AGTTGTCAGG   
  
  
- GTCTCTTTTC CCAACTTAGA CAGTTAGCGA GTTCCTCCTT CTTTTTAGCA TTTTCCCTAC TTCCACCAGT   
  
  
- ACTCCTTCCC TCCTCATTGT TCGTCGTTCG AAGTAGGTTA CTTCTAATAC AACTCTACTT CCTCAAACTA   
  
  
- CTACATGACG AGACGTTTCT TCTCTTTCTA CAATAACGTT TGACGTGGTT ACTAAGGAGT GGGCAGCTTC   
  
  
- GCTCACTATT CAACGTCTTC TTTCCCCCCT TCCCCTTTTG TAGCGCACCC TTCTTCGTCT TATCGTGTTA   
  
  
- TCTTCTCCAC CTAGACTCCT GAGAAGAGTG AGTGACACGA GTTCGTTAAA GTTTAAAACT AGAATCCTCA   
  
  
- CGTTTACTCG TAGAGTCCGT TTATTCCGTC GTAAGAAGTG TTATACCACT ATCGGAGGTC TCCGAACGGG   
  
  
- TAATAAAACG ATTACCAGAA CTCCGAGCGT ATCGACCGTG ACCAAGTTGT TAGAGACGAT TACAACAACT   
  
  
- ACGAGCTTAG TGCAGTAGAC TAAAGAATTT CCGAATATCC GATATATACA GTCGGCAAGG AAAGTTTTCC   
  
  
- TACAATATAA AAGAGCGGTT GTTCTGTTAT GCCTTCAACC GACTCTTCCG TTGGTCCTAG GTGTAGTAAC   
  
  
- TAAAACCTCA GAACAACCCA AATGTCACCG GGACAGAGTA TGTTTTGGAG AGTTTCTCTG GGTGACCGGG   
  
  
- CGGTTCGGAG GCATAATGAC CCTATCTGAT AGGGGTCGTA CCAAAGGCCG GTAGTCTTTC CCAACTTCGC   
  
  
- TGACCGGCAG CCAATCTTCC CATAACGCTC TCTAAATTAC ACGGCAAGTT CATATTCCGG TATCGATTTT   
  
  
- TAACCCTCTC ATATTTCGAT CTCCTAGATC TTTAACTACT CTTACTCTAC CACAAACAGT TAACGTACGA   
  
  
- AGCGAGACCT TGTGACGAAC TACTCTGTCA CCACCGTCTG TCAGGTTTCC TACGAAAGAA TTCCAATTAG   
  
  
- TCTCTCTATT TAGGGGCAGA AAAGTAAGTA CCCTGATAGT TACCTAGTAA GTTACGAGGT AAGAAGTAGT   
  
  
- GAGCCAAGTC CCTCCGCGAG AAGGTGATGA GAAGAAATAA ACTATAGAAA CTTCGTTGAT ACGGGGCACT   
  
  
- TCTAGTACTT TCCGAGGACC AACTCTCACT CTATGTCCCG TTTCTTCGAA ACTTACAGTA TCGTACGCTT   
  
  
- CCACGGCTCT CCTAAGTTTC CGGACTTTGT ATGTTCGTTA CCGTTCGTTC CTGTTGTTCC CGGCCCAAAT   
  
  
- CCGTCGAAGG TGATCTGGCC CTCGAACACT CGTCTCGTTT CCGATACCAC TTTCGTTTGA TGGTATTCCT   
  
  
- AAAACACCAC CTGCTCCTGT CCGTAACCTA CGATGTGCCA ACCTTTCCTT CTTAGAAGAC ACGTGAGAGA   
  
  
- CAAACCGTTG GGTTGAC

+     MYC

| Site Name | Organism | Position | Strand | Matrix score. | sequence | function |
| --- | --- | --- | --- | --- | --- | --- |
| MYC | Arabidopsis thaliana | 3563 | - | 6 | CAATTG |  |
| MYC | Arabidopsis thaliana | 1426 | - | 6 | CATGTG |  |
| MYC | Arabidopsis thaliana | 485 | + | 6 | CATGTG |  |
| MYC | Arabidopsis thaliana | 2520 | - | 6 | CAATTG |  |
| MYC | Arabidopsis thaliana | 1395 | + | 6 | CATTTG |  |
| MYC | Arabidopsis thaliana | 3016 | - | 6 | CATTTG |  |
| MYC | Arabidopsis thaliana | 1493 | + | 6 | CATTTG |  |

>HU07G02246.1   
+ -Up\_Stream \_Len000ATTTGA TGTGTATGCT GACTACTTTT GATGATGATG ATCACAGTGA TGCATATTTA   
  
  
+ GAGCTGGTAA ATTGGTCATT CGATTCGAGT ACGGGTCGGG TCAAGTTCTG ATTAAGTGAC ATTTCGCGTC   
  
  
+ GTTTTGGTTT AGTTCGGGTC GGATCGATTT CGGGTTAAAT AATTTTTGGT GGAATACGCT TGTCATGCCA   
  
  
+ AACACAAGCA ACTTTGTTAA AAATTTCGAT TCGGGTCAGG TCAATTCAGG TTTGGGGTCC ACTTTCGAGT   
  
  
+ GAGCATATTT CGGATGTCGG GTCGCGTATG GGTCCGGGTC ATTCGGTTTG AATTTCGGAT CTTGGATCAA   
  
  
+ TTTTGCTAAG TATGTGCATA TTCAACCCTT GATGACATAG GAAACGCTTT GTCATCTCCA CCAATCAGAT   
  
  
+ ATCGTTATCT CTTTAATCAT CGCCATAATA GCATGAGTAA GCATGACCAT CTTTTTCAAA CATGTGATAC   
  
  
+ AGTTAAGGCC TGTTCTTTTC GTTAATAAAT CCCAATTTAT CAATTTTAAT ATATACTTTA ACGAATTTTA   
  
  
+ ACAAAAAATT TTAATTTCAA TCCGCTATAA CAAAAAAAAT ACCAACTTCA ACTTATTATA TAAAAAAAAT   
  
  
+ ATACAATTTT TTTTCAATTT TAGCCAACTT CAACAAAAAA AAAAAAAATT TAACAAATCT CAATCAACTT   
  
  
+ CACCCATTTC TCAACTTATA TCTCCTAATG AAAAGAACAG ATCCTTAGCA AGACTTCTCG ATTCATTTAA   
  
  
+ AAGGATAGTA TGGTAAATTC ATTTTATACA AGTGTCAAGT GTGAATATCT TTTTCACATC AGGTGATATA   
  
  
+ TTAGTAGGAC TTTTCGATTC ACTTATCTTC CTCTTTATGT TTTGTTAATG TGATAGTAAC GATAGGATTG   
  
  
+ CATGTATTTT TTCCAACTAT TCATTTACAA CTATCAATAT CTATTGATAA ATATGTTAAA ATAATTATAT   
  
  
+ GTTATTTAAA TTATTATTTG TTACTTAAGG TGTCGACTTT GTCGAAAGCG ACTTAATTCT AATTAAGATA   
  
  
+ GCGGCAGAGA AAGTTACACC TGATGACCAA GTTAGATCTG GTAAAACTAA CCATAGATCT GAAACCCAAA   
  
  
+ CATAATGATG TGTATTCATA CCCGACCTGA CATCTGAAAT AAGTTTACTC AAAAGTGGTT CCGAAACTTG   
  
  
+ AATTGACTCG AACCGAACCG TATTTGACTC GAAATTTTCT ACAAAGTTGC TTATTTTTGC ATGAAGACAT   
  
  
+ ATTGAGCCAA AAATTAGTTG ATTCGAAGTC AACTTAAACA AAAATTCATT CGACTCAAAC TGAAATATCA   
  
  
+ CTTAACTAAA ACTATGACTC AACTCAAACT TGATCTGATC CGTATTCAAA TCAAATACTC CATTTGCTAG   
  
  
+ GTCTAGAACC AGCCACGACC CCACATGGAC TATGCGGTTC AATGAAACGG CCAAATATAT CGCAATTTCT   
  
  
+ GTCTCCATCA ATCACACCCA TTTGCTACAC CCTGTTAATA TTTGACTCGA CCCTGTGAAT TGCCTTCGCC   
  
  
+ AGTTTTGTTT AATCTCTCTG GTTGCTTCCC ATCCAAGTCT GAATAGTATA GTTTGTACAG TGTGTACTAC   
  
  
+ TAGAAAGCAC GTTCCTTGAA TCTTCTTTCA AAGTTGTTTT TAAAGGGTTG AGTGATCCAT TACGACTTTT   
  
  
+ GATCTGGGTT CATCGCCTCC TCTCCTAATC CCTCACCCGG GTACCCTCAA TTCTCTATCG TCTCTGTTGC   
  
  
+ TCAGCTAATT CAAATTCCTT TAGTTGGTCA TCATTGAAAT CTGAAATCAG GGTGTTTTTC TTTTTATTAT   
  
  
+ TATCTGGCAG TTGGTTTGTT TATCTCTTGG TTTTTGGGGC TTATTTGGGT GCTGGTTGAT GTTATCCTGC   
  
  
+ TTGGGTTGAA TCTTCATCAG GTTTATTTTT GATTCATAGT TGAGGGTGAA AATTATTTCT CATACCCACC   
  
  
+ ACCTGTTTGT TCTTTTGTCT CTGAGAAAAA CCAAAAAGCT TTCTCTTTTT CCTGATGCAT CCTGTGCTCG   
  
  
+ TTGATCCTGA GTTAGTGAAC CATGCGTCCA AATTCAAGCC TGATTTGCTC TCAAACTTTG ATAAACAACA   
  
  
+ AGAATTTAGC AACGGGATCG AACAAGATGA CATCTTGCAA AACCCTGATC TGTGTTTTGA TTCAGAAACC   
  
  
+ CCTTCAGTTG ATCTAACTGA GAGTGTCATC AGTTCTGATT CTGTAGAAGT GCCTGATTTC TCAGATGCTT   
  
  
+ GCCTCAAGTT CATAAGTGAG ATTCTCCTAG AAGAGGATTT GGATGAAAGT CCTGCCTCTG CTCAAGAATT   
  
  
+ TAGGGCTCTC CAAGCCACTG AGAAGTCCCT GTATGATGCT CTTGGAGAGG GCTACTCTTT TTCATCTGAT   
  
  
+ AACAGCCCAT CATCATTAGG ACAGAGTATT GAGCACCAAA ATGAGAATTT TGAATTCAGT TCCGGTTATC   
  
  
+ CTGGAATTGA GGGCTATGTT AATGGTGATA TCACGTTCGA GTCCAACTGG ATGTTCAACC TAAGCCAATT   
  
  
+ GGATCCTGTT CTTACTTTGG ATGATATTTC TCAGCCCTTG TCTGCATCAA ACTCCCGATC ATCTGGGTCG   
  
  
+ AGCAATGGCT TTGATGATTC AGGGGATGGG GCAGCCACAT CTCCTGGCAG TACAGTTACA TCAACAGTCC   
  
  
+ CAGAGAAAAG GGTTGAATCT GTCAATCGCT CAAGGAGGAA GAAAAATCGT AAAAGGGATG AAGGTGGTCA   
  
  
+ TGAGGAAGGG AGGAGTAACA AGCAGCAAGC TTCATCCAAT GAAGATTATG TTGAGATGAA GGAGTTTGAT   
  
  
+ GATGTACTGC TCTGCAAAGA AGAGAAAGAT GTTATTGCAA ACTGCACCAA TGATTCCTCA CCCGTCGAAG   
  
  
+ CGAGTGATAA GTTGCAGAAG AAAGGGGGGA AGGGGAAAAC ATCGCGTGGG AAGAAGCAGA ATAGCACAAT   
  
  
+ AGAAGAGGTG GATCTGAGGA CTCTTCTCAC TCACTGTGCT CAAGCAATTT CAAATTTTGA TCTTAGGAGT   
  
  
+ GCAAATGAGC ATCTCAGGCA AATAAGGCAG CATTCTTCAC AATATGGTGA TAGCCTCCAG AGGCTTGCCC   
  
  
+ ATTATTTTGC TAATGGTCTT GAGGCTCGCA TAGCTGGCAC TGGTTCAACA ATCTCTGCTA ATGTTGTTGA   
  
  
+ TGCTCGAATC ACGTCATCTG ATTTCTTAAA GGCTTATAGG CTATATATGT CAGCCGTTCC TTTCAAAAGG   
  
  
+ ATGTTATATT TTCTCGCCAA CAAGACAATA CGGAAGTTGG CTGAGAAGGC AACCAGGATC CACATCATTG   
  
  
+ ATTTTGGAGT CTTGTTGGGT TTACAGTGGC CCTGTCTCAT ACAAAACCTC TCAAAGAGAC CCACTGGCCC   
  
  
+ GCCAAGCCTC CGTATTACTG GGATAGACTA TCCCCAGCAT GGTTTCCGGC CATCAGAAAG GGTTGAAGCG   
  
  
+ ACTGGCCGTC GGTTAGAAGG GTATTGCGAG AGATTTAATG TGCCGTTCAA GTATAAGGCC ATAGCTAAAA   
  
  
+ ATTGGGAGAG TATAAAGCTA GAGGATCTAG AAATTGATGA GAATGAGATG GTGTTTGTCA ATTGCATGCT   
  
  
+ TCGCTCTGGA ACACTGCTTG ATGAGACAGT GGTGGCAGAC AGTCCAAAGG ATGCTTTCTT AAGGTTAATC   
  
  
+ AGAGAGATAA ATCCCCGTCT TTTCATTCAT GGGACTATCA ATGGATCATT CAATGCTCCA TTCTTCATCA   
  
  
+ CTCGGTTCAG GGAGGCGCTC TTCCACTACT CTTCTTTATT TGATATCTTT GAAGCAACTA TGCCCCGTGA   
  
  
+ AGATCATGAA AGGCTCCTGG TTGAGAGTGA GATACAGGGC AAAGAAGCTT TGAATGTCAT AGCATGCGAA   
  
  
+ GGTGCCGAGA GGATTCAAAG GCCTGAAACA TACAAGCAAT GGCAAGCAAG GACAACAAGG GCCGGGTTTA   
  
  
+ GGCAGCTTCC ACTAGACCGG GAGCTTGTGA GCAGAGCAAA GGCTATGGTG AAAGCAAACT ACCATAAGGA   
  
  
+ TTTTGTGGTG GACGAGGACA GGCATTGGAT GCTACACGGT TGGAAAGGAA GAATCTTCTG TGCACTCTCT   
  
  
+ GTTTGGCAAC CCAACTG  

- -Up\_Stream \_Len000TAAACT ACACATACGA CTGATGAAAA CTACTACTAC TAGTGTCACT ACGTATAAAT   
  
  
- CTCGACCATT TAACCAGTAA GCTAAGCTCA TGCCCAGCCC AGTTCAAGAC TAATTCACTG TAAAGCGCAG   
  
  
- CAAAACCAAA TCAAGCCCAG CCTAGCTAAA GCCCAATTTA TTAAAAACCA CCTTATGCGA ACAGTACGGT   
  
  
- TTGTGTTCGT TGAAACAATT TTTAAAGCTA AGCCCAGTCC AGTTAAGTCC AAACCCCAGG TGAAAGCTCA   
  
  
- CTCGTATAAA GCCTACAGCC CAGCGCATAC CCAGGCCCAG TAAGCCAAAC TTAAAGCCTA GAACCTAGTT   
  
  
- AAAACGATTC ATACACGTAT AAGTTGGGAA CTACTGTATC CTTTGCGAAA CAGTAGAGGT GGTTAGTCTA   
  
  
- TAGCAATAGA GAAATTAGTA GCGGTATTAT CGTACTCATT CGTACTGGTA GAAAAAGTTT GTACACTATG   
  
  
- TCAATTCCGG ACAAGAAAAG CAATTATTTA GGGTTAAATA GTTAAAATTA TATATGAAAT TGCTTAAAAT   
  
  
- TGTTTTTTAA AATTAAAGTT AGGCGATATT GTTTTTTTTA TGGTTGAAGT TGAATAATAT ATTTTTTTTA   
  
  
- TATGTTAAAA AAAAGTTAAA ATCGGTTGAA GTTGTTTTTT TTTTTTTTAA ATTGTTTAGA GTTAGTTGAA   
  
  
- GTGGGTAAAG AGTTGAATAT AGAGGATTAC TTTTCTTGTC TAGGAATCGT TCTGAAGAGC TAAGTAAATT   
  
  
- TTCCTATCAT ACCATTTAAG TAAAATATGT TCACAGTTCA CACTTATAGA AAAAGTGTAG TCCACTATAT   
  
  
- AATCATCCTG AAAAGCTAAG TGAATAGAAG GAGAAATACA AAACAATTAC ACTATCATTG CTATCCTAAC   
  
  
- GTACATAAAA AAGGTTGATA AGTAAATGTT GATAGTTATA GATAACTATT TATACAATTT TATTAATATA   
  
  
- CAATAAATTT AATAATAAAC AATGAATTCC ACAGCTGAAA CAGCTTTCGC TGAATTAAGA TTAATTCTAT   
  
  
- CGCCGTCTCT TTCAATGTGG ACTACTGGTT CAATCTAGAC CATTTTGATT GGTATCTAGA CTTTGGGTTT   
  
  
- GTATTACTAC ACATAAGTAT GGGCTGGACT GTAGACTTTA TTCAAATGAG TTTTCACCAA GGCTTTGAAC   
  
  
- TTAACTGAGC TTGGCTTGGC ATAAACTGAG CTTTAAAAGA TGTTTCAACG AATAAAAACG TACTTCTGTA   
  
  
- TAACTCGGTT TTTAATCAAC TAAGCTTCAG TTGAATTTGT TTTTAAGTAA GCTGAGTTTG ACTTTATAGT   
  
  
- GAATTGATTT TGATACTGAG TTGAGTTTGA ACTAGACTAG GCATAAGTTT AGTTTATGAG GTAAACGATC   
  
  
- CAGATCTTGG TCGGTGCTGG GGTGTACCTG ATACGCCAAG TTACTTTGCC GGTTTATATA GCGTTAAAGA   
  
  
- CAGAGGTAGT TAGTGTGGGT AAACGATGTG GGACAATTAT AAACTGAGCT GGGACACTTA ACGGAAGCGG   
  
  
- TCAAAACAAA TTAGAGAGAC CAACGAAGGG TAGGTTCAGA CTTATCATAT CAAACATGTC ACACATGATG   
  
  
- ATCTTTCGTG CAAGGAACTT AGAAGAAAGT TTCAACAAAA ATTTCCCAAC TCACTAGGTA ATGCTGAAAA   
  
  
- CTAGACCCAA GTAGCGGAGG AGAGGATTAG GGAGTGGGCC CATGGGAGTT AAGAGATAGC AGAGACAACG   
  
  
- AGTCGATTAA GTTTAAGGAA ATCAACCAGT AGTAACTTTA GACTTTAGTC CCACAAAAAG AAAAATAATA   
  
  
- ATAGACCGTC AACCAAACAA ATAGAGAACC AAAAACCCCG AATAAACCCA CGACCAACTA CAATAGGACG   
  
  
- AACCCAACTT AGAAGTAGTC CAAATAAAAA CTAAGTATCA ACTCCCACTT TTAATAAAGA GTATGGGTGG   
  
  
- TGGACAAACA AGAAAACAGA GACTCTTTTT GGTTTTTCGA AAGAGAAAAA GGACTACGTA GGACACGAGC   
  
  
- AACTAGGACT CAATCACTTG GTACGCAGGT TTAAGTTCGG ACTAAACGAG AGTTTGAAAC TATTTGTTGT   
  
  
- TCTTAAATCG TTGCCCTAGC TTGTTCTACT GTAGAACGTT TTGGGACTAG ACACAAAACT AAGTCTTTGG   
  
  
- GGAAGTCAAC TAGATTGACT CTCACAGTAG TCAAGACTAA GACATCTTCA CGGACTAAAG AGTCTACGAA   
  
  
- CGGAGTTCAA GTATTCACTC TAAGAGGATC TTCTCCTAAA CCTACTTTCA GGACGGAGAC GAGTTCTTAA   
  
  
- ATCCCGAGAG GTTCGGTGAC TCTTCAGGGA CATACTACGA GAACCTCTCC CGATGAGAAA AAGTAGACTA   
  
  
- TTGTCGGGTA GTAGTAATCC TGTCTCATAA CTCGTGGTTT TACTCTTAAA ACTTAAGTCA AGGCCAATAG   
  
  
- GACCTTAACT CCCGATACAA TTACCACTAT AGTGCAAGCT CAGGTTGACC TACAAGTTGG ATTCGGTTAA   
  
  
- CCTAGGACAA GAATGAAACC TACTATAAAG AGTCGGGAAC AGACGTAGTT TGAGGGCTAG TAGACCCAGC   
  
  
- TCGTTACCGA AACTACTAAG TCCCCTACCC CGTCGGTGTA GAGGACCGTC ATGTCAATGT AGTTGTCAGG   
  
  
- GTCTCTTTTC CCAACTTAGA CAGTTAGCGA GTTCCTCCTT CTTTTTAGCA TTTTCCCTAC TTCCACCAGT   
  
  
- ACTCCTTCCC TCCTCATTGT TCGTCGTTCG AAGTAGGTTA CTTCTAATAC AACTCTACTT CCTCAAACTA   
  
  
- CTACATGACG AGACGTTTCT TCTCTTTCTA CAATAACGTT TGACGTGGTT ACTAAGGAGT GGGCAGCTTC   
  
  
- GCTCACTATT CAACGTCTTC TTTCCCCCCT TCCCCTTTTG TAGCGCACCC TTCTTCGTCT TATCGTGTTA   
  
  
- TCTTCTCCAC CTAGACTCCT GAGAAGAGTG AGTGACACGA GTTCGTTAAA GTTTAAAACT AGAATCCTCA   
  
  
- CGTTTACTCG TAGAGTCCGT TTATTCCGTC GTAAGAAGTG TTATACCACT ATCGGAGGTC TCCGAACGGG   
  
  
- TAATAAAACG ATTACCAGAA CTCCGAGCGT ATCGACCGTG ACCAAGTTGT TAGAGACGAT TACAACAACT   
  
  
- ACGAGCTTAG TGCAGTAGAC TAAAGAATTT CCGAATATCC GATATATACA GTCGGCAAGG AAAGTTTTCC   
  
  
- TACAATATAA AAGAGCGGTT GTTCTGTTAT GCCTTCAACC GACTCTTCCG TTGGTCCTAG GTGTAGTAAC   
  
  
- TAAAACCTCA GAACAACCCA AATGTCACCG GGACAGAGTA TGTTTTGGAG AGTTTCTCTG GGTGACCGGG   
  
  
- CGGTTCGGAG GCATAATGAC CCTATCTGAT AGGGGTCGTA CCAAAGGCCG GTAGTCTTTC CCAACTTCGC   
  
  
- TGACCGGCAG CCAATCTTCC CATAACGCTC TCTAAATTAC ACGGCAAGTT CATATTCCGG TATCGATTTT   
  
  
- TAACCCTCTC ATATTTCGAT CTCCTAGATC TTTAACTACT CTTACTCTAC CACAAACAGT TAACGTACGA   
  
  
- AGCGAGACCT TGTGACGAAC TACTCTGTCA CCACCGTCTG TCAGGTTTCC TACGAAAGAA TTCCAATTAG   
  
  
- TCTCTCTATT TAGGGGCAGA AAAGTAAGTA CCCTGATAGT TACCTAGTAA GTTACGAGGT AAGAAGTAGT   
  
  
- GAGCCAAGTC CCTCCGCGAG AAGGTGATGA GAAGAAATAA ACTATAGAAA CTTCGTTGAT ACGGGGCACT   
  
  
- TCTAGTACTT TCCGAGGACC AACTCTCACT CTATGTCCCG TTTCTTCGAA ACTTACAGTA TCGTACGCTT   
  
  
- CCACGGCTCT CCTAAGTTTC CGGACTTTGT ATGTTCGTTA CCGTTCGTTC CTGTTGTTCC CGGCCCAAAT   
  
  
- CCGTCGAAGG TGATCTGGCC CTCGAACACT CGTCTCGTTT CCGATACCAC TTTCGTTTGA TGGTATTCCT   
  
  
- AAAACACCAC CTGCTCCTGT CCGTAACCTA CGATGTGCCA ACCTTTCCTT CTTAGAAGAC ACGTGAGAGA   
  
  
- CAAACCGTTG GGTTGAC

+     Myb

| Site Name | Organism | Position | Strand | Matrix score. | sequence | function |
| --- | --- | --- | --- | --- | --- | --- |
| Myb | Arabidopsis thaliana | 2188 | + | 6 | TAACTG |  |
| Myb | Arabidopsis thaliana | 4076 | + | 6 | CAACTG |  |
| Myb | Arabidopsis thaliana | 494 | - | 6 | TAACTG |  |
| Myb | Arabidopsis thaliana | 2498 | + | 6 | CAACTG |  |
| Myb | Arabidopsis thaliana | 2179 | - | 6 | CAACTG |  |
| Myb | Arabidopsis thaliana | 1832 | - | 6 | CAACTG |  |
| Myb | Arabidopsis thaliana | 2647 | - | 6 | TAACTG |  |

>HU07G02246.1   
+ -Up\_Stream \_Len000ATTTGA TGTGTATGCT GACTACTTTT GATGATGATG ATCACAGTGA TGCATATTTA   
  
  
+ GAGCTGGTAA ATTGGTCATT CGATTCGAGT ACGGGTCGGG TCAAGTTCTG ATTAAGTGAC ATTTCGCGTC   
  
  
+ GTTTTGGTTT AGTTCGGGTC GGATCGATTT CGGGTTAAAT AATTTTTGGT GGAATACGCT TGTCATGCCA   
  
  
+ AACACAAGCA ACTTTGTTAA AAATTTCGAT TCGGGTCAGG TCAATTCAGG TTTGGGGTCC ACTTTCGAGT   
  
  
+ GAGCATATTT CGGATGTCGG GTCGCGTATG GGTCCGGGTC ATTCGGTTTG AATTTCGGAT CTTGGATCAA   
  
  
+ TTTTGCTAAG TATGTGCATA TTCAACCCTT GATGACATAG GAAACGCTTT GTCATCTCCA CCAATCAGAT   
  
  
+ ATCGTTATCT CTTTAATCAT CGCCATAATA GCATGAGTAA GCATGACCAT CTTTTTCAAA CATGTGATAC   
  
  
+ AGTTAAGGCC TGTTCTTTTC GTTAATAAAT CCCAATTTAT CAATTTTAAT ATATACTTTA ACGAATTTTA   
  
  
+ ACAAAAAATT TTAATTTCAA TCCGCTATAA CAAAAAAAAT ACCAACTTCA ACTTATTATA TAAAAAAAAT   
  
  
+ ATACAATTTT TTTTCAATTT TAGCCAACTT CAACAAAAAA AAAAAAAATT TAACAAATCT CAATCAACTT   
  
  
+ CACCCATTTC TCAACTTATA TCTCCTAATG AAAAGAACAG ATCCTTAGCA AGACTTCTCG ATTCATTTAA   
  
  
+ AAGGATAGTA TGGTAAATTC ATTTTATACA AGTGTCAAGT GTGAATATCT TTTTCACATC AGGTGATATA   
  
  
+ TTAGTAGGAC TTTTCGATTC ACTTATCTTC CTCTTTATGT TTTGTTAATG TGATAGTAAC GATAGGATTG   
  
  
+ CATGTATTTT TTCCAACTAT TCATTTACAA CTATCAATAT CTATTGATAA ATATGTTAAA ATAATTATAT   
  
  
+ GTTATTTAAA TTATTATTTG TTACTTAAGG TGTCGACTTT GTCGAAAGCG ACTTAATTCT AATTAAGATA   
  
  
+ GCGGCAGAGA AAGTTACACC TGATGACCAA GTTAGATCTG GTAAAACTAA CCATAGATCT GAAACCCAAA   
  
  
+ CATAATGATG TGTATTCATA CCCGACCTGA CATCTGAAAT AAGTTTACTC AAAAGTGGTT CCGAAACTTG   
  
  
+ AATTGACTCG AACCGAACCG TATTTGACTC GAAATTTTCT ACAAAGTTGC TTATTTTTGC ATGAAGACAT   
  
  
+ ATTGAGCCAA AAATTAGTTG ATTCGAAGTC AACTTAAACA AAAATTCATT CGACTCAAAC TGAAATATCA   
  
  
+ CTTAACTAAA ACTATGACTC AACTCAAACT TGATCTGATC CGTATTCAAA TCAAATACTC CATTTGCTAG   
  
  
+ GTCTAGAACC AGCCACGACC CCACATGGAC TATGCGGTTC AATGAAACGG CCAAATATAT CGCAATTTCT   
  
  
+ GTCTCCATCA ATCACACCCA TTTGCTACAC CCTGTTAATA TTTGACTCGA CCCTGTGAAT TGCCTTCGCC   
  
  
+ AGTTTTGTTT AATCTCTCTG GTTGCTTCCC ATCCAAGTCT GAATAGTATA GTTTGTACAG TGTGTACTAC   
  
  
+ TAGAAAGCAC GTTCCTTGAA TCTTCTTTCA AAGTTGTTTT TAAAGGGTTG AGTGATCCAT TACGACTTTT   
  
  
+ GATCTGGGTT CATCGCCTCC TCTCCTAATC CCTCACCCGG GTACCCTCAA TTCTCTATCG TCTCTGTTGC   
  
  
+ TCAGCTAATT CAAATTCCTT TAGTTGGTCA TCATTGAAAT CTGAAATCAG GGTGTTTTTC TTTTTATTAT   
  
  
+ TATCTGGCAG TTGGTTTGTT TATCTCTTGG TTTTTGGGGC TTATTTGGGT GCTGGTTGAT GTTATCCTGC   
  
  
+ TTGGGTTGAA TCTTCATCAG GTTTATTTTT GATTCATAGT TGAGGGTGAA AATTATTTCT CATACCCACC   
  
  
+ ACCTGTTTGT TCTTTTGTCT CTGAGAAAAA CCAAAAAGCT TTCTCTTTTT CCTGATGCAT CCTGTGCTCG   
  
  
+ TTGATCCTGA GTTAGTGAAC CATGCGTCCA AATTCAAGCC TGATTTGCTC TCAAACTTTG ATAAACAACA   
  
  
+ AGAATTTAGC AACGGGATCG AACAAGATGA CATCTTGCAA AACCCTGATC TGTGTTTTGA TTCAGAAACC   
  
  
+ CCTTCAGTTG ATCTAACTGA GAGTGTCATC AGTTCTGATT CTGTAGAAGT GCCTGATTTC TCAGATGCTT   
  
  
+ GCCTCAAGTT CATAAGTGAG ATTCTCCTAG AAGAGGATTT GGATGAAAGT CCTGCCTCTG CTCAAGAATT   
  
  
+ TAGGGCTCTC CAAGCCACTG AGAAGTCCCT GTATGATGCT CTTGGAGAGG GCTACTCTTT TTCATCTGAT   
  
  
+ AACAGCCCAT CATCATTAGG ACAGAGTATT GAGCACCAAA ATGAGAATTT TGAATTCAGT TCCGGTTATC   
  
  
+ CTGGAATTGA GGGCTATGTT AATGGTGATA TCACGTTCGA GTCCAACTGG ATGTTCAACC TAAGCCAATT   
  
  
+ GGATCCTGTT CTTACTTTGG ATGATATTTC TCAGCCCTTG TCTGCATCAA ACTCCCGATC ATCTGGGTCG   
  
  
+ AGCAATGGCT TTGATGATTC AGGGGATGGG GCAGCCACAT CTCCTGGCAG TACAGTTACA TCAACAGTCC   
  
  
+ CAGAGAAAAG GGTTGAATCT GTCAATCGCT CAAGGAGGAA GAAAAATCGT AAAAGGGATG AAGGTGGTCA   
  
  
+ TGAGGAAGGG AGGAGTAACA AGCAGCAAGC TTCATCCAAT GAAGATTATG TTGAGATGAA GGAGTTTGAT   
  
  
+ GATGTACTGC TCTGCAAAGA AGAGAAAGAT GTTATTGCAA ACTGCACCAA TGATTCCTCA CCCGTCGAAG   
  
  
+ CGAGTGATAA GTTGCAGAAG AAAGGGGGGA AGGGGAAAAC ATCGCGTGGG AAGAAGCAGA ATAGCACAAT   
  
  
+ AGAAGAGGTG GATCTGAGGA CTCTTCTCAC TCACTGTGCT CAAGCAATTT CAAATTTTGA TCTTAGGAGT   
  
  
+ GCAAATGAGC ATCTCAGGCA AATAAGGCAG CATTCTTCAC AATATGGTGA TAGCCTCCAG AGGCTTGCCC   
  
  
+ ATTATTTTGC TAATGGTCTT GAGGCTCGCA TAGCTGGCAC TGGTTCAACA ATCTCTGCTA ATGTTGTTGA   
  
  
+ TGCTCGAATC ACGTCATCTG ATTTCTTAAA GGCTTATAGG CTATATATGT CAGCCGTTCC TTTCAAAAGG   
  
  
+ ATGTTATATT TTCTCGCCAA CAAGACAATA CGGAAGTTGG CTGAGAAGGC AACCAGGATC CACATCATTG   
  
  
+ ATTTTGGAGT CTTGTTGGGT TTACAGTGGC CCTGTCTCAT ACAAAACCTC TCAAAGAGAC CCACTGGCCC   
  
  
+ GCCAAGCCTC CGTATTACTG GGATAGACTA TCCCCAGCAT GGTTTCCGGC CATCAGAAAG GGTTGAAGCG   
  
  
+ ACTGGCCGTC GGTTAGAAGG GTATTGCGAG AGATTTAATG TGCCGTTCAA GTATAAGGCC ATAGCTAAAA   
  
  
+ ATTGGGAGAG TATAAAGCTA GAGGATCTAG AAATTGATGA GAATGAGATG GTGTTTGTCA ATTGCATGCT   
  
  
+ TCGCTCTGGA ACACTGCTTG ATGAGACAGT GGTGGCAGAC AGTCCAAAGG ATGCTTTCTT AAGGTTAATC   
  
  
+ AGAGAGATAA ATCCCCGTCT TTTCATTCAT GGGACTATCA ATGGATCATT CAATGCTCCA TTCTTCATCA   
  
  
+ CTCGGTTCAG GGAGGCGCTC TTCCACTACT CTTCTTTATT TGATATCTTT GAAGCAACTA TGCCCCGTGA   
  
  
+ AGATCATGAA AGGCTCCTGG TTGAGAGTGA GATACAGGGC AAAGAAGCTT TGAATGTCAT AGCATGCGAA   
  
  
+ GGTGCCGAGA GGATTCAAAG GCCTGAAACA TACAAGCAAT GGCAAGCAAG GACAACAAGG GCCGGGTTTA   
  
  
+ GGCAGCTTCC ACTAGACCGG GAGCTTGTGA GCAGAGCAAA GGCTATGGTG AAAGCAAACT ACCATAAGGA   
  
  
+ TTTTGTGGTG GACGAGGACA GGCATTGGAT GCTACACGGT TGGAAAGGAA GAATCTTCTG TGCACTCTCT   
  
  
+ GTTTGGCAAC CCAACTG  

- -Up\_Stream \_Len000TAAACT ACACATACGA CTGATGAAAA CTACTACTAC TAGTGTCACT ACGTATAAAT   
  
  
- CTCGACCATT TAACCAGTAA GCTAAGCTCA TGCCCAGCCC AGTTCAAGAC TAATTCACTG TAAAGCGCAG   
  
  
- CAAAACCAAA TCAAGCCCAG CCTAGCTAAA GCCCAATTTA TTAAAAACCA CCTTATGCGA ACAGTACGGT   
  
  
- TTGTGTTCGT TGAAACAATT TTTAAAGCTA AGCCCAGTCC AGTTAAGTCC AAACCCCAGG TGAAAGCTCA   
  
  
- CTCGTATAAA GCCTACAGCC CAGCGCATAC CCAGGCCCAG TAAGCCAAAC TTAAAGCCTA GAACCTAGTT   
  
  
- AAAACGATTC ATACACGTAT AAGTTGGGAA CTACTGTATC CTTTGCGAAA CAGTAGAGGT GGTTAGTCTA   
  
  
- TAGCAATAGA GAAATTAGTA GCGGTATTAT CGTACTCATT CGTACTGGTA GAAAAAGTTT GTACACTATG   
  
  
- TCAATTCCGG ACAAGAAAAG CAATTATTTA GGGTTAAATA GTTAAAATTA TATATGAAAT TGCTTAAAAT   
  
  
- TGTTTTTTAA AATTAAAGTT AGGCGATATT GTTTTTTTTA TGGTTGAAGT TGAATAATAT ATTTTTTTTA   
  
  
- TATGTTAAAA AAAAGTTAAA ATCGGTTGAA GTTGTTTTTT TTTTTTTTAA ATTGTTTAGA GTTAGTTGAA   
  
  
- GTGGGTAAAG AGTTGAATAT AGAGGATTAC TTTTCTTGTC TAGGAATCGT TCTGAAGAGC TAAGTAAATT   
  
  
- TTCCTATCAT ACCATTTAAG TAAAATATGT TCACAGTTCA CACTTATAGA AAAAGTGTAG TCCACTATAT   
  
  
- AATCATCCTG AAAAGCTAAG TGAATAGAAG GAGAAATACA AAACAATTAC ACTATCATTG CTATCCTAAC   
  
  
- GTACATAAAA AAGGTTGATA AGTAAATGTT GATAGTTATA GATAACTATT TATACAATTT TATTAATATA   
  
  
- CAATAAATTT AATAATAAAC AATGAATTCC ACAGCTGAAA CAGCTTTCGC TGAATTAAGA TTAATTCTAT   
  
  
- CGCCGTCTCT TTCAATGTGG ACTACTGGTT CAATCTAGAC CATTTTGATT GGTATCTAGA CTTTGGGTTT   
  
  
- GTATTACTAC ACATAAGTAT GGGCTGGACT GTAGACTTTA TTCAAATGAG TTTTCACCAA GGCTTTGAAC   
  
  
- TTAACTGAGC TTGGCTTGGC ATAAACTGAG CTTTAAAAGA TGTTTCAACG AATAAAAACG TACTTCTGTA   
  
  
- TAACTCGGTT TTTAATCAAC TAAGCTTCAG TTGAATTTGT TTTTAAGTAA GCTGAGTTTG ACTTTATAGT   
  
  
- GAATTGATTT TGATACTGAG TTGAGTTTGA ACTAGACTAG GCATAAGTTT AGTTTATGAG GTAAACGATC   
  
  
- CAGATCTTGG TCGGTGCTGG GGTGTACCTG ATACGCCAAG TTACTTTGCC GGTTTATATA GCGTTAAAGA   
  
  
- CAGAGGTAGT TAGTGTGGGT AAACGATGTG GGACAATTAT AAACTGAGCT GGGACACTTA ACGGAAGCGG   
  
  
- TCAAAACAAA TTAGAGAGAC CAACGAAGGG TAGGTTCAGA CTTATCATAT CAAACATGTC ACACATGATG   
  
  
- ATCTTTCGTG CAAGGAACTT AGAAGAAAGT TTCAACAAAA ATTTCCCAAC TCACTAGGTA ATGCTGAAAA   
  
  
- CTAGACCCAA GTAGCGGAGG AGAGGATTAG GGAGTGGGCC CATGGGAGTT AAGAGATAGC AGAGACAACG   
  
  
- AGTCGATTAA GTTTAAGGAA ATCAACCAGT AGTAACTTTA GACTTTAGTC CCACAAAAAG AAAAATAATA   
  
  
- ATAGACCGTC AACCAAACAA ATAGAGAACC AAAAACCCCG AATAAACCCA CGACCAACTA CAATAGGACG   
  
  
- AACCCAACTT AGAAGTAGTC CAAATAAAAA CTAAGTATCA ACTCCCACTT TTAATAAAGA GTATGGGTGG   
  
  
- TGGACAAACA AGAAAACAGA GACTCTTTTT GGTTTTTCGA AAGAGAAAAA GGACTACGTA GGACACGAGC   
  
  
- AACTAGGACT CAATCACTTG GTACGCAGGT TTAAGTTCGG ACTAAACGAG AGTTTGAAAC TATTTGTTGT   
  
  
- TCTTAAATCG TTGCCCTAGC TTGTTCTACT GTAGAACGTT TTGGGACTAG ACACAAAACT AAGTCTTTGG   
  
  
- GGAAGTCAAC TAGATTGACT CTCACAGTAG TCAAGACTAA GACATCTTCA CGGACTAAAG AGTCTACGAA   
  
  
- CGGAGTTCAA GTATTCACTC TAAGAGGATC TTCTCCTAAA CCTACTTTCA GGACGGAGAC GAGTTCTTAA   
  
  
- ATCCCGAGAG GTTCGGTGAC TCTTCAGGGA CATACTACGA GAACCTCTCC CGATGAGAAA AAGTAGACTA   
  
  
- TTGTCGGGTA GTAGTAATCC TGTCTCATAA CTCGTGGTTT TACTCTTAAA ACTTAAGTCA AGGCCAATAG   
  
  
- GACCTTAACT CCCGATACAA TTACCACTAT AGTGCAAGCT CAGGTTGACC TACAAGTTGG ATTCGGTTAA   
  
  
- CCTAGGACAA GAATGAAACC TACTATAAAG AGTCGGGAAC AGACGTAGTT TGAGGGCTAG TAGACCCAGC   
  
  
- TCGTTACCGA AACTACTAAG TCCCCTACCC CGTCGGTGTA GAGGACCGTC ATGTCAATGT AGTTGTCAGG   
  
  
- GTCTCTTTTC CCAACTTAGA CAGTTAGCGA GTTCCTCCTT CTTTTTAGCA TTTTCCCTAC TTCCACCAGT   
  
  
- ACTCCTTCCC TCCTCATTGT TCGTCGTTCG AAGTAGGTTA CTTCTAATAC AACTCTACTT CCTCAAACTA   
  
  
- CTACATGACG AGACGTTTCT TCTCTTTCTA CAATAACGTT TGACGTGGTT ACTAAGGAGT GGGCAGCTTC   
  
  
- GCTCACTATT CAACGTCTTC TTTCCCCCCT TCCCCTTTTG TAGCGCACCC TTCTTCGTCT TATCGTGTTA   
  
  
- TCTTCTCCAC CTAGACTCCT GAGAAGAGTG AGTGACACGA GTTCGTTAAA GTTTAAAACT AGAATCCTCA   
  
  
- CGTTTACTCG TAGAGTCCGT TTATTCCGTC GTAAGAAGTG TTATACCACT ATCGGAGGTC TCCGAACGGG   
  
  
- TAATAAAACG ATTACCAGAA CTCCGAGCGT ATCGACCGTG ACCAAGTTGT TAGAGACGAT TACAACAACT   
  
  
- ACGAGCTTAG TGCAGTAGAC TAAAGAATTT CCGAATATCC GATATATACA GTCGGCAAGG AAAGTTTTCC   
  
  
- TACAATATAA AAGAGCGGTT GTTCTGTTAT GCCTTCAACC GACTCTTCCG TTGGTCCTAG GTGTAGTAAC   
  
  
- TAAAACCTCA GAACAACCCA AATGTCACCG GGACAGAGTA TGTTTTGGAG AGTTTCTCTG GGTGACCGGG   
  
  
- CGGTTCGGAG GCATAATGAC CCTATCTGAT AGGGGTCGTA CCAAAGGCCG GTAGTCTTTC CCAACTTCGC   
  
  
- TGACCGGCAG CCAATCTTCC CATAACGCTC TCTAAATTAC ACGGCAAGTT CATATTCCGG TATCGATTTT   
  
  
- TAACCCTCTC ATATTTCGAT CTCCTAGATC TTTAACTACT CTTACTCTAC CACAAACAGT TAACGTACGA   
  
  
- AGCGAGACCT TGTGACGAAC TACTCTGTCA CCACCGTCTG TCAGGTTTCC TACGAAAGAA TTCCAATTAG   
  
  
- TCTCTCTATT TAGGGGCAGA AAAGTAAGTA CCCTGATAGT TACCTAGTAA GTTACGAGGT AAGAAGTAGT   
  
  
- GAGCCAAGTC CCTCCGCGAG AAGGTGATGA GAAGAAATAA ACTATAGAAA CTTCGTTGAT ACGGGGCACT   
  
  
- TCTAGTACTT TCCGAGGACC AACTCTCACT CTATGTCCCG TTTCTTCGAA ACTTACAGTA TCGTACGCTT   
  
  
- CCACGGCTCT CCTAAGTTTC CGGACTTTGT ATGTTCGTTA CCGTTCGTTC CTGTTGTTCC CGGCCCAAAT   
  
  
- CCGTCGAAGG TGATCTGGCC CTCGAACACT CGTCTCGTTT CCGATACCAC TTTCGTTTGA TGGTATTCCT   
  
  
- AAAACACCAC CTGCTCCTGT CCGTAACCTA CGATGTGCCA ACCTTTCCTT CTTAGAAGAC ACGTGAGAGA   
  
  
- CAAACCGTTG GGTTGAC

+     Myb-binding site

| Site Name | Organism | Position | Strand | Matrix score. | sequence | function |
| --- | --- | --- | --- | --- | --- | --- |
| Myb-binding site | Nicotiana tabacum | 2656 | + | 6 | CAACAG |  |
| Myb-binding site | Nicotiana tabacum | 1748 | - | 6 | CAACAG |  |

>HU07G02246.1   
+ -Up\_Stream \_Len000ATTTGA TGTGTATGCT GACTACTTTT GATGATGATG ATCACAGTGA TGCATATTTA   
  
  
+ GAGCTGGTAA ATTGGTCATT CGATTCGAGT ACGGGTCGGG TCAAGTTCTG ATTAAGTGAC ATTTCGCGTC   
  
  
+ GTTTTGGTTT AGTTCGGGTC GGATCGATTT CGGGTTAAAT AATTTTTGGT GGAATACGCT TGTCATGCCA   
  
  
+ AACACAAGCA ACTTTGTTAA AAATTTCGAT TCGGGTCAGG TCAATTCAGG TTTGGGGTCC ACTTTCGAGT   
  
  
+ GAGCATATTT CGGATGTCGG GTCGCGTATG GGTCCGGGTC ATTCGGTTTG AATTTCGGAT CTTGGATCAA   
  
  
+ TTTTGCTAAG TATGTGCATA TTCAACCCTT GATGACATAG GAAACGCTTT GTCATCTCCA CCAATCAGAT   
  
  
+ ATCGTTATCT CTTTAATCAT CGCCATAATA GCATGAGTAA GCATGACCAT CTTTTTCAAA CATGTGATAC   
  
  
+ AGTTAAGGCC TGTTCTTTTC GTTAATAAAT CCCAATTTAT CAATTTTAAT ATATACTTTA ACGAATTTTA   
  
  
+ ACAAAAAATT TTAATTTCAA TCCGCTATAA CAAAAAAAAT ACCAACTTCA ACTTATTATA TAAAAAAAAT   
  
  
+ ATACAATTTT TTTTCAATTT TAGCCAACTT CAACAAAAAA AAAAAAAATT TAACAAATCT CAATCAACTT   
  
  
+ CACCCATTTC TCAACTTATA TCTCCTAATG AAAAGAACAG ATCCTTAGCA AGACTTCTCG ATTCATTTAA   
  
  
+ AAGGATAGTA TGGTAAATTC ATTTTATACA AGTGTCAAGT GTGAATATCT TTTTCACATC AGGTGATATA   
  
  
+ TTAGTAGGAC TTTTCGATTC ACTTATCTTC CTCTTTATGT TTTGTTAATG TGATAGTAAC GATAGGATTG   
  
  
+ CATGTATTTT TTCCAACTAT TCATTTACAA CTATCAATAT CTATTGATAA ATATGTTAAA ATAATTATAT   
  
  
+ GTTATTTAAA TTATTATTTG TTACTTAAGG TGTCGACTTT GTCGAAAGCG ACTTAATTCT AATTAAGATA   
  
  
+ GCGGCAGAGA AAGTTACACC TGATGACCAA GTTAGATCTG GTAAAACTAA CCATAGATCT GAAACCCAAA   
  
  
+ CATAATGATG TGTATTCATA CCCGACCTGA CATCTGAAAT AAGTTTACTC AAAAGTGGTT CCGAAACTTG   
  
  
+ AATTGACTCG AACCGAACCG TATTTGACTC GAAATTTTCT ACAAAGTTGC TTATTTTTGC ATGAAGACAT   
  
  
+ ATTGAGCCAA AAATTAGTTG ATTCGAAGTC AACTTAAACA AAAATTCATT CGACTCAAAC TGAAATATCA   
  
  
+ CTTAACTAAA ACTATGACTC AACTCAAACT TGATCTGATC CGTATTCAAA TCAAATACTC CATTTGCTAG   
  
  
+ GTCTAGAACC AGCCACGACC CCACATGGAC TATGCGGTTC AATGAAACGG CCAAATATAT CGCAATTTCT   
  
  
+ GTCTCCATCA ATCACACCCA TTTGCTACAC CCTGTTAATA TTTGACTCGA CCCTGTGAAT TGCCTTCGCC   
  
  
+ AGTTTTGTTT AATCTCTCTG GTTGCTTCCC ATCCAAGTCT GAATAGTATA GTTTGTACAG TGTGTACTAC   
  
  
+ TAGAAAGCAC GTTCCTTGAA TCTTCTTTCA AAGTTGTTTT TAAAGGGTTG AGTGATCCAT TACGACTTTT   
  
  
+ GATCTGGGTT CATCGCCTCC TCTCCTAATC CCTCACCCGG GTACCCTCAA TTCTCTATCG TCTCTGTTGC   
  
  
+ TCAGCTAATT CAAATTCCTT TAGTTGGTCA TCATTGAAAT CTGAAATCAG GGTGTTTTTC TTTTTATTAT   
  
  
+ TATCTGGCAG TTGGTTTGTT TATCTCTTGG TTTTTGGGGC TTATTTGGGT GCTGGTTGAT GTTATCCTGC   
  
  
+ TTGGGTTGAA TCTTCATCAG GTTTATTTTT GATTCATAGT TGAGGGTGAA AATTATTTCT CATACCCACC   
  
  
+ ACCTGTTTGT TCTTTTGTCT CTGAGAAAAA CCAAAAAGCT TTCTCTTTTT CCTGATGCAT CCTGTGCTCG   
  
  
+ TTGATCCTGA GTTAGTGAAC CATGCGTCCA AATTCAAGCC TGATTTGCTC TCAAACTTTG ATAAACAACA   
  
  
+ AGAATTTAGC AACGGGATCG AACAAGATGA CATCTTGCAA AACCCTGATC TGTGTTTTGA TTCAGAAACC   
  
  
+ CCTTCAGTTG ATCTAACTGA GAGTGTCATC AGTTCTGATT CTGTAGAAGT GCCTGATTTC TCAGATGCTT   
  
  
+ GCCTCAAGTT CATAAGTGAG ATTCTCCTAG AAGAGGATTT GGATGAAAGT CCTGCCTCTG CTCAAGAATT   
  
  
+ TAGGGCTCTC CAAGCCACTG AGAAGTCCCT GTATGATGCT CTTGGAGAGG GCTACTCTTT TTCATCTGAT   
  
  
+ AACAGCCCAT CATCATTAGG ACAGAGTATT GAGCACCAAA ATGAGAATTT TGAATTCAGT TCCGGTTATC   
  
  
+ CTGGAATTGA GGGCTATGTT AATGGTGATA TCACGTTCGA GTCCAACTGG ATGTTCAACC TAAGCCAATT   
  
  
+ GGATCCTGTT CTTACTTTGG ATGATATTTC TCAGCCCTTG TCTGCATCAA ACTCCCGATC ATCTGGGTCG   
  
  
+ AGCAATGGCT TTGATGATTC AGGGGATGGG GCAGCCACAT CTCCTGGCAG TACAGTTACA TCAACAGTCC   
  
  
+ CAGAGAAAAG GGTTGAATCT GTCAATCGCT CAAGGAGGAA GAAAAATCGT AAAAGGGATG AAGGTGGTCA   
  
  
+ TGAGGAAGGG AGGAGTAACA AGCAGCAAGC TTCATCCAAT GAAGATTATG TTGAGATGAA GGAGTTTGAT   
  
  
+ GATGTACTGC TCTGCAAAGA AGAGAAAGAT GTTATTGCAA ACTGCACCAA TGATTCCTCA CCCGTCGAAG   
  
  
+ CGAGTGATAA GTTGCAGAAG AAAGGGGGGA AGGGGAAAAC ATCGCGTGGG AAGAAGCAGA ATAGCACAAT   
  
  
+ AGAAGAGGTG GATCTGAGGA CTCTTCTCAC TCACTGTGCT CAAGCAATTT CAAATTTTGA TCTTAGGAGT   
  
  
+ GCAAATGAGC ATCTCAGGCA AATAAGGCAG CATTCTTCAC AATATGGTGA TAGCCTCCAG AGGCTTGCCC   
  
  
+ ATTATTTTGC TAATGGTCTT GAGGCTCGCA TAGCTGGCAC TGGTTCAACA ATCTCTGCTA ATGTTGTTGA   
  
  
+ TGCTCGAATC ACGTCATCTG ATTTCTTAAA GGCTTATAGG CTATATATGT CAGCCGTTCC TTTCAAAAGG   
  
  
+ ATGTTATATT TTCTCGCCAA CAAGACAATA CGGAAGTTGG CTGAGAAGGC AACCAGGATC CACATCATTG   
  
  
+ ATTTTGGAGT CTTGTTGGGT TTACAGTGGC CCTGTCTCAT ACAAAACCTC TCAAAGAGAC CCACTGGCCC   
  
  
+ GCCAAGCCTC CGTATTACTG GGATAGACTA TCCCCAGCAT GGTTTCCGGC CATCAGAAAG GGTTGAAGCG   
  
  
+ ACTGGCCGTC GGTTAGAAGG GTATTGCGAG AGATTTAATG TGCCGTTCAA GTATAAGGCC ATAGCTAAAA   
  
  
+ ATTGGGAGAG TATAAAGCTA GAGGATCTAG AAATTGATGA GAATGAGATG GTGTTTGTCA ATTGCATGCT   
  
  
+ TCGCTCTGGA ACACTGCTTG ATGAGACAGT GGTGGCAGAC AGTCCAAAGG ATGCTTTCTT AAGGTTAATC   
  
  
+ AGAGAGATAA ATCCCCGTCT TTTCATTCAT GGGACTATCA ATGGATCATT CAATGCTCCA TTCTTCATCA   
  
  
+ CTCGGTTCAG GGAGGCGCTC TTCCACTACT CTTCTTTATT TGATATCTTT GAAGCAACTA TGCCCCGTGA   
  
  
+ AGATCATGAA AGGCTCCTGG TTGAGAGTGA GATACAGGGC AAAGAAGCTT TGAATGTCAT AGCATGCGAA   
  
  
+ GGTGCCGAGA GGATTCAAAG GCCTGAAACA TACAAGCAAT GGCAAGCAAG GACAACAAGG GCCGGGTTTA   
  
  
+ GGCAGCTTCC ACTAGACCGG GAGCTTGTGA GCAGAGCAAA GGCTATGGTG AAAGCAAACT ACCATAAGGA   
  
  
+ TTTTGTGGTG GACGAGGACA GGCATTGGAT GCTACACGGT TGGAAAGGAA GAATCTTCTG TGCACTCTCT   
  
  
+ GTTTGGCAAC CCAACTG  

- -Up\_Stream \_Len000TAAACT ACACATACGA CTGATGAAAA CTACTACTAC TAGTGTCACT ACGTATAAAT   
  
  
- CTCGACCATT TAACCAGTAA GCTAAGCTCA TGCCCAGCCC AGTTCAAGAC TAATTCACTG TAAAGCGCAG   
  
  
- CAAAACCAAA TCAAGCCCAG CCTAGCTAAA GCCCAATTTA TTAAAAACCA CCTTATGCGA ACAGTACGGT   
  
  
- TTGTGTTCGT TGAAACAATT TTTAAAGCTA AGCCCAGTCC AGTTAAGTCC AAACCCCAGG TGAAAGCTCA   
  
  
- CTCGTATAAA GCCTACAGCC CAGCGCATAC CCAGGCCCAG TAAGCCAAAC TTAAAGCCTA GAACCTAGTT   
  
  
- AAAACGATTC ATACACGTAT AAGTTGGGAA CTACTGTATC CTTTGCGAAA CAGTAGAGGT GGTTAGTCTA   
  
  
- TAGCAATAGA GAAATTAGTA GCGGTATTAT CGTACTCATT CGTACTGGTA GAAAAAGTTT GTACACTATG   
  
  
- TCAATTCCGG ACAAGAAAAG CAATTATTTA GGGTTAAATA GTTAAAATTA TATATGAAAT TGCTTAAAAT   
  
  
- TGTTTTTTAA AATTAAAGTT AGGCGATATT GTTTTTTTTA TGGTTGAAGT TGAATAATAT ATTTTTTTTA   
  
  
- TATGTTAAAA AAAAGTTAAA ATCGGTTGAA GTTGTTTTTT TTTTTTTTAA ATTGTTTAGA GTTAGTTGAA   
  
  
- GTGGGTAAAG AGTTGAATAT AGAGGATTAC TTTTCTTGTC TAGGAATCGT TCTGAAGAGC TAAGTAAATT   
  
  
- TTCCTATCAT ACCATTTAAG TAAAATATGT TCACAGTTCA CACTTATAGA AAAAGTGTAG TCCACTATAT   
  
  
- AATCATCCTG AAAAGCTAAG TGAATAGAAG GAGAAATACA AAACAATTAC ACTATCATTG CTATCCTAAC   
  
  
- GTACATAAAA AAGGTTGATA AGTAAATGTT GATAGTTATA GATAACTATT TATACAATTT TATTAATATA   
  
  
- CAATAAATTT AATAATAAAC AATGAATTCC ACAGCTGAAA CAGCTTTCGC TGAATTAAGA TTAATTCTAT   
  
  
- CGCCGTCTCT TTCAATGTGG ACTACTGGTT CAATCTAGAC CATTTTGATT GGTATCTAGA CTTTGGGTTT   
  
  
- GTATTACTAC ACATAAGTAT GGGCTGGACT GTAGACTTTA TTCAAATGAG TTTTCACCAA GGCTTTGAAC   
  
  
- TTAACTGAGC TTGGCTTGGC ATAAACTGAG CTTTAAAAGA TGTTTCAACG AATAAAAACG TACTTCTGTA   
  
  
- TAACTCGGTT TTTAATCAAC TAAGCTTCAG TTGAATTTGT TTTTAAGTAA GCTGAGTTTG ACTTTATAGT   
  
  
- GAATTGATTT TGATACTGAG TTGAGTTTGA ACTAGACTAG GCATAAGTTT AGTTTATGAG GTAAACGATC   
  
  
- CAGATCTTGG TCGGTGCTGG GGTGTACCTG ATACGCCAAG TTACTTTGCC GGTTTATATA GCGTTAAAGA   
  
  
- CAGAGGTAGT TAGTGTGGGT AAACGATGTG GGACAATTAT AAACTGAGCT GGGACACTTA ACGGAAGCGG   
  
  
- TCAAAACAAA TTAGAGAGAC CAACGAAGGG TAGGTTCAGA CTTATCATAT CAAACATGTC ACACATGATG   
  
  
- ATCTTTCGTG CAAGGAACTT AGAAGAAAGT TTCAACAAAA ATTTCCCAAC TCACTAGGTA ATGCTGAAAA   
  
  
- CTAGACCCAA GTAGCGGAGG AGAGGATTAG GGAGTGGGCC CATGGGAGTT AAGAGATAGC AGAGACAACG   
  
  
- AGTCGATTAA GTTTAAGGAA ATCAACCAGT AGTAACTTTA GACTTTAGTC CCACAAAAAG AAAAATAATA   
  
  
- ATAGACCGTC AACCAAACAA ATAGAGAACC AAAAACCCCG AATAAACCCA CGACCAACTA CAATAGGACG   
  
  
- AACCCAACTT AGAAGTAGTC CAAATAAAAA CTAAGTATCA ACTCCCACTT TTAATAAAGA GTATGGGTGG   
  
  
- TGGACAAACA AGAAAACAGA GACTCTTTTT GGTTTTTCGA AAGAGAAAAA GGACTACGTA GGACACGAGC   
  
  
- AACTAGGACT CAATCACTTG GTACGCAGGT TTAAGTTCGG ACTAAACGAG AGTTTGAAAC TATTTGTTGT   
  
  
- TCTTAAATCG TTGCCCTAGC TTGTTCTACT GTAGAACGTT TTGGGACTAG ACACAAAACT AAGTCTTTGG   
  
  
- GGAAGTCAAC TAGATTGACT CTCACAGTAG TCAAGACTAA GACATCTTCA CGGACTAAAG AGTCTACGAA   
  
  
- CGGAGTTCAA GTATTCACTC TAAGAGGATC TTCTCCTAAA CCTACTTTCA GGACGGAGAC GAGTTCTTAA   
  
  
- ATCCCGAGAG GTTCGGTGAC TCTTCAGGGA CATACTACGA GAACCTCTCC CGATGAGAAA AAGTAGACTA   
  
  
- TTGTCGGGTA GTAGTAATCC TGTCTCATAA CTCGTGGTTT TACTCTTAAA ACTTAAGTCA AGGCCAATAG   
  
  
- GACCTTAACT CCCGATACAA TTACCACTAT AGTGCAAGCT CAGGTTGACC TACAAGTTGG ATTCGGTTAA   
  
  
- CCTAGGACAA GAATGAAACC TACTATAAAG AGTCGGGAAC AGACGTAGTT TGAGGGCTAG TAGACCCAGC   
  
  
- TCGTTACCGA AACTACTAAG TCCCCTACCC CGTCGGTGTA GAGGACCGTC ATGTCAATGT AGTTGTCAGG   
  
  
- GTCTCTTTTC CCAACTTAGA CAGTTAGCGA GTTCCTCCTT CTTTTTAGCA TTTTCCCTAC TTCCACCAGT   
  
  
- ACTCCTTCCC TCCTCATTGT TCGTCGTTCG AAGTAGGTTA CTTCTAATAC AACTCTACTT CCTCAAACTA   
  
  
- CTACATGACG AGACGTTTCT TCTCTTTCTA CAATAACGTT TGACGTGGTT ACTAAGGAGT GGGCAGCTTC   
  
  
- GCTCACTATT CAACGTCTTC TTTCCCCCCT TCCCCTTTTG TAGCGCACCC TTCTTCGTCT TATCGTGTTA   
  
  
- TCTTCTCCAC CTAGACTCCT GAGAAGAGTG AGTGACACGA GTTCGTTAAA GTTTAAAACT AGAATCCTCA   
  
  
- CGTTTACTCG TAGAGTCCGT TTATTCCGTC GTAAGAAGTG TTATACCACT ATCGGAGGTC TCCGAACGGG   
  
  
- TAATAAAACG ATTACCAGAA CTCCGAGCGT ATCGACCGTG ACCAAGTTGT TAGAGACGAT TACAACAACT   
  
  
- ACGAGCTTAG TGCAGTAGAC TAAAGAATTT CCGAATATCC GATATATACA GTCGGCAAGG AAAGTTTTCC   
  
  
- TACAATATAA AAGAGCGGTT GTTCTGTTAT GCCTTCAACC GACTCTTCCG TTGGTCCTAG GTGTAGTAAC   
  
  
- TAAAACCTCA GAACAACCCA AATGTCACCG GGACAGAGTA TGTTTTGGAG AGTTTCTCTG GGTGACCGGG   
  
  
- CGGTTCGGAG GCATAATGAC CCTATCTGAT AGGGGTCGTA CCAAAGGCCG GTAGTCTTTC CCAACTTCGC   
  
  
- TGACCGGCAG CCAATCTTCC CATAACGCTC TCTAAATTAC ACGGCAAGTT CATATTCCGG TATCGATTTT   
  
  
- TAACCCTCTC ATATTTCGAT CTCCTAGATC TTTAACTACT CTTACTCTAC CACAAACAGT TAACGTACGA   
  
  
- AGCGAGACCT TGTGACGAAC TACTCTGTCA CCACCGTCTG TCAGGTTTCC TACGAAAGAA TTCCAATTAG   
  
  
- TCTCTCTATT TAGGGGCAGA AAAGTAAGTA CCCTGATAGT TACCTAGTAA GTTACGAGGT AAGAAGTAGT   
  
  
- GAGCCAAGTC CCTCCGCGAG AAGGTGATGA GAAGAAATAA ACTATAGAAA CTTCGTTGAT ACGGGGCACT   
  
  
- TCTAGTACTT TCCGAGGACC AACTCTCACT CTATGTCCCG TTTCTTCGAA ACTTACAGTA TCGTACGCTT   
  
  
- CCACGGCTCT CCTAAGTTTC CGGACTTTGT ATGTTCGTTA CCGTTCGTTC CTGTTGTTCC CGGCCCAAAT   
  
  
- CCGTCGAAGG TGATCTGGCC CTCGAACACT CGTCTCGTTT CCGATACCAC TTTCGTTTGA TGGTATTCCT   
  
  
- AAAACACCAC CTGCTCCTGT CCGTAACCTA CGATGTGCCA ACCTTTCCTT CTTAGAAGAC ACGTGAGAGA   
  
  
- CAAACCGTTG GGTTGAC

+     O2-site

| Site Name | Organism | Position | Strand | Matrix score. | sequence | function |
| --- | --- | --- | --- | --- | --- | --- |
| O2-site | Zea mays | 3284 | - | 9 | GATGATGTGG | cis-acting regulatory element involved in zein metabolism regulation |
| O2-site | Zea mays | 3163 | - | 9 | GTTGACGTGA | cis-acting regulatory element involved in zein metabolism regulation |
| O2-site | Zea mays | 385 | + | 9 | GATGACATGG | cis-acting regulatory element involved in zein metabolism regulation |

>HU07G02246.1   
+ -Up\_Stream \_Len000ATTTGA TGTGTATGCT GACTACTTTT GATGATGATG ATCACAGTGA TGCATATTTA   
  
  
+ GAGCTGGTAA ATTGGTCATT CGATTCGAGT ACGGGTCGGG TCAAGTTCTG ATTAAGTGAC ATTTCGCGTC   
  
  
+ GTTTTGGTTT AGTTCGGGTC GGATCGATTT CGGGTTAAAT AATTTTTGGT GGAATACGCT TGTCATGCCA   
  
  
+ AACACAAGCA ACTTTGTTAA AAATTTCGAT TCGGGTCAGG TCAATTCAGG TTTGGGGTCC ACTTTCGAGT   
  
  
+ GAGCATATTT CGGATGTCGG GTCGCGTATG GGTCCGGGTC ATTCGGTTTG AATTTCGGAT CTTGGATCAA   
  
  
+ TTTTGCTAAG TATGTGCATA TTCAACCCTT GATGACATAG GAAACGCTTT GTCATCTCCA CCAATCAGAT   
  
  
+ ATCGTTATCT CTTTAATCAT CGCCATAATA GCATGAGTAA GCATGACCAT CTTTTTCAAA CATGTGATAC   
  
  
+ AGTTAAGGCC TGTTCTTTTC GTTAATAAAT CCCAATTTAT CAATTTTAAT ATATACTTTA ACGAATTTTA   
  
  
+ ACAAAAAATT TTAATTTCAA TCCGCTATAA CAAAAAAAAT ACCAACTTCA ACTTATTATA TAAAAAAAAT   
  
  
+ ATACAATTTT TTTTCAATTT TAGCCAACTT CAACAAAAAA AAAAAAAATT TAACAAATCT CAATCAACTT   
  
  
+ CACCCATTTC TCAACTTATA TCTCCTAATG AAAAGAACAG ATCCTTAGCA AGACTTCTCG ATTCATTTAA   
  
  
+ AAGGATAGTA TGGTAAATTC ATTTTATACA AGTGTCAAGT GTGAATATCT TTTTCACATC AGGTGATATA   
  
  
+ TTAGTAGGAC TTTTCGATTC ACTTATCTTC CTCTTTATGT TTTGTTAATG TGATAGTAAC GATAGGATTG   
  
  
+ CATGTATTTT TTCCAACTAT TCATTTACAA CTATCAATAT CTATTGATAA ATATGTTAAA ATAATTATAT   
  
  
+ GTTATTTAAA TTATTATTTG TTACTTAAGG TGTCGACTTT GTCGAAAGCG ACTTAATTCT AATTAAGATA   
  
  
+ GCGGCAGAGA AAGTTACACC TGATGACCAA GTTAGATCTG GTAAAACTAA CCATAGATCT GAAACCCAAA   
  
  
+ CATAATGATG TGTATTCATA CCCGACCTGA CATCTGAAAT AAGTTTACTC AAAAGTGGTT CCGAAACTTG   
  
  
+ AATTGACTCG AACCGAACCG TATTTGACTC GAAATTTTCT ACAAAGTTGC TTATTTTTGC ATGAAGACAT   
  
  
+ ATTGAGCCAA AAATTAGTTG ATTCGAAGTC AACTTAAACA AAAATTCATT CGACTCAAAC TGAAATATCA   
  
  
+ CTTAACTAAA ACTATGACTC AACTCAAACT TGATCTGATC CGTATTCAAA TCAAATACTC CATTTGCTAG   
  
  
+ GTCTAGAACC AGCCACGACC CCACATGGAC TATGCGGTTC AATGAAACGG CCAAATATAT CGCAATTTCT   
  
  
+ GTCTCCATCA ATCACACCCA TTTGCTACAC CCTGTTAATA TTTGACTCGA CCCTGTGAAT TGCCTTCGCC   
  
  
+ AGTTTTGTTT AATCTCTCTG GTTGCTTCCC ATCCAAGTCT GAATAGTATA GTTTGTACAG TGTGTACTAC   
  
  
+ TAGAAAGCAC GTTCCTTGAA TCTTCTTTCA AAGTTGTTTT TAAAGGGTTG AGTGATCCAT TACGACTTTT   
  
  
+ GATCTGGGTT CATCGCCTCC TCTCCTAATC CCTCACCCGG GTACCCTCAA TTCTCTATCG TCTCTGTTGC   
  
  
+ TCAGCTAATT CAAATTCCTT TAGTTGGTCA TCATTGAAAT CTGAAATCAG GGTGTTTTTC TTTTTATTAT   
  
  
+ TATCTGGCAG TTGGTTTGTT TATCTCTTGG TTTTTGGGGC TTATTTGGGT GCTGGTTGAT GTTATCCTGC   
  
  
+ TTGGGTTGAA TCTTCATCAG GTTTATTTTT GATTCATAGT TGAGGGTGAA AATTATTTCT CATACCCACC   
  
  
+ ACCTGTTTGT TCTTTTGTCT CTGAGAAAAA CCAAAAAGCT TTCTCTTTTT CCTGATGCAT CCTGTGCTCG   
  
  
+ TTGATCCTGA GTTAGTGAAC CATGCGTCCA AATTCAAGCC TGATTTGCTC TCAAACTTTG ATAAACAACA   
  
  
+ AGAATTTAGC AACGGGATCG AACAAGATGA CATCTTGCAA AACCCTGATC TGTGTTTTGA TTCAGAAACC   
  
  
+ CCTTCAGTTG ATCTAACTGA GAGTGTCATC AGTTCTGATT CTGTAGAAGT GCCTGATTTC TCAGATGCTT   
  
  
+ GCCTCAAGTT CATAAGTGAG ATTCTCCTAG AAGAGGATTT GGATGAAAGT CCTGCCTCTG CTCAAGAATT   
  
  
+ TAGGGCTCTC CAAGCCACTG AGAAGTCCCT GTATGATGCT CTTGGAGAGG GCTACTCTTT TTCATCTGAT   
  
  
+ AACAGCCCAT CATCATTAGG ACAGAGTATT GAGCACCAAA ATGAGAATTT TGAATTCAGT TCCGGTTATC   
  
  
+ CTGGAATTGA GGGCTATGTT AATGGTGATA TCACGTTCGA GTCCAACTGG ATGTTCAACC TAAGCCAATT   
  
  
+ GGATCCTGTT CTTACTTTGG ATGATATTTC TCAGCCCTTG TCTGCATCAA ACTCCCGATC ATCTGGGTCG   
  
  
+ AGCAATGGCT TTGATGATTC AGGGGATGGG GCAGCCACAT CTCCTGGCAG TACAGTTACA TCAACAGTCC   
  
  
+ CAGAGAAAAG GGTTGAATCT GTCAATCGCT CAAGGAGGAA GAAAAATCGT AAAAGGGATG AAGGTGGTCA   
  
  
+ TGAGGAAGGG AGGAGTAACA AGCAGCAAGC TTCATCCAAT GAAGATTATG TTGAGATGAA GGAGTTTGAT   
  
  
+ GATGTACTGC TCTGCAAAGA AGAGAAAGAT GTTATTGCAA ACTGCACCAA TGATTCCTCA CCCGTCGAAG   
  
  
+ CGAGTGATAA GTTGCAGAAG AAAGGGGGGA AGGGGAAAAC ATCGCGTGGG AAGAAGCAGA ATAGCACAAT   
  
  
+ AGAAGAGGTG GATCTGAGGA CTCTTCTCAC TCACTGTGCT CAAGCAATTT CAAATTTTGA TCTTAGGAGT   
  
  
+ GCAAATGAGC ATCTCAGGCA AATAAGGCAG CATTCTTCAC AATATGGTGA TAGCCTCCAG AGGCTTGCCC   
  
  
+ ATTATTTTGC TAATGGTCTT GAGGCTCGCA TAGCTGGCAC TGGTTCAACA ATCTCTGCTA ATGTTGTTGA   
  
  
+ TGCTCGAATC ACGTCATCTG ATTTCTTAAA GGCTTATAGG CTATATATGT CAGCCGTTCC TTTCAAAAGG   
  
  
+ ATGTTATATT TTCTCGCCAA CAAGACAATA CGGAAGTTGG CTGAGAAGGC AACCAGGATC CACATCATTG   
  
  
+ ATTTTGGAGT CTTGTTGGGT TTACAGTGGC CCTGTCTCAT ACAAAACCTC TCAAAGAGAC CCACTGGCCC   
  
  
+ GCCAAGCCTC CGTATTACTG GGATAGACTA TCCCCAGCAT GGTTTCCGGC CATCAGAAAG GGTTGAAGCG   
  
  
+ ACTGGCCGTC GGTTAGAAGG GTATTGCGAG AGATTTAATG TGCCGTTCAA GTATAAGGCC ATAGCTAAAA   
  
  
+ ATTGGGAGAG TATAAAGCTA GAGGATCTAG AAATTGATGA GAATGAGATG GTGTTTGTCA ATTGCATGCT   
  
  
+ TCGCTCTGGA ACACTGCTTG ATGAGACAGT GGTGGCAGAC AGTCCAAAGG ATGCTTTCTT AAGGTTAATC   
  
  
+ AGAGAGATAA ATCCCCGTCT TTTCATTCAT GGGACTATCA ATGGATCATT CAATGCTCCA TTCTTCATCA   
  
  
+ CTCGGTTCAG GGAGGCGCTC TTCCACTACT CTTCTTTATT TGATATCTTT GAAGCAACTA TGCCCCGTGA   
  
  
+ AGATCATGAA AGGCTCCTGG TTGAGAGTGA GATACAGGGC AAAGAAGCTT TGAATGTCAT AGCATGCGAA   
  
  
+ GGTGCCGAGA GGATTCAAAG GCCTGAAACA TACAAGCAAT GGCAAGCAAG GACAACAAGG GCCGGGTTTA   
  
  
+ GGCAGCTTCC ACTAGACCGG GAGCTTGTGA GCAGAGCAAA GGCTATGGTG AAAGCAAACT ACCATAAGGA   
  
  
+ TTTTGTGGTG GACGAGGACA GGCATTGGAT GCTACACGGT TGGAAAGGAA GAATCTTCTG TGCACTCTCT   
  
  
+ GTTTGGCAAC CCAACTG  

- -Up\_Stream \_Len000TAAACT ACACATACGA CTGATGAAAA CTACTACTAC TAGTGTCACT ACGTATAAAT   
  
  
- CTCGACCATT TAACCAGTAA GCTAAGCTCA TGCCCAGCCC AGTTCAAGAC TAATTCACTG TAAAGCGCAG   
  
  
- CAAAACCAAA TCAAGCCCAG CCTAGCTAAA GCCCAATTTA TTAAAAACCA CCTTATGCGA ACAGTACGGT   
  
  
- TTGTGTTCGT TGAAACAATT TTTAAAGCTA AGCCCAGTCC AGTTAAGTCC AAACCCCAGG TGAAAGCTCA   
  
  
- CTCGTATAAA GCCTACAGCC CAGCGCATAC CCAGGCCCAG TAAGCCAAAC TTAAAGCCTA GAACCTAGTT   
  
  
- AAAACGATTC ATACACGTAT AAGTTGGGAA CTACTGTATC CTTTGCGAAA CAGTAGAGGT GGTTAGTCTA   
  
  
- TAGCAATAGA GAAATTAGTA GCGGTATTAT CGTACTCATT CGTACTGGTA GAAAAAGTTT GTACACTATG   
  
  
- TCAATTCCGG ACAAGAAAAG CAATTATTTA GGGTTAAATA GTTAAAATTA TATATGAAAT TGCTTAAAAT   
  
  
- TGTTTTTTAA AATTAAAGTT AGGCGATATT GTTTTTTTTA TGGTTGAAGT TGAATAATAT ATTTTTTTTA   
  
  
- TATGTTAAAA AAAAGTTAAA ATCGGTTGAA GTTGTTTTTT TTTTTTTTAA ATTGTTTAGA GTTAGTTGAA   
  
  
- GTGGGTAAAG AGTTGAATAT AGAGGATTAC TTTTCTTGTC TAGGAATCGT TCTGAAGAGC TAAGTAAATT   
  
  
- TTCCTATCAT ACCATTTAAG TAAAATATGT TCACAGTTCA CACTTATAGA AAAAGTGTAG TCCACTATAT   
  
  
- AATCATCCTG AAAAGCTAAG TGAATAGAAG GAGAAATACA AAACAATTAC ACTATCATTG CTATCCTAAC   
  
  
- GTACATAAAA AAGGTTGATA AGTAAATGTT GATAGTTATA GATAACTATT TATACAATTT TATTAATATA   
  
  
- CAATAAATTT AATAATAAAC AATGAATTCC ACAGCTGAAA CAGCTTTCGC TGAATTAAGA TTAATTCTAT   
  
  
- CGCCGTCTCT TTCAATGTGG ACTACTGGTT CAATCTAGAC CATTTTGATT GGTATCTAGA CTTTGGGTTT   
  
  
- GTATTACTAC ACATAAGTAT GGGCTGGACT GTAGACTTTA TTCAAATGAG TTTTCACCAA GGCTTTGAAC   
  
  
- TTAACTGAGC TTGGCTTGGC ATAAACTGAG CTTTAAAAGA TGTTTCAACG AATAAAAACG TACTTCTGTA   
  
  
- TAACTCGGTT TTTAATCAAC TAAGCTTCAG TTGAATTTGT TTTTAAGTAA GCTGAGTTTG ACTTTATAGT   
  
  
- GAATTGATTT TGATACTGAG TTGAGTTTGA ACTAGACTAG GCATAAGTTT AGTTTATGAG GTAAACGATC   
  
  
- CAGATCTTGG TCGGTGCTGG GGTGTACCTG ATACGCCAAG TTACTTTGCC GGTTTATATA GCGTTAAAGA   
  
  
- CAGAGGTAGT TAGTGTGGGT AAACGATGTG GGACAATTAT AAACTGAGCT GGGACACTTA ACGGAAGCGG   
  
  
- TCAAAACAAA TTAGAGAGAC CAACGAAGGG TAGGTTCAGA CTTATCATAT CAAACATGTC ACACATGATG   
  
  
- ATCTTTCGTG CAAGGAACTT AGAAGAAAGT TTCAACAAAA ATTTCCCAAC TCACTAGGTA ATGCTGAAAA   
  
  
- CTAGACCCAA GTAGCGGAGG AGAGGATTAG GGAGTGGGCC CATGGGAGTT AAGAGATAGC AGAGACAACG   
  
  
- AGTCGATTAA GTTTAAGGAA ATCAACCAGT AGTAACTTTA GACTTTAGTC CCACAAAAAG AAAAATAATA   
  
  
- ATAGACCGTC AACCAAACAA ATAGAGAACC AAAAACCCCG AATAAACCCA CGACCAACTA CAATAGGACG   
  
  
- AACCCAACTT AGAAGTAGTC CAAATAAAAA CTAAGTATCA ACTCCCACTT TTAATAAAGA GTATGGGTGG   
  
  
- TGGACAAACA AGAAAACAGA GACTCTTTTT GGTTTTTCGA AAGAGAAAAA GGACTACGTA GGACACGAGC   
  
  
- AACTAGGACT CAATCACTTG GTACGCAGGT TTAAGTTCGG ACTAAACGAG AGTTTGAAAC TATTTGTTGT   
  
  
- TCTTAAATCG TTGCCCTAGC TTGTTCTACT GTAGAACGTT TTGGGACTAG ACACAAAACT AAGTCTTTGG   
  
  
- GGAAGTCAAC TAGATTGACT CTCACAGTAG TCAAGACTAA GACATCTTCA CGGACTAAAG AGTCTACGAA   
  
  
- CGGAGTTCAA GTATTCACTC TAAGAGGATC TTCTCCTAAA CCTACTTTCA GGACGGAGAC GAGTTCTTAA   
  
  
- ATCCCGAGAG GTTCGGTGAC TCTTCAGGGA CATACTACGA GAACCTCTCC CGATGAGAAA AAGTAGACTA   
  
  
- TTGTCGGGTA GTAGTAATCC TGTCTCATAA CTCGTGGTTT TACTCTTAAA ACTTAAGTCA AGGCCAATAG   
  
  
- GACCTTAACT CCCGATACAA TTACCACTAT AGTGCAAGCT CAGGTTGACC TACAAGTTGG ATTCGGTTAA   
  
  
- CCTAGGACAA GAATGAAACC TACTATAAAG AGTCGGGAAC AGACGTAGTT TGAGGGCTAG TAGACCCAGC   
  
  
- TCGTTACCGA AACTACTAAG TCCCCTACCC CGTCGGTGTA GAGGACCGTC ATGTCAATGT AGTTGTCAGG   
  
  
- GTCTCTTTTC CCAACTTAGA CAGTTAGCGA GTTCCTCCTT CTTTTTAGCA TTTTCCCTAC TTCCACCAGT   
  
  
- ACTCCTTCCC TCCTCATTGT TCGTCGTTCG AAGTAGGTTA CTTCTAATAC AACTCTACTT CCTCAAACTA   
  
  
- CTACATGACG AGACGTTTCT TCTCTTTCTA CAATAACGTT TGACGTGGTT ACTAAGGAGT GGGCAGCTTC   
  
  
- GCTCACTATT CAACGTCTTC TTTCCCCCCT TCCCCTTTTG TAGCGCACCC TTCTTCGTCT TATCGTGTTA   
  
  
- TCTTCTCCAC CTAGACTCCT GAGAAGAGTG AGTGACACGA GTTCGTTAAA GTTTAAAACT AGAATCCTCA   
  
  
- CGTTTACTCG TAGAGTCCGT TTATTCCGTC GTAAGAAGTG TTATACCACT ATCGGAGGTC TCCGAACGGG   
  
  
- TAATAAAACG ATTACCAGAA CTCCGAGCGT ATCGACCGTG ACCAAGTTGT TAGAGACGAT TACAACAACT   
  
  
- ACGAGCTTAG TGCAGTAGAC TAAAGAATTT CCGAATATCC GATATATACA GTCGGCAAGG AAAGTTTTCC   
  
  
- TACAATATAA AAGAGCGGTT GTTCTGTTAT GCCTTCAACC GACTCTTCCG TTGGTCCTAG GTGTAGTAAC   
  
  
- TAAAACCTCA GAACAACCCA AATGTCACCG GGACAGAGTA TGTTTTGGAG AGTTTCTCTG GGTGACCGGG   
  
  
- CGGTTCGGAG GCATAATGAC CCTATCTGAT AGGGGTCGTA CCAAAGGCCG GTAGTCTTTC CCAACTTCGC   
  
  
- TGACCGGCAG CCAATCTTCC CATAACGCTC TCTAAATTAC ACGGCAAGTT CATATTCCGG TATCGATTTT   
  
  
- TAACCCTCTC ATATTTCGAT CTCCTAGATC TTTAACTACT CTTACTCTAC CACAAACAGT TAACGTACGA   
  
  
- AGCGAGACCT TGTGACGAAC TACTCTGTCA CCACCGTCTG TCAGGTTTCC TACGAAAGAA TTCCAATTAG   
  
  
- TCTCTCTATT TAGGGGCAGA AAAGTAAGTA CCCTGATAGT TACCTAGTAA GTTACGAGGT AAGAAGTAGT   
  
  
- GAGCCAAGTC CCTCCGCGAG AAGGTGATGA GAAGAAATAA ACTATAGAAA CTTCGTTGAT ACGGGGCACT   
  
  
- TCTAGTACTT TCCGAGGACC AACTCTCACT CTATGTCCCG TTTCTTCGAA ACTTACAGTA TCGTACGCTT   
  
  
- CCACGGCTCT CCTAAGTTTC CGGACTTTGT ATGTTCGTTA CCGTTCGTTC CTGTTGTTCC CGGCCCAAAT   
  
  
- CCGTCGAAGG TGATCTGGCC CTCGAACACT CGTCTCGTTT CCGATACCAC TTTCGTTTGA TGGTATTCCT   
  
  
- AAAACACCAC CTGCTCCTGT CCGTAACCTA CGATGTGCCA ACCTTTCCTT CTTAGAAGAC ACGTGAGAGA   
  
  
- CAAACCGTTG GGTTGAC

+     P-box

| Site Name | Organism | Position | Strand | Matrix score. | sequence | function |
| --- | --- | --- | --- | --- | --- | --- |
| P-box | Oryza sativa | 3218 | - | 7 | CCTTTTG | gibberellin-responsive element |

>HU07G02246.1   
+ -Up\_Stream \_Len000ATTTGA TGTGTATGCT GACTACTTTT GATGATGATG ATCACAGTGA TGCATATTTA   
  
  
+ GAGCTGGTAA ATTGGTCATT CGATTCGAGT ACGGGTCGGG TCAAGTTCTG ATTAAGTGAC ATTTCGCGTC   
  
  
+ GTTTTGGTTT AGTTCGGGTC GGATCGATTT CGGGTTAAAT AATTTTTGGT GGAATACGCT TGTCATGCCA   
  
  
+ AACACAAGCA ACTTTGTTAA AAATTTCGAT TCGGGTCAGG TCAATTCAGG TTTGGGGTCC ACTTTCGAGT   
  
  
+ GAGCATATTT CGGATGTCGG GTCGCGTATG GGTCCGGGTC ATTCGGTTTG AATTTCGGAT CTTGGATCAA   
  
  
+ TTTTGCTAAG TATGTGCATA TTCAACCCTT GATGACATAG GAAACGCTTT GTCATCTCCA CCAATCAGAT   
  
  
+ ATCGTTATCT CTTTAATCAT CGCCATAATA GCATGAGTAA GCATGACCAT CTTTTTCAAA CATGTGATAC   
  
  
+ AGTTAAGGCC TGTTCTTTTC GTTAATAAAT CCCAATTTAT CAATTTTAAT ATATACTTTA ACGAATTTTA   
  
  
+ ACAAAAAATT TTAATTTCAA TCCGCTATAA CAAAAAAAAT ACCAACTTCA ACTTATTATA TAAAAAAAAT   
  
  
+ ATACAATTTT TTTTCAATTT TAGCCAACTT CAACAAAAAA AAAAAAAATT TAACAAATCT CAATCAACTT   
  
  
+ CACCCATTTC TCAACTTATA TCTCCTAATG AAAAGAACAG ATCCTTAGCA AGACTTCTCG ATTCATTTAA   
  
  
+ AAGGATAGTA TGGTAAATTC ATTTTATACA AGTGTCAAGT GTGAATATCT TTTTCACATC AGGTGATATA   
  
  
+ TTAGTAGGAC TTTTCGATTC ACTTATCTTC CTCTTTATGT TTTGTTAATG TGATAGTAAC GATAGGATTG   
  
  
+ CATGTATTTT TTCCAACTAT TCATTTACAA CTATCAATAT CTATTGATAA ATATGTTAAA ATAATTATAT   
  
  
+ GTTATTTAAA TTATTATTTG TTACTTAAGG TGTCGACTTT GTCGAAAGCG ACTTAATTCT AATTAAGATA   
  
  
+ GCGGCAGAGA AAGTTACACC TGATGACCAA GTTAGATCTG GTAAAACTAA CCATAGATCT GAAACCCAAA   
  
  
+ CATAATGATG TGTATTCATA CCCGACCTGA CATCTGAAAT AAGTTTACTC AAAAGTGGTT CCGAAACTTG   
  
  
+ AATTGACTCG AACCGAACCG TATTTGACTC GAAATTTTCT ACAAAGTTGC TTATTTTTGC ATGAAGACAT   
  
  
+ ATTGAGCCAA AAATTAGTTG ATTCGAAGTC AACTTAAACA AAAATTCATT CGACTCAAAC TGAAATATCA   
  
  
+ CTTAACTAAA ACTATGACTC AACTCAAACT TGATCTGATC CGTATTCAAA TCAAATACTC CATTTGCTAG   
  
  
+ GTCTAGAACC AGCCACGACC CCACATGGAC TATGCGGTTC AATGAAACGG CCAAATATAT CGCAATTTCT   
  
  
+ GTCTCCATCA ATCACACCCA TTTGCTACAC CCTGTTAATA TTTGACTCGA CCCTGTGAAT TGCCTTCGCC   
  
  
+ AGTTTTGTTT AATCTCTCTG GTTGCTTCCC ATCCAAGTCT GAATAGTATA GTTTGTACAG TGTGTACTAC   
  
  
+ TAGAAAGCAC GTTCCTTGAA TCTTCTTTCA AAGTTGTTTT TAAAGGGTTG AGTGATCCAT TACGACTTTT   
  
  
+ GATCTGGGTT CATCGCCTCC TCTCCTAATC CCTCACCCGG GTACCCTCAA TTCTCTATCG TCTCTGTTGC   
  
  
+ TCAGCTAATT CAAATTCCTT TAGTTGGTCA TCATTGAAAT CTGAAATCAG GGTGTTTTTC TTTTTATTAT   
  
  
+ TATCTGGCAG TTGGTTTGTT TATCTCTTGG TTTTTGGGGC TTATTTGGGT GCTGGTTGAT GTTATCCTGC   
  
  
+ TTGGGTTGAA TCTTCATCAG GTTTATTTTT GATTCATAGT TGAGGGTGAA AATTATTTCT CATACCCACC   
  
  
+ ACCTGTTTGT TCTTTTGTCT CTGAGAAAAA CCAAAAAGCT TTCTCTTTTT CCTGATGCAT CCTGTGCTCG   
  
  
+ TTGATCCTGA GTTAGTGAAC CATGCGTCCA AATTCAAGCC TGATTTGCTC TCAAACTTTG ATAAACAACA   
  
  
+ AGAATTTAGC AACGGGATCG AACAAGATGA CATCTTGCAA AACCCTGATC TGTGTTTTGA TTCAGAAACC   
  
  
+ CCTTCAGTTG ATCTAACTGA GAGTGTCATC AGTTCTGATT CTGTAGAAGT GCCTGATTTC TCAGATGCTT   
  
  
+ GCCTCAAGTT CATAAGTGAG ATTCTCCTAG AAGAGGATTT GGATGAAAGT CCTGCCTCTG CTCAAGAATT   
  
  
+ TAGGGCTCTC CAAGCCACTG AGAAGTCCCT GTATGATGCT CTTGGAGAGG GCTACTCTTT TTCATCTGAT   
  
  
+ AACAGCCCAT CATCATTAGG ACAGAGTATT GAGCACCAAA ATGAGAATTT TGAATTCAGT TCCGGTTATC   
  
  
+ CTGGAATTGA GGGCTATGTT AATGGTGATA TCACGTTCGA GTCCAACTGG ATGTTCAACC TAAGCCAATT   
  
  
+ GGATCCTGTT CTTACTTTGG ATGATATTTC TCAGCCCTTG TCTGCATCAA ACTCCCGATC ATCTGGGTCG   
  
  
+ AGCAATGGCT TTGATGATTC AGGGGATGGG GCAGCCACAT CTCCTGGCAG TACAGTTACA TCAACAGTCC   
  
  
+ CAGAGAAAAG GGTTGAATCT GTCAATCGCT CAAGGAGGAA GAAAAATCGT AAAAGGGATG AAGGTGGTCA   
  
  
+ TGAGGAAGGG AGGAGTAACA AGCAGCAAGC TTCATCCAAT GAAGATTATG TTGAGATGAA GGAGTTTGAT   
  
  
+ GATGTACTGC TCTGCAAAGA AGAGAAAGAT GTTATTGCAA ACTGCACCAA TGATTCCTCA CCCGTCGAAG   
  
  
+ CGAGTGATAA GTTGCAGAAG AAAGGGGGGA AGGGGAAAAC ATCGCGTGGG AAGAAGCAGA ATAGCACAAT   
  
  
+ AGAAGAGGTG GATCTGAGGA CTCTTCTCAC TCACTGTGCT CAAGCAATTT CAAATTTTGA TCTTAGGAGT   
  
  
+ GCAAATGAGC ATCTCAGGCA AATAAGGCAG CATTCTTCAC AATATGGTGA TAGCCTCCAG AGGCTTGCCC   
  
  
+ ATTATTTTGC TAATGGTCTT GAGGCTCGCA TAGCTGGCAC TGGTTCAACA ATCTCTGCTA ATGTTGTTGA   
  
  
+ TGCTCGAATC ACGTCATCTG ATTTCTTAAA GGCTTATAGG CTATATATGT CAGCCGTTCC TTTCAAAAGG   
  
  
+ ATGTTATATT TTCTCGCCAA CAAGACAATA CGGAAGTTGG CTGAGAAGGC AACCAGGATC CACATCATTG   
  
  
+ ATTTTGGAGT CTTGTTGGGT TTACAGTGGC CCTGTCTCAT ACAAAACCTC TCAAAGAGAC CCACTGGCCC   
  
  
+ GCCAAGCCTC CGTATTACTG GGATAGACTA TCCCCAGCAT GGTTTCCGGC CATCAGAAAG GGTTGAAGCG   
  
  
+ ACTGGCCGTC GGTTAGAAGG GTATTGCGAG AGATTTAATG TGCCGTTCAA GTATAAGGCC ATAGCTAAAA   
  
  
+ ATTGGGAGAG TATAAAGCTA GAGGATCTAG AAATTGATGA GAATGAGATG GTGTTTGTCA ATTGCATGCT   
  
  
+ TCGCTCTGGA ACACTGCTTG ATGAGACAGT GGTGGCAGAC AGTCCAAAGG ATGCTTTCTT AAGGTTAATC   
  
  
+ AGAGAGATAA ATCCCCGTCT TTTCATTCAT GGGACTATCA ATGGATCATT CAATGCTCCA TTCTTCATCA   
  
  
+ CTCGGTTCAG GGAGGCGCTC TTCCACTACT CTTCTTTATT TGATATCTTT GAAGCAACTA TGCCCCGTGA   
  
  
+ AGATCATGAA AGGCTCCTGG TTGAGAGTGA GATACAGGGC AAAGAAGCTT TGAATGTCAT AGCATGCGAA   
  
  
+ GGTGCCGAGA GGATTCAAAG GCCTGAAACA TACAAGCAAT GGCAAGCAAG GACAACAAGG GCCGGGTTTA   
  
  
+ GGCAGCTTCC ACTAGACCGG GAGCTTGTGA GCAGAGCAAA GGCTATGGTG AAAGCAAACT ACCATAAGGA   
  
  
+ TTTTGTGGTG GACGAGGACA GGCATTGGAT GCTACACGGT TGGAAAGGAA GAATCTTCTG TGCACTCTCT   
  
  
+ GTTTGGCAAC CCAACTG  

- -Up\_Stream \_Len000TAAACT ACACATACGA CTGATGAAAA CTACTACTAC TAGTGTCACT ACGTATAAAT   
  
  
- CTCGACCATT TAACCAGTAA GCTAAGCTCA TGCCCAGCCC AGTTCAAGAC TAATTCACTG TAAAGCGCAG   
  
  
- CAAAACCAAA TCAAGCCCAG CCTAGCTAAA GCCCAATTTA TTAAAAACCA CCTTATGCGA ACAGTACGGT   
  
  
- TTGTGTTCGT TGAAACAATT TTTAAAGCTA AGCCCAGTCC AGTTAAGTCC AAACCCCAGG TGAAAGCTCA   
  
  
- CTCGTATAAA GCCTACAGCC CAGCGCATAC CCAGGCCCAG TAAGCCAAAC TTAAAGCCTA GAACCTAGTT   
  
  
- AAAACGATTC ATACACGTAT AAGTTGGGAA CTACTGTATC CTTTGCGAAA CAGTAGAGGT GGTTAGTCTA   
  
  
- TAGCAATAGA GAAATTAGTA GCGGTATTAT CGTACTCATT CGTACTGGTA GAAAAAGTTT GTACACTATG   
  
  
- TCAATTCCGG ACAAGAAAAG CAATTATTTA GGGTTAAATA GTTAAAATTA TATATGAAAT TGCTTAAAAT   
  
  
- TGTTTTTTAA AATTAAAGTT AGGCGATATT GTTTTTTTTA TGGTTGAAGT TGAATAATAT ATTTTTTTTA   
  
  
- TATGTTAAAA AAAAGTTAAA ATCGGTTGAA GTTGTTTTTT TTTTTTTTAA ATTGTTTAGA GTTAGTTGAA   
  
  
- GTGGGTAAAG AGTTGAATAT AGAGGATTAC TTTTCTTGTC TAGGAATCGT TCTGAAGAGC TAAGTAAATT   
  
  
- TTCCTATCAT ACCATTTAAG TAAAATATGT TCACAGTTCA CACTTATAGA AAAAGTGTAG TCCACTATAT   
  
  
- AATCATCCTG AAAAGCTAAG TGAATAGAAG GAGAAATACA AAACAATTAC ACTATCATTG CTATCCTAAC   
  
  
- GTACATAAAA AAGGTTGATA AGTAAATGTT GATAGTTATA GATAACTATT TATACAATTT TATTAATATA   
  
  
- CAATAAATTT AATAATAAAC AATGAATTCC ACAGCTGAAA CAGCTTTCGC TGAATTAAGA TTAATTCTAT   
  
  
- CGCCGTCTCT TTCAATGTGG ACTACTGGTT CAATCTAGAC CATTTTGATT GGTATCTAGA CTTTGGGTTT   
  
  
- GTATTACTAC ACATAAGTAT GGGCTGGACT GTAGACTTTA TTCAAATGAG TTTTCACCAA GGCTTTGAAC   
  
  
- TTAACTGAGC TTGGCTTGGC ATAAACTGAG CTTTAAAAGA TGTTTCAACG AATAAAAACG TACTTCTGTA   
  
  
- TAACTCGGTT TTTAATCAAC TAAGCTTCAG TTGAATTTGT TTTTAAGTAA GCTGAGTTTG ACTTTATAGT   
  
  
- GAATTGATTT TGATACTGAG TTGAGTTTGA ACTAGACTAG GCATAAGTTT AGTTTATGAG GTAAACGATC   
  
  
- CAGATCTTGG TCGGTGCTGG GGTGTACCTG ATACGCCAAG TTACTTTGCC GGTTTATATA GCGTTAAAGA   
  
  
- CAGAGGTAGT TAGTGTGGGT AAACGATGTG GGACAATTAT AAACTGAGCT GGGACACTTA ACGGAAGCGG   
  
  
- TCAAAACAAA TTAGAGAGAC CAACGAAGGG TAGGTTCAGA CTTATCATAT CAAACATGTC ACACATGATG   
  
  
- ATCTTTCGTG CAAGGAACTT AGAAGAAAGT TTCAACAAAA ATTTCCCAAC TCACTAGGTA ATGCTGAAAA   
  
  
- CTAGACCCAA GTAGCGGAGG AGAGGATTAG GGAGTGGGCC CATGGGAGTT AAGAGATAGC AGAGACAACG   
  
  
- AGTCGATTAA GTTTAAGGAA ATCAACCAGT AGTAACTTTA GACTTTAGTC CCACAAAAAG AAAAATAATA   
  
  
- ATAGACCGTC AACCAAACAA ATAGAGAACC AAAAACCCCG AATAAACCCA CGACCAACTA CAATAGGACG   
  
  
- AACCCAACTT AGAAGTAGTC CAAATAAAAA CTAAGTATCA ACTCCCACTT TTAATAAAGA GTATGGGTGG   
  
  
- TGGACAAACA AGAAAACAGA GACTCTTTTT GGTTTTTCGA AAGAGAAAAA GGACTACGTA GGACACGAGC   
  
  
- AACTAGGACT CAATCACTTG GTACGCAGGT TTAAGTTCGG ACTAAACGAG AGTTTGAAAC TATTTGTTGT   
  
  
- TCTTAAATCG TTGCCCTAGC TTGTTCTACT GTAGAACGTT TTGGGACTAG ACACAAAACT AAGTCTTTGG   
  
  
- GGAAGTCAAC TAGATTGACT CTCACAGTAG TCAAGACTAA GACATCTTCA CGGACTAAAG AGTCTACGAA   
  
  
- CGGAGTTCAA GTATTCACTC TAAGAGGATC TTCTCCTAAA CCTACTTTCA GGACGGAGAC GAGTTCTTAA   
  
  
- ATCCCGAGAG GTTCGGTGAC TCTTCAGGGA CATACTACGA GAACCTCTCC CGATGAGAAA AAGTAGACTA   
  
  
- TTGTCGGGTA GTAGTAATCC TGTCTCATAA CTCGTGGTTT TACTCTTAAA ACTTAAGTCA AGGCCAATAG   
  
  
- GACCTTAACT CCCGATACAA TTACCACTAT AGTGCAAGCT CAGGTTGACC TACAAGTTGG ATTCGGTTAA   
  
  
- CCTAGGACAA GAATGAAACC TACTATAAAG AGTCGGGAAC AGACGTAGTT TGAGGGCTAG TAGACCCAGC   
  
  
- TCGTTACCGA AACTACTAAG TCCCCTACCC CGTCGGTGTA GAGGACCGTC ATGTCAATGT AGTTGTCAGG   
  
  
- GTCTCTTTTC CCAACTTAGA CAGTTAGCGA GTTCCTCCTT CTTTTTAGCA TTTTCCCTAC TTCCACCAGT   
  
  
- ACTCCTTCCC TCCTCATTGT TCGTCGTTCG AAGTAGGTTA CTTCTAATAC AACTCTACTT CCTCAAACTA   
  
  
- CTACATGACG AGACGTTTCT TCTCTTTCTA CAATAACGTT TGACGTGGTT ACTAAGGAGT GGGCAGCTTC   
  
  
- GCTCACTATT CAACGTCTTC TTTCCCCCCT TCCCCTTTTG TAGCGCACCC TTCTTCGTCT TATCGTGTTA   
  
  
- TCTTCTCCAC CTAGACTCCT GAGAAGAGTG AGTGACACGA GTTCGTTAAA GTTTAAAACT AGAATCCTCA   
  
  
- CGTTTACTCG TAGAGTCCGT TTATTCCGTC GTAAGAAGTG TTATACCACT ATCGGAGGTC TCCGAACGGG   
  
  
- TAATAAAACG ATTACCAGAA CTCCGAGCGT ATCGACCGTG ACCAAGTTGT TAGAGACGAT TACAACAACT   
  
  
- ACGAGCTTAG TGCAGTAGAC TAAAGAATTT CCGAATATCC GATATATACA GTCGGCAAGG AAAGTTTTCC   
  
  
- TACAATATAA AAGAGCGGTT GTTCTGTTAT GCCTTCAACC GACTCTTCCG TTGGTCCTAG GTGTAGTAAC   
  
  
- TAAAACCTCA GAACAACCCA AATGTCACCG GGACAGAGTA TGTTTTGGAG AGTTTCTCTG GGTGACCGGG   
  
  
- CGGTTCGGAG GCATAATGAC CCTATCTGAT AGGGGTCGTA CCAAAGGCCG GTAGTCTTTC CCAACTTCGC   
  
  
- TGACCGGCAG CCAATCTTCC CATAACGCTC TCTAAATTAC ACGGCAAGTT CATATTCCGG TATCGATTTT   
  
  
- TAACCCTCTC ATATTTCGAT CTCCTAGATC TTTAACTACT CTTACTCTAC CACAAACAGT TAACGTACGA   
  
  
- AGCGAGACCT TGTGACGAAC TACTCTGTCA CCACCGTCTG TCAGGTTTCC TACGAAAGAA TTCCAATTAG   
  
  
- TCTCTCTATT TAGGGGCAGA AAAGTAAGTA CCCTGATAGT TACCTAGTAA GTTACGAGGT AAGAAGTAGT   
  
  
- GAGCCAAGTC CCTCCGCGAG AAGGTGATGA GAAGAAATAA ACTATAGAAA CTTCGTTGAT ACGGGGCACT   
  
  
- TCTAGTACTT TCCGAGGACC AACTCTCACT CTATGTCCCG TTTCTTCGAA ACTTACAGTA TCGTACGCTT   
  
  
- CCACGGCTCT CCTAAGTTTC CGGACTTTGT ATGTTCGTTA CCGTTCGTTC CTGTTGTTCC CGGCCCAAAT   
  
  
- CCGTCGAAGG TGATCTGGCC CTCGAACACT CGTCTCGTTT CCGATACCAC TTTCGTTTGA TGGTATTCCT   
  
  
- AAAACACCAC CTGCTCCTGT CCGTAACCTA CGATGTGCCA ACCTTTCCTT CTTAGAAGAC ACGTGAGAGA   
  
  
- CAAACCGTTG GGTTGAC

+     STRE

| Site Name | Organism | Position | Strand | Matrix score. | sequence | function |
| --- | --- | --- | --- | --- | --- | --- |
| STRE | Arabidopsis thaliana | 2615 | + | 5 | AGGGG |  |
| STRE | Arabidopsis thaliana | 2905 | + | 5 | AGGGG |  |
| STRE | Arabidopsis thaliana | 2897 | + | 5 | AGGGG |  |
| STRE | Arabidopsis thaliana | 2173 | - | 5 | AGGGG |  |

>HU07G02246.1   
+ -Up\_Stream \_Len000ATTTGA TGTGTATGCT GACTACTTTT GATGATGATG ATCACAGTGA TGCATATTTA   
  
  
+ GAGCTGGTAA ATTGGTCATT CGATTCGAGT ACGGGTCGGG TCAAGTTCTG ATTAAGTGAC ATTTCGCGTC   
  
  
+ GTTTTGGTTT AGTTCGGGTC GGATCGATTT CGGGTTAAAT AATTTTTGGT GGAATACGCT TGTCATGCCA   
  
  
+ AACACAAGCA ACTTTGTTAA AAATTTCGAT TCGGGTCAGG TCAATTCAGG TTTGGGGTCC ACTTTCGAGT   
  
  
+ GAGCATATTT CGGATGTCGG GTCGCGTATG GGTCCGGGTC ATTCGGTTTG AATTTCGGAT CTTGGATCAA   
  
  
+ TTTTGCTAAG TATGTGCATA TTCAACCCTT GATGACATAG GAAACGCTTT GTCATCTCCA CCAATCAGAT   
  
  
+ ATCGTTATCT CTTTAATCAT CGCCATAATA GCATGAGTAA GCATGACCAT CTTTTTCAAA CATGTGATAC   
  
  
+ AGTTAAGGCC TGTTCTTTTC GTTAATAAAT CCCAATTTAT CAATTTTAAT ATATACTTTA ACGAATTTTA   
  
  
+ ACAAAAAATT TTAATTTCAA TCCGCTATAA CAAAAAAAAT ACCAACTTCA ACTTATTATA TAAAAAAAAT   
  
  
+ ATACAATTTT TTTTCAATTT TAGCCAACTT CAACAAAAAA AAAAAAAATT TAACAAATCT CAATCAACTT   
  
  
+ CACCCATTTC TCAACTTATA TCTCCTAATG AAAAGAACAG ATCCTTAGCA AGACTTCTCG ATTCATTTAA   
  
  
+ AAGGATAGTA TGGTAAATTC ATTTTATACA AGTGTCAAGT GTGAATATCT TTTTCACATC AGGTGATATA   
  
  
+ TTAGTAGGAC TTTTCGATTC ACTTATCTTC CTCTTTATGT TTTGTTAATG TGATAGTAAC GATAGGATTG   
  
  
+ CATGTATTTT TTCCAACTAT TCATTTACAA CTATCAATAT CTATTGATAA ATATGTTAAA ATAATTATAT   
  
  
+ GTTATTTAAA TTATTATTTG TTACTTAAGG TGTCGACTTT GTCGAAAGCG ACTTAATTCT AATTAAGATA   
  
  
+ GCGGCAGAGA AAGTTACACC TGATGACCAA GTTAGATCTG GTAAAACTAA CCATAGATCT GAAACCCAAA   
  
  
+ CATAATGATG TGTATTCATA CCCGACCTGA CATCTGAAAT AAGTTTACTC AAAAGTGGTT CCGAAACTTG   
  
  
+ AATTGACTCG AACCGAACCG TATTTGACTC GAAATTTTCT ACAAAGTTGC TTATTTTTGC ATGAAGACAT   
  
  
+ ATTGAGCCAA AAATTAGTTG ATTCGAAGTC AACTTAAACA AAAATTCATT CGACTCAAAC TGAAATATCA   
  
  
+ CTTAACTAAA ACTATGACTC AACTCAAACT TGATCTGATC CGTATTCAAA TCAAATACTC CATTTGCTAG   
  
  
+ GTCTAGAACC AGCCACGACC CCACATGGAC TATGCGGTTC AATGAAACGG CCAAATATAT CGCAATTTCT   
  
  
+ GTCTCCATCA ATCACACCCA TTTGCTACAC CCTGTTAATA TTTGACTCGA CCCTGTGAAT TGCCTTCGCC   
  
  
+ AGTTTTGTTT AATCTCTCTG GTTGCTTCCC ATCCAAGTCT GAATAGTATA GTTTGTACAG TGTGTACTAC   
  
  
+ TAGAAAGCAC GTTCCTTGAA TCTTCTTTCA AAGTTGTTTT TAAAGGGTTG AGTGATCCAT TACGACTTTT   
  
  
+ GATCTGGGTT CATCGCCTCC TCTCCTAATC CCTCACCCGG GTACCCTCAA TTCTCTATCG TCTCTGTTGC   
  
  
+ TCAGCTAATT CAAATTCCTT TAGTTGGTCA TCATTGAAAT CTGAAATCAG GGTGTTTTTC TTTTTATTAT   
  
  
+ TATCTGGCAG TTGGTTTGTT TATCTCTTGG TTTTTGGGGC TTATTTGGGT GCTGGTTGAT GTTATCCTGC   
  
  
+ TTGGGTTGAA TCTTCATCAG GTTTATTTTT GATTCATAGT TGAGGGTGAA AATTATTTCT CATACCCACC   
  
  
+ ACCTGTTTGT TCTTTTGTCT CTGAGAAAAA CCAAAAAGCT TTCTCTTTTT CCTGATGCAT CCTGTGCTCG   
  
  
+ TTGATCCTGA GTTAGTGAAC CATGCGTCCA AATTCAAGCC TGATTTGCTC TCAAACTTTG ATAAACAACA   
  
  
+ AGAATTTAGC AACGGGATCG AACAAGATGA CATCTTGCAA AACCCTGATC TGTGTTTTGA TTCAGAAACC   
  
  
+ CCTTCAGTTG ATCTAACTGA GAGTGTCATC AGTTCTGATT CTGTAGAAGT GCCTGATTTC TCAGATGCTT   
  
  
+ GCCTCAAGTT CATAAGTGAG ATTCTCCTAG AAGAGGATTT GGATGAAAGT CCTGCCTCTG CTCAAGAATT   
  
  
+ TAGGGCTCTC CAAGCCACTG AGAAGTCCCT GTATGATGCT CTTGGAGAGG GCTACTCTTT TTCATCTGAT   
  
  
+ AACAGCCCAT CATCATTAGG ACAGAGTATT GAGCACCAAA ATGAGAATTT TGAATTCAGT TCCGGTTATC   
  
  
+ CTGGAATTGA GGGCTATGTT AATGGTGATA TCACGTTCGA GTCCAACTGG ATGTTCAACC TAAGCCAATT   
  
  
+ GGATCCTGTT CTTACTTTGG ATGATATTTC TCAGCCCTTG TCTGCATCAA ACTCCCGATC ATCTGGGTCG   
  
  
+ AGCAATGGCT TTGATGATTC AGGGGATGGG GCAGCCACAT CTCCTGGCAG TACAGTTACA TCAACAGTCC   
  
  
+ CAGAGAAAAG GGTTGAATCT GTCAATCGCT CAAGGAGGAA GAAAAATCGT AAAAGGGATG AAGGTGGTCA   
  
  
+ TGAGGAAGGG AGGAGTAACA AGCAGCAAGC TTCATCCAAT GAAGATTATG TTGAGATGAA GGAGTTTGAT   
  
  
+ GATGTACTGC TCTGCAAAGA AGAGAAAGAT GTTATTGCAA ACTGCACCAA TGATTCCTCA CCCGTCGAAG   
  
  
+ CGAGTGATAA GTTGCAGAAG AAAGGGGGGA AGGGGAAAAC ATCGCGTGGG AAGAAGCAGA ATAGCACAAT   
  
  
+ AGAAGAGGTG GATCTGAGGA CTCTTCTCAC TCACTGTGCT CAAGCAATTT CAAATTTTGA TCTTAGGAGT   
  
  
+ GCAAATGAGC ATCTCAGGCA AATAAGGCAG CATTCTTCAC AATATGGTGA TAGCCTCCAG AGGCTTGCCC   
  
  
+ ATTATTTTGC TAATGGTCTT GAGGCTCGCA TAGCTGGCAC TGGTTCAACA ATCTCTGCTA ATGTTGTTGA   
  
  
+ TGCTCGAATC ACGTCATCTG ATTTCTTAAA GGCTTATAGG CTATATATGT CAGCCGTTCC TTTCAAAAGG   
  
  
+ ATGTTATATT TTCTCGCCAA CAAGACAATA CGGAAGTTGG CTGAGAAGGC AACCAGGATC CACATCATTG   
  
  
+ ATTTTGGAGT CTTGTTGGGT TTACAGTGGC CCTGTCTCAT ACAAAACCTC TCAAAGAGAC CCACTGGCCC   
  
  
+ GCCAAGCCTC CGTATTACTG GGATAGACTA TCCCCAGCAT GGTTTCCGGC CATCAGAAAG GGTTGAAGCG   
  
  
+ ACTGGCCGTC GGTTAGAAGG GTATTGCGAG AGATTTAATG TGCCGTTCAA GTATAAGGCC ATAGCTAAAA   
  
  
+ ATTGGGAGAG TATAAAGCTA GAGGATCTAG AAATTGATGA GAATGAGATG GTGTTTGTCA ATTGCATGCT   
  
  
+ TCGCTCTGGA ACACTGCTTG ATGAGACAGT GGTGGCAGAC AGTCCAAAGG ATGCTTTCTT AAGGTTAATC   
  
  
+ AGAGAGATAA ATCCCCGTCT TTTCATTCAT GGGACTATCA ATGGATCATT CAATGCTCCA TTCTTCATCA   
  
  
+ CTCGGTTCAG GGAGGCGCTC TTCCACTACT CTTCTTTATT TGATATCTTT GAAGCAACTA TGCCCCGTGA   
  
  
+ AGATCATGAA AGGCTCCTGG TTGAGAGTGA GATACAGGGC AAAGAAGCTT TGAATGTCAT AGCATGCGAA   
  
  
+ GGTGCCGAGA GGATTCAAAG GCCTGAAACA TACAAGCAAT GGCAAGCAAG GACAACAAGG GCCGGGTTTA   
  
  
+ GGCAGCTTCC ACTAGACCGG GAGCTTGTGA GCAGAGCAAA GGCTATGGTG AAAGCAAACT ACCATAAGGA   
  
  
+ TTTTGTGGTG GACGAGGACA GGCATTGGAT GCTACACGGT TGGAAAGGAA GAATCTTCTG TGCACTCTCT   
  
  
+ GTTTGGCAAC CCAACTG  

- -Up\_Stream \_Len000TAAACT ACACATACGA CTGATGAAAA CTACTACTAC TAGTGTCACT ACGTATAAAT   
  
  
- CTCGACCATT TAACCAGTAA GCTAAGCTCA TGCCCAGCCC AGTTCAAGAC TAATTCACTG TAAAGCGCAG   
  
  
- CAAAACCAAA TCAAGCCCAG CCTAGCTAAA GCCCAATTTA TTAAAAACCA CCTTATGCGA ACAGTACGGT   
  
  
- TTGTGTTCGT TGAAACAATT TTTAAAGCTA AGCCCAGTCC AGTTAAGTCC AAACCCCAGG TGAAAGCTCA   
  
  
- CTCGTATAAA GCCTACAGCC CAGCGCATAC CCAGGCCCAG TAAGCCAAAC TTAAAGCCTA GAACCTAGTT   
  
  
- AAAACGATTC ATACACGTAT AAGTTGGGAA CTACTGTATC CTTTGCGAAA CAGTAGAGGT GGTTAGTCTA   
  
  
- TAGCAATAGA GAAATTAGTA GCGGTATTAT CGTACTCATT CGTACTGGTA GAAAAAGTTT GTACACTATG   
  
  
- TCAATTCCGG ACAAGAAAAG CAATTATTTA GGGTTAAATA GTTAAAATTA TATATGAAAT TGCTTAAAAT   
  
  
- TGTTTTTTAA AATTAAAGTT AGGCGATATT GTTTTTTTTA TGGTTGAAGT TGAATAATAT ATTTTTTTTA   
  
  
- TATGTTAAAA AAAAGTTAAA ATCGGTTGAA GTTGTTTTTT TTTTTTTTAA ATTGTTTAGA GTTAGTTGAA   
  
  
- GTGGGTAAAG AGTTGAATAT AGAGGATTAC TTTTCTTGTC TAGGAATCGT TCTGAAGAGC TAAGTAAATT   
  
  
- TTCCTATCAT ACCATTTAAG TAAAATATGT TCACAGTTCA CACTTATAGA AAAAGTGTAG TCCACTATAT   
  
  
- AATCATCCTG AAAAGCTAAG TGAATAGAAG GAGAAATACA AAACAATTAC ACTATCATTG CTATCCTAAC   
  
  
- GTACATAAAA AAGGTTGATA AGTAAATGTT GATAGTTATA GATAACTATT TATACAATTT TATTAATATA   
  
  
- CAATAAATTT AATAATAAAC AATGAATTCC ACAGCTGAAA CAGCTTTCGC TGAATTAAGA TTAATTCTAT   
  
  
- CGCCGTCTCT TTCAATGTGG ACTACTGGTT CAATCTAGAC CATTTTGATT GGTATCTAGA CTTTGGGTTT   
  
  
- GTATTACTAC ACATAAGTAT GGGCTGGACT GTAGACTTTA TTCAAATGAG TTTTCACCAA GGCTTTGAAC   
  
  
- TTAACTGAGC TTGGCTTGGC ATAAACTGAG CTTTAAAAGA TGTTTCAACG AATAAAAACG TACTTCTGTA   
  
  
- TAACTCGGTT TTTAATCAAC TAAGCTTCAG TTGAATTTGT TTTTAAGTAA GCTGAGTTTG ACTTTATAGT   
  
  
- GAATTGATTT TGATACTGAG TTGAGTTTGA ACTAGACTAG GCATAAGTTT AGTTTATGAG GTAAACGATC   
  
  
- CAGATCTTGG TCGGTGCTGG GGTGTACCTG ATACGCCAAG TTACTTTGCC GGTTTATATA GCGTTAAAGA   
  
  
- CAGAGGTAGT TAGTGTGGGT AAACGATGTG GGACAATTAT AAACTGAGCT GGGACACTTA ACGGAAGCGG   
  
  
- TCAAAACAAA TTAGAGAGAC CAACGAAGGG TAGGTTCAGA CTTATCATAT CAAACATGTC ACACATGATG   
  
  
- ATCTTTCGTG CAAGGAACTT AGAAGAAAGT TTCAACAAAA ATTTCCCAAC TCACTAGGTA ATGCTGAAAA   
  
  
- CTAGACCCAA GTAGCGGAGG AGAGGATTAG GGAGTGGGCC CATGGGAGTT AAGAGATAGC AGAGACAACG   
  
  
- AGTCGATTAA GTTTAAGGAA ATCAACCAGT AGTAACTTTA GACTTTAGTC CCACAAAAAG AAAAATAATA   
  
  
- ATAGACCGTC AACCAAACAA ATAGAGAACC AAAAACCCCG AATAAACCCA CGACCAACTA CAATAGGACG   
  
  
- AACCCAACTT AGAAGTAGTC CAAATAAAAA CTAAGTATCA ACTCCCACTT TTAATAAAGA GTATGGGTGG   
  
  
- TGGACAAACA AGAAAACAGA GACTCTTTTT GGTTTTTCGA AAGAGAAAAA GGACTACGTA GGACACGAGC   
  
  
- AACTAGGACT CAATCACTTG GTACGCAGGT TTAAGTTCGG ACTAAACGAG AGTTTGAAAC TATTTGTTGT   
  
  
- TCTTAAATCG TTGCCCTAGC TTGTTCTACT GTAGAACGTT TTGGGACTAG ACACAAAACT AAGTCTTTGG   
  
  
- GGAAGTCAAC TAGATTGACT CTCACAGTAG TCAAGACTAA GACATCTTCA CGGACTAAAG AGTCTACGAA   
  
  
- CGGAGTTCAA GTATTCACTC TAAGAGGATC TTCTCCTAAA CCTACTTTCA GGACGGAGAC GAGTTCTTAA   
  
  
- ATCCCGAGAG GTTCGGTGAC TCTTCAGGGA CATACTACGA GAACCTCTCC CGATGAGAAA AAGTAGACTA   
  
  
- TTGTCGGGTA GTAGTAATCC TGTCTCATAA CTCGTGGTTT TACTCTTAAA ACTTAAGTCA AGGCCAATAG   
  
  
- GACCTTAACT CCCGATACAA TTACCACTAT AGTGCAAGCT CAGGTTGACC TACAAGTTGG ATTCGGTTAA   
  
  
- CCTAGGACAA GAATGAAACC TACTATAAAG AGTCGGGAAC AGACGTAGTT TGAGGGCTAG TAGACCCAGC   
  
  
- TCGTTACCGA AACTACTAAG TCCCCTACCC CGTCGGTGTA GAGGACCGTC ATGTCAATGT AGTTGTCAGG   
  
  
- GTCTCTTTTC CCAACTTAGA CAGTTAGCGA GTTCCTCCTT CTTTTTAGCA TTTTCCCTAC TTCCACCAGT   
  
  
- ACTCCTTCCC TCCTCATTGT TCGTCGTTCG AAGTAGGTTA CTTCTAATAC AACTCTACTT CCTCAAACTA   
  
  
- CTACATGACG AGACGTTTCT TCTCTTTCTA CAATAACGTT TGACGTGGTT ACTAAGGAGT GGGCAGCTTC   
  
  
- GCTCACTATT CAACGTCTTC TTTCCCCCCT TCCCCTTTTG TAGCGCACCC TTCTTCGTCT TATCGTGTTA   
  
  
- TCTTCTCCAC CTAGACTCCT GAGAAGAGTG AGTGACACGA GTTCGTTAAA GTTTAAAACT AGAATCCTCA   
  
  
- CGTTTACTCG TAGAGTCCGT TTATTCCGTC GTAAGAAGTG TTATACCACT ATCGGAGGTC TCCGAACGGG   
  
  
- TAATAAAACG ATTACCAGAA CTCCGAGCGT ATCGACCGTG ACCAAGTTGT TAGAGACGAT TACAACAACT   
  
  
- ACGAGCTTAG TGCAGTAGAC TAAAGAATTT CCGAATATCC GATATATACA GTCGGCAAGG AAAGTTTTCC   
  
  
- TACAATATAA AAGAGCGGTT GTTCTGTTAT GCCTTCAACC GACTCTTCCG TTGGTCCTAG GTGTAGTAAC   
  
  
- TAAAACCTCA GAACAACCCA AATGTCACCG GGACAGAGTA TGTTTTGGAG AGTTTCTCTG GGTGACCGGG   
  
  
- CGGTTCGGAG GCATAATGAC CCTATCTGAT AGGGGTCGTA CCAAAGGCCG GTAGTCTTTC CCAACTTCGC   
  
  
- TGACCGGCAG CCAATCTTCC CATAACGCTC TCTAAATTAC ACGGCAAGTT CATATTCCGG TATCGATTTT   
  
  
- TAACCCTCTC ATATTTCGAT CTCCTAGATC TTTAACTACT CTTACTCTAC CACAAACAGT TAACGTACGA   
  
  
- AGCGAGACCT TGTGACGAAC TACTCTGTCA CCACCGTCTG TCAGGTTTCC TACGAAAGAA TTCCAATTAG   
  
  
- TCTCTCTATT TAGGGGCAGA AAAGTAAGTA CCCTGATAGT TACCTAGTAA GTTACGAGGT AAGAAGTAGT   
  
  
- GAGCCAAGTC CCTCCGCGAG AAGGTGATGA GAAGAAATAA ACTATAGAAA CTTCGTTGAT ACGGGGCACT   
  
  
- TCTAGTACTT TCCGAGGACC AACTCTCACT CTATGTCCCG TTTCTTCGAA ACTTACAGTA TCGTACGCTT   
  
  
- CCACGGCTCT CCTAAGTTTC CGGACTTTGT ATGTTCGTTA CCGTTCGTTC CTGTTGTTCC CGGCCCAAAT   
  
  
- CCGTCGAAGG TGATCTGGCC CTCGAACACT CGTCTCGTTT CCGATACCAC TTTCGTTTGA TGGTATTCCT   
  
  
- AAAACACCAC CTGCTCCTGT CCGTAACCTA CGATGTGCCA ACCTTTCCTT CTTAGAAGAC ACGTGAGAGA   
  
  
- CAAACCGTTG GGTTGAC

+     TATA

| Site Name | Organism | Position | Strand | Matrix score. | sequence | function |
| --- | --- | --- | --- | --- | --- | --- |
| TATA | Arabidopsis thaliana | 795 | - | 8 | TATAAAAT |  |

>HU07G02246.1   
+ -Up\_Stream \_Len000ATTTGA TGTGTATGCT GACTACTTTT GATGATGATG ATCACAGTGA TGCATATTTA   
  
  
+ GAGCTGGTAA ATTGGTCATT CGATTCGAGT ACGGGTCGGG TCAAGTTCTG ATTAAGTGAC ATTTCGCGTC   
  
  
+ GTTTTGGTTT AGTTCGGGTC GGATCGATTT CGGGTTAAAT AATTTTTGGT GGAATACGCT TGTCATGCCA   
  
  
+ AACACAAGCA ACTTTGTTAA AAATTTCGAT TCGGGTCAGG TCAATTCAGG TTTGGGGTCC ACTTTCGAGT   
  
  
+ GAGCATATTT CGGATGTCGG GTCGCGTATG GGTCCGGGTC ATTCGGTTTG AATTTCGGAT CTTGGATCAA   
  
  
+ TTTTGCTAAG TATGTGCATA TTCAACCCTT GATGACATAG GAAACGCTTT GTCATCTCCA CCAATCAGAT   
  
  
+ ATCGTTATCT CTTTAATCAT CGCCATAATA GCATGAGTAA GCATGACCAT CTTTTTCAAA CATGTGATAC   
  
  
+ AGTTAAGGCC TGTTCTTTTC GTTAATAAAT CCCAATTTAT CAATTTTAAT ATATACTTTA ACGAATTTTA   
  
  
+ ACAAAAAATT TTAATTTCAA TCCGCTATAA CAAAAAAAAT ACCAACTTCA ACTTATTATA TAAAAAAAAT   
  
  
+ ATACAATTTT TTTTCAATTT TAGCCAACTT CAACAAAAAA AAAAAAAATT TAACAAATCT CAATCAACTT   
  
  
+ CACCCATTTC TCAACTTATA TCTCCTAATG AAAAGAACAG ATCCTTAGCA AGACTTCTCG ATTCATTTAA   
  
  
+ AAGGATAGTA TGGTAAATTC ATTTTATACA AGTGTCAAGT GTGAATATCT TTTTCACATC AGGTGATATA   
  
  
+ TTAGTAGGAC TTTTCGATTC ACTTATCTTC CTCTTTATGT TTTGTTAATG TGATAGTAAC GATAGGATTG   
  
  
+ CATGTATTTT TTCCAACTAT TCATTTACAA CTATCAATAT CTATTGATAA ATATGTTAAA ATAATTATAT   
  
  
+ GTTATTTAAA TTATTATTTG TTACTTAAGG TGTCGACTTT GTCGAAAGCG ACTTAATTCT AATTAAGATA   
  
  
+ GCGGCAGAGA AAGTTACACC TGATGACCAA GTTAGATCTG GTAAAACTAA CCATAGATCT GAAACCCAAA   
  
  
+ CATAATGATG TGTATTCATA CCCGACCTGA CATCTGAAAT AAGTTTACTC AAAAGTGGTT CCGAAACTTG   
  
  
+ AATTGACTCG AACCGAACCG TATTTGACTC GAAATTTTCT ACAAAGTTGC TTATTTTTGC ATGAAGACAT   
  
  
+ ATTGAGCCAA AAATTAGTTG ATTCGAAGTC AACTTAAACA AAAATTCATT CGACTCAAAC TGAAATATCA   
  
  
+ CTTAACTAAA ACTATGACTC AACTCAAACT TGATCTGATC CGTATTCAAA TCAAATACTC CATTTGCTAG   
  
  
+ GTCTAGAACC AGCCACGACC CCACATGGAC TATGCGGTTC AATGAAACGG CCAAATATAT CGCAATTTCT   
  
  
+ GTCTCCATCA ATCACACCCA TTTGCTACAC CCTGTTAATA TTTGACTCGA CCCTGTGAAT TGCCTTCGCC   
  
  
+ AGTTTTGTTT AATCTCTCTG GTTGCTTCCC ATCCAAGTCT GAATAGTATA GTTTGTACAG TGTGTACTAC   
  
  
+ TAGAAAGCAC GTTCCTTGAA TCTTCTTTCA AAGTTGTTTT TAAAGGGTTG AGTGATCCAT TACGACTTTT   
  
  
+ GATCTGGGTT CATCGCCTCC TCTCCTAATC CCTCACCCGG GTACCCTCAA TTCTCTATCG TCTCTGTTGC   
  
  
+ TCAGCTAATT CAAATTCCTT TAGTTGGTCA TCATTGAAAT CTGAAATCAG GGTGTTTTTC TTTTTATTAT   
  
  
+ TATCTGGCAG TTGGTTTGTT TATCTCTTGG TTTTTGGGGC TTATTTGGGT GCTGGTTGAT GTTATCCTGC   
  
  
+ TTGGGTTGAA TCTTCATCAG GTTTATTTTT GATTCATAGT TGAGGGTGAA AATTATTTCT CATACCCACC   
  
  
+ ACCTGTTTGT TCTTTTGTCT CTGAGAAAAA CCAAAAAGCT TTCTCTTTTT CCTGATGCAT CCTGTGCTCG   
  
  
+ TTGATCCTGA GTTAGTGAAC CATGCGTCCA AATTCAAGCC TGATTTGCTC TCAAACTTTG ATAAACAACA   
  
  
+ AGAATTTAGC AACGGGATCG AACAAGATGA CATCTTGCAA AACCCTGATC TGTGTTTTGA TTCAGAAACC   
  
  
+ CCTTCAGTTG ATCTAACTGA GAGTGTCATC AGTTCTGATT CTGTAGAAGT GCCTGATTTC TCAGATGCTT   
  
  
+ GCCTCAAGTT CATAAGTGAG ATTCTCCTAG AAGAGGATTT GGATGAAAGT CCTGCCTCTG CTCAAGAATT   
  
  
+ TAGGGCTCTC CAAGCCACTG AGAAGTCCCT GTATGATGCT CTTGGAGAGG GCTACTCTTT TTCATCTGAT   
  
  
+ AACAGCCCAT CATCATTAGG ACAGAGTATT GAGCACCAAA ATGAGAATTT TGAATTCAGT TCCGGTTATC   
  
  
+ CTGGAATTGA GGGCTATGTT AATGGTGATA TCACGTTCGA GTCCAACTGG ATGTTCAACC TAAGCCAATT   
  
  
+ GGATCCTGTT CTTACTTTGG ATGATATTTC TCAGCCCTTG TCTGCATCAA ACTCCCGATC ATCTGGGTCG   
  
  
+ AGCAATGGCT TTGATGATTC AGGGGATGGG GCAGCCACAT CTCCTGGCAG TACAGTTACA TCAACAGTCC   
  
  
+ CAGAGAAAAG GGTTGAATCT GTCAATCGCT CAAGGAGGAA GAAAAATCGT AAAAGGGATG AAGGTGGTCA   
  
  
+ TGAGGAAGGG AGGAGTAACA AGCAGCAAGC TTCATCCAAT GAAGATTATG TTGAGATGAA GGAGTTTGAT   
  
  
+ GATGTACTGC TCTGCAAAGA AGAGAAAGAT GTTATTGCAA ACTGCACCAA TGATTCCTCA CCCGTCGAAG   
  
  
+ CGAGTGATAA GTTGCAGAAG AAAGGGGGGA AGGGGAAAAC ATCGCGTGGG AAGAAGCAGA ATAGCACAAT   
  
  
+ AGAAGAGGTG GATCTGAGGA CTCTTCTCAC TCACTGTGCT CAAGCAATTT CAAATTTTGA TCTTAGGAGT   
  
  
+ GCAAATGAGC ATCTCAGGCA AATAAGGCAG CATTCTTCAC AATATGGTGA TAGCCTCCAG AGGCTTGCCC   
  
  
+ ATTATTTTGC TAATGGTCTT GAGGCTCGCA TAGCTGGCAC TGGTTCAACA ATCTCTGCTA ATGTTGTTGA   
  
  
+ TGCTCGAATC ACGTCATCTG ATTTCTTAAA GGCTTATAGG CTATATATGT CAGCCGTTCC TTTCAAAAGG   
  
  
+ ATGTTATATT TTCTCGCCAA CAAGACAATA CGGAAGTTGG CTGAGAAGGC AACCAGGATC CACATCATTG   
  
  
+ ATTTTGGAGT CTTGTTGGGT TTACAGTGGC CCTGTCTCAT ACAAAACCTC TCAAAGAGAC CCACTGGCCC   
  
  
+ GCCAAGCCTC CGTATTACTG GGATAGACTA TCCCCAGCAT GGTTTCCGGC CATCAGAAAG GGTTGAAGCG   
  
  
+ ACTGGCCGTC GGTTAGAAGG GTATTGCGAG AGATTTAATG TGCCGTTCAA GTATAAGGCC ATAGCTAAAA   
  
  
+ ATTGGGAGAG TATAAAGCTA GAGGATCTAG AAATTGATGA GAATGAGATG GTGTTTGTCA ATTGCATGCT   
  
  
+ TCGCTCTGGA ACACTGCTTG ATGAGACAGT GGTGGCAGAC AGTCCAAAGG ATGCTTTCTT AAGGTTAATC   
  
  
+ AGAGAGATAA ATCCCCGTCT TTTCATTCAT GGGACTATCA ATGGATCATT CAATGCTCCA TTCTTCATCA   
  
  
+ CTCGGTTCAG GGAGGCGCTC TTCCACTACT CTTCTTTATT TGATATCTTT GAAGCAACTA TGCCCCGTGA   
  
  
+ AGATCATGAA AGGCTCCTGG TTGAGAGTGA GATACAGGGC AAAGAAGCTT TGAATGTCAT AGCATGCGAA   
  
  
+ GGTGCCGAGA GGATTCAAAG GCCTGAAACA TACAAGCAAT GGCAAGCAAG GACAACAAGG GCCGGGTTTA   
  
  
+ GGCAGCTTCC ACTAGACCGG GAGCTTGTGA GCAGAGCAAA GGCTATGGTG AAAGCAAACT ACCATAAGGA   
  
  
+ TTTTGTGGTG GACGAGGACA GGCATTGGAT GCTACACGGT TGGAAAGGAA GAATCTTCTG TGCACTCTCT   
  
  
+ GTTTGGCAAC CCAACTG  

- -Up\_Stream \_Len000TAAACT ACACATACGA CTGATGAAAA CTACTACTAC TAGTGTCACT ACGTATAAAT   
  
  
- CTCGACCATT TAACCAGTAA GCTAAGCTCA TGCCCAGCCC AGTTCAAGAC TAATTCACTG TAAAGCGCAG   
  
  
- CAAAACCAAA TCAAGCCCAG CCTAGCTAAA GCCCAATTTA TTAAAAACCA CCTTATGCGA ACAGTACGGT   
  
  
- TTGTGTTCGT TGAAACAATT TTTAAAGCTA AGCCCAGTCC AGTTAAGTCC AAACCCCAGG TGAAAGCTCA   
  
  
- CTCGTATAAA GCCTACAGCC CAGCGCATAC CCAGGCCCAG TAAGCCAAAC TTAAAGCCTA GAACCTAGTT   
  
  
- AAAACGATTC ATACACGTAT AAGTTGGGAA CTACTGTATC CTTTGCGAAA CAGTAGAGGT GGTTAGTCTA   
  
  
- TAGCAATAGA GAAATTAGTA GCGGTATTAT CGTACTCATT CGTACTGGTA GAAAAAGTTT GTACACTATG   
  
  
- TCAATTCCGG ACAAGAAAAG CAATTATTTA GGGTTAAATA GTTAAAATTA TATATGAAAT TGCTTAAAAT   
  
  
- TGTTTTTTAA AATTAAAGTT AGGCGATATT GTTTTTTTTA TGGTTGAAGT TGAATAATAT ATTTTTTTTA   
  
  
- TATGTTAAAA AAAAGTTAAA ATCGGTTGAA GTTGTTTTTT TTTTTTTTAA ATTGTTTAGA GTTAGTTGAA   
  
  
- GTGGGTAAAG AGTTGAATAT AGAGGATTAC TTTTCTTGTC TAGGAATCGT TCTGAAGAGC TAAGTAAATT   
  
  
- TTCCTATCAT ACCATTTAAG TAAAATATGT TCACAGTTCA CACTTATAGA AAAAGTGTAG TCCACTATAT   
  
  
- AATCATCCTG AAAAGCTAAG TGAATAGAAG GAGAAATACA AAACAATTAC ACTATCATTG CTATCCTAAC   
  
  
- GTACATAAAA AAGGTTGATA AGTAAATGTT GATAGTTATA GATAACTATT TATACAATTT TATTAATATA   
  
  
- CAATAAATTT AATAATAAAC AATGAATTCC ACAGCTGAAA CAGCTTTCGC TGAATTAAGA TTAATTCTAT   
  
  
- CGCCGTCTCT TTCAATGTGG ACTACTGGTT CAATCTAGAC CATTTTGATT GGTATCTAGA CTTTGGGTTT   
  
  
- GTATTACTAC ACATAAGTAT GGGCTGGACT GTAGACTTTA TTCAAATGAG TTTTCACCAA GGCTTTGAAC   
  
  
- TTAACTGAGC TTGGCTTGGC ATAAACTGAG CTTTAAAAGA TGTTTCAACG AATAAAAACG TACTTCTGTA   
  
  
- TAACTCGGTT TTTAATCAAC TAAGCTTCAG TTGAATTTGT TTTTAAGTAA GCTGAGTTTG ACTTTATAGT   
  
  
- GAATTGATTT TGATACTGAG TTGAGTTTGA ACTAGACTAG GCATAAGTTT AGTTTATGAG GTAAACGATC   
  
  
- CAGATCTTGG TCGGTGCTGG GGTGTACCTG ATACGCCAAG TTACTTTGCC GGTTTATATA GCGTTAAAGA   
  
  
- CAGAGGTAGT TAGTGTGGGT AAACGATGTG GGACAATTAT AAACTGAGCT GGGACACTTA ACGGAAGCGG   
  
  
- TCAAAACAAA TTAGAGAGAC CAACGAAGGG TAGGTTCAGA CTTATCATAT CAAACATGTC ACACATGATG   
  
  
- ATCTTTCGTG CAAGGAACTT AGAAGAAAGT TTCAACAAAA ATTTCCCAAC TCACTAGGTA ATGCTGAAAA   
  
  
- CTAGACCCAA GTAGCGGAGG AGAGGATTAG GGAGTGGGCC CATGGGAGTT AAGAGATAGC AGAGACAACG   
  
  
- AGTCGATTAA GTTTAAGGAA ATCAACCAGT AGTAACTTTA GACTTTAGTC CCACAAAAAG AAAAATAATA   
  
  
- ATAGACCGTC AACCAAACAA ATAGAGAACC AAAAACCCCG AATAAACCCA CGACCAACTA CAATAGGACG   
  
  
- AACCCAACTT AGAAGTAGTC CAAATAAAAA CTAAGTATCA ACTCCCACTT TTAATAAAGA GTATGGGTGG   
  
  
- TGGACAAACA AGAAAACAGA GACTCTTTTT GGTTTTTCGA AAGAGAAAAA GGACTACGTA GGACACGAGC   
  
  
- AACTAGGACT CAATCACTTG GTACGCAGGT TTAAGTTCGG ACTAAACGAG AGTTTGAAAC TATTTGTTGT   
  
  
- TCTTAAATCG TTGCCCTAGC TTGTTCTACT GTAGAACGTT TTGGGACTAG ACACAAAACT AAGTCTTTGG   
  
  
- GGAAGTCAAC TAGATTGACT CTCACAGTAG TCAAGACTAA GACATCTTCA CGGACTAAAG AGTCTACGAA   
  
  
- CGGAGTTCAA GTATTCACTC TAAGAGGATC TTCTCCTAAA CCTACTTTCA GGACGGAGAC GAGTTCTTAA   
  
  
- ATCCCGAGAG GTTCGGTGAC TCTTCAGGGA CATACTACGA GAACCTCTCC CGATGAGAAA AAGTAGACTA   
  
  
- TTGTCGGGTA GTAGTAATCC TGTCTCATAA CTCGTGGTTT TACTCTTAAA ACTTAAGTCA AGGCCAATAG   
  
  
- GACCTTAACT CCCGATACAA TTACCACTAT AGTGCAAGCT CAGGTTGACC TACAAGTTGG ATTCGGTTAA   
  
  
- CCTAGGACAA GAATGAAACC TACTATAAAG AGTCGGGAAC AGACGTAGTT TGAGGGCTAG TAGACCCAGC   
  
  
- TCGTTACCGA AACTACTAAG TCCCCTACCC CGTCGGTGTA GAGGACCGTC ATGTCAATGT AGTTGTCAGG   
  
  
- GTCTCTTTTC CCAACTTAGA CAGTTAGCGA GTTCCTCCTT CTTTTTAGCA TTTTCCCTAC TTCCACCAGT   
  
  
- ACTCCTTCCC TCCTCATTGT TCGTCGTTCG AAGTAGGTTA CTTCTAATAC AACTCTACTT CCTCAAACTA   
  
  
- CTACATGACG AGACGTTTCT TCTCTTTCTA CAATAACGTT TGACGTGGTT ACTAAGGAGT GGGCAGCTTC   
  
  
- GCTCACTATT CAACGTCTTC TTTCCCCCCT TCCCCTTTTG TAGCGCACCC TTCTTCGTCT TATCGTGTTA   
  
  
- TCTTCTCCAC CTAGACTCCT GAGAAGAGTG AGTGACACGA GTTCGTTAAA GTTTAAAACT AGAATCCTCA   
  
  
- CGTTTACTCG TAGAGTCCGT TTATTCCGTC GTAAGAAGTG TTATACCACT ATCGGAGGTC TCCGAACGGG   
  
  
- TAATAAAACG ATTACCAGAA CTCCGAGCGT ATCGACCGTG ACCAAGTTGT TAGAGACGAT TACAACAACT   
  
  
- ACGAGCTTAG TGCAGTAGAC TAAAGAATTT CCGAATATCC GATATATACA GTCGGCAAGG AAAGTTTTCC   
  
  
- TACAATATAA AAGAGCGGTT GTTCTGTTAT GCCTTCAACC GACTCTTCCG TTGGTCCTAG GTGTAGTAAC   
  
  
- TAAAACCTCA GAACAACCCA AATGTCACCG GGACAGAGTA TGTTTTGGAG AGTTTCTCTG GGTGACCGGG   
  
  
- CGGTTCGGAG GCATAATGAC CCTATCTGAT AGGGGTCGTA CCAAAGGCCG GTAGTCTTTC CCAACTTCGC   
  
  
- TGACCGGCAG CCAATCTTCC CATAACGCTC TCTAAATTAC ACGGCAAGTT CATATTCCGG TATCGATTTT   
  
  
- TAACCCTCTC ATATTTCGAT CTCCTAGATC TTTAACTACT CTTACTCTAC CACAAACAGT TAACGTACGA   
  
  
- AGCGAGACCT TGTGACGAAC TACTCTGTCA CCACCGTCTG TCAGGTTTCC TACGAAAGAA TTCCAATTAG   
  
  
- TCTCTCTATT TAGGGGCAGA AAAGTAAGTA CCCTGATAGT TACCTAGTAA GTTACGAGGT AAGAAGTAGT   
  
  
- GAGCCAAGTC CCTCCGCGAG AAGGTGATGA GAAGAAATAA ACTATAGAAA CTTCGTTGAT ACGGGGCACT   
  
  
- TCTAGTACTT TCCGAGGACC AACTCTCACT CTATGTCCCG TTTCTTCGAA ACTTACAGTA TCGTACGCTT   
  
  
- CCACGGCTCT CCTAAGTTTC CGGACTTTGT ATGTTCGTTA CCGTTCGTTC CTGTTGTTCC CGGCCCAAAT   
  
  
- CCGTCGAAGG TGATCTGGCC CTCGAACACT CGTCTCGTTT CCGATACCAC TTTCGTTTGA TGGTATTCCT   
  
  
- AAAACACCAC CTGCTCCTGT CCGTAACCTA CGATGTGCCA ACCTTTCCTT CTTAGAAGAC ACGTGAGAGA   
  
  
- CAAACCGTTG GGTTGAC

+     TATA-box

| Site Name | Organism | Position | Strand | Matrix score. | sequence | function |
| --- | --- | --- | --- | --- | --- | --- |
| TATA-box | Arabidopsis thaliana | 3515 | - | 4 | TATA | core promoter element around -30 of transcription start |
| TATA-box | Oryza sativa | 3334 | + | 7 | TACAAAA | core promoter element around -30 of transcription start |
| TATA-box | Arabidopsis thaliana | 623 | + | 4 | TATA | core promoter element around -30 of transcription start |
| TATA-box | Arabidopsis thaliana | 987 | + | 8 | TATTTAAA | core promoter element around -30 of transcription start |
| TATA-box | Arabidopsis thaliana | 3189 | - | 4 | TATA | core promoter element around -30 of transcription start |
| TATA-box | Arabidopsis thaliana | 1591 | + | 4 | TATA | core promoter element around -30 of transcription start |
| TATA-box | Arabidopsis thaliana | 546 | + | 4 | TATA | core promoter element around -30 of transcription start |
| TATA-box | Arabidopsis thaliana | 798 | - | 5 | TATAA | core promoter element around -30 of transcription start |
| TATA-box | Arabidopsis thaliana | 3229 | - | 4 | TATA | core promoter element around -30 of transcription start |
| TATA-box | Brassica napus | 3197 | - | 6 | ATATAT | core promoter element around -30 of transcription start |
| TATA-box | Brassica napus | 1459 | + | 6 | ATATAT | core promoter element around -30 of transcription start |
| TATA-box | Arabidopsis thaliana | 720 | - | 5 | TATAA | core promoter element around -30 of transcription start |
| TATA-box | Arabidopsis thaliana | 590 | + | 4 | TATA | core promoter element around -30 of transcription start |
| TATA-box | Arabidopsis thaliana | 980 | + | 4 | TATA | core promoter element around -30 of transcription start |
| TATA-box | Brassica napus | 840 | + | 6 | ATATAT | core promoter element around -30 of transcription start |
| TATA-box | Arabidopsis thaliana | 3196 | - | 6 | TATATA | core promoter element around -30 of transcription start |
| TATA-box | Arabidopsis thaliana | 721 | + | 4 | TATA | core promoter element around -30 of transcription start |
| TATA-box | Arabidopsis thaliana | 841 | + | 4 | TATA | core promoter element around -30 of transcription start |
| TATA-box | Arabidopsis thaliana | 1651 | - | 9 | ccTATAAAaa | core promoter element around -30 of transcription start |
| TATA-box | Helianthus annuus | 797 | - | 6 | TATAAA | core promoter element around -30 of transcription start |
| TATA-box | Arabidopsis thaliana | 979 | - | 5 | TATAA | core promoter element around -30 of transcription start |
| TATA-box | Arabidopsis thaliana | 621 | + | 6 | TATATA | core promoter element around -30 of transcription start |
| TATA-box | Arabidopsis thaliana | 3486 | - | 4 | TATA | core promoter element around -30 of transcription start |
| TATA-box | Brassica napus | 978 | + | 6 | ATTATA | core promoter element around -30 of transcription start |
| TATA-box | Arabidopsis thaliana | 3198 | - | 4 | TATA | core promoter element around -30 of transcription start |
| TATA-box | Arabidopsis thaliana | 3188 | - | 5 | TATAA | core promoter element around -30 of transcription start |
| TATA-box | Pisum sativum | 796 | - | 7 | TATAAAA | core promoter element around -30 of transcription start |
| TATA-box | Arabidopsis thaliana | 544 | + | 6 | TATATA | core promoter element around -30 of transcription start |
| TATA-box | Brassica napus | 543 | + | 6 | ATATAT | core promoter element around -30 of transcription start |
| TATA-box | Arabidopsis thaliana | 3228 | - | 5 | TATAA | core promoter element around -30 of transcription start |
| TATA-box | Arabidopsis thaliana | 799 | + | 4 | TATA | core promoter element around -30 of transcription start |
| TATA-box | Arabidopsis thaliana | 620 | - | 7 | TATATAA | core promoter element around -30 of transcription start |
| TATA-box | Arabidopsis thaliana | 1460 | + | 4 | TATA | core promoter element around -30 of transcription start |
| TATA-box | Brassica oleracea | 622 | + | 6 | ATATAA | core promoter element around -30 of transcription start |
| TATA-box | Brassica napus | 619 | + | 6 | ATTATA | core promoter element around -30 of transcription start |
| TATA-box | Arabidopsis thaliana | 634 | + | 4 | TATA | core promoter element around -30 of transcription start |

>HU07G02246.1   
+ -Up\_Stream \_Len000ATTTGA TGTGTATGCT GACTACTTTT GATGATGATG ATCACAGTGA TGCATATTTA   
  
  
+ GAGCTGGTAA ATTGGTCATT CGATTCGAGT ACGGGTCGGG TCAAGTTCTG ATTAAGTGAC ATTTCGCGTC   
  
  
+ GTTTTGGTTT AGTTCGGGTC GGATCGATTT CGGGTTAAAT AATTTTTGGT GGAATACGCT TGTCATGCCA   
  
  
+ AACACAAGCA ACTTTGTTAA AAATTTCGAT TCGGGTCAGG TCAATTCAGG TTTGGGGTCC ACTTTCGAGT   
  
  
+ GAGCATATTT CGGATGTCGG GTCGCGTATG GGTCCGGGTC ATTCGGTTTG AATTTCGGAT CTTGGATCAA   
  
  
+ TTTTGCTAAG TATGTGCATA TTCAACCCTT GATGACATAG GAAACGCTTT GTCATCTCCA CCAATCAGAT   
  
  
+ ATCGTTATCT CTTTAATCAT CGCCATAATA GCATGAGTAA GCATGACCAT CTTTTTCAAA CATGTGATAC   
  
  
+ AGTTAAGGCC TGTTCTTTTC GTTAATAAAT CCCAATTTAT CAATTTTAAT ATATACTTTA ACGAATTTTA   
  
  
+ ACAAAAAATT TTAATTTCAA TCCGCTATAA CAAAAAAAAT ACCAACTTCA ACTTATTATA TAAAAAAAAT   
  
  
+ ATACAATTTT TTTTCAATTT TAGCCAACTT CAACAAAAAA AAAAAAAATT TAACAAATCT CAATCAACTT   
  
  
+ CACCCATTTC TCAACTTATA TCTCCTAATG AAAAGAACAG ATCCTTAGCA AGACTTCTCG ATTCATTTAA   
  
  
+ AAGGATAGTA TGGTAAATTC ATTTTATACA AGTGTCAAGT GTGAATATCT TTTTCACATC AGGTGATATA   
  
  
+ TTAGTAGGAC TTTTCGATTC ACTTATCTTC CTCTTTATGT TTTGTTAATG TGATAGTAAC GATAGGATTG   
  
  
+ CATGTATTTT TTCCAACTAT TCATTTACAA CTATCAATAT CTATTGATAA ATATGTTAAA ATAATTATAT   
  
  
+ GTTATTTAAA TTATTATTTG TTACTTAAGG TGTCGACTTT GTCGAAAGCG ACTTAATTCT AATTAAGATA   
  
  
+ GCGGCAGAGA AAGTTACACC TGATGACCAA GTTAGATCTG GTAAAACTAA CCATAGATCT GAAACCCAAA   
  
  
+ CATAATGATG TGTATTCATA CCCGACCTGA CATCTGAAAT AAGTTTACTC AAAAGTGGTT CCGAAACTTG   
  
  
+ AATTGACTCG AACCGAACCG TATTTGACTC GAAATTTTCT ACAAAGTTGC TTATTTTTGC ATGAAGACAT   
  
  
+ ATTGAGCCAA AAATTAGTTG ATTCGAAGTC AACTTAAACA AAAATTCATT CGACTCAAAC TGAAATATCA   
  
  
+ CTTAACTAAA ACTATGACTC AACTCAAACT TGATCTGATC CGTATTCAAA TCAAATACTC CATTTGCTAG   
  
  
+ GTCTAGAACC AGCCACGACC CCACATGGAC TATGCGGTTC AATGAAACGG CCAAATATAT CGCAATTTCT   
  
  
+ GTCTCCATCA ATCACACCCA TTTGCTACAC CCTGTTAATA TTTGACTCGA CCCTGTGAAT TGCCTTCGCC   
  
  
+ AGTTTTGTTT AATCTCTCTG GTTGCTTCCC ATCCAAGTCT GAATAGTATA GTTTGTACAG TGTGTACTAC   
  
  
+ TAGAAAGCAC GTTCCTTGAA TCTTCTTTCA AAGTTGTTTT TAAAGGGTTG AGTGATCCAT TACGACTTTT   
  
  
+ GATCTGGGTT CATCGCCTCC TCTCCTAATC CCTCACCCGG GTACCCTCAA TTCTCTATCG TCTCTGTTGC   
  
  
+ TCAGCTAATT CAAATTCCTT TAGTTGGTCA TCATTGAAAT CTGAAATCAG GGTGTTTTTC TTTTTATTAT   
  
  
+ TATCTGGCAG TTGGTTTGTT TATCTCTTGG TTTTTGGGGC TTATTTGGGT GCTGGTTGAT GTTATCCTGC   
  
  
+ TTGGGTTGAA TCTTCATCAG GTTTATTTTT GATTCATAGT TGAGGGTGAA AATTATTTCT CATACCCACC   
  
  
+ ACCTGTTTGT TCTTTTGTCT CTGAGAAAAA CCAAAAAGCT TTCTCTTTTT CCTGATGCAT CCTGTGCTCG   
  
  
+ TTGATCCTGA GTTAGTGAAC CATGCGTCCA AATTCAAGCC TGATTTGCTC TCAAACTTTG ATAAACAACA   
  
  
+ AGAATTTAGC AACGGGATCG AACAAGATGA CATCTTGCAA AACCCTGATC TGTGTTTTGA TTCAGAAACC   
  
  
+ CCTTCAGTTG ATCTAACTGA GAGTGTCATC AGTTCTGATT CTGTAGAAGT GCCTGATTTC TCAGATGCTT   
  
  
+ GCCTCAAGTT CATAAGTGAG ATTCTCCTAG AAGAGGATTT GGATGAAAGT CCTGCCTCTG CTCAAGAATT   
  
  
+ TAGGGCTCTC CAAGCCACTG AGAAGTCCCT GTATGATGCT CTTGGAGAGG GCTACTCTTT TTCATCTGAT   
  
  
+ AACAGCCCAT CATCATTAGG ACAGAGTATT GAGCACCAAA ATGAGAATTT TGAATTCAGT TCCGGTTATC   
  
  
+ CTGGAATTGA GGGCTATGTT AATGGTGATA TCACGTTCGA GTCCAACTGG ATGTTCAACC TAAGCCAATT   
  
  
+ GGATCCTGTT CTTACTTTGG ATGATATTTC TCAGCCCTTG TCTGCATCAA ACTCCCGATC ATCTGGGTCG   
  
  
+ AGCAATGGCT TTGATGATTC AGGGGATGGG GCAGCCACAT CTCCTGGCAG TACAGTTACA TCAACAGTCC   
  
  
+ CAGAGAAAAG GGTTGAATCT GTCAATCGCT CAAGGAGGAA GAAAAATCGT AAAAGGGATG AAGGTGGTCA   
  
  
+ TGAGGAAGGG AGGAGTAACA AGCAGCAAGC TTCATCCAAT GAAGATTATG TTGAGATGAA GGAGTTTGAT   
  
  
+ GATGTACTGC TCTGCAAAGA AGAGAAAGAT GTTATTGCAA ACTGCACCAA TGATTCCTCA CCCGTCGAAG   
  
  
+ CGAGTGATAA GTTGCAGAAG AAAGGGGGGA AGGGGAAAAC ATCGCGTGGG AAGAAGCAGA ATAGCACAAT   
  
  
+ AGAAGAGGTG GATCTGAGGA CTCTTCTCAC TCACTGTGCT CAAGCAATTT CAAATTTTGA TCTTAGGAGT   
  
  
+ GCAAATGAGC ATCTCAGGCA AATAAGGCAG CATTCTTCAC AATATGGTGA TAGCCTCCAG AGGCTTGCCC   
  
  
+ ATTATTTTGC TAATGGTCTT GAGGCTCGCA TAGCTGGCAC TGGTTCAACA ATCTCTGCTA ATGTTGTTGA   
  
  
+ TGCTCGAATC ACGTCATCTG ATTTCTTAAA GGCTTATAGG CTATATATGT CAGCCGTTCC TTTCAAAAGG   
  
  
+ ATGTTATATT TTCTCGCCAA CAAGACAATA CGGAAGTTGG CTGAGAAGGC AACCAGGATC CACATCATTG   
  
  
+ ATTTTGGAGT CTTGTTGGGT TTACAGTGGC CCTGTCTCAT ACAAAACCTC TCAAAGAGAC CCACTGGCCC   
  
  
+ GCCAAGCCTC CGTATTACTG GGATAGACTA TCCCCAGCAT GGTTTCCGGC CATCAGAAAG GGTTGAAGCG   
  
  
+ ACTGGCCGTC GGTTAGAAGG GTATTGCGAG AGATTTAATG TGCCGTTCAA GTATAAGGCC ATAGCTAAAA   
  
  
+ ATTGGGAGAG TATAAAGCTA GAGGATCTAG AAATTGATGA GAATGAGATG GTGTTTGTCA ATTGCATGCT   
  
  
+ TCGCTCTGGA ACACTGCTTG ATGAGACAGT GGTGGCAGAC AGTCCAAAGG ATGCTTTCTT AAGGTTAATC   
  
  
+ AGAGAGATAA ATCCCCGTCT TTTCATTCAT GGGACTATCA ATGGATCATT CAATGCTCCA TTCTTCATCA   
  
  
+ CTCGGTTCAG GGAGGCGCTC TTCCACTACT CTTCTTTATT TGATATCTTT GAAGCAACTA TGCCCCGTGA   
  
  
+ AGATCATGAA AGGCTCCTGG TTGAGAGTGA GATACAGGGC AAAGAAGCTT TGAATGTCAT AGCATGCGAA   
  
  
+ GGTGCCGAGA GGATTCAAAG GCCTGAAACA TACAAGCAAT GGCAAGCAAG GACAACAAGG GCCGGGTTTA   
  
  
+ GGCAGCTTCC ACTAGACCGG GAGCTTGTGA GCAGAGCAAA GGCTATGGTG AAAGCAAACT ACCATAAGGA   
  
  
+ TTTTGTGGTG GACGAGGACA GGCATTGGAT GCTACACGGT TGGAAAGGAA GAATCTTCTG TGCACTCTCT   
  
  
+ GTTTGGCAAC CCAACTG  

- -Up\_Stream \_Len000TAAACT ACACATACGA CTGATGAAAA CTACTACTAC TAGTGTCACT ACGTATAAAT   
  
  
- CTCGACCATT TAACCAGTAA GCTAAGCTCA TGCCCAGCCC AGTTCAAGAC TAATTCACTG TAAAGCGCAG   
  
  
- CAAAACCAAA TCAAGCCCAG CCTAGCTAAA GCCCAATTTA TTAAAAACCA CCTTATGCGA ACAGTACGGT   
  
  
- TTGTGTTCGT TGAAACAATT TTTAAAGCTA AGCCCAGTCC AGTTAAGTCC AAACCCCAGG TGAAAGCTCA   
  
  
- CTCGTATAAA GCCTACAGCC CAGCGCATAC CCAGGCCCAG TAAGCCAAAC TTAAAGCCTA GAACCTAGTT   
  
  
- AAAACGATTC ATACACGTAT AAGTTGGGAA CTACTGTATC CTTTGCGAAA CAGTAGAGGT GGTTAGTCTA   
  
  
- TAGCAATAGA GAAATTAGTA GCGGTATTAT CGTACTCATT CGTACTGGTA GAAAAAGTTT GTACACTATG   
  
  
- TCAATTCCGG ACAAGAAAAG CAATTATTTA GGGTTAAATA GTTAAAATTA TATATGAAAT TGCTTAAAAT   
  
  
- TGTTTTTTAA AATTAAAGTT AGGCGATATT GTTTTTTTTA TGGTTGAAGT TGAATAATAT ATTTTTTTTA   
  
  
- TATGTTAAAA AAAAGTTAAA ATCGGTTGAA GTTGTTTTTT TTTTTTTTAA ATTGTTTAGA GTTAGTTGAA   
  
  
- GTGGGTAAAG AGTTGAATAT AGAGGATTAC TTTTCTTGTC TAGGAATCGT TCTGAAGAGC TAAGTAAATT   
  
  
- TTCCTATCAT ACCATTTAAG TAAAATATGT TCACAGTTCA CACTTATAGA AAAAGTGTAG TCCACTATAT   
  
  
- AATCATCCTG AAAAGCTAAG TGAATAGAAG GAGAAATACA AAACAATTAC ACTATCATTG CTATCCTAAC   
  
  
- GTACATAAAA AAGGTTGATA AGTAAATGTT GATAGTTATA GATAACTATT TATACAATTT TATTAATATA   
  
  
- CAATAAATTT AATAATAAAC AATGAATTCC ACAGCTGAAA CAGCTTTCGC TGAATTAAGA TTAATTCTAT   
  
  
- CGCCGTCTCT TTCAATGTGG ACTACTGGTT CAATCTAGAC CATTTTGATT GGTATCTAGA CTTTGGGTTT   
  
  
- GTATTACTAC ACATAAGTAT GGGCTGGACT GTAGACTTTA TTCAAATGAG TTTTCACCAA GGCTTTGAAC   
  
  
- TTAACTGAGC TTGGCTTGGC ATAAACTGAG CTTTAAAAGA TGTTTCAACG AATAAAAACG TACTTCTGTA   
  
  
- TAACTCGGTT TTTAATCAAC TAAGCTTCAG TTGAATTTGT TTTTAAGTAA GCTGAGTTTG ACTTTATAGT   
  
  
- GAATTGATTT TGATACTGAG TTGAGTTTGA ACTAGACTAG GCATAAGTTT AGTTTATGAG GTAAACGATC   
  
  
- CAGATCTTGG TCGGTGCTGG GGTGTACCTG ATACGCCAAG TTACTTTGCC GGTTTATATA GCGTTAAAGA   
  
  
- CAGAGGTAGT TAGTGTGGGT AAACGATGTG GGACAATTAT AAACTGAGCT GGGACACTTA ACGGAAGCGG   
  
  
- TCAAAACAAA TTAGAGAGAC CAACGAAGGG TAGGTTCAGA CTTATCATAT CAAACATGTC ACACATGATG   
  
  
- ATCTTTCGTG CAAGGAACTT AGAAGAAAGT TTCAACAAAA ATTTCCCAAC TCACTAGGTA ATGCTGAAAA   
  
  
- CTAGACCCAA GTAGCGGAGG AGAGGATTAG GGAGTGGGCC CATGGGAGTT AAGAGATAGC AGAGACAACG   
  
  
- AGTCGATTAA GTTTAAGGAA ATCAACCAGT AGTAACTTTA GACTTTAGTC CCACAAAAAG AAAAATAATA   
  
  
- ATAGACCGTC AACCAAACAA ATAGAGAACC AAAAACCCCG AATAAACCCA CGACCAACTA CAATAGGACG   
  
  
- AACCCAACTT AGAAGTAGTC CAAATAAAAA CTAAGTATCA ACTCCCACTT TTAATAAAGA GTATGGGTGG   
  
  
- TGGACAAACA AGAAAACAGA GACTCTTTTT GGTTTTTCGA AAGAGAAAAA GGACTACGTA GGACACGAGC   
  
  
- AACTAGGACT CAATCACTTG GTACGCAGGT TTAAGTTCGG ACTAAACGAG AGTTTGAAAC TATTTGTTGT   
  
  
- TCTTAAATCG TTGCCCTAGC TTGTTCTACT GTAGAACGTT TTGGGACTAG ACACAAAACT AAGTCTTTGG   
  
  
- GGAAGTCAAC TAGATTGACT CTCACAGTAG TCAAGACTAA GACATCTTCA CGGACTAAAG AGTCTACGAA   
  
  
- CGGAGTTCAA GTATTCACTC TAAGAGGATC TTCTCCTAAA CCTACTTTCA GGACGGAGAC GAGTTCTTAA   
  
  
- ATCCCGAGAG GTTCGGTGAC TCTTCAGGGA CATACTACGA GAACCTCTCC CGATGAGAAA AAGTAGACTA   
  
  
- TTGTCGGGTA GTAGTAATCC TGTCTCATAA CTCGTGGTTT TACTCTTAAA ACTTAAGTCA AGGCCAATAG   
  
  
- GACCTTAACT CCCGATACAA TTACCACTAT AGTGCAAGCT CAGGTTGACC TACAAGTTGG ATTCGGTTAA   
  
  
- CCTAGGACAA GAATGAAACC TACTATAAAG AGTCGGGAAC AGACGTAGTT TGAGGGCTAG TAGACCCAGC   
  
  
- TCGTTACCGA AACTACTAAG TCCCCTACCC CGTCGGTGTA GAGGACCGTC ATGTCAATGT AGTTGTCAGG   
  
  
- GTCTCTTTTC CCAACTTAGA CAGTTAGCGA GTTCCTCCTT CTTTTTAGCA TTTTCCCTAC TTCCACCAGT   
  
  
- ACTCCTTCCC TCCTCATTGT TCGTCGTTCG AAGTAGGTTA CTTCTAATAC AACTCTACTT CCTCAAACTA   
  
  
- CTACATGACG AGACGTTTCT TCTCTTTCTA CAATAACGTT TGACGTGGTT ACTAAGGAGT GGGCAGCTTC   
  
  
- GCTCACTATT CAACGTCTTC TTTCCCCCCT TCCCCTTTTG TAGCGCACCC TTCTTCGTCT TATCGTGTTA   
  
  
- TCTTCTCCAC CTAGACTCCT GAGAAGAGTG AGTGACACGA GTTCGTTAAA GTTTAAAACT AGAATCCTCA   
  
  
- CGTTTACTCG TAGAGTCCGT TTATTCCGTC GTAAGAAGTG TTATACCACT ATCGGAGGTC TCCGAACGGG   
  
  
- TAATAAAACG ATTACCAGAA CTCCGAGCGT ATCGACCGTG ACCAAGTTGT TAGAGACGAT TACAACAACT   
  
  
- ACGAGCTTAG TGCAGTAGAC TAAAGAATTT CCGAATATCC GATATATACA GTCGGCAAGG AAAGTTTTCC   
  
  
- TACAATATAA AAGAGCGGTT GTTCTGTTAT GCCTTCAACC GACTCTTCCG TTGGTCCTAG GTGTAGTAAC   
  
  
- TAAAACCTCA GAACAACCCA AATGTCACCG GGACAGAGTA TGTTTTGGAG AGTTTCTCTG GGTGACCGGG   
  
  
- CGGTTCGGAG GCATAATGAC CCTATCTGAT AGGGGTCGTA CCAAAGGCCG GTAGTCTTTC CCAACTTCGC   
  
  
- TGACCGGCAG CCAATCTTCC CATAACGCTC TCTAAATTAC ACGGCAAGTT CATATTCCGG TATCGATTTT   
  
  
- TAACCCTCTC ATATTTCGAT CTCCTAGATC TTTAACTACT CTTACTCTAC CACAAACAGT TAACGTACGA   
  
  
- AGCGAGACCT TGTGACGAAC TACTCTGTCA CCACCGTCTG TCAGGTTTCC TACGAAAGAA TTCCAATTAG   
  
  
- TCTCTCTATT TAGGGGCAGA AAAGTAAGTA CCCTGATAGT TACCTAGTAA GTTACGAGGT AAGAAGTAGT   
  
  
- GAGCCAAGTC CCTCCGCGAG AAGGTGATGA GAAGAAATAA ACTATAGAAA CTTCGTTGAT ACGGGGCACT   
  
  
- TCTAGTACTT TCCGAGGACC AACTCTCACT CTATGTCCCG TTTCTTCGAA ACTTACAGTA TCGTACGCTT   
  
  
- CCACGGCTCT CCTAAGTTTC CGGACTTTGT ATGTTCGTTA CCGTTCGTTC CTGTTGTTCC CGGCCCAAAT   
  
  
- CCGTCGAAGG TGATCTGGCC CTCGAACACT CGTCTCGTTT CCGATACCAC TTTCGTTTGA TGGTATTCCT   
  
  
- AAAACACCAC CTGCTCCTGT CCGTAACCTA CGATGTGCCA ACCTTTCCTT CTTAGAAGAC ACGTGAGAGA   
  
  
- CAAACCGTTG GGTTGAC

+     TATC-box

| Site Name | Organism | Position | Strand | Matrix score. | sequence | function |
| --- | --- | --- | --- | --- | --- | --- |
| TATC-box | Oryza sativa | 3383 | - | 7 | TATCCCA | cis-acting element involved in gibberellin-responsiveness |

>HU07G02246.1   
+ -Up\_Stream \_Len000ATTTGA TGTGTATGCT GACTACTTTT GATGATGATG ATCACAGTGA TGCATATTTA   
  
  
+ GAGCTGGTAA ATTGGTCATT CGATTCGAGT ACGGGTCGGG TCAAGTTCTG ATTAAGTGAC ATTTCGCGTC   
  
  
+ GTTTTGGTTT AGTTCGGGTC GGATCGATTT CGGGTTAAAT AATTTTTGGT GGAATACGCT TGTCATGCCA   
  
  
+ AACACAAGCA ACTTTGTTAA AAATTTCGAT TCGGGTCAGG TCAATTCAGG TTTGGGGTCC ACTTTCGAGT   
  
  
+ GAGCATATTT CGGATGTCGG GTCGCGTATG GGTCCGGGTC ATTCGGTTTG AATTTCGGAT CTTGGATCAA   
  
  
+ TTTTGCTAAG TATGTGCATA TTCAACCCTT GATGACATAG GAAACGCTTT GTCATCTCCA CCAATCAGAT   
  
  
+ ATCGTTATCT CTTTAATCAT CGCCATAATA GCATGAGTAA GCATGACCAT CTTTTTCAAA CATGTGATAC   
  
  
+ AGTTAAGGCC TGTTCTTTTC GTTAATAAAT CCCAATTTAT CAATTTTAAT ATATACTTTA ACGAATTTTA   
  
  
+ ACAAAAAATT TTAATTTCAA TCCGCTATAA CAAAAAAAAT ACCAACTTCA ACTTATTATA TAAAAAAAAT   
  
  
+ ATACAATTTT TTTTCAATTT TAGCCAACTT CAACAAAAAA AAAAAAAATT TAACAAATCT CAATCAACTT   
  
  
+ CACCCATTTC TCAACTTATA TCTCCTAATG AAAAGAACAG ATCCTTAGCA AGACTTCTCG ATTCATTTAA   
  
  
+ AAGGATAGTA TGGTAAATTC ATTTTATACA AGTGTCAAGT GTGAATATCT TTTTCACATC AGGTGATATA   
  
  
+ TTAGTAGGAC TTTTCGATTC ACTTATCTTC CTCTTTATGT TTTGTTAATG TGATAGTAAC GATAGGATTG   
  
  
+ CATGTATTTT TTCCAACTAT TCATTTACAA CTATCAATAT CTATTGATAA ATATGTTAAA ATAATTATAT   
  
  
+ GTTATTTAAA TTATTATTTG TTACTTAAGG TGTCGACTTT GTCGAAAGCG ACTTAATTCT AATTAAGATA   
  
  
+ GCGGCAGAGA AAGTTACACC TGATGACCAA GTTAGATCTG GTAAAACTAA CCATAGATCT GAAACCCAAA   
  
  
+ CATAATGATG TGTATTCATA CCCGACCTGA CATCTGAAAT AAGTTTACTC AAAAGTGGTT CCGAAACTTG   
  
  
+ AATTGACTCG AACCGAACCG TATTTGACTC GAAATTTTCT ACAAAGTTGC TTATTTTTGC ATGAAGACAT   
  
  
+ ATTGAGCCAA AAATTAGTTG ATTCGAAGTC AACTTAAACA AAAATTCATT CGACTCAAAC TGAAATATCA   
  
  
+ CTTAACTAAA ACTATGACTC AACTCAAACT TGATCTGATC CGTATTCAAA TCAAATACTC CATTTGCTAG   
  
  
+ GTCTAGAACC AGCCACGACC CCACATGGAC TATGCGGTTC AATGAAACGG CCAAATATAT CGCAATTTCT   
  
  
+ GTCTCCATCA ATCACACCCA TTTGCTACAC CCTGTTAATA TTTGACTCGA CCCTGTGAAT TGCCTTCGCC   
  
  
+ AGTTTTGTTT AATCTCTCTG GTTGCTTCCC ATCCAAGTCT GAATAGTATA GTTTGTACAG TGTGTACTAC   
  
  
+ TAGAAAGCAC GTTCCTTGAA TCTTCTTTCA AAGTTGTTTT TAAAGGGTTG AGTGATCCAT TACGACTTTT   
  
  
+ GATCTGGGTT CATCGCCTCC TCTCCTAATC CCTCACCCGG GTACCCTCAA TTCTCTATCG TCTCTGTTGC   
  
  
+ TCAGCTAATT CAAATTCCTT TAGTTGGTCA TCATTGAAAT CTGAAATCAG GGTGTTTTTC TTTTTATTAT   
  
  
+ TATCTGGCAG TTGGTTTGTT TATCTCTTGG TTTTTGGGGC TTATTTGGGT GCTGGTTGAT GTTATCCTGC   
  
  
+ TTGGGTTGAA TCTTCATCAG GTTTATTTTT GATTCATAGT TGAGGGTGAA AATTATTTCT CATACCCACC   
  
  
+ ACCTGTTTGT TCTTTTGTCT CTGAGAAAAA CCAAAAAGCT TTCTCTTTTT CCTGATGCAT CCTGTGCTCG   
  
  
+ TTGATCCTGA GTTAGTGAAC CATGCGTCCA AATTCAAGCC TGATTTGCTC TCAAACTTTG ATAAACAACA   
  
  
+ AGAATTTAGC AACGGGATCG AACAAGATGA CATCTTGCAA AACCCTGATC TGTGTTTTGA TTCAGAAACC   
  
  
+ CCTTCAGTTG ATCTAACTGA GAGTGTCATC AGTTCTGATT CTGTAGAAGT GCCTGATTTC TCAGATGCTT   
  
  
+ GCCTCAAGTT CATAAGTGAG ATTCTCCTAG AAGAGGATTT GGATGAAAGT CCTGCCTCTG CTCAAGAATT   
  
  
+ TAGGGCTCTC CAAGCCACTG AGAAGTCCCT GTATGATGCT CTTGGAGAGG GCTACTCTTT TTCATCTGAT   
  
  
+ AACAGCCCAT CATCATTAGG ACAGAGTATT GAGCACCAAA ATGAGAATTT TGAATTCAGT TCCGGTTATC   
  
  
+ CTGGAATTGA GGGCTATGTT AATGGTGATA TCACGTTCGA GTCCAACTGG ATGTTCAACC TAAGCCAATT   
  
  
+ GGATCCTGTT CTTACTTTGG ATGATATTTC TCAGCCCTTG TCTGCATCAA ACTCCCGATC ATCTGGGTCG   
  
  
+ AGCAATGGCT TTGATGATTC AGGGGATGGG GCAGCCACAT CTCCTGGCAG TACAGTTACA TCAACAGTCC   
  
  
+ CAGAGAAAAG GGTTGAATCT GTCAATCGCT CAAGGAGGAA GAAAAATCGT AAAAGGGATG AAGGTGGTCA   
  
  
+ TGAGGAAGGG AGGAGTAACA AGCAGCAAGC TTCATCCAAT GAAGATTATG TTGAGATGAA GGAGTTTGAT   
  
  
+ GATGTACTGC TCTGCAAAGA AGAGAAAGAT GTTATTGCAA ACTGCACCAA TGATTCCTCA CCCGTCGAAG   
  
  
+ CGAGTGATAA GTTGCAGAAG AAAGGGGGGA AGGGGAAAAC ATCGCGTGGG AAGAAGCAGA ATAGCACAAT   
  
  
+ AGAAGAGGTG GATCTGAGGA CTCTTCTCAC TCACTGTGCT CAAGCAATTT CAAATTTTGA TCTTAGGAGT   
  
  
+ GCAAATGAGC ATCTCAGGCA AATAAGGCAG CATTCTTCAC AATATGGTGA TAGCCTCCAG AGGCTTGCCC   
  
  
+ ATTATTTTGC TAATGGTCTT GAGGCTCGCA TAGCTGGCAC TGGTTCAACA ATCTCTGCTA ATGTTGTTGA   
  
  
+ TGCTCGAATC ACGTCATCTG ATTTCTTAAA GGCTTATAGG CTATATATGT CAGCCGTTCC TTTCAAAAGG   
  
  
+ ATGTTATATT TTCTCGCCAA CAAGACAATA CGGAAGTTGG CTGAGAAGGC AACCAGGATC CACATCATTG   
  
  
+ ATTTTGGAGT CTTGTTGGGT TTACAGTGGC CCTGTCTCAT ACAAAACCTC TCAAAGAGAC CCACTGGCCC   
  
  
+ GCCAAGCCTC CGTATTACTG GGATAGACTA TCCCCAGCAT GGTTTCCGGC CATCAGAAAG GGTTGAAGCG   
  
  
+ ACTGGCCGTC GGTTAGAAGG GTATTGCGAG AGATTTAATG TGCCGTTCAA GTATAAGGCC ATAGCTAAAA   
  
  
+ ATTGGGAGAG TATAAAGCTA GAGGATCTAG AAATTGATGA GAATGAGATG GTGTTTGTCA ATTGCATGCT   
  
  
+ TCGCTCTGGA ACACTGCTTG ATGAGACAGT GGTGGCAGAC AGTCCAAAGG ATGCTTTCTT AAGGTTAATC   
  
  
+ AGAGAGATAA ATCCCCGTCT TTTCATTCAT GGGACTATCA ATGGATCATT CAATGCTCCA TTCTTCATCA   
  
  
+ CTCGGTTCAG GGAGGCGCTC TTCCACTACT CTTCTTTATT TGATATCTTT GAAGCAACTA TGCCCCGTGA   
  
  
+ AGATCATGAA AGGCTCCTGG TTGAGAGTGA GATACAGGGC AAAGAAGCTT TGAATGTCAT AGCATGCGAA   
  
  
+ GGTGCCGAGA GGATTCAAAG GCCTGAAACA TACAAGCAAT GGCAAGCAAG GACAACAAGG GCCGGGTTTA   
  
  
+ GGCAGCTTCC ACTAGACCGG GAGCTTGTGA GCAGAGCAAA GGCTATGGTG AAAGCAAACT ACCATAAGGA   
  
  
+ TTTTGTGGTG GACGAGGACA GGCATTGGAT GCTACACGGT TGGAAAGGAA GAATCTTCTG TGCACTCTCT   
  
  
+ GTTTGGCAAC CCAACTG  

- -Up\_Stream \_Len000TAAACT ACACATACGA CTGATGAAAA CTACTACTAC TAGTGTCACT ACGTATAAAT   
  
  
- CTCGACCATT TAACCAGTAA GCTAAGCTCA TGCCCAGCCC AGTTCAAGAC TAATTCACTG TAAAGCGCAG   
  
  
- CAAAACCAAA TCAAGCCCAG CCTAGCTAAA GCCCAATTTA TTAAAAACCA CCTTATGCGA ACAGTACGGT   
  
  
- TTGTGTTCGT TGAAACAATT TTTAAAGCTA AGCCCAGTCC AGTTAAGTCC AAACCCCAGG TGAAAGCTCA   
  
  
- CTCGTATAAA GCCTACAGCC CAGCGCATAC CCAGGCCCAG TAAGCCAAAC TTAAAGCCTA GAACCTAGTT   
  
  
- AAAACGATTC ATACACGTAT AAGTTGGGAA CTACTGTATC CTTTGCGAAA CAGTAGAGGT GGTTAGTCTA   
  
  
- TAGCAATAGA GAAATTAGTA GCGGTATTAT CGTACTCATT CGTACTGGTA GAAAAAGTTT GTACACTATG   
  
  
- TCAATTCCGG ACAAGAAAAG CAATTATTTA GGGTTAAATA GTTAAAATTA TATATGAAAT TGCTTAAAAT   
  
  
- TGTTTTTTAA AATTAAAGTT AGGCGATATT GTTTTTTTTA TGGTTGAAGT TGAATAATAT ATTTTTTTTA   
  
  
- TATGTTAAAA AAAAGTTAAA ATCGGTTGAA GTTGTTTTTT TTTTTTTTAA ATTGTTTAGA GTTAGTTGAA   
  
  
- GTGGGTAAAG AGTTGAATAT AGAGGATTAC TTTTCTTGTC TAGGAATCGT TCTGAAGAGC TAAGTAAATT   
  
  
- TTCCTATCAT ACCATTTAAG TAAAATATGT TCACAGTTCA CACTTATAGA AAAAGTGTAG TCCACTATAT   
  
  
- AATCATCCTG AAAAGCTAAG TGAATAGAAG GAGAAATACA AAACAATTAC ACTATCATTG CTATCCTAAC   
  
  
- GTACATAAAA AAGGTTGATA AGTAAATGTT GATAGTTATA GATAACTATT TATACAATTT TATTAATATA   
  
  
- CAATAAATTT AATAATAAAC AATGAATTCC ACAGCTGAAA CAGCTTTCGC TGAATTAAGA TTAATTCTAT   
  
  
- CGCCGTCTCT TTCAATGTGG ACTACTGGTT CAATCTAGAC CATTTTGATT GGTATCTAGA CTTTGGGTTT   
  
  
- GTATTACTAC ACATAAGTAT GGGCTGGACT GTAGACTTTA TTCAAATGAG TTTTCACCAA GGCTTTGAAC   
  
  
- TTAACTGAGC TTGGCTTGGC ATAAACTGAG CTTTAAAAGA TGTTTCAACG AATAAAAACG TACTTCTGTA   
  
  
- TAACTCGGTT TTTAATCAAC TAAGCTTCAG TTGAATTTGT TTTTAAGTAA GCTGAGTTTG ACTTTATAGT   
  
  
- GAATTGATTT TGATACTGAG TTGAGTTTGA ACTAGACTAG GCATAAGTTT AGTTTATGAG GTAAACGATC   
  
  
- CAGATCTTGG TCGGTGCTGG GGTGTACCTG ATACGCCAAG TTACTTTGCC GGTTTATATA GCGTTAAAGA   
  
  
- CAGAGGTAGT TAGTGTGGGT AAACGATGTG GGACAATTAT AAACTGAGCT GGGACACTTA ACGGAAGCGG   
  
  
- TCAAAACAAA TTAGAGAGAC CAACGAAGGG TAGGTTCAGA CTTATCATAT CAAACATGTC ACACATGATG   
  
  
- ATCTTTCGTG CAAGGAACTT AGAAGAAAGT TTCAACAAAA ATTTCCCAAC TCACTAGGTA ATGCTGAAAA   
  
  
- CTAGACCCAA GTAGCGGAGG AGAGGATTAG GGAGTGGGCC CATGGGAGTT AAGAGATAGC AGAGACAACG   
  
  
- AGTCGATTAA GTTTAAGGAA ATCAACCAGT AGTAACTTTA GACTTTAGTC CCACAAAAAG AAAAATAATA   
  
  
- ATAGACCGTC AACCAAACAA ATAGAGAACC AAAAACCCCG AATAAACCCA CGACCAACTA CAATAGGACG   
  
  
- AACCCAACTT AGAAGTAGTC CAAATAAAAA CTAAGTATCA ACTCCCACTT TTAATAAAGA GTATGGGTGG   
  
  
- TGGACAAACA AGAAAACAGA GACTCTTTTT GGTTTTTCGA AAGAGAAAAA GGACTACGTA GGACACGAGC   
  
  
- AACTAGGACT CAATCACTTG GTACGCAGGT TTAAGTTCGG ACTAAACGAG AGTTTGAAAC TATTTGTTGT   
  
  
- TCTTAAATCG TTGCCCTAGC TTGTTCTACT GTAGAACGTT TTGGGACTAG ACACAAAACT AAGTCTTTGG   
  
  
- GGAAGTCAAC TAGATTGACT CTCACAGTAG TCAAGACTAA GACATCTTCA CGGACTAAAG AGTCTACGAA   
  
  
- CGGAGTTCAA GTATTCACTC TAAGAGGATC TTCTCCTAAA CCTACTTTCA GGACGGAGAC GAGTTCTTAA   
  
  
- ATCCCGAGAG GTTCGGTGAC TCTTCAGGGA CATACTACGA GAACCTCTCC CGATGAGAAA AAGTAGACTA   
  
  
- TTGTCGGGTA GTAGTAATCC TGTCTCATAA CTCGTGGTTT TACTCTTAAA ACTTAAGTCA AGGCCAATAG   
  
  
- GACCTTAACT CCCGATACAA TTACCACTAT AGTGCAAGCT CAGGTTGACC TACAAGTTGG ATTCGGTTAA   
  
  
- CCTAGGACAA GAATGAAACC TACTATAAAG AGTCGGGAAC AGACGTAGTT TGAGGGCTAG TAGACCCAGC   
  
  
- TCGTTACCGA AACTACTAAG TCCCCTACCC CGTCGGTGTA GAGGACCGTC ATGTCAATGT AGTTGTCAGG   
  
  
- GTCTCTTTTC CCAACTTAGA CAGTTAGCGA GTTCCTCCTT CTTTTTAGCA TTTTCCCTAC TTCCACCAGT   
  
  
- ACTCCTTCCC TCCTCATTGT TCGTCGTTCG AAGTAGGTTA CTTCTAATAC AACTCTACTT CCTCAAACTA   
  
  
- CTACATGACG AGACGTTTCT TCTCTTTCTA CAATAACGTT TGACGTGGTT ACTAAGGAGT GGGCAGCTTC   
  
  
- GCTCACTATT CAACGTCTTC TTTCCCCCCT TCCCCTTTTG TAGCGCACCC TTCTTCGTCT TATCGTGTTA   
  
  
- TCTTCTCCAC CTAGACTCCT GAGAAGAGTG AGTGACACGA GTTCGTTAAA GTTTAAAACT AGAATCCTCA   
  
  
- CGTTTACTCG TAGAGTCCGT TTATTCCGTC GTAAGAAGTG TTATACCACT ATCGGAGGTC TCCGAACGGG   
  
  
- TAATAAAACG ATTACCAGAA CTCCGAGCGT ATCGACCGTG ACCAAGTTGT TAGAGACGAT TACAACAACT   
  
  
- ACGAGCTTAG TGCAGTAGAC TAAAGAATTT CCGAATATCC GATATATACA GTCGGCAAGG AAAGTTTTCC   
  
  
- TACAATATAA AAGAGCGGTT GTTCTGTTAT GCCTTCAACC GACTCTTCCG TTGGTCCTAG GTGTAGTAAC   
  
  
- TAAAACCTCA GAACAACCCA AATGTCACCG GGACAGAGTA TGTTTTGGAG AGTTTCTCTG GGTGACCGGG   
  
  
- CGGTTCGGAG GCATAATGAC CCTATCTGAT AGGGGTCGTA CCAAAGGCCG GTAGTCTTTC CCAACTTCGC   
  
  
- TGACCGGCAG CCAATCTTCC CATAACGCTC TCTAAATTAC ACGGCAAGTT CATATTCCGG TATCGATTTT   
  
  
- TAACCCTCTC ATATTTCGAT CTCCTAGATC TTTAACTACT CTTACTCTAC CACAAACAGT TAACGTACGA   
  
  
- AGCGAGACCT TGTGACGAAC TACTCTGTCA CCACCGTCTG TCAGGTTTCC TACGAAAGAA TTCCAATTAG   
  
  
- TCTCTCTATT TAGGGGCAGA AAAGTAAGTA CCCTGATAGT TACCTAGTAA GTTACGAGGT AAGAAGTAGT   
  
  
- GAGCCAAGTC CCTCCGCGAG AAGGTGATGA GAAGAAATAA ACTATAGAAA CTTCGTTGAT ACGGGGCACT   
  
  
- TCTAGTACTT TCCGAGGACC AACTCTCACT CTATGTCCCG TTTCTTCGAA ACTTACAGTA TCGTACGCTT   
  
  
- CCACGGCTCT CCTAAGTTTC CGGACTTTGT ATGTTCGTTA CCGTTCGTTC CTGTTGTTCC CGGCCCAAAT   
  
  
- CCGTCGAAGG TGATCTGGCC CTCGAACACT CGTCTCGTTT CCGATACCAC TTTCGTTTGA TGGTATTCCT   
  
  
- AAAACACCAC CTGCTCCTGT CCGTAACCTA CGATGTGCCA ACCTTTCCTT CTTAGAAGAC ACGTGAGAGA   
  
  
- CAAACCGTTG GGTTGAC

+     TC-rich repeats

| Site Name | Organism | Position | Strand | Matrix score. | sequence | function |
| --- | --- | --- | --- | --- | --- | --- |
| TC-rich repeats | Nicotiana tabacum | 1734 | + | 9 | ATTCTCTAAC | cis-acting element involved in defense and stress responsiveness |

>HU07G02246.1   
+ -Up\_Stream \_Len000ATTTGA TGTGTATGCT GACTACTTTT GATGATGATG ATCACAGTGA TGCATATTTA   
  
  
+ GAGCTGGTAA ATTGGTCATT CGATTCGAGT ACGGGTCGGG TCAAGTTCTG ATTAAGTGAC ATTTCGCGTC   
  
  
+ GTTTTGGTTT AGTTCGGGTC GGATCGATTT CGGGTTAAAT AATTTTTGGT GGAATACGCT TGTCATGCCA   
  
  
+ AACACAAGCA ACTTTGTTAA AAATTTCGAT TCGGGTCAGG TCAATTCAGG TTTGGGGTCC ACTTTCGAGT   
  
  
+ GAGCATATTT CGGATGTCGG GTCGCGTATG GGTCCGGGTC ATTCGGTTTG AATTTCGGAT CTTGGATCAA   
  
  
+ TTTTGCTAAG TATGTGCATA TTCAACCCTT GATGACATAG GAAACGCTTT GTCATCTCCA CCAATCAGAT   
  
  
+ ATCGTTATCT CTTTAATCAT CGCCATAATA GCATGAGTAA GCATGACCAT CTTTTTCAAA CATGTGATAC   
  
  
+ AGTTAAGGCC TGTTCTTTTC GTTAATAAAT CCCAATTTAT CAATTTTAAT ATATACTTTA ACGAATTTTA   
  
  
+ ACAAAAAATT TTAATTTCAA TCCGCTATAA CAAAAAAAAT ACCAACTTCA ACTTATTATA TAAAAAAAAT   
  
  
+ ATACAATTTT TTTTCAATTT TAGCCAACTT CAACAAAAAA AAAAAAAATT TAACAAATCT CAATCAACTT   
  
  
+ CACCCATTTC TCAACTTATA TCTCCTAATG AAAAGAACAG ATCCTTAGCA AGACTTCTCG ATTCATTTAA   
  
  
+ AAGGATAGTA TGGTAAATTC ATTTTATACA AGTGTCAAGT GTGAATATCT TTTTCACATC AGGTGATATA   
  
  
+ TTAGTAGGAC TTTTCGATTC ACTTATCTTC CTCTTTATGT TTTGTTAATG TGATAGTAAC GATAGGATTG   
  
  
+ CATGTATTTT TTCCAACTAT TCATTTACAA CTATCAATAT CTATTGATAA ATATGTTAAA ATAATTATAT   
  
  
+ GTTATTTAAA TTATTATTTG TTACTTAAGG TGTCGACTTT GTCGAAAGCG ACTTAATTCT AATTAAGATA   
  
  
+ GCGGCAGAGA AAGTTACACC TGATGACCAA GTTAGATCTG GTAAAACTAA CCATAGATCT GAAACCCAAA   
  
  
+ CATAATGATG TGTATTCATA CCCGACCTGA CATCTGAAAT AAGTTTACTC AAAAGTGGTT CCGAAACTTG   
  
  
+ AATTGACTCG AACCGAACCG TATTTGACTC GAAATTTTCT ACAAAGTTGC TTATTTTTGC ATGAAGACAT   
  
  
+ ATTGAGCCAA AAATTAGTTG ATTCGAAGTC AACTTAAACA AAAATTCATT CGACTCAAAC TGAAATATCA   
  
  
+ CTTAACTAAA ACTATGACTC AACTCAAACT TGATCTGATC CGTATTCAAA TCAAATACTC CATTTGCTAG   
  
  
+ GTCTAGAACC AGCCACGACC CCACATGGAC TATGCGGTTC AATGAAACGG CCAAATATAT CGCAATTTCT   
  
  
+ GTCTCCATCA ATCACACCCA TTTGCTACAC CCTGTTAATA TTTGACTCGA CCCTGTGAAT TGCCTTCGCC   
  
  
+ AGTTTTGTTT AATCTCTCTG GTTGCTTCCC ATCCAAGTCT GAATAGTATA GTTTGTACAG TGTGTACTAC   
  
  
+ TAGAAAGCAC GTTCCTTGAA TCTTCTTTCA AAGTTGTTTT TAAAGGGTTG AGTGATCCAT TACGACTTTT   
  
  
+ GATCTGGGTT CATCGCCTCC TCTCCTAATC CCTCACCCGG GTACCCTCAA TTCTCTATCG TCTCTGTTGC   
  
  
+ TCAGCTAATT CAAATTCCTT TAGTTGGTCA TCATTGAAAT CTGAAATCAG GGTGTTTTTC TTTTTATTAT   
  
  
+ TATCTGGCAG TTGGTTTGTT TATCTCTTGG TTTTTGGGGC TTATTTGGGT GCTGGTTGAT GTTATCCTGC   
  
  
+ TTGGGTTGAA TCTTCATCAG GTTTATTTTT GATTCATAGT TGAGGGTGAA AATTATTTCT CATACCCACC   
  
  
+ ACCTGTTTGT TCTTTTGTCT CTGAGAAAAA CCAAAAAGCT TTCTCTTTTT CCTGATGCAT CCTGTGCTCG   
  
  
+ TTGATCCTGA GTTAGTGAAC CATGCGTCCA AATTCAAGCC TGATTTGCTC TCAAACTTTG ATAAACAACA   
  
  
+ AGAATTTAGC AACGGGATCG AACAAGATGA CATCTTGCAA AACCCTGATC TGTGTTTTGA TTCAGAAACC   
  
  
+ CCTTCAGTTG ATCTAACTGA GAGTGTCATC AGTTCTGATT CTGTAGAAGT GCCTGATTTC TCAGATGCTT   
  
  
+ GCCTCAAGTT CATAAGTGAG ATTCTCCTAG AAGAGGATTT GGATGAAAGT CCTGCCTCTG CTCAAGAATT   
  
  
+ TAGGGCTCTC CAAGCCACTG AGAAGTCCCT GTATGATGCT CTTGGAGAGG GCTACTCTTT TTCATCTGAT   
  
  
+ AACAGCCCAT CATCATTAGG ACAGAGTATT GAGCACCAAA ATGAGAATTT TGAATTCAGT TCCGGTTATC   
  
  
+ CTGGAATTGA GGGCTATGTT AATGGTGATA TCACGTTCGA GTCCAACTGG ATGTTCAACC TAAGCCAATT   
  
  
+ GGATCCTGTT CTTACTTTGG ATGATATTTC TCAGCCCTTG TCTGCATCAA ACTCCCGATC ATCTGGGTCG   
  
  
+ AGCAATGGCT TTGATGATTC AGGGGATGGG GCAGCCACAT CTCCTGGCAG TACAGTTACA TCAACAGTCC   
  
  
+ CAGAGAAAAG GGTTGAATCT GTCAATCGCT CAAGGAGGAA GAAAAATCGT AAAAGGGATG AAGGTGGTCA   
  
  
+ TGAGGAAGGG AGGAGTAACA AGCAGCAAGC TTCATCCAAT GAAGATTATG TTGAGATGAA GGAGTTTGAT   
  
  
+ GATGTACTGC TCTGCAAAGA AGAGAAAGAT GTTATTGCAA ACTGCACCAA TGATTCCTCA CCCGTCGAAG   
  
  
+ CGAGTGATAA GTTGCAGAAG AAAGGGGGGA AGGGGAAAAC ATCGCGTGGG AAGAAGCAGA ATAGCACAAT   
  
  
+ AGAAGAGGTG GATCTGAGGA CTCTTCTCAC TCACTGTGCT CAAGCAATTT CAAATTTTGA TCTTAGGAGT   
  
  
+ GCAAATGAGC ATCTCAGGCA AATAAGGCAG CATTCTTCAC AATATGGTGA TAGCCTCCAG AGGCTTGCCC   
  
  
+ ATTATTTTGC TAATGGTCTT GAGGCTCGCA TAGCTGGCAC TGGTTCAACA ATCTCTGCTA ATGTTGTTGA   
  
  
+ TGCTCGAATC ACGTCATCTG ATTTCTTAAA GGCTTATAGG CTATATATGT CAGCCGTTCC TTTCAAAAGG   
  
  
+ ATGTTATATT TTCTCGCCAA CAAGACAATA CGGAAGTTGG CTGAGAAGGC AACCAGGATC CACATCATTG   
  
  
+ ATTTTGGAGT CTTGTTGGGT TTACAGTGGC CCTGTCTCAT ACAAAACCTC TCAAAGAGAC CCACTGGCCC   
  
  
+ GCCAAGCCTC CGTATTACTG GGATAGACTA TCCCCAGCAT GGTTTCCGGC CATCAGAAAG GGTTGAAGCG   
  
  
+ ACTGGCCGTC GGTTAGAAGG GTATTGCGAG AGATTTAATG TGCCGTTCAA GTATAAGGCC ATAGCTAAAA   
  
  
+ ATTGGGAGAG TATAAAGCTA GAGGATCTAG AAATTGATGA GAATGAGATG GTGTTTGTCA ATTGCATGCT   
  
  
+ TCGCTCTGGA ACACTGCTTG ATGAGACAGT GGTGGCAGAC AGTCCAAAGG ATGCTTTCTT AAGGTTAATC   
  
  
+ AGAGAGATAA ATCCCCGTCT TTTCATTCAT GGGACTATCA ATGGATCATT CAATGCTCCA TTCTTCATCA   
  
  
+ CTCGGTTCAG GGAGGCGCTC TTCCACTACT CTTCTTTATT TGATATCTTT GAAGCAACTA TGCCCCGTGA   
  
  
+ AGATCATGAA AGGCTCCTGG TTGAGAGTGA GATACAGGGC AAAGAAGCTT TGAATGTCAT AGCATGCGAA   
  
  
+ GGTGCCGAGA GGATTCAAAG GCCTGAAACA TACAAGCAAT GGCAAGCAAG GACAACAAGG GCCGGGTTTA   
  
  
+ GGCAGCTTCC ACTAGACCGG GAGCTTGTGA GCAGAGCAAA GGCTATGGTG AAAGCAAACT ACCATAAGGA   
  
  
+ TTTTGTGGTG GACGAGGACA GGCATTGGAT GCTACACGGT TGGAAAGGAA GAATCTTCTG TGCACTCTCT   
  
  
+ GTTTGGCAAC CCAACTG  

- -Up\_Stream \_Len000TAAACT ACACATACGA CTGATGAAAA CTACTACTAC TAGTGTCACT ACGTATAAAT   
  
  
- CTCGACCATT TAACCAGTAA GCTAAGCTCA TGCCCAGCCC AGTTCAAGAC TAATTCACTG TAAAGCGCAG   
  
  
- CAAAACCAAA TCAAGCCCAG CCTAGCTAAA GCCCAATTTA TTAAAAACCA CCTTATGCGA ACAGTACGGT   
  
  
- TTGTGTTCGT TGAAACAATT TTTAAAGCTA AGCCCAGTCC AGTTAAGTCC AAACCCCAGG TGAAAGCTCA   
  
  
- CTCGTATAAA GCCTACAGCC CAGCGCATAC CCAGGCCCAG TAAGCCAAAC TTAAAGCCTA GAACCTAGTT   
  
  
- AAAACGATTC ATACACGTAT AAGTTGGGAA CTACTGTATC CTTTGCGAAA CAGTAGAGGT GGTTAGTCTA   
  
  
- TAGCAATAGA GAAATTAGTA GCGGTATTAT CGTACTCATT CGTACTGGTA GAAAAAGTTT GTACACTATG   
  
  
- TCAATTCCGG ACAAGAAAAG CAATTATTTA GGGTTAAATA GTTAAAATTA TATATGAAAT TGCTTAAAAT   
  
  
- TGTTTTTTAA AATTAAAGTT AGGCGATATT GTTTTTTTTA TGGTTGAAGT TGAATAATAT ATTTTTTTTA   
  
  
- TATGTTAAAA AAAAGTTAAA ATCGGTTGAA GTTGTTTTTT TTTTTTTTAA ATTGTTTAGA GTTAGTTGAA   
  
  
- GTGGGTAAAG AGTTGAATAT AGAGGATTAC TTTTCTTGTC TAGGAATCGT TCTGAAGAGC TAAGTAAATT   
  
  
- TTCCTATCAT ACCATTTAAG TAAAATATGT TCACAGTTCA CACTTATAGA AAAAGTGTAG TCCACTATAT   
  
  
- AATCATCCTG AAAAGCTAAG TGAATAGAAG GAGAAATACA AAACAATTAC ACTATCATTG CTATCCTAAC   
  
  
- GTACATAAAA AAGGTTGATA AGTAAATGTT GATAGTTATA GATAACTATT TATACAATTT TATTAATATA   
  
  
- CAATAAATTT AATAATAAAC AATGAATTCC ACAGCTGAAA CAGCTTTCGC TGAATTAAGA TTAATTCTAT   
  
  
- CGCCGTCTCT TTCAATGTGG ACTACTGGTT CAATCTAGAC CATTTTGATT GGTATCTAGA CTTTGGGTTT   
  
  
- GTATTACTAC ACATAAGTAT GGGCTGGACT GTAGACTTTA TTCAAATGAG TTTTCACCAA GGCTTTGAAC   
  
  
- TTAACTGAGC TTGGCTTGGC ATAAACTGAG CTTTAAAAGA TGTTTCAACG AATAAAAACG TACTTCTGTA   
  
  
- TAACTCGGTT TTTAATCAAC TAAGCTTCAG TTGAATTTGT TTTTAAGTAA GCTGAGTTTG ACTTTATAGT   
  
  
- GAATTGATTT TGATACTGAG TTGAGTTTGA ACTAGACTAG GCATAAGTTT AGTTTATGAG GTAAACGATC   
  
  
- CAGATCTTGG TCGGTGCTGG GGTGTACCTG ATACGCCAAG TTACTTTGCC GGTTTATATA GCGTTAAAGA   
  
  
- CAGAGGTAGT TAGTGTGGGT AAACGATGTG GGACAATTAT AAACTGAGCT GGGACACTTA ACGGAAGCGG   
  
  
- TCAAAACAAA TTAGAGAGAC CAACGAAGGG TAGGTTCAGA CTTATCATAT CAAACATGTC ACACATGATG   
  
  
- ATCTTTCGTG CAAGGAACTT AGAAGAAAGT TTCAACAAAA ATTTCCCAAC TCACTAGGTA ATGCTGAAAA   
  
  
- CTAGACCCAA GTAGCGGAGG AGAGGATTAG GGAGTGGGCC CATGGGAGTT AAGAGATAGC AGAGACAACG   
  
  
- AGTCGATTAA GTTTAAGGAA ATCAACCAGT AGTAACTTTA GACTTTAGTC CCACAAAAAG AAAAATAATA   
  
  
- ATAGACCGTC AACCAAACAA ATAGAGAACC AAAAACCCCG AATAAACCCA CGACCAACTA CAATAGGACG   
  
  
- AACCCAACTT AGAAGTAGTC CAAATAAAAA CTAAGTATCA ACTCCCACTT TTAATAAAGA GTATGGGTGG   
  
  
- TGGACAAACA AGAAAACAGA GACTCTTTTT GGTTTTTCGA AAGAGAAAAA GGACTACGTA GGACACGAGC   
  
  
- AACTAGGACT CAATCACTTG GTACGCAGGT TTAAGTTCGG ACTAAACGAG AGTTTGAAAC TATTTGTTGT   
  
  
- TCTTAAATCG TTGCCCTAGC TTGTTCTACT GTAGAACGTT TTGGGACTAG ACACAAAACT AAGTCTTTGG   
  
  
- GGAAGTCAAC TAGATTGACT CTCACAGTAG TCAAGACTAA GACATCTTCA CGGACTAAAG AGTCTACGAA   
  
  
- CGGAGTTCAA GTATTCACTC TAAGAGGATC TTCTCCTAAA CCTACTTTCA GGACGGAGAC GAGTTCTTAA   
  
  
- ATCCCGAGAG GTTCGGTGAC TCTTCAGGGA CATACTACGA GAACCTCTCC CGATGAGAAA AAGTAGACTA   
  
  
- TTGTCGGGTA GTAGTAATCC TGTCTCATAA CTCGTGGTTT TACTCTTAAA ACTTAAGTCA AGGCCAATAG   
  
  
- GACCTTAACT CCCGATACAA TTACCACTAT AGTGCAAGCT CAGGTTGACC TACAAGTTGG ATTCGGTTAA   
  
  
- CCTAGGACAA GAATGAAACC TACTATAAAG AGTCGGGAAC AGACGTAGTT TGAGGGCTAG TAGACCCAGC   
  
  
- TCGTTACCGA AACTACTAAG TCCCCTACCC CGTCGGTGTA GAGGACCGTC ATGTCAATGT AGTTGTCAGG   
  
  
- GTCTCTTTTC CCAACTTAGA CAGTTAGCGA GTTCCTCCTT CTTTTTAGCA TTTTCCCTAC TTCCACCAGT   
  
  
- ACTCCTTCCC TCCTCATTGT TCGTCGTTCG AAGTAGGTTA CTTCTAATAC AACTCTACTT CCTCAAACTA   
  
  
- CTACATGACG AGACGTTTCT TCTCTTTCTA CAATAACGTT TGACGTGGTT ACTAAGGAGT GGGCAGCTTC   
  
  
- GCTCACTATT CAACGTCTTC TTTCCCCCCT TCCCCTTTTG TAGCGCACCC TTCTTCGTCT TATCGTGTTA   
  
  
- TCTTCTCCAC CTAGACTCCT GAGAAGAGTG AGTGACACGA GTTCGTTAAA GTTTAAAACT AGAATCCTCA   
  
  
- CGTTTACTCG TAGAGTCCGT TTATTCCGTC GTAAGAAGTG TTATACCACT ATCGGAGGTC TCCGAACGGG   
  
  
- TAATAAAACG ATTACCAGAA CTCCGAGCGT ATCGACCGTG ACCAAGTTGT TAGAGACGAT TACAACAACT   
  
  
- ACGAGCTTAG TGCAGTAGAC TAAAGAATTT CCGAATATCC GATATATACA GTCGGCAAGG AAAGTTTTCC   
  
  
- TACAATATAA AAGAGCGGTT GTTCTGTTAT GCCTTCAACC GACTCTTCCG TTGGTCCTAG GTGTAGTAAC   
  
  
- TAAAACCTCA GAACAACCCA AATGTCACCG GGACAGAGTA TGTTTTGGAG AGTTTCTCTG GGTGACCGGG   
  
  
- CGGTTCGGAG GCATAATGAC CCTATCTGAT AGGGGTCGTA CCAAAGGCCG GTAGTCTTTC CCAACTTCGC   
  
  
- TGACCGGCAG CCAATCTTCC CATAACGCTC TCTAAATTAC ACGGCAAGTT CATATTCCGG TATCGATTTT   
  
  
- TAACCCTCTC ATATTTCGAT CTCCTAGATC TTTAACTACT CTTACTCTAC CACAAACAGT TAACGTACGA   
  
  
- AGCGAGACCT TGTGACGAAC TACTCTGTCA CCACCGTCTG TCAGGTTTCC TACGAAAGAA TTCCAATTAG   
  
  
- TCTCTCTATT TAGGGGCAGA AAAGTAAGTA CCCTGATAGT TACCTAGTAA GTTACGAGGT AAGAAGTAGT   
  
  
- GAGCCAAGTC CCTCCGCGAG AAGGTGATGA GAAGAAATAA ACTATAGAAA CTTCGTTGAT ACGGGGCACT   
  
  
- TCTAGTACTT TCCGAGGACC AACTCTCACT CTATGTCCCG TTTCTTCGAA ACTTACAGTA TCGTACGCTT   
  
  
- CCACGGCTCT CCTAAGTTTC CGGACTTTGT ATGTTCGTTA CCGTTCGTTC CTGTTGTTCC CGGCCCAAAT   
  
  
- CCGTCGAAGG TGATCTGGCC CTCGAACACT CGTCTCGTTT CCGATACCAC TTTCGTTTGA TGGTATTCCT   
  
  
- AAAACACCAC CTGCTCCTGT CCGTAACCTA CGATGTGCCA ACCTTTCCTT CTTAGAAGAC ACGTGAGAGA   
  
  
- CAAACCGTTG GGTTGAC

+     TCA

| Site Name | Organism | Position | Strand | Matrix score. | sequence | function |
| --- | --- | --- | --- | --- | --- | --- |
| TCA | Pisum sativum | 46 | - | 9 | TCATCTTCAT |  |
| TCA | Pisum sativum | 2773 | - | 9 | TCATCTTCAT |  |

>HU07G02246.1   
+ -Up\_Stream \_Len000ATTTGA TGTGTATGCT GACTACTTTT GATGATGATG ATCACAGTGA TGCATATTTA   
  
  
+ GAGCTGGTAA ATTGGTCATT CGATTCGAGT ACGGGTCGGG TCAAGTTCTG ATTAAGTGAC ATTTCGCGTC   
  
  
+ GTTTTGGTTT AGTTCGGGTC GGATCGATTT CGGGTTAAAT AATTTTTGGT GGAATACGCT TGTCATGCCA   
  
  
+ AACACAAGCA ACTTTGTTAA AAATTTCGAT TCGGGTCAGG TCAATTCAGG TTTGGGGTCC ACTTTCGAGT   
  
  
+ GAGCATATTT CGGATGTCGG GTCGCGTATG GGTCCGGGTC ATTCGGTTTG AATTTCGGAT CTTGGATCAA   
  
  
+ TTTTGCTAAG TATGTGCATA TTCAACCCTT GATGACATAG GAAACGCTTT GTCATCTCCA CCAATCAGAT   
  
  
+ ATCGTTATCT CTTTAATCAT CGCCATAATA GCATGAGTAA GCATGACCAT CTTTTTCAAA CATGTGATAC   
  
  
+ AGTTAAGGCC TGTTCTTTTC GTTAATAAAT CCCAATTTAT CAATTTTAAT ATATACTTTA ACGAATTTTA   
  
  
+ ACAAAAAATT TTAATTTCAA TCCGCTATAA CAAAAAAAAT ACCAACTTCA ACTTATTATA TAAAAAAAAT   
  
  
+ ATACAATTTT TTTTCAATTT TAGCCAACTT CAACAAAAAA AAAAAAAATT TAACAAATCT CAATCAACTT   
  
  
+ CACCCATTTC TCAACTTATA TCTCCTAATG AAAAGAACAG ATCCTTAGCA AGACTTCTCG ATTCATTTAA   
  
  
+ AAGGATAGTA TGGTAAATTC ATTTTATACA AGTGTCAAGT GTGAATATCT TTTTCACATC AGGTGATATA   
  
  
+ TTAGTAGGAC TTTTCGATTC ACTTATCTTC CTCTTTATGT TTTGTTAATG TGATAGTAAC GATAGGATTG   
  
  
+ CATGTATTTT TTCCAACTAT TCATTTACAA CTATCAATAT CTATTGATAA ATATGTTAAA ATAATTATAT   
  
  
+ GTTATTTAAA TTATTATTTG TTACTTAAGG TGTCGACTTT GTCGAAAGCG ACTTAATTCT AATTAAGATA   
  
  
+ GCGGCAGAGA AAGTTACACC TGATGACCAA GTTAGATCTG GTAAAACTAA CCATAGATCT GAAACCCAAA   
  
  
+ CATAATGATG TGTATTCATA CCCGACCTGA CATCTGAAAT AAGTTTACTC AAAAGTGGTT CCGAAACTTG   
  
  
+ AATTGACTCG AACCGAACCG TATTTGACTC GAAATTTTCT ACAAAGTTGC TTATTTTTGC ATGAAGACAT   
  
  
+ ATTGAGCCAA AAATTAGTTG ATTCGAAGTC AACTTAAACA AAAATTCATT CGACTCAAAC TGAAATATCA   
  
  
+ CTTAACTAAA ACTATGACTC AACTCAAACT TGATCTGATC CGTATTCAAA TCAAATACTC CATTTGCTAG   
  
  
+ GTCTAGAACC AGCCACGACC CCACATGGAC TATGCGGTTC AATGAAACGG CCAAATATAT CGCAATTTCT   
  
  
+ GTCTCCATCA ATCACACCCA TTTGCTACAC CCTGTTAATA TTTGACTCGA CCCTGTGAAT TGCCTTCGCC   
  
  
+ AGTTTTGTTT AATCTCTCTG GTTGCTTCCC ATCCAAGTCT GAATAGTATA GTTTGTACAG TGTGTACTAC   
  
  
+ TAGAAAGCAC GTTCCTTGAA TCTTCTTTCA AAGTTGTTTT TAAAGGGTTG AGTGATCCAT TACGACTTTT   
  
  
+ GATCTGGGTT CATCGCCTCC TCTCCTAATC CCTCACCCGG GTACCCTCAA TTCTCTATCG TCTCTGTTGC   
  
  
+ TCAGCTAATT CAAATTCCTT TAGTTGGTCA TCATTGAAAT CTGAAATCAG GGTGTTTTTC TTTTTATTAT   
  
  
+ TATCTGGCAG TTGGTTTGTT TATCTCTTGG TTTTTGGGGC TTATTTGGGT GCTGGTTGAT GTTATCCTGC   
  
  
+ TTGGGTTGAA TCTTCATCAG GTTTATTTTT GATTCATAGT TGAGGGTGAA AATTATTTCT CATACCCACC   
  
  
+ ACCTGTTTGT TCTTTTGTCT CTGAGAAAAA CCAAAAAGCT TTCTCTTTTT CCTGATGCAT CCTGTGCTCG   
  
  
+ TTGATCCTGA GTTAGTGAAC CATGCGTCCA AATTCAAGCC TGATTTGCTC TCAAACTTTG ATAAACAACA   
  
  
+ AGAATTTAGC AACGGGATCG AACAAGATGA CATCTTGCAA AACCCTGATC TGTGTTTTGA TTCAGAAACC   
  
  
+ CCTTCAGTTG ATCTAACTGA GAGTGTCATC AGTTCTGATT CTGTAGAAGT GCCTGATTTC TCAGATGCTT   
  
  
+ GCCTCAAGTT CATAAGTGAG ATTCTCCTAG AAGAGGATTT GGATGAAAGT CCTGCCTCTG CTCAAGAATT   
  
  
+ TAGGGCTCTC CAAGCCACTG AGAAGTCCCT GTATGATGCT CTTGGAGAGG GCTACTCTTT TTCATCTGAT   
  
  
+ AACAGCCCAT CATCATTAGG ACAGAGTATT GAGCACCAAA ATGAGAATTT TGAATTCAGT TCCGGTTATC   
  
  
+ CTGGAATTGA GGGCTATGTT AATGGTGATA TCACGTTCGA GTCCAACTGG ATGTTCAACC TAAGCCAATT   
  
  
+ GGATCCTGTT CTTACTTTGG ATGATATTTC TCAGCCCTTG TCTGCATCAA ACTCCCGATC ATCTGGGTCG   
  
  
+ AGCAATGGCT TTGATGATTC AGGGGATGGG GCAGCCACAT CTCCTGGCAG TACAGTTACA TCAACAGTCC   
  
  
+ CAGAGAAAAG GGTTGAATCT GTCAATCGCT CAAGGAGGAA GAAAAATCGT AAAAGGGATG AAGGTGGTCA   
  
  
+ TGAGGAAGGG AGGAGTAACA AGCAGCAAGC TTCATCCAAT GAAGATTATG TTGAGATGAA GGAGTTTGAT   
  
  
+ GATGTACTGC TCTGCAAAGA AGAGAAAGAT GTTATTGCAA ACTGCACCAA TGATTCCTCA CCCGTCGAAG   
  
  
+ CGAGTGATAA GTTGCAGAAG AAAGGGGGGA AGGGGAAAAC ATCGCGTGGG AAGAAGCAGA ATAGCACAAT   
  
  
+ AGAAGAGGTG GATCTGAGGA CTCTTCTCAC TCACTGTGCT CAAGCAATTT CAAATTTTGA TCTTAGGAGT   
  
  
+ GCAAATGAGC ATCTCAGGCA AATAAGGCAG CATTCTTCAC AATATGGTGA TAGCCTCCAG AGGCTTGCCC   
  
  
+ ATTATTTTGC TAATGGTCTT GAGGCTCGCA TAGCTGGCAC TGGTTCAACA ATCTCTGCTA ATGTTGTTGA   
  
  
+ TGCTCGAATC ACGTCATCTG ATTTCTTAAA GGCTTATAGG CTATATATGT CAGCCGTTCC TTTCAAAAGG   
  
  
+ ATGTTATATT TTCTCGCCAA CAAGACAATA CGGAAGTTGG CTGAGAAGGC AACCAGGATC CACATCATTG   
  
  
+ ATTTTGGAGT CTTGTTGGGT TTACAGTGGC CCTGTCTCAT ACAAAACCTC TCAAAGAGAC CCACTGGCCC   
  
  
+ GCCAAGCCTC CGTATTACTG GGATAGACTA TCCCCAGCAT GGTTTCCGGC CATCAGAAAG GGTTGAAGCG   
  
  
+ ACTGGCCGTC GGTTAGAAGG GTATTGCGAG AGATTTAATG TGCCGTTCAA GTATAAGGCC ATAGCTAAAA   
  
  
+ ATTGGGAGAG TATAAAGCTA GAGGATCTAG AAATTGATGA GAATGAGATG GTGTTTGTCA ATTGCATGCT   
  
  
+ TCGCTCTGGA ACACTGCTTG ATGAGACAGT GGTGGCAGAC AGTCCAAAGG ATGCTTTCTT AAGGTTAATC   
  
  
+ AGAGAGATAA ATCCCCGTCT TTTCATTCAT GGGACTATCA ATGGATCATT CAATGCTCCA TTCTTCATCA   
  
  
+ CTCGGTTCAG GGAGGCGCTC TTCCACTACT CTTCTTTATT TGATATCTTT GAAGCAACTA TGCCCCGTGA   
  
  
+ AGATCATGAA AGGCTCCTGG TTGAGAGTGA GATACAGGGC AAAGAAGCTT TGAATGTCAT AGCATGCGAA
[truncated: 133,403 more chars]
