# Supplementary material for: Genome-Wide Identification and Expression Pattern of the GRAS Gene Family in Pitaya (Selenicereus undatus L.)
Source: Biology (Basel). 2022 Dec 21;12(1):11. doi: 10.3390/biology12010011 (PMC9854919; doi:10.3390/biology12010011)
Supplement: Supplementary file 1 [file biology-12-00011-s001.zip › Supplementary file S5/HU07G02248.1_plantcare.html]

Content-Type: text/html; charset=ISO-8859-1


PlantCARE


Webmaster Firefox specific output  
To save the result:
click on the frame with the right mouse button and save the source code as a text file with extension .html  
REFERENCE:PlantCARE: a database of plant cis-acting regulatory elements and a portal to tools for in silico analysis of promoter sequences.  
Lescot, M., Déhais, P., Moreau, Y., De Moor, B., Rouzé ,P.,and Rombauts, S.  
Nucleic Acids Res., Database issue(2002), 30(1):325-327.   


---

>HU07G02248.1   
+ -Up\_Stream \_Len000CTGTTC AGCACTAGGG AATAAGGTTT TTGCCCTGCA TACTATGAAA TCTAACCAAA   
  
  
+ AACATTCCAT CTTGGAAGGC TGTAGCCTTG TGGATTTTGA ATTCGCTCCC AAGAACCAAT GAAGTAGCGC   
  
  
+ AGAAGGCCAA TACTCAATTT CAGAGTCCAC ATCAACTTGT TCAATGCCAG TATATTTCAG AACATTAATT   
  
  
+ ACATACTGTT TGGTTGGAGA AAAAATGGTT GTCTCGAAAA CTAGTTTTTT AATTTTTTGT TATTTAGCAC   
  
  
+ GGTAAAGAAT GGAAATTATT TTCATGCGAG AAAAAAGGAA GTTGGTTTTT CTTTTTTCAA CTCTTTTGAT   
  
  
+ GATATAATCA GTCGAAATTT AGGTGCACCA TCTGACTTCA ACTCTTGTAC GACCTCTGAT TGCTTGCTTA   
  
  
+ GAAGAACAAG AGGATTGACA GATAAGGAGG AGTGGGTGGT TTATACAGAG AATTGAGTGG ATGGTTATAA   
  
  
+ AGAAGTCGAT CTCACATAAG TAACTTCGTT ACGATTATAA ATATTTTCAT TGAAGAAAGA CGATTAGATA   
  
  
+ TAATTAAGAA GGTACATCTA AGCAAATTTG GCTGAGCAGA GTTGGAGGGC CACTAGGGTC ACATTGTGTC   
  
  
+ TTGCGCAACA AGCTTAGACC ATTCGCAACG GATGCCTCAC GTGGAGCAGG TGGGACCCGC GTGGAAGTTG   
  
  
+ CATGGCAAGA ATTCTCTGGC AGCAAGGAGA ATTGAGGGGG GACACGCGTG TGACCACGCC AGTGCGGTCC   
  
  
+ CCCTCTCGTG GCAGATTTGG TGATTTTTTT TTTGTTTTTT TTTGAAGTAA TATATAATTT TTATATGATT   
  
  
+ AAAAAAATAG GTTGTGTGTT ATAAAGTGGG TCAATGACAA ATATTTTTAT TGGATGATTA AAATTAAGTT   
  
  
+ CCATTTATTG GAGAAGTGAT GGAATGCAAG TTCCAAAATT GTGGAACTTT TGTTGACGTA GCAGGCTAGA   
  
  
+ GTTCCAAAAA TTAAAAAAAG GAAAGACATA TTGAGGTAAA GCCTTTTTAT TTTACAAATA GACGCACCAA   
  
  
+ GGTATAATAT AATTGACATG ATTTGAGGCG AGAGGAAGAT TCTTTTGCTA CCCAAACAAA ACACAATGAT   
  
  
+ CTTTTAAATG TAGCAATTTG GGATTGGCAA AATTTTGTCT AACCCGATTG TTGAAAGTTA TCCGATTTCA   
  
  
+ TAAAATTGGA TATCGTTATT AAATTTTCAT TCGAATATTT AAATTTGGAC ACAATGTTTT TCATATTTGG   
  
  
+ ATTTTCTAAA CTTGATTTTA ACTCAAATTT AAGTTTATTC AATTCATCTG ACCCAAAAAA TGGTTGCAAT   
  
  
+ AATGTATTTT CTTTTTGAAG TTAAATATTA TCGAATTCAT TTAACTTGTA GTTAACCCGT AATTGACCCA   
  
  
+ AACTCGACTT TTTGCTACCT TAGCAGCCAG GAAGAAGTCA TAGTATTGAC CCGTGGAACG CCTGAAGCCA   
  
  
+ ACATCACCAG ACCCACATAT TCCATCATCT TCTGATCTCC TCGTCAAAGA AGGAAGCTGA GCACAGCAAT   
  
  
+ AACTAGAAGA ACAGGAGGCA TCACACCCAA GCCAAAGGCC TTTATTTGAG CTCCCCACCT CATTCCTCGA   
  
  
+ AGCTTCCTCT TCACAATGTA AGACCATAAT CCTTCACCCC TTCTCTTTTT AAGCTCATCT TTACCTGCCC   
  
  
+ TAGTTCTCTC TCTCTCATGA GTCATCAGTA ATAGTTACCT CTTTACTTTC TCTTCCTTGA GGTATGCTTC   
  
  
+ AACTTTAATA TATATATGTA CCACTTGATT TCTCTTGTCA CTTTGCTATA ATACTTATTG TTTGGCTATC   
  
  
+ TTCCCCTGTT TTATACTCAT TTTCTTGCTT TCTATATCTG GGTTTGAATT GGGTTTCACT TATCCTCGTT   
  
  
+ GATTCTGTGG GAAATTAGTG AATTGGGTTT TGTACAGCAG GTTTATATGA ACTAGGGTTT CAAAGGTATC   
  
  
+ TACTTTTTCT CTTGTGATTA TTAGTTGATT AGGGATTTGG CCAAGATTAG CAGAATGGGT TCTGAATTTG   
  
  
+ GGGAATTCTC TGATGATGCT CTAAATGGGT ATGCTTACGT TGATATCCCT GCTTATGATG CGTCCTTAGA   
  
  
+ TTATGCCAAT TTGTTCAATT ACGAAGGCCT ATCTGAGGAT CTCACCTCAC TGAGCCTCCC AAGCCCCTTT   
  
  
+ TCTGACCCTT TGGCGTACAG TTTCACGTCC TCTTTGGGGC CGAGCCCTGG GGTTGATTCT AATGATGATA   
  
  
+ GTGATTCTGA TGATGTTCTC AAGTGCATTA GCCAAATGCT TATGGAAGAA GACATGGAGG CAAAGCCATG   
  
  
+ CATGTTTCAC GATCCTTTAG CACTTCAGGC TGCTGAGAAA CCCTTTTATG ATGCCTTAGG GAAGAAATAC   
  
  
+ CCAACTTCTC CGAACCAACA TCCTATAATT GATGATTGTT TGGATAATCC TGGTGAAAAT TCTTTGGGTT   
  
  
+ CTTCTAGTGA TTTTAGTGTT AGTCACTTTG GGTCTAGTTC AGCAAGCTCT ATTGGACCGA CAATTGTGTC   
  
  
+ TGATTTGAGT GAGCATTTTG AGCCACCCTT TGTTCAAGCA CTTCCAATTG AATCATATCC CCAACCATTG   
  
  
+ ACCCGTCCTC AATGGTCGTT TGGCCCTTCG GGTGCCTTAG ATTGCACGGC CTCTAATGGT TCAGTGATCT   
  
  
+ CATCCCTTGG TTTGCCGATG GATGTTATTA GTGTATTCAG TGAGAAAGAG TCCATGATTC AATTTCAGAA   
  
  
+ AGGGGTGGAA GAGGCTAGTA AGTTCCTTCC CAAGAATAAT AACCTTGTTA TTGATCTCAA GAACCTCACT   
  
  
+ TTTCCTAATG AAACGAAGGA GGATGATCGA GTGATGATGG TTAAGAAGGA AAAGAATGAT GTGAATTGGT   
  
  
+ CTAACTACTC AAGAGGGAGT AAGATTCACT ATCGTGAAGA CGAGGACTTT GAAGAAGGAA GGAGTAGCAA   
  
  
+ GCAGTCAGCT ATTTCTACTA CTGAGGAAGC TGAGTTGTGT GAAATTTTTG ACAAGTTTTT GCTTTGCAAT   
  
  
+ TGGTACCCTG TGAAACCTGA GGCTCATCCC ACCATGAGTT TGAACCCTGA GAAGGGCCAG TCACATGGAT   
  
  
+ TAGAAGGTGG GAGAAATGGG AAGGGTCACC CAAAGAAACA GGATAAAAGT AGTACCAACG TTGTGGATTT   
  
  
+ AAGGAATTTG CTGATGCTAT GTGCACAATC TACTGCATCT GATGACCGAA GAACTGCTGA TGGACTGCTA   
  
  
+ AAGAAAATCA GGGAGCACTC ATCTGCTGAG GGGGATGGAT TTCAAAGGTT GGCTCATTAC TTTGCTGATG   
  
  
+ CCCTAGAGGC ACGTTTAGCT GGAACTGGAT CTCACATTTA TACAGCCCTA AGTTCTCATA GGCCATCTGC   
  
  
+ TGTTGACGTG TTAAAAGCAT ATCAGTTCTA TGTTCGTGCT TGCCCATTTA AGAAGATCGT CATTCGTTGT   
  
  
+ GGTAAACATA TGATTCTAAA AGCTGCTGAA AAGGCATCAA AGCTTCATAT TATAGATTTT GGCATCCTCT   
  
  
+ ATGGATTCCA ATGGCCTAGC CTCATTCGAT GCCTCTCAGA GCGGTCTGGT GGACCTCCAA AACTGTTTAT   
  
  
+ TACAGGGATC GATCTCCCCC AGCCTGGGTT CAGGCCAGCA GAAAGAGTGG AAGCAACAGG GAGACGCTTG   
  
  
+ GCTAAGTACT GTGAGCGGTA TAATGTGCCA TTTGAGTATC ATGCCATTGC TCAGAAGTGG GAAACAATCA   
  
  
+ AACCAGGGGA TCTCAAGTTA GGAAGTAGGG ATGATGATGA AGTTGTCGTG GTGAACTGTC TCTGTAGGTT   
  
  
+ CAAGAACCTC CTTGACGAGA CAATGGTGGT GGATAGTCCA AGGAACACAG TTTTAAACCT GATTAGAAGG   
  
  
+ GTAAAACCCG ATATTTTTGT GCATGGCATT GTAAATGGTT CCTACAACAT CCCTTTCTTT GTGACACGTT   
  
  
+ TTAGAGAAGC CCTCTTTCAT TATTCCACTC TTTTCGACAT GTTAGATGCC AACGCCTCTA GGGAGGAGCC   
  
  
+ CGAGAGGTTG ATATTCGAGA AGGCATTCTA TGGGAGGGAG ATTATGAATG TGGTGGCATG TGAGGGCACA   
  
  
+ GAGAGGGTGG AAAGGCCAGA GACATACAAG CAATGGCACG TTAGGCATAG CAGGGCAGGG TTTCGGCAAC   
  
  
+ TACCATTGGA TCCCAAGTTG ATCGAGAAAA TGAGGTTTAA GGCCAAGGCA GACCACCACA AGGATTTCGT   
  
  
+ GATTGATGTG GATGGACATT GGGCAATTCA GGGATGGAAG GGGCGGATTG CCTGTGCCAT CTCTGCATGG   
  
  
+ GTTCTGGCTT G  

- -Up\_Stream \_Len000GACAAG TCGTGATCCC TTATTCCAAA AACGGGACGT ATGATACTTT AGATTGGTTT   
  
  
- TTGTAAGGTA GAACCTTCCG ACATCGGAAC ACCTAAAACT TAAGCGAGGG TTCTTGGTTA CTTCATCGCG   
  
  
- TCTTCCGGTT ATGAGTTAAA GTCTCAGGTG TAGTTGAACA AGTTACGGTC ATATAAAGTC TTGTAATTAA   
  
  
- TGTATGACAA ACCAACCTCT TTTTTACCAA CAGAGCTTTT GATCAAAAAA TTAAAAAACA ATAAATCGTG   
  
  
- CCATTTCTTA CCTTTAATAA AAGTACGCTC TTTTTTCCTT CAACCAAAAA GAAAAAAGTT GAGAAAACTA   
  
  
- CTATATTAGT CAGCTTTAAA TCCACGTGGT AGACTGAAGT TGAGAACATG CTGGAGACTA ACGAACGAAT   
  
  
- CTTCTTGTTC TCCTAACTGT CTATTCCTCC TCACCCACCA AATATGTCTC TTAACTCACC TACCAATATT   
  
  
- TCTTCAGCTA GAGTGTATTC ATTGAAGCAA TGCTAATATT TATAAAAGTA ACTTCTTTCT GCTAATCTAT   
  
  
- ATTAATTCTT CCATGTAGAT TCGTTTAAAC CGACTCGTCT CAACCTCCCG GTGATCCCAG TGTAACACAG   
  
  
- AACGCGTTGT TCGAATCTGG TAAGCGTTGC CTACGGAGTG CACCTCGTCC ACCCTGGGCG CACCTTCAAC   
  
  
- GTACCGTTCT TAAGAGACCG TCGTTCCTCT TAACTCCCCC CTGTGCGCAC ACTGGTGCGG TCACGCCAGG   
  
  
- GGGAGAGCAC CGTCTAAACC ACTAAAAAAA AAACAAAAAA AAACTTCATT ATATATTAAA AATATACTAA   
  
  
- TTTTTTTATC CAACACACAA TATTTCACCC AGTTACTGTT TATAAAAATA ACCTACTAAT TTTAATTCAA   
  
  
- GGTAAATAAC CTCTTCACTA CCTTACGTTC AAGGTTTTAA CACCTTGAAA ACAACTGCAT CGTCCGATCT   
  
  
- CAAGGTTTTT AATTTTTTTC CTTTCTGTAT AACTCCATTT CGGAAAAATA AAATGTTTAT CTGCGTGGTT   
  
  
- CCATATTATA TTAACTGTAC TAAACTCCGC TCTCCTTCTA AGAAAACGAT GGGTTTGTTT TGTGTTACTA   
  
  
- GAAAATTTAC ATCGTTAAAC CCTAACCGTT TTAAAACAGA TTGGGCTAAC AACTTTCAAT AGGCTAAAGT   
  
  
- ATTTTAACCT ATAGCAATAA TTTAAAAGTA AGCTTATAAA TTTAAACCTG TGTTACAAAA AGTATAAACC   
  
  
- TAAAAGATTT GAACTAAAAT TGAGTTTAAA TTCAAATAAG TTAAGTAGAC TGGGTTTTTT ACCAACGTTA   
  
  
- TTACATAAAA GAAAAACTTC AATTTATAAT AGCTTAAGTA AATTGAACAT CAATTGGGCA TTAACTGGGT   
  
  
- TTGAGCTGAA AAACGATGGA ATCGTCGGTC CTTCTTCAGT ATCATAACTG GGCACCTTGC GGACTTCGGT   
  
  
- TGTAGTGGTC TGGGTGTATA AGGTAGTAGA AGACTAGAGG AGCAGTTTCT TCCTTCGACT CGTGTCGTTA   
  
  
- TTGATCTTCT TGTCCTCCGT AGTGTGGGTT CGGTTTCCGG AAATAAACTC GAGGGGTGGA GTAAGGAGCT   
  
  
- TCGAAGGAGA AGTGTTACAT TCTGGTATTA GGAAGTGGGG AAGAGAAAAA TTCGAGTAGA AATGGACGGG   
  
  
- ATCAAGAGAG AGAGAGTACT CAGTAGTCAT TATCAATGGA GAAATGAAAG AGAAGGAACT CCATACGAAG   
  
  
- TTGAAATTAT ATATATACAT GGTGAACTAA AGAGAACAGT GAAACGATAT TATGAATAAC AAACCGATAG   
  
  
- AAGGGGACAA AATATGAGTA AAAGAACGAA AGATATAGAC CCAAACTTAA CCCAAAGTGA ATAGGAGCAA   
  
  
- CTAAGACACC CTTTAATCAC TTAACCCAAA ACATGTCGTC CAAATATACT TGATCCCAAA GTTTCCATAG   
  
  
- ATGAAAAAGA GAACACTAAT AATCAACTAA TCCCTAAACC GGTTCTAATC GTCTTACCCA AGACTTAAAC   
  
  
- CCCTTAAGAG ACTACTACGA GATTTACCCA TACGAATGCA ACTATAGGGA CGAATACTAC GCAGGAATCT   
  
  
- AATACGGTTA AACAAGTTAA TGCTTCCGGA TAGACTCCTA GAGTGGAGTG ACTCGGAGGG TTCGGGGAAA   
  
  
- AGACTGGGAA ACCGCATGTC AAAGTGCAGG AGAAACCCCG GCTCGGGACC CCAACTAAGA TTACTACTAT   
  
  
- CACTAAGACT ACTACAAGAG TTCACGTAAT CGGTTTACGA ATACCTTCTT CTGTACCTCC GTTTCGGTAC   
  
  
- GTACAAAGTG CTAGGAAATC GTGAAGTCCG ACGACTCTTT GGGAAAATAC TACGGAATCC CTTCTTTATG   
  
  
- GGTTGAAGAG GCTTGGTTGT AGGATATTAA CTACTAACAA ACCTATTAGG ACCACTTTTA AGAAACCCAA   
  
  
- GAAGATCACT AAAATCACAA TCAGTGAAAC CCAGATCAAG TCGTTCGAGA TAACCTGGCT GTTAACACAG   
  
  
- ACTAAACTCA CTCGTAAAAC TCGGTGGGAA ACAAGTTCGT GAAGGTTAAC TTAGTATAGG GGTTGGTAAC   
  
  
- TGGGCAGGAG TTACCAGCAA ACCGGGAAGC CCACGGAATC TAACGTGCCG GAGATTACCA AGTCACTAGA   
  
  
- GTAGGGAACC AAACGGCTAC CTACAATAAT CACATAAGTC ACTCTTTCTC AGGTACTAAG TTAAAGTCTT   
  
  
- TCCCCACCTT CTCCGATCAT TCAAGGAAGG GTTCTTATTA TTGGAACAAT AACTAGAGTT CTTGGAGTGA   
  
  
- AAAGGATTAC TTTGCTTCCT CCTACTAGCT CACTACTACC AATTCTTCCT TTTCTTACTA CACTTAACCA   
  
  
- GATTGATGAG TTCTCCCTCA TTCTAAGTGA TAGCACTTCT GCTCCTGAAA CTTCTTCCTT CCTCATCGTT   
  
  
- CGTCAGTCGA TAAAGATGAT GACTCCTTCG ACTCAACACA CTTTAAAAAC TGTTCAAAAA CGAAACGTTA   
  
  
- ACCATGGGAC ACTTTGGACT CCGAGTAGGG TGGTACTCAA ACTTGGGACT CTTCCCGGTC AGTGTACCTA   
  
  
- ATCTTCCACC CTCTTTACCC TTCCCAGTGG GTTTCTTTGT CCTATTTTCA TCATGGTTGC AACACCTAAA   
  
  
- TTCCTTAAAC GACTACGATA CACGTGTTAG ATGACGTAGA CTACTGGCTT CTTGACGACT ACCTGACGAT   
  
  
- TTCTTTTAGT CCCTCGTGAG TAGACGACTC CCCCTACCTA AAGTTTCCAA CCGAGTAATG AAACGACTAC   
  
  
- GGGATCTCCG TGCAAATCGA CCTTGACCTA GAGTGTAAAT ATGTCGGGAT TCAAGAGTAT CCGGTAGACG   
  
  
- ACAACTGCAC AATTTTCGTA TAGTCAAGAT ACAAGCACGA ACGGGTAAAT TCTTCTAGCA GTAAGCAACA   
  
  
- CCATTTGTAT ACTAAGATTT TCGACGACTT TTCCGTAGTT TCGAAGTATA ATATCTAAAA CCGTAGGAGA   
  
  
- TACCTAAGGT TACCGGATCG GAGTAAGCTA CGGAGAGTCT CGCCAGACCA CCTGGAGGTT TTGACAAATA   
  
  
- ATGTCCCTAG CTAGAGGGGG TCGGACCCAA GTCCGGTCGT CTTTCTCACC TTCGTTGTCC CTCTGCGAAC   
  
  
- CGATTCATGA CACTCGCCAT ATTACACGGT AAACTCATAG TACGGTAACG AGTCTTCACC CTTTGTTAGT   
  
  
- TTGGTCCCCT AGAGTTCAAT CCTTCATCCC TACTACTACT TCAACAGCAC CACTTGACAG AGACATCCAA   
  
  
- GTTCTTGGAG GAACTGCTCT GTTACCACCA CCTATCAGGT TCCTTGTGTC AAAATTTGGA CTAATCTTCC   
  
  
- CATTTTGGGC TATAAAAACA CGTACCGTAA CATTTACCAA GGATGTTGTA GGGAAAGAAA CACTGTGCAA   
  
  
- AATCTCTTCG GGAGAAAGTA ATAAGGTGAG AAAAGCTGTA CAATCTACGG TTGCGGAGAT CCCTCCTCGG   
  
  
- GCTCTCCAAC TATAAGCTCT TCCGTAAGAT ACCCTCCCTC TAATACTTAC ACCACCGTAC ACTCCCGTGT   
  
  
- CTCTCCCACC TTTCCGGTCT CTGTATGTTC GTTACCGTGC AATCCGTATC GTCCCGTCCC AAAGCCGTTG   
  
  
- ATGGTAACCT AGGGTTCAAC TAGCTCTTTT ACTCCAAATT CCGGTTCCGT CTGGTGGTGT TCCTAAAGCA   
  
  
- CTAACTACAC CTACCTGTAA CCCGTTAAGT CCCTACCTTC CCCGCCTAAC GGACACGGTA GAGACGTACC   
  
  
- CAAGACCGAA C

  
  
Motifs Found  

+   

| Site Name | Organism | Position | Strand | Matrix score. | sequence | function |
| --- | --- | --- | --- | --- | --- | --- |
|  | organism | 4218 | - | 4 | motif\_sequence | short\_function |
|  | organism | 3182 | + | 4 | motif\_sequence | short\_function |
|  | organism | 3173 | + | 4 | motif\_sequence | short\_function |
|  | organism | 3416 | - | 4 | motif\_sequence | short\_function |
|  | organism | 2257 | + | 4 | motif\_sequence | short\_function |
|  | organism | 1734 | + | 4 | motif\_sequence | short\_function |
|  | organism | 1962 | + | 4 | motif\_sequence | short\_function |
|  | organism | 4031 | - | 4 | motif\_sequence | short\_function |
|  | organism | 2189 | - | 4 | motif\_sequence | short\_function |
|  | organism | 1621 | + | 4 | motif\_sequence | short\_function |
|  | organism | 1822 | + | 4 | motif\_sequence | short\_function |
|  | organism | 4256 | + | 4 | motif\_sequence | short\_function |
|  | organism | 1770 | + | 4 | motif\_sequence | short\_function |
|  | organism | 204 | - | 4 | motif\_sequence | short\_function |
|  | organism | 4060 | - | 4 | motif\_sequence | short\_function |
|  | organism | 1501 | + | 4 | motif\_sequence | short\_function |
|  | organism | 1509 | + | 4 | motif\_sequence | short\_function |
|  | organism | 1089 | - | 4 | motif\_sequence | short\_function |
|  | organism | 2742 | - | 4 | motif\_sequence | short\_function |
|  | organism | 3245 | + | 4 | motif\_sequence | short\_function |
|  | organism | 1926 | - | 4 | motif\_sequence | short\_function |
|  | organism | 1553 | - | 4 | motif\_sequence | short\_function |
|  | organism | 3667 | + | 4 | motif\_sequence | short\_function |
|  | organism | 3359 | + | 4 | motif\_sequence | short\_function |
|  | organism | 1535 | - | 4 | motif\_sequence | short\_function |
|  | organism | 576 | - | 4 | motif\_sequence | short\_function |
|  | organism | 4265 | + | 4 | motif\_sequence | short\_function |
|  | organism | 785 | - | 4 | motif\_sequence | short\_function |
|  | organism | 3394 | + | 4 | motif\_sequence | short\_function |
|  | organism | 19 | + | 4 | motif\_sequence | short\_function |
|  | organism | 600 | - | 4 | motif\_sequence | short\_function |
|  | organism | 3586 | + | 4 | motif\_sequence | short\_function |

>HU07G02248.1   
+ -Up\_Stream \_Len000CTGTTC AGCACTAGGG AATAAGGTTT TTGCCCTGCA TACTATGAAA TCTAACCAAA   
  
  
+ AACATTCCAT CTTGGAAGGC TGTAGCCTTG TGGATTTTGA ATTCGCTCCC AAGAACCAAT GAAGTAGCGC   
  
  
+ AGAAGGCCAA TACTCAATTT CAGAGTCCAC ATCAACTTGT TCAATGCCAG TATATTTCAG AACATTAATT   
  
  
+ ACATACTGTT TGGTTGGAGA AAAAATGGTT GTCTCGAAAA CTAGTTTTTT AATTTTTTGT TATTTAGCAC   
  
  
+ GGTAAAGAAT GGAAATTATT TTCATGCGAG AAAAAAGGAA GTTGGTTTTT CTTTTTTCAA CTCTTTTGAT   
  
  
+ GATATAATCA GTCGAAATTT AGGTGCACCA TCTGACTTCA ACTCTTGTAC GACCTCTGAT TGCTTGCTTA   
  
  
+ GAAGAACAAG AGGATTGACA GATAAGGAGG AGTGGGTGGT TTATACAGAG AATTGAGTGG ATGGTTATAA   
  
  
+ AGAAGTCGAT CTCACATAAG TAACTTCGTT ACGATTATAA ATATTTTCAT TGAAGAAAGA CGATTAGATA   
  
  
+ TAATTAAGAA GGTACATCTA AGCAAATTTG GCTGAGCAGA GTTGGAGGGC CACTAGGGTC ACATTGTGTC   
  
  
+ TTGCGCAACA AGCTTAGACC ATTCGCAACG GATGCCTCAC GTGGAGCAGG TGGGACCCGC GTGGAAGTTG   
  
  
+ CATGGCAAGA ATTCTCTGGC AGCAAGGAGA ATTGAGGGGG GACACGCGTG TGACCACGCC AGTGCGGTCC   
  
  
+ CCCTCTCGTG GCAGATTTGG TGATTTTTTT TTTGTTTTTT TTTGAAGTAA TATATAATTT TTATATGATT   
  
  
+ AAAAAAATAG GTTGTGTGTT ATAAAGTGGG TCAATGACAA ATATTTTTAT TGGATGATTA AAATTAAGTT   
  
  
+ CCATTTATTG GAGAAGTGAT GGAATGCAAG TTCCAAAATT GTGGAACTTT TGTTGACGTA GCAGGCTAGA   
  
  
+ GTTCCAAAAA TTAAAAAAAG GAAAGACATA TTGAGGTAAA GCCTTTTTAT TTTACAAATA GACGCACCAA   
  
  
+ GGTATAATAT AATTGACATG ATTTGAGGCG AGAGGAAGAT TCTTTTGCTA CCCAAACAAA ACACAATGAT   
  
  
+ CTTTTAAATG TAGCAATTTG GGATTGGCAA AATTTTGTCT AACCCGATTG TTGAAAGTTA TCCGATTTCA   
  
  
+ TAAAATTGGA TATCGTTATT AAATTTTCAT TCGAATATTT AAATTTGGAC ACAATGTTTT TCATATTTGG   
  
  
+ ATTTTCTAAA CTTGATTTTA ACTCAAATTT AAGTTTATTC AATTCATCTG ACCCAAAAAA TGGTTGCAAT   
  
  
+ AATGTATTTT CTTTTTGAAG TTAAATATTA TCGAATTCAT TTAACTTGTA GTTAACCCGT AATTGACCCA   
  
  
+ AACTCGACTT TTTGCTACCT TAGCAGCCAG GAAGAAGTCA TAGTATTGAC CCGTGGAACG CCTGAAGCCA   
  
  
+ ACATCACCAG ACCCACATAT TCCATCATCT TCTGATCTCC TCGTCAAAGA AGGAAGCTGA GCACAGCAAT   
  
  
+ AACTAGAAGA ACAGGAGGCA TCACACCCAA GCCAAAGGCC TTTATTTGAG CTCCCCACCT CATTCCTCGA   
  
  
+ AGCTTCCTCT TCACAATGTA AGACCATAAT CCTTCACCCC TTCTCTTTTT AAGCTCATCT TTACCTGCCC   
  
  
+ TAGTTCTCTC TCTCTCATGA GTCATCAGTA ATAGTTACCT CTTTACTTTC TCTTCCTTGA GGTATGCTTC   
  
  
+ AACTTTAATA TATATATGTA CCACTTGATT TCTCTTGTCA CTTTGCTATA ATACTTATTG TTTGGCTATC   
  
  
+ TTCCCCTGTT TTATACTCAT TTTCTTGCTT TCTATATCTG GGTTTGAATT GGGTTTCACT TATCCTCGTT   
  
  
+ GATTCTGTGG GAAATTAGTG AATTGGGTTT TGTACAGCAG GTTTATATGA ACTAGGGTTT CAAAGGTATC   
  
  
+ TACTTTTTCT CTTGTGATTA TTAGTTGATT AGGGATTTGG CCAAGATTAG CAGAATGGGT TCTGAATTTG   
  
  
+ GGGAATTCTC TGATGATGCT CTAAATGGGT ATGCTTACGT TGATATCCCT GCTTATGATG CGTCCTTAGA   
  
  
+ TTATGCCAAT TTGTTCAATT ACGAAGGCCT ATCTGAGGAT CTCACCTCAC TGAGCCTCCC AAGCCCCTTT   
  
  
+ TCTGACCCTT TGGCGTACAG TTTCACGTCC TCTTTGGGGC CGAGCCCTGG GGTTGATTCT AATGATGATA   
  
  
+ GTGATTCTGA TGATGTTCTC AAGTGCATTA GCCAAATGCT TATGGAAGAA GACATGGAGG CAAAGCCATG   
  
  
+ CATGTTTCAC GATCCTTTAG CACTTCAGGC TGCTGAGAAA CCCTTTTATG ATGCCTTAGG GAAGAAATAC   
  
  
+ CCAACTTCTC CGAACCAACA TCCTATAATT GATGATTGTT TGGATAATCC TGGTGAAAAT TCTTTGGGTT   
  
  
+ CTTCTAGTGA TTTTAGTGTT AGTCACTTTG GGTCTAGTTC AGCAAGCTCT ATTGGACCGA CAATTGTGTC   
  
  
+ TGATTTGAGT GAGCATTTTG AGCCACCCTT TGTTCAAGCA CTTCCAATTG AATCATATCC CCAACCATTG   
  
  
+ ACCCGTCCTC AATGGTCGTT TGGCCCTTCG GGTGCCTTAG ATTGCACGGC CTCTAATGGT TCAGTGATCT   
  
  
+ CATCCCTTGG TTTGCCGATG GATGTTATTA GTGTATTCAG TGAGAAAGAG TCCATGATTC AATTTCAGAA   
  
  
+ AGGGGTGGAA GAGGCTAGTA AGTTCCTTCC CAAGAATAAT AACCTTGTTA TTGATCTCAA GAACCTCACT   
  
  
+ TTTCCTAATG AAACGAAGGA GGATGATCGA GTGATGATGG TTAAGAAGGA AAAGAATGAT GTGAATTGGT   
  
  
+ CTAACTACTC AAGAGGGAGT AAGATTCACT ATCGTGAAGA CGAGGACTTT GAAGAAGGAA GGAGTAGCAA   
  
  
+ GCAGTCAGCT ATTTCTACTA CTGAGGAAGC TGAGTTGTGT GAAATTTTTG ACAAGTTTTT GCTTTGCAAT   
  
  
+ TGGTACCCTG TGAAACCTGA GGCTCATCCC ACCATGAGTT TGAACCCTGA GAAGGGCCAG TCACATGGAT   
  
  
+ TAGAAGGTGG GAGAAATGGG AAGGGTCACC CAAAGAAACA GGATAAAAGT AGTACCAACG TTGTGGATTT   
  
  
+ AAGGAATTTG CTGATGCTAT GTGCACAATC TACTGCATCT GATGACCGAA GAACTGCTGA TGGACTGCTA   
  
  
+ AAGAAAATCA GGGAGCACTC ATCTGCTGAG GGGGATGGAT TTCAAAGGTT GGCTCATTAC TTTGCTGATG   
  
  
+ CCCTAGAGGC ACGTTTAGCT GGAACTGGAT CTCACATTTA TACAGCCCTA AGTTCTCATA GGCCATCTGC   
  
  
+ TGTTGACGTG TTAAAAGCAT ATCAGTTCTA TGTTCGTGCT TGCCCATTTA AGAAGATCGT CATTCGTTGT   
  
  
+ GGTAAACATA TGATTCTAAA AGCTGCTGAA AAGGCATCAA AGCTTCATAT TATAGATTTT GGCATCCTCT   
  
  
+ ATGGATTCCA ATGGCCTAGC CTCATTCGAT GCCTCTCAGA GCGGTCTGGT GGACCTCCAA AACTGTTTAT   
  
  
+ TACAGGGATC GATCTCCCCC AGCCTGGGTT CAGGCCAGCA GAAAGAGTGG AAGCAACAGG GAGACGCTTG   
  
  
+ GCTAAGTACT GTGAGCGGTA TAATGTGCCA TTTGAGTATC ATGCCATTGC TCAGAAGTGG GAAACAATCA   
  
  
+ AACCAGGGGA TCTCAAGTTA GGAAGTAGGG ATGATGATGA AGTTGTCGTG GTGAACTGTC TCTGTAGGTT   
  
  
+ CAAGAACCTC CTTGACGAGA CAATGGTGGT GGATAGTCCA AGGAACACAG TTTTAAACCT GATTAGAAGG   
  
  
+ GTAAAACCCG ATATTTTTGT GCATGGCATT GTAAATGGTT CCTACAACAT CCCTTTCTTT GTGACACGTT   
  
  
+ TTAGAGAAGC CCTCTTTCAT TATTCCACTC TTTTCGACAT GTTAGATGCC AACGCCTCTA GGGAGGAGCC   
  
  
+ CGAGAGGTTG ATATTCGAGA AGGCATTCTA TGGGAGGGAG ATTATGAATG TGGTGGCATG TGAGGGCACA   
  
  
+ GAGAGGGTGG AAAGGCCAGA GACATACAAG CAATGGCACG TTAGGCATAG CAGGGCAGGG TTTCGGCAAC   
  
  
+ TACCATTGGA TCCCAAGTTG ATCGAGAAAA TGAGGTTTAA GGCCAAGGCA GACCACCACA AGGATTTCGT   
  
  
+ GATTGATGTG GATGGACATT GGGCAATTCA GGGATGGAAG GGGCGGATTG CCTGTGCCAT CTCTGCATGG   
  
  
+ GTTCTGGCTT G  

- -Up\_Stream \_Len000GACAAG TCGTGATCCC TTATTCCAAA AACGGGACGT ATGATACTTT AGATTGGTTT   
  
  
- TTGTAAGGTA GAACCTTCCG ACATCGGAAC ACCTAAAACT TAAGCGAGGG TTCTTGGTTA CTTCATCGCG   
  
  
- TCTTCCGGTT ATGAGTTAAA GTCTCAGGTG TAGTTGAACA AGTTACGGTC ATATAAAGTC TTGTAATTAA   
  
  
- TGTATGACAA ACCAACCTCT TTTTTACCAA CAGAGCTTTT GATCAAAAAA TTAAAAAACA ATAAATCGTG   
  
  
- CCATTTCTTA CCTTTAATAA AAGTACGCTC TTTTTTCCTT CAACCAAAAA GAAAAAAGTT GAGAAAACTA   
  
  
- CTATATTAGT CAGCTTTAAA TCCACGTGGT AGACTGAAGT TGAGAACATG CTGGAGACTA ACGAACGAAT   
  
  
- CTTCTTGTTC TCCTAACTGT CTATTCCTCC TCACCCACCA AATATGTCTC TTAACTCACC TACCAATATT   
  
  
- TCTTCAGCTA GAGTGTATTC ATTGAAGCAA TGCTAATATT TATAAAAGTA ACTTCTTTCT GCTAATCTAT   
  
  
- ATTAATTCTT CCATGTAGAT TCGTTTAAAC CGACTCGTCT CAACCTCCCG GTGATCCCAG TGTAACACAG   
  
  
- AACGCGTTGT TCGAATCTGG TAAGCGTTGC CTACGGAGTG CACCTCGTCC ACCCTGGGCG CACCTTCAAC   
  
  
- GTACCGTTCT TAAGAGACCG TCGTTCCTCT TAACTCCCCC CTGTGCGCAC ACTGGTGCGG TCACGCCAGG   
  
  
- GGGAGAGCAC CGTCTAAACC ACTAAAAAAA AAACAAAAAA AAACTTCATT ATATATTAAA AATATACTAA   
  
  
- TTTTTTTATC CAACACACAA TATTTCACCC AGTTACTGTT TATAAAAATA ACCTACTAAT TTTAATTCAA   
  
  
- GGTAAATAAC CTCTTCACTA CCTTACGTTC AAGGTTTTAA CACCTTGAAA ACAACTGCAT CGTCCGATCT   
  
  
- CAAGGTTTTT AATTTTTTTC CTTTCTGTAT AACTCCATTT CGGAAAAATA AAATGTTTAT CTGCGTGGTT   
  
  
- CCATATTATA TTAACTGTAC TAAACTCCGC TCTCCTTCTA AGAAAACGAT GGGTTTGTTT TGTGTTACTA   
  
  
- GAAAATTTAC ATCGTTAAAC CCTAACCGTT TTAAAACAGA TTGGGCTAAC AACTTTCAAT AGGCTAAAGT   
  
  
- ATTTTAACCT ATAGCAATAA TTTAAAAGTA AGCTTATAAA TTTAAACCTG TGTTACAAAA AGTATAAACC   
  
  
- TAAAAGATTT GAACTAAAAT TGAGTTTAAA TTCAAATAAG TTAAGTAGAC TGGGTTTTTT ACCAACGTTA   
  
  
- TTACATAAAA GAAAAACTTC AATTTATAAT AGCTTAAGTA AATTGAACAT CAATTGGGCA TTAACTGGGT   
  
  
- TTGAGCTGAA AAACGATGGA ATCGTCGGTC CTTCTTCAGT ATCATAACTG GGCACCTTGC GGACTTCGGT   
  
  
- TGTAGTGGTC TGGGTGTATA AGGTAGTAGA AGACTAGAGG AGCAGTTTCT TCCTTCGACT CGTGTCGTTA   
  
  
- TTGATCTTCT TGTCCTCCGT AGTGTGGGTT CGGTTTCCGG AAATAAACTC GAGGGGTGGA GTAAGGAGCT   
  
  
- TCGAAGGAGA AGTGTTACAT TCTGGTATTA GGAAGTGGGG AAGAGAAAAA TTCGAGTAGA AATGGACGGG   
  
  
- ATCAAGAGAG AGAGAGTACT CAGTAGTCAT TATCAATGGA GAAATGAAAG AGAAGGAACT CCATACGAAG   
  
  
- TTGAAATTAT ATATATACAT GGTGAACTAA AGAGAACAGT GAAACGATAT TATGAATAAC AAACCGATAG   
  
  
- AAGGGGACAA AATATGAGTA AAAGAACGAA AGATATAGAC CCAAACTTAA CCCAAAGTGA ATAGGAGCAA   
  
  
- CTAAGACACC CTTTAATCAC TTAACCCAAA ACATGTCGTC CAAATATACT TGATCCCAAA GTTTCCATAG   
  
  
- ATGAAAAAGA GAACACTAAT AATCAACTAA TCCCTAAACC GGTTCTAATC GTCTTACCCA AGACTTAAAC   
  
  
- CCCTTAAGAG ACTACTACGA GATTTACCCA TACGAATGCA ACTATAGGGA CGAATACTAC GCAGGAATCT   
  
  
- AATACGGTTA AACAAGTTAA TGCTTCCGGA TAGACTCCTA GAGTGGAGTG ACTCGGAGGG TTCGGGGAAA   
  
  
- AGACTGGGAA ACCGCATGTC AAAGTGCAGG AGAAACCCCG GCTCGGGACC CCAACTAAGA TTACTACTAT   
  
  
- CACTAAGACT ACTACAAGAG TTCACGTAAT CGGTTTACGA ATACCTTCTT CTGTACCTCC GTTTCGGTAC   
  
  
- GTACAAAGTG CTAGGAAATC GTGAAGTCCG ACGACTCTTT GGGAAAATAC TACGGAATCC CTTCTTTATG   
  
  
- GGTTGAAGAG GCTTGGTTGT AGGATATTAA CTACTAACAA ACCTATTAGG ACCACTTTTA AGAAACCCAA   
  
  
- GAAGATCACT AAAATCACAA TCAGTGAAAC CCAGATCAAG TCGTTCGAGA TAACCTGGCT GTTAACACAG   
  
  
- ACTAAACTCA CTCGTAAAAC TCGGTGGGAA ACAAGTTCGT GAAGGTTAAC TTAGTATAGG GGTTGGTAAC   
  
  
- TGGGCAGGAG TTACCAGCAA ACCGGGAAGC CCACGGAATC TAACGTGCCG GAGATTACCA AGTCACTAGA   
  
  
- GTAGGGAACC AAACGGCTAC CTACAATAAT CACATAAGTC ACTCTTTCTC AGGTACTAAG TTAAAGTCTT   
  
  
- TCCCCACCTT CTCCGATCAT TCAAGGAAGG GTTCTTATTA TTGGAACAAT AACTAGAGTT CTTGGAGTGA   
  
  
- AAAGGATTAC TTTGCTTCCT CCTACTAGCT CACTACTACC AATTCTTCCT TTTCTTACTA CACTTAACCA   
  
  
- GATTGATGAG TTCTCCCTCA TTCTAAGTGA TAGCACTTCT GCTCCTGAAA CTTCTTCCTT CCTCATCGTT   
  
  
- CGTCAGTCGA TAAAGATGAT GACTCCTTCG ACTCAACACA CTTTAAAAAC TGTTCAAAAA CGAAACGTTA   
  
  
- ACCATGGGAC ACTTTGGACT CCGAGTAGGG TGGTACTCAA ACTTGGGACT CTTCCCGGTC AGTGTACCTA   
  
  
- ATCTTCCACC CTCTTTACCC TTCCCAGTGG GTTTCTTTGT CCTATTTTCA TCATGGTTGC AACACCTAAA   
  
  
- TTCCTTAAAC GACTACGATA CACGTGTTAG ATGACGTAGA CTACTGGCTT CTTGACGACT ACCTGACGAT   
  
  
- TTCTTTTAGT CCCTCGTGAG TAGACGACTC CCCCTACCTA AAGTTTCCAA CCGAGTAATG AAACGACTAC   
  
  
- GGGATCTCCG TGCAAATCGA CCTTGACCTA GAGTGTAAAT ATGTCGGGAT TCAAGAGTAT CCGGTAGACG   
  
  
- ACAACTGCAC AATTTTCGTA TAGTCAAGAT ACAAGCACGA ACGGGTAAAT TCTTCTAGCA GTAAGCAACA   
  
  
- CCATTTGTAT ACTAAGATTT TCGACGACTT TTCCGTAGTT TCGAAGTATA ATATCTAAAA CCGTAGGAGA   
  
  
- TACCTAAGGT TACCGGATCG GAGTAAGCTA CGGAGAGTCT CGCCAGACCA CCTGGAGGTT TTGACAAATA   
  
  
- ATGTCCCTAG CTAGAGGGGG TCGGACCCAA GTCCGGTCGT CTTTCTCACC TTCGTTGTCC CTCTGCGAAC   
  
  
- CGATTCATGA CACTCGCCAT ATTACACGGT AAACTCATAG TACGGTAACG AGTCTTCACC CTTTGTTAGT   
  
  
- TTGGTCCCCT AGAGTTCAAT CCTTCATCCC TACTACTACT TCAACAGCAC CACTTGACAG AGACATCCAA   
  
  
- GTTCTTGGAG GAACTGCTCT GTTACCACCA CCTATCAGGT TCCTTGTGTC AAAATTTGGA CTAATCTTCC   
  
  
- CATTTTGGGC TATAAAAACA CGTACCGTAA CATTTACCAA GGATGTTGTA GGGAAAGAAA CACTGTGCAA   
  
  
- AATCTCTTCG GGAGAAAGTA ATAAGGTGAG AAAAGCTGTA CAATCTACGG TTGCGGAGAT CCCTCCTCGG   
  
  
- GCTCTCCAAC TATAAGCTCT TCCGTAAGAT ACCCTCCCTC TAATACTTAC ACCACCGTAC ACTCCCGTGT   
  
  
- CTCTCCCACC TTTCCGGTCT CTGTATGTTC GTTACCGTGC AATCCGTATC GTCCCGTCCC AAAGCCGTTG   
  
  
- ATGGTAACCT AGGGTTCAAC TAGCTCTTTT ACTCCAAATT CCGGTTCCGT CTGGTGGTGT TCCTAAAGCA   
  
  
- CTAACTACAC CTACCTGTAA CCCGTTAAGT CCCTACCTTC CCCGCCTAAC GGACACGGTA GAGACGTACC   
  
  
- CAAGACCGAA C

+     A-box

| Site Name | Organism | Position | Strand | Matrix score. | sequence | function |
| --- | --- | --- | --- | --- | --- | --- |
| A-box | Petroselinum crispum | 2597 | + | 6 | CCGTCC | cis-acting regulatory element |

>HU07G02248.1   
+ -Up\_Stream \_Len000CTGTTC AGCACTAGGG AATAAGGTTT TTGCCCTGCA TACTATGAAA TCTAACCAAA   
  
  
+ AACATTCCAT CTTGGAAGGC TGTAGCCTTG TGGATTTTGA ATTCGCTCCC AAGAACCAAT GAAGTAGCGC   
  
  
+ AGAAGGCCAA TACTCAATTT CAGAGTCCAC ATCAACTTGT TCAATGCCAG TATATTTCAG AACATTAATT   
  
  
+ ACATACTGTT TGGTTGGAGA AAAAATGGTT GTCTCGAAAA CTAGTTTTTT AATTTTTTGT TATTTAGCAC   
  
  
+ GGTAAAGAAT GGAAATTATT TTCATGCGAG AAAAAAGGAA GTTGGTTTTT CTTTTTTCAA CTCTTTTGAT   
  
  
+ GATATAATCA GTCGAAATTT AGGTGCACCA TCTGACTTCA ACTCTTGTAC GACCTCTGAT TGCTTGCTTA   
  
  
+ GAAGAACAAG AGGATTGACA GATAAGGAGG AGTGGGTGGT TTATACAGAG AATTGAGTGG ATGGTTATAA   
  
  
+ AGAAGTCGAT CTCACATAAG TAACTTCGTT ACGATTATAA ATATTTTCAT TGAAGAAAGA CGATTAGATA   
  
  
+ TAATTAAGAA GGTACATCTA AGCAAATTTG GCTGAGCAGA GTTGGAGGGC CACTAGGGTC ACATTGTGTC   
  
  
+ TTGCGCAACA AGCTTAGACC ATTCGCAACG GATGCCTCAC GTGGAGCAGG TGGGACCCGC GTGGAAGTTG   
  
  
+ CATGGCAAGA ATTCTCTGGC AGCAAGGAGA ATTGAGGGGG GACACGCGTG TGACCACGCC AGTGCGGTCC   
  
  
+ CCCTCTCGTG GCAGATTTGG TGATTTTTTT TTTGTTTTTT TTTGAAGTAA TATATAATTT TTATATGATT   
  
  
+ AAAAAAATAG GTTGTGTGTT ATAAAGTGGG TCAATGACAA ATATTTTTAT TGGATGATTA AAATTAAGTT   
  
  
+ CCATTTATTG GAGAAGTGAT GGAATGCAAG TTCCAAAATT GTGGAACTTT TGTTGACGTA GCAGGCTAGA   
  
  
+ GTTCCAAAAA TTAAAAAAAG GAAAGACATA TTGAGGTAAA GCCTTTTTAT TTTACAAATA GACGCACCAA   
  
  
+ GGTATAATAT AATTGACATG ATTTGAGGCG AGAGGAAGAT TCTTTTGCTA CCCAAACAAA ACACAATGAT   
  
  
+ CTTTTAAATG TAGCAATTTG GGATTGGCAA AATTTTGTCT AACCCGATTG TTGAAAGTTA TCCGATTTCA   
  
  
+ TAAAATTGGA TATCGTTATT AAATTTTCAT TCGAATATTT AAATTTGGAC ACAATGTTTT TCATATTTGG   
  
  
+ ATTTTCTAAA CTTGATTTTA ACTCAAATTT AAGTTTATTC AATTCATCTG ACCCAAAAAA TGGTTGCAAT   
  
  
+ AATGTATTTT CTTTTTGAAG TTAAATATTA TCGAATTCAT TTAACTTGTA GTTAACCCGT AATTGACCCA   
  
  
+ AACTCGACTT TTTGCTACCT TAGCAGCCAG GAAGAAGTCA TAGTATTGAC CCGTGGAACG CCTGAAGCCA   
  
  
+ ACATCACCAG ACCCACATAT TCCATCATCT TCTGATCTCC TCGTCAAAGA AGGAAGCTGA GCACAGCAAT   
  
  
+ AACTAGAAGA ACAGGAGGCA TCACACCCAA GCCAAAGGCC TTTATTTGAG CTCCCCACCT CATTCCTCGA   
  
  
+ AGCTTCCTCT TCACAATGTA AGACCATAAT CCTTCACCCC TTCTCTTTTT AAGCTCATCT TTACCTGCCC   
  
  
+ TAGTTCTCTC TCTCTCATGA GTCATCAGTA ATAGTTACCT CTTTACTTTC TCTTCCTTGA GGTATGCTTC   
  
  
+ AACTTTAATA TATATATGTA CCACTTGATT TCTCTTGTCA CTTTGCTATA ATACTTATTG TTTGGCTATC   
  
  
+ TTCCCCTGTT TTATACTCAT TTTCTTGCTT TCTATATCTG GGTTTGAATT GGGTTTCACT TATCCTCGTT   
  
  
+ GATTCTGTGG GAAATTAGTG AATTGGGTTT TGTACAGCAG GTTTATATGA ACTAGGGTTT CAAAGGTATC   
  
  
+ TACTTTTTCT CTTGTGATTA TTAGTTGATT AGGGATTTGG CCAAGATTAG CAGAATGGGT TCTGAATTTG   
  
  
+ GGGAATTCTC TGATGATGCT CTAAATGGGT ATGCTTACGT TGATATCCCT GCTTATGATG CGTCCTTAGA   
  
  
+ TTATGCCAAT TTGTTCAATT ACGAAGGCCT ATCTGAGGAT CTCACCTCAC TGAGCCTCCC AAGCCCCTTT   
  
  
+ TCTGACCCTT TGGCGTACAG TTTCACGTCC TCTTTGGGGC CGAGCCCTGG GGTTGATTCT AATGATGATA   
  
  
+ GTGATTCTGA TGATGTTCTC AAGTGCATTA GCCAAATGCT TATGGAAGAA GACATGGAGG CAAAGCCATG   
  
  
+ CATGTTTCAC GATCCTTTAG CACTTCAGGC TGCTGAGAAA CCCTTTTATG ATGCCTTAGG GAAGAAATAC   
  
  
+ CCAACTTCTC CGAACCAACA TCCTATAATT GATGATTGTT TGGATAATCC TGGTGAAAAT TCTTTGGGTT   
  
  
+ CTTCTAGTGA TTTTAGTGTT AGTCACTTTG GGTCTAGTTC AGCAAGCTCT ATTGGACCGA CAATTGTGTC   
  
  
+ TGATTTGAGT GAGCATTTTG AGCCACCCTT TGTTCAAGCA CTTCCAATTG AATCATATCC CCAACCATTG   
  
  
+ ACCCGTCCTC AATGGTCGTT TGGCCCTTCG GGTGCCTTAG ATTGCACGGC CTCTAATGGT TCAGTGATCT   
  
  
+ CATCCCTTGG TTTGCCGATG GATGTTATTA GTGTATTCAG TGAGAAAGAG TCCATGATTC AATTTCAGAA   
  
  
+ AGGGGTGGAA GAGGCTAGTA AGTTCCTTCC CAAGAATAAT AACCTTGTTA TTGATCTCAA GAACCTCACT   
  
  
+ TTTCCTAATG AAACGAAGGA GGATGATCGA GTGATGATGG TTAAGAAGGA AAAGAATGAT GTGAATTGGT   
  
  
+ CTAACTACTC AAGAGGGAGT AAGATTCACT ATCGTGAAGA CGAGGACTTT GAAGAAGGAA GGAGTAGCAA   
  
  
+ GCAGTCAGCT ATTTCTACTA CTGAGGAAGC TGAGTTGTGT GAAATTTTTG ACAAGTTTTT GCTTTGCAAT   
  
  
+ TGGTACCCTG TGAAACCTGA GGCTCATCCC ACCATGAGTT TGAACCCTGA GAAGGGCCAG TCACATGGAT   
  
  
+ TAGAAGGTGG GAGAAATGGG AAGGGTCACC CAAAGAAACA GGATAAAAGT AGTACCAACG TTGTGGATTT   
  
  
+ AAGGAATTTG CTGATGCTAT GTGCACAATC TACTGCATCT GATGACCGAA GAACTGCTGA TGGACTGCTA   
  
  
+ AAGAAAATCA GGGAGCACTC ATCTGCTGAG GGGGATGGAT TTCAAAGGTT GGCTCATTAC TTTGCTGATG   
  
  
+ CCCTAGAGGC ACGTTTAGCT GGAACTGGAT CTCACATTTA TACAGCCCTA AGTTCTCATA GGCCATCTGC   
  
  
+ TGTTGACGTG TTAAAAGCAT ATCAGTTCTA TGTTCGTGCT TGCCCATTTA AGAAGATCGT CATTCGTTGT   
  
  
+ GGTAAACATA TGATTCTAAA AGCTGCTGAA AAGGCATCAA AGCTTCATAT TATAGATTTT GGCATCCTCT   
  
  
+ ATGGATTCCA ATGGCCTAGC CTCATTCGAT GCCTCTCAGA GCGGTCTGGT GGACCTCCAA AACTGTTTAT   
  
  
+ TACAGGGATC GATCTCCCCC AGCCTGGGTT CAGGCCAGCA GAAAGAGTGG AAGCAACAGG GAGACGCTTG   
  
  
+ GCTAAGTACT GTGAGCGGTA TAATGTGCCA TTTGAGTATC ATGCCATTGC TCAGAAGTGG GAAACAATCA   
  
  
+ AACCAGGGGA TCTCAAGTTA GGAAGTAGGG ATGATGATGA AGTTGTCGTG GTGAACTGTC TCTGTAGGTT   
  
  
+ CAAGAACCTC CTTGACGAGA CAATGGTGGT GGATAGTCCA AGGAACACAG TTTTAAACCT GATTAGAAGG   
  
  
+ GTAAAACCCG ATATTTTTGT GCATGGCATT GTAAATGGTT CCTACAACAT CCCTTTCTTT GTGACACGTT   
  
  
+ TTAGAGAAGC CCTCTTTCAT TATTCCACTC TTTTCGACAT GTTAGATGCC AACGCCTCTA GGGAGGAGCC   
  
  
+ CGAGAGGTTG ATATTCGAGA AGGCATTCTA TGGGAGGGAG ATTATGAATG TGGTGGCATG TGAGGGCACA   
  
  
+ GAGAGGGTGG AAAGGCCAGA GACATACAAG CAATGGCACG TTAGGCATAG CAGGGCAGGG TTTCGGCAAC   
  
  
+ TACCATTGGA TCCCAAGTTG ATCGAGAAAA TGAGGTTTAA GGCCAAGGCA GACCACCACA AGGATTTCGT   
  
  
+ GATTGATGTG GATGGACATT GGGCAATTCA GGGATGGAAG GGGCGGATTG CCTGTGCCAT CTCTGCATGG   
  
  
+ GTTCTGGCTT G  

- -Up\_Stream \_Len000GACAAG TCGTGATCCC TTATTCCAAA AACGGGACGT ATGATACTTT AGATTGGTTT   
  
  
- TTGTAAGGTA GAACCTTCCG ACATCGGAAC ACCTAAAACT TAAGCGAGGG TTCTTGGTTA CTTCATCGCG   
  
  
- TCTTCCGGTT ATGAGTTAAA GTCTCAGGTG TAGTTGAACA AGTTACGGTC ATATAAAGTC TTGTAATTAA   
  
  
- TGTATGACAA ACCAACCTCT TTTTTACCAA CAGAGCTTTT GATCAAAAAA TTAAAAAACA ATAAATCGTG   
  
  
- CCATTTCTTA CCTTTAATAA AAGTACGCTC TTTTTTCCTT CAACCAAAAA GAAAAAAGTT GAGAAAACTA   
  
  
- CTATATTAGT CAGCTTTAAA TCCACGTGGT AGACTGAAGT TGAGAACATG CTGGAGACTA ACGAACGAAT   
  
  
- CTTCTTGTTC TCCTAACTGT CTATTCCTCC TCACCCACCA AATATGTCTC TTAACTCACC TACCAATATT   
  
  
- TCTTCAGCTA GAGTGTATTC ATTGAAGCAA TGCTAATATT TATAAAAGTA ACTTCTTTCT GCTAATCTAT   
  
  
- ATTAATTCTT CCATGTAGAT TCGTTTAAAC CGACTCGTCT CAACCTCCCG GTGATCCCAG TGTAACACAG   
  
  
- AACGCGTTGT TCGAATCTGG TAAGCGTTGC CTACGGAGTG CACCTCGTCC ACCCTGGGCG CACCTTCAAC   
  
  
- GTACCGTTCT TAAGAGACCG TCGTTCCTCT TAACTCCCCC CTGTGCGCAC ACTGGTGCGG TCACGCCAGG   
  
  
- GGGAGAGCAC CGTCTAAACC ACTAAAAAAA AAACAAAAAA AAACTTCATT ATATATTAAA AATATACTAA   
  
  
- TTTTTTTATC CAACACACAA TATTTCACCC AGTTACTGTT TATAAAAATA ACCTACTAAT TTTAATTCAA   
  
  
- GGTAAATAAC CTCTTCACTA CCTTACGTTC AAGGTTTTAA CACCTTGAAA ACAACTGCAT CGTCCGATCT   
  
  
- CAAGGTTTTT AATTTTTTTC CTTTCTGTAT AACTCCATTT CGGAAAAATA AAATGTTTAT CTGCGTGGTT   
  
  
- CCATATTATA TTAACTGTAC TAAACTCCGC TCTCCTTCTA AGAAAACGAT GGGTTTGTTT TGTGTTACTA   
  
  
- GAAAATTTAC ATCGTTAAAC CCTAACCGTT TTAAAACAGA TTGGGCTAAC AACTTTCAAT AGGCTAAAGT   
  
  
- ATTTTAACCT ATAGCAATAA TTTAAAAGTA AGCTTATAAA TTTAAACCTG TGTTACAAAA AGTATAAACC   
  
  
- TAAAAGATTT GAACTAAAAT TGAGTTTAAA TTCAAATAAG TTAAGTAGAC TGGGTTTTTT ACCAACGTTA   
  
  
- TTACATAAAA GAAAAACTTC AATTTATAAT AGCTTAAGTA AATTGAACAT CAATTGGGCA TTAACTGGGT   
  
  
- TTGAGCTGAA AAACGATGGA ATCGTCGGTC CTTCTTCAGT ATCATAACTG GGCACCTTGC GGACTTCGGT   
  
  
- TGTAGTGGTC TGGGTGTATA AGGTAGTAGA AGACTAGAGG AGCAGTTTCT TCCTTCGACT CGTGTCGTTA   
  
  
- TTGATCTTCT TGTCCTCCGT AGTGTGGGTT CGGTTTCCGG AAATAAACTC GAGGGGTGGA GTAAGGAGCT   
  
  
- TCGAAGGAGA AGTGTTACAT TCTGGTATTA GGAAGTGGGG AAGAGAAAAA TTCGAGTAGA AATGGACGGG   
  
  
- ATCAAGAGAG AGAGAGTACT CAGTAGTCAT TATCAATGGA GAAATGAAAG AGAAGGAACT CCATACGAAG   
  
  
- TTGAAATTAT ATATATACAT GGTGAACTAA AGAGAACAGT GAAACGATAT TATGAATAAC AAACCGATAG   
  
  
- AAGGGGACAA AATATGAGTA AAAGAACGAA AGATATAGAC CCAAACTTAA CCCAAAGTGA ATAGGAGCAA   
  
  
- CTAAGACACC CTTTAATCAC TTAACCCAAA ACATGTCGTC CAAATATACT TGATCCCAAA GTTTCCATAG   
  
  
- ATGAAAAAGA GAACACTAAT AATCAACTAA TCCCTAAACC GGTTCTAATC GTCTTACCCA AGACTTAAAC   
  
  
- CCCTTAAGAG ACTACTACGA GATTTACCCA TACGAATGCA ACTATAGGGA CGAATACTAC GCAGGAATCT   
  
  
- AATACGGTTA AACAAGTTAA TGCTTCCGGA TAGACTCCTA GAGTGGAGTG ACTCGGAGGG TTCGGGGAAA   
  
  
- AGACTGGGAA ACCGCATGTC AAAGTGCAGG AGAAACCCCG GCTCGGGACC CCAACTAAGA TTACTACTAT   
  
  
- CACTAAGACT ACTACAAGAG TTCACGTAAT CGGTTTACGA ATACCTTCTT CTGTACCTCC GTTTCGGTAC   
  
  
- GTACAAAGTG CTAGGAAATC GTGAAGTCCG ACGACTCTTT GGGAAAATAC TACGGAATCC CTTCTTTATG   
  
  
- GGTTGAAGAG GCTTGGTTGT AGGATATTAA CTACTAACAA ACCTATTAGG ACCACTTTTA AGAAACCCAA   
  
  
- GAAGATCACT AAAATCACAA TCAGTGAAAC CCAGATCAAG TCGTTCGAGA TAACCTGGCT GTTAACACAG   
  
  
- ACTAAACTCA CTCGTAAAAC TCGGTGGGAA ACAAGTTCGT GAAGGTTAAC TTAGTATAGG GGTTGGTAAC   
  
  
- TGGGCAGGAG TTACCAGCAA ACCGGGAAGC CCACGGAATC TAACGTGCCG GAGATTACCA AGTCACTAGA   
  
  
- GTAGGGAACC AAACGGCTAC CTACAATAAT CACATAAGTC ACTCTTTCTC AGGTACTAAG TTAAAGTCTT   
  
  
- TCCCCACCTT CTCCGATCAT TCAAGGAAGG GTTCTTATTA TTGGAACAAT AACTAGAGTT CTTGGAGTGA   
  
  
- AAAGGATTAC TTTGCTTCCT CCTACTAGCT CACTACTACC AATTCTTCCT TTTCTTACTA CACTTAACCA   
  
  
- GATTGATGAG TTCTCCCTCA TTCTAAGTGA TAGCACTTCT GCTCCTGAAA CTTCTTCCTT CCTCATCGTT   
  
  
- CGTCAGTCGA TAAAGATGAT GACTCCTTCG ACTCAACACA CTTTAAAAAC TGTTCAAAAA CGAAACGTTA   
  
  
- ACCATGGGAC ACTTTGGACT CCGAGTAGGG TGGTACTCAA ACTTGGGACT CTTCCCGGTC AGTGTACCTA   
  
  
- ATCTTCCACC CTCTTTACCC TTCCCAGTGG GTTTCTTTGT CCTATTTTCA TCATGGTTGC AACACCTAAA   
  
  
- TTCCTTAAAC GACTACGATA CACGTGTTAG ATGACGTAGA CTACTGGCTT CTTGACGACT ACCTGACGAT   
  
  
- TTCTTTTAGT CCCTCGTGAG TAGACGACTC CCCCTACCTA AAGTTTCCAA CCGAGTAATG AAACGACTAC   
  
  
- GGGATCTCCG TGCAAATCGA CCTTGACCTA GAGTGTAAAT ATGTCGGGAT TCAAGAGTAT CCGGTAGACG   
  
  
- ACAACTGCAC AATTTTCGTA TAGTCAAGAT ACAAGCACGA ACGGGTAAAT TCTTCTAGCA GTAAGCAACA   
  
  
- CCATTTGTAT ACTAAGATTT TCGACGACTT TTCCGTAGTT TCGAAGTATA ATATCTAAAA CCGTAGGAGA   
  
  
- TACCTAAGGT TACCGGATCG GAGTAAGCTA CGGAGAGTCT CGCCAGACCA CCTGGAGGTT TTGACAAATA   
  
  
- ATGTCCCTAG CTAGAGGGGG TCGGACCCAA GTCCGGTCGT CTTTCTCACC TTCGTTGTCC CTCTGCGAAC   
  
  
- CGATTCATGA CACTCGCCAT ATTACACGGT AAACTCATAG TACGGTAACG AGTCTTCACC CTTTGTTAGT   
  
  
- TTGGTCCCCT AGAGTTCAAT CCTTCATCCC TACTACTACT TCAACAGCAC CACTTGACAG AGACATCCAA   
  
  
- GTTCTTGGAG GAACTGCTCT GTTACCACCA CCTATCAGGT TCCTTGTGTC AAAATTTGGA CTAATCTTCC   
  
  
- CATTTTGGGC TATAAAAACA CGTACCGTAA CATTTACCAA GGATGTTGTA GGGAAAGAAA CACTGTGCAA   
  
  
- AATCTCTTCG GGAGAAAGTA ATAAGGTGAG AAAAGCTGTA CAATCTACGG TTGCGGAGAT CCCTCCTCGG   
  
  
- GCTCTCCAAC TATAAGCTCT TCCGTAAGAT ACCCTCCCTC TAATACTTAC ACCACCGTAC ACTCCCGTGT   
  
  
- CTCTCCCACC TTTCCGGTCT CTGTATGTTC GTTACCGTGC AATCCGTATC GTCCCGTCCC AAAGCCGTTG   
  
  
- ATGGTAACCT AGGGTTCAAC TAGCTCTTTT ACTCCAAATT CCGGTTCCGT CTGGTGGTGT TCCTAAAGCA   
  
  
- CTAACTACAC CTACCTGTAA CCCGTTAAGT CCCTACCTTC CCCGCCTAAC GGACACGGTA GAGACGTACC   
  
  
- CAAGACCGAA C

+     AAGAA-motif

| Site Name | Organism | Position | Strand | Matrix score. | sequence | function |
| --- | --- | --- | --- | --- | --- | --- |
| AAGAA-motif | Avena sativa | 3221 | + | 9 | gGTAAAGAAA |  |
| AAGAA-motif | Avena sativa | 285 | + | 9 | gGTAAAGAAA |  |

>HU07G02248.1   
+ -Up\_Stream \_Len000CTGTTC AGCACTAGGG AATAAGGTTT TTGCCCTGCA TACTATGAAA TCTAACCAAA   
  
  
+ AACATTCCAT CTTGGAAGGC TGTAGCCTTG TGGATTTTGA ATTCGCTCCC AAGAACCAAT GAAGTAGCGC   
  
  
+ AGAAGGCCAA TACTCAATTT CAGAGTCCAC ATCAACTTGT TCAATGCCAG TATATTTCAG AACATTAATT   
  
  
+ ACATACTGTT TGGTTGGAGA AAAAATGGTT GTCTCGAAAA CTAGTTTTTT AATTTTTTGT TATTTAGCAC   
  
  
+ GGTAAAGAAT GGAAATTATT TTCATGCGAG AAAAAAGGAA GTTGGTTTTT CTTTTTTCAA CTCTTTTGAT   
  
  
+ GATATAATCA GTCGAAATTT AGGTGCACCA TCTGACTTCA ACTCTTGTAC GACCTCTGAT TGCTTGCTTA   
  
  
+ GAAGAACAAG AGGATTGACA GATAAGGAGG AGTGGGTGGT TTATACAGAG AATTGAGTGG ATGGTTATAA   
  
  
+ AGAAGTCGAT CTCACATAAG TAACTTCGTT ACGATTATAA ATATTTTCAT TGAAGAAAGA CGATTAGATA   
  
  
+ TAATTAAGAA GGTACATCTA AGCAAATTTG GCTGAGCAGA GTTGGAGGGC CACTAGGGTC ACATTGTGTC   
  
  
+ TTGCGCAACA AGCTTAGACC ATTCGCAACG GATGCCTCAC GTGGAGCAGG TGGGACCCGC GTGGAAGTTG   
  
  
+ CATGGCAAGA ATTCTCTGGC AGCAAGGAGA ATTGAGGGGG GACACGCGTG TGACCACGCC AGTGCGGTCC   
  
  
+ CCCTCTCGTG GCAGATTTGG TGATTTTTTT TTTGTTTTTT TTTGAAGTAA TATATAATTT TTATATGATT   
  
  
+ AAAAAAATAG GTTGTGTGTT ATAAAGTGGG TCAATGACAA ATATTTTTAT TGGATGATTA AAATTAAGTT   
  
  
+ CCATTTATTG GAGAAGTGAT GGAATGCAAG TTCCAAAATT GTGGAACTTT TGTTGACGTA GCAGGCTAGA   
  
  
+ GTTCCAAAAA TTAAAAAAAG GAAAGACATA TTGAGGTAAA GCCTTTTTAT TTTACAAATA GACGCACCAA   
  
  
+ GGTATAATAT AATTGACATG ATTTGAGGCG AGAGGAAGAT TCTTTTGCTA CCCAAACAAA ACACAATGAT   
  
  
+ CTTTTAAATG TAGCAATTTG GGATTGGCAA AATTTTGTCT AACCCGATTG TTGAAAGTTA TCCGATTTCA   
  
  
+ TAAAATTGGA TATCGTTATT AAATTTTCAT TCGAATATTT AAATTTGGAC ACAATGTTTT TCATATTTGG   
  
  
+ ATTTTCTAAA CTTGATTTTA ACTCAAATTT AAGTTTATTC AATTCATCTG ACCCAAAAAA TGGTTGCAAT   
  
  
+ AATGTATTTT CTTTTTGAAG TTAAATATTA TCGAATTCAT TTAACTTGTA GTTAACCCGT AATTGACCCA   
  
  
+ AACTCGACTT TTTGCTACCT TAGCAGCCAG GAAGAAGTCA TAGTATTGAC CCGTGGAACG CCTGAAGCCA   
  
  
+ ACATCACCAG ACCCACATAT TCCATCATCT TCTGATCTCC TCGTCAAAGA AGGAAGCTGA GCACAGCAAT   
  
  
+ AACTAGAAGA ACAGGAGGCA TCACACCCAA GCCAAAGGCC TTTATTTGAG CTCCCCACCT CATTCCTCGA   
  
  
+ AGCTTCCTCT TCACAATGTA AGACCATAAT CCTTCACCCC TTCTCTTTTT AAGCTCATCT TTACCTGCCC   
  
  
+ TAGTTCTCTC TCTCTCATGA GTCATCAGTA ATAGTTACCT CTTTACTTTC TCTTCCTTGA GGTATGCTTC   
  
  
+ AACTTTAATA TATATATGTA CCACTTGATT TCTCTTGTCA CTTTGCTATA ATACTTATTG TTTGGCTATC   
  
  
+ TTCCCCTGTT TTATACTCAT TTTCTTGCTT TCTATATCTG GGTTTGAATT GGGTTTCACT TATCCTCGTT   
  
  
+ GATTCTGTGG GAAATTAGTG AATTGGGTTT TGTACAGCAG GTTTATATGA ACTAGGGTTT CAAAGGTATC   
  
  
+ TACTTTTTCT CTTGTGATTA TTAGTTGATT AGGGATTTGG CCAAGATTAG CAGAATGGGT TCTGAATTTG   
  
  
+ GGGAATTCTC TGATGATGCT CTAAATGGGT ATGCTTACGT TGATATCCCT GCTTATGATG CGTCCTTAGA   
  
  
+ TTATGCCAAT TTGTTCAATT ACGAAGGCCT ATCTGAGGAT CTCACCTCAC TGAGCCTCCC AAGCCCCTTT   
  
  
+ TCTGACCCTT TGGCGTACAG TTTCACGTCC TCTTTGGGGC CGAGCCCTGG GGTTGATTCT AATGATGATA   
  
  
+ GTGATTCTGA TGATGTTCTC AAGTGCATTA GCCAAATGCT TATGGAAGAA GACATGGAGG CAAAGCCATG   
  
  
+ CATGTTTCAC GATCCTTTAG CACTTCAGGC TGCTGAGAAA CCCTTTTATG ATGCCTTAGG GAAGAAATAC   
  
  
+ CCAACTTCTC CGAACCAACA TCCTATAATT GATGATTGTT TGGATAATCC TGGTGAAAAT TCTTTGGGTT   
  
  
+ CTTCTAGTGA TTTTAGTGTT AGTCACTTTG GGTCTAGTTC AGCAAGCTCT ATTGGACCGA CAATTGTGTC   
  
  
+ TGATTTGAGT GAGCATTTTG AGCCACCCTT TGTTCAAGCA CTTCCAATTG AATCATATCC CCAACCATTG   
  
  
+ ACCCGTCCTC AATGGTCGTT TGGCCCTTCG GGTGCCTTAG ATTGCACGGC CTCTAATGGT TCAGTGATCT   
  
  
+ CATCCCTTGG TTTGCCGATG GATGTTATTA GTGTATTCAG TGAGAAAGAG TCCATGATTC AATTTCAGAA   
  
  
+ AGGGGTGGAA GAGGCTAGTA AGTTCCTTCC CAAGAATAAT AACCTTGTTA TTGATCTCAA GAACCTCACT   
  
  
+ TTTCCTAATG AAACGAAGGA GGATGATCGA GTGATGATGG TTAAGAAGGA AAAGAATGAT GTGAATTGGT   
  
  
+ CTAACTACTC AAGAGGGAGT AAGATTCACT ATCGTGAAGA CGAGGACTTT GAAGAAGGAA GGAGTAGCAA   
  
  
+ GCAGTCAGCT ATTTCTACTA CTGAGGAAGC TGAGTTGTGT GAAATTTTTG ACAAGTTTTT GCTTTGCAAT   
  
  
+ TGGTACCCTG TGAAACCTGA GGCTCATCCC ACCATGAGTT TGAACCCTGA GAAGGGCCAG TCACATGGAT   
  
  
+ TAGAAGGTGG GAGAAATGGG AAGGGTCACC CAAAGAAACA GGATAAAAGT AGTACCAACG TTGTGGATTT   
  
  
+ AAGGAATTTG CTGATGCTAT GTGCACAATC TACTGCATCT GATGACCGAA GAACTGCTGA TGGACTGCTA   
  
  
+ AAGAAAATCA GGGAGCACTC ATCTGCTGAG GGGGATGGAT TTCAAAGGTT GGCTCATTAC TTTGCTGATG   
  
  
+ CCCTAGAGGC ACGTTTAGCT GGAACTGGAT CTCACATTTA TACAGCCCTA AGTTCTCATA GGCCATCTGC   
  
  
+ TGTTGACGTG TTAAAAGCAT ATCAGTTCTA TGTTCGTGCT TGCCCATTTA AGAAGATCGT CATTCGTTGT   
  
  
+ GGTAAACATA TGATTCTAAA AGCTGCTGAA AAGGCATCAA AGCTTCATAT TATAGATTTT GGCATCCTCT   
  
  
+ ATGGATTCCA ATGGCCTAGC CTCATTCGAT GCCTCTCAGA GCGGTCTGGT GGACCTCCAA AACTGTTTAT   
  
  
+ TACAGGGATC GATCTCCCCC AGCCTGGGTT CAGGCCAGCA GAAAGAGTGG AAGCAACAGG GAGACGCTTG   
  
  
+ GCTAAGTACT GTGAGCGGTA TAATGTGCCA TTTGAGTATC ATGCCATTGC TCAGAAGTGG GAAACAATCA   
  
  
+ AACCAGGGGA TCTCAAGTTA GGAAGTAGGG ATGATGATGA AGTTGTCGTG GTGAACTGTC TCTGTAGGTT   
  
  
+ CAAGAACCTC CTTGACGAGA CAATGGTGGT GGATAGTCCA AGGAACACAG TTTTAAACCT GATTAGAAGG   
  
  
+ GTAAAACCCG ATATTTTTGT GCATGGCATT GTAAATGGTT CCTACAACAT CCCTTTCTTT GTGACACGTT   
  
  
+ TTAGAGAAGC CCTCTTTCAT TATTCCACTC TTTTCGACAT GTTAGATGCC AACGCCTCTA GGGAGGAGCC   
  
  
+ CGAGAGGTTG ATATTCGAGA AGGCATTCTA TGGGAGGGAG ATTATGAATG TGGTGGCATG TGAGGGCACA   
  
  
+ GAGAGGGTGG AAAGGCCAGA GACATACAAG CAATGGCACG TTAGGCATAG CAGGGCAGGG TTTCGGCAAC   
  
  
+ TACCATTGGA TCCCAAGTTG ATCGAGAAAA TGAGGTTTAA GGCCAAGGCA GACCACCACA AGGATTTCGT   
  
  
+ GATTGATGTG GATGGACATT GGGCAATTCA GGGATGGAAG GGGCGGATTG CCTGTGCCAT CTCTGCATGG   
  
  
+ GTTCTGGCTT G  

- -Up\_Stream \_Len000GACAAG TCGTGATCCC TTATTCCAAA AACGGGACGT ATGATACTTT AGATTGGTTT   
  
  
- TTGTAAGGTA GAACCTTCCG ACATCGGAAC ACCTAAAACT TAAGCGAGGG TTCTTGGTTA CTTCATCGCG   
  
  
- TCTTCCGGTT ATGAGTTAAA GTCTCAGGTG TAGTTGAACA AGTTACGGTC ATATAAAGTC TTGTAATTAA   
  
  
- TGTATGACAA ACCAACCTCT TTTTTACCAA CAGAGCTTTT GATCAAAAAA TTAAAAAACA ATAAATCGTG   
  
  
- CCATTTCTTA CCTTTAATAA AAGTACGCTC TTTTTTCCTT CAACCAAAAA GAAAAAAGTT GAGAAAACTA   
  
  
- CTATATTAGT CAGCTTTAAA TCCACGTGGT AGACTGAAGT TGAGAACATG CTGGAGACTA ACGAACGAAT   
  
  
- CTTCTTGTTC TCCTAACTGT CTATTCCTCC TCACCCACCA AATATGTCTC TTAACTCACC TACCAATATT   
  
  
- TCTTCAGCTA GAGTGTATTC ATTGAAGCAA TGCTAATATT TATAAAAGTA ACTTCTTTCT GCTAATCTAT   
  
  
- ATTAATTCTT CCATGTAGAT TCGTTTAAAC CGACTCGTCT CAACCTCCCG GTGATCCCAG TGTAACACAG   
  
  
- AACGCGTTGT TCGAATCTGG TAAGCGTTGC CTACGGAGTG CACCTCGTCC ACCCTGGGCG CACCTTCAAC   
  
  
- GTACCGTTCT TAAGAGACCG TCGTTCCTCT TAACTCCCCC CTGTGCGCAC ACTGGTGCGG TCACGCCAGG   
  
  
- GGGAGAGCAC CGTCTAAACC ACTAAAAAAA AAACAAAAAA AAACTTCATT ATATATTAAA AATATACTAA   
  
  
- TTTTTTTATC CAACACACAA TATTTCACCC AGTTACTGTT TATAAAAATA ACCTACTAAT TTTAATTCAA   
  
  
- GGTAAATAAC CTCTTCACTA CCTTACGTTC AAGGTTTTAA CACCTTGAAA ACAACTGCAT CGTCCGATCT   
  
  
- CAAGGTTTTT AATTTTTTTC CTTTCTGTAT AACTCCATTT CGGAAAAATA AAATGTTTAT CTGCGTGGTT   
  
  
- CCATATTATA TTAACTGTAC TAAACTCCGC TCTCCTTCTA AGAAAACGAT GGGTTTGTTT TGTGTTACTA   
  
  
- GAAAATTTAC ATCGTTAAAC CCTAACCGTT TTAAAACAGA TTGGGCTAAC AACTTTCAAT AGGCTAAAGT   
  
  
- ATTTTAACCT ATAGCAATAA TTTAAAAGTA AGCTTATAAA TTTAAACCTG TGTTACAAAA AGTATAAACC   
  
  
- TAAAAGATTT GAACTAAAAT TGAGTTTAAA TTCAAATAAG TTAAGTAGAC TGGGTTTTTT ACCAACGTTA   
  
  
- TTACATAAAA GAAAAACTTC AATTTATAAT AGCTTAAGTA AATTGAACAT CAATTGGGCA TTAACTGGGT   
  
  
- TTGAGCTGAA AAACGATGGA ATCGTCGGTC CTTCTTCAGT ATCATAACTG GGCACCTTGC GGACTTCGGT   
  
  
- TGTAGTGGTC TGGGTGTATA AGGTAGTAGA AGACTAGAGG AGCAGTTTCT TCCTTCGACT CGTGTCGTTA   
  
  
- TTGATCTTCT TGTCCTCCGT AGTGTGGGTT CGGTTTCCGG AAATAAACTC GAGGGGTGGA GTAAGGAGCT   
  
  
- TCGAAGGAGA AGTGTTACAT TCTGGTATTA GGAAGTGGGG AAGAGAAAAA TTCGAGTAGA AATGGACGGG   
  
  
- ATCAAGAGAG AGAGAGTACT CAGTAGTCAT TATCAATGGA GAAATGAAAG AGAAGGAACT CCATACGAAG   
  
  
- TTGAAATTAT ATATATACAT GGTGAACTAA AGAGAACAGT GAAACGATAT TATGAATAAC AAACCGATAG   
  
  
- AAGGGGACAA AATATGAGTA AAAGAACGAA AGATATAGAC CCAAACTTAA CCCAAAGTGA ATAGGAGCAA   
  
  
- CTAAGACACC CTTTAATCAC TTAACCCAAA ACATGTCGTC CAAATATACT TGATCCCAAA GTTTCCATAG   
  
  
- ATGAAAAAGA GAACACTAAT AATCAACTAA TCCCTAAACC GGTTCTAATC GTCTTACCCA AGACTTAAAC   
  
  
- CCCTTAAGAG ACTACTACGA GATTTACCCA TACGAATGCA ACTATAGGGA CGAATACTAC GCAGGAATCT   
  
  
- AATACGGTTA AACAAGTTAA TGCTTCCGGA TAGACTCCTA GAGTGGAGTG ACTCGGAGGG TTCGGGGAAA   
  
  
- AGACTGGGAA ACCGCATGTC AAAGTGCAGG AGAAACCCCG GCTCGGGACC CCAACTAAGA TTACTACTAT   
  
  
- CACTAAGACT ACTACAAGAG TTCACGTAAT CGGTTTACGA ATACCTTCTT CTGTACCTCC GTTTCGGTAC   
  
  
- GTACAAAGTG CTAGGAAATC GTGAAGTCCG ACGACTCTTT GGGAAAATAC TACGGAATCC CTTCTTTATG   
  
  
- GGTTGAAGAG GCTTGGTTGT AGGATATTAA CTACTAACAA ACCTATTAGG ACCACTTTTA AGAAACCCAA   
  
  
- GAAGATCACT AAAATCACAA TCAGTGAAAC CCAGATCAAG TCGTTCGAGA TAACCTGGCT GTTAACACAG   
  
  
- ACTAAACTCA CTCGTAAAAC TCGGTGGGAA ACAAGTTCGT GAAGGTTAAC TTAGTATAGG GGTTGGTAAC   
  
  
- TGGGCAGGAG TTACCAGCAA ACCGGGAAGC CCACGGAATC TAACGTGCCG GAGATTACCA AGTCACTAGA   
  
  
- GTAGGGAACC AAACGGCTAC CTACAATAAT CACATAAGTC ACTCTTTCTC AGGTACTAAG TTAAAGTCTT   
  
  
- TCCCCACCTT CTCCGATCAT TCAAGGAAGG GTTCTTATTA TTGGAACAAT AACTAGAGTT CTTGGAGTGA   
  
  
- AAAGGATTAC TTTGCTTCCT CCTACTAGCT CACTACTACC AATTCTTCCT TTTCTTACTA CACTTAACCA   
  
  
- GATTGATGAG TTCTCCCTCA TTCTAAGTGA TAGCACTTCT GCTCCTGAAA CTTCTTCCTT CCTCATCGTT   
  
  
- CGTCAGTCGA TAAAGATGAT GACTCCTTCG ACTCAACACA CTTTAAAAAC TGTTCAAAAA CGAAACGTTA   
  
  
- ACCATGGGAC ACTTTGGACT CCGAGTAGGG TGGTACTCAA ACTTGGGACT CTTCCCGGTC AGTGTACCTA   
  
  
- ATCTTCCACC CTCTTTACCC TTCCCAGTGG GTTTCTTTGT CCTATTTTCA TCATGGTTGC AACACCTAAA   
  
  
- TTCCTTAAAC GACTACGATA CACGTGTTAG ATGACGTAGA CTACTGGCTT CTTGACGACT ACCTGACGAT   
  
  
- TTCTTTTAGT CCCTCGTGAG TAGACGACTC CCCCTACCTA AAGTTTCCAA CCGAGTAATG AAACGACTAC   
  
  
- GGGATCTCCG TGCAAATCGA CCTTGACCTA GAGTGTAAAT ATGTCGGGAT TCAAGAGTAT CCGGTAGACG   
  
  
- ACAACTGCAC AATTTTCGTA TAGTCAAGAT ACAAGCACGA ACGGGTAAAT TCTTCTAGCA GTAAGCAACA   
  
  
- CCATTTGTAT ACTAAGATTT TCGACGACTT TTCCGTAGTT TCGAAGTATA ATATCTAAAA CCGTAGGAGA   
  
  
- TACCTAAGGT TACCGGATCG GAGTAAGCTA CGGAGAGTCT CGCCAGACCA CCTGGAGGTT TTGACAAATA   
  
  
- ATGTCCCTAG CTAGAGGGGG TCGGACCCAA GTCCGGTCGT CTTTCTCACC TTCGTTGTCC CTCTGCGAAC   
  
  
- CGATTCATGA CACTCGCCAT ATTACACGGT AAACTCATAG TACGGTAACG AGTCTTCACC CTTTGTTAGT   
  
  
- TTGGTCCCCT AGAGTTCAAT CCTTCATCCC TACTACTACT TCAACAGCAC CACTTGACAG AGACATCCAA   
  
  
- GTTCTTGGAG GAACTGCTCT GTTACCACCA CCTATCAGGT TCCTTGTGTC AAAATTTGGA CTAATCTTCC   
  
  
- CATTTTGGGC TATAAAAACA CGTACCGTAA CATTTACCAA GGATGTTGTA GGGAAAGAAA CACTGTGCAA   
  
  
- AATCTCTTCG GGAGAAAGTA ATAAGGTGAG AAAAGCTGTA CAATCTACGG TTGCGGAGAT CCCTCCTCGG   
  
  
- GCTCTCCAAC TATAAGCTCT TCCGTAAGAT ACCCTCCCTC TAATACTTAC ACCACCGTAC ACTCCCGTGT   
  
  
- CTCTCCCACC TTTCCGGTCT CTGTATGTTC GTTACCGTGC AATCCGTATC GTCCCGTCCC AAAGCCGTTG   
  
  
- ATGGTAACCT AGGGTTCAAC TAGCTCTTTT ACTCCAAATT CCGGTTCCGT CTGGTGGTGT TCCTAAAGCA   
  
  
- CTAACTACAC CTACCTGTAA CCCGTTAAGT CCCTACCTTC CCCGCCTAAC GGACACGGTA GAGACGTACC   
  
  
- CAAGACCGAA C

+     ABRE

| Site Name | Organism | Position | Strand | Matrix score. | sequence | function |
| --- | --- | --- | --- | --- | --- | --- |
| ABRE | Arabidopsis thaliana | 673 | + | 5 | ACGTG | cis-acting element involved in the abscisic acid responsiveness |
| ABRE | Arabidopsis thaliana | 672 | + | 6 | CACGTG | cis-acting element involved in the abscisic acid responsiveness |
| ABRE | Arabidopsis thaliana | 2198 | - | 5 | ACGTG | cis-acting element involved in the abscisic acid responsiveness |
| ABRE | Arabidopsis thaliana | 3304 | - | 5 | ACGTG | cis-acting element involved in the abscisic acid responsiveness |
| ABRE | Arabidopsis thaliana | 3919 | - | 5 | ACGTG | cis-acting element involved in the abscisic acid responsiveness |
| ABRE | Arabidopsis thaliana | 3370 | + | 5 | ACGTG | cis-acting element involved in the abscisic acid responsiveness |
| ABRE | Arabidopsis thaliana | 4101 | - | 5 | ACGTG | cis-acting element involved in the abscisic acid responsiveness |

>HU07G02248.1   
+ -Up\_Stream \_Len000CTGTTC AGCACTAGGG AATAAGGTTT TTGCCCTGCA TACTATGAAA TCTAACCAAA   
  
  
+ AACATTCCAT CTTGGAAGGC TGTAGCCTTG TGGATTTTGA ATTCGCTCCC AAGAACCAAT GAAGTAGCGC   
  
  
+ AGAAGGCCAA TACTCAATTT CAGAGTCCAC ATCAACTTGT TCAATGCCAG TATATTTCAG AACATTAATT   
  
  
+ ACATACTGTT TGGTTGGAGA AAAAATGGTT GTCTCGAAAA CTAGTTTTTT AATTTTTTGT TATTTAGCAC   
  
  
+ GGTAAAGAAT GGAAATTATT TTCATGCGAG AAAAAAGGAA GTTGGTTTTT CTTTTTTCAA CTCTTTTGAT   
  
  
+ GATATAATCA GTCGAAATTT AGGTGCACCA TCTGACTTCA ACTCTTGTAC GACCTCTGAT TGCTTGCTTA   
  
  
+ GAAGAACAAG AGGATTGACA GATAAGGAGG AGTGGGTGGT TTATACAGAG AATTGAGTGG ATGGTTATAA   
  
  
+ AGAAGTCGAT CTCACATAAG TAACTTCGTT ACGATTATAA ATATTTTCAT TGAAGAAAGA CGATTAGATA   
  
  
+ TAATTAAGAA GGTACATCTA AGCAAATTTG GCTGAGCAGA GTTGGAGGGC CACTAGGGTC ACATTGTGTC   
  
  
+ TTGCGCAACA AGCTTAGACC ATTCGCAACG GATGCCTCAC GTGGAGCAGG TGGGACCCGC GTGGAAGTTG   
  
  
+ CATGGCAAGA ATTCTCTGGC AGCAAGGAGA ATTGAGGGGG GACACGCGTG TGACCACGCC AGTGCGGTCC   
  
  
+ CCCTCTCGTG GCAGATTTGG TGATTTTTTT TTTGTTTTTT TTTGAAGTAA TATATAATTT TTATATGATT   
  
  
+ AAAAAAATAG GTTGTGTGTT ATAAAGTGGG TCAATGACAA ATATTTTTAT TGGATGATTA AAATTAAGTT   
  
  
+ CCATTTATTG GAGAAGTGAT GGAATGCAAG TTCCAAAATT GTGGAACTTT TGTTGACGTA GCAGGCTAGA   
  
  
+ GTTCCAAAAA TTAAAAAAAG GAAAGACATA TTGAGGTAAA GCCTTTTTAT TTTACAAATA GACGCACCAA   
  
  
+ GGTATAATAT AATTGACATG ATTTGAGGCG AGAGGAAGAT TCTTTTGCTA CCCAAACAAA ACACAATGAT   
  
  
+ CTTTTAAATG TAGCAATTTG GGATTGGCAA AATTTTGTCT AACCCGATTG TTGAAAGTTA TCCGATTTCA   
  
  
+ TAAAATTGGA TATCGTTATT AAATTTTCAT TCGAATATTT AAATTTGGAC ACAATGTTTT TCATATTTGG   
  
  
+ ATTTTCTAAA CTTGATTTTA ACTCAAATTT AAGTTTATTC AATTCATCTG ACCCAAAAAA TGGTTGCAAT   
  
  
+ AATGTATTTT CTTTTTGAAG TTAAATATTA TCGAATTCAT TTAACTTGTA GTTAACCCGT AATTGACCCA   
  
  
+ AACTCGACTT TTTGCTACCT TAGCAGCCAG GAAGAAGTCA TAGTATTGAC CCGTGGAACG CCTGAAGCCA   
  
  
+ ACATCACCAG ACCCACATAT TCCATCATCT TCTGATCTCC TCGTCAAAGA AGGAAGCTGA GCACAGCAAT   
  
  
+ AACTAGAAGA ACAGGAGGCA TCACACCCAA GCCAAAGGCC TTTATTTGAG CTCCCCACCT CATTCCTCGA   
  
  
+ AGCTTCCTCT TCACAATGTA AGACCATAAT CCTTCACCCC TTCTCTTTTT AAGCTCATCT TTACCTGCCC   
  
  
+ TAGTTCTCTC TCTCTCATGA GTCATCAGTA ATAGTTACCT CTTTACTTTC TCTTCCTTGA GGTATGCTTC   
  
  
+ AACTTTAATA TATATATGTA CCACTTGATT TCTCTTGTCA CTTTGCTATA ATACTTATTG TTTGGCTATC   
  
  
+ TTCCCCTGTT TTATACTCAT TTTCTTGCTT TCTATATCTG GGTTTGAATT GGGTTTCACT TATCCTCGTT   
  
  
+ GATTCTGTGG GAAATTAGTG AATTGGGTTT TGTACAGCAG GTTTATATGA ACTAGGGTTT CAAAGGTATC   
  
  
+ TACTTTTTCT CTTGTGATTA TTAGTTGATT AGGGATTTGG CCAAGATTAG CAGAATGGGT TCTGAATTTG   
  
  
+ GGGAATTCTC TGATGATGCT CTAAATGGGT ATGCTTACGT TGATATCCCT GCTTATGATG CGTCCTTAGA   
  
  
+ TTATGCCAAT TTGTTCAATT ACGAAGGCCT ATCTGAGGAT CTCACCTCAC TGAGCCTCCC AAGCCCCTTT   
  
  
+ TCTGACCCTT TGGCGTACAG TTTCACGTCC TCTTTGGGGC CGAGCCCTGG GGTTGATTCT AATGATGATA   
  
  
+ GTGATTCTGA TGATGTTCTC AAGTGCATTA GCCAAATGCT TATGGAAGAA GACATGGAGG CAAAGCCATG   
  
  
+ CATGTTTCAC GATCCTTTAG CACTTCAGGC TGCTGAGAAA CCCTTTTATG ATGCCTTAGG GAAGAAATAC   
  
  
+ CCAACTTCTC CGAACCAACA TCCTATAATT GATGATTGTT TGGATAATCC TGGTGAAAAT TCTTTGGGTT   
  
  
+ CTTCTAGTGA TTTTAGTGTT AGTCACTTTG GGTCTAGTTC AGCAAGCTCT ATTGGACCGA CAATTGTGTC   
  
  
+ TGATTTGAGT GAGCATTTTG AGCCACCCTT TGTTCAAGCA CTTCCAATTG AATCATATCC CCAACCATTG   
  
  
+ ACCCGTCCTC AATGGTCGTT TGGCCCTTCG GGTGCCTTAG ATTGCACGGC CTCTAATGGT TCAGTGATCT   
  
  
+ CATCCCTTGG TTTGCCGATG GATGTTATTA GTGTATTCAG TGAGAAAGAG TCCATGATTC AATTTCAGAA   
  
  
+ AGGGGTGGAA GAGGCTAGTA AGTTCCTTCC CAAGAATAAT AACCTTGTTA TTGATCTCAA GAACCTCACT   
  
  
+ TTTCCTAATG AAACGAAGGA GGATGATCGA GTGATGATGG TTAAGAAGGA AAAGAATGAT GTGAATTGGT   
  
  
+ CTAACTACTC AAGAGGGAGT AAGATTCACT ATCGTGAAGA CGAGGACTTT GAAGAAGGAA GGAGTAGCAA   
  
  
+ GCAGTCAGCT ATTTCTACTA CTGAGGAAGC TGAGTTGTGT GAAATTTTTG ACAAGTTTTT GCTTTGCAAT   
  
  
+ TGGTACCCTG TGAAACCTGA GGCTCATCCC ACCATGAGTT TGAACCCTGA GAAGGGCCAG TCACATGGAT   
  
  
+ TAGAAGGTGG GAGAAATGGG AAGGGTCACC CAAAGAAACA GGATAAAAGT AGTACCAACG TTGTGGATTT   
  
  
+ AAGGAATTTG CTGATGCTAT GTGCACAATC TACTGCATCT GATGACCGAA GAACTGCTGA TGGACTGCTA   
  
  
+ AAGAAAATCA GGGAGCACTC ATCTGCTGAG GGGGATGGAT TTCAAAGGTT GGCTCATTAC TTTGCTGATG   
  
  
+ CCCTAGAGGC ACGTTTAGCT GGAACTGGAT CTCACATTTA TACAGCCCTA AGTTCTCATA GGCCATCTGC   
  
  
+ TGTTGACGTG TTAAAAGCAT ATCAGTTCTA TGTTCGTGCT TGCCCATTTA AGAAGATCGT CATTCGTTGT   
  
  
+ GGTAAACATA TGATTCTAAA AGCTGCTGAA AAGGCATCAA AGCTTCATAT TATAGATTTT GGCATCCTCT   
  
  
+ ATGGATTCCA ATGGCCTAGC CTCATTCGAT GCCTCTCAGA GCGGTCTGGT GGACCTCCAA AACTGTTTAT   
  
  
+ TACAGGGATC GATCTCCCCC AGCCTGGGTT CAGGCCAGCA GAAAGAGTGG AAGCAACAGG GAGACGCTTG   
  
  
+ GCTAAGTACT GTGAGCGGTA TAATGTGCCA TTTGAGTATC ATGCCATTGC TCAGAAGTGG GAAACAATCA   
  
  
+ AACCAGGGGA TCTCAAGTTA GGAAGTAGGG ATGATGATGA AGTTGTCGTG GTGAACTGTC TCTGTAGGTT   
  
  
+ CAAGAACCTC CTTGACGAGA CAATGGTGGT GGATAGTCCA AGGAACACAG TTTTAAACCT GATTAGAAGG   
  
  
+ GTAAAACCCG ATATTTTTGT GCATGGCATT GTAAATGGTT CCTACAACAT CCCTTTCTTT GTGACACGTT   
  
  
+ TTAGAGAAGC CCTCTTTCAT TATTCCACTC TTTTCGACAT GTTAGATGCC AACGCCTCTA GGGAGGAGCC   
  
  
+ CGAGAGGTTG ATATTCGAGA AGGCATTCTA TGGGAGGGAG ATTATGAATG TGGTGGCATG TGAGGGCACA   
  
  
+ GAGAGGGTGG AAAGGCCAGA GACATACAAG CAATGGCACG TTAGGCATAG CAGGGCAGGG TTTCGGCAAC   
  
  
+ TACCATTGGA TCCCAAGTTG ATCGAGAAAA TGAGGTTTAA GGCCAAGGCA GACCACCACA AGGATTTCGT   
  
  
+ GATTGATGTG GATGGACATT GGGCAATTCA GGGATGGAAG GGGCGGATTG CCTGTGCCAT CTCTGCATGG   
  
  
+ GTTCTGGCTT G  

- -Up\_Stream \_Len000GACAAG TCGTGATCCC TTATTCCAAA AACGGGACGT ATGATACTTT AGATTGGTTT   
  
  
- TTGTAAGGTA GAACCTTCCG ACATCGGAAC ACCTAAAACT TAAGCGAGGG TTCTTGGTTA CTTCATCGCG   
  
  
- TCTTCCGGTT ATGAGTTAAA GTCTCAGGTG TAGTTGAACA AGTTACGGTC ATATAAAGTC TTGTAATTAA   
  
  
- TGTATGACAA ACCAACCTCT TTTTTACCAA CAGAGCTTTT GATCAAAAAA TTAAAAAACA ATAAATCGTG   
  
  
- CCATTTCTTA CCTTTAATAA AAGTACGCTC TTTTTTCCTT CAACCAAAAA GAAAAAAGTT GAGAAAACTA   
  
  
- CTATATTAGT CAGCTTTAAA TCCACGTGGT AGACTGAAGT TGAGAACATG CTGGAGACTA ACGAACGAAT   
  
  
- CTTCTTGTTC TCCTAACTGT CTATTCCTCC TCACCCACCA AATATGTCTC TTAACTCACC TACCAATATT   
  
  
- TCTTCAGCTA GAGTGTATTC ATTGAAGCAA TGCTAATATT TATAAAAGTA ACTTCTTTCT GCTAATCTAT   
  
  
- ATTAATTCTT CCATGTAGAT TCGTTTAAAC CGACTCGTCT CAACCTCCCG GTGATCCCAG TGTAACACAG   
  
  
- AACGCGTTGT TCGAATCTGG TAAGCGTTGC CTACGGAGTG CACCTCGTCC ACCCTGGGCG CACCTTCAAC   
  
  
- GTACCGTTCT TAAGAGACCG TCGTTCCTCT TAACTCCCCC CTGTGCGCAC ACTGGTGCGG TCACGCCAGG   
  
  
- GGGAGAGCAC CGTCTAAACC ACTAAAAAAA AAACAAAAAA AAACTTCATT ATATATTAAA AATATACTAA   
  
  
- TTTTTTTATC CAACACACAA TATTTCACCC AGTTACTGTT TATAAAAATA ACCTACTAAT TTTAATTCAA   
  
  
- GGTAAATAAC CTCTTCACTA CCTTACGTTC AAGGTTTTAA CACCTTGAAA ACAACTGCAT CGTCCGATCT   
  
  
- CAAGGTTTTT AATTTTTTTC CTTTCTGTAT AACTCCATTT CGGAAAAATA AAATGTTTAT CTGCGTGGTT   
  
  
- CCATATTATA TTAACTGTAC TAAACTCCGC TCTCCTTCTA AGAAAACGAT GGGTTTGTTT TGTGTTACTA   
  
  
- GAAAATTTAC ATCGTTAAAC CCTAACCGTT TTAAAACAGA TTGGGCTAAC AACTTTCAAT AGGCTAAAGT   
  
  
- ATTTTAACCT ATAGCAATAA TTTAAAAGTA AGCTTATAAA TTTAAACCTG TGTTACAAAA AGTATAAACC   
  
  
- TAAAAGATTT GAACTAAAAT TGAGTTTAAA TTCAAATAAG TTAAGTAGAC TGGGTTTTTT ACCAACGTTA   
  
  
- TTACATAAAA GAAAAACTTC AATTTATAAT AGCTTAAGTA AATTGAACAT CAATTGGGCA TTAACTGGGT   
  
  
- TTGAGCTGAA AAACGATGGA ATCGTCGGTC CTTCTTCAGT ATCATAACTG GGCACCTTGC GGACTTCGGT   
  
  
- TGTAGTGGTC TGGGTGTATA AGGTAGTAGA AGACTAGAGG AGCAGTTTCT TCCTTCGACT CGTGTCGTTA   
  
  
- TTGATCTTCT TGTCCTCCGT AGTGTGGGTT CGGTTTCCGG AAATAAACTC GAGGGGTGGA GTAAGGAGCT   
  
  
- TCGAAGGAGA AGTGTTACAT TCTGGTATTA GGAAGTGGGG AAGAGAAAAA TTCGAGTAGA AATGGACGGG   
  
  
- ATCAAGAGAG AGAGAGTACT CAGTAGTCAT TATCAATGGA GAAATGAAAG AGAAGGAACT CCATACGAAG   
  
  
- TTGAAATTAT ATATATACAT GGTGAACTAA AGAGAACAGT GAAACGATAT TATGAATAAC AAACCGATAG   
  
  
- AAGGGGACAA AATATGAGTA AAAGAACGAA AGATATAGAC CCAAACTTAA CCCAAAGTGA ATAGGAGCAA   
  
  
- CTAAGACACC CTTTAATCAC TTAACCCAAA ACATGTCGTC CAAATATACT TGATCCCAAA GTTTCCATAG   
  
  
- ATGAAAAAGA GAACACTAAT AATCAACTAA TCCCTAAACC GGTTCTAATC GTCTTACCCA AGACTTAAAC   
  
  
- CCCTTAAGAG ACTACTACGA GATTTACCCA TACGAATGCA ACTATAGGGA CGAATACTAC GCAGGAATCT   
  
  
- AATACGGTTA AACAAGTTAA TGCTTCCGGA TAGACTCCTA GAGTGGAGTG ACTCGGAGGG TTCGGGGAAA   
  
  
- AGACTGGGAA ACCGCATGTC AAAGTGCAGG AGAAACCCCG GCTCGGGACC CCAACTAAGA TTACTACTAT   
  
  
- CACTAAGACT ACTACAAGAG TTCACGTAAT CGGTTTACGA ATACCTTCTT CTGTACCTCC GTTTCGGTAC   
  
  
- GTACAAAGTG CTAGGAAATC GTGAAGTCCG ACGACTCTTT GGGAAAATAC TACGGAATCC CTTCTTTATG   
  
  
- GGTTGAAGAG GCTTGGTTGT AGGATATTAA CTACTAACAA ACCTATTAGG ACCACTTTTA AGAAACCCAA   
  
  
- GAAGATCACT AAAATCACAA TCAGTGAAAC CCAGATCAAG TCGTTCGAGA TAACCTGGCT GTTAACACAG   
  
  
- ACTAAACTCA CTCGTAAAAC TCGGTGGGAA ACAAGTTCGT GAAGGTTAAC TTAGTATAGG GGTTGGTAAC   
  
  
- TGGGCAGGAG TTACCAGCAA ACCGGGAAGC CCACGGAATC TAACGTGCCG GAGATTACCA AGTCACTAGA   
  
  
- GTAGGGAACC AAACGGCTAC CTACAATAAT CACATAAGTC ACTCTTTCTC AGGTACTAAG TTAAAGTCTT   
  
  
- TCCCCACCTT CTCCGATCAT TCAAGGAAGG GTTCTTATTA TTGGAACAAT AACTAGAGTT CTTGGAGTGA   
  
  
- AAAGGATTAC TTTGCTTCCT CCTACTAGCT CACTACTACC AATTCTTCCT TTTCTTACTA CACTTAACCA   
  
  
- GATTGATGAG TTCTCCCTCA TTCTAAGTGA TAGCACTTCT GCTCCTGAAA CTTCTTCCTT CCTCATCGTT   
  
  
- CGTCAGTCGA TAAAGATGAT GACTCCTTCG ACTCAACACA CTTTAAAAAC TGTTCAAAAA CGAAACGTTA   
  
  
- ACCATGGGAC ACTTTGGACT CCGAGTAGGG TGGTACTCAA ACTTGGGACT CTTCCCGGTC AGTGTACCTA   
  
  
- ATCTTCCACC CTCTTTACCC TTCCCAGTGG GTTTCTTTGT CCTATTTTCA TCATGGTTGC AACACCTAAA   
  
  
- TTCCTTAAAC GACTACGATA CACGTGTTAG ATGACGTAGA CTACTGGCTT CTTGACGACT ACCTGACGAT   
  
  
- TTCTTTTAGT CCCTCGTGAG TAGACGACTC CCCCTACCTA AAGTTTCCAA CCGAGTAATG AAACGACTAC   
  
  
- GGGATCTCCG TGCAAATCGA CCTTGACCTA GAGTGTAAAT ATGTCGGGAT TCAAGAGTAT CCGGTAGACG   
  
  
- ACAACTGCAC AATTTTCGTA TAGTCAAGAT ACAAGCACGA ACGGGTAAAT TCTTCTAGCA GTAAGCAACA   
  
  
- CCATTTGTAT ACTAAGATTT TCGACGACTT TTCCGTAGTT TCGAAGTATA ATATCTAAAA CCGTAGGAGA   
  
  
- TACCTAAGGT TACCGGATCG GAGTAAGCTA CGGAGAGTCT CGCCAGACCA CCTGGAGGTT TTGACAAATA   
  
  
- ATGTCCCTAG CTAGAGGGGG TCGGACCCAA GTCCGGTCGT CTTTCTCACC TTCGTTGTCC CTCTGCGAAC   
  
  
- CGATTCATGA CACTCGCCAT ATTACACGGT AAACTCATAG TACGGTAACG AGTCTTCACC CTTTGTTAGT   
  
  
- TTGGTCCCCT AGAGTTCAAT CCTTCATCCC TACTACTACT TCAACAGCAC CACTTGACAG AGACATCCAA   
  
  
- GTTCTTGGAG GAACTGCTCT GTTACCACCA CCTATCAGGT TCCTTGTGTC AAAATTTGGA CTAATCTTCC   
  
  
- CATTTTGGGC TATAAAAACA CGTACCGTAA CATTTACCAA GGATGTTGTA GGGAAAGAAA CACTGTGCAA   
  
  
- AATCTCTTCG GGAGAAAGTA ATAAGGTGAG AAAAGCTGTA CAATCTACGG TTGCGGAGAT CCCTCCTCGG   
  
  
- GCTCTCCAAC TATAAGCTCT TCCGTAAGAT ACCCTCCCTC TAATACTTAC ACCACCGTAC ACTCCCGTGT   
  
  
- CTCTCCCACC TTTCCGGTCT CTGTATGTTC GTTACCGTGC AATCCGTATC GTCCCGTCCC AAAGCCGTTG   
  
  
- ATGGTAACCT AGGGTTCAAC TAGCTCTTTT ACTCCAAATT CCGGTTCCGT CTGGTGGTGT TCCTAAAGCA   
  
  
- CTAACTACAC CTACCTGTAA CCCGTTAAGT CCCTACCTTC CCCGCCTAAC GGACACGGTA GAGACGTACC   
  
  
- CAAGACCGAA C

+     ARE

| Site Name | Organism | Position | Strand | Matrix score. | sequence | function |
| --- | --- | --- | --- | --- | --- | --- |
| ARE | Zea mays | 327 | - | 6 | AAACCA | cis-acting regulatory element essential for the anaerobic induction |
| ARE | Zea mays | 3714 | + | 6 | AAACCA | cis-acting regulatory element essential for the anaerobic induction |
| ARE | Zea mays | 461 | - | 6 | AAACCA | cis-acting regulatory element essential for the anaerobic induction |
| ARE | Zea mays | 2672 | - | 6 | AAACCA | cis-acting regulatory element essential for the anaerobic induction |

>HU07G02248.1   
+ -Up\_Stream \_Len000CTGTTC AGCACTAGGG AATAAGGTTT TTGCCCTGCA TACTATGAAA TCTAACCAAA   
  
  
+ AACATTCCAT CTTGGAAGGC TGTAGCCTTG TGGATTTTGA ATTCGCTCCC AAGAACCAAT GAAGTAGCGC   
  
  
+ AGAAGGCCAA TACTCAATTT CAGAGTCCAC ATCAACTTGT TCAATGCCAG TATATTTCAG AACATTAATT   
  
  
+ ACATACTGTT TGGTTGGAGA AAAAATGGTT GTCTCGAAAA CTAGTTTTTT AATTTTTTGT TATTTAGCAC   
  
  
+ GGTAAAGAAT GGAAATTATT TTCATGCGAG AAAAAAGGAA GTTGGTTTTT CTTTTTTCAA CTCTTTTGAT   
  
  
+ GATATAATCA GTCGAAATTT AGGTGCACCA TCTGACTTCA ACTCTTGTAC GACCTCTGAT TGCTTGCTTA   
  
  
+ GAAGAACAAG AGGATTGACA GATAAGGAGG AGTGGGTGGT TTATACAGAG AATTGAGTGG ATGGTTATAA   
  
  
+ AGAAGTCGAT CTCACATAAG TAACTTCGTT ACGATTATAA ATATTTTCAT TGAAGAAAGA CGATTAGATA   
  
  
+ TAATTAAGAA GGTACATCTA AGCAAATTTG GCTGAGCAGA GTTGGAGGGC CACTAGGGTC ACATTGTGTC   
  
  
+ TTGCGCAACA AGCTTAGACC ATTCGCAACG GATGCCTCAC GTGGAGCAGG TGGGACCCGC GTGGAAGTTG   
  
  
+ CATGGCAAGA ATTCTCTGGC AGCAAGGAGA ATTGAGGGGG GACACGCGTG TGACCACGCC AGTGCGGTCC   
  
  
+ CCCTCTCGTG GCAGATTTGG TGATTTTTTT TTTGTTTTTT TTTGAAGTAA TATATAATTT TTATATGATT   
  
  
+ AAAAAAATAG GTTGTGTGTT ATAAAGTGGG TCAATGACAA ATATTTTTAT TGGATGATTA AAATTAAGTT   
  
  
+ CCATTTATTG GAGAAGTGAT GGAATGCAAG TTCCAAAATT GTGGAACTTT TGTTGACGTA GCAGGCTAGA   
  
  
+ GTTCCAAAAA TTAAAAAAAG GAAAGACATA TTGAGGTAAA GCCTTTTTAT TTTACAAATA GACGCACCAA   
  
  
+ GGTATAATAT AATTGACATG ATTTGAGGCG AGAGGAAGAT TCTTTTGCTA CCCAAACAAA ACACAATGAT   
  
  
+ CTTTTAAATG TAGCAATTTG GGATTGGCAA AATTTTGTCT AACCCGATTG TTGAAAGTTA TCCGATTTCA   
  
  
+ TAAAATTGGA TATCGTTATT AAATTTTCAT TCGAATATTT AAATTTGGAC ACAATGTTTT TCATATTTGG   
  
  
+ ATTTTCTAAA CTTGATTTTA ACTCAAATTT AAGTTTATTC AATTCATCTG ACCCAAAAAA TGGTTGCAAT   
  
  
+ AATGTATTTT CTTTTTGAAG TTAAATATTA TCGAATTCAT TTAACTTGTA GTTAACCCGT AATTGACCCA   
  
  
+ AACTCGACTT TTTGCTACCT TAGCAGCCAG GAAGAAGTCA TAGTATTGAC CCGTGGAACG CCTGAAGCCA   
  
  
+ ACATCACCAG ACCCACATAT TCCATCATCT TCTGATCTCC TCGTCAAAGA AGGAAGCTGA GCACAGCAAT   
  
  
+ AACTAGAAGA ACAGGAGGCA TCACACCCAA GCCAAAGGCC TTTATTTGAG CTCCCCACCT CATTCCTCGA   
  
  
+ AGCTTCCTCT TCACAATGTA AGACCATAAT CCTTCACCCC TTCTCTTTTT AAGCTCATCT TTACCTGCCC   
  
  
+ TAGTTCTCTC TCTCTCATGA GTCATCAGTA ATAGTTACCT CTTTACTTTC TCTTCCTTGA GGTATGCTTC   
  
  
+ AACTTTAATA TATATATGTA CCACTTGATT TCTCTTGTCA CTTTGCTATA ATACTTATTG TTTGGCTATC   
  
  
+ TTCCCCTGTT TTATACTCAT TTTCTTGCTT TCTATATCTG GGTTTGAATT GGGTTTCACT TATCCTCGTT   
  
  
+ GATTCTGTGG GAAATTAGTG AATTGGGTTT TGTACAGCAG GTTTATATGA ACTAGGGTTT CAAAGGTATC   
  
  
+ TACTTTTTCT CTTGTGATTA TTAGTTGATT AGGGATTTGG CCAAGATTAG CAGAATGGGT TCTGAATTTG   
  
  
+ GGGAATTCTC TGATGATGCT CTAAATGGGT ATGCTTACGT TGATATCCCT GCTTATGATG CGTCCTTAGA   
  
  
+ TTATGCCAAT TTGTTCAATT ACGAAGGCCT ATCTGAGGAT CTCACCTCAC TGAGCCTCCC AAGCCCCTTT   
  
  
+ TCTGACCCTT TGGCGTACAG TTTCACGTCC TCTTTGGGGC CGAGCCCTGG GGTTGATTCT AATGATGATA   
  
  
+ GTGATTCTGA TGATGTTCTC AAGTGCATTA GCCAAATGCT TATGGAAGAA GACATGGAGG CAAAGCCATG   
  
  
+ CATGTTTCAC GATCCTTTAG CACTTCAGGC TGCTGAGAAA CCCTTTTATG ATGCCTTAGG GAAGAAATAC   
  
  
+ CCAACTTCTC CGAACCAACA TCCTATAATT GATGATTGTT TGGATAATCC TGGTGAAAAT TCTTTGGGTT   
  
  
+ CTTCTAGTGA TTTTAGTGTT AGTCACTTTG GGTCTAGTTC AGCAAGCTCT ATTGGACCGA CAATTGTGTC   
  
  
+ TGATTTGAGT GAGCATTTTG AGCCACCCTT TGTTCAAGCA CTTCCAATTG AATCATATCC CCAACCATTG   
  
  
+ ACCCGTCCTC AATGGTCGTT TGGCCCTTCG GGTGCCTTAG ATTGCACGGC CTCTAATGGT TCAGTGATCT   
  
  
+ CATCCCTTGG TTTGCCGATG GATGTTATTA GTGTATTCAG TGAGAAAGAG TCCATGATTC AATTTCAGAA   
  
  
+ AGGGGTGGAA GAGGCTAGTA AGTTCCTTCC CAAGAATAAT AACCTTGTTA TTGATCTCAA GAACCTCACT   
  
  
+ TTTCCTAATG AAACGAAGGA GGATGATCGA GTGATGATGG TTAAGAAGGA AAAGAATGAT GTGAATTGGT   
  
  
+ CTAACTACTC AAGAGGGAGT AAGATTCACT ATCGTGAAGA CGAGGACTTT GAAGAAGGAA GGAGTAGCAA   
  
  
+ GCAGTCAGCT ATTTCTACTA CTGAGGAAGC TGAGTTGTGT GAAATTTTTG ACAAGTTTTT GCTTTGCAAT   
  
  
+ TGGTACCCTG TGAAACCTGA GGCTCATCCC ACCATGAGTT TGAACCCTGA GAAGGGCCAG TCACATGGAT   
  
  
+ TAGAAGGTGG GAGAAATGGG AAGGGTCACC CAAAGAAACA GGATAAAAGT AGTACCAACG TTGTGGATTT   
  
  
+ AAGGAATTTG CTGATGCTAT GTGCACAATC TACTGCATCT GATGACCGAA GAACTGCTGA TGGACTGCTA   
  
  
+ AAGAAAATCA GGGAGCACTC ATCTGCTGAG GGGGATGGAT TTCAAAGGTT GGCTCATTAC TTTGCTGATG   
  
  
+ CCCTAGAGGC ACGTTTAGCT GGAACTGGAT CTCACATTTA TACAGCCCTA AGTTCTCATA GGCCATCTGC   
  
  
+ TGTTGACGTG TTAAAAGCAT ATCAGTTCTA TGTTCGTGCT TGCCCATTTA AGAAGATCGT CATTCGTTGT   
  
  
+ GGTAAACATA TGATTCTAAA AGCTGCTGAA AAGGCATCAA AGCTTCATAT TATAGATTTT GGCATCCTCT   
  
  
+ ATGGATTCCA ATGGCCTAGC CTCATTCGAT GCCTCTCAGA GCGGTCTGGT GGACCTCCAA AACTGTTTAT   
  
  
+ TACAGGGATC GATCTCCCCC AGCCTGGGTT CAGGCCAGCA GAAAGAGTGG AAGCAACAGG GAGACGCTTG   
  
  
+ GCTAAGTACT GTGAGCGGTA TAATGTGCCA TTTGAGTATC ATGCCATTGC TCAGAAGTGG GAAACAATCA   
  
  
+ AACCAGGGGA TCTCAAGTTA GGAAGTAGGG ATGATGATGA AGTTGTCGTG GTGAACTGTC TCTGTAGGTT   
  
  
+ CAAGAACCTC CTTGACGAGA CAATGGTGGT GGATAGTCCA AGGAACACAG TTTTAAACCT GATTAGAAGG   
  
  
+ GTAAAACCCG ATATTTTTGT GCATGGCATT GTAAATGGTT CCTACAACAT CCCTTTCTTT GTGACACGTT   
  
  
+ TTAGAGAAGC CCTCTTTCAT TATTCCACTC TTTTCGACAT GTTAGATGCC AACGCCTCTA GGGAGGAGCC   
  
  
+ CGAGAGGTTG ATATTCGAGA AGGCATTCTA TGGGAGGGAG ATTATGAATG TGGTGGCATG TGAGGGCACA   
  
  
+ GAGAGGGTGG AAAGGCCAGA GACATACAAG CAATGGCACG TTAGGCATAG CAGGGCAGGG TTTCGGCAAC   
  
  
+ TACCATTGGA TCCCAAGTTG ATCGAGAAAA TGAGGTTTAA GGCCAAGGCA GACCACCACA AGGATTTCGT   
  
  
+ GATTGATGTG GATGGACATT GGGCAATTCA GGGATGGAAG GGGCGGATTG CCTGTGCCAT CTCTGCATGG   
  
  
+ GTTCTGGCTT G  

- -Up\_Stream \_Len000GACAAG TCGTGATCCC TTATTCCAAA AACGGGACGT ATGATACTTT AGATTGGTTT   
  
  
- TTGTAAGGTA GAACCTTCCG ACATCGGAAC ACCTAAAACT TAAGCGAGGG TTCTTGGTTA CTTCATCGCG   
  
  
- TCTTCCGGTT ATGAGTTAAA GTCTCAGGTG TAGTTGAACA AGTTACGGTC ATATAAAGTC TTGTAATTAA   
  
  
- TGTATGACAA ACCAACCTCT TTTTTACCAA CAGAGCTTTT GATCAAAAAA TTAAAAAACA ATAAATCGTG   
  
  
- CCATTTCTTA CCTTTAATAA AAGTACGCTC TTTTTTCCTT CAACCAAAAA GAAAAAAGTT GAGAAAACTA   
  
  
- CTATATTAGT CAGCTTTAAA TCCACGTGGT AGACTGAAGT TGAGAACATG CTGGAGACTA ACGAACGAAT   
  
  
- CTTCTTGTTC TCCTAACTGT CTATTCCTCC TCACCCACCA AATATGTCTC TTAACTCACC TACCAATATT   
  
  
- TCTTCAGCTA GAGTGTATTC ATTGAAGCAA TGCTAATATT TATAAAAGTA ACTTCTTTCT GCTAATCTAT   
  
  
- ATTAATTCTT CCATGTAGAT TCGTTTAAAC CGACTCGTCT CAACCTCCCG GTGATCCCAG TGTAACACAG   
  
  
- AACGCGTTGT TCGAATCTGG TAAGCGTTGC CTACGGAGTG CACCTCGTCC ACCCTGGGCG CACCTTCAAC   
  
  
- GTACCGTTCT TAAGAGACCG TCGTTCCTCT TAACTCCCCC CTGTGCGCAC ACTGGTGCGG TCACGCCAGG   
  
  
- GGGAGAGCAC CGTCTAAACC ACTAAAAAAA AAACAAAAAA AAACTTCATT ATATATTAAA AATATACTAA   
  
  
- TTTTTTTATC CAACACACAA TATTTCACCC AGTTACTGTT TATAAAAATA ACCTACTAAT TTTAATTCAA   
  
  
- GGTAAATAAC CTCTTCACTA CCTTACGTTC AAGGTTTTAA CACCTTGAAA ACAACTGCAT CGTCCGATCT   
  
  
- CAAGGTTTTT AATTTTTTTC CTTTCTGTAT AACTCCATTT CGGAAAAATA AAATGTTTAT CTGCGTGGTT   
  
  
- CCATATTATA TTAACTGTAC TAAACTCCGC TCTCCTTCTA AGAAAACGAT GGGTTTGTTT TGTGTTACTA   
  
  
- GAAAATTTAC ATCGTTAAAC CCTAACCGTT TTAAAACAGA TTGGGCTAAC AACTTTCAAT AGGCTAAAGT   
  
  
- ATTTTAACCT ATAGCAATAA TTTAAAAGTA AGCTTATAAA TTTAAACCTG TGTTACAAAA AGTATAAACC   
  
  
- TAAAAGATTT GAACTAAAAT TGAGTTTAAA TTCAAATAAG TTAAGTAGAC TGGGTTTTTT ACCAACGTTA   
  
  
- TTACATAAAA GAAAAACTTC AATTTATAAT AGCTTAAGTA AATTGAACAT CAATTGGGCA TTAACTGGGT   
  
  
- TTGAGCTGAA AAACGATGGA ATCGTCGGTC CTTCTTCAGT ATCATAACTG GGCACCTTGC GGACTTCGGT   
  
  
- TGTAGTGGTC TGGGTGTATA AGGTAGTAGA AGACTAGAGG AGCAGTTTCT TCCTTCGACT CGTGTCGTTA   
  
  
- TTGATCTTCT TGTCCTCCGT AGTGTGGGTT CGGTTTCCGG AAATAAACTC GAGGGGTGGA GTAAGGAGCT   
  
  
- TCGAAGGAGA AGTGTTACAT TCTGGTATTA GGAAGTGGGG AAGAGAAAAA TTCGAGTAGA AATGGACGGG   
  
  
- ATCAAGAGAG AGAGAGTACT CAGTAGTCAT TATCAATGGA GAAATGAAAG AGAAGGAACT CCATACGAAG   
  
  
- TTGAAATTAT ATATATACAT GGTGAACTAA AGAGAACAGT GAAACGATAT TATGAATAAC AAACCGATAG   
  
  
- AAGGGGACAA AATATGAGTA AAAGAACGAA AGATATAGAC CCAAACTTAA CCCAAAGTGA ATAGGAGCAA   
  
  
- CTAAGACACC CTTTAATCAC TTAACCCAAA ACATGTCGTC CAAATATACT TGATCCCAAA GTTTCCATAG   
  
  
- ATGAAAAAGA GAACACTAAT AATCAACTAA TCCCTAAACC GGTTCTAATC GTCTTACCCA AGACTTAAAC   
  
  
- CCCTTAAGAG ACTACTACGA GATTTACCCA TACGAATGCA ACTATAGGGA CGAATACTAC GCAGGAATCT   
  
  
- AATACGGTTA AACAAGTTAA TGCTTCCGGA TAGACTCCTA GAGTGGAGTG ACTCGGAGGG TTCGGGGAAA   
  
  
- AGACTGGGAA ACCGCATGTC AAAGTGCAGG AGAAACCCCG GCTCGGGACC CCAACTAAGA TTACTACTAT   
  
  
- CACTAAGACT ACTACAAGAG TTCACGTAAT CGGTTTACGA ATACCTTCTT CTGTACCTCC GTTTCGGTAC   
  
  
- GTACAAAGTG CTAGGAAATC GTGAAGTCCG ACGACTCTTT GGGAAAATAC TACGGAATCC CTTCTTTATG   
  
  
- GGTTGAAGAG GCTTGGTTGT AGGATATTAA CTACTAACAA ACCTATTAGG ACCACTTTTA AGAAACCCAA   
  
  
- GAAGATCACT AAAATCACAA TCAGTGAAAC CCAGATCAAG TCGTTCGAGA TAACCTGGCT GTTAACACAG   
  
  
- ACTAAACTCA CTCGTAAAAC TCGGTGGGAA ACAAGTTCGT GAAGGTTAAC TTAGTATAGG GGTTGGTAAC   
  
  
- TGGGCAGGAG TTACCAGCAA ACCGGGAAGC CCACGGAATC TAACGTGCCG GAGATTACCA AGTCACTAGA   
  
  
- GTAGGGAACC AAACGGCTAC CTACAATAAT CACATAAGTC ACTCTTTCTC AGGTACTAAG TTAAAGTCTT   
  
  
- TCCCCACCTT CTCCGATCAT TCAAGGAAGG GTTCTTATTA TTGGAACAAT AACTAGAGTT CTTGGAGTGA   
  
  
- AAAGGATTAC TTTGCTTCCT CCTACTAGCT CACTACTACC AATTCTTCCT TTTCTTACTA CACTTAACCA   
  
  
- GATTGATGAG TTCTCCCTCA TTCTAAGTGA TAGCACTTCT GCTCCTGAAA CTTCTTCCTT CCTCATCGTT   
  
  
- CGTCAGTCGA TAAAGATGAT GACTCCTTCG ACTCAACACA CTTTAAAAAC TGTTCAAAAA CGAAACGTTA   
  
  
- ACCATGGGAC ACTTTGGACT CCGAGTAGGG TGGTACTCAA ACTTGGGACT CTTCCCGGTC AGTGTACCTA   
  
  
- ATCTTCCACC CTCTTTACCC TTCCCAGTGG GTTTCTTTGT CCTATTTTCA TCATGGTTGC AACACCTAAA   
  
  
- TTCCTTAAAC GACTACGATA CACGTGTTAG ATGACGTAGA CTACTGGCTT CTTGACGACT ACCTGACGAT   
  
  
- TTCTTTTAGT CCCTCGTGAG TAGACGACTC CCCCTACCTA AAGTTTCCAA CCGAGTAATG AAACGACTAC   
  
  
- GGGATCTCCG TGCAAATCGA CCTTGACCTA GAGTGTAAAT ATGTCGGGAT TCAAGAGTAT CCGGTAGACG   
  
  
- ACAACTGCAC AATTTTCGTA TAGTCAAGAT ACAAGCACGA ACGGGTAAAT TCTTCTAGCA GTAAGCAACA   
  
  
- CCATTTGTAT ACTAAGATTT TCGACGACTT TTCCGTAGTT TCGAAGTATA ATATCTAAAA CCGTAGGAGA   
  
  
- TACCTAAGGT TACCGGATCG GAGTAAGCTA CGGAGAGTCT CGCCAGACCA CCTGGAGGTT TTGACAAATA   
  
  
- ATGTCCCTAG CTAGAGGGGG TCGGACCCAA GTCCGGTCGT CTTTCTCACC TTCGTTGTCC CTCTGCGAAC   
  
  
- CGATTCATGA CACTCGCCAT ATTACACGGT AAACTCATAG TACGGTAACG AGTCTTCACC CTTTGTTAGT   
  
  
- TTGGTCCCCT AGAGTTCAAT CCTTCATCCC TACTACTACT TCAACAGCAC CACTTGACAG AGACATCCAA   
  
  
- GTTCTTGGAG GAACTGCTCT GTTACCACCA CCTATCAGGT TCCTTGTGTC AAAATTTGGA CTAATCTTCC   
  
  
- CATTTTGGGC TATAAAAACA CGTACCGTAA CATTTACCAA GGATGTTGTA GGGAAAGAAA CACTGTGCAA   
  
  
- AATCTCTTCG GGAGAAAGTA ATAAGGTGAG AAAAGCTGTA CAATCTACGG TTGCGGAGAT CCCTCCTCGG   
  
  
- GCTCTCCAAC TATAAGCTCT TCCGTAAGAT ACCCTCCCTC TAATACTTAC ACCACCGTAC ACTCCCGTGT   
  
  
- CTCTCCCACC TTTCCGGTCT CTGTATGTTC GTTACCGTGC AATCCGTATC GTCCCGTCCC AAAGCCGTTG   
  
  
- ATGGTAACCT AGGGTTCAAC TAGCTCTTTT ACTCCAAATT CCGGTTCCGT CTGGTGGTGT TCCTAAAGCA   
  
  
- CTAACTACAC CTACCTGTAA CCCGTTAAGT CCCTACCTTC CCCGCCTAAC GGACACGGTA GAGACGTACC   
  
  
- CAAGACCGAA C

+     AT-rich element

| Site Name | Organism | Position | Strand | Matrix score. | sequence | function |
| --- | --- | --- | --- | --- | --- | --- |
| AT-rich element | Glycine max | 1849 | - | 10 | ATAGAAATCAA | binding site of AT-rich DNA binding protein (ATBP-1) |
| AT-rich element | Glycine max | 1779 | - | 10 | ATAGAAATCAA | binding site of AT-rich DNA binding protein (ATBP-1) |

>HU07G02248.1   
+ -Up\_Stream \_Len000CTGTTC AGCACTAGGG AATAAGGTTT TTGCCCTGCA TACTATGAAA TCTAACCAAA   
  
  
+ AACATTCCAT CTTGGAAGGC TGTAGCCTTG TGGATTTTGA ATTCGCTCCC AAGAACCAAT GAAGTAGCGC   
  
  
+ AGAAGGCCAA TACTCAATTT CAGAGTCCAC ATCAACTTGT TCAATGCCAG TATATTTCAG AACATTAATT   
  
  
+ ACATACTGTT TGGTTGGAGA AAAAATGGTT GTCTCGAAAA CTAGTTTTTT AATTTTTTGT TATTTAGCAC   
  
  
+ GGTAAAGAAT GGAAATTATT TTCATGCGAG AAAAAAGGAA GTTGGTTTTT CTTTTTTCAA CTCTTTTGAT   
  
  
+ GATATAATCA GTCGAAATTT AGGTGCACCA TCTGACTTCA ACTCTTGTAC GACCTCTGAT TGCTTGCTTA   
  
  
+ GAAGAACAAG AGGATTGACA GATAAGGAGG AGTGGGTGGT TTATACAGAG AATTGAGTGG ATGGTTATAA   
  
  
+ AGAAGTCGAT CTCACATAAG TAACTTCGTT ACGATTATAA ATATTTTCAT TGAAGAAAGA CGATTAGATA   
  
  
+ TAATTAAGAA GGTACATCTA AGCAAATTTG GCTGAGCAGA GTTGGAGGGC CACTAGGGTC ACATTGTGTC   
  
  
+ TTGCGCAACA AGCTTAGACC ATTCGCAACG GATGCCTCAC GTGGAGCAGG TGGGACCCGC GTGGAAGTTG   
  
  
+ CATGGCAAGA ATTCTCTGGC AGCAAGGAGA ATTGAGGGGG GACACGCGTG TGACCACGCC AGTGCGGTCC   
  
  
+ CCCTCTCGTG GCAGATTTGG TGATTTTTTT TTTGTTTTTT TTTGAAGTAA TATATAATTT TTATATGATT   
  
  
+ AAAAAAATAG GTTGTGTGTT ATAAAGTGGG TCAATGACAA ATATTTTTAT TGGATGATTA AAATTAAGTT   
  
  
+ CCATTTATTG GAGAAGTGAT GGAATGCAAG TTCCAAAATT GTGGAACTTT TGTTGACGTA GCAGGCTAGA   
  
  
+ GTTCCAAAAA TTAAAAAAAG GAAAGACATA TTGAGGTAAA GCCTTTTTAT TTTACAAATA GACGCACCAA   
  
  
+ GGTATAATAT AATTGACATG ATTTGAGGCG AGAGGAAGAT TCTTTTGCTA CCCAAACAAA ACACAATGAT   
  
  
+ CTTTTAAATG TAGCAATTTG GGATTGGCAA AATTTTGTCT AACCCGATTG TTGAAAGTTA TCCGATTTCA   
  
  
+ TAAAATTGGA TATCGTTATT AAATTTTCAT TCGAATATTT AAATTTGGAC ACAATGTTTT TCATATTTGG   
  
  
+ ATTTTCTAAA CTTGATTTTA ACTCAAATTT AAGTTTATTC AATTCATCTG ACCCAAAAAA TGGTTGCAAT   
  
  
+ AATGTATTTT CTTTTTGAAG TTAAATATTA TCGAATTCAT TTAACTTGTA GTTAACCCGT AATTGACCCA   
  
  
+ AACTCGACTT TTTGCTACCT TAGCAGCCAG GAAGAAGTCA TAGTATTGAC CCGTGGAACG CCTGAAGCCA   
  
  
+ ACATCACCAG ACCCACATAT TCCATCATCT TCTGATCTCC TCGTCAAAGA AGGAAGCTGA GCACAGCAAT   
  
  
+ AACTAGAAGA ACAGGAGGCA TCACACCCAA GCCAAAGGCC TTTATTTGAG CTCCCCACCT CATTCCTCGA   
  
  
+ AGCTTCCTCT TCACAATGTA AGACCATAAT CCTTCACCCC TTCTCTTTTT AAGCTCATCT TTACCTGCCC   
  
  
+ TAGTTCTCTC TCTCTCATGA GTCATCAGTA ATAGTTACCT CTTTACTTTC TCTTCCTTGA GGTATGCTTC   
  
  
+ AACTTTAATA TATATATGTA CCACTTGATT TCTCTTGTCA CTTTGCTATA ATACTTATTG TTTGGCTATC   
  
  
+ TTCCCCTGTT TTATACTCAT TTTCTTGCTT TCTATATCTG GGTTTGAATT GGGTTTCACT TATCCTCGTT   
  
  
+ GATTCTGTGG GAAATTAGTG AATTGGGTTT TGTACAGCAG GTTTATATGA ACTAGGGTTT CAAAGGTATC   
  
  
+ TACTTTTTCT CTTGTGATTA TTAGTTGATT AGGGATTTGG CCAAGATTAG CAGAATGGGT TCTGAATTTG   
  
  
+ GGGAATTCTC TGATGATGCT CTAAATGGGT ATGCTTACGT TGATATCCCT GCTTATGATG CGTCCTTAGA   
  
  
+ TTATGCCAAT TTGTTCAATT ACGAAGGCCT ATCTGAGGAT CTCACCTCAC TGAGCCTCCC AAGCCCCTTT   
  
  
+ TCTGACCCTT TGGCGTACAG TTTCACGTCC TCTTTGGGGC CGAGCCCTGG GGTTGATTCT AATGATGATA   
  
  
+ GTGATTCTGA TGATGTTCTC AAGTGCATTA GCCAAATGCT TATGGAAGAA GACATGGAGG CAAAGCCATG   
  
  
+ CATGTTTCAC GATCCTTTAG CACTTCAGGC TGCTGAGAAA CCCTTTTATG ATGCCTTAGG GAAGAAATAC   
  
  
+ CCAACTTCTC CGAACCAACA TCCTATAATT GATGATTGTT TGGATAATCC TGGTGAAAAT TCTTTGGGTT   
  
  
+ CTTCTAGTGA TTTTAGTGTT AGTCACTTTG GGTCTAGTTC AGCAAGCTCT ATTGGACCGA CAATTGTGTC   
  
  
+ TGATTTGAGT GAGCATTTTG AGCCACCCTT TGTTCAAGCA CTTCCAATTG AATCATATCC CCAACCATTG   
  
  
+ ACCCGTCCTC AATGGTCGTT TGGCCCTTCG GGTGCCTTAG ATTGCACGGC CTCTAATGGT TCAGTGATCT   
  
  
+ CATCCCTTGG TTTGCCGATG GATGTTATTA GTGTATTCAG TGAGAAAGAG TCCATGATTC AATTTCAGAA   
  
  
+ AGGGGTGGAA GAGGCTAGTA AGTTCCTTCC CAAGAATAAT AACCTTGTTA TTGATCTCAA GAACCTCACT   
  
  
+ TTTCCTAATG AAACGAAGGA GGATGATCGA GTGATGATGG TTAAGAAGGA AAAGAATGAT GTGAATTGGT   
  
  
+ CTAACTACTC AAGAGGGAGT AAGATTCACT ATCGTGAAGA CGAGGACTTT GAAGAAGGAA GGAGTAGCAA   
  
  
+ GCAGTCAGCT ATTTCTACTA CTGAGGAAGC TGAGTTGTGT GAAATTTTTG ACAAGTTTTT GCTTTGCAAT   
  
  
+ TGGTACCCTG TGAAACCTGA GGCTCATCCC ACCATGAGTT TGAACCCTGA GAAGGGCCAG TCACATGGAT   
  
  
+ TAGAAGGTGG GAGAAATGGG AAGGGTCACC CAAAGAAACA GGATAAAAGT AGTACCAACG TTGTGGATTT   
  
  
+ AAGGAATTTG CTGATGCTAT GTGCACAATC TACTGCATCT GATGACCGAA GAACTGCTGA TGGACTGCTA   
  
  
+ AAGAAAATCA GGGAGCACTC ATCTGCTGAG GGGGATGGAT TTCAAAGGTT GGCTCATTAC TTTGCTGATG   
  
  
+ CCCTAGAGGC ACGTTTAGCT GGAACTGGAT CTCACATTTA TACAGCCCTA AGTTCTCATA GGCCATCTGC   
  
  
+ TGTTGACGTG TTAAAAGCAT ATCAGTTCTA TGTTCGTGCT TGCCCATTTA AGAAGATCGT CATTCGTTGT   
  
  
+ GGTAAACATA TGATTCTAAA AGCTGCTGAA AAGGCATCAA AGCTTCATAT TATAGATTTT GGCATCCTCT   
  
  
+ ATGGATTCCA ATGGCCTAGC CTCATTCGAT GCCTCTCAGA GCGGTCTGGT GGACCTCCAA AACTGTTTAT   
  
  
+ TACAGGGATC GATCTCCCCC AGCCTGGGTT CAGGCCAGCA GAAAGAGTGG AAGCAACAGG GAGACGCTTG   
  
  
+ GCTAAGTACT GTGAGCGGTA TAATGTGCCA TTTGAGTATC ATGCCATTGC TCAGAAGTGG GAAACAATCA   
  
  
+ AACCAGGGGA TCTCAAGTTA GGAAGTAGGG ATGATGATGA AGTTGTCGTG GTGAACTGTC TCTGTAGGTT   
  
  
+ CAAGAACCTC CTTGACGAGA CAATGGTGGT GGATAGTCCA AGGAACACAG TTTTAAACCT GATTAGAAGG   
  
  
+ GTAAAACCCG ATATTTTTGT GCATGGCATT GTAAATGGTT CCTACAACAT CCCTTTCTTT GTGACACGTT   
  
  
+ TTAGAGAAGC CCTCTTTCAT TATTCCACTC TTTTCGACAT GTTAGATGCC AACGCCTCTA GGGAGGAGCC   
  
  
+ CGAGAGGTTG ATATTCGAGA AGGCATTCTA TGGGAGGGAG ATTATGAATG TGGTGGCATG TGAGGGCACA   
  
  
+ GAGAGGGTGG AAAGGCCAGA GACATACAAG CAATGGCACG TTAGGCATAG CAGGGCAGGG TTTCGGCAAC   
  
  
+ TACCATTGGA TCCCAAGTTG ATCGAGAAAA TGAGGTTTAA GGCCAAGGCA GACCACCACA AGGATTTCGT   
  
  
+ GATTGATGTG GATGGACATT GGGCAATTCA GGGATGGAAG GGGCGGATTG CCTGTGCCAT CTCTGCATGG   
  
  
+ GTTCTGGCTT G  

- -Up\_Stream \_Len000GACAAG TCGTGATCCC TTATTCCAAA AACGGGACGT ATGATACTTT AGATTGGTTT   
  
  
- TTGTAAGGTA GAACCTTCCG ACATCGGAAC ACCTAAAACT TAAGCGAGGG TTCTTGGTTA CTTCATCGCG   
  
  
- TCTTCCGGTT ATGAGTTAAA GTCTCAGGTG TAGTTGAACA AGTTACGGTC ATATAAAGTC TTGTAATTAA   
  
  
- TGTATGACAA ACCAACCTCT TTTTTACCAA CAGAGCTTTT GATCAAAAAA TTAAAAAACA ATAAATCGTG   
  
  
- CCATTTCTTA CCTTTAATAA AAGTACGCTC TTTTTTCCTT CAACCAAAAA GAAAAAAGTT GAGAAAACTA   
  
  
- CTATATTAGT CAGCTTTAAA TCCACGTGGT AGACTGAAGT TGAGAACATG CTGGAGACTA ACGAACGAAT   
  
  
- CTTCTTGTTC TCCTAACTGT CTATTCCTCC TCACCCACCA AATATGTCTC TTAACTCACC TACCAATATT   
  
  
- TCTTCAGCTA GAGTGTATTC ATTGAAGCAA TGCTAATATT TATAAAAGTA ACTTCTTTCT GCTAATCTAT   
  
  
- ATTAATTCTT CCATGTAGAT TCGTTTAAAC CGACTCGTCT CAACCTCCCG GTGATCCCAG TGTAACACAG   
  
  
- AACGCGTTGT TCGAATCTGG TAAGCGTTGC CTACGGAGTG CACCTCGTCC ACCCTGGGCG CACCTTCAAC   
  
  
- GTACCGTTCT TAAGAGACCG TCGTTCCTCT TAACTCCCCC CTGTGCGCAC ACTGGTGCGG TCACGCCAGG   
  
  
- GGGAGAGCAC CGTCTAAACC ACTAAAAAAA AAACAAAAAA AAACTTCATT ATATATTAAA AATATACTAA   
  
  
- TTTTTTTATC CAACACACAA TATTTCACCC AGTTACTGTT TATAAAAATA ACCTACTAAT TTTAATTCAA   
  
  
- GGTAAATAAC CTCTTCACTA CCTTACGTTC AAGGTTTTAA CACCTTGAAA ACAACTGCAT CGTCCGATCT   
  
  
- CAAGGTTTTT AATTTTTTTC CTTTCTGTAT AACTCCATTT CGGAAAAATA AAATGTTTAT CTGCGTGGTT   
  
  
- CCATATTATA TTAACTGTAC TAAACTCCGC TCTCCTTCTA AGAAAACGAT GGGTTTGTTT TGTGTTACTA   
  
  
- GAAAATTTAC ATCGTTAAAC CCTAACCGTT TTAAAACAGA TTGGGCTAAC AACTTTCAAT AGGCTAAAGT   
  
  
- ATTTTAACCT ATAGCAATAA TTTAAAAGTA AGCTTATAAA TTTAAACCTG TGTTACAAAA AGTATAAACC   
  
  
- TAAAAGATTT GAACTAAAAT TGAGTTTAAA TTCAAATAAG TTAAGTAGAC TGGGTTTTTT ACCAACGTTA   
  
  
- TTACATAAAA GAAAAACTTC AATTTATAAT AGCTTAAGTA AATTGAACAT CAATTGGGCA TTAACTGGGT   
  
  
- TTGAGCTGAA AAACGATGGA ATCGTCGGTC CTTCTTCAGT ATCATAACTG GGCACCTTGC GGACTTCGGT   
  
  
- TGTAGTGGTC TGGGTGTATA AGGTAGTAGA AGACTAGAGG AGCAGTTTCT TCCTTCGACT CGTGTCGTTA   
  
  
- TTGATCTTCT TGTCCTCCGT AGTGTGGGTT CGGTTTCCGG AAATAAACTC GAGGGGTGGA GTAAGGAGCT   
  
  
- TCGAAGGAGA AGTGTTACAT TCTGGTATTA GGAAGTGGGG AAGAGAAAAA TTCGAGTAGA AATGGACGGG   
  
  
- ATCAAGAGAG AGAGAGTACT CAGTAGTCAT TATCAATGGA GAAATGAAAG AGAAGGAACT CCATACGAAG   
  
  
- TTGAAATTAT ATATATACAT GGTGAACTAA AGAGAACAGT GAAACGATAT TATGAATAAC AAACCGATAG   
  
  
- AAGGGGACAA AATATGAGTA AAAGAACGAA AGATATAGAC CCAAACTTAA CCCAAAGTGA ATAGGAGCAA   
  
  
- CTAAGACACC CTTTAATCAC TTAACCCAAA ACATGTCGTC CAAATATACT TGATCCCAAA GTTTCCATAG   
  
  
- ATGAAAAAGA GAACACTAAT AATCAACTAA TCCCTAAACC GGTTCTAATC GTCTTACCCA AGACTTAAAC   
  
  
- CCCTTAAGAG ACTACTACGA GATTTACCCA TACGAATGCA ACTATAGGGA CGAATACTAC GCAGGAATCT   
  
  
- AATACGGTTA AACAAGTTAA TGCTTCCGGA TAGACTCCTA GAGTGGAGTG ACTCGGAGGG TTCGGGGAAA   
  
  
- AGACTGGGAA ACCGCATGTC AAAGTGCAGG AGAAACCCCG GCTCGGGACC CCAACTAAGA TTACTACTAT   
  
  
- CACTAAGACT ACTACAAGAG TTCACGTAAT CGGTTTACGA ATACCTTCTT CTGTACCTCC GTTTCGGTAC   
  
  
- GTACAAAGTG CTAGGAAATC GTGAAGTCCG ACGACTCTTT GGGAAAATAC TACGGAATCC CTTCTTTATG   
  
  
- GGTTGAAGAG GCTTGGTTGT AGGATATTAA CTACTAACAA ACCTATTAGG ACCACTTTTA AGAAACCCAA   
  
  
- GAAGATCACT AAAATCACAA TCAGTGAAAC CCAGATCAAG TCGTTCGAGA TAACCTGGCT GTTAACACAG   
  
  
- ACTAAACTCA CTCGTAAAAC TCGGTGGGAA ACAAGTTCGT GAAGGTTAAC TTAGTATAGG GGTTGGTAAC   
  
  
- TGGGCAGGAG TTACCAGCAA ACCGGGAAGC CCACGGAATC TAACGTGCCG GAGATTACCA AGTCACTAGA   
  
  
- GTAGGGAACC AAACGGCTAC CTACAATAAT CACATAAGTC ACTCTTTCTC AGGTACTAAG TTAAAGTCTT   
  
  
- TCCCCACCTT CTCCGATCAT TCAAGGAAGG GTTCTTATTA TTGGAACAAT AACTAGAGTT CTTGGAGTGA   
  
  
- AAAGGATTAC TTTGCTTCCT CCTACTAGCT CACTACTACC AATTCTTCCT TTTCTTACTA CACTTAACCA   
  
  
- GATTGATGAG TTCTCCCTCA TTCTAAGTGA TAGCACTTCT GCTCCTGAAA CTTCTTCCTT CCTCATCGTT   
  
  
- CGTCAGTCGA TAAAGATGAT GACTCCTTCG ACTCAACACA CTTTAAAAAC TGTTCAAAAA CGAAACGTTA   
  
  
- ACCATGGGAC ACTTTGGACT CCGAGTAGGG TGGTACTCAA ACTTGGGACT CTTCCCGGTC AGTGTACCTA   
  
  
- ATCTTCCACC CTCTTTACCC TTCCCAGTGG GTTTCTTTGT CCTATTTTCA TCATGGTTGC AACACCTAAA   
  
  
- TTCCTTAAAC GACTACGATA CACGTGTTAG ATGACGTAGA CTACTGGCTT CTTGACGACT ACCTGACGAT   
  
  
- TTCTTTTAGT CCCTCGTGAG TAGACGACTC CCCCTACCTA AAGTTTCCAA CCGAGTAATG AAACGACTAC   
  
  
- GGGATCTCCG TGCAAATCGA CCTTGACCTA GAGTGTAAAT ATGTCGGGAT TCAAGAGTAT CCGGTAGACG   
  
  
- ACAACTGCAC AATTTTCGTA TAGTCAAGAT ACAAGCACGA ACGGGTAAAT TCTTCTAGCA GTAAGCAACA   
  
  
- CCATTTGTAT ACTAAGATTT TCGACGACTT TTCCGTAGTT TCGAAGTATA ATATCTAAAA CCGTAGGAGA   
  
  
- TACCTAAGGT TACCGGATCG GAGTAAGCTA CGGAGAGTCT CGCCAGACCA CCTGGAGGTT TTGACAAATA   
  
  
- ATGTCCCTAG CTAGAGGGGG TCGGACCCAA GTCCGGTCGT CTTTCTCACC TTCGTTGTCC CTCTGCGAAC   
  
  
- CGATTCATGA CACTCGCCAT ATTACACGGT AAACTCATAG TACGGTAACG AGTCTTCACC CTTTGTTAGT   
  
  
- TTGGTCCCCT AGAGTTCAAT CCTTCATCCC TACTACTACT TCAACAGCAC CACTTGACAG AGACATCCAA   
  
  
- GTTCTTGGAG GAACTGCTCT GTTACCACCA CCTATCAGGT TCCTTGTGTC AAAATTTGGA CTAATCTTCC   
  
  
- CATTTTGGGC TATAAAAACA CGTACCGTAA CATTTACCAA GGATGTTGTA GGGAAAGAAA CACTGTGCAA   
  
  
- AATCTCTTCG GGAGAAAGTA ATAAGGTGAG AAAAGCTGTA CAATCTACGG TTGCGGAGAT CCCTCCTCGG   
  
  
- GCTCTCCAAC TATAAGCTCT TCCGTAAGAT ACCCTCCCTC TAATACTTAC ACCACCGTAC ACTCCCGTGT   
  
  
- CTCTCCCACC TTTCCGGTCT CTGTATGTTC GTTACCGTGC AATCCGTATC GTCCCGTCCC AAAGCCGTTG   
  
  
- ATGGTAACCT AGGGTTCAAC TAGCTCTTTT ACTCCAAATT CCGGTTCCGT CTGGTGGTGT TCCTAAAGCA   
  
  
- CTAACTACAC CTACCTGTAA CCCGTTAAGT CCCTACCTTC CCCGCCTAAC GGACACGGTA GAGACGTACC   
  
  
- CAAGACCGAA C

+     AT~TATA-box

| Site Name | Organism | Position | Strand | Matrix score. | sequence | function |
| --- | --- | --- | --- | --- | --- | --- |
| AT~TATA-box | Arabidopsis thaliana | 1763 | + | 6 | TATATA |  |
| AT~TATA-box | Arabidopsis thaliana | 825 | + | 6 | TATATA |  |
| AT~TATA-box | Arabidopsis thaliana | 1765 | + | 6 | TATATA |  |

>HU07G02248.1   
+ -Up\_Stream \_Len000CTGTTC AGCACTAGGG AATAAGGTTT TTGCCCTGCA TACTATGAAA TCTAACCAAA   
  
  
+ AACATTCCAT CTTGGAAGGC TGTAGCCTTG TGGATTTTGA ATTCGCTCCC AAGAACCAAT GAAGTAGCGC   
  
  
+ AGAAGGCCAA TACTCAATTT CAGAGTCCAC ATCAACTTGT TCAATGCCAG TATATTTCAG AACATTAATT   
  
  
+ ACATACTGTT TGGTTGGAGA AAAAATGGTT GTCTCGAAAA CTAGTTTTTT AATTTTTTGT TATTTAGCAC   
  
  
+ GGTAAAGAAT GGAAATTATT TTCATGCGAG AAAAAAGGAA GTTGGTTTTT CTTTTTTCAA CTCTTTTGAT   
  
  
+ GATATAATCA GTCGAAATTT AGGTGCACCA TCTGACTTCA ACTCTTGTAC GACCTCTGAT TGCTTGCTTA   
  
  
+ GAAGAACAAG AGGATTGACA GATAAGGAGG AGTGGGTGGT TTATACAGAG AATTGAGTGG ATGGTTATAA   
  
  
+ AGAAGTCGAT CTCACATAAG TAACTTCGTT ACGATTATAA ATATTTTCAT TGAAGAAAGA CGATTAGATA   
  
  
+ TAATTAAGAA GGTACATCTA AGCAAATTTG GCTGAGCAGA GTTGGAGGGC CACTAGGGTC ACATTGTGTC   
  
  
+ TTGCGCAACA AGCTTAGACC ATTCGCAACG GATGCCTCAC GTGGAGCAGG TGGGACCCGC GTGGAAGTTG   
  
  
+ CATGGCAAGA ATTCTCTGGC AGCAAGGAGA ATTGAGGGGG GACACGCGTG TGACCACGCC AGTGCGGTCC   
  
  
+ CCCTCTCGTG GCAGATTTGG TGATTTTTTT TTTGTTTTTT TTTGAAGTAA TATATAATTT TTATATGATT   
  
  
+ AAAAAAATAG GTTGTGTGTT ATAAAGTGGG TCAATGACAA ATATTTTTAT TGGATGATTA AAATTAAGTT   
  
  
+ CCATTTATTG GAGAAGTGAT GGAATGCAAG TTCCAAAATT GTGGAACTTT TGTTGACGTA GCAGGCTAGA   
  
  
+ GTTCCAAAAA TTAAAAAAAG GAAAGACATA TTGAGGTAAA GCCTTTTTAT TTTACAAATA GACGCACCAA   
  
  
+ GGTATAATAT AATTGACATG ATTTGAGGCG AGAGGAAGAT TCTTTTGCTA CCCAAACAAA ACACAATGAT   
  
  
+ CTTTTAAATG TAGCAATTTG GGATTGGCAA AATTTTGTCT AACCCGATTG TTGAAAGTTA TCCGATTTCA   
  
  
+ TAAAATTGGA TATCGTTATT AAATTTTCAT TCGAATATTT AAATTTGGAC ACAATGTTTT TCATATTTGG   
  
  
+ ATTTTCTAAA CTTGATTTTA ACTCAAATTT AAGTTTATTC AATTCATCTG ACCCAAAAAA TGGTTGCAAT   
  
  
+ AATGTATTTT CTTTTTGAAG TTAAATATTA TCGAATTCAT TTAACTTGTA GTTAACCCGT AATTGACCCA   
  
  
+ AACTCGACTT TTTGCTACCT TAGCAGCCAG GAAGAAGTCA TAGTATTGAC CCGTGGAACG CCTGAAGCCA   
  
  
+ ACATCACCAG ACCCACATAT TCCATCATCT TCTGATCTCC TCGTCAAAGA AGGAAGCTGA GCACAGCAAT   
  
  
+ AACTAGAAGA ACAGGAGGCA TCACACCCAA GCCAAAGGCC TTTATTTGAG CTCCCCACCT CATTCCTCGA   
  
  
+ AGCTTCCTCT TCACAATGTA AGACCATAAT CCTTCACCCC TTCTCTTTTT AAGCTCATCT TTACCTGCCC   
  
  
+ TAGTTCTCTC TCTCTCATGA GTCATCAGTA ATAGTTACCT CTTTACTTTC TCTTCCTTGA GGTATGCTTC   
  
  
+ AACTTTAATA TATATATGTA CCACTTGATT TCTCTTGTCA CTTTGCTATA ATACTTATTG TTTGGCTATC   
  
  
+ TTCCCCTGTT TTATACTCAT TTTCTTGCTT TCTATATCTG GGTTTGAATT GGGTTTCACT TATCCTCGTT   
  
  
+ GATTCTGTGG GAAATTAGTG AATTGGGTTT TGTACAGCAG GTTTATATGA ACTAGGGTTT CAAAGGTATC   
  
  
+ TACTTTTTCT CTTGTGATTA TTAGTTGATT AGGGATTTGG CCAAGATTAG CAGAATGGGT TCTGAATTTG   
  
  
+ GGGAATTCTC TGATGATGCT CTAAATGGGT ATGCTTACGT TGATATCCCT GCTTATGATG CGTCCTTAGA   
  
  
+ TTATGCCAAT TTGTTCAATT ACGAAGGCCT ATCTGAGGAT CTCACCTCAC TGAGCCTCCC AAGCCCCTTT   
  
  
+ TCTGACCCTT TGGCGTACAG TTTCACGTCC TCTTTGGGGC CGAGCCCTGG GGTTGATTCT AATGATGATA   
  
  
+ GTGATTCTGA TGATGTTCTC AAGTGCATTA GCCAAATGCT TATGGAAGAA GACATGGAGG CAAAGCCATG   
  
  
+ CATGTTTCAC GATCCTTTAG CACTTCAGGC TGCTGAGAAA CCCTTTTATG ATGCCTTAGG GAAGAAATAC   
  
  
+ CCAACTTCTC CGAACCAACA TCCTATAATT GATGATTGTT TGGATAATCC TGGTGAAAAT TCTTTGGGTT   
  
  
+ CTTCTAGTGA TTTTAGTGTT AGTCACTTTG GGTCTAGTTC AGCAAGCTCT ATTGGACCGA CAATTGTGTC   
  
  
+ TGATTTGAGT GAGCATTTTG AGCCACCCTT TGTTCAAGCA CTTCCAATTG AATCATATCC CCAACCATTG   
  
  
+ ACCCGTCCTC AATGGTCGTT TGGCCCTTCG GGTGCCTTAG ATTGCACGGC CTCTAATGGT TCAGTGATCT   
  
  
+ CATCCCTTGG TTTGCCGATG GATGTTATTA GTGTATTCAG TGAGAAAGAG TCCATGATTC AATTTCAGAA   
  
  
+ AGGGGTGGAA GAGGCTAGTA AGTTCCTTCC CAAGAATAAT AACCTTGTTA TTGATCTCAA GAACCTCACT   
  
  
+ TTTCCTAATG AAACGAAGGA GGATGATCGA GTGATGATGG TTAAGAAGGA AAAGAATGAT GTGAATTGGT   
  
  
+ CTAACTACTC AAGAGGGAGT AAGATTCACT ATCGTGAAGA CGAGGACTTT GAAGAAGGAA GGAGTAGCAA   
  
  
+ GCAGTCAGCT ATTTCTACTA CTGAGGAAGC TGAGTTGTGT GAAATTTTTG ACAAGTTTTT GCTTTGCAAT   
  
  
+ TGGTACCCTG TGAAACCTGA GGCTCATCCC ACCATGAGTT TGAACCCTGA GAAGGGCCAG TCACATGGAT   
  
  
+ TAGAAGGTGG GAGAAATGGG AAGGGTCACC CAAAGAAACA GGATAAAAGT AGTACCAACG TTGTGGATTT   
  
  
+ AAGGAATTTG CTGATGCTAT GTGCACAATC TACTGCATCT GATGACCGAA GAACTGCTGA TGGACTGCTA   
  
  
+ AAGAAAATCA GGGAGCACTC ATCTGCTGAG GGGGATGGAT TTCAAAGGTT GGCTCATTAC TTTGCTGATG   
  
  
+ CCCTAGAGGC ACGTTTAGCT GGAACTGGAT CTCACATTTA TACAGCCCTA AGTTCTCATA GGCCATCTGC   
  
  
+ TGTTGACGTG TTAAAAGCAT ATCAGTTCTA TGTTCGTGCT TGCCCATTTA AGAAGATCGT CATTCGTTGT   
  
  
+ GGTAAACATA TGATTCTAAA AGCTGCTGAA AAGGCATCAA AGCTTCATAT TATAGATTTT GGCATCCTCT   
  
  
+ ATGGATTCCA ATGGCCTAGC CTCATTCGAT GCCTCTCAGA GCGGTCTGGT GGACCTCCAA AACTGTTTAT   
  
  
+ TACAGGGATC GATCTCCCCC AGCCTGGGTT CAGGCCAGCA GAAAGAGTGG AAGCAACAGG GAGACGCTTG   
  
  
+ GCTAAGTACT GTGAGCGGTA TAATGTGCCA TTTGAGTATC ATGCCATTGC TCAGAAGTGG GAAACAATCA   
  
  
+ AACCAGGGGA TCTCAAGTTA GGAAGTAGGG ATGATGATGA AGTTGTCGTG GTGAACTGTC TCTGTAGGTT   
  
  
+ CAAGAACCTC CTTGACGAGA CAATGGTGGT GGATAGTCCA AGGAACACAG TTTTAAACCT GATTAGAAGG   
  
  
+ GTAAAACCCG ATATTTTTGT GCATGGCATT GTAAATGGTT CCTACAACAT CCCTTTCTTT GTGACACGTT   
  
  
+ TTAGAGAAGC CCTCTTTCAT TATTCCACTC TTTTCGACAT GTTAGATGCC AACGCCTCTA GGGAGGAGCC   
  
  
+ CGAGAGGTTG ATATTCGAGA AGGCATTCTA TGGGAGGGAG ATTATGAATG TGGTGGCATG TGAGGGCACA   
  
  
+ GAGAGGGTGG AAAGGCCAGA GACATACAAG CAATGGCACG TTAGGCATAG CAGGGCAGGG TTTCGGCAAC   
  
  
+ TACCATTGGA TCCCAAGTTG ATCGAGAAAA TGAGGTTTAA GGCCAAGGCA GACCACCACA AGGATTTCGT   
  
  
+ GATTGATGTG GATGGACATT GGGCAATTCA GGGATGGAAG GGGCGGATTG CCTGTGCCAT CTCTGCATGG   
  
  
+ GTTCTGGCTT G  

- -Up\_Stream \_Len000GACAAG TCGTGATCCC TTATTCCAAA AACGGGACGT ATGATACTTT AGATTGGTTT   
  
  
- TTGTAAGGTA GAACCTTCCG ACATCGGAAC ACCTAAAACT TAAGCGAGGG TTCTTGGTTA CTTCATCGCG   
  
  
- TCTTCCGGTT ATGAGTTAAA GTCTCAGGTG TAGTTGAACA AGTTACGGTC ATATAAAGTC TTGTAATTAA   
  
  
- TGTATGACAA ACCAACCTCT TTTTTACCAA CAGAGCTTTT GATCAAAAAA TTAAAAAACA ATAAATCGTG   
  
  
- CCATTTCTTA CCTTTAATAA AAGTACGCTC TTTTTTCCTT CAACCAAAAA GAAAAAAGTT GAGAAAACTA   
  
  
- CTATATTAGT CAGCTTTAAA TCCACGTGGT AGACTGAAGT TGAGAACATG CTGGAGACTA ACGAACGAAT   
  
  
- CTTCTTGTTC TCCTAACTGT CTATTCCTCC TCACCCACCA AATATGTCTC TTAACTCACC TACCAATATT   
  
  
- TCTTCAGCTA GAGTGTATTC ATTGAAGCAA TGCTAATATT TATAAAAGTA ACTTCTTTCT GCTAATCTAT   
  
  
- ATTAATTCTT CCATGTAGAT TCGTTTAAAC CGACTCGTCT CAACCTCCCG GTGATCCCAG TGTAACACAG   
  
  
- AACGCGTTGT TCGAATCTGG TAAGCGTTGC CTACGGAGTG CACCTCGTCC ACCCTGGGCG CACCTTCAAC   
  
  
- GTACCGTTCT TAAGAGACCG TCGTTCCTCT TAACTCCCCC CTGTGCGCAC ACTGGTGCGG TCACGCCAGG   
  
  
- GGGAGAGCAC CGTCTAAACC ACTAAAAAAA AAACAAAAAA AAACTTCATT ATATATTAAA AATATACTAA   
  
  
- TTTTTTTATC CAACACACAA TATTTCACCC AGTTACTGTT TATAAAAATA ACCTACTAAT TTTAATTCAA   
  
  
- GGTAAATAAC CTCTTCACTA CCTTACGTTC AAGGTTTTAA CACCTTGAAA ACAACTGCAT CGTCCGATCT   
  
  
- CAAGGTTTTT AATTTTTTTC CTTTCTGTAT AACTCCATTT CGGAAAAATA AAATGTTTAT CTGCGTGGTT   
  
  
- CCATATTATA TTAACTGTAC TAAACTCCGC TCTCCTTCTA AGAAAACGAT GGGTTTGTTT TGTGTTACTA   
  
  
- GAAAATTTAC ATCGTTAAAC CCTAACCGTT TTAAAACAGA TTGGGCTAAC AACTTTCAAT AGGCTAAAGT   
  
  
- ATTTTAACCT ATAGCAATAA TTTAAAAGTA AGCTTATAAA TTTAAACCTG TGTTACAAAA AGTATAAACC   
  
  
- TAAAAGATTT GAACTAAAAT TGAGTTTAAA TTCAAATAAG TTAAGTAGAC TGGGTTTTTT ACCAACGTTA   
  
  
- TTACATAAAA GAAAAACTTC AATTTATAAT AGCTTAAGTA AATTGAACAT CAATTGGGCA TTAACTGGGT   
  
  
- TTGAGCTGAA AAACGATGGA ATCGTCGGTC CTTCTTCAGT ATCATAACTG GGCACCTTGC GGACTTCGGT   
  
  
- TGTAGTGGTC TGGGTGTATA AGGTAGTAGA AGACTAGAGG AGCAGTTTCT TCCTTCGACT CGTGTCGTTA   
  
  
- TTGATCTTCT TGTCCTCCGT AGTGTGGGTT CGGTTTCCGG AAATAAACTC GAGGGGTGGA GTAAGGAGCT   
  
  
- TCGAAGGAGA AGTGTTACAT TCTGGTATTA GGAAGTGGGG AAGAGAAAAA TTCGAGTAGA AATGGACGGG   
  
  
- ATCAAGAGAG AGAGAGTACT CAGTAGTCAT TATCAATGGA GAAATGAAAG AGAAGGAACT CCATACGAAG   
  
  
- TTGAAATTAT ATATATACAT GGTGAACTAA AGAGAACAGT GAAACGATAT TATGAATAAC AAACCGATAG   
  
  
- AAGGGGACAA AATATGAGTA AAAGAACGAA AGATATAGAC CCAAACTTAA CCCAAAGTGA ATAGGAGCAA   
  
  
- CTAAGACACC CTTTAATCAC TTAACCCAAA ACATGTCGTC CAAATATACT TGATCCCAAA GTTTCCATAG   
  
  
- ATGAAAAAGA GAACACTAAT AATCAACTAA TCCCTAAACC GGTTCTAATC GTCTTACCCA AGACTTAAAC   
  
  
- CCCTTAAGAG ACTACTACGA GATTTACCCA TACGAATGCA ACTATAGGGA CGAATACTAC GCAGGAATCT   
  
  
- AATACGGTTA AACAAGTTAA TGCTTCCGGA TAGACTCCTA GAGTGGAGTG ACTCGGAGGG TTCGGGGAAA   
  
  
- AGACTGGGAA ACCGCATGTC AAAGTGCAGG AGAAACCCCG GCTCGGGACC CCAACTAAGA TTACTACTAT   
  
  
- CACTAAGACT ACTACAAGAG TTCACGTAAT CGGTTTACGA ATACCTTCTT CTGTACCTCC GTTTCGGTAC   
  
  
- GTACAAAGTG CTAGGAAATC GTGAAGTCCG ACGACTCTTT GGGAAAATAC TACGGAATCC CTTCTTTATG   
  
  
- GGTTGAAGAG GCTTGGTTGT AGGATATTAA CTACTAACAA ACCTATTAGG ACCACTTTTA AGAAACCCAA   
  
  
- GAAGATCACT AAAATCACAA TCAGTGAAAC CCAGATCAAG TCGTTCGAGA TAACCTGGCT GTTAACACAG   
  
  
- ACTAAACTCA CTCGTAAAAC TCGGTGGGAA ACAAGTTCGT GAAGGTTAAC TTAGTATAGG GGTTGGTAAC   
  
  
- TGGGCAGGAG TTACCAGCAA ACCGGGAAGC CCACGGAATC TAACGTGCCG GAGATTACCA AGTCACTAGA   
  
  
- GTAGGGAACC AAACGGCTAC CTACAATAAT CACATAAGTC ACTCTTTCTC AGGTACTAAG TTAAAGTCTT   
  
  
- TCCCCACCTT CTCCGATCAT TCAAGGAAGG GTTCTTATTA TTGGAACAAT AACTAGAGTT CTTGGAGTGA   
  
  
- AAAGGATTAC TTTGCTTCCT CCTACTAGCT CACTACTACC AATTCTTCCT TTTCTTACTA CACTTAACCA   
  
  
- GATTGATGAG TTCTCCCTCA TTCTAAGTGA TAGCACTTCT GCTCCTGAAA CTTCTTCCTT CCTCATCGTT   
  
  
- CGTCAGTCGA TAAAGATGAT GACTCCTTCG ACTCAACACA CTTTAAAAAC TGTTCAAAAA CGAAACGTTA   
  
  
- ACCATGGGAC ACTTTGGACT CCGAGTAGGG TGGTACTCAA ACTTGGGACT CTTCCCGGTC AGTGTACCTA   
  
  
- ATCTTCCACC CTCTTTACCC TTCCCAGTGG GTTTCTTTGT CCTATTTTCA TCATGGTTGC AACACCTAAA   
  
  
- TTCCTTAAAC GACTACGATA CACGTGTTAG ATGACGTAGA CTACTGGCTT CTTGACGACT ACCTGACGAT   
  
  
- TTCTTTTAGT CCCTCGTGAG TAGACGACTC CCCCTACCTA AAGTTTCCAA CCGAGTAATG AAACGACTAC   
  
  
- GGGATCTCCG TGCAAATCGA CCTTGACCTA GAGTGTAAAT ATGTCGGGAT TCAAGAGTAT CCGGTAGACG   
  
  
- ACAACTGCAC AATTTTCGTA TAGTCAAGAT ACAAGCACGA ACGGGTAAAT TCTTCTAGCA GTAAGCAACA   
  
  
- CCATTTGTAT ACTAAGATTT TCGACGACTT TTCCGTAGTT TCGAAGTATA ATATCTAAAA CCGTAGGAGA   
  
  
- TACCTAAGGT TACCGGATCG GAGTAAGCTA CGGAGAGTCT CGCCAGACCA CCTGGAGGTT TTGACAAATA   
  
  
- ATGTCCCTAG CTAGAGGGGG TCGGACCCAA GTCCGGTCGT CTTTCTCACC TTCGTTGTCC CTCTGCGAAC   
  
  
- CGATTCATGA CACTCGCCAT ATTACACGGT AAACTCATAG TACGGTAACG AGTCTTCACC CTTTGTTAGT   
  
  
- TTGGTCCCCT AGAGTTCAAT CCTTCATCCC TACTACTACT TCAACAGCAC CACTTGACAG AGACATCCAA   
  
  
- GTTCTTGGAG GAACTGCTCT GTTACCACCA CCTATCAGGT TCCTTGTGTC AAAATTTGGA CTAATCTTCC   
  
  
- CATTTTGGGC TATAAAAACA CGTACCGTAA CATTTACCAA GGATGTTGTA GGGAAAGAAA CACTGTGCAA   
  
  
- AATCTCTTCG GGAGAAAGTA ATAAGGTGAG AAAAGCTGTA CAATCTACGG TTGCGGAGAT CCCTCCTCGG   
  
  
- GCTCTCCAAC TATAAGCTCT TCCGTAAGAT ACCCTCCCTC TAATACTTAC ACCACCGTAC ACTCCCGTGT   
  
  
- CTCTCCCACC TTTCCGGTCT CTGTATGTTC GTTACCGTGC AATCCGTATC GTCCCGTCCC AAAGCCGTTG   
  
  
- ATGGTAACCT AGGGTTCAAC TAGCTCTTTT ACTCCAAATT CCGGTTCCGT CTGGTGGTGT TCCTAAAGCA   
  
  
- CTAACTACAC CTACCTGTAA CCCGTTAAGT CCCTACCTTC CCCGCCTAAC GGACACGGTA GAGACGTACC   
  
  
- CAAGACCGAA C

+     Box 4

| Site Name | Organism | Position | Strand | Matrix score. | sequence | function |
| --- | --- | --- | --- | --- | --- | --- |
| Box 4 | Petroselinum crispum | 208 | + | 6 | ATTAAT | part of a conserved DNA module involved in light responsiveness |

>HU07G02248.1   
+ -Up\_Stream \_Len000CTGTTC AGCACTAGGG AATAAGGTTT TTGCCCTGCA TACTATGAAA TCTAACCAAA   
  
  
+ AACATTCCAT CTTGGAAGGC TGTAGCCTTG TGGATTTTGA ATTCGCTCCC AAGAACCAAT GAAGTAGCGC   
  
  
+ AGAAGGCCAA TACTCAATTT CAGAGTCCAC ATCAACTTGT TCAATGCCAG TATATTTCAG AACATTAATT   
  
  
+ ACATACTGTT TGGTTGGAGA AAAAATGGTT GTCTCGAAAA CTAGTTTTTT AATTTTTTGT TATTTAGCAC   
  
  
+ GGTAAAGAAT GGAAATTATT TTCATGCGAG AAAAAAGGAA GTTGGTTTTT CTTTTTTCAA CTCTTTTGAT   
  
  
+ GATATAATCA GTCGAAATTT AGGTGCACCA TCTGACTTCA ACTCTTGTAC GACCTCTGAT TGCTTGCTTA   
  
  
+ GAAGAACAAG AGGATTGACA GATAAGGAGG AGTGGGTGGT TTATACAGAG AATTGAGTGG ATGGTTATAA   
  
  
+ AGAAGTCGAT CTCACATAAG TAACTTCGTT ACGATTATAA ATATTTTCAT TGAAGAAAGA CGATTAGATA   
  
  
+ TAATTAAGAA GGTACATCTA AGCAAATTTG GCTGAGCAGA GTTGGAGGGC CACTAGGGTC ACATTGTGTC   
  
  
+ TTGCGCAACA AGCTTAGACC ATTCGCAACG GATGCCTCAC GTGGAGCAGG TGGGACCCGC GTGGAAGTTG   
  
  
+ CATGGCAAGA ATTCTCTGGC AGCAAGGAGA ATTGAGGGGG GACACGCGTG TGACCACGCC AGTGCGGTCC   
  
  
+ CCCTCTCGTG GCAGATTTGG TGATTTTTTT TTTGTTTTTT TTTGAAGTAA TATATAATTT TTATATGATT   
  
  
+ AAAAAAATAG GTTGTGTGTT ATAAAGTGGG TCAATGACAA ATATTTTTAT TGGATGATTA AAATTAAGTT   
  
  
+ CCATTTATTG GAGAAGTGAT GGAATGCAAG TTCCAAAATT GTGGAACTTT TGTTGACGTA GCAGGCTAGA   
  
  
+ GTTCCAAAAA TTAAAAAAAG GAAAGACATA TTGAGGTAAA GCCTTTTTAT TTTACAAATA GACGCACCAA   
  
  
+ GGTATAATAT AATTGACATG ATTTGAGGCG AGAGGAAGAT TCTTTTGCTA CCCAAACAAA ACACAATGAT   
  
  
+ CTTTTAAATG TAGCAATTTG GGATTGGCAA AATTTTGTCT AACCCGATTG TTGAAAGTTA TCCGATTTCA   
  
  
+ TAAAATTGGA TATCGTTATT AAATTTTCAT TCGAATATTT AAATTTGGAC ACAATGTTTT TCATATTTGG   
  
  
+ ATTTTCTAAA CTTGATTTTA ACTCAAATTT AAGTTTATTC AATTCATCTG ACCCAAAAAA TGGTTGCAAT   
  
  
+ AATGTATTTT CTTTTTGAAG TTAAATATTA TCGAATTCAT TTAACTTGTA GTTAACCCGT AATTGACCCA   
  
  
+ AACTCGACTT TTTGCTACCT TAGCAGCCAG GAAGAAGTCA TAGTATTGAC CCGTGGAACG CCTGAAGCCA   
  
  
+ ACATCACCAG ACCCACATAT TCCATCATCT TCTGATCTCC TCGTCAAAGA AGGAAGCTGA GCACAGCAAT   
  
  
+ AACTAGAAGA ACAGGAGGCA TCACACCCAA GCCAAAGGCC TTTATTTGAG CTCCCCACCT CATTCCTCGA   
  
  
+ AGCTTCCTCT TCACAATGTA AGACCATAAT CCTTCACCCC TTCTCTTTTT AAGCTCATCT TTACCTGCCC   
  
  
+ TAGTTCTCTC TCTCTCATGA GTCATCAGTA ATAGTTACCT CTTTACTTTC TCTTCCTTGA GGTATGCTTC   
  
  
+ AACTTTAATA TATATATGTA CCACTTGATT TCTCTTGTCA CTTTGCTATA ATACTTATTG TTTGGCTATC   
  
  
+ TTCCCCTGTT TTATACTCAT TTTCTTGCTT TCTATATCTG GGTTTGAATT GGGTTTCACT TATCCTCGTT   
  
  
+ GATTCTGTGG GAAATTAGTG AATTGGGTTT TGTACAGCAG GTTTATATGA ACTAGGGTTT CAAAGGTATC   
  
  
+ TACTTTTTCT CTTGTGATTA TTAGTTGATT AGGGATTTGG CCAAGATTAG CAGAATGGGT TCTGAATTTG   
  
  
+ GGGAATTCTC TGATGATGCT CTAAATGGGT ATGCTTACGT TGATATCCCT GCTTATGATG CGTCCTTAGA   
  
  
+ TTATGCCAAT TTGTTCAATT ACGAAGGCCT ATCTGAGGAT CTCACCTCAC TGAGCCTCCC AAGCCCCTTT   
  
  
+ TCTGACCCTT TGGCGTACAG TTTCACGTCC TCTTTGGGGC CGAGCCCTGG GGTTGATTCT AATGATGATA   
  
  
+ GTGATTCTGA TGATGTTCTC AAGTGCATTA GCCAAATGCT TATGGAAGAA GACATGGAGG CAAAGCCATG   
  
  
+ CATGTTTCAC GATCCTTTAG CACTTCAGGC TGCTGAGAAA CCCTTTTATG ATGCCTTAGG GAAGAAATAC   
  
  
+ CCAACTTCTC CGAACCAACA TCCTATAATT GATGATTGTT TGGATAATCC TGGTGAAAAT TCTTTGGGTT   
  
  
+ CTTCTAGTGA TTTTAGTGTT AGTCACTTTG GGTCTAGTTC AGCAAGCTCT ATTGGACCGA CAATTGTGTC   
  
  
+ TGATTTGAGT GAGCATTTTG AGCCACCCTT TGTTCAAGCA CTTCCAATTG AATCATATCC CCAACCATTG   
  
  
+ ACCCGTCCTC AATGGTCGTT TGGCCCTTCG GGTGCCTTAG ATTGCACGGC CTCTAATGGT TCAGTGATCT   
  
  
+ CATCCCTTGG TTTGCCGATG GATGTTATTA GTGTATTCAG TGAGAAAGAG TCCATGATTC AATTTCAGAA   
  
  
+ AGGGGTGGAA GAGGCTAGTA AGTTCCTTCC CAAGAATAAT AACCTTGTTA TTGATCTCAA GAACCTCACT   
  
  
+ TTTCCTAATG AAACGAAGGA GGATGATCGA GTGATGATGG TTAAGAAGGA AAAGAATGAT GTGAATTGGT   
  
  
+ CTAACTACTC AAGAGGGAGT AAGATTCACT ATCGTGAAGA CGAGGACTTT GAAGAAGGAA GGAGTAGCAA   
  
  
+ GCAGTCAGCT ATTTCTACTA CTGAGGAAGC TGAGTTGTGT GAAATTTTTG ACAAGTTTTT GCTTTGCAAT   
  
  
+ TGGTACCCTG TGAAACCTGA GGCTCATCCC ACCATGAGTT TGAACCCTGA GAAGGGCCAG TCACATGGAT   
  
  
+ TAGAAGGTGG GAGAAATGGG AAGGGTCACC CAAAGAAACA GGATAAAAGT AGTACCAACG TTGTGGATTT   
  
  
+ AAGGAATTTG CTGATGCTAT GTGCACAATC TACTGCATCT GATGACCGAA GAACTGCTGA TGGACTGCTA   
  
  
+ AAGAAAATCA GGGAGCACTC ATCTGCTGAG GGGGATGGAT TTCAAAGGTT GGCTCATTAC TTTGCTGATG   
  
  
+ CCCTAGAGGC ACGTTTAGCT GGAACTGGAT CTCACATTTA TACAGCCCTA AGTTCTCATA GGCCATCTGC   
  
  
+ TGTTGACGTG TTAAAAGCAT ATCAGTTCTA TGTTCGTGCT TGCCCATTTA AGAAGATCGT CATTCGTTGT   
  
  
+ GGTAAACATA TGATTCTAAA AGCTGCTGAA AAGGCATCAA AGCTTCATAT TATAGATTTT GGCATCCTCT   
  
  
+ ATGGATTCCA ATGGCCTAGC CTCATTCGAT GCCTCTCAGA GCGGTCTGGT GGACCTCCAA AACTGTTTAT   
  
  
+ TACAGGGATC GATCTCCCCC AGCCTGGGTT CAGGCCAGCA GAAAGAGTGG AAGCAACAGG GAGACGCTTG   
  
  
+ GCTAAGTACT GTGAGCGGTA TAATGTGCCA TTTGAGTATC ATGCCATTGC TCAGAAGTGG GAAACAATCA   
  
  
+ AACCAGGGGA TCTCAAGTTA GGAAGTAGGG ATGATGATGA AGTTGTCGTG GTGAACTGTC TCTGTAGGTT   
  
  
+ CAAGAACCTC CTTGACGAGA CAATGGTGGT GGATAGTCCA AGGAACACAG TTTTAAACCT GATTAGAAGG   
  
  
+ GTAAAACCCG ATATTTTTGT GCATGGCATT GTAAATGGTT CCTACAACAT CCCTTTCTTT GTGACACGTT   
  
  
+ TTAGAGAAGC CCTCTTTCAT TATTCCACTC TTTTCGACAT GTTAGATGCC AACGCCTCTA GGGAGGAGCC   
  
  
+ CGAGAGGTTG ATATTCGAGA AGGCATTCTA TGGGAGGGAG ATTATGAATG TGGTGGCATG TGAGGGCACA   
  
  
+ GAGAGGGTGG AAAGGCCAGA GACATACAAG CAATGGCACG TTAGGCATAG CAGGGCAGGG TTTCGGCAAC   
  
  
+ TACCATTGGA TCCCAAGTTG ATCGAGAAAA TGAGGTTTAA GGCCAAGGCA GACCACCACA AGGATTTCGT   
  
  
+ GATTGATGTG GATGGACATT GGGCAATTCA GGGATGGAAG GGGCGGATTG CCTGTGCCAT CTCTGCATGG   
  
  
+ GTTCTGGCTT G  

- -Up\_Stream \_Len000GACAAG TCGTGATCCC TTATTCCAAA AACGGGACGT ATGATACTTT AGATTGGTTT   
  
  
- TTGTAAGGTA GAACCTTCCG ACATCGGAAC ACCTAAAACT TAAGCGAGGG TTCTTGGTTA CTTCATCGCG   
  
  
- TCTTCCGGTT ATGAGTTAAA GTCTCAGGTG TAGTTGAACA AGTTACGGTC ATATAAAGTC TTGTAATTAA   
  
  
- TGTATGACAA ACCAACCTCT TTTTTACCAA CAGAGCTTTT GATCAAAAAA TTAAAAAACA ATAAATCGTG   
  
  
- CCATTTCTTA CCTTTAATAA AAGTACGCTC TTTTTTCCTT CAACCAAAAA GAAAAAAGTT GAGAAAACTA   
  
  
- CTATATTAGT CAGCTTTAAA TCCACGTGGT AGACTGAAGT TGAGAACATG CTGGAGACTA ACGAACGAAT   
  
  
- CTTCTTGTTC TCCTAACTGT CTATTCCTCC TCACCCACCA AATATGTCTC TTAACTCACC TACCAATATT   
  
  
- TCTTCAGCTA GAGTGTATTC ATTGAAGCAA TGCTAATATT TATAAAAGTA ACTTCTTTCT GCTAATCTAT   
  
  
- ATTAATTCTT CCATGTAGAT TCGTTTAAAC CGACTCGTCT CAACCTCCCG GTGATCCCAG TGTAACACAG   
  
  
- AACGCGTTGT TCGAATCTGG TAAGCGTTGC CTACGGAGTG CACCTCGTCC ACCCTGGGCG CACCTTCAAC   
  
  
- GTACCGTTCT TAAGAGACCG TCGTTCCTCT TAACTCCCCC CTGTGCGCAC ACTGGTGCGG TCACGCCAGG   
  
  
- GGGAGAGCAC CGTCTAAACC ACTAAAAAAA AAACAAAAAA AAACTTCATT ATATATTAAA AATATACTAA   
  
  
- TTTTTTTATC CAACACACAA TATTTCACCC AGTTACTGTT TATAAAAATA ACCTACTAAT TTTAATTCAA   
  
  
- GGTAAATAAC CTCTTCACTA CCTTACGTTC AAGGTTTTAA CACCTTGAAA ACAACTGCAT CGTCCGATCT   
  
  
- CAAGGTTTTT AATTTTTTTC CTTTCTGTAT AACTCCATTT CGGAAAAATA AAATGTTTAT CTGCGTGGTT   
  
  
- CCATATTATA TTAACTGTAC TAAACTCCGC TCTCCTTCTA AGAAAACGAT GGGTTTGTTT TGTGTTACTA   
  
  
- GAAAATTTAC ATCGTTAAAC CCTAACCGTT TTAAAACAGA TTGGGCTAAC AACTTTCAAT AGGCTAAAGT   
  
  
- ATTTTAACCT ATAGCAATAA TTTAAAAGTA AGCTTATAAA TTTAAACCTG TGTTACAAAA AGTATAAACC   
  
  
- TAAAAGATTT GAACTAAAAT TGAGTTTAAA TTCAAATAAG TTAAGTAGAC TGGGTTTTTT ACCAACGTTA   
  
  
- TTACATAAAA GAAAAACTTC AATTTATAAT AGCTTAAGTA AATTGAACAT CAATTGGGCA TTAACTGGGT   
  
  
- TTGAGCTGAA AAACGATGGA ATCGTCGGTC CTTCTTCAGT ATCATAACTG GGCACCTTGC GGACTTCGGT   
  
  
- TGTAGTGGTC TGGGTGTATA AGGTAGTAGA AGACTAGAGG AGCAGTTTCT TCCTTCGACT CGTGTCGTTA   
  
  
- TTGATCTTCT TGTCCTCCGT AGTGTGGGTT CGGTTTCCGG AAATAAACTC GAGGGGTGGA GTAAGGAGCT   
  
  
- TCGAAGGAGA AGTGTTACAT TCTGGTATTA GGAAGTGGGG AAGAGAAAAA TTCGAGTAGA AATGGACGGG   
  
  
- ATCAAGAGAG AGAGAGTACT CAGTAGTCAT TATCAATGGA GAAATGAAAG AGAAGGAACT CCATACGAAG   
  
  
- TTGAAATTAT ATATATACAT GGTGAACTAA AGAGAACAGT GAAACGATAT TATGAATAAC AAACCGATAG   
  
  
- AAGGGGACAA AATATGAGTA AAAGAACGAA AGATATAGAC CCAAACTTAA CCCAAAGTGA ATAGGAGCAA   
  
  
- CTAAGACACC CTTTAATCAC TTAACCCAAA ACATGTCGTC CAAATATACT TGATCCCAAA GTTTCCATAG   
  
  
- ATGAAAAAGA GAACACTAAT AATCAACTAA TCCCTAAACC GGTTCTAATC GTCTTACCCA AGACTTAAAC   
  
  
- CCCTTAAGAG ACTACTACGA GATTTACCCA TACGAATGCA ACTATAGGGA CGAATACTAC GCAGGAATCT   
  
  
- AATACGGTTA AACAAGTTAA TGCTTCCGGA TAGACTCCTA GAGTGGAGTG ACTCGGAGGG TTCGGGGAAA   
  
  
- AGACTGGGAA ACCGCATGTC AAAGTGCAGG AGAAACCCCG GCTCGGGACC CCAACTAAGA TTACTACTAT   
  
  
- CACTAAGACT ACTACAAGAG TTCACGTAAT CGGTTTACGA ATACCTTCTT CTGTACCTCC GTTTCGGTAC   
  
  
- GTACAAAGTG CTAGGAAATC GTGAAGTCCG ACGACTCTTT GGGAAAATAC TACGGAATCC CTTCTTTATG   
  
  
- GGTTGAAGAG GCTTGGTTGT AGGATATTAA CTACTAACAA ACCTATTAGG ACCACTTTTA AGAAACCCAA   
  
  
- GAAGATCACT AAAATCACAA TCAGTGAAAC CCAGATCAAG TCGTTCGAGA TAACCTGGCT GTTAACACAG   
  
  
- ACTAAACTCA CTCGTAAAAC TCGGTGGGAA ACAAGTTCGT GAAGGTTAAC TTAGTATAGG GGTTGGTAAC   
  
  
- TGGGCAGGAG TTACCAGCAA ACCGGGAAGC CCACGGAATC TAACGTGCCG GAGATTACCA AGTCACTAGA   
  
  
- GTAGGGAACC AAACGGCTAC CTACAATAAT CACATAAGTC ACTCTTTCTC AGGTACTAAG TTAAAGTCTT   
  
  
- TCCCCACCTT CTCCGATCAT TCAAGGAAGG GTTCTTATTA TTGGAACAAT AACTAGAGTT CTTGGAGTGA   
  
  
- AAAGGATTAC TTTGCTTCCT CCTACTAGCT CACTACTACC AATTCTTCCT TTTCTTACTA CACTTAACCA   
  
  
- GATTGATGAG TTCTCCCTCA TTCTAAGTGA TAGCACTTCT GCTCCTGAAA CTTCTTCCTT CCTCATCGTT   
  
  
- CGTCAGTCGA TAAAGATGAT GACTCCTTCG ACTCAACACA CTTTAAAAAC TGTTCAAAAA CGAAACGTTA   
  
  
- ACCATGGGAC ACTTTGGACT CCGAGTAGGG TGGTACTCAA ACTTGGGACT CTTCCCGGTC AGTGTACCTA   
  
  
- ATCTTCCACC CTCTTTACCC TTCCCAGTGG GTTTCTTTGT CCTATTTTCA TCATGGTTGC AACACCTAAA   
  
  
- TTCCTTAAAC GACTACGATA CACGTGTTAG ATGACGTAGA CTACTGGCTT CTTGACGACT ACCTGACGAT   
  
  
- TTCTTTTAGT CCCTCGTGAG TAGACGACTC CCCCTACCTA AAGTTTCCAA CCGAGTAATG AAACGACTAC   
  
  
- GGGATCTCCG TGCAAATCGA CCTTGACCTA GAGTGTAAAT ATGTCGGGAT TCAAGAGTAT CCGGTAGACG   
  
  
- ACAACTGCAC AATTTTCGTA TAGTCAAGAT ACAAGCACGA ACGGGTAAAT TCTTCTAGCA GTAAGCAACA   
  
  
- CCATTTGTAT ACTAAGATTT TCGACGACTT TTCCGTAGTT TCGAAGTATA ATATCTAAAA CCGTAGGAGA   
  
  
- TACCTAAGGT TACCGGATCG GAGTAAGCTA CGGAGAGTCT CGCCAGACCA CCTGGAGGTT TTGACAAATA   
  
  
- ATGTCCCTAG CTAGAGGGGG TCGGACCCAA GTCCGGTCGT CTTTCTCACC TTCGTTGTCC CTCTGCGAAC   
  
  
- CGATTCATGA CACTCGCCAT ATTACACGGT AAACTCATAG TACGGTAACG AGTCTTCACC CTTTGTTAGT   
  
  
- TTGGTCCCCT AGAGTTCAAT CCTTCATCCC TACTACTACT TCAACAGCAC CACTTGACAG AGACATCCAA   
  
  
- GTTCTTGGAG GAACTGCTCT GTTACCACCA CCTATCAGGT TCCTTGTGTC AAAATTTGGA CTAATCTTCC   
  
  
- CATTTTGGGC TATAAAAACA CGTACCGTAA CATTTACCAA GGATGTTGTA GGGAAAGAAA CACTGTGCAA   
  
  
- AATCTCTTCG GGAGAAAGTA ATAAGGTGAG AAAAGCTGTA CAATCTACGG TTGCGGAGAT CCCTCCTCGG   
  
  
- GCTCTCCAAC TATAAGCTCT TCCGTAAGAT ACCCTCCCTC TAATACTTAC ACCACCGTAC ACTCCCGTGT   
  
  
- CTCTCCCACC TTTCCGGTCT CTGTATGTTC GTTACCGTGC AATCCGTATC GTCCCGTCCC AAAGCCGTTG   
  
  
- ATGGTAACCT AGGGTTCAAC TAGCTCTTTT ACTCCAAATT CCGGTTCCGT CTGGTGGTGT TCCTAAAGCA   
  
  
- CTAACTACAC CTACCTGTAA CCCGTTAAGT CCCTACCTTC CCCGCCTAAC GGACACGGTA GAGACGTACC   
  
  
- CAAGACCGAA C

+     CAAT-box

| Site Name | Organism | Position | Strand | Matrix score. | sequence | function |
| --- | --- | --- | --- | --- | --- | --- |
| CAAT-box | Nicotiana glutinosa | 4228 | + | 4 | CAAT |  |
| CAAT-box | Arabidopsis thaliana | 2505 | - | 5 | CCAAT | common cis-acting element in promoter and enhancer regions |
| CAAT-box | Nicotiana glutinosa | 2111 | + | 4 | CAAT |  |
| CAAT-box | Arabidopsis thaliana | 2110 | + | 5 | CCAAT | common cis-acting element in promoter and enhancer regions |
| CAAT-box | Arabidopsis thaliana | 4139 | - | 5 | CCAAT | common cis-acting element in promoter and enhancer regions |
| CAAT-box | Nicotiana glutinosa | 3690 | - | 4 | CAAT |  |
| CAAT-box | Nicotiana glutinosa | 3513 | + | 4 | CAAT |  |
| CAAT-box | Arabidopsis thaliana | 2869 | - | 5 | CCAAT | common cis-acting element in promoter and enhancer regions |
| CAAT-box | Nicotiana glutinosa | 4251 | - | 4 | CAAT |  |
| CAAT-box | Nicotiana glutinosa | 4206 | - | 4 | CAAT |  |
| CAAT-box | Nicotiana glutinosa | 3882 | - | 4 | CAAT |  |
| CAAT-box | Nicotiana glutinosa | 3805 | + | 4 | CAAT |  |
| CAAT-box | Pisum sativum | 3160 | - | 5 | CAAAT | common cis-acting element in promoter and enhancer regions |
| CAAT-box | Arabidopsis thaliana | 1916 | - | 5 | CCAAT | common cis-acting element in promoter and enhancer regions |
| CAAT-box | Petunia hybrida | 3971 | + | 7 | TGCCAAC | common cis-acting element in promoter and enhancer regions |
| CAAT-box | Pisum sativum | 2527 | - | 5 | CAAAT | common cis-acting element in promoter and enhancer regions |
| CAAT-box | Nicotiana glutinosa | 2591 | - | 4 | CAAT |  |
| CAAT-box | Arabidopsis thaliana | 1199 | - | 5 | CCAAT | common cis-acting element in promoter and enhancer regions |
| CAAT-box | Nicotiana glutinosa | 3011 | + | 4 | CAAT |  |
| CAAT-box | Arabidopsis thaliana | 3512 | + | 5 | CCAAT | common cis-acting element in promoter and enhancer regions |
| CAAT-box | Nicotiana glutinosa | 1541 | + | 4 | CAAT |  |
| CAAT-box | Nicotiana glutinosa | 1304 | + | 4 | CAAT |  |
| CAAT-box | Nicotiana glutinosa | 1331 | + | 4 | CAAT |  |
| CAAT-box | Arabidopsis thaliana | 921 | - | 5 | CCAAT | common cis-acting element in promoter and enhancer regions |
| CAAT-box | Nicotiana glutinosa | 1246 | + | 4 | CAAT |  |
| CAAT-box | Arabidopsis thaliana | 1147 | - | 5 | CCAAT | common cis-acting element in promoter and enhancer regions |
| CAAT-box | Nicotiana glutinosa | 2515 | + | 4 | CAAT |  |
| CAAT-box | Nicotiana glutinosa | 876 | + | 4 | CAAT |  |
| CAAT-box | Nicotiana glutinosa | 2604 | + | 4 | CAAT |  |
| CAAT-box | Nicotiana glutinosa | 2517 | - | 4 | CAAT |  |
| CAAT-box | Pisum sativum | 882 | + | 5 | CAAAT | common cis-acting element in promoter and enhancer regions |
| CAAT-box | Nicotiana glutinosa | 1449 | - | 4 | CAAT |  |
| CAAT-box | Arabidopsis thaliana | 4222 | - | 5 | CCAAT | common cis-acting element in promoter and enhancer regions |
| CAAT-box | Nicotiana glutinosa | 1014 | - | 4 | CAAT |  |
| CAAT-box | Pisum sativum | 587 | + | 5 | CAAAT | common cis-acting element in promoter and enhancer regions |
| CAAT-box | Pisum sativum | 1140 | - | 5 | CAAAT | common cis-acting element in promoter and enhancer regions |
| CAAT-box | Nicotiana glutinosa | 2419 | - | 4 | CAAT |  |
| CAAT-box | Nicotiana glutinosa | 438 | - | 4 | CAAT |  |
| CAAT-box | Nicotiana glutinosa | 413 | - | 4 | CAAT |  |
| CAAT-box | Arabidopsis thaliana | 893 | - | 5 | CCAAT | common cis-acting element in promoter and enhancer regions |
| CAAT-box | Pisum sativum | 2277 | + | 5 | CAAAT | common cis-acting element in promoter and enhancer regions |
| CAAT-box | Nicotiana glutinosa | 1811 | - | 4 | CAAT |  |
| CAAT-box | Nicotiana glutinosa | 3180 | + | 4 | CAAT |  |
| CAAT-box | Arabidopsis thaliana | 1872 | - | 5 | CCAAT | common cis-acting element in promoter and enhancer regions |
| CAAT-box | Nicotiana glutinosa | 952 | - | 4 | CAAT |  |
| CAAT-box | Nicotiana glutinosa | 543 | - | 4 | CAAT |  |
| CAAT-box | Nicotiana glutinosa | 1171 | - | 4 | CAAT |  |
| CAAT-box | Nicotiana glutinosa | 476 | - | 4 | CAAT |  |
| CAAT-box | Nicotiana glutinosa | 3709 | + | 4 | CAAT |  |
| CAAT-box | Nicotiana glutinosa | 186 | + | 4 | CAAT |  |
| CAAT-box | Nicotiana glutinosa | 2724 | + | 4 | CAAT |  |
| CAAT-box | Nicotiana glutinosa | 1396 | - | 4 | CAAT |  |
| CAAT-box | Pisum sativum | 1039 | + | 5 | CAAAT | common cis-acting element in promoter and enhancer regions |
| CAAT-box | Pisum sativum | 3674 | - | 5 | CAAAT | common cis-acting element in promoter and enhancer regions |
| CAAT-box | Nicotiana glutinosa | 1066 | - | 4 | CAAT |  |
| CAAT-box | Nicotiana glutinosa | 159 | + | 4 | CAAT |  |
| CAAT-box | Nicotiana glutinosa | 2412 | - | 4 | CAAT |  |
| CAAT-box | Pisum sativum | 1588 | - | 5 | CAAAT | common cis-acting element in promoter and enhancer regions |
| CAAT-box | Nicotiana glutinosa | 1138 | + | 4 | CAAT |  |
| CAAT-box | Nicotiana glutinosa | 4095 | + | 4 | CAAT |  |
| CAAT-box | Pisum sativum | 590 | - | 5 | CAAAT | common cis-acting element in promoter and enhancer regions |
| CAAT-box | Nicotiana glutinosa | 2784 | - | 4 | CAAT |  |
| CAAT-box | Nicotiana glutinosa | 131 | + | 4 | CAAT |  |
| CAAT-box | Nicotiana glutinosa | 2569 | + | 4 | CAAT |  |
| CAAT-box | Pisum sativum | 1237 | - | 5 | CAAAT | common cis-acting element in promoter and enhancer regions |
| CAAT-box | Pisum sativum | 1999 | - | 5 | CAAAT | common cis-acting element in promoter and enhancer regions |
| CAAT-box | Arabidopsis thaliana | 130 | + | 5 | CCAAT | common cis-acting element in promoter and enhancer regions |
| CAAT-box | Pisum sativum | 1259 | - | 5 | CAAAT | common cis-acting element in promoter and enhancer regions |
| CAAT-box | Arabidopsis thaliana | 3013 | - | 5 | CCAAT | common cis-acting element in promoter and enhancer regions |
| CAAT-box | Pisum sativum | 789 | - | 5 | CAAAT | common cis-acting element in promoter and enhancer regions |
| CAAT-box | Nicotiana glutinosa | 735 | - | 4 | CAAT |  |
| CAAT-box | Arabidopsis thaliana | 151 | + | 5 | CCAAT | common cis-acting element in promoter and enhancer regions |
| CAAT-box | Pisum sativum | 2030 | - | 5 | CAAAT | common cis-acting element in promoter and enhancer regions |
| CAAT-box | Pisum sativum | 1075 | - | 5 | CAAAT | common cis-acting element in promoter and enhancer regions |
| CAAT-box | Pisum sativum | 2113 | - | 5 | CAAAT | common cis-acting element in promoter and enhancer regions |
| CAAT-box | Nicotiana glutinosa | 1118 | + | 4 | CAAT |  |
| CAAT-box | Nicotiana glutinosa | 2120 | + | 4 | CAAT |  |
| CAAT-box | Arabidopsis thaliana | 2568 | + | 5 | CCAAT | common cis-acting element in promoter and enhancer regions |
| CAAT-box | Nicotiana glutinosa | 1628 | + | 4 | CAAT |  |
| CAAT-box | Nicotiana glutinosa | 152 | + | 4 | CAAT |  |
| CAAT-box | Nicotiana glutinosa | 2635 | - | 4 | CAAT |  |
| CAAT-box | Nicotiana glutinosa | 2571 | - | 4 | CAAT |  |
| CAAT-box | Nicotiana glutinosa | 627 | - | 4 | CAAT |  |
| CAAT-box | Pisum sativum | 1288 | + | 5 | CAAAT | common cis-acting element in promoter and enhancer regions |

>HU07G02248.1   
+ -Up\_Stream \_Len000CTGTTC AGCACTAGGG AATAAGGTTT TTGCCCTGCA TACTATGAAA TCTAACCAAA   
  
  
+ AACATTCCAT CTTGGAAGGC TGTAGCCTTG TGGATTTTGA ATTCGCTCCC AAGAACCAAT GAAGTAGCGC   
  
  
+ AGAAGGCCAA TACTCAATTT CAGAGTCCAC ATCAACTTGT TCAATGCCAG TATATTTCAG AACATTAATT   
  
  
+ ACATACTGTT TGGTTGGAGA AAAAATGGTT GTCTCGAAAA CTAGTTTTTT AATTTTTTGT TATTTAGCAC   
  
  
+ GGTAAAGAAT GGAAATTATT TTCATGCGAG AAAAAAGGAA GTTGGTTTTT CTTTTTTCAA CTCTTTTGAT   
  
  
+ GATATAATCA GTCGAAATTT AGGTGCACCA TCTGACTTCA ACTCTTGTAC GACCTCTGAT TGCTTGCTTA   
  
  
+ GAAGAACAAG AGGATTGACA GATAAGGAGG AGTGGGTGGT TTATACAGAG AATTGAGTGG ATGGTTATAA   
  
  
+ AGAAGTCGAT CTCACATAAG TAACTTCGTT ACGATTATAA ATATTTTCAT TGAAGAAAGA CGATTAGATA   
  
  
+ TAATTAAGAA GGTACATCTA AGCAAATTTG GCTGAGCAGA GTTGGAGGGC CACTAGGGTC ACATTGTGTC   
  
  
+ TTGCGCAACA AGCTTAGACC ATTCGCAACG GATGCCTCAC GTGGAGCAGG TGGGACCCGC GTGGAAGTTG   
  
  
+ CATGGCAAGA ATTCTCTGGC AGCAAGGAGA ATTGAGGGGG GACACGCGTG TGACCACGCC AGTGCGGTCC   
  
  
+ CCCTCTCGTG GCAGATTTGG TGATTTTTTT TTTGTTTTTT TTTGAAGTAA TATATAATTT TTATATGATT   
  
  
+ AAAAAAATAG GTTGTGTGTT ATAAAGTGGG TCAATGACAA ATATTTTTAT TGGATGATTA AAATTAAGTT   
  
  
+ CCATTTATTG GAGAAGTGAT GGAATGCAAG TTCCAAAATT GTGGAACTTT TGTTGACGTA GCAGGCTAGA   
  
  
+ GTTCCAAAAA TTAAAAAAAG GAAAGACATA TTGAGGTAAA GCCTTTTTAT TTTACAAATA GACGCACCAA   
  
  
+ GGTATAATAT AATTGACATG ATTTGAGGCG AGAGGAAGAT TCTTTTGCTA CCCAAACAAA ACACAATGAT   
  
  
+ CTTTTAAATG TAGCAATTTG GGATTGGCAA AATTTTGTCT AACCCGATTG TTGAAAGTTA TCCGATTTCA   
  
  
+ TAAAATTGGA TATCGTTATT AAATTTTCAT TCGAATATTT AAATTTGGAC ACAATGTTTT TCATATTTGG   
  
  
+ ATTTTCTAAA CTTGATTTTA ACTCAAATTT AAGTTTATTC AATTCATCTG ACCCAAAAAA TGGTTGCAAT   
  
  
+ AATGTATTTT CTTTTTGAAG TTAAATATTA TCGAATTCAT TTAACTTGTA GTTAACCCGT AATTGACCCA   
  
  
+ AACTCGACTT TTTGCTACCT TAGCAGCCAG GAAGAAGTCA TAGTATTGAC CCGTGGAACG CCTGAAGCCA   
  
  
+ ACATCACCAG ACCCACATAT TCCATCATCT TCTGATCTCC TCGTCAAAGA AGGAAGCTGA GCACAGCAAT   
  
  
+ AACTAGAAGA ACAGGAGGCA TCACACCCAA GCCAAAGGCC TTTATTTGAG CTCCCCACCT CATTCCTCGA   
  
  
+ AGCTTCCTCT TCACAATGTA AGACCATAAT CCTTCACCCC TTCTCTTTTT AAGCTCATCT TTACCTGCCC   
  
  
+ TAGTTCTCTC TCTCTCATGA GTCATCAGTA ATAGTTACCT CTTTACTTTC TCTTCCTTGA GGTATGCTTC   
  
  
+ AACTTTAATA TATATATGTA CCACTTGATT TCTCTTGTCA CTTTGCTATA ATACTTATTG TTTGGCTATC   
  
  
+ TTCCCCTGTT TTATACTCAT TTTCTTGCTT TCTATATCTG GGTTTGAATT GGGTTTCACT TATCCTCGTT   
  
  
+ GATTCTGTGG GAAATTAGTG AATTGGGTTT TGTACAGCAG GTTTATATGA ACTAGGGTTT CAAAGGTATC   
  
  
+ TACTTTTTCT CTTGTGATTA TTAGTTGATT AGGGATTTGG CCAAGATTAG CAGAATGGGT TCTGAATTTG   
  
  
+ GGGAATTCTC TGATGATGCT CTAAATGGGT ATGCTTACGT TGATATCCCT GCTTATGATG CGTCCTTAGA   
  
  
+ TTATGCCAAT TTGTTCAATT ACGAAGGCCT ATCTGAGGAT CTCACCTCAC TGAGCCTCCC AAGCCCCTTT   
  
  
+ TCTGACCCTT TGGCGTACAG TTTCACGTCC TCTTTGGGGC CGAGCCCTGG GGTTGATTCT AATGATGATA   
  
  
+ GTGATTCTGA TGATGTTCTC AAGTGCATTA GCCAAATGCT TATGGAAGAA GACATGGAGG CAAAGCCATG   
  
  
+ CATGTTTCAC GATCCTTTAG CACTTCAGGC TGCTGAGAAA CCCTTTTATG ATGCCTTAGG GAAGAAATAC   
  
  
+ CCAACTTCTC CGAACCAACA TCCTATAATT GATGATTGTT TGGATAATCC TGGTGAAAAT TCTTTGGGTT   
  
  
+ CTTCTAGTGA TTTTAGTGTT AGTCACTTTG GGTCTAGTTC AGCAAGCTCT ATTGGACCGA CAATTGTGTC   
  
  
+ TGATTTGAGT GAGCATTTTG AGCCACCCTT TGTTCAAGCA CTTCCAATTG AATCATATCC CCAACCATTG   
  
  
+ ACCCGTCCTC AATGGTCGTT TGGCCCTTCG GGTGCCTTAG ATTGCACGGC CTCTAATGGT TCAGTGATCT   
  
  
+ CATCCCTTGG TTTGCCGATG GATGTTATTA GTGTATTCAG TGAGAAAGAG TCCATGATTC AATTTCAGAA   
  
  
+ AGGGGTGGAA GAGGCTAGTA AGTTCCTTCC CAAGAATAAT AACCTTGTTA TTGATCTCAA GAACCTCACT   
  
  
+ TTTCCTAATG AAACGAAGGA GGATGATCGA GTGATGATGG TTAAGAAGGA AAAGAATGAT GTGAATTGGT   
  
  
+ CTAACTACTC AAGAGGGAGT AAGATTCACT ATCGTGAAGA CGAGGACTTT GAAGAAGGAA GGAGTAGCAA   
  
  
+ GCAGTCAGCT ATTTCTACTA CTGAGGAAGC TGAGTTGTGT GAAATTTTTG ACAAGTTTTT GCTTTGCAAT   
  
  
+ TGGTACCCTG TGAAACCTGA GGCTCATCCC ACCATGAGTT TGAACCCTGA GAAGGGCCAG TCACATGGAT   
  
  
+ TAGAAGGTGG GAGAAATGGG AAGGGTCACC CAAAGAAACA GGATAAAAGT AGTACCAACG TTGTGGATTT   
  
  
+ AAGGAATTTG CTGATGCTAT GTGCACAATC TACTGCATCT GATGACCGAA GAACTGCTGA TGGACTGCTA   
  
  
+ AAGAAAATCA GGGAGCACTC ATCTGCTGAG GGGGATGGAT TTCAAAGGTT GGCTCATTAC TTTGCTGATG   
  
  
+ CCCTAGAGGC ACGTTTAGCT GGAACTGGAT CTCACATTTA TACAGCCCTA AGTTCTCATA GGCCATCTGC   
  
  
+ TGTTGACGTG TTAAAAGCAT ATCAGTTCTA TGTTCGTGCT TGCCCATTTA AGAAGATCGT CATTCGTTGT   
  
  
+ GGTAAACATA TGATTCTAAA AGCTGCTGAA AAGGCATCAA AGCTTCATAT TATAGATTTT GGCATCCTCT   
  
  
+ ATGGATTCCA ATGGCCTAGC CTCATTCGAT GCCTCTCAGA GCGGTCTGGT GGACCTCCAA AACTGTTTAT   
  
  
+ TACAGGGATC GATCTCCCCC AGCCTGGGTT CAGGCCAGCA GAAAGAGTGG AAGCAACAGG GAGACGCTTG   
  
  
+ GCTAAGTACT GTGAGCGGTA TAATGTGCCA TTTGAGTATC ATGCCATTGC TCAGAAGTGG GAAACAATCA   
  
  
+ AACCAGGGGA TCTCAAGTTA GGAAGTAGGG ATGATGATGA AGTTGTCGTG GTGAACTGTC TCTGTAGGTT   
  
  
+ CAAGAACCTC CTTGACGAGA CAATGGTGGT GGATAGTCCA AGGAACACAG TTTTAAACCT GATTAGAAGG   
  
  
+ GTAAAACCCG ATATTTTTGT GCATGGCATT GTAAATGGTT CCTACAACAT CCCTTTCTTT GTGACACGTT   
  
  
+ TTAGAGAAGC CCTCTTTCAT TATTCCACTC TTTTCGACAT GTTAGATGCC AACGCCTCTA GGGAGGAGCC   
  
  
+ CGAGAGGTTG ATATTCGAGA AGGCATTCTA TGGGAGGGAG ATTATGAATG TGGTGGCATG TGAGGGCACA   
  
  
+ GAGAGGGTGG AAAGGCCAGA GACATACAAG CAATGGCACG TTAGGCATAG CAGGGCAGGG TTTCGGCAAC   
  
  
+ TACCATTGGA TCCCAAGTTG ATCGAGAAAA TGAGGTTTAA GGCCAAGGCA GACCACCACA AGGATTTCGT   
  
  
+ GATTGATGTG GATGGACATT GGGCAATTCA GGGATGGAAG GGGCGGATTG CCTGTGCCAT CTCTGCATGG   
  
  
+ GTTCTGGCTT G  

- -Up\_Stream \_Len000GACAAG TCGTGATCCC TTATTCCAAA AACGGGACGT ATGATACTTT AGATTGGTTT   
  
  
- TTGTAAGGTA GAACCTTCCG ACATCGGAAC ACCTAAAACT TAAGCGAGGG TTCTTGGTTA CTTCATCGCG   
  
  
- TCTTCCGGTT ATGAGTTAAA GTCTCAGGTG TAGTTGAACA AGTTACGGTC ATATAAAGTC TTGTAATTAA   
  
  
- TGTATGACAA ACCAACCTCT TTTTTACCAA CAGAGCTTTT GATCAAAAAA TTAAAAAACA ATAAATCGTG   
  
  
- CCATTTCTTA CCTTTAATAA AAGTACGCTC TTTTTTCCTT CAACCAAAAA GAAAAAAGTT GAGAAAACTA   
  
  
- CTATATTAGT CAGCTTTAAA TCCACGTGGT AGACTGAAGT TGAGAACATG CTGGAGACTA ACGAACGAAT   
  
  
- CTTCTTGTTC TCCTAACTGT CTATTCCTCC TCACCCACCA AATATGTCTC TTAACTCACC TACCAATATT   
  
  
- TCTTCAGCTA GAGTGTATTC ATTGAAGCAA TGCTAATATT TATAAAAGTA ACTTCTTTCT GCTAATCTAT   
  
  
- ATTAATTCTT CCATGTAGAT TCGTTTAAAC CGACTCGTCT CAACCTCCCG GTGATCCCAG TGTAACACAG   
  
  
- AACGCGTTGT TCGAATCTGG TAAGCGTTGC CTACGGAGTG CACCTCGTCC ACCCTGGGCG CACCTTCAAC   
  
  
- GTACCGTTCT TAAGAGACCG TCGTTCCTCT TAACTCCCCC CTGTGCGCAC ACTGGTGCGG TCACGCCAGG   
  
  
- GGGAGAGCAC CGTCTAAACC ACTAAAAAAA AAACAAAAAA AAACTTCATT ATATATTAAA AATATACTAA   
  
  
- TTTTTTTATC CAACACACAA TATTTCACCC AGTTACTGTT TATAAAAATA ACCTACTAAT TTTAATTCAA   
  
  
- GGTAAATAAC CTCTTCACTA CCTTACGTTC AAGGTTTTAA CACCTTGAAA ACAACTGCAT CGTCCGATCT   
  
  
- CAAGGTTTTT AATTTTTTTC CTTTCTGTAT AACTCCATTT CGGAAAAATA AAATGTTTAT CTGCGTGGTT   
  
  
- CCATATTATA TTAACTGTAC TAAACTCCGC TCTCCTTCTA AGAAAACGAT GGGTTTGTTT TGTGTTACTA   
  
  
- GAAAATTTAC ATCGTTAAAC CCTAACCGTT TTAAAACAGA TTGGGCTAAC AACTTTCAAT AGGCTAAAGT   
  
  
- ATTTTAACCT ATAGCAATAA TTTAAAAGTA AGCTTATAAA TTTAAACCTG TGTTACAAAA AGTATAAACC   
  
  
- TAAAAGATTT GAACTAAAAT TGAGTTTAAA TTCAAATAAG TTAAGTAGAC TGGGTTTTTT ACCAACGTTA   
  
  
- TTACATAAAA GAAAAACTTC AATTTATAAT AGCTTAAGTA AATTGAACAT CAATTGGGCA TTAACTGGGT   
  
  
- TTGAGCTGAA AAACGATGGA ATCGTCGGTC CTTCTTCAGT ATCATAACTG GGCACCTTGC GGACTTCGGT   
  
  
- TGTAGTGGTC TGGGTGTATA AGGTAGTAGA AGACTAGAGG AGCAGTTTCT TCCTTCGACT CGTGTCGTTA   
  
  
- TTGATCTTCT TGTCCTCCGT AGTGTGGGTT CGGTTTCCGG AAATAAACTC GAGGGGTGGA GTAAGGAGCT   
  
  
- TCGAAGGAGA AGTGTTACAT TCTGGTATTA GGAAGTGGGG AAGAGAAAAA TTCGAGTAGA AATGGACGGG   
  
  
- ATCAAGAGAG AGAGAGTACT CAGTAGTCAT TATCAATGGA GAAATGAAAG AGAAGGAACT CCATACGAAG   
  
  
- TTGAAATTAT ATATATACAT GGTGAACTAA AGAGAACAGT GAAACGATAT TATGAATAAC AAACCGATAG   
  
  
- AAGGGGACAA AATATGAGTA AAAGAACGAA AGATATAGAC CCAAACTTAA CCCAAAGTGA ATAGGAGCAA   
  
  
- CTAAGACACC CTTTAATCAC TTAACCCAAA ACATGTCGTC CAAATATACT TGATCCCAAA GTTTCCATAG   
  
  
- ATGAAAAAGA GAACACTAAT AATCAACTAA TCCCTAAACC GGTTCTAATC GTCTTACCCA AGACTTAAAC   
  
  
- CCCTTAAGAG ACTACTACGA GATTTACCCA TACGAATGCA ACTATAGGGA CGAATACTAC GCAGGAATCT   
  
  
- AATACGGTTA AACAAGTTAA TGCTTCCGGA TAGACTCCTA GAGTGGAGTG ACTCGGAGGG TTCGGGGAAA   
  
  
- AGACTGGGAA ACCGCATGTC AAAGTGCAGG AGAAACCCCG GCTCGGGACC CCAACTAAGA TTACTACTAT   
  
  
- CACTAAGACT ACTACAAGAG TTCACGTAAT CGGTTTACGA ATACCTTCTT CTGTACCTCC GTTTCGGTAC   
  
  
- GTACAAAGTG CTAGGAAATC GTGAAGTCCG ACGACTCTTT GGGAAAATAC TACGGAATCC CTTCTTTATG   
  
  
- GGTTGAAGAG GCTTGGTTGT AGGATATTAA CTACTAACAA ACCTATTAGG ACCACTTTTA AGAAACCCAA   
  
  
- GAAGATCACT AAAATCACAA TCAGTGAAAC CCAGATCAAG TCGTTCGAGA TAACCTGGCT GTTAACACAG   
  
  
- ACTAAACTCA CTCGTAAAAC TCGGTGGGAA ACAAGTTCGT GAAGGTTAAC TTAGTATAGG GGTTGGTAAC   
  
  
- TGGGCAGGAG TTACCAGCAA ACCGGGAAGC CCACGGAATC TAACGTGCCG GAGATTACCA AGTCACTAGA   
  
  
- GTAGGGAACC AAACGGCTAC CTACAATAAT CACATAAGTC ACTCTTTCTC AGGTACTAAG TTAAAGTCTT   
  
  
- TCCCCACCTT CTCCGATCAT TCAAGGAAGG GTTCTTATTA TTGGAACAAT AACTAGAGTT CTTGGAGTGA   
  
  
- AAAGGATTAC TTTGCTTCCT CCTACTAGCT CACTACTACC AATTCTTCCT TTTCTTACTA CACTTAACCA   
  
  
- GATTGATGAG TTCTCCCTCA TTCTAAGTGA TAGCACTTCT GCTCCTGAAA CTTCTTCCTT CCTCATCGTT   
  
  
- CGTCAGTCGA TAAAGATGAT GACTCCTTCG ACTCAACACA CTTTAAAAAC TGTTCAAAAA CGAAACGTTA   
  
  
- ACCATGGGAC ACTTTGGACT CCGAGTAGGG TGGTACTCAA ACTTGGGACT CTTCCCGGTC AGTGTACCTA   
  
  
- ATCTTCCACC CTCTTTACCC TTCCCAGTGG GTTTCTTTGT CCTATTTTCA TCATGGTTGC AACACCTAAA   
  
  
- TTCCTTAAAC GACTACGATA CACGTGTTAG ATGACGTAGA CTACTGGCTT CTTGACGACT ACCTGACGAT   
  
  
- TTCTTTTAGT CCCTCGTGAG TAGACGACTC CCCCTACCTA AAGTTTCCAA CCGAGTAATG AAACGACTAC   
  
  
- GGGATCTCCG TGCAAATCGA CCTTGACCTA GAGTGTAAAT ATGTCGGGAT TCAAGAGTAT CCGGTAGACG   
  
  
- ACAACTGCAC AATTTTCGTA TAGTCAAGAT ACAAGCACGA ACGGGTAAAT TCTTCTAGCA GTAAGCAACA   
  
  
- CCATTTGTAT ACTAAGATTT TCGACGACTT TTCCGTAGTT TCGAAGTATA ATATCTAAAA CCGTAGGAGA   
  
  
- TACCTAAGGT TACCGGATCG GAGTAAGCTA CGGAGAGTCT CGCCAGACCA CCTGGAGGTT TTGACAAATA   
  
  
- ATGTCCCTAG CTAGAGGGGG TCGGACCCAA GTCCGGTCGT CTTTCTCACC TTCGTTGTCC CTCTGCGAAC   
  
  
- CGATTCATGA CACTCGCCAT ATTACACGGT AAACTCATAG TACGGTAACG AGTCTTCACC CTTTGTTAGT   
  
  
- TTGGTCCCCT AGAGTTCAAT CCTTCATCCC TACTACTACT TCAACAGCAC CACTTGACAG AGACATCCAA   
  
  
- GTTCTTGGAG GAACTGCTCT GTTACCACCA CCTATCAGGT TCCTTGTGTC AAAATTTGGA CTAATCTTCC   
  
  
- CATTTTGGGC TATAAAAACA CGTACCGTAA CATTTACCAA GGATGTTGTA GGGAAAGAAA CACTGTGCAA   
  
  
- AATCTCTTCG GGAGAAAGTA ATAAGGTGAG AAAAGCTGTA CAATCTACGG TTGCGGAGAT CCCTCCTCGG   
  
  
- GCTCTCCAAC TATAAGCTCT TCCGTAAGAT ACCCTCCCTC TAATACTTAC ACCACCGTAC ACTCCCGTGT   
  
  
- CTCTCCCACC TTTCCGGTCT CTGTATGTTC GTTACCGTGC AATCCGTATC GTCCCGTCCC AAAGCCGTTG   
  
  
- ATGGTAACCT AGGGTTCAAC TAGCTCTTTT ACTCCAAATT CCGGTTCCGT CTGGTGGTGT TCCTAAAGCA   
  
  
- CTAACTACAC CTACCTGTAA CCCGTTAAGT CCCTACCTTC CCCGCCTAAC GGACACGGTA GAGACGTACC   
  
  
- CAAGACCGAA C

+     CAT-box

| Site Name | Organism | Position | Strand | Matrix score. | sequence | function |
| --- | --- | --- | --- | --- | --- | --- |
| CAT-box | Arabidopsis thaliana | 613 | + | 6 | GCCACT | cis-acting regulatory element related to meristem expression |

>HU07G02248.1   
+ -Up\_Stream \_Len000CTGTTC AGCACTAGGG AATAAGGTTT TTGCCCTGCA TACTATGAAA TCTAACCAAA   
  
  
+ AACATTCCAT CTTGGAAGGC TGTAGCCTTG TGGATTTTGA ATTCGCTCCC AAGAACCAAT GAAGTAGCGC   
  
  
+ AGAAGGCCAA TACTCAATTT CAGAGTCCAC ATCAACTTGT TCAATGCCAG TATATTTCAG AACATTAATT   
  
  
+ ACATACTGTT TGGTTGGAGA AAAAATGGTT GTCTCGAAAA CTAGTTTTTT AATTTTTTGT TATTTAGCAC   
  
  
+ GGTAAAGAAT GGAAATTATT TTCATGCGAG AAAAAAGGAA GTTGGTTTTT CTTTTTTCAA CTCTTTTGAT   
  
  
+ GATATAATCA GTCGAAATTT AGGTGCACCA TCTGACTTCA ACTCTTGTAC GACCTCTGAT TGCTTGCTTA   
  
  
+ GAAGAACAAG AGGATTGACA GATAAGGAGG AGTGGGTGGT TTATACAGAG AATTGAGTGG ATGGTTATAA   
  
  
+ AGAAGTCGAT CTCACATAAG TAACTTCGTT ACGATTATAA ATATTTTCAT TGAAGAAAGA CGATTAGATA   
  
  
+ TAATTAAGAA GGTACATCTA AGCAAATTTG GCTGAGCAGA GTTGGAGGGC CACTAGGGTC ACATTGTGTC   
  
  
+ TTGCGCAACA AGCTTAGACC ATTCGCAACG GATGCCTCAC GTGGAGCAGG TGGGACCCGC GTGGAAGTTG   
  
  
+ CATGGCAAGA ATTCTCTGGC AGCAAGGAGA ATTGAGGGGG GACACGCGTG TGACCACGCC AGTGCGGTCC   
  
  
+ CCCTCTCGTG GCAGATTTGG TGATTTTTTT TTTGTTTTTT TTTGAAGTAA TATATAATTT TTATATGATT   
  
  
+ AAAAAAATAG GTTGTGTGTT ATAAAGTGGG TCAATGACAA ATATTTTTAT TGGATGATTA AAATTAAGTT   
  
  
+ CCATTTATTG GAGAAGTGAT GGAATGCAAG TTCCAAAATT GTGGAACTTT TGTTGACGTA GCAGGCTAGA   
  
  
+ GTTCCAAAAA TTAAAAAAAG GAAAGACATA TTGAGGTAAA GCCTTTTTAT TTTACAAATA GACGCACCAA   
  
  
+ GGTATAATAT AATTGACATG ATTTGAGGCG AGAGGAAGAT TCTTTTGCTA CCCAAACAAA ACACAATGAT   
  
  
+ CTTTTAAATG TAGCAATTTG GGATTGGCAA AATTTTGTCT AACCCGATTG TTGAAAGTTA TCCGATTTCA   
  
  
+ TAAAATTGGA TATCGTTATT AAATTTTCAT TCGAATATTT AAATTTGGAC ACAATGTTTT TCATATTTGG   
  
  
+ ATTTTCTAAA CTTGATTTTA ACTCAAATTT AAGTTTATTC AATTCATCTG ACCCAAAAAA TGGTTGCAAT   
  
  
+ AATGTATTTT CTTTTTGAAG TTAAATATTA TCGAATTCAT TTAACTTGTA GTTAACCCGT AATTGACCCA   
  
  
+ AACTCGACTT TTTGCTACCT TAGCAGCCAG GAAGAAGTCA TAGTATTGAC CCGTGGAACG CCTGAAGCCA   
  
  
+ ACATCACCAG ACCCACATAT TCCATCATCT TCTGATCTCC TCGTCAAAGA AGGAAGCTGA GCACAGCAAT   
  
  
+ AACTAGAAGA ACAGGAGGCA TCACACCCAA GCCAAAGGCC TTTATTTGAG CTCCCCACCT CATTCCTCGA   
  
  
+ AGCTTCCTCT TCACAATGTA AGACCATAAT CCTTCACCCC TTCTCTTTTT AAGCTCATCT TTACCTGCCC   
  
  
+ TAGTTCTCTC TCTCTCATGA GTCATCAGTA ATAGTTACCT CTTTACTTTC TCTTCCTTGA GGTATGCTTC   
  
  
+ AACTTTAATA TATATATGTA CCACTTGATT TCTCTTGTCA CTTTGCTATA ATACTTATTG TTTGGCTATC   
  
  
+ TTCCCCTGTT TTATACTCAT TTTCTTGCTT TCTATATCTG GGTTTGAATT GGGTTTCACT TATCCTCGTT   
  
  
+ GATTCTGTGG GAAATTAGTG AATTGGGTTT TGTACAGCAG GTTTATATGA ACTAGGGTTT CAAAGGTATC   
  
  
+ TACTTTTTCT CTTGTGATTA TTAGTTGATT AGGGATTTGG CCAAGATTAG CAGAATGGGT TCTGAATTTG   
  
  
+ GGGAATTCTC TGATGATGCT CTAAATGGGT ATGCTTACGT TGATATCCCT GCTTATGATG CGTCCTTAGA   
  
  
+ TTATGCCAAT TTGTTCAATT ACGAAGGCCT ATCTGAGGAT CTCACCTCAC TGAGCCTCCC AAGCCCCTTT   
  
  
+ TCTGACCCTT TGGCGTACAG TTTCACGTCC TCTTTGGGGC CGAGCCCTGG GGTTGATTCT AATGATGATA   
  
  
+ GTGATTCTGA TGATGTTCTC AAGTGCATTA GCCAAATGCT TATGGAAGAA GACATGGAGG CAAAGCCATG   
  
  
+ CATGTTTCAC GATCCTTTAG CACTTCAGGC TGCTGAGAAA CCCTTTTATG ATGCCTTAGG GAAGAAATAC   
  
  
+ CCAACTTCTC CGAACCAACA TCCTATAATT GATGATTGTT TGGATAATCC TGGTGAAAAT TCTTTGGGTT   
  
  
+ CTTCTAGTGA TTTTAGTGTT AGTCACTTTG GGTCTAGTTC AGCAAGCTCT ATTGGACCGA CAATTGTGTC   
  
  
+ TGATTTGAGT GAGCATTTTG AGCCACCCTT TGTTCAAGCA CTTCCAATTG AATCATATCC CCAACCATTG   
  
  
+ ACCCGTCCTC AATGGTCGTT TGGCCCTTCG GGTGCCTTAG ATTGCACGGC CTCTAATGGT TCAGTGATCT   
  
  
+ CATCCCTTGG TTTGCCGATG GATGTTATTA GTGTATTCAG TGAGAAAGAG TCCATGATTC AATTTCAGAA   
  
  
+ AGGGGTGGAA GAGGCTAGTA AGTTCCTTCC CAAGAATAAT AACCTTGTTA TTGATCTCAA GAACCTCACT   
  
  
+ TTTCCTAATG AAACGAAGGA GGATGATCGA GTGATGATGG TTAAGAAGGA AAAGAATGAT GTGAATTGGT   
  
  
+ CTAACTACTC AAGAGGGAGT AAGATTCACT ATCGTGAAGA CGAGGACTTT GAAGAAGGAA GGAGTAGCAA   
  
  
+ GCAGTCAGCT ATTTCTACTA CTGAGGAAGC TGAGTTGTGT GAAATTTTTG ACAAGTTTTT GCTTTGCAAT   
  
  
+ TGGTACCCTG TGAAACCTGA GGCTCATCCC ACCATGAGTT TGAACCCTGA GAAGGGCCAG TCACATGGAT   
  
  
+ TAGAAGGTGG GAGAAATGGG AAGGGTCACC CAAAGAAACA GGATAAAAGT AGTACCAACG TTGTGGATTT   
  
  
+ AAGGAATTTG CTGATGCTAT GTGCACAATC TACTGCATCT GATGACCGAA GAACTGCTGA TGGACTGCTA   
  
  
+ AAGAAAATCA GGGAGCACTC ATCTGCTGAG GGGGATGGAT TTCAAAGGTT GGCTCATTAC TTTGCTGATG   
  
  
+ CCCTAGAGGC ACGTTTAGCT GGAACTGGAT CTCACATTTA TACAGCCCTA AGTTCTCATA GGCCATCTGC   
  
  
+ TGTTGACGTG TTAAAAGCAT ATCAGTTCTA TGTTCGTGCT TGCCCATTTA AGAAGATCGT CATTCGTTGT   
  
  
+ GGTAAACATA TGATTCTAAA AGCTGCTGAA AAGGCATCAA AGCTTCATAT TATAGATTTT GGCATCCTCT   
  
  
+ ATGGATTCCA ATGGCCTAGC CTCATTCGAT GCCTCTCAGA GCGGTCTGGT GGACCTCCAA AACTGTTTAT   
  
  
+ TACAGGGATC GATCTCCCCC AGCCTGGGTT CAGGCCAGCA GAAAGAGTGG AAGCAACAGG GAGACGCTTG   
  
  
+ GCTAAGTACT GTGAGCGGTA TAATGTGCCA TTTGAGTATC ATGCCATTGC TCAGAAGTGG GAAACAATCA   
  
  
+ AACCAGGGGA TCTCAAGTTA GGAAGTAGGG ATGATGATGA AGTTGTCGTG GTGAACTGTC TCTGTAGGTT   
  
  
+ CAAGAACCTC CTTGACGAGA CAATGGTGGT GGATAGTCCA AGGAACACAG TTTTAAACCT GATTAGAAGG   
  
  
+ GTAAAACCCG ATATTTTTGT GCATGGCATT GTAAATGGTT CCTACAACAT CCCTTTCTTT GTGACACGTT   
  
  
+ TTAGAGAAGC CCTCTTTCAT TATTCCACTC TTTTCGACAT GTTAGATGCC AACGCCTCTA GGGAGGAGCC   
  
  
+ CGAGAGGTTG ATATTCGAGA AGGCATTCTA TGGGAGGGAG ATTATGAATG TGGTGGCATG TGAGGGCACA   
  
  
+ GAGAGGGTGG AAAGGCCAGA GACATACAAG CAATGGCACG TTAGGCATAG CAGGGCAGGG TTTCGGCAAC   
  
  
+ TACCATTGGA TCCCAAGTTG ATCGAGAAAA TGAGGTTTAA GGCCAAGGCA GACCACCACA AGGATTTCGT   
  
  
+ GATTGATGTG GATGGACATT GGGCAATTCA GGGATGGAAG GGGCGGATTG CCTGTGCCAT CTCTGCATGG   
  
  
+ GTTCTGGCTT G  

- -Up\_Stream \_Len000GACAAG TCGTGATCCC TTATTCCAAA AACGGGACGT ATGATACTTT AGATTGGTTT   
  
  
- TTGTAAGGTA GAACCTTCCG ACATCGGAAC ACCTAAAACT TAAGCGAGGG TTCTTGGTTA CTTCATCGCG   
  
  
- TCTTCCGGTT ATGAGTTAAA GTCTCAGGTG TAGTTGAACA AGTTACGGTC ATATAAAGTC TTGTAATTAA   
  
  
- TGTATGACAA ACCAACCTCT TTTTTACCAA CAGAGCTTTT GATCAAAAAA TTAAAAAACA ATAAATCGTG   
  
  
- CCATTTCTTA CCTTTAATAA AAGTACGCTC TTTTTTCCTT CAACCAAAAA GAAAAAAGTT GAGAAAACTA   
  
  
- CTATATTAGT CAGCTTTAAA TCCACGTGGT AGACTGAAGT TGAGAACATG CTGGAGACTA ACGAACGAAT   
  
  
- CTTCTTGTTC TCCTAACTGT CTATTCCTCC TCACCCACCA AATATGTCTC TTAACTCACC TACCAATATT   
  
  
- TCTTCAGCTA GAGTGTATTC ATTGAAGCAA TGCTAATATT TATAAAAGTA ACTTCTTTCT GCTAATCTAT   
  
  
- ATTAATTCTT CCATGTAGAT TCGTTTAAAC CGACTCGTCT CAACCTCCCG GTGATCCCAG TGTAACACAG   
  
  
- AACGCGTTGT TCGAATCTGG TAAGCGTTGC CTACGGAGTG CACCTCGTCC ACCCTGGGCG CACCTTCAAC   
  
  
- GTACCGTTCT TAAGAGACCG TCGTTCCTCT TAACTCCCCC CTGTGCGCAC ACTGGTGCGG TCACGCCAGG   
  
  
- GGGAGAGCAC CGTCTAAACC ACTAAAAAAA AAACAAAAAA AAACTTCATT ATATATTAAA AATATACTAA   
  
  
- TTTTTTTATC CAACACACAA TATTTCACCC AGTTACTGTT TATAAAAATA ACCTACTAAT TTTAATTCAA   
  
  
- GGTAAATAAC CTCTTCACTA CCTTACGTTC AAGGTTTTAA CACCTTGAAA ACAACTGCAT CGTCCGATCT   
  
  
- CAAGGTTTTT AATTTTTTTC CTTTCTGTAT AACTCCATTT CGGAAAAATA AAATGTTTAT CTGCGTGGTT   
  
  
- CCATATTATA TTAACTGTAC TAAACTCCGC TCTCCTTCTA AGAAAACGAT GGGTTTGTTT TGTGTTACTA   
  
  
- GAAAATTTAC ATCGTTAAAC CCTAACCGTT TTAAAACAGA TTGGGCTAAC AACTTTCAAT AGGCTAAAGT   
  
  
- ATTTTAACCT ATAGCAATAA TTTAAAAGTA AGCTTATAAA TTTAAACCTG TGTTACAAAA AGTATAAACC   
  
  
- TAAAAGATTT GAACTAAAAT TGAGTTTAAA TTCAAATAAG TTAAGTAGAC TGGGTTTTTT ACCAACGTTA   
  
  
- TTACATAAAA GAAAAACTTC AATTTATAAT AGCTTAAGTA AATTGAACAT CAATTGGGCA TTAACTGGGT   
  
  
- TTGAGCTGAA AAACGATGGA ATCGTCGGTC CTTCTTCAGT ATCATAACTG GGCACCTTGC GGACTTCGGT   
  
  
- TGTAGTGGTC TGGGTGTATA AGGTAGTAGA AGACTAGAGG AGCAGTTTCT TCCTTCGACT CGTGTCGTTA   
  
  
- TTGATCTTCT TGTCCTCCGT AGTGTGGGTT CGGTTTCCGG AAATAAACTC GAGGGGTGGA GTAAGGAGCT   
  
  
- TCGAAGGAGA AGTGTTACAT TCTGGTATTA GGAAGTGGGG AAGAGAAAAA TTCGAGTAGA AATGGACGGG   
  
  
- ATCAAGAGAG AGAGAGTACT CAGTAGTCAT TATCAATGGA GAAATGAAAG AGAAGGAACT CCATACGAAG   
  
  
- TTGAAATTAT ATATATACAT GGTGAACTAA AGAGAACAGT GAAACGATAT TATGAATAAC AAACCGATAG   
  
  
- AAGGGGACAA AATATGAGTA AAAGAACGAA AGATATAGAC CCAAACTTAA CCCAAAGTGA ATAGGAGCAA   
  
  
- CTAAGACACC CTTTAATCAC TTAACCCAAA ACATGTCGTC CAAATATACT TGATCCCAAA GTTTCCATAG   
  
  
- ATGAAAAAGA GAACACTAAT AATCAACTAA TCCCTAAACC GGTTCTAATC GTCTTACCCA AGACTTAAAC   
  
  
- CCCTTAAGAG ACTACTACGA GATTTACCCA TACGAATGCA ACTATAGGGA CGAATACTAC GCAGGAATCT   
  
  
- AATACGGTTA AACAAGTTAA TGCTTCCGGA TAGACTCCTA GAGTGGAGTG ACTCGGAGGG TTCGGGGAAA   
  
  
- AGACTGGGAA ACCGCATGTC AAAGTGCAGG AGAAACCCCG GCTCGGGACC CCAACTAAGA TTACTACTAT   
  
  
- CACTAAGACT ACTACAAGAG TTCACGTAAT CGGTTTACGA ATACCTTCTT CTGTACCTCC GTTTCGGTAC   
  
  
- GTACAAAGTG CTAGGAAATC GTGAAGTCCG ACGACTCTTT GGGAAAATAC TACGGAATCC CTTCTTTATG   
  
  
- GGTTGAAGAG GCTTGGTTGT AGGATATTAA CTACTAACAA ACCTATTAGG ACCACTTTTA AGAAACCCAA   
  
  
- GAAGATCACT AAAATCACAA TCAGTGAAAC CCAGATCAAG TCGTTCGAGA TAACCTGGCT GTTAACACAG   
  
  
- ACTAAACTCA CTCGTAAAAC TCGGTGGGAA ACAAGTTCGT GAAGGTTAAC TTAGTATAGG GGTTGGTAAC   
  
  
- TGGGCAGGAG TTACCAGCAA ACCGGGAAGC CCACGGAATC TAACGTGCCG GAGATTACCA AGTCACTAGA   
  
  
- GTAGGGAACC AAACGGCTAC CTACAATAAT CACATAAGTC ACTCTTTCTC AGGTACTAAG TTAAAGTCTT   
  
  
- TCCCCACCTT CTCCGATCAT TCAAGGAAGG GTTCTTATTA TTGGAACAAT AACTAGAGTT CTTGGAGTGA   
  
  
- AAAGGATTAC TTTGCTTCCT CCTACTAGCT CACTACTACC AATTCTTCCT TTTCTTACTA CACTTAACCA   
  
  
- GATTGATGAG TTCTCCCTCA TTCTAAGTGA TAGCACTTCT GCTCCTGAAA CTTCTTCCTT CCTCATCGTT   
  
  
- CGTCAGTCGA TAAAGATGAT GACTCCTTCG ACTCAACACA CTTTAAAAAC TGTTCAAAAA CGAAACGTTA   
  
  
- ACCATGGGAC ACTTTGGACT CCGAGTAGGG TGGTACTCAA ACTTGGGACT CTTCCCGGTC AGTGTACCTA   
  
  
- ATCTTCCACC CTCTTTACCC TTCCCAGTGG GTTTCTTTGT CCTATTTTCA TCATGGTTGC AACACCTAAA   
  
  
- TTCCTTAAAC GACTACGATA CACGTGTTAG ATGACGTAGA CTACTGGCTT CTTGACGACT ACCTGACGAT   
  
  
- TTCTTTTAGT CCCTCGTGAG TAGACGACTC CCCCTACCTA AAGTTTCCAA CCGAGTAATG AAACGACTAC   
  
  
- GGGATCTCCG TGCAAATCGA CCTTGACCTA GAGTGTAAAT ATGTCGGGAT TCAAGAGTAT CCGGTAGACG   
  
  
- ACAACTGCAC AATTTTCGTA TAGTCAAGAT ACAAGCACGA ACGGGTAAAT TCTTCTAGCA GTAAGCAACA   
  
  
- CCATTTGTAT ACTAAGATTT TCGACGACTT TTCCGTAGTT TCGAAGTATA ATATCTAAAA CCGTAGGAGA   
  
  
- TACCTAAGGT TACCGGATCG GAGTAAGCTA CGGAGAGTCT CGCCAGACCA CCTGGAGGTT TTGACAAATA   
  
  
- ATGTCCCTAG CTAGAGGGGG TCGGACCCAA GTCCGGTCGT CTTTCTCACC TTCGTTGTCC CTCTGCGAAC   
  
  
- CGATTCATGA CACTCGCCAT ATTACACGGT AAACTCATAG TACGGTAACG AGTCTTCACC CTTTGTTAGT   
  
  
- TTGGTCCCCT AGAGTTCAAT CCTTCATCCC TACTACTACT TCAACAGCAC CACTTGACAG AGACATCCAA   
  
  
- GTTCTTGGAG GAACTGCTCT GTTACCACCA CCTATCAGGT TCCTTGTGTC AAAATTTGGA CTAATCTTCC   
  
  
- CATTTTGGGC TATAAAAACA CGTACCGTAA CATTTACCAA GGATGTTGTA GGGAAAGAAA CACTGTGCAA   
  
  
- AATCTCTTCG GGAGAAAGTA ATAAGGTGAG AAAAGCTGTA CAATCTACGG TTGCGGAGAT CCCTCCTCGG   
  
  
- GCTCTCCAAC TATAAGCTCT TCCGTAAGAT ACCCTCCCTC TAATACTTAC ACCACCGTAC ACTCCCGTGT   
  
  
- CTCTCCCACC TTTCCGGTCT CTGTATGTTC GTTACCGTGC AATCCGTATC GTCCCGTCCC AAAGCCGTTG   
  
  
- ATGGTAACCT AGGGTTCAAC TAGCTCTTTT ACTCCAAATT CCGGTTCCGT CTGGTGGTGT TCCTAAAGCA   
  
  
- CTAACTACAC CTACCTGTAA CCCGTTAAGT CCCTACCTTC CCCGCCTAAC GGACACGGTA GAGACGTACC   
  
  
- CAAGACCGAA C

+     CCAAT-box

| Site Name | Organism | Position | Strand | Matrix score. | sequence | function |
| --- | --- | --- | --- | --- | --- | --- |
| CCAAT-box | Hordeum vulgare | 660 | + | 6 | CAACGG | MYBHv1 binding site |

>HU07G02248.1   
+ -Up\_Stream \_Len000CTGTTC AGCACTAGGG AATAAGGTTT TTGCCCTGCA TACTATGAAA TCTAACCAAA   
  
  
+ AACATTCCAT CTTGGAAGGC TGTAGCCTTG TGGATTTTGA ATTCGCTCCC AAGAACCAAT GAAGTAGCGC   
  
  
+ AGAAGGCCAA TACTCAATTT CAGAGTCCAC ATCAACTTGT TCAATGCCAG TATATTTCAG AACATTAATT   
  
  
+ ACATACTGTT TGGTTGGAGA AAAAATGGTT GTCTCGAAAA CTAGTTTTTT AATTTTTTGT TATTTAGCAC   
  
  
+ GGTAAAGAAT GGAAATTATT TTCATGCGAG AAAAAAGGAA GTTGGTTTTT CTTTTTTCAA CTCTTTTGAT   
  
  
+ GATATAATCA GTCGAAATTT AGGTGCACCA TCTGACTTCA ACTCTTGTAC GACCTCTGAT TGCTTGCTTA   
  
  
+ GAAGAACAAG AGGATTGACA GATAAGGAGG AGTGGGTGGT TTATACAGAG AATTGAGTGG ATGGTTATAA   
  
  
+ AGAAGTCGAT CTCACATAAG TAACTTCGTT ACGATTATAA ATATTTTCAT TGAAGAAAGA CGATTAGATA   
  
  
+ TAATTAAGAA GGTACATCTA AGCAAATTTG GCTGAGCAGA GTTGGAGGGC CACTAGGGTC ACATTGTGTC   
  
  
+ TTGCGCAACA AGCTTAGACC ATTCGCAACG GATGCCTCAC GTGGAGCAGG TGGGACCCGC GTGGAAGTTG   
  
  
+ CATGGCAAGA ATTCTCTGGC AGCAAGGAGA ATTGAGGGGG GACACGCGTG TGACCACGCC AGTGCGGTCC   
  
  
+ CCCTCTCGTG GCAGATTTGG TGATTTTTTT TTTGTTTTTT TTTGAAGTAA TATATAATTT TTATATGATT   
  
  
+ AAAAAAATAG GTTGTGTGTT ATAAAGTGGG TCAATGACAA ATATTTTTAT TGGATGATTA AAATTAAGTT   
  
  
+ CCATTTATTG GAGAAGTGAT GGAATGCAAG TTCCAAAATT GTGGAACTTT TGTTGACGTA GCAGGCTAGA   
  
  
+ GTTCCAAAAA TTAAAAAAAG GAAAGACATA TTGAGGTAAA GCCTTTTTAT TTTACAAATA GACGCACCAA   
  
  
+ GGTATAATAT AATTGACATG ATTTGAGGCG AGAGGAAGAT TCTTTTGCTA CCCAAACAAA ACACAATGAT   
  
  
+ CTTTTAAATG TAGCAATTTG GGATTGGCAA AATTTTGTCT AACCCGATTG TTGAAAGTTA TCCGATTTCA   
  
  
+ TAAAATTGGA TATCGTTATT AAATTTTCAT TCGAATATTT AAATTTGGAC ACAATGTTTT TCATATTTGG   
  
  
+ ATTTTCTAAA CTTGATTTTA ACTCAAATTT AAGTTTATTC AATTCATCTG ACCCAAAAAA TGGTTGCAAT   
  
  
+ AATGTATTTT CTTTTTGAAG TTAAATATTA TCGAATTCAT TTAACTTGTA GTTAACCCGT AATTGACCCA   
  
  
+ AACTCGACTT TTTGCTACCT TAGCAGCCAG GAAGAAGTCA TAGTATTGAC CCGTGGAACG CCTGAAGCCA   
  
  
+ ACATCACCAG ACCCACATAT TCCATCATCT TCTGATCTCC TCGTCAAAGA AGGAAGCTGA GCACAGCAAT   
  
  
+ AACTAGAAGA ACAGGAGGCA TCACACCCAA GCCAAAGGCC TTTATTTGAG CTCCCCACCT CATTCCTCGA   
  
  
+ AGCTTCCTCT TCACAATGTA AGACCATAAT CCTTCACCCC TTCTCTTTTT AAGCTCATCT TTACCTGCCC   
  
  
+ TAGTTCTCTC TCTCTCATGA GTCATCAGTA ATAGTTACCT CTTTACTTTC TCTTCCTTGA GGTATGCTTC   
  
  
+ AACTTTAATA TATATATGTA CCACTTGATT TCTCTTGTCA CTTTGCTATA ATACTTATTG TTTGGCTATC   
  
  
+ TTCCCCTGTT TTATACTCAT TTTCTTGCTT TCTATATCTG GGTTTGAATT GGGTTTCACT TATCCTCGTT   
  
  
+ GATTCTGTGG GAAATTAGTG AATTGGGTTT TGTACAGCAG GTTTATATGA ACTAGGGTTT CAAAGGTATC   
  
  
+ TACTTTTTCT CTTGTGATTA TTAGTTGATT AGGGATTTGG CCAAGATTAG CAGAATGGGT TCTGAATTTG   
  
  
+ GGGAATTCTC TGATGATGCT CTAAATGGGT ATGCTTACGT TGATATCCCT GCTTATGATG CGTCCTTAGA   
  
  
+ TTATGCCAAT TTGTTCAATT ACGAAGGCCT ATCTGAGGAT CTCACCTCAC TGAGCCTCCC AAGCCCCTTT   
  
  
+ TCTGACCCTT TGGCGTACAG TTTCACGTCC TCTTTGGGGC CGAGCCCTGG GGTTGATTCT AATGATGATA   
  
  
+ GTGATTCTGA TGATGTTCTC AAGTGCATTA GCCAAATGCT TATGGAAGAA GACATGGAGG CAAAGCCATG   
  
  
+ CATGTTTCAC GATCCTTTAG CACTTCAGGC TGCTGAGAAA CCCTTTTATG ATGCCTTAGG GAAGAAATAC   
  
  
+ CCAACTTCTC CGAACCAACA TCCTATAATT GATGATTGTT TGGATAATCC TGGTGAAAAT TCTTTGGGTT   
  
  
+ CTTCTAGTGA TTTTAGTGTT AGTCACTTTG GGTCTAGTTC AGCAAGCTCT ATTGGACCGA CAATTGTGTC   
  
  
+ TGATTTGAGT GAGCATTTTG AGCCACCCTT TGTTCAAGCA CTTCCAATTG AATCATATCC CCAACCATTG   
  
  
+ ACCCGTCCTC AATGGTCGTT TGGCCCTTCG GGTGCCTTAG ATTGCACGGC CTCTAATGGT TCAGTGATCT   
  
  
+ CATCCCTTGG TTTGCCGATG GATGTTATTA GTGTATTCAG TGAGAAAGAG TCCATGATTC AATTTCAGAA   
  
  
+ AGGGGTGGAA GAGGCTAGTA AGTTCCTTCC CAAGAATAAT AACCTTGTTA TTGATCTCAA GAACCTCACT   
  
  
+ TTTCCTAATG AAACGAAGGA GGATGATCGA GTGATGATGG TTAAGAAGGA AAAGAATGAT GTGAATTGGT   
  
  
+ CTAACTACTC AAGAGGGAGT AAGATTCACT ATCGTGAAGA CGAGGACTTT GAAGAAGGAA GGAGTAGCAA   
  
  
+ GCAGTCAGCT ATTTCTACTA CTGAGGAAGC TGAGTTGTGT GAAATTTTTG ACAAGTTTTT GCTTTGCAAT   
  
  
+ TGGTACCCTG TGAAACCTGA GGCTCATCCC ACCATGAGTT TGAACCCTGA GAAGGGCCAG TCACATGGAT   
  
  
+ TAGAAGGTGG GAGAAATGGG AAGGGTCACC CAAAGAAACA GGATAAAAGT AGTACCAACG TTGTGGATTT   
  
  
+ AAGGAATTTG CTGATGCTAT GTGCACAATC TACTGCATCT GATGACCGAA GAACTGCTGA TGGACTGCTA   
  
  
+ AAGAAAATCA GGGAGCACTC ATCTGCTGAG GGGGATGGAT TTCAAAGGTT GGCTCATTAC TTTGCTGATG   
  
  
+ CCCTAGAGGC ACGTTTAGCT GGAACTGGAT CTCACATTTA TACAGCCCTA AGTTCTCATA GGCCATCTGC   
  
  
+ TGTTGACGTG TTAAAAGCAT ATCAGTTCTA TGTTCGTGCT TGCCCATTTA AGAAGATCGT CATTCGTTGT   
  
  
+ GGTAAACATA TGATTCTAAA AGCTGCTGAA AAGGCATCAA AGCTTCATAT TATAGATTTT GGCATCCTCT   
  
  
+ ATGGATTCCA ATGGCCTAGC CTCATTCGAT GCCTCTCAGA GCGGTCTGGT GGACCTCCAA AACTGTTTAT   
  
  
+ TACAGGGATC GATCTCCCCC AGCCTGGGTT CAGGCCAGCA GAAAGAGTGG AAGCAACAGG GAGACGCTTG   
  
  
+ GCTAAGTACT GTGAGCGGTA TAATGTGCCA TTTGAGTATC ATGCCATTGC TCAGAAGTGG GAAACAATCA   
  
  
+ AACCAGGGGA TCTCAAGTTA GGAAGTAGGG ATGATGATGA AGTTGTCGTG GTGAACTGTC TCTGTAGGTT   
  
  
+ CAAGAACCTC CTTGACGAGA CAATGGTGGT GGATAGTCCA AGGAACACAG TTTTAAACCT GATTAGAAGG   
  
  
+ GTAAAACCCG ATATTTTTGT GCATGGCATT GTAAATGGTT CCTACAACAT CCCTTTCTTT GTGACACGTT   
  
  
+ TTAGAGAAGC CCTCTTTCAT TATTCCACTC TTTTCGACAT GTTAGATGCC AACGCCTCTA GGGAGGAGCC   
  
  
+ CGAGAGGTTG ATATTCGAGA AGGCATTCTA TGGGAGGGAG ATTATGAATG TGGTGGCATG TGAGGGCACA   
  
  
+ GAGAGGGTGG AAAGGCCAGA GACATACAAG CAATGGCACG TTAGGCATAG CAGGGCAGGG TTTCGGCAAC   
  
  
+ TACCATTGGA TCCCAAGTTG ATCGAGAAAA TGAGGTTTAA GGCCAAGGCA GACCACCACA AGGATTTCGT   
  
  
+ GATTGATGTG GATGGACATT GGGCAATTCA GGGATGGAAG GGGCGGATTG CCTGTGCCAT CTCTGCATGG   
  
  
+ GTTCTGGCTT G  

- -Up\_Stream \_Len000GACAAG TCGTGATCCC TTATTCCAAA AACGGGACGT ATGATACTTT AGATTGGTTT   
  
  
- TTGTAAGGTA GAACCTTCCG ACATCGGAAC ACCTAAAACT TAAGCGAGGG TTCTTGGTTA CTTCATCGCG   
  
  
- TCTTCCGGTT ATGAGTTAAA GTCTCAGGTG TAGTTGAACA AGTTACGGTC ATATAAAGTC TTGTAATTAA   
  
  
- TGTATGACAA ACCAACCTCT TTTTTACCAA CAGAGCTTTT GATCAAAAAA TTAAAAAACA ATAAATCGTG   
  
  
- CCATTTCTTA CCTTTAATAA AAGTACGCTC TTTTTTCCTT CAACCAAAAA GAAAAAAGTT GAGAAAACTA   
  
  
- CTATATTAGT CAGCTTTAAA TCCACGTGGT AGACTGAAGT TGAGAACATG CTGGAGACTA ACGAACGAAT   
  
  
- CTTCTTGTTC TCCTAACTGT CTATTCCTCC TCACCCACCA AATATGTCTC TTAACTCACC TACCAATATT   
  
  
- TCTTCAGCTA GAGTGTATTC ATTGAAGCAA TGCTAATATT TATAAAAGTA ACTTCTTTCT GCTAATCTAT   
  
  
- ATTAATTCTT CCATGTAGAT TCGTTTAAAC CGACTCGTCT CAACCTCCCG GTGATCCCAG TGTAACACAG   
  
  
- AACGCGTTGT TCGAATCTGG TAAGCGTTGC CTACGGAGTG CACCTCGTCC ACCCTGGGCG CACCTTCAAC   
  
  
- GTACCGTTCT TAAGAGACCG TCGTTCCTCT TAACTCCCCC CTGTGCGCAC ACTGGTGCGG TCACGCCAGG   
  
  
- GGGAGAGCAC CGTCTAAACC ACTAAAAAAA AAACAAAAAA AAACTTCATT ATATATTAAA AATATACTAA   
  
  
- TTTTTTTATC CAACACACAA TATTTCACCC AGTTACTGTT TATAAAAATA ACCTACTAAT TTTAATTCAA   
  
  
- GGTAAATAAC CTCTTCACTA CCTTACGTTC AAGGTTTTAA CACCTTGAAA ACAACTGCAT CGTCCGATCT   
  
  
- CAAGGTTTTT AATTTTTTTC CTTTCTGTAT AACTCCATTT CGGAAAAATA AAATGTTTAT CTGCGTGGTT   
  
  
- CCATATTATA TTAACTGTAC TAAACTCCGC TCTCCTTCTA AGAAAACGAT GGGTTTGTTT TGTGTTACTA   
  
  
- GAAAATTTAC ATCGTTAAAC CCTAACCGTT TTAAAACAGA TTGGGCTAAC AACTTTCAAT AGGCTAAAGT   
  
  
- ATTTTAACCT ATAGCAATAA TTTAAAAGTA AGCTTATAAA TTTAAACCTG TGTTACAAAA AGTATAAACC   
  
  
- TAAAAGATTT GAACTAAAAT TGAGTTTAAA TTCAAATAAG TTAAGTAGAC TGGGTTTTTT ACCAACGTTA   
  
  
- TTACATAAAA GAAAAACTTC AATTTATAAT AGCTTAAGTA AATTGAACAT CAATTGGGCA TTAACTGGGT   
  
  
- TTGAGCTGAA AAACGATGGA ATCGTCGGTC CTTCTTCAGT ATCATAACTG GGCACCTTGC GGACTTCGGT   
  
  
- TGTAGTGGTC TGGGTGTATA AGGTAGTAGA AGACTAGAGG AGCAGTTTCT TCCTTCGACT CGTGTCGTTA   
  
  
- TTGATCTTCT TGTCCTCCGT AGTGTGGGTT CGGTTTCCGG AAATAAACTC GAGGGGTGGA GTAAGGAGCT   
  
  
- TCGAAGGAGA AGTGTTACAT TCTGGTATTA GGAAGTGGGG AAGAGAAAAA TTCGAGTAGA AATGGACGGG   
  
  
- ATCAAGAGAG AGAGAGTACT CAGTAGTCAT TATCAATGGA GAAATGAAAG AGAAGGAACT CCATACGAAG   
  
  
- TTGAAATTAT ATATATACAT GGTGAACTAA AGAGAACAGT GAAACGATAT TATGAATAAC AAACCGATAG   
  
  
- AAGGGGACAA AATATGAGTA AAAGAACGAA AGATATAGAC CCAAACTTAA CCCAAAGTGA ATAGGAGCAA   
  
  
- CTAAGACACC CTTTAATCAC TTAACCCAAA ACATGTCGTC CAAATATACT TGATCCCAAA GTTTCCATAG   
  
  
- ATGAAAAAGA GAACACTAAT AATCAACTAA TCCCTAAACC GGTTCTAATC GTCTTACCCA AGACTTAAAC   
  
  
- CCCTTAAGAG ACTACTACGA GATTTACCCA TACGAATGCA ACTATAGGGA CGAATACTAC GCAGGAATCT   
  
  
- AATACGGTTA AACAAGTTAA TGCTTCCGGA TAGACTCCTA GAGTGGAGTG ACTCGGAGGG TTCGGGGAAA   
  
  
- AGACTGGGAA ACCGCATGTC AAAGTGCAGG AGAAACCCCG GCTCGGGACC CCAACTAAGA TTACTACTAT   
  
  
- CACTAAGACT ACTACAAGAG TTCACGTAAT CGGTTTACGA ATACCTTCTT CTGTACCTCC GTTTCGGTAC   
  
  
- GTACAAAGTG CTAGGAAATC GTGAAGTCCG ACGACTCTTT GGGAAAATAC TACGGAATCC CTTCTTTATG   
  
  
- GGTTGAAGAG GCTTGGTTGT AGGATATTAA CTACTAACAA ACCTATTAGG ACCACTTTTA AGAAACCCAA   
  
  
- GAAGATCACT AAAATCACAA TCAGTGAAAC CCAGATCAAG TCGTTCGAGA TAACCTGGCT GTTAACACAG   
  
  
- ACTAAACTCA CTCGTAAAAC TCGGTGGGAA ACAAGTTCGT GAAGGTTAAC TTAGTATAGG GGTTGGTAAC   
  
  
- TGGGCAGGAG TTACCAGCAA ACCGGGAAGC CCACGGAATC TAACGTGCCG GAGATTACCA AGTCACTAGA   
  
  
- GTAGGGAACC AAACGGCTAC CTACAATAAT CACATAAGTC ACTCTTTCTC AGGTACTAAG TTAAAGTCTT   
  
  
- TCCCCACCTT CTCCGATCAT TCAAGGAAGG GTTCTTATTA TTGGAACAAT AACTAGAGTT CTTGGAGTGA   
  
  
- AAAGGATTAC TTTGCTTCCT CCTACTAGCT CACTACTACC AATTCTTCCT TTTCTTACTA CACTTAACCA   
  
  
- GATTGATGAG TTCTCCCTCA TTCTAAGTGA TAGCACTTCT GCTCCTGAAA CTTCTTCCTT CCTCATCGTT   
  
  
- CGTCAGTCGA TAAAGATGAT GACTCCTTCG ACTCAACACA CTTTAAAAAC TGTTCAAAAA CGAAACGTTA   
  
  
- ACCATGGGAC ACTTTGGACT CCGAGTAGGG TGGTACTCAA ACTTGGGACT CTTCCCGGTC AGTGTACCTA   
  
  
- ATCTTCCACC CTCTTTACCC TTCCCAGTGG GTTTCTTTGT CCTATTTTCA TCATGGTTGC AACACCTAAA   
  
  
- TTCCTTAAAC GACTACGATA CACGTGTTAG ATGACGTAGA CTACTGGCTT CTTGACGACT ACCTGACGAT   
  
  
- TTCTTTTAGT CCCTCGTGAG TAGACGACTC CCCCTACCTA AAGTTTCCAA CCGAGTAATG AAACGACTAC   
  
  
- GGGATCTCCG TGCAAATCGA CCTTGACCTA GAGTGTAAAT ATGTCGGGAT TCAAGAGTAT CCGGTAGACG   
  
  
- ACAACTGCAC AATTTTCGTA TAGTCAAGAT ACAAGCACGA ACGGGTAAAT TCTTCTAGCA GTAAGCAACA   
  
  
- CCATTTGTAT ACTAAGATTT TCGACGACTT TTCCGTAGTT TCGAAGTATA ATATCTAAAA CCGTAGGAGA   
  
  
- TACCTAAGGT TACCGGATCG GAGTAAGCTA CGGAGAGTCT CGCCAGACCA CCTGGAGGTT TTGACAAATA   
  
  
- ATGTCCCTAG CTAGAGGGGG TCGGACCCAA GTCCGGTCGT CTTTCTCACC TTCGTTGTCC CTCTGCGAAC   
  
  
- CGATTCATGA CACTCGCCAT ATTACACGGT AAACTCATAG TACGGTAACG AGTCTTCACC CTTTGTTAGT   
  
  
- TTGGTCCCCT AGAGTTCAAT CCTTCATCCC TACTACTACT TCAACAGCAC CACTTGACAG AGACATCCAA   
  
  
- GTTCTTGGAG GAACTGCTCT GTTACCACCA CCTATCAGGT TCCTTGTGTC AAAATTTGGA CTAATCTTCC   
  
  
- CATTTTGGGC TATAAAAACA CGTACCGTAA CATTTACCAA GGATGTTGTA GGGAAAGAAA CACTGTGCAA   
  
  
- AATCTCTTCG GGAGAAAGTA ATAAGGTGAG AAAAGCTGTA CAATCTACGG TTGCGGAGAT CCCTCCTCGG   
  
  
- GCTCTCCAAC TATAAGCTCT TCCGTAAGAT ACCCTCCCTC TAATACTTAC ACCACCGTAC ACTCCCGTGT   
  
  
- CTCTCCCACC TTTCCGGTCT CTGTATGTTC GTTACCGTGC AATCCGTATC GTCCCGTCCC AAAGCCGTTG   
  
  
- ATGGTAACCT AGGGTTCAAC TAGCTCTTTT ACTCCAAATT CCGGTTCCGT CTGGTGGTGT TCCTAAAGCA   
  
  
- CTAACTACAC CTACCTGTAA CCCGTTAAGT CCCTACCTTC CCCGCCTAAC GGACACGGTA GAGACGTACC   
  
  
- CAAGACCGAA C

+     CCGTCC motif

| Site Name | Organism | Position | Strand | Matrix score. | sequence | function |
| --- | --- | --- | --- | --- | --- | --- |
| CCGTCC motif | Nicotiana tabacum | 2597 | + | 6 | CCGTCC |  |

>HU07G02248.1   
+ -Up\_Stream \_Len000CTGTTC AGCACTAGGG AATAAGGTTT TTGCCCTGCA TACTATGAAA TCTAACCAAA   
  
  
+ AACATTCCAT CTTGGAAGGC TGTAGCCTTG TGGATTTTGA ATTCGCTCCC AAGAACCAAT GAAGTAGCGC   
  
  
+ AGAAGGCCAA TACTCAATTT CAGAGTCCAC ATCAACTTGT TCAATGCCAG TATATTTCAG AACATTAATT   
  
  
+ ACATACTGTT TGGTTGGAGA AAAAATGGTT GTCTCGAAAA CTAGTTTTTT AATTTTTTGT TATTTAGCAC   
  
  
+ GGTAAAGAAT GGAAATTATT TTCATGCGAG AAAAAAGGAA GTTGGTTTTT CTTTTTTCAA CTCTTTTGAT   
  
  
+ GATATAATCA GTCGAAATTT AGGTGCACCA TCTGACTTCA ACTCTTGTAC GACCTCTGAT TGCTTGCTTA   
  
  
+ GAAGAACAAG AGGATTGACA GATAAGGAGG AGTGGGTGGT TTATACAGAG AATTGAGTGG ATGGTTATAA   
  
  
+ AGAAGTCGAT CTCACATAAG TAACTTCGTT ACGATTATAA ATATTTTCAT TGAAGAAAGA CGATTAGATA   
  
  
+ TAATTAAGAA GGTACATCTA AGCAAATTTG GCTGAGCAGA GTTGGAGGGC CACTAGGGTC ACATTGTGTC   
  
  
+ TTGCGCAACA AGCTTAGACC ATTCGCAACG GATGCCTCAC GTGGAGCAGG TGGGACCCGC GTGGAAGTTG   
  
  
+ CATGGCAAGA ATTCTCTGGC AGCAAGGAGA ATTGAGGGGG GACACGCGTG TGACCACGCC AGTGCGGTCC   
  
  
+ CCCTCTCGTG GCAGATTTGG TGATTTTTTT TTTGTTTTTT TTTGAAGTAA TATATAATTT TTATATGATT   
  
  
+ AAAAAAATAG GTTGTGTGTT ATAAAGTGGG TCAATGACAA ATATTTTTAT TGGATGATTA AAATTAAGTT   
  
  
+ CCATTTATTG GAGAAGTGAT GGAATGCAAG TTCCAAAATT GTGGAACTTT TGTTGACGTA GCAGGCTAGA   
  
  
+ GTTCCAAAAA TTAAAAAAAG GAAAGACATA TTGAGGTAAA GCCTTTTTAT TTTACAAATA GACGCACCAA   
  
  
+ GGTATAATAT AATTGACATG ATTTGAGGCG AGAGGAAGAT TCTTTTGCTA CCCAAACAAA ACACAATGAT   
  
  
+ CTTTTAAATG TAGCAATTTG GGATTGGCAA AATTTTGTCT AACCCGATTG TTGAAAGTTA TCCGATTTCA   
  
  
+ TAAAATTGGA TATCGTTATT AAATTTTCAT TCGAATATTT AAATTTGGAC ACAATGTTTT TCATATTTGG   
  
  
+ ATTTTCTAAA CTTGATTTTA ACTCAAATTT AAGTTTATTC AATTCATCTG ACCCAAAAAA TGGTTGCAAT   
  
  
+ AATGTATTTT CTTTTTGAAG TTAAATATTA TCGAATTCAT TTAACTTGTA GTTAACCCGT AATTGACCCA   
  
  
+ AACTCGACTT TTTGCTACCT TAGCAGCCAG GAAGAAGTCA TAGTATTGAC CCGTGGAACG CCTGAAGCCA   
  
  
+ ACATCACCAG ACCCACATAT TCCATCATCT TCTGATCTCC TCGTCAAAGA AGGAAGCTGA GCACAGCAAT   
  
  
+ AACTAGAAGA ACAGGAGGCA TCACACCCAA GCCAAAGGCC TTTATTTGAG CTCCCCACCT CATTCCTCGA   
  
  
+ AGCTTCCTCT TCACAATGTA AGACCATAAT CCTTCACCCC TTCTCTTTTT AAGCTCATCT TTACCTGCCC   
  
  
+ TAGTTCTCTC TCTCTCATGA GTCATCAGTA ATAGTTACCT CTTTACTTTC TCTTCCTTGA GGTATGCTTC   
  
  
+ AACTTTAATA TATATATGTA CCACTTGATT TCTCTTGTCA CTTTGCTATA ATACTTATTG TTTGGCTATC   
  
  
+ TTCCCCTGTT TTATACTCAT TTTCTTGCTT TCTATATCTG GGTTTGAATT GGGTTTCACT TATCCTCGTT   
  
  
+ GATTCTGTGG GAAATTAGTG AATTGGGTTT TGTACAGCAG GTTTATATGA ACTAGGGTTT CAAAGGTATC   
  
  
+ TACTTTTTCT CTTGTGATTA TTAGTTGATT AGGGATTTGG CCAAGATTAG CAGAATGGGT TCTGAATTTG   
  
  
+ GGGAATTCTC TGATGATGCT CTAAATGGGT ATGCTTACGT TGATATCCCT GCTTATGATG CGTCCTTAGA   
  
  
+ TTATGCCAAT TTGTTCAATT ACGAAGGCCT ATCTGAGGAT CTCACCTCAC TGAGCCTCCC AAGCCCCTTT   
  
  
+ TCTGACCCTT TGGCGTACAG TTTCACGTCC TCTTTGGGGC CGAGCCCTGG GGTTGATTCT AATGATGATA   
  
  
+ GTGATTCTGA TGATGTTCTC AAGTGCATTA GCCAAATGCT TATGGAAGAA GACATGGAGG CAAAGCCATG   
  
  
+ CATGTTTCAC GATCCTTTAG CACTTCAGGC TGCTGAGAAA CCCTTTTATG ATGCCTTAGG GAAGAAATAC   
  
  
+ CCAACTTCTC CGAACCAACA TCCTATAATT GATGATTGTT TGGATAATCC TGGTGAAAAT TCTTTGGGTT   
  
  
+ CTTCTAGTGA TTTTAGTGTT AGTCACTTTG GGTCTAGTTC AGCAAGCTCT ATTGGACCGA CAATTGTGTC   
  
  
+ TGATTTGAGT GAGCATTTTG AGCCACCCTT TGTTCAAGCA CTTCCAATTG AATCATATCC CCAACCATTG   
  
  
+ ACCCGTCCTC AATGGTCGTT TGGCCCTTCG GGTGCCTTAG ATTGCACGGC CTCTAATGGT TCAGTGATCT   
  
  
+ CATCCCTTGG TTTGCCGATG GATGTTATTA GTGTATTCAG TGAGAAAGAG TCCATGATTC AATTTCAGAA   
  
  
+ AGGGGTGGAA GAGGCTAGTA AGTTCCTTCC CAAGAATAAT AACCTTGTTA TTGATCTCAA GAACCTCACT   
  
  
+ TTTCCTAATG AAACGAAGGA GGATGATCGA GTGATGATGG TTAAGAAGGA AAAGAATGAT GTGAATTGGT   
  
  
+ CTAACTACTC AAGAGGGAGT AAGATTCACT ATCGTGAAGA CGAGGACTTT GAAGAAGGAA GGAGTAGCAA   
  
  
+ GCAGTCAGCT ATTTCTACTA CTGAGGAAGC TGAGTTGTGT GAAATTTTTG ACAAGTTTTT GCTTTGCAAT   
  
  
+ TGGTACCCTG TGAAACCTGA GGCTCATCCC ACCATGAGTT TGAACCCTGA GAAGGGCCAG TCACATGGAT   
  
  
+ TAGAAGGTGG GAGAAATGGG AAGGGTCACC CAAAGAAACA GGATAAAAGT AGTACCAACG TTGTGGATTT   
  
  
+ AAGGAATTTG CTGATGCTAT GTGCACAATC TACTGCATCT GATGACCGAA GAACTGCTGA TGGACTGCTA   
  
  
+ AAGAAAATCA GGGAGCACTC ATCTGCTGAG GGGGATGGAT TTCAAAGGTT GGCTCATTAC TTTGCTGATG   
  
  
+ CCCTAGAGGC ACGTTTAGCT GGAACTGGAT CTCACATTTA TACAGCCCTA AGTTCTCATA GGCCATCTGC   
  
  
+ TGTTGACGTG TTAAAAGCAT ATCAGTTCTA TGTTCGTGCT TGCCCATTTA AGAAGATCGT CATTCGTTGT   
  
  
+ GGTAAACATA TGATTCTAAA AGCTGCTGAA AAGGCATCAA AGCTTCATAT TATAGATTTT GGCATCCTCT   
  
  
+ ATGGATTCCA ATGGCCTAGC CTCATTCGAT GCCTCTCAGA GCGGTCTGGT GGACCTCCAA AACTGTTTAT   
  
  
+ TACAGGGATC GATCTCCCCC AGCCTGGGTT CAGGCCAGCA GAAAGAGTGG AAGCAACAGG GAGACGCTTG   
  
  
+ GCTAAGTACT GTGAGCGGTA TAATGTGCCA TTTGAGTATC ATGCCATTGC TCAGAAGTGG GAAACAATCA   
  
  
+ AACCAGGGGA TCTCAAGTTA GGAAGTAGGG ATGATGATGA AGTTGTCGTG GTGAACTGTC TCTGTAGGTT   
  
  
+ CAAGAACCTC CTTGACGAGA CAATGGTGGT GGATAGTCCA AGGAACACAG TTTTAAACCT GATTAGAAGG   
  
  
+ GTAAAACCCG ATATTTTTGT GCATGGCATT GTAAATGGTT CCTACAACAT CCCTTTCTTT GTGACACGTT   
  
  
+ TTAGAGAAGC CCTCTTTCAT TATTCCACTC TTTTCGACAT GTTAGATGCC AACGCCTCTA GGGAGGAGCC   
  
  
+ CGAGAGGTTG ATATTCGAGA AGGCATTCTA TGGGAGGGAG ATTATGAATG TGGTGGCATG TGAGGGCACA   
  
  
+ GAGAGGGTGG AAAGGCCAGA GACATACAAG CAATGGCACG TTAGGCATAG CAGGGCAGGG TTTCGGCAAC   
  
  
+ TACCATTGGA TCCCAAGTTG ATCGAGAAAA TGAGGTTTAA GGCCAAGGCA GACCACCACA AGGATTTCGT   
  
  
+ GATTGATGTG GATGGACATT GGGCAATTCA GGGATGGAAG GGGCGGATTG CCTGTGCCAT CTCTGCATGG   
  
  
+ GTTCTGGCTT G  

- -Up\_Stream \_Len000GACAAG TCGTGATCCC TTATTCCAAA AACGGGACGT ATGATACTTT AGATTGGTTT   
  
  
- TTGTAAGGTA GAACCTTCCG ACATCGGAAC ACCTAAAACT TAAGCGAGGG TTCTTGGTTA CTTCATCGCG   
  
  
- TCTTCCGGTT ATGAGTTAAA GTCTCAGGTG TAGTTGAACA AGTTACGGTC ATATAAAGTC TTGTAATTAA   
  
  
- TGTATGACAA ACCAACCTCT TTTTTACCAA CAGAGCTTTT GATCAAAAAA TTAAAAAACA ATAAATCGTG   
  
  
- CCATTTCTTA CCTTTAATAA AAGTACGCTC TTTTTTCCTT CAACCAAAAA GAAAAAAGTT GAGAAAACTA   
  
  
- CTATATTAGT CAGCTTTAAA TCCACGTGGT AGACTGAAGT TGAGAACATG CTGGAGACTA ACGAACGAAT   
  
  
- CTTCTTGTTC TCCTAACTGT CTATTCCTCC TCACCCACCA AATATGTCTC TTAACTCACC TACCAATATT   
  
  
- TCTTCAGCTA GAGTGTATTC ATTGAAGCAA TGCTAATATT TATAAAAGTA ACTTCTTTCT GCTAATCTAT   
  
  
- ATTAATTCTT CCATGTAGAT TCGTTTAAAC CGACTCGTCT CAACCTCCCG GTGATCCCAG TGTAACACAG   
  
  
- AACGCGTTGT TCGAATCTGG TAAGCGTTGC CTACGGAGTG CACCTCGTCC ACCCTGGGCG CACCTTCAAC   
  
  
- GTACCGTTCT TAAGAGACCG TCGTTCCTCT TAACTCCCCC CTGTGCGCAC ACTGGTGCGG TCACGCCAGG   
  
  
- GGGAGAGCAC CGTCTAAACC ACTAAAAAAA AAACAAAAAA AAACTTCATT ATATATTAAA AATATACTAA   
  
  
- TTTTTTTATC CAACACACAA TATTTCACCC AGTTACTGTT TATAAAAATA ACCTACTAAT TTTAATTCAA   
  
  
- GGTAAATAAC CTCTTCACTA CCTTACGTTC AAGGTTTTAA CACCTTGAAA ACAACTGCAT CGTCCGATCT   
  
  
- CAAGGTTTTT AATTTTTTTC CTTTCTGTAT AACTCCATTT CGGAAAAATA AAATGTTTAT CTGCGTGGTT   
  
  
- CCATATTATA TTAACTGTAC TAAACTCCGC TCTCCTTCTA AGAAAACGAT GGGTTTGTTT TGTGTTACTA   
  
  
- GAAAATTTAC ATCGTTAAAC CCTAACCGTT TTAAAACAGA TTGGGCTAAC AACTTTCAAT AGGCTAAAGT   
  
  
- ATTTTAACCT ATAGCAATAA TTTAAAAGTA AGCTTATAAA TTTAAACCTG TGTTACAAAA AGTATAAACC   
  
  
- TAAAAGATTT GAACTAAAAT TGAGTTTAAA TTCAAATAAG TTAAGTAGAC TGGGTTTTTT ACCAACGTTA   
  
  
- TTACATAAAA GAAAAACTTC AATTTATAAT AGCTTAAGTA AATTGAACAT CAATTGGGCA TTAACTGGGT   
  
  
- TTGAGCTGAA AAACGATGGA ATCGTCGGTC CTTCTTCAGT ATCATAACTG GGCACCTTGC GGACTTCGGT   
  
  
- TGTAGTGGTC TGGGTGTATA AGGTAGTAGA AGACTAGAGG AGCAGTTTCT TCCTTCGACT CGTGTCGTTA   
  
  
- TTGATCTTCT TGTCCTCCGT AGTGTGGGTT CGGTTTCCGG AAATAAACTC GAGGGGTGGA GTAAGGAGCT   
  
  
- TCGAAGGAGA AGTGTTACAT TCTGGTATTA GGAAGTGGGG AAGAGAAAAA TTCGAGTAGA AATGGACGGG   
  
  
- ATCAAGAGAG AGAGAGTACT CAGTAGTCAT TATCAATGGA GAAATGAAAG AGAAGGAACT CCATACGAAG   
  
  
- TTGAAATTAT ATATATACAT GGTGAACTAA AGAGAACAGT GAAACGATAT TATGAATAAC AAACCGATAG   
  
  
- AAGGGGACAA AATATGAGTA AAAGAACGAA AGATATAGAC CCAAACTTAA CCCAAAGTGA ATAGGAGCAA   
  
  
- CTAAGACACC CTTTAATCAC TTAACCCAAA ACATGTCGTC CAAATATACT TGATCCCAAA GTTTCCATAG   
  
  
- ATGAAAAAGA GAACACTAAT AATCAACTAA TCCCTAAACC GGTTCTAATC GTCTTACCCA AGACTTAAAC   
  
  
- CCCTTAAGAG ACTACTACGA GATTTACCCA TACGAATGCA ACTATAGGGA CGAATACTAC GCAGGAATCT   
  
  
- AATACGGTTA AACAAGTTAA TGCTTCCGGA TAGACTCCTA GAGTGGAGTG ACTCGGAGGG TTCGGGGAAA   
  
  
- AGACTGGGAA ACCGCATGTC AAAGTGCAGG AGAAACCCCG GCTCGGGACC CCAACTAAGA TTACTACTAT   
  
  
- CACTAAGACT ACTACAAGAG TTCACGTAAT CGGTTTACGA ATACCTTCTT CTGTACCTCC GTTTCGGTAC   
  
  
- GTACAAAGTG CTAGGAAATC GTGAAGTCCG ACGACTCTTT GGGAAAATAC TACGGAATCC CTTCTTTATG   
  
  
- GGTTGAAGAG GCTTGGTTGT AGGATATTAA CTACTAACAA ACCTATTAGG ACCACTTTTA AGAAACCCAA   
  
  
- GAAGATCACT AAAATCACAA TCAGTGAAAC CCAGATCAAG TCGTTCGAGA TAACCTGGCT GTTAACACAG   
  
  
- ACTAAACTCA CTCGTAAAAC TCGGTGGGAA ACAAGTTCGT GAAGGTTAAC TTAGTATAGG GGTTGGTAAC   
  
  
- TGGGCAGGAG TTACCAGCAA ACCGGGAAGC CCACGGAATC TAACGTGCCG GAGATTACCA AGTCACTAGA   
  
  
- GTAGGGAACC AAACGGCTAC CTACAATAAT CACATAAGTC ACTCTTTCTC AGGTACTAAG TTAAAGTCTT   
  
  
- TCCCCACCTT CTCCGATCAT TCAAGGAAGG GTTCTTATTA TTGGAACAAT AACTAGAGTT CTTGGAGTGA   
  
  
- AAAGGATTAC TTTGCTTCCT CCTACTAGCT CACTACTACC AATTCTTCCT TTTCTTACTA CACTTAACCA   
  
  
- GATTGATGAG TTCTCCCTCA TTCTAAGTGA TAGCACTTCT GCTCCTGAAA CTTCTTCCTT CCTCATCGTT   
  
  
- CGTCAGTCGA TAAAGATGAT GACTCCTTCG ACTCAACACA CTTTAAAAAC TGTTCAAAAA CGAAACGTTA   
  
  
- ACCATGGGAC ACTTTGGACT CCGAGTAGGG TGGTACTCAA ACTTGGGACT CTTCCCGGTC AGTGTACCTA   
  
  
- ATCTTCCACC CTCTTTACCC TTCCCAGTGG GTTTCTTTGT CCTATTTTCA TCATGGTTGC AACACCTAAA   
  
  
- TTCCTTAAAC GACTACGATA CACGTGTTAG ATGACGTAGA CTACTGGCTT CTTGACGACT ACCTGACGAT   
  
  
- TTCTTTTAGT CCCTCGTGAG TAGACGACTC CCCCTACCTA AAGTTTCCAA CCGAGTAATG AAACGACTAC   
  
  
- GGGATCTCCG TGCAAATCGA CCTTGACCTA GAGTGTAAAT ATGTCGGGAT TCAAGAGTAT CCGGTAGACG   
  
  
- ACAACTGCAC AATTTTCGTA TAGTCAAGAT ACAAGCACGA ACGGGTAAAT TCTTCTAGCA GTAAGCAACA   
  
  
- CCATTTGTAT ACTAAGATTT TCGACGACTT TTCCGTAGTT TCGAAGTATA ATATCTAAAA CCGTAGGAGA   
  
  
- TACCTAAGGT TACCGGATCG GAGTAAGCTA CGGAGAGTCT CGCCAGACCA CCTGGAGGTT TTGACAAATA   
  
  
- ATGTCCCTAG CTAGAGGGGG TCGGACCCAA GTCCGGTCGT CTTTCTCACC TTCGTTGTCC CTCTGCGAAC   
  
  
- CGATTCATGA CACTCGCCAT ATTACACGGT AAACTCATAG TACGGTAACG AGTCTTCACC CTTTGTTAGT   
  
  
- TTGGTCCCCT AGAGTTCAAT CCTTCATCCC TACTACTACT TCAACAGCAC CACTTGACAG AGACATCCAA   
  
  
- GTTCTTGGAG GAACTGCTCT GTTACCACCA CCTATCAGGT TCCTTGTGTC AAAATTTGGA CTAATCTTCC   
  
  
- CATTTTGGGC TATAAAAACA CGTACCGTAA CATTTACCAA GGATGTTGTA GGGAAAGAAA CACTGTGCAA   
  
  
- AATCTCTTCG GGAGAAAGTA ATAAGGTGAG AAAAGCTGTA CAATCTACGG TTGCGGAGAT CCCTCCTCGG   
  
  
- GCTCTCCAAC TATAAGCTCT TCCGTAAGAT ACCCTCCCTC TAATACTTAC ACCACCGTAC ACTCCCGTGT   
  
  
- CTCTCCCACC TTTCCGGTCT CTGTATGTTC GTTACCGTGC AATCCGTATC GTCCCGTCCC AAAGCCGTTG   
  
  
- ATGGTAACCT AGGGTTCAAC TAGCTCTTTT ACTCCAAATT CCGGTTCCGT CTGGTGGTGT TCCTAAAGCA   
  
  
- CTAACTACAC CTACCTGTAA CCCGTTAAGT CCCTACCTTC CCCGCCTAAC GGACACGGTA GAGACGTACC   
  
  
- CAAGACCGAA C

+     CCGTCC-box

| Site Name | Organism | Position | Strand | Matrix score. | sequence | function |
| --- | --- | --- | --- | --- | --- | --- |
| CCGTCC-box | Petroselinum hortense | 2597 | + | 6 | CCGTCC |  |

>HU07G02248.1   
+ -Up\_Stream \_Len000CTGTTC AGCACTAGGG AATAAGGTTT TTGCCCTGCA TACTATGAAA TCTAACCAAA   
  
  
+ AACATTCCAT CTTGGAAGGC TGTAGCCTTG TGGATTTTGA ATTCGCTCCC AAGAACCAAT GAAGTAGCGC   
  
  
+ AGAAGGCCAA TACTCAATTT CAGAGTCCAC ATCAACTTGT TCAATGCCAG TATATTTCAG AACATTAATT   
  
  
+ ACATACTGTT TGGTTGGAGA AAAAATGGTT GTCTCGAAAA CTAGTTTTTT AATTTTTTGT TATTTAGCAC   
  
  
+ GGTAAAGAAT GGAAATTATT TTCATGCGAG AAAAAAGGAA GTTGGTTTTT CTTTTTTCAA CTCTTTTGAT   
  
  
+ GATATAATCA GTCGAAATTT AGGTGCACCA TCTGACTTCA ACTCTTGTAC GACCTCTGAT TGCTTGCTTA   
  
  
+ GAAGAACAAG AGGATTGACA GATAAGGAGG AGTGGGTGGT TTATACAGAG AATTGAGTGG ATGGTTATAA   
  
  
+ AGAAGTCGAT CTCACATAAG TAACTTCGTT ACGATTATAA ATATTTTCAT TGAAGAAAGA CGATTAGATA   
  
  
+ TAATTAAGAA GGTACATCTA AGCAAATTTG GCTGAGCAGA GTTGGAGGGC CACTAGGGTC ACATTGTGTC   
  
  
+ TTGCGCAACA AGCTTAGACC ATTCGCAACG GATGCCTCAC GTGGAGCAGG TGGGACCCGC GTGGAAGTTG   
  
  
+ CATGGCAAGA ATTCTCTGGC AGCAAGGAGA ATTGAGGGGG GACACGCGTG TGACCACGCC AGTGCGGTCC   
  
  
+ CCCTCTCGTG GCAGATTTGG TGATTTTTTT TTTGTTTTTT TTTGAAGTAA TATATAATTT TTATATGATT   
  
  
+ AAAAAAATAG GTTGTGTGTT ATAAAGTGGG TCAATGACAA ATATTTTTAT TGGATGATTA AAATTAAGTT   
  
  
+ CCATTTATTG GAGAAGTGAT GGAATGCAAG TTCCAAAATT GTGGAACTTT TGTTGACGTA GCAGGCTAGA   
  
  
+ GTTCCAAAAA TTAAAAAAAG GAAAGACATA TTGAGGTAAA GCCTTTTTAT TTTACAAATA GACGCACCAA   
  
  
+ GGTATAATAT AATTGACATG ATTTGAGGCG AGAGGAAGAT TCTTTTGCTA CCCAAACAAA ACACAATGAT   
  
  
+ CTTTTAAATG TAGCAATTTG GGATTGGCAA AATTTTGTCT AACCCGATTG TTGAAAGTTA TCCGATTTCA   
  
  
+ TAAAATTGGA TATCGTTATT AAATTTTCAT TCGAATATTT AAATTTGGAC ACAATGTTTT TCATATTTGG   
  
  
+ ATTTTCTAAA CTTGATTTTA ACTCAAATTT AAGTTTATTC AATTCATCTG ACCCAAAAAA TGGTTGCAAT   
  
  
+ AATGTATTTT CTTTTTGAAG TTAAATATTA TCGAATTCAT TTAACTTGTA GTTAACCCGT AATTGACCCA   
  
  
+ AACTCGACTT TTTGCTACCT TAGCAGCCAG GAAGAAGTCA TAGTATTGAC CCGTGGAACG CCTGAAGCCA   
  
  
+ ACATCACCAG ACCCACATAT TCCATCATCT TCTGATCTCC TCGTCAAAGA AGGAAGCTGA GCACAGCAAT   
  
  
+ AACTAGAAGA ACAGGAGGCA TCACACCCAA GCCAAAGGCC TTTATTTGAG CTCCCCACCT CATTCCTCGA   
  
  
+ AGCTTCCTCT TCACAATGTA AGACCATAAT CCTTCACCCC TTCTCTTTTT AAGCTCATCT TTACCTGCCC   
  
  
+ TAGTTCTCTC TCTCTCATGA GTCATCAGTA ATAGTTACCT CTTTACTTTC TCTTCCTTGA GGTATGCTTC   
  
  
+ AACTTTAATA TATATATGTA CCACTTGATT TCTCTTGTCA CTTTGCTATA ATACTTATTG TTTGGCTATC   
  
  
+ TTCCCCTGTT TTATACTCAT TTTCTTGCTT TCTATATCTG GGTTTGAATT GGGTTTCACT TATCCTCGTT   
  
  
+ GATTCTGTGG GAAATTAGTG AATTGGGTTT TGTACAGCAG GTTTATATGA ACTAGGGTTT CAAAGGTATC   
  
  
+ TACTTTTTCT CTTGTGATTA TTAGTTGATT AGGGATTTGG CCAAGATTAG CAGAATGGGT TCTGAATTTG   
  
  
+ GGGAATTCTC TGATGATGCT CTAAATGGGT ATGCTTACGT TGATATCCCT GCTTATGATG CGTCCTTAGA   
  
  
+ TTATGCCAAT TTGTTCAATT ACGAAGGCCT ATCTGAGGAT CTCACCTCAC TGAGCCTCCC AAGCCCCTTT   
  
  
+ TCTGACCCTT TGGCGTACAG TTTCACGTCC TCTTTGGGGC CGAGCCCTGG GGTTGATTCT AATGATGATA   
  
  
+ GTGATTCTGA TGATGTTCTC AAGTGCATTA GCCAAATGCT TATGGAAGAA GACATGGAGG CAAAGCCATG   
  
  
+ CATGTTTCAC GATCCTTTAG CACTTCAGGC TGCTGAGAAA CCCTTTTATG ATGCCTTAGG GAAGAAATAC   
  
  
+ CCAACTTCTC CGAACCAACA TCCTATAATT GATGATTGTT TGGATAATCC TGGTGAAAAT TCTTTGGGTT   
  
  
+ CTTCTAGTGA TTTTAGTGTT AGTCACTTTG GGTCTAGTTC AGCAAGCTCT ATTGGACCGA CAATTGTGTC   
  
  
+ TGATTTGAGT GAGCATTTTG AGCCACCCTT TGTTCAAGCA CTTCCAATTG AATCATATCC CCAACCATTG   
  
  
+ ACCCGTCCTC AATGGTCGTT TGGCCCTTCG GGTGCCTTAG ATTGCACGGC CTCTAATGGT TCAGTGATCT   
  
  
+ CATCCCTTGG TTTGCCGATG GATGTTATTA GTGTATTCAG TGAGAAAGAG TCCATGATTC AATTTCAGAA   
  
  
+ AGGGGTGGAA GAGGCTAGTA AGTTCCTTCC CAAGAATAAT AACCTTGTTA TTGATCTCAA GAACCTCACT   
  
  
+ TTTCCTAATG AAACGAAGGA GGATGATCGA GTGATGATGG TTAAGAAGGA AAAGAATGAT GTGAATTGGT   
  
  
+ CTAACTACTC AAGAGGGAGT AAGATTCACT ATCGTGAAGA CGAGGACTTT GAAGAAGGAA GGAGTAGCAA   
  
  
+ GCAGTCAGCT ATTTCTACTA CTGAGGAAGC TGAGTTGTGT GAAATTTTTG ACAAGTTTTT GCTTTGCAAT   
  
  
+ TGGTACCCTG TGAAACCTGA GGCTCATCCC ACCATGAGTT TGAACCCTGA GAAGGGCCAG TCACATGGAT   
  
  
+ TAGAAGGTGG GAGAAATGGG AAGGGTCACC CAAAGAAACA GGATAAAAGT AGTACCAACG TTGTGGATTT   
  
  
+ AAGGAATTTG CTGATGCTAT GTGCACAATC TACTGCATCT GATGACCGAA GAACTGCTGA TGGACTGCTA   
  
  
+ AAGAAAATCA GGGAGCACTC ATCTGCTGAG GGGGATGGAT TTCAAAGGTT GGCTCATTAC TTTGCTGATG   
  
  
+ CCCTAGAGGC ACGTTTAGCT GGAACTGGAT CTCACATTTA TACAGCCCTA AGTTCTCATA GGCCATCTGC   
  
  
+ TGTTGACGTG TTAAAAGCAT ATCAGTTCTA TGTTCGTGCT TGCCCATTTA AGAAGATCGT CATTCGTTGT   
  
  
+ GGTAAACATA TGATTCTAAA AGCTGCTGAA AAGGCATCAA AGCTTCATAT TATAGATTTT GGCATCCTCT   
  
  
+ ATGGATTCCA ATGGCCTAGC CTCATTCGAT GCCTCTCAGA GCGGTCTGGT GGACCTCCAA AACTGTTTAT   
  
  
+ TACAGGGATC GATCTCCCCC AGCCTGGGTT CAGGCCAGCA GAAAGAGTGG AAGCAACAGG GAGACGCTTG   
  
  
+ GCTAAGTACT GTGAGCGGTA TAATGTGCCA TTTGAGTATC ATGCCATTGC TCAGAAGTGG GAAACAATCA   
  
  
+ AACCAGGGGA TCTCAAGTTA GGAAGTAGGG ATGATGATGA AGTTGTCGTG GTGAACTGTC TCTGTAGGTT   
  
  
+ CAAGAACCTC CTTGACGAGA CAATGGTGGT GGATAGTCCA AGGAACACAG TTTTAAACCT GATTAGAAGG   
  
  
+ GTAAAACCCG ATATTTTTGT GCATGGCATT GTAAATGGTT CCTACAACAT CCCTTTCTTT GTGACACGTT   
  
  
+ TTAGAGAAGC CCTCTTTCAT TATTCCACTC TTTTCGACAT GTTAGATGCC AACGCCTCTA GGGAGGAGCC   
  
  
+ CGAGAGGTTG ATATTCGAGA AGGCATTCTA TGGGAGGGAG ATTATGAATG TGGTGGCATG TGAGGGCACA   
  
  
+ GAGAGGGTGG AAAGGCCAGA GACATACAAG CAATGGCACG TTAGGCATAG CAGGGCAGGG TTTCGGCAAC   
  
  
+ TACCATTGGA TCCCAAGTTG ATCGAGAAAA TGAGGTTTAA GGCCAAGGCA GACCACCACA AGGATTTCGT   
  
  
+ GATTGATGTG GATGGACATT GGGCAATTCA GGGATGGAAG GGGCGGATTG CCTGTGCCAT CTCTGCATGG   
  
  
+ GTTCTGGCTT G  

- -Up\_Stream \_Len000GACAAG TCGTGATCCC TTATTCCAAA AACGGGACGT ATGATACTTT AGATTGGTTT   
  
  
- TTGTAAGGTA GAACCTTCCG ACATCGGAAC ACCTAAAACT TAAGCGAGGG TTCTTGGTTA CTTCATCGCG   
  
  
- TCTTCCGGTT ATGAGTTAAA GTCTCAGGTG TAGTTGAACA AGTTACGGTC ATATAAAGTC TTGTAATTAA   
  
  
- TGTATGACAA ACCAACCTCT TTTTTACCAA CAGAGCTTTT GATCAAAAAA TTAAAAAACA ATAAATCGTG   
  
  
- CCATTTCTTA CCTTTAATAA AAGTACGCTC TTTTTTCCTT CAACCAAAAA GAAAAAAGTT GAGAAAACTA   
  
  
- CTATATTAGT CAGCTTTAAA TCCACGTGGT AGACTGAAGT TGAGAACATG CTGGAGACTA ACGAACGAAT   
  
  
- CTTCTTGTTC TCCTAACTGT CTATTCCTCC TCACCCACCA AATATGTCTC TTAACTCACC TACCAATATT   
  
  
- TCTTCAGCTA GAGTGTATTC ATTGAAGCAA TGCTAATATT TATAAAAGTA ACTTCTTTCT GCTAATCTAT   
  
  
- ATTAATTCTT CCATGTAGAT TCGTTTAAAC CGACTCGTCT CAACCTCCCG GTGATCCCAG TGTAACACAG   
  
  
- AACGCGTTGT TCGAATCTGG TAAGCGTTGC CTACGGAGTG CACCTCGTCC ACCCTGGGCG CACCTTCAAC   
  
  
- GTACCGTTCT TAAGAGACCG TCGTTCCTCT TAACTCCCCC CTGTGCGCAC ACTGGTGCGG TCACGCCAGG   
  
  
- GGGAGAGCAC CGTCTAAACC ACTAAAAAAA AAACAAAAAA AAACTTCATT ATATATTAAA AATATACTAA   
  
  
- TTTTTTTATC CAACACACAA TATTTCACCC AGTTACTGTT TATAAAAATA ACCTACTAAT TTTAATTCAA   
  
  
- GGTAAATAAC CTCTTCACTA CCTTACGTTC AAGGTTTTAA CACCTTGAAA ACAACTGCAT CGTCCGATCT   
  
  
- CAAGGTTTTT AATTTTTTTC CTTTCTGTAT AACTCCATTT CGGAAAAATA AAATGTTTAT CTGCGTGGTT   
  
  
- CCATATTATA TTAACTGTAC TAAACTCCGC TCTCCTTCTA AGAAAACGAT GGGTTTGTTT TGTGTTACTA   
  
  
- GAAAATTTAC ATCGTTAAAC CCTAACCGTT TTAAAACAGA TTGGGCTAAC AACTTTCAAT AGGCTAAAGT   
  
  
- ATTTTAACCT ATAGCAATAA TTTAAAAGTA AGCTTATAAA TTTAAACCTG TGTTACAAAA AGTATAAACC   
  
  
- TAAAAGATTT GAACTAAAAT TGAGTTTAAA TTCAAATAAG TTAAGTAGAC TGGGTTTTTT ACCAACGTTA   
  
  
- TTACATAAAA GAAAAACTTC AATTTATAAT AGCTTAAGTA AATTGAACAT CAATTGGGCA TTAACTGGGT   
  
  
- TTGAGCTGAA AAACGATGGA ATCGTCGGTC CTTCTTCAGT ATCATAACTG GGCACCTTGC GGACTTCGGT   
  
  
- TGTAGTGGTC TGGGTGTATA AGGTAGTAGA AGACTAGAGG AGCAGTTTCT TCCTTCGACT CGTGTCGTTA   
  
  
- TTGATCTTCT TGTCCTCCGT AGTGTGGGTT CGGTTTCCGG AAATAAACTC GAGGGGTGGA GTAAGGAGCT   
  
  
- TCGAAGGAGA AGTGTTACAT TCTGGTATTA GGAAGTGGGG AAGAGAAAAA TTCGAGTAGA AATGGACGGG   
  
  
- ATCAAGAGAG AGAGAGTACT CAGTAGTCAT TATCAATGGA GAAATGAAAG AGAAGGAACT CCATACGAAG   
  
  
- TTGAAATTAT ATATATACAT GGTGAACTAA AGAGAACAGT GAAACGATAT TATGAATAAC AAACCGATAG   
  
  
- AAGGGGACAA AATATGAGTA AAAGAACGAA AGATATAGAC CCAAACTTAA CCCAAAGTGA ATAGGAGCAA   
  
  
- CTAAGACACC CTTTAATCAC TTAACCCAAA ACATGTCGTC CAAATATACT TGATCCCAAA GTTTCCATAG   
  
  
- ATGAAAAAGA GAACACTAAT AATCAACTAA TCCCTAAACC GGTTCTAATC GTCTTACCCA AGACTTAAAC   
  
  
- CCCTTAAGAG ACTACTACGA GATTTACCCA TACGAATGCA ACTATAGGGA CGAATACTAC GCAGGAATCT   
  
  
- AATACGGTTA AACAAGTTAA TGCTTCCGGA TAGACTCCTA GAGTGGAGTG ACTCGGAGGG TTCGGGGAAA   
  
  
- AGACTGGGAA ACCGCATGTC AAAGTGCAGG AGAAACCCCG GCTCGGGACC CCAACTAAGA TTACTACTAT   
  
  
- CACTAAGACT ACTACAAGAG TTCACGTAAT CGGTTTACGA ATACCTTCTT CTGTACCTCC GTTTCGGTAC   
  
  
- GTACAAAGTG CTAGGAAATC GTGAAGTCCG ACGACTCTTT GGGAAAATAC TACGGAATCC CTTCTTTATG   
  
  
- GGTTGAAGAG GCTTGGTTGT AGGATATTAA CTACTAACAA ACCTATTAGG ACCACTTTTA AGAAACCCAA   
  
  
- GAAGATCACT AAAATCACAA TCAGTGAAAC CCAGATCAAG TCGTTCGAGA TAACCTGGCT GTTAACACAG   
  
  
- ACTAAACTCA CTCGTAAAAC TCGGTGGGAA ACAAGTTCGT GAAGGTTAAC TTAGTATAGG GGTTGGTAAC   
  
  
- TGGGCAGGAG TTACCAGCAA ACCGGGAAGC CCACGGAATC TAACGTGCCG GAGATTACCA AGTCACTAGA   
  
  
- GTAGGGAACC AAACGGCTAC CTACAATAAT CACATAAGTC ACTCTTTCTC AGGTACTAAG TTAAAGTCTT   
  
  
- TCCCCACCTT CTCCGATCAT TCAAGGAAGG GTTCTTATTA TTGGAACAAT AACTAGAGTT CTTGGAGTGA   
  
  
- AAAGGATTAC TTTGCTTCCT CCTACTAGCT CACTACTACC AATTCTTCCT TTTCTTACTA CACTTAACCA   
  
  
- GATTGATGAG TTCTCCCTCA TTCTAAGTGA TAGCACTTCT GCTCCTGAAA CTTCTTCCTT CCTCATCGTT   
  
  
- CGTCAGTCGA TAAAGATGAT GACTCCTTCG ACTCAACACA CTTTAAAAAC TGTTCAAAAA CGAAACGTTA   
  
  
- ACCATGGGAC ACTTTGGACT CCGAGTAGGG TGGTACTCAA ACTTGGGACT CTTCCCGGTC AGTGTACCTA   
  
  
- ATCTTCCACC CTCTTTACCC TTCCCAGTGG GTTTCTTTGT CCTATTTTCA TCATGGTTGC AACACCTAAA   
  
  
- TTCCTTAAAC GACTACGATA CACGTGTTAG ATGACGTAGA CTACTGGCTT CTTGACGACT ACCTGACGAT   
  
  
- TTCTTTTAGT CCCTCGTGAG TAGACGACTC CCCCTACCTA AAGTTTCCAA CCGAGTAATG AAACGACTAC   
  
  
- GGGATCTCCG TGCAAATCGA CCTTGACCTA GAGTGTAAAT ATGTCGGGAT TCAAGAGTAT CCGGTAGACG   
  
  
- ACAACTGCAC AATTTTCGTA TAGTCAAGAT ACAAGCACGA ACGGGTAAAT TCTTCTAGCA GTAAGCAACA   
  
  
- CCATTTGTAT ACTAAGATTT TCGACGACTT TTCCGTAGTT TCGAAGTATA ATATCTAAAA CCGTAGGAGA   
  
  
- TACCTAAGGT TACCGGATCG GAGTAAGCTA CGGAGAGTCT CGCCAGACCA CCTGGAGGTT TTGACAAATA   
  
  
- ATGTCCCTAG CTAGAGGGGG TCGGACCCAA GTCCGGTCGT CTTTCTCACC TTCGTTGTCC CTCTGCGAAC   
  
  
- CGATTCATGA CACTCGCCAT ATTACACGGT AAACTCATAG TACGGTAACG AGTCTTCACC CTTTGTTAGT   
  
  
- TTGGTCCCCT AGAGTTCAAT CCTTCATCCC TACTACTACT TCAACAGCAC CACTTGACAG AGACATCCAA   
  
  
- GTTCTTGGAG GAACTGCTCT GTTACCACCA CCTATCAGGT TCCTTGTGTC AAAATTTGGA CTAATCTTCC   
  
  
- CATTTTGGGC TATAAAAACA CGTACCGTAA CATTTACCAA GGATGTTGTA GGGAAAGAAA CACTGTGCAA   
  
  
- AATCTCTTCG GGAGAAAGTA ATAAGGTGAG AAAAGCTGTA CAATCTACGG TTGCGGAGAT CCCTCCTCGG   
  
  
- GCTCTCCAAC TATAAGCTCT TCCGTAAGAT ACCCTCCCTC TAATACTTAC ACCACCGTAC ACTCCCGTGT   
  
  
- CTCTCCCACC TTTCCGGTCT CTGTATGTTC GTTACCGTGC AATCCGTATC GTCCCGTCCC AAAGCCGTTG   
  
  
- ATGGTAACCT AGGGTTCAAC TAGCTCTTTT ACTCCAAATT CCGGTTCCGT CTGGTGGTGT TCCTAAAGCA   
  
  
- CTAACTACAC CTACCTGTAA CCCGTTAAGT CCCTACCTTC CCCGCCTAAC GGACACGGTA GAGACGTACC   
  
  
- CAAGACCGAA C

+     CGTCA-motif

| Site Name | Organism | Position | Strand | Matrix score. | sequence | function |
| --- | --- | --- | --- | --- | --- | --- |
| CGTCA-motif | Hordeum vulgare | 3797 | - | 5 | CGTCA | cis-acting regulatory element involved in the MeJA-responsiveness |
| CGTCA-motif | Hordeum vulgare | 968 | - | 5 | CGTCA | cis-acting regulatory element involved in the MeJA-responsiveness |
| CGTCA-motif | Hordeum vulgare | 1516 | + | 5 | CGTCA | cis-acting regulatory element involved in the MeJA-responsiveness |
| CGTCA-motif | Hordeum vulgare | 3368 | - | 5 | CGTCA | cis-acting regulatory element involved in the MeJA-responsiveness |
| CGTCA-motif | Hordeum vulgare | 3422 | + | 5 | CGTCA | cis-acting regulatory element involved in the MeJA-responsiveness |

>HU07G02248.1   
+ -Up\_Stream \_Len000CTGTTC AGCACTAGGG AATAAGGTTT TTGCCCTGCA TACTATGAAA TCTAACCAAA   
  
  
+ AACATTCCAT CTTGGAAGGC TGTAGCCTTG TGGATTTTGA ATTCGCTCCC AAGAACCAAT GAAGTAGCGC   
  
  
+ AGAAGGCCAA TACTCAATTT CAGAGTCCAC ATCAACTTGT TCAATGCCAG TATATTTCAG AACATTAATT   
  
  
+ ACATACTGTT TGGTTGGAGA AAAAATGGTT GTCTCGAAAA CTAGTTTTTT AATTTTTTGT TATTTAGCAC   
  
  
+ GGTAAAGAAT GGAAATTATT TTCATGCGAG AAAAAAGGAA GTTGGTTTTT CTTTTTTCAA CTCTTTTGAT   
  
  
+ GATATAATCA GTCGAAATTT AGGTGCACCA TCTGACTTCA ACTCTTGTAC GACCTCTGAT TGCTTGCTTA   
  
  
+ GAAGAACAAG AGGATTGACA GATAAGGAGG AGTGGGTGGT TTATACAGAG AATTGAGTGG ATGGTTATAA   
  
  
+ AGAAGTCGAT CTCACATAAG TAACTTCGTT ACGATTATAA ATATTTTCAT TGAAGAAAGA CGATTAGATA   
  
  
+ TAATTAAGAA GGTACATCTA AGCAAATTTG GCTGAGCAGA GTTGGAGGGC CACTAGGGTC ACATTGTGTC   
  
  
+ TTGCGCAACA AGCTTAGACC ATTCGCAACG GATGCCTCAC GTGGAGCAGG TGGGACCCGC GTGGAAGTTG   
  
  
+ CATGGCAAGA ATTCTCTGGC AGCAAGGAGA ATTGAGGGGG GACACGCGTG TGACCACGCC AGTGCGGTCC   
  
  
+ CCCTCTCGTG GCAGATTTGG TGATTTTTTT TTTGTTTTTT TTTGAAGTAA TATATAATTT TTATATGATT   
  
  
+ AAAAAAATAG GTTGTGTGTT ATAAAGTGGG TCAATGACAA ATATTTTTAT TGGATGATTA AAATTAAGTT   
  
  
+ CCATTTATTG GAGAAGTGAT GGAATGCAAG TTCCAAAATT GTGGAACTTT TGTTGACGTA GCAGGCTAGA   
  
  
+ GTTCCAAAAA TTAAAAAAAG GAAAGACATA TTGAGGTAAA GCCTTTTTAT TTTACAAATA GACGCACCAA   
  
  
+ GGTATAATAT AATTGACATG ATTTGAGGCG AGAGGAAGAT TCTTTTGCTA CCCAAACAAA ACACAATGAT   
  
  
+ CTTTTAAATG TAGCAATTTG GGATTGGCAA AATTTTGTCT AACCCGATTG TTGAAAGTTA TCCGATTTCA   
  
  
+ TAAAATTGGA TATCGTTATT AAATTTTCAT TCGAATATTT AAATTTGGAC ACAATGTTTT TCATATTTGG   
  
  
+ ATTTTCTAAA CTTGATTTTA ACTCAAATTT AAGTTTATTC AATTCATCTG ACCCAAAAAA TGGTTGCAAT   
  
  
+ AATGTATTTT CTTTTTGAAG TTAAATATTA TCGAATTCAT TTAACTTGTA GTTAACCCGT AATTGACCCA   
  
  
+ AACTCGACTT TTTGCTACCT TAGCAGCCAG GAAGAAGTCA TAGTATTGAC CCGTGGAACG CCTGAAGCCA   
  
  
+ ACATCACCAG ACCCACATAT TCCATCATCT TCTGATCTCC TCGTCAAAGA AGGAAGCTGA GCACAGCAAT   
  
  
+ AACTAGAAGA ACAGGAGGCA TCACACCCAA GCCAAAGGCC TTTATTTGAG CTCCCCACCT CATTCCTCGA   
  
  
+ AGCTTCCTCT TCACAATGTA AGACCATAAT CCTTCACCCC TTCTCTTTTT AAGCTCATCT TTACCTGCCC   
  
  
+ TAGTTCTCTC TCTCTCATGA GTCATCAGTA ATAGTTACCT CTTTACTTTC TCTTCCTTGA GGTATGCTTC   
  
  
+ AACTTTAATA TATATATGTA CCACTTGATT TCTCTTGTCA CTTTGCTATA ATACTTATTG TTTGGCTATC   
  
  
+ TTCCCCTGTT TTATACTCAT TTTCTTGCTT TCTATATCTG GGTTTGAATT GGGTTTCACT TATCCTCGTT   
  
  
+ GATTCTGTGG GAAATTAGTG AATTGGGTTT TGTACAGCAG GTTTATATGA ACTAGGGTTT CAAAGGTATC   
  
  
+ TACTTTTTCT CTTGTGATTA TTAGTTGATT AGGGATTTGG CCAAGATTAG CAGAATGGGT TCTGAATTTG   
  
  
+ GGGAATTCTC TGATGATGCT CTAAATGGGT ATGCTTACGT TGATATCCCT GCTTATGATG CGTCCTTAGA   
  
  
+ TTATGCCAAT TTGTTCAATT ACGAAGGCCT ATCTGAGGAT CTCACCTCAC TGAGCCTCCC AAGCCCCTTT   
  
  
+ TCTGACCCTT TGGCGTACAG TTTCACGTCC TCTTTGGGGC CGAGCCCTGG GGTTGATTCT AATGATGATA   
  
  
+ GTGATTCTGA TGATGTTCTC AAGTGCATTA GCCAAATGCT TATGGAAGAA GACATGGAGG CAAAGCCATG   
  
  
+ CATGTTTCAC GATCCTTTAG CACTTCAGGC TGCTGAGAAA CCCTTTTATG ATGCCTTAGG GAAGAAATAC   
  
  
+ CCAACTTCTC CGAACCAACA TCCTATAATT GATGATTGTT TGGATAATCC TGGTGAAAAT TCTTTGGGTT   
  
  
+ CTTCTAGTGA TTTTAGTGTT AGTCACTTTG GGTCTAGTTC AGCAAGCTCT ATTGGACCGA CAATTGTGTC   
  
  
+ TGATTTGAGT GAGCATTTTG AGCCACCCTT TGTTCAAGCA CTTCCAATTG AATCATATCC CCAACCATTG   
  
  
+ ACCCGTCCTC AATGGTCGTT TGGCCCTTCG GGTGCCTTAG ATTGCACGGC CTCTAATGGT TCAGTGATCT   
  
  
+ CATCCCTTGG TTTGCCGATG GATGTTATTA GTGTATTCAG TGAGAAAGAG TCCATGATTC AATTTCAGAA   
  
  
+ AGGGGTGGAA GAGGCTAGTA AGTTCCTTCC CAAGAATAAT AACCTTGTTA TTGATCTCAA GAACCTCACT   
  
  
+ TTTCCTAATG AAACGAAGGA GGATGATCGA GTGATGATGG TTAAGAAGGA AAAGAATGAT GTGAATTGGT   
  
  
+ CTAACTACTC AAGAGGGAGT AAGATTCACT ATCGTGAAGA CGAGGACTTT GAAGAAGGAA GGAGTAGCAA   
  
  
+ GCAGTCAGCT ATTTCTACTA CTGAGGAAGC TGAGTTGTGT GAAATTTTTG ACAAGTTTTT GCTTTGCAAT   
  
  
+ TGGTACCCTG TGAAACCTGA GGCTCATCCC ACCATGAGTT TGAACCCTGA GAAGGGCCAG TCACATGGAT   
  
  
+ TAGAAGGTGG GAGAAATGGG AAGGGTCACC CAAAGAAACA GGATAAAAGT AGTACCAACG TTGTGGATTT   
  
  
+ AAGGAATTTG CTGATGCTAT GTGCACAATC TACTGCATCT GATGACCGAA GAACTGCTGA TGGACTGCTA   
  
  
+ AAGAAAATCA GGGAGCACTC ATCTGCTGAG GGGGATGGAT TTCAAAGGTT GGCTCATTAC TTTGCTGATG   
  
  
+ CCCTAGAGGC ACGTTTAGCT GGAACTGGAT CTCACATTTA TACAGCCCTA AGTTCTCATA GGCCATCTGC   
  
  
+ TGTTGACGTG TTAAAAGCAT ATCAGTTCTA TGTTCGTGCT TGCCCATTTA AGAAGATCGT CATTCGTTGT   
  
  
+ GGTAAACATA TGATTCTAAA AGCTGCTGAA AAGGCATCAA AGCTTCATAT TATAGATTTT GGCATCCTCT   
  
  
+ ATGGATTCCA ATGGCCTAGC CTCATTCGAT GCCTCTCAGA GCGGTCTGGT GGACCTCCAA AACTGTTTAT   
  
  
+ TACAGGGATC GATCTCCCCC AGCCTGGGTT CAGGCCAGCA GAAAGAGTGG AAGCAACAGG GAGACGCTTG   
  
  
+ GCTAAGTACT GTGAGCGGTA TAATGTGCCA TTTGAGTATC ATGCCATTGC TCAGAAGTGG GAAACAATCA   
  
  
+ AACCAGGGGA TCTCAAGTTA GGAAGTAGGG ATGATGATGA AGTTGTCGTG GTGAACTGTC TCTGTAGGTT   
  
  
+ CAAGAACCTC CTTGACGAGA CAATGGTGGT GGATAGTCCA AGGAACACAG TTTTAAACCT GATTAGAAGG   
  
  
+ GTAAAACCCG ATATTTTTGT GCATGGCATT GTAAATGGTT CCTACAACAT CCCTTTCTTT GTGACACGTT   
  
  
+ TTAGAGAAGC CCTCTTTCAT TATTCCACTC TTTTCGACAT GTTAGATGCC AACGCCTCTA GGGAGGAGCC   
  
  
+ CGAGAGGTTG ATATTCGAGA AGGCATTCTA TGGGAGGGAG ATTATGAATG TGGTGGCATG TGAGGGCACA   
  
  
+ GAGAGGGTGG AAAGGCCAGA GACATACAAG CAATGGCACG TTAGGCATAG CAGGGCAGGG TTTCGGCAAC   
  
  
+ TACCATTGGA TCCCAAGTTG ATCGAGAAAA TGAGGTTTAA GGCCAAGGCA GACCACCACA AGGATTTCGT   
  
  
+ GATTGATGTG GATGGACATT GGGCAATTCA GGGATGGAAG GGGCGGATTG CCTGTGCCAT CTCTGCATGG   
  
  
+ GTTCTGGCTT G  

- -Up\_Stream \_Len000GACAAG TCGTGATCCC TTATTCCAAA AACGGGACGT ATGATACTTT AGATTGGTTT   
  
  
- TTGTAAGGTA GAACCTTCCG ACATCGGAAC ACCTAAAACT TAAGCGAGGG TTCTTGGTTA CTTCATCGCG   
  
  
- TCTTCCGGTT ATGAGTTAAA GTCTCAGGTG TAGTTGAACA AGTTACGGTC ATATAAAGTC TTGTAATTAA   
  
  
- TGTATGACAA ACCAACCTCT TTTTTACCAA CAGAGCTTTT GATCAAAAAA TTAAAAAACA ATAAATCGTG   
  
  
- CCATTTCTTA CCTTTAATAA AAGTACGCTC TTTTTTCCTT CAACCAAAAA GAAAAAAGTT GAGAAAACTA   
  
  
- CTATATTAGT CAGCTTTAAA TCCACGTGGT AGACTGAAGT TGAGAACATG CTGGAGACTA ACGAACGAAT   
  
  
- CTTCTTGTTC TCCTAACTGT CTATTCCTCC TCACCCACCA AATATGTCTC TTAACTCACC TACCAATATT   
  
  
- TCTTCAGCTA GAGTGTATTC ATTGAAGCAA TGCTAATATT TATAAAAGTA ACTTCTTTCT GCTAATCTAT   
  
  
- ATTAATTCTT CCATGTAGAT TCGTTTAAAC CGACTCGTCT CAACCTCCCG GTGATCCCAG TGTAACACAG   
  
  
- AACGCGTTGT TCGAATCTGG TAAGCGTTGC CTACGGAGTG CACCTCGTCC ACCCTGGGCG CACCTTCAAC   
  
  
- GTACCGTTCT TAAGAGACCG TCGTTCCTCT TAACTCCCCC CTGTGCGCAC ACTGGTGCGG TCACGCCAGG   
  
  
- GGGAGAGCAC CGTCTAAACC ACTAAAAAAA AAACAAAAAA AAACTTCATT ATATATTAAA AATATACTAA   
  
  
- TTTTTTTATC CAACACACAA TATTTCACCC AGTTACTGTT TATAAAAATA ACCTACTAAT TTTAATTCAA   
  
  
- GGTAAATAAC CTCTTCACTA CCTTACGTTC AAGGTTTTAA CACCTTGAAA ACAACTGCAT CGTCCGATCT   
  
  
- CAAGGTTTTT AATTTTTTTC CTTTCTGTAT AACTCCATTT CGGAAAAATA AAATGTTTAT CTGCGTGGTT   
  
  
- CCATATTATA TTAACTGTAC TAAACTCCGC TCTCCTTCTA AGAAAACGAT GGGTTTGTTT TGTGTTACTA   
  
  
- GAAAATTTAC ATCGTTAAAC CCTAACCGTT TTAAAACAGA TTGGGCTAAC AACTTTCAAT AGGCTAAAGT   
  
  
- ATTTTAACCT ATAGCAATAA TTTAAAAGTA AGCTTATAAA TTTAAACCTG TGTTACAAAA AGTATAAACC   
  
  
- TAAAAGATTT GAACTAAAAT TGAGTTTAAA TTCAAATAAG TTAAGTAGAC TGGGTTTTTT ACCAACGTTA   
  
  
- TTACATAAAA GAAAAACTTC AATTTATAAT AGCTTAAGTA AATTGAACAT CAATTGGGCA TTAACTGGGT   
  
  
- TTGAGCTGAA AAACGATGGA ATCGTCGGTC CTTCTTCAGT ATCATAACTG GGCACCTTGC GGACTTCGGT   
  
  
- TGTAGTGGTC TGGGTGTATA AGGTAGTAGA AGACTAGAGG AGCAGTTTCT TCCTTCGACT CGTGTCGTTA   
  
  
- TTGATCTTCT TGTCCTCCGT AGTGTGGGTT CGGTTTCCGG AAATAAACTC GAGGGGTGGA GTAAGGAGCT   
  
  
- TCGAAGGAGA AGTGTTACAT TCTGGTATTA GGAAGTGGGG AAGAGAAAAA TTCGAGTAGA AATGGACGGG   
  
  
- ATCAAGAGAG AGAGAGTACT CAGTAGTCAT TATCAATGGA GAAATGAAAG AGAAGGAACT CCATACGAAG   
  
  
- TTGAAATTAT ATATATACAT GGTGAACTAA AGAGAACAGT GAAACGATAT TATGAATAAC AAACCGATAG   
  
  
- AAGGGGACAA AATATGAGTA AAAGAACGAA AGATATAGAC CCAAACTTAA CCCAAAGTGA ATAGGAGCAA   
  
  
- CTAAGACACC CTTTAATCAC TTAACCCAAA ACATGTCGTC CAAATATACT TGATCCCAAA GTTTCCATAG   
  
  
- ATGAAAAAGA GAACACTAAT AATCAACTAA TCCCTAAACC GGTTCTAATC GTCTTACCCA AGACTTAAAC   
  
  
- CCCTTAAGAG ACTACTACGA GATTTACCCA TACGAATGCA ACTATAGGGA CGAATACTAC GCAGGAATCT   
  
  
- AATACGGTTA AACAAGTTAA TGCTTCCGGA TAGACTCCTA GAGTGGAGTG ACTCGGAGGG TTCGGGGAAA   
  
  
- AGACTGGGAA ACCGCATGTC AAAGTGCAGG AGAAACCCCG GCTCGGGACC CCAACTAAGA TTACTACTAT   
  
  
- CACTAAGACT ACTACAAGAG TTCACGTAAT CGGTTTACGA ATACCTTCTT CTGTACCTCC GTTTCGGTAC   
  
  
- GTACAAAGTG CTAGGAAATC GTGAAGTCCG ACGACTCTTT GGGAAAATAC TACGGAATCC CTTCTTTATG   
  
  
- GGTTGAAGAG GCTTGGTTGT AGGATATTAA CTACTAACAA ACCTATTAGG ACCACTTTTA AGAAACCCAA   
  
  
- GAAGATCACT AAAATCACAA TCAGTGAAAC CCAGATCAAG TCGTTCGAGA TAACCTGGCT GTTAACACAG   
  
  
- ACTAAACTCA CTCGTAAAAC TCGGTGGGAA ACAAGTTCGT GAAGGTTAAC TTAGTATAGG GGTTGGTAAC   
  
  
- TGGGCAGGAG TTACCAGCAA ACCGGGAAGC CCACGGAATC TAACGTGCCG GAGATTACCA AGTCACTAGA   
  
  
- GTAGGGAACC AAACGGCTAC CTACAATAAT CACATAAGTC ACTCTTTCTC AGGTACTAAG TTAAAGTCTT   
  
  
- TCCCCACCTT CTCCGATCAT TCAAGGAAGG GTTCTTATTA TTGGAACAAT AACTAGAGTT CTTGGAGTGA   
  
  
- AAAGGATTAC TTTGCTTCCT CCTACTAGCT CACTACTACC AATTCTTCCT TTTCTTACTA CACTTAACCA   
  
  
- GATTGATGAG TTCTCCCTCA TTCTAAGTGA TAGCACTTCT GCTCCTGAAA CTTCTTCCTT CCTCATCGTT   
  
  
- CGTCAGTCGA TAAAGATGAT GACTCCTTCG ACTCAACACA CTTTAAAAAC TGTTCAAAAA CGAAACGTTA   
  
  
- ACCATGGGAC ACTTTGGACT CCGAGTAGGG TGGTACTCAA ACTTGGGACT CTTCCCGGTC AGTGTACCTA   
  
  
- ATCTTCCACC CTCTTTACCC TTCCCAGTGG GTTTCTTTGT CCTATTTTCA TCATGGTTGC AACACCTAAA   
  
  
- TTCCTTAAAC GACTACGATA CACGTGTTAG ATGACGTAGA CTACTGGCTT CTTGACGACT ACCTGACGAT   
  
  
- TTCTTTTAGT CCCTCGTGAG TAGACGACTC CCCCTACCTA AAGTTTCCAA CCGAGTAATG AAACGACTAC   
  
  
- GGGATCTCCG TGCAAATCGA CCTTGACCTA GAGTGTAAAT ATGTCGGGAT TCAAGAGTAT CCGGTAGACG   
  
  
- ACAACTGCAC AATTTTCGTA TAGTCAAGAT ACAAGCACGA ACGGGTAAAT TCTTCTAGCA GTAAGCAACA   
  
  
- CCATTTGTAT ACTAAGATTT TCGACGACTT TTCCGTAGTT TCGAAGTATA ATATCTAAAA CCGTAGGAGA   
  
  
- TACCTAAGGT TACCGGATCG GAGTAAGCTA CGGAGAGTCT CGCCAGACCA CCTGGAGGTT TTGACAAATA   
  
  
- ATGTCCCTAG CTAGAGGGGG TCGGACCCAA GTCCGGTCGT CTTTCTCACC TTCGTTGTCC CTCTGCGAAC   
  
  
- CGATTCATGA CACTCGCCAT ATTACACGGT AAACTCATAG TACGGTAACG AGTCTTCACC CTTTGTTAGT   
  
  
- TTGGTCCCCT AGAGTTCAAT CCTTCATCCC TACTACTACT TCAACAGCAC CACTTGACAG AGACATCCAA   
  
  
- GTTCTTGGAG GAACTGCTCT GTTACCACCA CCTATCAGGT TCCTTGTGTC AAAATTTGGA CTAATCTTCC   
  
  
- CATTTTGGGC TATAAAAACA CGTACCGTAA CATTTACCAA GGATGTTGTA GGGAAAGAAA CACTGTGCAA   
  
  
- AATCTCTTCG GGAGAAAGTA ATAAGGTGAG AAAAGCTGTA CAATCTACGG TTGCGGAGAT CCCTCCTCGG   
  
  
- GCTCTCCAAC TATAAGCTCT TCCGTAAGAT ACCCTCCCTC TAATACTTAC ACCACCGTAC ACTCCCGTGT   
  
  
- CTCTCCCACC TTTCCGGTCT CTGTATGTTC GTTACCGTGC AATCCGTATC GTCCCGTCCC AAAGCCGTTG   
  
  
- ATGGTAACCT AGGGTTCAAC TAGCTCTTTT ACTCCAAATT CCGGTTCCGT CTGGTGGTGT TCCTAAAGCA   
  
  
- CTAACTACAC CTACCTGTAA CCCGTTAAGT CCCTACCTTC CCCGCCTAAC GGACACGGTA GAGACGTACC   
  
  
- CAAGACCGAA C

+     CTAG-motif

| Site Name | Organism | Position | Strand | Matrix score. | sequence | function |
| --- | --- | --- | --- | --- | --- | --- |
| CTAG-motif | Avena sativa | 2453 | - | 9 | ACTAGCAGAA |  |
| CTAG-motif | Avena sativa | 2010 | + | 9 | ACTAGCAGAA |  |
| CTAG-motif | Avena sativa | 1546 | + | 9 | ACTAGCAGAA |  |

>HU07G02248.1   
+ -Up\_Stream \_Len000CTGTTC AGCACTAGGG AATAAGGTTT TTGCCCTGCA TACTATGAAA TCTAACCAAA   
  
  
+ AACATTCCAT CTTGGAAGGC TGTAGCCTTG TGGATTTTGA ATTCGCTCCC AAGAACCAAT GAAGTAGCGC   
  
  
+ AGAAGGCCAA TACTCAATTT CAGAGTCCAC ATCAACTTGT TCAATGCCAG TATATTTCAG AACATTAATT   
  
  
+ ACATACTGTT TGGTTGGAGA AAAAATGGTT GTCTCGAAAA CTAGTTTTTT AATTTTTTGT TATTTAGCAC   
  
  
+ GGTAAAGAAT GGAAATTATT TTCATGCGAG AAAAAAGGAA GTTGGTTTTT CTTTTTTCAA CTCTTTTGAT   
  
  
+ GATATAATCA GTCGAAATTT AGGTGCACCA TCTGACTTCA ACTCTTGTAC GACCTCTGAT TGCTTGCTTA   
  
  
+ GAAGAACAAG AGGATTGACA GATAAGGAGG AGTGGGTGGT TTATACAGAG AATTGAGTGG ATGGTTATAA   
  
  
+ AGAAGTCGAT CTCACATAAG TAACTTCGTT ACGATTATAA ATATTTTCAT TGAAGAAAGA CGATTAGATA   
  
  
+ TAATTAAGAA GGTACATCTA AGCAAATTTG GCTGAGCAGA GTTGGAGGGC CACTAGGGTC ACATTGTGTC   
  
  
+ TTGCGCAACA AGCTTAGACC ATTCGCAACG GATGCCTCAC GTGGAGCAGG TGGGACCCGC GTGGAAGTTG   
  
  
+ CATGGCAAGA ATTCTCTGGC AGCAAGGAGA ATTGAGGGGG GACACGCGTG TGACCACGCC AGTGCGGTCC   
  
  
+ CCCTCTCGTG GCAGATTTGG TGATTTTTTT TTTGTTTTTT TTTGAAGTAA TATATAATTT TTATATGATT   
  
  
+ AAAAAAATAG GTTGTGTGTT ATAAAGTGGG TCAATGACAA ATATTTTTAT TGGATGATTA AAATTAAGTT   
  
  
+ CCATTTATTG GAGAAGTGAT GGAATGCAAG TTCCAAAATT GTGGAACTTT TGTTGACGTA GCAGGCTAGA   
  
  
+ GTTCCAAAAA TTAAAAAAAG GAAAGACATA TTGAGGTAAA GCCTTTTTAT TTTACAAATA GACGCACCAA   
  
  
+ GGTATAATAT AATTGACATG ATTTGAGGCG AGAGGAAGAT TCTTTTGCTA CCCAAACAAA ACACAATGAT   
  
  
+ CTTTTAAATG TAGCAATTTG GGATTGGCAA AATTTTGTCT AACCCGATTG TTGAAAGTTA TCCGATTTCA   
  
  
+ TAAAATTGGA TATCGTTATT AAATTTTCAT TCGAATATTT AAATTTGGAC ACAATGTTTT TCATATTTGG   
  
  
+ ATTTTCTAAA CTTGATTTTA ACTCAAATTT AAGTTTATTC AATTCATCTG ACCCAAAAAA TGGTTGCAAT   
  
  
+ AATGTATTTT CTTTTTGAAG TTAAATATTA TCGAATTCAT TTAACTTGTA GTTAACCCGT AATTGACCCA   
  
  
+ AACTCGACTT TTTGCTACCT TAGCAGCCAG GAAGAAGTCA TAGTATTGAC CCGTGGAACG CCTGAAGCCA   
  
  
+ ACATCACCAG ACCCACATAT TCCATCATCT TCTGATCTCC TCGTCAAAGA AGGAAGCTGA GCACAGCAAT   
  
  
+ AACTAGAAGA ACAGGAGGCA TCACACCCAA GCCAAAGGCC TTTATTTGAG CTCCCCACCT CATTCCTCGA   
  
  
+ AGCTTCCTCT TCACAATGTA AGACCATAAT CCTTCACCCC TTCTCTTTTT AAGCTCATCT TTACCTGCCC   
  
  
+ TAGTTCTCTC TCTCTCATGA GTCATCAGTA ATAGTTACCT CTTTACTTTC TCTTCCTTGA GGTATGCTTC   
  
  
+ AACTTTAATA TATATATGTA CCACTTGATT TCTCTTGTCA CTTTGCTATA ATACTTATTG TTTGGCTATC   
  
  
+ TTCCCCTGTT TTATACTCAT TTTCTTGCTT TCTATATCTG GGTTTGAATT GGGTTTCACT TATCCTCGTT   
  
  
+ GATTCTGTGG GAAATTAGTG AATTGGGTTT TGTACAGCAG GTTTATATGA ACTAGGGTTT CAAAGGTATC   
  
  
+ TACTTTTTCT CTTGTGATTA TTAGTTGATT AGGGATTTGG CCAAGATTAG CAGAATGGGT TCTGAATTTG   
  
  
+ GGGAATTCTC TGATGATGCT CTAAATGGGT ATGCTTACGT TGATATCCCT GCTTATGATG CGTCCTTAGA   
  
  
+ TTATGCCAAT TTGTTCAATT ACGAAGGCCT ATCTGAGGAT CTCACCTCAC TGAGCCTCCC AAGCCCCTTT   
  
  
+ TCTGACCCTT TGGCGTACAG TTTCACGTCC TCTTTGGGGC CGAGCCCTGG GGTTGATTCT AATGATGATA   
  
  
+ GTGATTCTGA TGATGTTCTC AAGTGCATTA GCCAAATGCT TATGGAAGAA GACATGGAGG CAAAGCCATG   
  
  
+ CATGTTTCAC GATCCTTTAG CACTTCAGGC TGCTGAGAAA CCCTTTTATG ATGCCTTAGG GAAGAAATAC   
  
  
+ CCAACTTCTC CGAACCAACA TCCTATAATT GATGATTGTT TGGATAATCC TGGTGAAAAT TCTTTGGGTT   
  
  
+ CTTCTAGTGA TTTTAGTGTT AGTCACTTTG GGTCTAGTTC AGCAAGCTCT ATTGGACCGA CAATTGTGTC   
  
  
+ TGATTTGAGT GAGCATTTTG AGCCACCCTT TGTTCAAGCA CTTCCAATTG AATCATATCC CCAACCATTG   
  
  
+ ACCCGTCCTC AATGGTCGTT TGGCCCTTCG GGTGCCTTAG ATTGCACGGC CTCTAATGGT TCAGTGATCT   
  
  
+ CATCCCTTGG TTTGCCGATG GATGTTATTA GTGTATTCAG TGAGAAAGAG TCCATGATTC AATTTCAGAA   
  
  
+ AGGGGTGGAA GAGGCTAGTA AGTTCCTTCC CAAGAATAAT AACCTTGTTA TTGATCTCAA GAACCTCACT   
  
  
+ TTTCCTAATG AAACGAAGGA GGATGATCGA GTGATGATGG TTAAGAAGGA AAAGAATGAT GTGAATTGGT   
  
  
+ CTAACTACTC AAGAGGGAGT AAGATTCACT ATCGTGAAGA CGAGGACTTT GAAGAAGGAA GGAGTAGCAA   
  
  
+ GCAGTCAGCT ATTTCTACTA CTGAGGAAGC TGAGTTGTGT GAAATTTTTG ACAAGTTTTT GCTTTGCAAT   
  
  
+ TGGTACCCTG TGAAACCTGA GGCTCATCCC ACCATGAGTT TGAACCCTGA GAAGGGCCAG TCACATGGAT   
  
  
+ TAGAAGGTGG GAGAAATGGG AAGGGTCACC CAAAGAAACA GGATAAAAGT AGTACCAACG TTGTGGATTT   
  
  
+ AAGGAATTTG CTGATGCTAT GTGCACAATC TACTGCATCT GATGACCGAA GAACTGCTGA TGGACTGCTA   
  
  
+ AAGAAAATCA GGGAGCACTC ATCTGCTGAG GGGGATGGAT TTCAAAGGTT GGCTCATTAC TTTGCTGATG   
  
  
+ CCCTAGAGGC ACGTTTAGCT GGAACTGGAT CTCACATTTA TACAGCCCTA AGTTCTCATA GGCCATCTGC   
  
  
+ TGTTGACGTG TTAAAAGCAT ATCAGTTCTA TGTTCGTGCT TGCCCATTTA AGAAGATCGT CATTCGTTGT   
  
  
+ GGTAAACATA TGATTCTAAA AGCTGCTGAA AAGGCATCAA AGCTTCATAT TATAGATTTT GGCATCCTCT   
  
  
+ ATGGATTCCA ATGGCCTAGC CTCATTCGAT GCCTCTCAGA GCGGTCTGGT GGACCTCCAA AACTGTTTAT   
  
  
+ TACAGGGATC GATCTCCCCC AGCCTGGGTT CAGGCCAGCA GAAAGAGTGG AAGCAACAGG GAGACGCTTG   
  
  
+ GCTAAGTACT GTGAGCGGTA TAATGTGCCA TTTGAGTATC ATGCCATTGC TCAGAAGTGG GAAACAATCA   
  
  
+ AACCAGGGGA TCTCAAGTTA GGAAGTAGGG ATGATGATGA AGTTGTCGTG GTGAACTGTC TCTGTAGGTT   
  
  
+ CAAGAACCTC CTTGACGAGA CAATGGTGGT GGATAGTCCA AGGAACACAG TTTTAAACCT GATTAGAAGG   
  
  
+ GTAAAACCCG ATATTTTTGT GCATGGCATT GTAAATGGTT CCTACAACAT CCCTTTCTTT GTGACACGTT   
  
  
+ TTAGAGAAGC CCTCTTTCAT TATTCCACTC TTTTCGACAT GTTAGATGCC AACGCCTCTA GGGAGGAGCC   
  
  
+ CGAGAGGTTG ATATTCGAGA AGGCATTCTA TGGGAGGGAG ATTATGAATG TGGTGGCATG TGAGGGCACA   
  
  
+ GAGAGGGTGG AAAGGCCAGA GACATACAAG CAATGGCACG TTAGGCATAG CAGGGCAGGG TTTCGGCAAC   
  
  
+ TACCATTGGA TCCCAAGTTG ATCGAGAAAA TGAGGTTTAA GGCCAAGGCA GACCACCACA AGGATTTCGT   
  
  
+ GATTGATGTG GATGGACATT GGGCAATTCA GGGATGGAAG GGGCGGATTG CCTGTGCCAT CTCTGCATGG   
  
  
+ GTTCTGGCTT G  

- -Up\_Stream \_Len000GACAAG TCGTGATCCC TTATTCCAAA AACGGGACGT ATGATACTTT AGATTGGTTT   
  
  
- TTGTAAGGTA GAACCTTCCG ACATCGGAAC ACCTAAAACT TAAGCGAGGG TTCTTGGTTA CTTCATCGCG   
  
  
- TCTTCCGGTT ATGAGTTAAA GTCTCAGGTG TAGTTGAACA AGTTACGGTC ATATAAAGTC TTGTAATTAA   
  
  
- TGTATGACAA ACCAACCTCT TTTTTACCAA CAGAGCTTTT GATCAAAAAA TTAAAAAACA ATAAATCGTG   
  
  
- CCATTTCTTA CCTTTAATAA AAGTACGCTC TTTTTTCCTT CAACCAAAAA GAAAAAAGTT GAGAAAACTA   
  
  
- CTATATTAGT CAGCTTTAAA TCCACGTGGT AGACTGAAGT TGAGAACATG CTGGAGACTA ACGAACGAAT   
  
  
- CTTCTTGTTC TCCTAACTGT CTATTCCTCC TCACCCACCA AATATGTCTC TTAACTCACC TACCAATATT   
  
  
- TCTTCAGCTA GAGTGTATTC ATTGAAGCAA TGCTAATATT TATAAAAGTA ACTTCTTTCT GCTAATCTAT   
  
  
- ATTAATTCTT CCATGTAGAT TCGTTTAAAC CGACTCGTCT CAACCTCCCG GTGATCCCAG TGTAACACAG   
  
  
- AACGCGTTGT TCGAATCTGG TAAGCGTTGC CTACGGAGTG CACCTCGTCC ACCCTGGGCG CACCTTCAAC   
  
  
- GTACCGTTCT TAAGAGACCG TCGTTCCTCT TAACTCCCCC CTGTGCGCAC ACTGGTGCGG TCACGCCAGG   
  
  
- GGGAGAGCAC CGTCTAAACC ACTAAAAAAA AAACAAAAAA AAACTTCATT ATATATTAAA AATATACTAA   
  
  
- TTTTTTTATC CAACACACAA TATTTCACCC AGTTACTGTT TATAAAAATA ACCTACTAAT TTTAATTCAA   
  
  
- GGTAAATAAC CTCTTCACTA CCTTACGTTC AAGGTTTTAA CACCTTGAAA ACAACTGCAT CGTCCGATCT   
  
  
- CAAGGTTTTT AATTTTTTTC CTTTCTGTAT AACTCCATTT CGGAAAAATA AAATGTTTAT CTGCGTGGTT   
  
  
- CCATATTATA TTAACTGTAC TAAACTCCGC TCTCCTTCTA AGAAAACGAT GGGTTTGTTT TGTGTTACTA   
  
  
- GAAAATTTAC ATCGTTAAAC CCTAACCGTT TTAAAACAGA TTGGGCTAAC AACTTTCAAT AGGCTAAAGT   
  
  
- ATTTTAACCT ATAGCAATAA TTTAAAAGTA AGCTTATAAA TTTAAACCTG TGTTACAAAA AGTATAAACC   
  
  
- TAAAAGATTT GAACTAAAAT TGAGTTTAAA TTCAAATAAG TTAAGTAGAC TGGGTTTTTT ACCAACGTTA   
  
  
- TTACATAAAA GAAAAACTTC AATTTATAAT AGCTTAAGTA AATTGAACAT CAATTGGGCA TTAACTGGGT   
  
  
- TTGAGCTGAA AAACGATGGA ATCGTCGGTC CTTCTTCAGT ATCATAACTG GGCACCTTGC GGACTTCGGT   
  
  
- TGTAGTGGTC TGGGTGTATA AGGTAGTAGA AGACTAGAGG AGCAGTTTCT TCCTTCGACT CGTGTCGTTA   
  
  
- TTGATCTTCT TGTCCTCCGT AGTGTGGGTT CGGTTTCCGG AAATAAACTC GAGGGGTGGA GTAAGGAGCT   
  
  
- TCGAAGGAGA AGTGTTACAT TCTGGTATTA GGAAGTGGGG AAGAGAAAAA TTCGAGTAGA AATGGACGGG   
  
  
- ATCAAGAGAG AGAGAGTACT CAGTAGTCAT TATCAATGGA GAAATGAAAG AGAAGGAACT CCATACGAAG   
  
  
- TTGAAATTAT ATATATACAT GGTGAACTAA AGAGAACAGT GAAACGATAT TATGAATAAC AAACCGATAG   
  
  
- AAGGGGACAA AATATGAGTA AAAGAACGAA AGATATAGAC CCAAACTTAA CCCAAAGTGA ATAGGAGCAA   
  
  
- CTAAGACACC CTTTAATCAC TTAACCCAAA ACATGTCGTC CAAATATACT TGATCCCAAA GTTTCCATAG   
  
  
- ATGAAAAAGA GAACACTAAT AATCAACTAA TCCCTAAACC GGTTCTAATC GTCTTACCCA AGACTTAAAC   
  
  
- CCCTTAAGAG ACTACTACGA GATTTACCCA TACGAATGCA ACTATAGGGA CGAATACTAC GCAGGAATCT   
  
  
- AATACGGTTA AACAAGTTAA TGCTTCCGGA TAGACTCCTA GAGTGGAGTG ACTCGGAGGG TTCGGGGAAA   
  
  
- AGACTGGGAA ACCGCATGTC AAAGTGCAGG AGAAACCCCG GCTCGGGACC CCAACTAAGA TTACTACTAT   
  
  
- CACTAAGACT ACTACAAGAG TTCACGTAAT CGGTTTACGA ATACCTTCTT CTGTACCTCC GTTTCGGTAC   
  
  
- GTACAAAGTG CTAGGAAATC GTGAAGTCCG ACGACTCTTT GGGAAAATAC TACGGAATCC CTTCTTTATG   
  
  
- GGTTGAAGAG GCTTGGTTGT AGGATATTAA CTACTAACAA ACCTATTAGG ACCACTTTTA AGAAACCCAA   
  
  
- GAAGATCACT AAAATCACAA TCAGTGAAAC CCAGATCAAG TCGTTCGAGA TAACCTGGCT GTTAACACAG   
  
  
- ACTAAACTCA CTCGTAAAAC TCGGTGGGAA ACAAGTTCGT GAAGGTTAAC TTAGTATAGG GGTTGGTAAC   
  
  
- TGGGCAGGAG TTACCAGCAA ACCGGGAAGC CCACGGAATC TAACGTGCCG GAGATTACCA AGTCACTAGA   
  
  
- GTAGGGAACC AAACGGCTAC CTACAATAAT CACATAAGTC ACTCTTTCTC AGGTACTAAG TTAAAGTCTT   
  
  
- TCCCCACCTT CTCCGATCAT TCAAGGAAGG GTTCTTATTA TTGGAACAAT AACTAGAGTT CTTGGAGTGA   
  
  
- AAAGGATTAC TTTGCTTCCT CCTACTAGCT CACTACTACC AATTCTTCCT TTTCTTACTA CACTTAACCA   
  
  
- GATTGATGAG TTCTCCCTCA TTCTAAGTGA TAGCACTTCT GCTCCTGAAA CTTCTTCCTT CCTCATCGTT   
  
  
- CGTCAGTCGA TAAAGATGAT GACTCCTTCG ACTCAACACA CTTTAAAAAC TGTTCAAAAA CGAAACGTTA   
  
  
- ACCATGGGAC ACTTTGGACT CCGAGTAGGG TGGTACTCAA ACTTGGGACT CTTCCCGGTC AGTGTACCTA   
  
  
- ATCTTCCACC CTCTTTACCC TTCCCAGTGG GTTTCTTTGT CCTATTTTCA TCATGGTTGC AACACCTAAA   
  
  
- TTCCTTAAAC GACTACGATA CACGTGTTAG ATGACGTAGA CTACTGGCTT CTTGACGACT ACCTGACGAT   
  
  
- TTCTTTTAGT CCCTCGTGAG TAGACGACTC CCCCTACCTA AAGTTTCCAA CCGAGTAATG AAACGACTAC   
  
  
- GGGATCTCCG TGCAAATCGA CCTTGACCTA GAGTGTAAAT ATGTCGGGAT TCAAGAGTAT CCGGTAGACG   
  
  
- ACAACTGCAC AATTTTCGTA TAGTCAAGAT ACAAGCACGA ACGGGTAAAT TCTTCTAGCA GTAAGCAACA   
  
  
- CCATTTGTAT ACTAAGATTT TCGACGACTT TTCCGTAGTT TCGAAGTATA ATATCTAAAA CCGTAGGAGA   
  
  
- TACCTAAGGT TACCGGATCG GAGTAAGCTA CGGAGAGTCT CGCCAGACCA CCTGGAGGTT TTGACAAATA   
  
  
- ATGTCCCTAG CTAGAGGGGG TCGGACCCAA GTCCGGTCGT CTTTCTCACC TTCGTTGTCC CTCTGCGAAC   
  
  
- CGATTCATGA CACTCGCCAT ATTACACGGT AAACTCATAG TACGGTAACG AGTCTTCACC CTTTGTTAGT   
  
  
- TTGGTCCCCT AGAGTTCAAT CCTTCATCCC TACTACTACT TCAACAGCAC CACTTGACAG AGACATCCAA   
  
  
- GTTCTTGGAG GAACTGCTCT GTTACCACCA CCTATCAGGT TCCTTGTGTC AAAATTTGGA CTAATCTTCC   
  
  
- CATTTTGGGC TATAAAAACA CGTACCGTAA CATTTACCAA GGATGTTGTA GGGAAAGAAA CACTGTGCAA   
  
  
- AATCTCTTCG GGAGAAAGTA ATAAGGTGAG AAAAGCTGTA CAATCTACGG TTGCGGAGAT CCCTCCTCGG   
  
  
- GCTCTCCAAC TATAAGCTCT TCCGTAAGAT ACCCTCCCTC TAATACTTAC ACCACCGTAC ACTCCCGTGT   
  
  
- CTCTCCCACC TTTCCGGTCT CTGTATGTTC GTTACCGTGC AATCCGTATC GTCCCGTCCC AAAGCCGTTG   
  
  
- ATGGTAACCT AGGGTTCAAC TAGCTCTTTT ACTCCAAATT CCGGTTCCGT CTGGTGGTGT TCCTAAAGCA   
  
  
- CTAACTACAC CTACCTGTAA CCCGTTAAGT CCCTACCTTC CCCGCCTAAC GGACACGGTA GAGACGTACC   
  
  
- CAAGACCGAA C

+     ERE

| Site Name | Organism | Position | Strand | Matrix score. | sequence | function |
| --- | --- | --- | --- | --- | --- | --- |
| ERE | Nicotiana glutinos | 1189 | + | 8 | ATTTCATA |  |
| ERE | Nicotiana glutinos | 58 | - | 8 | ATTTCATA |  |

>HU07G02248.1   
+ -Up\_Stream \_Len000CTGTTC AGCACTAGGG AATAAGGTTT TTGCCCTGCA TACTATGAAA TCTAACCAAA   
  
  
+ AACATTCCAT CTTGGAAGGC TGTAGCCTTG TGGATTTTGA ATTCGCTCCC AAGAACCAAT GAAGTAGCGC   
  
  
+ AGAAGGCCAA TACTCAATTT CAGAGTCCAC ATCAACTTGT TCAATGCCAG TATATTTCAG AACATTAATT   
  
  
+ ACATACTGTT TGGTTGGAGA AAAAATGGTT GTCTCGAAAA CTAGTTTTTT AATTTTTTGT TATTTAGCAC   
  
  
+ GGTAAAGAAT GGAAATTATT TTCATGCGAG AAAAAAGGAA GTTGGTTTTT CTTTTTTCAA CTCTTTTGAT   
  
  
+ GATATAATCA GTCGAAATTT AGGTGCACCA TCTGACTTCA ACTCTTGTAC GACCTCTGAT TGCTTGCTTA   
  
  
+ GAAGAACAAG AGGATTGACA GATAAGGAGG AGTGGGTGGT TTATACAGAG AATTGAGTGG ATGGTTATAA   
  
  
+ AGAAGTCGAT CTCACATAAG TAACTTCGTT ACGATTATAA ATATTTTCAT TGAAGAAAGA CGATTAGATA   
  
  
+ TAATTAAGAA GGTACATCTA AGCAAATTTG GCTGAGCAGA GTTGGAGGGC CACTAGGGTC ACATTGTGTC   
  
  
+ TTGCGCAACA AGCTTAGACC ATTCGCAACG GATGCCTCAC GTGGAGCAGG TGGGACCCGC GTGGAAGTTG   
  
  
+ CATGGCAAGA ATTCTCTGGC AGCAAGGAGA ATTGAGGGGG GACACGCGTG TGACCACGCC AGTGCGGTCC   
  
  
+ CCCTCTCGTG GCAGATTTGG TGATTTTTTT TTTGTTTTTT TTTGAAGTAA TATATAATTT TTATATGATT   
  
  
+ AAAAAAATAG GTTGTGTGTT ATAAAGTGGG TCAATGACAA ATATTTTTAT TGGATGATTA AAATTAAGTT   
  
  
+ CCATTTATTG GAGAAGTGAT GGAATGCAAG TTCCAAAATT GTGGAACTTT TGTTGACGTA GCAGGCTAGA   
  
  
+ GTTCCAAAAA TTAAAAAAAG GAAAGACATA TTGAGGTAAA GCCTTTTTAT TTTACAAATA GACGCACCAA   
  
  
+ GGTATAATAT AATTGACATG ATTTGAGGCG AGAGGAAGAT TCTTTTGCTA CCCAAACAAA ACACAATGAT   
  
  
+ CTTTTAAATG TAGCAATTTG GGATTGGCAA AATTTTGTCT AACCCGATTG TTGAAAGTTA TCCGATTTCA   
  
  
+ TAAAATTGGA TATCGTTATT AAATTTTCAT TCGAATATTT AAATTTGGAC ACAATGTTTT TCATATTTGG   
  
  
+ ATTTTCTAAA CTTGATTTTA ACTCAAATTT AAGTTTATTC AATTCATCTG ACCCAAAAAA TGGTTGCAAT   
  
  
+ AATGTATTTT CTTTTTGAAG TTAAATATTA TCGAATTCAT TTAACTTGTA GTTAACCCGT AATTGACCCA   
  
  
+ AACTCGACTT TTTGCTACCT TAGCAGCCAG GAAGAAGTCA TAGTATTGAC CCGTGGAACG CCTGAAGCCA   
  
  
+ ACATCACCAG ACCCACATAT TCCATCATCT TCTGATCTCC TCGTCAAAGA AGGAAGCTGA GCACAGCAAT   
  
  
+ AACTAGAAGA ACAGGAGGCA TCACACCCAA GCCAAAGGCC TTTATTTGAG CTCCCCACCT CATTCCTCGA   
  
  
+ AGCTTCCTCT TCACAATGTA AGACCATAAT CCTTCACCCC TTCTCTTTTT AAGCTCATCT TTACCTGCCC   
  
  
+ TAGTTCTCTC TCTCTCATGA GTCATCAGTA ATAGTTACCT CTTTACTTTC TCTTCCTTGA GGTATGCTTC   
  
  
+ AACTTTAATA TATATATGTA CCACTTGATT TCTCTTGTCA CTTTGCTATA ATACTTATTG TTTGGCTATC   
  
  
+ TTCCCCTGTT TTATACTCAT TTTCTTGCTT TCTATATCTG GGTTTGAATT GGGTTTCACT TATCCTCGTT   
  
  
+ GATTCTGTGG GAAATTAGTG AATTGGGTTT TGTACAGCAG GTTTATATGA ACTAGGGTTT CAAAGGTATC   
  
  
+ TACTTTTTCT CTTGTGATTA TTAGTTGATT AGGGATTTGG CCAAGATTAG CAGAATGGGT TCTGAATTTG   
  
  
+ GGGAATTCTC TGATGATGCT CTAAATGGGT ATGCTTACGT TGATATCCCT GCTTATGATG CGTCCTTAGA   
  
  
+ TTATGCCAAT TTGTTCAATT ACGAAGGCCT ATCTGAGGAT CTCACCTCAC TGAGCCTCCC AAGCCCCTTT   
  
  
+ TCTGACCCTT TGGCGTACAG TTTCACGTCC TCTTTGGGGC CGAGCCCTGG GGTTGATTCT AATGATGATA   
  
  
+ GTGATTCTGA TGATGTTCTC AAGTGCATTA GCCAAATGCT TATGGAAGAA GACATGGAGG CAAAGCCATG   
  
  
+ CATGTTTCAC GATCCTTTAG CACTTCAGGC TGCTGAGAAA CCCTTTTATG ATGCCTTAGG GAAGAAATAC   
  
  
+ CCAACTTCTC CGAACCAACA TCCTATAATT GATGATTGTT TGGATAATCC TGGTGAAAAT TCTTTGGGTT   
  
  
+ CTTCTAGTGA TTTTAGTGTT AGTCACTTTG GGTCTAGTTC AGCAAGCTCT ATTGGACCGA CAATTGTGTC   
  
  
+ TGATTTGAGT GAGCATTTTG AGCCACCCTT TGTTCAAGCA CTTCCAATTG AATCATATCC CCAACCATTG   
  
  
+ ACCCGTCCTC AATGGTCGTT TGGCCCTTCG GGTGCCTTAG ATTGCACGGC CTCTAATGGT TCAGTGATCT   
  
  
+ CATCCCTTGG TTTGCCGATG GATGTTATTA GTGTATTCAG TGAGAAAGAG TCCATGATTC AATTTCAGAA   
  
  
+ AGGGGTGGAA GAGGCTAGTA AGTTCCTTCC CAAGAATAAT AACCTTGTTA TTGATCTCAA GAACCTCACT   
  
  
+ TTTCCTAATG AAACGAAGGA GGATGATCGA GTGATGATGG TTAAGAAGGA AAAGAATGAT GTGAATTGGT   
  
  
+ CTAACTACTC AAGAGGGAGT AAGATTCACT ATCGTGAAGA CGAGGACTTT GAAGAAGGAA GGAGTAGCAA   
  
  
+ GCAGTCAGCT ATTTCTACTA CTGAGGAAGC TGAGTTGTGT GAAATTTTTG ACAAGTTTTT GCTTTGCAAT   
  
  
+ TGGTACCCTG TGAAACCTGA GGCTCATCCC ACCATGAGTT TGAACCCTGA GAAGGGCCAG TCACATGGAT   
  
  
+ TAGAAGGTGG GAGAAATGGG AAGGGTCACC CAAAGAAACA GGATAAAAGT AGTACCAACG TTGTGGATTT   
  
  
+ AAGGAATTTG CTGATGCTAT GTGCACAATC TACTGCATCT GATGACCGAA GAACTGCTGA TGGACTGCTA   
  
  
+ AAGAAAATCA GGGAGCACTC ATCTGCTGAG GGGGATGGAT TTCAAAGGTT GGCTCATTAC TTTGCTGATG   
  
  
+ CCCTAGAGGC ACGTTTAGCT GGAACTGGAT CTCACATTTA TACAGCCCTA AGTTCTCATA GGCCATCTGC   
  
  
+ TGTTGACGTG TTAAAAGCAT ATCAGTTCTA TGTTCGTGCT TGCCCATTTA AGAAGATCGT CATTCGTTGT   
  
  
+ GGTAAACATA TGATTCTAAA AGCTGCTGAA AAGGCATCAA AGCTTCATAT TATAGATTTT GGCATCCTCT   
  
  
+ ATGGATTCCA ATGGCCTAGC CTCATTCGAT GCCTCTCAGA GCGGTCTGGT GGACCTCCAA AACTGTTTAT   
  
  
+ TACAGGGATC GATCTCCCCC AGCCTGGGTT CAGGCCAGCA GAAAGAGTGG AAGCAACAGG GAGACGCTTG   
  
  
+ GCTAAGTACT GTGAGCGGTA TAATGTGCCA TTTGAGTATC ATGCCATTGC TCAGAAGTGG GAAACAATCA   
  
  
+ AACCAGGGGA TCTCAAGTTA GGAAGTAGGG ATGATGATGA AGTTGTCGTG GTGAACTGTC TCTGTAGGTT   
  
  
+ CAAGAACCTC CTTGACGAGA CAATGGTGGT GGATAGTCCA AGGAACACAG TTTTAAACCT GATTAGAAGG   
  
  
+ GTAAAACCCG ATATTTTTGT GCATGGCATT GTAAATGGTT CCTACAACAT CCCTTTCTTT GTGACACGTT   
  
  
+ TTAGAGAAGC CCTCTTTCAT TATTCCACTC TTTTCGACAT GTTAGATGCC AACGCCTCTA GGGAGGAGCC   
  
  
+ CGAGAGGTTG ATATTCGAGA AGGCATTCTA TGGGAGGGAG ATTATGAATG TGGTGGCATG TGAGGGCACA   
  
  
+ GAGAGGGTGG AAAGGCCAGA GACATACAAG CAATGGCACG TTAGGCATAG CAGGGCAGGG TTTCGGCAAC   
  
  
+ TACCATTGGA TCCCAAGTTG ATCGAGAAAA TGAGGTTTAA GGCCAAGGCA GACCACCACA AGGATTTCGT   
  
  
+ GATTGATGTG GATGGACATT GGGCAATTCA GGGATGGAAG GGGCGGATTG CCTGTGCCAT CTCTGCATGG   
  
  
+ GTTCTGGCTT G  

- -Up\_Stream \_Len000GACAAG TCGTGATCCC TTATTCCAAA AACGGGACGT ATGATACTTT AGATTGGTTT   
  
  
- TTGTAAGGTA GAACCTTCCG ACATCGGAAC ACCTAAAACT TAAGCGAGGG TTCTTGGTTA CTTCATCGCG   
  
  
- TCTTCCGGTT ATGAGTTAAA GTCTCAGGTG TAGTTGAACA AGTTACGGTC ATATAAAGTC TTGTAATTAA   
  
  
- TGTATGACAA ACCAACCTCT TTTTTACCAA CAGAGCTTTT GATCAAAAAA TTAAAAAACA ATAAATCGTG   
  
  
- CCATTTCTTA CCTTTAATAA AAGTACGCTC TTTTTTCCTT CAACCAAAAA GAAAAAAGTT GAGAAAACTA   
  
  
- CTATATTAGT CAGCTTTAAA TCCACGTGGT AGACTGAAGT TGAGAACATG CTGGAGACTA ACGAACGAAT   
  
  
- CTTCTTGTTC TCCTAACTGT CTATTCCTCC TCACCCACCA AATATGTCTC TTAACTCACC TACCAATATT   
  
  
- TCTTCAGCTA GAGTGTATTC ATTGAAGCAA TGCTAATATT TATAAAAGTA ACTTCTTTCT GCTAATCTAT   
  
  
- ATTAATTCTT CCATGTAGAT TCGTTTAAAC CGACTCGTCT CAACCTCCCG GTGATCCCAG TGTAACACAG   
  
  
- AACGCGTTGT TCGAATCTGG TAAGCGTTGC CTACGGAGTG CACCTCGTCC ACCCTGGGCG CACCTTCAAC   
  
  
- GTACCGTTCT TAAGAGACCG TCGTTCCTCT TAACTCCCCC CTGTGCGCAC ACTGGTGCGG TCACGCCAGG   
  
  
- GGGAGAGCAC CGTCTAAACC ACTAAAAAAA AAACAAAAAA AAACTTCATT ATATATTAAA AATATACTAA   
  
  
- TTTTTTTATC CAACACACAA TATTTCACCC AGTTACTGTT TATAAAAATA ACCTACTAAT TTTAATTCAA   
  
  
- GGTAAATAAC CTCTTCACTA CCTTACGTTC AAGGTTTTAA CACCTTGAAA ACAACTGCAT CGTCCGATCT   
  
  
- CAAGGTTTTT AATTTTTTTC CTTTCTGTAT AACTCCATTT CGGAAAAATA AAATGTTTAT CTGCGTGGTT   
  
  
- CCATATTATA TTAACTGTAC TAAACTCCGC TCTCCTTCTA AGAAAACGAT GGGTTTGTTT TGTGTTACTA   
  
  
- GAAAATTTAC ATCGTTAAAC CCTAACCGTT TTAAAACAGA TTGGGCTAAC AACTTTCAAT AGGCTAAAGT   
  
  
- ATTTTAACCT ATAGCAATAA TTTAAAAGTA AGCTTATAAA TTTAAACCTG TGTTACAAAA AGTATAAACC   
  
  
- TAAAAGATTT GAACTAAAAT TGAGTTTAAA TTCAAATAAG TTAAGTAGAC TGGGTTTTTT ACCAACGTTA   
  
  
- TTACATAAAA GAAAAACTTC AATTTATAAT AGCTTAAGTA AATTGAACAT CAATTGGGCA TTAACTGGGT   
  
  
- TTGAGCTGAA AAACGATGGA ATCGTCGGTC CTTCTTCAGT ATCATAACTG GGCACCTTGC GGACTTCGGT   
  
  
- TGTAGTGGTC TGGGTGTATA AGGTAGTAGA AGACTAGAGG AGCAGTTTCT TCCTTCGACT CGTGTCGTTA   
  
  
- TTGATCTTCT TGTCCTCCGT AGTGTGGGTT CGGTTTCCGG AAATAAACTC GAGGGGTGGA GTAAGGAGCT   
  
  
- TCGAAGGAGA AGTGTTACAT TCTGGTATTA GGAAGTGGGG AAGAGAAAAA TTCGAGTAGA AATGGACGGG   
  
  
- ATCAAGAGAG AGAGAGTACT CAGTAGTCAT TATCAATGGA GAAATGAAAG AGAAGGAACT CCATACGAAG   
  
  
- TTGAAATTAT ATATATACAT GGTGAACTAA AGAGAACAGT GAAACGATAT TATGAATAAC AAACCGATAG   
  
  
- AAGGGGACAA AATATGAGTA AAAGAACGAA AGATATAGAC CCAAACTTAA CCCAAAGTGA ATAGGAGCAA   
  
  
- CTAAGACACC CTTTAATCAC TTAACCCAAA ACATGTCGTC CAAATATACT TGATCCCAAA GTTTCCATAG   
  
  
- ATGAAAAAGA GAACACTAAT AATCAACTAA TCCCTAAACC GGTTCTAATC GTCTTACCCA AGACTTAAAC   
  
  
- CCCTTAAGAG ACTACTACGA GATTTACCCA TACGAATGCA ACTATAGGGA CGAATACTAC GCAGGAATCT   
  
  
- AATACGGTTA AACAAGTTAA TGCTTCCGGA TAGACTCCTA GAGTGGAGTG ACTCGGAGGG TTCGGGGAAA   
  
  
- AGACTGGGAA ACCGCATGTC AAAGTGCAGG AGAAACCCCG GCTCGGGACC CCAACTAAGA TTACTACTAT   
  
  
- CACTAAGACT ACTACAAGAG TTCACGTAAT CGGTTTACGA ATACCTTCTT CTGTACCTCC GTTTCGGTAC   
  
  
- GTACAAAGTG CTAGGAAATC GTGAAGTCCG ACGACTCTTT GGGAAAATAC TACGGAATCC CTTCTTTATG   
  
  
- GGTTGAAGAG GCTTGGTTGT AGGATATTAA CTACTAACAA ACCTATTAGG ACCACTTTTA AGAAACCCAA   
  
  
- GAAGATCACT AAAATCACAA TCAGTGAAAC CCAGATCAAG TCGTTCGAGA TAACCTGGCT GTTAACACAG   
  
  
- ACTAAACTCA CTCGTAAAAC TCGGTGGGAA ACAAGTTCGT GAAGGTTAAC TTAGTATAGG GGTTGGTAAC   
  
  
- TGGGCAGGAG TTACCAGCAA ACCGGGAAGC CCACGGAATC TAACGTGCCG GAGATTACCA AGTCACTAGA   
  
  
- GTAGGGAACC AAACGGCTAC CTACAATAAT CACATAAGTC ACTCTTTCTC AGGTACTAAG TTAAAGTCTT   
  
  
- TCCCCACCTT CTCCGATCAT TCAAGGAAGG GTTCTTATTA TTGGAACAAT AACTAGAGTT CTTGGAGTGA   
  
  
- AAAGGATTAC TTTGCTTCCT CCTACTAGCT CACTACTACC AATTCTTCCT TTTCTTACTA CACTTAACCA   
  
  
- GATTGATGAG TTCTCCCTCA TTCTAAGTGA TAGCACTTCT GCTCCTGAAA CTTCTTCCTT CCTCATCGTT   
  
  
- CGTCAGTCGA TAAAGATGAT GACTCCTTCG ACTCAACACA CTTTAAAAAC TGTTCAAAAA CGAAACGTTA   
  
  
- ACCATGGGAC ACTTTGGACT CCGAGTAGGG TGGTACTCAA ACTTGGGACT CTTCCCGGTC AGTGTACCTA   
  
  
- ATCTTCCACC CTCTTTACCC TTCCCAGTGG GTTTCTTTGT CCTATTTTCA TCATGGTTGC AACACCTAAA   
  
  
- TTCCTTAAAC GACTACGATA CACGTGTTAG ATGACGTAGA CTACTGGCTT CTTGACGACT ACCTGACGAT   
  
  
- TTCTTTTAGT CCCTCGTGAG TAGACGACTC CCCCTACCTA AAGTTTCCAA CCGAGTAATG AAACGACTAC   
  
  
- GGGATCTCCG TGCAAATCGA CCTTGACCTA GAGTGTAAAT ATGTCGGGAT TCAAGAGTAT CCGGTAGACG   
  
  
- ACAACTGCAC AATTTTCGTA TAGTCAAGAT ACAAGCACGA ACGGGTAAAT TCTTCTAGCA GTAAGCAACA   
  
  
- CCATTTGTAT ACTAAGATTT TCGACGACTT TTCCGTAGTT TCGAAGTATA ATATCTAAAA CCGTAGGAGA   
  
  
- TACCTAAGGT TACCGGATCG GAGTAAGCTA CGGAGAGTCT CGCCAGACCA CCTGGAGGTT TTGACAAATA   
  
  
- ATGTCCCTAG CTAGAGGGGG TCGGACCCAA GTCCGGTCGT CTTTCTCACC TTCGTTGTCC CTCTGCGAAC   
  
  
- CGATTCATGA CACTCGCCAT ATTACACGGT AAACTCATAG TACGGTAACG AGTCTTCACC CTTTGTTAGT   
  
  
- TTGGTCCCCT AGAGTTCAAT CCTTCATCCC TACTACTACT TCAACAGCAC CACTTGACAG AGACATCCAA   
  
  
- GTTCTTGGAG GAACTGCTCT GTTACCACCA CCTATCAGGT TCCTTGTGTC AAAATTTGGA CTAATCTTCC   
  
  
- CATTTTGGGC TATAAAAACA CGTACCGTAA CATTTACCAA GGATGTTGTA GGGAAAGAAA CACTGTGCAA   
  
  
- AATCTCTTCG GGAGAAAGTA ATAAGGTGAG AAAAGCTGTA CAATCTACGG TTGCGGAGAT CCCTCCTCGG   
  
  
- GCTCTCCAAC TATAAGCTCT TCCGTAAGAT ACCCTCCCTC TAATACTTAC ACCACCGTAC ACTCCCGTGT   
  
  
- CTCTCCCACC TTTCCGGTCT CTGTATGTTC GTTACCGTGC AATCCGTATC GTCCCGTCCC AAAGCCGTTG   
  
  
- ATGGTAACCT AGGGTTCAAC TAGCTCTTTT ACTCCAAATT CCGGTTCCGT CTGGTGGTGT TCCTAAAGCA   
  
  
- CTAACTACAC CTACCTGTAA CCCGTTAAGT CCCTACCTTC CCCGCCTAAC GGACACGGTA GAGACGTACC   
  
  
- CAAGACCGAA C

+     G-Box

| Site Name | Organism | Position | Strand | Matrix score. | sequence | function |
| --- | --- | --- | --- | --- | --- | --- |
| G-Box | Pisum sativum | 672 | + | 6 | CACGTG | cis-acting regulatory element involved in light responsiveness |
| G-Box | Pisum sativum | 3919 | + | 6 | CACGTT | cis-acting regulatory element involved in light responsiveness |
| G-Box | Pisum sativum | 4101 | + | 6 | CACGTT | cis-acting regulatory element involved in light responsiveness |
| G-Box | Pisum sativum | 3304 | + | 6 | CACGTT | cis-acting regulatory element involved in light responsiveness |

>HU07G02248.1   
+ -Up\_Stream \_Len000CTGTTC AGCACTAGGG AATAAGGTTT TTGCCCTGCA TACTATGAAA TCTAACCAAA   
  
  
+ AACATTCCAT CTTGGAAGGC TGTAGCCTTG TGGATTTTGA ATTCGCTCCC AAGAACCAAT GAAGTAGCGC   
  
  
+ AGAAGGCCAA TACTCAATTT CAGAGTCCAC ATCAACTTGT TCAATGCCAG TATATTTCAG AACATTAATT   
  
  
+ ACATACTGTT TGGTTGGAGA AAAAATGGTT GTCTCGAAAA CTAGTTTTTT AATTTTTTGT TATTTAGCAC   
  
  
+ GGTAAAGAAT GGAAATTATT TTCATGCGAG AAAAAAGGAA GTTGGTTTTT CTTTTTTCAA CTCTTTTGAT   
  
  
+ GATATAATCA GTCGAAATTT AGGTGCACCA TCTGACTTCA ACTCTTGTAC GACCTCTGAT TGCTTGCTTA   
  
  
+ GAAGAACAAG AGGATTGACA GATAAGGAGG AGTGGGTGGT TTATACAGAG AATTGAGTGG ATGGTTATAA   
  
  
+ AGAAGTCGAT CTCACATAAG TAACTTCGTT ACGATTATAA ATATTTTCAT TGAAGAAAGA CGATTAGATA   
  
  
+ TAATTAAGAA GGTACATCTA AGCAAATTTG GCTGAGCAGA GTTGGAGGGC CACTAGGGTC ACATTGTGTC   
  
  
+ TTGCGCAACA AGCTTAGACC ATTCGCAACG GATGCCTCAC GTGGAGCAGG TGGGACCCGC GTGGAAGTTG   
  
  
+ CATGGCAAGA ATTCTCTGGC AGCAAGGAGA ATTGAGGGGG GACACGCGTG TGACCACGCC AGTGCGGTCC   
  
  
+ CCCTCTCGTG GCAGATTTGG TGATTTTTTT TTTGTTTTTT TTTGAAGTAA TATATAATTT TTATATGATT   
  
  
+ AAAAAAATAG GTTGTGTGTT ATAAAGTGGG TCAATGACAA ATATTTTTAT TGGATGATTA AAATTAAGTT   
  
  
+ CCATTTATTG GAGAAGTGAT GGAATGCAAG TTCCAAAATT GTGGAACTTT TGTTGACGTA GCAGGCTAGA   
  
  
+ GTTCCAAAAA TTAAAAAAAG GAAAGACATA TTGAGGTAAA GCCTTTTTAT TTTACAAATA GACGCACCAA   
  
  
+ GGTATAATAT AATTGACATG ATTTGAGGCG AGAGGAAGAT TCTTTTGCTA CCCAAACAAA ACACAATGAT   
  
  
+ CTTTTAAATG TAGCAATTTG GGATTGGCAA AATTTTGTCT AACCCGATTG TTGAAAGTTA TCCGATTTCA   
  
  
+ TAAAATTGGA TATCGTTATT AAATTTTCAT TCGAATATTT AAATTTGGAC ACAATGTTTT TCATATTTGG   
  
  
+ ATTTTCTAAA CTTGATTTTA ACTCAAATTT AAGTTTATTC AATTCATCTG ACCCAAAAAA TGGTTGCAAT   
  
  
+ AATGTATTTT CTTTTTGAAG TTAAATATTA TCGAATTCAT TTAACTTGTA GTTAACCCGT AATTGACCCA   
  
  
+ AACTCGACTT TTTGCTACCT TAGCAGCCAG GAAGAAGTCA TAGTATTGAC CCGTGGAACG CCTGAAGCCA   
  
  
+ ACATCACCAG ACCCACATAT TCCATCATCT TCTGATCTCC TCGTCAAAGA AGGAAGCTGA GCACAGCAAT   
  
  
+ AACTAGAAGA ACAGGAGGCA TCACACCCAA GCCAAAGGCC TTTATTTGAG CTCCCCACCT CATTCCTCGA   
  
  
+ AGCTTCCTCT TCACAATGTA AGACCATAAT CCTTCACCCC TTCTCTTTTT AAGCTCATCT TTACCTGCCC   
  
  
+ TAGTTCTCTC TCTCTCATGA GTCATCAGTA ATAGTTACCT CTTTACTTTC TCTTCCTTGA GGTATGCTTC   
  
  
+ AACTTTAATA TATATATGTA CCACTTGATT TCTCTTGTCA CTTTGCTATA ATACTTATTG TTTGGCTATC   
  
  
+ TTCCCCTGTT TTATACTCAT TTTCTTGCTT TCTATATCTG GGTTTGAATT GGGTTTCACT TATCCTCGTT   
  
  
+ GATTCTGTGG GAAATTAGTG AATTGGGTTT TGTACAGCAG GTTTATATGA ACTAGGGTTT CAAAGGTATC   
  
  
+ TACTTTTTCT CTTGTGATTA TTAGTTGATT AGGGATTTGG CCAAGATTAG CAGAATGGGT TCTGAATTTG   
  
  
+ GGGAATTCTC TGATGATGCT CTAAATGGGT ATGCTTACGT TGATATCCCT GCTTATGATG CGTCCTTAGA   
  
  
+ TTATGCCAAT TTGTTCAATT ACGAAGGCCT ATCTGAGGAT CTCACCTCAC TGAGCCTCCC AAGCCCCTTT   
  
  
+ TCTGACCCTT TGGCGTACAG TTTCACGTCC TCTTTGGGGC CGAGCCCTGG GGTTGATTCT AATGATGATA   
  
  
+ GTGATTCTGA TGATGTTCTC AAGTGCATTA GCCAAATGCT TATGGAAGAA GACATGGAGG CAAAGCCATG   
  
  
+ CATGTTTCAC GATCCTTTAG CACTTCAGGC TGCTGAGAAA CCCTTTTATG ATGCCTTAGG GAAGAAATAC   
  
  
+ CCAACTTCTC CGAACCAACA TCCTATAATT GATGATTGTT TGGATAATCC TGGTGAAAAT TCTTTGGGTT   
  
  
+ CTTCTAGTGA TTTTAGTGTT AGTCACTTTG GGTCTAGTTC AGCAAGCTCT ATTGGACCGA CAATTGTGTC   
  
  
+ TGATTTGAGT GAGCATTTTG AGCCACCCTT TGTTCAAGCA CTTCCAATTG AATCATATCC CCAACCATTG   
  
  
+ ACCCGTCCTC AATGGTCGTT TGGCCCTTCG GGTGCCTTAG ATTGCACGGC CTCTAATGGT TCAGTGATCT   
  
  
+ CATCCCTTGG TTTGCCGATG GATGTTATTA GTGTATTCAG TGAGAAAGAG TCCATGATTC AATTTCAGAA   
  
  
+ AGGGGTGGAA GAGGCTAGTA AGTTCCTTCC CAAGAATAAT AACCTTGTTA TTGATCTCAA GAACCTCACT   
  
  
+ TTTCCTAATG AAACGAAGGA GGATGATCGA GTGATGATGG TTAAGAAGGA AAAGAATGAT GTGAATTGGT   
  
  
+ CTAACTACTC AAGAGGGAGT AAGATTCACT ATCGTGAAGA CGAGGACTTT GAAGAAGGAA GGAGTAGCAA   
  
  
+ GCAGTCAGCT ATTTCTACTA CTGAGGAAGC TGAGTTGTGT GAAATTTTTG ACAAGTTTTT GCTTTGCAAT   
  
  
+ TGGTACCCTG TGAAACCTGA GGCTCATCCC ACCATGAGTT TGAACCCTGA GAAGGGCCAG TCACATGGAT   
  
  
+ TAGAAGGTGG GAGAAATGGG AAGGGTCACC CAAAGAAACA GGATAAAAGT AGTACCAACG TTGTGGATTT   
  
  
+ AAGGAATTTG CTGATGCTAT GTGCACAATC TACTGCATCT GATGACCGAA GAACTGCTGA TGGACTGCTA   
  
  
+ AAGAAAATCA GGGAGCACTC ATCTGCTGAG GGGGATGGAT TTCAAAGGTT GGCTCATTAC TTTGCTGATG   
  
  
+ CCCTAGAGGC ACGTTTAGCT GGAACTGGAT CTCACATTTA TACAGCCCTA AGTTCTCATA GGCCATCTGC   
  
  
+ TGTTGACGTG TTAAAAGCAT ATCAGTTCTA TGTTCGTGCT TGCCCATTTA AGAAGATCGT CATTCGTTGT   
  
  
+ GGTAAACATA TGATTCTAAA AGCTGCTGAA AAGGCATCAA AGCTTCATAT TATAGATTTT GGCATCCTCT   
  
  
+ ATGGATTCCA ATGGCCTAGC CTCATTCGAT GCCTCTCAGA GCGGTCTGGT GGACCTCCAA AACTGTTTAT   
  
  
+ TACAGGGATC GATCTCCCCC AGCCTGGGTT CAGGCCAGCA GAAAGAGTGG AAGCAACAGG GAGACGCTTG   
  
  
+ GCTAAGTACT GTGAGCGGTA TAATGTGCCA TTTGAGTATC ATGCCATTGC TCAGAAGTGG GAAACAATCA   
  
  
+ AACCAGGGGA TCTCAAGTTA GGAAGTAGGG ATGATGATGA AGTTGTCGTG GTGAACTGTC TCTGTAGGTT   
  
  
+ CAAGAACCTC CTTGACGAGA CAATGGTGGT GGATAGTCCA AGGAACACAG TTTTAAACCT GATTAGAAGG   
  
  
+ GTAAAACCCG ATATTTTTGT GCATGGCATT GTAAATGGTT CCTACAACAT CCCTTTCTTT GTGACACGTT   
  
  
+ TTAGAGAAGC CCTCTTTCAT TATTCCACTC TTTTCGACAT GTTAGATGCC AACGCCTCTA GGGAGGAGCC   
  
  
+ CGAGAGGTTG ATATTCGAGA AGGCATTCTA TGGGAGGGAG ATTATGAATG TGGTGGCATG TGAGGGCACA   
  
  
+ GAGAGGGTGG AAAGGCCAGA GACATACAAG CAATGGCACG TTAGGCATAG CAGGGCAGGG TTTCGGCAAC   
  
  
+ TACCATTGGA TCCCAAGTTG ATCGAGAAAA TGAGGTTTAA GGCCAAGGCA GACCACCACA AGGATTTCGT   
  
  
+ GATTGATGTG GATGGACATT GGGCAATTCA GGGATGGAAG GGGCGGATTG CCTGTGCCAT CTCTGCATGG   
  
  
+ GTTCTGGCTT G  

- -Up\_Stream \_Len000GACAAG TCGTGATCCC TTATTCCAAA AACGGGACGT ATGATACTTT AGATTGGTTT   
  
  
- TTGTAAGGTA GAACCTTCCG ACATCGGAAC ACCTAAAACT TAAGCGAGGG TTCTTGGTTA CTTCATCGCG   
  
  
- TCTTCCGGTT ATGAGTTAAA GTCTCAGGTG TAGTTGAACA AGTTACGGTC ATATAAAGTC TTGTAATTAA   
  
  
- TGTATGACAA ACCAACCTCT TTTTTACCAA CAGAGCTTTT GATCAAAAAA TTAAAAAACA ATAAATCGTG   
  
  
- CCATTTCTTA CCTTTAATAA AAGTACGCTC TTTTTTCCTT CAACCAAAAA GAAAAAAGTT GAGAAAACTA   
  
  
- CTATATTAGT CAGCTTTAAA TCCACGTGGT AGACTGAAGT TGAGAACATG CTGGAGACTA ACGAACGAAT   
  
  
- CTTCTTGTTC TCCTAACTGT CTATTCCTCC TCACCCACCA AATATGTCTC TTAACTCACC TACCAATATT   
  
  
- TCTTCAGCTA GAGTGTATTC ATTGAAGCAA TGCTAATATT TATAAAAGTA ACTTCTTTCT GCTAATCTAT   
  
  
- ATTAATTCTT CCATGTAGAT TCGTTTAAAC CGACTCGTCT CAACCTCCCG GTGATCCCAG TGTAACACAG   
  
  
- AACGCGTTGT TCGAATCTGG TAAGCGTTGC CTACGGAGTG CACCTCGTCC ACCCTGGGCG CACCTTCAAC   
  
  
- GTACCGTTCT TAAGAGACCG TCGTTCCTCT TAACTCCCCC CTGTGCGCAC ACTGGTGCGG TCACGCCAGG   
  
  
- GGGAGAGCAC CGTCTAAACC ACTAAAAAAA AAACAAAAAA AAACTTCATT ATATATTAAA AATATACTAA   
  
  
- TTTTTTTATC CAACACACAA TATTTCACCC AGTTACTGTT TATAAAAATA ACCTACTAAT TTTAATTCAA   
  
  
- GGTAAATAAC CTCTTCACTA CCTTACGTTC AAGGTTTTAA CACCTTGAAA ACAACTGCAT CGTCCGATCT   
  
  
- CAAGGTTTTT AATTTTTTTC CTTTCTGTAT AACTCCATTT CGGAAAAATA AAATGTTTAT CTGCGTGGTT   
  
  
- CCATATTATA TTAACTGTAC TAAACTCCGC TCTCCTTCTA AGAAAACGAT GGGTTTGTTT TGTGTTACTA   
  
  
- GAAAATTTAC ATCGTTAAAC CCTAACCGTT TTAAAACAGA TTGGGCTAAC AACTTTCAAT AGGCTAAAGT   
  
  
- ATTTTAACCT ATAGCAATAA TTTAAAAGTA AGCTTATAAA TTTAAACCTG TGTTACAAAA AGTATAAACC   
  
  
- TAAAAGATTT GAACTAAAAT TGAGTTTAAA TTCAAATAAG TTAAGTAGAC TGGGTTTTTT ACCAACGTTA   
  
  
- TTACATAAAA GAAAAACTTC AATTTATAAT AGCTTAAGTA AATTGAACAT CAATTGGGCA TTAACTGGGT   
  
  
- TTGAGCTGAA AAACGATGGA ATCGTCGGTC CTTCTTCAGT ATCATAACTG GGCACCTTGC GGACTTCGGT   
  
  
- TGTAGTGGTC TGGGTGTATA AGGTAGTAGA AGACTAGAGG AGCAGTTTCT TCCTTCGACT CGTGTCGTTA   
  
  
- TTGATCTTCT TGTCCTCCGT AGTGTGGGTT CGGTTTCCGG AAATAAACTC GAGGGGTGGA GTAAGGAGCT   
  
  
- TCGAAGGAGA AGTGTTACAT TCTGGTATTA GGAAGTGGGG AAGAGAAAAA TTCGAGTAGA AATGGACGGG   
  
  
- ATCAAGAGAG AGAGAGTACT CAGTAGTCAT TATCAATGGA GAAATGAAAG AGAAGGAACT CCATACGAAG   
  
  
- TTGAAATTAT ATATATACAT GGTGAACTAA AGAGAACAGT GAAACGATAT TATGAATAAC AAACCGATAG   
  
  
- AAGGGGACAA AATATGAGTA AAAGAACGAA AGATATAGAC CCAAACTTAA CCCAAAGTGA ATAGGAGCAA   
  
  
- CTAAGACACC CTTTAATCAC TTAACCCAAA ACATGTCGTC CAAATATACT TGATCCCAAA GTTTCCATAG   
  
  
- ATGAAAAAGA GAACACTAAT AATCAACTAA TCCCTAAACC GGTTCTAATC GTCTTACCCA AGACTTAAAC   
  
  
- CCCTTAAGAG ACTACTACGA GATTTACCCA TACGAATGCA ACTATAGGGA CGAATACTAC GCAGGAATCT   
  
  
- AATACGGTTA AACAAGTTAA TGCTTCCGGA TAGACTCCTA GAGTGGAGTG ACTCGGAGGG TTCGGGGAAA   
  
  
- AGACTGGGAA ACCGCATGTC AAAGTGCAGG AGAAACCCCG GCTCGGGACC CCAACTAAGA TTACTACTAT   
  
  
- CACTAAGACT ACTACAAGAG TTCACGTAAT CGGTTTACGA ATACCTTCTT CTGTACCTCC GTTTCGGTAC   
  
  
- GTACAAAGTG CTAGGAAATC GTGAAGTCCG ACGACTCTTT GGGAAAATAC TACGGAATCC CTTCTTTATG   
  
  
- GGTTGAAGAG GCTTGGTTGT AGGATATTAA CTACTAACAA ACCTATTAGG ACCACTTTTA AGAAACCCAA   
  
  
- GAAGATCACT AAAATCACAA TCAGTGAAAC CCAGATCAAG TCGTTCGAGA TAACCTGGCT GTTAACACAG   
  
  
- ACTAAACTCA CTCGTAAAAC TCGGTGGGAA ACAAGTTCGT GAAGGTTAAC TTAGTATAGG GGTTGGTAAC   
  
  
- TGGGCAGGAG TTACCAGCAA ACCGGGAAGC CCACGGAATC TAACGTGCCG GAGATTACCA AGTCACTAGA   
  
  
- GTAGGGAACC AAACGGCTAC CTACAATAAT CACATAAGTC ACTCTTTCTC AGGTACTAAG TTAAAGTCTT   
  
  
- TCCCCACCTT CTCCGATCAT TCAAGGAAGG GTTCTTATTA TTGGAACAAT AACTAGAGTT CTTGGAGTGA   
  
  
- AAAGGATTAC TTTGCTTCCT CCTACTAGCT CACTACTACC AATTCTTCCT TTTCTTACTA CACTTAACCA   
  
  
- GATTGATGAG TTCTCCCTCA TTCTAAGTGA TAGCACTTCT GCTCCTGAAA CTTCTTCCTT CCTCATCGTT   
  
  
- CGTCAGTCGA TAAAGATGAT GACTCCTTCG ACTCAACACA CTTTAAAAAC TGTTCAAAAA CGAAACGTTA   
  
  
- ACCATGGGAC ACTTTGGACT CCGAGTAGGG TGGTACTCAA ACTTGGGACT CTTCCCGGTC AGTGTACCTA   
  
  
- ATCTTCCACC CTCTTTACCC TTCCCAGTGG GTTTCTTTGT CCTATTTTCA TCATGGTTGC AACACCTAAA   
  
  
- TTCCTTAAAC GACTACGATA CACGTGTTAG ATGACGTAGA CTACTGGCTT CTTGACGACT ACCTGACGAT   
  
  
- TTCTTTTAGT CCCTCGTGAG TAGACGACTC CCCCTACCTA AAGTTTCCAA CCGAGTAATG AAACGACTAC   
  
  
- GGGATCTCCG TGCAAATCGA CCTTGACCTA GAGTGTAAAT ATGTCGGGAT TCAAGAGTAT CCGGTAGACG   
  
  
- ACAACTGCAC AATTTTCGTA TAGTCAAGAT ACAAGCACGA ACGGGTAAAT TCTTCTAGCA GTAAGCAACA   
  
  
- CCATTTGTAT ACTAAGATTT TCGACGACTT TTCCGTAGTT TCGAAGTATA ATATCTAAAA CCGTAGGAGA   
  
  
- TACCTAAGGT TACCGGATCG GAGTAAGCTA CGGAGAGTCT CGCCAGACCA CCTGGAGGTT TTGACAAATA   
  
  
- ATGTCCCTAG CTAGAGGGGG TCGGACCCAA GTCCGGTCGT CTTTCTCACC TTCGTTGTCC CTCTGCGAAC   
  
  
- CGATTCATGA CACTCGCCAT ATTACACGGT AAACTCATAG TACGGTAACG AGTCTTCACC CTTTGTTAGT   
  
  
- TTGGTCCCCT AGAGTTCAAT CCTTCATCCC TACTACTACT TCAACAGCAC CACTTGACAG AGACATCCAA   
  
  
- GTTCTTGGAG GAACTGCTCT GTTACCACCA CCTATCAGGT TCCTTGTGTC AAAATTTGGA CTAATCTTCC   
  
  
- CATTTTGGGC TATAAAAACA CGTACCGTAA CATTTACCAA GGATGTTGTA GGGAAAGAAA CACTGTGCAA   
  
  
- AATCTCTTCG GGAGAAAGTA ATAAGGTGAG AAAAGCTGTA CAATCTACGG TTGCGGAGAT CCCTCCTCGG   
  
  
- GCTCTCCAAC TATAAGCTCT TCCGTAAGAT ACCCTCCCTC TAATACTTAC ACCACCGTAC ACTCCCGTGT   
  
  
- CTCTCCCACC TTTCCGGTCT CTGTATGTTC GTTACCGTGC AATCCGTATC GTCCCGTCCC AAAGCCGTTG   
  
  
- ATGGTAACCT AGGGTTCAAC TAGCTCTTTT ACTCCAAATT CCGGTTCCGT CTGGTGGTGT TCCTAAAGCA   
  
  
- CTAACTACAC CTACCTGTAA CCCGTTAAGT CCCTACCTTC CCCGCCTAAC GGACACGGTA GAGACGTACC   
  
  
- CAAGACCGAA C

+     G-box

| Site Name | Organism | Position | Strand | Matrix score. | sequence | function |
| --- | --- | --- | --- | --- | --- | --- |
| G-box | Arabidopsis thaliana | 672 | + | 6 | CACGTG | cis-acting regulatory element involved in light responsiveness |
| G-box | Brassica oleracea | 3304 | - | 8 | TAAACGTG | cis-acting regulatory element involved in light responsiveness |
| G-box | Zea mays | 3759 | - | 6 | CACGAC | cis-acting regulatory element involved in light responsiveness |
| G-box | Zea mays | 2198 | + | 6 | CACGTC | cis-acting regulatory element involved in light responsiveness |
| G-box | Zea mays | 3369 | - | 6 | CACGTC | cis-acting regulatory element involved in light responsiveness |

>HU07G02248.1   
+ -Up\_Stream \_Len000CTGTTC AGCACTAGGG AATAAGGTTT TTGCCCTGCA TACTATGAAA TCTAACCAAA   
  
  
+ AACATTCCAT CTTGGAAGGC TGTAGCCTTG TGGATTTTGA ATTCGCTCCC AAGAACCAAT GAAGTAGCGC   
  
  
+ AGAAGGCCAA TACTCAATTT CAGAGTCCAC ATCAACTTGT TCAATGCCAG TATATTTCAG AACATTAATT   
  
  
+ ACATACTGTT TGGTTGGAGA AAAAATGGTT GTCTCGAAAA CTAGTTTTTT AATTTTTTGT TATTTAGCAC   
  
  
+ GGTAAAGAAT GGAAATTATT TTCATGCGAG AAAAAAGGAA GTTGGTTTTT CTTTTTTCAA CTCTTTTGAT   
  
  
+ GATATAATCA GTCGAAATTT AGGTGCACCA TCTGACTTCA ACTCTTGTAC GACCTCTGAT TGCTTGCTTA   
  
  
+ GAAGAACAAG AGGATTGACA GATAAGGAGG AGTGGGTGGT TTATACAGAG AATTGAGTGG ATGGTTATAA   
  
  
+ AGAAGTCGAT CTCACATAAG TAACTTCGTT ACGATTATAA ATATTTTCAT TGAAGAAAGA CGATTAGATA   
  
  
+ TAATTAAGAA GGTACATCTA AGCAAATTTG GCTGAGCAGA GTTGGAGGGC CACTAGGGTC ACATTGTGTC   
  
  
+ TTGCGCAACA AGCTTAGACC ATTCGCAACG GATGCCTCAC GTGGAGCAGG TGGGACCCGC GTGGAAGTTG   
  
  
+ CATGGCAAGA ATTCTCTGGC AGCAAGGAGA ATTGAGGGGG GACACGCGTG TGACCACGCC AGTGCGGTCC   
  
  
+ CCCTCTCGTG GCAGATTTGG TGATTTTTTT TTTGTTTTTT TTTGAAGTAA TATATAATTT TTATATGATT   
  
  
+ AAAAAAATAG GTTGTGTGTT ATAAAGTGGG TCAATGACAA ATATTTTTAT TGGATGATTA AAATTAAGTT   
  
  
+ CCATTTATTG GAGAAGTGAT GGAATGCAAG TTCCAAAATT GTGGAACTTT TGTTGACGTA GCAGGCTAGA   
  
  
+ GTTCCAAAAA TTAAAAAAAG GAAAGACATA TTGAGGTAAA GCCTTTTTAT TTTACAAATA GACGCACCAA   
  
  
+ GGTATAATAT AATTGACATG ATTTGAGGCG AGAGGAAGAT TCTTTTGCTA CCCAAACAAA ACACAATGAT   
  
  
+ CTTTTAAATG TAGCAATTTG GGATTGGCAA AATTTTGTCT AACCCGATTG TTGAAAGTTA TCCGATTTCA   
  
  
+ TAAAATTGGA TATCGTTATT AAATTTTCAT TCGAATATTT AAATTTGGAC ACAATGTTTT TCATATTTGG   
  
  
+ ATTTTCTAAA CTTGATTTTA ACTCAAATTT AAGTTTATTC AATTCATCTG ACCCAAAAAA TGGTTGCAAT   
  
  
+ AATGTATTTT CTTTTTGAAG TTAAATATTA TCGAATTCAT TTAACTTGTA GTTAACCCGT AATTGACCCA   
  
  
+ AACTCGACTT TTTGCTACCT TAGCAGCCAG GAAGAAGTCA TAGTATTGAC CCGTGGAACG CCTGAAGCCA   
  
  
+ ACATCACCAG ACCCACATAT TCCATCATCT TCTGATCTCC TCGTCAAAGA AGGAAGCTGA GCACAGCAAT   
  
  
+ AACTAGAAGA ACAGGAGGCA TCACACCCAA GCCAAAGGCC TTTATTTGAG CTCCCCACCT CATTCCTCGA   
  
  
+ AGCTTCCTCT TCACAATGTA AGACCATAAT CCTTCACCCC TTCTCTTTTT AAGCTCATCT TTACCTGCCC   
  
  
+ TAGTTCTCTC TCTCTCATGA GTCATCAGTA ATAGTTACCT CTTTACTTTC TCTTCCTTGA GGTATGCTTC   
  
  
+ AACTTTAATA TATATATGTA CCACTTGATT TCTCTTGTCA CTTTGCTATA ATACTTATTG TTTGGCTATC   
  
  
+ TTCCCCTGTT TTATACTCAT TTTCTTGCTT TCTATATCTG GGTTTGAATT GGGTTTCACT TATCCTCGTT   
  
  
+ GATTCTGTGG GAAATTAGTG AATTGGGTTT TGTACAGCAG GTTTATATGA ACTAGGGTTT CAAAGGTATC   
  
  
+ TACTTTTTCT CTTGTGATTA TTAGTTGATT AGGGATTTGG CCAAGATTAG CAGAATGGGT TCTGAATTTG   
  
  
+ GGGAATTCTC TGATGATGCT CTAAATGGGT ATGCTTACGT TGATATCCCT GCTTATGATG CGTCCTTAGA   
  
  
+ TTATGCCAAT TTGTTCAATT ACGAAGGCCT ATCTGAGGAT CTCACCTCAC TGAGCCTCCC AAGCCCCTTT   
  
  
+ TCTGACCCTT TGGCGTACAG TTTCACGTCC TCTTTGGGGC CGAGCCCTGG GGTTGATTCT AATGATGATA   
  
  
+ GTGATTCTGA TGATGTTCTC AAGTGCATTA GCCAAATGCT TATGGAAGAA GACATGGAGG CAAAGCCATG   
  
  
+ CATGTTTCAC GATCCTTTAG CACTTCAGGC TGCTGAGAAA CCCTTTTATG ATGCCTTAGG GAAGAAATAC   
  
  
+ CCAACTTCTC CGAACCAACA TCCTATAATT GATGATTGTT TGGATAATCC TGGTGAAAAT TCTTTGGGTT   
  
  
+ CTTCTAGTGA TTTTAGTGTT AGTCACTTTG GGTCTAGTTC AGCAAGCTCT ATTGGACCGA CAATTGTGTC   
  
  
+ TGATTTGAGT GAGCATTTTG AGCCACCCTT TGTTCAAGCA CTTCCAATTG AATCATATCC CCAACCATTG   
  
  
+ ACCCGTCCTC AATGGTCGTT TGGCCCTTCG GGTGCCTTAG ATTGCACGGC CTCTAATGGT TCAGTGATCT   
  
  
+ CATCCCTTGG TTTGCCGATG GATGTTATTA GTGTATTCAG TGAGAAAGAG TCCATGATTC AATTTCAGAA   
  
  
+ AGGGGTGGAA GAGGCTAGTA AGTTCCTTCC CAAGAATAAT AACCTTGTTA TTGATCTCAA GAACCTCACT   
  
  
+ TTTCCTAATG AAACGAAGGA GGATGATCGA GTGATGATGG TTAAGAAGGA AAAGAATGAT GTGAATTGGT   
  
  
+ CTAACTACTC AAGAGGGAGT AAGATTCACT ATCGTGAAGA CGAGGACTTT GAAGAAGGAA GGAGTAGCAA   
  
  
+ GCAGTCAGCT ATTTCTACTA CTGAGGAAGC TGAGTTGTGT GAAATTTTTG ACAAGTTTTT GCTTTGCAAT   
  
  
+ TGGTACCCTG TGAAACCTGA GGCTCATCCC ACCATGAGTT TGAACCCTGA GAAGGGCCAG TCACATGGAT   
  
  
+ TAGAAGGTGG GAGAAATGGG AAGGGTCACC CAAAGAAACA GGATAAAAGT AGTACCAACG TTGTGGATTT   
  
  
+ AAGGAATTTG CTGATGCTAT GTGCACAATC TACTGCATCT GATGACCGAA GAACTGCTGA TGGACTGCTA   
  
  
+ AAGAAAATCA GGGAGCACTC ATCTGCTGAG GGGGATGGAT TTCAAAGGTT GGCTCATTAC TTTGCTGATG   
  
  
+ CCCTAGAGGC ACGTTTAGCT GGAACTGGAT CTCACATTTA TACAGCCCTA AGTTCTCATA GGCCATCTGC   
  
  
+ TGTTGACGTG TTAAAAGCAT ATCAGTTCTA TGTTCGTGCT TGCCCATTTA AGAAGATCGT CATTCGTTGT   
  
  
+ GGTAAACATA TGATTCTAAA AGCTGCTGAA AAGGCATCAA AGCTTCATAT TATAGATTTT GGCATCCTCT   
  
  
+ ATGGATTCCA ATGGCCTAGC CTCATTCGAT GCCTCTCAGA GCGGTCTGGT GGACCTCCAA AACTGTTTAT   
  
  
+ TACAGGGATC GATCTCCCCC AGCCTGGGTT CAGGCCAGCA GAAAGAGTGG AAGCAACAGG GAGACGCTTG   
  
  
+ GCTAAGTACT GTGAGCGGTA TAATGTGCCA TTTGAGTATC ATGCCATTGC TCAGAAGTGG GAAACAATCA   
  
  
+ AACCAGGGGA TCTCAAGTTA GGAAGTAGGG ATGATGATGA AGTTGTCGTG GTGAACTGTC TCTGTAGGTT   
  
  
+ CAAGAACCTC CTTGACGAGA CAATGGTGGT GGATAGTCCA AGGAACACAG TTTTAAACCT GATTAGAAGG   
  
  
+ GTAAAACCCG ATATTTTTGT GCATGGCATT GTAAATGGTT CCTACAACAT CCCTTTCTTT GTGACACGTT   
  
  
+ TTAGAGAAGC CCTCTTTCAT TATTCCACTC TTTTCGACAT GTTAGATGCC AACGCCTCTA GGGAGGAGCC   
  
  
+ CGAGAGGTTG ATATTCGAGA AGGCATTCTA TGGGAGGGAG ATTATGAATG TGGTGGCATG TGAGGGCACA   
  
  
+ GAGAGGGTGG AAAGGCCAGA GACATACAAG CAATGGCACG TTAGGCATAG CAGGGCAGGG TTTCGGCAAC   
  
  
+ TACCATTGGA TCCCAAGTTG ATCGAGAAAA TGAGGTTTAA GGCCAAGGCA GACCACCACA AGGATTTCGT   
  
  
+ GATTGATGTG GATGGACATT GGGCAATTCA GGGATGGAAG GGGCGGATTG CCTGTGCCAT CTCTGCATGG   
  
  
+ GTTCTGGCTT G  

- -Up\_Stream \_Len000GACAAG TCGTGATCCC TTATTCCAAA AACGGGACGT ATGATACTTT AGATTGGTTT   
  
  
- TTGTAAGGTA GAACCTTCCG ACATCGGAAC ACCTAAAACT TAAGCGAGGG TTCTTGGTTA CTTCATCGCG   
  
  
- TCTTCCGGTT ATGAGTTAAA GTCTCAGGTG TAGTTGAACA AGTTACGGTC ATATAAAGTC TTGTAATTAA   
  
  
- TGTATGACAA ACCAACCTCT TTTTTACCAA CAGAGCTTTT GATCAAAAAA TTAAAAAACA ATAAATCGTG   
  
  
- CCATTTCTTA CCTTTAATAA AAGTACGCTC TTTTTTCCTT CAACCAAAAA GAAAAAAGTT GAGAAAACTA   
  
  
- CTATATTAGT CAGCTTTAAA TCCACGTGGT AGACTGAAGT TGAGAACATG CTGGAGACTA ACGAACGAAT   
  
  
- CTTCTTGTTC TCCTAACTGT CTATTCCTCC TCACCCACCA AATATGTCTC TTAACTCACC TACCAATATT   
  
  
- TCTTCAGCTA GAGTGTATTC ATTGAAGCAA TGCTAATATT TATAAAAGTA ACTTCTTTCT GCTAATCTAT   
  
  
- ATTAATTCTT CCATGTAGAT TCGTTTAAAC CGACTCGTCT CAACCTCCCG GTGATCCCAG TGTAACACAG   
  
  
- AACGCGTTGT TCGAATCTGG TAAGCGTTGC CTACGGAGTG CACCTCGTCC ACCCTGGGCG CACCTTCAAC   
  
  
- GTACCGTTCT TAAGAGACCG TCGTTCCTCT TAACTCCCCC CTGTGCGCAC ACTGGTGCGG TCACGCCAGG   
  
  
- GGGAGAGCAC CGTCTAAACC ACTAAAAAAA AAACAAAAAA AAACTTCATT ATATATTAAA AATATACTAA   
  
  
- TTTTTTTATC CAACACACAA TATTTCACCC AGTTACTGTT TATAAAAATA ACCTACTAAT TTTAATTCAA   
  
  
- GGTAAATAAC CTCTTCACTA CCTTACGTTC AAGGTTTTAA CACCTTGAAA ACAACTGCAT CGTCCGATCT   
  
  
- CAAGGTTTTT AATTTTTTTC CTTTCTGTAT AACTCCATTT CGGAAAAATA AAATGTTTAT CTGCGTGGTT   
  
  
- CCATATTATA TTAACTGTAC TAAACTCCGC TCTCCTTCTA AGAAAACGAT GGGTTTGTTT TGTGTTACTA   
  
  
- GAAAATTTAC ATCGTTAAAC CCTAACCGTT TTAAAACAGA TTGGGCTAAC AACTTTCAAT AGGCTAAAGT   
  
  
- ATTTTAACCT ATAGCAATAA TTTAAAAGTA AGCTTATAAA TTTAAACCTG TGTTACAAAA AGTATAAACC   
  
  
- TAAAAGATTT GAACTAAAAT TGAGTTTAAA TTCAAATAAG TTAAGTAGAC TGGGTTTTTT ACCAACGTTA   
  
  
- TTACATAAAA GAAAAACTTC AATTTATAAT AGCTTAAGTA AATTGAACAT CAATTGGGCA TTAACTGGGT   
  
  
- TTGAGCTGAA AAACGATGGA ATCGTCGGTC CTTCTTCAGT ATCATAACTG GGCACCTTGC GGACTTCGGT   
  
  
- TGTAGTGGTC TGGGTGTATA AGGTAGTAGA AGACTAGAGG AGCAGTTTCT TCCTTCGACT CGTGTCGTTA   
  
  
- TTGATCTTCT TGTCCTCCGT AGTGTGGGTT CGGTTTCCGG AAATAAACTC GAGGGGTGGA GTAAGGAGCT   
  
  
- TCGAAGGAGA AGTGTTACAT TCTGGTATTA GGAAGTGGGG AAGAGAAAAA TTCGAGTAGA AATGGACGGG   
  
  
- ATCAAGAGAG AGAGAGTACT CAGTAGTCAT TATCAATGGA GAAATGAAAG AGAAGGAACT CCATACGAAG   
  
  
- TTGAAATTAT ATATATACAT GGTGAACTAA AGAGAACAGT GAAACGATAT TATGAATAAC AAACCGATAG   
  
  
- AAGGGGACAA AATATGAGTA AAAGAACGAA AGATATAGAC CCAAACTTAA CCCAAAGTGA ATAGGAGCAA   
  
  
- CTAAGACACC CTTTAATCAC TTAACCCAAA ACATGTCGTC CAAATATACT TGATCCCAAA GTTTCCATAG   
  
  
- ATGAAAAAGA GAACACTAAT AATCAACTAA TCCCTAAACC GGTTCTAATC GTCTTACCCA AGACTTAAAC   
  
  
- CCCTTAAGAG ACTACTACGA GATTTACCCA TACGAATGCA ACTATAGGGA CGAATACTAC GCAGGAATCT   
  
  
- AATACGGTTA AACAAGTTAA TGCTTCCGGA TAGACTCCTA GAGTGGAGTG ACTCGGAGGG TTCGGGGAAA   
  
  
- AGACTGGGAA ACCGCATGTC AAAGTGCAGG AGAAACCCCG GCTCGGGACC CCAACTAAGA TTACTACTAT   
  
  
- CACTAAGACT ACTACAAGAG TTCACGTAAT CGGTTTACGA ATACCTTCTT CTGTACCTCC GTTTCGGTAC   
  
  
- GTACAAAGTG CTAGGAAATC GTGAAGTCCG ACGACTCTTT GGGAAAATAC TACGGAATCC CTTCTTTATG   
  
  
- GGTTGAAGAG GCTTGGTTGT AGGATATTAA CTACTAACAA ACCTATTAGG ACCACTTTTA AGAAACCCAA   
  
  
- GAAGATCACT AAAATCACAA TCAGTGAAAC CCAGATCAAG TCGTTCGAGA TAACCTGGCT GTTAACACAG   
  
  
- ACTAAACTCA CTCGTAAAAC TCGGTGGGAA ACAAGTTCGT GAAGGTTAAC TTAGTATAGG GGTTGGTAAC   
  
  
- TGGGCAGGAG TTACCAGCAA ACCGGGAAGC CCACGGAATC TAACGTGCCG GAGATTACCA AGTCACTAGA   
  
  
- GTAGGGAACC AAACGGCTAC CTACAATAAT CACATAAGTC ACTCTTTCTC AGGTACTAAG TTAAAGTCTT   
  
  
- TCCCCACCTT CTCCGATCAT TCAAGGAAGG GTTCTTATTA TTGGAACAAT AACTAGAGTT CTTGGAGTGA   
  
  
- AAAGGATTAC TTTGCTTCCT CCTACTAGCT CACTACTACC AATTCTTCCT TTTCTTACTA CACTTAACCA   
  
  
- GATTGATGAG TTCTCCCTCA TTCTAAGTGA TAGCACTTCT GCTCCTGAAA CTTCTTCCTT CCTCATCGTT   
  
  
- CGTCAGTCGA TAAAGATGAT GACTCCTTCG ACTCAACACA CTTTAAAAAC TGTTCAAAAA CGAAACGTTA   
  
  
- ACCATGGGAC ACTTTGGACT CCGAGTAGGG TGGTACTCAA ACTTGGGACT CTTCCCGGTC AGTGTACCTA   
  
  
- ATCTTCCACC CTCTTTACCC TTCCCAGTGG GTTTCTTTGT CCTATTTTCA TCATGGTTGC AACACCTAAA   
  
  
- TTCCTTAAAC GACTACGATA CACGTGTTAG ATGACGTAGA CTACTGGCTT CTTGACGACT ACCTGACGAT   
  
  
- TTCTTTTAGT CCCTCGTGAG TAGACGACTC CCCCTACCTA AAGTTTCCAA CCGAGTAATG AAACGACTAC   
  
  
- GGGATCTCCG TGCAAATCGA CCTTGACCTA GAGTGTAAAT ATGTCGGGAT TCAAGAGTAT CCGGTAGACG   
  
  
- ACAACTGCAC AATTTTCGTA TAGTCAAGAT ACAAGCACGA ACGGGTAAAT TCTTCTAGCA GTAAGCAACA   
  
  
- CCATTTGTAT ACTAAGATTT TCGACGACTT TTCCGTAGTT TCGAAGTATA ATATCTAAAA CCGTAGGAGA   
  
  
- TACCTAAGGT TACCGGATCG GAGTAAGCTA CGGAGAGTCT CGCCAGACCA CCTGGAGGTT TTGACAAATA   
  
  
- ATGTCCCTAG CTAGAGGGGG TCGGACCCAA GTCCGGTCGT CTTTCTCACC TTCGTTGTCC CTCTGCGAAC   
  
  
- CGATTCATGA CACTCGCCAT ATTACACGGT AAACTCATAG TACGGTAACG AGTCTTCACC CTTTGTTAGT   
  
  
- TTGGTCCCCT AGAGTTCAAT CCTTCATCCC TACTACTACT TCAACAGCAC CACTTGACAG AGACATCCAA   
  
  
- GTTCTTGGAG GAACTGCTCT GTTACCACCA CCTATCAGGT TCCTTGTGTC AAAATTTGGA CTAATCTTCC   
  
  
- CATTTTGGGC TATAAAAACA CGTACCGTAA CATTTACCAA GGATGTTGTA GGGAAAGAAA CACTGTGCAA   
  
  
- AATCTCTTCG GGAGAAAGTA ATAAGGTGAG AAAAGCTGTA CAATCTACGG TTGCGGAGAT CCCTCCTCGG   
  
  
- GCTCTCCAAC TATAAGCTCT TCCGTAAGAT ACCCTCCCTC TAATACTTAC ACCACCGTAC ACTCCCGTGT   
  
  
- CTCTCCCACC TTTCCGGTCT CTGTATGTTC GTTACCGTGC AATCCGTATC GTCCCGTCCC AAAGCCGTTG   
  
  
- ATGGTAACCT AGGGTTCAAC TAGCTCTTTT ACTCCAAATT CCGGTTCCGT CTGGTGGTGT TCCTAAAGCA   
  
  
- CTAACTACAC CTACCTGTAA CCCGTTAAGT CCCTACCTTC CCCGCCTAAC GGACACGGTA GAGACGTACC   
  
  
- CAAGACCGAA C

+     GCN4\_motif

| Site Name | Organism | Position | Strand | Matrix score. | sequence | function |
| --- | --- | --- | --- | --- | --- | --- |
| GCN4\_motif | Oryza sativa | 1702 | + | 7 | TGAGTCA | cis-regulatory element involved in endosperm expression |

>HU07G02248.1   
+ -Up\_Stream \_Len000CTGTTC AGCACTAGGG AATAAGGTTT TTGCCCTGCA TACTATGAAA TCTAACCAAA   
  
  
+ AACATTCCAT CTTGGAAGGC TGTAGCCTTG TGGATTTTGA ATTCGCTCCC AAGAACCAAT GAAGTAGCGC   
  
  
+ AGAAGGCCAA TACTCAATTT CAGAGTCCAC ATCAACTTGT TCAATGCCAG TATATTTCAG AACATTAATT   
  
  
+ ACATACTGTT TGGTTGGAGA AAAAATGGTT GTCTCGAAAA CTAGTTTTTT AATTTTTTGT TATTTAGCAC   
  
  
+ GGTAAAGAAT GGAAATTATT TTCATGCGAG AAAAAAGGAA GTTGGTTTTT CTTTTTTCAA CTCTTTTGAT   
  
  
+ GATATAATCA GTCGAAATTT AGGTGCACCA TCTGACTTCA ACTCTTGTAC GACCTCTGAT TGCTTGCTTA   
  
  
+ GAAGAACAAG AGGATTGACA GATAAGGAGG AGTGGGTGGT TTATACAGAG AATTGAGTGG ATGGTTATAA   
  
  
+ AGAAGTCGAT CTCACATAAG TAACTTCGTT ACGATTATAA ATATTTTCAT TGAAGAAAGA CGATTAGATA   
  
  
+ TAATTAAGAA GGTACATCTA AGCAAATTTG GCTGAGCAGA GTTGGAGGGC CACTAGGGTC ACATTGTGTC   
  
  
+ TTGCGCAACA AGCTTAGACC ATTCGCAACG GATGCCTCAC GTGGAGCAGG TGGGACCCGC GTGGAAGTTG   
  
  
+ CATGGCAAGA ATTCTCTGGC AGCAAGGAGA ATTGAGGGGG GACACGCGTG TGACCACGCC AGTGCGGTCC   
  
  
+ CCCTCTCGTG GCAGATTTGG TGATTTTTTT TTTGTTTTTT TTTGAAGTAA TATATAATTT TTATATGATT   
  
  
+ AAAAAAATAG GTTGTGTGTT ATAAAGTGGG TCAATGACAA ATATTTTTAT TGGATGATTA AAATTAAGTT   
  
  
+ CCATTTATTG GAGAAGTGAT GGAATGCAAG TTCCAAAATT GTGGAACTTT TGTTGACGTA GCAGGCTAGA   
  
  
+ GTTCCAAAAA TTAAAAAAAG GAAAGACATA TTGAGGTAAA GCCTTTTTAT TTTACAAATA GACGCACCAA   
  
  
+ GGTATAATAT AATTGACATG ATTTGAGGCG AGAGGAAGAT TCTTTTGCTA CCCAAACAAA ACACAATGAT   
  
  
+ CTTTTAAATG TAGCAATTTG GGATTGGCAA AATTTTGTCT AACCCGATTG TTGAAAGTTA TCCGATTTCA   
  
  
+ TAAAATTGGA TATCGTTATT AAATTTTCAT TCGAATATTT AAATTTGGAC ACAATGTTTT TCATATTTGG   
  
  
+ ATTTTCTAAA CTTGATTTTA ACTCAAATTT AAGTTTATTC AATTCATCTG ACCCAAAAAA TGGTTGCAAT   
  
  
+ AATGTATTTT CTTTTTGAAG TTAAATATTA TCGAATTCAT TTAACTTGTA GTTAACCCGT AATTGACCCA   
  
  
+ AACTCGACTT TTTGCTACCT TAGCAGCCAG GAAGAAGTCA TAGTATTGAC CCGTGGAACG CCTGAAGCCA   
  
  
+ ACATCACCAG ACCCACATAT TCCATCATCT TCTGATCTCC TCGTCAAAGA AGGAAGCTGA GCACAGCAAT   
  
  
+ AACTAGAAGA ACAGGAGGCA TCACACCCAA GCCAAAGGCC TTTATTTGAG CTCCCCACCT CATTCCTCGA   
  
  
+ AGCTTCCTCT TCACAATGTA AGACCATAAT CCTTCACCCC TTCTCTTTTT AAGCTCATCT TTACCTGCCC   
  
  
+ TAGTTCTCTC TCTCTCATGA GTCATCAGTA ATAGTTACCT CTTTACTTTC TCTTCCTTGA GGTATGCTTC   
  
  
+ AACTTTAATA TATATATGTA CCACTTGATT TCTCTTGTCA CTTTGCTATA ATACTTATTG TTTGGCTATC   
  
  
+ TTCCCCTGTT TTATACTCAT TTTCTTGCTT TCTATATCTG GGTTTGAATT GGGTTTCACT TATCCTCGTT   
  
  
+ GATTCTGTGG GAAATTAGTG AATTGGGTTT TGTACAGCAG GTTTATATGA ACTAGGGTTT CAAAGGTATC   
  
  
+ TACTTTTTCT CTTGTGATTA TTAGTTGATT AGGGATTTGG CCAAGATTAG CAGAATGGGT TCTGAATTTG   
  
  
+ GGGAATTCTC TGATGATGCT CTAAATGGGT ATGCTTACGT TGATATCCCT GCTTATGATG CGTCCTTAGA   
  
  
+ TTATGCCAAT TTGTTCAATT ACGAAGGCCT ATCTGAGGAT CTCACCTCAC TGAGCCTCCC AAGCCCCTTT   
  
  
+ TCTGACCCTT TGGCGTACAG TTTCACGTCC TCTTTGGGGC CGAGCCCTGG GGTTGATTCT AATGATGATA   
  
  
+ GTGATTCTGA TGATGTTCTC AAGTGCATTA GCCAAATGCT TATGGAAGAA GACATGGAGG CAAAGCCATG   
  
  
+ CATGTTTCAC GATCCTTTAG CACTTCAGGC TGCTGAGAAA CCCTTTTATG ATGCCTTAGG GAAGAAATAC   
  
  
+ CCAACTTCTC CGAACCAACA TCCTATAATT GATGATTGTT TGGATAATCC TGGTGAAAAT TCTTTGGGTT   
  
  
+ CTTCTAGTGA TTTTAGTGTT AGTCACTTTG GGTCTAGTTC AGCAAGCTCT ATTGGACCGA CAATTGTGTC   
  
  
+ TGATTTGAGT GAGCATTTTG AGCCACCCTT TGTTCAAGCA CTTCCAATTG AATCATATCC CCAACCATTG   
  
  
+ ACCCGTCCTC AATGGTCGTT TGGCCCTTCG GGTGCCTTAG ATTGCACGGC CTCTAATGGT TCAGTGATCT   
  
  
+ CATCCCTTGG TTTGCCGATG GATGTTATTA GTGTATTCAG TGAGAAAGAG TCCATGATTC AATTTCAGAA   
  
  
+ AGGGGTGGAA GAGGCTAGTA AGTTCCTTCC CAAGAATAAT AACCTTGTTA TTGATCTCAA GAACCTCACT   
  
  
+ TTTCCTAATG AAACGAAGGA GGATGATCGA GTGATGATGG TTAAGAAGGA AAAGAATGAT GTGAATTGGT   
  
  
+ CTAACTACTC AAGAGGGAGT AAGATTCACT ATCGTGAAGA CGAGGACTTT GAAGAAGGAA GGAGTAGCAA   
  
  
+ GCAGTCAGCT ATTTCTACTA CTGAGGAAGC TGAGTTGTGT GAAATTTTTG ACAAGTTTTT GCTTTGCAAT   
  
  
+ TGGTACCCTG TGAAACCTGA GGCTCATCCC ACCATGAGTT TGAACCCTGA GAAGGGCCAG TCACATGGAT   
  
  
+ TAGAAGGTGG GAGAAATGGG AAGGGTCACC CAAAGAAACA GGATAAAAGT AGTACCAACG TTGTGGATTT   
  
  
+ AAGGAATTTG CTGATGCTAT GTGCACAATC TACTGCATCT GATGACCGAA GAACTGCTGA TGGACTGCTA   
  
  
+ AAGAAAATCA GGGAGCACTC ATCTGCTGAG GGGGATGGAT TTCAAAGGTT GGCTCATTAC TTTGCTGATG   
  
  
+ CCCTAGAGGC ACGTTTAGCT GGAACTGGAT CTCACATTTA TACAGCCCTA AGTTCTCATA GGCCATCTGC   
  
  
+ TGTTGACGTG TTAAAAGCAT ATCAGTTCTA TGTTCGTGCT TGCCCATTTA AGAAGATCGT CATTCGTTGT   
  
  
+ GGTAAACATA TGATTCTAAA AGCTGCTGAA AAGGCATCAA AGCTTCATAT TATAGATTTT GGCATCCTCT   
  
  
+ ATGGATTCCA ATGGCCTAGC CTCATTCGAT GCCTCTCAGA GCGGTCTGGT GGACCTCCAA AACTGTTTAT   
  
  
+ TACAGGGATC GATCTCCCCC AGCCTGGGTT CAGGCCAGCA GAAAGAGTGG AAGCAACAGG GAGACGCTTG   
  
  
+ GCTAAGTACT GTGAGCGGTA TAATGTGCCA TTTGAGTATC ATGCCATTGC TCAGAAGTGG GAAACAATCA   
  
  
+ AACCAGGGGA TCTCAAGTTA GGAAGTAGGG ATGATGATGA AGTTGTCGTG GTGAACTGTC TCTGTAGGTT   
  
  
+ CAAGAACCTC CTTGACGAGA CAATGGTGGT GGATAGTCCA AGGAACACAG TTTTAAACCT GATTAGAAGG   
  
  
+ GTAAAACCCG ATATTTTTGT GCATGGCATT GTAAATGGTT CCTACAACAT CCCTTTCTTT GTGACACGTT   
  
  
+ TTAGAGAAGC CCTCTTTCAT TATTCCACTC TTTTCGACAT GTTAGATGCC AACGCCTCTA GGGAGGAGCC   
  
  
+ CGAGAGGTTG ATATTCGAGA AGGCATTCTA TGGGAGGGAG ATTATGAATG TGGTGGCATG TGAGGGCACA   
  
  
+ GAGAGGGTGG AAAGGCCAGA GACATACAAG CAATGGCACG TTAGGCATAG CAGGGCAGGG TTTCGGCAAC   
  
  
+ TACCATTGGA TCCCAAGTTG ATCGAGAAAA TGAGGTTTAA GGCCAAGGCA GACCACCACA AGGATTTCGT   
  
  
+ GATTGATGTG GATGGACATT GGGCAATTCA GGGATGGAAG GGGCGGATTG CCTGTGCCAT CTCTGCATGG   
  
  
+ GTTCTGGCTT G  

- -Up\_Stream \_Len000GACAAG TCGTGATCCC TTATTCCAAA AACGGGACGT ATGATACTTT AGATTGGTTT   
  
  
- TTGTAAGGTA GAACCTTCCG ACATCGGAAC ACCTAAAACT TAAGCGAGGG TTCTTGGTTA CTTCATCGCG   
  
  
- TCTTCCGGTT ATGAGTTAAA GTCTCAGGTG TAGTTGAACA AGTTACGGTC ATATAAAGTC TTGTAATTAA   
  
  
- TGTATGACAA ACCAACCTCT TTTTTACCAA CAGAGCTTTT GATCAAAAAA TTAAAAAACA ATAAATCGTG   
  
  
- CCATTTCTTA CCTTTAATAA AAGTACGCTC TTTTTTCCTT CAACCAAAAA GAAAAAAGTT GAGAAAACTA   
  
  
- CTATATTAGT CAGCTTTAAA TCCACGTGGT AGACTGAAGT TGAGAACATG CTGGAGACTA ACGAACGAAT   
  
  
- CTTCTTGTTC TCCTAACTGT CTATTCCTCC TCACCCACCA AATATGTCTC TTAACTCACC TACCAATATT   
  
  
- TCTTCAGCTA GAGTGTATTC ATTGAAGCAA TGCTAATATT TATAAAAGTA ACTTCTTTCT GCTAATCTAT   
  
  
- ATTAATTCTT CCATGTAGAT TCGTTTAAAC CGACTCGTCT CAACCTCCCG GTGATCCCAG TGTAACACAG   
  
  
- AACGCGTTGT TCGAATCTGG TAAGCGTTGC CTACGGAGTG CACCTCGTCC ACCCTGGGCG CACCTTCAAC   
  
  
- GTACCGTTCT TAAGAGACCG TCGTTCCTCT TAACTCCCCC CTGTGCGCAC ACTGGTGCGG TCACGCCAGG   
  
  
- GGGAGAGCAC CGTCTAAACC ACTAAAAAAA AAACAAAAAA AAACTTCATT ATATATTAAA AATATACTAA   
  
  
- TTTTTTTATC CAACACACAA TATTTCACCC AGTTACTGTT TATAAAAATA ACCTACTAAT TTTAATTCAA   
  
  
- GGTAAATAAC CTCTTCACTA CCTTACGTTC AAGGTTTTAA CACCTTGAAA ACAACTGCAT CGTCCGATCT   
  
  
- CAAGGTTTTT AATTTTTTTC CTTTCTGTAT AACTCCATTT CGGAAAAATA AAATGTTTAT CTGCGTGGTT   
  
  
- CCATATTATA TTAACTGTAC TAAACTCCGC TCTCCTTCTA AGAAAACGAT GGGTTTGTTT TGTGTTACTA   
  
  
- GAAAATTTAC ATCGTTAAAC CCTAACCGTT TTAAAACAGA TTGGGCTAAC AACTTTCAAT AGGCTAAAGT   
  
  
- ATTTTAACCT ATAGCAATAA TTTAAAAGTA AGCTTATAAA TTTAAACCTG TGTTACAAAA AGTATAAACC   
  
  
- TAAAAGATTT GAACTAAAAT TGAGTTTAAA TTCAAATAAG TTAAGTAGAC TGGGTTTTTT ACCAACGTTA   
  
  
- TTACATAAAA GAAAAACTTC AATTTATAAT AGCTTAAGTA AATTGAACAT CAATTGGGCA TTAACTGGGT   
  
  
- TTGAGCTGAA AAACGATGGA ATCGTCGGTC CTTCTTCAGT ATCATAACTG GGCACCTTGC GGACTTCGGT   
  
  
- TGTAGTGGTC TGGGTGTATA AGGTAGTAGA AGACTAGAGG AGCAGTTTCT TCCTTCGACT CGTGTCGTTA   
  
  
- TTGATCTTCT TGTCCTCCGT AGTGTGGGTT CGGTTTCCGG AAATAAACTC GAGGGGTGGA GTAAGGAGCT   
  
  
- TCGAAGGAGA AGTGTTACAT TCTGGTATTA GGAAGTGGGG AAGAGAAAAA TTCGAGTAGA AATGGACGGG   
  
  
- ATCAAGAGAG AGAGAGTACT CAGTAGTCAT TATCAATGGA GAAATGAAAG AGAAGGAACT CCATACGAAG   
  
  
- TTGAAATTAT ATATATACAT GGTGAACTAA AGAGAACAGT GAAACGATAT TATGAATAAC AAACCGATAG   
  
  
- AAGGGGACAA AATATGAGTA AAAGAACGAA AGATATAGAC CCAAACTTAA CCCAAAGTGA ATAGGAGCAA   
  
  
- CTAAGACACC CTTTAATCAC TTAACCCAAA ACATGTCGTC CAAATATACT TGATCCCAAA GTTTCCATAG   
  
  
- ATGAAAAAGA GAACACTAAT AATCAACTAA TCCCTAAACC GGTTCTAATC GTCTTACCCA AGACTTAAAC   
  
  
- CCCTTAAGAG ACTACTACGA GATTTACCCA TACGAATGCA ACTATAGGGA CGAATACTAC GCAGGAATCT   
  
  
- AATACGGTTA AACAAGTTAA TGCTTCCGGA TAGACTCCTA GAGTGGAGTG ACTCGGAGGG TTCGGGGAAA   
  
  
- AGACTGGGAA ACCGCATGTC AAAGTGCAGG AGAAACCCCG GCTCGGGACC CCAACTAAGA TTACTACTAT   
  
  
- CACTAAGACT ACTACAAGAG TTCACGTAAT CGGTTTACGA ATACCTTCTT CTGTACCTCC GTTTCGGTAC   
  
  
- GTACAAAGTG CTAGGAAATC GTGAAGTCCG ACGACTCTTT GGGAAAATAC TACGGAATCC CTTCTTTATG   
  
  
- GGTTGAAGAG GCTTGGTTGT AGGATATTAA CTACTAACAA ACCTATTAGG ACCACTTTTA AGAAACCCAA   
  
  
- GAAGATCACT AAAATCACAA TCAGTGAAAC CCAGATCAAG TCGTTCGAGA TAACCTGGCT GTTAACACAG   
  
  
- ACTAAACTCA CTCGTAAAAC TCGGTGGGAA ACAAGTTCGT GAAGGTTAAC TTAGTATAGG GGTTGGTAAC   
  
  
- TGGGCAGGAG TTACCAGCAA ACCGGGAAGC CCACGGAATC TAACGTGCCG GAGATTACCA AGTCACTAGA   
  
  
- GTAGGGAACC AAACGGCTAC CTACAATAAT CACATAAGTC ACTCTTTCTC AGGTACTAAG TTAAAGTCTT   
  
  
- TCCCCACCTT CTCCGATCAT TCAAGGAAGG GTTCTTATTA TTGGAACAAT AACTAGAGTT CTTGGAGTGA   
  
  
- AAAGGATTAC TTTGCTTCCT CCTACTAGCT CACTACTACC AATTCTTCCT TTTCTTACTA CACTTAACCA   
  
  
- GATTGATGAG TTCTCCCTCA TTCTAAGTGA TAGCACTTCT GCTCCTGAAA CTTCTTCCTT CCTCATCGTT   
  
  
- CGTCAGTCGA TAAAGATGAT GACTCCTTCG ACTCAACACA CTTTAAAAAC TGTTCAAAAA CGAAACGTTA   
  
  
- ACCATGGGAC ACTTTGGACT CCGAGTAGGG TGGTACTCAA ACTTGGGACT CTTCCCGGTC AGTGTACCTA   
  
  
- ATCTTCCACC CTCTTTACCC TTCCCAGTGG GTTTCTTTGT CCTATTTTCA TCATGGTTGC AACACCTAAA   
  
  
- TTCCTTAAAC GACTACGATA CACGTGTTAG ATGACGTAGA CTACTGGCTT CTTGACGACT ACCTGACGAT   
  
  
- TTCTTTTAGT CCCTCGTGAG TAGACGACTC CCCCTACCTA AAGTTTCCAA CCGAGTAATG AAACGACTAC   
  
  
- GGGATCTCCG TGCAAATCGA CCTTGACCTA GAGTGTAAAT ATGTCGGGAT TCAAGAGTAT CCGGTAGACG   
  
  
- ACAACTGCAC AATTTTCGTA TAGTCAAGAT ACAAGCACGA ACGGGTAAAT TCTTCTAGCA GTAAGCAACA   
  
  
- CCATTTGTAT ACTAAGATTT TCGACGACTT TTCCGTAGTT TCGAAGTATA ATATCTAAAA CCGTAGGAGA   
  
  
- TACCTAAGGT TACCGGATCG GAGTAAGCTA CGGAGAGTCT CGCCAGACCA CCTGGAGGTT TTGACAAATA   
  
  
- ATGTCCCTAG CTAGAGGGGG TCGGACCCAA GTCCGGTCGT CTTTCTCACC TTCGTTGTCC CTCTGCGAAC   
  
  
- CGATTCATGA CACTCGCCAT ATTACACGGT AAACTCATAG TACGGTAACG AGTCTTCACC CTTTGTTAGT   
  
  
- TTGGTCCCCT AGAGTTCAAT CCTTCATCCC TACTACTACT TCAACAGCAC CACTTGACAG AGACATCCAA   
  
  
- GTTCTTGGAG GAACTGCTCT GTTACCACCA CCTATCAGGT TCCTTGTGTC AAAATTTGGA CTAATCTTCC   
  
  
- CATTTTGGGC TATAAAAACA CGTACCGTAA CATTTACCAA GGATGTTGTA GGGAAAGAAA CACTGTGCAA   
  
  
- AATCTCTTCG GGAGAAAGTA ATAAGGTGAG AAAAGCTGTA CAATCTACGG TTGCGGAGAT CCCTCCTCGG   
  
  
- GCTCTCCAAC TATAAGCTCT TCCGTAAGAT ACCCTCCCTC TAATACTTAC ACCACCGTAC ACTCCCGTGT   
  
  
- CTCTCCCACC TTTCCGGTCT CTGTATGTTC GTTACCGTGC AATCCGTATC GTCCCGTCCC AAAGCCGTTG   
  
  
- ATGGTAACCT AGGGTTCAAC TAGCTCTTTT ACTCCAAATT CCGGTTCCGT CTGGTGGTGT TCCTAAAGCA   
  
  
- CTAACTACAC CTACCTGTAA CCCGTTAAGT CCCTACCTTC CCCGCCTAAC GGACACGGTA GAGACGTACC   
  
  
- CAAGACCGAA C

+     GT1-motif

| Site Name | Organism | Position | Strand | Matrix score. | sequence | function |
| --- | --- | --- | --- | --- | --- | --- |
| GT1-motif | Arabidopsis thaliana | 2843 | + | 6 | GGTTAA | light responsive element |
| GT1-motif | Arabidopsis thaliana | 1386 | - | 6 | GGTTAA | light responsive element |

>HU07G02248.1   
+ -Up\_Stream \_Len000CTGTTC AGCACTAGGG AATAAGGTTT TTGCCCTGCA TACTATGAAA TCTAACCAAA   
  
  
+ AACATTCCAT CTTGGAAGGC TGTAGCCTTG TGGATTTTGA ATTCGCTCCC AAGAACCAAT GAAGTAGCGC   
  
  
+ AGAAGGCCAA TACTCAATTT CAGAGTCCAC ATCAACTTGT TCAATGCCAG TATATTTCAG AACATTAATT   
  
  
+ ACATACTGTT TGGTTGGAGA AAAAATGGTT GTCTCGAAAA CTAGTTTTTT AATTTTTTGT TATTTAGCAC   
  
  
+ GGTAAAGAAT GGAAATTATT TTCATGCGAG AAAAAAGGAA GTTGGTTTTT CTTTTTTCAA CTCTTTTGAT   
  
  
+ GATATAATCA GTCGAAATTT AGGTGCACCA TCTGACTTCA ACTCTTGTAC GACCTCTGAT TGCTTGCTTA   
  
  
+ GAAGAACAAG AGGATTGACA GATAAGGAGG AGTGGGTGGT TTATACAGAG AATTGAGTGG ATGGTTATAA   
  
  
+ AGAAGTCGAT CTCACATAAG TAACTTCGTT ACGATTATAA ATATTTTCAT TGAAGAAAGA CGATTAGATA   
  
  
+ TAATTAAGAA GGTACATCTA AGCAAATTTG GCTGAGCAGA GTTGGAGGGC CACTAGGGTC ACATTGTGTC   
  
  
+ TTGCGCAACA AGCTTAGACC ATTCGCAACG GATGCCTCAC GTGGAGCAGG TGGGACCCGC GTGGAAGTTG   
  
  
+ CATGGCAAGA ATTCTCTGGC AGCAAGGAGA ATTGAGGGGG GACACGCGTG TGACCACGCC AGTGCGGTCC   
  
  
+ CCCTCTCGTG GCAGATTTGG TGATTTTTTT TTTGTTTTTT TTTGAAGTAA TATATAATTT TTATATGATT   
  
  
+ AAAAAAATAG GTTGTGTGTT ATAAAGTGGG TCAATGACAA ATATTTTTAT TGGATGATTA AAATTAAGTT   
  
  
+ CCATTTATTG GAGAAGTGAT GGAATGCAAG TTCCAAAATT GTGGAACTTT TGTTGACGTA GCAGGCTAGA   
  
  
+ GTTCCAAAAA TTAAAAAAAG GAAAGACATA TTGAGGTAAA GCCTTTTTAT TTTACAAATA GACGCACCAA   
  
  
+ GGTATAATAT AATTGACATG ATTTGAGGCG AGAGGAAGAT TCTTTTGCTA CCCAAACAAA ACACAATGAT   
  
  
+ CTTTTAAATG TAGCAATTTG GGATTGGCAA AATTTTGTCT AACCCGATTG TTGAAAGTTA TCCGATTTCA   
  
  
+ TAAAATTGGA TATCGTTATT AAATTTTCAT TCGAATATTT AAATTTGGAC ACAATGTTTT TCATATTTGG   
  
  
+ ATTTTCTAAA CTTGATTTTA ACTCAAATTT AAGTTTATTC AATTCATCTG ACCCAAAAAA TGGTTGCAAT   
  
  
+ AATGTATTTT CTTTTTGAAG TTAAATATTA TCGAATTCAT TTAACTTGTA GTTAACCCGT AATTGACCCA   
  
  
+ AACTCGACTT TTTGCTACCT TAGCAGCCAG GAAGAAGTCA TAGTATTGAC CCGTGGAACG CCTGAAGCCA   
  
  
+ ACATCACCAG ACCCACATAT TCCATCATCT TCTGATCTCC TCGTCAAAGA AGGAAGCTGA GCACAGCAAT   
  
  
+ AACTAGAAGA ACAGGAGGCA TCACACCCAA GCCAAAGGCC TTTATTTGAG CTCCCCACCT CATTCCTCGA   
  
  
+ AGCTTCCTCT TCACAATGTA AGACCATAAT CCTTCACCCC TTCTCTTTTT AAGCTCATCT TTACCTGCCC   
  
  
+ TAGTTCTCTC TCTCTCATGA GTCATCAGTA ATAGTTACCT CTTTACTTTC TCTTCCTTGA GGTATGCTTC   
  
  
+ AACTTTAATA TATATATGTA CCACTTGATT TCTCTTGTCA CTTTGCTATA ATACTTATTG TTTGGCTATC   
  
  
+ TTCCCCTGTT TTATACTCAT TTTCTTGCTT TCTATATCTG GGTTTGAATT GGGTTTCACT TATCCTCGTT   
  
  
+ GATTCTGTGG GAAATTAGTG AATTGGGTTT TGTACAGCAG GTTTATATGA ACTAGGGTTT CAAAGGTATC   
  
  
+ TACTTTTTCT CTTGTGATTA TTAGTTGATT AGGGATTTGG CCAAGATTAG CAGAATGGGT TCTGAATTTG   
  
  
+ GGGAATTCTC TGATGATGCT CTAAATGGGT ATGCTTACGT TGATATCCCT GCTTATGATG CGTCCTTAGA   
  
  
+ TTATGCCAAT TTGTTCAATT ACGAAGGCCT ATCTGAGGAT CTCACCTCAC TGAGCCTCCC AAGCCCCTTT   
  
  
+ TCTGACCCTT TGGCGTACAG TTTCACGTCC TCTTTGGGGC CGAGCCCTGG GGTTGATTCT AATGATGATA   
  
  
+ GTGATTCTGA TGATGTTCTC AAGTGCATTA GCCAAATGCT TATGGAAGAA GACATGGAGG CAAAGCCATG   
  
  
+ CATGTTTCAC GATCCTTTAG CACTTCAGGC TGCTGAGAAA CCCTTTTATG ATGCCTTAGG GAAGAAATAC   
  
  
+ CCAACTTCTC CGAACCAACA TCCTATAATT GATGATTGTT TGGATAATCC TGGTGAAAAT TCTTTGGGTT   
  
  
+ CTTCTAGTGA TTTTAGTGTT AGTCACTTTG GGTCTAGTTC AGCAAGCTCT ATTGGACCGA CAATTGTGTC   
  
  
+ TGATTTGAGT GAGCATTTTG AGCCACCCTT TGTTCAAGCA CTTCCAATTG AATCATATCC CCAACCATTG   
  
  
+ ACCCGTCCTC AATGGTCGTT TGGCCCTTCG GGTGCCTTAG ATTGCACGGC CTCTAATGGT TCAGTGATCT   
  
  
+ CATCCCTTGG TTTGCCGATG GATGTTATTA GTGTATTCAG TGAGAAAGAG TCCATGATTC AATTTCAGAA   
  
  
+ AGGGGTGGAA GAGGCTAGTA AGTTCCTTCC CAAGAATAAT AACCTTGTTA TTGATCTCAA GAACCTCACT   
  
  
+ TTTCCTAATG AAACGAAGGA GGATGATCGA GTGATGATGG TTAAGAAGGA AAAGAATGAT GTGAATTGGT   
  
  
+ CTAACTACTC AAGAGGGAGT AAGATTCACT ATCGTGAAGA CGAGGACTTT GAAGAAGGAA GGAGTAGCAA   
  
  
+ GCAGTCAGCT ATTTCTACTA CTGAGGAAGC TGAGTTGTGT GAAATTTTTG ACAAGTTTTT GCTTTGCAAT   
  
  
+ TGGTACCCTG TGAAACCTGA GGCTCATCCC ACCATGAGTT TGAACCCTGA GAAGGGCCAG TCACATGGAT   
  
  
+ TAGAAGGTGG GAGAAATGGG AAGGGTCACC CAAAGAAACA GGATAAAAGT AGTACCAACG TTGTGGATTT   
  
  
+ AAGGAATTTG CTGATGCTAT GTGCACAATC TACTGCATCT GATGACCGAA GAACTGCTGA TGGACTGCTA   
  
  
+ AAGAAAATCA GGGAGCACTC ATCTGCTGAG GGGGATGGAT TTCAAAGGTT GGCTCATTAC TTTGCTGATG   
  
  
+ CCCTAGAGGC ACGTTTAGCT GGAACTGGAT CTCACATTTA TACAGCCCTA AGTTCTCATA GGCCATCTGC   
  
  
+ TGTTGACGTG TTAAAAGCAT ATCAGTTCTA TGTTCGTGCT TGCCCATTTA AGAAGATCGT CATTCGTTGT   
  
  
+ GGTAAACATA TGATTCTAAA AGCTGCTGAA AAGGCATCAA AGCTTCATAT TATAGATTTT GGCATCCTCT   
  
  
+ ATGGATTCCA ATGGCCTAGC CTCATTCGAT GCCTCTCAGA GCGGTCTGGT GGACCTCCAA AACTGTTTAT   
  
  
+ TACAGGGATC GATCTCCCCC AGCCTGGGTT CAGGCCAGCA GAAAGAGTGG AAGCAACAGG GAGACGCTTG   
  
  
+ GCTAAGTACT GTGAGCGGTA TAATGTGCCA TTTGAGTATC ATGCCATTGC TCAGAAGTGG GAAACAATCA   
  
  
+ AACCAGGGGA TCTCAAGTTA GGAAGTAGGG ATGATGATGA AGTTGTCGTG GTGAACTGTC TCTGTAGGTT   
  
  
+ CAAGAACCTC CTTGACGAGA CAATGGTGGT GGATAGTCCA AGGAACACAG TTTTAAACCT GATTAGAAGG   
  
  
+ GTAAAACCCG ATATTTTTGT GCATGGCATT GTAAATGGTT CCTACAACAT CCCTTTCTTT GTGACACGTT   
  
  
+ TTAGAGAAGC CCTCTTTCAT TATTCCACTC TTTTCGACAT GTTAGATGCC AACGCCTCTA GGGAGGAGCC   
  
  
+ CGAGAGGTTG ATATTCGAGA AGGCATTCTA TGGGAGGGAG ATTATGAATG TGGTGGCATG TGAGGGCACA   
  
  
+ GAGAGGGTGG AAAGGCCAGA GACATACAAG CAATGGCACG TTAGGCATAG CAGGGCAGGG TTTCGGCAAC   
  
  
+ TACCATTGGA TCCCAAGTTG ATCGAGAAAA TGAGGTTTAA GGCCAAGGCA GACCACCACA AGGATTTCGT   
  
  
+ GATTGATGTG GATGGACATT GGGCAATTCA GGGATGGAAG GGGCGGATTG CCTGTGCCAT CTCTGCATGG   
  
  
+ GTTCTGGCTT G  

- -Up\_Stream \_Len000GACAAG TCGTGATCCC TTATTCCAAA AACGGGACGT ATGATACTTT AGATTGGTTT   
  
  
- TTGTAAGGTA GAACCTTCCG ACATCGGAAC ACCTAAAACT TAAGCGAGGG TTCTTGGTTA CTTCATCGCG   
  
  
- TCTTCCGGTT ATGAGTTAAA GTCTCAGGTG TAGTTGAACA AGTTACGGTC ATATAAAGTC TTGTAATTAA   
  
  
- TGTATGACAA ACCAACCTCT TTTTTACCAA CAGAGCTTTT GATCAAAAAA TTAAAAAACA ATAAATCGTG   
  
  
- CCATTTCTTA CCTTTAATAA AAGTACGCTC TTTTTTCCTT CAACCAAAAA GAAAAAAGTT GAGAAAACTA   
  
  
- CTATATTAGT CAGCTTTAAA TCCACGTGGT AGACTGAAGT TGAGAACATG CTGGAGACTA ACGAACGAAT   
  
  
- CTTCTTGTTC TCCTAACTGT CTATTCCTCC TCACCCACCA AATATGTCTC TTAACTCACC TACCAATATT   
  
  
- TCTTCAGCTA GAGTGTATTC ATTGAAGCAA TGCTAATATT TATAAAAGTA ACTTCTTTCT GCTAATCTAT   
  
  
- ATTAATTCTT CCATGTAGAT TCGTTTAAAC CGACTCGTCT CAACCTCCCG GTGATCCCAG TGTAACACAG   
  
  
- AACGCGTTGT TCGAATCTGG TAAGCGTTGC CTACGGAGTG CACCTCGTCC ACCCTGGGCG CACCTTCAAC   
  
  
- GTACCGTTCT TAAGAGACCG TCGTTCCTCT TAACTCCCCC CTGTGCGCAC ACTGGTGCGG TCACGCCAGG   
  
  
- GGGAGAGCAC CGTCTAAACC ACTAAAAAAA AAACAAAAAA AAACTTCATT ATATATTAAA AATATACTAA   
  
  
- TTTTTTTATC CAACACACAA TATTTCACCC AGTTACTGTT TATAAAAATA ACCTACTAAT TTTAATTCAA   
  
  
- GGTAAATAAC CTCTTCACTA CCTTACGTTC AAGGTTTTAA CACCTTGAAA ACAACTGCAT CGTCCGATCT   
  
  
- CAAGGTTTTT AATTTTTTTC CTTTCTGTAT AACTCCATTT CGGAAAAATA AAATGTTTAT CTGCGTGGTT   
  
  
- CCATATTATA TTAACTGTAC TAAACTCCGC TCTCCTTCTA AGAAAACGAT GGGTTTGTTT TGTGTTACTA   
  
  
- GAAAATTTAC ATCGTTAAAC CCTAACCGTT TTAAAACAGA TTGGGCTAAC AACTTTCAAT AGGCTAAAGT   
  
  
- ATTTTAACCT ATAGCAATAA TTTAAAAGTA AGCTTATAAA TTTAAACCTG TGTTACAAAA AGTATAAACC   
  
  
- TAAAAGATTT GAACTAAAAT TGAGTTTAAA TTCAAATAAG TTAAGTAGAC TGGGTTTTTT ACCAACGTTA   
  
  
- TTACATAAAA GAAAAACTTC AATTTATAAT AGCTTAAGTA AATTGAACAT CAATTGGGCA TTAACTGGGT   
  
  
- TTGAGCTGAA AAACGATGGA ATCGTCGGTC CTTCTTCAGT ATCATAACTG GGCACCTTGC GGACTTCGGT   
  
  
- TGTAGTGGTC TGGGTGTATA AGGTAGTAGA AGACTAGAGG AGCAGTTTCT TCCTTCGACT CGTGTCGTTA   
  
  
- TTGATCTTCT TGTCCTCCGT AGTGTGGGTT CGGTTTCCGG AAATAAACTC GAGGGGTGGA GTAAGGAGCT   
  
  
- TCGAAGGAGA AGTGTTACAT TCTGGTATTA GGAAGTGGGG AAGAGAAAAA TTCGAGTAGA AATGGACGGG   
  
  
- ATCAAGAGAG AGAGAGTACT CAGTAGTCAT TATCAATGGA GAAATGAAAG AGAAGGAACT CCATACGAAG   
  
  
- TTGAAATTAT ATATATACAT GGTGAACTAA AGAGAACAGT GAAACGATAT TATGAATAAC AAACCGATAG   
  
  
- AAGGGGACAA AATATGAGTA AAAGAACGAA AGATATAGAC CCAAACTTAA CCCAAAGTGA ATAGGAGCAA   
  
  
- CTAAGACACC CTTTAATCAC TTAACCCAAA ACATGTCGTC CAAATATACT TGATCCCAAA GTTTCCATAG   
  
  
- ATGAAAAAGA GAACACTAAT AATCAACTAA TCCCTAAACC GGTTCTAATC GTCTTACCCA AGACTTAAAC   
  
  
- CCCTTAAGAG ACTACTACGA GATTTACCCA TACGAATGCA ACTATAGGGA CGAATACTAC GCAGGAATCT   
  
  
- AATACGGTTA AACAAGTTAA TGCTTCCGGA TAGACTCCTA GAGTGGAGTG ACTCGGAGGG TTCGGGGAAA   
  
  
- AGACTGGGAA ACCGCATGTC AAAGTGCAGG AGAAACCCCG GCTCGGGACC CCAACTAAGA TTACTACTAT   
  
  
- CACTAAGACT ACTACAAGAG TTCACGTAAT CGGTTTACGA ATACCTTCTT CTGTACCTCC GTTTCGGTAC   
  
  
- GTACAAAGTG CTAGGAAATC GTGAAGTCCG ACGACTCTTT GGGAAAATAC TACGGAATCC CTTCTTTATG   
  
  
- GGTTGAAGAG GCTTGGTTGT AGGATATTAA CTACTAACAA ACCTATTAGG ACCACTTTTA AGAAACCCAA   
  
  
- GAAGATCACT AAAATCACAA TCAGTGAAAC CCAGATCAAG TCGTTCGAGA TAACCTGGCT GTTAACACAG   
  
  
- ACTAAACTCA CTCGTAAAAC TCGGTGGGAA ACAAGTTCGT GAAGGTTAAC TTAGTATAGG GGTTGGTAAC   
  
  
- TGGGCAGGAG TTACCAGCAA ACCGGGAAGC CCACGGAATC TAACGTGCCG GAGATTACCA AGTCACTAGA   
  
  
- GTAGGGAACC AAACGGCTAC CTACAATAAT CACATAAGTC ACTCTTTCTC AGGTACTAAG TTAAAGTCTT   
  
  
- TCCCCACCTT CTCCGATCAT TCAAGGAAGG GTTCTTATTA TTGGAACAAT AACTAGAGTT CTTGGAGTGA   
  
  
- AAAGGATTAC TTTGCTTCCT CCTACTAGCT CACTACTACC AATTCTTCCT TTTCTTACTA CACTTAACCA   
  
  
- GATTGATGAG TTCTCCCTCA TTCTAAGTGA TAGCACTTCT GCTCCTGAAA CTTCTTCCTT CCTCATCGTT   
  
  
- CGTCAGTCGA TAAAGATGAT GACTCCTTCG ACTCAACACA CTTTAAAAAC TGTTCAAAAA CGAAACGTTA   
  
  
- ACCATGGGAC ACTTTGGACT CCGAGTAGGG TGGTACTCAA ACTTGGGACT CTTCCCGGTC AGTGTACCTA   
  
  
- ATCTTCCACC CTCTTTACCC TTCCCAGTGG GTTTCTTTGT CCTATTTTCA TCATGGTTGC AACACCTAAA   
  
  
- TTCCTTAAAC GACTACGATA CACGTGTTAG ATGACGTAGA CTACTGGCTT CTTGACGACT ACCTGACGAT   
  
  
- TTCTTTTAGT CCCTCGTGAG TAGACGACTC CCCCTACCTA AAGTTTCCAA CCGAGTAATG AAACGACTAC   
  
  
- GGGATCTCCG TGCAAATCGA CCTTGACCTA GAGTGTAAAT ATGTCGGGAT TCAAGAGTAT CCGGTAGACG   
  
  
- ACAACTGCAC AATTTTCGTA TAGTCAAGAT ACAAGCACGA ACGGGTAAAT TCTTCTAGCA GTAAGCAACA   
  
  
- CCATTTGTAT ACTAAGATTT TCGACGACTT TTCCGTAGTT TCGAAGTATA ATATCTAAAA CCGTAGGAGA   
  
  
- TACCTAAGGT TACCGGATCG GAGTAAGCTA CGGAGAGTCT CGCCAGACCA CCTGGAGGTT TTGACAAATA   
  
  
- ATGTCCCTAG CTAGAGGGGG TCGGACCCAA GTCCGGTCGT CTTTCTCACC TTCGTTGTCC CTCTGCGAAC   
  
  
- CGATTCATGA CACTCGCCAT ATTACACGGT AAACTCATAG TACGGTAACG AGTCTTCACC CTTTGTTAGT   
  
  
- TTGGTCCCCT AGAGTTCAAT CCTTCATCCC TACTACTACT TCAACAGCAC CACTTGACAG AGACATCCAA   
  
  
- GTTCTTGGAG GAACTGCTCT GTTACCACCA CCTATCAGGT TCCTTGTGTC AAAATTTGGA CTAATCTTCC   
  
  
- CATTTTGGGC TATAAAAACA CGTACCGTAA CATTTACCAA GGATGTTGTA GGGAAAGAAA CACTGTGCAA   
  
  
- AATCTCTTCG GGAGAAAGTA ATAAGGTGAG AAAAGCTGTA CAATCTACGG TTGCGGAGAT CCCTCCTCGG   
  
  
- GCTCTCCAAC TATAAGCTCT TCCGTAAGAT ACCCTCCCTC TAATACTTAC ACCACCGTAC ACTCCCGTGT   
  
  
- CTCTCCCACC TTTCCGGTCT CTGTATGTTC GTTACCGTGC AATCCGTATC GTCCCGTCCC AAAGCCGTTG   
  
  
- ATGGTAACCT AGGGTTCAAC TAGCTCTTTT ACTCCAAATT CCGGTTCCGT CTGGTGGTGT TCCTAAAGCA   
  
  
- CTAACTACAC CTACCTGTAA CCCGTTAAGT CCCTACCTTC CCCGCCTAAC GGACACGGTA GAGACGTACC   
  
  
- CAAGACCGAA C

+     GTGGC-motif

| Site Name | Organism | Position | Strand | Matrix score. | sequence | function |
| --- | --- | --- | --- | --- | --- | --- |
| GTGGC-motif | Spinacia oleracea | 1895 | + | 10 | GATTCTGTGGC | part of a light responsive element |

>HU07G02248.1   
+ -Up\_Stream \_Len000CTGTTC AGCACTAGGG AATAAGGTTT TTGCCCTGCA TACTATGAAA TCTAACCAAA   
  
  
+ AACATTCCAT CTTGGAAGGC TGTAGCCTTG TGGATTTTGA ATTCGCTCCC AAGAACCAAT GAAGTAGCGC   
  
  
+ AGAAGGCCAA TACTCAATTT CAGAGTCCAC ATCAACTTGT TCAATGCCAG TATATTTCAG AACATTAATT   
  
  
+ ACATACTGTT TGGTTGGAGA AAAAATGGTT GTCTCGAAAA CTAGTTTTTT AATTTTTTGT TATTTAGCAC   
  
  
+ GGTAAAGAAT GGAAATTATT TTCATGCGAG AAAAAAGGAA GTTGGTTTTT CTTTTTTCAA CTCTTTTGAT   
  
  
+ GATATAATCA GTCGAAATTT AGGTGCACCA TCTGACTTCA ACTCTTGTAC GACCTCTGAT TGCTTGCTTA   
  
  
+ GAAGAACAAG AGGATTGACA GATAAGGAGG AGTGGGTGGT TTATACAGAG AATTGAGTGG ATGGTTATAA   
  
  
+ AGAAGTCGAT CTCACATAAG TAACTTCGTT ACGATTATAA ATATTTTCAT TGAAGAAAGA CGATTAGATA   
  
  
+ TAATTAAGAA GGTACATCTA AGCAAATTTG GCTGAGCAGA GTTGGAGGGC CACTAGGGTC ACATTGTGTC   
  
  
+ TTGCGCAACA AGCTTAGACC ATTCGCAACG GATGCCTCAC GTGGAGCAGG TGGGACCCGC GTGGAAGTTG   
  
  
+ CATGGCAAGA ATTCTCTGGC AGCAAGGAGA ATTGAGGGGG GACACGCGTG TGACCACGCC AGTGCGGTCC   
  
  
+ CCCTCTCGTG GCAGATTTGG TGATTTTTTT TTTGTTTTTT TTTGAAGTAA TATATAATTT TTATATGATT   
  
  
+ AAAAAAATAG GTTGTGTGTT ATAAAGTGGG TCAATGACAA ATATTTTTAT TGGATGATTA AAATTAAGTT   
  
  
+ CCATTTATTG GAGAAGTGAT GGAATGCAAG TTCCAAAATT GTGGAACTTT TGTTGACGTA GCAGGCTAGA   
  
  
+ GTTCCAAAAA TTAAAAAAAG GAAAGACATA TTGAGGTAAA GCCTTTTTAT TTTACAAATA GACGCACCAA   
  
  
+ GGTATAATAT AATTGACATG ATTTGAGGCG AGAGGAAGAT TCTTTTGCTA CCCAAACAAA ACACAATGAT   
  
  
+ CTTTTAAATG TAGCAATTTG GGATTGGCAA AATTTTGTCT AACCCGATTG TTGAAAGTTA TCCGATTTCA   
  
  
+ TAAAATTGGA TATCGTTATT AAATTTTCAT TCGAATATTT AAATTTGGAC ACAATGTTTT TCATATTTGG   
  
  
+ ATTTTCTAAA CTTGATTTTA ACTCAAATTT AAGTTTATTC AATTCATCTG ACCCAAAAAA TGGTTGCAAT   
  
  
+ AATGTATTTT CTTTTTGAAG TTAAATATTA TCGAATTCAT TTAACTTGTA GTTAACCCGT AATTGACCCA   
  
  
+ AACTCGACTT TTTGCTACCT TAGCAGCCAG GAAGAAGTCA TAGTATTGAC CCGTGGAACG CCTGAAGCCA   
  
  
+ ACATCACCAG ACCCACATAT TCCATCATCT TCTGATCTCC TCGTCAAAGA AGGAAGCTGA GCACAGCAAT   
  
  
+ AACTAGAAGA ACAGGAGGCA TCACACCCAA GCCAAAGGCC TTTATTTGAG CTCCCCACCT CATTCCTCGA   
  
  
+ AGCTTCCTCT TCACAATGTA AGACCATAAT CCTTCACCCC TTCTCTTTTT AAGCTCATCT TTACCTGCCC   
  
  
+ TAGTTCTCTC TCTCTCATGA GTCATCAGTA ATAGTTACCT CTTTACTTTC TCTTCCTTGA GGTATGCTTC   
  
  
+ AACTTTAATA TATATATGTA CCACTTGATT TCTCTTGTCA CTTTGCTATA ATACTTATTG TTTGGCTATC   
  
  
+ TTCCCCTGTT TTATACTCAT TTTCTTGCTT TCTATATCTG GGTTTGAATT GGGTTTCACT TATCCTCGTT   
  
  
+ GATTCTGTGG GAAATTAGTG AATTGGGTTT TGTACAGCAG GTTTATATGA ACTAGGGTTT CAAAGGTATC   
  
  
+ TACTTTTTCT CTTGTGATTA TTAGTTGATT AGGGATTTGG CCAAGATTAG CAGAATGGGT TCTGAATTTG   
  
  
+ GGGAATTCTC TGATGATGCT CTAAATGGGT ATGCTTACGT TGATATCCCT GCTTATGATG CGTCCTTAGA   
  
  
+ TTATGCCAAT TTGTTCAATT ACGAAGGCCT ATCTGAGGAT CTCACCTCAC TGAGCCTCCC AAGCCCCTTT   
  
  
+ TCTGACCCTT TGGCGTACAG TTTCACGTCC TCTTTGGGGC CGAGCCCTGG GGTTGATTCT AATGATGATA   
  
  
+ GTGATTCTGA TGATGTTCTC AAGTGCATTA GCCAAATGCT TATGGAAGAA GACATGGAGG CAAAGCCATG   
  
  
+ CATGTTTCAC GATCCTTTAG CACTTCAGGC TGCTGAGAAA CCCTTTTATG ATGCCTTAGG GAAGAAATAC   
  
  
+ CCAACTTCTC CGAACCAACA TCCTATAATT GATGATTGTT TGGATAATCC TGGTGAAAAT TCTTTGGGTT   
  
  
+ CTTCTAGTGA TTTTAGTGTT AGTCACTTTG GGTCTAGTTC AGCAAGCTCT ATTGGACCGA CAATTGTGTC   
  
  
+ TGATTTGAGT GAGCATTTTG AGCCACCCTT TGTTCAAGCA CTTCCAATTG AATCATATCC CCAACCATTG   
  
  
+ ACCCGTCCTC AATGGTCGTT TGGCCCTTCG GGTGCCTTAG ATTGCACGGC CTCTAATGGT TCAGTGATCT   
  
  
+ CATCCCTTGG TTTGCCGATG GATGTTATTA GTGTATTCAG TGAGAAAGAG TCCATGATTC AATTTCAGAA   
  
  
+ AGGGGTGGAA GAGGCTAGTA AGTTCCTTCC CAAGAATAAT AACCTTGTTA TTGATCTCAA GAACCTCACT   
  
  
+ TTTCCTAATG AAACGAAGGA GGATGATCGA GTGATGATGG TTAAGAAGGA AAAGAATGAT GTGAATTGGT   
  
  
+ CTAACTACTC AAGAGGGAGT AAGATTCACT ATCGTGAAGA CGAGGACTTT GAAGAAGGAA GGAGTAGCAA   
  
  
+ GCAGTCAGCT ATTTCTACTA CTGAGGAAGC TGAGTTGTGT GAAATTTTTG ACAAGTTTTT GCTTTGCAAT   
  
  
+ TGGTACCCTG TGAAACCTGA GGCTCATCCC ACCATGAGTT TGAACCCTGA GAAGGGCCAG TCACATGGAT   
  
  
+ TAGAAGGTGG GAGAAATGGG AAGGGTCACC CAAAGAAACA GGATAAAAGT AGTACCAACG TTGTGGATTT   
  
  
+ AAGGAATTTG CTGATGCTAT GTGCACAATC TACTGCATCT GATGACCGAA GAACTGCTGA TGGACTGCTA   
  
  
+ AAGAAAATCA GGGAGCACTC ATCTGCTGAG GGGGATGGAT TTCAAAGGTT GGCTCATTAC TTTGCTGATG   
  
  
+ CCCTAGAGGC ACGTTTAGCT GGAACTGGAT CTCACATTTA TACAGCCCTA AGTTCTCATA GGCCATCTGC   
  
  
+ TGTTGACGTG TTAAAAGCAT ATCAGTTCTA TGTTCGTGCT TGCCCATTTA AGAAGATCGT CATTCGTTGT   
  
  
+ GGTAAACATA TGATTCTAAA AGCTGCTGAA AAGGCATCAA AGCTTCATAT TATAGATTTT GGCATCCTCT   
  
  
+ ATGGATTCCA ATGGCCTAGC CTCATTCGAT GCCTCTCAGA GCGGTCTGGT GGACCTCCAA AACTGTTTAT   
  
  
+ TACAGGGATC GATCTCCCCC AGCCTGGGTT CAGGCCAGCA GAAAGAGTGG AAGCAACAGG GAGACGCTTG   
  
  
+ GCTAAGTACT GTGAGCGGTA TAATGTGCCA TTTGAGTATC ATGCCATTGC TCAGAAGTGG GAAACAATCA   
  
  
+ AACCAGGGGA TCTCAAGTTA GGAAGTAGGG ATGATGATGA AGTTGTCGTG GTGAACTGTC TCTGTAGGTT   
  
  
+ CAAGAACCTC CTTGACGAGA CAATGGTGGT GGATAGTCCA AGGAACACAG TTTTAAACCT GATTAGAAGG   
  
  
+ GTAAAACCCG ATATTTTTGT GCATGGCATT GTAAATGGTT CCTACAACAT CCCTTTCTTT GTGACACGTT   
  
  
+ TTAGAGAAGC CCTCTTTCAT TATTCCACTC TTTTCGACAT GTTAGATGCC AACGCCTCTA GGGAGGAGCC   
  
  
+ CGAGAGGTTG ATATTCGAGA AGGCATTCTA TGGGAGGGAG ATTATGAATG TGGTGGCATG TGAGGGCACA   
  
  
+ GAGAGGGTGG AAAGGCCAGA GACATACAAG CAATGGCACG TTAGGCATAG CAGGGCAGGG TTTCGGCAAC   
  
  
+ TACCATTGGA TCCCAAGTTG ATCGAGAAAA TGAGGTTTAA GGCCAAGGCA GACCACCACA AGGATTTCGT   
  
  
+ GATTGATGTG GATGGACATT GGGCAATTCA GGGATGGAAG GGGCGGATTG CCTGTGCCAT CTCTGCATGG   
  
  
+ GTTCTGGCTT G  

- -Up\_Stream \_Len000GACAAG TCGTGATCCC TTATTCCAAA AACGGGACGT ATGATACTTT AGATTGGTTT   
  
  
- TTGTAAGGTA GAACCTTCCG ACATCGGAAC ACCTAAAACT TAAGCGAGGG TTCTTGGTTA CTTCATCGCG   
  
  
- TCTTCCGGTT ATGAGTTAAA GTCTCAGGTG TAGTTGAACA AGTTACGGTC ATATAAAGTC TTGTAATTAA   
  
  
- TGTATGACAA ACCAACCTCT TTTTTACCAA CAGAGCTTTT GATCAAAAAA TTAAAAAACA ATAAATCGTG   
  
  
- CCATTTCTTA CCTTTAATAA AAGTACGCTC TTTTTTCCTT CAACCAAAAA GAAAAAAGTT GAGAAAACTA   
  
  
- CTATATTAGT CAGCTTTAAA TCCACGTGGT AGACTGAAGT TGAGAACATG CTGGAGACTA ACGAACGAAT   
  
  
- CTTCTTGTTC TCCTAACTGT CTATTCCTCC TCACCCACCA AATATGTCTC TTAACTCACC TACCAATATT   
  
  
- TCTTCAGCTA GAGTGTATTC ATTGAAGCAA TGCTAATATT TATAAAAGTA ACTTCTTTCT GCTAATCTAT   
  
  
- ATTAATTCTT CCATGTAGAT TCGTTTAAAC CGACTCGTCT CAACCTCCCG GTGATCCCAG TGTAACACAG   
  
  
- AACGCGTTGT TCGAATCTGG TAAGCGTTGC CTACGGAGTG CACCTCGTCC ACCCTGGGCG CACCTTCAAC   
  
  
- GTACCGTTCT TAAGAGACCG TCGTTCCTCT TAACTCCCCC CTGTGCGCAC ACTGGTGCGG TCACGCCAGG   
  
  
- GGGAGAGCAC CGTCTAAACC ACTAAAAAAA AAACAAAAAA AAACTTCATT ATATATTAAA AATATACTAA   
  
  
- TTTTTTTATC CAACACACAA TATTTCACCC AGTTACTGTT TATAAAAATA ACCTACTAAT TTTAATTCAA   
  
  
- GGTAAATAAC CTCTTCACTA CCTTACGTTC AAGGTTTTAA CACCTTGAAA ACAACTGCAT CGTCCGATCT   
  
  
- CAAGGTTTTT AATTTTTTTC CTTTCTGTAT AACTCCATTT CGGAAAAATA AAATGTTTAT CTGCGTGGTT   
  
  
- CCATATTATA TTAACTGTAC TAAACTCCGC TCTCCTTCTA AGAAAACGAT GGGTTTGTTT TGTGTTACTA   
  
  
- GAAAATTTAC ATCGTTAAAC CCTAACCGTT TTAAAACAGA TTGGGCTAAC AACTTTCAAT AGGCTAAAGT   
  
  
- ATTTTAACCT ATAGCAATAA TTTAAAAGTA AGCTTATAAA TTTAAACCTG TGTTACAAAA AGTATAAACC   
  
  
- TAAAAGATTT GAACTAAAAT TGAGTTTAAA TTCAAATAAG TTAAGTAGAC TGGGTTTTTT ACCAACGTTA   
  
  
- TTACATAAAA GAAAAACTTC AATTTATAAT AGCTTAAGTA AATTGAACAT CAATTGGGCA TTAACTGGGT   
  
  
- TTGAGCTGAA AAACGATGGA ATCGTCGGTC CTTCTTCAGT ATCATAACTG GGCACCTTGC GGACTTCGGT   
  
  
- TGTAGTGGTC TGGGTGTATA AGGTAGTAGA AGACTAGAGG AGCAGTTTCT TCCTTCGACT CGTGTCGTTA   
  
  
- TTGATCTTCT TGTCCTCCGT AGTGTGGGTT CGGTTTCCGG AAATAAACTC GAGGGGTGGA GTAAGGAGCT   
  
  
- TCGAAGGAGA AGTGTTACAT TCTGGTATTA GGAAGTGGGG AAGAGAAAAA TTCGAGTAGA AATGGACGGG   
  
  
- ATCAAGAGAG AGAGAGTACT CAGTAGTCAT TATCAATGGA GAAATGAAAG AGAAGGAACT CCATACGAAG   
  
  
- TTGAAATTAT ATATATACAT GGTGAACTAA AGAGAACAGT GAAACGATAT TATGAATAAC AAACCGATAG   
  
  
- AAGGGGACAA AATATGAGTA AAAGAACGAA AGATATAGAC CCAAACTTAA CCCAAAGTGA ATAGGAGCAA   
  
  
- CTAAGACACC CTTTAATCAC TTAACCCAAA ACATGTCGTC CAAATATACT TGATCCCAAA GTTTCCATAG   
  
  
- ATGAAAAAGA GAACACTAAT AATCAACTAA TCCCTAAACC GGTTCTAATC GTCTTACCCA AGACTTAAAC   
  
  
- CCCTTAAGAG ACTACTACGA GATTTACCCA TACGAATGCA ACTATAGGGA CGAATACTAC GCAGGAATCT   
  
  
- AATACGGTTA AACAAGTTAA TGCTTCCGGA TAGACTCCTA GAGTGGAGTG ACTCGGAGGG TTCGGGGAAA   
  
  
- AGACTGGGAA ACCGCATGTC AAAGTGCAGG AGAAACCCCG GCTCGGGACC CCAACTAAGA TTACTACTAT   
  
  
- CACTAAGACT ACTACAAGAG TTCACGTAAT CGGTTTACGA ATACCTTCTT CTGTACCTCC GTTTCGGTAC   
  
  
- GTACAAAGTG CTAGGAAATC GTGAAGTCCG ACGACTCTTT GGGAAAATAC TACGGAATCC CTTCTTTATG   
  
  
- GGTTGAAGAG GCTTGGTTGT AGGATATTAA CTACTAACAA ACCTATTAGG ACCACTTTTA AGAAACCCAA   
  
  
- GAAGATCACT AAAATCACAA TCAGTGAAAC CCAGATCAAG TCGTTCGAGA TAACCTGGCT GTTAACACAG   
  
  
- ACTAAACTCA CTCGTAAAAC TCGGTGGGAA ACAAGTTCGT GAAGGTTAAC TTAGTATAGG GGTTGGTAAC   
  
  
- TGGGCAGGAG TTACCAGCAA ACCGGGAAGC CCACGGAATC TAACGTGCCG GAGATTACCA AGTCACTAGA   
  
  
- GTAGGGAACC AAACGGCTAC CTACAATAAT CACATAAGTC ACTCTTTCTC AGGTACTAAG TTAAAGTCTT   
  
  
- TCCCCACCTT CTCCGATCAT TCAAGGAAGG GTTCTTATTA TTGGAACAAT AACTAGAGTT CTTGGAGTGA   
  
  
- AAAGGATTAC TTTGCTTCCT CCTACTAGCT CACTACTACC AATTCTTCCT TTTCTTACTA CACTTAACCA   
  
  
- GATTGATGAG TTCTCCCTCA TTCTAAGTGA TAGCACTTCT GCTCCTGAAA CTTCTTCCTT CCTCATCGTT   
  
  
- CGTCAGTCGA TAAAGATGAT GACTCCTTCG ACTCAACACA CTTTAAAAAC TGTTCAAAAA CGAAACGTTA   
  
  
- ACCATGGGAC ACTTTGGACT CCGAGTAGGG TGGTACTCAA ACTTGGGACT CTTCCCGGTC AGTGTACCTA   
  
  
- ATCTTCCACC CTCTTTACCC TTCCCAGTGG GTTTCTTTGT CCTATTTTCA TCATGGTTGC AACACCTAAA   
  
  
- TTCCTTAAAC GACTACGATA CACGTGTTAG ATGACGTAGA CTACTGGCTT CTTGACGACT ACCTGACGAT   
  
  
- TTCTTTTAGT CCCTCGTGAG TAGACGACTC CCCCTACCTA AAGTTTCCAA CCGAGTAATG AAACGACTAC   
  
  
- GGGATCTCCG TGCAAATCGA CCTTGACCTA GAGTGTAAAT ATGTCGGGAT TCAAGAGTAT CCGGTAGACG   
  
  
- ACAACTGCAC AATTTTCGTA TAGTCAAGAT ACAAGCACGA ACGGGTAAAT TCTTCTAGCA GTAAGCAACA   
  
  
- CCATTTGTAT ACTAAGATTT TCGACGACTT TTCCGTAGTT TCGAAGTATA ATATCTAAAA CCGTAGGAGA   
  
  
- TACCTAAGGT TACCGGATCG GAGTAAGCTA CGGAGAGTCT CGCCAGACCA CCTGGAGGTT TTGACAAATA   
  
  
- ATGTCCCTAG CTAGAGGGGG TCGGACCCAA GTCCGGTCGT CTTTCTCACC TTCGTTGTCC CTCTGCGAAC   
  
  
- CGATTCATGA CACTCGCCAT ATTACACGGT AAACTCATAG TACGGTAACG AGTCTTCACC CTTTGTTAGT   
  
  
- TTGGTCCCCT AGAGTTCAAT CCTTCATCCC TACTACTACT TCAACAGCAC CACTTGACAG AGACATCCAA   
  
  
- GTTCTTGGAG GAACTGCTCT GTTACCACCA CCTATCAGGT TCCTTGTGTC AAAATTTGGA CTAATCTTCC   
  
  
- CATTTTGGGC TATAAAAACA CGTACCGTAA CATTTACCAA GGATGTTGTA GGGAAAGAAA CACTGTGCAA   
  
  
- AATCTCTTCG GGAGAAAGTA ATAAGGTGAG AAAAGCTGTA CAATCTACGG TTGCGGAGAT CCCTCCTCGG   
  
  
- GCTCTCCAAC TATAAGCTCT TCCGTAAGAT ACCCTCCCTC TAATACTTAC ACCACCGTAC ACTCCCGTGT   
  
  
- CTCTCCCACC TTTCCGGTCT CTGTATGTTC GTTACCGTGC AATCCGTATC GTCCCGTCCC AAAGCCGTTG   
  
  
- ATGGTAACCT AGGGTTCAAC TAGCTCTTTT ACTCCAAATT CCGGTTCCGT CTGGTGGTGT TCCTAAAGCA   
  
  
- CTAACTACAC CTACCTGTAA CCCGTTAAGT CCCTACCTTC CCCGCCTAAC GGACACGGTA GAGACGTACC   
  
  
- CAAGACCGAA C

+     I-box

| Site Name | Organism | Position | Strand | Matrix score. | sequence | function |
| --- | --- | --- | --- | --- | --- | --- |
| I-box | Flaveria trinervia | 1199 | - | 10 | cCATATCCAAT | part of a light responsive element |
| I-box | Larix laricina | 4169 | + | 9 | GTATAAGGCC | part of a light responsive element |
| I-box | Triticum aestivum | 444 | + | 8 | AGATAAGG | part of a light responsive element |

>HU07G02248.1   
+ -Up\_Stream \_Len000CTGTTC AGCACTAGGG AATAAGGTTT TTGCCCTGCA TACTATGAAA TCTAACCAAA   
  
  
+ AACATTCCAT CTTGGAAGGC TGTAGCCTTG TGGATTTTGA ATTCGCTCCC AAGAACCAAT GAAGTAGCGC   
  
  
+ AGAAGGCCAA TACTCAATTT CAGAGTCCAC ATCAACTTGT TCAATGCCAG TATATTTCAG AACATTAATT   
  
  
+ ACATACTGTT TGGTTGGAGA AAAAATGGTT GTCTCGAAAA CTAGTTTTTT AATTTTTTGT TATTTAGCAC   
  
  
+ GGTAAAGAAT GGAAATTATT TTCATGCGAG AAAAAAGGAA GTTGGTTTTT CTTTTTTCAA CTCTTTTGAT   
  
  
+ GATATAATCA GTCGAAATTT AGGTGCACCA TCTGACTTCA ACTCTTGTAC GACCTCTGAT TGCTTGCTTA   
  
  
+ GAAGAACAAG AGGATTGACA GATAAGGAGG AGTGGGTGGT TTATACAGAG AATTGAGTGG ATGGTTATAA   
  
  
+ AGAAGTCGAT CTCACATAAG TAACTTCGTT ACGATTATAA ATATTTTCAT TGAAGAAAGA CGATTAGATA   
  
  
+ TAATTAAGAA GGTACATCTA AGCAAATTTG GCTGAGCAGA GTTGGAGGGC CACTAGGGTC ACATTGTGTC   
  
  
+ TTGCGCAACA AGCTTAGACC ATTCGCAACG GATGCCTCAC GTGGAGCAGG TGGGACCCGC GTGGAAGTTG   
  
  
+ CATGGCAAGA ATTCTCTGGC AGCAAGGAGA ATTGAGGGGG GACACGCGTG TGACCACGCC AGTGCGGTCC   
  
  
+ CCCTCTCGTG GCAGATTTGG TGATTTTTTT TTTGTTTTTT TTTGAAGTAA TATATAATTT TTATATGATT   
  
  
+ AAAAAAATAG GTTGTGTGTT ATAAAGTGGG TCAATGACAA ATATTTTTAT TGGATGATTA AAATTAAGTT   
  
  
+ CCATTTATTG GAGAAGTGAT GGAATGCAAG TTCCAAAATT GTGGAACTTT TGTTGACGTA GCAGGCTAGA   
  
  
+ GTTCCAAAAA TTAAAAAAAG GAAAGACATA TTGAGGTAAA GCCTTTTTAT TTTACAAATA GACGCACCAA   
  
  
+ GGTATAATAT AATTGACATG ATTTGAGGCG AGAGGAAGAT TCTTTTGCTA CCCAAACAAA ACACAATGAT   
  
  
+ CTTTTAAATG TAGCAATTTG GGATTGGCAA AATTTTGTCT AACCCGATTG TTGAAAGTTA TCCGATTTCA   
  
  
+ TAAAATTGGA TATCGTTATT AAATTTTCAT TCGAATATTT AAATTTGGAC ACAATGTTTT TCATATTTGG   
  
  
+ ATTTTCTAAA CTTGATTTTA ACTCAAATTT AAGTTTATTC AATTCATCTG ACCCAAAAAA TGGTTGCAAT   
  
  
+ AATGTATTTT CTTTTTGAAG TTAAATATTA TCGAATTCAT TTAACTTGTA GTTAACCCGT AATTGACCCA   
  
  
+ AACTCGACTT TTTGCTACCT TAGCAGCCAG GAAGAAGTCA TAGTATTGAC CCGTGGAACG CCTGAAGCCA   
  
  
+ ACATCACCAG ACCCACATAT TCCATCATCT TCTGATCTCC TCGTCAAAGA AGGAAGCTGA GCACAGCAAT   
  
  
+ AACTAGAAGA ACAGGAGGCA TCACACCCAA GCCAAAGGCC TTTATTTGAG CTCCCCACCT CATTCCTCGA   
  
  
+ AGCTTCCTCT TCACAATGTA AGACCATAAT CCTTCACCCC TTCTCTTTTT AAGCTCATCT TTACCTGCCC   
  
  
+ TAGTTCTCTC TCTCTCATGA GTCATCAGTA ATAGTTACCT CTTTACTTTC TCTTCCTTGA GGTATGCTTC   
  
  
+ AACTTTAATA TATATATGTA CCACTTGATT TCTCTTGTCA CTTTGCTATA ATACTTATTG TTTGGCTATC   
  
  
+ TTCCCCTGTT TTATACTCAT TTTCTTGCTT TCTATATCTG GGTTTGAATT GGGTTTCACT TATCCTCGTT   
  
  
+ GATTCTGTGG GAAATTAGTG AATTGGGTTT TGTACAGCAG GTTTATATGA ACTAGGGTTT CAAAGGTATC   
  
  
+ TACTTTTTCT CTTGTGATTA TTAGTTGATT AGGGATTTGG CCAAGATTAG CAGAATGGGT TCTGAATTTG   
  
  
+ GGGAATTCTC TGATGATGCT CTAAATGGGT ATGCTTACGT TGATATCCCT GCTTATGATG CGTCCTTAGA   
  
  
+ TTATGCCAAT TTGTTCAATT ACGAAGGCCT ATCTGAGGAT CTCACCTCAC TGAGCCTCCC AAGCCCCTTT   
  
  
+ TCTGACCCTT TGGCGTACAG TTTCACGTCC TCTTTGGGGC CGAGCCCTGG GGTTGATTCT AATGATGATA   
  
  
+ GTGATTCTGA TGATGTTCTC AAGTGCATTA GCCAAATGCT TATGGAAGAA GACATGGAGG CAAAGCCATG   
  
  
+ CATGTTTCAC GATCCTTTAG CACTTCAGGC TGCTGAGAAA CCCTTTTATG ATGCCTTAGG GAAGAAATAC   
  
  
+ CCAACTTCTC CGAACCAACA TCCTATAATT GATGATTGTT TGGATAATCC TGGTGAAAAT TCTTTGGGTT   
  
  
+ CTTCTAGTGA TTTTAGTGTT AGTCACTTTG GGTCTAGTTC AGCAAGCTCT ATTGGACCGA CAATTGTGTC   
  
  
+ TGATTTGAGT GAGCATTTTG AGCCACCCTT TGTTCAAGCA CTTCCAATTG AATCATATCC CCAACCATTG   
  
  
+ ACCCGTCCTC AATGGTCGTT TGGCCCTTCG GGTGCCTTAG ATTGCACGGC CTCTAATGGT TCAGTGATCT   
  
  
+ CATCCCTTGG TTTGCCGATG GATGTTATTA GTGTATTCAG TGAGAAAGAG TCCATGATTC AATTTCAGAA   
  
  
+ AGGGGTGGAA GAGGCTAGTA AGTTCCTTCC CAAGAATAAT AACCTTGTTA TTGATCTCAA GAACCTCACT   
  
  
+ TTTCCTAATG AAACGAAGGA GGATGATCGA GTGATGATGG TTAAGAAGGA AAAGAATGAT GTGAATTGGT   
  
  
+ CTAACTACTC AAGAGGGAGT AAGATTCACT ATCGTGAAGA CGAGGACTTT GAAGAAGGAA GGAGTAGCAA   
  
  
+ GCAGTCAGCT ATTTCTACTA CTGAGGAAGC TGAGTTGTGT GAAATTTTTG ACAAGTTTTT GCTTTGCAAT   
  
  
+ TGGTACCCTG TGAAACCTGA GGCTCATCCC ACCATGAGTT TGAACCCTGA GAAGGGCCAG TCACATGGAT   
  
  
+ TAGAAGGTGG GAGAAATGGG AAGGGTCACC CAAAGAAACA GGATAAAAGT AGTACCAACG TTGTGGATTT   
  
  
+ AAGGAATTTG CTGATGCTAT GTGCACAATC TACTGCATCT GATGACCGAA GAACTGCTGA TGGACTGCTA   
  
  
+ AAGAAAATCA GGGAGCACTC ATCTGCTGAG GGGGATGGAT TTCAAAGGTT GGCTCATTAC TTTGCTGATG   
  
  
+ CCCTAGAGGC ACGTTTAGCT GGAACTGGAT CTCACATTTA TACAGCCCTA AGTTCTCATA GGCCATCTGC   
  
  
+ TGTTGACGTG TTAAAAGCAT ATCAGTTCTA TGTTCGTGCT TGCCCATTTA AGAAGATCGT CATTCGTTGT   
  
  
+ GGTAAACATA TGATTCTAAA AGCTGCTGAA AAGGCATCAA AGCTTCATAT TATAGATTTT GGCATCCTCT   
  
  
+ ATGGATTCCA ATGGCCTAGC CTCATTCGAT GCCTCTCAGA GCGGTCTGGT GGACCTCCAA AACTGTTTAT   
  
  
+ TACAGGGATC GATCTCCCCC AGCCTGGGTT CAGGCCAGCA GAAAGAGTGG AAGCAACAGG GAGACGCTTG   
  
  
+ GCTAAGTACT GTGAGCGGTA TAATGTGCCA TTTGAGTATC ATGCCATTGC TCAGAAGTGG GAAACAATCA   
  
  
+ AACCAGGGGA TCTCAAGTTA GGAAGTAGGG ATGATGATGA AGTTGTCGTG GTGAACTGTC TCTGTAGGTT   
  
  
+ CAAGAACCTC CTTGACGAGA CAATGGTGGT GGATAGTCCA AGGAACACAG TTTTAAACCT GATTAGAAGG   
  
  
+ GTAAAACCCG ATATTTTTGT GCATGGCATT GTAAATGGTT CCTACAACAT CCCTTTCTTT GTGACACGTT   
  
  
+ TTAGAGAAGC CCTCTTTCAT TATTCCACTC TTTTCGACAT GTTAGATGCC AACGCCTCTA GGGAGGAGCC   
  
  
+ CGAGAGGTTG ATATTCGAGA AGGCATTCTA TGGGAGGGAG ATTATGAATG TGGTGGCATG TGAGGGCACA   
  
  
+ GAGAGGGTGG AAAGGCCAGA GACATACAAG CAATGGCACG TTAGGCATAG CAGGGCAGGG TTTCGGCAAC   
  
  
+ TACCATTGGA TCCCAAGTTG ATCGAGAAAA TGAGGTTTAA GGCCAAGGCA GACCACCACA AGGATTTCGT   
  
  
+ GATTGATGTG GATGGACATT GGGCAATTCA GGGATGGAAG GGGCGGATTG CCTGTGCCAT CTCTGCATGG   
  
  
+ GTTCTGGCTT G  

- -Up\_Stream \_Len000GACAAG TCGTGATCCC TTATTCCAAA AACGGGACGT ATGATACTTT AGATTGGTTT   
  
  
- TTGTAAGGTA GAACCTTCCG ACATCGGAAC ACCTAAAACT TAAGCGAGGG TTCTTGGTTA CTTCATCGCG   
  
  
- TCTTCCGGTT ATGAGTTAAA GTCTCAGGTG TAGTTGAACA AGTTACGGTC ATATAAAGTC TTGTAATTAA   
  
  
- TGTATGACAA ACCAACCTCT TTTTTACCAA CAGAGCTTTT GATCAAAAAA TTAAAAAACA ATAAATCGTG   
  
  
- CCATTTCTTA CCTTTAATAA AAGTACGCTC TTTTTTCCTT CAACCAAAAA GAAAAAAGTT GAGAAAACTA   
  
  
- CTATATTAGT CAGCTTTAAA TCCACGTGGT AGACTGAAGT TGAGAACATG CTGGAGACTA ACGAACGAAT   
  
  
- CTTCTTGTTC TCCTAACTGT CTATTCCTCC TCACCCACCA AATATGTCTC TTAACTCACC TACCAATATT   
  
  
- TCTTCAGCTA GAGTGTATTC ATTGAAGCAA TGCTAATATT TATAAAAGTA ACTTCTTTCT GCTAATCTAT   
  
  
- ATTAATTCTT CCATGTAGAT TCGTTTAAAC CGACTCGTCT CAACCTCCCG GTGATCCCAG TGTAACACAG   
  
  
- AACGCGTTGT TCGAATCTGG TAAGCGTTGC CTACGGAGTG CACCTCGTCC ACCCTGGGCG CACCTTCAAC   
  
  
- GTACCGTTCT TAAGAGACCG TCGTTCCTCT TAACTCCCCC CTGTGCGCAC ACTGGTGCGG TCACGCCAGG   
  
  
- GGGAGAGCAC CGTCTAAACC ACTAAAAAAA AAACAAAAAA AAACTTCATT ATATATTAAA AATATACTAA   
  
  
- TTTTTTTATC CAACACACAA TATTTCACCC AGTTACTGTT TATAAAAATA ACCTACTAAT TTTAATTCAA   
  
  
- GGTAAATAAC CTCTTCACTA CCTTACGTTC AAGGTTTTAA CACCTTGAAA ACAACTGCAT CGTCCGATCT   
  
  
- CAAGGTTTTT AATTTTTTTC CTTTCTGTAT AACTCCATTT CGGAAAAATA AAATGTTTAT CTGCGTGGTT   
  
  
- CCATATTATA TTAACTGTAC TAAACTCCGC TCTCCTTCTA AGAAAACGAT GGGTTTGTTT TGTGTTACTA   
  
  
- GAAAATTTAC ATCGTTAAAC CCTAACCGTT TTAAAACAGA TTGGGCTAAC AACTTTCAAT AGGCTAAAGT   
  
  
- ATTTTAACCT ATAGCAATAA TTTAAAAGTA AGCTTATAAA TTTAAACCTG TGTTACAAAA AGTATAAACC   
  
  
- TAAAAGATTT GAACTAAAAT TGAGTTTAAA TTCAAATAAG TTAAGTAGAC TGGGTTTTTT ACCAACGTTA   
  
  
- TTACATAAAA GAAAAACTTC AATTTATAAT AGCTTAAGTA AATTGAACAT CAATTGGGCA TTAACTGGGT   
  
  
- TTGAGCTGAA AAACGATGGA ATCGTCGGTC CTTCTTCAGT ATCATAACTG GGCACCTTGC GGACTTCGGT   
  
  
- TGTAGTGGTC TGGGTGTATA AGGTAGTAGA AGACTAGAGG AGCAGTTTCT TCCTTCGACT CGTGTCGTTA   
  
  
- TTGATCTTCT TGTCCTCCGT AGTGTGGGTT CGGTTTCCGG AAATAAACTC GAGGGGTGGA GTAAGGAGCT   
  
  
- TCGAAGGAGA AGTGTTACAT TCTGGTATTA GGAAGTGGGG AAGAGAAAAA TTCGAGTAGA AATGGACGGG   
  
  
- ATCAAGAGAG AGAGAGTACT CAGTAGTCAT TATCAATGGA GAAATGAAAG AGAAGGAACT CCATACGAAG   
  
  
- TTGAAATTAT ATATATACAT GGTGAACTAA AGAGAACAGT GAAACGATAT TATGAATAAC AAACCGATAG   
  
  
- AAGGGGACAA AATATGAGTA AAAGAACGAA AGATATAGAC CCAAACTTAA CCCAAAGTGA ATAGGAGCAA   
  
  
- CTAAGACACC CTTTAATCAC TTAACCCAAA ACATGTCGTC CAAATATACT TGATCCCAAA GTTTCCATAG   
  
  
- ATGAAAAAGA GAACACTAAT AATCAACTAA TCCCTAAACC GGTTCTAATC GTCTTACCCA AGACTTAAAC   
  
  
- CCCTTAAGAG ACTACTACGA GATTTACCCA TACGAATGCA ACTATAGGGA CGAATACTAC GCAGGAATCT   
  
  
- AATACGGTTA AACAAGTTAA TGCTTCCGGA TAGACTCCTA GAGTGGAGTG ACTCGGAGGG TTCGGGGAAA   
  
  
- AGACTGGGAA ACCGCATGTC AAAGTGCAGG AGAAACCCCG GCTCGGGACC CCAACTAAGA TTACTACTAT   
  
  
- CACTAAGACT ACTACAAGAG TTCACGTAAT CGGTTTACGA ATACCTTCTT CTGTACCTCC GTTTCGGTAC   
  
  
- GTACAAAGTG CTAGGAAATC GTGAAGTCCG ACGACTCTTT GGGAAAATAC TACGGAATCC CTTCTTTATG   
  
  
- GGTTGAAGAG GCTTGGTTGT AGGATATTAA CTACTAACAA ACCTATTAGG ACCACTTTTA AGAAACCCAA   
  
  
- GAAGATCACT AAAATCACAA TCAGTGAAAC CCAGATCAAG TCGTTCGAGA TAACCTGGCT GTTAACACAG   
  
  
- ACTAAACTCA CTCGTAAAAC TCGGTGGGAA ACAAGTTCGT GAAGGTTAAC TTAGTATAGG GGTTGGTAAC   
  
  
- TGGGCAGGAG TTACCAGCAA ACCGGGAAGC CCACGGAATC TAACGTGCCG GAGATTACCA AGTCACTAGA   
  
  
- GTAGGGAACC AAACGGCTAC CTACAATAAT CACATAAGTC ACTCTTTCTC AGGTACTAAG TTAAAGTCTT   
  
  
- TCCCCACCTT CTCCGATCAT TCAAGGAAGG GTTCTTATTA TTGGAACAAT AACTAGAGTT CTTGGAGTGA   
  
  
- AAAGGATTAC TTTGCTTCCT CCTACTAGCT CACTACTACC AATTCTTCCT TTTCTTACTA CACTTAACCA   
  
  
- GATTGATGAG TTCTCCCTCA TTCTAAGTGA TAGCACTTCT GCTCCTGAAA CTTCTTCCTT CCTCATCGTT   
  
  
- CGTCAGTCGA TAAAGATGAT GACTCCTTCG ACTCAACACA CTTTAAAAAC TGTTCAAAAA CGAAACGTTA   
  
  
- ACCATGGGAC ACTTTGGACT CCGAGTAGGG TGGTACTCAA ACTTGGGACT CTTCCCGGTC AGTGTACCTA   
  
  
- ATCTTCCACC CTCTTTACCC TTCCCAGTGG GTTTCTTTGT CCTATTTTCA TCATGGTTGC AACACCTAAA   
  
  
- TTCCTTAAAC GACTACGATA CACGTGTTAG ATGACGTAGA CTACTGGCTT CTTGACGACT ACCTGACGAT   
  
  
- TTCTTTTAGT CCCTCGTGAG TAGACGACTC CCCCTACCTA AAGTTTCCAA CCGAGTAATG AAACGACTAC   
  
  
- GGGATCTCCG TGCAAATCGA CCTTGACCTA GAGTGTAAAT ATGTCGGGAT TCAAGAGTAT CCGGTAGACG   
  
  
- ACAACTGCAC AATTTTCGTA TAGTCAAGAT ACAAGCACGA ACGGGTAAAT TCTTCTAGCA GTAAGCAACA   
  
  
- CCATTTGTAT ACTAAGATTT TCGACGACTT TTCCGTAGTT TCGAAGTATA ATATCTAAAA CCGTAGGAGA   
  
  
- TACCTAAGGT TACCGGATCG GAGTAAGCTA CGGAGAGTCT CGCCAGACCA CCTGGAGGTT TTGACAAATA   
  
  
- ATGTCCCTAG CTAGAGGGGG TCGGACCCAA GTCCGGTCGT CTTTCTCACC TTCGTTGTCC CTCTGCGAAC   
  
  
- CGATTCATGA CACTCGCCAT ATTACACGGT AAACTCATAG TACGGTAACG AGTCTTCACC CTTTGTTAGT   
  
  
- TTGGTCCCCT AGAGTTCAAT CCTTCATCCC TACTACTACT TCAACAGCAC CACTTGACAG AGACATCCAA   
  
  
- GTTCTTGGAG GAACTGCTCT GTTACCACCA CCTATCAGGT TCCTTGTGTC AAAATTTGGA CTAATCTTCC   
  
  
- CATTTTGGGC TATAAAAACA CGTACCGTAA CATTTACCAA GGATGTTGTA GGGAAAGAAA CACTGTGCAA   
  
  
- AATCTCTTCG GGAGAAAGTA ATAAGGTGAG AAAAGCTGTA CAATCTACGG TTGCGGAGAT CCCTCCTCGG   
  
  
- GCTCTCCAAC TATAAGCTCT TCCGTAAGAT ACCCTCCCTC TAATACTTAC ACCACCGTAC ACTCCCGTGT   
  
  
- CTCTCCCACC TTTCCGGTCT CTGTATGTTC GTTACCGTGC AATCCGTATC GTCCCGTCCC AAAGCCGTTG   
  
  
- ATGGTAACCT AGGGTTCAAC TAGCTCTTTT ACTCCAAATT CCGGTTCCGT CTGGTGGTGT TCCTAAAGCA   
  
  
- CTAACTACAC CTACCTGTAA CCCGTTAAGT CCCTACCTTC CCCGCCTAAC GGACACGGTA GAGACGTACC   
  
  
- CAAGACCGAA C

+     LTR

| Site Name | Organism | Position | Strand | Matrix score. | sequence | function |
| --- | --- | --- | --- | --- | --- | --- |
| LTR | Hordeum vulgare | 4125 | - | 6 | CCGAAA | cis-acting element involved in low-temperature responsiveness |

>HU07G02248.1   
+ -Up\_Stream \_Len000CTGTTC AGCACTAGGG AATAAGGTTT TTGCCCTGCA TACTATGAAA TCTAACCAAA   
  
  
+ AACATTCCAT CTTGGAAGGC TGTAGCCTTG TGGATTTTGA ATTCGCTCCC AAGAACCAAT GAAGTAGCGC   
  
  
+ AGAAGGCCAA TACTCAATTT CAGAGTCCAC ATCAACTTGT TCAATGCCAG TATATTTCAG AACATTAATT   
  
  
+ ACATACTGTT TGGTTGGAGA AAAAATGGTT GTCTCGAAAA CTAGTTTTTT AATTTTTTGT TATTTAGCAC   
  
  
+ GGTAAAGAAT GGAAATTATT TTCATGCGAG AAAAAAGGAA GTTGGTTTTT CTTTTTTCAA CTCTTTTGAT   
  
  
+ GATATAATCA GTCGAAATTT AGGTGCACCA TCTGACTTCA ACTCTTGTAC GACCTCTGAT TGCTTGCTTA   
  
  
+ GAAGAACAAG AGGATTGACA GATAAGGAGG AGTGGGTGGT TTATACAGAG AATTGAGTGG ATGGTTATAA   
  
  
+ AGAAGTCGAT CTCACATAAG TAACTTCGTT ACGATTATAA ATATTTTCAT TGAAGAAAGA CGATTAGATA   
  
  
+ TAATTAAGAA GGTACATCTA AGCAAATTTG GCTGAGCAGA GTTGGAGGGC CACTAGGGTC ACATTGTGTC   
  
  
+ TTGCGCAACA AGCTTAGACC ATTCGCAACG GATGCCTCAC GTGGAGCAGG TGGGACCCGC GTGGAAGTTG   
  
  
+ CATGGCAAGA ATTCTCTGGC AGCAAGGAGA ATTGAGGGGG GACACGCGTG TGACCACGCC AGTGCGGTCC   
  
  
+ CCCTCTCGTG GCAGATTTGG TGATTTTTTT TTTGTTTTTT TTTGAAGTAA TATATAATTT TTATATGATT   
  
  
+ AAAAAAATAG GTTGTGTGTT ATAAAGTGGG TCAATGACAA ATATTTTTAT TGGATGATTA AAATTAAGTT   
  
  
+ CCATTTATTG GAGAAGTGAT GGAATGCAAG TTCCAAAATT GTGGAACTTT TGTTGACGTA GCAGGCTAGA   
  
  
+ GTTCCAAAAA TTAAAAAAAG GAAAGACATA TTGAGGTAAA GCCTTTTTAT TTTACAAATA GACGCACCAA   
  
  
+ GGTATAATAT AATTGACATG ATTTGAGGCG AGAGGAAGAT TCTTTTGCTA CCCAAACAAA ACACAATGAT   
  
  
+ CTTTTAAATG TAGCAATTTG GGATTGGCAA AATTTTGTCT AACCCGATTG TTGAAAGTTA TCCGATTTCA   
  
  
+ TAAAATTGGA TATCGTTATT AAATTTTCAT TCGAATATTT AAATTTGGAC ACAATGTTTT TCATATTTGG   
  
  
+ ATTTTCTAAA CTTGATTTTA ACTCAAATTT AAGTTTATTC AATTCATCTG ACCCAAAAAA TGGTTGCAAT   
  
  
+ AATGTATTTT CTTTTTGAAG TTAAATATTA TCGAATTCAT TTAACTTGTA GTTAACCCGT AATTGACCCA   
  
  
+ AACTCGACTT TTTGCTACCT TAGCAGCCAG GAAGAAGTCA TAGTATTGAC CCGTGGAACG CCTGAAGCCA   
  
  
+ ACATCACCAG ACCCACATAT TCCATCATCT TCTGATCTCC TCGTCAAAGA AGGAAGCTGA GCACAGCAAT   
  
  
+ AACTAGAAGA ACAGGAGGCA TCACACCCAA GCCAAAGGCC TTTATTTGAG CTCCCCACCT CATTCCTCGA   
  
  
+ AGCTTCCTCT TCACAATGTA AGACCATAAT CCTTCACCCC TTCTCTTTTT AAGCTCATCT TTACCTGCCC   
  
  
+ TAGTTCTCTC TCTCTCATGA GTCATCAGTA ATAGTTACCT CTTTACTTTC TCTTCCTTGA GGTATGCTTC   
  
  
+ AACTTTAATA TATATATGTA CCACTTGATT TCTCTTGTCA CTTTGCTATA ATACTTATTG TTTGGCTATC   
  
  
+ TTCCCCTGTT TTATACTCAT TTTCTTGCTT TCTATATCTG GGTTTGAATT GGGTTTCACT TATCCTCGTT   
  
  
+ GATTCTGTGG GAAATTAGTG AATTGGGTTT TGTACAGCAG GTTTATATGA ACTAGGGTTT CAAAGGTATC   
  
  
+ TACTTTTTCT CTTGTGATTA TTAGTTGATT AGGGATTTGG CCAAGATTAG CAGAATGGGT TCTGAATTTG   
  
  
+ GGGAATTCTC TGATGATGCT CTAAATGGGT ATGCTTACGT TGATATCCCT GCTTATGATG CGTCCTTAGA   
  
  
+ TTATGCCAAT TTGTTCAATT ACGAAGGCCT ATCTGAGGAT CTCACCTCAC TGAGCCTCCC AAGCCCCTTT   
  
  
+ TCTGACCCTT TGGCGTACAG TTTCACGTCC TCTTTGGGGC CGAGCCCTGG GGTTGATTCT AATGATGATA   
  
  
+ GTGATTCTGA TGATGTTCTC AAGTGCATTA GCCAAATGCT TATGGAAGAA GACATGGAGG CAAAGCCATG   
  
  
+ CATGTTTCAC GATCCTTTAG CACTTCAGGC TGCTGAGAAA CCCTTTTATG ATGCCTTAGG GAAGAAATAC   
  
  
+ CCAACTTCTC CGAACCAACA TCCTATAATT GATGATTGTT TGGATAATCC TGGTGAAAAT TCTTTGGGTT   
  
  
+ CTTCTAGTGA TTTTAGTGTT AGTCACTTTG GGTCTAGTTC AGCAAGCTCT ATTGGACCGA CAATTGTGTC   
  
  
+ TGATTTGAGT GAGCATTTTG AGCCACCCTT TGTTCAAGCA CTTCCAATTG AATCATATCC CCAACCATTG   
  
  
+ ACCCGTCCTC AATGGTCGTT TGGCCCTTCG GGTGCCTTAG ATTGCACGGC CTCTAATGGT TCAGTGATCT   
  
  
+ CATCCCTTGG TTTGCCGATG GATGTTATTA GTGTATTCAG TGAGAAAGAG TCCATGATTC AATTTCAGAA   
  
  
+ AGGGGTGGAA GAGGCTAGTA AGTTCCTTCC CAAGAATAAT AACCTTGTTA TTGATCTCAA GAACCTCACT   
  
  
+ TTTCCTAATG AAACGAAGGA GGATGATCGA GTGATGATGG TTAAGAAGGA AAAGAATGAT GTGAATTGGT   
  
  
+ CTAACTACTC AAGAGGGAGT AAGATTCACT ATCGTGAAGA CGAGGACTTT GAAGAAGGAA GGAGTAGCAA   
  
  
+ GCAGTCAGCT ATTTCTACTA CTGAGGAAGC TGAGTTGTGT GAAATTTTTG ACAAGTTTTT GCTTTGCAAT   
  
  
+ TGGTACCCTG TGAAACCTGA GGCTCATCCC ACCATGAGTT TGAACCCTGA GAAGGGCCAG TCACATGGAT   
  
  
+ TAGAAGGTGG GAGAAATGGG AAGGGTCACC CAAAGAAACA GGATAAAAGT AGTACCAACG TTGTGGATTT   
  
  
+ AAGGAATTTG CTGATGCTAT GTGCACAATC TACTGCATCT GATGACCGAA GAACTGCTGA TGGACTGCTA   
  
  
+ AAGAAAATCA GGGAGCACTC ATCTGCTGAG GGGGATGGAT TTCAAAGGTT GGCTCATTAC TTTGCTGATG   
  
  
+ CCCTAGAGGC ACGTTTAGCT GGAACTGGAT CTCACATTTA TACAGCCCTA AGTTCTCATA GGCCATCTGC   
  
  
+ TGTTGACGTG TTAAAAGCAT ATCAGTTCTA TGTTCGTGCT TGCCCATTTA AGAAGATCGT CATTCGTTGT   
  
  
+ GGTAAACATA TGATTCTAAA AGCTGCTGAA AAGGCATCAA AGCTTCATAT TATAGATTTT GGCATCCTCT   
  
  
+ ATGGATTCCA ATGGCCTAGC CTCATTCGAT GCCTCTCAGA GCGGTCTGGT GGACCTCCAA AACTGTTTAT   
  
  
+ TACAGGGATC GATCTCCCCC AGCCTGGGTT CAGGCCAGCA GAAAGAGTGG AAGCAACAGG GAGACGCTTG   
  
  
+ GCTAAGTACT GTGAGCGGTA TAATGTGCCA TTTGAGTATC ATGCCATTGC TCAGAAGTGG GAAACAATCA   
  
  
+ AACCAGGGGA TCTCAAGTTA GGAAGTAGGG ATGATGATGA AGTTGTCGTG GTGAACTGTC TCTGTAGGTT   
  
  
+ CAAGAACCTC CTTGACGAGA CAATGGTGGT GGATAGTCCA AGGAACACAG TTTTAAACCT GATTAGAAGG   
  
  
+ GTAAAACCCG ATATTTTTGT GCATGGCATT GTAAATGGTT CCTACAACAT CCCTTTCTTT GTGACACGTT   
  
  
+ TTAGAGAAGC CCTCTTTCAT TATTCCACTC TTTTCGACAT GTTAGATGCC AACGCCTCTA GGGAGGAGCC   
  
  
+ CGAGAGGTTG ATATTCGAGA AGGCATTCTA TGGGAGGGAG ATTATGAATG TGGTGGCATG TGAGGGCACA   
  
  
+ GAGAGGGTGG AAAGGCCAGA GACATACAAG CAATGGCACG TTAGGCATAG CAGGGCAGGG TTTCGGCAAC   
  
  
+ TACCATTGGA TCCCAAGTTG ATCGAGAAAA TGAGGTTTAA GGCCAAGGCA GACCACCACA AGGATTTCGT   
  
  
+ GATTGATGTG GATGGACATT GGGCAATTCA GGGATGGAAG GGGCGGATTG CCTGTGCCAT CTCTGCATGG   
  
  
+ GTTCTGGCTT G  

- -Up\_Stream \_Len000GACAAG TCGTGATCCC TTATTCCAAA AACGGGACGT ATGATACTTT AGATTGGTTT   
  
  
- TTGTAAGGTA GAACCTTCCG ACATCGGAAC ACCTAAAACT TAAGCGAGGG TTCTTGGTTA CTTCATCGCG   
  
  
- TCTTCCGGTT ATGAGTTAAA GTCTCAGGTG TAGTTGAACA AGTTACGGTC ATATAAAGTC TTGTAATTAA   
  
  
- TGTATGACAA ACCAACCTCT TTTTTACCAA CAGAGCTTTT GATCAAAAAA TTAAAAAACA ATAAATCGTG   
  
  
- CCATTTCTTA CCTTTAATAA AAGTACGCTC TTTTTTCCTT CAACCAAAAA GAAAAAAGTT GAGAAAACTA   
  
  
- CTATATTAGT CAGCTTTAAA TCCACGTGGT AGACTGAAGT TGAGAACATG CTGGAGACTA ACGAACGAAT   
  
  
- CTTCTTGTTC TCCTAACTGT CTATTCCTCC TCACCCACCA AATATGTCTC TTAACTCACC TACCAATATT   
  
  
- TCTTCAGCTA GAGTGTATTC ATTGAAGCAA TGCTAATATT TATAAAAGTA ACTTCTTTCT GCTAATCTAT   
  
  
- ATTAATTCTT CCATGTAGAT TCGTTTAAAC CGACTCGTCT CAACCTCCCG GTGATCCCAG TGTAACACAG   
  
  
- AACGCGTTGT TCGAATCTGG TAAGCGTTGC CTACGGAGTG CACCTCGTCC ACCCTGGGCG CACCTTCAAC   
  
  
- GTACCGTTCT TAAGAGACCG TCGTTCCTCT TAACTCCCCC CTGTGCGCAC ACTGGTGCGG TCACGCCAGG   
  
  
- GGGAGAGCAC CGTCTAAACC ACTAAAAAAA AAACAAAAAA AAACTTCATT ATATATTAAA AATATACTAA   
  
  
- TTTTTTTATC CAACACACAA TATTTCACCC AGTTACTGTT TATAAAAATA ACCTACTAAT TTTAATTCAA   
  
  
- GGTAAATAAC CTCTTCACTA CCTTACGTTC AAGGTTTTAA CACCTTGAAA ACAACTGCAT CGTCCGATCT   
  
  
- CAAGGTTTTT AATTTTTTTC CTTTCTGTAT AACTCCATTT CGGAAAAATA AAATGTTTAT CTGCGTGGTT   
  
  
- CCATATTATA TTAACTGTAC TAAACTCCGC TCTCCTTCTA AGAAAACGAT GGGTTTGTTT TGTGTTACTA   
  
  
- GAAAATTTAC ATCGTTAAAC CCTAACCGTT TTAAAACAGA TTGGGCTAAC AACTTTCAAT AGGCTAAAGT   
  
  
- ATTTTAACCT ATAGCAATAA TTTAAAAGTA AGCTTATAAA TTTAAACCTG TGTTACAAAA AGTATAAACC   
  
  
- TAAAAGATTT GAACTAAAAT TGAGTTTAAA TTCAAATAAG TTAAGTAGAC TGGGTTTTTT ACCAACGTTA   
  
  
- TTACATAAAA GAAAAACTTC AATTTATAAT AGCTTAAGTA AATTGAACAT CAATTGGGCA TTAACTGGGT   
  
  
- TTGAGCTGAA AAACGATGGA ATCGTCGGTC CTTCTTCAGT ATCATAACTG GGCACCTTGC GGACTTCGGT   
  
  
- TGTAGTGGTC TGGGTGTATA AGGTAGTAGA AGACTAGAGG AGCAGTTTCT TCCTTCGACT CGTGTCGTTA   
  
  
- TTGATCTTCT TGTCCTCCGT AGTGTGGGTT CGGTTTCCGG AAATAAACTC GAGGGGTGGA GTAAGGAGCT   
  
  
- TCGAAGGAGA AGTGTTACAT TCTGGTATTA GGAAGTGGGG AAGAGAAAAA TTCGAGTAGA AATGGACGGG   
  
  
- ATCAAGAGAG AGAGAGTACT CAGTAGTCAT TATCAATGGA GAAATGAAAG AGAAGGAACT CCATACGAAG   
  
  
- TTGAAATTAT ATATATACAT GGTGAACTAA AGAGAACAGT GAAACGATAT TATGAATAAC AAACCGATAG   
  
  
- AAGGGGACAA AATATGAGTA AAAGAACGAA AGATATAGAC CCAAACTTAA CCCAAAGTGA ATAGGAGCAA   
  
  
- CTAAGACACC CTTTAATCAC TTAACCCAAA ACATGTCGTC CAAATATACT TGATCCCAAA GTTTCCATAG   
  
  
- ATGAAAAAGA GAACACTAAT AATCAACTAA TCCCTAAACC GGTTCTAATC GTCTTACCCA AGACTTAAAC   
  
  
- CCCTTAAGAG ACTACTACGA GATTTACCCA TACGAATGCA ACTATAGGGA CGAATACTAC GCAGGAATCT   
  
  
- AATACGGTTA AACAAGTTAA TGCTTCCGGA TAGACTCCTA GAGTGGAGTG ACTCGGAGGG TTCGGGGAAA   
  
  
- AGACTGGGAA ACCGCATGTC AAAGTGCAGG AGAAACCCCG GCTCGGGACC CCAACTAAGA TTACTACTAT   
  
  
- CACTAAGACT ACTACAAGAG TTCACGTAAT CGGTTTACGA ATACCTTCTT CTGTACCTCC GTTTCGGTAC   
  
  
- GTACAAAGTG CTAGGAAATC GTGAAGTCCG ACGACTCTTT GGGAAAATAC TACGGAATCC CTTCTTTATG   
  
  
- GGTTGAAGAG GCTTGGTTGT AGGATATTAA CTACTAACAA ACCTATTAGG ACCACTTTTA AGAAACCCAA   
  
  
- GAAGATCACT AAAATCACAA TCAGTGAAAC CCAGATCAAG TCGTTCGAGA TAACCTGGCT GTTAACACAG   
  
  
- ACTAAACTCA CTCGTAAAAC TCGGTGGGAA ACAAGTTCGT GAAGGTTAAC TTAGTATAGG GGTTGGTAAC   
  
  
- TGGGCAGGAG TTACCAGCAA ACCGGGAAGC CCACGGAATC TAACGTGCCG GAGATTACCA AGTCACTAGA   
  
  
- GTAGGGAACC AAACGGCTAC CTACAATAAT CACATAAGTC ACTCTTTCTC AGGTACTAAG TTAAAGTCTT   
  
  
- TCCCCACCTT CTCCGATCAT TCAAGGAAGG GTTCTTATTA TTGGAACAAT AACTAGAGTT CTTGGAGTGA   
  
  
- AAAGGATTAC TTTGCTTCCT CCTACTAGCT CACTACTACC AATTCTTCCT TTTCTTACTA CACTTAACCA   
  
  
- GATTGATGAG TTCTCCCTCA TTCTAAGTGA TAGCACTTCT GCTCCTGAAA CTTCTTCCTT CCTCATCGTT   
  
  
- CGTCAGTCGA TAAAGATGAT GACTCCTTCG ACTCAACACA CTTTAAAAAC TGTTCAAAAA CGAAACGTTA   
  
  
- ACCATGGGAC ACTTTGGACT CCGAGTAGGG TGGTACTCAA ACTTGGGACT CTTCCCGGTC AGTGTACCTA   
  
  
- ATCTTCCACC CTCTTTACCC TTCCCAGTGG GTTTCTTTGT CCTATTTTCA TCATGGTTGC AACACCTAAA   
  
  
- TTCCTTAAAC GACTACGATA CACGTGTTAG ATGACGTAGA CTACTGGCTT CTTGACGACT ACCTGACGAT   
  
  
- TTCTTTTAGT CCCTCGTGAG TAGACGACTC CCCCTACCTA AAGTTTCCAA CCGAGTAATG AAACGACTAC   
  
  
- GGGATCTCCG TGCAAATCGA CCTTGACCTA GAGTGTAAAT ATGTCGGGAT TCAAGAGTAT CCGGTAGACG   
  
  
- ACAACTGCAC AATTTTCGTA TAGTCAAGAT ACAAGCACGA ACGGGTAAAT TCTTCTAGCA GTAAGCAACA   
  
  
- CCATTTGTAT ACTAAGATTT TCGACGACTT TTCCGTAGTT TCGAAGTATA ATATCTAAAA CCGTAGGAGA   
  
  
- TACCTAAGGT TACCGGATCG GAGTAAGCTA CGGAGAGTCT CGCCAGACCA CCTGGAGGTT TTGACAAATA   
  
  
- ATGTCCCTAG CTAGAGGGGG TCGGACCCAA GTCCGGTCGT CTTTCTCACC TTCGTTGTCC CTCTGCGAAC   
  
  
- CGATTCATGA CACTCGCCAT ATTACACGGT AAACTCATAG TACGGTAACG AGTCTTCACC CTTTGTTAGT   
  
  
- TTGGTCCCCT AGAGTTCAAT CCTTCATCCC TACTACTACT TCAACAGCAC CACTTGACAG AGACATCCAA   
  
  
- GTTCTTGGAG GAACTGCTCT GTTACCACCA CCTATCAGGT TCCTTGTGTC AAAATTTGGA CTAATCTTCC   
  
  
- CATTTTGGGC TATAAAAACA CGTACCGTAA CATTTACCAA GGATGTTGTA GGGAAAGAAA CACTGTGCAA   
  
  
- AATCTCTTCG GGAGAAAGTA ATAAGGTGAG AAAAGCTGTA CAATCTACGG TTGCGGAGAT CCCTCCTCGG   
  
  
- GCTCTCCAAC TATAAGCTCT TCCGTAAGAT ACCCTCCCTC TAATACTTAC ACCACCGTAC ACTCCCGTGT   
  
  
- CTCTCCCACC TTTCCGGTCT CTGTATGTTC GTTACCGTGC AATCCGTATC GTCCCGTCCC AAAGCCGTTG   
  
  
- ATGGTAACCT AGGGTTCAAC TAGCTCTTTT ACTCCAAATT CCGGTTCCGT CTGGTGGTGT TCCTAAAGCA   
  
  
- CTAACTACAC CTACCTGTAA CCCGTTAAGT CCCTACCTTC CCCGCCTAAC GGACACGGTA GAGACGTACC   
  
  
- CAAGACCGAA C

+     MYB

| Site Name | Organism | Position | Strand | Matrix score. | sequence | function |
| --- | --- | --- | --- | --- | --- | --- |
| MYB | Arabidopsis thaliana | 3364 | - | 6 | CAACAG |  |
| MYB | Arabidopsis thaliana | 2842 | - | 6 | TAACCA |  |
| MYB | Arabidopsis thaliana | 1325 | - | 6 | CAACCA |  |
| MYB | Arabidopsis thaliana | 240 | - | 6 | CAACCA |  |
| MYB | Arabidopsis thaliana | 225 | - | 6 | CAACCA |  |
| MYB | Arabidopsis thaliana | 3628 | + | 6 | CAACAG |  |
| MYB | Arabidopsis thaliana | 2586 | + | 6 | CAACCA |  |
| MYB | Arabidopsis thaliana | 67 | + | 6 | TAACCA |  |
| MYB | Arabidopsis thaliana | 486 | - | 6 | TAACCA |  |

>HU07G02248.1   
+ -Up\_Stream \_Len000CTGTTC AGCACTAGGG AATAAGGTTT TTGCCCTGCA TACTATGAAA TCTAACCAAA   
  
  
+ AACATTCCAT CTTGGAAGGC TGTAGCCTTG TGGATTTTGA ATTCGCTCCC AAGAACCAAT GAAGTAGCGC   
  
  
+ AGAAGGCCAA TACTCAATTT CAGAGTCCAC ATCAACTTGT TCAATGCCAG TATATTTCAG AACATTAATT   
  
  
+ ACATACTGTT TGGTTGGAGA AAAAATGGTT GTCTCGAAAA CTAGTTTTTT AATTTTTTGT TATTTAGCAC   
  
  
+ GGTAAAGAAT GGAAATTATT TTCATGCGAG AAAAAAGGAA GTTGGTTTTT CTTTTTTCAA CTCTTTTGAT   
  
  
+ GATATAATCA GTCGAAATTT AGGTGCACCA TCTGACTTCA ACTCTTGTAC GACCTCTGAT TGCTTGCTTA   
  
  
+ GAAGAACAAG AGGATTGACA GATAAGGAGG AGTGGGTGGT TTATACAGAG AATTGAGTGG ATGGTTATAA   
  
  
+ AGAAGTCGAT CTCACATAAG TAACTTCGTT ACGATTATAA ATATTTTCAT TGAAGAAAGA CGATTAGATA   
  
  
+ TAATTAAGAA GGTACATCTA AGCAAATTTG GCTGAGCAGA GTTGGAGGGC CACTAGGGTC ACATTGTGTC   
  
  
+ TTGCGCAACA AGCTTAGACC ATTCGCAACG GATGCCTCAC GTGGAGCAGG TGGGACCCGC GTGGAAGTTG   
  
  
+ CATGGCAAGA ATTCTCTGGC AGCAAGGAGA ATTGAGGGGG GACACGCGTG TGACCACGCC AGTGCGGTCC   
  
  
+ CCCTCTCGTG GCAGATTTGG TGATTTTTTT TTTGTTTTTT TTTGAAGTAA TATATAATTT TTATATGATT   
  
  
+ AAAAAAATAG GTTGTGTGTT ATAAAGTGGG TCAATGACAA ATATTTTTAT TGGATGATTA AAATTAAGTT   
  
  
+ CCATTTATTG GAGAAGTGAT GGAATGCAAG TTCCAAAATT GTGGAACTTT TGTTGACGTA GCAGGCTAGA   
  
  
+ GTTCCAAAAA TTAAAAAAAG GAAAGACATA TTGAGGTAAA GCCTTTTTAT TTTACAAATA GACGCACCAA   
  
  
+ GGTATAATAT AATTGACATG ATTTGAGGCG AGAGGAAGAT TCTTTTGCTA CCCAAACAAA ACACAATGAT   
  
  
+ CTTTTAAATG TAGCAATTTG GGATTGGCAA AATTTTGTCT AACCCGATTG TTGAAAGTTA TCCGATTTCA   
  
  
+ TAAAATTGGA TATCGTTATT AAATTTTCAT TCGAATATTT AAATTTGGAC ACAATGTTTT TCATATTTGG   
  
  
+ ATTTTCTAAA CTTGATTTTA ACTCAAATTT AAGTTTATTC AATTCATCTG ACCCAAAAAA TGGTTGCAAT   
  
  
+ AATGTATTTT CTTTTTGAAG TTAAATATTA TCGAATTCAT TTAACTTGTA GTTAACCCGT AATTGACCCA   
  
  
+ AACTCGACTT TTTGCTACCT TAGCAGCCAG GAAGAAGTCA TAGTATTGAC CCGTGGAACG CCTGAAGCCA   
  
  
+ ACATCACCAG ACCCACATAT TCCATCATCT TCTGATCTCC TCGTCAAAGA AGGAAGCTGA GCACAGCAAT   
  
  
+ AACTAGAAGA ACAGGAGGCA TCACACCCAA GCCAAAGGCC TTTATTTGAG CTCCCCACCT CATTCCTCGA   
  
  
+ AGCTTCCTCT TCACAATGTA AGACCATAAT CCTTCACCCC TTCTCTTTTT AAGCTCATCT TTACCTGCCC   
  
  
+ TAGTTCTCTC TCTCTCATGA GTCATCAGTA ATAGTTACCT CTTTACTTTC TCTTCCTTGA GGTATGCTTC   
  
  
+ AACTTTAATA TATATATGTA CCACTTGATT TCTCTTGTCA CTTTGCTATA ATACTTATTG TTTGGCTATC   
  
  
+ TTCCCCTGTT TTATACTCAT TTTCTTGCTT TCTATATCTG GGTTTGAATT GGGTTTCACT TATCCTCGTT   
  
  
+ GATTCTGTGG GAAATTAGTG AATTGGGTTT TGTACAGCAG GTTTATATGA ACTAGGGTTT CAAAGGTATC   
  
  
+ TACTTTTTCT CTTGTGATTA TTAGTTGATT AGGGATTTGG CCAAGATTAG CAGAATGGGT TCTGAATTTG   
  
  
+ GGGAATTCTC TGATGATGCT CTAAATGGGT ATGCTTACGT TGATATCCCT GCTTATGATG CGTCCTTAGA   
  
  
+ TTATGCCAAT TTGTTCAATT ACGAAGGCCT ATCTGAGGAT CTCACCTCAC TGAGCCTCCC AAGCCCCTTT   
  
  
+ TCTGACCCTT TGGCGTACAG TTTCACGTCC TCTTTGGGGC CGAGCCCTGG GGTTGATTCT AATGATGATA   
  
  
+ GTGATTCTGA TGATGTTCTC AAGTGCATTA GCCAAATGCT TATGGAAGAA GACATGGAGG CAAAGCCATG   
  
  
+ CATGTTTCAC GATCCTTTAG CACTTCAGGC TGCTGAGAAA CCCTTTTATG ATGCCTTAGG GAAGAAATAC   
  
  
+ CCAACTTCTC CGAACCAACA TCCTATAATT GATGATTGTT TGGATAATCC TGGTGAAAAT TCTTTGGGTT   
  
  
+ CTTCTAGTGA TTTTAGTGTT AGTCACTTTG GGTCTAGTTC AGCAAGCTCT ATTGGACCGA CAATTGTGTC   
  
  
+ TGATTTGAGT GAGCATTTTG AGCCACCCTT TGTTCAAGCA CTTCCAATTG AATCATATCC CCAACCATTG   
  
  
+ ACCCGTCCTC AATGGTCGTT TGGCCCTTCG GGTGCCTTAG ATTGCACGGC CTCTAATGGT TCAGTGATCT   
  
  
+ CATCCCTTGG TTTGCCGATG GATGTTATTA GTGTATTCAG TGAGAAAGAG TCCATGATTC AATTTCAGAA   
  
  
+ AGGGGTGGAA GAGGCTAGTA AGTTCCTTCC CAAGAATAAT AACCTTGTTA TTGATCTCAA GAACCTCACT   
  
  
+ TTTCCTAATG AAACGAAGGA GGATGATCGA GTGATGATGG TTAAGAAGGA AAAGAATGAT GTGAATTGGT   
  
  
+ CTAACTACTC AAGAGGGAGT AAGATTCACT ATCGTGAAGA CGAGGACTTT GAAGAAGGAA GGAGTAGCAA   
  
  
+ GCAGTCAGCT ATTTCTACTA CTGAGGAAGC TGAGTTGTGT GAAATTTTTG ACAAGTTTTT GCTTTGCAAT   
  
  
+ TGGTACCCTG TGAAACCTGA GGCTCATCCC ACCATGAGTT TGAACCCTGA GAAGGGCCAG TCACATGGAT   
  
  
+ TAGAAGGTGG GAGAAATGGG AAGGGTCACC CAAAGAAACA GGATAAAAGT AGTACCAACG TTGTGGATTT   
  
  
+ AAGGAATTTG CTGATGCTAT GTGCACAATC TACTGCATCT GATGACCGAA GAACTGCTGA TGGACTGCTA   
  
  
+ AAGAAAATCA GGGAGCACTC ATCTGCTGAG GGGGATGGAT TTCAAAGGTT GGCTCATTAC TTTGCTGATG   
  
  
+ CCCTAGAGGC ACGTTTAGCT GGAACTGGAT CTCACATTTA TACAGCCCTA AGTTCTCATA GGCCATCTGC   
  
  
+ TGTTGACGTG TTAAAAGCAT ATCAGTTCTA TGTTCGTGCT TGCCCATTTA AGAAGATCGT CATTCGTTGT   
  
  
+ GGTAAACATA TGATTCTAAA AGCTGCTGAA AAGGCATCAA AGCTTCATAT TATAGATTTT GGCATCCTCT   
  
  
+ ATGGATTCCA ATGGCCTAGC CTCATTCGAT GCCTCTCAGA GCGGTCTGGT GGACCTCCAA AACTGTTTAT   
  
  
+ TACAGGGATC GATCTCCCCC AGCCTGGGTT CAGGCCAGCA GAAAGAGTGG AAGCAACAGG GAGACGCTTG   
  
  
+ GCTAAGTACT GTGAGCGGTA TAATGTGCCA TTTGAGTATC ATGCCATTGC TCAGAAGTGG GAAACAATCA   
  
  
+ AACCAGGGGA TCTCAAGTTA GGAAGTAGGG ATGATGATGA AGTTGTCGTG GTGAACTGTC TCTGTAGGTT   
  
  
+ CAAGAACCTC CTTGACGAGA CAATGGTGGT GGATAGTCCA AGGAACACAG TTTTAAACCT GATTAGAAGG   
  
  
+ GTAAAACCCG ATATTTTTGT GCATGGCATT GTAAATGGTT CCTACAACAT CCCTTTCTTT GTGACACGTT   
  
  
+ TTAGAGAAGC CCTCTTTCAT TATTCCACTC TTTTCGACAT GTTAGATGCC AACGCCTCTA GGGAGGAGCC   
  
  
+ CGAGAGGTTG ATATTCGAGA AGGCATTCTA TGGGAGGGAG ATTATGAATG TGGTGGCATG TGAGGGCACA   
  
  
+ GAGAGGGTGG AAAGGCCAGA GACATACAAG CAATGGCACG TTAGGCATAG CAGGGCAGGG TTTCGGCAAC   
  
  
+ TACCATTGGA TCCCAAGTTG ATCGAGAAAA TGAGGTTTAA GGCCAAGGCA GACCACCACA AGGATTTCGT   
  
  
+ GATTGATGTG GATGGACATT GGGCAATTCA GGGATGGAAG GGGCGGATTG CCTGTGCCAT CTCTGCATGG   
  
  
+ GTTCTGGCTT G  

- -Up\_Stream \_Len000GACAAG TCGTGATCCC TTATTCCAAA AACGGGACGT ATGATACTTT AGATTGGTTT   
  
  
- TTGTAAGGTA GAACCTTCCG ACATCGGAAC ACCTAAAACT TAAGCGAGGG TTCTTGGTTA CTTCATCGCG   
  
  
- TCTTCCGGTT ATGAGTTAAA GTCTCAGGTG TAGTTGAACA AGTTACGGTC ATATAAAGTC TTGTAATTAA   
  
  
- TGTATGACAA ACCAACCTCT TTTTTACCAA CAGAGCTTTT GATCAAAAAA TTAAAAAACA ATAAATCGTG   
  
  
- CCATTTCTTA CCTTTAATAA AAGTACGCTC TTTTTTCCTT CAACCAAAAA GAAAAAAGTT GAGAAAACTA   
  
  
- CTATATTAGT CAGCTTTAAA TCCACGTGGT AGACTGAAGT TGAGAACATG CTGGAGACTA ACGAACGAAT   
  
  
- CTTCTTGTTC TCCTAACTGT CTATTCCTCC TCACCCACCA AATATGTCTC TTAACTCACC TACCAATATT   
  
  
- TCTTCAGCTA GAGTGTATTC ATTGAAGCAA TGCTAATATT TATAAAAGTA ACTTCTTTCT GCTAATCTAT   
  
  
- ATTAATTCTT CCATGTAGAT TCGTTTAAAC CGACTCGTCT CAACCTCCCG GTGATCCCAG TGTAACACAG   
  
  
- AACGCGTTGT TCGAATCTGG TAAGCGTTGC CTACGGAGTG CACCTCGTCC ACCCTGGGCG CACCTTCAAC   
  
  
- GTACCGTTCT TAAGAGACCG TCGTTCCTCT TAACTCCCCC CTGTGCGCAC ACTGGTGCGG TCACGCCAGG   
  
  
- GGGAGAGCAC CGTCTAAACC ACTAAAAAAA AAACAAAAAA AAACTTCATT ATATATTAAA AATATACTAA   
  
  
- TTTTTTTATC CAACACACAA TATTTCACCC AGTTACTGTT TATAAAAATA ACCTACTAAT TTTAATTCAA   
  
  
- GGTAAATAAC CTCTTCACTA CCTTACGTTC AAGGTTTTAA CACCTTGAAA ACAACTGCAT CGTCCGATCT   
  
  
- CAAGGTTTTT AATTTTTTTC CTTTCTGTAT AACTCCATTT CGGAAAAATA AAATGTTTAT CTGCGTGGTT   
  
  
- CCATATTATA TTAACTGTAC TAAACTCCGC TCTCCTTCTA AGAAAACGAT GGGTTTGTTT TGTGTTACTA   
  
  
- GAAAATTTAC ATCGTTAAAC CCTAACCGTT TTAAAACAGA TTGGGCTAAC AACTTTCAAT AGGCTAAAGT   
  
  
- ATTTTAACCT ATAGCAATAA TTTAAAAGTA AGCTTATAAA TTTAAACCTG TGTTACAAAA AGTATAAACC   
  
  
- TAAAAGATTT GAACTAAAAT TGAGTTTAAA TTCAAATAAG TTAAGTAGAC TGGGTTTTTT ACCAACGTTA   
  
  
- TTACATAAAA GAAAAACTTC AATTTATAAT AGCTTAAGTA AATTGAACAT CAATTGGGCA TTAACTGGGT   
  
  
- TTGAGCTGAA AAACGATGGA ATCGTCGGTC CTTCTTCAGT ATCATAACTG GGCACCTTGC GGACTTCGGT   
  
  
- TGTAGTGGTC TGGGTGTATA AGGTAGTAGA AGACTAGAGG AGCAGTTTCT TCCTTCGACT CGTGTCGTTA   
  
  
- TTGATCTTCT TGTCCTCCGT AGTGTGGGTT CGGTTTCCGG AAATAAACTC GAGGGGTGGA GTAAGGAGCT   
  
  
- TCGAAGGAGA AGTGTTACAT TCTGGTATTA GGAAGTGGGG AAGAGAAAAA TTCGAGTAGA AATGGACGGG   
  
  
- ATCAAGAGAG AGAGAGTACT CAGTAGTCAT TATCAATGGA GAAATGAAAG AGAAGGAACT CCATACGAAG   
  
  
- TTGAAATTAT ATATATACAT GGTGAACTAA AGAGAACAGT GAAACGATAT TATGAATAAC AAACCGATAG   
  
  
- AAGGGGACAA AATATGAGTA AAAGAACGAA AGATATAGAC CCAAACTTAA CCCAAAGTGA ATAGGAGCAA   
  
  
- CTAAGACACC CTTTAATCAC TTAACCCAAA ACATGTCGTC CAAATATACT TGATCCCAAA GTTTCCATAG   
  
  
- ATGAAAAAGA GAACACTAAT AATCAACTAA TCCCTAAACC GGTTCTAATC GTCTTACCCA AGACTTAAAC   
  
  
- CCCTTAAGAG ACTACTACGA GATTTACCCA TACGAATGCA ACTATAGGGA CGAATACTAC GCAGGAATCT   
  
  
- AATACGGTTA AACAAGTTAA TGCTTCCGGA TAGACTCCTA GAGTGGAGTG ACTCGGAGGG TTCGGGGAAA   
  
  
- AGACTGGGAA ACCGCATGTC AAAGTGCAGG AGAAACCCCG GCTCGGGACC CCAACTAAGA TTACTACTAT   
  
  
- CACTAAGACT ACTACAAGAG TTCACGTAAT CGGTTTACGA ATACCTTCTT CTGTACCTCC GTTTCGGTAC   
  
  
- GTACAAAGTG CTAGGAAATC GTGAAGTCCG ACGACTCTTT GGGAAAATAC TACGGAATCC CTTCTTTATG   
  
  
- GGTTGAAGAG GCTTGGTTGT AGGATATTAA CTACTAACAA ACCTATTAGG ACCACTTTTA AGAAACCCAA   
  
  
- GAAGATCACT AAAATCACAA TCAGTGAAAC CCAGATCAAG TCGTTCGAGA TAACCTGGCT GTTAACACAG   
  
  
- ACTAAACTCA CTCGTAAAAC TCGGTGGGAA ACAAGTTCGT GAAGGTTAAC TTAGTATAGG GGTTGGTAAC   
  
  
- TGGGCAGGAG TTACCAGCAA ACCGGGAAGC CCACGGAATC TAACGTGCCG GAGATTACCA AGTCACTAGA   
  
  
- GTAGGGAACC AAACGGCTAC CTACAATAAT CACATAAGTC ACTCTTTCTC AGGTACTAAG TTAAAGTCTT   
  
  
- TCCCCACCTT CTCCGATCAT TCAAGGAAGG GTTCTTATTA TTGGAACAAT AACTAGAGTT CTTGGAGTGA   
  
  
- AAAGGATTAC TTTGCTTCCT CCTACTAGCT CACTACTACC AATTCTTCCT TTTCTTACTA CACTTAACCA   
  
  
- GATTGATGAG TTCTCCCTCA TTCTAAGTGA TAGCACTTCT GCTCCTGAAA CTTCTTCCTT CCTCATCGTT   
  
  
- CGTCAGTCGA TAAAGATGAT GACTCCTTCG ACTCAACACA CTTTAAAAAC TGTTCAAAAA CGAAACGTTA   
  
  
- ACCATGGGAC ACTTTGGACT CCGAGTAGGG TGGTACTCAA ACTTGGGACT CTTCCCGGTC AGTGTACCTA   
  
  
- ATCTTCCACC CTCTTTACCC TTCCCAGTGG GTTTCTTTGT CCTATTTTCA TCATGGTTGC AACACCTAAA   
  
  
- TTCCTTAAAC GACTACGATA CACGTGTTAG ATGACGTAGA CTACTGGCTT CTTGACGACT ACCTGACGAT   
  
  
- TTCTTTTAGT CCCTCGTGAG TAGACGACTC CCCCTACCTA AAGTTTCCAA CCGAGTAATG AAACGACTAC   
  
  
- GGGATCTCCG TGCAAATCGA CCTTGACCTA GAGTGTAAAT ATGTCGGGAT TCAAGAGTAT CCGGTAGACG   
  
  
- ACAACTGCAC AATTTTCGTA TAGTCAAGAT ACAAGCACGA ACGGGTAAAT TCTTCTAGCA GTAAGCAACA   
  
  
- CCATTTGTAT ACTAAGATTT TCGACGACTT TTCCGTAGTT TCGAAGTATA ATATCTAAAA CCGTAGGAGA   
  
  
- TACCTAAGGT TACCGGATCG GAGTAAGCTA CGGAGAGTCT CGCCAGACCA CCTGGAGGTT TTGACAAATA   
  
  
- ATGTCCCTAG CTAGAGGGGG TCGGACCCAA GTCCGGTCGT CTTTCTCACC TTCGTTGTCC CTCTGCGAAC   
  
  
- CGATTCATGA CACTCGCCAT ATTACACGGT AAACTCATAG TACGGTAACG AGTCTTCACC CTTTGTTAGT   
  
  
- TTGGTCCCCT AGAGTTCAAT CCTTCATCCC TACTACTACT TCAACAGCAC CACTTGACAG AGACATCCAA   
  
  
- GTTCTTGGAG GAACTGCTCT GTTACCACCA CCTATCAGGT TCCTTGTGTC AAAATTTGGA CTAATCTTCC   
  
  
- CATTTTGGGC TATAAAAACA CGTACCGTAA CATTTACCAA GGATGTTGTA GGGAAAGAAA CACTGTGCAA   
  
  
- AATCTCTTCG GGAGAAAGTA ATAAGGTGAG AAAAGCTGTA CAATCTACGG TTGCGGAGAT CCCTCCTCGG   
  
  
- GCTCTCCAAC TATAAGCTCT TCCGTAAGAT ACCCTCCCTC TAATACTTAC ACCACCGTAC ACTCCCGTGT   
  
  
- CTCTCCCACC TTTCCGGTCT CTGTATGTTC GTTACCGTGC AATCCGTATC GTCCCGTCCC AAAGCCGTTG   
  
  
- ATGGTAACCT AGGGTTCAAC TAGCTCTTTT ACTCCAAATT CCGGTTCCGT CTGGTGGTGT TCCTAAAGCA   
  
  
- CTAACTACAC CTACCTGTAA CCCGTTAAGT CCCTACCTTC CCCGCCTAAC GGACACGGTA GAGACGTACC   
  
  
- CAAGACCGAA C

+     MYB recognition site

| Site Name | Organism | Position | Strand | Matrix score. | sequence | function |
| --- | --- | --- | --- | --- | --- | --- |
| MYB recognition site | Arabidopsis thaliana | 660 | - | 6 | CCGTTG |  |

>HU07G02248.1   
+ -Up\_Stream \_Len000CTGTTC AGCACTAGGG AATAAGGTTT TTGCCCTGCA TACTATGAAA TCTAACCAAA   
  
  
+ AACATTCCAT CTTGGAAGGC TGTAGCCTTG TGGATTTTGA ATTCGCTCCC AAGAACCAAT GAAGTAGCGC   
  
  
+ AGAAGGCCAA TACTCAATTT CAGAGTCCAC ATCAACTTGT TCAATGCCAG TATATTTCAG AACATTAATT   
  
  
+ ACATACTGTT TGGTTGGAGA AAAAATGGTT GTCTCGAAAA CTAGTTTTTT AATTTTTTGT TATTTAGCAC   
  
  
+ GGTAAAGAAT GGAAATTATT TTCATGCGAG AAAAAAGGAA GTTGGTTTTT CTTTTTTCAA CTCTTTTGAT   
  
  
+ GATATAATCA GTCGAAATTT AGGTGCACCA TCTGACTTCA ACTCTTGTAC GACCTCTGAT TGCTTGCTTA   
  
  
+ GAAGAACAAG AGGATTGACA GATAAGGAGG AGTGGGTGGT TTATACAGAG AATTGAGTGG ATGGTTATAA   
  
  
+ AGAAGTCGAT CTCACATAAG TAACTTCGTT ACGATTATAA ATATTTTCAT TGAAGAAAGA CGATTAGATA   
  
  
+ TAATTAAGAA GGTACATCTA AGCAAATTTG GCTGAGCAGA GTTGGAGGGC CACTAGGGTC ACATTGTGTC   
  
  
+ TTGCGCAACA AGCTTAGACC ATTCGCAACG GATGCCTCAC GTGGAGCAGG TGGGACCCGC GTGGAAGTTG   
  
  
+ CATGGCAAGA ATTCTCTGGC AGCAAGGAGA ATTGAGGGGG GACACGCGTG TGACCACGCC AGTGCGGTCC   
  
  
+ CCCTCTCGTG GCAGATTTGG TGATTTTTTT TTTGTTTTTT TTTGAAGTAA TATATAATTT TTATATGATT   
  
  
+ AAAAAAATAG GTTGTGTGTT ATAAAGTGGG TCAATGACAA ATATTTTTAT TGGATGATTA AAATTAAGTT   
  
  
+ CCATTTATTG GAGAAGTGAT GGAATGCAAG TTCCAAAATT GTGGAACTTT TGTTGACGTA GCAGGCTAGA   
  
  
+ GTTCCAAAAA TTAAAAAAAG GAAAGACATA TTGAGGTAAA GCCTTTTTAT TTTACAAATA GACGCACCAA   
  
  
+ GGTATAATAT AATTGACATG ATTTGAGGCG AGAGGAAGAT TCTTTTGCTA CCCAAACAAA ACACAATGAT   
  
  
+ CTTTTAAATG TAGCAATTTG GGATTGGCAA AATTTTGTCT AACCCGATTG TTGAAAGTTA TCCGATTTCA   
  
  
+ TAAAATTGGA TATCGTTATT AAATTTTCAT TCGAATATTT AAATTTGGAC ACAATGTTTT TCATATTTGG   
  
  
+ ATTTTCTAAA CTTGATTTTA ACTCAAATTT AAGTTTATTC AATTCATCTG ACCCAAAAAA TGGTTGCAAT   
  
  
+ AATGTATTTT CTTTTTGAAG TTAAATATTA TCGAATTCAT TTAACTTGTA GTTAACCCGT AATTGACCCA   
  
  
+ AACTCGACTT TTTGCTACCT TAGCAGCCAG GAAGAAGTCA TAGTATTGAC CCGTGGAACG CCTGAAGCCA   
  
  
+ ACATCACCAG ACCCACATAT TCCATCATCT TCTGATCTCC TCGTCAAAGA AGGAAGCTGA GCACAGCAAT   
  
  
+ AACTAGAAGA ACAGGAGGCA TCACACCCAA GCCAAAGGCC TTTATTTGAG CTCCCCACCT CATTCCTCGA   
  
  
+ AGCTTCCTCT TCACAATGTA AGACCATAAT CCTTCACCCC TTCTCTTTTT AAGCTCATCT TTACCTGCCC   
  
  
+ TAGTTCTCTC TCTCTCATGA GTCATCAGTA ATAGTTACCT CTTTACTTTC TCTTCCTTGA GGTATGCTTC   
  
  
+ AACTTTAATA TATATATGTA CCACTTGATT TCTCTTGTCA CTTTGCTATA ATACTTATTG TTTGGCTATC   
  
  
+ TTCCCCTGTT TTATACTCAT TTTCTTGCTT TCTATATCTG GGTTTGAATT GGGTTTCACT TATCCTCGTT   
  
  
+ GATTCTGTGG GAAATTAGTG AATTGGGTTT TGTACAGCAG GTTTATATGA ACTAGGGTTT CAAAGGTATC   
  
  
+ TACTTTTTCT CTTGTGATTA TTAGTTGATT AGGGATTTGG CCAAGATTAG CAGAATGGGT TCTGAATTTG   
  
  
+ GGGAATTCTC TGATGATGCT CTAAATGGGT ATGCTTACGT TGATATCCCT GCTTATGATG CGTCCTTAGA   
  
  
+ TTATGCCAAT TTGTTCAATT ACGAAGGCCT ATCTGAGGAT CTCACCTCAC TGAGCCTCCC AAGCCCCTTT   
  
  
+ TCTGACCCTT TGGCGTACAG TTTCACGTCC TCTTTGGGGC CGAGCCCTGG GGTTGATTCT AATGATGATA   
  
  
+ GTGATTCTGA TGATGTTCTC AAGTGCATTA GCCAAATGCT TATGGAAGAA GACATGGAGG CAAAGCCATG   
  
  
+ CATGTTTCAC GATCCTTTAG CACTTCAGGC TGCTGAGAAA CCCTTTTATG ATGCCTTAGG GAAGAAATAC   
  
  
+ CCAACTTCTC CGAACCAACA TCCTATAATT GATGATTGTT TGGATAATCC TGGTGAAAAT TCTTTGGGTT   
  
  
+ CTTCTAGTGA TTTTAGTGTT AGTCACTTTG GGTCTAGTTC AGCAAGCTCT ATTGGACCGA CAATTGTGTC   
  
  
+ TGATTTGAGT GAGCATTTTG AGCCACCCTT TGTTCAAGCA CTTCCAATTG AATCATATCC CCAACCATTG   
  
  
+ ACCCGTCCTC AATGGTCGTT TGGCCCTTCG GGTGCCTTAG ATTGCACGGC CTCTAATGGT TCAGTGATCT   
  
  
+ CATCCCTTGG TTTGCCGATG GATGTTATTA GTGTATTCAG TGAGAAAGAG TCCATGATTC AATTTCAGAA   
  
  
+ AGGGGTGGAA GAGGCTAGTA AGTTCCTTCC CAAGAATAAT AACCTTGTTA TTGATCTCAA GAACCTCACT   
  
  
+ TTTCCTAATG AAACGAAGGA GGATGATCGA GTGATGATGG TTAAGAAGGA AAAGAATGAT GTGAATTGGT   
  
  
+ CTAACTACTC AAGAGGGAGT AAGATTCACT ATCGTGAAGA CGAGGACTTT GAAGAAGGAA GGAGTAGCAA   
  
  
+ GCAGTCAGCT ATTTCTACTA CTGAGGAAGC TGAGTTGTGT GAAATTTTTG ACAAGTTTTT GCTTTGCAAT   
  
  
+ TGGTACCCTG TGAAACCTGA GGCTCATCCC ACCATGAGTT TGAACCCTGA GAAGGGCCAG TCACATGGAT   
  
  
+ TAGAAGGTGG GAGAAATGGG AAGGGTCACC CAAAGAAACA GGATAAAAGT AGTACCAACG TTGTGGATTT   
  
  
+ AAGGAATTTG CTGATGCTAT GTGCACAATC TACTGCATCT GATGACCGAA GAACTGCTGA TGGACTGCTA   
  
  
+ AAGAAAATCA GGGAGCACTC ATCTGCTGAG GGGGATGGAT TTCAAAGGTT GGCTCATTAC TTTGCTGATG   
  
  
+ CCCTAGAGGC ACGTTTAGCT GGAACTGGAT CTCACATTTA TACAGCCCTA AGTTCTCATA GGCCATCTGC   
  
  
+ TGTTGACGTG TTAAAAGCAT ATCAGTTCTA TGTTCGTGCT TGCCCATTTA AGAAGATCGT CATTCGTTGT   
  
  
+ GGTAAACATA TGATTCTAAA AGCTGCTGAA AAGGCATCAA AGCTTCATAT TATAGATTTT GGCATCCTCT   
  
  
+ ATGGATTCCA ATGGCCTAGC CTCATTCGAT GCCTCTCAGA GCGGTCTGGT GGACCTCCAA AACTGTTTAT   
  
  
+ TACAGGGATC GATCTCCCCC AGCCTGGGTT CAGGCCAGCA GAAAGAGTGG AAGCAACAGG GAGACGCTTG   
  
  
+ GCTAAGTACT GTGAGCGGTA TAATGTGCCA TTTGAGTATC ATGCCATTGC TCAGAAGTGG GAAACAATCA   
  
  
+ AACCAGGGGA TCTCAAGTTA GGAAGTAGGG ATGATGATGA AGTTGTCGTG GTGAACTGTC TCTGTAGGTT   
  
  
+ CAAGAACCTC CTTGACGAGA CAATGGTGGT GGATAGTCCA AGGAACACAG TTTTAAACCT GATTAGAAGG   
  
  
+ GTAAAACCCG ATATTTTTGT GCATGGCATT GTAAATGGTT CCTACAACAT CCCTTTCTTT GTGACACGTT   
  
  
+ TTAGAGAAGC CCTCTTTCAT TATTCCACTC TTTTCGACAT GTTAGATGCC AACGCCTCTA GGGAGGAGCC   
  
  
+ CGAGAGGTTG ATATTCGAGA AGGCATTCTA TGGGAGGGAG ATTATGAATG TGGTGGCATG TGAGGGCACA   
  
  
+ GAGAGGGTGG AAAGGCCAGA GACATACAAG CAATGGCACG TTAGGCATAG CAGGGCAGGG TTTCGGCAAC   
  
  
+ TACCATTGGA TCCCAAGTTG ATCGAGAAAA TGAGGTTTAA GGCCAAGGCA GACCACCACA AGGATTTCGT   
  
  
+ GATTGATGTG GATGGACATT GGGCAATTCA GGGATGGAAG GGGCGGATTG CCTGTGCCAT CTCTGCATGG   
  
  
+ GTTCTGGCTT G  

- -Up\_Stream \_Len000GACAAG TCGTGATCCC TTATTCCAAA AACGGGACGT ATGATACTTT AGATTGGTTT   
  
  
- TTGTAAGGTA GAACCTTCCG ACATCGGAAC ACCTAAAACT TAAGCGAGGG TTCTTGGTTA CTTCATCGCG   
  
  
- TCTTCCGGTT ATGAGTTAAA GTCTCAGGTG TAGTTGAACA AGTTACGGTC ATATAAAGTC TTGTAATTAA   
  
  
- TGTATGACAA ACCAACCTCT TTTTTACCAA CAGAGCTTTT GATCAAAAAA TTAAAAAACA ATAAATCGTG   
  
  
- CCATTTCTTA CCTTTAATAA AAGTACGCTC TTTTTTCCTT CAACCAAAAA GAAAAAAGTT GAGAAAACTA   
  
  
- CTATATTAGT CAGCTTTAAA TCCACGTGGT AGACTGAAGT TGAGAACATG CTGGAGACTA ACGAACGAAT   
  
  
- CTTCTTGTTC TCCTAACTGT CTATTCCTCC TCACCCACCA AATATGTCTC TTAACTCACC TACCAATATT   
  
  
- TCTTCAGCTA GAGTGTATTC ATTGAAGCAA TGCTAATATT TATAAAAGTA ACTTCTTTCT GCTAATCTAT   
  
  
- ATTAATTCTT CCATGTAGAT TCGTTTAAAC CGACTCGTCT CAACCTCCCG GTGATCCCAG TGTAACACAG   
  
  
- AACGCGTTGT TCGAATCTGG TAAGCGTTGC CTACGGAGTG CACCTCGTCC ACCCTGGGCG CACCTTCAAC   
  
  
- GTACCGTTCT TAAGAGACCG TCGTTCCTCT TAACTCCCCC CTGTGCGCAC ACTGGTGCGG TCACGCCAGG   
  
  
- GGGAGAGCAC CGTCTAAACC ACTAAAAAAA AAACAAAAAA AAACTTCATT ATATATTAAA AATATACTAA   
  
  
- TTTTTTTATC CAACACACAA TATTTCACCC AGTTACTGTT TATAAAAATA ACCTACTAAT TTTAATTCAA   
  
  
- GGTAAATAAC CTCTTCACTA CCTTACGTTC AAGGTTTTAA CACCTTGAAA ACAACTGCAT CGTCCGATCT   
  
  
- CAAGGTTTTT AATTTTTTTC CTTTCTGTAT AACTCCATTT CGGAAAAATA AAATGTTTAT CTGCGTGGTT   
  
  
- CCATATTATA TTAACTGTAC TAAACTCCGC TCTCCTTCTA AGAAAACGAT GGGTTTGTTT TGTGTTACTA   
  
  
- GAAAATTTAC ATCGTTAAAC CCTAACCGTT TTAAAACAGA TTGGGCTAAC AACTTTCAAT AGGCTAAAGT   
  
  
- ATTTTAACCT ATAGCAATAA TTTAAAAGTA AGCTTATAAA TTTAAACCTG TGTTACAAAA AGTATAAACC   
  
  
- TAAAAGATTT GAACTAAAAT TGAGTTTAAA TTCAAATAAG TTAAGTAGAC TGGGTTTTTT ACCAACGTTA   
  
  
- TTACATAAAA GAAAAACTTC AATTTATAAT AGCTTAAGTA AATTGAACAT CAATTGGGCA TTAACTGGGT   
  
  
- TTGAGCTGAA AAACGATGGA ATCGTCGGTC CTTCTTCAGT ATCATAACTG GGCACCTTGC GGACTTCGGT   
  
  
- TGTAGTGGTC TGGGTGTATA AGGTAGTAGA AGACTAGAGG AGCAGTTTCT TCCTTCGACT CGTGTCGTTA   
  
  
- TTGATCTTCT TGTCCTCCGT AGTGTGGGTT CGGTTTCCGG AAATAAACTC GAGGGGTGGA GTAAGGAGCT   
  
  
- TCGAAGGAGA AGTGTTACAT TCTGGTATTA GGAAGTGGGG AAGAGAAAAA TTCGAGTAGA AATGGACGGG   
  
  
- ATCAAGAGAG AGAGAGTACT CAGTAGTCAT TATCAATGGA GAAATGAAAG AGAAGGAACT CCATACGAAG   
  
  
- TTGAAATTAT ATATATACAT GGTGAACTAA AGAGAACAGT GAAACGATAT TATGAATAAC AAACCGATAG   
  
  
- AAGGGGACAA AATATGAGTA AAAGAACGAA AGATATAGAC CCAAACTTAA CCCAAAGTGA ATAGGAGCAA   
  
  
- CTAAGACACC CTTTAATCAC TTAACCCAAA ACATGTCGTC CAAATATACT TGATCCCAAA GTTTCCATAG   
  
  
- ATGAAAAAGA GAACACTAAT AATCAACTAA TCCCTAAACC GGTTCTAATC GTCTTACCCA AGACTTAAAC   
  
  
- CCCTTAAGAG ACTACTACGA GATTTACCCA TACGAATGCA ACTATAGGGA CGAATACTAC GCAGGAATCT   
  
  
- AATACGGTTA AACAAGTTAA TGCTTCCGGA TAGACTCCTA GAGTGGAGTG ACTCGGAGGG TTCGGGGAAA   
  
  
- AGACTGGGAA ACCGCATGTC AAAGTGCAGG AGAAACCCCG GCTCGGGACC CCAACTAAGA TTACTACTAT   
  
  
- CACTAAGACT ACTACAAGAG TTCACGTAAT CGGTTTACGA ATACCTTCTT CTGTACCTCC GTTTCGGTAC   
  
  
- GTACAAAGTG CTAGGAAATC GTGAAGTCCG ACGACTCTTT GGGAAAATAC TACGGAATCC CTTCTTTATG   
  
  
- GGTTGAAGAG GCTTGGTTGT AGGATATTAA CTACTAACAA ACCTATTAGG ACCACTTTTA AGAAACCCAA   
  
  
- GAAGATCACT AAAATCACAA TCAGTGAAAC CCAGATCAAG TCGTTCGAGA TAACCTGGCT GTTAACACAG   
  
  
- ACTAAACTCA CTCGTAAAAC TCGGTGGGAA ACAAGTTCGT GAAGGTTAAC TTAGTATAGG GGTTGGTAAC   
  
  
- TGGGCAGGAG TTACCAGCAA ACCGGGAAGC CCACGGAATC TAACGTGCCG GAGATTACCA AGTCACTAGA   
  
  
- GTAGGGAACC AAACGGCTAC CTACAATAAT CACATAAGTC ACTCTTTCTC AGGTACTAAG TTAAAGTCTT   
  
  
- TCCCCACCTT CTCCGATCAT TCAAGGAAGG GTTCTTATTA TTGGAACAAT AACTAGAGTT CTTGGAGTGA   
  
  
- AAAGGATTAC TTTGCTTCCT CCTACTAGCT CACTACTACC AATTCTTCCT TTTCTTACTA CACTTAACCA   
  
  
- GATTGATGAG TTCTCCCTCA TTCTAAGTGA TAGCACTTCT GCTCCTGAAA CTTCTTCCTT CCTCATCGTT   
  
  
- CGTCAGTCGA TAAAGATGAT GACTCCTTCG ACTCAACACA CTTTAAAAAC TGTTCAAAAA CGAAACGTTA   
  
  
- ACCATGGGAC ACTTTGGACT CCGAGTAGGG TGGTACTCAA ACTTGGGACT CTTCCCGGTC AGTGTACCTA   
  
  
- ATCTTCCACC CTCTTTACCC TTCCCAGTGG GTTTCTTTGT CCTATTTTCA TCATGGTTGC AACACCTAAA   
  
  
- TTCCTTAAAC GACTACGATA CACGTGTTAG ATGACGTAGA CTACTGGCTT CTTGACGACT ACCTGACGAT   
  
  
- TTCTTTTAGT CCCTCGTGAG TAGACGACTC CCCCTACCTA AAGTTTCCAA CCGAGTAATG AAACGACTAC   
  
  
- GGGATCTCCG TGCAAATCGA CCTTGACCTA GAGTGTAAAT ATGTCGGGAT TCAAGAGTAT CCGGTAGACG   
  
  
- ACAACTGCAC AATTTTCGTA TAGTCAAGAT ACAAGCACGA ACGGGTAAAT TCTTCTAGCA GTAAGCAACA   
  
  
- CCATTTGTAT ACTAAGATTT TCGACGACTT TTCCGTAGTT TCGAAGTATA ATATCTAAAA CCGTAGGAGA   
  
  
- TACCTAAGGT TACCGGATCG GAGTAAGCTA CGGAGAGTCT CGCCAGACCA CCTGGAGGTT TTGACAAATA   
  
  
- ATGTCCCTAG CTAGAGGGGG TCGGACCCAA GTCCGGTCGT CTTTCTCACC TTCGTTGTCC CTCTGCGAAC   
  
  
- CGATTCATGA CACTCGCCAT ATTACACGGT AAACTCATAG TACGGTAACG AGTCTTCACC CTTTGTTAGT   
  
  
- TTGGTCCCCT AGAGTTCAAT CCTTCATCCC TACTACTACT TCAACAGCAC CACTTGACAG AGACATCCAA   
  
  
- GTTCTTGGAG GAACTGCTCT GTTACCACCA CCTATCAGGT TCCTTGTGTC AAAATTTGGA CTAATCTTCC   
  
  
- CATTTTGGGC TATAAAAACA CGTACCGTAA CATTTACCAA GGATGTTGTA GGGAAAGAAA CACTGTGCAA   
  
  
- AATCTCTTCG GGAGAAAGTA ATAAGGTGAG AAAAGCTGTA CAATCTACGG TTGCGGAGAT CCCTCCTCGG   
  
  
- GCTCTCCAAC TATAAGCTCT TCCGTAAGAT ACCCTCCCTC TAATACTTAC ACCACCGTAC ACTCCCGTGT   
  
  
- CTCTCCCACC TTTCCGGTCT CTGTATGTTC GTTACCGTGC AATCCGTATC GTCCCGTCCC AAAGCCGTTG   
  
  
- ATGGTAACCT AGGGTTCAAC TAGCTCTTTT ACTCCAAATT CCGGTTCCGT CTGGTGGTGT TCCTAAAGCA   
  
  
- CTAACTACAC CTACCTGTAA CCCGTTAAGT CCCTACCTTC CCCGCCTAAC GGACACGGTA GAGACGTACC   
  
  
- CAAGACCGAA C

+     MYB-like sequence

| Site Name | Organism | Position | Strand | Matrix score. | sequence | function |
| --- | --- | --- | --- | --- | --- | --- |
| MYB-like sequence | Arabidopsis thaliana | 2842 | - | 6 | TAACCA |  |
| MYB-like sequence | Arabidopsis thaliana | 486 | - | 6 | TAACCA |  |
| MYB-like sequence | Arabidopsis thaliana | 67 | + | 6 | TAACCA |  |

>HU07G02248.1   
+ -Up\_Stream \_Len000CTGTTC AGCACTAGGG AATAAGGTTT TTGCCCTGCA TACTATGAAA TCTAACCAAA   
  
  
+ AACATTCCAT CTTGGAAGGC TGTAGCCTTG TGGATTTTGA ATTCGCTCCC AAGAACCAAT GAAGTAGCGC   
  
  
+ AGAAGGCCAA TACTCAATTT CAGAGTCCAC ATCAACTTGT TCAATGCCAG TATATTTCAG AACATTAATT   
  
  
+ ACATACTGTT TGGTTGGAGA AAAAATGGTT GTCTCGAAAA CTAGTTTTTT AATTTTTTGT TATTTAGCAC   
  
  
+ GGTAAAGAAT GGAAATTATT TTCATGCGAG AAAAAAGGAA GTTGGTTTTT CTTTTTTCAA CTCTTTTGAT   
  
  
+ GATATAATCA GTCGAAATTT AGGTGCACCA TCTGACTTCA ACTCTTGTAC GACCTCTGAT TGCTTGCTTA   
  
  
+ GAAGAACAAG AGGATTGACA GATAAGGAGG AGTGGGTGGT TTATACAGAG AATTGAGTGG ATGGTTATAA   
  
  
+ AGAAGTCGAT CTCACATAAG TAACTTCGTT ACGATTATAA ATATTTTCAT TGAAGAAAGA CGATTAGATA   
  
  
+ TAATTAAGAA GGTACATCTA AGCAAATTTG GCTGAGCAGA GTTGGAGGGC CACTAGGGTC ACATTGTGTC   
  
  
+ TTGCGCAACA AGCTTAGACC ATTCGCAACG GATGCCTCAC GTGGAGCAGG TGGGACCCGC GTGGAAGTTG   
  
  
+ CATGGCAAGA ATTCTCTGGC AGCAAGGAGA ATTGAGGGGG GACACGCGTG TGACCACGCC AGTGCGGTCC   
  
  
+ CCCTCTCGTG GCAGATTTGG TGATTTTTTT TTTGTTTTTT TTTGAAGTAA TATATAATTT TTATATGATT   
  
  
+ AAAAAAATAG GTTGTGTGTT ATAAAGTGGG TCAATGACAA ATATTTTTAT TGGATGATTA AAATTAAGTT   
  
  
+ CCATTTATTG GAGAAGTGAT GGAATGCAAG TTCCAAAATT GTGGAACTTT TGTTGACGTA GCAGGCTAGA   
  
  
+ GTTCCAAAAA TTAAAAAAAG GAAAGACATA TTGAGGTAAA GCCTTTTTAT TTTACAAATA GACGCACCAA   
  
  
+ GGTATAATAT AATTGACATG ATTTGAGGCG AGAGGAAGAT TCTTTTGCTA CCCAAACAAA ACACAATGAT   
  
  
+ CTTTTAAATG TAGCAATTTG GGATTGGCAA AATTTTGTCT AACCCGATTG TTGAAAGTTA TCCGATTTCA   
  
  
+ TAAAATTGGA TATCGTTATT AAATTTTCAT TCGAATATTT AAATTTGGAC ACAATGTTTT TCATATTTGG   
  
  
+ ATTTTCTAAA CTTGATTTTA ACTCAAATTT AAGTTTATTC AATTCATCTG ACCCAAAAAA TGGTTGCAAT   
  
  
+ AATGTATTTT CTTTTTGAAG TTAAATATTA TCGAATTCAT TTAACTTGTA GTTAACCCGT AATTGACCCA   
  
  
+ AACTCGACTT TTTGCTACCT TAGCAGCCAG GAAGAAGTCA TAGTATTGAC CCGTGGAACG CCTGAAGCCA   
  
  
+ ACATCACCAG ACCCACATAT TCCATCATCT TCTGATCTCC TCGTCAAAGA AGGAAGCTGA GCACAGCAAT   
  
  
+ AACTAGAAGA ACAGGAGGCA TCACACCCAA GCCAAAGGCC TTTATTTGAG CTCCCCACCT CATTCCTCGA   
  
  
+ AGCTTCCTCT TCACAATGTA AGACCATAAT CCTTCACCCC TTCTCTTTTT AAGCTCATCT TTACCTGCCC   
  
  
+ TAGTTCTCTC TCTCTCATGA GTCATCAGTA ATAGTTACCT CTTTACTTTC TCTTCCTTGA GGTATGCTTC   
  
  
+ AACTTTAATA TATATATGTA CCACTTGATT TCTCTTGTCA CTTTGCTATA ATACTTATTG TTTGGCTATC   
  
  
+ TTCCCCTGTT TTATACTCAT TTTCTTGCTT TCTATATCTG GGTTTGAATT GGGTTTCACT TATCCTCGTT   
  
  
+ GATTCTGTGG GAAATTAGTG AATTGGGTTT TGTACAGCAG GTTTATATGA ACTAGGGTTT CAAAGGTATC   
  
  
+ TACTTTTTCT CTTGTGATTA TTAGTTGATT AGGGATTTGG CCAAGATTAG CAGAATGGGT TCTGAATTTG   
  
  
+ GGGAATTCTC TGATGATGCT CTAAATGGGT ATGCTTACGT TGATATCCCT GCTTATGATG CGTCCTTAGA   
  
  
+ TTATGCCAAT TTGTTCAATT ACGAAGGCCT ATCTGAGGAT CTCACCTCAC TGAGCCTCCC AAGCCCCTTT   
  
  
+ TCTGACCCTT TGGCGTACAG TTTCACGTCC TCTTTGGGGC CGAGCCCTGG GGTTGATTCT AATGATGATA   
  
  
+ GTGATTCTGA TGATGTTCTC AAGTGCATTA GCCAAATGCT TATGGAAGAA GACATGGAGG CAAAGCCATG   
  
  
+ CATGTTTCAC GATCCTTTAG CACTTCAGGC TGCTGAGAAA CCCTTTTATG ATGCCTTAGG GAAGAAATAC   
  
  
+ CCAACTTCTC CGAACCAACA TCCTATAATT GATGATTGTT TGGATAATCC TGGTGAAAAT TCTTTGGGTT   
  
  
+ CTTCTAGTGA TTTTAGTGTT AGTCACTTTG GGTCTAGTTC AGCAAGCTCT ATTGGACCGA CAATTGTGTC   
  
  
+ TGATTTGAGT GAGCATTTTG AGCCACCCTT TGTTCAAGCA CTTCCAATTG AATCATATCC CCAACCATTG   
  
  
+ ACCCGTCCTC AATGGTCGTT TGGCCCTTCG GGTGCCTTAG ATTGCACGGC CTCTAATGGT TCAGTGATCT   
  
  
+ CATCCCTTGG TTTGCCGATG GATGTTATTA GTGTATTCAG TGAGAAAGAG TCCATGATTC AATTTCAGAA   
  
  
+ AGGGGTGGAA GAGGCTAGTA AGTTCCTTCC CAAGAATAAT AACCTTGTTA TTGATCTCAA GAACCTCACT   
  
  
+ TTTCCTAATG AAACGAAGGA GGATGATCGA GTGATGATGG TTAAGAAGGA AAAGAATGAT GTGAATTGGT   
  
  
+ CTAACTACTC AAGAGGGAGT AAGATTCACT ATCGTGAAGA CGAGGACTTT GAAGAAGGAA GGAGTAGCAA   
  
  
+ GCAGTCAGCT ATTTCTACTA CTGAGGAAGC TGAGTTGTGT GAAATTTTTG ACAAGTTTTT GCTTTGCAAT   
  
  
+ TGGTACCCTG TGAAACCTGA GGCTCATCCC ACCATGAGTT TGAACCCTGA GAAGGGCCAG TCACATGGAT   
  
  
+ TAGAAGGTGG GAGAAATGGG AAGGGTCACC CAAAGAAACA GGATAAAAGT AGTACCAACG TTGTGGATTT   
  
  
+ AAGGAATTTG CTGATGCTAT GTGCACAATC TACTGCATCT GATGACCGAA GAACTGCTGA TGGACTGCTA   
  
  
+ AAGAAAATCA GGGAGCACTC ATCTGCTGAG GGGGATGGAT TTCAAAGGTT GGCTCATTAC TTTGCTGATG   
  
  
+ CCCTAGAGGC ACGTTTAGCT GGAACTGGAT CTCACATTTA TACAGCCCTA AGTTCTCATA GGCCATCTGC   
  
  
+ TGTTGACGTG TTAAAAGCAT ATCAGTTCTA TGTTCGTGCT TGCCCATTTA AGAAGATCGT CATTCGTTGT   
  
  
+ GGTAAACATA TGATTCTAAA AGCTGCTGAA AAGGCATCAA AGCTTCATAT TATAGATTTT GGCATCCTCT   
  
  
+ ATGGATTCCA ATGGCCTAGC CTCATTCGAT GCCTCTCAGA GCGGTCTGGT GGACCTCCAA AACTGTTTAT   
  
  
+ TACAGGGATC GATCTCCCCC AGCCTGGGTT CAGGCCAGCA GAAAGAGTGG AAGCAACAGG GAGACGCTTG   
  
  
+ GCTAAGTACT GTGAGCGGTA TAATGTGCCA TTTGAGTATC ATGCCATTGC TCAGAAGTGG GAAACAATCA   
  
  
+ AACCAGGGGA TCTCAAGTTA GGAAGTAGGG ATGATGATGA AGTTGTCGTG GTGAACTGTC TCTGTAGGTT   
  
  
+ CAAGAACCTC CTTGACGAGA CAATGGTGGT GGATAGTCCA AGGAACACAG TTTTAAACCT GATTAGAAGG   
  
  
+ GTAAAACCCG ATATTTTTGT GCATGGCATT GTAAATGGTT CCTACAACAT CCCTTTCTTT GTGACACGTT   
  
  
+ TTAGAGAAGC CCTCTTTCAT TATTCCACTC TTTTCGACAT GTTAGATGCC AACGCCTCTA GGGAGGAGCC   
  
  
+ CGAGAGGTTG ATATTCGAGA AGGCATTCTA TGGGAGGGAG ATTATGAATG TGGTGGCATG TGAGGGCACA   
  
  
+ GAGAGGGTGG AAAGGCCAGA GACATACAAG CAATGGCACG TTAGGCATAG CAGGGCAGGG TTTCGGCAAC   
  
  
+ TACCATTGGA TCCCAAGTTG ATCGAGAAAA TGAGGTTTAA GGCCAAGGCA GACCACCACA AGGATTTCGT   
  
  
+ GATTGATGTG GATGGACATT GGGCAATTCA GGGATGGAAG GGGCGGATTG CCTGTGCCAT CTCTGCATGG   
  
  
+ GTTCTGGCTT G  

- -Up\_Stream \_Len000GACAAG TCGTGATCCC TTATTCCAAA AACGGGACGT ATGATACTTT AGATTGGTTT   
  
  
- TTGTAAGGTA GAACCTTCCG ACATCGGAAC ACCTAAAACT TAAGCGAGGG TTCTTGGTTA CTTCATCGCG   
  
  
- TCTTCCGGTT ATGAGTTAAA GTCTCAGGTG TAGTTGAACA AGTTACGGTC ATATAAAGTC TTGTAATTAA   
  
  
- TGTATGACAA ACCAACCTCT TTTTTACCAA CAGAGCTTTT GATCAAAAAA TTAAAAAACA ATAAATCGTG   
  
  
- CCATTTCTTA CCTTTAATAA AAGTACGCTC TTTTTTCCTT CAACCAAAAA GAAAAAAGTT GAGAAAACTA   
  
  
- CTATATTAGT CAGCTTTAAA TCCACGTGGT AGACTGAAGT TGAGAACATG CTGGAGACTA ACGAACGAAT   
  
  
- CTTCTTGTTC TCCTAACTGT CTATTCCTCC TCACCCACCA AATATGTCTC TTAACTCACC TACCAATATT   
  
  
- TCTTCAGCTA GAGTGTATTC ATTGAAGCAA TGCTAATATT TATAAAAGTA ACTTCTTTCT GCTAATCTAT   
  
  
- ATTAATTCTT CCATGTAGAT TCGTTTAAAC CGACTCGTCT CAACCTCCCG GTGATCCCAG TGTAACACAG   
  
  
- AACGCGTTGT TCGAATCTGG TAAGCGTTGC CTACGGAGTG CACCTCGTCC ACCCTGGGCG CACCTTCAAC   
  
  
- GTACCGTTCT TAAGAGACCG TCGTTCCTCT TAACTCCCCC CTGTGCGCAC ACTGGTGCGG TCACGCCAGG   
  
  
- GGGAGAGCAC CGTCTAAACC ACTAAAAAAA AAACAAAAAA AAACTTCATT ATATATTAAA AATATACTAA   
  
  
- TTTTTTTATC CAACACACAA TATTTCACCC AGTTACTGTT TATAAAAATA ACCTACTAAT TTTAATTCAA   
  
  
- GGTAAATAAC CTCTTCACTA CCTTACGTTC AAGGTTTTAA CACCTTGAAA ACAACTGCAT CGTCCGATCT   
  
  
- CAAGGTTTTT AATTTTTTTC CTTTCTGTAT AACTCCATTT CGGAAAAATA AAATGTTTAT CTGCGTGGTT   
  
  
- CCATATTATA TTAACTGTAC TAAACTCCGC TCTCCTTCTA AGAAAACGAT GGGTTTGTTT TGTGTTACTA   
  
  
- GAAAATTTAC ATCGTTAAAC CCTAACCGTT TTAAAACAGA TTGGGCTAAC AACTTTCAAT AGGCTAAAGT   
  
  
- ATTTTAACCT ATAGCAATAA TTTAAAAGTA AGCTTATAAA TTTAAACCTG TGTTACAAAA AGTATAAACC   
  
  
- TAAAAGATTT GAACTAAAAT TGAGTTTAAA TTCAAATAAG TTAAGTAGAC TGGGTTTTTT ACCAACGTTA   
  
  
- TTACATAAAA GAAAAACTTC AATTTATAAT AGCTTAAGTA AATTGAACAT CAATTGGGCA TTAACTGGGT   
  
  
- TTGAGCTGAA AAACGATGGA ATCGTCGGTC CTTCTTCAGT ATCATAACTG GGCACCTTGC GGACTTCGGT   
  
  
- TGTAGTGGTC TGGGTGTATA AGGTAGTAGA AGACTAGAGG AGCAGTTTCT TCCTTCGACT CGTGTCGTTA   
  
  
- TTGATCTTCT TGTCCTCCGT AGTGTGGGTT CGGTTTCCGG AAATAAACTC GAGGGGTGGA GTAAGGAGCT   
  
  
- TCGAAGGAGA AGTGTTACAT TCTGGTATTA GGAAGTGGGG AAGAGAAAAA TTCGAGTAGA AATGGACGGG   
  
  
- ATCAAGAGAG AGAGAGTACT CAGTAGTCAT TATCAATGGA GAAATGAAAG AGAAGGAACT CCATACGAAG   
  
  
- TTGAAATTAT ATATATACAT GGTGAACTAA AGAGAACAGT GAAACGATAT TATGAATAAC AAACCGATAG   
  
  
- AAGGGGACAA AATATGAGTA AAAGAACGAA AGATATAGAC CCAAACTTAA CCCAAAGTGA ATAGGAGCAA   
  
  
- CTAAGACACC CTTTAATCAC TTAACCCAAA ACATGTCGTC CAAATATACT TGATCCCAAA GTTTCCATAG   
  
  
- ATGAAAAAGA GAACACTAAT AATCAACTAA TCCCTAAACC GGTTCTAATC GTCTTACCCA AGACTTAAAC   
  
  
- CCCTTAAGAG ACTACTACGA GATTTACCCA TACGAATGCA ACTATAGGGA CGAATACTAC GCAGGAATCT   
  
  
- AATACGGTTA AACAAGTTAA TGCTTCCGGA TAGACTCCTA GAGTGGAGTG ACTCGGAGGG TTCGGGGAAA   
  
  
- AGACTGGGAA ACCGCATGTC AAAGTGCAGG AGAAACCCCG GCTCGGGACC CCAACTAAGA TTACTACTAT   
  
  
- CACTAAGACT ACTACAAGAG TTCACGTAAT CGGTTTACGA ATACCTTCTT CTGTACCTCC GTTTCGGTAC   
  
  
- GTACAAAGTG CTAGGAAATC GTGAAGTCCG ACGACTCTTT GGGAAAATAC TACGGAATCC CTTCTTTATG   
  
  
- GGTTGAAGAG GCTTGGTTGT AGGATATTAA CTACTAACAA ACCTATTAGG ACCACTTTTA AGAAACCCAA   
  
  
- GAAGATCACT AAAATCACAA TCAGTGAAAC CCAGATCAAG TCGTTCGAGA TAACCTGGCT GTTAACACAG   
  
  
- ACTAAACTCA CTCGTAAAAC TCGGTGGGAA ACAAGTTCGT GAAGGTTAAC TTAGTATAGG GGTTGGTAAC   
  
  
- TGGGCAGGAG TTACCAGCAA ACCGGGAAGC CCACGGAATC TAACGTGCCG GAGATTACCA AGTCACTAGA   
  
  
- GTAGGGAACC AAACGGCTAC CTACAATAAT CACATAAGTC ACTCTTTCTC AGGTACTAAG TTAAAGTCTT   
  
  
- TCCCCACCTT CTCCGATCAT TCAAGGAAGG GTTCTTATTA TTGGAACAAT AACTAGAGTT CTTGGAGTGA   
  
  
- AAAGGATTAC TTTGCTTCCT CCTACTAGCT CACTACTACC AATTCTTCCT TTTCTTACTA CACTTAACCA   
  
  
- GATTGATGAG TTCTCCCTCA TTCTAAGTGA TAGCACTTCT GCTCCTGAAA CTTCTTCCTT CCTCATCGTT   
  
  
- CGTCAGTCGA TAAAGATGAT GACTCCTTCG ACTCAACACA CTTTAAAAAC TGTTCAAAAA CGAAACGTTA   
  
  
- ACCATGGGAC ACTTTGGACT CCGAGTAGGG TGGTACTCAA ACTTGGGACT CTTCCCGGTC AGTGTACCTA   
  
  
- ATCTTCCACC CTCTTTACCC TTCCCAGTGG GTTTCTTTGT CCTATTTTCA TCATGGTTGC AACACCTAAA   
  
  
- TTCCTTAAAC GACTACGATA CACGTGTTAG ATGACGTAGA CTACTGGCTT CTTGACGACT ACCTGACGAT   
  
  
- TTCTTTTAGT CCCTCGTGAG TAGACGACTC CCCCTACCTA AAGTTTCCAA CCGAGTAATG AAACGACTAC   
  
  
- GGGATCTCCG TGCAAATCGA CCTTGACCTA GAGTGTAAAT ATGTCGGGAT TCAAGAGTAT CCGGTAGACG   
  
  
- ACAACTGCAC AATTTTCGTA TAGTCAAGAT ACAAGCACGA ACGGGTAAAT TCTTCTAGCA GTAAGCAACA   
  
  
- CCATTTGTAT ACTAAGATTT TCGACGACTT TTCCGTAGTT TCGAAGTATA ATATCTAAAA CCGTAGGAGA   
  
  
- TACCTAAGGT TACCGGATCG GAGTAAGCTA CGGAGAGTCT CGCCAGACCA CCTGGAGGTT TTGACAAATA   
  
  
- ATGTCCCTAG CTAGAGGGGG TCGGACCCAA GTCCGGTCGT CTTTCTCACC TTCGTTGTCC CTCTGCGAAC   
  
  
- CGATTCATGA CACTCGCCAT ATTACACGGT AAACTCATAG TACGGTAACG AGTCTTCACC CTTTGTTAGT   
  
  
- TTGGTCCCCT AGAGTTCAAT CCTTCATCCC TACTACTACT TCAACAGCAC CACTTGACAG AGACATCCAA   
  
  
- GTTCTTGGAG GAACTGCTCT GTTACCACCA CCTATCAGGT TCCTTGTGTC AAAATTTGGA CTAATCTTCC   
  
  
- CATTTTGGGC TATAAAAACA CGTACCGTAA CATTTACCAA GGATGTTGTA GGGAAAGAAA CACTGTGCAA   
  
  
- AATCTCTTCG GGAGAAAGTA ATAAGGTGAG AAAAGCTGTA CAATCTACGG TTGCGGAGAT CCCTCCTCGG   
  
  
- GCTCTCCAAC TATAAGCTCT TCCGTAAGAT ACCCTCCCTC TAATACTTAC ACCACCGTAC ACTCCCGTGT   
  
  
- CTCTCCCACC TTTCCGGTCT CTGTATGTTC GTTACCGTGC AATCCGTATC GTCCCGTCCC AAAGCCGTTG   
  
  
- ATGGTAACCT AGGGTTCAAC TAGCTCTTTT ACTCCAAATT CCGGTTCCGT CTGGTGGTGT TCCTAAAGCA   
  
  
- CTAACTACAC CTACCTGTAA CCCGTTAAGT CCCTACCTTC CCCGCCTAAC GGACACGGTA GAGACGTACC   
  
  
- CAAGACCGAA C

+     MYC

| Site Name | Organism | Position | Strand | Matrix score. | sequence | function |
| --- | --- | --- | --- | --- | --- | --- |
| MYC | Arabidopsis thaliana | 4051 | + | 6 | CATGTG |  |
| MYC | Arabidopsis thaliana | 2277 | - | 6 | CATTTG |  |
| MYC | Arabidopsis thaliana | 2515 | - | 6 | CAATTG |  |
| MYC | Arabidopsis thaliana | 2569 | - | 6 | CAATTG |  |
| MYC | Arabidopsis thaliana | 3673 | + | 6 | CATTTG |  |
| MYC | Arabidopsis thaliana | 3076 | - | 6 | CATGTG |  |
| MYC | Arabidopsis thaliana | 3011 | - | 6 | CAATTG |  |

>HU07G02248.1   
+ -Up\_Stream \_Len000CTGTTC AGCACTAGGG AATAAGGTTT TTGCCCTGCA TACTATGAAA TCTAACCAAA   
  
  
+ AACATTCCAT CTTGGAAGGC TGTAGCCTTG TGGATTTTGA ATTCGCTCCC AAGAACCAAT GAAGTAGCGC   
  
  
+ AGAAGGCCAA TACTCAATTT CAGAGTCCAC ATCAACTTGT TCAATGCCAG TATATTTCAG AACATTAATT   
  
  
+ ACATACTGTT TGGTTGGAGA AAAAATGGTT GTCTCGAAAA CTAGTTTTTT AATTTTTTGT TATTTAGCAC   
  
  
+ GGTAAAGAAT GGAAATTATT TTCATGCGAG AAAAAAGGAA GTTGGTTTTT CTTTTTTCAA CTCTTTTGAT   
  
  
+ GATATAATCA GTCGAAATTT AGGTGCACCA TCTGACTTCA ACTCTTGTAC GACCTCTGAT TGCTTGCTTA   
  
  
+ GAAGAACAAG AGGATTGACA GATAAGGAGG AGTGGGTGGT TTATACAGAG AATTGAGTGG ATGGTTATAA   
  
  
+ AGAAGTCGAT CTCACATAAG TAACTTCGTT ACGATTATAA ATATTTTCAT TGAAGAAAGA CGATTAGATA   
  
  
+ TAATTAAGAA GGTACATCTA AGCAAATTTG GCTGAGCAGA GTTGGAGGGC CACTAGGGTC ACATTGTGTC   
  
  
+ TTGCGCAACA AGCTTAGACC ATTCGCAACG GATGCCTCAC GTGGAGCAGG TGGGACCCGC GTGGAAGTTG   
  
  
+ CATGGCAAGA ATTCTCTGGC AGCAAGGAGA ATTGAGGGGG GACACGCGTG TGACCACGCC AGTGCGGTCC   
  
  
+ CCCTCTCGTG GCAGATTTGG TGATTTTTTT TTTGTTTTTT TTTGAAGTAA TATATAATTT TTATATGATT   
  
  
+ AAAAAAATAG GTTGTGTGTT ATAAAGTGGG TCAATGACAA ATATTTTTAT TGGATGATTA AAATTAAGTT   
  
  
+ CCATTTATTG GAGAAGTGAT GGAATGCAAG TTCCAAAATT GTGGAACTTT TGTTGACGTA GCAGGCTAGA   
  
  
+ GTTCCAAAAA TTAAAAAAAG GAAAGACATA TTGAGGTAAA GCCTTTTTAT TTTACAAATA GACGCACCAA   
  
  
+ GGTATAATAT AATTGACATG ATTTGAGGCG AGAGGAAGAT TCTTTTGCTA CCCAAACAAA ACACAATGAT   
  
  
+ CTTTTAAATG TAGCAATTTG GGATTGGCAA AATTTTGTCT AACCCGATTG TTGAAAGTTA TCCGATTTCA   
  
  
+ TAAAATTGGA TATCGTTATT AAATTTTCAT TCGAATATTT AAATTTGGAC ACAATGTTTT TCATATTTGG   
  
  
+ ATTTTCTAAA CTTGATTTTA ACTCAAATTT AAGTTTATTC AATTCATCTG ACCCAAAAAA TGGTTGCAAT   
  
  
+ AATGTATTTT CTTTTTGAAG TTAAATATTA TCGAATTCAT TTAACTTGTA GTTAACCCGT AATTGACCCA   
  
  
+ AACTCGACTT TTTGCTACCT TAGCAGCCAG GAAGAAGTCA TAGTATTGAC CCGTGGAACG CCTGAAGCCA   
  
  
+ ACATCACCAG ACCCACATAT TCCATCATCT TCTGATCTCC TCGTCAAAGA AGGAAGCTGA GCACAGCAAT   
  
  
+ AACTAGAAGA ACAGGAGGCA TCACACCCAA GCCAAAGGCC TTTATTTGAG CTCCCCACCT CATTCCTCGA   
  
  
+ AGCTTCCTCT TCACAATGTA AGACCATAAT CCTTCACCCC TTCTCTTTTT AAGCTCATCT TTACCTGCCC   
  
  
+ TAGTTCTCTC TCTCTCATGA GTCATCAGTA ATAGTTACCT CTTTACTTTC TCTTCCTTGA GGTATGCTTC   
  
  
+ AACTTTAATA TATATATGTA CCACTTGATT TCTCTTGTCA CTTTGCTATA ATACTTATTG TTTGGCTATC   
  
  
+ TTCCCCTGTT TTATACTCAT TTTCTTGCTT TCTATATCTG GGTTTGAATT GGGTTTCACT TATCCTCGTT   
  
  
+ GATTCTGTGG GAAATTAGTG AATTGGGTTT TGTACAGCAG GTTTATATGA ACTAGGGTTT CAAAGGTATC   
  
  
+ TACTTTTTCT CTTGTGATTA TTAGTTGATT AGGGATTTGG CCAAGATTAG CAGAATGGGT TCTGAATTTG   
  
  
+ GGGAATTCTC TGATGATGCT CTAAATGGGT ATGCTTACGT TGATATCCCT GCTTATGATG CGTCCTTAGA   
  
  
+ TTATGCCAAT TTGTTCAATT ACGAAGGCCT ATCTGAGGAT CTCACCTCAC TGAGCCTCCC AAGCCCCTTT   
  
  
+ TCTGACCCTT TGGCGTACAG TTTCACGTCC TCTTTGGGGC CGAGCCCTGG GGTTGATTCT AATGATGATA   
  
  
+ GTGATTCTGA TGATGTTCTC AAGTGCATTA GCCAAATGCT TATGGAAGAA GACATGGAGG CAAAGCCATG   
  
  
+ CATGTTTCAC GATCCTTTAG CACTTCAGGC TGCTGAGAAA CCCTTTTATG ATGCCTTAGG GAAGAAATAC   
  
  
+ CCAACTTCTC CGAACCAACA TCCTATAATT GATGATTGTT TGGATAATCC TGGTGAAAAT TCTTTGGGTT   
  
  
+ CTTCTAGTGA TTTTAGTGTT AGTCACTTTG GGTCTAGTTC AGCAAGCTCT ATTGGACCGA CAATTGTGTC   
  
  
+ TGATTTGAGT GAGCATTTTG AGCCACCCTT TGTTCAAGCA CTTCCAATTG AATCATATCC CCAACCATTG   
  
  
+ ACCCGTCCTC AATGGTCGTT TGGCCCTTCG GGTGCCTTAG ATTGCACGGC CTCTAATGGT TCAGTGATCT   
  
  
+ CATCCCTTGG TTTGCCGATG GATGTTATTA GTGTATTCAG TGAGAAAGAG TCCATGATTC AATTTCAGAA   
  
  
+ AGGGGTGGAA GAGGCTAGTA AGTTCCTTCC CAAGAATAAT AACCTTGTTA TTGATCTCAA GAACCTCACT   
  
  
+ TTTCCTAATG AAACGAAGGA GGATGATCGA GTGATGATGG TTAAGAAGGA AAAGAATGAT GTGAATTGGT   
  
  
+ CTAACTACTC AAGAGGGAGT AAGATTCACT ATCGTGAAGA CGAGGACTTT GAAGAAGGAA GGAGTAGCAA   
  
  
+ GCAGTCAGCT ATTTCTACTA CTGAGGAAGC TGAGTTGTGT GAAATTTTTG ACAAGTTTTT GCTTTGCAAT   
  
  
+ TGGTACCCTG TGAAACCTGA GGCTCATCCC ACCATGAGTT TGAACCCTGA GAAGGGCCAG TCACATGGAT   
  
  
+ TAGAAGGTGG GAGAAATGGG AAGGGTCACC CAAAGAAACA GGATAAAAGT AGTACCAACG TTGTGGATTT   
  
  
+ AAGGAATTTG CTGATGCTAT GTGCACAATC TACTGCATCT GATGACCGAA GAACTGCTGA TGGACTGCTA   
  
  
+ AAGAAAATCA GGGAGCACTC ATCTGCTGAG GGGGATGGAT TTCAAAGGTT GGCTCATTAC TTTGCTGATG   
  
  
+ CCCTAGAGGC ACGTTTAGCT GGAACTGGAT CTCACATTTA TACAGCCCTA AGTTCTCATA GGCCATCTGC   
  
  
+ TGTTGACGTG TTAAAAGCAT ATCAGTTCTA TGTTCGTGCT TGCCCATTTA AGAAGATCGT CATTCGTTGT   
  
  
+ GGTAAACATA TGATTCTAAA AGCTGCTGAA AAGGCATCAA AGCTTCATAT TATAGATTTT GGCATCCTCT   
  
  
+ ATGGATTCCA ATGGCCTAGC CTCATTCGAT GCCTCTCAGA GCGGTCTGGT GGACCTCCAA AACTGTTTAT   
  
  
+ TACAGGGATC GATCTCCCCC AGCCTGGGTT CAGGCCAGCA GAAAGAGTGG AAGCAACAGG GAGACGCTTG   
  
  
+ GCTAAGTACT GTGAGCGGTA TAATGTGCCA TTTGAGTATC ATGCCATTGC TCAGAAGTGG GAAACAATCA   
  
  
+ AACCAGGGGA TCTCAAGTTA GGAAGTAGGG ATGATGATGA AGTTGTCGTG GTGAACTGTC TCTGTAGGTT   
  
  
+ CAAGAACCTC CTTGACGAGA CAATGGTGGT GGATAGTCCA AGGAACACAG TTTTAAACCT GATTAGAAGG   
  
  
+ GTAAAACCCG ATATTTTTGT GCATGGCATT GTAAATGGTT CCTACAACAT CCCTTTCTTT GTGACACGTT   
  
  
+ TTAGAGAAGC CCTCTTTCAT TATTCCACTC TTTTCGACAT GTTAGATGCC AACGCCTCTA GGGAGGAGCC   
  
  
+ CGAGAGGTTG ATATTCGAGA AGGCATTCTA TGGGAGGGAG ATTATGAATG TGGTGGCATG TGAGGGCACA   
  
  
+ GAGAGGGTGG AAAGGCCAGA GACATACAAG CAATGGCACG TTAGGCATAG CAGGGCAGGG TTTCGGCAAC   
  
  
+ TACCATTGGA TCCCAAGTTG ATCGAGAAAA TGAGGTTTAA GGCCAAGGCA GACCACCACA AGGATTTCGT   
  
  
+ GATTGATGTG GATGGACATT GGGCAATTCA GGGATGGAAG GGGCGGATTG CCTGTGCCAT CTCTGCATGG   
  
  
+ GTTCTGGCTT G  

- -Up\_Stream \_Len000GACAAG TCGTGATCCC TTATTCCAAA AACGGGACGT ATGATACTTT AGATTGGTTT   
  
  
- TTGTAAGGTA GAACCTTCCG ACATCGGAAC ACCTAAAACT TAAGCGAGGG TTCTTGGTTA CTTCATCGCG   
  
  
- TCTTCCGGTT ATGAGTTAAA GTCTCAGGTG TAGTTGAACA AGTTACGGTC ATATAAAGTC TTGTAATTAA   
  
  
- TGTATGACAA ACCAACCTCT TTTTTACCAA CAGAGCTTTT GATCAAAAAA TTAAAAAACA ATAAATCGTG   
  
  
- CCATTTCTTA CCTTTAATAA AAGTACGCTC TTTTTTCCTT CAACCAAAAA GAAAAAAGTT GAGAAAACTA   
  
  
- CTATATTAGT CAGCTTTAAA TCCACGTGGT AGACTGAAGT TGAGAACATG CTGGAGACTA ACGAACGAAT   
  
  
- CTTCTTGTTC TCCTAACTGT CTATTCCTCC TCACCCACCA AATATGTCTC TTAACTCACC TACCAATATT   
  
  
- TCTTCAGCTA GAGTGTATTC ATTGAAGCAA TGCTAATATT TATAAAAGTA ACTTCTTTCT GCTAATCTAT   
  
  
- ATTAATTCTT CCATGTAGAT TCGTTTAAAC CGACTCGTCT CAACCTCCCG GTGATCCCAG TGTAACACAG   
  
  
- AACGCGTTGT TCGAATCTGG TAAGCGTTGC CTACGGAGTG CACCTCGTCC ACCCTGGGCG CACCTTCAAC   
  
  
- GTACCGTTCT TAAGAGACCG TCGTTCCTCT TAACTCCCCC CTGTGCGCAC ACTGGTGCGG TCACGCCAGG   
  
  
- GGGAGAGCAC CGTCTAAACC ACTAAAAAAA AAACAAAAAA AAACTTCATT ATATATTAAA AATATACTAA   
  
  
- TTTTTTTATC CAACACACAA TATTTCACCC AGTTACTGTT TATAAAAATA ACCTACTAAT TTTAATTCAA   
  
  
- GGTAAATAAC CTCTTCACTA CCTTACGTTC AAGGTTTTAA CACCTTGAAA ACAACTGCAT CGTCCGATCT   
  
  
- CAAGGTTTTT AATTTTTTTC CTTTCTGTAT AACTCCATTT CGGAAAAATA AAATGTTTAT CTGCGTGGTT   
  
  
- CCATATTATA TTAACTGTAC TAAACTCCGC TCTCCTTCTA AGAAAACGAT GGGTTTGTTT TGTGTTACTA   
  
  
- GAAAATTTAC ATCGTTAAAC CCTAACCGTT TTAAAACAGA TTGGGCTAAC AACTTTCAAT AGGCTAAAGT   
  
  
- ATTTTAACCT ATAGCAATAA TTTAAAAGTA AGCTTATAAA TTTAAACCTG TGTTACAAAA AGTATAAACC   
  
  
- TAAAAGATTT GAACTAAAAT TGAGTTTAAA TTCAAATAAG TTAAGTAGAC TGGGTTTTTT ACCAACGTTA   
  
  
- TTACATAAAA GAAAAACTTC AATTTATAAT AGCTTAAGTA AATTGAACAT CAATTGGGCA TTAACTGGGT   
  
  
- TTGAGCTGAA AAACGATGGA ATCGTCGGTC CTTCTTCAGT ATCATAACTG GGCACCTTGC GGACTTCGGT   
  
  
- TGTAGTGGTC TGGGTGTATA AGGTAGTAGA AGACTAGAGG AGCAGTTTCT TCCTTCGACT CGTGTCGTTA   
  
  
- TTGATCTTCT TGTCCTCCGT AGTGTGGGTT CGGTTTCCGG AAATAAACTC GAGGGGTGGA GTAAGGAGCT   
  
  
- TCGAAGGAGA AGTGTTACAT TCTGGTATTA GGAAGTGGGG AAGAGAAAAA TTCGAGTAGA AATGGACGGG   
  
  
- ATCAAGAGAG AGAGAGTACT CAGTAGTCAT TATCAATGGA GAAATGAAAG AGAAGGAACT CCATACGAAG   
  
  
- TTGAAATTAT ATATATACAT GGTGAACTAA AGAGAACAGT GAAACGATAT TATGAATAAC AAACCGATAG   
  
  
- AAGGGGACAA AATATGAGTA AAAGAACGAA AGATATAGAC CCAAACTTAA CCCAAAGTGA ATAGGAGCAA   
  
  
- CTAAGACACC CTTTAATCAC TTAACCCAAA ACATGTCGTC CAAATATACT TGATCCCAAA GTTTCCATAG   
  
  
- ATGAAAAAGA GAACACTAAT AATCAACTAA TCCCTAAACC GGTTCTAATC GTCTTACCCA AGACTTAAAC   
  
  
- CCCTTAAGAG ACTACTACGA GATTTACCCA TACGAATGCA ACTATAGGGA CGAATACTAC GCAGGAATCT   
  
  
- AATACGGTTA AACAAGTTAA TGCTTCCGGA TAGACTCCTA GAGTGGAGTG ACTCGGAGGG TTCGGGGAAA   
  
  
- AGACTGGGAA ACCGCATGTC AAAGTGCAGG AGAAACCCCG GCTCGGGACC CCAACTAAGA TTACTACTAT   
  
  
- CACTAAGACT ACTACAAGAG TTCACGTAAT CGGTTTACGA ATACCTTCTT CTGTACCTCC GTTTCGGTAC   
  
  
- GTACAAAGTG CTAGGAAATC GTGAAGTCCG ACGACTCTTT GGGAAAATAC TACGGAATCC CTTCTTTATG   
  
  
- GGTTGAAGAG GCTTGGTTGT AGGATATTAA CTACTAACAA ACCTATTAGG ACCACTTTTA AGAAACCCAA   
  
  
- GAAGATCACT AAAATCACAA TCAGTGAAAC CCAGATCAAG TCGTTCGAGA TAACCTGGCT GTTAACACAG   
  
  
- ACTAAACTCA CTCGTAAAAC TCGGTGGGAA ACAAGTTCGT GAAGGTTAAC TTAGTATAGG GGTTGGTAAC   
  
  
- TGGGCAGGAG TTACCAGCAA ACCGGGAAGC CCACGGAATC TAACGTGCCG GAGATTACCA AGTCACTAGA   
  
  
- GTAGGGAACC AAACGGCTAC CTACAATAAT CACATAAGTC ACTCTTTCTC AGGTACTAAG TTAAAGTCTT   
  
  
- TCCCCACCTT CTCCGATCAT TCAAGGAAGG GTTCTTATTA TTGGAACAAT AACTAGAGTT CTTGGAGTGA   
  
  
- AAAGGATTAC TTTGCTTCCT CCTACTAGCT CACTACTACC AATTCTTCCT TTTCTTACTA CACTTAACCA   
  
  
- GATTGATGAG TTCTCCCTCA TTCTAAGTGA TAGCACTTCT GCTCCTGAAA CTTCTTCCTT CCTCATCGTT   
  
  
- CGTCAGTCGA TAAAGATGAT GACTCCTTCG ACTCAACACA CTTTAAAAAC TGTTCAAAAA CGAAACGTTA   
  
  
- ACCATGGGAC ACTTTGGACT CCGAGTAGGG TGGTACTCAA ACTTGGGACT CTTCCCGGTC AGTGTACCTA   
  
  
- ATCTTCCACC CTCTTTACCC TTCCCAGTGG GTTTCTTTGT CCTATTTTCA TCATGGTTGC AACACCTAAA   
  
  
- TTCCTTAAAC GACTACGATA CACGTGTTAG ATGACGTAGA CTACTGGCTT CTTGACGACT ACCTGACGAT   
  
  
- TTCTTTTAGT CCCTCGTGAG TAGACGACTC CCCCTACCTA AAGTTTCCAA CCGAGTAATG AAACGACTAC   
  
  
- GGGATCTCCG TGCAAATCGA CCTTGACCTA GAGTGTAAAT ATGTCGGGAT TCAAGAGTAT CCGGTAGACG   
  
  
- ACAACTGCAC AATTTTCGTA TAGTCAAGAT ACAAGCACGA ACGGGTAAAT TCTTCTAGCA GTAAGCAACA   
  
  
- CCATTTGTAT ACTAAGATTT TCGACGACTT TTCCGTAGTT TCGAAGTATA ATATCTAAAA CCGTAGGAGA   
  
  
- TACCTAAGGT TACCGGATCG GAGTAAGCTA CGGAGAGTCT CGCCAGACCA CCTGGAGGTT TTGACAAATA   
  
  
- ATGTCCCTAG CTAGAGGGGG TCGGACCCAA GTCCGGTCGT CTTTCTCACC TTCGTTGTCC CTCTGCGAAC   
  
  
- CGATTCATGA CACTCGCCAT ATTACACGGT AAACTCATAG TACGGTAACG AGTCTTCACC CTTTGTTAGT   
  
  
- TTGGTCCCCT AGAGTTCAAT CCTTCATCCC TACTACTACT TCAACAGCAC CACTTGACAG AGACATCCAA   
  
  
- GTTCTTGGAG GAACTGCTCT GTTACCACCA CCTATCAGGT TCCTTGTGTC AAAATTTGGA CTAATCTTCC   
  
  
- CATTTTGGGC TATAAAAACA CGTACCGTAA CATTTACCAA GGATGTTGTA GGGAAAGAAA CACTGTGCAA   
  
  
- AATCTCTTCG GGAGAAAGTA ATAAGGTGAG AAAAGCTGTA CAATCTACGG TTGCGGAGAT CCCTCCTCGG   
  
  
- GCTCTCCAAC TATAAGCTCT TCCGTAAGAT ACCCTCCCTC TAATACTTAC ACCACCGTAC ACTCCCGTGT   
  
  
- CTCTCCCACC TTTCCGGTCT CTGTATGTTC GTTACCGTGC AATCCGTATC GTCCCGTCCC AAAGCCGTTG   
  
  
- ATGGTAACCT AGGGTTCAAC TAGCTCTTTT ACTCCAAATT CCGGTTCCGT CTGGTGGTGT TCCTAAAGCA   
  
  
- CTAACTACAC CTACCTGTAA CCCGTTAAGT CCCTACCTTC CCCGCCTAAC GGACACGGTA GAGACGTACC   
  
  
- CAAGACCGAA C

+     Myb-binding site

| Site Name | Organism | Position | Strand | Matrix score. | sequence | function |
| --- | --- | --- | --- | --- | --- | --- |
| Myb-binding site | Nicotiana tabacum | 3364 | - | 6 | CAACAG |  |
| Myb-binding site | Nicotiana tabacum | 3628 | + | 6 | CAACAG |  |

>HU07G02248.1   
+ -Up\_Stream \_Len000CTGTTC AGCACTAGGG AATAAGGTTT TTGCCCTGCA TACTATGAAA TCTAACCAAA   
  
  
+ AACATTCCAT CTTGGAAGGC TGTAGCCTTG TGGATTTTGA ATTCGCTCCC AAGAACCAAT GAAGTAGCGC   
  
  
+ AGAAGGCCAA TACTCAATTT CAGAGTCCAC ATCAACTTGT TCAATGCCAG TATATTTCAG AACATTAATT   
  
  
+ ACATACTGTT TGGTTGGAGA AAAAATGGTT GTCTCGAAAA CTAGTTTTTT AATTTTTTGT TATTTAGCAC   
  
  
+ GGTAAAGAAT GGAAATTATT TTCATGCGAG AAAAAAGGAA GTTGGTTTTT CTTTTTTCAA CTCTTTTGAT   
  
  
+ GATATAATCA GTCGAAATTT AGGTGCACCA TCTGACTTCA ACTCTTGTAC GACCTCTGAT TGCTTGCTTA   
  
  
+ GAAGAACAAG AGGATTGACA GATAAGGAGG AGTGGGTGGT TTATACAGAG AATTGAGTGG ATGGTTATAA   
  
  
+ AGAAGTCGAT CTCACATAAG TAACTTCGTT ACGATTATAA ATATTTTCAT TGAAGAAAGA CGATTAGATA   
  
  
+ TAATTAAGAA GGTACATCTA AGCAAATTTG GCTGAGCAGA GTTGGAGGGC CACTAGGGTC ACATTGTGTC   
  
  
+ TTGCGCAACA AGCTTAGACC ATTCGCAACG GATGCCTCAC GTGGAGCAGG TGGGACCCGC GTGGAAGTTG   
  
  
+ CATGGCAAGA ATTCTCTGGC AGCAAGGAGA ATTGAGGGGG GACACGCGTG TGACCACGCC AGTGCGGTCC   
  
  
+ CCCTCTCGTG GCAGATTTGG TGATTTTTTT TTTGTTTTTT TTTGAAGTAA TATATAATTT TTATATGATT   
  
  
+ AAAAAAATAG GTTGTGTGTT ATAAAGTGGG TCAATGACAA ATATTTTTAT TGGATGATTA AAATTAAGTT   
  
  
+ CCATTTATTG GAGAAGTGAT GGAATGCAAG TTCCAAAATT GTGGAACTTT TGTTGACGTA GCAGGCTAGA   
  
  
+ GTTCCAAAAA TTAAAAAAAG GAAAGACATA TTGAGGTAAA GCCTTTTTAT TTTACAAATA GACGCACCAA   
  
  
+ GGTATAATAT AATTGACATG ATTTGAGGCG AGAGGAAGAT TCTTTTGCTA CCCAAACAAA ACACAATGAT   
  
  
+ CTTTTAAATG TAGCAATTTG GGATTGGCAA AATTTTGTCT AACCCGATTG TTGAAAGTTA TCCGATTTCA   
  
  
+ TAAAATTGGA TATCGTTATT AAATTTTCAT TCGAATATTT AAATTTGGAC ACAATGTTTT TCATATTTGG   
  
  
+ ATTTTCTAAA CTTGATTTTA ACTCAAATTT AAGTTTATTC AATTCATCTG ACCCAAAAAA TGGTTGCAAT   
  
  
+ AATGTATTTT CTTTTTGAAG TTAAATATTA TCGAATTCAT TTAACTTGTA GTTAACCCGT AATTGACCCA   
  
  
+ AACTCGACTT TTTGCTACCT TAGCAGCCAG GAAGAAGTCA TAGTATTGAC CCGTGGAACG CCTGAAGCCA   
  
  
+ ACATCACCAG ACCCACATAT TCCATCATCT TCTGATCTCC TCGTCAAAGA AGGAAGCTGA GCACAGCAAT   
  
  
+ AACTAGAAGA ACAGGAGGCA TCACACCCAA GCCAAAGGCC TTTATTTGAG CTCCCCACCT CATTCCTCGA   
  
  
+ AGCTTCCTCT TCACAATGTA AGACCATAAT CCTTCACCCC TTCTCTTTTT AAGCTCATCT TTACCTGCCC   
  
  
+ TAGTTCTCTC TCTCTCATGA GTCATCAGTA ATAGTTACCT CTTTACTTTC TCTTCCTTGA GGTATGCTTC   
  
  
+ AACTTTAATA TATATATGTA CCACTTGATT TCTCTTGTCA CTTTGCTATA ATACTTATTG TTTGGCTATC   
  
  
+ TTCCCCTGTT TTATACTCAT TTTCTTGCTT TCTATATCTG GGTTTGAATT GGGTTTCACT TATCCTCGTT   
  
  
+ GATTCTGTGG GAAATTAGTG AATTGGGTTT TGTACAGCAG GTTTATATGA ACTAGGGTTT CAAAGGTATC   
  
  
+ TACTTTTTCT CTTGTGATTA TTAGTTGATT AGGGATTTGG CCAAGATTAG CAGAATGGGT TCTGAATTTG   
  
  
+ GGGAATTCTC TGATGATGCT CTAAATGGGT ATGCTTACGT TGATATCCCT GCTTATGATG CGTCCTTAGA   
  
  
+ TTATGCCAAT TTGTTCAATT ACGAAGGCCT ATCTGAGGAT CTCACCTCAC TGAGCCTCCC AAGCCCCTTT   
  
  
+ TCTGACCCTT TGGCGTACAG TTTCACGTCC TCTTTGGGGC CGAGCCCTGG GGTTGATTCT AATGATGATA   
  
  
+ GTGATTCTGA TGATGTTCTC AAGTGCATTA GCCAAATGCT TATGGAAGAA GACATGGAGG CAAAGCCATG   
  
  
+ CATGTTTCAC GATCCTTTAG CACTTCAGGC TGCTGAGAAA CCCTTTTATG ATGCCTTAGG GAAGAAATAC   
  
  
+ CCAACTTCTC CGAACCAACA TCCTATAATT GATGATTGTT TGGATAATCC TGGTGAAAAT TCTTTGGGTT   
  
  
+ CTTCTAGTGA TTTTAGTGTT AGTCACTTTG GGTCTAGTTC AGCAAGCTCT ATTGGACCGA CAATTGTGTC   
  
  
+ TGATTTGAGT GAGCATTTTG AGCCACCCTT TGTTCAAGCA CTTCCAATTG AATCATATCC CCAACCATTG   
  
  
+ ACCCGTCCTC AATGGTCGTT TGGCCCTTCG GGTGCCTTAG ATTGCACGGC CTCTAATGGT TCAGTGATCT   
  
  
+ CATCCCTTGG TTTGCCGATG GATGTTATTA GTGTATTCAG TGAGAAAGAG TCCATGATTC AATTTCAGAA   
  
  
+ AGGGGTGGAA GAGGCTAGTA AGTTCCTTCC CAAGAATAAT AACCTTGTTA TTGATCTCAA GAACCTCACT   
  
  
+ TTTCCTAATG AAACGAAGGA GGATGATCGA GTGATGATGG TTAAGAAGGA AAAGAATGAT GTGAATTGGT   
  
  
+ CTAACTACTC AAGAGGGAGT AAGATTCACT ATCGTGAAGA CGAGGACTTT GAAGAAGGAA GGAGTAGCAA   
  
  
+ GCAGTCAGCT ATTTCTACTA CTGAGGAAGC TGAGTTGTGT GAAATTTTTG ACAAGTTTTT GCTTTGCAAT   
  
  
+ TGGTACCCTG TGAAACCTGA GGCTCATCCC ACCATGAGTT TGAACCCTGA GAAGGGCCAG TCACATGGAT   
  
  
+ TAGAAGGTGG GAGAAATGGG AAGGGTCACC CAAAGAAACA GGATAAAAGT AGTACCAACG TTGTGGATTT   
  
  
+ AAGGAATTTG CTGATGCTAT GTGCACAATC TACTGCATCT GATGACCGAA GAACTGCTGA TGGACTGCTA   
  
  
+ AAGAAAATCA GGGAGCACTC ATCTGCTGAG GGGGATGGAT TTCAAAGGTT GGCTCATTAC TTTGCTGATG   
  
  
+ CCCTAGAGGC ACGTTTAGCT GGAACTGGAT CTCACATTTA TACAGCCCTA AGTTCTCATA GGCCATCTGC   
  
  
+ TGTTGACGTG TTAAAAGCAT ATCAGTTCTA TGTTCGTGCT TGCCCATTTA AGAAGATCGT CATTCGTTGT   
  
  
+ GGTAAACATA TGATTCTAAA AGCTGCTGAA AAGGCATCAA AGCTTCATAT TATAGATTTT GGCATCCTCT   
  
  
+ ATGGATTCCA ATGGCCTAGC CTCATTCGAT GCCTCTCAGA GCGGTCTGGT GGACCTCCAA AACTGTTTAT   
  
  
+ TACAGGGATC GATCTCCCCC AGCCTGGGTT CAGGCCAGCA GAAAGAGTGG AAGCAACAGG GAGACGCTTG   
  
  
+ GCTAAGTACT GTGAGCGGTA TAATGTGCCA TTTGAGTATC ATGCCATTGC TCAGAAGTGG GAAACAATCA   
  
  
+ AACCAGGGGA TCTCAAGTTA GGAAGTAGGG ATGATGATGA AGTTGTCGTG GTGAACTGTC TCTGTAGGTT   
  
  
+ CAAGAACCTC CTTGACGAGA CAATGGTGGT GGATAGTCCA AGGAACACAG TTTTAAACCT GATTAGAAGG   
  
  
+ GTAAAACCCG ATATTTTTGT GCATGGCATT GTAAATGGTT CCTACAACAT CCCTTTCTTT GTGACACGTT   
  
  
+ TTAGAGAAGC CCTCTTTCAT TATTCCACTC TTTTCGACAT GTTAGATGCC AACGCCTCTA GGGAGGAGCC   
  
  
+ CGAGAGGTTG ATATTCGAGA AGGCATTCTA TGGGAGGGAG ATTATGAATG TGGTGGCATG TGAGGGCACA   
  
  
+ GAGAGGGTGG AAAGGCCAGA GACATACAAG CAATGGCACG TTAGGCATAG CAGGGCAGGG TTTCGGCAAC   
  
  
+ TACCATTGGA TCCCAAGTTG ATCGAGAAAA TGAGGTTTAA GGCCAAGGCA GACCACCACA AGGATTTCGT   
  
  
+ GATTGATGTG GATGGACATT GGGCAATTCA GGGATGGAAG GGGCGGATTG CCTGTGCCAT CTCTGCATGG   
  
  
+ GTTCTGGCTT G  

- -Up\_Stream \_Len000GACAAG TCGTGATCCC TTATTCCAAA AACGGGACGT ATGATACTTT AGATTGGTTT   
  
  
- TTGTAAGGTA GAACCTTCCG ACATCGGAAC ACCTAAAACT TAAGCGAGGG TTCTTGGTTA CTTCATCGCG   
  
  
- TCTTCCGGTT ATGAGTTAAA GTCTCAGGTG TAGTTGAACA AGTTACGGTC ATATAAAGTC TTGTAATTAA   
  
  
- TGTATGACAA ACCAACCTCT TTTTTACCAA CAGAGCTTTT GATCAAAAAA TTAAAAAACA ATAAATCGTG   
  
  
- CCATTTCTTA CCTTTAATAA AAGTACGCTC TTTTTTCCTT CAACCAAAAA GAAAAAAGTT GAGAAAACTA   
  
  
- CTATATTAGT CAGCTTTAAA TCCACGTGGT AGACTGAAGT TGAGAACATG CTGGAGACTA ACGAACGAAT   
  
  
- CTTCTTGTTC TCCTAACTGT CTATTCCTCC TCACCCACCA AATATGTCTC TTAACTCACC TACCAATATT   
  
  
- TCTTCAGCTA GAGTGTATTC ATTGAAGCAA TGCTAATATT TATAAAAGTA ACTTCTTTCT GCTAATCTAT   
  
  
- ATTAATTCTT CCATGTAGAT TCGTTTAAAC CGACTCGTCT CAACCTCCCG GTGATCCCAG TGTAACACAG   
  
  
- AACGCGTTGT TCGAATCTGG TAAGCGTTGC CTACGGAGTG CACCTCGTCC ACCCTGGGCG CACCTTCAAC   
  
  
- GTACCGTTCT TAAGAGACCG TCGTTCCTCT TAACTCCCCC CTGTGCGCAC ACTGGTGCGG TCACGCCAGG   
  
  
- GGGAGAGCAC CGTCTAAACC ACTAAAAAAA AAACAAAAAA AAACTTCATT ATATATTAAA AATATACTAA   
  
  
- TTTTTTTATC CAACACACAA TATTTCACCC AGTTACTGTT TATAAAAATA ACCTACTAAT TTTAATTCAA   
  
  
- GGTAAATAAC CTCTTCACTA CCTTACGTTC AAGGTTTTAA CACCTTGAAA ACAACTGCAT CGTCCGATCT   
  
  
- CAAGGTTTTT AATTTTTTTC CTTTCTGTAT AACTCCATTT CGGAAAAATA AAATGTTTAT CTGCGTGGTT   
  
  
- CCATATTATA TTAACTGTAC TAAACTCCGC TCTCCTTCTA AGAAAACGAT GGGTTTGTTT TGTGTTACTA   
  
  
- GAAAATTTAC ATCGTTAAAC CCTAACCGTT TTAAAACAGA TTGGGCTAAC AACTTTCAAT AGGCTAAAGT   
  
  
- ATTTTAACCT ATAGCAATAA TTTAAAAGTA AGCTTATAAA TTTAAACCTG TGTTACAAAA AGTATAAACC   
  
  
- TAAAAGATTT GAACTAAAAT TGAGTTTAAA TTCAAATAAG TTAAGTAGAC TGGGTTTTTT ACCAACGTTA   
  
  
- TTACATAAAA GAAAAACTTC AATTTATAAT AGCTTAAGTA AATTGAACAT CAATTGGGCA TTAACTGGGT   
  
  
- TTGAGCTGAA AAACGATGGA ATCGTCGGTC CTTCTTCAGT ATCATAACTG GGCACCTTGC GGACTTCGGT   
  
  
- TGTAGTGGTC TGGGTGTATA AGGTAGTAGA AGACTAGAGG AGCAGTTTCT TCCTTCGACT CGTGTCGTTA   
  
  
- TTGATCTTCT TGTCCTCCGT AGTGTGGGTT CGGTTTCCGG AAATAAACTC GAGGGGTGGA GTAAGGAGCT   
  
  
- TCGAAGGAGA AGTGTTACAT TCTGGTATTA GGAAGTGGGG AAGAGAAAAA TTCGAGTAGA AATGGACGGG   
  
  
- ATCAAGAGAG AGAGAGTACT CAGTAGTCAT TATCAATGGA GAAATGAAAG AGAAGGAACT CCATACGAAG   
  
  
- TTGAAATTAT ATATATACAT GGTGAACTAA AGAGAACAGT GAAACGATAT TATGAATAAC AAACCGATAG   
  
  
- AAGGGGACAA AATATGAGTA AAAGAACGAA AGATATAGAC CCAAACTTAA CCCAAAGTGA ATAGGAGCAA   
  
  
- CTAAGACACC CTTTAATCAC TTAACCCAAA ACATGTCGTC CAAATATACT TGATCCCAAA GTTTCCATAG   
  
  
- ATGAAAAAGA GAACACTAAT AATCAACTAA TCCCTAAACC GGTTCTAATC GTCTTACCCA AGACTTAAAC   
  
  
- CCCTTAAGAG ACTACTACGA GATTTACCCA TACGAATGCA ACTATAGGGA CGAATACTAC GCAGGAATCT   
  
  
- AATACGGTTA AACAAGTTAA TGCTTCCGGA TAGACTCCTA GAGTGGAGTG ACTCGGAGGG TTCGGGGAAA   
  
  
- AGACTGGGAA ACCGCATGTC AAAGTGCAGG AGAAACCCCG GCTCGGGACC CCAACTAAGA TTACTACTAT   
  
  
- CACTAAGACT ACTACAAGAG TTCACGTAAT CGGTTTACGA ATACCTTCTT CTGTACCTCC GTTTCGGTAC   
  
  
- GTACAAAGTG CTAGGAAATC GTGAAGTCCG ACGACTCTTT GGGAAAATAC TACGGAATCC CTTCTTTATG   
  
  
- GGTTGAAGAG GCTTGGTTGT AGGATATTAA CTACTAACAA ACCTATTAGG ACCACTTTTA AGAAACCCAA   
  
  
- GAAGATCACT AAAATCACAA TCAGTGAAAC CCAGATCAAG TCGTTCGAGA TAACCTGGCT GTTAACACAG   
  
  
- ACTAAACTCA CTCGTAAAAC TCGGTGGGAA ACAAGTTCGT GAAGGTTAAC TTAGTATAGG GGTTGGTAAC   
  
  
- TGGGCAGGAG TTACCAGCAA ACCGGGAAGC CCACGGAATC TAACGTGCCG GAGATTACCA AGTCACTAGA   
  
  
- GTAGGGAACC AAACGGCTAC CTACAATAAT CACATAAGTC ACTCTTTCTC AGGTACTAAG TTAAAGTCTT   
  
  
- TCCCCACCTT CTCCGATCAT TCAAGGAAGG GTTCTTATTA TTGGAACAAT AACTAGAGTT CTTGGAGTGA   
  
  
- AAAGGATTAC TTTGCTTCCT CCTACTAGCT CACTACTACC AATTCTTCCT TTTCTTACTA CACTTAACCA   
  
  
- GATTGATGAG TTCTCCCTCA TTCTAAGTGA TAGCACTTCT GCTCCTGAAA CTTCTTCCTT CCTCATCGTT   
  
  
- CGTCAGTCGA TAAAGATGAT GACTCCTTCG ACTCAACACA CTTTAAAAAC TGTTCAAAAA CGAAACGTTA   
  
  
- ACCATGGGAC ACTTTGGACT CCGAGTAGGG TGGTACTCAA ACTTGGGACT CTTCCCGGTC AGTGTACCTA   
  
  
- ATCTTCCACC CTCTTTACCC TTCCCAGTGG GTTTCTTTGT CCTATTTTCA TCATGGTTGC AACACCTAAA   
  
  
- TTCCTTAAAC GACTACGATA CACGTGTTAG ATGACGTAGA CTACTGGCTT CTTGACGACT ACCTGACGAT   
  
  
- TTCTTTTAGT CCCTCGTGAG TAGACGACTC CCCCTACCTA AAGTTTCCAA CCGAGTAATG AAACGACTAC   
  
  
- GGGATCTCCG TGCAAATCGA CCTTGACCTA GAGTGTAAAT ATGTCGGGAT TCAAGAGTAT CCGGTAGACG   
  
  
- ACAACTGCAC AATTTTCGTA TAGTCAAGAT ACAAGCACGA ACGGGTAAAT TCTTCTAGCA GTAAGCAACA   
  
  
- CCATTTGTAT ACTAAGATTT TCGACGACTT TTCCGTAGTT TCGAAGTATA ATATCTAAAA CCGTAGGAGA   
  
  
- TACCTAAGGT TACCGGATCG GAGTAAGCTA CGGAGAGTCT CGCCAGACCA CCTGGAGGTT TTGACAAATA   
  
  
- ATGTCCCTAG CTAGAGGGGG TCGGACCCAA GTCCGGTCGT CTTTCTCACC TTCGTTGTCC CTCTGCGAAC   
  
  
- CGATTCATGA CACTCGCCAT ATTACACGGT AAACTCATAG TACGGTAACG AGTCTTCACC CTTTGTTAGT   
  
  
- TTGGTCCCCT AGAGTTCAAT CCTTCATCCC TACTACTACT TCAACAGCAC CACTTGACAG AGACATCCAA   
  
  
- GTTCTTGGAG GAACTGCTCT GTTACCACCA CCTATCAGGT TCCTTGTGTC AAAATTTGGA CTAATCTTCC   
  
  
- CATTTTGGGC TATAAAAACA CGTACCGTAA CATTTACCAA GGATGTTGTA GGGAAAGAAA CACTGTGCAA   
  
  
- AATCTCTTCG GGAGAAAGTA ATAAGGTGAG AAAAGCTGTA CAATCTACGG TTGCGGAGAT CCCTCCTCGG   
  
  
- GCTCTCCAAC TATAAGCTCT TCCGTAAGAT ACCCTCCCTC TAATACTTAC ACCACCGTAC ACTCCCGTGT   
  
  
- CTCTCCCACC TTTCCGGTCT CTGTATGTTC GTTACCGTGC AATCCGTATC GTCCCGTCCC AAAGCCGTTG   
  
  
- ATGGTAACCT AGGGTTCAAC TAGCTCTTTT ACTCCAAATT CCGGTTCCGT CTGGTGGTGT TCCTAAAGCA   
  
  
- CTAACTACAC CTACCTGTAA CCCGTTAAGT CCCTACCTTC CCCGCCTAAC GGACACGGTA GAGACGTACC   
  
  
- CAAGACCGAA C

+     O2-site

| Site Name | Organism | Position | Strand | Matrix score. | sequence | function |
| --- | --- | --- | --- | --- | --- | --- |
| O2-site | Zea mays | 171 | - | 9 | GATGATGTGG | cis-acting regulatory element involved in zein metabolism regulation |
| O2-site | Zea mays | 3366 | + | 9 | GTTGACGTGA | cis-acting regulatory element involved in zein metabolism regulation |
| O2-site | Zea mays | 2292 | + | 9 | GATGACATGG | cis-acting regulatory element involved in zein metabolism regulation |

>HU07G02248.1   
+ -Up\_Stream \_Len000CTGTTC AGCACTAGGG AATAAGGTTT TTGCCCTGCA TACTATGAAA TCTAACCAAA   
  
  
+ AACATTCCAT CTTGGAAGGC TGTAGCCTTG TGGATTTTGA ATTCGCTCCC AAGAACCAAT GAAGTAGCGC   
  
  
+ AGAAGGCCAA TACTCAATTT CAGAGTCCAC ATCAACTTGT TCAATGCCAG TATATTTCAG AACATTAATT   
  
  
+ ACATACTGTT TGGTTGGAGA AAAAATGGTT GTCTCGAAAA CTAGTTTTTT AATTTTTTGT TATTTAGCAC   
  
  
+ GGTAAAGAAT GGAAATTATT TTCATGCGAG AAAAAAGGAA GTTGGTTTTT CTTTTTTCAA CTCTTTTGAT   
  
  
+ GATATAATCA GTCGAAATTT AGGTGCACCA TCTGACTTCA ACTCTTGTAC GACCTCTGAT TGCTTGCTTA   
  
  
+ GAAGAACAAG AGGATTGACA GATAAGGAGG AGTGGGTGGT TTATACAGAG AATTGAGTGG ATGGTTATAA   
  
  
+ AGAAGTCGAT CTCACATAAG TAACTTCGTT ACGATTATAA ATATTTTCAT TGAAGAAAGA CGATTAGATA   
  
  
+ TAATTAAGAA GGTACATCTA AGCAAATTTG GCTGAGCAGA GTTGGAGGGC CACTAGGGTC ACATTGTGTC   
  
  
+ TTGCGCAACA AGCTTAGACC ATTCGCAACG GATGCCTCAC GTGGAGCAGG TGGGACCCGC GTGGAAGTTG   
  
  
+ CATGGCAAGA ATTCTCTGGC AGCAAGGAGA ATTGAGGGGG GACACGCGTG TGACCACGCC AGTGCGGTCC   
  
  
+ CCCTCTCGTG GCAGATTTGG TGATTTTTTT TTTGTTTTTT TTTGAAGTAA TATATAATTT TTATATGATT   
  
  
+ AAAAAAATAG GTTGTGTGTT ATAAAGTGGG TCAATGACAA ATATTTTTAT TGGATGATTA AAATTAAGTT   
  
  
+ CCATTTATTG GAGAAGTGAT GGAATGCAAG TTCCAAAATT GTGGAACTTT TGTTGACGTA GCAGGCTAGA   
  
  
+ GTTCCAAAAA TTAAAAAAAG GAAAGACATA TTGAGGTAAA GCCTTTTTAT TTTACAAATA GACGCACCAA   
  
  
+ GGTATAATAT AATTGACATG ATTTGAGGCG AGAGGAAGAT TCTTTTGCTA CCCAAACAAA ACACAATGAT   
  
  
+ CTTTTAAATG TAGCAATTTG GGATTGGCAA AATTTTGTCT AACCCGATTG TTGAAAGTTA TCCGATTTCA   
  
  
+ TAAAATTGGA TATCGTTATT AAATTTTCAT TCGAATATTT AAATTTGGAC ACAATGTTTT TCATATTTGG   
  
  
+ ATTTTCTAAA CTTGATTTTA ACTCAAATTT AAGTTTATTC AATTCATCTG ACCCAAAAAA TGGTTGCAAT   
  
  
+ AATGTATTTT CTTTTTGAAG TTAAATATTA TCGAATTCAT TTAACTTGTA GTTAACCCGT AATTGACCCA   
  
  
+ AACTCGACTT TTTGCTACCT TAGCAGCCAG GAAGAAGTCA TAGTATTGAC CCGTGGAACG CCTGAAGCCA   
  
  
+ ACATCACCAG ACCCACATAT TCCATCATCT TCTGATCTCC TCGTCAAAGA AGGAAGCTGA GCACAGCAAT   
  
  
+ AACTAGAAGA ACAGGAGGCA TCACACCCAA GCCAAAGGCC TTTATTTGAG CTCCCCACCT CATTCCTCGA   
  
  
+ AGCTTCCTCT TCACAATGTA AGACCATAAT CCTTCACCCC TTCTCTTTTT AAGCTCATCT TTACCTGCCC   
  
  
+ TAGTTCTCTC TCTCTCATGA GTCATCAGTA ATAGTTACCT CTTTACTTTC TCTTCCTTGA GGTATGCTTC   
  
  
+ AACTTTAATA TATATATGTA CCACTTGATT TCTCTTGTCA CTTTGCTATA ATACTTATTG TTTGGCTATC   
  
  
+ TTCCCCTGTT TTATACTCAT TTTCTTGCTT TCTATATCTG GGTTTGAATT GGGTTTCACT TATCCTCGTT   
  
  
+ GATTCTGTGG GAAATTAGTG AATTGGGTTT TGTACAGCAG GTTTATATGA ACTAGGGTTT CAAAGGTATC   
  
  
+ TACTTTTTCT CTTGTGATTA TTAGTTGATT AGGGATTTGG CCAAGATTAG CAGAATGGGT TCTGAATTTG   
  
  
+ GGGAATTCTC TGATGATGCT CTAAATGGGT ATGCTTACGT TGATATCCCT GCTTATGATG CGTCCTTAGA   
  
  
+ TTATGCCAAT TTGTTCAATT ACGAAGGCCT ATCTGAGGAT CTCACCTCAC TGAGCCTCCC AAGCCCCTTT   
  
  
+ TCTGACCCTT TGGCGTACAG TTTCACGTCC TCTTTGGGGC CGAGCCCTGG GGTTGATTCT AATGATGATA   
  
  
+ GTGATTCTGA TGATGTTCTC AAGTGCATTA GCCAAATGCT TATGGAAGAA GACATGGAGG CAAAGCCATG   
  
  
+ CATGTTTCAC GATCCTTTAG CACTTCAGGC TGCTGAGAAA CCCTTTTATG ATGCCTTAGG GAAGAAATAC   
  
  
+ CCAACTTCTC CGAACCAACA TCCTATAATT GATGATTGTT TGGATAATCC TGGTGAAAAT TCTTTGGGTT   
  
  
+ CTTCTAGTGA TTTTAGTGTT AGTCACTTTG GGTCTAGTTC AGCAAGCTCT ATTGGACCGA CAATTGTGTC   
  
  
+ TGATTTGAGT GAGCATTTTG AGCCACCCTT TGTTCAAGCA CTTCCAATTG AATCATATCC CCAACCATTG   
  
  
+ ACCCGTCCTC AATGGTCGTT TGGCCCTTCG GGTGCCTTAG ATTGCACGGC CTCTAATGGT TCAGTGATCT   
  
  
+ CATCCCTTGG TTTGCCGATG GATGTTATTA GTGTATTCAG TGAGAAAGAG TCCATGATTC AATTTCAGAA   
  
  
+ AGGGGTGGAA GAGGCTAGTA AGTTCCTTCC CAAGAATAAT AACCTTGTTA TTGATCTCAA GAACCTCACT   
  
  
+ TTTCCTAATG AAACGAAGGA GGATGATCGA GTGATGATGG TTAAGAAGGA AAAGAATGAT GTGAATTGGT   
  
  
+ CTAACTACTC AAGAGGGAGT AAGATTCACT ATCGTGAAGA CGAGGACTTT GAAGAAGGAA GGAGTAGCAA   
  
  
+ GCAGTCAGCT ATTTCTACTA CTGAGGAAGC TGAGTTGTGT GAAATTTTTG ACAAGTTTTT GCTTTGCAAT   
  
  
+ TGGTACCCTG TGAAACCTGA GGCTCATCCC ACCATGAGTT TGAACCCTGA GAAGGGCCAG TCACATGGAT   
  
  
+ TAGAAGGTGG GAGAAATGGG AAGGGTCACC CAAAGAAACA GGATAAAAGT AGTACCAACG TTGTGGATTT   
  
  
+ AAGGAATTTG CTGATGCTAT GTGCACAATC TACTGCATCT GATGACCGAA GAACTGCTGA TGGACTGCTA   
  
  
+ AAGAAAATCA GGGAGCACTC ATCTGCTGAG GGGGATGGAT TTCAAAGGTT GGCTCATTAC TTTGCTGATG   
  
  
+ CCCTAGAGGC ACGTTTAGCT GGAACTGGAT CTCACATTTA TACAGCCCTA AGTTCTCATA GGCCATCTGC   
  
  
+ TGTTGACGTG TTAAAAGCAT ATCAGTTCTA TGTTCGTGCT TGCCCATTTA AGAAGATCGT CATTCGTTGT   
  
  
+ GGTAAACATA TGATTCTAAA AGCTGCTGAA AAGGCATCAA AGCTTCATAT TATAGATTTT GGCATCCTCT   
  
  
+ ATGGATTCCA ATGGCCTAGC CTCATTCGAT GCCTCTCAGA GCGGTCTGGT GGACCTCCAA AACTGTTTAT   
  
  
+ TACAGGGATC GATCTCCCCC AGCCTGGGTT CAGGCCAGCA GAAAGAGTGG AAGCAACAGG GAGACGCTTG   
  
  
+ GCTAAGTACT GTGAGCGGTA TAATGTGCCA TTTGAGTATC ATGCCATTGC TCAGAAGTGG GAAACAATCA   
  
  
+ AACCAGGGGA TCTCAAGTTA GGAAGTAGGG ATGATGATGA AGTTGTCGTG GTGAACTGTC TCTGTAGGTT   
  
  
+ CAAGAACCTC CTTGACGAGA CAATGGTGGT GGATAGTCCA AGGAACACAG TTTTAAACCT GATTAGAAGG   
  
  
+ GTAAAACCCG ATATTTTTGT GCATGGCATT GTAAATGGTT CCTACAACAT CCCTTTCTTT GTGACACGTT   
  
  
+ TTAGAGAAGC CCTCTTTCAT TATTCCACTC TTTTCGACAT GTTAGATGCC AACGCCTCTA GGGAGGAGCC   
  
  
+ CGAGAGGTTG ATATTCGAGA AGGCATTCTA TGGGAGGGAG ATTATGAATG TGGTGGCATG TGAGGGCACA   
  
  
+ GAGAGGGTGG AAAGGCCAGA GACATACAAG CAATGGCACG TTAGGCATAG CAGGGCAGGG TTTCGGCAAC   
  
  
+ TACCATTGGA TCCCAAGTTG ATCGAGAAAA TGAGGTTTAA GGCCAAGGCA GACCACCACA AGGATTTCGT   
  
  
+ GATTGATGTG GATGGACATT GGGCAATTCA GGGATGGAAG GGGCGGATTG CCTGTGCCAT CTCTGCATGG   
  
  
+ GTTCTGGCTT G  

- -Up\_Stream \_Len000GACAAG TCGTGATCCC TTATTCCAAA AACGGGACGT ATGATACTTT AGATTGGTTT   
  
  
- TTGTAAGGTA GAACCTTCCG ACATCGGAAC ACCTAAAACT TAAGCGAGGG TTCTTGGTTA CTTCATCGCG   
  
  
- TCTTCCGGTT ATGAGTTAAA GTCTCAGGTG TAGTTGAACA AGTTACGGTC ATATAAAGTC TTGTAATTAA   
  
  
- TGTATGACAA ACCAACCTCT TTTTTACCAA CAGAGCTTTT GATCAAAAAA TTAAAAAACA ATAAATCGTG   
  
  
- CCATTTCTTA CCTTTAATAA AAGTACGCTC TTTTTTCCTT CAACCAAAAA GAAAAAAGTT GAGAAAACTA   
  
  
- CTATATTAGT CAGCTTTAAA TCCACGTGGT AGACTGAAGT TGAGAACATG CTGGAGACTA ACGAACGAAT   
  
  
- CTTCTTGTTC TCCTAACTGT CTATTCCTCC TCACCCACCA AATATGTCTC TTAACTCACC TACCAATATT   
  
  
- TCTTCAGCTA GAGTGTATTC ATTGAAGCAA TGCTAATATT TATAAAAGTA ACTTCTTTCT GCTAATCTAT   
  
  
- ATTAATTCTT CCATGTAGAT TCGTTTAAAC CGACTCGTCT CAACCTCCCG GTGATCCCAG TGTAACACAG   
  
  
- AACGCGTTGT TCGAATCTGG TAAGCGTTGC CTACGGAGTG CACCTCGTCC ACCCTGGGCG CACCTTCAAC   
  
  
- GTACCGTTCT TAAGAGACCG TCGTTCCTCT TAACTCCCCC CTGTGCGCAC ACTGGTGCGG TCACGCCAGG   
  
  
- GGGAGAGCAC CGTCTAAACC ACTAAAAAAA AAACAAAAAA AAACTTCATT ATATATTAAA AATATACTAA   
  
  
- TTTTTTTATC CAACACACAA TATTTCACCC AGTTACTGTT TATAAAAATA ACCTACTAAT TTTAATTCAA   
  
  
- GGTAAATAAC CTCTTCACTA CCTTACGTTC AAGGTTTTAA CACCTTGAAA ACAACTGCAT CGTCCGATCT   
  
  
- CAAGGTTTTT AATTTTTTTC CTTTCTGTAT AACTCCATTT CGGAAAAATA AAATGTTTAT CTGCGTGGTT   
  
  
- CCATATTATA TTAACTGTAC TAAACTCCGC TCTCCTTCTA AGAAAACGAT GGGTTTGTTT TGTGTTACTA   
  
  
- GAAAATTTAC ATCGTTAAAC CCTAACCGTT TTAAAACAGA TTGGGCTAAC AACTTTCAAT AGGCTAAAGT   
  
  
- ATTTTAACCT ATAGCAATAA TTTAAAAGTA AGCTTATAAA TTTAAACCTG TGTTACAAAA AGTATAAACC   
  
  
- TAAAAGATTT GAACTAAAAT TGAGTTTAAA TTCAAATAAG TTAAGTAGAC TGGGTTTTTT ACCAACGTTA   
  
  
- TTACATAAAA GAAAAACTTC AATTTATAAT AGCTTAAGTA AATTGAACAT CAATTGGGCA TTAACTGGGT   
  
  
- TTGAGCTGAA AAACGATGGA ATCGTCGGTC CTTCTTCAGT ATCATAACTG GGCACCTTGC GGACTTCGGT   
  
  
- TGTAGTGGTC TGGGTGTATA AGGTAGTAGA AGACTAGAGG AGCAGTTTCT TCCTTCGACT CGTGTCGTTA   
  
  
- TTGATCTTCT TGTCCTCCGT AGTGTGGGTT CGGTTTCCGG AAATAAACTC GAGGGGTGGA GTAAGGAGCT   
  
  
- TCGAAGGAGA AGTGTTACAT TCTGGTATTA GGAAGTGGGG AAGAGAAAAA TTCGAGTAGA AATGGACGGG   
  
  
- ATCAAGAGAG AGAGAGTACT CAGTAGTCAT TATCAATGGA GAAATGAAAG AGAAGGAACT CCATACGAAG   
  
  
- TTGAAATTAT ATATATACAT GGTGAACTAA AGAGAACAGT GAAACGATAT TATGAATAAC AAACCGATAG   
  
  
- AAGGGGACAA AATATGAGTA AAAGAACGAA AGATATAGAC CCAAACTTAA CCCAAAGTGA ATAGGAGCAA   
  
  
- CTAAGACACC CTTTAATCAC TTAACCCAAA ACATGTCGTC CAAATATACT TGATCCCAAA GTTTCCATAG   
  
  
- ATGAAAAAGA GAACACTAAT AATCAACTAA TCCCTAAACC GGTTCTAATC GTCTTACCCA AGACTTAAAC   
  
  
- CCCTTAAGAG ACTACTACGA GATTTACCCA TACGAATGCA ACTATAGGGA CGAATACTAC GCAGGAATCT   
  
  
- AATACGGTTA AACAAGTTAA TGCTTCCGGA TAGACTCCTA GAGTGGAGTG ACTCGGAGGG TTCGGGGAAA   
  
  
- AGACTGGGAA ACCGCATGTC AAAGTGCAGG AGAAACCCCG GCTCGGGACC CCAACTAAGA TTACTACTAT   
  
  
- CACTAAGACT ACTACAAGAG TTCACGTAAT CGGTTTACGA ATACCTTCTT CTGTACCTCC GTTTCGGTAC   
  
  
- GTACAAAGTG CTAGGAAATC GTGAAGTCCG ACGACTCTTT GGGAAAATAC TACGGAATCC CTTCTTTATG   
  
  
- GGTTGAAGAG GCTTGGTTGT AGGATATTAA CTACTAACAA ACCTATTAGG ACCACTTTTA AGAAACCCAA   
  
  
- GAAGATCACT AAAATCACAA TCAGTGAAAC CCAGATCAAG TCGTTCGAGA TAACCTGGCT GTTAACACAG   
  
  
- ACTAAACTCA CTCGTAAAAC TCGGTGGGAA ACAAGTTCGT GAAGGTTAAC TTAGTATAGG GGTTGGTAAC   
  
  
- TGGGCAGGAG TTACCAGCAA ACCGGGAAGC CCACGGAATC TAACGTGCCG GAGATTACCA AGTCACTAGA   
  
  
- GTAGGGAACC AAACGGCTAC CTACAATAAT CACATAAGTC ACTCTTTCTC AGGTACTAAG TTAAAGTCTT   
  
  
- TCCCCACCTT CTCCGATCAT TCAAGGAAGG GTTCTTATTA TTGGAACAAT AACTAGAGTT CTTGGAGTGA   
  
  
- AAAGGATTAC TTTGCTTCCT CCTACTAGCT CACTACTACC AATTCTTCCT TTTCTTACTA CACTTAACCA   
  
  
- GATTGATGAG TTCTCCCTCA TTCTAAGTGA TAGCACTTCT GCTCCTGAAA CTTCTTCCTT CCTCATCGTT   
  
  
- CGTCAGTCGA TAAAGATGAT GACTCCTTCG ACTCAACACA CTTTAAAAAC TGTTCAAAAA CGAAACGTTA   
  
  
- ACCATGGGAC ACTTTGGACT CCGAGTAGGG TGGTACTCAA ACTTGGGACT CTTCCCGGTC AGTGTACCTA   
  
  
- ATCTTCCACC CTCTTTACCC TTCCCAGTGG GTTTCTTTGT CCTATTTTCA TCATGGTTGC AACACCTAAA   
  
  
- TTCCTTAAAC GACTACGATA CACGTGTTAG ATGACGTAGA CTACTGGCTT CTTGACGACT ACCTGACGAT   
  
  
- TTCTTTTAGT CCCTCGTGAG TAGACGACTC CCCCTACCTA AAGTTTCCAA CCGAGTAATG AAACGACTAC   
  
  
- GGGATCTCCG TGCAAATCGA CCTTGACCTA GAGTGTAAAT ATGTCGGGAT TCAAGAGTAT CCGGTAGACG   
  
  
- ACAACTGCAC AATTTTCGTA TAGTCAAGAT ACAAGCACGA ACGGGTAAAT TCTTCTAGCA GTAAGCAACA   
  
  
- CCATTTGTAT ACTAAGATTT TCGACGACTT TTCCGTAGTT TCGAAGTATA ATATCTAAAA CCGTAGGAGA   
  
  
- TACCTAAGGT TACCGGATCG GAGTAAGCTA CGGAGAGTCT CGCCAGACCA CCTGGAGGTT TTGACAAATA   
  
  
- ATGTCCCTAG CTAGAGGGGG TCGGACCCAA GTCCGGTCGT CTTTCTCACC TTCGTTGTCC CTCTGCGAAC   
  
  
- CGATTCATGA CACTCGCCAT ATTACACGGT AAACTCATAG TACGGTAACG AGTCTTCACC CTTTGTTAGT   
  
  
- TTGGTCCCCT AGAGTTCAAT CCTTCATCCC TACTACTACT TCAACAGCAC CACTTGACAG AGACATCCAA   
  
  
- GTTCTTGGAG GAACTGCTCT GTTACCACCA CCTATCAGGT TCCTTGTGTC AAAATTTGGA CTAATCTTCC   
  
  
- CATTTTGGGC TATAAAAACA CGTACCGTAA CATTTACCAA GGATGTTGTA GGGAAAGAAA CACTGTGCAA   
  
  
- AATCTCTTCG GGAGAAAGTA ATAAGGTGAG AAAAGCTGTA CAATCTACGG TTGCGGAGAT CCCTCCTCGG   
  
  
- GCTCTCCAAC TATAAGCTCT TCCGTAAGAT ACCCTCCCTC TAATACTTAC ACCACCGTAC ACTCCCGTGT   
  
  
- CTCTCCCACC TTTCCGGTCT CTGTATGTTC GTTACCGTGC AATCCGTATC GTCCCGTCCC AAAGCCGTTG   
  
  
- ATGGTAACCT AGGGTTCAAC TAGCTCTTTT ACTCCAAATT CCGGTTCCGT CTGGTGGTGT TCCTAAAGCA   
  
  
- CTAACTACAC CTACCTGTAA CCCGTTAAGT CCCTACCTTC CCCGCCTAAC GGACACGGTA GAGACGTACC   
  
  
- CAAGACCGAA C

+     RY-element

| Site Name | Organism | Position | Strand | Matrix score. | sequence | function |
| --- | --- | --- | --- | --- | --- | --- |
| RY-element | Helianthus annuus | 2311 | - | 8 | CATGCATG | cis-acting regulatory element involved in seed-specific regulation |

>HU07G02248.1   
+ -Up\_Stream \_Len000CTGTTC AGCACTAGGG AATAAGGTTT TTGCCCTGCA TACTATGAAA TCTAACCAAA   
  
  
+ AACATTCCAT CTTGGAAGGC TGTAGCCTTG TGGATTTTGA ATTCGCTCCC AAGAACCAAT GAAGTAGCGC   
  
  
+ AGAAGGCCAA TACTCAATTT CAGAGTCCAC ATCAACTTGT TCAATGCCAG TATATTTCAG AACATTAATT   
  
  
+ ACATACTGTT TGGTTGGAGA AAAAATGGTT GTCTCGAAAA CTAGTTTTTT AATTTTTTGT TATTTAGCAC   
  
  
+ GGTAAAGAAT GGAAATTATT TTCATGCGAG AAAAAAGGAA GTTGGTTTTT CTTTTTTCAA CTCTTTTGAT   
  
  
+ GATATAATCA GTCGAAATTT AGGTGCACCA TCTGACTTCA ACTCTTGTAC GACCTCTGAT TGCTTGCTTA   
  
  
+ GAAGAACAAG AGGATTGACA GATAAGGAGG AGTGGGTGGT TTATACAGAG AATTGAGTGG ATGGTTATAA   
  
  
+ AGAAGTCGAT CTCACATAAG TAACTTCGTT ACGATTATAA ATATTTTCAT TGAAGAAAGA CGATTAGATA   
  
  
+ TAATTAAGAA GGTACATCTA AGCAAATTTG GCTGAGCAGA GTTGGAGGGC CACTAGGGTC ACATTGTGTC   
  
  
+ TTGCGCAACA AGCTTAGACC ATTCGCAACG GATGCCTCAC GTGGAGCAGG TGGGACCCGC GTGGAAGTTG   
  
  
+ CATGGCAAGA ATTCTCTGGC AGCAAGGAGA ATTGAGGGGG GACACGCGTG TGACCACGCC AGTGCGGTCC   
  
  
+ CCCTCTCGTG GCAGATTTGG TGATTTTTTT TTTGTTTTTT TTTGAAGTAA TATATAATTT TTATATGATT   
  
  
+ AAAAAAATAG GTTGTGTGTT ATAAAGTGGG TCAATGACAA ATATTTTTAT TGGATGATTA AAATTAAGTT   
  
  
+ CCATTTATTG GAGAAGTGAT GGAATGCAAG TTCCAAAATT GTGGAACTTT TGTTGACGTA GCAGGCTAGA   
  
  
+ GTTCCAAAAA TTAAAAAAAG GAAAGACATA TTGAGGTAAA GCCTTTTTAT TTTACAAATA GACGCACCAA   
  
  
+ GGTATAATAT AATTGACATG ATTTGAGGCG AGAGGAAGAT TCTTTTGCTA CCCAAACAAA ACACAATGAT   
  
  
+ CTTTTAAATG TAGCAATTTG GGATTGGCAA AATTTTGTCT AACCCGATTG TTGAAAGTTA TCCGATTTCA   
  
  
+ TAAAATTGGA TATCGTTATT AAATTTTCAT TCGAATATTT AAATTTGGAC ACAATGTTTT TCATATTTGG   
  
  
+ ATTTTCTAAA CTTGATTTTA ACTCAAATTT AAGTTTATTC AATTCATCTG ACCCAAAAAA TGGTTGCAAT   
  
  
+ AATGTATTTT CTTTTTGAAG TTAAATATTA TCGAATTCAT TTAACTTGTA GTTAACCCGT AATTGACCCA   
  
  
+ AACTCGACTT TTTGCTACCT TAGCAGCCAG GAAGAAGTCA TAGTATTGAC CCGTGGAACG CCTGAAGCCA   
  
  
+ ACATCACCAG ACCCACATAT TCCATCATCT TCTGATCTCC TCGTCAAAGA AGGAAGCTGA GCACAGCAAT   
  
  
+ AACTAGAAGA ACAGGAGGCA TCACACCCAA GCCAAAGGCC TTTATTTGAG CTCCCCACCT CATTCCTCGA   
  
  
+ AGCTTCCTCT TCACAATGTA AGACCATAAT CCTTCACCCC TTCTCTTTTT AAGCTCATCT TTACCTGCCC   
  
  
+ TAGTTCTCTC TCTCTCATGA GTCATCAGTA ATAGTTACCT CTTTACTTTC TCTTCCTTGA GGTATGCTTC   
  
  
+ AACTTTAATA TATATATGTA CCACTTGATT TCTCTTGTCA CTTTGCTATA ATACTTATTG TTTGGCTATC   
  
  
+ TTCCCCTGTT TTATACTCAT TTTCTTGCTT TCTATATCTG GGTTTGAATT GGGTTTCACT TATCCTCGTT   
  
  
+ GATTCTGTGG GAAATTAGTG AATTGGGTTT TGTACAGCAG GTTTATATGA ACTAGGGTTT CAAAGGTATC   
  
  
+ TACTTTTTCT CTTGTGATTA TTAGTTGATT AGGGATTTGG CCAAGATTAG CAGAATGGGT TCTGAATTTG   
  
  
+ GGGAATTCTC TGATGATGCT CTAAATGGGT ATGCTTACGT TGATATCCCT GCTTATGATG CGTCCTTAGA   
  
  
+ TTATGCCAAT TTGTTCAATT ACGAAGGCCT ATCTGAGGAT CTCACCTCAC TGAGCCTCCC AAGCCCCTTT   
  
  
+ TCTGACCCTT TGGCGTACAG TTTCACGTCC TCTTTGGGGC CGAGCCCTGG GGTTGATTCT AATGATGATA   
  
  
+ GTGATTCTGA TGATGTTCTC AAGTGCATTA GCCAAATGCT TATGGAAGAA GACATGGAGG CAAAGCCATG   
  
  
+ CATGTTTCAC GATCCTTTAG CACTTCAGGC TGCTGAGAAA CCCTTTTATG ATGCCTTAGG GAAGAAATAC   
  
  
+ CCAACTTCTC CGAACCAACA TCCTATAATT GATGATTGTT TGGATAATCC TGGTGAAAAT TCTTTGGGTT   
  
  
+ CTTCTAGTGA TTTTAGTGTT AGTCACTTTG GGTCTAGTTC AGCAAGCTCT ATTGGACCGA CAATTGTGTC   
  
  
+ TGATTTGAGT GAGCATTTTG AGCCACCCTT TGTTCAAGCA CTTCCAATTG AATCATATCC CCAACCATTG   
  
  
+ ACCCGTCCTC AATGGTCGTT TGGCCCTTCG GGTGCCTTAG ATTGCACGGC CTCTAATGGT TCAGTGATCT   
  
  
+ CATCCCTTGG TTTGCCGATG GATGTTATTA GTGTATTCAG TGAGAAAGAG TCCATGATTC AATTTCAGAA   
  
  
+ AGGGGTGGAA GAGGCTAGTA AGTTCCTTCC CAAGAATAAT AACCTTGTTA TTGATCTCAA GAACCTCACT   
  
  
+ TTTCCTAATG AAACGAAGGA GGATGATCGA GTGATGATGG TTAAGAAGGA AAAGAATGAT GTGAATTGGT   
  
  
+ CTAACTACTC AAGAGGGAGT AAGATTCACT ATCGTGAAGA CGAGGACTTT GAAGAAGGAA GGAGTAGCAA   
  
  
+ GCAGTCAGCT ATTTCTACTA CTGAGGAAGC TGAGTTGTGT GAAATTTTTG ACAAGTTTTT GCTTTGCAAT   
  
  
+ TGGTACCCTG TGAAACCTGA GGCTCATCCC ACCATGAGTT TGAACCCTGA GAAGGGCCAG TCACATGGAT   
  
  
+ TAGAAGGTGG GAGAAATGGG AAGGGTCACC CAAAGAAACA GGATAAAAGT AGTACCAACG TTGTGGATTT   
  
  
+ AAGGAATTTG CTGATGCTAT GTGCACAATC TACTGCATCT GATGACCGAA GAACTGCTGA TGGACTGCTA   
  
  
+ AAGAAAATCA GGGAGCACTC ATCTGCTGAG GGGGATGGAT TTCAAAGGTT GGCTCATTAC TTTGCTGATG   
  
  
+ CCCTAGAGGC ACGTTTAGCT GGAACTGGAT CTCACATTTA TACAGCCCTA AGTTCTCATA GGCCATCTGC   
  
  
+ TGTTGACGTG TTAAAAGCAT ATCAGTTCTA TGTTCGTGCT TGCCCATTTA AGAAGATCGT CATTCGTTGT   
  
  
+ GGTAAACATA TGATTCTAAA AGCTGCTGAA AAGGCATCAA AGCTTCATAT TATAGATTTT GGCATCCTCT   
  
  
+ ATGGATTCCA ATGGCCTAGC CTCATTCGAT GCCTCTCAGA GCGGTCTGGT GGACCTCCAA AACTGTTTAT   
  
  
+ TACAGGGATC GATCTCCCCC AGCCTGGGTT CAGGCCAGCA GAAAGAGTGG AAGCAACAGG GAGACGCTTG   
  
  
+ GCTAAGTACT GTGAGCGGTA TAATGTGCCA TTTGAGTATC ATGCCATTGC TCAGAAGTGG GAAACAATCA   
  
  
+ AACCAGGGGA TCTCAAGTTA GGAAGTAGGG ATGATGATGA AGTTGTCGTG GTGAACTGTC TCTGTAGGTT   
  
  
+ CAAGAACCTC CTTGACGAGA CAATGGTGGT GGATAGTCCA AGGAACACAG TTTTAAACCT GATTAGAAGG   
  
  
+ GTAAAACCCG ATATTTTTGT GCATGGCATT GTAAATGGTT CCTACAACAT CCCTTTCTTT GTGACACGTT   
  
  
+ TTAGAGAAGC CCTCTTTCAT TATTCCACTC TTTTCGACAT GTTAGATGCC AACGCCTCTA GGGAGGAGCC   
  
  
+ CGAGAGGTTG ATATTCGAGA AGGCATTCTA TGGGAGGGAG ATTATGAATG TGGTGGCATG TGAGGGCACA   
  
  
+ GAGAGGGTGG AAAGGCCAGA GACATACAAG CAATGGCACG TTAGGCATAG CAGGGCAGGG TTTCGGCAAC   
  
  
+ TACCATTGGA TCCCAAGTTG ATCGAGAAAA TGAGGTTTAA GGCCAAGGCA GACCACCACA AGGATTTCGT   
  
  
+ GATTGATGTG GATGGACATT GGGCAATTCA GGGATGGAAG GGGCGGATTG CCTGTGCCAT CTCTGCATGG   
  
  
+ GTTCTGGCTT G  

- -Up\_Stream \_Len000GACAAG TCGTGATCCC TTATTCCAAA AACGGGACGT ATGATACTTT AGATTGGTTT   
  
  
- TTGTAAGGTA GAACCTTCCG ACATCGGAAC ACCTAAAACT TAAGCGAGGG TTCTTGGTTA CTTCATCGCG   
  
  
- TCTTCCGGTT ATGAGTTAAA GTCTCAGGTG TAGTTGAACA AGTTACGGTC ATATAAAGTC TTGTAATTAA   
  
  
- TGTATGACAA ACCAACCTCT TTTTTACCAA CAGAGCTTTT GATCAAAAAA TTAAAAAACA ATAAATCGTG   
  
  
- CCATTTCTTA CCTTTAATAA AAGTACGCTC TTTTTTCCTT CAACCAAAAA GAAAAAAGTT GAGAAAACTA   
  
  
- CTATATTAGT CAGCTTTAAA TCCACGTGGT AGACTGAAGT TGAGAACATG CTGGAGACTA ACGAACGAAT   
  
  
- CTTCTTGTTC TCCTAACTGT CTATTCCTCC TCACCCACCA AATATGTCTC TTAACTCACC TACCAATATT   
  
  
- TCTTCAGCTA GAGTGTATTC ATTGAAGCAA TGCTAATATT TATAAAAGTA ACTTCTTTCT GCTAATCTAT   
  
  
- ATTAATTCTT CCATGTAGAT TCGTTTAAAC CGACTCGTCT CAACCTCCCG GTGATCCCAG TGTAACACAG   
  
  
- AACGCGTTGT TCGAATCTGG TAAGCGTTGC CTACGGAGTG CACCTCGTCC ACCCTGGGCG CACCTTCAAC   
  
  
- GTACCGTTCT TAAGAGACCG TCGTTCCTCT TAACTCCCCC CTGTGCGCAC ACTGGTGCGG TCACGCCAGG   
  
  
- GGGAGAGCAC CGTCTAAACC ACTAAAAAAA AAACAAAAAA AAACTTCATT ATATATTAAA AATATACTAA   
  
  
- TTTTTTTATC CAACACACAA TATTTCACCC AGTTACTGTT TATAAAAATA ACCTACTAAT TTTAATTCAA   
  
  
- GGTAAATAAC CTCTTCACTA CCTTACGTTC AAGGTTTTAA CACCTTGAAA ACAACTGCAT CGTCCGATCT   
  
  
- CAAGGTTTTT AATTTTTTTC CTTTCTGTAT AACTCCATTT CGGAAAAATA AAATGTTTAT CTGCGTGGTT   
  
  
- CCATATTATA TTAACTGTAC TAAACTCCGC TCTCCTTCTA AGAAAACGAT GGGTTTGTTT TGTGTTACTA   
  
  
- GAAAATTTAC ATCGTTAAAC CCTAACCGTT TTAAAACAGA TTGGGCTAAC AACTTTCAAT AGGCTAAAGT   
  
  
- ATTTTAACCT ATAGCAATAA TTTAAAAGTA AGCTTATAAA TTTAAACCTG TGTTACAAAA AGTATAAACC   
  
  
- TAAAAGATTT GAACTAAAAT TGAGTTTAAA TTCAAATAAG TTAAGTAGAC TGGGTTTTTT ACCAACGTTA   
  
  
- TTACATAAAA GAAAAACTTC AATTTATAAT AGCTTAAGTA AATTGAACAT CAATTGGGCA TTAACTGGGT   
  
  
- TTGAGCTGAA AAACGATGGA ATCGTCGGTC CTTCTTCAGT ATCATAACTG GGCACCTTGC GGACTTCGGT   
  
  
- TGTAGTGGTC TGGGTGTATA AGGTAGTAGA AGACTAGAGG AGCAGTTTCT TCCTTCGACT CGTGTCGTTA   
  
  
- TTGATCTTCT TGTCCTCCGT AGTGTGGGTT CGGTTTCCGG AAATAAACTC GAGGGGTGGA GTAAGGAGCT   
  
  
- TCGAAGGAGA AGTGTTACAT TCTGGTATTA GGAAGTGGGG AAGAGAAAAA TTCGAGTAGA AATGGACGGG   
  
  
- ATCAAGAGAG AGAGAGTACT CAGTAGTCAT TATCAATGGA GAAATGAAAG AGAAGGAACT CCATACGAAG   
  
  
- TTGAAATTAT ATATATACAT GGTGAACTAA AGAGAACAGT GAAACGATAT TATGAATAAC AAACCGATAG   
  
  
- AAGGGGACAA AATATGAGTA AAAGAACGAA AGATATAGAC CCAAACTTAA CCCAAAGTGA ATAGGAGCAA   
  
  
- CTAAGACACC CTTTAATCAC TTAACCCAAA ACATGTCGTC CAAATATACT TGATCCCAAA GTTTCCATAG   
  
  
- ATGAAAAAGA GAACACTAAT AATCAACTAA TCCCTAAACC GGTTCTAATC GTCTTACCCA AGACTTAAAC   
  
  
- CCCTTAAGAG ACTACTACGA GATTTACCCA TACGAATGCA ACTATAGGGA CGAATACTAC GCAGGAATCT   
  
  
- AATACGGTTA AACAAGTTAA TGCTTCCGGA TAGACTCCTA GAGTGGAGTG ACTCGGAGGG TTCGGGGAAA   
  
  
- AGACTGGGAA ACCGCATGTC AAAGTGCAGG AGAAACCCCG GCTCGGGACC CCAACTAAGA TTACTACTAT   
  
  
- CACTAAGACT ACTACAAGAG TTCACGTAAT CGGTTTACGA ATACCTTCTT CTGTACCTCC GTTTCGGTAC   
  
  
- GTACAAAGTG CTAGGAAATC GTGAAGTCCG ACGACTCTTT GGGAAAATAC TACGGAATCC CTTCTTTATG   
  
  
- GGTTGAAGAG GCTTGGTTGT AGGATATTAA CTACTAACAA ACCTATTAGG ACCACTTTTA AGAAACCCAA   
  
  
- GAAGATCACT AAAATCACAA TCAGTGAAAC CCAGATCAAG TCGTTCGAGA TAACCTGGCT GTTAACACAG   
  
  
- ACTAAACTCA CTCGTAAAAC TCGGTGGGAA ACAAGTTCGT GAAGGTTAAC TTAGTATAGG GGTTGGTAAC   
  
  
- TGGGCAGGAG TTACCAGCAA ACCGGGAAGC CCACGGAATC TAACGTGCCG GAGATTACCA AGTCACTAGA   
  
  
- GTAGGGAACC AAACGGCTAC CTACAATAAT CACATAAGTC ACTCTTTCTC AGGTACTAAG TTAAAGTCTT   
  
  
- TCCCCACCTT CTCCGATCAT TCAAGGAAGG GTTCTTATTA TTGGAACAAT AACTAGAGTT CTTGGAGTGA   
  
  
- AAAGGATTAC TTTGCTTCCT CCTACTAGCT CACTACTACC AATTCTTCCT TTTCTTACTA CACTTAACCA   
  
  
- GATTGATGAG TTCTCCCTCA TTCTAAGTGA TAGCACTTCT GCTCCTGAAA CTTCTTCCTT CCTCATCGTT   
  
  
- CGTCAGTCGA TAAAGATGAT GACTCCTTCG ACTCAACACA CTTTAAAAAC TGTTCAAAAA CGAAACGTTA   
  
  
- ACCATGGGAC ACTTTGGACT CCGAGTAGGG TGGTACTCAA ACTTGGGACT CTTCCCGGTC AGTGTACCTA   
  
  
- ATCTTCCACC CTCTTTACCC TTCCCAGTGG GTTTCTTTGT CCTATTTTCA TCATGGTTGC AACACCTAAA   
  
  
- TTCCTTAAAC GACTACGATA CACGTGTTAG ATGACGTAGA CTACTGGCTT CTTGACGACT ACCTGACGAT   
  
  
- TTCTTTTAGT CCCTCGTGAG TAGACGACTC CCCCTACCTA AAGTTTCCAA CCGAGTAATG AAACGACTAC   
  
  
- GGGATCTCCG TGCAAATCGA CCTTGACCTA GAGTGTAAAT ATGTCGGGAT TCAAGAGTAT CCGGTAGACG   
  
  
- ACAACTGCAC AATTTTCGTA TAGTCAAGAT ACAAGCACGA ACGGGTAAAT TCTTCTAGCA GTAAGCAACA   
  
  
- CCATTTGTAT ACTAAGATTT TCGACGACTT TTCCGTAGTT TCGAAGTATA ATATCTAAAA CCGTAGGAGA   
  
  
- TACCTAAGGT TACCGGATCG GAGTAAGCTA CGGAGAGTCT CGCCAGACCA CCTGGAGGTT TTGACAAATA   
  
  
- ATGTCCCTAG CTAGAGGGGG TCGGACCCAA GTCCGGTCGT CTTTCTCACC TTCGTTGTCC CTCTGCGAAC   
  
  
- CGATTCATGA CACTCGCCAT ATTACACGGT AAACTCATAG TACGGTAACG AGTCTTCACC CTTTGTTAGT   
  
  
- TTGGTCCCCT AGAGTTCAAT CCTTCATCCC TACTACTACT TCAACAGCAC CACTTGACAG AGACATCCAA   
  
  
- GTTCTTGGAG GAACTGCTCT GTTACCACCA CCTATCAGGT TCCTTGTGTC AAAATTTGGA CTAATCTTCC   
  
  
- CATTTTGGGC TATAAAAACA CGTACCGTAA CATTTACCAA GGATGTTGTA GGGAAAGAAA CACTGTGCAA   
  
  
- AATCTCTTCG GGAGAAAGTA ATAAGGTGAG AAAAGCTGTA CAATCTACGG TTGCGGAGAT CCCTCCTCGG   
  
  
- GCTCTCCAAC TATAAGCTCT TCCGTAAGAT ACCCTCCCTC TAATACTTAC ACCACCGTAC ACTCCCGTGT   
  
  
- CTCTCCCACC TTTCCGGTCT CTGTATGTTC GTTACCGTGC AATCCGTATC GTCCCGTCCC AAAGCCGTTG   
  
  
- ATGGTAACCT AGGGTTCAAC TAGCTCTTTT ACTCCAAATT CCGGTTCCGT CTGGTGGTGT TCCTAAAGCA   
  
  
- CTAACTACAC CTACCTGTAA CCCGTTAAGT CCCTACCTTC CCCGCCTAAC GGACACGGTA GAGACGTACC   
  
  
- CAAGACCGAA C

+     STRE

| Site Name | Organism | Position | Strand | Matrix score. | sequence | function |
| --- | --- | --- | --- | --- | --- | --- |
| STRE | Arabidopsis thaliana | 3719 | + | 5 | AGGGG |  |
| STRE | Arabidopsis thaliana | 4243 | + | 5 | AGGGG |  |
| STRE | Arabidopsis thaliana | 3253 | + | 5 | AGGGG |  |
| STRE | Arabidopsis thaliana | 2735 | + | 5 | AGGGG |  |
| STRE | Arabidopsis thaliana | 2168 | - | 5 | AGGGG |  |
| STRE | Arabidopsis thaliana | 774 | - | 5 | AGGGG |  |
| STRE | Arabidopsis thaliana | 739 | + | 5 | AGGGG |  |
| STRE | Arabidopsis thaliana | 1827 | - | 5 | AGGGG |  |
| STRE | Arabidopsis thaliana | 1651 | - | 5 | AGGGG |  |

>HU07G02248.1   
+ -Up\_Stream \_Len000CTGTTC AGCACTAGGG AATAAGGTTT TTGCCCTGCA TACTATGAAA TCTAACCAAA   
  
  
+ AACATTCCAT CTTGGAAGGC TGTAGCCTTG TGGATTTTGA ATTCGCTCCC AAGAACCAAT GAAGTAGCGC   
  
  
+ AGAAGGCCAA TACTCAATTT CAGAGTCCAC ATCAACTTGT TCAATGCCAG TATATTTCAG AACATTAATT   
  
  
+ ACATACTGTT TGGTTGGAGA AAAAATGGTT GTCTCGAAAA CTAGTTTTTT AATTTTTTGT TATTTAGCAC   
  
  
+ GGTAAAGAAT GGAAATTATT TTCATGCGAG AAAAAAGGAA GTTGGTTTTT CTTTTTTCAA CTCTTTTGAT   
  
  
+ GATATAATCA GTCGAAATTT AGGTGCACCA TCTGACTTCA ACTCTTGTAC GACCTCTGAT TGCTTGCTTA   
  
  
+ GAAGAACAAG AGGATTGACA GATAAGGAGG AGTGGGTGGT TTATACAGAG AATTGAGTGG ATGGTTATAA   
  
  
+ AGAAGTCGAT CTCACATAAG TAACTTCGTT ACGATTATAA ATATTTTCAT TGAAGAAAGA CGATTAGATA   
  
  
+ TAATTAAGAA GGTACATCTA AGCAAATTTG GCTGAGCAGA GTTGGAGGGC CACTAGGGTC ACATTGTGTC   
  
  
+ TTGCGCAACA AGCTTAGACC ATTCGCAACG GATGCCTCAC GTGGAGCAGG TGGGACCCGC GTGGAAGTTG   
  
  
+ CATGGCAAGA ATTCTCTGGC AGCAAGGAGA ATTGAGGGGG GACACGCGTG TGACCACGCC AGTGCGGTCC   
  
  
+ CCCTCTCGTG GCAGATTTGG TGATTTTTTT TTTGTTTTTT TTTGAAGTAA TATATAATTT TTATATGATT   
  
  
+ AAAAAAATAG GTTGTGTGTT ATAAAGTGGG TCAATGACAA ATATTTTTAT TGGATGATTA AAATTAAGTT   
  
  
+ CCATTTATTG GAGAAGTGAT GGAATGCAAG TTCCAAAATT GTGGAACTTT TGTTGACGTA GCAGGCTAGA   
  
  
+ GTTCCAAAAA TTAAAAAAAG GAAAGACATA TTGAGGTAAA GCCTTTTTAT TTTACAAATA GACGCACCAA   
  
  
+ GGTATAATAT AATTGACATG ATTTGAGGCG AGAGGAAGAT TCTTTTGCTA CCCAAACAAA ACACAATGAT   
  
  
+ CTTTTAAATG TAGCAATTTG GGATTGGCAA AATTTTGTCT AACCCGATTG TTGAAAGTTA TCCGATTTCA   
  
  
+ TAAAATTGGA TATCGTTATT AAATTTTCAT TCGAATATTT AAATTTGGAC ACAATGTTTT TCATATTTGG   
  
  
+ ATTTTCTAAA CTTGATTTTA ACTCAAATTT AAGTTTATTC AATTCATCTG ACCCAAAAAA TGGTTGCAAT   
  
  
+ AATGTATTTT CTTTTTGAAG TTAAATATTA TCGAATTCAT TTAACTTGTA GTTAACCCGT AATTGACCCA   
  
  
+ AACTCGACTT TTTGCTACCT TAGCAGCCAG GAAGAAGTCA TAGTATTGAC CCGTGGAACG CCTGAAGCCA   
  
  
+ ACATCACCAG ACCCACATAT TCCATCATCT TCTGATCTCC TCGTCAAAGA AGGAAGCTGA GCACAGCAAT   
  
  
+ AACTAGAAGA ACAGGAGGCA TCACACCCAA GCCAAAGGCC TTTATTTGAG CTCCCCACCT CATTCCTCGA   
  
  
+ AGCTTCCTCT TCACAATGTA AGACCATAAT CCTTCACCCC TTCTCTTTTT AAGCTCATCT TTACCTGCCC   
  
  
+ TAGTTCTCTC TCTCTCATGA GTCATCAGTA ATAGTTACCT CTTTACTTTC TCTTCCTTGA GGTATGCTTC   
  
  
+ AACTTTAATA TATATATGTA CCACTTGATT TCTCTTGTCA CTTTGCTATA ATACTTATTG TTTGGCTATC   
  
  
+ TTCCCCTGTT TTATACTCAT TTTCTTGCTT TCTATATCTG GGTTTGAATT GGGTTTCACT TATCCTCGTT   
  
  
+ GATTCTGTGG GAAATTAGTG AATTGGGTTT TGTACAGCAG GTTTATATGA ACTAGGGTTT CAAAGGTATC   
  
  
+ TACTTTTTCT CTTGTGATTA TTAGTTGATT AGGGATTTGG CCAAGATTAG CAGAATGGGT TCTGAATTTG   
  
  
+ GGGAATTCTC TGATGATGCT CTAAATGGGT ATGCTTACGT TGATATCCCT GCTTATGATG CGTCCTTAGA   
  
  
+ TTATGCCAAT TTGTTCAATT ACGAAGGCCT ATCTGAGGAT CTCACCTCAC TGAGCCTCCC AAGCCCCTTT   
  
  
+ TCTGACCCTT TGGCGTACAG TTTCACGTCC TCTTTGGGGC CGAGCCCTGG GGTTGATTCT AATGATGATA   
  
  
+ GTGATTCTGA TGATGTTCTC AAGTGCATTA GCCAAATGCT TATGGAAGAA GACATGGAGG CAAAGCCATG   
  
  
+ CATGTTTCAC GATCCTTTAG CACTTCAGGC TGCTGAGAAA CCCTTTTATG ATGCCTTAGG GAAGAAATAC   
  
  
+ CCAACTTCTC CGAACCAACA TCCTATAATT GATGATTGTT TGGATAATCC TGGTGAAAAT TCTTTGGGTT   
  
  
+ CTTCTAGTGA TTTTAGTGTT AGTCACTTTG GGTCTAGTTC AGCAAGCTCT ATTGGACCGA CAATTGTGTC   
  
  
+ TGATTTGAGT GAGCATTTTG AGCCACCCTT TGTTCAAGCA CTTCCAATTG AATCATATCC CCAACCATTG   
  
  
+ ACCCGTCCTC AATGGTCGTT TGGCCCTTCG GGTGCCTTAG ATTGCACGGC CTCTAATGGT TCAGTGATCT   
  
  
+ CATCCCTTGG TTTGCCGATG GATGTTATTA GTGTATTCAG TGAGAAAGAG TCCATGATTC AATTTCAGAA   
  
  
+ AGGGGTGGAA GAGGCTAGTA AGTTCCTTCC CAAGAATAAT AACCTTGTTA TTGATCTCAA GAACCTCACT   
  
  
+ TTTCCTAATG AAACGAAGGA GGATGATCGA GTGATGATGG TTAAGAAGGA AAAGAATGAT GTGAATTGGT   
  
  
+ CTAACTACTC AAGAGGGAGT AAGATTCACT ATCGTGAAGA CGAGGACTTT GAAGAAGGAA GGAGTAGCAA   
  
  
+ GCAGTCAGCT ATTTCTACTA CTGAGGAAGC TGAGTTGTGT GAAATTTTTG ACAAGTTTTT GCTTTGCAAT   
  
  
+ TGGTACCCTG TGAAACCTGA GGCTCATCCC ACCATGAGTT TGAACCCTGA GAAGGGCCAG TCACATGGAT   
  
  
+ TAGAAGGTGG GAGAAATGGG AAGGGTCACC CAAAGAAACA GGATAAAAGT AGTACCAACG TTGTGGATTT   
  
  
+ AAGGAATTTG CTGATGCTAT GTGCACAATC TACTGCATCT GATGACCGAA GAACTGCTGA TGGACTGCTA   
  
  
+ AAGAAAATCA GGGAGCACTC ATCTGCTGAG GGGGATGGAT TTCAAAGGTT GGCTCATTAC TTTGCTGATG   
  
  
+ CCCTAGAGGC ACGTTTAGCT GGAACTGGAT CTCACATTTA TACAGCCCTA AGTTCTCATA GGCCATCTGC   
  
  
+ TGTTGACGTG TTAAAAGCAT ATCAGTTCTA TGTTCGTGCT TGCCCATTTA AGAAGATCGT CATTCGTTGT   
  
  
+ GGTAAACATA TGATTCTAAA AGCTGCTGAA AAGGCATCAA AGCTTCATAT TATAGATTTT GGCATCCTCT   
  
  
+ ATGGATTCCA ATGGCCTAGC CTCATTCGAT GCCTCTCAGA GCGGTCTGGT GGACCTCCAA AACTGTTTAT   
  
  
+ TACAGGGATC GATCTCCCCC AGCCTGGGTT CAGGCCAGCA GAAAGAGTGG AAGCAACAGG GAGACGCTTG   
  
  
+ GCTAAGTACT GTGAGCGGTA TAATGTGCCA TTTGAGTATC ATGCCATTGC TCAGAAGTGG GAAACAATCA   
  
  
+ AACCAGGGGA TCTCAAGTTA GGAAGTAGGG ATGATGATGA AGTTGTCGTG GTGAACTGTC TCTGTAGGTT   
  
  
+ CAAGAACCTC CTTGACGAGA CAATGGTGGT GGATAGTCCA AGGAACACAG TTTTAAACCT GATTAGAAGG   
  
  
+ GTAAAACCCG ATATTTTTGT GCATGGCATT GTAAATGGTT CCTACAACAT CCCTTTCTTT GTGACACGTT   
  
  
+ TTAGAGAAGC CCTCTTTCAT TATTCCACTC TTTTCGACAT GTTAGATGCC AACGCCTCTA GGGAGGAGCC   
  
  
+ CGAGAGGTTG ATATTCGAGA AGGCATTCTA TGGGAGGGAG ATTATGAATG TGGTGGCATG TGAGGGCACA   
  
  
+ GAGAGGGTGG AAAGGCCAGA GACATACAAG CAATGGCACG TTAGGCATAG CAGGGCAGGG TTTCGGCAAC   
  
  
+ TACCATTGGA TCCCAAGTTG ATCGAGAAAA TGAGGTTTAA GGCCAAGGCA GACCACCACA AGGATTTCGT   
  
  
+ GATTGATGTG GATGGACATT GGGCAATTCA GGGATGGAAG GGGCGGATTG CCTGTGCCAT CTCTGCATGG   
  
  
+ GTTCTGGCTT G  

- -Up\_Stream \_Len000GACAAG TCGTGATCCC TTATTCCAAA AACGGGACGT ATGATACTTT AGATTGGTTT   
  
  
- TTGTAAGGTA GAACCTTCCG ACATCGGAAC ACCTAAAACT TAAGCGAGGG TTCTTGGTTA CTTCATCGCG   
  
  
- TCTTCCGGTT ATGAGTTAAA GTCTCAGGTG TAGTTGAACA AGTTACGGTC ATATAAAGTC TTGTAATTAA   
  
  
- TGTATGACAA ACCAACCTCT TTTTTACCAA CAGAGCTTTT GATCAAAAAA TTAAAAAACA ATAAATCGTG   
  
  
- CCATTTCTTA CCTTTAATAA AAGTACGCTC TTTTTTCCTT CAACCAAAAA GAAAAAAGTT GAGAAAACTA   
  
  
- CTATATTAGT CAGCTTTAAA TCCACGTGGT AGACTGAAGT TGAGAACATG CTGGAGACTA ACGAACGAAT   
  
  
- CTTCTTGTTC TCCTAACTGT CTATTCCTCC TCACCCACCA AATATGTCTC TTAACTCACC TACCAATATT   
  
  
- TCTTCAGCTA GAGTGTATTC ATTGAAGCAA TGCTAATATT TATAAAAGTA ACTTCTTTCT GCTAATCTAT   
  
  
- ATTAATTCTT CCATGTAGAT TCGTTTAAAC CGACTCGTCT CAACCTCCCG GTGATCCCAG TGTAACACAG   
  
  
- AACGCGTTGT TCGAATCTGG TAAGCGTTGC CTACGGAGTG CACCTCGTCC ACCCTGGGCG CACCTTCAAC   
  
  
- GTACCGTTCT TAAGAGACCG TCGTTCCTCT TAACTCCCCC CTGTGCGCAC ACTGGTGCGG TCACGCCAGG   
  
  
- GGGAGAGCAC CGTCTAAACC ACTAAAAAAA AAACAAAAAA AAACTTCATT ATATATTAAA AATATACTAA   
  
  
- TTTTTTTATC CAACACACAA TATTTCACCC AGTTACTGTT TATAAAAATA ACCTACTAAT TTTAATTCAA   
  
  
- GGTAAATAAC CTCTTCACTA CCTTACGTTC AAGGTTTTAA CACCTTGAAA ACAACTGCAT CGTCCGATCT   
  
  
- CAAGGTTTTT AATTTTTTTC CTTTCTGTAT AACTCCATTT CGGAAAAATA AAATGTTTAT CTGCGTGGTT   
  
  
- CCATATTATA TTAACTGTAC TAAACTCCGC TCTCCTTCTA AGAAAACGAT GGGTTTGTTT TGTGTTACTA   
  
  
- GAAAATTTAC ATCGTTAAAC CCTAACCGTT TTAAAACAGA TTGGGCTAAC AACTTTCAAT AGGCTAAAGT   
  
  
- ATTTTAACCT ATAGCAATAA TTTAAAAGTA AGCTTATAAA TTTAAACCTG TGTTACAAAA AGTATAAACC   
  
  
- TAAAAGATTT GAACTAAAAT TGAGTTTAAA TTCAAATAAG TTAAGTAGAC TGGGTTTTTT ACCAACGTTA   
  
  
- TTACATAAAA GAAAAACTTC AATTTATAAT AGCTTAAGTA AATTGAACAT CAATTGGGCA TTAACTGGGT   
  
  
- TTGAGCTGAA AAACGATGGA ATCGTCGGTC CTTCTTCAGT ATCATAACTG GGCACCTTGC GGACTTCGGT   
  
  
- TGTAGTGGTC TGGGTGTATA AGGTAGTAGA AGACTAGAGG AGCAGTTTCT TCCTTCGACT CGTGTCGTTA   
  
  
- TTGATCTTCT TGTCCTCCGT AGTGTGGGTT CGGTTTCCGG AAATAAACTC GAGGGGTGGA GTAAGGAGCT   
  
  
- TCGAAGGAGA AGTGTTACAT TCTGGTATTA GGAAGTGGGG AAGAGAAAAA TTCGAGTAGA AATGGACGGG   
  
  
- ATCAAGAGAG AGAGAGTACT CAGTAGTCAT TATCAATGGA GAAATGAAAG AGAAGGAACT CCATACGAAG   
  
  
- TTGAAATTAT ATATATACAT GGTGAACTAA AGAGAACAGT GAAACGATAT TATGAATAAC AAACCGATAG   
  
  
- AAGGGGACAA AATATGAGTA AAAGAACGAA AGATATAGAC CCAAACTTAA CCCAAAGTGA ATAGGAGCAA   
  
  
- CTAAGACACC CTTTAATCAC TTAACCCAAA ACATGTCGTC CAAATATACT TGATCCCAAA GTTTCCATAG   
  
  
- ATGAAAAAGA GAACACTAAT AATCAACTAA TCCCTAAACC GGTTCTAATC GTCTTACCCA AGACTTAAAC   
  
  
- CCCTTAAGAG ACTACTACGA GATTTACCCA TACGAATGCA ACTATAGGGA CGAATACTAC GCAGGAATCT   
  
  
- AATACGGTTA AACAAGTTAA TGCTTCCGGA TAGACTCCTA GAGTGGAGTG ACTCGGAGGG TTCGGGGAAA   
  
  
- AGACTGGGAA ACCGCATGTC AAAGTGCAGG AGAAACCCCG GCTCGGGACC CCAACTAAGA TTACTACTAT   
  
  
- CACTAAGACT ACTACAAGAG TTCACGTAAT CGGTTTACGA ATACCTTCTT CTGTACCTCC GTTTCGGTAC   
  
  
- GTACAAAGTG CTAGGAAATC GTGAAGTCCG ACGACTCTTT GGGAAAATAC TACGGAATCC CTTCTTTATG   
  
  
- GGTTGAAGAG GCTTGGTTGT AGGATATTAA CTACTAACAA ACCTATTAGG ACCACTTTTA AGAAACCCAA   
  
  
- GAAGATCACT AAAATCACAA TCAGTGAAAC CCAGATCAAG TCGTTCGAGA TAACCTGGCT GTTAACACAG   
  
  
- ACTAAACTCA CTCGTAAAAC TCGGTGGGAA ACAAGTTCGT GAAGGTTAAC TTAGTATAGG GGTTGGTAAC   
  
  
- TGGGCAGGAG TTACCAGCAA ACCGGGAAGC CCACGGAATC TAACGTGCCG GAGATTACCA AGTCACTAGA   
  
  
- GTAGGGAACC AAACGGCTAC CTACAATAAT CACATAAGTC ACTCTTTCTC AGGTACTAAG TTAAAGTCTT   
  
  
- TCCCCACCTT CTCCGATCAT TCAAGGAAGG GTTCTTATTA TTGGAACAAT AACTAGAGTT CTTGGAGTGA   
  
  
- AAAGGATTAC TTTGCTTCCT CCTACTAGCT CACTACTACC AATTCTTCCT TTTCTTACTA CACTTAACCA   
  
  
- GATTGATGAG TTCTCCCTCA TTCTAAGTGA TAGCACTTCT GCTCCTGAAA CTTCTTCCTT CCTCATCGTT   
  
  
- CGTCAGTCGA TAAAGATGAT GACTCCTTCG ACTCAACACA CTTTAAAAAC TGTTCAAAAA CGAAACGTTA   
  
  
- ACCATGGGAC ACTTTGGACT CCGAGTAGGG TGGTACTCAA ACTTGGGACT CTTCCCGGTC AGTGTACCTA   
  
  
- ATCTTCCACC CTCTTTACCC TTCCCAGTGG GTTTCTTTGT CCTATTTTCA TCATGGTTGC AACACCTAAA   
  
  
- TTCCTTAAAC GACTACGATA CACGTGTTAG ATGACGTAGA CTACTGGCTT CTTGACGACT ACCTGACGAT   
  
  
- TTCTTTTAGT CCCTCGTGAG TAGACGACTC CCCCTACCTA AAGTTTCCAA CCGAGTAATG AAACGACTAC   
  
  
- GGGATCTCCG TGCAAATCGA CCTTGACCTA GAGTGTAAAT ATGTCGGGAT TCAAGAGTAT CCGGTAGACG   
  
  
- ACAACTGCAC AATTTTCGTA TAGTCAAGAT ACAAGCACGA ACGGGTAAAT TCTTCTAGCA GTAAGCAACA   
  
  
- CCATTTGTAT ACTAAGATTT TCGACGACTT TTCCGTAGTT TCGAAGTATA ATATCTAAAA CCGTAGGAGA   
  
  
- TACCTAAGGT TACCGGATCG GAGTAAGCTA CGGAGAGTCT CGCCAGACCA CCTGGAGGTT TTGACAAATA   
  
  
- ATGTCCCTAG CTAGAGGGGG TCGGACCCAA GTCCGGTCGT CTTTCTCACC TTCGTTGTCC CTCTGCGAAC   
  
  
- CGATTCATGA CACTCGCCAT ATTACACGGT AAACTCATAG TACGGTAACG AGTCTTCACC CTTTGTTAGT   
  
  
- TTGGTCCCCT AGAGTTCAAT CCTTCATCCC TACTACTACT TCAACAGCAC CACTTGACAG AGACATCCAA   
  
  
- GTTCTTGGAG GAACTGCTCT GTTACCACCA CCTATCAGGT TCCTTGTGTC AAAATTTGGA CTAATCTTCC   
  
  
- CATTTTGGGC TATAAAAACA CGTACCGTAA CATTTACCAA GGATGTTGTA GGGAAAGAAA CACTGTGCAA   
  
  
- AATCTCTTCG GGAGAAAGTA ATAAGGTGAG AAAAGCTGTA CAATCTACGG TTGCGGAGAT CCCTCCTCGG   
  
  
- GCTCTCCAAC TATAAGCTCT TCCGTAAGAT ACCCTCCCTC TAATACTTAC ACCACCGTAC ACTCCCGTGT   
  
  
- CTCTCCCACC TTTCCGGTCT CTGTATGTTC GTTACCGTGC AATCCGTATC GTCCCGTCCC AAAGCCGTTG   
  
  
- ATGGTAACCT AGGGTTCAAC TAGCTCTTTT ACTCCAAATT CCGGTTCCGT CTGGTGGTGT TCCTAAAGCA   
  
  
- CTAACTACAC CTACCTGTAA CCCGTTAAGT CCCTACCTTC CCCGCCTAAC GGACACGGTA GAGACGTACC   
  
  
- CAAGACCGAA C

+     Sp1

| Site Name | Organism | Position | Strand | Matrix score. | sequence | function |
| --- | --- | --- | --- | --- | --- | --- |
| Sp1 | Oryza sativa | 4245 | + | 6 | GGGCGG | light responsive element |

>HU07G02248.1   
+ -Up\_Stream \_Len000CTGTTC AGCACTAGGG AATAAGGTTT TTGCCCTGCA TACTATGAAA TCTAACCAAA   
  
  
+ AACATTCCAT CTTGGAAGGC TGTAGCCTTG TGGATTTTGA ATTCGCTCCC AAGAACCAAT GAAGTAGCGC   
  
  
+ AGAAGGCCAA TACTCAATTT CAGAGTCCAC ATCAACTTGT TCAATGCCAG TATATTTCAG AACATTAATT   
  
  
+ ACATACTGTT TGGTTGGAGA AAAAATGGTT GTCTCGAAAA CTAGTTTTTT AATTTTTTGT TATTTAGCAC   
  
  
+ GGTAAAGAAT GGAAATTATT TTCATGCGAG AAAAAAGGAA GTTGGTTTTT CTTTTTTCAA CTCTTTTGAT   
  
  
+ GATATAATCA GTCGAAATTT AGGTGCACCA TCTGACTTCA ACTCTTGTAC GACCTCTGAT TGCTTGCTTA   
  
  
+ GAAGAACAAG AGGATTGACA GATAAGGAGG AGTGGGTGGT TTATACAGAG AATTGAGTGG ATGGTTATAA   
  
  
+ AGAAGTCGAT CTCACATAAG TAACTTCGTT ACGATTATAA ATATTTTCAT TGAAGAAAGA CGATTAGATA   
  
  
+ TAATTAAGAA GGTACATCTA AGCAAATTTG GCTGAGCAGA GTTGGAGGGC CACTAGGGTC ACATTGTGTC   
  
  
+ TTGCGCAACA AGCTTAGACC ATTCGCAACG GATGCCTCAC GTGGAGCAGG TGGGACCCGC GTGGAAGTTG   
  
  
+ CATGGCAAGA ATTCTCTGGC AGCAAGGAGA ATTGAGGGGG GACACGCGTG TGACCACGCC AGTGCGGTCC   
  
  
+ CCCTCTCGTG GCAGATTTGG TGATTTTTTT TTTGTTTTTT TTTGAAGTAA TATATAATTT TTATATGATT   
  
  
+ AAAAAAATAG GTTGTGTGTT ATAAAGTGGG TCAATGACAA ATATTTTTAT TGGATGATTA AAATTAAGTT   
  
  
+ CCATTTATTG GAGAAGTGAT GGAATGCAAG TTCCAAAATT GTGGAACTTT TGTTGACGTA GCAGGCTAGA   
  
  
+ GTTCCAAAAA TTAAAAAAAG GAAAGACATA TTGAGGTAAA GCCTTTTTAT TTTACAAATA GACGCACCAA   
  
  
+ GGTATAATAT AATTGACATG ATTTGAGGCG AGAGGAAGAT TCTTTTGCTA CCCAAACAAA ACACAATGAT   
  
  
+ CTTTTAAATG TAGCAATTTG GGATTGGCAA AATTTTGTCT AACCCGATTG TTGAAAGTTA TCCGATTTCA   
  
  
+ TAAAATTGGA TATCGTTATT AAATTTTCAT TCGAATATTT AAATTTGGAC ACAATGTTTT TCATATTTGG   
  
  
+ ATTTTCTAAA CTTGATTTTA ACTCAAATTT AAGTTTATTC AATTCATCTG ACCCAAAAAA TGGTTGCAAT   
  
  
+ AATGTATTTT CTTTTTGAAG TTAAATATTA TCGAATTCAT TTAACTTGTA GTTAACCCGT AATTGACCCA   
  
  
+ AACTCGACTT TTTGCTACCT TAGCAGCCAG GAAGAAGTCA TAGTATTGAC CCGTGGAACG CCTGAAGCCA   
  
  
+ ACATCACCAG ACCCACATAT TCCATCATCT TCTGATCTCC TCGTCAAAGA AGGAAGCTGA GCACAGCAAT   
  
  
+ AACTAGAAGA ACAGGAGGCA TCACACCCAA GCCAAAGGCC TTTATTTGAG CTCCCCACCT CATTCCTCGA   
  
  
+ AGCTTCCTCT TCACAATGTA AGACCATAAT CCTTCACCCC TTCTCTTTTT AAGCTCATCT TTACCTGCCC   
  
  
+ TAGTTCTCTC TCTCTCATGA GTCATCAGTA ATAGTTACCT CTTTACTTTC TCTTCCTTGA GGTATGCTTC   
  
  
+ AACTTTAATA TATATATGTA CCACTTGATT TCTCTTGTCA CTTTGCTATA ATACTTATTG TTTGGCTATC   
  
  
+ TTCCCCTGTT TTATACTCAT TTTCTTGCTT TCTATATCTG GGTTTGAATT GGGTTTCACT TATCCTCGTT   
  
  
+ GATTCTGTGG GAAATTAGTG AATTGGGTTT TGTACAGCAG GTTTATATGA ACTAGGGTTT CAAAGGTATC   
  
  
+ TACTTTTTCT CTTGTGATTA TTAGTTGATT AGGGATTTGG CCAAGATTAG CAGAATGGGT TCTGAATTTG   
  
  
+ GGGAATTCTC TGATGATGCT CTAAATGGGT ATGCTTACGT TGATATCCCT GCTTATGATG CGTCCTTAGA   
  
  
+ TTATGCCAAT TTGTTCAATT ACGAAGGCCT ATCTGAGGAT CTCACCTCAC TGAGCCTCCC AAGCCCCTTT   
  
  
+ TCTGACCCTT TGGCGTACAG TTTCACGTCC TCTTTGGGGC CGAGCCCTGG GGTTGATTCT AATGATGATA   
  
  
+ GTGATTCTGA TGATGTTCTC AAGTGCATTA GCCAAATGCT TATGGAAGAA GACATGGAGG CAAAGCCATG   
  
  
+ CATGTTTCAC GATCCTTTAG CACTTCAGGC TGCTGAGAAA CCCTTTTATG ATGCCTTAGG GAAGAAATAC   
  
  
+ CCAACTTCTC CGAACCAACA TCCTATAATT GATGATTGTT TGGATAATCC TGGTGAAAAT TCTTTGGGTT   
  
  
+ CTTCTAGTGA TTTTAGTGTT AGTCACTTTG GGTCTAGTTC AGCAAGCTCT ATTGGACCGA CAATTGTGTC   
  
  
+ TGATTTGAGT GAGCATTTTG AGCCACCCTT TGTTCAAGCA CTTCCAATTG AATCATATCC CCAACCATTG   
  
  
+ ACCCGTCCTC AATGGTCGTT TGGCCCTTCG GGTGCCTTAG ATTGCACGGC CTCTAATGGT TCAGTGATCT   
  
  
+ CATCCCTTGG TTTGCCGATG GATGTTATTA GTGTATTCAG TGAGAAAGAG TCCATGATTC AATTTCAGAA   
  
  
+ AGGGGTGGAA GAGGCTAGTA AGTTCCTTCC CAAGAATAAT AACCTTGTTA TTGATCTCAA GAACCTCACT   
  
  
+ TTTCCTAATG AAACGAAGGA GGATGATCGA GTGATGATGG TTAAGAAGGA AAAGAATGAT GTGAATTGGT   
  
  
+ CTAACTACTC AAGAGGGAGT AAGATTCACT ATCGTGAAGA CGAGGACTTT GAAGAAGGAA GGAGTAGCAA   
  
  
+ GCAGTCAGCT ATTTCTACTA CTGAGGAAGC TGAGTTGTGT GAAATTTTTG ACAAGTTTTT GCTTTGCAAT   
  
  
+ TGGTACCCTG TGAAACCTGA GGCTCATCCC ACCATGAGTT TGAACCCTGA GAAGGGCCAG TCACATGGAT   
  
  
+ TAGAAGGTGG GAGAAATGGG AAGGGTCACC CAAAGAAACA GGATAAAAGT AGTACCAACG TTGTGGATTT   
  
  
+ AAGGAATTTG CTGATGCTAT GTGCACAATC TACTGCATCT GATGACCGAA GAACTGCTGA TGGACTGCTA   
  
  
+ AAGAAAATCA GGGAGCACTC ATCTGCTGAG GGGGATGGAT TTCAAAGGTT GGCTCATTAC TTTGCTGATG   
  
  
+ CCCTAGAGGC ACGTTTAGCT GGAACTGGAT CTCACATTTA TACAGCCCTA AGTTCTCATA GGCCATCTGC   
  
  
+ TGTTGACGTG TTAAAAGCAT ATCAGTTCTA TGTTCGTGCT TGCCCATTTA AGAAGATCGT CATTCGTTGT   
  
  
+ GGTAAACATA TGATTCTAAA AGCTGCTGAA AAGGCATCAA AGCTTCATAT TATAGATTTT GGCATCCTCT   
  
  
+ ATGGATTCCA ATGGCCTAGC CTCATTCGAT GCCTCTCAGA GCGGTCTGGT GGACCTCCAA AACTGTTTAT   
  
  
+ TACAGGGATC GATCTCCCCC AGCCTGGGTT CAGGCCAGCA GAAAGAGTGG AAGCAACAGG GAGACGCTTG   
  
  
+ GCTAAGTACT GTGAGCGGTA TAATGTGCCA TTTGAGTATC ATGCCATTGC TCAGAAGTGG GAAACAATCA   
  
  
+ AACCAGGGGA TCTCAAGTTA GGAAGTAGGG ATGATGATGA AGTTGTCGTG GTGAACTGTC TCTGTAGGTT   
  
  
+ CAAGAACCTC CTTGACGAGA CAATGGTGGT GGATAGTCCA AGGAACACAG TTTTAAACCT GATTAGAAGG   
  
  
+ GTAAAACCCG ATATTTTTGT GCATGGCATT GTAAATGGTT CCTACAACAT CCCTTTCTTT GTGACACGTT   
  
  
+ TTAGAGAAGC CCTCTTTCAT TATTCCACTC TTTTCGACAT GTTAGATGCC AACGCCTCTA GGGAGGAGCC   
  
  
+ CGAGAGGTTG ATATTCGAGA AGGCATTCTA TGGGAGGGAG ATTATGAATG TGGTGGCATG TGAGGGCACA   
  
  
+ GAGAGGGTGG AAAGGCCAGA GACATACAAG CAATGGCACG TTAGGCATAG CAGGGCAGGG TTTCGGCAAC   
  
  
+ TACCATTGGA TCCCAAGTTG ATCGAGAAAA TGAGGTTTAA GGCCAAGGCA GACCACCACA AGGATTTCGT   
  
  
+ GATTGATGTG GATGGACATT GGGCAATTCA GGGATGGAAG GGGCGGATTG CCTGTGCCAT CTCTGCATGG   
  
  
+ GTTCTGGCTT G  

- -Up\_Stream \_Len000GACAAG TCGTGATCCC TTATTCCAAA AACGGGACGT ATGATACTTT AGATTGGTTT   
  
  
- TTGTAAGGTA GAACCTTCCG ACATCGGAAC ACCTAAAACT TAAGCGAGGG TTCTTGGTTA CTTCATCGCG   
  
  
- TCTTCCGGTT ATGAGTTAAA GTCTCAGGTG TAGTTGAACA AGTTACGGTC ATATAAAGTC TTGTAATTAA   
  
  
- TGTATGACAA ACCAACCTCT TTTTTACCAA CAGAGCTTTT GATCAAAAAA TTAAAAAACA ATAAATCGTG   
  
  
- CCATTTCTTA CCTTTAATAA AAGTACGCTC TTTTTTCCTT CAACCAAAAA GAAAAAAGTT GAGAAAACTA   
  
  
- CTATATTAGT CAGCTTTAAA TCCACGTGGT AGACTGAAGT TGAGAACATG CTGGAGACTA ACGAACGAAT   
  
  
- CTTCTTGTTC TCCTAACTGT CTATTCCTCC TCACCCACCA AATATGTCTC TTAACTCACC TACCAATATT   
  
  
- TCTTCAGCTA GAGTGTATTC ATTGAAGCAA TGCTAATATT TATAAAAGTA ACTTCTTTCT GCTAATCTAT   
  
  
- ATTAATTCTT CCATGTAGAT TCGTTTAAAC CGACTCGTCT CAACCTCCCG GTGATCCCAG TGTAACACAG   
  
  
- AACGCGTTGT TCGAATCTGG TAAGCGTTGC CTACGGAGTG CACCTCGTCC ACCCTGGGCG CACCTTCAAC   
  
  
- GTACCGTTCT TAAGAGACCG TCGTTCCTCT TAACTCCCCC CTGTGCGCAC ACTGGTGCGG TCACGCCAGG   
  
  
- GGGAGAGCAC CGTCTAAACC ACTAAAAAAA AAACAAAAAA AAACTTCATT ATATATTAAA AATATACTAA   
  
  
- TTTTTTTATC CAACACACAA TATTTCACCC AGTTACTGTT TATAAAAATA ACCTACTAAT TTTAATTCAA   
  
  
- GGTAAATAAC CTCTTCACTA CCTTACGTTC AAGGTTTTAA CACCTTGAAA ACAACTGCAT CGTCCGATCT   
  
  
- CAAGGTTTTT AATTTTTTTC CTTTCTGTAT AACTCCATTT CGGAAAAATA AAATGTTTAT CTGCGTGGTT   
  
  
- CCATATTATA TTAACTGTAC TAAACTCCGC TCTCCTTCTA AGAAAACGAT GGGTTTGTTT TGTGTTACTA   
  
  
- GAAAATTTAC ATCGTTAAAC CCTAACCGTT TTAAAACAGA TTGGGCTAAC AACTTTCAAT AGGCTAAAGT   
  
  
- ATTTTAACCT ATAGCAATAA TTTAAAAGTA AGCTTATAAA TTTAAACCTG TGTTACAAAA AGTATAAACC   
  
  
- TAAAAGATTT GAACTAAAAT TGAGTTTAAA TTCAAATAAG TTAAGTAGAC TGGGTTTTTT ACCAACGTTA   
  
  
- TTACATAAAA GAAAAACTTC AATTTATAAT AGCTTAAGTA AATTGAACAT CAATTGGGCA TTAACTGGGT   
  
  
- TTGAGCTGAA AAACGATGGA ATCGTCGGTC CTTCTTCAGT ATCATAACTG GGCACCTTGC GGACTTCGGT   
  
  
- TGTAGTGGTC TGGGTGTATA AGGTAGTAGA AGACTAGAGG AGCAGTTTCT TCCTTCGACT CGTGTCGTTA   
  
  
- TTGATCTTCT TGTCCTCCGT AGTGTGGGTT CGGTTTCCGG AAATAAACTC GAGGGGTGGA GTAAGGAGCT   
  
  
- TCGAAGGAGA AGTGTTACAT TCTGGTATTA GGAAGTGGGG AAGAGAAAAA TTCGAGTAGA AATGGACGGG   
  
  
- ATCAAGAGAG AGAGAGTACT CAGTAGTCAT TATCAATGGA GAAATGAAAG AGAAGGAACT CCATACGAAG   
  
  
- TTGAAATTAT ATATATACAT GGTGAACTAA AGAGAACAGT GAAACGATAT TATGAATAAC AAACCGATAG   
  
  
- AAGGGGACAA AATATGAGTA AAAGAACGAA AGATATAGAC CCAAACTTAA CCCAAAGTGA ATAGGAGCAA   
  
  
- CTAAGACACC CTTTAATCAC TTAACCCAAA ACATGTCGTC CAAATATACT TGATCCCAAA GTTTCCATAG   
  
  
- ATGAAAAAGA GAACACTAAT AATCAACTAA TCCCTAAACC GGTTCTAATC GTCTTACCCA AGACTTAAAC   
  
  
- CCCTTAAGAG ACTACTACGA GATTTACCCA TACGAATGCA ACTATAGGGA CGAATACTAC GCAGGAATCT   
  
  
- AATACGGTTA AACAAGTTAA TGCTTCCGGA TAGACTCCTA GAGTGGAGTG ACTCGGAGGG TTCGGGGAAA   
  
  
- AGACTGGGAA ACCGCATGTC AAAGTGCAGG AGAAACCCCG GCTCGGGACC CCAACTAAGA TTACTACTAT   
  
  
- CACTAAGACT ACTACAAGAG TTCACGTAAT CGGTTTACGA ATACCTTCTT CTGTACCTCC GTTTCGGTAC   
  
  
- GTACAAAGTG CTAGGAAATC GTGAAGTCCG ACGACTCTTT GGGAAAATAC TACGGAATCC CTTCTTTATG   
  
  
- GGTTGAAGAG GCTTGGTTGT AGGATATTAA CTACTAACAA ACCTATTAGG ACCACTTTTA AGAAACCCAA   
  
  
- GAAGATCACT AAAATCACAA TCAGTGAAAC CCAGATCAAG TCGTTCGAGA TAACCTGGCT GTTAACACAG   
  
  
- ACTAAACTCA CTCGTAAAAC TCGGTGGGAA ACAAGTTCGT GAAGGTTAAC TTAGTATAGG GGTTGGTAAC   
  
  
- TGGGCAGGAG TTACCAGCAA ACCGGGAAGC CCACGGAATC TAACGTGCCG GAGATTACCA AGTCACTAGA   
  
  
- GTAGGGAACC AAACGGCTAC CTACAATAAT CACATAAGTC ACTCTTTCTC AGGTACTAAG TTAAAGTCTT   
  
  
- TCCCCACCTT CTCCGATCAT TCAAGGAAGG GTTCTTATTA TTGGAACAAT AACTAGAGTT CTTGGAGTGA   
  
  
- AAAGGATTAC TTTGCTTCCT CCTACTAGCT CACTACTACC AATTCTTCCT TTTCTTACTA CACTTAACCA   
  
  
- GATTGATGAG TTCTCCCTCA TTCTAAGTGA TAGCACTTCT GCTCCTGAAA CTTCTTCCTT CCTCATCGTT   
  
  
- CGTCAGTCGA TAAAGATGAT GACTCCTTCG ACTCAACACA CTTTAAAAAC TGTTCAAAAA CGAAACGTTA   
  
  
- ACCATGGGAC ACTTTGGACT CCGAGTAGGG TGGTACTCAA ACTTGGGACT CTTCCCGGTC AGTGTACCTA   
  
  
- ATCTTCCACC CTCTTTACCC TTCCCAGTGG GTTTCTTTGT CCTATTTTCA TCATGGTTGC AACACCTAAA   
  
  
- TTCCTTAAAC GACTACGATA CACGTGTTAG ATGACGTAGA CTACTGGCTT CTTGACGACT ACCTGACGAT   
  
  
- TTCTTTTAGT CCCTCGTGAG TAGACGACTC CCCCTACCTA AAGTTTCCAA CCGAGTAATG AAACGACTAC   
  
  
- GGGATCTCCG TGCAAATCGA CCTTGACCTA GAGTGTAAAT ATGTCGGGAT TCAAGAGTAT CCGGTAGACG   
  
  
- ACAACTGCAC AATTTTCGTA TAGTCAAGAT ACAAGCACGA ACGGGTAAAT TCTTCTAGCA GTAAGCAACA   
  
  
- CCATTTGTAT ACTAAGATTT TCGACGACTT TTCCGTAGTT TCGAAGTATA ATATCTAAAA CCGTAGGAGA   
  
  
- TACCTAAGGT TACCGGATCG GAGTAAGCTA CGGAGAGTCT CGCCAGACCA CCTGGAGGTT TTGACAAATA   
  
  
- ATGTCCCTAG CTAGAGGGGG TCGGACCCAA GTCCGGTCGT CTTTCTCACC TTCGTTGTCC CTCTGCGAAC   
  
  
- CGATTCATGA CACTCGCCAT ATTACACGGT AAACTCATAG TACGGTAACG AGTCTTCACC CTTTGTTAGT   
  
  
- TTGGTCCCCT AGAGTTCAAT CCTTCATCCC TACTACTACT TCAACAGCAC CACTTGACAG AGACATCCAA   
  
  
- GTTCTTGGAG GAACTGCTCT GTTACCACCA CCTATCAGGT TCCTTGTGTC AAAATTTGGA CTAATCTTCC   
  
  
- CATTTTGGGC TATAAAAACA CGTACCGTAA CATTTACCAA GGATGTTGTA GGGAAAGAAA CACTGTGCAA   
  
  
- AATCTCTTCG GGAGAAAGTA ATAAGGTGAG AAAAGCTGTA CAATCTACGG TTGCGGAGAT CCCTCCTCGG   
  
  
- GCTCTCCAAC TATAAGCTCT TCCGTAAGAT ACCCTCCCTC TAATACTTAC ACCACCGTAC ACTCCCGTGT   
  
  
- CTCTCCCACC TTTCCGGTCT CTGTATGTTC GTTACCGTGC AATCCGTATC GTCCCGTCCC AAAGCCGTTG   
  
  
- ATGGTAACCT AGGGTTCAAC TAGCTCTTTT ACTCCAAATT CCGGTTCCGT CTGGTGGTGT TCCTAAAGCA   
  
  
- CTAACTACAC CTACCTGTAA CCCGTTAAGT CCCTACCTTC CCCGCCTAAC GGACACGGTA GAGACGTACC   
  
  
- CAAGACCGAA C

+     TATA-box

| Site Name | Organism | Position | Strand | Matrix score. | sequence | function |
| --- | --- | --- | --- | --- | --- | --- |
| TATA-box | Arabidopsis thaliana | 3663 | - | 4 | TATA | core promoter element around -30 of transcription start |
| TATA-box | Helianthus annuus | 3331 | - | 6 | TATAAA | core promoter element around -30 of transcription start |
| TATA-box | Arabidopsis thaliana | 3484 | - | 5 | TATAA | core promoter element around -30 of transcription start |
| TATA-box | Arabidopsis thaliana | 3332 | - | 5 | TATAA | core promoter element around -30 of transcription start |
| TATA-box | Brassica oleracea | 1061 | + | 6 | ATATAA | core promoter element around -30 of transcription start |
| TATA-box | Brassica juncea | 3330 | - | 7 | TATAAAT | core promoter element around -30 of transcription start |
| TATA-box | Arabidopsis thaliana | 1938 | + | 4 | TATA | core promoter element around -30 of transcription start |
| TATA-box | Brassica napus | 1766 | + | 6 | ATATAT | core promoter element around -30 of transcription start |
| TATA-box | Brassica napus | 824 | + | 6 | ATATAT | core promoter element around -30 of transcription start |
| TATA-box | Arabidopsis thaliana | 465 | - | 5 | TATAA | core promoter element around -30 of transcription start |
| TATA-box | Arabidopsis thaliana | 563 | + | 4 | TATA | core promoter element around -30 of transcription start |
| TATA-box | Arabidopsis thaliana | 357 | + | 4 | TATA | core promoter element around -30 of transcription start |
| TATA-box | Arabidopsis thaliana | 1765 | + | 6 | TATATA | core promoter element around -30 of transcription start |
| TATA-box | Arabidopsis thaliana | 3485 | - | 4 | TATA | core promoter element around -30 of transcription start |
| TATA-box | Brassica napus | 3483 | + | 6 | ATTATA | core promoter element around -30 of transcription start |
| TATA-box | Helianthus annuus | 834 | - | 6 | TATAAA | core promoter element around -30 of transcription start |
| TATA-box | Arabidopsis thaliana | 490 | + | 4 | TATA | core promoter element around -30 of transcription start |
| TATA-box | Arabidopsis thaliana | 489 | - | 5 | TATAA | core promoter element around -30 of transcription start |
| TATA-box | Arabidopsis thaliana | 1857 | + | 4 | TATA | core promoter element around -30 of transcription start |
| TATA-box | Oryza sativa | 1922 | - | 7 | TACAAAA | core promoter element around -30 of transcription start |
| TATA-box | Helianthus annuus | 1834 | - | 6 | TATAAA | core promoter element around -30 of transcription start |
| TATA-box | Arabidopsis thaliana | 835 | - | 5 | TATAA | core promoter element around -30 of transcription start |
| TATA-box | Arabidopsis thaliana | 466 | + | 4 | TATA | core promoter element around -30 of transcription start |
| TATA-box | Arabidopsis thaliana | 1937 | - | 5 | TATAA | core promoter element around -30 of transcription start |
| TATA-box | Brassica oleracea | 356 | + | 6 | ATATAA | core promoter element around -30 of transcription start |
| TATA-box | Arabidopsis thaliana | 1767 | + | 4 | TATA | core promoter element around -30 of transcription start |
| TATA-box | Helianthus annuus | 1936 | - | 6 | TATAAA | core promoter element around -30 of transcription start |
| TATA-box | Arabidopsis thaliana | 3333 | - | 4 | TATA | core promoter element around -30 of transcription start |
| TATA-box | Arabidopsis thaliana | 1836 | + | 4 | TATA | core promoter element around -30 of transcription start |
| TATA-box | Arabidopsis thaliana | 1835 | - | 5 | TATAA | core promoter element around -30 of transcription start |
| TATA-box | Brassica napus | 1762 | + | 6 | ATATAT | core promoter element around -30 of transcription start |
| TATA-box | Brassica oleracea | 562 | + | 6 | ATATAA | core promoter element around -30 of transcription start |
| TATA-box | Brassica napus | 528 | + | 6 | ATTATA | core promoter element around -30 of transcription start |
| TATA-box | Arabidopsis thaliana | 195 | + | 4 | TATA | core promoter element around -30 of transcription start |
| TATA-box | Arabidopsis thaliana | 529 | - | 5 | TATAA | core promoter element around -30 of transcription start |
| TATA-box | Arabidopsis thaliana | 2408 | - | 4 | TATA | core promoter element around -30 of transcription start |
| TATA-box | Arabidopsis thaliana | 1230 | + | 8 | TATTTAAA | core promoter element around -30 of transcription start |
| TATA-box | Pisum sativum | 1833 | - | 7 | TATAAAA | core promoter element around -30 of transcription start |
| TATA-box | Brassica napus | 1764 | + | 6 | ATATAT | core promoter element around -30 of transcription start |
| TATA-box | Arabidopsis thaliana | 1062 | + | 4 | TATA | core promoter element around -30 of transcription start |
| TATA-box | Arabidopsis thaliana | 864 | + | 4 | TATA | core promoter element around -30 of transcription start |
| TATA-box | Brassica oleracea | 826 | + | 6 | ATATAA | core promoter element around -30 of transcription start |
| TATA-box | Arabidopsis thaliana | 1801 | + | 4 | TATA | core promoter element around -30 of transcription start |
| TATA-box | Helianthus annuus | 464 | - | 6 | TATAAA | core promoter element around -30 of transcription start |
| TATA-box | Arabidopsis thaliana | 863 | - | 5 | TATAA | core promoter element around -30 of transcription start |
| TATA-box | Arabidopsis thaliana | 1763 | + | 6 | TATATA | core promoter element around -30 of transcription start |
| TATA-box | Arabidopsis thaliana | 530 | + | 4 | TATA | core promoter element around -30 of transcription start |
| TATA-box | Arabidopsis thaliana | 888 | - | 9 | ccTATAAAaa | core promoter element around -30 of transcription start |
| TATA-box | Arabidopsis thaliana | 836 | + | 4 | TATA | core promoter element around -30 of transcription start |
| TATA-box | Arabidopsis thaliana | 1057 | + | 4 | TATA | core promoter element around -30 of transcription start |
| TATA-box | Arabidopsis thaliana | 825 | + | 6 | TATATA | core promoter element around -30 of transcription start |
| TATA-box | Arabidopsis thaliana | 827 | + | 4 | TATA | core promoter element around -30 of transcription start |
| TATA-box | Arabidopsis thaliana | 832 | - | 9 | ccTATAAAaa | core promoter element around -30 of transcription start |
| TATA-box | Pisum sativum | 833 | - | 7 | TATAAAA | core promoter element around -30 of transcription start |

>HU07G02248.1   
+ -Up\_Stream \_Len000CTGTTC AGCACTAGGG AATAAGGTTT TTGCCCTGCA TACTATGAAA TCTAACCAAA   
  
  
+ AACATTCCAT CTTGGAAGGC TGTAGCCTTG TGGATTTTGA ATTCGCTCCC AAGAACCAAT GAAGTAGCGC   
  
  
+ AGAAGGCCAA TACTCAATTT CAGAGTCCAC ATCAACTTGT TCAATGCCAG TATATTTCAG AACATTAATT   
  
  
+ ACATACTGTT TGGTTGGAGA AAAAATGGTT GTCTCGAAAA CTAGTTTTTT AATTTTTTGT TATTTAGCAC   
  
  
+ GGTAAAGAAT GGAAATTATT TTCATGCGAG AAAAAAGGAA GTTGGTTTTT CTTTTTTCAA CTCTTTTGAT   
  
  
+ GATATAATCA GTCGAAATTT AGGTGCACCA TCTGACTTCA ACTCTTGTAC GACCTCTGAT TGCTTGCTTA   
  
  
+ GAAGAACAAG AGGATTGACA GATAAGGAGG AGTGGGTGGT TTATACAGAG AATTGAGTGG ATGGTTATAA   
  
  
+ AGAAGTCGAT CTCACATAAG TAACTTCGTT ACGATTATAA ATATTTTCAT TGAAGAAAGA CGATTAGATA   
  
  
+ TAATTAAGAA GGTACATCTA AGCAAATTTG GCTGAGCAGA GTTGGAGGGC CACTAGGGTC ACATTGTGTC   
  
  
+ TTGCGCAACA AGCTTAGACC ATTCGCAACG GATGCCTCAC GTGGAGCAGG TGGGACCCGC GTGGAAGTTG   
  
  
+ CATGGCAAGA ATTCTCTGGC AGCAAGGAGA ATTGAGGGGG GACACGCGTG TGACCACGCC AGTGCGGTCC   
  
  
+ CCCTCTCGTG GCAGATTTGG TGATTTTTTT TTTGTTTTTT TTTGAAGTAA TATATAATTT TTATATGATT   
  
  
+ AAAAAAATAG GTTGTGTGTT ATAAAGTGGG TCAATGACAA ATATTTTTAT TGGATGATTA AAATTAAGTT   
  
  
+ CCATTTATTG GAGAAGTGAT GGAATGCAAG TTCCAAAATT GTGGAACTTT TGTTGACGTA GCAGGCTAGA   
  
  
+ GTTCCAAAAA TTAAAAAAAG GAAAGACATA TTGAGGTAAA GCCTTTTTAT TTTACAAATA GACGCACCAA   
  
  
+ GGTATAATAT AATTGACATG ATTTGAGGCG AGAGGAAGAT TCTTTTGCTA CCCAAACAAA ACACAATGAT   
  
  
+ CTTTTAAATG TAGCAATTTG GGATTGGCAA AATTTTGTCT AACCCGATTG TTGAAAGTTA TCCGATTTCA   
  
  
+ TAAAATTGGA TATCGTTATT AAATTTTCAT TCGAATATTT AAATTTGGAC ACAATGTTTT TCATATTTGG   
  
  
+ ATTTTCTAAA CTTGATTTTA ACTCAAATTT AAGTTTATTC AATTCATCTG ACCCAAAAAA TGGTTGCAAT   
  
  
+ AATGTATTTT CTTTTTGAAG TTAAATATTA TCGAATTCAT TTAACTTGTA GTTAACCCGT AATTGACCCA   
  
  
+ AACTCGACTT TTTGCTACCT TAGCAGCCAG GAAGAAGTCA TAGTATTGAC CCGTGGAACG CCTGAAGCCA   
  
  
+ ACATCACCAG ACCCACATAT TCCATCATCT TCTGATCTCC TCGTCAAAGA AGGAAGCTGA GCACAGCAAT   
  
  
+ AACTAGAAGA ACAGGAGGCA TCACACCCAA GCCAAAGGCC TTTATTTGAG CTCCCCACCT CATTCCTCGA   
  
  
+ AGCTTCCTCT TCACAATGTA AGACCATAAT CCTTCACCCC TTCTCTTTTT AAGCTCATCT TTACCTGCCC   
  
  
+ TAGTTCTCTC TCTCTCATGA GTCATCAGTA ATAGTTACCT CTTTACTTTC TCTTCCTTGA GGTATGCTTC   
  
  
+ AACTTTAATA TATATATGTA CCACTTGATT TCTCTTGTCA CTTTGCTATA ATACTTATTG TTTGGCTATC   
  
  
+ TTCCCCTGTT TTATACTCAT TTTCTTGCTT TCTATATCTG GGTTTGAATT GGGTTTCACT TATCCTCGTT   
  
  
+ GATTCTGTGG GAAATTAGTG AATTGGGTTT TGTACAGCAG GTTTATATGA ACTAGGGTTT CAAAGGTATC   
  
  
+ TACTTTTTCT CTTGTGATTA TTAGTTGATT AGGGATTTGG CCAAGATTAG CAGAATGGGT TCTGAATTTG   
  
  
+ GGGAATTCTC TGATGATGCT CTAAATGGGT ATGCTTACGT TGATATCCCT GCTTATGATG CGTCCTTAGA   
  
  
+ TTATGCCAAT TTGTTCAATT ACGAAGGCCT ATCTGAGGAT CTCACCTCAC TGAGCCTCCC AAGCCCCTTT   
  
  
+ TCTGACCCTT TGGCGTACAG TTTCACGTCC TCTTTGGGGC CGAGCCCTGG GGTTGATTCT AATGATGATA   
  
  
+ GTGATTCTGA TGATGTTCTC AAGTGCATTA GCCAAATGCT TATGGAAGAA GACATGGAGG CAAAGCCATG   
  
  
+ CATGTTTCAC GATCCTTTAG CACTTCAGGC TGCTGAGAAA CCCTTTTATG ATGCCTTAGG GAAGAAATAC   
  
  
+ CCAACTTCTC CGAACCAACA TCCTATAATT GATGATTGTT TGGATAATCC TGGTGAAAAT TCTTTGGGTT   
  
  
+ CTTCTAGTGA TTTTAGTGTT AGTCACTTTG GGTCTAGTTC AGCAAGCTCT ATTGGACCGA CAATTGTGTC   
  
  
+ TGATTTGAGT GAGCATTTTG AGCCACCCTT TGTTCAAGCA CTTCCAATTG AATCATATCC CCAACCATTG   
  
  
+ ACCCGTCCTC AATGGTCGTT TGGCCCTTCG GGTGCCTTAG ATTGCACGGC CTCTAATGGT TCAGTGATCT   
  
  
+ CATCCCTTGG TTTGCCGATG GATGTTATTA GTGTATTCAG TGAGAAAGAG TCCATGATTC AATTTCAGAA   
  
  
+ AGGGGTGGAA GAGGCTAGTA AGTTCCTTCC CAAGAATAAT AACCTTGTTA TTGATCTCAA GAACCTCACT   
  
  
+ TTTCCTAATG AAACGAAGGA GGATGATCGA GTGATGATGG TTAAGAAGGA AAAGAATGAT GTGAATTGGT   
  
  
+ CTAACTACTC AAGAGGGAGT AAGATTCACT ATCGTGAAGA CGAGGACTTT GAAGAAGGAA GGAGTAGCAA   
  
  
+ GCAGTCAGCT ATTTCTACTA CTGAGGAAGC TGAGTTGTGT GAAATTTTTG ACAAGTTTTT GCTTTGCAAT   
  
  
+ TGGTACCCTG TGAAACCTGA GGCTCATCCC ACCATGAGTT TGAACCCTGA GAAGGGCCAG TCACATGGAT   
  
  
+ TAGAAGGTGG GAGAAATGGG AAGGGTCACC CAAAGAAACA GGATAAAAGT AGTACCAACG TTGTGGATTT   
  
  
+ AAGGAATTTG CTGATGCTAT GTGCACAATC TACTGCATCT GATGACCGAA GAACTGCTGA TGGACTGCTA   
  
  
+ AAGAAAATCA GGGAGCACTC ATCTGCTGAG GGGGATGGAT TTCAAAGGTT GGCTCATTAC TTTGCTGATG   
  
  
+ CCCTAGAGGC ACGTTTAGCT GGAACTGGAT CTCACATTTA TACAGCCCTA AGTTCTCATA GGCCATCTGC   
  
  
+ TGTTGACGTG TTAAAAGCAT ATCAGTTCTA TGTTCGTGCT TGCCCATTTA AGAAGATCGT CATTCGTTGT   
  
  
+ GGTAAACATA TGATTCTAAA AGCTGCTGAA AAGGCATCAA AGCTTCATAT TATAGATTTT GGCATCCTCT   
  
  
+ ATGGATTCCA ATGGCCTAGC CTCATTCGAT GCCTCTCAGA GCGGTCTGGT GGACCTCCAA AACTGTTTAT   
  
  
+ TACAGGGATC GATCTCCCCC AGCCTGGGTT CAGGCCAGCA GAAAGAGTGG AAGCAACAGG GAGACGCTTG   
  
  
+ GCTAAGTACT GTGAGCGGTA TAATGTGCCA TTTGAGTATC ATGCCATTGC TCAGAAGTGG GAAACAATCA   
  
  
+ AACCAGGGGA TCTCAAGTTA GGAAGTAGGG ATGATGATGA AGTTGTCGTG GTGAACTGTC TCTGTAGGTT   
  
  
+ CAAGAACCTC CTTGACGAGA CAATGGTGGT GGATAGTCCA AGGAACACAG TTTTAAACCT GATTAGAAGG   
  
  
+ GTAAAACCCG ATATTTTTGT GCATGGCATT GTAAATGGTT CCTACAACAT CCCTTTCTTT GTGACACGTT   
  
  
+ TTAGAGAAGC CCTCTTTCAT TATTCCACTC TTTTCGACAT GTTAGATGCC AACGCCTCTA GGGAGGAGCC   
  
  
+ CGAGAGGTTG ATATTCGAGA AGGCATTCTA TGGGAGGGAG ATTATGAATG TGGTGGCATG TGAGGGCACA   
  
  
+ GAGAGGGTGG AAAGGCCAGA GACATACAAG CAATGGCACG TTAGGCATAG CAGGGCAGGG TTTCGGCAAC   
  
  
+ TACCATTGGA TCCCAAGTTG ATCGAGAAAA TGAGGTTTAA GGCCAAGGCA GACCACCACA AGGATTTCGT   
  
  
+ GATTGATGTG GATGGACATT GGGCAATTCA GGGATGGAAG GGGCGGATTG CCTGTGCCAT CTCTGCATGG   
  
  
+ GTTCTGGCTT G  

- -Up\_Stream \_Len000GACAAG TCGTGATCCC TTATTCCAAA AACGGGACGT ATGATACTTT AGATTGGTTT   
  
  
- TTGTAAGGTA GAACCTTCCG ACATCGGAAC ACCTAAAACT TAAGCGAGGG TTCTTGGTTA CTTCATCGCG   
  
  
- TCTTCCGGTT ATGAGTTAAA GTCTCAGGTG TAGTTGAACA AGTTACGGTC ATATAAAGTC TTGTAATTAA   
  
  
- TGTATGACAA ACCAACCTCT TTTTTACCAA CAGAGCTTTT GATCAAAAAA TTAAAAAACA ATAAATCGTG   
  
  
- CCATTTCTTA CCTTTAATAA AAGTACGCTC TTTTTTCCTT CAACCAAAAA GAAAAAAGTT GAGAAAACTA   
  
  
- CTATATTAGT CAGCTTTAAA TCCACGTGGT AGACTGAAGT TGAGAACATG CTGGAGACTA ACGAACGAAT   
  
  
- CTTCTTGTTC TCCTAACTGT CTATTCCTCC TCACCCACCA AATATGTCTC TTAACTCACC TACCAATATT   
  
  
- TCTTCAGCTA GAGTGTATTC ATTGAAGCAA TGCTAATATT TATAAAAGTA ACTTCTTTCT GCTAATCTAT   
  
  
- ATTAATTCTT CCATGTAGAT TCGTTTAAAC CGACTCGTCT CAACCTCCCG GTGATCCCAG TGTAACACAG   
  
  
- AACGCGTTGT TCGAATCTGG TAAGCGTTGC CTACGGAGTG CACCTCGTCC ACCCTGGGCG CACCTTCAAC   
  
  
- GTACCGTTCT TAAGAGACCG TCGTTCCTCT TAACTCCCCC CTGTGCGCAC ACTGGTGCGG TCACGCCAGG   
  
  
- GGGAGAGCAC CGTCTAAACC ACTAAAAAAA AAACAAAAAA AAACTTCATT ATATATTAAA AATATACTAA   
  
  
- TTTTTTTATC CAACACACAA TATTTCACCC AGTTACTGTT TATAAAAATA ACCTACTAAT TTTAATTCAA   
  
  
- GGTAAATAAC CTCTTCACTA CCTTACGTTC AAGGTTTTAA CACCTTGAAA ACAACTGCAT CGTCCGATCT   
  
  
- CAAGGTTTTT AATTTTTTTC CTTTCTGTAT AACTCCATTT CGGAAAAATA AAATGTTTAT CTGCGTGGTT   
  
  
- CCATATTATA TTAACTGTAC TAAACTCCGC TCTCCTTCTA AGAAAACGAT GGGTTTGTTT TGTGTTACTA   
  
  
- GAAAATTTAC ATCGTTAAAC CCTAACCGTT TTAAAACAGA TTGGGCTAAC AACTTTCAAT AGGCTAAAGT   
  
  
- ATTTTAACCT ATAGCAATAA TTTAAAAGTA AGCTTATAAA TTTAAACCTG TGTTACAAAA AGTATAAACC   
  
  
- TAAAAGATTT GAACTAAAAT TGAGTTTAAA TTCAAATAAG TTAAGTAGAC TGGGTTTTTT ACCAACGTTA   
  
  
- TTACATAAAA GAAAAACTTC AATTTATAAT AGCTTAAGTA AATTGAACAT CAATTGGGCA TTAACTGGGT   
  
  
- TTGAGCTGAA AAACGATGGA ATCGTCGGTC CTTCTTCAGT ATCATAACTG GGCACCTTGC GGACTTCGGT   
  
  
- TGTAGTGGTC TGGGTGTATA AGGTAGTAGA AGACTAGAGG AGCAGTTTCT TCCTTCGACT CGTGTCGTTA   
  
  
- TTGATCTTCT TGTCCTCCGT AGTGTGGGTT CGGTTTCCGG AAATAAACTC GAGGGGTGGA GTAAGGAGCT   
  
  
- TCGAAGGAGA AGTGTTACAT TCTGGTATTA GGAAGTGGGG AAGAGAAAAA TTCGAGTAGA AATGGACGGG   
  
  
- ATCAAGAGAG AGAGAGTACT CAGTAGTCAT TATCAATGGA GAAATGAAAG AGAAGGAACT CCATACGAAG   
  
  
- TTGAAATTAT ATATATACAT GGTGAACTAA AGAGAACAGT GAAACGATAT TATGAATAAC AAACCGATAG   
  
  
- AAGGGGACAA AATATGAGTA AAAGAACGAA AGATATAGAC CCAAACTTAA CCCAAAGTGA ATAGGAGCAA   
  
  
- CTAAGACACC CTTTAATCAC TTAACCCAAA ACATGTCGTC CAAATATACT TGATCCCAAA GTTTCCATAG   
  
  
- ATGAAAAAGA GAACACTAAT AATCAACTAA TCCCTAAACC GGTTCTAATC GTCTTACCCA AGACTTAAAC   
  
  
- CCCTTAAGAG ACTACTACGA GATTTACCCA TACGAATGCA ACTATAGGGA CGAATACTAC GCAGGAATCT   
  
  
- AATACGGTTA AACAAGTTAA TGCTTCCGGA TAGACTCCTA GAGTGGAGTG ACTCGGAGGG TTCGGGGAAA   
  
  
- AGACTGGGAA ACCGCATGTC AAAGTGCAGG AGAAACCCCG GCTCGGGACC CCAACTAAGA TTACTACTAT   
  
  
- CACTAAGACT ACTACAAGAG TTCACGTAAT CGGTTTACGA ATACCTTCTT CTGTACCTCC GTTTCGGTAC   
  
  
- GTACAAAGTG CTAGGAAATC GTGAAGTCCG ACGACTCTTT GGGAAAATAC TACGGAATCC CTTCTTTATG   
  
  
- GGTTGAAGAG GCTTGGTTGT AGGATATTAA CTACTAACAA ACCTATTAGG ACCACTTTTA AGAAACCCAA   
  
  
- GAAGATCACT AAAATCACAA TCAGTGAAAC CCAGATCAAG TCGTTCGAGA TAACCTGGCT GTTAACACAG   
  
  
- ACTAAACTCA CTCGTAAAAC TCGGTGGGAA ACAAGTTCGT GAAGGTTAAC TTAGTATAGG GGTTGGTAAC   
  
  
- TGGGCAGGAG TTACCAGCAA ACCGGGAAGC CCACGGAATC TAACGTGCCG GAGATTACCA AGTCACTAGA   
  
  
- GTAGGGAACC AAACGGCTAC CTACAATAAT CACATAAGTC ACTCTTTCTC AGGTACTAAG TTAAAGTCTT   
  
  
- TCCCCACCTT CTCCGATCAT TCAAGGAAGG GTTCTTATTA TTGGAACAAT AACTAGAGTT CTTGGAGTGA   
  
  
- AAAGGATTAC TTTGCTTCCT CCTACTAGCT CACTACTACC AATTCTTCCT TTTCTTACTA CACTTAACCA   
  
  
- GATTGATGAG TTCTCCCTCA TTCTAAGTGA TAGCACTTCT GCTCCTGAAA CTTCTTCCTT CCTCATCGTT   
  
  
- CGTCAGTCGA TAAAGATGAT GACTCCTTCG ACTCAACACA CTTTAAAAAC TGTTCAAAAA CGAAACGTTA   
  
  
- ACCATGGGAC ACTTTGGACT CCGAGTAGGG TGGTACTCAA ACTTGGGACT CTTCCCGGTC AGTGTACCTA   
  
  
- ATCTTCCACC CTCTTTACCC TTCCCAGTGG GTTTCTTTGT CCTATTTTCA TCATGGTTGC AACACCTAAA   
  
  
- TTCCTTAAAC GACTACGATA CACGTGTTAG ATGACGTAGA CTACTGGCTT CTTGACGACT ACCTGACGAT   
  
  
- TTCTTTTAGT CCCTCGTGAG TAGACGACTC CCCCTACCTA AAGTTTCCAA CCGAGTAATG AAACGACTAC   
  
  
- GGGATCTCCG TGCAAATCGA CCTTGACCTA GAGTGTAAAT ATGTCGGGAT TCAAGAGTAT CCGGTAGACG   
  
  
- ACAACTGCAC AATTTTCGTA TAGTCAAGAT ACAAGCACGA ACGGGTAAAT TCTTCTAGCA GTAAGCAACA   
  
  
- CCATTTGTAT ACTAAGATTT TCGACGACTT TTCCGTAGTT TCGAAGTATA ATATCTAAAA CCGTAGGAGA   
  
  
- TACCTAAGGT TACCGGATCG GAGTAAGCTA CGGAGAGTCT CGCCAGACCA CCTGGAGGTT TTGACAAATA   
  
  
- ATGTCCCTAG CTAGAGGGGG TCGGACCCAA GTCCGGTCGT CTTTCTCACC TTCGTTGTCC CTCTGCGAAC   
  
  
- CGATTCATGA CACTCGCCAT ATTACACGGT AAACTCATAG TACGGTAACG AGTCTTCACC CTTTGTTAGT   
  
  
- TTGGTCCCCT AGAGTTCAAT CCTTCATCCC TACTACTACT TCAACAGCAC CACTTGACAG AGACATCCAA   
  
  
- GTTCTTGGAG GAACTGCTCT GTTACCACCA CCTATCAGGT TCCTTGTGTC AAAATTTGGA CTAATCTTCC   
  
  
- CATTTTGGGC TATAAAAACA CGTACCGTAA CATTTACCAA GGATGTTGTA GGGAAAGAAA CACTGTGCAA   
  
  
- AATCTCTTCG GGAGAAAGTA ATAAGGTGAG AAAAGCTGTA CAATCTACGG TTGCGGAGAT CCCTCCTCGG   
  
  
- GCTCTCCAAC TATAAGCTCT TCCGTAAGAT ACCCTCCCTC TAATACTTAC ACCACCGTAC ACTCCCGTGT   
  
  
- CTCTCCCACC TTTCCGGTCT CTGTATGTTC GTTACCGTGC AATCCGTATC GTCCCGTCCC AAAGCCGTTG   
  
  
- ATGGTAACCT AGGGTTCAAC TAGCTCTTTT ACTCCAAATT CCGGTTCCGT CTGGTGGTGT TCCTAAAGCA   
  
  
- CTAACTACAC CTACCTGTAA CCCGTTAAGT CCCTACCTTC CCCGCCTAAC GGACACGGTA GAGACGTACC   
  
  
- CAAGACCGAA C

+     TCA

| Site Name | Organism | Position | Strand | Matrix score. | sequence | function |
| --- | --- | --- | --- | --- | --- | --- |
| TCA | Pisum sativum | 3745 | - | 9 | TCATCTTCAT |  |

>HU07G02248.1   
+ -Up\_Stream \_Len000CTGTTC AGCACTAGGG AATAAGGTTT TTGCCCTGCA TACTATGAAA TCTAACCAAA   
  
  
+ AACATTCCAT CTTGGAAGGC TGTAGCCTTG TGGATTTTGA ATTCGCTCCC AAGAACCAAT GAAGTAGCGC   
  
  
+ AGAAGGCCAA TACTCAATTT CAGAGTCCAC ATCAACTTGT TCAATGCCAG TATATTTCAG AACATTAATT   
  
  
+ ACATACTGTT TGGTTGGAGA AAAAATGGTT GTCTCGAAAA CTAGTTTTTT AATTTTTTGT TATTTAGCAC   
  
  
+ GGTAAAGAAT GGAAATTATT TTCATGCGAG AAAAAAGGAA GTTGGTTTTT CTTTTTTCAA CTCTTTTGAT   
  
  
+ GATATAATCA GTCGAAATTT AGGTGCACCA TCTGACTTCA ACTCTTGTAC GACCTCTGAT TGCTTGCTTA   
  
  
+ GAAGAACAAG AGGATTGACA GATAAGGAGG AGTGGGTGGT TTATACAGAG AATTGAGTGG ATGGTTATAA   
  
  
+ AGAAGTCGAT CTCACATAAG TAACTTCGTT ACGATTATAA ATATTTTCAT TGAAGAAAGA CGATTAGATA   
  
  
+ TAATTAAGAA GGTACATCTA AGCAAATTTG GCTGAGCAGA GTTGGAGGGC CACTAGGGTC ACATTGTGTC   
  
  
+ TTGCGCAACA AGCTTAGACC ATTCGCAACG GATGCCTCAC GTGGAGCAGG TGGGACCCGC GTGGAAGTTG   
  
  
+ CATGGCAAGA ATTCTCTGGC AGCAAGGAGA ATTGAGGGGG GACACGCGTG TGACCACGCC AGTGCGGTCC   
  
  
+ CCCTCTCGTG GCAGATTTGG TGATTTTTTT TTTGTTTTTT TTTGAAGTAA TATATAATTT TTATATGATT   
  
  
+ AAAAAAATAG GTTGTGTGTT ATAAAGTGGG TCAATGACAA ATATTTTTAT TGGATGATTA AAATTAAGTT   
  
  
+ CCATTTATTG GAGAAGTGAT GGAATGCAAG TTCCAAAATT GTGGAACTTT TGTTGACGTA GCAGGCTAGA   
  
  
+ GTTCCAAAAA TTAAAAAAAG GAAAGACATA TTGAGGTAAA GCCTTTTTAT TTTACAAATA GACGCACCAA   
  
  
+ GGTATAATAT AATTGACATG ATTTGAGGCG AGAGGAAGAT TCTTTTGCTA CCCAAACAAA ACACAATGAT   
  
  
+ CTTTTAAATG TAGCAATTTG GGATTGGCAA AATTTTGTCT AACCCGATTG TTGAAAGTTA TCCGATTTCA   
  
  
+ TAAAATTGGA TATCGTTATT AAATTTTCAT TCGAATATTT AAATTTGGAC ACAATGTTTT TCATATTTGG   
  
  
+ ATTTTCTAAA CTTGATTTTA ACTCAAATTT AAGTTTATTC AATTCATCTG ACCCAAAAAA TGGTTGCAAT   
  
  
+ AATGTATTTT CTTTTTGAAG TTAAATATTA TCGAATTCAT TTAACTTGTA GTTAACCCGT AATTGACCCA   
  
  
+ AACTCGACTT TTTGCTACCT TAGCAGCCAG GAAGAAGTCA TAGTATTGAC CCGTGGAACG CCTGAAGCCA   
  
  
+ ACATCACCAG ACCCACATAT TCCATCATCT TCTGATCTCC TCGTCAAAGA AGGAAGCTGA GCACAGCAAT   
  
  
+ AACTAGAAGA ACAGGAGGCA TCACACCCAA GCCAAAGGCC TTTATTTGAG CTCCCCACCT CATTCCTCGA   
  
  
+ AGCTTCCTCT TCACAATGTA AGACCATAAT CCTTCACCCC TTCTCTTTTT AAGCTCATCT TTACCTGCCC   
  
  
+ TAGTTCTCTC TCTCTCATGA GTCATCAGTA ATAGTTACCT CTTTACTTTC TCTTCCTTGA GGTATGCTTC   
  
  
+ AACTTTAATA TATATATGTA CCACTTGATT TCTCTTGTCA CTTTGCTATA ATACTTATTG TTTGGCTATC   
  
  
+ TTCCCCTGTT TTATACTCAT TTTCTTGCTT TCTATATCTG GGTTTGAATT GGGTTTCACT TATCCTCGTT   
  
  
+ GATTCTGTGG GAAATTAGTG AATTGGGTTT TGTACAGCAG GTTTATATGA ACTAGGGTTT CAAAGGTATC   
  
  
+ TACTTTTTCT CTTGTGATTA TTAGTTGATT AGGGATTTGG CCAAGATTAG CAGAATGGGT TCTGAATTTG   
  
  
+ GGGAATTCTC TGATGATGCT CTAAATGGGT ATGCTTACGT TGATATCCCT GCTTATGATG CGTCCTTAGA   
  
  
+ TTATGCCAAT TTGTTCAATT ACGAAGGCCT ATCTGAGGAT CTCACCTCAC TGAGCCTCCC AAGCCCCTTT   
  
  
+ TCTGACCCTT TGGCGTACAG TTTCACGTCC TCTTTGGGGC CGAGCCCTGG GGTTGATTCT AATGATGATA   
  
  
+ GTGATTCTGA TGATGTTCTC AAGTGCATTA GCCAAATGCT TATGGAAGAA GACATGGAGG CAAAGCCATG   
  
  
+ CATGTTTCAC GATCCTTTAG CACTTCAGGC TGCTGAGAAA CCCTTTTATG ATGCCTTAGG GAAGAAATAC   
  
  
+ CCAACTTCTC CGAACCAACA TCCTATAATT GATGATTGTT TGGATAATCC TGGTGAAAAT TCTTTGGGTT   
  
  
+ CTTCTAGTGA TTTTAGTGTT AGTCACTTTG GGTCTAGTTC AGCAAGCTCT ATTGGACCGA CAATTGTGTC   
  
  
+ TGATTTGAGT GAGCATTTTG AGCCACCCTT TGTTCAAGCA CTTCCAATTG AATCATATCC CCAACCATTG   
  
  
+ ACCCGTCCTC AATGGTCGTT TGGCCCTTCG GGTGCCTTAG ATTGCACGGC CTCTAATGGT TCAGTGATCT   
  
  
+ CATCCCTTGG TTTGCCGATG GATGTTATTA GTGTATTCAG TGAGAAAGAG TCCATGATTC AATTTCAGAA   
  
  
+ AGGGGTGGAA GAGGCTAGTA AGTTCCTTCC CAAGAATAAT AACCTTGTTA TTGATCTCAA GAACCTCACT   
  
  
+ TTTCCTAATG AAACGAAGGA GGATGATCGA GTGATGATGG TTAAGAAGGA AAAGAATGAT GTGAATTGGT   
  
  
+ CTAACTACTC AAGAGGGAGT AAGATTCACT ATCGTGAAGA CGAGGACTTT GAAGAAGGAA GGAGTAGCAA   
  
  
+ GCAGTCAGCT ATTTCTACTA CTGAGGAAGC TGAGTTGTGT GAAATTTTTG ACAAGTTTTT GCTTTGCAAT   
  
  
+ TGGTACCCTG TGAAACCTGA GGCTCATCCC ACCATGAGTT TGAACCCTGA GAAGGGCCAG TCACATGGAT   
  
  
+ TAGAAGGTGG GAGAAATGGG AAGGGTCACC CAAAGAAACA GGATAAAAGT AGTACCAACG TTGTGGATTT   
  
  
+ AAGGAATTTG CTGATGCTAT GTGCACAATC TACTGCATCT GATGACCGAA GAACTGCTGA TGGACTGCTA   
  
  
+ AAGAAAATCA GGGAGCACTC ATCTGCTGAG GGGGATGGAT TTCAAAGGTT GGCTCATTAC TTTGCTGATG   
  
  
+ CCCTAGAGGC ACGTTTAGCT GGAACTGGAT CTCACATTTA TACAGCCCTA AGTTCTCATA GGCCATCTGC   
  
  
+ TGTTGACGTG TTAAAAGCAT ATCAGTTCTA TGTTCGTGCT TGCCCATTTA AGAAGATCGT CATTCGTTGT   
  
  
+ GGTAAACATA TGATTCTAAA AGCTGCTGAA AAGGCATCAA AGCTTCATAT TATAGATTTT GGCATCCTCT   
  
  
+ ATGGATTCCA ATGGCCTAGC CTCATTCGAT GCCTCTCAGA GCGGTCTGGT GGACCTCCAA AACTGTTTAT
[truncated: 153,176 more chars]
