# Supplementary material for: Genome-Wide Identification and Expression Pattern of the GRAS Gene Family in Pitaya (Selenicereus undatus L.)
Source: Biology (Basel). 2022 Dec 21;12(1):11. doi: 10.3390/biology12010011 (PMC9854919; doi:10.3390/biology12010011)
Supplement: Supplementary file 1 [file biology-12-00011-s001.zip › Supplementary file S5/HU07G02249.1_plantcare.html]

Content-Type: text/html; charset=ISO-8859-1


PlantCARE


Webmaster Firefox specific output  
To save the result:
click on the frame with the right mouse button and save the source code as a text file with extension .html  
REFERENCE:PlantCARE: a database of plant cis-acting regulatory elements and a portal to tools for in silico analysis of promoter sequences.  
Lescot, M., Déhais, P., Moreau, Y., De Moor, B., Rouzé ,P.,and Rombauts, S.  
Nucleic Acids Res., Database issue(2002), 30(1):325-327.   


---

>HU07G02249.1   
+ -Up\_Stream \_Len000GATTTG ATTGTCGAAA TTTGTCCGAT TTTATAAAAT TGAAAATTGT TATTGGACTA   
  
  
+ TAATCTACTG TAGTTTAGAG GTGTTGGATA ATACTCTCAA CTTTGAACTT GGACGTCCAT GCACCACACT   
  
  
+ ATGGGCGTAT TTACTGTTTA TATACCAAGA ATAATTTTGA AGATGTTCTT AGTTGGTGAT GTAAGTGTAT   
  
  
+ ATTCAACATA TGTCATTACA TAAACTTGAA AATTCTTGAA TTTTAGACAT ATACAATTGC AAATCATTGT   
  
  
+ AAAATTGTTG CATTACATTT TGATAAAAAT GCATCCAAAC TTTGTCTTGG TATAAACAGT AAATAGGCCC   
  
  
+ ATAGGTCCAT AGTGTAGTGC ATGGACGTCC AAGTTTAAAG GTGGGAGTAT TATTGCCCAA CGCCCCTAAA   
  
  
+ GTACAGTATA TTATAGTCCA ATATCCCAAT TAATTTTTCA TTTGAATATT TAAATTTGGA CATAATATTT   
  
  
+ TTCATATTTG AATTCTCTAA ACCTGATTTT AACCTGAATT TAAGTTGATT TAATTCATCC GACCAAAAAA   
  
  
+ ATAAACACAA TTATTTATTT TTCTATTCGA AGTTAGATAG TAGCCAATTC ATTTGACATA TAATTAACCC   
  
  
+ GTGATTGACA CAAACTCGAG TTTTTGCTTA GAGCTAGGAA AAGAGTCGGG TTAGGTCGAC CCATTTAAGA   
  
  
+ AATGGGTCGA CTTCATGTCG GCCTTCAAAA TAGTTAAGAT TTGCTCAAAA CTAAAAAATA ACAAGTTCAC   
  
  
+ ACTAAACGGT CAAATCGTGT CGACTTCGGA TTGAAATTGG TTCAGTTTGG GTCGGCTTCG TATTTCTTTC   
  
  
+ AGTAACTTCG AACCGAATTC GTATCGTGTT ATCGTGTCAG GTCAGACTTT CTCAGTTCTA TTTTTGCTAC   
  
  
+ CTTATGTTAC GGATAAAAAA AGTCTCACAT TGGAAAAAGT GTGGGAGGGT CCTGGGCTTA TAAATAGTGG   
  
  
+ CGCTGCACAC CTCATCAGAC CGACCTTTTG GGGAGAGGGT AATGACCCCG TGACAAGTGG TATCAGAGTC   
  
  
+ AACCCAGACC CGACTCAGAT GTCCTGCCAA GCGCTCCGAC GGGCGGGGTA GGCAGGGGGC CCATTTGTGA   
  
  
+ TGAGGACGTC ACAGAATTGG GCGGGGGAGA GTGTCACGGA TAAAAAAAGT CCCACATCGA AAAAAGTGTG   
  
  
+ GGAGGGTCCT GGGCTTATAA ACGGTAGCGC CGCACACCCC ATCAGATCGA CTTTTTGGGG AGAGGGTAAT   
  
  
+ GACCCCATTT CAAGTGGTAT CAGAGCCAAC CCAGACCCGA CTCCAATGTC CCACCAAGCG CTCTGATGGG   
  
  
+ CGGGGTAGGC AAGGGGCTCC ATATGTGACG AGGGCGTCAC GGAATTGGAT GGGAGAGAGT ACCACGGATA   
  
  
+ AAAAAAGTCC CACATCAGAA AAAGTGTGGA CTTAGCAACT AGGAAGTAAA GTTACAGTAT TAGCCGAGAG   
  
  
+ CCCATGGAAC GCCTGAAACT GACATCACCA GACCCACATA TTTCACCATC ATCTGATCTC CCCGTCAAAG   
  
  
+ GAGGAAGCTG AGCACAGCAA GAACTAGAAG AACAGGCGGC ATCACACCCA AGCCAAAATT CTCTATTTGA   
  
  
+ CCTCCCTCTC CTCATTCCTC TGAGCTTCCT CTTCACAATG TAAGACCATA ATCCTTAACA CCCTTCTTTT   
  
  
+ TTAAGCTCAT CTTTACCTGC TCTGGTTCTC TCTCTCATCG TCACTAATAG TTACCTCTTT ACTTTCTCTT   
  
  
+ CCCTGAGGTA TGCTTCAACT TTAACATATA TATCCCACTT GATTTCTCTT GTCTTTTTGC TATAATACTT   
  
  
+ GTTGTTTGGC TATCATCCCC TGTTTTATAC TCATTTTCTT GCTTTCTATT TCTGGGTTTG AATTGGGTTT   
  
  
+ CACTTATCCT CGTCAATTCT ATGGGTAATT AGTGAATTGG GTTTATATGA ACTAGGGTTG CAAAAGTATC   
  
  
+ TACTTTTTCT CTTGTGATTA TTAGTTGATT AGGGATTTGG CCAATATTAG CAGAATGGGT TCTGAATTTG   
  
  
+ CGGAATTCTC TGATGATGCT CTAAATGGGT ATGCTTATGT TGATATCCCT GCTTATGATG CATCCTTAGA   
  
  
+ TTATGCCAAT TTGTTCAATT ACGAAGGCCC ATCTGAGGAT CGCACCTCAC TGAGCCTCCC AAGACCCTTT   
  
  
+ TCTGACCCTT TGGCTTACAG TTTCACGTCC TCTTCTGAGC TGGGCCCTGG GGTTGATTCT AATGATGATA   
  
  
+ GTGATTCTGG CGATGTTCTC AAGTACATTA GCCAAATGCT TATGGAAGAA GACATGGAGG CAAAGCCATG   
  
  
+ CATGTTTCAT GATCCTTTAG CACTTCAGGC TGCTGAGAAA CCCTTTTATG ATGCCTTAGG GGAGCAATGC   
  
  
+ CCAACTTCTC CTGACCAACA TCCTATAATT GATCATTATT TGGATAGTCC TGATGAAAAT TCTTTGAGTT   
  
  
+ CAACTGGTGA TTTTAGTGTT AGTCATTCTG GGTCTAGTTC AACAAACTCC GTTGGACCGA TAATAGTGTC   
  
  
+ TGATTTGAGT GAGCATTTTG AGCCACCCTT TGTTGAAACA CTTCCAATTG AATCATATCA CCAACCATTG   
  
  
+ ACCCGTCCTC AATGGTCATT TGGCTCTTCG GGTGCCTTAG ATGGCACGGC CTCTAATGGT TCGGTGATCT   
  
  
+ CATCCCTTGG TTTGCCAGTG GATGTGATTA GCGTATTTAG GGAGAAAGAG TCCATGATTC AATTTCAGAA   
  
  
+ AGGGGTGGAG GAGGCCAGTA AGTTCCTTCC CAAGAATAAT AACCTTGTTA TTGATCTCGA GAACCTCACT   
  
  
+ TTTCCTAATG AAACAAAGGA GGATGATCGA ATGATGATGG TTAAGAAGGA AAAGGATGAC GTGAATTGGT   
  
  
+ CTAACTACTC AAGAGGGAGT AAGATTCACT ATTGTGAAGA CGAGGCCTTT GAAGAAGGAA GGAGTGGCAA   
  
  
+ GCAGTCAGCT ATTTCTTCTA CTGAGGAAGC TGAGTTTTCT GACATTTTTG ACAAGGTTTT GCTTTGCGAT   
  
  
+ TGCTACCCTG TGAAACCTGA GGCTCATCCC ACCATGAGTT TGAACCCTGA GAAGGGCCAG TCACATGGAT   
  
  
+ TAGAAGGTGG GAGAAATGGG AAGGCTCGCC CTAAGAAACA GGATAATAGT AGCACAAATA TTGTGGATTT   
  
  
+ AAGGAATTTG CTGATACTAT GCGCACAATC TACTGCATCT GATGACCGAA GAACTGCTGA TGGACTGCTA   
  
  
+ AAGCAAATCA GGGAGCACTC GTCTGCTGAG GGGGATGGAT CTCAAAGGTT GGCGCATTAC TTTGCTGATG   
  
  
+ CCCTAGAGGC ACGTTTAGCT GGAACTGGAT CTCGCATTTA TACGGCCCTA TGTTCTAATA GGCCATCTGT   
  
  
+ CACTGACATG ATAAAAGCAT ATCAGTTCTA TATTCGTGCT TGCCCATTTA CGAAGATCGT CATTGGTTGT   
  
  
+ GGTACCCATA TGATTCTAAA AGCAGCTGAG AAGGCATCAA AGCTTCATAT TATAGATTTT GGCATCCTCT   
  
  
+ ATGGTTGCCA ATGGCCCAAC CTCATTCAAC GCCTCTCAGA GCGATCTGGT GGACCTCCAA AACTGTTTAT   
  
  
+ TACAGGGATC GATCTCCCCC AGCCTGGGTT CAGGCCAGCA GAAAGAGTGG AAGCAACAGG GAGACGCTTG   
  
  
+ GCCAAGTACT GTGAGCGGTA TAATGTGCCA TTTGAGTATC ATGCCATTGC TCAGGAGTGG GAAACAATCA   
  
  
+ AACCAGGGGA TCTCAAGATA GGAAGTAGGA ATGATGAAGT TGTTGCGGTG AACTGTCTCT GTAGGTTCAA   
  
  
+ GAACCTCCTT GACGAGACAG TGGTGGTGGA TAGTCCAAGG AACACAGTTT TAAACCTGAT TACAAGGGTA   
  
  
+ AAGCCTGATA TTTTTGTGCA TGGCGTTGTA AATGGTTCCT ACAACATCCC TTTCTTTGTG ACACGTTTTA   
  
  
+ GAGAAGCCCT CTTTCATTAT TCCACTCTTT TCGACATGTT AGATGCCAAC GCCTCTAGGG AGGAGCCCGA   
  
  
+ GAGGTTGATA TTCGAGAAGG CATTCTATGG GAGGGAGATT ATGAATGTGG TGGCCTGTGA GGGCACAGAG   
  
  
+ AGGGTGGAGA GGCCGGAGAC ATACAAGCAA TGGCATGTTA GGCATAGCAG GGCAGGGTTT CGGCAAGTAC   
  
  
+ CATTGGATCC CAAGTTGATC GAGAAAATGA GGTTTAAGGC CAAGGCAGAC CACCACAAGG ATTTCATGAT   
  
  
+ CGATGTGGAT GGACATTGGG CAATTCAGGG ATGGAAGGGG CGGATTGCAC ATGCGATCTC TGCATGGGTT   
  
  
+ CCGGCTTG  

- -Up\_Stream \_Len000CTAAAC TAACAGCTTT AAACAGGCTA AAATATTTTA ACTTTTAACA ATAACCTGAT   
  
  
- ATTAGATGAC ATCAAATCTC CACAACCTAT TATGAGAGTT GAAACTTGAA CCTGCAGGTA CGTGGTGTGA   
  
  
- TACCCGCATA AATGACAAAT ATATGGTTCT TATTAAAACT TCTACAAGAA TCAACCACTA CATTCACATA   
  
  
- TAAGTTGTAT ACAGTAATGT ATTTGAACTT TTAAGAACTT AAAATCTGTA TATGTTAACG TTTAGTAACA   
  
  
- TTTTAACAAC GTAATGTAAA ACTATTTTTA CGTAGGTTTG AAACAGAACC ATATTTGTCA TTTATCCGGG   
  
  
- TATCCAGGTA TCACATCACG TACCTGCAGG TTCAAATTTC CACCCTCATA ATAACGGGTT GCGGGGATTT   
  
  
- CATGTCATAT AATATCAGGT TATAGGGTTA ATTAAAAAGT AAACTTATAA ATTTAAACCT GTATTATAAA   
  
  
- AAGTATAAAC TTAAGAGATT TGGACTAAAA TTGGACTTAA ATTCAACTAA ATTAAGTAGG CTGGTTTTTT   
  
  
- TATTTGTGTT AATAAATAAA AAGATAAGCT TCAATCTATC ATCGGTTAAG TAAACTGTAT ATTAATTGGG   
  
  
- CACTAACTGT GTTTGAGCTC AAAAACGAAT CTCGATCCTT TTCTCAGCCC AATCCAGCTG GGTAAATTCT   
  
  
- TTACCCAGCT GAAGTACAGC CGGAAGTTTT ATCAATTCTA AACGAGTTTT GATTTTTTAT TGTTCAAGTG   
  
  
- TGATTTGCCA GTTTAGCACA GCTGAAGCCT AACTTTAACC AAGTCAAACC CAGCCGAAGC ATAAAGAAAG   
  
  
- TCATTGAAGC TTGGCTTAAG CATAGCACAA TAGCACAGTC CAGTCTGAAA GAGTCAAGAT AAAAACGATG   
  
  
- GAATACAATG CCTATTTTTT TCAGAGTGTA ACCTTTTTCA CACCCTCCCA GGACCCGAAT ATTTATCACC   
  
  
- GCGACGTGTG GAGTAGTCTG GCTGGAAAAC CCCTCTCCCA TTACTGGGGC ACTGTTCACC ATAGTCTCAG   
  
  
- TTGGGTCTGG GCTGAGTCTA CAGGACGGTT CGCGAGGCTG CCCGCCCCAT CCGTCCCCCG GGTAAACACT   
  
  
- ACTCCTGCAG TGTCTTAACC CGCCCCCTCT CACAGTGCCT ATTTTTTTCA GGGTGTAGCT TTTTTCACAC   
  
  
- CCTCCCAGGA CCCGAATATT TGCCATCGCG GCGTGTGGGG TAGTCTAGCT GAAAAACCCC TCTCCCATTA   
  
  
- CTGGGGTAAA GTTCACCATA GTCTCGGTTG GGTCTGGGCT GAGGTTACAG GGTGGTTCGC GAGACTACCC   
  
  
- GCCCCATCCG TTCCCCGAGG TATACACTGC TCCCGCAGTG CCTTAACCTA CCCTCTCTCA TGGTGCCTAT   
  
  
- TTTTTTCAGG GTGTAGTCTT TTTCACACCT GAATCGTTGA TCCTTCATTT CAATGTCATA ATCGGCTCTC   
  
  
- GGGTACCTTG CGGACTTTGA CTGTAGTGGT CTGGGTGTAT AAAGTGGTAG TAGACTAGAG GGGCAGTTTC   
  
  
- CTCCTTCGAC TCGTGTCGTT CTTGATCTTC TTGTCCGCCG TAGTGTGGGT TCGGTTTTAA GAGATAAACT   
  
  
- GGAGGGAGAG GAGTAAGGAG ACTCGAAGGA GAAGTGTTAC ATTCTGGTAT TAGGAATTGT GGGAAGAAAA   
  
  
- AATTCGAGTA GAAATGGACG AGACCAAGAG AGAGAGTAGC AGTGATTATC AATGGAGAAA TGAAAGAGAA   
  
  
- GGGACTCCAT ACGAAGTTGA AATTGTATAT ATAGGGTGAA CTAAAGAGAA CAGAAAAACG ATATTATGAA   
  
  
- CAACAAACCG ATAGTAGGGG ACAAAATATG AGTAAAAGAA CGAAAGATAA AGACCCAAAC TTAACCCAAA   
  
  
- GTGAATAGGA GCAGTTAAGA TACCCATTAA TCACTTAACC CAAATATACT TGATCCCAAC GTTTTCATAG   
  
  
- ATGAAAAAGA GAACACTAAT AATCAACTAA TCCCTAAACC GGTTATAATC GTCTTACCCA AGACTTAAAC   
  
  
- GCCTTAAGAG ACTACTACGA GATTTACCCA TACGAATACA ACTATAGGGA CGAATACTAC GTAGGAATCT   
  
  
- AATACGGTTA AACAAGTTAA TGCTTCCGGG TAGACTCCTA GCGTGGAGTG ACTCGGAGGG TTCTGGGAAA   
  
  
- AGACTGGGAA ACCGAATGTC AAAGTGCAGG AGAAGACTCG ACCCGGGACC CCAACTAAGA TTACTACTAT   
  
  
- CACTAAGACC GCTACAAGAG TTCATGTAAT CGGTTTACGA ATACCTTCTT CTGTACCTCC GTTTCGGTAC   
  
  
- GTACAAAGTA CTAGGAAATC GTGAAGTCCG ACGACTCTTT GGGAAAATAC TACGGAATCC CCTCGTTACG   
  
  
- GGTTGAAGAG GACTGGTTGT AGGATATTAA CTAGTAATAA ACCTATCAGG ACTACTTTTA AGAAACTCAA   
  
  
- GTTGACCACT AAAATCACAA TCAGTAAGAC CCAGATCAAG TTGTTTGAGG CAACCTGGCT ATTATCACAG   
  
  
- ACTAAACTCA CTCGTAAAAC TCGGTGGGAA ACAACTTTGT GAAGGTTAAC TTAGTATAGT GGTTGGTAAC   
  
  
- TGGGCAGGAG TTACCAGTAA ACCGAGAAGC CCACGGAATC TACCGTGCCG GAGATTACCA AGCCACTAGA   
  
  
- GTAGGGAACC AAACGGTCAC CTACACTAAT CGCATAAATC CCTCTTTCTC AGGTACTAAG TTAAAGTCTT   
  
  
- TCCCCACCTC CTCCGGTCAT TCAAGGAAGG GTTCTTATTA TTGGAACAAT AACTAGAGCT CTTGGAGTGA   
  
  
- AAAGGATTAC TTTGTTTCCT CCTACTAGCT TACTACTACC AATTCTTCCT TTTCCTACTG CACTTAACCA   
  
  
- GATTGATGAG TTCTCCCTCA TTCTAAGTGA TAACACTTCT GCTCCGGAAA CTTCTTCCTT CCTCACCGTT   
  
  
- CGTCAGTCGA TAAAGAAGAT GACTCCTTCG ACTCAAAAGA CTGTAAAAAC TGTTCCAAAA CGAAACGCTA   
  
  
- ACGATGGGAC ACTTTGGACT CCGAGTAGGG TGGTACTCAA ACTTGGGACT CTTCCCGGTC AGTGTACCTA   
  
  
- ATCTTCCACC CTCTTTACCC TTCCGAGCGG GATTCTTTGT CCTATTATCA TCGTGTTTAT AACACCTAAA   
  
  
- TTCCTTAAAC GACTATGATA CGCGTGTTAG ATGACGTAGA CTACTGGCTT CTTGACGACT ACCTGACGAT   
  
  
- TTCGTTTAGT CCCTCGTGAG CAGACGACTC CCCCTACCTA GAGTTTCCAA CCGCGTAATG AAACGACTAC   
  
  
- GGGATCTCCG TGCAAATCGA CCTTGACCTA GAGCGTAAAT ATGCCGGGAT ACAAGATTAT CCGGTAGACA   
  
  
- GTGACTGTAC TATTTTCGTA TAGTCAAGAT ATAAGCACGA ACGGGTAAAT GCTTCTAGCA GTAACCAACA   
  
  
- CCATGGGTAT ACTAAGATTT TCGTCGACTC TTCCGTAGTT TCGAAGTATA ATATCTAAAA CCGTAGGAGA   
  
  
- TACCAACGGT TACCGGGTTG GAGTAAGTTG CGGAGAGTCT CGCTAGACCA CCTGGAGGTT TTGACAAATA   
  
  
- ATGTCCCTAG CTAGAGGGGG TCGGACCCAA GTCCGGTCGT CTTTCTCACC TTCGTTGTCC CTCTGCGAAC   
  
  
- CGGTTCATGA CACTCGCCAT ATTACACGGT AAACTCATAG TACGGTAACG AGTCCTCACC CTTTGTTAGT   
  
  
- TTGGTCCCCT AGAGTTCTAT CCTTCATCCT TACTACTTCA ACAACGCCAC TTGACAGAGA CATCCAAGTT   
  
  
- CTTGGAGGAA CTGCTCTGTC ACCACCACCT ATCAGGTTCC TTGTGTCAAA ATTTGGACTA ATGTTCCCAT   
  
  
- TTCGGACTAT AAAAACACGT ACCGCAACAT TTACCAAGGA TGTTGTAGGG AAAGAAACAC TGTGCAAAAT   
  
  
- CTCTTCGGGA GAAAGTAATA AGGTGAGAAA AGCTGTACAA TCTACGGTTG CGGAGATCCC TCCTCGGGCT   
  
  
- CTCCAACTAT AAGCTCTTCC GTAAGATACC CTCCCTCTAA TACTTACACC ACCGGACACT CCCGTGTCTC   
  
  
- TCCCACCTCT CCGGCCTCTG TATGTTCGTT ACCGTACAAT CCGTATCGTC CCGTCCCAAA GCCGTTCATG   
  
  
- GTAACCTAGG GTTCAACTAG CTCTTTTACT CCAAATTCCG GTTCCGTCTG GTGGTGTTCC TAAAGTACTA   
  
  
- GCTACACCTA CCTGTAACCC GTTAAGTCCC TACCTTCCCC GCCTAACGTG TACGCTAGAG ACGTACCCAA   
  
  
- GGCCGAAC

  
  
Motifs Found  

+   

| Site Name | Organism | Position | Strand | Matrix score. | sequence | function |
| --- | --- | --- | --- | --- | --- | --- |
|  | organism | 2204 | + | 4 | motif\_sequence | short\_function |
|  | organism | 482 | - | 4 | motif\_sequence | short\_function |
|  | organism | 1016 | - | 4 | motif\_sequence | short\_function |
|  | organism | 187 | + | 4 | motif\_sequence | short\_function |
|  | organism | 2618 | + | 4 | motif\_sequence | short\_function |
|  | organism | 1253 | - | 4 | motif\_sequence | short\_function |
|  | organism | 1073 | + | 4 | motif\_sequence | short\_function |
|  | organism | 3182 | + | 4 | motif\_sequence | short\_function |
|  | organism | 1386 | - | 4 | motif\_sequence | short\_function |
|  | organism | 2257 | + | 4 | motif\_sequence | short\_function |
|  | organism | 3586 | + | 4 | motif\_sequence | short\_function |
|  | organism | 4251 | - | 4 | motif\_sequence | short\_function |
|  | organism | 4215 | - | 4 | motif\_sequence | short\_function |
|  | organism | 3667 | + | 4 | motif\_sequence | short\_function |
|  | organism | 3416 | - | 4 | motif\_sequence | short\_function |
|  | organism | 4070 | - | 4 | motif\_sequence | short\_function |
|  | organism | 1962 | + | 4 | motif\_sequence | short\_function |
|  | organism | 1620 | + | 4 | motif\_sequence | short\_function |
|  | organism | 3344 | + | 4 | motif\_sequence | short\_function |
|  | organism | 425 | - | 4 | motif\_sequence | short\_function |
|  | organism | 183 | - | 4 | motif\_sequence | short\_function |
|  | organism | 77 | + | 4 | motif\_sequence | short\_function |
|  | organism | 2267 | - | 4 | motif\_sequence | short\_function |
|  | organism | 1556 | - | 4 | motif\_sequence | short\_function |
|  | organism | 1310 | + | 4 | motif\_sequence | short\_function |
|  | organism | 4057 | - | 4 | motif\_sequence | short\_function |
|  | organism | 1574 | - | 4 | motif\_sequence | short\_function |
|  | organism | 1150 | - | 4 | motif\_sequence | short\_function |
|  | organism | 4262 | + | 4 | motif\_sequence | short\_function |
|  | organism | 1643 | + | 4 | motif\_sequence | short\_function |
|  | organism | 4028 | - | 4 | motif\_sequence | short\_function |
|  | organism | 1530 | + | 4 | motif\_sequence | short\_function |
|  | organism | 1750 | + | 4 | motif\_sequence | short\_function |

>HU07G02249.1   
+ -Up\_Stream \_Len000GATTTG ATTGTCGAAA TTTGTCCGAT TTTATAAAAT TGAAAATTGT TATTGGACTA   
  
  
+ TAATCTACTG TAGTTTAGAG GTGTTGGATA ATACTCTCAA CTTTGAACTT GGACGTCCAT GCACCACACT   
  
  
+ ATGGGCGTAT TTACTGTTTA TATACCAAGA ATAATTTTGA AGATGTTCTT AGTTGGTGAT GTAAGTGTAT   
  
  
+ ATTCAACATA TGTCATTACA TAAACTTGAA AATTCTTGAA TTTTAGACAT ATACAATTGC AAATCATTGT   
  
  
+ AAAATTGTTG CATTACATTT TGATAAAAAT GCATCCAAAC TTTGTCTTGG TATAAACAGT AAATAGGCCC   
  
  
+ ATAGGTCCAT AGTGTAGTGC ATGGACGTCC AAGTTTAAAG GTGGGAGTAT TATTGCCCAA CGCCCCTAAA   
  
  
+ GTACAGTATA TTATAGTCCA ATATCCCAAT TAATTTTTCA TTTGAATATT TAAATTTGGA CATAATATTT   
  
  
+ TTCATATTTG AATTCTCTAA ACCTGATTTT AACCTGAATT TAAGTTGATT TAATTCATCC GACCAAAAAA   
  
  
+ ATAAACACAA TTATTTATTT TTCTATTCGA AGTTAGATAG TAGCCAATTC ATTTGACATA TAATTAACCC   
  
  
+ GTGATTGACA CAAACTCGAG TTTTTGCTTA GAGCTAGGAA AAGAGTCGGG TTAGGTCGAC CCATTTAAGA   
  
  
+ AATGGGTCGA CTTCATGTCG GCCTTCAAAA TAGTTAAGAT TTGCTCAAAA CTAAAAAATA ACAAGTTCAC   
  
  
+ ACTAAACGGT CAAATCGTGT CGACTTCGGA TTGAAATTGG TTCAGTTTGG GTCGGCTTCG TATTTCTTTC   
  
  
+ AGTAACTTCG AACCGAATTC GTATCGTGTT ATCGTGTCAG GTCAGACTTT CTCAGTTCTA TTTTTGCTAC   
  
  
+ CTTATGTTAC GGATAAAAAA AGTCTCACAT TGGAAAAAGT GTGGGAGGGT CCTGGGCTTA TAAATAGTGG   
  
  
+ CGCTGCACAC CTCATCAGAC CGACCTTTTG GGGAGAGGGT AATGACCCCG TGACAAGTGG TATCAGAGTC   
  
  
+ AACCCAGACC CGACTCAGAT GTCCTGCCAA GCGCTCCGAC GGGCGGGGTA GGCAGGGGGC CCATTTGTGA   
  
  
+ TGAGGACGTC ACAGAATTGG GCGGGGGAGA GTGTCACGGA TAAAAAAAGT CCCACATCGA AAAAAGTGTG   
  
  
+ GGAGGGTCCT GGGCTTATAA ACGGTAGCGC CGCACACCCC ATCAGATCGA CTTTTTGGGG AGAGGGTAAT   
  
  
+ GACCCCATTT CAAGTGGTAT CAGAGCCAAC CCAGACCCGA CTCCAATGTC CCACCAAGCG CTCTGATGGG   
  
  
+ CGGGGTAGGC AAGGGGCTCC ATATGTGACG AGGGCGTCAC GGAATTGGAT GGGAGAGAGT ACCACGGATA   
  
  
+ AAAAAAGTCC CACATCAGAA AAAGTGTGGA CTTAGCAACT AGGAAGTAAA GTTACAGTAT TAGCCGAGAG   
  
  
+ CCCATGGAAC GCCTGAAACT GACATCACCA GACCCACATA TTTCACCATC ATCTGATCTC CCCGTCAAAG   
  
  
+ GAGGAAGCTG AGCACAGCAA GAACTAGAAG AACAGGCGGC ATCACACCCA AGCCAAAATT CTCTATTTGA   
  
  
+ CCTCCCTCTC CTCATTCCTC TGAGCTTCCT CTTCACAATG TAAGACCATA ATCCTTAACA CCCTTCTTTT   
  
  
+ TTAAGCTCAT CTTTACCTGC TCTGGTTCTC TCTCTCATCG TCACTAATAG TTACCTCTTT ACTTTCTCTT   
  
  
+ CCCTGAGGTA TGCTTCAACT TTAACATATA TATCCCACTT GATTTCTCTT GTCTTTTTGC TATAATACTT   
  
  
+ GTTGTTTGGC TATCATCCCC TGTTTTATAC TCATTTTCTT GCTTTCTATT TCTGGGTTTG AATTGGGTTT   
  
  
+ CACTTATCCT CGTCAATTCT ATGGGTAATT AGTGAATTGG GTTTATATGA ACTAGGGTTG CAAAAGTATC   
  
  
+ TACTTTTTCT CTTGTGATTA TTAGTTGATT AGGGATTTGG CCAATATTAG CAGAATGGGT TCTGAATTTG   
  
  
+ CGGAATTCTC TGATGATGCT CTAAATGGGT ATGCTTATGT TGATATCCCT GCTTATGATG CATCCTTAGA   
  
  
+ TTATGCCAAT TTGTTCAATT ACGAAGGCCC ATCTGAGGAT CGCACCTCAC TGAGCCTCCC AAGACCCTTT   
  
  
+ TCTGACCCTT TGGCTTACAG TTTCACGTCC TCTTCTGAGC TGGGCCCTGG GGTTGATTCT AATGATGATA   
  
  
+ GTGATTCTGG CGATGTTCTC AAGTACATTA GCCAAATGCT TATGGAAGAA GACATGGAGG CAAAGCCATG   
  
  
+ CATGTTTCAT GATCCTTTAG CACTTCAGGC TGCTGAGAAA CCCTTTTATG ATGCCTTAGG GGAGCAATGC   
  
  
+ CCAACTTCTC CTGACCAACA TCCTATAATT GATCATTATT TGGATAGTCC TGATGAAAAT TCTTTGAGTT   
  
  
+ CAACTGGTGA TTTTAGTGTT AGTCATTCTG GGTCTAGTTC AACAAACTCC GTTGGACCGA TAATAGTGTC   
  
  
+ TGATTTGAGT GAGCATTTTG AGCCACCCTT TGTTGAAACA CTTCCAATTG AATCATATCA CCAACCATTG   
  
  
+ ACCCGTCCTC AATGGTCATT TGGCTCTTCG GGTGCCTTAG ATGGCACGGC CTCTAATGGT TCGGTGATCT   
  
  
+ CATCCCTTGG TTTGCCAGTG GATGTGATTA GCGTATTTAG GGAGAAAGAG TCCATGATTC AATTTCAGAA   
  
  
+ AGGGGTGGAG GAGGCCAGTA AGTTCCTTCC CAAGAATAAT AACCTTGTTA TTGATCTCGA GAACCTCACT   
  
  
+ TTTCCTAATG AAACAAAGGA GGATGATCGA ATGATGATGG TTAAGAAGGA AAAGGATGAC GTGAATTGGT   
  
  
+ CTAACTACTC AAGAGGGAGT AAGATTCACT ATTGTGAAGA CGAGGCCTTT GAAGAAGGAA GGAGTGGCAA   
  
  
+ GCAGTCAGCT ATTTCTTCTA CTGAGGAAGC TGAGTTTTCT GACATTTTTG ACAAGGTTTT GCTTTGCGAT   
  
  
+ TGCTACCCTG TGAAACCTGA GGCTCATCCC ACCATGAGTT TGAACCCTGA GAAGGGCCAG TCACATGGAT   
  
  
+ TAGAAGGTGG GAGAAATGGG AAGGCTCGCC CTAAGAAACA GGATAATAGT AGCACAAATA TTGTGGATTT   
  
  
+ AAGGAATTTG CTGATACTAT GCGCACAATC TACTGCATCT GATGACCGAA GAACTGCTGA TGGACTGCTA   
  
  
+ AAGCAAATCA GGGAGCACTC GTCTGCTGAG GGGGATGGAT CTCAAAGGTT GGCGCATTAC TTTGCTGATG   
  
  
+ CCCTAGAGGC ACGTTTAGCT GGAACTGGAT CTCGCATTTA TACGGCCCTA TGTTCTAATA GGCCATCTGT   
  
  
+ CACTGACATG ATAAAAGCAT ATCAGTTCTA TATTCGTGCT TGCCCATTTA CGAAGATCGT CATTGGTTGT   
  
  
+ GGTACCCATA TGATTCTAAA AGCAGCTGAG AAGGCATCAA AGCTTCATAT TATAGATTTT GGCATCCTCT   
  
  
+ ATGGTTGCCA ATGGCCCAAC CTCATTCAAC GCCTCTCAGA GCGATCTGGT GGACCTCCAA AACTGTTTAT   
  
  
+ TACAGGGATC GATCTCCCCC AGCCTGGGTT CAGGCCAGCA GAAAGAGTGG AAGCAACAGG GAGACGCTTG   
  
  
+ GCCAAGTACT GTGAGCGGTA TAATGTGCCA TTTGAGTATC ATGCCATTGC TCAGGAGTGG GAAACAATCA   
  
  
+ AACCAGGGGA TCTCAAGATA GGAAGTAGGA ATGATGAAGT TGTTGCGGTG AACTGTCTCT GTAGGTTCAA   
  
  
+ GAACCTCCTT GACGAGACAG TGGTGGTGGA TAGTCCAAGG AACACAGTTT TAAACCTGAT TACAAGGGTA   
  
  
+ AAGCCTGATA TTTTTGTGCA TGGCGTTGTA AATGGTTCCT ACAACATCCC TTTCTTTGTG ACACGTTTTA   
  
  
+ GAGAAGCCCT CTTTCATTAT TCCACTCTTT TCGACATGTT AGATGCCAAC GCCTCTAGGG AGGAGCCCGA   
  
  
+ GAGGTTGATA TTCGAGAAGG CATTCTATGG GAGGGAGATT ATGAATGTGG TGGCCTGTGA GGGCACAGAG   
  
  
+ AGGGTGGAGA GGCCGGAGAC ATACAAGCAA TGGCATGTTA GGCATAGCAG GGCAGGGTTT CGGCAAGTAC   
  
  
+ CATTGGATCC CAAGTTGATC GAGAAAATGA GGTTTAAGGC CAAGGCAGAC CACCACAAGG ATTTCATGAT   
  
  
+ CGATGTGGAT GGACATTGGG CAATTCAGGG ATGGAAGGGG CGGATTGCAC ATGCGATCTC TGCATGGGTT   
  
  
+ CCGGCTTG  

- -Up\_Stream \_Len000CTAAAC TAACAGCTTT AAACAGGCTA AAATATTTTA ACTTTTAACA ATAACCTGAT   
  
  
- ATTAGATGAC ATCAAATCTC CACAACCTAT TATGAGAGTT GAAACTTGAA CCTGCAGGTA CGTGGTGTGA   
  
  
- TACCCGCATA AATGACAAAT ATATGGTTCT TATTAAAACT TCTACAAGAA TCAACCACTA CATTCACATA   
  
  
- TAAGTTGTAT ACAGTAATGT ATTTGAACTT TTAAGAACTT AAAATCTGTA TATGTTAACG TTTAGTAACA   
  
  
- TTTTAACAAC GTAATGTAAA ACTATTTTTA CGTAGGTTTG AAACAGAACC ATATTTGTCA TTTATCCGGG   
  
  
- TATCCAGGTA TCACATCACG TACCTGCAGG TTCAAATTTC CACCCTCATA ATAACGGGTT GCGGGGATTT   
  
  
- CATGTCATAT AATATCAGGT TATAGGGTTA ATTAAAAAGT AAACTTATAA ATTTAAACCT GTATTATAAA   
  
  
- AAGTATAAAC TTAAGAGATT TGGACTAAAA TTGGACTTAA ATTCAACTAA ATTAAGTAGG CTGGTTTTTT   
  
  
- TATTTGTGTT AATAAATAAA AAGATAAGCT TCAATCTATC ATCGGTTAAG TAAACTGTAT ATTAATTGGG   
  
  
- CACTAACTGT GTTTGAGCTC AAAAACGAAT CTCGATCCTT TTCTCAGCCC AATCCAGCTG GGTAAATTCT   
  
  
- TTACCCAGCT GAAGTACAGC CGGAAGTTTT ATCAATTCTA AACGAGTTTT GATTTTTTAT TGTTCAAGTG   
  
  
- TGATTTGCCA GTTTAGCACA GCTGAAGCCT AACTTTAACC AAGTCAAACC CAGCCGAAGC ATAAAGAAAG   
  
  
- TCATTGAAGC TTGGCTTAAG CATAGCACAA TAGCACAGTC CAGTCTGAAA GAGTCAAGAT AAAAACGATG   
  
  
- GAATACAATG CCTATTTTTT TCAGAGTGTA ACCTTTTTCA CACCCTCCCA GGACCCGAAT ATTTATCACC   
  
  
- GCGACGTGTG GAGTAGTCTG GCTGGAAAAC CCCTCTCCCA TTACTGGGGC ACTGTTCACC ATAGTCTCAG   
  
  
- TTGGGTCTGG GCTGAGTCTA CAGGACGGTT CGCGAGGCTG CCCGCCCCAT CCGTCCCCCG GGTAAACACT   
  
  
- ACTCCTGCAG TGTCTTAACC CGCCCCCTCT CACAGTGCCT ATTTTTTTCA GGGTGTAGCT TTTTTCACAC   
  
  
- CCTCCCAGGA CCCGAATATT TGCCATCGCG GCGTGTGGGG TAGTCTAGCT GAAAAACCCC TCTCCCATTA   
  
  
- CTGGGGTAAA GTTCACCATA GTCTCGGTTG GGTCTGGGCT GAGGTTACAG GGTGGTTCGC GAGACTACCC   
  
  
- GCCCCATCCG TTCCCCGAGG TATACACTGC TCCCGCAGTG CCTTAACCTA CCCTCTCTCA TGGTGCCTAT   
  
  
- TTTTTTCAGG GTGTAGTCTT TTTCACACCT GAATCGTTGA TCCTTCATTT CAATGTCATA ATCGGCTCTC   
  
  
- GGGTACCTTG CGGACTTTGA CTGTAGTGGT CTGGGTGTAT AAAGTGGTAG TAGACTAGAG GGGCAGTTTC   
  
  
- CTCCTTCGAC TCGTGTCGTT CTTGATCTTC TTGTCCGCCG TAGTGTGGGT TCGGTTTTAA GAGATAAACT   
  
  
- GGAGGGAGAG GAGTAAGGAG ACTCGAAGGA GAAGTGTTAC ATTCTGGTAT TAGGAATTGT GGGAAGAAAA   
  
  
- AATTCGAGTA GAAATGGACG AGACCAAGAG AGAGAGTAGC AGTGATTATC AATGGAGAAA TGAAAGAGAA   
  
  
- GGGACTCCAT ACGAAGTTGA AATTGTATAT ATAGGGTGAA CTAAAGAGAA CAGAAAAACG ATATTATGAA   
  
  
- CAACAAACCG ATAGTAGGGG ACAAAATATG AGTAAAAGAA CGAAAGATAA AGACCCAAAC TTAACCCAAA   
  
  
- GTGAATAGGA GCAGTTAAGA TACCCATTAA TCACTTAACC CAAATATACT TGATCCCAAC GTTTTCATAG   
  
  
- ATGAAAAAGA GAACACTAAT AATCAACTAA TCCCTAAACC GGTTATAATC GTCTTACCCA AGACTTAAAC   
  
  
- GCCTTAAGAG ACTACTACGA GATTTACCCA TACGAATACA ACTATAGGGA CGAATACTAC GTAGGAATCT   
  
  
- AATACGGTTA AACAAGTTAA TGCTTCCGGG TAGACTCCTA GCGTGGAGTG ACTCGGAGGG TTCTGGGAAA   
  
  
- AGACTGGGAA ACCGAATGTC AAAGTGCAGG AGAAGACTCG ACCCGGGACC CCAACTAAGA TTACTACTAT   
  
  
- CACTAAGACC GCTACAAGAG TTCATGTAAT CGGTTTACGA ATACCTTCTT CTGTACCTCC GTTTCGGTAC   
  
  
- GTACAAAGTA CTAGGAAATC GTGAAGTCCG ACGACTCTTT GGGAAAATAC TACGGAATCC CCTCGTTACG   
  
  
- GGTTGAAGAG GACTGGTTGT AGGATATTAA CTAGTAATAA ACCTATCAGG ACTACTTTTA AGAAACTCAA   
  
  
- GTTGACCACT AAAATCACAA TCAGTAAGAC CCAGATCAAG TTGTTTGAGG CAACCTGGCT ATTATCACAG   
  
  
- ACTAAACTCA CTCGTAAAAC TCGGTGGGAA ACAACTTTGT GAAGGTTAAC TTAGTATAGT GGTTGGTAAC   
  
  
- TGGGCAGGAG TTACCAGTAA ACCGAGAAGC CCACGGAATC TACCGTGCCG GAGATTACCA AGCCACTAGA   
  
  
- GTAGGGAACC AAACGGTCAC CTACACTAAT CGCATAAATC CCTCTTTCTC AGGTACTAAG TTAAAGTCTT   
  
  
- TCCCCACCTC CTCCGGTCAT TCAAGGAAGG GTTCTTATTA TTGGAACAAT AACTAGAGCT CTTGGAGTGA   
  
  
- AAAGGATTAC TTTGTTTCCT CCTACTAGCT TACTACTACC AATTCTTCCT TTTCCTACTG CACTTAACCA   
  
  
- GATTGATGAG TTCTCCCTCA TTCTAAGTGA TAACACTTCT GCTCCGGAAA CTTCTTCCTT CCTCACCGTT   
  
  
- CGTCAGTCGA TAAAGAAGAT GACTCCTTCG ACTCAAAAGA CTGTAAAAAC TGTTCCAAAA CGAAACGCTA   
  
  
- ACGATGGGAC ACTTTGGACT CCGAGTAGGG TGGTACTCAA ACTTGGGACT CTTCCCGGTC AGTGTACCTA   
  
  
- ATCTTCCACC CTCTTTACCC TTCCGAGCGG GATTCTTTGT CCTATTATCA TCGTGTTTAT AACACCTAAA   
  
  
- TTCCTTAAAC GACTATGATA CGCGTGTTAG ATGACGTAGA CTACTGGCTT CTTGACGACT ACCTGACGAT   
  
  
- TTCGTTTAGT CCCTCGTGAG CAGACGACTC CCCCTACCTA GAGTTTCCAA CCGCGTAATG AAACGACTAC   
  
  
- GGGATCTCCG TGCAAATCGA CCTTGACCTA GAGCGTAAAT ATGCCGGGAT ACAAGATTAT CCGGTAGACA   
  
  
- GTGACTGTAC TATTTTCGTA TAGTCAAGAT ATAAGCACGA ACGGGTAAAT GCTTCTAGCA GTAACCAACA   
  
  
- CCATGGGTAT ACTAAGATTT TCGTCGACTC TTCCGTAGTT TCGAAGTATA ATATCTAAAA CCGTAGGAGA   
  
  
- TACCAACGGT TACCGGGTTG GAGTAAGTTG CGGAGAGTCT CGCTAGACCA CCTGGAGGTT TTGACAAATA   
  
  
- ATGTCCCTAG CTAGAGGGGG TCGGACCCAA GTCCGGTCGT CTTTCTCACC TTCGTTGTCC CTCTGCGAAC   
  
  
- CGGTTCATGA CACTCGCCAT ATTACACGGT AAACTCATAG TACGGTAACG AGTCCTCACC CTTTGTTAGT   
  
  
- TTGGTCCCCT AGAGTTCTAT CCTTCATCCT TACTACTTCA ACAACGCCAC TTGACAGAGA CATCCAAGTT   
  
  
- CTTGGAGGAA CTGCTCTGTC ACCACCACCT ATCAGGTTCC TTGTGTCAAA ATTTGGACTA ATGTTCCCAT   
  
  
- TTCGGACTAT AAAAACACGT ACCGCAACAT TTACCAAGGA TGTTGTAGGG AAAGAAACAC TGTGCAAAAT   
  
  
- CTCTTCGGGA GAAAGTAATA AGGTGAGAAA AGCTGTACAA TCTACGGTTG CGGAGATCCC TCCTCGGGCT   
  
  
- CTCCAACTAT AAGCTCTTCC GTAAGATACC CTCCCTCTAA TACTTACACC ACCGGACACT CCCGTGTCTC   
  
  
- TCCCACCTCT CCGGCCTCTG TATGTTCGTT ACCGTACAAT CCGTATCGTC CCGTCCCAAA GCCGTTCATG   
  
  
- GTAACCTAGG GTTCAACTAG CTCTTTTACT CCAAATTCCG GTTCCGTCTG GTGGTGTTCC TAAAGTACTA   
  
  
- GCTACACCTA CCTGTAACCC GTTAAGTCCC TACCTTCCCC GCCTAACGTG TACGCTAGAG ACGTACCCAA   
  
  
- GGCCGAAC

+     3-AF3 binding site

| Site Name | Organism | Position | Strand | Matrix score. | sequence | function |
| --- | --- | --- | --- | --- | --- | --- |
| 3-AF3 binding site | Pisum sativum | 596 | - | 10 | CACTATCTAAC | part of a conserved DNA module array (CMA3) |

>HU07G02249.1   
+ -Up\_Stream \_Len000GATTTG ATTGTCGAAA TTTGTCCGAT TTTATAAAAT TGAAAATTGT TATTGGACTA   
  
  
+ TAATCTACTG TAGTTTAGAG GTGTTGGATA ATACTCTCAA CTTTGAACTT GGACGTCCAT GCACCACACT   
  
  
+ ATGGGCGTAT TTACTGTTTA TATACCAAGA ATAATTTTGA AGATGTTCTT AGTTGGTGAT GTAAGTGTAT   
  
  
+ ATTCAACATA TGTCATTACA TAAACTTGAA AATTCTTGAA TTTTAGACAT ATACAATTGC AAATCATTGT   
  
  
+ AAAATTGTTG CATTACATTT TGATAAAAAT GCATCCAAAC TTTGTCTTGG TATAAACAGT AAATAGGCCC   
  
  
+ ATAGGTCCAT AGTGTAGTGC ATGGACGTCC AAGTTTAAAG GTGGGAGTAT TATTGCCCAA CGCCCCTAAA   
  
  
+ GTACAGTATA TTATAGTCCA ATATCCCAAT TAATTTTTCA TTTGAATATT TAAATTTGGA CATAATATTT   
  
  
+ TTCATATTTG AATTCTCTAA ACCTGATTTT AACCTGAATT TAAGTTGATT TAATTCATCC GACCAAAAAA   
  
  
+ ATAAACACAA TTATTTATTT TTCTATTCGA AGTTAGATAG TAGCCAATTC ATTTGACATA TAATTAACCC   
  
  
+ GTGATTGACA CAAACTCGAG TTTTTGCTTA GAGCTAGGAA AAGAGTCGGG TTAGGTCGAC CCATTTAAGA   
  
  
+ AATGGGTCGA CTTCATGTCG GCCTTCAAAA TAGTTAAGAT TTGCTCAAAA CTAAAAAATA ACAAGTTCAC   
  
  
+ ACTAAACGGT CAAATCGTGT CGACTTCGGA TTGAAATTGG TTCAGTTTGG GTCGGCTTCG TATTTCTTTC   
  
  
+ AGTAACTTCG AACCGAATTC GTATCGTGTT ATCGTGTCAG GTCAGACTTT CTCAGTTCTA TTTTTGCTAC   
  
  
+ CTTATGTTAC GGATAAAAAA AGTCTCACAT TGGAAAAAGT GTGGGAGGGT CCTGGGCTTA TAAATAGTGG   
  
  
+ CGCTGCACAC CTCATCAGAC CGACCTTTTG GGGAGAGGGT AATGACCCCG TGACAAGTGG TATCAGAGTC   
  
  
+ AACCCAGACC CGACTCAGAT GTCCTGCCAA GCGCTCCGAC GGGCGGGGTA GGCAGGGGGC CCATTTGTGA   
  
  
+ TGAGGACGTC ACAGAATTGG GCGGGGGAGA GTGTCACGGA TAAAAAAAGT CCCACATCGA AAAAAGTGTG   
  
  
+ GGAGGGTCCT GGGCTTATAA ACGGTAGCGC CGCACACCCC ATCAGATCGA CTTTTTGGGG AGAGGGTAAT   
  
  
+ GACCCCATTT CAAGTGGTAT CAGAGCCAAC CCAGACCCGA CTCCAATGTC CCACCAAGCG CTCTGATGGG   
  
  
+ CGGGGTAGGC AAGGGGCTCC ATATGTGACG AGGGCGTCAC GGAATTGGAT GGGAGAGAGT ACCACGGATA   
  
  
+ AAAAAAGTCC CACATCAGAA AAAGTGTGGA CTTAGCAACT AGGAAGTAAA GTTACAGTAT TAGCCGAGAG   
  
  
+ CCCATGGAAC GCCTGAAACT GACATCACCA GACCCACATA TTTCACCATC ATCTGATCTC CCCGTCAAAG   
  
  
+ GAGGAAGCTG AGCACAGCAA GAACTAGAAG AACAGGCGGC ATCACACCCA AGCCAAAATT CTCTATTTGA   
  
  
+ CCTCCCTCTC CTCATTCCTC TGAGCTTCCT CTTCACAATG TAAGACCATA ATCCTTAACA CCCTTCTTTT   
  
  
+ TTAAGCTCAT CTTTACCTGC TCTGGTTCTC TCTCTCATCG TCACTAATAG TTACCTCTTT ACTTTCTCTT   
  
  
+ CCCTGAGGTA TGCTTCAACT TTAACATATA TATCCCACTT GATTTCTCTT GTCTTTTTGC TATAATACTT   
  
  
+ GTTGTTTGGC TATCATCCCC TGTTTTATAC TCATTTTCTT GCTTTCTATT TCTGGGTTTG AATTGGGTTT   
  
  
+ CACTTATCCT CGTCAATTCT ATGGGTAATT AGTGAATTGG GTTTATATGA ACTAGGGTTG CAAAAGTATC   
  
  
+ TACTTTTTCT CTTGTGATTA TTAGTTGATT AGGGATTTGG CCAATATTAG CAGAATGGGT TCTGAATTTG   
  
  
+ CGGAATTCTC TGATGATGCT CTAAATGGGT ATGCTTATGT TGATATCCCT GCTTATGATG CATCCTTAGA   
  
  
+ TTATGCCAAT TTGTTCAATT ACGAAGGCCC ATCTGAGGAT CGCACCTCAC TGAGCCTCCC AAGACCCTTT   
  
  
+ TCTGACCCTT TGGCTTACAG TTTCACGTCC TCTTCTGAGC TGGGCCCTGG GGTTGATTCT AATGATGATA   
  
  
+ GTGATTCTGG CGATGTTCTC AAGTACATTA GCCAAATGCT TATGGAAGAA GACATGGAGG CAAAGCCATG   
  
  
+ CATGTTTCAT GATCCTTTAG CACTTCAGGC TGCTGAGAAA CCCTTTTATG ATGCCTTAGG GGAGCAATGC   
  
  
+ CCAACTTCTC CTGACCAACA TCCTATAATT GATCATTATT TGGATAGTCC TGATGAAAAT TCTTTGAGTT   
  
  
+ CAACTGGTGA TTTTAGTGTT AGTCATTCTG GGTCTAGTTC AACAAACTCC GTTGGACCGA TAATAGTGTC   
  
  
+ TGATTTGAGT GAGCATTTTG AGCCACCCTT TGTTGAAACA CTTCCAATTG AATCATATCA CCAACCATTG   
  
  
+ ACCCGTCCTC AATGGTCATT TGGCTCTTCG GGTGCCTTAG ATGGCACGGC CTCTAATGGT TCGGTGATCT   
  
  
+ CATCCCTTGG TTTGCCAGTG GATGTGATTA GCGTATTTAG GGAGAAAGAG TCCATGATTC AATTTCAGAA   
  
  
+ AGGGGTGGAG GAGGCCAGTA AGTTCCTTCC CAAGAATAAT AACCTTGTTA TTGATCTCGA GAACCTCACT   
  
  
+ TTTCCTAATG AAACAAAGGA GGATGATCGA ATGATGATGG TTAAGAAGGA AAAGGATGAC GTGAATTGGT   
  
  
+ CTAACTACTC AAGAGGGAGT AAGATTCACT ATTGTGAAGA CGAGGCCTTT GAAGAAGGAA GGAGTGGCAA   
  
  
+ GCAGTCAGCT ATTTCTTCTA CTGAGGAAGC TGAGTTTTCT GACATTTTTG ACAAGGTTTT GCTTTGCGAT   
  
  
+ TGCTACCCTG TGAAACCTGA GGCTCATCCC ACCATGAGTT TGAACCCTGA GAAGGGCCAG TCACATGGAT   
  
  
+ TAGAAGGTGG GAGAAATGGG AAGGCTCGCC CTAAGAAACA GGATAATAGT AGCACAAATA TTGTGGATTT   
  
  
+ AAGGAATTTG CTGATACTAT GCGCACAATC TACTGCATCT GATGACCGAA GAACTGCTGA TGGACTGCTA   
  
  
+ AAGCAAATCA GGGAGCACTC GTCTGCTGAG GGGGATGGAT CTCAAAGGTT GGCGCATTAC TTTGCTGATG   
  
  
+ CCCTAGAGGC ACGTTTAGCT GGAACTGGAT CTCGCATTTA TACGGCCCTA TGTTCTAATA GGCCATCTGT   
  
  
+ CACTGACATG ATAAAAGCAT ATCAGTTCTA TATTCGTGCT TGCCCATTTA CGAAGATCGT CATTGGTTGT   
  
  
+ GGTACCCATA TGATTCTAAA AGCAGCTGAG AAGGCATCAA AGCTTCATAT TATAGATTTT GGCATCCTCT   
  
  
+ ATGGTTGCCA ATGGCCCAAC CTCATTCAAC GCCTCTCAGA GCGATCTGGT GGACCTCCAA AACTGTTTAT   
  
  
+ TACAGGGATC GATCTCCCCC AGCCTGGGTT CAGGCCAGCA GAAAGAGTGG AAGCAACAGG GAGACGCTTG   
  
  
+ GCCAAGTACT GTGAGCGGTA TAATGTGCCA TTTGAGTATC ATGCCATTGC TCAGGAGTGG GAAACAATCA   
  
  
+ AACCAGGGGA TCTCAAGATA GGAAGTAGGA ATGATGAAGT TGTTGCGGTG AACTGTCTCT GTAGGTTCAA   
  
  
+ GAACCTCCTT GACGAGACAG TGGTGGTGGA TAGTCCAAGG AACACAGTTT TAAACCTGAT TACAAGGGTA   
  
  
+ AAGCCTGATA TTTTTGTGCA TGGCGTTGTA AATGGTTCCT ACAACATCCC TTTCTTTGTG ACACGTTTTA   
  
  
+ GAGAAGCCCT CTTTCATTAT TCCACTCTTT TCGACATGTT AGATGCCAAC GCCTCTAGGG AGGAGCCCGA   
  
  
+ GAGGTTGATA TTCGAGAAGG CATTCTATGG GAGGGAGATT ATGAATGTGG TGGCCTGTGA GGGCACAGAG   
  
  
+ AGGGTGGAGA GGCCGGAGAC ATACAAGCAA TGGCATGTTA GGCATAGCAG GGCAGGGTTT CGGCAAGTAC   
  
  
+ CATTGGATCC CAAGTTGATC GAGAAAATGA GGTTTAAGGC CAAGGCAGAC CACCACAAGG ATTTCATGAT   
  
  
+ CGATGTGGAT GGACATTGGG CAATTCAGGG ATGGAAGGGG CGGATTGCAC ATGCGATCTC TGCATGGGTT   
  
  
+ CCGGCTTG  

- -Up\_Stream \_Len000CTAAAC TAACAGCTTT AAACAGGCTA AAATATTTTA ACTTTTAACA ATAACCTGAT   
  
  
- ATTAGATGAC ATCAAATCTC CACAACCTAT TATGAGAGTT GAAACTTGAA CCTGCAGGTA CGTGGTGTGA   
  
  
- TACCCGCATA AATGACAAAT ATATGGTTCT TATTAAAACT TCTACAAGAA TCAACCACTA CATTCACATA   
  
  
- TAAGTTGTAT ACAGTAATGT ATTTGAACTT TTAAGAACTT AAAATCTGTA TATGTTAACG TTTAGTAACA   
  
  
- TTTTAACAAC GTAATGTAAA ACTATTTTTA CGTAGGTTTG AAACAGAACC ATATTTGTCA TTTATCCGGG   
  
  
- TATCCAGGTA TCACATCACG TACCTGCAGG TTCAAATTTC CACCCTCATA ATAACGGGTT GCGGGGATTT   
  
  
- CATGTCATAT AATATCAGGT TATAGGGTTA ATTAAAAAGT AAACTTATAA ATTTAAACCT GTATTATAAA   
  
  
- AAGTATAAAC TTAAGAGATT TGGACTAAAA TTGGACTTAA ATTCAACTAA ATTAAGTAGG CTGGTTTTTT   
  
  
- TATTTGTGTT AATAAATAAA AAGATAAGCT TCAATCTATC ATCGGTTAAG TAAACTGTAT ATTAATTGGG   
  
  
- CACTAACTGT GTTTGAGCTC AAAAACGAAT CTCGATCCTT TTCTCAGCCC AATCCAGCTG GGTAAATTCT   
  
  
- TTACCCAGCT GAAGTACAGC CGGAAGTTTT ATCAATTCTA AACGAGTTTT GATTTTTTAT TGTTCAAGTG   
  
  
- TGATTTGCCA GTTTAGCACA GCTGAAGCCT AACTTTAACC AAGTCAAACC CAGCCGAAGC ATAAAGAAAG   
  
  
- TCATTGAAGC TTGGCTTAAG CATAGCACAA TAGCACAGTC CAGTCTGAAA GAGTCAAGAT AAAAACGATG   
  
  
- GAATACAATG CCTATTTTTT TCAGAGTGTA ACCTTTTTCA CACCCTCCCA GGACCCGAAT ATTTATCACC   
  
  
- GCGACGTGTG GAGTAGTCTG GCTGGAAAAC CCCTCTCCCA TTACTGGGGC ACTGTTCACC ATAGTCTCAG   
  
  
- TTGGGTCTGG GCTGAGTCTA CAGGACGGTT CGCGAGGCTG CCCGCCCCAT CCGTCCCCCG GGTAAACACT   
  
  
- ACTCCTGCAG TGTCTTAACC CGCCCCCTCT CACAGTGCCT ATTTTTTTCA GGGTGTAGCT TTTTTCACAC   
  
  
- CCTCCCAGGA CCCGAATATT TGCCATCGCG GCGTGTGGGG TAGTCTAGCT GAAAAACCCC TCTCCCATTA   
  
  
- CTGGGGTAAA GTTCACCATA GTCTCGGTTG GGTCTGGGCT GAGGTTACAG GGTGGTTCGC GAGACTACCC   
  
  
- GCCCCATCCG TTCCCCGAGG TATACACTGC TCCCGCAGTG CCTTAACCTA CCCTCTCTCA TGGTGCCTAT   
  
  
- TTTTTTCAGG GTGTAGTCTT TTTCACACCT GAATCGTTGA TCCTTCATTT CAATGTCATA ATCGGCTCTC   
  
  
- GGGTACCTTG CGGACTTTGA CTGTAGTGGT CTGGGTGTAT AAAGTGGTAG TAGACTAGAG GGGCAGTTTC   
  
  
- CTCCTTCGAC TCGTGTCGTT CTTGATCTTC TTGTCCGCCG TAGTGTGGGT TCGGTTTTAA GAGATAAACT   
  
  
- GGAGGGAGAG GAGTAAGGAG ACTCGAAGGA GAAGTGTTAC ATTCTGGTAT TAGGAATTGT GGGAAGAAAA   
  
  
- AATTCGAGTA GAAATGGACG AGACCAAGAG AGAGAGTAGC AGTGATTATC AATGGAGAAA TGAAAGAGAA   
  
  
- GGGACTCCAT ACGAAGTTGA AATTGTATAT ATAGGGTGAA CTAAAGAGAA CAGAAAAACG ATATTATGAA   
  
  
- CAACAAACCG ATAGTAGGGG ACAAAATATG AGTAAAAGAA CGAAAGATAA AGACCCAAAC TTAACCCAAA   
  
  
- GTGAATAGGA GCAGTTAAGA TACCCATTAA TCACTTAACC CAAATATACT TGATCCCAAC GTTTTCATAG   
  
  
- ATGAAAAAGA GAACACTAAT AATCAACTAA TCCCTAAACC GGTTATAATC GTCTTACCCA AGACTTAAAC   
  
  
- GCCTTAAGAG ACTACTACGA GATTTACCCA TACGAATACA ACTATAGGGA CGAATACTAC GTAGGAATCT   
  
  
- AATACGGTTA AACAAGTTAA TGCTTCCGGG TAGACTCCTA GCGTGGAGTG ACTCGGAGGG TTCTGGGAAA   
  
  
- AGACTGGGAA ACCGAATGTC AAAGTGCAGG AGAAGACTCG ACCCGGGACC CCAACTAAGA TTACTACTAT   
  
  
- CACTAAGACC GCTACAAGAG TTCATGTAAT CGGTTTACGA ATACCTTCTT CTGTACCTCC GTTTCGGTAC   
  
  
- GTACAAAGTA CTAGGAAATC GTGAAGTCCG ACGACTCTTT GGGAAAATAC TACGGAATCC CCTCGTTACG   
  
  
- GGTTGAAGAG GACTGGTTGT AGGATATTAA CTAGTAATAA ACCTATCAGG ACTACTTTTA AGAAACTCAA   
  
  
- GTTGACCACT AAAATCACAA TCAGTAAGAC CCAGATCAAG TTGTTTGAGG CAACCTGGCT ATTATCACAG   
  
  
- ACTAAACTCA CTCGTAAAAC TCGGTGGGAA ACAACTTTGT GAAGGTTAAC TTAGTATAGT GGTTGGTAAC   
  
  
- TGGGCAGGAG TTACCAGTAA ACCGAGAAGC CCACGGAATC TACCGTGCCG GAGATTACCA AGCCACTAGA   
  
  
- GTAGGGAACC AAACGGTCAC CTACACTAAT CGCATAAATC CCTCTTTCTC AGGTACTAAG TTAAAGTCTT   
  
  
- TCCCCACCTC CTCCGGTCAT TCAAGGAAGG GTTCTTATTA TTGGAACAAT AACTAGAGCT CTTGGAGTGA   
  
  
- AAAGGATTAC TTTGTTTCCT CCTACTAGCT TACTACTACC AATTCTTCCT TTTCCTACTG CACTTAACCA   
  
  
- GATTGATGAG TTCTCCCTCA TTCTAAGTGA TAACACTTCT GCTCCGGAAA CTTCTTCCTT CCTCACCGTT   
  
  
- CGTCAGTCGA TAAAGAAGAT GACTCCTTCG ACTCAAAAGA CTGTAAAAAC TGTTCCAAAA CGAAACGCTA   
  
  
- ACGATGGGAC ACTTTGGACT CCGAGTAGGG TGGTACTCAA ACTTGGGACT CTTCCCGGTC AGTGTACCTA   
  
  
- ATCTTCCACC CTCTTTACCC TTCCGAGCGG GATTCTTTGT CCTATTATCA TCGTGTTTAT AACACCTAAA   
  
  
- TTCCTTAAAC GACTATGATA CGCGTGTTAG ATGACGTAGA CTACTGGCTT CTTGACGACT ACCTGACGAT   
  
  
- TTCGTTTAGT CCCTCGTGAG CAGACGACTC CCCCTACCTA GAGTTTCCAA CCGCGTAATG AAACGACTAC   
  
  
- GGGATCTCCG TGCAAATCGA CCTTGACCTA GAGCGTAAAT ATGCCGGGAT ACAAGATTAT CCGGTAGACA   
  
  
- GTGACTGTAC TATTTTCGTA TAGTCAAGAT ATAAGCACGA ACGGGTAAAT GCTTCTAGCA GTAACCAACA   
  
  
- CCATGGGTAT ACTAAGATTT TCGTCGACTC TTCCGTAGTT TCGAAGTATA ATATCTAAAA CCGTAGGAGA   
  
  
- TACCAACGGT TACCGGGTTG GAGTAAGTTG CGGAGAGTCT CGCTAGACCA CCTGGAGGTT TTGACAAATA   
  
  
- ATGTCCCTAG CTAGAGGGGG TCGGACCCAA GTCCGGTCGT CTTTCTCACC TTCGTTGTCC CTCTGCGAAC   
  
  
- CGGTTCATGA CACTCGCCAT ATTACACGGT AAACTCATAG TACGGTAACG AGTCCTCACC CTTTGTTAGT   
  
  
- TTGGTCCCCT AGAGTTCTAT CCTTCATCCT TACTACTTCA ACAACGCCAC TTGACAGAGA CATCCAAGTT   
  
  
- CTTGGAGGAA CTGCTCTGTC ACCACCACCT ATCAGGTTCC TTGTGTCAAA ATTTGGACTA ATGTTCCCAT   
  
  
- TTCGGACTAT AAAAACACGT ACCGCAACAT TTACCAAGGA TGTTGTAGGG AAAGAAACAC TGTGCAAAAT   
  
  
- CTCTTCGGGA GAAAGTAATA AGGTGAGAAA AGCTGTACAA TCTACGGTTG CGGAGATCCC TCCTCGGGCT   
  
  
- CTCCAACTAT AAGCTCTTCC GTAAGATACC CTCCCTCTAA TACTTACACC ACCGGACACT CCCGTGTCTC   
  
  
- TCCCACCTCT CCGGCCTCTG TATGTTCGTT ACCGTACAAT CCGTATCGTC CCGTCCCAAA GCCGTTCATG   
  
  
- GTAACCTAGG GTTCAACTAG CTCTTTTACT CCAAATTCCG GTTCCGTCTG GTGGTGTTCC TAAAGTACTA   
  
  
- GCTACACCTA CCTGTAACCC GTTAAGTCCC TACCTTCCCC GCCTAACGTG TACGCTAGAG ACGTACCCAA   
  
  
- GGCCGAAC

+     A-box

| Site Name | Organism | Position | Strand | Matrix score. | sequence | function |
| --- | --- | --- | --- | --- | --- | --- |
| A-box | Petroselinum crispum | 2597 | + | 6 | CCGTCC | cis-acting regulatory element |

>HU07G02249.1   
+ -Up\_Stream \_Len000GATTTG ATTGTCGAAA TTTGTCCGAT TTTATAAAAT TGAAAATTGT TATTGGACTA   
  
  
+ TAATCTACTG TAGTTTAGAG GTGTTGGATA ATACTCTCAA CTTTGAACTT GGACGTCCAT GCACCACACT   
  
  
+ ATGGGCGTAT TTACTGTTTA TATACCAAGA ATAATTTTGA AGATGTTCTT AGTTGGTGAT GTAAGTGTAT   
  
  
+ ATTCAACATA TGTCATTACA TAAACTTGAA AATTCTTGAA TTTTAGACAT ATACAATTGC AAATCATTGT   
  
  
+ AAAATTGTTG CATTACATTT TGATAAAAAT GCATCCAAAC TTTGTCTTGG TATAAACAGT AAATAGGCCC   
  
  
+ ATAGGTCCAT AGTGTAGTGC ATGGACGTCC AAGTTTAAAG GTGGGAGTAT TATTGCCCAA CGCCCCTAAA   
  
  
+ GTACAGTATA TTATAGTCCA ATATCCCAAT TAATTTTTCA TTTGAATATT TAAATTTGGA CATAATATTT   
  
  
+ TTCATATTTG AATTCTCTAA ACCTGATTTT AACCTGAATT TAAGTTGATT TAATTCATCC GACCAAAAAA   
  
  
+ ATAAACACAA TTATTTATTT TTCTATTCGA AGTTAGATAG TAGCCAATTC ATTTGACATA TAATTAACCC   
  
  
+ GTGATTGACA CAAACTCGAG TTTTTGCTTA GAGCTAGGAA AAGAGTCGGG TTAGGTCGAC CCATTTAAGA   
  
  
+ AATGGGTCGA CTTCATGTCG GCCTTCAAAA TAGTTAAGAT TTGCTCAAAA CTAAAAAATA ACAAGTTCAC   
  
  
+ ACTAAACGGT CAAATCGTGT CGACTTCGGA TTGAAATTGG TTCAGTTTGG GTCGGCTTCG TATTTCTTTC   
  
  
+ AGTAACTTCG AACCGAATTC GTATCGTGTT ATCGTGTCAG GTCAGACTTT CTCAGTTCTA TTTTTGCTAC   
  
  
+ CTTATGTTAC GGATAAAAAA AGTCTCACAT TGGAAAAAGT GTGGGAGGGT CCTGGGCTTA TAAATAGTGG   
  
  
+ CGCTGCACAC CTCATCAGAC CGACCTTTTG GGGAGAGGGT AATGACCCCG TGACAAGTGG TATCAGAGTC   
  
  
+ AACCCAGACC CGACTCAGAT GTCCTGCCAA GCGCTCCGAC GGGCGGGGTA GGCAGGGGGC CCATTTGTGA   
  
  
+ TGAGGACGTC ACAGAATTGG GCGGGGGAGA GTGTCACGGA TAAAAAAAGT CCCACATCGA AAAAAGTGTG   
  
  
+ GGAGGGTCCT GGGCTTATAA ACGGTAGCGC CGCACACCCC ATCAGATCGA CTTTTTGGGG AGAGGGTAAT   
  
  
+ GACCCCATTT CAAGTGGTAT CAGAGCCAAC CCAGACCCGA CTCCAATGTC CCACCAAGCG CTCTGATGGG   
  
  
+ CGGGGTAGGC AAGGGGCTCC ATATGTGACG AGGGCGTCAC GGAATTGGAT GGGAGAGAGT ACCACGGATA   
  
  
+ AAAAAAGTCC CACATCAGAA AAAGTGTGGA CTTAGCAACT AGGAAGTAAA GTTACAGTAT TAGCCGAGAG   
  
  
+ CCCATGGAAC GCCTGAAACT GACATCACCA GACCCACATA TTTCACCATC ATCTGATCTC CCCGTCAAAG   
  
  
+ GAGGAAGCTG AGCACAGCAA GAACTAGAAG AACAGGCGGC ATCACACCCA AGCCAAAATT CTCTATTTGA   
  
  
+ CCTCCCTCTC CTCATTCCTC TGAGCTTCCT CTTCACAATG TAAGACCATA ATCCTTAACA CCCTTCTTTT   
  
  
+ TTAAGCTCAT CTTTACCTGC TCTGGTTCTC TCTCTCATCG TCACTAATAG TTACCTCTTT ACTTTCTCTT   
  
  
+ CCCTGAGGTA TGCTTCAACT TTAACATATA TATCCCACTT GATTTCTCTT GTCTTTTTGC TATAATACTT   
  
  
+ GTTGTTTGGC TATCATCCCC TGTTTTATAC TCATTTTCTT GCTTTCTATT TCTGGGTTTG AATTGGGTTT   
  
  
+ CACTTATCCT CGTCAATTCT ATGGGTAATT AGTGAATTGG GTTTATATGA ACTAGGGTTG CAAAAGTATC   
  
  
+ TACTTTTTCT CTTGTGATTA TTAGTTGATT AGGGATTTGG CCAATATTAG CAGAATGGGT TCTGAATTTG   
  
  
+ CGGAATTCTC TGATGATGCT CTAAATGGGT ATGCTTATGT TGATATCCCT GCTTATGATG CATCCTTAGA   
  
  
+ TTATGCCAAT TTGTTCAATT ACGAAGGCCC ATCTGAGGAT CGCACCTCAC TGAGCCTCCC AAGACCCTTT   
  
  
+ TCTGACCCTT TGGCTTACAG TTTCACGTCC TCTTCTGAGC TGGGCCCTGG GGTTGATTCT AATGATGATA   
  
  
+ GTGATTCTGG CGATGTTCTC AAGTACATTA GCCAAATGCT TATGGAAGAA GACATGGAGG CAAAGCCATG   
  
  
+ CATGTTTCAT GATCCTTTAG CACTTCAGGC TGCTGAGAAA CCCTTTTATG ATGCCTTAGG GGAGCAATGC   
  
  
+ CCAACTTCTC CTGACCAACA TCCTATAATT GATCATTATT TGGATAGTCC TGATGAAAAT TCTTTGAGTT   
  
  
+ CAACTGGTGA TTTTAGTGTT AGTCATTCTG GGTCTAGTTC AACAAACTCC GTTGGACCGA TAATAGTGTC   
  
  
+ TGATTTGAGT GAGCATTTTG AGCCACCCTT TGTTGAAACA CTTCCAATTG AATCATATCA CCAACCATTG   
  
  
+ ACCCGTCCTC AATGGTCATT TGGCTCTTCG GGTGCCTTAG ATGGCACGGC CTCTAATGGT TCGGTGATCT   
  
  
+ CATCCCTTGG TTTGCCAGTG GATGTGATTA GCGTATTTAG GGAGAAAGAG TCCATGATTC AATTTCAGAA   
  
  
+ AGGGGTGGAG GAGGCCAGTA AGTTCCTTCC CAAGAATAAT AACCTTGTTA TTGATCTCGA GAACCTCACT   
  
  
+ TTTCCTAATG AAACAAAGGA GGATGATCGA ATGATGATGG TTAAGAAGGA AAAGGATGAC GTGAATTGGT   
  
  
+ CTAACTACTC AAGAGGGAGT AAGATTCACT ATTGTGAAGA CGAGGCCTTT GAAGAAGGAA GGAGTGGCAA   
  
  
+ GCAGTCAGCT ATTTCTTCTA CTGAGGAAGC TGAGTTTTCT GACATTTTTG ACAAGGTTTT GCTTTGCGAT   
  
  
+ TGCTACCCTG TGAAACCTGA GGCTCATCCC ACCATGAGTT TGAACCCTGA GAAGGGCCAG TCACATGGAT   
  
  
+ TAGAAGGTGG GAGAAATGGG AAGGCTCGCC CTAAGAAACA GGATAATAGT AGCACAAATA TTGTGGATTT   
  
  
+ AAGGAATTTG CTGATACTAT GCGCACAATC TACTGCATCT GATGACCGAA GAACTGCTGA TGGACTGCTA   
  
  
+ AAGCAAATCA GGGAGCACTC GTCTGCTGAG GGGGATGGAT CTCAAAGGTT GGCGCATTAC TTTGCTGATG   
  
  
+ CCCTAGAGGC ACGTTTAGCT GGAACTGGAT CTCGCATTTA TACGGCCCTA TGTTCTAATA GGCCATCTGT   
  
  
+ CACTGACATG ATAAAAGCAT ATCAGTTCTA TATTCGTGCT TGCCCATTTA CGAAGATCGT CATTGGTTGT   
  
  
+ GGTACCCATA TGATTCTAAA AGCAGCTGAG AAGGCATCAA AGCTTCATAT TATAGATTTT GGCATCCTCT   
  
  
+ ATGGTTGCCA ATGGCCCAAC CTCATTCAAC GCCTCTCAGA GCGATCTGGT GGACCTCCAA AACTGTTTAT   
  
  
+ TACAGGGATC GATCTCCCCC AGCCTGGGTT CAGGCCAGCA GAAAGAGTGG AAGCAACAGG GAGACGCTTG   
  
  
+ GCCAAGTACT GTGAGCGGTA TAATGTGCCA TTTGAGTATC ATGCCATTGC TCAGGAGTGG GAAACAATCA   
  
  
+ AACCAGGGGA TCTCAAGATA GGAAGTAGGA ATGATGAAGT TGTTGCGGTG AACTGTCTCT GTAGGTTCAA   
  
  
+ GAACCTCCTT GACGAGACAG TGGTGGTGGA TAGTCCAAGG AACACAGTTT TAAACCTGAT TACAAGGGTA   
  
  
+ AAGCCTGATA TTTTTGTGCA TGGCGTTGTA AATGGTTCCT ACAACATCCC TTTCTTTGTG ACACGTTTTA   
  
  
+ GAGAAGCCCT CTTTCATTAT TCCACTCTTT TCGACATGTT AGATGCCAAC GCCTCTAGGG AGGAGCCCGA   
  
  
+ GAGGTTGATA TTCGAGAAGG CATTCTATGG GAGGGAGATT ATGAATGTGG TGGCCTGTGA GGGCACAGAG   
  
  
+ AGGGTGGAGA GGCCGGAGAC ATACAAGCAA TGGCATGTTA GGCATAGCAG GGCAGGGTTT CGGCAAGTAC   
  
  
+ CATTGGATCC CAAGTTGATC GAGAAAATGA GGTTTAAGGC CAAGGCAGAC CACCACAAGG ATTTCATGAT   
  
  
+ CGATGTGGAT GGACATTGGG CAATTCAGGG ATGGAAGGGG CGGATTGCAC ATGCGATCTC TGCATGGGTT   
  
  
+ CCGGCTTG  

- -Up\_Stream \_Len000CTAAAC TAACAGCTTT AAACAGGCTA AAATATTTTA ACTTTTAACA ATAACCTGAT   
  
  
- ATTAGATGAC ATCAAATCTC CACAACCTAT TATGAGAGTT GAAACTTGAA CCTGCAGGTA CGTGGTGTGA   
  
  
- TACCCGCATA AATGACAAAT ATATGGTTCT TATTAAAACT TCTACAAGAA TCAACCACTA CATTCACATA   
  
  
- TAAGTTGTAT ACAGTAATGT ATTTGAACTT TTAAGAACTT AAAATCTGTA TATGTTAACG TTTAGTAACA   
  
  
- TTTTAACAAC GTAATGTAAA ACTATTTTTA CGTAGGTTTG AAACAGAACC ATATTTGTCA TTTATCCGGG   
  
  
- TATCCAGGTA TCACATCACG TACCTGCAGG TTCAAATTTC CACCCTCATA ATAACGGGTT GCGGGGATTT   
  
  
- CATGTCATAT AATATCAGGT TATAGGGTTA ATTAAAAAGT AAACTTATAA ATTTAAACCT GTATTATAAA   
  
  
- AAGTATAAAC TTAAGAGATT TGGACTAAAA TTGGACTTAA ATTCAACTAA ATTAAGTAGG CTGGTTTTTT   
  
  
- TATTTGTGTT AATAAATAAA AAGATAAGCT TCAATCTATC ATCGGTTAAG TAAACTGTAT ATTAATTGGG   
  
  
- CACTAACTGT GTTTGAGCTC AAAAACGAAT CTCGATCCTT TTCTCAGCCC AATCCAGCTG GGTAAATTCT   
  
  
- TTACCCAGCT GAAGTACAGC CGGAAGTTTT ATCAATTCTA AACGAGTTTT GATTTTTTAT TGTTCAAGTG   
  
  
- TGATTTGCCA GTTTAGCACA GCTGAAGCCT AACTTTAACC AAGTCAAACC CAGCCGAAGC ATAAAGAAAG   
  
  
- TCATTGAAGC TTGGCTTAAG CATAGCACAA TAGCACAGTC CAGTCTGAAA GAGTCAAGAT AAAAACGATG   
  
  
- GAATACAATG CCTATTTTTT TCAGAGTGTA ACCTTTTTCA CACCCTCCCA GGACCCGAAT ATTTATCACC   
  
  
- GCGACGTGTG GAGTAGTCTG GCTGGAAAAC CCCTCTCCCA TTACTGGGGC ACTGTTCACC ATAGTCTCAG   
  
  
- TTGGGTCTGG GCTGAGTCTA CAGGACGGTT CGCGAGGCTG CCCGCCCCAT CCGTCCCCCG GGTAAACACT   
  
  
- ACTCCTGCAG TGTCTTAACC CGCCCCCTCT CACAGTGCCT ATTTTTTTCA GGGTGTAGCT TTTTTCACAC   
  
  
- CCTCCCAGGA CCCGAATATT TGCCATCGCG GCGTGTGGGG TAGTCTAGCT GAAAAACCCC TCTCCCATTA   
  
  
- CTGGGGTAAA GTTCACCATA GTCTCGGTTG GGTCTGGGCT GAGGTTACAG GGTGGTTCGC GAGACTACCC   
  
  
- GCCCCATCCG TTCCCCGAGG TATACACTGC TCCCGCAGTG CCTTAACCTA CCCTCTCTCA TGGTGCCTAT   
  
  
- TTTTTTCAGG GTGTAGTCTT TTTCACACCT GAATCGTTGA TCCTTCATTT CAATGTCATA ATCGGCTCTC   
  
  
- GGGTACCTTG CGGACTTTGA CTGTAGTGGT CTGGGTGTAT AAAGTGGTAG TAGACTAGAG GGGCAGTTTC   
  
  
- CTCCTTCGAC TCGTGTCGTT CTTGATCTTC TTGTCCGCCG TAGTGTGGGT TCGGTTTTAA GAGATAAACT   
  
  
- GGAGGGAGAG GAGTAAGGAG ACTCGAAGGA GAAGTGTTAC ATTCTGGTAT TAGGAATTGT GGGAAGAAAA   
  
  
- AATTCGAGTA GAAATGGACG AGACCAAGAG AGAGAGTAGC AGTGATTATC AATGGAGAAA TGAAAGAGAA   
  
  
- GGGACTCCAT ACGAAGTTGA AATTGTATAT ATAGGGTGAA CTAAAGAGAA CAGAAAAACG ATATTATGAA   
  
  
- CAACAAACCG ATAGTAGGGG ACAAAATATG AGTAAAAGAA CGAAAGATAA AGACCCAAAC TTAACCCAAA   
  
  
- GTGAATAGGA GCAGTTAAGA TACCCATTAA TCACTTAACC CAAATATACT TGATCCCAAC GTTTTCATAG   
  
  
- ATGAAAAAGA GAACACTAAT AATCAACTAA TCCCTAAACC GGTTATAATC GTCTTACCCA AGACTTAAAC   
  
  
- GCCTTAAGAG ACTACTACGA GATTTACCCA TACGAATACA ACTATAGGGA CGAATACTAC GTAGGAATCT   
  
  
- AATACGGTTA AACAAGTTAA TGCTTCCGGG TAGACTCCTA GCGTGGAGTG ACTCGGAGGG TTCTGGGAAA   
  
  
- AGACTGGGAA ACCGAATGTC AAAGTGCAGG AGAAGACTCG ACCCGGGACC CCAACTAAGA TTACTACTAT   
  
  
- CACTAAGACC GCTACAAGAG TTCATGTAAT CGGTTTACGA ATACCTTCTT CTGTACCTCC GTTTCGGTAC   
  
  
- GTACAAAGTA CTAGGAAATC GTGAAGTCCG ACGACTCTTT GGGAAAATAC TACGGAATCC CCTCGTTACG   
  
  
- GGTTGAAGAG GACTGGTTGT AGGATATTAA CTAGTAATAA ACCTATCAGG ACTACTTTTA AGAAACTCAA   
  
  
- GTTGACCACT AAAATCACAA TCAGTAAGAC CCAGATCAAG TTGTTTGAGG CAACCTGGCT ATTATCACAG   
  
  
- ACTAAACTCA CTCGTAAAAC TCGGTGGGAA ACAACTTTGT GAAGGTTAAC TTAGTATAGT GGTTGGTAAC   
  
  
- TGGGCAGGAG TTACCAGTAA ACCGAGAAGC CCACGGAATC TACCGTGCCG GAGATTACCA AGCCACTAGA   
  
  
- GTAGGGAACC AAACGGTCAC CTACACTAAT CGCATAAATC CCTCTTTCTC AGGTACTAAG TTAAAGTCTT   
  
  
- TCCCCACCTC CTCCGGTCAT TCAAGGAAGG GTTCTTATTA TTGGAACAAT AACTAGAGCT CTTGGAGTGA   
  
  
- AAAGGATTAC TTTGTTTCCT CCTACTAGCT TACTACTACC AATTCTTCCT TTTCCTACTG CACTTAACCA   
  
  
- GATTGATGAG TTCTCCCTCA TTCTAAGTGA TAACACTTCT GCTCCGGAAA CTTCTTCCTT CCTCACCGTT   
  
  
- CGTCAGTCGA TAAAGAAGAT GACTCCTTCG ACTCAAAAGA CTGTAAAAAC TGTTCCAAAA CGAAACGCTA   
  
  
- ACGATGGGAC ACTTTGGACT CCGAGTAGGG TGGTACTCAA ACTTGGGACT CTTCCCGGTC AGTGTACCTA   
  
  
- ATCTTCCACC CTCTTTACCC TTCCGAGCGG GATTCTTTGT CCTATTATCA TCGTGTTTAT AACACCTAAA   
  
  
- TTCCTTAAAC GACTATGATA CGCGTGTTAG ATGACGTAGA CTACTGGCTT CTTGACGACT ACCTGACGAT   
  
  
- TTCGTTTAGT CCCTCGTGAG CAGACGACTC CCCCTACCTA GAGTTTCCAA CCGCGTAATG AAACGACTAC   
  
  
- GGGATCTCCG TGCAAATCGA CCTTGACCTA GAGCGTAAAT ATGCCGGGAT ACAAGATTAT CCGGTAGACA   
  
  
- GTGACTGTAC TATTTTCGTA TAGTCAAGAT ATAAGCACGA ACGGGTAAAT GCTTCTAGCA GTAACCAACA   
  
  
- CCATGGGTAT ACTAAGATTT TCGTCGACTC TTCCGTAGTT TCGAAGTATA ATATCTAAAA CCGTAGGAGA   
  
  
- TACCAACGGT TACCGGGTTG GAGTAAGTTG CGGAGAGTCT CGCTAGACCA CCTGGAGGTT TTGACAAATA   
  
  
- ATGTCCCTAG CTAGAGGGGG TCGGACCCAA GTCCGGTCGT CTTTCTCACC TTCGTTGTCC CTCTGCGAAC   
  
  
- CGGTTCATGA CACTCGCCAT ATTACACGGT AAACTCATAG TACGGTAACG AGTCCTCACC CTTTGTTAGT   
  
  
- TTGGTCCCCT AGAGTTCTAT CCTTCATCCT TACTACTTCA ACAACGCCAC TTGACAGAGA CATCCAAGTT   
  
  
- CTTGGAGGAA CTGCTCTGTC ACCACCACCT ATCAGGTTCC TTGTGTCAAA ATTTGGACTA ATGTTCCCAT   
  
  
- TTCGGACTAT AAAAACACGT ACCGCAACAT TTACCAAGGA TGTTGTAGGG AAAGAAACAC TGTGCAAAAT   
  
  
- CTCTTCGGGA GAAAGTAATA AGGTGAGAAA AGCTGTACAA TCTACGGTTG CGGAGATCCC TCCTCGGGCT   
  
  
- CTCCAACTAT AAGCTCTTCC GTAAGATACC CTCCCTCTAA TACTTACACC ACCGGACACT CCCGTGTCTC   
  
  
- TCCCACCTCT CCGGCCTCTG TATGTTCGTT ACCGTACAAT CCGTATCGTC CCGTCCCAAA GCCGTTCATG   
  
  
- GTAACCTAGG GTTCAACTAG CTCTTTTACT CCAAATTCCG GTTCCGTCTG GTGGTGTTCC TAAAGTACTA   
  
  
- GCTACACCTA CCTGTAACCC GTTAAGTCCC TACCTTCCCC GCCTAACGTG TACGCTAGAG ACGTACCCAA   
  
  
- GGCCGAAC

+     AAGAA-motif

| Site Name | Organism | Position | Strand | Matrix score. | sequence | function |
| --- | --- | --- | --- | --- | --- | --- |
| AAGAA-motif | Avena sativa | 838 | - | 7 | GAAAGAA |  |

>HU07G02249.1   
+ -Up\_Stream \_Len000GATTTG ATTGTCGAAA TTTGTCCGAT TTTATAAAAT TGAAAATTGT TATTGGACTA   
  
  
+ TAATCTACTG TAGTTTAGAG GTGTTGGATA ATACTCTCAA CTTTGAACTT GGACGTCCAT GCACCACACT   
  
  
+ ATGGGCGTAT TTACTGTTTA TATACCAAGA ATAATTTTGA AGATGTTCTT AGTTGGTGAT GTAAGTGTAT   
  
  
+ ATTCAACATA TGTCATTACA TAAACTTGAA AATTCTTGAA TTTTAGACAT ATACAATTGC AAATCATTGT   
  
  
+ AAAATTGTTG CATTACATTT TGATAAAAAT GCATCCAAAC TTTGTCTTGG TATAAACAGT AAATAGGCCC   
  
  
+ ATAGGTCCAT AGTGTAGTGC ATGGACGTCC AAGTTTAAAG GTGGGAGTAT TATTGCCCAA CGCCCCTAAA   
  
  
+ GTACAGTATA TTATAGTCCA ATATCCCAAT TAATTTTTCA TTTGAATATT TAAATTTGGA CATAATATTT   
  
  
+ TTCATATTTG AATTCTCTAA ACCTGATTTT AACCTGAATT TAAGTTGATT TAATTCATCC GACCAAAAAA   
  
  
+ ATAAACACAA TTATTTATTT TTCTATTCGA AGTTAGATAG TAGCCAATTC ATTTGACATA TAATTAACCC   
  
  
+ GTGATTGACA CAAACTCGAG TTTTTGCTTA GAGCTAGGAA AAGAGTCGGG TTAGGTCGAC CCATTTAAGA   
  
  
+ AATGGGTCGA CTTCATGTCG GCCTTCAAAA TAGTTAAGAT TTGCTCAAAA CTAAAAAATA ACAAGTTCAC   
  
  
+ ACTAAACGGT CAAATCGTGT CGACTTCGGA TTGAAATTGG TTCAGTTTGG GTCGGCTTCG TATTTCTTTC   
  
  
+ AGTAACTTCG AACCGAATTC GTATCGTGTT ATCGTGTCAG GTCAGACTTT CTCAGTTCTA TTTTTGCTAC   
  
  
+ CTTATGTTAC GGATAAAAAA AGTCTCACAT TGGAAAAAGT GTGGGAGGGT CCTGGGCTTA TAAATAGTGG   
  
  
+ CGCTGCACAC CTCATCAGAC CGACCTTTTG GGGAGAGGGT AATGACCCCG TGACAAGTGG TATCAGAGTC   
  
  
+ AACCCAGACC CGACTCAGAT GTCCTGCCAA GCGCTCCGAC GGGCGGGGTA GGCAGGGGGC CCATTTGTGA   
  
  
+ TGAGGACGTC ACAGAATTGG GCGGGGGAGA GTGTCACGGA TAAAAAAAGT CCCACATCGA AAAAAGTGTG   
  
  
+ GGAGGGTCCT GGGCTTATAA ACGGTAGCGC CGCACACCCC ATCAGATCGA CTTTTTGGGG AGAGGGTAAT   
  
  
+ GACCCCATTT CAAGTGGTAT CAGAGCCAAC CCAGACCCGA CTCCAATGTC CCACCAAGCG CTCTGATGGG   
  
  
+ CGGGGTAGGC AAGGGGCTCC ATATGTGACG AGGGCGTCAC GGAATTGGAT GGGAGAGAGT ACCACGGATA   
  
  
+ AAAAAAGTCC CACATCAGAA AAAGTGTGGA CTTAGCAACT AGGAAGTAAA GTTACAGTAT TAGCCGAGAG   
  
  
+ CCCATGGAAC GCCTGAAACT GACATCACCA GACCCACATA TTTCACCATC ATCTGATCTC CCCGTCAAAG   
  
  
+ GAGGAAGCTG AGCACAGCAA GAACTAGAAG AACAGGCGGC ATCACACCCA AGCCAAAATT CTCTATTTGA   
  
  
+ CCTCCCTCTC CTCATTCCTC TGAGCTTCCT CTTCACAATG TAAGACCATA ATCCTTAACA CCCTTCTTTT   
  
  
+ TTAAGCTCAT CTTTACCTGC TCTGGTTCTC TCTCTCATCG TCACTAATAG TTACCTCTTT ACTTTCTCTT   
  
  
+ CCCTGAGGTA TGCTTCAACT TTAACATATA TATCCCACTT GATTTCTCTT GTCTTTTTGC TATAATACTT   
  
  
+ GTTGTTTGGC TATCATCCCC TGTTTTATAC TCATTTTCTT GCTTTCTATT TCTGGGTTTG AATTGGGTTT   
  
  
+ CACTTATCCT CGTCAATTCT ATGGGTAATT AGTGAATTGG GTTTATATGA ACTAGGGTTG CAAAAGTATC   
  
  
+ TACTTTTTCT CTTGTGATTA TTAGTTGATT AGGGATTTGG CCAATATTAG CAGAATGGGT TCTGAATTTG   
  
  
+ CGGAATTCTC TGATGATGCT CTAAATGGGT ATGCTTATGT TGATATCCCT GCTTATGATG CATCCTTAGA   
  
  
+ TTATGCCAAT TTGTTCAATT ACGAAGGCCC ATCTGAGGAT CGCACCTCAC TGAGCCTCCC AAGACCCTTT   
  
  
+ TCTGACCCTT TGGCTTACAG TTTCACGTCC TCTTCTGAGC TGGGCCCTGG GGTTGATTCT AATGATGATA   
  
  
+ GTGATTCTGG CGATGTTCTC AAGTACATTA GCCAAATGCT TATGGAAGAA GACATGGAGG CAAAGCCATG   
  
  
+ CATGTTTCAT GATCCTTTAG CACTTCAGGC TGCTGAGAAA CCCTTTTATG ATGCCTTAGG GGAGCAATGC   
  
  
+ CCAACTTCTC CTGACCAACA TCCTATAATT GATCATTATT TGGATAGTCC TGATGAAAAT TCTTTGAGTT   
  
  
+ CAACTGGTGA TTTTAGTGTT AGTCATTCTG GGTCTAGTTC AACAAACTCC GTTGGACCGA TAATAGTGTC   
  
  
+ TGATTTGAGT GAGCATTTTG AGCCACCCTT TGTTGAAACA CTTCCAATTG AATCATATCA CCAACCATTG   
  
  
+ ACCCGTCCTC AATGGTCATT TGGCTCTTCG GGTGCCTTAG ATGGCACGGC CTCTAATGGT TCGGTGATCT   
  
  
+ CATCCCTTGG TTTGCCAGTG GATGTGATTA GCGTATTTAG GGAGAAAGAG TCCATGATTC AATTTCAGAA   
  
  
+ AGGGGTGGAG GAGGCCAGTA AGTTCCTTCC CAAGAATAAT AACCTTGTTA TTGATCTCGA GAACCTCACT   
  
  
+ TTTCCTAATG AAACAAAGGA GGATGATCGA ATGATGATGG TTAAGAAGGA AAAGGATGAC GTGAATTGGT   
  
  
+ CTAACTACTC AAGAGGGAGT AAGATTCACT ATTGTGAAGA CGAGGCCTTT GAAGAAGGAA GGAGTGGCAA   
  
  
+ GCAGTCAGCT ATTTCTTCTA CTGAGGAAGC TGAGTTTTCT GACATTTTTG ACAAGGTTTT GCTTTGCGAT   
  
  
+ TGCTACCCTG TGAAACCTGA GGCTCATCCC ACCATGAGTT TGAACCCTGA GAAGGGCCAG TCACATGGAT   
  
  
+ TAGAAGGTGG GAGAAATGGG AAGGCTCGCC CTAAGAAACA GGATAATAGT AGCACAAATA TTGTGGATTT   
  
  
+ AAGGAATTTG CTGATACTAT GCGCACAATC TACTGCATCT GATGACCGAA GAACTGCTGA TGGACTGCTA   
  
  
+ AAGCAAATCA GGGAGCACTC GTCTGCTGAG GGGGATGGAT CTCAAAGGTT GGCGCATTAC TTTGCTGATG   
  
  
+ CCCTAGAGGC ACGTTTAGCT GGAACTGGAT CTCGCATTTA TACGGCCCTA TGTTCTAATA GGCCATCTGT   
  
  
+ CACTGACATG ATAAAAGCAT ATCAGTTCTA TATTCGTGCT TGCCCATTTA CGAAGATCGT CATTGGTTGT   
  
  
+ GGTACCCATA TGATTCTAAA AGCAGCTGAG AAGGCATCAA AGCTTCATAT TATAGATTTT GGCATCCTCT   
  
  
+ ATGGTTGCCA ATGGCCCAAC CTCATTCAAC GCCTCTCAGA GCGATCTGGT GGACCTCCAA AACTGTTTAT   
  
  
+ TACAGGGATC GATCTCCCCC AGCCTGGGTT CAGGCCAGCA GAAAGAGTGG AAGCAACAGG GAGACGCTTG   
  
  
+ GCCAAGTACT GTGAGCGGTA TAATGTGCCA TTTGAGTATC ATGCCATTGC TCAGGAGTGG GAAACAATCA   
  
  
+ AACCAGGGGA TCTCAAGATA GGAAGTAGGA ATGATGAAGT TGTTGCGGTG AACTGTCTCT GTAGGTTCAA   
  
  
+ GAACCTCCTT GACGAGACAG TGGTGGTGGA TAGTCCAAGG AACACAGTTT TAAACCTGAT TACAAGGGTA   
  
  
+ AAGCCTGATA TTTTTGTGCA TGGCGTTGTA AATGGTTCCT ACAACATCCC TTTCTTTGTG ACACGTTTTA   
  
  
+ GAGAAGCCCT CTTTCATTAT TCCACTCTTT TCGACATGTT AGATGCCAAC GCCTCTAGGG AGGAGCCCGA   
  
  
+ GAGGTTGATA TTCGAGAAGG CATTCTATGG GAGGGAGATT ATGAATGTGG TGGCCTGTGA GGGCACAGAG   
  
  
+ AGGGTGGAGA GGCCGGAGAC ATACAAGCAA TGGCATGTTA GGCATAGCAG GGCAGGGTTT CGGCAAGTAC   
  
  
+ CATTGGATCC CAAGTTGATC GAGAAAATGA GGTTTAAGGC CAAGGCAGAC CACCACAAGG ATTTCATGAT   
  
  
+ CGATGTGGAT GGACATTGGG CAATTCAGGG ATGGAAGGGG CGGATTGCAC ATGCGATCTC TGCATGGGTT   
  
  
+ CCGGCTTG  

- -Up\_Stream \_Len000CTAAAC TAACAGCTTT AAACAGGCTA AAATATTTTA ACTTTTAACA ATAACCTGAT   
  
  
- ATTAGATGAC ATCAAATCTC CACAACCTAT TATGAGAGTT GAAACTTGAA CCTGCAGGTA CGTGGTGTGA   
  
  
- TACCCGCATA AATGACAAAT ATATGGTTCT TATTAAAACT TCTACAAGAA TCAACCACTA CATTCACATA   
  
  
- TAAGTTGTAT ACAGTAATGT ATTTGAACTT TTAAGAACTT AAAATCTGTA TATGTTAACG TTTAGTAACA   
  
  
- TTTTAACAAC GTAATGTAAA ACTATTTTTA CGTAGGTTTG AAACAGAACC ATATTTGTCA TTTATCCGGG   
  
  
- TATCCAGGTA TCACATCACG TACCTGCAGG TTCAAATTTC CACCCTCATA ATAACGGGTT GCGGGGATTT   
  
  
- CATGTCATAT AATATCAGGT TATAGGGTTA ATTAAAAAGT AAACTTATAA ATTTAAACCT GTATTATAAA   
  
  
- AAGTATAAAC TTAAGAGATT TGGACTAAAA TTGGACTTAA ATTCAACTAA ATTAAGTAGG CTGGTTTTTT   
  
  
- TATTTGTGTT AATAAATAAA AAGATAAGCT TCAATCTATC ATCGGTTAAG TAAACTGTAT ATTAATTGGG   
  
  
- CACTAACTGT GTTTGAGCTC AAAAACGAAT CTCGATCCTT TTCTCAGCCC AATCCAGCTG GGTAAATTCT   
  
  
- TTACCCAGCT GAAGTACAGC CGGAAGTTTT ATCAATTCTA AACGAGTTTT GATTTTTTAT TGTTCAAGTG   
  
  
- TGATTTGCCA GTTTAGCACA GCTGAAGCCT AACTTTAACC AAGTCAAACC CAGCCGAAGC ATAAAGAAAG   
  
  
- TCATTGAAGC TTGGCTTAAG CATAGCACAA TAGCACAGTC CAGTCTGAAA GAGTCAAGAT AAAAACGATG   
  
  
- GAATACAATG CCTATTTTTT TCAGAGTGTA ACCTTTTTCA CACCCTCCCA GGACCCGAAT ATTTATCACC   
  
  
- GCGACGTGTG GAGTAGTCTG GCTGGAAAAC CCCTCTCCCA TTACTGGGGC ACTGTTCACC ATAGTCTCAG   
  
  
- TTGGGTCTGG GCTGAGTCTA CAGGACGGTT CGCGAGGCTG CCCGCCCCAT CCGTCCCCCG GGTAAACACT   
  
  
- ACTCCTGCAG TGTCTTAACC CGCCCCCTCT CACAGTGCCT ATTTTTTTCA GGGTGTAGCT TTTTTCACAC   
  
  
- CCTCCCAGGA CCCGAATATT TGCCATCGCG GCGTGTGGGG TAGTCTAGCT GAAAAACCCC TCTCCCATTA   
  
  
- CTGGGGTAAA GTTCACCATA GTCTCGGTTG GGTCTGGGCT GAGGTTACAG GGTGGTTCGC GAGACTACCC   
  
  
- GCCCCATCCG TTCCCCGAGG TATACACTGC TCCCGCAGTG CCTTAACCTA CCCTCTCTCA TGGTGCCTAT   
  
  
- TTTTTTCAGG GTGTAGTCTT TTTCACACCT GAATCGTTGA TCCTTCATTT CAATGTCATA ATCGGCTCTC   
  
  
- GGGTACCTTG CGGACTTTGA CTGTAGTGGT CTGGGTGTAT AAAGTGGTAG TAGACTAGAG GGGCAGTTTC   
  
  
- CTCCTTCGAC TCGTGTCGTT CTTGATCTTC TTGTCCGCCG TAGTGTGGGT TCGGTTTTAA GAGATAAACT   
  
  
- GGAGGGAGAG GAGTAAGGAG ACTCGAAGGA GAAGTGTTAC ATTCTGGTAT TAGGAATTGT GGGAAGAAAA   
  
  
- AATTCGAGTA GAAATGGACG AGACCAAGAG AGAGAGTAGC AGTGATTATC AATGGAGAAA TGAAAGAGAA   
  
  
- GGGACTCCAT ACGAAGTTGA AATTGTATAT ATAGGGTGAA CTAAAGAGAA CAGAAAAACG ATATTATGAA   
  
  
- CAACAAACCG ATAGTAGGGG ACAAAATATG AGTAAAAGAA CGAAAGATAA AGACCCAAAC TTAACCCAAA   
  
  
- GTGAATAGGA GCAGTTAAGA TACCCATTAA TCACTTAACC CAAATATACT TGATCCCAAC GTTTTCATAG   
  
  
- ATGAAAAAGA GAACACTAAT AATCAACTAA TCCCTAAACC GGTTATAATC GTCTTACCCA AGACTTAAAC   
  
  
- GCCTTAAGAG ACTACTACGA GATTTACCCA TACGAATACA ACTATAGGGA CGAATACTAC GTAGGAATCT   
  
  
- AATACGGTTA AACAAGTTAA TGCTTCCGGG TAGACTCCTA GCGTGGAGTG ACTCGGAGGG TTCTGGGAAA   
  
  
- AGACTGGGAA ACCGAATGTC AAAGTGCAGG AGAAGACTCG ACCCGGGACC CCAACTAAGA TTACTACTAT   
  
  
- CACTAAGACC GCTACAAGAG TTCATGTAAT CGGTTTACGA ATACCTTCTT CTGTACCTCC GTTTCGGTAC   
  
  
- GTACAAAGTA CTAGGAAATC GTGAAGTCCG ACGACTCTTT GGGAAAATAC TACGGAATCC CCTCGTTACG   
  
  
- GGTTGAAGAG GACTGGTTGT AGGATATTAA CTAGTAATAA ACCTATCAGG ACTACTTTTA AGAAACTCAA   
  
  
- GTTGACCACT AAAATCACAA TCAGTAAGAC CCAGATCAAG TTGTTTGAGG CAACCTGGCT ATTATCACAG   
  
  
- ACTAAACTCA CTCGTAAAAC TCGGTGGGAA ACAACTTTGT GAAGGTTAAC TTAGTATAGT GGTTGGTAAC   
  
  
- TGGGCAGGAG TTACCAGTAA ACCGAGAAGC CCACGGAATC TACCGTGCCG GAGATTACCA AGCCACTAGA   
  
  
- GTAGGGAACC AAACGGTCAC CTACACTAAT CGCATAAATC CCTCTTTCTC AGGTACTAAG TTAAAGTCTT   
  
  
- TCCCCACCTC CTCCGGTCAT TCAAGGAAGG GTTCTTATTA TTGGAACAAT AACTAGAGCT CTTGGAGTGA   
  
  
- AAAGGATTAC TTTGTTTCCT CCTACTAGCT TACTACTACC AATTCTTCCT TTTCCTACTG CACTTAACCA   
  
  
- GATTGATGAG TTCTCCCTCA TTCTAAGTGA TAACACTTCT GCTCCGGAAA CTTCTTCCTT CCTCACCGTT   
  
  
- CGTCAGTCGA TAAAGAAGAT GACTCCTTCG ACTCAAAAGA CTGTAAAAAC TGTTCCAAAA CGAAACGCTA   
  
  
- ACGATGGGAC ACTTTGGACT CCGAGTAGGG TGGTACTCAA ACTTGGGACT CTTCCCGGTC AGTGTACCTA   
  
  
- ATCTTCCACC CTCTTTACCC TTCCGAGCGG GATTCTTTGT CCTATTATCA TCGTGTTTAT AACACCTAAA   
  
  
- TTCCTTAAAC GACTATGATA CGCGTGTTAG ATGACGTAGA CTACTGGCTT CTTGACGACT ACCTGACGAT   
  
  
- TTCGTTTAGT CCCTCGTGAG CAGACGACTC CCCCTACCTA GAGTTTCCAA CCGCGTAATG AAACGACTAC   
  
  
- GGGATCTCCG TGCAAATCGA CCTTGACCTA GAGCGTAAAT ATGCCGGGAT ACAAGATTAT CCGGTAGACA   
  
  
- GTGACTGTAC TATTTTCGTA TAGTCAAGAT ATAAGCACGA ACGGGTAAAT GCTTCTAGCA GTAACCAACA   
  
  
- CCATGGGTAT ACTAAGATTT TCGTCGACTC TTCCGTAGTT TCGAAGTATA ATATCTAAAA CCGTAGGAGA   
  
  
- TACCAACGGT TACCGGGTTG GAGTAAGTTG CGGAGAGTCT CGCTAGACCA CCTGGAGGTT TTGACAAATA   
  
  
- ATGTCCCTAG CTAGAGGGGG TCGGACCCAA GTCCGGTCGT CTTTCTCACC TTCGTTGTCC CTCTGCGAAC   
  
  
- CGGTTCATGA CACTCGCCAT ATTACACGGT AAACTCATAG TACGGTAACG AGTCCTCACC CTTTGTTAGT   
  
  
- TTGGTCCCCT AGAGTTCTAT CCTTCATCCT TACTACTTCA ACAACGCCAC TTGACAGAGA CATCCAAGTT   
  
  
- CTTGGAGGAA CTGCTCTGTC ACCACCACCT ATCAGGTTCC TTGTGTCAAA ATTTGGACTA ATGTTCCCAT   
  
  
- TTCGGACTAT AAAAACACGT ACCGCAACAT TTACCAAGGA TGTTGTAGGG AAAGAAACAC TGTGCAAAAT   
  
  
- CTCTTCGGGA GAAAGTAATA AGGTGAGAAA AGCTGTACAA TCTACGGTTG CGGAGATCCC TCCTCGGGCT   
  
  
- CTCCAACTAT AAGCTCTTCC GTAAGATACC CTCCCTCTAA TACTTACACC ACCGGACACT CCCGTGTCTC   
  
  
- TCCCACCTCT CCGGCCTCTG TATGTTCGTT ACCGTACAAT CCGTATCGTC CCGTCCCAAA GCCGTTCATG   
  
  
- GTAACCTAGG GTTCAACTAG CTCTTTTACT CCAAATTCCG GTTCCGTCTG GTGGTGTTCC TAAAGTACTA   
  
  
- GCTACACCTA CCTGTAACCC GTTAAGTCCC TACCTTCCCC GCCTAACGTG TACGCTAGAG ACGTACCCAA   
  
  
- GGCCGAAC

+     ABRE

| Site Name | Organism | Position | Strand | Matrix score. | sequence | function |
| --- | --- | --- | --- | --- | --- | --- |
| ABRE | Arabidopsis thaliana | 3916 | - | 5 | ACGTG | cis-acting element involved in the abscisic acid responsiveness |
| ABRE | Arabidopsis thaliana | 2863 | + | 5 | ACGTG | cis-acting element involved in the abscisic acid responsiveness |
| ABRE | Arabidopsis thaliana | 2198 | - | 5 | ACGTG | cis-acting element involved in the abscisic acid responsiveness |
| ABRE | Arabidopsis thaliana | 3304 | - | 5 | ACGTG | cis-acting element involved in the abscisic acid responsiveness |

>HU07G02249.1   
+ -Up\_Stream \_Len000GATTTG ATTGTCGAAA TTTGTCCGAT TTTATAAAAT TGAAAATTGT TATTGGACTA   
  
  
+ TAATCTACTG TAGTTTAGAG GTGTTGGATA ATACTCTCAA CTTTGAACTT GGACGTCCAT GCACCACACT   
  
  
+ ATGGGCGTAT TTACTGTTTA TATACCAAGA ATAATTTTGA AGATGTTCTT AGTTGGTGAT GTAAGTGTAT   
  
  
+ ATTCAACATA TGTCATTACA TAAACTTGAA AATTCTTGAA TTTTAGACAT ATACAATTGC AAATCATTGT   
  
  
+ AAAATTGTTG CATTACATTT TGATAAAAAT GCATCCAAAC TTTGTCTTGG TATAAACAGT AAATAGGCCC   
  
  
+ ATAGGTCCAT AGTGTAGTGC ATGGACGTCC AAGTTTAAAG GTGGGAGTAT TATTGCCCAA CGCCCCTAAA   
  
  
+ GTACAGTATA TTATAGTCCA ATATCCCAAT TAATTTTTCA TTTGAATATT TAAATTTGGA CATAATATTT   
  
  
+ TTCATATTTG AATTCTCTAA ACCTGATTTT AACCTGAATT TAAGTTGATT TAATTCATCC GACCAAAAAA   
  
  
+ ATAAACACAA TTATTTATTT TTCTATTCGA AGTTAGATAG TAGCCAATTC ATTTGACATA TAATTAACCC   
  
  
+ GTGATTGACA CAAACTCGAG TTTTTGCTTA GAGCTAGGAA AAGAGTCGGG TTAGGTCGAC CCATTTAAGA   
  
  
+ AATGGGTCGA CTTCATGTCG GCCTTCAAAA TAGTTAAGAT TTGCTCAAAA CTAAAAAATA ACAAGTTCAC   
  
  
+ ACTAAACGGT CAAATCGTGT CGACTTCGGA TTGAAATTGG TTCAGTTTGG GTCGGCTTCG TATTTCTTTC   
  
  
+ AGTAACTTCG AACCGAATTC GTATCGTGTT ATCGTGTCAG GTCAGACTTT CTCAGTTCTA TTTTTGCTAC   
  
  
+ CTTATGTTAC GGATAAAAAA AGTCTCACAT TGGAAAAAGT GTGGGAGGGT CCTGGGCTTA TAAATAGTGG   
  
  
+ CGCTGCACAC CTCATCAGAC CGACCTTTTG GGGAGAGGGT AATGACCCCG TGACAAGTGG TATCAGAGTC   
  
  
+ AACCCAGACC CGACTCAGAT GTCCTGCCAA GCGCTCCGAC GGGCGGGGTA GGCAGGGGGC CCATTTGTGA   
  
  
+ TGAGGACGTC ACAGAATTGG GCGGGGGAGA GTGTCACGGA TAAAAAAAGT CCCACATCGA AAAAAGTGTG   
  
  
+ GGAGGGTCCT GGGCTTATAA ACGGTAGCGC CGCACACCCC ATCAGATCGA CTTTTTGGGG AGAGGGTAAT   
  
  
+ GACCCCATTT CAAGTGGTAT CAGAGCCAAC CCAGACCCGA CTCCAATGTC CCACCAAGCG CTCTGATGGG   
  
  
+ CGGGGTAGGC AAGGGGCTCC ATATGTGACG AGGGCGTCAC GGAATTGGAT GGGAGAGAGT ACCACGGATA   
  
  
+ AAAAAAGTCC CACATCAGAA AAAGTGTGGA CTTAGCAACT AGGAAGTAAA GTTACAGTAT TAGCCGAGAG   
  
  
+ CCCATGGAAC GCCTGAAACT GACATCACCA GACCCACATA TTTCACCATC ATCTGATCTC CCCGTCAAAG   
  
  
+ GAGGAAGCTG AGCACAGCAA GAACTAGAAG AACAGGCGGC ATCACACCCA AGCCAAAATT CTCTATTTGA   
  
  
+ CCTCCCTCTC CTCATTCCTC TGAGCTTCCT CTTCACAATG TAAGACCATA ATCCTTAACA CCCTTCTTTT   
  
  
+ TTAAGCTCAT CTTTACCTGC TCTGGTTCTC TCTCTCATCG TCACTAATAG TTACCTCTTT ACTTTCTCTT   
  
  
+ CCCTGAGGTA TGCTTCAACT TTAACATATA TATCCCACTT GATTTCTCTT GTCTTTTTGC TATAATACTT   
  
  
+ GTTGTTTGGC TATCATCCCC TGTTTTATAC TCATTTTCTT GCTTTCTATT TCTGGGTTTG AATTGGGTTT   
  
  
+ CACTTATCCT CGTCAATTCT ATGGGTAATT AGTGAATTGG GTTTATATGA ACTAGGGTTG CAAAAGTATC   
  
  
+ TACTTTTTCT CTTGTGATTA TTAGTTGATT AGGGATTTGG CCAATATTAG CAGAATGGGT TCTGAATTTG   
  
  
+ CGGAATTCTC TGATGATGCT CTAAATGGGT ATGCTTATGT TGATATCCCT GCTTATGATG CATCCTTAGA   
  
  
+ TTATGCCAAT TTGTTCAATT ACGAAGGCCC ATCTGAGGAT CGCACCTCAC TGAGCCTCCC AAGACCCTTT   
  
  
+ TCTGACCCTT TGGCTTACAG TTTCACGTCC TCTTCTGAGC TGGGCCCTGG GGTTGATTCT AATGATGATA   
  
  
+ GTGATTCTGG CGATGTTCTC AAGTACATTA GCCAAATGCT TATGGAAGAA GACATGGAGG CAAAGCCATG   
  
  
+ CATGTTTCAT GATCCTTTAG CACTTCAGGC TGCTGAGAAA CCCTTTTATG ATGCCTTAGG GGAGCAATGC   
  
  
+ CCAACTTCTC CTGACCAACA TCCTATAATT GATCATTATT TGGATAGTCC TGATGAAAAT TCTTTGAGTT   
  
  
+ CAACTGGTGA TTTTAGTGTT AGTCATTCTG GGTCTAGTTC AACAAACTCC GTTGGACCGA TAATAGTGTC   
  
  
+ TGATTTGAGT GAGCATTTTG AGCCACCCTT TGTTGAAACA CTTCCAATTG AATCATATCA CCAACCATTG   
  
  
+ ACCCGTCCTC AATGGTCATT TGGCTCTTCG GGTGCCTTAG ATGGCACGGC CTCTAATGGT TCGGTGATCT   
  
  
+ CATCCCTTGG TTTGCCAGTG GATGTGATTA GCGTATTTAG GGAGAAAGAG TCCATGATTC AATTTCAGAA   
  
  
+ AGGGGTGGAG GAGGCCAGTA AGTTCCTTCC CAAGAATAAT AACCTTGTTA TTGATCTCGA GAACCTCACT   
  
  
+ TTTCCTAATG AAACAAAGGA GGATGATCGA ATGATGATGG TTAAGAAGGA AAAGGATGAC GTGAATTGGT   
  
  
+ CTAACTACTC AAGAGGGAGT AAGATTCACT ATTGTGAAGA CGAGGCCTTT GAAGAAGGAA GGAGTGGCAA   
  
  
+ GCAGTCAGCT ATTTCTTCTA CTGAGGAAGC TGAGTTTTCT GACATTTTTG ACAAGGTTTT GCTTTGCGAT   
  
  
+ TGCTACCCTG TGAAACCTGA GGCTCATCCC ACCATGAGTT TGAACCCTGA GAAGGGCCAG TCACATGGAT   
  
  
+ TAGAAGGTGG GAGAAATGGG AAGGCTCGCC CTAAGAAACA GGATAATAGT AGCACAAATA TTGTGGATTT   
  
  
+ AAGGAATTTG CTGATACTAT GCGCACAATC TACTGCATCT GATGACCGAA GAACTGCTGA TGGACTGCTA   
  
  
+ AAGCAAATCA GGGAGCACTC GTCTGCTGAG GGGGATGGAT CTCAAAGGTT GGCGCATTAC TTTGCTGATG   
  
  
+ CCCTAGAGGC ACGTTTAGCT GGAACTGGAT CTCGCATTTA TACGGCCCTA TGTTCTAATA GGCCATCTGT   
  
  
+ CACTGACATG ATAAAAGCAT ATCAGTTCTA TATTCGTGCT TGCCCATTTA CGAAGATCGT CATTGGTTGT   
  
  
+ GGTACCCATA TGATTCTAAA AGCAGCTGAG AAGGCATCAA AGCTTCATAT TATAGATTTT GGCATCCTCT   
  
  
+ ATGGTTGCCA ATGGCCCAAC CTCATTCAAC GCCTCTCAGA GCGATCTGGT GGACCTCCAA AACTGTTTAT   
  
  
+ TACAGGGATC GATCTCCCCC AGCCTGGGTT CAGGCCAGCA GAAAGAGTGG AAGCAACAGG GAGACGCTTG   
  
  
+ GCCAAGTACT GTGAGCGGTA TAATGTGCCA TTTGAGTATC ATGCCATTGC TCAGGAGTGG GAAACAATCA   
  
  
+ AACCAGGGGA TCTCAAGATA GGAAGTAGGA ATGATGAAGT TGTTGCGGTG AACTGTCTCT GTAGGTTCAA   
  
  
+ GAACCTCCTT GACGAGACAG TGGTGGTGGA TAGTCCAAGG AACACAGTTT TAAACCTGAT TACAAGGGTA   
  
  
+ AAGCCTGATA TTTTTGTGCA TGGCGTTGTA AATGGTTCCT ACAACATCCC TTTCTTTGTG ACACGTTTTA   
  
  
+ GAGAAGCCCT CTTTCATTAT TCCACTCTTT TCGACATGTT AGATGCCAAC GCCTCTAGGG AGGAGCCCGA   
  
  
+ GAGGTTGATA TTCGAGAAGG CATTCTATGG GAGGGAGATT ATGAATGTGG TGGCCTGTGA GGGCACAGAG   
  
  
+ AGGGTGGAGA GGCCGGAGAC ATACAAGCAA TGGCATGTTA GGCATAGCAG GGCAGGGTTT CGGCAAGTAC   
  
  
+ CATTGGATCC CAAGTTGATC GAGAAAATGA GGTTTAAGGC CAAGGCAGAC CACCACAAGG ATTTCATGAT   
  
  
+ CGATGTGGAT GGACATTGGG CAATTCAGGG ATGGAAGGGG CGGATTGCAC ATGCGATCTC TGCATGGGTT   
  
  
+ CCGGCTTG  

- -Up\_Stream \_Len000CTAAAC TAACAGCTTT AAACAGGCTA AAATATTTTA ACTTTTAACA ATAACCTGAT   
  
  
- ATTAGATGAC ATCAAATCTC CACAACCTAT TATGAGAGTT GAAACTTGAA CCTGCAGGTA CGTGGTGTGA   
  
  
- TACCCGCATA AATGACAAAT ATATGGTTCT TATTAAAACT TCTACAAGAA TCAACCACTA CATTCACATA   
  
  
- TAAGTTGTAT ACAGTAATGT ATTTGAACTT TTAAGAACTT AAAATCTGTA TATGTTAACG TTTAGTAACA   
  
  
- TTTTAACAAC GTAATGTAAA ACTATTTTTA CGTAGGTTTG AAACAGAACC ATATTTGTCA TTTATCCGGG   
  
  
- TATCCAGGTA TCACATCACG TACCTGCAGG TTCAAATTTC CACCCTCATA ATAACGGGTT GCGGGGATTT   
  
  
- CATGTCATAT AATATCAGGT TATAGGGTTA ATTAAAAAGT AAACTTATAA ATTTAAACCT GTATTATAAA   
  
  
- AAGTATAAAC TTAAGAGATT TGGACTAAAA TTGGACTTAA ATTCAACTAA ATTAAGTAGG CTGGTTTTTT   
  
  
- TATTTGTGTT AATAAATAAA AAGATAAGCT TCAATCTATC ATCGGTTAAG TAAACTGTAT ATTAATTGGG   
  
  
- CACTAACTGT GTTTGAGCTC AAAAACGAAT CTCGATCCTT TTCTCAGCCC AATCCAGCTG GGTAAATTCT   
  
  
- TTACCCAGCT GAAGTACAGC CGGAAGTTTT ATCAATTCTA AACGAGTTTT GATTTTTTAT TGTTCAAGTG   
  
  
- TGATTTGCCA GTTTAGCACA GCTGAAGCCT AACTTTAACC AAGTCAAACC CAGCCGAAGC ATAAAGAAAG   
  
  
- TCATTGAAGC TTGGCTTAAG CATAGCACAA TAGCACAGTC CAGTCTGAAA GAGTCAAGAT AAAAACGATG   
  
  
- GAATACAATG CCTATTTTTT TCAGAGTGTA ACCTTTTTCA CACCCTCCCA GGACCCGAAT ATTTATCACC   
  
  
- GCGACGTGTG GAGTAGTCTG GCTGGAAAAC CCCTCTCCCA TTACTGGGGC ACTGTTCACC ATAGTCTCAG   
  
  
- TTGGGTCTGG GCTGAGTCTA CAGGACGGTT CGCGAGGCTG CCCGCCCCAT CCGTCCCCCG GGTAAACACT   
  
  
- ACTCCTGCAG TGTCTTAACC CGCCCCCTCT CACAGTGCCT ATTTTTTTCA GGGTGTAGCT TTTTTCACAC   
  
  
- CCTCCCAGGA CCCGAATATT TGCCATCGCG GCGTGTGGGG TAGTCTAGCT GAAAAACCCC TCTCCCATTA   
  
  
- CTGGGGTAAA GTTCACCATA GTCTCGGTTG GGTCTGGGCT GAGGTTACAG GGTGGTTCGC GAGACTACCC   
  
  
- GCCCCATCCG TTCCCCGAGG TATACACTGC TCCCGCAGTG CCTTAACCTA CCCTCTCTCA TGGTGCCTAT   
  
  
- TTTTTTCAGG GTGTAGTCTT TTTCACACCT GAATCGTTGA TCCTTCATTT CAATGTCATA ATCGGCTCTC   
  
  
- GGGTACCTTG CGGACTTTGA CTGTAGTGGT CTGGGTGTAT AAAGTGGTAG TAGACTAGAG GGGCAGTTTC   
  
  
- CTCCTTCGAC TCGTGTCGTT CTTGATCTTC TTGTCCGCCG TAGTGTGGGT TCGGTTTTAA GAGATAAACT   
  
  
- GGAGGGAGAG GAGTAAGGAG ACTCGAAGGA GAAGTGTTAC ATTCTGGTAT TAGGAATTGT GGGAAGAAAA   
  
  
- AATTCGAGTA GAAATGGACG AGACCAAGAG AGAGAGTAGC AGTGATTATC AATGGAGAAA TGAAAGAGAA   
  
  
- GGGACTCCAT ACGAAGTTGA AATTGTATAT ATAGGGTGAA CTAAAGAGAA CAGAAAAACG ATATTATGAA   
  
  
- CAACAAACCG ATAGTAGGGG ACAAAATATG AGTAAAAGAA CGAAAGATAA AGACCCAAAC TTAACCCAAA   
  
  
- GTGAATAGGA GCAGTTAAGA TACCCATTAA TCACTTAACC CAAATATACT TGATCCCAAC GTTTTCATAG   
  
  
- ATGAAAAAGA GAACACTAAT AATCAACTAA TCCCTAAACC GGTTATAATC GTCTTACCCA AGACTTAAAC   
  
  
- GCCTTAAGAG ACTACTACGA GATTTACCCA TACGAATACA ACTATAGGGA CGAATACTAC GTAGGAATCT   
  
  
- AATACGGTTA AACAAGTTAA TGCTTCCGGG TAGACTCCTA GCGTGGAGTG ACTCGGAGGG TTCTGGGAAA   
  
  
- AGACTGGGAA ACCGAATGTC AAAGTGCAGG AGAAGACTCG ACCCGGGACC CCAACTAAGA TTACTACTAT   
  
  
- CACTAAGACC GCTACAAGAG TTCATGTAAT CGGTTTACGA ATACCTTCTT CTGTACCTCC GTTTCGGTAC   
  
  
- GTACAAAGTA CTAGGAAATC GTGAAGTCCG ACGACTCTTT GGGAAAATAC TACGGAATCC CCTCGTTACG   
  
  
- GGTTGAAGAG GACTGGTTGT AGGATATTAA CTAGTAATAA ACCTATCAGG ACTACTTTTA AGAAACTCAA   
  
  
- GTTGACCACT AAAATCACAA TCAGTAAGAC CCAGATCAAG TTGTTTGAGG CAACCTGGCT ATTATCACAG   
  
  
- ACTAAACTCA CTCGTAAAAC TCGGTGGGAA ACAACTTTGT GAAGGTTAAC TTAGTATAGT GGTTGGTAAC   
  
  
- TGGGCAGGAG TTACCAGTAA ACCGAGAAGC CCACGGAATC TACCGTGCCG GAGATTACCA AGCCACTAGA   
  
  
- GTAGGGAACC AAACGGTCAC CTACACTAAT CGCATAAATC CCTCTTTCTC AGGTACTAAG TTAAAGTCTT   
  
  
- TCCCCACCTC CTCCGGTCAT TCAAGGAAGG GTTCTTATTA TTGGAACAAT AACTAGAGCT CTTGGAGTGA   
  
  
- AAAGGATTAC TTTGTTTCCT CCTACTAGCT TACTACTACC AATTCTTCCT TTTCCTACTG CACTTAACCA   
  
  
- GATTGATGAG TTCTCCCTCA TTCTAAGTGA TAACACTTCT GCTCCGGAAA CTTCTTCCTT CCTCACCGTT   
  
  
- CGTCAGTCGA TAAAGAAGAT GACTCCTTCG ACTCAAAAGA CTGTAAAAAC TGTTCCAAAA CGAAACGCTA   
  
  
- ACGATGGGAC ACTTTGGACT CCGAGTAGGG TGGTACTCAA ACTTGGGACT CTTCCCGGTC AGTGTACCTA   
  
  
- ATCTTCCACC CTCTTTACCC TTCCGAGCGG GATTCTTTGT CCTATTATCA TCGTGTTTAT AACACCTAAA   
  
  
- TTCCTTAAAC GACTATGATA CGCGTGTTAG ATGACGTAGA CTACTGGCTT CTTGACGACT ACCTGACGAT   
  
  
- TTCGTTTAGT CCCTCGTGAG CAGACGACTC CCCCTACCTA GAGTTTCCAA CCGCGTAATG AAACGACTAC   
  
  
- GGGATCTCCG TGCAAATCGA CCTTGACCTA GAGCGTAAAT ATGCCGGGAT ACAAGATTAT CCGGTAGACA   
  
  
- GTGACTGTAC TATTTTCGTA TAGTCAAGAT ATAAGCACGA ACGGGTAAAT GCTTCTAGCA GTAACCAACA   
  
  
- CCATGGGTAT ACTAAGATTT TCGTCGACTC TTCCGTAGTT TCGAAGTATA ATATCTAAAA CCGTAGGAGA   
  
  
- TACCAACGGT TACCGGGTTG GAGTAAGTTG CGGAGAGTCT CGCTAGACCA CCTGGAGGTT TTGACAAATA   
  
  
- ATGTCCCTAG CTAGAGGGGG TCGGACCCAA GTCCGGTCGT CTTTCTCACC TTCGTTGTCC CTCTGCGAAC   
  
  
- CGGTTCATGA CACTCGCCAT ATTACACGGT AAACTCATAG TACGGTAACG AGTCCTCACC CTTTGTTAGT   
  
  
- TTGGTCCCCT AGAGTTCTAT CCTTCATCCT TACTACTTCA ACAACGCCAC TTGACAGAGA CATCCAAGTT   
  
  
- CTTGGAGGAA CTGCTCTGTC ACCACCACCT ATCAGGTTCC TTGTGTCAAA ATTTGGACTA ATGTTCCCAT   
  
  
- TTCGGACTAT AAAAACACGT ACCGCAACAT TTACCAAGGA TGTTGTAGGG AAAGAAACAC TGTGCAAAAT   
  
  
- CTCTTCGGGA GAAAGTAATA AGGTGAGAAA AGCTGTACAA TCTACGGTTG CGGAGATCCC TCCTCGGGCT   
  
  
- CTCCAACTAT AAGCTCTTCC GTAAGATACC CTCCCTCTAA TACTTACACC ACCGGACACT CCCGTGTCTC   
  
  
- TCCCACCTCT CCGGCCTCTG TATGTTCGTT ACCGTACAAT CCGTATCGTC CCGTCCCAAA GCCGTTCATG   
  
  
- GTAACCTAGG GTTCAACTAG CTCTTTTACT CCAAATTCCG GTTCCGTCTG GTGGTGTTCC TAAAGTACTA   
  
  
- GCTACACCTA CCTGTAACCC GTTAAGTCCC TACCTTCCCC GCCTAACGTG TACGCTAGAG ACGTACCCAA   
  
  
- GGCCGAAC

+     ARE

| Site Name | Organism | Position | Strand | Matrix score. | sequence | function |
| --- | --- | --- | --- | --- | --- | --- |
| ARE | Zea mays | 3714 | + | 6 | AAACCA | cis-acting regulatory element essential for the anaerobic induction |
| ARE | Zea mays | 2672 | - | 6 | AAACCA | cis-acting regulatory element essential for the anaerobic induction |

>HU07G02249.1   
+ -Up\_Stream \_Len000GATTTG ATTGTCGAAA TTTGTCCGAT TTTATAAAAT TGAAAATTGT TATTGGACTA   
  
  
+ TAATCTACTG TAGTTTAGAG GTGTTGGATA ATACTCTCAA CTTTGAACTT GGACGTCCAT GCACCACACT   
  
  
+ ATGGGCGTAT TTACTGTTTA TATACCAAGA ATAATTTTGA AGATGTTCTT AGTTGGTGAT GTAAGTGTAT   
  
  
+ ATTCAACATA TGTCATTACA TAAACTTGAA AATTCTTGAA TTTTAGACAT ATACAATTGC AAATCATTGT   
  
  
+ AAAATTGTTG CATTACATTT TGATAAAAAT GCATCCAAAC TTTGTCTTGG TATAAACAGT AAATAGGCCC   
  
  
+ ATAGGTCCAT AGTGTAGTGC ATGGACGTCC AAGTTTAAAG GTGGGAGTAT TATTGCCCAA CGCCCCTAAA   
  
  
+ GTACAGTATA TTATAGTCCA ATATCCCAAT TAATTTTTCA TTTGAATATT TAAATTTGGA CATAATATTT   
  
  
+ TTCATATTTG AATTCTCTAA ACCTGATTTT AACCTGAATT TAAGTTGATT TAATTCATCC GACCAAAAAA   
  
  
+ ATAAACACAA TTATTTATTT TTCTATTCGA AGTTAGATAG TAGCCAATTC ATTTGACATA TAATTAACCC   
  
  
+ GTGATTGACA CAAACTCGAG TTTTTGCTTA GAGCTAGGAA AAGAGTCGGG TTAGGTCGAC CCATTTAAGA   
  
  
+ AATGGGTCGA CTTCATGTCG GCCTTCAAAA TAGTTAAGAT TTGCTCAAAA CTAAAAAATA ACAAGTTCAC   
  
  
+ ACTAAACGGT CAAATCGTGT CGACTTCGGA TTGAAATTGG TTCAGTTTGG GTCGGCTTCG TATTTCTTTC   
  
  
+ AGTAACTTCG AACCGAATTC GTATCGTGTT ATCGTGTCAG GTCAGACTTT CTCAGTTCTA TTTTTGCTAC   
  
  
+ CTTATGTTAC GGATAAAAAA AGTCTCACAT TGGAAAAAGT GTGGGAGGGT CCTGGGCTTA TAAATAGTGG   
  
  
+ CGCTGCACAC CTCATCAGAC CGACCTTTTG GGGAGAGGGT AATGACCCCG TGACAAGTGG TATCAGAGTC   
  
  
+ AACCCAGACC CGACTCAGAT GTCCTGCCAA GCGCTCCGAC GGGCGGGGTA GGCAGGGGGC CCATTTGTGA   
  
  
+ TGAGGACGTC ACAGAATTGG GCGGGGGAGA GTGTCACGGA TAAAAAAAGT CCCACATCGA AAAAAGTGTG   
  
  
+ GGAGGGTCCT GGGCTTATAA ACGGTAGCGC CGCACACCCC ATCAGATCGA CTTTTTGGGG AGAGGGTAAT   
  
  
+ GACCCCATTT CAAGTGGTAT CAGAGCCAAC CCAGACCCGA CTCCAATGTC CCACCAAGCG CTCTGATGGG   
  
  
+ CGGGGTAGGC AAGGGGCTCC ATATGTGACG AGGGCGTCAC GGAATTGGAT GGGAGAGAGT ACCACGGATA   
  
  
+ AAAAAAGTCC CACATCAGAA AAAGTGTGGA CTTAGCAACT AGGAAGTAAA GTTACAGTAT TAGCCGAGAG   
  
  
+ CCCATGGAAC GCCTGAAACT GACATCACCA GACCCACATA TTTCACCATC ATCTGATCTC CCCGTCAAAG   
  
  
+ GAGGAAGCTG AGCACAGCAA GAACTAGAAG AACAGGCGGC ATCACACCCA AGCCAAAATT CTCTATTTGA   
  
  
+ CCTCCCTCTC CTCATTCCTC TGAGCTTCCT CTTCACAATG TAAGACCATA ATCCTTAACA CCCTTCTTTT   
  
  
+ TTAAGCTCAT CTTTACCTGC TCTGGTTCTC TCTCTCATCG TCACTAATAG TTACCTCTTT ACTTTCTCTT   
  
  
+ CCCTGAGGTA TGCTTCAACT TTAACATATA TATCCCACTT GATTTCTCTT GTCTTTTTGC TATAATACTT   
  
  
+ GTTGTTTGGC TATCATCCCC TGTTTTATAC TCATTTTCTT GCTTTCTATT TCTGGGTTTG AATTGGGTTT   
  
  
+ CACTTATCCT CGTCAATTCT ATGGGTAATT AGTGAATTGG GTTTATATGA ACTAGGGTTG CAAAAGTATC   
  
  
+ TACTTTTTCT CTTGTGATTA TTAGTTGATT AGGGATTTGG CCAATATTAG CAGAATGGGT TCTGAATTTG   
  
  
+ CGGAATTCTC TGATGATGCT CTAAATGGGT ATGCTTATGT TGATATCCCT GCTTATGATG CATCCTTAGA   
  
  
+ TTATGCCAAT TTGTTCAATT ACGAAGGCCC ATCTGAGGAT CGCACCTCAC TGAGCCTCCC AAGACCCTTT   
  
  
+ TCTGACCCTT TGGCTTACAG TTTCACGTCC TCTTCTGAGC TGGGCCCTGG GGTTGATTCT AATGATGATA   
  
  
+ GTGATTCTGG CGATGTTCTC AAGTACATTA GCCAAATGCT TATGGAAGAA GACATGGAGG CAAAGCCATG   
  
  
+ CATGTTTCAT GATCCTTTAG CACTTCAGGC TGCTGAGAAA CCCTTTTATG ATGCCTTAGG GGAGCAATGC   
  
  
+ CCAACTTCTC CTGACCAACA TCCTATAATT GATCATTATT TGGATAGTCC TGATGAAAAT TCTTTGAGTT   
  
  
+ CAACTGGTGA TTTTAGTGTT AGTCATTCTG GGTCTAGTTC AACAAACTCC GTTGGACCGA TAATAGTGTC   
  
  
+ TGATTTGAGT GAGCATTTTG AGCCACCCTT TGTTGAAACA CTTCCAATTG AATCATATCA CCAACCATTG   
  
  
+ ACCCGTCCTC AATGGTCATT TGGCTCTTCG GGTGCCTTAG ATGGCACGGC CTCTAATGGT TCGGTGATCT   
  
  
+ CATCCCTTGG TTTGCCAGTG GATGTGATTA GCGTATTTAG GGAGAAAGAG TCCATGATTC AATTTCAGAA   
  
  
+ AGGGGTGGAG GAGGCCAGTA AGTTCCTTCC CAAGAATAAT AACCTTGTTA TTGATCTCGA GAACCTCACT   
  
  
+ TTTCCTAATG AAACAAAGGA GGATGATCGA ATGATGATGG TTAAGAAGGA AAAGGATGAC GTGAATTGGT   
  
  
+ CTAACTACTC AAGAGGGAGT AAGATTCACT ATTGTGAAGA CGAGGCCTTT GAAGAAGGAA GGAGTGGCAA   
  
  
+ GCAGTCAGCT ATTTCTTCTA CTGAGGAAGC TGAGTTTTCT GACATTTTTG ACAAGGTTTT GCTTTGCGAT   
  
  
+ TGCTACCCTG TGAAACCTGA GGCTCATCCC ACCATGAGTT TGAACCCTGA GAAGGGCCAG TCACATGGAT   
  
  
+ TAGAAGGTGG GAGAAATGGG AAGGCTCGCC CTAAGAAACA GGATAATAGT AGCACAAATA TTGTGGATTT   
  
  
+ AAGGAATTTG CTGATACTAT GCGCACAATC TACTGCATCT GATGACCGAA GAACTGCTGA TGGACTGCTA   
  
  
+ AAGCAAATCA GGGAGCACTC GTCTGCTGAG GGGGATGGAT CTCAAAGGTT GGCGCATTAC TTTGCTGATG   
  
  
+ CCCTAGAGGC ACGTTTAGCT GGAACTGGAT CTCGCATTTA TACGGCCCTA TGTTCTAATA GGCCATCTGT   
  
  
+ CACTGACATG ATAAAAGCAT ATCAGTTCTA TATTCGTGCT TGCCCATTTA CGAAGATCGT CATTGGTTGT   
  
  
+ GGTACCCATA TGATTCTAAA AGCAGCTGAG AAGGCATCAA AGCTTCATAT TATAGATTTT GGCATCCTCT   
  
  
+ ATGGTTGCCA ATGGCCCAAC CTCATTCAAC GCCTCTCAGA GCGATCTGGT GGACCTCCAA AACTGTTTAT   
  
  
+ TACAGGGATC GATCTCCCCC AGCCTGGGTT CAGGCCAGCA GAAAGAGTGG AAGCAACAGG GAGACGCTTG   
  
  
+ GCCAAGTACT GTGAGCGGTA TAATGTGCCA TTTGAGTATC ATGCCATTGC TCAGGAGTGG GAAACAATCA   
  
  
+ AACCAGGGGA TCTCAAGATA GGAAGTAGGA ATGATGAAGT TGTTGCGGTG AACTGTCTCT GTAGGTTCAA   
  
  
+ GAACCTCCTT GACGAGACAG TGGTGGTGGA TAGTCCAAGG AACACAGTTT TAAACCTGAT TACAAGGGTA   
  
  
+ AAGCCTGATA TTTTTGTGCA TGGCGTTGTA AATGGTTCCT ACAACATCCC TTTCTTTGTG ACACGTTTTA   
  
  
+ GAGAAGCCCT CTTTCATTAT TCCACTCTTT TCGACATGTT AGATGCCAAC GCCTCTAGGG AGGAGCCCGA   
  
  
+ GAGGTTGATA TTCGAGAAGG CATTCTATGG GAGGGAGATT ATGAATGTGG TGGCCTGTGA GGGCACAGAG   
  
  
+ AGGGTGGAGA GGCCGGAGAC ATACAAGCAA TGGCATGTTA GGCATAGCAG GGCAGGGTTT CGGCAAGTAC   
  
  
+ CATTGGATCC CAAGTTGATC GAGAAAATGA GGTTTAAGGC CAAGGCAGAC CACCACAAGG ATTTCATGAT   
  
  
+ CGATGTGGAT GGACATTGGG CAATTCAGGG ATGGAAGGGG CGGATTGCAC ATGCGATCTC TGCATGGGTT   
  
  
+ CCGGCTTG  

- -Up\_Stream \_Len000CTAAAC TAACAGCTTT AAACAGGCTA AAATATTTTA ACTTTTAACA ATAACCTGAT   
  
  
- ATTAGATGAC ATCAAATCTC CACAACCTAT TATGAGAGTT GAAACTTGAA CCTGCAGGTA CGTGGTGTGA   
  
  
- TACCCGCATA AATGACAAAT ATATGGTTCT TATTAAAACT TCTACAAGAA TCAACCACTA CATTCACATA   
  
  
- TAAGTTGTAT ACAGTAATGT ATTTGAACTT TTAAGAACTT AAAATCTGTA TATGTTAACG TTTAGTAACA   
  
  
- TTTTAACAAC GTAATGTAAA ACTATTTTTA CGTAGGTTTG AAACAGAACC ATATTTGTCA TTTATCCGGG   
  
  
- TATCCAGGTA TCACATCACG TACCTGCAGG TTCAAATTTC CACCCTCATA ATAACGGGTT GCGGGGATTT   
  
  
- CATGTCATAT AATATCAGGT TATAGGGTTA ATTAAAAAGT AAACTTATAA ATTTAAACCT GTATTATAAA   
  
  
- AAGTATAAAC TTAAGAGATT TGGACTAAAA TTGGACTTAA ATTCAACTAA ATTAAGTAGG CTGGTTTTTT   
  
  
- TATTTGTGTT AATAAATAAA AAGATAAGCT TCAATCTATC ATCGGTTAAG TAAACTGTAT ATTAATTGGG   
  
  
- CACTAACTGT GTTTGAGCTC AAAAACGAAT CTCGATCCTT TTCTCAGCCC AATCCAGCTG GGTAAATTCT   
  
  
- TTACCCAGCT GAAGTACAGC CGGAAGTTTT ATCAATTCTA AACGAGTTTT GATTTTTTAT TGTTCAAGTG   
  
  
- TGATTTGCCA GTTTAGCACA GCTGAAGCCT AACTTTAACC AAGTCAAACC CAGCCGAAGC ATAAAGAAAG   
  
  
- TCATTGAAGC TTGGCTTAAG CATAGCACAA TAGCACAGTC CAGTCTGAAA GAGTCAAGAT AAAAACGATG   
  
  
- GAATACAATG CCTATTTTTT TCAGAGTGTA ACCTTTTTCA CACCCTCCCA GGACCCGAAT ATTTATCACC   
  
  
- GCGACGTGTG GAGTAGTCTG GCTGGAAAAC CCCTCTCCCA TTACTGGGGC ACTGTTCACC ATAGTCTCAG   
  
  
- TTGGGTCTGG GCTGAGTCTA CAGGACGGTT CGCGAGGCTG CCCGCCCCAT CCGTCCCCCG GGTAAACACT   
  
  
- ACTCCTGCAG TGTCTTAACC CGCCCCCTCT CACAGTGCCT ATTTTTTTCA GGGTGTAGCT TTTTTCACAC   
  
  
- CCTCCCAGGA CCCGAATATT TGCCATCGCG GCGTGTGGGG TAGTCTAGCT GAAAAACCCC TCTCCCATTA   
  
  
- CTGGGGTAAA GTTCACCATA GTCTCGGTTG GGTCTGGGCT GAGGTTACAG GGTGGTTCGC GAGACTACCC   
  
  
- GCCCCATCCG TTCCCCGAGG TATACACTGC TCCCGCAGTG CCTTAACCTA CCCTCTCTCA TGGTGCCTAT   
  
  
- TTTTTTCAGG GTGTAGTCTT TTTCACACCT GAATCGTTGA TCCTTCATTT CAATGTCATA ATCGGCTCTC   
  
  
- GGGTACCTTG CGGACTTTGA CTGTAGTGGT CTGGGTGTAT AAAGTGGTAG TAGACTAGAG GGGCAGTTTC   
  
  
- CTCCTTCGAC TCGTGTCGTT CTTGATCTTC TTGTCCGCCG TAGTGTGGGT TCGGTTTTAA GAGATAAACT   
  
  
- GGAGGGAGAG GAGTAAGGAG ACTCGAAGGA GAAGTGTTAC ATTCTGGTAT TAGGAATTGT GGGAAGAAAA   
  
  
- AATTCGAGTA GAAATGGACG AGACCAAGAG AGAGAGTAGC AGTGATTATC AATGGAGAAA TGAAAGAGAA   
  
  
- GGGACTCCAT ACGAAGTTGA AATTGTATAT ATAGGGTGAA CTAAAGAGAA CAGAAAAACG ATATTATGAA   
  
  
- CAACAAACCG ATAGTAGGGG ACAAAATATG AGTAAAAGAA CGAAAGATAA AGACCCAAAC TTAACCCAAA   
  
  
- GTGAATAGGA GCAGTTAAGA TACCCATTAA TCACTTAACC CAAATATACT TGATCCCAAC GTTTTCATAG   
  
  
- ATGAAAAAGA GAACACTAAT AATCAACTAA TCCCTAAACC GGTTATAATC GTCTTACCCA AGACTTAAAC   
  
  
- GCCTTAAGAG ACTACTACGA GATTTACCCA TACGAATACA ACTATAGGGA CGAATACTAC GTAGGAATCT   
  
  
- AATACGGTTA AACAAGTTAA TGCTTCCGGG TAGACTCCTA GCGTGGAGTG ACTCGGAGGG TTCTGGGAAA   
  
  
- AGACTGGGAA ACCGAATGTC AAAGTGCAGG AGAAGACTCG ACCCGGGACC CCAACTAAGA TTACTACTAT   
  
  
- CACTAAGACC GCTACAAGAG TTCATGTAAT CGGTTTACGA ATACCTTCTT CTGTACCTCC GTTTCGGTAC   
  
  
- GTACAAAGTA CTAGGAAATC GTGAAGTCCG ACGACTCTTT GGGAAAATAC TACGGAATCC CCTCGTTACG   
  
  
- GGTTGAAGAG GACTGGTTGT AGGATATTAA CTAGTAATAA ACCTATCAGG ACTACTTTTA AGAAACTCAA   
  
  
- GTTGACCACT AAAATCACAA TCAGTAAGAC CCAGATCAAG TTGTTTGAGG CAACCTGGCT ATTATCACAG   
  
  
- ACTAAACTCA CTCGTAAAAC TCGGTGGGAA ACAACTTTGT GAAGGTTAAC TTAGTATAGT GGTTGGTAAC   
  
  
- TGGGCAGGAG TTACCAGTAA ACCGAGAAGC CCACGGAATC TACCGTGCCG GAGATTACCA AGCCACTAGA   
  
  
- GTAGGGAACC AAACGGTCAC CTACACTAAT CGCATAAATC CCTCTTTCTC AGGTACTAAG TTAAAGTCTT   
  
  
- TCCCCACCTC CTCCGGTCAT TCAAGGAAGG GTTCTTATTA TTGGAACAAT AACTAGAGCT CTTGGAGTGA   
  
  
- AAAGGATTAC TTTGTTTCCT CCTACTAGCT TACTACTACC AATTCTTCCT TTTCCTACTG CACTTAACCA   
  
  
- GATTGATGAG TTCTCCCTCA TTCTAAGTGA TAACACTTCT GCTCCGGAAA CTTCTTCCTT CCTCACCGTT   
  
  
- CGTCAGTCGA TAAAGAAGAT GACTCCTTCG ACTCAAAAGA CTGTAAAAAC TGTTCCAAAA CGAAACGCTA   
  
  
- ACGATGGGAC ACTTTGGACT CCGAGTAGGG TGGTACTCAA ACTTGGGACT CTTCCCGGTC AGTGTACCTA   
  
  
- ATCTTCCACC CTCTTTACCC TTCCGAGCGG GATTCTTTGT CCTATTATCA TCGTGTTTAT AACACCTAAA   
  
  
- TTCCTTAAAC GACTATGATA CGCGTGTTAG ATGACGTAGA CTACTGGCTT CTTGACGACT ACCTGACGAT   
  
  
- TTCGTTTAGT CCCTCGTGAG CAGACGACTC CCCCTACCTA GAGTTTCCAA CCGCGTAATG AAACGACTAC   
  
  
- GGGATCTCCG TGCAAATCGA CCTTGACCTA GAGCGTAAAT ATGCCGGGAT ACAAGATTAT CCGGTAGACA   
  
  
- GTGACTGTAC TATTTTCGTA TAGTCAAGAT ATAAGCACGA ACGGGTAAAT GCTTCTAGCA GTAACCAACA   
  
  
- CCATGGGTAT ACTAAGATTT TCGTCGACTC TTCCGTAGTT TCGAAGTATA ATATCTAAAA CCGTAGGAGA   
  
  
- TACCAACGGT TACCGGGTTG GAGTAAGTTG CGGAGAGTCT CGCTAGACCA CCTGGAGGTT TTGACAAATA   
  
  
- ATGTCCCTAG CTAGAGGGGG TCGGACCCAA GTCCGGTCGT CTTTCTCACC TTCGTTGTCC CTCTGCGAAC   
  
  
- CGGTTCATGA CACTCGCCAT ATTACACGGT AAACTCATAG TACGGTAACG AGTCCTCACC CTTTGTTAGT   
  
  
- TTGGTCCCCT AGAGTTCTAT CCTTCATCCT TACTACTTCA ACAACGCCAC TTGACAGAGA CATCCAAGTT   
  
  
- CTTGGAGGAA CTGCTCTGTC ACCACCACCT ATCAGGTTCC TTGTGTCAAA ATTTGGACTA ATGTTCCCAT   
  
  
- TTCGGACTAT AAAAACACGT ACCGCAACAT TTACCAAGGA TGTTGTAGGG AAAGAAACAC TGTGCAAAAT   
  
  
- CTCTTCGGGA GAAAGTAATA AGGTGAGAAA AGCTGTACAA TCTACGGTTG CGGAGATCCC TCCTCGGGCT   
  
  
- CTCCAACTAT AAGCTCTTCC GTAAGATACC CTCCCTCTAA TACTTACACC ACCGGACACT CCCGTGTCTC   
  
  
- TCCCACCTCT CCGGCCTCTG TATGTTCGTT ACCGTACAAT CCGTATCGTC CCGTCCCAAA GCCGTTCATG   
  
  
- GTAACCTAGG GTTCAACTAG CTCTTTTACT CCAAATTCCG GTTCCGTCTG GTGGTGTTCC TAAAGTACTA   
  
  
- GCTACACCTA CCTGTAACCC GTTAAGTCCC TACCTTCCCC GCCTAACGTG TACGCTAGAG ACGTACCCAA   
  
  
- GGCCGAAC

+     AT-rich element

| Site Name | Organism | Position | Strand | Matrix score. | sequence | function |
| --- | --- | --- | --- | --- | --- | --- |
| AT-rich element | Glycine max | 1863 | - | 10 | ATAGAAATCAA | binding site of AT-rich DNA binding protein (ATBP-1) |
| AT-rich element | Glycine max | 1793 | - | 10 | ATAGAAATCAA | binding site of AT-rich DNA binding protein (ATBP-1) |

>HU07G02249.1   
+ -Up\_Stream \_Len000GATTTG ATTGTCGAAA TTTGTCCGAT TTTATAAAAT TGAAAATTGT TATTGGACTA   
  
  
+ TAATCTACTG TAGTTTAGAG GTGTTGGATA ATACTCTCAA CTTTGAACTT GGACGTCCAT GCACCACACT   
  
  
+ ATGGGCGTAT TTACTGTTTA TATACCAAGA ATAATTTTGA AGATGTTCTT AGTTGGTGAT GTAAGTGTAT   
  
  
+ ATTCAACATA TGTCATTACA TAAACTTGAA AATTCTTGAA TTTTAGACAT ATACAATTGC AAATCATTGT   
  
  
+ AAAATTGTTG CATTACATTT TGATAAAAAT GCATCCAAAC TTTGTCTTGG TATAAACAGT AAATAGGCCC   
  
  
+ ATAGGTCCAT AGTGTAGTGC ATGGACGTCC AAGTTTAAAG GTGGGAGTAT TATTGCCCAA CGCCCCTAAA   
  
  
+ GTACAGTATA TTATAGTCCA ATATCCCAAT TAATTTTTCA TTTGAATATT TAAATTTGGA CATAATATTT   
  
  
+ TTCATATTTG AATTCTCTAA ACCTGATTTT AACCTGAATT TAAGTTGATT TAATTCATCC GACCAAAAAA   
  
  
+ ATAAACACAA TTATTTATTT TTCTATTCGA AGTTAGATAG TAGCCAATTC ATTTGACATA TAATTAACCC   
  
  
+ GTGATTGACA CAAACTCGAG TTTTTGCTTA GAGCTAGGAA AAGAGTCGGG TTAGGTCGAC CCATTTAAGA   
  
  
+ AATGGGTCGA CTTCATGTCG GCCTTCAAAA TAGTTAAGAT TTGCTCAAAA CTAAAAAATA ACAAGTTCAC   
  
  
+ ACTAAACGGT CAAATCGTGT CGACTTCGGA TTGAAATTGG TTCAGTTTGG GTCGGCTTCG TATTTCTTTC   
  
  
+ AGTAACTTCG AACCGAATTC GTATCGTGTT ATCGTGTCAG GTCAGACTTT CTCAGTTCTA TTTTTGCTAC   
  
  
+ CTTATGTTAC GGATAAAAAA AGTCTCACAT TGGAAAAAGT GTGGGAGGGT CCTGGGCTTA TAAATAGTGG   
  
  
+ CGCTGCACAC CTCATCAGAC CGACCTTTTG GGGAGAGGGT AATGACCCCG TGACAAGTGG TATCAGAGTC   
  
  
+ AACCCAGACC CGACTCAGAT GTCCTGCCAA GCGCTCCGAC GGGCGGGGTA GGCAGGGGGC CCATTTGTGA   
  
  
+ TGAGGACGTC ACAGAATTGG GCGGGGGAGA GTGTCACGGA TAAAAAAAGT CCCACATCGA AAAAAGTGTG   
  
  
+ GGAGGGTCCT GGGCTTATAA ACGGTAGCGC CGCACACCCC ATCAGATCGA CTTTTTGGGG AGAGGGTAAT   
  
  
+ GACCCCATTT CAAGTGGTAT CAGAGCCAAC CCAGACCCGA CTCCAATGTC CCACCAAGCG CTCTGATGGG   
  
  
+ CGGGGTAGGC AAGGGGCTCC ATATGTGACG AGGGCGTCAC GGAATTGGAT GGGAGAGAGT ACCACGGATA   
  
  
+ AAAAAAGTCC CACATCAGAA AAAGTGTGGA CTTAGCAACT AGGAAGTAAA GTTACAGTAT TAGCCGAGAG   
  
  
+ CCCATGGAAC GCCTGAAACT GACATCACCA GACCCACATA TTTCACCATC ATCTGATCTC CCCGTCAAAG   
  
  
+ GAGGAAGCTG AGCACAGCAA GAACTAGAAG AACAGGCGGC ATCACACCCA AGCCAAAATT CTCTATTTGA   
  
  
+ CCTCCCTCTC CTCATTCCTC TGAGCTTCCT CTTCACAATG TAAGACCATA ATCCTTAACA CCCTTCTTTT   
  
  
+ TTAAGCTCAT CTTTACCTGC TCTGGTTCTC TCTCTCATCG TCACTAATAG TTACCTCTTT ACTTTCTCTT   
  
  
+ CCCTGAGGTA TGCTTCAACT TTAACATATA TATCCCACTT GATTTCTCTT GTCTTTTTGC TATAATACTT   
  
  
+ GTTGTTTGGC TATCATCCCC TGTTTTATAC TCATTTTCTT GCTTTCTATT TCTGGGTTTG AATTGGGTTT   
  
  
+ CACTTATCCT CGTCAATTCT ATGGGTAATT AGTGAATTGG GTTTATATGA ACTAGGGTTG CAAAAGTATC   
  
  
+ TACTTTTTCT CTTGTGATTA TTAGTTGATT AGGGATTTGG CCAATATTAG CAGAATGGGT TCTGAATTTG   
  
  
+ CGGAATTCTC TGATGATGCT CTAAATGGGT ATGCTTATGT TGATATCCCT GCTTATGATG CATCCTTAGA   
  
  
+ TTATGCCAAT TTGTTCAATT ACGAAGGCCC ATCTGAGGAT CGCACCTCAC TGAGCCTCCC AAGACCCTTT   
  
  
+ TCTGACCCTT TGGCTTACAG TTTCACGTCC TCTTCTGAGC TGGGCCCTGG GGTTGATTCT AATGATGATA   
  
  
+ GTGATTCTGG CGATGTTCTC AAGTACATTA GCCAAATGCT TATGGAAGAA GACATGGAGG CAAAGCCATG   
  
  
+ CATGTTTCAT GATCCTTTAG CACTTCAGGC TGCTGAGAAA CCCTTTTATG ATGCCTTAGG GGAGCAATGC   
  
  
+ CCAACTTCTC CTGACCAACA TCCTATAATT GATCATTATT TGGATAGTCC TGATGAAAAT TCTTTGAGTT   
  
  
+ CAACTGGTGA TTTTAGTGTT AGTCATTCTG GGTCTAGTTC AACAAACTCC GTTGGACCGA TAATAGTGTC   
  
  
+ TGATTTGAGT GAGCATTTTG AGCCACCCTT TGTTGAAACA CTTCCAATTG AATCATATCA CCAACCATTG   
  
  
+ ACCCGTCCTC AATGGTCATT TGGCTCTTCG GGTGCCTTAG ATGGCACGGC CTCTAATGGT TCGGTGATCT   
  
  
+ CATCCCTTGG TTTGCCAGTG GATGTGATTA GCGTATTTAG GGAGAAAGAG TCCATGATTC AATTTCAGAA   
  
  
+ AGGGGTGGAG GAGGCCAGTA AGTTCCTTCC CAAGAATAAT AACCTTGTTA TTGATCTCGA GAACCTCACT   
  
  
+ TTTCCTAATG AAACAAAGGA GGATGATCGA ATGATGATGG TTAAGAAGGA AAAGGATGAC GTGAATTGGT   
  
  
+ CTAACTACTC AAGAGGGAGT AAGATTCACT ATTGTGAAGA CGAGGCCTTT GAAGAAGGAA GGAGTGGCAA   
  
  
+ GCAGTCAGCT ATTTCTTCTA CTGAGGAAGC TGAGTTTTCT GACATTTTTG ACAAGGTTTT GCTTTGCGAT   
  
  
+ TGCTACCCTG TGAAACCTGA GGCTCATCCC ACCATGAGTT TGAACCCTGA GAAGGGCCAG TCACATGGAT   
  
  
+ TAGAAGGTGG GAGAAATGGG AAGGCTCGCC CTAAGAAACA GGATAATAGT AGCACAAATA TTGTGGATTT   
  
  
+ AAGGAATTTG CTGATACTAT GCGCACAATC TACTGCATCT GATGACCGAA GAACTGCTGA TGGACTGCTA   
  
  
+ AAGCAAATCA GGGAGCACTC GTCTGCTGAG GGGGATGGAT CTCAAAGGTT GGCGCATTAC TTTGCTGATG   
  
  
+ CCCTAGAGGC ACGTTTAGCT GGAACTGGAT CTCGCATTTA TACGGCCCTA TGTTCTAATA GGCCATCTGT   
  
  
+ CACTGACATG ATAAAAGCAT ATCAGTTCTA TATTCGTGCT TGCCCATTTA CGAAGATCGT CATTGGTTGT   
  
  
+ GGTACCCATA TGATTCTAAA AGCAGCTGAG AAGGCATCAA AGCTTCATAT TATAGATTTT GGCATCCTCT   
  
  
+ ATGGTTGCCA ATGGCCCAAC CTCATTCAAC GCCTCTCAGA GCGATCTGGT GGACCTCCAA AACTGTTTAT   
  
  
+ TACAGGGATC GATCTCCCCC AGCCTGGGTT CAGGCCAGCA GAAAGAGTGG AAGCAACAGG GAGACGCTTG   
  
  
+ GCCAAGTACT GTGAGCGGTA TAATGTGCCA TTTGAGTATC ATGCCATTGC TCAGGAGTGG GAAACAATCA   
  
  
+ AACCAGGGGA TCTCAAGATA GGAAGTAGGA ATGATGAAGT TGTTGCGGTG AACTGTCTCT GTAGGTTCAA   
  
  
+ GAACCTCCTT GACGAGACAG TGGTGGTGGA TAGTCCAAGG AACACAGTTT TAAACCTGAT TACAAGGGTA   
  
  
+ AAGCCTGATA TTTTTGTGCA TGGCGTTGTA AATGGTTCCT ACAACATCCC TTTCTTTGTG ACACGTTTTA   
  
  
+ GAGAAGCCCT CTTTCATTAT TCCACTCTTT TCGACATGTT AGATGCCAAC GCCTCTAGGG AGGAGCCCGA   
  
  
+ GAGGTTGATA TTCGAGAAGG CATTCTATGG GAGGGAGATT ATGAATGTGG TGGCCTGTGA GGGCACAGAG   
  
  
+ AGGGTGGAGA GGCCGGAGAC ATACAAGCAA TGGCATGTTA GGCATAGCAG GGCAGGGTTT CGGCAAGTAC   
  
  
+ CATTGGATCC CAAGTTGATC GAGAAAATGA GGTTTAAGGC CAAGGCAGAC CACCACAAGG ATTTCATGAT   
  
  
+ CGATGTGGAT GGACATTGGG CAATTCAGGG ATGGAAGGGG CGGATTGCAC ATGCGATCTC TGCATGGGTT   
  
  
+ CCGGCTTG  

- -Up\_Stream \_Len000CTAAAC TAACAGCTTT AAACAGGCTA AAATATTTTA ACTTTTAACA ATAACCTGAT   
  
  
- ATTAGATGAC ATCAAATCTC CACAACCTAT TATGAGAGTT GAAACTTGAA CCTGCAGGTA CGTGGTGTGA   
  
  
- TACCCGCATA AATGACAAAT ATATGGTTCT TATTAAAACT TCTACAAGAA TCAACCACTA CATTCACATA   
  
  
- TAAGTTGTAT ACAGTAATGT ATTTGAACTT TTAAGAACTT AAAATCTGTA TATGTTAACG TTTAGTAACA   
  
  
- TTTTAACAAC GTAATGTAAA ACTATTTTTA CGTAGGTTTG AAACAGAACC ATATTTGTCA TTTATCCGGG   
  
  
- TATCCAGGTA TCACATCACG TACCTGCAGG TTCAAATTTC CACCCTCATA ATAACGGGTT GCGGGGATTT   
  
  
- CATGTCATAT AATATCAGGT TATAGGGTTA ATTAAAAAGT AAACTTATAA ATTTAAACCT GTATTATAAA   
  
  
- AAGTATAAAC TTAAGAGATT TGGACTAAAA TTGGACTTAA ATTCAACTAA ATTAAGTAGG CTGGTTTTTT   
  
  
- TATTTGTGTT AATAAATAAA AAGATAAGCT TCAATCTATC ATCGGTTAAG TAAACTGTAT ATTAATTGGG   
  
  
- CACTAACTGT GTTTGAGCTC AAAAACGAAT CTCGATCCTT TTCTCAGCCC AATCCAGCTG GGTAAATTCT   
  
  
- TTACCCAGCT GAAGTACAGC CGGAAGTTTT ATCAATTCTA AACGAGTTTT GATTTTTTAT TGTTCAAGTG   
  
  
- TGATTTGCCA GTTTAGCACA GCTGAAGCCT AACTTTAACC AAGTCAAACC CAGCCGAAGC ATAAAGAAAG   
  
  
- TCATTGAAGC TTGGCTTAAG CATAGCACAA TAGCACAGTC CAGTCTGAAA GAGTCAAGAT AAAAACGATG   
  
  
- GAATACAATG CCTATTTTTT TCAGAGTGTA ACCTTTTTCA CACCCTCCCA GGACCCGAAT ATTTATCACC   
  
  
- GCGACGTGTG GAGTAGTCTG GCTGGAAAAC CCCTCTCCCA TTACTGGGGC ACTGTTCACC ATAGTCTCAG   
  
  
- TTGGGTCTGG GCTGAGTCTA CAGGACGGTT CGCGAGGCTG CCCGCCCCAT CCGTCCCCCG GGTAAACACT   
  
  
- ACTCCTGCAG TGTCTTAACC CGCCCCCTCT CACAGTGCCT ATTTTTTTCA GGGTGTAGCT TTTTTCACAC   
  
  
- CCTCCCAGGA CCCGAATATT TGCCATCGCG GCGTGTGGGG TAGTCTAGCT GAAAAACCCC TCTCCCATTA   
  
  
- CTGGGGTAAA GTTCACCATA GTCTCGGTTG GGTCTGGGCT GAGGTTACAG GGTGGTTCGC GAGACTACCC   
  
  
- GCCCCATCCG TTCCCCGAGG TATACACTGC TCCCGCAGTG CCTTAACCTA CCCTCTCTCA TGGTGCCTAT   
  
  
- TTTTTTCAGG GTGTAGTCTT TTTCACACCT GAATCGTTGA TCCTTCATTT CAATGTCATA ATCGGCTCTC   
  
  
- GGGTACCTTG CGGACTTTGA CTGTAGTGGT CTGGGTGTAT AAAGTGGTAG TAGACTAGAG GGGCAGTTTC   
  
  
- CTCCTTCGAC TCGTGTCGTT CTTGATCTTC TTGTCCGCCG TAGTGTGGGT TCGGTTTTAA GAGATAAACT   
  
  
- GGAGGGAGAG GAGTAAGGAG ACTCGAAGGA GAAGTGTTAC ATTCTGGTAT TAGGAATTGT GGGAAGAAAA   
  
  
- AATTCGAGTA GAAATGGACG AGACCAAGAG AGAGAGTAGC AGTGATTATC AATGGAGAAA TGAAAGAGAA   
  
  
- GGGACTCCAT ACGAAGTTGA AATTGTATAT ATAGGGTGAA CTAAAGAGAA CAGAAAAACG ATATTATGAA   
  
  
- CAACAAACCG ATAGTAGGGG ACAAAATATG AGTAAAAGAA CGAAAGATAA AGACCCAAAC TTAACCCAAA   
  
  
- GTGAATAGGA GCAGTTAAGA TACCCATTAA TCACTTAACC CAAATATACT TGATCCCAAC GTTTTCATAG   
  
  
- ATGAAAAAGA GAACACTAAT AATCAACTAA TCCCTAAACC GGTTATAATC GTCTTACCCA AGACTTAAAC   
  
  
- GCCTTAAGAG ACTACTACGA GATTTACCCA TACGAATACA ACTATAGGGA CGAATACTAC GTAGGAATCT   
  
  
- AATACGGTTA AACAAGTTAA TGCTTCCGGG TAGACTCCTA GCGTGGAGTG ACTCGGAGGG TTCTGGGAAA   
  
  
- AGACTGGGAA ACCGAATGTC AAAGTGCAGG AGAAGACTCG ACCCGGGACC CCAACTAAGA TTACTACTAT   
  
  
- CACTAAGACC GCTACAAGAG TTCATGTAAT CGGTTTACGA ATACCTTCTT CTGTACCTCC GTTTCGGTAC   
  
  
- GTACAAAGTA CTAGGAAATC GTGAAGTCCG ACGACTCTTT GGGAAAATAC TACGGAATCC CCTCGTTACG   
  
  
- GGTTGAAGAG GACTGGTTGT AGGATATTAA CTAGTAATAA ACCTATCAGG ACTACTTTTA AGAAACTCAA   
  
  
- GTTGACCACT AAAATCACAA TCAGTAAGAC CCAGATCAAG TTGTTTGAGG CAACCTGGCT ATTATCACAG   
  
  
- ACTAAACTCA CTCGTAAAAC TCGGTGGGAA ACAACTTTGT GAAGGTTAAC TTAGTATAGT GGTTGGTAAC   
  
  
- TGGGCAGGAG TTACCAGTAA ACCGAGAAGC CCACGGAATC TACCGTGCCG GAGATTACCA AGCCACTAGA   
  
  
- GTAGGGAACC AAACGGTCAC CTACACTAAT CGCATAAATC CCTCTTTCTC AGGTACTAAG TTAAAGTCTT   
  
  
- TCCCCACCTC CTCCGGTCAT TCAAGGAAGG GTTCTTATTA TTGGAACAAT AACTAGAGCT CTTGGAGTGA   
  
  
- AAAGGATTAC TTTGTTTCCT CCTACTAGCT TACTACTACC AATTCTTCCT TTTCCTACTG CACTTAACCA   
  
  
- GATTGATGAG TTCTCCCTCA TTCTAAGTGA TAACACTTCT GCTCCGGAAA CTTCTTCCTT CCTCACCGTT   
  
  
- CGTCAGTCGA TAAAGAAGAT GACTCCTTCG ACTCAAAAGA CTGTAAAAAC TGTTCCAAAA CGAAACGCTA   
  
  
- ACGATGGGAC ACTTTGGACT CCGAGTAGGG TGGTACTCAA ACTTGGGACT CTTCCCGGTC AGTGTACCTA   
  
  
- ATCTTCCACC CTCTTTACCC TTCCGAGCGG GATTCTTTGT CCTATTATCA TCGTGTTTAT AACACCTAAA   
  
  
- TTCCTTAAAC GACTATGATA CGCGTGTTAG ATGACGTAGA CTACTGGCTT CTTGACGACT ACCTGACGAT   
  
  
- TTCGTTTAGT CCCTCGTGAG CAGACGACTC CCCCTACCTA GAGTTTCCAA CCGCGTAATG AAACGACTAC   
  
  
- GGGATCTCCG TGCAAATCGA CCTTGACCTA GAGCGTAAAT ATGCCGGGAT ACAAGATTAT CCGGTAGACA   
  
  
- GTGACTGTAC TATTTTCGTA TAGTCAAGAT ATAAGCACGA ACGGGTAAAT GCTTCTAGCA GTAACCAACA   
  
  
- CCATGGGTAT ACTAAGATTT TCGTCGACTC TTCCGTAGTT TCGAAGTATA ATATCTAAAA CCGTAGGAGA   
  
  
- TACCAACGGT TACCGGGTTG GAGTAAGTTG CGGAGAGTCT CGCTAGACCA CCTGGAGGTT TTGACAAATA   
  
  
- ATGTCCCTAG CTAGAGGGGG TCGGACCCAA GTCCGGTCGT CTTTCTCACC TTCGTTGTCC CTCTGCGAAC   
  
  
- CGGTTCATGA CACTCGCCAT ATTACACGGT AAACTCATAG TACGGTAACG AGTCCTCACC CTTTGTTAGT   
  
  
- TTGGTCCCCT AGAGTTCTAT CCTTCATCCT TACTACTTCA ACAACGCCAC TTGACAGAGA CATCCAAGTT   
  
  
- CTTGGAGGAA CTGCTCTGTC ACCACCACCT ATCAGGTTCC TTGTGTCAAA ATTTGGACTA ATGTTCCCAT   
  
  
- TTCGGACTAT AAAAACACGT ACCGCAACAT TTACCAAGGA TGTTGTAGGG AAAGAAACAC TGTGCAAAAT   
  
  
- CTCTTCGGGA GAAAGTAATA AGGTGAGAAA AGCTGTACAA TCTACGGTTG CGGAGATCCC TCCTCGGGCT   
  
  
- CTCCAACTAT AAGCTCTTCC GTAAGATACC CTCCCTCTAA TACTTACACC ACCGGACACT CCCGTGTCTC   
  
  
- TCCCACCTCT CCGGCCTCTG TATGTTCGTT ACCGTACAAT CCGTATCGTC CCGTCCCAAA GCCGTTCATG   
  
  
- GTAACCTAGG GTTCAACTAG CTCTTTTACT CCAAATTCCG GTTCCGTCTG GTGGTGTTCC TAAAGTACTA   
  
  
- GCTACACCTA CCTGTAACCC GTTAAGTCCC TACCTTCCCC GCCTAACGTG TACGCTAGAG ACGTACCCAA   
  
  
- GGCCGAAC

+     AT~TATA-box

| Site Name | Organism | Position | Strand | Matrix score. | sequence | function |
| --- | --- | --- | --- | --- | --- | --- |
| AT~TATA-box | Arabidopsis thaliana | 163 | + | 6 | TATATA |  |
| AT~TATA-box | Arabidopsis thaliana | 1781 | + | 6 | TATATA |  |
| AT~TATA-box | Arabidopsis thaliana | 161 | - | 8 | TATATAAA |  |

>HU07G02249.1   
+ -Up\_Stream \_Len000GATTTG ATTGTCGAAA TTTGTCCGAT TTTATAAAAT TGAAAATTGT TATTGGACTA   
  
  
+ TAATCTACTG TAGTTTAGAG GTGTTGGATA ATACTCTCAA CTTTGAACTT GGACGTCCAT GCACCACACT   
  
  
+ ATGGGCGTAT TTACTGTTTA TATACCAAGA ATAATTTTGA AGATGTTCTT AGTTGGTGAT GTAAGTGTAT   
  
  
+ ATTCAACATA TGTCATTACA TAAACTTGAA AATTCTTGAA TTTTAGACAT ATACAATTGC AAATCATTGT   
  
  
+ AAAATTGTTG CATTACATTT TGATAAAAAT GCATCCAAAC TTTGTCTTGG TATAAACAGT AAATAGGCCC   
  
  
+ ATAGGTCCAT AGTGTAGTGC ATGGACGTCC AAGTTTAAAG GTGGGAGTAT TATTGCCCAA CGCCCCTAAA   
  
  
+ GTACAGTATA TTATAGTCCA ATATCCCAAT TAATTTTTCA TTTGAATATT TAAATTTGGA CATAATATTT   
  
  
+ TTCATATTTG AATTCTCTAA ACCTGATTTT AACCTGAATT TAAGTTGATT TAATTCATCC GACCAAAAAA   
  
  
+ ATAAACACAA TTATTTATTT TTCTATTCGA AGTTAGATAG TAGCCAATTC ATTTGACATA TAATTAACCC   
  
  
+ GTGATTGACA CAAACTCGAG TTTTTGCTTA GAGCTAGGAA AAGAGTCGGG TTAGGTCGAC CCATTTAAGA   
  
  
+ AATGGGTCGA CTTCATGTCG GCCTTCAAAA TAGTTAAGAT TTGCTCAAAA CTAAAAAATA ACAAGTTCAC   
  
  
+ ACTAAACGGT CAAATCGTGT CGACTTCGGA TTGAAATTGG TTCAGTTTGG GTCGGCTTCG TATTTCTTTC   
  
  
+ AGTAACTTCG AACCGAATTC GTATCGTGTT ATCGTGTCAG GTCAGACTTT CTCAGTTCTA TTTTTGCTAC   
  
  
+ CTTATGTTAC GGATAAAAAA AGTCTCACAT TGGAAAAAGT GTGGGAGGGT CCTGGGCTTA TAAATAGTGG   
  
  
+ CGCTGCACAC CTCATCAGAC CGACCTTTTG GGGAGAGGGT AATGACCCCG TGACAAGTGG TATCAGAGTC   
  
  
+ AACCCAGACC CGACTCAGAT GTCCTGCCAA GCGCTCCGAC GGGCGGGGTA GGCAGGGGGC CCATTTGTGA   
  
  
+ TGAGGACGTC ACAGAATTGG GCGGGGGAGA GTGTCACGGA TAAAAAAAGT CCCACATCGA AAAAAGTGTG   
  
  
+ GGAGGGTCCT GGGCTTATAA ACGGTAGCGC CGCACACCCC ATCAGATCGA CTTTTTGGGG AGAGGGTAAT   
  
  
+ GACCCCATTT CAAGTGGTAT CAGAGCCAAC CCAGACCCGA CTCCAATGTC CCACCAAGCG CTCTGATGGG   
  
  
+ CGGGGTAGGC AAGGGGCTCC ATATGTGACG AGGGCGTCAC GGAATTGGAT GGGAGAGAGT ACCACGGATA   
  
  
+ AAAAAAGTCC CACATCAGAA AAAGTGTGGA CTTAGCAACT AGGAAGTAAA GTTACAGTAT TAGCCGAGAG   
  
  
+ CCCATGGAAC GCCTGAAACT GACATCACCA GACCCACATA TTTCACCATC ATCTGATCTC CCCGTCAAAG   
  
  
+ GAGGAAGCTG AGCACAGCAA GAACTAGAAG AACAGGCGGC ATCACACCCA AGCCAAAATT CTCTATTTGA   
  
  
+ CCTCCCTCTC CTCATTCCTC TGAGCTTCCT CTTCACAATG TAAGACCATA ATCCTTAACA CCCTTCTTTT   
  
  
+ TTAAGCTCAT CTTTACCTGC TCTGGTTCTC TCTCTCATCG TCACTAATAG TTACCTCTTT ACTTTCTCTT   
  
  
+ CCCTGAGGTA TGCTTCAACT TTAACATATA TATCCCACTT GATTTCTCTT GTCTTTTTGC TATAATACTT   
  
  
+ GTTGTTTGGC TATCATCCCC TGTTTTATAC TCATTTTCTT GCTTTCTATT TCTGGGTTTG AATTGGGTTT   
  
  
+ CACTTATCCT CGTCAATTCT ATGGGTAATT AGTGAATTGG GTTTATATGA ACTAGGGTTG CAAAAGTATC   
  
  
+ TACTTTTTCT CTTGTGATTA TTAGTTGATT AGGGATTTGG CCAATATTAG CAGAATGGGT TCTGAATTTG   
  
  
+ CGGAATTCTC TGATGATGCT CTAAATGGGT ATGCTTATGT TGATATCCCT GCTTATGATG CATCCTTAGA   
  
  
+ TTATGCCAAT TTGTTCAATT ACGAAGGCCC ATCTGAGGAT CGCACCTCAC TGAGCCTCCC AAGACCCTTT   
  
  
+ TCTGACCCTT TGGCTTACAG TTTCACGTCC TCTTCTGAGC TGGGCCCTGG GGTTGATTCT AATGATGATA   
  
  
+ GTGATTCTGG CGATGTTCTC AAGTACATTA GCCAAATGCT TATGGAAGAA GACATGGAGG CAAAGCCATG   
  
  
+ CATGTTTCAT GATCCTTTAG CACTTCAGGC TGCTGAGAAA CCCTTTTATG ATGCCTTAGG GGAGCAATGC   
  
  
+ CCAACTTCTC CTGACCAACA TCCTATAATT GATCATTATT TGGATAGTCC TGATGAAAAT TCTTTGAGTT   
  
  
+ CAACTGGTGA TTTTAGTGTT AGTCATTCTG GGTCTAGTTC AACAAACTCC GTTGGACCGA TAATAGTGTC   
  
  
+ TGATTTGAGT GAGCATTTTG AGCCACCCTT TGTTGAAACA CTTCCAATTG AATCATATCA CCAACCATTG   
  
  
+ ACCCGTCCTC AATGGTCATT TGGCTCTTCG GGTGCCTTAG ATGGCACGGC CTCTAATGGT TCGGTGATCT   
  
  
+ CATCCCTTGG TTTGCCAGTG GATGTGATTA GCGTATTTAG GGAGAAAGAG TCCATGATTC AATTTCAGAA   
  
  
+ AGGGGTGGAG GAGGCCAGTA AGTTCCTTCC CAAGAATAAT AACCTTGTTA TTGATCTCGA GAACCTCACT   
  
  
+ TTTCCTAATG AAACAAAGGA GGATGATCGA ATGATGATGG TTAAGAAGGA AAAGGATGAC GTGAATTGGT   
  
  
+ CTAACTACTC AAGAGGGAGT AAGATTCACT ATTGTGAAGA CGAGGCCTTT GAAGAAGGAA GGAGTGGCAA   
  
  
+ GCAGTCAGCT ATTTCTTCTA CTGAGGAAGC TGAGTTTTCT GACATTTTTG ACAAGGTTTT GCTTTGCGAT   
  
  
+ TGCTACCCTG TGAAACCTGA GGCTCATCCC ACCATGAGTT TGAACCCTGA GAAGGGCCAG TCACATGGAT   
  
  
+ TAGAAGGTGG GAGAAATGGG AAGGCTCGCC CTAAGAAACA GGATAATAGT AGCACAAATA TTGTGGATTT   
  
  
+ AAGGAATTTG CTGATACTAT GCGCACAATC TACTGCATCT GATGACCGAA GAACTGCTGA TGGACTGCTA   
  
  
+ AAGCAAATCA GGGAGCACTC GTCTGCTGAG GGGGATGGAT CTCAAAGGTT GGCGCATTAC TTTGCTGATG   
  
  
+ CCCTAGAGGC ACGTTTAGCT GGAACTGGAT CTCGCATTTA TACGGCCCTA TGTTCTAATA GGCCATCTGT   
  
  
+ CACTGACATG ATAAAAGCAT ATCAGTTCTA TATTCGTGCT TGCCCATTTA CGAAGATCGT CATTGGTTGT   
  
  
+ GGTACCCATA TGATTCTAAA AGCAGCTGAG AAGGCATCAA AGCTTCATAT TATAGATTTT GGCATCCTCT   
  
  
+ ATGGTTGCCA ATGGCCCAAC CTCATTCAAC GCCTCTCAGA GCGATCTGGT GGACCTCCAA AACTGTTTAT   
  
  
+ TACAGGGATC GATCTCCCCC AGCCTGGGTT CAGGCCAGCA GAAAGAGTGG AAGCAACAGG GAGACGCTTG   
  
  
+ GCCAAGTACT GTGAGCGGTA TAATGTGCCA TTTGAGTATC ATGCCATTGC TCAGGAGTGG GAAACAATCA   
  
  
+ AACCAGGGGA TCTCAAGATA GGAAGTAGGA ATGATGAAGT TGTTGCGGTG AACTGTCTCT GTAGGTTCAA   
  
  
+ GAACCTCCTT GACGAGACAG TGGTGGTGGA TAGTCCAAGG AACACAGTTT TAAACCTGAT TACAAGGGTA   
  
  
+ AAGCCTGATA TTTTTGTGCA TGGCGTTGTA AATGGTTCCT ACAACATCCC TTTCTTTGTG ACACGTTTTA   
  
  
+ GAGAAGCCCT CTTTCATTAT TCCACTCTTT TCGACATGTT AGATGCCAAC GCCTCTAGGG AGGAGCCCGA   
  
  
+ GAGGTTGATA TTCGAGAAGG CATTCTATGG GAGGGAGATT ATGAATGTGG TGGCCTGTGA GGGCACAGAG   
  
  
+ AGGGTGGAGA GGCCGGAGAC ATACAAGCAA TGGCATGTTA GGCATAGCAG GGCAGGGTTT CGGCAAGTAC   
  
  
+ CATTGGATCC CAAGTTGATC GAGAAAATGA GGTTTAAGGC CAAGGCAGAC CACCACAAGG ATTTCATGAT   
  
  
+ CGATGTGGAT GGACATTGGG CAATTCAGGG ATGGAAGGGG CGGATTGCAC ATGCGATCTC TGCATGGGTT   
  
  
+ CCGGCTTG  

- -Up\_Stream \_Len000CTAAAC TAACAGCTTT AAACAGGCTA AAATATTTTA ACTTTTAACA ATAACCTGAT   
  
  
- ATTAGATGAC ATCAAATCTC CACAACCTAT TATGAGAGTT GAAACTTGAA CCTGCAGGTA CGTGGTGTGA   
  
  
- TACCCGCATA AATGACAAAT ATATGGTTCT TATTAAAACT TCTACAAGAA TCAACCACTA CATTCACATA   
  
  
- TAAGTTGTAT ACAGTAATGT ATTTGAACTT TTAAGAACTT AAAATCTGTA TATGTTAACG TTTAGTAACA   
  
  
- TTTTAACAAC GTAATGTAAA ACTATTTTTA CGTAGGTTTG AAACAGAACC ATATTTGTCA TTTATCCGGG   
  
  
- TATCCAGGTA TCACATCACG TACCTGCAGG TTCAAATTTC CACCCTCATA ATAACGGGTT GCGGGGATTT   
  
  
- CATGTCATAT AATATCAGGT TATAGGGTTA ATTAAAAAGT AAACTTATAA ATTTAAACCT GTATTATAAA   
  
  
- AAGTATAAAC TTAAGAGATT TGGACTAAAA TTGGACTTAA ATTCAACTAA ATTAAGTAGG CTGGTTTTTT   
  
  
- TATTTGTGTT AATAAATAAA AAGATAAGCT TCAATCTATC ATCGGTTAAG TAAACTGTAT ATTAATTGGG   
  
  
- CACTAACTGT GTTTGAGCTC AAAAACGAAT CTCGATCCTT TTCTCAGCCC AATCCAGCTG GGTAAATTCT   
  
  
- TTACCCAGCT GAAGTACAGC CGGAAGTTTT ATCAATTCTA AACGAGTTTT GATTTTTTAT TGTTCAAGTG   
  
  
- TGATTTGCCA GTTTAGCACA GCTGAAGCCT AACTTTAACC AAGTCAAACC CAGCCGAAGC ATAAAGAAAG   
  
  
- TCATTGAAGC TTGGCTTAAG CATAGCACAA TAGCACAGTC CAGTCTGAAA GAGTCAAGAT AAAAACGATG   
  
  
- GAATACAATG CCTATTTTTT TCAGAGTGTA ACCTTTTTCA CACCCTCCCA GGACCCGAAT ATTTATCACC   
  
  
- GCGACGTGTG GAGTAGTCTG GCTGGAAAAC CCCTCTCCCA TTACTGGGGC ACTGTTCACC ATAGTCTCAG   
  
  
- TTGGGTCTGG GCTGAGTCTA CAGGACGGTT CGCGAGGCTG CCCGCCCCAT CCGTCCCCCG GGTAAACACT   
  
  
- ACTCCTGCAG TGTCTTAACC CGCCCCCTCT CACAGTGCCT ATTTTTTTCA GGGTGTAGCT TTTTTCACAC   
  
  
- CCTCCCAGGA CCCGAATATT TGCCATCGCG GCGTGTGGGG TAGTCTAGCT GAAAAACCCC TCTCCCATTA   
  
  
- CTGGGGTAAA GTTCACCATA GTCTCGGTTG GGTCTGGGCT GAGGTTACAG GGTGGTTCGC GAGACTACCC   
  
  
- GCCCCATCCG TTCCCCGAGG TATACACTGC TCCCGCAGTG CCTTAACCTA CCCTCTCTCA TGGTGCCTAT   
  
  
- TTTTTTCAGG GTGTAGTCTT TTTCACACCT GAATCGTTGA TCCTTCATTT CAATGTCATA ATCGGCTCTC   
  
  
- GGGTACCTTG CGGACTTTGA CTGTAGTGGT CTGGGTGTAT AAAGTGGTAG TAGACTAGAG GGGCAGTTTC   
  
  
- CTCCTTCGAC TCGTGTCGTT CTTGATCTTC TTGTCCGCCG TAGTGTGGGT TCGGTTTTAA GAGATAAACT   
  
  
- GGAGGGAGAG GAGTAAGGAG ACTCGAAGGA GAAGTGTTAC ATTCTGGTAT TAGGAATTGT GGGAAGAAAA   
  
  
- AATTCGAGTA GAAATGGACG AGACCAAGAG AGAGAGTAGC AGTGATTATC AATGGAGAAA TGAAAGAGAA   
  
  
- GGGACTCCAT ACGAAGTTGA AATTGTATAT ATAGGGTGAA CTAAAGAGAA CAGAAAAACG ATATTATGAA   
  
  
- CAACAAACCG ATAGTAGGGG ACAAAATATG AGTAAAAGAA CGAAAGATAA AGACCCAAAC TTAACCCAAA   
  
  
- GTGAATAGGA GCAGTTAAGA TACCCATTAA TCACTTAACC CAAATATACT TGATCCCAAC GTTTTCATAG   
  
  
- ATGAAAAAGA GAACACTAAT AATCAACTAA TCCCTAAACC GGTTATAATC GTCTTACCCA AGACTTAAAC   
  
  
- GCCTTAAGAG ACTACTACGA GATTTACCCA TACGAATACA ACTATAGGGA CGAATACTAC GTAGGAATCT   
  
  
- AATACGGTTA AACAAGTTAA TGCTTCCGGG TAGACTCCTA GCGTGGAGTG ACTCGGAGGG TTCTGGGAAA   
  
  
- AGACTGGGAA ACCGAATGTC AAAGTGCAGG AGAAGACTCG ACCCGGGACC CCAACTAAGA TTACTACTAT   
  
  
- CACTAAGACC GCTACAAGAG TTCATGTAAT CGGTTTACGA ATACCTTCTT CTGTACCTCC GTTTCGGTAC   
  
  
- GTACAAAGTA CTAGGAAATC GTGAAGTCCG ACGACTCTTT GGGAAAATAC TACGGAATCC CCTCGTTACG   
  
  
- GGTTGAAGAG GACTGGTTGT AGGATATTAA CTAGTAATAA ACCTATCAGG ACTACTTTTA AGAAACTCAA   
  
  
- GTTGACCACT AAAATCACAA TCAGTAAGAC CCAGATCAAG TTGTTTGAGG CAACCTGGCT ATTATCACAG   
  
  
- ACTAAACTCA CTCGTAAAAC TCGGTGGGAA ACAACTTTGT GAAGGTTAAC TTAGTATAGT GGTTGGTAAC   
  
  
- TGGGCAGGAG TTACCAGTAA ACCGAGAAGC CCACGGAATC TACCGTGCCG GAGATTACCA AGCCACTAGA   
  
  
- GTAGGGAACC AAACGGTCAC CTACACTAAT CGCATAAATC CCTCTTTCTC AGGTACTAAG TTAAAGTCTT   
  
  
- TCCCCACCTC CTCCGGTCAT TCAAGGAAGG GTTCTTATTA TTGGAACAAT AACTAGAGCT CTTGGAGTGA   
  
  
- AAAGGATTAC TTTGTTTCCT CCTACTAGCT TACTACTACC AATTCTTCCT TTTCCTACTG CACTTAACCA   
  
  
- GATTGATGAG TTCTCCCTCA TTCTAAGTGA TAACACTTCT GCTCCGGAAA CTTCTTCCTT CCTCACCGTT   
  
  
- CGTCAGTCGA TAAAGAAGAT GACTCCTTCG ACTCAAAAGA CTGTAAAAAC TGTTCCAAAA CGAAACGCTA   
  
  
- ACGATGGGAC ACTTTGGACT CCGAGTAGGG TGGTACTCAA ACTTGGGACT CTTCCCGGTC AGTGTACCTA   
  
  
- ATCTTCCACC CTCTTTACCC TTCCGAGCGG GATTCTTTGT CCTATTATCA TCGTGTTTAT AACACCTAAA   
  
  
- TTCCTTAAAC GACTATGATA CGCGTGTTAG ATGACGTAGA CTACTGGCTT CTTGACGACT ACCTGACGAT   
  
  
- TTCGTTTAGT CCCTCGTGAG CAGACGACTC CCCCTACCTA GAGTTTCCAA CCGCGTAATG AAACGACTAC   
  
  
- GGGATCTCCG TGCAAATCGA CCTTGACCTA GAGCGTAAAT ATGCCGGGAT ACAAGATTAT CCGGTAGACA   
  
  
- GTGACTGTAC TATTTTCGTA TAGTCAAGAT ATAAGCACGA ACGGGTAAAT GCTTCTAGCA GTAACCAACA   
  
  
- CCATGGGTAT ACTAAGATTT TCGTCGACTC TTCCGTAGTT TCGAAGTATA ATATCTAAAA CCGTAGGAGA   
  
  
- TACCAACGGT TACCGGGTTG GAGTAAGTTG CGGAGAGTCT CGCTAGACCA CCTGGAGGTT TTGACAAATA   
  
  
- ATGTCCCTAG CTAGAGGGGG TCGGACCCAA GTCCGGTCGT CTTTCTCACC TTCGTTGTCC CTCTGCGAAC   
  
  
- CGGTTCATGA CACTCGCCAT ATTACACGGT AAACTCATAG TACGGTAACG AGTCCTCACC CTTTGTTAGT   
  
  
- TTGGTCCCCT AGAGTTCTAT CCTTCATCCT TACTACTTCA ACAACGCCAC TTGACAGAGA CATCCAAGTT   
  
  
- CTTGGAGGAA CTGCTCTGTC ACCACCACCT ATCAGGTTCC TTGTGTCAAA ATTTGGACTA ATGTTCCCAT   
  
  
- TTCGGACTAT AAAAACACGT ACCGCAACAT TTACCAAGGA TGTTGTAGGG AAAGAAACAC TGTGCAAAAT   
  
  
- CTCTTCGGGA GAAAGTAATA AGGTGAGAAA AGCTGTACAA TCTACGGTTG CGGAGATCCC TCCTCGGGCT   
  
  
- CTCCAACTAT AAGCTCTTCC GTAAGATACC CTCCCTCTAA TACTTACACC ACCGGACACT CCCGTGTCTC   
  
  
- TCCCACCTCT CCGGCCTCTG TATGTTCGTT ACCGTACAAT CCGTATCGTC CCGTCCCAAA GCCGTTCATG   
  
  
- GTAACCTAGG GTTCAACTAG CTCTTTTACT CCAAATTCCG GTTCCGTCTG GTGGTGTTCC TAAAGTACTA   
  
  
- GCTACACCTA CCTGTAACCC GTTAAGTCCC TACCTTCCCC GCCTAACGTG TACGCTAGAG ACGTACCCAA   
  
  
- GGCCGAAC

+     AuxRR-core

| Site Name | Organism | Position | Strand | Matrix score. | sequence | function |
| --- | --- | --- | --- | --- | --- | --- |
| AuxRR-core | Nicotiana tabacum | 358 | + | 7 | GGTCCAT | cis-acting regulatory element involved in auxin responsiveness |

>HU07G02249.1   
+ -Up\_Stream \_Len000GATTTG ATTGTCGAAA TTTGTCCGAT TTTATAAAAT TGAAAATTGT TATTGGACTA   
  
  
+ TAATCTACTG TAGTTTAGAG GTGTTGGATA ATACTCTCAA CTTTGAACTT GGACGTCCAT GCACCACACT   
  
  
+ ATGGGCGTAT TTACTGTTTA TATACCAAGA ATAATTTTGA AGATGTTCTT AGTTGGTGAT GTAAGTGTAT   
  
  
+ ATTCAACATA TGTCATTACA TAAACTTGAA AATTCTTGAA TTTTAGACAT ATACAATTGC AAATCATTGT   
  
  
+ AAAATTGTTG CATTACATTT TGATAAAAAT GCATCCAAAC TTTGTCTTGG TATAAACAGT AAATAGGCCC   
  
  
+ ATAGGTCCAT AGTGTAGTGC ATGGACGTCC AAGTTTAAAG GTGGGAGTAT TATTGCCCAA CGCCCCTAAA   
  
  
+ GTACAGTATA TTATAGTCCA ATATCCCAAT TAATTTTTCA TTTGAATATT TAAATTTGGA CATAATATTT   
  
  
+ TTCATATTTG AATTCTCTAA ACCTGATTTT AACCTGAATT TAAGTTGATT TAATTCATCC GACCAAAAAA   
  
  
+ ATAAACACAA TTATTTATTT TTCTATTCGA AGTTAGATAG TAGCCAATTC ATTTGACATA TAATTAACCC   
  
  
+ GTGATTGACA CAAACTCGAG TTTTTGCTTA GAGCTAGGAA AAGAGTCGGG TTAGGTCGAC CCATTTAAGA   
  
  
+ AATGGGTCGA CTTCATGTCG GCCTTCAAAA TAGTTAAGAT TTGCTCAAAA CTAAAAAATA ACAAGTTCAC   
  
  
+ ACTAAACGGT CAAATCGTGT CGACTTCGGA TTGAAATTGG TTCAGTTTGG GTCGGCTTCG TATTTCTTTC   
  
  
+ AGTAACTTCG AACCGAATTC GTATCGTGTT ATCGTGTCAG GTCAGACTTT CTCAGTTCTA TTTTTGCTAC   
  
  
+ CTTATGTTAC GGATAAAAAA AGTCTCACAT TGGAAAAAGT GTGGGAGGGT CCTGGGCTTA TAAATAGTGG   
  
  
+ CGCTGCACAC CTCATCAGAC CGACCTTTTG GGGAGAGGGT AATGACCCCG TGACAAGTGG TATCAGAGTC   
  
  
+ AACCCAGACC CGACTCAGAT GTCCTGCCAA GCGCTCCGAC GGGCGGGGTA GGCAGGGGGC CCATTTGTGA   
  
  
+ TGAGGACGTC ACAGAATTGG GCGGGGGAGA GTGTCACGGA TAAAAAAAGT CCCACATCGA AAAAAGTGTG   
  
  
+ GGAGGGTCCT GGGCTTATAA ACGGTAGCGC CGCACACCCC ATCAGATCGA CTTTTTGGGG AGAGGGTAAT   
  
  
+ GACCCCATTT CAAGTGGTAT CAGAGCCAAC CCAGACCCGA CTCCAATGTC CCACCAAGCG CTCTGATGGG   
  
  
+ CGGGGTAGGC AAGGGGCTCC ATATGTGACG AGGGCGTCAC GGAATTGGAT GGGAGAGAGT ACCACGGATA   
  
  
+ AAAAAAGTCC CACATCAGAA AAAGTGTGGA CTTAGCAACT AGGAAGTAAA GTTACAGTAT TAGCCGAGAG   
  
  
+ CCCATGGAAC GCCTGAAACT GACATCACCA GACCCACATA TTTCACCATC ATCTGATCTC CCCGTCAAAG   
  
  
+ GAGGAAGCTG AGCACAGCAA GAACTAGAAG AACAGGCGGC ATCACACCCA AGCCAAAATT CTCTATTTGA   
  
  
+ CCTCCCTCTC CTCATTCCTC TGAGCTTCCT CTTCACAATG TAAGACCATA ATCCTTAACA CCCTTCTTTT   
  
  
+ TTAAGCTCAT CTTTACCTGC TCTGGTTCTC TCTCTCATCG TCACTAATAG TTACCTCTTT ACTTTCTCTT   
  
  
+ CCCTGAGGTA TGCTTCAACT TTAACATATA TATCCCACTT GATTTCTCTT GTCTTTTTGC TATAATACTT   
  
  
+ GTTGTTTGGC TATCATCCCC TGTTTTATAC TCATTTTCTT GCTTTCTATT TCTGGGTTTG AATTGGGTTT   
  
  
+ CACTTATCCT CGTCAATTCT ATGGGTAATT AGTGAATTGG GTTTATATGA ACTAGGGTTG CAAAAGTATC   
  
  
+ TACTTTTTCT CTTGTGATTA TTAGTTGATT AGGGATTTGG CCAATATTAG CAGAATGGGT TCTGAATTTG   
  
  
+ CGGAATTCTC TGATGATGCT CTAAATGGGT ATGCTTATGT TGATATCCCT GCTTATGATG CATCCTTAGA   
  
  
+ TTATGCCAAT TTGTTCAATT ACGAAGGCCC ATCTGAGGAT CGCACCTCAC TGAGCCTCCC AAGACCCTTT   
  
  
+ TCTGACCCTT TGGCTTACAG TTTCACGTCC TCTTCTGAGC TGGGCCCTGG GGTTGATTCT AATGATGATA   
  
  
+ GTGATTCTGG CGATGTTCTC AAGTACATTA GCCAAATGCT TATGGAAGAA GACATGGAGG CAAAGCCATG   
  
  
+ CATGTTTCAT GATCCTTTAG CACTTCAGGC TGCTGAGAAA CCCTTTTATG ATGCCTTAGG GGAGCAATGC   
  
  
+ CCAACTTCTC CTGACCAACA TCCTATAATT GATCATTATT TGGATAGTCC TGATGAAAAT TCTTTGAGTT   
  
  
+ CAACTGGTGA TTTTAGTGTT AGTCATTCTG GGTCTAGTTC AACAAACTCC GTTGGACCGA TAATAGTGTC   
  
  
+ TGATTTGAGT GAGCATTTTG AGCCACCCTT TGTTGAAACA CTTCCAATTG AATCATATCA CCAACCATTG   
  
  
+ ACCCGTCCTC AATGGTCATT TGGCTCTTCG GGTGCCTTAG ATGGCACGGC CTCTAATGGT TCGGTGATCT   
  
  
+ CATCCCTTGG TTTGCCAGTG GATGTGATTA GCGTATTTAG GGAGAAAGAG TCCATGATTC AATTTCAGAA   
  
  
+ AGGGGTGGAG GAGGCCAGTA AGTTCCTTCC CAAGAATAAT AACCTTGTTA TTGATCTCGA GAACCTCACT   
  
  
+ TTTCCTAATG AAACAAAGGA GGATGATCGA ATGATGATGG TTAAGAAGGA AAAGGATGAC GTGAATTGGT   
  
  
+ CTAACTACTC AAGAGGGAGT AAGATTCACT ATTGTGAAGA CGAGGCCTTT GAAGAAGGAA GGAGTGGCAA   
  
  
+ GCAGTCAGCT ATTTCTTCTA CTGAGGAAGC TGAGTTTTCT GACATTTTTG ACAAGGTTTT GCTTTGCGAT   
  
  
+ TGCTACCCTG TGAAACCTGA GGCTCATCCC ACCATGAGTT TGAACCCTGA GAAGGGCCAG TCACATGGAT   
  
  
+ TAGAAGGTGG GAGAAATGGG AAGGCTCGCC CTAAGAAACA GGATAATAGT AGCACAAATA TTGTGGATTT   
  
  
+ AAGGAATTTG CTGATACTAT GCGCACAATC TACTGCATCT GATGACCGAA GAACTGCTGA TGGACTGCTA   
  
  
+ AAGCAAATCA GGGAGCACTC GTCTGCTGAG GGGGATGGAT CTCAAAGGTT GGCGCATTAC TTTGCTGATG   
  
  
+ CCCTAGAGGC ACGTTTAGCT GGAACTGGAT CTCGCATTTA TACGGCCCTA TGTTCTAATA GGCCATCTGT   
  
  
+ CACTGACATG ATAAAAGCAT ATCAGTTCTA TATTCGTGCT TGCCCATTTA CGAAGATCGT CATTGGTTGT   
  
  
+ GGTACCCATA TGATTCTAAA AGCAGCTGAG AAGGCATCAA AGCTTCATAT TATAGATTTT GGCATCCTCT   
  
  
+ ATGGTTGCCA ATGGCCCAAC CTCATTCAAC GCCTCTCAGA GCGATCTGGT GGACCTCCAA AACTGTTTAT   
  
  
+ TACAGGGATC GATCTCCCCC AGCCTGGGTT CAGGCCAGCA GAAAGAGTGG AAGCAACAGG GAGACGCTTG   
  
  
+ GCCAAGTACT GTGAGCGGTA TAATGTGCCA TTTGAGTATC ATGCCATTGC TCAGGAGTGG GAAACAATCA   
  
  
+ AACCAGGGGA TCTCAAGATA GGAAGTAGGA ATGATGAAGT TGTTGCGGTG AACTGTCTCT GTAGGTTCAA   
  
  
+ GAACCTCCTT GACGAGACAG TGGTGGTGGA TAGTCCAAGG AACACAGTTT TAAACCTGAT TACAAGGGTA   
  
  
+ AAGCCTGATA TTTTTGTGCA TGGCGTTGTA AATGGTTCCT ACAACATCCC TTTCTTTGTG ACACGTTTTA   
  
  
+ GAGAAGCCCT CTTTCATTAT TCCACTCTTT TCGACATGTT AGATGCCAAC GCCTCTAGGG AGGAGCCCGA   
  
  
+ GAGGTTGATA TTCGAGAAGG CATTCTATGG GAGGGAGATT ATGAATGTGG TGGCCTGTGA GGGCACAGAG   
  
  
+ AGGGTGGAGA GGCCGGAGAC ATACAAGCAA TGGCATGTTA GGCATAGCAG GGCAGGGTTT CGGCAAGTAC   
  
  
+ CATTGGATCC CAAGTTGATC GAGAAAATGA GGTTTAAGGC CAAGGCAGAC CACCACAAGG ATTTCATGAT   
  
  
+ CGATGTGGAT GGACATTGGG CAATTCAGGG ATGGAAGGGG CGGATTGCAC ATGCGATCTC TGCATGGGTT   
  
  
+ CCGGCTTG  

- -Up\_Stream \_Len000CTAAAC TAACAGCTTT AAACAGGCTA AAATATTTTA ACTTTTAACA ATAACCTGAT   
  
  
- ATTAGATGAC ATCAAATCTC CACAACCTAT TATGAGAGTT GAAACTTGAA CCTGCAGGTA CGTGGTGTGA   
  
  
- TACCCGCATA AATGACAAAT ATATGGTTCT TATTAAAACT TCTACAAGAA TCAACCACTA CATTCACATA   
  
  
- TAAGTTGTAT ACAGTAATGT ATTTGAACTT TTAAGAACTT AAAATCTGTA TATGTTAACG TTTAGTAACA   
  
  
- TTTTAACAAC GTAATGTAAA ACTATTTTTA CGTAGGTTTG AAACAGAACC ATATTTGTCA TTTATCCGGG   
  
  
- TATCCAGGTA TCACATCACG TACCTGCAGG TTCAAATTTC CACCCTCATA ATAACGGGTT GCGGGGATTT   
  
  
- CATGTCATAT AATATCAGGT TATAGGGTTA ATTAAAAAGT AAACTTATAA ATTTAAACCT GTATTATAAA   
  
  
- AAGTATAAAC TTAAGAGATT TGGACTAAAA TTGGACTTAA ATTCAACTAA ATTAAGTAGG CTGGTTTTTT   
  
  
- TATTTGTGTT AATAAATAAA AAGATAAGCT TCAATCTATC ATCGGTTAAG TAAACTGTAT ATTAATTGGG   
  
  
- CACTAACTGT GTTTGAGCTC AAAAACGAAT CTCGATCCTT TTCTCAGCCC AATCCAGCTG GGTAAATTCT   
  
  
- TTACCCAGCT GAAGTACAGC CGGAAGTTTT ATCAATTCTA AACGAGTTTT GATTTTTTAT TGTTCAAGTG   
  
  
- TGATTTGCCA GTTTAGCACA GCTGAAGCCT AACTTTAACC AAGTCAAACC CAGCCGAAGC ATAAAGAAAG   
  
  
- TCATTGAAGC TTGGCTTAAG CATAGCACAA TAGCACAGTC CAGTCTGAAA GAGTCAAGAT AAAAACGATG   
  
  
- GAATACAATG CCTATTTTTT TCAGAGTGTA ACCTTTTTCA CACCCTCCCA GGACCCGAAT ATTTATCACC   
  
  
- GCGACGTGTG GAGTAGTCTG GCTGGAAAAC CCCTCTCCCA TTACTGGGGC ACTGTTCACC ATAGTCTCAG   
  
  
- TTGGGTCTGG GCTGAGTCTA CAGGACGGTT CGCGAGGCTG CCCGCCCCAT CCGTCCCCCG GGTAAACACT   
  
  
- ACTCCTGCAG TGTCTTAACC CGCCCCCTCT CACAGTGCCT ATTTTTTTCA GGGTGTAGCT TTTTTCACAC   
  
  
- CCTCCCAGGA CCCGAATATT TGCCATCGCG GCGTGTGGGG TAGTCTAGCT GAAAAACCCC TCTCCCATTA   
  
  
- CTGGGGTAAA GTTCACCATA GTCTCGGTTG GGTCTGGGCT GAGGTTACAG GGTGGTTCGC GAGACTACCC   
  
  
- GCCCCATCCG TTCCCCGAGG TATACACTGC TCCCGCAGTG CCTTAACCTA CCCTCTCTCA TGGTGCCTAT   
  
  
- TTTTTTCAGG GTGTAGTCTT TTTCACACCT GAATCGTTGA TCCTTCATTT CAATGTCATA ATCGGCTCTC   
  
  
- GGGTACCTTG CGGACTTTGA CTGTAGTGGT CTGGGTGTAT AAAGTGGTAG TAGACTAGAG GGGCAGTTTC   
  
  
- CTCCTTCGAC TCGTGTCGTT CTTGATCTTC TTGTCCGCCG TAGTGTGGGT TCGGTTTTAA GAGATAAACT   
  
  
- GGAGGGAGAG GAGTAAGGAG ACTCGAAGGA GAAGTGTTAC ATTCTGGTAT TAGGAATTGT GGGAAGAAAA   
  
  
- AATTCGAGTA GAAATGGACG AGACCAAGAG AGAGAGTAGC AGTGATTATC AATGGAGAAA TGAAAGAGAA   
  
  
- GGGACTCCAT ACGAAGTTGA AATTGTATAT ATAGGGTGAA CTAAAGAGAA CAGAAAAACG ATATTATGAA   
  
  
- CAACAAACCG ATAGTAGGGG ACAAAATATG AGTAAAAGAA CGAAAGATAA AGACCCAAAC TTAACCCAAA   
  
  
- GTGAATAGGA GCAGTTAAGA TACCCATTAA TCACTTAACC CAAATATACT TGATCCCAAC GTTTTCATAG   
  
  
- ATGAAAAAGA GAACACTAAT AATCAACTAA TCCCTAAACC GGTTATAATC GTCTTACCCA AGACTTAAAC   
  
  
- GCCTTAAGAG ACTACTACGA GATTTACCCA TACGAATACA ACTATAGGGA CGAATACTAC GTAGGAATCT   
  
  
- AATACGGTTA AACAAGTTAA TGCTTCCGGG TAGACTCCTA GCGTGGAGTG ACTCGGAGGG TTCTGGGAAA   
  
  
- AGACTGGGAA ACCGAATGTC AAAGTGCAGG AGAAGACTCG ACCCGGGACC CCAACTAAGA TTACTACTAT   
  
  
- CACTAAGACC GCTACAAGAG TTCATGTAAT CGGTTTACGA ATACCTTCTT CTGTACCTCC GTTTCGGTAC   
  
  
- GTACAAAGTA CTAGGAAATC GTGAAGTCCG ACGACTCTTT GGGAAAATAC TACGGAATCC CCTCGTTACG   
  
  
- GGTTGAAGAG GACTGGTTGT AGGATATTAA CTAGTAATAA ACCTATCAGG ACTACTTTTA AGAAACTCAA   
  
  
- GTTGACCACT AAAATCACAA TCAGTAAGAC CCAGATCAAG TTGTTTGAGG CAACCTGGCT ATTATCACAG   
  
  
- ACTAAACTCA CTCGTAAAAC TCGGTGGGAA ACAACTTTGT GAAGGTTAAC TTAGTATAGT GGTTGGTAAC   
  
  
- TGGGCAGGAG TTACCAGTAA ACCGAGAAGC CCACGGAATC TACCGTGCCG GAGATTACCA AGCCACTAGA   
  
  
- GTAGGGAACC AAACGGTCAC CTACACTAAT CGCATAAATC CCTCTTTCTC AGGTACTAAG TTAAAGTCTT   
  
  
- TCCCCACCTC CTCCGGTCAT TCAAGGAAGG GTTCTTATTA TTGGAACAAT AACTAGAGCT CTTGGAGTGA   
  
  
- AAAGGATTAC TTTGTTTCCT CCTACTAGCT TACTACTACC AATTCTTCCT TTTCCTACTG CACTTAACCA   
  
  
- GATTGATGAG TTCTCCCTCA TTCTAAGTGA TAACACTTCT GCTCCGGAAA CTTCTTCCTT CCTCACCGTT   
  
  
- CGTCAGTCGA TAAAGAAGAT GACTCCTTCG ACTCAAAAGA CTGTAAAAAC TGTTCCAAAA CGAAACGCTA   
  
  
- ACGATGGGAC ACTTTGGACT CCGAGTAGGG TGGTACTCAA ACTTGGGACT CTTCCCGGTC AGTGTACCTA   
  
  
- ATCTTCCACC CTCTTTACCC TTCCGAGCGG GATTCTTTGT CCTATTATCA TCGTGTTTAT AACACCTAAA   
  
  
- TTCCTTAAAC GACTATGATA CGCGTGTTAG ATGACGTAGA CTACTGGCTT CTTGACGACT ACCTGACGAT   
  
  
- TTCGTTTAGT CCCTCGTGAG CAGACGACTC CCCCTACCTA GAGTTTCCAA CCGCGTAATG AAACGACTAC   
  
  
- GGGATCTCCG TGCAAATCGA CCTTGACCTA GAGCGTAAAT ATGCCGGGAT ACAAGATTAT CCGGTAGACA   
  
  
- GTGACTGTAC TATTTTCGTA TAGTCAAGAT ATAAGCACGA ACGGGTAAAT GCTTCTAGCA GTAACCAACA   
  
  
- CCATGGGTAT ACTAAGATTT TCGTCGACTC TTCCGTAGTT TCGAAGTATA ATATCTAAAA CCGTAGGAGA   
  
  
- TACCAACGGT TACCGGGTTG GAGTAAGTTG CGGAGAGTCT CGCTAGACCA CCTGGAGGTT TTGACAAATA   
  
  
- ATGTCCCTAG CTAGAGGGGG TCGGACCCAA GTCCGGTCGT CTTTCTCACC TTCGTTGTCC CTCTGCGAAC   
  
  
- CGGTTCATGA CACTCGCCAT ATTACACGGT AAACTCATAG TACGGTAACG AGTCCTCACC CTTTGTTAGT   
  
  
- TTGGTCCCCT AGAGTTCTAT CCTTCATCCT TACTACTTCA ACAACGCCAC TTGACAGAGA CATCCAAGTT   
  
  
- CTTGGAGGAA CTGCTCTGTC ACCACCACCT ATCAGGTTCC TTGTGTCAAA ATTTGGACTA ATGTTCCCAT   
  
  
- TTCGGACTAT AAAAACACGT ACCGCAACAT TTACCAAGGA TGTTGTAGGG AAAGAAACAC TGTGCAAAAT   
  
  
- CTCTTCGGGA GAAAGTAATA AGGTGAGAAA AGCTGTACAA TCTACGGTTG CGGAGATCCC TCCTCGGGCT   
  
  
- CTCCAACTAT AAGCTCTTCC GTAAGATACC CTCCCTCTAA TACTTACACC ACCGGACACT CCCGTGTCTC   
  
  
- TCCCACCTCT CCGGCCTCTG TATGTTCGTT ACCGTACAAT CCGTATCGTC CCGTCCCAAA GCCGTTCATG   
  
  
- GTAACCTAGG GTTCAACTAG CTCTTTTACT CCAAATTCCG GTTCCGTCTG GTGGTGTTCC TAAAGTACTA   
  
  
- GCTACACCTA CCTGTAACCC GTTAAGTCCC TACCTTCCCC GCCTAACGTG TACGCTAGAG ACGTACCCAA   
  
  
- GGCCGAAC

+     Box 4

| Site Name | Organism | Position | Strand | Matrix score. | sequence | function |
| --- | --- | --- | --- | --- | --- | --- |
| Box 4 | Petroselinum crispum | 453 | + | 6 | ATTAAT | part of a conserved DNA module involved in light responsiveness |

>HU07G02249.1   
+ -Up\_Stream \_Len000GATTTG ATTGTCGAAA TTTGTCCGAT TTTATAAAAT TGAAAATTGT TATTGGACTA   
  
  
+ TAATCTACTG TAGTTTAGAG GTGTTGGATA ATACTCTCAA CTTTGAACTT GGACGTCCAT GCACCACACT   
  
  
+ ATGGGCGTAT TTACTGTTTA TATACCAAGA ATAATTTTGA AGATGTTCTT AGTTGGTGAT GTAAGTGTAT   
  
  
+ ATTCAACATA TGTCATTACA TAAACTTGAA AATTCTTGAA TTTTAGACAT ATACAATTGC AAATCATTGT   
  
  
+ AAAATTGTTG CATTACATTT TGATAAAAAT GCATCCAAAC TTTGTCTTGG TATAAACAGT AAATAGGCCC   
  
  
+ ATAGGTCCAT AGTGTAGTGC ATGGACGTCC AAGTTTAAAG GTGGGAGTAT TATTGCCCAA CGCCCCTAAA   
  
  
+ GTACAGTATA TTATAGTCCA ATATCCCAAT TAATTTTTCA TTTGAATATT TAAATTTGGA CATAATATTT   
  
  
+ TTCATATTTG AATTCTCTAA ACCTGATTTT AACCTGAATT TAAGTTGATT TAATTCATCC GACCAAAAAA   
  
  
+ ATAAACACAA TTATTTATTT TTCTATTCGA AGTTAGATAG TAGCCAATTC ATTTGACATA TAATTAACCC   
  
  
+ GTGATTGACA CAAACTCGAG TTTTTGCTTA GAGCTAGGAA AAGAGTCGGG TTAGGTCGAC CCATTTAAGA   
  
  
+ AATGGGTCGA CTTCATGTCG GCCTTCAAAA TAGTTAAGAT TTGCTCAAAA CTAAAAAATA ACAAGTTCAC   
  
  
+ ACTAAACGGT CAAATCGTGT CGACTTCGGA TTGAAATTGG TTCAGTTTGG GTCGGCTTCG TATTTCTTTC   
  
  
+ AGTAACTTCG AACCGAATTC GTATCGTGTT ATCGTGTCAG GTCAGACTTT CTCAGTTCTA TTTTTGCTAC   
  
  
+ CTTATGTTAC GGATAAAAAA AGTCTCACAT TGGAAAAAGT GTGGGAGGGT CCTGGGCTTA TAAATAGTGG   
  
  
+ CGCTGCACAC CTCATCAGAC CGACCTTTTG GGGAGAGGGT AATGACCCCG TGACAAGTGG TATCAGAGTC   
  
  
+ AACCCAGACC CGACTCAGAT GTCCTGCCAA GCGCTCCGAC GGGCGGGGTA GGCAGGGGGC CCATTTGTGA   
  
  
+ TGAGGACGTC ACAGAATTGG GCGGGGGAGA GTGTCACGGA TAAAAAAAGT CCCACATCGA AAAAAGTGTG   
  
  
+ GGAGGGTCCT GGGCTTATAA ACGGTAGCGC CGCACACCCC ATCAGATCGA CTTTTTGGGG AGAGGGTAAT   
  
  
+ GACCCCATTT CAAGTGGTAT CAGAGCCAAC CCAGACCCGA CTCCAATGTC CCACCAAGCG CTCTGATGGG   
  
  
+ CGGGGTAGGC AAGGGGCTCC ATATGTGACG AGGGCGTCAC GGAATTGGAT GGGAGAGAGT ACCACGGATA   
  
  
+ AAAAAAGTCC CACATCAGAA AAAGTGTGGA CTTAGCAACT AGGAAGTAAA GTTACAGTAT TAGCCGAGAG   
  
  
+ CCCATGGAAC GCCTGAAACT GACATCACCA GACCCACATA TTTCACCATC ATCTGATCTC CCCGTCAAAG   
  
  
+ GAGGAAGCTG AGCACAGCAA GAACTAGAAG AACAGGCGGC ATCACACCCA AGCCAAAATT CTCTATTTGA   
  
  
+ CCTCCCTCTC CTCATTCCTC TGAGCTTCCT CTTCACAATG TAAGACCATA ATCCTTAACA CCCTTCTTTT   
  
  
+ TTAAGCTCAT CTTTACCTGC TCTGGTTCTC TCTCTCATCG TCACTAATAG TTACCTCTTT ACTTTCTCTT   
  
  
+ CCCTGAGGTA TGCTTCAACT TTAACATATA TATCCCACTT GATTTCTCTT GTCTTTTTGC TATAATACTT   
  
  
+ GTTGTTTGGC TATCATCCCC TGTTTTATAC TCATTTTCTT GCTTTCTATT TCTGGGTTTG AATTGGGTTT   
  
  
+ CACTTATCCT CGTCAATTCT ATGGGTAATT AGTGAATTGG GTTTATATGA ACTAGGGTTG CAAAAGTATC   
  
  
+ TACTTTTTCT CTTGTGATTA TTAGTTGATT AGGGATTTGG CCAATATTAG CAGAATGGGT TCTGAATTTG   
  
  
+ CGGAATTCTC TGATGATGCT CTAAATGGGT ATGCTTATGT TGATATCCCT GCTTATGATG CATCCTTAGA   
  
  
+ TTATGCCAAT TTGTTCAATT ACGAAGGCCC ATCTGAGGAT CGCACCTCAC TGAGCCTCCC AAGACCCTTT   
  
  
+ TCTGACCCTT TGGCTTACAG TTTCACGTCC TCTTCTGAGC TGGGCCCTGG GGTTGATTCT AATGATGATA   
  
  
+ GTGATTCTGG CGATGTTCTC AAGTACATTA GCCAAATGCT TATGGAAGAA GACATGGAGG CAAAGCCATG   
  
  
+ CATGTTTCAT GATCCTTTAG CACTTCAGGC TGCTGAGAAA CCCTTTTATG ATGCCTTAGG GGAGCAATGC   
  
  
+ CCAACTTCTC CTGACCAACA TCCTATAATT GATCATTATT TGGATAGTCC TGATGAAAAT TCTTTGAGTT   
  
  
+ CAACTGGTGA TTTTAGTGTT AGTCATTCTG GGTCTAGTTC AACAAACTCC GTTGGACCGA TAATAGTGTC   
  
  
+ TGATTTGAGT GAGCATTTTG AGCCACCCTT TGTTGAAACA CTTCCAATTG AATCATATCA CCAACCATTG   
  
  
+ ACCCGTCCTC AATGGTCATT TGGCTCTTCG GGTGCCTTAG ATGGCACGGC CTCTAATGGT TCGGTGATCT   
  
  
+ CATCCCTTGG TTTGCCAGTG GATGTGATTA GCGTATTTAG GGAGAAAGAG TCCATGATTC AATTTCAGAA   
  
  
+ AGGGGTGGAG GAGGCCAGTA AGTTCCTTCC CAAGAATAAT AACCTTGTTA TTGATCTCGA GAACCTCACT   
  
  
+ TTTCCTAATG AAACAAAGGA GGATGATCGA ATGATGATGG TTAAGAAGGA AAAGGATGAC GTGAATTGGT   
  
  
+ CTAACTACTC AAGAGGGAGT AAGATTCACT ATTGTGAAGA CGAGGCCTTT GAAGAAGGAA GGAGTGGCAA   
  
  
+ GCAGTCAGCT ATTTCTTCTA CTGAGGAAGC TGAGTTTTCT GACATTTTTG ACAAGGTTTT GCTTTGCGAT   
  
  
+ TGCTACCCTG TGAAACCTGA GGCTCATCCC ACCATGAGTT TGAACCCTGA GAAGGGCCAG TCACATGGAT   
  
  
+ TAGAAGGTGG GAGAAATGGG AAGGCTCGCC CTAAGAAACA GGATAATAGT AGCACAAATA TTGTGGATTT   
  
  
+ AAGGAATTTG CTGATACTAT GCGCACAATC TACTGCATCT GATGACCGAA GAACTGCTGA TGGACTGCTA   
  
  
+ AAGCAAATCA GGGAGCACTC GTCTGCTGAG GGGGATGGAT CTCAAAGGTT GGCGCATTAC TTTGCTGATG   
  
  
+ CCCTAGAGGC ACGTTTAGCT GGAACTGGAT CTCGCATTTA TACGGCCCTA TGTTCTAATA GGCCATCTGT   
  
  
+ CACTGACATG ATAAAAGCAT ATCAGTTCTA TATTCGTGCT TGCCCATTTA CGAAGATCGT CATTGGTTGT   
  
  
+ GGTACCCATA TGATTCTAAA AGCAGCTGAG AAGGCATCAA AGCTTCATAT TATAGATTTT GGCATCCTCT   
  
  
+ ATGGTTGCCA ATGGCCCAAC CTCATTCAAC GCCTCTCAGA GCGATCTGGT GGACCTCCAA AACTGTTTAT   
  
  
+ TACAGGGATC GATCTCCCCC AGCCTGGGTT CAGGCCAGCA GAAAGAGTGG AAGCAACAGG GAGACGCTTG   
  
  
+ GCCAAGTACT GTGAGCGGTA TAATGTGCCA TTTGAGTATC ATGCCATTGC TCAGGAGTGG GAAACAATCA   
  
  
+ AACCAGGGGA TCTCAAGATA GGAAGTAGGA ATGATGAAGT TGTTGCGGTG AACTGTCTCT GTAGGTTCAA   
  
  
+ GAACCTCCTT GACGAGACAG TGGTGGTGGA TAGTCCAAGG AACACAGTTT TAAACCTGAT TACAAGGGTA   
  
  
+ AAGCCTGATA TTTTTGTGCA TGGCGTTGTA AATGGTTCCT ACAACATCCC TTTCTTTGTG ACACGTTTTA   
  
  
+ GAGAAGCCCT CTTTCATTAT TCCACTCTTT TCGACATGTT AGATGCCAAC GCCTCTAGGG AGGAGCCCGA   
  
  
+ GAGGTTGATA TTCGAGAAGG CATTCTATGG GAGGGAGATT ATGAATGTGG TGGCCTGTGA GGGCACAGAG   
  
  
+ AGGGTGGAGA GGCCGGAGAC ATACAAGCAA TGGCATGTTA GGCATAGCAG GGCAGGGTTT CGGCAAGTAC   
  
  
+ CATTGGATCC CAAGTTGATC GAGAAAATGA GGTTTAAGGC CAAGGCAGAC CACCACAAGG ATTTCATGAT   
  
  
+ CGATGTGGAT GGACATTGGG CAATTCAGGG ATGGAAGGGG CGGATTGCAC ATGCGATCTC TGCATGGGTT   
  
  
+ CCGGCTTG  

- -Up\_Stream \_Len000CTAAAC TAACAGCTTT AAACAGGCTA AAATATTTTA ACTTTTAACA ATAACCTGAT   
  
  
- ATTAGATGAC ATCAAATCTC CACAACCTAT TATGAGAGTT GAAACTTGAA CCTGCAGGTA CGTGGTGTGA   
  
  
- TACCCGCATA AATGACAAAT ATATGGTTCT TATTAAAACT TCTACAAGAA TCAACCACTA CATTCACATA   
  
  
- TAAGTTGTAT ACAGTAATGT ATTTGAACTT TTAAGAACTT AAAATCTGTA TATGTTAACG TTTAGTAACA   
  
  
- TTTTAACAAC GTAATGTAAA ACTATTTTTA CGTAGGTTTG AAACAGAACC ATATTTGTCA TTTATCCGGG   
  
  
- TATCCAGGTA TCACATCACG TACCTGCAGG TTCAAATTTC CACCCTCATA ATAACGGGTT GCGGGGATTT   
  
  
- CATGTCATAT AATATCAGGT TATAGGGTTA ATTAAAAAGT AAACTTATAA ATTTAAACCT GTATTATAAA   
  
  
- AAGTATAAAC TTAAGAGATT TGGACTAAAA TTGGACTTAA ATTCAACTAA ATTAAGTAGG CTGGTTTTTT   
  
  
- TATTTGTGTT AATAAATAAA AAGATAAGCT TCAATCTATC ATCGGTTAAG TAAACTGTAT ATTAATTGGG   
  
  
- CACTAACTGT GTTTGAGCTC AAAAACGAAT CTCGATCCTT TTCTCAGCCC AATCCAGCTG GGTAAATTCT   
  
  
- TTACCCAGCT GAAGTACAGC CGGAAGTTTT ATCAATTCTA AACGAGTTTT GATTTTTTAT TGTTCAAGTG   
  
  
- TGATTTGCCA GTTTAGCACA GCTGAAGCCT AACTTTAACC AAGTCAAACC CAGCCGAAGC ATAAAGAAAG   
  
  
- TCATTGAAGC TTGGCTTAAG CATAGCACAA TAGCACAGTC CAGTCTGAAA GAGTCAAGAT AAAAACGATG   
  
  
- GAATACAATG CCTATTTTTT TCAGAGTGTA ACCTTTTTCA CACCCTCCCA GGACCCGAAT ATTTATCACC   
  
  
- GCGACGTGTG GAGTAGTCTG GCTGGAAAAC CCCTCTCCCA TTACTGGGGC ACTGTTCACC ATAGTCTCAG   
  
  
- TTGGGTCTGG GCTGAGTCTA CAGGACGGTT CGCGAGGCTG CCCGCCCCAT CCGTCCCCCG GGTAAACACT   
  
  
- ACTCCTGCAG TGTCTTAACC CGCCCCCTCT CACAGTGCCT ATTTTTTTCA GGGTGTAGCT TTTTTCACAC   
  
  
- CCTCCCAGGA CCCGAATATT TGCCATCGCG GCGTGTGGGG TAGTCTAGCT GAAAAACCCC TCTCCCATTA   
  
  
- CTGGGGTAAA GTTCACCATA GTCTCGGTTG GGTCTGGGCT GAGGTTACAG GGTGGTTCGC GAGACTACCC   
  
  
- GCCCCATCCG TTCCCCGAGG TATACACTGC TCCCGCAGTG CCTTAACCTA CCCTCTCTCA TGGTGCCTAT   
  
  
- TTTTTTCAGG GTGTAGTCTT TTTCACACCT GAATCGTTGA TCCTTCATTT CAATGTCATA ATCGGCTCTC   
  
  
- GGGTACCTTG CGGACTTTGA CTGTAGTGGT CTGGGTGTAT AAAGTGGTAG TAGACTAGAG GGGCAGTTTC   
  
  
- CTCCTTCGAC TCGTGTCGTT CTTGATCTTC TTGTCCGCCG TAGTGTGGGT TCGGTTTTAA GAGATAAACT   
  
  
- GGAGGGAGAG GAGTAAGGAG ACTCGAAGGA GAAGTGTTAC ATTCTGGTAT TAGGAATTGT GGGAAGAAAA   
  
  
- AATTCGAGTA GAAATGGACG AGACCAAGAG AGAGAGTAGC AGTGATTATC AATGGAGAAA TGAAAGAGAA   
  
  
- GGGACTCCAT ACGAAGTTGA AATTGTATAT ATAGGGTGAA CTAAAGAGAA CAGAAAAACG ATATTATGAA   
  
  
- CAACAAACCG ATAGTAGGGG ACAAAATATG AGTAAAAGAA CGAAAGATAA AGACCCAAAC TTAACCCAAA   
  
  
- GTGAATAGGA GCAGTTAAGA TACCCATTAA TCACTTAACC CAAATATACT TGATCCCAAC GTTTTCATAG   
  
  
- ATGAAAAAGA GAACACTAAT AATCAACTAA TCCCTAAACC GGTTATAATC GTCTTACCCA AGACTTAAAC   
  
  
- GCCTTAAGAG ACTACTACGA GATTTACCCA TACGAATACA ACTATAGGGA CGAATACTAC GTAGGAATCT   
  
  
- AATACGGTTA AACAAGTTAA TGCTTCCGGG TAGACTCCTA GCGTGGAGTG ACTCGGAGGG TTCTGGGAAA   
  
  
- AGACTGGGAA ACCGAATGTC AAAGTGCAGG AGAAGACTCG ACCCGGGACC CCAACTAAGA TTACTACTAT   
  
  
- CACTAAGACC GCTACAAGAG TTCATGTAAT CGGTTTACGA ATACCTTCTT CTGTACCTCC GTTTCGGTAC   
  
  
- GTACAAAGTA CTAGGAAATC GTGAAGTCCG ACGACTCTTT GGGAAAATAC TACGGAATCC CCTCGTTACG   
  
  
- GGTTGAAGAG GACTGGTTGT AGGATATTAA CTAGTAATAA ACCTATCAGG ACTACTTTTA AGAAACTCAA   
  
  
- GTTGACCACT AAAATCACAA TCAGTAAGAC CCAGATCAAG TTGTTTGAGG CAACCTGGCT ATTATCACAG   
  
  
- ACTAAACTCA CTCGTAAAAC TCGGTGGGAA ACAACTTTGT GAAGGTTAAC TTAGTATAGT GGTTGGTAAC   
  
  
- TGGGCAGGAG TTACCAGTAA ACCGAGAAGC CCACGGAATC TACCGTGCCG GAGATTACCA AGCCACTAGA   
  
  
- GTAGGGAACC AAACGGTCAC CTACACTAAT CGCATAAATC CCTCTTTCTC AGGTACTAAG TTAAAGTCTT   
  
  
- TCCCCACCTC CTCCGGTCAT TCAAGGAAGG GTTCTTATTA TTGGAACAAT AACTAGAGCT CTTGGAGTGA   
  
  
- AAAGGATTAC TTTGTTTCCT CCTACTAGCT TACTACTACC AATTCTTCCT TTTCCTACTG CACTTAACCA   
  
  
- GATTGATGAG TTCTCCCTCA TTCTAAGTGA TAACACTTCT GCTCCGGAAA CTTCTTCCTT CCTCACCGTT   
  
  
- CGTCAGTCGA TAAAGAAGAT GACTCCTTCG ACTCAAAAGA CTGTAAAAAC TGTTCCAAAA CGAAACGCTA   
  
  
- ACGATGGGAC ACTTTGGACT CCGAGTAGGG TGGTACTCAA ACTTGGGACT CTTCCCGGTC AGTGTACCTA   
  
  
- ATCTTCCACC CTCTTTACCC TTCCGAGCGG GATTCTTTGT CCTATTATCA TCGTGTTTAT AACACCTAAA   
  
  
- TTCCTTAAAC GACTATGATA CGCGTGTTAG ATGACGTAGA CTACTGGCTT CTTGACGACT ACCTGACGAT   
  
  
- TTCGTTTAGT CCCTCGTGAG CAGACGACTC CCCCTACCTA GAGTTTCCAA CCGCGTAATG AAACGACTAC   
  
  
- GGGATCTCCG TGCAAATCGA CCTTGACCTA GAGCGTAAAT ATGCCGGGAT ACAAGATTAT CCGGTAGACA   
  
  
- GTGACTGTAC TATTTTCGTA TAGTCAAGAT ATAAGCACGA ACGGGTAAAT GCTTCTAGCA GTAACCAACA   
  
  
- CCATGGGTAT ACTAAGATTT TCGTCGACTC TTCCGTAGTT TCGAAGTATA ATATCTAAAA CCGTAGGAGA   
  
  
- TACCAACGGT TACCGGGTTG GAGTAAGTTG CGGAGAGTCT CGCTAGACCA CCTGGAGGTT TTGACAAATA   
  
  
- ATGTCCCTAG CTAGAGGGGG TCGGACCCAA GTCCGGTCGT CTTTCTCACC TTCGTTGTCC CTCTGCGAAC   
  
  
- CGGTTCATGA CACTCGCCAT ATTACACGGT AAACTCATAG TACGGTAACG AGTCCTCACC CTTTGTTAGT   
  
  
- TTGGTCCCCT AGAGTTCTAT CCTTCATCCT TACTACTTCA ACAACGCCAC TTGACAGAGA CATCCAAGTT   
  
  
- CTTGGAGGAA CTGCTCTGTC ACCACCACCT ATCAGGTTCC TTGTGTCAAA ATTTGGACTA ATGTTCCCAT   
  
  
- TTCGGACTAT AAAAACACGT ACCGCAACAT TTACCAAGGA TGTTGTAGGG AAAGAAACAC TGTGCAAAAT   
  
  
- CTCTTCGGGA GAAAGTAATA AGGTGAGAAA AGCTGTACAA TCTACGGTTG CGGAGATCCC TCCTCGGGCT   
  
  
- CTCCAACTAT AAGCTCTTCC GTAAGATACC CTCCCTCTAA TACTTACACC ACCGGACACT CCCGTGTCTC   
  
  
- TCCCACCTCT CCGGCCTCTG TATGTTCGTT ACCGTACAAT CCGTATCGTC CCGTCCCAAA GCCGTTCATG   
  
  
- GTAACCTAGG GTTCAACTAG CTCTTTTACT CCAAATTCCG GTTCCGTCTG GTGGTGTTCC TAAAGTACTA   
  
  
- GCTACACCTA CCTGTAACCC GTTAAGTCCC TACCTTCCCC GCCTAACGTG TACGCTAGAG ACGTACCCAA   
  
  
- GGCCGAAC

+     CAAT-box

| Site Name | Organism | Position | Strand | Matrix score. | sequence | function |
| --- | --- | --- | --- | --- | --- | --- |
| CAAT-box | Nicotiana glutinosa | 3513 | + | 4 | CAAT |  |
| CAAT-box | Arabidopsis thaliana | 2494 | + | 10 | CAACCAACTCC | common cis-acting element in promoter and enhancer regions |
| CAAT-box | Nicotiana glutinosa | 2591 | - | 4 | CAAT |  |
| CAAT-box | Pisum sativum | 2527 | - | 5 | CAAAT | common cis-acting element in promoter and enhancer regions |
| CAAT-box | Pisum sativum | 2422 | - | 5 | CAAAT | common cis-acting element in promoter and enhancer regions |
| CAAT-box | Nicotiana glutinosa | 3709 | + | 4 | CAAT |  |
| CAAT-box | Nicotiana glutinosa | 2604 | + | 4 | CAAT |  |
| CAAT-box | Nicotiana glutinosa | 2412 | - | 4 | CAAT |  |
| CAAT-box | Arabidopsis thaliana | 2869 | - | 5 | CCAAT | common cis-acting element in promoter and enhancer regions |
| CAAT-box | Pisum sativum | 2277 | + | 5 | CAAAT | common cis-acting element in promoter and enhancer regions |
| CAAT-box | Nicotiana glutinosa | 2724 | + | 4 | CAAT |  |
| CAAT-box | Pisum sativum | 2612 | - | 5 | CAAAT | common cis-acting element in promoter and enhancer regions |
| CAAT-box | Nicotiana glutinosa | 2120 | + | 4 | CAAT |  |
| CAAT-box | Pisum sativum | 3674 | - | 5 | CAAAT | common cis-acting element in promoter and enhancer regions |
| CAAT-box | Nicotiana glutinosa | 4248 | - | 4 | CAAT |  |
| CAAT-box | Nicotiana glutinosa | 2111 | + | 4 | CAAT |  |
| CAAT-box | Arabidopsis thaliana | 608 | + | 5 | CCAAT | common cis-acting element in promoter and enhancer regions |
| CAAT-box | Pisum sativum | 3228 | + | 5 | CAAAT | common cis-acting element in promoter and enhancer regions |
| CAAT-box | Nicotiana glutinosa | 2571 | - | 4 | CAAT |  |
| CAAT-box | Nicotiana glutinosa | 572 | + | 4 | CAAT |  |
| CAAT-box | Nicotiana glutinosa | 2569 | + | 4 | CAAT |  |
| CAAT-box | Arabidopsis thaliana | 66 | - | 5 | CCAAT | common cis-acting element in promoter and enhancer regions |
| CAAT-box | Nicotiana glutinosa | 3180 | + | 4 | CAAT |  |
| CAAT-box | Arabidopsis thaliana | 4219 | - | 5 | CCAAT | common cis-acting element in promoter and enhancer regions |
| CAAT-box | Arabidopsis thaliana | 3426 | - | 5 | CCAAT | common cis-acting element in promoter and enhancer regions |
| CAAT-box | Nicotiana glutinosa | 1308 | + | 4 | CAAT |  |
| CAAT-box | Arabidopsis thaliana | 1140 | - | 5 | CCAAT | common cis-acting element in promoter and enhancer regions |
| CAAT-box | Arabidopsis thaliana | 943 | - | 5 | CCAAT | common cis-acting element in promoter and enhancer regions |
| CAAT-box | Pisum sativum | 34 | - | 5 | CAAAT | common cis-acting element in promoter and enhancer regions |
| CAAT-box | Nicotiana glutinosa | 60 | - | 4 | CAAT |  |
| CAAT-box | Nicotiana glutinosa | 25 | - | 4 | CAAT |  |
| CAAT-box | Nicotiana glutinosa | 2379 | + | 4 | CAAT |  |
| CAAT-box | Nicotiana glutinosa | 4225 | + | 4 | CAAT |  |
| CAAT-box | Nicotiana glutinosa | 4092 | + | 4 | CAAT |  |
| CAAT-box | Nicotiana glutinosa | 2006 | + | 4 | CAAT |  |
| CAAT-box | Arabidopsis thaliana | 2005 | + | 5 | CCAAT | common cis-acting element in promoter and enhancer regions |
| CAAT-box | Nicotiana glutinosa | 2905 | - | 4 | CAAT |  |
| CAAT-box | Pisum sativum | 1609 | - | 5 | CAAAT | common cis-acting element in promoter and enhancer regions |
| CAAT-box | Arabidopsis thaliana | 2568 | + | 5 | CCAAT | common cis-acting element in promoter and enhancer regions |
| CAAT-box | Pisum sativum | 3160 | - | 5 | CAAAT | common cis-acting element in promoter and enhancer regions |
| CAAT-box | Pisum sativum | 2113 | - | 5 | CAAAT | common cis-acting element in promoter and enhancer regions |
| CAAT-box | Arabidopsis thaliana | 4136 | - | 5 | CCAAT | common cis-acting element in promoter and enhancer regions |
| CAAT-box | Nicotiana glutinosa | 804 | - | 4 | CAAT |  |
| CAAT-box | Nicotiana glutinosa | 2784 | - | 4 | CAAT |  |
| CAAT-box | Arabidopsis thaliana | 2110 | + | 5 | CCAAT | common cis-acting element in promoter and enhancer regions |
| CAAT-box | Pisum sativum | 2030 | - | 5 | CAAAT | common cis-acting element in promoter and enhancer regions |
| CAAT-box | Pisum sativum | 785 | + | 5 | CAAAT | common cis-acting element in promoter and enhancer regions |
| CAAT-box | Pisum sativum | 274 | + | 5 | CAAAT | common cis-acting element in promoter and enhancer regions |
| CAAT-box | Pisum sativum | 500 | - | 5 | CAAAT | common cis-acting element in promoter and enhancer regions |
| CAAT-box | Arabidopsis thaliana | 3512 | + | 5 | CCAAT | common cis-acting element in promoter and enhancer regions |
| CAAT-box | Arabidopsis thaliana | 1930 | - | 5 | CCAAT | common cis-acting element in promoter and enhancer regions |
| CAAT-box | Nicotiana glutinosa | 3144 | - | 4 | CAAT |  |
| CAAT-box | Pisum sativum | 20 | - | 5 | CAAAT | common cis-acting element in promoter and enhancer regions |
| CAAT-box | Nicotiana glutinosa | 3690 | - | 4 | CAAT |  |
| CAAT-box | Nicotiana glutinosa | 3013 | - | 4 | CAAT |  |
| CAAT-box | Pisum sativum | 3139 | + | 5 | CAAAT | common cis-acting element in promoter and enhancer regions |
| CAAT-box | Petunia hybrida | 3968 | + | 7 | TGCCAAC | common cis-acting element in promoter and enhancer regions |
| CAAT-box | Arabidopsis thaliana | 1307 | + | 5 | CCAAT | common cis-acting element in promoter and enhancer regions |
| CAAT-box | Nicotiana glutinosa | 268 | + | 4 | CAAT |  |
| CAAT-box | Arabidopsis thaliana | 1886 | - | 5 | CCAAT | common cis-acting element in promoter and enhancer regions |
| CAAT-box | Arabidopsis thaliana | 810 | - | 5 | CCAAT | common cis-acting element in promoter and enhancer regions |
| CAAT-box | Pisum sativum | 743 | - | 5 | CAAAT | common cis-acting element in promoter and enhancer regions |
| CAAT-box | Nicotiana glutinosa | 443 | + | 4 | CAAT |  |
| CAAT-box | Nicotiana glutinosa | 53 | - | 4 | CAAT |  |
| CAAT-box | Pisum sativum | 615 | - | 5 | CAAAT | common cis-acting element in promoter and enhancer regions |
| CAAT-box | Nicotiana glutinosa | 638 | - | 4 | CAAT |  |
| CAAT-box | Pisum sativum | 1117 | - | 5 | CAAAT | common cis-acting element in promoter and enhancer regions |
| CAAT-box | Arabidopsis thaliana | 450 | + | 5 | CCAAT | common cis-acting element in promoter and enhancer regions |
| CAAT-box | Nicotiana glutinosa | 288 | - | 4 | CAAT |  |
| CAAT-box | Nicotiana glutinosa | 280 | - | 4 | CAAT |  |
| CAAT-box | Nicotiana glutinosa | 1650 | + | 4 | CAAT |  |
| CAAT-box | Nicotiana glutinosa | 270 | - | 4 | CAAT |  |
| CAAT-box | Nicotiana glutinosa | 406 | - | 4 | CAAT |  |
| CAAT-box | Pisum sativum | 1999 | - | 5 | CAAAT | common cis-acting element in promoter and enhancer regions |
| CAAT-box | Arabidopsis thaliana | 1378 | - | 5 | CCAAT | common cis-acting element in promoter and enhancer regions |
| CAAT-box | Nicotiana glutinosa | 609 | + | 4 | CAAT |  |
| CAAT-box | Nicotiana glutinosa | 1908 | + | 4 | CAAT |  |
| CAAT-box | Arabidopsis thaliana | 442 | + | 5 | CCAAT | common cis-acting element in promoter and enhancer regions |
| CAAT-box | Pisum sativum | 478 | - | 5 | CAAAT | common cis-acting element in promoter and enhancer regions |
| CAAT-box | Pisum sativum | 464 | - | 5 | CAAAT | common cis-acting element in promoter and enhancer regions |
| CAAT-box | Nicotiana glutinosa | 451 | + | 4 | CAAT |  |

>HU07G02249.1   
+ -Up\_Stream \_Len000GATTTG ATTGTCGAAA TTTGTCCGAT TTTATAAAAT TGAAAATTGT TATTGGACTA   
  
  
+ TAATCTACTG TAGTTTAGAG GTGTTGGATA ATACTCTCAA CTTTGAACTT GGACGTCCAT GCACCACACT   
  
  
+ ATGGGCGTAT TTACTGTTTA TATACCAAGA ATAATTTTGA AGATGTTCTT AGTTGGTGAT GTAAGTGTAT   
  
  
+ ATTCAACATA TGTCATTACA TAAACTTGAA AATTCTTGAA TTTTAGACAT ATACAATTGC AAATCATTGT   
  
  
+ AAAATTGTTG CATTACATTT TGATAAAAAT GCATCCAAAC TTTGTCTTGG TATAAACAGT AAATAGGCCC   
  
  
+ ATAGGTCCAT AGTGTAGTGC ATGGACGTCC AAGTTTAAAG GTGGGAGTAT TATTGCCCAA CGCCCCTAAA   
  
  
+ GTACAGTATA TTATAGTCCA ATATCCCAAT TAATTTTTCA TTTGAATATT TAAATTTGGA CATAATATTT   
  
  
+ TTCATATTTG AATTCTCTAA ACCTGATTTT AACCTGAATT TAAGTTGATT TAATTCATCC GACCAAAAAA   
  
  
+ ATAAACACAA TTATTTATTT TTCTATTCGA AGTTAGATAG TAGCCAATTC ATTTGACATA TAATTAACCC   
  
  
+ GTGATTGACA CAAACTCGAG TTTTTGCTTA GAGCTAGGAA AAGAGTCGGG TTAGGTCGAC CCATTTAAGA   
  
  
+ AATGGGTCGA CTTCATGTCG GCCTTCAAAA TAGTTAAGAT TTGCTCAAAA CTAAAAAATA ACAAGTTCAC   
  
  
+ ACTAAACGGT CAAATCGTGT CGACTTCGGA TTGAAATTGG TTCAGTTTGG GTCGGCTTCG TATTTCTTTC   
  
  
+ AGTAACTTCG AACCGAATTC GTATCGTGTT ATCGTGTCAG GTCAGACTTT CTCAGTTCTA TTTTTGCTAC   
  
  
+ CTTATGTTAC GGATAAAAAA AGTCTCACAT TGGAAAAAGT GTGGGAGGGT CCTGGGCTTA TAAATAGTGG   
  
  
+ CGCTGCACAC CTCATCAGAC CGACCTTTTG GGGAGAGGGT AATGACCCCG TGACAAGTGG TATCAGAGTC   
  
  
+ AACCCAGACC CGACTCAGAT GTCCTGCCAA GCGCTCCGAC GGGCGGGGTA GGCAGGGGGC CCATTTGTGA   
  
  
+ TGAGGACGTC ACAGAATTGG GCGGGGGAGA GTGTCACGGA TAAAAAAAGT CCCACATCGA AAAAAGTGTG   
  
  
+ GGAGGGTCCT GGGCTTATAA ACGGTAGCGC CGCACACCCC ATCAGATCGA CTTTTTGGGG AGAGGGTAAT   
  
  
+ GACCCCATTT CAAGTGGTAT CAGAGCCAAC CCAGACCCGA CTCCAATGTC CCACCAAGCG CTCTGATGGG   
  
  
+ CGGGGTAGGC AAGGGGCTCC ATATGTGACG AGGGCGTCAC GGAATTGGAT GGGAGAGAGT ACCACGGATA   
  
  
+ AAAAAAGTCC CACATCAGAA AAAGTGTGGA CTTAGCAACT AGGAAGTAAA GTTACAGTAT TAGCCGAGAG   
  
  
+ CCCATGGAAC GCCTGAAACT GACATCACCA GACCCACATA TTTCACCATC ATCTGATCTC CCCGTCAAAG   
  
  
+ GAGGAAGCTG AGCACAGCAA GAACTAGAAG AACAGGCGGC ATCACACCCA AGCCAAAATT CTCTATTTGA   
  
  
+ CCTCCCTCTC CTCATTCCTC TGAGCTTCCT CTTCACAATG TAAGACCATA ATCCTTAACA CCCTTCTTTT   
  
  
+ TTAAGCTCAT CTTTACCTGC TCTGGTTCTC TCTCTCATCG TCACTAATAG TTACCTCTTT ACTTTCTCTT   
  
  
+ CCCTGAGGTA TGCTTCAACT TTAACATATA TATCCCACTT GATTTCTCTT GTCTTTTTGC TATAATACTT   
  
  
+ GTTGTTTGGC TATCATCCCC TGTTTTATAC TCATTTTCTT GCTTTCTATT TCTGGGTTTG AATTGGGTTT   
  
  
+ CACTTATCCT CGTCAATTCT ATGGGTAATT AGTGAATTGG GTTTATATGA ACTAGGGTTG CAAAAGTATC   
  
  
+ TACTTTTTCT CTTGTGATTA TTAGTTGATT AGGGATTTGG CCAATATTAG CAGAATGGGT TCTGAATTTG   
  
  
+ CGGAATTCTC TGATGATGCT CTAAATGGGT ATGCTTATGT TGATATCCCT GCTTATGATG CATCCTTAGA   
  
  
+ TTATGCCAAT TTGTTCAATT ACGAAGGCCC ATCTGAGGAT CGCACCTCAC TGAGCCTCCC AAGACCCTTT   
  
  
+ TCTGACCCTT TGGCTTACAG TTTCACGTCC TCTTCTGAGC TGGGCCCTGG GGTTGATTCT AATGATGATA   
  
  
+ GTGATTCTGG CGATGTTCTC AAGTACATTA GCCAAATGCT TATGGAAGAA GACATGGAGG CAAAGCCATG   
  
  
+ CATGTTTCAT GATCCTTTAG CACTTCAGGC TGCTGAGAAA CCCTTTTATG ATGCCTTAGG GGAGCAATGC   
  
  
+ CCAACTTCTC CTGACCAACA TCCTATAATT GATCATTATT TGGATAGTCC TGATGAAAAT TCTTTGAGTT   
  
  
+ CAACTGGTGA TTTTAGTGTT AGTCATTCTG GGTCTAGTTC AACAAACTCC GTTGGACCGA TAATAGTGTC   
  
  
+ TGATTTGAGT GAGCATTTTG AGCCACCCTT TGTTGAAACA CTTCCAATTG AATCATATCA CCAACCATTG   
  
  
+ ACCCGTCCTC AATGGTCATT TGGCTCTTCG GGTGCCTTAG ATGGCACGGC CTCTAATGGT TCGGTGATCT   
  
  
+ CATCCCTTGG TTTGCCAGTG GATGTGATTA GCGTATTTAG GGAGAAAGAG TCCATGATTC AATTTCAGAA   
  
  
+ AGGGGTGGAG GAGGCCAGTA AGTTCCTTCC CAAGAATAAT AACCTTGTTA TTGATCTCGA GAACCTCACT   
  
  
+ TTTCCTAATG AAACAAAGGA GGATGATCGA ATGATGATGG TTAAGAAGGA AAAGGATGAC GTGAATTGGT   
  
  
+ CTAACTACTC AAGAGGGAGT AAGATTCACT ATTGTGAAGA CGAGGCCTTT GAAGAAGGAA GGAGTGGCAA   
  
  
+ GCAGTCAGCT ATTTCTTCTA CTGAGGAAGC TGAGTTTTCT GACATTTTTG ACAAGGTTTT GCTTTGCGAT   
  
  
+ TGCTACCCTG TGAAACCTGA GGCTCATCCC ACCATGAGTT TGAACCCTGA GAAGGGCCAG TCACATGGAT   
  
  
+ TAGAAGGTGG GAGAAATGGG AAGGCTCGCC CTAAGAAACA GGATAATAGT AGCACAAATA TTGTGGATTT   
  
  
+ AAGGAATTTG CTGATACTAT GCGCACAATC TACTGCATCT GATGACCGAA GAACTGCTGA TGGACTGCTA   
  
  
+ AAGCAAATCA GGGAGCACTC GTCTGCTGAG GGGGATGGAT CTCAAAGGTT GGCGCATTAC TTTGCTGATG   
  
  
+ CCCTAGAGGC ACGTTTAGCT GGAACTGGAT CTCGCATTTA TACGGCCCTA TGTTCTAATA GGCCATCTGT   
  
  
+ CACTGACATG ATAAAAGCAT ATCAGTTCTA TATTCGTGCT TGCCCATTTA CGAAGATCGT CATTGGTTGT   
  
  
+ GGTACCCATA TGATTCTAAA AGCAGCTGAG AAGGCATCAA AGCTTCATAT TATAGATTTT GGCATCCTCT   
  
  
+ ATGGTTGCCA ATGGCCCAAC CTCATTCAAC GCCTCTCAGA GCGATCTGGT GGACCTCCAA AACTGTTTAT   
  
  
+ TACAGGGATC GATCTCCCCC AGCCTGGGTT CAGGCCAGCA GAAAGAGTGG AAGCAACAGG GAGACGCTTG   
  
  
+ GCCAAGTACT GTGAGCGGTA TAATGTGCCA TTTGAGTATC ATGCCATTGC TCAGGAGTGG GAAACAATCA   
  
  
+ AACCAGGGGA TCTCAAGATA GGAAGTAGGA ATGATGAAGT TGTTGCGGTG AACTGTCTCT GTAGGTTCAA   
  
  
+ GAACCTCCTT GACGAGACAG TGGTGGTGGA TAGTCCAAGG AACACAGTTT TAAACCTGAT TACAAGGGTA   
  
  
+ AAGCCTGATA TTTTTGTGCA TGGCGTTGTA AATGGTTCCT ACAACATCCC TTTCTTTGTG ACACGTTTTA   
  
  
+ GAGAAGCCCT CTTTCATTAT TCCACTCTTT TCGACATGTT AGATGCCAAC GCCTCTAGGG AGGAGCCCGA   
  
  
+ GAGGTTGATA TTCGAGAAGG CATTCTATGG GAGGGAGATT ATGAATGTGG TGGCCTGTGA GGGCACAGAG   
  
  
+ AGGGTGGAGA GGCCGGAGAC ATACAAGCAA TGGCATGTTA GGCATAGCAG GGCAGGGTTT CGGCAAGTAC   
  
  
+ CATTGGATCC CAAGTTGATC GAGAAAATGA GGTTTAAGGC CAAGGCAGAC CACCACAAGG ATTTCATGAT   
  
  
+ CGATGTGGAT GGACATTGGG CAATTCAGGG ATGGAAGGGG CGGATTGCAC ATGCGATCTC TGCATGGGTT   
  
  
+ CCGGCTTG  

- -Up\_Stream \_Len000CTAAAC TAACAGCTTT AAACAGGCTA AAATATTTTA ACTTTTAACA ATAACCTGAT   
  
  
- ATTAGATGAC ATCAAATCTC CACAACCTAT TATGAGAGTT GAAACTTGAA CCTGCAGGTA CGTGGTGTGA   
  
  
- TACCCGCATA AATGACAAAT ATATGGTTCT TATTAAAACT TCTACAAGAA TCAACCACTA CATTCACATA   
  
  
- TAAGTTGTAT ACAGTAATGT ATTTGAACTT TTAAGAACTT AAAATCTGTA TATGTTAACG TTTAGTAACA   
  
  
- TTTTAACAAC GTAATGTAAA ACTATTTTTA CGTAGGTTTG AAACAGAACC ATATTTGTCA TTTATCCGGG   
  
  
- TATCCAGGTA TCACATCACG TACCTGCAGG TTCAAATTTC CACCCTCATA ATAACGGGTT GCGGGGATTT   
  
  
- CATGTCATAT AATATCAGGT TATAGGGTTA ATTAAAAAGT AAACTTATAA ATTTAAACCT GTATTATAAA   
  
  
- AAGTATAAAC TTAAGAGATT TGGACTAAAA TTGGACTTAA ATTCAACTAA ATTAAGTAGG CTGGTTTTTT   
  
  
- TATTTGTGTT AATAAATAAA AAGATAAGCT TCAATCTATC ATCGGTTAAG TAAACTGTAT ATTAATTGGG   
  
  
- CACTAACTGT GTTTGAGCTC AAAAACGAAT CTCGATCCTT TTCTCAGCCC AATCCAGCTG GGTAAATTCT   
  
  
- TTACCCAGCT GAAGTACAGC CGGAAGTTTT ATCAATTCTA AACGAGTTTT GATTTTTTAT TGTTCAAGTG   
  
  
- TGATTTGCCA GTTTAGCACA GCTGAAGCCT AACTTTAACC AAGTCAAACC CAGCCGAAGC ATAAAGAAAG   
  
  
- TCATTGAAGC TTGGCTTAAG CATAGCACAA TAGCACAGTC CAGTCTGAAA GAGTCAAGAT AAAAACGATG   
  
  
- GAATACAATG CCTATTTTTT TCAGAGTGTA ACCTTTTTCA CACCCTCCCA GGACCCGAAT ATTTATCACC   
  
  
- GCGACGTGTG GAGTAGTCTG GCTGGAAAAC CCCTCTCCCA TTACTGGGGC ACTGTTCACC ATAGTCTCAG   
  
  
- TTGGGTCTGG GCTGAGTCTA CAGGACGGTT CGCGAGGCTG CCCGCCCCAT CCGTCCCCCG GGTAAACACT   
  
  
- ACTCCTGCAG TGTCTTAACC CGCCCCCTCT CACAGTGCCT ATTTTTTTCA GGGTGTAGCT TTTTTCACAC   
  
  
- CCTCCCAGGA CCCGAATATT TGCCATCGCG GCGTGTGGGG TAGTCTAGCT GAAAAACCCC TCTCCCATTA   
  
  
- CTGGGGTAAA GTTCACCATA GTCTCGGTTG GGTCTGGGCT GAGGTTACAG GGTGGTTCGC GAGACTACCC   
  
  
- GCCCCATCCG TTCCCCGAGG TATACACTGC TCCCGCAGTG CCTTAACCTA CCCTCTCTCA TGGTGCCTAT   
  
  
- TTTTTTCAGG GTGTAGTCTT TTTCACACCT GAATCGTTGA TCCTTCATTT CAATGTCATA ATCGGCTCTC   
  
  
- GGGTACCTTG CGGACTTTGA CTGTAGTGGT CTGGGTGTAT AAAGTGGTAG TAGACTAGAG GGGCAGTTTC   
  
  
- CTCCTTCGAC TCGTGTCGTT CTTGATCTTC TTGTCCGCCG TAGTGTGGGT TCGGTTTTAA GAGATAAACT   
  
  
- GGAGGGAGAG GAGTAAGGAG ACTCGAAGGA GAAGTGTTAC ATTCTGGTAT TAGGAATTGT GGGAAGAAAA   
  
  
- AATTCGAGTA GAAATGGACG AGACCAAGAG AGAGAGTAGC AGTGATTATC AATGGAGAAA TGAAAGAGAA   
  
  
- GGGACTCCAT ACGAAGTTGA AATTGTATAT ATAGGGTGAA CTAAAGAGAA CAGAAAAACG ATATTATGAA   
  
  
- CAACAAACCG ATAGTAGGGG ACAAAATATG AGTAAAAGAA CGAAAGATAA AGACCCAAAC TTAACCCAAA   
  
  
- GTGAATAGGA GCAGTTAAGA TACCCATTAA TCACTTAACC CAAATATACT TGATCCCAAC GTTTTCATAG   
  
  
- ATGAAAAAGA GAACACTAAT AATCAACTAA TCCCTAAACC GGTTATAATC GTCTTACCCA AGACTTAAAC   
  
  
- GCCTTAAGAG ACTACTACGA GATTTACCCA TACGAATACA ACTATAGGGA CGAATACTAC GTAGGAATCT   
  
  
- AATACGGTTA AACAAGTTAA TGCTTCCGGG TAGACTCCTA GCGTGGAGTG ACTCGGAGGG TTCTGGGAAA   
  
  
- AGACTGGGAA ACCGAATGTC AAAGTGCAGG AGAAGACTCG ACCCGGGACC CCAACTAAGA TTACTACTAT   
  
  
- CACTAAGACC GCTACAAGAG TTCATGTAAT CGGTTTACGA ATACCTTCTT CTGTACCTCC GTTTCGGTAC   
  
  
- GTACAAAGTA CTAGGAAATC GTGAAGTCCG ACGACTCTTT GGGAAAATAC TACGGAATCC CCTCGTTACG   
  
  
- GGTTGAAGAG GACTGGTTGT AGGATATTAA CTAGTAATAA ACCTATCAGG ACTACTTTTA AGAAACTCAA   
  
  
- GTTGACCACT AAAATCACAA TCAGTAAGAC CCAGATCAAG TTGTTTGAGG CAACCTGGCT ATTATCACAG   
  
  
- ACTAAACTCA CTCGTAAAAC TCGGTGGGAA ACAACTTTGT GAAGGTTAAC TTAGTATAGT GGTTGGTAAC   
  
  
- TGGGCAGGAG TTACCAGTAA ACCGAGAAGC CCACGGAATC TACCGTGCCG GAGATTACCA AGCCACTAGA   
  
  
- GTAGGGAACC AAACGGTCAC CTACACTAAT CGCATAAATC CCTCTTTCTC AGGTACTAAG TTAAAGTCTT   
  
  
- TCCCCACCTC CTCCGGTCAT TCAAGGAAGG GTTCTTATTA TTGGAACAAT AACTAGAGCT CTTGGAGTGA   
  
  
- AAAGGATTAC TTTGTTTCCT CCTACTAGCT TACTACTACC AATTCTTCCT TTTCCTACTG CACTTAACCA   
  
  
- GATTGATGAG TTCTCCCTCA TTCTAAGTGA TAACACTTCT GCTCCGGAAA CTTCTTCCTT CCTCACCGTT   
  
  
- CGTCAGTCGA TAAAGAAGAT GACTCCTTCG ACTCAAAAGA CTGTAAAAAC TGTTCCAAAA CGAAACGCTA   
  
  
- ACGATGGGAC ACTTTGGACT CCGAGTAGGG TGGTACTCAA ACTTGGGACT CTTCCCGGTC AGTGTACCTA   
  
  
- ATCTTCCACC CTCTTTACCC TTCCGAGCGG GATTCTTTGT CCTATTATCA TCGTGTTTAT AACACCTAAA   
  
  
- TTCCTTAAAC GACTATGATA CGCGTGTTAG ATGACGTAGA CTACTGGCTT CTTGACGACT ACCTGACGAT   
  
  
- TTCGTTTAGT CCCTCGTGAG CAGACGACTC CCCCTACCTA GAGTTTCCAA CCGCGTAATG AAACGACTAC   
  
  
- GGGATCTCCG TGCAAATCGA CCTTGACCTA GAGCGTAAAT ATGCCGGGAT ACAAGATTAT CCGGTAGACA   
  
  
- GTGACTGTAC TATTTTCGTA TAGTCAAGAT ATAAGCACGA ACGGGTAAAT GCTTCTAGCA GTAACCAACA   
  
  
- CCATGGGTAT ACTAAGATTT TCGTCGACTC TTCCGTAGTT TCGAAGTATA ATATCTAAAA CCGTAGGAGA   
  
  
- TACCAACGGT TACCGGGTTG GAGTAAGTTG CGGAGAGTCT CGCTAGACCA CCTGGAGGTT TTGACAAATA   
  
  
- ATGTCCCTAG CTAGAGGGGG TCGGACCCAA GTCCGGTCGT CTTTCTCACC TTCGTTGTCC CTCTGCGAAC   
  
  
- CGGTTCATGA CACTCGCCAT ATTACACGGT AAACTCATAG TACGGTAACG AGTCCTCACC CTTTGTTAGT   
  
  
- TTGGTCCCCT AGAGTTCTAT CCTTCATCCT TACTACTTCA ACAACGCCAC TTGACAGAGA CATCCAAGTT   
  
  
- CTTGGAGGAA CTGCTCTGTC ACCACCACCT ATCAGGTTCC TTGTGTCAAA ATTTGGACTA ATGTTCCCAT   
  
  
- TTCGGACTAT AAAAACACGT ACCGCAACAT TTACCAAGGA TGTTGTAGGG AAAGAAACAC TGTGCAAAAT   
  
  
- CTCTTCGGGA GAAAGTAATA AGGTGAGAAA AGCTGTACAA TCTACGGTTG CGGAGATCCC TCCTCGGGCT   
  
  
- CTCCAACTAT AAGCTCTTCC GTAAGATACC CTCCCTCTAA TACTTACACC ACCGGACACT CCCGTGTCTC   
  
  
- TCCCACCTCT CCGGCCTCTG TATGTTCGTT ACCGTACAAT CCGTATCGTC CCGTCCCAAA GCCGTTCATG   
  
  
- GTAACCTAGG GTTCAACTAG CTCTTTTACT CCAAATTCCG GTTCCGTCTG GTGGTGTTCC TAAAGTACTA   
  
  
- GCTACACCTA CCTGTAACCC GTTAAGTCCC TACCTTCCCC GCCTAACGTG TACGCTAGAG ACGTACCCAA   
  
  
- GGCCGAAC

+     CAT-box

| Site Name | Organism | Position | Strand | Matrix score. | sequence | function |
| --- | --- | --- | --- | --- | --- | --- |
| CAT-box | Arabidopsis thaliana | 2937 | - | 6 | GCCACT | cis-acting regulatory element related to meristem expression |
| CAT-box | Arabidopsis thaliana | 980 | - | 6 | GCCACT | cis-acting regulatory element related to meristem expression |

>HU07G02249.1   
+ -Up\_Stream \_Len000GATTTG ATTGTCGAAA TTTGTCCGAT TTTATAAAAT TGAAAATTGT TATTGGACTA   
  
  
+ TAATCTACTG TAGTTTAGAG GTGTTGGATA ATACTCTCAA CTTTGAACTT GGACGTCCAT GCACCACACT   
  
  
+ ATGGGCGTAT TTACTGTTTA TATACCAAGA ATAATTTTGA AGATGTTCTT AGTTGGTGAT GTAAGTGTAT   
  
  
+ ATTCAACATA TGTCATTACA TAAACTTGAA AATTCTTGAA TTTTAGACAT ATACAATTGC AAATCATTGT   
  
  
+ AAAATTGTTG CATTACATTT TGATAAAAAT GCATCCAAAC TTTGTCTTGG TATAAACAGT AAATAGGCCC   
  
  
+ ATAGGTCCAT AGTGTAGTGC ATGGACGTCC AAGTTTAAAG GTGGGAGTAT TATTGCCCAA CGCCCCTAAA   
  
  
+ GTACAGTATA TTATAGTCCA ATATCCCAAT TAATTTTTCA TTTGAATATT TAAATTTGGA CATAATATTT   
  
  
+ TTCATATTTG AATTCTCTAA ACCTGATTTT AACCTGAATT TAAGTTGATT TAATTCATCC GACCAAAAAA   
  
  
+ ATAAACACAA TTATTTATTT TTCTATTCGA AGTTAGATAG TAGCCAATTC ATTTGACATA TAATTAACCC   
  
  
+ GTGATTGACA CAAACTCGAG TTTTTGCTTA GAGCTAGGAA AAGAGTCGGG TTAGGTCGAC CCATTTAAGA   
  
  
+ AATGGGTCGA CTTCATGTCG GCCTTCAAAA TAGTTAAGAT TTGCTCAAAA CTAAAAAATA ACAAGTTCAC   
  
  
+ ACTAAACGGT CAAATCGTGT CGACTTCGGA TTGAAATTGG TTCAGTTTGG GTCGGCTTCG TATTTCTTTC   
  
  
+ AGTAACTTCG AACCGAATTC GTATCGTGTT ATCGTGTCAG GTCAGACTTT CTCAGTTCTA TTTTTGCTAC   
  
  
+ CTTATGTTAC GGATAAAAAA AGTCTCACAT TGGAAAAAGT GTGGGAGGGT CCTGGGCTTA TAAATAGTGG   
  
  
+ CGCTGCACAC CTCATCAGAC CGACCTTTTG GGGAGAGGGT AATGACCCCG TGACAAGTGG TATCAGAGTC   
  
  
+ AACCCAGACC CGACTCAGAT GTCCTGCCAA GCGCTCCGAC GGGCGGGGTA GGCAGGGGGC CCATTTGTGA   
  
  
+ TGAGGACGTC ACAGAATTGG GCGGGGGAGA GTGTCACGGA TAAAAAAAGT CCCACATCGA AAAAAGTGTG   
  
  
+ GGAGGGTCCT GGGCTTATAA ACGGTAGCGC CGCACACCCC ATCAGATCGA CTTTTTGGGG AGAGGGTAAT   
  
  
+ GACCCCATTT CAAGTGGTAT CAGAGCCAAC CCAGACCCGA CTCCAATGTC CCACCAAGCG CTCTGATGGG   
  
  
+ CGGGGTAGGC AAGGGGCTCC ATATGTGACG AGGGCGTCAC GGAATTGGAT GGGAGAGAGT ACCACGGATA   
  
  
+ AAAAAAGTCC CACATCAGAA AAAGTGTGGA CTTAGCAACT AGGAAGTAAA GTTACAGTAT TAGCCGAGAG   
  
  
+ CCCATGGAAC GCCTGAAACT GACATCACCA GACCCACATA TTTCACCATC ATCTGATCTC CCCGTCAAAG   
  
  
+ GAGGAAGCTG AGCACAGCAA GAACTAGAAG AACAGGCGGC ATCACACCCA AGCCAAAATT CTCTATTTGA   
  
  
+ CCTCCCTCTC CTCATTCCTC TGAGCTTCCT CTTCACAATG TAAGACCATA ATCCTTAACA CCCTTCTTTT   
  
  
+ TTAAGCTCAT CTTTACCTGC TCTGGTTCTC TCTCTCATCG TCACTAATAG TTACCTCTTT ACTTTCTCTT   
  
  
+ CCCTGAGGTA TGCTTCAACT TTAACATATA TATCCCACTT GATTTCTCTT GTCTTTTTGC TATAATACTT   
  
  
+ GTTGTTTGGC TATCATCCCC TGTTTTATAC TCATTTTCTT GCTTTCTATT TCTGGGTTTG AATTGGGTTT   
  
  
+ CACTTATCCT CGTCAATTCT ATGGGTAATT AGTGAATTGG GTTTATATGA ACTAGGGTTG CAAAAGTATC   
  
  
+ TACTTTTTCT CTTGTGATTA TTAGTTGATT AGGGATTTGG CCAATATTAG CAGAATGGGT TCTGAATTTG   
  
  
+ CGGAATTCTC TGATGATGCT CTAAATGGGT ATGCTTATGT TGATATCCCT GCTTATGATG CATCCTTAGA   
  
  
+ TTATGCCAAT TTGTTCAATT ACGAAGGCCC ATCTGAGGAT CGCACCTCAC TGAGCCTCCC AAGACCCTTT   
  
  
+ TCTGACCCTT TGGCTTACAG TTTCACGTCC TCTTCTGAGC TGGGCCCTGG GGTTGATTCT AATGATGATA   
  
  
+ GTGATTCTGG CGATGTTCTC AAGTACATTA GCCAAATGCT TATGGAAGAA GACATGGAGG CAAAGCCATG   
  
  
+ CATGTTTCAT GATCCTTTAG CACTTCAGGC TGCTGAGAAA CCCTTTTATG ATGCCTTAGG GGAGCAATGC   
  
  
+ CCAACTTCTC CTGACCAACA TCCTATAATT GATCATTATT TGGATAGTCC TGATGAAAAT TCTTTGAGTT   
  
  
+ CAACTGGTGA TTTTAGTGTT AGTCATTCTG GGTCTAGTTC AACAAACTCC GTTGGACCGA TAATAGTGTC   
  
  
+ TGATTTGAGT GAGCATTTTG AGCCACCCTT TGTTGAAACA CTTCCAATTG AATCATATCA CCAACCATTG   
  
  
+ ACCCGTCCTC AATGGTCATT TGGCTCTTCG GGTGCCTTAG ATGGCACGGC CTCTAATGGT TCGGTGATCT   
  
  
+ CATCCCTTGG TTTGCCAGTG GATGTGATTA GCGTATTTAG GGAGAAAGAG TCCATGATTC AATTTCAGAA   
  
  
+ AGGGGTGGAG GAGGCCAGTA AGTTCCTTCC CAAGAATAAT AACCTTGTTA TTGATCTCGA GAACCTCACT   
  
  
+ TTTCCTAATG AAACAAAGGA GGATGATCGA ATGATGATGG TTAAGAAGGA AAAGGATGAC GTGAATTGGT   
  
  
+ CTAACTACTC AAGAGGGAGT AAGATTCACT ATTGTGAAGA CGAGGCCTTT GAAGAAGGAA GGAGTGGCAA   
  
  
+ GCAGTCAGCT ATTTCTTCTA CTGAGGAAGC TGAGTTTTCT GACATTTTTG ACAAGGTTTT GCTTTGCGAT   
  
  
+ TGCTACCCTG TGAAACCTGA GGCTCATCCC ACCATGAGTT TGAACCCTGA GAAGGGCCAG TCACATGGAT   
  
  
+ TAGAAGGTGG GAGAAATGGG AAGGCTCGCC CTAAGAAACA GGATAATAGT AGCACAAATA TTGTGGATTT   
  
  
+ AAGGAATTTG CTGATACTAT GCGCACAATC TACTGCATCT GATGACCGAA GAACTGCTGA TGGACTGCTA   
  
  
+ AAGCAAATCA GGGAGCACTC GTCTGCTGAG GGGGATGGAT CTCAAAGGTT GGCGCATTAC TTTGCTGATG   
  
  
+ CCCTAGAGGC ACGTTTAGCT GGAACTGGAT CTCGCATTTA TACGGCCCTA TGTTCTAATA GGCCATCTGT   
  
  
+ CACTGACATG ATAAAAGCAT ATCAGTTCTA TATTCGTGCT TGCCCATTTA CGAAGATCGT CATTGGTTGT   
  
  
+ GGTACCCATA TGATTCTAAA AGCAGCTGAG AAGGCATCAA AGCTTCATAT TATAGATTTT GGCATCCTCT   
  
  
+ ATGGTTGCCA ATGGCCCAAC CTCATTCAAC GCCTCTCAGA GCGATCTGGT GGACCTCCAA AACTGTTTAT   
  
  
+ TACAGGGATC GATCTCCCCC AGCCTGGGTT CAGGCCAGCA GAAAGAGTGG AAGCAACAGG GAGACGCTTG   
  
  
+ GCCAAGTACT GTGAGCGGTA TAATGTGCCA TTTGAGTATC ATGCCATTGC TCAGGAGTGG GAAACAATCA   
  
  
+ AACCAGGGGA TCTCAAGATA GGAAGTAGGA ATGATGAAGT TGTTGCGGTG AACTGTCTCT GTAGGTTCAA   
  
  
+ GAACCTCCTT GACGAGACAG TGGTGGTGGA TAGTCCAAGG AACACAGTTT TAAACCTGAT TACAAGGGTA   
  
  
+ AAGCCTGATA TTTTTGTGCA TGGCGTTGTA AATGGTTCCT ACAACATCCC TTTCTTTGTG ACACGTTTTA   
  
  
+ GAGAAGCCCT CTTTCATTAT TCCACTCTTT TCGACATGTT AGATGCCAAC GCCTCTAGGG AGGAGCCCGA   
  
  
+ GAGGTTGATA TTCGAGAAGG CATTCTATGG GAGGGAGATT ATGAATGTGG TGGCCTGTGA GGGCACAGAG   
  
  
+ AGGGTGGAGA GGCCGGAGAC ATACAAGCAA TGGCATGTTA GGCATAGCAG GGCAGGGTTT CGGCAAGTAC   
  
  
+ CATTGGATCC CAAGTTGATC GAGAAAATGA GGTTTAAGGC CAAGGCAGAC CACCACAAGG ATTTCATGAT   
  
  
+ CGATGTGGAT GGACATTGGG CAATTCAGGG ATGGAAGGGG CGGATTGCAC ATGCGATCTC TGCATGGGTT   
  
  
+ CCGGCTTG  

- -Up\_Stream \_Len000CTAAAC TAACAGCTTT AAACAGGCTA AAATATTTTA ACTTTTAACA ATAACCTGAT   
  
  
- ATTAGATGAC ATCAAATCTC CACAACCTAT TATGAGAGTT GAAACTTGAA CCTGCAGGTA CGTGGTGTGA   
  
  
- TACCCGCATA AATGACAAAT ATATGGTTCT TATTAAAACT TCTACAAGAA TCAACCACTA CATTCACATA   
  
  
- TAAGTTGTAT ACAGTAATGT ATTTGAACTT TTAAGAACTT AAAATCTGTA TATGTTAACG TTTAGTAACA   
  
  
- TTTTAACAAC GTAATGTAAA ACTATTTTTA CGTAGGTTTG AAACAGAACC ATATTTGTCA TTTATCCGGG   
  
  
- TATCCAGGTA TCACATCACG TACCTGCAGG TTCAAATTTC CACCCTCATA ATAACGGGTT GCGGGGATTT   
  
  
- CATGTCATAT AATATCAGGT TATAGGGTTA ATTAAAAAGT AAACTTATAA ATTTAAACCT GTATTATAAA   
  
  
- AAGTATAAAC TTAAGAGATT TGGACTAAAA TTGGACTTAA ATTCAACTAA ATTAAGTAGG CTGGTTTTTT   
  
  
- TATTTGTGTT AATAAATAAA AAGATAAGCT TCAATCTATC ATCGGTTAAG TAAACTGTAT ATTAATTGGG   
  
  
- CACTAACTGT GTTTGAGCTC AAAAACGAAT CTCGATCCTT TTCTCAGCCC AATCCAGCTG GGTAAATTCT   
  
  
- TTACCCAGCT GAAGTACAGC CGGAAGTTTT ATCAATTCTA AACGAGTTTT GATTTTTTAT TGTTCAAGTG   
  
  
- TGATTTGCCA GTTTAGCACA GCTGAAGCCT AACTTTAACC AAGTCAAACC CAGCCGAAGC ATAAAGAAAG   
  
  
- TCATTGAAGC TTGGCTTAAG CATAGCACAA TAGCACAGTC CAGTCTGAAA GAGTCAAGAT AAAAACGATG   
  
  
- GAATACAATG CCTATTTTTT TCAGAGTGTA ACCTTTTTCA CACCCTCCCA GGACCCGAAT ATTTATCACC   
  
  
- GCGACGTGTG GAGTAGTCTG GCTGGAAAAC CCCTCTCCCA TTACTGGGGC ACTGTTCACC ATAGTCTCAG   
  
  
- TTGGGTCTGG GCTGAGTCTA CAGGACGGTT CGCGAGGCTG CCCGCCCCAT CCGTCCCCCG GGTAAACACT   
  
  
- ACTCCTGCAG TGTCTTAACC CGCCCCCTCT CACAGTGCCT ATTTTTTTCA GGGTGTAGCT TTTTTCACAC   
  
  
- CCTCCCAGGA CCCGAATATT TGCCATCGCG GCGTGTGGGG TAGTCTAGCT GAAAAACCCC TCTCCCATTA   
  
  
- CTGGGGTAAA GTTCACCATA GTCTCGGTTG GGTCTGGGCT GAGGTTACAG GGTGGTTCGC GAGACTACCC   
  
  
- GCCCCATCCG TTCCCCGAGG TATACACTGC TCCCGCAGTG CCTTAACCTA CCCTCTCTCA TGGTGCCTAT   
  
  
- TTTTTTCAGG GTGTAGTCTT TTTCACACCT GAATCGTTGA TCCTTCATTT CAATGTCATA ATCGGCTCTC   
  
  
- GGGTACCTTG CGGACTTTGA CTGTAGTGGT CTGGGTGTAT AAAGTGGTAG TAGACTAGAG GGGCAGTTTC   
  
  
- CTCCTTCGAC TCGTGTCGTT CTTGATCTTC TTGTCCGCCG TAGTGTGGGT TCGGTTTTAA GAGATAAACT   
  
  
- GGAGGGAGAG GAGTAAGGAG ACTCGAAGGA GAAGTGTTAC ATTCTGGTAT TAGGAATTGT GGGAAGAAAA   
  
  
- AATTCGAGTA GAAATGGACG AGACCAAGAG AGAGAGTAGC AGTGATTATC AATGGAGAAA TGAAAGAGAA   
  
  
- GGGACTCCAT ACGAAGTTGA AATTGTATAT ATAGGGTGAA CTAAAGAGAA CAGAAAAACG ATATTATGAA   
  
  
- CAACAAACCG ATAGTAGGGG ACAAAATATG AGTAAAAGAA CGAAAGATAA AGACCCAAAC TTAACCCAAA   
  
  
- GTGAATAGGA GCAGTTAAGA TACCCATTAA TCACTTAACC CAAATATACT TGATCCCAAC GTTTTCATAG   
  
  
- ATGAAAAAGA GAACACTAAT AATCAACTAA TCCCTAAACC GGTTATAATC GTCTTACCCA AGACTTAAAC   
  
  
- GCCTTAAGAG ACTACTACGA GATTTACCCA TACGAATACA ACTATAGGGA CGAATACTAC GTAGGAATCT   
  
  
- AATACGGTTA AACAAGTTAA TGCTTCCGGG TAGACTCCTA GCGTGGAGTG ACTCGGAGGG TTCTGGGAAA   
  
  
- AGACTGGGAA ACCGAATGTC AAAGTGCAGG AGAAGACTCG ACCCGGGACC CCAACTAAGA TTACTACTAT   
  
  
- CACTAAGACC GCTACAAGAG TTCATGTAAT CGGTTTACGA ATACCTTCTT CTGTACCTCC GTTTCGGTAC   
  
  
- GTACAAAGTA CTAGGAAATC GTGAAGTCCG ACGACTCTTT GGGAAAATAC TACGGAATCC CCTCGTTACG   
  
  
- GGTTGAAGAG GACTGGTTGT AGGATATTAA CTAGTAATAA ACCTATCAGG ACTACTTTTA AGAAACTCAA   
  
  
- GTTGACCACT AAAATCACAA TCAGTAAGAC CCAGATCAAG TTGTTTGAGG CAACCTGGCT ATTATCACAG   
  
  
- ACTAAACTCA CTCGTAAAAC TCGGTGGGAA ACAACTTTGT GAAGGTTAAC TTAGTATAGT GGTTGGTAAC   
  
  
- TGGGCAGGAG TTACCAGTAA ACCGAGAAGC CCACGGAATC TACCGTGCCG GAGATTACCA AGCCACTAGA   
  
  
- GTAGGGAACC AAACGGTCAC CTACACTAAT CGCATAAATC CCTCTTTCTC AGGTACTAAG TTAAAGTCTT   
  
  
- TCCCCACCTC CTCCGGTCAT TCAAGGAAGG GTTCTTATTA TTGGAACAAT AACTAGAGCT CTTGGAGTGA   
  
  
- AAAGGATTAC TTTGTTTCCT CCTACTAGCT TACTACTACC AATTCTTCCT TTTCCTACTG CACTTAACCA   
  
  
- GATTGATGAG TTCTCCCTCA TTCTAAGTGA TAACACTTCT GCTCCGGAAA CTTCTTCCTT CCTCACCGTT   
  
  
- CGTCAGTCGA TAAAGAAGAT GACTCCTTCG ACTCAAAAGA CTGTAAAAAC TGTTCCAAAA CGAAACGCTA   
  
  
- ACGATGGGAC ACTTTGGACT CCGAGTAGGG TGGTACTCAA ACTTGGGACT CTTCCCGGTC AGTGTACCTA   
  
  
- ATCTTCCACC CTCTTTACCC TTCCGAGCGG GATTCTTTGT CCTATTATCA TCGTGTTTAT AACACCTAAA   
  
  
- TTCCTTAAAC GACTATGATA CGCGTGTTAG ATGACGTAGA CTACTGGCTT CTTGACGACT ACCTGACGAT   
  
  
- TTCGTTTAGT CCCTCGTGAG CAGACGACTC CCCCTACCTA GAGTTTCCAA CCGCGTAATG AAACGACTAC   
  
  
- GGGATCTCCG TGCAAATCGA CCTTGACCTA GAGCGTAAAT ATGCCGGGAT ACAAGATTAT CCGGTAGACA   
  
  
- GTGACTGTAC TATTTTCGTA TAGTCAAGAT ATAAGCACGA ACGGGTAAAT GCTTCTAGCA GTAACCAACA   
  
  
- CCATGGGTAT ACTAAGATTT TCGTCGACTC TTCCGTAGTT TCGAAGTATA ATATCTAAAA CCGTAGGAGA   
  
  
- TACCAACGGT TACCGGGTTG GAGTAAGTTG CGGAGAGTCT CGCTAGACCA CCTGGAGGTT TTGACAAATA   
  
  
- ATGTCCCTAG CTAGAGGGGG TCGGACCCAA GTCCGGTCGT CTTTCTCACC TTCGTTGTCC CTCTGCGAAC   
  
  
- CGGTTCATGA CACTCGCCAT ATTACACGGT AAACTCATAG TACGGTAACG AGTCCTCACC CTTTGTTAGT   
  
  
- TTGGTCCCCT AGAGTTCTAT CCTTCATCCT TACTACTTCA ACAACGCCAC TTGACAGAGA CATCCAAGTT   
  
  
- CTTGGAGGAA CTGCTCTGTC ACCACCACCT ATCAGGTTCC TTGTGTCAAA ATTTGGACTA ATGTTCCCAT   
  
  
- TTCGGACTAT AAAAACACGT ACCGCAACAT TTACCAAGGA TGTTGTAGGG AAAGAAACAC TGTGCAAAAT   
  
  
- CTCTTCGGGA GAAAGTAATA AGGTGAGAAA AGCTGTACAA TCTACGGTTG CGGAGATCCC TCCTCGGGCT   
  
  
- CTCCAACTAT AAGCTCTTCC GTAAGATACC CTCCCTCTAA TACTTACACC ACCGGACACT CCCGTGTCTC   
  
  
- TCCCACCTCT CCGGCCTCTG TATGTTCGTT ACCGTACAAT CCGTATCGTC CCGTCCCAAA GCCGTTCATG   
  
  
- GTAACCTAGG GTTCAACTAG CTCTTTTACT CCAAATTCCG GTTCCGTCTG GTGGTGTTCC TAAAGTACTA   
  
  
- GCTACACCTA CCTGTAACCC GTTAAGTCCC TACCTTCCCC GCCTAACGTG TACGCTAGAG ACGTACCCAA   
  
  
- GGCCGAAC

+     CCAAT-box

| Site Name | Organism | Position | Strand | Matrix score. | sequence | function |
| --- | --- | --- | --- | --- | --- | --- |
| CCAAT-box | Hordeum vulgare | 2503 | - | 6 | CAACGG | MYBHv1 binding site |

>HU07G02249.1   
+ -Up\_Stream \_Len000GATTTG ATTGTCGAAA TTTGTCCGAT TTTATAAAAT TGAAAATTGT TATTGGACTA   
  
  
+ TAATCTACTG TAGTTTAGAG GTGTTGGATA ATACTCTCAA CTTTGAACTT GGACGTCCAT GCACCACACT   
  
  
+ ATGGGCGTAT TTACTGTTTA TATACCAAGA ATAATTTTGA AGATGTTCTT AGTTGGTGAT GTAAGTGTAT   
  
  
+ ATTCAACATA TGTCATTACA TAAACTTGAA AATTCTTGAA TTTTAGACAT ATACAATTGC AAATCATTGT   
  
  
+ AAAATTGTTG CATTACATTT TGATAAAAAT GCATCCAAAC TTTGTCTTGG TATAAACAGT AAATAGGCCC   
  
  
+ ATAGGTCCAT AGTGTAGTGC ATGGACGTCC AAGTTTAAAG GTGGGAGTAT TATTGCCCAA CGCCCCTAAA   
  
  
+ GTACAGTATA TTATAGTCCA ATATCCCAAT TAATTTTTCA TTTGAATATT TAAATTTGGA CATAATATTT   
  
  
+ TTCATATTTG AATTCTCTAA ACCTGATTTT AACCTGAATT TAAGTTGATT TAATTCATCC GACCAAAAAA   
  
  
+ ATAAACACAA TTATTTATTT TTCTATTCGA AGTTAGATAG TAGCCAATTC ATTTGACATA TAATTAACCC   
  
  
+ GTGATTGACA CAAACTCGAG TTTTTGCTTA GAGCTAGGAA AAGAGTCGGG TTAGGTCGAC CCATTTAAGA   
  
  
+ AATGGGTCGA CTTCATGTCG GCCTTCAAAA TAGTTAAGAT TTGCTCAAAA CTAAAAAATA ACAAGTTCAC   
  
  
+ ACTAAACGGT CAAATCGTGT CGACTTCGGA TTGAAATTGG TTCAGTTTGG GTCGGCTTCG TATTTCTTTC   
  
  
+ AGTAACTTCG AACCGAATTC GTATCGTGTT ATCGTGTCAG GTCAGACTTT CTCAGTTCTA TTTTTGCTAC   
  
  
+ CTTATGTTAC GGATAAAAAA AGTCTCACAT TGGAAAAAGT GTGGGAGGGT CCTGGGCTTA TAAATAGTGG   
  
  
+ CGCTGCACAC CTCATCAGAC CGACCTTTTG GGGAGAGGGT AATGACCCCG TGACAAGTGG TATCAGAGTC   
  
  
+ AACCCAGACC CGACTCAGAT GTCCTGCCAA GCGCTCCGAC GGGCGGGGTA GGCAGGGGGC CCATTTGTGA   
  
  
+ TGAGGACGTC ACAGAATTGG GCGGGGGAGA GTGTCACGGA TAAAAAAAGT CCCACATCGA AAAAAGTGTG   
  
  
+ GGAGGGTCCT GGGCTTATAA ACGGTAGCGC CGCACACCCC ATCAGATCGA CTTTTTGGGG AGAGGGTAAT   
  
  
+ GACCCCATTT CAAGTGGTAT CAGAGCCAAC CCAGACCCGA CTCCAATGTC CCACCAAGCG CTCTGATGGG   
  
  
+ CGGGGTAGGC AAGGGGCTCC ATATGTGACG AGGGCGTCAC GGAATTGGAT GGGAGAGAGT ACCACGGATA   
  
  
+ AAAAAAGTCC CACATCAGAA AAAGTGTGGA CTTAGCAACT AGGAAGTAAA GTTACAGTAT TAGCCGAGAG   
  
  
+ CCCATGGAAC GCCTGAAACT GACATCACCA GACCCACATA TTTCACCATC ATCTGATCTC CCCGTCAAAG   
  
  
+ GAGGAAGCTG AGCACAGCAA GAACTAGAAG AACAGGCGGC ATCACACCCA AGCCAAAATT CTCTATTTGA   
  
  
+ CCTCCCTCTC CTCATTCCTC TGAGCTTCCT CTTCACAATG TAAGACCATA ATCCTTAACA CCCTTCTTTT   
  
  
+ TTAAGCTCAT CTTTACCTGC TCTGGTTCTC TCTCTCATCG TCACTAATAG TTACCTCTTT ACTTTCTCTT   
  
  
+ CCCTGAGGTA TGCTTCAACT TTAACATATA TATCCCACTT GATTTCTCTT GTCTTTTTGC TATAATACTT   
  
  
+ GTTGTTTGGC TATCATCCCC TGTTTTATAC TCATTTTCTT GCTTTCTATT TCTGGGTTTG AATTGGGTTT   
  
  
+ CACTTATCCT CGTCAATTCT ATGGGTAATT AGTGAATTGG GTTTATATGA ACTAGGGTTG CAAAAGTATC   
  
  
+ TACTTTTTCT CTTGTGATTA TTAGTTGATT AGGGATTTGG CCAATATTAG CAGAATGGGT TCTGAATTTG   
  
  
+ CGGAATTCTC TGATGATGCT CTAAATGGGT ATGCTTATGT TGATATCCCT GCTTATGATG CATCCTTAGA   
  
  
+ TTATGCCAAT TTGTTCAATT ACGAAGGCCC ATCTGAGGAT CGCACCTCAC TGAGCCTCCC AAGACCCTTT   
  
  
+ TCTGACCCTT TGGCTTACAG TTTCACGTCC TCTTCTGAGC TGGGCCCTGG GGTTGATTCT AATGATGATA   
  
  
+ GTGATTCTGG CGATGTTCTC AAGTACATTA GCCAAATGCT TATGGAAGAA GACATGGAGG CAAAGCCATG   
  
  
+ CATGTTTCAT GATCCTTTAG CACTTCAGGC TGCTGAGAAA CCCTTTTATG ATGCCTTAGG GGAGCAATGC   
  
  
+ CCAACTTCTC CTGACCAACA TCCTATAATT GATCATTATT TGGATAGTCC TGATGAAAAT TCTTTGAGTT   
  
  
+ CAACTGGTGA TTTTAGTGTT AGTCATTCTG GGTCTAGTTC AACAAACTCC GTTGGACCGA TAATAGTGTC   
  
  
+ TGATTTGAGT GAGCATTTTG AGCCACCCTT TGTTGAAACA CTTCCAATTG AATCATATCA CCAACCATTG   
  
  
+ ACCCGTCCTC AATGGTCATT TGGCTCTTCG GGTGCCTTAG ATGGCACGGC CTCTAATGGT TCGGTGATCT   
  
  
+ CATCCCTTGG TTTGCCAGTG GATGTGATTA GCGTATTTAG GGAGAAAGAG TCCATGATTC AATTTCAGAA   
  
  
+ AGGGGTGGAG GAGGCCAGTA AGTTCCTTCC CAAGAATAAT AACCTTGTTA TTGATCTCGA GAACCTCACT   
  
  
+ TTTCCTAATG AAACAAAGGA GGATGATCGA ATGATGATGG TTAAGAAGGA AAAGGATGAC GTGAATTGGT   
  
  
+ CTAACTACTC AAGAGGGAGT AAGATTCACT ATTGTGAAGA CGAGGCCTTT GAAGAAGGAA GGAGTGGCAA   
  
  
+ GCAGTCAGCT ATTTCTTCTA CTGAGGAAGC TGAGTTTTCT GACATTTTTG ACAAGGTTTT GCTTTGCGAT   
  
  
+ TGCTACCCTG TGAAACCTGA GGCTCATCCC ACCATGAGTT TGAACCCTGA GAAGGGCCAG TCACATGGAT   
  
  
+ TAGAAGGTGG GAGAAATGGG AAGGCTCGCC CTAAGAAACA GGATAATAGT AGCACAAATA TTGTGGATTT   
  
  
+ AAGGAATTTG CTGATACTAT GCGCACAATC TACTGCATCT GATGACCGAA GAACTGCTGA TGGACTGCTA   
  
  
+ AAGCAAATCA GGGAGCACTC GTCTGCTGAG GGGGATGGAT CTCAAAGGTT GGCGCATTAC TTTGCTGATG   
  
  
+ CCCTAGAGGC ACGTTTAGCT GGAACTGGAT CTCGCATTTA TACGGCCCTA TGTTCTAATA GGCCATCTGT   
  
  
+ CACTGACATG ATAAAAGCAT ATCAGTTCTA TATTCGTGCT TGCCCATTTA CGAAGATCGT CATTGGTTGT   
  
  
+ GGTACCCATA TGATTCTAAA AGCAGCTGAG AAGGCATCAA AGCTTCATAT TATAGATTTT GGCATCCTCT   
  
  
+ ATGGTTGCCA ATGGCCCAAC CTCATTCAAC GCCTCTCAGA GCGATCTGGT GGACCTCCAA AACTGTTTAT   
  
  
+ TACAGGGATC GATCTCCCCC AGCCTGGGTT CAGGCCAGCA GAAAGAGTGG AAGCAACAGG GAGACGCTTG   
  
  
+ GCCAAGTACT GTGAGCGGTA TAATGTGCCA TTTGAGTATC ATGCCATTGC TCAGGAGTGG GAAACAATCA   
  
  
+ AACCAGGGGA TCTCAAGATA GGAAGTAGGA ATGATGAAGT TGTTGCGGTG AACTGTCTCT GTAGGTTCAA   
  
  
+ GAACCTCCTT GACGAGACAG TGGTGGTGGA TAGTCCAAGG AACACAGTTT TAAACCTGAT TACAAGGGTA   
  
  
+ AAGCCTGATA TTTTTGTGCA TGGCGTTGTA AATGGTTCCT ACAACATCCC TTTCTTTGTG ACACGTTTTA   
  
  
+ GAGAAGCCCT CTTTCATTAT TCCACTCTTT TCGACATGTT AGATGCCAAC GCCTCTAGGG AGGAGCCCGA   
  
  
+ GAGGTTGATA TTCGAGAAGG CATTCTATGG GAGGGAGATT ATGAATGTGG TGGCCTGTGA GGGCACAGAG   
  
  
+ AGGGTGGAGA GGCCGGAGAC ATACAAGCAA TGGCATGTTA GGCATAGCAG GGCAGGGTTT CGGCAAGTAC   
  
  
+ CATTGGATCC CAAGTTGATC GAGAAAATGA GGTTTAAGGC CAAGGCAGAC CACCACAAGG ATTTCATGAT   
  
  
+ CGATGTGGAT GGACATTGGG CAATTCAGGG ATGGAAGGGG CGGATTGCAC ATGCGATCTC TGCATGGGTT   
  
  
+ CCGGCTTG  

- -Up\_Stream \_Len000CTAAAC TAACAGCTTT AAACAGGCTA AAATATTTTA ACTTTTAACA ATAACCTGAT   
  
  
- ATTAGATGAC ATCAAATCTC CACAACCTAT TATGAGAGTT GAAACTTGAA CCTGCAGGTA CGTGGTGTGA   
  
  
- TACCCGCATA AATGACAAAT ATATGGTTCT TATTAAAACT TCTACAAGAA TCAACCACTA CATTCACATA   
  
  
- TAAGTTGTAT ACAGTAATGT ATTTGAACTT TTAAGAACTT AAAATCTGTA TATGTTAACG TTTAGTAACA   
  
  
- TTTTAACAAC GTAATGTAAA ACTATTTTTA CGTAGGTTTG AAACAGAACC ATATTTGTCA TTTATCCGGG   
  
  
- TATCCAGGTA TCACATCACG TACCTGCAGG TTCAAATTTC CACCCTCATA ATAACGGGTT GCGGGGATTT   
  
  
- CATGTCATAT AATATCAGGT TATAGGGTTA ATTAAAAAGT AAACTTATAA ATTTAAACCT GTATTATAAA   
  
  
- AAGTATAAAC TTAAGAGATT TGGACTAAAA TTGGACTTAA ATTCAACTAA ATTAAGTAGG CTGGTTTTTT   
  
  
- TATTTGTGTT AATAAATAAA AAGATAAGCT TCAATCTATC ATCGGTTAAG TAAACTGTAT ATTAATTGGG   
  
  
- CACTAACTGT GTTTGAGCTC AAAAACGAAT CTCGATCCTT TTCTCAGCCC AATCCAGCTG GGTAAATTCT   
  
  
- TTACCCAGCT GAAGTACAGC CGGAAGTTTT ATCAATTCTA AACGAGTTTT GATTTTTTAT TGTTCAAGTG   
  
  
- TGATTTGCCA GTTTAGCACA GCTGAAGCCT AACTTTAACC AAGTCAAACC CAGCCGAAGC ATAAAGAAAG   
  
  
- TCATTGAAGC TTGGCTTAAG CATAGCACAA TAGCACAGTC CAGTCTGAAA GAGTCAAGAT AAAAACGATG   
  
  
- GAATACAATG CCTATTTTTT TCAGAGTGTA ACCTTTTTCA CACCCTCCCA GGACCCGAAT ATTTATCACC   
  
  
- GCGACGTGTG GAGTAGTCTG GCTGGAAAAC CCCTCTCCCA TTACTGGGGC ACTGTTCACC ATAGTCTCAG   
  
  
- TTGGGTCTGG GCTGAGTCTA CAGGACGGTT CGCGAGGCTG CCCGCCCCAT CCGTCCCCCG GGTAAACACT   
  
  
- ACTCCTGCAG TGTCTTAACC CGCCCCCTCT CACAGTGCCT ATTTTTTTCA GGGTGTAGCT TTTTTCACAC   
  
  
- CCTCCCAGGA CCCGAATATT TGCCATCGCG GCGTGTGGGG TAGTCTAGCT GAAAAACCCC TCTCCCATTA   
  
  
- CTGGGGTAAA GTTCACCATA GTCTCGGTTG GGTCTGGGCT GAGGTTACAG GGTGGTTCGC GAGACTACCC   
  
  
- GCCCCATCCG TTCCCCGAGG TATACACTGC TCCCGCAGTG CCTTAACCTA CCCTCTCTCA TGGTGCCTAT   
  
  
- TTTTTTCAGG GTGTAGTCTT TTTCACACCT GAATCGTTGA TCCTTCATTT CAATGTCATA ATCGGCTCTC   
  
  
- GGGTACCTTG CGGACTTTGA CTGTAGTGGT CTGGGTGTAT AAAGTGGTAG TAGACTAGAG GGGCAGTTTC   
  
  
- CTCCTTCGAC TCGTGTCGTT CTTGATCTTC TTGTCCGCCG TAGTGTGGGT TCGGTTTTAA GAGATAAACT   
  
  
- GGAGGGAGAG GAGTAAGGAG ACTCGAAGGA GAAGTGTTAC ATTCTGGTAT TAGGAATTGT GGGAAGAAAA   
  
  
- AATTCGAGTA GAAATGGACG AGACCAAGAG AGAGAGTAGC AGTGATTATC AATGGAGAAA TGAAAGAGAA   
  
  
- GGGACTCCAT ACGAAGTTGA AATTGTATAT ATAGGGTGAA CTAAAGAGAA CAGAAAAACG ATATTATGAA   
  
  
- CAACAAACCG ATAGTAGGGG ACAAAATATG AGTAAAAGAA CGAAAGATAA AGACCCAAAC TTAACCCAAA   
  
  
- GTGAATAGGA GCAGTTAAGA TACCCATTAA TCACTTAACC CAAATATACT TGATCCCAAC GTTTTCATAG   
  
  
- ATGAAAAAGA GAACACTAAT AATCAACTAA TCCCTAAACC GGTTATAATC GTCTTACCCA AGACTTAAAC   
  
  
- GCCTTAAGAG ACTACTACGA GATTTACCCA TACGAATACA ACTATAGGGA CGAATACTAC GTAGGAATCT   
  
  
- AATACGGTTA AACAAGTTAA TGCTTCCGGG TAGACTCCTA GCGTGGAGTG ACTCGGAGGG TTCTGGGAAA   
  
  
- AGACTGGGAA ACCGAATGTC AAAGTGCAGG AGAAGACTCG ACCCGGGACC CCAACTAAGA TTACTACTAT   
  
  
- CACTAAGACC GCTACAAGAG TTCATGTAAT CGGTTTACGA ATACCTTCTT CTGTACCTCC GTTTCGGTAC   
  
  
- GTACAAAGTA CTAGGAAATC GTGAAGTCCG ACGACTCTTT GGGAAAATAC TACGGAATCC CCTCGTTACG   
  
  
- GGTTGAAGAG GACTGGTTGT AGGATATTAA CTAGTAATAA ACCTATCAGG ACTACTTTTA AGAAACTCAA   
  
  
- GTTGACCACT AAAATCACAA TCAGTAAGAC CCAGATCAAG TTGTTTGAGG CAACCTGGCT ATTATCACAG   
  
  
- ACTAAACTCA CTCGTAAAAC TCGGTGGGAA ACAACTTTGT GAAGGTTAAC TTAGTATAGT GGTTGGTAAC   
  
  
- TGGGCAGGAG TTACCAGTAA ACCGAGAAGC CCACGGAATC TACCGTGCCG GAGATTACCA AGCCACTAGA   
  
  
- GTAGGGAACC AAACGGTCAC CTACACTAAT CGCATAAATC CCTCTTTCTC AGGTACTAAG TTAAAGTCTT   
  
  
- TCCCCACCTC CTCCGGTCAT TCAAGGAAGG GTTCTTATTA TTGGAACAAT AACTAGAGCT CTTGGAGTGA   
  
  
- AAAGGATTAC TTTGTTTCCT CCTACTAGCT TACTACTACC AATTCTTCCT TTTCCTACTG CACTTAACCA   
  
  
- GATTGATGAG TTCTCCCTCA TTCTAAGTGA TAACACTTCT GCTCCGGAAA CTTCTTCCTT CCTCACCGTT   
  
  
- CGTCAGTCGA TAAAGAAGAT GACTCCTTCG ACTCAAAAGA CTGTAAAAAC TGTTCCAAAA CGAAACGCTA   
  
  
- ACGATGGGAC ACTTTGGACT CCGAGTAGGG TGGTACTCAA ACTTGGGACT CTTCCCGGTC AGTGTACCTA   
  
  
- ATCTTCCACC CTCTTTACCC TTCCGAGCGG GATTCTTTGT CCTATTATCA TCGTGTTTAT AACACCTAAA   
  
  
- TTCCTTAAAC GACTATGATA CGCGTGTTAG ATGACGTAGA CTACTGGCTT CTTGACGACT ACCTGACGAT   
  
  
- TTCGTTTAGT CCCTCGTGAG CAGACGACTC CCCCTACCTA GAGTTTCCAA CCGCGTAATG AAACGACTAC   
  
  
- GGGATCTCCG TGCAAATCGA CCTTGACCTA GAGCGTAAAT ATGCCGGGAT ACAAGATTAT CCGGTAGACA   
  
  
- GTGACTGTAC TATTTTCGTA TAGTCAAGAT ATAAGCACGA ACGGGTAAAT GCTTCTAGCA GTAACCAACA   
  
  
- CCATGGGTAT ACTAAGATTT TCGTCGACTC TTCCGTAGTT TCGAAGTATA ATATCTAAAA CCGTAGGAGA   
  
  
- TACCAACGGT TACCGGGTTG GAGTAAGTTG CGGAGAGTCT CGCTAGACCA CCTGGAGGTT TTGACAAATA   
  
  
- ATGTCCCTAG CTAGAGGGGG TCGGACCCAA GTCCGGTCGT CTTTCTCACC TTCGTTGTCC CTCTGCGAAC   
  
  
- CGGTTCATGA CACTCGCCAT ATTACACGGT AAACTCATAG TACGGTAACG AGTCCTCACC CTTTGTTAGT   
  
  
- TTGGTCCCCT AGAGTTCTAT CCTTCATCCT TACTACTTCA ACAACGCCAC TTGACAGAGA CATCCAAGTT   
  
  
- CTTGGAGGAA CTGCTCTGTC ACCACCACCT ATCAGGTTCC TTGTGTCAAA ATTTGGACTA ATGTTCCCAT   
  
  
- TTCGGACTAT AAAAACACGT ACCGCAACAT TTACCAAGGA TGTTGTAGGG AAAGAAACAC TGTGCAAAAT   
  
  
- CTCTTCGGGA GAAAGTAATA AGGTGAGAAA AGCTGTACAA TCTACGGTTG CGGAGATCCC TCCTCGGGCT   
  
  
- CTCCAACTAT AAGCTCTTCC GTAAGATACC CTCCCTCTAA TACTTACACC ACCGGACACT CCCGTGTCTC   
  
  
- TCCCACCTCT CCGGCCTCTG TATGTTCGTT ACCGTACAAT CCGTATCGTC CCGTCCCAAA GCCGTTCATG   
  
  
- GTAACCTAGG GTTCAACTAG CTCTTTTACT CCAAATTCCG GTTCCGTCTG GTGGTGTTCC TAAAGTACTA   
  
  
- GCTACACCTA CCTGTAACCC GTTAAGTCCC TACCTTCCCC GCCTAACGTG TACGCTAGAG ACGTACCCAA   
  
  
- GGCCGAAC

+     CCGTCC motif

| Site Name | Organism | Position | Strand | Matrix score. | sequence | function |
| --- | --- | --- | --- | --- | --- | --- |
| CCGTCC motif | Nicotiana tabacum | 2597 | + | 6 | CCGTCC |  |

>HU07G02249.1   
+ -Up\_Stream \_Len000GATTTG ATTGTCGAAA TTTGTCCGAT TTTATAAAAT TGAAAATTGT TATTGGACTA   
  
  
+ TAATCTACTG TAGTTTAGAG GTGTTGGATA ATACTCTCAA CTTTGAACTT GGACGTCCAT GCACCACACT   
  
  
+ ATGGGCGTAT TTACTGTTTA TATACCAAGA ATAATTTTGA AGATGTTCTT AGTTGGTGAT GTAAGTGTAT   
  
  
+ ATTCAACATA TGTCATTACA TAAACTTGAA AATTCTTGAA TTTTAGACAT ATACAATTGC AAATCATTGT   
  
  
+ AAAATTGTTG CATTACATTT TGATAAAAAT GCATCCAAAC TTTGTCTTGG TATAAACAGT AAATAGGCCC   
  
  
+ ATAGGTCCAT AGTGTAGTGC ATGGACGTCC AAGTTTAAAG GTGGGAGTAT TATTGCCCAA CGCCCCTAAA   
  
  
+ GTACAGTATA TTATAGTCCA ATATCCCAAT TAATTTTTCA TTTGAATATT TAAATTTGGA CATAATATTT   
  
  
+ TTCATATTTG AATTCTCTAA ACCTGATTTT AACCTGAATT TAAGTTGATT TAATTCATCC GACCAAAAAA   
  
  
+ ATAAACACAA TTATTTATTT TTCTATTCGA AGTTAGATAG TAGCCAATTC ATTTGACATA TAATTAACCC   
  
  
+ GTGATTGACA CAAACTCGAG TTTTTGCTTA GAGCTAGGAA AAGAGTCGGG TTAGGTCGAC CCATTTAAGA   
  
  
+ AATGGGTCGA CTTCATGTCG GCCTTCAAAA TAGTTAAGAT TTGCTCAAAA CTAAAAAATA ACAAGTTCAC   
  
  
+ ACTAAACGGT CAAATCGTGT CGACTTCGGA TTGAAATTGG TTCAGTTTGG GTCGGCTTCG TATTTCTTTC   
  
  
+ AGTAACTTCG AACCGAATTC GTATCGTGTT ATCGTGTCAG GTCAGACTTT CTCAGTTCTA TTTTTGCTAC   
  
  
+ CTTATGTTAC GGATAAAAAA AGTCTCACAT TGGAAAAAGT GTGGGAGGGT CCTGGGCTTA TAAATAGTGG   
  
  
+ CGCTGCACAC CTCATCAGAC CGACCTTTTG GGGAGAGGGT AATGACCCCG TGACAAGTGG TATCAGAGTC   
  
  
+ AACCCAGACC CGACTCAGAT GTCCTGCCAA GCGCTCCGAC GGGCGGGGTA GGCAGGGGGC CCATTTGTGA   
  
  
+ TGAGGACGTC ACAGAATTGG GCGGGGGAGA GTGTCACGGA TAAAAAAAGT CCCACATCGA AAAAAGTGTG   
  
  
+ GGAGGGTCCT GGGCTTATAA ACGGTAGCGC CGCACACCCC ATCAGATCGA CTTTTTGGGG AGAGGGTAAT   
  
  
+ GACCCCATTT CAAGTGGTAT CAGAGCCAAC CCAGACCCGA CTCCAATGTC CCACCAAGCG CTCTGATGGG   
  
  
+ CGGGGTAGGC AAGGGGCTCC ATATGTGACG AGGGCGTCAC GGAATTGGAT GGGAGAGAGT ACCACGGATA   
  
  
+ AAAAAAGTCC CACATCAGAA AAAGTGTGGA CTTAGCAACT AGGAAGTAAA GTTACAGTAT TAGCCGAGAG   
  
  
+ CCCATGGAAC GCCTGAAACT GACATCACCA GACCCACATA TTTCACCATC ATCTGATCTC CCCGTCAAAG   
  
  
+ GAGGAAGCTG AGCACAGCAA GAACTAGAAG AACAGGCGGC ATCACACCCA AGCCAAAATT CTCTATTTGA   
  
  
+ CCTCCCTCTC CTCATTCCTC TGAGCTTCCT CTTCACAATG TAAGACCATA ATCCTTAACA CCCTTCTTTT   
  
  
+ TTAAGCTCAT CTTTACCTGC TCTGGTTCTC TCTCTCATCG TCACTAATAG TTACCTCTTT ACTTTCTCTT   
  
  
+ CCCTGAGGTA TGCTTCAACT TTAACATATA TATCCCACTT GATTTCTCTT GTCTTTTTGC TATAATACTT   
  
  
+ GTTGTTTGGC TATCATCCCC TGTTTTATAC TCATTTTCTT GCTTTCTATT TCTGGGTTTG AATTGGGTTT   
  
  
+ CACTTATCCT CGTCAATTCT ATGGGTAATT AGTGAATTGG GTTTATATGA ACTAGGGTTG CAAAAGTATC   
  
  
+ TACTTTTTCT CTTGTGATTA TTAGTTGATT AGGGATTTGG CCAATATTAG CAGAATGGGT TCTGAATTTG   
  
  
+ CGGAATTCTC TGATGATGCT CTAAATGGGT ATGCTTATGT TGATATCCCT GCTTATGATG CATCCTTAGA   
  
  
+ TTATGCCAAT TTGTTCAATT ACGAAGGCCC ATCTGAGGAT CGCACCTCAC TGAGCCTCCC AAGACCCTTT   
  
  
+ TCTGACCCTT TGGCTTACAG TTTCACGTCC TCTTCTGAGC TGGGCCCTGG GGTTGATTCT AATGATGATA   
  
  
+ GTGATTCTGG CGATGTTCTC AAGTACATTA GCCAAATGCT TATGGAAGAA GACATGGAGG CAAAGCCATG   
  
  
+ CATGTTTCAT GATCCTTTAG CACTTCAGGC TGCTGAGAAA CCCTTTTATG ATGCCTTAGG GGAGCAATGC   
  
  
+ CCAACTTCTC CTGACCAACA TCCTATAATT GATCATTATT TGGATAGTCC TGATGAAAAT TCTTTGAGTT   
  
  
+ CAACTGGTGA TTTTAGTGTT AGTCATTCTG GGTCTAGTTC AACAAACTCC GTTGGACCGA TAATAGTGTC   
  
  
+ TGATTTGAGT GAGCATTTTG AGCCACCCTT TGTTGAAACA CTTCCAATTG AATCATATCA CCAACCATTG   
  
  
+ ACCCGTCCTC AATGGTCATT TGGCTCTTCG GGTGCCTTAG ATGGCACGGC CTCTAATGGT TCGGTGATCT   
  
  
+ CATCCCTTGG TTTGCCAGTG GATGTGATTA GCGTATTTAG GGAGAAAGAG TCCATGATTC AATTTCAGAA   
  
  
+ AGGGGTGGAG GAGGCCAGTA AGTTCCTTCC CAAGAATAAT AACCTTGTTA TTGATCTCGA GAACCTCACT   
  
  
+ TTTCCTAATG AAACAAAGGA GGATGATCGA ATGATGATGG TTAAGAAGGA AAAGGATGAC GTGAATTGGT   
  
  
+ CTAACTACTC AAGAGGGAGT AAGATTCACT ATTGTGAAGA CGAGGCCTTT GAAGAAGGAA GGAGTGGCAA   
  
  
+ GCAGTCAGCT ATTTCTTCTA CTGAGGAAGC TGAGTTTTCT GACATTTTTG ACAAGGTTTT GCTTTGCGAT   
  
  
+ TGCTACCCTG TGAAACCTGA GGCTCATCCC ACCATGAGTT TGAACCCTGA GAAGGGCCAG TCACATGGAT   
  
  
+ TAGAAGGTGG GAGAAATGGG AAGGCTCGCC CTAAGAAACA GGATAATAGT AGCACAAATA TTGTGGATTT   
  
  
+ AAGGAATTTG CTGATACTAT GCGCACAATC TACTGCATCT GATGACCGAA GAACTGCTGA TGGACTGCTA   
  
  
+ AAGCAAATCA GGGAGCACTC GTCTGCTGAG GGGGATGGAT CTCAAAGGTT GGCGCATTAC TTTGCTGATG   
  
  
+ CCCTAGAGGC ACGTTTAGCT GGAACTGGAT CTCGCATTTA TACGGCCCTA TGTTCTAATA GGCCATCTGT   
  
  
+ CACTGACATG ATAAAAGCAT ATCAGTTCTA TATTCGTGCT TGCCCATTTA CGAAGATCGT CATTGGTTGT   
  
  
+ GGTACCCATA TGATTCTAAA AGCAGCTGAG AAGGCATCAA AGCTTCATAT TATAGATTTT GGCATCCTCT   
  
  
+ ATGGTTGCCA ATGGCCCAAC CTCATTCAAC GCCTCTCAGA GCGATCTGGT GGACCTCCAA AACTGTTTAT   
  
  
+ TACAGGGATC GATCTCCCCC AGCCTGGGTT CAGGCCAGCA GAAAGAGTGG AAGCAACAGG GAGACGCTTG   
  
  
+ GCCAAGTACT GTGAGCGGTA TAATGTGCCA TTTGAGTATC ATGCCATTGC TCAGGAGTGG GAAACAATCA   
  
  
+ AACCAGGGGA TCTCAAGATA GGAAGTAGGA ATGATGAAGT TGTTGCGGTG AACTGTCTCT GTAGGTTCAA   
  
  
+ GAACCTCCTT GACGAGACAG TGGTGGTGGA TAGTCCAAGG AACACAGTTT TAAACCTGAT TACAAGGGTA   
  
  
+ AAGCCTGATA TTTTTGTGCA TGGCGTTGTA AATGGTTCCT ACAACATCCC TTTCTTTGTG ACACGTTTTA   
  
  
+ GAGAAGCCCT CTTTCATTAT TCCACTCTTT TCGACATGTT AGATGCCAAC GCCTCTAGGG AGGAGCCCGA   
  
  
+ GAGGTTGATA TTCGAGAAGG CATTCTATGG GAGGGAGATT ATGAATGTGG TGGCCTGTGA GGGCACAGAG   
  
  
+ AGGGTGGAGA GGCCGGAGAC ATACAAGCAA TGGCATGTTA GGCATAGCAG GGCAGGGTTT CGGCAAGTAC   
  
  
+ CATTGGATCC CAAGTTGATC GAGAAAATGA GGTTTAAGGC CAAGGCAGAC CACCACAAGG ATTTCATGAT   
  
  
+ CGATGTGGAT GGACATTGGG CAATTCAGGG ATGGAAGGGG CGGATTGCAC ATGCGATCTC TGCATGGGTT   
  
  
+ CCGGCTTG  

- -Up\_Stream \_Len000CTAAAC TAACAGCTTT AAACAGGCTA AAATATTTTA ACTTTTAACA ATAACCTGAT   
  
  
- ATTAGATGAC ATCAAATCTC CACAACCTAT TATGAGAGTT GAAACTTGAA CCTGCAGGTA CGTGGTGTGA   
  
  
- TACCCGCATA AATGACAAAT ATATGGTTCT TATTAAAACT TCTACAAGAA TCAACCACTA CATTCACATA   
  
  
- TAAGTTGTAT ACAGTAATGT ATTTGAACTT TTAAGAACTT AAAATCTGTA TATGTTAACG TTTAGTAACA   
  
  
- TTTTAACAAC GTAATGTAAA ACTATTTTTA CGTAGGTTTG AAACAGAACC ATATTTGTCA TTTATCCGGG   
  
  
- TATCCAGGTA TCACATCACG TACCTGCAGG TTCAAATTTC CACCCTCATA ATAACGGGTT GCGGGGATTT   
  
  
- CATGTCATAT AATATCAGGT TATAGGGTTA ATTAAAAAGT AAACTTATAA ATTTAAACCT GTATTATAAA   
  
  
- AAGTATAAAC TTAAGAGATT TGGACTAAAA TTGGACTTAA ATTCAACTAA ATTAAGTAGG CTGGTTTTTT   
  
  
- TATTTGTGTT AATAAATAAA AAGATAAGCT TCAATCTATC ATCGGTTAAG TAAACTGTAT ATTAATTGGG   
  
  
- CACTAACTGT GTTTGAGCTC AAAAACGAAT CTCGATCCTT TTCTCAGCCC AATCCAGCTG GGTAAATTCT   
  
  
- TTACCCAGCT GAAGTACAGC CGGAAGTTTT ATCAATTCTA AACGAGTTTT GATTTTTTAT TGTTCAAGTG   
  
  
- TGATTTGCCA GTTTAGCACA GCTGAAGCCT AACTTTAACC AAGTCAAACC CAGCCGAAGC ATAAAGAAAG   
  
  
- TCATTGAAGC TTGGCTTAAG CATAGCACAA TAGCACAGTC CAGTCTGAAA GAGTCAAGAT AAAAACGATG   
  
  
- GAATACAATG CCTATTTTTT TCAGAGTGTA ACCTTTTTCA CACCCTCCCA GGACCCGAAT ATTTATCACC   
  
  
- GCGACGTGTG GAGTAGTCTG GCTGGAAAAC CCCTCTCCCA TTACTGGGGC ACTGTTCACC ATAGTCTCAG   
  
  
- TTGGGTCTGG GCTGAGTCTA CAGGACGGTT CGCGAGGCTG CCCGCCCCAT CCGTCCCCCG GGTAAACACT   
  
  
- ACTCCTGCAG TGTCTTAACC CGCCCCCTCT CACAGTGCCT ATTTTTTTCA GGGTGTAGCT TTTTTCACAC   
  
  
- CCTCCCAGGA CCCGAATATT TGCCATCGCG GCGTGTGGGG TAGTCTAGCT GAAAAACCCC TCTCCCATTA   
  
  
- CTGGGGTAAA GTTCACCATA GTCTCGGTTG GGTCTGGGCT GAGGTTACAG GGTGGTTCGC GAGACTACCC   
  
  
- GCCCCATCCG TTCCCCGAGG TATACACTGC TCCCGCAGTG CCTTAACCTA CCCTCTCTCA TGGTGCCTAT   
  
  
- TTTTTTCAGG GTGTAGTCTT TTTCACACCT GAATCGTTGA TCCTTCATTT CAATGTCATA ATCGGCTCTC   
  
  
- GGGTACCTTG CGGACTTTGA CTGTAGTGGT CTGGGTGTAT AAAGTGGTAG TAGACTAGAG GGGCAGTTTC   
  
  
- CTCCTTCGAC TCGTGTCGTT CTTGATCTTC TTGTCCGCCG TAGTGTGGGT TCGGTTTTAA GAGATAAACT   
  
  
- GGAGGGAGAG GAGTAAGGAG ACTCGAAGGA GAAGTGTTAC ATTCTGGTAT TAGGAATTGT GGGAAGAAAA   
  
  
- AATTCGAGTA GAAATGGACG AGACCAAGAG AGAGAGTAGC AGTGATTATC AATGGAGAAA TGAAAGAGAA   
  
  
- GGGACTCCAT ACGAAGTTGA AATTGTATAT ATAGGGTGAA CTAAAGAGAA CAGAAAAACG ATATTATGAA   
  
  
- CAACAAACCG ATAGTAGGGG ACAAAATATG AGTAAAAGAA CGAAAGATAA AGACCCAAAC TTAACCCAAA   
  
  
- GTGAATAGGA GCAGTTAAGA TACCCATTAA TCACTTAACC CAAATATACT TGATCCCAAC GTTTTCATAG   
  
  
- ATGAAAAAGA GAACACTAAT AATCAACTAA TCCCTAAACC GGTTATAATC GTCTTACCCA AGACTTAAAC   
  
  
- GCCTTAAGAG ACTACTACGA GATTTACCCA TACGAATACA ACTATAGGGA CGAATACTAC GTAGGAATCT   
  
  
- AATACGGTTA AACAAGTTAA TGCTTCCGGG TAGACTCCTA GCGTGGAGTG ACTCGGAGGG TTCTGGGAAA   
  
  
- AGACTGGGAA ACCGAATGTC AAAGTGCAGG AGAAGACTCG ACCCGGGACC CCAACTAAGA TTACTACTAT   
  
  
- CACTAAGACC GCTACAAGAG TTCATGTAAT CGGTTTACGA ATACCTTCTT CTGTACCTCC GTTTCGGTAC   
  
  
- GTACAAAGTA CTAGGAAATC GTGAAGTCCG ACGACTCTTT GGGAAAATAC TACGGAATCC CCTCGTTACG   
  
  
- GGTTGAAGAG GACTGGTTGT AGGATATTAA CTAGTAATAA ACCTATCAGG ACTACTTTTA AGAAACTCAA   
  
  
- GTTGACCACT AAAATCACAA TCAGTAAGAC CCAGATCAAG TTGTTTGAGG CAACCTGGCT ATTATCACAG   
  
  
- ACTAAACTCA CTCGTAAAAC TCGGTGGGAA ACAACTTTGT GAAGGTTAAC TTAGTATAGT GGTTGGTAAC   
  
  
- TGGGCAGGAG TTACCAGTAA ACCGAGAAGC CCACGGAATC TACCGTGCCG GAGATTACCA AGCCACTAGA   
  
  
- GTAGGGAACC AAACGGTCAC CTACACTAAT CGCATAAATC CCTCTTTCTC AGGTACTAAG TTAAAGTCTT   
  
  
- TCCCCACCTC CTCCGGTCAT TCAAGGAAGG GTTCTTATTA TTGGAACAAT AACTAGAGCT CTTGGAGTGA   
  
  
- AAAGGATTAC TTTGTTTCCT CCTACTAGCT TACTACTACC AATTCTTCCT TTTCCTACTG CACTTAACCA   
  
  
- GATTGATGAG TTCTCCCTCA TTCTAAGTGA TAACACTTCT GCTCCGGAAA CTTCTTCCTT CCTCACCGTT   
  
  
- CGTCAGTCGA TAAAGAAGAT GACTCCTTCG ACTCAAAAGA CTGTAAAAAC TGTTCCAAAA CGAAACGCTA   
  
  
- ACGATGGGAC ACTTTGGACT CCGAGTAGGG TGGTACTCAA ACTTGGGACT CTTCCCGGTC AGTGTACCTA   
  
  
- ATCTTCCACC CTCTTTACCC TTCCGAGCGG GATTCTTTGT CCTATTATCA TCGTGTTTAT AACACCTAAA   
  
  
- TTCCTTAAAC GACTATGATA CGCGTGTTAG ATGACGTAGA CTACTGGCTT CTTGACGACT ACCTGACGAT   
  
  
- TTCGTTTAGT CCCTCGTGAG CAGACGACTC CCCCTACCTA GAGTTTCCAA CCGCGTAATG AAACGACTAC   
  
  
- GGGATCTCCG TGCAAATCGA CCTTGACCTA GAGCGTAAAT ATGCCGGGAT ACAAGATTAT CCGGTAGACA   
  
  
- GTGACTGTAC TATTTTCGTA TAGTCAAGAT ATAAGCACGA ACGGGTAAAT GCTTCTAGCA GTAACCAACA   
  
  
- CCATGGGTAT ACTAAGATTT TCGTCGACTC TTCCGTAGTT TCGAAGTATA ATATCTAAAA CCGTAGGAGA   
  
  
- TACCAACGGT TACCGGGTTG GAGTAAGTTG CGGAGAGTCT CGCTAGACCA CCTGGAGGTT TTGACAAATA   
  
  
- ATGTCCCTAG CTAGAGGGGG TCGGACCCAA GTCCGGTCGT CTTTCTCACC TTCGTTGTCC CTCTGCGAAC   
  
  
- CGGTTCATGA CACTCGCCAT ATTACACGGT AAACTCATAG TACGGTAACG AGTCCTCACC CTTTGTTAGT   
  
  
- TTGGTCCCCT AGAGTTCTAT CCTTCATCCT TACTACTTCA ACAACGCCAC TTGACAGAGA CATCCAAGTT   
  
  
- CTTGGAGGAA CTGCTCTGTC ACCACCACCT ATCAGGTTCC TTGTGTCAAA ATTTGGACTA ATGTTCCCAT   
  
  
- TTCGGACTAT AAAAACACGT ACCGCAACAT TTACCAAGGA TGTTGTAGGG AAAGAAACAC TGTGCAAAAT   
  
  
- CTCTTCGGGA GAAAGTAATA AGGTGAGAAA AGCTGTACAA TCTACGGTTG CGGAGATCCC TCCTCGGGCT   
  
  
- CTCCAACTAT AAGCTCTTCC GTAAGATACC CTCCCTCTAA TACTTACACC ACCGGACACT CCCGTGTCTC   
  
  
- TCCCACCTCT CCGGCCTCTG TATGTTCGTT ACCGTACAAT CCGTATCGTC CCGTCCCAAA GCCGTTCATG   
  
  
- GTAACCTAGG GTTCAACTAG CTCTTTTACT CCAAATTCCG GTTCCGTCTG GTGGTGTTCC TAAAGTACTA   
  
  
- GCTACACCTA CCTGTAACCC GTTAAGTCCC TACCTTCCCC GCCTAACGTG TACGCTAGAG ACGTACCCAA   
  
  
- GGCCGAAC

+     CCGTCC-box

| Site Name | Organism | Position | Strand | Matrix score. | sequence | function |
| --- | --- | --- | --- | --- | --- | --- |
| CCGTCC-box | Petroselinum hortense | 2597 | + | 6 | CCGTCC |  |

>HU07G02249.1   
+ -Up\_Stream \_Len000GATTTG ATTGTCGAAA TTTGTCCGAT TTTATAAAAT TGAAAATTGT TATTGGACTA   
  
  
+ TAATCTACTG TAGTTTAGAG GTGTTGGATA ATACTCTCAA CTTTGAACTT GGACGTCCAT GCACCACACT   
  
  
+ ATGGGCGTAT TTACTGTTTA TATACCAAGA ATAATTTTGA AGATGTTCTT AGTTGGTGAT GTAAGTGTAT   
  
  
+ ATTCAACATA TGTCATTACA TAAACTTGAA AATTCTTGAA TTTTAGACAT ATACAATTGC AAATCATTGT   
  
  
+ AAAATTGTTG CATTACATTT TGATAAAAAT GCATCCAAAC TTTGTCTTGG TATAAACAGT AAATAGGCCC   
  
  
+ ATAGGTCCAT AGTGTAGTGC ATGGACGTCC AAGTTTAAAG GTGGGAGTAT TATTGCCCAA CGCCCCTAAA   
  
  
+ GTACAGTATA TTATAGTCCA ATATCCCAAT TAATTTTTCA TTTGAATATT TAAATTTGGA CATAATATTT   
  
  
+ TTCATATTTG AATTCTCTAA ACCTGATTTT AACCTGAATT TAAGTTGATT TAATTCATCC GACCAAAAAA   
  
  
+ ATAAACACAA TTATTTATTT TTCTATTCGA AGTTAGATAG TAGCCAATTC ATTTGACATA TAATTAACCC   
  
  
+ GTGATTGACA CAAACTCGAG TTTTTGCTTA GAGCTAGGAA AAGAGTCGGG TTAGGTCGAC CCATTTAAGA   
  
  
+ AATGGGTCGA CTTCATGTCG GCCTTCAAAA TAGTTAAGAT TTGCTCAAAA CTAAAAAATA ACAAGTTCAC   
  
  
+ ACTAAACGGT CAAATCGTGT CGACTTCGGA TTGAAATTGG TTCAGTTTGG GTCGGCTTCG TATTTCTTTC   
  
  
+ AGTAACTTCG AACCGAATTC GTATCGTGTT ATCGTGTCAG GTCAGACTTT CTCAGTTCTA TTTTTGCTAC   
  
  
+ CTTATGTTAC GGATAAAAAA AGTCTCACAT TGGAAAAAGT GTGGGAGGGT CCTGGGCTTA TAAATAGTGG   
  
  
+ CGCTGCACAC CTCATCAGAC CGACCTTTTG GGGAGAGGGT AATGACCCCG TGACAAGTGG TATCAGAGTC   
  
  
+ AACCCAGACC CGACTCAGAT GTCCTGCCAA GCGCTCCGAC GGGCGGGGTA GGCAGGGGGC CCATTTGTGA   
  
  
+ TGAGGACGTC ACAGAATTGG GCGGGGGAGA GTGTCACGGA TAAAAAAAGT CCCACATCGA AAAAAGTGTG   
  
  
+ GGAGGGTCCT GGGCTTATAA ACGGTAGCGC CGCACACCCC ATCAGATCGA CTTTTTGGGG AGAGGGTAAT   
  
  
+ GACCCCATTT CAAGTGGTAT CAGAGCCAAC CCAGACCCGA CTCCAATGTC CCACCAAGCG CTCTGATGGG   
  
  
+ CGGGGTAGGC AAGGGGCTCC ATATGTGACG AGGGCGTCAC GGAATTGGAT GGGAGAGAGT ACCACGGATA   
  
  
+ AAAAAAGTCC CACATCAGAA AAAGTGTGGA CTTAGCAACT AGGAAGTAAA GTTACAGTAT TAGCCGAGAG   
  
  
+ CCCATGGAAC GCCTGAAACT GACATCACCA GACCCACATA TTTCACCATC ATCTGATCTC CCCGTCAAAG   
  
  
+ GAGGAAGCTG AGCACAGCAA GAACTAGAAG AACAGGCGGC ATCACACCCA AGCCAAAATT CTCTATTTGA   
  
  
+ CCTCCCTCTC CTCATTCCTC TGAGCTTCCT CTTCACAATG TAAGACCATA ATCCTTAACA CCCTTCTTTT   
  
  
+ TTAAGCTCAT CTTTACCTGC TCTGGTTCTC TCTCTCATCG TCACTAATAG TTACCTCTTT ACTTTCTCTT   
  
  
+ CCCTGAGGTA TGCTTCAACT TTAACATATA TATCCCACTT GATTTCTCTT GTCTTTTTGC TATAATACTT   
  
  
+ GTTGTTTGGC TATCATCCCC TGTTTTATAC TCATTTTCTT GCTTTCTATT TCTGGGTTTG AATTGGGTTT   
  
  
+ CACTTATCCT CGTCAATTCT ATGGGTAATT AGTGAATTGG GTTTATATGA ACTAGGGTTG CAAAAGTATC   
  
  
+ TACTTTTTCT CTTGTGATTA TTAGTTGATT AGGGATTTGG CCAATATTAG CAGAATGGGT TCTGAATTTG   
  
  
+ CGGAATTCTC TGATGATGCT CTAAATGGGT ATGCTTATGT TGATATCCCT GCTTATGATG CATCCTTAGA   
  
  
+ TTATGCCAAT TTGTTCAATT ACGAAGGCCC ATCTGAGGAT CGCACCTCAC TGAGCCTCCC AAGACCCTTT   
  
  
+ TCTGACCCTT TGGCTTACAG TTTCACGTCC TCTTCTGAGC TGGGCCCTGG GGTTGATTCT AATGATGATA   
  
  
+ GTGATTCTGG CGATGTTCTC AAGTACATTA GCCAAATGCT TATGGAAGAA GACATGGAGG CAAAGCCATG   
  
  
+ CATGTTTCAT GATCCTTTAG CACTTCAGGC TGCTGAGAAA CCCTTTTATG ATGCCTTAGG GGAGCAATGC   
  
  
+ CCAACTTCTC CTGACCAACA TCCTATAATT GATCATTATT TGGATAGTCC TGATGAAAAT TCTTTGAGTT   
  
  
+ CAACTGGTGA TTTTAGTGTT AGTCATTCTG GGTCTAGTTC AACAAACTCC GTTGGACCGA TAATAGTGTC   
  
  
+ TGATTTGAGT GAGCATTTTG AGCCACCCTT TGTTGAAACA CTTCCAATTG AATCATATCA CCAACCATTG   
  
  
+ ACCCGTCCTC AATGGTCATT TGGCTCTTCG GGTGCCTTAG ATGGCACGGC CTCTAATGGT TCGGTGATCT   
  
  
+ CATCCCTTGG TTTGCCAGTG GATGTGATTA GCGTATTTAG GGAGAAAGAG TCCATGATTC AATTTCAGAA   
  
  
+ AGGGGTGGAG GAGGCCAGTA AGTTCCTTCC CAAGAATAAT AACCTTGTTA TTGATCTCGA GAACCTCACT   
  
  
+ TTTCCTAATG AAACAAAGGA GGATGATCGA ATGATGATGG TTAAGAAGGA AAAGGATGAC GTGAATTGGT   
  
  
+ CTAACTACTC AAGAGGGAGT AAGATTCACT ATTGTGAAGA CGAGGCCTTT GAAGAAGGAA GGAGTGGCAA   
  
  
+ GCAGTCAGCT ATTTCTTCTA CTGAGGAAGC TGAGTTTTCT GACATTTTTG ACAAGGTTTT GCTTTGCGAT   
  
  
+ TGCTACCCTG TGAAACCTGA GGCTCATCCC ACCATGAGTT TGAACCCTGA GAAGGGCCAG TCACATGGAT   
  
  
+ TAGAAGGTGG GAGAAATGGG AAGGCTCGCC CTAAGAAACA GGATAATAGT AGCACAAATA TTGTGGATTT   
  
  
+ AAGGAATTTG CTGATACTAT GCGCACAATC TACTGCATCT GATGACCGAA GAACTGCTGA TGGACTGCTA   
  
  
+ AAGCAAATCA GGGAGCACTC GTCTGCTGAG GGGGATGGAT CTCAAAGGTT GGCGCATTAC TTTGCTGATG   
  
  
+ CCCTAGAGGC ACGTTTAGCT GGAACTGGAT CTCGCATTTA TACGGCCCTA TGTTCTAATA GGCCATCTGT   
  
  
+ CACTGACATG ATAAAAGCAT ATCAGTTCTA TATTCGTGCT TGCCCATTTA CGAAGATCGT CATTGGTTGT   
  
  
+ GGTACCCATA TGATTCTAAA AGCAGCTGAG AAGGCATCAA AGCTTCATAT TATAGATTTT GGCATCCTCT   
  
  
+ ATGGTTGCCA ATGGCCCAAC CTCATTCAAC GCCTCTCAGA GCGATCTGGT GGACCTCCAA AACTGTTTAT   
  
  
+ TACAGGGATC GATCTCCCCC AGCCTGGGTT CAGGCCAGCA GAAAGAGTGG AAGCAACAGG GAGACGCTTG   
  
  
+ GCCAAGTACT GTGAGCGGTA TAATGTGCCA TTTGAGTATC ATGCCATTGC TCAGGAGTGG GAAACAATCA   
  
  
+ AACCAGGGGA TCTCAAGATA GGAAGTAGGA ATGATGAAGT TGTTGCGGTG AACTGTCTCT GTAGGTTCAA   
  
  
+ GAACCTCCTT GACGAGACAG TGGTGGTGGA TAGTCCAAGG AACACAGTTT TAAACCTGAT TACAAGGGTA   
  
  
+ AAGCCTGATA TTTTTGTGCA TGGCGTTGTA AATGGTTCCT ACAACATCCC TTTCTTTGTG ACACGTTTTA   
  
  
+ GAGAAGCCCT CTTTCATTAT TCCACTCTTT TCGACATGTT AGATGCCAAC GCCTCTAGGG AGGAGCCCGA   
  
  
+ GAGGTTGATA TTCGAGAAGG CATTCTATGG GAGGGAGATT ATGAATGTGG TGGCCTGTGA GGGCACAGAG   
  
  
+ AGGGTGGAGA GGCCGGAGAC ATACAAGCAA TGGCATGTTA GGCATAGCAG GGCAGGGTTT CGGCAAGTAC   
  
  
+ CATTGGATCC CAAGTTGATC GAGAAAATGA GGTTTAAGGC CAAGGCAGAC CACCACAAGG ATTTCATGAT   
  
  
+ CGATGTGGAT GGACATTGGG CAATTCAGGG ATGGAAGGGG CGGATTGCAC ATGCGATCTC TGCATGGGTT   
  
  
+ CCGGCTTG  

- -Up\_Stream \_Len000CTAAAC TAACAGCTTT AAACAGGCTA AAATATTTTA ACTTTTAACA ATAACCTGAT   
  
  
- ATTAGATGAC ATCAAATCTC CACAACCTAT TATGAGAGTT GAAACTTGAA CCTGCAGGTA CGTGGTGTGA   
  
  
- TACCCGCATA AATGACAAAT ATATGGTTCT TATTAAAACT TCTACAAGAA TCAACCACTA CATTCACATA   
  
  
- TAAGTTGTAT ACAGTAATGT ATTTGAACTT TTAAGAACTT AAAATCTGTA TATGTTAACG TTTAGTAACA   
  
  
- TTTTAACAAC GTAATGTAAA ACTATTTTTA CGTAGGTTTG AAACAGAACC ATATTTGTCA TTTATCCGGG   
  
  
- TATCCAGGTA TCACATCACG TACCTGCAGG TTCAAATTTC CACCCTCATA ATAACGGGTT GCGGGGATTT   
  
  
- CATGTCATAT AATATCAGGT TATAGGGTTA ATTAAAAAGT AAACTTATAA ATTTAAACCT GTATTATAAA   
  
  
- AAGTATAAAC TTAAGAGATT TGGACTAAAA TTGGACTTAA ATTCAACTAA ATTAAGTAGG CTGGTTTTTT   
  
  
- TATTTGTGTT AATAAATAAA AAGATAAGCT TCAATCTATC ATCGGTTAAG TAAACTGTAT ATTAATTGGG   
  
  
- CACTAACTGT GTTTGAGCTC AAAAACGAAT CTCGATCCTT TTCTCAGCCC AATCCAGCTG GGTAAATTCT   
  
  
- TTACCCAGCT GAAGTACAGC CGGAAGTTTT ATCAATTCTA AACGAGTTTT GATTTTTTAT TGTTCAAGTG   
  
  
- TGATTTGCCA GTTTAGCACA GCTGAAGCCT AACTTTAACC AAGTCAAACC CAGCCGAAGC ATAAAGAAAG   
  
  
- TCATTGAAGC TTGGCTTAAG CATAGCACAA TAGCACAGTC CAGTCTGAAA GAGTCAAGAT AAAAACGATG   
  
  
- GAATACAATG CCTATTTTTT TCAGAGTGTA ACCTTTTTCA CACCCTCCCA GGACCCGAAT ATTTATCACC   
  
  
- GCGACGTGTG GAGTAGTCTG GCTGGAAAAC CCCTCTCCCA TTACTGGGGC ACTGTTCACC ATAGTCTCAG   
  
  
- TTGGGTCTGG GCTGAGTCTA CAGGACGGTT CGCGAGGCTG CCCGCCCCAT CCGTCCCCCG GGTAAACACT   
  
  
- ACTCCTGCAG TGTCTTAACC CGCCCCCTCT CACAGTGCCT ATTTTTTTCA GGGTGTAGCT TTTTTCACAC   
  
  
- CCTCCCAGGA CCCGAATATT TGCCATCGCG GCGTGTGGGG TAGTCTAGCT GAAAAACCCC TCTCCCATTA   
  
  
- CTGGGGTAAA GTTCACCATA GTCTCGGTTG GGTCTGGGCT GAGGTTACAG GGTGGTTCGC GAGACTACCC   
  
  
- GCCCCATCCG TTCCCCGAGG TATACACTGC TCCCGCAGTG CCTTAACCTA CCCTCTCTCA TGGTGCCTAT   
  
  
- TTTTTTCAGG GTGTAGTCTT TTTCACACCT GAATCGTTGA TCCTTCATTT CAATGTCATA ATCGGCTCTC   
  
  
- GGGTACCTTG CGGACTTTGA CTGTAGTGGT CTGGGTGTAT AAAGTGGTAG TAGACTAGAG GGGCAGTTTC   
  
  
- CTCCTTCGAC TCGTGTCGTT CTTGATCTTC TTGTCCGCCG TAGTGTGGGT TCGGTTTTAA GAGATAAACT   
  
  
- GGAGGGAGAG GAGTAAGGAG ACTCGAAGGA GAAGTGTTAC ATTCTGGTAT TAGGAATTGT GGGAAGAAAA   
  
  
- AATTCGAGTA GAAATGGACG AGACCAAGAG AGAGAGTAGC AGTGATTATC AATGGAGAAA TGAAAGAGAA   
  
  
- GGGACTCCAT ACGAAGTTGA AATTGTATAT ATAGGGTGAA CTAAAGAGAA CAGAAAAACG ATATTATGAA   
  
  
- CAACAAACCG ATAGTAGGGG ACAAAATATG AGTAAAAGAA CGAAAGATAA AGACCCAAAC TTAACCCAAA   
  
  
- GTGAATAGGA GCAGTTAAGA TACCCATTAA TCACTTAACC CAAATATACT TGATCCCAAC GTTTTCATAG   
  
  
- ATGAAAAAGA GAACACTAAT AATCAACTAA TCCCTAAACC GGTTATAATC GTCTTACCCA AGACTTAAAC   
  
  
- GCCTTAAGAG ACTACTACGA GATTTACCCA TACGAATACA ACTATAGGGA CGAATACTAC GTAGGAATCT   
  
  
- AATACGGTTA AACAAGTTAA TGCTTCCGGG TAGACTCCTA GCGTGGAGTG ACTCGGAGGG TTCTGGGAAA   
  
  
- AGACTGGGAA ACCGAATGTC AAAGTGCAGG AGAAGACTCG ACCCGGGACC CCAACTAAGA TTACTACTAT   
  
  
- CACTAAGACC GCTACAAGAG TTCATGTAAT CGGTTTACGA ATACCTTCTT CTGTACCTCC GTTTCGGTAC   
  
  
- GTACAAAGTA CTAGGAAATC GTGAAGTCCG ACGACTCTTT GGGAAAATAC TACGGAATCC CCTCGTTACG   
  
  
- GGTTGAAGAG GACTGGTTGT AGGATATTAA CTAGTAATAA ACCTATCAGG ACTACTTTTA AGAAACTCAA   
  
  
- GTTGACCACT AAAATCACAA TCAGTAAGAC CCAGATCAAG TTGTTTGAGG CAACCTGGCT ATTATCACAG   
  
  
- ACTAAACTCA CTCGTAAAAC TCGGTGGGAA ACAACTTTGT GAAGGTTAAC TTAGTATAGT GGTTGGTAAC   
  
  
- TGGGCAGGAG TTACCAGTAA ACCGAGAAGC CCACGGAATC TACCGTGCCG GAGATTACCA AGCCACTAGA   
  
  
- GTAGGGAACC AAACGGTCAC CTACACTAAT CGCATAAATC CCTCTTTCTC AGGTACTAAG TTAAAGTCTT   
  
  
- TCCCCACCTC CTCCGGTCAT TCAAGGAAGG GTTCTTATTA TTGGAACAAT AACTAGAGCT CTTGGAGTGA   
  
  
- AAAGGATTAC TTTGTTTCCT CCTACTAGCT TACTACTACC AATTCTTCCT TTTCCTACTG CACTTAACCA   
  
  
- GATTGATGAG TTCTCCCTCA TTCTAAGTGA TAACACTTCT GCTCCGGAAA CTTCTTCCTT CCTCACCGTT   
  
  
- CGTCAGTCGA TAAAGAAGAT GACTCCTTCG ACTCAAAAGA CTGTAAAAAC TGTTCCAAAA CGAAACGCTA   
  
  
- ACGATGGGAC ACTTTGGACT CCGAGTAGGG TGGTACTCAA ACTTGGGACT CTTCCCGGTC AGTGTACCTA   
  
  
- ATCTTCCACC CTCTTTACCC TTCCGAGCGG GATTCTTTGT CCTATTATCA TCGTGTTTAT AACACCTAAA   
  
  
- TTCCTTAAAC GACTATGATA CGCGTGTTAG ATGACGTAGA CTACTGGCTT CTTGACGACT ACCTGACGAT   
  
  
- TTCGTTTAGT CCCTCGTGAG CAGACGACTC CCCCTACCTA GAGTTTCCAA CCGCGTAATG AAACGACTAC   
  
  
- GGGATCTCCG TGCAAATCGA CCTTGACCTA GAGCGTAAAT ATGCCGGGAT ACAAGATTAT CCGGTAGACA   
  
  
- GTGACTGTAC TATTTTCGTA TAGTCAAGAT ATAAGCACGA ACGGGTAAAT GCTTCTAGCA GTAACCAACA   
  
  
- CCATGGGTAT ACTAAGATTT TCGTCGACTC TTCCGTAGTT TCGAAGTATA ATATCTAAAA CCGTAGGAGA   
  
  
- TACCAACGGT TACCGGGTTG GAGTAAGTTG CGGAGAGTCT CGCTAGACCA CCTGGAGGTT TTGACAAATA   
  
  
- ATGTCCCTAG CTAGAGGGGG TCGGACCCAA GTCCGGTCGT CTTTCTCACC TTCGTTGTCC CTCTGCGAAC   
  
  
- CGGTTCATGA CACTCGCCAT ATTACACGGT AAACTCATAG TACGGTAACG AGTCCTCACC CTTTGTTAGT   
  
  
- TTGGTCCCCT AGAGTTCTAT CCTTCATCCT TACTACTTCA ACAACGCCAC TTGACAGAGA CATCCAAGTT   
  
  
- CTTGGAGGAA CTGCTCTGTC ACCACCACCT ATCAGGTTCC TTGTGTCAAA ATTTGGACTA ATGTTCCCAT   
  
  
- TTCGGACTAT AAAAACACGT ACCGCAACAT TTACCAAGGA TGTTGTAGGG AAAGAAACAC TGTGCAAAAT   
  
  
- CTCTTCGGGA GAAAGTAATA AGGTGAGAAA AGCTGTACAA TCTACGGTTG CGGAGATCCC TCCTCGGGCT   
  
  
- CTCCAACTAT AAGCTCTTCC GTAAGATACC CTCCCTCTAA TACTTACACC ACCGGACACT CCCGTGTCTC   
  
  
- TCCCACCTCT CCGGCCTCTG TATGTTCGTT ACCGTACAAT CCGTATCGTC CCGTCCCAAA GCCGTTCATG   
  
  
- GTAACCTAGG GTTCAACTAG CTCTTTTACT CCAAATTCCG GTTCCGTCTG GTGGTGTTCC TAAAGTACTA   
  
  
- GCTACACCTA CCTGTAACCC GTTAAGTCCC TACCTTCCCC GCCTAACGTG TACGCTAGAG ACGTACCCAA   
  
  
- GGCCGAAC

+     CGTCA-motif

| Site Name | Organism | Position | Strand | Matrix score. | sequence | function |
| --- | --- | --- | --- | --- | --- | --- |
| CGTCA-motif | Hordeum vulgare | 3794 | - | 5 | CGTCA | cis-acting regulatory element involved in the MeJA-responsiveness |
| CGTCA-motif | Hordeum vulgare | 2861 | - | 5 | CGTCA | cis-acting regulatory element involved in the MeJA-responsiveness |
| CGTCA-motif | Hordeum vulgare | 1723 | + | 5 | CGTCA | cis-acting regulatory element involved in the MeJA-responsiveness |
| CGTCA-motif | Hordeum vulgare | 1360 | - | 5 | CGTCA | cis-acting regulatory element involved in the MeJA-responsiveness |
| CGTCA-motif | Hordeum vulgare | 1131 | + | 5 | CGTCA | cis-acting regulatory element involved in the MeJA-responsiveness |
| CGTCA-motif | Hordeum vulgare | 3422 | + | 5 | CGTCA | cis-acting regulatory element involved in the MeJA-responsiveness |
| CGTCA-motif | Hordeum vulgare | 1905 | + | 5 | CGTCA | cis-acting regulatory element involved in the MeJA-responsiveness |
| CGTCA-motif | Hordeum vulgare | 1369 | + | 5 | CGTCA | cis-acting regulatory element involved in the MeJA-responsiveness |
| CGTCA-motif | Hordeum vulgare | 1537 | + | 5 | CGTCA | cis-acting regulatory element involved in the MeJA-responsiveness |

>HU07G02249.1   
+ -Up\_Stream \_Len000GATTTG ATTGTCGAAA TTTGTCCGAT TTTATAAAAT TGAAAATTGT TATTGGACTA   
  
  
+ TAATCTACTG TAGTTTAGAG GTGTTGGATA ATACTCTCAA CTTTGAACTT GGACGTCCAT GCACCACACT   
  
  
+ ATGGGCGTAT TTACTGTTTA TATACCAAGA ATAATTTTGA AGATGTTCTT AGTTGGTGAT GTAAGTGTAT   
  
  
+ ATTCAACATA TGTCATTACA TAAACTTGAA AATTCTTGAA TTTTAGACAT ATACAATTGC AAATCATTGT   
  
  
+ AAAATTGTTG CATTACATTT TGATAAAAAT GCATCCAAAC TTTGTCTTGG TATAAACAGT AAATAGGCCC   
  
  
+ ATAGGTCCAT AGTGTAGTGC ATGGACGTCC AAGTTTAAAG GTGGGAGTAT TATTGCCCAA CGCCCCTAAA   
  
  
+ GTACAGTATA TTATAGTCCA ATATCCCAAT TAATTTTTCA TTTGAATATT TAAATTTGGA CATAATATTT   
  
  
+ TTCATATTTG AATTCTCTAA ACCTGATTTT AACCTGAATT TAAGTTGATT TAATTCATCC GACCAAAAAA   
  
  
+ ATAAACACAA TTATTTATTT TTCTATTCGA AGTTAGATAG TAGCCAATTC ATTTGACATA TAATTAACCC   
  
  
+ GTGATTGACA CAAACTCGAG TTTTTGCTTA GAGCTAGGAA AAGAGTCGGG TTAGGTCGAC CCATTTAAGA   
  
  
+ AATGGGTCGA CTTCATGTCG GCCTTCAAAA TAGTTAAGAT TTGCTCAAAA CTAAAAAATA ACAAGTTCAC   
  
  
+ ACTAAACGGT CAAATCGTGT CGACTTCGGA TTGAAATTGG TTCAGTTTGG GTCGGCTTCG TATTTCTTTC   
  
  
+ AGTAACTTCG AACCGAATTC GTATCGTGTT ATCGTGTCAG GTCAGACTTT CTCAGTTCTA TTTTTGCTAC   
  
  
+ CTTATGTTAC GGATAAAAAA AGTCTCACAT TGGAAAAAGT GTGGGAGGGT CCTGGGCTTA TAAATAGTGG   
  
  
+ CGCTGCACAC CTCATCAGAC CGACCTTTTG GGGAGAGGGT AATGACCCCG TGACAAGTGG TATCAGAGTC   
  
  
+ AACCCAGACC CGACTCAGAT GTCCTGCCAA GCGCTCCGAC GGGCGGGGTA GGCAGGGGGC CCATTTGTGA   
  
  
+ TGAGGACGTC ACAGAATTGG GCGGGGGAGA GTGTCACGGA TAAAAAAAGT CCCACATCGA AAAAAGTGTG   
  
  
+ GGAGGGTCCT GGGCTTATAA ACGGTAGCGC CGCACACCCC ATCAGATCGA CTTTTTGGGG AGAGGGTAAT   
  
  
+ GACCCCATTT CAAGTGGTAT CAGAGCCAAC CCAGACCCGA CTCCAATGTC CCACCAAGCG CTCTGATGGG   
  
  
+ CGGGGTAGGC AAGGGGCTCC ATATGTGACG AGGGCGTCAC GGAATTGGAT GGGAGAGAGT ACCACGGATA   
  
  
+ AAAAAAGTCC CACATCAGAA AAAGTGTGGA CTTAGCAACT AGGAAGTAAA GTTACAGTAT TAGCCGAGAG   
  
  
+ CCCATGGAAC GCCTGAAACT GACATCACCA GACCCACATA TTTCACCATC ATCTGATCTC CCCGTCAAAG   
  
  
+ GAGGAAGCTG AGCACAGCAA GAACTAGAAG AACAGGCGGC ATCACACCCA AGCCAAAATT CTCTATTTGA   
  
  
+ CCTCCCTCTC CTCATTCCTC TGAGCTTCCT CTTCACAATG TAAGACCATA ATCCTTAACA CCCTTCTTTT   
  
  
+ TTAAGCTCAT CTTTACCTGC TCTGGTTCTC TCTCTCATCG TCACTAATAG TTACCTCTTT ACTTTCTCTT   
  
  
+ CCCTGAGGTA TGCTTCAACT TTAACATATA TATCCCACTT GATTTCTCTT GTCTTTTTGC TATAATACTT   
  
  
+ GTTGTTTGGC TATCATCCCC TGTTTTATAC TCATTTTCTT GCTTTCTATT TCTGGGTTTG AATTGGGTTT   
  
  
+ CACTTATCCT CGTCAATTCT ATGGGTAATT AGTGAATTGG GTTTATATGA ACTAGGGTTG CAAAAGTATC   
  
  
+ TACTTTTTCT CTTGTGATTA TTAGTTGATT AGGGATTTGG CCAATATTAG CAGAATGGGT TCTGAATTTG   
  
  
+ CGGAATTCTC TGATGATGCT CTAAATGGGT ATGCTTATGT TGATATCCCT GCTTATGATG CATCCTTAGA   
  
  
+ TTATGCCAAT TTGTTCAATT ACGAAGGCCC ATCTGAGGAT CGCACCTCAC TGAGCCTCCC AAGACCCTTT   
  
  
+ TCTGACCCTT TGGCTTACAG TTTCACGTCC TCTTCTGAGC TGGGCCCTGG GGTTGATTCT AATGATGATA   
  
  
+ GTGATTCTGG CGATGTTCTC AAGTACATTA GCCAAATGCT TATGGAAGAA GACATGGAGG CAAAGCCATG   
  
  
+ CATGTTTCAT GATCCTTTAG CACTTCAGGC TGCTGAGAAA CCCTTTTATG ATGCCTTAGG GGAGCAATGC   
  
  
+ CCAACTTCTC CTGACCAACA TCCTATAATT GATCATTATT TGGATAGTCC TGATGAAAAT TCTTTGAGTT   
  
  
+ CAACTGGTGA TTTTAGTGTT AGTCATTCTG GGTCTAGTTC AACAAACTCC GTTGGACCGA TAATAGTGTC   
  
  
+ TGATTTGAGT GAGCATTTTG AGCCACCCTT TGTTGAAACA CTTCCAATTG AATCATATCA CCAACCATTG   
  
  
+ ACCCGTCCTC AATGGTCATT TGGCTCTTCG GGTGCCTTAG ATGGCACGGC CTCTAATGGT TCGGTGATCT   
  
  
+ CATCCCTTGG TTTGCCAGTG GATGTGATTA GCGTATTTAG GGAGAAAGAG TCCATGATTC AATTTCAGAA   
  
  
+ AGGGGTGGAG GAGGCCAGTA AGTTCCTTCC CAAGAATAAT AACCTTGTTA TTGATCTCGA GAACCTCACT   
  
  
+ TTTCCTAATG AAACAAAGGA GGATGATCGA ATGATGATGG TTAAGAAGGA AAAGGATGAC GTGAATTGGT   
  
  
+ CTAACTACTC AAGAGGGAGT AAGATTCACT ATTGTGAAGA CGAGGCCTTT GAAGAAGGAA GGAGTGGCAA   
  
  
+ GCAGTCAGCT ATTTCTTCTA CTGAGGAAGC TGAGTTTTCT GACATTTTTG ACAAGGTTTT GCTTTGCGAT   
  
  
+ TGCTACCCTG TGAAACCTGA GGCTCATCCC ACCATGAGTT TGAACCCTGA GAAGGGCCAG TCACATGGAT   
  
  
+ TAGAAGGTGG GAGAAATGGG AAGGCTCGCC CTAAGAAACA GGATAATAGT AGCACAAATA TTGTGGATTT   
  
  
+ AAGGAATTTG CTGATACTAT GCGCACAATC TACTGCATCT GATGACCGAA GAACTGCTGA TGGACTGCTA   
  
  
+ AAGCAAATCA GGGAGCACTC GTCTGCTGAG GGGGATGGAT CTCAAAGGTT GGCGCATTAC TTTGCTGATG   
  
  
+ CCCTAGAGGC ACGTTTAGCT GGAACTGGAT CTCGCATTTA TACGGCCCTA TGTTCTAATA GGCCATCTGT   
  
  
+ CACTGACATG ATAAAAGCAT ATCAGTTCTA TATTCGTGCT TGCCCATTTA CGAAGATCGT CATTGGTTGT   
  
  
+ GGTACCCATA TGATTCTAAA AGCAGCTGAG AAGGCATCAA AGCTTCATAT TATAGATTTT GGCATCCTCT   
  
  
+ ATGGTTGCCA ATGGCCCAAC CTCATTCAAC GCCTCTCAGA GCGATCTGGT GGACCTCCAA AACTGTTTAT   
  
  
+ TACAGGGATC GATCTCCCCC AGCCTGGGTT CAGGCCAGCA GAAAGAGTGG AAGCAACAGG GAGACGCTTG   
  
  
+ GCCAAGTACT GTGAGCGGTA TAATGTGCCA TTTGAGTATC ATGCCATTGC TCAGGAGTGG GAAACAATCA   
  
  
+ AACCAGGGGA TCTCAAGATA GGAAGTAGGA ATGATGAAGT TGTTGCGGTG AACTGTCTCT GTAGGTTCAA   
  
  
+ GAACCTCCTT GACGAGACAG TGGTGGTGGA TAGTCCAAGG AACACAGTTT TAAACCTGAT TACAAGGGTA   
  
  
+ AAGCCTGATA TTTTTGTGCA TGGCGTTGTA AATGGTTCCT ACAACATCCC TTTCTTTGTG ACACGTTTTA   
  
  
+ GAGAAGCCCT CTTTCATTAT TCCACTCTTT TCGACATGTT AGATGCCAAC GCCTCTAGGG AGGAGCCCGA   
  
  
+ GAGGTTGATA TTCGAGAAGG CATTCTATGG GAGGGAGATT ATGAATGTGG TGGCCTGTGA GGGCACAGAG   
  
  
+ AGGGTGGAGA GGCCGGAGAC ATACAAGCAA TGGCATGTTA GGCATAGCAG GGCAGGGTTT CGGCAAGTAC   
  
  
+ CATTGGATCC CAAGTTGATC GAGAAAATGA GGTTTAAGGC CAAGGCAGAC CACCACAAGG ATTTCATGAT   
  
  
+ CGATGTGGAT GGACATTGGG CAATTCAGGG ATGGAAGGGG CGGATTGCAC ATGCGATCTC TGCATGGGTT   
  
  
+ CCGGCTTG  

- -Up\_Stream \_Len000CTAAAC TAACAGCTTT AAACAGGCTA AAATATTTTA ACTTTTAACA ATAACCTGAT   
  
  
- ATTAGATGAC ATCAAATCTC CACAACCTAT TATGAGAGTT GAAACTTGAA CCTGCAGGTA CGTGGTGTGA   
  
  
- TACCCGCATA AATGACAAAT ATATGGTTCT TATTAAAACT TCTACAAGAA TCAACCACTA CATTCACATA   
  
  
- TAAGTTGTAT ACAGTAATGT ATTTGAACTT TTAAGAACTT AAAATCTGTA TATGTTAACG TTTAGTAACA   
  
  
- TTTTAACAAC GTAATGTAAA ACTATTTTTA CGTAGGTTTG AAACAGAACC ATATTTGTCA TTTATCCGGG   
  
  
- TATCCAGGTA TCACATCACG TACCTGCAGG TTCAAATTTC CACCCTCATA ATAACGGGTT GCGGGGATTT   
  
  
- CATGTCATAT AATATCAGGT TATAGGGTTA ATTAAAAAGT AAACTTATAA ATTTAAACCT GTATTATAAA   
  
  
- AAGTATAAAC TTAAGAGATT TGGACTAAAA TTGGACTTAA ATTCAACTAA ATTAAGTAGG CTGGTTTTTT   
  
  
- TATTTGTGTT AATAAATAAA AAGATAAGCT TCAATCTATC ATCGGTTAAG TAAACTGTAT ATTAATTGGG   
  
  
- CACTAACTGT GTTTGAGCTC AAAAACGAAT CTCGATCCTT TTCTCAGCCC AATCCAGCTG GGTAAATTCT   
  
  
- TTACCCAGCT GAAGTACAGC CGGAAGTTTT ATCAATTCTA AACGAGTTTT GATTTTTTAT TGTTCAAGTG   
  
  
- TGATTTGCCA GTTTAGCACA GCTGAAGCCT AACTTTAACC AAGTCAAACC CAGCCGAAGC ATAAAGAAAG   
  
  
- TCATTGAAGC TTGGCTTAAG CATAGCACAA TAGCACAGTC CAGTCTGAAA GAGTCAAGAT AAAAACGATG   
  
  
- GAATACAATG CCTATTTTTT TCAGAGTGTA ACCTTTTTCA CACCCTCCCA GGACCCGAAT ATTTATCACC   
  
  
- GCGACGTGTG GAGTAGTCTG GCTGGAAAAC CCCTCTCCCA TTACTGGGGC ACTGTTCACC ATAGTCTCAG   
  
  
- TTGGGTCTGG GCTGAGTCTA CAGGACGGTT CGCGAGGCTG CCCGCCCCAT CCGTCCCCCG GGTAAACACT   
  
  
- ACTCCTGCAG TGTCTTAACC CGCCCCCTCT CACAGTGCCT ATTTTTTTCA GGGTGTAGCT TTTTTCACAC   
  
  
- CCTCCCAGGA CCCGAATATT TGCCATCGCG GCGTGTGGGG TAGTCTAGCT GAAAAACCCC TCTCCCATTA   
  
  
- CTGGGGTAAA GTTCACCATA GTCTCGGTTG GGTCTGGGCT GAGGTTACAG GGTGGTTCGC GAGACTACCC   
  
  
- GCCCCATCCG TTCCCCGAGG TATACACTGC TCCCGCAGTG CCTTAACCTA CCCTCTCTCA TGGTGCCTAT   
  
  
- TTTTTTCAGG GTGTAGTCTT TTTCACACCT GAATCGTTGA TCCTTCATTT CAATGTCATA ATCGGCTCTC   
  
  
- GGGTACCTTG CGGACTTTGA CTGTAGTGGT CTGGGTGTAT AAAGTGGTAG TAGACTAGAG GGGCAGTTTC   
  
  
- CTCCTTCGAC TCGTGTCGTT CTTGATCTTC TTGTCCGCCG TAGTGTGGGT TCGGTTTTAA GAGATAAACT   
  
  
- GGAGGGAGAG GAGTAAGGAG ACTCGAAGGA GAAGTGTTAC ATTCTGGTAT TAGGAATTGT GGGAAGAAAA   
  
  
- AATTCGAGTA GAAATGGACG AGACCAAGAG AGAGAGTAGC AGTGATTATC AATGGAGAAA TGAAAGAGAA   
  
  
- GGGACTCCAT ACGAAGTTGA AATTGTATAT ATAGGGTGAA CTAAAGAGAA CAGAAAAACG ATATTATGAA   
  
  
- CAACAAACCG ATAGTAGGGG ACAAAATATG AGTAAAAGAA CGAAAGATAA AGACCCAAAC TTAACCCAAA   
  
  
- GTGAATAGGA GCAGTTAAGA TACCCATTAA TCACTTAACC CAAATATACT TGATCCCAAC GTTTTCATAG   
  
  
- ATGAAAAAGA GAACACTAAT AATCAACTAA TCCCTAAACC GGTTATAATC GTCTTACCCA AGACTTAAAC   
  
  
- GCCTTAAGAG ACTACTACGA GATTTACCCA TACGAATACA ACTATAGGGA CGAATACTAC GTAGGAATCT   
  
  
- AATACGGTTA AACAAGTTAA TGCTTCCGGG TAGACTCCTA GCGTGGAGTG ACTCGGAGGG TTCTGGGAAA   
  
  
- AGACTGGGAA ACCGAATGTC AAAGTGCAGG AGAAGACTCG ACCCGGGACC CCAACTAAGA TTACTACTAT   
  
  
- CACTAAGACC GCTACAAGAG TTCATGTAAT CGGTTTACGA ATACCTTCTT CTGTACCTCC GTTTCGGTAC   
  
  
- GTACAAAGTA CTAGGAAATC GTGAAGTCCG ACGACTCTTT GGGAAAATAC TACGGAATCC CCTCGTTACG   
  
  
- GGTTGAAGAG GACTGGTTGT AGGATATTAA CTAGTAATAA ACCTATCAGG ACTACTTTTA AGAAACTCAA   
  
  
- GTTGACCACT AAAATCACAA TCAGTAAGAC CCAGATCAAG TTGTTTGAGG CAACCTGGCT ATTATCACAG   
  
  
- ACTAAACTCA CTCGTAAAAC TCGGTGGGAA ACAACTTTGT GAAGGTTAAC TTAGTATAGT GGTTGGTAAC   
  
  
- TGGGCAGGAG TTACCAGTAA ACCGAGAAGC CCACGGAATC TACCGTGCCG GAGATTACCA AGCCACTAGA   
  
  
- GTAGGGAACC AAACGGTCAC CTACACTAAT CGCATAAATC CCTCTTTCTC AGGTACTAAG TTAAAGTCTT   
  
  
- TCCCCACCTC CTCCGGTCAT TCAAGGAAGG GTTCTTATTA TTGGAACAAT AACTAGAGCT CTTGGAGTGA   
  
  
- AAAGGATTAC TTTGTTTCCT CCTACTAGCT TACTACTACC AATTCTTCCT TTTCCTACTG CACTTAACCA   
  
  
- GATTGATGAG TTCTCCCTCA TTCTAAGTGA TAACACTTCT GCTCCGGAAA CTTCTTCCTT CCTCACCGTT   
  
  
- CGTCAGTCGA TAAAGAAGAT GACTCCTTCG ACTCAAAAGA CTGTAAAAAC TGTTCCAAAA CGAAACGCTA   
  
  
- ACGATGGGAC ACTTTGGACT CCGAGTAGGG TGGTACTCAA ACTTGGGACT CTTCCCGGTC AGTGTACCTA   
  
  
- ATCTTCCACC CTCTTTACCC TTCCGAGCGG GATTCTTTGT CCTATTATCA TCGTGTTTAT AACACCTAAA   
  
  
- TTCCTTAAAC GACTATGATA CGCGTGTTAG ATGACGTAGA CTACTGGCTT CTTGACGACT ACCTGACGAT   
  
  
- TTCGTTTAGT CCCTCGTGAG CAGACGACTC CCCCTACCTA GAGTTTCCAA CCGCGTAATG AAACGACTAC   
  
  
- GGGATCTCCG TGCAAATCGA CCTTGACCTA GAGCGTAAAT ATGCCGGGAT ACAAGATTAT CCGGTAGACA   
  
  
- GTGACTGTAC TATTTTCGTA TAGTCAAGAT ATAAGCACGA ACGGGTAAAT GCTTCTAGCA GTAACCAACA   
  
  
- CCATGGGTAT ACTAAGATTT TCGTCGACTC TTCCGTAGTT TCGAAGTATA ATATCTAAAA CCGTAGGAGA   
  
  
- TACCAACGGT TACCGGGTTG GAGTAAGTTG CGGAGAGTCT CGCTAGACCA CCTGGAGGTT TTGACAAATA   
  
  
- ATGTCCCTAG CTAGAGGGGG TCGGACCCAA GTCCGGTCGT CTTTCTCACC TTCGTTGTCC CTCTGCGAAC   
  
  
- CGGTTCATGA CACTCGCCAT ATTACACGGT AAACTCATAG TACGGTAACG AGTCCTCACC CTTTGTTAGT   
  
  
- TTGGTCCCCT AGAGTTCTAT CCTTCATCCT TACTACTTCA ACAACGCCAC TTGACAGAGA CATCCAAGTT   
  
  
- CTTGGAGGAA CTGCTCTGTC ACCACCACCT ATCAGGTTCC TTGTGTCAAA ATTTGGACTA ATGTTCCCAT   
  
  
- TTCGGACTAT AAAAACACGT ACCGCAACAT TTACCAAGGA TGTTGTAGGG AAAGAAACAC TGTGCAAAAT   
  
  
- CTCTTCGGGA GAAAGTAATA AGGTGAGAAA AGCTGTACAA TCTACGGTTG CGGAGATCCC TCCTCGGGCT   
  
  
- CTCCAACTAT AAGCTCTTCC GTAAGATACC CTCCCTCTAA TACTTACACC ACCGGACACT CCCGTGTCTC   
  
  
- TCCCACCTCT CCGGCCTCTG TATGTTCGTT ACCGTACAAT CCGTATCGTC CCGTCCCAAA GCCGTTCATG   
  
  
- GTAACCTAGG GTTCAACTAG CTCTTTTACT CCAAATTCCG GTTCCGTCTG GTGGTGTTCC TAAAGTACTA   
  
  
- GCTACACCTA CCTGTAACCC GTTAAGTCCC TACCTTCCCC GCCTAACGTG TACGCTAGAG ACGTACCCAA   
  
  
- GGCCGAAC

+     CTAG-motif

| Site Name | Organism | Position | Strand | Matrix score. | sequence | function |
| --- | --- | --- | --- | --- | --- | --- |
| CTAG-motif | Avena sativa | 2010 | + | 9 | ACTAGCAGAA |  |
| CTAG-motif | Avena sativa | 1567 | + | 9 | ACTAGCAGAA |  |

>HU07G02249.1   
+ -Up\_Stream \_Len000GATTTG ATTGTCGAAA TTTGTCCGAT TTTATAAAAT TGAAAATTGT TATTGGACTA   
  
  
+ TAATCTACTG TAGTTTAGAG GTGTTGGATA ATACTCTCAA CTTTGAACTT GGACGTCCAT GCACCACACT   
  
  
+ ATGGGCGTAT TTACTGTTTA TATACCAAGA ATAATTTTGA AGATGTTCTT AGTTGGTGAT GTAAGTGTAT   
  
  
+ ATTCAACATA TGTCATTACA TAAACTTGAA AATTCTTGAA TTTTAGACAT ATACAATTGC AAATCATTGT   
  
  
+ AAAATTGTTG CATTACATTT TGATAAAAAT GCATCCAAAC TTTGTCTTGG TATAAACAGT AAATAGGCCC   
  
  
+ ATAGGTCCAT AGTGTAGTGC ATGGACGTCC AAGTTTAAAG GTGGGAGTAT TATTGCCCAA CGCCCCTAAA   
  
  
+ GTACAGTATA TTATAGTCCA ATATCCCAAT TAATTTTTCA TTTGAATATT TAAATTTGGA CATAATATTT   
  
  
+ TTCATATTTG AATTCTCTAA ACCTGATTTT AACCTGAATT TAAGTTGATT TAATTCATCC GACCAAAAAA   
  
  
+ ATAAACACAA TTATTTATTT TTCTATTCGA AGTTAGATAG TAGCCAATTC ATTTGACATA TAATTAACCC   
  
  
+ GTGATTGACA CAAACTCGAG TTTTTGCTTA GAGCTAGGAA AAGAGTCGGG TTAGGTCGAC CCATTTAAGA   
  
  
+ AATGGGTCGA CTTCATGTCG GCCTTCAAAA TAGTTAAGAT TTGCTCAAAA CTAAAAAATA ACAAGTTCAC   
  
  
+ ACTAAACGGT CAAATCGTGT CGACTTCGGA TTGAAATTGG TTCAGTTTGG GTCGGCTTCG TATTTCTTTC   
  
  
+ AGTAACTTCG AACCGAATTC GTATCGTGTT ATCGTGTCAG GTCAGACTTT CTCAGTTCTA TTTTTGCTAC   
  
  
+ CTTATGTTAC GGATAAAAAA AGTCTCACAT TGGAAAAAGT GTGGGAGGGT CCTGGGCTTA TAAATAGTGG   
  
  
+ CGCTGCACAC CTCATCAGAC CGACCTTTTG GGGAGAGGGT AATGACCCCG TGACAAGTGG TATCAGAGTC   
  
  
+ AACCCAGACC CGACTCAGAT GTCCTGCCAA GCGCTCCGAC GGGCGGGGTA GGCAGGGGGC CCATTTGTGA   
  
  
+ TGAGGACGTC ACAGAATTGG GCGGGGGAGA GTGTCACGGA TAAAAAAAGT CCCACATCGA AAAAAGTGTG   
  
  
+ GGAGGGTCCT GGGCTTATAA ACGGTAGCGC CGCACACCCC ATCAGATCGA CTTTTTGGGG AGAGGGTAAT   
  
  
+ GACCCCATTT CAAGTGGTAT CAGAGCCAAC CCAGACCCGA CTCCAATGTC CCACCAAGCG CTCTGATGGG   
  
  
+ CGGGGTAGGC AAGGGGCTCC ATATGTGACG AGGGCGTCAC GGAATTGGAT GGGAGAGAGT ACCACGGATA   
  
  
+ AAAAAAGTCC CACATCAGAA AAAGTGTGGA CTTAGCAACT AGGAAGTAAA GTTACAGTAT TAGCCGAGAG   
  
  
+ CCCATGGAAC GCCTGAAACT GACATCACCA GACCCACATA TTTCACCATC ATCTGATCTC CCCGTCAAAG   
  
  
+ GAGGAAGCTG AGCACAGCAA GAACTAGAAG AACAGGCGGC ATCACACCCA AGCCAAAATT CTCTATTTGA   
  
  
+ CCTCCCTCTC CTCATTCCTC TGAGCTTCCT CTTCACAATG TAAGACCATA ATCCTTAACA CCCTTCTTTT   
  
  
+ TTAAGCTCAT CTTTACCTGC TCTGGTTCTC TCTCTCATCG TCACTAATAG TTACCTCTTT ACTTTCTCTT   
  
  
+ CCCTGAGGTA TGCTTCAACT TTAACATATA TATCCCACTT GATTTCTCTT GTCTTTTTGC TATAATACTT   
  
  
+ GTTGTTTGGC TATCATCCCC TGTTTTATAC TCATTTTCTT GCTTTCTATT TCTGGGTTTG AATTGGGTTT   
  
  
+ CACTTATCCT CGTCAATTCT ATGGGTAATT AGTGAATTGG GTTTATATGA ACTAGGGTTG CAAAAGTATC   
  
  
+ TACTTTTTCT CTTGTGATTA TTAGTTGATT AGGGATTTGG CCAATATTAG CAGAATGGGT TCTGAATTTG   
  
  
+ CGGAATTCTC TGATGATGCT CTAAATGGGT ATGCTTATGT TGATATCCCT GCTTATGATG CATCCTTAGA   
  
  
+ TTATGCCAAT TTGTTCAATT ACGAAGGCCC ATCTGAGGAT CGCACCTCAC TGAGCCTCCC AAGACCCTTT   
  
  
+ TCTGACCCTT TGGCTTACAG TTTCACGTCC TCTTCTGAGC TGGGCCCTGG GGTTGATTCT AATGATGATA   
  
  
+ GTGATTCTGG CGATGTTCTC AAGTACATTA GCCAAATGCT TATGGAAGAA GACATGGAGG CAAAGCCATG   
  
  
+ CATGTTTCAT GATCCTTTAG CACTTCAGGC TGCTGAGAAA CCCTTTTATG ATGCCTTAGG GGAGCAATGC   
  
  
+ CCAACTTCTC CTGACCAACA TCCTATAATT GATCATTATT TGGATAGTCC TGATGAAAAT TCTTTGAGTT   
  
  
+ CAACTGGTGA TTTTAGTGTT AGTCATTCTG GGTCTAGTTC AACAAACTCC GTTGGACCGA TAATAGTGTC   
  
  
+ TGATTTGAGT GAGCATTTTG AGCCACCCTT TGTTGAAACA CTTCCAATTG AATCATATCA CCAACCATTG   
  
  
+ ACCCGTCCTC AATGGTCATT TGGCTCTTCG GGTGCCTTAG ATGGCACGGC CTCTAATGGT TCGGTGATCT   
  
  
+ CATCCCTTGG TTTGCCAGTG GATGTGATTA GCGTATTTAG GGAGAAAGAG TCCATGATTC AATTTCAGAA   
  
  
+ AGGGGTGGAG GAGGCCAGTA AGTTCCTTCC CAAGAATAAT AACCTTGTTA TTGATCTCGA GAACCTCACT   
  
  
+ TTTCCTAATG AAACAAAGGA GGATGATCGA ATGATGATGG TTAAGAAGGA AAAGGATGAC GTGAATTGGT   
  
  
+ CTAACTACTC AAGAGGGAGT AAGATTCACT ATTGTGAAGA CGAGGCCTTT GAAGAAGGAA GGAGTGGCAA   
  
  
+ GCAGTCAGCT ATTTCTTCTA CTGAGGAAGC TGAGTTTTCT GACATTTTTG ACAAGGTTTT GCTTTGCGAT   
  
  
+ TGCTACCCTG TGAAACCTGA GGCTCATCCC ACCATGAGTT TGAACCCTGA GAAGGGCCAG TCACATGGAT   
  
  
+ TAGAAGGTGG GAGAAATGGG AAGGCTCGCC CTAAGAAACA GGATAATAGT AGCACAAATA TTGTGGATTT   
  
  
+ AAGGAATTTG CTGATACTAT GCGCACAATC TACTGCATCT GATGACCGAA GAACTGCTGA TGGACTGCTA   
  
  
+ AAGCAAATCA GGGAGCACTC GTCTGCTGAG GGGGATGGAT CTCAAAGGTT GGCGCATTAC TTTGCTGATG   
  
  
+ CCCTAGAGGC ACGTTTAGCT GGAACTGGAT CTCGCATTTA TACGGCCCTA TGTTCTAATA GGCCATCTGT   
  
  
+ CACTGACATG ATAAAAGCAT ATCAGTTCTA TATTCGTGCT TGCCCATTTA CGAAGATCGT CATTGGTTGT   
  
  
+ GGTACCCATA TGATTCTAAA AGCAGCTGAG AAGGCATCAA AGCTTCATAT TATAGATTTT GGCATCCTCT   
  
  
+ ATGGTTGCCA ATGGCCCAAC CTCATTCAAC GCCTCTCAGA GCGATCTGGT GGACCTCCAA AACTGTTTAT   
  
  
+ TACAGGGATC GATCTCCCCC AGCCTGGGTT CAGGCCAGCA GAAAGAGTGG AAGCAACAGG GAGACGCTTG   
  
  
+ GCCAAGTACT GTGAGCGGTA TAATGTGCCA TTTGAGTATC ATGCCATTGC TCAGGAGTGG GAAACAATCA   
  
  
+ AACCAGGGGA TCTCAAGATA GGAAGTAGGA ATGATGAAGT TGTTGCGGTG AACTGTCTCT GTAGGTTCAA   
  
  
+ GAACCTCCTT GACGAGACAG TGGTGGTGGA TAGTCCAAGG AACACAGTTT TAAACCTGAT TACAAGGGTA   
  
  
+ AAGCCTGATA TTTTTGTGCA TGGCGTTGTA AATGGTTCCT ACAACATCCC TTTCTTTGTG ACACGTTTTA   
  
  
+ GAGAAGCCCT CTTTCATTAT TCCACTCTTT TCGACATGTT AGATGCCAAC GCCTCTAGGG AGGAGCCCGA   
  
  
+ GAGGTTGATA TTCGAGAAGG CATTCTATGG GAGGGAGATT ATGAATGTGG TGGCCTGTGA GGGCACAGAG   
  
  
+ AGGGTGGAGA GGCCGGAGAC ATACAAGCAA TGGCATGTTA GGCATAGCAG GGCAGGGTTT CGGCAAGTAC   
  
  
+ CATTGGATCC CAAGTTGATC GAGAAAATGA GGTTTAAGGC CAAGGCAGAC CACCACAAGG ATTTCATGAT   
  
  
+ CGATGTGGAT GGACATTGGG CAATTCAGGG ATGGAAGGGG CGGATTGCAC ATGCGATCTC TGCATGGGTT   
  
  
+ CCGGCTTG  

- -Up\_Stream \_Len000CTAAAC TAACAGCTTT AAACAGGCTA AAATATTTTA ACTTTTAACA ATAACCTGAT   
  
  
- ATTAGATGAC ATCAAATCTC CACAACCTAT TATGAGAGTT GAAACTTGAA CCTGCAGGTA CGTGGTGTGA   
  
  
- TACCCGCATA AATGACAAAT ATATGGTTCT TATTAAAACT TCTACAAGAA TCAACCACTA CATTCACATA   
  
  
- TAAGTTGTAT ACAGTAATGT ATTTGAACTT TTAAGAACTT AAAATCTGTA TATGTTAACG TTTAGTAACA   
  
  
- TTTTAACAAC GTAATGTAAA ACTATTTTTA CGTAGGTTTG AAACAGAACC ATATTTGTCA TTTATCCGGG   
  
  
- TATCCAGGTA TCACATCACG TACCTGCAGG TTCAAATTTC CACCCTCATA ATAACGGGTT GCGGGGATTT   
  
  
- CATGTCATAT AATATCAGGT TATAGGGTTA ATTAAAAAGT AAACTTATAA ATTTAAACCT GTATTATAAA   
  
  
- AAGTATAAAC TTAAGAGATT TGGACTAAAA TTGGACTTAA ATTCAACTAA ATTAAGTAGG CTGGTTTTTT   
  
  
- TATTTGTGTT AATAAATAAA AAGATAAGCT TCAATCTATC ATCGGTTAAG TAAACTGTAT ATTAATTGGG   
  
  
- CACTAACTGT GTTTGAGCTC AAAAACGAAT CTCGATCCTT TTCTCAGCCC AATCCAGCTG GGTAAATTCT   
  
  
- TTACCCAGCT GAAGTACAGC CGGAAGTTTT ATCAATTCTA AACGAGTTTT GATTTTTTAT TGTTCAAGTG   
  
  
- TGATTTGCCA GTTTAGCACA GCTGAAGCCT AACTTTAACC AAGTCAAACC CAGCCGAAGC ATAAAGAAAG   
  
  
- TCATTGAAGC TTGGCTTAAG CATAGCACAA TAGCACAGTC CAGTCTGAAA GAGTCAAGAT AAAAACGATG   
  
  
- GAATACAATG CCTATTTTTT TCAGAGTGTA ACCTTTTTCA CACCCTCCCA GGACCCGAAT ATTTATCACC   
  
  
- GCGACGTGTG GAGTAGTCTG GCTGGAAAAC CCCTCTCCCA TTACTGGGGC ACTGTTCACC ATAGTCTCAG   
  
  
- TTGGGTCTGG GCTGAGTCTA CAGGACGGTT CGCGAGGCTG CCCGCCCCAT CCGTCCCCCG GGTAAACACT   
  
  
- ACTCCTGCAG TGTCTTAACC CGCCCCCTCT CACAGTGCCT ATTTTTTTCA GGGTGTAGCT TTTTTCACAC   
  
  
- CCTCCCAGGA CCCGAATATT TGCCATCGCG GCGTGTGGGG TAGTCTAGCT GAAAAACCCC TCTCCCATTA   
  
  
- CTGGGGTAAA GTTCACCATA GTCTCGGTTG GGTCTGGGCT GAGGTTACAG GGTGGTTCGC GAGACTACCC   
  
  
- GCCCCATCCG TTCCCCGAGG TATACACTGC TCCCGCAGTG CCTTAACCTA CCCTCTCTCA TGGTGCCTAT   
  
  
- TTTTTTCAGG GTGTAGTCTT TTTCACACCT GAATCGTTGA TCCTTCATTT CAATGTCATA ATCGGCTCTC   
  
  
- GGGTACCTTG CGGACTTTGA CTGTAGTGGT CTGGGTGTAT AAAGTGGTAG TAGACTAGAG GGGCAGTTTC   
  
  
- CTCCTTCGAC TCGTGTCGTT CTTGATCTTC TTGTCCGCCG TAGTGTGGGT TCGGTTTTAA GAGATAAACT   
  
  
- GGAGGGAGAG GAGTAAGGAG ACTCGAAGGA GAAGTGTTAC ATTCTGGTAT TAGGAATTGT GGGAAGAAAA   
  
  
- AATTCGAGTA GAAATGGACG AGACCAAGAG AGAGAGTAGC AGTGATTATC AATGGAGAAA TGAAAGAGAA   
  
  
- GGGACTCCAT ACGAAGTTGA AATTGTATAT ATAGGGTGAA CTAAAGAGAA CAGAAAAACG ATATTATGAA   
  
  
- CAACAAACCG ATAGTAGGGG ACAAAATATG AGTAAAAGAA CGAAAGATAA AGACCCAAAC TTAACCCAAA   
  
  
- GTGAATAGGA GCAGTTAAGA TACCCATTAA TCACTTAACC CAAATATACT TGATCCCAAC GTTTTCATAG   
  
  
- ATGAAAAAGA GAACACTAAT AATCAACTAA TCCCTAAACC GGTTATAATC GTCTTACCCA AGACTTAAAC   
  
  
- GCCTTAAGAG ACTACTACGA GATTTACCCA TACGAATACA ACTATAGGGA CGAATACTAC GTAGGAATCT   
  
  
- AATACGGTTA AACAAGTTAA TGCTTCCGGG TAGACTCCTA GCGTGGAGTG ACTCGGAGGG TTCTGGGAAA   
  
  
- AGACTGGGAA ACCGAATGTC AAAGTGCAGG AGAAGACTCG ACCCGGGACC CCAACTAAGA TTACTACTAT   
  
  
- CACTAAGACC GCTACAAGAG TTCATGTAAT CGGTTTACGA ATACCTTCTT CTGTACCTCC GTTTCGGTAC   
  
  
- GTACAAAGTA CTAGGAAATC GTGAAGTCCG ACGACTCTTT GGGAAAATAC TACGGAATCC CCTCGTTACG   
  
  
- GGTTGAAGAG GACTGGTTGT AGGATATTAA CTAGTAATAA ACCTATCAGG ACTACTTTTA AGAAACTCAA   
  
  
- GTTGACCACT AAAATCACAA TCAGTAAGAC CCAGATCAAG TTGTTTGAGG CAACCTGGCT ATTATCACAG   
  
  
- ACTAAACTCA CTCGTAAAAC TCGGTGGGAA ACAACTTTGT GAAGGTTAAC TTAGTATAGT GGTTGGTAAC   
  
  
- TGGGCAGGAG TTACCAGTAA ACCGAGAAGC CCACGGAATC TACCGTGCCG GAGATTACCA AGCCACTAGA   
  
  
- GTAGGGAACC AAACGGTCAC CTACACTAAT CGCATAAATC CCTCTTTCTC AGGTACTAAG TTAAAGTCTT   
  
  
- TCCCCACCTC CTCCGGTCAT TCAAGGAAGG GTTCTTATTA TTGGAACAAT AACTAGAGCT CTTGGAGTGA   
  
  
- AAAGGATTAC TTTGTTTCCT CCTACTAGCT TACTACTACC AATTCTTCCT TTTCCTACTG CACTTAACCA   
  
  
- GATTGATGAG TTCTCCCTCA TTCTAAGTGA TAACACTTCT GCTCCGGAAA CTTCTTCCTT CCTCACCGTT   
  
  
- CGTCAGTCGA TAAAGAAGAT GACTCCTTCG ACTCAAAAGA CTGTAAAAAC TGTTCCAAAA CGAAACGCTA   
  
  
- ACGATGGGAC ACTTTGGACT CCGAGTAGGG TGGTACTCAA ACTTGGGACT CTTCCCGGTC AGTGTACCTA   
  
  
- ATCTTCCACC CTCTTTACCC TTCCGAGCGG GATTCTTTGT CCTATTATCA TCGTGTTTAT AACACCTAAA   
  
  
- TTCCTTAAAC GACTATGATA CGCGTGTTAG ATGACGTAGA CTACTGGCTT CTTGACGACT ACCTGACGAT   
  
  
- TTCGTTTAGT CCCTCGTGAG CAGACGACTC CCCCTACCTA GAGTTTCCAA CCGCGTAATG AAACGACTAC   
  
  
- GGGATCTCCG TGCAAATCGA CCTTGACCTA GAGCGTAAAT ATGCCGGGAT ACAAGATTAT CCGGTAGACA   
  
  
- GTGACTGTAC TATTTTCGTA TAGTCAAGAT ATAAGCACGA ACGGGTAAAT GCTTCTAGCA GTAACCAACA   
  
  
- CCATGGGTAT ACTAAGATTT TCGTCGACTC TTCCGTAGTT TCGAAGTATA ATATCTAAAA CCGTAGGAGA   
  
  
- TACCAACGGT TACCGGGTTG GAGTAAGTTG CGGAGAGTCT CGCTAGACCA CCTGGAGGTT TTGACAAATA   
  
  
- ATGTCCCTAG CTAGAGGGGG TCGGACCCAA GTCCGGTCGT CTTTCTCACC TTCGTTGTCC CTCTGCGAAC   
  
  
- CGGTTCATGA CACTCGCCAT ATTACACGGT AAACTCATAG TACGGTAACG AGTCCTCACC CTTTGTTAGT   
  
  
- TTGGTCCCCT AGAGTTCTAT CCTTCATCCT TACTACTTCA ACAACGCCAC TTGACAGAGA CATCCAAGTT   
  
  
- CTTGGAGGAA CTGCTCTGTC ACCACCACCT ATCAGGTTCC TTGTGTCAAA ATTTGGACTA ATGTTCCCAT   
  
  
- TTCGGACTAT AAAAACACGT ACCGCAACAT TTACCAAGGA TGTTGTAGGG AAAGAAACAC TGTGCAAAAT   
  
  
- CTCTTCGGGA GAAAGTAATA AGGTGAGAAA AGCTGTACAA TCTACGGTTG CGGAGATCCC TCCTCGGGCT   
  
  
- CTCCAACTAT AAGCTCTTCC GTAAGATACC CTCCCTCTAA TACTTACACC ACCGGACACT CCCGTGTCTC   
  
  
- TCCCACCTCT CCGGCCTCTG TATGTTCGTT ACCGTACAAT CCGTATCGTC CCGTCCCAAA GCCGTTCATG   
  
  
- GTAACCTAGG GTTCAACTAG CTCTTTTACT CCAAATTCCG GTTCCGTCTG GTGGTGTTCC TAAAGTACTA   
  
  
- GCTACACCTA CCTGTAACCC GTTAAGTCCC TACCTTCCCC GCCTAACGTG TACGCTAGAG ACGTACCCAA   
  
  
- GGCCGAAC

+     DRE core

| Site Name | Organism | Position | Strand | Matrix score. | sequence | function |
| --- | --- | --- | --- | --- | --- | --- |
| DRE core | Arabidopsis thaliana | 825 | - | 6 | GCCGAC |  |
| DRE core | Arabidopsis thaliana | 721 | - | 6 | GCCGAC |  |

>HU07G02249.1   
+ -Up\_Stream \_Len000GATTTG ATTGTCGAAA TTTGTCCGAT TTTATAAAAT TGAAAATTGT TATTGGACTA   
  
  
+ TAATCTACTG TAGTTTAGAG GTGTTGGATA ATACTCTCAA CTTTGAACTT GGACGTCCAT GCACCACACT   
  
  
+ ATGGGCGTAT TTACTGTTTA TATACCAAGA ATAATTTTGA AGATGTTCTT AGTTGGTGAT GTAAGTGTAT   
  
  
+ ATTCAACATA TGTCATTACA TAAACTTGAA AATTCTTGAA TTTTAGACAT ATACAATTGC AAATCATTGT   
  
  
+ AAAATTGTTG CATTACATTT TGATAAAAAT GCATCCAAAC TTTGTCTTGG TATAAACAGT AAATAGGCCC   
  
  
+ ATAGGTCCAT AGTGTAGTGC ATGGACGTCC AAGTTTAAAG GTGGGAGTAT TATTGCCCAA CGCCCCTAAA   
  
  
+ GTACAGTATA TTATAGTCCA ATATCCCAAT TAATTTTTCA TTTGAATATT TAAATTTGGA CATAATATTT   
  
  
+ TTCATATTTG AATTCTCTAA ACCTGATTTT AACCTGAATT TAAGTTGATT TAATTCATCC GACCAAAAAA   
  
  
+ ATAAACACAA TTATTTATTT TTCTATTCGA AGTTAGATAG TAGCCAATTC ATTTGACATA TAATTAACCC   
  
  
+ GTGATTGACA CAAACTCGAG TTTTTGCTTA GAGCTAGGAA AAGAGTCGGG TTAGGTCGAC CCATTTAAGA   
  
  
+ AATGGGTCGA CTTCATGTCG GCCTTCAAAA TAGTTAAGAT TTGCTCAAAA CTAAAAAATA ACAAGTTCAC   
  
  
+ ACTAAACGGT CAAATCGTGT CGACTTCGGA TTGAAATTGG TTCAGTTTGG GTCGGCTTCG TATTTCTTTC   
  
  
+ AGTAACTTCG AACCGAATTC GTATCGTGTT ATCGTGTCAG GTCAGACTTT CTCAGTTCTA TTTTTGCTAC   
  
  
+ CTTATGTTAC GGATAAAAAA AGTCTCACAT TGGAAAAAGT GTGGGAGGGT CCTGGGCTTA TAAATAGTGG   
  
  
+ CGCTGCACAC CTCATCAGAC CGACCTTTTG GGGAGAGGGT AATGACCCCG TGACAAGTGG TATCAGAGTC   
  
  
+ AACCCAGACC CGACTCAGAT GTCCTGCCAA GCGCTCCGAC GGGCGGGGTA GGCAGGGGGC CCATTTGTGA   
  
  
+ TGAGGACGTC ACAGAATTGG GCGGGGGAGA GTGTCACGGA TAAAAAAAGT CCCACATCGA AAAAAGTGTG   
  
  
+ GGAGGGTCCT GGGCTTATAA ACGGTAGCGC CGCACACCCC ATCAGATCGA CTTTTTGGGG AGAGGGTAAT   
  
  
+ GACCCCATTT CAAGTGGTAT CAGAGCCAAC CCAGACCCGA CTCCAATGTC CCACCAAGCG CTCTGATGGG   
  
  
+ CGGGGTAGGC AAGGGGCTCC ATATGTGACG AGGGCGTCAC GGAATTGGAT GGGAGAGAGT ACCACGGATA   
  
  
+ AAAAAAGTCC CACATCAGAA AAAGTGTGGA CTTAGCAACT AGGAAGTAAA GTTACAGTAT TAGCCGAGAG   
  
  
+ CCCATGGAAC GCCTGAAACT GACATCACCA GACCCACATA TTTCACCATC ATCTGATCTC CCCGTCAAAG   
  
  
+ GAGGAAGCTG AGCACAGCAA GAACTAGAAG AACAGGCGGC ATCACACCCA AGCCAAAATT CTCTATTTGA   
  
  
+ CCTCCCTCTC CTCATTCCTC TGAGCTTCCT CTTCACAATG TAAGACCATA ATCCTTAACA CCCTTCTTTT   
  
  
+ TTAAGCTCAT CTTTACCTGC TCTGGTTCTC TCTCTCATCG TCACTAATAG TTACCTCTTT ACTTTCTCTT   
  
  
+ CCCTGAGGTA TGCTTCAACT TTAACATATA TATCCCACTT GATTTCTCTT GTCTTTTTGC TATAATACTT   
  
  
+ GTTGTTTGGC TATCATCCCC TGTTTTATAC TCATTTTCTT GCTTTCTATT TCTGGGTTTG AATTGGGTTT   
  
  
+ CACTTATCCT CGTCAATTCT ATGGGTAATT AGTGAATTGG GTTTATATGA ACTAGGGTTG CAAAAGTATC   
  
  
+ TACTTTTTCT CTTGTGATTA TTAGTTGATT AGGGATTTGG CCAATATTAG CAGAATGGGT TCTGAATTTG   
  
  
+ CGGAATTCTC TGATGATGCT CTAAATGGGT ATGCTTATGT TGATATCCCT GCTTATGATG CATCCTTAGA   
  
  
+ TTATGCCAAT TTGTTCAATT ACGAAGGCCC ATCTGAGGAT CGCACCTCAC TGAGCCTCCC AAGACCCTTT   
  
  
+ TCTGACCCTT TGGCTTACAG TTTCACGTCC TCTTCTGAGC TGGGCCCTGG GGTTGATTCT AATGATGATA   
  
  
+ GTGATTCTGG CGATGTTCTC AAGTACATTA GCCAAATGCT TATGGAAGAA GACATGGAGG CAAAGCCATG   
  
  
+ CATGTTTCAT GATCCTTTAG CACTTCAGGC TGCTGAGAAA CCCTTTTATG ATGCCTTAGG GGAGCAATGC   
  
  
+ CCAACTTCTC CTGACCAACA TCCTATAATT GATCATTATT TGGATAGTCC TGATGAAAAT TCTTTGAGTT   
  
  
+ CAACTGGTGA TTTTAGTGTT AGTCATTCTG GGTCTAGTTC AACAAACTCC GTTGGACCGA TAATAGTGTC   
  
  
+ TGATTTGAGT GAGCATTTTG AGCCACCCTT TGTTGAAACA CTTCCAATTG AATCATATCA CCAACCATTG   
  
  
+ ACCCGTCCTC AATGGTCATT TGGCTCTTCG GGTGCCTTAG ATGGCACGGC CTCTAATGGT TCGGTGATCT   
  
  
+ CATCCCTTGG TTTGCCAGTG GATGTGATTA GCGTATTTAG GGAGAAAGAG TCCATGATTC AATTTCAGAA   
  
  
+ AGGGGTGGAG GAGGCCAGTA AGTTCCTTCC CAAGAATAAT AACCTTGTTA TTGATCTCGA GAACCTCACT   
  
  
+ TTTCCTAATG AAACAAAGGA GGATGATCGA ATGATGATGG TTAAGAAGGA AAAGGATGAC GTGAATTGGT   
  
  
+ CTAACTACTC AAGAGGGAGT AAGATTCACT ATTGTGAAGA CGAGGCCTTT GAAGAAGGAA GGAGTGGCAA   
  
  
+ GCAGTCAGCT ATTTCTTCTA CTGAGGAAGC TGAGTTTTCT GACATTTTTG ACAAGGTTTT GCTTTGCGAT   
  
  
+ TGCTACCCTG TGAAACCTGA GGCTCATCCC ACCATGAGTT TGAACCCTGA GAAGGGCCAG TCACATGGAT   
  
  
+ TAGAAGGTGG GAGAAATGGG AAGGCTCGCC CTAAGAAACA GGATAATAGT AGCACAAATA TTGTGGATTT   
  
  
+ AAGGAATTTG CTGATACTAT GCGCACAATC TACTGCATCT GATGACCGAA GAACTGCTGA TGGACTGCTA   
  
  
+ AAGCAAATCA GGGAGCACTC GTCTGCTGAG GGGGATGGAT CTCAAAGGTT GGCGCATTAC TTTGCTGATG   
  
  
+ CCCTAGAGGC ACGTTTAGCT GGAACTGGAT CTCGCATTTA TACGGCCCTA TGTTCTAATA GGCCATCTGT   
  
  
+ CACTGACATG ATAAAAGCAT ATCAGTTCTA TATTCGTGCT TGCCCATTTA CGAAGATCGT CATTGGTTGT   
  
  
+ GGTACCCATA TGATTCTAAA AGCAGCTGAG AAGGCATCAA AGCTTCATAT TATAGATTTT GGCATCCTCT   
  
  
+ ATGGTTGCCA ATGGCCCAAC CTCATTCAAC GCCTCTCAGA GCGATCTGGT GGACCTCCAA AACTGTTTAT   
  
  
+ TACAGGGATC GATCTCCCCC AGCCTGGGTT CAGGCCAGCA GAAAGAGTGG AAGCAACAGG GAGACGCTTG   
  
  
+ GCCAAGTACT GTGAGCGGTA TAATGTGCCA TTTGAGTATC ATGCCATTGC TCAGGAGTGG GAAACAATCA   
  
  
+ AACCAGGGGA TCTCAAGATA GGAAGTAGGA ATGATGAAGT TGTTGCGGTG AACTGTCTCT GTAGGTTCAA   
  
  
+ GAACCTCCTT GACGAGACAG TGGTGGTGGA TAGTCCAAGG AACACAGTTT TAAACCTGAT TACAAGGGTA   
  
  
+ AAGCCTGATA TTTTTGTGCA TGGCGTTGTA AATGGTTCCT ACAACATCCC TTTCTTTGTG ACACGTTTTA   
  
  
+ GAGAAGCCCT CTTTCATTAT TCCACTCTTT TCGACATGTT AGATGCCAAC GCCTCTAGGG AGGAGCCCGA   
  
  
+ GAGGTTGATA TTCGAGAAGG CATTCTATGG GAGGGAGATT ATGAATGTGG TGGCCTGTGA GGGCACAGAG   
  
  
+ AGGGTGGAGA GGCCGGAGAC ATACAAGCAA TGGCATGTTA GGCATAGCAG GGCAGGGTTT CGGCAAGTAC   
  
  
+ CATTGGATCC CAAGTTGATC GAGAAAATGA GGTTTAAGGC CAAGGCAGAC CACCACAAGG ATTTCATGAT   
  
  
+ CGATGTGGAT GGACATTGGG CAATTCAGGG ATGGAAGGGG CGGATTGCAC ATGCGATCTC TGCATGGGTT   
  
  
+ CCGGCTTG  

- -Up\_Stream \_Len000CTAAAC TAACAGCTTT AAACAGGCTA AAATATTTTA ACTTTTAACA ATAACCTGAT   
  
  
- ATTAGATGAC ATCAAATCTC CACAACCTAT TATGAGAGTT GAAACTTGAA CCTGCAGGTA CGTGGTGTGA   
  
  
- TACCCGCATA AATGACAAAT ATATGGTTCT TATTAAAACT TCTACAAGAA TCAACCACTA CATTCACATA   
  
  
- TAAGTTGTAT ACAGTAATGT ATTTGAACTT TTAAGAACTT AAAATCTGTA TATGTTAACG TTTAGTAACA   
  
  
- TTTTAACAAC GTAATGTAAA ACTATTTTTA CGTAGGTTTG AAACAGAACC ATATTTGTCA TTTATCCGGG   
  
  
- TATCCAGGTA TCACATCACG TACCTGCAGG TTCAAATTTC CACCCTCATA ATAACGGGTT GCGGGGATTT   
  
  
- CATGTCATAT AATATCAGGT TATAGGGTTA ATTAAAAAGT AAACTTATAA ATTTAAACCT GTATTATAAA   
  
  
- AAGTATAAAC TTAAGAGATT TGGACTAAAA TTGGACTTAA ATTCAACTAA ATTAAGTAGG CTGGTTTTTT   
  
  
- TATTTGTGTT AATAAATAAA AAGATAAGCT TCAATCTATC ATCGGTTAAG TAAACTGTAT ATTAATTGGG   
  
  
- CACTAACTGT GTTTGAGCTC AAAAACGAAT CTCGATCCTT TTCTCAGCCC AATCCAGCTG GGTAAATTCT   
  
  
- TTACCCAGCT GAAGTACAGC CGGAAGTTTT ATCAATTCTA AACGAGTTTT GATTTTTTAT TGTTCAAGTG   
  
  
- TGATTTGCCA GTTTAGCACA GCTGAAGCCT AACTTTAACC AAGTCAAACC CAGCCGAAGC ATAAAGAAAG   
  
  
- TCATTGAAGC TTGGCTTAAG CATAGCACAA TAGCACAGTC CAGTCTGAAA GAGTCAAGAT AAAAACGATG   
  
  
- GAATACAATG CCTATTTTTT TCAGAGTGTA ACCTTTTTCA CACCCTCCCA GGACCCGAAT ATTTATCACC   
  
  
- GCGACGTGTG GAGTAGTCTG GCTGGAAAAC CCCTCTCCCA TTACTGGGGC ACTGTTCACC ATAGTCTCAG   
  
  
- TTGGGTCTGG GCTGAGTCTA CAGGACGGTT CGCGAGGCTG CCCGCCCCAT CCGTCCCCCG GGTAAACACT   
  
  
- ACTCCTGCAG TGTCTTAACC CGCCCCCTCT CACAGTGCCT ATTTTTTTCA GGGTGTAGCT TTTTTCACAC   
  
  
- CCTCCCAGGA CCCGAATATT TGCCATCGCG GCGTGTGGGG TAGTCTAGCT GAAAAACCCC TCTCCCATTA   
  
  
- CTGGGGTAAA GTTCACCATA GTCTCGGTTG GGTCTGGGCT GAGGTTACAG GGTGGTTCGC GAGACTACCC   
  
  
- GCCCCATCCG TTCCCCGAGG TATACACTGC TCCCGCAGTG CCTTAACCTA CCCTCTCTCA TGGTGCCTAT   
  
  
- TTTTTTCAGG GTGTAGTCTT TTTCACACCT GAATCGTTGA TCCTTCATTT CAATGTCATA ATCGGCTCTC   
  
  
- GGGTACCTTG CGGACTTTGA CTGTAGTGGT CTGGGTGTAT AAAGTGGTAG TAGACTAGAG GGGCAGTTTC   
  
  
- CTCCTTCGAC TCGTGTCGTT CTTGATCTTC TTGTCCGCCG TAGTGTGGGT TCGGTTTTAA GAGATAAACT   
  
  
- GGAGGGAGAG GAGTAAGGAG ACTCGAAGGA GAAGTGTTAC ATTCTGGTAT TAGGAATTGT GGGAAGAAAA   
  
  
- AATTCGAGTA GAAATGGACG AGACCAAGAG AGAGAGTAGC AGTGATTATC AATGGAGAAA TGAAAGAGAA   
  
  
- GGGACTCCAT ACGAAGTTGA AATTGTATAT ATAGGGTGAA CTAAAGAGAA CAGAAAAACG ATATTATGAA   
  
  
- CAACAAACCG ATAGTAGGGG ACAAAATATG AGTAAAAGAA CGAAAGATAA AGACCCAAAC TTAACCCAAA   
  
  
- GTGAATAGGA GCAGTTAAGA TACCCATTAA TCACTTAACC CAAATATACT TGATCCCAAC GTTTTCATAG   
  
  
- ATGAAAAAGA GAACACTAAT AATCAACTAA TCCCTAAACC GGTTATAATC GTCTTACCCA AGACTTAAAC   
  
  
- GCCTTAAGAG ACTACTACGA GATTTACCCA TACGAATACA ACTATAGGGA CGAATACTAC GTAGGAATCT   
  
  
- AATACGGTTA AACAAGTTAA TGCTTCCGGG TAGACTCCTA GCGTGGAGTG ACTCGGAGGG TTCTGGGAAA   
  
  
- AGACTGGGAA ACCGAATGTC AAAGTGCAGG AGAAGACTCG ACCCGGGACC CCAACTAAGA TTACTACTAT   
  
  
- CACTAAGACC GCTACAAGAG TTCATGTAAT CGGTTTACGA ATACCTTCTT CTGTACCTCC GTTTCGGTAC   
  
  
- GTACAAAGTA CTAGGAAATC GTGAAGTCCG ACGACTCTTT GGGAAAATAC TACGGAATCC CCTCGTTACG   
  
  
- GGTTGAAGAG GACTGGTTGT AGGATATTAA CTAGTAATAA ACCTATCAGG ACTACTTTTA AGAAACTCAA   
  
  
- GTTGACCACT AAAATCACAA TCAGTAAGAC CCAGATCAAG TTGTTTGAGG CAACCTGGCT ATTATCACAG   
  
  
- ACTAAACTCA CTCGTAAAAC TCGGTGGGAA ACAACTTTGT GAAGGTTAAC TTAGTATAGT GGTTGGTAAC   
  
  
- TGGGCAGGAG TTACCAGTAA ACCGAGAAGC CCACGGAATC TACCGTGCCG GAGATTACCA AGCCACTAGA   
  
  
- GTAGGGAACC AAACGGTCAC CTACACTAAT CGCATAAATC CCTCTTTCTC AGGTACTAAG TTAAAGTCTT   
  
  
- TCCCCACCTC CTCCGGTCAT TCAAGGAAGG GTTCTTATTA TTGGAACAAT AACTAGAGCT CTTGGAGTGA   
  
  
- AAAGGATTAC TTTGTTTCCT CCTACTAGCT TACTACTACC AATTCTTCCT TTTCCTACTG CACTTAACCA   
  
  
- GATTGATGAG TTCTCCCTCA TTCTAAGTGA TAACACTTCT GCTCCGGAAA CTTCTTCCTT CCTCACCGTT   
  
  
- CGTCAGTCGA TAAAGAAGAT GACTCCTTCG ACTCAAAAGA CTGTAAAAAC TGTTCCAAAA CGAAACGCTA   
  
  
- ACGATGGGAC ACTTTGGACT CCGAGTAGGG TGGTACTCAA ACTTGGGACT CTTCCCGGTC AGTGTACCTA   
  
  
- ATCTTCCACC CTCTTTACCC TTCCGAGCGG GATTCTTTGT CCTATTATCA TCGTGTTTAT AACACCTAAA   
  
  
- TTCCTTAAAC GACTATGATA CGCGTGTTAG ATGACGTAGA CTACTGGCTT CTTGACGACT ACCTGACGAT   
  
  
- TTCGTTTAGT CCCTCGTGAG CAGACGACTC CCCCTACCTA GAGTTTCCAA CCGCGTAATG AAACGACTAC   
  
  
- GGGATCTCCG TGCAAATCGA CCTTGACCTA GAGCGTAAAT ATGCCGGGAT ACAAGATTAT CCGGTAGACA   
  
  
- GTGACTGTAC TATTTTCGTA TAGTCAAGAT ATAAGCACGA ACGGGTAAAT GCTTCTAGCA GTAACCAACA   
  
  
- CCATGGGTAT ACTAAGATTT TCGTCGACTC TTCCGTAGTT TCGAAGTATA ATATCTAAAA CCGTAGGAGA   
  
  
- TACCAACGGT TACCGGGTTG GAGTAAGTTG CGGAGAGTCT CGCTAGACCA CCTGGAGGTT TTGACAAATA   
  
  
- ATGTCCCTAG CTAGAGGGGG TCGGACCCAA GTCCGGTCGT CTTTCTCACC TTCGTTGTCC CTCTGCGAAC   
  
  
- CGGTTCATGA CACTCGCCAT ATTACACGGT AAACTCATAG TACGGTAACG AGTCCTCACC CTTTGTTAGT   
  
  
- TTGGTCCCCT AGAGTTCTAT CCTTCATCCT TACTACTTCA ACAACGCCAC TTGACAGAGA CATCCAAGTT   
  
  
- CTTGGAGGAA CTGCTCTGTC ACCACCACCT ATCAGGTTCC TTGTGTCAAA ATTTGGACTA ATGTTCCCAT   
  
  
- TTCGGACTAT AAAAACACGT ACCGCAACAT TTACCAAGGA TGTTGTAGGG AAAGAAACAC TGTGCAAAAT   
  
  
- CTCTTCGGGA GAAAGTAATA AGGTGAGAAA AGCTGTACAA TCTACGGTTG CGGAGATCCC TCCTCGGGCT   
  
  
- CTCCAACTAT AAGCTCTTCC GTAAGATACC CTCCCTCTAA TACTTACACC ACCGGACACT CCCGTGTCTC   
  
  
- TCCCACCTCT CCGGCCTCTG TATGTTCGTT ACCGTACAAT CCGTATCGTC CCGTCCCAAA GCCGTTCATG   
  
  
- GTAACCTAGG GTTCAACTAG CTCTTTTACT CCAAATTCCG GTTCCGTCTG GTGGTGTTCC TAAAGTACTA   
  
  
- GCTACACCTA CCTGTAACCC GTTAAGTCCC TACCTTCCCC GCCTAACGTG TACGCTAGAG ACGTACCCAA   
  
  
- GGCCGAAC

+     G-Box

| Site Name | Organism | Position | Strand | Matrix score. | sequence | function |
| --- | --- | --- | --- | --- | --- | --- |
| G-Box | Pisum sativum | 3916 | + | 6 | CACGTT | cis-acting regulatory element involved in light responsiveness |
| G-Box | Pisum sativum | 3304 | + | 6 | CACGTT | cis-acting regulatory element involved in light responsiveness |

>HU07G02249.1   
+ -Up\_Stream \_Len000GATTTG ATTGTCGAAA TTTGTCCGAT TTTATAAAAT TGAAAATTGT TATTGGACTA   
  
  
+ TAATCTACTG TAGTTTAGAG GTGTTGGATA ATACTCTCAA CTTTGAACTT GGACGTCCAT GCACCACACT   
  
  
+ ATGGGCGTAT TTACTGTTTA TATACCAAGA ATAATTTTGA AGATGTTCTT AGTTGGTGAT GTAAGTGTAT   
  
  
+ ATTCAACATA TGTCATTACA TAAACTTGAA AATTCTTGAA TTTTAGACAT ATACAATTGC AAATCATTGT   
  
  
+ AAAATTGTTG CATTACATTT TGATAAAAAT GCATCCAAAC TTTGTCTTGG TATAAACAGT AAATAGGCCC   
  
  
+ ATAGGTCCAT AGTGTAGTGC ATGGACGTCC AAGTTTAAAG GTGGGAGTAT TATTGCCCAA CGCCCCTAAA   
  
  
+ GTACAGTATA TTATAGTCCA ATATCCCAAT TAATTTTTCA TTTGAATATT TAAATTTGGA CATAATATTT   
  
  
+ TTCATATTTG AATTCTCTAA ACCTGATTTT AACCTGAATT TAAGTTGATT TAATTCATCC GACCAAAAAA   
  
  
+ ATAAACACAA TTATTTATTT TTCTATTCGA AGTTAGATAG TAGCCAATTC ATTTGACATA TAATTAACCC   
  
  
+ GTGATTGACA CAAACTCGAG TTTTTGCTTA GAGCTAGGAA AAGAGTCGGG TTAGGTCGAC CCATTTAAGA   
  
  
+ AATGGGTCGA CTTCATGTCG GCCTTCAAAA TAGTTAAGAT TTGCTCAAAA CTAAAAAATA ACAAGTTCAC   
  
  
+ ACTAAACGGT CAAATCGTGT CGACTTCGGA TTGAAATTGG TTCAGTTTGG GTCGGCTTCG TATTTCTTTC   
  
  
+ AGTAACTTCG AACCGAATTC GTATCGTGTT ATCGTGTCAG GTCAGACTTT CTCAGTTCTA TTTTTGCTAC   
  
  
+ CTTATGTTAC GGATAAAAAA AGTCTCACAT TGGAAAAAGT GTGGGAGGGT CCTGGGCTTA TAAATAGTGG   
  
  
+ CGCTGCACAC CTCATCAGAC CGACCTTTTG GGGAGAGGGT AATGACCCCG TGACAAGTGG TATCAGAGTC   
  
  
+ AACCCAGACC CGACTCAGAT GTCCTGCCAA GCGCTCCGAC GGGCGGGGTA GGCAGGGGGC CCATTTGTGA   
  
  
+ TGAGGACGTC ACAGAATTGG GCGGGGGAGA GTGTCACGGA TAAAAAAAGT CCCACATCGA AAAAAGTGTG   
  
  
+ GGAGGGTCCT GGGCTTATAA ACGGTAGCGC CGCACACCCC ATCAGATCGA CTTTTTGGGG AGAGGGTAAT   
  
  
+ GACCCCATTT CAAGTGGTAT CAGAGCCAAC CCAGACCCGA CTCCAATGTC CCACCAAGCG CTCTGATGGG   
  
  
+ CGGGGTAGGC AAGGGGCTCC ATATGTGACG AGGGCGTCAC GGAATTGGAT GGGAGAGAGT ACCACGGATA   
  
  
+ AAAAAAGTCC CACATCAGAA AAAGTGTGGA CTTAGCAACT AGGAAGTAAA GTTACAGTAT TAGCCGAGAG   
  
  
+ CCCATGGAAC GCCTGAAACT GACATCACCA GACCCACATA TTTCACCATC ATCTGATCTC CCCGTCAAAG   
  
  
+ GAGGAAGCTG AGCACAGCAA GAACTAGAAG AACAGGCGGC ATCACACCCA AGCCAAAATT CTCTATTTGA   
  
  
+ CCTCCCTCTC CTCATTCCTC TGAGCTTCCT CTTCACAATG TAAGACCATA ATCCTTAACA CCCTTCTTTT   
  
  
+ TTAAGCTCAT CTTTACCTGC TCTGGTTCTC TCTCTCATCG TCACTAATAG TTACCTCTTT ACTTTCTCTT   
  
  
+ CCCTGAGGTA TGCTTCAACT TTAACATATA TATCCCACTT GATTTCTCTT GTCTTTTTGC TATAATACTT   
  
  
+ GTTGTTTGGC TATCATCCCC TGTTTTATAC TCATTTTCTT GCTTTCTATT TCTGGGTTTG AATTGGGTTT   
  
  
+ CACTTATCCT CGTCAATTCT ATGGGTAATT AGTGAATTGG GTTTATATGA ACTAGGGTTG CAAAAGTATC   
  
  
+ TACTTTTTCT CTTGTGATTA TTAGTTGATT AGGGATTTGG CCAATATTAG CAGAATGGGT TCTGAATTTG   
  
  
+ CGGAATTCTC TGATGATGCT CTAAATGGGT ATGCTTATGT TGATATCCCT GCTTATGATG CATCCTTAGA   
  
  
+ TTATGCCAAT TTGTTCAATT ACGAAGGCCC ATCTGAGGAT CGCACCTCAC TGAGCCTCCC AAGACCCTTT   
  
  
+ TCTGACCCTT TGGCTTACAG TTTCACGTCC TCTTCTGAGC TGGGCCCTGG GGTTGATTCT AATGATGATA   
  
  
+ GTGATTCTGG CGATGTTCTC AAGTACATTA GCCAAATGCT TATGGAAGAA GACATGGAGG CAAAGCCATG   
  
  
+ CATGTTTCAT GATCCTTTAG CACTTCAGGC TGCTGAGAAA CCCTTTTATG ATGCCTTAGG GGAGCAATGC   
  
  
+ CCAACTTCTC CTGACCAACA TCCTATAATT GATCATTATT TGGATAGTCC TGATGAAAAT TCTTTGAGTT   
  
  
+ CAACTGGTGA TTTTAGTGTT AGTCATTCTG GGTCTAGTTC AACAAACTCC GTTGGACCGA TAATAGTGTC   
  
  
+ TGATTTGAGT GAGCATTTTG AGCCACCCTT TGTTGAAACA CTTCCAATTG AATCATATCA CCAACCATTG   
  
  
+ ACCCGTCCTC AATGGTCATT TGGCTCTTCG GGTGCCTTAG ATGGCACGGC CTCTAATGGT TCGGTGATCT   
  
  
+ CATCCCTTGG TTTGCCAGTG GATGTGATTA GCGTATTTAG GGAGAAAGAG TCCATGATTC AATTTCAGAA   
  
  
+ AGGGGTGGAG GAGGCCAGTA AGTTCCTTCC CAAGAATAAT AACCTTGTTA TTGATCTCGA GAACCTCACT   
  
  
+ TTTCCTAATG AAACAAAGGA GGATGATCGA ATGATGATGG TTAAGAAGGA AAAGGATGAC GTGAATTGGT   
  
  
+ CTAACTACTC AAGAGGGAGT AAGATTCACT ATTGTGAAGA CGAGGCCTTT GAAGAAGGAA GGAGTGGCAA   
  
  
+ GCAGTCAGCT ATTTCTTCTA CTGAGGAAGC TGAGTTTTCT GACATTTTTG ACAAGGTTTT GCTTTGCGAT   
  
  
+ TGCTACCCTG TGAAACCTGA GGCTCATCCC ACCATGAGTT TGAACCCTGA GAAGGGCCAG TCACATGGAT   
  
  
+ TAGAAGGTGG GAGAAATGGG AAGGCTCGCC CTAAGAAACA GGATAATAGT AGCACAAATA TTGTGGATTT   
  
  
+ AAGGAATTTG CTGATACTAT GCGCACAATC TACTGCATCT GATGACCGAA GAACTGCTGA TGGACTGCTA   
  
  
+ AAGCAAATCA GGGAGCACTC GTCTGCTGAG GGGGATGGAT CTCAAAGGTT GGCGCATTAC TTTGCTGATG   
  
  
+ CCCTAGAGGC ACGTTTAGCT GGAACTGGAT CTCGCATTTA TACGGCCCTA TGTTCTAATA GGCCATCTGT   
  
  
+ CACTGACATG ATAAAAGCAT ATCAGTTCTA TATTCGTGCT TGCCCATTTA CGAAGATCGT CATTGGTTGT   
  
  
+ GGTACCCATA TGATTCTAAA AGCAGCTGAG AAGGCATCAA AGCTTCATAT TATAGATTTT GGCATCCTCT   
  
  
+ ATGGTTGCCA ATGGCCCAAC CTCATTCAAC GCCTCTCAGA GCGATCTGGT GGACCTCCAA AACTGTTTAT   
  
  
+ TACAGGGATC GATCTCCCCC AGCCTGGGTT CAGGCCAGCA GAAAGAGTGG AAGCAACAGG GAGACGCTTG   
  
  
+ GCCAAGTACT GTGAGCGGTA TAATGTGCCA TTTGAGTATC ATGCCATTGC TCAGGAGTGG GAAACAATCA   
  
  
+ AACCAGGGGA TCTCAAGATA GGAAGTAGGA ATGATGAAGT TGTTGCGGTG AACTGTCTCT GTAGGTTCAA   
  
  
+ GAACCTCCTT GACGAGACAG TGGTGGTGGA TAGTCCAAGG AACACAGTTT TAAACCTGAT TACAAGGGTA   
  
  
+ AAGCCTGATA TTTTTGTGCA TGGCGTTGTA AATGGTTCCT ACAACATCCC TTTCTTTGTG ACACGTTTTA   
  
  
+ GAGAAGCCCT CTTTCATTAT TCCACTCTTT TCGACATGTT AGATGCCAAC GCCTCTAGGG AGGAGCCCGA   
  
  
+ GAGGTTGATA TTCGAGAAGG CATTCTATGG GAGGGAGATT ATGAATGTGG TGGCCTGTGA GGGCACAGAG   
  
  
+ AGGGTGGAGA GGCCGGAGAC ATACAAGCAA TGGCATGTTA GGCATAGCAG GGCAGGGTTT CGGCAAGTAC   
  
  
+ CATTGGATCC CAAGTTGATC GAGAAAATGA GGTTTAAGGC CAAGGCAGAC CACCACAAGG ATTTCATGAT   
  
  
+ CGATGTGGAT GGACATTGGG CAATTCAGGG ATGGAAGGGG CGGATTGCAC ATGCGATCTC TGCATGGGTT   
  
  
+ CCGGCTTG  

- -Up\_Stream \_Len000CTAAAC TAACAGCTTT AAACAGGCTA AAATATTTTA ACTTTTAACA ATAACCTGAT   
  
  
- ATTAGATGAC ATCAAATCTC CACAACCTAT TATGAGAGTT GAAACTTGAA CCTGCAGGTA CGTGGTGTGA   
  
  
- TACCCGCATA AATGACAAAT ATATGGTTCT TATTAAAACT TCTACAAGAA TCAACCACTA CATTCACATA   
  
  
- TAAGTTGTAT ACAGTAATGT ATTTGAACTT TTAAGAACTT AAAATCTGTA TATGTTAACG TTTAGTAACA   
  
  
- TTTTAACAAC GTAATGTAAA ACTATTTTTA CGTAGGTTTG AAACAGAACC ATATTTGTCA TTTATCCGGG   
  
  
- TATCCAGGTA TCACATCACG TACCTGCAGG TTCAAATTTC CACCCTCATA ATAACGGGTT GCGGGGATTT   
  
  
- CATGTCATAT AATATCAGGT TATAGGGTTA ATTAAAAAGT AAACTTATAA ATTTAAACCT GTATTATAAA   
  
  
- AAGTATAAAC TTAAGAGATT TGGACTAAAA TTGGACTTAA ATTCAACTAA ATTAAGTAGG CTGGTTTTTT   
  
  
- TATTTGTGTT AATAAATAAA AAGATAAGCT TCAATCTATC ATCGGTTAAG TAAACTGTAT ATTAATTGGG   
  
  
- CACTAACTGT GTTTGAGCTC AAAAACGAAT CTCGATCCTT TTCTCAGCCC AATCCAGCTG GGTAAATTCT   
  
  
- TTACCCAGCT GAAGTACAGC CGGAAGTTTT ATCAATTCTA AACGAGTTTT GATTTTTTAT TGTTCAAGTG   
  
  
- TGATTTGCCA GTTTAGCACA GCTGAAGCCT AACTTTAACC AAGTCAAACC CAGCCGAAGC ATAAAGAAAG   
  
  
- TCATTGAAGC TTGGCTTAAG CATAGCACAA TAGCACAGTC CAGTCTGAAA GAGTCAAGAT AAAAACGATG   
  
  
- GAATACAATG CCTATTTTTT TCAGAGTGTA ACCTTTTTCA CACCCTCCCA GGACCCGAAT ATTTATCACC   
  
  
- GCGACGTGTG GAGTAGTCTG GCTGGAAAAC CCCTCTCCCA TTACTGGGGC ACTGTTCACC ATAGTCTCAG   
  
  
- TTGGGTCTGG GCTGAGTCTA CAGGACGGTT CGCGAGGCTG CCCGCCCCAT CCGTCCCCCG GGTAAACACT   
  
  
- ACTCCTGCAG TGTCTTAACC CGCCCCCTCT CACAGTGCCT ATTTTTTTCA GGGTGTAGCT TTTTTCACAC   
  
  
- CCTCCCAGGA CCCGAATATT TGCCATCGCG GCGTGTGGGG TAGTCTAGCT GAAAAACCCC TCTCCCATTA   
  
  
- CTGGGGTAAA GTTCACCATA GTCTCGGTTG GGTCTGGGCT GAGGTTACAG GGTGGTTCGC GAGACTACCC   
  
  
- GCCCCATCCG TTCCCCGAGG TATACACTGC TCCCGCAGTG CCTTAACCTA CCCTCTCTCA TGGTGCCTAT   
  
  
- TTTTTTCAGG GTGTAGTCTT TTTCACACCT GAATCGTTGA TCCTTCATTT CAATGTCATA ATCGGCTCTC   
  
  
- GGGTACCTTG CGGACTTTGA CTGTAGTGGT CTGGGTGTAT AAAGTGGTAG TAGACTAGAG GGGCAGTTTC   
  
  
- CTCCTTCGAC TCGTGTCGTT CTTGATCTTC TTGTCCGCCG TAGTGTGGGT TCGGTTTTAA GAGATAAACT   
  
  
- GGAGGGAGAG GAGTAAGGAG ACTCGAAGGA GAAGTGTTAC ATTCTGGTAT TAGGAATTGT GGGAAGAAAA   
  
  
- AATTCGAGTA GAAATGGACG AGACCAAGAG AGAGAGTAGC AGTGATTATC AATGGAGAAA TGAAAGAGAA   
  
  
- GGGACTCCAT ACGAAGTTGA AATTGTATAT ATAGGGTGAA CTAAAGAGAA CAGAAAAACG ATATTATGAA   
  
  
- CAACAAACCG ATAGTAGGGG ACAAAATATG AGTAAAAGAA CGAAAGATAA AGACCCAAAC TTAACCCAAA   
  
  
- GTGAATAGGA GCAGTTAAGA TACCCATTAA TCACTTAACC CAAATATACT TGATCCCAAC GTTTTCATAG   
  
  
- ATGAAAAAGA GAACACTAAT AATCAACTAA TCCCTAAACC GGTTATAATC GTCTTACCCA AGACTTAAAC   
  
  
- GCCTTAAGAG ACTACTACGA GATTTACCCA TACGAATACA ACTATAGGGA CGAATACTAC GTAGGAATCT   
  
  
- AATACGGTTA AACAAGTTAA TGCTTCCGGG TAGACTCCTA GCGTGGAGTG ACTCGGAGGG TTCTGGGAAA   
  
  
- AGACTGGGAA ACCGAATGTC AAAGTGCAGG AGAAGACTCG ACCCGGGACC CCAACTAAGA TTACTACTAT   
  
  
- CACTAAGACC GCTACAAGAG TTCATGTAAT CGGTTTACGA ATACCTTCTT CTGTACCTCC GTTTCGGTAC   
  
  
- GTACAAAGTA CTAGGAAATC GTGAAGTCCG ACGACTCTTT GGGAAAATAC TACGGAATCC CCTCGTTACG   
  
  
- GGTTGAAGAG GACTGGTTGT AGGATATTAA CTAGTAATAA ACCTATCAGG ACTACTTTTA AGAAACTCAA   
  
  
- GTTGACCACT AAAATCACAA TCAGTAAGAC CCAGATCAAG TTGTTTGAGG CAACCTGGCT ATTATCACAG   
  
  
- ACTAAACTCA CTCGTAAAAC TCGGTGGGAA ACAACTTTGT GAAGGTTAAC TTAGTATAGT GGTTGGTAAC   
  
  
- TGGGCAGGAG TTACCAGTAA ACCGAGAAGC CCACGGAATC TACCGTGCCG GAGATTACCA AGCCACTAGA   
  
  
- GTAGGGAACC AAACGGTCAC CTACACTAAT CGCATAAATC CCTCTTTCTC AGGTACTAAG TTAAAGTCTT   
  
  
- TCCCCACCTC CTCCGGTCAT TCAAGGAAGG GTTCTTATTA TTGGAACAAT AACTAGAGCT CTTGGAGTGA   
  
  
- AAAGGATTAC TTTGTTTCCT CCTACTAGCT TACTACTACC AATTCTTCCT TTTCCTACTG CACTTAACCA   
  
  
- GATTGATGAG TTCTCCCTCA TTCTAAGTGA TAACACTTCT GCTCCGGAAA CTTCTTCCTT CCTCACCGTT   
  
  
- CGTCAGTCGA TAAAGAAGAT GACTCCTTCG ACTCAAAAGA CTGTAAAAAC TGTTCCAAAA CGAAACGCTA   
  
  
- ACGATGGGAC ACTTTGGACT CCGAGTAGGG TGGTACTCAA ACTTGGGACT CTTCCCGGTC AGTGTACCTA   
  
  
- ATCTTCCACC CTCTTTACCC TTCCGAGCGG GATTCTTTGT CCTATTATCA TCGTGTTTAT AACACCTAAA   
  
  
- TTCCTTAAAC GACTATGATA CGCGTGTTAG ATGACGTAGA CTACTGGCTT CTTGACGACT ACCTGACGAT   
  
  
- TTCGTTTAGT CCCTCGTGAG CAGACGACTC CCCCTACCTA GAGTTTCCAA CCGCGTAATG AAACGACTAC   
  
  
- GGGATCTCCG TGCAAATCGA CCTTGACCTA GAGCGTAAAT ATGCCGGGAT ACAAGATTAT CCGGTAGACA   
  
  
- GTGACTGTAC TATTTTCGTA TAGTCAAGAT ATAAGCACGA ACGGGTAAAT GCTTCTAGCA GTAACCAACA   
  
  
- CCATGGGTAT ACTAAGATTT TCGTCGACTC TTCCGTAGTT TCGAAGTATA ATATCTAAAA CCGTAGGAGA   
  
  
- TACCAACGGT TACCGGGTTG GAGTAAGTTG CGGAGAGTCT CGCTAGACCA CCTGGAGGTT TTGACAAATA   
  
  
- ATGTCCCTAG CTAGAGGGGG TCGGACCCAA GTCCGGTCGT CTTTCTCACC TTCGTTGTCC CTCTGCGAAC   
  
  
- CGGTTCATGA CACTCGCCAT ATTACACGGT AAACTCATAG TACGGTAACG AGTCCTCACC CTTTGTTAGT   
  
  
- TTGGTCCCCT AGAGTTCTAT CCTTCATCCT TACTACTTCA ACAACGCCAC TTGACAGAGA CATCCAAGTT   
  
  
- CTTGGAGGAA CTGCTCTGTC ACCACCACCT ATCAGGTTCC TTGTGTCAAA ATTTGGACTA ATGTTCCCAT   
  
  
- TTCGGACTAT AAAAACACGT ACCGCAACAT TTACCAAGGA TGTTGTAGGG AAAGAAACAC TGTGCAAAAT   
  
  
- CTCTTCGGGA GAAAGTAATA AGGTGAGAAA AGCTGTACAA TCTACGGTTG CGGAGATCCC TCCTCGGGCT   
  
  
- CTCCAACTAT AAGCTCTTCC GTAAGATACC CTCCCTCTAA TACTTACACC ACCGGACACT CCCGTGTCTC   
  
  
- TCCCACCTCT CCGGCCTCTG TATGTTCGTT ACCGTACAAT CCGTATCGTC CCGTCCCAAA GCCGTTCATG   
  
  
- GTAACCTAGG GTTCAACTAG CTCTTTTACT CCAAATTCCG GTTCCGTCTG GTGGTGTTCC TAAAGTACTA   
  
  
- GCTACACCTA CCTGTAACCC GTTAAGTCCC TACCTTCCCC GCCTAACGTG TACGCTAGAG ACGTACCCAA   
  
  
- GGCCGAAC

+     G-box

| Site Name | Organism | Position | Strand | Matrix score. | sequence | function |
| --- | --- | --- | --- | --- | --- | --- |
| G-box | Zea mays | 2862 | - | 6 | CACGTC | cis-acting regulatory element involved in light responsiveness |
| G-box | Brassica oleracea | 3304 | - | 8 | TAAACGTG | cis-acting regulatory element involved in light responsiveness |
| G-box | Zea mays | 2198 | + | 6 | CACGTC | cis-acting regulatory element involved in light responsiveness |

>HU07G02249.1   
+ -Up\_Stream \_Len000GATTTG ATTGTCGAAA TTTGTCCGAT TTTATAAAAT TGAAAATTGT TATTGGACTA   
  
  
+ TAATCTACTG TAGTTTAGAG GTGTTGGATA ATACTCTCAA CTTTGAACTT GGACGTCCAT GCACCACACT   
  
  
+ ATGGGCGTAT TTACTGTTTA TATACCAAGA ATAATTTTGA AGATGTTCTT AGTTGGTGAT GTAAGTGTAT   
  
  
+ ATTCAACATA TGTCATTACA TAAACTTGAA AATTCTTGAA TTTTAGACAT ATACAATTGC AAATCATTGT   
  
  
+ AAAATTGTTG CATTACATTT TGATAAAAAT GCATCCAAAC TTTGTCTTGG TATAAACAGT AAATAGGCCC   
  
  
+ ATAGGTCCAT AGTGTAGTGC ATGGACGTCC AAGTTTAAAG GTGGGAGTAT TATTGCCCAA CGCCCCTAAA   
  
  
+ GTACAGTATA TTATAGTCCA ATATCCCAAT TAATTTTTCA TTTGAATATT TAAATTTGGA CATAATATTT   
  
  
+ TTCATATTTG AATTCTCTAA ACCTGATTTT AACCTGAATT TAAGTTGATT TAATTCATCC GACCAAAAAA   
  
  
+ ATAAACACAA TTATTTATTT TTCTATTCGA AGTTAGATAG TAGCCAATTC ATTTGACATA TAATTAACCC   
  
  
+ GTGATTGACA CAAACTCGAG TTTTTGCTTA GAGCTAGGAA AAGAGTCGGG TTAGGTCGAC CCATTTAAGA   
  
  
+ AATGGGTCGA CTTCATGTCG GCCTTCAAAA TAGTTAAGAT TTGCTCAAAA CTAAAAAATA ACAAGTTCAC   
  
  
+ ACTAAACGGT CAAATCGTGT CGACTTCGGA TTGAAATTGG TTCAGTTTGG GTCGGCTTCG TATTTCTTTC   
  
  
+ AGTAACTTCG AACCGAATTC GTATCGTGTT ATCGTGTCAG GTCAGACTTT CTCAGTTCTA TTTTTGCTAC   
  
  
+ CTTATGTTAC GGATAAAAAA AGTCTCACAT TGGAAAAAGT GTGGGAGGGT CCTGGGCTTA TAAATAGTGG   
  
  
+ CGCTGCACAC CTCATCAGAC CGACCTTTTG GGGAGAGGGT AATGACCCCG TGACAAGTGG TATCAGAGTC   
  
  
+ AACCCAGACC CGACTCAGAT GTCCTGCCAA GCGCTCCGAC GGGCGGGGTA GGCAGGGGGC CCATTTGTGA   
  
  
+ TGAGGACGTC ACAGAATTGG GCGGGGGAGA GTGTCACGGA TAAAAAAAGT CCCACATCGA AAAAAGTGTG   
  
  
+ GGAGGGTCCT GGGCTTATAA ACGGTAGCGC CGCACACCCC ATCAGATCGA CTTTTTGGGG AGAGGGTAAT   
  
  
+ GACCCCATTT CAAGTGGTAT CAGAGCCAAC CCAGACCCGA CTCCAATGTC CCACCAAGCG CTCTGATGGG   
  
  
+ CGGGGTAGGC AAGGGGCTCC ATATGTGACG AGGGCGTCAC GGAATTGGAT GGGAGAGAGT ACCACGGATA   
  
  
+ AAAAAAGTCC CACATCAGAA AAAGTGTGGA CTTAGCAACT AGGAAGTAAA GTTACAGTAT TAGCCGAGAG   
  
  
+ CCCATGGAAC GCCTGAAACT GACATCACCA GACCCACATA TTTCACCATC ATCTGATCTC CCCGTCAAAG   
  
  
+ GAGGAAGCTG AGCACAGCAA GAACTAGAAG AACAGGCGGC ATCACACCCA AGCCAAAATT CTCTATTTGA   
  
  
+ CCTCCCTCTC CTCATTCCTC TGAGCTTCCT CTTCACAATG TAAGACCATA ATCCTTAACA CCCTTCTTTT   
  
  
+ TTAAGCTCAT CTTTACCTGC TCTGGTTCTC TCTCTCATCG TCACTAATAG TTACCTCTTT ACTTTCTCTT   
  
  
+ CCCTGAGGTA TGCTTCAACT TTAACATATA TATCCCACTT GATTTCTCTT GTCTTTTTGC TATAATACTT   
  
  
+ GTTGTTTGGC TATCATCCCC TGTTTTATAC TCATTTTCTT GCTTTCTATT TCTGGGTTTG AATTGGGTTT   
  
  
+ CACTTATCCT CGTCAATTCT ATGGGTAATT AGTGAATTGG GTTTATATGA ACTAGGGTTG CAAAAGTATC   
  
  
+ TACTTTTTCT CTTGTGATTA TTAGTTGATT AGGGATTTGG CCAATATTAG CAGAATGGGT TCTGAATTTG   
  
  
+ CGGAATTCTC TGATGATGCT CTAAATGGGT ATGCTTATGT TGATATCCCT GCTTATGATG CATCCTTAGA   
  
  
+ TTATGCCAAT TTGTTCAATT ACGAAGGCCC ATCTGAGGAT CGCACCTCAC TGAGCCTCCC AAGACCCTTT   
  
  
+ TCTGACCCTT TGGCTTACAG TTTCACGTCC TCTTCTGAGC TGGGCCCTGG GGTTGATTCT AATGATGATA   
  
  
+ GTGATTCTGG CGATGTTCTC AAGTACATTA GCCAAATGCT TATGGAAGAA GACATGGAGG CAAAGCCATG   
  
  
+ CATGTTTCAT GATCCTTTAG CACTTCAGGC TGCTGAGAAA CCCTTTTATG ATGCCTTAGG GGAGCAATGC   
  
  
+ CCAACTTCTC CTGACCAACA TCCTATAATT GATCATTATT TGGATAGTCC TGATGAAAAT TCTTTGAGTT   
  
  
+ CAACTGGTGA TTTTAGTGTT AGTCATTCTG GGTCTAGTTC AACAAACTCC GTTGGACCGA TAATAGTGTC   
  
  
+ TGATTTGAGT GAGCATTTTG AGCCACCCTT TGTTGAAACA CTTCCAATTG AATCATATCA CCAACCATTG   
  
  
+ ACCCGTCCTC AATGGTCATT TGGCTCTTCG GGTGCCTTAG ATGGCACGGC CTCTAATGGT TCGGTGATCT   
  
  
+ CATCCCTTGG TTTGCCAGTG GATGTGATTA GCGTATTTAG GGAGAAAGAG TCCATGATTC AATTTCAGAA   
  
  
+ AGGGGTGGAG GAGGCCAGTA AGTTCCTTCC CAAGAATAAT AACCTTGTTA TTGATCTCGA GAACCTCACT   
  
  
+ TTTCCTAATG AAACAAAGGA GGATGATCGA ATGATGATGG TTAAGAAGGA AAAGGATGAC GTGAATTGGT   
  
  
+ CTAACTACTC AAGAGGGAGT AAGATTCACT ATTGTGAAGA CGAGGCCTTT GAAGAAGGAA GGAGTGGCAA   
  
  
+ GCAGTCAGCT ATTTCTTCTA CTGAGGAAGC TGAGTTTTCT GACATTTTTG ACAAGGTTTT GCTTTGCGAT   
  
  
+ TGCTACCCTG TGAAACCTGA GGCTCATCCC ACCATGAGTT TGAACCCTGA GAAGGGCCAG TCACATGGAT   
  
  
+ TAGAAGGTGG GAGAAATGGG AAGGCTCGCC CTAAGAAACA GGATAATAGT AGCACAAATA TTGTGGATTT   
  
  
+ AAGGAATTTG CTGATACTAT GCGCACAATC TACTGCATCT GATGACCGAA GAACTGCTGA TGGACTGCTA   
  
  
+ AAGCAAATCA GGGAGCACTC GTCTGCTGAG GGGGATGGAT CTCAAAGGTT GGCGCATTAC TTTGCTGATG   
  
  
+ CCCTAGAGGC ACGTTTAGCT GGAACTGGAT CTCGCATTTA TACGGCCCTA TGTTCTAATA GGCCATCTGT   
  
  
+ CACTGACATG ATAAAAGCAT ATCAGTTCTA TATTCGTGCT TGCCCATTTA CGAAGATCGT CATTGGTTGT   
  
  
+ GGTACCCATA TGATTCTAAA AGCAGCTGAG AAGGCATCAA AGCTTCATAT TATAGATTTT GGCATCCTCT   
  
  
+ ATGGTTGCCA ATGGCCCAAC CTCATTCAAC GCCTCTCAGA GCGATCTGGT GGACCTCCAA AACTGTTTAT   
  
  
+ TACAGGGATC GATCTCCCCC AGCCTGGGTT CAGGCCAGCA GAAAGAGTGG AAGCAACAGG GAGACGCTTG   
  
  
+ GCCAAGTACT GTGAGCGGTA TAATGTGCCA TTTGAGTATC ATGCCATTGC TCAGGAGTGG GAAACAATCA   
  
  
+ AACCAGGGGA TCTCAAGATA GGAAGTAGGA ATGATGAAGT TGTTGCGGTG AACTGTCTCT GTAGGTTCAA   
  
  
+ GAACCTCCTT GACGAGACAG TGGTGGTGGA TAGTCCAAGG AACACAGTTT TAAACCTGAT TACAAGGGTA   
  
  
+ AAGCCTGATA TTTTTGTGCA TGGCGTTGTA AATGGTTCCT ACAACATCCC TTTCTTTGTG ACACGTTTTA   
  
  
+ GAGAAGCCCT CTTTCATTAT TCCACTCTTT TCGACATGTT AGATGCCAAC GCCTCTAGGG AGGAGCCCGA   
  
  
+ GAGGTTGATA TTCGAGAAGG CATTCTATGG GAGGGAGATT ATGAATGTGG TGGCCTGTGA GGGCACAGAG   
  
  
+ AGGGTGGAGA GGCCGGAGAC ATACAAGCAA TGGCATGTTA GGCATAGCAG GGCAGGGTTT CGGCAAGTAC   
  
  
+ CATTGGATCC CAAGTTGATC GAGAAAATGA GGTTTAAGGC CAAGGCAGAC CACCACAAGG ATTTCATGAT   
  
  
+ CGATGTGGAT GGACATTGGG CAATTCAGGG ATGGAAGGGG CGGATTGCAC ATGCGATCTC TGCATGGGTT   
  
  
+ CCGGCTTG  

- -Up\_Stream \_Len000CTAAAC TAACAGCTTT AAACAGGCTA AAATATTTTA ACTTTTAACA ATAACCTGAT   
  
  
- ATTAGATGAC ATCAAATCTC CACAACCTAT TATGAGAGTT GAAACTTGAA CCTGCAGGTA CGTGGTGTGA   
  
  
- TACCCGCATA AATGACAAAT ATATGGTTCT TATTAAAACT TCTACAAGAA TCAACCACTA CATTCACATA   
  
  
- TAAGTTGTAT ACAGTAATGT ATTTGAACTT TTAAGAACTT AAAATCTGTA TATGTTAACG TTTAGTAACA   
  
  
- TTTTAACAAC GTAATGTAAA ACTATTTTTA CGTAGGTTTG AAACAGAACC ATATTTGTCA TTTATCCGGG   
  
  
- TATCCAGGTA TCACATCACG TACCTGCAGG TTCAAATTTC CACCCTCATA ATAACGGGTT GCGGGGATTT   
  
  
- CATGTCATAT AATATCAGGT TATAGGGTTA ATTAAAAAGT AAACTTATAA ATTTAAACCT GTATTATAAA   
  
  
- AAGTATAAAC TTAAGAGATT TGGACTAAAA TTGGACTTAA ATTCAACTAA ATTAAGTAGG CTGGTTTTTT   
  
  
- TATTTGTGTT AATAAATAAA AAGATAAGCT TCAATCTATC ATCGGTTAAG TAAACTGTAT ATTAATTGGG   
  
  
- CACTAACTGT GTTTGAGCTC AAAAACGAAT CTCGATCCTT TTCTCAGCCC AATCCAGCTG GGTAAATTCT   
  
  
- TTACCCAGCT GAAGTACAGC CGGAAGTTTT ATCAATTCTA AACGAGTTTT GATTTTTTAT TGTTCAAGTG   
  
  
- TGATTTGCCA GTTTAGCACA GCTGAAGCCT AACTTTAACC AAGTCAAACC CAGCCGAAGC ATAAAGAAAG   
  
  
- TCATTGAAGC TTGGCTTAAG CATAGCACAA TAGCACAGTC CAGTCTGAAA GAGTCAAGAT AAAAACGATG   
  
  
- GAATACAATG CCTATTTTTT TCAGAGTGTA ACCTTTTTCA CACCCTCCCA GGACCCGAAT ATTTATCACC   
  
  
- GCGACGTGTG GAGTAGTCTG GCTGGAAAAC CCCTCTCCCA TTACTGGGGC ACTGTTCACC ATAGTCTCAG   
  
  
- TTGGGTCTGG GCTGAGTCTA CAGGACGGTT CGCGAGGCTG CCCGCCCCAT CCGTCCCCCG GGTAAACACT   
  
  
- ACTCCTGCAG TGTCTTAACC CGCCCCCTCT CACAGTGCCT ATTTTTTTCA GGGTGTAGCT TTTTTCACAC   
  
  
- CCTCCCAGGA CCCGAATATT TGCCATCGCG GCGTGTGGGG TAGTCTAGCT GAAAAACCCC TCTCCCATTA   
  
  
- CTGGGGTAAA GTTCACCATA GTCTCGGTTG GGTCTGGGCT GAGGTTACAG GGTGGTTCGC GAGACTACCC   
  
  
- GCCCCATCCG TTCCCCGAGG TATACACTGC TCCCGCAGTG CCTTAACCTA CCCTCTCTCA TGGTGCCTAT   
  
  
- TTTTTTCAGG GTGTAGTCTT TTTCACACCT GAATCGTTGA TCCTTCATTT CAATGTCATA ATCGGCTCTC   
  
  
- GGGTACCTTG CGGACTTTGA CTGTAGTGGT CTGGGTGTAT AAAGTGGTAG TAGACTAGAG GGGCAGTTTC   
  
  
- CTCCTTCGAC TCGTGTCGTT CTTGATCTTC TTGTCCGCCG TAGTGTGGGT TCGGTTTTAA GAGATAAACT   
  
  
- GGAGGGAGAG GAGTAAGGAG ACTCGAAGGA GAAGTGTTAC ATTCTGGTAT TAGGAATTGT GGGAAGAAAA   
  
  
- AATTCGAGTA GAAATGGACG AGACCAAGAG AGAGAGTAGC AGTGATTATC AATGGAGAAA TGAAAGAGAA   
  
  
- GGGACTCCAT ACGAAGTTGA AATTGTATAT ATAGGGTGAA CTAAAGAGAA CAGAAAAACG ATATTATGAA   
  
  
- CAACAAACCG ATAGTAGGGG ACAAAATATG AGTAAAAGAA CGAAAGATAA AGACCCAAAC TTAACCCAAA   
  
  
- GTGAATAGGA GCAGTTAAGA TACCCATTAA TCACTTAACC CAAATATACT TGATCCCAAC GTTTTCATAG   
  
  
- ATGAAAAAGA GAACACTAAT AATCAACTAA TCCCTAAACC GGTTATAATC GTCTTACCCA AGACTTAAAC   
  
  
- GCCTTAAGAG ACTACTACGA GATTTACCCA TACGAATACA ACTATAGGGA CGAATACTAC GTAGGAATCT   
  
  
- AATACGGTTA AACAAGTTAA TGCTTCCGGG TAGACTCCTA GCGTGGAGTG ACTCGGAGGG TTCTGGGAAA   
  
  
- AGACTGGGAA ACCGAATGTC AAAGTGCAGG AGAAGACTCG ACCCGGGACC CCAACTAAGA TTACTACTAT   
  
  
- CACTAAGACC GCTACAAGAG TTCATGTAAT CGGTTTACGA ATACCTTCTT CTGTACCTCC GTTTCGGTAC   
  
  
- GTACAAAGTA CTAGGAAATC GTGAAGTCCG ACGACTCTTT GGGAAAATAC TACGGAATCC CCTCGTTACG   
  
  
- GGTTGAAGAG GACTGGTTGT AGGATATTAA CTAGTAATAA ACCTATCAGG ACTACTTTTA AGAAACTCAA   
  
  
- GTTGACCACT AAAATCACAA TCAGTAAGAC CCAGATCAAG TTGTTTGAGG CAACCTGGCT ATTATCACAG   
  
  
- ACTAAACTCA CTCGTAAAAC TCGGTGGGAA ACAACTTTGT GAAGGTTAAC TTAGTATAGT GGTTGGTAAC   
  
  
- TGGGCAGGAG TTACCAGTAA ACCGAGAAGC CCACGGAATC TACCGTGCCG GAGATTACCA AGCCACTAGA   
  
  
- GTAGGGAACC AAACGGTCAC CTACACTAAT CGCATAAATC CCTCTTTCTC AGGTACTAAG TTAAAGTCTT   
  
  
- TCCCCACCTC CTCCGGTCAT TCAAGGAAGG GTTCTTATTA TTGGAACAAT AACTAGAGCT CTTGGAGTGA   
  
  
- AAAGGATTAC TTTGTTTCCT CCTACTAGCT TACTACTACC AATTCTTCCT TTTCCTACTG CACTTAACCA   
  
  
- GATTGATGAG TTCTCCCTCA TTCTAAGTGA TAACACTTCT GCTCCGGAAA CTTCTTCCTT CCTCACCGTT   
  
  
- CGTCAGTCGA TAAAGAAGAT GACTCCTTCG ACTCAAAAGA CTGTAAAAAC TGTTCCAAAA CGAAACGCTA   
  
  
- ACGATGGGAC ACTTTGGACT CCGAGTAGGG TGGTACTCAA ACTTGGGACT CTTCCCGGTC AGTGTACCTA   
  
  
- ATCTTCCACC CTCTTTACCC TTCCGAGCGG GATTCTTTGT CCTATTATCA TCGTGTTTAT AACACCTAAA   
  
  
- TTCCTTAAAC GACTATGATA CGCGTGTTAG ATGACGTAGA CTACTGGCTT CTTGACGACT ACCTGACGAT   
  
  
- TTCGTTTAGT CCCTCGTGAG CAGACGACTC CCCCTACCTA GAGTTTCCAA CCGCGTAATG AAACGACTAC   
  
  
- GGGATCTCCG TGCAAATCGA CCTTGACCTA GAGCGTAAAT ATGCCGGGAT ACAAGATTAT CCGGTAGACA   
  
  
- GTGACTGTAC TATTTTCGTA TAGTCAAGAT ATAAGCACGA ACGGGTAAAT GCTTCTAGCA GTAACCAACA   
  
  
- CCATGGGTAT ACTAAGATTT TCGTCGACTC TTCCGTAGTT TCGAAGTATA ATATCTAAAA CCGTAGGAGA   
  
  
- TACCAACGGT TACCGGGTTG GAGTAAGTTG CGGAGAGTCT CGCTAGACCA CCTGGAGGTT TTGACAAATA   
  
  
- ATGTCCCTAG CTAGAGGGGG TCGGACCCAA GTCCGGTCGT CTTTCTCACC TTCGTTGTCC CTCTGCGAAC   
  
  
- CGGTTCATGA CACTCGCCAT ATTACACGGT AAACTCATAG TACGGTAACG AGTCCTCACC CTTTGTTAGT   
  
  
- TTGGTCCCCT AGAGTTCTAT CCTTCATCCT TACTACTTCA ACAACGCCAC TTGACAGAGA CATCCAAGTT   
  
  
- CTTGGAGGAA CTGCTCTGTC ACCACCACCT ATCAGGTTCC TTGTGTCAAA ATTTGGACTA ATGTTCCCAT   
  
  
- TTCGGACTAT AAAAACACGT ACCGCAACAT TTACCAAGGA TGTTGTAGGG AAAGAAACAC TGTGCAAAAT   
  
  
- CTCTTCGGGA GAAAGTAATA AGGTGAGAAA AGCTGTACAA TCTACGGTTG CGGAGATCCC TCCTCGGGCT   
  
  
- CTCCAACTAT AAGCTCTTCC GTAAGATACC CTCCCTCTAA TACTTACACC ACCGGACACT CCCGTGTCTC   
  
  
- TCCCACCTCT CCGGCCTCTG TATGTTCGTT ACCGTACAAT CCGTATCGTC CCGTCCCAAA GCCGTTCATG   
  
  
- GTAACCTAGG GTTCAACTAG CTCTTTTACT CCAAATTCCG GTTCCGTCTG GTGGTGTTCC TAAAGTACTA   
  
  
- GCTACACCTA CCTGTAACCC GTTAAGTCCC TACCTTCCCC GCCTAACGTG TACGCTAGAG ACGTACCCAA   
  
  
- GGCCGAAC

+     GATA-motif

| Site Name | Organism | Position | Strand | Matrix score. | sequence | function |
| --- | --- | --- | --- | --- | --- | --- |
| GATA-motif | Arabidopsis thaliana | 3731 | + | 7 | GATAGGA | part of a light responsive element |
| GATA-motif | Solanum tuberosum | 2850 | + | 9 | AAGGATAAGG | part of a light responsive element |

>HU07G02249.1   
+ -Up\_Stream \_Len000GATTTG ATTGTCGAAA TTTGTCCGAT TTTATAAAAT TGAAAATTGT TATTGGACTA   
  
  
+ TAATCTACTG TAGTTTAGAG GTGTTGGATA ATACTCTCAA CTTTGAACTT GGACGTCCAT GCACCACACT   
  
  
+ ATGGGCGTAT TTACTGTTTA TATACCAAGA ATAATTTTGA AGATGTTCTT AGTTGGTGAT GTAAGTGTAT   
  
  
+ ATTCAACATA TGTCATTACA TAAACTTGAA AATTCTTGAA TTTTAGACAT ATACAATTGC AAATCATTGT   
  
  
+ AAAATTGTTG CATTACATTT TGATAAAAAT GCATCCAAAC TTTGTCTTGG TATAAACAGT AAATAGGCCC   
  
  
+ ATAGGTCCAT AGTGTAGTGC ATGGACGTCC AAGTTTAAAG GTGGGAGTAT TATTGCCCAA CGCCCCTAAA   
  
  
+ GTACAGTATA TTATAGTCCA ATATCCCAAT TAATTTTTCA TTTGAATATT TAAATTTGGA CATAATATTT   
  
  
+ TTCATATTTG AATTCTCTAA ACCTGATTTT AACCTGAATT TAAGTTGATT TAATTCATCC GACCAAAAAA   
  
  
+ ATAAACACAA TTATTTATTT TTCTATTCGA AGTTAGATAG TAGCCAATTC ATTTGACATA TAATTAACCC   
  
  
+ GTGATTGACA CAAACTCGAG TTTTTGCTTA GAGCTAGGAA AAGAGTCGGG TTAGGTCGAC CCATTTAAGA   
  
  
+ AATGGGTCGA CTTCATGTCG GCCTTCAAAA TAGTTAAGAT TTGCTCAAAA CTAAAAAATA ACAAGTTCAC   
  
  
+ ACTAAACGGT CAAATCGTGT CGACTTCGGA TTGAAATTGG TTCAGTTTGG GTCGGCTTCG TATTTCTTTC   
  
  
+ AGTAACTTCG AACCGAATTC GTATCGTGTT ATCGTGTCAG GTCAGACTTT CTCAGTTCTA TTTTTGCTAC   
  
  
+ CTTATGTTAC GGATAAAAAA AGTCTCACAT TGGAAAAAGT GTGGGAGGGT CCTGGGCTTA TAAATAGTGG   
  
  
+ CGCTGCACAC CTCATCAGAC CGACCTTTTG GGGAGAGGGT AATGACCCCG TGACAAGTGG TATCAGAGTC   
  
  
+ AACCCAGACC CGACTCAGAT GTCCTGCCAA GCGCTCCGAC GGGCGGGGTA GGCAGGGGGC CCATTTGTGA   
  
  
+ TGAGGACGTC ACAGAATTGG GCGGGGGAGA GTGTCACGGA TAAAAAAAGT CCCACATCGA AAAAAGTGTG   
  
  
+ GGAGGGTCCT GGGCTTATAA ACGGTAGCGC CGCACACCCC ATCAGATCGA CTTTTTGGGG AGAGGGTAAT   
  
  
+ GACCCCATTT CAAGTGGTAT CAGAGCCAAC CCAGACCCGA CTCCAATGTC CCACCAAGCG CTCTGATGGG   
  
  
+ CGGGGTAGGC AAGGGGCTCC ATATGTGACG AGGGCGTCAC GGAATTGGAT GGGAGAGAGT ACCACGGATA   
  
  
+ AAAAAAGTCC CACATCAGAA AAAGTGTGGA CTTAGCAACT AGGAAGTAAA GTTACAGTAT TAGCCGAGAG   
  
  
+ CCCATGGAAC GCCTGAAACT GACATCACCA GACCCACATA TTTCACCATC ATCTGATCTC CCCGTCAAAG   
  
  
+ GAGGAAGCTG AGCACAGCAA GAACTAGAAG AACAGGCGGC ATCACACCCA AGCCAAAATT CTCTATTTGA   
  
  
+ CCTCCCTCTC CTCATTCCTC TGAGCTTCCT CTTCACAATG TAAGACCATA ATCCTTAACA CCCTTCTTTT   
  
  
+ TTAAGCTCAT CTTTACCTGC TCTGGTTCTC TCTCTCATCG TCACTAATAG TTACCTCTTT ACTTTCTCTT   
  
  
+ CCCTGAGGTA TGCTTCAACT TTAACATATA TATCCCACTT GATTTCTCTT GTCTTTTTGC TATAATACTT   
  
  
+ GTTGTTTGGC TATCATCCCC TGTTTTATAC TCATTTTCTT GCTTTCTATT TCTGGGTTTG AATTGGGTTT   
  
  
+ CACTTATCCT CGTCAATTCT ATGGGTAATT AGTGAATTGG GTTTATATGA ACTAGGGTTG CAAAAGTATC   
  
  
+ TACTTTTTCT CTTGTGATTA TTAGTTGATT AGGGATTTGG CCAATATTAG CAGAATGGGT TCTGAATTTG   
  
  
+ CGGAATTCTC TGATGATGCT CTAAATGGGT ATGCTTATGT TGATATCCCT GCTTATGATG CATCCTTAGA   
  
  
+ TTATGCCAAT TTGTTCAATT ACGAAGGCCC ATCTGAGGAT CGCACCTCAC TGAGCCTCCC AAGACCCTTT   
  
  
+ TCTGACCCTT TGGCTTACAG TTTCACGTCC TCTTCTGAGC TGGGCCCTGG GGTTGATTCT AATGATGATA   
  
  
+ GTGATTCTGG CGATGTTCTC AAGTACATTA GCCAAATGCT TATGGAAGAA GACATGGAGG CAAAGCCATG   
  
  
+ CATGTTTCAT GATCCTTTAG CACTTCAGGC TGCTGAGAAA CCCTTTTATG ATGCCTTAGG GGAGCAATGC   
  
  
+ CCAACTTCTC CTGACCAACA TCCTATAATT GATCATTATT TGGATAGTCC TGATGAAAAT TCTTTGAGTT   
  
  
+ CAACTGGTGA TTTTAGTGTT AGTCATTCTG GGTCTAGTTC AACAAACTCC GTTGGACCGA TAATAGTGTC   
  
  
+ TGATTTGAGT GAGCATTTTG AGCCACCCTT TGTTGAAACA CTTCCAATTG AATCATATCA CCAACCATTG   
  
  
+ ACCCGTCCTC AATGGTCATT TGGCTCTTCG GGTGCCTTAG ATGGCACGGC CTCTAATGGT TCGGTGATCT   
  
  
+ CATCCCTTGG TTTGCCAGTG GATGTGATTA GCGTATTTAG GGAGAAAGAG TCCATGATTC AATTTCAGAA   
  
  
+ AGGGGTGGAG GAGGCCAGTA AGTTCCTTCC CAAGAATAAT AACCTTGTTA TTGATCTCGA GAACCTCACT   
  
  
+ TTTCCTAATG AAACAAAGGA GGATGATCGA ATGATGATGG TTAAGAAGGA AAAGGATGAC GTGAATTGGT   
  
  
+ CTAACTACTC AAGAGGGAGT AAGATTCACT ATTGTGAAGA CGAGGCCTTT GAAGAAGGAA GGAGTGGCAA   
  
  
+ GCAGTCAGCT ATTTCTTCTA CTGAGGAAGC TGAGTTTTCT GACATTTTTG ACAAGGTTTT GCTTTGCGAT   
  
  
+ TGCTACCCTG TGAAACCTGA GGCTCATCCC ACCATGAGTT TGAACCCTGA GAAGGGCCAG TCACATGGAT   
  
  
+ TAGAAGGTGG GAGAAATGGG AAGGCTCGCC CTAAGAAACA GGATAATAGT AGCACAAATA TTGTGGATTT   
  
  
+ AAGGAATTTG CTGATACTAT GCGCACAATC TACTGCATCT GATGACCGAA GAACTGCTGA TGGACTGCTA   
  
  
+ AAGCAAATCA GGGAGCACTC GTCTGCTGAG GGGGATGGAT CTCAAAGGTT GGCGCATTAC TTTGCTGATG   
  
  
+ CCCTAGAGGC ACGTTTAGCT GGAACTGGAT CTCGCATTTA TACGGCCCTA TGTTCTAATA GGCCATCTGT   
  
  
+ CACTGACATG ATAAAAGCAT ATCAGTTCTA TATTCGTGCT TGCCCATTTA CGAAGATCGT CATTGGTTGT   
  
  
+ GGTACCCATA TGATTCTAAA AGCAGCTGAG AAGGCATCAA AGCTTCATAT TATAGATTTT GGCATCCTCT   
  
  
+ ATGGTTGCCA ATGGCCCAAC CTCATTCAAC GCCTCTCAGA GCGATCTGGT GGACCTCCAA AACTGTTTAT   
  
  
+ TACAGGGATC GATCTCCCCC AGCCTGGGTT CAGGCCAGCA GAAAGAGTGG AAGCAACAGG GAGACGCTTG   
  
  
+ GCCAAGTACT GTGAGCGGTA TAATGTGCCA TTTGAGTATC ATGCCATTGC TCAGGAGTGG GAAACAATCA   
  
  
+ AACCAGGGGA TCTCAAGATA GGAAGTAGGA ATGATGAAGT TGTTGCGGTG AACTGTCTCT GTAGGTTCAA   
  
  
+ GAACCTCCTT GACGAGACAG TGGTGGTGGA TAGTCCAAGG AACACAGTTT TAAACCTGAT TACAAGGGTA   
  
  
+ AAGCCTGATA TTTTTGTGCA TGGCGTTGTA AATGGTTCCT ACAACATCCC TTTCTTTGTG ACACGTTTTA   
  
  
+ GAGAAGCCCT CTTTCATTAT TCCACTCTTT TCGACATGTT AGATGCCAAC GCCTCTAGGG AGGAGCCCGA   
  
  
+ GAGGTTGATA TTCGAGAAGG CATTCTATGG GAGGGAGATT ATGAATGTGG TGGCCTGTGA GGGCACAGAG   
  
  
+ AGGGTGGAGA GGCCGGAGAC ATACAAGCAA TGGCATGTTA GGCATAGCAG GGCAGGGTTT CGGCAAGTAC   
  
  
+ CATTGGATCC CAAGTTGATC GAGAAAATGA GGTTTAAGGC CAAGGCAGAC CACCACAAGG ATTTCATGAT   
  
  
+ CGATGTGGAT GGACATTGGG CAATTCAGGG ATGGAAGGGG CGGATTGCAC ATGCGATCTC TGCATGGGTT   
  
  
+ CCGGCTTG  

- -Up\_Stream \_Len000CTAAAC TAACAGCTTT AAACAGGCTA AAATATTTTA ACTTTTAACA ATAACCTGAT   
  
  
- ATTAGATGAC ATCAAATCTC CACAACCTAT TATGAGAGTT GAAACTTGAA CCTGCAGGTA CGTGGTGTGA   
  
  
- TACCCGCATA AATGACAAAT ATATGGTTCT TATTAAAACT TCTACAAGAA TCAACCACTA CATTCACATA   
  
  
- TAAGTTGTAT ACAGTAATGT ATTTGAACTT TTAAGAACTT AAAATCTGTA TATGTTAACG TTTAGTAACA   
  
  
- TTTTAACAAC GTAATGTAAA ACTATTTTTA CGTAGGTTTG AAACAGAACC ATATTTGTCA TTTATCCGGG   
  
  
- TATCCAGGTA TCACATCACG TACCTGCAGG TTCAAATTTC CACCCTCATA ATAACGGGTT GCGGGGATTT   
  
  
- CATGTCATAT AATATCAGGT TATAGGGTTA ATTAAAAAGT AAACTTATAA ATTTAAACCT GTATTATAAA   
  
  
- AAGTATAAAC TTAAGAGATT TGGACTAAAA TTGGACTTAA ATTCAACTAA ATTAAGTAGG CTGGTTTTTT   
  
  
- TATTTGTGTT AATAAATAAA AAGATAAGCT TCAATCTATC ATCGGTTAAG TAAACTGTAT ATTAATTGGG   
  
  
- CACTAACTGT GTTTGAGCTC AAAAACGAAT CTCGATCCTT TTCTCAGCCC AATCCAGCTG GGTAAATTCT   
  
  
- TTACCCAGCT GAAGTACAGC CGGAAGTTTT ATCAATTCTA AACGAGTTTT GATTTTTTAT TGTTCAAGTG   
  
  
- TGATTTGCCA GTTTAGCACA GCTGAAGCCT AACTTTAACC AAGTCAAACC CAGCCGAAGC ATAAAGAAAG   
  
  
- TCATTGAAGC TTGGCTTAAG CATAGCACAA TAGCACAGTC CAGTCTGAAA GAGTCAAGAT AAAAACGATG   
  
  
- GAATACAATG CCTATTTTTT TCAGAGTGTA ACCTTTTTCA CACCCTCCCA GGACCCGAAT ATTTATCACC   
  
  
- GCGACGTGTG GAGTAGTCTG GCTGGAAAAC CCCTCTCCCA TTACTGGGGC ACTGTTCACC ATAGTCTCAG   
  
  
- TTGGGTCTGG GCTGAGTCTA CAGGACGGTT CGCGAGGCTG CCCGCCCCAT CCGTCCCCCG GGTAAACACT   
  
  
- ACTCCTGCAG TGTCTTAACC CGCCCCCTCT CACAGTGCCT ATTTTTTTCA GGGTGTAGCT TTTTTCACAC   
  
  
- CCTCCCAGGA CCCGAATATT TGCCATCGCG GCGTGTGGGG TAGTCTAGCT GAAAAACCCC TCTCCCATTA   
  
  
- CTGGGGTAAA GTTCACCATA GTCTCGGTTG GGTCTGGGCT GAGGTTACAG GGTGGTTCGC GAGACTACCC   
  
  
- GCCCCATCCG TTCCCCGAGG TATACACTGC TCCCGCAGTG CCTTAACCTA CCCTCTCTCA TGGTGCCTAT   
  
  
- TTTTTTCAGG GTGTAGTCTT TTTCACACCT GAATCGTTGA TCCTTCATTT CAATGTCATA ATCGGCTCTC   
  
  
- GGGTACCTTG CGGACTTTGA CTGTAGTGGT CTGGGTGTAT AAAGTGGTAG TAGACTAGAG GGGCAGTTTC   
  
  
- CTCCTTCGAC TCGTGTCGTT CTTGATCTTC TTGTCCGCCG TAGTGTGGGT TCGGTTTTAA GAGATAAACT   
  
  
- GGAGGGAGAG GAGTAAGGAG ACTCGAAGGA GAAGTGTTAC ATTCTGGTAT TAGGAATTGT GGGAAGAAAA   
  
  
- AATTCGAGTA GAAATGGACG AGACCAAGAG AGAGAGTAGC AGTGATTATC AATGGAGAAA TGAAAGAGAA   
  
  
- GGGACTCCAT ACGAAGTTGA AATTGTATAT ATAGGGTGAA CTAAAGAGAA CAGAAAAACG ATATTATGAA   
  
  
- CAACAAACCG ATAGTAGGGG ACAAAATATG AGTAAAAGAA CGAAAGATAA AGACCCAAAC TTAACCCAAA   
  
  
- GTGAATAGGA GCAGTTAAGA TACCCATTAA TCACTTAACC CAAATATACT TGATCCCAAC GTTTTCATAG   
  
  
- ATGAAAAAGA GAACACTAAT AATCAACTAA TCCCTAAACC GGTTATAATC GTCTTACCCA AGACTTAAAC   
  
  
- GCCTTAAGAG ACTACTACGA GATTTACCCA TACGAATACA ACTATAGGGA CGAATACTAC GTAGGAATCT   
  
  
- AATACGGTTA AACAAGTTAA TGCTTCCGGG TAGACTCCTA GCGTGGAGTG ACTCGGAGGG TTCTGGGAAA   
  
  
- AGACTGGGAA ACCGAATGTC AAAGTGCAGG AGAAGACTCG ACCCGGGACC CCAACTAAGA TTACTACTAT   
  
  
- CACTAAGACC GCTACAAGAG TTCATGTAAT CGGTTTACGA ATACCTTCTT CTGTACCTCC GTTTCGGTAC   
  
  
- GTACAAAGTA CTAGGAAATC GTGAAGTCCG ACGACTCTTT GGGAAAATAC TACGGAATCC CCTCGTTACG   
  
  
- GGTTGAAGAG GACTGGTTGT AGGATATTAA CTAGTAATAA ACCTATCAGG ACTACTTTTA AGAAACTCAA   
  
  
- GTTGACCACT AAAATCACAA TCAGTAAGAC CCAGATCAAG TTGTTTGAGG CAACCTGGCT ATTATCACAG   
  
  
- ACTAAACTCA CTCGTAAAAC TCGGTGGGAA ACAACTTTGT GAAGGTTAAC TTAGTATAGT GGTTGGTAAC   
  
  
- TGGGCAGGAG TTACCAGTAA ACCGAGAAGC CCACGGAATC TACCGTGCCG GAGATTACCA AGCCACTAGA   
  
  
- GTAGGGAACC AAACGGTCAC CTACACTAAT CGCATAAATC CCTCTTTCTC AGGTACTAAG TTAAAGTCTT   
  
  
- TCCCCACCTC CTCCGGTCAT TCAAGGAAGG GTTCTTATTA TTGGAACAAT AACTAGAGCT CTTGGAGTGA   
  
  
- AAAGGATTAC TTTGTTTCCT CCTACTAGCT TACTACTACC AATTCTTCCT TTTCCTACTG CACTTAACCA   
  
  
- GATTGATGAG TTCTCCCTCA TTCTAAGTGA TAACACTTCT GCTCCGGAAA CTTCTTCCTT CCTCACCGTT   
  
  
- CGTCAGTCGA TAAAGAAGAT GACTCCTTCG ACTCAAAAGA CTGTAAAAAC TGTTCCAAAA CGAAACGCTA   
  
  
- ACGATGGGAC ACTTTGGACT CCGAGTAGGG TGGTACTCAA ACTTGGGACT CTTCCCGGTC AGTGTACCTA   
  
  
- ATCTTCCACC CTCTTTACCC TTCCGAGCGG GATTCTTTGT CCTATTATCA TCGTGTTTAT AACACCTAAA   
  
  
- TTCCTTAAAC GACTATGATA CGCGTGTTAG ATGACGTAGA CTACTGGCTT CTTGACGACT ACCTGACGAT   
  
  
- TTCGTTTAGT CCCTCGTGAG CAGACGACTC CCCCTACCTA GAGTTTCCAA CCGCGTAATG AAACGACTAC   
  
  
- GGGATCTCCG TGCAAATCGA CCTTGACCTA GAGCGTAAAT ATGCCGGGAT ACAAGATTAT CCGGTAGACA   
  
  
- GTGACTGTAC TATTTTCGTA TAGTCAAGAT ATAAGCACGA ACGGGTAAAT GCTTCTAGCA GTAACCAACA   
  
  
- CCATGGGTAT ACTAAGATTT TCGTCGACTC TTCCGTAGTT TCGAAGTATA ATATCTAAAA CCGTAGGAGA   
  
  
- TACCAACGGT TACCGGGTTG GAGTAAGTTG CGGAGAGTCT CGCTAGACCA CCTGGAGGTT TTGACAAATA   
  
  
- ATGTCCCTAG CTAGAGGGGG TCGGACCCAA GTCCGGTCGT CTTTCTCACC TTCGTTGTCC CTCTGCGAAC   
  
  
- CGGTTCATGA CACTCGCCAT ATTACACGGT AAACTCATAG TACGGTAACG AGTCCTCACC CTTTGTTAGT   
  
  
- TTGGTCCCCT AGAGTTCTAT CCTTCATCCT TACTACTTCA ACAACGCCAC TTGACAGAGA CATCCAAGTT   
  
  
- CTTGGAGGAA CTGCTCTGTC ACCACCACCT ATCAGGTTCC TTGTGTCAAA ATTTGGACTA ATGTTCCCAT   
  
  
- TTCGGACTAT AAAAACACGT ACCGCAACAT TTACCAAGGA TGTTGTAGGG AAAGAAACAC TGTGCAAAAT   
  
  
- CTCTTCGGGA GAAAGTAATA AGGTGAGAAA AGCTGTACAA TCTACGGTTG CGGAGATCCC TCCTCGGGCT   
  
  
- CTCCAACTAT AAGCTCTTCC GTAAGATACC CTCCCTCTAA TACTTACACC ACCGGACACT CCCGTGTCTC   
  
  
- TCCCACCTCT CCGGCCTCTG TATGTTCGTT ACCGTACAAT CCGTATCGTC CCGTCCCAAA GCCGTTCATG   
  
  
- GTAACCTAGG GTTCAACTAG CTCTTTTACT CCAAATTCCG GTTCCGTCTG GTGGTGTTCC TAAAGTACTA   
  
  
- GCTACACCTA CCTGTAACCC GTTAAGTCCC TACCTTCCCC GCCTAACGTG TACGCTAGAG ACGTACCCAA   
  
  
- GGCCGAAC

+     GC-motif

| Site Name | Organism | Position | Strand | Matrix score. | sequence | function |
| --- | --- | --- | --- | --- | --- | --- |
| GC-motif | Zea mays | 1146 | - | 6 | CCCCCG | enhancer-like element involved in anoxic specific inducibility |

>HU07G02249.1   
+ -Up\_Stream \_Len000GATTTG ATTGTCGAAA TTTGTCCGAT TTTATAAAAT TGAAAATTGT TATTGGACTA   
  
  
+ TAATCTACTG TAGTTTAGAG GTGTTGGATA ATACTCTCAA CTTTGAACTT GGACGTCCAT GCACCACACT   
  
  
+ ATGGGCGTAT TTACTGTTTA TATACCAAGA ATAATTTTGA AGATGTTCTT AGTTGGTGAT GTAAGTGTAT   
  
  
+ ATTCAACATA TGTCATTACA TAAACTTGAA AATTCTTGAA TTTTAGACAT ATACAATTGC AAATCATTGT   
  
  
+ AAAATTGTTG CATTACATTT TGATAAAAAT GCATCCAAAC TTTGTCTTGG TATAAACAGT AAATAGGCCC   
  
  
+ ATAGGTCCAT AGTGTAGTGC ATGGACGTCC AAGTTTAAAG GTGGGAGTAT TATTGCCCAA CGCCCCTAAA   
  
  
+ GTACAGTATA TTATAGTCCA ATATCCCAAT TAATTTTTCA TTTGAATATT TAAATTTGGA CATAATATTT   
  
  
+ TTCATATTTG AATTCTCTAA ACCTGATTTT AACCTGAATT TAAGTTGATT TAATTCATCC GACCAAAAAA   
  
  
+ ATAAACACAA TTATTTATTT TTCTATTCGA AGTTAGATAG TAGCCAATTC ATTTGACATA TAATTAACCC   
  
  
+ GTGATTGACA CAAACTCGAG TTTTTGCTTA GAGCTAGGAA AAGAGTCGGG TTAGGTCGAC CCATTTAAGA   
  
  
+ AATGGGTCGA CTTCATGTCG GCCTTCAAAA TAGTTAAGAT TTGCTCAAAA CTAAAAAATA ACAAGTTCAC   
  
  
+ ACTAAACGGT CAAATCGTGT CGACTTCGGA TTGAAATTGG TTCAGTTTGG GTCGGCTTCG TATTTCTTTC   
  
  
+ AGTAACTTCG AACCGAATTC GTATCGTGTT ATCGTGTCAG GTCAGACTTT CTCAGTTCTA TTTTTGCTAC   
  
  
+ CTTATGTTAC GGATAAAAAA AGTCTCACAT TGGAAAAAGT GTGGGAGGGT CCTGGGCTTA TAAATAGTGG   
  
  
+ CGCTGCACAC CTCATCAGAC CGACCTTTTG GGGAGAGGGT AATGACCCCG TGACAAGTGG TATCAGAGTC   
  
  
+ AACCCAGACC CGACTCAGAT GTCCTGCCAA GCGCTCCGAC GGGCGGGGTA GGCAGGGGGC CCATTTGTGA   
  
  
+ TGAGGACGTC ACAGAATTGG GCGGGGGAGA GTGTCACGGA TAAAAAAAGT CCCACATCGA AAAAAGTGTG   
  
  
+ GGAGGGTCCT GGGCTTATAA ACGGTAGCGC CGCACACCCC ATCAGATCGA CTTTTTGGGG AGAGGGTAAT   
  
  
+ GACCCCATTT CAAGTGGTAT CAGAGCCAAC CCAGACCCGA CTCCAATGTC CCACCAAGCG CTCTGATGGG   
  
  
+ CGGGGTAGGC AAGGGGCTCC ATATGTGACG AGGGCGTCAC GGAATTGGAT GGGAGAGAGT ACCACGGATA   
  
  
+ AAAAAAGTCC CACATCAGAA AAAGTGTGGA CTTAGCAACT AGGAAGTAAA GTTACAGTAT TAGCCGAGAG   
  
  
+ CCCATGGAAC GCCTGAAACT GACATCACCA GACCCACATA TTTCACCATC ATCTGATCTC CCCGTCAAAG   
  
  
+ GAGGAAGCTG AGCACAGCAA GAACTAGAAG AACAGGCGGC ATCACACCCA AGCCAAAATT CTCTATTTGA   
  
  
+ CCTCCCTCTC CTCATTCCTC TGAGCTTCCT CTTCACAATG TAAGACCATA ATCCTTAACA CCCTTCTTTT   
  
  
+ TTAAGCTCAT CTTTACCTGC TCTGGTTCTC TCTCTCATCG TCACTAATAG TTACCTCTTT ACTTTCTCTT   
  
  
+ CCCTGAGGTA TGCTTCAACT TTAACATATA TATCCCACTT GATTTCTCTT GTCTTTTTGC TATAATACTT   
  
  
+ GTTGTTTGGC TATCATCCCC TGTTTTATAC TCATTTTCTT GCTTTCTATT TCTGGGTTTG AATTGGGTTT   
  
  
+ CACTTATCCT CGTCAATTCT ATGGGTAATT AGTGAATTGG GTTTATATGA ACTAGGGTTG CAAAAGTATC   
  
  
+ TACTTTTTCT CTTGTGATTA TTAGTTGATT AGGGATTTGG CCAATATTAG CAGAATGGGT TCTGAATTTG   
  
  
+ CGGAATTCTC TGATGATGCT CTAAATGGGT ATGCTTATGT TGATATCCCT GCTTATGATG CATCCTTAGA   
  
  
+ TTATGCCAAT TTGTTCAATT ACGAAGGCCC ATCTGAGGAT CGCACCTCAC TGAGCCTCCC AAGACCCTTT   
  
  
+ TCTGACCCTT TGGCTTACAG TTTCACGTCC TCTTCTGAGC TGGGCCCTGG GGTTGATTCT AATGATGATA   
  
  
+ GTGATTCTGG CGATGTTCTC AAGTACATTA GCCAAATGCT TATGGAAGAA GACATGGAGG CAAAGCCATG   
  
  
+ CATGTTTCAT GATCCTTTAG CACTTCAGGC TGCTGAGAAA CCCTTTTATG ATGCCTTAGG GGAGCAATGC   
  
  
+ CCAACTTCTC CTGACCAACA TCCTATAATT GATCATTATT TGGATAGTCC TGATGAAAAT TCTTTGAGTT   
  
  
+ CAACTGGTGA TTTTAGTGTT AGTCATTCTG GGTCTAGTTC AACAAACTCC GTTGGACCGA TAATAGTGTC   
  
  
+ TGATTTGAGT GAGCATTTTG AGCCACCCTT TGTTGAAACA CTTCCAATTG AATCATATCA CCAACCATTG   
  
  
+ ACCCGTCCTC AATGGTCATT TGGCTCTTCG GGTGCCTTAG ATGGCACGGC CTCTAATGGT TCGGTGATCT   
  
  
+ CATCCCTTGG TTTGCCAGTG GATGTGATTA GCGTATTTAG GGAGAAAGAG TCCATGATTC AATTTCAGAA   
  
  
+ AGGGGTGGAG GAGGCCAGTA AGTTCCTTCC CAAGAATAAT AACCTTGTTA TTGATCTCGA GAACCTCACT   
  
  
+ TTTCCTAATG AAACAAAGGA GGATGATCGA ATGATGATGG TTAAGAAGGA AAAGGATGAC GTGAATTGGT   
  
  
+ CTAACTACTC AAGAGGGAGT AAGATTCACT ATTGTGAAGA CGAGGCCTTT GAAGAAGGAA GGAGTGGCAA   
  
  
+ GCAGTCAGCT ATTTCTTCTA CTGAGGAAGC TGAGTTTTCT GACATTTTTG ACAAGGTTTT GCTTTGCGAT   
  
  
+ TGCTACCCTG TGAAACCTGA GGCTCATCCC ACCATGAGTT TGAACCCTGA GAAGGGCCAG TCACATGGAT   
  
  
+ TAGAAGGTGG GAGAAATGGG AAGGCTCGCC CTAAGAAACA GGATAATAGT AGCACAAATA TTGTGGATTT   
  
  
+ AAGGAATTTG CTGATACTAT GCGCACAATC TACTGCATCT GATGACCGAA GAACTGCTGA TGGACTGCTA   
  
  
+ AAGCAAATCA GGGAGCACTC GTCTGCTGAG GGGGATGGAT CTCAAAGGTT GGCGCATTAC TTTGCTGATG   
  
  
+ CCCTAGAGGC ACGTTTAGCT GGAACTGGAT CTCGCATTTA TACGGCCCTA TGTTCTAATA GGCCATCTGT   
  
  
+ CACTGACATG ATAAAAGCAT ATCAGTTCTA TATTCGTGCT TGCCCATTTA CGAAGATCGT CATTGGTTGT   
  
  
+ GGTACCCATA TGATTCTAAA AGCAGCTGAG AAGGCATCAA AGCTTCATAT TATAGATTTT GGCATCCTCT   
  
  
+ ATGGTTGCCA ATGGCCCAAC CTCATTCAAC GCCTCTCAGA GCGATCTGGT GGACCTCCAA AACTGTTTAT   
  
  
+ TACAGGGATC GATCTCCCCC AGCCTGGGTT CAGGCCAGCA GAAAGAGTGG AAGCAACAGG GAGACGCTTG   
  
  
+ GCCAAGTACT GTGAGCGGTA TAATGTGCCA TTTGAGTATC ATGCCATTGC TCAGGAGTGG GAAACAATCA   
  
  
+ AACCAGGGGA TCTCAAGATA GGAAGTAGGA ATGATGAAGT TGTTGCGGTG AACTGTCTCT GTAGGTTCAA   
  
  
+ GAACCTCCTT GACGAGACAG TGGTGGTGGA TAGTCCAAGG AACACAGTTT TAAACCTGAT TACAAGGGTA   
  
  
+ AAGCCTGATA TTTTTGTGCA TGGCGTTGTA AATGGTTCCT ACAACATCCC TTTCTTTGTG ACACGTTTTA   
  
  
+ GAGAAGCCCT CTTTCATTAT TCCACTCTTT TCGACATGTT AGATGCCAAC GCCTCTAGGG AGGAGCCCGA   
  
  
+ GAGGTTGATA TTCGAGAAGG CATTCTATGG GAGGGAGATT ATGAATGTGG TGGCCTGTGA GGGCACAGAG   
  
  
+ AGGGTGGAGA GGCCGGAGAC ATACAAGCAA TGGCATGTTA GGCATAGCAG GGCAGGGTTT CGGCAAGTAC   
  
  
+ CATTGGATCC CAAGTTGATC GAGAAAATGA GGTTTAAGGC CAAGGCAGAC CACCACAAGG ATTTCATGAT   
  
  
+ CGATGTGGAT GGACATTGGG CAATTCAGGG ATGGAAGGGG CGGATTGCAC ATGCGATCTC TGCATGGGTT   
  
  
+ CCGGCTTG  

- -Up\_Stream \_Len000CTAAAC TAACAGCTTT AAACAGGCTA AAATATTTTA ACTTTTAACA ATAACCTGAT   
  
  
- ATTAGATGAC ATCAAATCTC CACAACCTAT TATGAGAGTT GAAACTTGAA CCTGCAGGTA CGTGGTGTGA   
  
  
- TACCCGCATA AATGACAAAT ATATGGTTCT TATTAAAACT TCTACAAGAA TCAACCACTA CATTCACATA   
  
  
- TAAGTTGTAT ACAGTAATGT ATTTGAACTT TTAAGAACTT AAAATCTGTA TATGTTAACG TTTAGTAACA   
  
  
- TTTTAACAAC GTAATGTAAA ACTATTTTTA CGTAGGTTTG AAACAGAACC ATATTTGTCA TTTATCCGGG   
  
  
- TATCCAGGTA TCACATCACG TACCTGCAGG TTCAAATTTC CACCCTCATA ATAACGGGTT GCGGGGATTT   
  
  
- CATGTCATAT AATATCAGGT TATAGGGTTA ATTAAAAAGT AAACTTATAA ATTTAAACCT GTATTATAAA   
  
  
- AAGTATAAAC TTAAGAGATT TGGACTAAAA TTGGACTTAA ATTCAACTAA ATTAAGTAGG CTGGTTTTTT   
  
  
- TATTTGTGTT AATAAATAAA AAGATAAGCT TCAATCTATC ATCGGTTAAG TAAACTGTAT ATTAATTGGG   
  
  
- CACTAACTGT GTTTGAGCTC AAAAACGAAT CTCGATCCTT TTCTCAGCCC AATCCAGCTG GGTAAATTCT   
  
  
- TTACCCAGCT GAAGTACAGC CGGAAGTTTT ATCAATTCTA AACGAGTTTT GATTTTTTAT TGTTCAAGTG   
  
  
- TGATTTGCCA GTTTAGCACA GCTGAAGCCT AACTTTAACC AAGTCAAACC CAGCCGAAGC ATAAAGAAAG   
  
  
- TCATTGAAGC TTGGCTTAAG CATAGCACAA TAGCACAGTC CAGTCTGAAA GAGTCAAGAT AAAAACGATG   
  
  
- GAATACAATG CCTATTTTTT TCAGAGTGTA ACCTTTTTCA CACCCTCCCA GGACCCGAAT ATTTATCACC   
  
  
- GCGACGTGTG GAGTAGTCTG GCTGGAAAAC CCCTCTCCCA TTACTGGGGC ACTGTTCACC ATAGTCTCAG   
  
  
- TTGGGTCTGG GCTGAGTCTA CAGGACGGTT CGCGAGGCTG CCCGCCCCAT CCGTCCCCCG GGTAAACACT   
  
  
- ACTCCTGCAG TGTCTTAACC CGCCCCCTCT CACAGTGCCT ATTTTTTTCA GGGTGTAGCT TTTTTCACAC   
  
  
- CCTCCCAGGA CCCGAATATT TGCCATCGCG GCGTGTGGGG TAGTCTAGCT GAAAAACCCC TCTCCCATTA   
  
  
- CTGGGGTAAA GTTCACCATA GTCTCGGTTG GGTCTGGGCT GAGGTTACAG GGTGGTTCGC GAGACTACCC   
  
  
- GCCCCATCCG TTCCCCGAGG TATACACTGC TCCCGCAGTG CCTTAACCTA CCCTCTCTCA TGGTGCCTAT   
  
  
- TTTTTTCAGG GTGTAGTCTT TTTCACACCT GAATCGTTGA TCCTTCATTT CAATGTCATA ATCGGCTCTC   
  
  
- GGGTACCTTG CGGACTTTGA CTGTAGTGGT CTGGGTGTAT AAAGTGGTAG TAGACTAGAG GGGCAGTTTC   
  
  
- CTCCTTCGAC TCGTGTCGTT CTTGATCTTC TTGTCCGCCG TAGTGTGGGT TCGGTTTTAA GAGATAAACT   
  
  
- GGAGGGAGAG GAGTAAGGAG ACTCGAAGGA GAAGTGTTAC ATTCTGGTAT TAGGAATTGT GGGAAGAAAA   
  
  
- AATTCGAGTA GAAATGGACG AGACCAAGAG AGAGAGTAGC AGTGATTATC AATGGAGAAA TGAAAGAGAA   
  
  
- GGGACTCCAT ACGAAGTTGA AATTGTATAT ATAGGGTGAA CTAAAGAGAA CAGAAAAACG ATATTATGAA   
  
  
- CAACAAACCG ATAGTAGGGG ACAAAATATG AGTAAAAGAA CGAAAGATAA AGACCCAAAC TTAACCCAAA   
  
  
- GTGAATAGGA GCAGTTAAGA TACCCATTAA TCACTTAACC CAAATATACT TGATCCCAAC GTTTTCATAG   
  
  
- ATGAAAAAGA GAACACTAAT AATCAACTAA TCCCTAAACC GGTTATAATC GTCTTACCCA AGACTTAAAC   
  
  
- GCCTTAAGAG ACTACTACGA GATTTACCCA TACGAATACA ACTATAGGGA CGAATACTAC GTAGGAATCT   
  
  
- AATACGGTTA AACAAGTTAA TGCTTCCGGG TAGACTCCTA GCGTGGAGTG ACTCGGAGGG TTCTGGGAAA   
  
  
- AGACTGGGAA ACCGAATGTC AAAGTGCAGG AGAAGACTCG ACCCGGGACC CCAACTAAGA TTACTACTAT   
  
  
- CACTAAGACC GCTACAAGAG TTCATGTAAT CGGTTTACGA ATACCTTCTT CTGTACCTCC GTTTCGGTAC   
  
  
- GTACAAAGTA CTAGGAAATC GTGAAGTCCG ACGACTCTTT GGGAAAATAC TACGGAATCC CCTCGTTACG   
  
  
- GGTTGAAGAG GACTGGTTGT AGGATATTAA CTAGTAATAA ACCTATCAGG ACTACTTTTA AGAAACTCAA   
  
  
- GTTGACCACT AAAATCACAA TCAGTAAGAC CCAGATCAAG TTGTTTGAGG CAACCTGGCT ATTATCACAG   
  
  
- ACTAAACTCA CTCGTAAAAC TCGGTGGGAA ACAACTTTGT GAAGGTTAAC TTAGTATAGT GGTTGGTAAC   
  
  
- TGGGCAGGAG TTACCAGTAA ACCGAGAAGC CCACGGAATC TACCGTGCCG GAGATTACCA AGCCACTAGA   
  
  
- GTAGGGAACC AAACGGTCAC CTACACTAAT CGCATAAATC CCTCTTTCTC AGGTACTAAG TTAAAGTCTT   
  
  
- TCCCCACCTC CTCCGGTCAT TCAAGGAAGG GTTCTTATTA TTGGAACAAT AACTAGAGCT CTTGGAGTGA   
  
  
- AAAGGATTAC TTTGTTTCCT CCTACTAGCT TACTACTACC AATTCTTCCT TTTCCTACTG CACTTAACCA   
  
  
- GATTGATGAG TTCTCCCTCA TTCTAAGTGA TAACACTTCT GCTCCGGAAA CTTCTTCCTT CCTCACCGTT   
  
  
- CGTCAGTCGA TAAAGAAGAT GACTCCTTCG ACTCAAAAGA CTGTAAAAAC TGTTCCAAAA CGAAACGCTA   
  
  
- ACGATGGGAC ACTTTGGACT CCGAGTAGGG TGGTACTCAA ACTTGGGACT CTTCCCGGTC AGTGTACCTA   
  
  
- ATCTTCCACC CTCTTTACCC TTCCGAGCGG GATTCTTTGT CCTATTATCA TCGTGTTTAT AACACCTAAA   
  
  
- TTCCTTAAAC GACTATGATA CGCGTGTTAG ATGACGTAGA CTACTGGCTT CTTGACGACT ACCTGACGAT   
  
  
- TTCGTTTAGT CCCTCGTGAG CAGACGACTC CCCCTACCTA GAGTTTCCAA CCGCGTAATG AAACGACTAC   
  
  
- GGGATCTCCG TGCAAATCGA CCTTGACCTA GAGCGTAAAT ATGCCGGGAT ACAAGATTAT CCGGTAGACA   
  
  
- GTGACTGTAC TATTTTCGTA TAGTCAAGAT ATAAGCACGA ACGGGTAAAT GCTTCTAGCA GTAACCAACA   
  
  
- CCATGGGTAT ACTAAGATTT TCGTCGACTC TTCCGTAGTT TCGAAGTATA ATATCTAAAA CCGTAGGAGA   
  
  
- TACCAACGGT TACCGGGTTG GAGTAAGTTG CGGAGAGTCT CGCTAGACCA CCTGGAGGTT TTGACAAATA   
  
  
- ATGTCCCTAG CTAGAGGGGG TCGGACCCAA GTCCGGTCGT CTTTCTCACC TTCGTTGTCC CTCTGCGAAC   
  
  
- CGGTTCATGA CACTCGCCAT ATTACACGGT AAACTCATAG TACGGTAACG AGTCCTCACC CTTTGTTAGT   
  
  
- TTGGTCCCCT AGAGTTCTAT CCTTCATCCT TACTACTTCA ACAACGCCAC TTGACAGAGA CATCCAAGTT   
  
  
- CTTGGAGGAA CTGCTCTGTC ACCACCACCT ATCAGGTTCC TTGTGTCAAA ATTTGGACTA ATGTTCCCAT   
  
  
- TTCGGACTAT AAAAACACGT ACCGCAACAT TTACCAAGGA TGTTGTAGGG AAAGAAACAC TGTGCAAAAT   
  
  
- CTCTTCGGGA GAAAGTAATA AGGTGAGAAA AGCTGTACAA TCTACGGTTG CGGAGATCCC TCCTCGGGCT   
  
  
- CTCCAACTAT AAGCTCTTCC GTAAGATACC CTCCCTCTAA TACTTACACC ACCGGACACT CCCGTGTCTC   
  
  
- TCCCACCTCT CCGGCCTCTG TATGTTCGTT ACCGTACAAT CCGTATCGTC CCGTCCCAAA GCCGTTCATG   
  
  
- GTAACCTAGG GTTCAACTAG CTCTTTTACT CCAAATTCCG GTTCCGTCTG GTGGTGTTCC TAAAGTACTA   
  
  
- GCTACACCTA CCTGTAACCC GTTAAGTCCC TACCTTCCCC GCCTAACGTG TACGCTAGAG ACGTACCCAA   
  
  
- GGCCGAAC

+     GT1-motif

| Site Name | Organism | Position | Strand | Matrix score. | sequence | function |
| --- | --- | --- | --- | --- | --- | --- |
| GT1-motif | Avena sativa | 627 | - | 7 | GGTTAAT | light responsive element |
| GT1-motif | Arabidopsis thaliana | 523 | - | 6 | GGTTAA | light responsive element |
| GT1-motif | Arabidopsis thaliana | 628 | - | 6 | GGTTAA | light responsive element |
| GT1-motif | Arabidopsis thaliana | 2843 | + | 6 | GGTTAA | light responsive element |

>HU07G02249.1   
+ -Up\_Stream \_Len000GATTTG ATTGTCGAAA TTTGTCCGAT TTTATAAAAT TGAAAATTGT TATTGGACTA   
  
  
+ TAATCTACTG TAGTTTAGAG GTGTTGGATA ATACTCTCAA CTTTGAACTT GGACGTCCAT GCACCACACT   
  
  
+ ATGGGCGTAT TTACTGTTTA TATACCAAGA ATAATTTTGA AGATGTTCTT AGTTGGTGAT GTAAGTGTAT   
  
  
+ ATTCAACATA TGTCATTACA TAAACTTGAA AATTCTTGAA TTTTAGACAT ATACAATTGC AAATCATTGT   
  
  
+ AAAATTGTTG CATTACATTT TGATAAAAAT GCATCCAAAC TTTGTCTTGG TATAAACAGT AAATAGGCCC   
  
  
+ ATAGGTCCAT AGTGTAGTGC ATGGACGTCC AAGTTTAAAG GTGGGAGTAT TATTGCCCAA CGCCCCTAAA   
  
  
+ GTACAGTATA TTATAGTCCA ATATCCCAAT TAATTTTTCA TTTGAATATT TAAATTTGGA CATAATATTT   
  
  
+ TTCATATTTG AATTCTCTAA ACCTGATTTT AACCTGAATT TAAGTTGATT TAATTCATCC GACCAAAAAA   
  
  
+ ATAAACACAA TTATTTATTT TTCTATTCGA AGTTAGATAG TAGCCAATTC ATTTGACATA TAATTAACCC   
  
  
+ GTGATTGACA CAAACTCGAG TTTTTGCTTA GAGCTAGGAA AAGAGTCGGG TTAGGTCGAC CCATTTAAGA   
  
  
+ AATGGGTCGA CTTCATGTCG GCCTTCAAAA TAGTTAAGAT TTGCTCAAAA CTAAAAAATA ACAAGTTCAC   
  
  
+ ACTAAACGGT CAAATCGTGT CGACTTCGGA TTGAAATTGG TTCAGTTTGG GTCGGCTTCG TATTTCTTTC   
  
  
+ AGTAACTTCG AACCGAATTC GTATCGTGTT ATCGTGTCAG GTCAGACTTT CTCAGTTCTA TTTTTGCTAC   
  
  
+ CTTATGTTAC GGATAAAAAA AGTCTCACAT TGGAAAAAGT GTGGGAGGGT CCTGGGCTTA TAAATAGTGG   
  
  
+ CGCTGCACAC CTCATCAGAC CGACCTTTTG GGGAGAGGGT AATGACCCCG TGACAAGTGG TATCAGAGTC   
  
  
+ AACCCAGACC CGACTCAGAT GTCCTGCCAA GCGCTCCGAC GGGCGGGGTA GGCAGGGGGC CCATTTGTGA   
  
  
+ TGAGGACGTC ACAGAATTGG GCGGGGGAGA GTGTCACGGA TAAAAAAAGT CCCACATCGA AAAAAGTGTG   
  
  
+ GGAGGGTCCT GGGCTTATAA ACGGTAGCGC CGCACACCCC ATCAGATCGA CTTTTTGGGG AGAGGGTAAT   
  
  
+ GACCCCATTT CAAGTGGTAT CAGAGCCAAC CCAGACCCGA CTCCAATGTC CCACCAAGCG CTCTGATGGG   
  
  
+ CGGGGTAGGC AAGGGGCTCC ATATGTGACG AGGGCGTCAC GGAATTGGAT GGGAGAGAGT ACCACGGATA   
  
  
+ AAAAAAGTCC CACATCAGAA AAAGTGTGGA CTTAGCAACT AGGAAGTAAA GTTACAGTAT TAGCCGAGAG   
  
  
+ CCCATGGAAC GCCTGAAACT GACATCACCA GACCCACATA TTTCACCATC ATCTGATCTC CCCGTCAAAG   
  
  
+ GAGGAAGCTG AGCACAGCAA GAACTAGAAG AACAGGCGGC ATCACACCCA AGCCAAAATT CTCTATTTGA   
  
  
+ CCTCCCTCTC CTCATTCCTC TGAGCTTCCT CTTCACAATG TAAGACCATA ATCCTTAACA CCCTTCTTTT   
  
  
+ TTAAGCTCAT CTTTACCTGC TCTGGTTCTC TCTCTCATCG TCACTAATAG TTACCTCTTT ACTTTCTCTT   
  
  
+ CCCTGAGGTA TGCTTCAACT TTAACATATA TATCCCACTT GATTTCTCTT GTCTTTTTGC TATAATACTT   
  
  
+ GTTGTTTGGC TATCATCCCC TGTTTTATAC TCATTTTCTT GCTTTCTATT TCTGGGTTTG AATTGGGTTT   
  
  
+ CACTTATCCT CGTCAATTCT ATGGGTAATT AGTGAATTGG GTTTATATGA ACTAGGGTTG CAAAAGTATC   
  
  
+ TACTTTTTCT CTTGTGATTA TTAGTTGATT AGGGATTTGG CCAATATTAG CAGAATGGGT TCTGAATTTG   
  
  
+ CGGAATTCTC TGATGATGCT CTAAATGGGT ATGCTTATGT TGATATCCCT GCTTATGATG CATCCTTAGA   
  
  
+ TTATGCCAAT TTGTTCAATT ACGAAGGCCC ATCTGAGGAT CGCACCTCAC TGAGCCTCCC AAGACCCTTT   
  
  
+ TCTGACCCTT TGGCTTACAG TTTCACGTCC TCTTCTGAGC TGGGCCCTGG GGTTGATTCT AATGATGATA   
  
  
+ GTGATTCTGG CGATGTTCTC AAGTACATTA GCCAAATGCT TATGGAAGAA GACATGGAGG CAAAGCCATG   
  
  
+ CATGTTTCAT GATCCTTTAG CACTTCAGGC TGCTGAGAAA CCCTTTTATG ATGCCTTAGG GGAGCAATGC   
  
  
+ CCAACTTCTC CTGACCAACA TCCTATAATT GATCATTATT TGGATAGTCC TGATGAAAAT TCTTTGAGTT   
  
  
+ CAACTGGTGA TTTTAGTGTT AGTCATTCTG GGTCTAGTTC AACAAACTCC GTTGGACCGA TAATAGTGTC   
  
  
+ TGATTTGAGT GAGCATTTTG AGCCACCCTT TGTTGAAACA CTTCCAATTG AATCATATCA CCAACCATTG   
  
  
+ ACCCGTCCTC AATGGTCATT TGGCTCTTCG GGTGCCTTAG ATGGCACGGC CTCTAATGGT TCGGTGATCT   
  
  
+ CATCCCTTGG TTTGCCAGTG GATGTGATTA GCGTATTTAG GGAGAAAGAG TCCATGATTC AATTTCAGAA   
  
  
+ AGGGGTGGAG GAGGCCAGTA AGTTCCTTCC CAAGAATAAT AACCTTGTTA TTGATCTCGA GAACCTCACT   
  
  
+ TTTCCTAATG AAACAAAGGA GGATGATCGA ATGATGATGG TTAAGAAGGA AAAGGATGAC GTGAATTGGT   
  
  
+ CTAACTACTC AAGAGGGAGT AAGATTCACT ATTGTGAAGA CGAGGCCTTT GAAGAAGGAA GGAGTGGCAA   
  
  
+ GCAGTCAGCT ATTTCTTCTA CTGAGGAAGC TGAGTTTTCT GACATTTTTG ACAAGGTTTT GCTTTGCGAT   
  
  
+ TGCTACCCTG TGAAACCTGA GGCTCATCCC ACCATGAGTT TGAACCCTGA GAAGGGCCAG TCACATGGAT   
  
  
+ TAGAAGGTGG GAGAAATGGG AAGGCTCGCC CTAAGAAACA GGATAATAGT AGCACAAATA TTGTGGATTT   
  
  
+ AAGGAATTTG CTGATACTAT GCGCACAATC TACTGCATCT GATGACCGAA GAACTGCTGA TGGACTGCTA   
  
  
+ AAGCAAATCA GGGAGCACTC GTCTGCTGAG GGGGATGGAT CTCAAAGGTT GGCGCATTAC TTTGCTGATG   
  
  
+ CCCTAGAGGC ACGTTTAGCT GGAACTGGAT CTCGCATTTA TACGGCCCTA TGTTCTAATA GGCCATCTGT   
  
  
+ CACTGACATG ATAAAAGCAT ATCAGTTCTA TATTCGTGCT TGCCCATTTA CGAAGATCGT CATTGGTTGT   
  
  
+ GGTACCCATA TGATTCTAAA AGCAGCTGAG AAGGCATCAA AGCTTCATAT TATAGATTTT GGCATCCTCT   
  
  
+ ATGGTTGCCA ATGGCCCAAC CTCATTCAAC GCCTCTCAGA GCGATCTGGT GGACCTCCAA AACTGTTTAT   
  
  
+ TACAGGGATC GATCTCCCCC AGCCTGGGTT CAGGCCAGCA GAAAGAGTGG AAGCAACAGG GAGACGCTTG   
  
  
+ GCCAAGTACT GTGAGCGGTA TAATGTGCCA TTTGAGTATC ATGCCATTGC TCAGGAGTGG GAAACAATCA   
  
  
+ AACCAGGGGA TCTCAAGATA GGAAGTAGGA ATGATGAAGT TGTTGCGGTG AACTGTCTCT GTAGGTTCAA   
  
  
+ GAACCTCCTT GACGAGACAG TGGTGGTGGA TAGTCCAAGG AACACAGTTT TAAACCTGAT TACAAGGGTA   
  
  
+ AAGCCTGATA TTTTTGTGCA TGGCGTTGTA AATGGTTCCT ACAACATCCC TTTCTTTGTG ACACGTTTTA   
  
  
+ GAGAAGCCCT CTTTCATTAT TCCACTCTTT TCGACATGTT AGATGCCAAC GCCTCTAGGG AGGAGCCCGA   
  
  
+ GAGGTTGATA TTCGAGAAGG CATTCTATGG GAGGGAGATT ATGAATGTGG TGGCCTGTGA GGGCACAGAG   
  
  
+ AGGGTGGAGA GGCCGGAGAC ATACAAGCAA TGGCATGTTA GGCATAGCAG GGCAGGGTTT CGGCAAGTAC   
  
  
+ CATTGGATCC CAAGTTGATC GAGAAAATGA GGTTTAAGGC CAAGGCAGAC CACCACAAGG ATTTCATGAT   
  
  
+ CGATGTGGAT GGACATTGGG CAATTCAGGG ATGGAAGGGG CGGATTGCAC ATGCGATCTC TGCATGGGTT   
  
  
+ CCGGCTTG  

- -Up\_Stream \_Len000CTAAAC TAACAGCTTT AAACAGGCTA AAATATTTTA ACTTTTAACA ATAACCTGAT   
  
  
- ATTAGATGAC ATCAAATCTC CACAACCTAT TATGAGAGTT GAAACTTGAA CCTGCAGGTA CGTGGTGTGA   
  
  
- TACCCGCATA AATGACAAAT ATATGGTTCT TATTAAAACT TCTACAAGAA TCAACCACTA CATTCACATA   
  
  
- TAAGTTGTAT ACAGTAATGT ATTTGAACTT TTAAGAACTT AAAATCTGTA TATGTTAACG TTTAGTAACA   
  
  
- TTTTAACAAC GTAATGTAAA ACTATTTTTA CGTAGGTTTG AAACAGAACC ATATTTGTCA TTTATCCGGG   
  
  
- TATCCAGGTA TCACATCACG TACCTGCAGG TTCAAATTTC CACCCTCATA ATAACGGGTT GCGGGGATTT   
  
  
- CATGTCATAT AATATCAGGT TATAGGGTTA ATTAAAAAGT AAACTTATAA ATTTAAACCT GTATTATAAA   
  
  
- AAGTATAAAC TTAAGAGATT TGGACTAAAA TTGGACTTAA ATTCAACTAA ATTAAGTAGG CTGGTTTTTT   
  
  
- TATTTGTGTT AATAAATAAA AAGATAAGCT TCAATCTATC ATCGGTTAAG TAAACTGTAT ATTAATTGGG   
  
  
- CACTAACTGT GTTTGAGCTC AAAAACGAAT CTCGATCCTT TTCTCAGCCC AATCCAGCTG GGTAAATTCT   
  
  
- TTACCCAGCT GAAGTACAGC CGGAAGTTTT ATCAATTCTA AACGAGTTTT GATTTTTTAT TGTTCAAGTG   
  
  
- TGATTTGCCA GTTTAGCACA GCTGAAGCCT AACTTTAACC AAGTCAAACC CAGCCGAAGC ATAAAGAAAG   
  
  
- TCATTGAAGC TTGGCTTAAG CATAGCACAA TAGCACAGTC CAGTCTGAAA GAGTCAAGAT AAAAACGATG   
  
  
- GAATACAATG CCTATTTTTT TCAGAGTGTA ACCTTTTTCA CACCCTCCCA GGACCCGAAT ATTTATCACC   
  
  
- GCGACGTGTG GAGTAGTCTG GCTGGAAAAC CCCTCTCCCA TTACTGGGGC ACTGTTCACC ATAGTCTCAG   
  
  
- TTGGGTCTGG GCTGAGTCTA CAGGACGGTT CGCGAGGCTG CCCGCCCCAT CCGTCCCCCG GGTAAACACT   
  
  
- ACTCCTGCAG TGTCTTAACC CGCCCCCTCT CACAGTGCCT ATTTTTTTCA GGGTGTAGCT TTTTTCACAC   
  
  
- CCTCCCAGGA CCCGAATATT TGCCATCGCG GCGTGTGGGG TAGTCTAGCT GAAAAACCCC TCTCCCATTA   
  
  
- CTGGGGTAAA GTTCACCATA GTCTCGGTTG GGTCTGGGCT GAGGTTACAG GGTGGTTCGC GAGACTACCC   
  
  
- GCCCCATCCG TTCCCCGAGG TATACACTGC TCCCGCAGTG CCTTAACCTA CCCTCTCTCA TGGTGCCTAT   
  
  
- TTTTTTCAGG GTGTAGTCTT TTTCACACCT GAATCGTTGA TCCTTCATTT CAATGTCATA ATCGGCTCTC   
  
  
- GGGTACCTTG CGGACTTTGA CTGTAGTGGT CTGGGTGTAT AAAGTGGTAG TAGACTAGAG GGGCAGTTTC   
  
  
- CTCCTTCGAC TCGTGTCGTT CTTGATCTTC TTGTCCGCCG TAGTGTGGGT TCGGTTTTAA GAGATAAACT   
  
  
- GGAGGGAGAG GAGTAAGGAG ACTCGAAGGA GAAGTGTTAC ATTCTGGTAT TAGGAATTGT GGGAAGAAAA   
  
  
- AATTCGAGTA GAAATGGACG AGACCAAGAG AGAGAGTAGC AGTGATTATC AATGGAGAAA TGAAAGAGAA   
  
  
- GGGACTCCAT ACGAAGTTGA AATTGTATAT ATAGGGTGAA CTAAAGAGAA CAGAAAAACG ATATTATGAA   
  
  
- CAACAAACCG ATAGTAGGGG ACAAAATATG AGTAAAAGAA CGAAAGATAA AGACCCAAAC TTAACCCAAA   
  
  
- GTGAATAGGA GCAGTTAAGA TACCCATTAA TCACTTAACC CAAATATACT TGATCCCAAC GTTTTCATAG   
  
  
- ATGAAAAAGA GAACACTAAT AATCAACTAA TCCCTAAACC GGTTATAATC GTCTTACCCA AGACTTAAAC   
  
  
- GCCTTAAGAG ACTACTACGA GATTTACCCA TACGAATACA ACTATAGGGA CGAATACTAC GTAGGAATCT   
  
  
- AATACGGTTA AACAAGTTAA TGCTTCCGGG TAGACTCCTA GCGTGGAGTG ACTCGGAGGG TTCTGGGAAA   
  
  
- AGACTGGGAA ACCGAATGTC AAAGTGCAGG AGAAGACTCG ACCCGGGACC CCAACTAAGA TTACTACTAT   
  
  
- CACTAAGACC GCTACAAGAG TTCATGTAAT CGGTTTACGA ATACCTTCTT CTGTACCTCC GTTTCGGTAC   
  
  
- GTACAAAGTA CTAGGAAATC GTGAAGTCCG ACGACTCTTT GGGAAAATAC TACGGAATCC CCTCGTTACG   
  
  
- GGTTGAAGAG GACTGGTTGT AGGATATTAA CTAGTAATAA ACCTATCAGG ACTACTTTTA AGAAACTCAA   
  
  
- GTTGACCACT AAAATCACAA TCAGTAAGAC CCAGATCAAG TTGTTTGAGG CAACCTGGCT ATTATCACAG   
  
  
- ACTAAACTCA CTCGTAAAAC TCGGTGGGAA ACAACTTTGT GAAGGTTAAC TTAGTATAGT GGTTGGTAAC   
  
  
- TGGGCAGGAG TTACCAGTAA ACCGAGAAGC CCACGGAATC TACCGTGCCG GAGATTACCA AGCCACTAGA   
  
  
- GTAGGGAACC AAACGGTCAC CTACACTAAT CGCATAAATC CCTCTTTCTC AGGTACTAAG TTAAAGTCTT   
  
  
- TCCCCACCTC CTCCGGTCAT TCAAGGAAGG GTTCTTATTA TTGGAACAAT AACTAGAGCT CTTGGAGTGA   
  
  
- AAAGGATTAC TTTGTTTCCT CCTACTAGCT TACTACTACC AATTCTTCCT TTTCCTACTG CACTTAACCA   
  
  
- GATTGATGAG TTCTCCCTCA TTCTAAGTGA TAACACTTCT GCTCCGGAAA CTTCTTCCTT CCTCACCGTT   
  
  
- CGTCAGTCGA TAAAGAAGAT GACTCCTTCG ACTCAAAAGA CTGTAAAAAC TGTTCCAAAA CGAAACGCTA   
  
  
- ACGATGGGAC ACTTTGGACT CCGAGTAGGG TGGTACTCAA ACTTGGGACT CTTCCCGGTC AGTGTACCTA   
  
  
- ATCTTCCACC CTCTTTACCC TTCCGAGCGG GATTCTTTGT CCTATTATCA TCGTGTTTAT AACACCTAAA   
  
  
- TTCCTTAAAC GACTATGATA CGCGTGTTAG ATGACGTAGA CTACTGGCTT CTTGACGACT ACCTGACGAT   
  
  
- TTCGTTTAGT CCCTCGTGAG CAGACGACTC CCCCTACCTA GAGTTTCCAA CCGCGTAATG AAACGACTAC   
  
  
- GGGATCTCCG TGCAAATCGA CCTTGACCTA GAGCGTAAAT ATGCCGGGAT ACAAGATTAT CCGGTAGACA   
  
  
- GTGACTGTAC TATTTTCGTA TAGTCAAGAT ATAAGCACGA ACGGGTAAAT GCTTCTAGCA GTAACCAACA   
  
  
- CCATGGGTAT ACTAAGATTT TCGTCGACTC TTCCGTAGTT TCGAAGTATA ATATCTAAAA CCGTAGGAGA   
  
  
- TACCAACGGT TACCGGGTTG GAGTAAGTTG CGGAGAGTCT CGCTAGACCA CCTGGAGGTT TTGACAAATA   
  
  
- ATGTCCCTAG CTAGAGGGGG TCGGACCCAA GTCCGGTCGT CTTTCTCACC TTCGTTGTCC CTCTGCGAAC   
  
  
- CGGTTCATGA CACTCGCCAT ATTACACGGT AAACTCATAG TACGGTAACG AGTCCTCACC CTTTGTTAGT   
  
  
- TTGGTCCCCT AGAGTTCTAT CCTTCATCCT TACTACTTCA ACAACGCCAC TTGACAGAGA CATCCAAGTT   
  
  
- CTTGGAGGAA CTGCTCTGTC ACCACCACCT ATCAGGTTCC TTGTGTCAAA ATTTGGACTA ATGTTCCCAT   
  
  
- TTCGGACTAT AAAAACACGT ACCGCAACAT TTACCAAGGA TGTTGTAGGG AAAGAAACAC TGTGCAAAAT   
  
  
- CTCTTCGGGA GAAAGTAATA AGGTGAGAAA AGCTGTACAA TCTACGGTTG CGGAGATCCC TCCTCGGGCT   
  
  
- CTCCAACTAT AAGCTCTTCC GTAAGATACC CTCCCTCTAA TACTTACACC ACCGGACACT CCCGTGTCTC   
  
  
- TCCCACCTCT CCGGCCTCTG TATGTTCGTT ACCGTACAAT CCGTATCGTC CCGTCCCAAA GCCGTTCATG   
  
  
- GTAACCTAGG GTTCAACTAG CTCTTTTACT CCAAATTCCG GTTCCGTCTG GTGGTGTTCC TAAAGTACTA   
  
  
- GCTACACCTA CCTGTAACCC GTTAAGTCCC TACCTTCCCC GCCTAACGTG TACGCTAGAG ACGTACCCAA   
  
  
- GGCCGAAC

+     Gap-box

| Site Name | Organism | Position | Strand | Matrix score. | sequence | function |
| --- | --- | --- | --- | --- | --- | --- |
| Gap-box | Arabidopsis thaliana | 459 | - | 9.5 | CAAATGAA(A/G)A | part of a light responsive element |

>HU07G02249.1   
+ -Up\_Stream \_Len000GATTTG ATTGTCGAAA TTTGTCCGAT TTTATAAAAT TGAAAATTGT TATTGGACTA   
  
  
+ TAATCTACTG TAGTTTAGAG GTGTTGGATA ATACTCTCAA CTTTGAACTT GGACGTCCAT GCACCACACT   
  
  
+ ATGGGCGTAT TTACTGTTTA TATACCAAGA ATAATTTTGA AGATGTTCTT AGTTGGTGAT GTAAGTGTAT   
  
  
+ ATTCAACATA TGTCATTACA TAAACTTGAA AATTCTTGAA TTTTAGACAT ATACAATTGC AAATCATTGT   
  
  
+ AAAATTGTTG CATTACATTT TGATAAAAAT GCATCCAAAC TTTGTCTTGG TATAAACAGT AAATAGGCCC   
  
  
+ ATAGGTCCAT AGTGTAGTGC ATGGACGTCC AAGTTTAAAG GTGGGAGTAT TATTGCCCAA CGCCCCTAAA   
  
  
+ GTACAGTATA TTATAGTCCA ATATCCCAAT TAATTTTTCA TTTGAATATT TAAATTTGGA CATAATATTT   
  
  
+ TTCATATTTG AATTCTCTAA ACCTGATTTT AACCTGAATT TAAGTTGATT TAATTCATCC GACCAAAAAA   
  
  
+ ATAAACACAA TTATTTATTT TTCTATTCGA AGTTAGATAG TAGCCAATTC ATTTGACATA TAATTAACCC   
  
  
+ GTGATTGACA CAAACTCGAG TTTTTGCTTA GAGCTAGGAA AAGAGTCGGG TTAGGTCGAC CCATTTAAGA   
  
  
+ AATGGGTCGA CTTCATGTCG GCCTTCAAAA TAGTTAAGAT TTGCTCAAAA CTAAAAAATA ACAAGTTCAC   
  
  
+ ACTAAACGGT CAAATCGTGT CGACTTCGGA TTGAAATTGG TTCAGTTTGG GTCGGCTTCG TATTTCTTTC   
  
  
+ AGTAACTTCG AACCGAATTC GTATCGTGTT ATCGTGTCAG GTCAGACTTT CTCAGTTCTA TTTTTGCTAC   
  
  
+ CTTATGTTAC GGATAAAAAA AGTCTCACAT TGGAAAAAGT GTGGGAGGGT CCTGGGCTTA TAAATAGTGG   
  
  
+ CGCTGCACAC CTCATCAGAC CGACCTTTTG GGGAGAGGGT AATGACCCCG TGACAAGTGG TATCAGAGTC   
  
  
+ AACCCAGACC CGACTCAGAT GTCCTGCCAA GCGCTCCGAC GGGCGGGGTA GGCAGGGGGC CCATTTGTGA   
  
  
+ TGAGGACGTC ACAGAATTGG GCGGGGGAGA GTGTCACGGA TAAAAAAAGT CCCACATCGA AAAAAGTGTG   
  
  
+ GGAGGGTCCT GGGCTTATAA ACGGTAGCGC CGCACACCCC ATCAGATCGA CTTTTTGGGG AGAGGGTAAT   
  
  
+ GACCCCATTT CAAGTGGTAT CAGAGCCAAC CCAGACCCGA CTCCAATGTC CCACCAAGCG CTCTGATGGG   
  
  
+ CGGGGTAGGC AAGGGGCTCC ATATGTGACG AGGGCGTCAC GGAATTGGAT GGGAGAGAGT ACCACGGATA   
  
  
+ AAAAAAGTCC CACATCAGAA AAAGTGTGGA CTTAGCAACT AGGAAGTAAA GTTACAGTAT TAGCCGAGAG   
  
  
+ CCCATGGAAC GCCTGAAACT GACATCACCA GACCCACATA TTTCACCATC ATCTGATCTC CCCGTCAAAG   
  
  
+ GAGGAAGCTG AGCACAGCAA GAACTAGAAG AACAGGCGGC ATCACACCCA AGCCAAAATT CTCTATTTGA   
  
  
+ CCTCCCTCTC CTCATTCCTC TGAGCTTCCT CTTCACAATG TAAGACCATA ATCCTTAACA CCCTTCTTTT   
  
  
+ TTAAGCTCAT CTTTACCTGC TCTGGTTCTC TCTCTCATCG TCACTAATAG TTACCTCTTT ACTTTCTCTT   
  
  
+ CCCTGAGGTA TGCTTCAACT TTAACATATA TATCCCACTT GATTTCTCTT GTCTTTTTGC TATAATACTT   
  
  
+ GTTGTTTGGC TATCATCCCC TGTTTTATAC TCATTTTCTT GCTTTCTATT TCTGGGTTTG AATTGGGTTT   
  
  
+ CACTTATCCT CGTCAATTCT ATGGGTAATT AGTGAATTGG GTTTATATGA ACTAGGGTTG CAAAAGTATC   
  
  
+ TACTTTTTCT CTTGTGATTA TTAGTTGATT AGGGATTTGG CCAATATTAG CAGAATGGGT TCTGAATTTG   
  
  
+ CGGAATTCTC TGATGATGCT CTAAATGGGT ATGCTTATGT TGATATCCCT GCTTATGATG CATCCTTAGA   
  
  
+ TTATGCCAAT TTGTTCAATT ACGAAGGCCC ATCTGAGGAT CGCACCTCAC TGAGCCTCCC AAGACCCTTT   
  
  
+ TCTGACCCTT TGGCTTACAG TTTCACGTCC TCTTCTGAGC TGGGCCCTGG GGTTGATTCT AATGATGATA   
  
  
+ GTGATTCTGG CGATGTTCTC AAGTACATTA GCCAAATGCT TATGGAAGAA GACATGGAGG CAAAGCCATG   
  
  
+ CATGTTTCAT GATCCTTTAG CACTTCAGGC TGCTGAGAAA CCCTTTTATG ATGCCTTAGG GGAGCAATGC   
  
  
+ CCAACTTCTC CTGACCAACA TCCTATAATT GATCATTATT TGGATAGTCC TGATGAAAAT TCTTTGAGTT   
  
  
+ CAACTGGTGA TTTTAGTGTT AGTCATTCTG GGTCTAGTTC AACAAACTCC GTTGGACCGA TAATAGTGTC   
  
  
+ TGATTTGAGT GAGCATTTTG AGCCACCCTT TGTTGAAACA CTTCCAATTG AATCATATCA CCAACCATTG   
  
  
+ ACCCGTCCTC AATGGTCATT TGGCTCTTCG GGTGCCTTAG ATGGCACGGC CTCTAATGGT TCGGTGATCT   
  
  
+ CATCCCTTGG TTTGCCAGTG GATGTGATTA GCGTATTTAG GGAGAAAGAG TCCATGATTC AATTTCAGAA   
  
  
+ AGGGGTGGAG GAGGCCAGTA AGTTCCTTCC CAAGAATAAT AACCTTGTTA TTGATCTCGA GAACCTCACT   
  
  
+ TTTCCTAATG AAACAAAGGA GGATGATCGA ATGATGATGG TTAAGAAGGA AAAGGATGAC GTGAATTGGT   
  
  
+ CTAACTACTC AAGAGGGAGT AAGATTCACT ATTGTGAAGA CGAGGCCTTT GAAGAAGGAA GGAGTGGCAA   
  
  
+ GCAGTCAGCT ATTTCTTCTA CTGAGGAAGC TGAGTTTTCT GACATTTTTG ACAAGGTTTT GCTTTGCGAT   
  
  
+ TGCTACCCTG TGAAACCTGA GGCTCATCCC ACCATGAGTT TGAACCCTGA GAAGGGCCAG TCACATGGAT   
  
  
+ TAGAAGGTGG GAGAAATGGG AAGGCTCGCC CTAAGAAACA GGATAATAGT AGCACAAATA TTGTGGATTT   
  
  
+ AAGGAATTTG CTGATACTAT GCGCACAATC TACTGCATCT GATGACCGAA GAACTGCTGA TGGACTGCTA   
  
  
+ AAGCAAATCA GGGAGCACTC GTCTGCTGAG GGGGATGGAT CTCAAAGGTT GGCGCATTAC TTTGCTGATG   
  
  
+ CCCTAGAGGC ACGTTTAGCT GGAACTGGAT CTCGCATTTA TACGGCCCTA TGTTCTAATA GGCCATCTGT   
  
  
+ CACTGACATG ATAAAAGCAT ATCAGTTCTA TATTCGTGCT TGCCCATTTA CGAAGATCGT CATTGGTTGT   
  
  
+ GGTACCCATA TGATTCTAAA AGCAGCTGAG AAGGCATCAA AGCTTCATAT TATAGATTTT GGCATCCTCT   
  
  
+ ATGGTTGCCA ATGGCCCAAC CTCATTCAAC GCCTCTCAGA GCGATCTGGT GGACCTCCAA AACTGTTTAT   
  
  
+ TACAGGGATC GATCTCCCCC AGCCTGGGTT CAGGCCAGCA GAAAGAGTGG AAGCAACAGG GAGACGCTTG   
  
  
+ GCCAAGTACT GTGAGCGGTA TAATGTGCCA TTTGAGTATC ATGCCATTGC TCAGGAGTGG GAAACAATCA   
  
  
+ AACCAGGGGA TCTCAAGATA GGAAGTAGGA ATGATGAAGT TGTTGCGGTG AACTGTCTCT GTAGGTTCAA   
  
  
+ GAACCTCCTT GACGAGACAG TGGTGGTGGA TAGTCCAAGG AACACAGTTT TAAACCTGAT TACAAGGGTA   
  
  
+ AAGCCTGATA TTTTTGTGCA TGGCGTTGTA AATGGTTCCT ACAACATCCC TTTCTTTGTG ACACGTTTTA   
  
  
+ GAGAAGCCCT CTTTCATTAT TCCACTCTTT TCGACATGTT AGATGCCAAC GCCTCTAGGG AGGAGCCCGA   
  
  
+ GAGGTTGATA TTCGAGAAGG CATTCTATGG GAGGGAGATT ATGAATGTGG TGGCCTGTGA GGGCACAGAG   
  
  
+ AGGGTGGAGA GGCCGGAGAC ATACAAGCAA TGGCATGTTA GGCATAGCAG GGCAGGGTTT CGGCAAGTAC   
  
  
+ CATTGGATCC CAAGTTGATC GAGAAAATGA GGTTTAAGGC CAAGGCAGAC CACCACAAGG ATTTCATGAT   
  
  
+ CGATGTGGAT GGACATTGGG CAATTCAGGG ATGGAAGGGG CGGATTGCAC ATGCGATCTC TGCATGGGTT   
  
  
+ CCGGCTTG  

- -Up\_Stream \_Len000CTAAAC TAACAGCTTT AAACAGGCTA AAATATTTTA ACTTTTAACA ATAACCTGAT   
  
  
- ATTAGATGAC ATCAAATCTC CACAACCTAT TATGAGAGTT GAAACTTGAA CCTGCAGGTA CGTGGTGTGA   
  
  
- TACCCGCATA AATGACAAAT ATATGGTTCT TATTAAAACT TCTACAAGAA TCAACCACTA CATTCACATA   
  
  
- TAAGTTGTAT ACAGTAATGT ATTTGAACTT TTAAGAACTT AAAATCTGTA TATGTTAACG TTTAGTAACA   
  
  
- TTTTAACAAC GTAATGTAAA ACTATTTTTA CGTAGGTTTG AAACAGAACC ATATTTGTCA TTTATCCGGG   
  
  
- TATCCAGGTA TCACATCACG TACCTGCAGG TTCAAATTTC CACCCTCATA ATAACGGGTT GCGGGGATTT   
  
  
- CATGTCATAT AATATCAGGT TATAGGGTTA ATTAAAAAGT AAACTTATAA ATTTAAACCT GTATTATAAA   
  
  
- AAGTATAAAC TTAAGAGATT TGGACTAAAA TTGGACTTAA ATTCAACTAA ATTAAGTAGG CTGGTTTTTT   
  
  
- TATTTGTGTT AATAAATAAA AAGATAAGCT TCAATCTATC ATCGGTTAAG TAAACTGTAT ATTAATTGGG   
  
  
- CACTAACTGT GTTTGAGCTC AAAAACGAAT CTCGATCCTT TTCTCAGCCC AATCCAGCTG GGTAAATTCT   
  
  
- TTACCCAGCT GAAGTACAGC CGGAAGTTTT ATCAATTCTA AACGAGTTTT GATTTTTTAT TGTTCAAGTG   
  
  
- TGATTTGCCA GTTTAGCACA GCTGAAGCCT AACTTTAACC AAGTCAAACC CAGCCGAAGC ATAAAGAAAG   
  
  
- TCATTGAAGC TTGGCTTAAG CATAGCACAA TAGCACAGTC CAGTCTGAAA GAGTCAAGAT AAAAACGATG   
  
  
- GAATACAATG CCTATTTTTT TCAGAGTGTA ACCTTTTTCA CACCCTCCCA GGACCCGAAT ATTTATCACC   
  
  
- GCGACGTGTG GAGTAGTCTG GCTGGAAAAC CCCTCTCCCA TTACTGGGGC ACTGTTCACC ATAGTCTCAG   
  
  
- TTGGGTCTGG GCTGAGTCTA CAGGACGGTT CGCGAGGCTG CCCGCCCCAT CCGTCCCCCG GGTAAACACT   
  
  
- ACTCCTGCAG TGTCTTAACC CGCCCCCTCT CACAGTGCCT ATTTTTTTCA GGGTGTAGCT TTTTTCACAC   
  
  
- CCTCCCAGGA CCCGAATATT TGCCATCGCG GCGTGTGGGG TAGTCTAGCT GAAAAACCCC TCTCCCATTA   
  
  
- CTGGGGTAAA GTTCACCATA GTCTCGGTTG GGTCTGGGCT GAGGTTACAG GGTGGTTCGC GAGACTACCC   
  
  
- GCCCCATCCG TTCCCCGAGG TATACACTGC TCCCGCAGTG CCTTAACCTA CCCTCTCTCA TGGTGCCTAT   
  
  
- TTTTTTCAGG GTGTAGTCTT TTTCACACCT GAATCGTTGA TCCTTCATTT CAATGTCATA ATCGGCTCTC   
  
  
- GGGTACCTTG CGGACTTTGA CTGTAGTGGT CTGGGTGTAT AAAGTGGTAG TAGACTAGAG GGGCAGTTTC   
  
  
- CTCCTTCGAC TCGTGTCGTT CTTGATCTTC TTGTCCGCCG TAGTGTGGGT TCGGTTTTAA GAGATAAACT   
  
  
- GGAGGGAGAG GAGTAAGGAG ACTCGAAGGA GAAGTGTTAC ATTCTGGTAT TAGGAATTGT GGGAAGAAAA   
  
  
- AATTCGAGTA GAAATGGACG AGACCAAGAG AGAGAGTAGC AGTGATTATC AATGGAGAAA TGAAAGAGAA   
  
  
- GGGACTCCAT ACGAAGTTGA AATTGTATAT ATAGGGTGAA CTAAAGAGAA CAGAAAAACG ATATTATGAA   
  
  
- CAACAAACCG ATAGTAGGGG ACAAAATATG AGTAAAAGAA CGAAAGATAA AGACCCAAAC TTAACCCAAA   
  
  
- GTGAATAGGA GCAGTTAAGA TACCCATTAA TCACTTAACC CAAATATACT TGATCCCAAC GTTTTCATAG   
  
  
- ATGAAAAAGA GAACACTAAT AATCAACTAA TCCCTAAACC GGTTATAATC GTCTTACCCA AGACTTAAAC   
  
  
- GCCTTAAGAG ACTACTACGA GATTTACCCA TACGAATACA ACTATAGGGA CGAATACTAC GTAGGAATCT   
  
  
- AATACGGTTA AACAAGTTAA TGCTTCCGGG TAGACTCCTA GCGTGGAGTG ACTCGGAGGG TTCTGGGAAA   
  
  
- AGACTGGGAA ACCGAATGTC AAAGTGCAGG AGAAGACTCG ACCCGGGACC CCAACTAAGA TTACTACTAT   
  
  
- CACTAAGACC GCTACAAGAG TTCATGTAAT CGGTTTACGA ATACCTTCTT CTGTACCTCC GTTTCGGTAC   
  
  
- GTACAAAGTA CTAGGAAATC GTGAAGTCCG ACGACTCTTT GGGAAAATAC TACGGAATCC CCTCGTTACG   
  
  
- GGTTGAAGAG GACTGGTTGT AGGATATTAA CTAGTAATAA ACCTATCAGG ACTACTTTTA AGAAACTCAA   
  
  
- GTTGACCACT AAAATCACAA TCAGTAAGAC CCAGATCAAG TTGTTTGAGG CAACCTGGCT ATTATCACAG   
  
  
- ACTAAACTCA CTCGTAAAAC TCGGTGGGAA ACAACTTTGT GAAGGTTAAC TTAGTATAGT GGTTGGTAAC   
  
  
- TGGGCAGGAG TTACCAGTAA ACCGAGAAGC CCACGGAATC TACCGTGCCG GAGATTACCA AGCCACTAGA   
  
  
- GTAGGGAACC AAACGGTCAC CTACACTAAT CGCATAAATC CCTCTTTCTC AGGTACTAAG TTAAAGTCTT   
  
  
- TCCCCACCTC CTCCGGTCAT TCAAGGAAGG GTTCTTATTA TTGGAACAAT AACTAGAGCT CTTGGAGTGA   
  
  
- AAAGGATTAC TTTGTTTCCT CCTACTAGCT TACTACTACC AATTCTTCCT TTTCCTACTG CACTTAACCA   
  
  
- GATTGATGAG TTCTCCCTCA TTCTAAGTGA TAACACTTCT GCTCCGGAAA CTTCTTCCTT CCTCACCGTT   
  
  
- CGTCAGTCGA TAAAGAAGAT GACTCCTTCG ACTCAAAAGA CTGTAAAAAC TGTTCCAAAA CGAAACGCTA   
  
  
- ACGATGGGAC ACTTTGGACT CCGAGTAGGG TGGTACTCAA ACTTGGGACT CTTCCCGGTC AGTGTACCTA   
  
  
- ATCTTCCACC CTCTTTACCC TTCCGAGCGG GATTCTTTGT CCTATTATCA TCGTGTTTAT AACACCTAAA   
  
  
- TTCCTTAAAC GACTATGATA CGCGTGTTAG ATGACGTAGA CTACTGGCTT CTTGACGACT ACCTGACGAT   
  
  
- TTCGTTTAGT CCCTCGTGAG CAGACGACTC CCCCTACCTA GAGTTTCCAA CCGCGTAATG AAACGACTAC   
  
  
- GGGATCTCCG TGCAAATCGA CCTTGACCTA GAGCGTAAAT ATGCCGGGAT ACAAGATTAT CCGGTAGACA   
  
  
- GTGACTGTAC TATTTTCGTA TAGTCAAGAT ATAAGCACGA ACGGGTAAAT GCTTCTAGCA GTAACCAACA   
  
  
- CCATGGGTAT ACTAAGATTT TCGTCGACTC TTCCGTAGTT TCGAAGTATA ATATCTAAAA CCGTAGGAGA   
  
  
- TACCAACGGT TACCGGGTTG GAGTAAGTTG CGGAGAGTCT CGCTAGACCA CCTGGAGGTT TTGACAAATA   
  
  
- ATGTCCCTAG CTAGAGGGGG TCGGACCCAA GTCCGGTCGT CTTTCTCACC TTCGTTGTCC CTCTGCGAAC   
  
  
- CGGTTCATGA CACTCGCCAT ATTACACGGT AAACTCATAG TACGGTAACG AGTCCTCACC CTTTGTTAGT   
  
  
- TTGGTCCCCT AGAGTTCTAT CCTTCATCCT TACTACTTCA ACAACGCCAC TTGACAGAGA CATCCAAGTT   
  
  
- CTTGGAGGAA CTGCTCTGTC ACCACCACCT ATCAGGTTCC TTGTGTCAAA ATTTGGACTA ATGTTCCCAT   
  
  
- TTCGGACTAT AAAAACACGT ACCGCAACAT TTACCAAGGA TGTTGTAGGG AAAGAAACAC TGTGCAAAAT   
  
  
- CTCTTCGGGA GAAAGTAATA AGGTGAGAAA AGCTGTACAA TCTACGGTTG CGGAGATCCC TCCTCGGGCT   
  
  
- CTCCAACTAT AAGCTCTTCC GTAAGATACC CTCCCTCTAA TACTTACACC ACCGGACACT CCCGTGTCTC   
  
  
- TCCCACCTCT CCGGCCTCTG TATGTTCGTT ACCGTACAAT CCGTATCGTC CCGTCCCAAA GCCGTTCATG   
  
  
- GTAACCTAGG GTTCAACTAG CTCTTTTACT CCAAATTCCG GTTCCGTCTG GTGGTGTTCC TAAAGTACTA   
  
  
- GCTACACCTA CCTGTAACCC GTTAAGTCCC TACCTTCCCC GCCTAACGTG TACGCTAGAG ACGTACCCAA   
  
  
- GGCCGAAC

+     I-box

| Site Name | Organism | Position | Strand | Matrix score. | sequence | function |
| --- | --- | --- | --- | --- | --- | --- |
| I-box | Larix laricina | 4166 | + | 9 | GTATAAGGCC | part of a light responsive element |

>HU07G02249.1   
+ -Up\_Stream \_Len000GATTTG ATTGTCGAAA TTTGTCCGAT TTTATAAAAT TGAAAATTGT TATTGGACTA   
  
  
+ TAATCTACTG TAGTTTAGAG GTGTTGGATA ATACTCTCAA CTTTGAACTT GGACGTCCAT GCACCACACT   
  
  
+ ATGGGCGTAT TTACTGTTTA TATACCAAGA ATAATTTTGA AGATGTTCTT AGTTGGTGAT GTAAGTGTAT   
  
  
+ ATTCAACATA TGTCATTACA TAAACTTGAA AATTCTTGAA TTTTAGACAT ATACAATTGC AAATCATTGT   
  
  
+ AAAATTGTTG CATTACATTT TGATAAAAAT GCATCCAAAC TTTGTCTTGG TATAAACAGT AAATAGGCCC   
  
  
+ ATAGGTCCAT AGTGTAGTGC ATGGACGTCC AAGTTTAAAG GTGGGAGTAT TATTGCCCAA CGCCCCTAAA   
  
  
+ GTACAGTATA TTATAGTCCA ATATCCCAAT TAATTTTTCA TTTGAATATT TAAATTTGGA CATAATATTT   
  
  
+ TTCATATTTG AATTCTCTAA ACCTGATTTT AACCTGAATT TAAGTTGATT TAATTCATCC GACCAAAAAA   
  
  
+ ATAAACACAA TTATTTATTT TTCTATTCGA AGTTAGATAG TAGCCAATTC ATTTGACATA TAATTAACCC   
  
  
+ GTGATTGACA CAAACTCGAG TTTTTGCTTA GAGCTAGGAA AAGAGTCGGG TTAGGTCGAC CCATTTAAGA   
  
  
+ AATGGGTCGA CTTCATGTCG GCCTTCAAAA TAGTTAAGAT TTGCTCAAAA CTAAAAAATA ACAAGTTCAC   
  
  
+ ACTAAACGGT CAAATCGTGT CGACTTCGGA TTGAAATTGG TTCAGTTTGG GTCGGCTTCG TATTTCTTTC   
  
  
+ AGTAACTTCG AACCGAATTC GTATCGTGTT ATCGTGTCAG GTCAGACTTT CTCAGTTCTA TTTTTGCTAC   
  
  
+ CTTATGTTAC GGATAAAAAA AGTCTCACAT TGGAAAAAGT GTGGGAGGGT CCTGGGCTTA TAAATAGTGG   
  
  
+ CGCTGCACAC CTCATCAGAC CGACCTTTTG GGGAGAGGGT AATGACCCCG TGACAAGTGG TATCAGAGTC   
  
  
+ AACCCAGACC CGACTCAGAT GTCCTGCCAA GCGCTCCGAC GGGCGGGGTA GGCAGGGGGC CCATTTGTGA   
  
  
+ TGAGGACGTC ACAGAATTGG GCGGGGGAGA GTGTCACGGA TAAAAAAAGT CCCACATCGA AAAAAGTGTG   
  
  
+ GGAGGGTCCT GGGCTTATAA ACGGTAGCGC CGCACACCCC ATCAGATCGA CTTTTTGGGG AGAGGGTAAT   
  
  
+ GACCCCATTT CAAGTGGTAT CAGAGCCAAC CCAGACCCGA CTCCAATGTC CCACCAAGCG CTCTGATGGG   
  
  
+ CGGGGTAGGC AAGGGGCTCC ATATGTGACG AGGGCGTCAC GGAATTGGAT GGGAGAGAGT ACCACGGATA   
  
  
+ AAAAAAGTCC CACATCAGAA AAAGTGTGGA CTTAGCAACT AGGAAGTAAA GTTACAGTAT TAGCCGAGAG   
  
  
+ CCCATGGAAC GCCTGAAACT GACATCACCA GACCCACATA TTTCACCATC ATCTGATCTC CCCGTCAAAG   
  
  
+ GAGGAAGCTG AGCACAGCAA GAACTAGAAG AACAGGCGGC ATCACACCCA AGCCAAAATT CTCTATTTGA   
  
  
+ CCTCCCTCTC CTCATTCCTC TGAGCTTCCT CTTCACAATG TAAGACCATA ATCCTTAACA CCCTTCTTTT   
  
  
+ TTAAGCTCAT CTTTACCTGC TCTGGTTCTC TCTCTCATCG TCACTAATAG TTACCTCTTT ACTTTCTCTT   
  
  
+ CCCTGAGGTA TGCTTCAACT TTAACATATA TATCCCACTT GATTTCTCTT GTCTTTTTGC TATAATACTT   
  
  
+ GTTGTTTGGC TATCATCCCC TGTTTTATAC TCATTTTCTT GCTTTCTATT TCTGGGTTTG AATTGGGTTT   
  
  
+ CACTTATCCT CGTCAATTCT ATGGGTAATT AGTGAATTGG GTTTATATGA ACTAGGGTTG CAAAAGTATC   
  
  
+ TACTTTTTCT CTTGTGATTA TTAGTTGATT AGGGATTTGG CCAATATTAG CAGAATGGGT TCTGAATTTG   
  
  
+ CGGAATTCTC TGATGATGCT CTAAATGGGT ATGCTTATGT TGATATCCCT GCTTATGATG CATCCTTAGA   
  
  
+ TTATGCCAAT TTGTTCAATT ACGAAGGCCC ATCTGAGGAT CGCACCTCAC TGAGCCTCCC AAGACCCTTT   
  
  
+ TCTGACCCTT TGGCTTACAG TTTCACGTCC TCTTCTGAGC TGGGCCCTGG GGTTGATTCT AATGATGATA   
  
  
+ GTGATTCTGG CGATGTTCTC AAGTACATTA GCCAAATGCT TATGGAAGAA GACATGGAGG CAAAGCCATG   
  
  
+ CATGTTTCAT GATCCTTTAG CACTTCAGGC TGCTGAGAAA CCCTTTTATG ATGCCTTAGG GGAGCAATGC   
  
  
+ CCAACTTCTC CTGACCAACA TCCTATAATT GATCATTATT TGGATAGTCC TGATGAAAAT TCTTTGAGTT   
  
  
+ CAACTGGTGA TTTTAGTGTT AGTCATTCTG GGTCTAGTTC AACAAACTCC GTTGGACCGA TAATAGTGTC   
  
  
+ TGATTTGAGT GAGCATTTTG AGCCACCCTT TGTTGAAACA CTTCCAATTG AATCATATCA CCAACCATTG   
  
  
+ ACCCGTCCTC AATGGTCATT TGGCTCTTCG GGTGCCTTAG ATGGCACGGC CTCTAATGGT TCGGTGATCT   
  
  
+ CATCCCTTGG TTTGCCAGTG GATGTGATTA GCGTATTTAG GGAGAAAGAG TCCATGATTC AATTTCAGAA   
  
  
+ AGGGGTGGAG GAGGCCAGTA AGTTCCTTCC CAAGAATAAT AACCTTGTTA TTGATCTCGA GAACCTCACT   
  
  
+ TTTCCTAATG AAACAAAGGA GGATGATCGA ATGATGATGG TTAAGAAGGA AAAGGATGAC GTGAATTGGT   
  
  
+ CTAACTACTC AAGAGGGAGT AAGATTCACT ATTGTGAAGA CGAGGCCTTT GAAGAAGGAA GGAGTGGCAA   
  
  
+ GCAGTCAGCT ATTTCTTCTA CTGAGGAAGC TGAGTTTTCT GACATTTTTG ACAAGGTTTT GCTTTGCGAT   
  
  
+ TGCTACCCTG TGAAACCTGA GGCTCATCCC ACCATGAGTT TGAACCCTGA GAAGGGCCAG TCACATGGAT   
  
  
+ TAGAAGGTGG GAGAAATGGG AAGGCTCGCC CTAAGAAACA GGATAATAGT AGCACAAATA TTGTGGATTT   
  
  
+ AAGGAATTTG CTGATACTAT GCGCACAATC TACTGCATCT GATGACCGAA GAACTGCTGA TGGACTGCTA   
  
  
+ AAGCAAATCA GGGAGCACTC GTCTGCTGAG GGGGATGGAT CTCAAAGGTT GGCGCATTAC TTTGCTGATG   
  
  
+ CCCTAGAGGC ACGTTTAGCT GGAACTGGAT CTCGCATTTA TACGGCCCTA TGTTCTAATA GGCCATCTGT   
  
  
+ CACTGACATG ATAAAAGCAT ATCAGTTCTA TATTCGTGCT TGCCCATTTA CGAAGATCGT CATTGGTTGT   
  
  
+ GGTACCCATA TGATTCTAAA AGCAGCTGAG AAGGCATCAA AGCTTCATAT TATAGATTTT GGCATCCTCT   
  
  
+ ATGGTTGCCA ATGGCCCAAC CTCATTCAAC GCCTCTCAGA GCGATCTGGT GGACCTCCAA AACTGTTTAT   
  
  
+ TACAGGGATC GATCTCCCCC AGCCTGGGTT CAGGCCAGCA GAAAGAGTGG AAGCAACAGG GAGACGCTTG   
  
  
+ GCCAAGTACT GTGAGCGGTA TAATGTGCCA TTTGAGTATC ATGCCATTGC TCAGGAGTGG GAAACAATCA   
  
  
+ AACCAGGGGA TCTCAAGATA GGAAGTAGGA ATGATGAAGT TGTTGCGGTG AACTGTCTCT GTAGGTTCAA   
  
  
+ GAACCTCCTT GACGAGACAG TGGTGGTGGA TAGTCCAAGG AACACAGTTT TAAACCTGAT TACAAGGGTA   
  
  
+ AAGCCTGATA TTTTTGTGCA TGGCGTTGTA AATGGTTCCT ACAACATCCC TTTCTTTGTG ACACGTTTTA   
  
  
+ GAGAAGCCCT CTTTCATTAT TCCACTCTTT TCGACATGTT AGATGCCAAC GCCTCTAGGG AGGAGCCCGA   
  
  
+ GAGGTTGATA TTCGAGAAGG CATTCTATGG GAGGGAGATT ATGAATGTGG TGGCCTGTGA GGGCACAGAG   
  
  
+ AGGGTGGAGA GGCCGGAGAC ATACAAGCAA TGGCATGTTA GGCATAGCAG GGCAGGGTTT CGGCAAGTAC   
  
  
+ CATTGGATCC CAAGTTGATC GAGAAAATGA GGTTTAAGGC CAAGGCAGAC CACCACAAGG ATTTCATGAT   
  
  
+ CGATGTGGAT GGACATTGGG CAATTCAGGG ATGGAAGGGG CGGATTGCAC ATGCGATCTC TGCATGGGTT   
  
  
+ CCGGCTTG  

- -Up\_Stream \_Len000CTAAAC TAACAGCTTT AAACAGGCTA AAATATTTTA ACTTTTAACA ATAACCTGAT   
  
  
- ATTAGATGAC ATCAAATCTC CACAACCTAT TATGAGAGTT GAAACTTGAA CCTGCAGGTA CGTGGTGTGA   
  
  
- TACCCGCATA AATGACAAAT ATATGGTTCT TATTAAAACT TCTACAAGAA TCAACCACTA CATTCACATA   
  
  
- TAAGTTGTAT ACAGTAATGT ATTTGAACTT TTAAGAACTT AAAATCTGTA TATGTTAACG TTTAGTAACA   
  
  
- TTTTAACAAC GTAATGTAAA ACTATTTTTA CGTAGGTTTG AAACAGAACC ATATTTGTCA TTTATCCGGG   
  
  
- TATCCAGGTA TCACATCACG TACCTGCAGG TTCAAATTTC CACCCTCATA ATAACGGGTT GCGGGGATTT   
  
  
- CATGTCATAT AATATCAGGT TATAGGGTTA ATTAAAAAGT AAACTTATAA ATTTAAACCT GTATTATAAA   
  
  
- AAGTATAAAC TTAAGAGATT TGGACTAAAA TTGGACTTAA ATTCAACTAA ATTAAGTAGG CTGGTTTTTT   
  
  
- TATTTGTGTT AATAAATAAA AAGATAAGCT TCAATCTATC ATCGGTTAAG TAAACTGTAT ATTAATTGGG   
  
  
- CACTAACTGT GTTTGAGCTC AAAAACGAAT CTCGATCCTT TTCTCAGCCC AATCCAGCTG GGTAAATTCT   
  
  
- TTACCCAGCT GAAGTACAGC CGGAAGTTTT ATCAATTCTA AACGAGTTTT GATTTTTTAT TGTTCAAGTG   
  
  
- TGATTTGCCA GTTTAGCACA GCTGAAGCCT AACTTTAACC AAGTCAAACC CAGCCGAAGC ATAAAGAAAG   
  
  
- TCATTGAAGC TTGGCTTAAG CATAGCACAA TAGCACAGTC CAGTCTGAAA GAGTCAAGAT AAAAACGATG   
  
  
- GAATACAATG CCTATTTTTT TCAGAGTGTA ACCTTTTTCA CACCCTCCCA GGACCCGAAT ATTTATCACC   
  
  
- GCGACGTGTG GAGTAGTCTG GCTGGAAAAC CCCTCTCCCA TTACTGGGGC ACTGTTCACC ATAGTCTCAG   
  
  
- TTGGGTCTGG GCTGAGTCTA CAGGACGGTT CGCGAGGCTG CCCGCCCCAT CCGTCCCCCG GGTAAACACT   
  
  
- ACTCCTGCAG TGTCTTAACC CGCCCCCTCT CACAGTGCCT ATTTTTTTCA GGGTGTAGCT TTTTTCACAC   
  
  
- CCTCCCAGGA CCCGAATATT TGCCATCGCG GCGTGTGGGG TAGTCTAGCT GAAAAACCCC TCTCCCATTA   
  
  
- CTGGGGTAAA GTTCACCATA GTCTCGGTTG GGTCTGGGCT GAGGTTACAG GGTGGTTCGC GAGACTACCC   
  
  
- GCCCCATCCG TTCCCCGAGG TATACACTGC TCCCGCAGTG CCTTAACCTA CCCTCTCTCA TGGTGCCTAT   
  
  
- TTTTTTCAGG GTGTAGTCTT TTTCACACCT GAATCGTTGA TCCTTCATTT CAATGTCATA ATCGGCTCTC   
  
  
- GGGTACCTTG CGGACTTTGA CTGTAGTGGT CTGGGTGTAT AAAGTGGTAG TAGACTAGAG GGGCAGTTTC   
  
  
- CTCCTTCGAC TCGTGTCGTT CTTGATCTTC TTGTCCGCCG TAGTGTGGGT TCGGTTTTAA GAGATAAACT   
  
  
- GGAGGGAGAG GAGTAAGGAG ACTCGAAGGA GAAGTGTTAC ATTCTGGTAT TAGGAATTGT GGGAAGAAAA   
  
  
- AATTCGAGTA GAAATGGACG AGACCAAGAG AGAGAGTAGC AGTGATTATC AATGGAGAAA TGAAAGAGAA   
  
  
- GGGACTCCAT ACGAAGTTGA AATTGTATAT ATAGGGTGAA CTAAAGAGAA CAGAAAAACG ATATTATGAA   
  
  
- CAACAAACCG ATAGTAGGGG ACAAAATATG AGTAAAAGAA CGAAAGATAA AGACCCAAAC TTAACCCAAA   
  
  
- GTGAATAGGA GCAGTTAAGA TACCCATTAA TCACTTAACC CAAATATACT TGATCCCAAC GTTTTCATAG   
  
  
- ATGAAAAAGA GAACACTAAT AATCAACTAA TCCCTAAACC GGTTATAATC GTCTTACCCA AGACTTAAAC   
  
  
- GCCTTAAGAG ACTACTACGA GATTTACCCA TACGAATACA ACTATAGGGA CGAATACTAC GTAGGAATCT   
  
  
- AATACGGTTA AACAAGTTAA TGCTTCCGGG TAGACTCCTA GCGTGGAGTG ACTCGGAGGG TTCTGGGAAA   
  
  
- AGACTGGGAA ACCGAATGTC AAAGTGCAGG AGAAGACTCG ACCCGGGACC CCAACTAAGA TTACTACTAT   
  
  
- CACTAAGACC GCTACAAGAG TTCATGTAAT CGGTTTACGA ATACCTTCTT CTGTACCTCC GTTTCGGTAC   
  
  
- GTACAAAGTA CTAGGAAATC GTGAAGTCCG ACGACTCTTT GGGAAAATAC TACGGAATCC CCTCGTTACG   
  
  
- GGTTGAAGAG GACTGGTTGT AGGATATTAA CTAGTAATAA ACCTATCAGG ACTACTTTTA AGAAACTCAA   
  
  
- GTTGACCACT AAAATCACAA TCAGTAAGAC CCAGATCAAG TTGTTTGAGG CAACCTGGCT ATTATCACAG   
  
  
- ACTAAACTCA CTCGTAAAAC TCGGTGGGAA ACAACTTTGT GAAGGTTAAC TTAGTATAGT GGTTGGTAAC   
  
  
- TGGGCAGGAG TTACCAGTAA ACCGAGAAGC CCACGGAATC TACCGTGCCG GAGATTACCA AGCCACTAGA   
  
  
- GTAGGGAACC AAACGGTCAC CTACACTAAT CGCATAAATC CCTCTTTCTC AGGTACTAAG TTAAAGTCTT   
  
  
- TCCCCACCTC CTCCGGTCAT TCAAGGAAGG GTTCTTATTA TTGGAACAAT AACTAGAGCT CTTGGAGTGA   
  
  
- AAAGGATTAC TTTGTTTCCT CCTACTAGCT TACTACTACC AATTCTTCCT TTTCCTACTG CACTTAACCA   
  
  
- GATTGATGAG TTCTCCCTCA TTCTAAGTGA TAACACTTCT GCTCCGGAAA CTTCTTCCTT CCTCACCGTT   
  
  
- CGTCAGTCGA TAAAGAAGAT GACTCCTTCG ACTCAAAAGA CTGTAAAAAC TGTTCCAAAA CGAAACGCTA   
  
  
- ACGATGGGAC ACTTTGGACT CCGAGTAGGG TGGTACTCAA ACTTGGGACT CTTCCCGGTC AGTGTACCTA   
  
  
- ATCTTCCACC CTCTTTACCC TTCCGAGCGG GATTCTTTGT CCTATTATCA TCGTGTTTAT AACACCTAAA   
  
  
- TTCCTTAAAC GACTATGATA CGCGTGTTAG ATGACGTAGA CTACTGGCTT CTTGACGACT ACCTGACGAT   
  
  
- TTCGTTTAGT CCCTCGTGAG CAGACGACTC CCCCTACCTA GAGTTTCCAA CCGCGTAATG AAACGACTAC   
  
  
- GGGATCTCCG TGCAAATCGA CCTTGACCTA GAGCGTAAAT ATGCCGGGAT ACAAGATTAT CCGGTAGACA   
  
  
- GTGACTGTAC TATTTTCGTA TAGTCAAGAT ATAAGCACGA ACGGGTAAAT GCTTCTAGCA GTAACCAACA   
  
  
- CCATGGGTAT ACTAAGATTT TCGTCGACTC TTCCGTAGTT TCGAAGTATA ATATCTAAAA CCGTAGGAGA   
  
  
- TACCAACGGT TACCGGGTTG GAGTAAGTTG CGGAGAGTCT CGCTAGACCA CCTGGAGGTT TTGACAAATA   
  
  
- ATGTCCCTAG CTAGAGGGGG TCGGACCCAA GTCCGGTCGT CTTTCTCACC TTCGTTGTCC CTCTGCGAAC   
  
  
- CGGTTCATGA CACTCGCCAT ATTACACGGT AAACTCATAG TACGGTAACG AGTCCTCACC CTTTGTTAGT   
  
  
- TTGGTCCCCT AGAGTTCTAT CCTTCATCCT TACTACTTCA ACAACGCCAC TTGACAGAGA CATCCAAGTT   
  
  
- CTTGGAGGAA CTGCTCTGTC ACCACCACCT ATCAGGTTCC TTGTGTCAAA ATTTGGACTA ATGTTCCCAT   
  
  
- TTCGGACTAT AAAAACACGT ACCGCAACAT TTACCAAGGA TGTTGTAGGG AAAGAAACAC TGTGCAAAAT   
  
  
- CTCTTCGGGA GAAAGTAATA AGGTGAGAAA AGCTGTACAA TCTACGGTTG CGGAGATCCC TCCTCGGGCT   
  
  
- CTCCAACTAT AAGCTCTTCC GTAAGATACC CTCCCTCTAA TACTTACACC ACCGGACACT CCCGTGTCTC   
  
  
- TCCCACCTCT CCGGCCTCTG TATGTTCGTT ACCGTACAAT CCGTATCGTC CCGTCCCAAA GCCGTTCATG   
  
  
- GTAACCTAGG GTTCAACTAG CTCTTTTACT CCAAATTCCG GTTCCGTCTG GTGGTGTTCC TAAAGTACTA   
  
  
- GCTACACCTA CCTGTAACCC GTTAAGTCCC TACCTTCCCC GCCTAACGTG TACGCTAGAG ACGTACCCAA   
  
  
- GGCCGAAC

+     LTR

| Site Name | Organism | Position | Strand | Matrix score. | sequence | function |
| --- | --- | --- | --- | --- | --- | --- |
| LTR | Hordeum vulgare | 4122 | - | 6 | CCGAAA | cis-acting element involved in low-temperature responsiveness |

>HU07G02249.1   
+ -Up\_Stream \_Len000GATTTG ATTGTCGAAA TTTGTCCGAT TTTATAAAAT TGAAAATTGT TATTGGACTA   
  
  
+ TAATCTACTG TAGTTTAGAG GTGTTGGATA ATACTCTCAA CTTTGAACTT GGACGTCCAT GCACCACACT   
  
  
+ ATGGGCGTAT TTACTGTTTA TATACCAAGA ATAATTTTGA AGATGTTCTT AGTTGGTGAT GTAAGTGTAT   
  
  
+ ATTCAACATA TGTCATTACA TAAACTTGAA AATTCTTGAA TTTTAGACAT ATACAATTGC AAATCATTGT   
  
  
+ AAAATTGTTG CATTACATTT TGATAAAAAT GCATCCAAAC TTTGTCTTGG TATAAACAGT AAATAGGCCC   
  
  
+ ATAGGTCCAT AGTGTAGTGC ATGGACGTCC AAGTTTAAAG GTGGGAGTAT TATTGCCCAA CGCCCCTAAA   
  
  
+ GTACAGTATA TTATAGTCCA ATATCCCAAT TAATTTTTCA TTTGAATATT TAAATTTGGA CATAATATTT   
  
  
+ TTCATATTTG AATTCTCTAA ACCTGATTTT AACCTGAATT TAAGTTGATT TAATTCATCC GACCAAAAAA   
  
  
+ ATAAACACAA TTATTTATTT TTCTATTCGA AGTTAGATAG TAGCCAATTC ATTTGACATA TAATTAACCC   
  
  
+ GTGATTGACA CAAACTCGAG TTTTTGCTTA GAGCTAGGAA AAGAGTCGGG TTAGGTCGAC CCATTTAAGA   
  
  
+ AATGGGTCGA CTTCATGTCG GCCTTCAAAA TAGTTAAGAT TTGCTCAAAA CTAAAAAATA ACAAGTTCAC   
  
  
+ ACTAAACGGT CAAATCGTGT CGACTTCGGA TTGAAATTGG TTCAGTTTGG GTCGGCTTCG TATTTCTTTC   
  
  
+ AGTAACTTCG AACCGAATTC GTATCGTGTT ATCGTGTCAG GTCAGACTTT CTCAGTTCTA TTTTTGCTAC   
  
  
+ CTTATGTTAC GGATAAAAAA AGTCTCACAT TGGAAAAAGT GTGGGAGGGT CCTGGGCTTA TAAATAGTGG   
  
  
+ CGCTGCACAC CTCATCAGAC CGACCTTTTG GGGAGAGGGT AATGACCCCG TGACAAGTGG TATCAGAGTC   
  
  
+ AACCCAGACC CGACTCAGAT GTCCTGCCAA GCGCTCCGAC GGGCGGGGTA GGCAGGGGGC CCATTTGTGA   
  
  
+ TGAGGACGTC ACAGAATTGG GCGGGGGAGA GTGTCACGGA TAAAAAAAGT CCCACATCGA AAAAAGTGTG   
  
  
+ GGAGGGTCCT GGGCTTATAA ACGGTAGCGC CGCACACCCC ATCAGATCGA CTTTTTGGGG AGAGGGTAAT   
  
  
+ GACCCCATTT CAAGTGGTAT CAGAGCCAAC CCAGACCCGA CTCCAATGTC CCACCAAGCG CTCTGATGGG   
  
  
+ CGGGGTAGGC AAGGGGCTCC ATATGTGACG AGGGCGTCAC GGAATTGGAT GGGAGAGAGT ACCACGGATA   
  
  
+ AAAAAAGTCC CACATCAGAA AAAGTGTGGA CTTAGCAACT AGGAAGTAAA GTTACAGTAT TAGCCGAGAG   
  
  
+ CCCATGGAAC GCCTGAAACT GACATCACCA GACCCACATA TTTCACCATC ATCTGATCTC CCCGTCAAAG   
  
  
+ GAGGAAGCTG AGCACAGCAA GAACTAGAAG AACAGGCGGC ATCACACCCA AGCCAAAATT CTCTATTTGA   
  
  
+ CCTCCCTCTC CTCATTCCTC TGAGCTTCCT CTTCACAATG TAAGACCATA ATCCTTAACA CCCTTCTTTT   
  
  
+ TTAAGCTCAT CTTTACCTGC TCTGGTTCTC TCTCTCATCG TCACTAATAG TTACCTCTTT ACTTTCTCTT   
  
  
+ CCCTGAGGTA TGCTTCAACT TTAACATATA TATCCCACTT GATTTCTCTT GTCTTTTTGC TATAATACTT   
  
  
+ GTTGTTTGGC TATCATCCCC TGTTTTATAC TCATTTTCTT GCTTTCTATT TCTGGGTTTG AATTGGGTTT   
  
  
+ CACTTATCCT CGTCAATTCT ATGGGTAATT AGTGAATTGG GTTTATATGA ACTAGGGTTG CAAAAGTATC   
  
  
+ TACTTTTTCT CTTGTGATTA TTAGTTGATT AGGGATTTGG CCAATATTAG CAGAATGGGT TCTGAATTTG   
  
  
+ CGGAATTCTC TGATGATGCT CTAAATGGGT ATGCTTATGT TGATATCCCT GCTTATGATG CATCCTTAGA   
  
  
+ TTATGCCAAT TTGTTCAATT ACGAAGGCCC ATCTGAGGAT CGCACCTCAC TGAGCCTCCC AAGACCCTTT   
  
  
+ TCTGACCCTT TGGCTTACAG TTTCACGTCC TCTTCTGAGC TGGGCCCTGG GGTTGATTCT AATGATGATA   
  
  
+ GTGATTCTGG CGATGTTCTC AAGTACATTA GCCAAATGCT TATGGAAGAA GACATGGAGG CAAAGCCATG   
  
  
+ CATGTTTCAT GATCCTTTAG CACTTCAGGC TGCTGAGAAA CCCTTTTATG ATGCCTTAGG GGAGCAATGC   
  
  
+ CCAACTTCTC CTGACCAACA TCCTATAATT GATCATTATT TGGATAGTCC TGATGAAAAT TCTTTGAGTT   
  
  
+ CAACTGGTGA TTTTAGTGTT AGTCATTCTG GGTCTAGTTC AACAAACTCC GTTGGACCGA TAATAGTGTC   
  
  
+ TGATTTGAGT GAGCATTTTG AGCCACCCTT TGTTGAAACA CTTCCAATTG AATCATATCA CCAACCATTG   
  
  
+ ACCCGTCCTC AATGGTCATT TGGCTCTTCG GGTGCCTTAG ATGGCACGGC CTCTAATGGT TCGGTGATCT   
  
  
+ CATCCCTTGG TTTGCCAGTG GATGTGATTA GCGTATTTAG GGAGAAAGAG TCCATGATTC AATTTCAGAA   
  
  
+ AGGGGTGGAG GAGGCCAGTA AGTTCCTTCC CAAGAATAAT AACCTTGTTA TTGATCTCGA GAACCTCACT   
  
  
+ TTTCCTAATG AAACAAAGGA GGATGATCGA ATGATGATGG TTAAGAAGGA AAAGGATGAC GTGAATTGGT   
  
  
+ CTAACTACTC AAGAGGGAGT AAGATTCACT ATTGTGAAGA CGAGGCCTTT GAAGAAGGAA GGAGTGGCAA   
  
  
+ GCAGTCAGCT ATTTCTTCTA CTGAGGAAGC TGAGTTTTCT GACATTTTTG ACAAGGTTTT GCTTTGCGAT   
  
  
+ TGCTACCCTG TGAAACCTGA GGCTCATCCC ACCATGAGTT TGAACCCTGA GAAGGGCCAG TCACATGGAT   
  
  
+ TAGAAGGTGG GAGAAATGGG AAGGCTCGCC CTAAGAAACA GGATAATAGT AGCACAAATA TTGTGGATTT   
  
  
+ AAGGAATTTG CTGATACTAT GCGCACAATC TACTGCATCT GATGACCGAA GAACTGCTGA TGGACTGCTA   
  
  
+ AAGCAAATCA GGGAGCACTC GTCTGCTGAG GGGGATGGAT CTCAAAGGTT GGCGCATTAC TTTGCTGATG   
  
  
+ CCCTAGAGGC ACGTTTAGCT GGAACTGGAT CTCGCATTTA TACGGCCCTA TGTTCTAATA GGCCATCTGT   
  
  
+ CACTGACATG ATAAAAGCAT ATCAGTTCTA TATTCGTGCT TGCCCATTTA CGAAGATCGT CATTGGTTGT   
  
  
+ GGTACCCATA TGATTCTAAA AGCAGCTGAG AAGGCATCAA AGCTTCATAT TATAGATTTT GGCATCCTCT   
  
  
+ ATGGTTGCCA ATGGCCCAAC CTCATTCAAC GCCTCTCAGA GCGATCTGGT GGACCTCCAA AACTGTTTAT   
  
  
+ TACAGGGATC GATCTCCCCC AGCCTGGGTT CAGGCCAGCA GAAAGAGTGG AAGCAACAGG GAGACGCTTG   
  
  
+ GCCAAGTACT GTGAGCGGTA TAATGTGCCA TTTGAGTATC ATGCCATTGC TCAGGAGTGG GAAACAATCA   
  
  
+ AACCAGGGGA TCTCAAGATA GGAAGTAGGA ATGATGAAGT TGTTGCGGTG AACTGTCTCT GTAGGTTCAA   
  
  
+ GAACCTCCTT GACGAGACAG TGGTGGTGGA TAGTCCAAGG AACACAGTTT TAAACCTGAT TACAAGGGTA   
  
  
+ AAGCCTGATA TTTTTGTGCA TGGCGTTGTA AATGGTTCCT ACAACATCCC TTTCTTTGTG ACACGTTTTA   
  
  
+ GAGAAGCCCT CTTTCATTAT TCCACTCTTT TCGACATGTT AGATGCCAAC GCCTCTAGGG AGGAGCCCGA   
  
  
+ GAGGTTGATA TTCGAGAAGG CATTCTATGG GAGGGAGATT ATGAATGTGG TGGCCTGTGA GGGCACAGAG   
  
  
+ AGGGTGGAGA GGCCGGAGAC ATACAAGCAA TGGCATGTTA GGCATAGCAG GGCAGGGTTT CGGCAAGTAC   
  
  
+ CATTGGATCC CAAGTTGATC GAGAAAATGA GGTTTAAGGC CAAGGCAGAC CACCACAAGG ATTTCATGAT   
  
  
+ CGATGTGGAT GGACATTGGG CAATTCAGGG ATGGAAGGGG CGGATTGCAC ATGCGATCTC TGCATGGGTT   
  
  
+ CCGGCTTG  

- -Up\_Stream \_Len000CTAAAC TAACAGCTTT AAACAGGCTA AAATATTTTA ACTTTTAACA ATAACCTGAT   
  
  
- ATTAGATGAC ATCAAATCTC CACAACCTAT TATGAGAGTT GAAACTTGAA CCTGCAGGTA CGTGGTGTGA   
  
  
- TACCCGCATA AATGACAAAT ATATGGTTCT TATTAAAACT TCTACAAGAA TCAACCACTA CATTCACATA   
  
  
- TAAGTTGTAT ACAGTAATGT ATTTGAACTT TTAAGAACTT AAAATCTGTA TATGTTAACG TTTAGTAACA   
  
  
- TTTTAACAAC GTAATGTAAA ACTATTTTTA CGTAGGTTTG AAACAGAACC ATATTTGTCA TTTATCCGGG   
  
  
- TATCCAGGTA TCACATCACG TACCTGCAGG TTCAAATTTC CACCCTCATA ATAACGGGTT GCGGGGATTT   
  
  
- CATGTCATAT AATATCAGGT TATAGGGTTA ATTAAAAAGT AAACTTATAA ATTTAAACCT GTATTATAAA   
  
  
- AAGTATAAAC TTAAGAGATT TGGACTAAAA TTGGACTTAA ATTCAACTAA ATTAAGTAGG CTGGTTTTTT   
  
  
- TATTTGTGTT AATAAATAAA AAGATAAGCT TCAATCTATC ATCGGTTAAG TAAACTGTAT ATTAATTGGG   
  
  
- CACTAACTGT GTTTGAGCTC AAAAACGAAT CTCGATCCTT TTCTCAGCCC AATCCAGCTG GGTAAATTCT   
  
  
- TTACCCAGCT GAAGTACAGC CGGAAGTTTT ATCAATTCTA AACGAGTTTT GATTTTTTAT TGTTCAAGTG   
  
  
- TGATTTGCCA GTTTAGCACA GCTGAAGCCT AACTTTAACC AAGTCAAACC CAGCCGAAGC ATAAAGAAAG   
  
  
- TCATTGAAGC TTGGCTTAAG CATAGCACAA TAGCACAGTC CAGTCTGAAA GAGTCAAGAT AAAAACGATG   
  
  
- GAATACAATG CCTATTTTTT TCAGAGTGTA ACCTTTTTCA CACCCTCCCA GGACCCGAAT ATTTATCACC   
  
  
- GCGACGTGTG GAGTAGTCTG GCTGGAAAAC CCCTCTCCCA TTACTGGGGC ACTGTTCACC ATAGTCTCAG   
  
  
- TTGGGTCTGG GCTGAGTCTA CAGGACGGTT CGCGAGGCTG CCCGCCCCAT CCGTCCCCCG GGTAAACACT   
  
  
- ACTCCTGCAG TGTCTTAACC CGCCCCCTCT CACAGTGCCT ATTTTTTTCA GGGTGTAGCT TTTTTCACAC   
  
  
- CCTCCCAGGA CCCGAATATT TGCCATCGCG GCGTGTGGGG TAGTCTAGCT GAAAAACCCC TCTCCCATTA   
  
  
- CTGGGGTAAA GTTCACCATA GTCTCGGTTG GGTCTGGGCT GAGGTTACAG GGTGGTTCGC GAGACTACCC   
  
  
- GCCCCATCCG TTCCCCGAGG TATACACTGC TCCCGCAGTG CCTTAACCTA CCCTCTCTCA TGGTGCCTAT   
  
  
- TTTTTTCAGG GTGTAGTCTT TTTCACACCT GAATCGTTGA TCCTTCATTT CAATGTCATA ATCGGCTCTC   
  
  
- GGGTACCTTG CGGACTTTGA CTGTAGTGGT CTGGGTGTAT AAAGTGGTAG TAGACTAGAG GGGCAGTTTC   
  
  
- CTCCTTCGAC TCGTGTCGTT CTTGATCTTC TTGTCCGCCG TAGTGTGGGT TCGGTTTTAA GAGATAAACT   
  
  
- GGAGGGAGAG GAGTAAGGAG ACTCGAAGGA GAAGTGTTAC ATTCTGGTAT TAGGAATTGT GGGAAGAAAA   
  
  
- AATTCGAGTA GAAATGGACG AGACCAAGAG AGAGAGTAGC AGTGATTATC AATGGAGAAA TGAAAGAGAA   
  
  
- GGGACTCCAT ACGAAGTTGA AATTGTATAT ATAGGGTGAA CTAAAGAGAA CAGAAAAACG ATATTATGAA   
  
  
- CAACAAACCG ATAGTAGGGG ACAAAATATG AGTAAAAGAA CGAAAGATAA AGACCCAAAC TTAACCCAAA   
  
  
- GTGAATAGGA GCAGTTAAGA TACCCATTAA TCACTTAACC CAAATATACT TGATCCCAAC GTTTTCATAG   
  
  
- ATGAAAAAGA GAACACTAAT AATCAACTAA TCCCTAAACC GGTTATAATC GTCTTACCCA AGACTTAAAC   
  
  
- GCCTTAAGAG ACTACTACGA GATTTACCCA TACGAATACA ACTATAGGGA CGAATACTAC GTAGGAATCT   
  
  
- AATACGGTTA AACAAGTTAA TGCTTCCGGG TAGACTCCTA GCGTGGAGTG ACTCGGAGGG TTCTGGGAAA   
  
  
- AGACTGGGAA ACCGAATGTC AAAGTGCAGG AGAAGACTCG ACCCGGGACC CCAACTAAGA TTACTACTAT   
  
  
- CACTAAGACC GCTACAAGAG TTCATGTAAT CGGTTTACGA ATACCTTCTT CTGTACCTCC GTTTCGGTAC   
  
  
- GTACAAAGTA CTAGGAAATC GTGAAGTCCG ACGACTCTTT GGGAAAATAC TACGGAATCC CCTCGTTACG   
  
  
- GGTTGAAGAG GACTGGTTGT AGGATATTAA CTAGTAATAA ACCTATCAGG ACTACTTTTA AGAAACTCAA   
  
  
- GTTGACCACT AAAATCACAA TCAGTAAGAC CCAGATCAAG TTGTTTGAGG CAACCTGGCT ATTATCACAG   
  
  
- ACTAAACTCA CTCGTAAAAC TCGGTGGGAA ACAACTTTGT GAAGGTTAAC TTAGTATAGT GGTTGGTAAC   
  
  
- TGGGCAGGAG TTACCAGTAA ACCGAGAAGC CCACGGAATC TACCGTGCCG GAGATTACCA AGCCACTAGA   
  
  
- GTAGGGAACC AAACGGTCAC CTACACTAAT CGCATAAATC CCTCTTTCTC AGGTACTAAG TTAAAGTCTT   
  
  
- TCCCCACCTC CTCCGGTCAT TCAAGGAAGG GTTCTTATTA TTGGAACAAT AACTAGAGCT CTTGGAGTGA   
  
  
- AAAGGATTAC TTTGTTTCCT CCTACTAGCT TACTACTACC AATTCTTCCT TTTCCTACTG CACTTAACCA   
  
  
- GATTGATGAG TTCTCCCTCA TTCTAAGTGA TAACACTTCT GCTCCGGAAA CTTCTTCCTT CCTCACCGTT   
  
  
- CGTCAGTCGA TAAAGAAGAT GACTCCTTCG ACTCAAAAGA CTGTAAAAAC TGTTCCAAAA CGAAACGCTA   
  
  
- ACGATGGGAC ACTTTGGACT CCGAGTAGGG TGGTACTCAA ACTTGGGACT CTTCCCGGTC AGTGTACCTA   
  
  
- ATCTTCCACC CTCTTTACCC TTCCGAGCGG GATTCTTTGT CCTATTATCA TCGTGTTTAT AACACCTAAA   
  
  
- TTCCTTAAAC GACTATGATA CGCGTGTTAG ATGACGTAGA CTACTGGCTT CTTGACGACT ACCTGACGAT   
  
  
- TTCGTTTAGT CCCTCGTGAG CAGACGACTC CCCCTACCTA GAGTTTCCAA CCGCGTAATG AAACGACTAC   
  
  
- GGGATCTCCG TGCAAATCGA CCTTGACCTA GAGCGTAAAT ATGCCGGGAT ACAAGATTAT CCGGTAGACA   
  
  
- GTGACTGTAC TATTTTCGTA TAGTCAAGAT ATAAGCACGA ACGGGTAAAT GCTTCTAGCA GTAACCAACA   
  
  
- CCATGGGTAT ACTAAGATTT TCGTCGACTC TTCCGTAGTT TCGAAGTATA ATATCTAAAA CCGTAGGAGA   
  
  
- TACCAACGGT TACCGGGTTG GAGTAAGTTG CGGAGAGTCT CGCTAGACCA CCTGGAGGTT TTGACAAATA   
  
  
- ATGTCCCTAG CTAGAGGGGG TCGGACCCAA GTCCGGTCGT CTTTCTCACC TTCGTTGTCC CTCTGCGAAC   
  
  
- CGGTTCATGA CACTCGCCAT ATTACACGGT AAACTCATAG TACGGTAACG AGTCCTCACC CTTTGTTAGT   
  
  
- TTGGTCCCCT AGAGTTCTAT CCTTCATCCT TACTACTTCA ACAACGCCAC TTGACAGAGA CATCCAAGTT   
  
  
- CTTGGAGGAA CTGCTCTGTC ACCACCACCT ATCAGGTTCC TTGTGTCAAA ATTTGGACTA ATGTTCCCAT   
  
  
- TTCGGACTAT AAAAACACGT ACCGCAACAT TTACCAAGGA TGTTGTAGGG AAAGAAACAC TGTGCAAAAT   
  
  
- CTCTTCGGGA GAAAGTAATA AGGTGAGAAA AGCTGTACAA TCTACGGTTG CGGAGATCCC TCCTCGGGCT   
  
  
- CTCCAACTAT AAGCTCTTCC GTAAGATACC CTCCCTCTAA TACTTACACC ACCGGACACT CCCGTGTCTC   
  
  
- TCCCACCTCT CCGGCCTCTG TATGTTCGTT ACCGTACAAT CCGTATCGTC CCGTCCCAAA GCCGTTCATG   
  
  
- GTAACCTAGG GTTCAACTAG CTCTTTTACT CCAAATTCCG GTTCCGTCTG GTGGTGTTCC TAAAGTACTA   
  
  
- GCTACACCTA CCTGTAACCC GTTAAGTCCC TACCTTCCCC GCCTAACGTG TACGCTAGAG ACGTACCCAA   
  
  
- GGCCGAAC

+     MBS

| Site Name | Organism | Position | Strand | Matrix score. | sequence | function |
| --- | --- | --- | --- | --- | --- | --- |
| MBS | Arabidopsis thaliana | 2455 | + | 6 | CAACTG | MYB binding site involved in drought-inducibility |

>HU07G02249.1   
+ -Up\_Stream \_Len000GATTTG ATTGTCGAAA TTTGTCCGAT TTTATAAAAT TGAAAATTGT TATTGGACTA   
  
  
+ TAATCTACTG TAGTTTAGAG GTGTTGGATA ATACTCTCAA CTTTGAACTT GGACGTCCAT GCACCACACT   
  
  
+ ATGGGCGTAT TTACTGTTTA TATACCAAGA ATAATTTTGA AGATGTTCTT AGTTGGTGAT GTAAGTGTAT   
  
  
+ ATTCAACATA TGTCATTACA TAAACTTGAA AATTCTTGAA TTTTAGACAT ATACAATTGC AAATCATTGT   
  
  
+ AAAATTGTTG CATTACATTT TGATAAAAAT GCATCCAAAC TTTGTCTTGG TATAAACAGT AAATAGGCCC   
  
  
+ ATAGGTCCAT AGTGTAGTGC ATGGACGTCC AAGTTTAAAG GTGGGAGTAT TATTGCCCAA CGCCCCTAAA   
  
  
+ GTACAGTATA TTATAGTCCA ATATCCCAAT TAATTTTTCA TTTGAATATT TAAATTTGGA CATAATATTT   
  
  
+ TTCATATTTG AATTCTCTAA ACCTGATTTT AACCTGAATT TAAGTTGATT TAATTCATCC GACCAAAAAA   
  
  
+ ATAAACACAA TTATTTATTT TTCTATTCGA AGTTAGATAG TAGCCAATTC ATTTGACATA TAATTAACCC   
  
  
+ GTGATTGACA CAAACTCGAG TTTTTGCTTA GAGCTAGGAA AAGAGTCGGG TTAGGTCGAC CCATTTAAGA   
  
  
+ AATGGGTCGA CTTCATGTCG GCCTTCAAAA TAGTTAAGAT TTGCTCAAAA CTAAAAAATA ACAAGTTCAC   
  
  
+ ACTAAACGGT CAAATCGTGT CGACTTCGGA TTGAAATTGG TTCAGTTTGG GTCGGCTTCG TATTTCTTTC   
  
  
+ AGTAACTTCG AACCGAATTC GTATCGTGTT ATCGTGTCAG GTCAGACTTT CTCAGTTCTA TTTTTGCTAC   
  
  
+ CTTATGTTAC GGATAAAAAA AGTCTCACAT TGGAAAAAGT GTGGGAGGGT CCTGGGCTTA TAAATAGTGG   
  
  
+ CGCTGCACAC CTCATCAGAC CGACCTTTTG GGGAGAGGGT AATGACCCCG TGACAAGTGG TATCAGAGTC   
  
  
+ AACCCAGACC CGACTCAGAT GTCCTGCCAA GCGCTCCGAC GGGCGGGGTA GGCAGGGGGC CCATTTGTGA   
  
  
+ TGAGGACGTC ACAGAATTGG GCGGGGGAGA GTGTCACGGA TAAAAAAAGT CCCACATCGA AAAAAGTGTG   
  
  
+ GGAGGGTCCT GGGCTTATAA ACGGTAGCGC CGCACACCCC ATCAGATCGA CTTTTTGGGG AGAGGGTAAT   
  
  
+ GACCCCATTT CAAGTGGTAT CAGAGCCAAC CCAGACCCGA CTCCAATGTC CCACCAAGCG CTCTGATGGG   
  
  
+ CGGGGTAGGC AAGGGGCTCC ATATGTGACG AGGGCGTCAC GGAATTGGAT GGGAGAGAGT ACCACGGATA   
  
  
+ AAAAAAGTCC CACATCAGAA AAAGTGTGGA CTTAGCAACT AGGAAGTAAA GTTACAGTAT TAGCCGAGAG   
  
  
+ CCCATGGAAC GCCTGAAACT GACATCACCA GACCCACATA TTTCACCATC ATCTGATCTC CCCGTCAAAG   
  
  
+ GAGGAAGCTG AGCACAGCAA GAACTAGAAG AACAGGCGGC ATCACACCCA AGCCAAAATT CTCTATTTGA   
  
  
+ CCTCCCTCTC CTCATTCCTC TGAGCTTCCT CTTCACAATG TAAGACCATA ATCCTTAACA CCCTTCTTTT   
  
  
+ TTAAGCTCAT CTTTACCTGC TCTGGTTCTC TCTCTCATCG TCACTAATAG TTACCTCTTT ACTTTCTCTT   
  
  
+ CCCTGAGGTA TGCTTCAACT TTAACATATA TATCCCACTT GATTTCTCTT GTCTTTTTGC TATAATACTT   
  
  
+ GTTGTTTGGC TATCATCCCC TGTTTTATAC TCATTTTCTT GCTTTCTATT TCTGGGTTTG AATTGGGTTT   
  
  
+ CACTTATCCT CGTCAATTCT ATGGGTAATT AGTGAATTGG GTTTATATGA ACTAGGGTTG CAAAAGTATC   
  
  
+ TACTTTTTCT CTTGTGATTA TTAGTTGATT AGGGATTTGG CCAATATTAG CAGAATGGGT TCTGAATTTG   
  
  
+ CGGAATTCTC TGATGATGCT CTAAATGGGT ATGCTTATGT TGATATCCCT GCTTATGATG CATCCTTAGA   
  
  
+ TTATGCCAAT TTGTTCAATT ACGAAGGCCC ATCTGAGGAT CGCACCTCAC TGAGCCTCCC AAGACCCTTT   
  
  
+ TCTGACCCTT TGGCTTACAG TTTCACGTCC TCTTCTGAGC TGGGCCCTGG GGTTGATTCT AATGATGATA   
  
  
+ GTGATTCTGG CGATGTTCTC AAGTACATTA GCCAAATGCT TATGGAAGAA GACATGGAGG CAAAGCCATG   
  
  
+ CATGTTTCAT GATCCTTTAG CACTTCAGGC TGCTGAGAAA CCCTTTTATG ATGCCTTAGG GGAGCAATGC   
  
  
+ CCAACTTCTC CTGACCAACA TCCTATAATT GATCATTATT TGGATAGTCC TGATGAAAAT TCTTTGAGTT   
  
  
+ CAACTGGTGA TTTTAGTGTT AGTCATTCTG GGTCTAGTTC AACAAACTCC GTTGGACCGA TAATAGTGTC   
  
  
+ TGATTTGAGT GAGCATTTTG AGCCACCCTT TGTTGAAACA CTTCCAATTG AATCATATCA CCAACCATTG   
  
  
+ ACCCGTCCTC AATGGTCATT TGGCTCTTCG GGTGCCTTAG ATGGCACGGC CTCTAATGGT TCGGTGATCT   
  
  
+ CATCCCTTGG TTTGCCAGTG GATGTGATTA GCGTATTTAG GGAGAAAGAG TCCATGATTC AATTTCAGAA   
  
  
+ AGGGGTGGAG GAGGCCAGTA AGTTCCTTCC CAAGAATAAT AACCTTGTTA TTGATCTCGA GAACCTCACT   
  
  
+ TTTCCTAATG AAACAAAGGA GGATGATCGA ATGATGATGG TTAAGAAGGA AAAGGATGAC GTGAATTGGT   
  
  
+ CTAACTACTC AAGAGGGAGT AAGATTCACT ATTGTGAAGA CGAGGCCTTT GAAGAAGGAA GGAGTGGCAA   
  
  
+ GCAGTCAGCT ATTTCTTCTA CTGAGGAAGC TGAGTTTTCT GACATTTTTG ACAAGGTTTT GCTTTGCGAT   
  
  
+ TGCTACCCTG TGAAACCTGA GGCTCATCCC ACCATGAGTT TGAACCCTGA GAAGGGCCAG TCACATGGAT   
  
  
+ TAGAAGGTGG GAGAAATGGG AAGGCTCGCC CTAAGAAACA GGATAATAGT AGCACAAATA TTGTGGATTT   
  
  
+ AAGGAATTTG CTGATACTAT GCGCACAATC TACTGCATCT GATGACCGAA GAACTGCTGA TGGACTGCTA   
  
  
+ AAGCAAATCA GGGAGCACTC GTCTGCTGAG GGGGATGGAT CTCAAAGGTT GGCGCATTAC TTTGCTGATG   
  
  
+ CCCTAGAGGC ACGTTTAGCT GGAACTGGAT CTCGCATTTA TACGGCCCTA TGTTCTAATA GGCCATCTGT   
  
  
+ CACTGACATG ATAAAAGCAT ATCAGTTCTA TATTCGTGCT TGCCCATTTA CGAAGATCGT CATTGGTTGT   
  
  
+ GGTACCCATA TGATTCTAAA AGCAGCTGAG AAGGCATCAA AGCTTCATAT TATAGATTTT GGCATCCTCT   
  
  
+ ATGGTTGCCA ATGGCCCAAC CTCATTCAAC GCCTCTCAGA GCGATCTGGT GGACCTCCAA AACTGTTTAT   
  
  
+ TACAGGGATC GATCTCCCCC AGCCTGGGTT CAGGCCAGCA GAAAGAGTGG AAGCAACAGG GAGACGCTTG   
  
  
+ GCCAAGTACT GTGAGCGGTA TAATGTGCCA TTTGAGTATC ATGCCATTGC TCAGGAGTGG GAAACAATCA   
  
  
+ AACCAGGGGA TCTCAAGATA GGAAGTAGGA ATGATGAAGT TGTTGCGGTG AACTGTCTCT GTAGGTTCAA   
  
  
+ GAACCTCCTT GACGAGACAG TGGTGGTGGA TAGTCCAAGG AACACAGTTT TAAACCTGAT TACAAGGGTA   
  
  
+ AAGCCTGATA TTTTTGTGCA TGGCGTTGTA AATGGTTCCT ACAACATCCC TTTCTTTGTG ACACGTTTTA   
  
  
+ GAGAAGCCCT CTTTCATTAT TCCACTCTTT TCGACATGTT AGATGCCAAC GCCTCTAGGG AGGAGCCCGA   
  
  
+ GAGGTTGATA TTCGAGAAGG CATTCTATGG GAGGGAGATT ATGAATGTGG TGGCCTGTGA GGGCACAGAG   
  
  
+ AGGGTGGAGA GGCCGGAGAC ATACAAGCAA TGGCATGTTA GGCATAGCAG GGCAGGGTTT CGGCAAGTAC   
  
  
+ CATTGGATCC CAAGTTGATC GAGAAAATGA GGTTTAAGGC CAAGGCAGAC CACCACAAGG ATTTCATGAT   
  
  
+ CGATGTGGAT GGACATTGGG CAATTCAGGG ATGGAAGGGG CGGATTGCAC ATGCGATCTC TGCATGGGTT   
  
  
+ CCGGCTTG  

- -Up\_Stream \_Len000CTAAAC TAACAGCTTT AAACAGGCTA AAATATTTTA ACTTTTAACA ATAACCTGAT   
  
  
- ATTAGATGAC ATCAAATCTC CACAACCTAT TATGAGAGTT GAAACTTGAA CCTGCAGGTA CGTGGTGTGA   
  
  
- TACCCGCATA AATGACAAAT ATATGGTTCT TATTAAAACT TCTACAAGAA TCAACCACTA CATTCACATA   
  
  
- TAAGTTGTAT ACAGTAATGT ATTTGAACTT TTAAGAACTT AAAATCTGTA TATGTTAACG TTTAGTAACA   
  
  
- TTTTAACAAC GTAATGTAAA ACTATTTTTA CGTAGGTTTG AAACAGAACC ATATTTGTCA TTTATCCGGG   
  
  
- TATCCAGGTA TCACATCACG TACCTGCAGG TTCAAATTTC CACCCTCATA ATAACGGGTT GCGGGGATTT   
  
  
- CATGTCATAT AATATCAGGT TATAGGGTTA ATTAAAAAGT AAACTTATAA ATTTAAACCT GTATTATAAA   
  
  
- AAGTATAAAC TTAAGAGATT TGGACTAAAA TTGGACTTAA ATTCAACTAA ATTAAGTAGG CTGGTTTTTT   
  
  
- TATTTGTGTT AATAAATAAA AAGATAAGCT TCAATCTATC ATCGGTTAAG TAAACTGTAT ATTAATTGGG   
  
  
- CACTAACTGT GTTTGAGCTC AAAAACGAAT CTCGATCCTT TTCTCAGCCC AATCCAGCTG GGTAAATTCT   
  
  
- TTACCCAGCT GAAGTACAGC CGGAAGTTTT ATCAATTCTA AACGAGTTTT GATTTTTTAT TGTTCAAGTG   
  
  
- TGATTTGCCA GTTTAGCACA GCTGAAGCCT AACTTTAACC AAGTCAAACC CAGCCGAAGC ATAAAGAAAG   
  
  
- TCATTGAAGC TTGGCTTAAG CATAGCACAA TAGCACAGTC CAGTCTGAAA GAGTCAAGAT AAAAACGATG   
  
  
- GAATACAATG CCTATTTTTT TCAGAGTGTA ACCTTTTTCA CACCCTCCCA GGACCCGAAT ATTTATCACC   
  
  
- GCGACGTGTG GAGTAGTCTG GCTGGAAAAC CCCTCTCCCA TTACTGGGGC ACTGTTCACC ATAGTCTCAG   
  
  
- TTGGGTCTGG GCTGAGTCTA CAGGACGGTT CGCGAGGCTG CCCGCCCCAT CCGTCCCCCG GGTAAACACT   
  
  
- ACTCCTGCAG TGTCTTAACC CGCCCCCTCT CACAGTGCCT ATTTTTTTCA GGGTGTAGCT TTTTTCACAC   
  
  
- CCTCCCAGGA CCCGAATATT TGCCATCGCG GCGTGTGGGG TAGTCTAGCT GAAAAACCCC TCTCCCATTA   
  
  
- CTGGGGTAAA GTTCACCATA GTCTCGGTTG GGTCTGGGCT GAGGTTACAG GGTGGTTCGC GAGACTACCC   
  
  
- GCCCCATCCG TTCCCCGAGG TATACACTGC TCCCGCAGTG CCTTAACCTA CCCTCTCTCA TGGTGCCTAT   
  
  
- TTTTTTCAGG GTGTAGTCTT TTTCACACCT GAATCGTTGA TCCTTCATTT CAATGTCATA ATCGGCTCTC   
  
  
- GGGTACCTTG CGGACTTTGA CTGTAGTGGT CTGGGTGTAT AAAGTGGTAG TAGACTAGAG GGGCAGTTTC   
  
  
- CTCCTTCGAC TCGTGTCGTT CTTGATCTTC TTGTCCGCCG TAGTGTGGGT TCGGTTTTAA GAGATAAACT   
  
  
- GGAGGGAGAG GAGTAAGGAG ACTCGAAGGA GAAGTGTTAC ATTCTGGTAT TAGGAATTGT GGGAAGAAAA   
  
  
- AATTCGAGTA GAAATGGACG AGACCAAGAG AGAGAGTAGC AGTGATTATC AATGGAGAAA TGAAAGAGAA   
  
  
- GGGACTCCAT ACGAAGTTGA AATTGTATAT ATAGGGTGAA CTAAAGAGAA CAGAAAAACG ATATTATGAA   
  
  
- CAACAAACCG ATAGTAGGGG ACAAAATATG AGTAAAAGAA CGAAAGATAA AGACCCAAAC TTAACCCAAA   
  
  
- GTGAATAGGA GCAGTTAAGA TACCCATTAA TCACTTAACC CAAATATACT TGATCCCAAC GTTTTCATAG   
  
  
- ATGAAAAAGA GAACACTAAT AATCAACTAA TCCCTAAACC GGTTATAATC GTCTTACCCA AGACTTAAAC   
  
  
- GCCTTAAGAG ACTACTACGA GATTTACCCA TACGAATACA ACTATAGGGA CGAATACTAC GTAGGAATCT   
  
  
- AATACGGTTA AACAAGTTAA TGCTTCCGGG TAGACTCCTA GCGTGGAGTG ACTCGGAGGG TTCTGGGAAA   
  
  
- AGACTGGGAA ACCGAATGTC AAAGTGCAGG AGAAGACTCG ACCCGGGACC CCAACTAAGA TTACTACTAT   
  
  
- CACTAAGACC GCTACAAGAG TTCATGTAAT CGGTTTACGA ATACCTTCTT CTGTACCTCC GTTTCGGTAC   
  
  
- GTACAAAGTA CTAGGAAATC GTGAAGTCCG ACGACTCTTT GGGAAAATAC TACGGAATCC CCTCGTTACG   
  
  
- GGTTGAAGAG GACTGGTTGT AGGATATTAA CTAGTAATAA ACCTATCAGG ACTACTTTTA AGAAACTCAA   
  
  
- GTTGACCACT AAAATCACAA TCAGTAAGAC CCAGATCAAG TTGTTTGAGG CAACCTGGCT ATTATCACAG   
  
  
- ACTAAACTCA CTCGTAAAAC TCGGTGGGAA ACAACTTTGT GAAGGTTAAC TTAGTATAGT GGTTGGTAAC   
  
  
- TGGGCAGGAG TTACCAGTAA ACCGAGAAGC CCACGGAATC TACCGTGCCG GAGATTACCA AGCCACTAGA   
  
  
- GTAGGGAACC AAACGGTCAC CTACACTAAT CGCATAAATC CCTCTTTCTC AGGTACTAAG TTAAAGTCTT   
  
  
- TCCCCACCTC CTCCGGTCAT TCAAGGAAGG GTTCTTATTA TTGGAACAAT AACTAGAGCT CTTGGAGTGA   
  
  
- AAAGGATTAC TTTGTTTCCT CCTACTAGCT TACTACTACC AATTCTTCCT TTTCCTACTG CACTTAACCA   
  
  
- GATTGATGAG TTCTCCCTCA TTCTAAGTGA TAACACTTCT GCTCCGGAAA CTTCTTCCTT CCTCACCGTT   
  
  
- CGTCAGTCGA TAAAGAAGAT GACTCCTTCG ACTCAAAAGA CTGTAAAAAC TGTTCCAAAA CGAAACGCTA   
  
  
- ACGATGGGAC ACTTTGGACT CCGAGTAGGG TGGTACTCAA ACTTGGGACT CTTCCCGGTC AGTGTACCTA   
  
  
- ATCTTCCACC CTCTTTACCC TTCCGAGCGG GATTCTTTGT CCTATTATCA TCGTGTTTAT AACACCTAAA   
  
  
- TTCCTTAAAC GACTATGATA CGCGTGTTAG ATGACGTAGA CTACTGGCTT CTTGACGACT ACCTGACGAT   
  
  
- TTCGTTTAGT CCCTCGTGAG CAGACGACTC CCCCTACCTA GAGTTTCCAA CCGCGTAATG AAACGACTAC   
  
  
- GGGATCTCCG TGCAAATCGA CCTTGACCTA GAGCGTAAAT ATGCCGGGAT ACAAGATTAT CCGGTAGACA   
  
  
- GTGACTGTAC TATTTTCGTA TAGTCAAGAT ATAAGCACGA ACGGGTAAAT GCTTCTAGCA GTAACCAACA   
  
  
- CCATGGGTAT ACTAAGATTT TCGTCGACTC TTCCGTAGTT TCGAAGTATA ATATCTAAAA CCGTAGGAGA   
  
  
- TACCAACGGT TACCGGGTTG GAGTAAGTTG CGGAGAGTCT CGCTAGACCA CCTGGAGGTT TTGACAAATA   
  
  
- ATGTCCCTAG CTAGAGGGGG TCGGACCCAA GTCCGGTCGT CTTTCTCACC TTCGTTGTCC CTCTGCGAAC   
  
  
- CGGTTCATGA CACTCGCCAT ATTACACGGT AAACTCATAG TACGGTAACG AGTCCTCACC CTTTGTTAGT   
  
  
- TTGGTCCCCT AGAGTTCTAT CCTTCATCCT TACTACTTCA ACAACGCCAC TTGACAGAGA CATCCAAGTT   
  
  
- CTTGGAGGAA CTGCTCTGTC ACCACCACCT ATCAGGTTCC TTGTGTCAAA ATTTGGACTA ATGTTCCCAT   
  
  
- TTCGGACTAT AAAAACACGT ACCGCAACAT TTACCAAGGA TGTTGTAGGG AAAGAAACAC TGTGCAAAAT   
  
  
- CTCTTCGGGA GAAAGTAATA AGGTGAGAAA AGCTGTACAA TCTACGGTTG CGGAGATCCC TCCTCGGGCT   
  
  
- CTCCAACTAT AAGCTCTTCC GTAAGATACC CTCCCTCTAA TACTTACACC ACCGGACACT CCCGTGTCTC   
  
  
- TCCCACCTCT CCGGCCTCTG TATGTTCGTT ACCGTACAAT CCGTATCGTC CCGTCCCAAA GCCGTTCATG   
  
  
- GTAACCTAGG GTTCAACTAG CTCTTTTACT CCAAATTCCG GTTCCGTCTG GTGGTGTTCC TAAAGTACTA   
  
  
- GCTACACCTA CCTGTAACCC GTTAAGTCCC TACCTTCCCC GCCTAACGTG TACGCTAGAG ACGTACCCAA   
  
  
- GGCCGAAC

+     MYB

| Site Name | Organism | Position | Strand | Matrix score. | sequence | function |
| --- | --- | --- | --- | --- | --- | --- |
| MYB | Arabidopsis thaliana | 3628 | + | 6 | CAACAG |  |
| MYB | Arabidopsis thaliana | 3506 | - | 6 | CAACCA |  |
| MYB | Arabidopsis thaliana | 2586 | + | 6 | CAACCA |  |
| MYB | Arabidopsis thaliana | 3428 | - | 6 | CAACCA |  |
| MYB | Arabidopsis thaliana | 2842 | - | 6 | TAACCA |  |

>HU07G02249.1   
+ -Up\_Stream \_Len000GATTTG ATTGTCGAAA TTTGTCCGAT TTTATAAAAT TGAAAATTGT TATTGGACTA   
  
  
+ TAATCTACTG TAGTTTAGAG GTGTTGGATA ATACTCTCAA CTTTGAACTT GGACGTCCAT GCACCACACT   
  
  
+ ATGGGCGTAT TTACTGTTTA TATACCAAGA ATAATTTTGA AGATGTTCTT AGTTGGTGAT GTAAGTGTAT   
  
  
+ ATTCAACATA TGTCATTACA TAAACTTGAA AATTCTTGAA TTTTAGACAT ATACAATTGC AAATCATTGT   
  
  
+ AAAATTGTTG CATTACATTT TGATAAAAAT GCATCCAAAC TTTGTCTTGG TATAAACAGT AAATAGGCCC   
  
  
+ ATAGGTCCAT AGTGTAGTGC ATGGACGTCC AAGTTTAAAG GTGGGAGTAT TATTGCCCAA CGCCCCTAAA   
  
  
+ GTACAGTATA TTATAGTCCA ATATCCCAAT TAATTTTTCA TTTGAATATT TAAATTTGGA CATAATATTT   
  
  
+ TTCATATTTG AATTCTCTAA ACCTGATTTT AACCTGAATT TAAGTTGATT TAATTCATCC GACCAAAAAA   
  
  
+ ATAAACACAA TTATTTATTT TTCTATTCGA AGTTAGATAG TAGCCAATTC ATTTGACATA TAATTAACCC   
  
  
+ GTGATTGACA CAAACTCGAG TTTTTGCTTA GAGCTAGGAA AAGAGTCGGG TTAGGTCGAC CCATTTAAGA   
  
  
+ AATGGGTCGA CTTCATGTCG GCCTTCAAAA TAGTTAAGAT TTGCTCAAAA CTAAAAAATA ACAAGTTCAC   
  
  
+ ACTAAACGGT CAAATCGTGT CGACTTCGGA TTGAAATTGG TTCAGTTTGG GTCGGCTTCG TATTTCTTTC   
  
  
+ AGTAACTTCG AACCGAATTC GTATCGTGTT ATCGTGTCAG GTCAGACTTT CTCAGTTCTA TTTTTGCTAC   
  
  
+ CTTATGTTAC GGATAAAAAA AGTCTCACAT TGGAAAAAGT GTGGGAGGGT CCTGGGCTTA TAAATAGTGG   
  
  
+ CGCTGCACAC CTCATCAGAC CGACCTTTTG GGGAGAGGGT AATGACCCCG TGACAAGTGG TATCAGAGTC   
  
  
+ AACCCAGACC CGACTCAGAT GTCCTGCCAA GCGCTCCGAC GGGCGGGGTA GGCAGGGGGC CCATTTGTGA   
  
  
+ TGAGGACGTC ACAGAATTGG GCGGGGGAGA GTGTCACGGA TAAAAAAAGT CCCACATCGA AAAAAGTGTG   
  
  
+ GGAGGGTCCT GGGCTTATAA ACGGTAGCGC CGCACACCCC ATCAGATCGA CTTTTTGGGG AGAGGGTAAT   
  
  
+ GACCCCATTT CAAGTGGTAT CAGAGCCAAC CCAGACCCGA CTCCAATGTC CCACCAAGCG CTCTGATGGG   
  
  
+ CGGGGTAGGC AAGGGGCTCC ATATGTGACG AGGGCGTCAC GGAATTGGAT GGGAGAGAGT ACCACGGATA   
  
  
+ AAAAAAGTCC CACATCAGAA AAAGTGTGGA CTTAGCAACT AGGAAGTAAA GTTACAGTAT TAGCCGAGAG   
  
  
+ CCCATGGAAC GCCTGAAACT GACATCACCA GACCCACATA TTTCACCATC ATCTGATCTC CCCGTCAAAG   
  
  
+ GAGGAAGCTG AGCACAGCAA GAACTAGAAG AACAGGCGGC ATCACACCCA AGCCAAAATT CTCTATTTGA   
  
  
+ CCTCCCTCTC CTCATTCCTC TGAGCTTCCT CTTCACAATG TAAGACCATA ATCCTTAACA CCCTTCTTTT   
  
  
+ TTAAGCTCAT CTTTACCTGC TCTGGTTCTC TCTCTCATCG TCACTAATAG TTACCTCTTT ACTTTCTCTT   
  
  
+ CCCTGAGGTA TGCTTCAACT TTAACATATA TATCCCACTT GATTTCTCTT GTCTTTTTGC TATAATACTT   
  
  
+ GTTGTTTGGC TATCATCCCC TGTTTTATAC TCATTTTCTT GCTTTCTATT TCTGGGTTTG AATTGGGTTT   
  
  
+ CACTTATCCT CGTCAATTCT ATGGGTAATT AGTGAATTGG GTTTATATGA ACTAGGGTTG CAAAAGTATC   
  
  
+ TACTTTTTCT CTTGTGATTA TTAGTTGATT AGGGATTTGG CCAATATTAG CAGAATGGGT TCTGAATTTG   
  
  
+ CGGAATTCTC TGATGATGCT CTAAATGGGT ATGCTTATGT TGATATCCCT GCTTATGATG CATCCTTAGA   
  
  
+ TTATGCCAAT TTGTTCAATT ACGAAGGCCC ATCTGAGGAT CGCACCTCAC TGAGCCTCCC AAGACCCTTT   
  
  
+ TCTGACCCTT TGGCTTACAG TTTCACGTCC TCTTCTGAGC TGGGCCCTGG GGTTGATTCT AATGATGATA   
  
  
+ GTGATTCTGG CGATGTTCTC AAGTACATTA GCCAAATGCT TATGGAAGAA GACATGGAGG CAAAGCCATG   
  
  
+ CATGTTTCAT GATCCTTTAG CACTTCAGGC TGCTGAGAAA CCCTTTTATG ATGCCTTAGG GGAGCAATGC   
  
  
+ CCAACTTCTC CTGACCAACA TCCTATAATT GATCATTATT TGGATAGTCC TGATGAAAAT TCTTTGAGTT   
  
  
+ CAACTGGTGA TTTTAGTGTT AGTCATTCTG GGTCTAGTTC AACAAACTCC GTTGGACCGA TAATAGTGTC   
  
  
+ TGATTTGAGT GAGCATTTTG AGCCACCCTT TGTTGAAACA CTTCCAATTG AATCATATCA CCAACCATTG   
  
  
+ ACCCGTCCTC AATGGTCATT TGGCTCTTCG GGTGCCTTAG ATGGCACGGC CTCTAATGGT TCGGTGATCT   
  
  
+ CATCCCTTGG TTTGCCAGTG GATGTGATTA GCGTATTTAG GGAGAAAGAG TCCATGATTC AATTTCAGAA   
  
  
+ AGGGGTGGAG GAGGCCAGTA AGTTCCTTCC CAAGAATAAT AACCTTGTTA TTGATCTCGA GAACCTCACT   
  
  
+ TTTCCTAATG AAACAAAGGA GGATGATCGA ATGATGATGG TTAAGAAGGA AAAGGATGAC GTGAATTGGT   
  
  
+ CTAACTACTC AAGAGGGAGT AAGATTCACT ATTGTGAAGA CGAGGCCTTT GAAGAAGGAA GGAGTGGCAA   
  
  
+ GCAGTCAGCT ATTTCTTCTA CTGAGGAAGC TGAGTTTTCT GACATTTTTG ACAAGGTTTT GCTTTGCGAT   
  
  
+ TGCTACCCTG TGAAACCTGA GGCTCATCCC ACCATGAGTT TGAACCCTGA GAAGGGCCAG TCACATGGAT   
  
  
+ TAGAAGGTGG GAGAAATGGG AAGGCTCGCC CTAAGAAACA GGATAATAGT AGCACAAATA TTGTGGATTT   
  
  
+ AAGGAATTTG CTGATACTAT GCGCACAATC TACTGCATCT GATGACCGAA GAACTGCTGA TGGACTGCTA   
  
  
+ AAGCAAATCA GGGAGCACTC GTCTGCTGAG GGGGATGGAT CTCAAAGGTT GGCGCATTAC TTTGCTGATG   
  
  
+ CCCTAGAGGC ACGTTTAGCT GGAACTGGAT CTCGCATTTA TACGGCCCTA TGTTCTAATA GGCCATCTGT   
  
  
+ CACTGACATG ATAAAAGCAT ATCAGTTCTA TATTCGTGCT TGCCCATTTA CGAAGATCGT CATTGGTTGT   
  
  
+ GGTACCCATA TGATTCTAAA AGCAGCTGAG AAGGCATCAA AGCTTCATAT TATAGATTTT GGCATCCTCT   
  
  
+ ATGGTTGCCA ATGGCCCAAC CTCATTCAAC GCCTCTCAGA GCGATCTGGT GGACCTCCAA AACTGTTTAT   
  
  
+ TACAGGGATC GATCTCCCCC AGCCTGGGTT CAGGCCAGCA GAAAGAGTGG AAGCAACAGG GAGACGCTTG   
  
  
+ GCCAAGTACT GTGAGCGGTA TAATGTGCCA TTTGAGTATC ATGCCATTGC TCAGGAGTGG GAAACAATCA   
  
  
+ AACCAGGGGA TCTCAAGATA GGAAGTAGGA ATGATGAAGT TGTTGCGGTG AACTGTCTCT GTAGGTTCAA   
  
  
+ GAACCTCCTT GACGAGACAG TGGTGGTGGA TAGTCCAAGG AACACAGTTT TAAACCTGAT TACAAGGGTA   
  
  
+ AAGCCTGATA TTTTTGTGCA TGGCGTTGTA AATGGTTCCT ACAACATCCC TTTCTTTGTG ACACGTTTTA   
  
  
+ GAGAAGCCCT CTTTCATTAT TCCACTCTTT TCGACATGTT AGATGCCAAC GCCTCTAGGG AGGAGCCCGA   
  
  
+ GAGGTTGATA TTCGAGAAGG CATTCTATGG GAGGGAGATT ATGAATGTGG TGGCCTGTGA GGGCACAGAG   
  
  
+ AGGGTGGAGA GGCCGGAGAC ATACAAGCAA TGGCATGTTA GGCATAGCAG GGCAGGGTTT CGGCAAGTAC   
  
  
+ CATTGGATCC CAAGTTGATC GAGAAAATGA GGTTTAAGGC CAAGGCAGAC CACCACAAGG ATTTCATGAT   
  
  
+ CGATGTGGAT GGACATTGGG CAATTCAGGG ATGGAAGGGG CGGATTGCAC ATGCGATCTC TGCATGGGTT   
  
  
+ CCGGCTTG  

- -Up\_Stream \_Len000CTAAAC TAACAGCTTT AAACAGGCTA AAATATTTTA ACTTTTAACA ATAACCTGAT   
  
  
- ATTAGATGAC ATCAAATCTC CACAACCTAT TATGAGAGTT GAAACTTGAA CCTGCAGGTA CGTGGTGTGA   
  
  
- TACCCGCATA AATGACAAAT ATATGGTTCT TATTAAAACT TCTACAAGAA TCAACCACTA CATTCACATA   
  
  
- TAAGTTGTAT ACAGTAATGT ATTTGAACTT TTAAGAACTT AAAATCTGTA TATGTTAACG TTTAGTAACA   
  
  
- TTTTAACAAC GTAATGTAAA ACTATTTTTA CGTAGGTTTG AAACAGAACC ATATTTGTCA TTTATCCGGG   
  
  
- TATCCAGGTA TCACATCACG TACCTGCAGG TTCAAATTTC CACCCTCATA ATAACGGGTT GCGGGGATTT   
  
  
- CATGTCATAT AATATCAGGT TATAGGGTTA ATTAAAAAGT AAACTTATAA ATTTAAACCT GTATTATAAA   
  
  
- AAGTATAAAC TTAAGAGATT TGGACTAAAA TTGGACTTAA ATTCAACTAA ATTAAGTAGG CTGGTTTTTT   
  
  
- TATTTGTGTT AATAAATAAA AAGATAAGCT TCAATCTATC ATCGGTTAAG TAAACTGTAT ATTAATTGGG   
  
  
- CACTAACTGT GTTTGAGCTC AAAAACGAAT CTCGATCCTT TTCTCAGCCC AATCCAGCTG GGTAAATTCT   
  
  
- TTACCCAGCT GAAGTACAGC CGGAAGTTTT ATCAATTCTA AACGAGTTTT GATTTTTTAT TGTTCAAGTG   
  
  
- TGATTTGCCA GTTTAGCACA GCTGAAGCCT AACTTTAACC AAGTCAAACC CAGCCGAAGC ATAAAGAAAG   
  
  
- TCATTGAAGC TTGGCTTAAG CATAGCACAA TAGCACAGTC CAGTCTGAAA GAGTCAAGAT AAAAACGATG   
  
  
- GAATACAATG CCTATTTTTT TCAGAGTGTA ACCTTTTTCA CACCCTCCCA GGACCCGAAT ATTTATCACC   
  
  
- GCGACGTGTG GAGTAGTCTG GCTGGAAAAC CCCTCTCCCA TTACTGGGGC ACTGTTCACC ATAGTCTCAG   
  
  
- TTGGGTCTGG GCTGAGTCTA CAGGACGGTT CGCGAGGCTG CCCGCCCCAT CCGTCCCCCG GGTAAACACT   
  
  
- ACTCCTGCAG TGTCTTAACC CGCCCCCTCT CACAGTGCCT ATTTTTTTCA GGGTGTAGCT TTTTTCACAC   
  
  
- CCTCCCAGGA CCCGAATATT TGCCATCGCG GCGTGTGGGG TAGTCTAGCT GAAAAACCCC TCTCCCATTA   
  
  
- CTGGGGTAAA GTTCACCATA GTCTCGGTTG GGTCTGGGCT GAGGTTACAG GGTGGTTCGC GAGACTACCC   
  
  
- GCCCCATCCG TTCCCCGAGG TATACACTGC TCCCGCAGTG CCTTAACCTA CCCTCTCTCA TGGTGCCTAT   
  
  
- TTTTTTCAGG GTGTAGTCTT TTTCACACCT GAATCGTTGA TCCTTCATTT CAATGTCATA ATCGGCTCTC   
  
  
- GGGTACCTTG CGGACTTTGA CTGTAGTGGT CTGGGTGTAT AAAGTGGTAG TAGACTAGAG GGGCAGTTTC   
  
  
- CTCCTTCGAC TCGTGTCGTT CTTGATCTTC TTGTCCGCCG TAGTGTGGGT TCGGTTTTAA GAGATAAACT   
  
  
- GGAGGGAGAG GAGTAAGGAG ACTCGAAGGA GAAGTGTTAC ATTCTGGTAT TAGGAATTGT GGGAAGAAAA   
  
  
- AATTCGAGTA GAAATGGACG AGACCAAGAG AGAGAGTAGC AGTGATTATC AATGGAGAAA TGAAAGAGAA   
  
  
- GGGACTCCAT ACGAAGTTGA AATTGTATAT ATAGGGTGAA CTAAAGAGAA CAGAAAAACG ATATTATGAA   
  
  
- CAACAAACCG ATAGTAGGGG ACAAAATATG AGTAAAAGAA CGAAAGATAA AGACCCAAAC TTAACCCAAA   
  
  
- GTGAATAGGA GCAGTTAAGA TACCCATTAA TCACTTAACC CAAATATACT TGATCCCAAC GTTTTCATAG   
  
  
- ATGAAAAAGA GAACACTAAT AATCAACTAA TCCCTAAACC GGTTATAATC GTCTTACCCA AGACTTAAAC   
  
  
- GCCTTAAGAG ACTACTACGA GATTTACCCA TACGAATACA ACTATAGGGA CGAATACTAC GTAGGAATCT   
  
  
- AATACGGTTA AACAAGTTAA TGCTTCCGGG TAGACTCCTA GCGTGGAGTG ACTCGGAGGG TTCTGGGAAA   
  
  
- AGACTGGGAA ACCGAATGTC AAAGTGCAGG AGAAGACTCG ACCCGGGACC CCAACTAAGA TTACTACTAT   
  
  
- CACTAAGACC GCTACAAGAG TTCATGTAAT CGGTTTACGA ATACCTTCTT CTGTACCTCC GTTTCGGTAC   
  
  
- GTACAAAGTA CTAGGAAATC GTGAAGTCCG ACGACTCTTT GGGAAAATAC TACGGAATCC CCTCGTTACG   
  
  
- GGTTGAAGAG GACTGGTTGT AGGATATTAA CTAGTAATAA ACCTATCAGG ACTACTTTTA AGAAACTCAA   
  
  
- GTTGACCACT AAAATCACAA TCAGTAAGAC CCAGATCAAG TTGTTTGAGG CAACCTGGCT ATTATCACAG   
  
  
- ACTAAACTCA CTCGTAAAAC TCGGTGGGAA ACAACTTTGT GAAGGTTAAC TTAGTATAGT GGTTGGTAAC   
  
  
- TGGGCAGGAG TTACCAGTAA ACCGAGAAGC CCACGGAATC TACCGTGCCG GAGATTACCA AGCCACTAGA   
  
  
- GTAGGGAACC AAACGGTCAC CTACACTAAT CGCATAAATC CCTCTTTCTC AGGTACTAAG TTAAAGTCTT   
  
  
- TCCCCACCTC CTCCGGTCAT TCAAGGAAGG GTTCTTATTA TTGGAACAAT AACTAGAGCT CTTGGAGTGA   
  
  
- AAAGGATTAC TTTGTTTCCT CCTACTAGCT TACTACTACC AATTCTTCCT TTTCCTACTG CACTTAACCA   
  
  
- GATTGATGAG TTCTCCCTCA TTCTAAGTGA TAACACTTCT GCTCCGGAAA CTTCTTCCTT CCTCACCGTT   
  
  
- CGTCAGTCGA TAAAGAAGAT GACTCCTTCG ACTCAAAAGA CTGTAAAAAC TGTTCCAAAA CGAAACGCTA   
  
  
- ACGATGGGAC ACTTTGGACT CCGAGTAGGG TGGTACTCAA ACTTGGGACT CTTCCCGGTC AGTGTACCTA   
  
  
- ATCTTCCACC CTCTTTACCC TTCCGAGCGG GATTCTTTGT CCTATTATCA TCGTGTTTAT AACACCTAAA   
  
  
- TTCCTTAAAC GACTATGATA CGCGTGTTAG ATGACGTAGA CTACTGGCTT CTTGACGACT ACCTGACGAT   
  
  
- TTCGTTTAGT CCCTCGTGAG CAGACGACTC CCCCTACCTA GAGTTTCCAA CCGCGTAATG AAACGACTAC   
  
  
- GGGATCTCCG TGCAAATCGA CCTTGACCTA GAGCGTAAAT ATGCCGGGAT ACAAGATTAT CCGGTAGACA   
  
  
- GTGACTGTAC TATTTTCGTA TAGTCAAGAT ATAAGCACGA ACGGGTAAAT GCTTCTAGCA GTAACCAACA   
  
  
- CCATGGGTAT ACTAAGATTT TCGTCGACTC TTCCGTAGTT TCGAAGTATA ATATCTAAAA CCGTAGGAGA   
  
  
- TACCAACGGT TACCGGGTTG GAGTAAGTTG CGGAGAGTCT CGCTAGACCA CCTGGAGGTT TTGACAAATA   
  
  
- ATGTCCCTAG CTAGAGGGGG TCGGACCCAA GTCCGGTCGT CTTTCTCACC TTCGTTGTCC CTCTGCGAAC   
  
  
- CGGTTCATGA CACTCGCCAT ATTACACGGT AAACTCATAG TACGGTAACG AGTCCTCACC CTTTGTTAGT   
  
  
- TTGGTCCCCT AGAGTTCTAT CCTTCATCCT TACTACTTCA ACAACGCCAC TTGACAGAGA CATCCAAGTT   
  
  
- CTTGGAGGAA CTGCTCTGTC ACCACCACCT ATCAGGTTCC TTGTGTCAAA ATTTGGACTA ATGTTCCCAT   
  
  
- TTCGGACTAT AAAAACACGT ACCGCAACAT TTACCAAGGA TGTTGTAGGG AAAGAAACAC TGTGCAAAAT   
  
  
- CTCTTCGGGA GAAAGTAATA AGGTGAGAAA AGCTGTACAA TCTACGGTTG CGGAGATCCC TCCTCGGGCT   
  
  
- CTCCAACTAT AAGCTCTTCC GTAAGATACC CTCCCTCTAA TACTTACACC ACCGGACACT CCCGTGTCTC   
  
  
- TCCCACCTCT CCGGCCTCTG TATGTTCGTT ACCGTACAAT CCGTATCGTC CCGTCCCAAA GCCGTTCATG   
  
  
- GTAACCTAGG GTTCAACTAG CTCTTTTACT CCAAATTCCG GTTCCGTCTG GTGGTGTTCC TAAAGTACTA   
  
  
- GCTACACCTA CCTGTAACCC GTTAAGTCCC TACCTTCCCC GCCTAACGTG TACGCTAGAG ACGTACCCAA   
  
  
- GGCCGAAC

+     MYB recognition site

| Site Name | Organism | Position | Strand | Matrix score. | sequence | function |
| --- | --- | --- | --- | --- | --- | --- |
| MYB recognition site | Arabidopsis thaliana | 2503 | + | 6 | CCGTTG |  |

>HU07G02249.1   
+ -Up\_Stream \_Len000GATTTG ATTGTCGAAA TTTGTCCGAT TTTATAAAAT TGAAAATTGT TATTGGACTA   
  
  
+ TAATCTACTG TAGTTTAGAG GTGTTGGATA ATACTCTCAA CTTTGAACTT GGACGTCCAT GCACCACACT   
  
  
+ ATGGGCGTAT TTACTGTTTA TATACCAAGA ATAATTTTGA AGATGTTCTT AGTTGGTGAT GTAAGTGTAT   
  
  
+ ATTCAACATA TGTCATTACA TAAACTTGAA AATTCTTGAA TTTTAGACAT ATACAATTGC AAATCATTGT   
  
  
+ AAAATTGTTG CATTACATTT TGATAAAAAT GCATCCAAAC TTTGTCTTGG TATAAACAGT AAATAGGCCC   
  
  
+ ATAGGTCCAT AGTGTAGTGC ATGGACGTCC AAGTTTAAAG GTGGGAGTAT TATTGCCCAA CGCCCCTAAA   
  
  
+ GTACAGTATA TTATAGTCCA ATATCCCAAT TAATTTTTCA TTTGAATATT TAAATTTGGA CATAATATTT   
  
  
+ TTCATATTTG AATTCTCTAA ACCTGATTTT AACCTGAATT TAAGTTGATT TAATTCATCC GACCAAAAAA   
  
  
+ ATAAACACAA TTATTTATTT TTCTATTCGA AGTTAGATAG TAGCCAATTC ATTTGACATA TAATTAACCC   
  
  
+ GTGATTGACA CAAACTCGAG TTTTTGCTTA GAGCTAGGAA AAGAGTCGGG TTAGGTCGAC CCATTTAAGA   
  
  
+ AATGGGTCGA CTTCATGTCG GCCTTCAAAA TAGTTAAGAT TTGCTCAAAA CTAAAAAATA ACAAGTTCAC   
  
  
+ ACTAAACGGT CAAATCGTGT CGACTTCGGA TTGAAATTGG TTCAGTTTGG GTCGGCTTCG TATTTCTTTC   
  
  
+ AGTAACTTCG AACCGAATTC GTATCGTGTT ATCGTGTCAG GTCAGACTTT CTCAGTTCTA TTTTTGCTAC   
  
  
+ CTTATGTTAC GGATAAAAAA AGTCTCACAT TGGAAAAAGT GTGGGAGGGT CCTGGGCTTA TAAATAGTGG   
  
  
+ CGCTGCACAC CTCATCAGAC CGACCTTTTG GGGAGAGGGT AATGACCCCG TGACAAGTGG TATCAGAGTC   
  
  
+ AACCCAGACC CGACTCAGAT GTCCTGCCAA GCGCTCCGAC GGGCGGGGTA GGCAGGGGGC CCATTTGTGA   
  
  
+ TGAGGACGTC ACAGAATTGG GCGGGGGAGA GTGTCACGGA TAAAAAAAGT CCCACATCGA AAAAAGTGTG   
  
  
+ GGAGGGTCCT GGGCTTATAA ACGGTAGCGC CGCACACCCC ATCAGATCGA CTTTTTGGGG AGAGGGTAAT   
  
  
+ GACCCCATTT CAAGTGGTAT CAGAGCCAAC CCAGACCCGA CTCCAATGTC CCACCAAGCG CTCTGATGGG   
  
  
+ CGGGGTAGGC AAGGGGCTCC ATATGTGACG AGGGCGTCAC GGAATTGGAT GGGAGAGAGT ACCACGGATA   
  
  
+ AAAAAAGTCC CACATCAGAA AAAGTGTGGA CTTAGCAACT AGGAAGTAAA GTTACAGTAT TAGCCGAGAG   
  
  
+ CCCATGGAAC GCCTGAAACT GACATCACCA GACCCACATA TTTCACCATC ATCTGATCTC CCCGTCAAAG   
  
  
+ GAGGAAGCTG AGCACAGCAA GAACTAGAAG AACAGGCGGC ATCACACCCA AGCCAAAATT CTCTATTTGA   
  
  
+ CCTCCCTCTC CTCATTCCTC TGAGCTTCCT CTTCACAATG TAAGACCATA ATCCTTAACA CCCTTCTTTT   
  
  
+ TTAAGCTCAT CTTTACCTGC TCTGGTTCTC TCTCTCATCG TCACTAATAG TTACCTCTTT ACTTTCTCTT   
  
  
+ CCCTGAGGTA TGCTTCAACT TTAACATATA TATCCCACTT GATTTCTCTT GTCTTTTTGC TATAATACTT   
  
  
+ GTTGTTTGGC TATCATCCCC TGTTTTATAC TCATTTTCTT GCTTTCTATT TCTGGGTTTG AATTGGGTTT   
  
  
+ CACTTATCCT CGTCAATTCT ATGGGTAATT AGTGAATTGG GTTTATATGA ACTAGGGTTG CAAAAGTATC   
  
  
+ TACTTTTTCT CTTGTGATTA TTAGTTGATT AGGGATTTGG CCAATATTAG CAGAATGGGT TCTGAATTTG   
  
  
+ CGGAATTCTC TGATGATGCT CTAAATGGGT ATGCTTATGT TGATATCCCT GCTTATGATG CATCCTTAGA   
  
  
+ TTATGCCAAT TTGTTCAATT ACGAAGGCCC ATCTGAGGAT CGCACCTCAC TGAGCCTCCC AAGACCCTTT   
  
  
+ TCTGACCCTT TGGCTTACAG TTTCACGTCC TCTTCTGAGC TGGGCCCTGG GGTTGATTCT AATGATGATA   
  
  
+ GTGATTCTGG CGATGTTCTC AAGTACATTA GCCAAATGCT TATGGAAGAA GACATGGAGG CAAAGCCATG   
  
  
+ CATGTTTCAT GATCCTTTAG CACTTCAGGC TGCTGAGAAA CCCTTTTATG ATGCCTTAGG GGAGCAATGC   
  
  
+ CCAACTTCTC CTGACCAACA TCCTATAATT GATCATTATT TGGATAGTCC TGATGAAAAT TCTTTGAGTT   
  
  
+ CAACTGGTGA TTTTAGTGTT AGTCATTCTG GGTCTAGTTC AACAAACTCC GTTGGACCGA TAATAGTGTC   
  
  
+ TGATTTGAGT GAGCATTTTG AGCCACCCTT TGTTGAAACA CTTCCAATTG AATCATATCA CCAACCATTG   
  
  
+ ACCCGTCCTC AATGGTCATT TGGCTCTTCG GGTGCCTTAG ATGGCACGGC CTCTAATGGT TCGGTGATCT   
  
  
+ CATCCCTTGG TTTGCCAGTG GATGTGATTA GCGTATTTAG GGAGAAAGAG TCCATGATTC AATTTCAGAA   
  
  
+ AGGGGTGGAG GAGGCCAGTA AGTTCCTTCC CAAGAATAAT AACCTTGTTA TTGATCTCGA GAACCTCACT   
  
  
+ TTTCCTAATG AAACAAAGGA GGATGATCGA ATGATGATGG TTAAGAAGGA AAAGGATGAC GTGAATTGGT   
  
  
+ CTAACTACTC AAGAGGGAGT AAGATTCACT ATTGTGAAGA CGAGGCCTTT GAAGAAGGAA GGAGTGGCAA   
  
  
+ GCAGTCAGCT ATTTCTTCTA CTGAGGAAGC TGAGTTTTCT GACATTTTTG ACAAGGTTTT GCTTTGCGAT   
  
  
+ TGCTACCCTG TGAAACCTGA GGCTCATCCC ACCATGAGTT TGAACCCTGA GAAGGGCCAG TCACATGGAT   
  
  
+ TAGAAGGTGG GAGAAATGGG AAGGCTCGCC CTAAGAAACA GGATAATAGT AGCACAAATA TTGTGGATTT   
  
  
+ AAGGAATTTG CTGATACTAT GCGCACAATC TACTGCATCT GATGACCGAA GAACTGCTGA TGGACTGCTA   
  
  
+ AAGCAAATCA GGGAGCACTC GTCTGCTGAG GGGGATGGAT CTCAAAGGTT GGCGCATTAC TTTGCTGATG   
  
  
+ CCCTAGAGGC ACGTTTAGCT GGAACTGGAT CTCGCATTTA TACGGCCCTA TGTTCTAATA GGCCATCTGT   
  
  
+ CACTGACATG ATAAAAGCAT ATCAGTTCTA TATTCGTGCT TGCCCATTTA CGAAGATCGT CATTGGTTGT   
  
  
+ GGTACCCATA TGATTCTAAA AGCAGCTGAG AAGGCATCAA AGCTTCATAT TATAGATTTT GGCATCCTCT   
  
  
+ ATGGTTGCCA ATGGCCCAAC CTCATTCAAC GCCTCTCAGA GCGATCTGGT GGACCTCCAA AACTGTTTAT   
  
  
+ TACAGGGATC GATCTCCCCC AGCCTGGGTT CAGGCCAGCA GAAAGAGTGG AAGCAACAGG GAGACGCTTG   
  
  
+ GCCAAGTACT GTGAGCGGTA TAATGTGCCA TTTGAGTATC ATGCCATTGC TCAGGAGTGG GAAACAATCA   
  
  
+ AACCAGGGGA TCTCAAGATA GGAAGTAGGA ATGATGAAGT TGTTGCGGTG AACTGTCTCT GTAGGTTCAA   
  
  
+ GAACCTCCTT GACGAGACAG TGGTGGTGGA TAGTCCAAGG AACACAGTTT TAAACCTGAT TACAAGGGTA   
  
  
+ AAGCCTGATA TTTTTGTGCA TGGCGTTGTA AATGGTTCCT ACAACATCCC TTTCTTTGTG ACACGTTTTA   
  
  
+ GAGAAGCCCT CTTTCATTAT TCCACTCTTT TCGACATGTT AGATGCCAAC GCCTCTAGGG AGGAGCCCGA   
  
  
+ GAGGTTGATA TTCGAGAAGG CATTCTATGG GAGGGAGATT ATGAATGTGG TGGCCTGTGA GGGCACAGAG   
  
  
+ AGGGTGGAGA GGCCGGAGAC ATACAAGCAA TGGCATGTTA GGCATAGCAG GGCAGGGTTT CGGCAAGTAC   
  
  
+ CATTGGATCC CAAGTTGATC GAGAAAATGA GGTTTAAGGC CAAGGCAGAC CACCACAAGG ATTTCATGAT   
  
  
+ CGATGTGGAT GGACATTGGG CAATTCAGGG ATGGAAGGGG CGGATTGCAC ATGCGATCTC TGCATGGGTT   
  
  
+ CCGGCTTG  

- -Up\_Stream \_Len000CTAAAC TAACAGCTTT AAACAGGCTA AAATATTTTA ACTTTTAACA ATAACCTGAT   
  
  
- ATTAGATGAC ATCAAATCTC CACAACCTAT TATGAGAGTT GAAACTTGAA CCTGCAGGTA CGTGGTGTGA   
  
  
- TACCCGCATA AATGACAAAT ATATGGTTCT TATTAAAACT TCTACAAGAA TCAACCACTA CATTCACATA   
  
  
- TAAGTTGTAT ACAGTAATGT ATTTGAACTT TTAAGAACTT AAAATCTGTA TATGTTAACG TTTAGTAACA   
  
  
- TTTTAACAAC GTAATGTAAA ACTATTTTTA CGTAGGTTTG AAACAGAACC ATATTTGTCA TTTATCCGGG   
  
  
- TATCCAGGTA TCACATCACG TACCTGCAGG TTCAAATTTC CACCCTCATA ATAACGGGTT GCGGGGATTT   
  
  
- CATGTCATAT AATATCAGGT TATAGGGTTA ATTAAAAAGT AAACTTATAA ATTTAAACCT GTATTATAAA   
  
  
- AAGTATAAAC TTAAGAGATT TGGACTAAAA TTGGACTTAA ATTCAACTAA ATTAAGTAGG CTGGTTTTTT   
  
  
- TATTTGTGTT AATAAATAAA AAGATAAGCT TCAATCTATC ATCGGTTAAG TAAACTGTAT ATTAATTGGG   
  
  
- CACTAACTGT GTTTGAGCTC AAAAACGAAT CTCGATCCTT TTCTCAGCCC AATCCAGCTG GGTAAATTCT   
  
  
- TTACCCAGCT GAAGTACAGC CGGAAGTTTT ATCAATTCTA AACGAGTTTT GATTTTTTAT TGTTCAAGTG   
  
  
- TGATTTGCCA GTTTAGCACA GCTGAAGCCT AACTTTAACC AAGTCAAACC CAGCCGAAGC ATAAAGAAAG   
  
  
- TCATTGAAGC TTGGCTTAAG CATAGCACAA TAGCACAGTC CAGTCTGAAA GAGTCAAGAT AAAAACGATG   
  
  
- GAATACAATG CCTATTTTTT TCAGAGTGTA ACCTTTTTCA CACCCTCCCA GGACCCGAAT ATTTATCACC   
  
  
- GCGACGTGTG GAGTAGTCTG GCTGGAAAAC CCCTCTCCCA TTACTGGGGC ACTGTTCACC ATAGTCTCAG   
  
  
- TTGGGTCTGG GCTGAGTCTA CAGGACGGTT CGCGAGGCTG CCCGCCCCAT CCGTCCCCCG GGTAAACACT   
  
  
- ACTCCTGCAG TGTCTTAACC CGCCCCCTCT CACAGTGCCT ATTTTTTTCA GGGTGTAGCT TTTTTCACAC   
  
  
- CCTCCCAGGA CCCGAATATT TGCCATCGCG GCGTGTGGGG TAGTCTAGCT GAAAAACCCC TCTCCCATTA   
  
  
- CTGGGGTAAA GTTCACCATA GTCTCGGTTG GGTCTGGGCT GAGGTTACAG GGTGGTTCGC GAGACTACCC   
  
  
- GCCCCATCCG TTCCCCGAGG TATACACTGC TCCCGCAGTG CCTTAACCTA CCCTCTCTCA TGGTGCCTAT   
  
  
- TTTTTTCAGG GTGTAGTCTT TTTCACACCT GAATCGTTGA TCCTTCATTT CAATGTCATA ATCGGCTCTC   
  
  
- GGGTACCTTG CGGACTTTGA CTGTAGTGGT CTGGGTGTAT AAAGTGGTAG TAGACTAGAG GGGCAGTTTC   
  
  
- CTCCTTCGAC TCGTGTCGTT CTTGATCTTC TTGTCCGCCG TAGTGTGGGT TCGGTTTTAA GAGATAAACT   
  
  
- GGAGGGAGAG GAGTAAGGAG ACTCGAAGGA GAAGTGTTAC ATTCTGGTAT TAGGAATTGT GGGAAGAAAA   
  
  
- AATTCGAGTA GAAATGGACG AGACCAAGAG AGAGAGTAGC AGTGATTATC AATGGAGAAA TGAAAGAGAA   
  
  
- GGGACTCCAT ACGAAGTTGA AATTGTATAT ATAGGGTGAA CTAAAGAGAA CAGAAAAACG ATATTATGAA   
  
  
- CAACAAACCG ATAGTAGGGG ACAAAATATG AGTAAAAGAA CGAAAGATAA AGACCCAAAC TTAACCCAAA   
  
  
- GTGAATAGGA GCAGTTAAGA TACCCATTAA TCACTTAACC CAAATATACT TGATCCCAAC GTTTTCATAG   
  
  
- ATGAAAAAGA GAACACTAAT AATCAACTAA TCCCTAAACC GGTTATAATC GTCTTACCCA AGACTTAAAC   
  
  
- GCCTTAAGAG ACTACTACGA GATTTACCCA TACGAATACA ACTATAGGGA CGAATACTAC GTAGGAATCT   
  
  
- AATACGGTTA AACAAGTTAA TGCTTCCGGG TAGACTCCTA GCGTGGAGTG ACTCGGAGGG TTCTGGGAAA   
  
  
- AGACTGGGAA ACCGAATGTC AAAGTGCAGG AGAAGACTCG ACCCGGGACC CCAACTAAGA TTACTACTAT   
  
  
- CACTAAGACC GCTACAAGAG TTCATGTAAT CGGTTTACGA ATACCTTCTT CTGTACCTCC GTTTCGGTAC   
  
  
- GTACAAAGTA CTAGGAAATC GTGAAGTCCG ACGACTCTTT GGGAAAATAC TACGGAATCC CCTCGTTACG   
  
  
- GGTTGAAGAG GACTGGTTGT AGGATATTAA CTAGTAATAA ACCTATCAGG ACTACTTTTA AGAAACTCAA   
  
  
- GTTGACCACT AAAATCACAA TCAGTAAGAC CCAGATCAAG TTGTTTGAGG CAACCTGGCT ATTATCACAG   
  
  
- ACTAAACTCA CTCGTAAAAC TCGGTGGGAA ACAACTTTGT GAAGGTTAAC TTAGTATAGT GGTTGGTAAC   
  
  
- TGGGCAGGAG TTACCAGTAA ACCGAGAAGC CCACGGAATC TACCGTGCCG GAGATTACCA AGCCACTAGA   
  
  
- GTAGGGAACC AAACGGTCAC CTACACTAAT CGCATAAATC CCTCTTTCTC AGGTACTAAG TTAAAGTCTT   
  
  
- TCCCCACCTC CTCCGGTCAT TCAAGGAAGG GTTCTTATTA TTGGAACAAT AACTAGAGCT CTTGGAGTGA   
  
  
- AAAGGATTAC TTTGTTTCCT CCTACTAGCT TACTACTACC AATTCTTCCT TTTCCTACTG CACTTAACCA   
  
  
- GATTGATGAG TTCTCCCTCA TTCTAAGTGA TAACACTTCT GCTCCGGAAA CTTCTTCCTT CCTCACCGTT   
  
  
- CGTCAGTCGA TAAAGAAGAT GACTCCTTCG ACTCAAAAGA CTGTAAAAAC TGTTCCAAAA CGAAACGCTA   
  
  
- ACGATGGGAC ACTTTGGACT CCGAGTAGGG TGGTACTCAA ACTTGGGACT CTTCCCGGTC AGTGTACCTA   
  
  
- ATCTTCCACC CTCTTTACCC TTCCGAGCGG GATTCTTTGT CCTATTATCA TCGTGTTTAT AACACCTAAA   
  
  
- TTCCTTAAAC GACTATGATA CGCGTGTTAG ATGACGTAGA CTACTGGCTT CTTGACGACT ACCTGACGAT   
  
  
- TTCGTTTAGT CCCTCGTGAG CAGACGACTC CCCCTACCTA GAGTTTCCAA CCGCGTAATG AAACGACTAC   
  
  
- GGGATCTCCG TGCAAATCGA CCTTGACCTA GAGCGTAAAT ATGCCGGGAT ACAAGATTAT CCGGTAGACA   
  
  
- GTGACTGTAC TATTTTCGTA TAGTCAAGAT ATAAGCACGA ACGGGTAAAT GCTTCTAGCA GTAACCAACA   
  
  
- CCATGGGTAT ACTAAGATTT TCGTCGACTC TTCCGTAGTT TCGAAGTATA ATATCTAAAA CCGTAGGAGA   
  
  
- TACCAACGGT TACCGGGTTG GAGTAAGTTG CGGAGAGTCT CGCTAGACCA CCTGGAGGTT TTGACAAATA   
  
  
- ATGTCCCTAG CTAGAGGGGG TCGGACCCAA GTCCGGTCGT CTTTCTCACC TTCGTTGTCC CTCTGCGAAC   
  
  
- CGGTTCATGA CACTCGCCAT ATTACACGGT AAACTCATAG TACGGTAACG AGTCCTCACC CTTTGTTAGT   
  
  
- TTGGTCCCCT AGAGTTCTAT CCTTCATCCT TACTACTTCA ACAACGCCAC TTGACAGAGA CATCCAAGTT   
  
  
- CTTGGAGGAA CTGCTCTGTC ACCACCACCT ATCAGGTTCC TTGTGTCAAA ATTTGGACTA ATGTTCCCAT   
  
  
- TTCGGACTAT AAAAACACGT ACCGCAACAT TTACCAAGGA TGTTGTAGGG AAAGAAACAC TGTGCAAAAT   
  
  
- CTCTTCGGGA GAAAGTAATA AGGTGAGAAA AGCTGTACAA TCTACGGTTG CGGAGATCCC TCCTCGGGCT   
  
  
- CTCCAACTAT AAGCTCTTCC GTAAGATACC CTCCCTCTAA TACTTACACC ACCGGACACT CCCGTGTCTC   
  
  
- TCCCACCTCT CCGGCCTCTG TATGTTCGTT ACCGTACAAT CCGTATCGTC CCGTCCCAAA GCCGTTCATG   
  
  
- GTAACCTAGG GTTCAACTAG CTCTTTTACT CCAAATTCCG GTTCCGTCTG GTGGTGTTCC TAAAGTACTA   
  
  
- GCTACACCTA CCTGTAACCC GTTAAGTCCC TACCTTCCCC GCCTAACGTG TACGCTAGAG ACGTACCCAA   
  
  
- GGCCGAAC

+     MYB-like sequence

| Site Name | Organism | Position | Strand | Matrix score. | sequence | function |
| --- | --- | --- | --- | --- | --- | --- |
| MYB-like sequence | Arabidopsis thaliana | 2842 | - | 6 | TAACCA |  |

>HU07G02249.1   
+ -Up\_Stream \_Len000GATTTG ATTGTCGAAA TTTGTCCGAT TTTATAAAAT TGAAAATTGT TATTGGACTA   
  
  
+ TAATCTACTG TAGTTTAGAG GTGTTGGATA ATACTCTCAA CTTTGAACTT GGACGTCCAT GCACCACACT   
  
  
+ ATGGGCGTAT TTACTGTTTA TATACCAAGA ATAATTTTGA AGATGTTCTT AGTTGGTGAT GTAAGTGTAT   
  
  
+ ATTCAACATA TGTCATTACA TAAACTTGAA AATTCTTGAA TTTTAGACAT ATACAATTGC AAATCATTGT   
  
  
+ AAAATTGTTG CATTACATTT TGATAAAAAT GCATCCAAAC TTTGTCTTGG TATAAACAGT AAATAGGCCC   
  
  
+ ATAGGTCCAT AGTGTAGTGC ATGGACGTCC AAGTTTAAAG GTGGGAGTAT TATTGCCCAA CGCCCCTAAA   
  
  
+ GTACAGTATA TTATAGTCCA ATATCCCAAT TAATTTTTCA TTTGAATATT TAAATTTGGA CATAATATTT   
  
  
+ TTCATATTTG AATTCTCTAA ACCTGATTTT AACCTGAATT TAAGTTGATT TAATTCATCC GACCAAAAAA   
  
  
+ ATAAACACAA TTATTTATTT TTCTATTCGA AGTTAGATAG TAGCCAATTC ATTTGACATA TAATTAACCC   
  
  
+ GTGATTGACA CAAACTCGAG TTTTTGCTTA GAGCTAGGAA AAGAGTCGGG TTAGGTCGAC CCATTTAAGA   
  
  
+ AATGGGTCGA CTTCATGTCG GCCTTCAAAA TAGTTAAGAT TTGCTCAAAA CTAAAAAATA ACAAGTTCAC   
  
  
+ ACTAAACGGT CAAATCGTGT CGACTTCGGA TTGAAATTGG TTCAGTTTGG GTCGGCTTCG TATTTCTTTC   
  
  
+ AGTAACTTCG AACCGAATTC GTATCGTGTT ATCGTGTCAG GTCAGACTTT CTCAGTTCTA TTTTTGCTAC   
  
  
+ CTTATGTTAC GGATAAAAAA AGTCTCACAT TGGAAAAAGT GTGGGAGGGT CCTGGGCTTA TAAATAGTGG   
  
  
+ CGCTGCACAC CTCATCAGAC CGACCTTTTG GGGAGAGGGT AATGACCCCG TGACAAGTGG TATCAGAGTC   
  
  
+ AACCCAGACC CGACTCAGAT GTCCTGCCAA GCGCTCCGAC GGGCGGGGTA GGCAGGGGGC CCATTTGTGA   
  
  
+ TGAGGACGTC ACAGAATTGG GCGGGGGAGA GTGTCACGGA TAAAAAAAGT CCCACATCGA AAAAAGTGTG   
  
  
+ GGAGGGTCCT GGGCTTATAA ACGGTAGCGC CGCACACCCC ATCAGATCGA CTTTTTGGGG AGAGGGTAAT   
  
  
+ GACCCCATTT CAAGTGGTAT CAGAGCCAAC CCAGACCCGA CTCCAATGTC CCACCAAGCG CTCTGATGGG   
  
  
+ CGGGGTAGGC AAGGGGCTCC ATATGTGACG AGGGCGTCAC GGAATTGGAT GGGAGAGAGT ACCACGGATA   
  
  
+ AAAAAAGTCC CACATCAGAA AAAGTGTGGA CTTAGCAACT AGGAAGTAAA GTTACAGTAT TAGCCGAGAG   
  
  
+ CCCATGGAAC GCCTGAAACT GACATCACCA GACCCACATA TTTCACCATC ATCTGATCTC CCCGTCAAAG   
  
  
+ GAGGAAGCTG AGCACAGCAA GAACTAGAAG AACAGGCGGC ATCACACCCA AGCCAAAATT CTCTATTTGA   
  
  
+ CCTCCCTCTC CTCATTCCTC TGAGCTTCCT CTTCACAATG TAAGACCATA ATCCTTAACA CCCTTCTTTT   
  
  
+ TTAAGCTCAT CTTTACCTGC TCTGGTTCTC TCTCTCATCG TCACTAATAG TTACCTCTTT ACTTTCTCTT   
  
  
+ CCCTGAGGTA TGCTTCAACT TTAACATATA TATCCCACTT GATTTCTCTT GTCTTTTTGC TATAATACTT   
  
  
+ GTTGTTTGGC TATCATCCCC TGTTTTATAC TCATTTTCTT GCTTTCTATT TCTGGGTTTG AATTGGGTTT   
  
  
+ CACTTATCCT CGTCAATTCT ATGGGTAATT AGTGAATTGG GTTTATATGA ACTAGGGTTG CAAAAGTATC   
  
  
+ TACTTTTTCT CTTGTGATTA TTAGTTGATT AGGGATTTGG CCAATATTAG CAGAATGGGT TCTGAATTTG   
  
  
+ CGGAATTCTC TGATGATGCT CTAAATGGGT ATGCTTATGT TGATATCCCT GCTTATGATG CATCCTTAGA   
  
  
+ TTATGCCAAT TTGTTCAATT ACGAAGGCCC ATCTGAGGAT CGCACCTCAC TGAGCCTCCC AAGACCCTTT   
  
  
+ TCTGACCCTT TGGCTTACAG TTTCACGTCC TCTTCTGAGC TGGGCCCTGG GGTTGATTCT AATGATGATA   
  
  
+ GTGATTCTGG CGATGTTCTC AAGTACATTA GCCAAATGCT TATGGAAGAA GACATGGAGG CAAAGCCATG   
  
  
+ CATGTTTCAT GATCCTTTAG CACTTCAGGC TGCTGAGAAA CCCTTTTATG ATGCCTTAGG GGAGCAATGC   
  
  
+ CCAACTTCTC CTGACCAACA TCCTATAATT GATCATTATT TGGATAGTCC TGATGAAAAT TCTTTGAGTT   
  
  
+ CAACTGGTGA TTTTAGTGTT AGTCATTCTG GGTCTAGTTC AACAAACTCC GTTGGACCGA TAATAGTGTC   
  
  
+ TGATTTGAGT GAGCATTTTG AGCCACCCTT TGTTGAAACA CTTCCAATTG AATCATATCA CCAACCATTG   
  
  
+ ACCCGTCCTC AATGGTCATT TGGCTCTTCG GGTGCCTTAG ATGGCACGGC CTCTAATGGT TCGGTGATCT   
  
  
+ CATCCCTTGG TTTGCCAGTG GATGTGATTA GCGTATTTAG GGAGAAAGAG TCCATGATTC AATTTCAGAA   
  
  
+ AGGGGTGGAG GAGGCCAGTA AGTTCCTTCC CAAGAATAAT AACCTTGTTA TTGATCTCGA GAACCTCACT   
  
  
+ TTTCCTAATG AAACAAAGGA GGATGATCGA ATGATGATGG TTAAGAAGGA AAAGGATGAC GTGAATTGGT   
  
  
+ CTAACTACTC AAGAGGGAGT AAGATTCACT ATTGTGAAGA CGAGGCCTTT GAAGAAGGAA GGAGTGGCAA   
  
  
+ GCAGTCAGCT ATTTCTTCTA CTGAGGAAGC TGAGTTTTCT GACATTTTTG ACAAGGTTTT GCTTTGCGAT   
  
  
+ TGCTACCCTG TGAAACCTGA GGCTCATCCC ACCATGAGTT TGAACCCTGA GAAGGGCCAG TCACATGGAT   
  
  
+ TAGAAGGTGG GAGAAATGGG AAGGCTCGCC CTAAGAAACA GGATAATAGT AGCACAAATA TTGTGGATTT   
  
  
+ AAGGAATTTG CTGATACTAT GCGCACAATC TACTGCATCT GATGACCGAA GAACTGCTGA TGGACTGCTA   
  
  
+ AAGCAAATCA GGGAGCACTC GTCTGCTGAG GGGGATGGAT CTCAAAGGTT GGCGCATTAC TTTGCTGATG   
  
  
+ CCCTAGAGGC ACGTTTAGCT GGAACTGGAT CTCGCATTTA TACGGCCCTA TGTTCTAATA GGCCATCTGT   
  
  
+ CACTGACATG ATAAAAGCAT ATCAGTTCTA TATTCGTGCT TGCCCATTTA CGAAGATCGT CATTGGTTGT   
  
  
+ GGTACCCATA TGATTCTAAA AGCAGCTGAG AAGGCATCAA AGCTTCATAT TATAGATTTT GGCATCCTCT   
  
  
+ ATGGTTGCCA ATGGCCCAAC CTCATTCAAC GCCTCTCAGA GCGATCTGGT GGACCTCCAA AACTGTTTAT   
  
  
+ TACAGGGATC GATCTCCCCC AGCCTGGGTT CAGGCCAGCA GAAAGAGTGG AAGCAACAGG GAGACGCTTG   
  
  
+ GCCAAGTACT GTGAGCGGTA TAATGTGCCA TTTGAGTATC ATGCCATTGC TCAGGAGTGG GAAACAATCA   
  
  
+ AACCAGGGGA TCTCAAGATA GGAAGTAGGA ATGATGAAGT TGTTGCGGTG AACTGTCTCT GTAGGTTCAA   
  
  
+ GAACCTCCTT GACGAGACAG TGGTGGTGGA TAGTCCAAGG AACACAGTTT TAAACCTGAT TACAAGGGTA   
  
  
+ AAGCCTGATA TTTTTGTGCA TGGCGTTGTA AATGGTTCCT ACAACATCCC TTTCTTTGTG ACACGTTTTA   
  
  
+ GAGAAGCCCT CTTTCATTAT TCCACTCTTT TCGACATGTT AGATGCCAAC GCCTCTAGGG AGGAGCCCGA   
  
  
+ GAGGTTGATA TTCGAGAAGG CATTCTATGG GAGGGAGATT ATGAATGTGG TGGCCTGTGA GGGCACAGAG   
  
  
+ AGGGTGGAGA GGCCGGAGAC ATACAAGCAA TGGCATGTTA GGCATAGCAG GGCAGGGTTT CGGCAAGTAC   
  
  
+ CATTGGATCC CAAGTTGATC GAGAAAATGA GGTTTAAGGC CAAGGCAGAC CACCACAAGG ATTTCATGAT   
  
  
+ CGATGTGGAT GGACATTGGG CAATTCAGGG ATGGAAGGGG CGGATTGCAC ATGCGATCTC TGCATGGGTT   
  
  
+ CCGGCTTG  

- -Up\_Stream \_Len000CTAAAC TAACAGCTTT AAACAGGCTA AAATATTTTA ACTTTTAACA ATAACCTGAT   
  
  
- ATTAGATGAC ATCAAATCTC CACAACCTAT TATGAGAGTT GAAACTTGAA CCTGCAGGTA CGTGGTGTGA   
  
  
- TACCCGCATA AATGACAAAT ATATGGTTCT TATTAAAACT TCTACAAGAA TCAACCACTA CATTCACATA   
  
  
- TAAGTTGTAT ACAGTAATGT ATTTGAACTT TTAAGAACTT AAAATCTGTA TATGTTAACG TTTAGTAACA   
  
  
- TTTTAACAAC GTAATGTAAA ACTATTTTTA CGTAGGTTTG AAACAGAACC ATATTTGTCA TTTATCCGGG   
  
  
- TATCCAGGTA TCACATCACG TACCTGCAGG TTCAAATTTC CACCCTCATA ATAACGGGTT GCGGGGATTT   
  
  
- CATGTCATAT AATATCAGGT TATAGGGTTA ATTAAAAAGT AAACTTATAA ATTTAAACCT GTATTATAAA   
  
  
- AAGTATAAAC TTAAGAGATT TGGACTAAAA TTGGACTTAA ATTCAACTAA ATTAAGTAGG CTGGTTTTTT   
  
  
- TATTTGTGTT AATAAATAAA AAGATAAGCT TCAATCTATC ATCGGTTAAG TAAACTGTAT ATTAATTGGG   
  
  
- CACTAACTGT GTTTGAGCTC AAAAACGAAT CTCGATCCTT TTCTCAGCCC AATCCAGCTG GGTAAATTCT   
  
  
- TTACCCAGCT GAAGTACAGC CGGAAGTTTT ATCAATTCTA AACGAGTTTT GATTTTTTAT TGTTCAAGTG   
  
  
- TGATTTGCCA GTTTAGCACA GCTGAAGCCT AACTTTAACC AAGTCAAACC CAGCCGAAGC ATAAAGAAAG   
  
  
- TCATTGAAGC TTGGCTTAAG CATAGCACAA TAGCACAGTC CAGTCTGAAA GAGTCAAGAT AAAAACGATG   
  
  
- GAATACAATG CCTATTTTTT TCAGAGTGTA ACCTTTTTCA CACCCTCCCA GGACCCGAAT ATTTATCACC   
  
  
- GCGACGTGTG GAGTAGTCTG GCTGGAAAAC CCCTCTCCCA TTACTGGGGC ACTGTTCACC ATAGTCTCAG   
  
  
- TTGGGTCTGG GCTGAGTCTA CAGGACGGTT CGCGAGGCTG CCCGCCCCAT CCGTCCCCCG GGTAAACACT   
  
  
- ACTCCTGCAG TGTCTTAACC CGCCCCCTCT CACAGTGCCT ATTTTTTTCA GGGTGTAGCT TTTTTCACAC   
  
  
- CCTCCCAGGA CCCGAATATT TGCCATCGCG GCGTGTGGGG TAGTCTAGCT GAAAAACCCC TCTCCCATTA   
  
  
- CTGGGGTAAA GTTCACCATA GTCTCGGTTG GGTCTGGGCT GAGGTTACAG GGTGGTTCGC GAGACTACCC   
  
  
- GCCCCATCCG TTCCCCGAGG TATACACTGC TCCCGCAGTG CCTTAACCTA CCCTCTCTCA TGGTGCCTAT   
  
  
- TTTTTTCAGG GTGTAGTCTT TTTCACACCT GAATCGTTGA TCCTTCATTT CAATGTCATA ATCGGCTCTC   
  
  
- GGGTACCTTG CGGACTTTGA CTGTAGTGGT CTGGGTGTAT AAAGTGGTAG TAGACTAGAG GGGCAGTTTC   
  
  
- CTCCTTCGAC TCGTGTCGTT CTTGATCTTC TTGTCCGCCG TAGTGTGGGT TCGGTTTTAA GAGATAAACT   
  
  
- GGAGGGAGAG GAGTAAGGAG ACTCGAAGGA GAAGTGTTAC ATTCTGGTAT TAGGAATTGT GGGAAGAAAA   
  
  
- AATTCGAGTA GAAATGGACG AGACCAAGAG AGAGAGTAGC AGTGATTATC AATGGAGAAA TGAAAGAGAA   
  
  
- GGGACTCCAT ACGAAGTTGA AATTGTATAT ATAGGGTGAA CTAAAGAGAA CAGAAAAACG ATATTATGAA   
  
  
- CAACAAACCG ATAGTAGGGG ACAAAATATG AGTAAAAGAA CGAAAGATAA AGACCCAAAC TTAACCCAAA   
  
  
- GTGAATAGGA GCAGTTAAGA TACCCATTAA TCACTTAACC CAAATATACT TGATCCCAAC GTTTTCATAG   
  
  
- ATGAAAAAGA GAACACTAAT AATCAACTAA TCCCTAAACC GGTTATAATC GTCTTACCCA AGACTTAAAC   
  
  
- GCCTTAAGAG ACTACTACGA GATTTACCCA TACGAATACA ACTATAGGGA CGAATACTAC GTAGGAATCT   
  
  
- AATACGGTTA AACAAGTTAA TGCTTCCGGG TAGACTCCTA GCGTGGAGTG ACTCGGAGGG TTCTGGGAAA   
  
  
- AGACTGGGAA ACCGAATGTC AAAGTGCAGG AGAAGACTCG ACCCGGGACC CCAACTAAGA TTACTACTAT   
  
  
- CACTAAGACC GCTACAAGAG TTCATGTAAT CGGTTTACGA ATACCTTCTT CTGTACCTCC GTTTCGGTAC   
  
  
- GTACAAAGTA CTAGGAAATC GTGAAGTCCG ACGACTCTTT GGGAAAATAC TACGGAATCC CCTCGTTACG   
  
  
- GGTTGAAGAG GACTGGTTGT AGGATATTAA CTAGTAATAA ACCTATCAGG ACTACTTTTA AGAAACTCAA   
  
  
- GTTGACCACT AAAATCACAA TCAGTAAGAC CCAGATCAAG TTGTTTGAGG CAACCTGGCT ATTATCACAG   
  
  
- ACTAAACTCA CTCGTAAAAC TCGGTGGGAA ACAACTTTGT GAAGGTTAAC TTAGTATAGT GGTTGGTAAC   
  
  
- TGGGCAGGAG TTACCAGTAA ACCGAGAAGC CCACGGAATC TACCGTGCCG GAGATTACCA AGCCACTAGA   
  
  
- GTAGGGAACC AAACGGTCAC CTACACTAAT CGCATAAATC CCTCTTTCTC AGGTACTAAG TTAAAGTCTT   
  
  
- TCCCCACCTC CTCCGGTCAT TCAAGGAAGG GTTCTTATTA TTGGAACAAT AACTAGAGCT CTTGGAGTGA   
  
  
- AAAGGATTAC TTTGTTTCCT CCTACTAGCT TACTACTACC AATTCTTCCT TTTCCTACTG CACTTAACCA   
  
  
- GATTGATGAG TTCTCCCTCA TTCTAAGTGA TAACACTTCT GCTCCGGAAA CTTCTTCCTT CCTCACCGTT   
  
  
- CGTCAGTCGA TAAAGAAGAT GACTCCTTCG ACTCAAAAGA CTGTAAAAAC TGTTCCAAAA CGAAACGCTA   
  
  
- ACGATGGGAC ACTTTGGACT CCGAGTAGGG TGGTACTCAA ACTTGGGACT CTTCCCGGTC AGTGTACCTA   
  
  
- ATCTTCCACC CTCTTTACCC TTCCGAGCGG GATTCTTTGT CCTATTATCA TCGTGTTTAT AACACCTAAA   
  
  
- TTCCTTAAAC GACTATGATA CGCGTGTTAG ATGACGTAGA CTACTGGCTT CTTGACGACT ACCTGACGAT   
  
  
- TTCGTTTAGT CCCTCGTGAG CAGACGACTC CCCCTACCTA GAGTTTCCAA CCGCGTAATG AAACGACTAC   
  
  
- GGGATCTCCG TGCAAATCGA CCTTGACCTA GAGCGTAAAT ATGCCGGGAT ACAAGATTAT CCGGTAGACA   
  
  
- GTGACTGTAC TATTTTCGTA TAGTCAAGAT ATAAGCACGA ACGGGTAAAT GCTTCTAGCA GTAACCAACA   
  
  
- CCATGGGTAT ACTAAGATTT TCGTCGACTC TTCCGTAGTT TCGAAGTATA ATATCTAAAA CCGTAGGAGA   
  
  
- TACCAACGGT TACCGGGTTG GAGTAAGTTG CGGAGAGTCT CGCTAGACCA CCTGGAGGTT TTGACAAATA   
  
  
- ATGTCCCTAG CTAGAGGGGG TCGGACCCAA GTCCGGTCGT CTTTCTCACC TTCGTTGTCC CTCTGCGAAC   
  
  
- CGGTTCATGA CACTCGCCAT ATTACACGGT AAACTCATAG TACGGTAACG AGTCCTCACC CTTTGTTAGT   
  
  
- TTGGTCCCCT AGAGTTCTAT CCTTCATCCT TACTACTTCA ACAACGCCAC TTGACAGAGA CATCCAAGTT   
  
  
- CTTGGAGGAA CTGCTCTGTC ACCACCACCT ATCAGGTTCC TTGTGTCAAA ATTTGGACTA ATGTTCCCAT   
  
  
- TTCGGACTAT AAAAACACGT ACCGCAACAT TTACCAAGGA TGTTGTAGGG AAAGAAACAC TGTGCAAAAT   
  
  
- CTCTTCGGGA GAAAGTAATA AGGTGAGAAA AGCTGTACAA TCTACGGTTG CGGAGATCCC TCCTCGGGCT   
  
  
- CTCCAACTAT AAGCTCTTCC GTAAGATACC CTCCCTCTAA TACTTACACC ACCGGACACT CCCGTGTCTC   
  
  
- TCCCACCTCT CCGGCCTCTG TATGTTCGTT ACCGTACAAT CCGTATCGTC CCGTCCCAAA GCCGTTCATG   
  
  
- GTAACCTAGG GTTCAACTAG CTCTTTTACT CCAAATTCCG GTTCCGTCTG GTGGTGTTCC TAAAGTACTA   
  
  
- GCTACACCTA CCTGTAACCC GTTAAGTCCC TACCTTCCCC GCCTAACGTG TACGCTAGAG ACGTACCCAA   
  
  
- GGCCGAAC

+     MYC

| Site Name | Organism | Position | Strand | Matrix score. | sequence | function |
| --- | --- | --- | --- | --- | --- | --- |
| MYC | Arabidopsis thaliana | 4252 | - | 6 | CATGTG |  |
| MYC | Arabidopsis thaliana | 2569 | - | 6 | CAATTG |  |
| MYC | Arabidopsis thaliana | 2277 | - | 6 | CATTTG |  |
| MYC | Arabidopsis thaliana | 1116 | + | 6 | CATTTG |  |
| MYC | Arabidopsis thaliana | 614 | + | 6 | CATTTG |  |
| MYC | Arabidopsis thaliana | 3076 | - | 6 | CATGTG |  |
| MYC | Arabidopsis thaliana | 2611 | + | 6 | CATTTG |  |
| MYC | Arabidopsis thaliana | 3673 | + | 6 | CATTTG |  |
| MYC | Arabidopsis thaliana | 463 | + | 6 | CATTTG |  |
| MYC | Arabidopsis thaliana | 268 | + | 6 | CAATTG |  |

>HU07G02249.1   
+ -Up\_Stream \_Len000GATTTG ATTGTCGAAA TTTGTCCGAT TTTATAAAAT TGAAAATTGT TATTGGACTA   
  
  
+ TAATCTACTG TAGTTTAGAG GTGTTGGATA ATACTCTCAA CTTTGAACTT GGACGTCCAT GCACCACACT   
  
  
+ ATGGGCGTAT TTACTGTTTA TATACCAAGA ATAATTTTGA AGATGTTCTT AGTTGGTGAT GTAAGTGTAT   
  
  
+ ATTCAACATA TGTCATTACA TAAACTTGAA AATTCTTGAA TTTTAGACAT ATACAATTGC AAATCATTGT   
  
  
+ AAAATTGTTG CATTACATTT TGATAAAAAT GCATCCAAAC TTTGTCTTGG TATAAACAGT AAATAGGCCC   
  
  
+ ATAGGTCCAT AGTGTAGTGC ATGGACGTCC AAGTTTAAAG GTGGGAGTAT TATTGCCCAA CGCCCCTAAA   
  
  
+ GTACAGTATA TTATAGTCCA ATATCCCAAT TAATTTTTCA TTTGAATATT TAAATTTGGA CATAATATTT   
  
  
+ TTCATATTTG AATTCTCTAA ACCTGATTTT AACCTGAATT TAAGTTGATT TAATTCATCC GACCAAAAAA   
  
  
+ ATAAACACAA TTATTTATTT TTCTATTCGA AGTTAGATAG TAGCCAATTC ATTTGACATA TAATTAACCC   
  
  
+ GTGATTGACA CAAACTCGAG TTTTTGCTTA GAGCTAGGAA AAGAGTCGGG TTAGGTCGAC CCATTTAAGA   
  
  
+ AATGGGTCGA CTTCATGTCG GCCTTCAAAA TAGTTAAGAT TTGCTCAAAA CTAAAAAATA ACAAGTTCAC   
  
  
+ ACTAAACGGT CAAATCGTGT CGACTTCGGA TTGAAATTGG TTCAGTTTGG GTCGGCTTCG TATTTCTTTC   
  
  
+ AGTAACTTCG AACCGAATTC GTATCGTGTT ATCGTGTCAG GTCAGACTTT CTCAGTTCTA TTTTTGCTAC   
  
  
+ CTTATGTTAC GGATAAAAAA AGTCTCACAT TGGAAAAAGT GTGGGAGGGT CCTGGGCTTA TAAATAGTGG   
  
  
+ CGCTGCACAC CTCATCAGAC CGACCTTTTG GGGAGAGGGT AATGACCCCG TGACAAGTGG TATCAGAGTC   
  
  
+ AACCCAGACC CGACTCAGAT GTCCTGCCAA GCGCTCCGAC GGGCGGGGTA GGCAGGGGGC CCATTTGTGA   
  
  
+ TGAGGACGTC ACAGAATTGG GCGGGGGAGA GTGTCACGGA TAAAAAAAGT CCCACATCGA AAAAAGTGTG   
  
  
+ GGAGGGTCCT GGGCTTATAA ACGGTAGCGC CGCACACCCC ATCAGATCGA CTTTTTGGGG AGAGGGTAAT   
  
  
+ GACCCCATTT CAAGTGGTAT CAGAGCCAAC CCAGACCCGA CTCCAATGTC CCACCAAGCG CTCTGATGGG   
  
  
+ CGGGGTAGGC AAGGGGCTCC ATATGTGACG AGGGCGTCAC GGAATTGGAT GGGAGAGAGT ACCACGGATA   
  
  
+ AAAAAAGTCC CACATCAGAA AAAGTGTGGA CTTAGCAACT AGGAAGTAAA GTTACAGTAT TAGCCGAGAG   
  
  
+ CCCATGGAAC GCCTGAAACT GACATCACCA GACCCACATA TTTCACCATC ATCTGATCTC CCCGTCAAAG   
  
  
+ GAGGAAGCTG AGCACAGCAA GAACTAGAAG AACAGGCGGC ATCACACCCA AGCCAAAATT CTCTATTTGA   
  
  
+ CCTCCCTCTC CTCATTCCTC TGAGCTTCCT CTTCACAATG TAAGACCATA ATCCTTAACA CCCTTCTTTT   
  
  
+ TTAAGCTCAT CTTTACCTGC TCTGGTTCTC TCTCTCATCG TCACTAATAG TTACCTCTTT ACTTTCTCTT   
  
  
+ CCCTGAGGTA TGCTTCAACT TTAACATATA TATCCCACTT GATTTCTCTT GTCTTTTTGC TATAATACTT   
  
  
+ GTTGTTTGGC TATCATCCCC TGTTTTATAC TCATTTTCTT GCTTTCTATT TCTGGGTTTG AATTGGGTTT   
  
  
+ CACTTATCCT CGTCAATTCT ATGGGTAATT AGTGAATTGG GTTTATATGA ACTAGGGTTG CAAAAGTATC   
  
  
+ TACTTTTTCT CTTGTGATTA TTAGTTGATT AGGGATTTGG CCAATATTAG CAGAATGGGT TCTGAATTTG   
  
  
+ CGGAATTCTC TGATGATGCT CTAAATGGGT ATGCTTATGT TGATATCCCT GCTTATGATG CATCCTTAGA   
  
  
+ TTATGCCAAT TTGTTCAATT ACGAAGGCCC ATCTGAGGAT CGCACCTCAC TGAGCCTCCC AAGACCCTTT   
  
  
+ TCTGACCCTT TGGCTTACAG TTTCACGTCC TCTTCTGAGC TGGGCCCTGG GGTTGATTCT AATGATGATA   
  
  
+ GTGATTCTGG CGATGTTCTC AAGTACATTA GCCAAATGCT TATGGAAGAA GACATGGAGG CAAAGCCATG   
  
  
+ CATGTTTCAT GATCCTTTAG CACTTCAGGC TGCTGAGAAA CCCTTTTATG ATGCCTTAGG GGAGCAATGC   
  
  
+ CCAACTTCTC CTGACCAACA TCCTATAATT GATCATTATT TGGATAGTCC TGATGAAAAT TCTTTGAGTT   
  
  
+ CAACTGGTGA TTTTAGTGTT AGTCATTCTG GGTCTAGTTC AACAAACTCC GTTGGACCGA TAATAGTGTC   
  
  
+ TGATTTGAGT GAGCATTTTG AGCCACCCTT TGTTGAAACA CTTCCAATTG AATCATATCA CCAACCATTG   
  
  
+ ACCCGTCCTC AATGGTCATT TGGCTCTTCG GGTGCCTTAG ATGGCACGGC CTCTAATGGT TCGGTGATCT   
  
  
+ CATCCCTTGG TTTGCCAGTG GATGTGATTA GCGTATTTAG GGAGAAAGAG TCCATGATTC AATTTCAGAA   
  
  
+ AGGGGTGGAG GAGGCCAGTA AGTTCCTTCC CAAGAATAAT AACCTTGTTA TTGATCTCGA GAACCTCACT   
  
  
+ TTTCCTAATG AAACAAAGGA GGATGATCGA ATGATGATGG TTAAGAAGGA AAAGGATGAC GTGAATTGGT   
  
  
+ CTAACTACTC AAGAGGGAGT AAGATTCACT ATTGTGAAGA CGAGGCCTTT GAAGAAGGAA GGAGTGGCAA   
  
  
+ GCAGTCAGCT ATTTCTTCTA CTGAGGAAGC TGAGTTTTCT GACATTTTTG ACAAGGTTTT GCTTTGCGAT   
  
  
+ TGCTACCCTG TGAAACCTGA GGCTCATCCC ACCATGAGTT TGAACCCTGA GAAGGGCCAG TCACATGGAT   
  
  
+ TAGAAGGTGG GAGAAATGGG AAGGCTCGCC CTAAGAAACA GGATAATAGT AGCACAAATA TTGTGGATTT   
  
  
+ AAGGAATTTG CTGATACTAT GCGCACAATC TACTGCATCT GATGACCGAA GAACTGCTGA TGGACTGCTA   
  
  
+ AAGCAAATCA GGGAGCACTC GTCTGCTGAG GGGGATGGAT CTCAAAGGTT GGCGCATTAC TTTGCTGATG   
  
  
+ CCCTAGAGGC ACGTTTAGCT GGAACTGGAT CTCGCATTTA TACGGCCCTA TGTTCTAATA GGCCATCTGT   
  
  
+ CACTGACATG ATAAAAGCAT ATCAGTTCTA TATTCGTGCT TGCCCATTTA CGAAGATCGT CATTGGTTGT   
  
  
+ GGTACCCATA TGATTCTAAA AGCAGCTGAG AAGGCATCAA AGCTTCATAT TATAGATTTT GGCATCCTCT   
  
  
+ ATGGTTGCCA ATGGCCCAAC CTCATTCAAC GCCTCTCAGA GCGATCTGGT GGACCTCCAA AACTGTTTAT   
  
  
+ TACAGGGATC GATCTCCCCC AGCCTGGGTT CAGGCCAGCA GAAAGAGTGG AAGCAACAGG GAGACGCTTG   
  
  
+ GCCAAGTACT GTGAGCGGTA TAATGTGCCA TTTGAGTATC ATGCCATTGC TCAGGAGTGG GAAACAATCA   
  
  
+ AACCAGGGGA TCTCAAGATA GGAAGTAGGA ATGATGAAGT TGTTGCGGTG AACTGTCTCT GTAGGTTCAA   
  
  
+ GAACCTCCTT GACGAGACAG TGGTGGTGGA TAGTCCAAGG AACACAGTTT TAAACCTGAT TACAAGGGTA   
  
  
+ AAGCCTGATA TTTTTGTGCA TGGCGTTGTA AATGGTTCCT ACAACATCCC TTTCTTTGTG ACACGTTTTA   
  
  
+ GAGAAGCCCT CTTTCATTAT TCCACTCTTT TCGACATGTT AGATGCCAAC GCCTCTAGGG AGGAGCCCGA   
  
  
+ GAGGTTGATA TTCGAGAAGG CATTCTATGG GAGGGAGATT ATGAATGTGG TGGCCTGTGA GGGCACAGAG   
  
  
+ AGGGTGGAGA GGCCGGAGAC ATACAAGCAA TGGCATGTTA GGCATAGCAG GGCAGGGTTT CGGCAAGTAC   
  
  
+ CATTGGATCC CAAGTTGATC GAGAAAATGA GGTTTAAGGC CAAGGCAGAC CACCACAAGG ATTTCATGAT   
  
  
+ CGATGTGGAT GGACATTGGG CAATTCAGGG ATGGAAGGGG CGGATTGCAC ATGCGATCTC TGCATGGGTT   
  
  
+ CCGGCTTG  

- -Up\_Stream \_Len000CTAAAC TAACAGCTTT AAACAGGCTA AAATATTTTA ACTTTTAACA ATAACCTGAT   
  
  
- ATTAGATGAC ATCAAATCTC CACAACCTAT TATGAGAGTT GAAACTTGAA CCTGCAGGTA CGTGGTGTGA   
  
  
- TACCCGCATA AATGACAAAT ATATGGTTCT TATTAAAACT TCTACAAGAA TCAACCACTA CATTCACATA   
  
  
- TAAGTTGTAT ACAGTAATGT ATTTGAACTT TTAAGAACTT AAAATCTGTA TATGTTAACG TTTAGTAACA   
  
  
- TTTTAACAAC GTAATGTAAA ACTATTTTTA CGTAGGTTTG AAACAGAACC ATATTTGTCA TTTATCCGGG   
  
  
- TATCCAGGTA TCACATCACG TACCTGCAGG TTCAAATTTC CACCCTCATA ATAACGGGTT GCGGGGATTT   
  
  
- CATGTCATAT AATATCAGGT TATAGGGTTA ATTAAAAAGT AAACTTATAA ATTTAAACCT GTATTATAAA   
  
  
- AAGTATAAAC TTAAGAGATT TGGACTAAAA TTGGACTTAA ATTCAACTAA ATTAAGTAGG CTGGTTTTTT   
  
  
- TATTTGTGTT AATAAATAAA AAGATAAGCT TCAATCTATC ATCGGTTAAG TAAACTGTAT ATTAATTGGG   
  
  
- CACTAACTGT GTTTGAGCTC AAAAACGAAT CTCGATCCTT TTCTCAGCCC AATCCAGCTG GGTAAATTCT   
  
  
- TTACCCAGCT GAAGTACAGC CGGAAGTTTT ATCAATTCTA AACGAGTTTT GATTTTTTAT TGTTCAAGTG   
  
  
- TGATTTGCCA GTTTAGCACA GCTGAAGCCT AACTTTAACC AAGTCAAACC CAGCCGAAGC ATAAAGAAAG   
  
  
- TCATTGAAGC TTGGCTTAAG CATAGCACAA TAGCACAGTC CAGTCTGAAA GAGTCAAGAT AAAAACGATG   
  
  
- GAATACAATG CCTATTTTTT TCAGAGTGTA ACCTTTTTCA CACCCTCCCA GGACCCGAAT ATTTATCACC   
  
  
- GCGACGTGTG GAGTAGTCTG GCTGGAAAAC CCCTCTCCCA TTACTGGGGC ACTGTTCACC ATAGTCTCAG   
  
  
- TTGGGTCTGG GCTGAGTCTA CAGGACGGTT CGCGAGGCTG CCCGCCCCAT CCGTCCCCCG GGTAAACACT   
  
  
- ACTCCTGCAG TGTCTTAACC CGCCCCCTCT CACAGTGCCT ATTTTTTTCA GGGTGTAGCT TTTTTCACAC   
  
  
- CCTCCCAGGA CCCGAATATT TGCCATCGCG GCGTGTGGGG TAGTCTAGCT GAAAAACCCC TCTCCCATTA   
  
  
- CTGGGGTAAA GTTCACCATA GTCTCGGTTG GGTCTGGGCT GAGGTTACAG GGTGGTTCGC GAGACTACCC   
  
  
- GCCCCATCCG TTCCCCGAGG TATACACTGC TCCCGCAGTG CCTTAACCTA CCCTCTCTCA TGGTGCCTAT   
  
  
- TTTTTTCAGG GTGTAGTCTT TTTCACACCT GAATCGTTGA TCCTTCATTT CAATGTCATA ATCGGCTCTC   
  
  
- GGGTACCTTG CGGACTTTGA CTGTAGTGGT CTGGGTGTAT AAAGTGGTAG TAGACTAGAG GGGCAGTTTC   
  
  
- CTCCTTCGAC TCGTGTCGTT CTTGATCTTC TTGTCCGCCG TAGTGTGGGT TCGGTTTTAA GAGATAAACT   
  
  
- GGAGGGAGAG GAGTAAGGAG ACTCGAAGGA GAAGTGTTAC ATTCTGGTAT TAGGAATTGT GGGAAGAAAA   
  
  
- AATTCGAGTA GAAATGGACG AGACCAAGAG AGAGAGTAGC AGTGATTATC AATGGAGAAA TGAAAGAGAA   
  
  
- GGGACTCCAT ACGAAGTTGA AATTGTATAT ATAGGGTGAA CTAAAGAGAA CAGAAAAACG ATATTATGAA   
  
  
- CAACAAACCG ATAGTAGGGG ACAAAATATG AGTAAAAGAA CGAAAGATAA AGACCCAAAC TTAACCCAAA   
  
  
- GTGAATAGGA GCAGTTAAGA TACCCATTAA TCACTTAACC CAAATATACT TGATCCCAAC GTTTTCATAG   
  
  
- ATGAAAAAGA GAACACTAAT AATCAACTAA TCCCTAAACC GGTTATAATC GTCTTACCCA AGACTTAAAC   
  
  
- GCCTTAAGAG ACTACTACGA GATTTACCCA TACGAATACA ACTATAGGGA CGAATACTAC GTAGGAATCT   
  
  
- AATACGGTTA AACAAGTTAA TGCTTCCGGG TAGACTCCTA GCGTGGAGTG ACTCGGAGGG TTCTGGGAAA   
  
  
- AGACTGGGAA ACCGAATGTC AAAGTGCAGG AGAAGACTCG ACCCGGGACC CCAACTAAGA TTACTACTAT   
  
  
- CACTAAGACC GCTACAAGAG TTCATGTAAT CGGTTTACGA ATACCTTCTT CTGTACCTCC GTTTCGGTAC   
  
  
- GTACAAAGTA CTAGGAAATC GTGAAGTCCG ACGACTCTTT GGGAAAATAC TACGGAATCC CCTCGTTACG   
  
  
- GGTTGAAGAG GACTGGTTGT AGGATATTAA CTAGTAATAA ACCTATCAGG ACTACTTTTA AGAAACTCAA   
  
  
- GTTGACCACT AAAATCACAA TCAGTAAGAC CCAGATCAAG TTGTTTGAGG CAACCTGGCT ATTATCACAG   
  
  
- ACTAAACTCA CTCGTAAAAC TCGGTGGGAA ACAACTTTGT GAAGGTTAAC TTAGTATAGT GGTTGGTAAC   
  
  
- TGGGCAGGAG TTACCAGTAA ACCGAGAAGC CCACGGAATC TACCGTGCCG GAGATTACCA AGCCACTAGA   
  
  
- GTAGGGAACC AAACGGTCAC CTACACTAAT CGCATAAATC CCTCTTTCTC AGGTACTAAG TTAAAGTCTT   
  
  
- TCCCCACCTC CTCCGGTCAT TCAAGGAAGG GTTCTTATTA TTGGAACAAT AACTAGAGCT CTTGGAGTGA   
  
  
- AAAGGATTAC TTTGTTTCCT CCTACTAGCT TACTACTACC AATTCTTCCT TTTCCTACTG CACTTAACCA   
  
  
- GATTGATGAG TTCTCCCTCA TTCTAAGTGA TAACACTTCT GCTCCGGAAA CTTCTTCCTT CCTCACCGTT   
  
  
- CGTCAGTCGA TAAAGAAGAT GACTCCTTCG ACTCAAAAGA CTGTAAAAAC TGTTCCAAAA CGAAACGCTA   
  
  
- ACGATGGGAC ACTTTGGACT CCGAGTAGGG TGGTACTCAA ACTTGGGACT CTTCCCGGTC AGTGTACCTA   
  
  
- ATCTTCCACC CTCTTTACCC TTCCGAGCGG GATTCTTTGT CCTATTATCA TCGTGTTTAT AACACCTAAA   
  
  
- TTCCTTAAAC GACTATGATA CGCGTGTTAG ATGACGTAGA CTACTGGCTT CTTGACGACT ACCTGACGAT   
  
  
- TTCGTTTAGT CCCTCGTGAG CAGACGACTC CCCCTACCTA GAGTTTCCAA CCGCGTAATG AAACGACTAC   
  
  
- GGGATCTCCG TGCAAATCGA CCTTGACCTA GAGCGTAAAT ATGCCGGGAT ACAAGATTAT CCGGTAGACA   
  
  
- GTGACTGTAC TATTTTCGTA TAGTCAAGAT ATAAGCACGA ACGGGTAAAT GCTTCTAGCA GTAACCAACA   
  
  
- CCATGGGTAT ACTAAGATTT TCGTCGACTC TTCCGTAGTT TCGAAGTATA ATATCTAAAA CCGTAGGAGA   
  
  
- TACCAACGGT TACCGGGTTG GAGTAAGTTG CGGAGAGTCT CGCTAGACCA CCTGGAGGTT TTGACAAATA   
  
  
- ATGTCCCTAG CTAGAGGGGG TCGGACCCAA GTCCGGTCGT CTTTCTCACC TTCGTTGTCC CTCTGCGAAC   
  
  
- CGGTTCATGA CACTCGCCAT ATTACACGGT AAACTCATAG TACGGTAACG AGTCCTCACC CTTTGTTAGT   
  
  
- TTGGTCCCCT AGAGTTCTAT CCTTCATCCT TACTACTTCA ACAACGCCAC TTGACAGAGA CATCCAAGTT   
  
  
- CTTGGAGGAA CTGCTCTGTC ACCACCACCT ATCAGGTTCC TTGTGTCAAA ATTTGGACTA ATGTTCCCAT   
  
  
- TTCGGACTAT AAAAACACGT ACCGCAACAT TTACCAAGGA TGTTGTAGGG AAAGAAACAC TGTGCAAAAT   
  
  
- CTCTTCGGGA GAAAGTAATA AGGTGAGAAA AGCTGTACAA TCTACGGTTG CGGAGATCCC TCCTCGGGCT   
  
  
- CTCCAACTAT AAGCTCTTCC GTAAGATACC CTCCCTCTAA TACTTACACC ACCGGACACT CCCGTGTCTC   
  
  
- TCCCACCTCT CCGGCCTCTG TATGTTCGTT ACCGTACAAT CCGTATCGTC CCGTCCCAAA GCCGTTCATG   
  
  
- GTAACCTAGG GTTCAACTAG CTCTTTTACT CCAAATTCCG GTTCCGTCTG GTGGTGTTCC TAAAGTACTA   
  
  
- GCTACACCTA CCTGTAACCC GTTAAGTCCC TACCTTCCCC GCCTAACGTG TACGCTAGAG ACGTACCCAA   
  
  
- GGCCGAAC

+     Myb

| Site Name | Organism | Position | Strand | Matrix score. | sequence | function |
| --- | --- | --- | --- | --- | --- | --- |
| Myb | Arabidopsis thaliana | 2455 | + | 6 | CAACTG |  |

>HU07G02249.1   
+ -Up\_Stream \_Len000GATTTG ATTGTCGAAA TTTGTCCGAT TTTATAAAAT TGAAAATTGT TATTGGACTA   
  
  
+ TAATCTACTG TAGTTTAGAG GTGTTGGATA ATACTCTCAA CTTTGAACTT GGACGTCCAT GCACCACACT   
  
  
+ ATGGGCGTAT TTACTGTTTA TATACCAAGA ATAATTTTGA AGATGTTCTT AGTTGGTGAT GTAAGTGTAT   
  
  
+ ATTCAACATA TGTCATTACA TAAACTTGAA AATTCTTGAA TTTTAGACAT ATACAATTGC AAATCATTGT   
  
  
+ AAAATTGTTG CATTACATTT TGATAAAAAT GCATCCAAAC TTTGTCTTGG TATAAACAGT AAATAGGCCC   
  
  
+ ATAGGTCCAT AGTGTAGTGC ATGGACGTCC AAGTTTAAAG GTGGGAGTAT TATTGCCCAA CGCCCCTAAA   
  
  
+ GTACAGTATA TTATAGTCCA ATATCCCAAT TAATTTTTCA TTTGAATATT TAAATTTGGA CATAATATTT   
  
  
+ TTCATATTTG AATTCTCTAA ACCTGATTTT AACCTGAATT TAAGTTGATT TAATTCATCC GACCAAAAAA   
  
  
+ ATAAACACAA TTATTTATTT TTCTATTCGA AGTTAGATAG TAGCCAATTC ATTTGACATA TAATTAACCC   
  
  
+ GTGATTGACA CAAACTCGAG TTTTTGCTTA GAGCTAGGAA AAGAGTCGGG TTAGGTCGAC CCATTTAAGA   
  
  
+ AATGGGTCGA CTTCATGTCG GCCTTCAAAA TAGTTAAGAT TTGCTCAAAA CTAAAAAATA ACAAGTTCAC   
  
  
+ ACTAAACGGT CAAATCGTGT CGACTTCGGA TTGAAATTGG TTCAGTTTGG GTCGGCTTCG TATTTCTTTC   
  
  
+ AGTAACTTCG AACCGAATTC GTATCGTGTT ATCGTGTCAG GTCAGACTTT CTCAGTTCTA TTTTTGCTAC   
  
  
+ CTTATGTTAC GGATAAAAAA AGTCTCACAT TGGAAAAAGT GTGGGAGGGT CCTGGGCTTA TAAATAGTGG   
  
  
+ CGCTGCACAC CTCATCAGAC CGACCTTTTG GGGAGAGGGT AATGACCCCG TGACAAGTGG TATCAGAGTC   
  
  
+ AACCCAGACC CGACTCAGAT GTCCTGCCAA GCGCTCCGAC GGGCGGGGTA GGCAGGGGGC CCATTTGTGA   
  
  
+ TGAGGACGTC ACAGAATTGG GCGGGGGAGA GTGTCACGGA TAAAAAAAGT CCCACATCGA AAAAAGTGTG   
  
  
+ GGAGGGTCCT GGGCTTATAA ACGGTAGCGC CGCACACCCC ATCAGATCGA CTTTTTGGGG AGAGGGTAAT   
  
  
+ GACCCCATTT CAAGTGGTAT CAGAGCCAAC CCAGACCCGA CTCCAATGTC CCACCAAGCG CTCTGATGGG   
  
  
+ CGGGGTAGGC AAGGGGCTCC ATATGTGACG AGGGCGTCAC GGAATTGGAT GGGAGAGAGT ACCACGGATA   
  
  
+ AAAAAAGTCC CACATCAGAA AAAGTGTGGA CTTAGCAACT AGGAAGTAAA GTTACAGTAT TAGCCGAGAG   
  
  
+ CCCATGGAAC GCCTGAAACT GACATCACCA GACCCACATA TTTCACCATC ATCTGATCTC CCCGTCAAAG   
  
  
+ GAGGAAGCTG AGCACAGCAA GAACTAGAAG AACAGGCGGC ATCACACCCA AGCCAAAATT CTCTATTTGA   
  
  
+ CCTCCCTCTC CTCATTCCTC TGAGCTTCCT CTTCACAATG TAAGACCATA ATCCTTAACA CCCTTCTTTT   
  
  
+ TTAAGCTCAT CTTTACCTGC TCTGGTTCTC TCTCTCATCG TCACTAATAG TTACCTCTTT ACTTTCTCTT   
  
  
+ CCCTGAGGTA TGCTTCAACT TTAACATATA TATCCCACTT GATTTCTCTT GTCTTTTTGC TATAATACTT   
  
  
+ GTTGTTTGGC TATCATCCCC TGTTTTATAC TCATTTTCTT GCTTTCTATT TCTGGGTTTG AATTGGGTTT   
  
  
+ CACTTATCCT CGTCAATTCT ATGGGTAATT AGTGAATTGG GTTTATATGA ACTAGGGTTG CAAAAGTATC   
  
  
+ TACTTTTTCT CTTGTGATTA TTAGTTGATT AGGGATTTGG CCAATATTAG CAGAATGGGT TCTGAATTTG   
  
  
+ CGGAATTCTC TGATGATGCT CTAAATGGGT ATGCTTATGT TGATATCCCT GCTTATGATG CATCCTTAGA   
  
  
+ TTATGCCAAT TTGTTCAATT ACGAAGGCCC ATCTGAGGAT CGCACCTCAC TGAGCCTCCC AAGACCCTTT   
  
  
+ TCTGACCCTT TGGCTTACAG TTTCACGTCC TCTTCTGAGC TGGGCCCTGG GGTTGATTCT AATGATGATA   
  
  
+ GTGATTCTGG CGATGTTCTC AAGTACATTA GCCAAATGCT TATGGAAGAA GACATGGAGG CAAAGCCATG   
  
  
+ CATGTTTCAT GATCCTTTAG CACTTCAGGC TGCTGAGAAA CCCTTTTATG ATGCCTTAGG GGAGCAATGC   
  
  
+ CCAACTTCTC CTGACCAACA TCCTATAATT GATCATTATT TGGATAGTCC TGATGAAAAT TCTTTGAGTT   
  
  
+ CAACTGGTGA TTTTAGTGTT AGTCATTCTG GGTCTAGTTC AACAAACTCC GTTGGACCGA TAATAGTGTC   
  
  
+ TGATTTGAGT GAGCATTTTG AGCCACCCTT TGTTGAAACA CTTCCAATTG AATCATATCA CCAACCATTG   
  
  
+ ACCCGTCCTC AATGGTCATT TGGCTCTTCG GGTGCCTTAG ATGGCACGGC CTCTAATGGT TCGGTGATCT   
  
  
+ CATCCCTTGG TTTGCCAGTG GATGTGATTA GCGTATTTAG GGAGAAAGAG TCCATGATTC AATTTCAGAA   
  
  
+ AGGGGTGGAG GAGGCCAGTA AGTTCCTTCC CAAGAATAAT AACCTTGTTA TTGATCTCGA GAACCTCACT   
  
  
+ TTTCCTAATG AAACAAAGGA GGATGATCGA ATGATGATGG TTAAGAAGGA AAAGGATGAC GTGAATTGGT   
  
  
+ CTAACTACTC AAGAGGGAGT AAGATTCACT ATTGTGAAGA CGAGGCCTTT GAAGAAGGAA GGAGTGGCAA   
  
  
+ GCAGTCAGCT ATTTCTTCTA CTGAGGAAGC TGAGTTTTCT GACATTTTTG ACAAGGTTTT GCTTTGCGAT   
  
  
+ TGCTACCCTG TGAAACCTGA GGCTCATCCC ACCATGAGTT TGAACCCTGA GAAGGGCCAG TCACATGGAT   
  
  
+ TAGAAGGTGG GAGAAATGGG AAGGCTCGCC CTAAGAAACA GGATAATAGT AGCACAAATA TTGTGGATTT   
  
  
+ AAGGAATTTG CTGATACTAT GCGCACAATC TACTGCATCT GATGACCGAA GAACTGCTGA TGGACTGCTA   
  
  
+ AAGCAAATCA GGGAGCACTC GTCTGCTGAG GGGGATGGAT CTCAAAGGTT GGCGCATTAC TTTGCTGATG   
  
  
+ CCCTAGAGGC ACGTTTAGCT GGAACTGGAT CTCGCATTTA TACGGCCCTA TGTTCTAATA GGCCATCTGT   
  
  
+ CACTGACATG ATAAAAGCAT ATCAGTTCTA TATTCGTGCT TGCCCATTTA CGAAGATCGT CATTGGTTGT   
  
  
+ GGTACCCATA TGATTCTAAA AGCAGCTGAG AAGGCATCAA AGCTTCATAT TATAGATTTT GGCATCCTCT   
  
  
+ ATGGTTGCCA ATGGCCCAAC CTCATTCAAC GCCTCTCAGA GCGATCTGGT GGACCTCCAA AACTGTTTAT   
  
  
+ TACAGGGATC GATCTCCCCC AGCCTGGGTT CAGGCCAGCA GAAAGAGTGG AAGCAACAGG GAGACGCTTG   
  
  
+ GCCAAGTACT GTGAGCGGTA TAATGTGCCA TTTGAGTATC ATGCCATTGC TCAGGAGTGG GAAACAATCA   
  
  
+ AACCAGGGGA TCTCAAGATA GGAAGTAGGA ATGATGAAGT TGTTGCGGTG AACTGTCTCT GTAGGTTCAA   
  
  
+ GAACCTCCTT GACGAGACAG TGGTGGTGGA TAGTCCAAGG AACACAGTTT TAAACCTGAT TACAAGGGTA   
  
  
+ AAGCCTGATA TTTTTGTGCA TGGCGTTGTA AATGGTTCCT ACAACATCCC TTTCTTTGTG ACACGTTTTA   
  
  
+ GAGAAGCCCT CTTTCATTAT TCCACTCTTT TCGACATGTT AGATGCCAAC GCCTCTAGGG AGGAGCCCGA   
  
  
+ GAGGTTGATA TTCGAGAAGG CATTCTATGG GAGGGAGATT ATGAATGTGG TGGCCTGTGA GGGCACAGAG   
  
  
+ AGGGTGGAGA GGCCGGAGAC ATACAAGCAA TGGCATGTTA GGCATAGCAG GGCAGGGTTT CGGCAAGTAC   
  
  
+ CATTGGATCC CAAGTTGATC GAGAAAATGA GGTTTAAGGC CAAGGCAGAC CACCACAAGG ATTTCATGAT   
  
  
+ CGATGTGGAT GGACATTGGG CAATTCAGGG ATGGAAGGGG CGGATTGCAC ATGCGATCTC TGCATGGGTT   
  
  
+ CCGGCTTG  

- -Up\_Stream \_Len000CTAAAC TAACAGCTTT AAACAGGCTA AAATATTTTA ACTTTTAACA ATAACCTGAT   
  
  
- ATTAGATGAC ATCAAATCTC CACAACCTAT TATGAGAGTT GAAACTTGAA CCTGCAGGTA CGTGGTGTGA   
  
  
- TACCCGCATA AATGACAAAT ATATGGTTCT TATTAAAACT TCTACAAGAA TCAACCACTA CATTCACATA   
  
  
- TAAGTTGTAT ACAGTAATGT ATTTGAACTT TTAAGAACTT AAAATCTGTA TATGTTAACG TTTAGTAACA   
  
  
- TTTTAACAAC GTAATGTAAA ACTATTTTTA CGTAGGTTTG AAACAGAACC ATATTTGTCA TTTATCCGGG   
  
  
- TATCCAGGTA TCACATCACG TACCTGCAGG TTCAAATTTC CACCCTCATA ATAACGGGTT GCGGGGATTT   
  
  
- CATGTCATAT AATATCAGGT TATAGGGTTA ATTAAAAAGT AAACTTATAA ATTTAAACCT GTATTATAAA   
  
  
- AAGTATAAAC TTAAGAGATT TGGACTAAAA TTGGACTTAA ATTCAACTAA ATTAAGTAGG CTGGTTTTTT   
  
  
- TATTTGTGTT AATAAATAAA AAGATAAGCT TCAATCTATC ATCGGTTAAG TAAACTGTAT ATTAATTGGG   
  
  
- CACTAACTGT GTTTGAGCTC AAAAACGAAT CTCGATCCTT TTCTCAGCCC AATCCAGCTG GGTAAATTCT   
  
  
- TTACCCAGCT GAAGTACAGC CGGAAGTTTT ATCAATTCTA AACGAGTTTT GATTTTTTAT TGTTCAAGTG   
  
  
- TGATTTGCCA GTTTAGCACA GCTGAAGCCT AACTTTAACC AAGTCAAACC CAGCCGAAGC ATAAAGAAAG   
  
  
- TCATTGAAGC TTGGCTTAAG CATAGCACAA TAGCACAGTC CAGTCTGAAA GAGTCAAGAT AAAAACGATG   
  
  
- GAATACAATG CCTATTTTTT TCAGAGTGTA ACCTTTTTCA CACCCTCCCA GGACCCGAAT ATTTATCACC   
  
  
- GCGACGTGTG GAGTAGTCTG GCTGGAAAAC CCCTCTCCCA TTACTGGGGC ACTGTTCACC ATAGTCTCAG   
  
  
- TTGGGTCTGG GCTGAGTCTA CAGGACGGTT CGCGAGGCTG CCCGCCCCAT CCGTCCCCCG GGTAAACACT   
  
  
- ACTCCTGCAG TGTCTTAACC CGCCCCCTCT CACAGTGCCT ATTTTTTTCA GGGTGTAGCT TTTTTCACAC   
  
  
- CCTCCCAGGA CCCGAATATT TGCCATCGCG GCGTGTGGGG TAGTCTAGCT GAAAAACCCC TCTCCCATTA   
  
  
- CTGGGGTAAA GTTCACCATA GTCTCGGTTG GGTCTGGGCT GAGGTTACAG GGTGGTTCGC GAGACTACCC   
  
  
- GCCCCATCCG TTCCCCGAGG TATACACTGC TCCCGCAGTG CCTTAACCTA CCCTCTCTCA TGGTGCCTAT   
  
  
- TTTTTTCAGG GTGTAGTCTT TTTCACACCT GAATCGTTGA TCCTTCATTT CAATGTCATA ATCGGCTCTC   
  
  
- GGGTACCTTG CGGACTTTGA CTGTAGTGGT CTGGGTGTAT AAAGTGGTAG TAGACTAGAG GGGCAGTTTC   
  
  
- CTCCTTCGAC TCGTGTCGTT CTTGATCTTC TTGTCCGCCG TAGTGTGGGT TCGGTTTTAA GAGATAAACT   
  
  
- GGAGGGAGAG GAGTAAGGAG ACTCGAAGGA GAAGTGTTAC ATTCTGGTAT TAGGAATTGT GGGAAGAAAA   
  
  
- AATTCGAGTA GAAATGGACG AGACCAAGAG AGAGAGTAGC AGTGATTATC AATGGAGAAA TGAAAGAGAA   
  
  
- GGGACTCCAT ACGAAGTTGA AATTGTATAT ATAGGGTGAA CTAAAGAGAA CAGAAAAACG ATATTATGAA   
  
  
- CAACAAACCG ATAGTAGGGG ACAAAATATG AGTAAAAGAA CGAAAGATAA AGACCCAAAC TTAACCCAAA   
  
  
- GTGAATAGGA GCAGTTAAGA TACCCATTAA TCACTTAACC CAAATATACT TGATCCCAAC GTTTTCATAG   
  
  
- ATGAAAAAGA GAACACTAAT AATCAACTAA TCCCTAAACC GGTTATAATC GTCTTACCCA AGACTTAAAC   
  
  
- GCCTTAAGAG ACTACTACGA GATTTACCCA TACGAATACA ACTATAGGGA CGAATACTAC GTAGGAATCT   
  
  
- AATACGGTTA AACAAGTTAA TGCTTCCGGG TAGACTCCTA GCGTGGAGTG ACTCGGAGGG TTCTGGGAAA   
  
  
- AGACTGGGAA ACCGAATGTC AAAGTGCAGG AGAAGACTCG ACCCGGGACC CCAACTAAGA TTACTACTAT   
  
  
- CACTAAGACC GCTACAAGAG TTCATGTAAT CGGTTTACGA ATACCTTCTT CTGTACCTCC GTTTCGGTAC   
  
  
- GTACAAAGTA CTAGGAAATC GTGAAGTCCG ACGACTCTTT GGGAAAATAC TACGGAATCC CCTCGTTACG   
  
  
- GGTTGAAGAG GACTGGTTGT AGGATATTAA CTAGTAATAA ACCTATCAGG ACTACTTTTA AGAAACTCAA   
  
  
- GTTGACCACT AAAATCACAA TCAGTAAGAC CCAGATCAAG TTGTTTGAGG CAACCTGGCT ATTATCACAG   
  
  
- ACTAAACTCA CTCGTAAAAC TCGGTGGGAA ACAACTTTGT GAAGGTTAAC TTAGTATAGT GGTTGGTAAC   
  
  
- TGGGCAGGAG TTACCAGTAA ACCGAGAAGC CCACGGAATC TACCGTGCCG GAGATTACCA AGCCACTAGA   
  
  
- GTAGGGAACC AAACGGTCAC CTACACTAAT CGCATAAATC CCTCTTTCTC AGGTACTAAG TTAAAGTCTT   
  
  
- TCCCCACCTC CTCCGGTCAT TCAAGGAAGG GTTCTTATTA TTGGAACAAT AACTAGAGCT CTTGGAGTGA   
  
  
- AAAGGATTAC TTTGTTTCCT CCTACTAGCT TACTACTACC AATTCTTCCT TTTCCTACTG CACTTAACCA   
  
  
- GATTGATGAG TTCTCCCTCA TTCTAAGTGA TAACACTTCT GCTCCGGAAA CTTCTTCCTT CCTCACCGTT   
  
  
- CGTCAGTCGA TAAAGAAGAT GACTCCTTCG ACTCAAAAGA CTGTAAAAAC TGTTCCAAAA CGAAACGCTA   
  
  
- ACGATGGGAC ACTTTGGACT CCGAGTAGGG TGGTACTCAA ACTTGGGACT CTTCCCGGTC AGTGTACCTA   
  
  
- ATCTTCCACC CTCTTTACCC TTCCGAGCGG GATTCTTTGT CCTATTATCA TCGTGTTTAT AACACCTAAA   
  
  
- TTCCTTAAAC GACTATGATA CGCGTGTTAG ATGACGTAGA CTACTGGCTT CTTGACGACT ACCTGACGAT   
  
  
- TTCGTTTAGT CCCTCGTGAG CAGACGACTC CCCCTACCTA GAGTTTCCAA CCGCGTAATG AAACGACTAC   
  
  
- GGGATCTCCG TGCAAATCGA CCTTGACCTA GAGCGTAAAT ATGCCGGGAT ACAAGATTAT CCGGTAGACA   
  
  
- GTGACTGTAC TATTTTCGTA TAGTCAAGAT ATAAGCACGA ACGGGTAAAT GCTTCTAGCA GTAACCAACA   
  
  
- CCATGGGTAT ACTAAGATTT TCGTCGACTC TTCCGTAGTT TCGAAGTATA ATATCTAAAA CCGTAGGAGA   
  
  
- TACCAACGGT TACCGGGTTG GAGTAAGTTG CGGAGAGTCT CGCTAGACCA CCTGGAGGTT TTGACAAATA   
  
  
- ATGTCCCTAG CTAGAGGGGG TCGGACCCAA GTCCGGTCGT CTTTCTCACC TTCGTTGTCC CTCTGCGAAC   
  
  
- CGGTTCATGA CACTCGCCAT ATTACACGGT AAACTCATAG TACGGTAACG AGTCCTCACC CTTTGTTAGT   
  
  
- TTGGTCCCCT AGAGTTCTAT CCTTCATCCT TACTACTTCA ACAACGCCAC TTGACAGAGA CATCCAAGTT   
  
  
- CTTGGAGGAA CTGCTCTGTC ACCACCACCT ATCAGGTTCC TTGTGTCAAA ATTTGGACTA ATGTTCCCAT   
  
  
- TTCGGACTAT AAAAACACGT ACCGCAACAT TTACCAAGGA TGTTGTAGGG AAAGAAACAC TGTGCAAAAT   
  
  
- CTCTTCGGGA GAAAGTAATA AGGTGAGAAA AGCTGTACAA TCTACGGTTG CGGAGATCCC TCCTCGGGCT   
  
  
- CTCCAACTAT AAGCTCTTCC GTAAGATACC CTCCCTCTAA TACTTACACC ACCGGACACT CCCGTGTCTC   
  
  
- TCCCACCTCT CCGGCCTCTG TATGTTCGTT ACCGTACAAT CCGTATCGTC CCGTCCCAAA GCCGTTCATG   
  
  
- GTAACCTAGG GTTCAACTAG CTCTTTTACT CCAAATTCCG GTTCCGTCTG GTGGTGTTCC TAAAGTACTA   
  
  
- GCTACACCTA CCTGTAACCC GTTAAGTCCC TACCTTCCCC GCCTAACGTG TACGCTAGAG ACGTACCCAA   
  
  
- GGCCGAAC

+     Myb-binding site

| Site Name | Organism | Position | Strand | Matrix score. | sequence | function |
| --- | --- | --- | --- | --- | --- | --- |
| Myb-binding site | Nicotiana tabacum | 3628 | + | 6 | CAACAG |  |

>HU07G02249.1   
+ -Up\_Stream \_Len000GATTTG ATTGTCGAAA TTTGTCCGAT TTTATAAAAT TGAAAATTGT TATTGGACTA   
  
  
+ TAATCTACTG TAGTTTAGAG GTGTTGGATA ATACTCTCAA CTTTGAACTT GGACGTCCAT GCACCACACT   
  
  
+ ATGGGCGTAT TTACTGTTTA TATACCAAGA ATAATTTTGA AGATGTTCTT AGTTGGTGAT GTAAGTGTAT   
  
  
+ ATTCAACATA TGTCATTACA TAAACTTGAA AATTCTTGAA TTTTAGACAT ATACAATTGC AAATCATTGT   
  
  
+ AAAATTGTTG CATTACATTT TGATAAAAAT GCATCCAAAC TTTGTCTTGG TATAAACAGT AAATAGGCCC   
  
  
+ ATAGGTCCAT AGTGTAGTGC ATGGACGTCC AAGTTTAAAG GTGGGAGTAT TATTGCCCAA CGCCCCTAAA   
  
  
+ GTACAGTATA TTATAGTCCA ATATCCCAAT TAATTTTTCA TTTGAATATT TAAATTTGGA CATAATATTT   
  
  
+ TTCATATTTG AATTCTCTAA ACCTGATTTT AACCTGAATT TAAGTTGATT TAATTCATCC GACCAAAAAA   
  
  
+ ATAAACACAA TTATTTATTT TTCTATTCGA AGTTAGATAG TAGCCAATTC ATTTGACATA TAATTAACCC   
  
  
+ GTGATTGACA CAAACTCGAG TTTTTGCTTA GAGCTAGGAA AAGAGTCGGG TTAGGTCGAC CCATTTAAGA   
  
  
+ AATGGGTCGA CTTCATGTCG GCCTTCAAAA TAGTTAAGAT TTGCTCAAAA CTAAAAAATA ACAAGTTCAC   
  
  
+ ACTAAACGGT CAAATCGTGT CGACTTCGGA TTGAAATTGG TTCAGTTTGG GTCGGCTTCG TATTTCTTTC   
  
  
+ AGTAACTTCG AACCGAATTC GTATCGTGTT ATCGTGTCAG GTCAGACTTT CTCAGTTCTA TTTTTGCTAC   
  
  
+ CTTATGTTAC GGATAAAAAA AGTCTCACAT TGGAAAAAGT GTGGGAGGGT CCTGGGCTTA TAAATAGTGG   
  
  
+ CGCTGCACAC CTCATCAGAC CGACCTTTTG GGGAGAGGGT AATGACCCCG TGACAAGTGG TATCAGAGTC   
  
  
+ AACCCAGACC CGACTCAGAT GTCCTGCCAA GCGCTCCGAC GGGCGGGGTA GGCAGGGGGC CCATTTGTGA   
  
  
+ TGAGGACGTC ACAGAATTGG GCGGGGGAGA GTGTCACGGA TAAAAAAAGT CCCACATCGA AAAAAGTGTG   
  
  
+ GGAGGGTCCT GGGCTTATAA ACGGTAGCGC CGCACACCCC ATCAGATCGA CTTTTTGGGG AGAGGGTAAT   
  
  
+ GACCCCATTT CAAGTGGTAT CAGAGCCAAC CCAGACCCGA CTCCAATGTC CCACCAAGCG CTCTGATGGG   
  
  
+ CGGGGTAGGC AAGGGGCTCC ATATGTGACG AGGGCGTCAC GGAATTGGAT GGGAGAGAGT ACCACGGATA   
  
  
+ AAAAAAGTCC CACATCAGAA AAAGTGTGGA CTTAGCAACT AGGAAGTAAA GTTACAGTAT TAGCCGAGAG   
  
  
+ CCCATGGAAC GCCTGAAACT GACATCACCA GACCCACATA TTTCACCATC ATCTGATCTC CCCGTCAAAG   
  
  
+ GAGGAAGCTG AGCACAGCAA GAACTAGAAG AACAGGCGGC ATCACACCCA AGCCAAAATT CTCTATTTGA   
  
  
+ CCTCCCTCTC CTCATTCCTC TGAGCTTCCT CTTCACAATG TAAGACCATA ATCCTTAACA CCCTTCTTTT   
  
  
+ TTAAGCTCAT CTTTACCTGC TCTGGTTCTC TCTCTCATCG TCACTAATAG TTACCTCTTT ACTTTCTCTT   
  
  
+ CCCTGAGGTA TGCTTCAACT TTAACATATA TATCCCACTT GATTTCTCTT GTCTTTTTGC TATAATACTT   
  
  
+ GTTGTTTGGC TATCATCCCC TGTTTTATAC TCATTTTCTT GCTTTCTATT TCTGGGTTTG AATTGGGTTT   
  
  
+ CACTTATCCT CGTCAATTCT ATGGGTAATT AGTGAATTGG GTTTATATGA ACTAGGGTTG CAAAAGTATC   
  
  
+ TACTTTTTCT CTTGTGATTA TTAGTTGATT AGGGATTTGG CCAATATTAG CAGAATGGGT TCTGAATTTG   
  
  
+ CGGAATTCTC TGATGATGCT CTAAATGGGT ATGCTTATGT TGATATCCCT GCTTATGATG CATCCTTAGA   
  
  
+ TTATGCCAAT TTGTTCAATT ACGAAGGCCC ATCTGAGGAT CGCACCTCAC TGAGCCTCCC AAGACCCTTT   
  
  
+ TCTGACCCTT TGGCTTACAG TTTCACGTCC TCTTCTGAGC TGGGCCCTGG GGTTGATTCT AATGATGATA   
  
  
+ GTGATTCTGG CGATGTTCTC AAGTACATTA GCCAAATGCT TATGGAAGAA GACATGGAGG CAAAGCCATG   
  
  
+ CATGTTTCAT GATCCTTTAG CACTTCAGGC TGCTGAGAAA CCCTTTTATG ATGCCTTAGG GGAGCAATGC   
  
  
+ CCAACTTCTC CTGACCAACA TCCTATAATT GATCATTATT TGGATAGTCC TGATGAAAAT TCTTTGAGTT   
  
  
+ CAACTGGTGA TTTTAGTGTT AGTCATTCTG GGTCTAGTTC AACAAACTCC GTTGGACCGA TAATAGTGTC   
  
  
+ TGATTTGAGT GAGCATTTTG AGCCACCCTT TGTTGAAACA CTTCCAATTG AATCATATCA CCAACCATTG   
  
  
+ ACCCGTCCTC AATGGTCATT TGGCTCTTCG GGTGCCTTAG ATGGCACGGC CTCTAATGGT TCGGTGATCT   
  
  
+ CATCCCTTGG TTTGCCAGTG GATGTGATTA GCGTATTTAG GGAGAAAGAG TCCATGATTC AATTTCAGAA   
  
  
+ AGGGGTGGAG GAGGCCAGTA AGTTCCTTCC CAAGAATAAT AACCTTGTTA TTGATCTCGA GAACCTCACT   
  
  
+ TTTCCTAATG AAACAAAGGA GGATGATCGA ATGATGATGG TTAAGAAGGA AAAGGATGAC GTGAATTGGT   
  
  
+ CTAACTACTC AAGAGGGAGT AAGATTCACT ATTGTGAAGA CGAGGCCTTT GAAGAAGGAA GGAGTGGCAA   
  
  
+ GCAGTCAGCT ATTTCTTCTA CTGAGGAAGC TGAGTTTTCT GACATTTTTG ACAAGGTTTT GCTTTGCGAT   
  
  
+ TGCTACCCTG TGAAACCTGA GGCTCATCCC ACCATGAGTT TGAACCCTGA GAAGGGCCAG TCACATGGAT   
  
  
+ TAGAAGGTGG GAGAAATGGG AAGGCTCGCC CTAAGAAACA GGATAATAGT AGCACAAATA TTGTGGATTT   
  
  
+ AAGGAATTTG CTGATACTAT GCGCACAATC TACTGCATCT GATGACCGAA GAACTGCTGA TGGACTGCTA   
  
  
+ AAGCAAATCA GGGAGCACTC GTCTGCTGAG GGGGATGGAT CTCAAAGGTT GGCGCATTAC TTTGCTGATG   
  
  
+ CCCTAGAGGC ACGTTTAGCT GGAACTGGAT CTCGCATTTA TACGGCCCTA TGTTCTAATA GGCCATCTGT   
  
  
+ CACTGACATG ATAAAAGCAT ATCAGTTCTA TATTCGTGCT TGCCCATTTA CGAAGATCGT CATTGGTTGT   
  
  
+ GGTACCCATA TGATTCTAAA AGCAGCTGAG AAGGCATCAA AGCTTCATAT TATAGATTTT GGCATCCTCT   
  
  
+ ATGGTTGCCA ATGGCCCAAC CTCATTCAAC GCCTCTCAGA GCGATCTGGT GGACCTCCAA AACTGTTTAT   
  
  
+ TACAGGGATC GATCTCCCCC AGCCTGGGTT CAGGCCAGCA GAAAGAGTGG AAGCAACAGG GAGACGCTTG   
  
  
+ GCCAAGTACT GTGAGCGGTA TAATGTGCCA TTTGAGTATC ATGCCATTGC TCAGGAGTGG GAAACAATCA   
  
  
+ AACCAGGGGA TCTCAAGATA GGAAGTAGGA ATGATGAAGT TGTTGCGGTG AACTGTCTCT GTAGGTTCAA   
  
  
+ GAACCTCCTT GACGAGACAG TGGTGGTGGA TAGTCCAAGG AACACAGTTT TAAACCTGAT TACAAGGGTA   
  
  
+ AAGCCTGATA TTTTTGTGCA TGGCGTTGTA AATGGTTCCT ACAACATCCC TTTCTTTGTG ACACGTTTTA   
  
  
+ GAGAAGCCCT CTTTCATTAT TCCACTCTTT TCGACATGTT AGATGCCAAC GCCTCTAGGG AGGAGCCCGA   
  
  
+ GAGGTTGATA TTCGAGAAGG CATTCTATGG GAGGGAGATT ATGAATGTGG TGGCCTGTGA GGGCACAGAG   
  
  
+ AGGGTGGAGA GGCCGGAGAC ATACAAGCAA TGGCATGTTA GGCATAGCAG GGCAGGGTTT CGGCAAGTAC   
  
  
+ CATTGGATCC CAAGTTGATC GAGAAAATGA GGTTTAAGGC CAAGGCAGAC CACCACAAGG ATTTCATGAT   
  
  
+ CGATGTGGAT GGACATTGGG CAATTCAGGG ATGGAAGGGG CGGATTGCAC ATGCGATCTC TGCATGGGTT   
  
  
+ CCGGCTTG  

- -Up\_Stream \_Len000CTAAAC TAACAGCTTT AAACAGGCTA AAATATTTTA ACTTTTAACA ATAACCTGAT   
  
  
- ATTAGATGAC ATCAAATCTC CACAACCTAT TATGAGAGTT GAAACTTGAA CCTGCAGGTA CGTGGTGTGA   
  
  
- TACCCGCATA AATGACAAAT ATATGGTTCT TATTAAAACT TCTACAAGAA TCAACCACTA CATTCACATA   
  
  
- TAAGTTGTAT ACAGTAATGT ATTTGAACTT TTAAGAACTT AAAATCTGTA TATGTTAACG TTTAGTAACA   
  
  
- TTTTAACAAC GTAATGTAAA ACTATTTTTA CGTAGGTTTG AAACAGAACC ATATTTGTCA TTTATCCGGG   
  
  
- TATCCAGGTA TCACATCACG TACCTGCAGG TTCAAATTTC CACCCTCATA ATAACGGGTT GCGGGGATTT   
  
  
- CATGTCATAT AATATCAGGT TATAGGGTTA ATTAAAAAGT AAACTTATAA ATTTAAACCT GTATTATAAA   
  
  
- AAGTATAAAC TTAAGAGATT TGGACTAAAA TTGGACTTAA ATTCAACTAA ATTAAGTAGG CTGGTTTTTT   
  
  
- TATTTGTGTT AATAAATAAA AAGATAAGCT TCAATCTATC ATCGGTTAAG TAAACTGTAT ATTAATTGGG   
  
  
- CACTAACTGT GTTTGAGCTC AAAAACGAAT CTCGATCCTT TTCTCAGCCC AATCCAGCTG GGTAAATTCT   
  
  
- TTACCCAGCT GAAGTACAGC CGGAAGTTTT ATCAATTCTA AACGAGTTTT GATTTTTTAT TGTTCAAGTG   
  
  
- TGATTTGCCA GTTTAGCACA GCTGAAGCCT AACTTTAACC AAGTCAAACC CAGCCGAAGC ATAAAGAAAG   
  
  
- TCATTGAAGC TTGGCTTAAG CATAGCACAA TAGCACAGTC CAGTCTGAAA GAGTCAAGAT AAAAACGATG   
  
  
- GAATACAATG CCTATTTTTT TCAGAGTGTA ACCTTTTTCA CACCCTCCCA GGACCCGAAT ATTTATCACC   
  
  
- GCGACGTGTG GAGTAGTCTG GCTGGAAAAC CCCTCTCCCA TTACTGGGGC ACTGTTCACC ATAGTCTCAG   
  
  
- TTGGGTCTGG GCTGAGTCTA CAGGACGGTT CGCGAGGCTG CCCGCCCCAT CCGTCCCCCG GGTAAACACT   
  
  
- ACTCCTGCAG TGTCTTAACC CGCCCCCTCT CACAGTGCCT ATTTTTTTCA GGGTGTAGCT TTTTTCACAC   
  
  
- CCTCCCAGGA CCCGAATATT TGCCATCGCG GCGTGTGGGG TAGTCTAGCT GAAAAACCCC TCTCCCATTA   
  
  
- CTGGGGTAAA GTTCACCATA GTCTCGGTTG GGTCTGGGCT GAGGTTACAG GGTGGTTCGC GAGACTACCC   
  
  
- GCCCCATCCG TTCCCCGAGG TATACACTGC TCCCGCAGTG CCTTAACCTA CCCTCTCTCA TGGTGCCTAT   
  
  
- TTTTTTCAGG GTGTAGTCTT TTTCACACCT GAATCGTTGA TCCTTCATTT CAATGTCATA ATCGGCTCTC   
  
  
- GGGTACCTTG CGGACTTTGA CTGTAGTGGT CTGGGTGTAT AAAGTGGTAG TAGACTAGAG GGGCAGTTTC   
  
  
- CTCCTTCGAC TCGTGTCGTT CTTGATCTTC TTGTCCGCCG TAGTGTGGGT TCGGTTTTAA GAGATAAACT   
  
  
- GGAGGGAGAG GAGTAAGGAG ACTCGAAGGA GAAGTGTTAC ATTCTGGTAT TAGGAATTGT GGGAAGAAAA   
  
  
- AATTCGAGTA GAAATGGACG AGACCAAGAG AGAGAGTAGC AGTGATTATC AATGGAGAAA TGAAAGAGAA   
  
  
- GGGACTCCAT ACGAAGTTGA AATTGTATAT ATAGGGTGAA CTAAAGAGAA CAGAAAAACG ATATTATGAA   
  
  
- CAACAAACCG ATAGTAGGGG ACAAAATATG AGTAAAAGAA CGAAAGATAA AGACCCAAAC TTAACCCAAA   
  
  
- GTGAATAGGA GCAGTTAAGA TACCCATTAA TCACTTAACC CAAATATACT TGATCCCAAC GTTTTCATAG   
  
  
- ATGAAAAAGA GAACACTAAT AATCAACTAA TCCCTAAACC GGTTATAATC GTCTTACCCA AGACTTAAAC   
  
  
- GCCTTAAGAG ACTACTACGA GATTTACCCA TACGAATACA ACTATAGGGA CGAATACTAC GTAGGAATCT   
  
  
- AATACGGTTA AACAAGTTAA TGCTTCCGGG TAGACTCCTA GCGTGGAGTG ACTCGGAGGG TTCTGGGAAA   
  
  
- AGACTGGGAA ACCGAATGTC AAAGTGCAGG AGAAGACTCG ACCCGGGACC CCAACTAAGA TTACTACTAT   
  
  
- CACTAAGACC GCTACAAGAG TTCATGTAAT CGGTTTACGA ATACCTTCTT CTGTACCTCC GTTTCGGTAC   
  
  
- GTACAAAGTA CTAGGAAATC GTGAAGTCCG ACGACTCTTT GGGAAAATAC TACGGAATCC CCTCGTTACG   
  
  
- GGTTGAAGAG GACTGGTTGT AGGATATTAA CTAGTAATAA ACCTATCAGG ACTACTTTTA AGAAACTCAA   
  
  
- GTTGACCACT AAAATCACAA TCAGTAAGAC CCAGATCAAG TTGTTTGAGG CAACCTGGCT ATTATCACAG   
  
  
- ACTAAACTCA CTCGTAAAAC TCGGTGGGAA ACAACTTTGT GAAGGTTAAC TTAGTATAGT GGTTGGTAAC   
  
  
- TGGGCAGGAG TTACCAGTAA ACCGAGAAGC CCACGGAATC TACCGTGCCG GAGATTACCA AGCCACTAGA   
  
  
- GTAGGGAACC AAACGGTCAC CTACACTAAT CGCATAAATC CCTCTTTCTC AGGTACTAAG TTAAAGTCTT   
  
  
- TCCCCACCTC CTCCGGTCAT TCAAGGAAGG GTTCTTATTA TTGGAACAAT AACTAGAGCT CTTGGAGTGA   
  
  
- AAAGGATTAC TTTGTTTCCT CCTACTAGCT TACTACTACC AATTCTTCCT TTTCCTACTG CACTTAACCA   
  
  
- GATTGATGAG TTCTCCCTCA TTCTAAGTGA TAACACTTCT GCTCCGGAAA CTTCTTCCTT CCTCACCGTT   
  
  
- CGTCAGTCGA TAAAGAAGAT GACTCCTTCG ACTCAAAAGA CTGTAAAAAC TGTTCCAAAA CGAAACGCTA   
  
  
- ACGATGGGAC ACTTTGGACT CCGAGTAGGG TGGTACTCAA ACTTGGGACT CTTCCCGGTC AGTGTACCTA   
  
  
- ATCTTCCACC CTCTTTACCC TTCCGAGCGG GATTCTTTGT CCTATTATCA TCGTGTTTAT AACACCTAAA   
  
  
- TTCCTTAAAC GACTATGATA CGCGTGTTAG ATGACGTAGA CTACTGGCTT CTTGACGACT ACCTGACGAT   
  
  
- TTCGTTTAGT CCCTCGTGAG CAGACGACTC CCCCTACCTA GAGTTTCCAA CCGCGTAATG AAACGACTAC   
  
  
- GGGATCTCCG TGCAAATCGA CCTTGACCTA GAGCGTAAAT ATGCCGGGAT ACAAGATTAT CCGGTAGACA   
  
  
- GTGACTGTAC TATTTTCGTA TAGTCAAGAT ATAAGCACGA ACGGGTAAAT GCTTCTAGCA GTAACCAACA   
  
  
- CCATGGGTAT ACTAAGATTT TCGTCGACTC TTCCGTAGTT TCGAAGTATA ATATCTAAAA CCGTAGGAGA   
  
  
- TACCAACGGT TACCGGGTTG GAGTAAGTTG CGGAGAGTCT CGCTAGACCA CCTGGAGGTT TTGACAAATA   
  
  
- ATGTCCCTAG CTAGAGGGGG TCGGACCCAA GTCCGGTCGT CTTTCTCACC TTCGTTGTCC CTCTGCGAAC   
  
  
- CGGTTCATGA CACTCGCCAT ATTACACGGT AAACTCATAG TACGGTAACG AGTCCTCACC CTTTGTTAGT   
  
  
- TTGGTCCCCT AGAGTTCTAT CCTTCATCCT TACTACTTCA ACAACGCCAC TTGACAGAGA CATCCAAGTT   
  
  
- CTTGGAGGAA CTGCTCTGTC ACCACCACCT ATCAGGTTCC TTGTGTCAAA ATTTGGACTA ATGTTCCCAT   
  
  
- TTCGGACTAT AAAAACACGT ACCGCAACAT TTACCAAGGA TGTTGTAGGG AAAGAAACAC TGTGCAAAAT   
  
  
- CTCTTCGGGA GAAAGTAATA AGGTGAGAAA AGCTGTACAA TCTACGGTTG CGGAGATCCC TCCTCGGGCT   
  
  
- CTCCAACTAT AAGCTCTTCC GTAAGATACC CTCCCTCTAA TACTTACACC ACCGGACACT CCCGTGTCTC   
  
  
- TCCCACCTCT CCGGCCTCTG TATGTTCGTT ACCGTACAAT CCGTATCGTC CCGTCCCAAA GCCGTTCATG   
  
  
- GTAACCTAGG GTTCAACTAG CTCTTTTACT CCAAATTCCG GTTCCGTCTG GTGGTGTTCC TAAAGTACTA   
  
  
- GCTACACCTA CCTGTAACCC GTTAAGTCCC TACCTTCCCC GCCTAACGTG TACGCTAGAG ACGTACCCAA   
  
  
- GGCCGAAC

+     O2-site

| Site Name | Organism | Position | Strand | Matrix score. | sequence | function |
| --- | --- | --- | --- | --- | --- | --- |
| O2-site | Zea mays | 2859 | + | 9 | GTTGACGTGA | cis-acting regulatory element involved in zein metabolism regulation |
| O2-site | Zea mays | 2292 | + | 9 | GATGACATGG | cis-acting regulatory element involved in zein metabolism regulation |

>HU07G02249.1   
+ -Up\_Stream \_Len000GATTTG ATTGTCGAAA TTTGTCCGAT TTTATAAAAT TGAAAATTGT TATTGGACTA   
  
  
+ TAATCTACTG TAGTTTAGAG GTGTTGGATA ATACTCTCAA CTTTGAACTT GGACGTCCAT GCACCACACT   
  
  
+ ATGGGCGTAT TTACTGTTTA TATACCAAGA ATAATTTTGA AGATGTTCTT AGTTGGTGAT GTAAGTGTAT   
  
  
+ ATTCAACATA TGTCATTACA TAAACTTGAA AATTCTTGAA TTTTAGACAT ATACAATTGC AAATCATTGT   
  
  
+ AAAATTGTTG CATTACATTT TGATAAAAAT GCATCCAAAC TTTGTCTTGG TATAAACAGT AAATAGGCCC   
  
  
+ ATAGGTCCAT AGTGTAGTGC ATGGACGTCC AAGTTTAAAG GTGGGAGTAT TATTGCCCAA CGCCCCTAAA   
  
  
+ GTACAGTATA TTATAGTCCA ATATCCCAAT TAATTTTTCA TTTGAATATT TAAATTTGGA CATAATATTT   
  
  
+ TTCATATTTG AATTCTCTAA ACCTGATTTT AACCTGAATT TAAGTTGATT TAATTCATCC GACCAAAAAA   
  
  
+ ATAAACACAA TTATTTATTT TTCTATTCGA AGTTAGATAG TAGCCAATTC ATTTGACATA TAATTAACCC   
  
  
+ GTGATTGACA CAAACTCGAG TTTTTGCTTA GAGCTAGGAA AAGAGTCGGG TTAGGTCGAC CCATTTAAGA   
  
  
+ AATGGGTCGA CTTCATGTCG GCCTTCAAAA TAGTTAAGAT TTGCTCAAAA CTAAAAAATA ACAAGTTCAC   
  
  
+ ACTAAACGGT CAAATCGTGT CGACTTCGGA TTGAAATTGG TTCAGTTTGG GTCGGCTTCG TATTTCTTTC   
  
  
+ AGTAACTTCG AACCGAATTC GTATCGTGTT ATCGTGTCAG GTCAGACTTT CTCAGTTCTA TTTTTGCTAC   
  
  
+ CTTATGTTAC GGATAAAAAA AGTCTCACAT TGGAAAAAGT GTGGGAGGGT CCTGGGCTTA TAAATAGTGG   
  
  
+ CGCTGCACAC CTCATCAGAC CGACCTTTTG GGGAGAGGGT AATGACCCCG TGACAAGTGG TATCAGAGTC   
  
  
+ AACCCAGACC CGACTCAGAT GTCCTGCCAA GCGCTCCGAC GGGCGGGGTA GGCAGGGGGC CCATTTGTGA   
  
  
+ TGAGGACGTC ACAGAATTGG GCGGGGGAGA GTGTCACGGA TAAAAAAAGT CCCACATCGA AAAAAGTGTG   
  
  
+ GGAGGGTCCT GGGCTTATAA ACGGTAGCGC CGCACACCCC ATCAGATCGA CTTTTTGGGG AGAGGGTAAT   
  
  
+ GACCCCATTT CAAGTGGTAT CAGAGCCAAC CCAGACCCGA CTCCAATGTC CCACCAAGCG CTCTGATGGG   
  
  
+ CGGGGTAGGC AAGGGGCTCC ATATGTGACG AGGGCGTCAC GGAATTGGAT GGGAGAGAGT ACCACGGATA   
  
  
+ AAAAAAGTCC CACATCAGAA AAAGTGTGGA CTTAGCAACT AGGAAGTAAA GTTACAGTAT TAGCCGAGAG   
  
  
+ CCCATGGAAC GCCTGAAACT GACATCACCA GACCCACATA TTTCACCATC ATCTGATCTC CCCGTCAAAG   
  
  
+ GAGGAAGCTG AGCACAGCAA GAACTAGAAG AACAGGCGGC ATCACACCCA AGCCAAAATT CTCTATTTGA   
  
  
+ CCTCCCTCTC CTCATTCCTC TGAGCTTCCT CTTCACAATG TAAGACCATA ATCCTTAACA CCCTTCTTTT   
  
  
+ TTAAGCTCAT CTTTACCTGC TCTGGTTCTC TCTCTCATCG TCACTAATAG TTACCTCTTT ACTTTCTCTT   
  
  
+ CCCTGAGGTA TGCTTCAACT TTAACATATA TATCCCACTT GATTTCTCTT GTCTTTTTGC TATAATACTT   
  
  
+ GTTGTTTGGC TATCATCCCC TGTTTTATAC TCATTTTCTT GCTTTCTATT TCTGGGTTTG AATTGGGTTT   
  
  
+ CACTTATCCT CGTCAATTCT ATGGGTAATT AGTGAATTGG GTTTATATGA ACTAGGGTTG CAAAAGTATC   
  
  
+ TACTTTTTCT CTTGTGATTA TTAGTTGATT AGGGATTTGG CCAATATTAG CAGAATGGGT TCTGAATTTG   
  
  
+ CGGAATTCTC TGATGATGCT CTAAATGGGT ATGCTTATGT TGATATCCCT GCTTATGATG CATCCTTAGA   
  
  
+ TTATGCCAAT TTGTTCAATT ACGAAGGCCC ATCTGAGGAT CGCACCTCAC TGAGCCTCCC AAGACCCTTT   
  
  
+ TCTGACCCTT TGGCTTACAG TTTCACGTCC TCTTCTGAGC TGGGCCCTGG GGTTGATTCT AATGATGATA   
  
  
+ GTGATTCTGG CGATGTTCTC AAGTACATTA GCCAAATGCT TATGGAAGAA GACATGGAGG CAAAGCCATG   
  
  
+ CATGTTTCAT GATCCTTTAG CACTTCAGGC TGCTGAGAAA CCCTTTTATG ATGCCTTAGG GGAGCAATGC   
  
  
+ CCAACTTCTC CTGACCAACA TCCTATAATT GATCATTATT TGGATAGTCC TGATGAAAAT TCTTTGAGTT   
  
  
+ CAACTGGTGA TTTTAGTGTT AGTCATTCTG GGTCTAGTTC AACAAACTCC GTTGGACCGA TAATAGTGTC   
  
  
+ TGATTTGAGT GAGCATTTTG AGCCACCCTT TGTTGAAACA CTTCCAATTG AATCATATCA CCAACCATTG   
  
  
+ ACCCGTCCTC AATGGTCATT TGGCTCTTCG GGTGCCTTAG ATGGCACGGC CTCTAATGGT TCGGTGATCT   
  
  
+ CATCCCTTGG TTTGCCAGTG GATGTGATTA GCGTATTTAG GGAGAAAGAG TCCATGATTC AATTTCAGAA   
  
  
+ AGGGGTGGAG GAGGCCAGTA AGTTCCTTCC CAAGAATAAT AACCTTGTTA TTGATCTCGA GAACCTCACT   
  
  
+ TTTCCTAATG AAACAAAGGA GGATGATCGA ATGATGATGG TTAAGAAGGA AAAGGATGAC GTGAATTGGT   
  
  
+ CTAACTACTC AAGAGGGAGT AAGATTCACT ATTGTGAAGA CGAGGCCTTT GAAGAAGGAA GGAGTGGCAA   
  
  
+ GCAGTCAGCT ATTTCTTCTA CTGAGGAAGC TGAGTTTTCT GACATTTTTG ACAAGGTTTT GCTTTGCGAT   
  
  
+ TGCTACCCTG TGAAACCTGA GGCTCATCCC ACCATGAGTT TGAACCCTGA GAAGGGCCAG TCACATGGAT   
  
  
+ TAGAAGGTGG GAGAAATGGG AAGGCTCGCC CTAAGAAACA GGATAATAGT AGCACAAATA TTGTGGATTT   
  
  
+ AAGGAATTTG CTGATACTAT GCGCACAATC TACTGCATCT GATGACCGAA GAACTGCTGA TGGACTGCTA   
  
  
+ AAGCAAATCA GGGAGCACTC GTCTGCTGAG GGGGATGGAT CTCAAAGGTT GGCGCATTAC TTTGCTGATG   
  
  
+ CCCTAGAGGC ACGTTTAGCT GGAACTGGAT CTCGCATTTA TACGGCCCTA TGTTCTAATA GGCCATCTGT   
  
  
+ CACTGACATG ATAAAAGCAT ATCAGTTCTA TATTCGTGCT TGCCCATTTA CGAAGATCGT CATTGGTTGT   
  
  
+ GGTACCCATA TGATTCTAAA AGCAGCTGAG AAGGCATCAA AGCTTCATAT TATAGATTTT GGCATCCTCT   
  
  
+ ATGGTTGCCA ATGGCCCAAC CTCATTCAAC GCCTCTCAGA GCGATCTGGT GGACCTCCAA AACTGTTTAT   
  
  
+ TACAGGGATC GATCTCCCCC AGCCTGGGTT CAGGCCAGCA GAAAGAGTGG AAGCAACAGG GAGACGCTTG   
  
  
+ GCCAAGTACT GTGAGCGGTA TAATGTGCCA TTTGAGTATC ATGCCATTGC TCAGGAGTGG GAAACAATCA   
  
  
+ AACCAGGGGA TCTCAAGATA GGAAGTAGGA ATGATGAAGT TGTTGCGGTG AACTGTCTCT GTAGGTTCAA   
  
  
+ GAACCTCCTT GACGAGACAG TGGTGGTGGA TAGTCCAAGG AACACAGTTT TAAACCTGAT TACAAGGGTA   
  
  
+ AAGCCTGATA TTTTTGTGCA TGGCGTTGTA AATGGTTCCT ACAACATCCC TTTCTTTGTG ACACGTTTTA   
  
  
+ GAGAAGCCCT CTTTCATTAT TCCACTCTTT TCGACATGTT AGATGCCAAC GCCTCTAGGG AGGAGCCCGA   
  
  
+ GAGGTTGATA TTCGAGAAGG CATTCTATGG GAGGGAGATT ATGAATGTGG TGGCCTGTGA GGGCACAGAG   
  
  
+ AGGGTGGAGA GGCCGGAGAC ATACAAGCAA TGGCATGTTA GGCATAGCAG GGCAGGGTTT CGGCAAGTAC   
  
  
+ CATTGGATCC CAAGTTGATC GAGAAAATGA GGTTTAAGGC CAAGGCAGAC CACCACAAGG ATTTCATGAT   
  
  
+ CGATGTGGAT GGACATTGGG CAATTCAGGG ATGGAAGGGG CGGATTGCAC ATGCGATCTC TGCATGGGTT   
  
  
+ CCGGCTTG  

- -Up\_Stream \_Len000CTAAAC TAACAGCTTT AAACAGGCTA AAATATTTTA ACTTTTAACA ATAACCTGAT   
  
  
- ATTAGATGAC ATCAAATCTC CACAACCTAT TATGAGAGTT GAAACTTGAA CCTGCAGGTA CGTGGTGTGA   
  
  
- TACCCGCATA AATGACAAAT ATATGGTTCT TATTAAAACT TCTACAAGAA TCAACCACTA CATTCACATA   
  
  
- TAAGTTGTAT ACAGTAATGT ATTTGAACTT TTAAGAACTT AAAATCTGTA TATGTTAACG TTTAGTAACA   
  
  
- TTTTAACAAC GTAATGTAAA ACTATTTTTA CGTAGGTTTG AAACAGAACC ATATTTGTCA TTTATCCGGG   
  
  
- TATCCAGGTA TCACATCACG TACCTGCAGG TTCAAATTTC CACCCTCATA ATAACGGGTT GCGGGGATTT   
  
  
- CATGTCATAT AATATCAGGT TATAGGGTTA ATTAAAAAGT AAACTTATAA ATTTAAACCT GTATTATAAA   
  
  
- AAGTATAAAC TTAAGAGATT TGGACTAAAA TTGGACTTAA ATTCAACTAA ATTAAGTAGG CTGGTTTTTT   
  
  
- TATTTGTGTT AATAAATAAA AAGATAAGCT TCAATCTATC ATCGGTTAAG TAAACTGTAT ATTAATTGGG   
  
  
- CACTAACTGT GTTTGAGCTC AAAAACGAAT CTCGATCCTT TTCTCAGCCC AATCCAGCTG GGTAAATTCT   
  
  
- TTACCCAGCT GAAGTACAGC CGGAAGTTTT ATCAATTCTA AACGAGTTTT GATTTTTTAT TGTTCAAGTG   
  
  
- TGATTTGCCA GTTTAGCACA GCTGAAGCCT AACTTTAACC AAGTCAAACC CAGCCGAAGC ATAAAGAAAG   
  
  
- TCATTGAAGC TTGGCTTAAG CATAGCACAA TAGCACAGTC CAGTCTGAAA GAGTCAAGAT AAAAACGATG   
  
  
- GAATACAATG CCTATTTTTT TCAGAGTGTA ACCTTTTTCA CACCCTCCCA GGACCCGAAT ATTTATCACC   
  
  
- GCGACGTGTG GAGTAGTCTG GCTGGAAAAC CCCTCTCCCA TTACTGGGGC ACTGTTCACC ATAGTCTCAG   
  
  
- TTGGGTCTGG GCTGAGTCTA CAGGACGGTT CGCGAGGCTG CCCGCCCCAT CCGTCCCCCG GGTAAACACT   
  
  
- ACTCCTGCAG TGTCTTAACC CGCCCCCTCT CACAGTGCCT ATTTTTTTCA GGGTGTAGCT TTTTTCACAC   
  
  
- CCTCCCAGGA CCCGAATATT TGCCATCGCG GCGTGTGGGG TAGTCTAGCT GAAAAACCCC TCTCCCATTA   
  
  
- CTGGGGTAAA GTTCACCATA GTCTCGGTTG GGTCTGGGCT GAGGTTACAG GGTGGTTCGC GAGACTACCC   
  
  
- GCCCCATCCG TTCCCCGAGG TATACACTGC TCCCGCAGTG CCTTAACCTA CCCTCTCTCA TGGTGCCTAT   
  
  
- TTTTTTCAGG GTGTAGTCTT TTTCACACCT GAATCGTTGA TCCTTCATTT CAATGTCATA ATCGGCTCTC   
  
  
- GGGTACCTTG CGGACTTTGA CTGTAGTGGT CTGGGTGTAT AAAGTGGTAG TAGACTAGAG GGGCAGTTTC   
  
  
- CTCCTTCGAC TCGTGTCGTT CTTGATCTTC TTGTCCGCCG TAGTGTGGGT TCGGTTTTAA GAGATAAACT   
  
  
- GGAGGGAGAG GAGTAAGGAG ACTCGAAGGA GAAGTGTTAC ATTCTGGTAT TAGGAATTGT GGGAAGAAAA   
  
  
- AATTCGAGTA GAAATGGACG AGACCAAGAG AGAGAGTAGC AGTGATTATC AATGGAGAAA TGAAAGAGAA   
  
  
- GGGACTCCAT ACGAAGTTGA AATTGTATAT ATAGGGTGAA CTAAAGAGAA CAGAAAAACG ATATTATGAA   
  
  
- CAACAAACCG ATAGTAGGGG ACAAAATATG AGTAAAAGAA CGAAAGATAA AGACCCAAAC TTAACCCAAA   
  
  
- GTGAATAGGA GCAGTTAAGA TACCCATTAA TCACTTAACC CAAATATACT TGATCCCAAC GTTTTCATAG   
  
  
- ATGAAAAAGA GAACACTAAT AATCAACTAA TCCCTAAACC GGTTATAATC GTCTTACCCA AGACTTAAAC   
  
  
- GCCTTAAGAG ACTACTACGA GATTTACCCA TACGAATACA ACTATAGGGA CGAATACTAC GTAGGAATCT   
  
  
- AATACGGTTA AACAAGTTAA TGCTTCCGGG TAGACTCCTA GCGTGGAGTG ACTCGGAGGG TTCTGGGAAA   
  
  
- AGACTGGGAA ACCGAATGTC AAAGTGCAGG AGAAGACTCG ACCCGGGACC CCAACTAAGA TTACTACTAT   
  
  
- CACTAAGACC GCTACAAGAG TTCATGTAAT CGGTTTACGA ATACCTTCTT CTGTACCTCC GTTTCGGTAC   
  
  
- GTACAAAGTA CTAGGAAATC GTGAAGTCCG ACGACTCTTT GGGAAAATAC TACGGAATCC CCTCGTTACG   
  
  
- GGTTGAAGAG GACTGGTTGT AGGATATTAA CTAGTAATAA ACCTATCAGG ACTACTTTTA AGAAACTCAA   
  
  
- GTTGACCACT AAAATCACAA TCAGTAAGAC CCAGATCAAG TTGTTTGAGG CAACCTGGCT ATTATCACAG   
  
  
- ACTAAACTCA CTCGTAAAAC TCGGTGGGAA ACAACTTTGT GAAGGTTAAC TTAGTATAGT GGTTGGTAAC   
  
  
- TGGGCAGGAG TTACCAGTAA ACCGAGAAGC CCACGGAATC TACCGTGCCG GAGATTACCA AGCCACTAGA   
  
  
- GTAGGGAACC AAACGGTCAC CTACACTAAT CGCATAAATC CCTCTTTCTC AGGTACTAAG TTAAAGTCTT   
  
  
- TCCCCACCTC CTCCGGTCAT TCAAGGAAGG GTTCTTATTA TTGGAACAAT AACTAGAGCT CTTGGAGTGA   
  
  
- AAAGGATTAC TTTGTTTCCT CCTACTAGCT TACTACTACC AATTCTTCCT TTTCCTACTG CACTTAACCA   
  
  
- GATTGATGAG TTCTCCCTCA TTCTAAGTGA TAACACTTCT GCTCCGGAAA CTTCTTCCTT CCTCACCGTT   
  
  
- CGTCAGTCGA TAAAGAAGAT GACTCCTTCG ACTCAAAAGA CTGTAAAAAC TGTTCCAAAA CGAAACGCTA   
  
  
- ACGATGGGAC ACTTTGGACT CCGAGTAGGG TGGTACTCAA ACTTGGGACT CTTCCCGGTC AGTGTACCTA   
  
  
- ATCTTCCACC CTCTTTACCC TTCCGAGCGG GATTCTTTGT CCTATTATCA TCGTGTTTAT AACACCTAAA   
  
  
- TTCCTTAAAC GACTATGATA CGCGTGTTAG ATGACGTAGA CTACTGGCTT CTTGACGACT ACCTGACGAT   
  
  
- TTCGTTTAGT CCCTCGTGAG CAGACGACTC CCCCTACCTA GAGTTTCCAA CCGCGTAATG AAACGACTAC   
  
  
- GGGATCTCCG TGCAAATCGA CCTTGACCTA GAGCGTAAAT ATGCCGGGAT ACAAGATTAT CCGGTAGACA   
  
  
- GTGACTGTAC TATTTTCGTA TAGTCAAGAT ATAAGCACGA ACGGGTAAAT GCTTCTAGCA GTAACCAACA   
  
  
- CCATGGGTAT ACTAAGATTT TCGTCGACTC TTCCGTAGTT TCGAAGTATA ATATCTAAAA CCGTAGGAGA   
  
  
- TACCAACGGT TACCGGGTTG GAGTAAGTTG CGGAGAGTCT CGCTAGACCA CCTGGAGGTT TTGACAAATA   
  
  
- ATGTCCCTAG CTAGAGGGGG TCGGACCCAA GTCCGGTCGT CTTTCTCACC TTCGTTGTCC CTCTGCGAAC   
  
  
- CGGTTCATGA CACTCGCCAT ATTACACGGT AAACTCATAG TACGGTAACG AGTCCTCACC CTTTGTTAGT   
  
  
- TTGGTCCCCT AGAGTTCTAT CCTTCATCCT TACTACTTCA ACAACGCCAC TTGACAGAGA CATCCAAGTT   
  
  
- CTTGGAGGAA CTGCTCTGTC ACCACCACCT ATCAGGTTCC TTGTGTCAAA ATTTGGACTA ATGTTCCCAT   
  
  
- TTCGGACTAT AAAAACACGT ACCGCAACAT TTACCAAGGA TGTTGTAGGG AAAGAAACAC TGTGCAAAAT   
  
  
- CTCTTCGGGA GAAAGTAATA AGGTGAGAAA AGCTGTACAA TCTACGGTTG CGGAGATCCC TCCTCGGGCT   
  
  
- CTCCAACTAT AAGCTCTTCC GTAAGATACC CTCCCTCTAA TACTTACACC ACCGGACACT CCCGTGTCTC   
  
  
- TCCCACCTCT CCGGCCTCTG TATGTTCGTT ACCGTACAAT CCGTATCGTC CCGTCCCAAA GCCGTTCATG   
  
  
- GTAACCTAGG GTTCAACTAG CTCTTTTACT CCAAATTCCG GTTCCGTCTG GTGGTGTTCC TAAAGTACTA   
  
  
- GCTACACCTA CCTGTAACCC GTTAAGTCCC TACCTTCCCC GCCTAACGTG TACGCTAGAG ACGTACCCAA   
  
  
- GGCCGAAC

+     P-box

| Site Name | Organism | Position | Strand | Matrix score. | sequence | function |
| --- | --- | --- | --- | --- | --- | --- |
| P-box | Oryza sativa | 1008 | + | 7 | CCTTTTG | gibberellin-responsive element |

>HU07G02249.1   
+ -Up\_Stream \_Len000GATTTG ATTGTCGAAA TTTGTCCGAT TTTATAAAAT TGAAAATTGT TATTGGACTA   
  
  
+ TAATCTACTG TAGTTTAGAG GTGTTGGATA ATACTCTCAA CTTTGAACTT GGACGTCCAT GCACCACACT   
  
  
+ ATGGGCGTAT TTACTGTTTA TATACCAAGA ATAATTTTGA AGATGTTCTT AGTTGGTGAT GTAAGTGTAT   
  
  
+ ATTCAACATA TGTCATTACA TAAACTTGAA AATTCTTGAA TTTTAGACAT ATACAATTGC AAATCATTGT   
  
  
+ AAAATTGTTG CATTACATTT TGATAAAAAT GCATCCAAAC TTTGTCTTGG TATAAACAGT AAATAGGCCC   
  
  
+ ATAGGTCCAT AGTGTAGTGC ATGGACGTCC AAGTTTAAAG GTGGGAGTAT TATTGCCCAA CGCCCCTAAA   
  
  
+ GTACAGTATA TTATAGTCCA ATATCCCAAT TAATTTTTCA TTTGAATATT TAAATTTGGA CATAATATTT   
  
  
+ TTCATATTTG AATTCTCTAA ACCTGATTTT AACCTGAATT TAAGTTGATT TAATTCATCC GACCAAAAAA
[truncated: 241,410 more chars]
