# Supplementary material for: Genome-Wide Identification and Expression Pattern of the GRAS Gene Family in Pitaya (Selenicereus undatus L.)
Source: Biology (Basel). 2022 Dec 21;12(1):11. doi: 10.3390/biology12010011 (PMC9854919; doi:10.3390/biology12010011)
Supplement: Supplementary file 1 [file biology-12-00011-s001.zip › Supplementary file S5/HU08G00014.1_plantcare.html]

Content-Type: text/html; charset=ISO-8859-1


PlantCARE


Webmaster Firefox specific output  
To save the result:
click on the frame with the right mouse button and save the source code as a text file with extension .html  
REFERENCE:PlantCARE: a database of plant cis-acting regulatory elements and a portal to tools for in silico analysis of promoter sequences.  
Lescot, M., Déhais, P., Moreau, Y., De Moor, B., Rouzé ,P.,and Rombauts, S.  
Nucleic Acids Res., Database issue(2002), 30(1):325-327.   


---

>HU08G00014.1   
+ +Up\_Stream \_Len000TCCTCT TTTGGGCTTC TTTCTTTCTA TCCTGTTTTT TTTTGGGTCG AAAAGTTGGA   
  
  
+ GGTTGAATTG AAGCATCCGA ATTCGAGCAG GATGTTTCTC TGCGTAGTGA TTCTTTCTGG GTTTGTGATT   
  
  
+ TTGAATTCTG GGTAATCGGT TGTTTTGCTA ATTTTGAGGA CAGAGTTCCT TTTCTGAATT AAAATTTCGT   
  
  
+ TTTTTTATCG GGAAAATCTT CAGTATTTGA GAAAAAAGAA CGAAATTTGG ATGATTTGCT GTCTAGATTT   
  
  
+ TGCTTTCATA TTCCTGGGTG TGGATTGGTT TATTAATTGC ATGTGAGGAA GTACAAGCTT CTGGTCAATG   
  
  
+ GTTGGGTTTT TTTTTTTTTT TTGTGGGTAT AAAGTTGGAA ATTGATTACG AGTCAAATTT CTCTTTCTAG   
  
  
+ AAGAGGGGGG GGGGGGGGGG TTGGTTTAAA ACTCCATATT TTCTTTGAAT GGAAAGCTTA ATTTGCTGAC   
  
  
+ TATCTTGGTT AGTAGCATAA GCTTGATCAG AAAACAAAAC GCTCTCTGCC CTGCAAAAAC TTTATTTGTC   
  
  
+ TTTTAAGGAA AATTTTGGAA TTTGAGAGTG AAAATTTTGG TTGTGAATCT TGTCTTACTA TGTGGTTCCT   
  
  
+ TCTGACAAAA GTTTGAATCT GAGTATGAGA GTTCATCAAT CTCTTTTACT TTCCTACTTT CTTTATTGCT   
  
  
+ TGCAAATTGT TGTGAACTTA CTTGGATTTT CTGGTAGAGG AGAGTTCTGT CCTGGAATTT GGTGGAAAGT   
  
  
+ GAGACATACT TTGGGATTTA ATTAGCCATC AATTTGGTTT CACACCTTAT CAGTTTGGAC TTCTGGTGTG   
  
  
+ TTATTGTATG TGTTCTTAGC TTGGGATTAT TCTTGTATCG GAAGAGAGGG GGGGGGGGGT GTTTCTTTTA   
  
  
+ GCTGAATGTT AGAACATTTT AAGGGATCTA GATATCTAGA GAAGAGCCTA GGAAATGAAG CATTTTTCCC   
  
  
+ TTATAATCTG ACACAACTTT ATGAATTGAT TAGGAACAAA GAACGATTAG TTCAGAAAGG TTACAACTCC   
  
  
+ TCTAGGTGTT ATACTAAAGA GATGTGAGTT AATAAGCAAT TGCTTACTAA CTGTTTAGGC TGTTTCATTG   
  
  
+ CTTGTAGAGG CTTTTTTTTT GGGGGGGGGG GGGGGTTTGG GGTTCATAAA TGAGAAGGTT GATTTGTTGT   
  
  
+ CTGCTCTGCT CTTGATTCGA TAGTAATTCT CTCAGAAAAG GGTAAAATGA CATGGCCTTC CATCGCCTCC   
  
  
+ CATGAGGCTT TTTGCCTCTG CTTGTGTATC TTTCATCTCT TGGTTTGGTG GGTTGGGGGA GGGGAGGGGG   
  
  
+ GGAAGGGGGT TTTGATTCGA TAGATCATCA CTTTCAGAAA TGGCCTCTCT GGCCAATGGT AAGGGGTAAG   
  
  
+ GTTAGTACAT GTGGACTTTC CCATGTGGAT TTTTCCAGAG TCCGTTGTGT GATTGTTATT GCTGTTGTTG   
  
  
+ TAACTTTCTG TCACTCGGAA AACATGAATC CATATGCAAC AAGAACATAA TAATTGTGAA TGCGACTTTA   
  
  
+ TTTAGGAATG TCTTACATAG TACGTGCACA TTCTTTAATT ATCTGTTTAT CTGTTGGTTT GTTATTTTCA   
  
  
+ AGGGGATGGG GAGTGGGTGG ATGCTTAGTA TGTAATCTTT TTATATAGGG AAAAATCATA TAGCTTGAAA   
  
  
+ GTAAAACTTT CATTGCCAGG ATTCCAAGGT AGGGGTAGGG TTGTCTACAC CTTGATCTCC CTAGAAGCAT   
  
  
+ GGCGATCCTT CCTCGGATTG TGTTATAGAA TAAAGAATGT GGTTCTGCTG TAATGAGACT TATGTTCTAT   
  
  
+ CAAATGATGA ATCTGTTTAG AACTTCTTGC TTATTTTTTC TGCAAAATTT TAGGAGACTT GATAAACTAT   
  
  
+ AGTTCTCTTG AATTTACAGG TGGATATTGG AGTTCACAGA ATAGTATTCA GATCAAAGTG CTTGTGCTTA   
  
  
+ ATTGGAAGGA CAACTTGCTT GAGCTGTTTA TAGAGCTCTA ACTGATGCTA TCTCATGGAC TCACATCAGT   
  
  
+ TTTTTGGATT TAATCTTACC AGTGTTGATC CGTCGTACAT TTCCGCTCAG TACAGTCCGC CGTCAGTGAC   
  
  
+ AAATAGGATG TTTGCATCAC TGAAGCTCGA CTCTAGAGGT TCTCCTGTGT CACCCTTCTC AACTCAGTTT   
  
  
+ GATTGTGATA CGGTTACTAC ATTGAGTGAT AGTCAAGAGC ATCACAGCTC GACGGGGAGT CTATCAACAA   
  
  
+ GAAGCCCTTC TTGTAATTCT CCCCTTGAAA CGAGCAGTTA TCATCATTTA TCGACGAATG GCCCGTCTTG   
  
  
+ TAATTCTGCC CCTGAAACTA GCAGTTATCG TCATCGGTTC AACGCGAGTC CCGTGGGATA TTCCCATCAA   
  
  
+ GGCACTGATT ATGGAGTGAA TATGAAGAGC GCTCTGCAGG AGCTAGAGAC TACTCTAATG GGTGCAGATG   
  
  
+ GTGAGGAAGT ATCTGCTGCT AATCAACCTA TGGGGGGAAG TCGTCAGTCC GGGATCCCAA GTCAGAGATC   
  
  
+ AAAATCATTG AGCGAAGATC CACAGGGTTC GCATCCTACT CAGCCTGATT CATCATCTCT TTCTAGGGCA   
  
  
+ AGAAGATCAG GAGATGAAAG CCAGAGAGAG AAACGGCACA AGGCAATGGA AGAACCAACG GAACTACCAA   
  
  
+ GTTTGCCACC TGGTGATTTG AAGCAGTTGC TAATTGAATG TGCGAGGGCT TTATCGGATA ACCGAATAGA   
  
  
+ TGACTTTGAG AGTTTGGTTA AACGGGCGAG GAAAGAGGTC TCTATCTCGG GTGAGCCTAT CCAACGTCTC   
  
  
+ GGTGCCTACA TGATCGAAGG GCTTGTGGCA AGGAAGCAGT CTTCGGGGAC TAGCATCTAT CGGGCTCTGA   
  
  
+ AGTGTAAAGA GCCTCTTGGA AAAGACTTGC TCTCCTACAT GCACATCCTT TATGAAATAT GCCCTTATCT   
  
  
+ CAAGTTTGGT TATATGGCTG CGAATGGAGC GATAGCTGAA GCTTGTAGAA ATGAGGATAA CATACATATT   
  
  
+ ATAGATTTCC AGATTGCACA GGGCACTCAG TGGGTTACTC TATTACAAGC CCTAGCAGCA AGACCTGGTG   
  
  
+ GGCCACCTAA GGTGCGAATT ACAGGCATTG ATGATCCTGT TTCTAAGTAT GCCCGTGGTG CTAGCTTGGA   
  
  
+ GGCTGTTGGG AAACGGTTAG CGTCTCTATC TGAAAAGTTC AAAATACCCG TCGAGTTCAA TGCGTTGCCC   
  
  
+ GTTTATGGAC CCGATGTCAG GCGGGAAATG CTGGATGTGA GGCCCGGGGA GGCTTTGGCC GTTAATTTTC   
  
  
+ CATTGCAGCT CCACCACACT CCTGACGAGA GTGTCGATGT GAACAACCCT AGGGATGGGC TTCTCAGAAT   
  
  
+ GGTGAAATCA CTTGGTCCTA AGGTAACCAC TTTGGTTGAG CAAGAATCAA ACACCAACAC TACCCCTTTC   
  
  
+ TTGACCCGGT TCATAGAGAC CCTTGACTAC TACTCAGCCA TGTTTGAGTC TATAGACGTG ACCATGCCAA   
  
  
+ GAGACCGGAA GGAGAGGATC AATGTTGAGC AGCATTGTTT GGCTAAGGAC ATTGTGAATA TCATAGCTTG   
  
  
+ CGAGGGCAAG GAGAGGGTGG AGCGTCATGA ACTTTTTGGG AAATGGAAGT CAAGGTTTAC CATGGCAGGG   
  
  
+ TTCAGGCAGT ACCCGTTGAG CTCATACGTA AACTCTGTGA TAAGAAGCCT ACTCCGGTGT TACTCCGAGC   
  
  
+ ATTATACTCT GATAGAGAAG GATGGTGCCA TGCTTCTGGG TTGGAAGGGC CGAATGCTGA TTTCAGCTTC   
  
  
+ GGCATGGCAT TG  

- +Up\_Stream \_Len000AGGAGA AAACCCGAAG AAAGAAAGAT AGGACAAAAA AAAACCCAGC TTTTCAACCT   
  
  
- CCAACTTAAC TTCGTAGGCT TAAGCTCGTC CTACAAAGAG ACGCATCACT AAGAAAGACC CAAACACTAA   
  
  
- AACTTAAGAC CCATTAGCCA ACAAAACGAT TAAAACTCCT GTCTCAAGGA AAAGACTTAA TTTTAAAGCA   
  
  
- AAAAAATAGC CCTTTTAGAA GTCATAAACT CTTTTTTCTT GCTTTAAACC TACTAAACGA CAGATCTAAA   
  
  
- ACGAAAGTAT AAGGACCCAC ACCTAACCAA ATAATTAACG TACACTCCTT CATGTTCGAA GACCAGTTAC   
  
  
- CAACCCAAAA AAAAAAAAAA AACACCCATA TTTCAACCTT TAACTAATGC TCAGTTTAAA GAGAAAGATC   
  
  
- TTCTCCCCCC CCCCCCCCCC AACCAAATTT TGAGGTATAA AAGAAACTTA CCTTTCGAAT TAAACGACTG   
  
  
- ATAGAACCAA TCATCGTATT CGAACTAGTC TTTTGTTTTG CGAGAGACGG GACGTTTTTG AAATAAACAG   
  
  
- AAAATTCCTT TTAAAACCTT AAACTCTCAC TTTTAAAACC AACACTTAGA ACAGAATGAT ACACCAAGGA   
  
  
- AGACTGTTTT CAAACTTAGA CTCATACTCT CAAGTAGTTA GAGAAAATGA AAGGATGAAA GAAATAACGA   
  
  
- ACGTTTAACA ACACTTGAAT GAACCTAAAA GACCATCTCC TCTCAAGACA GGACCTTAAA CCACCTTTCA   
  
  
- CTCTGTATGA AACCCTAAAT TAATCGGTAG TTAAACCAAA GTGTGGAATA GTCAAACCTG AAGACCACAC   
  
  
- AATAACATAC ACAAGAATCG AACCCTAATA AGAACATAGC CTTCTCTCCC CCCCCCCCCA CAAAGAAAAT   
  
  
- CGACTTACAA TCTTGTAAAA TTCCCTAGAT CTATAGATCT CTTCTCGGAT CCTTTACTTC GTAAAAAGGG   
  
  
- AATATTAGAC TGTGTTGAAA TACTTAACTA ATCCTTGTTT CTTGCTAATC AAGTCTTTCC AATGTTGAGG   
  
  
- AGATCCACAA TATGATTTCT CTACACTCAA TTATTCGTTA ACGAATGATT GACAAATCCG ACAAAGTAAC   
  
  
- GAACATCTCC GAAAAAAAAA CCCCCCCCCC CCCCCAAACC CCAAGTATTT ACTCTTCCAA CTAAACAACA   
  
  
- GACGAGACGA GAACTAAGCT ATCATTAAGA GAGTCTTTTC CCATTTTACT GTACCGGAAG GTAGCGGAGG   
  
  
- GTACTCCGAA AAACGGAGAC GAACACATAG AAAGTAGAGA ACCAAACCAC CCAACCCCCT CCCCTCCCCC   
  
  
- CCTTCCCCCA AAACTAAGCT ATCTAGTAGT GAAAGTCTTT ACCGGAGAGA CCGGTTACCA TTCCCCATTC   
  
  
- CAATCATGTA CACCTGAAAG GGTACACCTA AAAAGGTCTC AGGCAACACA CTAACAATAA CGACAACAAC   
  
  
- ATTGAAAGAC AGTGAGCCTT TTGTACTTAG GTATACGTTG TTCTTGTATT ATTAACACTT ACGCTGAAAT   
  
  
- AAATCCTTAC AGAATGTATC ATGCACGTGT AAGAAATTAA TAGACAAATA GACAACCAAA CAATAAAAGT   
  
  
- TCCCCTACCC CTCACCCACC TACGAATCAT ACATTAGAAA AATATATCCC TTTTTAGTAT ATCGAACTTT   
  
  
- CATTTTGAAA GTAACGGTCC TAAGGTTCCA TCCCCATCCC AACAGATGTG GAACTAGAGG GATCTTCGTA   
  
  
- CCGCTAGGAA GGAGCCTAAC ACAATATCTT ATTTCTTACA CCAAGACGAC ATTACTCTGA ATACAAGATA   
  
  
- GTTTACTACT TAGACAAATC TTGAAGAACG AATAAAAAAG ACGTTTTAAA ATCCTCTGAA CTATTTGATA   
  
  
- TCAAGAGAAC TTAAATGTCC ACCTATAACC TCAAGTGTCT TATCATAAGT CTAGTTTCAC GAACACGAAT   
  
  
- TAACCTTCCT GTTGAACGAA CTCGACAAAT ATCTCGAGAT TGACTACGAT AGAGTACCTG AGTGTAGTCA   
  
  
- AAAAACCTAA ATTAGAATGG TCACAACTAG GCAGCATGTA AAGGCGAGTC ATGTCAGGCG GCAGTCACTG   
  
  
- TTTATCCTAC AAACGTAGTG ACTTCGAGCT GAGATCTCCA AGAGGACACA GTGGGAAGAG TTGAGTCAAA   
  
  
- CTAACACTAT GCCAATGATG TAACTCACTA TCAGTTCTCG TAGTGTCGAG CTGCCCCTCA GATAGTTGTT   
  
  
- CTTCGGGAAG AACATTAAGA GGGGAACTTT GCTCGTCAAT AGTAGTAAAT AGCTGCTTAC CGGGCAGAAC   
  
  
- ATTAAGACGG GGACTTTGAT CGTCAATAGC AGTAGCCAAG TTGCGCTCAG GGCACCCTAT AAGGGTAGTT   
  
  
- CCGTGACTAA TACCTCACTT ATACTTCTCG CGAGACGTCC TCGATCTCTG ATGAGATTAC CCACGTCTAC   
  
  
- CACTCCTTCA TAGACGACGA TTAGTTGGAT ACCCCCCTTC AGCAGTCAGG CCCTAGGGTT CAGTCTCTAG   
  
  
- TTTTAGTAAC TCGCTTCTAG GTGTCCCAAG CGTAGGATGA GTCGGACTAA GTAGTAGAGA AAGATCCCGT   
  
  
- TCTTCTAGTC CTCTACTTTC GGTCTCTCTC TTTGCCGTGT TCCGTTACCT TCTTGGTTGC CTTGATGGTT   
  
  
- CAAACGGTGG ACCACTAAAC TTCGTCAACG ATTAACTTAC ACGCTCCCGA AATAGCCTAT TGGCTTATCT   
  
  
- ACTGAAACTC TCAAACCAAT TTGCCCGCTC CTTTCTCCAG AGATAGAGCC CACTCGGATA GGTTGCAGAG   
  
  
- CCACGGATGT ACTAGCTTCC CGAACACCGT TCCTTCGTCA GAAGCCCCTG ATCGTAGATA GCCCGAGACT   
  
  
- TCACATTTCT CGGAGAACCT TTTCTGAACG AGAGGATGTA CGTGTAGGAA ATACTTTATA CGGGAATAGA   
  
  
- GTTCAAACCA ATATACCGAC GCTTACCTCG CTATCGACTT CGAACATCTT TACTCCTATT GTATGTATAA   
  
  
- TATCTAAAGG TCTAACGTGT CCCGTGAGTC ACCCAATGAG ATAATGTTCG GGATCGTCGT TCTGGACCAC   
  
  
- CCGGTGGATT CCACGCTTAA TGTCCGTAAC TACTAGGACA AAGATTCATA CGGGCACCAC GATCGAACCT   
  
  
- CCGACAACCC TTTGCCAATC GCAGAGATAG ACTTTTCAAG TTTTATGGGC AGCTCAAGTT ACGCAACGGG   
  
  
- CAAATACCTG GGCTACAGTC CGCCCTTTAC GACCTACACT CCGGGCCCCT CCGAAACCGG CAATTAAAAG   
  
  
- GTAACGTCGA GGTGGTGTGA GGACTGCTCT CACAGCTACA CTTGTTGGGA TCCCTACCCG AAGAGTCTTA   
  
  
- CCACTTTAGT GAACCAGGAT TCCATTGGTG AAACCAACTC GTTCTTAGTT TGTGGTTGTG ATGGGGAAAG   
  
  
- AACTGGGCCA AGTATCTCTG GGAACTGATG ATGAGTCGGT ACAAACTCAG ATATCTGCAC TGGTACGGTT   
  
  
- CTCTGGCCTT CCTCTCCTAG TTACAACTCG TCGTAACAAA CCGATTCCTG TAACACTTAT AGTATCGAAC   
  
  
- GCTCCCGTTC CTCTCCCACC TCGCAGTACT TGAAAAACCC TTTACCTTCA GTTCCAAATG GTACCGTCCC   
  
  
- AAGTCCGTCA TGGGCAACTC GAGTATGCAT TTGAGACACT ATTCTTCGGA TGAGGCCACA ATGAGGCTCG   
  
  
- TAATATGAGA CTATCTCTTC CTACCACGGT ACGAAGACCC AACCTTCCCG GCTTACGACT AAAGTCGAAG   
  
  
- CCGTACCGTA AC

  
  
Motifs Found  

+   

| Site Name | Organism | Position | Strand | Matrix score. | sequence | function |
| --- | --- | --- | --- | --- | --- | --- |
|  | organism | 538 | + | 4 | motif\_sequence | short\_function |
|  | organism | 2408 | - | 4 | motif\_sequence | short\_function |
|  | organism | 2465 | + | 4 | motif\_sequence | short\_function |
|  | organism | 3515 | - | 4 | motif\_sequence | short\_function |
|  | organism | 2596 | - | 4 | motif\_sequence | short\_function |
|  | organism | 2084 | - | 4 | motif\_sequence | short\_function |
|  | organism | 3030 | - | 4 | motif\_sequence | short\_function |
|  | organism | 2904 | + | 4 | motif\_sequence | short\_function |
|  | organism | 2069 | - | 4 | motif\_sequence | short\_function |
|  | organism | 2604 | - | 4 | motif\_sequence | short\_function |
|  | organism | 1739 | + | 4 | motif\_sequence | short\_function |
|  | organism | 112 | + | 4 | motif\_sequence | short\_function |
|  | organism | 1128 | - | 4 | motif\_sequence | short\_function |
|  | organism | 1570 | - | 4 | motif\_sequence | short\_function |
|  | organism | 955 | - | 4 | motif\_sequence | short\_function |
|  | organism | 424 | - | 4 | motif\_sequence | short\_function |
|  | organism | 3584 | - | 4 | motif\_sequence | short\_function |
|  | organism | 2702 | + | 4 | motif\_sequence | short\_function |
|  | organism | 1409 | - | 4 | motif\_sequence | short\_function |
|  | organism | 751 | + | 4 | motif\_sequence | short\_function |
|  | organism | 738 | - | 4 | motif\_sequence | short\_function |
|  | organism | 2416 | + | 4 | motif\_sequence | short\_function |
|  | organism | 743 | - | 4 | motif\_sequence | short\_function |
|  | organism | 926 | - | 4 | motif\_sequence | short\_function |
|  | organism | 885 | - | 4 | motif\_sequence | short\_function |
|  | organism | 1198 | + | 4 | motif\_sequence | short\_function |
|  | organism | 182 | - | 4 | motif\_sequence | short\_function |
|  | organism | 2538 | - | 4 | motif\_sequence | short\_function |
|  | organism | 1280 | + | 4 | motif\_sequence | short\_function |
|  | organism | 3551 | - | 4 | motif\_sequence | short\_function |
|  | organism | 230 | + | 4 | motif\_sequence | short\_function |
|  | organism | 2448 | - | 4 | motif\_sequence | short\_function |
|  | organism | 1517 | - | 4 | motif\_sequence | short\_function |
|  | organism | 2915 | - | 4 | motif\_sequence | short\_function |
|  | organism | 1816 | + | 4 | motif\_sequence | short\_function |

>HU08G00014.1   
+ +Up\_Stream \_Len000TCCTCT TTTGGGCTTC TTTCTTTCTA TCCTGTTTTT TTTTGGGTCG AAAAGTTGGA   
  
  
+ GGTTGAATTG AAGCATCCGA ATTCGAGCAG GATGTTTCTC TGCGTAGTGA TTCTTTCTGG GTTTGTGATT   
  
  
+ TTGAATTCTG GGTAATCGGT TGTTTTGCTA ATTTTGAGGA CAGAGTTCCT TTTCTGAATT AAAATTTCGT   
  
  
+ TTTTTTATCG GGAAAATCTT CAGTATTTGA GAAAAAAGAA CGAAATTTGG ATGATTTGCT GTCTAGATTT   
  
  
+ TGCTTTCATA TTCCTGGGTG TGGATTGGTT TATTAATTGC ATGTGAGGAA GTACAAGCTT CTGGTCAATG   
  
  
+ GTTGGGTTTT TTTTTTTTTT TTGTGGGTAT AAAGTTGGAA ATTGATTACG AGTCAAATTT CTCTTTCTAG   
  
  
+ AAGAGGGGGG GGGGGGGGGG TTGGTTTAAA ACTCCATATT TTCTTTGAAT GGAAAGCTTA ATTTGCTGAC   
  
  
+ TATCTTGGTT AGTAGCATAA GCTTGATCAG AAAACAAAAC GCTCTCTGCC CTGCAAAAAC TTTATTTGTC   
  
  
+ TTTTAAGGAA AATTTTGGAA TTTGAGAGTG AAAATTTTGG TTGTGAATCT TGTCTTACTA TGTGGTTCCT   
  
  
+ TCTGACAAAA GTTTGAATCT GAGTATGAGA GTTCATCAAT CTCTTTTACT TTCCTACTTT CTTTATTGCT   
  
  
+ TGCAAATTGT TGTGAACTTA CTTGGATTTT CTGGTAGAGG AGAGTTCTGT CCTGGAATTT GGTGGAAAGT   
  
  
+ GAGACATACT TTGGGATTTA ATTAGCCATC AATTTGGTTT CACACCTTAT CAGTTTGGAC TTCTGGTGTG   
  
  
+ TTATTGTATG TGTTCTTAGC TTGGGATTAT TCTTGTATCG GAAGAGAGGG GGGGGGGGGT GTTTCTTTTA   
  
  
+ GCTGAATGTT AGAACATTTT AAGGGATCTA GATATCTAGA GAAGAGCCTA GGAAATGAAG CATTTTTCCC   
  
  
+ TTATAATCTG ACACAACTTT ATGAATTGAT TAGGAACAAA GAACGATTAG TTCAGAAAGG TTACAACTCC   
  
  
+ TCTAGGTGTT ATACTAAAGA GATGTGAGTT AATAAGCAAT TGCTTACTAA CTGTTTAGGC TGTTTCATTG   
  
  
+ CTTGTAGAGG CTTTTTTTTT GGGGGGGGGG GGGGGTTTGG GGTTCATAAA TGAGAAGGTT GATTTGTTGT   
  
  
+ CTGCTCTGCT CTTGATTCGA TAGTAATTCT CTCAGAAAAG GGTAAAATGA CATGGCCTTC CATCGCCTCC   
  
  
+ CATGAGGCTT TTTGCCTCTG CTTGTGTATC TTTCATCTCT TGGTTTGGTG GGTTGGGGGA GGGGAGGGGG   
  
  
+ GGAAGGGGGT TTTGATTCGA TAGATCATCA CTTTCAGAAA TGGCCTCTCT GGCCAATGGT AAGGGGTAAG   
  
  
+ GTTAGTACAT GTGGACTTTC CCATGTGGAT TTTTCCAGAG TCCGTTGTGT GATTGTTATT GCTGTTGTTG   
  
  
+ TAACTTTCTG TCACTCGGAA AACATGAATC CATATGCAAC AAGAACATAA TAATTGTGAA TGCGACTTTA   
  
  
+ TTTAGGAATG TCTTACATAG TACGTGCACA TTCTTTAATT ATCTGTTTAT CTGTTGGTTT GTTATTTTCA   
  
  
+ AGGGGATGGG GAGTGGGTGG ATGCTTAGTA TGTAATCTTT TTATATAGGG AAAAATCATA TAGCTTGAAA   
  
  
+ GTAAAACTTT CATTGCCAGG ATTCCAAGGT AGGGGTAGGG TTGTCTACAC CTTGATCTCC CTAGAAGCAT   
  
  
+ GGCGATCCTT CCTCGGATTG TGTTATAGAA TAAAGAATGT GGTTCTGCTG TAATGAGACT TATGTTCTAT   
  
  
+ CAAATGATGA ATCTGTTTAG AACTTCTTGC TTATTTTTTC TGCAAAATTT TAGGAGACTT GATAAACTAT   
  
  
+ AGTTCTCTTG AATTTACAGG TGGATATTGG AGTTCACAGA ATAGTATTCA GATCAAAGTG CTTGTGCTTA   
  
  
+ ATTGGAAGGA CAACTTGCTT GAGCTGTTTA TAGAGCTCTA ACTGATGCTA TCTCATGGAC TCACATCAGT   
  
  
+ TTTTTGGATT TAATCTTACC AGTGTTGATC CGTCGTACAT TTCCGCTCAG TACAGTCCGC CGTCAGTGAC   
  
  
+ AAATAGGATG TTTGCATCAC TGAAGCTCGA CTCTAGAGGT TCTCCTGTGT CACCCTTCTC AACTCAGTTT   
  
  
+ GATTGTGATA CGGTTACTAC ATTGAGTGAT AGTCAAGAGC ATCACAGCTC GACGGGGAGT CTATCAACAA   
  
  
+ GAAGCCCTTC TTGTAATTCT CCCCTTGAAA CGAGCAGTTA TCATCATTTA TCGACGAATG GCCCGTCTTG   
  
  
+ TAATTCTGCC CCTGAAACTA GCAGTTATCG TCATCGGTTC AACGCGAGTC CCGTGGGATA TTCCCATCAA   
  
  
+ GGCACTGATT ATGGAGTGAA TATGAAGAGC GCTCTGCAGG AGCTAGAGAC TACTCTAATG GGTGCAGATG   
  
  
+ GTGAGGAAGT ATCTGCTGCT AATCAACCTA TGGGGGGAAG TCGTCAGTCC GGGATCCCAA GTCAGAGATC   
  
  
+ AAAATCATTG AGCGAAGATC CACAGGGTTC GCATCCTACT CAGCCTGATT CATCATCTCT TTCTAGGGCA   
  
  
+ AGAAGATCAG GAGATGAAAG CCAGAGAGAG AAACGGCACA AGGCAATGGA AGAACCAACG GAACTACCAA   
  
  
+ GTTTGCCACC TGGTGATTTG AAGCAGTTGC TAATTGAATG TGCGAGGGCT TTATCGGATA ACCGAATAGA   
  
  
+ TGACTTTGAG AGTTTGGTTA AACGGGCGAG GAAAGAGGTC TCTATCTCGG GTGAGCCTAT CCAACGTCTC   
  
  
+ GGTGCCTACA TGATCGAAGG GCTTGTGGCA AGGAAGCAGT CTTCGGGGAC TAGCATCTAT CGGGCTCTGA   
  
  
+ AGTGTAAAGA GCCTCTTGGA AAAGACTTGC TCTCCTACAT GCACATCCTT TATGAAATAT GCCCTTATCT   
  
  
+ CAAGTTTGGT TATATGGCTG CGAATGGAGC GATAGCTGAA GCTTGTAGAA ATGAGGATAA CATACATATT   
  
  
+ ATAGATTTCC AGATTGCACA GGGCACTCAG TGGGTTACTC TATTACAAGC CCTAGCAGCA AGACCTGGTG   
  
  
+ GGCCACCTAA GGTGCGAATT ACAGGCATTG ATGATCCTGT TTCTAAGTAT GCCCGTGGTG CTAGCTTGGA   
  
  
+ GGCTGTTGGG AAACGGTTAG CGTCTCTATC TGAAAAGTTC AAAATACCCG TCGAGTTCAA TGCGTTGCCC   
  
  
+ GTTTATGGAC CCGATGTCAG GCGGGAAATG CTGGATGTGA GGCCCGGGGA GGCTTTGGCC GTTAATTTTC   
  
  
+ CATTGCAGCT CCACCACACT CCTGACGAGA GTGTCGATGT GAACAACCCT AGGGATGGGC TTCTCAGAAT   
  
  
+ GGTGAAATCA CTTGGTCCTA AGGTAACCAC TTTGGTTGAG CAAGAATCAA ACACCAACAC TACCCCTTTC   
  
  
+ TTGACCCGGT TCATAGAGAC CCTTGACTAC TACTCAGCCA TGTTTGAGTC TATAGACGTG ACCATGCCAA   
  
  
+ GAGACCGGAA GGAGAGGATC AATGTTGAGC AGCATTGTTT GGCTAAGGAC ATTGTGAATA TCATAGCTTG   
  
  
+ CGAGGGCAAG GAGAGGGTGG AGCGTCATGA ACTTTTTGGG AAATGGAAGT CAAGGTTTAC CATGGCAGGG   
  
  
+ TTCAGGCAGT ACCCGTTGAG CTCATACGTA AACTCTGTGA TAAGAAGCCT ACTCCGGTGT TACTCCGAGC   
  
  
+ ATTATACTCT GATAGAGAAG GATGGTGCCA TGCTTCTGGG TTGGAAGGGC CGAATGCTGA TTTCAGCTTC   
  
  
+ GGCATGGCAT TG  

- +Up\_Stream \_Len000AGGAGA AAACCCGAAG AAAGAAAGAT AGGACAAAAA AAAACCCAGC TTTTCAACCT   
  
  
- CCAACTTAAC TTCGTAGGCT TAAGCTCGTC CTACAAAGAG ACGCATCACT AAGAAAGACC CAAACACTAA   
  
  
- AACTTAAGAC CCATTAGCCA ACAAAACGAT TAAAACTCCT GTCTCAAGGA AAAGACTTAA TTTTAAAGCA   
  
  
- AAAAAATAGC CCTTTTAGAA GTCATAAACT CTTTTTTCTT GCTTTAAACC TACTAAACGA CAGATCTAAA   
  
  
- ACGAAAGTAT AAGGACCCAC ACCTAACCAA ATAATTAACG TACACTCCTT CATGTTCGAA GACCAGTTAC   
  
  
- CAACCCAAAA AAAAAAAAAA AACACCCATA TTTCAACCTT TAACTAATGC TCAGTTTAAA GAGAAAGATC   
  
  
- TTCTCCCCCC CCCCCCCCCC AACCAAATTT TGAGGTATAA AAGAAACTTA CCTTTCGAAT TAAACGACTG   
  
  
- ATAGAACCAA TCATCGTATT CGAACTAGTC TTTTGTTTTG CGAGAGACGG GACGTTTTTG AAATAAACAG   
  
  
- AAAATTCCTT TTAAAACCTT AAACTCTCAC TTTTAAAACC AACACTTAGA ACAGAATGAT ACACCAAGGA   
  
  
- AGACTGTTTT CAAACTTAGA CTCATACTCT CAAGTAGTTA GAGAAAATGA AAGGATGAAA GAAATAACGA   
  
  
- ACGTTTAACA ACACTTGAAT GAACCTAAAA GACCATCTCC TCTCAAGACA GGACCTTAAA CCACCTTTCA   
  
  
- CTCTGTATGA AACCCTAAAT TAATCGGTAG TTAAACCAAA GTGTGGAATA GTCAAACCTG AAGACCACAC   
  
  
- AATAACATAC ACAAGAATCG AACCCTAATA AGAACATAGC CTTCTCTCCC CCCCCCCCCA CAAAGAAAAT   
  
  
- CGACTTACAA TCTTGTAAAA TTCCCTAGAT CTATAGATCT CTTCTCGGAT CCTTTACTTC GTAAAAAGGG   
  
  
- AATATTAGAC TGTGTTGAAA TACTTAACTA ATCCTTGTTT CTTGCTAATC AAGTCTTTCC AATGTTGAGG   
  
  
- AGATCCACAA TATGATTTCT CTACACTCAA TTATTCGTTA ACGAATGATT GACAAATCCG ACAAAGTAAC   
  
  
- GAACATCTCC GAAAAAAAAA CCCCCCCCCC CCCCCAAACC CCAAGTATTT ACTCTTCCAA CTAAACAACA   
  
  
- GACGAGACGA GAACTAAGCT ATCATTAAGA GAGTCTTTTC CCATTTTACT GTACCGGAAG GTAGCGGAGG   
  
  
- GTACTCCGAA AAACGGAGAC GAACACATAG AAAGTAGAGA ACCAAACCAC CCAACCCCCT CCCCTCCCCC   
  
  
- CCTTCCCCCA AAACTAAGCT ATCTAGTAGT GAAAGTCTTT ACCGGAGAGA CCGGTTACCA TTCCCCATTC   
  
  
- CAATCATGTA CACCTGAAAG GGTACACCTA AAAAGGTCTC AGGCAACACA CTAACAATAA CGACAACAAC   
  
  
- ATTGAAAGAC AGTGAGCCTT TTGTACTTAG GTATACGTTG TTCTTGTATT ATTAACACTT ACGCTGAAAT   
  
  
- AAATCCTTAC AGAATGTATC ATGCACGTGT AAGAAATTAA TAGACAAATA GACAACCAAA CAATAAAAGT   
  
  
- TCCCCTACCC CTCACCCACC TACGAATCAT ACATTAGAAA AATATATCCC TTTTTAGTAT ATCGAACTTT   
  
  
- CATTTTGAAA GTAACGGTCC TAAGGTTCCA TCCCCATCCC AACAGATGTG GAACTAGAGG GATCTTCGTA   
  
  
- CCGCTAGGAA GGAGCCTAAC ACAATATCTT ATTTCTTACA CCAAGACGAC ATTACTCTGA ATACAAGATA   
  
  
- GTTTACTACT TAGACAAATC TTGAAGAACG AATAAAAAAG ACGTTTTAAA ATCCTCTGAA CTATTTGATA   
  
  
- TCAAGAGAAC TTAAATGTCC ACCTATAACC TCAAGTGTCT TATCATAAGT CTAGTTTCAC GAACACGAAT   
  
  
- TAACCTTCCT GTTGAACGAA CTCGACAAAT ATCTCGAGAT TGACTACGAT AGAGTACCTG AGTGTAGTCA   
  
  
- AAAAACCTAA ATTAGAATGG TCACAACTAG GCAGCATGTA AAGGCGAGTC ATGTCAGGCG GCAGTCACTG   
  
  
- TTTATCCTAC AAACGTAGTG ACTTCGAGCT GAGATCTCCA AGAGGACACA GTGGGAAGAG TTGAGTCAAA   
  
  
- CTAACACTAT GCCAATGATG TAACTCACTA TCAGTTCTCG TAGTGTCGAG CTGCCCCTCA GATAGTTGTT   
  
  
- CTTCGGGAAG AACATTAAGA GGGGAACTTT GCTCGTCAAT AGTAGTAAAT AGCTGCTTAC CGGGCAGAAC   
  
  
- ATTAAGACGG GGACTTTGAT CGTCAATAGC AGTAGCCAAG TTGCGCTCAG GGCACCCTAT AAGGGTAGTT   
  
  
- CCGTGACTAA TACCTCACTT ATACTTCTCG CGAGACGTCC TCGATCTCTG ATGAGATTAC CCACGTCTAC   
  
  
- CACTCCTTCA TAGACGACGA TTAGTTGGAT ACCCCCCTTC AGCAGTCAGG CCCTAGGGTT CAGTCTCTAG   
  
  
- TTTTAGTAAC TCGCTTCTAG GTGTCCCAAG CGTAGGATGA GTCGGACTAA GTAGTAGAGA AAGATCCCGT   
  
  
- TCTTCTAGTC CTCTACTTTC GGTCTCTCTC TTTGCCGTGT TCCGTTACCT TCTTGGTTGC CTTGATGGTT   
  
  
- CAAACGGTGG ACCACTAAAC TTCGTCAACG ATTAACTTAC ACGCTCCCGA AATAGCCTAT TGGCTTATCT   
  
  
- ACTGAAACTC TCAAACCAAT TTGCCCGCTC CTTTCTCCAG AGATAGAGCC CACTCGGATA GGTTGCAGAG   
  
  
- CCACGGATGT ACTAGCTTCC CGAACACCGT TCCTTCGTCA GAAGCCCCTG ATCGTAGATA GCCCGAGACT   
  
  
- TCACATTTCT CGGAGAACCT TTTCTGAACG AGAGGATGTA CGTGTAGGAA ATACTTTATA CGGGAATAGA   
  
  
- GTTCAAACCA ATATACCGAC GCTTACCTCG CTATCGACTT CGAACATCTT TACTCCTATT GTATGTATAA   
  
  
- TATCTAAAGG TCTAACGTGT CCCGTGAGTC ACCCAATGAG ATAATGTTCG GGATCGTCGT TCTGGACCAC   
  
  
- CCGGTGGATT CCACGCTTAA TGTCCGTAAC TACTAGGACA AAGATTCATA CGGGCACCAC GATCGAACCT   
  
  
- CCGACAACCC TTTGCCAATC GCAGAGATAG ACTTTTCAAG TTTTATGGGC AGCTCAAGTT ACGCAACGGG   
  
  
- CAAATACCTG GGCTACAGTC CGCCCTTTAC GACCTACACT CCGGGCCCCT CCGAAACCGG CAATTAAAAG   
  
  
- GTAACGTCGA GGTGGTGTGA GGACTGCTCT CACAGCTACA CTTGTTGGGA TCCCTACCCG AAGAGTCTTA   
  
  
- CCACTTTAGT GAACCAGGAT TCCATTGGTG AAACCAACTC GTTCTTAGTT TGTGGTTGTG ATGGGGAAAG   
  
  
- AACTGGGCCA AGTATCTCTG GGAACTGATG ATGAGTCGGT ACAAACTCAG ATATCTGCAC TGGTACGGTT   
  
  
- CTCTGGCCTT CCTCTCCTAG TTACAACTCG TCGTAACAAA CCGATTCCTG TAACACTTAT AGTATCGAAC   
  
  
- GCTCCCGTTC CTCTCCCACC TCGCAGTACT TGAAAAACCC TTTACCTTCA GTTCCAAATG GTACCGTCCC   
  
  
- AAGTCCGTCA TGGGCAACTC GAGTATGCAT TTGAGACACT ATTCTTCGGA TGAGGCCACA ATGAGGCTCG   
  
  
- TAATATGAGA CTATCTCTTC CTACCACGGT ACGAAGACCC AACCTTCCCG GCTTACGACT AAAGTCGAAG   
  
  
- CCGTACCGTA AC

+     AAGAA-motif

| Site Name | Organism | Position | Strand | Matrix score. | sequence | function |
| --- | --- | --- | --- | --- | --- | --- |
| AAGAA-motif | Avena sativa | 3431 | - | 9 | gGTAAAGAAA |  |
| AAGAA-motif | Avena sativa | 32 | - | 7 | GAAAGAA |  |
| AAGAA-motif | Avena sativa | 125 | - | 7 | GAAAGAA |  |
| AAGAA-motif | Avena sativa | 36 | - | 7 | GAAAGAA |  |

>HU08G00014.1   
+ +Up\_Stream \_Len000TCCTCT TTTGGGCTTC TTTCTTTCTA TCCTGTTTTT TTTTGGGTCG AAAAGTTGGA   
  
  
+ GGTTGAATTG AAGCATCCGA ATTCGAGCAG GATGTTTCTC TGCGTAGTGA TTCTTTCTGG GTTTGTGATT   
  
  
+ TTGAATTCTG GGTAATCGGT TGTTTTGCTA ATTTTGAGGA CAGAGTTCCT TTTCTGAATT AAAATTTCGT   
  
  
+ TTTTTTATCG GGAAAATCTT CAGTATTTGA GAAAAAAGAA CGAAATTTGG ATGATTTGCT GTCTAGATTT   
  
  
+ TGCTTTCATA TTCCTGGGTG TGGATTGGTT TATTAATTGC ATGTGAGGAA GTACAAGCTT CTGGTCAATG   
  
  
+ GTTGGGTTTT TTTTTTTTTT TTGTGGGTAT AAAGTTGGAA ATTGATTACG AGTCAAATTT CTCTTTCTAG   
  
  
+ AAGAGGGGGG GGGGGGGGGG TTGGTTTAAA ACTCCATATT TTCTTTGAAT GGAAAGCTTA ATTTGCTGAC   
  
  
+ TATCTTGGTT AGTAGCATAA GCTTGATCAG AAAACAAAAC GCTCTCTGCC CTGCAAAAAC TTTATTTGTC   
  
  
+ TTTTAAGGAA AATTTTGGAA TTTGAGAGTG AAAATTTTGG TTGTGAATCT TGTCTTACTA TGTGGTTCCT   
  
  
+ TCTGACAAAA GTTTGAATCT GAGTATGAGA GTTCATCAAT CTCTTTTACT TTCCTACTTT CTTTATTGCT   
  
  
+ TGCAAATTGT TGTGAACTTA CTTGGATTTT CTGGTAGAGG AGAGTTCTGT CCTGGAATTT GGTGGAAAGT   
  
  
+ GAGACATACT TTGGGATTTA ATTAGCCATC AATTTGGTTT CACACCTTAT CAGTTTGGAC TTCTGGTGTG   
  
  
+ TTATTGTATG TGTTCTTAGC TTGGGATTAT TCTTGTATCG GAAGAGAGGG GGGGGGGGGT GTTTCTTTTA   
  
  
+ GCTGAATGTT AGAACATTTT AAGGGATCTA GATATCTAGA GAAGAGCCTA GGAAATGAAG CATTTTTCCC   
  
  
+ TTATAATCTG ACACAACTTT ATGAATTGAT TAGGAACAAA GAACGATTAG TTCAGAAAGG TTACAACTCC   
  
  
+ TCTAGGTGTT ATACTAAAGA GATGTGAGTT AATAAGCAAT TGCTTACTAA CTGTTTAGGC TGTTTCATTG   
  
  
+ CTTGTAGAGG CTTTTTTTTT GGGGGGGGGG GGGGGTTTGG GGTTCATAAA TGAGAAGGTT GATTTGTTGT   
  
  
+ CTGCTCTGCT CTTGATTCGA TAGTAATTCT CTCAGAAAAG GGTAAAATGA CATGGCCTTC CATCGCCTCC   
  
  
+ CATGAGGCTT TTTGCCTCTG CTTGTGTATC TTTCATCTCT TGGTTTGGTG GGTTGGGGGA GGGGAGGGGG   
  
  
+ GGAAGGGGGT TTTGATTCGA TAGATCATCA CTTTCAGAAA TGGCCTCTCT GGCCAATGGT AAGGGGTAAG   
  
  
+ GTTAGTACAT GTGGACTTTC CCATGTGGAT TTTTCCAGAG TCCGTTGTGT GATTGTTATT GCTGTTGTTG   
  
  
+ TAACTTTCTG TCACTCGGAA AACATGAATC CATATGCAAC AAGAACATAA TAATTGTGAA TGCGACTTTA   
  
  
+ TTTAGGAATG TCTTACATAG TACGTGCACA TTCTTTAATT ATCTGTTTAT CTGTTGGTTT GTTATTTTCA   
  
  
+ AGGGGATGGG GAGTGGGTGG ATGCTTAGTA TGTAATCTTT TTATATAGGG AAAAATCATA TAGCTTGAAA   
  
  
+ GTAAAACTTT CATTGCCAGG ATTCCAAGGT AGGGGTAGGG TTGTCTACAC CTTGATCTCC CTAGAAGCAT   
  
  
+ GGCGATCCTT CCTCGGATTG TGTTATAGAA TAAAGAATGT GGTTCTGCTG TAATGAGACT TATGTTCTAT   
  
  
+ CAAATGATGA ATCTGTTTAG AACTTCTTGC TTATTTTTTC TGCAAAATTT TAGGAGACTT GATAAACTAT   
  
  
+ AGTTCTCTTG AATTTACAGG TGGATATTGG AGTTCACAGA ATAGTATTCA GATCAAAGTG CTTGTGCTTA   
  
  
+ ATTGGAAGGA CAACTTGCTT GAGCTGTTTA TAGAGCTCTA ACTGATGCTA TCTCATGGAC TCACATCAGT   
  
  
+ TTTTTGGATT TAATCTTACC AGTGTTGATC CGTCGTACAT TTCCGCTCAG TACAGTCCGC CGTCAGTGAC   
  
  
+ AAATAGGATG TTTGCATCAC TGAAGCTCGA CTCTAGAGGT TCTCCTGTGT CACCCTTCTC AACTCAGTTT   
  
  
+ GATTGTGATA CGGTTACTAC ATTGAGTGAT AGTCAAGAGC ATCACAGCTC GACGGGGAGT CTATCAACAA   
  
  
+ GAAGCCCTTC TTGTAATTCT CCCCTTGAAA CGAGCAGTTA TCATCATTTA TCGACGAATG GCCCGTCTTG   
  
  
+ TAATTCTGCC CCTGAAACTA GCAGTTATCG TCATCGGTTC AACGCGAGTC CCGTGGGATA TTCCCATCAA   
  
  
+ GGCACTGATT ATGGAGTGAA TATGAAGAGC GCTCTGCAGG AGCTAGAGAC TACTCTAATG GGTGCAGATG   
  
  
+ GTGAGGAAGT ATCTGCTGCT AATCAACCTA TGGGGGGAAG TCGTCAGTCC GGGATCCCAA GTCAGAGATC   
  
  
+ AAAATCATTG AGCGAAGATC CACAGGGTTC GCATCCTACT CAGCCTGATT CATCATCTCT TTCTAGGGCA   
  
  
+ AGAAGATCAG GAGATGAAAG CCAGAGAGAG AAACGGCACA AGGCAATGGA AGAACCAACG GAACTACCAA   
  
  
+ GTTTGCCACC TGGTGATTTG AAGCAGTTGC TAATTGAATG TGCGAGGGCT TTATCGGATA ACCGAATAGA   
  
  
+ TGACTTTGAG AGTTTGGTTA AACGGGCGAG GAAAGAGGTC TCTATCTCGG GTGAGCCTAT CCAACGTCTC   
  
  
+ GGTGCCTACA TGATCGAAGG GCTTGTGGCA AGGAAGCAGT CTTCGGGGAC TAGCATCTAT CGGGCTCTGA   
  
  
+ AGTGTAAAGA GCCTCTTGGA AAAGACTTGC TCTCCTACAT GCACATCCTT TATGAAATAT GCCCTTATCT   
  
  
+ CAAGTTTGGT TATATGGCTG CGAATGGAGC GATAGCTGAA GCTTGTAGAA ATGAGGATAA CATACATATT   
  
  
+ ATAGATTTCC AGATTGCACA GGGCACTCAG TGGGTTACTC TATTACAAGC CCTAGCAGCA AGACCTGGTG   
  
  
+ GGCCACCTAA GGTGCGAATT ACAGGCATTG ATGATCCTGT TTCTAAGTAT GCCCGTGGTG CTAGCTTGGA   
  
  
+ GGCTGTTGGG AAACGGTTAG CGTCTCTATC TGAAAAGTTC AAAATACCCG TCGAGTTCAA TGCGTTGCCC   
  
  
+ GTTTATGGAC CCGATGTCAG GCGGGAAATG CTGGATGTGA GGCCCGGGGA GGCTTTGGCC GTTAATTTTC   
  
  
+ CATTGCAGCT CCACCACACT CCTGACGAGA GTGTCGATGT GAACAACCCT AGGGATGGGC TTCTCAGAAT   
  
  
+ GGTGAAATCA CTTGGTCCTA AGGTAACCAC TTTGGTTGAG CAAGAATCAA ACACCAACAC TACCCCTTTC   
  
  
+ TTGACCCGGT TCATAGAGAC CCTTGACTAC TACTCAGCCA TGTTTGAGTC TATAGACGTG ACCATGCCAA   
  
  
+ GAGACCGGAA GGAGAGGATC AATGTTGAGC AGCATTGTTT GGCTAAGGAC ATTGTGAATA TCATAGCTTG   
  
  
+ CGAGGGCAAG GAGAGGGTGG AGCGTCATGA ACTTTTTGGG AAATGGAAGT CAAGGTTTAC CATGGCAGGG   
  
  
+ TTCAGGCAGT ACCCGTTGAG CTCATACGTA AACTCTGTGA TAAGAAGCCT ACTCCGGTGT TACTCCGAGC   
  
  
+ ATTATACTCT GATAGAGAAG GATGGTGCCA TGCTTCTGGG TTGGAAGGGC CGAATGCTGA TTTCAGCTTC   
  
  
+ GGCATGGCAT TG  

- +Up\_Stream \_Len000AGGAGA AAACCCGAAG AAAGAAAGAT AGGACAAAAA AAAACCCAGC TTTTCAACCT   
  
  
- CCAACTTAAC TTCGTAGGCT TAAGCTCGTC CTACAAAGAG ACGCATCACT AAGAAAGACC CAAACACTAA   
  
  
- AACTTAAGAC CCATTAGCCA ACAAAACGAT TAAAACTCCT GTCTCAAGGA AAAGACTTAA TTTTAAAGCA   
  
  
- AAAAAATAGC CCTTTTAGAA GTCATAAACT CTTTTTTCTT GCTTTAAACC TACTAAACGA CAGATCTAAA   
  
  
- ACGAAAGTAT AAGGACCCAC ACCTAACCAA ATAATTAACG TACACTCCTT CATGTTCGAA GACCAGTTAC   
  
  
- CAACCCAAAA AAAAAAAAAA AACACCCATA TTTCAACCTT TAACTAATGC TCAGTTTAAA GAGAAAGATC   
  
  
- TTCTCCCCCC CCCCCCCCCC AACCAAATTT TGAGGTATAA AAGAAACTTA CCTTTCGAAT TAAACGACTG   
  
  
- ATAGAACCAA TCATCGTATT CGAACTAGTC TTTTGTTTTG CGAGAGACGG GACGTTTTTG AAATAAACAG   
  
  
- AAAATTCCTT TTAAAACCTT AAACTCTCAC TTTTAAAACC AACACTTAGA ACAGAATGAT ACACCAAGGA   
  
  
- AGACTGTTTT CAAACTTAGA CTCATACTCT CAAGTAGTTA GAGAAAATGA AAGGATGAAA GAAATAACGA   
  
  
- ACGTTTAACA ACACTTGAAT GAACCTAAAA GACCATCTCC TCTCAAGACA GGACCTTAAA CCACCTTTCA   
  
  
- CTCTGTATGA AACCCTAAAT TAATCGGTAG TTAAACCAAA GTGTGGAATA GTCAAACCTG AAGACCACAC   
  
  
- AATAACATAC ACAAGAATCG AACCCTAATA AGAACATAGC CTTCTCTCCC CCCCCCCCCA CAAAGAAAAT   
  
  
- CGACTTACAA TCTTGTAAAA TTCCCTAGAT CTATAGATCT CTTCTCGGAT CCTTTACTTC GTAAAAAGGG   
  
  
- AATATTAGAC TGTGTTGAAA TACTTAACTA ATCCTTGTTT CTTGCTAATC AAGTCTTTCC AATGTTGAGG   
  
  
- AGATCCACAA TATGATTTCT CTACACTCAA TTATTCGTTA ACGAATGATT GACAAATCCG ACAAAGTAAC   
  
  
- GAACATCTCC GAAAAAAAAA CCCCCCCCCC CCCCCAAACC CCAAGTATTT ACTCTTCCAA CTAAACAACA   
  
  
- GACGAGACGA GAACTAAGCT ATCATTAAGA GAGTCTTTTC CCATTTTACT GTACCGGAAG GTAGCGGAGG   
  
  
- GTACTCCGAA AAACGGAGAC GAACACATAG AAAGTAGAGA ACCAAACCAC CCAACCCCCT CCCCTCCCCC   
  
  
- CCTTCCCCCA AAACTAAGCT ATCTAGTAGT GAAAGTCTTT ACCGGAGAGA CCGGTTACCA TTCCCCATTC   
  
  
- CAATCATGTA CACCTGAAAG GGTACACCTA AAAAGGTCTC AGGCAACACA CTAACAATAA CGACAACAAC   
  
  
- ATTGAAAGAC AGTGAGCCTT TTGTACTTAG GTATACGTTG TTCTTGTATT ATTAACACTT ACGCTGAAAT   
  
  
- AAATCCTTAC AGAATGTATC ATGCACGTGT AAGAAATTAA TAGACAAATA GACAACCAAA CAATAAAAGT   
  
  
- TCCCCTACCC CTCACCCACC TACGAATCAT ACATTAGAAA AATATATCCC TTTTTAGTAT ATCGAACTTT   
  
  
- CATTTTGAAA GTAACGGTCC TAAGGTTCCA TCCCCATCCC AACAGATGTG GAACTAGAGG GATCTTCGTA   
  
  
- CCGCTAGGAA GGAGCCTAAC ACAATATCTT ATTTCTTACA CCAAGACGAC ATTACTCTGA ATACAAGATA   
  
  
- GTTTACTACT TAGACAAATC TTGAAGAACG AATAAAAAAG ACGTTTTAAA ATCCTCTGAA CTATTTGATA   
  
  
- TCAAGAGAAC TTAAATGTCC ACCTATAACC TCAAGTGTCT TATCATAAGT CTAGTTTCAC GAACACGAAT   
  
  
- TAACCTTCCT GTTGAACGAA CTCGACAAAT ATCTCGAGAT TGACTACGAT AGAGTACCTG AGTGTAGTCA   
  
  
- AAAAACCTAA ATTAGAATGG TCACAACTAG GCAGCATGTA AAGGCGAGTC ATGTCAGGCG GCAGTCACTG   
  
  
- TTTATCCTAC AAACGTAGTG ACTTCGAGCT GAGATCTCCA AGAGGACACA GTGGGAAGAG TTGAGTCAAA   
  
  
- CTAACACTAT GCCAATGATG TAACTCACTA TCAGTTCTCG TAGTGTCGAG CTGCCCCTCA GATAGTTGTT   
  
  
- CTTCGGGAAG AACATTAAGA GGGGAACTTT GCTCGTCAAT AGTAGTAAAT AGCTGCTTAC CGGGCAGAAC   
  
  
- ATTAAGACGG GGACTTTGAT CGTCAATAGC AGTAGCCAAG TTGCGCTCAG GGCACCCTAT AAGGGTAGTT   
  
  
- CCGTGACTAA TACCTCACTT ATACTTCTCG CGAGACGTCC TCGATCTCTG ATGAGATTAC CCACGTCTAC   
  
  
- CACTCCTTCA TAGACGACGA TTAGTTGGAT ACCCCCCTTC AGCAGTCAGG CCCTAGGGTT CAGTCTCTAG   
  
  
- TTTTAGTAAC TCGCTTCTAG GTGTCCCAAG CGTAGGATGA GTCGGACTAA GTAGTAGAGA AAGATCCCGT   
  
  
- TCTTCTAGTC CTCTACTTTC GGTCTCTCTC TTTGCCGTGT TCCGTTACCT TCTTGGTTGC CTTGATGGTT   
  
  
- CAAACGGTGG ACCACTAAAC TTCGTCAACG ATTAACTTAC ACGCTCCCGA AATAGCCTAT TGGCTTATCT   
  
  
- ACTGAAACTC TCAAACCAAT TTGCCCGCTC CTTTCTCCAG AGATAGAGCC CACTCGGATA GGTTGCAGAG   
  
  
- CCACGGATGT ACTAGCTTCC CGAACACCGT TCCTTCGTCA GAAGCCCCTG ATCGTAGATA GCCCGAGACT   
  
  
- TCACATTTCT CGGAGAACCT TTTCTGAACG AGAGGATGTA CGTGTAGGAA ATACTTTATA CGGGAATAGA   
  
  
- GTTCAAACCA ATATACCGAC GCTTACCTCG CTATCGACTT CGAACATCTT TACTCCTATT GTATGTATAA   
  
  
- TATCTAAAGG TCTAACGTGT CCCGTGAGTC ACCCAATGAG ATAATGTTCG GGATCGTCGT TCTGGACCAC   
  
  
- CCGGTGGATT CCACGCTTAA TGTCCGTAAC TACTAGGACA AAGATTCATA CGGGCACCAC GATCGAACCT   
  
  
- CCGACAACCC TTTGCCAATC GCAGAGATAG ACTTTTCAAG TTTTATGGGC AGCTCAAGTT ACGCAACGGG   
  
  
- CAAATACCTG GGCTACAGTC CGCCCTTTAC GACCTACACT CCGGGCCCCT CCGAAACCGG CAATTAAAAG   
  
  
- GTAACGTCGA GGTGGTGTGA GGACTGCTCT CACAGCTACA CTTGTTGGGA TCCCTACCCG AAGAGTCTTA   
  
  
- CCACTTTAGT GAACCAGGAT TCCATTGGTG AAACCAACTC GTTCTTAGTT TGTGGTTGTG ATGGGGAAAG   
  
  
- AACTGGGCCA AGTATCTCTG GGAACTGATG ATGAGTCGGT ACAAACTCAG ATATCTGCAC TGGTACGGTT   
  
  
- CTCTGGCCTT CCTCTCCTAG TTACAACTCG TCGTAACAAA CCGATTCCTG TAACACTTAT AGTATCGAAC   
  
  
- GCTCCCGTTC CTCTCCCACC TCGCAGTACT TGAAAAACCC TTTACCTTCA GTTCCAAATG GTACCGTCCC   
  
  
- AAGTCCGTCA TGGGCAACTC GAGTATGCAT TTGAGACACT ATTCTTCGGA TGAGGCCACA ATGAGGCTCG   
  
  
- TAATATGAGA CTATCTCTTC CTACCACGGT ACGAAGACCC AACCTTCCCG GCTTACGACT AAAGTCGAAG   
  
  
- CCGTACCGTA AC

+     ABRE

| Site Name | Organism | Position | Strand | Matrix score. | sequence | function |
| --- | --- | --- | --- | --- | --- | --- |
| ABRE | Arabidopsis thaliana | 3490 | + | 5 | ACGTG | cis-acting element involved in the abscisic acid responsiveness |
| ABRE | Arabidopsis thaliana | 1566 | + | 5 | ACGTG | cis-acting element involved in the abscisic acid responsiveness |
| ABRE | Hordeum vulgare | 1563 | + | 9 | CGTACGTGCA | cis-acting element involved in the abscisic acid responsiveness |

>HU08G00014.1   
+ +Up\_Stream \_Len000TCCTCT TTTGGGCTTC TTTCTTTCTA TCCTGTTTTT TTTTGGGTCG AAAAGTTGGA   
  
  
+ GGTTGAATTG AAGCATCCGA ATTCGAGCAG GATGTTTCTC TGCGTAGTGA TTCTTTCTGG GTTTGTGATT   
  
  
+ TTGAATTCTG GGTAATCGGT TGTTTTGCTA ATTTTGAGGA CAGAGTTCCT TTTCTGAATT AAAATTTCGT   
  
  
+ TTTTTTATCG GGAAAATCTT CAGTATTTGA GAAAAAAGAA CGAAATTTGG ATGATTTGCT GTCTAGATTT   
  
  
+ TGCTTTCATA TTCCTGGGTG TGGATTGGTT TATTAATTGC ATGTGAGGAA GTACAAGCTT CTGGTCAATG   
  
  
+ GTTGGGTTTT TTTTTTTTTT TTGTGGGTAT AAAGTTGGAA ATTGATTACG AGTCAAATTT CTCTTTCTAG   
  
  
+ AAGAGGGGGG GGGGGGGGGG TTGGTTTAAA ACTCCATATT TTCTTTGAAT GGAAAGCTTA ATTTGCTGAC   
  
  
+ TATCTTGGTT AGTAGCATAA GCTTGATCAG AAAACAAAAC GCTCTCTGCC CTGCAAAAAC TTTATTTGTC   
  
  
+ TTTTAAGGAA AATTTTGGAA TTTGAGAGTG AAAATTTTGG TTGTGAATCT TGTCTTACTA TGTGGTTCCT   
  
  
+ TCTGACAAAA GTTTGAATCT GAGTATGAGA GTTCATCAAT CTCTTTTACT TTCCTACTTT CTTTATTGCT   
  
  
+ TGCAAATTGT TGTGAACTTA CTTGGATTTT CTGGTAGAGG AGAGTTCTGT CCTGGAATTT GGTGGAAAGT   
  
  
+ GAGACATACT TTGGGATTTA ATTAGCCATC AATTTGGTTT CACACCTTAT CAGTTTGGAC TTCTGGTGTG   
  
  
+ TTATTGTATG TGTTCTTAGC TTGGGATTAT TCTTGTATCG GAAGAGAGGG GGGGGGGGGT GTTTCTTTTA   
  
  
+ GCTGAATGTT AGAACATTTT AAGGGATCTA GATATCTAGA GAAGAGCCTA GGAAATGAAG CATTTTTCCC   
  
  
+ TTATAATCTG ACACAACTTT ATGAATTGAT TAGGAACAAA GAACGATTAG TTCAGAAAGG TTACAACTCC   
  
  
+ TCTAGGTGTT ATACTAAAGA GATGTGAGTT AATAAGCAAT TGCTTACTAA CTGTTTAGGC TGTTTCATTG   
  
  
+ CTTGTAGAGG CTTTTTTTTT GGGGGGGGGG GGGGGTTTGG GGTTCATAAA TGAGAAGGTT GATTTGTTGT   
  
  
+ CTGCTCTGCT CTTGATTCGA TAGTAATTCT CTCAGAAAAG GGTAAAATGA CATGGCCTTC CATCGCCTCC   
  
  
+ CATGAGGCTT TTTGCCTCTG CTTGTGTATC TTTCATCTCT TGGTTTGGTG GGTTGGGGGA GGGGAGGGGG   
  
  
+ GGAAGGGGGT TTTGATTCGA TAGATCATCA CTTTCAGAAA TGGCCTCTCT GGCCAATGGT AAGGGGTAAG   
  
  
+ GTTAGTACAT GTGGACTTTC CCATGTGGAT TTTTCCAGAG TCCGTTGTGT GATTGTTATT GCTGTTGTTG   
  
  
+ TAACTTTCTG TCACTCGGAA AACATGAATC CATATGCAAC AAGAACATAA TAATTGTGAA TGCGACTTTA   
  
  
+ TTTAGGAATG TCTTACATAG TACGTGCACA TTCTTTAATT ATCTGTTTAT CTGTTGGTTT GTTATTTTCA   
  
  
+ AGGGGATGGG GAGTGGGTGG ATGCTTAGTA TGTAATCTTT TTATATAGGG AAAAATCATA TAGCTTGAAA   
  
  
+ GTAAAACTTT CATTGCCAGG ATTCCAAGGT AGGGGTAGGG TTGTCTACAC CTTGATCTCC CTAGAAGCAT   
  
  
+ GGCGATCCTT CCTCGGATTG TGTTATAGAA TAAAGAATGT GGTTCTGCTG TAATGAGACT TATGTTCTAT   
  
  
+ CAAATGATGA ATCTGTTTAG AACTTCTTGC TTATTTTTTC TGCAAAATTT TAGGAGACTT GATAAACTAT   
  
  
+ AGTTCTCTTG AATTTACAGG TGGATATTGG AGTTCACAGA ATAGTATTCA GATCAAAGTG CTTGTGCTTA   
  
  
+ ATTGGAAGGA CAACTTGCTT GAGCTGTTTA TAGAGCTCTA ACTGATGCTA TCTCATGGAC TCACATCAGT   
  
  
+ TTTTTGGATT TAATCTTACC AGTGTTGATC CGTCGTACAT TTCCGCTCAG TACAGTCCGC CGTCAGTGAC   
  
  
+ AAATAGGATG TTTGCATCAC TGAAGCTCGA CTCTAGAGGT TCTCCTGTGT CACCCTTCTC AACTCAGTTT   
  
  
+ GATTGTGATA CGGTTACTAC ATTGAGTGAT AGTCAAGAGC ATCACAGCTC GACGGGGAGT CTATCAACAA   
  
  
+ GAAGCCCTTC TTGTAATTCT CCCCTTGAAA CGAGCAGTTA TCATCATTTA TCGACGAATG GCCCGTCTTG   
  
  
+ TAATTCTGCC CCTGAAACTA GCAGTTATCG TCATCGGTTC AACGCGAGTC CCGTGGGATA TTCCCATCAA   
  
  
+ GGCACTGATT ATGGAGTGAA TATGAAGAGC GCTCTGCAGG AGCTAGAGAC TACTCTAATG GGTGCAGATG   
  
  
+ GTGAGGAAGT ATCTGCTGCT AATCAACCTA TGGGGGGAAG TCGTCAGTCC GGGATCCCAA GTCAGAGATC   
  
  
+ AAAATCATTG AGCGAAGATC CACAGGGTTC GCATCCTACT CAGCCTGATT CATCATCTCT TTCTAGGGCA   
  
  
+ AGAAGATCAG GAGATGAAAG CCAGAGAGAG AAACGGCACA AGGCAATGGA AGAACCAACG GAACTACCAA   
  
  
+ GTTTGCCACC TGGTGATTTG AAGCAGTTGC TAATTGAATG TGCGAGGGCT TTATCGGATA ACCGAATAGA   
  
  
+ TGACTTTGAG AGTTTGGTTA AACGGGCGAG GAAAGAGGTC TCTATCTCGG GTGAGCCTAT CCAACGTCTC   
  
  
+ GGTGCCTACA TGATCGAAGG GCTTGTGGCA AGGAAGCAGT CTTCGGGGAC TAGCATCTAT CGGGCTCTGA   
  
  
+ AGTGTAAAGA GCCTCTTGGA AAAGACTTGC TCTCCTACAT GCACATCCTT TATGAAATAT GCCCTTATCT   
  
  
+ CAAGTTTGGT TATATGGCTG CGAATGGAGC GATAGCTGAA GCTTGTAGAA ATGAGGATAA CATACATATT   
  
  
+ ATAGATTTCC AGATTGCACA GGGCACTCAG TGGGTTACTC TATTACAAGC CCTAGCAGCA AGACCTGGTG   
  
  
+ GGCCACCTAA GGTGCGAATT ACAGGCATTG ATGATCCTGT TTCTAAGTAT GCCCGTGGTG CTAGCTTGGA   
  
  
+ GGCTGTTGGG AAACGGTTAG CGTCTCTATC TGAAAAGTTC AAAATACCCG TCGAGTTCAA TGCGTTGCCC   
  
  
+ GTTTATGGAC CCGATGTCAG GCGGGAAATG CTGGATGTGA GGCCCGGGGA GGCTTTGGCC GTTAATTTTC   
  
  
+ CATTGCAGCT CCACCACACT CCTGACGAGA GTGTCGATGT GAACAACCCT AGGGATGGGC TTCTCAGAAT   
  
  
+ GGTGAAATCA CTTGGTCCTA AGGTAACCAC TTTGGTTGAG CAAGAATCAA ACACCAACAC TACCCCTTTC   
  
  
+ TTGACCCGGT TCATAGAGAC CCTTGACTAC TACTCAGCCA TGTTTGAGTC TATAGACGTG ACCATGCCAA   
  
  
+ GAGACCGGAA GGAGAGGATC AATGTTGAGC AGCATTGTTT GGCTAAGGAC ATTGTGAATA TCATAGCTTG   
  
  
+ CGAGGGCAAG GAGAGGGTGG AGCGTCATGA ACTTTTTGGG AAATGGAAGT CAAGGTTTAC CATGGCAGGG   
  
  
+ TTCAGGCAGT ACCCGTTGAG CTCATACGTA AACTCTGTGA TAAGAAGCCT ACTCCGGTGT TACTCCGAGC   
  
  
+ ATTATACTCT GATAGAGAAG GATGGTGCCA TGCTTCTGGG TTGGAAGGGC CGAATGCTGA TTTCAGCTTC   
  
  
+ GGCATGGCAT TG  

- +Up\_Stream \_Len000AGGAGA AAACCCGAAG AAAGAAAGAT AGGACAAAAA AAAACCCAGC TTTTCAACCT   
  
  
- CCAACTTAAC TTCGTAGGCT TAAGCTCGTC CTACAAAGAG ACGCATCACT AAGAAAGACC CAAACACTAA   
  
  
- AACTTAAGAC CCATTAGCCA ACAAAACGAT TAAAACTCCT GTCTCAAGGA AAAGACTTAA TTTTAAAGCA   
  
  
- AAAAAATAGC CCTTTTAGAA GTCATAAACT CTTTTTTCTT GCTTTAAACC TACTAAACGA CAGATCTAAA   
  
  
- ACGAAAGTAT AAGGACCCAC ACCTAACCAA ATAATTAACG TACACTCCTT CATGTTCGAA GACCAGTTAC   
  
  
- CAACCCAAAA AAAAAAAAAA AACACCCATA TTTCAACCTT TAACTAATGC TCAGTTTAAA GAGAAAGATC   
  
  
- TTCTCCCCCC CCCCCCCCCC AACCAAATTT TGAGGTATAA AAGAAACTTA CCTTTCGAAT TAAACGACTG   
  
  
- ATAGAACCAA TCATCGTATT CGAACTAGTC TTTTGTTTTG CGAGAGACGG GACGTTTTTG AAATAAACAG   
  
  
- AAAATTCCTT TTAAAACCTT AAACTCTCAC TTTTAAAACC AACACTTAGA ACAGAATGAT ACACCAAGGA   
  
  
- AGACTGTTTT CAAACTTAGA CTCATACTCT CAAGTAGTTA GAGAAAATGA AAGGATGAAA GAAATAACGA   
  
  
- ACGTTTAACA ACACTTGAAT GAACCTAAAA GACCATCTCC TCTCAAGACA GGACCTTAAA CCACCTTTCA   
  
  
- CTCTGTATGA AACCCTAAAT TAATCGGTAG TTAAACCAAA GTGTGGAATA GTCAAACCTG AAGACCACAC   
  
  
- AATAACATAC ACAAGAATCG AACCCTAATA AGAACATAGC CTTCTCTCCC CCCCCCCCCA CAAAGAAAAT   
  
  
- CGACTTACAA TCTTGTAAAA TTCCCTAGAT CTATAGATCT CTTCTCGGAT CCTTTACTTC GTAAAAAGGG   
  
  
- AATATTAGAC TGTGTTGAAA TACTTAACTA ATCCTTGTTT CTTGCTAATC AAGTCTTTCC AATGTTGAGG   
  
  
- AGATCCACAA TATGATTTCT CTACACTCAA TTATTCGTTA ACGAATGATT GACAAATCCG ACAAAGTAAC   
  
  
- GAACATCTCC GAAAAAAAAA CCCCCCCCCC CCCCCAAACC CCAAGTATTT ACTCTTCCAA CTAAACAACA   
  
  
- GACGAGACGA GAACTAAGCT ATCATTAAGA GAGTCTTTTC CCATTTTACT GTACCGGAAG GTAGCGGAGG   
  
  
- GTACTCCGAA AAACGGAGAC GAACACATAG AAAGTAGAGA ACCAAACCAC CCAACCCCCT CCCCTCCCCC   
  
  
- CCTTCCCCCA AAACTAAGCT ATCTAGTAGT GAAAGTCTTT ACCGGAGAGA CCGGTTACCA TTCCCCATTC   
  
  
- CAATCATGTA CACCTGAAAG GGTACACCTA AAAAGGTCTC AGGCAACACA CTAACAATAA CGACAACAAC   
  
  
- ATTGAAAGAC AGTGAGCCTT TTGTACTTAG GTATACGTTG TTCTTGTATT ATTAACACTT ACGCTGAAAT   
  
  
- AAATCCTTAC AGAATGTATC ATGCACGTGT AAGAAATTAA TAGACAAATA GACAACCAAA CAATAAAAGT   
  
  
- TCCCCTACCC CTCACCCACC TACGAATCAT ACATTAGAAA AATATATCCC TTTTTAGTAT ATCGAACTTT   
  
  
- CATTTTGAAA GTAACGGTCC TAAGGTTCCA TCCCCATCCC AACAGATGTG GAACTAGAGG GATCTTCGTA   
  
  
- CCGCTAGGAA GGAGCCTAAC ACAATATCTT ATTTCTTACA CCAAGACGAC ATTACTCTGA ATACAAGATA   
  
  
- GTTTACTACT TAGACAAATC TTGAAGAACG AATAAAAAAG ACGTTTTAAA ATCCTCTGAA CTATTTGATA   
  
  
- TCAAGAGAAC TTAAATGTCC ACCTATAACC TCAAGTGTCT TATCATAAGT CTAGTTTCAC GAACACGAAT   
  
  
- TAACCTTCCT GTTGAACGAA CTCGACAAAT ATCTCGAGAT TGACTACGAT AGAGTACCTG AGTGTAGTCA   
  
  
- AAAAACCTAA ATTAGAATGG TCACAACTAG GCAGCATGTA AAGGCGAGTC ATGTCAGGCG GCAGTCACTG   
  
  
- TTTATCCTAC AAACGTAGTG ACTTCGAGCT GAGATCTCCA AGAGGACACA GTGGGAAGAG TTGAGTCAAA   
  
  
- CTAACACTAT GCCAATGATG TAACTCACTA TCAGTTCTCG TAGTGTCGAG CTGCCCCTCA GATAGTTGTT   
  
  
- CTTCGGGAAG AACATTAAGA GGGGAACTTT GCTCGTCAAT AGTAGTAAAT AGCTGCTTAC CGGGCAGAAC   
  
  
- ATTAAGACGG GGACTTTGAT CGTCAATAGC AGTAGCCAAG TTGCGCTCAG GGCACCCTAT AAGGGTAGTT   
  
  
- CCGTGACTAA TACCTCACTT ATACTTCTCG CGAGACGTCC TCGATCTCTG ATGAGATTAC CCACGTCTAC   
  
  
- CACTCCTTCA TAGACGACGA TTAGTTGGAT ACCCCCCTTC AGCAGTCAGG CCCTAGGGTT CAGTCTCTAG   
  
  
- TTTTAGTAAC TCGCTTCTAG GTGTCCCAAG CGTAGGATGA GTCGGACTAA GTAGTAGAGA AAGATCCCGT   
  
  
- TCTTCTAGTC CTCTACTTTC GGTCTCTCTC TTTGCCGTGT TCCGTTACCT TCTTGGTTGC CTTGATGGTT   
  
  
- CAAACGGTGG ACCACTAAAC TTCGTCAACG ATTAACTTAC ACGCTCCCGA AATAGCCTAT TGGCTTATCT   
  
  
- ACTGAAACTC TCAAACCAAT TTGCCCGCTC CTTTCTCCAG AGATAGAGCC CACTCGGATA GGTTGCAGAG   
  
  
- CCACGGATGT ACTAGCTTCC CGAACACCGT TCCTTCGTCA GAAGCCCCTG ATCGTAGATA GCCCGAGACT   
  
  
- TCACATTTCT CGGAGAACCT TTTCTGAACG AGAGGATGTA CGTGTAGGAA ATACTTTATA CGGGAATAGA   
  
  
- GTTCAAACCA ATATACCGAC GCTTACCTCG CTATCGACTT CGAACATCTT TACTCCTATT GTATGTATAA   
  
  
- TATCTAAAGG TCTAACGTGT CCCGTGAGTC ACCCAATGAG ATAATGTTCG GGATCGTCGT TCTGGACCAC   
  
  
- CCGGTGGATT CCACGCTTAA TGTCCGTAAC TACTAGGACA AAGATTCATA CGGGCACCAC GATCGAACCT   
  
  
- CCGACAACCC TTTGCCAATC GCAGAGATAG ACTTTTCAAG TTTTATGGGC AGCTCAAGTT ACGCAACGGG   
  
  
- CAAATACCTG GGCTACAGTC CGCCCTTTAC GACCTACACT CCGGGCCCCT CCGAAACCGG CAATTAAAAG   
  
  
- GTAACGTCGA GGTGGTGTGA GGACTGCTCT CACAGCTACA CTTGTTGGGA TCCCTACCCG AAGAGTCTTA   
  
  
- CCACTTTAGT GAACCAGGAT TCCATTGGTG AAACCAACTC GTTCTTAGTT TGTGGTTGTG ATGGGGAAAG   
  
  
- AACTGGGCCA AGTATCTCTG GGAACTGATG ATGAGTCGGT ACAAACTCAG ATATCTGCAC TGGTACGGTT   
  
  
- CTCTGGCCTT CCTCTCCTAG TTACAACTCG TCGTAACAAA CCGATTCCTG TAACACTTAT AGTATCGAAC   
  
  
- GCTCCCGTTC CTCTCCCACC TCGCAGTACT TGAAAAACCC TTTACCTTCA GTTCCAAATG GTACCGTCCC   
  
  
- AAGTCCGTCA TGGGCAACTC GAGTATGCAT TTGAGACACT ATTCTTCGGA TGAGGCCACA ATGAGGCTCG   
  
  
- TAATATGAGA CTATCTCTTC CTACCACGGT ACGAAGACCC AACCTTCCCG GCTTACGACT AAAGTCGAAG   
  
  
- CCGTACCGTA AC

+     ABRE3a

| Site Name | Organism | Position | Strand | Matrix score. | sequence | function |
| --- | --- | --- | --- | --- | --- | --- |
| ABRE3a | Zea mays | 1565 | + | 6 | TACGTG |  |

>HU08G00014.1   
+ +Up\_Stream \_Len000TCCTCT TTTGGGCTTC TTTCTTTCTA TCCTGTTTTT TTTTGGGTCG AAAAGTTGGA   
  
  
+ GGTTGAATTG AAGCATCCGA ATTCGAGCAG GATGTTTCTC TGCGTAGTGA TTCTTTCTGG GTTTGTGATT   
  
  
+ TTGAATTCTG GGTAATCGGT TGTTTTGCTA ATTTTGAGGA CAGAGTTCCT TTTCTGAATT AAAATTTCGT   
  
  
+ TTTTTTATCG GGAAAATCTT CAGTATTTGA GAAAAAAGAA CGAAATTTGG ATGATTTGCT GTCTAGATTT   
  
  
+ TGCTTTCATA TTCCTGGGTG TGGATTGGTT TATTAATTGC ATGTGAGGAA GTACAAGCTT CTGGTCAATG   
  
  
+ GTTGGGTTTT TTTTTTTTTT TTGTGGGTAT AAAGTTGGAA ATTGATTACG AGTCAAATTT CTCTTTCTAG   
  
  
+ AAGAGGGGGG GGGGGGGGGG TTGGTTTAAA ACTCCATATT TTCTTTGAAT GGAAAGCTTA ATTTGCTGAC   
  
  
+ TATCTTGGTT AGTAGCATAA GCTTGATCAG AAAACAAAAC GCTCTCTGCC CTGCAAAAAC TTTATTTGTC   
  
  
+ TTTTAAGGAA AATTTTGGAA TTTGAGAGTG AAAATTTTGG TTGTGAATCT TGTCTTACTA TGTGGTTCCT   
  
  
+ TCTGACAAAA GTTTGAATCT GAGTATGAGA GTTCATCAAT CTCTTTTACT TTCCTACTTT CTTTATTGCT   
  
  
+ TGCAAATTGT TGTGAACTTA CTTGGATTTT CTGGTAGAGG AGAGTTCTGT CCTGGAATTT GGTGGAAAGT   
  
  
+ GAGACATACT TTGGGATTTA ATTAGCCATC AATTTGGTTT CACACCTTAT CAGTTTGGAC TTCTGGTGTG   
  
  
+ TTATTGTATG TGTTCTTAGC TTGGGATTAT TCTTGTATCG GAAGAGAGGG GGGGGGGGGT GTTTCTTTTA   
  
  
+ GCTGAATGTT AGAACATTTT AAGGGATCTA GATATCTAGA GAAGAGCCTA GGAAATGAAG CATTTTTCCC   
  
  
+ TTATAATCTG ACACAACTTT ATGAATTGAT TAGGAACAAA GAACGATTAG TTCAGAAAGG TTACAACTCC   
  
  
+ TCTAGGTGTT ATACTAAAGA GATGTGAGTT AATAAGCAAT TGCTTACTAA CTGTTTAGGC TGTTTCATTG   
  
  
+ CTTGTAGAGG CTTTTTTTTT GGGGGGGGGG GGGGGTTTGG GGTTCATAAA TGAGAAGGTT GATTTGTTGT   
  
  
+ CTGCTCTGCT CTTGATTCGA TAGTAATTCT CTCAGAAAAG GGTAAAATGA CATGGCCTTC CATCGCCTCC   
  
  
+ CATGAGGCTT TTTGCCTCTG CTTGTGTATC TTTCATCTCT TGGTTTGGTG GGTTGGGGGA GGGGAGGGGG   
  
  
+ GGAAGGGGGT TTTGATTCGA TAGATCATCA CTTTCAGAAA TGGCCTCTCT GGCCAATGGT AAGGGGTAAG   
  
  
+ GTTAGTACAT GTGGACTTTC CCATGTGGAT TTTTCCAGAG TCCGTTGTGT GATTGTTATT GCTGTTGTTG   
  
  
+ TAACTTTCTG TCACTCGGAA AACATGAATC CATATGCAAC AAGAACATAA TAATTGTGAA TGCGACTTTA   
  
  
+ TTTAGGAATG TCTTACATAG TACGTGCACA TTCTTTAATT ATCTGTTTAT CTGTTGGTTT GTTATTTTCA   
  
  
+ AGGGGATGGG GAGTGGGTGG ATGCTTAGTA TGTAATCTTT TTATATAGGG AAAAATCATA TAGCTTGAAA   
  
  
+ GTAAAACTTT CATTGCCAGG ATTCCAAGGT AGGGGTAGGG TTGTCTACAC CTTGATCTCC CTAGAAGCAT   
  
  
+ GGCGATCCTT CCTCGGATTG TGTTATAGAA TAAAGAATGT GGTTCTGCTG TAATGAGACT TATGTTCTAT   
  
  
+ CAAATGATGA ATCTGTTTAG AACTTCTTGC TTATTTTTTC TGCAAAATTT TAGGAGACTT GATAAACTAT   
  
  
+ AGTTCTCTTG AATTTACAGG TGGATATTGG AGTTCACAGA ATAGTATTCA GATCAAAGTG CTTGTGCTTA   
  
  
+ ATTGGAAGGA CAACTTGCTT GAGCTGTTTA TAGAGCTCTA ACTGATGCTA TCTCATGGAC TCACATCAGT   
  
  
+ TTTTTGGATT TAATCTTACC AGTGTTGATC CGTCGTACAT TTCCGCTCAG TACAGTCCGC CGTCAGTGAC   
  
  
+ AAATAGGATG TTTGCATCAC TGAAGCTCGA CTCTAGAGGT TCTCCTGTGT CACCCTTCTC AACTCAGTTT   
  
  
+ GATTGTGATA CGGTTACTAC ATTGAGTGAT AGTCAAGAGC ATCACAGCTC GACGGGGAGT CTATCAACAA   
  
  
+ GAAGCCCTTC TTGTAATTCT CCCCTTGAAA CGAGCAGTTA TCATCATTTA TCGACGAATG GCCCGTCTTG   
  
  
+ TAATTCTGCC CCTGAAACTA GCAGTTATCG TCATCGGTTC AACGCGAGTC CCGTGGGATA TTCCCATCAA   
  
  
+ GGCACTGATT ATGGAGTGAA TATGAAGAGC GCTCTGCAGG AGCTAGAGAC TACTCTAATG GGTGCAGATG   
  
  
+ GTGAGGAAGT ATCTGCTGCT AATCAACCTA TGGGGGGAAG TCGTCAGTCC GGGATCCCAA GTCAGAGATC   
  
  
+ AAAATCATTG AGCGAAGATC CACAGGGTTC GCATCCTACT CAGCCTGATT CATCATCTCT TTCTAGGGCA   
  
  
+ AGAAGATCAG GAGATGAAAG CCAGAGAGAG AAACGGCACA AGGCAATGGA AGAACCAACG GAACTACCAA   
  
  
+ GTTTGCCACC TGGTGATTTG AAGCAGTTGC TAATTGAATG TGCGAGGGCT TTATCGGATA ACCGAATAGA   
  
  
+ TGACTTTGAG AGTTTGGTTA AACGGGCGAG GAAAGAGGTC TCTATCTCGG GTGAGCCTAT CCAACGTCTC   
  
  
+ GGTGCCTACA TGATCGAAGG GCTTGTGGCA AGGAAGCAGT CTTCGGGGAC TAGCATCTAT CGGGCTCTGA   
  
  
+ AGTGTAAAGA GCCTCTTGGA AAAGACTTGC TCTCCTACAT GCACATCCTT TATGAAATAT GCCCTTATCT   
  
  
+ CAAGTTTGGT TATATGGCTG CGAATGGAGC GATAGCTGAA GCTTGTAGAA ATGAGGATAA CATACATATT   
  
  
+ ATAGATTTCC AGATTGCACA GGGCACTCAG TGGGTTACTC TATTACAAGC CCTAGCAGCA AGACCTGGTG   
  
  
+ GGCCACCTAA GGTGCGAATT ACAGGCATTG ATGATCCTGT TTCTAAGTAT GCCCGTGGTG CTAGCTTGGA   
  
  
+ GGCTGTTGGG AAACGGTTAG CGTCTCTATC TGAAAAGTTC AAAATACCCG TCGAGTTCAA TGCGTTGCCC   
  
  
+ GTTTATGGAC CCGATGTCAG GCGGGAAATG CTGGATGTGA GGCCCGGGGA GGCTTTGGCC GTTAATTTTC   
  
  
+ CATTGCAGCT CCACCACACT CCTGACGAGA GTGTCGATGT GAACAACCCT AGGGATGGGC TTCTCAGAAT   
  
  
+ GGTGAAATCA CTTGGTCCTA AGGTAACCAC TTTGGTTGAG CAAGAATCAA ACACCAACAC TACCCCTTTC   
  
  
+ TTGACCCGGT TCATAGAGAC CCTTGACTAC TACTCAGCCA TGTTTGAGTC TATAGACGTG ACCATGCCAA   
  
  
+ GAGACCGGAA GGAGAGGATC AATGTTGAGC AGCATTGTTT GGCTAAGGAC ATTGTGAATA TCATAGCTTG   
  
  
+ CGAGGGCAAG GAGAGGGTGG AGCGTCATGA ACTTTTTGGG AAATGGAAGT CAAGGTTTAC CATGGCAGGG   
  
  
+ TTCAGGCAGT ACCCGTTGAG CTCATACGTA AACTCTGTGA TAAGAAGCCT ACTCCGGTGT TACTCCGAGC   
  
  
+ ATTATACTCT GATAGAGAAG GATGGTGCCA TGCTTCTGGG TTGGAAGGGC CGAATGCTGA TTTCAGCTTC   
  
  
+ GGCATGGCAT TG  

- +Up\_Stream \_Len000AGGAGA AAACCCGAAG AAAGAAAGAT AGGACAAAAA AAAACCCAGC TTTTCAACCT   
  
  
- CCAACTTAAC TTCGTAGGCT TAAGCTCGTC CTACAAAGAG ACGCATCACT AAGAAAGACC CAAACACTAA   
  
  
- AACTTAAGAC CCATTAGCCA ACAAAACGAT TAAAACTCCT GTCTCAAGGA AAAGACTTAA TTTTAAAGCA   
  
  
- AAAAAATAGC CCTTTTAGAA GTCATAAACT CTTTTTTCTT GCTTTAAACC TACTAAACGA CAGATCTAAA   
  
  
- ACGAAAGTAT AAGGACCCAC ACCTAACCAA ATAATTAACG TACACTCCTT CATGTTCGAA GACCAGTTAC   
  
  
- CAACCCAAAA AAAAAAAAAA AACACCCATA TTTCAACCTT TAACTAATGC TCAGTTTAAA GAGAAAGATC   
  
  
- TTCTCCCCCC CCCCCCCCCC AACCAAATTT TGAGGTATAA AAGAAACTTA CCTTTCGAAT TAAACGACTG   
  
  
- ATAGAACCAA TCATCGTATT CGAACTAGTC TTTTGTTTTG CGAGAGACGG GACGTTTTTG AAATAAACAG   
  
  
- AAAATTCCTT TTAAAACCTT AAACTCTCAC TTTTAAAACC AACACTTAGA ACAGAATGAT ACACCAAGGA   
  
  
- AGACTGTTTT CAAACTTAGA CTCATACTCT CAAGTAGTTA GAGAAAATGA AAGGATGAAA GAAATAACGA   
  
  
- ACGTTTAACA ACACTTGAAT GAACCTAAAA GACCATCTCC TCTCAAGACA GGACCTTAAA CCACCTTTCA   
  
  
- CTCTGTATGA AACCCTAAAT TAATCGGTAG TTAAACCAAA GTGTGGAATA GTCAAACCTG AAGACCACAC   
  
  
- AATAACATAC ACAAGAATCG AACCCTAATA AGAACATAGC CTTCTCTCCC CCCCCCCCCA CAAAGAAAAT   
  
  
- CGACTTACAA TCTTGTAAAA TTCCCTAGAT CTATAGATCT CTTCTCGGAT CCTTTACTTC GTAAAAAGGG   
  
  
- AATATTAGAC TGTGTTGAAA TACTTAACTA ATCCTTGTTT CTTGCTAATC AAGTCTTTCC AATGTTGAGG   
  
  
- AGATCCACAA TATGATTTCT CTACACTCAA TTATTCGTTA ACGAATGATT GACAAATCCG ACAAAGTAAC   
  
  
- GAACATCTCC GAAAAAAAAA CCCCCCCCCC CCCCCAAACC CCAAGTATTT ACTCTTCCAA CTAAACAACA   
  
  
- GACGAGACGA GAACTAAGCT ATCATTAAGA GAGTCTTTTC CCATTTTACT GTACCGGAAG GTAGCGGAGG   
  
  
- GTACTCCGAA AAACGGAGAC GAACACATAG AAAGTAGAGA ACCAAACCAC CCAACCCCCT CCCCTCCCCC   
  
  
- CCTTCCCCCA AAACTAAGCT ATCTAGTAGT GAAAGTCTTT ACCGGAGAGA CCGGTTACCA TTCCCCATTC   
  
  
- CAATCATGTA CACCTGAAAG GGTACACCTA AAAAGGTCTC AGGCAACACA CTAACAATAA CGACAACAAC   
  
  
- ATTGAAAGAC AGTGAGCCTT TTGTACTTAG GTATACGTTG TTCTTGTATT ATTAACACTT ACGCTGAAAT   
  
  
- AAATCCTTAC AGAATGTATC ATGCACGTGT AAGAAATTAA TAGACAAATA GACAACCAAA CAATAAAAGT   
  
  
- TCCCCTACCC CTCACCCACC TACGAATCAT ACATTAGAAA AATATATCCC TTTTTAGTAT ATCGAACTTT   
  
  
- CATTTTGAAA GTAACGGTCC TAAGGTTCCA TCCCCATCCC AACAGATGTG GAACTAGAGG GATCTTCGTA   
  
  
- CCGCTAGGAA GGAGCCTAAC ACAATATCTT ATTTCTTACA CCAAGACGAC ATTACTCTGA ATACAAGATA   
  
  
- GTTTACTACT TAGACAAATC TTGAAGAACG AATAAAAAAG ACGTTTTAAA ATCCTCTGAA CTATTTGATA   
  
  
- TCAAGAGAAC TTAAATGTCC ACCTATAACC TCAAGTGTCT TATCATAAGT CTAGTTTCAC GAACACGAAT   
  
  
- TAACCTTCCT GTTGAACGAA CTCGACAAAT ATCTCGAGAT TGACTACGAT AGAGTACCTG AGTGTAGTCA   
  
  
- AAAAACCTAA ATTAGAATGG TCACAACTAG GCAGCATGTA AAGGCGAGTC ATGTCAGGCG GCAGTCACTG   
  
  
- TTTATCCTAC AAACGTAGTG ACTTCGAGCT GAGATCTCCA AGAGGACACA GTGGGAAGAG TTGAGTCAAA   
  
  
- CTAACACTAT GCCAATGATG TAACTCACTA TCAGTTCTCG TAGTGTCGAG CTGCCCCTCA GATAGTTGTT   
  
  
- CTTCGGGAAG AACATTAAGA GGGGAACTTT GCTCGTCAAT AGTAGTAAAT AGCTGCTTAC CGGGCAGAAC   
  
  
- ATTAAGACGG GGACTTTGAT CGTCAATAGC AGTAGCCAAG TTGCGCTCAG GGCACCCTAT AAGGGTAGTT   
  
  
- CCGTGACTAA TACCTCACTT ATACTTCTCG CGAGACGTCC TCGATCTCTG ATGAGATTAC CCACGTCTAC   
  
  
- CACTCCTTCA TAGACGACGA TTAGTTGGAT ACCCCCCTTC AGCAGTCAGG CCCTAGGGTT CAGTCTCTAG   
  
  
- TTTTAGTAAC TCGCTTCTAG GTGTCCCAAG CGTAGGATGA GTCGGACTAA GTAGTAGAGA AAGATCCCGT   
  
  
- TCTTCTAGTC CTCTACTTTC GGTCTCTCTC TTTGCCGTGT TCCGTTACCT TCTTGGTTGC CTTGATGGTT   
  
  
- CAAACGGTGG ACCACTAAAC TTCGTCAACG ATTAACTTAC ACGCTCCCGA AATAGCCTAT TGGCTTATCT   
  
  
- ACTGAAACTC TCAAACCAAT TTGCCCGCTC CTTTCTCCAG AGATAGAGCC CACTCGGATA GGTTGCAGAG   
  
  
- CCACGGATGT ACTAGCTTCC CGAACACCGT TCCTTCGTCA GAAGCCCCTG ATCGTAGATA GCCCGAGACT   
  
  
- TCACATTTCT CGGAGAACCT TTTCTGAACG AGAGGATGTA CGTGTAGGAA ATACTTTATA CGGGAATAGA   
  
  
- GTTCAAACCA ATATACCGAC GCTTACCTCG CTATCGACTT CGAACATCTT TACTCCTATT GTATGTATAA   
  
  
- TATCTAAAGG TCTAACGTGT CCCGTGAGTC ACCCAATGAG ATAATGTTCG GGATCGTCGT TCTGGACCAC   
  
  
- CCGGTGGATT CCACGCTTAA TGTCCGTAAC TACTAGGACA AAGATTCATA CGGGCACCAC GATCGAACCT   
  
  
- CCGACAACCC TTTGCCAATC GCAGAGATAG ACTTTTCAAG TTTTATGGGC AGCTCAAGTT ACGCAACGGG   
  
  
- CAAATACCTG GGCTACAGTC CGCCCTTTAC GACCTACACT CCGGGCCCCT CCGAAACCGG CAATTAAAAG   
  
  
- GTAACGTCGA GGTGGTGTGA GGACTGCTCT CACAGCTACA CTTGTTGGGA TCCCTACCCG AAGAGTCTTA   
  
  
- CCACTTTAGT GAACCAGGAT TCCATTGGTG AAACCAACTC GTTCTTAGTT TGTGGTTGTG ATGGGGAAAG   
  
  
- AACTGGGCCA AGTATCTCTG GGAACTGATG ATGAGTCGGT ACAAACTCAG ATATCTGCAC TGGTACGGTT   
  
  
- CTCTGGCCTT CCTCTCCTAG TTACAACTCG TCGTAACAAA CCGATTCCTG TAACACTTAT AGTATCGAAC   
  
  
- GCTCCCGTTC CTCTCCCACC TCGCAGTACT TGAAAAACCC TTTACCTTCA GTTCCAAATG GTACCGTCCC   
  
  
- AAGTCCGTCA TGGGCAACTC GAGTATGCAT TTGAGACACT ATTCTTCGGA TGAGGCCACA ATGAGGCTCG   
  
  
- TAATATGAGA CTATCTCTTC CTACCACGGT ACGAAGACCC AACCTTCCCG GCTTACGACT AAAGTCGAAG   
  
  
- CCGTACCGTA AC

+     ABRE4

| Site Name | Organism | Position | Strand | Matrix score. | sequence | function |
| --- | --- | --- | --- | --- | --- | --- |
| ABRE4 | Zea mays | 1565 | - | 6 | CACGTA |  |

>HU08G00014.1   
+ +Up\_Stream \_Len000TCCTCT TTTGGGCTTC TTTCTTTCTA TCCTGTTTTT TTTTGGGTCG AAAAGTTGGA   
  
  
+ GGTTGAATTG AAGCATCCGA ATTCGAGCAG GATGTTTCTC TGCGTAGTGA TTCTTTCTGG GTTTGTGATT   
  
  
+ TTGAATTCTG GGTAATCGGT TGTTTTGCTA ATTTTGAGGA CAGAGTTCCT TTTCTGAATT AAAATTTCGT   
  
  
+ TTTTTTATCG GGAAAATCTT CAGTATTTGA GAAAAAAGAA CGAAATTTGG ATGATTTGCT GTCTAGATTT   
  
  
+ TGCTTTCATA TTCCTGGGTG TGGATTGGTT TATTAATTGC ATGTGAGGAA GTACAAGCTT CTGGTCAATG   
  
  
+ GTTGGGTTTT TTTTTTTTTT TTGTGGGTAT AAAGTTGGAA ATTGATTACG AGTCAAATTT CTCTTTCTAG   
  
  
+ AAGAGGGGGG GGGGGGGGGG TTGGTTTAAA ACTCCATATT TTCTTTGAAT GGAAAGCTTA ATTTGCTGAC   
  
  
+ TATCTTGGTT AGTAGCATAA GCTTGATCAG AAAACAAAAC GCTCTCTGCC CTGCAAAAAC TTTATTTGTC   
  
  
+ TTTTAAGGAA AATTTTGGAA TTTGAGAGTG AAAATTTTGG TTGTGAATCT TGTCTTACTA TGTGGTTCCT   
  
  
+ TCTGACAAAA GTTTGAATCT GAGTATGAGA GTTCATCAAT CTCTTTTACT TTCCTACTTT CTTTATTGCT   
  
  
+ TGCAAATTGT TGTGAACTTA CTTGGATTTT CTGGTAGAGG AGAGTTCTGT CCTGGAATTT GGTGGAAAGT   
  
  
+ GAGACATACT TTGGGATTTA ATTAGCCATC AATTTGGTTT CACACCTTAT CAGTTTGGAC TTCTGGTGTG   
  
  
+ TTATTGTATG TGTTCTTAGC TTGGGATTAT TCTTGTATCG GAAGAGAGGG GGGGGGGGGT GTTTCTTTTA   
  
  
+ GCTGAATGTT AGAACATTTT AAGGGATCTA GATATCTAGA GAAGAGCCTA GGAAATGAAG CATTTTTCCC   
  
  
+ TTATAATCTG ACACAACTTT ATGAATTGAT TAGGAACAAA GAACGATTAG TTCAGAAAGG TTACAACTCC   
  
  
+ TCTAGGTGTT ATACTAAAGA GATGTGAGTT AATAAGCAAT TGCTTACTAA CTGTTTAGGC TGTTTCATTG   
  
  
+ CTTGTAGAGG CTTTTTTTTT GGGGGGGGGG GGGGGTTTGG GGTTCATAAA TGAGAAGGTT GATTTGTTGT   
  
  
+ CTGCTCTGCT CTTGATTCGA TAGTAATTCT CTCAGAAAAG GGTAAAATGA CATGGCCTTC CATCGCCTCC   
  
  
+ CATGAGGCTT TTTGCCTCTG CTTGTGTATC TTTCATCTCT TGGTTTGGTG GGTTGGGGGA GGGGAGGGGG   
  
  
+ GGAAGGGGGT TTTGATTCGA TAGATCATCA CTTTCAGAAA TGGCCTCTCT GGCCAATGGT AAGGGGTAAG   
  
  
+ GTTAGTACAT GTGGACTTTC CCATGTGGAT TTTTCCAGAG TCCGTTGTGT GATTGTTATT GCTGTTGTTG   
  
  
+ TAACTTTCTG TCACTCGGAA AACATGAATC CATATGCAAC AAGAACATAA TAATTGTGAA TGCGACTTTA   
  
  
+ TTTAGGAATG TCTTACATAG TACGTGCACA TTCTTTAATT ATCTGTTTAT CTGTTGGTTT GTTATTTTCA   
  
  
+ AGGGGATGGG GAGTGGGTGG ATGCTTAGTA TGTAATCTTT TTATATAGGG AAAAATCATA TAGCTTGAAA   
  
  
+ GTAAAACTTT CATTGCCAGG ATTCCAAGGT AGGGGTAGGG TTGTCTACAC CTTGATCTCC CTAGAAGCAT   
  
  
+ GGCGATCCTT CCTCGGATTG TGTTATAGAA TAAAGAATGT GGTTCTGCTG TAATGAGACT TATGTTCTAT   
  
  
+ CAAATGATGA ATCTGTTTAG AACTTCTTGC TTATTTTTTC TGCAAAATTT TAGGAGACTT GATAAACTAT   
  
  
+ AGTTCTCTTG AATTTACAGG TGGATATTGG AGTTCACAGA ATAGTATTCA GATCAAAGTG CTTGTGCTTA   
  
  
+ ATTGGAAGGA CAACTTGCTT GAGCTGTTTA TAGAGCTCTA ACTGATGCTA TCTCATGGAC TCACATCAGT   
  
  
+ TTTTTGGATT TAATCTTACC AGTGTTGATC CGTCGTACAT TTCCGCTCAG TACAGTCCGC CGTCAGTGAC   
  
  
+ AAATAGGATG TTTGCATCAC TGAAGCTCGA CTCTAGAGGT TCTCCTGTGT CACCCTTCTC AACTCAGTTT   
  
  
+ GATTGTGATA CGGTTACTAC ATTGAGTGAT AGTCAAGAGC ATCACAGCTC GACGGGGAGT CTATCAACAA   
  
  
+ GAAGCCCTTC TTGTAATTCT CCCCTTGAAA CGAGCAGTTA TCATCATTTA TCGACGAATG GCCCGTCTTG   
  
  
+ TAATTCTGCC CCTGAAACTA GCAGTTATCG TCATCGGTTC AACGCGAGTC CCGTGGGATA TTCCCATCAA   
  
  
+ GGCACTGATT ATGGAGTGAA TATGAAGAGC GCTCTGCAGG AGCTAGAGAC TACTCTAATG GGTGCAGATG   
  
  
+ GTGAGGAAGT ATCTGCTGCT AATCAACCTA TGGGGGGAAG TCGTCAGTCC GGGATCCCAA GTCAGAGATC   
  
  
+ AAAATCATTG AGCGAAGATC CACAGGGTTC GCATCCTACT CAGCCTGATT CATCATCTCT TTCTAGGGCA   
  
  
+ AGAAGATCAG GAGATGAAAG CCAGAGAGAG AAACGGCACA AGGCAATGGA AGAACCAACG GAACTACCAA   
  
  
+ GTTTGCCACC TGGTGATTTG AAGCAGTTGC TAATTGAATG TGCGAGGGCT TTATCGGATA ACCGAATAGA   
  
  
+ TGACTTTGAG AGTTTGGTTA AACGGGCGAG GAAAGAGGTC TCTATCTCGG GTGAGCCTAT CCAACGTCTC   
  
  
+ GGTGCCTACA TGATCGAAGG GCTTGTGGCA AGGAAGCAGT CTTCGGGGAC TAGCATCTAT CGGGCTCTGA   
  
  
+ AGTGTAAAGA GCCTCTTGGA AAAGACTTGC TCTCCTACAT GCACATCCTT TATGAAATAT GCCCTTATCT   
  
  
+ CAAGTTTGGT TATATGGCTG CGAATGGAGC GATAGCTGAA GCTTGTAGAA ATGAGGATAA CATACATATT   
  
  
+ ATAGATTTCC AGATTGCACA GGGCACTCAG TGGGTTACTC TATTACAAGC CCTAGCAGCA AGACCTGGTG   
  
  
+ GGCCACCTAA GGTGCGAATT ACAGGCATTG ATGATCCTGT TTCTAAGTAT GCCCGTGGTG CTAGCTTGGA   
  
  
+ GGCTGTTGGG AAACGGTTAG CGTCTCTATC TGAAAAGTTC AAAATACCCG TCGAGTTCAA TGCGTTGCCC   
  
  
+ GTTTATGGAC CCGATGTCAG GCGGGAAATG CTGGATGTGA GGCCCGGGGA GGCTTTGGCC GTTAATTTTC   
  
  
+ CATTGCAGCT CCACCACACT CCTGACGAGA GTGTCGATGT GAACAACCCT AGGGATGGGC TTCTCAGAAT   
  
  
+ GGTGAAATCA CTTGGTCCTA AGGTAACCAC TTTGGTTGAG CAAGAATCAA ACACCAACAC TACCCCTTTC   
  
  
+ TTGACCCGGT TCATAGAGAC CCTTGACTAC TACTCAGCCA TGTTTGAGTC TATAGACGTG ACCATGCCAA   
  
  
+ GAGACCGGAA GGAGAGGATC AATGTTGAGC AGCATTGTTT GGCTAAGGAC ATTGTGAATA TCATAGCTTG   
  
  
+ CGAGGGCAAG GAGAGGGTGG AGCGTCATGA ACTTTTTGGG AAATGGAAGT CAAGGTTTAC CATGGCAGGG   
  
  
+ TTCAGGCAGT ACCCGTTGAG CTCATACGTA AACTCTGTGA TAAGAAGCCT ACTCCGGTGT TACTCCGAGC   
  
  
+ ATTATACTCT GATAGAGAAG GATGGTGCCA TGCTTCTGGG TTGGAAGGGC CGAATGCTGA TTTCAGCTTC   
  
  
+ GGCATGGCAT TG  

- +Up\_Stream \_Len000AGGAGA AAACCCGAAG AAAGAAAGAT AGGACAAAAA AAAACCCAGC TTTTCAACCT   
  
  
- CCAACTTAAC TTCGTAGGCT TAAGCTCGTC CTACAAAGAG ACGCATCACT AAGAAAGACC CAAACACTAA   
  
  
- AACTTAAGAC CCATTAGCCA ACAAAACGAT TAAAACTCCT GTCTCAAGGA AAAGACTTAA TTTTAAAGCA   
  
  
- AAAAAATAGC CCTTTTAGAA GTCATAAACT CTTTTTTCTT GCTTTAAACC TACTAAACGA CAGATCTAAA   
  
  
- ACGAAAGTAT AAGGACCCAC ACCTAACCAA ATAATTAACG TACACTCCTT CATGTTCGAA GACCAGTTAC   
  
  
- CAACCCAAAA AAAAAAAAAA AACACCCATA TTTCAACCTT TAACTAATGC TCAGTTTAAA GAGAAAGATC   
  
  
- TTCTCCCCCC CCCCCCCCCC AACCAAATTT TGAGGTATAA AAGAAACTTA CCTTTCGAAT TAAACGACTG   
  
  
- ATAGAACCAA TCATCGTATT CGAACTAGTC TTTTGTTTTG CGAGAGACGG GACGTTTTTG AAATAAACAG   
  
  
- AAAATTCCTT TTAAAACCTT AAACTCTCAC TTTTAAAACC AACACTTAGA ACAGAATGAT ACACCAAGGA   
  
  
- AGACTGTTTT CAAACTTAGA CTCATACTCT CAAGTAGTTA GAGAAAATGA AAGGATGAAA GAAATAACGA   
  
  
- ACGTTTAACA ACACTTGAAT GAACCTAAAA GACCATCTCC TCTCAAGACA GGACCTTAAA CCACCTTTCA   
  
  
- CTCTGTATGA AACCCTAAAT TAATCGGTAG TTAAACCAAA GTGTGGAATA GTCAAACCTG AAGACCACAC   
  
  
- AATAACATAC ACAAGAATCG AACCCTAATA AGAACATAGC CTTCTCTCCC CCCCCCCCCA CAAAGAAAAT   
  
  
- CGACTTACAA TCTTGTAAAA TTCCCTAGAT CTATAGATCT CTTCTCGGAT CCTTTACTTC GTAAAAAGGG   
  
  
- AATATTAGAC TGTGTTGAAA TACTTAACTA ATCCTTGTTT CTTGCTAATC AAGTCTTTCC AATGTTGAGG   
  
  
- AGATCCACAA TATGATTTCT CTACACTCAA TTATTCGTTA ACGAATGATT GACAAATCCG ACAAAGTAAC   
  
  
- GAACATCTCC GAAAAAAAAA CCCCCCCCCC CCCCCAAACC CCAAGTATTT ACTCTTCCAA CTAAACAACA   
  
  
- GACGAGACGA GAACTAAGCT ATCATTAAGA GAGTCTTTTC CCATTTTACT GTACCGGAAG GTAGCGGAGG   
  
  
- GTACTCCGAA AAACGGAGAC GAACACATAG AAAGTAGAGA ACCAAACCAC CCAACCCCCT CCCCTCCCCC   
  
  
- CCTTCCCCCA AAACTAAGCT ATCTAGTAGT GAAAGTCTTT ACCGGAGAGA CCGGTTACCA TTCCCCATTC   
  
  
- CAATCATGTA CACCTGAAAG GGTACACCTA AAAAGGTCTC AGGCAACACA CTAACAATAA CGACAACAAC   
  
  
- ATTGAAAGAC AGTGAGCCTT TTGTACTTAG GTATACGTTG TTCTTGTATT ATTAACACTT ACGCTGAAAT   
  
  
- AAATCCTTAC AGAATGTATC ATGCACGTGT AAGAAATTAA TAGACAAATA GACAACCAAA CAATAAAAGT   
  
  
- TCCCCTACCC CTCACCCACC TACGAATCAT ACATTAGAAA AATATATCCC TTTTTAGTAT ATCGAACTTT   
  
  
- CATTTTGAAA GTAACGGTCC TAAGGTTCCA TCCCCATCCC AACAGATGTG GAACTAGAGG GATCTTCGTA   
  
  
- CCGCTAGGAA GGAGCCTAAC ACAATATCTT ATTTCTTACA CCAAGACGAC ATTACTCTGA ATACAAGATA   
  
  
- GTTTACTACT TAGACAAATC TTGAAGAACG AATAAAAAAG ACGTTTTAAA ATCCTCTGAA CTATTTGATA   
  
  
- TCAAGAGAAC TTAAATGTCC ACCTATAACC TCAAGTGTCT TATCATAAGT CTAGTTTCAC GAACACGAAT   
  
  
- TAACCTTCCT GTTGAACGAA CTCGACAAAT ATCTCGAGAT TGACTACGAT AGAGTACCTG AGTGTAGTCA   
  
  
- AAAAACCTAA ATTAGAATGG TCACAACTAG GCAGCATGTA AAGGCGAGTC ATGTCAGGCG GCAGTCACTG   
  
  
- TTTATCCTAC AAACGTAGTG ACTTCGAGCT GAGATCTCCA AGAGGACACA GTGGGAAGAG TTGAGTCAAA   
  
  
- CTAACACTAT GCCAATGATG TAACTCACTA TCAGTTCTCG TAGTGTCGAG CTGCCCCTCA GATAGTTGTT   
  
  
- CTTCGGGAAG AACATTAAGA GGGGAACTTT GCTCGTCAAT AGTAGTAAAT AGCTGCTTAC CGGGCAGAAC   
  
  
- ATTAAGACGG GGACTTTGAT CGTCAATAGC AGTAGCCAAG TTGCGCTCAG GGCACCCTAT AAGGGTAGTT   
  
  
- CCGTGACTAA TACCTCACTT ATACTTCTCG CGAGACGTCC TCGATCTCTG ATGAGATTAC CCACGTCTAC   
  
  
- CACTCCTTCA TAGACGACGA TTAGTTGGAT ACCCCCCTTC AGCAGTCAGG CCCTAGGGTT CAGTCTCTAG   
  
  
- TTTTAGTAAC TCGCTTCTAG GTGTCCCAAG CGTAGGATGA GTCGGACTAA GTAGTAGAGA AAGATCCCGT   
  
  
- TCTTCTAGTC CTCTACTTTC GGTCTCTCTC TTTGCCGTGT TCCGTTACCT TCTTGGTTGC CTTGATGGTT   
  
  
- CAAACGGTGG ACCACTAAAC TTCGTCAACG ATTAACTTAC ACGCTCCCGA AATAGCCTAT TGGCTTATCT   
  
  
- ACTGAAACTC TCAAACCAAT TTGCCCGCTC CTTTCTCCAG AGATAGAGCC CACTCGGATA GGTTGCAGAG   
  
  
- CCACGGATGT ACTAGCTTCC CGAACACCGT TCCTTCGTCA GAAGCCCCTG ATCGTAGATA GCCCGAGACT   
  
  
- TCACATTTCT CGGAGAACCT TTTCTGAACG AGAGGATGTA CGTGTAGGAA ATACTTTATA CGGGAATAGA   
  
  
- GTTCAAACCA ATATACCGAC GCTTACCTCG CTATCGACTT CGAACATCTT TACTCCTATT GTATGTATAA   
  
  
- TATCTAAAGG TCTAACGTGT CCCGTGAGTC ACCCAATGAG ATAATGTTCG GGATCGTCGT TCTGGACCAC   
  
  
- CCGGTGGATT CCACGCTTAA TGTCCGTAAC TACTAGGACA AAGATTCATA CGGGCACCAC GATCGAACCT   
  
  
- CCGACAACCC TTTGCCAATC GCAGAGATAG ACTTTTCAAG TTTTATGGGC AGCTCAAGTT ACGCAACGGG   
  
  
- CAAATACCTG GGCTACAGTC CGCCCTTTAC GACCTACACT CCGGGCCCCT CCGAAACCGG CAATTAAAAG   
  
  
- GTAACGTCGA GGTGGTGTGA GGACTGCTCT CACAGCTACA CTTGTTGGGA TCCCTACCCG AAGAGTCTTA   
  
  
- CCACTTTAGT GAACCAGGAT TCCATTGGTG AAACCAACTC GTTCTTAGTT TGTGGTTGTG ATGGGGAAAG   
  
  
- AACTGGGCCA AGTATCTCTG GGAACTGATG ATGAGTCGGT ACAAACTCAG ATATCTGCAC TGGTACGGTT   
  
  
- CTCTGGCCTT CCTCTCCTAG TTACAACTCG TCGTAACAAA CCGATTCCTG TAACACTTAT AGTATCGAAC   
  
  
- GCTCCCGTTC CTCTCCCACC TCGCAGTACT TGAAAAACCC TTTACCTTCA GTTCCAAATG GTACCGTCCC   
  
  
- AAGTCCGTCA TGGGCAACTC GAGTATGCAT TTGAGACACT ATTCTTCGGA TGAGGCCACA ATGAGGCTCG   
  
  
- TAATATGAGA CTATCTCTTC CTACCACGGT ACGAAGACCC AACCTTCCCG GCTTACGACT AAAGTCGAAG   
  
  
- CCGTACCGTA AC

+     AC-I

| Site Name | Organism | Position | Strand | Matrix score. | sequence | function |
| --- | --- | --- | --- | --- | --- | --- |
| AC-I | Phaseolus vulgaris | 1311 | - | 8.5 | (T/C)C(T/C)(C/T)ACC(T/C)ACC |  |

>HU08G00014.1   
+ +Up\_Stream \_Len000TCCTCT TTTGGGCTTC TTTCTTTCTA TCCTGTTTTT TTTTGGGTCG AAAAGTTGGA   
  
  
+ GGTTGAATTG AAGCATCCGA ATTCGAGCAG GATGTTTCTC TGCGTAGTGA TTCTTTCTGG GTTTGTGATT   
  
  
+ TTGAATTCTG GGTAATCGGT TGTTTTGCTA ATTTTGAGGA CAGAGTTCCT TTTCTGAATT AAAATTTCGT   
  
  
+ TTTTTTATCG GGAAAATCTT CAGTATTTGA GAAAAAAGAA CGAAATTTGG ATGATTTGCT GTCTAGATTT   
  
  
+ TGCTTTCATA TTCCTGGGTG TGGATTGGTT TATTAATTGC ATGTGAGGAA GTACAAGCTT CTGGTCAATG   
  
  
+ GTTGGGTTTT TTTTTTTTTT TTGTGGGTAT AAAGTTGGAA ATTGATTACG AGTCAAATTT CTCTTTCTAG   
  
  
+ AAGAGGGGGG GGGGGGGGGG TTGGTTTAAA ACTCCATATT TTCTTTGAAT GGAAAGCTTA ATTTGCTGAC   
  
  
+ TATCTTGGTT AGTAGCATAA GCTTGATCAG AAAACAAAAC GCTCTCTGCC CTGCAAAAAC TTTATTTGTC   
  
  
+ TTTTAAGGAA AATTTTGGAA TTTGAGAGTG AAAATTTTGG TTGTGAATCT TGTCTTACTA TGTGGTTCCT   
  
  
+ TCTGACAAAA GTTTGAATCT GAGTATGAGA GTTCATCAAT CTCTTTTACT TTCCTACTTT CTTTATTGCT   
  
  
+ TGCAAATTGT TGTGAACTTA CTTGGATTTT CTGGTAGAGG AGAGTTCTGT CCTGGAATTT GGTGGAAAGT   
  
  
+ GAGACATACT TTGGGATTTA ATTAGCCATC AATTTGGTTT CACACCTTAT CAGTTTGGAC TTCTGGTGTG   
  
  
+ TTATTGTATG TGTTCTTAGC TTGGGATTAT TCTTGTATCG GAAGAGAGGG GGGGGGGGGT GTTTCTTTTA   
  
  
+ GCTGAATGTT AGAACATTTT AAGGGATCTA GATATCTAGA GAAGAGCCTA GGAAATGAAG CATTTTTCCC   
  
  
+ TTATAATCTG ACACAACTTT ATGAATTGAT TAGGAACAAA GAACGATTAG TTCAGAAAGG TTACAACTCC   
  
  
+ TCTAGGTGTT ATACTAAAGA GATGTGAGTT AATAAGCAAT TGCTTACTAA CTGTTTAGGC TGTTTCATTG   
  
  
+ CTTGTAGAGG CTTTTTTTTT GGGGGGGGGG GGGGGTTTGG GGTTCATAAA TGAGAAGGTT GATTTGTTGT   
  
  
+ CTGCTCTGCT CTTGATTCGA TAGTAATTCT CTCAGAAAAG GGTAAAATGA CATGGCCTTC CATCGCCTCC   
  
  
+ CATGAGGCTT TTTGCCTCTG CTTGTGTATC TTTCATCTCT TGGTTTGGTG GGTTGGGGGA GGGGAGGGGG   
  
  
+ GGAAGGGGGT TTTGATTCGA TAGATCATCA CTTTCAGAAA TGGCCTCTCT GGCCAATGGT AAGGGGTAAG   
  
  
+ GTTAGTACAT GTGGACTTTC CCATGTGGAT TTTTCCAGAG TCCGTTGTGT GATTGTTATT GCTGTTGTTG   
  
  
+ TAACTTTCTG TCACTCGGAA AACATGAATC CATATGCAAC AAGAACATAA TAATTGTGAA TGCGACTTTA   
  
  
+ TTTAGGAATG TCTTACATAG TACGTGCACA TTCTTTAATT ATCTGTTTAT CTGTTGGTTT GTTATTTTCA   
  
  
+ AGGGGATGGG GAGTGGGTGG ATGCTTAGTA TGTAATCTTT TTATATAGGG AAAAATCATA TAGCTTGAAA   
  
  
+ GTAAAACTTT CATTGCCAGG ATTCCAAGGT AGGGGTAGGG TTGTCTACAC CTTGATCTCC CTAGAAGCAT   
  
  
+ GGCGATCCTT CCTCGGATTG TGTTATAGAA TAAAGAATGT GGTTCTGCTG TAATGAGACT TATGTTCTAT   
  
  
+ CAAATGATGA ATCTGTTTAG AACTTCTTGC TTATTTTTTC TGCAAAATTT TAGGAGACTT GATAAACTAT   
  
  
+ AGTTCTCTTG AATTTACAGG TGGATATTGG AGTTCACAGA ATAGTATTCA GATCAAAGTG CTTGTGCTTA   
  
  
+ ATTGGAAGGA CAACTTGCTT GAGCTGTTTA TAGAGCTCTA ACTGATGCTA TCTCATGGAC TCACATCAGT   
  
  
+ TTTTTGGATT TAATCTTACC AGTGTTGATC CGTCGTACAT TTCCGCTCAG TACAGTCCGC CGTCAGTGAC   
  
  
+ AAATAGGATG TTTGCATCAC TGAAGCTCGA CTCTAGAGGT TCTCCTGTGT CACCCTTCTC AACTCAGTTT   
  
  
+ GATTGTGATA CGGTTACTAC ATTGAGTGAT AGTCAAGAGC ATCACAGCTC GACGGGGAGT CTATCAACAA   
  
  
+ GAAGCCCTTC TTGTAATTCT CCCCTTGAAA CGAGCAGTTA TCATCATTTA TCGACGAATG GCCCGTCTTG   
  
  
+ TAATTCTGCC CCTGAAACTA GCAGTTATCG TCATCGGTTC AACGCGAGTC CCGTGGGATA TTCCCATCAA   
  
  
+ GGCACTGATT ATGGAGTGAA TATGAAGAGC GCTCTGCAGG AGCTAGAGAC TACTCTAATG GGTGCAGATG   
  
  
+ GTGAGGAAGT ATCTGCTGCT AATCAACCTA TGGGGGGAAG TCGTCAGTCC GGGATCCCAA GTCAGAGATC   
  
  
+ AAAATCATTG AGCGAAGATC CACAGGGTTC GCATCCTACT CAGCCTGATT CATCATCTCT TTCTAGGGCA   
  
  
+ AGAAGATCAG GAGATGAAAG CCAGAGAGAG AAACGGCACA AGGCAATGGA AGAACCAACG GAACTACCAA   
  
  
+ GTTTGCCACC TGGTGATTTG AAGCAGTTGC TAATTGAATG TGCGAGGGCT TTATCGGATA ACCGAATAGA   
  
  
+ TGACTTTGAG AGTTTGGTTA AACGGGCGAG GAAAGAGGTC TCTATCTCGG GTGAGCCTAT CCAACGTCTC   
  
  
+ GGTGCCTACA TGATCGAAGG GCTTGTGGCA AGGAAGCAGT CTTCGGGGAC TAGCATCTAT CGGGCTCTGA   
  
  
+ AGTGTAAAGA GCCTCTTGGA AAAGACTTGC TCTCCTACAT GCACATCCTT TATGAAATAT GCCCTTATCT   
  
  
+ CAAGTTTGGT TATATGGCTG CGAATGGAGC GATAGCTGAA GCTTGTAGAA ATGAGGATAA CATACATATT   
  
  
+ ATAGATTTCC AGATTGCACA GGGCACTCAG TGGGTTACTC TATTACAAGC CCTAGCAGCA AGACCTGGTG   
  
  
+ GGCCACCTAA GGTGCGAATT ACAGGCATTG ATGATCCTGT TTCTAAGTAT GCCCGTGGTG CTAGCTTGGA   
  
  
+ GGCTGTTGGG AAACGGTTAG CGTCTCTATC TGAAAAGTTC AAAATACCCG TCGAGTTCAA TGCGTTGCCC   
  
  
+ GTTTATGGAC CCGATGTCAG GCGGGAAATG CTGGATGTGA GGCCCGGGGA GGCTTTGGCC GTTAATTTTC   
  
  
+ CATTGCAGCT CCACCACACT CCTGACGAGA GTGTCGATGT GAACAACCCT AGGGATGGGC TTCTCAGAAT   
  
  
+ GGTGAAATCA CTTGGTCCTA AGGTAACCAC TTTGGTTGAG CAAGAATCAA ACACCAACAC TACCCCTTTC   
  
  
+ TTGACCCGGT TCATAGAGAC CCTTGACTAC TACTCAGCCA TGTTTGAGTC TATAGACGTG ACCATGCCAA   
  
  
+ GAGACCGGAA GGAGAGGATC AATGTTGAGC AGCATTGTTT GGCTAAGGAC ATTGTGAATA TCATAGCTTG   
  
  
+ CGAGGGCAAG GAGAGGGTGG AGCGTCATGA ACTTTTTGGG AAATGGAAGT CAAGGTTTAC CATGGCAGGG   
  
  
+ TTCAGGCAGT ACCCGTTGAG CTCATACGTA AACTCTGTGA TAAGAAGCCT ACTCCGGTGT TACTCCGAGC   
  
  
+ ATTATACTCT GATAGAGAAG GATGGTGCCA TGCTTCTGGG TTGGAAGGGC CGAATGCTGA TTTCAGCTTC   
  
  
+ GGCATGGCAT TG  

- +Up\_Stream \_Len000AGGAGA AAACCCGAAG AAAGAAAGAT AGGACAAAAA AAAACCCAGC TTTTCAACCT   
  
  
- CCAACTTAAC TTCGTAGGCT TAAGCTCGTC CTACAAAGAG ACGCATCACT AAGAAAGACC CAAACACTAA   
  
  
- AACTTAAGAC CCATTAGCCA ACAAAACGAT TAAAACTCCT GTCTCAAGGA AAAGACTTAA TTTTAAAGCA   
  
  
- AAAAAATAGC CCTTTTAGAA GTCATAAACT CTTTTTTCTT GCTTTAAACC TACTAAACGA CAGATCTAAA   
  
  
- ACGAAAGTAT AAGGACCCAC ACCTAACCAA ATAATTAACG TACACTCCTT CATGTTCGAA GACCAGTTAC   
  
  
- CAACCCAAAA AAAAAAAAAA AACACCCATA TTTCAACCTT TAACTAATGC TCAGTTTAAA GAGAAAGATC   
  
  
- TTCTCCCCCC CCCCCCCCCC AACCAAATTT TGAGGTATAA AAGAAACTTA CCTTTCGAAT TAAACGACTG   
  
  
- ATAGAACCAA TCATCGTATT CGAACTAGTC TTTTGTTTTG CGAGAGACGG GACGTTTTTG AAATAAACAG   
  
  
- AAAATTCCTT TTAAAACCTT AAACTCTCAC TTTTAAAACC AACACTTAGA ACAGAATGAT ACACCAAGGA   
  
  
- AGACTGTTTT CAAACTTAGA CTCATACTCT CAAGTAGTTA GAGAAAATGA AAGGATGAAA GAAATAACGA   
  
  
- ACGTTTAACA ACACTTGAAT GAACCTAAAA GACCATCTCC TCTCAAGACA GGACCTTAAA CCACCTTTCA   
  
  
- CTCTGTATGA AACCCTAAAT TAATCGGTAG TTAAACCAAA GTGTGGAATA GTCAAACCTG AAGACCACAC   
  
  
- AATAACATAC ACAAGAATCG AACCCTAATA AGAACATAGC CTTCTCTCCC CCCCCCCCCA CAAAGAAAAT   
  
  
- CGACTTACAA TCTTGTAAAA TTCCCTAGAT CTATAGATCT CTTCTCGGAT CCTTTACTTC GTAAAAAGGG   
  
  
- AATATTAGAC TGTGTTGAAA TACTTAACTA ATCCTTGTTT CTTGCTAATC AAGTCTTTCC AATGTTGAGG   
  
  
- AGATCCACAA TATGATTTCT CTACACTCAA TTATTCGTTA ACGAATGATT GACAAATCCG ACAAAGTAAC   
  
  
- GAACATCTCC GAAAAAAAAA CCCCCCCCCC CCCCCAAACC CCAAGTATTT ACTCTTCCAA CTAAACAACA   
  
  
- GACGAGACGA GAACTAAGCT ATCATTAAGA GAGTCTTTTC CCATTTTACT GTACCGGAAG GTAGCGGAGG   
  
  
- GTACTCCGAA AAACGGAGAC GAACACATAG AAAGTAGAGA ACCAAACCAC CCAACCCCCT CCCCTCCCCC   
  
  
- CCTTCCCCCA AAACTAAGCT ATCTAGTAGT GAAAGTCTTT ACCGGAGAGA CCGGTTACCA TTCCCCATTC   
  
  
- CAATCATGTA CACCTGAAAG GGTACACCTA AAAAGGTCTC AGGCAACACA CTAACAATAA CGACAACAAC   
  
  
- ATTGAAAGAC AGTGAGCCTT TTGTACTTAG GTATACGTTG TTCTTGTATT ATTAACACTT ACGCTGAAAT   
  
  
- AAATCCTTAC AGAATGTATC ATGCACGTGT AAGAAATTAA TAGACAAATA GACAACCAAA CAATAAAAGT   
  
  
- TCCCCTACCC CTCACCCACC TACGAATCAT ACATTAGAAA AATATATCCC TTTTTAGTAT ATCGAACTTT   
  
  
- CATTTTGAAA GTAACGGTCC TAAGGTTCCA TCCCCATCCC AACAGATGTG GAACTAGAGG GATCTTCGTA   
  
  
- CCGCTAGGAA GGAGCCTAAC ACAATATCTT ATTTCTTACA CCAAGACGAC ATTACTCTGA ATACAAGATA   
  
  
- GTTTACTACT TAGACAAATC TTGAAGAACG AATAAAAAAG ACGTTTTAAA ATCCTCTGAA CTATTTGATA   
  
  
- TCAAGAGAAC TTAAATGTCC ACCTATAACC TCAAGTGTCT TATCATAAGT CTAGTTTCAC GAACACGAAT   
  
  
- TAACCTTCCT GTTGAACGAA CTCGACAAAT ATCTCGAGAT TGACTACGAT AGAGTACCTG AGTGTAGTCA   
  
  
- AAAAACCTAA ATTAGAATGG TCACAACTAG GCAGCATGTA AAGGCGAGTC ATGTCAGGCG GCAGTCACTG   
  
  
- TTTATCCTAC AAACGTAGTG ACTTCGAGCT GAGATCTCCA AGAGGACACA GTGGGAAGAG TTGAGTCAAA   
  
  
- CTAACACTAT GCCAATGATG TAACTCACTA TCAGTTCTCG TAGTGTCGAG CTGCCCCTCA GATAGTTGTT   
  
  
- CTTCGGGAAG AACATTAAGA GGGGAACTTT GCTCGTCAAT AGTAGTAAAT AGCTGCTTAC CGGGCAGAAC   
  
  
- ATTAAGACGG GGACTTTGAT CGTCAATAGC AGTAGCCAAG TTGCGCTCAG GGCACCCTAT AAGGGTAGTT   
  
  
- CCGTGACTAA TACCTCACTT ATACTTCTCG CGAGACGTCC TCGATCTCTG ATGAGATTAC CCACGTCTAC   
  
  
- CACTCCTTCA TAGACGACGA TTAGTTGGAT ACCCCCCTTC AGCAGTCAGG CCCTAGGGTT CAGTCTCTAG   
  
  
- TTTTAGTAAC TCGCTTCTAG GTGTCCCAAG CGTAGGATGA GTCGGACTAA GTAGTAGAGA AAGATCCCGT   
  
  
- TCTTCTAGTC CTCTACTTTC GGTCTCTCTC TTTGCCGTGT TCCGTTACCT TCTTGGTTGC CTTGATGGTT   
  
  
- CAAACGGTGG ACCACTAAAC TTCGTCAACG ATTAACTTAC ACGCTCCCGA AATAGCCTAT TGGCTTATCT   
  
  
- ACTGAAACTC TCAAACCAAT TTGCCCGCTC CTTTCTCCAG AGATAGAGCC CACTCGGATA GGTTGCAGAG   
  
  
- CCACGGATGT ACTAGCTTCC CGAACACCGT TCCTTCGTCA GAAGCCCCTG ATCGTAGATA GCCCGAGACT   
  
  
- TCACATTTCT CGGAGAACCT TTTCTGAACG AGAGGATGTA CGTGTAGGAA ATACTTTATA CGGGAATAGA   
  
  
- GTTCAAACCA ATATACCGAC GCTTACCTCG CTATCGACTT CGAACATCTT TACTCCTATT GTATGTATAA   
  
  
- TATCTAAAGG TCTAACGTGT CCCGTGAGTC ACCCAATGAG ATAATGTTCG GGATCGTCGT TCTGGACCAC   
  
  
- CCGGTGGATT CCACGCTTAA TGTCCGTAAC TACTAGGACA AAGATTCATA CGGGCACCAC GATCGAACCT   
  
  
- CCGACAACCC TTTGCCAATC GCAGAGATAG ACTTTTCAAG TTTTATGGGC AGCTCAAGTT ACGCAACGGG   
  
  
- CAAATACCTG GGCTACAGTC CGCCCTTTAC GACCTACACT CCGGGCCCCT CCGAAACCGG CAATTAAAAG   
  
  
- GTAACGTCGA GGTGGTGTGA GGACTGCTCT CACAGCTACA CTTGTTGGGA TCCCTACCCG AAGAGTCTTA   
  
  
- CCACTTTAGT GAACCAGGAT TCCATTGGTG AAACCAACTC GTTCTTAGTT TGTGGTTGTG ATGGGGAAAG   
  
  
- AACTGGGCCA AGTATCTCTG GGAACTGATG ATGAGTCGGT ACAAACTCAG ATATCTGCAC TGGTACGGTT   
  
  
- CTCTGGCCTT CCTCTCCTAG TTACAACTCG TCGTAACAAA CCGATTCCTG TAACACTTAT AGTATCGAAC   
  
  
- GCTCCCGTTC CTCTCCCACC TCGCAGTACT TGAAAAACCC TTTACCTTCA GTTCCAAATG GTACCGTCCC   
  
  
- AAGTCCGTCA TGGGCAACTC GAGTATGCAT TTGAGACACT ATTCTTCGGA TGAGGCCACA ATGAGGCTCG   
  
  
- TAATATGAGA CTATCTCTTC CTACCACGGT ACGAAGACCC AACCTTCCCG GCTTACGACT AAAGTCGAAG   
  
  
- CCGTACCGTA AC

+     ACA-motif

| Site Name | Organism | Position | Strand | Matrix score. | sequence | function |
| --- | --- | --- | --- | --- | --- | --- |
| ACA-motif | Pisum sativum | 3101 | + | 12 | AATTACAGCCATT | part of gapA in (gapA-CMA1) involved with light responsiveness |

>HU08G00014.1   
+ +Up\_Stream \_Len000TCCTCT TTTGGGCTTC TTTCTTTCTA TCCTGTTTTT TTTTGGGTCG AAAAGTTGGA   
  
  
+ GGTTGAATTG AAGCATCCGA ATTCGAGCAG GATGTTTCTC TGCGTAGTGA TTCTTTCTGG GTTTGTGATT   
  
  
+ TTGAATTCTG GGTAATCGGT TGTTTTGCTA ATTTTGAGGA CAGAGTTCCT TTTCTGAATT AAAATTTCGT   
  
  
+ TTTTTTATCG GGAAAATCTT CAGTATTTGA GAAAAAAGAA CGAAATTTGG ATGATTTGCT GTCTAGATTT   
  
  
+ TGCTTTCATA TTCCTGGGTG TGGATTGGTT TATTAATTGC ATGTGAGGAA GTACAAGCTT CTGGTCAATG   
  
  
+ GTTGGGTTTT TTTTTTTTTT TTGTGGGTAT AAAGTTGGAA ATTGATTACG AGTCAAATTT CTCTTTCTAG   
  
  
+ AAGAGGGGGG GGGGGGGGGG TTGGTTTAAA ACTCCATATT TTCTTTGAAT GGAAAGCTTA ATTTGCTGAC   
  
  
+ TATCTTGGTT AGTAGCATAA GCTTGATCAG AAAACAAAAC GCTCTCTGCC CTGCAAAAAC TTTATTTGTC   
  
  
+ TTTTAAGGAA AATTTTGGAA TTTGAGAGTG AAAATTTTGG TTGTGAATCT TGTCTTACTA TGTGGTTCCT   
  
  
+ TCTGACAAAA GTTTGAATCT GAGTATGAGA GTTCATCAAT CTCTTTTACT TTCCTACTTT CTTTATTGCT   
  
  
+ TGCAAATTGT TGTGAACTTA CTTGGATTTT CTGGTAGAGG AGAGTTCTGT CCTGGAATTT GGTGGAAAGT   
  
  
+ GAGACATACT TTGGGATTTA ATTAGCCATC AATTTGGTTT CACACCTTAT CAGTTTGGAC TTCTGGTGTG   
  
  
+ TTATTGTATG TGTTCTTAGC TTGGGATTAT TCTTGTATCG GAAGAGAGGG GGGGGGGGGT GTTTCTTTTA   
  
  
+ GCTGAATGTT AGAACATTTT AAGGGATCTA GATATCTAGA GAAGAGCCTA GGAAATGAAG CATTTTTCCC   
  
  
+ TTATAATCTG ACACAACTTT ATGAATTGAT TAGGAACAAA GAACGATTAG TTCAGAAAGG TTACAACTCC   
  
  
+ TCTAGGTGTT ATACTAAAGA GATGTGAGTT AATAAGCAAT TGCTTACTAA CTGTTTAGGC TGTTTCATTG   
  
  
+ CTTGTAGAGG CTTTTTTTTT GGGGGGGGGG GGGGGTTTGG GGTTCATAAA TGAGAAGGTT GATTTGTTGT   
  
  
+ CTGCTCTGCT CTTGATTCGA TAGTAATTCT CTCAGAAAAG GGTAAAATGA CATGGCCTTC CATCGCCTCC   
  
  
+ CATGAGGCTT TTTGCCTCTG CTTGTGTATC TTTCATCTCT TGGTTTGGTG GGTTGGGGGA GGGGAGGGGG   
  
  
+ GGAAGGGGGT TTTGATTCGA TAGATCATCA CTTTCAGAAA TGGCCTCTCT GGCCAATGGT AAGGGGTAAG   
  
  
+ GTTAGTACAT GTGGACTTTC CCATGTGGAT TTTTCCAGAG TCCGTTGTGT GATTGTTATT GCTGTTGTTG   
  
  
+ TAACTTTCTG TCACTCGGAA AACATGAATC CATATGCAAC AAGAACATAA TAATTGTGAA TGCGACTTTA   
  
  
+ TTTAGGAATG TCTTACATAG TACGTGCACA TTCTTTAATT ATCTGTTTAT CTGTTGGTTT GTTATTTTCA   
  
  
+ AGGGGATGGG GAGTGGGTGG ATGCTTAGTA TGTAATCTTT TTATATAGGG AAAAATCATA TAGCTTGAAA   
  
  
+ GTAAAACTTT CATTGCCAGG ATTCCAAGGT AGGGGTAGGG TTGTCTACAC CTTGATCTCC CTAGAAGCAT   
  
  
+ GGCGATCCTT CCTCGGATTG TGTTATAGAA TAAAGAATGT GGTTCTGCTG TAATGAGACT TATGTTCTAT   
  
  
+ CAAATGATGA ATCTGTTTAG AACTTCTTGC TTATTTTTTC TGCAAAATTT TAGGAGACTT GATAAACTAT   
  
  
+ AGTTCTCTTG AATTTACAGG TGGATATTGG AGTTCACAGA ATAGTATTCA GATCAAAGTG CTTGTGCTTA   
  
  
+ ATTGGAAGGA CAACTTGCTT GAGCTGTTTA TAGAGCTCTA ACTGATGCTA TCTCATGGAC TCACATCAGT   
  
  
+ TTTTTGGATT TAATCTTACC AGTGTTGATC CGTCGTACAT TTCCGCTCAG TACAGTCCGC CGTCAGTGAC   
  
  
+ AAATAGGATG TTTGCATCAC TGAAGCTCGA CTCTAGAGGT TCTCCTGTGT CACCCTTCTC AACTCAGTTT   
  
  
+ GATTGTGATA CGGTTACTAC ATTGAGTGAT AGTCAAGAGC ATCACAGCTC GACGGGGAGT CTATCAACAA   
  
  
+ GAAGCCCTTC TTGTAATTCT CCCCTTGAAA CGAGCAGTTA TCATCATTTA TCGACGAATG GCCCGTCTTG   
  
  
+ TAATTCTGCC CCTGAAACTA GCAGTTATCG TCATCGGTTC AACGCGAGTC CCGTGGGATA TTCCCATCAA   
  
  
+ GGCACTGATT ATGGAGTGAA TATGAAGAGC GCTCTGCAGG AGCTAGAGAC TACTCTAATG GGTGCAGATG   
  
  
+ GTGAGGAAGT ATCTGCTGCT AATCAACCTA TGGGGGGAAG TCGTCAGTCC GGGATCCCAA GTCAGAGATC   
  
  
+ AAAATCATTG AGCGAAGATC CACAGGGTTC GCATCCTACT CAGCCTGATT CATCATCTCT TTCTAGGGCA   
  
  
+ AGAAGATCAG GAGATGAAAG CCAGAGAGAG AAACGGCACA AGGCAATGGA AGAACCAACG GAACTACCAA   
  
  
+ GTTTGCCACC TGGTGATTTG AAGCAGTTGC TAATTGAATG TGCGAGGGCT TTATCGGATA ACCGAATAGA   
  
  
+ TGACTTTGAG AGTTTGGTTA AACGGGCGAG GAAAGAGGTC TCTATCTCGG GTGAGCCTAT CCAACGTCTC   
  
  
+ GGTGCCTACA TGATCGAAGG GCTTGTGGCA AGGAAGCAGT CTTCGGGGAC TAGCATCTAT CGGGCTCTGA   
  
  
+ AGTGTAAAGA GCCTCTTGGA AAAGACTTGC TCTCCTACAT GCACATCCTT TATGAAATAT GCCCTTATCT   
  
  
+ CAAGTTTGGT TATATGGCTG CGAATGGAGC GATAGCTGAA GCTTGTAGAA ATGAGGATAA CATACATATT   
  
  
+ ATAGATTTCC AGATTGCACA GGGCACTCAG TGGGTTACTC TATTACAAGC CCTAGCAGCA AGACCTGGTG   
  
  
+ GGCCACCTAA GGTGCGAATT ACAGGCATTG ATGATCCTGT TTCTAAGTAT GCCCGTGGTG CTAGCTTGGA   
  
  
+ GGCTGTTGGG AAACGGTTAG CGTCTCTATC TGAAAAGTTC AAAATACCCG TCGAGTTCAA TGCGTTGCCC   
  
  
+ GTTTATGGAC CCGATGTCAG GCGGGAAATG CTGGATGTGA GGCCCGGGGA GGCTTTGGCC GTTAATTTTC   
  
  
+ CATTGCAGCT CCACCACACT CCTGACGAGA GTGTCGATGT GAACAACCCT AGGGATGGGC TTCTCAGAAT   
  
  
+ GGTGAAATCA CTTGGTCCTA AGGTAACCAC TTTGGTTGAG CAAGAATCAA ACACCAACAC TACCCCTTTC   
  
  
+ TTGACCCGGT TCATAGAGAC CCTTGACTAC TACTCAGCCA TGTTTGAGTC TATAGACGTG ACCATGCCAA   
  
  
+ GAGACCGGAA GGAGAGGATC AATGTTGAGC AGCATTGTTT GGCTAAGGAC ATTGTGAATA TCATAGCTTG   
  
  
+ CGAGGGCAAG GAGAGGGTGG AGCGTCATGA ACTTTTTGGG AAATGGAAGT CAAGGTTTAC CATGGCAGGG   
  
  
+ TTCAGGCAGT ACCCGTTGAG CTCATACGTA AACTCTGTGA TAAGAAGCCT ACTCCGGTGT TACTCCGAGC   
  
  
+ ATTATACTCT GATAGAGAAG GATGGTGCCA TGCTTCTGGG TTGGAAGGGC CGAATGCTGA TTTCAGCTTC   
  
  
+ GGCATGGCAT TG  

- +Up\_Stream \_Len000AGGAGA AAACCCGAAG AAAGAAAGAT AGGACAAAAA AAAACCCAGC TTTTCAACCT   
  
  
- CCAACTTAAC TTCGTAGGCT TAAGCTCGTC CTACAAAGAG ACGCATCACT AAGAAAGACC CAAACACTAA   
  
  
- AACTTAAGAC CCATTAGCCA ACAAAACGAT TAAAACTCCT GTCTCAAGGA AAAGACTTAA TTTTAAAGCA   
  
  
- AAAAAATAGC CCTTTTAGAA GTCATAAACT CTTTTTTCTT GCTTTAAACC TACTAAACGA CAGATCTAAA   
  
  
- ACGAAAGTAT AAGGACCCAC ACCTAACCAA ATAATTAACG TACACTCCTT CATGTTCGAA GACCAGTTAC   
  
  
- CAACCCAAAA AAAAAAAAAA AACACCCATA TTTCAACCTT TAACTAATGC TCAGTTTAAA GAGAAAGATC   
  
  
- TTCTCCCCCC CCCCCCCCCC AACCAAATTT TGAGGTATAA AAGAAACTTA CCTTTCGAAT TAAACGACTG   
  
  
- ATAGAACCAA TCATCGTATT CGAACTAGTC TTTTGTTTTG CGAGAGACGG GACGTTTTTG AAATAAACAG   
  
  
- AAAATTCCTT TTAAAACCTT AAACTCTCAC TTTTAAAACC AACACTTAGA ACAGAATGAT ACACCAAGGA   
  
  
- AGACTGTTTT CAAACTTAGA CTCATACTCT CAAGTAGTTA GAGAAAATGA AAGGATGAAA GAAATAACGA   
  
  
- ACGTTTAACA ACACTTGAAT GAACCTAAAA GACCATCTCC TCTCAAGACA GGACCTTAAA CCACCTTTCA   
  
  
- CTCTGTATGA AACCCTAAAT TAATCGGTAG TTAAACCAAA GTGTGGAATA GTCAAACCTG AAGACCACAC   
  
  
- AATAACATAC ACAAGAATCG AACCCTAATA AGAACATAGC CTTCTCTCCC CCCCCCCCCA CAAAGAAAAT   
  
  
- CGACTTACAA TCTTGTAAAA TTCCCTAGAT CTATAGATCT CTTCTCGGAT CCTTTACTTC GTAAAAAGGG   
  
  
- AATATTAGAC TGTGTTGAAA TACTTAACTA ATCCTTGTTT CTTGCTAATC AAGTCTTTCC AATGTTGAGG   
  
  
- AGATCCACAA TATGATTTCT CTACACTCAA TTATTCGTTA ACGAATGATT GACAAATCCG ACAAAGTAAC   
  
  
- GAACATCTCC GAAAAAAAAA CCCCCCCCCC CCCCCAAACC CCAAGTATTT ACTCTTCCAA CTAAACAACA   
  
  
- GACGAGACGA GAACTAAGCT ATCATTAAGA GAGTCTTTTC CCATTTTACT GTACCGGAAG GTAGCGGAGG   
  
  
- GTACTCCGAA AAACGGAGAC GAACACATAG AAAGTAGAGA ACCAAACCAC CCAACCCCCT CCCCTCCCCC   
  
  
- CCTTCCCCCA AAACTAAGCT ATCTAGTAGT GAAAGTCTTT ACCGGAGAGA CCGGTTACCA TTCCCCATTC   
  
  
- CAATCATGTA CACCTGAAAG GGTACACCTA AAAAGGTCTC AGGCAACACA CTAACAATAA CGACAACAAC   
  
  
- ATTGAAAGAC AGTGAGCCTT TTGTACTTAG GTATACGTTG TTCTTGTATT ATTAACACTT ACGCTGAAAT   
  
  
- AAATCCTTAC AGAATGTATC ATGCACGTGT AAGAAATTAA TAGACAAATA GACAACCAAA CAATAAAAGT   
  
  
- TCCCCTACCC CTCACCCACC TACGAATCAT ACATTAGAAA AATATATCCC TTTTTAGTAT ATCGAACTTT   
  
  
- CATTTTGAAA GTAACGGTCC TAAGGTTCCA TCCCCATCCC AACAGATGTG GAACTAGAGG GATCTTCGTA   
  
  
- CCGCTAGGAA GGAGCCTAAC ACAATATCTT ATTTCTTACA CCAAGACGAC ATTACTCTGA ATACAAGATA   
  
  
- GTTTACTACT TAGACAAATC TTGAAGAACG AATAAAAAAG ACGTTTTAAA ATCCTCTGAA CTATTTGATA   
  
  
- TCAAGAGAAC TTAAATGTCC ACCTATAACC TCAAGTGTCT TATCATAAGT CTAGTTTCAC GAACACGAAT   
  
  
- TAACCTTCCT GTTGAACGAA CTCGACAAAT ATCTCGAGAT TGACTACGAT AGAGTACCTG AGTGTAGTCA   
  
  
- AAAAACCTAA ATTAGAATGG TCACAACTAG GCAGCATGTA AAGGCGAGTC ATGTCAGGCG GCAGTCACTG   
  
  
- TTTATCCTAC AAACGTAGTG ACTTCGAGCT GAGATCTCCA AGAGGACACA GTGGGAAGAG TTGAGTCAAA   
  
  
- CTAACACTAT GCCAATGATG TAACTCACTA TCAGTTCTCG TAGTGTCGAG CTGCCCCTCA GATAGTTGTT   
  
  
- CTTCGGGAAG AACATTAAGA GGGGAACTTT GCTCGTCAAT AGTAGTAAAT AGCTGCTTAC CGGGCAGAAC   
  
  
- ATTAAGACGG GGACTTTGAT CGTCAATAGC AGTAGCCAAG TTGCGCTCAG GGCACCCTAT AAGGGTAGTT   
  
  
- CCGTGACTAA TACCTCACTT ATACTTCTCG CGAGACGTCC TCGATCTCTG ATGAGATTAC CCACGTCTAC   
  
  
- CACTCCTTCA TAGACGACGA TTAGTTGGAT ACCCCCCTTC AGCAGTCAGG CCCTAGGGTT CAGTCTCTAG   
  
  
- TTTTAGTAAC TCGCTTCTAG GTGTCCCAAG CGTAGGATGA GTCGGACTAA GTAGTAGAGA AAGATCCCGT   
  
  
- TCTTCTAGTC CTCTACTTTC GGTCTCTCTC TTTGCCGTGT TCCGTTACCT TCTTGGTTGC CTTGATGGTT   
  
  
- CAAACGGTGG ACCACTAAAC TTCGTCAACG ATTAACTTAC ACGCTCCCGA AATAGCCTAT TGGCTTATCT   
  
  
- ACTGAAACTC TCAAACCAAT TTGCCCGCTC CTTTCTCCAG AGATAGAGCC CACTCGGATA GGTTGCAGAG   
  
  
- CCACGGATGT ACTAGCTTCC CGAACACCGT TCCTTCGTCA GAAGCCCCTG ATCGTAGATA GCCCGAGACT   
  
  
- TCACATTTCT CGGAGAACCT TTTCTGAACG AGAGGATGTA CGTGTAGGAA ATACTTTATA CGGGAATAGA   
  
  
- GTTCAAACCA ATATACCGAC GCTTACCTCG CTATCGACTT CGAACATCTT TACTCCTATT GTATGTATAA   
  
  
- TATCTAAAGG TCTAACGTGT CCCGTGAGTC ACCCAATGAG ATAATGTTCG GGATCGTCGT TCTGGACCAC   
  
  
- CCGGTGGATT CCACGCTTAA TGTCCGTAAC TACTAGGACA AAGATTCATA CGGGCACCAC GATCGAACCT   
  
  
- CCGACAACCC TTTGCCAATC GCAGAGATAG ACTTTTCAAG TTTTATGGGC AGCTCAAGTT ACGCAACGGG   
  
  
- CAAATACCTG GGCTACAGTC CGCCCTTTAC GACCTACACT CCGGGCCCCT CCGAAACCGG CAATTAAAAG   
  
  
- GTAACGTCGA GGTGGTGTGA GGACTGCTCT CACAGCTACA CTTGTTGGGA TCCCTACCCG AAGAGTCTTA   
  
  
- CCACTTTAGT GAACCAGGAT TCCATTGGTG AAACCAACTC GTTCTTAGTT TGTGGTTGTG ATGGGGAAAG   
  
  
- AACTGGGCCA AGTATCTCTG GGAACTGATG ATGAGTCGGT ACAAACTCAG ATATCTGCAC TGGTACGGTT   
  
  
- CTCTGGCCTT CCTCTCCTAG TTACAACTCG TCGTAACAAA CCGATTCCTG TAACACTTAT AGTATCGAAC   
  
  
- GCTCCCGTTC CTCTCCCACC TCGCAGTACT TGAAAAACCC TTTACCTTCA GTTCCAAATG GTACCGTCCC   
  
  
- AAGTCCGTCA TGGGCAACTC GAGTATGCAT TTGAGACACT ATTCTTCGGA TGAGGCCACA ATGAGGCTCG   
  
  
- TAATATGAGA CTATCTCTTC CTACCACGGT ACGAAGACCC AACCTTCCCG GCTTACGACT AAAGTCGAAG   
  
  
- CCGTACCGTA AC

+     ARE

| Site Name | Organism | Position | Strand | Matrix score. | sequence | function |
| --- | --- | --- | --- | --- | --- | --- |
| ARE | Zea mays | 809 | - | 6 | AAACCA | cis-acting regulatory element essential for the anaerobic induction |
| ARE | Zea mays | 1305 | - | 6 | AAACCA | cis-acting regulatory element essential for the anaerobic induction |
| ARE | Zea mays | 1599 | - | 6 | AAACCA | cis-acting regulatory element essential for the anaerobic induction |
| ARE | Zea mays | 310 | - | 6 | AAACCA | cis-acting regulatory element essential for the anaerobic induction |
| ARE | Zea mays | 446 | - | 6 | AAACCA | cis-acting regulatory element essential for the anaerobic induction |

>HU08G00014.1   
+ +Up\_Stream \_Len000TCCTCT TTTGGGCTTC TTTCTTTCTA TCCTGTTTTT TTTTGGGTCG AAAAGTTGGA   
  
  
+ GGTTGAATTG AAGCATCCGA ATTCGAGCAG GATGTTTCTC TGCGTAGTGA TTCTTTCTGG GTTTGTGATT   
  
  
+ TTGAATTCTG GGTAATCGGT TGTTTTGCTA ATTTTGAGGA CAGAGTTCCT TTTCTGAATT AAAATTTCGT   
  
  
+ TTTTTTATCG GGAAAATCTT CAGTATTTGA GAAAAAAGAA CGAAATTTGG ATGATTTGCT GTCTAGATTT   
  
  
+ TGCTTTCATA TTCCTGGGTG TGGATTGGTT TATTAATTGC ATGTGAGGAA GTACAAGCTT CTGGTCAATG   
  
  
+ GTTGGGTTTT TTTTTTTTTT TTGTGGGTAT AAAGTTGGAA ATTGATTACG AGTCAAATTT CTCTTTCTAG   
  
  
+ AAGAGGGGGG GGGGGGGGGG TTGGTTTAAA ACTCCATATT TTCTTTGAAT GGAAAGCTTA ATTTGCTGAC   
  
  
+ TATCTTGGTT AGTAGCATAA GCTTGATCAG AAAACAAAAC GCTCTCTGCC CTGCAAAAAC TTTATTTGTC   
  
  
+ TTTTAAGGAA AATTTTGGAA TTTGAGAGTG AAAATTTTGG TTGTGAATCT TGTCTTACTA TGTGGTTCCT   
  
  
+ TCTGACAAAA GTTTGAATCT GAGTATGAGA GTTCATCAAT CTCTTTTACT TTCCTACTTT CTTTATTGCT   
  
  
+ TGCAAATTGT TGTGAACTTA CTTGGATTTT CTGGTAGAGG AGAGTTCTGT CCTGGAATTT GGTGGAAAGT   
  
  
+ GAGACATACT TTGGGATTTA ATTAGCCATC AATTTGGTTT CACACCTTAT CAGTTTGGAC TTCTGGTGTG   
  
  
+ TTATTGTATG TGTTCTTAGC TTGGGATTAT TCTTGTATCG GAAGAGAGGG GGGGGGGGGT GTTTCTTTTA   
  
  
+ GCTGAATGTT AGAACATTTT AAGGGATCTA GATATCTAGA GAAGAGCCTA GGAAATGAAG CATTTTTCCC   
  
  
+ TTATAATCTG ACACAACTTT ATGAATTGAT TAGGAACAAA GAACGATTAG TTCAGAAAGG TTACAACTCC   
  
  
+ TCTAGGTGTT ATACTAAAGA GATGTGAGTT AATAAGCAAT TGCTTACTAA CTGTTTAGGC TGTTTCATTG   
  
  
+ CTTGTAGAGG CTTTTTTTTT GGGGGGGGGG GGGGGTTTGG GGTTCATAAA TGAGAAGGTT GATTTGTTGT   
  
  
+ CTGCTCTGCT CTTGATTCGA TAGTAATTCT CTCAGAAAAG GGTAAAATGA CATGGCCTTC CATCGCCTCC   
  
  
+ CATGAGGCTT TTTGCCTCTG CTTGTGTATC TTTCATCTCT TGGTTTGGTG GGTTGGGGGA GGGGAGGGGG   
  
  
+ GGAAGGGGGT TTTGATTCGA TAGATCATCA CTTTCAGAAA TGGCCTCTCT GGCCAATGGT AAGGGGTAAG   
  
  
+ GTTAGTACAT GTGGACTTTC CCATGTGGAT TTTTCCAGAG TCCGTTGTGT GATTGTTATT GCTGTTGTTG   
  
  
+ TAACTTTCTG TCACTCGGAA AACATGAATC CATATGCAAC AAGAACATAA TAATTGTGAA TGCGACTTTA   
  
  
+ TTTAGGAATG TCTTACATAG TACGTGCACA TTCTTTAATT ATCTGTTTAT CTGTTGGTTT GTTATTTTCA   
  
  
+ AGGGGATGGG GAGTGGGTGG ATGCTTAGTA TGTAATCTTT TTATATAGGG AAAAATCATA TAGCTTGAAA   
  
  
+ GTAAAACTTT CATTGCCAGG ATTCCAAGGT AGGGGTAGGG TTGTCTACAC CTTGATCTCC CTAGAAGCAT   
  
  
+ GGCGATCCTT CCTCGGATTG TGTTATAGAA TAAAGAATGT GGTTCTGCTG TAATGAGACT TATGTTCTAT   
  
  
+ CAAATGATGA ATCTGTTTAG AACTTCTTGC TTATTTTTTC TGCAAAATTT TAGGAGACTT GATAAACTAT   
  
  
+ AGTTCTCTTG AATTTACAGG TGGATATTGG AGTTCACAGA ATAGTATTCA GATCAAAGTG CTTGTGCTTA   
  
  
+ ATTGGAAGGA CAACTTGCTT GAGCTGTTTA TAGAGCTCTA ACTGATGCTA TCTCATGGAC TCACATCAGT   
  
  
+ TTTTTGGATT TAATCTTACC AGTGTTGATC CGTCGTACAT TTCCGCTCAG TACAGTCCGC CGTCAGTGAC   
  
  
+ AAATAGGATG TTTGCATCAC TGAAGCTCGA CTCTAGAGGT TCTCCTGTGT CACCCTTCTC AACTCAGTTT   
  
  
+ GATTGTGATA CGGTTACTAC ATTGAGTGAT AGTCAAGAGC ATCACAGCTC GACGGGGAGT CTATCAACAA   
  
  
+ GAAGCCCTTC TTGTAATTCT CCCCTTGAAA CGAGCAGTTA TCATCATTTA TCGACGAATG GCCCGTCTTG   
  
  
+ TAATTCTGCC CCTGAAACTA GCAGTTATCG TCATCGGTTC AACGCGAGTC CCGTGGGATA TTCCCATCAA   
  
  
+ GGCACTGATT ATGGAGTGAA TATGAAGAGC GCTCTGCAGG AGCTAGAGAC TACTCTAATG GGTGCAGATG   
  
  
+ GTGAGGAAGT ATCTGCTGCT AATCAACCTA TGGGGGGAAG TCGTCAGTCC GGGATCCCAA GTCAGAGATC   
  
  
+ AAAATCATTG AGCGAAGATC CACAGGGTTC GCATCCTACT CAGCCTGATT CATCATCTCT TTCTAGGGCA   
  
  
+ AGAAGATCAG GAGATGAAAG CCAGAGAGAG AAACGGCACA AGGCAATGGA AGAACCAACG GAACTACCAA   
  
  
+ GTTTGCCACC TGGTGATTTG AAGCAGTTGC TAATTGAATG TGCGAGGGCT TTATCGGATA ACCGAATAGA   
  
  
+ TGACTTTGAG AGTTTGGTTA AACGGGCGAG GAAAGAGGTC TCTATCTCGG GTGAGCCTAT CCAACGTCTC   
  
  
+ GGTGCCTACA TGATCGAAGG GCTTGTGGCA AGGAAGCAGT CTTCGGGGAC TAGCATCTAT CGGGCTCTGA   
  
  
+ AGTGTAAAGA GCCTCTTGGA AAAGACTTGC TCTCCTACAT GCACATCCTT TATGAAATAT GCCCTTATCT   
  
  
+ CAAGTTTGGT TATATGGCTG CGAATGGAGC GATAGCTGAA GCTTGTAGAA ATGAGGATAA CATACATATT   
  
  
+ ATAGATTTCC AGATTGCACA GGGCACTCAG TGGGTTACTC TATTACAAGC CCTAGCAGCA AGACCTGGTG   
  
  
+ GGCCACCTAA GGTGCGAATT ACAGGCATTG ATGATCCTGT TTCTAAGTAT GCCCGTGGTG CTAGCTTGGA   
  
  
+ GGCTGTTGGG AAACGGTTAG CGTCTCTATC TGAAAAGTTC AAAATACCCG TCGAGTTCAA TGCGTTGCCC   
  
  
+ GTTTATGGAC CCGATGTCAG GCGGGAAATG CTGGATGTGA GGCCCGGGGA GGCTTTGGCC GTTAATTTTC   
  
  
+ CATTGCAGCT CCACCACACT CCTGACGAGA GTGTCGATGT GAACAACCCT AGGGATGGGC TTCTCAGAAT   
  
  
+ GGTGAAATCA CTTGGTCCTA AGGTAACCAC TTTGGTTGAG CAAGAATCAA ACACCAACAC TACCCCTTTC   
  
  
+ TTGACCCGGT TCATAGAGAC CCTTGACTAC TACTCAGCCA TGTTTGAGTC TATAGACGTG ACCATGCCAA   
  
  
+ GAGACCGGAA GGAGAGGATC AATGTTGAGC AGCATTGTTT GGCTAAGGAC ATTGTGAATA TCATAGCTTG   
  
  
+ CGAGGGCAAG GAGAGGGTGG AGCGTCATGA ACTTTTTGGG AAATGGAAGT CAAGGTTTAC CATGGCAGGG   
  
  
+ TTCAGGCAGT ACCCGTTGAG CTCATACGTA AACTCTGTGA TAAGAAGCCT ACTCCGGTGT TACTCCGAGC   
  
  
+ ATTATACTCT GATAGAGAAG GATGGTGCCA TGCTTCTGGG TTGGAAGGGC CGAATGCTGA TTTCAGCTTC   
  
  
+ GGCATGGCAT TG  

- +Up\_Stream \_Len000AGGAGA AAACCCGAAG AAAGAAAGAT AGGACAAAAA AAAACCCAGC TTTTCAACCT   
  
  
- CCAACTTAAC TTCGTAGGCT TAAGCTCGTC CTACAAAGAG ACGCATCACT AAGAAAGACC CAAACACTAA   
  
  
- AACTTAAGAC CCATTAGCCA ACAAAACGAT TAAAACTCCT GTCTCAAGGA AAAGACTTAA TTTTAAAGCA   
  
  
- AAAAAATAGC CCTTTTAGAA GTCATAAACT CTTTTTTCTT GCTTTAAACC TACTAAACGA CAGATCTAAA   
  
  
- ACGAAAGTAT AAGGACCCAC ACCTAACCAA ATAATTAACG TACACTCCTT CATGTTCGAA GACCAGTTAC   
  
  
- CAACCCAAAA AAAAAAAAAA AACACCCATA TTTCAACCTT TAACTAATGC TCAGTTTAAA GAGAAAGATC   
  
  
- TTCTCCCCCC CCCCCCCCCC AACCAAATTT TGAGGTATAA AAGAAACTTA CCTTTCGAAT TAAACGACTG   
  
  
- ATAGAACCAA TCATCGTATT CGAACTAGTC TTTTGTTTTG CGAGAGACGG GACGTTTTTG AAATAAACAG   
  
  
- AAAATTCCTT TTAAAACCTT AAACTCTCAC TTTTAAAACC AACACTTAGA ACAGAATGAT ACACCAAGGA   
  
  
- AGACTGTTTT CAAACTTAGA CTCATACTCT CAAGTAGTTA GAGAAAATGA AAGGATGAAA GAAATAACGA   
  
  
- ACGTTTAACA ACACTTGAAT GAACCTAAAA GACCATCTCC TCTCAAGACA GGACCTTAAA CCACCTTTCA   
  
  
- CTCTGTATGA AACCCTAAAT TAATCGGTAG TTAAACCAAA GTGTGGAATA GTCAAACCTG AAGACCACAC   
  
  
- AATAACATAC ACAAGAATCG AACCCTAATA AGAACATAGC CTTCTCTCCC CCCCCCCCCA CAAAGAAAAT   
  
  
- CGACTTACAA TCTTGTAAAA TTCCCTAGAT CTATAGATCT CTTCTCGGAT CCTTTACTTC GTAAAAAGGG   
  
  
- AATATTAGAC TGTGTTGAAA TACTTAACTA ATCCTTGTTT CTTGCTAATC AAGTCTTTCC AATGTTGAGG   
  
  
- AGATCCACAA TATGATTTCT CTACACTCAA TTATTCGTTA ACGAATGATT GACAAATCCG ACAAAGTAAC   
  
  
- GAACATCTCC GAAAAAAAAA CCCCCCCCCC CCCCCAAACC CCAAGTATTT ACTCTTCCAA CTAAACAACA   
  
  
- GACGAGACGA GAACTAAGCT ATCATTAAGA GAGTCTTTTC CCATTTTACT GTACCGGAAG GTAGCGGAGG   
  
  
- GTACTCCGAA AAACGGAGAC GAACACATAG AAAGTAGAGA ACCAAACCAC CCAACCCCCT CCCCTCCCCC   
  
  
- CCTTCCCCCA AAACTAAGCT ATCTAGTAGT GAAAGTCTTT ACCGGAGAGA CCGGTTACCA TTCCCCATTC   
  
  
- CAATCATGTA CACCTGAAAG GGTACACCTA AAAAGGTCTC AGGCAACACA CTAACAATAA CGACAACAAC   
  
  
- ATTGAAAGAC AGTGAGCCTT TTGTACTTAG GTATACGTTG TTCTTGTATT ATTAACACTT ACGCTGAAAT   
  
  
- AAATCCTTAC AGAATGTATC ATGCACGTGT AAGAAATTAA TAGACAAATA GACAACCAAA CAATAAAAGT   
  
  
- TCCCCTACCC CTCACCCACC TACGAATCAT ACATTAGAAA AATATATCCC TTTTTAGTAT ATCGAACTTT   
  
  
- CATTTTGAAA GTAACGGTCC TAAGGTTCCA TCCCCATCCC AACAGATGTG GAACTAGAGG GATCTTCGTA   
  
  
- CCGCTAGGAA GGAGCCTAAC ACAATATCTT ATTTCTTACA CCAAGACGAC ATTACTCTGA ATACAAGATA   
  
  
- GTTTACTACT TAGACAAATC TTGAAGAACG AATAAAAAAG ACGTTTTAAA ATCCTCTGAA CTATTTGATA   
  
  
- TCAAGAGAAC TTAAATGTCC ACCTATAACC TCAAGTGTCT TATCATAAGT CTAGTTTCAC GAACACGAAT   
  
  
- TAACCTTCCT GTTGAACGAA CTCGACAAAT ATCTCGAGAT TGACTACGAT AGAGTACCTG AGTGTAGTCA   
  
  
- AAAAACCTAA ATTAGAATGG TCACAACTAG GCAGCATGTA AAGGCGAGTC ATGTCAGGCG GCAGTCACTG   
  
  
- TTTATCCTAC AAACGTAGTG ACTTCGAGCT GAGATCTCCA AGAGGACACA GTGGGAAGAG TTGAGTCAAA   
  
  
- CTAACACTAT GCCAATGATG TAACTCACTA TCAGTTCTCG TAGTGTCGAG CTGCCCCTCA GATAGTTGTT   
  
  
- CTTCGGGAAG AACATTAAGA GGGGAACTTT GCTCGTCAAT AGTAGTAAAT AGCTGCTTAC CGGGCAGAAC   
  
  
- ATTAAGACGG GGACTTTGAT CGTCAATAGC AGTAGCCAAG TTGCGCTCAG GGCACCCTAT AAGGGTAGTT   
  
  
- CCGTGACTAA TACCTCACTT ATACTTCTCG CGAGACGTCC TCGATCTCTG ATGAGATTAC CCACGTCTAC   
  
  
- CACTCCTTCA TAGACGACGA TTAGTTGGAT ACCCCCCTTC AGCAGTCAGG CCCTAGGGTT CAGTCTCTAG   
  
  
- TTTTAGTAAC TCGCTTCTAG GTGTCCCAAG CGTAGGATGA GTCGGACTAA GTAGTAGAGA AAGATCCCGT   
  
  
- TCTTCTAGTC CTCTACTTTC GGTCTCTCTC TTTGCCGTGT TCCGTTACCT TCTTGGTTGC CTTGATGGTT   
  
  
- CAAACGGTGG ACCACTAAAC TTCGTCAACG ATTAACTTAC ACGCTCCCGA AATAGCCTAT TGGCTTATCT   
  
  
- ACTGAAACTC TCAAACCAAT TTGCCCGCTC CTTTCTCCAG AGATAGAGCC CACTCGGATA GGTTGCAGAG   
  
  
- CCACGGATGT ACTAGCTTCC CGAACACCGT TCCTTCGTCA GAAGCCCCTG ATCGTAGATA GCCCGAGACT   
  
  
- TCACATTTCT CGGAGAACCT TTTCTGAACG AGAGGATGTA CGTGTAGGAA ATACTTTATA CGGGAATAGA   
  
  
- GTTCAAACCA ATATACCGAC GCTTACCTCG CTATCGACTT CGAACATCTT TACTCCTATT GTATGTATAA   
  
  
- TATCTAAAGG TCTAACGTGT CCCGTGAGTC ACCCAATGAG ATAATGTTCG GGATCGTCGT TCTGGACCAC   
  
  
- CCGGTGGATT CCACGCTTAA TGTCCGTAAC TACTAGGACA AAGATTCATA CGGGCACCAC GATCGAACCT   
  
  
- CCGACAACCC TTTGCCAATC GCAGAGATAG ACTTTTCAAG TTTTATGGGC AGCTCAAGTT ACGCAACGGG   
  
  
- CAAATACCTG GGCTACAGTC CGCCCTTTAC GACCTACACT CCGGGCCCCT CCGAAACCGG CAATTAAAAG   
  
  
- GTAACGTCGA GGTGGTGTGA GGACTGCTCT CACAGCTACA CTTGTTGGGA TCCCTACCCG AAGAGTCTTA   
  
  
- CCACTTTAGT GAACCAGGAT TCCATTGGTG AAACCAACTC GTTCTTAGTT TGTGGTTGTG ATGGGGAAAG   
  
  
- AACTGGGCCA AGTATCTCTG GGAACTGATG ATGAGTCGGT ACAAACTCAG ATATCTGCAC TGGTACGGTT   
  
  
- CTCTGGCCTT CCTCTCCTAG TTACAACTCG TCGTAACAAA CCGATTCCTG TAACACTTAT AGTATCGAAC   
  
  
- GCTCCCGTTC CTCTCCCACC TCGCAGTACT TGAAAAACCC TTTACCTTCA GTTCCAAATG GTACCGTCCC   
  
  
- AAGTCCGTCA TGGGCAACTC GAGTATGCAT TTGAGACACT ATTCTTCGGA TGAGGCCACA ATGAGGCTCG   
  
  
- TAATATGAGA CTATCTCTTC CTACCACGGT ACGAAGACCC AACCTTCCCG GCTTACGACT AAAGTCGAAG   
  
  
- CCGTACCGTA AC

+     AT~TATA-box

| Site Name | Organism | Position | Strand | Matrix score. | sequence | function |
| --- | --- | --- | --- | --- | --- | --- |
| AT~TATA-box | Arabidopsis thaliana | 1654 | - | 8 | TATATAAA |  |
| AT~TATA-box | Arabidopsis thaliana | 1656 | + | 6 | TATATA |  |

>HU08G00014.1   
+ +Up\_Stream \_Len000TCCTCT TTTGGGCTTC TTTCTTTCTA TCCTGTTTTT TTTTGGGTCG AAAAGTTGGA   
  
  
+ GGTTGAATTG AAGCATCCGA ATTCGAGCAG GATGTTTCTC TGCGTAGTGA TTCTTTCTGG GTTTGTGATT   
  
  
+ TTGAATTCTG GGTAATCGGT TGTTTTGCTA ATTTTGAGGA CAGAGTTCCT TTTCTGAATT AAAATTTCGT   
  
  
+ TTTTTTATCG GGAAAATCTT CAGTATTTGA GAAAAAAGAA CGAAATTTGG ATGATTTGCT GTCTAGATTT   
  
  
+ TGCTTTCATA TTCCTGGGTG TGGATTGGTT TATTAATTGC ATGTGAGGAA GTACAAGCTT CTGGTCAATG   
  
  
+ GTTGGGTTTT TTTTTTTTTT TTGTGGGTAT AAAGTTGGAA ATTGATTACG AGTCAAATTT CTCTTTCTAG   
  
  
+ AAGAGGGGGG GGGGGGGGGG TTGGTTTAAA ACTCCATATT TTCTTTGAAT GGAAAGCTTA ATTTGCTGAC   
  
  
+ TATCTTGGTT AGTAGCATAA GCTTGATCAG AAAACAAAAC GCTCTCTGCC CTGCAAAAAC TTTATTTGTC   
  
  
+ TTTTAAGGAA AATTTTGGAA TTTGAGAGTG AAAATTTTGG TTGTGAATCT TGTCTTACTA TGTGGTTCCT   
  
  
+ TCTGACAAAA GTTTGAATCT GAGTATGAGA GTTCATCAAT CTCTTTTACT TTCCTACTTT CTTTATTGCT   
  
  
+ TGCAAATTGT TGTGAACTTA CTTGGATTTT CTGGTAGAGG AGAGTTCTGT CCTGGAATTT GGTGGAAAGT   
  
  
+ GAGACATACT TTGGGATTTA ATTAGCCATC AATTTGGTTT CACACCTTAT CAGTTTGGAC TTCTGGTGTG   
  
  
+ TTATTGTATG TGTTCTTAGC TTGGGATTAT TCTTGTATCG GAAGAGAGGG GGGGGGGGGT GTTTCTTTTA   
  
  
+ GCTGAATGTT AGAACATTTT AAGGGATCTA GATATCTAGA GAAGAGCCTA GGAAATGAAG CATTTTTCCC   
  
  
+ TTATAATCTG ACACAACTTT ATGAATTGAT TAGGAACAAA GAACGATTAG TTCAGAAAGG TTACAACTCC   
  
  
+ TCTAGGTGTT ATACTAAAGA GATGTGAGTT AATAAGCAAT TGCTTACTAA CTGTTTAGGC TGTTTCATTG   
  
  
+ CTTGTAGAGG CTTTTTTTTT GGGGGGGGGG GGGGGTTTGG GGTTCATAAA TGAGAAGGTT GATTTGTTGT   
  
  
+ CTGCTCTGCT CTTGATTCGA TAGTAATTCT CTCAGAAAAG GGTAAAATGA CATGGCCTTC CATCGCCTCC   
  
  
+ CATGAGGCTT TTTGCCTCTG CTTGTGTATC TTTCATCTCT TGGTTTGGTG GGTTGGGGGA GGGGAGGGGG   
  
  
+ GGAAGGGGGT TTTGATTCGA TAGATCATCA CTTTCAGAAA TGGCCTCTCT GGCCAATGGT AAGGGGTAAG   
  
  
+ GTTAGTACAT GTGGACTTTC CCATGTGGAT TTTTCCAGAG TCCGTTGTGT GATTGTTATT GCTGTTGTTG   
  
  
+ TAACTTTCTG TCACTCGGAA AACATGAATC CATATGCAAC AAGAACATAA TAATTGTGAA TGCGACTTTA   
  
  
+ TTTAGGAATG TCTTACATAG TACGTGCACA TTCTTTAATT ATCTGTTTAT CTGTTGGTTT GTTATTTTCA   
  
  
+ AGGGGATGGG GAGTGGGTGG ATGCTTAGTA TGTAATCTTT TTATATAGGG AAAAATCATA TAGCTTGAAA   
  
  
+ GTAAAACTTT CATTGCCAGG ATTCCAAGGT AGGGGTAGGG TTGTCTACAC CTTGATCTCC CTAGAAGCAT   
  
  
+ GGCGATCCTT CCTCGGATTG TGTTATAGAA TAAAGAATGT GGTTCTGCTG TAATGAGACT TATGTTCTAT   
  
  
+ CAAATGATGA ATCTGTTTAG AACTTCTTGC TTATTTTTTC TGCAAAATTT TAGGAGACTT GATAAACTAT   
  
  
+ AGTTCTCTTG AATTTACAGG TGGATATTGG AGTTCACAGA ATAGTATTCA GATCAAAGTG CTTGTGCTTA   
  
  
+ ATTGGAAGGA CAACTTGCTT GAGCTGTTTA TAGAGCTCTA ACTGATGCTA TCTCATGGAC TCACATCAGT   
  
  
+ TTTTTGGATT TAATCTTACC AGTGTTGATC CGTCGTACAT TTCCGCTCAG TACAGTCCGC CGTCAGTGAC   
  
  
+ AAATAGGATG TTTGCATCAC TGAAGCTCGA CTCTAGAGGT TCTCCTGTGT CACCCTTCTC AACTCAGTTT   
  
  
+ GATTGTGATA CGGTTACTAC ATTGAGTGAT AGTCAAGAGC ATCACAGCTC GACGGGGAGT CTATCAACAA   
  
  
+ GAAGCCCTTC TTGTAATTCT CCCCTTGAAA CGAGCAGTTA TCATCATTTA TCGACGAATG GCCCGTCTTG   
  
  
+ TAATTCTGCC CCTGAAACTA GCAGTTATCG TCATCGGTTC AACGCGAGTC CCGTGGGATA TTCCCATCAA   
  
  
+ GGCACTGATT ATGGAGTGAA TATGAAGAGC GCTCTGCAGG AGCTAGAGAC TACTCTAATG GGTGCAGATG   
  
  
+ GTGAGGAAGT ATCTGCTGCT AATCAACCTA TGGGGGGAAG TCGTCAGTCC GGGATCCCAA GTCAGAGATC   
  
  
+ AAAATCATTG AGCGAAGATC CACAGGGTTC GCATCCTACT CAGCCTGATT CATCATCTCT TTCTAGGGCA   
  
  
+ AGAAGATCAG GAGATGAAAG CCAGAGAGAG AAACGGCACA AGGCAATGGA AGAACCAACG GAACTACCAA   
  
  
+ GTTTGCCACC TGGTGATTTG AAGCAGTTGC TAATTGAATG TGCGAGGGCT TTATCGGATA ACCGAATAGA   
  
  
+ TGACTTTGAG AGTTTGGTTA AACGGGCGAG GAAAGAGGTC TCTATCTCGG GTGAGCCTAT CCAACGTCTC   
  
  
+ GGTGCCTACA TGATCGAAGG GCTTGTGGCA AGGAAGCAGT CTTCGGGGAC TAGCATCTAT CGGGCTCTGA   
  
  
+ AGTGTAAAGA GCCTCTTGGA AAAGACTTGC TCTCCTACAT GCACATCCTT TATGAAATAT GCCCTTATCT   
  
  
+ CAAGTTTGGT TATATGGCTG CGAATGGAGC GATAGCTGAA GCTTGTAGAA ATGAGGATAA CATACATATT   
  
  
+ ATAGATTTCC AGATTGCACA GGGCACTCAG TGGGTTACTC TATTACAAGC CCTAGCAGCA AGACCTGGTG   
  
  
+ GGCCACCTAA GGTGCGAATT ACAGGCATTG ATGATCCTGT TTCTAAGTAT GCCCGTGGTG CTAGCTTGGA   
  
  
+ GGCTGTTGGG AAACGGTTAG CGTCTCTATC TGAAAAGTTC AAAATACCCG TCGAGTTCAA TGCGTTGCCC   
  
  
+ GTTTATGGAC CCGATGTCAG GCGGGAAATG CTGGATGTGA GGCCCGGGGA GGCTTTGGCC GTTAATTTTC   
  
  
+ CATTGCAGCT CCACCACACT CCTGACGAGA GTGTCGATGT GAACAACCCT AGGGATGGGC TTCTCAGAAT   
  
  
+ GGTGAAATCA CTTGGTCCTA AGGTAACCAC TTTGGTTGAG CAAGAATCAA ACACCAACAC TACCCCTTTC   
  
  
+ TTGACCCGGT TCATAGAGAC CCTTGACTAC TACTCAGCCA TGTTTGAGTC TATAGACGTG ACCATGCCAA   
  
  
+ GAGACCGGAA GGAGAGGATC AATGTTGAGC AGCATTGTTT GGCTAAGGAC ATTGTGAATA TCATAGCTTG   
  
  
+ CGAGGGCAAG GAGAGGGTGG AGCGTCATGA ACTTTTTGGG AAATGGAAGT CAAGGTTTAC CATGGCAGGG   
  
  
+ TTCAGGCAGT ACCCGTTGAG CTCATACGTA AACTCTGTGA TAAGAAGCCT ACTCCGGTGT TACTCCGAGC   
  
  
+ ATTATACTCT GATAGAGAAG GATGGTGCCA TGCTTCTGGG TTGGAAGGGC CGAATGCTGA TTTCAGCTTC   
  
  
+ GGCATGGCAT TG  

- +Up\_Stream \_Len000AGGAGA AAACCCGAAG AAAGAAAGAT AGGACAAAAA AAAACCCAGC TTTTCAACCT   
  
  
- CCAACTTAAC TTCGTAGGCT TAAGCTCGTC CTACAAAGAG ACGCATCACT AAGAAAGACC CAAACACTAA   
  
  
- AACTTAAGAC CCATTAGCCA ACAAAACGAT TAAAACTCCT GTCTCAAGGA AAAGACTTAA TTTTAAAGCA   
  
  
- AAAAAATAGC CCTTTTAGAA GTCATAAACT CTTTTTTCTT GCTTTAAACC TACTAAACGA CAGATCTAAA   
  
  
- ACGAAAGTAT AAGGACCCAC ACCTAACCAA ATAATTAACG TACACTCCTT CATGTTCGAA GACCAGTTAC   
  
  
- CAACCCAAAA AAAAAAAAAA AACACCCATA TTTCAACCTT TAACTAATGC TCAGTTTAAA GAGAAAGATC   
  
  
- TTCTCCCCCC CCCCCCCCCC AACCAAATTT TGAGGTATAA AAGAAACTTA CCTTTCGAAT TAAACGACTG   
  
  
- ATAGAACCAA TCATCGTATT CGAACTAGTC TTTTGTTTTG CGAGAGACGG GACGTTTTTG AAATAAACAG   
  
  
- AAAATTCCTT TTAAAACCTT AAACTCTCAC TTTTAAAACC AACACTTAGA ACAGAATGAT ACACCAAGGA   
  
  
- AGACTGTTTT CAAACTTAGA CTCATACTCT CAAGTAGTTA GAGAAAATGA AAGGATGAAA GAAATAACGA   
  
  
- ACGTTTAACA ACACTTGAAT GAACCTAAAA GACCATCTCC TCTCAAGACA GGACCTTAAA CCACCTTTCA   
  
  
- CTCTGTATGA AACCCTAAAT TAATCGGTAG TTAAACCAAA GTGTGGAATA GTCAAACCTG AAGACCACAC   
  
  
- AATAACATAC ACAAGAATCG AACCCTAATA AGAACATAGC CTTCTCTCCC CCCCCCCCCA CAAAGAAAAT   
  
  
- CGACTTACAA TCTTGTAAAA TTCCCTAGAT CTATAGATCT CTTCTCGGAT CCTTTACTTC GTAAAAAGGG   
  
  
- AATATTAGAC TGTGTTGAAA TACTTAACTA ATCCTTGTTT CTTGCTAATC AAGTCTTTCC AATGTTGAGG   
  
  
- AGATCCACAA TATGATTTCT CTACACTCAA TTATTCGTTA ACGAATGATT GACAAATCCG ACAAAGTAAC   
  
  
- GAACATCTCC GAAAAAAAAA CCCCCCCCCC CCCCCAAACC CCAAGTATTT ACTCTTCCAA CTAAACAACA   
  
  
- GACGAGACGA GAACTAAGCT ATCATTAAGA GAGTCTTTTC CCATTTTACT GTACCGGAAG GTAGCGGAGG   
  
  
- GTACTCCGAA AAACGGAGAC GAACACATAG AAAGTAGAGA ACCAAACCAC CCAACCCCCT CCCCTCCCCC   
  
  
- CCTTCCCCCA AAACTAAGCT ATCTAGTAGT GAAAGTCTTT ACCGGAGAGA CCGGTTACCA TTCCCCATTC   
  
  
- CAATCATGTA CACCTGAAAG GGTACACCTA AAAAGGTCTC AGGCAACACA CTAACAATAA CGACAACAAC   
  
  
- ATTGAAAGAC AGTGAGCCTT TTGTACTTAG GTATACGTTG TTCTTGTATT ATTAACACTT ACGCTGAAAT   
  
  
- AAATCCTTAC AGAATGTATC ATGCACGTGT AAGAAATTAA TAGACAAATA GACAACCAAA CAATAAAAGT   
  
  
- TCCCCTACCC CTCACCCACC TACGAATCAT ACATTAGAAA AATATATCCC TTTTTAGTAT ATCGAACTTT   
  
  
- CATTTTGAAA GTAACGGTCC TAAGGTTCCA TCCCCATCCC AACAGATGTG GAACTAGAGG GATCTTCGTA   
  
  
- CCGCTAGGAA GGAGCCTAAC ACAATATCTT ATTTCTTACA CCAAGACGAC ATTACTCTGA ATACAAGATA   
  
  
- GTTTACTACT TAGACAAATC TTGAAGAACG AATAAAAAAG ACGTTTTAAA ATCCTCTGAA CTATTTGATA   
  
  
- TCAAGAGAAC TTAAATGTCC ACCTATAACC TCAAGTGTCT TATCATAAGT CTAGTTTCAC GAACACGAAT   
  
  
- TAACCTTCCT GTTGAACGAA CTCGACAAAT ATCTCGAGAT TGACTACGAT AGAGTACCTG AGTGTAGTCA   
  
  
- AAAAACCTAA ATTAGAATGG TCACAACTAG GCAGCATGTA AAGGCGAGTC ATGTCAGGCG GCAGTCACTG   
  
  
- TTTATCCTAC AAACGTAGTG ACTTCGAGCT GAGATCTCCA AGAGGACACA GTGGGAAGAG TTGAGTCAAA   
  
  
- CTAACACTAT GCCAATGATG TAACTCACTA TCAGTTCTCG TAGTGTCGAG CTGCCCCTCA GATAGTTGTT   
  
  
- CTTCGGGAAG AACATTAAGA GGGGAACTTT GCTCGTCAAT AGTAGTAAAT AGCTGCTTAC CGGGCAGAAC   
  
  
- ATTAAGACGG GGACTTTGAT CGTCAATAGC AGTAGCCAAG TTGCGCTCAG GGCACCCTAT AAGGGTAGTT   
  
  
- CCGTGACTAA TACCTCACTT ATACTTCTCG CGAGACGTCC TCGATCTCTG ATGAGATTAC CCACGTCTAC   
  
  
- CACTCCTTCA TAGACGACGA TTAGTTGGAT ACCCCCCTTC AGCAGTCAGG CCCTAGGGTT CAGTCTCTAG   
  
  
- TTTTAGTAAC TCGCTTCTAG GTGTCCCAAG CGTAGGATGA GTCGGACTAA GTAGTAGAGA AAGATCCCGT   
  
  
- TCTTCTAGTC CTCTACTTTC GGTCTCTCTC TTTGCCGTGT TCCGTTACCT TCTTGGTTGC CTTGATGGTT   
  
  
- CAAACGGTGG ACCACTAAAC TTCGTCAACG ATTAACTTAC ACGCTCCCGA AATAGCCTAT TGGCTTATCT   
  
  
- ACTGAAACTC TCAAACCAAT TTGCCCGCTC CTTTCTCCAG AGATAGAGCC CACTCGGATA GGTTGCAGAG   
  
  
- CCACGGATGT ACTAGCTTCC CGAACACCGT TCCTTCGTCA GAAGCCCCTG ATCGTAGATA GCCCGAGACT   
  
  
- TCACATTTCT CGGAGAACCT TTTCTGAACG AGAGGATGTA CGTGTAGGAA ATACTTTATA CGGGAATAGA   
  
  
- GTTCAAACCA ATATACCGAC GCTTACCTCG CTATCGACTT CGAACATCTT TACTCCTATT GTATGTATAA   
  
  
- TATCTAAAGG TCTAACGTGT CCCGTGAGTC ACCCAATGAG ATAATGTTCG GGATCGTCGT TCTGGACCAC   
  
  
- CCGGTGGATT CCACGCTTAA TGTCCGTAAC TACTAGGACA AAGATTCATA CGGGCACCAC GATCGAACCT   
  
  
- CCGACAACCC TTTGCCAATC GCAGAGATAG ACTTTTCAAG TTTTATGGGC AGCTCAAGTT ACGCAACGGG   
  
  
- CAAATACCTG GGCTACAGTC CGCCCTTTAC GACCTACACT CCGGGCCCCT CCGAAACCGG CAATTAAAAG   
  
  
- GTAACGTCGA GGTGGTGTGA GGACTGCTCT CACAGCTACA CTTGTTGGGA TCCCTACCCG AAGAGTCTTA   
  
  
- CCACTTTAGT GAACCAGGAT TCCATTGGTG AAACCAACTC GTTCTTAGTT TGTGGTTGTG ATGGGGAAAG   
  
  
- AACTGGGCCA AGTATCTCTG GGAACTGATG ATGAGTCGGT ACAAACTCAG ATATCTGCAC TGGTACGGTT   
  
  
- CTCTGGCCTT CCTCTCCTAG TTACAACTCG TCGTAACAAA CCGATTCCTG TAACACTTAT AGTATCGAAC   
  
  
- GCTCCCGTTC CTCTCCCACC TCGCAGTACT TGAAAAACCC TTTACCTTCA GTTCCAAATG GTACCGTCCC   
  
  
- AAGTCCGTCA TGGGCAACTC GAGTATGCAT TTGAGACACT ATTCTTCGGA TGAGGCCACA ATGAGGCTCG   
  
  
- TAATATGAGA CTATCTCTTC CTACCACGGT ACGAAGACCC AACCTTCCCG GCTTACGACT AAAGTCGAAG   
  
  
- CCGTACCGTA AC

+     AuxRR-core

| Site Name | Organism | Position | Strand | Matrix score. | sequence | function |
| --- | --- | --- | --- | --- | --- | --- |
| AuxRR-core | Nicotiana tabacum | 3229 | - | 7 | GGTCCAT | cis-acting regulatory element involved in auxin responsiveness |

>HU08G00014.1   
+ +Up\_Stream \_Len000TCCTCT TTTGGGCTTC TTTCTTTCTA TCCTGTTTTT TTTTGGGTCG AAAAGTTGGA   
  
  
+ GGTTGAATTG AAGCATCCGA ATTCGAGCAG GATGTTTCTC TGCGTAGTGA TTCTTTCTGG GTTTGTGATT   
  
  
+ TTGAATTCTG GGTAATCGGT TGTTTTGCTA ATTTTGAGGA CAGAGTTCCT TTTCTGAATT AAAATTTCGT   
  
  
+ TTTTTTATCG GGAAAATCTT CAGTATTTGA GAAAAAAGAA CGAAATTTGG ATGATTTGCT GTCTAGATTT   
  
  
+ TGCTTTCATA TTCCTGGGTG TGGATTGGTT TATTAATTGC ATGTGAGGAA GTACAAGCTT CTGGTCAATG   
  
  
+ GTTGGGTTTT TTTTTTTTTT TTGTGGGTAT AAAGTTGGAA ATTGATTACG AGTCAAATTT CTCTTTCTAG   
  
  
+ AAGAGGGGGG GGGGGGGGGG TTGGTTTAAA ACTCCATATT TTCTTTGAAT GGAAAGCTTA ATTTGCTGAC   
  
  
+ TATCTTGGTT AGTAGCATAA GCTTGATCAG AAAACAAAAC GCTCTCTGCC CTGCAAAAAC TTTATTTGTC   
  
  
+ TTTTAAGGAA AATTTTGGAA TTTGAGAGTG AAAATTTTGG TTGTGAATCT TGTCTTACTA TGTGGTTCCT   
  
  
+ TCTGACAAAA GTTTGAATCT GAGTATGAGA GTTCATCAAT CTCTTTTACT TTCCTACTTT CTTTATTGCT   
  
  
+ TGCAAATTGT TGTGAACTTA CTTGGATTTT CTGGTAGAGG AGAGTTCTGT CCTGGAATTT GGTGGAAAGT   
  
  
+ GAGACATACT TTGGGATTTA ATTAGCCATC AATTTGGTTT CACACCTTAT CAGTTTGGAC TTCTGGTGTG   
  
  
+ TTATTGTATG TGTTCTTAGC TTGGGATTAT TCTTGTATCG GAAGAGAGGG GGGGGGGGGT GTTTCTTTTA   
  
  
+ GCTGAATGTT AGAACATTTT AAGGGATCTA GATATCTAGA GAAGAGCCTA GGAAATGAAG CATTTTTCCC   
  
  
+ TTATAATCTG ACACAACTTT ATGAATTGAT TAGGAACAAA GAACGATTAG TTCAGAAAGG TTACAACTCC   
  
  
+ TCTAGGTGTT ATACTAAAGA GATGTGAGTT AATAAGCAAT TGCTTACTAA CTGTTTAGGC TGTTTCATTG   
  
  
+ CTTGTAGAGG CTTTTTTTTT GGGGGGGGGG GGGGGTTTGG GGTTCATAAA TGAGAAGGTT GATTTGTTGT   
  
  
+ CTGCTCTGCT CTTGATTCGA TAGTAATTCT CTCAGAAAAG GGTAAAATGA CATGGCCTTC CATCGCCTCC   
  
  
+ CATGAGGCTT TTTGCCTCTG CTTGTGTATC TTTCATCTCT TGGTTTGGTG GGTTGGGGGA GGGGAGGGGG   
  
  
+ GGAAGGGGGT TTTGATTCGA TAGATCATCA CTTTCAGAAA TGGCCTCTCT GGCCAATGGT AAGGGGTAAG   
  
  
+ GTTAGTACAT GTGGACTTTC CCATGTGGAT TTTTCCAGAG TCCGTTGTGT GATTGTTATT GCTGTTGTTG   
  
  
+ TAACTTTCTG TCACTCGGAA AACATGAATC CATATGCAAC AAGAACATAA TAATTGTGAA TGCGACTTTA   
  
  
+ TTTAGGAATG TCTTACATAG TACGTGCACA TTCTTTAATT ATCTGTTTAT CTGTTGGTTT GTTATTTTCA   
  
  
+ AGGGGATGGG GAGTGGGTGG ATGCTTAGTA TGTAATCTTT TTATATAGGG AAAAATCATA TAGCTTGAAA   
  
  
+ GTAAAACTTT CATTGCCAGG ATTCCAAGGT AGGGGTAGGG TTGTCTACAC CTTGATCTCC CTAGAAGCAT   
  
  
+ GGCGATCCTT CCTCGGATTG TGTTATAGAA TAAAGAATGT GGTTCTGCTG TAATGAGACT TATGTTCTAT   
  
  
+ CAAATGATGA ATCTGTTTAG AACTTCTTGC TTATTTTTTC TGCAAAATTT TAGGAGACTT GATAAACTAT   
  
  
+ AGTTCTCTTG AATTTACAGG TGGATATTGG AGTTCACAGA ATAGTATTCA GATCAAAGTG CTTGTGCTTA   
  
  
+ ATTGGAAGGA CAACTTGCTT GAGCTGTTTA TAGAGCTCTA ACTGATGCTA TCTCATGGAC TCACATCAGT   
  
  
+ TTTTTGGATT TAATCTTACC AGTGTTGATC CGTCGTACAT TTCCGCTCAG TACAGTCCGC CGTCAGTGAC   
  
  
+ AAATAGGATG TTTGCATCAC TGAAGCTCGA CTCTAGAGGT TCTCCTGTGT CACCCTTCTC AACTCAGTTT   
  
  
+ GATTGTGATA CGGTTACTAC ATTGAGTGAT AGTCAAGAGC ATCACAGCTC GACGGGGAGT CTATCAACAA   
  
  
+ GAAGCCCTTC TTGTAATTCT CCCCTTGAAA CGAGCAGTTA TCATCATTTA TCGACGAATG GCCCGTCTTG   
  
  
+ TAATTCTGCC CCTGAAACTA GCAGTTATCG TCATCGGTTC AACGCGAGTC CCGTGGGATA TTCCCATCAA   
  
  
+ GGCACTGATT ATGGAGTGAA TATGAAGAGC GCTCTGCAGG AGCTAGAGAC TACTCTAATG GGTGCAGATG   
  
  
+ GTGAGGAAGT ATCTGCTGCT AATCAACCTA TGGGGGGAAG TCGTCAGTCC GGGATCCCAA GTCAGAGATC   
  
  
+ AAAATCATTG AGCGAAGATC CACAGGGTTC GCATCCTACT CAGCCTGATT CATCATCTCT TTCTAGGGCA   
  
  
+ AGAAGATCAG GAGATGAAAG CCAGAGAGAG AAACGGCACA AGGCAATGGA AGAACCAACG GAACTACCAA   
  
  
+ GTTTGCCACC TGGTGATTTG AAGCAGTTGC TAATTGAATG TGCGAGGGCT TTATCGGATA ACCGAATAGA   
  
  
+ TGACTTTGAG AGTTTGGTTA AACGGGCGAG GAAAGAGGTC TCTATCTCGG GTGAGCCTAT CCAACGTCTC   
  
  
+ GGTGCCTACA TGATCGAAGG GCTTGTGGCA AGGAAGCAGT CTTCGGGGAC TAGCATCTAT CGGGCTCTGA   
  
  
+ AGTGTAAAGA GCCTCTTGGA AAAGACTTGC TCTCCTACAT GCACATCCTT TATGAAATAT GCCCTTATCT   
  
  
+ CAAGTTTGGT TATATGGCTG CGAATGGAGC GATAGCTGAA GCTTGTAGAA ATGAGGATAA CATACATATT   
  
  
+ ATAGATTTCC AGATTGCACA GGGCACTCAG TGGGTTACTC TATTACAAGC CCTAGCAGCA AGACCTGGTG   
  
  
+ GGCCACCTAA GGTGCGAATT ACAGGCATTG ATGATCCTGT TTCTAAGTAT GCCCGTGGTG CTAGCTTGGA   
  
  
+ GGCTGTTGGG AAACGGTTAG CGTCTCTATC TGAAAAGTTC AAAATACCCG TCGAGTTCAA TGCGTTGCCC   
  
  
+ GTTTATGGAC CCGATGTCAG GCGGGAAATG CTGGATGTGA GGCCCGGGGA GGCTTTGGCC GTTAATTTTC   
  
  
+ CATTGCAGCT CCACCACACT CCTGACGAGA GTGTCGATGT GAACAACCCT AGGGATGGGC TTCTCAGAAT   
  
  
+ GGTGAAATCA CTTGGTCCTA AGGTAACCAC TTTGGTTGAG CAAGAATCAA ACACCAACAC TACCCCTTTC   
  
  
+ TTGACCCGGT TCATAGAGAC CCTTGACTAC TACTCAGCCA TGTTTGAGTC TATAGACGTG ACCATGCCAA   
  
  
+ GAGACCGGAA GGAGAGGATC AATGTTGAGC AGCATTGTTT GGCTAAGGAC ATTGTGAATA TCATAGCTTG   
  
  
+ CGAGGGCAAG GAGAGGGTGG AGCGTCATGA ACTTTTTGGG AAATGGAAGT CAAGGTTTAC CATGGCAGGG   
  
  
+ TTCAGGCAGT ACCCGTTGAG CTCATACGTA AACTCTGTGA TAAGAAGCCT ACTCCGGTGT TACTCCGAGC   
  
  
+ ATTATACTCT GATAGAGAAG GATGGTGCCA TGCTTCTGGG TTGGAAGGGC CGAATGCTGA TTTCAGCTTC   
  
  
+ GGCATGGCAT TG  

- +Up\_Stream \_Len000AGGAGA AAACCCGAAG AAAGAAAGAT AGGACAAAAA AAAACCCAGC TTTTCAACCT   
  
  
- CCAACTTAAC TTCGTAGGCT TAAGCTCGTC CTACAAAGAG ACGCATCACT AAGAAAGACC CAAACACTAA   
  
  
- AACTTAAGAC CCATTAGCCA ACAAAACGAT TAAAACTCCT GTCTCAAGGA AAAGACTTAA TTTTAAAGCA   
  
  
- AAAAAATAGC CCTTTTAGAA GTCATAAACT CTTTTTTCTT GCTTTAAACC TACTAAACGA CAGATCTAAA   
  
  
- ACGAAAGTAT AAGGACCCAC ACCTAACCAA ATAATTAACG TACACTCCTT CATGTTCGAA GACCAGTTAC   
  
  
- CAACCCAAAA AAAAAAAAAA AACACCCATA TTTCAACCTT TAACTAATGC TCAGTTTAAA GAGAAAGATC   
  
  
- TTCTCCCCCC CCCCCCCCCC AACCAAATTT TGAGGTATAA AAGAAACTTA CCTTTCGAAT TAAACGACTG   
  
  
- ATAGAACCAA TCATCGTATT CGAACTAGTC TTTTGTTTTG CGAGAGACGG GACGTTTTTG AAATAAACAG   
  
  
- AAAATTCCTT TTAAAACCTT AAACTCTCAC TTTTAAAACC AACACTTAGA ACAGAATGAT ACACCAAGGA   
  
  
- AGACTGTTTT CAAACTTAGA CTCATACTCT CAAGTAGTTA GAGAAAATGA AAGGATGAAA GAAATAACGA   
  
  
- ACGTTTAACA ACACTTGAAT GAACCTAAAA GACCATCTCC TCTCAAGACA GGACCTTAAA CCACCTTTCA   
  
  
- CTCTGTATGA AACCCTAAAT TAATCGGTAG TTAAACCAAA GTGTGGAATA GTCAAACCTG AAGACCACAC   
  
  
- AATAACATAC ACAAGAATCG AACCCTAATA AGAACATAGC CTTCTCTCCC CCCCCCCCCA CAAAGAAAAT   
  
  
- CGACTTACAA TCTTGTAAAA TTCCCTAGAT CTATAGATCT CTTCTCGGAT CCTTTACTTC GTAAAAAGGG   
  
  
- AATATTAGAC TGTGTTGAAA TACTTAACTA ATCCTTGTTT CTTGCTAATC AAGTCTTTCC AATGTTGAGG   
  
  
- AGATCCACAA TATGATTTCT CTACACTCAA TTATTCGTTA ACGAATGATT GACAAATCCG ACAAAGTAAC   
  
  
- GAACATCTCC GAAAAAAAAA CCCCCCCCCC CCCCCAAACC CCAAGTATTT ACTCTTCCAA CTAAACAACA   
  
  
- GACGAGACGA GAACTAAGCT ATCATTAAGA GAGTCTTTTC CCATTTTACT GTACCGGAAG GTAGCGGAGG   
  
  
- GTACTCCGAA AAACGGAGAC GAACACATAG AAAGTAGAGA ACCAAACCAC CCAACCCCCT CCCCTCCCCC   
  
  
- CCTTCCCCCA AAACTAAGCT ATCTAGTAGT GAAAGTCTTT ACCGGAGAGA CCGGTTACCA TTCCCCATTC   
  
  
- CAATCATGTA CACCTGAAAG GGTACACCTA AAAAGGTCTC AGGCAACACA CTAACAATAA CGACAACAAC   
  
  
- ATTGAAAGAC AGTGAGCCTT TTGTACTTAG GTATACGTTG TTCTTGTATT ATTAACACTT ACGCTGAAAT   
  
  
- AAATCCTTAC AGAATGTATC ATGCACGTGT AAGAAATTAA TAGACAAATA GACAACCAAA CAATAAAAGT   
  
  
- TCCCCTACCC CTCACCCACC TACGAATCAT ACATTAGAAA AATATATCCC TTTTTAGTAT ATCGAACTTT   
  
  
- CATTTTGAAA GTAACGGTCC TAAGGTTCCA TCCCCATCCC AACAGATGTG GAACTAGAGG GATCTTCGTA   
  
  
- CCGCTAGGAA GGAGCCTAAC ACAATATCTT ATTTCTTACA CCAAGACGAC ATTACTCTGA ATACAAGATA   
  
  
- GTTTACTACT TAGACAAATC TTGAAGAACG AATAAAAAAG ACGTTTTAAA ATCCTCTGAA CTATTTGATA   
  
  
- TCAAGAGAAC TTAAATGTCC ACCTATAACC TCAAGTGTCT TATCATAAGT CTAGTTTCAC GAACACGAAT   
  
  
- TAACCTTCCT GTTGAACGAA CTCGACAAAT ATCTCGAGAT TGACTACGAT AGAGTACCTG AGTGTAGTCA   
  
  
- AAAAACCTAA ATTAGAATGG TCACAACTAG GCAGCATGTA AAGGCGAGTC ATGTCAGGCG GCAGTCACTG   
  
  
- TTTATCCTAC AAACGTAGTG ACTTCGAGCT GAGATCTCCA AGAGGACACA GTGGGAAGAG TTGAGTCAAA   
  
  
- CTAACACTAT GCCAATGATG TAACTCACTA TCAGTTCTCG TAGTGTCGAG CTGCCCCTCA GATAGTTGTT   
  
  
- CTTCGGGAAG AACATTAAGA GGGGAACTTT GCTCGTCAAT AGTAGTAAAT AGCTGCTTAC CGGGCAGAAC   
  
  
- ATTAAGACGG GGACTTTGAT CGTCAATAGC AGTAGCCAAG TTGCGCTCAG GGCACCCTAT AAGGGTAGTT   
  
  
- CCGTGACTAA TACCTCACTT ATACTTCTCG CGAGACGTCC TCGATCTCTG ATGAGATTAC CCACGTCTAC   
  
  
- CACTCCTTCA TAGACGACGA TTAGTTGGAT ACCCCCCTTC AGCAGTCAGG CCCTAGGGTT CAGTCTCTAG   
  
  
- TTTTAGTAAC TCGCTTCTAG GTGTCCCAAG CGTAGGATGA GTCGGACTAA GTAGTAGAGA AAGATCCCGT   
  
  
- TCTTCTAGTC CTCTACTTTC GGTCTCTCTC TTTGCCGTGT TCCGTTACCT TCTTGGTTGC CTTGATGGTT   
  
  
- CAAACGGTGG ACCACTAAAC TTCGTCAACG ATTAACTTAC ACGCTCCCGA AATAGCCTAT TGGCTTATCT   
  
  
- ACTGAAACTC TCAAACCAAT TTGCCCGCTC CTTTCTCCAG AGATAGAGCC CACTCGGATA GGTTGCAGAG   
  
  
- CCACGGATGT ACTAGCTTCC CGAACACCGT TCCTTCGTCA GAAGCCCCTG ATCGTAGATA GCCCGAGACT   
  
  
- TCACATTTCT CGGAGAACCT TTTCTGAACG AGAGGATGTA CGTGTAGGAA ATACTTTATA CGGGAATAGA   
  
  
- GTTCAAACCA ATATACCGAC GCTTACCTCG CTATCGACTT CGAACATCTT TACTCCTATT GTATGTATAA   
  
  
- TATCTAAAGG TCTAACGTGT CCCGTGAGTC ACCCAATGAG ATAATGTTCG GGATCGTCGT TCTGGACCAC   
  
  
- CCGGTGGATT CCACGCTTAA TGTCCGTAAC TACTAGGACA AAGATTCATA CGGGCACCAC GATCGAACCT   
  
  
- CCGACAACCC TTTGCCAATC GCAGAGATAG ACTTTTCAAG TTTTATGGGC AGCTCAAGTT ACGCAACGGG   
  
  
- CAAATACCTG GGCTACAGTC CGCCCTTTAC GACCTACACT CCGGGCCCCT CCGAAACCGG CAATTAAAAG   
  
  
- GTAACGTCGA GGTGGTGTGA GGACTGCTCT CACAGCTACA CTTGTTGGGA TCCCTACCCG AAGAGTCTTA   
  
  
- CCACTTTAGT GAACCAGGAT TCCATTGGTG AAACCAACTC GTTCTTAGTT TGTGGTTGTG ATGGGGAAAG   
  
  
- AACTGGGCCA AGTATCTCTG GGAACTGATG ATGAGTCGGT ACAAACTCAG ATATCTGCAC TGGTACGGTT   
  
  
- CTCTGGCCTT CCTCTCCTAG TTACAACTCG TCGTAACAAA CCGATTCCTG TAACACTTAT AGTATCGAAC   
  
  
- GCTCCCGTTC CTCTCCCACC TCGCAGTACT TGAAAAACCC TTTACCTTCA GTTCCAAATG GTACCGTCCC   
  
  
- AAGTCCGTCA TGGGCAACTC GAGTATGCAT TTGAGACACT ATTCTTCGGA TGAGGCCACA ATGAGGCTCG   
  
  
- TAATATGAGA CTATCTCTTC CTACCACGGT ACGAAGACCC AACCTTCCCG GCTTACGACT AAAGTCGAAG   
  
  
- CCGTACCGTA AC

+     Box 4

| Site Name | Organism | Position | Strand | Matrix score. | sequence | function |
| --- | --- | --- | --- | --- | --- | --- |
| Box 4 | Petroselinum crispum | 316 | + | 6 | ATTAAT | part of a conserved DNA module involved in light responsiveness |

>HU08G00014.1   
+ +Up\_Stream \_Len000TCCTCT TTTGGGCTTC TTTCTTTCTA TCCTGTTTTT TTTTGGGTCG AAAAGTTGGA   
  
  
+ GGTTGAATTG AAGCATCCGA ATTCGAGCAG GATGTTTCTC TGCGTAGTGA TTCTTTCTGG GTTTGTGATT   
  
  
+ TTGAATTCTG GGTAATCGGT TGTTTTGCTA ATTTTGAGGA CAGAGTTCCT TTTCTGAATT AAAATTTCGT   
  
  
+ TTTTTTATCG GGAAAATCTT CAGTATTTGA GAAAAAAGAA CGAAATTTGG ATGATTTGCT GTCTAGATTT   
  
  
+ TGCTTTCATA TTCCTGGGTG TGGATTGGTT TATTAATTGC ATGTGAGGAA GTACAAGCTT CTGGTCAATG   
  
  
+ GTTGGGTTTT TTTTTTTTTT TTGTGGGTAT AAAGTTGGAA ATTGATTACG AGTCAAATTT CTCTTTCTAG   
  
  
+ AAGAGGGGGG GGGGGGGGGG TTGGTTTAAA ACTCCATATT TTCTTTGAAT GGAAAGCTTA ATTTGCTGAC   
  
  
+ TATCTTGGTT AGTAGCATAA GCTTGATCAG AAAACAAAAC GCTCTCTGCC CTGCAAAAAC TTTATTTGTC   
  
  
+ TTTTAAGGAA AATTTTGGAA TTTGAGAGTG AAAATTTTGG TTGTGAATCT TGTCTTACTA TGTGGTTCCT   
  
  
+ TCTGACAAAA GTTTGAATCT GAGTATGAGA GTTCATCAAT CTCTTTTACT TTCCTACTTT CTTTATTGCT   
  
  
+ TGCAAATTGT TGTGAACTTA CTTGGATTTT CTGGTAGAGG AGAGTTCTGT CCTGGAATTT GGTGGAAAGT   
  
  
+ GAGACATACT TTGGGATTTA ATTAGCCATC AATTTGGTTT CACACCTTAT CAGTTTGGAC TTCTGGTGTG   
  
  
+ TTATTGTATG TGTTCTTAGC TTGGGATTAT TCTTGTATCG GAAGAGAGGG GGGGGGGGGT GTTTCTTTTA   
  
  
+ GCTGAATGTT AGAACATTTT AAGGGATCTA GATATCTAGA GAAGAGCCTA GGAAATGAAG CATTTTTCCC   
  
  
+ TTATAATCTG ACACAACTTT ATGAATTGAT TAGGAACAAA GAACGATTAG TTCAGAAAGG TTACAACTCC   
  
  
+ TCTAGGTGTT ATACTAAAGA GATGTGAGTT AATAAGCAAT TGCTTACTAA CTGTTTAGGC TGTTTCATTG   
  
  
+ CTTGTAGAGG CTTTTTTTTT GGGGGGGGGG GGGGGTTTGG GGTTCATAAA TGAGAAGGTT GATTTGTTGT   
  
  
+ CTGCTCTGCT CTTGATTCGA TAGTAATTCT CTCAGAAAAG GGTAAAATGA CATGGCCTTC CATCGCCTCC   
  
  
+ CATGAGGCTT TTTGCCTCTG CTTGTGTATC TTTCATCTCT TGGTTTGGTG GGTTGGGGGA GGGGAGGGGG   
  
  
+ GGAAGGGGGT TTTGATTCGA TAGATCATCA CTTTCAGAAA TGGCCTCTCT GGCCAATGGT AAGGGGTAAG   
  
  
+ GTTAGTACAT GTGGACTTTC CCATGTGGAT TTTTCCAGAG TCCGTTGTGT GATTGTTATT GCTGTTGTTG   
  
  
+ TAACTTTCTG TCACTCGGAA AACATGAATC CATATGCAAC AAGAACATAA TAATTGTGAA TGCGACTTTA   
  
  
+ TTTAGGAATG TCTTACATAG TACGTGCACA TTCTTTAATT ATCTGTTTAT CTGTTGGTTT GTTATTTTCA   
  
  
+ AGGGGATGGG GAGTGGGTGG ATGCTTAGTA TGTAATCTTT TTATATAGGG AAAAATCATA TAGCTTGAAA   
  
  
+ GTAAAACTTT CATTGCCAGG ATTCCAAGGT AGGGGTAGGG TTGTCTACAC CTTGATCTCC CTAGAAGCAT   
  
  
+ GGCGATCCTT CCTCGGATTG TGTTATAGAA TAAAGAATGT GGTTCTGCTG TAATGAGACT TATGTTCTAT   
  
  
+ CAAATGATGA ATCTGTTTAG AACTTCTTGC TTATTTTTTC TGCAAAATTT TAGGAGACTT GATAAACTAT   
  
  
+ AGTTCTCTTG AATTTACAGG TGGATATTGG AGTTCACAGA ATAGTATTCA GATCAAAGTG CTTGTGCTTA   
  
  
+ ATTGGAAGGA CAACTTGCTT GAGCTGTTTA TAGAGCTCTA ACTGATGCTA TCTCATGGAC TCACATCAGT   
  
  
+ TTTTTGGATT TAATCTTACC AGTGTTGATC CGTCGTACAT TTCCGCTCAG TACAGTCCGC CGTCAGTGAC   
  
  
+ AAATAGGATG TTTGCATCAC TGAAGCTCGA CTCTAGAGGT TCTCCTGTGT CACCCTTCTC AACTCAGTTT   
  
  
+ GATTGTGATA CGGTTACTAC ATTGAGTGAT AGTCAAGAGC ATCACAGCTC GACGGGGAGT CTATCAACAA   
  
  
+ GAAGCCCTTC TTGTAATTCT CCCCTTGAAA CGAGCAGTTA TCATCATTTA TCGACGAATG GCCCGTCTTG   
  
  
+ TAATTCTGCC CCTGAAACTA GCAGTTATCG TCATCGGTTC AACGCGAGTC CCGTGGGATA TTCCCATCAA   
  
  
+ GGCACTGATT ATGGAGTGAA TATGAAGAGC GCTCTGCAGG AGCTAGAGAC TACTCTAATG GGTGCAGATG   
  
  
+ GTGAGGAAGT ATCTGCTGCT AATCAACCTA TGGGGGGAAG TCGTCAGTCC GGGATCCCAA GTCAGAGATC   
  
  
+ AAAATCATTG AGCGAAGATC CACAGGGTTC GCATCCTACT CAGCCTGATT CATCATCTCT TTCTAGGGCA   
  
  
+ AGAAGATCAG GAGATGAAAG CCAGAGAGAG AAACGGCACA AGGCAATGGA AGAACCAACG GAACTACCAA   
  
  
+ GTTTGCCACC TGGTGATTTG AAGCAGTTGC TAATTGAATG TGCGAGGGCT TTATCGGATA ACCGAATAGA   
  
  
+ TGACTTTGAG AGTTTGGTTA AACGGGCGAG GAAAGAGGTC TCTATCTCGG GTGAGCCTAT CCAACGTCTC   
  
  
+ GGTGCCTACA TGATCGAAGG GCTTGTGGCA AGGAAGCAGT CTTCGGGGAC TAGCATCTAT CGGGCTCTGA   
  
  
+ AGTGTAAAGA GCCTCTTGGA AAAGACTTGC TCTCCTACAT GCACATCCTT TATGAAATAT GCCCTTATCT   
  
  
+ CAAGTTTGGT TATATGGCTG CGAATGGAGC GATAGCTGAA GCTTGTAGAA ATGAGGATAA CATACATATT   
  
  
+ ATAGATTTCC AGATTGCACA GGGCACTCAG TGGGTTACTC TATTACAAGC CCTAGCAGCA AGACCTGGTG   
  
  
+ GGCCACCTAA GGTGCGAATT ACAGGCATTG ATGATCCTGT TTCTAAGTAT GCCCGTGGTG CTAGCTTGGA   
  
  
+ GGCTGTTGGG AAACGGTTAG CGTCTCTATC TGAAAAGTTC AAAATACCCG TCGAGTTCAA TGCGTTGCCC   
  
  
+ GTTTATGGAC CCGATGTCAG GCGGGAAATG CTGGATGTGA GGCCCGGGGA GGCTTTGGCC GTTAATTTTC   
  
  
+ CATTGCAGCT CCACCACACT CCTGACGAGA GTGTCGATGT GAACAACCCT AGGGATGGGC TTCTCAGAAT   
  
  
+ GGTGAAATCA CTTGGTCCTA AGGTAACCAC TTTGGTTGAG CAAGAATCAA ACACCAACAC TACCCCTTTC   
  
  
+ TTGACCCGGT TCATAGAGAC CCTTGACTAC TACTCAGCCA TGTTTGAGTC TATAGACGTG ACCATGCCAA   
  
  
+ GAGACCGGAA GGAGAGGATC AATGTTGAGC AGCATTGTTT GGCTAAGGAC ATTGTGAATA TCATAGCTTG   
  
  
+ CGAGGGCAAG GAGAGGGTGG AGCGTCATGA ACTTTTTGGG AAATGGAAGT CAAGGTTTAC CATGGCAGGG   
  
  
+ TTCAGGCAGT ACCCGTTGAG CTCATACGTA AACTCTGTGA TAAGAAGCCT ACTCCGGTGT TACTCCGAGC   
  
  
+ ATTATACTCT GATAGAGAAG GATGGTGCCA TGCTTCTGGG TTGGAAGGGC CGAATGCTGA TTTCAGCTTC   
  
  
+ GGCATGGCAT TG  

- +Up\_Stream \_Len000AGGAGA AAACCCGAAG AAAGAAAGAT AGGACAAAAA AAAACCCAGC TTTTCAACCT   
  
  
- CCAACTTAAC TTCGTAGGCT TAAGCTCGTC CTACAAAGAG ACGCATCACT AAGAAAGACC CAAACACTAA   
  
  
- AACTTAAGAC CCATTAGCCA ACAAAACGAT TAAAACTCCT GTCTCAAGGA AAAGACTTAA TTTTAAAGCA   
  
  
- AAAAAATAGC CCTTTTAGAA GTCATAAACT CTTTTTTCTT GCTTTAAACC TACTAAACGA CAGATCTAAA   
  
  
- ACGAAAGTAT AAGGACCCAC ACCTAACCAA ATAATTAACG TACACTCCTT CATGTTCGAA GACCAGTTAC   
  
  
- CAACCCAAAA AAAAAAAAAA AACACCCATA TTTCAACCTT TAACTAATGC TCAGTTTAAA GAGAAAGATC   
  
  
- TTCTCCCCCC CCCCCCCCCC AACCAAATTT TGAGGTATAA AAGAAACTTA CCTTTCGAAT TAAACGACTG   
  
  
- ATAGAACCAA TCATCGTATT CGAACTAGTC TTTTGTTTTG CGAGAGACGG GACGTTTTTG AAATAAACAG   
  
  
- AAAATTCCTT TTAAAACCTT AAACTCTCAC TTTTAAAACC AACACTTAGA ACAGAATGAT ACACCAAGGA   
  
  
- AGACTGTTTT CAAACTTAGA CTCATACTCT CAAGTAGTTA GAGAAAATGA AAGGATGAAA GAAATAACGA   
  
  
- ACGTTTAACA ACACTTGAAT GAACCTAAAA GACCATCTCC TCTCAAGACA GGACCTTAAA CCACCTTTCA   
  
  
- CTCTGTATGA AACCCTAAAT TAATCGGTAG TTAAACCAAA GTGTGGAATA GTCAAACCTG AAGACCACAC   
  
  
- AATAACATAC ACAAGAATCG AACCCTAATA AGAACATAGC CTTCTCTCCC CCCCCCCCCA CAAAGAAAAT   
  
  
- CGACTTACAA TCTTGTAAAA TTCCCTAGAT CTATAGATCT CTTCTCGGAT CCTTTACTTC GTAAAAAGGG   
  
  
- AATATTAGAC TGTGTTGAAA TACTTAACTA ATCCTTGTTT CTTGCTAATC AAGTCTTTCC AATGTTGAGG   
  
  
- AGATCCACAA TATGATTTCT CTACACTCAA TTATTCGTTA ACGAATGATT GACAAATCCG ACAAAGTAAC   
  
  
- GAACATCTCC GAAAAAAAAA CCCCCCCCCC CCCCCAAACC CCAAGTATTT ACTCTTCCAA CTAAACAACA   
  
  
- GACGAGACGA GAACTAAGCT ATCATTAAGA GAGTCTTTTC CCATTTTACT GTACCGGAAG GTAGCGGAGG   
  
  
- GTACTCCGAA AAACGGAGAC GAACACATAG AAAGTAGAGA ACCAAACCAC CCAACCCCCT CCCCTCCCCC   
  
  
- CCTTCCCCCA AAACTAAGCT ATCTAGTAGT GAAAGTCTTT ACCGGAGAGA CCGGTTACCA TTCCCCATTC   
  
  
- CAATCATGTA CACCTGAAAG GGTACACCTA AAAAGGTCTC AGGCAACACA CTAACAATAA CGACAACAAC   
  
  
- ATTGAAAGAC AGTGAGCCTT TTGTACTTAG GTATACGTTG TTCTTGTATT ATTAACACTT ACGCTGAAAT   
  
  
- AAATCCTTAC AGAATGTATC ATGCACGTGT AAGAAATTAA TAGACAAATA GACAACCAAA CAATAAAAGT   
  
  
- TCCCCTACCC CTCACCCACC TACGAATCAT ACATTAGAAA AATATATCCC TTTTTAGTAT ATCGAACTTT   
  
  
- CATTTTGAAA GTAACGGTCC TAAGGTTCCA TCCCCATCCC AACAGATGTG GAACTAGAGG GATCTTCGTA   
  
  
- CCGCTAGGAA GGAGCCTAAC ACAATATCTT ATTTCTTACA CCAAGACGAC ATTACTCTGA ATACAAGATA   
  
  
- GTTTACTACT TAGACAAATC TTGAAGAACG AATAAAAAAG ACGTTTTAAA ATCCTCTGAA CTATTTGATA   
  
  
- TCAAGAGAAC TTAAATGTCC ACCTATAACC TCAAGTGTCT TATCATAAGT CTAGTTTCAC GAACACGAAT   
  
  
- TAACCTTCCT GTTGAACGAA CTCGACAAAT ATCTCGAGAT TGACTACGAT AGAGTACCTG AGTGTAGTCA   
  
  
- AAAAACCTAA ATTAGAATGG TCACAACTAG GCAGCATGTA AAGGCGAGTC ATGTCAGGCG GCAGTCACTG   
  
  
- TTTATCCTAC AAACGTAGTG ACTTCGAGCT GAGATCTCCA AGAGGACACA GTGGGAAGAG TTGAGTCAAA   
  
  
- CTAACACTAT GCCAATGATG TAACTCACTA TCAGTTCTCG TAGTGTCGAG CTGCCCCTCA GATAGTTGTT   
  
  
- CTTCGGGAAG AACATTAAGA GGGGAACTTT GCTCGTCAAT AGTAGTAAAT AGCTGCTTAC CGGGCAGAAC   
  
  
- ATTAAGACGG GGACTTTGAT CGTCAATAGC AGTAGCCAAG TTGCGCTCAG GGCACCCTAT AAGGGTAGTT   
  
  
- CCGTGACTAA TACCTCACTT ATACTTCTCG CGAGACGTCC TCGATCTCTG ATGAGATTAC CCACGTCTAC   
  
  
- CACTCCTTCA TAGACGACGA TTAGTTGGAT ACCCCCCTTC AGCAGTCAGG CCCTAGGGTT CAGTCTCTAG   
  
  
- TTTTAGTAAC TCGCTTCTAG GTGTCCCAAG CGTAGGATGA GTCGGACTAA GTAGTAGAGA AAGATCCCGT   
  
  
- TCTTCTAGTC CTCTACTTTC GGTCTCTCTC TTTGCCGTGT TCCGTTACCT TCTTGGTTGC CTTGATGGTT   
  
  
- CAAACGGTGG ACCACTAAAC TTCGTCAACG ATTAACTTAC ACGCTCCCGA AATAGCCTAT TGGCTTATCT   
  
  
- ACTGAAACTC TCAAACCAAT TTGCCCGCTC CTTTCTCCAG AGATAGAGCC CACTCGGATA GGTTGCAGAG   
  
  
- CCACGGATGT ACTAGCTTCC CGAACACCGT TCCTTCGTCA GAAGCCCCTG ATCGTAGATA GCCCGAGACT   
  
  
- TCACATTTCT CGGAGAACCT TTTCTGAACG AGAGGATGTA CGTGTAGGAA ATACTTTATA CGGGAATAGA   
  
  
- GTTCAAACCA ATATACCGAC GCTTACCTCG CTATCGACTT CGAACATCTT TACTCCTATT GTATGTATAA   
  
  
- TATCTAAAGG TCTAACGTGT CCCGTGAGTC ACCCAATGAG ATAATGTTCG GGATCGTCGT TCTGGACCAC   
  
  
- CCGGTGGATT CCACGCTTAA TGTCCGTAAC TACTAGGACA AAGATTCATA CGGGCACCAC GATCGAACCT   
  
  
- CCGACAACCC TTTGCCAATC GCAGAGATAG ACTTTTCAAG TTTTATGGGC AGCTCAAGTT ACGCAACGGG   
  
  
- CAAATACCTG GGCTACAGTC CGCCCTTTAC GACCTACACT CCGGGCCCCT CCGAAACCGG CAATTAAAAG   
  
  
- GTAACGTCGA GGTGGTGTGA GGACTGCTCT CACAGCTACA CTTGTTGGGA TCCCTACCCG AAGAGTCTTA   
  
  
- CCACTTTAGT GAACCAGGAT TCCATTGGTG AAACCAACTC GTTCTTAGTT TGTGGTTGTG ATGGGGAAAG   
  
  
- AACTGGGCCA AGTATCTCTG GGAACTGATG ATGAGTCGGT ACAAACTCAG ATATCTGCAC TGGTACGGTT   
  
  
- CTCTGGCCTT CCTCTCCTAG TTACAACTCG TCGTAACAAA CCGATTCCTG TAACACTTAT AGTATCGAAC   
  
  
- GCTCCCGTTC CTCTCCCACC TCGCAGTACT TGAAAAACCC TTTACCTTCA GTTCCAAATG GTACCGTCCC   
  
  
- AAGTCCGTCA TGGGCAACTC GAGTATGCAT TTGAGACACT ATTCTTCGGA TGAGGCCACA ATGAGGCTCG   
  
  
- TAATATGAGA CTATCTCTTC CTACCACGGT ACGAAGACCC AACCTTCCCG GCTTACGACT AAAGTCGAAG   
  
  
- CCGTACCGTA AC

+     CAAT-box

| Site Name | Organism | Position | Strand | Matrix score. | sequence | function |
| --- | --- | --- | --- | --- | --- | --- |
| CAAT-box | Nicotiana glutinosa | 3296 | - | 4 | CAAT |  |
| CAAT-box | Nicotiana glutinosa | 3111 | - | 4 | CAAT |  |
| CAAT-box | Nicotiana glutinosa | 3212 | + | 4 | CAAT |  |
| CAAT-box | Nicotiana glutinosa | 3793 | - | 4 | CAAT |  |
| CAAT-box | Pisum sativum | 1186 | - | 5 | CAAAT | common cis-acting element in promoter and enhancer regions |
| CAAT-box | Nicotiana glutinosa | 1527 | - | 4 | CAAT |  |
| CAAT-box | Nicotiana glutinosa | 1009 | - | 4 | CAAT |  |
| CAAT-box | Nicotiana glutinosa | 1091 | + | 4 | CAAT |  |
| CAAT-box | Nicotiana glutinosa | 847 | - | 4 | CAAT |  |
| CAAT-box | Pisum sativum | 408 | + | 5 | CAAAT | common cis-acting element in promoter and enhancer regions |
| CAAT-box | Pisum sativum | 1825 | + | 5 | CAAAT | common cis-acting element in promoter and enhancer regions |
| CAAT-box | Nicotiana glutinosa | 350 | + | 4 | CAAT |  |
| CAAT-box | Nicotiana glutinosa | 3027 | - | 4 | CAAT |  |
| CAAT-box | Nicotiana glutinosa | 2195 | - | 4 | CAAT |  |
| CAAT-box | Nicotiana glutinosa | 804 | + | 4 | CAAT |  |
| CAAT-box | Nicotiana glutinosa | 395 | - | 4 | CAAT |  |
| CAAT-box | Nicotiana glutinosa | 1093 | - | 4 | CAAT |  |
| CAAT-box | Nicotiana glutinosa | 3524 | + | 4 | CAAT |  |
| CAAT-box | Nicotiana glutinosa | 671 | + | 4 | CAAT |  |
| CAAT-box | Nicotiana glutinosa | 320 | - | 4 | CAAT |  |
| CAAT-box | Nicotiana glutinosa | 81 | - | 4 | CAAT |  |
| CAAT-box | Nicotiana glutinosa | 1696 | - | 4 | CAAT |  |
| CAAT-box | Pisum sativum | 707 | + | 5 | CAAAT | common cis-acting element in promoter and enhancer regions |
| CAAT-box | Pisum sativum | 761 | - | 5 | CAAAT | common cis-acting element in promoter and enhancer regions |
| CAAT-box | Nicotiana glutinosa | 1121 | - | 4 | CAAT |  |
| CAAT-box | Nicotiana glutinosa | 710 | - | 4 | CAAT |  |
| CAAT-box | Nicotiana glutinosa | 2531 | - | 4 | CAAT |  |
| CAAT-box | Nicotiana glutinosa | 1771 | - | 4 | CAAT |  |
| CAAT-box | Nicotiana glutinosa | 1456 | - | 4 | CAAT |  |
| CAAT-box | Nicotiana glutinosa | 1388 | + | 4 | CAAT |  |
| CAAT-box | Nicotiana glutinosa | 3555 | - | 4 | CAAT |  |
| CAAT-box | Pisum sativum | 259 | - | 5 | CAAAT | common cis-acting element in promoter and enhancer regions |
| CAAT-box | Pisum sativum | 2104 | + | 5 | CAAAT | common cis-acting element in promoter and enhancer regions |
| CAAT-box | Pisum sativum | 584 | - | 5 | CAAAT | common cis-acting element in promoter and enhancer regions |
| CAAT-box | Pisum sativum | 558 | - | 5 | CAAAT | common cis-acting element in promoter and enhancer regions |
| CAAT-box | Nicotiana glutinosa | 3538 | - | 4 | CAAT |  |
| CAAT-box | Pisum sativum | 2680 | - | 5 | CAAAT | common cis-acting element in promoter and enhancer regions |
| CAAT-box | Arabidopsis thaliana | 1387 | + | 5 | CCAAT | common cis-acting element in promoter and enhancer regions |
| CAAT-box | Pisum sativum | 806 | - | 5 | CAAAT | common cis-acting element in promoter and enhancer regions |
| CAAT-box | Nicotiana glutinosa | 1462 | - | 4 | CAAT |  |
| CAAT-box | Pisum sativum | 239 | - | 5 | CAAAT | common cis-acting element in promoter and enhancer regions |
| CAAT-box | Pisum sativum | 485 | - | 5 | CAAAT | common cis-acting element in promoter and enhancer regions |
| CAAT-box | Nicotiana glutinosa | 2176 | - | 4 | CAAT |  |
| CAAT-box | Arabidopsis thaliana | 308 | - | 5 | CCAAT | common cis-acting element in promoter and enhancer regions |
| CAAT-box | Pisum sativum | 268 | - | 5 | CAAAT | common cis-acting element in promoter and enhancer regions |
| CAAT-box | Arabidopsis thaliana | 1965 | - | 5 | CCAAT | common cis-acting element in promoter and enhancer regions |
| CAAT-box | Nicotiana glutinosa | 699 | - | 4 | CAAT |  |
| CAAT-box | Nicotiana glutinosa | 2697 | - | 4 | CAAT |  |
| CAAT-box | Arabidopsis thaliana | 1920 | - | 5 | CCAAT | common cis-acting element in promoter and enhancer regions |
| CAAT-box | Nicotiana glutinosa | 2638 | + | 4 | CAAT |  |

>HU08G00014.1   
+ +Up\_Stream \_Len000TCCTCT TTTGGGCTTC TTTCTTTCTA TCCTGTTTTT TTTTGGGTCG AAAAGTTGGA   
  
  
+ GGTTGAATTG AAGCATCCGA ATTCGAGCAG GATGTTTCTC TGCGTAGTGA TTCTTTCTGG GTTTGTGATT   
  
  
+ TTGAATTCTG GGTAATCGGT TGTTTTGCTA ATTTTGAGGA CAGAGTTCCT TTTCTGAATT AAAATTTCGT   
  
  
+ TTTTTTATCG GGAAAATCTT CAGTATTTGA GAAAAAAGAA CGAAATTTGG ATGATTTGCT GTCTAGATTT   
  
  
+ TGCTTTCATA TTCCTGGGTG TGGATTGGTT TATTAATTGC ATGTGAGGAA GTACAAGCTT CTGGTCAATG   
  
  
+ GTTGGGTTTT TTTTTTTTTT TTGTGGGTAT AAAGTTGGAA ATTGATTACG AGTCAAATTT CTCTTTCTAG   
  
  
+ AAGAGGGGGG GGGGGGGGGG TTGGTTTAAA ACTCCATATT TTCTTTGAAT GGAAAGCTTA ATTTGCTGAC   
  
  
+ TATCTTGGTT AGTAGCATAA GCTTGATCAG AAAACAAAAC GCTCTCTGCC CTGCAAAAAC TTTATTTGTC   
  
  
+ TTTTAAGGAA AATTTTGGAA TTTGAGAGTG AAAATTTTGG TTGTGAATCT TGTCTTACTA TGTGGTTCCT   
  
  
+ TCTGACAAAA GTTTGAATCT GAGTATGAGA GTTCATCAAT CTCTTTTACT TTCCTACTTT CTTTATTGCT   
  
  
+ TGCAAATTGT TGTGAACTTA CTTGGATTTT CTGGTAGAGG AGAGTTCTGT CCTGGAATTT GGTGGAAAGT   
  
  
+ GAGACATACT TTGGGATTTA ATTAGCCATC AATTTGGTTT CACACCTTAT CAGTTTGGAC TTCTGGTGTG   
  
  
+ TTATTGTATG TGTTCTTAGC TTGGGATTAT TCTTGTATCG GAAGAGAGGG GGGGGGGGGT GTTTCTTTTA   
  
  
+ GCTGAATGTT AGAACATTTT AAGGGATCTA GATATCTAGA GAAGAGCCTA GGAAATGAAG CATTTTTCCC   
  
  
+ TTATAATCTG ACACAACTTT ATGAATTGAT TAGGAACAAA GAACGATTAG TTCAGAAAGG TTACAACTCC   
  
  
+ TCTAGGTGTT ATACTAAAGA GATGTGAGTT AATAAGCAAT TGCTTACTAA CTGTTTAGGC TGTTTCATTG   
  
  
+ CTTGTAGAGG CTTTTTTTTT GGGGGGGGGG GGGGGTTTGG GGTTCATAAA TGAGAAGGTT GATTTGTTGT   
  
  
+ CTGCTCTGCT CTTGATTCGA TAGTAATTCT CTCAGAAAAG GGTAAAATGA CATGGCCTTC CATCGCCTCC   
  
  
+ CATGAGGCTT TTTGCCTCTG CTTGTGTATC TTTCATCTCT TGGTTTGGTG GGTTGGGGGA GGGGAGGGGG   
  
  
+ GGAAGGGGGT TTTGATTCGA TAGATCATCA CTTTCAGAAA TGGCCTCTCT GGCCAATGGT AAGGGGTAAG   
  
  
+ GTTAGTACAT GTGGACTTTC CCATGTGGAT TTTTCCAGAG TCCGTTGTGT GATTGTTATT GCTGTTGTTG   
  
  
+ TAACTTTCTG TCACTCGGAA AACATGAATC CATATGCAAC AAGAACATAA TAATTGTGAA TGCGACTTTA   
  
  
+ TTTAGGAATG TCTTACATAG TACGTGCACA TTCTTTAATT ATCTGTTTAT CTGTTGGTTT GTTATTTTCA   
  
  
+ AGGGGATGGG GAGTGGGTGG ATGCTTAGTA TGTAATCTTT TTATATAGGG AAAAATCATA TAGCTTGAAA   
  
  
+ GTAAAACTTT CATTGCCAGG ATTCCAAGGT AGGGGTAGGG TTGTCTACAC CTTGATCTCC CTAGAAGCAT   
  
  
+ GGCGATCCTT CCTCGGATTG TGTTATAGAA TAAAGAATGT GGTTCTGCTG TAATGAGACT TATGTTCTAT   
  
  
+ CAAATGATGA ATCTGTTTAG AACTTCTTGC TTATTTTTTC TGCAAAATTT TAGGAGACTT GATAAACTAT   
  
  
+ AGTTCTCTTG AATTTACAGG TGGATATTGG AGTTCACAGA ATAGTATTCA GATCAAAGTG CTTGTGCTTA   
  
  
+ ATTGGAAGGA CAACTTGCTT GAGCTGTTTA TAGAGCTCTA ACTGATGCTA TCTCATGGAC TCACATCAGT   
  
  
+ TTTTTGGATT TAATCTTACC AGTGTTGATC CGTCGTACAT TTCCGCTCAG TACAGTCCGC CGTCAGTGAC   
  
  
+ AAATAGGATG TTTGCATCAC TGAAGCTCGA CTCTAGAGGT TCTCCTGTGT CACCCTTCTC AACTCAGTTT   
  
  
+ GATTGTGATA CGGTTACTAC ATTGAGTGAT AGTCAAGAGC ATCACAGCTC GACGGGGAGT CTATCAACAA   
  
  
+ GAAGCCCTTC TTGTAATTCT CCCCTTGAAA CGAGCAGTTA TCATCATTTA TCGACGAATG GCCCGTCTTG   
  
  
+ TAATTCTGCC CCTGAAACTA GCAGTTATCG TCATCGGTTC AACGCGAGTC CCGTGGGATA TTCCCATCAA   
  
  
+ GGCACTGATT ATGGAGTGAA TATGAAGAGC GCTCTGCAGG AGCTAGAGAC TACTCTAATG GGTGCAGATG   
  
  
+ GTGAGGAAGT ATCTGCTGCT AATCAACCTA TGGGGGGAAG TCGTCAGTCC GGGATCCCAA GTCAGAGATC   
  
  
+ AAAATCATTG AGCGAAGATC CACAGGGTTC GCATCCTACT CAGCCTGATT CATCATCTCT TTCTAGGGCA   
  
  
+ AGAAGATCAG GAGATGAAAG CCAGAGAGAG AAACGGCACA AGGCAATGGA AGAACCAACG GAACTACCAA   
  
  
+ GTTTGCCACC TGGTGATTTG AAGCAGTTGC TAATTGAATG TGCGAGGGCT TTATCGGATA ACCGAATAGA   
  
  
+ TGACTTTGAG AGTTTGGTTA AACGGGCGAG GAAAGAGGTC TCTATCTCGG GTGAGCCTAT CCAACGTCTC   
  
  
+ GGTGCCTACA TGATCGAAGG GCTTGTGGCA AGGAAGCAGT CTTCGGGGAC TAGCATCTAT CGGGCTCTGA   
  
  
+ AGTGTAAAGA GCCTCTTGGA AAAGACTTGC TCTCCTACAT GCACATCCTT TATGAAATAT GCCCTTATCT   
  
  
+ CAAGTTTGGT TATATGGCTG CGAATGGAGC GATAGCTGAA GCTTGTAGAA ATGAGGATAA CATACATATT   
  
  
+ ATAGATTTCC AGATTGCACA GGGCACTCAG TGGGTTACTC TATTACAAGC CCTAGCAGCA AGACCTGGTG   
  
  
+ GGCCACCTAA GGTGCGAATT ACAGGCATTG ATGATCCTGT TTCTAAGTAT GCCCGTGGTG CTAGCTTGGA   
  
  
+ GGCTGTTGGG AAACGGTTAG CGTCTCTATC TGAAAAGTTC AAAATACCCG TCGAGTTCAA TGCGTTGCCC   
  
  
+ GTTTATGGAC CCGATGTCAG GCGGGAAATG CTGGATGTGA GGCCCGGGGA GGCTTTGGCC GTTAATTTTC   
  
  
+ CATTGCAGCT CCACCACACT CCTGACGAGA GTGTCGATGT GAACAACCCT AGGGATGGGC TTCTCAGAAT   
  
  
+ GGTGAAATCA CTTGGTCCTA AGGTAACCAC TTTGGTTGAG CAAGAATCAA ACACCAACAC TACCCCTTTC   
  
  
+ TTGACCCGGT TCATAGAGAC CCTTGACTAC TACTCAGCCA TGTTTGAGTC TATAGACGTG ACCATGCCAA   
  
  
+ GAGACCGGAA GGAGAGGATC AATGTTGAGC AGCATTGTTT GGCTAAGGAC ATTGTGAATA TCATAGCTTG   
  
  
+ CGAGGGCAAG GAGAGGGTGG AGCGTCATGA ACTTTTTGGG AAATGGAAGT CAAGGTTTAC CATGGCAGGG   
  
  
+ TTCAGGCAGT ACCCGTTGAG CTCATACGTA AACTCTGTGA TAAGAAGCCT ACTCCGGTGT TACTCCGAGC   
  
  
+ ATTATACTCT GATAGAGAAG GATGGTGCCA TGCTTCTGGG TTGGAAGGGC CGAATGCTGA TTTCAGCTTC   
  
  
+ GGCATGGCAT TG  

- +Up\_Stream \_Len000AGGAGA AAACCCGAAG AAAGAAAGAT AGGACAAAAA AAAACCCAGC TTTTCAACCT   
  
  
- CCAACTTAAC TTCGTAGGCT TAAGCTCGTC CTACAAAGAG ACGCATCACT AAGAAAGACC CAAACACTAA   
  
  
- AACTTAAGAC CCATTAGCCA ACAAAACGAT TAAAACTCCT GTCTCAAGGA AAAGACTTAA TTTTAAAGCA   
  
  
- AAAAAATAGC CCTTTTAGAA GTCATAAACT CTTTTTTCTT GCTTTAAACC TACTAAACGA CAGATCTAAA   
  
  
- ACGAAAGTAT AAGGACCCAC ACCTAACCAA ATAATTAACG TACACTCCTT CATGTTCGAA GACCAGTTAC   
  
  
- CAACCCAAAA AAAAAAAAAA AACACCCATA TTTCAACCTT TAACTAATGC TCAGTTTAAA GAGAAAGATC   
  
  
- TTCTCCCCCC CCCCCCCCCC AACCAAATTT TGAGGTATAA AAGAAACTTA CCTTTCGAAT TAAACGACTG   
  
  
- ATAGAACCAA TCATCGTATT CGAACTAGTC TTTTGTTTTG CGAGAGACGG GACGTTTTTG AAATAAACAG   
  
  
- AAAATTCCTT TTAAAACCTT AAACTCTCAC TTTTAAAACC AACACTTAGA ACAGAATGAT ACACCAAGGA   
  
  
- AGACTGTTTT CAAACTTAGA CTCATACTCT CAAGTAGTTA GAGAAAATGA AAGGATGAAA GAAATAACGA   
  
  
- ACGTTTAACA ACACTTGAAT GAACCTAAAA GACCATCTCC TCTCAAGACA GGACCTTAAA CCACCTTTCA   
  
  
- CTCTGTATGA AACCCTAAAT TAATCGGTAG TTAAACCAAA GTGTGGAATA GTCAAACCTG AAGACCACAC   
  
  
- AATAACATAC ACAAGAATCG AACCCTAATA AGAACATAGC CTTCTCTCCC CCCCCCCCCA CAAAGAAAAT   
  
  
- CGACTTACAA TCTTGTAAAA TTCCCTAGAT CTATAGATCT CTTCTCGGAT CCTTTACTTC GTAAAAAGGG   
  
  
- AATATTAGAC TGTGTTGAAA TACTTAACTA ATCCTTGTTT CTTGCTAATC AAGTCTTTCC AATGTTGAGG   
  
  
- AGATCCACAA TATGATTTCT CTACACTCAA TTATTCGTTA ACGAATGATT GACAAATCCG ACAAAGTAAC   
  
  
- GAACATCTCC GAAAAAAAAA CCCCCCCCCC CCCCCAAACC CCAAGTATTT ACTCTTCCAA CTAAACAACA   
  
  
- GACGAGACGA GAACTAAGCT ATCATTAAGA GAGTCTTTTC CCATTTTACT GTACCGGAAG GTAGCGGAGG   
  
  
- GTACTCCGAA AAACGGAGAC GAACACATAG AAAGTAGAGA ACCAAACCAC CCAACCCCCT CCCCTCCCCC   
  
  
- CCTTCCCCCA AAACTAAGCT ATCTAGTAGT GAAAGTCTTT ACCGGAGAGA CCGGTTACCA TTCCCCATTC   
  
  
- CAATCATGTA CACCTGAAAG GGTACACCTA AAAAGGTCTC AGGCAACACA CTAACAATAA CGACAACAAC   
  
  
- ATTGAAAGAC AGTGAGCCTT TTGTACTTAG GTATACGTTG TTCTTGTATT ATTAACACTT ACGCTGAAAT   
  
  
- AAATCCTTAC AGAATGTATC ATGCACGTGT AAGAAATTAA TAGACAAATA GACAACCAAA CAATAAAAGT   
  
  
- TCCCCTACCC CTCACCCACC TACGAATCAT ACATTAGAAA AATATATCCC TTTTTAGTAT ATCGAACTTT   
  
  
- CATTTTGAAA GTAACGGTCC TAAGGTTCCA TCCCCATCCC AACAGATGTG GAACTAGAGG GATCTTCGTA   
  
  
- CCGCTAGGAA GGAGCCTAAC ACAATATCTT ATTTCTTACA CCAAGACGAC ATTACTCTGA ATACAAGATA   
  
  
- GTTTACTACT TAGACAAATC TTGAAGAACG AATAAAAAAG ACGTTTTAAA ATCCTCTGAA CTATTTGATA   
  
  
- TCAAGAGAAC TTAAATGTCC ACCTATAACC TCAAGTGTCT TATCATAAGT CTAGTTTCAC GAACACGAAT   
  
  
- TAACCTTCCT GTTGAACGAA CTCGACAAAT ATCTCGAGAT TGACTACGAT AGAGTACCTG AGTGTAGTCA   
  
  
- AAAAACCTAA ATTAGAATGG TCACAACTAG GCAGCATGTA AAGGCGAGTC ATGTCAGGCG GCAGTCACTG   
  
  
- TTTATCCTAC AAACGTAGTG ACTTCGAGCT GAGATCTCCA AGAGGACACA GTGGGAAGAG TTGAGTCAAA   
  
  
- CTAACACTAT GCCAATGATG TAACTCACTA TCAGTTCTCG TAGTGTCGAG CTGCCCCTCA GATAGTTGTT   
  
  
- CTTCGGGAAG AACATTAAGA GGGGAACTTT GCTCGTCAAT AGTAGTAAAT AGCTGCTTAC CGGGCAGAAC   
  
  
- ATTAAGACGG GGACTTTGAT CGTCAATAGC AGTAGCCAAG TTGCGCTCAG GGCACCCTAT AAGGGTAGTT   
  
  
- CCGTGACTAA TACCTCACTT ATACTTCTCG CGAGACGTCC TCGATCTCTG ATGAGATTAC CCACGTCTAC   
  
  
- CACTCCTTCA TAGACGACGA TTAGTTGGAT ACCCCCCTTC AGCAGTCAGG CCCTAGGGTT CAGTCTCTAG   
  
  
- TTTTAGTAAC TCGCTTCTAG GTGTCCCAAG CGTAGGATGA GTCGGACTAA GTAGTAGAGA AAGATCCCGT   
  
  
- TCTTCTAGTC CTCTACTTTC GGTCTCTCTC TTTGCCGTGT TCCGTTACCT TCTTGGTTGC CTTGATGGTT   
  
  
- CAAACGGTGG ACCACTAAAC TTCGTCAACG ATTAACTTAC ACGCTCCCGA AATAGCCTAT TGGCTTATCT   
  
  
- ACTGAAACTC TCAAACCAAT TTGCCCGCTC CTTTCTCCAG AGATAGAGCC CACTCGGATA GGTTGCAGAG   
  
  
- CCACGGATGT ACTAGCTTCC CGAACACCGT TCCTTCGTCA GAAGCCCCTG ATCGTAGATA GCCCGAGACT   
  
  
- TCACATTTCT CGGAGAACCT TTTCTGAACG AGAGGATGTA CGTGTAGGAA ATACTTTATA CGGGAATAGA   
  
  
- GTTCAAACCA ATATACCGAC GCTTACCTCG CTATCGACTT CGAACATCTT TACTCCTATT GTATGTATAA   
  
  
- TATCTAAAGG TCTAACGTGT CCCGTGAGTC ACCCAATGAG ATAATGTTCG GGATCGTCGT TCTGGACCAC   
  
  
- CCGGTGGATT CCACGCTTAA TGTCCGTAAC TACTAGGACA AAGATTCATA CGGGCACCAC GATCGAACCT   
  
  
- CCGACAACCC TTTGCCAATC GCAGAGATAG ACTTTTCAAG TTTTATGGGC AGCTCAAGTT ACGCAACGGG   
  
  
- CAAATACCTG GGCTACAGTC CGCCCTTTAC GACCTACACT CCGGGCCCCT CCGAAACCGG CAATTAAAAG   
  
  
- GTAACGTCGA GGTGGTGTGA GGACTGCTCT CACAGCTACA CTTGTTGGGA TCCCTACCCG AAGAGTCTTA   
  
  
- CCACTTTAGT GAACCAGGAT TCCATTGGTG AAACCAACTC GTTCTTAGTT TGTGGTTGTG ATGGGGAAAG   
  
  
- AACTGGGCCA AGTATCTCTG GGAACTGATG ATGAGTCGGT ACAAACTCAG ATATCTGCAC TGGTACGGTT   
  
  
- CTCTGGCCTT CCTCTCCTAG TTACAACTCG TCGTAACAAA CCGATTCCTG TAACACTTAT AGTATCGAAC   
  
  
- GCTCCCGTTC CTCTCCCACC TCGCAGTACT TGAAAAACCC TTTACCTTCA GTTCCAAATG GTACCGTCCC   
  
  
- AAGTCCGTCA TGGGCAACTC GAGTATGCAT TTGAGACACT ATTCTTCGGA TGAGGCCACA ATGAGGCTCG   
  
  
- TAATATGAGA CTATCTCTTC CTACCACGGT ACGAAGACCC AACCTTCCCG GCTTACGACT AAAGTCGAAG   
  
  
- CCGTACCGTA AC

+     CCAAT-box

| Site Name | Organism | Position | Strand | Matrix score. | sequence | function |
| --- | --- | --- | --- | --- | --- | --- |
| CCAAT-box | Hordeum vulgare | 2650 | + | 6 | CAACGG | MYBHv1 binding site |
| CCAAT-box | Hordeum vulgare | 1446 | - | 6 | CAACGG | MYBHv1 binding site |
| CCAAT-box | Hordeum vulgare | 3657 | - | 6 | CAACGG | MYBHv1 binding site |

>HU08G00014.1   
+ +Up\_Stream \_Len000TCCTCT TTTGGGCTTC TTTCTTTCTA TCCTGTTTTT TTTTGGGTCG AAAAGTTGGA   
  
  
+ GGTTGAATTG AAGCATCCGA ATTCGAGCAG GATGTTTCTC TGCGTAGTGA TTCTTTCTGG GTTTGTGATT   
  
  
+ TTGAATTCTG GGTAATCGGT TGTTTTGCTA ATTTTGAGGA CAGAGTTCCT TTTCTGAATT AAAATTTCGT   
  
  
+ TTTTTTATCG GGAAAATCTT CAGTATTTGA GAAAAAAGAA CGAAATTTGG ATGATTTGCT GTCTAGATTT   
  
  
+ TGCTTTCATA TTCCTGGGTG TGGATTGGTT TATTAATTGC ATGTGAGGAA GTACAAGCTT CTGGTCAATG   
  
  
+ GTTGGGTTTT TTTTTTTTTT TTGTGGGTAT AAAGTTGGAA ATTGATTACG AGTCAAATTT CTCTTTCTAG   
  
  
+ AAGAGGGGGG GGGGGGGGGG TTGGTTTAAA ACTCCATATT TTCTTTGAAT GGAAAGCTTA ATTTGCTGAC   
  
  
+ TATCTTGGTT AGTAGCATAA GCTTGATCAG AAAACAAAAC GCTCTCTGCC CTGCAAAAAC TTTATTTGTC   
  
  
+ TTTTAAGGAA AATTTTGGAA TTTGAGAGTG AAAATTTTGG TTGTGAATCT TGTCTTACTA TGTGGTTCCT   
  
  
+ TCTGACAAAA GTTTGAATCT GAGTATGAGA GTTCATCAAT CTCTTTTACT TTCCTACTTT CTTTATTGCT   
  
  
+ TGCAAATTGT TGTGAACTTA CTTGGATTTT CTGGTAGAGG AGAGTTCTGT CCTGGAATTT GGTGGAAAGT   
  
  
+ GAGACATACT TTGGGATTTA ATTAGCCATC AATTTGGTTT CACACCTTAT CAGTTTGGAC TTCTGGTGTG   
  
  
+ TTATTGTATG TGTTCTTAGC TTGGGATTAT TCTTGTATCG GAAGAGAGGG GGGGGGGGGT GTTTCTTTTA   
  
  
+ GCTGAATGTT AGAACATTTT AAGGGATCTA GATATCTAGA GAAGAGCCTA GGAAATGAAG CATTTTTCCC   
  
  
+ TTATAATCTG ACACAACTTT ATGAATTGAT TAGGAACAAA GAACGATTAG TTCAGAAAGG TTACAACTCC   
  
  
+ TCTAGGTGTT ATACTAAAGA GATGTGAGTT AATAAGCAAT TGCTTACTAA CTGTTTAGGC TGTTTCATTG   
  
  
+ CTTGTAGAGG CTTTTTTTTT GGGGGGGGGG GGGGGTTTGG GGTTCATAAA TGAGAAGGTT GATTTGTTGT   
  
  
+ CTGCTCTGCT CTTGATTCGA TAGTAATTCT CTCAGAAAAG GGTAAAATGA CATGGCCTTC CATCGCCTCC   
  
  
+ CATGAGGCTT TTTGCCTCTG CTTGTGTATC TTTCATCTCT TGGTTTGGTG GGTTGGGGGA GGGGAGGGGG   
  
  
+ GGAAGGGGGT TTTGATTCGA TAGATCATCA CTTTCAGAAA TGGCCTCTCT GGCCAATGGT AAGGGGTAAG   
  
  
+ GTTAGTACAT GTGGACTTTC CCATGTGGAT TTTTCCAGAG TCCGTTGTGT GATTGTTATT GCTGTTGTTG   
  
  
+ TAACTTTCTG TCACTCGGAA AACATGAATC CATATGCAAC AAGAACATAA TAATTGTGAA TGCGACTTTA   
  
  
+ TTTAGGAATG TCTTACATAG TACGTGCACA TTCTTTAATT ATCTGTTTAT CTGTTGGTTT GTTATTTTCA   
  
  
+ AGGGGATGGG GAGTGGGTGG ATGCTTAGTA TGTAATCTTT TTATATAGGG AAAAATCATA TAGCTTGAAA   
  
  
+ GTAAAACTTT CATTGCCAGG ATTCCAAGGT AGGGGTAGGG TTGTCTACAC CTTGATCTCC CTAGAAGCAT   
  
  
+ GGCGATCCTT CCTCGGATTG TGTTATAGAA TAAAGAATGT GGTTCTGCTG TAATGAGACT TATGTTCTAT   
  
  
+ CAAATGATGA ATCTGTTTAG AACTTCTTGC TTATTTTTTC TGCAAAATTT TAGGAGACTT GATAAACTAT   
  
  
+ AGTTCTCTTG AATTTACAGG TGGATATTGG AGTTCACAGA ATAGTATTCA GATCAAAGTG CTTGTGCTTA   
  
  
+ ATTGGAAGGA CAACTTGCTT GAGCTGTTTA TAGAGCTCTA ACTGATGCTA TCTCATGGAC TCACATCAGT   
  
  
+ TTTTTGGATT TAATCTTACC AGTGTTGATC CGTCGTACAT TTCCGCTCAG TACAGTCCGC CGTCAGTGAC   
  
  
+ AAATAGGATG TTTGCATCAC TGAAGCTCGA CTCTAGAGGT TCTCCTGTGT CACCCTTCTC AACTCAGTTT   
  
  
+ GATTGTGATA CGGTTACTAC ATTGAGTGAT AGTCAAGAGC ATCACAGCTC GACGGGGAGT CTATCAACAA   
  
  
+ GAAGCCCTTC TTGTAATTCT CCCCTTGAAA CGAGCAGTTA TCATCATTTA TCGACGAATG GCCCGTCTTG   
  
  
+ TAATTCTGCC CCTGAAACTA GCAGTTATCG TCATCGGTTC AACGCGAGTC CCGTGGGATA TTCCCATCAA   
  
  
+ GGCACTGATT ATGGAGTGAA TATGAAGAGC GCTCTGCAGG AGCTAGAGAC TACTCTAATG GGTGCAGATG   
  
  
+ GTGAGGAAGT ATCTGCTGCT AATCAACCTA TGGGGGGAAG TCGTCAGTCC GGGATCCCAA GTCAGAGATC   
  
  
+ AAAATCATTG AGCGAAGATC CACAGGGTTC GCATCCTACT CAGCCTGATT CATCATCTCT TTCTAGGGCA   
  
  
+ AGAAGATCAG GAGATGAAAG CCAGAGAGAG AAACGGCACA AGGCAATGGA AGAACCAACG GAACTACCAA   
  
  
+ GTTTGCCACC TGGTGATTTG AAGCAGTTGC TAATTGAATG TGCGAGGGCT TTATCGGATA ACCGAATAGA   
  
  
+ TGACTTTGAG AGTTTGGTTA AACGGGCGAG GAAAGAGGTC TCTATCTCGG GTGAGCCTAT CCAACGTCTC   
  
  
+ GGTGCCTACA TGATCGAAGG GCTTGTGGCA AGGAAGCAGT CTTCGGGGAC TAGCATCTAT CGGGCTCTGA   
  
  
+ AGTGTAAAGA GCCTCTTGGA AAAGACTTGC TCTCCTACAT GCACATCCTT TATGAAATAT GCCCTTATCT   
  
  
+ CAAGTTTGGT TATATGGCTG CGAATGGAGC GATAGCTGAA GCTTGTAGAA ATGAGGATAA CATACATATT   
  
  
+ ATAGATTTCC AGATTGCACA GGGCACTCAG TGGGTTACTC TATTACAAGC CCTAGCAGCA AGACCTGGTG   
  
  
+ GGCCACCTAA GGTGCGAATT ACAGGCATTG ATGATCCTGT TTCTAAGTAT GCCCGTGGTG CTAGCTTGGA   
  
  
+ GGCTGTTGGG AAACGGTTAG CGTCTCTATC TGAAAAGTTC AAAATACCCG TCGAGTTCAA TGCGTTGCCC   
  
  
+ GTTTATGGAC CCGATGTCAG GCGGGAAATG CTGGATGTGA GGCCCGGGGA GGCTTTGGCC GTTAATTTTC   
  
  
+ CATTGCAGCT CCACCACACT CCTGACGAGA GTGTCGATGT GAACAACCCT AGGGATGGGC TTCTCAGAAT   
  
  
+ GGTGAAATCA CTTGGTCCTA AGGTAACCAC TTTGGTTGAG CAAGAATCAA ACACCAACAC TACCCCTTTC   
  
  
+ TTGACCCGGT TCATAGAGAC CCTTGACTAC TACTCAGCCA TGTTTGAGTC TATAGACGTG ACCATGCCAA   
  
  
+ GAGACCGGAA GGAGAGGATC AATGTTGAGC AGCATTGTTT GGCTAAGGAC ATTGTGAATA TCATAGCTTG   
  
  
+ CGAGGGCAAG GAGAGGGTGG AGCGTCATGA ACTTTTTGGG AAATGGAAGT CAAGGTTTAC CATGGCAGGG   
  
  
+ TTCAGGCAGT ACCCGTTGAG CTCATACGTA AACTCTGTGA TAAGAAGCCT ACTCCGGTGT TACTCCGAGC   
  
  
+ ATTATACTCT GATAGAGAAG GATGGTGCCA TGCTTCTGGG TTGGAAGGGC CGAATGCTGA TTTCAGCTTC   
  
  
+ GGCATGGCAT TG  

- +Up\_Stream \_Len000AGGAGA AAACCCGAAG AAAGAAAGAT AGGACAAAAA AAAACCCAGC TTTTCAACCT   
  
  
- CCAACTTAAC TTCGTAGGCT TAAGCTCGTC CTACAAAGAG ACGCATCACT AAGAAAGACC CAAACACTAA   
  
  
- AACTTAAGAC CCATTAGCCA ACAAAACGAT TAAAACTCCT GTCTCAAGGA AAAGACTTAA TTTTAAAGCA   
  
  
- AAAAAATAGC CCTTTTAGAA GTCATAAACT CTTTTTTCTT GCTTTAAACC TACTAAACGA CAGATCTAAA   
  
  
- ACGAAAGTAT AAGGACCCAC ACCTAACCAA ATAATTAACG TACACTCCTT CATGTTCGAA GACCAGTTAC   
  
  
- CAACCCAAAA AAAAAAAAAA AACACCCATA TTTCAACCTT TAACTAATGC TCAGTTTAAA GAGAAAGATC   
  
  
- TTCTCCCCCC CCCCCCCCCC AACCAAATTT TGAGGTATAA AAGAAACTTA CCTTTCGAAT TAAACGACTG   
  
  
- ATAGAACCAA TCATCGTATT CGAACTAGTC TTTTGTTTTG CGAGAGACGG GACGTTTTTG AAATAAACAG   
  
  
- AAAATTCCTT TTAAAACCTT AAACTCTCAC TTTTAAAACC AACACTTAGA ACAGAATGAT ACACCAAGGA   
  
  
- AGACTGTTTT CAAACTTAGA CTCATACTCT CAAGTAGTTA GAGAAAATGA AAGGATGAAA GAAATAACGA   
  
  
- ACGTTTAACA ACACTTGAAT GAACCTAAAA GACCATCTCC TCTCAAGACA GGACCTTAAA CCACCTTTCA   
  
  
- CTCTGTATGA AACCCTAAAT TAATCGGTAG TTAAACCAAA GTGTGGAATA GTCAAACCTG AAGACCACAC   
  
  
- AATAACATAC ACAAGAATCG AACCCTAATA AGAACATAGC CTTCTCTCCC CCCCCCCCCA CAAAGAAAAT   
  
  
- CGACTTACAA TCTTGTAAAA TTCCCTAGAT CTATAGATCT CTTCTCGGAT CCTTTACTTC GTAAAAAGGG   
  
  
- AATATTAGAC TGTGTTGAAA TACTTAACTA ATCCTTGTTT CTTGCTAATC AAGTCTTTCC AATGTTGAGG   
  
  
- AGATCCACAA TATGATTTCT CTACACTCAA TTATTCGTTA ACGAATGATT GACAAATCCG ACAAAGTAAC   
  
  
- GAACATCTCC GAAAAAAAAA CCCCCCCCCC CCCCCAAACC CCAAGTATTT ACTCTTCCAA CTAAACAACA   
  
  
- GACGAGACGA GAACTAAGCT ATCATTAAGA GAGTCTTTTC CCATTTTACT GTACCGGAAG GTAGCGGAGG   
  
  
- GTACTCCGAA AAACGGAGAC GAACACATAG AAAGTAGAGA ACCAAACCAC CCAACCCCCT CCCCTCCCCC   
  
  
- CCTTCCCCCA AAACTAAGCT ATCTAGTAGT GAAAGTCTTT ACCGGAGAGA CCGGTTACCA TTCCCCATTC   
  
  
- CAATCATGTA CACCTGAAAG GGTACACCTA AAAAGGTCTC AGGCAACACA CTAACAATAA CGACAACAAC   
  
  
- ATTGAAAGAC AGTGAGCCTT TTGTACTTAG GTATACGTTG TTCTTGTATT ATTAACACTT ACGCTGAAAT   
  
  
- AAATCCTTAC AGAATGTATC ATGCACGTGT AAGAAATTAA TAGACAAATA GACAACCAAA CAATAAAAGT   
  
  
- TCCCCTACCC CTCACCCACC TACGAATCAT ACATTAGAAA AATATATCCC TTTTTAGTAT ATCGAACTTT   
  
  
- CATTTTGAAA GTAACGGTCC TAAGGTTCCA TCCCCATCCC AACAGATGTG GAACTAGAGG GATCTTCGTA   
  
  
- CCGCTAGGAA GGAGCCTAAC ACAATATCTT ATTTCTTACA CCAAGACGAC ATTACTCTGA ATACAAGATA   
  
  
- GTTTACTACT TAGACAAATC TTGAAGAACG AATAAAAAAG ACGTTTTAAA ATCCTCTGAA CTATTTGATA   
  
  
- TCAAGAGAAC TTAAATGTCC ACCTATAACC TCAAGTGTCT TATCATAAGT CTAGTTTCAC GAACACGAAT   
  
  
- TAACCTTCCT GTTGAACGAA CTCGACAAAT ATCTCGAGAT TGACTACGAT AGAGTACCTG AGTGTAGTCA   
  
  
- AAAAACCTAA ATTAGAATGG TCACAACTAG GCAGCATGTA AAGGCGAGTC ATGTCAGGCG GCAGTCACTG   
  
  
- TTTATCCTAC AAACGTAGTG ACTTCGAGCT GAGATCTCCA AGAGGACACA GTGGGAAGAG TTGAGTCAAA   
  
  
- CTAACACTAT GCCAATGATG TAACTCACTA TCAGTTCTCG TAGTGTCGAG CTGCCCCTCA GATAGTTGTT   
  
  
- CTTCGGGAAG AACATTAAGA GGGGAACTTT GCTCGTCAAT AGTAGTAAAT AGCTGCTTAC CGGGCAGAAC   
  
  
- ATTAAGACGG GGACTTTGAT CGTCAATAGC AGTAGCCAAG TTGCGCTCAG GGCACCCTAT AAGGGTAGTT   
  
  
- CCGTGACTAA TACCTCACTT ATACTTCTCG CGAGACGTCC TCGATCTCTG ATGAGATTAC CCACGTCTAC   
  
  
- CACTCCTTCA TAGACGACGA TTAGTTGGAT ACCCCCCTTC AGCAGTCAGG CCCTAGGGTT CAGTCTCTAG   
  
  
- TTTTAGTAAC TCGCTTCTAG GTGTCCCAAG CGTAGGATGA GTCGGACTAA GTAGTAGAGA AAGATCCCGT   
  
  
- TCTTCTAGTC CTCTACTTTC GGTCTCTCTC TTTGCCGTGT TCCGTTACCT TCTTGGTTGC CTTGATGGTT   
  
  
- CAAACGGTGG ACCACTAAAC TTCGTCAACG ATTAACTTAC ACGCTCCCGA AATAGCCTAT TGGCTTATCT   
  
  
- ACTGAAACTC TCAAACCAAT TTGCCCGCTC CTTTCTCCAG AGATAGAGCC CACTCGGATA GGTTGCAGAG   
  
  
- CCACGGATGT ACTAGCTTCC CGAACACCGT TCCTTCGTCA GAAGCCCCTG ATCGTAGATA GCCCGAGACT   
  
  
- TCACATTTCT CGGAGAACCT TTTCTGAACG AGAGGATGTA CGTGTAGGAA ATACTTTATA CGGGAATAGA   
  
  
- GTTCAAACCA ATATACCGAC GCTTACCTCG CTATCGACTT CGAACATCTT TACTCCTATT GTATGTATAA   
  
  
- TATCTAAAGG TCTAACGTGT CCCGTGAGTC ACCCAATGAG ATAATGTTCG GGATCGTCGT TCTGGACCAC   
  
  
- CCGGTGGATT CCACGCTTAA TGTCCGTAAC TACTAGGACA AAGATTCATA CGGGCACCAC GATCGAACCT   
  
  
- CCGACAACCC TTTGCCAATC GCAGAGATAG ACTTTTCAAG TTTTATGGGC AGCTCAAGTT ACGCAACGGG   
  
  
- CAAATACCTG GGCTACAGTC CGCCCTTTAC GACCTACACT CCGGGCCCCT CCGAAACCGG CAATTAAAAG   
  
  
- GTAACGTCGA GGTGGTGTGA GGACTGCTCT CACAGCTACA CTTGTTGGGA TCCCTACCCG AAGAGTCTTA   
  
  
- CCACTTTAGT GAACCAGGAT TCCATTGGTG AAACCAACTC GTTCTTAGTT TGTGGTTGTG ATGGGGAAAG   
  
  
- AACTGGGCCA AGTATCTCTG GGAACTGATG ATGAGTCGGT ACAAACTCAG ATATCTGCAC TGGTACGGTT   
  
  
- CTCTGGCCTT CCTCTCCTAG TTACAACTCG TCGTAACAAA CCGATTCCTG TAACACTTAT AGTATCGAAC   
  
  
- GCTCCCGTTC CTCTCCCACC TCGCAGTACT TGAAAAACCC TTTACCTTCA GTTCCAAATG GTACCGTCCC   
  
  
- AAGTCCGTCA TGGGCAACTC GAGTATGCAT TTGAGACACT ATTCTTCGGA TGAGGCCACA ATGAGGCTCG   
  
  
- TAATATGAGA CTATCTCTTC CTACCACGGT ACGAAGACCC AACCTTCCCG GCTTACGACT AAAGTCGAAG   
  
  
- CCGTACCGTA AC

+     CGTCA-motif

| Site Name | Organism | Position | Strand | Matrix score. | sequence | function |
| --- | --- | --- | --- | --- | --- | --- |
| CGTCA-motif | Hordeum vulgare | 3597 | + | 5 | CGTCA | cis-acting regulatory element involved in the MeJA-responsiveness |
| CGTCA-motif | Hordeum vulgare | 2343 | + | 5 | CGTCA | cis-acting regulatory element involved in the MeJA-responsiveness |
| CGTCA-motif | Hordeum vulgare | 2095 | + | 5 | CGTCA | cis-acting regulatory element involved in the MeJA-responsiveness |
| CGTCA-motif | Hordeum vulgare | 2496 | + | 5 | CGTCA | cis-acting regulatory element involved in the MeJA-responsiveness |
| CGTCA-motif | Hordeum vulgare | 3317 | - | 5 | CGTCA | cis-acting regulatory element involved in the MeJA-responsiveness |

>HU08G00014.1   
+ +Up\_Stream \_Len000TCCTCT TTTGGGCTTC TTTCTTTCTA TCCTGTTTTT TTTTGGGTCG AAAAGTTGGA   
  
  
+ GGTTGAATTG AAGCATCCGA ATTCGAGCAG GATGTTTCTC TGCGTAGTGA TTCTTTCTGG GTTTGTGATT   
  
  
+ TTGAATTCTG GGTAATCGGT TGTTTTGCTA ATTTTGAGGA CAGAGTTCCT TTTCTGAATT AAAATTTCGT   
  
  
+ TTTTTTATCG GGAAAATCTT CAGTATTTGA GAAAAAAGAA CGAAATTTGG ATGATTTGCT GTCTAGATTT   
  
  
+ TGCTTTCATA TTCCTGGGTG TGGATTGGTT TATTAATTGC ATGTGAGGAA GTACAAGCTT CTGGTCAATG   
  
  
+ GTTGGGTTTT TTTTTTTTTT TTGTGGGTAT AAAGTTGGAA ATTGATTACG AGTCAAATTT CTCTTTCTAG   
  
  
+ AAGAGGGGGG GGGGGGGGGG TTGGTTTAAA ACTCCATATT TTCTTTGAAT GGAAAGCTTA ATTTGCTGAC   
  
  
+ TATCTTGGTT AGTAGCATAA GCTTGATCAG AAAACAAAAC GCTCTCTGCC CTGCAAAAAC TTTATTTGTC   
  
  
+ TTTTAAGGAA AATTTTGGAA TTTGAGAGTG AAAATTTTGG TTGTGAATCT TGTCTTACTA TGTGGTTCCT   
  
  
+ TCTGACAAAA GTTTGAATCT GAGTATGAGA GTTCATCAAT CTCTTTTACT TTCCTACTTT CTTTATTGCT   
  
  
+ TGCAAATTGT TGTGAACTTA CTTGGATTTT CTGGTAGAGG AGAGTTCTGT CCTGGAATTT GGTGGAAAGT   
  
  
+ GAGACATACT TTGGGATTTA ATTAGCCATC AATTTGGTTT CACACCTTAT CAGTTTGGAC TTCTGGTGTG   
  
  
+ TTATTGTATG TGTTCTTAGC TTGGGATTAT TCTTGTATCG GAAGAGAGGG GGGGGGGGGT GTTTCTTTTA   
  
  
+ GCTGAATGTT AGAACATTTT AAGGGATCTA GATATCTAGA GAAGAGCCTA GGAAATGAAG CATTTTTCCC   
  
  
+ TTATAATCTG ACACAACTTT ATGAATTGAT TAGGAACAAA GAACGATTAG TTCAGAAAGG TTACAACTCC   
  
  
+ TCTAGGTGTT ATACTAAAGA GATGTGAGTT AATAAGCAAT TGCTTACTAA CTGTTTAGGC TGTTTCATTG   
  
  
+ CTTGTAGAGG CTTTTTTTTT GGGGGGGGGG GGGGGTTTGG GGTTCATAAA TGAGAAGGTT GATTTGTTGT   
  
  
+ CTGCTCTGCT CTTGATTCGA TAGTAATTCT CTCAGAAAAG GGTAAAATGA CATGGCCTTC CATCGCCTCC   
  
  
+ CATGAGGCTT TTTGCCTCTG CTTGTGTATC TTTCATCTCT TGGTTTGGTG GGTTGGGGGA GGGGAGGGGG   
  
  
+ GGAAGGGGGT TTTGATTCGA TAGATCATCA CTTTCAGAAA TGGCCTCTCT GGCCAATGGT AAGGGGTAAG   
  
  
+ GTTAGTACAT GTGGACTTTC CCATGTGGAT TTTTCCAGAG TCCGTTGTGT GATTGTTATT GCTGTTGTTG   
  
  
+ TAACTTTCTG TCACTCGGAA AACATGAATC CATATGCAAC AAGAACATAA TAATTGTGAA TGCGACTTTA   
  
  
+ TTTAGGAATG TCTTACATAG TACGTGCACA TTCTTTAATT ATCTGTTTAT CTGTTGGTTT GTTATTTTCA   
  
  
+ AGGGGATGGG GAGTGGGTGG ATGCTTAGTA TGTAATCTTT TTATATAGGG AAAAATCATA TAGCTTGAAA   
  
  
+ GTAAAACTTT CATTGCCAGG ATTCCAAGGT AGGGGTAGGG TTGTCTACAC CTTGATCTCC CTAGAAGCAT   
  
  
+ GGCGATCCTT CCTCGGATTG TGTTATAGAA TAAAGAATGT GGTTCTGCTG TAATGAGACT TATGTTCTAT   
  
  
+ CAAATGATGA ATCTGTTTAG AACTTCTTGC TTATTTTTTC TGCAAAATTT TAGGAGACTT GATAAACTAT   
  
  
+ AGTTCTCTTG AATTTACAGG TGGATATTGG AGTTCACAGA ATAGTATTCA GATCAAAGTG CTTGTGCTTA   
  
  
+ ATTGGAAGGA CAACTTGCTT GAGCTGTTTA TAGAGCTCTA ACTGATGCTA TCTCATGGAC TCACATCAGT   
  
  
+ TTTTTGGATT TAATCTTACC AGTGTTGATC CGTCGTACAT TTCCGCTCAG TACAGTCCGC CGTCAGTGAC   
  
  
+ AAATAGGATG TTTGCATCAC TGAAGCTCGA CTCTAGAGGT TCTCCTGTGT CACCCTTCTC AACTCAGTTT   
  
  
+ GATTGTGATA CGGTTACTAC ATTGAGTGAT AGTCAAGAGC ATCACAGCTC GACGGGGAGT CTATCAACAA   
  
  
+ GAAGCCCTTC TTGTAATTCT CCCCTTGAAA CGAGCAGTTA TCATCATTTA TCGACGAATG GCCCGTCTTG   
  
  
+ TAATTCTGCC CCTGAAACTA GCAGTTATCG TCATCGGTTC AACGCGAGTC CCGTGGGATA TTCCCATCAA   
  
  
+ GGCACTGATT ATGGAGTGAA TATGAAGAGC GCTCTGCAGG AGCTAGAGAC TACTCTAATG GGTGCAGATG   
  
  
+ GTGAGGAAGT ATCTGCTGCT AATCAACCTA TGGGGGGAAG TCGTCAGTCC GGGATCCCAA GTCAGAGATC   
  
  
+ AAAATCATTG AGCGAAGATC CACAGGGTTC GCATCCTACT CAGCCTGATT CATCATCTCT TTCTAGGGCA   
  
  
+ AGAAGATCAG GAGATGAAAG CCAGAGAGAG AAACGGCACA AGGCAATGGA AGAACCAACG GAACTACCAA   
  
  
+ GTTTGCCACC TGGTGATTTG AAGCAGTTGC TAATTGAATG TGCGAGGGCT TTATCGGATA ACCGAATAGA   
  
  
+ TGACTTTGAG AGTTTGGTTA AACGGGCGAG GAAAGAGGTC TCTATCTCGG GTGAGCCTAT CCAACGTCTC   
  
  
+ GGTGCCTACA TGATCGAAGG GCTTGTGGCA AGGAAGCAGT CTTCGGGGAC TAGCATCTAT CGGGCTCTGA   
  
  
+ AGTGTAAAGA GCCTCTTGGA AAAGACTTGC TCTCCTACAT GCACATCCTT TATGAAATAT GCCCTTATCT   
  
  
+ CAAGTTTGGT TATATGGCTG CGAATGGAGC GATAGCTGAA GCTTGTAGAA ATGAGGATAA CATACATATT   
  
  
+ ATAGATTTCC AGATTGCACA GGGCACTCAG TGGGTTACTC TATTACAAGC CCTAGCAGCA AGACCTGGTG   
  
  
+ GGCCACCTAA GGTGCGAATT ACAGGCATTG ATGATCCTGT TTCTAAGTAT GCCCGTGGTG CTAGCTTGGA   
  
  
+ GGCTGTTGGG AAACGGTTAG CGTCTCTATC TGAAAAGTTC AAAATACCCG TCGAGTTCAA TGCGTTGCCC   
  
  
+ GTTTATGGAC CCGATGTCAG GCGGGAAATG CTGGATGTGA GGCCCGGGGA GGCTTTGGCC GTTAATTTTC   
  
  
+ CATTGCAGCT CCACCACACT CCTGACGAGA GTGTCGATGT GAACAACCCT AGGGATGGGC TTCTCAGAAT   
  
  
+ GGTGAAATCA CTTGGTCCTA AGGTAACCAC TTTGGTTGAG CAAGAATCAA ACACCAACAC TACCCCTTTC   
  
  
+ TTGACCCGGT TCATAGAGAC CCTTGACTAC TACTCAGCCA TGTTTGAGTC TATAGACGTG ACCATGCCAA   
  
  
+ GAGACCGGAA GGAGAGGATC AATGTTGAGC AGCATTGTTT GGCTAAGGAC ATTGTGAATA TCATAGCTTG   
  
  
+ CGAGGGCAAG GAGAGGGTGG AGCGTCATGA ACTTTTTGGG AAATGGAAGT CAAGGTTTAC CATGGCAGGG   
  
  
+ TTCAGGCAGT ACCCGTTGAG CTCATACGTA AACTCTGTGA TAAGAAGCCT ACTCCGGTGT TACTCCGAGC   
  
  
+ ATTATACTCT GATAGAGAAG GATGGTGCCA TGCTTCTGGG TTGGAAGGGC CGAATGCTGA TTTCAGCTTC   
  
  
+ GGCATGGCAT TG  

- +Up\_Stream \_Len000AGGAGA AAACCCGAAG AAAGAAAGAT AGGACAAAAA AAAACCCAGC TTTTCAACCT   
  
  
- CCAACTTAAC TTCGTAGGCT TAAGCTCGTC CTACAAAGAG ACGCATCACT AAGAAAGACC CAAACACTAA   
  
  
- AACTTAAGAC CCATTAGCCA ACAAAACGAT TAAAACTCCT GTCTCAAGGA AAAGACTTAA TTTTAAAGCA   
  
  
- AAAAAATAGC CCTTTTAGAA GTCATAAACT CTTTTTTCTT GCTTTAAACC TACTAAACGA CAGATCTAAA   
  
  
- ACGAAAGTAT AAGGACCCAC ACCTAACCAA ATAATTAACG TACACTCCTT CATGTTCGAA GACCAGTTAC   
  
  
- CAACCCAAAA AAAAAAAAAA AACACCCATA TTTCAACCTT TAACTAATGC TCAGTTTAAA GAGAAAGATC   
  
  
- TTCTCCCCCC CCCCCCCCCC AACCAAATTT TGAGGTATAA AAGAAACTTA CCTTTCGAAT TAAACGACTG   
  
  
- ATAGAACCAA TCATCGTATT CGAACTAGTC TTTTGTTTTG CGAGAGACGG GACGTTTTTG AAATAAACAG   
  
  
- AAAATTCCTT TTAAAACCTT AAACTCTCAC TTTTAAAACC AACACTTAGA ACAGAATGAT ACACCAAGGA   
  
  
- AGACTGTTTT CAAACTTAGA CTCATACTCT CAAGTAGTTA GAGAAAATGA AAGGATGAAA GAAATAACGA   
  
  
- ACGTTTAACA ACACTTGAAT GAACCTAAAA GACCATCTCC TCTCAAGACA GGACCTTAAA CCACCTTTCA   
  
  
- CTCTGTATGA AACCCTAAAT TAATCGGTAG TTAAACCAAA GTGTGGAATA GTCAAACCTG AAGACCACAC   
  
  
- AATAACATAC ACAAGAATCG AACCCTAATA AGAACATAGC CTTCTCTCCC CCCCCCCCCA CAAAGAAAAT   
  
  
- CGACTTACAA TCTTGTAAAA TTCCCTAGAT CTATAGATCT CTTCTCGGAT CCTTTACTTC GTAAAAAGGG   
  
  
- AATATTAGAC TGTGTTGAAA TACTTAACTA ATCCTTGTTT CTTGCTAATC AAGTCTTTCC AATGTTGAGG   
  
  
- AGATCCACAA TATGATTTCT CTACACTCAA TTATTCGTTA ACGAATGATT GACAAATCCG ACAAAGTAAC   
  
  
- GAACATCTCC GAAAAAAAAA CCCCCCCCCC CCCCCAAACC CCAAGTATTT ACTCTTCCAA CTAAACAACA   
  
  
- GACGAGACGA GAACTAAGCT ATCATTAAGA GAGTCTTTTC CCATTTTACT GTACCGGAAG GTAGCGGAGG   
  
  
- GTACTCCGAA AAACGGAGAC GAACACATAG AAAGTAGAGA ACCAAACCAC CCAACCCCCT CCCCTCCCCC   
  
  
- CCTTCCCCCA AAACTAAGCT ATCTAGTAGT GAAAGTCTTT ACCGGAGAGA CCGGTTACCA TTCCCCATTC   
  
  
- CAATCATGTA CACCTGAAAG GGTACACCTA AAAAGGTCTC AGGCAACACA CTAACAATAA CGACAACAAC   
  
  
- ATTGAAAGAC AGTGAGCCTT TTGTACTTAG GTATACGTTG TTCTTGTATT ATTAACACTT ACGCTGAAAT   
  
  
- AAATCCTTAC AGAATGTATC ATGCACGTGT AAGAAATTAA TAGACAAATA GACAACCAAA CAATAAAAGT   
  
  
- TCCCCTACCC CTCACCCACC TACGAATCAT ACATTAGAAA AATATATCCC TTTTTAGTAT ATCGAACTTT   
  
  
- CATTTTGAAA GTAACGGTCC TAAGGTTCCA TCCCCATCCC AACAGATGTG GAACTAGAGG GATCTTCGTA   
  
  
- CCGCTAGGAA GGAGCCTAAC ACAATATCTT ATTTCTTACA CCAAGACGAC ATTACTCTGA ATACAAGATA   
  
  
- GTTTACTACT TAGACAAATC TTGAAGAACG AATAAAAAAG ACGTTTTAAA ATCCTCTGAA CTATTTGATA   
  
  
- TCAAGAGAAC TTAAATGTCC ACCTATAACC TCAAGTGTCT TATCATAAGT CTAGTTTCAC GAACACGAAT   
  
  
- TAACCTTCCT GTTGAACGAA CTCGACAAAT ATCTCGAGAT TGACTACGAT AGAGTACCTG AGTGTAGTCA   
  
  
- AAAAACCTAA ATTAGAATGG TCACAACTAG GCAGCATGTA AAGGCGAGTC ATGTCAGGCG GCAGTCACTG   
  
  
- TTTATCCTAC AAACGTAGTG ACTTCGAGCT GAGATCTCCA AGAGGACACA GTGGGAAGAG TTGAGTCAAA   
  
  
- CTAACACTAT GCCAATGATG TAACTCACTA TCAGTTCTCG TAGTGTCGAG CTGCCCCTCA GATAGTTGTT   
  
  
- CTTCGGGAAG AACATTAAGA GGGGAACTTT GCTCGTCAAT AGTAGTAAAT AGCTGCTTAC CGGGCAGAAC   
  
  
- ATTAAGACGG GGACTTTGAT CGTCAATAGC AGTAGCCAAG TTGCGCTCAG GGCACCCTAT AAGGGTAGTT   
  
  
- CCGTGACTAA TACCTCACTT ATACTTCTCG CGAGACGTCC TCGATCTCTG ATGAGATTAC CCACGTCTAC   
  
  
- CACTCCTTCA TAGACGACGA TTAGTTGGAT ACCCCCCTTC AGCAGTCAGG CCCTAGGGTT CAGTCTCTAG   
  
  
- TTTTAGTAAC TCGCTTCTAG GTGTCCCAAG CGTAGGATGA GTCGGACTAA GTAGTAGAGA AAGATCCCGT   
  
  
- TCTTCTAGTC CTCTACTTTC GGTCTCTCTC TTTGCCGTGT TCCGTTACCT TCTTGGTTGC CTTGATGGTT   
  
  
- CAAACGGTGG ACCACTAAAC TTCGTCAACG ATTAACTTAC ACGCTCCCGA AATAGCCTAT TGGCTTATCT   
  
  
- ACTGAAACTC TCAAACCAAT TTGCCCGCTC CTTTCTCCAG AGATAGAGCC CACTCGGATA GGTTGCAGAG   
  
  
- CCACGGATGT ACTAGCTTCC CGAACACCGT TCCTTCGTCA GAAGCCCCTG ATCGTAGATA GCCCGAGACT   
  
  
- TCACATTTCT CGGAGAACCT TTTCTGAACG AGAGGATGTA CGTGTAGGAA ATACTTTATA CGGGAATAGA   
  
  
- GTTCAAACCA ATATACCGAC GCTTACCTCG CTATCGACTT CGAACATCTT TACTCCTATT GTATGTATAA   
  
  
- TATCTAAAGG TCTAACGTGT CCCGTGAGTC ACCCAATGAG ATAATGTTCG GGATCGTCGT TCTGGACCAC   
  
  
- CCGGTGGATT CCACGCTTAA TGTCCGTAAC TACTAGGACA AAGATTCATA CGGGCACCAC GATCGAACCT   
  
  
- CCGACAACCC TTTGCCAATC GCAGAGATAG ACTTTTCAAG TTTTATGGGC AGCTCAAGTT ACGCAACGGG   
  
  
- CAAATACCTG GGCTACAGTC CGCCCTTTAC GACCTACACT CCGGGCCCCT CCGAAACCGG CAATTAAAAG   
  
  
- GTAACGTCGA GGTGGTGTGA GGACTGCTCT CACAGCTACA CTTGTTGGGA TCCCTACCCG AAGAGTCTTA   
  
  
- CCACTTTAGT GAACCAGGAT TCCATTGGTG AAACCAACTC GTTCTTAGTT TGTGGTTGTG ATGGGGAAAG   
  
  
- AACTGGGCCA AGTATCTCTG GGAACTGATG ATGAGTCGGT ACAAACTCAG ATATCTGCAC TGGTACGGTT   
  
  
- CTCTGGCCTT CCTCTCCTAG TTACAACTCG TCGTAACAAA CCGATTCCTG TAACACTTAT AGTATCGAAC   
  
  
- GCTCCCGTTC CTCTCCCACC TCGCAGTACT TGAAAAACCC TTTACCTTCA GTTCCAAATG GTACCGTCCC   
  
  
- AAGTCCGTCA TGGGCAACTC GAGTATGCAT TTGAGACACT ATTCTTCGGA TGAGGCCACA ATGAGGCTCG   
  
  
- TAATATGAGA CTATCTCTTC CTACCACGGT ACGAAGACCC AACCTTCCCG GCTTACGACT AAAGTCGAAG   
  
  
- CCGTACCGTA AC

+     DRE1

| Site Name | Organism | Position | Strand | Matrix score. | sequence | function |
| --- | --- | --- | --- | --- | --- | --- |
| DRE1 | Zea mays | 2801 | - | 7 | ACCGAGA |  |

>HU08G00014.1   
+ +Up\_Stream \_Len000TCCTCT TTTGGGCTTC TTTCTTTCTA TCCTGTTTTT TTTTGGGTCG AAAAGTTGGA   
  
  
+ GGTTGAATTG AAGCATCCGA ATTCGAGCAG GATGTTTCTC TGCGTAGTGA TTCTTTCTGG GTTTGTGATT   
  
  
+ TTGAATTCTG GGTAATCGGT TGTTTTGCTA ATTTTGAGGA CAGAGTTCCT TTTCTGAATT AAAATTTCGT   
  
  
+ TTTTTTATCG GGAAAATCTT CAGTATTTGA GAAAAAAGAA CGAAATTTGG ATGATTTGCT GTCTAGATTT   
  
  
+ TGCTTTCATA TTCCTGGGTG TGGATTGGTT TATTAATTGC ATGTGAGGAA GTACAAGCTT CTGGTCAATG   
  
  
+ GTTGGGTTTT TTTTTTTTTT TTGTGGGTAT AAAGTTGGAA ATTGATTACG AGTCAAATTT CTCTTTCTAG   
  
  
+ AAGAGGGGGG GGGGGGGGGG TTGGTTTAAA ACTCCATATT TTCTTTGAAT GGAAAGCTTA ATTTGCTGAC   
  
  
+ TATCTTGGTT AGTAGCATAA GCTTGATCAG AAAACAAAAC GCTCTCTGCC CTGCAAAAAC TTTATTTGTC   
  
  
+ TTTTAAGGAA AATTTTGGAA TTTGAGAGTG AAAATTTTGG TTGTGAATCT TGTCTTACTA TGTGGTTCCT   
  
  
+ TCTGACAAAA GTTTGAATCT GAGTATGAGA GTTCATCAAT CTCTTTTACT TTCCTACTTT CTTTATTGCT   
  
  
+ TGCAAATTGT TGTGAACTTA CTTGGATTTT CTGGTAGAGG AGAGTTCTGT CCTGGAATTT GGTGGAAAGT   
  
  
+ GAGACATACT TTGGGATTTA ATTAGCCATC AATTTGGTTT CACACCTTAT CAGTTTGGAC TTCTGGTGTG   
  
  
+ TTATTGTATG TGTTCTTAGC TTGGGATTAT TCTTGTATCG GAAGAGAGGG GGGGGGGGGT GTTTCTTTTA   
  
  
+ GCTGAATGTT AGAACATTTT AAGGGATCTA GATATCTAGA GAAGAGCCTA GGAAATGAAG CATTTTTCCC   
  
  
+ TTATAATCTG ACACAACTTT ATGAATTGAT TAGGAACAAA GAACGATTAG TTCAGAAAGG TTACAACTCC   
  
  
+ TCTAGGTGTT ATACTAAAGA GATGTGAGTT AATAAGCAAT TGCTTACTAA CTGTTTAGGC TGTTTCATTG   
  
  
+ CTTGTAGAGG CTTTTTTTTT GGGGGGGGGG GGGGGTTTGG GGTTCATAAA TGAGAAGGTT GATTTGTTGT   
  
  
+ CTGCTCTGCT CTTGATTCGA TAGTAATTCT CTCAGAAAAG GGTAAAATGA CATGGCCTTC CATCGCCTCC   
  
  
+ CATGAGGCTT TTTGCCTCTG CTTGTGTATC TTTCATCTCT TGGTTTGGTG GGTTGGGGGA GGGGAGGGGG   
  
  
+ GGAAGGGGGT TTTGATTCGA TAGATCATCA CTTTCAGAAA TGGCCTCTCT GGCCAATGGT AAGGGGTAAG   
  
  
+ GTTAGTACAT GTGGACTTTC CCATGTGGAT TTTTCCAGAG TCCGTTGTGT GATTGTTATT GCTGTTGTTG   
  
  
+ TAACTTTCTG TCACTCGGAA AACATGAATC CATATGCAAC AAGAACATAA TAATTGTGAA TGCGACTTTA   
  
  
+ TTTAGGAATG TCTTACATAG TACGTGCACA TTCTTTAATT ATCTGTTTAT CTGTTGGTTT GTTATTTTCA   
  
  
+ AGGGGATGGG GAGTGGGTGG ATGCTTAGTA TGTAATCTTT TTATATAGGG AAAAATCATA TAGCTTGAAA   
  
  
+ GTAAAACTTT CATTGCCAGG ATTCCAAGGT AGGGGTAGGG TTGTCTACAC CTTGATCTCC CTAGAAGCAT   
  
  
+ GGCGATCCTT CCTCGGATTG TGTTATAGAA TAAAGAATGT GGTTCTGCTG TAATGAGACT TATGTTCTAT   
  
  
+ CAAATGATGA ATCTGTTTAG AACTTCTTGC TTATTTTTTC TGCAAAATTT TAGGAGACTT GATAAACTAT   
  
  
+ AGTTCTCTTG AATTTACAGG TGGATATTGG AGTTCACAGA ATAGTATTCA GATCAAAGTG CTTGTGCTTA   
  
  
+ ATTGGAAGGA CAACTTGCTT GAGCTGTTTA TAGAGCTCTA ACTGATGCTA TCTCATGGAC TCACATCAGT   
  
  
+ TTTTTGGATT TAATCTTACC AGTGTTGATC CGTCGTACAT TTCCGCTCAG TACAGTCCGC CGTCAGTGAC   
  
  
+ AAATAGGATG TTTGCATCAC TGAAGCTCGA CTCTAGAGGT TCTCCTGTGT CACCCTTCTC AACTCAGTTT   
  
  
+ GATTGTGATA CGGTTACTAC ATTGAGTGAT AGTCAAGAGC ATCACAGCTC GACGGGGAGT CTATCAACAA   
  
  
+ GAAGCCCTTC TTGTAATTCT CCCCTTGAAA CGAGCAGTTA TCATCATTTA TCGACGAATG GCCCGTCTTG   
  
  
+ TAATTCTGCC CCTGAAACTA GCAGTTATCG TCATCGGTTC AACGCGAGTC CCGTGGGATA TTCCCATCAA   
  
  
+ GGCACTGATT ATGGAGTGAA TATGAAGAGC GCTCTGCAGG AGCTAGAGAC TACTCTAATG GGTGCAGATG   
  
  
+ GTGAGGAAGT ATCTGCTGCT AATCAACCTA TGGGGGGAAG TCGTCAGTCC GGGATCCCAA GTCAGAGATC   
  
  
+ AAAATCATTG AGCGAAGATC CACAGGGTTC GCATCCTACT CAGCCTGATT CATCATCTCT TTCTAGGGCA   
  
  
+ AGAAGATCAG GAGATGAAAG CCAGAGAGAG AAACGGCACA AGGCAATGGA AGAACCAACG GAACTACCAA   
  
  
+ GTTTGCCACC TGGTGATTTG AAGCAGTTGC TAATTGAATG TGCGAGGGCT TTATCGGATA ACCGAATAGA   
  
  
+ TGACTTTGAG AGTTTGGTTA AACGGGCGAG GAAAGAGGTC TCTATCTCGG GTGAGCCTAT CCAACGTCTC   
  
  
+ GGTGCCTACA TGATCGAAGG GCTTGTGGCA AGGAAGCAGT CTTCGGGGAC TAGCATCTAT CGGGCTCTGA   
  
  
+ AGTGTAAAGA GCCTCTTGGA AAAGACTTGC TCTCCTACAT GCACATCCTT TATGAAATAT GCCCTTATCT   
  
  
+ CAAGTTTGGT TATATGGCTG CGAATGGAGC GATAGCTGAA GCTTGTAGAA ATGAGGATAA CATACATATT   
  
  
+ ATAGATTTCC AGATTGCACA GGGCACTCAG TGGGTTACTC TATTACAAGC CCTAGCAGCA AGACCTGGTG   
  
  
+ GGCCACCTAA GGTGCGAATT ACAGGCATTG ATGATCCTGT TTCTAAGTAT GCCCGTGGTG CTAGCTTGGA   
  
  
+ GGCTGTTGGG AAACGGTTAG CGTCTCTATC TGAAAAGTTC AAAATACCCG TCGAGTTCAA TGCGTTGCCC   
  
  
+ GTTTATGGAC CCGATGTCAG GCGGGAAATG CTGGATGTGA GGCCCGGGGA GGCTTTGGCC GTTAATTTTC   
  
  
+ CATTGCAGCT CCACCACACT CCTGACGAGA GTGTCGATGT GAACAACCCT AGGGATGGGC TTCTCAGAAT   
  
  
+ GGTGAAATCA CTTGGTCCTA AGGTAACCAC TTTGGTTGAG CAAGAATCAA ACACCAACAC TACCCCTTTC   
  
  
+ TTGACCCGGT TCATAGAGAC CCTTGACTAC TACTCAGCCA TGTTTGAGTC TATAGACGTG ACCATGCCAA   
  
  
+ GAGACCGGAA GGAGAGGATC AATGTTGAGC AGCATTGTTT GGCTAAGGAC ATTGTGAATA TCATAGCTTG   
  
  
+ CGAGGGCAAG GAGAGGGTGG AGCGTCATGA ACTTTTTGGG AAATGGAAGT CAAGGTTTAC CATGGCAGGG   
  
  
+ TTCAGGCAGT ACCCGTTGAG CTCATACGTA AACTCTGTGA TAAGAAGCCT ACTCCGGTGT TACTCCGAGC   
  
  
+ ATTATACTCT GATAGAGAAG GATGGTGCCA TGCTTCTGGG TTGGAAGGGC CGAATGCTGA TTTCAGCTTC   
  
  
+ GGCATGGCAT TG  

- +Up\_Stream \_Len000AGGAGA AAACCCGAAG AAAGAAAGAT AGGACAAAAA AAAACCCAGC TTTTCAACCT   
  
  
- CCAACTTAAC TTCGTAGGCT TAAGCTCGTC CTACAAAGAG ACGCATCACT AAGAAAGACC CAAACACTAA   
  
  
- AACTTAAGAC CCATTAGCCA ACAAAACGAT TAAAACTCCT GTCTCAAGGA AAAGACTTAA TTTTAAAGCA   
  
  
- AAAAAATAGC CCTTTTAGAA GTCATAAACT CTTTTTTCTT GCTTTAAACC TACTAAACGA CAGATCTAAA   
  
  
- ACGAAAGTAT AAGGACCCAC ACCTAACCAA ATAATTAACG TACACTCCTT CATGTTCGAA GACCAGTTAC   
  
  
- CAACCCAAAA AAAAAAAAAA AACACCCATA TTTCAACCTT TAACTAATGC TCAGTTTAAA GAGAAAGATC   
  
  
- TTCTCCCCCC CCCCCCCCCC AACCAAATTT TGAGGTATAA AAGAAACTTA CCTTTCGAAT TAAACGACTG   
  
  
- ATAGAACCAA TCATCGTATT CGAACTAGTC TTTTGTTTTG CGAGAGACGG GACGTTTTTG AAATAAACAG   
  
  
- AAAATTCCTT TTAAAACCTT AAACTCTCAC TTTTAAAACC AACACTTAGA ACAGAATGAT ACACCAAGGA   
  
  
- AGACTGTTTT CAAACTTAGA CTCATACTCT CAAGTAGTTA GAGAAAATGA AAGGATGAAA GAAATAACGA   
  
  
- ACGTTTAACA ACACTTGAAT GAACCTAAAA GACCATCTCC TCTCAAGACA GGACCTTAAA CCACCTTTCA   
  
  
- CTCTGTATGA AACCCTAAAT TAATCGGTAG TTAAACCAAA GTGTGGAATA GTCAAACCTG AAGACCACAC   
  
  
- AATAACATAC ACAAGAATCG AACCCTAATA AGAACATAGC CTTCTCTCCC CCCCCCCCCA CAAAGAAAAT   
  
  
- CGACTTACAA TCTTGTAAAA TTCCCTAGAT CTATAGATCT CTTCTCGGAT CCTTTACTTC GTAAAAAGGG   
  
  
- AATATTAGAC TGTGTTGAAA TACTTAACTA ATCCTTGTTT CTTGCTAATC AAGTCTTTCC AATGTTGAGG   
  
  
- AGATCCACAA TATGATTTCT CTACACTCAA TTATTCGTTA ACGAATGATT GACAAATCCG ACAAAGTAAC   
  
  
- GAACATCTCC GAAAAAAAAA CCCCCCCCCC CCCCCAAACC CCAAGTATTT ACTCTTCCAA CTAAACAACA   
  
  
- GACGAGACGA GAACTAAGCT ATCATTAAGA GAGTCTTTTC CCATTTTACT GTACCGGAAG GTAGCGGAGG   
  
  
- GTACTCCGAA AAACGGAGAC GAACACATAG AAAGTAGAGA ACCAAACCAC CCAACCCCCT CCCCTCCCCC   
  
  
- CCTTCCCCCA AAACTAAGCT ATCTAGTAGT GAAAGTCTTT ACCGGAGAGA CCGGTTACCA TTCCCCATTC   
  
  
- CAATCATGTA CACCTGAAAG GGTACACCTA AAAAGGTCTC AGGCAACACA CTAACAATAA CGACAACAAC   
  
  
- ATTGAAAGAC AGTGAGCCTT TTGTACTTAG GTATACGTTG TTCTTGTATT ATTAACACTT ACGCTGAAAT   
  
  
- AAATCCTTAC AGAATGTATC ATGCACGTGT AAGAAATTAA TAGACAAATA GACAACCAAA CAATAAAAGT   
  
  
- TCCCCTACCC CTCACCCACC TACGAATCAT ACATTAGAAA AATATATCCC TTTTTAGTAT ATCGAACTTT   
  
  
- CATTTTGAAA GTAACGGTCC TAAGGTTCCA TCCCCATCCC AACAGATGTG GAACTAGAGG GATCTTCGTA   
  
  
- CCGCTAGGAA GGAGCCTAAC ACAATATCTT ATTTCTTACA CCAAGACGAC ATTACTCTGA ATACAAGATA   
  
  
- GTTTACTACT TAGACAAATC TTGAAGAACG AATAAAAAAG ACGTTTTAAA ATCCTCTGAA CTATTTGATA   
  
  
- TCAAGAGAAC TTAAATGTCC ACCTATAACC TCAAGTGTCT TATCATAAGT CTAGTTTCAC GAACACGAAT   
  
  
- TAACCTTCCT GTTGAACGAA CTCGACAAAT ATCTCGAGAT TGACTACGAT AGAGTACCTG AGTGTAGTCA   
  
  
- AAAAACCTAA ATTAGAATGG TCACAACTAG GCAGCATGTA AAGGCGAGTC ATGTCAGGCG GCAGTCACTG   
  
  
- TTTATCCTAC AAACGTAGTG ACTTCGAGCT GAGATCTCCA AGAGGACACA GTGGGAAGAG TTGAGTCAAA   
  
  
- CTAACACTAT GCCAATGATG TAACTCACTA TCAGTTCTCG TAGTGTCGAG CTGCCCCTCA GATAGTTGTT   
  
  
- CTTCGGGAAG AACATTAAGA GGGGAACTTT GCTCGTCAAT AGTAGTAAAT AGCTGCTTAC CGGGCAGAAC   
  
  
- ATTAAGACGG GGACTTTGAT CGTCAATAGC AGTAGCCAAG TTGCGCTCAG GGCACCCTAT AAGGGTAGTT   
  
  
- CCGTGACTAA TACCTCACTT ATACTTCTCG CGAGACGTCC TCGATCTCTG ATGAGATTAC CCACGTCTAC   
  
  
- CACTCCTTCA TAGACGACGA TTAGTTGGAT ACCCCCCTTC AGCAGTCAGG CCCTAGGGTT CAGTCTCTAG   
  
  
- TTTTAGTAAC TCGCTTCTAG GTGTCCCAAG CGTAGGATGA GTCGGACTAA GTAGTAGAGA AAGATCCCGT   
  
  
- TCTTCTAGTC CTCTACTTTC GGTCTCTCTC TTTGCCGTGT TCCGTTACCT TCTTGGTTGC CTTGATGGTT   
  
  
- CAAACGGTGG ACCACTAAAC TTCGTCAACG ATTAACTTAC ACGCTCCCGA AATAGCCTAT TGGCTTATCT   
  
  
- ACTGAAACTC TCAAACCAAT TTGCCCGCTC CTTTCTCCAG AGATAGAGCC CACTCGGATA GGTTGCAGAG   
  
  
- CCACGGATGT ACTAGCTTCC CGAACACCGT TCCTTCGTCA GAAGCCCCTG ATCGTAGATA GCCCGAGACT   
  
  
- TCACATTTCT CGGAGAACCT TTTCTGAACG AGAGGATGTA CGTGTAGGAA ATACTTTATA CGGGAATAGA   
  
  
- GTTCAAACCA ATATACCGAC GCTTACCTCG CTATCGACTT CGAACATCTT TACTCCTATT GTATGTATAA   
  
  
- TATCTAAAGG TCTAACGTGT CCCGTGAGTC ACCCAATGAG ATAATGTTCG GGATCGTCGT TCTGGACCAC   
  
  
- CCGGTGGATT CCACGCTTAA TGTCCGTAAC TACTAGGACA AAGATTCATA CGGGCACCAC GATCGAACCT   
  
  
- CCGACAACCC TTTGCCAATC GCAGAGATAG ACTTTTCAAG TTTTATGGGC AGCTCAAGTT ACGCAACGGG   
  
  
- CAAATACCTG GGCTACAGTC CGCCCTTTAC GACCTACACT CCGGGCCCCT CCGAAACCGG CAATTAAAAG   
  
  
- GTAACGTCGA GGTGGTGTGA GGACTGCTCT CACAGCTACA CTTGTTGGGA TCCCTACCCG AAGAGTCTTA   
  
  
- CCACTTTAGT GAACCAGGAT TCCATTGGTG AAACCAACTC GTTCTTAGTT TGTGGTTGTG ATGGGGAAAG   
  
  
- AACTGGGCCA AGTATCTCTG GGAACTGATG ATGAGTCGGT ACAAACTCAG ATATCTGCAC TGGTACGGTT   
  
  
- CTCTGGCCTT CCTCTCCTAG TTACAACTCG TCGTAACAAA CCGATTCCTG TAACACTTAT AGTATCGAAC   
  
  
- GCTCCCGTTC CTCTCCCACC TCGCAGTACT TGAAAAACCC TTTACCTTCA GTTCCAAATG GTACCGTCCC   
  
  
- AAGTCCGTCA TGGGCAACTC GAGTATGCAT TTGAGACACT ATTCTTCGGA TGAGGCCACA ATGAGGCTCG   
  
  
- TAATATGAGA CTATCTCTTC CTACCACGGT ACGAAGACCC AACCTTCCCG GCTTACGACT AAAGTCGAAG   
  
  
- CCGTACCGTA AC

+     ERE

| Site Name | Organism | Position | Strand | Matrix score. | sequence | function |
| --- | --- | --- | --- | --- | --- | --- |
| ERE | Nicotiana glutinos | 2925 | - | 8 | ATTTCATA |  |

>HU08G00014.1   
+ +Up\_Stream \_Len000TCCTCT TTTGGGCTTC TTTCTTTCTA TCCTGTTTTT TTTTGGGTCG AAAAGTTGGA   
  
  
+ GGTTGAATTG AAGCATCCGA ATTCGAGCAG GATGTTTCTC TGCGTAGTGA TTCTTTCTGG GTTTGTGATT   
  
  
+ TTGAATTCTG GGTAATCGGT TGTTTTGCTA ATTTTGAGGA CAGAGTTCCT TTTCTGAATT AAAATTTCGT   
  
  
+ TTTTTTATCG GGAAAATCTT CAGTATTTGA GAAAAAAGAA CGAAATTTGG ATGATTTGCT GTCTAGATTT   
  
  
+ TGCTTTCATA TTCCTGGGTG TGGATTGGTT TATTAATTGC ATGTGAGGAA GTACAAGCTT CTGGTCAATG   
  
  
+ GTTGGGTTTT TTTTTTTTTT TTGTGGGTAT AAAGTTGGAA ATTGATTACG AGTCAAATTT CTCTTTCTAG   
  
  
+ AAGAGGGGGG GGGGGGGGGG TTGGTTTAAA ACTCCATATT TTCTTTGAAT GGAAAGCTTA ATTTGCTGAC   
  
  
+ TATCTTGGTT AGTAGCATAA GCTTGATCAG AAAACAAAAC GCTCTCTGCC CTGCAAAAAC TTTATTTGTC   
  
  
+ TTTTAAGGAA AATTTTGGAA TTTGAGAGTG AAAATTTTGG TTGTGAATCT TGTCTTACTA TGTGGTTCCT   
  
  
+ TCTGACAAAA GTTTGAATCT GAGTATGAGA GTTCATCAAT CTCTTTTACT TTCCTACTTT CTTTATTGCT   
  
  
+ TGCAAATTGT TGTGAACTTA CTTGGATTTT CTGGTAGAGG AGAGTTCTGT CCTGGAATTT GGTGGAAAGT   
  
  
+ GAGACATACT TTGGGATTTA ATTAGCCATC AATTTGGTTT CACACCTTAT CAGTTTGGAC TTCTGGTGTG   
  
  
+ TTATTGTATG TGTTCTTAGC TTGGGATTAT TCTTGTATCG GAAGAGAGGG GGGGGGGGGT GTTTCTTTTA   
  
  
+ GCTGAATGTT AGAACATTTT AAGGGATCTA GATATCTAGA GAAGAGCCTA GGAAATGAAG CATTTTTCCC   
  
  
+ TTATAATCTG ACACAACTTT ATGAATTGAT TAGGAACAAA GAACGATTAG TTCAGAAAGG TTACAACTCC   
  
  
+ TCTAGGTGTT ATACTAAAGA GATGTGAGTT AATAAGCAAT TGCTTACTAA CTGTTTAGGC TGTTTCATTG   
  
  
+ CTTGTAGAGG CTTTTTTTTT GGGGGGGGGG GGGGGTTTGG GGTTCATAAA TGAGAAGGTT GATTTGTTGT   
  
  
+ CTGCTCTGCT CTTGATTCGA TAGTAATTCT CTCAGAAAAG GGTAAAATGA CATGGCCTTC CATCGCCTCC   
  
  
+ CATGAGGCTT TTTGCCTCTG CTTGTGTATC TTTCATCTCT TGGTTTGGTG GGTTGGGGGA GGGGAGGGGG   
  
  
+ GGAAGGGGGT TTTGATTCGA TAGATCATCA CTTTCAGAAA TGGCCTCTCT GGCCAATGGT AAGGGGTAAG   
  
  
+ GTTAGTACAT GTGGACTTTC CCATGTGGAT TTTTCCAGAG TCCGTTGTGT GATTGTTATT GCTGTTGTTG   
  
  
+ TAACTTTCTG TCACTCGGAA AACATGAATC CATATGCAAC AAGAACATAA TAATTGTGAA TGCGACTTTA   
  
  
+ TTTAGGAATG TCTTACATAG TACGTGCACA TTCTTTAATT ATCTGTTTAT CTGTTGGTTT GTTATTTTCA   
  
  
+ AGGGGATGGG GAGTGGGTGG ATGCTTAGTA TGTAATCTTT TTATATAGGG AAAAATCATA TAGCTTGAAA   
  
  
+ GTAAAACTTT CATTGCCAGG ATTCCAAGGT AGGGGTAGGG TTGTCTACAC CTTGATCTCC CTAGAAGCAT   
  
  
+ GGCGATCCTT CCTCGGATTG TGTTATAGAA TAAAGAATGT GGTTCTGCTG TAATGAGACT TATGTTCTAT   
  
  
+ CAAATGATGA ATCTGTTTAG AACTTCTTGC TTATTTTTTC TGCAAAATTT TAGGAGACTT GATAAACTAT   
  
  
+ AGTTCTCTTG AATTTACAGG TGGATATTGG AGTTCACAGA ATAGTATTCA GATCAAAGTG CTTGTGCTTA   
  
  
+ ATTGGAAGGA CAACTTGCTT GAGCTGTTTA TAGAGCTCTA ACTGATGCTA TCTCATGGAC TCACATCAGT   
  
  
+ TTTTTGGATT TAATCTTACC AGTGTTGATC CGTCGTACAT TTCCGCTCAG TACAGTCCGC CGTCAGTGAC   
  
  
+ AAATAGGATG TTTGCATCAC TGAAGCTCGA CTCTAGAGGT TCTCCTGTGT CACCCTTCTC AACTCAGTTT   
  
  
+ GATTGTGATA CGGTTACTAC ATTGAGTGAT AGTCAAGAGC ATCACAGCTC GACGGGGAGT CTATCAACAA   
  
  
+ GAAGCCCTTC TTGTAATTCT CCCCTTGAAA CGAGCAGTTA TCATCATTTA TCGACGAATG GCCCGTCTTG   
  
  
+ TAATTCTGCC CCTGAAACTA GCAGTTATCG TCATCGGTTC AACGCGAGTC CCGTGGGATA TTCCCATCAA   
  
  
+ GGCACTGATT ATGGAGTGAA TATGAAGAGC GCTCTGCAGG AGCTAGAGAC TACTCTAATG GGTGCAGATG   
  
  
+ GTGAGGAAGT ATCTGCTGCT AATCAACCTA TGGGGGGAAG TCGTCAGTCC GGGATCCCAA GTCAGAGATC   
  
  
+ AAAATCATTG AGCGAAGATC CACAGGGTTC GCATCCTACT CAGCCTGATT CATCATCTCT TTCTAGGGCA   
  
  
+ AGAAGATCAG GAGATGAAAG CCAGAGAGAG AAACGGCACA AGGCAATGGA AGAACCAACG GAACTACCAA   
  
  
+ GTTTGCCACC TGGTGATTTG AAGCAGTTGC TAATTGAATG TGCGAGGGCT TTATCGGATA ACCGAATAGA   
  
  
+ TGACTTTGAG AGTTTGGTTA AACGGGCGAG GAAAGAGGTC TCTATCTCGG GTGAGCCTAT CCAACGTCTC   
  
  
+ GGTGCCTACA TGATCGAAGG GCTTGTGGCA AGGAAGCAGT CTTCGGGGAC TAGCATCTAT CGGGCTCTGA   
  
  
+ AGTGTAAAGA GCCTCTTGGA AAAGACTTGC TCTCCTACAT GCACATCCTT TATGAAATAT GCCCTTATCT   
  
  
+ CAAGTTTGGT TATATGGCTG CGAATGGAGC GATAGCTGAA GCTTGTAGAA ATGAGGATAA CATACATATT   
  
  
+ ATAGATTTCC AGATTGCACA GGGCACTCAG TGGGTTACTC TATTACAAGC CCTAGCAGCA AGACCTGGTG   
  
  
+ GGCCACCTAA GGTGCGAATT ACAGGCATTG ATGATCCTGT TTCTAAGTAT GCCCGTGGTG CTAGCTTGGA   
  
  
+ GGCTGTTGGG AAACGGTTAG CGTCTCTATC TGAAAAGTTC AAAATACCCG TCGAGTTCAA TGCGTTGCCC   
  
  
+ GTTTATGGAC CCGATGTCAG GCGGGAAATG CTGGATGTGA GGCCCGGGGA GGCTTTGGCC GTTAATTTTC   
  
  
+ CATTGCAGCT CCACCACACT CCTGACGAGA GTGTCGATGT GAACAACCCT AGGGATGGGC TTCTCAGAAT   
  
  
+ GGTGAAATCA CTTGGTCCTA AGGTAACCAC TTTGGTTGAG CAAGAATCAA ACACCAACAC TACCCCTTTC   
  
  
+ TTGACCCGGT TCATAGAGAC CCTTGACTAC TACTCAGCCA TGTTTGAGTC TATAGACGTG ACCATGCCAA   
  
  
+ GAGACCGGAA GGAGAGGATC AATGTTGAGC AGCATTGTTT GGCTAAGGAC ATTGTGAATA TCATAGCTTG   
  
  
+ CGAGGGCAAG GAGAGGGTGG AGCGTCATGA ACTTTTTGGG AAATGGAAGT CAAGGTTTAC CATGGCAGGG   
  
  
+ TTCAGGCAGT ACCCGTTGAG CTCATACGTA AACTCTGTGA TAAGAAGCCT ACTCCGGTGT TACTCCGAGC   
  
  
+ ATTATACTCT GATAGAGAAG GATGGTGCCA TGCTTCTGGG TTGGAAGGGC CGAATGCTGA TTTCAGCTTC   
  
  
+ GGCATGGCAT TG  

- +Up\_Stream \_Len000AGGAGA AAACCCGAAG AAAGAAAGAT AGGACAAAAA AAAACCCAGC TTTTCAACCT   
  
  
- CCAACTTAAC TTCGTAGGCT TAAGCTCGTC CTACAAAGAG ACGCATCACT AAGAAAGACC CAAACACTAA   
  
  
- AACTTAAGAC CCATTAGCCA ACAAAACGAT TAAAACTCCT GTCTCAAGGA AAAGACTTAA TTTTAAAGCA   
  
  
- AAAAAATAGC CCTTTTAGAA GTCATAAACT CTTTTTTCTT GCTTTAAACC TACTAAACGA CAGATCTAAA   
  
  
- ACGAAAGTAT AAGGACCCAC ACCTAACCAA ATAATTAACG TACACTCCTT CATGTTCGAA GACCAGTTAC   
  
  
- CAACCCAAAA AAAAAAAAAA AACACCCATA TTTCAACCTT TAACTAATGC TCAGTTTAAA GAGAAAGATC   
  
  
- TTCTCCCCCC CCCCCCCCCC AACCAAATTT TGAGGTATAA AAGAAACTTA CCTTTCGAAT TAAACGACTG   
  
  
- ATAGAACCAA TCATCGTATT CGAACTAGTC TTTTGTTTTG CGAGAGACGG GACGTTTTTG AAATAAACAG   
  
  
- AAAATTCCTT TTAAAACCTT AAACTCTCAC TTTTAAAACC AACACTTAGA ACAGAATGAT ACACCAAGGA   
  
  
- AGACTGTTTT CAAACTTAGA CTCATACTCT CAAGTAGTTA GAGAAAATGA AAGGATGAAA GAAATAACGA   
  
  
- ACGTTTAACA ACACTTGAAT GAACCTAAAA GACCATCTCC TCTCAAGACA GGACCTTAAA CCACCTTTCA   
  
  
- CTCTGTATGA AACCCTAAAT TAATCGGTAG TTAAACCAAA GTGTGGAATA GTCAAACCTG AAGACCACAC   
  
  
- AATAACATAC ACAAGAATCG AACCCTAATA AGAACATAGC CTTCTCTCCC CCCCCCCCCA CAAAGAAAAT   
  
  
- CGACTTACAA TCTTGTAAAA TTCCCTAGAT CTATAGATCT CTTCTCGGAT CCTTTACTTC GTAAAAAGGG   
  
  
- AATATTAGAC TGTGTTGAAA TACTTAACTA ATCCTTGTTT CTTGCTAATC AAGTCTTTCC AATGTTGAGG   
  
  
- AGATCCACAA TATGATTTCT CTACACTCAA TTATTCGTTA ACGAATGATT GACAAATCCG ACAAAGTAAC   
  
  
- GAACATCTCC GAAAAAAAAA CCCCCCCCCC CCCCCAAACC CCAAGTATTT ACTCTTCCAA CTAAACAACA   
  
  
- GACGAGACGA GAACTAAGCT ATCATTAAGA GAGTCTTTTC CCATTTTACT GTACCGGAAG GTAGCGGAGG   
  
  
- GTACTCCGAA AAACGGAGAC GAACACATAG AAAGTAGAGA ACCAAACCAC CCAACCCCCT CCCCTCCCCC   
  
  
- CCTTCCCCCA AAACTAAGCT ATCTAGTAGT GAAAGTCTTT ACCGGAGAGA CCGGTTACCA TTCCCCATTC   
  
  
- CAATCATGTA CACCTGAAAG GGTACACCTA AAAAGGTCTC AGGCAACACA CTAACAATAA CGACAACAAC   
  
  
- ATTGAAAGAC AGTGAGCCTT TTGTACTTAG GTATACGTTG TTCTTGTATT ATTAACACTT ACGCTGAAAT   
  
  
- AAATCCTTAC AGAATGTATC ATGCACGTGT AAGAAATTAA TAGACAAATA GACAACCAAA CAATAAAAGT   
  
  
- TCCCCTACCC CTCACCCACC TACGAATCAT ACATTAGAAA AATATATCCC TTTTTAGTAT ATCGAACTTT   
  
  
- CATTTTGAAA GTAACGGTCC TAAGGTTCCA TCCCCATCCC AACAGATGTG GAACTAGAGG GATCTTCGTA   
  
  
- CCGCTAGGAA GGAGCCTAAC ACAATATCTT ATTTCTTACA CCAAGACGAC ATTACTCTGA ATACAAGATA   
  
  
- GTTTACTACT TAGACAAATC TTGAAGAACG AATAAAAAAG ACGTTTTAAA ATCCTCTGAA CTATTTGATA   
  
  
- TCAAGAGAAC TTAAATGTCC ACCTATAACC TCAAGTGTCT TATCATAAGT CTAGTTTCAC GAACACGAAT   
  
  
- TAACCTTCCT GTTGAACGAA CTCGACAAAT ATCTCGAGAT TGACTACGAT AGAGTACCTG AGTGTAGTCA   
  
  
- AAAAACCTAA ATTAGAATGG TCACAACTAG GCAGCATGTA AAGGCGAGTC ATGTCAGGCG GCAGTCACTG   
  
  
- TTTATCCTAC AAACGTAGTG ACTTCGAGCT GAGATCTCCA AGAGGACACA GTGGGAAGAG TTGAGTCAAA   
  
  
- CTAACACTAT GCCAATGATG TAACTCACTA TCAGTTCTCG TAGTGTCGAG CTGCCCCTCA GATAGTTGTT   
  
  
- CTTCGGGAAG AACATTAAGA GGGGAACTTT GCTCGTCAAT AGTAGTAAAT AGCTGCTTAC CGGGCAGAAC   
  
  
- ATTAAGACGG GGACTTTGAT CGTCAATAGC AGTAGCCAAG TTGCGCTCAG GGCACCCTAT AAGGGTAGTT   
  
  
- CCGTGACTAA TACCTCACTT ATACTTCTCG CGAGACGTCC TCGATCTCTG ATGAGATTAC CCACGTCTAC   
  
  
- CACTCCTTCA TAGACGACGA TTAGTTGGAT ACCCCCCTTC AGCAGTCAGG CCCTAGGGTT CAGTCTCTAG   
  
  
- TTTTAGTAAC TCGCTTCTAG GTGTCCCAAG CGTAGGATGA GTCGGACTAA GTAGTAGAGA AAGATCCCGT   
  
  
- TCTTCTAGTC CTCTACTTTC GGTCTCTCTC TTTGCCGTGT TCCGTTACCT TCTTGGTTGC CTTGATGGTT   
  
  
- CAAACGGTGG ACCACTAAAC TTCGTCAACG ATTAACTTAC ACGCTCCCGA AATAGCCTAT TGGCTTATCT   
  
  
- ACTGAAACTC TCAAACCAAT TTGCCCGCTC CTTTCTCCAG AGATAGAGCC CACTCGGATA GGTTGCAGAG   
  
  
- CCACGGATGT ACTAGCTTCC CGAACACCGT TCCTTCGTCA GAAGCCCCTG ATCGTAGATA GCCCGAGACT   
  
  
- TCACATTTCT CGGAGAACCT TTTCTGAACG AGAGGATGTA CGTGTAGGAA ATACTTTATA CGGGAATAGA   
  
  
- GTTCAAACCA ATATACCGAC GCTTACCTCG CTATCGACTT CGAACATCTT TACTCCTATT GTATGTATAA   
  
  
- TATCTAAAGG TCTAACGTGT CCCGTGAGTC ACCCAATGAG ATAATGTTCG GGATCGTCGT TCTGGACCAC   
  
  
- CCGGTGGATT CCACGCTTAA TGTCCGTAAC TACTAGGACA AAGATTCATA CGGGCACCAC GATCGAACCT   
  
  
- CCGACAACCC TTTGCCAATC GCAGAGATAG ACTTTTCAAG TTTTATGGGC AGCTCAAGTT ACGCAACGGG   
  
  
- CAAATACCTG GGCTACAGTC CGCCCTTTAC GACCTACACT CCGGGCCCCT CCGAAACCGG CAATTAAAAG   
  
  
- GTAACGTCGA GGTGGTGTGA GGACTGCTCT CACAGCTACA CTTGTTGGGA TCCCTACCCG AAGAGTCTTA   
  
  
- CCACTTTAGT GAACCAGGAT TCCATTGGTG AAACCAACTC GTTCTTAGTT TGTGGTTGTG ATGGGGAAAG   
  
  
- AACTGGGCCA AGTATCTCTG GGAACTGATG ATGAGTCGGT ACAAACTCAG ATATCTGCAC TGGTACGGTT   
  
  
- CTCTGGCCTT CCTCTCCTAG TTACAACTCG TCGTAACAAA CCGATTCCTG TAACACTTAT AGTATCGAAC   
  
  
- GCTCCCGTTC CTCTCCCACC TCGCAGTACT TGAAAAACCC TTTACCTTCA GTTCCAAATG GTACCGTCCC   
  
  
- AAGTCCGTCA TGGGCAACTC GAGTATGCAT TTGAGACACT ATTCTTCGGA TGAGGCCACA ATGAGGCTCG   
  
  
- TAATATGAGA CTATCTCTTC CTACCACGGT ACGAAGACCC AACCTTCCCG GCTTACGACT AAAGTCGAAG   
  
  
- CCGTACCGTA AC

+     G-Box

| Site Name | Organism | Position | Strand | Matrix score. | sequence | function |
| --- | --- | --- | --- | --- | --- | --- |
| G-Box | Triticum aestivum | 1423 | - | 10 | TCCACATGGCA | cis-acting regulatory element involved in light responsiveness |

>HU08G00014.1   
+ +Up\_Stream \_Len000TCCTCT TTTGGGCTTC TTTCTTTCTA TCCTGTTTTT TTTTGGGTCG AAAAGTTGGA   
  
  
+ GGTTGAATTG AAGCATCCGA ATTCGAGCAG GATGTTTCTC TGCGTAGTGA TTCTTTCTGG GTTTGTGATT   
  
  
+ TTGAATTCTG GGTAATCGGT TGTTTTGCTA ATTTTGAGGA CAGAGTTCCT TTTCTGAATT AAAATTTCGT   
  
  
+ TTTTTTATCG GGAAAATCTT CAGTATTTGA GAAAAAAGAA CGAAATTTGG ATGATTTGCT GTCTAGATTT   
  
  
+ TGCTTTCATA TTCCTGGGTG TGGATTGGTT TATTAATTGC ATGTGAGGAA GTACAAGCTT CTGGTCAATG   
  
  
+ GTTGGGTTTT TTTTTTTTTT TTGTGGGTAT AAAGTTGGAA ATTGATTACG AGTCAAATTT CTCTTTCTAG   
  
  
+ AAGAGGGGGG GGGGGGGGGG TTGGTTTAAA ACTCCATATT TTCTTTGAAT GGAAAGCTTA ATTTGCTGAC   
  
  
+ TATCTTGGTT AGTAGCATAA GCTTGATCAG AAAACAAAAC GCTCTCTGCC CTGCAAAAAC TTTATTTGTC   
  
  
+ TTTTAAGGAA AATTTTGGAA TTTGAGAGTG AAAATTTTGG TTGTGAATCT TGTCTTACTA TGTGGTTCCT   
  
  
+ TCTGACAAAA GTTTGAATCT GAGTATGAGA GTTCATCAAT CTCTTTTACT TTCCTACTTT CTTTATTGCT   
  
  
+ TGCAAATTGT TGTGAACTTA CTTGGATTTT CTGGTAGAGG AGAGTTCTGT CCTGGAATTT GGTGGAAAGT   
  
  
+ GAGACATACT TTGGGATTTA ATTAGCCATC AATTTGGTTT CACACCTTAT CAGTTTGGAC TTCTGGTGTG   
  
  
+ TTATTGTATG TGTTCTTAGC TTGGGATTAT TCTTGTATCG GAAGAGAGGG GGGGGGGGGT GTTTCTTTTA   
  
  
+ GCTGAATGTT AGAACATTTT AAGGGATCTA GATATCTAGA GAAGAGCCTA GGAAATGAAG CATTTTTCCC   
  
  
+ TTATAATCTG ACACAACTTT ATGAATTGAT TAGGAACAAA GAACGATTAG TTCAGAAAGG TTACAACTCC   
  
  
+ TCTAGGTGTT ATACTAAAGA GATGTGAGTT AATAAGCAAT TGCTTACTAA CTGTTTAGGC TGTTTCATTG   
  
  
+ CTTGTAGAGG CTTTTTTTTT GGGGGGGGGG GGGGGTTTGG GGTTCATAAA TGAGAAGGTT GATTTGTTGT   
  
  
+ CTGCTCTGCT CTTGATTCGA TAGTAATTCT CTCAGAAAAG GGTAAAATGA CATGGCCTTC CATCGCCTCC   
  
  
+ CATGAGGCTT TTTGCCTCTG CTTGTGTATC TTTCATCTCT TGGTTTGGTG GGTTGGGGGA GGGGAGGGGG   
  
  
+ GGAAGGGGGT TTTGATTCGA TAGATCATCA CTTTCAGAAA TGGCCTCTCT GGCCAATGGT AAGGGGTAAG   
  
  
+ GTTAGTACAT GTGGACTTTC CCATGTGGAT TTTTCCAGAG TCCGTTGTGT GATTGTTATT GCTGTTGTTG   
  
  
+ TAACTTTCTG TCACTCGGAA AACATGAATC CATATGCAAC AAGAACATAA TAATTGTGAA TGCGACTTTA   
  
  
+ TTTAGGAATG TCTTACATAG TACGTGCACA TTCTTTAATT ATCTGTTTAT CTGTTGGTTT GTTATTTTCA   
  
  
+ AGGGGATGGG GAGTGGGTGG ATGCTTAGTA TGTAATCTTT TTATATAGGG AAAAATCATA TAGCTTGAAA   
  
  
+ GTAAAACTTT CATTGCCAGG ATTCCAAGGT AGGGGTAGGG TTGTCTACAC CTTGATCTCC CTAGAAGCAT   
  
  
+ GGCGATCCTT CCTCGGATTG TGTTATAGAA TAAAGAATGT GGTTCTGCTG TAATGAGACT TATGTTCTAT   
  
  
+ CAAATGATGA ATCTGTTTAG AACTTCTTGC TTATTTTTTC TGCAAAATTT TAGGAGACTT GATAAACTAT   
  
  
+ AGTTCTCTTG AATTTACAGG TGGATATTGG AGTTCACAGA ATAGTATTCA GATCAAAGTG CTTGTGCTTA   
  
  
+ ATTGGAAGGA CAACTTGCTT GAGCTGTTTA TAGAGCTCTA ACTGATGCTA TCTCATGGAC TCACATCAGT   
  
  
+ TTTTTGGATT TAATCTTACC AGTGTTGATC CGTCGTACAT TTCCGCTCAG TACAGTCCGC CGTCAGTGAC   
  
  
+ AAATAGGATG TTTGCATCAC TGAAGCTCGA CTCTAGAGGT TCTCCTGTGT CACCCTTCTC AACTCAGTTT   
  
  
+ GATTGTGATA CGGTTACTAC ATTGAGTGAT AGTCAAGAGC ATCACAGCTC GACGGGGAGT CTATCAACAA   
  
  
+ GAAGCCCTTC TTGTAATTCT CCCCTTGAAA CGAGCAGTTA TCATCATTTA TCGACGAATG GCCCGTCTTG   
  
  
+ TAATTCTGCC CCTGAAACTA GCAGTTATCG TCATCGGTTC AACGCGAGTC CCGTGGGATA TTCCCATCAA   
  
  
+ GGCACTGATT ATGGAGTGAA TATGAAGAGC GCTCTGCAGG AGCTAGAGAC TACTCTAATG GGTGCAGATG   
  
  
+ GTGAGGAAGT ATCTGCTGCT AATCAACCTA TGGGGGGAAG TCGTCAGTCC GGGATCCCAA GTCAGAGATC   
  
  
+ AAAATCATTG AGCGAAGATC CACAGGGTTC GCATCCTACT CAGCCTGATT CATCATCTCT TTCTAGGGCA   
  
  
+ AGAAGATCAG GAGATGAAAG CCAGAGAGAG AAACGGCACA AGGCAATGGA AGAACCAACG GAACTACCAA   
  
  
+ GTTTGCCACC TGGTGATTTG AAGCAGTTGC TAATTGAATG TGCGAGGGCT TTATCGGATA ACCGAATAGA   
  
  
+ TGACTTTGAG AGTTTGGTTA AACGGGCGAG GAAAGAGGTC TCTATCTCGG GTGAGCCTAT CCAACGTCTC   
  
  
+ GGTGCCTACA TGATCGAAGG GCTTGTGGCA AGGAAGCAGT CTTCGGGGAC TAGCATCTAT CGGGCTCTGA   
  
  
+ AGTGTAAAGA GCCTCTTGGA AAAGACTTGC TCTCCTACAT GCACATCCTT TATGAAATAT GCCCTTATCT   
  
  
+ CAAGTTTGGT TATATGGCTG CGAATGGAGC GATAGCTGAA GCTTGTAGAA ATGAGGATAA CATACATATT   
  
  
+ ATAGATTTCC AGATTGCACA GGGCACTCAG TGGGTTACTC TATTACAAGC CCTAGCAGCA AGACCTGGTG   
  
  
+ GGCCACCTAA GGTGCGAATT ACAGGCATTG ATGATCCTGT TTCTAAGTAT GCCCGTGGTG CTAGCTTGGA   
  
  
+ GGCTGTTGGG AAACGGTTAG CGTCTCTATC TGAAAAGTTC AAAATACCCG TCGAGTTCAA TGCGTTGCCC   
  
  
+ GTTTATGGAC CCGATGTCAG GCGGGAAATG CTGGATGTGA GGCCCGGGGA GGCTTTGGCC GTTAATTTTC   
  
  
+ CATTGCAGCT CCACCACACT CCTGACGAGA GTGTCGATGT GAACAACCCT AGGGATGGGC TTCTCAGAAT   
  
  
+ GGTGAAATCA CTTGGTCCTA AGGTAACCAC TTTGGTTGAG CAAGAATCAA ACACCAACAC TACCCCTTTC   
  
  
+ TTGACCCGGT TCATAGAGAC CCTTGACTAC TACTCAGCCA TGTTTGAGTC TATAGACGTG ACCATGCCAA   
  
  
+ GAGACCGGAA GGAGAGGATC AATGTTGAGC AGCATTGTTT GGCTAAGGAC ATTGTGAATA TCATAGCTTG   
  
  
+ CGAGGGCAAG GAGAGGGTGG AGCGTCATGA ACTTTTTGGG AAATGGAAGT CAAGGTTTAC CATGGCAGGG   
  
  
+ TTCAGGCAGT ACCCGTTGAG CTCATACGTA AACTCTGTGA TAAGAAGCCT ACTCCGGTGT TACTCCGAGC   
  
  
+ ATTATACTCT GATAGAGAAG GATGGTGCCA TGCTTCTGGG TTGGAAGGGC CGAATGCTGA TTTCAGCTTC   
  
  
+ GGCATGGCAT TG  

- +Up\_Stream \_Len000AGGAGA AAACCCGAAG AAAGAAAGAT AGGACAAAAA AAAACCCAGC TTTTCAACCT   
  
  
- CCAACTTAAC TTCGTAGGCT TAAGCTCGTC CTACAAAGAG ACGCATCACT AAGAAAGACC CAAACACTAA   
  
  
- AACTTAAGAC CCATTAGCCA ACAAAACGAT TAAAACTCCT GTCTCAAGGA AAAGACTTAA TTTTAAAGCA   
  
  
- AAAAAATAGC CCTTTTAGAA GTCATAAACT CTTTTTTCTT GCTTTAAACC TACTAAACGA CAGATCTAAA   
  
  
- ACGAAAGTAT AAGGACCCAC ACCTAACCAA ATAATTAACG TACACTCCTT CATGTTCGAA GACCAGTTAC   
  
  
- CAACCCAAAA AAAAAAAAAA AACACCCATA TTTCAACCTT TAACTAATGC TCAGTTTAAA GAGAAAGATC   
  
  
- TTCTCCCCCC CCCCCCCCCC AACCAAATTT TGAGGTATAA AAGAAACTTA CCTTTCGAAT TAAACGACTG   
  
  
- ATAGAACCAA TCATCGTATT CGAACTAGTC TTTTGTTTTG CGAGAGACGG GACGTTTTTG AAATAAACAG   
  
  
- AAAATTCCTT TTAAAACCTT AAACTCTCAC TTTTAAAACC AACACTTAGA ACAGAATGAT ACACCAAGGA   
  
  
- AGACTGTTTT CAAACTTAGA CTCATACTCT CAAGTAGTTA GAGAAAATGA AAGGATGAAA GAAATAACGA   
  
  
- ACGTTTAACA ACACTTGAAT GAACCTAAAA GACCATCTCC TCTCAAGACA GGACCTTAAA CCACCTTTCA   
  
  
- CTCTGTATGA AACCCTAAAT TAATCGGTAG TTAAACCAAA GTGTGGAATA GTCAAACCTG AAGACCACAC   
  
  
- AATAACATAC ACAAGAATCG AACCCTAATA AGAACATAGC CTTCTCTCCC CCCCCCCCCA CAAAGAAAAT   
  
  
- CGACTTACAA TCTTGTAAAA TTCCCTAGAT CTATAGATCT CTTCTCGGAT CCTTTACTTC GTAAAAAGGG   
  
  
- AATATTAGAC TGTGTTGAAA TACTTAACTA ATCCTTGTTT CTTGCTAATC AAGTCTTTCC AATGTTGAGG   
  
  
- AGATCCACAA TATGATTTCT CTACACTCAA TTATTCGTTA ACGAATGATT GACAAATCCG ACAAAGTAAC   
  
  
- GAACATCTCC GAAAAAAAAA CCCCCCCCCC CCCCCAAACC CCAAGTATTT ACTCTTCCAA CTAAACAACA   
  
  
- GACGAGACGA GAACTAAGCT ATCATTAAGA GAGTCTTTTC CCATTTTACT GTACCGGAAG GTAGCGGAGG   
  
  
- GTACTCCGAA AAACGGAGAC GAACACATAG AAAGTAGAGA ACCAAACCAC CCAACCCCCT CCCCTCCCCC   
  
  
- CCTTCCCCCA AAACTAAGCT ATCTAGTAGT GAAAGTCTTT ACCGGAGAGA CCGGTTACCA TTCCCCATTC   
  
  
- CAATCATGTA CACCTGAAAG GGTACACCTA AAAAGGTCTC AGGCAACACA CTAACAATAA CGACAACAAC   
  
  
- ATTGAAAGAC AGTGAGCCTT TTGTACTTAG GTATACGTTG TTCTTGTATT ATTAACACTT ACGCTGAAAT   
  
  
- AAATCCTTAC AGAATGTATC ATGCACGTGT AAGAAATTAA TAGACAAATA GACAACCAAA CAATAAAAGT   
  
  
- TCCCCTACCC CTCACCCACC TACGAATCAT ACATTAGAAA AATATATCCC TTTTTAGTAT ATCGAACTTT   
  
  
- CATTTTGAAA GTAACGGTCC TAAGGTTCCA TCCCCATCCC AACAGATGTG GAACTAGAGG GATCTTCGTA   
  
  
- CCGCTAGGAA GGAGCCTAAC ACAATATCTT ATTTCTTACA CCAAGACGAC ATTACTCTGA ATACAAGATA   
  
  
- GTTTACTACT TAGACAAATC TTGAAGAACG AATAAAAAAG ACGTTTTAAA ATCCTCTGAA CTATTTGATA   
  
  
- TCAAGAGAAC TTAAATGTCC ACCTATAACC TCAAGTGTCT TATCATAAGT CTAGTTTCAC GAACACGAAT   
  
  
- TAACCTTCCT GTTGAACGAA CTCGACAAAT ATCTCGAGAT TGACTACGAT AGAGTACCTG AGTGTAGTCA   
  
  
- AAAAACCTAA ATTAGAATGG TCACAACTAG GCAGCATGTA AAGGCGAGTC ATGTCAGGCG GCAGTCACTG   
  
  
- TTTATCCTAC AAACGTAGTG ACTTCGAGCT GAGATCTCCA AGAGGACACA GTGGGAAGAG TTGAGTCAAA   
  
  
- CTAACACTAT GCCAATGATG TAACTCACTA TCAGTTCTCG TAGTGTCGAG CTGCCCCTCA GATAGTTGTT   
  
  
- CTTCGGGAAG AACATTAAGA GGGGAACTTT GCTCGTCAAT AGTAGTAAAT AGCTGCTTAC CGGGCAGAAC   
  
  
- ATTAAGACGG GGACTTTGAT CGTCAATAGC AGTAGCCAAG TTGCGCTCAG GGCACCCTAT AAGGGTAGTT   
  
  
- CCGTGACTAA TACCTCACTT ATACTTCTCG CGAGACGTCC TCGATCTCTG ATGAGATTAC CCACGTCTAC   
  
  
- CACTCCTTCA TAGACGACGA TTAGTTGGAT ACCCCCCTTC AGCAGTCAGG CCCTAGGGTT CAGTCTCTAG   
  
  
- TTTTAGTAAC TCGCTTCTAG GTGTCCCAAG CGTAGGATGA GTCGGACTAA GTAGTAGAGA AAGATCCCGT   
  
  
- TCTTCTAGTC CTCTACTTTC GGTCTCTCTC TTTGCCGTGT TCCGTTACCT TCTTGGTTGC CTTGATGGTT   
  
  
- CAAACGGTGG ACCACTAAAC TTCGTCAACG ATTAACTTAC ACGCTCCCGA AATAGCCTAT TGGCTTATCT   
  
  
- ACTGAAACTC TCAAACCAAT TTGCCCGCTC CTTTCTCCAG AGATAGAGCC CACTCGGATA GGTTGCAGAG   
  
  
- CCACGGATGT ACTAGCTTCC CGAACACCGT TCCTTCGTCA GAAGCCCCTG ATCGTAGATA GCCCGAGACT   
  
  
- TCACATTTCT CGGAGAACCT TTTCTGAACG AGAGGATGTA CGTGTAGGAA ATACTTTATA CGGGAATAGA   
  
  
- GTTCAAACCA ATATACCGAC GCTTACCTCG CTATCGACTT CGAACATCTT TACTCCTATT GTATGTATAA   
  
  
- TATCTAAAGG TCTAACGTGT CCCGTGAGTC ACCCAATGAG ATAATGTTCG GGATCGTCGT TCTGGACCAC   
  
  
- CCGGTGGATT CCACGCTTAA TGTCCGTAAC TACTAGGACA AAGATTCATA CGGGCACCAC GATCGAACCT   
  
  
- CCGACAACCC TTTGCCAATC GCAGAGATAG ACTTTTCAAG TTTTATGGGC AGCTCAAGTT ACGCAACGGG   
  
  
- CAAATACCTG GGCTACAGTC CGCCCTTTAC GACCTACACT CCGGGCCCCT CCGAAACCGG CAATTAAAAG   
  
  
- GTAACGTCGA GGTGGTGTGA GGACTGCTCT CACAGCTACA CTTGTTGGGA TCCCTACCCG AAGAGTCTTA   
  
  
- CCACTTTAGT GAACCAGGAT TCCATTGGTG AAACCAACTC GTTCTTAGTT TGTGGTTGTG ATGGGGAAAG   
  
  
- AACTGGGCCA AGTATCTCTG GGAACTGATG ATGAGTCGGT ACAAACTCAG ATATCTGCAC TGGTACGGTT   
  
  
- CTCTGGCCTT CCTCTCCTAG TTACAACTCG TCGTAACAAA CCGATTCCTG TAACACTTAT AGTATCGAAC   
  
  
- GCTCCCGTTC CTCTCCCACC TCGCAGTACT TGAAAAACCC TTTACCTTCA GTTCCAAATG GTACCGTCCC   
  
  
- AAGTCCGTCA TGGGCAACTC GAGTATGCAT TTGAGACACT ATTCTTCGGA TGAGGCCACA ATGAGGCTCG   
  
  
- TAATATGAGA CTATCTCTTC CTACCACGGT ACGAAGACCC AACCTTCCCG GCTTACGACT AAAGTCGAAG   
  
  
- CCGTACCGTA AC

+     G-box

| Site Name | Organism | Position | Strand | Matrix score. | sequence | function |
| --- | --- | --- | --- | --- | --- | --- |
| G-box | Brassica oleracea | 1056 | - | 9 | TAACACGTAG | cis-acting regulatory element involved in light responsiveness |
| G-box | Arabidopsis thaliana | 1565 | + | 6 | TACGTG | cis-acting regulatory element involved in light responsiveness |
| G-box | Zea mays | 3489 | - | 6 | CACGTC | cis-acting regulatory element involved in light responsiveness |

>HU08G00014.1   
+ +Up\_Stream \_Len000TCCTCT TTTGGGCTTC TTTCTTTCTA TCCTGTTTTT TTTTGGGTCG AAAAGTTGGA   
  
  
+ GGTTGAATTG AAGCATCCGA ATTCGAGCAG GATGTTTCTC TGCGTAGTGA TTCTTTCTGG GTTTGTGATT   
  
  
+ TTGAATTCTG GGTAATCGGT TGTTTTGCTA ATTTTGAGGA CAGAGTTCCT TTTCTGAATT AAAATTTCGT   
  
  
+ TTTTTTATCG GGAAAATCTT CAGTATTTGA GAAAAAAGAA CGAAATTTGG ATGATTTGCT GTCTAGATTT   
  
  
+ TGCTTTCATA TTCCTGGGTG TGGATTGGTT TATTAATTGC ATGTGAGGAA GTACAAGCTT CTGGTCAATG   
  
  
+ GTTGGGTTTT TTTTTTTTTT TTGTGGGTAT AAAGTTGGAA ATTGATTACG AGTCAAATTT CTCTTTCTAG   
  
  
+ AAGAGGGGGG GGGGGGGGGG TTGGTTTAAA ACTCCATATT TTCTTTGAAT GGAAAGCTTA ATTTGCTGAC   
  
  
+ TATCTTGGTT AGTAGCATAA GCTTGATCAG AAAACAAAAC GCTCTCTGCC CTGCAAAAAC TTTATTTGTC   
  
  
+ TTTTAAGGAA AATTTTGGAA TTTGAGAGTG AAAATTTTGG TTGTGAATCT TGTCTTACTA TGTGGTTCCT   
  
  
+ TCTGACAAAA GTTTGAATCT GAGTATGAGA GTTCATCAAT CTCTTTTACT TTCCTACTTT CTTTATTGCT   
  
  
+ TGCAAATTGT TGTGAACTTA CTTGGATTTT CTGGTAGAGG AGAGTTCTGT CCTGGAATTT GGTGGAAAGT   
  
  
+ GAGACATACT TTGGGATTTA ATTAGCCATC AATTTGGTTT CACACCTTAT CAGTTTGGAC TTCTGGTGTG   
  
  
+ TTATTGTATG TGTTCTTAGC TTGGGATTAT TCTTGTATCG GAAGAGAGGG GGGGGGGGGT GTTTCTTTTA   
  
  
+ GCTGAATGTT AGAACATTTT AAGGGATCTA GATATCTAGA GAAGAGCCTA GGAAATGAAG CATTTTTCCC   
  
  
+ TTATAATCTG ACACAACTTT ATGAATTGAT TAGGAACAAA GAACGATTAG TTCAGAAAGG TTACAACTCC   
  
  
+ TCTAGGTGTT ATACTAAAGA GATGTGAGTT AATAAGCAAT TGCTTACTAA CTGTTTAGGC TGTTTCATTG   
  
  
+ CTTGTAGAGG CTTTTTTTTT GGGGGGGGGG GGGGGTTTGG GGTTCATAAA TGAGAAGGTT GATTTGTTGT   
  
  
+ CTGCTCTGCT CTTGATTCGA TAGTAATTCT CTCAGAAAAG GGTAAAATGA CATGGCCTTC CATCGCCTCC   
  
  
+ CATGAGGCTT TTTGCCTCTG CTTGTGTATC TTTCATCTCT TGGTTTGGTG GGTTGGGGGA GGGGAGGGGG   
  
  
+ GGAAGGGGGT TTTGATTCGA TAGATCATCA CTTTCAGAAA TGGCCTCTCT GGCCAATGGT AAGGGGTAAG   
  
  
+ GTTAGTACAT GTGGACTTTC CCATGTGGAT TTTTCCAGAG TCCGTTGTGT GATTGTTATT GCTGTTGTTG   
  
  
+ TAACTTTCTG TCACTCGGAA AACATGAATC CATATGCAAC AAGAACATAA TAATTGTGAA TGCGACTTTA   
  
  
+ TTTAGGAATG TCTTACATAG TACGTGCACA TTCTTTAATT ATCTGTTTAT CTGTTGGTTT GTTATTTTCA   
  
  
+ AGGGGATGGG GAGTGGGTGG ATGCTTAGTA TGTAATCTTT TTATATAGGG AAAAATCATA TAGCTTGAAA   
  
  
+ GTAAAACTTT CATTGCCAGG ATTCCAAGGT AGGGGTAGGG TTGTCTACAC CTTGATCTCC CTAGAAGCAT   
  
  
+ GGCGATCCTT CCTCGGATTG TGTTATAGAA TAAAGAATGT GGTTCTGCTG TAATGAGACT TATGTTCTAT   
  
  
+ CAAATGATGA ATCTGTTTAG AACTTCTTGC TTATTTTTTC TGCAAAATTT TAGGAGACTT GATAAACTAT   
  
  
+ AGTTCTCTTG AATTTACAGG TGGATATTGG AGTTCACAGA ATAGTATTCA GATCAAAGTG CTTGTGCTTA   
  
  
+ ATTGGAAGGA CAACTTGCTT GAGCTGTTTA TAGAGCTCTA ACTGATGCTA TCTCATGGAC TCACATCAGT   
  
  
+ TTTTTGGATT TAATCTTACC AGTGTTGATC CGTCGTACAT TTCCGCTCAG TACAGTCCGC CGTCAGTGAC   
  
  
+ AAATAGGATG TTTGCATCAC TGAAGCTCGA CTCTAGAGGT TCTCCTGTGT CACCCTTCTC AACTCAGTTT   
  
  
+ GATTGTGATA CGGTTACTAC ATTGAGTGAT AGTCAAGAGC ATCACAGCTC GACGGGGAGT CTATCAACAA   
  
  
+ GAAGCCCTTC TTGTAATTCT CCCCTTGAAA CGAGCAGTTA TCATCATTTA TCGACGAATG GCCCGTCTTG   
  
  
+ TAATTCTGCC CCTGAAACTA GCAGTTATCG TCATCGGTTC AACGCGAGTC CCGTGGGATA TTCCCATCAA   
  
  
+ GGCACTGATT ATGGAGTGAA TATGAAGAGC GCTCTGCAGG AGCTAGAGAC TACTCTAATG GGTGCAGATG   
  
  
+ GTGAGGAAGT ATCTGCTGCT AATCAACCTA TGGGGGGAAG TCGTCAGTCC GGGATCCCAA GTCAGAGATC   
  
  
+ AAAATCATTG AGCGAAGATC CACAGGGTTC GCATCCTACT CAGCCTGATT CATCATCTCT TTCTAGGGCA   
  
  
+ AGAAGATCAG GAGATGAAAG CCAGAGAGAG AAACGGCACA AGGCAATGGA AGAACCAACG GAACTACCAA   
  
  
+ GTTTGCCACC TGGTGATTTG AAGCAGTTGC TAATTGAATG TGCGAGGGCT TTATCGGATA ACCGAATAGA   
  
  
+ TGACTTTGAG AGTTTGGTTA AACGGGCGAG GAAAGAGGTC TCTATCTCGG GTGAGCCTAT CCAACGTCTC   
  
  
+ GGTGCCTACA TGATCGAAGG GCTTGTGGCA AGGAAGCAGT CTTCGGGGAC TAGCATCTAT CGGGCTCTGA   
  
  
+ AGTGTAAAGA GCCTCTTGGA AAAGACTTGC TCTCCTACAT GCACATCCTT TATGAAATAT GCCCTTATCT   
  
  
+ CAAGTTTGGT TATATGGCTG CGAATGGAGC GATAGCTGAA GCTTGTAGAA ATGAGGATAA CATACATATT   
  
  
+ ATAGATTTCC AGATTGCACA GGGCACTCAG TGGGTTACTC TATTACAAGC CCTAGCAGCA AGACCTGGTG   
  
  
+ GGCCACCTAA GGTGCGAATT ACAGGCATTG ATGATCCTGT TTCTAAGTAT GCCCGTGGTG CTAGCTTGGA   
  
  
+ GGCTGTTGGG AAACGGTTAG CGTCTCTATC TGAAAAGTTC AAAATACCCG TCGAGTTCAA TGCGTTGCCC   
  
  
+ GTTTATGGAC CCGATGTCAG GCGGGAAATG CTGGATGTGA GGCCCGGGGA GGCTTTGGCC GTTAATTTTC   
  
  
+ CATTGCAGCT CCACCACACT CCTGACGAGA GTGTCGATGT GAACAACCCT AGGGATGGGC TTCTCAGAAT   
  
  
+ GGTGAAATCA CTTGGTCCTA AGGTAACCAC TTTGGTTGAG CAAGAATCAA ACACCAACAC TACCCCTTTC   
  
  
+ TTGACCCGGT TCATAGAGAC CCTTGACTAC TACTCAGCCA TGTTTGAGTC TATAGACGTG ACCATGCCAA   
  
  
+ GAGACCGGAA GGAGAGGATC AATGTTGAGC AGCATTGTTT GGCTAAGGAC ATTGTGAATA TCATAGCTTG   
  
  
+ CGAGGGCAAG GAGAGGGTGG AGCGTCATGA ACTTTTTGGG AAATGGAAGT CAAGGTTTAC CATGGCAGGG   
  
  
+ TTCAGGCAGT ACCCGTTGAG CTCATACGTA AACTCTGTGA TAAGAAGCCT ACTCCGGTGT TACTCCGAGC   
  
  
+ ATTATACTCT GATAGAGAAG GATGGTGCCA TGCTTCTGGG TTGGAAGGGC CGAATGCTGA TTTCAGCTTC   
  
  
+ GGCATGGCAT TG  

- +Up\_Stream \_Len000AGGAGA AAACCCGAAG AAAGAAAGAT AGGACAAAAA AAAACCCAGC TTTTCAACCT   
  
  
- CCAACTTAAC TTCGTAGGCT TAAGCTCGTC CTACAAAGAG ACGCATCACT AAGAAAGACC CAAACACTAA   
  
  
- AACTTAAGAC CCATTAGCCA ACAAAACGAT TAAAACTCCT GTCTCAAGGA AAAGACTTAA TTTTAAAGCA   
  
  
- AAAAAATAGC CCTTTTAGAA GTCATAAACT CTTTTTTCTT GCTTTAAACC TACTAAACGA CAGATCTAAA   
  
  
- ACGAAAGTAT AAGGACCCAC ACCTAACCAA ATAATTAACG TACACTCCTT CATGTTCGAA GACCAGTTAC   
  
  
- CAACCCAAAA AAAAAAAAAA AACACCCATA TTTCAACCTT TAACTAATGC TCAGTTTAAA GAGAAAGATC   
  
  
- TTCTCCCCCC CCCCCCCCCC AACCAAATTT TGAGGTATAA AAGAAACTTA CCTTTCGAAT TAAACGACTG   
  
  
- ATAGAACCAA TCATCGTATT CGAACTAGTC TTTTGTTTTG CGAGAGACGG GACGTTTTTG AAATAAACAG   
  
  
- AAAATTCCTT TTAAAACCTT AAACTCTCAC TTTTAAAACC AACACTTAGA ACAGAATGAT ACACCAAGGA   
  
  
- AGACTGTTTT CAAACTTAGA CTCATACTCT CAAGTAGTTA GAGAAAATGA AAGGATGAAA GAAATAACGA   
  
  
- ACGTTTAACA ACACTTGAAT GAACCTAAAA GACCATCTCC TCTCAAGACA GGACCTTAAA CCACCTTTCA   
  
  
- CTCTGTATGA AACCCTAAAT TAATCGGTAG TTAAACCAAA GTGTGGAATA GTCAAACCTG AAGACCACAC   
  
  
- AATAACATAC ACAAGAATCG AACCCTAATA AGAACATAGC CTTCTCTCCC CCCCCCCCCA CAAAGAAAAT   
  
  
- CGACTTACAA TCTTGTAAAA TTCCCTAGAT CTATAGATCT CTTCTCGGAT CCTTTACTTC GTAAAAAGGG   
  
  
- AATATTAGAC TGTGTTGAAA TACTTAACTA ATCCTTGTTT CTTGCTAATC AAGTCTTTCC AATGTTGAGG   
  
  
- AGATCCACAA TATGATTTCT CTACACTCAA TTATTCGTTA ACGAATGATT GACAAATCCG ACAAAGTAAC   
  
  
- GAACATCTCC GAAAAAAAAA CCCCCCCCCC CCCCCAAACC CCAAGTATTT ACTCTTCCAA CTAAACAACA   
  
  
- GACGAGACGA GAACTAAGCT ATCATTAAGA GAGTCTTTTC CCATTTTACT GTACCGGAAG GTAGCGGAGG   
  
  
- GTACTCCGAA AAACGGAGAC GAACACATAG AAAGTAGAGA ACCAAACCAC CCAACCCCCT CCCCTCCCCC   
  
  
- CCTTCCCCCA AAACTAAGCT ATCTAGTAGT GAAAGTCTTT ACCGGAGAGA CCGGTTACCA TTCCCCATTC   
  
  
- CAATCATGTA CACCTGAAAG GGTACACCTA AAAAGGTCTC AGGCAACACA CTAACAATAA CGACAACAAC   
  
  
- ATTGAAAGAC AGTGAGCCTT TTGTACTTAG GTATACGTTG TTCTTGTATT ATTAACACTT ACGCTGAAAT   
  
  
- AAATCCTTAC AGAATGTATC ATGCACGTGT AAGAAATTAA TAGACAAATA GACAACCAAA CAATAAAAGT   
  
  
- TCCCCTACCC CTCACCCACC TACGAATCAT ACATTAGAAA AATATATCCC TTTTTAGTAT ATCGAACTTT   
  
  
- CATTTTGAAA GTAACGGTCC TAAGGTTCCA TCCCCATCCC AACAGATGTG GAACTAGAGG GATCTTCGTA   
  
  
- CCGCTAGGAA GGAGCCTAAC ACAATATCTT ATTTCTTACA CCAAGACGAC ATTACTCTGA ATACAAGATA   
  
  
- GTTTACTACT TAGACAAATC TTGAAGAACG AATAAAAAAG ACGTTTTAAA ATCCTCTGAA CTATTTGATA   
  
  
- TCAAGAGAAC TTAAATGTCC ACCTATAACC TCAAGTGTCT TATCATAAGT CTAGTTTCAC GAACACGAAT   
  
  
- TAACCTTCCT GTTGAACGAA CTCGACAAAT ATCTCGAGAT TGACTACGAT AGAGTACCTG AGTGTAGTCA   
  
  
- AAAAACCTAA ATTAGAATGG TCACAACTAG GCAGCATGTA AAGGCGAGTC ATGTCAGGCG GCAGTCACTG   
  
  
- TTTATCCTAC AAACGTAGTG ACTTCGAGCT GAGATCTCCA AGAGGACACA GTGGGAAGAG TTGAGTCAAA   
  
  
- CTAACACTAT GCCAATGATG TAACTCACTA TCAGTTCTCG TAGTGTCGAG CTGCCCCTCA GATAGTTGTT   
  
  
- CTTCGGGAAG AACATTAAGA GGGGAACTTT GCTCGTCAAT AGTAGTAAAT AGCTGCTTAC CGGGCAGAAC   
  
  
- ATTAAGACGG GGACTTTGAT CGTCAATAGC AGTAGCCAAG TTGCGCTCAG GGCACCCTAT AAGGGTAGTT   
  
  
- CCGTGACTAA TACCTCACTT ATACTTCTCG CGAGACGTCC TCGATCTCTG ATGAGATTAC CCACGTCTAC   
  
  
- CACTCCTTCA TAGACGACGA TTAGTTGGAT ACCCCCCTTC AGCAGTCAGG CCCTAGGGTT CAGTCTCTAG   
  
  
- TTTTAGTAAC TCGCTTCTAG GTGTCCCAAG CGTAGGATGA GTCGGACTAA GTAGTAGAGA AAGATCCCGT   
  
  
- TCTTCTAGTC CTCTACTTTC GGTCTCTCTC TTTGCCGTGT TCCGTTACCT TCTTGGTTGC CTTGATGGTT   
  
  
- CAAACGGTGG ACCACTAAAC TTCGTCAACG ATTAACTTAC ACGCTCCCGA AATAGCCTAT TGGCTTATCT   
  
  
- ACTGAAACTC TCAAACCAAT TTGCCCGCTC CTTTCTCCAG AGATAGAGCC CACTCGGATA GGTTGCAGAG   
  
  
- CCACGGATGT ACTAGCTTCC CGAACACCGT TCCTTCGTCA GAAGCCCCTG ATCGTAGATA GCCCGAGACT   
  
  
- TCACATTTCT CGGAGAACCT TTTCTGAACG AGAGGATGTA CGTGTAGGAA ATACTTTATA CGGGAATAGA   
  
  
- GTTCAAACCA ATATACCGAC GCTTACCTCG CTATCGACTT CGAACATCTT TACTCCTATT GTATGTATAA   
  
  
- TATCTAAAGG TCTAACGTGT CCCGTGAGTC ACCCAATGAG ATAATGTTCG GGATCGTCGT TCTGGACCAC   
  
  
- CCGGTGGATT CCACGCTTAA TGTCCGTAAC TACTAGGACA AAGATTCATA CGGGCACCAC GATCGAACCT   
  
  
- CCGACAACCC TTTGCCAATC GCAGAGATAG ACTTTTCAAG TTTTATGGGC AGCTCAAGTT ACGCAACGGG   
  
  
- CAAATACCTG GGCTACAGTC CGCCCTTTAC GACCTACACT CCGGGCCCCT CCGAAACCGG CAATTAAAAG   
  
  
- GTAACGTCGA GGTGGTGTGA GGACTGCTCT CACAGCTACA CTTGTTGGGA TCCCTACCCG AAGAGTCTTA   
  
  
- CCACTTTAGT GAACCAGGAT TCCATTGGTG AAACCAACTC GTTCTTAGTT TGTGGTTGTG ATGGGGAAAG   
  
  
- AACTGGGCCA AGTATCTCTG GGAACTGATG ATGAGTCGGT ACAAACTCAG ATATCTGCAC TGGTACGGTT   
  
  
- CTCTGGCCTT CCTCTCCTAG TTACAACTCG TCGTAACAAA CCGATTCCTG TAACACTTAT AGTATCGAAC   
  
  
- GCTCCCGTTC CTCTCCCACC TCGCAGTACT TGAAAAACCC TTTACCTTCA GTTCCAAATG GTACCGTCCC   
  
  
- AAGTCCGTCA TGGGCAACTC GAGTATGCAT TTGAGACACT ATTCTTCGGA TGAGGCCACA ATGAGGCTCG   
  
  
- TAATATGAGA CTATCTCTTC CTACCACGGT ACGAAGACCC AACCTTCCCG GCTTACGACT AAAGTCGAAG   
  
  
- CCGTACCGTA AC

+     GARE-motif

| Site Name | Organism | Position | Strand | Matrix score. | sequence | function |
| --- | --- | --- | --- | --- | --- | --- |
| GARE-motif | Brassica oleracea | 1594 | + | 7 | TCTGTTG | gibberellin-responsive element |

>HU08G00014.1   
+ +Up\_Stream \_Len000TCCTCT TTTGGGCTTC TTTCTTTCTA TCCTGTTTTT TTTTGGGTCG AAAAGTTGGA   
  
  
+ GGTTGAATTG AAGCATCCGA ATTCGAGCAG GATGTTTCTC TGCGTAGTGA TTCTTTCTGG GTTTGTGATT   
  
  
+ TTGAATTCTG GGTAATCGGT TGTTTTGCTA ATTTTGAGGA CAGAGTTCCT TTTCTGAATT AAAATTTCGT   
  
  
+ TTTTTTATCG GGAAAATCTT CAGTATTTGA GAAAAAAGAA CGAAATTTGG ATGATTTGCT GTCTAGATTT   
  
  
+ TGCTTTCATA TTCCTGGGTG TGGATTGGTT TATTAATTGC ATGTGAGGAA GTACAAGCTT CTGGTCAATG   
  
  
+ GTTGGGTTTT TTTTTTTTTT TTGTGGGTAT AAAGTTGGAA ATTGATTACG AGTCAAATTT CTCTTTCTAG   
  
  
+ AAGAGGGGGG GGGGGGGGGG TTGGTTTAAA ACTCCATATT TTCTTTGAAT GGAAAGCTTA ATTTGCTGAC   
  
  
+ TATCTTGGTT AGTAGCATAA GCTTGATCAG AAAACAAAAC GCTCTCTGCC CTGCAAAAAC TTTATTTGTC   
  
  
+ TTTTAAGGAA AATTTTGGAA TTTGAGAGTG AAAATTTTGG TTGTGAATCT TGTCTTACTA TGTGGTTCCT   
  
  
+ TCTGACAAAA GTTTGAATCT GAGTATGAGA GTTCATCAAT CTCTTTTACT TTCCTACTTT CTTTATTGCT   
  
  
+ TGCAAATTGT TGTGAACTTA CTTGGATTTT CTGGTAGAGG AGAGTTCTGT CCTGGAATTT GGTGGAAAGT   
  
  
+ GAGACATACT TTGGGATTTA ATTAGCCATC AATTTGGTTT CACACCTTAT CAGTTTGGAC TTCTGGTGTG   
  
  
+ TTATTGTATG TGTTCTTAGC TTGGGATTAT TCTTGTATCG GAAGAGAGGG GGGGGGGGGT GTTTCTTTTA   
  
  
+ GCTGAATGTT AGAACATTTT AAGGGATCTA GATATCTAGA GAAGAGCCTA GGAAATGAAG CATTTTTCCC   
  
  
+ TTATAATCTG ACACAACTTT ATGAATTGAT TAGGAACAAA GAACGATTAG TTCAGAAAGG TTACAACTCC   
  
  
+ TCTAGGTGTT ATACTAAAGA GATGTGAGTT AATAAGCAAT TGCTTACTAA CTGTTTAGGC TGTTTCATTG   
  
  
+ CTTGTAGAGG CTTTTTTTTT GGGGGGGGGG GGGGGTTTGG GGTTCATAAA TGAGAAGGTT GATTTGTTGT   
  
  
+ CTGCTCTGCT CTTGATTCGA TAGTAATTCT CTCAGAAAAG GGTAAAATGA CATGGCCTTC CATCGCCTCC   
  
  
+ CATGAGGCTT TTTGCCTCTG CTTGTGTATC TTTCATCTCT TGGTTTGGTG GGTTGGGGGA GGGGAGGGGG   
  
  
+ GGAAGGGGGT TTTGATTCGA TAGATCATCA CTTTCAGAAA TGGCCTCTCT GGCCAATGGT AAGGGGTAAG   
  
  
+ GTTAGTACAT GTGGACTTTC CCATGTGGAT TTTTCCAGAG TCCGTTGTGT GATTGTTATT GCTGTTGTTG   
  
  
+ TAACTTTCTG TCACTCGGAA AACATGAATC CATATGCAAC AAGAACATAA TAATTGTGAA TGCGACTTTA   
  
  
+ TTTAGGAATG TCTTACATAG TACGTGCACA TTCTTTAATT ATCTGTTTAT CTGTTGGTTT GTTATTTTCA   
  
  
+ AGGGGATGGG GAGTGGGTGG ATGCTTAGTA TGTAATCTTT TTATATAGGG AAAAATCATA TAGCTTGAAA   
  
  
+ GTAAAACTTT CATTGCCAGG ATTCCAAGGT AGGGGTAGGG TTGTCTACAC CTTGATCTCC CTAGAAGCAT   
  
  
+ GGCGATCCTT CCTCGGATTG TGTTATAGAA TAAAGAATGT GGTTCTGCTG TAATGAGACT TATGTTCTAT   
  
  
+ CAAATGATGA ATCTGTTTAG AACTTCTTGC TTATTTTTTC TGCAAAATTT TAGGAGACTT GATAAACTAT   
  
  
+ AGTTCTCTTG AATTTACAGG TGGATATTGG AGTTCACAGA ATAGTATTCA GATCAAAGTG CTTGTGCTTA   
  
  
+ ATTGGAAGGA CAACTTGCTT GAGCTGTTTA TAGAGCTCTA ACTGATGCTA TCTCATGGAC TCACATCAGT   
  
  
+ TTTTTGGATT TAATCTTACC AGTGTTGATC CGTCGTACAT TTCCGCTCAG TACAGTCCGC CGTCAGTGAC   
  
  
+ AAATAGGATG TTTGCATCAC TGAAGCTCGA CTCTAGAGGT TCTCCTGTGT CACCCTTCTC AACTCAGTTT   
  
  
+ GATTGTGATA CGGTTACTAC ATTGAGTGAT AGTCAAGAGC ATCACAGCTC GACGGGGAGT CTATCAACAA   
  
  
+ GAAGCCCTTC TTGTAATTCT CCCCTTGAAA CGAGCAGTTA TCATCATTTA TCGACGAATG GCCCGTCTTG   
  
  
+ TAATTCTGCC CCTGAAACTA GCAGTTATCG TCATCGGTTC AACGCGAGTC CCGTGGGATA TTCCCATCAA   
  
  
+ GGCACTGATT ATGGAGTGAA TATGAAGAGC GCTCTGCAGG AGCTAGAGAC TACTCTAATG GGTGCAGATG   
  
  
+ GTGAGGAAGT ATCTGCTGCT AATCAACCTA TGGGGGGAAG TCGTCAGTCC GGGATCCCAA GTCAGAGATC   
  
  
+ AAAATCATTG AGCGAAGATC CACAGGGTTC GCATCCTACT CAGCCTGATT CATCATCTCT TTCTAGGGCA   
  
  
+ AGAAGATCAG GAGATGAAAG CCAGAGAGAG AAACGGCACA AGGCAATGGA AGAACCAACG GAACTACCAA   
  
  
+ GTTTGCCACC TGGTGATTTG AAGCAGTTGC TAATTGAATG TGCGAGGGCT TTATCGGATA ACCGAATAGA   
  
  
+ TGACTTTGAG AGTTTGGTTA AACGGGCGAG GAAAGAGGTC TCTATCTCGG GTGAGCCTAT CCAACGTCTC   
  
  
+ GGTGCCTACA TGATCGAAGG GCTTGTGGCA AGGAAGCAGT CTTCGGGGAC TAGCATCTAT CGGGCTCTGA   
  
  
+ AGTGTAAAGA GCCTCTTGGA AAAGACTTGC TCTCCTACAT GCACATCCTT TATGAAATAT GCCCTTATCT   
  
  
+ CAAGTTTGGT TATATGGCTG CGAATGGAGC GATAGCTGAA GCTTGTAGAA ATGAGGATAA CATACATATT   
  
  
+ ATAGATTTCC AGATTGCACA GGGCACTCAG TGGGTTACTC TATTACAAGC CCTAGCAGCA AGACCTGGTG   
  
  
+ GGCCACCTAA GGTGCGAATT ACAGGCATTG ATGATCCTGT TTCTAAGTAT GCCCGTGGTG CTAGCTTGGA   
  
  
+ GGCTGTTGGG AAACGGTTAG CGTCTCTATC TGAAAAGTTC AAAATACCCG TCGAGTTCAA TGCGTTGCCC   
  
  
+ GTTTATGGAC CCGATGTCAG GCGGGAAATG CTGGATGTGA GGCCCGGGGA GGCTTTGGCC GTTAATTTTC   
  
  
+ CATTGCAGCT CCACCACACT CCTGACGAGA GTGTCGATGT GAACAACCCT AGGGATGGGC TTCTCAGAAT   
  
  
+ GGTGAAATCA CTTGGTCCTA AGGTAACCAC TTTGGTTGAG CAAGAATCAA ACACCAACAC TACCCCTTTC   
  
  
+ TTGACCCGGT TCATAGAGAC CCTTGACTAC TACTCAGCCA TGTTTGAGTC TATAGACGTG ACCATGCCAA   
  
  
+ GAGACCGGAA GGAGAGGATC AATGTTGAGC AGCATTGTTT GGCTAAGGAC ATTGTGAATA TCATAGCTTG   
  
  
+ CGAGGGCAAG GAGAGGGTGG AGCGTCATGA ACTTTTTGGG AAATGGAAGT CAAGGTTTAC CATGGCAGGG   
  
  
+ TTCAGGCAGT ACCCGTTGAG CTCATACGTA AACTCTGTGA TAAGAAGCCT ACTCCGGTGT TACTCCGAGC   
  
  
+ ATTATACTCT GATAGAGAAG GATGGTGCCA TGCTTCTGGG TTGGAAGGGC CGAATGCTGA TTTCAGCTTC   
  
  
+ GGCATGGCAT TG  

- +Up\_Stream \_Len000AGGAGA AAACCCGAAG AAAGAAAGAT AGGACAAAAA AAAACCCAGC TTTTCAACCT   
  
  
- CCAACTTAAC TTCGTAGGCT TAAGCTCGTC CTACAAAGAG ACGCATCACT AAGAAAGACC CAAACACTAA   
  
  
- AACTTAAGAC CCATTAGCCA ACAAAACGAT TAAAACTCCT GTCTCAAGGA AAAGACTTAA TTTTAAAGCA   
  
  
- AAAAAATAGC CCTTTTAGAA GTCATAAACT CTTTTTTCTT GCTTTAAACC TACTAAACGA CAGATCTAAA   
  
  
- ACGAAAGTAT AAGGACCCAC ACCTAACCAA ATAATTAACG TACACTCCTT CATGTTCGAA GACCAGTTAC   
  
  
- CAACCCAAAA AAAAAAAAAA AACACCCATA TTTCAACCTT TAACTAATGC TCAGTTTAAA GAGAAAGATC   
  
  
- TTCTCCCCCC CCCCCCCCCC AACCAAATTT TGAGGTATAA AAGAAACTTA CCTTTCGAAT TAAACGACTG   
  
  
- ATAGAACCAA TCATCGTATT CGAACTAGTC TTTTGTTTTG CGAGAGACGG GACGTTTTTG AAATAAACAG   
  
  
- AAAATTCCTT TTAAAACCTT AAACTCTCAC TTTTAAAACC AACACTTAGA ACAGAATGAT ACACCAAGGA   
  
  
- AGACTGTTTT CAAACTTAGA CTCATACTCT CAAGTAGTTA GAGAAAATGA AAGGATGAAA GAAATAACGA   
  
  
- ACGTTTAACA ACACTTGAAT GAACCTAAAA GACCATCTCC TCTCAAGACA GGACCTTAAA CCACCTTTCA   
  
  
- CTCTGTATGA AACCCTAAAT TAATCGGTAG TTAAACCAAA GTGTGGAATA GTCAAACCTG AAGACCACAC   
  
  
- AATAACATAC ACAAGAATCG AACCCTAATA AGAACATAGC CTTCTCTCCC CCCCCCCCCA CAAAGAAAAT   
  
  
- CGACTTACAA TCTTGTAAAA TTCCCTAGAT CTATAGATCT CTTCTCGGAT CCTTTACTTC GTAAAAAGGG   
  
  
- AATATTAGAC TGTGTTGAAA TACTTAACTA ATCCTTGTTT CTTGCTAATC AAGTCTTTCC AATGTTGAGG   
  
  
- AGATCCACAA TATGATTTCT CTACACTCAA TTATTCGTTA ACGAATGATT GACAAATCCG ACAAAGTAAC   
  
  
- GAACATCTCC GAAAAAAAAA CCCCCCCCCC CCCCCAAACC CCAAGTATTT ACTCTTCCAA CTAAACAACA   
  
  
- GACGAGACGA GAACTAAGCT ATCATTAAGA GAGTCTTTTC CCATTTTACT GTACCGGAAG GTAGCGGAGG   
  
  
- GTACTCCGAA AAACGGAGAC GAACACATAG AAAGTAGAGA ACCAAACCAC CCAACCCCCT CCCCTCCCCC   
  
  
- CCTTCCCCCA AAACTAAGCT ATCTAGTAGT GAAAGTCTTT ACCGGAGAGA CCGGTTACCA TTCCCCATTC   
  
  
- CAATCATGTA CACCTGAAAG GGTACACCTA AAAAGGTCTC AGGCAACACA CTAACAATAA CGACAACAAC   
  
  
- ATTGAAAGAC AGTGAGCCTT TTGTACTTAG GTATACGTTG TTCTTGTATT ATTAACACTT ACGCTGAAAT   
  
  
- AAATCCTTAC AGAATGTATC ATGCACGTGT AAGAAATTAA TAGACAAATA GACAACCAAA CAATAAAAGT   
  
  
- TCCCCTACCC CTCACCCACC TACGAATCAT ACATTAGAAA AATATATCCC TTTTTAGTAT ATCGAACTTT   
  
  
- CATTTTGAAA GTAACGGTCC TAAGGTTCCA TCCCCATCCC AACAGATGTG GAACTAGAGG GATCTTCGTA   
  
  
- CCGCTAGGAA GGAGCCTAAC ACAATATCTT ATTTCTTACA CCAAGACGAC ATTACTCTGA ATACAAGATA   
  
  
- GTTTACTACT TAGACAAATC TTGAAGAACG AATAAAAAAG ACGTTTTAAA ATCCTCTGAA CTATTTGATA   
  
  
- TCAAGAGAAC TTAAATGTCC ACCTATAACC TCAAGTGTCT TATCATAAGT CTAGTTTCAC GAACACGAAT   
  
  
- TAACCTTCCT GTTGAACGAA CTCGACAAAT ATCTCGAGAT TGACTACGAT AGAGTACCTG AGTGTAGTCA   
  
  
- AAAAACCTAA ATTAGAATGG TCACAACTAG GCAGCATGTA AAGGCGAGTC ATGTCAGGCG GCAGTCACTG   
  
  
- TTTATCCTAC AAACGTAGTG ACTTCGAGCT GAGATCTCCA AGAGGACACA GTGGGAAGAG TTGAGTCAAA   
  
  
- CTAACACTAT GCCAATGATG TAACTCACTA TCAGTTCTCG TAGTGTCGAG CTGCCCCTCA GATAGTTGTT   
  
  
- CTTCGGGAAG AACATTAAGA GGGGAACTTT GCTCGTCAAT AGTAGTAAAT AGCTGCTTAC CGGGCAGAAC   
  
  
- ATTAAGACGG GGACTTTGAT CGTCAATAGC AGTAGCCAAG TTGCGCTCAG GGCACCCTAT AAGGGTAGTT   
  
  
- CCGTGACTAA TACCTCACTT ATACTTCTCG CGAGACGTCC TCGATCTCTG ATGAGATTAC CCACGTCTAC   
  
  
- CACTCCTTCA TAGACGACGA TTAGTTGGAT ACCCCCCTTC AGCAGTCAGG CCCTAGGGTT CAGTCTCTAG   
  
  
- TTTTAGTAAC TCGCTTCTAG GTGTCCCAAG CGTAGGATGA GTCGGACTAA GTAGTAGAGA AAGATCCCGT   
  
  
- TCTTCTAGTC CTCTACTTTC GGTCTCTCTC TTTGCCGTGT TCCGTTACCT TCTTGGTTGC CTTGATGGTT   
  
  
- CAAACGGTGG ACCACTAAAC TTCGTCAACG ATTAACTTAC ACGCTCCCGA AATAGCCTAT TGGCTTATCT   
  
  
- ACTGAAACTC TCAAACCAAT TTGCCCGCTC CTTTCTCCAG AGATAGAGCC CACTCGGATA GGTTGCAGAG   
  
  
- CCACGGATGT ACTAGCTTCC CGAACACCGT TCCTTCGTCA GAAGCCCCTG ATCGTAGATA GCCCGAGACT   
  
  
- TCACATTTCT CGGAGAACCT TTTCTGAACG AGAGGATGTA CGTGTAGGAA ATACTTTATA CGGGAATAGA   
  
  
- GTTCAAACCA ATATACCGAC GCTTACCTCG CTATCGACTT CGAACATCTT TACTCCTATT GTATGTATAA   
  
  
- TATCTAAAGG TCTAACGTGT CCCGTGAGTC ACCCAATGAG ATAATGTTCG GGATCGTCGT TCTGGACCAC   
  
  
- CCGGTGGATT CCACGCTTAA TGTCCGTAAC TACTAGGACA AAGATTCATA CGGGCACCAC GATCGAACCT   
  
  
- CCGACAACCC TTTGCCAATC GCAGAGATAG ACTTTTCAAG TTTTATGGGC AGCTCAAGTT ACGCAACGGG   
  
  
- CAAATACCTG GGCTACAGTC CGCCCTTTAC GACCTACACT CCGGGCCCCT CCGAAACCGG CAATTAAAAG   
  
  
- GTAACGTCGA GGTGGTGTGA GGACTGCTCT CACAGCTACA CTTGTTGGGA TCCCTACCCG AAGAGTCTTA   
  
  
- CCACTTTAGT GAACCAGGAT TCCATTGGTG AAACCAACTC GTTCTTAGTT TGTGGTTGTG ATGGGGAAAG   
  
  
- AACTGGGCCA AGTATCTCTG GGAACTGATG ATGAGTCGGT ACAAACTCAG ATATCTGCAC TGGTACGGTT   
  
  
- CTCTGGCCTT CCTCTCCTAG TTACAACTCG TCGTAACAAA CCGATTCCTG TAACACTTAT AGTATCGAAC   
  
  
- GCTCCCGTTC CTCTCCCACC TCGCAGTACT TGAAAAACCC TTTACCTTCA GTTCCAAATG GTACCGTCCC   
  
  
- AAGTCCGTCA TGGGCAACTC GAGTATGCAT TTGAGACACT ATTCTTCGGA TGAGGCCACA ATGAGGCTCG   
  
  
- TAATATGAGA CTATCTCTTC CTACCACGGT ACGAAGACCC AACCTTCCCG GCTTACGACT AAAGTCGAAG   
  
  
- CCGTACCGTA AC

+     GATA-motif

| Site Name | Organism | Position | Strand | Matrix score. | sequence | function |
| --- | --- | --- | --- | --- | --- | --- |
| GATA-motif | Arabidopsis thaliana | 610 | - | 10 | AAGATAAGATT | part of a light responsive element |

>HU08G00014.1   
+ +Up\_Stream \_Len000TCCTCT TTTGGGCTTC TTTCTTTCTA TCCTGTTTTT TTTTGGGTCG AAAAGTTGGA   
  
  
+ GGTTGAATTG AAGCATCCGA ATTCGAGCAG GATGTTTCTC TGCGTAGTGA TTCTTTCTGG GTTTGTGATT   
  
  
+ TTGAATTCTG GGTAATCGGT TGTTTTGCTA ATTTTGAGGA CAGAGTTCCT TTTCTGAATT AAAATTTCGT   
  
  
+ TTTTTTATCG GGAAAATCTT CAGTATTTGA GAAAAAAGAA CGAAATTTGG ATGATTTGCT GTCTAGATTT   
  
  
+ TGCTTTCATA TTCCTGGGTG TGGATTGGTT TATTAATTGC ATGTGAGGAA GTACAAGCTT CTGGTCAATG   
  
  
+ GTTGGGTTTT TTTTTTTTTT TTGTGGGTAT AAAGTTGGAA ATTGATTACG AGTCAAATTT CTCTTTCTAG   
  
  
+ AAGAGGGGGG GGGGGGGGGG TTGGTTTAAA ACTCCATATT TTCTTTGAAT GGAAAGCTTA ATTTGCTGAC   
  
  
+ TATCTTGGTT AGTAGCATAA GCTTGATCAG AAAACAAAAC GCTCTCTGCC CTGCAAAAAC TTTATTTGTC   
  
  
+ TTTTAAGGAA AATTTTGGAA TTTGAGAGTG AAAATTTTGG TTGTGAATCT TGTCTTACTA TGTGGTTCCT   
  
  
+ TCTGACAAAA GTTTGAATCT GAGTATGAGA GTTCATCAAT CTCTTTTACT TTCCTACTTT CTTTATTGCT   
  
  
+ TGCAAATTGT TGTGAACTTA CTTGGATTTT CTGGTAGAGG AGAGTTCTGT CCTGGAATTT GGTGGAAAGT   
  
  
+ GAGACATACT TTGGGATTTA ATTAGCCATC AATTTGGTTT CACACCTTAT CAGTTTGGAC TTCTGGTGTG   
  
  
+ TTATTGTATG TGTTCTTAGC TTGGGATTAT TCTTGTATCG GAAGAGAGGG GGGGGGGGGT GTTTCTTTTA   
  
  
+ GCTGAATGTT AGAACATTTT AAGGGATCTA GATATCTAGA GAAGAGCCTA GGAAATGAAG CATTTTTCCC   
  
  
+ TTATAATCTG ACACAACTTT ATGAATTGAT TAGGAACAAA GAACGATTAG TTCAGAAAGG TTACAACTCC   
  
  
+ TCTAGGTGTT ATACTAAAGA GATGTGAGTT AATAAGCAAT TGCTTACTAA CTGTTTAGGC TGTTTCATTG   
  
  
+ CTTGTAGAGG CTTTTTTTTT GGGGGGGGGG GGGGGTTTGG GGTTCATAAA TGAGAAGGTT GATTTGTTGT   
  
  
+ CTGCTCTGCT CTTGATTCGA TAGTAATTCT CTCAGAAAAG GGTAAAATGA CATGGCCTTC CATCGCCTCC   
  
  
+ CATGAGGCTT TTTGCCTCTG CTTGTGTATC TTTCATCTCT TGGTTTGGTG GGTTGGGGGA GGGGAGGGGG   
  
  
+ GGAAGGGGGT TTTGATTCGA TAGATCATCA CTTTCAGAAA TGGCCTCTCT GGCCAATGGT AAGGGGTAAG   
  
  
+ GTTAGTACAT GTGGACTTTC CCATGTGGAT TTTTCCAGAG TCCGTTGTGT GATTGTTATT GCTGTTGTTG   
  
  
+ TAACTTTCTG TCACTCGGAA AACATGAATC CATATGCAAC AAGAACATAA TAATTGTGAA TGCGACTTTA   
  
  
+ TTTAGGAATG TCTTACATAG TACGTGCACA TTCTTTAATT ATCTGTTTAT CTGTTGGTTT GTTATTTTCA   
  
  
+ AGGGGATGGG GAGTGGGTGG ATGCTTAGTA TGTAATCTTT TTATATAGGG AAAAATCATA TAGCTTGAAA   
  
  
+ GTAAAACTTT CATTGCCAGG ATTCCAAGGT AGGGGTAGGG TTGTCTACAC CTTGATCTCC CTAGAAGCAT   
  
  
+ GGCGATCCTT CCTCGGATTG TGTTATAGAA TAAAGAATGT GGTTCTGCTG TAATGAGACT TATGTTCTAT   
  
  
+ CAAATGATGA ATCTGTTTAG AACTTCTTGC TTATTTTTTC TGCAAAATTT TAGGAGACTT GATAAACTAT   
  
  
+ AGTTCTCTTG AATTTACAGG TGGATATTGG AGTTCACAGA ATAGTATTCA GATCAAAGTG CTTGTGCTTA   
  
  
+ ATTGGAAGGA CAACTTGCTT GAGCTGTTTA TAGAGCTCTA ACTGATGCTA TCTCATGGAC TCACATCAGT   
  
  
+ TTTTTGGATT TAATCTTACC AGTGTTGATC CGTCGTACAT TTCCGCTCAG TACAGTCCGC CGTCAGTGAC   
  
  
+ AAATAGGATG TTTGCATCAC TGAAGCTCGA CTCTAGAGGT TCTCCTGTGT CACCCTTCTC AACTCAGTTT   
  
  
+ GATTGTGATA CGGTTACTAC ATTGAGTGAT AGTCAAGAGC ATCACAGCTC GACGGGGAGT CTATCAACAA   
  
  
+ GAAGCCCTTC TTGTAATTCT CCCCTTGAAA CGAGCAGTTA TCATCATTTA TCGACGAATG GCCCGTCTTG   
  
  
+ TAATTCTGCC CCTGAAACTA GCAGTTATCG TCATCGGTTC AACGCGAGTC CCGTGGGATA TTCCCATCAA   
  
  
+ GGCACTGATT ATGGAGTGAA TATGAAGAGC GCTCTGCAGG AGCTAGAGAC TACTCTAATG GGTGCAGATG   
  
  
+ GTGAGGAAGT ATCTGCTGCT AATCAACCTA TGGGGGGAAG TCGTCAGTCC GGGATCCCAA GTCAGAGATC   
  
  
+ AAAATCATTG AGCGAAGATC CACAGGGTTC GCATCCTACT CAGCCTGATT CATCATCTCT TTCTAGGGCA   
  
  
+ AGAAGATCAG GAGATGAAAG CCAGAGAGAG AAACGGCACA AGGCAATGGA AGAACCAACG GAACTACCAA   
  
  
+ GTTTGCCACC TGGTGATTTG AAGCAGTTGC TAATTGAATG TGCGAGGGCT TTATCGGATA ACCGAATAGA   
  
  
+ TGACTTTGAG AGTTTGGTTA AACGGGCGAG GAAAGAGGTC TCTATCTCGG GTGAGCCTAT CCAACGTCTC   
  
  
+ GGTGCCTACA TGATCGAAGG GCTTGTGGCA AGGAAGCAGT CTTCGGGGAC TAGCATCTAT CGGGCTCTGA   
  
  
+ AGTGTAAAGA GCCTCTTGGA AAAGACTTGC TCTCCTACAT GCACATCCTT TATGAAATAT GCCCTTATCT   
  
  
+ CAAGTTTGGT TATATGGCTG CGAATGGAGC GATAGCTGAA GCTTGTAGAA ATGAGGATAA CATACATATT   
  
  
+ ATAGATTTCC AGATTGCACA GGGCACTCAG TGGGTTACTC TATTACAAGC CCTAGCAGCA AGACCTGGTG   
  
  
+ GGCCACCTAA GGTGCGAATT ACAGGCATTG ATGATCCTGT TTCTAAGTAT GCCCGTGGTG CTAGCTTGGA   
  
  
+ GGCTGTTGGG AAACGGTTAG CGTCTCTATC TGAAAAGTTC AAAATACCCG TCGAGTTCAA TGCGTTGCCC   
  
  
+ GTTTATGGAC CCGATGTCAG GCGGGAAATG CTGGATGTGA GGCCCGGGGA GGCTTTGGCC GTTAATTTTC   
  
  
+ CATTGCAGCT CCACCACACT CCTGACGAGA GTGTCGATGT GAACAACCCT AGGGATGGGC TTCTCAGAAT   
  
  
+ GGTGAAATCA CTTGGTCCTA AGGTAACCAC TTTGGTTGAG CAAGAATCAA ACACCAACAC TACCCCTTTC   
  
  
+ TTGACCCGGT TCATAGAGAC CCTTGACTAC TACTCAGCCA TGTTTGAGTC TATAGACGTG ACCATGCCAA   
  
  
+ GAGACCGGAA GGAGAGGATC AATGTTGAGC AGCATTGTTT GGCTAAGGAC ATTGTGAATA TCATAGCTTG   
  
  
+ CGAGGGCAAG GAGAGGGTGG AGCGTCATGA ACTTTTTGGG AAATGGAAGT CAAGGTTTAC CATGGCAGGG   
  
  
+ TTCAGGCAGT ACCCGTTGAG CTCATACGTA AACTCTGTGA TAAGAAGCCT ACTCCGGTGT TACTCCGAGC   
  
  
+ ATTATACTCT GATAGAGAAG GATGGTGCCA TGCTTCTGGG TTGGAAGGGC CGAATGCTGA TTTCAGCTTC   
  
  
+ GGCATGGCAT TG  

- +Up\_Stream \_Len000AGGAGA AAACCCGAAG AAAGAAAGAT AGGACAAAAA AAAACCCAGC TTTTCAACCT   
  
  
- CCAACTTAAC TTCGTAGGCT TAAGCTCGTC CTACAAAGAG ACGCATCACT AAGAAAGACC CAAACACTAA   
  
  
- AACTTAAGAC CCATTAGCCA ACAAAACGAT TAAAACTCCT GTCTCAAGGA AAAGACTTAA TTTTAAAGCA   
  
  
- AAAAAATAGC CCTTTTAGAA GTCATAAACT CTTTTTTCTT GCTTTAAACC TACTAAACGA CAGATCTAAA   
  
  
- ACGAAAGTAT AAGGACCCAC ACCTAACCAA ATAATTAACG TACACTCCTT CATGTTCGAA GACCAGTTAC   
  
  
- CAACCCAAAA AAAAAAAAAA AACACCCATA TTTCAACCTT TAACTAATGC TCAGTTTAAA GAGAAAGATC   
  
  
- TTCTCCCCCC CCCCCCCCCC AACCAAATTT TGAGGTATAA AAGAAACTTA CCTTTCGAAT TAAACGACTG   
  
  
- ATAGAACCAA TCATCGTATT CGAACTAGTC TTTTGTTTTG CGAGAGACGG GACGTTTTTG AAATAAACAG   
  
  
- AAAATTCCTT TTAAAACCTT AAACTCTCAC TTTTAAAACC AACACTTAGA ACAGAATGAT ACACCAAGGA   
  
  
- AGACTGTTTT CAAACTTAGA CTCATACTCT CAAGTAGTTA GAGAAAATGA AAGGATGAAA GAAATAACGA   
  
  
- ACGTTTAACA ACACTTGAAT GAACCTAAAA GACCATCTCC TCTCAAGACA GGACCTTAAA CCACCTTTCA   
  
  
- CTCTGTATGA AACCCTAAAT TAATCGGTAG TTAAACCAAA GTGTGGAATA GTCAAACCTG AAGACCACAC   
  
  
- AATAACATAC ACAAGAATCG AACCCTAATA AGAACATAGC CTTCTCTCCC CCCCCCCCCA CAAAGAAAAT   
  
  
- CGACTTACAA TCTTGTAAAA TTCCCTAGAT CTATAGATCT CTTCTCGGAT CCTTTACTTC GTAAAAAGGG   
  
  
- AATATTAGAC TGTGTTGAAA TACTTAACTA ATCCTTGTTT CTTGCTAATC AAGTCTTTCC AATGTTGAGG   
  
  
- AGATCCACAA TATGATTTCT CTACACTCAA TTATTCGTTA ACGAATGATT GACAAATCCG ACAAAGTAAC   
  
  
- GAACATCTCC GAAAAAAAAA CCCCCCCCCC CCCCCAAACC CCAAGTATTT ACTCTTCCAA CTAAACAACA   
  
  
- GACGAGACGA GAACTAAGCT ATCATTAAGA GAGTCTTTTC CCATTTTACT GTACCGGAAG GTAGCGGAGG   
  
  
- GTACTCCGAA AAACGGAGAC GAACACATAG AAAGTAGAGA ACCAAACCAC CCAACCCCCT CCCCTCCCCC   
  
  
- CCTTCCCCCA AAACTAAGCT ATCTAGTAGT GAAAGTCTTT ACCGGAGAGA CCGGTTACCA TTCCCCATTC   
  
  
- CAATCATGTA CACCTGAAAG GGTACACCTA AAAAGGTCTC AGGCAACACA CTAACAATAA CGACAACAAC   
  
  
- ATTGAAAGAC AGTGAGCCTT TTGTACTTAG GTATACGTTG TTCTTGTATT ATTAACACTT ACGCTGAAAT   
  
  
- AAATCCTTAC AGAATGTATC ATGCACGTGT AAGAAATTAA TAGACAAATA GACAACCAAA CAATAAAAGT   
  
  
- TCCCCTACCC CTCACCCACC TACGAATCAT ACATTAGAAA AATATATCCC TTTTTAGTAT ATCGAACTTT   
  
  
- CATTTTGAAA GTAACGGTCC TAAGGTTCCA TCCCCATCCC AACAGATGTG GAACTAGAGG GATCTTCGTA   
  
  
- CCGCTAGGAA GGAGCCTAAC ACAATATCTT ATTTCTTACA CCAAGACGAC ATTACTCTGA ATACAAGATA   
  
  
- GTTTACTACT TAGACAAATC TTGAAGAACG AATAAAAAAG ACGTTTTAAA ATCCTCTGAA CTATTTGATA   
  
  
- TCAAGAGAAC TTAAATGTCC ACCTATAACC TCAAGTGTCT TATCATAAGT CTAGTTTCAC GAACACGAAT   
  
  
- TAACCTTCCT GTTGAACGAA CTCGACAAAT ATCTCGAGAT TGACTACGAT AGAGTACCTG AGTGTAGTCA   
  
  
- AAAAACCTAA ATTAGAATGG TCACAACTAG GCAGCATGTA AAGGCGAGTC ATGTCAGGCG GCAGTCACTG   
  
  
- TTTATCCTAC AAACGTAGTG ACTTCGAGCT GAGATCTCCA AGAGGACACA GTGGGAAGAG TTGAGTCAAA   
  
  
- CTAACACTAT GCCAATGATG TAACTCACTA TCAGTTCTCG TAGTGTCGAG CTGCCCCTCA GATAGTTGTT   
  
  
- CTTCGGGAAG AACATTAAGA GGGGAACTTT GCTCGTCAAT AGTAGTAAAT AGCTGCTTAC CGGGCAGAAC   
  
  
- ATTAAGACGG GGACTTTGAT CGTCAATAGC AGTAGCCAAG TTGCGCTCAG GGCACCCTAT AAGGGTAGTT   
  
  
- CCGTGACTAA TACCTCACTT ATACTTCTCG CGAGACGTCC TCGATCTCTG ATGAGATTAC CCACGTCTAC   
  
  
- CACTCCTTCA TAGACGACGA TTAGTTGGAT ACCCCCCTTC AGCAGTCAGG CCCTAGGGTT CAGTCTCTAG   
  
  
- TTTTAGTAAC TCGCTTCTAG GTGTCCCAAG CGTAGGATGA GTCGGACTAA GTAGTAGAGA AAGATCCCGT   
  
  
- TCTTCTAGTC CTCTACTTTC GGTCTCTCTC TTTGCCGTGT TCCGTTACCT TCTTGGTTGC CTTGATGGTT   
  
  
- CAAACGGTGG ACCACTAAAC TTCGTCAACG ATTAACTTAC ACGCTCCCGA AATAGCCTAT TGGCTTATCT   
  
  
- ACTGAAACTC TCAAACCAAT TTGCCCGCTC CTTTCTCCAG AGATAGAGCC CACTCGGATA GGTTGCAGAG   
  
  
- CCACGGATGT ACTAGCTTCC CGAACACCGT TCCTTCGTCA GAAGCCCCTG ATCGTAGATA GCCCGAGACT   
  
  
- TCACATTTCT CGGAGAACCT TTTCTGAACG AGAGGATGTA CGTGTAGGAA ATACTTTATA CGGGAATAGA   
  
  
- GTTCAAACCA ATATACCGAC GCTTACCTCG CTATCGACTT CGAACATCTT TACTCCTATT GTATGTATAA   
  
  
- TATCTAAAGG TCTAACGTGT CCCGTGAGTC ACCCAATGAG ATAATGTTCG GGATCGTCGT TCTGGACCAC   
  
  
- CCGGTGGATT CCACGCTTAA TGTCCGTAAC TACTAGGACA AAGATTCATA CGGGCACCAC GATCGAACCT   
  
  
- CCGACAACCC TTTGCCAATC GCAGAGATAG ACTTTTCAAG TTTTATGGGC AGCTCAAGTT ACGCAACGGG   
  
  
- CAAATACCTG GGCTACAGTC CGCCCTTTAC GACCTACACT CCGGGCCCCT CCGAAACCGG CAATTAAAAG   
  
  
- GTAACGTCGA GGTGGTGTGA GGACTGCTCT CACAGCTACA CTTGTTGGGA TCCCTACCCG AAGAGTCTTA   
  
  
- CCACTTTAGT GAACCAGGAT TCCATTGGTG AAACCAACTC GTTCTTAGTT TGTGGTTGTG ATGGGGAAAG   
  
  
- AACTGGGCCA AGTATCTCTG GGAACTGATG ATGAGTCGGT ACAAACTCAG ATATCTGCAC TGGTACGGTT   
  
  
- CTCTGGCCTT CCTCTCCTAG TTACAACTCG TCGTAACAAA CCGATTCCTG TAACACTTAT AGTATCGAAC   
  
  
- GCTCCCGTTC CTCTCCCACC TCGCAGTACT TGAAAAACCC TTTACCTTCA GTTCCAAATG GTACCGTCCC   
  
  
- AAGTCCGTCA TGGGCAACTC GAGTATGCAT TTGAGACACT ATTCTTCGGA TGAGGCCACA ATGAGGCTCG   
  
  
- TAATATGAGA CTATCTCTTC CTACCACGGT ACGAAGACCC AACCTTCCCG GCTTACGACT AAAGTCGAAG   
  
  
- CCGTACCGTA AC

+     GT1-motif

| Site Name | Organism | Position | Strand | Matrix score. | sequence | function |
| --- | --- | --- | --- | --- | --- | --- |
| GT1-motif | Arabidopsis thaliana | 2750 | + | 6 | GGTTAA | light responsive element |

>HU08G00014.1   
+ +Up\_Stream \_Len000TCCTCT TTTGGGCTTC TTTCTTTCTA TCCTGTTTTT TTTTGGGTCG AAAAGTTGGA   
  
  
+ GGTTGAATTG AAGCATCCGA ATTCGAGCAG GATGTTTCTC TGCGTAGTGA TTCTTTCTGG GTTTGTGATT   
  
  
+ TTGAATTCTG GGTAATCGGT TGTTTTGCTA ATTTTGAGGA CAGAGTTCCT TTTCTGAATT AAAATTTCGT   
  
  
+ TTTTTTATCG GGAAAATCTT CAGTATTTGA GAAAAAAGAA CGAAATTTGG ATGATTTGCT GTCTAGATTT   
  
  
+ TGCTTTCATA TTCCTGGGTG TGGATTGGTT TATTAATTGC ATGTGAGGAA GTACAAGCTT CTGGTCAATG   
  
  
+ GTTGGGTTTT TTTTTTTTTT TTGTGGGTAT AAAGTTGGAA ATTGATTACG AGTCAAATTT CTCTTTCTAG   
  
  
+ AAGAGGGGGG GGGGGGGGGG TTGGTTTAAA ACTCCATATT TTCTTTGAAT GGAAAGCTTA ATTTGCTGAC   
  
  
+ TATCTTGGTT AGTAGCATAA GCTTGATCAG AAAACAAAAC GCTCTCTGCC CTGCAAAAAC TTTATTTGTC   
  
  
+ TTTTAAGGAA AATTTTGGAA TTTGAGAGTG AAAATTTTGG TTGTGAATCT TGTCTTACTA TGTGGTTCCT   
  
  
+ TCTGACAAAA GTTTGAATCT GAGTATGAGA GTTCATCAAT CTCTTTTACT TTCCTACTTT CTTTATTGCT   
  
  
+ TGCAAATTGT TGTGAACTTA CTTGGATTTT CTGGTAGAGG AGAGTTCTGT CCTGGAATTT GGTGGAAAGT   
  
  
+ GAGACATACT TTGGGATTTA ATTAGCCATC AATTTGGTTT CACACCTTAT CAGTTTGGAC TTCTGGTGTG   
  
  
+ TTATTGTATG TGTTCTTAGC TTGGGATTAT TCTTGTATCG GAAGAGAGGG GGGGGGGGGT GTTTCTTTTA   
  
  
+ GCTGAATGTT AGAACATTTT AAGGGATCTA GATATCTAGA GAAGAGCCTA GGAAATGAAG CATTTTTCCC   
  
  
+ TTATAATCTG ACACAACTTT ATGAATTGAT TAGGAACAAA GAACGATTAG TTCAGAAAGG TTACAACTCC   
  
  
+ TCTAGGTGTT ATACTAAAGA GATGTGAGTT AATAAGCAAT TGCTTACTAA CTGTTTAGGC TGTTTCATTG   
  
  
+ CTTGTAGAGG CTTTTTTTTT GGGGGGGGGG GGGGGTTTGG GGTTCATAAA TGAGAAGGTT GATTTGTTGT   
  
  
+ CTGCTCTGCT CTTGATTCGA TAGTAATTCT CTCAGAAAAG GGTAAAATGA CATGGCCTTC CATCGCCTCC   
  
  
+ CATGAGGCTT TTTGCCTCTG CTTGTGTATC TTTCATCTCT TGGTTTGGTG GGTTGGGGGA GGGGAGGGGG   
  
  
+ GGAAGGGGGT TTTGATTCGA TAGATCATCA CTTTCAGAAA TGGCCTCTCT GGCCAATGGT AAGGGGTAAG   
  
  
+ GTTAGTACAT GTGGACTTTC CCATGTGGAT TTTTCCAGAG TCCGTTGTGT GATTGTTATT GCTGTTGTTG   
  
  
+ TAACTTTCTG TCACTCGGAA AACATGAATC CATATGCAAC AAGAACATAA TAATTGTGAA TGCGACTTTA   
  
  
+ TTTAGGAATG TCTTACATAG TACGTGCACA TTCTTTAATT ATCTGTTTAT CTGTTGGTTT GTTATTTTCA   
  
  
+ AGGGGATGGG GAGTGGGTGG ATGCTTAGTA TGTAATCTTT TTATATAGGG AAAAATCATA TAGCTTGAAA   
  
  
+ GTAAAACTTT CATTGCCAGG ATTCCAAGGT AGGGGTAGGG TTGTCTACAC CTTGATCTCC CTAGAAGCAT   
  
  
+ GGCGATCCTT CCTCGGATTG TGTTATAGAA TAAAGAATGT GGTTCTGCTG TAATGAGACT TATGTTCTAT   
  
  
+ CAAATGATGA ATCTGTTTAG AACTTCTTGC TTATTTTTTC TGCAAAATTT TAGGAGACTT GATAAACTAT   
  
  
+ AGTTCTCTTG AATTTACAGG TGGATATTGG AGTTCACAGA ATAGTATTCA GATCAAAGTG CTTGTGCTTA   
  
  
+ ATTGGAAGGA CAACTTGCTT GAGCTGTTTA TAGAGCTCTA ACTGATGCTA TCTCATGGAC TCACATCAGT   
  
  
+ TTTTTGGATT TAATCTTACC AGTGTTGATC CGTCGTACAT TTCCGCTCAG TACAGTCCGC CGTCAGTGAC   
  
  
+ AAATAGGATG TTTGCATCAC TGAAGCTCGA CTCTAGAGGT TCTCCTGTGT CACCCTTCTC AACTCAGTTT   
  
  
+ GATTGTGATA CGGTTACTAC ATTGAGTGAT AGTCAAGAGC ATCACAGCTC GACGGGGAGT CTATCAACAA   
  
  
+ GAAGCCCTTC TTGTAATTCT CCCCTTGAAA CGAGCAGTTA TCATCATTTA TCGACGAATG GCCCGTCTTG   
  
  
+ TAATTCTGCC CCTGAAACTA GCAGTTATCG TCATCGGTTC AACGCGAGTC CCGTGGGATA TTCCCATCAA   
  
  
+ GGCACTGATT ATGGAGTGAA TATGAAGAGC GCTCTGCAGG AGCTAGAGAC TACTCTAATG GGTGCAGATG   
  
  
+ GTGAGGAAGT ATCTGCTGCT AATCAACCTA TGGGGGGAAG TCGTCAGTCC GGGATCCCAA GTCAGAGATC   
  
  
+ AAAATCATTG AGCGAAGATC CACAGGGTTC GCATCCTACT CAGCCTGATT CATCATCTCT TTCTAGGGCA   
  
  
+ AGAAGATCAG GAGATGAAAG CCAGAGAGAG AAACGGCACA AGGCAATGGA AGAACCAACG GAACTACCAA   
  
  
+ GTTTGCCACC TGGTGATTTG AAGCAGTTGC TAATTGAATG TGCGAGGGCT TTATCGGATA ACCGAATAGA   
  
  
+ TGACTTTGAG AGTTTGGTTA AACGGGCGAG GAAAGAGGTC TCTATCTCGG GTGAGCCTAT CCAACGTCTC   
  
  
+ GGTGCCTACA TGATCGAAGG GCTTGTGGCA AGGAAGCAGT CTTCGGGGAC TAGCATCTAT CGGGCTCTGA   
  
  
+ AGTGTAAAGA GCCTCTTGGA AAAGACTTGC TCTCCTACAT GCACATCCTT TATGAAATAT GCCCTTATCT   
  
  
+ CAAGTTTGGT TATATGGCTG CGAATGGAGC GATAGCTGAA GCTTGTAGAA ATGAGGATAA CATACATATT   
  
  
+ ATAGATTTCC AGATTGCACA GGGCACTCAG TGGGTTACTC TATTACAAGC CCTAGCAGCA AGACCTGGTG   
  
  
+ GGCCACCTAA GGTGCGAATT ACAGGCATTG ATGATCCTGT TTCTAAGTAT GCCCGTGGTG CTAGCTTGGA   
  
  
+ GGCTGTTGGG AAACGGTTAG CGTCTCTATC TGAAAAGTTC AAAATACCCG TCGAGTTCAA TGCGTTGCCC   
  
  
+ GTTTATGGAC CCGATGTCAG GCGGGAAATG CTGGATGTGA GGCCCGGGGA GGCTTTGGCC GTTAATTTTC   
  
  
+ CATTGCAGCT CCACCACACT CCTGACGAGA GTGTCGATGT GAACAACCCT AGGGATGGGC TTCTCAGAAT   
  
  
+ GGTGAAATCA CTTGGTCCTA AGGTAACCAC TTTGGTTGAG CAAGAATCAA ACACCAACAC TACCCCTTTC   
  
  
+ TTGACCCGGT TCATAGAGAC CCTTGACTAC TACTCAGCCA TGTTTGAGTC TATAGACGTG ACCATGCCAA   
  
  
+ GAGACCGGAA GGAGAGGATC AATGTTGAGC AGCATTGTTT GGCTAAGGAC ATTGTGAATA TCATAGCTTG   
  
  
+ CGAGGGCAAG GAGAGGGTGG AGCGTCATGA ACTTTTTGGG AAATGGAAGT CAAGGTTTAC CATGGCAGGG   
  
  
+ TTCAGGCAGT ACCCGTTGAG CTCATACGTA AACTCTGTGA TAAGAAGCCT ACTCCGGTGT TACTCCGAGC   
  
  
+ ATTATACTCT GATAGAGAAG GATGGTGCCA TGCTTCTGGG TTGGAAGGGC CGAATGCTGA TTTCAGCTTC   
  
  
+ GGCATGGCAT TG  

- +Up\_Stream \_Len000AGGAGA AAACCCGAAG AAAGAAAGAT AGGACAAAAA AAAACCCAGC TTTTCAACCT   
  
  
- CCAACTTAAC TTCGTAGGCT TAAGCTCGTC CTACAAAGAG ACGCATCACT AAGAAAGACC CAAACACTAA   
  
  
- AACTTAAGAC CCATTAGCCA ACAAAACGAT TAAAACTCCT GTCTCAAGGA AAAGACTTAA TTTTAAAGCA   
  
  
- AAAAAATAGC CCTTTTAGAA GTCATAAACT CTTTTTTCTT GCTTTAAACC TACTAAACGA CAGATCTAAA   
  
  
- ACGAAAGTAT AAGGACCCAC ACCTAACCAA ATAATTAACG TACACTCCTT CATGTTCGAA GACCAGTTAC   
  
  
- CAACCCAAAA AAAAAAAAAA AACACCCATA TTTCAACCTT TAACTAATGC TCAGTTTAAA GAGAAAGATC   
  
  
- TTCTCCCCCC CCCCCCCCCC AACCAAATTT TGAGGTATAA AAGAAACTTA CCTTTCGAAT TAAACGACTG   
  
  
- ATAGAACCAA TCATCGTATT CGAACTAGTC TTTTGTTTTG CGAGAGACGG GACGTTTTTG AAATAAACAG   
  
  
- AAAATTCCTT TTAAAACCTT AAACTCTCAC TTTTAAAACC AACACTTAGA ACAGAATGAT ACACCAAGGA   
  
  
- AGACTGTTTT CAAACTTAGA CTCATACTCT CAAGTAGTTA GAGAAAATGA AAGGATGAAA GAAATAACGA   
  
  
- ACGTTTAACA ACACTTGAAT GAACCTAAAA GACCATCTCC TCTCAAGACA GGACCTTAAA CCACCTTTCA   
  
  
- CTCTGTATGA AACCCTAAAT TAATCGGTAG TTAAACCAAA GTGTGGAATA GTCAAACCTG AAGACCACAC   
  
  
- AATAACATAC ACAAGAATCG AACCCTAATA AGAACATAGC CTTCTCTCCC CCCCCCCCCA CAAAGAAAAT   
  
  
- CGACTTACAA TCTTGTAAAA TTCCCTAGAT CTATAGATCT CTTCTCGGAT CCTTTACTTC GTAAAAAGGG   
  
  
- AATATTAGAC TGTGTTGAAA TACTTAACTA ATCCTTGTTT CTTGCTAATC AAGTCTTTCC AATGTTGAGG   
  
  
- AGATCCACAA TATGATTTCT CTACACTCAA TTATTCGTTA ACGAATGATT GACAAATCCG ACAAAGTAAC   
  
  
- GAACATCTCC GAAAAAAAAA CCCCCCCCCC CCCCCAAACC CCAAGTATTT ACTCTTCCAA CTAAACAACA   
  
  
- GACGAGACGA GAACTAAGCT ATCATTAAGA GAGTCTTTTC CCATTTTACT GTACCGGAAG GTAGCGGAGG   
  
  
- GTACTCCGAA AAACGGAGAC GAACACATAG AAAGTAGAGA ACCAAACCAC CCAACCCCCT CCCCTCCCCC   
  
  
- CCTTCCCCCA AAACTAAGCT ATCTAGTAGT GAAAGTCTTT ACCGGAGAGA CCGGTTACCA TTCCCCATTC   
  
  
- CAATCATGTA CACCTGAAAG GGTACACCTA AAAAGGTCTC AGGCAACACA CTAACAATAA CGACAACAAC   
  
  
- ATTGAAAGAC AGTGAGCCTT TTGTACTTAG GTATACGTTG TTCTTGTATT ATTAACACTT ACGCTGAAAT   
  
  
- AAATCCTTAC AGAATGTATC ATGCACGTGT AAGAAATTAA TAGACAAATA GACAACCAAA CAATAAAAGT   
  
  
- TCCCCTACCC CTCACCCACC TACGAATCAT ACATTAGAAA AATATATCCC TTTTTAGTAT ATCGAACTTT   
  
  
- CATTTTGAAA GTAACGGTCC TAAGGTTCCA TCCCCATCCC AACAGATGTG GAACTAGAGG GATCTTCGTA   
  
  
- CCGCTAGGAA GGAGCCTAAC ACAATATCTT ATTTCTTACA CCAAGACGAC ATTACTCTGA ATACAAGATA   
  
  
- GTTTACTACT TAGACAAATC TTGAAGAACG AATAAAAAAG ACGTTTTAAA ATCCTCTGAA CTATTTGATA   
  
  
- TCAAGAGAAC TTAAATGTCC ACCTATAACC TCAAGTGTCT TATCATAAGT CTAGTTTCAC GAACACGAAT   
  
  
- TAACCTTCCT GTTGAACGAA CTCGACAAAT ATCTCGAGAT TGACTACGAT AGAGTACCTG AGTGTAGTCA   
  
  
- AAAAACCTAA ATTAGAATGG TCACAACTAG GCAGCATGTA AAGGCGAGTC ATGTCAGGCG GCAGTCACTG   
  
  
- TTTATCCTAC AAACGTAGTG ACTTCGAGCT GAGATCTCCA AGAGGACACA GTGGGAAGAG TTGAGTCAAA   
  
  
- CTAACACTAT GCCAATGATG TAACTCACTA TCAGTTCTCG TAGTGTCGAG CTGCCCCTCA GATAGTTGTT   
  
  
- CTTCGGGAAG AACATTAAGA GGGGAACTTT GCTCGTCAAT AGTAGTAAAT AGCTGCTTAC CGGGCAGAAC   
  
  
- ATTAAGACGG GGACTTTGAT CGTCAATAGC AGTAGCCAAG TTGCGCTCAG GGCACCCTAT AAGGGTAGTT   
  
  
- CCGTGACTAA TACCTCACTT ATACTTCTCG CGAGACGTCC TCGATCTCTG ATGAGATTAC CCACGTCTAC   
  
  
- CACTCCTTCA TAGACGACGA TTAGTTGGAT ACCCCCCTTC AGCAGTCAGG CCCTAGGGTT CAGTCTCTAG   
  
  
- TTTTAGTAAC TCGCTTCTAG GTGTCCCAAG CGTAGGATGA GTCGGACTAA GTAGTAGAGA AAGATCCCGT   
  
  
- TCTTCTAGTC CTCTACTTTC GGTCTCTCTC TTTGCCGTGT TCCGTTACCT TCTTGGTTGC CTTGATGGTT   
  
  
- CAAACGGTGG ACCACTAAAC TTCGTCAACG ATTAACTTAC ACGCTCCCGA AATAGCCTAT TGGCTTATCT   
  
  
- ACTGAAACTC TCAAACCAAT TTGCCCGCTC CTTTCTCCAG AGATAGAGCC CACTCGGATA GGTTGCAGAG   
  
  
- CCACGGATGT ACTAGCTTCC CGAACACCGT TCCTTCGTCA GAAGCCCCTG ATCGTAGATA GCCCGAGACT   
  
  
- TCACATTTCT CGGAGAACCT TTTCTGAACG AGAGGATGTA CGTGTAGGAA ATACTTTATA CGGGAATAGA   
  
  
- GTTCAAACCA ATATACCGAC GCTTACCTCG CTATCGACTT CGAACATCTT TACTCCTATT GTATGTATAA   
  
  
- TATCTAAAGG TCTAACGTGT CCCGTGAGTC ACCCAATGAG ATAATGTTCG GGATCGTCGT TCTGGACCAC   
  
  
- CCGGTGGATT CCACGCTTAA TGTCCGTAAC TACTAGGACA AAGATTCATA CGGGCACCAC GATCGAACCT   
  
  
- CCGACAACCC TTTGCCAATC GCAGAGATAG ACTTTTCAAG TTTTATGGGC AGCTCAAGTT ACGCAACGGG   
  
  
- CAAATACCTG GGCTACAGTC CGCCCTTTAC GACCTACACT CCGGGCCCCT CCGAAACCGG CAATTAAAAG   
  
  
- GTAACGTCGA GGTGGTGTGA GGACTGCTCT CACAGCTACA CTTGTTGGGA TCCCTACCCG AAGAGTCTTA   
  
  
- CCACTTTAGT GAACCAGGAT TCCATTGGTG AAACCAACTC GTTCTTAGTT TGTGGTTGTG ATGGGGAAAG   
  
  
- AACTGGGCCA AGTATCTCTG GGAACTGATG ATGAGTCGGT ACAAACTCAG ATATCTGCAC TGGTACGGTT   
  
  
- CTCTGGCCTT CCTCTCCTAG TTACAACTCG TCGTAACAAA CCGATTCCTG TAACACTTAT AGTATCGAAC   
  
  
- GCTCCCGTTC CTCTCCCACC TCGCAGTACT TGAAAAACCC TTTACCTTCA GTTCCAAATG GTACCGTCCC   
  
  
- AAGTCCGTCA TGGGCAACTC GAGTATGCAT TTGAGACACT ATTCTTCGGA TGAGGCCACA ATGAGGCTCG   
  
  
- TAATATGAGA CTATCTCTTC CTACCACGGT ACGAAGACCC AACCTTCCCG GCTTACGACT AAAGTCGAAG   
  
  
- CCGTACCGTA AC

+     I-box

| Site Name | Organism | Position | Strand | Matrix score. | sequence | function |
| --- | --- | --- | --- | --- | --- | --- |
| I-box | Triticum aestivum | 2937 | - | 8 | AGATAAGG | part of a light responsive element |
| I-box | Zea mays | 817 | - | 9 | gGATAAGGTG | part of a light responsive element |

>HU08G00014.1   
+ +Up\_Stream \_Len000TCCTCT TTTGGGCTTC TTTCTTTCTA TCCTGTTTTT TTTTGGGTCG AAAAGTTGGA   
  
  
+ GGTTGAATTG AAGCATCCGA ATTCGAGCAG GATGTTTCTC TGCGTAGTGA TTCTTTCTGG GTTTGTGATT   
  
  
+ TTGAATTCTG GGTAATCGGT TGTTTTGCTA ATTTTGAGGA CAGAGTTCCT TTTCTGAATT AAAATTTCGT   
  
  
+ TTTTTTATCG GGAAAATCTT CAGTATTTGA GAAAAAAGAA CGAAATTTGG ATGATTTGCT GTCTAGATTT   
  
  
+ TGCTTTCATA TTCCTGGGTG TGGATTGGTT TATTAATTGC ATGTGAGGAA GTACAAGCTT CTGGTCAATG   
  
  
+ GTTGGGTTTT TTTTTTTTTT TTGTGGGTAT AAAGTTGGAA ATTGATTACG AGTCAAATTT CTCTTTCTAG   
  
  
+ AAGAGGGGGG GGGGGGGGGG TTGGTTTAAA ACTCCATATT TTCTTTGAAT GGAAAGCTTA ATTTGCTGAC   
  
  
+ TATCTTGGTT AGTAGCATAA GCTTGATCAG AAAACAAAAC GCTCTCTGCC CTGCAAAAAC TTTATTTGTC   
  
  
+ TTTTAAGGAA AATTTTGGAA TTTGAGAGTG AAAATTTTGG TTGTGAATCT TGTCTTACTA TGTGGTTCCT   
  
  
+ TCTGACAAAA GTTTGAATCT GAGTATGAGA GTTCATCAAT CTCTTTTACT TTCCTACTTT CTTTATTGCT   
  
  
+ TGCAAATTGT TGTGAACTTA CTTGGATTTT CTGGTAGAGG AGAGTTCTGT CCTGGAATTT GGTGGAAAGT   
  
  
+ GAGACATACT TTGGGATTTA ATTAGCCATC AATTTGGTTT CACACCTTAT CAGTTTGGAC TTCTGGTGTG   
  
  
+ TTATTGTATG TGTTCTTAGC TTGGGATTAT TCTTGTATCG GAAGAGAGGG GGGGGGGGGT GTTTCTTTTA   
  
  
+ GCTGAATGTT AGAACATTTT AAGGGATCTA GATATCTAGA GAAGAGCCTA GGAAATGAAG CATTTTTCCC   
  
  
+ TTATAATCTG ACACAACTTT ATGAATTGAT TAGGAACAAA GAACGATTAG TTCAGAAAGG TTACAACTCC   
  
  
+ TCTAGGTGTT ATACTAAAGA GATGTGAGTT AATAAGCAAT TGCTTACTAA CTGTTTAGGC TGTTTCATTG   
  
  
+ CTTGTAGAGG CTTTTTTTTT GGGGGGGGGG GGGGGTTTGG GGTTCATAAA TGAGAAGGTT GATTTGTTGT   
  
  
+ CTGCTCTGCT CTTGATTCGA TAGTAATTCT CTCAGAAAAG GGTAAAATGA CATGGCCTTC CATCGCCTCC   
  
  
+ CATGAGGCTT TTTGCCTCTG CTTGTGTATC TTTCATCTCT TGGTTTGGTG GGTTGGGGGA GGGGAGGGGG   
  
  
+ GGAAGGGGGT TTTGATTCGA TAGATCATCA CTTTCAGAAA TGGCCTCTCT GGCCAATGGT AAGGGGTAAG   
  
  
+ GTTAGTACAT GTGGACTTTC CCATGTGGAT TTTTCCAGAG TCCGTTGTGT GATTGTTATT GCTGTTGTTG   
  
  
+ TAACTTTCTG TCACTCGGAA AACATGAATC CATATGCAAC AAGAACATAA TAATTGTGAA TGCGACTTTA   
  
  
+ TTTAGGAATG TCTTACATAG TACGTGCACA TTCTTTAATT ATCTGTTTAT CTGTTGGTTT GTTATTTTCA   
  
  
+ AGGGGATGGG GAGTGGGTGG ATGCTTAGTA TGTAATCTTT TTATATAGGG AAAAATCATA TAGCTTGAAA   
  
  
+ GTAAAACTTT CATTGCCAGG ATTCCAAGGT AGGGGTAGGG TTGTCTACAC CTTGATCTCC CTAGAAGCAT   
  
  
+ GGCGATCCTT CCTCGGATTG TGTTATAGAA TAAAGAATGT GGTTCTGCTG TAATGAGACT TATGTTCTAT   
  
  
+ CAAATGATGA ATCTGTTTAG AACTTCTTGC TTATTTTTTC TGCAAAATTT TAGGAGACTT GATAAACTAT   
  
  
+ AGTTCTCTTG AATTTACAGG TGGATATTGG AGTTCACAGA ATAGTATTCA GATCAAAGTG CTTGTGCTTA   
  
  
+ ATTGGAAGGA CAACTTGCTT GAGCTGTTTA TAGAGCTCTA ACTGATGCTA TCTCATGGAC TCACATCAGT   
  
  
+ TTTTTGGATT TAATCTTACC AGTGTTGATC CGTCGTACAT TTCCGCTCAG TACAGTCCGC CGTCAGTGAC   
  
  
+ AAATAGGATG TTTGCATCAC TGAAGCTCGA CTCTAGAGGT TCTCCTGTGT CACCCTTCTC AACTCAGTTT   
  
  
+ GATTGTGATA CGGTTACTAC ATTGAGTGAT AGTCAAGAGC ATCACAGCTC GACGGGGAGT CTATCAACAA   
  
  
+ GAAGCCCTTC TTGTAATTCT CCCCTTGAAA CGAGCAGTTA TCATCATTTA TCGACGAATG GCCCGTCTTG   
  
  
+ TAATTCTGCC CCTGAAACTA GCAGTTATCG TCATCGGTTC AACGCGAGTC CCGTGGGATA TTCCCATCAA   
  
  
+ GGCACTGATT ATGGAGTGAA TATGAAGAGC GCTCTGCAGG AGCTAGAGAC TACTCTAATG GGTGCAGATG   
  
  
+ GTGAGGAAGT ATCTGCTGCT AATCAACCTA TGGGGGGAAG TCGTCAGTCC GGGATCCCAA GTCAGAGATC   
  
  
+ AAAATCATTG AGCGAAGATC CACAGGGTTC GCATCCTACT CAGCCTGATT CATCATCTCT TTCTAGGGCA   
  
  
+ AGAAGATCAG GAGATGAAAG CCAGAGAGAG AAACGGCACA AGGCAATGGA AGAACCAACG GAACTACCAA   
  
  
+ GTTTGCCACC TGGTGATTTG AAGCAGTTGC TAATTGAATG TGCGAGGGCT TTATCGGATA ACCGAATAGA   
  
  
+ TGACTTTGAG AGTTTGGTTA AACGGGCGAG GAAAGAGGTC TCTATCTCGG GTGAGCCTAT CCAACGTCTC   
  
  
+ GGTGCCTACA TGATCGAAGG GCTTGTGGCA AGGAAGCAGT CTTCGGGGAC TAGCATCTAT CGGGCTCTGA   
  
  
+ AGTGTAAAGA GCCTCTTGGA AAAGACTTGC TCTCCTACAT GCACATCCTT TATGAAATAT GCCCTTATCT   
  
  
+ CAAGTTTGGT TATATGGCTG CGAATGGAGC GATAGCTGAA GCTTGTAGAA ATGAGGATAA CATACATATT   
  
  
+ ATAGATTTCC AGATTGCACA GGGCACTCAG TGGGTTACTC TATTACAAGC CCTAGCAGCA AGACCTGGTG   
  
  
+ GGCCACCTAA GGTGCGAATT ACAGGCATTG ATGATCCTGT TTCTAAGTAT GCCCGTGGTG CTAGCTTGGA   
  
  
+ GGCTGTTGGG AAACGGTTAG CGTCTCTATC TGAAAAGTTC AAAATACCCG TCGAGTTCAA TGCGTTGCCC   
  
  
+ GTTTATGGAC CCGATGTCAG GCGGGAAATG CTGGATGTGA GGCCCGGGGA GGCTTTGGCC GTTAATTTTC   
  
  
+ CATTGCAGCT CCACCACACT CCTGACGAGA GTGTCGATGT GAACAACCCT AGGGATGGGC TTCTCAGAAT   
  
  
+ GGTGAAATCA CTTGGTCCTA AGGTAACCAC TTTGGTTGAG CAAGAATCAA ACACCAACAC TACCCCTTTC   
  
  
+ TTGACCCGGT TCATAGAGAC CCTTGACTAC TACTCAGCCA TGTTTGAGTC TATAGACGTG ACCATGCCAA   
  
  
+ GAGACCGGAA GGAGAGGATC AATGTTGAGC AGCATTGTTT GGCTAAGGAC ATTGTGAATA TCATAGCTTG   
  
  
+ CGAGGGCAAG GAGAGGGTGG AGCGTCATGA ACTTTTTGGG AAATGGAAGT CAAGGTTTAC CATGGCAGGG   
  
  
+ TTCAGGCAGT ACCCGTTGAG CTCATACGTA AACTCTGTGA TAAGAAGCCT ACTCCGGTGT TACTCCGAGC   
  
  
+ ATTATACTCT GATAGAGAAG GATGGTGCCA TGCTTCTGGG TTGGAAGGGC CGAATGCTGA TTTCAGCTTC   
  
  
+ GGCATGGCAT TG  

- +Up\_Stream \_Len000AGGAGA AAACCCGAAG AAAGAAAGAT AGGACAAAAA AAAACCCAGC TTTTCAACCT   
  
  
- CCAACTTAAC TTCGTAGGCT TAAGCTCGTC CTACAAAGAG ACGCATCACT AAGAAAGACC CAAACACTAA   
  
  
- AACTTAAGAC CCATTAGCCA ACAAAACGAT TAAAACTCCT GTCTCAAGGA AAAGACTTAA TTTTAAAGCA   
  
  
- AAAAAATAGC CCTTTTAGAA GTCATAAACT CTTTTTTCTT GCTTTAAACC TACTAAACGA CAGATCTAAA   
  
  
- ACGAAAGTAT AAGGACCCAC ACCTAACCAA ATAATTAACG TACACTCCTT CATGTTCGAA GACCAGTTAC   
  
  
- CAACCCAAAA AAAAAAAAAA AACACCCATA TTTCAACCTT TAACTAATGC TCAGTTTAAA GAGAAAGATC   
  
  
- TTCTCCCCCC CCCCCCCCCC AACCAAATTT TGAGGTATAA AAGAAACTTA CCTTTCGAAT TAAACGACTG   
  
  
- ATAGAACCAA TCATCGTATT CGAACTAGTC TTTTGTTTTG CGAGAGACGG GACGTTTTTG AAATAAACAG   
  
  
- AAAATTCCTT TTAAAACCTT AAACTCTCAC TTTTAAAACC AACACTTAGA ACAGAATGAT ACACCAAGGA   
  
  
- AGACTGTTTT CAAACTTAGA CTCATACTCT CAAGTAGTTA GAGAAAATGA AAGGATGAAA GAAATAACGA   
  
  
- ACGTTTAACA ACACTTGAAT GAACCTAAAA GACCATCTCC TCTCAAGACA GGACCTTAAA CCACCTTTCA   
  
  
- CTCTGTATGA AACCCTAAAT TAATCGGTAG TTAAACCAAA GTGTGGAATA GTCAAACCTG AAGACCACAC   
  
  
- AATAACATAC ACAAGAATCG AACCCTAATA AGAACATAGC CTTCTCTCCC CCCCCCCCCA CAAAGAAAAT   
  
  
- CGACTTACAA TCTTGTAAAA TTCCCTAGAT CTATAGATCT CTTCTCGGAT CCTTTACTTC GTAAAAAGGG   
  
  
- AATATTAGAC TGTGTTGAAA TACTTAACTA ATCCTTGTTT CTTGCTAATC AAGTCTTTCC AATGTTGAGG   
  
  
- AGATCCACAA TATGATTTCT CTACACTCAA TTATTCGTTA ACGAATGATT GACAAATCCG ACAAAGTAAC   
  
  
- GAACATCTCC GAAAAAAAAA CCCCCCCCCC CCCCCAAACC CCAAGTATTT ACTCTTCCAA CTAAACAACA   
  
  
- GACGAGACGA GAACTAAGCT ATCATTAAGA GAGTCTTTTC CCATTTTACT GTACCGGAAG GTAGCGGAGG   
  
  
- GTACTCCGAA AAACGGAGAC GAACACATAG AAAGTAGAGA ACCAAACCAC CCAACCCCCT CCCCTCCCCC   
  
  
- CCTTCCCCCA AAACTAAGCT ATCTAGTAGT GAAAGTCTTT ACCGGAGAGA CCGGTTACCA TTCCCCATTC   
  
  
- CAATCATGTA CACCTGAAAG GGTACACCTA AAAAGGTCTC AGGCAACACA CTAACAATAA CGACAACAAC   
  
  
- ATTGAAAGAC AGTGAGCCTT TTGTACTTAG GTATACGTTG TTCTTGTATT ATTAACACTT ACGCTGAAAT   
  
  
- AAATCCTTAC AGAATGTATC ATGCACGTGT AAGAAATTAA TAGACAAATA GACAACCAAA CAATAAAAGT   
  
  
- TCCCCTACCC CTCACCCACC TACGAATCAT ACATTAGAAA AATATATCCC TTTTTAGTAT ATCGAACTTT   
  
  
- CATTTTGAAA GTAACGGTCC TAAGGTTCCA TCCCCATCCC AACAGATGTG GAACTAGAGG GATCTTCGTA   
  
  
- CCGCTAGGAA GGAGCCTAAC ACAATATCTT ATTTCTTACA CCAAGACGAC ATTACTCTGA ATACAAGATA   
  
  
- GTTTACTACT TAGACAAATC TTGAAGAACG AATAAAAAAG ACGTTTTAAA ATCCTCTGAA CTATTTGATA   
  
  
- TCAAGAGAAC TTAAATGTCC ACCTATAACC TCAAGTGTCT TATCATAAGT CTAGTTTCAC GAACACGAAT   
  
  
- TAACCTTCCT GTTGAACGAA CTCGACAAAT ATCTCGAGAT TGACTACGAT AGAGTACCTG AGTGTAGTCA   
  
  
- AAAAACCTAA ATTAGAATGG TCACAACTAG GCAGCATGTA AAGGCGAGTC ATGTCAGGCG GCAGTCACTG   
  
  
- TTTATCCTAC AAACGTAGTG ACTTCGAGCT GAGATCTCCA AGAGGACACA GTGGGAAGAG TTGAGTCAAA   
  
  
- CTAACACTAT GCCAATGATG TAACTCACTA TCAGTTCTCG TAGTGTCGAG CTGCCCCTCA GATAGTTGTT   
  
  
- CTTCGGGAAG AACATTAAGA GGGGAACTTT GCTCGTCAAT AGTAGTAAAT AGCTGCTTAC CGGGCAGAAC   
  
  
- ATTAAGACGG GGACTTTGAT CGTCAATAGC AGTAGCCAAG TTGCGCTCAG GGCACCCTAT AAGGGTAGTT   
  
  
- CCGTGACTAA TACCTCACTT ATACTTCTCG CGAGACGTCC TCGATCTCTG ATGAGATTAC CCACGTCTAC   
  
  
- CACTCCTTCA TAGACGACGA TTAGTTGGAT ACCCCCCTTC AGCAGTCAGG CCCTAGGGTT CAGTCTCTAG   
  
  
- TTTTAGTAAC TCGCTTCTAG GTGTCCCAAG CGTAGGATGA GTCGGACTAA GTAGTAGAGA AAGATCCCGT   
  
  
- TCTTCTAGTC CTCTACTTTC GGTCTCTCTC TTTGCCGTGT TCCGTTACCT TCTTGGTTGC CTTGATGGTT   
  
  
- CAAACGGTGG ACCACTAAAC TTCGTCAACG ATTAACTTAC ACGCTCCCGA AATAGCCTAT TGGCTTATCT   
  
  
- ACTGAAACTC TCAAACCAAT TTGCCCGCTC CTTTCTCCAG AGATAGAGCC CACTCGGATA GGTTGCAGAG   
  
  
- CCACGGATGT ACTAGCTTCC CGAACACCGT TCCTTCGTCA GAAGCCCCTG ATCGTAGATA GCCCGAGACT   
  
  
- TCACATTTCT CGGAGAACCT TTTCTGAACG AGAGGATGTA CGTGTAGGAA ATACTTTATA CGGGAATAGA   
  
  
- GTTCAAACCA ATATACCGAC GCTTACCTCG CTATCGACTT CGAACATCTT TACTCCTATT GTATGTATAA   
  
  
- TATCTAAAGG TCTAACGTGT CCCGTGAGTC ACCCAATGAG ATAATGTTCG GGATCGTCGT TCTGGACCAC   
  
  
- CCGGTGGATT CCACGCTTAA TGTCCGTAAC TACTAGGACA AAGATTCATA CGGGCACCAC GATCGAACCT   
  
  
- CCGACAACCC TTTGCCAATC GCAGAGATAG ACTTTTCAAG TTTTATGGGC AGCTCAAGTT ACGCAACGGG   
  
  
- CAAATACCTG GGCTACAGTC CGCCCTTTAC GACCTACACT CCGGGCCCCT CCGAAACCGG CAATTAAAAG   
  
  
- GTAACGTCGA GGTGGTGTGA GGACTGCTCT CACAGCTACA CTTGTTGGGA TCCCTACCCG AAGAGTCTTA   
  
  
- CCACTTTAGT GAACCAGGAT TCCATTGGTG AAACCAACTC GTTCTTAGTT TGTGGTTGTG ATGGGGAAAG   
  
  
- AACTGGGCCA AGTATCTCTG GGAACTGATG ATGAGTCGGT ACAAACTCAG ATATCTGCAC TGGTACGGTT   
  
  
- CTCTGGCCTT CCTCTCCTAG TTACAACTCG TCGTAACAAA CCGATTCCTG TAACACTTAT AGTATCGAAC   
  
  
- GCTCCCGTTC CTCTCCCACC TCGCAGTACT TGAAAAACCC TTTACCTTCA GTTCCAAATG GTACCGTCCC   
  
  
- AAGTCCGTCA TGGGCAACTC GAGTATGCAT TTGAGACACT ATTCTTCGGA TGAGGCCACA ATGAGGCTCG   
  
  
- TAATATGAGA CTATCTCTTC CTACCACGGT ACGAAGACCC AACCTTCCCG GCTTACGACT AAAGTCGAAG   
  
  
- CCGTACCGTA AC

+     MBS

| Site Name | Organism | Position | Strand | Matrix score. | sequence | function |
| --- | --- | --- | --- | --- | --- | --- |
| MBS | Arabidopsis thaliana | 2688 | - | 6 | CAACTG | MYB binding site involved in drought-inducibility |

>HU08G00014.1   
+ +Up\_Stream \_Len000TCCTCT TTTGGGCTTC TTTCTTTCTA TCCTGTTTTT TTTTGGGTCG AAAAGTTGGA   
  
  
+ GGTTGAATTG AAGCATCCGA ATTCGAGCAG GATGTTTCTC TGCGTAGTGA TTCTTTCTGG GTTTGTGATT   
  
  
+ TTGAATTCTG GGTAATCGGT TGTTTTGCTA ATTTTGAGGA CAGAGTTCCT TTTCTGAATT AAAATTTCGT   
  
  
+ TTTTTTATCG GGAAAATCTT CAGTATTTGA GAAAAAAGAA CGAAATTTGG ATGATTTGCT GTCTAGATTT   
  
  
+ TGCTTTCATA TTCCTGGGTG TGGATTGGTT TATTAATTGC ATGTGAGGAA GTACAAGCTT CTGGTCAATG   
  
  
+ GTTGGGTTTT TTTTTTTTTT TTGTGGGTAT AAAGTTGGAA ATTGATTACG AGTCAAATTT CTCTTTCTAG   
  
  
+ AAGAGGGGGG GGGGGGGGGG TTGGTTTAAA ACTCCATATT TTCTTTGAAT GGAAAGCTTA ATTTGCTGAC   
  
  
+ TATCTTGGTT AGTAGCATAA GCTTGATCAG AAAACAAAAC GCTCTCTGCC CTGCAAAAAC TTTATTTGTC   
  
  
+ TTTTAAGGAA AATTTTGGAA TTTGAGAGTG AAAATTTTGG TTGTGAATCT TGTCTTACTA TGTGGTTCCT   
  
  
+ TCTGACAAAA GTTTGAATCT GAGTATGAGA GTTCATCAAT CTCTTTTACT TTCCTACTTT CTTTATTGCT   
  
  
+ TGCAAATTGT TGTGAACTTA CTTGGATTTT CTGGTAGAGG AGAGTTCTGT CCTGGAATTT GGTGGAAAGT   
  
  
+ GAGACATACT TTGGGATTTA ATTAGCCATC AATTTGGTTT CACACCTTAT CAGTTTGGAC TTCTGGTGTG   
  
  
+ TTATTGTATG TGTTCTTAGC TTGGGATTAT TCTTGTATCG GAAGAGAGGG GGGGGGGGGT GTTTCTTTTA   
  
  
+ GCTGAATGTT AGAACATTTT AAGGGATCTA GATATCTAGA GAAGAGCCTA GGAAATGAAG CATTTTTCCC   
  
  
+ TTATAATCTG ACACAACTTT ATGAATTGAT TAGGAACAAA GAACGATTAG TTCAGAAAGG TTACAACTCC   
  
  
+ TCTAGGTGTT ATACTAAAGA GATGTGAGTT AATAAGCAAT TGCTTACTAA CTGTTTAGGC TGTTTCATTG   
  
  
+ CTTGTAGAGG CTTTTTTTTT GGGGGGGGGG GGGGGTTTGG GGTTCATAAA TGAGAAGGTT GATTTGTTGT   
  
  
+ CTGCTCTGCT CTTGATTCGA TAGTAATTCT CTCAGAAAAG GGTAAAATGA CATGGCCTTC CATCGCCTCC   
  
  
+ CATGAGGCTT TTTGCCTCTG CTTGTGTATC TTTCATCTCT TGGTTTGGTG GGTTGGGGGA GGGGAGGGGG   
  
  
+ GGAAGGGGGT TTTGATTCGA TAGATCATCA CTTTCAGAAA TGGCCTCTCT GGCCAATGGT AAGGGGTAAG   
  
  
+ GTTAGTACAT GTGGACTTTC CCATGTGGAT TTTTCCAGAG TCCGTTGTGT GATTGTTATT GCTGTTGTTG   
  
  
+ TAACTTTCTG TCACTCGGAA AACATGAATC CATATGCAAC AAGAACATAA TAATTGTGAA TGCGACTTTA   
  
  
+ TTTAGGAATG TCTTACATAG TACGTGCACA TTCTTTAATT ATCTGTTTAT CTGTTGGTTT GTTATTTTCA   
  
  
+ AGGGGATGGG GAGTGGGTGG ATGCTTAGTA TGTAATCTTT TTATATAGGG AAAAATCATA TAGCTTGAAA   
  
  
+ GTAAAACTTT CATTGCCAGG ATTCCAAGGT AGGGGTAGGG TTGTCTACAC CTTGATCTCC CTAGAAGCAT   
  
  
+ GGCGATCCTT CCTCGGATTG TGTTATAGAA TAAAGAATGT GGTTCTGCTG TAATGAGACT TATGTTCTAT   
  
  
+ CAAATGATGA ATCTGTTTAG AACTTCTTGC TTATTTTTTC TGCAAAATTT TAGGAGACTT GATAAACTAT   
  
  
+ AGTTCTCTTG AATTTACAGG TGGATATTGG AGTTCACAGA ATAGTATTCA GATCAAAGTG CTTGTGCTTA   
  
  
+ ATTGGAAGGA CAACTTGCTT GAGCTGTTTA TAGAGCTCTA ACTGATGCTA TCTCATGGAC TCACATCAGT   
  
  
+ TTTTTGGATT TAATCTTACC AGTGTTGATC CGTCGTACAT TTCCGCTCAG TACAGTCCGC CGTCAGTGAC   
  
  
+ AAATAGGATG TTTGCATCAC TGAAGCTCGA CTCTAGAGGT TCTCCTGTGT CACCCTTCTC AACTCAGTTT   
  
  
+ GATTGTGATA CGGTTACTAC ATTGAGTGAT AGTCAAGAGC ATCACAGCTC GACGGGGAGT CTATCAACAA   
  
  
+ GAAGCCCTTC TTGTAATTCT CCCCTTGAAA CGAGCAGTTA TCATCATTTA TCGACGAATG GCCCGTCTTG   
  
  
+ TAATTCTGCC CCTGAAACTA GCAGTTATCG TCATCGGTTC AACGCGAGTC CCGTGGGATA TTCCCATCAA   
  
  
+ GGCACTGATT ATGGAGTGAA TATGAAGAGC GCTCTGCAGG AGCTAGAGAC TACTCTAATG GGTGCAGATG   
  
  
+ GTGAGGAAGT ATCTGCTGCT AATCAACCTA TGGGGGGAAG TCGTCAGTCC GGGATCCCAA GTCAGAGATC   
  
  
+ AAAATCATTG AGCGAAGATC CACAGGGTTC GCATCCTACT CAGCCTGATT CATCATCTCT TTCTAGGGCA   
  
  
+ AGAAGATCAG GAGATGAAAG CCAGAGAGAG AAACGGCACA AGGCAATGGA AGAACCAACG GAACTACCAA   
  
  
+ GTTTGCCACC TGGTGATTTG AAGCAGTTGC TAATTGAATG TGCGAGGGCT TTATCGGATA ACCGAATAGA   
  
  
+ TGACTTTGAG AGTTTGGTTA AACGGGCGAG GAAAGAGGTC TCTATCTCGG GTGAGCCTAT CCAACGTCTC   
  
  
+ GGTGCCTACA TGATCGAAGG GCTTGTGGCA AGGAAGCAGT CTTCGGGGAC TAGCATCTAT CGGGCTCTGA   
  
  
+ AGTGTAAAGA GCCTCTTGGA AAAGACTTGC TCTCCTACAT GCACATCCTT TATGAAATAT GCCCTTATCT   
  
  
+ CAAGTTTGGT TATATGGCTG CGAATGGAGC GATAGCTGAA GCTTGTAGAA ATGAGGATAA CATACATATT   
  
  
+ ATAGATTTCC AGATTGCACA GGGCACTCAG TGGGTTACTC TATTACAAGC CCTAGCAGCA AGACCTGGTG   
  
  
+ GGCCACCTAA GGTGCGAATT ACAGGCATTG ATGATCCTGT TTCTAAGTAT GCCCGTGGTG CTAGCTTGGA   
  
  
+ GGCTGTTGGG AAACGGTTAG CGTCTCTATC TGAAAAGTTC AAAATACCCG TCGAGTTCAA TGCGTTGCCC   
  
  
+ GTTTATGGAC CCGATGTCAG GCGGGAAATG CTGGATGTGA GGCCCGGGGA GGCTTTGGCC GTTAATTTTC   
  
  
+ CATTGCAGCT CCACCACACT CCTGACGAGA GTGTCGATGT GAACAACCCT AGGGATGGGC TTCTCAGAAT   
  
  
+ GGTGAAATCA CTTGGTCCTA AGGTAACCAC TTTGGTTGAG CAAGAATCAA ACACCAACAC TACCCCTTTC   
  
  
+ TTGACCCGGT TCATAGAGAC CCTTGACTAC TACTCAGCCA TGTTTGAGTC TATAGACGTG ACCATGCCAA   
  
  
+ GAGACCGGAA GGAGAGGATC AATGTTGAGC AGCATTGTTT GGCTAAGGAC ATTGTGAATA TCATAGCTTG   
  
  
+ CGAGGGCAAG GAGAGGGTGG AGCGTCATGA ACTTTTTGGG AAATGGAAGT CAAGGTTTAC CATGGCAGGG   
  
  
+ TTCAGGCAGT ACCCGTTGAG CTCATACGTA AACTCTGTGA TAAGAAGCCT ACTCCGGTGT TACTCCGAGC   
  
  
+ ATTATACTCT GATAGAGAAG GATGGTGCCA TGCTTCTGGG TTGGAAGGGC CGAATGCTGA TTTCAGCTTC   
  
  
+ GGCATGGCAT TG  

- +Up\_Stream \_Len000AGGAGA AAACCCGAAG AAAGAAAGAT AGGACAAAAA AAAACCCAGC TTTTCAACCT   
  
  
- CCAACTTAAC TTCGTAGGCT TAAGCTCGTC CTACAAAGAG ACGCATCACT AAGAAAGACC CAAACACTAA   
  
  
- AACTTAAGAC CCATTAGCCA ACAAAACGAT TAAAACTCCT GTCTCAAGGA AAAGACTTAA TTTTAAAGCA   
  
  
- AAAAAATAGC CCTTTTAGAA GTCATAAACT CTTTTTTCTT GCTTTAAACC TACTAAACGA CAGATCTAAA   
  
  
- ACGAAAGTAT AAGGACCCAC ACCTAACCAA ATAATTAACG TACACTCCTT CATGTTCGAA GACCAGTTAC   
  
  
- CAACCCAAAA AAAAAAAAAA AACACCCATA TTTCAACCTT TAACTAATGC TCAGTTTAAA GAGAAAGATC   
  
  
- TTCTCCCCCC CCCCCCCCCC AACCAAATTT TGAGGTATAA AAGAAACTTA CCTTTCGAAT TAAACGACTG   
  
  
- ATAGAACCAA TCATCGTATT CGAACTAGTC TTTTGTTTTG CGAGAGACGG GACGTTTTTG AAATAAACAG   
  
  
- AAAATTCCTT TTAAAACCTT AAACTCTCAC TTTTAAAACC AACACTTAGA ACAGAATGAT ACACCAAGGA   
  
  
- AGACTGTTTT CAAACTTAGA CTCATACTCT CAAGTAGTTA GAGAAAATGA AAGGATGAAA GAAATAACGA   
  
  
- ACGTTTAACA ACACTTGAAT GAACCTAAAA GACCATCTCC TCTCAAGACA GGACCTTAAA CCACCTTTCA   
  
  
- CTCTGTATGA AACCCTAAAT TAATCGGTAG TTAAACCAAA GTGTGGAATA GTCAAACCTG AAGACCACAC   
  
  
- AATAACATAC ACAAGAATCG AACCCTAATA AGAACATAGC CTTCTCTCCC CCCCCCCCCA CAAAGAAAAT   
  
  
- CGACTTACAA TCTTGTAAAA TTCCCTAGAT CTATAGATCT CTTCTCGGAT CCTTTACTTC GTAAAAAGGG   
  
  
- AATATTAGAC TGTGTTGAAA TACTTAACTA ATCCTTGTTT CTTGCTAATC AAGTCTTTCC AATGTTGAGG   
  
  
- AGATCCACAA TATGATTTCT CTACACTCAA TTATTCGTTA ACGAATGATT GACAAATCCG ACAAAGTAAC   
  
  
- GAACATCTCC GAAAAAAAAA CCCCCCCCCC CCCCCAAACC CCAAGTATTT ACTCTTCCAA CTAAACAACA   
  
  
- GACGAGACGA GAACTAAGCT ATCATTAAGA GAGTCTTTTC CCATTTTACT GTACCGGAAG GTAGCGGAGG   
  
  
- GTACTCCGAA AAACGGAGAC GAACACATAG AAAGTAGAGA ACCAAACCAC CCAACCCCCT CCCCTCCCCC   
  
  
- CCTTCCCCCA AAACTAAGCT ATCTAGTAGT GAAAGTCTTT ACCGGAGAGA CCGGTTACCA TTCCCCATTC   
  
  
- CAATCATGTA CACCTGAAAG GGTACACCTA AAAAGGTCTC AGGCAACACA CTAACAATAA CGACAACAAC   
  
  
- ATTGAAAGAC AGTGAGCCTT TTGTACTTAG GTATACGTTG TTCTTGTATT ATTAACACTT ACGCTGAAAT   
  
  
- AAATCCTTAC AGAATGTATC ATGCACGTGT AAGAAATTAA TAGACAAATA GACAACCAAA CAATAAAAGT   
  
  
- TCCCCTACCC CTCACCCACC TACGAATCAT ACATTAGAAA AATATATCCC TTTTTAGTAT ATCGAACTTT   
  
  
- CATTTTGAAA GTAACGGTCC TAAGGTTCCA TCCCCATCCC AACAGATGTG GAACTAGAGG GATCTTCGTA   
  
  
- CCGCTAGGAA GGAGCCTAAC ACAATATCTT ATTTCTTACA CCAAGACGAC ATTACTCTGA ATACAAGATA   
  
  
- GTTTACTACT TAGACAAATC TTGAAGAACG AATAAAAAAG ACGTTTTAAA ATCCTCTGAA CTATTTGATA   
  
  
- TCAAGAGAAC TTAAATGTCC ACCTATAACC TCAAGTGTCT TATCATAAGT CTAGTTTCAC GAACACGAAT   
  
  
- TAACCTTCCT GTTGAACGAA CTCGACAAAT ATCTCGAGAT TGACTACGAT AGAGTACCTG AGTGTAGTCA   
  
  
- AAAAACCTAA ATTAGAATGG TCACAACTAG GCAGCATGTA AAGGCGAGTC ATGTCAGGCG GCAGTCACTG   
  
  
- TTTATCCTAC AAACGTAGTG ACTTCGAGCT GAGATCTCCA AGAGGACACA GTGGGAAGAG TTGAGTCAAA   
  
  
- CTAACACTAT GCCAATGATG TAACTCACTA TCAGTTCTCG TAGTGTCGAG CTGCCCCTCA GATAGTTGTT   
  
  
- CTTCGGGAAG AACATTAAGA GGGGAACTTT GCTCGTCAAT AGTAGTAAAT AGCTGCTTAC CGGGCAGAAC   
  
  
- ATTAAGACGG GGACTTTGAT CGTCAATAGC AGTAGCCAAG TTGCGCTCAG GGCACCCTAT AAGGGTAGTT   
  
  
- CCGTGACTAA TACCTCACTT ATACTTCTCG CGAGACGTCC TCGATCTCTG ATGAGATTAC CCACGTCTAC   
  
  
- CACTCCTTCA TAGACGACGA TTAGTTGGAT ACCCCCCTTC AGCAGTCAGG CCCTAGGGTT CAGTCTCTAG   
  
  
- TTTTAGTAAC TCGCTTCTAG GTGTCCCAAG CGTAGGATGA GTCGGACTAA GTAGTAGAGA AAGATCCCGT   
  
  
- TCTTCTAGTC CTCTACTTTC GGTCTCTCTC TTTGCCGTGT TCCGTTACCT TCTTGGTTGC CTTGATGGTT   
  
  
- CAAACGGTGG ACCACTAAAC TTCGTCAACG ATTAACTTAC ACGCTCCCGA AATAGCCTAT TGGCTTATCT   
  
  
- ACTGAAACTC TCAAACCAAT TTGCCCGCTC CTTTCTCCAG AGATAGAGCC CACTCGGATA GGTTGCAGAG   
  
  
- CCACGGATGT ACTAGCTTCC CGAACACCGT TCCTTCGTCA GAAGCCCCTG ATCGTAGATA GCCCGAGACT   
  
  
- TCACATTTCT CGGAGAACCT TTTCTGAACG AGAGGATGTA CGTGTAGGAA ATACTTTATA CGGGAATAGA   
  
  
- GTTCAAACCA ATATACCGAC GCTTACCTCG CTATCGACTT CGAACATCTT TACTCCTATT GTATGTATAA   
  
  
- TATCTAAAGG TCTAACGTGT CCCGTGAGTC ACCCAATGAG ATAATGTTCG GGATCGTCGT TCTGGACCAC   
  
  
- CCGGTGGATT CCACGCTTAA TGTCCGTAAC TACTAGGACA AAGATTCATA CGGGCACCAC GATCGAACCT   
  
  
- CCGACAACCC TTTGCCAATC GCAGAGATAG ACTTTTCAAG TTTTATGGGC AGCTCAAGTT ACGCAACGGG   
  
  
- CAAATACCTG GGCTACAGTC CGCCCTTTAC GACCTACACT CCGGGCCCCT CCGAAACCGG CAATTAAAAG   
  
  
- GTAACGTCGA GGTGGTGTGA GGACTGCTCT CACAGCTACA CTTGTTGGGA TCCCTACCCG AAGAGTCTTA   
  
  
- CCACTTTAGT GAACCAGGAT TCCATTGGTG AAACCAACTC GTTCTTAGTT TGTGGTTGTG ATGGGGAAAG   
  
  
- AACTGGGCCA AGTATCTCTG GGAACTGATG ATGAGTCGGT ACAAACTCAG ATATCTGCAC TGGTACGGTT   
  
  
- CTCTGGCCTT CCTCTCCTAG TTACAACTCG TCGTAACAAA CCGATTCCTG TAACACTTAT AGTATCGAAC   
  
  
- GCTCCCGTTC CTCTCCCACC TCGCAGTACT TGAAAAACCC TTTACCTTCA GTTCCAAATG GTACCGTCCC   
  
  
- AAGTCCGTCA TGGGCAACTC GAGTATGCAT TTGAGACACT ATTCTTCGGA TGAGGCCACA ATGAGGCTCG   
  
  
- TAATATGAGA CTATCTCTTC CTACCACGGT ACGAAGACCC AACCTTCCCG GCTTACGACT AAAGTCGAAG   
  
  
- CCGTACCGTA AC

+     MYB

| Site Name | Organism | Position | Strand | Matrix score. | sequence | function |
| --- | --- | --- | --- | --- | --- | --- |
| MYB | Arabidopsis thaliana | 1466 | - | 6 | CAACAG |  |
| MYB | Arabidopsis thaliana | 3388 | + | 6 | TAACCA |  |
| MYB | Arabidopsis thaliana | 1595 | - | 6 | CAACAG |  |
| MYB | Arabidopsis thaliana | 3397 | - | 6 | CAACCA |  |
| MYB | Arabidopsis thaliana | 602 | - | 6 | CAACCA |  |
| MYB | Arabidopsis thaliana | 2951 | - | 6 | TAACCA |  |
| MYB | Arabidopsis thaliana | 3157 | - | 6 | CAACAG |  |
| MYB | Arabidopsis thaliana | 353 | - | 6 | CAACCA |  |
| MYB | Arabidopsis thaliana | 2749 | - | 6 | TAACCA |  |
| MYB | Arabidopsis thaliana | 500 | - | 6 | TAACCA |  |

>HU08G00014.1   
+ +Up\_Stream \_Len000TCCTCT TTTGGGCTTC TTTCTTTCTA TCCTGTTTTT TTTTGGGTCG AAAAGTTGGA   
  
  
+ GGTTGAATTG AAGCATCCGA ATTCGAGCAG GATGTTTCTC TGCGTAGTGA TTCTTTCTGG GTTTGTGATT   
  
  
+ TTGAATTCTG GGTAATCGGT TGTTTTGCTA ATTTTGAGGA CAGAGTTCCT TTTCTGAATT AAAATTTCGT   
  
  
+ TTTTTTATCG GGAAAATCTT CAGTATTTGA GAAAAAAGAA CGAAATTTGG ATGATTTGCT GTCTAGATTT   
  
  
+ TGCTTTCATA TTCCTGGGTG TGGATTGGTT TATTAATTGC ATGTGAGGAA GTACAAGCTT CTGGTCAATG   
  
  
+ GTTGGGTTTT TTTTTTTTTT TTGTGGGTAT AAAGTTGGAA ATTGATTACG AGTCAAATTT CTCTTTCTAG   
  
  
+ AAGAGGGGGG GGGGGGGGGG TTGGTTTAAA ACTCCATATT TTCTTTGAAT GGAAAGCTTA ATTTGCTGAC   
  
  
+ TATCTTGGTT AGTAGCATAA GCTTGATCAG AAAACAAAAC GCTCTCTGCC CTGCAAAAAC TTTATTTGTC   
  
  
+ TTTTAAGGAA AATTTTGGAA TTTGAGAGTG AAAATTTTGG TTGTGAATCT TGTCTTACTA TGTGGTTCCT   
  
  
+ TCTGACAAAA GTTTGAATCT GAGTATGAGA GTTCATCAAT CTCTTTTACT TTCCTACTTT CTTTATTGCT   
  
  
+ TGCAAATTGT TGTGAACTTA CTTGGATTTT CTGGTAGAGG AGAGTTCTGT CCTGGAATTT GGTGGAAAGT   
  
  
+ GAGACATACT TTGGGATTTA ATTAGCCATC AATTTGGTTT CACACCTTAT CAGTTTGGAC TTCTGGTGTG   
  
  
+ TTATTGTATG TGTTCTTAGC TTGGGATTAT TCTTGTATCG GAAGAGAGGG GGGGGGGGGT GTTTCTTTTA   
  
  
+ GCTGAATGTT AGAACATTTT AAGGGATCTA GATATCTAGA GAAGAGCCTA GGAAATGAAG CATTTTTCCC   
  
  
+ TTATAATCTG ACACAACTTT ATGAATTGAT TAGGAACAAA GAACGATTAG TTCAGAAAGG TTACAACTCC   
  
  
+ TCTAGGTGTT ATACTAAAGA GATGTGAGTT AATAAGCAAT TGCTTACTAA CTGTTTAGGC TGTTTCATTG   
  
  
+ CTTGTAGAGG CTTTTTTTTT GGGGGGGGGG GGGGGTTTGG GGTTCATAAA TGAGAAGGTT GATTTGTTGT   
  
  
+ CTGCTCTGCT CTTGATTCGA TAGTAATTCT CTCAGAAAAG GGTAAAATGA CATGGCCTTC CATCGCCTCC   
  
  
+ CATGAGGCTT TTTGCCTCTG CTTGTGTATC TTTCATCTCT TGGTTTGGTG GGTTGGGGGA GGGGAGGGGG   
  
  
+ GGAAGGGGGT TTTGATTCGA TAGATCATCA CTTTCAGAAA TGGCCTCTCT GGCCAATGGT AAGGGGTAAG   
  
  
+ GTTAGTACAT GTGGACTTTC CCATGTGGAT TTTTCCAGAG TCCGTTGTGT GATTGTTATT GCTGTTGTTG   
  
  
+ TAACTTTCTG TCACTCGGAA AACATGAATC CATATGCAAC AAGAACATAA TAATTGTGAA TGCGACTTTA   
  
  
+ TTTAGGAATG TCTTACATAG TACGTGCACA TTCTTTAATT ATCTGTTTAT CTGTTGGTTT GTTATTTTCA   
  
  
+ AGGGGATGGG GAGTGGGTGG ATGCTTAGTA TGTAATCTTT TTATATAGGG AAAAATCATA TAGCTTGAAA   
  
  
+ GTAAAACTTT CATTGCCAGG ATTCCAAGGT AGGGGTAGGG TTGTCTACAC CTTGATCTCC CTAGAAGCAT   
  
  
+ GGCGATCCTT CCTCGGATTG TGTTATAGAA TAAAGAATGT GGTTCTGCTG TAATGAGACT TATGTTCTAT   
  
  
+ CAAATGATGA ATCTGTTTAG AACTTCTTGC TTATTTTTTC TGCAAAATTT TAGGAGACTT GATAAACTAT   
  
  
+ AGTTCTCTTG AATTTACAGG TGGATATTGG AGTTCACAGA ATAGTATTCA GATCAAAGTG CTTGTGCTTA   
  
  
+ ATTGGAAGGA CAACTTGCTT GAGCTGTTTA TAGAGCTCTA ACTGATGCTA TCTCATGGAC TCACATCAGT   
  
  
+ TTTTTGGATT TAATCTTACC AGTGTTGATC CGTCGTACAT TTCCGCTCAG TACAGTCCGC CGTCAGTGAC   
  
  
+ AAATAGGATG TTTGCATCAC TGAAGCTCGA CTCTAGAGGT TCTCCTGTGT CACCCTTCTC AACTCAGTTT   
  
  
+ GATTGTGATA CGGTTACTAC ATTGAGTGAT AGTCAAGAGC ATCACAGCTC GACGGGGAGT CTATCAACAA   
  
  
+ GAAGCCCTTC TTGTAATTCT CCCCTTGAAA CGAGCAGTTA TCATCATTTA TCGACGAATG GCCCGTCTTG   
  
  
+ TAATTCTGCC CCTGAAACTA GCAGTTATCG TCATCGGTTC AACGCGAGTC CCGTGGGATA TTCCCATCAA   
  
  
+ GGCACTGATT ATGGAGTGAA TATGAAGAGC GCTCTGCAGG AGCTAGAGAC TACTCTAATG GGTGCAGATG   
  
  
+ GTGAGGAAGT ATCTGCTGCT AATCAACCTA TGGGGGGAAG TCGTCAGTCC GGGATCCCAA GTCAGAGATC   
  
  
+ AAAATCATTG AGCGAAGATC CACAGGGTTC GCATCCTACT CAGCCTGATT CATCATCTCT TTCTAGGGCA   
  
  
+ AGAAGATCAG GAGATGAAAG CCAGAGAGAG AAACGGCACA AGGCAATGGA AGAACCAACG GAACTACCAA   
  
  
+ GTTTGCCACC TGGTGATTTG AAGCAGTTGC TAATTGAATG TGCGAGGGCT TTATCGGATA ACCGAATAGA   
  
  
+ TGACTTTGAG AGTTTGGTTA AACGGGCGAG GAAAGAGGTC TCTATCTCGG GTGAGCCTAT CCAACGTCTC   
  
  
+ GGTGCCTACA TGATCGAAGG GCTTGTGGCA AGGAAGCAGT CTTCGGGGAC TAGCATCTAT CGGGCTCTGA   
  
  
+ AGTGTAAAGA GCCTCTTGGA AAAGACTTGC TCTCCTACAT GCACATCCTT TATGAAATAT GCCCTTATCT   
  
  
+ CAAGTTTGGT TATATGGCTG CGAATGGAGC GATAGCTGAA GCTTGTAGAA ATGAGGATAA CATACATATT   
  
  
+ ATAGATTTCC AGATTGCACA GGGCACTCAG TGGGTTACTC TATTACAAGC CCTAGCAGCA AGACCTGGTG   
  
  
+ GGCCACCTAA GGTGCGAATT ACAGGCATTG ATGATCCTGT TTCTAAGTAT GCCCGTGGTG CTAGCTTGGA   
  
  
+ GGCTGTTGGG AAACGGTTAG CGTCTCTATC TGAAAAGTTC AAAATACCCG TCGAGTTCAA TGCGTTGCCC   
  
  
+ GTTTATGGAC CCGATGTCAG GCGGGAAATG CTGGATGTGA GGCCCGGGGA GGCTTTGGCC GTTAATTTTC   
  
  
+ CATTGCAGCT CCACCACACT CCTGACGAGA GTGTCGATGT GAACAACCCT AGGGATGGGC TTCTCAGAAT   
  
  
+ GGTGAAATCA CTTGGTCCTA AGGTAACCAC TTTGGTTGAG CAAGAATCAA ACACCAACAC TACCCCTTTC   
  
  
+ TTGACCCGGT TCATAGAGAC CCTTGACTAC TACTCAGCCA TGTTTGAGTC TATAGACGTG ACCATGCCAA   
  
  
+ GAGACCGGAA GGAGAGGATC AATGTTGAGC AGCATTGTTT GGCTAAGGAC ATTGTGAATA TCATAGCTTG   
  
  
+ CGAGGGCAAG GAGAGGGTGG AGCGTCATGA ACTTTTTGGG AAATGGAAGT CAAGGTTTAC CATGGCAGGG   
  
  
+ TTCAGGCAGT ACCCGTTGAG CTCATACGTA AACTCTGTGA TAAGAAGCCT ACTCCGGTGT TACTCCGAGC   
  
  
+ ATTATACTCT GATAGAGAAG GATGGTGCCA TGCTTCTGGG TTGGAAGGGC CGAATGCTGA TTTCAGCTTC   
  
  
+ GGCATGGCAT TG  

- +Up\_Stream \_Len000AGGAGA AAACCCGAAG AAAGAAAGAT AGGACAAAAA AAAACCCAGC TTTTCAACCT   
  
  
- CCAACTTAAC TTCGTAGGCT TAAGCTCGTC CTACAAAGAG ACGCATCACT AAGAAAGACC CAAACACTAA   
  
  
- AACTTAAGAC CCATTAGCCA ACAAAACGAT TAAAACTCCT GTCTCAAGGA AAAGACTTAA TTTTAAAGCA   
  
  
- AAAAAATAGC CCTTTTAGAA GTCATAAACT CTTTTTTCTT GCTTTAAACC TACTAAACGA CAGATCTAAA   
  
  
- ACGAAAGTAT AAGGACCCAC ACCTAACCAA ATAATTAACG TACACTCCTT CATGTTCGAA GACCAGTTAC   
  
  
- CAACCCAAAA AAAAAAAAAA AACACCCATA TTTCAACCTT TAACTAATGC TCAGTTTAAA GAGAAAGATC   
  
  
- TTCTCCCCCC CCCCCCCCCC AACCAAATTT TGAGGTATAA AAGAAACTTA CCTTTCGAAT TAAACGACTG   
  
  
- ATAGAACCAA TCATCGTATT CGAACTAGTC TTTTGTTTTG CGAGAGACGG GACGTTTTTG AAATAAACAG   
  
  
- AAAATTCCTT TTAAAACCTT AAACTCTCAC TTTTAAAACC AACACTTAGA ACAGAATGAT ACACCAAGGA   
  
  
- AGACTGTTTT CAAACTTAGA CTCATACTCT CAAGTAGTTA GAGAAAATGA AAGGATGAAA GAAATAACGA   
  
  
- ACGTTTAACA ACACTTGAAT GAACCTAAAA GACCATCTCC TCTCAAGACA GGACCTTAAA CCACCTTTCA   
  
  
- CTCTGTATGA AACCCTAAAT TAATCGGTAG TTAAACCAAA GTGTGGAATA GTCAAACCTG AAGACCACAC   
  
  
- AATAACATAC ACAAGAATCG AACCCTAATA AGAACATAGC CTTCTCTCCC CCCCCCCCCA CAAAGAAAAT   
  
  
- CGACTTACAA TCTTGTAAAA TTCCCTAGAT CTATAGATCT CTTCTCGGAT CCTTTACTTC GTAAAAAGGG   
  
  
- AATATTAGAC TGTGTTGAAA TACTTAACTA ATCCTTGTTT CTTGCTAATC AAGTCTTTCC AATGTTGAGG   
  
  
- AGATCCACAA TATGATTTCT CTACACTCAA TTATTCGTTA ACGAATGATT GACAAATCCG ACAAAGTAAC   
  
  
- GAACATCTCC GAAAAAAAAA CCCCCCCCCC CCCCCAAACC CCAAGTATTT ACTCTTCCAA CTAAACAACA   
  
  
- GACGAGACGA GAACTAAGCT ATCATTAAGA GAGTCTTTTC CCATTTTACT GTACCGGAAG GTAGCGGAGG   
  
  
- GTACTCCGAA AAACGGAGAC GAACACATAG AAAGTAGAGA ACCAAACCAC CCAACCCCCT CCCCTCCCCC   
  
  
- CCTTCCCCCA AAACTAAGCT ATCTAGTAGT GAAAGTCTTT ACCGGAGAGA CCGGTTACCA TTCCCCATTC   
  
  
- CAATCATGTA CACCTGAAAG GGTACACCTA AAAAGGTCTC AGGCAACACA CTAACAATAA CGACAACAAC   
  
  
- ATTGAAAGAC AGTGAGCCTT TTGTACTTAG GTATACGTTG TTCTTGTATT ATTAACACTT ACGCTGAAAT   
  
  
- AAATCCTTAC AGAATGTATC ATGCACGTGT AAGAAATTAA TAGACAAATA GACAACCAAA CAATAAAAGT   
  
  
- TCCCCTACCC CTCACCCACC TACGAATCAT ACATTAGAAA AATATATCCC TTTTTAGTAT ATCGAACTTT   
  
  
- CATTTTGAAA GTAACGGTCC TAAGGTTCCA TCCCCATCCC AACAGATGTG GAACTAGAGG GATCTTCGTA   
  
  
- CCGCTAGGAA GGAGCCTAAC ACAATATCTT ATTTCTTACA CCAAGACGAC ATTACTCTGA ATACAAGATA   
  
  
- GTTTACTACT TAGACAAATC TTGAAGAACG AATAAAAAAG ACGTTTTAAA ATCCTCTGAA CTATTTGATA   
  
  
- TCAAGAGAAC TTAAATGTCC ACCTATAACC TCAAGTGTCT TATCATAAGT CTAGTTTCAC GAACACGAAT   
  
  
- TAACCTTCCT GTTGAACGAA CTCGACAAAT ATCTCGAGAT TGACTACGAT AGAGTACCTG AGTGTAGTCA   
  
  
- AAAAACCTAA ATTAGAATGG TCACAACTAG GCAGCATGTA AAGGCGAGTC ATGTCAGGCG GCAGTCACTG   
  
  
- TTTATCCTAC AAACGTAGTG ACTTCGAGCT GAGATCTCCA AGAGGACACA GTGGGAAGAG TTGAGTCAAA   
  
  
- CTAACACTAT GCCAATGATG TAACTCACTA TCAGTTCTCG TAGTGTCGAG CTGCCCCTCA GATAGTTGTT   
  
  
- CTTCGGGAAG AACATTAAGA GGGGAACTTT GCTCGTCAAT AGTAGTAAAT AGCTGCTTAC CGGGCAGAAC   
  
  
- ATTAAGACGG GGACTTTGAT CGTCAATAGC AGTAGCCAAG TTGCGCTCAG GGCACCCTAT AAGGGTAGTT   
  
  
- CCGTGACTAA TACCTCACTT ATACTTCTCG CGAGACGTCC TCGATCTCTG ATGAGATTAC CCACGTCTAC   
  
  
- CACTCCTTCA TAGACGACGA TTAGTTGGAT ACCCCCCTTC AGCAGTCAGG CCCTAGGGTT CAGTCTCTAG   
  
  
- TTTTAGTAAC TCGCTTCTAG GTGTCCCAAG CGTAGGATGA GTCGGACTAA GTAGTAGAGA AAGATCCCGT   
  
  
- TCTTCTAGTC CTCTACTTTC GGTCTCTCTC TTTGCCGTGT TCCGTTACCT TCTTGGTTGC CTTGATGGTT   
  
  
- CAAACGGTGG ACCACTAAAC TTCGTCAACG ATTAACTTAC ACGCTCCCGA AATAGCCTAT TGGCTTATCT   
  
  
- ACTGAAACTC TCAAACCAAT TTGCCCGCTC CTTTCTCCAG AGATAGAGCC CACTCGGATA GGTTGCAGAG   
  
  
- CCACGGATGT ACTAGCTTCC CGAACACCGT TCCTTCGTCA GAAGCCCCTG ATCGTAGATA GCCCGAGACT   
  
  
- TCACATTTCT CGGAGAACCT TTTCTGAACG AGAGGATGTA CGTGTAGGAA ATACTTTATA CGGGAATAGA   
  
  
- GTTCAAACCA ATATACCGAC GCTTACCTCG CTATCGACTT CGAACATCTT TACTCCTATT GTATGTATAA   
  
  
- TATCTAAAGG TCTAACGTGT CCCGTGAGTC ACCCAATGAG ATAATGTTCG GGATCGTCGT TCTGGACCAC   
  
  
- CCGGTGGATT CCACGCTTAA TGTCCGTAAC TACTAGGACA AAGATTCATA CGGGCACCAC GATCGAACCT   
  
  
- CCGACAACCC TTTGCCAATC GCAGAGATAG ACTTTTCAAG TTTTATGGGC AGCTCAAGTT ACGCAACGGG   
  
  
- CAAATACCTG GGCTACAGTC CGCCCTTTAC GACCTACACT CCGGGCCCCT CCGAAACCGG CAATTAAAAG   
  
  
- GTAACGTCGA GGTGGTGTGA GGACTGCTCT CACAGCTACA CTTGTTGGGA TCCCTACCCG AAGAGTCTTA   
  
  
- CCACTTTAGT GAACCAGGAT TCCATTGGTG AAACCAACTC GTTCTTAGTT TGTGGTTGTG ATGGGGAAAG   
  
  
- AACTGGGCCA AGTATCTCTG GGAACTGATG ATGAGTCGGT ACAAACTCAG ATATCTGCAC TGGTACGGTT   
  
  
- CTCTGGCCTT CCTCTCCTAG TTACAACTCG TCGTAACAAA CCGATTCCTG TAACACTTAT AGTATCGAAC   
  
  
- GCTCCCGTTC CTCTCCCACC TCGCAGTACT TGAAAAACCC TTTACCTTCA GTTCCAAATG GTACCGTCCC   
  
  
- AAGTCCGTCA TGGGCAACTC GAGTATGCAT TTGAGACACT ATTCTTCGGA TGAGGCCACA ATGAGGCTCG   
  
  
- TAATATGAGA CTATCTCTTC CTACCACGGT ACGAAGACCC AACCTTCCCG GCTTACGACT AAAGTCGAAG   
  
  
- CCGTACCGTA AC

+     MYB recognition site

| Site Name | Organism | Position | Strand | Matrix score. | sequence | function |
| --- | --- | --- | --- | --- | --- | --- |
| MYB recognition site | Arabidopsis thaliana | 1446 | + | 6 | CCGTTG |  |
| MYB recognition site | Arabidopsis thaliana | 3657 | + | 6 | CCGTTG |  |
| MYB recognition site | Arabidopsis thaliana | 2650 | - | 6 | CCGTTG |  |

>HU08G00014.1   
+ +Up\_Stream \_Len000TCCTCT TTTGGGCTTC TTTCTTTCTA TCCTGTTTTT TTTTGGGTCG AAAAGTTGGA   
  
  
+ GGTTGAATTG AAGCATCCGA ATTCGAGCAG GATGTTTCTC TGCGTAGTGA TTCTTTCTGG GTTTGTGATT   
  
  
+ TTGAATTCTG GGTAATCGGT TGTTTTGCTA ATTTTGAGGA CAGAGTTCCT TTTCTGAATT AAAATTTCGT   
  
  
+ TTTTTTATCG GGAAAATCTT CAGTATTTGA GAAAAAAGAA CGAAATTTGG ATGATTTGCT GTCTAGATTT   
  
  
+ TGCTTTCATA TTCCTGGGTG TGGATTGGTT TATTAATTGC ATGTGAGGAA GTACAAGCTT CTGGTCAATG   
  
  
+ GTTGGGTTTT TTTTTTTTTT TTGTGGGTAT AAAGTTGGAA ATTGATTACG AGTCAAATTT CTCTTTCTAG   
  
  
+ AAGAGGGGGG GGGGGGGGGG TTGGTTTAAA ACTCCATATT TTCTTTGAAT GGAAAGCTTA ATTTGCTGAC   
  
  
+ TATCTTGGTT AGTAGCATAA GCTTGATCAG AAAACAAAAC GCTCTCTGCC CTGCAAAAAC TTTATTTGTC   
  
  
+ TTTTAAGGAA AATTTTGGAA TTTGAGAGTG AAAATTTTGG TTGTGAATCT TGTCTTACTA TGTGGTTCCT   
  
  
+ TCTGACAAAA GTTTGAATCT GAGTATGAGA GTTCATCAAT CTCTTTTACT TTCCTACTTT CTTTATTGCT   
  
  
+ TGCAAATTGT TGTGAACTTA CTTGGATTTT CTGGTAGAGG AGAGTTCTGT CCTGGAATTT GGTGGAAAGT   
  
  
+ GAGACATACT TTGGGATTTA ATTAGCCATC AATTTGGTTT CACACCTTAT CAGTTTGGAC TTCTGGTGTG   
  
  
+ TTATTGTATG TGTTCTTAGC TTGGGATTAT TCTTGTATCG GAAGAGAGGG GGGGGGGGGT GTTTCTTTTA   
  
  
+ GCTGAATGTT AGAACATTTT AAGGGATCTA GATATCTAGA GAAGAGCCTA GGAAATGAAG CATTTTTCCC   
  
  
+ TTATAATCTG ACACAACTTT ATGAATTGAT TAGGAACAAA GAACGATTAG TTCAGAAAGG TTACAACTCC   
  
  
+ TCTAGGTGTT ATACTAAAGA GATGTGAGTT AATAAGCAAT TGCTTACTAA CTGTTTAGGC TGTTTCATTG   
  
  
+ CTTGTAGAGG CTTTTTTTTT GGGGGGGGGG GGGGGTTTGG GGTTCATAAA TGAGAAGGTT GATTTGTTGT   
  
  
+ CTGCTCTGCT CTTGATTCGA TAGTAATTCT CTCAGAAAAG GGTAAAATGA CATGGCCTTC CATCGCCTCC   
  
  
+ CATGAGGCTT TTTGCCTCTG CTTGTGTATC TTTCATCTCT TGGTTTGGTG GGTTGGGGGA GGGGAGGGGG   
  
  
+ GGAAGGGGGT TTTGATTCGA TAGATCATCA CTTTCAGAAA TGGCCTCTCT GGCCAATGGT AAGGGGTAAG   
  
  
+ GTTAGTACAT GTGGACTTTC CCATGTGGAT TTTTCCAGAG TCCGTTGTGT GATTGTTATT GCTGTTGTTG   
  
  
+ TAACTTTCTG TCACTCGGAA AACATGAATC CATATGCAAC AAGAACATAA TAATTGTGAA TGCGACTTTA   
  
  
+ TTTAGGAATG TCTTACATAG TACGTGCACA TTCTTTAATT ATCTGTTTAT CTGTTGGTTT GTTATTTTCA   
  
  
+ AGGGGATGGG GAGTGGGTGG ATGCTTAGTA TGTAATCTTT TTATATAGGG AAAAATCATA TAGCTTGAAA   
  
  
+ GTAAAACTTT CATTGCCAGG ATTCCAAGGT AGGGGTAGGG TTGTCTACAC CTTGATCTCC CTAGAAGCAT   
  
  
+ GGCGATCCTT CCTCGGATTG TGTTATAGAA TAAAGAATGT GGTTCTGCTG TAATGAGACT TATGTTCTAT   
  
  
+ CAAATGATGA ATCTGTTTAG AACTTCTTGC TTATTTTTTC TGCAAAATTT TAGGAGACTT GATAAACTAT   
  
  
+ AGTTCTCTTG AATTTACAGG TGGATATTGG AGTTCACAGA ATAGTATTCA GATCAAAGTG CTTGTGCTTA   
  
  
+ ATTGGAAGGA CAACTTGCTT GAGCTGTTTA TAGAGCTCTA ACTGATGCTA TCTCATGGAC TCACATCAGT   
  
  
+ TTTTTGGATT TAATCTTACC AGTGTTGATC CGTCGTACAT TTCCGCTCAG TACAGTCCGC CGTCAGTGAC   
  
  
+ AAATAGGATG TTTGCATCAC TGAAGCTCGA CTCTAGAGGT TCTCCTGTGT CACCCTTCTC AACTCAGTTT   
  
  
+ GATTGTGATA CGGTTACTAC ATTGAGTGAT AGTCAAGAGC ATCACAGCTC GACGGGGAGT CTATCAACAA   
  
  
+ GAAGCCCTTC TTGTAATTCT CCCCTTGAAA CGAGCAGTTA TCATCATTTA TCGACGAATG GCCCGTCTTG   
  
  
+ TAATTCTGCC CCTGAAACTA GCAGTTATCG TCATCGGTTC AACGCGAGTC CCGTGGGATA TTCCCATCAA   
  
  
+ GGCACTGATT ATGGAGTGAA TATGAAGAGC GCTCTGCAGG AGCTAGAGAC TACTCTAATG GGTGCAGATG   
  
  
+ GTGAGGAAGT ATCTGCTGCT AATCAACCTA TGGGGGGAAG TCGTCAGTCC GGGATCCCAA GTCAGAGATC   
  
  
+ AAAATCATTG AGCGAAGATC CACAGGGTTC GCATCCTACT CAGCCTGATT CATCATCTCT TTCTAGGGCA   
  
  
+ AGAAGATCAG GAGATGAAAG CCAGAGAGAG AAACGGCACA AGGCAATGGA AGAACCAACG GAACTACCAA   
  
  
+ GTTTGCCACC TGGTGATTTG AAGCAGTTGC TAATTGAATG TGCGAGGGCT TTATCGGATA ACCGAATAGA   
  
  
+ TGACTTTGAG AGTTTGGTTA AACGGGCGAG GAAAGAGGTC TCTATCTCGG GTGAGCCTAT CCAACGTCTC   
  
  
+ GGTGCCTACA TGATCGAAGG GCTTGTGGCA AGGAAGCAGT CTTCGGGGAC TAGCATCTAT CGGGCTCTGA   
  
  
+ AGTGTAAAGA GCCTCTTGGA AAAGACTTGC TCTCCTACAT GCACATCCTT TATGAAATAT GCCCTTATCT   
  
  
+ CAAGTTTGGT TATATGGCTG CGAATGGAGC GATAGCTGAA GCTTGTAGAA ATGAGGATAA CATACATATT   
  
  
+ ATAGATTTCC AGATTGCACA GGGCACTCAG TGGGTTACTC TATTACAAGC CCTAGCAGCA AGACCTGGTG   
  
  
+ GGCCACCTAA GGTGCGAATT ACAGGCATTG ATGATCCTGT TTCTAAGTAT GCCCGTGGTG CTAGCTTGGA   
  
  
+ GGCTGTTGGG AAACGGTTAG CGTCTCTATC TGAAAAGTTC AAAATACCCG TCGAGTTCAA TGCGTTGCCC   
  
  
+ GTTTATGGAC CCGATGTCAG GCGGGAAATG CTGGATGTGA GGCCCGGGGA GGCTTTGGCC GTTAATTTTC   
  
  
+ CATTGCAGCT CCACCACACT CCTGACGAGA GTGTCGATGT GAACAACCCT AGGGATGGGC TTCTCAGAAT   
  
  
+ GGTGAAATCA CTTGGTCCTA AGGTAACCAC TTTGGTTGAG CAAGAATCAA ACACCAACAC TACCCCTTTC   
  
  
+ TTGACCCGGT TCATAGAGAC CCTTGACTAC TACTCAGCCA TGTTTGAGTC TATAGACGTG ACCATGCCAA   
  
  
+ GAGACCGGAA GGAGAGGATC AATGTTGAGC AGCATTGTTT GGCTAAGGAC ATTGTGAATA TCATAGCTTG   
  
  
+ CGAGGGCAAG GAGAGGGTGG AGCGTCATGA ACTTTTTGGG AAATGGAAGT CAAGGTTTAC CATGGCAGGG   
  
  
+ TTCAGGCAGT ACCCGTTGAG CTCATACGTA AACTCTGTGA TAAGAAGCCT ACTCCGGTGT TACTCCGAGC   
  
  
+ ATTATACTCT GATAGAGAAG GATGGTGCCA TGCTTCTGGG TTGGAAGGGC CGAATGCTGA TTTCAGCTTC   
  
  
+ GGCATGGCAT TG  

- +Up\_Stream \_Len000AGGAGA AAACCCGAAG AAAGAAAGAT AGGACAAAAA AAAACCCAGC TTTTCAACCT   
  
  
- CCAACTTAAC TTCGTAGGCT TAAGCTCGTC CTACAAAGAG ACGCATCACT AAGAAAGACC CAAACACTAA   
  
  
- AACTTAAGAC CCATTAGCCA ACAAAACGAT TAAAACTCCT GTCTCAAGGA AAAGACTTAA TTTTAAAGCA   
  
  
- AAAAAATAGC CCTTTTAGAA GTCATAAACT CTTTTTTCTT GCTTTAAACC TACTAAACGA CAGATCTAAA   
  
  
- ACGAAAGTAT AAGGACCCAC ACCTAACCAA ATAATTAACG TACACTCCTT CATGTTCGAA GACCAGTTAC   
  
  
- CAACCCAAAA AAAAAAAAAA AACACCCATA TTTCAACCTT TAACTAATGC TCAGTTTAAA GAGAAAGATC   
  
  
- TTCTCCCCCC CCCCCCCCCC AACCAAATTT TGAGGTATAA AAGAAACTTA CCTTTCGAAT TAAACGACTG   
  
  
- ATAGAACCAA TCATCGTATT CGAACTAGTC TTTTGTTTTG CGAGAGACGG GACGTTTTTG AAATAAACAG   
  
  
- AAAATTCCTT TTAAAACCTT AAACTCTCAC TTTTAAAACC AACACTTAGA ACAGAATGAT ACACCAAGGA   
  
  
- AGACTGTTTT CAAACTTAGA CTCATACTCT CAAGTAGTTA GAGAAAATGA AAGGATGAAA GAAATAACGA   
  
  
- ACGTTTAACA ACACTTGAAT GAACCTAAAA GACCATCTCC TCTCAAGACA GGACCTTAAA CCACCTTTCA   
  
  
- CTCTGTATGA AACCCTAAAT TAATCGGTAG TTAAACCAAA GTGTGGAATA GTCAAACCTG AAGACCACAC   
  
  
- AATAACATAC ACAAGAATCG AACCCTAATA AGAACATAGC CTTCTCTCCC CCCCCCCCCA CAAAGAAAAT   
  
  
- CGACTTACAA TCTTGTAAAA TTCCCTAGAT CTATAGATCT CTTCTCGGAT CCTTTACTTC GTAAAAAGGG   
  
  
- AATATTAGAC TGTGTTGAAA TACTTAACTA ATCCTTGTTT CTTGCTAATC AAGTCTTTCC AATGTTGAGG   
  
  
- AGATCCACAA TATGATTTCT CTACACTCAA TTATTCGTTA ACGAATGATT GACAAATCCG ACAAAGTAAC   
  
  
- GAACATCTCC GAAAAAAAAA CCCCCCCCCC CCCCCAAACC CCAAGTATTT ACTCTTCCAA CTAAACAACA   
  
  
- GACGAGACGA GAACTAAGCT ATCATTAAGA GAGTCTTTTC CCATTTTACT GTACCGGAAG GTAGCGGAGG   
  
  
- GTACTCCGAA AAACGGAGAC GAACACATAG AAAGTAGAGA ACCAAACCAC CCAACCCCCT CCCCTCCCCC   
  
  
- CCTTCCCCCA AAACTAAGCT ATCTAGTAGT GAAAGTCTTT ACCGGAGAGA CCGGTTACCA TTCCCCATTC   
  
  
- CAATCATGTA CACCTGAAAG GGTACACCTA AAAAGGTCTC AGGCAACACA CTAACAATAA CGACAACAAC   
  
  
- ATTGAAAGAC AGTGAGCCTT TTGTACTTAG GTATACGTTG TTCTTGTATT ATTAACACTT ACGCTGAAAT   
  
  
- AAATCCTTAC AGAATGTATC ATGCACGTGT AAGAAATTAA TAGACAAATA GACAACCAAA CAATAAAAGT   
  
  
- TCCCCTACCC CTCACCCACC TACGAATCAT ACATTAGAAA AATATATCCC TTTTTAGTAT ATCGAACTTT   
  
  
- CATTTTGAAA GTAACGGTCC TAAGGTTCCA TCCCCATCCC AACAGATGTG GAACTAGAGG GATCTTCGTA   
  
  
- CCGCTAGGAA GGAGCCTAAC ACAATATCTT ATTTCTTACA CCAAGACGAC ATTACTCTGA ATACAAGATA   
  
  
- GTTTACTACT TAGACAAATC TTGAAGAACG AATAAAAAAG ACGTTTTAAA ATCCTCTGAA CTATTTGATA   
  
  
- TCAAGAGAAC TTAAATGTCC ACCTATAACC TCAAGTGTCT TATCATAAGT CTAGTTTCAC GAACACGAAT   
  
  
- TAACCTTCCT GTTGAACGAA CTCGACAAAT ATCTCGAGAT TGACTACGAT AGAGTACCTG AGTGTAGTCA   
  
  
- AAAAACCTAA ATTAGAATGG TCACAACTAG GCAGCATGTA AAGGCGAGTC ATGTCAGGCG GCAGTCACTG   
  
  
- TTTATCCTAC AAACGTAGTG ACTTCGAGCT GAGATCTCCA AGAGGACACA GTGGGAAGAG TTGAGTCAAA   
  
  
- CTAACACTAT GCCAATGATG TAACTCACTA TCAGTTCTCG TAGTGTCGAG CTGCCCCTCA GATAGTTGTT   
  
  
- CTTCGGGAAG AACATTAAGA GGGGAACTTT GCTCGTCAAT AGTAGTAAAT AGCTGCTTAC CGGGCAGAAC   
  
  
- ATTAAGACGG GGACTTTGAT CGTCAATAGC AGTAGCCAAG TTGCGCTCAG GGCACCCTAT AAGGGTAGTT   
  
  
- CCGTGACTAA TACCTCACTT ATACTTCTCG CGAGACGTCC TCGATCTCTG ATGAGATTAC CCACGTCTAC   
  
  
- CACTCCTTCA TAGACGACGA TTAGTTGGAT ACCCCCCTTC AGCAGTCAGG CCCTAGGGTT CAGTCTCTAG   
  
  
- TTTTAGTAAC TCGCTTCTAG GTGTCCCAAG CGTAGGATGA GTCGGACTAA GTAGTAGAGA AAGATCCCGT   
  
  
- TCTTCTAGTC CTCTACTTTC GGTCTCTCTC TTTGCCGTGT TCCGTTACCT TCTTGGTTGC CTTGATGGTT   
  
  
- CAAACGGTGG ACCACTAAAC TTCGTCAACG ATTAACTTAC ACGCTCCCGA AATAGCCTAT TGGCTTATCT   
  
  
- ACTGAAACTC TCAAACCAAT TTGCCCGCTC CTTTCTCCAG AGATAGAGCC CACTCGGATA GGTTGCAGAG   
  
  
- CCACGGATGT ACTAGCTTCC CGAACACCGT TCCTTCGTCA GAAGCCCCTG ATCGTAGATA GCCCGAGACT   
  
  
- TCACATTTCT CGGAGAACCT TTTCTGAACG AGAGGATGTA CGTGTAGGAA ATACTTTATA CGGGAATAGA   
  
  
- GTTCAAACCA ATATACCGAC GCTTACCTCG CTATCGACTT CGAACATCTT TACTCCTATT GTATGTATAA   
  
  
- TATCTAAAGG TCTAACGTGT CCCGTGAGTC ACCCAATGAG ATAATGTTCG GGATCGTCGT TCTGGACCAC   
  
  
- CCGGTGGATT CCACGCTTAA TGTCCGTAAC TACTAGGACA AAGATTCATA CGGGCACCAC GATCGAACCT   
  
  
- CCGACAACCC TTTGCCAATC GCAGAGATAG ACTTTTCAAG TTTTATGGGC AGCTCAAGTT ACGCAACGGG   
  
  
- CAAATACCTG GGCTACAGTC CGCCCTTTAC GACCTACACT CCGGGCCCCT CCGAAACCGG CAATTAAAAG   
  
  
- GTAACGTCGA GGTGGTGTGA GGACTGCTCT CACAGCTACA CTTGTTGGGA TCCCTACCCG AAGAGTCTTA   
  
  
- CCACTTTAGT GAACCAGGAT TCCATTGGTG AAACCAACTC GTTCTTAGTT TGTGGTTGTG ATGGGGAAAG   
  
  
- AACTGGGCCA AGTATCTCTG GGAACTGATG ATGAGTCGGT ACAAACTCAG ATATCTGCAC TGGTACGGTT   
  
  
- CTCTGGCCTT CCTCTCCTAG TTACAACTCG TCGTAACAAA CCGATTCCTG TAACACTTAT AGTATCGAAC   
  
  
- GCTCCCGTTC CTCTCCCACC TCGCAGTACT TGAAAAACCC TTTACCTTCA GTTCCAAATG GTACCGTCCC   
  
  
- AAGTCCGTCA TGGGCAACTC GAGTATGCAT TTGAGACACT ATTCTTCGGA TGAGGCCACA ATGAGGCTCG   
  
  
- TAATATGAGA CTATCTCTTC CTACCACGGT ACGAAGACCC AACCTTCCCG GCTTACGACT AAAGTCGAAG   
  
  
- CCGTACCGTA AC

+     MYB-like sequence

| Site Name | Organism | Position | Strand | Matrix score. | sequence | function |
| --- | --- | --- | --- | --- | --- | --- |
| MYB-like sequence | Arabidopsis thaliana | 3388 | + | 6 | TAACCA |  |
| MYB-like sequence | Arabidopsis thaliana | 2951 | - | 6 | TAACCA |  |
| MYB-like sequence | Arabidopsis thaliana | 500 | - | 6 | TAACCA |  |
| MYB-like sequence | Arabidopsis thaliana | 2749 | - | 6 | TAACCA |  |

>HU08G00014.1   
+ +Up\_Stream \_Len000TCCTCT TTTGGGCTTC TTTCTTTCTA TCCTGTTTTT TTTTGGGTCG AAAAGTTGGA   
  
  
+ GGTTGAATTG AAGCATCCGA ATTCGAGCAG GATGTTTCTC TGCGTAGTGA TTCTTTCTGG GTTTGTGATT   
  
  
+ TTGAATTCTG GGTAATCGGT TGTTTTGCTA ATTTTGAGGA CAGAGTTCCT TTTCTGAATT AAAATTTCGT   
  
  
+ TTTTTTATCG GGAAAATCTT CAGTATTTGA GAAAAAAGAA CGAAATTTGG ATGATTTGCT GTCTAGATTT   
  
  
+ TGCTTTCATA TTCCTGGGTG TGGATTGGTT TATTAATTGC ATGTGAGGAA GTACAAGCTT CTGGTCAATG   
  
  
+ GTTGGGTTTT TTTTTTTTTT TTGTGGGTAT AAAGTTGGAA ATTGATTACG AGTCAAATTT CTCTTTCTAG   
  
  
+ AAGAGGGGGG GGGGGGGGGG TTGGTTTAAA ACTCCATATT TTCTTTGAAT GGAAAGCTTA ATTTGCTGAC   
  
  
+ TATCTTGGTT AGTAGCATAA GCTTGATCAG AAAACAAAAC GCTCTCTGCC CTGCAAAAAC TTTATTTGTC   
  
  
+ TTTTAAGGAA AATTTTGGAA TTTGAGAGTG AAAATTTTGG TTGTGAATCT TGTCTTACTA TGTGGTTCCT   
  
  
+ TCTGACAAAA GTTTGAATCT GAGTATGAGA GTTCATCAAT CTCTTTTACT TTCCTACTTT CTTTATTGCT   
  
  
+ TGCAAATTGT TGTGAACTTA CTTGGATTTT CTGGTAGAGG AGAGTTCTGT CCTGGAATTT GGTGGAAAGT   
  
  
+ GAGACATACT TTGGGATTTA ATTAGCCATC AATTTGGTTT CACACCTTAT CAGTTTGGAC TTCTGGTGTG   
  
  
+ TTATTGTATG TGTTCTTAGC TTGGGATTAT TCTTGTATCG GAAGAGAGGG GGGGGGGGGT GTTTCTTTTA   
  
  
+ GCTGAATGTT AGAACATTTT AAGGGATCTA GATATCTAGA GAAGAGCCTA GGAAATGAAG CATTTTTCCC   
  
  
+ TTATAATCTG ACACAACTTT ATGAATTGAT TAGGAACAAA GAACGATTAG TTCAGAAAGG TTACAACTCC   
  
  
+ TCTAGGTGTT ATACTAAAGA GATGTGAGTT AATAAGCAAT TGCTTACTAA CTGTTTAGGC TGTTTCATTG   
  
  
+ CTTGTAGAGG CTTTTTTTTT GGGGGGGGGG GGGGGTTTGG GGTTCATAAA TGAGAAGGTT GATTTGTTGT   
  
  
+ CTGCTCTGCT CTTGATTCGA TAGTAATTCT CTCAGAAAAG GGTAAAATGA CATGGCCTTC CATCGCCTCC   
  
  
+ CATGAGGCTT TTTGCCTCTG CTTGTGTATC TTTCATCTCT TGGTTTGGTG GGTTGGGGGA GGGGAGGGGG   
  
  
+ GGAAGGGGGT TTTGATTCGA TAGATCATCA CTTTCAGAAA TGGCCTCTCT GGCCAATGGT AAGGGGTAAG   
  
  
+ GTTAGTACAT GTGGACTTTC CCATGTGGAT TTTTCCAGAG TCCGTTGTGT GATTGTTATT GCTGTTGTTG   
  
  
+ TAACTTTCTG TCACTCGGAA AACATGAATC CATATGCAAC AAGAACATAA TAATTGTGAA TGCGACTTTA   
  
  
+ TTTAGGAATG TCTTACATAG TACGTGCACA TTCTTTAATT ATCTGTTTAT CTGTTGGTTT GTTATTTTCA   
  
  
+ AGGGGATGGG GAGTGGGTGG ATGCTTAGTA TGTAATCTTT TTATATAGGG AAAAATCATA TAGCTTGAAA   
  
  
+ GTAAAACTTT CATTGCCAGG ATTCCAAGGT AGGGGTAGGG TTGTCTACAC CTTGATCTCC CTAGAAGCAT   
  
  
+ GGCGATCCTT CCTCGGATTG TGTTATAGAA TAAAGAATGT GGTTCTGCTG TAATGAGACT TATGTTCTAT   
  
  
+ CAAATGATGA ATCTGTTTAG AACTTCTTGC TTATTTTTTC TGCAAAATTT TAGGAGACTT GATAAACTAT   
  
  
+ AGTTCTCTTG AATTTACAGG TGGATATTGG AGTTCACAGA ATAGTATTCA GATCAAAGTG CTTGTGCTTA   
  
  
+ ATTGGAAGGA CAACTTGCTT GAGCTGTTTA TAGAGCTCTA ACTGATGCTA TCTCATGGAC TCACATCAGT   
  
  
+ TTTTTGGATT TAATCTTACC AGTGTTGATC CGTCGTACAT TTCCGCTCAG TACAGTCCGC CGTCAGTGAC   
  
  
+ AAATAGGATG TTTGCATCAC TGAAGCTCGA CTCTAGAGGT TCTCCTGTGT CACCCTTCTC AACTCAGTTT   
  
  
+ GATTGTGATA CGGTTACTAC ATTGAGTGAT AGTCAAGAGC ATCACAGCTC GACGGGGAGT CTATCAACAA   
  
  
+ GAAGCCCTTC TTGTAATTCT CCCCTTGAAA CGAGCAGTTA TCATCATTTA TCGACGAATG GCCCGTCTTG   
  
  
+ TAATTCTGCC CCTGAAACTA GCAGTTATCG TCATCGGTTC AACGCGAGTC CCGTGGGATA TTCCCATCAA   
  
  
+ GGCACTGATT ATGGAGTGAA TATGAAGAGC GCTCTGCAGG AGCTAGAGAC TACTCTAATG GGTGCAGATG   
  
  
+ GTGAGGAAGT ATCTGCTGCT AATCAACCTA TGGGGGGAAG TCGTCAGTCC GGGATCCCAA GTCAGAGATC   
  
  
+ AAAATCATTG AGCGAAGATC CACAGGGTTC GCATCCTACT CAGCCTGATT CATCATCTCT TTCTAGGGCA   
  
  
+ AGAAGATCAG GAGATGAAAG CCAGAGAGAG AAACGGCACA AGGCAATGGA AGAACCAACG GAACTACCAA   
  
  
+ GTTTGCCACC TGGTGATTTG AAGCAGTTGC TAATTGAATG TGCGAGGGCT TTATCGGATA ACCGAATAGA   
  
  
+ TGACTTTGAG AGTTTGGTTA AACGGGCGAG GAAAGAGGTC TCTATCTCGG GTGAGCCTAT CCAACGTCTC   
  
  
+ GGTGCCTACA TGATCGAAGG GCTTGTGGCA AGGAAGCAGT CTTCGGGGAC TAGCATCTAT CGGGCTCTGA   
  
  
+ AGTGTAAAGA GCCTCTTGGA AAAGACTTGC TCTCCTACAT GCACATCCTT TATGAAATAT GCCCTTATCT   
  
  
+ CAAGTTTGGT TATATGGCTG CGAATGGAGC GATAGCTGAA GCTTGTAGAA ATGAGGATAA CATACATATT   
  
  
+ ATAGATTTCC AGATTGCACA GGGCACTCAG TGGGTTACTC TATTACAAGC CCTAGCAGCA AGACCTGGTG   
  
  
+ GGCCACCTAA GGTGCGAATT ACAGGCATTG ATGATCCTGT TTCTAAGTAT GCCCGTGGTG CTAGCTTGGA   
  
  
+ GGCTGTTGGG AAACGGTTAG CGTCTCTATC TGAAAAGTTC AAAATACCCG TCGAGTTCAA TGCGTTGCCC   
  
  
+ GTTTATGGAC CCGATGTCAG GCGGGAAATG CTGGATGTGA GGCCCGGGGA GGCTTTGGCC GTTAATTTTC   
  
  
+ CATTGCAGCT CCACCACACT CCTGACGAGA GTGTCGATGT GAACAACCCT AGGGATGGGC TTCTCAGAAT   
  
  
+ GGTGAAATCA CTTGGTCCTA AGGTAACCAC TTTGGTTGAG CAAGAATCAA ACACCAACAC TACCCCTTTC   
  
  
+ TTGACCCGGT TCATAGAGAC CCTTGACTAC TACTCAGCCA TGTTTGAGTC TATAGACGTG ACCATGCCAA   
  
  
+ GAGACCGGAA GGAGAGGATC AATGTTGAGC AGCATTGTTT GGCTAAGGAC ATTGTGAATA TCATAGCTTG   
  
  
+ CGAGGGCAAG GAGAGGGTGG AGCGTCATGA ACTTTTTGGG AAATGGAAGT CAAGGTTTAC CATGGCAGGG   
  
  
+ TTCAGGCAGT ACCCGTTGAG CTCATACGTA AACTCTGTGA TAAGAAGCCT ACTCCGGTGT TACTCCGAGC   
  
  
+ ATTATACTCT GATAGAGAAG GATGGTGCCA TGCTTCTGGG TTGGAAGGGC CGAATGCTGA TTTCAGCTTC   
  
  
+ GGCATGGCAT TG  

- +Up\_Stream \_Len000AGGAGA AAACCCGAAG AAAGAAAGAT AGGACAAAAA AAAACCCAGC TTTTCAACCT   
  
  
- CCAACTTAAC TTCGTAGGCT TAAGCTCGTC CTACAAAGAG ACGCATCACT AAGAAAGACC CAAACACTAA   
  
  
- AACTTAAGAC CCATTAGCCA ACAAAACGAT TAAAACTCCT GTCTCAAGGA AAAGACTTAA TTTTAAAGCA   
  
  
- AAAAAATAGC CCTTTTAGAA GTCATAAACT CTTTTTTCTT GCTTTAAACC TACTAAACGA CAGATCTAAA   
  
  
- ACGAAAGTAT AAGGACCCAC ACCTAACCAA ATAATTAACG TACACTCCTT CATGTTCGAA GACCAGTTAC   
  
  
- CAACCCAAAA AAAAAAAAAA AACACCCATA TTTCAACCTT TAACTAATGC TCAGTTTAAA GAGAAAGATC   
  
  
- TTCTCCCCCC CCCCCCCCCC AACCAAATTT TGAGGTATAA AAGAAACTTA CCTTTCGAAT TAAACGACTG   
  
  
- ATAGAACCAA TCATCGTATT CGAACTAGTC TTTTGTTTTG CGAGAGACGG GACGTTTTTG AAATAAACAG   
  
  
- AAAATTCCTT TTAAAACCTT AAACTCTCAC TTTTAAAACC AACACTTAGA ACAGAATGAT ACACCAAGGA   
  
  
- AGACTGTTTT CAAACTTAGA CTCATACTCT CAAGTAGTTA GAGAAAATGA AAGGATGAAA GAAATAACGA   
  
  
- ACGTTTAACA ACACTTGAAT GAACCTAAAA GACCATCTCC TCTCAAGACA GGACCTTAAA CCACCTTTCA   
  
  
- CTCTGTATGA AACCCTAAAT TAATCGGTAG TTAAACCAAA GTGTGGAATA GTCAAACCTG AAGACCACAC   
  
  
- AATAACATAC ACAAGAATCG AACCCTAATA AGAACATAGC CTTCTCTCCC CCCCCCCCCA CAAAGAAAAT   
  
  
- CGACTTACAA TCTTGTAAAA TTCCCTAGAT CTATAGATCT CTTCTCGGAT CCTTTACTTC GTAAAAAGGG   
  
  
- AATATTAGAC TGTGTTGAAA TACTTAACTA ATCCTTGTTT CTTGCTAATC AAGTCTTTCC AATGTTGAGG   
  
  
- AGATCCACAA TATGATTTCT CTACACTCAA TTATTCGTTA ACGAATGATT GACAAATCCG ACAAAGTAAC   
  
  
- GAACATCTCC GAAAAAAAAA CCCCCCCCCC CCCCCAAACC CCAAGTATTT ACTCTTCCAA CTAAACAACA   
  
  
- GACGAGACGA GAACTAAGCT ATCATTAAGA GAGTCTTTTC CCATTTTACT GTACCGGAAG GTAGCGGAGG   
  
  
- GTACTCCGAA AAACGGAGAC GAACACATAG AAAGTAGAGA ACCAAACCAC CCAACCCCCT CCCCTCCCCC   
  
  
- CCTTCCCCCA AAACTAAGCT ATCTAGTAGT GAAAGTCTTT ACCGGAGAGA CCGGTTACCA TTCCCCATTC   
  
  
- CAATCATGTA CACCTGAAAG GGTACACCTA AAAAGGTCTC AGGCAACACA CTAACAATAA CGACAACAAC   
  
  
- ATTGAAAGAC AGTGAGCCTT TTGTACTTAG GTATACGTTG TTCTTGTATT ATTAACACTT ACGCTGAAAT   
  
  
- AAATCCTTAC AGAATGTATC ATGCACGTGT AAGAAATTAA TAGACAAATA GACAACCAAA CAATAAAAGT   
  
  
- TCCCCTACCC CTCACCCACC TACGAATCAT ACATTAGAAA AATATATCCC TTTTTAGTAT ATCGAACTTT   
  
  
- CATTTTGAAA GTAACGGTCC TAAGGTTCCA TCCCCATCCC AACAGATGTG GAACTAGAGG GATCTTCGTA   
  
  
- CCGCTAGGAA GGAGCCTAAC ACAATATCTT ATTTCTTACA CCAAGACGAC ATTACTCTGA ATACAAGATA   
  
  
- GTTTACTACT TAGACAAATC TTGAAGAACG AATAAAAAAG ACGTTTTAAA ATCCTCTGAA CTATTTGATA   
  
  
- TCAAGAGAAC TTAAATGTCC ACCTATAACC TCAAGTGTCT TATCATAAGT CTAGTTTCAC GAACACGAAT   
  
  
- TAACCTTCCT GTTGAACGAA CTCGACAAAT ATCTCGAGAT TGACTACGAT AGAGTACCTG AGTGTAGTCA   
  
  
- AAAAACCTAA ATTAGAATGG TCACAACTAG GCAGCATGTA AAGGCGAGTC ATGTCAGGCG GCAGTCACTG   
  
  
- TTTATCCTAC AAACGTAGTG ACTTCGAGCT GAGATCTCCA AGAGGACACA GTGGGAAGAG TTGAGTCAAA   
  
  
- CTAACACTAT GCCAATGATG TAACTCACTA TCAGTTCTCG TAGTGTCGAG CTGCCCCTCA GATAGTTGTT   
  
  
- CTTCGGGAAG AACATTAAGA GGGGAACTTT GCTCGTCAAT AGTAGTAAAT AGCTGCTTAC CGGGCAGAAC   
  
  
- ATTAAGACGG GGACTTTGAT CGTCAATAGC AGTAGCCAAG TTGCGCTCAG GGCACCCTAT AAGGGTAGTT   
  
  
- CCGTGACTAA TACCTCACTT ATACTTCTCG CGAGACGTCC TCGATCTCTG ATGAGATTAC CCACGTCTAC   
  
  
- CACTCCTTCA TAGACGACGA TTAGTTGGAT ACCCCCCTTC AGCAGTCAGG CCCTAGGGTT CAGTCTCTAG   
  
  
- TTTTAGTAAC TCGCTTCTAG GTGTCCCAAG CGTAGGATGA GTCGGACTAA GTAGTAGAGA AAGATCCCGT   
  
  
- TCTTCTAGTC CTCTACTTTC GGTCTCTCTC TTTGCCGTGT TCCGTTACCT TCTTGGTTGC CTTGATGGTT   
  
  
- CAAACGGTGG ACCACTAAAC TTCGTCAACG ATTAACTTAC ACGCTCCCGA AATAGCCTAT TGGCTTATCT   
  
  
- ACTGAAACTC TCAAACCAAT TTGCCCGCTC CTTTCTCCAG AGATAGAGCC CACTCGGATA GGTTGCAGAG   
  
  
- CCACGGATGT ACTAGCTTCC CGAACACCGT TCCTTCGTCA GAAGCCCCTG ATCGTAGATA GCCCGAGACT   
  
  
- TCACATTTCT CGGAGAACCT TTTCTGAACG AGAGGATGTA CGTGTAGGAA ATACTTTATA CGGGAATAGA   
  
  
- GTTCAAACCA ATATACCGAC GCTTACCTCG CTATCGACTT CGAACATCTT TACTCCTATT GTATGTATAA   
  
  
- TATCTAAAGG TCTAACGTGT CCCGTGAGTC ACCCAATGAG ATAATGTTCG GGATCGTCGT TCTGGACCAC   
  
  
- CCGGTGGATT CCACGCTTAA TGTCCGTAAC TACTAGGACA AAGATTCATA CGGGCACCAC GATCGAACCT   
  
  
- CCGACAACCC TTTGCCAATC GCAGAGATAG ACTTTTCAAG TTTTATGGGC AGCTCAAGTT ACGCAACGGG   
  
  
- CAAATACCTG GGCTACAGTC CGCCCTTTAC GACCTACACT CCGGGCCCCT CCGAAACCGG CAATTAAAAG   
  
  
- GTAACGTCGA GGTGGTGTGA GGACTGCTCT CACAGCTACA CTTGTTGGGA TCCCTACCCG AAGAGTCTTA   
  
  
- CCACTTTAGT GAACCAGGAT TCCATTGGTG AAACCAACTC GTTCTTAGTT TGTGGTTGTG ATGGGGAAAG   
  
  
- AACTGGGCCA AGTATCTCTG GGAACTGATG ATGAGTCGGT ACAAACTCAG ATATCTGCAC TGGTACGGTT   
  
  
- CTCTGGCCTT CCTCTCCTAG TTACAACTCG TCGTAACAAA CCGATTCCTG TAACACTTAT AGTATCGAAC   
  
  
- GCTCCCGTTC CTCTCCCACC TCGCAGTACT TGAAAAACCC TTTACCTTCA GTTCCAAATG GTACCGTCCC   
  
  
- AAGTCCGTCA TGGGCAACTC GAGTATGCAT TTGAGACACT ATTCTTCGGA TGAGGCCACA ATGAGGCTCG   
  
  
- TAATATGAGA CTATCTCTTC CTACCACGGT ACGAAGACCC AACCTTCCCG GCTTACGACT AAAGTCGAAG   
  
  
- CCGTACCGTA AC

+     MYC

| Site Name | Organism | Position | Strand | Matrix score. | sequence | function |
| --- | --- | --- | --- | --- | --- | --- |
| MYC | Arabidopsis thaliana | 1091 | + | 6 | CAATTG |  |
| MYC | Arabidopsis thaliana | 1426 | + | 6 | CATGTG |  |
| MYC | Arabidopsis thaliana | 1825 | - | 6 | CATTTG |  |
| MYC | Arabidopsis thaliana | 324 | + | 6 | CATGTG |  |
| MYC | Arabidopsis thaliana | 1412 | + | 6 | CATGTG |  |

>HU08G00014.1   
+ +Up\_Stream \_Len000TCCTCT TTTGGGCTTC TTTCTTTCTA TCCTGTTTTT TTTTGGGTCG AAAAGTTGGA   
  
  
+ GGTTGAATTG AAGCATCCGA ATTCGAGCAG GATGTTTCTC TGCGTAGTGA TTCTTTCTGG GTTTGTGATT   
  
  
+ TTGAATTCTG GGTAATCGGT TGTTTTGCTA ATTTTGAGGA CAGAGTTCCT TTTCTGAATT AAAATTTCGT   
  
  
+ TTTTTTATCG GGAAAATCTT CAGTATTTGA GAAAAAAGAA CGAAATTTGG ATGATTTGCT GTCTAGATTT   
  
  
+ TGCTTTCATA TTCCTGGGTG TGGATTGGTT TATTAATTGC ATGTGAGGAA GTACAAGCTT CTGGTCAATG   
  
  
+ GTTGGGTTTT TTTTTTTTTT TTGTGGGTAT AAAGTTGGAA ATTGATTACG AGTCAAATTT CTCTTTCTAG   
  
  
+ AAGAGGGGGG GGGGGGGGGG TTGGTTTAAA ACTCCATATT TTCTTTGAAT GGAAAGCTTA ATTTGCTGAC   
  
  
+ TATCTTGGTT AGTAGCATAA GCTTGATCAG AAAACAAAAC GCTCTCTGCC CTGCAAAAAC TTTATTTGTC   
  
  
+ TTTTAAGGAA AATTTTGGAA TTTGAGAGTG AAAATTTTGG TTGTGAATCT TGTCTTACTA TGTGGTTCCT   
  
  
+ TCTGACAAAA GTTTGAATCT GAGTATGAGA GTTCATCAAT CTCTTTTACT TTCCTACTTT CTTTATTGCT   
  
  
+ TGCAAATTGT TGTGAACTTA CTTGGATTTT CTGGTAGAGG AGAGTTCTGT CCTGGAATTT GGTGGAAAGT   
  
  
+ GAGACATACT TTGGGATTTA ATTAGCCATC AATTTGGTTT CACACCTTAT CAGTTTGGAC TTCTGGTGTG   
  
  
+ TTATTGTATG TGTTCTTAGC TTGGGATTAT TCTTGTATCG GAAGAGAGGG GGGGGGGGGT GTTTCTTTTA   
  
  
+ GCTGAATGTT AGAACATTTT AAGGGATCTA GATATCTAGA GAAGAGCCTA GGAAATGAAG CATTTTTCCC   
  
  
+ TTATAATCTG ACACAACTTT ATGAATTGAT TAGGAACAAA GAACGATTAG TTCAGAAAGG TTACAACTCC   
  
  
+ TCTAGGTGTT ATACTAAAGA GATGTGAGTT AATAAGCAAT TGCTTACTAA CTGTTTAGGC TGTTTCATTG   
  
  
+ CTTGTAGAGG CTTTTTTTTT GGGGGGGGGG GGGGGTTTGG GGTTCATAAA TGAGAAGGTT GATTTGTTGT   
  
  
+ CTGCTCTGCT CTTGATTCGA TAGTAATTCT CTCAGAAAAG GGTAAAATGA CATGGCCTTC CATCGCCTCC   
  
  
+ CATGAGGCTT TTTGCCTCTG CTTGTGTATC TTTCATCTCT TGGTTTGGTG GGTTGGGGGA GGGGAGGGGG   
  
  
+ GGAAGGGGGT TTTGATTCGA TAGATCATCA CTTTCAGAAA TGGCCTCTCT GGCCAATGGT AAGGGGTAAG   
  
  
+ GTTAGTACAT GTGGACTTTC CCATGTGGAT TTTTCCAGAG TCCGTTGTGT GATTGTTATT GCTGTTGTTG   
  
  
+ TAACTTTCTG TCACTCGGAA AACATGAATC CATATGCAAC AAGAACATAA TAATTGTGAA TGCGACTTTA   
  
  
+ TTTAGGAATG TCTTACATAG TACGTGCACA TTCTTTAATT ATCTGTTTAT CTGTTGGTTT GTTATTTTCA   
  
  
+ AGGGGATGGG GAGTGGGTGG ATGCTTAGTA TGTAATCTTT TTATATAGGG AAAAATCATA TAGCTTGAAA   
  
  
+ GTAAAACTTT CATTGCCAGG ATTCCAAGGT AGGGGTAGGG TTGTCTACAC CTTGATCTCC CTAGAAGCAT   
  
  
+ GGCGATCCTT CCTCGGATTG TGTTATAGAA TAAAGAATGT GGTTCTGCTG TAATGAGACT TATGTTCTAT   
  
  
+ CAAATGATGA ATCTGTTTAG AACTTCTTGC TTATTTTTTC TGCAAAATTT TAGGAGACTT GATAAACTAT   
  
  
+ AGTTCTCTTG AATTTACAGG TGGATATTGG AGTTCACAGA ATAGTATTCA GATCAAAGTG CTTGTGCTTA   
  
  
+ ATTGGAAGGA CAACTTGCTT GAGCTGTTTA TAGAGCTCTA ACTGATGCTA TCTCATGGAC TCACATCAGT   
  
  
+ TTTTTGGATT TAATCTTACC AGTGTTGATC CGTCGTACAT TTCCGCTCAG TACAGTCCGC CGTCAGTGAC   
  
  
+ AAATAGGATG TTTGCATCAC TGAAGCTCGA CTCTAGAGGT TCTCCTGTGT CACCCTTCTC AACTCAGTTT   
  
  
+ GATTGTGATA CGGTTACTAC ATTGAGTGAT AGTCAAGAGC ATCACAGCTC GACGGGGAGT CTATCAACAA   
  
  
+ GAAGCCCTTC TTGTAATTCT CCCCTTGAAA CGAGCAGTTA TCATCATTTA TCGACGAATG GCCCGTCTTG   
  
  
+ TAATTCTGCC CCTGAAACTA GCAGTTATCG TCATCGGTTC AACGCGAGTC CCGTGGGATA TTCCCATCAA   
  
  
+ GGCACTGATT ATGGAGTGAA TATGAAGAGC GCTCTGCAGG AGCTAGAGAC TACTCTAATG GGTGCAGATG   
  
  
+ GTGAGGAAGT ATCTGCTGCT AATCAACCTA TGGGGGGAAG TCGTCAGTCC GGGATCCCAA GTCAGAGATC   
  
  
+ AAAATCATTG AGCGAAGATC CACAGGGTTC GCATCCTACT CAGCCTGATT CATCATCTCT TTCTAGGGCA   
  
  
+ AGAAGATCAG GAGATGAAAG CCAGAGAGAG AAACGGCACA AGGCAATGGA AGAACCAACG GAACTACCAA   
  
  
+ GTTTGCCACC TGGTGATTTG AAGCAGTTGC TAATTGAATG TGCGAGGGCT TTATCGGATA ACCGAATAGA   
  
  
+ TGACTTTGAG AGTTTGGTTA AACGGGCGAG GAAAGAGGTC TCTATCTCGG GTGAGCCTAT CCAACGTCTC   
  
  
+ GGTGCCTACA TGATCGAAGG GCTTGTGGCA AGGAAGCAGT CTTCGGGGAC TAGCATCTAT CGGGCTCTGA   
  
  
+ AGTGTAAAGA GCCTCTTGGA AAAGACTTGC TCTCCTACAT GCACATCCTT TATGAAATAT GCCCTTATCT   
  
  
+ CAAGTTTGGT TATATGGCTG CGAATGGAGC GATAGCTGAA GCTTGTAGAA ATGAGGATAA CATACATATT   
  
  
+ ATAGATTTCC AGATTGCACA GGGCACTCAG TGGGTTACTC TATTACAAGC CCTAGCAGCA AGACCTGGTG   
  
  
+ GGCCACCTAA GGTGCGAATT ACAGGCATTG ATGATCCTGT TTCTAAGTAT GCCCGTGGTG CTAGCTTGGA   
  
  
+ GGCTGTTGGG AAACGGTTAG CGTCTCTATC TGAAAAGTTC AAAATACCCG TCGAGTTCAA TGCGTTGCCC   
  
  
+ GTTTATGGAC CCGATGTCAG GCGGGAAATG CTGGATGTGA GGCCCGGGGA GGCTTTGGCC GTTAATTTTC   
  
  
+ CATTGCAGCT CCACCACACT CCTGACGAGA GTGTCGATGT GAACAACCCT AGGGATGGGC TTCTCAGAAT   
  
  
+ GGTGAAATCA CTTGGTCCTA AGGTAACCAC TTTGGTTGAG CAAGAATCAA ACACCAACAC TACCCCTTTC   
  
  
+ TTGACCCGGT TCATAGAGAC CCTTGACTAC TACTCAGCCA TGTTTGAGTC TATAGACGTG ACCATGCCAA   
  
  
+ GAGACCGGAA GGAGAGGATC AATGTTGAGC AGCATTGTTT GGCTAAGGAC ATTGTGAATA TCATAGCTTG   
  
  
+ CGAGGGCAAG GAGAGGGTGG AGCGTCATGA ACTTTTTGGG AAATGGAAGT CAAGGTTTAC CATGGCAGGG   
  
  
+ TTCAGGCAGT ACCCGTTGAG CTCATACGTA AACTCTGTGA TAAGAAGCCT ACTCCGGTGT TACTCCGAGC   
  
  
+ ATTATACTCT GATAGAGAAG GATGGTGCCA TGCTTCTGGG TTGGAAGGGC CGAATGCTGA TTTCAGCTTC   
  
  
+ GGCATGGCAT TG  

- +Up\_Stream \_Len000AGGAGA AAACCCGAAG AAAGAAAGAT AGGACAAAAA AAAACCCAGC TTTTCAACCT   
  
  
- CCAACTTAAC TTCGTAGGCT TAAGCTCGTC CTACAAAGAG ACGCATCACT AAGAAAGACC CAAACACTAA   
  
  
- AACTTAAGAC CCATTAGCCA ACAAAACGAT TAAAACTCCT GTCTCAAGGA AAAGACTTAA TTTTAAAGCA   
  
  
- AAAAAATAGC CCTTTTAGAA GTCATAAACT CTTTTTTCTT GCTTTAAACC TACTAAACGA CAGATCTAAA   
  
  
- ACGAAAGTAT AAGGACCCAC ACCTAACCAA ATAATTAACG TACACTCCTT CATGTTCGAA GACCAGTTAC   
  
  
- CAACCCAAAA AAAAAAAAAA AACACCCATA TTTCAACCTT TAACTAATGC TCAGTTTAAA GAGAAAGATC   
  
  
- TTCTCCCCCC CCCCCCCCCC AACCAAATTT TGAGGTATAA AAGAAACTTA CCTTTCGAAT TAAACGACTG   
  
  
- ATAGAACCAA TCATCGTATT CGAACTAGTC TTTTGTTTTG CGAGAGACGG GACGTTTTTG AAATAAACAG   
  
  
- AAAATTCCTT TTAAAACCTT AAACTCTCAC TTTTAAAACC AACACTTAGA ACAGAATGAT ACACCAAGGA   
  
  
- AGACTGTTTT CAAACTTAGA CTCATACTCT CAAGTAGTTA GAGAAAATGA AAGGATGAAA GAAATAACGA   
  
  
- ACGTTTAACA ACACTTGAAT GAACCTAAAA GACCATCTCC TCTCAAGACA GGACCTTAAA CCACCTTTCA   
  
  
- CTCTGTATGA AACCCTAAAT TAATCGGTAG TTAAACCAAA GTGTGGAATA GTCAAACCTG AAGACCACAC   
  
  
- AATAACATAC ACAAGAATCG AACCCTAATA AGAACATAGC CTTCTCTCCC CCCCCCCCCA CAAAGAAAAT   
  
  
- CGACTTACAA TCTTGTAAAA TTCCCTAGAT CTATAGATCT CTTCTCGGAT CCTTTACTTC GTAAAAAGGG   
  
  
- AATATTAGAC TGTGTTGAAA TACTTAACTA ATCCTTGTTT CTTGCTAATC AAGTCTTTCC AATGTTGAGG   
  
  
- AGATCCACAA TATGATTTCT CTACACTCAA TTATTCGTTA ACGAATGATT GACAAATCCG ACAAAGTAAC   
  
  
- GAACATCTCC GAAAAAAAAA CCCCCCCCCC CCCCCAAACC CCAAGTATTT ACTCTTCCAA CTAAACAACA   
  
  
- GACGAGACGA GAACTAAGCT ATCATTAAGA GAGTCTTTTC CCATTTTACT GTACCGGAAG GTAGCGGAGG   
  
  
- GTACTCCGAA AAACGGAGAC GAACACATAG AAAGTAGAGA ACCAAACCAC CCAACCCCCT CCCCTCCCCC   
  
  
- CCTTCCCCCA AAACTAAGCT ATCTAGTAGT GAAAGTCTTT ACCGGAGAGA CCGGTTACCA TTCCCCATTC   
  
  
- CAATCATGTA CACCTGAAAG GGTACACCTA AAAAGGTCTC AGGCAACACA CTAACAATAA CGACAACAAC   
  
  
- ATTGAAAGAC AGTGAGCCTT TTGTACTTAG GTATACGTTG TTCTTGTATT ATTAACACTT ACGCTGAAAT   
  
  
- AAATCCTTAC AGAATGTATC ATGCACGTGT AAGAAATTAA TAGACAAATA GACAACCAAA CAATAAAAGT   
  
  
- TCCCCTACCC CTCACCCACC TACGAATCAT ACATTAGAAA AATATATCCC TTTTTAGTAT ATCGAACTTT   
  
  
- CATTTTGAAA GTAACGGTCC TAAGGTTCCA TCCCCATCCC AACAGATGTG GAACTAGAGG GATCTTCGTA   
  
  
- CCGCTAGGAA GGAGCCTAAC ACAATATCTT ATTTCTTACA CCAAGACGAC ATTACTCTGA ATACAAGATA   
  
  
- GTTTACTACT TAGACAAATC TTGAAGAACG AATAAAAAAG ACGTTTTAAA ATCCTCTGAA CTATTTGATA   
  
  
- TCAAGAGAAC TTAAATGTCC ACCTATAACC TCAAGTGTCT TATCATAAGT CTAGTTTCAC GAACACGAAT   
  
  
- TAACCTTCCT GTTGAACGAA CTCGACAAAT ATCTCGAGAT TGACTACGAT AGAGTACCTG AGTGTAGTCA   
  
  
- AAAAACCTAA ATTAGAATGG TCACAACTAG GCAGCATGTA AAGGCGAGTC ATGTCAGGCG GCAGTCACTG   
  
  
- TTTATCCTAC AAACGTAGTG ACTTCGAGCT GAGATCTCCA AGAGGACACA GTGGGAAGAG TTGAGTCAAA   
  
  
- CTAACACTAT GCCAATGATG TAACTCACTA TCAGTTCTCG TAGTGTCGAG CTGCCCCTCA GATAGTTGTT   
  
  
- CTTCGGGAAG AACATTAAGA GGGGAACTTT GCTCGTCAAT AGTAGTAAAT AGCTGCTTAC CGGGCAGAAC   
  
  
- ATTAAGACGG GGACTTTGAT CGTCAATAGC AGTAGCCAAG TTGCGCTCAG GGCACCCTAT AAGGGTAGTT   
  
  
- CCGTGACTAA TACCTCACTT ATACTTCTCG CGAGACGTCC TCGATCTCTG ATGAGATTAC CCACGTCTAC   
  
  
- CACTCCTTCA TAGACGACGA TTAGTTGGAT ACCCCCCTTC AGCAGTCAGG CCCTAGGGTT CAGTCTCTAG   
  
  
- TTTTAGTAAC TCGCTTCTAG GTGTCCCAAG CGTAGGATGA GTCGGACTAA GTAGTAGAGA AAGATCCCGT   
  
  
- TCTTCTAGTC CTCTACTTTC GGTCTCTCTC TTTGCCGTGT TCCGTTACCT TCTTGGTTGC CTTGATGGTT   
  
  
- CAAACGGTGG ACCACTAAAC TTCGTCAACG ATTAACTTAC ACGCTCCCGA AATAGCCTAT TGGCTTATCT   
  
  
- ACTGAAACTC TCAAACCAAT TTGCCCGCTC CTTTCTCCAG AGATAGAGCC CACTCGGATA GGTTGCAGAG   
  
  
- CCACGGATGT ACTAGCTTCC CGAACACCGT TCCTTCGTCA GAAGCCCCTG ATCGTAGATA GCCCGAGACT   
  
  
- TCACATTTCT CGGAGAACCT TTTCTGAACG AGAGGATGTA CGTGTAGGAA ATACTTTATA CGGGAATAGA   
  
  
- GTTCAAACCA ATATACCGAC GCTTACCTCG CTATCGACTT CGAACATCTT TACTCCTATT GTATGTATAA   
  
  
- TATCTAAAGG TCTAACGTGT CCCGTGAGTC ACCCAATGAG ATAATGTTCG GGATCGTCGT TCTGGACCAC   
  
  
- CCGGTGGATT CCACGCTTAA TGTCCGTAAC TACTAGGACA AAGATTCATA CGGGCACCAC GATCGAACCT   
  
  
- CCGACAACCC TTTGCCAATC GCAGAGATAG ACTTTTCAAG TTTTATGGGC AGCTCAAGTT ACGCAACGGG   
  
  
- CAAATACCTG GGCTACAGTC CGCCCTTTAC GACCTACACT CCGGGCCCCT CCGAAACCGG CAATTAAAAG   
  
  
- GTAACGTCGA GGTGGTGTGA GGACTGCTCT CACAGCTACA CTTGTTGGGA TCCCTACCCG AAGAGTCTTA   
  
  
- CCACTTTAGT GAACCAGGAT TCCATTGGTG AAACCAACTC GTTCTTAGTT TGTGGTTGTG ATGGGGAAAG   
  
  
- AACTGGGCCA AGTATCTCTG GGAACTGATG ATGAGTCGGT ACAAACTCAG ATATCTGCAC TGGTACGGTT   
  
  
- CTCTGGCCTT CCTCTCCTAG TTACAACTCG TCGTAACAAA CCGATTCCTG TAACACTTAT AGTATCGAAC   
  
  
- GCTCCCGTTC CTCTCCCACC TCGCAGTACT TGAAAAACCC TTTACCTTCA GTTCCAAATG GTACCGTCCC   
  
  
- AAGTCCGTCA TGGGCAACTC GAGTATGCAT TTGAGACACT ATTCTTCGGA TGAGGCCACA ATGAGGCTCG   
  
  
- TAATATGAGA CTATCTCTTC CTACCACGGT ACGAAGACCC AACCTTCCCG GCTTACGACT AAAGTCGAAG   
  
  
- CCGTACCGTA AC

+     Myb

| Site Name | Organism | Position | Strand | Matrix score. | sequence | function |
| --- | --- | --- | --- | --- | --- | --- |
| Myb | Arabidopsis thaliana | 2688 | - | 6 | CAACTG |  |
| Myb | Arabidopsis thaliana | 2279 | - | 6 | TAACTG |  |
| Myb | Arabidopsis thaliana | 2003 | + | 6 | TAACTG |  |
| Myb | Arabidopsis thaliana | 2336 | - | 6 | TAACTG |  |
| Myb | Arabidopsis thaliana | 1102 | + | 6 | TAACTG |  |

>HU08G00014.1   
+ +Up\_Stream \_Len000TCCTCT TTTGGGCTTC TTTCTTTCTA TCCTGTTTTT TTTTGGGTCG AAAAGTTGGA   
  
  
+ GGTTGAATTG AAGCATCCGA ATTCGAGCAG GATGTTTCTC TGCGTAGTGA TTCTTTCTGG GTTTGTGATT   
  
  
+ TTGAATTCTG GGTAATCGGT TGTTTTGCTA ATTTTGAGGA CAGAGTTCCT TTTCTGAATT AAAATTTCGT   
  
  
+ TTTTTTATCG GGAAAATCTT CAGTATTTGA GAAAAAAGAA CGAAATTTGG ATGATTTGCT GTCTAGATTT   
  
  
+ TGCTTTCATA TTCCTGGGTG TGGATTGGTT TATTAATTGC ATGTGAGGAA GTACAAGCTT CTGGTCAATG   
  
  
+ GTTGGGTTTT TTTTTTTTTT TTGTGGGTAT AAAGTTGGAA ATTGATTACG AGTCAAATTT CTCTTTCTAG   
  
  
+ AAGAGGGGGG GGGGGGGGGG TTGGTTTAAA ACTCCATATT TTCTTTGAAT GGAAAGCTTA ATTTGCTGAC   
  
  
+ TATCTTGGTT AGTAGCATAA GCTTGATCAG AAAACAAAAC GCTCTCTGCC CTGCAAAAAC TTTATTTGTC   
  
  
+ TTTTAAGGAA AATTTTGGAA TTTGAGAGTG AAAATTTTGG TTGTGAATCT TGTCTTACTA TGTGGTTCCT   
  
  
+ TCTGACAAAA GTTTGAATCT GAGTATGAGA GTTCATCAAT CTCTTTTACT TTCCTACTTT CTTTATTGCT   
  
  
+ TGCAAATTGT TGTGAACTTA CTTGGATTTT CTGGTAGAGG AGAGTTCTGT CCTGGAATTT GGTGGAAAGT   
  
  
+ GAGACATACT TTGGGATTTA ATTAGCCATC AATTTGGTTT CACACCTTAT CAGTTTGGAC TTCTGGTGTG   
  
  
+ TTATTGTATG TGTTCTTAGC TTGGGATTAT TCTTGTATCG GAAGAGAGGG GGGGGGGGGT GTTTCTTTTA   
  
  
+ GCTGAATGTT AGAACATTTT AAGGGATCTA GATATCTAGA GAAGAGCCTA GGAAATGAAG CATTTTTCCC   
  
  
+ TTATAATCTG ACACAACTTT ATGAATTGAT TAGGAACAAA GAACGATTAG TTCAGAAAGG TTACAACTCC   
  
  
+ TCTAGGTGTT ATACTAAAGA GATGTGAGTT AATAAGCAAT TGCTTACTAA CTGTTTAGGC TGTTTCATTG   
  
  
+ CTTGTAGAGG CTTTTTTTTT GGGGGGGGGG GGGGGTTTGG GGTTCATAAA TGAGAAGGTT GATTTGTTGT   
  
  
+ CTGCTCTGCT CTTGATTCGA TAGTAATTCT CTCAGAAAAG GGTAAAATGA CATGGCCTTC CATCGCCTCC   
  
  
+ CATGAGGCTT TTTGCCTCTG CTTGTGTATC TTTCATCTCT TGGTTTGGTG GGTTGGGGGA GGGGAGGGGG   
  
  
+ GGAAGGGGGT TTTGATTCGA TAGATCATCA CTTTCAGAAA TGGCCTCTCT GGCCAATGGT AAGGGGTAAG   
  
  
+ GTTAGTACAT GTGGACTTTC CCATGTGGAT TTTTCCAGAG TCCGTTGTGT GATTGTTATT GCTGTTGTTG   
  
  
+ TAACTTTCTG TCACTCGGAA AACATGAATC CATATGCAAC AAGAACATAA TAATTGTGAA TGCGACTTTA   
  
  
+ TTTAGGAATG TCTTACATAG TACGTGCACA TTCTTTAATT ATCTGTTTAT CTGTTGGTTT GTTATTTTCA   
  
  
+ AGGGGATGGG GAGTGGGTGG ATGCTTAGTA TGTAATCTTT TTATATAGGG AAAAATCATA TAGCTTGAAA   
  
  
+ GTAAAACTTT CATTGCCAGG ATTCCAAGGT AGGGGTAGGG TTGTCTACAC CTTGATCTCC CTAGAAGCAT   
  
  
+ GGCGATCCTT CCTCGGATTG TGTTATAGAA TAAAGAATGT GGTTCTGCTG TAATGAGACT TATGTTCTAT   
  
  
+ CAAATGATGA ATCTGTTTAG AACTTCTTGC TTATTTTTTC TGCAAAATTT TAGGAGACTT GATAAACTAT   
  
  
+ AGTTCTCTTG AATTTACAGG TGGATATTGG AGTTCACAGA ATAGTATTCA GATCAAAGTG CTTGTGCTTA   
  
  
+ ATTGGAAGGA CAACTTGCTT GAGCTGTTTA TAGAGCTCTA ACTGATGCTA TCTCATGGAC TCACATCAGT   
  
  
+ TTTTTGGATT TAATCTTACC AGTGTTGATC CGTCGTACAT TTCCGCTCAG TACAGTCCGC CGTCAGTGAC   
  
  
+ AAATAGGATG TTTGCATCAC TGAAGCTCGA CTCTAGAGGT TCTCCTGTGT CACCCTTCTC AACTCAGTTT   
  
  
+ GATTGTGATA CGGTTACTAC ATTGAGTGAT AGTCAAGAGC ATCACAGCTC GACGGGGAGT CTATCAACAA   
  
  
+ GAAGCCCTTC TTGTAATTCT CCCCTTGAAA CGAGCAGTTA TCATCATTTA TCGACGAATG GCCCGTCTTG   
  
  
+ TAATTCTGCC CCTGAAACTA GCAGTTATCG TCATCGGTTC AACGCGAGTC CCGTGGGATA TTCCCATCAA   
  
  
+ GGCACTGATT ATGGAGTGAA TATGAAGAGC GCTCTGCAGG AGCTAGAGAC TACTCTAATG GGTGCAGATG   
  
  
+ GTGAGGAAGT ATCTGCTGCT AATCAACCTA TGGGGGGAAG TCGTCAGTCC GGGATCCCAA GTCAGAGATC   
  
  
+ AAAATCATTG AGCGAAGATC CACAGGGTTC GCATCCTACT CAGCCTGATT CATCATCTCT TTCTAGGGCA   
  
  
+ AGAAGATCAG GAGATGAAAG CCAGAGAGAG AAACGGCACA AGGCAATGGA AGAACCAACG GAACTACCAA   
  
  
+ GTTTGCCACC TGGTGATTTG AAGCAGTTGC TAATTGAATG TGCGAGGGCT TTATCGGATA ACCGAATAGA   
  
  
+ TGACTTTGAG AGTTTGGTTA AACGGGCGAG GAAAGAGGTC TCTATCTCGG GTGAGCCTAT CCAACGTCTC   
  
  
+ GGTGCCTACA TGATCGAAGG GCTTGTGGCA AGGAAGCAGT CTTCGGGGAC TAGCATCTAT CGGGCTCTGA   
  
  
+ AGTGTAAAGA GCCTCTTGGA AAAGACTTGC TCTCCTACAT GCACATCCTT TATGAAATAT GCCCTTATCT   
  
  
+ CAAGTTTGGT TATATGGCTG CGAATGGAGC GATAGCTGAA GCTTGTAGAA ATGAGGATAA CATACATATT   
  
  
+ ATAGATTTCC AGATTGCACA GGGCACTCAG TGGGTTACTC TATTACAAGC CCTAGCAGCA AGACCTGGTG   
  
  
+ GGCCACCTAA GGTGCGAATT ACAGGCATTG ATGATCCTGT TTCTAAGTAT GCCCGTGGTG CTAGCTTGGA   
  
  
+ GGCTGTTGGG AAACGGTTAG CGTCTCTATC TGAAAAGTTC AAAATACCCG TCGAGTTCAA TGCGTTGCCC   
  
  
+ GTTTATGGAC CCGATGTCAG GCGGGAAATG CTGGATGTGA GGCCCGGGGA GGCTTTGGCC GTTAATTTTC   
  
  
+ CATTGCAGCT CCACCACACT CCTGACGAGA GTGTCGATGT GAACAACCCT AGGGATGGGC TTCTCAGAAT   
  
  
+ GGTGAAATCA CTTGGTCCTA AGGTAACCAC TTTGGTTGAG CAAGAATCAA ACACCAACAC TACCCCTTTC   
  
  
+ TTGACCCGGT TCATAGAGAC CCTTGACTAC TACTCAGCCA TGTTTGAGTC TATAGACGTG ACCATGCCAA   
  
  
+ GAGACCGGAA GGAGAGGATC AATGTTGAGC AGCATTGTTT GGCTAAGGAC ATTGTGAATA TCATAGCTTG   
  
  
+ CGAGGGCAAG GAGAGGGTGG AGCGTCATGA ACTTTTTGGG AAATGGAAGT CAAGGTTTAC CATGGCAGGG   
  
  
+ TTCAGGCAGT ACCCGTTGAG CTCATACGTA AACTCTGTGA TAAGAAGCCT ACTCCGGTGT TACTCCGAGC   
  
  
+ ATTATACTCT GATAGAGAAG GATGGTGCCA TGCTTCTGGG TTGGAAGGGC CGAATGCTGA TTTCAGCTTC   
  
  
+ GGCATGGCAT TG  

- +Up\_Stream \_Len000AGGAGA AAACCCGAAG AAAGAAAGAT AGGACAAAAA AAAACCCAGC TTTTCAACCT   
  
  
- CCAACTTAAC TTCGTAGGCT TAAGCTCGTC CTACAAAGAG ACGCATCACT AAGAAAGACC CAAACACTAA   
  
  
- AACTTAAGAC CCATTAGCCA ACAAAACGAT TAAAACTCCT GTCTCAAGGA AAAGACTTAA TTTTAAAGCA   
  
  
- AAAAAATAGC CCTTTTAGAA GTCATAAACT CTTTTTTCTT GCTTTAAACC TACTAAACGA CAGATCTAAA   
  
  
- ACGAAAGTAT AAGGACCCAC ACCTAACCAA ATAATTAACG TACACTCCTT CATGTTCGAA GACCAGTTAC   
  
  
- CAACCCAAAA AAAAAAAAAA AACACCCATA TTTCAACCTT TAACTAATGC TCAGTTTAAA GAGAAAGATC   
  
  
- TTCTCCCCCC CCCCCCCCCC AACCAAATTT TGAGGTATAA AAGAAACTTA CCTTTCGAAT TAAACGACTG   
  
  
- ATAGAACCAA TCATCGTATT CGAACTAGTC TTTTGTTTTG CGAGAGACGG GACGTTTTTG AAATAAACAG   
  
  
- AAAATTCCTT TTAAAACCTT AAACTCTCAC TTTTAAAACC AACACTTAGA ACAGAATGAT ACACCAAGGA   
  
  
- AGACTGTTTT CAAACTTAGA CTCATACTCT CAAGTAGTTA GAGAAAATGA AAGGATGAAA GAAATAACGA   
  
  
- ACGTTTAACA ACACTTGAAT GAACCTAAAA GACCATCTCC TCTCAAGACA GGACCTTAAA CCACCTTTCA   
  
  
- CTCTGTATGA AACCCTAAAT TAATCGGTAG TTAAACCAAA GTGTGGAATA GTCAAACCTG AAGACCACAC   
  
  
- AATAACATAC ACAAGAATCG AACCCTAATA AGAACATAGC CTTCTCTCCC CCCCCCCCCA CAAAGAAAAT   
  
  
- CGACTTACAA TCTTGTAAAA TTCCCTAGAT CTATAGATCT CTTCTCGGAT CCTTTACTTC GTAAAAAGGG   
  
  
- AATATTAGAC TGTGTTGAAA TACTTAACTA ATCCTTGTTT CTTGCTAATC AAGTCTTTCC AATGTTGAGG   
  
  
- AGATCCACAA TATGATTTCT CTACACTCAA TTATTCGTTA ACGAATGATT GACAAATCCG ACAAAGTAAC   
  
  
- GAACATCTCC GAAAAAAAAA CCCCCCCCCC CCCCCAAACC CCAAGTATTT ACTCTTCCAA CTAAACAACA   
  
  
- GACGAGACGA GAACTAAGCT ATCATTAAGA GAGTCTTTTC CCATTTTACT GTACCGGAAG GTAGCGGAGG   
  
  
- GTACTCCGAA AAACGGAGAC GAACACATAG AAAGTAGAGA ACCAAACCAC CCAACCCCCT CCCCTCCCCC   
  
  
- CCTTCCCCCA AAACTAAGCT ATCTAGTAGT GAAAGTCTTT ACCGGAGAGA CCGGTTACCA TTCCCCATTC   
  
  
- CAATCATGTA CACCTGAAAG GGTACACCTA AAAAGGTCTC AGGCAACACA CTAACAATAA CGACAACAAC   
  
  
- ATTGAAAGAC AGTGAGCCTT TTGTACTTAG GTATACGTTG TTCTTGTATT ATTAACACTT ACGCTGAAAT   
  
  
- AAATCCTTAC AGAATGTATC ATGCACGTGT AAGAAATTAA TAGACAAATA GACAACCAAA CAATAAAAGT   
  
  
- TCCCCTACCC CTCACCCACC TACGAATCAT ACATTAGAAA AATATATCCC TTTTTAGTAT ATCGAACTTT   
  
  
- CATTTTGAAA GTAACGGTCC TAAGGTTCCA TCCCCATCCC AACAGATGTG GAACTAGAGG GATCTTCGTA   
  
  
- CCGCTAGGAA GGAGCCTAAC ACAATATCTT ATTTCTTACA CCAAGACGAC ATTACTCTGA ATACAAGATA   
  
  
- GTTTACTACT TAGACAAATC TTGAAGAACG AATAAAAAAG ACGTTTTAAA ATCCTCTGAA CTATTTGATA   
  
  
- TCAAGAGAAC TTAAATGTCC ACCTATAACC TCAAGTGTCT TATCATAAGT CTAGTTTCAC GAACACGAAT   
  
  
- TAACCTTCCT GTTGAACGAA CTCGACAAAT ATCTCGAGAT TGACTACGAT AGAGTACCTG AGTGTAGTCA   
  
  
- AAAAACCTAA ATTAGAATGG TCACAACTAG GCAGCATGTA AAGGCGAGTC ATGTCAGGCG GCAGTCACTG   
  
  
- TTTATCCTAC AAACGTAGTG ACTTCGAGCT GAGATCTCCA AGAGGACACA GTGGGAAGAG TTGAGTCAAA   
  
  
- CTAACACTAT GCCAATGATG TAACTCACTA TCAGTTCTCG TAGTGTCGAG CTGCCCCTCA GATAGTTGTT   
  
  
- CTTCGGGAAG AACATTAAGA GGGGAACTTT GCTCGTCAAT AGTAGTAAAT AGCTGCTTAC CGGGCAGAAC   
  
  
- ATTAAGACGG GGACTTTGAT CGTCAATAGC AGTAGCCAAG TTGCGCTCAG GGCACCCTAT AAGGGTAGTT   
  
  
- CCGTGACTAA TACCTCACTT ATACTTCTCG CGAGACGTCC TCGATCTCTG ATGAGATTAC CCACGTCTAC   
  
  
- CACTCCTTCA TAGACGACGA TTAGTTGGAT ACCCCCCTTC AGCAGTCAGG CCCTAGGGTT CAGTCTCTAG   
  
  
- TTTTAGTAAC TCGCTTCTAG GTGTCCCAAG CGTAGGATGA GTCGGACTAA GTAGTAGAGA AAGATCCCGT   
  
  
- TCTTCTAGTC CTCTACTTTC GGTCTCTCTC TTTGCCGTGT TCCGTTACCT TCTTGGTTGC CTTGATGGTT   
  
  
- CAAACGGTGG ACCACTAAAC TTCGTCAACG ATTAACTTAC ACGCTCCCGA AATAGCCTAT TGGCTTATCT   
  
  
- ACTGAAACTC TCAAACCAAT TTGCCCGCTC CTTTCTCCAG AGATAGAGCC CACTCGGATA GGTTGCAGAG   
  
  
- CCACGGATGT ACTAGCTTCC CGAACACCGT TCCTTCGTCA GAAGCCCCTG ATCGTAGATA GCCCGAGACT   
  
  
- TCACATTTCT CGGAGAACCT TTTCTGAACG AGAGGATGTA CGTGTAGGAA ATACTTTATA CGGGAATAGA   
  
  
- GTTCAAACCA ATATACCGAC GCTTACCTCG CTATCGACTT CGAACATCTT TACTCCTATT GTATGTATAA   
  
  
- TATCTAAAGG TCTAACGTGT CCCGTGAGTC ACCCAATGAG ATAATGTTCG GGATCGTCGT TCTGGACCAC   
  
  
- CCGGTGGATT CCACGCTTAA TGTCCGTAAC TACTAGGACA AAGATTCATA CGGGCACCAC GATCGAACCT   
  
  
- CCGACAACCC TTTGCCAATC GCAGAGATAG ACTTTTCAAG TTTTATGGGC AGCTCAAGTT ACGCAACGGG   
  
  
- CAAATACCTG GGCTACAGTC CGCCCTTTAC GACCTACACT CCGGGCCCCT CCGAAACCGG CAATTAAAAG   
  
  
- GTAACGTCGA GGTGGTGTGA GGACTGCTCT CACAGCTACA CTTGTTGGGA TCCCTACCCG AAGAGTCTTA   
  
  
- CCACTTTAGT GAACCAGGAT TCCATTGGTG AAACCAACTC GTTCTTAGTT TGTGGTTGTG ATGGGGAAAG   
  
  
- AACTGGGCCA AGTATCTCTG GGAACTGATG ATGAGTCGGT ACAAACTCAG ATATCTGCAC TGGTACGGTT   
  
  
- CTCTGGCCTT CCTCTCCTAG TTACAACTCG TCGTAACAAA CCGATTCCTG TAACACTTAT AGTATCGAAC   
  
  
- GCTCCCGTTC CTCTCCCACC TCGCAGTACT TGAAAAACCC TTTACCTTCA GTTCCAAATG GTACCGTCCC   
  
  
- AAGTCCGTCA TGGGCAACTC GAGTATGCAT TTGAGACACT ATTCTTCGGA TGAGGCCACA ATGAGGCTCG   
  
  
- TAATATGAGA CTATCTCTTC CTACCACGGT ACGAAGACCC AACCTTCCCG GCTTACGACT AAAGTCGAAG   
  
  
- CCGTACCGTA AC

+     Myb-binding site

| Site Name | Organism | Position | Strand | Matrix score. | sequence | function |
| --- | --- | --- | --- | --- | --- | --- |
| Myb-binding site | Nicotiana tabacum | 1466 | - | 6 | CAACAG |  |
| Myb-binding site | Nicotiana tabacum | 1595 | - | 6 | CAACAG |  |
| Myb-binding site | Nicotiana tabacum | 3157 | - | 6 | CAACAG |  |

>HU08G00014.1   
+ +Up\_Stream \_Len000TCCTCT TTTGGGCTTC TTTCTTTCTA TCCTGTTTTT TTTTGGGTCG AAAAGTTGGA   
  
  
+ GGTTGAATTG AAGCATCCGA ATTCGAGCAG GATGTTTCTC TGCGTAGTGA TTCTTTCTGG GTTTGTGATT   
  
  
+ TTGAATTCTG GGTAATCGGT TGTTTTGCTA ATTTTGAGGA CAGAGTTCCT TTTCTGAATT AAAATTTCGT   
  
  
+ TTTTTTATCG GGAAAATCTT CAGTATTTGA GAAAAAAGAA CGAAATTTGG ATGATTTGCT GTCTAGATTT   
  
  
+ TGCTTTCATA TTCCTGGGTG TGGATTGGTT TATTAATTGC ATGTGAGGAA GTACAAGCTT CTGGTCAATG   
  
  
+ GTTGGGTTTT TTTTTTTTTT TTGTGGGTAT AAAGTTGGAA ATTGATTACG AGTCAAATTT CTCTTTCTAG   
  
  
+ AAGAGGGGGG GGGGGGGGGG TTGGTTTAAA ACTCCATATT TTCTTTGAAT GGAAAGCTTA ATTTGCTGAC   
  
  
+ TATCTTGGTT AGTAGCATAA GCTTGATCAG AAAACAAAAC GCTCTCTGCC CTGCAAAAAC TTTATTTGTC   
  
  
+ TTTTAAGGAA AATTTTGGAA TTTGAGAGTG AAAATTTTGG TTGTGAATCT TGTCTTACTA TGTGGTTCCT   
  
  
+ TCTGACAAAA GTTTGAATCT GAGTATGAGA GTTCATCAAT CTCTTTTACT TTCCTACTTT CTTTATTGCT   
  
  
+ TGCAAATTGT TGTGAACTTA CTTGGATTTT CTGGTAGAGG AGAGTTCTGT CCTGGAATTT GGTGGAAAGT   
  
  
+ GAGACATACT TTGGGATTTA ATTAGCCATC AATTTGGTTT CACACCTTAT CAGTTTGGAC TTCTGGTGTG   
  
  
+ TTATTGTATG TGTTCTTAGC TTGGGATTAT TCTTGTATCG GAAGAGAGGG GGGGGGGGGT GTTTCTTTTA   
  
  
+ GCTGAATGTT AGAACATTTT AAGGGATCTA GATATCTAGA GAAGAGCCTA GGAAATGAAG CATTTTTCCC   
  
  
+ TTATAATCTG ACACAACTTT ATGAATTGAT TAGGAACAAA GAACGATTAG TTCAGAAAGG TTACAACTCC   
  
  
+ TCTAGGTGTT ATACTAAAGA GATGTGAGTT AATAAGCAAT TGCTTACTAA CTGTTTAGGC TGTTTCATTG   
  
  
+ CTTGTAGAGG CTTTTTTTTT GGGGGGGGGG GGGGGTTTGG GGTTCATAAA TGAGAAGGTT GATTTGTTGT   
  
  
+ CTGCTCTGCT CTTGATTCGA TAGTAATTCT CTCAGAAAAG GGTAAAATGA CATGGCCTTC CATCGCCTCC   
  
  
+ CATGAGGCTT TTTGCCTCTG CTTGTGTATC TTTCATCTCT TGGTTTGGTG GGTTGGGGGA GGGGAGGGGG   
  
  
+ GGAAGGGGGT TTTGATTCGA TAGATCATCA CTTTCAGAAA TGGCCTCTCT GGCCAATGGT AAGGGGTAAG   
  
  
+ GTTAGTACAT GTGGACTTTC CCATGTGGAT TTTTCCAGAG TCCGTTGTGT GATTGTTATT GCTGTTGTTG   
  
  
+ TAACTTTCTG TCACTCGGAA AACATGAATC CATATGCAAC AAGAACATAA TAATTGTGAA TGCGACTTTA   
  
  
+ TTTAGGAATG TCTTACATAG TACGTGCACA TTCTTTAATT ATCTGTTTAT CTGTTGGTTT GTTATTTTCA   
  
  
+ AGGGGATGGG GAGTGGGTGG ATGCTTAGTA TGTAATCTTT TTATATAGGG AAAAATCATA TAGCTTGAAA   
  
  
+ GTAAAACTTT CATTGCCAGG ATTCCAAGGT AGGGGTAGGG TTGTCTACAC CTTGATCTCC CTAGAAGCAT   
  
  
+ GGCGATCCTT CCTCGGATTG TGTTATAGAA TAAAGAATGT GGTTCTGCTG TAATGAGACT TATGTTCTAT   
  
  
+ CAAATGATGA ATCTGTTTAG AACTTCTTGC TTATTTTTTC TGCAAAATTT TAGGAGACTT GATAAACTAT   
  
  
+ AGTTCTCTTG AATTTACAGG TGGATATTGG AGTTCACAGA ATAGTATTCA GATCAAAGTG CTTGTGCTTA   
  
  
+ ATTGGAAGGA CAACTTGCTT GAGCTGTTTA TAGAGCTCTA ACTGATGCTA TCTCATGGAC TCACATCAGT   
  
  
+ TTTTTGGATT TAATCTTACC AGTGTTGATC CGTCGTACAT TTCCGCTCAG TACAGTCCGC CGTCAGTGAC   
  
  
+ AAATAGGATG TTTGCATCAC TGAAGCTCGA CTCTAGAGGT TCTCCTGTGT CACCCTTCTC AACTCAGTTT   
  
  
+ GATTGTGATA CGGTTACTAC ATTGAGTGAT AGTCAAGAGC ATCACAGCTC GACGGGGAGT CTATCAACAA   
  
  
+ GAAGCCCTTC TTGTAATTCT CCCCTTGAAA CGAGCAGTTA TCATCATTTA TCGACGAATG GCCCGTCTTG   
  
  
+ TAATTCTGCC CCTGAAACTA GCAGTTATCG TCATCGGTTC AACGCGAGTC CCGTGGGATA TTCCCATCAA   
  
  
+ GGCACTGATT ATGGAGTGAA TATGAAGAGC GCTCTGCAGG AGCTAGAGAC TACTCTAATG GGTGCAGATG   
  
  
+ GTGAGGAAGT ATCTGCTGCT AATCAACCTA TGGGGGGAAG TCGTCAGTCC GGGATCCCAA GTCAGAGATC   
  
  
+ AAAATCATTG AGCGAAGATC CACAGGGTTC GCATCCTACT CAGCCTGATT CATCATCTCT TTCTAGGGCA   
  
  
+ AGAAGATCAG GAGATGAAAG CCAGAGAGAG AAACGGCACA AGGCAATGGA AGAACCAACG GAACTACCAA   
  
  
+ GTTTGCCACC TGGTGATTTG AAGCAGTTGC TAATTGAATG TGCGAGGGCT TTATCGGATA ACCGAATAGA   
  
  
+ TGACTTTGAG AGTTTGGTTA AACGGGCGAG GAAAGAGGTC TCTATCTCGG GTGAGCCTAT CCAACGTCTC   
  
  
+ GGTGCCTACA TGATCGAAGG GCTTGTGGCA AGGAAGCAGT CTTCGGGGAC TAGCATCTAT CGGGCTCTGA   
  
  
+ AGTGTAAAGA GCCTCTTGGA AAAGACTTGC TCTCCTACAT GCACATCCTT TATGAAATAT GCCCTTATCT   
  
  
+ CAAGTTTGGT TATATGGCTG CGAATGGAGC GATAGCTGAA GCTTGTAGAA ATGAGGATAA CATACATATT   
  
  
+ ATAGATTTCC AGATTGCACA GGGCACTCAG TGGGTTACTC TATTACAAGC CCTAGCAGCA AGACCTGGTG   
  
  
+ GGCCACCTAA GGTGCGAATT ACAGGCATTG ATGATCCTGT TTCTAAGTAT GCCCGTGGTG CTAGCTTGGA   
  
  
+ GGCTGTTGGG AAACGGTTAG CGTCTCTATC TGAAAAGTTC AAAATACCCG TCGAGTTCAA TGCGTTGCCC   
  
  
+ GTTTATGGAC CCGATGTCAG GCGGGAAATG CTGGATGTGA GGCCCGGGGA GGCTTTGGCC GTTAATTTTC   
  
  
+ CATTGCAGCT CCACCACACT CCTGACGAGA GTGTCGATGT GAACAACCCT AGGGATGGGC TTCTCAGAAT   
  
  
+ GGTGAAATCA CTTGGTCCTA AGGTAACCAC TTTGGTTGAG CAAGAATCAA ACACCAACAC TACCCCTTTC   
  
  
+ TTGACCCGGT TCATAGAGAC CCTTGACTAC TACTCAGCCA TGTTTGAGTC TATAGACGTG ACCATGCCAA   
  
  
+ GAGACCGGAA GGAGAGGATC AATGTTGAGC AGCATTGTTT GGCTAAGGAC ATTGTGAATA TCATAGCTTG   
  
  
+ CGAGGGCAAG GAGAGGGTGG AGCGTCATGA ACTTTTTGGG AAATGGAAGT CAAGGTTTAC CATGGCAGGG   
  
  
+ TTCAGGCAGT ACCCGTTGAG CTCATACGTA AACTCTGTGA TAAGAAGCCT ACTCCGGTGT TACTCCGAGC   
  
  
+ ATTATACTCT GATAGAGAAG GATGGTGCCA TGCTTCTGGG TTGGAAGGGC CGAATGCTGA TTTCAGCTTC   
  
  
+ GGCATGGCAT TG  

- +Up\_Stream \_Len000AGGAGA AAACCCGAAG AAAGAAAGAT AGGACAAAAA AAAACCCAGC TTTTCAACCT   
  
  
- CCAACTTAAC TTCGTAGGCT TAAGCTCGTC CTACAAAGAG ACGCATCACT AAGAAAGACC CAAACACTAA   
  
  
- AACTTAAGAC CCATTAGCCA ACAAAACGAT TAAAACTCCT GTCTCAAGGA AAAGACTTAA TTTTAAAGCA   
  
  
- AAAAAATAGC CCTTTTAGAA GTCATAAACT CTTTTTTCTT GCTTTAAACC TACTAAACGA CAGATCTAAA   
  
  
- ACGAAAGTAT AAGGACCCAC ACCTAACCAA ATAATTAACG TACACTCCTT CATGTTCGAA GACCAGTTAC   
  
  
- CAACCCAAAA AAAAAAAAAA AACACCCATA TTTCAACCTT TAACTAATGC TCAGTTTAAA GAGAAAGATC   
  
  
- TTCTCCCCCC CCCCCCCCCC AACCAAATTT TGAGGTATAA AAGAAACTTA CCTTTCGAAT TAAACGACTG   
  
  
- ATAGAACCAA TCATCGTATT CGAACTAGTC TTTTGTTTTG CGAGAGACGG GACGTTTTTG AAATAAACAG   
  
  
- AAAATTCCTT TTAAAACCTT AAACTCTCAC TTTTAAAACC AACACTTAGA ACAGAATGAT ACACCAAGGA   
  
  
- AGACTGTTTT CAAACTTAGA CTCATACTCT CAAGTAGTTA GAGAAAATGA AAGGATGAAA GAAATAACGA   
  
  
- ACGTTTAACA ACACTTGAAT GAACCTAAAA GACCATCTCC TCTCAAGACA GGACCTTAAA CCACCTTTCA   
  
  
- CTCTGTATGA AACCCTAAAT TAATCGGTAG TTAAACCAAA GTGTGGAATA GTCAAACCTG AAGACCACAC   
  
  
- AATAACATAC ACAAGAATCG AACCCTAATA AGAACATAGC CTTCTCTCCC CCCCCCCCCA CAAAGAAAAT   
  
  
- CGACTTACAA TCTTGTAAAA TTCCCTAGAT CTATAGATCT CTTCTCGGAT CCTTTACTTC GTAAAAAGGG   
  
  
- AATATTAGAC TGTGTTGAAA TACTTAACTA ATCCTTGTTT CTTGCTAATC AAGTCTTTCC AATGTTGAGG   
  
  
- AGATCCACAA TATGATTTCT CTACACTCAA TTATTCGTTA ACGAATGATT GACAAATCCG ACAAAGTAAC   
  
  
- GAACATCTCC GAAAAAAAAA CCCCCCCCCC CCCCCAAACC CCAAGTATTT ACTCTTCCAA CTAAACAACA   
  
  
- GACGAGACGA GAACTAAGCT ATCATTAAGA GAGTCTTTTC CCATTTTACT GTACCGGAAG GTAGCGGAGG   
  
  
- GTACTCCGAA AAACGGAGAC GAACACATAG AAAGTAGAGA ACCAAACCAC CCAACCCCCT CCCCTCCCCC   
  
  
- CCTTCCCCCA AAACTAAGCT ATCTAGTAGT GAAAGTCTTT ACCGGAGAGA CCGGTTACCA TTCCCCATTC   
  
  
- CAATCATGTA CACCTGAAAG GGTACACCTA AAAAGGTCTC AGGCAACACA CTAACAATAA CGACAACAAC   
  
  
- ATTGAAAGAC AGTGAGCCTT TTGTACTTAG GTATACGTTG TTCTTGTATT ATTAACACTT ACGCTGAAAT   
  
  
- AAATCCTTAC AGAATGTATC ATGCACGTGT AAGAAATTAA TAGACAAATA GACAACCAAA CAATAAAAGT   
  
  
- TCCCCTACCC CTCACCCACC TACGAATCAT ACATTAGAAA AATATATCCC TTTTTAGTAT ATCGAACTTT   
  
  
- CATTTTGAAA GTAACGGTCC TAAGGTTCCA TCCCCATCCC AACAGATGTG GAACTAGAGG GATCTTCGTA   
  
  
- CCGCTAGGAA GGAGCCTAAC ACAATATCTT ATTTCTTACA CCAAGACGAC ATTACTCTGA ATACAAGATA   
  
  
- GTTTACTACT TAGACAAATC TTGAAGAACG AATAAAAAAG ACGTTTTAAA ATCCTCTGAA CTATTTGATA   
  
  
- TCAAGAGAAC TTAAATGTCC ACCTATAACC TCAAGTGTCT TATCATAAGT CTAGTTTCAC GAACACGAAT   
  
  
- TAACCTTCCT GTTGAACGAA CTCGACAAAT ATCTCGAGAT TGACTACGAT AGAGTACCTG AGTGTAGTCA   
  
  
- AAAAACCTAA ATTAGAATGG TCACAACTAG GCAGCATGTA AAGGCGAGTC ATGTCAGGCG GCAGTCACTG   
  
  
- TTTATCCTAC AAACGTAGTG ACTTCGAGCT GAGATCTCCA AGAGGACACA GTGGGAAGAG TTGAGTCAAA   
  
  
- CTAACACTAT GCCAATGATG TAACTCACTA TCAGTTCTCG TAGTGTCGAG CTGCCCCTCA GATAGTTGTT   
  
  
- CTTCGGGAAG AACATTAAGA GGGGAACTTT GCTCGTCAAT AGTAGTAAAT AGCTGCTTAC CGGGCAGAAC   
  
  
- ATTAAGACGG GGACTTTGAT CGTCAATAGC AGTAGCCAAG TTGCGCTCAG GGCACCCTAT AAGGGTAGTT   
  
  
- CCGTGACTAA TACCTCACTT ATACTTCTCG CGAGACGTCC TCGATCTCTG ATGAGATTAC CCACGTCTAC   
  
  
- CACTCCTTCA TAGACGACGA TTAGTTGGAT ACCCCCCTTC AGCAGTCAGG CCCTAGGGTT CAGTCTCTAG   
  
  
- TTTTAGTAAC TCGCTTCTAG GTGTCCCAAG CGTAGGATGA GTCGGACTAA GTAGTAGAGA AAGATCCCGT   
  
  
- TCTTCTAGTC CTCTACTTTC GGTCTCTCTC TTTGCCGTGT TCCGTTACCT TCTTGGTTGC CTTGATGGTT   
  
  
- CAAACGGTGG ACCACTAAAC TTCGTCAACG ATTAACTTAC ACGCTCCCGA AATAGCCTAT TGGCTTATCT   
  
  
- ACTGAAACTC TCAAACCAAT TTGCCCGCTC CTTTCTCCAG AGATAGAGCC CACTCGGATA GGTTGCAGAG   
  
  
- CCACGGATGT ACTAGCTTCC CGAACACCGT TCCTTCGTCA GAAGCCCCTG ATCGTAGATA GCCCGAGACT   
  
  
- TCACATTTCT CGGAGAACCT TTTCTGAACG AGAGGATGTA CGTGTAGGAA ATACTTTATA CGGGAATAGA   
  
  
- GTTCAAACCA ATATACCGAC GCTTACCTCG CTATCGACTT CGAACATCTT TACTCCTATT GTATGTATAA   
  
  
- TATCTAAAGG TCTAACGTGT CCCGTGAGTC ACCCAATGAG ATAATGTTCG GGATCGTCGT TCTGGACCAC   
  
  
- CCGGTGGATT CCACGCTTAA TGTCCGTAAC TACTAGGACA AAGATTCATA CGGGCACCAC GATCGAACCT   
  
  
- CCGACAACCC TTTGCCAATC GCAGAGATAG ACTTTTCAAG TTTTATGGGC AGCTCAAGTT ACGCAACGGG   
  
  
- CAAATACCTG GGCTACAGTC CGCCCTTTAC GACCTACACT CCGGGCCCCT CCGAAACCGG CAATTAAAAG   
  
  
- GTAACGTCGA GGTGGTGTGA GGACTGCTCT CACAGCTACA CTTGTTGGGA TCCCTACCCG AAGAGTCTTA   
  
  
- CCACTTTAGT GAACCAGGAT TCCATTGGTG AAACCAACTC GTTCTTAGTT TGTGGTTGTG ATGGGGAAAG   
  
  
- AACTGGGCCA AGTATCTCTG GGAACTGATG ATGAGTCGGT ACAAACTCAG ATATCTGCAC TGGTACGGTT   
  
  
- CTCTGGCCTT CCTCTCCTAG TTACAACTCG TCGTAACAAA CCGATTCCTG TAACACTTAT AGTATCGAAC   
  
  
- GCTCCCGTTC CTCTCCCACC TCGCAGTACT TGAAAAACCC TTTACCTTCA GTTCCAAATG GTACCGTCCC   
  
  
- AAGTCCGTCA TGGGCAACTC GAGTATGCAT TTGAGACACT ATTCTTCGGA TGAGGCCACA ATGAGGCTCG   
  
  
- TAATATGAGA CTATCTCTTC CTACCACGGT ACGAAGACCC AACCTTCCCG GCTTACGACT AAAGTCGAAG   
  
  
- CCGTACCGTA AC

+     O2-site

| Site Name | Organism | Position | Strand | Matrix score. | sequence | function |
| --- | --- | --- | --- | --- | --- | --- |
| O2-site | Zea mays | 1240 | + | 9 | GATGACATGG | cis-acting regulatory element involved in zein metabolism regulation |

>HU08G00014.1   
+ +Up\_Stream \_Len000TCCTCT TTTGGGCTTC TTTCTTTCTA TCCTGTTTTT TTTTGGGTCG AAAAGTTGGA   
  
  
+ GGTTGAATTG AAGCATCCGA ATTCGAGCAG GATGTTTCTC TGCGTAGTGA TTCTTTCTGG GTTTGTGATT   
  
  
+ TTGAATTCTG GGTAATCGGT TGTTTTGCTA ATTTTGAGGA CAGAGTTCCT TTTCTGAATT AAAATTTCGT   
  
  
+ TTTTTTATCG GGAAAATCTT CAGTATTTGA GAAAAAAGAA CGAAATTTGG ATGATTTGCT GTCTAGATTT   
  
  
+ TGCTTTCATA TTCCTGGGTG TGGATTGGTT TATTAATTGC ATGTGAGGAA GTACAAGCTT CTGGTCAATG   
  
  
+ GTTGGGTTTT TTTTTTTTTT TTGTGGGTAT AAAGTTGGAA ATTGATTACG AGTCAAATTT CTCTTTCTAG   
  
  
+ AAGAGGGGGG GGGGGGGGGG TTGGTTTAAA ACTCCATATT TTCTTTGAAT GGAAAGCTTA ATTTGCTGAC   
  
  
+ TATCTTGGTT AGTAGCATAA GCTTGATCAG AAAACAAAAC GCTCTCTGCC CTGCAAAAAC TTTATTTGTC   
  
  
+ TTTTAAGGAA AATTTTGGAA TTTGAGAGTG AAAATTTTGG TTGTGAATCT TGTCTTACTA TGTGGTTCCT   
  
  
+ TCTGACAAAA GTTTGAATCT GAGTATGAGA GTTCATCAAT CTCTTTTACT TTCCTACTTT CTTTATTGCT   
  
  
+ TGCAAATTGT TGTGAACTTA CTTGGATTTT CTGGTAGAGG AGAGTTCTGT CCTGGAATTT GGTGGAAAGT   
  
  
+ GAGACATACT TTGGGATTTA ATTAGCCATC AATTTGGTTT CACACCTTAT CAGTTTGGAC TTCTGGTGTG   
  
  
+ TTATTGTATG TGTTCTTAGC TTGGGATTAT TCTTGTATCG GAAGAGAGGG GGGGGGGGGT GTTTCTTTTA   
  
  
+ GCTGAATGTT AGAACATTTT AAGGGATCTA GATATCTAGA GAAGAGCCTA GGAAATGAAG CATTTTTCCC   
  
  
+ TTATAATCTG ACACAACTTT ATGAATTGAT TAGGAACAAA GAACGATTAG TTCAGAAAGG TTACAACTCC   
  
  
+ TCTAGGTGTT ATACTAAAGA GATGTGAGTT AATAAGCAAT TGCTTACTAA CTGTTTAGGC TGTTTCATTG   
  
  
+ CTTGTAGAGG CTTTTTTTTT GGGGGGGGGG GGGGGTTTGG GGTTCATAAA TGAGAAGGTT GATTTGTTGT   
  
  
+ CTGCTCTGCT CTTGATTCGA TAGTAATTCT CTCAGAAAAG GGTAAAATGA CATGGCCTTC CATCGCCTCC   
  
  
+ CATGAGGCTT TTTGCCTCTG CTTGTGTATC TTTCATCTCT TGGTTTGGTG GGTTGGGGGA GGGGAGGGGG   
  
  
+ GGAAGGGGGT TTTGATTCGA TAGATCATCA CTTTCAGAAA TGGCCTCTCT GGCCAATGGT AAGGGGTAAG   
  
  
+ GTTAGTACAT GTGGACTTTC CCATGTGGAT TTTTCCAGAG TCCGTTGTGT GATTGTTATT GCTGTTGTTG   
  
  
+ TAACTTTCTG TCACTCGGAA AACATGAATC CATATGCAAC AAGAACATAA TAATTGTGAA TGCGACTTTA   
  
  
+ TTTAGGAATG TCTTACATAG TACGTGCACA TTCTTTAATT ATCTGTTTAT CTGTTGGTTT GTTATTTTCA   
  
  
+ AGGGGATGGG GAGTGGGTGG ATGCTTAGTA TGTAATCTTT TTATATAGGG AAAAATCATA TAGCTTGAAA   
  
  
+ GTAAAACTTT CATTGCCAGG ATTCCAAGGT AGGGGTAGGG TTGTCTACAC CTTGATCTCC CTAGAAGCAT   
  
  
+ GGCGATCCTT CCTCGGATTG TGTTATAGAA TAAAGAATGT GGTTCTGCTG TAATGAGACT TATGTTCTAT   
  
  
+ CAAATGATGA ATCTGTTTAG AACTTCTTGC TTATTTTTTC TGCAAAATTT TAGGAGACTT GATAAACTAT   
  
  
+ AGTTCTCTTG AATTTACAGG TGGATATTGG AGTTCACAGA ATAGTATTCA GATCAAAGTG CTTGTGCTTA   
  
  
+ ATTGGAAGGA CAACTTGCTT GAGCTGTTTA TAGAGCTCTA ACTGATGCTA TCTCATGGAC TCACATCAGT   
  
  
+ TTTTTGGATT TAATCTTACC AGTGTTGATC CGTCGTACAT TTCCGCTCAG TACAGTCCGC CGTCAGTGAC   
  
  
+ AAATAGGATG TTTGCATCAC TGAAGCTCGA CTCTAGAGGT TCTCCTGTGT CACCCTTCTC AACTCAGTTT   
  
  
+ GATTGTGATA CGGTTACTAC ATTGAGTGAT AGTCAAGAGC ATCACAGCTC GACGGGGAGT CTATCAACAA   
  
  
+ GAAGCCCTTC TTGTAATTCT CCCCTTGAAA CGAGCAGTTA TCATCATTTA TCGACGAATG GCCCGTCTTG   
  
  
+ TAATTCTGCC CCTGAAACTA GCAGTTATCG TCATCGGTTC AACGCGAGTC CCGTGGGATA TTCCCATCAA   
  
  
+ GGCACTGATT ATGGAGTGAA TATGAAGAGC GCTCTGCAGG AGCTAGAGAC TACTCTAATG GGTGCAGATG   
  
  
+ GTGAGGAAGT ATCTGCTGCT AATCAACCTA TGGGGGGAAG TCGTCAGTCC GGGATCCCAA GTCAGAGATC   
  
  
+ AAAATCATTG AGCGAAGATC CACAGGGTTC GCATCCTACT CAGCCTGATT CATCATCTCT TTCTAGGGCA   
  
  
+ AGAAGATCAG GAGATGAAAG CCAGAGAGAG AAACGGCACA AGGCAATGGA AGAACCAACG GAACTACCAA   
  
  
+ GTTTGCCACC TGGTGATTTG AAGCAGTTGC TAATTGAATG TGCGAGGGCT TTATCGGATA ACCGAATAGA   
  
  
+ TGACTTTGAG AGTTTGGTTA AACGGGCGAG GAAAGAGGTC TCTATCTCGG GTGAGCCTAT CCAACGTCTC   
  
  
+ GGTGCCTACA TGATCGAAGG GCTTGTGGCA AGGAAGCAGT CTTCGGGGAC TAGCATCTAT CGGGCTCTGA   
  
  
+ AGTGTAAAGA GCCTCTTGGA AAAGACTTGC TCTCCTACAT GCACATCCTT TATGAAATAT GCCCTTATCT   
  
  
+ CAAGTTTGGT TATATGGCTG CGAATGGAGC GATAGCTGAA GCTTGTAGAA ATGAGGATAA CATACATATT   
  
  
+ ATAGATTTCC AGATTGCACA GGGCACTCAG TGGGTTACTC TATTACAAGC CCTAGCAGCA AGACCTGGTG   
  
  
+ GGCCACCTAA GGTGCGAATT ACAGGCATTG ATGATCCTGT TTCTAAGTAT GCCCGTGGTG CTAGCTTGGA   
  
  
+ GGCTGTTGGG AAACGGTTAG CGTCTCTATC TGAAAAGTTC AAAATACCCG TCGAGTTCAA TGCGTTGCCC   
  
  
+ GTTTATGGAC CCGATGTCAG GCGGGAAATG CTGGATGTGA GGCCCGGGGA GGCTTTGGCC GTTAATTTTC   
  
  
+ CATTGCAGCT CCACCACACT CCTGACGAGA GTGTCGATGT GAACAACCCT AGGGATGGGC TTCTCAGAAT   
  
  
+ GGTGAAATCA CTTGGTCCTA AGGTAACCAC TTTGGTTGAG CAAGAATCAA ACACCAACAC TACCCCTTTC   
  
  
+ TTGACCCGGT TCATAGAGAC CCTTGACTAC TACTCAGCCA TGTTTGAGTC TATAGACGTG ACCATGCCAA   
  
  
+ GAGACCGGAA GGAGAGGATC AATGTTGAGC AGCATTGTTT GGCTAAGGAC ATTGTGAATA TCATAGCTTG   
  
  
+ CGAGGGCAAG GAGAGGGTGG AGCGTCATGA ACTTTTTGGG AAATGGAAGT CAAGGTTTAC CATGGCAGGG   
  
  
+ TTCAGGCAGT ACCCGTTGAG CTCATACGTA AACTCTGTGA TAAGAAGCCT ACTCCGGTGT TACTCCGAGC   
  
  
+ ATTATACTCT GATAGAGAAG GATGGTGCCA TGCTTCTGGG TTGGAAGGGC CGAATGCTGA TTTCAGCTTC   
  
  
+ GGCATGGCAT TG  

- +Up\_Stream \_Len000AGGAGA AAACCCGAAG AAAGAAAGAT AGGACAAAAA AAAACCCAGC TTTTCAACCT   
  
  
- CCAACTTAAC TTCGTAGGCT TAAGCTCGTC CTACAAAGAG ACGCATCACT AAGAAAGACC CAAACACTAA   
  
  
- AACTTAAGAC CCATTAGCCA ACAAAACGAT TAAAACTCCT GTCTCAAGGA AAAGACTTAA TTTTAAAGCA   
  
  
- AAAAAATAGC CCTTTTAGAA GTCATAAACT CTTTTTTCTT GCTTTAAACC TACTAAACGA CAGATCTAAA   
  
  
- ACGAAAGTAT AAGGACCCAC ACCTAACCAA ATAATTAACG TACACTCCTT CATGTTCGAA GACCAGTTAC   
  
  
- CAACCCAAAA AAAAAAAAAA AACACCCATA TTTCAACCTT TAACTAATGC TCAGTTTAAA GAGAAAGATC   
  
  
- TTCTCCCCCC CCCCCCCCCC AACCAAATTT TGAGGTATAA AAGAAACTTA CCTTTCGAAT TAAACGACTG   
  
  
- ATAGAACCAA TCATCGTATT CGAACTAGTC TTTTGTTTTG CGAGAGACGG GACGTTTTTG AAATAAACAG   
  
  
- AAAATTCCTT TTAAAACCTT AAACTCTCAC TTTTAAAACC AACACTTAGA ACAGAATGAT ACACCAAGGA   
  
  
- AGACTGTTTT CAAACTTAGA CTCATACTCT CAAGTAGTTA GAGAAAATGA AAGGATGAAA GAAATAACGA   
  
  
- ACGTTTAACA ACACTTGAAT GAACCTAAAA GACCATCTCC TCTCAAGACA GGACCTTAAA CCACCTTTCA   
  
  
- CTCTGTATGA AACCCTAAAT TAATCGGTAG TTAAACCAAA GTGTGGAATA GTCAAACCTG AAGACCACAC   
  
  
- AATAACATAC ACAAGAATCG AACCCTAATA AGAACATAGC CTTCTCTCCC CCCCCCCCCA CAAAGAAAAT   
  
  
- CGACTTACAA TCTTGTAAAA TTCCCTAGAT CTATAGATCT CTTCTCGGAT CCTTTACTTC GTAAAAAGGG   
  
  
- AATATTAGAC TGTGTTGAAA TACTTAACTA ATCCTTGTTT CTTGCTAATC AAGTCTTTCC AATGTTGAGG   
  
  
- AGATCCACAA TATGATTTCT CTACACTCAA TTATTCGTTA ACGAATGATT GACAAATCCG ACAAAGTAAC   
  
  
- GAACATCTCC GAAAAAAAAA CCCCCCCCCC CCCCCAAACC CCAAGTATTT ACTCTTCCAA CTAAACAACA   
  
  
- GACGAGACGA GAACTAAGCT ATCATTAAGA GAGTCTTTTC CCATTTTACT GTACCGGAAG GTAGCGGAGG   
  
  
- GTACTCCGAA AAACGGAGAC GAACACATAG AAAGTAGAGA ACCAAACCAC CCAACCCCCT CCCCTCCCCC   
  
  
- CCTTCCCCCA AAACTAAGCT ATCTAGTAGT GAAAGTCTTT ACCGGAGAGA CCGGTTACCA TTCCCCATTC   
  
  
- CAATCATGTA CACCTGAAAG GGTACACCTA AAAAGGTCTC AGGCAACACA CTAACAATAA CGACAACAAC   
  
  
- ATTGAAAGAC AGTGAGCCTT TTGTACTTAG GTATACGTTG TTCTTGTATT ATTAACACTT ACGCTGAAAT   
  
  
- AAATCCTTAC AGAATGTATC ATGCACGTGT AAGAAATTAA TAGACAAATA GACAACCAAA CAATAAAAGT   
  
  
- TCCCCTACCC CTCACCCACC TACGAATCAT ACATTAGAAA AATATATCCC TTTTTAGTAT ATCGAACTTT   
  
  
- CATTTTGAAA GTAACGGTCC TAAGGTTCCA TCCCCATCCC AACAGATGTG GAACTAGAGG GATCTTCGTA   
  
  
- CCGCTAGGAA GGAGCCTAAC ACAATATCTT ATTTCTTACA CCAAGACGAC ATTACTCTGA ATACAAGATA   
  
  
- GTTTACTACT TAGACAAATC TTGAAGAACG AATAAAAAAG ACGTTTTAAA ATCCTCTGAA CTATTTGATA   
  
  
- TCAAGAGAAC TTAAATGTCC ACCTATAACC TCAAGTGTCT TATCATAAGT CTAGTTTCAC GAACACGAAT   
  
  
- TAACCTTCCT GTTGAACGAA CTCGACAAAT ATCTCGAGAT TGACTACGAT AGAGTACCTG AGTGTAGTCA   
  
  
- AAAAACCTAA ATTAGAATGG TCACAACTAG GCAGCATGTA AAGGCGAGTC ATGTCAGGCG GCAGTCACTG   
  
  
- TTTATCCTAC AAACGTAGTG ACTTCGAGCT GAGATCTCCA AGAGGACACA GTGGGAAGAG TTGAGTCAAA   
  
  
- CTAACACTAT GCCAATGATG TAACTCACTA TCAGTTCTCG TAGTGTCGAG CTGCCCCTCA GATAGTTGTT   
  
  
- CTTCGGGAAG AACATTAAGA GGGGAACTTT GCTCGTCAAT AGTAGTAAAT AGCTGCTTAC CGGGCAGAAC   
  
  
- ATTAAGACGG GGACTTTGAT CGTCAATAGC AGTAGCCAAG TTGCGCTCAG GGCACCCTAT AAGGGTAGTT   
  
  
- CCGTGACTAA TACCTCACTT ATACTTCTCG CGAGACGTCC TCGATCTCTG ATGAGATTAC CCACGTCTAC   
  
  
- CACTCCTTCA TAGACGACGA TTAGTTGGAT ACCCCCCTTC AGCAGTCAGG CCCTAGGGTT CAGTCTCTAG   
  
  
- TTTTAGTAAC TCGCTTCTAG GTGTCCCAAG CGTAGGATGA GTCGGACTAA GTAGTAGAGA AAGATCCCGT   
  
  
- TCTTCTAGTC CTCTACTTTC GGTCTCTCTC TTTGCCGTGT TCCGTTACCT TCTTGGTTGC CTTGATGGTT   
  
  
- CAAACGGTGG ACCACTAAAC TTCGTCAACG ATTAACTTAC ACGCTCCCGA AATAGCCTAT TGGCTTATCT   
  
  
- ACTGAAACTC TCAAACCAAT TTGCCCGCTC CTTTCTCCAG AGATAGAGCC CACTCGGATA GGTTGCAGAG   
  
  
- CCACGGATGT ACTAGCTTCC CGAACACCGT TCCTTCGTCA GAAGCCCCTG ATCGTAGATA GCCCGAGACT   
  
  
- TCACATTTCT CGGAGAACCT TTTCTGAACG AGAGGATGTA CGTGTAGGAA ATACTTTATA CGGGAATAGA   
  
  
- GTTCAAACCA ATATACCGAC GCTTACCTCG CTATCGACTT CGAACATCTT TACTCCTATT GTATGTATAA   
  
  
- TATCTAAAGG TCTAACGTGT CCCGTGAGTC ACCCAATGAG ATAATGTTCG GGATCGTCGT TCTGGACCAC   
  
  
- CCGGTGGATT CCACGCTTAA TGTCCGTAAC TACTAGGACA AAGATTCATA CGGGCACCAC GATCGAACCT   
  
  
- CCGACAACCC TTTGCCAATC GCAGAGATAG ACTTTTCAAG TTTTATGGGC AGCTCAAGTT ACGCAACGGG   
  
  
- CAAATACCTG GGCTACAGTC CGCCCTTTAC GACCTACACT CCGGGCCCCT CCGAAACCGG CAATTAAAAG   
  
  
- GTAACGTCGA GGTGGTGTGA GGACTGCTCT CACAGCTACA CTTGTTGGGA TCCCTACCCG AAGAGTCTTA   
  
  
- CCACTTTAGT GAACCAGGAT TCCATTGGTG AAACCAACTC GTTCTTAGTT TGTGGTTGTG ATGGGGAAAG   
  
  
- AACTGGGCCA AGTATCTCTG GGAACTGATG ATGAGTCGGT ACAAACTCAG ATATCTGCAC TGGTACGGTT   
  
  
- CTCTGGCCTT CCTCTCCTAG TTACAACTCG TCGTAACAAA CCGATTCCTG TAACACTTAT AGTATCGAAC   
  
  
- GCTCCCGTTC CTCTCCCACC TCGCAGTACT TGAAAAACCC TTTACCTTCA GTTCCAAATG GTACCGTCCC   
  
  
- AAGTCCGTCA TGGGCAACTC GAGTATGCAT TTGAGACACT ATTCTTCGGA TGAGGCCACA ATGAGGCTCG   
  
  
- TAATATGAGA CTATCTCTTC CTACCACGGT ACGAAGACCC AACCTTCCCG GCTTACGACT AAAGTCGAAG   
  
  
- CCGTACCGTA AC

+     STRE

| Site Name | Organism | Position | Strand | Matrix score. | sequence | function |
| --- | --- | --- | --- | --- | --- | --- |
| STRE | Arabidopsis thaliana | 2265 | - | 5 | AGGGG |  |
| STRE | Arabidopsis thaliana | 1329 | + | 5 | AGGGG |  |
| STRE | Arabidopsis thaliana | 1615 | + | 5 | AGGGG |  |
| STRE | Arabidopsis thaliana | 1715 | + | 5 | AGGGG |  |
| STRE | Arabidopsis thaliana | 3427 | - | 5 | AGGGG |  |
| STRE | Arabidopsis thaliana | 1396 | + | 5 | AGGGG |  |
| STRE | Arabidopsis thaliana | 1338 | + | 5 | AGGGG |  |
| STRE | Arabidopsis thaliana | 2323 | - | 5 | AGGGG |  |
| STRE | Arabidopsis thaliana | 428 | + | 5 | AGGGG |  |
| STRE | Arabidopsis thaliana | 891 | + | 5 | AGGGG |  |
| STRE | Arabidopsis thaliana | 1324 | + | 5 | AGGGG |  |

>HU08G00014.1   
+ +Up\_Stream \_Len000TCCTCT TTTGGGCTTC TTTCTTTCTA TCCTGTTTTT TTTTGGGTCG AAAAGTTGGA   
  
  
+ GGTTGAATTG AAGCATCCGA ATTCGAGCAG GATGTTTCTC TGCGTAGTGA TTCTTTCTGG GTTTGTGATT   
  
  
+ TTGAATTCTG GGTAATCGGT TGTTTTGCTA ATTTTGAGGA CAGAGTTCCT TTTCTGAATT AAAATTTCGT   
  
  
+ TTTTTTATCG GGAAAATCTT CAGTATTTGA GAAAAAAGAA CGAAATTTGG ATGATTTGCT GTCTAGATTT   
  
  
+ TGCTTTCATA TTCCTGGGTG TGGATTGGTT TATTAATTGC ATGTGAGGAA GTACAAGCTT CTGGTCAATG   
  
  
+ GTTGGGTTTT TTTTTTTTTT TTGTGGGTAT AAAGTTGGAA ATTGATTACG AGTCAAATTT CTCTTTCTAG   
  
  
+ AAGAGGGGGG GGGGGGGGGG TTGGTTTAAA ACTCCATATT TTCTTTGAAT GGAAAGCTTA ATTTGCTGAC   
  
  
+ TATCTTGGTT AGTAGCATAA GCTTGATCAG AAAACAAAAC GCTCTCTGCC CTGCAAAAAC TTTATTTGTC   
  
  
+ TTTTAAGGAA AATTTTGGAA TTTGAGAGTG AAAATTTTGG TTGTGAATCT TGTCTTACTA TGTGGTTCCT   
  
  
+ TCTGACAAAA GTTTGAATCT GAGTATGAGA GTTCATCAAT CTCTTTTACT TTCCTACTTT CTTTATTGCT   
  
  
+ TGCAAATTGT TGTGAACTTA CTTGGATTTT CTGGTAGAGG AGAGTTCTGT CCTGGAATTT GGTGGAAAGT   
  
  
+ GAGACATACT TTGGGATTTA ATTAGCCATC AATTTGGTTT CACACCTTAT CAGTTTGGAC TTCTGGTGTG   
  
  
+ TTATTGTATG TGTTCTTAGC TTGGGATTAT TCTTGTATCG GAAGAGAGGG GGGGGGGGGT GTTTCTTTTA   
  
  
+ GCTGAATGTT AGAACATTTT AAGGGATCTA GATATCTAGA GAAGAGCCTA GGAAATGAAG CATTTTTCCC   
  
  
+ TTATAATCTG ACACAACTTT ATGAATTGAT TAGGAACAAA GAACGATTAG TTCAGAAAGG TTACAACTCC   
  
  
+ TCTAGGTGTT ATACTAAAGA GATGTGAGTT AATAAGCAAT TGCTTACTAA CTGTTTAGGC TGTTTCATTG   
  
  
+ CTTGTAGAGG CTTTTTTTTT GGGGGGGGGG GGGGGTTTGG GGTTCATAAA TGAGAAGGTT GATTTGTTGT   
  
  
+ CTGCTCTGCT CTTGATTCGA TAGTAATTCT CTCAGAAAAG GGTAAAATGA CATGGCCTTC CATCGCCTCC   
  
  
+ CATGAGGCTT TTTGCCTCTG CTTGTGTATC TTTCATCTCT TGGTTTGGTG GGTTGGGGGA GGGGAGGGGG   
  
  
+ GGAAGGGGGT TTTGATTCGA TAGATCATCA CTTTCAGAAA TGGCCTCTCT GGCCAATGGT AAGGGGTAAG   
  
  
+ GTTAGTACAT GTGGACTTTC CCATGTGGAT TTTTCCAGAG TCCGTTGTGT GATTGTTATT GCTGTTGTTG   
  
  
+ TAACTTTCTG TCACTCGGAA AACATGAATC CATATGCAAC AAGAACATAA TAATTGTGAA TGCGACTTTA   
  
  
+ TTTAGGAATG TCTTACATAG TACGTGCACA TTCTTTAATT ATCTGTTTAT CTGTTGGTTT GTTATTTTCA   
  
  
+ AGGGGATGGG GAGTGGGTGG ATGCTTAGTA TGTAATCTTT TTATATAGGG AAAAATCATA TAGCTTGAAA   
  
  
+ GTAAAACTTT CATTGCCAGG ATTCCAAGGT AGGGGTAGGG TTGTCTACAC CTTGATCTCC CTAGAAGCAT   
  
  
+ GGCGATCCTT CCTCGGATTG TGTTATAGAA TAAAGAATGT GGTTCTGCTG TAATGAGACT TATGTTCTAT   
  
  
+ CAAATGATGA ATCTGTTTAG AACTTCTTGC TTATTTTTTC TGCAAAATTT TAGGAGACTT GATAAACTAT   
  
  
+ AGTTCTCTTG AATTTACAGG TGGATATTGG AGTTCACAGA ATAGTATTCA GATCAAAGTG CTTGTGCTTA   
  
  
+ ATTGGAAGGA CAACTTGCTT GAGCTGTTTA TAGAGCTCTA ACTGATGCTA TCTCATGGAC TCACATCAGT   
  
  
+ TTTTTGGATT TAATCTTACC AGTGTTGATC CGTCGTACAT TTCCGCTCAG TACAGTCCGC CGTCAGTGAC   
  
  
+ AAATAGGATG TTTGCATCAC TGAAGCTCGA CTCTAGAGGT TCTCCTGTGT CACCCTTCTC AACTCAGTTT   
  
  
+ GATTGTGATA CGGTTACTAC ATTGAGTGAT AGTCAAGAGC ATCACAGCTC GACGGGGAGT CTATCAACAA   
  
  
+ GAAGCCCTTC TTGTAATTCT CCCCTTGAAA CGAGCAGTTA TCATCATTTA TCGACGAATG GCCCGTCTTG   
  
  
+ TAATTCTGCC CCTGAAACTA GCAGTTATCG TCATCGGTTC AACGCGAGTC CCGTGGGATA TTCCCATCAA   
  
  
+ GGCACTGATT ATGGAGTGAA TATGAAGAGC GCTCTGCAGG AGCTAGAGAC TACTCTAATG GGTGCAGATG   
  
  
+ GTGAGGAAGT ATCTGCTGCT AATCAACCTA TGGGGGGAAG TCGTCAGTCC GGGATCCCAA GTCAGAGATC   
  
  
+ AAAATCATTG AGCGAAGATC CACAGGGTTC GCATCCTACT CAGCCTGATT CATCATCTCT TTCTAGGGCA   
  
  
+ AGAAGATCAG GAGATGAAAG CCAGAGAGAG AAACGGCACA AGGCAATGGA AGAACCAACG GAACTACCAA   
  
  
+ GTTTGCCACC TGGTGATTTG AAGCAGTTGC TAATTGAATG TGCGAGGGCT TTATCGGATA ACCGAATAGA   
  
  
+ TGACTTTGAG AGTTTGGTTA AACGGGCGAG GAAAGAGGTC TCTATCTCGG GTGAGCCTAT CCAACGTCTC   
  
  
+ GGTGCCTACA TGATCGAAGG GCTTGTGGCA AGGAAGCAGT CTTCGGGGAC TAGCATCTAT CGGGCTCTGA   
  
  
+ AGTGTAAAGA GCCTCTTGGA AAAGACTTGC TCTCCTACAT GCACATCCTT TATGAAATAT GCCCTTATCT   
  
  
+ CAAGTTTGGT TATATGGCTG CGAATGGAGC GATAGCTGAA GCTTGTAGAA ATGAGGATAA CATACATATT   
  
  
+ ATAGATTTCC AGATTGCACA GGGCACTCAG TGGGTTACTC TATTACAAGC CCTAGCAGCA AGACCTGGTG   
  
  
+ GGCCACCTAA GGTGCGAATT ACAGGCATTG ATGATCCTGT TTCTAAGTAT GCCCGTGGTG CTAGCTTGGA   
  
  
+ GGCTGTTGGG AAACGGTTAG CGTCTCTATC TGAAAAGTTC AAAATACCCG TCGAGTTCAA TGCGTTGCCC   
  
  
+ GTTTATGGAC CCGATGTCAG GCGGGAAATG CTGGATGTGA GGCCCGGGGA GGCTTTGGCC GTTAATTTTC   
  
  
+ CATTGCAGCT CCACCACACT CCTGACGAGA GTGTCGATGT GAACAACCCT AGGGATGGGC TTCTCAGAAT   
  
  
+ GGTGAAATCA CTTGGTCCTA AGGTAACCAC TTTGGTTGAG CAAGAATCAA ACACCAACAC TACCCCTTTC   
  
  
+ TTGACCCGGT TCATAGAGAC CCTTGACTAC TACTCAGCCA TGTTTGAGTC TATAGACGTG ACCATGCCAA   
  
  
+ GAGACCGGAA GGAGAGGATC AATGTTGAGC AGCATTGTTT GGCTAAGGAC ATTGTGAATA TCATAGCTTG   
  
  
+ CGAGGGCAAG GAGAGGGTGG AGCGTCATGA ACTTTTTGGG AAATGGAAGT CAAGGTTTAC CATGGCAGGG   
  
  
+ TTCAGGCAGT ACCCGTTGAG CTCATACGTA AACTCTGTGA TAAGAAGCCT ACTCCGGTGT TACTCCGAGC   
  
  
+ ATTATACTCT GATAGAGAAG GATGGTGCCA TGCTTCTGGG TTGGAAGGGC CGAATGCTGA TTTCAGCTTC   
  
  
+ GGCATGGCAT TG  

- +Up\_Stream \_Len000AGGAGA AAACCCGAAG AAAGAAAGAT AGGACAAAAA AAAACCCAGC TTTTCAACCT   
  
  
- CCAACTTAAC TTCGTAGGCT TAAGCTCGTC CTACAAAGAG ACGCATCACT AAGAAAGACC CAAACACTAA   
  
  
- AACTTAAGAC CCATTAGCCA ACAAAACGAT TAAAACTCCT GTCTCAAGGA AAAGACTTAA TTTTAAAGCA   
  
  
- AAAAAATAGC CCTTTTAGAA GTCATAAACT CTTTTTTCTT GCTTTAAACC TACTAAACGA CAGATCTAAA   
  
  
- ACGAAAGTAT AAGGACCCAC ACCTAACCAA ATAATTAACG TACACTCCTT CATGTTCGAA GACCAGTTAC   
  
  
- CAACCCAAAA AAAAAAAAAA AACACCCATA TTTCAACCTT TAACTAATGC TCAGTTTAAA GAGAAAGATC   
  
  
- TTCTCCCCCC CCCCCCCCCC AACCAAATTT TGAGGTATAA AAGAAACTTA CCTTTCGAAT TAAACGACTG   
  
  
- ATAGAACCAA TCATCGTATT CGAACTAGTC TTTTGTTTTG CGAGAGACGG GACGTTTTTG AAATAAACAG   
  
  
- AAAATTCCTT TTAAAACCTT AAACTCTCAC TTTTAAAACC AACACTTAGA ACAGAATGAT ACACCAAGGA   
  
  
- AGACTGTTTT CAAACTTAGA CTCATACTCT CAAGTAGTTA GAGAAAATGA AAGGATGAAA GAAATAACGA   
  
  
- ACGTTTAACA ACACTTGAAT GAACCTAAAA GACCATCTCC TCTCAAGACA GGACCTTAAA CCACCTTTCA   
  
  
- CTCTGTATGA AACCCTAAAT TAATCGGTAG TTAAACCAAA GTGTGGAATA GTCAAACCTG AAGACCACAC   
  
  
- AATAACATAC ACAAGAATCG AACCCTAATA AGAACATAGC CTTCTCTCCC CCCCCCCCCA CAAAGAAAAT   
  
  
- CGACTTACAA TCTTGTAAAA TTCCCTAGAT CTATAGATCT CTTCTCGGAT CCTTTACTTC GTAAAAAGGG   
  
  
- AATATTAGAC TGTGTTGAAA TACTTAACTA ATCCTTGTTT CTTGCTAATC AAGTCTTTCC AATGTTGAGG   
  
  
- AGATCCACAA TATGATTTCT CTACACTCAA TTATTCGTTA ACGAATGATT GACAAATCCG ACAAAGTAAC   
  
  
- GAACATCTCC GAAAAAAAAA CCCCCCCCCC CCCCCAAACC CCAAGTATTT ACTCTTCCAA CTAAACAACA   
  
  
- GACGAGACGA GAACTAAGCT ATCATTAAGA GAGTCTTTTC CCATTTTACT GTACCGGAAG GTAGCGGAGG   
  
  
- GTACTCCGAA AAACGGAGAC GAACACATAG AAAGTAGAGA ACCAAACCAC CCAACCCCCT CCCCTCCCCC   
  
  
- CCTTCCCCCA AAACTAAGCT ATCTAGTAGT GAAAGTCTTT ACCGGAGAGA CCGGTTACCA TTCCCCATTC   
  
  
- CAATCATGTA CACCTGAAAG GGTACACCTA AAAAGGTCTC AGGCAACACA CTAACAATAA CGACAACAAC   
  
  
- ATTGAAAGAC AGTGAGCCTT TTGTACTTAG GTATACGTTG TTCTTGTATT ATTAACACTT ACGCTGAAAT   
  
  
- AAATCCTTAC AGAATGTATC ATGCACGTGT AAGAAATTAA TAGACAAATA GACAACCAAA CAATAAAAGT   
  
  
- TCCCCTACCC CTCACCCACC TACGAATCAT ACATTAGAAA AATATATCCC TTTTTAGTAT ATCGAACTTT   
  
  
- CATTTTGAAA GTAACGGTCC TAAGGTTCCA TCCCCATCCC AACAGATGTG GAACTAGAGG GATCTTCGTA   
  
  
- CCGCTAGGAA GGAGCCTAAC ACAATATCTT ATTTCTTACA CCAAGACGAC ATTACTCTGA ATACAAGATA   
  
  
- GTTTACTACT TAGACAAATC TTGAAGAACG AATAAAAAAG ACGTTTTAAA ATCCTCTGAA CTATTTGATA   
  
  
- TCAAGAGAAC TTAAATGTCC ACCTATAACC TCAAGTGTCT TATCATAAGT CTAGTTTCAC GAACACGAAT   
  
  
- TAACCTTCCT GTTGAACGAA CTCGACAAAT ATCTCGAGAT TGACTACGAT AGAGTACCTG AGTGTAGTCA   
  
  
- AAAAACCTAA ATTAGAATGG TCACAACTAG GCAGCATGTA AAGGCGAGTC ATGTCAGGCG GCAGTCACTG   
  
  
- TTTATCCTAC AAACGTAGTG ACTTCGAGCT GAGATCTCCA AGAGGACACA GTGGGAAGAG TTGAGTCAAA   
  
  
- CTAACACTAT GCCAATGATG TAACTCACTA TCAGTTCTCG TAGTGTCGAG CTGCCCCTCA GATAGTTGTT   
  
  
- CTTCGGGAAG AACATTAAGA GGGGAACTTT GCTCGTCAAT AGTAGTAAAT AGCTGCTTAC CGGGCAGAAC   
  
  
- ATTAAGACGG GGACTTTGAT CGTCAATAGC AGTAGCCAAG TTGCGCTCAG GGCACCCTAT AAGGGTAGTT   
  
  
- CCGTGACTAA TACCTCACTT ATACTTCTCG CGAGACGTCC TCGATCTCTG ATGAGATTAC CCACGTCTAC   
  
  
- CACTCCTTCA TAGACGACGA TTAGTTGGAT ACCCCCCTTC AGCAGTCAGG CCCTAGGGTT CAGTCTCTAG   
  
  
- TTTTAGTAAC TCGCTTCTAG GTGTCCCAAG CGTAGGATGA GTCGGACTAA GTAGTAGAGA AAGATCCCGT   
  
  
- TCTTCTAGTC CTCTACTTTC GGTCTCTCTC TTTGCCGTGT TCCGTTACCT TCTTGGTTGC CTTGATGGTT   
  
  
- CAAACGGTGG ACCACTAAAC TTCGTCAACG ATTAACTTAC ACGCTCCCGA AATAGCCTAT TGGCTTATCT   
  
  
- ACTGAAACTC TCAAACCAAT TTGCCCGCTC CTTTCTCCAG AGATAGAGCC CACTCGGATA GGTTGCAGAG   
  
  
- CCACGGATGT ACTAGCTTCC CGAACACCGT TCCTTCGTCA GAAGCCCCTG ATCGTAGATA GCCCGAGACT   
  
  
- TCACATTTCT CGGAGAACCT TTTCTGAACG AGAGGATGTA CGTGTAGGAA ATACTTTATA CGGGAATAGA   
  
  
- GTTCAAACCA ATATACCGAC GCTTACCTCG CTATCGACTT CGAACATCTT TACTCCTATT GTATGTATAA   
  
  
- TATCTAAAGG TCTAACGTGT CCCGTGAGTC ACCCAATGAG ATAATGTTCG GGATCGTCGT TCTGGACCAC   
  
  
- CCGGTGGATT CCACGCTTAA TGTCCGTAAC TACTAGGACA AAGATTCATA CGGGCACCAC GATCGAACCT   
  
  
- CCGACAACCC TTTGCCAATC GCAGAGATAG ACTTTTCAAG TTTTATGGGC AGCTCAAGTT ACGCAACGGG   
  
  
- CAAATACCTG GGCTACAGTC CGCCCTTTAC GACCTACACT CCGGGCCCCT CCGAAACCGG CAATTAAAAG   
  
  
- GTAACGTCGA GGTGGTGTGA GGACTGCTCT CACAGCTACA CTTGTTGGGA TCCCTACCCG AAGAGTCTTA   
  
  
- CCACTTTAGT GAACCAGGAT TCCATTGGTG AAACCAACTC GTTCTTAGTT TGTGGTTGTG ATGGGGAAAG   
  
  
- AACTGGGCCA AGTATCTCTG GGAACTGATG ATGAGTCGGT ACAAACTCAG ATATCTGCAC TGGTACGGTT   
  
  
- CTCTGGCCTT CCTCTCCTAG TTACAACTCG TCGTAACAAA CCGATTCCTG TAACACTTAT AGTATCGAAC   
  
  
- GCTCCCGTTC CTCTCCCACC TCGCAGTACT TGAAAAACCC TTTACCTTCA GTTCCAAATG GTACCGTCCC   
  
  
- AAGTCCGTCA TGGGCAACTC GAGTATGCAT TTGAGACACT ATTCTTCGGA TGAGGCCACA ATGAGGCTCG   
  
  
- TAATATGAGA CTATCTCTTC CTACCACGGT ACGAAGACCC AACCTTCCCG GCTTACGACT AAAGTCGAAG   
  
  
- CCGTACCGTA AC

+     TATA-box

| Site Name | Organism | Position | Strand | Matrix score. | sequence | function |
| --- | --- | --- | --- | --- | --- | --- |
| TATA-box | Arabidopsis thaliana | 1778 | + | 4 | TATA | core promoter element around -30 of transcription start |
| TATA-box | Arabidopsis thaliana | 1673 | + | 4 | TATA | core promoter element around -30 of transcription start |
| TATA-box | Arabidopsis thaliana | 1892 | + | 4 | TATA | core promoter element around -30 of transcription start |
| TATA-box | Arabidopsis thaliana | 3717 | - | 4 | TATA | core promoter element around -30 of transcription start |
| TATA-box | Arabidopsis thaliana | 3485 | - | 4 | TATA | core promoter element around -30 of transcription start |
| TATA-box | Arabidopsis thaliana | 1777 | - | 5 | TATAA | core promoter element around -30 of transcription start |
| TATA-box | Arabidopsis thaliana | 1658 | + | 4 | TATA | core promoter element around -30 of transcription start |
| TATA-box | Arabidopsis thaliana | 1655 | - | 7 | TATATAA | core promoter element around -30 of transcription start |
| TATA-box | Arabidopsis thaliana | 986 | + | 4 | TATA | core promoter element around -30 of transcription start |
| TATA-box | Arabidopsis thaliana | 1656 | + | 6 | TATATA | core promoter element around -30 of transcription start |
| TATA-box | Arabidopsis thaliana | 2954 | - | 5 | TATAA | core promoter element around -30 of transcription start |
| TATA-box | Arabidopsis thaliana | 1064 | + | 4 | TATA | core promoter element around -30 of transcription start |
| TATA-box | Arabidopsis thaliana | 382 | + | 4 | TATA | core promoter element around -30 of transcription start |
| TATA-box | Helianthus annuus | 1991 | - | 6 | TATAAA | core promoter element around -30 of transcription start |
| TATA-box | Arabidopsis thaliana | 1654 | - | 9 | ccTATAAAaa | core promoter element around -30 of transcription start |
| TATA-box | Brassica napus | 3012 | + | 6 | ATTATA | core promoter element around -30 of transcription start |
| TATA-box | Arabidopsis thaliana | 1993 | - | 4 | TATA | core promoter element around -30 of transcription start |
| TATA-box | Arabidopsis thaliana | 985 | - | 5 | TATAA | core promoter element around -30 of transcription start |
| TATA-box | Arabidopsis thaliana | 1063 | - | 5 | TATAA | core promoter element around -30 of transcription start |
| TATA-box | Brassica napus | 3715 | + | 6 | ATTATA | core promoter element around -30 of transcription start |
| TATA-box | Arabidopsis thaliana | 216 | - | 9 | ccTATAAAaa | core promoter element around -30 of transcription start |
| TATA-box | Pisum sativum | 1653 | - | 7 | TATAAAA | core promoter element around -30 of transcription start |
| TATA-box | Arabidopsis thaliana | 2955 | - | 4 | TATA | core promoter element around -30 of transcription start |
| TATA-box | Arabidopsis thaliana | 3014 | - | 4 | TATA | core promoter element around -30 of transcription start |
| TATA-box | Arabidopsis thaliana | 3716 | - | 5 | TATAA | core promoter element around -30 of transcription start |
| TATA-box | Arabidopsis thaliana | 1992 | - | 5 | TATAA | core promoter element around -30 of transcription start |
| TATA-box | Arabidopsis thaliana | 3013 | - | 5 | TATAA | core promoter element around -30 of transcription start |

>HU08G00014.1   
+ +Up\_Stream \_Len000TCCTCT TTTGGGCTTC TTTCTTTCTA TCCTGTTTTT TTTTGGGTCG AAAAGTTGGA   
  
  
+ GGTTGAATTG AAGCATCCGA ATTCGAGCAG GATGTTTCTC TGCGTAGTGA TTCTTTCTGG GTTTGTGATT   
  
  
+ TTGAATTCTG GGTAATCGGT TGTTTTGCTA ATTTTGAGGA CAGAGTTCCT TTTCTGAATT AAAATTTCGT   
  
  
+ TTTTTTATCG GGAAAATCTT CAGTATTTGA GAAAAAAGAA CGAAATTTGG ATGATTTGCT GTCTAGATTT   
  
  
+ TGCTTTCATA TTCCTGGGTG TGGATTGGTT TATTAATTGC ATGTGAGGAA GTACAAGCTT CTGGTCAATG   
  
  
+ GTTGGGTTTT TTTTTTTTTT TTGTGGGTAT AAAGTTGGAA ATTGATTACG AGTCAAATTT CTCTTTCTAG   
  
  
+ AAGAGGGGGG GGGGGGGGGG TTGGTTTAAA ACTCCATATT TTCTTTGAAT GGAAAGCTTA ATTTGCTGAC   
  
  
+ TATCTTGGTT AGTAGCATAA GCTTGATCAG AAAACAAAAC GCTCTCTGCC CTGCAAAAAC TTTATTTGTC   
  
  
+ TTTTAAGGAA AATTTTGGAA TTTGAGAGTG AAAATTTTGG TTGTGAATCT TGTCTTACTA TGTGGTTCCT   
  
  
+ TCTGACAAAA GTTTGAATCT GAGTATGAGA GTTCATCAAT CTCTTTTACT TTCCTACTTT CTTTATTGCT   
  
  
+ TGCAAATTGT TGTGAACTTA CTTGGATTTT CTGGTAGAGG AGAGTTCTGT CCTGGAATTT GGTGGAAAGT   
  
  
+ GAGACATACT TTGGGATTTA ATTAGCCATC AATTTGGTTT CACACCTTAT CAGTTTGGAC TTCTGGTGTG   
  
  
+ TTATTGTATG TGTTCTTAGC TTGGGATTAT TCTTGTATCG GAAGAGAGGG GGGGGGGGGT GTTTCTTTTA   
  
  
+ GCTGAATGTT AGAACATTTT AAGGGATCTA GATATCTAGA GAAGAGCCTA GGAAATGAAG CATTTTTCCC   
  
  
+ TTATAATCTG ACACAACTTT ATGAATTGAT TAGGAACAAA GAACGATTAG TTCAGAAAGG TTACAACTCC   
  
  
+ TCTAGGTGTT ATACTAAAGA GATGTGAGTT AATAAGCAAT TGCTTACTAA CTGTTTAGGC TGTTTCATTG   
  
  
+ CTTGTAGAGG CTTTTTTTTT GGGGGGGGGG GGGGGTTTGG GGTTCATAAA TGAGAAGGTT GATTTGTTGT   
  
  
+ CTGCTCTGCT CTTGATTCGA TAGTAATTCT CTCAGAAAAG GGTAAAATGA CATGGCCTTC CATCGCCTCC   
  
  
+ CATGAGGCTT TTTGCCTCTG CTTGTGTATC TTTCATCTCT TGGTTTGGTG GGTTGGGGGA GGGGAGGGGG   
  
  
+ GGAAGGGGGT TTTGATTCGA TAGATCATCA CTTTCAGAAA TGGCCTCTCT GGCCAATGGT AAGGGGTAAG   
  
  
+ GTTAGTACAT GTGGACTTTC CCATGTGGAT TTTTCCAGAG TCCGTTGTGT GATTGTTATT GCTGTTGTTG   
  
  
+ TAACTTTCTG TCACTCGGAA AACATGAATC CATATGCAAC AAGAACATAA TAATTGTGAA TGCGACTTTA   
  
  
+ TTTAGGAATG TCTTACATAG TACGTGCACA TTCTTTAATT ATCTGTTTAT CTGTTGGTTT GTTATTTTCA   
  
  
+ AGGGGATGGG GAGTGGGTGG ATGCTTAGTA TGTAATCTTT TTATATAGGG AAAAATCATA TAGCTTGAAA   
  
  
+ GTAAAACTTT CATTGCCAGG ATTCCAAGGT AGGGGTAGGG TTGTCTACAC CTTGATCTCC CTAGAAGCAT   
  
  
+ GGCGATCCTT CCTCGGATTG TGTTATAGAA TAAAGAATGT GGTTCTGCTG TAATGAGACT TATGTTCTAT   
  
  
+ CAAATGATGA ATCTGTTTAG AACTTCTTGC TTATTTTTTC TGCAAAATTT TAGGAGACTT GATAAACTAT   
  
  
+ AGTTCTCTTG AATTTACAGG TGGATATTGG AGTTCACAGA ATAGTATTCA GATCAAAGTG CTTGTGCTTA   
  
  
+ ATTGGAAGGA CAACTTGCTT GAGCTGTTTA TAGAGCTCTA ACTGATGCTA TCTCATGGAC TCACATCAGT   
  
  
+ TTTTTGGATT TAATCTTACC AGTGTTGATC CGTCGTACAT TTCCGCTCAG TACAGTCCGC CGTCAGTGAC   
  
  
+ AAATAGGATG TTTGCATCAC TGAAGCTCGA CTCTAGAGGT TCTCCTGTGT CACCCTTCTC AACTCAGTTT   
  
  
+ GATTGTGATA CGGTTACTAC ATTGAGTGAT AGTCAAGAGC ATCACAGCTC GACGGGGAGT CTATCAACAA   
  
  
+ GAAGCCCTTC TTGTAATTCT CCCCTTGAAA CGAGCAGTTA TCATCATTTA TCGACGAATG GCCCGTCTTG   
  
  
+ TAATTCTGCC CCTGAAACTA GCAGTTATCG TCATCGGTTC AACGCGAGTC CCGTGGGATA TTCCCATCAA   
  
  
+ GGCACTGATT ATGGAGTGAA TATGAAGAGC GCTCTGCAGG AGCTAGAGAC TACTCTAATG GGTGCAGATG   
  
  
+ GTGAGGAAGT ATCTGCTGCT AATCAACCTA TGGGGGGAAG TCGTCAGTCC GGGATCCCAA GTCAGAGATC   
  
  
+ AAAATCATTG AGCGAAGATC CACAGGGTTC GCATCCTACT CAGCCTGATT CATCATCTCT TTCTAGGGCA   
  
  
+ AGAAGATCAG GAGATGAAAG CCAGAGAGAG AAACGGCACA AGGCAATGGA AGAACCAACG GAACTACCAA   
  
  
+ GTTTGCCACC TGGTGATTTG AAGCAGTTGC TAATTGAATG TGCGAGGGCT TTATCGGATA ACCGAATAGA   
  
  
+ TGACTTTGAG AGTTTGGTTA AACGGGCGAG GAAAGAGGTC TCTATCTCGG GTGAGCCTAT CCAACGTCTC   
  
  
+ GGTGCCTACA TGATCGAAGG GCTTGTGGCA AGGAAGCAGT CTTCGGGGAC TAGCATCTAT CGGGCTCTGA   
  
  
+ AGTGTAAAGA GCCTCTTGGA AAAGACTTGC TCTCCTACAT GCACATCCTT TATGAAATAT GCCCTTATCT   
  
  
+ CAAGTTTGGT TATATGGCTG CGAATGGAGC GATAGCTGAA GCTTGTAGAA ATGAGGATAA CATACATATT   
  
  
+ ATAGATTTCC AGATTGCACA GGGCACTCAG TGGGTTACTC TATTACAAGC CCTAGCAGCA AGACCTGGTG   
  
  
+ GGCCACCTAA GGTGCGAATT ACAGGCATTG ATGATCCTGT TTCTAAGTAT GCCCGTGGTG CTAGCTTGGA   
  
  
+ GGCTGTTGGG AAACGGTTAG CGTCTCTATC TGAAAAGTTC AAAATACCCG TCGAGTTCAA TGCGTTGCCC   
  
  
+ GTTTATGGAC CCGATGTCAG GCGGGAAATG CTGGATGTGA GGCCCGGGGA GGCTTTGGCC GTTAATTTTC   
  
  
+ CATTGCAGCT CCACCACACT CCTGACGAGA GTGTCGATGT GAACAACCCT AGGGATGGGC TTCTCAGAAT   
  
  
+ GGTGAAATCA CTTGGTCCTA AGGTAACCAC TTTGGTTGAG CAAGAATCAA ACACCAACAC TACCCCTTTC   
  
  
+ TTGACCCGGT TCATAGAGAC CCTTGACTAC TACTCAGCCA TGTTTGAGTC TATAGACGTG ACCATGCCAA   
  
  
+ GAGACCGGAA GGAGAGGATC AATGTTGAGC AGCATTGTTT GGCTAAGGAC ATTGTGAATA TCATAGCTTG   
  
  
+ CGAGGGCAAG GAGAGGGTGG AGCGTCATGA ACTTTTTGGG AAATGGAAGT CAAGGTTTAC CATGGCAGGG   
  
  
+ TTCAGGCAGT ACCCGTTGAG CTCATACGTA AACTCTGTGA TAAGAAGCCT ACTCCGGTGT TACTCCGAGC   
  
  
+ ATTATACTCT GATAGAGAAG GATGGTGCCA TGCTTCTGGG TTGGAAGGGC CGAATGCTGA TTTCAGCTTC   
  
  
+ GGCATGGCAT TG  

- +Up\_Stream \_Len000AGGAGA AAACCCGAAG AAAGAAAGAT AGGACAAAAA AAAACCCAGC TTTTCAACCT   
  
  
- CCAACTTAAC TTCGTAGGCT TAAGCTCGTC CTACAAAGAG ACGCATCACT AAGAAAGACC CAAACACTAA   
  
  
- AACTTAAGAC CCATTAGCCA ACAAAACGAT TAAAACTCCT GTCTCAAGGA AAAGACTTAA TTTTAAAGCA   
  
  
- AAAAAATAGC CCTTTTAGAA GTCATAAACT CTTTTTTCTT GCTTTAAACC TACTAAACGA CAGATCTAAA   
  
  
- ACGAAAGTAT AAGGACCCAC ACCTAACCAA ATAATTAACG TACACTCCTT CATGTTCGAA GACCAGTTAC   
  
  
- CAACCCAAAA AAAAAAAAAA AACACCCATA TTTCAACCTT TAACTAATGC TCAGTTTAAA GAGAAAGATC   
  
  
- TTCTCCCCCC CCCCCCCCCC AACCAAATTT TGAGGTATAA AAGAAACTTA CCTTTCGAAT TAAACGACTG   
  
  
- ATAGAACCAA TCATCGTATT CGAACTAGTC TTTTGTTTTG CGAGAGACGG GACGTTTTTG AAATAAACAG   
  
  
- AAAATTCCTT TTAAAACCTT AAACTCTCAC TTTTAAAACC AACACTTAGA ACAGAATGAT ACACCAAGGA   
  
  
- AGACTGTTTT CAAACTTAGA CTCATACTCT CAAGTAGTTA GAGAAAATGA AAGGATGAAA GAAATAACGA   
  
  
- ACGTTTAACA ACACTTGAAT GAACCTAAAA GACCATCTCC TCTCAAGACA GGACCTTAAA CCACCTTTCA   
  
  
- CTCTGTATGA AACCCTAAAT TAATCGGTAG TTAAACCAAA GTGTGGAATA GTCAAACCTG AAGACCACAC   
  
  
- AATAACATAC ACAAGAATCG AACCCTAATA AGAACATAGC CTTCTCTCCC CCCCCCCCCA CAAAGAAAAT   
  
  
- CGACTTACAA TCTTGTAAAA TTCCCTAGAT CTATAGATCT CTTCTCGGAT CCTTTACTTC GTAAAAAGGG   
  
  
- AATATTAGAC TGTGTTGAAA TACTTAACTA ATCCTTGTTT CTTGCTAATC AAGTCTTTCC AATGTTGAGG   
  
  
- AGATCCACAA TATGATTTCT CTACACTCAA TTATTCGTTA ACGAATGATT GACAAATCCG ACAAAGTAAC   
  
  
- GAACATCTCC GAAAAAAAAA CCCCCCCCCC CCCCCAAACC CCAAGTATTT ACTCTTCCAA CTAAACAACA   
  
  
- GACGAGACGA GAACTAAGCT ATCATTAAGA GAGTCTTTTC CCATTTTACT GTACCGGAAG GTAGCGGAGG   
  
  
- GTACTCCGAA AAACGGAGAC GAACACATAG AAAGTAGAGA ACCAAACCAC CCAACCCCCT CCCCTCCCCC   
  
  
- CCTTCCCCCA AAACTAAGCT ATCTAGTAGT GAAAGTCTTT ACCGGAGAGA CCGGTTACCA TTCCCCATTC   
  
  
- CAATCATGTA CACCTGAAAG GGTACACCTA AAAAGGTCTC AGGCAACACA CTAACAATAA CGACAACAAC   
  
  
- ATTGAAAGAC AGTGAGCCTT TTGTACTTAG GTATACGTTG TTCTTGTATT ATTAACACTT ACGCTGAAAT   
  
  
- AAATCCTTAC AGAATGTATC ATGCACGTGT AAGAAATTAA TAGACAAATA GACAACCAAA CAATAAAAGT   
  
  
- TCCCCTACCC CTCACCCACC TACGAATCAT ACATTAGAAA AATATATCCC TTTTTAGTAT ATCGAACTTT   
  
  
- CATTTTGAAA GTAACGGTCC TAAGGTTCCA TCCCCATCCC AACAGATGTG GAACTAGAGG GATCTTCGTA   
  
  
- CCGCTAGGAA GGAGCCTAAC ACAATATCTT ATTTCTTACA CCAAGACGAC ATTACTCTGA ATACAAGATA   
  
  
- GTTTACTACT TAGACAAATC TTGAAGAACG AATAAAAAAG ACGTTTTAAA ATCCTCTGAA CTATTTGATA   
  
  
- TCAAGAGAAC TTAAATGTCC ACCTATAACC TCAAGTGTCT TATCATAAGT CTAGTTTCAC GAACACGAAT   
  
  
- TAACCTTCCT GTTGAACGAA CTCGACAAAT ATCTCGAGAT TGACTACGAT AGAGTACCTG AGTGTAGTCA   
  
  
- AAAAACCTAA ATTAGAATGG TCACAACTAG GCAGCATGTA AAGGCGAGTC ATGTCAGGCG GCAGTCACTG   
  
  
- TTTATCCTAC AAACGTAGTG ACTTCGAGCT GAGATCTCCA AGAGGACACA GTGGGAAGAG TTGAGTCAAA   
  
  
- CTAACACTAT GCCAATGATG TAACTCACTA TCAGTTCTCG TAGTGTCGAG CTGCCCCTCA GATAGTTGTT   
  
  
- CTTCGGGAAG AACATTAAGA GGGGAACTTT GCTCGTCAAT AGTAGTAAAT AGCTGCTTAC CGGGCAGAAC   
  
  
- ATTAAGACGG GGACTTTGAT CGTCAATAGC AGTAGCCAAG TTGCGCTCAG GGCACCCTAT AAGGGTAGTT   
  
  
- CCGTGACTAA TACCTCACTT ATACTTCTCG CGAGACGTCC TCGATCTCTG ATGAGATTAC CCACGTCTAC   
  
  
- CACTCCTTCA TAGACGACGA TTAGTTGGAT ACCCCCCTTC AGCAGTCAGG CCCTAGGGTT CAGTCTCTAG   
  
  
- TTTTAGTAAC TCGCTTCTAG GTGTCCCAAG CGTAGGATGA GTCGGACTAA GTAGTAGAGA AAGATCCCGT   
  
  
- TCTTCTAGTC CTCTACTTTC GGTCTCTCTC TTTGCCGTGT TCCGTTACCT TCTTGGTTGC CTTGATGGTT   
  
  
- CAAACGGTGG ACCACTAAAC TTCGTCAACG ATTAACTTAC ACGCTCCCGA AATAGCCTAT TGGCTTATCT   
  
  
- ACTGAAACTC TCAAACCAAT TTGCCCGCTC CTTTCTCCAG AGATAGAGCC CACTCGGATA GGTTGCAGAG   
  
  
- CCACGGATGT ACTAGCTTCC CGAACACCGT TCCTTCGTCA GAAGCCCCTG ATCGTAGATA GCCCGAGACT   
  
  
- TCACATTTCT CGGAGAACCT TTTCTGAACG AGAGGATGTA CGTGTAGGAA ATACTTTATA CGGGAATAGA   
  
  
- GTTCAAACCA ATATACCGAC GCTTACCTCG CTATCGACTT CGAACATCTT TACTCCTATT GTATGTATAA   
  
  
- TATCTAAAGG TCTAACGTGT CCCGTGAGTC ACCCAATGAG ATAATGTTCG GGATCGTCGT TCTGGACCAC   
  
  
- CCGGTGGATT CCACGCTTAA TGTCCGTAAC TACTAGGACA AAGATTCATA CGGGCACCAC GATCGAACCT   
  
  
- CCGACAACCC TTTGCCAATC GCAGAGATAG ACTTTTCAAG TTTTATGGGC AGCTCAAGTT ACGCAACGGG   
  
  
- CAAATACCTG GGCTACAGTC CGCCCTTTAC GACCTACACT CCGGGCCCCT CCGAAACCGG CAATTAAAAG   
  
  
- GTAACGTCGA GGTGGTGTGA GGACTGCTCT CACAGCTACA CTTGTTGGGA TCCCTACCCG AAGAGTCTTA   
  
  
- CCACTTTAGT GAACCAGGAT TCCATTGGTG AAACCAACTC GTTCTTAGTT TGTGGTTGTG ATGGGGAAAG   
  
  
- AACTGGGCCA AGTATCTCTG GGAACTGATG ATGAGTCGGT ACAAACTCAG ATATCTGCAC TGGTACGGTT   
  
  
- CTCTGGCCTT CCTCTCCTAG TTACAACTCG TCGTAACAAA CCGATTCCTG TAACACTTAT AGTATCGAAC   
  
  
- GCTCCCGTTC CTCTCCCACC TCGCAGTACT TGAAAAACCC TTTACCTTCA GTTCCAAATG GTACCGTCCC   
  
  
- AAGTCCGTCA TGGGCAACTC GAGTATGCAT TTGAGACACT ATTCTTCGGA TGAGGCCACA ATGAGGCTCG   
  
  
- TAATATGAGA CTATCTCTTC CTACCACGGT ACGAAGACCC AACCTTCCCG GCTTACGACT AAAGTCGAAG   
  
  
- CCGTACCGTA AC

+     TATC-box

| Site Name | Organism | Position | Strand | Matrix score. | sequence | function |
| --- | --- | --- | --- | --- | --- | --- |
| TATC-box | Oryza sativa | 2368 | - | 7 | TATCCCA | cis-acting element involved in gibberellin-responsiveness |

>HU08G00014.1   
+ +Up\_Stream \_Len000TCCTCT TTTGGGCTTC TTTCTTTCTA TCCTGTTTTT TTTTGGGTCG AAAAGTTGGA   
  
  
+ GGTTGAATTG AAGCATCCGA ATTCGAGCAG GATGTTTCTC TGCGTAGTGA TTCTTTCTGG GTTTGTGATT   
  
  
+ TTGAATTCTG GGTAATCGGT TGTTTTGCTA ATTTTGAGGA CAGAGTTCCT TTTCTGAATT AAAATTTCGT   
  
  
+ TTTTTTATCG GGAAAATCTT CAGTATTTGA GAAAAAAGAA CGAAATTTGG ATGATTTGCT GTCTAGATTT   
  
  
+ TGCTTTCATA TTCCTGGGTG TGGATTGGTT TATTAATTGC ATGTGAGGAA GTACAAGCTT CTGGTCAATG   
  
  
+ GTTGGGTTTT TTTTTTTTTT TTGTGGGTAT AAAGTTGGAA ATTGATTACG AGTCAAATTT CTCTTTCTAG   
  
  
+ AAGAGGGGGG GGGGGGGGGG TTGGTTTAAA ACTCCATATT TTCTTTGAAT GGAAAGCTTA ATTTGCTGAC   
  
  
+ TATCTTGGTT AGTAGCATAA GCTTGATCAG AAAACAAAAC GCTCTCTGCC CTGCAAAAAC TTTATTTGTC   
  
  
+ TTTTAAGGAA AATTTTGGAA TTTGAGAGTG AAAATTTTGG TTGTGAATCT TGTCTTACTA TGTGGTTCCT   
  
  
+ TCTGACAAAA GTTTGAATCT GAGTATGAGA GTTCATCAAT CTCTTTTACT TTCCTACTTT CTTTATTGCT   
  
  
+ TGCAAATTGT TGTGAACTTA CTTGGATTTT CTGGTAGAGG AGAGTTCTGT CCTGGAATTT GGTGGAAAGT   
  
  
+ GAGACATACT TTGGGATTTA ATTAGCCATC AATTTGGTTT CACACCTTAT CAGTTTGGAC TTCTGGTGTG   
  
  
+ TTATTGTATG TGTTCTTAGC TTGGGATTAT TCTTGTATCG GAAGAGAGGG GGGGGGGGGT GTTTCTTTTA   
  
  
+ GCTGAATGTT AGAACATTTT AAGGGATCTA GATATCTAGA GAAGAGCCTA GGAAATGAAG CATTTTTCCC   
  
  
+ TTATAATCTG ACACAACTTT ATGAATTGAT TAGGAACAAA GAACGATTAG TTCAGAAAGG TTACAACTCC   
  
  
+ TCTAGGTGTT ATACTAAAGA GATGTGAGTT AATAAGCAAT TGCTTACTAA CTGTTTAGGC TGTTTCATTG   
  
  
+ CTTGTAGAGG CTTTTTTTTT GGGGGGGGGG GGGGGTTTGG GGTTCATAAA TGAGAAGGTT GATTTGTTGT   
  
  
+ CTGCTCTGCT CTTGATTCGA TAGTAATTCT CTCAGAAAAG GGTAAAATGA CATGGCCTTC CATCGCCTCC   
  
  
+ CATGAGGCTT TTTGCCTCTG CTTGTGTATC TTTCATCTCT TGGTTTGGTG GGTTGGGGGA GGGGAGGGGG   
  
  
+ GGAAGGGGGT TTTGATTCGA TAGATCATCA CTTTCAGAAA TGGCCTCTCT GGCCAATGGT AAGGGGTAAG   
  
  
+ GTTAGTACAT GTGGACTTTC CCATGTGGAT TTTTCCAGAG TCCGTTGTGT GATTGTTATT GCTGTTGTTG   
  
  
+ TAACTTTCTG TCACTCGGAA AACATGAATC CATATGCAAC AAGAACATAA TAATTGTGAA TGCGACTTTA   
  
  
+ TTTAGGAATG TCTTACATAG TACGTGCACA TTCTTTAATT ATCTGTTTAT CTGTTGGTTT GTTATTTTCA   
  
  
+ AGGGGATGGG GAGTGGGTGG ATGCTTAGTA TGTAATCTTT TTATATAGGG AAAAATCATA TAGCTTGAAA   
  
  
+ GTAAAACTTT CATTGCCAGG ATTCCAAGGT AGGGGTAGGG TTGTCTACAC CTTGATCTCC CTAGAAGCAT   
  
  
+ GGCGATCCTT CCTCGGATTG TGTTATAGAA TAAAGAATGT GGTTCTGCTG TAATGAGACT TATGTTCTAT   
  
  
+ CAAATGATGA ATCTGTTTAG AACTTCTTGC TTATTTTTTC TGCAAAATTT TAGGAGACTT GATAAACTAT   
  
  
+ AGTTCTCTTG AATTTACAGG TGGATATTGG AGTTCACAGA ATAGTATTCA GATCAAAGTG CTTGTGCTTA   
  
  
+ ATTGGAAGGA CAACTTGCTT GAGCTGTTTA TAGAGCTCTA ACTGATGCTA TCTCATGGAC TCACATCAGT   
  
  
+ TTTTTGGATT TAATCTTACC AGTGTTGATC CGTCGTACAT TTCCGCTCAG TACAGTCCGC CGTCAGTGAC   
  
  
+ AAATAGGATG TTTGCATCAC TGAAGCTCGA CTCTAGAGGT TCTCCTGTGT CACCCTTCTC AACTCAGTTT   
  
  
+ GATTGTGATA CGGTTACTAC ATTGAGTGAT AGTCAAGAGC ATCACAGCTC GACGGGGAGT CTATCAACAA   
  
  
+ GAAGCCCTTC TTGTAATTCT CCCCTTGAAA CGAGCAGTTA TCATCATTTA TCGACGAATG GCCCGTCTTG   
  
  
+ TAATTCTGCC CCTGAAACTA GCAGTTATCG TCATCGGTTC AACGCGAGTC CCGTGGGATA TTCCCATCAA   
  
  
+ GGCACTGATT ATGGAGTGAA TATGAAGAGC GCTCTGCAGG AGCTAGAGAC TACTCTAATG GGTGCAGATG   
  
  
+ GTGAGGAAGT ATCTGCTGCT AATCAACCTA TGGGGGGAAG TCGTCAGTCC GGGATCCCAA GTCAGAGATC   
  
  
+ AAAATCATTG AGCGAAGATC CACAGGGTTC GCATCCTACT CAGCCTGATT CATCATCTCT TTCTAGGGCA   
  
  
+ AGAAGATCAG GAGATGAAAG CCAGAGAGAG AAACGGCACA AGGCAATGGA AGAACCAACG GAACTACCAA   
  
  
+ GTTTGCCACC TGGTGATTTG AAGCAGTTGC TAATTGAATG TGCGAGGGCT TTATCGGATA ACCGAATAGA   
  
  
+ TGACTTTGAG AGTTTGGTTA AACGGGCGAG GAAAGAGGTC TCTATCTCGG GTGAGCCTAT CCAACGTCTC   
  
  
+ GGTGCCTACA TGATCGAAGG GCTTGTGGCA AGGAAGCAGT CTTCGGGGAC TAGCATCTAT CGGGCTCTGA   
  
  
+ AGTGTAAAGA GCCTCTTGGA AAAGACTTGC TCTCCTACAT GCACATCCTT TATGAAATAT GCCCTTATCT   
  
  
+ CAAGTTTGGT TATATGGCTG CGAATGGAGC GATAGCTGAA GCTTGTAGAA ATGAGGATAA CATACATATT   
  
  
+ ATAGATTTCC AGATTGCACA GGGCACTCAG TGGGTTACTC TATTACAAGC CCTAGCAGCA AGACCTGGTG   
  
  
+ GGCCACCTAA GGTGCGAATT ACAGGCATTG ATGATCCTGT TTCTAAGTAT GCCCGTGGTG CTAGCTTGGA   
  
  
+ GGCTGTTGGG AAACGGTTAG CGTCTCTATC TGAAAAGTTC AAAATACCCG TCGAGTTCAA TGCGTTGCCC   
  
  
+ GTTTATGGAC CCGATGTCAG GCGGGAAATG CTGGATGTGA GGCCCGGGGA GGCTTTGGCC GTTAATTTTC   
  
  
+ CATTGCAGCT CCACCACACT CCTGACGAGA GTGTCGATGT GAACAACCCT AGGGATGGGC TTCTCAGAAT   
  
  
+ GGTGAAATCA CTTGGTCCTA AGGTAACCAC TTTGGTTGAG CAAGAATCAA ACACCAACAC TACCCCTTTC   
  
  
+ TTGACCCGGT TCATAGAGAC CCTTGACTAC TACTCAGCCA TGTTTGAGTC TATAGACGTG ACCATGCCAA   
  
  
+ GAGACCGGAA GGAGAGGATC AATGTTGAGC AGCATTGTTT GGCTAAGGAC ATTGTGAATA TCATAGCTTG   
  
  
+ CGAGGGCAAG GAGAGGGTGG AGCGTCATGA ACTTTTTGGG AAATGGAAGT CAAGGTTTAC CATGGCAGGG   
  
  
+ TTCAGGCAGT ACCCGTTGAG CTCATACGTA AACTCTGTGA TAAGAAGCCT ACTCCGGTGT TACTCCGAGC   
  
  
+ ATTATACTCT GATAGAGAAG GATGGTGCCA TGCTTCTGGG TTGGAAGGGC CGAATGCTGA TTTCAGCTTC   
  
  
+ GGCATGGCAT TG  

- +Up\_Stream \_Len000AGGAGA AAACCCGAAG AAAGAAAGAT AGGACAAAAA AAAACCCAGC TTTTCAACCT   
  
  
- CCAACTTAAC TTCGTAGGCT TAAGCTCGTC CTACAAAGAG ACGCATCACT AAGAAAGACC CAAACACTAA   
  
  
- AACTTAAGAC CCATTAGCCA ACAAAACGAT TAAAACTCCT GTCTCAAGGA AAAGACTTAA TTTTAAAGCA   
  
  
- AAAAAATAGC CCTTTTAGAA GTCATAAACT CTTTTTTCTT GCTTTAAACC TACTAAACGA CAGATCTAAA   
  
  
- ACGAAAGTAT AAGGACCCAC ACCTAACCAA ATAATTAACG TACACTCCTT CATGTTCGAA GACCAGTTAC   
  
  
- CAACCCAAAA AAAAAAAAAA AACACCCATA TTTCAACCTT TAACTAATGC TCAGTTTAAA GAGAAAGATC   
  
  
- TTCTCCCCCC CCCCCCCCCC AACCAAATTT TGAGGTATAA AAGAAACTTA CCTTTCGAAT TAAACGACTG   
  
  
- ATAGAACCAA TCATCGTATT CGAACTAGTC TTTTGTTTTG CGAGAGACGG GACGTTTTTG AAATAAACAG   
  
  
- AAAATTCCTT TTAAAACCTT AAACTCTCAC TTTTAAAACC AACACTTAGA ACAGAATGAT ACACCAAGGA   
  
  
- AGACTGTTTT CAAACTTAGA CTCATACTCT CAAGTAGTTA GAGAAAATGA AAGGATGAAA GAAATAACGA   
  
  
- ACGTTTAACA ACACTTGAAT GAACCTAAAA GACCATCTCC TCTCAAGACA GGACCTTAAA CCACCTTTCA   
  
  
- CTCTGTATGA AACCCTAAAT TAATCGGTAG TTAAACCAAA GTGTGGAATA GTCAAACCTG AAGACCACAC   
  
  
- AATAACATAC ACAAGAATCG AACCCTAATA AGAACATAGC CTTCTCTCCC CCCCCCCCCA CAAAGAAAAT   
  
  
- CGACTTACAA TCTTGTAAAA TTCCCTAGAT CTATAGATCT CTTCTCGGAT CCTTTACTTC GTAAAAAGGG   
  
  
- AATATTAGAC TGTGTTGAAA TACTTAACTA ATCCTTGTTT CTTGCTAATC AAGTCTTTCC AATGTTGAGG   
  
  
- AGATCCACAA TATGATTTCT CTACACTCAA TTATTCGTTA ACGAATGATT GACAAATCCG ACAAAGTAAC   
  
  
- GAACATCTCC GAAAAAAAAA CCCCCCCCCC CCCCCAAACC CCAAGTATTT ACTCTTCCAA CTAAACAACA   
  
  
- GACGAGACGA GAACTAAGCT ATCATTAAGA GAGTCTTTTC CCATTTTACT GTACCGGAAG GTAGCGGAGG   
  
  
- GTACTCCGAA AAACGGAGAC GAACACATAG AAAGTAGAGA ACCAAACCAC CCAACCCCCT CCCCTCCCCC   
  
  
- CCTTCCCCCA AAACTAAGCT ATCTAGTAGT GAAAGTCTTT ACCGGAGAGA CCGGTTACCA TTCCCCATTC   
  
  
- CAATCATGTA CACCTGAAAG GGTACACCTA AAAAGGTCTC AGGCAACACA CTAACAATAA CGACAACAAC   
  
  
- ATTGAAAGAC AGTGAGCCTT TTGTACTTAG GTATACGTTG TTCTTGTATT ATTAACACTT ACGCTGAAAT   
  
  
- AAATCCTTAC AGAATGTATC ATGCACGTGT AAGAAATTAA TAGACAAATA GACAACCAAA CAATAAAAGT   
  
  
- TCCCCTACCC CTCACCCACC TACGAATCAT ACATTAGAAA AATATATCCC TTTTTAGTAT ATCGAACTTT   
  
  
- CATTTTGAAA GTAACGGTCC TAAGGTTCCA TCCCCATCCC AACAGATGTG GAACTAGAGG GATCTTCGTA   
  
  
- CCGCTAGGAA GGAGCCTAAC ACAATATCTT ATTTCTTACA CCAAGACGAC ATTACTCTGA ATACAAGATA   
  
  
- GTTTACTACT TAGACAAATC TTGAAGAACG AATAAAAAAG ACGTTTTAAA ATCCTCTGAA CTATTTGATA   
  
  
- TCAAGAGAAC TTAAATGTCC ACCTATAACC TCAAGTGTCT TATCATAAGT CTAGTTTCAC GAACACGAAT   
  
  
- TAACCTTCCT GTTGAACGAA CTCGACAAAT ATCTCGAGAT TGACTACGAT AGAGTACCTG AGTGTAGTCA   
  
  
- AAAAACCTAA ATTAGAATGG TCACAACTAG GCAGCATGTA AAGGCGAGTC ATGTCAGGCG GCAGTCACTG   
  
  
- TTTATCCTAC AAACGTAGTG ACTTCGAGCT GAGATCTCCA AGAGGACACA GTGGGAAGAG TTGAGTCAAA   
  
  
- CTAACACTAT GCCAATGATG TAACTCACTA TCAGTTCTCG TAGTGTCGAG CTGCCCCTCA GATAGTTGTT   
  
  
- CTTCGGGAAG AACATTAAGA GGGGAACTTT GCTCGTCAAT AGTAGTAAAT AGCTGCTTAC CGGGCAGAAC   
  
  
- ATTAAGACGG GGACTTTGAT CGTCAATAGC AGTAGCCAAG TTGCGCTCAG GGCACCCTAT AAGGGTAGTT   
  
  
- CCGTGACTAA TACCTCACTT ATACTTCTCG CGAGACGTCC TCGATCTCTG ATGAGATTAC CCACGTCTAC   
  
  
- CACTCCTTCA TAGACGACGA TTAGTTGGAT ACCCCCCTTC AGCAGTCAGG CCCTAGGGTT CAGTCTCTAG   
  
  
- TTTTAGTAAC TCGCTTCTAG GTGTCCCAAG CGTAGGATGA GTCGGACTAA GTAGTAGAGA AAGATCCCGT   
  
  
- TCTTCTAGTC CTCTACTTTC GGTCTCTCTC TTTGCCGTGT TCCGTTACCT TCTTGGTTGC CTTGATGGTT   
  
  
- CAAACGGTGG ACCACTAAAC TTCGTCAACG ATTAACTTAC ACGCTCCCGA AATAGCCTAT TGGCTTATCT   
  
  
- ACTGAAACTC TCAAACCAAT TTGCCCGCTC CTTTCTCCAG AGATAGAGCC CACTCGGATA GGTTGCAGAG   
  
  
- CCACGGATGT ACTAGCTTCC CGAACACCGT TCCTTCGTCA GAAGCCCCTG ATCGTAGATA GCCCGAGACT   
  
  
- TCACATTTCT CGGAGAACCT TTTCTGAACG AGAGGATGTA CGTGTAGGAA ATACTTTATA CGGGAATAGA   
  
  
- GTTCAAACCA ATATACCGAC GCTTACCTCG CTATCGACTT CGAACATCTT TACTCCTATT GTATGTATAA   
  
  
- TATCTAAAGG TCTAACGTGT CCCGTGAGTC ACCCAATGAG ATAATGTTCG GGATCGTCGT TCTGGACCAC   
  
  
- CCGGTGGATT CCACGCTTAA TGTCCGTAAC TACTAGGACA AAGATTCATA CGGGCACCAC GATCGAACCT   
  
  
- CCGACAACCC TTTGCCAATC GCAGAGATAG ACTTTTCAAG TTTTATGGGC AGCTCAAGTT ACGCAACGGG   
  
  
- CAAATACCTG GGCTACAGTC CGCCCTTTAC GACCTACACT CCGGGCCCCT CCGAAACCGG CAATTAAAAG   
  
  
- GTAACGTCGA GGTGGTGTGA GGACTGCTCT CACAGCTACA CTTGTTGGGA TCCCTACCCG AAGAGTCTTA   
  
  
- CCACTTTAGT GAACCAGGAT TCCATTGGTG AAACCAACTC GTTCTTAGTT TGTGGTTGTG ATGGGGAAAG   
  
  
- AACTGGGCCA AGTATCTCTG GGAACTGATG ATGAGTCGGT ACAAACTCAG ATATCTGCAC TGGTACGGTT   
  
  
- CTCTGGCCTT CCTCTCCTAG TTACAACTCG TCGTAACAAA CCGATTCCTG TAACACTTAT AGTATCGAAC   
  
  
- GCTCCCGTTC CTCTCCCACC TCGCAGTACT TGAAAAACCC TTTACCTTCA GTTCCAAATG GTACCGTCCC   
  
  
- AAGTCCGTCA TGGGCAACTC GAGTATGCAT TTGAGACACT ATTCTTCGGA TGAGGCCACA ATGAGGCTCG   
  
  
- TAATATGAGA CTATCTCTTC CTACCACGGT ACGAAGACCC AACCTTCCCG GCTTACGACT AAAGTCGAAG   
  
  
- CCGTACCGTA AC

+     TC-rich repeats

| Site Name | Organism | Position | Strand | Matrix score. | sequence | function |
| --- | --- | --- | --- | --- | --- | --- |
| TC-rich repeats | Nicotiana tabacum | 1776 | - | 9 | ATTCTCTAAC | cis-acting element involved in defense and stress responsiveness |

>HU08G00014.1   
+ +Up\_Stream \_Len000TCCTCT TTTGGGCTTC TTTCTTTCTA TCCTGTTTTT TTTTGGGTCG AAAAGTTGGA   
  
  
+ GGTTGAATTG AAGCATCCGA ATTCGAGCAG GATGTTTCTC TGCGTAGTGA TTCTTTCTGG GTTTGTGATT   
  
  
+ TTGAATTCTG GGTAATCGGT TGTTTTGCTA ATTTTGAGGA CAGAGTTCCT TTTCTGAATT AAAATTTCGT   
  
  
+ TTTTTTATCG GGAAAATCTT CAGTATTTGA GAAAAAAGAA CGAAATTTGG ATGATTTGCT GTCTAGATTT   
  
  
+ TGCTTTCATA TTCCTGGGTG TGGATTGGTT TATTAATTGC ATGTGAGGAA GTACAAGCTT CTGGTCAATG   
  
  
+ GTTGGGTTTT TTTTTTTTTT TTGTGGGTAT AAAGTTGGAA ATTGATTACG AGTCAAATTT CTCTTTCTAG   
  
  
+ AAGAGGGGGG GGGGGGGGGG TTGGTTTAAA ACTCCATATT TTCTTTGAAT GGAAAGCTTA ATTTGCTGAC   
  
  
+ TATCTTGGTT AGTAGCATAA GCTTGATCAG AAAACAAAAC GCTCTCTGCC CTGCAAAAAC TTTATTTGTC   
  
  
+ TTTTAAGGAA AATTTTGGAA TTTGAGAGTG AAAATTTTGG TTGTGAATCT TGTCTTACTA TGTGGTTCCT   
  
  
+ TCTGACAAAA GTTTGAATCT GAGTATGAGA GTTCATCAAT CTCTTTTACT TTCCTACTTT CTTTATTGCT   
  
  
+ TGCAAATTGT TGTGAACTTA CTTGGATTTT CTGGTAGAGG AGAGTTCTGT CCTGGAATTT GGTGGAAAGT   
  
  
+ GAGACATACT TTGGGATTTA ATTAGCCATC AATTTGGTTT CACACCTTAT CAGTTTGGAC TTCTGGTGTG   
  
  
+ TTATTGTATG TGTTCTTAGC TTGGGATTAT TCTTGTATCG GAAGAGAGGG GGGGGGGGGT GTTTCTTTTA   
  
  
+ GCTGAATGTT AGAACATTTT AAGGGATCTA GATATCTAGA GAAGAGCCTA GGAAATGAAG CATTTTTCCC   
  
  
+ TTATAATCTG ACACAACTTT ATGAATTGAT TAGGAACAAA GAACGATTAG TTCAGAAAGG TTACAACTCC   
  
  
+ TCTAGGTGTT ATACTAAAGA GATGTGAGTT AATAAGCAAT TGCTTACTAA CTGTTTAGGC TGTTTCATTG   
  
  
+ CTTGTAGAGG CTTTTTTTTT GGGGGGGGGG GGGGGTTTGG GGTTCATAAA TGAGAAGGTT GATTTGTTGT   
  
  
+ CTGCTCTGCT CTTGATTCGA TAGTAATTCT CTCAGAAAAG GGTAAAATGA CATGGCCTTC CATCGCCTCC   
  
  
+ CATGAGGCTT TTTGCCTCTG CTTGTGTATC TTTCATCTCT TGGTTTGGTG GGTTGGGGGA GGGGAGGGGG   
  
  
+ GGAAGGGGGT TTTGATTCGA TAGATCATCA CTTTCAGAAA TGGCCTCTCT GGCCAATGGT AAGGGGTAAG   
  
  
+ GTTAGTACAT GTGGACTTTC CCATGTGGAT TTTTCCAGAG TCCGTTGTGT GATTGTTATT GCTGTTGTTG   
  
  
+ TAACTTTCTG TCACTCGGAA AACATGAATC CATATGCAAC AAGAACATAA TAATTGTGAA TGCGACTTTA   
  
  
+ TTTAGGAATG TCTTACATAG TACGTGCACA TTCTTTAATT ATCTGTTTAT CTGTTGGTTT GTTATTTTCA   
  
  
+ AGGGGATGGG GAGTGGGTGG ATGCTTAGTA TGTAATCTTT TTATATAGGG AAAAATCATA TAGCTTGAAA   
  
  
+ GTAAAACTTT CATTGCCAGG ATTCCAAGGT AGGGGTAGGG TTGTCTACAC CTTGATCTCC CTAGAAGCAT   
  
  
+ GGCGATCCTT CCTCGGATTG TGTTATAGAA TAAAGAATGT GGTTCTGCTG TAATGAGACT TATGTTCTAT   
  
  
+ CAAATGATGA ATCTGTTTAG AACTTCTTGC TTATTTTTTC TGCAAAATTT TAGGAGACTT GATAAACTAT   
  
  
+ AGTTCTCTTG AATTTACAGG TGGATATTGG AGTTCACAGA ATAGTATTCA GATCAAAGTG CTTGTGCTTA   
  
  
+ ATTGGAAGGA CAACTTGCTT GAGCTGTTTA TAGAGCTCTA ACTGATGCTA TCTCATGGAC TCACATCAGT   
  
  
+ TTTTTGGATT TAATCTTACC AGTGTTGATC CGTCGTACAT TTCCGCTCAG TACAGTCCGC CGTCAGTGAC   
  
  
+ AAATAGGATG TTTGCATCAC TGAAGCTCGA CTCTAGAGGT TCTCCTGTGT CACCCTTCTC AACTCAGTTT   
  
  
+ GATTGTGATA CGGTTACTAC ATTGAGTGAT AGTCAAGAGC ATCACAGCTC GACGGGGAGT CTATCAACAA   
  
  
+ GAAGCCCTTC TTGTAATTCT CCCCTTGAAA CGAGCAGTTA TCATCATTTA TCGACGAATG GCCCGTCTTG   
  
  
+ TAATTCTGCC CCTGAAACTA GCAGTTATCG TCATCGGTTC AACGCGAGTC CCGTGGGATA TTCCCATCAA   
  
  
+ GGCACTGATT ATGGAGTGAA TATGAAGAGC GCTCTGCAGG AGCTAGAGAC TACTCTAATG GGTGCAGATG   
  
  
+ GTGAGGAAGT ATCTGCTGCT AATCAACCTA TGGGGGGAAG TCGTCAGTCC GGGATCCCAA GTCAGAGATC   
  
  
+ AAAATCATTG AGCGAAGATC CACAGGGTTC GCATCCTACT CAGCCTGATT CATCATCTCT TTCTAGGGCA   
  
  
+ AGAAGATCAG GAGATGAAAG CCAGAGAGAG AAACGGCACA AGGCAATGGA AGAACCAACG GAACTACCAA   
  
  
+ GTTTGCCACC TGGTGATTTG AAGCAGTTGC TAATTGAATG TGCGAGGGCT TTATCGGATA ACCGAATAGA   
  
  
+ TGACTTTGAG AGTTTGGTTA AACGGGCGAG GAAAGAGGTC TCTATCTCGG GTGAGCCTAT CCAACGTCTC   
  
  
+ GGTGCCTACA TGATCGAAGG GCTTGTGGCA AGGAAGCAGT CTTCGGGGAC TAGCATCTAT CGGGCTCTGA   
  
  
+ AGTGTAAAGA GCCTCTTGGA AAAGACTTGC TCTCCTACAT GCACATCCTT TATGAAATAT GCCCTTATCT   
  
  
+ CAAGTTTGGT TATATGGCTG CGAATGGAGC GATAGCTGAA GCTTGTAGAA ATGAGGATAA CATACATATT   
  
  
+ ATAGATTTCC AGATTGCACA GGGCACTCAG TGGGTTACTC TATTACAAGC CCTAGCAGCA AGACCTGGTG   
  
  
+ GGCCACCTAA GGTGCGAATT ACAGGCATTG ATGATCCTGT TTCTAAGTAT GCCCGTGGTG CTAGCTTGGA   
  
  
+ GGCTGTTGGG AAACGGTTAG CGTCTCTATC TGAAAAGTTC AAAATACCCG TCGAGTTCAA TGCGTTGCCC   
  
  
+ GTTTATGGAC CCGATGTCAG GCGGGAAATG CTGGATGTGA GGCCCGGGGA GGCTTTGGCC GTTAATTTTC   
  
  
+ CATTGCAGCT CCACCACACT CCTGACGAGA GTGTCGATGT GAACAACCCT AGGGATGGGC TTCTCAGAAT   
  
  
+ GGTGAAATCA CTTGGTCCTA AGGTAACCAC TTTGGTTGAG CAAGAATCAA ACACCAACAC TACCCCTTTC   
  
  
+ TTGACCCGGT TCATAGAGAC CCTTGACTAC TACTCAGCCA TGTTTGAGTC TATAGACGTG ACCATGCCAA   
  
  
+ GAGACCGGAA GGAGAGGATC AATGTTGAGC AGCATTGTTT GGCTAAGGAC ATTGTGAATA TCATAGCTTG   
  
  
+ CGAGGGCAAG GAGAGGGTGG AGCGTCATGA ACTTTTTGGG AAATGGAAGT CAAGGTTTAC CATGGCAGGG   
  
  
+ TTCAGGCAGT ACCCGTTGAG CTCATACGTA AACTCTGTGA TAAGAAGCCT ACTCCGGTGT TACTCCGAGC   
  
  
+ ATTATACTCT GATAGAGAAG GATGGTGCCA TGCTTCTGGG TTGGAAGGGC CGAATGCTGA TTTCAGCTTC   
  
  
+ GGCATGGCAT TG  

- +Up\_Stream \_Len000AGGAGA AAACCCGAAG AAAGAAAGAT AGGACAAAAA AAAACCCAGC TTTTCAACCT   
  
  
- CCAACTTAAC TTCGTAGGCT TAAGCTCGTC CTACAAAGAG ACGCATCACT AAGAAAGACC CAAACACTAA   
  
  
- AACTTAAGAC CCATTAGCCA ACAAAACGAT TAAAACTCCT GTCTCAAGGA AAAGACTTAA TTTTAAAGCA   
  
  
- AAAAAATAGC CCTTTTAGAA GTCATAAACT CTTTTTTCTT GCTTTAAACC TACTAAACGA CAGATCTAAA   
  
  
- ACGAAAGTAT AAGGACCCAC ACCTAACCAA ATAATTAACG TACACTCCTT CATGTTCGAA GACCAGTTAC   
  
  
- CAACCCAAAA AAAAAAAAAA AACACCCATA TTTCAACCTT TAACTAATGC TCAGTTTAAA GAGAAAGATC   
  
  
- TTCTCCCCCC CCCCCCCCCC AACCAAATTT TGAGGTATAA AAGAAACTTA CCTTTCGAAT TAAACGACTG   
  
  
- ATAGAACCAA TCATCGTATT CGAACTAGTC TTTTGTTTTG CGAGAGACGG GACGTTTTTG AAATAAACAG   
  
  
- AAAATTCCTT TTAAAACCTT AAACTCTCAC TTTTAAAACC AACACTTAGA ACAGAATGAT ACACCAAGGA   
  
  
- AGACTGTTTT CAAACTTAGA CTCATACTCT CAAGTAGTTA GAGAAAATGA AAGGATGAAA GAAATAACGA   
  
  
- ACGTTTAACA ACACTTGAAT GAACCTAAAA GACCATCTCC TCTCAAGACA GGACCTTAAA CCACCTTTCA   
  
  
- CTCTGTATGA AACCCTAAAT TAATCGGTAG TTAAACCAAA GTGTGGAATA GTCAAACCTG AAGACCACAC   
  
  
- AATAACATAC ACAAGAATCG AACCCTAATA AGAACATAGC CTTCTCTCCC CCCCCCCCCA CAAAGAAAAT   
  
  
- CGACTTACAA TCTTGTAAAA TTCCCTAGAT CTATAGATCT CTTCTCGGAT CCTTTACTTC GTAAAAAGGG   
  
  
- AATATTAGAC TGTGTTGAAA TACTTAACTA ATCCTTGTTT CTTGCTAATC AAGTCTTTCC AATGTTGAGG   
  
  
- AGATCCACAA TATGATTTCT CTACACTCAA TTATTCGTTA ACGAATGATT GACAAATCCG ACAAAGTAAC   
  
  
- GAACATCTCC GAAAAAAAAA CCCCCCCCCC CCCCCAAACC CCAAGTATTT ACTCTTCCAA CTAAACAACA   
  
  
- GACGAGACGA GAACTAAGCT ATCATTAAGA GAGTCTTTTC CCATTTTACT GTACCGGAAG GTAGCGGAGG   
  
  
- GTACTCCGAA AAACGGAGAC GAACACATAG AAAGTAGAGA ACCAAACCAC CCAACCCCCT CCCCTCCCCC   
  
  
- CCTTCCCCCA AAACTAAGCT ATCTAGTAGT GAAAGTCTTT ACCGGAGAGA CCGGTTACCA TTCCCCATTC   
  
  
- CAATCATGTA CACCTGAAAG GGTACACCTA AAAAGGTCTC AGGCAACACA CTAACAATAA CGACAACAAC   
  
  
- ATTGAAAGAC AGTGAGCCTT TTGTACTTAG GTATACGTTG TTCTTGTATT ATTAACACTT ACGCTGAAAT   
  
  
- AAATCCTTAC AGAATGTATC ATGCACGTGT AAGAAATTAA TAGACAAATA GACAACCAAA CAATAAAAGT   
  
  
- TCCCCTACCC CTCACCCACC TACGAATCAT ACATTAGAAA AATATATCCC TTTTTAGTAT ATCGAACTTT   
  
  
- CATTTTGAAA GTAACGGTCC TAAGGTTCCA TCCCCATCCC AACAGATGTG GAACTAGAGG GATCTTCGTA   
  
  
- CCGCTAGGAA GGAGCCTAAC ACAATATCTT ATTTCTTACA CCAAGACGAC ATTACTCTGA ATACAAGATA   
  
  
- GTTTACTACT TAGACAAATC TTGAAGAACG AATAAAAAAG ACGTTTTAAA ATCCTCTGAA CTATTTGATA   
  
  
- TCAAGAGAAC TTAAATGTCC ACCTATAACC TCAAGTGTCT TATCATAAGT CTAGTTTCAC GAACACGAAT   
  
  
- TAACCTTCCT GTTGAACGAA CTCGACAAAT ATCTCGAGAT TGACTACGAT AGAGTACCTG AGTGTAGTCA   
  
  
- AAAAACCTAA ATTAGAATGG TCACAACTAG GCAGCATGTA AAGGCGAGTC ATGTCAGGCG GCAGTCACTG   
  
  
- TTTATCCTAC AAACGTAGTG ACTTCGAGCT GAGATCTCCA AGAGGACACA GTGGGAAGAG TTGAGTCAAA   
  
  
- CTAACACTAT GCCAATGATG TAACTCACTA TCAGTTCTCG TAGTGTCGAG CTGCCCCTCA GATAGTTGTT   
  
  
- CTTCGGGAAG AACATTAAGA GGGGAACTTT GCTCGTCAAT AGTAGTAAAT AGCTGCTTAC CGGGCAGAAC   
  
  
- ATTAAGACGG GGACTTTGAT CGTCAATAGC AGTAGCCAAG TTGCGCTCAG GGCACCCTAT AAGGGTAGTT   
  
  
- CCGTGACTAA TACCTCACTT ATACTTCTCG CGAGACGTCC TCGATCTCTG ATGAGATTAC CCACGTCTAC   
  
  
- CACTCCTTCA TAGACGACGA TTAGTTGGAT ACCCCCCTTC AGCAGTCAGG CCCTAGGGTT CAGTCTCTAG   
  
  
- TTTTAGTAAC TCGCTTCTAG GTGTCCCAAG CGTAGGATGA GTCGGACTAA GTAGTAGAGA AAGATCCCGT   
  
  
- TCTTCTAGTC CTCTACTTTC GGTCTCTCTC TTTGCCGTGT TCCGTTACCT TCTTGGTTGC CTTGATGGTT   
  
  
- CAAACGGTGG ACCACTAAAC TTCGTCAACG ATTAACTTAC ACGCTCCCGA AATAGCCTAT TGGCTTATCT   
  
  
- ACTGAAACTC TCAAACCAAT TTGCCCGCTC CTTTCTCCAG AGATAGAGCC CACTCGGATA GGTTGCAGAG   
  
  
- CCACGGATGT ACTAGCTTCC CGAACACCGT TCCTTCGTCA GAAGCCCCTG ATCGTAGATA GCCCGAGACT   
  
  
- TCACATTTCT CGGAGAACCT TTTCTGAACG AGAGGATGTA CGTGTAGGAA ATACTTTATA CGGGAATAGA   
  
  
- GTTCAAACCA ATATACCGAC GCTTACCTCG CTATCGACTT CGAACATCTT TACTCCTATT GTATGTATAA   
  
  
- TATCTAAAGG TCTAACGTGT CCCGTGAGTC ACCCAATGAG ATAATGTTCG GGATCGTCGT TCTGGACCAC   
  
  
- CCGGTGGATT CCACGCTTAA TGTCCGTAAC TACTAGGACA AAGATTCATA CGGGCACCAC GATCGAACCT   
  
  
- CCGACAACCC TTTGCCAATC GCAGAGATAG ACTTTTCAAG TTTTATGGGC AGCTCAAGTT ACGCAACGGG   
  
  
- CAAATACCTG GGCTACAGTC CGCCCTTTAC GACCTACACT CCGGGCCCCT CCGAAACCGG CAATTAAAAG   
  
  
- GTAACGTCGA GGTGGTGTGA GGACTGCTCT CACAGCTACA CTTGTTGGGA TCCCTACCCG AAGAGTCTTA   
  
  
- CCACTTTAGT GAACCAGGAT TCCATTGGTG AAACCAACTC GTTCTTAGTT TGTGGTTGTG ATGGGGAAAG   
  
  
- AACTGGGCCA AGTATCTCTG GGAACTGATG ATGAGTCGGT ACAAACTCAG ATATCTGCAC TGGTACGGTT   
  
  
- CTCTGGCCTT CCTCTCCTAG TTACAACTCG TCGTAACAAA CCGATTCCTG TAACACTTAT AGTATCGAAC   
  
  
- GCTCCCGTTC CTCTCCCACC TCGCAGTACT TGAAAAACCC TTTACCTTCA GTTCCAAATG GTACCGTCCC   
  
  
- AAGTCCGTCA TGGGCAACTC GAGTATGCAT TTGAGACACT ATTCTTCGGA TGAGGCCACA ATGAGGCTCG   
  
  
- TAATATGAGA CTATCTCTTC CTACCACGGT ACGAAGACCC AACCTTCCCG GCTTACGACT AAAGTCGAAG   
  
  
- CCGTACCGTA AC

+     TCA-element

| Site Name | Organism | Position | Strand | Matrix score. | sequence | function |
| --- | --- | --- | --- | --- | --- | --- |
| TCA-element | Brassica oleracea | 192 | - | 9 | TCAGAAGAGG | cis-acting element involved in salicylic acid responsiveness |
| TCA-element | Brassica oleracea | 1226 | + | 9 | TCAGAAGAGG | cis-acting element involved in salicylic acid responsiveness |

>HU08G00014.1   
+ +Up\_Stream \_Len000TCCTCT TTTGGGCTTC TTTCTTTCTA TCCTGTTTTT TTTTGGGTCG AAAAGTTGGA   
  
  
+ GGTTGAATTG AAGCATCCGA ATTCGAGCAG GATGTTTCTC TGCGTAGTGA TTCTTTCTGG GTTTGTGATT   
  
  
+ TTGAATTCTG GGTAATCGGT TGTTTTGCTA ATTTTGAGGA CAGAGTTCCT TTTCTGAATT AAAATTTCGT   
  
  
+ TTTTTTATCG GGAAAATCTT CAGTATTTGA GAAAAAAGAA CGAAATTTGG ATGATTTGCT GTCTAGATTT   
  
  
+ TGCTTTCATA TTCCTGGGTG TGGATTGGTT TATTAATTGC ATGTGAGGAA GTACAAGCTT CTGGTCAATG   
  
  
+ GTTGGGTTTT TTTTTTTTTT TTGTGGGTAT AAAGTTGGAA ATTGATTACG AGTCAAATTT CTCTTTCTAG   
  
  
+ AAGAGGGGGG GGGGGGGGGG TTGGTTTAAA ACTCCATATT TTCTTTGAAT GGAAAGCTTA ATTTGCTGAC   
  
  
+ TATCTTGGTT AGTAGCATAA GCTTGATCAG AAAACAAAAC GCTCTCTGCC CTGCAAAAAC TTTATTTGTC   
  
  
+ TTTTAAGGAA AATTTTGGAA TTTGAGAGTG AAAATTTTGG TTGTGAATCT TGTCTTACTA TGTGGTTCCT   
  
  
+ TCTGACAAAA GTTTGAATCT GAGTATGAGA GTTCATCAAT CTCTTTTACT TTCCTACTTT CTTTATTGCT   
  
  
+ TGCAAATTGT TGTGAACTTA CTTGGATTTT CTGGTAGAGG AGAGTTCTGT CCTGGAATTT GGTGGAAAGT   
  
  
+ GAGACATACT TTGGGATTTA ATTAGCCATC AATTTGGTTT CACACCTTAT CAGTTTGGAC TTCTGGTGTG   
  
  
+ TTATTGTATG TGTTCTTAGC TTGGGATTAT TCTTGTATCG GAAGAGAGGG GGGGGGGGGT GTTTCTTTTA   
  
  
+ GCTGAATGTT AGAACATTTT AAGGGATCTA GATATCTAGA GAAGAGCCTA GGAAATGAAG CATTTTTCCC   
  
  
+ TTATAATCTG ACACAACTTT ATGAATTGAT TAGGAACAAA GAACGATTAG TTCAGAAAGG TTACAACTCC   
  
  
+ TCTAGGTGTT ATACTAAAGA GATGTGAGTT AATAAGCAAT TGCTTACTAA CTGTTTAGGC TGTTTCATTG   
  
  
+ CTTGTAGAGG CTTTTTTTTT GGGGGGGGGG GGGGGTTTGG GGTTCATAAA TGAGAAGGTT GATTTGTTGT   
  
  
+ CTGCTCTGCT CTTGATTCGA TAGTAATTCT CTCAGAAAAG GGTAAAATGA CATGGCCTTC CATCGCCTCC   
  
  
+ CATGAGGCTT TTTGCCTCTG CTTGTGTATC TTTCATCTCT TGGTTTGGTG GGTTGGGGGA GGGGAGGGGG   
  
  
+ GGAAGGGGGT TTTGATTCGA TAGATCATCA CTTTCAGAAA TGGCCTCTCT GGCCAATGGT AAGGGGTAAG   
  
  
+ GTTAGTACAT GTGGACTTTC CCATGTGGAT TTTTCCAGAG TCCGTTGTGT GATTGTTATT GCTGTTGTTG   
  
  
+ TAACTTTCTG TCACTCGGAA AACATGAATC CATATGCAAC AAGAACATAA TAATTGTGAA TGCGACTTTA   
  
  
+ TTTAGGAATG TCTTACATAG TACGTGCACA TTCTTTAATT ATCTGTTTAT CTGTTGGTTT GTTATTTTCA   
  
  
+ AGGGGATGGG GAGTGGGTGG ATGCTTAGTA TGTAATCTTT TTATATAGGG AAAAATCATA TAGCTTGAAA   
  
  
+ GTAAAACTTT CATTGCCAGG ATTCCAAGGT AGGGGTAGGG TTGTCTACAC CTTGATCTCC CTAGAAGCAT   
  
  
+ GGCGATCCTT CCTCGGATTG TGTTATAGAA TAAAGAATGT GGTTCTGCTG TAATGAGACT TATGTTCTAT   
  
  
+ CAAATGATGA ATCTGTTTAG AACTTCTTGC TTATTTTTTC TGCAAAATTT TAGGAGACTT GATAAACTAT   
  
  
+ AGTTCTCTTG AATTTACAGG TGGATATTGG AGTTCACAGA ATAGTATTCA GATCAAAGTG CTTGTGCTTA   
  
  
+ ATTGGAAGGA CAACTTGCTT GAGCTGTTTA TAGAGCTCTA ACTGATGCTA TCTCATGGAC TCACATCAGT   
  
  
+ TTTTTGGATT TAATCTTACC AGTGTTGATC CGTCGTACAT TTCCGCTCAG TACAGTCCGC CGTCAGTGAC   
  
  
+ AAATAGGATG TTTGCATCAC TGAAGCTCGA CTCTAGAGGT TCTCCTGTGT CACCCTTCTC AACTCAGTTT   
  
  
+ GATTGTGATA CGGTTACTAC ATTGAGTGAT AGTCAAGAGC ATCACAGCTC GACGGGGAGT CTATCAACAA   
  
  
+ GAAGCCCTTC TTGTAATTCT CCCCTTGAAA CGAGCAGTTA TCATCATTTA TCGACGAATG GCCCGTCTTG   
  
  
+ TAATTCTGCC CCTGAAACTA GCAGTTATCG TCATCGGTTC AACGCGAGTC CCGTGGGATA TTCCCATCAA   
  
  
+ GGCACTGATT ATGGAGTGAA TATGAAGAGC GCTCTGCAGG AGCTAGAGAC TACTCTAATG GGTGCAGATG   
  
  
+ GTGAGGAAGT ATCTGCTGCT AATCAACCTA TGGGGGGAAG TCGTCAGTCC GGGATCCCAA GTCAGAGATC   
  
  
+ AAAATCATTG AGCGAAGATC CACAGGGTTC GCATCCTACT CAGCCTGATT CATCATCTCT TTCTAGGGCA   
  
  
+ AGAAGATCAG GAGATGAAAG CCAGAGAGAG AAACGGCACA AGGCAATGGA AGAACCAACG GAACTACCAA   
  
  
+ GTTTGCCACC TGGTGATTTG AAGCAGTTGC TAATTGAATG TGCGAGGGCT TTATCGGATA ACCGAATAGA   
  
  
+ TGACTTTGAG AGTTTGGTTA AACGGGCGAG GAAAGAGGTC TCTATCTCGG GTGAGCCTAT CCAACGTCTC   
  
  
+ GGTGCCTACA TGATCGAAGG GCTTGTGGCA AGGAAGCAGT CTTCGGGGAC TAGCATCTAT CGGGCTCTGA   
  
  
+ AGTGTAAAGA GCCTCTTGGA AAAGACTTGC TCTCCTACAT GCACATCCTT TATGAAATAT GCCCTTATCT   
  
  
+ CAAGTTTGGT TATATGGCTG CGAATGGAGC GATAGCTGAA GCTTGTAGAA ATGAGGATAA CATACATATT   
  
  
+ ATAGATTTCC AGATTGCACA GGGCACTCAG TGGGTTACTC TATTACAAGC CCTAGCAGCA AGACCTGGTG   
  
  
+ GGCCACCTAA GGTGCGAATT ACAGGCATTG ATGATCCTGT TTCTAAGTAT GCCCGTGGTG CTAGCTTGGA   
  
  
+ GGCTGTTGGG AAACGGTTAG CGTCTCTATC TGAAAAGTTC AAAATACCCG TCGAGTTCAA TGCGTTGCCC   
  
  
+ GTTTATGGAC CCGATGTCAG GCGGGAAATG CTGGATGTGA GGCCCGGGGA GGCTTTGGCC GTTAATTTTC   
  
  
+ CATTGCAGCT CCACCACACT CCTGACGAGA GTGTCGATGT GAACAACCCT AGGGATGGGC TTCTCAGAAT   
  
  
+ GGTGAAATCA CTTGGTCCTA AGGTAACCAC TTTGGTTGAG CAAGAATCAA ACACCAACAC TACCCCTTTC   
  
  
+ TTGACCCGGT TCATAGAGAC CCTTGACTAC TACTCAGCCA TGTTTGAGTC TATAGACGTG ACCATGCCAA   
  
  
+ GAGACCGGAA GGAGAGGATC AATGTTGAGC AGCATTGTTT GGCTAAGGAC ATTGTGAATA TCATAGCTTG   
  
  
+ CGAGGGCAAG GAGAGGGTGG AGCGTCATGA ACTTTTTGGG AAATGGAAGT CAAGGTTTAC CATGGCAGGG   
  
  
+ TTCAGGCAGT ACCCGTTGAG CTCATACGTA AACTCTGTGA TAAGAAGCCT ACTCCGGTGT TACTCCGAGC   
  
  
+ ATTATACTCT GATAGAGAAG GATGGTGCCA TGCTTCTGGG TTGGAAGGGC CGAATGCTGA TTTCAGCTTC   
  
  
+ GGCATGGCAT TG  

- +Up\_Stream \_Len000AGGAGA AAACCCGAAG AAAGAAAGAT AGGACAAAAA AAAACCCAGC TTTTCAACCT   
  
  
- CCAACTTAAC TTCGTAGGCT TAAGCTCGTC CTACAAAGAG ACGCATCACT AAGAAAGACC CAAACACTAA   
  
  
- AACTTAAGAC CCATTAGCCA ACAAAACGAT TAAAACTCCT GTCTCAAGGA AAAGACTTAA TTTTAAAGCA   
  
  
- AAAAAATAGC CCTTTTAGAA GTCATAAACT CTTTTTTCTT GCTTTAAACC TACTAAACGA CAGATCTAAA   
  
  
- ACGAAAGTAT AAGGACCCAC ACCTAACCAA ATAATTAACG TACACTCCTT CATGTTCGAA GACCAGTTAC   
  
  
- CAACCCAAAA AAAAAAAAAA AACACCCATA TTTCAACCTT TAACTAATGC TCAGTTTAAA GAGAAAGATC   
  
  
- TTCTCCCCCC CCCCCCCCCC AACCAAATTT TGAGGTATAA AAGAAACTTA CCTTTCGAAT TAAACGACTG   
  
  
- ATAGAACCAA TCATCGTATT CGAACTAGTC TTTTGTTTTG CGAGAGACGG GACGTTTTTG AAATAAACAG   
  
  
- AAAATTCCTT TTAAAACCTT AAACTCTCAC TTTTAAAACC AACACTTAGA ACAGAATGAT ACACCAAGGA   
  
  
- AGACTGTTTT CAAACTTAGA CTCATACTCT CAAGTAGTTA GAGAAAATGA AAGGATGAAA GAAATAACGA   
  
  
- ACGTTTAACA ACACTTGAAT GAACCTAAAA GACCATCTCC TCTCAAGACA GGACCTTAAA CCACCTTTCA   
  
  
- CTCTGTATGA AACCCTAAAT TAATCGGTAG TTAAACCAAA GTGTGGAATA GTCAAACCTG AAGACCACAC   
  
  
- AATAACATAC ACAAGAATCG AACCCTAATA AGAACATAGC CTTCTCTCCC CCCCCCCCCA CAAAGAAAAT   
  
  
- CGACTTACAA TCTTGTAAAA TTCCCTAGAT CTATAGATCT CTTCTCGGAT CCTTTACTTC GTAAAAAGGG   
  
  
- AATATTAGAC TGTGTTGAAA TACTTAACTA ATCCTTGTTT CTTGCTAATC AAGTCTTTCC AATGTTGAGG   
  
  
- AGATCCACAA TATGATTTCT CTACACTCAA TTATTCGTTA ACGAATGATT GACAAATCCG ACAAAGTAAC   
  
  
- GAACATCTCC GAAAAAAAAA CCCCCCCCCC CCCCCAAACC CCAAGTATTT ACTCTTCCAA CTAAACAACA   
  
  
- GACGAGACGA GAACTAAGCT ATCATTAAGA GAGTCTTTTC CCATTTTACT GTACCGGAAG GTAGCGGAGG   
  
  
- GTACTCCGAA AAACGGAGAC GAACACATAG AAAGTAGAGA ACCAAACCAC CCAACCCCCT CCCCTCCCCC   
  
  
- CCTTCCCCCA AAACTAAGCT ATCTAGTAGT GAAAGTCTTT ACCGGAGAGA CCGGTTACCA TTCCCCATTC   
  
  
- CAATCATGTA CACCTGAAAG GGTACACCTA AAAAGGTCTC AGGCAACACA CTAACAATAA CGACAACAAC   
  
  
- ATTGAAAGAC AGTGAGCCTT TTGTACTTAG GTATACGTTG TTCTTGTATT ATTAACACTT ACGCTGAAAT   
  
  
- AAATCCTTAC AGAATGTATC ATGCACGTGT AAGAAATTAA TAGACAAATA GACAACCAAA CAATAAAAGT   
  
  
- TCCCCTACCC CTCACCCACC TACGAATCAT ACATTAGAAA AATATATCCC TTTTTAGTAT ATCGAACTTT   
  
  
- CATTTTGAAA GTAACGGTCC TAAGGTTCCA TCCCCATCCC AACAGATGTG GAACTAGAGG GATCTTCGTA   
  
  
- CCGCTAGGAA GGAGCCTAAC ACAATATCTT ATTTCTTACA CCAAGACGAC ATTACTCTGA ATACAAGATA   
  
  
- GTTTACTACT TAGACAAATC TTGAAGAACG AATAAAAAAG ACGTTTTAAA ATCCTCTGAA CTATTTGATA   
  
  
- TCAAGAGAAC TTAAATGTCC ACCTATAACC TCAAGTGTCT TATCATAAGT CTAGTTTCAC GAACACGAAT   
  
  
- TAACCTTCCT GTTGAACGAA CTCGACAAAT ATCTCGAGAT TGACTACGAT AGAGTACCTG AGTGTAGTCA   
  
  
- AAAAACCTAA ATTAGAATGG TCACAACTAG GCAGCATGTA AAGGCGAGTC ATGTCAGGCG GCAGTCACTG   
  
  
- TTTATCCTAC AAACGTAGTG ACTTCGAGCT GAGATCTCCA AGAGGACACA GTGGGAAGAG TTGAGTCAAA   
  
  
- CTAACACTAT GCCAATGATG TAACTCACTA TCAGTTCTCG TAGTGTCGAG CTGCCCCTCA GATAGTTGTT   
  
  
- CTTCGGGAAG AACATTAAGA GGGGAACTTT GCTCGTCAAT AGTAGTAAAT AGCTGCTTAC CGGGCAGAAC   
  
  
- ATTAAGACGG GGACTTTGAT CGTCAATAGC AGTAGCCAAG TTGCGCTCAG GGCACCCTAT AAGGGTAGTT   
  
  
- CCGTGACTAA TACCTCACTT ATACTTCTCG CGAGACGTCC TCGATCTCTG ATGAGATTAC CCACGTCTAC   
  
  
- CACTCCTTCA TAGACGACGA TTAGTTGGAT ACCCCCCTTC AGCAGTCAGG CCCTAGGGTT CAGTCTCTAG   
  
  
- TTTTAGTAAC TCGCTTCTAG GTGTCCCAAG CGTAGGATGA GTCGGACTAA GTAGTAGAGA AAGATCCCGT   
  
  
- TCTTCTAGTC CTCTACTTTC GGTCTCTCTC TTTGCCGTGT TCCGTTACCT TCTTGGTTGC CTTGATGGTT   
  
  
- CAAACGGTGG ACCACTAAAC TTCGTCAACG ATTAACTTAC ACGCTCCCGA AATAGCCTAT TGGCTTATCT   
  
  
- ACTGAAACTC TCAAACCAAT TTGCCCGCTC CTTTCTCCAG AGATAGAGCC CACTCGGATA GGTTGCAGAG   
  
  
- CCACGGATGT ACTAGCTTCC CGAACACCGT TCCTTCGTCA GAAGCCCCTG ATCGTAGATA GCCCGAGACT   
  
  
- TCACATTTCT CGGAGAACCT TTTCTGAACG AGAGGATGTA CGTGTAGGAA ATACTTTATA CGGGAATAGA   
  
  
- GTTCAAACCA ATATACCGAC GCTTACCTCG CTATCGACTT CGAACATCTT TACTCCTATT GTATGTATAA   
  
  
- TATCTAAAGG TCTAACGTGT CCCGTGAGTC ACCCAATGAG ATAATGTTCG GGATCGTCGT TCTGGACCAC   
  
  
- CCGGTGGATT CCACGCTTAA TGTCCGTAAC TACTAGGACA AAGATTCATA CGGGCACCAC GATCGAACCT   
  
  
- CCGACAACCC TTTGCCAATC GCAGAGATAG ACTTTTCAAG TTTTATGGGC AGCTCAAGTT ACGCAACGGG   
  
  
- CAAATACCTG GGCTACAGTC CGCCCTTTAC GACCTACACT CCGGGCCCCT CCGAAACCGG CAATTAAAAG   
  
  
- GTAACGTCGA GGTGGTGTGA GGACTGCTCT CACAGCTACA CTTGTTGGGA TCCCTACCCG AAGAGTCTTA   
  
  
- CCACTTTAGT GAACCAGGAT TCCATTGGTG AAACCAACTC GTTCTTAGTT TGTGGTTGTG ATGGGGAAAG   
  
  
- AACTGGGCCA AGTATCTCTG GGAACTGATG ATGAGTCGGT ACAAACTCAG ATATCTGCAC TGGTACGGTT   
  
  
- CTCTGGCCTT CCTCTCCTAG TTACAACTCG TCGTAACAAA CCGATTCCTG TAACACTTAT AGTATCGAAC   
  
  
- GCTCCCGTTC CTCTCCCACC TCGCAGTACT TGAAAAACCC TTTACCTTCA GTTCCAAATG GTACCGTCCC   
  
  
- AAGTCCGTCA TGGGCAACTC GAGTATGCAT TTGAGACACT ATTCTTCGGA TGAGGCCACA ATGAGGCTCG   
  
  
- TAATATGAGA CTATCTCTTC CTACCACGGT ACGAAGACCC AACCTTCCCG GCTTACGACT AAAGTCGAAG   
  
  
- CCGTACCGTA AC

+     TCCC-motif

| Site Name | Organism | Position | Strand | Matrix score. | sequence | function |
| --- | --- | --- | --- | --- | --- | --- |
| TCCC-motif | Spinacia oleracea | 1740 | + | 7 | TCTCCCT | part of a light responsive element |

>HU08G00014.1   
+ +Up\_Stream \_Len000TCCTCT TTTGGGCTTC TTTCTTTCTA TCCTGTTTTT TTTTGGGTCG AAAAGTTGGA   
  
  
+ GGTTGAATTG AAGCATCCGA ATTCGAGCAG GATGTTTCTC TGCGTAGTGA TTCTTTCTGG GTTTGTGATT   
  
  
+ TTGAATTCTG GGTAATCGGT TGTTTTGCTA ATTTTGAGGA CAGAGTTCCT TTTCTGAATT AAAATTTCGT   
  
  
+ TTTTTTATCG GGAAAATCTT CAGTATTTGA GAAAAAAGAA CGAAATTTGG ATGATTTGCT GTCTAGATTT   
  
  
+ TGCTTTCATA TTCCTGGGTG TGGATTGGTT TATTAATTGC ATGTGAGGAA GTACAAGCTT CTGGTCAATG   
  
  
+ GTTGGGTTTT TTTTTTTTTT TTGTGGGTAT AAAGTTGGAA ATTGATTACG AGTCAAATTT CTCTTTCTAG   
  
  
+ AAGAGGGGGG GGGGGGGGGG TTGGTTTAAA ACTCCATATT TTCTTTGAAT GGAAAGCTTA ATTTGCTGAC   
  
  
+ TATCTTGGTT AGTAGCATAA GCTTGATCAG AAAACAAAAC GCTCTCTGCC CTGCAAAAAC TTTATTTGTC   
  
  
+ TTTTAAGGAA AATTTTGGAA TTTGAGAGTG AAAATTTTGG TTGTGAATCT TGTCTTACTA TGTGGTTCCT   
  
  
+ TCTGACAAAA GTTTGAATCT GAGTATGAGA GTTCATCAAT CTCTTTTACT TTCCTACTTT CTTTATTGCT   
  
  
+ TGCAAATTGT TGTGAACTTA CTTGGATTTT CTGGTAGAGG AGAGTTCTGT CCTGGAATTT GGTGGAAAGT   
  
  
+ GAGACATACT TTGGGATTTA ATTAGCCATC AATTTGGTTT CACACCTTAT CAGTTTGGAC TTCTGGTGTG   
  
  
+ TTATTGTATG TGTTCTTAGC TTGGGATTAT TCTTGTATCG GAAGAGAGGG GGGGGGGGGT GTTTCTTTTA   
  
  
+ GCTGAATGTT AGAACATTTT AAGGGATCTA GATATCTAGA GAAGAGCCTA GGAAATGAAG CATTTTTCCC   
  
  
+ TTATAATCTG ACACAACTTT ATGAATTGAT TAGGAACAAA GAACGATTAG TTCAGAAAGG TTACAACTCC   
  
  
+ TCTAGGTGTT ATACTAAAGA GATGTGAGTT AATAAGCAAT TGCTTACTAA CTGTTTAGGC TGTTTCATTG   
  
  
+ CTTGTAGAGG CTTTTTTTTT GGGGGGGGGG GGGGGTTTGG GGTTCATAAA TGAGAAGGTT GATTTGTTGT   
  
  
+ CTGCTCTGCT CTTGATTCGA TAGTAATTCT CTCAGAAAAG GGTAAAATGA CATGGCCTTC CATCGCCTCC   
  
  
+ CATGAGGCTT TTTGCCTCTG CTTGTGTATC TTTCATCTCT TGGTTTGGTG GGTTGGGGGA GGGGAGGGGG   
  
  
+ GGAAGGGGGT TTTGATTCGA TAGATCATCA CTTTCAGAAA TGGCCTCTCT GGCCAATGGT AAGGGGTAAG   
  
  
+ GTTAGTACAT GTGGACTTTC CCATGTGGAT TTTTCCAGAG TCCGTTGTGT GATTGTTATT GCTGTTGTTG   
  
  
+ TAACTTTCTG TCACTCGGAA AACATGAATC CATATGCAAC AAGAACATAA TAATTGTGAA TGCGACTTTA   
  
  
+ TTTAGGAATG TCTTACATAG TACGTGCACA TTCTTTAATT ATCTGTTTAT CTGTTGGTTT GTTATTTTCA   
  
  
+ AGGGGATGGG GAGTGGGTGG ATGCTTAGTA TGTAATCTTT TTATATAGGG AAAAATCATA TAGCTTGAAA   
  
  
+ GTAAAACTTT CATTGCCAGG ATTCCAAGGT AGGGGTAGGG TTGTCTACAC CTTGATCTCC CTAGAAGCAT   
  
  
+ GGCGATCCTT CCTCGGATTG TGTTATAGAA TAAAGAATGT GGTTCTGCTG TAATGAGACT TATGTTCTAT   
  
  
+ CAAATGATGA ATCTGTTTAG AACTTCTTGC TTATTTTTTC TGCAAAATTT TAGGAGACTT GATAAACTAT   
  
  
+ AGTTCTCTTG AATTTACAGG TGGATATTGG AGTTCACAGA ATAGTATTCA GATCAAAGTG CTTGTGCTTA   
  
  
+ ATTGGAAGGA CAACTTGCTT GAGCTGTTTA TAGAGCTCTA ACTGATGCTA TCTCATGGAC TCACATCAGT   
  
  
+ TTTTTGGATT TAATCTTACC AGTGTTGATC CGTCGTACAT TTCCGCTCAG TACAGTCCGC CGTCAGTGAC   
  
  
+ AAATAGGATG TTTGCATCAC TGAAGCTCGA CTCTAGAGGT TCTCCTGTGT CACCCTTCTC AACTCAGTTT   
  
  
+ GATTGTGATA CGGTTACTAC ATTGAGTGAT AGTCAAGAGC ATCACAGCTC GACGGGGAGT CTATCAACAA   
  
  
+ GAAGCCCTTC TTGTAATTCT CCCCTTGAAA CGAGCAGTTA TCATCATTTA TCGACGAATG GCCCGTCTTG   
  
  
+ TAATTCTGCC CCTGAAACTA GCAGTTATCG TCATCGGTTC AACGCGAGTC CCGTGGGATA TTCCCATCAA   
  
  
+ GGCACTGATT ATGGAGTGAA TATGAAGAGC GCTCTGCAGG AGCTAGAGAC TACTCTAATG GGTGCAGATG   
  
  
+ GTGAGGAAGT ATCTGCTGCT AATCAACCTA TGGGGGGAAG TCGTCAGTCC GGGATCCCAA GTCAGAGATC   
  
  
+ AAAATCATTG AGCGAAGATC CACAGGGTTC GCATCCTACT CAGCCTGATT CATCATCTCT TTCTAGGGCA   
  
  
+ AGAAGATCAG GAGATGAAAG CCAGAGAGAG AAACGGCACA AGGCAATGGA AGAACCAACG GAACTACCAA   
  
  
+ GTTTGCCACC TGGTGATTTG AAGCAGTTGC TAATTGAATG TGCGAGGGCT TTATCGGATA ACCGAATAGA   
  
  
+ TGACTTTGAG AGTTTGGTTA AACGGGCGAG GAAAGAGGTC TCTATCTCGG GTGAGCCTAT CCAACGTCTC   
  
  
+ GGTGCCTACA TGATCGAAGG GCTTGTGGCA AGGAAGCAGT CTTCGGGGAC TAGCATCTAT CGGGCTCTGA   
  
  
+ AGTGTAAAGA GCCTCTTGGA AAAGACTTGC TCTCCTACAT GCACATCCTT TATGAAATAT GCCCTTATCT   
  
  
+ CAAGTTTGGT TATATGGCTG CGAATGGAGC GATAGCTGAA GCTTGTAGAA ATGAGGATAA CATACATATT   
  
  
+ ATAGATTTCC AGATTGCACA GGGCACTCAG TGGGTTACTC TATTACAAGC CCTAGCAGCA AGACCTGGTG   
  
  
+ GGCCACCTAA GGTGCGAATT ACAGGCATTG ATGATCCTGT TTCTAAGTAT GCCCGTGGTG CTAGCTTGGA   
  
  
+ GGCTGTTGGG AAACGGTTAG CGTCTCTATC TGAAAAGTTC AAAATACCCG TCGAGTTCAA TGCGTTGCCC   
  
  
+ GTTTATGGAC CCGATGTCAG GCGGGAAATG CTGGATGTGA GGCCCGGGGA GGCTTTGGCC GTTAATTTTC   
  
  
+ CATTGCAGCT CCACCACACT CCTGACGAGA GTGTCGATGT GAACAACCCT AGGGATGGGC TTCTCAGAAT   
  
  
+ GGTGAAATCA CTTGGTCCTA AGGTAACCAC TTTGGTTGAG CAAGAATCAA ACACCAACAC TACCCCTTTC   
  
  
+ TTGACCCGGT TCATAGAGAC CCTTGACTAC TACTCAGCCA TGTTTGAGTC TATAGACGTG ACCATGCCAA   
  
  
+ GAGACCGGAA GGAGAGGATC AATGTTGAGC AGCATTGTTT GGCTAAGGAC ATTGTGAATA TCATAGCTTG   
  
  
+ CGAGGGCAAG GAGAGGGTGG AGCGTCATGA ACTTTTTGGG AAATGGAAGT CAAGGTTTAC CATGGCAGGG   
  
  
+ TTCAGGCAGT ACCCGTTGAG CTCATACGTA AACTCTGTGA TAAGAAGCCT ACTCCGGTGT TACTCCGAGC   
  
  
+ ATTATACTCT GATAGAGAAG GATGGTGCCA TGCTTCTGGG TTGGAAGGGC CGAATGCTGA TTTCAGCTTC   
  
  
+ GGCATGGCAT TG  

- +Up\_Stream \_Len000AGGAGA AAACCCGAAG AAAGAAAGAT AGGACAAAAA AAAACCCAGC TTTTCAACCT   
  
  
- CCAACTTAAC TTCGTAGGCT TAAGCTCGTC CTACAAAGAG ACGCATCACT AAGAAAGACC CAAACACTAA   
  
  
- AACTTAAGAC CCATTAGCCA ACAAAACGAT TAAAACTCCT GTCTCAAGGA AAAGACTTAA TTTTAAAGCA   
  
  
- AAAAAATAGC CCTTTTAGAA GTCATAAACT CTTTTTTCTT GCTTTAAACC TACTAAACGA CAGATCTAAA   
  
  
- ACGAAAGTAT AAGGACCCAC ACCTAACCAA ATAATTAACG TACACTCCTT CATGTTCGAA GACCAGTTAC   
  
  
- CAACCCAAAA AAAAAAAAAA AACACCCATA TTTCAACCTT TAACTAATGC TCAGTTTAAA GAGAAAGATC   
  
  
- TTCTCCCCCC CCCCCCCCCC AACCAAATTT TGAGGTATAA AAGAAACTTA CCTTTCGAAT TAAACGACTG   
  
  
- ATAGAACCAA TCATCGTATT CGAACTAGTC TTTTGTTTTG CGAGAGACGG GACGTTTTTG AAATAAACAG   
  
  
- AAAATTCCTT TTAAAACCTT AAACTCTCAC TTTTAAAACC AACACTTAGA ACAGAATGAT ACACCAAGGA   
  
  
- AGACTGTTTT CAAACTTAGA CTCATACTCT CAAGTAGTTA GAGAAAATGA AAGGATGAAA GAAATAACGA   
  
  
- ACGTTTAACA ACACTTGAAT GAACCTAAAA GACCATCTCC TCTCAAGACA GGACCTTAAA CCACCTTTCA   
  
  
- CTCTGTATGA AACCCTAAAT TAATCGGTAG TTAAACCAAA GTGTGGAATA GTCAAACCTG AAGACCACAC   
  
  
- AATAACATAC ACAAGAATCG AACCCTAATA AGAACATAGC CTTCTCTCCC CCCCCCCCCA CAAAGAAAAT   
  
  
- CGACTTACAA TCTTGTAAAA TTCCCTAGAT CTATAGATCT CTTCTCGGAT CCTTTACTTC GTAAAAAGGG   
  
  
- AATATTAGAC TGTGTTGAAA TACTTAACTA ATCCTTGTTT CTTGCTAATC AAGTCTTTCC AATGTTGAGG   
  
  
- AGATCCACAA TATGATTTCT CTACACTCAA TTATTCGTTA ACGAATGATT GACAAATCCG ACAAAGTAAC   
  
  
- GAACATCTCC GAAAAAAAAA CCCCCCCCCC CCCCCAAACC CCAAGTATTT ACTCTTCCAA CTAAACAACA   
  
  
- GACGAGACGA GAACTAAGCT ATCATTAAGA GAGTCTTTTC CCATTTTACT GTACCGGAAG GTAGCGGAGG   
  
  
- GTACTCCGAA AAACGGAGAC GAACACATAG AAAGTAGAGA ACCAAACCAC CCAACCCCCT CCCCTCCCCC   
  
  
- CCTTCCCCCA AAACTAAGCT ATCTAGTAGT GAAAGTCTTT ACCGGAGAGA CCGGTTACCA TTCCCCATTC   
  
  
- CAATCATGTA CACCTGAAAG GGTACACCTA AAAAGGTCTC AGGCAACACA CTAACAATAA CGACAACAAC   
  
  
- ATTGAAAGAC AGTGAGCCTT TTGTACTTAG GTATACGTTG TTCTTGTATT ATTAACACTT ACGCTGAAAT   
  
  
- AAATCCTTAC AGAATGTATC ATGCACGTGT AAGAAATTAA TAGACAAATA GACAACCAAA CAATAAAAGT   
  
  
- TCCCCTACCC CTCACCCACC TACGAATCAT ACATTAGAAA AATATATCCC TTTTTAGTAT ATCGAACTTT   
  
  
- CATTTTGAAA GTAACGGTCC TAAGGTTCCA TCCCCATCCC AACAGATGTG GAACTAGAGG GATCTTCGTA   
  
  
- CCGCTAGGAA GGAGCCTAAC ACAATATCTT ATTTCTTACA CCAAGACGAC ATTACTCTGA ATACAAGATA   
  
  
- GTTTACTACT TAGACAAATC TTGAAGAACG AATAAAAAAG ACGTTTTAAA ATCCTCTGAA CTATTTGATA   
  
  
- TCAAGAGAAC TTAAATGTCC ACCTATAACC TCAAGTGTCT TATCATAAGT CTAGTTTCAC GAACACGAAT   
  
  
- TAACCTTCCT GTTGAACGAA CTCGACAAAT ATCTCGAGAT TGACTACGAT AGAGTACCTG AGTGTAGTCA   
  
  
- AAAAACCTAA ATTAGAATGG TCACAACTAG GCAGCATGTA AAGGCGAGTC ATGTCAGGCG GCAGTCACTG   
  
  
- TTTATCCTAC AAACGTAGTG ACTTCGAGCT GAGATCTCCA AGAGGACACA GTGGGAAGAG TTGAGTCAAA   
  
  
- CTAACACTAT GCCAATGATG TAACTCACTA TCAGTTCTCG TAGTGTCGAG CTGCCCCTCA GATAGTTGTT   
  
  
- CTTCGGGAAG AACATTAAGA GGGGAACTTT GCTCGTCAAT AGTAGTAAAT AGCTGCTTAC CGGGCAGAAC   
  
  
- ATTAAGACGG GGACTTTGAT CGTCAATAGC AGTAGCCAAG TTGCGCTCAG GGCACCCTAT AAGGGTAGTT   
  
  
- CCGTGACTAA TACCTCACTT ATACTTCTCG CGAGACGTCC TCGATCTCTG ATGAGATTAC CCACGTCTAC   
  
  
- CACTCCTTCA TAGACGACGA TTAGTTGGAT ACCCCCCTTC AGCAGTCAGG CCCTAGGGTT CAGTCTCTAG   
  
  
- TTTTAGTAAC TCGCTTCTAG GTGTCCCAAG CGTAGGATGA GTCGGACTAA GTAGTAGAGA AAGATCCCGT   
  
  
- TCTTCTAGTC CTCTACTTTC GGTCTCTCTC TTTGCCGTGT TCCGTTACCT TCTTGGTTGC CTTGATGGTT   
  
  
- CAAACGGTGG ACCACTAAAC TTCGTCAACG ATTAACTTAC ACGCTCCCGA AATAGCCTAT TGGCTTATCT   
  
  
- ACTGAAACTC TCAAACCAAT TTGCCCGCTC CTTTCTCCAG AGATAGAGCC CACTCGGATA GGTTGCAGAG   
  
  
- CCACGGATGT ACTAGCTTCC CGAACACCGT TCCTTCGTCA GAAGCCCCTG ATCGTAGATA GCCCGAGACT   
  
  
- TCACATTTCT CGGAGAACCT TTTCTGAACG AGAGGATGTA CGTGTAGGAA ATACTTTATA CGGGAATAGA   
  
  
- GTTCAAACCA ATATACCGAC GCTTACCTCG CTATCGACTT CGAACATCTT TACTCCTATT GTATGTATAA   
  
  
- TATCTAAAGG TCTAACGTGT CCCGTGAGTC ACCCAATGAG ATAATGTTCG GGATCGTCGT TCTGGACCAC   
  
  
- CCGGTGGATT CCACGCTTAA TGTCCGTAAC TACTAGGACA AAGATTCATA CGGGCACCAC GATCGAACCT   
  
  
- CCGACAACCC TTTGCCAATC GCAGAGATAG ACTTTTCAAG TTTTATGGGC AGCTCAAGTT ACGCAACGGG   
  
  
- CAAATACCTG GGCTACAGTC CGCCCTTTAC GACCTACACT CCGGGCCCCT CCGAAACCGG CAATTAAAAG   
  
  
- GTAACGTCGA GGTGGTGTGA GGACTGCTCT CACAGCTACA CTTGTTGGGA TCCCTACCCG AAGAGTCTTA   
  
  
- CCACTTTAGT GAACCAGGAT TCCATTGGTG AAACCAACTC GTTCTTAGTT TGTGGTTGTG ATGGGGAAAG   
  
  
- AACTGGGCCA AGTATCTCTG GGAACTGATG ATGAGTCGGT ACAAACTCAG ATATCTGCAC TGGTACGGTT   
  
  
- CTCTGGCCTT CCTCTCCTAG TTACAACTCG TCGTAACAAA CCGATTCCTG TAACACTTAT AGTATCGAAC   
  
  
- GCTCCCGTTC CTCTCCCACC TCGCAGTACT TGAAAAACCC TTTACCTTCA GTTCCAAATG GTACCGTCCC   
  
  
- AAGTCCGTCA TGGGCAACTC GAGTATGCAT TTGAGACACT ATTCTTCGGA TGAGGCCACA ATGAGGCTCG   
  
  
- TAATATGAGA CTATCTCTTC CTACCACGGT ACGAAGACCC AACCTTCCCG GCTTACGACT AAAGTCGAAG   
  
  
- CCGTACCGTA AC

+     TCT-motif

| Site Name | Organism | Position | Strand | Matrix score. | sequence | function |
| --- | --- | --- | --- | --- | --- | --- |
| TCT-motif | Arabidopsis thaliana | 2048 | + | 6 | TCTTAC | part of a light responsive element |
| TCT-motif | Arabidopsis thaliana | 617 | + | 6 | TCTTAC | part of a light responsive element |
| TCT-motif | Arabidopsis thaliana | 1555 | + | 6 | TCTTAC | part of a light responsive element |

>HU08G00014.1   
+ +Up\_Stream \_Len000TCCTCT TTTGGGCTTC TTTCTTTCTA TCCTGTTTTT TTTTGGGTCG AAAAGTTGGA   
  
  
+ GGTTGAATTG AAGCATCCGA ATTCGAGCAG GATGTTTCTC TGCGTAGTGA TTCTTTCTGG GTTTGTGATT   
  
  
+ TTGAATTCTG GGTAATCGGT TGTTTTGCTA ATTTTGAGGA CAGAGTTCCT TTTCTGAATT AAAATTTCGT   
  
  
+ TTTTTTATCG GGAAAATCTT CAGTATTTGA GAAAAAAGAA CGAAATTTGG ATGATTTGCT GTCTAGATTT   
  
  
+ TGCTTTCATA TTCCTGGGTG TGGATTGGTT TATTAATTGC ATGTGAGGAA GTACAAGCTT CTGGTCAATG   
  
  
+ GTTGGGTTTT TTTTTTTTTT TTGTGGGTAT AAAGTTGGAA ATTGATTACG AGTCAAATTT CTCTTTCTAG   
  
  
+ AAGAGGGGGG GGGGGGGGGG TTGGTTTAAA ACTCCATATT TTCTTTGAAT GGAAAGCTTA ATTTGCTGAC   
  
  
+ TATCTTGGTT AGTAGCATAA GCTTGATCAG AAAACAAAAC GCTCTCTGCC CTGCAAAAAC TTTATTTGTC   
  
  
+ TTTTAAGGAA AATTTTGGAA TTTGAGAGTG AAAATTTTGG TTGTGAATCT TGTCTTACTA TGTGGTTCCT   
  
  
+ TCTGACAAAA GTTTGAATCT GAGTATGAGA GTTCATCAAT CTCTTTTACT TTCCTACTTT CTTTATTGCT   
  
  
+ TGCAAATTGT TGTGAACTTA CTTGGATTTT CTGGTAGAGG AGAGTTCTGT CCTGGAATTT GGTGGAAAGT   
  
  
+ GAGACATACT TTGGGATTTA ATTAGCCATC AATTTGGTTT CACACCTTAT CAGTTTGGAC TTCTGGTGTG   
  
  
+ TTATTGTATG TGTTCTTAGC TTGGGATTAT TCTTGTATCG GAAGAGAGGG GGGGGGGGGT GTTTCTTTTA   
  
  
+ GCTGAATGTT AGAACATTTT AAGGGATCTA GATATCTAGA GAAGAGCCTA GGAAATGAAG CATTTTTCCC   
  
  
+ TTATAATCTG ACACAACTTT ATGAATTGAT TAGGAACAAA GAACGATTAG TTCAGAAAGG TTACAACTCC   
  
  
+ TCTAGGTGTT ATACTAAAGA GATGTGAGTT AATAAGCAAT TGCTTACTAA CTGTTTAGGC TGTTTCATTG   
  
  
+ CTTGTAGAGG CTTTTTTTTT GGGGGGGGGG GGGGGTTTGG GGTTCATAAA TGAGAAGGTT GATTTGTTGT   
  
  
+ CTGCTCTGCT CTTGATTCGA TAGTAATTCT CTCAGAAAAG GGTAAAATGA CATGGCCTTC CATCGCCTCC   
  
  
+ CATGAGGCTT TTTGCCTCTG CTTGTGTATC TTTCATCTCT TGGTTTGGTG GGTTGGGGGA GGGGAGGGGG   
  
  
+ GGAAGGGGGT TTTGATTCGA TAGATCATCA CTTTCAGAAA TGGCCTCTCT GGCCAATGGT AAGGGGTAAG   
  
  
+ GTTAGTACAT GTGGACTTTC CCATGTGGAT TTTTCCAGAG TCCGTTGTGT GATTGTTATT GCTGTTGTTG   
  
  
+ TAACTTTCTG TCACTCGGAA AACATGAATC CATATGCAAC AAGAACATAA TAATTGTGAA TGCGACTTTA   
  
  
+ TTTAGGAATG TCTTACATAG TACGTGCACA TTCTTTAATT ATCTGTTTAT CTGTTGGTTT GTTATTTTCA   
  
  
+ AGGGGATGGG GAGTGGGTGG ATGCTTAGTA TGTAATCTTT TTATATAGGG AAAAATCATA TAGCTTGAAA   
  
  
+ GTAAAACTTT CATTGCCAGG ATTCCAAGGT AGGGGTAGGG TTGTCTACAC CTTGATCTCC CTAGAAGCAT   
  
  
+ GGCGATCCTT CCTCGGATTG TGTTATAGAA TAAAGAATGT GGTTCTGCTG TAATGAGACT TATGTTCTAT   
  
  
+ CAAATGATGA ATCTGTTTAG AACTTCTTGC TTATTTTTTC TGCAAAATTT TAGGAGACTT GATAAACTAT   
  
  
+ AGTTCTCTTG AATTTACAGG TGGATATTGG AGTTCACAGA ATAGTATTCA GATCAAAGTG CTTGTGCTTA   
  
  
+ ATTGGAAGGA CAACTTGCTT GAGCTGTTTA TAGAGCTCTA ACTGATGCTA TCTCATGGAC TCACATCAGT   
  
  
+ TTTTTGGATT TAATCTTACC AGTGTTGATC CGTCGTACAT TTCCGCTCAG TACAGTCCGC CGTCAGTGAC   
  
  
+ AAATAGGATG TTTGCATCAC TGAAGCTCGA CTCTAGAGGT TCTCCTGTGT CACCCTTCTC AACTCAGTTT   
  
  
+ GATTGTGATA CGGTTACTAC ATTGAGTGAT AGTCAAGAGC ATCACAGCTC GACGGGGAGT CTATCAACAA   
  
  
+ GAAGCCCTTC TTGTAATTCT CCCCTTGAAA CGAGCAGTTA TCATCATTTA TCGACGAATG GCCCGTCTTG   
  
  
+ TAATTCTGCC CCTGAAACTA GCAGTTATCG TCATCGGTTC AACGCGAGTC CCGTGGGATA TTCCCATCAA   
  
  
+ GGCACTGATT ATGGAGTGAA TATGAAGAGC GCTCTGCAGG AGCTAGAGAC TACTCTAATG GGTGCAGATG   
  
  
+ GTGAGGAAGT ATCTGCTGCT AATCAACCTA TGGGGGGAAG TCGTCAGTCC GGGATCCCAA GTCAGAGATC   
  
  
+ AAAATCATTG AGCGAAGATC CACAGGGTTC GCATCCTACT CAGCCTGATT CATCATCTCT TTCTAGGGCA   
  
  
+ AGAAGATCAG GAGATGAAAG CCAGAGAGAG AAACGGCACA AGGCAATGGA AGAACCAACG GAACTACCAA   
  
  
+ GTTTGCCACC TGGTGATTTG AAGCAGTTGC TAATTGAATG TGCGAGGGCT TTATCGGATA ACCGAATAGA   
  
  
+ TGACTTTGAG AGTTTGGTTA AACGGGCGAG GAAAGAGGTC TCTATCTCGG GTGAGCCTAT CCAACGTCTC   
  
  
+ GGTGCCTACA TGATCGAAGG GCTTGTGGCA AGGAAGCAGT CTTCGGGGAC TAGCATCTAT CGGGCTCTGA   
  
  
+ AGTGTAAAGA GCCTCTTGGA AAAGACTTGC TCTCCTACAT GCACATCCTT TATGAAATAT GCCCTTATCT   
  
  
+ CAAGTTTGGT TATATGGCTG CGAATGGAGC GATAGCTGAA GCTTGTAGAA ATGAGGATAA CATACATATT   
  
  
+ ATAGATTTCC AGATTGCACA GGGCACTCAG TGGGTTACTC TATTACAAGC CCTAGCAGCA AGACCTGGTG   
  
  
+ GGCCACCTAA GGTGCGAATT ACAGGCATTG ATGATCCTGT TTCTAAGTAT GCCCGTGGTG CTAGCTTGGA   
  
  
+ GGCTGTTGGG AAACGGTTAG CGTCTCTATC TGAAAAGTTC AAAATACCCG TCGAGTTCAA TGCGTTGCCC   
  
  
+ GTTTATGGAC CCGATGTCAG GCGGGAAATG CTGGATGTGA GGCCCGGGGA GGCTTTGGCC GTTAATTTTC   
  
  
+ CATTGCAGCT CCACCACACT CCTGACGAGA GTGTCGATGT GAACAACCCT AGGGATGGGC TTCTCAGAAT   
  
  
+ GGTGAAATCA CTTGGTCCTA AGGTAACCAC TTTGGTTGAG CAAGAATCAA ACACCAACAC TACCCCTTTC   
  
  
+ TTGACCCGGT TCATAGAGAC CCTTGACTAC TACTCAGCCA TGTTTGAGTC TATAGACGTG ACCATGCCAA   
  
  
+ GAGACCGGAA GGAGAGGATC AATGTTGAGC AGCATTGTTT GGCTAAGGAC ATTGTGAATA TCATAGCTTG   
  
  
+ CGAGGGCAAG GAGAGGGTGG AGCGTCATGA ACTTTTTGGG AAATGGAAGT CAAGGTTTAC CATGGCAGGG   
  
  
+ TTCAGGCAGT ACCCGTTGAG CTCATACGTA AACTCTGTGA TAAGAAGCCT ACTCCGGTGT TACTCCGAGC   
  
  
+ ATTATACTCT GATAGAGAAG GATGGTGCCA TGCTTCTGGG TTGGAAGGGC CGAATGCTGA TTTCAGCTTC   
  
  
+ GGCATGGCAT TG  

- +Up\_Stream \_Len000AGGAGA AAACCCGAAG AAAGAAAGAT AGGACAAAAA AAAACCCAGC TTTTCAACCT   
  
  
- CCAACTTAAC TTCGTAGGCT TAAGCTCGTC CTACAAAGAG ACGCATCACT AAGAAAGACC CAAACACTAA   
  
  
- AACTTAAGAC CCATTAGCCA ACAAAACGAT TAAAACTCCT GTCTCAAGGA AAAGACTTAA TTTTAAAGCA   
  
  
- AAAAAATAGC CCTTTTAGAA GTCATAAACT CTTTTTTCTT GCTTTAAACC TACTAAACGA CAGATCTAAA   
  
  
- ACGAAAGTAT AAGGACCCAC ACCTAACCAA ATAATTAACG TACACTCCTT CATGTTCGAA GACCAGTTAC   
  
  
- CAACCCAAAA AAAAAAAAAA AACACCCATA TTTCAACCTT TAACTAATGC TCAGTTTAAA GAGAAAGATC   
  
  
- TTCTCCCCCC CCCCCCCCCC AACCAAATTT TGAGGTATAA AAGAAACTTA CCTTTCGAAT TAAACGACTG   
  
  
- ATAGAACCAA TCATCGTATT CGAACTAGTC TTTTGTTTTG CGAGAGACGG GACGTTTTTG AAATAAACAG   
  
  
- AAAATTCCTT TTAAAACCTT AAACTCTCAC TTTTAAAACC AACACTTAGA ACAGAATGAT ACACCAAGGA   
  
  
- AGACTGTTTT CAAACTTAGA CTCATACTCT CAAGTAGTTA GAGAAAATGA AAGGATGAAA GAAATAACGA   
  
  
- ACGTTTAACA ACACTTGAAT GAACCTAAAA GACCATCTCC TCTCAAGACA GGACCTTAAA CCACCTTTCA   
  
  
- CTCTGTATGA AACCCTAAAT TAATCGGTAG TTAAACCAAA GTGTGGAATA GTCAAACCTG AAGACCACAC   
  
  
- AATAACATAC ACAAGAATCG AACCCTAATA AGAACATAGC CTTCTCTCCC CCCCCCCCCA CAAAGAAAAT   
  
  
- CGACTTACAA TCTTGTAAAA TTCCCTAGAT CTATAGATCT CTTCTCGGAT CCTTTACTTC GTAAAAAGGG   
  
  
- AATATTAGAC TGTGTTGAAA TACTTAACTA ATCCTTGTTT CTTGCTAATC AAGTCTTTCC AATGTTGAGG   
  
  
- AGATCCACAA TATGATTTCT CTACACTCAA TTATTCGTTA ACGAATGATT GACAAATCCG ACAAAGTAAC   
  
  
- GAACATCTCC GAAAAAAAAA CCCCCCCCCC CCCCCAAACC CCAAGTATTT ACTCTTCCAA CTAAACAACA   
  
  
- GACGAGACGA GAACTAAGCT ATCATTAAGA GAGTCTTTTC CCATTTTACT GTACCGGAAG GTAGCGGAGG   
  
  
- GTACTCCGAA AAACGGAGAC GAACACATAG AAAGTAGAGA ACCAAACCAC CCAACCCCCT CCCCTCCCCC   
  
  
- CCTTCCCCCA AAACTAAGCT ATCTAGTAGT GAAAGTCTTT ACCGGAGAGA CCGGTTACCA TTCCCCATTC   
  
  
- CAATCATGTA CACCTGAAAG GGTACACCTA AAAAGGTCTC AGGCAACACA CTAACAATAA CGACAACAAC   
  
  
- ATTGAAAGAC AGTGAGCCTT TTGTACTTAG GTATACGTTG TTCTTGTATT ATTAACACTT ACGCTGAAAT   
  
  
- AAATCCTTAC AGAATGTATC ATGCACGTGT AAGAAATTAA TAGACAAATA GACAACCAAA CAATAAAAGT   
  
  
- TCCCCTACCC CTCACCCACC TACGAATCAT ACATTAGAAA AATATATCCC TTTTTAGTAT ATCGAACTTT   
  
  
- CATTTTGAAA GTAACGGTCC TAAGGTTCCA TCCCCATCCC AACAGATGTG GAACTAGAGG GATCTTCGTA   
  
  
- CCGCTAGGAA GGAGCCTAAC ACAATATCTT ATTTCTTACA CCAAGACGAC ATTACTCTGA ATACAAGATA   
  
  
- GTTTACTACT TAGACAAATC TTGAAGAACG AATAAAAAAG ACGTTTTAAA ATCCTCTGAA CTATTTGATA   
  
  
- TCAAGAGAAC TTAAATGTCC ACCTATAACC TCAAGTGTCT TATCATAAGT CTAGTTTCAC GAACACGAAT   
  
  
- TAACCTTCCT GTTGAACGAA CTCGACAAAT ATCTCGAGAT TGACTACGAT AGAGTACCTG AGTGTAGTCA   
  
  
- AAAAACCTAA ATTAGAATGG TCACAACTAG GCAGCATGTA AAGGCGAGTC ATGTCAGGCG GCAGTCACTG   
  
  
- TTTATCCTAC AAACGTAGTG ACTTCGAGCT GAGATCTCCA AGAGGACACA GTGGGAAGAG TTGAGTCAAA   
  
  
- CTAACACTAT GCCAATGATG TAACTCACTA TCAGTTCTCG TAGTGTCGAG CTGCCCCTCA GATAGTTGTT   
  
  
- CTTCGGGAAG AACATTAAGA GGGGAACTTT GCTCGTCAAT AGTAGTAAAT AGCTGCTTAC CGGGCAGAAC   
  
  
- ATTAAGACGG GGACTTTGAT CGTCAATAGC AGTAGCCAAG TTGCGCTCAG GGCACCCTAT AAGGGTAGTT   
  
  
- CCGTGACTAA TACCTCACTT ATACTTCTCG CGAGACGTCC TCGATCTCTG ATGAGATTAC CCACGTCTAC   
  
  
- CACTCCTTCA TAGACGACGA TTAGTTGGAT ACCCCCCTTC AGCAGTCAGG CCCTAGGGTT CAGTCTCTAG   
  
  
- TTTTAGTAAC TCGCTTCTAG GTGTCCCAAG CGTAGGATGA GTCGGACTAA GTAGTAGAGA AAGATCCCGT   
  
  
- TCTTCTAGTC CTCTACTTTC GGTCTCTCTC TTTGCCGTGT TCCGTTACCT TCTTGGTTGC CTTGATGGTT   
  
  
- CAAACGGTGG ACCACTAAAC TTCGTCAACG ATTAACTTAC ACGCTCCCGA AATAGCCTAT TGGCTTATCT   
  
  
- ACTGAAACTC TCAAACCAAT TTGCCCGCTC CTTTCTCCAG AGATAGAGCC CACTCGGATA GGTTGCAGAG   
  
  
- CCACGGATGT ACTAGCTTCC CGAACACCGT TCCTTCGTCA GAAGCCCCTG ATCGTAGATA GCCCGAGACT   
  
  
- TCACATTTCT CGGAGAACCT TTTCTGAACG AGAGGATGTA CGTGTAGGAA ATACTTTATA CGGGAATAGA   
  
  
- GTTCAAACCA ATATACCGAC GCTTACCTCG CTATCGACTT CGAACATCTT TACTCCTATT GTATGTATAA   
  
  
- TATCTAAAGG TCTAACGTGT CCCGTGAGTC ACCCAATGAG ATAATGTTCG GGATCGTCGT TCTGGACCAC   
  
  
- CCGGTGGATT CCACGCTTAA TGTCCGTAAC TACTAGGACA AAGATTCATA CGGGCACCAC GATCGAACCT   
  
  
- CCGACAACCC TTTGCCAATC GCAGAGATAG ACTTTTCAAG TTTTATGGGC AGCTCAAGTT ACGCAACGGG   
  
  
- CAAATACCTG GGCTACAGTC CGCCCTTTAC GACCTACACT CCGGGCCCCT CCGAAACCGG CAATTAAAAG   
  
  
- GTAACGTCGA GGTGGTGTGA GGACTGCTCT CACAGCTACA CTTGTTGGGA TCCCTACCCG AAGAGTCTTA   
  
  
- CCACTTTAGT GAACCAGGAT TCCATTGGTG AAACCAACTC GTTCTTAGTT TGTGGTTGTG ATGGGGAAAG   
  
  
- AACTGGGCCA AGTATCTCTG GGAACTGATG ATGAGTCGGT ACAAACTCAG ATATCTGCAC TGGTACGGTT   
  
  
- CTCTGGCCTT CCTCTCCTAG TTACAACTCG TCGTAACAAA CCGATTCCTG TAACACTTAT AGTATCGAAC   
  
  
- GCTCCCGTTC CTCTCCCACC TCGCAGTACT TGAAAAACCC TTTACCTTCA GTTCCAAATG GTACCGTCCC   
  
  
- AAGTCCGTCA TGGGCAACTC GAGTATGCAT TTGAGACACT ATTCTTCGGA TGAGGCCACA ATGAGGCTCG   
  
  
- TAATATGAGA CTATCTCTTC CTACCACGGT ACGAAGACCC AACCTTCCCG GCTTACGACT AAAGTCGAAG   
  
  
- CCGTACCGTA AC

+     TGACG-motif

| Site Name | Organism | Position | Strand | Matrix score. | sequence | function |
| --- | --- | --- | --- | --- | --- | --- |
| TGACG-motif | Hordeum vulgare | 2095 | - | 5 | TGACG | cis-acting regulatory element involved in the MeJA-responsiveness |
| TGACG-motif | Hordeum vulgare | 3597 | - | 5 | TGACG | cis-acting regulatory element involved in the MeJA-responsiveness |
| TGACG-motif | Hordeum vulgare | 3317 | + | 5 | TGACG | cis-acting regulatory element involved in the MeJA-responsiveness |
| TGACG-motif | Hordeum vulgare | 2343 | - | 5 | TGACG | cis-acting regulatory element involved in the MeJA-responsiveness |
| TGACG-motif | Hordeum vulgare | 2496 | - | 5 | TGACG | cis-acting regulatory element involved in the MeJA-responsiveness |

>HU08G00014.1   
+ +Up\_Stream \_Len000TCCTCT TTTGGGCTTC TTTCTTTCTA TCCTGTTTTT TTTTGGGTCG AAAAGTTGGA   
  
  
+ GGTTGAATTG AAGCATCCGA ATTCGAGCAG GATGTTTCTC TGCGTAGTGA TTCTTTCTGG GTTTGTGATT   
  
  
+ TTGAATTCTG GGTAATCGGT TGTTTTGCTA ATTTTGAGGA CAGAGTTCCT TTTCTGAATT AAAATTTCGT   
  
  
+ TTTTTTATCG GGAAAATCTT CAGTATTTGA GAAAAAAGAA CGAAATTTGG ATGATTTGCT GTCTAGATTT   
  
  
+ TGCTTTCATA TTCCTGGGTG TGGATTGGTT TATTAATTGC ATGTGAGGAA GTACAAGCTT CTGGTCAATG   
  
  
+ GTTGGGTTTT TTTTTTTTTT TTGTGGGTAT AAAGTTGGAA ATTGATTACG AGTCAAATTT CTCTTTCTAG   
  
  
+ AAGAGGGGGG GGGGGGGGGG TTGGTTTAAA ACTCCATATT TTCTTTGAAT GGAAAGCTTA ATTTGCTGAC   
  
  
+ TATCTTGGTT AGTAGCATAA GCTTGATCAG AAAACAAAAC GCTCTCTGCC CTGCAAAAAC TTTATTTGTC   
  
  
+ TTTTAAGGAA AATTTTGGAA TTTGAGAGTG AAAATTTTGG TTGTGAATCT TGTCTTACTA TGTGGTTCCT   
  
  
+ TCTGACAAAA GTTTGAATCT GAGTATGAGA GTTCATCAAT CTCTTTTACT TTCCTACTTT CTTTATTGCT   
  
  
+ TGCAAATTGT TGTGAACTTA CTTGGATTTT CTGGTAGAGG AGAGTTCTGT CCTGGAATTT GGTGGAAAGT   
  
  
+ GAGACATACT TTGGGATTTA ATTAGCCATC AATTTGGTTT CACACCTTAT CAGTTTGGAC TTCTGGTGTG   
  
  
+ TTATTGTATG TGTTCTTAGC TTGGGATTAT TCTTGTATCG GAAGAGAGGG GGGGGGGGGT GTTTCTTTTA   
  
  
+ GCTGAATGTT AGAACATTTT AAGGGATCTA GATATCTAGA GAAGAGCCTA GGAAATGAAG CATTTTTCCC   
  
  
+ TTATAATCTG ACACAACTTT ATGAATTGAT TAGGAACAAA GAACGATTAG TTCAGAAAGG TTACAACTCC   
  
  
+ TCTAGGTGTT ATACTAAAGA GATGTGAGTT AATAAGCAAT TGCTTACTAA CTGTTTAGGC TGTTTCATTG   
  
  
+ CTTGTAGAGG CTTTTTTTTT GGGGGGGGGG GGGGGTTTGG GGTTCATAAA TGAGAAGGTT GATTTGTTGT   
  
  
+ CTGCTCTGCT CTTGATTCGA TAGTAATTCT CTCAGAAAAG GGTAAAATGA CATGGCCTTC CATCGCCTCC   
  
  
+ CATGAGGCTT TTTGCCTCTG CTTGTGTATC TTTCATCTCT TGGTTTGGTG GGTTGGGGGA GGGGAGGGGG   
  
  
+ GGAAGGGGGT TTTGATTCGA TAGATCATCA CTTTCAGAAA TGGCCTCTCT GGCCAATGGT AAGGGGTAAG   
  
  
+ GTTAGTACAT GTGGACTTTC CCATGTGGAT TTTTCCAGAG TCCGTTGTGT GATTGTTATT GCTGTTGTTG   
  
  
+ TAACTTTCTG TCACTCGGAA AACATGAATC CATATGCAAC AAGAACATAA TAATTGTGAA TGCGACTTTA   
  
  
+ TTTAGGAATG TCTTACATAG TACGTGCACA TTCTTTAATT ATCTGTTTAT CTGTTGGTTT GTTATTTTCA   
  
  
+ AGGGGATGGG GAGTGGGTGG ATGCTTAGTA TGTAATCTTT TTATATAGGG AAAAATCATA TAGCTTGAAA   
  
  
+ GTAAAACTTT CATTGCCAGG ATTCCAAGGT AGGGGTAGGG TTGTCTACAC CTTGATCTCC CTAGAAGCAT   
  
  
+ GGCGATCCTT CCTCGGATTG TGTTATAGAA TAAAGAATGT GGTTCTGCTG TAATGAGACT TATGTTCTAT   
  
  
+ CAAATGATGA ATCTGTTTAG AACTTCTTGC TTATTTTTTC TGCAAAATTT TAGGAGACTT GATAAACTAT   
  
  
+ AGTTCTCTTG AATTTACAGG TGGATATTGG AGTTCACAGA ATAGTATTCA GATCAAAGTG CTTGTGCTTA   
  
  
+ ATTGGAAGGA CAACTTGCTT GAGCTGTTTA TAGAGCTCTA ACTGATGCTA TCTCATGGAC TCACATCAGT   
  
  
+ TTTTTGGATT TAATCTTACC AGTGTTGATC CGTCGTACAT TTCCGCTCAG TACAGTCCGC CGTCAGTGAC   
  
  
+ AAATAGGATG TTTGCATCAC TGAAGCTCGA CTCTAGAGGT TCTCCTGTGT CACCCTTCTC AACTCAGTTT   
  
  
+ GATTGTGATA CGGTTACTAC ATTGAGTGAT AGTCAAGAGC ATCACAGCTC GACGGGGAGT CTATCAACAA   
  
  
+ GAAGCCCTTC TTGTAATTCT CCCCTTGAAA CGAGCAGTTA TCATCATTTA TCGACGAATG GCCCGTCTTG   
  
  
+ TAATTCTGCC CCTGAAACTA GCAGTTATCG TCATCGGTTC AACGCGAGTC CCGTGGGATA TTCCCATCAA   
  
  
+ GGCACTGATT ATGGAGTGAA TATGAAGAGC GCTCTGCAGG AGCTAGAGAC TACTCTAATG GGTGCAGATG   
  
  
+ GTGAGGAAGT ATCTGCTGCT AATCAACCTA TGGGGGGAAG TCGTCAGTCC GGGATCCCAA GTCAGAGATC   
  
  
+ AAAATCATTG AGCGAAGATC CACAGGGTTC GCATCCTACT CAGCCTGATT CATCATCTCT TTCTAGGGCA   
  
  
+ AGAAGATCAG GAGATGAAAG CCAGAGAGAG AAACGGCACA AGGCAATGGA AGAACCAACG GAACTACCAA   
  
  
+ GTTTGCCACC TGGTGATTTG AAGCAGTTGC TAATTGAATG TGCGAGGGCT TTATCGGATA ACCGAATAGA   
  
  
+ TGACTTTGAG AGTTTGGTTA AACGGGCGAG GAAAGAGGTC TCTATCTCGG GTGAGCCTAT CCAACGTCTC   
  
  
+ GGTGCCTACA TGATCGAAGG GCTTGTGGCA AGGAAGCAGT CTTCGGGGAC TAGCATCTAT CGGGCTCTGA   
  
  
+ AGTGTAAAGA GCCTCTTGGA AAAGACTTGC TCTCCTACAT GCACATCCTT TATGAAATAT GCCCTTATCT   
  
  
+ CAAGTTTGGT TATATGGCTG CGAATGGAGC GATAGCTGAA GCTTGTAGAA ATGAGGATAA CATACATATT   
  
  
+ ATAGATTTCC AGATTGCACA GGGCACTCAG TGGGTTACTC TATTACAAGC CCTAGCAGCA AGACCTGGTG   
  
  
+ GGCCACCTAA GGTGCGAATT ACAGGCATTG ATGATCCTGT TTCTAAGTAT GCCCGTGGTG CTAGCTTGGA   
  
  
+ GGCTGTTGGG AAACGGTTAG CGTCTCTATC TGAAAAGTTC AAAATACCCG TCGAGTTCAA TGCGTTGCCC   
  
  
+ GTTTATGGAC CCGATGTCAG GCGGGAAATG CTGGATGTGA GGCCCGGGGA GGCTTTGGCC GTTAATTTTC   
  
  
+ CATTGCAGCT CCACCACACT CCTGACGAGA GTGTCGATGT GAACAACCCT AGGGATGGGC TTCTCAGAAT   
  
  
+ GGTGAAATCA CTTGGTCCTA AGGTAACCAC TTTGGTTGAG CAAGAATCAA ACACCAACAC TACCCCTTTC   
  
  
+ TTGACCCGGT TCATAGAGAC CCTTGACTAC TACTCAGCCA TGTTTGAGTC TATAGACGTG ACCATGCCAA   
  
  
+ GAGACCGGAA GGAGAGGATC AATGTTGAGC AGCATTGTTT GGCTAAGGAC ATTGTGAATA TCATAGCTTG   
  
  
+ CGAGGGCAAG GAGAGGGTGG AGCGTCATGA ACTTTTTGGG AAATGGAAGT CAAGGTTTAC CATGGCAGGG   
  
  
+ TTCAGGCAGT ACCCGTTGAG CTCATACGTA AACTCTGTGA TAAGAAGCCT ACTCCGGTGT TACTCCGAGC   
  
  
+ ATTATACTCT GATAGAGAAG GATGGTGCCA TGCTTCTGGG TTGGAAGGGC CGAATGCTGA TTTCAGCTTC   
  
  
+ GGCATGGCAT TG  

- +Up\_Stream \_Len000AGGAGA AAACCCGAAG AAAGAAAGAT AGGACAAAAA AAAACCCAGC TTTTCAACCT   
  
  
- CCAACTTAAC TTCGTAGGCT TAAGCTCGTC CTACAAAGAG ACGCATCACT AAGAAAGACC CAAACACTAA   
  
  
- AACTTAAGAC CCATTAGCCA ACAAAACGAT TAAAACTCCT GTCTCAAGGA AAAGACTTAA TTTTAAAGCA   
  
  
- AAAAAATAGC CCTTTTAGAA GTCATAAACT CTTTTTTCTT GCTTTAAACC TACTAAACGA CAGATCTAAA   
  
  
- ACGAAAGTAT AAGGACCCAC ACCTAACCAA ATAATTAACG TACACTCCTT CATGTTCGAA GACCAGTTAC   
  
  
- CAACCCAAAA AAAAAAAAAA AACACCCATA TTTCAACCTT TAACTAATGC TCAGTTTAAA GAGAAAGATC   
  
  
- TTCTCCCCCC CCCCCCCCCC AACCAAATTT TGAGGTATAA AAGAAACTTA CCTTTCGAAT TAAACGACTG   
  
  
- ATAGAACCAA TCATCGTATT CGAACTAGTC TTTTGTTTTG CGAGAGACGG GACGTTTTTG AAATAAACAG   
  
  
- AAAATTCCTT TTAAAACCTT AAACTCTCAC TTTTAAAACC AACACTTAGA ACAGAATGAT ACACCAAGGA   
  
  
- AGACTGTTTT CAAACTTAGA CTCATACTCT CAAGTAGTTA GAGAAAATGA AAGGATGAAA GAAATAACGA   
  
  
- ACGTTTAACA ACACTTGAAT GAACCTAAAA GACCATCTCC TCTCAAGACA GGACCTTAAA CCACCTTTCA   
  
  
- CTCTGTATGA AACCCTAAAT TAATCGGTAG TTAAACCAAA GTGTGGAATA GTCAAACCTG AAGACCACAC   
  
  
- AATAACATAC ACAAGAATCG AACCCTAATA AGAACATAGC CTTCTCTCCC CCCCCCCCCA CAAAGAAAAT   
  
  
- CGACTTACAA TCTTGTAAAA TTCCCTAGAT CTATAGATCT CTTCTCGGAT CCTTTACTTC GTAAAAAGGG   
  
  
- AATATTAGAC TGTGTTGAAA TACTTAACTA ATCCTTGTTT CTTGCTAATC AAGTCTTTCC AATGTTGAGG   
  
  
- AGATCCACAA TATGATTTCT CTACACTCAA TTATTCGTTA ACGAATGATT GACAAATCCG ACAAAGTAAC   
  
  
- GAACATCTCC GAAAAAAAAA CCCCCCCCCC CCCCCAAACC CCAAGTATTT ACTCTTCCAA CTAAACAACA   
  
  
- GACGAGACGA GAACTAAGCT ATCATTAAGA GAGTCTTTTC CCATTTTACT GTACCGGAAG GTAGCGGAGG   
  
  
- GTACTCCGAA AAACGGAGAC GAACACATAG AAAGTAGAGA ACCAAACCAC CCAACCCCCT CCCCTCCCCC   
  
  
- CCTTCCCCCA AAACTAAGCT ATCTAGTAGT GAAAGTCTTT ACCGGAGAGA CCGGTTACCA TTCCCCATTC   
  
  
- CAATCATGTA CACCTGAAAG GGTACACCTA AAAAGGTCTC AGGCAACACA CTAACAATAA CGACAACAAC   
  
  
- ATTGAAAGAC AGTGAGCCTT TTGTACTTAG GTATACGTTG TTCTTGTATT ATTAACACTT ACGCTGAAAT   
  
  
- AAATCCTTAC AGAATGTATC ATGCACGTGT AAGAAATTAA TAGACAAATA GACAACCAAA CAATAAAAGT   
  
  
- TCCCCTACCC CTCACCCACC TACGAATCAT ACATTAGAAA AATATATCCC TTTTTAGTAT ATCGAACTTT   
  
  
- CATTTTGAAA GTAACGGTCC TAAGGTTCCA TCCCCATCCC AACAGATGTG GAACTAGAGG GATCTTCGTA   
  
  
- CCGCTAGGAA GGAGCCTAAC ACAATATCTT ATTTCTTACA CCAAGACGAC ATTACTCTGA ATACAAGATA   
  
  
- GTTTACTACT TAGACAAATC TTGAAGAACG AATAAAAAAG ACGTTTTAAA ATCCTCTGAA CTATTTGATA   
  
  
- TCAAGAGAAC TTAAATGTCC ACCTATAACC TCAAGTGTCT TATCATAAGT CTAGTTTCAC GAACACGAAT   
  
  
- TAACCTTCCT GTTGAACGAA CTCGACAAAT ATCTCGAGAT TGACTACGAT AGAGTACCTG AGTGTAGTCA   
  
  
- AAAAACCTAA ATTAGAATGG TCACAACTAG GCAGCATGTA AAGGCGAGTC ATGTCAGGCG GCAGTCACTG   
  
  
- TTTATCCTAC AAACGTAGTG ACTTCGAGCT GAGATCTCCA AGAGGACACA GTGGGAAGAG TTGAGTCAAA   
  
  
- CTAACACTAT GCCAATGATG TAACTCACTA TCAGTTCTCG TAGTGTCGAG CTGCCCCTCA GATAGTTGTT   
  
  
- CTTCGGGAAG AACATTAAGA GGGGAACTTT GCTCGTCAAT AGTAGTAAAT AGCTGCTTAC CGGGCAGAAC   
  
  
- ATTAAGACGG GGACTTTGAT CGTCAATAGC AGTAGCCAAG TTGCGCTCAG GGCACCCTAT AAGGGTAGTT   
  
  
- CCGTGACTAA TACCTCACTT ATACTTCTCG CGAGACGTCC TCGATCTCTG ATGAGATTAC CCACGTCTAC   
  
  
- CACTCCTTCA TAGACGACGA TTAGTTGGAT ACCCCCCTTC AGCAGTCAGG CCCTAGGGTT CAGTCTCTAG   
  
  
- TTTTAGTAAC TCGCTTCTAG GTGTCCCAAG CGTAGGATGA GTCGGACTAA GTAGTAGAGA AAGATCCCGT   
  
  
- TCTTCTAGTC CTCTACTTTC GGTCTCTCTC TTTGCCGTGT TCCGTTACCT TCTTGGTTGC CTTGATGGTT   
  
  
- CAAACGGTGG ACCACTAAAC TTCGTCAACG ATTAACTTAC ACGCTCCCGA AATAGCCTAT TGGCTTATCT   
  
  
- ACTGAAACTC TCAAACCAAT TTGCCCGCTC CTTTCTCCAG AGATAGAGCC CACTCGGATA GGTTGCAGAG   
  
  
- CCACGGATGT ACTAGCTTCC CGAACACCGT TCCTTCGTCA GAAGCCCCTG ATCGTAGATA GCCCGAGACT   
  
  
- TCACATTTCT CGGAGAACCT TTTCTGAACG AGAGGATGTA CGTGTAGGAA ATACTTTATA CGGGAATAGA   
  
  
- GTTCAAACCA ATATACCGAC GCTTACCTCG CTATCGACTT CGAACATCTT TACTCCTATT GTATGTATAA   
  
  
- TATCTAAAGG TCTAACGTGT CCCGTGAGTC ACCCAATGAG ATAATGTTCG GGATCGTCGT TCTGGACCAC   
  
  
- CCGGTGGATT CCACGCTTAA TGTCCGTAAC TACTAGGACA AAGATTCATA CGGGCACCAC GATCGAACCT   
  
  
- CCGACAACCC TTTGCCAATC GCAGAGATAG ACTTTTCAAG TTTTATGGGC AGCTCAAGTT ACGCAACGGG   
  
  
- CAAATACCTG GGCTACAGTC CGCCCTTTAC GACCTACACT CCGGGCCCCT CCGAAACCGG CAATTAAAAG   
  
  
- GTAACGTCGA GGTGGTGTGA GGACTGCTCT CACAGCTACA CTTGTTGGGA TCCCTACCCG AAGAGTCTTA   
  
  
- CCACTTTAGT GAACCAGGAT TCCATTGGTG AAACCAACTC GTTCTTAGTT TGTGGTTGTG ATGGGGAAAG   
  
  
- AACTGGGCCA AGTATCTCTG GGAACTGATG ATGAGTCGGT ACAAACTCAG ATATCTGCAC TGGTACGGTT   
  
  
- CTCTGGCCTT CCTCTCCTAG TTACAACTCG TCGTAACAAA CCGATTCCTG TAACACTTAT AGTATCGAAC   
  
  
- GCTCCCGTTC CTCTCCCACC TCGCAGTACT TGAAAAACCC TTTACCTTCA GTTCCAAATG GTACCGTCCC   
  
  
- AAGTCCGTCA TGGGCAACTC GAGTATGCAT TTGAGACACT ATTCTTCGGA TGAGGCCACA ATGAGGCTCG   
  
  
- TAATATGAGA CTATCTCTTC CTACCACGGT ACGAAGACCC AACCTTCCCG GCTTACGACT AAAGTCGAAG   
  
  
- CCGTACCGTA AC

+     Unnamed\_\_1

| Site Name | Organism | Position | Strand | Matrix score. | sequence | function |
| --- | --- | --- | --- | --- | --- | --- |
| Unnamed\_\_1 | Zea mays | 3138 | + | 5 | CGTGG |  |
| Unnamed\_\_1 | Zea mays | 2366 | + | 5 | CGTGG |  |

>HU08G00014.1   
+ +Up\_Stream \_Len000TCCTCT TTTGGGCTTC TTTCTTTCTA TCCTGTTTTT TTTTGGGTCG AAAAGTTGGA   
  
  
+ GGTTGAATTG AAGCATCCGA ATTCGAGCAG GATGTTTCTC TGCGTAGTGA TTCTTTCTGG GTTTGTGATT   
  
  
+ TTGAATTCTG GGTAATCGGT TGTTTTGCTA ATTTTGAGGA CAGAGTTCCT TTTCTGAATT AAAATTTCGT   
  
  
+ TTTTTTATCG GGAAAATCTT CAGTATTTGA GAAAAAAGAA CGAAATTTGG ATGATTTGCT GTCTAGATTT   
  
  
+ TGCTTTCATA TTCCTGGGTG TGGATTGGTT TATTAATTGC ATGTGAGGAA GTACAAGCTT CTGGTCAATG   
  
  
+ GTTGGGTTTT TTTTTTTTTT TTGTGGGTAT AAAGTTGGAA ATTGATTACG AGTCAAATTT CTCTTTCTAG   
  
  
+ AAGAGGGGGG GGGGGGGGGG TTGGTTTAAA ACTCCATATT TTCTTTGAAT GGAAAGCTTA ATTTGCTGAC   
  
  
+ TATCTTGGTT AGTAGCATAA GCTTGATCAG AAAACAAAAC GCTCTCTGCC CTGCAAAAAC TTTATTTGTC   
  
  
+ TTTTAAGGAA AATTTTGGAA TTTGAGAGTG AAAATTTTGG TTGTGAATCT TGTCTTACTA TGTGGTTCCT   
  
  
+ TCTGACAAAA GTTTGAATCT GAGTATGAGA GTTCATCAAT CTCTTTTACT TTCCTACTTT CTTTATTGCT   
  
  
+ TGCAAATTGT TGTGAACTTA CTTGGATTTT CTGGTAGAGG AGAGTTCTGT CCTGGAATTT GGTGGAAAGT   
  
  
+ GAGACATACT TTGGGATTTA ATTAGCCATC AATTTGGTTT CACACCTTAT CAGTTTGGAC TTCTGGTGTG   
  
  
+ TTATTGTATG TGTTCTTAGC TTGGGATTAT TCTTGTATCG GAAGAGAGGG GGGGGGGGGT GTTTCTTTTA   
  
  
+ GCTGAATGTT AGAACATTTT AAGGGATCTA GATATCTAGA GAAGAGCCTA GGAAATGAAG CATTTTTCCC   
  
  
+ TTATAATCTG ACACAACTTT ATGAATTGAT TAGGAACAAA GAACGATTAG TTCAGAAAGG TTACAACTCC   
  
  
+ TCTAGGTGTT ATACTAAAGA GATGTGAGTT AATAAGCAAT TGCTTACTAA CTGTTTAGGC TGTTTCATTG   
  
  
+ CTTGTAGAGG CTTTTTTTTT GGGGGGGGGG GGGGGTTTGG GGTTCATAAA TGAGAAGGTT GATTTGTTGT   
  
  
+ CTGCTCTGCT CTTGATTCGA TAGTAATTCT CTCAGAAAAG GGTAAAATGA CATGGCCTTC CATCGCCTCC   
  
  
+ CATGAGGCTT TTTGCCTCTG CTTGTGTATC TTTCATCTCT TGGTTTGGTG GGTTGGGGGA GGGGAGGGGG   
  
  
+ GGAAGGGGGT TTTGATTCGA TAGATCATCA CTTTCAGAAA TGGCCTCTCT GGCCAATGGT AAGGGGTAAG   
  
  
+ GTTAGTACAT GTGGACTTTC CCATGTGGAT TTTTCCAGAG TCCGTTGTGT GATTGTTATT GCTGTTGTTG   
  
  
+ TAACTTTCTG TCACTCGGAA AACATGAATC CATATGCAAC AAGAACATAA TAATTGTGAA TGCGACTTTA   
  
  
+ TTTAGGAATG TCTTACATAG TACGTGCACA TTCTTTAATT ATCTGTTTAT CTGTTGGTTT GTTATTTTCA   
  
  
+ AGGGGATGGG GAGTGGGTGG ATGCTTAGTA TGTAATCTTT TTATATAGGG AAAAATCATA TAGCTTGAAA   
  
  
+ GTAAAACTTT CATTGCCAGG ATTCCAAGGT AGGGGTAGGG TTGTCTACAC CTTGATCTCC CTAGAAGCAT   
  
  
+ GGCGATCCTT CCTCGGATTG TGTTATAGAA TAAAGAATGT GGTTCTGCTG TAATGAGACT TATGTTCTAT   
  
  
+ CAAATGATGA ATCTGTTTAG AACTTCTTGC TTATTTTTTC TGCAAAATTT TAGGAGACTT GATAAACTAT   
  
  
+ AGTTCTCTTG AATTTACAGG TGGATATTGG AGTTCACAGA ATAGTATTCA GATCAAAGTG CTTGTGCTTA   
  
  
+ ATTGGAAGGA CAACTTGCTT GAGCTGTTTA TAGAGCTCTA ACTGATGCTA TCTCATGGAC TCACATCAGT   
  
  
+ TTTTTGGATT TAATCTTACC AGTGTTGATC CGTCGTACAT TTCCGCTCAG TACAGTCCGC CGTCAGTGAC   
  
  
+ AAATAGGATG TTTGCATCAC TGAAGCTCGA CTCTAGAGGT TCTCCTGTGT CACCCTTCTC AACTCAGTTT   
  
  
+ GATTGTGATA CGGTTACTAC ATTGAGTGAT AGTCAAGAGC ATCACAGCTC GACGGGGAGT CTATCAACAA   
  
  
+ GAAGCCCTTC TTGTAATTCT CCCCTTGAAA CGAGCAGTTA TCATCATTTA TCGACGAATG GCCCGTCTTG   
  
  
+ TAATTCTGCC CCTGAAACTA GCAGTTATCG TCATCGGTTC AACGCGAGTC CCGTGGGATA TTCCCATCAA   
  
  
+ GGCACTGATT ATGGAGTGAA TATGAAGAGC GCTCTGCAGG AGCTAGAGAC TACTCTAATG GGTGCAGATG   
  
  
+ GTGAGGAAGT ATCTGCTGCT AATCAACCTA TGGGGGGAAG TCGTCAGTCC GGGATCCCAA GTCAGAGATC
[truncated: 67,156 more chars]
