# Supplementary material for: Genome-Wide Identification and Expression Pattern of the GRAS Gene Family in Pitaya (Selenicereus undatus L.)
Source: Biology (Basel). 2022 Dec 21;12(1):11. doi: 10.3390/biology12010011 (PMC9854919; doi:10.3390/biology12010011)
Supplement: Supplementary file 1 [file biology-12-00011-s001.zip › Supplementary file S5/HU08G00019.1_plantcare.html]

Content-Type: text/html; charset=ISO-8859-1


PlantCARE


Webmaster Firefox specific output  
To save the result:
click on the frame with the right mouse button and save the source code as a text file with extension .html  
REFERENCE:PlantCARE: a database of plant cis-acting regulatory elements and a portal to tools for in silico analysis of promoter sequences.  
Lescot, M., Déhais, P., Moreau, Y., De Moor, B., Rouzé ,P.,and Rombauts, S.  
Nucleic Acids Res., Database issue(2002), 30(1):325-327.   


---

>HU08G00019.1   
+ +Up\_Stream \_Len000AGTTGT AGCAAGCACA TTAAAGTTGA TTATCATTTT GTCCGTGAAA CGGTGAGCAA   
  
  
+ ACGTCATCTC CAGGTCAAAT TCATTCGTAC TCAATCACAA GTTGCTGACA TTTTTACCAA AGGCTAATCG   
  
  
+ TCGCAAAAGT TTCTTTAGTT TAAGGCCAAT CTCTCCGTTG TTTCACCCCA TACAGATTGA GGGGTTGTAA   
  
  
+ TAGTGTATAT ATATAGCCTT GTGTTAGCGG GGCTTGGGCA TTAGACTTTT ACTCTTGTAA ACCCTAATGT   
  
  
+ GTTATTATAA ATATAGATCT AGCCACCCAT GTTGAGGTAG GCACTCTAAT TCTGACACAG GTAGGATAAG   
  
  
+ TTTTCATAGT TTATGTTGTG AATCTTGTCT TACTTTGTGG TTCCTATTGA CAAAAGTTTG AATCCGAGCA   
  
  
+ TGAGAGTTCA TCAATCTCTT TTTCTTTCCT ATTTTATTTC TTGTGGACTT AATTGGATTT TCTGGTAGAG   
  
  
+ GAAAGTTCCT TGCTGGAATT TGGTCGAAAG TGATATACAT TTTGGGATTT AATTAGCCAT AAATTTGGTT   
  
  
+ TTACACCTTG TTAGTTTGGA GTTCTGGTGT GTTATTGTAT GTGTTCTGGT GGGTTTTTCT TTTAGCTAAA   
  
  
+ TGTTGGAGCA TTTTAAGGGA TCTAGATATC TGAAGAAGTG CTTGGGAAAT GAAGCATATT TCCCTTATAG   
  
  
+ TCTGACATAA CTTTAATGAA TTGATCATGA ACAAAGGGCA ATTAGTTCAG AAAGGTTCAA CTCCTCTAGG   
  
  
+ TGTTGTGCTA AAGAGATGTG AGTTAGTAAC AATTGCTTAC TAACTATATA GGTTGTTTTA TTACTTGTAG   
  
  
+ AGGTTTTTTT TTTGGGATAT CTTTTTGGGG TTTATAAATG AGAAGGTTGA TTTGTTGTCT ACTCTTCTTT   
  
  
+ CGATTCAATA GTAATTCTCT CAGAGAAGGC TAAAATGACA TGGCATTCCA TCGCCTCTCA TGAGGCTTTT   
  
  
+ TGCATGTGCT TATGTATCTT TCATCTCTTA CTTTGGTGGG GTGGCATTGG GGAGGAAAAG GGGGTTTCGA   
  
  
+ TTCGATAGAT CGTCACTTTC GGAAATGACG TCTCTGACCA ATGGTAAGGG TAAGGTTAAT ACATGTGGAT   
  
  
+ ATTTCCAGAG TTAGTTGTGA TTGTTATTGT TGTTGTTGTA ACTTTATGTC ACGGGGAAAA CATAATAATT   
  
  
+ ATCTGTTTAT CTGTTTGTTT GTTATTTTCA AGGGGAAGGG GAGTGGGCGG ATGCTTAGTA TGTAATCATT   
  
  
+ TTATATAGGG AAAATTCATA TAGCATGAAA GTAAAACTTT CATTGCCAGC ATTCTAAGCT AGGGGTAGGG   
  
  
+ TTGTCTACAC CTTGATCTCC CTAGAAGCAT GGTGATCATT CCTCAGATTG TGTTATAGAA TAAAGAATGT   
  
  
+ GGTTCTGCTG TAATGAGACT TATGTATTAT CAAATGATGA ATCTGTTTAG AACTTCCTGT TTATTTTTTC   
  
  
+ TGCAAAATTT TAAGAGACTA GCATCTATCG GGCTCGACTC TAGAGGTTCT CTTGTGTTAC CCTTCTCAAC   
  
  
+ TCAGTTTGAT TGTGATACAG TTACTATATT GAGTGATAGT CGAGAGCATC ACAACCCGCT AAGGAGTCTA   
  
  
+ TCAACAAGAA GCCCTTCTTG TAATTCTCCC CTTGAAACTA GCAGTTATCA TCACTTGTCA TCGAATAGCC   
  
  
+ CTTTTTGTAA TTCTACCCTT GAAACTAGCA GTTATTGTCA TCAGTTCAAT GTGAGTCCCG TTAGATATTC   
  
  
+ CCATCAAGGC ATAGATTATG GAGTGAGCAT GAAGAACGCT TTGCACGAGC TAGAGACTGC TCTAATTGGT   
  
  
+ GTAGATGGCG AGAAAGCATC TGTTGCTAAT CAACCTATGG GGGGAATTCA TTAGTCCGGG ATCCCAAGTT   
  
  
+ AGAGATCGAA GTCATTGAGT GAAGATCCAC AGGGTTCACA TCCTCCTCAG CTTGATTCAT CATCTTTTTC   
  
  
+ AAGGGTGAAA AGATTCGGAG ATGAAAGCCA GAGAGAGAGA AATGCCACAA GGCAATGGAA GAACCAGCGG   
  
  
+ AACTACTAAG TTTCTCACCA GGTGATTTAA AGCAGTTGCT AATTGAATGT GCGAGGGCTT TATCAGATAA   
  
  
+ CCGAATAGAT GACTTTGAGA GTTTGGCTAA ACGGCCAAGG AAAGAGGTCT CCATCTCAGG TGAGCCTGTC   
  
  
+ CAACGTCTCG GTACTTGTAT GATCGAAGGG CTTGTGGCAA GGAAACAGTC TTCGGGGACT AACATCTATC   
  
  
+ GGGCTCTTAA GTACTGTAAA GAGCCTCTTG GAAAATACTT GCTCTCCTAC GGCACTCAGT GGGTTACTCT   
  
  
+ ATCGCAAGCC ATAGCAGCAA GGCTTGGTGG GCCTCCTAAA GTGCGACTTA CAGGCATTGA TGATCCTGTT   
  
  
+ TCTAAGTATA CTTGTGATGC TAGCTTGGAG GCTATTGGGA AACGATTAGC GTCTCTATTT GAAAAGTTTA   
  
  
+ AAATACTCGT CGAGTTCAAT GCATTGCCCG TTTATGGACC TGATGTCAGG TGGGAAATGC TGGATGTGAG   
  
  
+ GCCCAGGGAG GCTTTGGCCG TTAATTGTCC ATTACAGCTC CATCACACTC CTGACGAGAG TGTCGATGTG   
  
  
+ AGCAACCCTA GGGATAGGCT TCTCAGAATG GTGAAATTGC TCGGTCCTAA GGTATGCACT TTGGTTGAGC   
  
  
+ AAGAATCAAA CACCAACACT ACACGTTTCT TGACCCGGTT CATAGAGACC CTTGACTACT ACTCGATCAG   
  
  
+ CCATGTTTG  

- +Up\_Stream \_Len000TCAACA TCGTTCGTGT AATTTCAACT AATAGTAAAA CAGGCACTTT GCCACTCGTT   
  
  
- TGCAGTAGAG GTCCAGTTTA AGTAAGCATG AGTTAGTGTT CAACGACTGT AAAAATGGTT TCCGATTAGC   
  
  
- AGCGTTTTCA AAGAAATCAA ATTCCGGTTA GAGAGGCAAC AAAGTGGGGT ATGTCTAACT CCCCAACATT   
  
  
- ATCACATATA TATATCGGAA CACAATCGCC CCGAACCCGT AATCTGAAAA TGAGAACATT TGGGATTACA   
  
  
- CAATAATATT TATATCTAGA TCGGTGGGTA CAACTCCATC CGTGAGATTA AGACTGTGTC CATCCTATTC   
  
  
- AAAAGTATCA AATACAACAC TTAGAACAGA ATGAAACACC AAGGATAACT GTTTTCAAAC TTAGGCTCGT   
  
  
- ACTCTCAAGT AGTTAGAGAA AAAGAAAGGA TAAAATAAAG AACACCTGAA TTAACCTAAA AGACCATCTC   
  
  
- CTTTCAAGGA ACGACCTTAA ACCAGCTTTC ACTATATGTA AAACCCTAAA TTAATCGGTA TTTAAACCAA   
  
  
- AATGTGGAAC AATCAAACCT CAAGACCACA CAATAACATA CACAAGACCA CCCAAAAAGA AAATCGATTT   
  
  
- ACAACCTCGT AAAATTCCCT AGATCTATAG ACTTCTTCAC GAACCCTTTA CTTCGTATAA AGGGAATATC   
  
  
- AGACTGTATT GAAATTACTT AACTAGTACT TGTTTCCCGT TAATCAAGTC TTTCCAAGTT GAGGAGATCC   
  
  
- ACAACACGAT TTCTCTACAC TCAATCATTG TTAACGAATG ATTGATATAT CCAACAAAAT AATGAACATC   
  
  
- TCCAAAAAAA AAACCCTATA GAAAAACCCC AAATATTTAC TCTTCCAACT AAACAACAGA TGAGAAGAAA   
  
  
- GCTAAGTTAT CATTAAGAGA GTCTCTTCCG ATTTTACTGT ACCGTAAGGT AGCGGAGAGT ACTCCGAAAA   
  
  
- ACGTACACGA ATACATAGAA AGTAGAGAAT GAAACCACCC CACCGTAACC CCTCCTTTTC CCCCAAAGCT   
  
  
- AAGCTATCTA GCAGTGAAAG CCTTTACTGC AGAGACTGGT TACCATTCCC ATTCCAATTA TGTACACCTA   
  
  
- TAAAGGTCTC AATCAACACT AACAATAACA ACAACAACAT TGAAATACAG TGCCCCTTTT GTATTATTAA   
  
  
- TAGACAAATA GACAAACAAA CAATAAAAGT TCCCCTTCCC CTCACCCGCC TACGAATCAT ACATTAGTAA   
  
  
- AATATATCCC TTTTAAGTAT ATCGTACTTT CATTTTGAAA GTAACGGTCG TAAGATTCGA TCCCCATCCC   
  
  
- AACAGATGTG GAACTAGAGG GATCTTCGTA CCACTAGTAA GGAGTCTAAC ACAATATCTT ATTTCTTACA   
  
  
- CCAAGACGAC ATTACTCTGA ATACATAATA GTTTACTACT TAGACAAATC TTGAAGGACA AATAAAAAAG   
  
  
- ACGTTTTAAA ATTCTCTGAT CGTAGATAGC CCGAGCTGAG ATCTCCAAGA GAACACAATG GGAAGAGTTG   
  
  
- AGTCAAACTA ACACTATGTC AATGATATAA CTCACTATCA GCTCTCGTAG TGTTGGGCGA TTCCTCAGAT   
  
  
- AGTTGTTCTT CGGGAAGAAC ATTAAGAGGG GAACTTTGAT CGTCAATAGT AGTGAACAGT AGCTTATCGG   
  
  
- GAAAAACATT AAGATGGGAA CTTTGATCGT CAATAACAGT AGTCAAGTTA CACTCAGGGC AATCTATAAG   
  
  
- GGTAGTTCCG TATCTAATAC CTCACTCGTA CTTCTTGCGA AACGTGCTCG ATCTCTGACG AGATTAACCA   
  
  
- CATCTACCGC TCTTTCGTAG ACAACGATTA GTTGGATACC CCCCTTAAGT AATCAGGCCC TAGGGTTCAA   
  
  
- TCTCTAGCTT CAGTAACTCA CTTCTAGGTG TCCCAAGTGT AGGAGGAGTC GAACTAAGTA GTAGAAAAAG   
  
  
- TTCCCACTTT TCTAAGCCTC TACTTTCGGT CTCTCTCTCT TTACGGTGTT CCGTTACCTT CTTGGTCGCC   
  
  
- TTGATGATTC AAAGAGTGGT CCACTAAATT TCGTCAACGA TTAACTTACA CGCTCCCGAA ATAGTCTATT   
  
  
- GGCTTATCTA CTGAAACTCT CAAACCGATT TGCCGGTTCC TTTCTCCAGA GGTAGAGTCC ACTCGGACAG   
  
  
- GTTGCAGAGC CATGAACATA CTAGCTTCCC GAACACCGTT CCTTTGTCAG AAGCCCCTGA TTGTAGATAG   
  
  
- CCCGAGAATT CATGACATTT CTCGGAGAAC CTTTTATGAA CGAGAGGATG CCGTGAGTCA CCCAATGAGA   
  
  
- TAGCGTTCGG TATCGTCGTT CCGAACCACC CGGAGGATTT CACGCTGAAT GTCCGTAACT ACTAGGACAA   
  
  
- AGATTCATAT GAACACTACG ATCGAACCTC CGATAACCCT TTGCTAATCG CAGAGATAAA CTTTTCAAAT   
  
  
- TTTATGAGCA GCTCAAGTTA CGTAACGGGC AAATACCTGG ACTACAGTCC ACCCTTTACG ACCTACACTC   
  
  
- CGGGTCCCTC CGAAACCGGC AATTAACAGG TAATGTCGAG GTAGTGTGAG GACTGCTCTC ACAGCTACAC   
  
  
- TCGTTGGGAT CCCTATCCGA AGAGTCTTAC CACTTTAACG AGCCAGGATT CCATACGTGA AACCAACTCG   
  
  
- TTCTTAGTTT GTGGTTGTGA TGTGCAAAGA ACTGGGCCAA GTATCTCTGG GAACTGATGA TGAGCTAGTC   
  
  
- GGTACAAAC

  
  
Motifs Found  

+   

| Site Name | Organism | Position | Strand | Matrix score. | sequence | function |
| --- | --- | --- | --- | --- | --- | --- |
|  | organism | 1915 | - | 4 | motif\_sequence | short\_function |
|  | organism | 1825 | - | 4 | motif\_sequence | short\_function |
|  | organism | 2286 | + | 4 | motif\_sequence | short\_function |
|  | organism | 2170 | + | 4 | motif\_sequence | short\_function |
|  | organism | 1981 | - | 4 | motif\_sequence | short\_function |
|  | organism | 841 | - | 4 | motif\_sequence | short\_function |
|  | organism | 2081 | + | 4 | motif\_sequence | short\_function |
|  | organism | 175 | + | 4 | motif\_sequence | short\_function |
|  | organism | 489 | - | 4 | motif\_sequence | short\_function |
|  | organism | 80 | + | 4 | motif\_sequence | short\_function |
|  | organism | 988 | + | 4 | motif\_sequence | short\_function |
|  | organism | 30 | - | 4 | motif\_sequence | short\_function |
|  | organism | 906 | + | 4 | motif\_sequence | short\_function |
|  | organism | 1349 | + | 4 | motif\_sequence | short\_function |

>HU08G00019.1   
+ +Up\_Stream \_Len000AGTTGT AGCAAGCACA TTAAAGTTGA TTATCATTTT GTCCGTGAAA CGGTGAGCAA   
  
  
+ ACGTCATCTC CAGGTCAAAT TCATTCGTAC TCAATCACAA GTTGCTGACA TTTTTACCAA AGGCTAATCG   
  
  
+ TCGCAAAAGT TTCTTTAGTT TAAGGCCAAT CTCTCCGTTG TTTCACCCCA TACAGATTGA GGGGTTGTAA   
  
  
+ TAGTGTATAT ATATAGCCTT GTGTTAGCGG GGCTTGGGCA TTAGACTTTT ACTCTTGTAA ACCCTAATGT   
  
  
+ GTTATTATAA ATATAGATCT AGCCACCCAT GTTGAGGTAG GCACTCTAAT TCTGACACAG GTAGGATAAG   
  
  
+ TTTTCATAGT TTATGTTGTG AATCTTGTCT TACTTTGTGG TTCCTATTGA CAAAAGTTTG AATCCGAGCA   
  
  
+ TGAGAGTTCA TCAATCTCTT TTTCTTTCCT ATTTTATTTC TTGTGGACTT AATTGGATTT TCTGGTAGAG   
  
  
+ GAAAGTTCCT TGCTGGAATT TGGTCGAAAG TGATATACAT TTTGGGATTT AATTAGCCAT AAATTTGGTT   
  
  
+ TTACACCTTG TTAGTTTGGA GTTCTGGTGT GTTATTGTAT GTGTTCTGGT GGGTTTTTCT TTTAGCTAAA   
  
  
+ TGTTGGAGCA TTTTAAGGGA TCTAGATATC TGAAGAAGTG CTTGGGAAAT GAAGCATATT TCCCTTATAG   
  
  
+ TCTGACATAA CTTTAATGAA TTGATCATGA ACAAAGGGCA ATTAGTTCAG AAAGGTTCAA CTCCTCTAGG   
  
  
+ TGTTGTGCTA AAGAGATGTG AGTTAGTAAC AATTGCTTAC TAACTATATA GGTTGTTTTA TTACTTGTAG   
  
  
+ AGGTTTTTTT TTTGGGATAT CTTTTTGGGG TTTATAAATG AGAAGGTTGA TTTGTTGTCT ACTCTTCTTT   
  
  
+ CGATTCAATA GTAATTCTCT CAGAGAAGGC TAAAATGACA TGGCATTCCA TCGCCTCTCA TGAGGCTTTT   
  
  
+ TGCATGTGCT TATGTATCTT TCATCTCTTA CTTTGGTGGG GTGGCATTGG GGAGGAAAAG GGGGTTTCGA   
  
  
+ TTCGATAGAT CGTCACTTTC GGAAATGACG TCTCTGACCA ATGGTAAGGG TAAGGTTAAT ACATGTGGAT   
  
  
+ ATTTCCAGAG TTAGTTGTGA TTGTTATTGT TGTTGTTGTA ACTTTATGTC ACGGGGAAAA CATAATAATT   
  
  
+ ATCTGTTTAT CTGTTTGTTT GTTATTTTCA AGGGGAAGGG GAGTGGGCGG ATGCTTAGTA TGTAATCATT   
  
  
+ TTATATAGGG AAAATTCATA TAGCATGAAA GTAAAACTTT CATTGCCAGC ATTCTAAGCT AGGGGTAGGG   
  
  
+ TTGTCTACAC CTTGATCTCC CTAGAAGCAT GGTGATCATT CCTCAGATTG TGTTATAGAA TAAAGAATGT   
  
  
+ GGTTCTGCTG TAATGAGACT TATGTATTAT CAAATGATGA ATCTGTTTAG AACTTCCTGT TTATTTTTTC   
  
  
+ TGCAAAATTT TAAGAGACTA GCATCTATCG GGCTCGACTC TAGAGGTTCT CTTGTGTTAC CCTTCTCAAC   
  
  
+ TCAGTTTGAT TGTGATACAG TTACTATATT GAGTGATAGT CGAGAGCATC ACAACCCGCT AAGGAGTCTA   
  
  
+ TCAACAAGAA GCCCTTCTTG TAATTCTCCC CTTGAAACTA GCAGTTATCA TCACTTGTCA TCGAATAGCC   
  
  
+ CTTTTTGTAA TTCTACCCTT GAAACTAGCA GTTATTGTCA TCAGTTCAAT GTGAGTCCCG TTAGATATTC   
  
  
+ CCATCAAGGC ATAGATTATG GAGTGAGCAT GAAGAACGCT TTGCACGAGC TAGAGACTGC TCTAATTGGT   
  
  
+ GTAGATGGCG AGAAAGCATC TGTTGCTAAT CAACCTATGG GGGGAATTCA TTAGTCCGGG ATCCCAAGTT   
  
  
+ AGAGATCGAA GTCATTGAGT GAAGATCCAC AGGGTTCACA TCCTCCTCAG CTTGATTCAT CATCTTTTTC   
  
  
+ AAGGGTGAAA AGATTCGGAG ATGAAAGCCA GAGAGAGAGA AATGCCACAA GGCAATGGAA GAACCAGCGG   
  
  
+ AACTACTAAG TTTCTCACCA GGTGATTTAA AGCAGTTGCT AATTGAATGT GCGAGGGCTT TATCAGATAA   
  
  
+ CCGAATAGAT GACTTTGAGA GTTTGGCTAA ACGGCCAAGG AAAGAGGTCT CCATCTCAGG TGAGCCTGTC   
  
  
+ CAACGTCTCG GTACTTGTAT GATCGAAGGG CTTGTGGCAA GGAAACAGTC TTCGGGGACT AACATCTATC   
  
  
+ GGGCTCTTAA GTACTGTAAA GAGCCTCTTG GAAAATACTT GCTCTCCTAC GGCACTCAGT GGGTTACTCT   
  
  
+ ATCGCAAGCC ATAGCAGCAA GGCTTGGTGG GCCTCCTAAA GTGCGACTTA CAGGCATTGA TGATCCTGTT   
  
  
+ TCTAAGTATA CTTGTGATGC TAGCTTGGAG GCTATTGGGA AACGATTAGC GTCTCTATTT GAAAAGTTTA   
  
  
+ AAATACTCGT CGAGTTCAAT GCATTGCCCG TTTATGGACC TGATGTCAGG TGGGAAATGC TGGATGTGAG   
  
  
+ GCCCAGGGAG GCTTTGGCCG TTAATTGTCC ATTACAGCTC CATCACACTC CTGACGAGAG TGTCGATGTG   
  
  
+ AGCAACCCTA GGGATAGGCT TCTCAGAATG GTGAAATTGC TCGGTCCTAA GGTATGCACT TTGGTTGAGC   
  
  
+ AAGAATCAAA CACCAACACT ACACGTTTCT TGACCCGGTT CATAGAGACC CTTGACTACT ACTCGATCAG   
  
  
+ CCATGTTTG  

- +Up\_Stream \_Len000TCAACA TCGTTCGTGT AATTTCAACT AATAGTAAAA CAGGCACTTT GCCACTCGTT   
  
  
- TGCAGTAGAG GTCCAGTTTA AGTAAGCATG AGTTAGTGTT CAACGACTGT AAAAATGGTT TCCGATTAGC   
  
  
- AGCGTTTTCA AAGAAATCAA ATTCCGGTTA GAGAGGCAAC AAAGTGGGGT ATGTCTAACT CCCCAACATT   
  
  
- ATCACATATA TATATCGGAA CACAATCGCC CCGAACCCGT AATCTGAAAA TGAGAACATT TGGGATTACA   
  
  
- CAATAATATT TATATCTAGA TCGGTGGGTA CAACTCCATC CGTGAGATTA AGACTGTGTC CATCCTATTC   
  
  
- AAAAGTATCA AATACAACAC TTAGAACAGA ATGAAACACC AAGGATAACT GTTTTCAAAC TTAGGCTCGT   
  
  
- ACTCTCAAGT AGTTAGAGAA AAAGAAAGGA TAAAATAAAG AACACCTGAA TTAACCTAAA AGACCATCTC   
  
  
- CTTTCAAGGA ACGACCTTAA ACCAGCTTTC ACTATATGTA AAACCCTAAA TTAATCGGTA TTTAAACCAA   
  
  
- AATGTGGAAC AATCAAACCT CAAGACCACA CAATAACATA CACAAGACCA CCCAAAAAGA AAATCGATTT   
  
  
- ACAACCTCGT AAAATTCCCT AGATCTATAG ACTTCTTCAC GAACCCTTTA CTTCGTATAA AGGGAATATC   
  
  
- AGACTGTATT GAAATTACTT AACTAGTACT TGTTTCCCGT TAATCAAGTC TTTCCAAGTT GAGGAGATCC   
  
  
- ACAACACGAT TTCTCTACAC TCAATCATTG TTAACGAATG ATTGATATAT CCAACAAAAT AATGAACATC   
  
  
- TCCAAAAAAA AAACCCTATA GAAAAACCCC AAATATTTAC TCTTCCAACT AAACAACAGA TGAGAAGAAA   
  
  
- GCTAAGTTAT CATTAAGAGA GTCTCTTCCG ATTTTACTGT ACCGTAAGGT AGCGGAGAGT ACTCCGAAAA   
  
  
- ACGTACACGA ATACATAGAA AGTAGAGAAT GAAACCACCC CACCGTAACC CCTCCTTTTC CCCCAAAGCT   
  
  
- AAGCTATCTA GCAGTGAAAG CCTTTACTGC AGAGACTGGT TACCATTCCC ATTCCAATTA TGTACACCTA   
  
  
- TAAAGGTCTC AATCAACACT AACAATAACA ACAACAACAT TGAAATACAG TGCCCCTTTT GTATTATTAA   
  
  
- TAGACAAATA GACAAACAAA CAATAAAAGT TCCCCTTCCC CTCACCCGCC TACGAATCAT ACATTAGTAA   
  
  
- AATATATCCC TTTTAAGTAT ATCGTACTTT CATTTTGAAA GTAACGGTCG TAAGATTCGA TCCCCATCCC   
  
  
- AACAGATGTG GAACTAGAGG GATCTTCGTA CCACTAGTAA GGAGTCTAAC ACAATATCTT ATTTCTTACA   
  
  
- CCAAGACGAC ATTACTCTGA ATACATAATA GTTTACTACT TAGACAAATC TTGAAGGACA AATAAAAAAG   
  
  
- ACGTTTTAAA ATTCTCTGAT CGTAGATAGC CCGAGCTGAG ATCTCCAAGA GAACACAATG GGAAGAGTTG   
  
  
- AGTCAAACTA ACACTATGTC AATGATATAA CTCACTATCA GCTCTCGTAG TGTTGGGCGA TTCCTCAGAT   
  
  
- AGTTGTTCTT CGGGAAGAAC ATTAAGAGGG GAACTTTGAT CGTCAATAGT AGTGAACAGT AGCTTATCGG   
  
  
- GAAAAACATT AAGATGGGAA CTTTGATCGT CAATAACAGT AGTCAAGTTA CACTCAGGGC AATCTATAAG   
  
  
- GGTAGTTCCG TATCTAATAC CTCACTCGTA CTTCTTGCGA AACGTGCTCG ATCTCTGACG AGATTAACCA   
  
  
- CATCTACCGC TCTTTCGTAG ACAACGATTA GTTGGATACC CCCCTTAAGT AATCAGGCCC TAGGGTTCAA   
  
  
- TCTCTAGCTT CAGTAACTCA CTTCTAGGTG TCCCAAGTGT AGGAGGAGTC GAACTAAGTA GTAGAAAAAG   
  
  
- TTCCCACTTT TCTAAGCCTC TACTTTCGGT CTCTCTCTCT TTACGGTGTT CCGTTACCTT CTTGGTCGCC   
  
  
- TTGATGATTC AAAGAGTGGT CCACTAAATT TCGTCAACGA TTAACTTACA CGCTCCCGAA ATAGTCTATT   
  
  
- GGCTTATCTA CTGAAACTCT CAAACCGATT TGCCGGTTCC TTTCTCCAGA GGTAGAGTCC ACTCGGACAG   
  
  
- GTTGCAGAGC CATGAACATA CTAGCTTCCC GAACACCGTT CCTTTGTCAG AAGCCCCTGA TTGTAGATAG   
  
  
- CCCGAGAATT CATGACATTT CTCGGAGAAC CTTTTATGAA CGAGAGGATG CCGTGAGTCA CCCAATGAGA   
  
  
- TAGCGTTCGG TATCGTCGTT CCGAACCACC CGGAGGATTT CACGCTGAAT GTCCGTAACT ACTAGGACAA   
  
  
- AGATTCATAT GAACACTACG ATCGAACCTC CGATAACCCT TTGCTAATCG CAGAGATAAA CTTTTCAAAT   
  
  
- TTTATGAGCA GCTCAAGTTA CGTAACGGGC AAATACCTGG ACTACAGTCC ACCCTTTACG ACCTACACTC   
  
  
- CGGGTCCCTC CGAAACCGGC AATTAACAGG TAATGTCGAG GTAGTGTGAG GACTGCTCTC ACAGCTACAC   
  
  
- TCGTTGGGAT CCCTATCCGA AGAGTCTTAC CACTTTAACG AGCCAGGATT CCATACGTGA AACCAACTCG   
  
  
- TTCTTAGTTT GTGGTTGTGA TGTGCAAAGA ACTGGGCCAA GTATCTCTGG GAACTGATGA TGAGCTAGTC   
  
  
- GGTACAAAC

+     3-AF1 binding site

| Site Name | Organism | Position | Strand | Matrix score. | sequence | function |
| --- | --- | --- | --- | --- | --- | --- |
| 3-AF1 binding site | Solanum tuberosum | 1004 | - | 10 | TAAGAGAGGAA | light responsive element |

>HU08G00019.1   
+ +Up\_Stream \_Len000AGTTGT AGCAAGCACA TTAAAGTTGA TTATCATTTT GTCCGTGAAA CGGTGAGCAA   
  
  
+ ACGTCATCTC CAGGTCAAAT TCATTCGTAC TCAATCACAA GTTGCTGACA TTTTTACCAA AGGCTAATCG   
  
  
+ TCGCAAAAGT TTCTTTAGTT TAAGGCCAAT CTCTCCGTTG TTTCACCCCA TACAGATTGA GGGGTTGTAA   
  
  
+ TAGTGTATAT ATATAGCCTT GTGTTAGCGG GGCTTGGGCA TTAGACTTTT ACTCTTGTAA ACCCTAATGT   
  
  
+ GTTATTATAA ATATAGATCT AGCCACCCAT GTTGAGGTAG GCACTCTAAT TCTGACACAG GTAGGATAAG   
  
  
+ TTTTCATAGT TTATGTTGTG AATCTTGTCT TACTTTGTGG TTCCTATTGA CAAAAGTTTG AATCCGAGCA   
  
  
+ TGAGAGTTCA TCAATCTCTT TTTCTTTCCT ATTTTATTTC TTGTGGACTT AATTGGATTT TCTGGTAGAG   
  
  
+ GAAAGTTCCT TGCTGGAATT TGGTCGAAAG TGATATACAT TTTGGGATTT AATTAGCCAT AAATTTGGTT   
  
  
+ TTACACCTTG TTAGTTTGGA GTTCTGGTGT GTTATTGTAT GTGTTCTGGT GGGTTTTTCT TTTAGCTAAA   
  
  
+ TGTTGGAGCA TTTTAAGGGA TCTAGATATC TGAAGAAGTG CTTGGGAAAT GAAGCATATT TCCCTTATAG   
  
  
+ TCTGACATAA CTTTAATGAA TTGATCATGA ACAAAGGGCA ATTAGTTCAG AAAGGTTCAA CTCCTCTAGG   
  
  
+ TGTTGTGCTA AAGAGATGTG AGTTAGTAAC AATTGCTTAC TAACTATATA GGTTGTTTTA TTACTTGTAG   
  
  
+ AGGTTTTTTT TTTGGGATAT CTTTTTGGGG TTTATAAATG AGAAGGTTGA TTTGTTGTCT ACTCTTCTTT   
  
  
+ CGATTCAATA GTAATTCTCT CAGAGAAGGC TAAAATGACA TGGCATTCCA TCGCCTCTCA TGAGGCTTTT   
  
  
+ TGCATGTGCT TATGTATCTT TCATCTCTTA CTTTGGTGGG GTGGCATTGG GGAGGAAAAG GGGGTTTCGA   
  
  
+ TTCGATAGAT CGTCACTTTC GGAAATGACG TCTCTGACCA ATGGTAAGGG TAAGGTTAAT ACATGTGGAT   
  
  
+ ATTTCCAGAG TTAGTTGTGA TTGTTATTGT TGTTGTTGTA ACTTTATGTC ACGGGGAAAA CATAATAATT   
  
  
+ ATCTGTTTAT CTGTTTGTTT GTTATTTTCA AGGGGAAGGG GAGTGGGCGG ATGCTTAGTA TGTAATCATT   
  
  
+ TTATATAGGG AAAATTCATA TAGCATGAAA GTAAAACTTT CATTGCCAGC ATTCTAAGCT AGGGGTAGGG   
  
  
+ TTGTCTACAC CTTGATCTCC CTAGAAGCAT GGTGATCATT CCTCAGATTG TGTTATAGAA TAAAGAATGT   
  
  
+ GGTTCTGCTG TAATGAGACT TATGTATTAT CAAATGATGA ATCTGTTTAG AACTTCCTGT TTATTTTTTC   
  
  
+ TGCAAAATTT TAAGAGACTA GCATCTATCG GGCTCGACTC TAGAGGTTCT CTTGTGTTAC CCTTCTCAAC   
  
  
+ TCAGTTTGAT TGTGATACAG TTACTATATT GAGTGATAGT CGAGAGCATC ACAACCCGCT AAGGAGTCTA   
  
  
+ TCAACAAGAA GCCCTTCTTG TAATTCTCCC CTTGAAACTA GCAGTTATCA TCACTTGTCA TCGAATAGCC   
  
  
+ CTTTTTGTAA TTCTACCCTT GAAACTAGCA GTTATTGTCA TCAGTTCAAT GTGAGTCCCG TTAGATATTC   
  
  
+ CCATCAAGGC ATAGATTATG GAGTGAGCAT GAAGAACGCT TTGCACGAGC TAGAGACTGC TCTAATTGGT   
  
  
+ GTAGATGGCG AGAAAGCATC TGTTGCTAAT CAACCTATGG GGGGAATTCA TTAGTCCGGG ATCCCAAGTT   
  
  
+ AGAGATCGAA GTCATTGAGT GAAGATCCAC AGGGTTCACA TCCTCCTCAG CTTGATTCAT CATCTTTTTC   
  
  
+ AAGGGTGAAA AGATTCGGAG ATGAAAGCCA GAGAGAGAGA AATGCCACAA GGCAATGGAA GAACCAGCGG   
  
  
+ AACTACTAAG TTTCTCACCA GGTGATTTAA AGCAGTTGCT AATTGAATGT GCGAGGGCTT TATCAGATAA   
  
  
+ CCGAATAGAT GACTTTGAGA GTTTGGCTAA ACGGCCAAGG AAAGAGGTCT CCATCTCAGG TGAGCCTGTC   
  
  
+ CAACGTCTCG GTACTTGTAT GATCGAAGGG CTTGTGGCAA GGAAACAGTC TTCGGGGACT AACATCTATC   
  
  
+ GGGCTCTTAA GTACTGTAAA GAGCCTCTTG GAAAATACTT GCTCTCCTAC GGCACTCAGT GGGTTACTCT   
  
  
+ ATCGCAAGCC ATAGCAGCAA GGCTTGGTGG GCCTCCTAAA GTGCGACTTA CAGGCATTGA TGATCCTGTT   
  
  
+ TCTAAGTATA CTTGTGATGC TAGCTTGGAG GCTATTGGGA AACGATTAGC GTCTCTATTT GAAAAGTTTA   
  
  
+ AAATACTCGT CGAGTTCAAT GCATTGCCCG TTTATGGACC TGATGTCAGG TGGGAAATGC TGGATGTGAG   
  
  
+ GCCCAGGGAG GCTTTGGCCG TTAATTGTCC ATTACAGCTC CATCACACTC CTGACGAGAG TGTCGATGTG   
  
  
+ AGCAACCCTA GGGATAGGCT TCTCAGAATG GTGAAATTGC TCGGTCCTAA GGTATGCACT TTGGTTGAGC   
  
  
+ AAGAATCAAA CACCAACACT ACACGTTTCT TGACCCGGTT CATAGAGACC CTTGACTACT ACTCGATCAG   
  
  
+ CCATGTTTG  

- +Up\_Stream \_Len000TCAACA TCGTTCGTGT AATTTCAACT AATAGTAAAA CAGGCACTTT GCCACTCGTT   
  
  
- TGCAGTAGAG GTCCAGTTTA AGTAAGCATG AGTTAGTGTT CAACGACTGT AAAAATGGTT TCCGATTAGC   
  
  
- AGCGTTTTCA AAGAAATCAA ATTCCGGTTA GAGAGGCAAC AAAGTGGGGT ATGTCTAACT CCCCAACATT   
  
  
- ATCACATATA TATATCGGAA CACAATCGCC CCGAACCCGT AATCTGAAAA TGAGAACATT TGGGATTACA   
  
  
- CAATAATATT TATATCTAGA TCGGTGGGTA CAACTCCATC CGTGAGATTA AGACTGTGTC CATCCTATTC   
  
  
- AAAAGTATCA AATACAACAC TTAGAACAGA ATGAAACACC AAGGATAACT GTTTTCAAAC TTAGGCTCGT   
  
  
- ACTCTCAAGT AGTTAGAGAA AAAGAAAGGA TAAAATAAAG AACACCTGAA TTAACCTAAA AGACCATCTC   
  
  
- CTTTCAAGGA ACGACCTTAA ACCAGCTTTC ACTATATGTA AAACCCTAAA TTAATCGGTA TTTAAACCAA   
  
  
- AATGTGGAAC AATCAAACCT CAAGACCACA CAATAACATA CACAAGACCA CCCAAAAAGA AAATCGATTT   
  
  
- ACAACCTCGT AAAATTCCCT AGATCTATAG ACTTCTTCAC GAACCCTTTA CTTCGTATAA AGGGAATATC   
  
  
- AGACTGTATT GAAATTACTT AACTAGTACT TGTTTCCCGT TAATCAAGTC TTTCCAAGTT GAGGAGATCC   
  
  
- ACAACACGAT TTCTCTACAC TCAATCATTG TTAACGAATG ATTGATATAT CCAACAAAAT AATGAACATC   
  
  
- TCCAAAAAAA AAACCCTATA GAAAAACCCC AAATATTTAC TCTTCCAACT AAACAACAGA TGAGAAGAAA   
  
  
- GCTAAGTTAT CATTAAGAGA GTCTCTTCCG ATTTTACTGT ACCGTAAGGT AGCGGAGAGT ACTCCGAAAA   
  
  
- ACGTACACGA ATACATAGAA AGTAGAGAAT GAAACCACCC CACCGTAACC CCTCCTTTTC CCCCAAAGCT   
  
  
- AAGCTATCTA GCAGTGAAAG CCTTTACTGC AGAGACTGGT TACCATTCCC ATTCCAATTA TGTACACCTA   
  
  
- TAAAGGTCTC AATCAACACT AACAATAACA ACAACAACAT TGAAATACAG TGCCCCTTTT GTATTATTAA   
  
  
- TAGACAAATA GACAAACAAA CAATAAAAGT TCCCCTTCCC CTCACCCGCC TACGAATCAT ACATTAGTAA   
  
  
- AATATATCCC TTTTAAGTAT ATCGTACTTT CATTTTGAAA GTAACGGTCG TAAGATTCGA TCCCCATCCC   
  
  
- AACAGATGTG GAACTAGAGG GATCTTCGTA CCACTAGTAA GGAGTCTAAC ACAATATCTT ATTTCTTACA   
  
  
- CCAAGACGAC ATTACTCTGA ATACATAATA GTTTACTACT TAGACAAATC TTGAAGGACA AATAAAAAAG   
  
  
- ACGTTTTAAA ATTCTCTGAT CGTAGATAGC CCGAGCTGAG ATCTCCAAGA GAACACAATG GGAAGAGTTG   
  
  
- AGTCAAACTA ACACTATGTC AATGATATAA CTCACTATCA GCTCTCGTAG TGTTGGGCGA TTCCTCAGAT   
  
  
- AGTTGTTCTT CGGGAAGAAC ATTAAGAGGG GAACTTTGAT CGTCAATAGT AGTGAACAGT AGCTTATCGG   
  
  
- GAAAAACATT AAGATGGGAA CTTTGATCGT CAATAACAGT AGTCAAGTTA CACTCAGGGC AATCTATAAG   
  
  
- GGTAGTTCCG TATCTAATAC CTCACTCGTA CTTCTTGCGA AACGTGCTCG ATCTCTGACG AGATTAACCA   
  
  
- CATCTACCGC TCTTTCGTAG ACAACGATTA GTTGGATACC CCCCTTAAGT AATCAGGCCC TAGGGTTCAA   
  
  
- TCTCTAGCTT CAGTAACTCA CTTCTAGGTG TCCCAAGTGT AGGAGGAGTC GAACTAAGTA GTAGAAAAAG   
  
  
- TTCCCACTTT TCTAAGCCTC TACTTTCGGT CTCTCTCTCT TTACGGTGTT CCGTTACCTT CTTGGTCGCC   
  
  
- TTGATGATTC AAAGAGTGGT CCACTAAATT TCGTCAACGA TTAACTTACA CGCTCCCGAA ATAGTCTATT   
  
  
- GGCTTATCTA CTGAAACTCT CAAACCGATT TGCCGGTTCC TTTCTCCAGA GGTAGAGTCC ACTCGGACAG   
  
  
- GTTGCAGAGC CATGAACATA CTAGCTTCCC GAACACCGTT CCTTTGTCAG AAGCCCCTGA TTGTAGATAG   
  
  
- CCCGAGAATT CATGACATTT CTCGGAGAAC CTTTTATGAA CGAGAGGATG CCGTGAGTCA CCCAATGAGA   
  
  
- TAGCGTTCGG TATCGTCGTT CCGAACCACC CGGAGGATTT CACGCTGAAT GTCCGTAACT ACTAGGACAA   
  
  
- AGATTCATAT GAACACTACG ATCGAACCTC CGATAACCCT TTGCTAATCG CAGAGATAAA CTTTTCAAAT   
  
  
- TTTATGAGCA GCTCAAGTTA CGTAACGGGC AAATACCTGG ACTACAGTCC ACCCTTTACG ACCTACACTC   
  
  
- CGGGTCCCTC CGAAACCGGC AATTAACAGG TAATGTCGAG GTAGTGTGAG GACTGCTCTC ACAGCTACAC   
  
  
- TCGTTGGGAT CCCTATCCGA AGAGTCTTAC CACTTTAACG AGCCAGGATT CCATACGTGA AACCAACTCG   
  
  
- TTCTTAGTTT GTGGTTGTGA TGTGCAAAGA ACTGGGCCAA GTATCTCTGG GAACTGATGA TGAGCTAGTC   
  
  
- GGTACAAAC

+     AAGAA-motif

| Site Name | Organism | Position | Strand | Matrix score. | sequence | function |
| --- | --- | --- | --- | --- | --- | --- |
| AAGAA-motif | Avena sativa | 2690 | - | 9 | gGTAAAGAAA |  |
| AAGAA-motif | Avena sativa | 909 | - | 7 | GAAAGAA |  |
| AAGAA-motif | Avena sativa | 446 | - | 7 | GAAAGAA |  |

>HU08G00019.1   
+ +Up\_Stream \_Len000AGTTGT AGCAAGCACA TTAAAGTTGA TTATCATTTT GTCCGTGAAA CGGTGAGCAA   
  
  
+ ACGTCATCTC CAGGTCAAAT TCATTCGTAC TCAATCACAA GTTGCTGACA TTTTTACCAA AGGCTAATCG   
  
  
+ TCGCAAAAGT TTCTTTAGTT TAAGGCCAAT CTCTCCGTTG TTTCACCCCA TACAGATTGA GGGGTTGTAA   
  
  
+ TAGTGTATAT ATATAGCCTT GTGTTAGCGG GGCTTGGGCA TTAGACTTTT ACTCTTGTAA ACCCTAATGT   
  
  
+ GTTATTATAA ATATAGATCT AGCCACCCAT GTTGAGGTAG GCACTCTAAT TCTGACACAG GTAGGATAAG   
  
  
+ TTTTCATAGT TTATGTTGTG AATCTTGTCT TACTTTGTGG TTCCTATTGA CAAAAGTTTG AATCCGAGCA   
  
  
+ TGAGAGTTCA TCAATCTCTT TTTCTTTCCT ATTTTATTTC TTGTGGACTT AATTGGATTT TCTGGTAGAG   
  
  
+ GAAAGTTCCT TGCTGGAATT TGGTCGAAAG TGATATACAT TTTGGGATTT AATTAGCCAT AAATTTGGTT   
  
  
+ TTACACCTTG TTAGTTTGGA GTTCTGGTGT GTTATTGTAT GTGTTCTGGT GGGTTTTTCT TTTAGCTAAA   
  
  
+ TGTTGGAGCA TTTTAAGGGA TCTAGATATC TGAAGAAGTG CTTGGGAAAT GAAGCATATT TCCCTTATAG   
  
  
+ TCTGACATAA CTTTAATGAA TTGATCATGA ACAAAGGGCA ATTAGTTCAG AAAGGTTCAA CTCCTCTAGG   
  
  
+ TGTTGTGCTA AAGAGATGTG AGTTAGTAAC AATTGCTTAC TAACTATATA GGTTGTTTTA TTACTTGTAG   
  
  
+ AGGTTTTTTT TTTGGGATAT CTTTTTGGGG TTTATAAATG AGAAGGTTGA TTTGTTGTCT ACTCTTCTTT   
  
  
+ CGATTCAATA GTAATTCTCT CAGAGAAGGC TAAAATGACA TGGCATTCCA TCGCCTCTCA TGAGGCTTTT   
  
  
+ TGCATGTGCT TATGTATCTT TCATCTCTTA CTTTGGTGGG GTGGCATTGG GGAGGAAAAG GGGGTTTCGA   
  
  
+ TTCGATAGAT CGTCACTTTC GGAAATGACG TCTCTGACCA ATGGTAAGGG TAAGGTTAAT ACATGTGGAT   
  
  
+ ATTTCCAGAG TTAGTTGTGA TTGTTATTGT TGTTGTTGTA ACTTTATGTC ACGGGGAAAA CATAATAATT   
  
  
+ ATCTGTTTAT CTGTTTGTTT GTTATTTTCA AGGGGAAGGG GAGTGGGCGG ATGCTTAGTA TGTAATCATT   
  
  
+ TTATATAGGG AAAATTCATA TAGCATGAAA GTAAAACTTT CATTGCCAGC ATTCTAAGCT AGGGGTAGGG   
  
  
+ TTGTCTACAC CTTGATCTCC CTAGAAGCAT GGTGATCATT CCTCAGATTG TGTTATAGAA TAAAGAATGT   
  
  
+ GGTTCTGCTG TAATGAGACT TATGTATTAT CAAATGATGA ATCTGTTTAG AACTTCCTGT TTATTTTTTC   
  
  
+ TGCAAAATTT TAAGAGACTA GCATCTATCG GGCTCGACTC TAGAGGTTCT CTTGTGTTAC CCTTCTCAAC   
  
  
+ TCAGTTTGAT TGTGATACAG TTACTATATT GAGTGATAGT CGAGAGCATC ACAACCCGCT AAGGAGTCTA   
  
  
+ TCAACAAGAA GCCCTTCTTG TAATTCTCCC CTTGAAACTA GCAGTTATCA TCACTTGTCA TCGAATAGCC   
  
  
+ CTTTTTGTAA TTCTACCCTT GAAACTAGCA GTTATTGTCA TCAGTTCAAT GTGAGTCCCG TTAGATATTC   
  
  
+ CCATCAAGGC ATAGATTATG GAGTGAGCAT GAAGAACGCT TTGCACGAGC TAGAGACTGC TCTAATTGGT   
  
  
+ GTAGATGGCG AGAAAGCATC TGTTGCTAAT CAACCTATGG GGGGAATTCA TTAGTCCGGG ATCCCAAGTT   
  
  
+ AGAGATCGAA GTCATTGAGT GAAGATCCAC AGGGTTCACA TCCTCCTCAG CTTGATTCAT CATCTTTTTC   
  
  
+ AAGGGTGAAA AGATTCGGAG ATGAAAGCCA GAGAGAGAGA AATGCCACAA GGCAATGGAA GAACCAGCGG   
  
  
+ AACTACTAAG TTTCTCACCA GGTGATTTAA AGCAGTTGCT AATTGAATGT GCGAGGGCTT TATCAGATAA   
  
  
+ CCGAATAGAT GACTTTGAGA GTTTGGCTAA ACGGCCAAGG AAAGAGGTCT CCATCTCAGG TGAGCCTGTC   
  
  
+ CAACGTCTCG GTACTTGTAT GATCGAAGGG CTTGTGGCAA GGAAACAGTC TTCGGGGACT AACATCTATC   
  
  
+ GGGCTCTTAA GTACTGTAAA GAGCCTCTTG GAAAATACTT GCTCTCCTAC GGCACTCAGT GGGTTACTCT   
  
  
+ ATCGCAAGCC ATAGCAGCAA GGCTTGGTGG GCCTCCTAAA GTGCGACTTA CAGGCATTGA TGATCCTGTT   
  
  
+ TCTAAGTATA CTTGTGATGC TAGCTTGGAG GCTATTGGGA AACGATTAGC GTCTCTATTT GAAAAGTTTA   
  
  
+ AAATACTCGT CGAGTTCAAT GCATTGCCCG TTTATGGACC TGATGTCAGG TGGGAAATGC TGGATGTGAG   
  
  
+ GCCCAGGGAG GCTTTGGCCG TTAATTGTCC ATTACAGCTC CATCACACTC CTGACGAGAG TGTCGATGTG   
  
  
+ AGCAACCCTA GGGATAGGCT TCTCAGAATG GTGAAATTGC TCGGTCCTAA GGTATGCACT TTGGTTGAGC   
  
  
+ AAGAATCAAA CACCAACACT ACACGTTTCT TGACCCGGTT CATAGAGACC CTTGACTACT ACTCGATCAG   
  
  
+ CCATGTTTG  

- +Up\_Stream \_Len000TCAACA TCGTTCGTGT AATTTCAACT AATAGTAAAA CAGGCACTTT GCCACTCGTT   
  
  
- TGCAGTAGAG GTCCAGTTTA AGTAAGCATG AGTTAGTGTT CAACGACTGT AAAAATGGTT TCCGATTAGC   
  
  
- AGCGTTTTCA AAGAAATCAA ATTCCGGTTA GAGAGGCAAC AAAGTGGGGT ATGTCTAACT CCCCAACATT   
  
  
- ATCACATATA TATATCGGAA CACAATCGCC CCGAACCCGT AATCTGAAAA TGAGAACATT TGGGATTACA   
  
  
- CAATAATATT TATATCTAGA TCGGTGGGTA CAACTCCATC CGTGAGATTA AGACTGTGTC CATCCTATTC   
  
  
- AAAAGTATCA AATACAACAC TTAGAACAGA ATGAAACACC AAGGATAACT GTTTTCAAAC TTAGGCTCGT   
  
  
- ACTCTCAAGT AGTTAGAGAA AAAGAAAGGA TAAAATAAAG AACACCTGAA TTAACCTAAA AGACCATCTC   
  
  
- CTTTCAAGGA ACGACCTTAA ACCAGCTTTC ACTATATGTA AAACCCTAAA TTAATCGGTA TTTAAACCAA   
  
  
- AATGTGGAAC AATCAAACCT CAAGACCACA CAATAACATA CACAAGACCA CCCAAAAAGA AAATCGATTT   
  
  
- ACAACCTCGT AAAATTCCCT AGATCTATAG ACTTCTTCAC GAACCCTTTA CTTCGTATAA AGGGAATATC   
  
  
- AGACTGTATT GAAATTACTT AACTAGTACT TGTTTCCCGT TAATCAAGTC TTTCCAAGTT GAGGAGATCC   
  
  
- ACAACACGAT TTCTCTACAC TCAATCATTG TTAACGAATG ATTGATATAT CCAACAAAAT AATGAACATC   
  
  
- TCCAAAAAAA AAACCCTATA GAAAAACCCC AAATATTTAC TCTTCCAACT AAACAACAGA TGAGAAGAAA   
  
  
- GCTAAGTTAT CATTAAGAGA GTCTCTTCCG ATTTTACTGT ACCGTAAGGT AGCGGAGAGT ACTCCGAAAA   
  
  
- ACGTACACGA ATACATAGAA AGTAGAGAAT GAAACCACCC CACCGTAACC CCTCCTTTTC CCCCAAAGCT   
  
  
- AAGCTATCTA GCAGTGAAAG CCTTTACTGC AGAGACTGGT TACCATTCCC ATTCCAATTA TGTACACCTA   
  
  
- TAAAGGTCTC AATCAACACT AACAATAACA ACAACAACAT TGAAATACAG TGCCCCTTTT GTATTATTAA   
  
  
- TAGACAAATA GACAAACAAA CAATAAAAGT TCCCCTTCCC CTCACCCGCC TACGAATCAT ACATTAGTAA   
  
  
- AATATATCCC TTTTAAGTAT ATCGTACTTT CATTTTGAAA GTAACGGTCG TAAGATTCGA TCCCCATCCC   
  
  
- AACAGATGTG GAACTAGAGG GATCTTCGTA CCACTAGTAA GGAGTCTAAC ACAATATCTT ATTTCTTACA   
  
  
- CCAAGACGAC ATTACTCTGA ATACATAATA GTTTACTACT TAGACAAATC TTGAAGGACA AATAAAAAAG   
  
  
- ACGTTTTAAA ATTCTCTGAT CGTAGATAGC CCGAGCTGAG ATCTCCAAGA GAACACAATG GGAAGAGTTG   
  
  
- AGTCAAACTA ACACTATGTC AATGATATAA CTCACTATCA GCTCTCGTAG TGTTGGGCGA TTCCTCAGAT   
  
  
- AGTTGTTCTT CGGGAAGAAC ATTAAGAGGG GAACTTTGAT CGTCAATAGT AGTGAACAGT AGCTTATCGG   
  
  
- GAAAAACATT AAGATGGGAA CTTTGATCGT CAATAACAGT AGTCAAGTTA CACTCAGGGC AATCTATAAG   
  
  
- GGTAGTTCCG TATCTAATAC CTCACTCGTA CTTCTTGCGA AACGTGCTCG ATCTCTGACG AGATTAACCA   
  
  
- CATCTACCGC TCTTTCGTAG ACAACGATTA GTTGGATACC CCCCTTAAGT AATCAGGCCC TAGGGTTCAA   
  
  
- TCTCTAGCTT CAGTAACTCA CTTCTAGGTG TCCCAAGTGT AGGAGGAGTC GAACTAAGTA GTAGAAAAAG   
  
  
- TTCCCACTTT TCTAAGCCTC TACTTTCGGT CTCTCTCTCT TTACGGTGTT CCGTTACCTT CTTGGTCGCC   
  
  
- TTGATGATTC AAAGAGTGGT CCACTAAATT TCGTCAACGA TTAACTTACA CGCTCCCGAA ATAGTCTATT   
  
  
- GGCTTATCTA CTGAAACTCT CAAACCGATT TGCCGGTTCC TTTCTCCAGA GGTAGAGTCC ACTCGGACAG   
  
  
- GTTGCAGAGC CATGAACATA CTAGCTTCCC GAACACCGTT CCTTTGTCAG AAGCCCCTGA TTGTAGATAG   
  
  
- CCCGAGAATT CATGACATTT CTCGGAGAAC CTTTTATGAA CGAGAGGATG CCGTGAGTCA CCCAATGAGA   
  
  
- TAGCGTTCGG TATCGTCGTT CCGAACCACC CGGAGGATTT CACGCTGAAT GTCCGTAACT ACTAGGACAA   
  
  
- AGATTCATAT GAACACTACG ATCGAACCTC CGATAACCCT TTGCTAATCG CAGAGATAAA CTTTTCAAAT   
  
  
- TTTATGAGCA GCTCAAGTTA CGTAACGGGC AAATACCTGG ACTACAGTCC ACCCTTTACG ACCTACACTC   
  
  
- CGGGTCCCTC CGAAACCGGC AATTAACAGG TAATGTCGAG GTAGTGTGAG GACTGCTCTC ACAGCTACAC   
  
  
- TCGTTGGGAT CCCTATCCGA AGAGTCTTAC CACTTTAACG AGCCAGGATT CCATACGTGA AACCAACTCG   
  
  
- TTCTTAGTTT GTGGTTGTGA TGTGCAAAGA ACTGGGCCAA GTATCTCTGG GAACTGATGA TGAGCTAGTC   
  
  
- GGTACAAAC

+     ABRE

| Site Name | Organism | Position | Strand | Matrix score. | sequence | function |
| --- | --- | --- | --- | --- | --- | --- |
| ABRE | Arabidopsis thaliana | 2686 | - | 5 | ACGTG | cis-acting element involved in the abscisic acid responsiveness |

>HU08G00019.1   
+ +Up\_Stream \_Len000AGTTGT AGCAAGCACA TTAAAGTTGA TTATCATTTT GTCCGTGAAA CGGTGAGCAA   
  
  
+ ACGTCATCTC CAGGTCAAAT TCATTCGTAC TCAATCACAA GTTGCTGACA TTTTTACCAA AGGCTAATCG   
  
  
+ TCGCAAAAGT TTCTTTAGTT TAAGGCCAAT CTCTCCGTTG TTTCACCCCA TACAGATTGA GGGGTTGTAA   
  
  
+ TAGTGTATAT ATATAGCCTT GTGTTAGCGG GGCTTGGGCA TTAGACTTTT ACTCTTGTAA ACCCTAATGT   
  
  
+ GTTATTATAA ATATAGATCT AGCCACCCAT GTTGAGGTAG GCACTCTAAT TCTGACACAG GTAGGATAAG   
  
  
+ TTTTCATAGT TTATGTTGTG AATCTTGTCT TACTTTGTGG TTCCTATTGA CAAAAGTTTG AATCCGAGCA   
  
  
+ TGAGAGTTCA TCAATCTCTT TTTCTTTCCT ATTTTATTTC TTGTGGACTT AATTGGATTT TCTGGTAGAG   
  
  
+ GAAAGTTCCT TGCTGGAATT TGGTCGAAAG TGATATACAT TTTGGGATTT AATTAGCCAT AAATTTGGTT   
  
  
+ TTACACCTTG TTAGTTTGGA GTTCTGGTGT GTTATTGTAT GTGTTCTGGT GGGTTTTTCT TTTAGCTAAA   
  
  
+ TGTTGGAGCA TTTTAAGGGA TCTAGATATC TGAAGAAGTG CTTGGGAAAT GAAGCATATT TCCCTTATAG   
  
  
+ TCTGACATAA CTTTAATGAA TTGATCATGA ACAAAGGGCA ATTAGTTCAG AAAGGTTCAA CTCCTCTAGG   
  
  
+ TGTTGTGCTA AAGAGATGTG AGTTAGTAAC AATTGCTTAC TAACTATATA GGTTGTTTTA TTACTTGTAG   
  
  
+ AGGTTTTTTT TTTGGGATAT CTTTTTGGGG TTTATAAATG AGAAGGTTGA TTTGTTGTCT ACTCTTCTTT   
  
  
+ CGATTCAATA GTAATTCTCT CAGAGAAGGC TAAAATGACA TGGCATTCCA TCGCCTCTCA TGAGGCTTTT   
  
  
+ TGCATGTGCT TATGTATCTT TCATCTCTTA CTTTGGTGGG GTGGCATTGG GGAGGAAAAG GGGGTTTCGA   
  
  
+ TTCGATAGAT CGTCACTTTC GGAAATGACG TCTCTGACCA ATGGTAAGGG TAAGGTTAAT ACATGTGGAT   
  
  
+ ATTTCCAGAG TTAGTTGTGA TTGTTATTGT TGTTGTTGTA ACTTTATGTC ACGGGGAAAA CATAATAATT   
  
  
+ ATCTGTTTAT CTGTTTGTTT GTTATTTTCA AGGGGAAGGG GAGTGGGCGG ATGCTTAGTA TGTAATCATT   
  
  
+ TTATATAGGG AAAATTCATA TAGCATGAAA GTAAAACTTT CATTGCCAGC ATTCTAAGCT AGGGGTAGGG   
  
  
+ TTGTCTACAC CTTGATCTCC CTAGAAGCAT GGTGATCATT CCTCAGATTG TGTTATAGAA TAAAGAATGT   
  
  
+ GGTTCTGCTG TAATGAGACT TATGTATTAT CAAATGATGA ATCTGTTTAG AACTTCCTGT TTATTTTTTC   
  
  
+ TGCAAAATTT TAAGAGACTA GCATCTATCG GGCTCGACTC TAGAGGTTCT CTTGTGTTAC CCTTCTCAAC   
  
  
+ TCAGTTTGAT TGTGATACAG TTACTATATT GAGTGATAGT CGAGAGCATC ACAACCCGCT AAGGAGTCTA   
  
  
+ TCAACAAGAA GCCCTTCTTG TAATTCTCCC CTTGAAACTA GCAGTTATCA TCACTTGTCA TCGAATAGCC   
  
  
+ CTTTTTGTAA TTCTACCCTT GAAACTAGCA GTTATTGTCA TCAGTTCAAT GTGAGTCCCG TTAGATATTC   
  
  
+ CCATCAAGGC ATAGATTATG GAGTGAGCAT GAAGAACGCT TTGCACGAGC TAGAGACTGC TCTAATTGGT   
  
  
+ GTAGATGGCG AGAAAGCATC TGTTGCTAAT CAACCTATGG GGGGAATTCA TTAGTCCGGG ATCCCAAGTT   
  
  
+ AGAGATCGAA GTCATTGAGT GAAGATCCAC AGGGTTCACA TCCTCCTCAG CTTGATTCAT CATCTTTTTC   
  
  
+ AAGGGTGAAA AGATTCGGAG ATGAAAGCCA GAGAGAGAGA AATGCCACAA GGCAATGGAA GAACCAGCGG   
  
  
+ AACTACTAAG TTTCTCACCA GGTGATTTAA AGCAGTTGCT AATTGAATGT GCGAGGGCTT TATCAGATAA   
  
  
+ CCGAATAGAT GACTTTGAGA GTTTGGCTAA ACGGCCAAGG AAAGAGGTCT CCATCTCAGG TGAGCCTGTC   
  
  
+ CAACGTCTCG GTACTTGTAT GATCGAAGGG CTTGTGGCAA GGAAACAGTC TTCGGGGACT AACATCTATC   
  
  
+ GGGCTCTTAA GTACTGTAAA GAGCCTCTTG GAAAATACTT GCTCTCCTAC GGCACTCAGT GGGTTACTCT   
  
  
+ ATCGCAAGCC ATAGCAGCAA GGCTTGGTGG GCCTCCTAAA GTGCGACTTA CAGGCATTGA TGATCCTGTT   
  
  
+ TCTAAGTATA CTTGTGATGC TAGCTTGGAG GCTATTGGGA AACGATTAGC GTCTCTATTT GAAAAGTTTA   
  
  
+ AAATACTCGT CGAGTTCAAT GCATTGCCCG TTTATGGACC TGATGTCAGG TGGGAAATGC TGGATGTGAG   
  
  
+ GCCCAGGGAG GCTTTGGCCG TTAATTGTCC ATTACAGCTC CATCACACTC CTGACGAGAG TGTCGATGTG   
  
  
+ AGCAACCCTA GGGATAGGCT TCTCAGAATG GTGAAATTGC TCGGTCCTAA GGTATGCACT TTGGTTGAGC   
  
  
+ AAGAATCAAA CACCAACACT ACACGTTTCT TGACCCGGTT CATAGAGACC CTTGACTACT ACTCGATCAG   
  
  
+ CCATGTTTG  

- +Up\_Stream \_Len000TCAACA TCGTTCGTGT AATTTCAACT AATAGTAAAA CAGGCACTTT GCCACTCGTT   
  
  
- TGCAGTAGAG GTCCAGTTTA AGTAAGCATG AGTTAGTGTT CAACGACTGT AAAAATGGTT TCCGATTAGC   
  
  
- AGCGTTTTCA AAGAAATCAA ATTCCGGTTA GAGAGGCAAC AAAGTGGGGT ATGTCTAACT CCCCAACATT   
  
  
- ATCACATATA TATATCGGAA CACAATCGCC CCGAACCCGT AATCTGAAAA TGAGAACATT TGGGATTACA   
  
  
- CAATAATATT TATATCTAGA TCGGTGGGTA CAACTCCATC CGTGAGATTA AGACTGTGTC CATCCTATTC   
  
  
- AAAAGTATCA AATACAACAC TTAGAACAGA ATGAAACACC AAGGATAACT GTTTTCAAAC TTAGGCTCGT   
  
  
- ACTCTCAAGT AGTTAGAGAA AAAGAAAGGA TAAAATAAAG AACACCTGAA TTAACCTAAA AGACCATCTC   
  
  
- CTTTCAAGGA ACGACCTTAA ACCAGCTTTC ACTATATGTA AAACCCTAAA TTAATCGGTA TTTAAACCAA   
  
  
- AATGTGGAAC AATCAAACCT CAAGACCACA CAATAACATA CACAAGACCA CCCAAAAAGA AAATCGATTT   
  
  
- ACAACCTCGT AAAATTCCCT AGATCTATAG ACTTCTTCAC GAACCCTTTA CTTCGTATAA AGGGAATATC   
  
  
- AGACTGTATT GAAATTACTT AACTAGTACT TGTTTCCCGT TAATCAAGTC TTTCCAAGTT GAGGAGATCC   
  
  
- ACAACACGAT TTCTCTACAC TCAATCATTG TTAACGAATG ATTGATATAT CCAACAAAAT AATGAACATC   
  
  
- TCCAAAAAAA AAACCCTATA GAAAAACCCC AAATATTTAC TCTTCCAACT AAACAACAGA TGAGAAGAAA   
  
  
- GCTAAGTTAT CATTAAGAGA GTCTCTTCCG ATTTTACTGT ACCGTAAGGT AGCGGAGAGT ACTCCGAAAA   
  
  
- ACGTACACGA ATACATAGAA AGTAGAGAAT GAAACCACCC CACCGTAACC CCTCCTTTTC CCCCAAAGCT   
  
  
- AAGCTATCTA GCAGTGAAAG CCTTTACTGC AGAGACTGGT TACCATTCCC ATTCCAATTA TGTACACCTA   
  
  
- TAAAGGTCTC AATCAACACT AACAATAACA ACAACAACAT TGAAATACAG TGCCCCTTTT GTATTATTAA   
  
  
- TAGACAAATA GACAAACAAA CAATAAAAGT TCCCCTTCCC CTCACCCGCC TACGAATCAT ACATTAGTAA   
  
  
- AATATATCCC TTTTAAGTAT ATCGTACTTT CATTTTGAAA GTAACGGTCG TAAGATTCGA TCCCCATCCC   
  
  
- AACAGATGTG GAACTAGAGG GATCTTCGTA CCACTAGTAA GGAGTCTAAC ACAATATCTT ATTTCTTACA   
  
  
- CCAAGACGAC ATTACTCTGA ATACATAATA GTTTACTACT TAGACAAATC TTGAAGGACA AATAAAAAAG   
  
  
- ACGTTTTAAA ATTCTCTGAT CGTAGATAGC CCGAGCTGAG ATCTCCAAGA GAACACAATG GGAAGAGTTG   
  
  
- AGTCAAACTA ACACTATGTC AATGATATAA CTCACTATCA GCTCTCGTAG TGTTGGGCGA TTCCTCAGAT   
  
  
- AGTTGTTCTT CGGGAAGAAC ATTAAGAGGG GAACTTTGAT CGTCAATAGT AGTGAACAGT AGCTTATCGG   
  
  
- GAAAAACATT AAGATGGGAA CTTTGATCGT CAATAACAGT AGTCAAGTTA CACTCAGGGC AATCTATAAG   
  
  
- GGTAGTTCCG TATCTAATAC CTCACTCGTA CTTCTTGCGA AACGTGCTCG ATCTCTGACG AGATTAACCA   
  
  
- CATCTACCGC TCTTTCGTAG ACAACGATTA GTTGGATACC CCCCTTAAGT AATCAGGCCC TAGGGTTCAA   
  
  
- TCTCTAGCTT CAGTAACTCA CTTCTAGGTG TCCCAAGTGT AGGAGGAGTC GAACTAAGTA GTAGAAAAAG   
  
  
- TTCCCACTTT TCTAAGCCTC TACTTTCGGT CTCTCTCTCT TTACGGTGTT CCGTTACCTT CTTGGTCGCC   
  
  
- TTGATGATTC AAAGAGTGGT CCACTAAATT TCGTCAACGA TTAACTTACA CGCTCCCGAA ATAGTCTATT   
  
  
- GGCTTATCTA CTGAAACTCT CAAACCGATT TGCCGGTTCC TTTCTCCAGA GGTAGAGTCC ACTCGGACAG   
  
  
- GTTGCAGAGC CATGAACATA CTAGCTTCCC GAACACCGTT CCTTTGTCAG AAGCCCCTGA TTGTAGATAG   
  
  
- CCCGAGAATT CATGACATTT CTCGGAGAAC CTTTTATGAA CGAGAGGATG CCGTGAGTCA CCCAATGAGA   
  
  
- TAGCGTTCGG TATCGTCGTT CCGAACCACC CGGAGGATTT CACGCTGAAT GTCCGTAACT ACTAGGACAA   
  
  
- AGATTCATAT GAACACTACG ATCGAACCTC CGATAACCCT TTGCTAATCG CAGAGATAAA CTTTTCAAAT   
  
  
- TTTATGAGCA GCTCAAGTTA CGTAACGGGC AAATACCTGG ACTACAGTCC ACCCTTTACG ACCTACACTC   
  
  
- CGGGTCCCTC CGAAACCGGC AATTAACAGG TAATGTCGAG GTAGTGTGAG GACTGCTCTC ACAGCTACAC   
  
  
- TCGTTGGGAT CCCTATCCGA AGAGTCTTAC CACTTTAACG AGCCAGGATT CCATACGTGA AACCAACTCG   
  
  
- TTCTTAGTTT GTGGTTGTGA TGTGCAAAGA ACTGGGCCAA GTATCTCTGG GAACTGATGA TGAGCTAGTC   
  
  
- GGTACAAAC

+     AE-box

| Site Name | Organism | Position | Strand | Matrix score. | sequence | function |
| --- | --- | --- | --- | --- | --- | --- |
| AE-box | Arabidopsis thaliana | 151 | - | 8 | AGAAACTT | part of a module for light response |
| AE-box | Arabidopsis thaliana | 2042 | - | 8 | AGAAACTT | part of a module for light response |

>HU08G00019.1   
+ +Up\_Stream \_Len000AGTTGT AGCAAGCACA TTAAAGTTGA TTATCATTTT GTCCGTGAAA CGGTGAGCAA   
  
  
+ ACGTCATCTC CAGGTCAAAT TCATTCGTAC TCAATCACAA GTTGCTGACA TTTTTACCAA AGGCTAATCG   
  
  
+ TCGCAAAAGT TTCTTTAGTT TAAGGCCAAT CTCTCCGTTG TTTCACCCCA TACAGATTGA GGGGTTGTAA   
  
  
+ TAGTGTATAT ATATAGCCTT GTGTTAGCGG GGCTTGGGCA TTAGACTTTT ACTCTTGTAA ACCCTAATGT   
  
  
+ GTTATTATAA ATATAGATCT AGCCACCCAT GTTGAGGTAG GCACTCTAAT TCTGACACAG GTAGGATAAG   
  
  
+ TTTTCATAGT TTATGTTGTG AATCTTGTCT TACTTTGTGG TTCCTATTGA CAAAAGTTTG AATCCGAGCA   
  
  
+ TGAGAGTTCA TCAATCTCTT TTTCTTTCCT ATTTTATTTC TTGTGGACTT AATTGGATTT TCTGGTAGAG   
  
  
+ GAAAGTTCCT TGCTGGAATT TGGTCGAAAG TGATATACAT TTTGGGATTT AATTAGCCAT AAATTTGGTT   
  
  
+ TTACACCTTG TTAGTTTGGA GTTCTGGTGT GTTATTGTAT GTGTTCTGGT GGGTTTTTCT TTTAGCTAAA   
  
  
+ TGTTGGAGCA TTTTAAGGGA TCTAGATATC TGAAGAAGTG CTTGGGAAAT GAAGCATATT TCCCTTATAG   
  
  
+ TCTGACATAA CTTTAATGAA TTGATCATGA ACAAAGGGCA ATTAGTTCAG AAAGGTTCAA CTCCTCTAGG   
  
  
+ TGTTGTGCTA AAGAGATGTG AGTTAGTAAC AATTGCTTAC TAACTATATA GGTTGTTTTA TTACTTGTAG   
  
  
+ AGGTTTTTTT TTTGGGATAT CTTTTTGGGG TTTATAAATG AGAAGGTTGA TTTGTTGTCT ACTCTTCTTT   
  
  
+ CGATTCAATA GTAATTCTCT CAGAGAAGGC TAAAATGACA TGGCATTCCA TCGCCTCTCA TGAGGCTTTT   
  
  
+ TGCATGTGCT TATGTATCTT TCATCTCTTA CTTTGGTGGG GTGGCATTGG GGAGGAAAAG GGGGTTTCGA   
  
  
+ TTCGATAGAT CGTCACTTTC GGAAATGACG TCTCTGACCA ATGGTAAGGG TAAGGTTAAT ACATGTGGAT   
  
  
+ ATTTCCAGAG TTAGTTGTGA TTGTTATTGT TGTTGTTGTA ACTTTATGTC ACGGGGAAAA CATAATAATT   
  
  
+ ATCTGTTTAT CTGTTTGTTT GTTATTTTCA AGGGGAAGGG GAGTGGGCGG ATGCTTAGTA TGTAATCATT   
  
  
+ TTATATAGGG AAAATTCATA TAGCATGAAA GTAAAACTTT CATTGCCAGC ATTCTAAGCT AGGGGTAGGG   
  
  
+ TTGTCTACAC CTTGATCTCC CTAGAAGCAT GGTGATCATT CCTCAGATTG TGTTATAGAA TAAAGAATGT   
  
  
+ GGTTCTGCTG TAATGAGACT TATGTATTAT CAAATGATGA ATCTGTTTAG AACTTCCTGT TTATTTTTTC   
  
  
+ TGCAAAATTT TAAGAGACTA GCATCTATCG GGCTCGACTC TAGAGGTTCT CTTGTGTTAC CCTTCTCAAC   
  
  
+ TCAGTTTGAT TGTGATACAG TTACTATATT GAGTGATAGT CGAGAGCATC ACAACCCGCT AAGGAGTCTA   
  
  
+ TCAACAAGAA GCCCTTCTTG TAATTCTCCC CTTGAAACTA GCAGTTATCA TCACTTGTCA TCGAATAGCC   
  
  
+ CTTTTTGTAA TTCTACCCTT GAAACTAGCA GTTATTGTCA TCAGTTCAAT GTGAGTCCCG TTAGATATTC   
  
  
+ CCATCAAGGC ATAGATTATG GAGTGAGCAT GAAGAACGCT TTGCACGAGC TAGAGACTGC TCTAATTGGT   
  
  
+ GTAGATGGCG AGAAAGCATC TGTTGCTAAT CAACCTATGG GGGGAATTCA TTAGTCCGGG ATCCCAAGTT   
  
  
+ AGAGATCGAA GTCATTGAGT GAAGATCCAC AGGGTTCACA TCCTCCTCAG CTTGATTCAT CATCTTTTTC   
  
  
+ AAGGGTGAAA AGATTCGGAG ATGAAAGCCA GAGAGAGAGA AATGCCACAA GGCAATGGAA GAACCAGCGG   
  
  
+ AACTACTAAG TTTCTCACCA GGTGATTTAA AGCAGTTGCT AATTGAATGT GCGAGGGCTT TATCAGATAA   
  
  
+ CCGAATAGAT GACTTTGAGA GTTTGGCTAA ACGGCCAAGG AAAGAGGTCT CCATCTCAGG TGAGCCTGTC   
  
  
+ CAACGTCTCG GTACTTGTAT GATCGAAGGG CTTGTGGCAA GGAAACAGTC TTCGGGGACT AACATCTATC   
  
  
+ GGGCTCTTAA GTACTGTAAA GAGCCTCTTG GAAAATACTT GCTCTCCTAC GGCACTCAGT GGGTTACTCT   
  
  
+ ATCGCAAGCC ATAGCAGCAA GGCTTGGTGG GCCTCCTAAA GTGCGACTTA CAGGCATTGA TGATCCTGTT   
  
  
+ TCTAAGTATA CTTGTGATGC TAGCTTGGAG GCTATTGGGA AACGATTAGC GTCTCTATTT GAAAAGTTTA   
  
  
+ AAATACTCGT CGAGTTCAAT GCATTGCCCG TTTATGGACC TGATGTCAGG TGGGAAATGC TGGATGTGAG   
  
  
+ GCCCAGGGAG GCTTTGGCCG TTAATTGTCC ATTACAGCTC CATCACACTC CTGACGAGAG TGTCGATGTG   
  
  
+ AGCAACCCTA GGGATAGGCT TCTCAGAATG GTGAAATTGC TCGGTCCTAA GGTATGCACT TTGGTTGAGC   
  
  
+ AAGAATCAAA CACCAACACT ACACGTTTCT TGACCCGGTT CATAGAGACC CTTGACTACT ACTCGATCAG   
  
  
+ CCATGTTTG  

- +Up\_Stream \_Len000TCAACA TCGTTCGTGT AATTTCAACT AATAGTAAAA CAGGCACTTT GCCACTCGTT   
  
  
- TGCAGTAGAG GTCCAGTTTA AGTAAGCATG AGTTAGTGTT CAACGACTGT AAAAATGGTT TCCGATTAGC   
  
  
- AGCGTTTTCA AAGAAATCAA ATTCCGGTTA GAGAGGCAAC AAAGTGGGGT ATGTCTAACT CCCCAACATT   
  
  
- ATCACATATA TATATCGGAA CACAATCGCC CCGAACCCGT AATCTGAAAA TGAGAACATT TGGGATTACA   
  
  
- CAATAATATT TATATCTAGA TCGGTGGGTA CAACTCCATC CGTGAGATTA AGACTGTGTC CATCCTATTC   
  
  
- AAAAGTATCA AATACAACAC TTAGAACAGA ATGAAACACC AAGGATAACT GTTTTCAAAC TTAGGCTCGT   
  
  
- ACTCTCAAGT AGTTAGAGAA AAAGAAAGGA TAAAATAAAG AACACCTGAA TTAACCTAAA AGACCATCTC   
  
  
- CTTTCAAGGA ACGACCTTAA ACCAGCTTTC ACTATATGTA AAACCCTAAA TTAATCGGTA TTTAAACCAA   
  
  
- AATGTGGAAC AATCAAACCT CAAGACCACA CAATAACATA CACAAGACCA CCCAAAAAGA AAATCGATTT   
  
  
- ACAACCTCGT AAAATTCCCT AGATCTATAG ACTTCTTCAC GAACCCTTTA CTTCGTATAA AGGGAATATC   
  
  
- AGACTGTATT GAAATTACTT AACTAGTACT TGTTTCCCGT TAATCAAGTC TTTCCAAGTT GAGGAGATCC   
  
  
- ACAACACGAT TTCTCTACAC TCAATCATTG TTAACGAATG ATTGATATAT CCAACAAAAT AATGAACATC   
  
  
- TCCAAAAAAA AAACCCTATA GAAAAACCCC AAATATTTAC TCTTCCAACT AAACAACAGA TGAGAAGAAA   
  
  
- GCTAAGTTAT CATTAAGAGA GTCTCTTCCG ATTTTACTGT ACCGTAAGGT AGCGGAGAGT ACTCCGAAAA   
  
  
- ACGTACACGA ATACATAGAA AGTAGAGAAT GAAACCACCC CACCGTAACC CCTCCTTTTC CCCCAAAGCT   
  
  
- AAGCTATCTA GCAGTGAAAG CCTTTACTGC AGAGACTGGT TACCATTCCC ATTCCAATTA TGTACACCTA   
  
  
- TAAAGGTCTC AATCAACACT AACAATAACA ACAACAACAT TGAAATACAG TGCCCCTTTT GTATTATTAA   
  
  
- TAGACAAATA GACAAACAAA CAATAAAAGT TCCCCTTCCC CTCACCCGCC TACGAATCAT ACATTAGTAA   
  
  
- AATATATCCC TTTTAAGTAT ATCGTACTTT CATTTTGAAA GTAACGGTCG TAAGATTCGA TCCCCATCCC   
  
  
- AACAGATGTG GAACTAGAGG GATCTTCGTA CCACTAGTAA GGAGTCTAAC ACAATATCTT ATTTCTTACA   
  
  
- CCAAGACGAC ATTACTCTGA ATACATAATA GTTTACTACT TAGACAAATC TTGAAGGACA AATAAAAAAG   
  
  
- ACGTTTTAAA ATTCTCTGAT CGTAGATAGC CCGAGCTGAG ATCTCCAAGA GAACACAATG GGAAGAGTTG   
  
  
- AGTCAAACTA ACACTATGTC AATGATATAA CTCACTATCA GCTCTCGTAG TGTTGGGCGA TTCCTCAGAT   
  
  
- AGTTGTTCTT CGGGAAGAAC ATTAAGAGGG GAACTTTGAT CGTCAATAGT AGTGAACAGT AGCTTATCGG   
  
  
- GAAAAACATT AAGATGGGAA CTTTGATCGT CAATAACAGT AGTCAAGTTA CACTCAGGGC AATCTATAAG   
  
  
- GGTAGTTCCG TATCTAATAC CTCACTCGTA CTTCTTGCGA AACGTGCTCG ATCTCTGACG AGATTAACCA   
  
  
- CATCTACCGC TCTTTCGTAG ACAACGATTA GTTGGATACC CCCCTTAAGT AATCAGGCCC TAGGGTTCAA   
  
  
- TCTCTAGCTT CAGTAACTCA CTTCTAGGTG TCCCAAGTGT AGGAGGAGTC GAACTAAGTA GTAGAAAAAG   
  
  
- TTCCCACTTT TCTAAGCCTC TACTTTCGGT CTCTCTCTCT TTACGGTGTT CCGTTACCTT CTTGGTCGCC   
  
  
- TTGATGATTC AAAGAGTGGT CCACTAAATT TCGTCAACGA TTAACTTACA CGCTCCCGAA ATAGTCTATT   
  
  
- GGCTTATCTA CTGAAACTCT CAAACCGATT TGCCGGTTCC TTTCTCCAGA GGTAGAGTCC ACTCGGACAG   
  
  
- GTTGCAGAGC CATGAACATA CTAGCTTCCC GAACACCGTT CCTTTGTCAG AAGCCCCTGA TTGTAGATAG   
  
  
- CCCGAGAATT CATGACATTT CTCGGAGAAC CTTTTATGAA CGAGAGGATG CCGTGAGTCA CCCAATGAGA   
  
  
- TAGCGTTCGG TATCGTCGTT CCGAACCACC CGGAGGATTT CACGCTGAAT GTCCGTAACT ACTAGGACAA   
  
  
- AGATTCATAT GAACACTACG ATCGAACCTC CGATAACCCT TTGCTAATCG CAGAGATAAA CTTTTCAAAT   
  
  
- TTTATGAGCA GCTCAAGTTA CGTAACGGGC AAATACCTGG ACTACAGTCC ACCCTTTACG ACCTACACTC   
  
  
- CGGGTCCCTC CGAAACCGGC AATTAACAGG TAATGTCGAG GTAGTGTGAG GACTGCTCTC ACAGCTACAC   
  
  
- TCGTTGGGAT CCCTATCCGA AGAGTCTTAC CACTTTAACG AGCCAGGATT CCATACGTGA AACCAACTCG   
  
  
- TTCTTAGTTT GTGGTTGTGA TGTGCAAAGA ACTGGGCCAA GTATCTCTGG GAACTGATGA TGAGCTAGTC   
  
  
- GGTACAAAC

+     AP-1

| Site Name | Organism | Position | Strand | Matrix score. | sequence | function |
| --- | --- | --- | --- | --- | --- | --- |
| AP-1 | Arabidopsis thaliana | 793 | + | 8 | TGAGTTAG |  |

>HU08G00019.1   
+ +Up\_Stream \_Len000AGTTGT AGCAAGCACA TTAAAGTTGA TTATCATTTT GTCCGTGAAA CGGTGAGCAA   
  
  
+ ACGTCATCTC CAGGTCAAAT TCATTCGTAC TCAATCACAA GTTGCTGACA TTTTTACCAA AGGCTAATCG   
  
  
+ TCGCAAAAGT TTCTTTAGTT TAAGGCCAAT CTCTCCGTTG TTTCACCCCA TACAGATTGA GGGGTTGTAA   
  
  
+ TAGTGTATAT ATATAGCCTT GTGTTAGCGG GGCTTGGGCA TTAGACTTTT ACTCTTGTAA ACCCTAATGT   
  
  
+ GTTATTATAA ATATAGATCT AGCCACCCAT GTTGAGGTAG GCACTCTAAT TCTGACACAG GTAGGATAAG   
  
  
+ TTTTCATAGT TTATGTTGTG AATCTTGTCT TACTTTGTGG TTCCTATTGA CAAAAGTTTG AATCCGAGCA   
  
  
+ TGAGAGTTCA TCAATCTCTT TTTCTTTCCT ATTTTATTTC TTGTGGACTT AATTGGATTT TCTGGTAGAG   
  
  
+ GAAAGTTCCT TGCTGGAATT TGGTCGAAAG TGATATACAT TTTGGGATTT AATTAGCCAT AAATTTGGTT   
  
  
+ TTACACCTTG TTAGTTTGGA GTTCTGGTGT GTTATTGTAT GTGTTCTGGT GGGTTTTTCT TTTAGCTAAA   
  
  
+ TGTTGGAGCA TTTTAAGGGA TCTAGATATC TGAAGAAGTG CTTGGGAAAT GAAGCATATT TCCCTTATAG   
  
  
+ TCTGACATAA CTTTAATGAA TTGATCATGA ACAAAGGGCA ATTAGTTCAG AAAGGTTCAA CTCCTCTAGG   
  
  
+ TGTTGTGCTA AAGAGATGTG AGTTAGTAAC AATTGCTTAC TAACTATATA GGTTGTTTTA TTACTTGTAG   
  
  
+ AGGTTTTTTT TTTGGGATAT CTTTTTGGGG TTTATAAATG AGAAGGTTGA TTTGTTGTCT ACTCTTCTTT   
  
  
+ CGATTCAATA GTAATTCTCT CAGAGAAGGC TAAAATGACA TGGCATTCCA TCGCCTCTCA TGAGGCTTTT   
  
  
+ TGCATGTGCT TATGTATCTT TCATCTCTTA CTTTGGTGGG GTGGCATTGG GGAGGAAAAG GGGGTTTCGA   
  
  
+ TTCGATAGAT CGTCACTTTC GGAAATGACG TCTCTGACCA ATGGTAAGGG TAAGGTTAAT ACATGTGGAT   
  
  
+ ATTTCCAGAG TTAGTTGTGA TTGTTATTGT TGTTGTTGTA ACTTTATGTC ACGGGGAAAA CATAATAATT   
  
  
+ ATCTGTTTAT CTGTTTGTTT GTTATTTTCA AGGGGAAGGG GAGTGGGCGG ATGCTTAGTA TGTAATCATT   
  
  
+ TTATATAGGG AAAATTCATA TAGCATGAAA GTAAAACTTT CATTGCCAGC ATTCTAAGCT AGGGGTAGGG   
  
  
+ TTGTCTACAC CTTGATCTCC CTAGAAGCAT GGTGATCATT CCTCAGATTG TGTTATAGAA TAAAGAATGT   
  
  
+ GGTTCTGCTG TAATGAGACT TATGTATTAT CAAATGATGA ATCTGTTTAG AACTTCCTGT TTATTTTTTC   
  
  
+ TGCAAAATTT TAAGAGACTA GCATCTATCG GGCTCGACTC TAGAGGTTCT CTTGTGTTAC CCTTCTCAAC   
  
  
+ TCAGTTTGAT TGTGATACAG TTACTATATT GAGTGATAGT CGAGAGCATC ACAACCCGCT AAGGAGTCTA   
  
  
+ TCAACAAGAA GCCCTTCTTG TAATTCTCCC CTTGAAACTA GCAGTTATCA TCACTTGTCA TCGAATAGCC   
  
  
+ CTTTTTGTAA TTCTACCCTT GAAACTAGCA GTTATTGTCA TCAGTTCAAT GTGAGTCCCG TTAGATATTC   
  
  
+ CCATCAAGGC ATAGATTATG GAGTGAGCAT GAAGAACGCT TTGCACGAGC TAGAGACTGC TCTAATTGGT   
  
  
+ GTAGATGGCG AGAAAGCATC TGTTGCTAAT CAACCTATGG GGGGAATTCA TTAGTCCGGG ATCCCAAGTT   
  
  
+ AGAGATCGAA GTCATTGAGT GAAGATCCAC AGGGTTCACA TCCTCCTCAG CTTGATTCAT CATCTTTTTC   
  
  
+ AAGGGTGAAA AGATTCGGAG ATGAAAGCCA GAGAGAGAGA AATGCCACAA GGCAATGGAA GAACCAGCGG   
  
  
+ AACTACTAAG TTTCTCACCA GGTGATTTAA AGCAGTTGCT AATTGAATGT GCGAGGGCTT TATCAGATAA   
  
  
+ CCGAATAGAT GACTTTGAGA GTTTGGCTAA ACGGCCAAGG AAAGAGGTCT CCATCTCAGG TGAGCCTGTC   
  
  
+ CAACGTCTCG GTACTTGTAT GATCGAAGGG CTTGTGGCAA GGAAACAGTC TTCGGGGACT AACATCTATC   
  
  
+ GGGCTCTTAA GTACTGTAAA GAGCCTCTTG GAAAATACTT GCTCTCCTAC GGCACTCAGT GGGTTACTCT   
  
  
+ ATCGCAAGCC ATAGCAGCAA GGCTTGGTGG GCCTCCTAAA GTGCGACTTA CAGGCATTGA TGATCCTGTT   
  
  
+ TCTAAGTATA CTTGTGATGC TAGCTTGGAG GCTATTGGGA AACGATTAGC GTCTCTATTT GAAAAGTTTA   
  
  
+ AAATACTCGT CGAGTTCAAT GCATTGCCCG TTTATGGACC TGATGTCAGG TGGGAAATGC TGGATGTGAG   
  
  
+ GCCCAGGGAG GCTTTGGCCG TTAATTGTCC ATTACAGCTC CATCACACTC CTGACGAGAG TGTCGATGTG   
  
  
+ AGCAACCCTA GGGATAGGCT TCTCAGAATG GTGAAATTGC TCGGTCCTAA GGTATGCACT TTGGTTGAGC   
  
  
+ AAGAATCAAA CACCAACACT ACACGTTTCT TGACCCGGTT CATAGAGACC CTTGACTACT ACTCGATCAG   
  
  
+ CCATGTTTG  

- +Up\_Stream \_Len000TCAACA TCGTTCGTGT AATTTCAACT AATAGTAAAA CAGGCACTTT GCCACTCGTT   
  
  
- TGCAGTAGAG GTCCAGTTTA AGTAAGCATG AGTTAGTGTT CAACGACTGT AAAAATGGTT TCCGATTAGC   
  
  
- AGCGTTTTCA AAGAAATCAA ATTCCGGTTA GAGAGGCAAC AAAGTGGGGT ATGTCTAACT CCCCAACATT   
  
  
- ATCACATATA TATATCGGAA CACAATCGCC CCGAACCCGT AATCTGAAAA TGAGAACATT TGGGATTACA   
  
  
- CAATAATATT TATATCTAGA TCGGTGGGTA CAACTCCATC CGTGAGATTA AGACTGTGTC CATCCTATTC   
  
  
- AAAAGTATCA AATACAACAC TTAGAACAGA ATGAAACACC AAGGATAACT GTTTTCAAAC TTAGGCTCGT   
  
  
- ACTCTCAAGT AGTTAGAGAA AAAGAAAGGA TAAAATAAAG AACACCTGAA TTAACCTAAA AGACCATCTC   
  
  
- CTTTCAAGGA ACGACCTTAA ACCAGCTTTC ACTATATGTA AAACCCTAAA TTAATCGGTA TTTAAACCAA   
  
  
- AATGTGGAAC AATCAAACCT CAAGACCACA CAATAACATA CACAAGACCA CCCAAAAAGA AAATCGATTT   
  
  
- ACAACCTCGT AAAATTCCCT AGATCTATAG ACTTCTTCAC GAACCCTTTA CTTCGTATAA AGGGAATATC   
  
  
- AGACTGTATT GAAATTACTT AACTAGTACT TGTTTCCCGT TAATCAAGTC TTTCCAAGTT GAGGAGATCC   
  
  
- ACAACACGAT TTCTCTACAC TCAATCATTG TTAACGAATG ATTGATATAT CCAACAAAAT AATGAACATC   
  
  
- TCCAAAAAAA AAACCCTATA GAAAAACCCC AAATATTTAC TCTTCCAACT AAACAACAGA TGAGAAGAAA   
  
  
- GCTAAGTTAT CATTAAGAGA GTCTCTTCCG ATTTTACTGT ACCGTAAGGT AGCGGAGAGT ACTCCGAAAA   
  
  
- ACGTACACGA ATACATAGAA AGTAGAGAAT GAAACCACCC CACCGTAACC CCTCCTTTTC CCCCAAAGCT   
  
  
- AAGCTATCTA GCAGTGAAAG CCTTTACTGC AGAGACTGGT TACCATTCCC ATTCCAATTA TGTACACCTA   
  
  
- TAAAGGTCTC AATCAACACT AACAATAACA ACAACAACAT TGAAATACAG TGCCCCTTTT GTATTATTAA   
  
  
- TAGACAAATA GACAAACAAA CAATAAAAGT TCCCCTTCCC CTCACCCGCC TACGAATCAT ACATTAGTAA   
  
  
- AATATATCCC TTTTAAGTAT ATCGTACTTT CATTTTGAAA GTAACGGTCG TAAGATTCGA TCCCCATCCC   
  
  
- AACAGATGTG GAACTAGAGG GATCTTCGTA CCACTAGTAA GGAGTCTAAC ACAATATCTT ATTTCTTACA   
  
  
- CCAAGACGAC ATTACTCTGA ATACATAATA GTTTACTACT TAGACAAATC TTGAAGGACA AATAAAAAAG   
  
  
- ACGTTTTAAA ATTCTCTGAT CGTAGATAGC CCGAGCTGAG ATCTCCAAGA GAACACAATG GGAAGAGTTG   
  
  
- AGTCAAACTA ACACTATGTC AATGATATAA CTCACTATCA GCTCTCGTAG TGTTGGGCGA TTCCTCAGAT   
  
  
- AGTTGTTCTT CGGGAAGAAC ATTAAGAGGG GAACTTTGAT CGTCAATAGT AGTGAACAGT AGCTTATCGG   
  
  
- GAAAAACATT AAGATGGGAA CTTTGATCGT CAATAACAGT AGTCAAGTTA CACTCAGGGC AATCTATAAG   
  
  
- GGTAGTTCCG TATCTAATAC CTCACTCGTA CTTCTTGCGA AACGTGCTCG ATCTCTGACG AGATTAACCA   
  
  
- CATCTACCGC TCTTTCGTAG ACAACGATTA GTTGGATACC CCCCTTAAGT AATCAGGCCC TAGGGTTCAA   
  
  
- TCTCTAGCTT CAGTAACTCA CTTCTAGGTG TCCCAAGTGT AGGAGGAGTC GAACTAAGTA GTAGAAAAAG   
  
  
- TTCCCACTTT TCTAAGCCTC TACTTTCGGT CTCTCTCTCT TTACGGTGTT CCGTTACCTT CTTGGTCGCC   
  
  
- TTGATGATTC AAAGAGTGGT CCACTAAATT TCGTCAACGA TTAACTTACA CGCTCCCGAA ATAGTCTATT   
  
  
- GGCTTATCTA CTGAAACTCT CAAACCGATT TGCCGGTTCC TTTCTCCAGA GGTAGAGTCC ACTCGGACAG   
  
  
- GTTGCAGAGC CATGAACATA CTAGCTTCCC GAACACCGTT CCTTTGTCAG AAGCCCCTGA TTGTAGATAG   
  
  
- CCCGAGAATT CATGACATTT CTCGGAGAAC CTTTTATGAA CGAGAGGATG CCGTGAGTCA CCCAATGAGA   
  
  
- TAGCGTTCGG TATCGTCGTT CCGAACCACC CGGAGGATTT CACGCTGAAT GTCCGTAACT ACTAGGACAA   
  
  
- AGATTCATAT GAACACTACG ATCGAACCTC CGATAACCCT TTGCTAATCG CAGAGATAAA CTTTTCAAAT   
  
  
- TTTATGAGCA GCTCAAGTTA CGTAACGGGC AAATACCTGG ACTACAGTCC ACCCTTTACG ACCTACACTC   
  
  
- CGGGTCCCTC CGAAACCGGC AATTAACAGG TAATGTCGAG GTAGTGTGAG GACTGCTCTC ACAGCTACAC   
  
  
- TCGTTGGGAT CCCTATCCGA AGAGTCTTAC CACTTTAACG AGCCAGGATT CCATACGTGA AACCAACTCG   
  
  
- TTCTTAGTTT GTGGTTGTGA TGTGCAAAGA ACTGGGCCAA GTATCTCTGG GAACTGATGA TGAGCTAGTC   
  
  
- GGTACAAAC

+     ARE

| Site Name | Organism | Position | Strand | Matrix score. | sequence | function |
| --- | --- | --- | --- | --- | --- | --- |
| ARE | Zea mays | 560 | - | 6 | AAACCA | cis-acting regulatory element essential for the anaerobic induction |

>HU08G00019.1   
+ +Up\_Stream \_Len000AGTTGT AGCAAGCACA TTAAAGTTGA TTATCATTTT GTCCGTGAAA CGGTGAGCAA   
  
  
+ ACGTCATCTC CAGGTCAAAT TCATTCGTAC TCAATCACAA GTTGCTGACA TTTTTACCAA AGGCTAATCG   
  
  
+ TCGCAAAAGT TTCTTTAGTT TAAGGCCAAT CTCTCCGTTG TTTCACCCCA TACAGATTGA GGGGTTGTAA   
  
  
+ TAGTGTATAT ATATAGCCTT GTGTTAGCGG GGCTTGGGCA TTAGACTTTT ACTCTTGTAA ACCCTAATGT   
  
  
+ GTTATTATAA ATATAGATCT AGCCACCCAT GTTGAGGTAG GCACTCTAAT TCTGACACAG GTAGGATAAG   
  
  
+ TTTTCATAGT TTATGTTGTG AATCTTGTCT TACTTTGTGG TTCCTATTGA CAAAAGTTTG AATCCGAGCA   
  
  
+ TGAGAGTTCA TCAATCTCTT TTTCTTTCCT ATTTTATTTC TTGTGGACTT AATTGGATTT TCTGGTAGAG   
  
  
+ GAAAGTTCCT TGCTGGAATT TGGTCGAAAG TGATATACAT TTTGGGATTT AATTAGCCAT AAATTTGGTT   
  
  
+ TTACACCTTG TTAGTTTGGA GTTCTGGTGT GTTATTGTAT GTGTTCTGGT GGGTTTTTCT TTTAGCTAAA   
  
  
+ TGTTGGAGCA TTTTAAGGGA TCTAGATATC TGAAGAAGTG CTTGGGAAAT GAAGCATATT TCCCTTATAG   
  
  
+ TCTGACATAA CTTTAATGAA TTGATCATGA ACAAAGGGCA ATTAGTTCAG AAAGGTTCAA CTCCTCTAGG   
  
  
+ TGTTGTGCTA AAGAGATGTG AGTTAGTAAC AATTGCTTAC TAACTATATA GGTTGTTTTA TTACTTGTAG   
  
  
+ AGGTTTTTTT TTTGGGATAT CTTTTTGGGG TTTATAAATG AGAAGGTTGA TTTGTTGTCT ACTCTTCTTT   
  
  
+ CGATTCAATA GTAATTCTCT CAGAGAAGGC TAAAATGACA TGGCATTCCA TCGCCTCTCA TGAGGCTTTT   
  
  
+ TGCATGTGCT TATGTATCTT TCATCTCTTA CTTTGGTGGG GTGGCATTGG GGAGGAAAAG GGGGTTTCGA   
  
  
+ TTCGATAGAT CGTCACTTTC GGAAATGACG TCTCTGACCA ATGGTAAGGG TAAGGTTAAT ACATGTGGAT   
  
  
+ ATTTCCAGAG TTAGTTGTGA TTGTTATTGT TGTTGTTGTA ACTTTATGTC ACGGGGAAAA CATAATAATT   
  
  
+ ATCTGTTTAT CTGTTTGTTT GTTATTTTCA AGGGGAAGGG GAGTGGGCGG ATGCTTAGTA TGTAATCATT   
  
  
+ TTATATAGGG AAAATTCATA TAGCATGAAA GTAAAACTTT CATTGCCAGC ATTCTAAGCT AGGGGTAGGG   
  
  
+ TTGTCTACAC CTTGATCTCC CTAGAAGCAT GGTGATCATT CCTCAGATTG TGTTATAGAA TAAAGAATGT   
  
  
+ GGTTCTGCTG TAATGAGACT TATGTATTAT CAAATGATGA ATCTGTTTAG AACTTCCTGT TTATTTTTTC   
  
  
+ TGCAAAATTT TAAGAGACTA GCATCTATCG GGCTCGACTC TAGAGGTTCT CTTGTGTTAC CCTTCTCAAC   
  
  
+ TCAGTTTGAT TGTGATACAG TTACTATATT GAGTGATAGT CGAGAGCATC ACAACCCGCT AAGGAGTCTA   
  
  
+ TCAACAAGAA GCCCTTCTTG TAATTCTCCC CTTGAAACTA GCAGTTATCA TCACTTGTCA TCGAATAGCC   
  
  
+ CTTTTTGTAA TTCTACCCTT GAAACTAGCA GTTATTGTCA TCAGTTCAAT GTGAGTCCCG TTAGATATTC   
  
  
+ CCATCAAGGC ATAGATTATG GAGTGAGCAT GAAGAACGCT TTGCACGAGC TAGAGACTGC TCTAATTGGT   
  
  
+ GTAGATGGCG AGAAAGCATC TGTTGCTAAT CAACCTATGG GGGGAATTCA TTAGTCCGGG ATCCCAAGTT   
  
  
+ AGAGATCGAA GTCATTGAGT GAAGATCCAC AGGGTTCACA TCCTCCTCAG CTTGATTCAT CATCTTTTTC   
  
  
+ AAGGGTGAAA AGATTCGGAG ATGAAAGCCA GAGAGAGAGA AATGCCACAA GGCAATGGAA GAACCAGCGG   
  
  
+ AACTACTAAG TTTCTCACCA GGTGATTTAA AGCAGTTGCT AATTGAATGT GCGAGGGCTT TATCAGATAA   
  
  
+ CCGAATAGAT GACTTTGAGA GTTTGGCTAA ACGGCCAAGG AAAGAGGTCT CCATCTCAGG TGAGCCTGTC   
  
  
+ CAACGTCTCG GTACTTGTAT GATCGAAGGG CTTGTGGCAA GGAAACAGTC TTCGGGGACT AACATCTATC   
  
  
+ GGGCTCTTAA GTACTGTAAA GAGCCTCTTG GAAAATACTT GCTCTCCTAC GGCACTCAGT GGGTTACTCT   
  
  
+ ATCGCAAGCC ATAGCAGCAA GGCTTGGTGG GCCTCCTAAA GTGCGACTTA CAGGCATTGA TGATCCTGTT   
  
  
+ TCTAAGTATA CTTGTGATGC TAGCTTGGAG GCTATTGGGA AACGATTAGC GTCTCTATTT GAAAAGTTTA   
  
  
+ AAATACTCGT CGAGTTCAAT GCATTGCCCG TTTATGGACC TGATGTCAGG TGGGAAATGC TGGATGTGAG   
  
  
+ GCCCAGGGAG GCTTTGGCCG TTAATTGTCC ATTACAGCTC CATCACACTC CTGACGAGAG TGTCGATGTG   
  
  
+ AGCAACCCTA GGGATAGGCT TCTCAGAATG GTGAAATTGC TCGGTCCTAA GGTATGCACT TTGGTTGAGC   
  
  
+ AAGAATCAAA CACCAACACT ACACGTTTCT TGACCCGGTT CATAGAGACC CTTGACTACT ACTCGATCAG   
  
  
+ CCATGTTTG  

- +Up\_Stream \_Len000TCAACA TCGTTCGTGT AATTTCAACT AATAGTAAAA CAGGCACTTT GCCACTCGTT   
  
  
- TGCAGTAGAG GTCCAGTTTA AGTAAGCATG AGTTAGTGTT CAACGACTGT AAAAATGGTT TCCGATTAGC   
  
  
- AGCGTTTTCA AAGAAATCAA ATTCCGGTTA GAGAGGCAAC AAAGTGGGGT ATGTCTAACT CCCCAACATT   
  
  
- ATCACATATA TATATCGGAA CACAATCGCC CCGAACCCGT AATCTGAAAA TGAGAACATT TGGGATTACA   
  
  
- CAATAATATT TATATCTAGA TCGGTGGGTA CAACTCCATC CGTGAGATTA AGACTGTGTC CATCCTATTC   
  
  
- AAAAGTATCA AATACAACAC TTAGAACAGA ATGAAACACC AAGGATAACT GTTTTCAAAC TTAGGCTCGT   
  
  
- ACTCTCAAGT AGTTAGAGAA AAAGAAAGGA TAAAATAAAG AACACCTGAA TTAACCTAAA AGACCATCTC   
  
  
- CTTTCAAGGA ACGACCTTAA ACCAGCTTTC ACTATATGTA AAACCCTAAA TTAATCGGTA TTTAAACCAA   
  
  
- AATGTGGAAC AATCAAACCT CAAGACCACA CAATAACATA CACAAGACCA CCCAAAAAGA AAATCGATTT   
  
  
- ACAACCTCGT AAAATTCCCT AGATCTATAG ACTTCTTCAC GAACCCTTTA CTTCGTATAA AGGGAATATC   
  
  
- AGACTGTATT GAAATTACTT AACTAGTACT TGTTTCCCGT TAATCAAGTC TTTCCAAGTT GAGGAGATCC   
  
  
- ACAACACGAT TTCTCTACAC TCAATCATTG TTAACGAATG ATTGATATAT CCAACAAAAT AATGAACATC   
  
  
- TCCAAAAAAA AAACCCTATA GAAAAACCCC AAATATTTAC TCTTCCAACT AAACAACAGA TGAGAAGAAA   
  
  
- GCTAAGTTAT CATTAAGAGA GTCTCTTCCG ATTTTACTGT ACCGTAAGGT AGCGGAGAGT ACTCCGAAAA   
  
  
- ACGTACACGA ATACATAGAA AGTAGAGAAT GAAACCACCC CACCGTAACC CCTCCTTTTC CCCCAAAGCT   
  
  
- AAGCTATCTA GCAGTGAAAG CCTTTACTGC AGAGACTGGT TACCATTCCC ATTCCAATTA TGTACACCTA   
  
  
- TAAAGGTCTC AATCAACACT AACAATAACA ACAACAACAT TGAAATACAG TGCCCCTTTT GTATTATTAA   
  
  
- TAGACAAATA GACAAACAAA CAATAAAAGT TCCCCTTCCC CTCACCCGCC TACGAATCAT ACATTAGTAA   
  
  
- AATATATCCC TTTTAAGTAT ATCGTACTTT CATTTTGAAA GTAACGGTCG TAAGATTCGA TCCCCATCCC   
  
  
- AACAGATGTG GAACTAGAGG GATCTTCGTA CCACTAGTAA GGAGTCTAAC ACAATATCTT ATTTCTTACA   
  
  
- CCAAGACGAC ATTACTCTGA ATACATAATA GTTTACTACT TAGACAAATC TTGAAGGACA AATAAAAAAG   
  
  
- ACGTTTTAAA ATTCTCTGAT CGTAGATAGC CCGAGCTGAG ATCTCCAAGA GAACACAATG GGAAGAGTTG   
  
  
- AGTCAAACTA ACACTATGTC AATGATATAA CTCACTATCA GCTCTCGTAG TGTTGGGCGA TTCCTCAGAT   
  
  
- AGTTGTTCTT CGGGAAGAAC ATTAAGAGGG GAACTTTGAT CGTCAATAGT AGTGAACAGT AGCTTATCGG   
  
  
- GAAAAACATT AAGATGGGAA CTTTGATCGT CAATAACAGT AGTCAAGTTA CACTCAGGGC AATCTATAAG   
  
  
- GGTAGTTCCG TATCTAATAC CTCACTCGTA CTTCTTGCGA AACGTGCTCG ATCTCTGACG AGATTAACCA   
  
  
- CATCTACCGC TCTTTCGTAG ACAACGATTA GTTGGATACC CCCCTTAAGT AATCAGGCCC TAGGGTTCAA   
  
  
- TCTCTAGCTT CAGTAACTCA CTTCTAGGTG TCCCAAGTGT AGGAGGAGTC GAACTAAGTA GTAGAAAAAG   
  
  
- TTCCCACTTT TCTAAGCCTC TACTTTCGGT CTCTCTCTCT TTACGGTGTT CCGTTACCTT CTTGGTCGCC   
  
  
- TTGATGATTC AAAGAGTGGT CCACTAAATT TCGTCAACGA TTAACTTACA CGCTCCCGAA ATAGTCTATT   
  
  
- GGCTTATCTA CTGAAACTCT CAAACCGATT TGCCGGTTCC TTTCTCCAGA GGTAGAGTCC ACTCGGACAG   
  
  
- GTTGCAGAGC CATGAACATA CTAGCTTCCC GAACACCGTT CCTTTGTCAG AAGCCCCTGA TTGTAGATAG   
  
  
- CCCGAGAATT CATGACATTT CTCGGAGAAC CTTTTATGAA CGAGAGGATG CCGTGAGTCA CCCAATGAGA   
  
  
- TAGCGTTCGG TATCGTCGTT CCGAACCACC CGGAGGATTT CACGCTGAAT GTCCGTAACT ACTAGGACAA   
  
  
- AGATTCATAT GAACACTACG ATCGAACCTC CGATAACCCT TTGCTAATCG CAGAGATAAA CTTTTCAAAT   
  
  
- TTTATGAGCA GCTCAAGTTA CGTAACGGGC AAATACCTGG ACTACAGTCC ACCCTTTACG ACCTACACTC   
  
  
- CGGGTCCCTC CGAAACCGGC AATTAACAGG TAATGTCGAG GTAGTGTGAG GACTGCTCTC ACAGCTACAC   
  
  
- TCGTTGGGAT CCCTATCCGA AGAGTCTTAC CACTTTAACG AGCCAGGATT CCATACGTGA AACCAACTCG   
  
  
- TTCTTAGTTT GTGGTTGTGA TGTGCAAAGA ACTGGGCCAA GTATCTCTGG GAACTGATGA TGAGCTAGTC   
  
  
- GGTACAAAC

+     AT-rich sequence

| Site Name | Organism | Position | Strand | Matrix score. | sequence | function |
| --- | --- | --- | --- | --- | --- | --- |
| AT-rich sequence | Pisum sativum | 2453 | + | 9 | TAAAATACT | element for maximal elicitor-mediated activation (2copies) |

>HU08G00019.1   
+ +Up\_Stream \_Len000AGTTGT AGCAAGCACA TTAAAGTTGA TTATCATTTT GTCCGTGAAA CGGTGAGCAA   
  
  
+ ACGTCATCTC CAGGTCAAAT TCATTCGTAC TCAATCACAA GTTGCTGACA TTTTTACCAA AGGCTAATCG   
  
  
+ TCGCAAAAGT TTCTTTAGTT TAAGGCCAAT CTCTCCGTTG TTTCACCCCA TACAGATTGA GGGGTTGTAA   
  
  
+ TAGTGTATAT ATATAGCCTT GTGTTAGCGG GGCTTGGGCA TTAGACTTTT ACTCTTGTAA ACCCTAATGT   
  
  
+ GTTATTATAA ATATAGATCT AGCCACCCAT GTTGAGGTAG GCACTCTAAT TCTGACACAG GTAGGATAAG   
  
  
+ TTTTCATAGT TTATGTTGTG AATCTTGTCT TACTTTGTGG TTCCTATTGA CAAAAGTTTG AATCCGAGCA   
  
  
+ TGAGAGTTCA TCAATCTCTT TTTCTTTCCT ATTTTATTTC TTGTGGACTT AATTGGATTT TCTGGTAGAG   
  
  
+ GAAAGTTCCT TGCTGGAATT TGGTCGAAAG TGATATACAT TTTGGGATTT AATTAGCCAT AAATTTGGTT   
  
  
+ TTACACCTTG TTAGTTTGGA GTTCTGGTGT GTTATTGTAT GTGTTCTGGT GGGTTTTTCT TTTAGCTAAA   
  
  
+ TGTTGGAGCA TTTTAAGGGA TCTAGATATC TGAAGAAGTG CTTGGGAAAT GAAGCATATT TCCCTTATAG   
  
  
+ TCTGACATAA CTTTAATGAA TTGATCATGA ACAAAGGGCA ATTAGTTCAG AAAGGTTCAA CTCCTCTAGG   
  
  
+ TGTTGTGCTA AAGAGATGTG AGTTAGTAAC AATTGCTTAC TAACTATATA GGTTGTTTTA TTACTTGTAG   
  
  
+ AGGTTTTTTT TTTGGGATAT CTTTTTGGGG TTTATAAATG AGAAGGTTGA TTTGTTGTCT ACTCTTCTTT   
  
  
+ CGATTCAATA GTAATTCTCT CAGAGAAGGC TAAAATGACA TGGCATTCCA TCGCCTCTCA TGAGGCTTTT   
  
  
+ TGCATGTGCT TATGTATCTT TCATCTCTTA CTTTGGTGGG GTGGCATTGG GGAGGAAAAG GGGGTTTCGA   
  
  
+ TTCGATAGAT CGTCACTTTC GGAAATGACG TCTCTGACCA ATGGTAAGGG TAAGGTTAAT ACATGTGGAT   
  
  
+ ATTTCCAGAG TTAGTTGTGA TTGTTATTGT TGTTGTTGTA ACTTTATGTC ACGGGGAAAA CATAATAATT   
  
  
+ ATCTGTTTAT CTGTTTGTTT GTTATTTTCA AGGGGAAGGG GAGTGGGCGG ATGCTTAGTA TGTAATCATT   
  
  
+ TTATATAGGG AAAATTCATA TAGCATGAAA GTAAAACTTT CATTGCCAGC ATTCTAAGCT AGGGGTAGGG   
  
  
+ TTGTCTACAC CTTGATCTCC CTAGAAGCAT GGTGATCATT CCTCAGATTG TGTTATAGAA TAAAGAATGT   
  
  
+ GGTTCTGCTG TAATGAGACT TATGTATTAT CAAATGATGA ATCTGTTTAG AACTTCCTGT TTATTTTTTC   
  
  
+ TGCAAAATTT TAAGAGACTA GCATCTATCG GGCTCGACTC TAGAGGTTCT CTTGTGTTAC CCTTCTCAAC   
  
  
+ TCAGTTTGAT TGTGATACAG TTACTATATT GAGTGATAGT CGAGAGCATC ACAACCCGCT AAGGAGTCTA   
  
  
+ TCAACAAGAA GCCCTTCTTG TAATTCTCCC CTTGAAACTA GCAGTTATCA TCACTTGTCA TCGAATAGCC   
  
  
+ CTTTTTGTAA TTCTACCCTT GAAACTAGCA GTTATTGTCA TCAGTTCAAT GTGAGTCCCG TTAGATATTC   
  
  
+ CCATCAAGGC ATAGATTATG GAGTGAGCAT GAAGAACGCT TTGCACGAGC TAGAGACTGC TCTAATTGGT   
  
  
+ GTAGATGGCG AGAAAGCATC TGTTGCTAAT CAACCTATGG GGGGAATTCA TTAGTCCGGG ATCCCAAGTT   
  
  
+ AGAGATCGAA GTCATTGAGT GAAGATCCAC AGGGTTCACA TCCTCCTCAG CTTGATTCAT CATCTTTTTC   
  
  
+ AAGGGTGAAA AGATTCGGAG ATGAAAGCCA GAGAGAGAGA AATGCCACAA GGCAATGGAA GAACCAGCGG   
  
  
+ AACTACTAAG TTTCTCACCA GGTGATTTAA AGCAGTTGCT AATTGAATGT GCGAGGGCTT TATCAGATAA   
  
  
+ CCGAATAGAT GACTTTGAGA GTTTGGCTAA ACGGCCAAGG AAAGAGGTCT CCATCTCAGG TGAGCCTGTC   
  
  
+ CAACGTCTCG GTACTTGTAT GATCGAAGGG CTTGTGGCAA GGAAACAGTC TTCGGGGACT AACATCTATC   
  
  
+ GGGCTCTTAA GTACTGTAAA GAGCCTCTTG GAAAATACTT GCTCTCCTAC GGCACTCAGT GGGTTACTCT   
  
  
+ ATCGCAAGCC ATAGCAGCAA GGCTTGGTGG GCCTCCTAAA GTGCGACTTA CAGGCATTGA TGATCCTGTT   
  
  
+ TCTAAGTATA CTTGTGATGC TAGCTTGGAG GCTATTGGGA AACGATTAGC GTCTCTATTT GAAAAGTTTA   
  
  
+ AAATACTCGT CGAGTTCAAT GCATTGCCCG TTTATGGACC TGATGTCAGG TGGGAAATGC TGGATGTGAG   
  
  
+ GCCCAGGGAG GCTTTGGCCG TTAATTGTCC ATTACAGCTC CATCACACTC CTGACGAGAG TGTCGATGTG   
  
  
+ AGCAACCCTA GGGATAGGCT TCTCAGAATG GTGAAATTGC TCGGTCCTAA GGTATGCACT TTGGTTGAGC   
  
  
+ AAGAATCAAA CACCAACACT ACACGTTTCT TGACCCGGTT CATAGAGACC CTTGACTACT ACTCGATCAG   
  
  
+ CCATGTTTG  

- +Up\_Stream \_Len000TCAACA TCGTTCGTGT AATTTCAACT AATAGTAAAA CAGGCACTTT GCCACTCGTT   
  
  
- TGCAGTAGAG GTCCAGTTTA AGTAAGCATG AGTTAGTGTT CAACGACTGT AAAAATGGTT TCCGATTAGC   
  
  
- AGCGTTTTCA AAGAAATCAA ATTCCGGTTA GAGAGGCAAC AAAGTGGGGT ATGTCTAACT CCCCAACATT   
  
  
- ATCACATATA TATATCGGAA CACAATCGCC CCGAACCCGT AATCTGAAAA TGAGAACATT TGGGATTACA   
  
  
- CAATAATATT TATATCTAGA TCGGTGGGTA CAACTCCATC CGTGAGATTA AGACTGTGTC CATCCTATTC   
  
  
- AAAAGTATCA AATACAACAC TTAGAACAGA ATGAAACACC AAGGATAACT GTTTTCAAAC TTAGGCTCGT   
  
  
- ACTCTCAAGT AGTTAGAGAA AAAGAAAGGA TAAAATAAAG AACACCTGAA TTAACCTAAA AGACCATCTC   
  
  
- CTTTCAAGGA ACGACCTTAA ACCAGCTTTC ACTATATGTA AAACCCTAAA TTAATCGGTA TTTAAACCAA   
  
  
- AATGTGGAAC AATCAAACCT CAAGACCACA CAATAACATA CACAAGACCA CCCAAAAAGA AAATCGATTT   
  
  
- ACAACCTCGT AAAATTCCCT AGATCTATAG ACTTCTTCAC GAACCCTTTA CTTCGTATAA AGGGAATATC   
  
  
- AGACTGTATT GAAATTACTT AACTAGTACT TGTTTCCCGT TAATCAAGTC TTTCCAAGTT GAGGAGATCC   
  
  
- ACAACACGAT TTCTCTACAC TCAATCATTG TTAACGAATG ATTGATATAT CCAACAAAAT AATGAACATC   
  
  
- TCCAAAAAAA AAACCCTATA GAAAAACCCC AAATATTTAC TCTTCCAACT AAACAACAGA TGAGAAGAAA   
  
  
- GCTAAGTTAT CATTAAGAGA GTCTCTTCCG ATTTTACTGT ACCGTAAGGT AGCGGAGAGT ACTCCGAAAA   
  
  
- ACGTACACGA ATACATAGAA AGTAGAGAAT GAAACCACCC CACCGTAACC CCTCCTTTTC CCCCAAAGCT   
  
  
- AAGCTATCTA GCAGTGAAAG CCTTTACTGC AGAGACTGGT TACCATTCCC ATTCCAATTA TGTACACCTA   
  
  
- TAAAGGTCTC AATCAACACT AACAATAACA ACAACAACAT TGAAATACAG TGCCCCTTTT GTATTATTAA   
  
  
- TAGACAAATA GACAAACAAA CAATAAAAGT TCCCCTTCCC CTCACCCGCC TACGAATCAT ACATTAGTAA   
  
  
- AATATATCCC TTTTAAGTAT ATCGTACTTT CATTTTGAAA GTAACGGTCG TAAGATTCGA TCCCCATCCC   
  
  
- AACAGATGTG GAACTAGAGG GATCTTCGTA CCACTAGTAA GGAGTCTAAC ACAATATCTT ATTTCTTACA   
  
  
- CCAAGACGAC ATTACTCTGA ATACATAATA GTTTACTACT TAGACAAATC TTGAAGGACA AATAAAAAAG   
  
  
- ACGTTTTAAA ATTCTCTGAT CGTAGATAGC CCGAGCTGAG ATCTCCAAGA GAACACAATG GGAAGAGTTG   
  
  
- AGTCAAACTA ACACTATGTC AATGATATAA CTCACTATCA GCTCTCGTAG TGTTGGGCGA TTCCTCAGAT   
  
  
- AGTTGTTCTT CGGGAAGAAC ATTAAGAGGG GAACTTTGAT CGTCAATAGT AGTGAACAGT AGCTTATCGG   
  
  
- GAAAAACATT AAGATGGGAA CTTTGATCGT CAATAACAGT AGTCAAGTTA CACTCAGGGC AATCTATAAG   
  
  
- GGTAGTTCCG TATCTAATAC CTCACTCGTA CTTCTTGCGA AACGTGCTCG ATCTCTGACG AGATTAACCA   
  
  
- CATCTACCGC TCTTTCGTAG ACAACGATTA GTTGGATACC CCCCTTAAGT AATCAGGCCC TAGGGTTCAA   
  
  
- TCTCTAGCTT CAGTAACTCA CTTCTAGGTG TCCCAAGTGT AGGAGGAGTC GAACTAAGTA GTAGAAAAAG   
  
  
- TTCCCACTTT TCTAAGCCTC TACTTTCGGT CTCTCTCTCT TTACGGTGTT CCGTTACCTT CTTGGTCGCC   
  
  
- TTGATGATTC AAAGAGTGGT CCACTAAATT TCGTCAACGA TTAACTTACA CGCTCCCGAA ATAGTCTATT   
  
  
- GGCTTATCTA CTGAAACTCT CAAACCGATT TGCCGGTTCC TTTCTCCAGA GGTAGAGTCC ACTCGGACAG   
  
  
- GTTGCAGAGC CATGAACATA CTAGCTTCCC GAACACCGTT CCTTTGTCAG AAGCCCCTGA TTGTAGATAG   
  
  
- CCCGAGAATT CATGACATTT CTCGGAGAAC CTTTTATGAA CGAGAGGATG CCGTGAGTCA CCCAATGAGA   
  
  
- TAGCGTTCGG TATCGTCGTT CCGAACCACC CGGAGGATTT CACGCTGAAT GTCCGTAACT ACTAGGACAA   
  
  
- AGATTCATAT GAACACTACG ATCGAACCTC CGATAACCCT TTGCTAATCG CAGAGATAAA CTTTTCAAAT   
  
  
- TTTATGAGCA GCTCAAGTTA CGTAACGGGC AAATACCTGG ACTACAGTCC ACCCTTTACG ACCTACACTC   
  
  
- CGGGTCCCTC CGAAACCGGC AATTAACAGG TAATGTCGAG GTAGTGTGAG GACTGCTCTC ACAGCTACAC   
  
  
- TCGTTGGGAT CCCTATCCGA AGAGTCTTAC CACTTTAACG AGCCAGGATT CCATACGTGA AACCAACTCG   
  
  
- TTCTTAGTTT GTGGTTGTGA TGTGCAAAGA ACTGGGCCAA GTATCTCTGG GAACTGATGA TGAGCTAGTC   
  
  
- GGTACAAAC

+     AT~TATA-box

| Site Name | Organism | Position | Strand | Matrix score. | sequence | function |
| --- | --- | --- | --- | --- | --- | --- |
| AT~TATA-box | Arabidopsis thaliana | 1266 | + | 6 | TATATA |  |
| AT~TATA-box | Arabidopsis thaliana | 1264 | - | 8 | TATATAAA |  |
| AT~TATA-box | Arabidopsis thaliana | 220 | + | 6 | TATATA |  |
| AT~TATA-box | Arabidopsis thaliana | 224 | + | 6 | TATATA |  |
| AT~TATA-box | Arabidopsis thaliana | 222 | + | 6 | TATATA |  |
| AT~TATA-box | Arabidopsis thaliana | 819 | + | 6 | TATATA |  |

>HU08G00019.1   
+ +Up\_Stream \_Len000AGTTGT AGCAAGCACA TTAAAGTTGA TTATCATTTT GTCCGTGAAA CGGTGAGCAA   
  
  
+ ACGTCATCTC CAGGTCAAAT TCATTCGTAC TCAATCACAA GTTGCTGACA TTTTTACCAA AGGCTAATCG   
  
  
+ TCGCAAAAGT TTCTTTAGTT TAAGGCCAAT CTCTCCGTTG TTTCACCCCA TACAGATTGA GGGGTTGTAA   
  
  
+ TAGTGTATAT ATATAGCCTT GTGTTAGCGG GGCTTGGGCA TTAGACTTTT ACTCTTGTAA ACCCTAATGT   
  
  
+ GTTATTATAA ATATAGATCT AGCCACCCAT GTTGAGGTAG GCACTCTAAT TCTGACACAG GTAGGATAAG   
  
  
+ TTTTCATAGT TTATGTTGTG AATCTTGTCT TACTTTGTGG TTCCTATTGA CAAAAGTTTG AATCCGAGCA   
  
  
+ TGAGAGTTCA TCAATCTCTT TTTCTTTCCT ATTTTATTTC TTGTGGACTT AATTGGATTT TCTGGTAGAG   
  
  
+ GAAAGTTCCT TGCTGGAATT TGGTCGAAAG TGATATACAT TTTGGGATTT AATTAGCCAT AAATTTGGTT   
  
  
+ TTACACCTTG TTAGTTTGGA GTTCTGGTGT GTTATTGTAT GTGTTCTGGT GGGTTTTTCT TTTAGCTAAA   
  
  
+ TGTTGGAGCA TTTTAAGGGA TCTAGATATC TGAAGAAGTG CTTGGGAAAT GAAGCATATT TCCCTTATAG   
  
  
+ TCTGACATAA CTTTAATGAA TTGATCATGA ACAAAGGGCA ATTAGTTCAG AAAGGTTCAA CTCCTCTAGG   
  
  
+ TGTTGTGCTA AAGAGATGTG AGTTAGTAAC AATTGCTTAC TAACTATATA GGTTGTTTTA TTACTTGTAG   
  
  
+ AGGTTTTTTT TTTGGGATAT CTTTTTGGGG TTTATAAATG AGAAGGTTGA TTTGTTGTCT ACTCTTCTTT   
  
  
+ CGATTCAATA GTAATTCTCT CAGAGAAGGC TAAAATGACA TGGCATTCCA TCGCCTCTCA TGAGGCTTTT   
  
  
+ TGCATGTGCT TATGTATCTT TCATCTCTTA CTTTGGTGGG GTGGCATTGG GGAGGAAAAG GGGGTTTCGA   
  
  
+ TTCGATAGAT CGTCACTTTC GGAAATGACG TCTCTGACCA ATGGTAAGGG TAAGGTTAAT ACATGTGGAT   
  
  
+ ATTTCCAGAG TTAGTTGTGA TTGTTATTGT TGTTGTTGTA ACTTTATGTC ACGGGGAAAA CATAATAATT   
  
  
+ ATCTGTTTAT CTGTTTGTTT GTTATTTTCA AGGGGAAGGG GAGTGGGCGG ATGCTTAGTA TGTAATCATT   
  
  
+ TTATATAGGG AAAATTCATA TAGCATGAAA GTAAAACTTT CATTGCCAGC ATTCTAAGCT AGGGGTAGGG   
  
  
+ TTGTCTACAC CTTGATCTCC CTAGAAGCAT GGTGATCATT CCTCAGATTG TGTTATAGAA TAAAGAATGT   
  
  
+ GGTTCTGCTG TAATGAGACT TATGTATTAT CAAATGATGA ATCTGTTTAG AACTTCCTGT TTATTTTTTC   
  
  
+ TGCAAAATTT TAAGAGACTA GCATCTATCG GGCTCGACTC TAGAGGTTCT CTTGTGTTAC CCTTCTCAAC   
  
  
+ TCAGTTTGAT TGTGATACAG TTACTATATT GAGTGATAGT CGAGAGCATC ACAACCCGCT AAGGAGTCTA   
  
  
+ TCAACAAGAA GCCCTTCTTG TAATTCTCCC CTTGAAACTA GCAGTTATCA TCACTTGTCA TCGAATAGCC   
  
  
+ CTTTTTGTAA TTCTACCCTT GAAACTAGCA GTTATTGTCA TCAGTTCAAT GTGAGTCCCG TTAGATATTC   
  
  
+ CCATCAAGGC ATAGATTATG GAGTGAGCAT GAAGAACGCT TTGCACGAGC TAGAGACTGC TCTAATTGGT   
  
  
+ GTAGATGGCG AGAAAGCATC TGTTGCTAAT CAACCTATGG GGGGAATTCA TTAGTCCGGG ATCCCAAGTT   
  
  
+ AGAGATCGAA GTCATTGAGT GAAGATCCAC AGGGTTCACA TCCTCCTCAG CTTGATTCAT CATCTTTTTC   
  
  
+ AAGGGTGAAA AGATTCGGAG ATGAAAGCCA GAGAGAGAGA AATGCCACAA GGCAATGGAA GAACCAGCGG   
  
  
+ AACTACTAAG TTTCTCACCA GGTGATTTAA AGCAGTTGCT AATTGAATGT GCGAGGGCTT TATCAGATAA   
  
  
+ CCGAATAGAT GACTTTGAGA GTTTGGCTAA ACGGCCAAGG AAAGAGGTCT CCATCTCAGG TGAGCCTGTC   
  
  
+ CAACGTCTCG GTACTTGTAT GATCGAAGGG CTTGTGGCAA GGAAACAGTC TTCGGGGACT AACATCTATC   
  
  
+ GGGCTCTTAA GTACTGTAAA GAGCCTCTTG GAAAATACTT GCTCTCCTAC GGCACTCAGT GGGTTACTCT   
  
  
+ ATCGCAAGCC ATAGCAGCAA GGCTTGGTGG GCCTCCTAAA GTGCGACTTA CAGGCATTGA TGATCCTGTT   
  
  
+ TCTAAGTATA CTTGTGATGC TAGCTTGGAG GCTATTGGGA AACGATTAGC GTCTCTATTT GAAAAGTTTA   
  
  
+ AAATACTCGT CGAGTTCAAT GCATTGCCCG TTTATGGACC TGATGTCAGG TGGGAAATGC TGGATGTGAG   
  
  
+ GCCCAGGGAG GCTTTGGCCG TTAATTGTCC ATTACAGCTC CATCACACTC CTGACGAGAG TGTCGATGTG   
  
  
+ AGCAACCCTA GGGATAGGCT TCTCAGAATG GTGAAATTGC TCGGTCCTAA GGTATGCACT TTGGTTGAGC   
  
  
+ AAGAATCAAA CACCAACACT ACACGTTTCT TGACCCGGTT CATAGAGACC CTTGACTACT ACTCGATCAG   
  
  
+ CCATGTTTG  

- +Up\_Stream \_Len000TCAACA TCGTTCGTGT AATTTCAACT AATAGTAAAA CAGGCACTTT GCCACTCGTT   
  
  
- TGCAGTAGAG GTCCAGTTTA AGTAAGCATG AGTTAGTGTT CAACGACTGT AAAAATGGTT TCCGATTAGC   
  
  
- AGCGTTTTCA AAGAAATCAA ATTCCGGTTA GAGAGGCAAC AAAGTGGGGT ATGTCTAACT CCCCAACATT   
  
  
- ATCACATATA TATATCGGAA CACAATCGCC CCGAACCCGT AATCTGAAAA TGAGAACATT TGGGATTACA   
  
  
- CAATAATATT TATATCTAGA TCGGTGGGTA CAACTCCATC CGTGAGATTA AGACTGTGTC CATCCTATTC   
  
  
- AAAAGTATCA AATACAACAC TTAGAACAGA ATGAAACACC AAGGATAACT GTTTTCAAAC TTAGGCTCGT   
  
  
- ACTCTCAAGT AGTTAGAGAA AAAGAAAGGA TAAAATAAAG AACACCTGAA TTAACCTAAA AGACCATCTC   
  
  
- CTTTCAAGGA ACGACCTTAA ACCAGCTTTC ACTATATGTA AAACCCTAAA TTAATCGGTA TTTAAACCAA   
  
  
- AATGTGGAAC AATCAAACCT CAAGACCACA CAATAACATA CACAAGACCA CCCAAAAAGA AAATCGATTT   
  
  
- ACAACCTCGT AAAATTCCCT AGATCTATAG ACTTCTTCAC GAACCCTTTA CTTCGTATAA AGGGAATATC   
  
  
- AGACTGTATT GAAATTACTT AACTAGTACT TGTTTCCCGT TAATCAAGTC TTTCCAAGTT GAGGAGATCC   
  
  
- ACAACACGAT TTCTCTACAC TCAATCATTG TTAACGAATG ATTGATATAT CCAACAAAAT AATGAACATC   
  
  
- TCCAAAAAAA AAACCCTATA GAAAAACCCC AAATATTTAC TCTTCCAACT AAACAACAGA TGAGAAGAAA   
  
  
- GCTAAGTTAT CATTAAGAGA GTCTCTTCCG ATTTTACTGT ACCGTAAGGT AGCGGAGAGT ACTCCGAAAA   
  
  
- ACGTACACGA ATACATAGAA AGTAGAGAAT GAAACCACCC CACCGTAACC CCTCCTTTTC CCCCAAAGCT   
  
  
- AAGCTATCTA GCAGTGAAAG CCTTTACTGC AGAGACTGGT TACCATTCCC ATTCCAATTA TGTACACCTA   
  
  
- TAAAGGTCTC AATCAACACT AACAATAACA ACAACAACAT TGAAATACAG TGCCCCTTTT GTATTATTAA   
  
  
- TAGACAAATA GACAAACAAA CAATAAAAGT TCCCCTTCCC CTCACCCGCC TACGAATCAT ACATTAGTAA   
  
  
- AATATATCCC TTTTAAGTAT ATCGTACTTT CATTTTGAAA GTAACGGTCG TAAGATTCGA TCCCCATCCC   
  
  
- AACAGATGTG GAACTAGAGG GATCTTCGTA CCACTAGTAA GGAGTCTAAC ACAATATCTT ATTTCTTACA   
  
  
- CCAAGACGAC ATTACTCTGA ATACATAATA GTTTACTACT TAGACAAATC TTGAAGGACA AATAAAAAAG   
  
  
- ACGTTTTAAA ATTCTCTGAT CGTAGATAGC CCGAGCTGAG ATCTCCAAGA GAACACAATG GGAAGAGTTG   
  
  
- AGTCAAACTA ACACTATGTC AATGATATAA CTCACTATCA GCTCTCGTAG TGTTGGGCGA TTCCTCAGAT   
  
  
- AGTTGTTCTT CGGGAAGAAC ATTAAGAGGG GAACTTTGAT CGTCAATAGT AGTGAACAGT AGCTTATCGG   
  
  
- GAAAAACATT AAGATGGGAA CTTTGATCGT CAATAACAGT AGTCAAGTTA CACTCAGGGC AATCTATAAG   
  
  
- GGTAGTTCCG TATCTAATAC CTCACTCGTA CTTCTTGCGA AACGTGCTCG ATCTCTGACG AGATTAACCA   
  
  
- CATCTACCGC TCTTTCGTAG ACAACGATTA GTTGGATACC CCCCTTAAGT AATCAGGCCC TAGGGTTCAA   
  
  
- TCTCTAGCTT CAGTAACTCA CTTCTAGGTG TCCCAAGTGT AGGAGGAGTC GAACTAAGTA GTAGAAAAAG   
  
  
- TTCCCACTTT TCTAAGCCTC TACTTTCGGT CTCTCTCTCT TTACGGTGTT CCGTTACCTT CTTGGTCGCC   
  
  
- TTGATGATTC AAAGAGTGGT CCACTAAATT TCGTCAACGA TTAACTTACA CGCTCCCGAA ATAGTCTATT   
  
  
- GGCTTATCTA CTGAAACTCT CAAACCGATT TGCCGGTTCC TTTCTCCAGA GGTAGAGTCC ACTCGGACAG   
  
  
- GTTGCAGAGC CATGAACATA CTAGCTTCCC GAACACCGTT CCTTTGTCAG AAGCCCCTGA TTGTAGATAG   
  
  
- CCCGAGAATT CATGACATTT CTCGGAGAAC CTTTTATGAA CGAGAGGATG CCGTGAGTCA CCCAATGAGA   
  
  
- TAGCGTTCGG TATCGTCGTT CCGAACCACC CGGAGGATTT CACGCTGAAT GTCCGTAACT ACTAGGACAA   
  
  
- AGATTCATAT GAACACTACG ATCGAACCTC CGATAACCCT TTGCTAATCG CAGAGATAAA CTTTTCAAAT   
  
  
- TTTATGAGCA GCTCAAGTTA CGTAACGGGC AAATACCTGG ACTACAGTCC ACCCTTTACG ACCTACACTC   
  
  
- CGGGTCCCTC CGAAACCGGC AATTAACAGG TAATGTCGAG GTAGTGTGAG GACTGCTCTC ACAGCTACAC   
  
  
- TCGTTGGGAT CCCTATCCGA AGAGTCTTAC CACTTTAACG AGCCAGGATT CCATACGTGA AACCAACTCG   
  
  
- TTCTTAGTTT GTGGTTGTGA TGTGCAAAGA ACTGGGCCAA GTATCTCTGG GAACTGATGA TGAGCTAGTC   
  
  
- GGTACAAAC

+     AuxRR-core

| Site Name | Organism | Position | Strand | Matrix score. | sequence | function |
| --- | --- | --- | --- | --- | --- | --- |
| AuxRR-core | Nicotiana tabacum | 2488 | - | 7 | GGTCCAT | cis-acting regulatory element involved in auxin responsiveness |

>HU08G00019.1   
+ +Up\_Stream \_Len000AGTTGT AGCAAGCACA TTAAAGTTGA TTATCATTTT GTCCGTGAAA CGGTGAGCAA   
  
  
+ ACGTCATCTC CAGGTCAAAT TCATTCGTAC TCAATCACAA GTTGCTGACA TTTTTACCAA AGGCTAATCG   
  
  
+ TCGCAAAAGT TTCTTTAGTT TAAGGCCAAT CTCTCCGTTG TTTCACCCCA TACAGATTGA GGGGTTGTAA   
  
  
+ TAGTGTATAT ATATAGCCTT GTGTTAGCGG GGCTTGGGCA TTAGACTTTT ACTCTTGTAA ACCCTAATGT   
  
  
+ GTTATTATAA ATATAGATCT AGCCACCCAT GTTGAGGTAG GCACTCTAAT TCTGACACAG GTAGGATAAG   
  
  
+ TTTTCATAGT TTATGTTGTG AATCTTGTCT TACTTTGTGG TTCCTATTGA CAAAAGTTTG AATCCGAGCA   
  
  
+ TGAGAGTTCA TCAATCTCTT TTTCTTTCCT ATTTTATTTC TTGTGGACTT AATTGGATTT TCTGGTAGAG   
  
  
+ GAAAGTTCCT TGCTGGAATT TGGTCGAAAG TGATATACAT TTTGGGATTT AATTAGCCAT AAATTTGGTT   
  
  
+ TTACACCTTG TTAGTTTGGA GTTCTGGTGT GTTATTGTAT GTGTTCTGGT GGGTTTTTCT TTTAGCTAAA   
  
  
+ TGTTGGAGCA TTTTAAGGGA TCTAGATATC TGAAGAAGTG CTTGGGAAAT GAAGCATATT TCCCTTATAG   
  
  
+ TCTGACATAA CTTTAATGAA TTGATCATGA ACAAAGGGCA ATTAGTTCAG AAAGGTTCAA CTCCTCTAGG   
  
  
+ TGTTGTGCTA AAGAGATGTG AGTTAGTAAC AATTGCTTAC TAACTATATA GGTTGTTTTA TTACTTGTAG   
  
  
+ AGGTTTTTTT TTTGGGATAT CTTTTTGGGG TTTATAAATG AGAAGGTTGA TTTGTTGTCT ACTCTTCTTT   
  
  
+ CGATTCAATA GTAATTCTCT CAGAGAAGGC TAAAATGACA TGGCATTCCA TCGCCTCTCA TGAGGCTTTT   
  
  
+ TGCATGTGCT TATGTATCTT TCATCTCTTA CTTTGGTGGG GTGGCATTGG GGAGGAAAAG GGGGTTTCGA   
  
  
+ TTCGATAGAT CGTCACTTTC GGAAATGACG TCTCTGACCA ATGGTAAGGG TAAGGTTAAT ACATGTGGAT   
  
  
+ ATTTCCAGAG TTAGTTGTGA TTGTTATTGT TGTTGTTGTA ACTTTATGTC ACGGGGAAAA CATAATAATT   
  
  
+ ATCTGTTTAT CTGTTTGTTT GTTATTTTCA AGGGGAAGGG GAGTGGGCGG ATGCTTAGTA TGTAATCATT   
  
  
+ TTATATAGGG AAAATTCATA TAGCATGAAA GTAAAACTTT CATTGCCAGC ATTCTAAGCT AGGGGTAGGG   
  
  
+ TTGTCTACAC CTTGATCTCC CTAGAAGCAT GGTGATCATT CCTCAGATTG TGTTATAGAA TAAAGAATGT   
  
  
+ GGTTCTGCTG TAATGAGACT TATGTATTAT CAAATGATGA ATCTGTTTAG AACTTCCTGT TTATTTTTTC   
  
  
+ TGCAAAATTT TAAGAGACTA GCATCTATCG GGCTCGACTC TAGAGGTTCT CTTGTGTTAC CCTTCTCAAC   
  
  
+ TCAGTTTGAT TGTGATACAG TTACTATATT GAGTGATAGT CGAGAGCATC ACAACCCGCT AAGGAGTCTA   
  
  
+ TCAACAAGAA GCCCTTCTTG TAATTCTCCC CTTGAAACTA GCAGTTATCA TCACTTGTCA TCGAATAGCC   
  
  
+ CTTTTTGTAA TTCTACCCTT GAAACTAGCA GTTATTGTCA TCAGTTCAAT GTGAGTCCCG TTAGATATTC   
  
  
+ CCATCAAGGC ATAGATTATG GAGTGAGCAT GAAGAACGCT TTGCACGAGC TAGAGACTGC TCTAATTGGT   
  
  
+ GTAGATGGCG AGAAAGCATC TGTTGCTAAT CAACCTATGG GGGGAATTCA TTAGTCCGGG ATCCCAAGTT   
  
  
+ AGAGATCGAA GTCATTGAGT GAAGATCCAC AGGGTTCACA TCCTCCTCAG CTTGATTCAT CATCTTTTTC   
  
  
+ AAGGGTGAAA AGATTCGGAG ATGAAAGCCA GAGAGAGAGA AATGCCACAA GGCAATGGAA GAACCAGCGG   
  
  
+ AACTACTAAG TTTCTCACCA GGTGATTTAA AGCAGTTGCT AATTGAATGT GCGAGGGCTT TATCAGATAA   
  
  
+ CCGAATAGAT GACTTTGAGA GTTTGGCTAA ACGGCCAAGG AAAGAGGTCT CCATCTCAGG TGAGCCTGTC   
  
  
+ CAACGTCTCG GTACTTGTAT GATCGAAGGG CTTGTGGCAA GGAAACAGTC TTCGGGGACT AACATCTATC   
  
  
+ GGGCTCTTAA GTACTGTAAA GAGCCTCTTG GAAAATACTT GCTCTCCTAC GGCACTCAGT GGGTTACTCT   
  
  
+ ATCGCAAGCC ATAGCAGCAA GGCTTGGTGG GCCTCCTAAA GTGCGACTTA CAGGCATTGA TGATCCTGTT   
  
  
+ TCTAAGTATA CTTGTGATGC TAGCTTGGAG GCTATTGGGA AACGATTAGC GTCTCTATTT GAAAAGTTTA   
  
  
+ AAATACTCGT CGAGTTCAAT GCATTGCCCG TTTATGGACC TGATGTCAGG TGGGAAATGC TGGATGTGAG   
  
  
+ GCCCAGGGAG GCTTTGGCCG TTAATTGTCC ATTACAGCTC CATCACACTC CTGACGAGAG TGTCGATGTG   
  
  
+ AGCAACCCTA GGGATAGGCT TCTCAGAATG GTGAAATTGC TCGGTCCTAA GGTATGCACT TTGGTTGAGC   
  
  
+ AAGAATCAAA CACCAACACT ACACGTTTCT TGACCCGGTT CATAGAGACC CTTGACTACT ACTCGATCAG   
  
  
+ CCATGTTTG  

- +Up\_Stream \_Len000TCAACA TCGTTCGTGT AATTTCAACT AATAGTAAAA CAGGCACTTT GCCACTCGTT   
  
  
- TGCAGTAGAG GTCCAGTTTA AGTAAGCATG AGTTAGTGTT CAACGACTGT AAAAATGGTT TCCGATTAGC   
  
  
- AGCGTTTTCA AAGAAATCAA ATTCCGGTTA GAGAGGCAAC AAAGTGGGGT ATGTCTAACT CCCCAACATT   
  
  
- ATCACATATA TATATCGGAA CACAATCGCC CCGAACCCGT AATCTGAAAA TGAGAACATT TGGGATTACA   
  
  
- CAATAATATT TATATCTAGA TCGGTGGGTA CAACTCCATC CGTGAGATTA AGACTGTGTC CATCCTATTC   
  
  
- AAAAGTATCA AATACAACAC TTAGAACAGA ATGAAACACC AAGGATAACT GTTTTCAAAC TTAGGCTCGT   
  
  
- ACTCTCAAGT AGTTAGAGAA AAAGAAAGGA TAAAATAAAG AACACCTGAA TTAACCTAAA AGACCATCTC   
  
  
- CTTTCAAGGA ACGACCTTAA ACCAGCTTTC ACTATATGTA AAACCCTAAA TTAATCGGTA TTTAAACCAA   
  
  
- AATGTGGAAC AATCAAACCT CAAGACCACA CAATAACATA CACAAGACCA CCCAAAAAGA AAATCGATTT   
  
  
- ACAACCTCGT AAAATTCCCT AGATCTATAG ACTTCTTCAC GAACCCTTTA CTTCGTATAA AGGGAATATC   
  
  
- AGACTGTATT GAAATTACTT AACTAGTACT TGTTTCCCGT TAATCAAGTC TTTCCAAGTT GAGGAGATCC   
  
  
- ACAACACGAT TTCTCTACAC TCAATCATTG TTAACGAATG ATTGATATAT CCAACAAAAT AATGAACATC   
  
  
- TCCAAAAAAA AAACCCTATA GAAAAACCCC AAATATTTAC TCTTCCAACT AAACAACAGA TGAGAAGAAA   
  
  
- GCTAAGTTAT CATTAAGAGA GTCTCTTCCG ATTTTACTGT ACCGTAAGGT AGCGGAGAGT ACTCCGAAAA   
  
  
- ACGTACACGA ATACATAGAA AGTAGAGAAT GAAACCACCC CACCGTAACC CCTCCTTTTC CCCCAAAGCT   
  
  
- AAGCTATCTA GCAGTGAAAG CCTTTACTGC AGAGACTGGT TACCATTCCC ATTCCAATTA TGTACACCTA   
  
  
- TAAAGGTCTC AATCAACACT AACAATAACA ACAACAACAT TGAAATACAG TGCCCCTTTT GTATTATTAA   
  
  
- TAGACAAATA GACAAACAAA CAATAAAAGT TCCCCTTCCC CTCACCCGCC TACGAATCAT ACATTAGTAA   
  
  
- AATATATCCC TTTTAAGTAT ATCGTACTTT CATTTTGAAA GTAACGGTCG TAAGATTCGA TCCCCATCCC   
  
  
- AACAGATGTG GAACTAGAGG GATCTTCGTA CCACTAGTAA GGAGTCTAAC ACAATATCTT ATTTCTTACA   
  
  
- CCAAGACGAC ATTACTCTGA ATACATAATA GTTTACTACT TAGACAAATC TTGAAGGACA AATAAAAAAG   
  
  
- ACGTTTTAAA ATTCTCTGAT CGTAGATAGC CCGAGCTGAG ATCTCCAAGA GAACACAATG GGAAGAGTTG   
  
  
- AGTCAAACTA ACACTATGTC AATGATATAA CTCACTATCA GCTCTCGTAG TGTTGGGCGA TTCCTCAGAT   
  
  
- AGTTGTTCTT CGGGAAGAAC ATTAAGAGGG GAACTTTGAT CGTCAATAGT AGTGAACAGT AGCTTATCGG   
  
  
- GAAAAACATT AAGATGGGAA CTTTGATCGT CAATAACAGT AGTCAAGTTA CACTCAGGGC AATCTATAAG   
  
  
- GGTAGTTCCG TATCTAATAC CTCACTCGTA CTTCTTGCGA AACGTGCTCG ATCTCTGACG AGATTAACCA   
  
  
- CATCTACCGC TCTTTCGTAG ACAACGATTA GTTGGATACC CCCCTTAAGT AATCAGGCCC TAGGGTTCAA   
  
  
- TCTCTAGCTT CAGTAACTCA CTTCTAGGTG TCCCAAGTGT AGGAGGAGTC GAACTAAGTA GTAGAAAAAG   
  
  
- TTCCCACTTT TCTAAGCCTC TACTTTCGGT CTCTCTCTCT TTACGGTGTT CCGTTACCTT CTTGGTCGCC   
  
  
- TTGATGATTC AAAGAGTGGT CCACTAAATT TCGTCAACGA TTAACTTACA CGCTCCCGAA ATAGTCTATT   
  
  
- GGCTTATCTA CTGAAACTCT CAAACCGATT TGCCGGTTCC TTTCTCCAGA GGTAGAGTCC ACTCGGACAG   
  
  
- GTTGCAGAGC CATGAACATA CTAGCTTCCC GAACACCGTT CCTTTGTCAG AAGCCCCTGA TTGTAGATAG   
  
  
- CCCGAGAATT CATGACATTT CTCGGAGAAC CTTTTATGAA CGAGAGGATG CCGTGAGTCA CCCAATGAGA   
  
  
- TAGCGTTCGG TATCGTCGTT CCGAACCACC CGGAGGATTT CACGCTGAAT GTCCGTAACT ACTAGGACAA   
  
  
- AGATTCATAT GAACACTACG ATCGAACCTC CGATAACCCT TTGCTAATCG CAGAGATAAA CTTTTCAAAT   
  
  
- TTTATGAGCA GCTCAAGTTA CGTAACGGGC AAATACCTGG ACTACAGTCC ACCCTTTACG ACCTACACTC   
  
  
- CGGGTCCCTC CGAAACCGGC AATTAACAGG TAATGTCGAG GTAGTGTGAG GACTGCTCTC ACAGCTACAC   
  
  
- TCGTTGGGAT CCCTATCCGA AGAGTCTTAC CACTTTAACG AGCCAGGATT CCATACGTGA AACCAACTCG   
  
  
- TTCTTAGTTT GTGGTTGTGA TGTGCAAAGA ACTGGGCCAA GTATCTCTGG GAACTGATGA TGAGCTAGTC   
  
  
- GGTACAAAC

+     CAAT-box

| Site Name | Organism | Position | Strand | Matrix score. | sequence | function |
| --- | --- | --- | --- | --- | --- | --- |
| CAAT-box | Nicotiana glutinosa | 2630 | - | 4 | CAAT |  |
| CAAT-box | Nicotiana glutinosa | 2477 | - | 4 | CAAT |  |
| CAAT-box | Arabidopsis thaliana | 2418 | - | 5 | CCAAT | common cis-acting element in promoter and enhancer regions |
| CAAT-box | Arabidopsis thaliana | 1819 | - | 5 | CCAAT | common cis-acting element in promoter and enhancer regions |
| CAAT-box | Nicotiana glutinosa | 2471 | + | 4 | CAAT |  |
| CAAT-box | Nicotiana glutinosa | 2548 | - | 4 | CAAT |  |
| CAAT-box | Nicotiana glutinosa | 2076 | - | 4 | CAAT |  |
| CAAT-box | Nicotiana glutinosa | 2017 | + | 4 | CAAT |  |
| CAAT-box | Nicotiana glutinosa | 1908 | - | 4 | CAAT |  |
| CAAT-box | Pisum sativum | 2441 | - | 5 | CAAAT | common cis-acting element in promoter and enhancer regions |
| CAAT-box | Nicotiana glutinosa | 2370 | - | 4 | CAAT |  |
| CAAT-box | Nicotiana glutinosa | 1731 | + | 4 | CAAT |  |
| CAAT-box | Nicotiana glutinosa | 1572 | - | 4 | CAAT |  |
| CAAT-box | Nicotiana glutinosa | 1381 | - | 4 | CAAT |  |
| CAAT-box | Nicotiana glutinosa | 1144 | - | 4 | CAAT |  |
| CAAT-box | Nicotiana glutinosa | 1718 | - | 4 | CAAT |  |
| CAAT-box | Pisum sativum | 1435 | + | 5 | CAAAT | common cis-acting element in promoter and enhancer regions |
| CAAT-box | Nicotiana glutinosa | 1553 | - | 4 | CAAT |  |
| CAAT-box | Nicotiana glutinosa | 1150 | - | 4 | CAAT |  |
| CAAT-box | Nicotiana glutinosa | 1306 | - | 4 | CAAT |  |
| CAAT-box | Nicotiana glutinosa | 920 | + | 4 | CAAT |  |
| CAAT-box | Nicotiana glutinosa | 598 | - | 4 | CAAT |  |
| CAAT-box | Nicotiana glutinosa | 724 | - | 4 | CAAT |  |
| CAAT-box | Nicotiana glutinosa | 1093 | + | 4 | CAAT |  |
| CAAT-box | Pisum sativum | 557 | - | 5 | CAAAT | common cis-acting element in promoter and enhancer regions |
| CAAT-box | Nicotiana glutinosa | 200 | - | 4 | CAAT |  |
| CAAT-box | Arabidopsis thaliana | 476 | - | 5 | CCAAT | common cis-acting element in promoter and enhancer regions |
| CAAT-box | Nicotiana glutinosa | 106 | + | 4 | CAAT |  |
| CAAT-box | Nicotiana glutinosa | 743 | + | 4 | CAAT |  |
| CAAT-box | Pisum sativum | 894 | - | 5 | CAAAT | common cis-acting element in promoter and enhancer regions |
| CAAT-box | Nicotiana glutinosa | 806 | - | 4 | CAAT |  |
| CAAT-box | Pisum sativum | 512 | - | 5 | CAAAT | common cis-acting element in promoter and enhancer regions |
| CAAT-box | Nicotiana glutinosa | 436 | + | 4 | CAAT |  |
| CAAT-box | Pisum sativum | 90 | + | 5 | CAAAT | common cis-acting element in promoter and enhancer regions |
| CAAT-box | Arabidopsis thaliana | 1030 | - | 5 | CCAAT | common cis-acting element in promoter and enhancer regions |
| CAAT-box | Nicotiana glutinosa | 400 | - | 4 | CAAT |  |
| CAAT-box | Arabidopsis thaliana | 170 | + | 5 | CCAAT | common cis-acting element in promoter and enhancer regions |
| CAAT-box | Nicotiana glutinosa | 171 | + | 4 | CAAT |  |
| CAAT-box | Nicotiana glutinosa | 804 | + | 4 | CAAT |  |
| CAAT-box | Arabidopsis thaliana | 1092 | + | 5 | CCAAT | common cis-acting element in promoter and enhancer regions |

>HU08G00019.1   
+ +Up\_Stream \_Len000AGTTGT AGCAAGCACA TTAAAGTTGA TTATCATTTT GTCCGTGAAA CGGTGAGCAA   
  
  
+ ACGTCATCTC CAGGTCAAAT TCATTCGTAC TCAATCACAA GTTGCTGACA TTTTTACCAA AGGCTAATCG   
  
  
+ TCGCAAAAGT TTCTTTAGTT TAAGGCCAAT CTCTCCGTTG TTTCACCCCA TACAGATTGA GGGGTTGTAA   
  
  
+ TAGTGTATAT ATATAGCCTT GTGTTAGCGG GGCTTGGGCA TTAGACTTTT ACTCTTGTAA ACCCTAATGT   
  
  
+ GTTATTATAA ATATAGATCT AGCCACCCAT GTTGAGGTAG GCACTCTAAT TCTGACACAG GTAGGATAAG   
  
  
+ TTTTCATAGT TTATGTTGTG AATCTTGTCT TACTTTGTGG TTCCTATTGA CAAAAGTTTG AATCCGAGCA   
  
  
+ TGAGAGTTCA TCAATCTCTT TTTCTTTCCT ATTTTATTTC TTGTGGACTT AATTGGATTT TCTGGTAGAG   
  
  
+ GAAAGTTCCT TGCTGGAATT TGGTCGAAAG TGATATACAT TTTGGGATTT AATTAGCCAT AAATTTGGTT   
  
  
+ TTACACCTTG TTAGTTTGGA GTTCTGGTGT GTTATTGTAT GTGTTCTGGT GGGTTTTTCT TTTAGCTAAA   
  
  
+ TGTTGGAGCA TTTTAAGGGA TCTAGATATC TGAAGAAGTG CTTGGGAAAT GAAGCATATT TCCCTTATAG   
  
  
+ TCTGACATAA CTTTAATGAA TTGATCATGA ACAAAGGGCA ATTAGTTCAG AAAGGTTCAA CTCCTCTAGG   
  
  
+ TGTTGTGCTA AAGAGATGTG AGTTAGTAAC AATTGCTTAC TAACTATATA GGTTGTTTTA TTACTTGTAG   
  
  
+ AGGTTTTTTT TTTGGGATAT CTTTTTGGGG TTTATAAATG AGAAGGTTGA TTTGTTGTCT ACTCTTCTTT   
  
  
+ CGATTCAATA GTAATTCTCT CAGAGAAGGC TAAAATGACA TGGCATTCCA TCGCCTCTCA TGAGGCTTTT   
  
  
+ TGCATGTGCT TATGTATCTT TCATCTCTTA CTTTGGTGGG GTGGCATTGG GGAGGAAAAG GGGGTTTCGA   
  
  
+ TTCGATAGAT CGTCACTTTC GGAAATGACG TCTCTGACCA ATGGTAAGGG TAAGGTTAAT ACATGTGGAT   
  
  
+ ATTTCCAGAG TTAGTTGTGA TTGTTATTGT TGTTGTTGTA ACTTTATGTC ACGGGGAAAA CATAATAATT   
  
  
+ ATCTGTTTAT CTGTTTGTTT GTTATTTTCA AGGGGAAGGG GAGTGGGCGG ATGCTTAGTA TGTAATCATT   
  
  
+ TTATATAGGG AAAATTCATA TAGCATGAAA GTAAAACTTT CATTGCCAGC ATTCTAAGCT AGGGGTAGGG   
  
  
+ TTGTCTACAC CTTGATCTCC CTAGAAGCAT GGTGATCATT CCTCAGATTG TGTTATAGAA TAAAGAATGT   
  
  
+ GGTTCTGCTG TAATGAGACT TATGTATTAT CAAATGATGA ATCTGTTTAG AACTTCCTGT TTATTTTTTC   
  
  
+ TGCAAAATTT TAAGAGACTA GCATCTATCG GGCTCGACTC TAGAGGTTCT CTTGTGTTAC CCTTCTCAAC   
  
  
+ TCAGTTTGAT TGTGATACAG TTACTATATT GAGTGATAGT CGAGAGCATC ACAACCCGCT AAGGAGTCTA   
  
  
+ TCAACAAGAA GCCCTTCTTG TAATTCTCCC CTTGAAACTA GCAGTTATCA TCACTTGTCA TCGAATAGCC   
  
  
+ CTTTTTGTAA TTCTACCCTT GAAACTAGCA GTTATTGTCA TCAGTTCAAT GTGAGTCCCG TTAGATATTC   
  
  
+ CCATCAAGGC ATAGATTATG GAGTGAGCAT GAAGAACGCT TTGCACGAGC TAGAGACTGC TCTAATTGGT   
  
  
+ GTAGATGGCG AGAAAGCATC TGTTGCTAAT CAACCTATGG GGGGAATTCA TTAGTCCGGG ATCCCAAGTT   
  
  
+ AGAGATCGAA GTCATTGAGT GAAGATCCAC AGGGTTCACA TCCTCCTCAG CTTGATTCAT CATCTTTTTC   
  
  
+ AAGGGTGAAA AGATTCGGAG ATGAAAGCCA GAGAGAGAGA AATGCCACAA GGCAATGGAA GAACCAGCGG   
  
  
+ AACTACTAAG TTTCTCACCA GGTGATTTAA AGCAGTTGCT AATTGAATGT GCGAGGGCTT TATCAGATAA   
  
  
+ CCGAATAGAT GACTTTGAGA GTTTGGCTAA ACGGCCAAGG AAAGAGGTCT CCATCTCAGG TGAGCCTGTC   
  
  
+ CAACGTCTCG GTACTTGTAT GATCGAAGGG CTTGTGGCAA GGAAACAGTC TTCGGGGACT AACATCTATC   
  
  
+ GGGCTCTTAA GTACTGTAAA GAGCCTCTTG GAAAATACTT GCTCTCCTAC GGCACTCAGT GGGTTACTCT   
  
  
+ ATCGCAAGCC ATAGCAGCAA GGCTTGGTGG GCCTCCTAAA GTGCGACTTA CAGGCATTGA TGATCCTGTT   
  
  
+ TCTAAGTATA CTTGTGATGC TAGCTTGGAG GCTATTGGGA AACGATTAGC GTCTCTATTT GAAAAGTTTA   
  
  
+ AAATACTCGT CGAGTTCAAT GCATTGCCCG TTTATGGACC TGATGTCAGG TGGGAAATGC TGGATGTGAG   
  
  
+ GCCCAGGGAG GCTTTGGCCG TTAATTGTCC ATTACAGCTC CATCACACTC CTGACGAGAG TGTCGATGTG   
  
  
+ AGCAACCCTA GGGATAGGCT TCTCAGAATG GTGAAATTGC TCGGTCCTAA GGTATGCACT TTGGTTGAGC   
  
  
+ AAGAATCAAA CACCAACACT ACACGTTTCT TGACCCGGTT CATAGAGACC CTTGACTACT ACTCGATCAG   
  
  
+ CCATGTTTG  

- +Up\_Stream \_Len000TCAACA TCGTTCGTGT AATTTCAACT AATAGTAAAA CAGGCACTTT GCCACTCGTT   
  
  
- TGCAGTAGAG GTCCAGTTTA AGTAAGCATG AGTTAGTGTT CAACGACTGT AAAAATGGTT TCCGATTAGC   
  
  
- AGCGTTTTCA AAGAAATCAA ATTCCGGTTA GAGAGGCAAC AAAGTGGGGT ATGTCTAACT CCCCAACATT   
  
  
- ATCACATATA TATATCGGAA CACAATCGCC CCGAACCCGT AATCTGAAAA TGAGAACATT TGGGATTACA   
  
  
- CAATAATATT TATATCTAGA TCGGTGGGTA CAACTCCATC CGTGAGATTA AGACTGTGTC CATCCTATTC   
  
  
- AAAAGTATCA AATACAACAC TTAGAACAGA ATGAAACACC AAGGATAACT GTTTTCAAAC TTAGGCTCGT   
  
  
- ACTCTCAAGT AGTTAGAGAA AAAGAAAGGA TAAAATAAAG AACACCTGAA TTAACCTAAA AGACCATCTC   
  
  
- CTTTCAAGGA ACGACCTTAA ACCAGCTTTC ACTATATGTA AAACCCTAAA TTAATCGGTA TTTAAACCAA   
  
  
- AATGTGGAAC AATCAAACCT CAAGACCACA CAATAACATA CACAAGACCA CCCAAAAAGA AAATCGATTT   
  
  
- ACAACCTCGT AAAATTCCCT AGATCTATAG ACTTCTTCAC GAACCCTTTA CTTCGTATAA AGGGAATATC   
  
  
- AGACTGTATT GAAATTACTT AACTAGTACT TGTTTCCCGT TAATCAAGTC TTTCCAAGTT GAGGAGATCC   
  
  
- ACAACACGAT TTCTCTACAC TCAATCATTG TTAACGAATG ATTGATATAT CCAACAAAAT AATGAACATC   
  
  
- TCCAAAAAAA AAACCCTATA GAAAAACCCC AAATATTTAC TCTTCCAACT AAACAACAGA TGAGAAGAAA   
  
  
- GCTAAGTTAT CATTAAGAGA GTCTCTTCCG ATTTTACTGT ACCGTAAGGT AGCGGAGAGT ACTCCGAAAA   
  
  
- ACGTACACGA ATACATAGAA AGTAGAGAAT GAAACCACCC CACCGTAACC CCTCCTTTTC CCCCAAAGCT   
  
  
- AAGCTATCTA GCAGTGAAAG CCTTTACTGC AGAGACTGGT TACCATTCCC ATTCCAATTA TGTACACCTA   
  
  
- TAAAGGTCTC AATCAACACT AACAATAACA ACAACAACAT TGAAATACAG TGCCCCTTTT GTATTATTAA   
  
  
- TAGACAAATA GACAAACAAA CAATAAAAGT TCCCCTTCCC CTCACCCGCC TACGAATCAT ACATTAGTAA   
  
  
- AATATATCCC TTTTAAGTAT ATCGTACTTT CATTTTGAAA GTAACGGTCG TAAGATTCGA TCCCCATCCC   
  
  
- AACAGATGTG GAACTAGAGG GATCTTCGTA CCACTAGTAA GGAGTCTAAC ACAATATCTT ATTTCTTACA   
  
  
- CCAAGACGAC ATTACTCTGA ATACATAATA GTTTACTACT TAGACAAATC TTGAAGGACA AATAAAAAAG   
  
  
- ACGTTTTAAA ATTCTCTGAT CGTAGATAGC CCGAGCTGAG ATCTCCAAGA GAACACAATG GGAAGAGTTG   
  
  
- AGTCAAACTA ACACTATGTC AATGATATAA CTCACTATCA GCTCTCGTAG TGTTGGGCGA TTCCTCAGAT   
  
  
- AGTTGTTCTT CGGGAAGAAC ATTAAGAGGG GAACTTTGAT CGTCAATAGT AGTGAACAGT AGCTTATCGG   
  
  
- GAAAAACATT AAGATGGGAA CTTTGATCGT CAATAACAGT AGTCAAGTTA CACTCAGGGC AATCTATAAG   
  
  
- GGTAGTTCCG TATCTAATAC CTCACTCGTA CTTCTTGCGA AACGTGCTCG ATCTCTGACG AGATTAACCA   
  
  
- CATCTACCGC TCTTTCGTAG ACAACGATTA GTTGGATACC CCCCTTAAGT AATCAGGCCC TAGGGTTCAA   
  
  
- TCTCTAGCTT CAGTAACTCA CTTCTAGGTG TCCCAAGTGT AGGAGGAGTC GAACTAAGTA GTAGAAAAAG   
  
  
- TTCCCACTTT TCTAAGCCTC TACTTTCGGT CTCTCTCTCT TTACGGTGTT CCGTTACCTT CTTGGTCGCC   
  
  
- TTGATGATTC AAAGAGTGGT CCACTAAATT TCGTCAACGA TTAACTTACA CGCTCCCGAA ATAGTCTATT   
  
  
- GGCTTATCTA CTGAAACTCT CAAACCGATT TGCCGGTTCC TTTCTCCAGA GGTAGAGTCC ACTCGGACAG   
  
  
- GTTGCAGAGC CATGAACATA CTAGCTTCCC GAACACCGTT CCTTTGTCAG AAGCCCCTGA TTGTAGATAG   
  
  
- CCCGAGAATT CATGACATTT CTCGGAGAAC CTTTTATGAA CGAGAGGATG CCGTGAGTCA CCCAATGAGA   
  
  
- TAGCGTTCGG TATCGTCGTT CCGAACCACC CGGAGGATTT CACGCTGAAT GTCCGTAACT ACTAGGACAA   
  
  
- AGATTCATAT GAACACTACG ATCGAACCTC CGATAACCCT TTGCTAATCG CAGAGATAAA CTTTTCAAAT   
  
  
- TTTATGAGCA GCTCAAGTTA CGTAACGGGC AAATACCTGG ACTACAGTCC ACCCTTTACG ACCTACACTC   
  
  
- CGGGTCCCTC CGAAACCGGC AATTAACAGG TAATGTCGAG GTAGTGTGAG GACTGCTCTC ACAGCTACAC   
  
  
- TCGTTGGGAT CCCTATCCGA AGAGTCTTAC CACTTTAACG AGCCAGGATT CCATACGTGA AACCAACTCG   
  
  
- TTCTTAGTTT GTGGTTGTGA TGTGCAAAGA ACTGGGCCAA GTATCTCTGG GAACTGATGA TGAGCTAGTC   
  
  
- GGTACAAAC

+     CCAAT-box

| Site Name | Organism | Position | Strand | Matrix score. | sequence | function |
| --- | --- | --- | --- | --- | --- | --- |
| CCAAT-box | Hordeum vulgare | 179 | - | 6 | CAACGG | MYBHv1 binding site |

>HU08G00019.1   
+ +Up\_Stream \_Len000AGTTGT AGCAAGCACA TTAAAGTTGA TTATCATTTT GTCCGTGAAA CGGTGAGCAA   
  
  
+ ACGTCATCTC CAGGTCAAAT TCATTCGTAC TCAATCACAA GTTGCTGACA TTTTTACCAA AGGCTAATCG   
  
  
+ TCGCAAAAGT TTCTTTAGTT TAAGGCCAAT CTCTCCGTTG TTTCACCCCA TACAGATTGA GGGGTTGTAA   
  
  
+ TAGTGTATAT ATATAGCCTT GTGTTAGCGG GGCTTGGGCA TTAGACTTTT ACTCTTGTAA ACCCTAATGT   
  
  
+ GTTATTATAA ATATAGATCT AGCCACCCAT GTTGAGGTAG GCACTCTAAT TCTGACACAG GTAGGATAAG   
  
  
+ TTTTCATAGT TTATGTTGTG AATCTTGTCT TACTTTGTGG TTCCTATTGA CAAAAGTTTG AATCCGAGCA   
  
  
+ TGAGAGTTCA TCAATCTCTT TTTCTTTCCT ATTTTATTTC TTGTGGACTT AATTGGATTT TCTGGTAGAG   
  
  
+ GAAAGTTCCT TGCTGGAATT TGGTCGAAAG TGATATACAT TTTGGGATTT AATTAGCCAT AAATTTGGTT   
  
  
+ TTACACCTTG TTAGTTTGGA GTTCTGGTGT GTTATTGTAT GTGTTCTGGT GGGTTTTTCT TTTAGCTAAA   
  
  
+ TGTTGGAGCA TTTTAAGGGA TCTAGATATC TGAAGAAGTG CTTGGGAAAT GAAGCATATT TCCCTTATAG   
  
  
+ TCTGACATAA CTTTAATGAA TTGATCATGA ACAAAGGGCA ATTAGTTCAG AAAGGTTCAA CTCCTCTAGG   
  
  
+ TGTTGTGCTA AAGAGATGTG AGTTAGTAAC AATTGCTTAC TAACTATATA GGTTGTTTTA TTACTTGTAG   
  
  
+ AGGTTTTTTT TTTGGGATAT CTTTTTGGGG TTTATAAATG AGAAGGTTGA TTTGTTGTCT ACTCTTCTTT   
  
  
+ CGATTCAATA GTAATTCTCT CAGAGAAGGC TAAAATGACA TGGCATTCCA TCGCCTCTCA TGAGGCTTTT   
  
  
+ TGCATGTGCT TATGTATCTT TCATCTCTTA CTTTGGTGGG GTGGCATTGG GGAGGAAAAG GGGGTTTCGA   
  
  
+ TTCGATAGAT CGTCACTTTC GGAAATGACG TCTCTGACCA ATGGTAAGGG TAAGGTTAAT ACATGTGGAT   
  
  
+ ATTTCCAGAG TTAGTTGTGA TTGTTATTGT TGTTGTTGTA ACTTTATGTC ACGGGGAAAA CATAATAATT   
  
  
+ ATCTGTTTAT CTGTTTGTTT GTTATTTTCA AGGGGAAGGG GAGTGGGCGG ATGCTTAGTA TGTAATCATT   
  
  
+ TTATATAGGG AAAATTCATA TAGCATGAAA GTAAAACTTT CATTGCCAGC ATTCTAAGCT AGGGGTAGGG   
  
  
+ TTGTCTACAC CTTGATCTCC CTAGAAGCAT GGTGATCATT CCTCAGATTG TGTTATAGAA TAAAGAATGT   
  
  
+ GGTTCTGCTG TAATGAGACT TATGTATTAT CAAATGATGA ATCTGTTTAG AACTTCCTGT TTATTTTTTC   
  
  
+ TGCAAAATTT TAAGAGACTA GCATCTATCG GGCTCGACTC TAGAGGTTCT CTTGTGTTAC CCTTCTCAAC   
  
  
+ TCAGTTTGAT TGTGATACAG TTACTATATT GAGTGATAGT CGAGAGCATC ACAACCCGCT AAGGAGTCTA   
  
  
+ TCAACAAGAA GCCCTTCTTG TAATTCTCCC CTTGAAACTA GCAGTTATCA TCACTTGTCA TCGAATAGCC   
  
  
+ CTTTTTGTAA TTCTACCCTT GAAACTAGCA GTTATTGTCA TCAGTTCAAT GTGAGTCCCG TTAGATATTC   
  
  
+ CCATCAAGGC ATAGATTATG GAGTGAGCAT GAAGAACGCT TTGCACGAGC TAGAGACTGC TCTAATTGGT   
  
  
+ GTAGATGGCG AGAAAGCATC TGTTGCTAAT CAACCTATGG GGGGAATTCA TTAGTCCGGG ATCCCAAGTT   
  
  
+ AGAGATCGAA GTCATTGAGT GAAGATCCAC AGGGTTCACA TCCTCCTCAG CTTGATTCAT CATCTTTTTC   
  
  
+ AAGGGTGAAA AGATTCGGAG ATGAAAGCCA GAGAGAGAGA AATGCCACAA GGCAATGGAA GAACCAGCGG   
  
  
+ AACTACTAAG TTTCTCACCA GGTGATTTAA AGCAGTTGCT AATTGAATGT GCGAGGGCTT TATCAGATAA   
  
  
+ CCGAATAGAT GACTTTGAGA GTTTGGCTAA ACGGCCAAGG AAAGAGGTCT CCATCTCAGG TGAGCCTGTC   
  
  
+ CAACGTCTCG GTACTTGTAT GATCGAAGGG CTTGTGGCAA GGAAACAGTC TTCGGGGACT AACATCTATC   
  
  
+ GGGCTCTTAA GTACTGTAAA GAGCCTCTTG GAAAATACTT GCTCTCCTAC GGCACTCAGT GGGTTACTCT   
  
  
+ ATCGCAAGCC ATAGCAGCAA GGCTTGGTGG GCCTCCTAAA GTGCGACTTA CAGGCATTGA TGATCCTGTT   
  
  
+ TCTAAGTATA CTTGTGATGC TAGCTTGGAG GCTATTGGGA AACGATTAGC GTCTCTATTT GAAAAGTTTA   
  
  
+ AAATACTCGT CGAGTTCAAT GCATTGCCCG TTTATGGACC TGATGTCAGG TGGGAAATGC TGGATGTGAG   
  
  
+ GCCCAGGGAG GCTTTGGCCG TTAATTGTCC ATTACAGCTC CATCACACTC CTGACGAGAG TGTCGATGTG   
  
  
+ AGCAACCCTA GGGATAGGCT TCTCAGAATG GTGAAATTGC TCGGTCCTAA GGTATGCACT TTGGTTGAGC   
  
  
+ AAGAATCAAA CACCAACACT ACACGTTTCT TGACCCGGTT CATAGAGACC CTTGACTACT ACTCGATCAG   
  
  
+ CCATGTTTG  

- +Up\_Stream \_Len000TCAACA TCGTTCGTGT AATTTCAACT AATAGTAAAA CAGGCACTTT GCCACTCGTT   
  
  
- TGCAGTAGAG GTCCAGTTTA AGTAAGCATG AGTTAGTGTT CAACGACTGT AAAAATGGTT TCCGATTAGC   
  
  
- AGCGTTTTCA AAGAAATCAA ATTCCGGTTA GAGAGGCAAC AAAGTGGGGT ATGTCTAACT CCCCAACATT   
  
  
- ATCACATATA TATATCGGAA CACAATCGCC CCGAACCCGT AATCTGAAAA TGAGAACATT TGGGATTACA   
  
  
- CAATAATATT TATATCTAGA TCGGTGGGTA CAACTCCATC CGTGAGATTA AGACTGTGTC CATCCTATTC   
  
  
- AAAAGTATCA AATACAACAC TTAGAACAGA ATGAAACACC AAGGATAACT GTTTTCAAAC TTAGGCTCGT   
  
  
- ACTCTCAAGT AGTTAGAGAA AAAGAAAGGA TAAAATAAAG AACACCTGAA TTAACCTAAA AGACCATCTC   
  
  
- CTTTCAAGGA ACGACCTTAA ACCAGCTTTC ACTATATGTA AAACCCTAAA TTAATCGGTA TTTAAACCAA   
  
  
- AATGTGGAAC AATCAAACCT CAAGACCACA CAATAACATA CACAAGACCA CCCAAAAAGA AAATCGATTT   
  
  
- ACAACCTCGT AAAATTCCCT AGATCTATAG ACTTCTTCAC GAACCCTTTA CTTCGTATAA AGGGAATATC   
  
  
- AGACTGTATT GAAATTACTT AACTAGTACT TGTTTCCCGT TAATCAAGTC TTTCCAAGTT GAGGAGATCC   
  
  
- ACAACACGAT TTCTCTACAC TCAATCATTG TTAACGAATG ATTGATATAT CCAACAAAAT AATGAACATC   
  
  
- TCCAAAAAAA AAACCCTATA GAAAAACCCC AAATATTTAC TCTTCCAACT AAACAACAGA TGAGAAGAAA   
  
  
- GCTAAGTTAT CATTAAGAGA GTCTCTTCCG ATTTTACTGT ACCGTAAGGT AGCGGAGAGT ACTCCGAAAA   
  
  
- ACGTACACGA ATACATAGAA AGTAGAGAAT GAAACCACCC CACCGTAACC CCTCCTTTTC CCCCAAAGCT   
  
  
- AAGCTATCTA GCAGTGAAAG CCTTTACTGC AGAGACTGGT TACCATTCCC ATTCCAATTA TGTACACCTA   
  
  
- TAAAGGTCTC AATCAACACT AACAATAACA ACAACAACAT TGAAATACAG TGCCCCTTTT GTATTATTAA   
  
  
- TAGACAAATA GACAAACAAA CAATAAAAGT TCCCCTTCCC CTCACCCGCC TACGAATCAT ACATTAGTAA   
  
  
- AATATATCCC TTTTAAGTAT ATCGTACTTT CATTTTGAAA GTAACGGTCG TAAGATTCGA TCCCCATCCC   
  
  
- AACAGATGTG GAACTAGAGG GATCTTCGTA CCACTAGTAA GGAGTCTAAC ACAATATCTT ATTTCTTACA   
  
  
- CCAAGACGAC ATTACTCTGA ATACATAATA GTTTACTACT TAGACAAATC TTGAAGGACA AATAAAAAAG   
  
  
- ACGTTTTAAA ATTCTCTGAT CGTAGATAGC CCGAGCTGAG ATCTCCAAGA GAACACAATG GGAAGAGTTG   
  
  
- AGTCAAACTA ACACTATGTC AATGATATAA CTCACTATCA GCTCTCGTAG TGTTGGGCGA TTCCTCAGAT   
  
  
- AGTTGTTCTT CGGGAAGAAC ATTAAGAGGG GAACTTTGAT CGTCAATAGT AGTGAACAGT AGCTTATCGG   
  
  
- GAAAAACATT AAGATGGGAA CTTTGATCGT CAATAACAGT AGTCAAGTTA CACTCAGGGC AATCTATAAG   
  
  
- GGTAGTTCCG TATCTAATAC CTCACTCGTA CTTCTTGCGA AACGTGCTCG ATCTCTGACG AGATTAACCA   
  
  
- CATCTACCGC TCTTTCGTAG ACAACGATTA GTTGGATACC CCCCTTAAGT AATCAGGCCC TAGGGTTCAA   
  
  
- TCTCTAGCTT CAGTAACTCA CTTCTAGGTG TCCCAAGTGT AGGAGGAGTC GAACTAAGTA GTAGAAAAAG   
  
  
- TTCCCACTTT TCTAAGCCTC TACTTTCGGT CTCTCTCTCT TTACGGTGTT CCGTTACCTT CTTGGTCGCC   
  
  
- TTGATGATTC AAAGAGTGGT CCACTAAATT TCGTCAACGA TTAACTTACA CGCTCCCGAA ATAGTCTATT   
  
  
- GGCTTATCTA CTGAAACTCT CAAACCGATT TGCCGGTTCC TTTCTCCAGA GGTAGAGTCC ACTCGGACAG   
  
  
- GTTGCAGAGC CATGAACATA CTAGCTTCCC GAACACCGTT CCTTTGTCAG AAGCCCCTGA TTGTAGATAG   
  
  
- CCCGAGAATT CATGACATTT CTCGGAGAAC CTTTTATGAA CGAGAGGATG CCGTGAGTCA CCCAATGAGA   
  
  
- TAGCGTTCGG TATCGTCGTT CCGAACCACC CGGAGGATTT CACGCTGAAT GTCCGTAACT ACTAGGACAA   
  
  
- AGATTCATAT GAACACTACG ATCGAACCTC CGATAACCCT TTGCTAATCG CAGAGATAAA CTTTTCAAAT   
  
  
- TTTATGAGCA GCTCAAGTTA CGTAACGGGC AAATACCTGG ACTACAGTCC ACCCTTTACG ACCTACACTC   
  
  
- CGGGTCCCTC CGAAACCGGC AATTAACAGG TAATGTCGAG GTAGTGTGAG GACTGCTCTC ACAGCTACAC   
  
  
- TCGTTGGGAT CCCTATCCGA AGAGTCTTAC CACTTTAACG AGCCAGGATT CCATACGTGA AACCAACTCG   
  
  
- TTCTTAGTTT GTGGTTGTGA TGTGCAAAGA ACTGGGCCAA GTATCTCTGG GAACTGATGA TGAGCTAGTC   
  
  
- GGTACAAAC

+     CGTCA-motif

| Site Name | Organism | Position | Strand | Matrix score. | sequence | function |
| --- | --- | --- | --- | --- | --- | --- |
| CGTCA-motif | Hordeum vulgare | 1080 | - | 5 | CGTCA | cis-acting regulatory element involved in the MeJA-responsiveness |
| CGTCA-motif | Hordeum vulgare | 76 | + | 5 | CGTCA | cis-acting regulatory element involved in the MeJA-responsiveness |
| CGTCA-motif | Hordeum vulgare | 1065 | + | 5 | CGTCA | cis-acting regulatory element involved in the MeJA-responsiveness |
| CGTCA-motif | Hordeum vulgare | 2576 | - | 5 | CGTCA | cis-acting regulatory element involved in the MeJA-responsiveness |

>HU08G00019.1   
+ +Up\_Stream \_Len000AGTTGT AGCAAGCACA TTAAAGTTGA TTATCATTTT GTCCGTGAAA CGGTGAGCAA   
  
  
+ ACGTCATCTC CAGGTCAAAT TCATTCGTAC TCAATCACAA GTTGCTGACA TTTTTACCAA AGGCTAATCG   
  
  
+ TCGCAAAAGT TTCTTTAGTT TAAGGCCAAT CTCTCCGTTG TTTCACCCCA TACAGATTGA GGGGTTGTAA   
  
  
+ TAGTGTATAT ATATAGCCTT GTGTTAGCGG GGCTTGGGCA TTAGACTTTT ACTCTTGTAA ACCCTAATGT   
  
  
+ GTTATTATAA ATATAGATCT AGCCACCCAT GTTGAGGTAG GCACTCTAAT TCTGACACAG GTAGGATAAG   
  
  
+ TTTTCATAGT TTATGTTGTG AATCTTGTCT TACTTTGTGG TTCCTATTGA CAAAAGTTTG AATCCGAGCA   
  
  
+ TGAGAGTTCA TCAATCTCTT TTTCTTTCCT ATTTTATTTC TTGTGGACTT AATTGGATTT TCTGGTAGAG   
  
  
+ GAAAGTTCCT TGCTGGAATT TGGTCGAAAG TGATATACAT TTTGGGATTT AATTAGCCAT AAATTTGGTT   
  
  
+ TTACACCTTG TTAGTTTGGA GTTCTGGTGT GTTATTGTAT GTGTTCTGGT GGGTTTTTCT TTTAGCTAAA   
  
  
+ TGTTGGAGCA TTTTAAGGGA TCTAGATATC TGAAGAAGTG CTTGGGAAAT GAAGCATATT TCCCTTATAG   
  
  
+ TCTGACATAA CTTTAATGAA TTGATCATGA ACAAAGGGCA ATTAGTTCAG AAAGGTTCAA CTCCTCTAGG   
  
  
+ TGTTGTGCTA AAGAGATGTG AGTTAGTAAC AATTGCTTAC TAACTATATA GGTTGTTTTA TTACTTGTAG   
  
  
+ AGGTTTTTTT TTTGGGATAT CTTTTTGGGG TTTATAAATG AGAAGGTTGA TTTGTTGTCT ACTCTTCTTT   
  
  
+ CGATTCAATA GTAATTCTCT CAGAGAAGGC TAAAATGACA TGGCATTCCA TCGCCTCTCA TGAGGCTTTT   
  
  
+ TGCATGTGCT TATGTATCTT TCATCTCTTA CTTTGGTGGG GTGGCATTGG GGAGGAAAAG GGGGTTTCGA   
  
  
+ TTCGATAGAT CGTCACTTTC GGAAATGACG TCTCTGACCA ATGGTAAGGG TAAGGTTAAT ACATGTGGAT   
  
  
+ ATTTCCAGAG TTAGTTGTGA TTGTTATTGT TGTTGTTGTA ACTTTATGTC ACGGGGAAAA CATAATAATT   
  
  
+ ATCTGTTTAT CTGTTTGTTT GTTATTTTCA AGGGGAAGGG GAGTGGGCGG ATGCTTAGTA TGTAATCATT   
  
  
+ TTATATAGGG AAAATTCATA TAGCATGAAA GTAAAACTTT CATTGCCAGC ATTCTAAGCT AGGGGTAGGG   
  
  
+ TTGTCTACAC CTTGATCTCC CTAGAAGCAT GGTGATCATT CCTCAGATTG TGTTATAGAA TAAAGAATGT   
  
  
+ GGTTCTGCTG TAATGAGACT TATGTATTAT CAAATGATGA ATCTGTTTAG AACTTCCTGT TTATTTTTTC   
  
  
+ TGCAAAATTT TAAGAGACTA GCATCTATCG GGCTCGACTC TAGAGGTTCT CTTGTGTTAC CCTTCTCAAC   
  
  
+ TCAGTTTGAT TGTGATACAG TTACTATATT GAGTGATAGT CGAGAGCATC ACAACCCGCT AAGGAGTCTA   
  
  
+ TCAACAAGAA GCCCTTCTTG TAATTCTCCC CTTGAAACTA GCAGTTATCA TCACTTGTCA TCGAATAGCC   
  
  
+ CTTTTTGTAA TTCTACCCTT GAAACTAGCA GTTATTGTCA TCAGTTCAAT GTGAGTCCCG TTAGATATTC   
  
  
+ CCATCAAGGC ATAGATTATG GAGTGAGCAT GAAGAACGCT TTGCACGAGC TAGAGACTGC TCTAATTGGT   
  
  
+ GTAGATGGCG AGAAAGCATC TGTTGCTAAT CAACCTATGG GGGGAATTCA TTAGTCCGGG ATCCCAAGTT   
  
  
+ AGAGATCGAA GTCATTGAGT GAAGATCCAC AGGGTTCACA TCCTCCTCAG CTTGATTCAT CATCTTTTTC   
  
  
+ AAGGGTGAAA AGATTCGGAG ATGAAAGCCA GAGAGAGAGA AATGCCACAA GGCAATGGAA GAACCAGCGG   
  
  
+ AACTACTAAG TTTCTCACCA GGTGATTTAA AGCAGTTGCT AATTGAATGT GCGAGGGCTT TATCAGATAA   
  
  
+ CCGAATAGAT GACTTTGAGA GTTTGGCTAA ACGGCCAAGG AAAGAGGTCT CCATCTCAGG TGAGCCTGTC   
  
  
+ CAACGTCTCG GTACTTGTAT GATCGAAGGG CTTGTGGCAA GGAAACAGTC TTCGGGGACT AACATCTATC   
  
  
+ GGGCTCTTAA GTACTGTAAA GAGCCTCTTG GAAAATACTT GCTCTCCTAC GGCACTCAGT GGGTTACTCT   
  
  
+ ATCGCAAGCC ATAGCAGCAA GGCTTGGTGG GCCTCCTAAA GTGCGACTTA CAGGCATTGA TGATCCTGTT   
  
  
+ TCTAAGTATA CTTGTGATGC TAGCTTGGAG GCTATTGGGA AACGATTAGC GTCTCTATTT GAAAAGTTTA   
  
  
+ AAATACTCGT CGAGTTCAAT GCATTGCCCG TTTATGGACC TGATGTCAGG TGGGAAATGC TGGATGTGAG   
  
  
+ GCCCAGGGAG GCTTTGGCCG TTAATTGTCC ATTACAGCTC CATCACACTC CTGACGAGAG TGTCGATGTG   
  
  
+ AGCAACCCTA GGGATAGGCT TCTCAGAATG GTGAAATTGC TCGGTCCTAA GGTATGCACT TTGGTTGAGC   
  
  
+ AAGAATCAAA CACCAACACT ACACGTTTCT TGACCCGGTT CATAGAGACC CTTGACTACT ACTCGATCAG   
  
  
+ CCATGTTTG  

- +Up\_Stream \_Len000TCAACA TCGTTCGTGT AATTTCAACT AATAGTAAAA CAGGCACTTT GCCACTCGTT   
  
  
- TGCAGTAGAG GTCCAGTTTA AGTAAGCATG AGTTAGTGTT CAACGACTGT AAAAATGGTT TCCGATTAGC   
  
  
- AGCGTTTTCA AAGAAATCAA ATTCCGGTTA GAGAGGCAAC AAAGTGGGGT ATGTCTAACT CCCCAACATT   
  
  
- ATCACATATA TATATCGGAA CACAATCGCC CCGAACCCGT AATCTGAAAA TGAGAACATT TGGGATTACA   
  
  
- CAATAATATT TATATCTAGA TCGGTGGGTA CAACTCCATC CGTGAGATTA AGACTGTGTC CATCCTATTC   
  
  
- AAAAGTATCA AATACAACAC TTAGAACAGA ATGAAACACC AAGGATAACT GTTTTCAAAC TTAGGCTCGT   
  
  
- ACTCTCAAGT AGTTAGAGAA AAAGAAAGGA TAAAATAAAG AACACCTGAA TTAACCTAAA AGACCATCTC   
  
  
- CTTTCAAGGA ACGACCTTAA ACCAGCTTTC ACTATATGTA AAACCCTAAA TTAATCGGTA TTTAAACCAA   
  
  
- AATGTGGAAC AATCAAACCT CAAGACCACA CAATAACATA CACAAGACCA CCCAAAAAGA AAATCGATTT   
  
  
- ACAACCTCGT AAAATTCCCT AGATCTATAG ACTTCTTCAC GAACCCTTTA CTTCGTATAA AGGGAATATC   
  
  
- AGACTGTATT GAAATTACTT AACTAGTACT TGTTTCCCGT TAATCAAGTC TTTCCAAGTT GAGGAGATCC   
  
  
- ACAACACGAT TTCTCTACAC TCAATCATTG TTAACGAATG ATTGATATAT CCAACAAAAT AATGAACATC   
  
  
- TCCAAAAAAA AAACCCTATA GAAAAACCCC AAATATTTAC TCTTCCAACT AAACAACAGA TGAGAAGAAA   
  
  
- GCTAAGTTAT CATTAAGAGA GTCTCTTCCG ATTTTACTGT ACCGTAAGGT AGCGGAGAGT ACTCCGAAAA   
  
  
- ACGTACACGA ATACATAGAA AGTAGAGAAT GAAACCACCC CACCGTAACC CCTCCTTTTC CCCCAAAGCT   
  
  
- AAGCTATCTA GCAGTGAAAG CCTTTACTGC AGAGACTGGT TACCATTCCC ATTCCAATTA TGTACACCTA   
  
  
- TAAAGGTCTC AATCAACACT AACAATAACA ACAACAACAT TGAAATACAG TGCCCCTTTT GTATTATTAA   
  
  
- TAGACAAATA GACAAACAAA CAATAAAAGT TCCCCTTCCC CTCACCCGCC TACGAATCAT ACATTAGTAA   
  
  
- AATATATCCC TTTTAAGTAT ATCGTACTTT CATTTTGAAA GTAACGGTCG TAAGATTCGA TCCCCATCCC   
  
  
- AACAGATGTG GAACTAGAGG GATCTTCGTA CCACTAGTAA GGAGTCTAAC ACAATATCTT ATTTCTTACA   
  
  
- CCAAGACGAC ATTACTCTGA ATACATAATA GTTTACTACT TAGACAAATC TTGAAGGACA AATAAAAAAG   
  
  
- ACGTTTTAAA ATTCTCTGAT CGTAGATAGC CCGAGCTGAG ATCTCCAAGA GAACACAATG GGAAGAGTTG   
  
  
- AGTCAAACTA ACACTATGTC AATGATATAA CTCACTATCA GCTCTCGTAG TGTTGGGCGA TTCCTCAGAT   
  
  
- AGTTGTTCTT CGGGAAGAAC ATTAAGAGGG GAACTTTGAT CGTCAATAGT AGTGAACAGT AGCTTATCGG   
  
  
- GAAAAACATT AAGATGGGAA CTTTGATCGT CAATAACAGT AGTCAAGTTA CACTCAGGGC AATCTATAAG   
  
  
- GGTAGTTCCG TATCTAATAC CTCACTCGTA CTTCTTGCGA AACGTGCTCG ATCTCTGACG AGATTAACCA   
  
  
- CATCTACCGC TCTTTCGTAG ACAACGATTA GTTGGATACC CCCCTTAAGT AATCAGGCCC TAGGGTTCAA   
  
  
- TCTCTAGCTT CAGTAACTCA CTTCTAGGTG TCCCAAGTGT AGGAGGAGTC GAACTAAGTA GTAGAAAAAG   
  
  
- TTCCCACTTT TCTAAGCCTC TACTTTCGGT CTCTCTCTCT TTACGGTGTT CCGTTACCTT CTTGGTCGCC   
  
  
- TTGATGATTC AAAGAGTGGT CCACTAAATT TCGTCAACGA TTAACTTACA CGCTCCCGAA ATAGTCTATT   
  
  
- GGCTTATCTA CTGAAACTCT CAAACCGATT TGCCGGTTCC TTTCTCCAGA GGTAGAGTCC ACTCGGACAG   
  
  
- GTTGCAGAGC CATGAACATA CTAGCTTCCC GAACACCGTT CCTTTGTCAG AAGCCCCTGA TTGTAGATAG   
  
  
- CCCGAGAATT CATGACATTT CTCGGAGAAC CTTTTATGAA CGAGAGGATG CCGTGAGTCA CCCAATGAGA   
  
  
- TAGCGTTCGG TATCGTCGTT CCGAACCACC CGGAGGATTT CACGCTGAAT GTCCGTAACT ACTAGGACAA   
  
  
- AGATTCATAT GAACACTACG ATCGAACCTC CGATAACCCT TTGCTAATCG CAGAGATAAA CTTTTCAAAT   
  
  
- TTTATGAGCA GCTCAAGTTA CGTAACGGGC AAATACCTGG ACTACAGTCC ACCCTTTACG ACCTACACTC   
  
  
- CGGGTCCCTC CGAAACCGGC AATTAACAGG TAATGTCGAG GTAGTGTGAG GACTGCTCTC ACAGCTACAC   
  
  
- TCGTTGGGAT CCCTATCCGA AGAGTCTTAC CACTTTAACG AGCCAGGATT CCATACGTGA AACCAACTCG   
  
  
- TTCTTAGTTT GTGGTTGTGA TGTGCAAAGA ACTGGGCCAA GTATCTCTGG GAACTGATGA TGAGCTAGTC   
  
  
- GGTACAAAC

+     DRE1

| Site Name | Organism | Position | Strand | Matrix score. | sequence | function |
| --- | --- | --- | --- | --- | --- | --- |
| DRE1 | Zea mays | 2180 | - | 7 | ACCGAGA |  |

>HU08G00019.1   
+ +Up\_Stream \_Len000AGTTGT AGCAAGCACA TTAAAGTTGA TTATCATTTT GTCCGTGAAA CGGTGAGCAA   
  
  
+ ACGTCATCTC CAGGTCAAAT TCATTCGTAC TCAATCACAA GTTGCTGACA TTTTTACCAA AGGCTAATCG   
  
  
+ TCGCAAAAGT TTCTTTAGTT TAAGGCCAAT CTCTCCGTTG TTTCACCCCA TACAGATTGA GGGGTTGTAA   
  
  
+ TAGTGTATAT ATATAGCCTT GTGTTAGCGG GGCTTGGGCA TTAGACTTTT ACTCTTGTAA ACCCTAATGT   
  
  
+ GTTATTATAA ATATAGATCT AGCCACCCAT GTTGAGGTAG GCACTCTAAT TCTGACACAG GTAGGATAAG   
  
  
+ TTTTCATAGT TTATGTTGTG AATCTTGTCT TACTTTGTGG TTCCTATTGA CAAAAGTTTG AATCCGAGCA   
  
  
+ TGAGAGTTCA TCAATCTCTT TTTCTTTCCT ATTTTATTTC TTGTGGACTT AATTGGATTT TCTGGTAGAG   
  
  
+ GAAAGTTCCT TGCTGGAATT TGGTCGAAAG TGATATACAT TTTGGGATTT AATTAGCCAT AAATTTGGTT   
  
  
+ TTACACCTTG TTAGTTTGGA GTTCTGGTGT GTTATTGTAT GTGTTCTGGT GGGTTTTTCT TTTAGCTAAA   
  
  
+ TGTTGGAGCA TTTTAAGGGA TCTAGATATC TGAAGAAGTG CTTGGGAAAT GAAGCATATT TCCCTTATAG   
  
  
+ TCTGACATAA CTTTAATGAA TTGATCATGA ACAAAGGGCA ATTAGTTCAG AAAGGTTCAA CTCCTCTAGG   
  
  
+ TGTTGTGCTA AAGAGATGTG AGTTAGTAAC AATTGCTTAC TAACTATATA GGTTGTTTTA TTACTTGTAG   
  
  
+ AGGTTTTTTT TTTGGGATAT CTTTTTGGGG TTTATAAATG AGAAGGTTGA TTTGTTGTCT ACTCTTCTTT   
  
  
+ CGATTCAATA GTAATTCTCT CAGAGAAGGC TAAAATGACA TGGCATTCCA TCGCCTCTCA TGAGGCTTTT   
  
  
+ TGCATGTGCT TATGTATCTT TCATCTCTTA CTTTGGTGGG GTGGCATTGG GGAGGAAAAG GGGGTTTCGA   
  
  
+ TTCGATAGAT CGTCACTTTC GGAAATGACG TCTCTGACCA ATGGTAAGGG TAAGGTTAAT ACATGTGGAT   
  
  
+ ATTTCCAGAG TTAGTTGTGA TTGTTATTGT TGTTGTTGTA ACTTTATGTC ACGGGGAAAA CATAATAATT   
  
  
+ ATCTGTTTAT CTGTTTGTTT GTTATTTTCA AGGGGAAGGG GAGTGGGCGG ATGCTTAGTA TGTAATCATT   
  
  
+ TTATATAGGG AAAATTCATA TAGCATGAAA GTAAAACTTT CATTGCCAGC ATTCTAAGCT AGGGGTAGGG   
  
  
+ TTGTCTACAC CTTGATCTCC CTAGAAGCAT GGTGATCATT CCTCAGATTG TGTTATAGAA TAAAGAATGT   
  
  
+ GGTTCTGCTG TAATGAGACT TATGTATTAT CAAATGATGA ATCTGTTTAG AACTTCCTGT TTATTTTTTC   
  
  
+ TGCAAAATTT TAAGAGACTA GCATCTATCG GGCTCGACTC TAGAGGTTCT CTTGTGTTAC CCTTCTCAAC   
  
  
+ TCAGTTTGAT TGTGATACAG TTACTATATT GAGTGATAGT CGAGAGCATC ACAACCCGCT AAGGAGTCTA   
  
  
+ TCAACAAGAA GCCCTTCTTG TAATTCTCCC CTTGAAACTA GCAGTTATCA TCACTTGTCA TCGAATAGCC   
  
  
+ CTTTTTGTAA TTCTACCCTT GAAACTAGCA GTTATTGTCA TCAGTTCAAT GTGAGTCCCG TTAGATATTC   
  
  
+ CCATCAAGGC ATAGATTATG GAGTGAGCAT GAAGAACGCT TTGCACGAGC TAGAGACTGC TCTAATTGGT   
  
  
+ GTAGATGGCG AGAAAGCATC TGTTGCTAAT CAACCTATGG GGGGAATTCA TTAGTCCGGG ATCCCAAGTT   
  
  
+ AGAGATCGAA GTCATTGAGT GAAGATCCAC AGGGTTCACA TCCTCCTCAG CTTGATTCAT CATCTTTTTC   
  
  
+ AAGGGTGAAA AGATTCGGAG ATGAAAGCCA GAGAGAGAGA AATGCCACAA GGCAATGGAA GAACCAGCGG   
  
  
+ AACTACTAAG TTTCTCACCA GGTGATTTAA AGCAGTTGCT AATTGAATGT GCGAGGGCTT TATCAGATAA   
  
  
+ CCGAATAGAT GACTTTGAGA GTTTGGCTAA ACGGCCAAGG AAAGAGGTCT CCATCTCAGG TGAGCCTGTC   
  
  
+ CAACGTCTCG GTACTTGTAT GATCGAAGGG CTTGTGGCAA GGAAACAGTC TTCGGGGACT AACATCTATC   
  
  
+ GGGCTCTTAA GTACTGTAAA GAGCCTCTTG GAAAATACTT GCTCTCCTAC GGCACTCAGT GGGTTACTCT   
  
  
+ ATCGCAAGCC ATAGCAGCAA GGCTTGGTGG GCCTCCTAAA GTGCGACTTA CAGGCATTGA TGATCCTGTT   
  
  
+ TCTAAGTATA CTTGTGATGC TAGCTTGGAG GCTATTGGGA AACGATTAGC GTCTCTATTT GAAAAGTTTA   
  
  
+ AAATACTCGT CGAGTTCAAT GCATTGCCCG TTTATGGACC TGATGTCAGG TGGGAAATGC TGGATGTGAG   
  
  
+ GCCCAGGGAG GCTTTGGCCG TTAATTGTCC ATTACAGCTC CATCACACTC CTGACGAGAG TGTCGATGTG   
  
  
+ AGCAACCCTA GGGATAGGCT TCTCAGAATG GTGAAATTGC TCGGTCCTAA GGTATGCACT TTGGTTGAGC   
  
  
+ AAGAATCAAA CACCAACACT ACACGTTTCT TGACCCGGTT CATAGAGACC CTTGACTACT ACTCGATCAG   
  
  
+ CCATGTTTG  

- +Up\_Stream \_Len000TCAACA TCGTTCGTGT AATTTCAACT AATAGTAAAA CAGGCACTTT GCCACTCGTT   
  
  
- TGCAGTAGAG GTCCAGTTTA AGTAAGCATG AGTTAGTGTT CAACGACTGT AAAAATGGTT TCCGATTAGC   
  
  
- AGCGTTTTCA AAGAAATCAA ATTCCGGTTA GAGAGGCAAC AAAGTGGGGT ATGTCTAACT CCCCAACATT   
  
  
- ATCACATATA TATATCGGAA CACAATCGCC CCGAACCCGT AATCTGAAAA TGAGAACATT TGGGATTACA   
  
  
- CAATAATATT TATATCTAGA TCGGTGGGTA CAACTCCATC CGTGAGATTA AGACTGTGTC CATCCTATTC   
  
  
- AAAAGTATCA AATACAACAC TTAGAACAGA ATGAAACACC AAGGATAACT GTTTTCAAAC TTAGGCTCGT   
  
  
- ACTCTCAAGT AGTTAGAGAA AAAGAAAGGA TAAAATAAAG AACACCTGAA TTAACCTAAA AGACCATCTC   
  
  
- CTTTCAAGGA ACGACCTTAA ACCAGCTTTC ACTATATGTA AAACCCTAAA TTAATCGGTA TTTAAACCAA   
  
  
- AATGTGGAAC AATCAAACCT CAAGACCACA CAATAACATA CACAAGACCA CCCAAAAAGA AAATCGATTT   
  
  
- ACAACCTCGT AAAATTCCCT AGATCTATAG ACTTCTTCAC GAACCCTTTA CTTCGTATAA AGGGAATATC   
  
  
- AGACTGTATT GAAATTACTT AACTAGTACT TGTTTCCCGT TAATCAAGTC TTTCCAAGTT GAGGAGATCC   
  
  
- ACAACACGAT TTCTCTACAC TCAATCATTG TTAACGAATG ATTGATATAT CCAACAAAAT AATGAACATC   
  
  
- TCCAAAAAAA AAACCCTATA GAAAAACCCC AAATATTTAC TCTTCCAACT AAACAACAGA TGAGAAGAAA   
  
  
- GCTAAGTTAT CATTAAGAGA GTCTCTTCCG ATTTTACTGT ACCGTAAGGT AGCGGAGAGT ACTCCGAAAA   
  
  
- ACGTACACGA ATACATAGAA AGTAGAGAAT GAAACCACCC CACCGTAACC CCTCCTTTTC CCCCAAAGCT   
  
  
- AAGCTATCTA GCAGTGAAAG CCTTTACTGC AGAGACTGGT TACCATTCCC ATTCCAATTA TGTACACCTA   
  
  
- TAAAGGTCTC AATCAACACT AACAATAACA ACAACAACAT TGAAATACAG TGCCCCTTTT GTATTATTAA   
  
  
- TAGACAAATA GACAAACAAA CAATAAAAGT TCCCCTTCCC CTCACCCGCC TACGAATCAT ACATTAGTAA   
  
  
- AATATATCCC TTTTAAGTAT ATCGTACTTT CATTTTGAAA GTAACGGTCG TAAGATTCGA TCCCCATCCC   
  
  
- AACAGATGTG GAACTAGAGG GATCTTCGTA CCACTAGTAA GGAGTCTAAC ACAATATCTT ATTTCTTACA   
  
  
- CCAAGACGAC ATTACTCTGA ATACATAATA GTTTACTACT TAGACAAATC TTGAAGGACA AATAAAAAAG   
  
  
- ACGTTTTAAA ATTCTCTGAT CGTAGATAGC CCGAGCTGAG ATCTCCAAGA GAACACAATG GGAAGAGTTG   
  
  
- AGTCAAACTA ACACTATGTC AATGATATAA CTCACTATCA GCTCTCGTAG TGTTGGGCGA TTCCTCAGAT   
  
  
- AGTTGTTCTT CGGGAAGAAC ATTAAGAGGG GAACTTTGAT CGTCAATAGT AGTGAACAGT AGCTTATCGG   
  
  
- GAAAAACATT AAGATGGGAA CTTTGATCGT CAATAACAGT AGTCAAGTTA CACTCAGGGC AATCTATAAG   
  
  
- GGTAGTTCCG TATCTAATAC CTCACTCGTA CTTCTTGCGA AACGTGCTCG ATCTCTGACG AGATTAACCA   
  
  
- CATCTACCGC TCTTTCGTAG ACAACGATTA GTTGGATACC CCCCTTAAGT AATCAGGCCC TAGGGTTCAA   
  
  
- TCTCTAGCTT CAGTAACTCA CTTCTAGGTG TCCCAAGTGT AGGAGGAGTC GAACTAAGTA GTAGAAAAAG   
  
  
- TTCCCACTTT TCTAAGCCTC TACTTTCGGT CTCTCTCTCT TTACGGTGTT CCGTTACCTT CTTGGTCGCC   
  
  
- TTGATGATTC AAAGAGTGGT CCACTAAATT TCGTCAACGA TTAACTTACA CGCTCCCGAA ATAGTCTATT   
  
  
- GGCTTATCTA CTGAAACTCT CAAACCGATT TGCCGGTTCC TTTCTCCAGA GGTAGAGTCC ACTCGGACAG   
  
  
- GTTGCAGAGC CATGAACATA CTAGCTTCCC GAACACCGTT CCTTTGTCAG AAGCCCCTGA TTGTAGATAG   
  
  
- CCCGAGAATT CATGACATTT CTCGGAGAAC CTTTTATGAA CGAGAGGATG CCGTGAGTCA CCCAATGAGA   
  
  
- TAGCGTTCGG TATCGTCGTT CCGAACCACC CGGAGGATTT CACGCTGAAT GTCCGTAACT ACTAGGACAA   
  
  
- AGATTCATAT GAACACTACG ATCGAACCTC CGATAACCCT TTGCTAATCG CAGAGATAAA CTTTTCAAAT   
  
  
- TTTATGAGCA GCTCAAGTTA CGTAACGGGC AAATACCTGG ACTACAGTCC ACCCTTTACG ACCTACACTC   
  
  
- CGGGTCCCTC CGAAACCGGC AATTAACAGG TAATGTCGAG GTAGTGTGAG GACTGCTCTC ACAGCTACAC   
  
  
- TCGTTGGGAT CCCTATCCGA AGAGTCTTAC CACTTTAACG AGCCAGGATT CCATACGTGA AACCAACTCG   
  
  
- TTCTTAGTTT GTGGTTGTGA TGTGCAAAGA ACTGGGCCAA GTATCTCTGG GAACTGATGA TGAGCTAGTC   
  
  
- GGTACAAAC

+     ERE

| Site Name | Organism | Position | Strand | Matrix score. | sequence | function |
| --- | --- | --- | --- | --- | --- | --- |
| ERE | Nicotiana glutinos | 2451 | - | 8 | ATTTTAAA |  |

>HU08G00019.1   
+ +Up\_Stream \_Len000AGTTGT AGCAAGCACA TTAAAGTTGA TTATCATTTT GTCCGTGAAA CGGTGAGCAA   
  
  
+ ACGTCATCTC CAGGTCAAAT TCATTCGTAC TCAATCACAA GTTGCTGACA TTTTTACCAA AGGCTAATCG   
  
  
+ TCGCAAAAGT TTCTTTAGTT TAAGGCCAAT CTCTCCGTTG TTTCACCCCA TACAGATTGA GGGGTTGTAA   
  
  
+ TAGTGTATAT ATATAGCCTT GTGTTAGCGG GGCTTGGGCA TTAGACTTTT ACTCTTGTAA ACCCTAATGT   
  
  
+ GTTATTATAA ATATAGATCT AGCCACCCAT GTTGAGGTAG GCACTCTAAT TCTGACACAG GTAGGATAAG   
  
  
+ TTTTCATAGT TTATGTTGTG AATCTTGTCT TACTTTGTGG TTCCTATTGA CAAAAGTTTG AATCCGAGCA   
  
  
+ TGAGAGTTCA TCAATCTCTT TTTCTTTCCT ATTTTATTTC TTGTGGACTT AATTGGATTT TCTGGTAGAG   
  
  
+ GAAAGTTCCT TGCTGGAATT TGGTCGAAAG TGATATACAT TTTGGGATTT AATTAGCCAT AAATTTGGTT   
  
  
+ TTACACCTTG TTAGTTTGGA GTTCTGGTGT GTTATTGTAT GTGTTCTGGT GGGTTTTTCT TTTAGCTAAA   
  
  
+ TGTTGGAGCA TTTTAAGGGA TCTAGATATC TGAAGAAGTG CTTGGGAAAT GAAGCATATT TCCCTTATAG   
  
  
+ TCTGACATAA CTTTAATGAA TTGATCATGA ACAAAGGGCA ATTAGTTCAG AAAGGTTCAA CTCCTCTAGG   
  
  
+ TGTTGTGCTA AAGAGATGTG AGTTAGTAAC AATTGCTTAC TAACTATATA GGTTGTTTTA TTACTTGTAG   
  
  
+ AGGTTTTTTT TTTGGGATAT CTTTTTGGGG TTTATAAATG AGAAGGTTGA TTTGTTGTCT ACTCTTCTTT   
  
  
+ CGATTCAATA GTAATTCTCT CAGAGAAGGC TAAAATGACA TGGCATTCCA TCGCCTCTCA TGAGGCTTTT   
  
  
+ TGCATGTGCT TATGTATCTT TCATCTCTTA CTTTGGTGGG GTGGCATTGG GGAGGAAAAG GGGGTTTCGA   
  
  
+ TTCGATAGAT CGTCACTTTC GGAAATGACG TCTCTGACCA ATGGTAAGGG TAAGGTTAAT ACATGTGGAT   
  
  
+ ATTTCCAGAG TTAGTTGTGA TTGTTATTGT TGTTGTTGTA ACTTTATGTC ACGGGGAAAA CATAATAATT   
  
  
+ ATCTGTTTAT CTGTTTGTTT GTTATTTTCA AGGGGAAGGG GAGTGGGCGG ATGCTTAGTA TGTAATCATT   
  
  
+ TTATATAGGG AAAATTCATA TAGCATGAAA GTAAAACTTT CATTGCCAGC ATTCTAAGCT AGGGGTAGGG   
  
  
+ TTGTCTACAC CTTGATCTCC CTAGAAGCAT GGTGATCATT CCTCAGATTG TGTTATAGAA TAAAGAATGT   
  
  
+ GGTTCTGCTG TAATGAGACT TATGTATTAT CAAATGATGA ATCTGTTTAG AACTTCCTGT TTATTTTTTC   
  
  
+ TGCAAAATTT TAAGAGACTA GCATCTATCG GGCTCGACTC TAGAGGTTCT CTTGTGTTAC CCTTCTCAAC   
  
  
+ TCAGTTTGAT TGTGATACAG TTACTATATT GAGTGATAGT CGAGAGCATC ACAACCCGCT AAGGAGTCTA   
  
  
+ TCAACAAGAA GCCCTTCTTG TAATTCTCCC CTTGAAACTA GCAGTTATCA TCACTTGTCA TCGAATAGCC   
  
  
+ CTTTTTGTAA TTCTACCCTT GAAACTAGCA GTTATTGTCA TCAGTTCAAT GTGAGTCCCG TTAGATATTC   
  
  
+ CCATCAAGGC ATAGATTATG GAGTGAGCAT GAAGAACGCT TTGCACGAGC TAGAGACTGC TCTAATTGGT   
  
  
+ GTAGATGGCG AGAAAGCATC TGTTGCTAAT CAACCTATGG GGGGAATTCA TTAGTCCGGG ATCCCAAGTT   
  
  
+ AGAGATCGAA GTCATTGAGT GAAGATCCAC AGGGTTCACA TCCTCCTCAG CTTGATTCAT CATCTTTTTC   
  
  
+ AAGGGTGAAA AGATTCGGAG ATGAAAGCCA GAGAGAGAGA AATGCCACAA GGCAATGGAA GAACCAGCGG   
  
  
+ AACTACTAAG TTTCTCACCA GGTGATTTAA AGCAGTTGCT AATTGAATGT GCGAGGGCTT TATCAGATAA   
  
  
+ CCGAATAGAT GACTTTGAGA GTTTGGCTAA ACGGCCAAGG AAAGAGGTCT CCATCTCAGG TGAGCCTGTC   
  
  
+ CAACGTCTCG GTACTTGTAT GATCGAAGGG CTTGTGGCAA GGAAACAGTC TTCGGGGACT AACATCTATC   
  
  
+ GGGCTCTTAA GTACTGTAAA GAGCCTCTTG GAAAATACTT GCTCTCCTAC GGCACTCAGT GGGTTACTCT   
  
  
+ ATCGCAAGCC ATAGCAGCAA GGCTTGGTGG GCCTCCTAAA GTGCGACTTA CAGGCATTGA TGATCCTGTT   
  
  
+ TCTAAGTATA CTTGTGATGC TAGCTTGGAG GCTATTGGGA AACGATTAGC GTCTCTATTT GAAAAGTTTA   
  
  
+ AAATACTCGT CGAGTTCAAT GCATTGCCCG TTTATGGACC TGATGTCAGG TGGGAAATGC TGGATGTGAG   
  
  
+ GCCCAGGGAG GCTTTGGCCG TTAATTGTCC ATTACAGCTC CATCACACTC CTGACGAGAG TGTCGATGTG   
  
  
+ AGCAACCCTA GGGATAGGCT TCTCAGAATG GTGAAATTGC TCGGTCCTAA GGTATGCACT TTGGTTGAGC   
  
  
+ AAGAATCAAA CACCAACACT ACACGTTTCT TGACCCGGTT CATAGAGACC CTTGACTACT ACTCGATCAG   
  
  
+ CCATGTTTG  

- +Up\_Stream \_Len000TCAACA TCGTTCGTGT AATTTCAACT AATAGTAAAA CAGGCACTTT GCCACTCGTT   
  
  
- TGCAGTAGAG GTCCAGTTTA AGTAAGCATG AGTTAGTGTT CAACGACTGT AAAAATGGTT TCCGATTAGC   
  
  
- AGCGTTTTCA AAGAAATCAA ATTCCGGTTA GAGAGGCAAC AAAGTGGGGT ATGTCTAACT CCCCAACATT   
  
  
- ATCACATATA TATATCGGAA CACAATCGCC CCGAACCCGT AATCTGAAAA TGAGAACATT TGGGATTACA   
  
  
- CAATAATATT TATATCTAGA TCGGTGGGTA CAACTCCATC CGTGAGATTA AGACTGTGTC CATCCTATTC   
  
  
- AAAAGTATCA AATACAACAC TTAGAACAGA ATGAAACACC AAGGATAACT GTTTTCAAAC TTAGGCTCGT   
  
  
- ACTCTCAAGT AGTTAGAGAA AAAGAAAGGA TAAAATAAAG AACACCTGAA TTAACCTAAA AGACCATCTC   
  
  
- CTTTCAAGGA ACGACCTTAA ACCAGCTTTC ACTATATGTA AAACCCTAAA TTAATCGGTA TTTAAACCAA   
  
  
- AATGTGGAAC AATCAAACCT CAAGACCACA CAATAACATA CACAAGACCA CCCAAAAAGA AAATCGATTT   
  
  
- ACAACCTCGT AAAATTCCCT AGATCTATAG ACTTCTTCAC GAACCCTTTA CTTCGTATAA AGGGAATATC   
  
  
- AGACTGTATT GAAATTACTT AACTAGTACT TGTTTCCCGT TAATCAAGTC TTTCCAAGTT GAGGAGATCC   
  
  
- ACAACACGAT TTCTCTACAC TCAATCATTG TTAACGAATG ATTGATATAT CCAACAAAAT AATGAACATC   
  
  
- TCCAAAAAAA AAACCCTATA GAAAAACCCC AAATATTTAC TCTTCCAACT AAACAACAGA TGAGAAGAAA   
  
  
- GCTAAGTTAT CATTAAGAGA GTCTCTTCCG ATTTTACTGT ACCGTAAGGT AGCGGAGAGT ACTCCGAAAA   
  
  
- ACGTACACGA ATACATAGAA AGTAGAGAAT GAAACCACCC CACCGTAACC CCTCCTTTTC CCCCAAAGCT   
  
  
- AAGCTATCTA GCAGTGAAAG CCTTTACTGC AGAGACTGGT TACCATTCCC ATTCCAATTA TGTACACCTA   
  
  
- TAAAGGTCTC AATCAACACT AACAATAACA ACAACAACAT TGAAATACAG TGCCCCTTTT GTATTATTAA   
  
  
- TAGACAAATA GACAAACAAA CAATAAAAGT TCCCCTTCCC CTCACCCGCC TACGAATCAT ACATTAGTAA   
  
  
- AATATATCCC TTTTAAGTAT ATCGTACTTT CATTTTGAAA GTAACGGTCG TAAGATTCGA TCCCCATCCC   
  
  
- AACAGATGTG GAACTAGAGG GATCTTCGTA CCACTAGTAA GGAGTCTAAC ACAATATCTT ATTTCTTACA   
  
  
- CCAAGACGAC ATTACTCTGA ATACATAATA GTTTACTACT TAGACAAATC TTGAAGGACA AATAAAAAAG   
  
  
- ACGTTTTAAA ATTCTCTGAT CGTAGATAGC CCGAGCTGAG ATCTCCAAGA GAACACAATG GGAAGAGTTG   
  
  
- AGTCAAACTA ACACTATGTC AATGATATAA CTCACTATCA GCTCTCGTAG TGTTGGGCGA TTCCTCAGAT   
  
  
- AGTTGTTCTT CGGGAAGAAC ATTAAGAGGG GAACTTTGAT CGTCAATAGT AGTGAACAGT AGCTTATCGG   
  
  
- GAAAAACATT AAGATGGGAA CTTTGATCGT CAATAACAGT AGTCAAGTTA CACTCAGGGC AATCTATAAG   
  
  
- GGTAGTTCCG TATCTAATAC CTCACTCGTA CTTCTTGCGA AACGTGCTCG ATCTCTGACG AGATTAACCA   
  
  
- CATCTACCGC TCTTTCGTAG ACAACGATTA GTTGGATACC CCCCTTAAGT AATCAGGCCC TAGGGTTCAA   
  
  
- TCTCTAGCTT CAGTAACTCA CTTCTAGGTG TCCCAAGTGT AGGAGGAGTC GAACTAAGTA GTAGAAAAAG   
  
  
- TTCCCACTTT TCTAAGCCTC TACTTTCGGT CTCTCTCTCT TTACGGTGTT CCGTTACCTT CTTGGTCGCC   
  
  
- TTGATGATTC AAAGAGTGGT CCACTAAATT TCGTCAACGA TTAACTTACA CGCTCCCGAA ATAGTCTATT   
  
  
- GGCTTATCTA CTGAAACTCT CAAACCGATT TGCCGGTTCC TTTCTCCAGA GGTAGAGTCC ACTCGGACAG   
  
  
- GTTGCAGAGC CATGAACATA CTAGCTTCCC GAACACCGTT CCTTTGTCAG AAGCCCCTGA TTGTAGATAG   
  
  
- CCCGAGAATT CATGACATTT CTCGGAGAAC CTTTTATGAA CGAGAGGATG CCGTGAGTCA CCCAATGAGA   
  
  
- TAGCGTTCGG TATCGTCGTT CCGAACCACC CGGAGGATTT CACGCTGAAT GTCCGTAACT ACTAGGACAA   
  
  
- AGATTCATAT GAACACTACG ATCGAACCTC CGATAACCCT TTGCTAATCG CAGAGATAAA CTTTTCAAAT   
  
  
- TTTATGAGCA GCTCAAGTTA CGTAACGGGC AAATACCTGG ACTACAGTCC ACCCTTTACG ACCTACACTC   
  
  
- CGGGTCCCTC CGAAACCGGC AATTAACAGG TAATGTCGAG GTAGTGTGAG GACTGCTCTC ACAGCTACAC   
  
  
- TCGTTGGGAT CCCTATCCGA AGAGTCTTAC CACTTTAACG AGCCAGGATT CCATACGTGA AACCAACTCG   
  
  
- TTCTTAGTTT GTGGTTGTGA TGTGCAAAGA ACTGGGCCAA GTATCTCTGG GAACTGATGA TGAGCTAGTC   
  
  
- GGTACAAAC

+     G-Box

| Site Name | Organism | Position | Strand | Matrix score. | sequence | function |
| --- | --- | --- | --- | --- | --- | --- |
| G-Box | Pisum sativum | 2686 | + | 6 | CACGTT | cis-acting regulatory element involved in light responsiveness |

>HU08G00019.1   
+ +Up\_Stream \_Len000AGTTGT AGCAAGCACA TTAAAGTTGA TTATCATTTT GTCCGTGAAA CGGTGAGCAA   
  
  
+ ACGTCATCTC CAGGTCAAAT TCATTCGTAC TCAATCACAA GTTGCTGACA TTTTTACCAA AGGCTAATCG   
  
  
+ TCGCAAAAGT TTCTTTAGTT TAAGGCCAAT CTCTCCGTTG TTTCACCCCA TACAGATTGA GGGGTTGTAA   
  
  
+ TAGTGTATAT ATATAGCCTT GTGTTAGCGG GGCTTGGGCA TTAGACTTTT ACTCTTGTAA ACCCTAATGT   
  
  
+ GTTATTATAA ATATAGATCT AGCCACCCAT GTTGAGGTAG GCACTCTAAT TCTGACACAG GTAGGATAAG   
  
  
+ TTTTCATAGT TTATGTTGTG AATCTTGTCT TACTTTGTGG TTCCTATTGA CAAAAGTTTG AATCCGAGCA   
  
  
+ TGAGAGTTCA TCAATCTCTT TTTCTTTCCT ATTTTATTTC TTGTGGACTT AATTGGATTT TCTGGTAGAG   
  
  
+ GAAAGTTCCT TGCTGGAATT TGGTCGAAAG TGATATACAT TTTGGGATTT AATTAGCCAT AAATTTGGTT   
  
  
+ TTACACCTTG TTAGTTTGGA GTTCTGGTGT GTTATTGTAT GTGTTCTGGT GGGTTTTTCT TTTAGCTAAA   
  
  
+ TGTTGGAGCA TTTTAAGGGA TCTAGATATC TGAAGAAGTG CTTGGGAAAT GAAGCATATT TCCCTTATAG   
  
  
+ TCTGACATAA CTTTAATGAA TTGATCATGA ACAAAGGGCA ATTAGTTCAG AAAGGTTCAA CTCCTCTAGG   
  
  
+ TGTTGTGCTA AAGAGATGTG AGTTAGTAAC AATTGCTTAC TAACTATATA GGTTGTTTTA TTACTTGTAG   
  
  
+ AGGTTTTTTT TTTGGGATAT CTTTTTGGGG TTTATAAATG AGAAGGTTGA TTTGTTGTCT ACTCTTCTTT   
  
  
+ CGATTCAATA GTAATTCTCT CAGAGAAGGC TAAAATGACA TGGCATTCCA TCGCCTCTCA TGAGGCTTTT   
  
  
+ TGCATGTGCT TATGTATCTT TCATCTCTTA CTTTGGTGGG GTGGCATTGG GGAGGAAAAG GGGGTTTCGA   
  
  
+ TTCGATAGAT CGTCACTTTC GGAAATGACG TCTCTGACCA ATGGTAAGGG TAAGGTTAAT ACATGTGGAT   
  
  
+ ATTTCCAGAG TTAGTTGTGA TTGTTATTGT TGTTGTTGTA ACTTTATGTC ACGGGGAAAA CATAATAATT   
  
  
+ ATCTGTTTAT CTGTTTGTTT GTTATTTTCA AGGGGAAGGG GAGTGGGCGG ATGCTTAGTA TGTAATCATT   
  
  
+ TTATATAGGG AAAATTCATA TAGCATGAAA GTAAAACTTT CATTGCCAGC ATTCTAAGCT AGGGGTAGGG   
  
  
+ TTGTCTACAC CTTGATCTCC CTAGAAGCAT GGTGATCATT CCTCAGATTG TGTTATAGAA TAAAGAATGT   
  
  
+ GGTTCTGCTG TAATGAGACT TATGTATTAT CAAATGATGA ATCTGTTTAG AACTTCCTGT TTATTTTTTC   
  
  
+ TGCAAAATTT TAAGAGACTA GCATCTATCG GGCTCGACTC TAGAGGTTCT CTTGTGTTAC CCTTCTCAAC   
  
  
+ TCAGTTTGAT TGTGATACAG TTACTATATT GAGTGATAGT CGAGAGCATC ACAACCCGCT AAGGAGTCTA   
  
  
+ TCAACAAGAA GCCCTTCTTG TAATTCTCCC CTTGAAACTA GCAGTTATCA TCACTTGTCA TCGAATAGCC   
  
  
+ CTTTTTGTAA TTCTACCCTT GAAACTAGCA GTTATTGTCA TCAGTTCAAT GTGAGTCCCG TTAGATATTC   
  
  
+ CCATCAAGGC ATAGATTATG GAGTGAGCAT GAAGAACGCT TTGCACGAGC TAGAGACTGC TCTAATTGGT   
  
  
+ GTAGATGGCG AGAAAGCATC TGTTGCTAAT CAACCTATGG GGGGAATTCA TTAGTCCGGG ATCCCAAGTT   
  
  
+ AGAGATCGAA GTCATTGAGT GAAGATCCAC AGGGTTCACA TCCTCCTCAG CTTGATTCAT CATCTTTTTC   
  
  
+ AAGGGTGAAA AGATTCGGAG ATGAAAGCCA GAGAGAGAGA AATGCCACAA GGCAATGGAA GAACCAGCGG   
  
  
+ AACTACTAAG TTTCTCACCA GGTGATTTAA AGCAGTTGCT AATTGAATGT GCGAGGGCTT TATCAGATAA   
  
  
+ CCGAATAGAT GACTTTGAGA GTTTGGCTAA ACGGCCAAGG AAAGAGGTCT CCATCTCAGG TGAGCCTGTC   
  
  
+ CAACGTCTCG GTACTTGTAT GATCGAAGGG CTTGTGGCAA GGAAACAGTC TTCGGGGACT AACATCTATC   
  
  
+ GGGCTCTTAA GTACTGTAAA GAGCCTCTTG GAAAATACTT GCTCTCCTAC GGCACTCAGT GGGTTACTCT   
  
  
+ ATCGCAAGCC ATAGCAGCAA GGCTTGGTGG GCCTCCTAAA GTGCGACTTA CAGGCATTGA TGATCCTGTT   
  
  
+ TCTAAGTATA CTTGTGATGC TAGCTTGGAG GCTATTGGGA AACGATTAGC GTCTCTATTT GAAAAGTTTA   
  
  
+ AAATACTCGT CGAGTTCAAT GCATTGCCCG TTTATGGACC TGATGTCAGG TGGGAAATGC TGGATGTGAG   
  
  
+ GCCCAGGGAG GCTTTGGCCG TTAATTGTCC ATTACAGCTC CATCACACTC CTGACGAGAG TGTCGATGTG   
  
  
+ AGCAACCCTA GGGATAGGCT TCTCAGAATG GTGAAATTGC TCGGTCCTAA GGTATGCACT TTGGTTGAGC   
  
  
+ AAGAATCAAA CACCAACACT ACACGTTTCT TGACCCGGTT CATAGAGACC CTTGACTACT ACTCGATCAG   
  
  
+ CCATGTTTG  

- +Up\_Stream \_Len000TCAACA TCGTTCGTGT AATTTCAACT AATAGTAAAA CAGGCACTTT GCCACTCGTT   
  
  
- TGCAGTAGAG GTCCAGTTTA AGTAAGCATG AGTTAGTGTT CAACGACTGT AAAAATGGTT TCCGATTAGC   
  
  
- AGCGTTTTCA AAGAAATCAA ATTCCGGTTA GAGAGGCAAC AAAGTGGGGT ATGTCTAACT CCCCAACATT   
  
  
- ATCACATATA TATATCGGAA CACAATCGCC CCGAACCCGT AATCTGAAAA TGAGAACATT TGGGATTACA   
  
  
- CAATAATATT TATATCTAGA TCGGTGGGTA CAACTCCATC CGTGAGATTA AGACTGTGTC CATCCTATTC   
  
  
- AAAAGTATCA AATACAACAC TTAGAACAGA ATGAAACACC AAGGATAACT GTTTTCAAAC TTAGGCTCGT   
  
  
- ACTCTCAAGT AGTTAGAGAA AAAGAAAGGA TAAAATAAAG AACACCTGAA TTAACCTAAA AGACCATCTC   
  
  
- CTTTCAAGGA ACGACCTTAA ACCAGCTTTC ACTATATGTA AAACCCTAAA TTAATCGGTA TTTAAACCAA   
  
  
- AATGTGGAAC AATCAAACCT CAAGACCACA CAATAACATA CACAAGACCA CCCAAAAAGA AAATCGATTT   
  
  
- ACAACCTCGT AAAATTCCCT AGATCTATAG ACTTCTTCAC GAACCCTTTA CTTCGTATAA AGGGAATATC   
  
  
- AGACTGTATT GAAATTACTT AACTAGTACT TGTTTCCCGT TAATCAAGTC TTTCCAAGTT GAGGAGATCC   
  
  
- ACAACACGAT TTCTCTACAC TCAATCATTG TTAACGAATG ATTGATATAT CCAACAAAAT AATGAACATC   
  
  
- TCCAAAAAAA AAACCCTATA GAAAAACCCC AAATATTTAC TCTTCCAACT AAACAACAGA TGAGAAGAAA   
  
  
- GCTAAGTTAT CATTAAGAGA GTCTCTTCCG ATTTTACTGT ACCGTAAGGT AGCGGAGAGT ACTCCGAAAA   
  
  
- ACGTACACGA ATACATAGAA AGTAGAGAAT GAAACCACCC CACCGTAACC CCTCCTTTTC CCCCAAAGCT   
  
  
- AAGCTATCTA GCAGTGAAAG CCTTTACTGC AGAGACTGGT TACCATTCCC ATTCCAATTA TGTACACCTA   
  
  
- TAAAGGTCTC AATCAACACT AACAATAACA ACAACAACAT TGAAATACAG TGCCCCTTTT GTATTATTAA   
  
  
- TAGACAAATA GACAAACAAA CAATAAAAGT TCCCCTTCCC CTCACCCGCC TACGAATCAT ACATTAGTAA   
  
  
- AATATATCCC TTTTAAGTAT ATCGTACTTT CATTTTGAAA GTAACGGTCG TAAGATTCGA TCCCCATCCC   
  
  
- AACAGATGTG GAACTAGAGG GATCTTCGTA CCACTAGTAA GGAGTCTAAC ACAATATCTT ATTTCTTACA   
  
  
- CCAAGACGAC ATTACTCTGA ATACATAATA GTTTACTACT TAGACAAATC TTGAAGGACA AATAAAAAAG   
  
  
- ACGTTTTAAA ATTCTCTGAT CGTAGATAGC CCGAGCTGAG ATCTCCAAGA GAACACAATG GGAAGAGTTG   
  
  
- AGTCAAACTA ACACTATGTC AATGATATAA CTCACTATCA GCTCTCGTAG TGTTGGGCGA TTCCTCAGAT   
  
  
- AGTTGTTCTT CGGGAAGAAC ATTAAGAGGG GAACTTTGAT CGTCAATAGT AGTGAACAGT AGCTTATCGG   
  
  
- GAAAAACATT AAGATGGGAA CTTTGATCGT CAATAACAGT AGTCAAGTTA CACTCAGGGC AATCTATAAG   
  
  
- GGTAGTTCCG TATCTAATAC CTCACTCGTA CTTCTTGCGA AACGTGCTCG ATCTCTGACG AGATTAACCA   
  
  
- CATCTACCGC TCTTTCGTAG ACAACGATTA GTTGGATACC CCCCTTAAGT AATCAGGCCC TAGGGTTCAA   
  
  
- TCTCTAGCTT CAGTAACTCA CTTCTAGGTG TCCCAAGTGT AGGAGGAGTC GAACTAAGTA GTAGAAAAAG   
  
  
- TTCCCACTTT TCTAAGCCTC TACTTTCGGT CTCTCTCTCT TTACGGTGTT CCGTTACCTT CTTGGTCGCC   
  
  
- TTGATGATTC AAAGAGTGGT CCACTAAATT TCGTCAACGA TTAACTTACA CGCTCCCGAA ATAGTCTATT   
  
  
- GGCTTATCTA CTGAAACTCT CAAACCGATT TGCCGGTTCC TTTCTCCAGA GGTAGAGTCC ACTCGGACAG   
  
  
- GTTGCAGAGC CATGAACATA CTAGCTTCCC GAACACCGTT CCTTTGTCAG AAGCCCCTGA TTGTAGATAG   
  
  
- CCCGAGAATT CATGACATTT CTCGGAGAAC CTTTTATGAA CGAGAGGATG CCGTGAGTCA CCCAATGAGA   
  
  
- TAGCGTTCGG TATCGTCGTT CCGAACCACC CGGAGGATTT CACGCTGAAT GTCCGTAACT ACTAGGACAA   
  
  
- AGATTCATAT GAACACTACG ATCGAACCTC CGATAACCCT TTGCTAATCG CAGAGATAAA CTTTTCAAAT   
  
  
- TTTATGAGCA GCTCAAGTTA CGTAACGGGC AAATACCTGG ACTACAGTCC ACCCTTTACG ACCTACACTC   
  
  
- CGGGTCCCTC CGAAACCGGC AATTAACAGG TAATGTCGAG GTAGTGTGAG GACTGCTCTC ACAGCTACAC   
  
  
- TCGTTGGGAT CCCTATCCGA AGAGTCTTAC CACTTTAACG AGCCAGGATT CCATACGTGA AACCAACTCG   
  
  
- TTCTTAGTTT GTGGTTGTGA TGTGCAAAGA ACTGGGCCAA GTATCTCTGG GAACTGATGA TGAGCTAGTC   
  
  
- GGTACAAAC

+     GARE-motif

| Site Name | Organism | Position | Strand | Matrix score. | sequence | function |
| --- | --- | --- | --- | --- | --- | --- |
| GARE-motif | Brassica oleracea | 1843 | + | 7 | TCTGTTG | gibberellin-responsive element |

>HU08G00019.1   
+ +Up\_Stream \_Len000AGTTGT AGCAAGCACA TTAAAGTTGA TTATCATTTT GTCCGTGAAA CGGTGAGCAA   
  
  
+ ACGTCATCTC CAGGTCAAAT TCATTCGTAC TCAATCACAA GTTGCTGACA TTTTTACCAA AGGCTAATCG   
  
  
+ TCGCAAAAGT TTCTTTAGTT TAAGGCCAAT CTCTCCGTTG TTTCACCCCA TACAGATTGA GGGGTTGTAA   
  
  
+ TAGTGTATAT ATATAGCCTT GTGTTAGCGG GGCTTGGGCA TTAGACTTTT ACTCTTGTAA ACCCTAATGT   
  
  
+ GTTATTATAA ATATAGATCT AGCCACCCAT GTTGAGGTAG GCACTCTAAT TCTGACACAG GTAGGATAAG   
  
  
+ TTTTCATAGT TTATGTTGTG AATCTTGTCT TACTTTGTGG TTCCTATTGA CAAAAGTTTG AATCCGAGCA   
  
  
+ TGAGAGTTCA TCAATCTCTT TTTCTTTCCT ATTTTATTTC TTGTGGACTT AATTGGATTT TCTGGTAGAG   
  
  
+ GAAAGTTCCT TGCTGGAATT TGGTCGAAAG TGATATACAT TTTGGGATTT AATTAGCCAT AAATTTGGTT   
  
  
+ TTACACCTTG TTAGTTTGGA GTTCTGGTGT GTTATTGTAT GTGTTCTGGT GGGTTTTTCT TTTAGCTAAA   
  
  
+ TGTTGGAGCA TTTTAAGGGA TCTAGATATC TGAAGAAGTG CTTGGGAAAT GAAGCATATT TCCCTTATAG   
  
  
+ TCTGACATAA CTTTAATGAA TTGATCATGA ACAAAGGGCA ATTAGTTCAG AAAGGTTCAA CTCCTCTAGG   
  
  
+ TGTTGTGCTA AAGAGATGTG AGTTAGTAAC AATTGCTTAC TAACTATATA GGTTGTTTTA TTACTTGTAG   
  
  
+ AGGTTTTTTT TTTGGGATAT CTTTTTGGGG TTTATAAATG AGAAGGTTGA TTTGTTGTCT ACTCTTCTTT   
  
  
+ CGATTCAATA GTAATTCTCT CAGAGAAGGC TAAAATGACA TGGCATTCCA TCGCCTCTCA TGAGGCTTTT   
  
  
+ TGCATGTGCT TATGTATCTT TCATCTCTTA CTTTGGTGGG GTGGCATTGG GGAGGAAAAG GGGGTTTCGA   
  
  
+ TTCGATAGAT CGTCACTTTC GGAAATGACG TCTCTGACCA ATGGTAAGGG TAAGGTTAAT ACATGTGGAT   
  
  
+ ATTTCCAGAG TTAGTTGTGA TTGTTATTGT TGTTGTTGTA ACTTTATGTC ACGGGGAAAA CATAATAATT   
  
  
+ ATCTGTTTAT CTGTTTGTTT GTTATTTTCA AGGGGAAGGG GAGTGGGCGG ATGCTTAGTA TGTAATCATT   
  
  
+ TTATATAGGG AAAATTCATA TAGCATGAAA GTAAAACTTT CATTGCCAGC ATTCTAAGCT AGGGGTAGGG   
  
  
+ TTGTCTACAC CTTGATCTCC CTAGAAGCAT GGTGATCATT CCTCAGATTG TGTTATAGAA TAAAGAATGT   
  
  
+ GGTTCTGCTG TAATGAGACT TATGTATTAT CAAATGATGA ATCTGTTTAG AACTTCCTGT TTATTTTTTC   
  
  
+ TGCAAAATTT TAAGAGACTA GCATCTATCG GGCTCGACTC TAGAGGTTCT CTTGTGTTAC CCTTCTCAAC   
  
  
+ TCAGTTTGAT TGTGATACAG TTACTATATT GAGTGATAGT CGAGAGCATC ACAACCCGCT AAGGAGTCTA   
  
  
+ TCAACAAGAA GCCCTTCTTG TAATTCTCCC CTTGAAACTA GCAGTTATCA TCACTTGTCA TCGAATAGCC   
  
  
+ CTTTTTGTAA TTCTACCCTT GAAACTAGCA GTTATTGTCA TCAGTTCAAT GTGAGTCCCG TTAGATATTC   
  
  
+ CCATCAAGGC ATAGATTATG GAGTGAGCAT GAAGAACGCT TTGCACGAGC TAGAGACTGC TCTAATTGGT   
  
  
+ GTAGATGGCG AGAAAGCATC TGTTGCTAAT CAACCTATGG GGGGAATTCA TTAGTCCGGG ATCCCAAGTT   
  
  
+ AGAGATCGAA GTCATTGAGT GAAGATCCAC AGGGTTCACA TCCTCCTCAG CTTGATTCAT CATCTTTTTC   
  
  
+ AAGGGTGAAA AGATTCGGAG ATGAAAGCCA GAGAGAGAGA AATGCCACAA GGCAATGGAA GAACCAGCGG   
  
  
+ AACTACTAAG TTTCTCACCA GGTGATTTAA AGCAGTTGCT AATTGAATGT GCGAGGGCTT TATCAGATAA   
  
  
+ CCGAATAGAT GACTTTGAGA GTTTGGCTAA ACGGCCAAGG AAAGAGGTCT CCATCTCAGG TGAGCCTGTC   
  
  
+ CAACGTCTCG GTACTTGTAT GATCGAAGGG CTTGTGGCAA GGAAACAGTC TTCGGGGACT AACATCTATC   
  
  
+ GGGCTCTTAA GTACTGTAAA GAGCCTCTTG GAAAATACTT GCTCTCCTAC GGCACTCAGT GGGTTACTCT   
  
  
+ ATCGCAAGCC ATAGCAGCAA GGCTTGGTGG GCCTCCTAAA GTGCGACTTA CAGGCATTGA TGATCCTGTT   
  
  
+ TCTAAGTATA CTTGTGATGC TAGCTTGGAG GCTATTGGGA AACGATTAGC GTCTCTATTT GAAAAGTTTA   
  
  
+ AAATACTCGT CGAGTTCAAT GCATTGCCCG TTTATGGACC TGATGTCAGG TGGGAAATGC TGGATGTGAG   
  
  
+ GCCCAGGGAG GCTTTGGCCG TTAATTGTCC ATTACAGCTC CATCACACTC CTGACGAGAG TGTCGATGTG   
  
  
+ AGCAACCCTA GGGATAGGCT TCTCAGAATG GTGAAATTGC TCGGTCCTAA GGTATGCACT TTGGTTGAGC   
  
  
+ AAGAATCAAA CACCAACACT ACACGTTTCT TGACCCGGTT CATAGAGACC CTTGACTACT ACTCGATCAG   
  
  
+ CCATGTTTG  

- +Up\_Stream \_Len000TCAACA TCGTTCGTGT AATTTCAACT AATAGTAAAA CAGGCACTTT GCCACTCGTT   
  
  
- TGCAGTAGAG GTCCAGTTTA AGTAAGCATG AGTTAGTGTT CAACGACTGT AAAAATGGTT TCCGATTAGC   
  
  
- AGCGTTTTCA AAGAAATCAA ATTCCGGTTA GAGAGGCAAC AAAGTGGGGT ATGTCTAACT CCCCAACATT   
  
  
- ATCACATATA TATATCGGAA CACAATCGCC CCGAACCCGT AATCTGAAAA TGAGAACATT TGGGATTACA   
  
  
- CAATAATATT TATATCTAGA TCGGTGGGTA CAACTCCATC CGTGAGATTA AGACTGTGTC CATCCTATTC   
  
  
- AAAAGTATCA AATACAACAC TTAGAACAGA ATGAAACACC AAGGATAACT GTTTTCAAAC TTAGGCTCGT   
  
  
- ACTCTCAAGT AGTTAGAGAA AAAGAAAGGA TAAAATAAAG AACACCTGAA TTAACCTAAA AGACCATCTC   
  
  
- CTTTCAAGGA ACGACCTTAA ACCAGCTTTC ACTATATGTA AAACCCTAAA TTAATCGGTA TTTAAACCAA   
  
  
- AATGTGGAAC AATCAAACCT CAAGACCACA CAATAACATA CACAAGACCA CCCAAAAAGA AAATCGATTT   
  
  
- ACAACCTCGT AAAATTCCCT AGATCTATAG ACTTCTTCAC GAACCCTTTA CTTCGTATAA AGGGAATATC   
  
  
- AGACTGTATT GAAATTACTT AACTAGTACT TGTTTCCCGT TAATCAAGTC TTTCCAAGTT GAGGAGATCC   
  
  
- ACAACACGAT TTCTCTACAC TCAATCATTG TTAACGAATG ATTGATATAT CCAACAAAAT AATGAACATC   
  
  
- TCCAAAAAAA AAACCCTATA GAAAAACCCC AAATATTTAC TCTTCCAACT AAACAACAGA TGAGAAGAAA   
  
  
- GCTAAGTTAT CATTAAGAGA GTCTCTTCCG ATTTTACTGT ACCGTAAGGT AGCGGAGAGT ACTCCGAAAA   
  
  
- ACGTACACGA ATACATAGAA AGTAGAGAAT GAAACCACCC CACCGTAACC CCTCCTTTTC CCCCAAAGCT   
  
  
- AAGCTATCTA GCAGTGAAAG CCTTTACTGC AGAGACTGGT TACCATTCCC ATTCCAATTA TGTACACCTA   
  
  
- TAAAGGTCTC AATCAACACT AACAATAACA ACAACAACAT TGAAATACAG TGCCCCTTTT GTATTATTAA   
  
  
- TAGACAAATA GACAAACAAA CAATAAAAGT TCCCCTTCCC CTCACCCGCC TACGAATCAT ACATTAGTAA   
  
  
- AATATATCCC TTTTAAGTAT ATCGTACTTT CATTTTGAAA GTAACGGTCG TAAGATTCGA TCCCCATCCC   
  
  
- AACAGATGTG GAACTAGAGG GATCTTCGTA CCACTAGTAA GGAGTCTAAC ACAATATCTT ATTTCTTACA   
  
  
- CCAAGACGAC ATTACTCTGA ATACATAATA GTTTACTACT TAGACAAATC TTGAAGGACA AATAAAAAAG   
  
  
- ACGTTTTAAA ATTCTCTGAT CGTAGATAGC CCGAGCTGAG ATCTCCAAGA GAACACAATG GGAAGAGTTG   
  
  
- AGTCAAACTA ACACTATGTC AATGATATAA CTCACTATCA GCTCTCGTAG TGTTGGGCGA TTCCTCAGAT   
  
  
- AGTTGTTCTT CGGGAAGAAC ATTAAGAGGG GAACTTTGAT CGTCAATAGT AGTGAACAGT AGCTTATCGG   
  
  
- GAAAAACATT AAGATGGGAA CTTTGATCGT CAATAACAGT AGTCAAGTTA CACTCAGGGC AATCTATAAG   
  
  
- GGTAGTTCCG TATCTAATAC CTCACTCGTA CTTCTTGCGA AACGTGCTCG ATCTCTGACG AGATTAACCA   
  
  
- CATCTACCGC TCTTTCGTAG ACAACGATTA GTTGGATACC CCCCTTAAGT AATCAGGCCC TAGGGTTCAA   
  
  
- TCTCTAGCTT CAGTAACTCA CTTCTAGGTG TCCCAAGTGT AGGAGGAGTC GAACTAAGTA GTAGAAAAAG   
  
  
- TTCCCACTTT TCTAAGCCTC TACTTTCGGT CTCTCTCTCT TTACGGTGTT CCGTTACCTT CTTGGTCGCC   
  
  
- TTGATGATTC AAAGAGTGGT CCACTAAATT TCGTCAACGA TTAACTTACA CGCTCCCGAA ATAGTCTATT   
  
  
- GGCTTATCTA CTGAAACTCT CAAACCGATT TGCCGGTTCC TTTCTCCAGA GGTAGAGTCC ACTCGGACAG   
  
  
- GTTGCAGAGC CATGAACATA CTAGCTTCCC GAACACCGTT CCTTTGTCAG AAGCCCCTGA TTGTAGATAG   
  
  
- CCCGAGAATT CATGACATTT CTCGGAGAAC CTTTTATGAA CGAGAGGATG CCGTGAGTCA CCCAATGAGA   
  
  
- TAGCGTTCGG TATCGTCGTT CCGAACCACC CGGAGGATTT CACGCTGAAT GTCCGTAACT ACTAGGACAA   
  
  
- AGATTCATAT GAACACTACG ATCGAACCTC CGATAACCCT TTGCTAATCG CAGAGATAAA CTTTTCAAAT   
  
  
- TTTATGAGCA GCTCAAGTTA CGTAACGGGC AAATACCTGG ACTACAGTCC ACCCTTTACG ACCTACACTC   
  
  
- CGGGTCCCTC CGAAACCGGC AATTAACAGG TAATGTCGAG GTAGTGTGAG GACTGCTCTC ACAGCTACAC   
  
  
- TCGTTGGGAT CCCTATCCGA AGAGTCTTAC CACTTTAACG AGCCAGGATT CCATACGTGA AACCAACTCG   
  
  
- TTCTTAGTTT GTGGTTGTGA TGTGCAAAGA ACTGGGCCAA GTATCTCTGG GAACTGATGA TGAGCTAGTC   
  
  
- GGTACAAAC

+     GATA-motif

| Site Name | Organism | Position | Strand | Matrix score. | sequence | function |
| --- | --- | --- | --- | --- | --- | --- |
| GATA-motif | Arabidopsis thaliana | 375 | - | 10 | AAGATAAGATT | part of a light responsive element |
| GATA-motif | Solanum tuberosum | 1100 | + | 9 | AAGGATAAGG | part of a light responsive element |

>HU08G00019.1   
+ +Up\_Stream \_Len000AGTTGT AGCAAGCACA TTAAAGTTGA TTATCATTTT GTCCGTGAAA CGGTGAGCAA   
  
  
+ ACGTCATCTC CAGGTCAAAT TCATTCGTAC TCAATCACAA GTTGCTGACA TTTTTACCAA AGGCTAATCG   
  
  
+ TCGCAAAAGT TTCTTTAGTT TAAGGCCAAT CTCTCCGTTG TTTCACCCCA TACAGATTGA GGGGTTGTAA   
  
  
+ TAGTGTATAT ATATAGCCTT GTGTTAGCGG GGCTTGGGCA TTAGACTTTT ACTCTTGTAA ACCCTAATGT   
  
  
+ GTTATTATAA ATATAGATCT AGCCACCCAT GTTGAGGTAG GCACTCTAAT TCTGACACAG GTAGGATAAG   
  
  
+ TTTTCATAGT TTATGTTGTG AATCTTGTCT TACTTTGTGG TTCCTATTGA CAAAAGTTTG AATCCGAGCA   
  
  
+ TGAGAGTTCA TCAATCTCTT TTTCTTTCCT ATTTTATTTC TTGTGGACTT AATTGGATTT TCTGGTAGAG   
  
  
+ GAAAGTTCCT TGCTGGAATT TGGTCGAAAG TGATATACAT TTTGGGATTT AATTAGCCAT AAATTTGGTT   
  
  
+ TTACACCTTG TTAGTTTGGA GTTCTGGTGT GTTATTGTAT GTGTTCTGGT GGGTTTTTCT TTTAGCTAAA   
  
  
+ TGTTGGAGCA TTTTAAGGGA TCTAGATATC TGAAGAAGTG CTTGGGAAAT GAAGCATATT TCCCTTATAG   
  
  
+ TCTGACATAA CTTTAATGAA TTGATCATGA ACAAAGGGCA ATTAGTTCAG AAAGGTTCAA CTCCTCTAGG   
  
  
+ TGTTGTGCTA AAGAGATGTG AGTTAGTAAC AATTGCTTAC TAACTATATA GGTTGTTTTA TTACTTGTAG   
  
  
+ AGGTTTTTTT TTTGGGATAT CTTTTTGGGG TTTATAAATG AGAAGGTTGA TTTGTTGTCT ACTCTTCTTT   
  
  
+ CGATTCAATA GTAATTCTCT CAGAGAAGGC TAAAATGACA TGGCATTCCA TCGCCTCTCA TGAGGCTTTT   
  
  
+ TGCATGTGCT TATGTATCTT TCATCTCTTA CTTTGGTGGG GTGGCATTGG GGAGGAAAAG GGGGTTTCGA   
  
  
+ TTCGATAGAT CGTCACTTTC GGAAATGACG TCTCTGACCA ATGGTAAGGG TAAGGTTAAT ACATGTGGAT   
  
  
+ ATTTCCAGAG TTAGTTGTGA TTGTTATTGT TGTTGTTGTA ACTTTATGTC ACGGGGAAAA CATAATAATT   
  
  
+ ATCTGTTTAT CTGTTTGTTT GTTATTTTCA AGGGGAAGGG GAGTGGGCGG ATGCTTAGTA TGTAATCATT   
  
  
+ TTATATAGGG AAAATTCATA TAGCATGAAA GTAAAACTTT CATTGCCAGC ATTCTAAGCT AGGGGTAGGG   
  
  
+ TTGTCTACAC CTTGATCTCC CTAGAAGCAT GGTGATCATT CCTCAGATTG TGTTATAGAA TAAAGAATGT   
  
  
+ GGTTCTGCTG TAATGAGACT TATGTATTAT CAAATGATGA ATCTGTTTAG AACTTCCTGT TTATTTTTTC   
  
  
+ TGCAAAATTT TAAGAGACTA GCATCTATCG GGCTCGACTC TAGAGGTTCT CTTGTGTTAC CCTTCTCAAC   
  
  
+ TCAGTTTGAT TGTGATACAG TTACTATATT GAGTGATAGT CGAGAGCATC ACAACCCGCT AAGGAGTCTA   
  
  
+ TCAACAAGAA GCCCTTCTTG TAATTCTCCC CTTGAAACTA GCAGTTATCA TCACTTGTCA TCGAATAGCC   
  
  
+ CTTTTTGTAA TTCTACCCTT GAAACTAGCA GTTATTGTCA TCAGTTCAAT GTGAGTCCCG TTAGATATTC   
  
  
+ CCATCAAGGC ATAGATTATG GAGTGAGCAT GAAGAACGCT TTGCACGAGC TAGAGACTGC TCTAATTGGT   
  
  
+ GTAGATGGCG AGAAAGCATC TGTTGCTAAT CAACCTATGG GGGGAATTCA TTAGTCCGGG ATCCCAAGTT   
  
  
+ AGAGATCGAA GTCATTGAGT GAAGATCCAC AGGGTTCACA TCCTCCTCAG CTTGATTCAT CATCTTTTTC   
  
  
+ AAGGGTGAAA AGATTCGGAG ATGAAAGCCA GAGAGAGAGA AATGCCACAA GGCAATGGAA GAACCAGCGG   
  
  
+ AACTACTAAG TTTCTCACCA GGTGATTTAA AGCAGTTGCT AATTGAATGT GCGAGGGCTT TATCAGATAA   
  
  
+ CCGAATAGAT GACTTTGAGA GTTTGGCTAA ACGGCCAAGG AAAGAGGTCT CCATCTCAGG TGAGCCTGTC   
  
  
+ CAACGTCTCG GTACTTGTAT GATCGAAGGG CTTGTGGCAA GGAAACAGTC TTCGGGGACT AACATCTATC   
  
  
+ GGGCTCTTAA GTACTGTAAA GAGCCTCTTG GAAAATACTT GCTCTCCTAC GGCACTCAGT GGGTTACTCT   
  
  
+ ATCGCAAGCC ATAGCAGCAA GGCTTGGTGG GCCTCCTAAA GTGCGACTTA CAGGCATTGA TGATCCTGTT   
  
  
+ TCTAAGTATA CTTGTGATGC TAGCTTGGAG GCTATTGGGA AACGATTAGC GTCTCTATTT GAAAAGTTTA   
  
  
+ AAATACTCGT CGAGTTCAAT GCATTGCCCG TTTATGGACC TGATGTCAGG TGGGAAATGC TGGATGTGAG   
  
  
+ GCCCAGGGAG GCTTTGGCCG TTAATTGTCC ATTACAGCTC CATCACACTC CTGACGAGAG TGTCGATGTG   
  
  
+ AGCAACCCTA GGGATAGGCT TCTCAGAATG GTGAAATTGC TCGGTCCTAA GGTATGCACT TTGGTTGAGC   
  
  
+ AAGAATCAAA CACCAACACT ACACGTTTCT TGACCCGGTT CATAGAGACC CTTGACTACT ACTCGATCAG   
  
  
+ CCATGTTTG  

- +Up\_Stream \_Len000TCAACA TCGTTCGTGT AATTTCAACT AATAGTAAAA CAGGCACTTT GCCACTCGTT   
  
  
- TGCAGTAGAG GTCCAGTTTA AGTAAGCATG AGTTAGTGTT CAACGACTGT AAAAATGGTT TCCGATTAGC   
  
  
- AGCGTTTTCA AAGAAATCAA ATTCCGGTTA GAGAGGCAAC AAAGTGGGGT ATGTCTAACT CCCCAACATT   
  
  
- ATCACATATA TATATCGGAA CACAATCGCC CCGAACCCGT AATCTGAAAA TGAGAACATT TGGGATTACA   
  
  
- CAATAATATT TATATCTAGA TCGGTGGGTA CAACTCCATC CGTGAGATTA AGACTGTGTC CATCCTATTC   
  
  
- AAAAGTATCA AATACAACAC TTAGAACAGA ATGAAACACC AAGGATAACT GTTTTCAAAC TTAGGCTCGT   
  
  
- ACTCTCAAGT AGTTAGAGAA AAAGAAAGGA TAAAATAAAG AACACCTGAA TTAACCTAAA AGACCATCTC   
  
  
- CTTTCAAGGA ACGACCTTAA ACCAGCTTTC ACTATATGTA AAACCCTAAA TTAATCGGTA TTTAAACCAA   
  
  
- AATGTGGAAC AATCAAACCT CAAGACCACA CAATAACATA CACAAGACCA CCCAAAAAGA AAATCGATTT   
  
  
- ACAACCTCGT AAAATTCCCT AGATCTATAG ACTTCTTCAC GAACCCTTTA CTTCGTATAA AGGGAATATC   
  
  
- AGACTGTATT GAAATTACTT AACTAGTACT TGTTTCCCGT TAATCAAGTC TTTCCAAGTT GAGGAGATCC   
  
  
- ACAACACGAT TTCTCTACAC TCAATCATTG TTAACGAATG ATTGATATAT CCAACAAAAT AATGAACATC   
  
  
- TCCAAAAAAA AAACCCTATA GAAAAACCCC AAATATTTAC TCTTCCAACT AAACAACAGA TGAGAAGAAA   
  
  
- GCTAAGTTAT CATTAAGAGA GTCTCTTCCG ATTTTACTGT ACCGTAAGGT AGCGGAGAGT ACTCCGAAAA   
  
  
- ACGTACACGA ATACATAGAA AGTAGAGAAT GAAACCACCC CACCGTAACC CCTCCTTTTC CCCCAAAGCT   
  
  
- AAGCTATCTA GCAGTGAAAG CCTTTACTGC AGAGACTGGT TACCATTCCC ATTCCAATTA TGTACACCTA   
  
  
- TAAAGGTCTC AATCAACACT AACAATAACA ACAACAACAT TGAAATACAG TGCCCCTTTT GTATTATTAA   
  
  
- TAGACAAATA GACAAACAAA CAATAAAAGT TCCCCTTCCC CTCACCCGCC TACGAATCAT ACATTAGTAA   
  
  
- AATATATCCC TTTTAAGTAT ATCGTACTTT CATTTTGAAA GTAACGGTCG TAAGATTCGA TCCCCATCCC   
  
  
- AACAGATGTG GAACTAGAGG GATCTTCGTA CCACTAGTAA GGAGTCTAAC ACAATATCTT ATTTCTTACA   
  
  
- CCAAGACGAC ATTACTCTGA ATACATAATA GTTTACTACT TAGACAAATC TTGAAGGACA AATAAAAAAG   
  
  
- ACGTTTTAAA ATTCTCTGAT CGTAGATAGC CCGAGCTGAG ATCTCCAAGA GAACACAATG GGAAGAGTTG   
  
  
- AGTCAAACTA ACACTATGTC AATGATATAA CTCACTATCA GCTCTCGTAG TGTTGGGCGA TTCCTCAGAT   
  
  
- AGTTGTTCTT CGGGAAGAAC ATTAAGAGGG GAACTTTGAT CGTCAATAGT AGTGAACAGT AGCTTATCGG   
  
  
- GAAAAACATT AAGATGGGAA CTTTGATCGT CAATAACAGT AGTCAAGTTA CACTCAGGGC AATCTATAAG   
  
  
- GGTAGTTCCG TATCTAATAC CTCACTCGTA CTTCTTGCGA AACGTGCTCG ATCTCTGACG AGATTAACCA   
  
  
- CATCTACCGC TCTTTCGTAG ACAACGATTA GTTGGATACC CCCCTTAAGT AATCAGGCCC TAGGGTTCAA   
  
  
- TCTCTAGCTT CAGTAACTCA CTTCTAGGTG TCCCAAGTGT AGGAGGAGTC GAACTAAGTA GTAGAAAAAG   
  
  
- TTCCCACTTT TCTAAGCCTC TACTTTCGGT CTCTCTCTCT TTACGGTGTT CCGTTACCTT CTTGGTCGCC   
  
  
- TTGATGATTC AAAGAGTGGT CCACTAAATT TCGTCAACGA TTAACTTACA CGCTCCCGAA ATAGTCTATT   
  
  
- GGCTTATCTA CTGAAACTCT CAAACCGATT TGCCGGTTCC TTTCTCCAGA GGTAGAGTCC ACTCGGACAG   
  
  
- GTTGCAGAGC CATGAACATA CTAGCTTCCC GAACACCGTT CCTTTGTCAG AAGCCCCTGA TTGTAGATAG   
  
  
- CCCGAGAATT CATGACATTT CTCGGAGAAC CTTTTATGAA CGAGAGGATG CCGTGAGTCA CCCAATGAGA   
  
  
- TAGCGTTCGG TATCGTCGTT CCGAACCACC CGGAGGATTT CACGCTGAAT GTCCGTAACT ACTAGGACAA   
  
  
- AGATTCATAT GAACACTACG ATCGAACCTC CGATAACCCT TTGCTAATCG CAGAGATAAA CTTTTCAAAT   
  
  
- TTTATGAGCA GCTCAAGTTA CGTAACGGGC AAATACCTGG ACTACAGTCC ACCCTTTACG ACCTACACTC   
  
  
- CGGGTCCCTC CGAAACCGGC AATTAACAGG TAATGTCGAG GTAGTGTGAG GACTGCTCTC ACAGCTACAC   
  
  
- TCGTTGGGAT CCCTATCCGA AGAGTCTTAC CACTTTAACG AGCCAGGATT CCATACGTGA AACCAACTCG   
  
  
- TTCTTAGTTT GTGGTTGTGA TGTGCAAAGA ACTGGGCCAA GTATCTCTGG GAACTGATGA TGAGCTAGTC   
  
  
- GGTACAAAC

+     GT1-motif

| Site Name | Organism | Position | Strand | Matrix score. | sequence | function |
| --- | --- | --- | --- | --- | --- | --- |
| GT1-motif | Avena sativa | 1108 | + | 7 | GGTTAAT | light responsive element |

>HU08G00019.1   
+ +Up\_Stream \_Len000AGTTGT AGCAAGCACA TTAAAGTTGA TTATCATTTT GTCCGTGAAA CGGTGAGCAA   
  
  
+ ACGTCATCTC CAGGTCAAAT TCATTCGTAC TCAATCACAA GTTGCTGACA TTTTTACCAA AGGCTAATCG   
  
  
+ TCGCAAAAGT TTCTTTAGTT TAAGGCCAAT CTCTCCGTTG TTTCACCCCA TACAGATTGA GGGGTTGTAA   
  
  
+ TAGTGTATAT ATATAGCCTT GTGTTAGCGG GGCTTGGGCA TTAGACTTTT ACTCTTGTAA ACCCTAATGT   
  
  
+ GTTATTATAA ATATAGATCT AGCCACCCAT GTTGAGGTAG GCACTCTAAT TCTGACACAG GTAGGATAAG   
  
  
+ TTTTCATAGT TTATGTTGTG AATCTTGTCT TACTTTGTGG TTCCTATTGA CAAAAGTTTG AATCCGAGCA   
  
  
+ TGAGAGTTCA TCAATCTCTT TTTCTTTCCT ATTTTATTTC TTGTGGACTT AATTGGATTT TCTGGTAGAG   
  
  
+ GAAAGTTCCT TGCTGGAATT TGGTCGAAAG TGATATACAT TTTGGGATTT AATTAGCCAT AAATTTGGTT   
  
  
+ TTACACCTTG TTAGTTTGGA GTTCTGGTGT GTTATTGTAT GTGTTCTGGT GGGTTTTTCT TTTAGCTAAA   
  
  
+ TGTTGGAGCA TTTTAAGGGA TCTAGATATC TGAAGAAGTG CTTGGGAAAT GAAGCATATT TCCCTTATAG   
  
  
+ TCTGACATAA CTTTAATGAA TTGATCATGA ACAAAGGGCA ATTAGTTCAG AAAGGTTCAA CTCCTCTAGG   
  
  
+ TGTTGTGCTA AAGAGATGTG AGTTAGTAAC AATTGCTTAC TAACTATATA GGTTGTTTTA TTACTTGTAG   
  
  
+ AGGTTTTTTT TTTGGGATAT CTTTTTGGGG TTTATAAATG AGAAGGTTGA TTTGTTGTCT ACTCTTCTTT   
  
  
+ CGATTCAATA GTAATTCTCT CAGAGAAGGC TAAAATGACA TGGCATTCCA TCGCCTCTCA TGAGGCTTTT   
  
  
+ TGCATGTGCT TATGTATCTT TCATCTCTTA CTTTGGTGGG GTGGCATTGG GGAGGAAAAG GGGGTTTCGA   
  
  
+ TTCGATAGAT CGTCACTTTC GGAAATGACG TCTCTGACCA ATGGTAAGGG TAAGGTTAAT ACATGTGGAT   
  
  
+ ATTTCCAGAG TTAGTTGTGA TTGTTATTGT TGTTGTTGTA ACTTTATGTC ACGGGGAAAA CATAATAATT   
  
  
+ ATCTGTTTAT CTGTTTGTTT GTTATTTTCA AGGGGAAGGG GAGTGGGCGG ATGCTTAGTA TGTAATCATT   
  
  
+ TTATATAGGG AAAATTCATA TAGCATGAAA GTAAAACTTT CATTGCCAGC ATTCTAAGCT AGGGGTAGGG   
  
  
+ TTGTCTACAC CTTGATCTCC CTAGAAGCAT GGTGATCATT CCTCAGATTG TGTTATAGAA TAAAGAATGT   
  
  
+ GGTTCTGCTG TAATGAGACT TATGTATTAT CAAATGATGA ATCTGTTTAG AACTTCCTGT TTATTTTTTC   
  
  
+ TGCAAAATTT TAAGAGACTA GCATCTATCG GGCTCGACTC TAGAGGTTCT CTTGTGTTAC CCTTCTCAAC   
  
  
+ TCAGTTTGAT TGTGATACAG TTACTATATT GAGTGATAGT CGAGAGCATC ACAACCCGCT AAGGAGTCTA   
  
  
+ TCAACAAGAA GCCCTTCTTG TAATTCTCCC CTTGAAACTA GCAGTTATCA TCACTTGTCA TCGAATAGCC   
  
  
+ CTTTTTGTAA TTCTACCCTT GAAACTAGCA GTTATTGTCA TCAGTTCAAT GTGAGTCCCG TTAGATATTC   
  
  
+ CCATCAAGGC ATAGATTATG GAGTGAGCAT GAAGAACGCT TTGCACGAGC TAGAGACTGC TCTAATTGGT   
  
  
+ GTAGATGGCG AGAAAGCATC TGTTGCTAAT CAACCTATGG GGGGAATTCA TTAGTCCGGG ATCCCAAGTT   
  
  
+ AGAGATCGAA GTCATTGAGT GAAGATCCAC AGGGTTCACA TCCTCCTCAG CTTGATTCAT CATCTTTTTC   
  
  
+ AAGGGTGAAA AGATTCGGAG ATGAAAGCCA GAGAGAGAGA AATGCCACAA GGCAATGGAA GAACCAGCGG   
  
  
+ AACTACTAAG TTTCTCACCA GGTGATTTAA AGCAGTTGCT AATTGAATGT GCGAGGGCTT TATCAGATAA   
  
  
+ CCGAATAGAT GACTTTGAGA GTTTGGCTAA ACGGCCAAGG AAAGAGGTCT CCATCTCAGG TGAGCCTGTC   
  
  
+ CAACGTCTCG GTACTTGTAT GATCGAAGGG CTTGTGGCAA GGAAACAGTC TTCGGGGACT AACATCTATC   
  
  
+ GGGCTCTTAA GTACTGTAAA GAGCCTCTTG GAAAATACTT GCTCTCCTAC GGCACTCAGT GGGTTACTCT   
  
  
+ ATCGCAAGCC ATAGCAGCAA GGCTTGGTGG GCCTCCTAAA GTGCGACTTA CAGGCATTGA TGATCCTGTT   
  
  
+ TCTAAGTATA CTTGTGATGC TAGCTTGGAG GCTATTGGGA AACGATTAGC GTCTCTATTT GAAAAGTTTA   
  
  
+ AAATACTCGT CGAGTTCAAT GCATTGCCCG TTTATGGACC TGATGTCAGG TGGGAAATGC TGGATGTGAG   
  
  
+ GCCCAGGGAG GCTTTGGCCG TTAATTGTCC ATTACAGCTC CATCACACTC CTGACGAGAG TGTCGATGTG   
  
  
+ AGCAACCCTA GGGATAGGCT TCTCAGAATG GTGAAATTGC TCGGTCCTAA GGTATGCACT TTGGTTGAGC   
  
  
+ AAGAATCAAA CACCAACACT ACACGTTTCT TGACCCGGTT CATAGAGACC CTTGACTACT ACTCGATCAG   
  
  
+ CCATGTTTG  

- +Up\_Stream \_Len000TCAACA TCGTTCGTGT AATTTCAACT AATAGTAAAA CAGGCACTTT GCCACTCGTT   
  
  
- TGCAGTAGAG GTCCAGTTTA AGTAAGCATG AGTTAGTGTT CAACGACTGT AAAAATGGTT TCCGATTAGC   
  
  
- AGCGTTTTCA AAGAAATCAA ATTCCGGTTA GAGAGGCAAC AAAGTGGGGT ATGTCTAACT CCCCAACATT   
  
  
- ATCACATATA TATATCGGAA CACAATCGCC CCGAACCCGT AATCTGAAAA TGAGAACATT TGGGATTACA   
  
  
- CAATAATATT TATATCTAGA TCGGTGGGTA CAACTCCATC CGTGAGATTA AGACTGTGTC CATCCTATTC   
  
  
- AAAAGTATCA AATACAACAC TTAGAACAGA ATGAAACACC AAGGATAACT GTTTTCAAAC TTAGGCTCGT   
  
  
- ACTCTCAAGT AGTTAGAGAA AAAGAAAGGA TAAAATAAAG AACACCTGAA TTAACCTAAA AGACCATCTC   
  
  
- CTTTCAAGGA ACGACCTTAA ACCAGCTTTC ACTATATGTA AAACCCTAAA TTAATCGGTA TTTAAACCAA   
  
  
- AATGTGGAAC AATCAAACCT CAAGACCACA CAATAACATA CACAAGACCA CCCAAAAAGA AAATCGATTT   
  
  
- ACAACCTCGT AAAATTCCCT AGATCTATAG ACTTCTTCAC GAACCCTTTA CTTCGTATAA AGGGAATATC   
  
  
- AGACTGTATT GAAATTACTT AACTAGTACT TGTTTCCCGT TAATCAAGTC TTTCCAAGTT GAGGAGATCC   
  
  
- ACAACACGAT TTCTCTACAC TCAATCATTG TTAACGAATG ATTGATATAT CCAACAAAAT AATGAACATC   
  
  
- TCCAAAAAAA AAACCCTATA GAAAAACCCC AAATATTTAC TCTTCCAACT AAACAACAGA TGAGAAGAAA   
  
  
- GCTAAGTTAT CATTAAGAGA GTCTCTTCCG ATTTTACTGT ACCGTAAGGT AGCGGAGAGT ACTCCGAAAA   
  
  
- ACGTACACGA ATACATAGAA AGTAGAGAAT GAAACCACCC CACCGTAACC CCTCCTTTTC CCCCAAAGCT   
  
  
- AAGCTATCTA GCAGTGAAAG CCTTTACTGC AGAGACTGGT TACCATTCCC ATTCCAATTA TGTACACCTA   
  
  
- TAAAGGTCTC AATCAACACT AACAATAACA ACAACAACAT TGAAATACAG TGCCCCTTTT GTATTATTAA   
  
  
- TAGACAAATA GACAAACAAA CAATAAAAGT TCCCCTTCCC CTCACCCGCC TACGAATCAT ACATTAGTAA   
  
  
- AATATATCCC TTTTAAGTAT ATCGTACTTT CATTTTGAAA GTAACGGTCG TAAGATTCGA TCCCCATCCC   
  
  
- AACAGATGTG GAACTAGAGG GATCTTCGTA CCACTAGTAA GGAGTCTAAC ACAATATCTT ATTTCTTACA   
  
  
- CCAAGACGAC ATTACTCTGA ATACATAATA GTTTACTACT TAGACAAATC TTGAAGGACA AATAAAAAAG   
  
  
- ACGTTTTAAA ATTCTCTGAT CGTAGATAGC CCGAGCTGAG ATCTCCAAGA GAACACAATG GGAAGAGTTG   
  
  
- AGTCAAACTA ACACTATGTC AATGATATAA CTCACTATCA GCTCTCGTAG TGTTGGGCGA TTCCTCAGAT   
  
  
- AGTTGTTCTT CGGGAAGAAC ATTAAGAGGG GAACTTTGAT CGTCAATAGT AGTGAACAGT AGCTTATCGG   
  
  
- GAAAAACATT AAGATGGGAA CTTTGATCGT CAATAACAGT AGTCAAGTTA CACTCAGGGC AATCTATAAG   
  
  
- GGTAGTTCCG TATCTAATAC CTCACTCGTA CTTCTTGCGA AACGTGCTCG ATCTCTGACG AGATTAACCA   
  
  
- CATCTACCGC TCTTTCGTAG ACAACGATTA GTTGGATACC CCCCTTAAGT AATCAGGCCC TAGGGTTCAA   
  
  
- TCTCTAGCTT CAGTAACTCA CTTCTAGGTG TCCCAAGTGT AGGAGGAGTC GAACTAAGTA GTAGAAAAAG   
  
  
- TTCCCACTTT TCTAAGCCTC TACTTTCGGT CTCTCTCTCT TTACGGTGTT CCGTTACCTT CTTGGTCGCC   
  
  
- TTGATGATTC AAAGAGTGGT CCACTAAATT TCGTCAACGA TTAACTTACA CGCTCCCGAA ATAGTCTATT   
  
  
- GGCTTATCTA CTGAAACTCT CAAACCGATT TGCCGGTTCC TTTCTCCAGA GGTAGAGTCC ACTCGGACAG   
  
  
- GTTGCAGAGC CATGAACATA CTAGCTTCCC GAACACCGTT CCTTTGTCAG AAGCCCCTGA TTGTAGATAG   
  
  
- CCCGAGAATT CATGACATTT CTCGGAGAAC CTTTTATGAA CGAGAGGATG CCGTGAGTCA CCCAATGAGA   
  
  
- TAGCGTTCGG TATCGTCGTT CCGAACCACC CGGAGGATTT CACGCTGAAT GTCCGTAACT ACTAGGACAA   
  
  
- AGATTCATAT GAACACTACG ATCGAACCTC CGATAACCCT TTGCTAATCG CAGAGATAAA CTTTTCAAAT   
  
  
- TTTATGAGCA GCTCAAGTTA CGTAACGGGC AAATACCTGG ACTACAGTCC ACCCTTTACG ACCTACACTC   
  
  
- CGGGTCCCTC CGAAACCGGC AATTAACAGG TAATGTCGAG GTAGTGTGAG GACTGCTCTC ACAGCTACAC   
  
  
- TCGTTGGGAT CCCTATCCGA AGAGTCTTAC CACTTTAACG AGCCAGGATT CCATACGTGA AACCAACTCG   
  
  
- TTCTTAGTTT GTGGTTGTGA TGTGCAAAGA ACTGGGCCAA GTATCTCTGG GAACTGATGA TGAGCTAGTC   
  
  
- GGTACAAAC

+     I-box

| Site Name | Organism | Position | Strand | Matrix score. | sequence | function |
| --- | --- | --- | --- | --- | --- | --- |
| I-box | Larix laricina | 162 | + | 9 | GTATAAGGCC | part of a light responsive element |

>HU08G00019.1   
+ +Up\_Stream \_Len000AGTTGT AGCAAGCACA TTAAAGTTGA TTATCATTTT GTCCGTGAAA CGGTGAGCAA   
  
  
+ ACGTCATCTC CAGGTCAAAT TCATTCGTAC TCAATCACAA GTTGCTGACA TTTTTACCAA AGGCTAATCG   
  
  
+ TCGCAAAAGT TTCTTTAGTT TAAGGCCAAT CTCTCCGTTG TTTCACCCCA TACAGATTGA GGGGTTGTAA   
  
  
+ TAGTGTATAT ATATAGCCTT GTGTTAGCGG GGCTTGGGCA TTAGACTTTT ACTCTTGTAA ACCCTAATGT   
  
  
+ GTTATTATAA ATATAGATCT AGCCACCCAT GTTGAGGTAG GCACTCTAAT TCTGACACAG GTAGGATAAG   
  
  
+ TTTTCATAGT TTATGTTGTG AATCTTGTCT TACTTTGTGG TTCCTATTGA CAAAAGTTTG AATCCGAGCA   
  
  
+ TGAGAGTTCA TCAATCTCTT TTTCTTTCCT ATTTTATTTC TTGTGGACTT AATTGGATTT TCTGGTAGAG   
  
  
+ GAAAGTTCCT TGCTGGAATT TGGTCGAAAG TGATATACAT TTTGGGATTT AATTAGCCAT AAATTTGGTT   
  
  
+ TTACACCTTG TTAGTTTGGA GTTCTGGTGT GTTATTGTAT GTGTTCTGGT GGGTTTTTCT TTTAGCTAAA   
  
  
+ TGTTGGAGCA TTTTAAGGGA TCTAGATATC TGAAGAAGTG CTTGGGAAAT GAAGCATATT TCCCTTATAG   
  
  
+ TCTGACATAA CTTTAATGAA TTGATCATGA ACAAAGGGCA ATTAGTTCAG AAAGGTTCAA CTCCTCTAGG   
  
  
+ TGTTGTGCTA AAGAGATGTG AGTTAGTAAC AATTGCTTAC TAACTATATA GGTTGTTTTA TTACTTGTAG   
  
  
+ AGGTTTTTTT TTTGGGATAT CTTTTTGGGG TTTATAAATG AGAAGGTTGA TTTGTTGTCT ACTCTTCTTT   
  
  
+ CGATTCAATA GTAATTCTCT CAGAGAAGGC TAAAATGACA TGGCATTCCA TCGCCTCTCA TGAGGCTTTT   
  
  
+ TGCATGTGCT TATGTATCTT TCATCTCTTA CTTTGGTGGG GTGGCATTGG GGAGGAAAAG GGGGTTTCGA   
  
  
+ TTCGATAGAT CGTCACTTTC GGAAATGACG TCTCTGACCA ATGGTAAGGG TAAGGTTAAT ACATGTGGAT   
  
  
+ ATTTCCAGAG TTAGTTGTGA TTGTTATTGT TGTTGTTGTA ACTTTATGTC ACGGGGAAAA CATAATAATT   
  
  
+ ATCTGTTTAT CTGTTTGTTT GTTATTTTCA AGGGGAAGGG GAGTGGGCGG ATGCTTAGTA TGTAATCATT   
  
  
+ TTATATAGGG AAAATTCATA TAGCATGAAA GTAAAACTTT CATTGCCAGC ATTCTAAGCT AGGGGTAGGG   
  
  
+ TTGTCTACAC CTTGATCTCC CTAGAAGCAT GGTGATCATT CCTCAGATTG TGTTATAGAA TAAAGAATGT   
  
  
+ GGTTCTGCTG TAATGAGACT TATGTATTAT CAAATGATGA ATCTGTTTAG AACTTCCTGT TTATTTTTTC   
  
  
+ TGCAAAATTT TAAGAGACTA GCATCTATCG GGCTCGACTC TAGAGGTTCT CTTGTGTTAC CCTTCTCAAC   
  
  
+ TCAGTTTGAT TGTGATACAG TTACTATATT GAGTGATAGT CGAGAGCATC ACAACCCGCT AAGGAGTCTA   
  
  
+ TCAACAAGAA GCCCTTCTTG TAATTCTCCC CTTGAAACTA GCAGTTATCA TCACTTGTCA TCGAATAGCC   
  
  
+ CTTTTTGTAA TTCTACCCTT GAAACTAGCA GTTATTGTCA TCAGTTCAAT GTGAGTCCCG TTAGATATTC   
  
  
+ CCATCAAGGC ATAGATTATG GAGTGAGCAT GAAGAACGCT TTGCACGAGC TAGAGACTGC TCTAATTGGT   
  
  
+ GTAGATGGCG AGAAAGCATC TGTTGCTAAT CAACCTATGG GGGGAATTCA TTAGTCCGGG ATCCCAAGTT   
  
  
+ AGAGATCGAA GTCATTGAGT GAAGATCCAC AGGGTTCACA TCCTCCTCAG CTTGATTCAT CATCTTTTTC   
  
  
+ AAGGGTGAAA AGATTCGGAG ATGAAAGCCA GAGAGAGAGA AATGCCACAA GGCAATGGAA GAACCAGCGG   
  
  
+ AACTACTAAG TTTCTCACCA GGTGATTTAA AGCAGTTGCT AATTGAATGT GCGAGGGCTT TATCAGATAA   
  
  
+ CCGAATAGAT GACTTTGAGA GTTTGGCTAA ACGGCCAAGG AAAGAGGTCT CCATCTCAGG TGAGCCTGTC   
  
  
+ CAACGTCTCG GTACTTGTAT GATCGAAGGG CTTGTGGCAA GGAAACAGTC TTCGGGGACT AACATCTATC   
  
  
+ GGGCTCTTAA GTACTGTAAA GAGCCTCTTG GAAAATACTT GCTCTCCTAC GGCACTCAGT GGGTTACTCT   
  
  
+ ATCGCAAGCC ATAGCAGCAA GGCTTGGTGG GCCTCCTAAA GTGCGACTTA CAGGCATTGA TGATCCTGTT   
  
  
+ TCTAAGTATA CTTGTGATGC TAGCTTGGAG GCTATTGGGA AACGATTAGC GTCTCTATTT GAAAAGTTTA   
  
  
+ AAATACTCGT CGAGTTCAAT GCATTGCCCG TTTATGGACC TGATGTCAGG TGGGAAATGC TGGATGTGAG   
  
  
+ GCCCAGGGAG GCTTTGGCCG TTAATTGTCC ATTACAGCTC CATCACACTC CTGACGAGAG TGTCGATGTG   
  
  
+ AGCAACCCTA GGGATAGGCT TCTCAGAATG GTGAAATTGC TCGGTCCTAA GGTATGCACT TTGGTTGAGC   
  
  
+ AAGAATCAAA CACCAACACT ACACGTTTCT TGACCCGGTT CATAGAGACC CTTGACTACT ACTCGATCAG   
  
  
+ CCATGTTTG  

- +Up\_Stream \_Len000TCAACA TCGTTCGTGT AATTTCAACT AATAGTAAAA CAGGCACTTT GCCACTCGTT   
  
  
- TGCAGTAGAG GTCCAGTTTA AGTAAGCATG AGTTAGTGTT CAACGACTGT AAAAATGGTT TCCGATTAGC   
  
  
- AGCGTTTTCA AAGAAATCAA ATTCCGGTTA GAGAGGCAAC AAAGTGGGGT ATGTCTAACT CCCCAACATT   
  
  
- ATCACATATA TATATCGGAA CACAATCGCC CCGAACCCGT AATCTGAAAA TGAGAACATT TGGGATTACA   
  
  
- CAATAATATT TATATCTAGA TCGGTGGGTA CAACTCCATC CGTGAGATTA AGACTGTGTC CATCCTATTC   
  
  
- AAAAGTATCA AATACAACAC TTAGAACAGA ATGAAACACC AAGGATAACT GTTTTCAAAC TTAGGCTCGT   
  
  
- ACTCTCAAGT AGTTAGAGAA AAAGAAAGGA TAAAATAAAG AACACCTGAA TTAACCTAAA AGACCATCTC   
  
  
- CTTTCAAGGA ACGACCTTAA ACCAGCTTTC ACTATATGTA AAACCCTAAA TTAATCGGTA TTTAAACCAA   
  
  
- AATGTGGAAC AATCAAACCT CAAGACCACA CAATAACATA CACAAGACCA CCCAAAAAGA AAATCGATTT   
  
  
- ACAACCTCGT AAAATTCCCT AGATCTATAG ACTTCTTCAC GAACCCTTTA CTTCGTATAA AGGGAATATC   
  
  
- AGACTGTATT GAAATTACTT AACTAGTACT TGTTTCCCGT TAATCAAGTC TTTCCAAGTT GAGGAGATCC   
  
  
- ACAACACGAT TTCTCTACAC TCAATCATTG TTAACGAATG ATTGATATAT CCAACAAAAT AATGAACATC   
  
  
- TCCAAAAAAA AAACCCTATA GAAAAACCCC AAATATTTAC TCTTCCAACT AAACAACAGA TGAGAAGAAA   
  
  
- GCTAAGTTAT CATTAAGAGA GTCTCTTCCG ATTTTACTGT ACCGTAAGGT AGCGGAGAGT ACTCCGAAAA   
  
  
- ACGTACACGA ATACATAGAA AGTAGAGAAT GAAACCACCC CACCGTAACC CCTCCTTTTC CCCCAAAGCT   
  
  
- AAGCTATCTA GCAGTGAAAG CCTTTACTGC AGAGACTGGT TACCATTCCC ATTCCAATTA TGTACACCTA   
  
  
- TAAAGGTCTC AATCAACACT AACAATAACA ACAACAACAT TGAAATACAG TGCCCCTTTT GTATTATTAA   
  
  
- TAGACAAATA GACAAACAAA CAATAAAAGT TCCCCTTCCC CTCACCCGCC TACGAATCAT ACATTAGTAA   
  
  
- AATATATCCC TTTTAAGTAT ATCGTACTTT CATTTTGAAA GTAACGGTCG TAAGATTCGA TCCCCATCCC   
  
  
- AACAGATGTG GAACTAGAGG GATCTTCGTA CCACTAGTAA GGAGTCTAAC ACAATATCTT ATTTCTTACA   
  
  
- CCAAGACGAC ATTACTCTGA ATACATAATA GTTTACTACT TAGACAAATC TTGAAGGACA AATAAAAAAG   
  
  
- ACGTTTTAAA ATTCTCTGAT CGTAGATAGC CCGAGCTGAG ATCTCCAAGA GAACACAATG GGAAGAGTTG   
  
  
- AGTCAAACTA ACACTATGTC AATGATATAA CTCACTATCA GCTCTCGTAG TGTTGGGCGA TTCCTCAGAT   
  
  
- AGTTGTTCTT CGGGAAGAAC ATTAAGAGGG GAACTTTGAT CGTCAATAGT AGTGAACAGT AGCTTATCGG   
  
  
- GAAAAACATT AAGATGGGAA CTTTGATCGT CAATAACAGT AGTCAAGTTA CACTCAGGGC AATCTATAAG   
  
  
- GGTAGTTCCG TATCTAATAC CTCACTCGTA CTTCTTGCGA AACGTGCTCG ATCTCTGACG AGATTAACCA   
  
  
- CATCTACCGC TCTTTCGTAG ACAACGATTA GTTGGATACC CCCCTTAAGT AATCAGGCCC TAGGGTTCAA   
  
  
- TCTCTAGCTT CAGTAACTCA CTTCTAGGTG TCCCAAGTGT AGGAGGAGTC GAACTAAGTA GTAGAAAAAG   
  
  
- TTCCCACTTT TCTAAGCCTC TACTTTCGGT CTCTCTCTCT TTACGGTGTT CCGTTACCTT CTTGGTCGCC   
  
  
- TTGATGATTC AAAGAGTGGT CCACTAAATT TCGTCAACGA TTAACTTACA CGCTCCCGAA ATAGTCTATT   
  
  
- GGCTTATCTA CTGAAACTCT CAAACCGATT TGCCGGTTCC TTTCTCCAGA GGTAGAGTCC ACTCGGACAG   
  
  
- GTTGCAGAGC CATGAACATA CTAGCTTCCC GAACACCGTT CCTTTGTCAG AAGCCCCTGA TTGTAGATAG   
  
  
- CCCGAGAATT CATGACATTT CTCGGAGAAC CTTTTATGAA CGAGAGGATG CCGTGAGTCA CCCAATGAGA   
  
  
- TAGCGTTCGG TATCGTCGTT CCGAACCACC CGGAGGATTT CACGCTGAAT GTCCGTAACT ACTAGGACAA   
  
  
- AGATTCATAT GAACACTACG ATCGAACCTC CGATAACCCT TTGCTAATCG CAGAGATAAA CTTTTCAAAT   
  
  
- TTTATGAGCA GCTCAAGTTA CGTAACGGGC AAATACCTGG ACTACAGTCC ACCCTTTACG ACCTACACTC   
  
  
- CGGGTCCCTC CGAAACCGGC AATTAACAGG TAATGTCGAG GTAGTGTGAG GACTGCTCTC ACAGCTACAC   
  
  
- TCGTTGGGAT CCCTATCCGA AGAGTCTTAC CACTTTAACG AGCCAGGATT CCATACGTGA AACCAACTCG   
  
  
- TTCTTAGTTT GTGGTTGTGA TGTGCAAAGA ACTGGGCCAA GTATCTCTGG GAACTGATGA TGAGCTAGTC   
  
  
- GGTACAAAC

+     LAMP-element

| Site Name | Organism | Position | Strand | Matrix score. | sequence | function |
| --- | --- | --- | --- | --- | --- | --- |
| LAMP-element | Pisum sativum | 2092 | + | 8 | CTTTATCA | part of a light responsive element |

>HU08G00019.1   
+ +Up\_Stream \_Len000AGTTGT AGCAAGCACA TTAAAGTTGA TTATCATTTT GTCCGTGAAA CGGTGAGCAA   
  
  
+ ACGTCATCTC CAGGTCAAAT TCATTCGTAC TCAATCACAA GTTGCTGACA TTTTTACCAA AGGCTAATCG   
  
  
+ TCGCAAAAGT TTCTTTAGTT TAAGGCCAAT CTCTCCGTTG TTTCACCCCA TACAGATTGA GGGGTTGTAA   
  
  
+ TAGTGTATAT ATATAGCCTT GTGTTAGCGG GGCTTGGGCA TTAGACTTTT ACTCTTGTAA ACCCTAATGT   
  
  
+ GTTATTATAA ATATAGATCT AGCCACCCAT GTTGAGGTAG GCACTCTAAT TCTGACACAG GTAGGATAAG   
  
  
+ TTTTCATAGT TTATGTTGTG AATCTTGTCT TACTTTGTGG TTCCTATTGA CAAAAGTTTG AATCCGAGCA   
  
  
+ TGAGAGTTCA TCAATCTCTT TTTCTTTCCT ATTTTATTTC TTGTGGACTT AATTGGATTT TCTGGTAGAG   
  
  
+ GAAAGTTCCT TGCTGGAATT TGGTCGAAAG TGATATACAT TTTGGGATTT AATTAGCCAT AAATTTGGTT   
  
  
+ TTACACCTTG TTAGTTTGGA GTTCTGGTGT GTTATTGTAT GTGTTCTGGT GGGTTTTTCT TTTAGCTAAA   
  
  
+ TGTTGGAGCA TTTTAAGGGA TCTAGATATC TGAAGAAGTG CTTGGGAAAT GAAGCATATT TCCCTTATAG   
  
  
+ TCTGACATAA CTTTAATGAA TTGATCATGA ACAAAGGGCA ATTAGTTCAG AAAGGTTCAA CTCCTCTAGG   
  
  
+ TGTTGTGCTA AAGAGATGTG AGTTAGTAAC AATTGCTTAC TAACTATATA GGTTGTTTTA TTACTTGTAG   
  
  
+ AGGTTTTTTT TTTGGGATAT CTTTTTGGGG TTTATAAATG AGAAGGTTGA TTTGTTGTCT ACTCTTCTTT   
  
  
+ CGATTCAATA GTAATTCTCT CAGAGAAGGC TAAAATGACA TGGCATTCCA TCGCCTCTCA TGAGGCTTTT   
  
  
+ TGCATGTGCT TATGTATCTT TCATCTCTTA CTTTGGTGGG GTGGCATTGG GGAGGAAAAG GGGGTTTCGA   
  
  
+ TTCGATAGAT CGTCACTTTC GGAAATGACG TCTCTGACCA ATGGTAAGGG TAAGGTTAAT ACATGTGGAT   
  
  
+ ATTTCCAGAG TTAGTTGTGA TTGTTATTGT TGTTGTTGTA ACTTTATGTC ACGGGGAAAA CATAATAATT   
  
  
+ ATCTGTTTAT CTGTTTGTTT GTTATTTTCA AGGGGAAGGG GAGTGGGCGG ATGCTTAGTA TGTAATCATT   
  
  
+ TTATATAGGG AAAATTCATA TAGCATGAAA GTAAAACTTT CATTGCCAGC ATTCTAAGCT AGGGGTAGGG   
  
  
+ TTGTCTACAC CTTGATCTCC CTAGAAGCAT GGTGATCATT CCTCAGATTG TGTTATAGAA TAAAGAATGT   
  
  
+ GGTTCTGCTG TAATGAGACT TATGTATTAT CAAATGATGA ATCTGTTTAG AACTTCCTGT TTATTTTTTC   
  
  
+ TGCAAAATTT TAAGAGACTA GCATCTATCG GGCTCGACTC TAGAGGTTCT CTTGTGTTAC CCTTCTCAAC   
  
  
+ TCAGTTTGAT TGTGATACAG TTACTATATT GAGTGATAGT CGAGAGCATC ACAACCCGCT AAGGAGTCTA   
  
  
+ TCAACAAGAA GCCCTTCTTG TAATTCTCCC CTTGAAACTA GCAGTTATCA TCACTTGTCA TCGAATAGCC   
  
  
+ CTTTTTGTAA TTCTACCCTT GAAACTAGCA GTTATTGTCA TCAGTTCAAT GTGAGTCCCG TTAGATATTC   
  
  
+ CCATCAAGGC ATAGATTATG GAGTGAGCAT GAAGAACGCT TTGCACGAGC TAGAGACTGC TCTAATTGGT   
  
  
+ GTAGATGGCG AGAAAGCATC TGTTGCTAAT CAACCTATGG GGGGAATTCA TTAGTCCGGG ATCCCAAGTT   
  
  
+ AGAGATCGAA GTCATTGAGT GAAGATCCAC AGGGTTCACA TCCTCCTCAG CTTGATTCAT CATCTTTTTC   
  
  
+ AAGGGTGAAA AGATTCGGAG ATGAAAGCCA GAGAGAGAGA AATGCCACAA GGCAATGGAA GAACCAGCGG   
  
  
+ AACTACTAAG TTTCTCACCA GGTGATTTAA AGCAGTTGCT AATTGAATGT GCGAGGGCTT TATCAGATAA   
  
  
+ CCGAATAGAT GACTTTGAGA GTTTGGCTAA ACGGCCAAGG AAAGAGGTCT CCATCTCAGG TGAGCCTGTC   
  
  
+ CAACGTCTCG GTACTTGTAT GATCGAAGGG CTTGTGGCAA GGAAACAGTC TTCGGGGACT AACATCTATC   
  
  
+ GGGCTCTTAA GTACTGTAAA GAGCCTCTTG GAAAATACTT GCTCTCCTAC GGCACTCAGT GGGTTACTCT   
  
  
+ ATCGCAAGCC ATAGCAGCAA GGCTTGGTGG GCCTCCTAAA GTGCGACTTA CAGGCATTGA TGATCCTGTT   
  
  
+ TCTAAGTATA CTTGTGATGC TAGCTTGGAG GCTATTGGGA AACGATTAGC GTCTCTATTT GAAAAGTTTA   
  
  
+ AAATACTCGT CGAGTTCAAT GCATTGCCCG TTTATGGACC TGATGTCAGG TGGGAAATGC TGGATGTGAG   
  
  
+ GCCCAGGGAG GCTTTGGCCG TTAATTGTCC ATTACAGCTC CATCACACTC CTGACGAGAG TGTCGATGTG   
  
  
+ AGCAACCCTA GGGATAGGCT TCTCAGAATG GTGAAATTGC TCGGTCCTAA GGTATGCACT TTGGTTGAGC   
  
  
+ AAGAATCAAA CACCAACACT ACACGTTTCT TGACCCGGTT CATAGAGACC CTTGACTACT ACTCGATCAG   
  
  
+ CCATGTTTG  

- +Up\_Stream \_Len000TCAACA TCGTTCGTGT AATTTCAACT AATAGTAAAA CAGGCACTTT GCCACTCGTT   
  
  
- TGCAGTAGAG GTCCAGTTTA AGTAAGCATG AGTTAGTGTT CAACGACTGT AAAAATGGTT TCCGATTAGC   
  
  
- AGCGTTTTCA AAGAAATCAA ATTCCGGTTA GAGAGGCAAC AAAGTGGGGT ATGTCTAACT CCCCAACATT   
  
  
- ATCACATATA TATATCGGAA CACAATCGCC CCGAACCCGT AATCTGAAAA TGAGAACATT TGGGATTACA   
  
  
- CAATAATATT TATATCTAGA TCGGTGGGTA CAACTCCATC CGTGAGATTA AGACTGTGTC CATCCTATTC   
  
  
- AAAAGTATCA AATACAACAC TTAGAACAGA ATGAAACACC AAGGATAACT GTTTTCAAAC TTAGGCTCGT   
  
  
- ACTCTCAAGT AGTTAGAGAA AAAGAAAGGA TAAAATAAAG AACACCTGAA TTAACCTAAA AGACCATCTC   
  
  
- CTTTCAAGGA ACGACCTTAA ACCAGCTTTC ACTATATGTA AAACCCTAAA TTAATCGGTA TTTAAACCAA   
  
  
- AATGTGGAAC AATCAAACCT CAAGACCACA CAATAACATA CACAAGACCA CCCAAAAAGA AAATCGATTT   
  
  
- ACAACCTCGT AAAATTCCCT AGATCTATAG ACTTCTTCAC GAACCCTTTA CTTCGTATAA AGGGAATATC   
  
  
- AGACTGTATT GAAATTACTT AACTAGTACT TGTTTCCCGT TAATCAAGTC TTTCCAAGTT GAGGAGATCC   
  
  
- ACAACACGAT TTCTCTACAC TCAATCATTG TTAACGAATG ATTGATATAT CCAACAAAAT AATGAACATC   
  
  
- TCCAAAAAAA AAACCCTATA GAAAAACCCC AAATATTTAC TCTTCCAACT AAACAACAGA TGAGAAGAAA   
  
  
- GCTAAGTTAT CATTAAGAGA GTCTCTTCCG ATTTTACTGT ACCGTAAGGT AGCGGAGAGT ACTCCGAAAA   
  
  
- ACGTACACGA ATACATAGAA AGTAGAGAAT GAAACCACCC CACCGTAACC CCTCCTTTTC CCCCAAAGCT   
  
  
- AAGCTATCTA GCAGTGAAAG CCTTTACTGC AGAGACTGGT TACCATTCCC ATTCCAATTA TGTACACCTA   
  
  
- TAAAGGTCTC AATCAACACT AACAATAACA ACAACAACAT TGAAATACAG TGCCCCTTTT GTATTATTAA   
  
  
- TAGACAAATA GACAAACAAA CAATAAAAGT TCCCCTTCCC CTCACCCGCC TACGAATCAT ACATTAGTAA   
  
  
- AATATATCCC TTTTAAGTAT ATCGTACTTT CATTTTGAAA GTAACGGTCG TAAGATTCGA TCCCCATCCC   
  
  
- AACAGATGTG GAACTAGAGG GATCTTCGTA CCACTAGTAA GGAGTCTAAC ACAATATCTT ATTTCTTACA   
  
  
- CCAAGACGAC ATTACTCTGA ATACATAATA GTTTACTACT TAGACAAATC TTGAAGGACA AATAAAAAAG   
  
  
- ACGTTTTAAA ATTCTCTGAT CGTAGATAGC CCGAGCTGAG ATCTCCAAGA GAACACAATG GGAAGAGTTG   
  
  
- AGTCAAACTA ACACTATGTC AATGATATAA CTCACTATCA GCTCTCGTAG TGTTGGGCGA TTCCTCAGAT   
  
  
- AGTTGTTCTT CGGGAAGAAC ATTAAGAGGG GAACTTTGAT CGTCAATAGT AGTGAACAGT AGCTTATCGG   
  
  
- GAAAAACATT AAGATGGGAA CTTTGATCGT CAATAACAGT AGTCAAGTTA CACTCAGGGC AATCTATAAG   
  
  
- GGTAGTTCCG TATCTAATAC CTCACTCGTA CTTCTTGCGA AACGTGCTCG ATCTCTGACG AGATTAACCA   
  
  
- CATCTACCGC TCTTTCGTAG ACAACGATTA GTTGGATACC CCCCTTAAGT AATCAGGCCC TAGGGTTCAA   
  
  
- TCTCTAGCTT CAGTAACTCA CTTCTAGGTG TCCCAAGTGT AGGAGGAGTC GAACTAAGTA GTAGAAAAAG   
  
  
- TTCCCACTTT TCTAAGCCTC TACTTTCGGT CTCTCTCTCT TTACGGTGTT CCGTTACCTT CTTGGTCGCC   
  
  
- TTGATGATTC AAAGAGTGGT CCACTAAATT TCGTCAACGA TTAACTTACA CGCTCCCGAA ATAGTCTATT   
  
  
- GGCTTATCTA CTGAAACTCT CAAACCGATT TGCCGGTTCC TTTCTCCAGA GGTAGAGTCC ACTCGGACAG   
  
  
- GTTGCAGAGC CATGAACATA CTAGCTTCCC GAACACCGTT CCTTTGTCAG AAGCCCCTGA TTGTAGATAG   
  
  
- CCCGAGAATT CATGACATTT CTCGGAGAAC CTTTTATGAA CGAGAGGATG CCGTGAGTCA CCCAATGAGA   
  
  
- TAGCGTTCGG TATCGTCGTT CCGAACCACC CGGAGGATTT CACGCTGAAT GTCCGTAACT ACTAGGACAA   
  
  
- AGATTCATAT GAACACTACG ATCGAACCTC CGATAACCCT TTGCTAATCG CAGAGATAAA CTTTTCAAAT   
  
  
- TTTATGAGCA GCTCAAGTTA CGTAACGGGC AAATACCTGG ACTACAGTCC ACCCTTTACG ACCTACACTC   
  
  
- CGGGTCCCTC CGAAACCGGC AATTAACAGG TAATGTCGAG GTAGTGTGAG GACTGCTCTC ACAGCTACAC   
  
  
- TCGTTGGGAT CCCTATCCGA AGAGTCTTAC CACTTTAACG AGCCAGGATT CCATACGTGA AACCAACTCG   
  
  
- TTCTTAGTTT GTGGTTGTGA TGTGCAAAGA ACTGGGCCAA GTATCTCTGG GAACTGATGA TGAGCTAGTC   
  
  
- GGTACAAAC

+     LTR

| Site Name | Organism | Position | Strand | Matrix score. | sequence | function |
| --- | --- | --- | --- | --- | --- | --- |
| LTR | Hordeum vulgare | 1071 | - | 6 | CCGAAA | cis-acting element involved in low-temperature responsiveness |

>HU08G00019.1   
+ +Up\_Stream \_Len000AGTTGT AGCAAGCACA TTAAAGTTGA TTATCATTTT GTCCGTGAAA CGGTGAGCAA   
  
  
+ ACGTCATCTC CAGGTCAAAT TCATTCGTAC TCAATCACAA GTTGCTGACA TTTTTACCAA AGGCTAATCG   
  
  
+ TCGCAAAAGT TTCTTTAGTT TAAGGCCAAT CTCTCCGTTG TTTCACCCCA TACAGATTGA GGGGTTGTAA   
  
  
+ TAGTGTATAT ATATAGCCTT GTGTTAGCGG GGCTTGGGCA TTAGACTTTT ACTCTTGTAA ACCCTAATGT   
  
  
+ GTTATTATAA ATATAGATCT AGCCACCCAT GTTGAGGTAG GCACTCTAAT TCTGACACAG GTAGGATAAG   
  
  
+ TTTTCATAGT TTATGTTGTG AATCTTGTCT TACTTTGTGG TTCCTATTGA CAAAAGTTTG AATCCGAGCA   
  
  
+ TGAGAGTTCA TCAATCTCTT TTTCTTTCCT ATTTTATTTC TTGTGGACTT AATTGGATTT TCTGGTAGAG   
  
  
+ GAAAGTTCCT TGCTGGAATT TGGTCGAAAG TGATATACAT TTTGGGATTT AATTAGCCAT AAATTTGGTT   
  
  
+ TTACACCTTG TTAGTTTGGA GTTCTGGTGT GTTATTGTAT GTGTTCTGGT GGGTTTTTCT TTTAGCTAAA   
  
  
+ TGTTGGAGCA TTTTAAGGGA TCTAGATATC TGAAGAAGTG CTTGGGAAAT GAAGCATATT TCCCTTATAG   
  
  
+ TCTGACATAA CTTTAATGAA TTGATCATGA ACAAAGGGCA ATTAGTTCAG AAAGGTTCAA CTCCTCTAGG   
  
  
+ TGTTGTGCTA AAGAGATGTG AGTTAGTAAC AATTGCTTAC TAACTATATA GGTTGTTTTA TTACTTGTAG   
  
  
+ AGGTTTTTTT TTTGGGATAT CTTTTTGGGG TTTATAAATG AGAAGGTTGA TTTGTTGTCT ACTCTTCTTT   
  
  
+ CGATTCAATA GTAATTCTCT CAGAGAAGGC TAAAATGACA TGGCATTCCA TCGCCTCTCA TGAGGCTTTT   
  
  
+ TGCATGTGCT TATGTATCTT TCATCTCTTA CTTTGGTGGG GTGGCATTGG GGAGGAAAAG GGGGTTTCGA   
  
  
+ TTCGATAGAT CGTCACTTTC GGAAATGACG TCTCTGACCA ATGGTAAGGG TAAGGTTAAT ACATGTGGAT   
  
  
+ ATTTCCAGAG TTAGTTGTGA TTGTTATTGT TGTTGTTGTA ACTTTATGTC ACGGGGAAAA CATAATAATT   
  
  
+ ATCTGTTTAT CTGTTTGTTT GTTATTTTCA AGGGGAAGGG GAGTGGGCGG ATGCTTAGTA TGTAATCATT   
  
  
+ TTATATAGGG AAAATTCATA TAGCATGAAA GTAAAACTTT CATTGCCAGC ATTCTAAGCT AGGGGTAGGG   
  
  
+ TTGTCTACAC CTTGATCTCC CTAGAAGCAT GGTGATCATT CCTCAGATTG TGTTATAGAA TAAAGAATGT   
  
  
+ GGTTCTGCTG TAATGAGACT TATGTATTAT CAAATGATGA ATCTGTTTAG AACTTCCTGT TTATTTTTTC   
  
  
+ TGCAAAATTT TAAGAGACTA GCATCTATCG GGCTCGACTC TAGAGGTTCT CTTGTGTTAC CCTTCTCAAC   
  
  
+ TCAGTTTGAT TGTGATACAG TTACTATATT GAGTGATAGT CGAGAGCATC ACAACCCGCT AAGGAGTCTA   
  
  
+ TCAACAAGAA GCCCTTCTTG TAATTCTCCC CTTGAAACTA GCAGTTATCA TCACTTGTCA TCGAATAGCC   
  
  
+ CTTTTTGTAA TTCTACCCTT GAAACTAGCA GTTATTGTCA TCAGTTCAAT GTGAGTCCCG TTAGATATTC   
  
  
+ CCATCAAGGC ATAGATTATG GAGTGAGCAT GAAGAACGCT TTGCACGAGC TAGAGACTGC TCTAATTGGT   
  
  
+ GTAGATGGCG AGAAAGCATC TGTTGCTAAT CAACCTATGG GGGGAATTCA TTAGTCCGGG ATCCCAAGTT   
  
  
+ AGAGATCGAA GTCATTGAGT GAAGATCCAC AGGGTTCACA TCCTCCTCAG CTTGATTCAT CATCTTTTTC   
  
  
+ AAGGGTGAAA AGATTCGGAG ATGAAAGCCA GAGAGAGAGA AATGCCACAA GGCAATGGAA GAACCAGCGG   
  
  
+ AACTACTAAG TTTCTCACCA GGTGATTTAA AGCAGTTGCT AATTGAATGT GCGAGGGCTT TATCAGATAA   
  
  
+ CCGAATAGAT GACTTTGAGA GTTTGGCTAA ACGGCCAAGG AAAGAGGTCT CCATCTCAGG TGAGCCTGTC   
  
  
+ CAACGTCTCG GTACTTGTAT GATCGAAGGG CTTGTGGCAA GGAAACAGTC TTCGGGGACT AACATCTATC   
  
  
+ GGGCTCTTAA GTACTGTAAA GAGCCTCTTG GAAAATACTT GCTCTCCTAC GGCACTCAGT GGGTTACTCT   
  
  
+ ATCGCAAGCC ATAGCAGCAA GGCTTGGTGG GCCTCCTAAA GTGCGACTTA CAGGCATTGA TGATCCTGTT   
  
  
+ TCTAAGTATA CTTGTGATGC TAGCTTGGAG GCTATTGGGA AACGATTAGC GTCTCTATTT GAAAAGTTTA   
  
  
+ AAATACTCGT CGAGTTCAAT GCATTGCCCG TTTATGGACC TGATGTCAGG TGGGAAATGC TGGATGTGAG   
  
  
+ GCCCAGGGAG GCTTTGGCCG TTAATTGTCC ATTACAGCTC CATCACACTC CTGACGAGAG TGTCGATGTG   
  
  
+ AGCAACCCTA GGGATAGGCT TCTCAGAATG GTGAAATTGC TCGGTCCTAA GGTATGCACT TTGGTTGAGC   
  
  
+ AAGAATCAAA CACCAACACT ACACGTTTCT TGACCCGGTT CATAGAGACC CTTGACTACT ACTCGATCAG   
  
  
+ CCATGTTTG  

- +Up\_Stream \_Len000TCAACA TCGTTCGTGT AATTTCAACT AATAGTAAAA CAGGCACTTT GCCACTCGTT   
  
  
- TGCAGTAGAG GTCCAGTTTA AGTAAGCATG AGTTAGTGTT CAACGACTGT AAAAATGGTT TCCGATTAGC   
  
  
- AGCGTTTTCA AAGAAATCAA ATTCCGGTTA GAGAGGCAAC AAAGTGGGGT ATGTCTAACT CCCCAACATT   
  
  
- ATCACATATA TATATCGGAA CACAATCGCC CCGAACCCGT AATCTGAAAA TGAGAACATT TGGGATTACA   
  
  
- CAATAATATT TATATCTAGA TCGGTGGGTA CAACTCCATC CGTGAGATTA AGACTGTGTC CATCCTATTC   
  
  
- AAAAGTATCA AATACAACAC TTAGAACAGA ATGAAACACC AAGGATAACT GTTTTCAAAC TTAGGCTCGT   
  
  
- ACTCTCAAGT AGTTAGAGAA AAAGAAAGGA TAAAATAAAG AACACCTGAA TTAACCTAAA AGACCATCTC   
  
  
- CTTTCAAGGA ACGACCTTAA ACCAGCTTTC ACTATATGTA AAACCCTAAA TTAATCGGTA TTTAAACCAA   
  
  
- AATGTGGAAC AATCAAACCT CAAGACCACA CAATAACATA CACAAGACCA CCCAAAAAGA AAATCGATTT   
  
  
- ACAACCTCGT AAAATTCCCT AGATCTATAG ACTTCTTCAC GAACCCTTTA CTTCGTATAA AGGGAATATC   
  
  
- AGACTGTATT GAAATTACTT AACTAGTACT TGTTTCCCGT TAATCAAGTC TTTCCAAGTT GAGGAGATCC   
  
  
- ACAACACGAT TTCTCTACAC TCAATCATTG TTAACGAATG ATTGATATAT CCAACAAAAT AATGAACATC   
  
  
- TCCAAAAAAA AAACCCTATA GAAAAACCCC AAATATTTAC TCTTCCAACT AAACAACAGA TGAGAAGAAA   
  
  
- GCTAAGTTAT CATTAAGAGA GTCTCTTCCG ATTTTACTGT ACCGTAAGGT AGCGGAGAGT ACTCCGAAAA   
  
  
- ACGTACACGA ATACATAGAA AGTAGAGAAT GAAACCACCC CACCGTAACC CCTCCTTTTC CCCCAAAGCT   
  
  
- AAGCTATCTA GCAGTGAAAG CCTTTACTGC AGAGACTGGT TACCATTCCC ATTCCAATTA TGTACACCTA   
  
  
- TAAAGGTCTC AATCAACACT AACAATAACA ACAACAACAT TGAAATACAG TGCCCCTTTT GTATTATTAA   
  
  
- TAGACAAATA GACAAACAAA CAATAAAAGT TCCCCTTCCC CTCACCCGCC TACGAATCAT ACATTAGTAA   
  
  
- AATATATCCC TTTTAAGTAT ATCGTACTTT CATTTTGAAA GTAACGGTCG TAAGATTCGA TCCCCATCCC   
  
  
- AACAGATGTG GAACTAGAGG GATCTTCGTA CCACTAGTAA GGAGTCTAAC ACAATATCTT ATTTCTTACA   
  
  
- CCAAGACGAC ATTACTCTGA ATACATAATA GTTTACTACT TAGACAAATC TTGAAGGACA AATAAAAAAG   
  
  
- ACGTTTTAAA ATTCTCTGAT CGTAGATAGC CCGAGCTGAG ATCTCCAAGA GAACACAATG GGAAGAGTTG   
  
  
- AGTCAAACTA ACACTATGTC AATGATATAA CTCACTATCA GCTCTCGTAG TGTTGGGCGA TTCCTCAGAT   
  
  
- AGTTGTTCTT CGGGAAGAAC ATTAAGAGGG GAACTTTGAT CGTCAATAGT AGTGAACAGT AGCTTATCGG   
  
  
- GAAAAACATT AAGATGGGAA CTTTGATCGT CAATAACAGT AGTCAAGTTA CACTCAGGGC AATCTATAAG   
  
  
- GGTAGTTCCG TATCTAATAC CTCACTCGTA CTTCTTGCGA AACGTGCTCG ATCTCTGACG AGATTAACCA   
  
  
- CATCTACCGC TCTTTCGTAG ACAACGATTA GTTGGATACC CCCCTTAAGT AATCAGGCCC TAGGGTTCAA   
  
  
- TCTCTAGCTT CAGTAACTCA CTTCTAGGTG TCCCAAGTGT AGGAGGAGTC GAACTAAGTA GTAGAAAAAG   
  
  
- TTCCCACTTT TCTAAGCCTC TACTTTCGGT CTCTCTCTCT TTACGGTGTT CCGTTACCTT CTTGGTCGCC   
  
  
- TTGATGATTC AAAGAGTGGT CCACTAAATT TCGTCAACGA TTAACTTACA CGCTCCCGAA ATAGTCTATT   
  
  
- GGCTTATCTA CTGAAACTCT CAAACCGATT TGCCGGTTCC TTTCTCCAGA GGTAGAGTCC ACTCGGACAG   
  
  
- GTTGCAGAGC CATGAACATA CTAGCTTCCC GAACACCGTT CCTTTGTCAG AAGCCCCTGA TTGTAGATAG   
  
  
- CCCGAGAATT CATGACATTT CTCGGAGAAC CTTTTATGAA CGAGAGGATG CCGTGAGTCA CCCAATGAGA   
  
  
- TAGCGTTCGG TATCGTCGTT CCGAACCACC CGGAGGATTT CACGCTGAAT GTCCGTAACT ACTAGGACAA   
  
  
- AGATTCATAT GAACACTACG ATCGAACCTC CGATAACCCT TTGCTAATCG CAGAGATAAA CTTTTCAAAT   
  
  
- TTTATGAGCA GCTCAAGTTA CGTAACGGGC AAATACCTGG ACTACAGTCC ACCCTTTACG ACCTACACTC   
  
  
- CGGGTCCCTC CGAAACCGGC AATTAACAGG TAATGTCGAG GTAGTGTGAG GACTGCTCTC ACAGCTACAC   
  
  
- TCGTTGGGAT CCCTATCCGA AGAGTCTTAC CACTTTAACG AGCCAGGATT CCATACGTGA AACCAACTCG   
  
  
- TTCTTAGTTT GTGGTTGTGA TGTGCAAAGA ACTGGGCCAA GTATCTCTGG GAACTGATGA TGAGCTAGTC   
  
  
- GGTACAAAC

+     MBS

| Site Name | Organism | Position | Strand | Matrix score. | sequence | function |
| --- | --- | --- | --- | --- | --- | --- |
| MBS | Arabidopsis thaliana | 2067 | - | 6 | CAACTG | MYB binding site involved in drought-inducibility |

>HU08G00019.1   
+ +Up\_Stream \_Len000AGTTGT AGCAAGCACA TTAAAGTTGA TTATCATTTT GTCCGTGAAA CGGTGAGCAA   
  
  
+ ACGTCATCTC CAGGTCAAAT TCATTCGTAC TCAATCACAA GTTGCTGACA TTTTTACCAA AGGCTAATCG   
  
  
+ TCGCAAAAGT TTCTTTAGTT TAAGGCCAAT CTCTCCGTTG TTTCACCCCA TACAGATTGA GGGGTTGTAA   
  
  
+ TAGTGTATAT ATATAGCCTT GTGTTAGCGG GGCTTGGGCA TTAGACTTTT ACTCTTGTAA ACCCTAATGT   
  
  
+ GTTATTATAA ATATAGATCT AGCCACCCAT GTTGAGGTAG GCACTCTAAT TCTGACACAG GTAGGATAAG   
  
  
+ TTTTCATAGT TTATGTTGTG AATCTTGTCT TACTTTGTGG TTCCTATTGA CAAAAGTTTG AATCCGAGCA   
  
  
+ TGAGAGTTCA TCAATCTCTT TTTCTTTCCT ATTTTATTTC TTGTGGACTT AATTGGATTT TCTGGTAGAG   
  
  
+ GAAAGTTCCT TGCTGGAATT TGGTCGAAAG TGATATACAT TTTGGGATTT AATTAGCCAT AAATTTGGTT   
  
  
+ TTACACCTTG TTAGTTTGGA GTTCTGGTGT GTTATTGTAT GTGTTCTGGT GGGTTTTTCT TTTAGCTAAA   
  
  
+ TGTTGGAGCA TTTTAAGGGA TCTAGATATC TGAAGAAGTG CTTGGGAAAT GAAGCATATT TCCCTTATAG   
  
  
+ TCTGACATAA CTTTAATGAA TTGATCATGA ACAAAGGGCA ATTAGTTCAG AAAGGTTCAA CTCCTCTAGG   
  
  
+ TGTTGTGCTA AAGAGATGTG AGTTAGTAAC AATTGCTTAC TAACTATATA GGTTGTTTTA TTACTTGTAG   
  
  
+ AGGTTTTTTT TTTGGGATAT CTTTTTGGGG TTTATAAATG AGAAGGTTGA TTTGTTGTCT ACTCTTCTTT   
  
  
+ CGATTCAATA GTAATTCTCT CAGAGAAGGC TAAAATGACA TGGCATTCCA TCGCCTCTCA TGAGGCTTTT   
  
  
+ TGCATGTGCT TATGTATCTT TCATCTCTTA CTTTGGTGGG GTGGCATTGG GGAGGAAAAG GGGGTTTCGA   
  
  
+ TTCGATAGAT CGTCACTTTC GGAAATGACG TCTCTGACCA ATGGTAAGGG TAAGGTTAAT ACATGTGGAT   
  
  
+ ATTTCCAGAG TTAGTTGTGA TTGTTATTGT TGTTGTTGTA ACTTTATGTC ACGGGGAAAA CATAATAATT   
  
  
+ ATCTGTTTAT CTGTTTGTTT GTTATTTTCA AGGGGAAGGG GAGTGGGCGG ATGCTTAGTA TGTAATCATT   
  
  
+ TTATATAGGG AAAATTCATA TAGCATGAAA GTAAAACTTT CATTGCCAGC ATTCTAAGCT AGGGGTAGGG   
  
  
+ TTGTCTACAC CTTGATCTCC CTAGAAGCAT GGTGATCATT CCTCAGATTG TGTTATAGAA TAAAGAATGT   
  
  
+ GGTTCTGCTG TAATGAGACT TATGTATTAT CAAATGATGA ATCTGTTTAG AACTTCCTGT TTATTTTTTC   
  
  
+ TGCAAAATTT TAAGAGACTA GCATCTATCG GGCTCGACTC TAGAGGTTCT CTTGTGTTAC CCTTCTCAAC   
  
  
+ TCAGTTTGAT TGTGATACAG TTACTATATT GAGTGATAGT CGAGAGCATC ACAACCCGCT AAGGAGTCTA   
  
  
+ TCAACAAGAA GCCCTTCTTG TAATTCTCCC CTTGAAACTA GCAGTTATCA TCACTTGTCA TCGAATAGCC   
  
  
+ CTTTTTGTAA TTCTACCCTT GAAACTAGCA GTTATTGTCA TCAGTTCAAT GTGAGTCCCG TTAGATATTC   
  
  
+ CCATCAAGGC ATAGATTATG GAGTGAGCAT GAAGAACGCT TTGCACGAGC TAGAGACTGC TCTAATTGGT   
  
  
+ GTAGATGGCG AGAAAGCATC TGTTGCTAAT CAACCTATGG GGGGAATTCA TTAGTCCGGG ATCCCAAGTT   
  
  
+ AGAGATCGAA GTCATTGAGT GAAGATCCAC AGGGTTCACA TCCTCCTCAG CTTGATTCAT CATCTTTTTC   
  
  
+ AAGGGTGAAA AGATTCGGAG ATGAAAGCCA GAGAGAGAGA AATGCCACAA GGCAATGGAA GAACCAGCGG   
  
  
+ AACTACTAAG TTTCTCACCA GGTGATTTAA AGCAGTTGCT AATTGAATGT GCGAGGGCTT TATCAGATAA   
  
  
+ CCGAATAGAT GACTTTGAGA GTTTGGCTAA ACGGCCAAGG AAAGAGGTCT CCATCTCAGG TGAGCCTGTC   
  
  
+ CAACGTCTCG GTACTTGTAT GATCGAAGGG CTTGTGGCAA GGAAACAGTC TTCGGGGACT AACATCTATC   
  
  
+ GGGCTCTTAA GTACTGTAAA GAGCCTCTTG GAAAATACTT GCTCTCCTAC GGCACTCAGT GGGTTACTCT   
  
  
+ ATCGCAAGCC ATAGCAGCAA GGCTTGGTGG GCCTCCTAAA GTGCGACTTA CAGGCATTGA TGATCCTGTT   
  
  
+ TCTAAGTATA CTTGTGATGC TAGCTTGGAG GCTATTGGGA AACGATTAGC GTCTCTATTT GAAAAGTTTA   
  
  
+ AAATACTCGT CGAGTTCAAT GCATTGCCCG TTTATGGACC TGATGTCAGG TGGGAAATGC TGGATGTGAG   
  
  
+ GCCCAGGGAG GCTTTGGCCG TTAATTGTCC ATTACAGCTC CATCACACTC CTGACGAGAG TGTCGATGTG   
  
  
+ AGCAACCCTA GGGATAGGCT TCTCAGAATG GTGAAATTGC TCGGTCCTAA GGTATGCACT TTGGTTGAGC   
  
  
+ AAGAATCAAA CACCAACACT ACACGTTTCT TGACCCGGTT CATAGAGACC CTTGACTACT ACTCGATCAG   
  
  
+ CCATGTTTG  

- +Up\_Stream \_Len000TCAACA TCGTTCGTGT AATTTCAACT AATAGTAAAA CAGGCACTTT GCCACTCGTT   
  
  
- TGCAGTAGAG GTCCAGTTTA AGTAAGCATG AGTTAGTGTT CAACGACTGT AAAAATGGTT TCCGATTAGC   
  
  
- AGCGTTTTCA AAGAAATCAA ATTCCGGTTA GAGAGGCAAC AAAGTGGGGT ATGTCTAACT CCCCAACATT   
  
  
- ATCACATATA TATATCGGAA CACAATCGCC CCGAACCCGT AATCTGAAAA TGAGAACATT TGGGATTACA   
  
  
- CAATAATATT TATATCTAGA TCGGTGGGTA CAACTCCATC CGTGAGATTA AGACTGTGTC CATCCTATTC   
  
  
- AAAAGTATCA AATACAACAC TTAGAACAGA ATGAAACACC AAGGATAACT GTTTTCAAAC TTAGGCTCGT   
  
  
- ACTCTCAAGT AGTTAGAGAA AAAGAAAGGA TAAAATAAAG AACACCTGAA TTAACCTAAA AGACCATCTC   
  
  
- CTTTCAAGGA ACGACCTTAA ACCAGCTTTC ACTATATGTA AAACCCTAAA TTAATCGGTA TTTAAACCAA   
  
  
- AATGTGGAAC AATCAAACCT CAAGACCACA CAATAACATA CACAAGACCA CCCAAAAAGA AAATCGATTT   
  
  
- ACAACCTCGT AAAATTCCCT AGATCTATAG ACTTCTTCAC GAACCCTTTA CTTCGTATAA AGGGAATATC   
  
  
- AGACTGTATT GAAATTACTT AACTAGTACT TGTTTCCCGT TAATCAAGTC TTTCCAAGTT GAGGAGATCC   
  
  
- ACAACACGAT TTCTCTACAC TCAATCATTG TTAACGAATG ATTGATATAT CCAACAAAAT AATGAACATC   
  
  
- TCCAAAAAAA AAACCCTATA GAAAAACCCC AAATATTTAC TCTTCCAACT AAACAACAGA TGAGAAGAAA   
  
  
- GCTAAGTTAT CATTAAGAGA GTCTCTTCCG ATTTTACTGT ACCGTAAGGT AGCGGAGAGT ACTCCGAAAA   
  
  
- ACGTACACGA ATACATAGAA AGTAGAGAAT GAAACCACCC CACCGTAACC CCTCCTTTTC CCCCAAAGCT   
  
  
- AAGCTATCTA GCAGTGAAAG CCTTTACTGC AGAGACTGGT TACCATTCCC ATTCCAATTA TGTACACCTA   
  
  
- TAAAGGTCTC AATCAACACT AACAATAACA ACAACAACAT TGAAATACAG TGCCCCTTTT GTATTATTAA   
  
  
- TAGACAAATA GACAAACAAA CAATAAAAGT TCCCCTTCCC CTCACCCGCC TACGAATCAT ACATTAGTAA   
  
  
- AATATATCCC TTTTAAGTAT ATCGTACTTT CATTTTGAAA GTAACGGTCG TAAGATTCGA TCCCCATCCC   
  
  
- AACAGATGTG GAACTAGAGG GATCTTCGTA CCACTAGTAA GGAGTCTAAC ACAATATCTT ATTTCTTACA   
  
  
- CCAAGACGAC ATTACTCTGA ATACATAATA GTTTACTACT TAGACAAATC TTGAAGGACA AATAAAAAAG   
  
  
- ACGTTTTAAA ATTCTCTGAT CGTAGATAGC CCGAGCTGAG ATCTCCAAGA GAACACAATG GGAAGAGTTG   
  
  
- AGTCAAACTA ACACTATGTC AATGATATAA CTCACTATCA GCTCTCGTAG TGTTGGGCGA TTCCTCAGAT   
  
  
- AGTTGTTCTT CGGGAAGAAC ATTAAGAGGG GAACTTTGAT CGTCAATAGT AGTGAACAGT AGCTTATCGG   
  
  
- GAAAAACATT AAGATGGGAA CTTTGATCGT CAATAACAGT AGTCAAGTTA CACTCAGGGC AATCTATAAG   
  
  
- GGTAGTTCCG TATCTAATAC CTCACTCGTA CTTCTTGCGA AACGTGCTCG ATCTCTGACG AGATTAACCA   
  
  
- CATCTACCGC TCTTTCGTAG ACAACGATTA GTTGGATACC CCCCTTAAGT AATCAGGCCC TAGGGTTCAA   
  
  
- TCTCTAGCTT CAGTAACTCA CTTCTAGGTG TCCCAAGTGT AGGAGGAGTC GAACTAAGTA GTAGAAAAAG   
  
  
- TTCCCACTTT TCTAAGCCTC TACTTTCGGT CTCTCTCTCT TTACGGTGTT CCGTTACCTT CTTGGTCGCC   
  
  
- TTGATGATTC AAAGAGTGGT CCACTAAATT TCGTCAACGA TTAACTTACA CGCTCCCGAA ATAGTCTATT   
  
  
- GGCTTATCTA CTGAAACTCT CAAACCGATT TGCCGGTTCC TTTCTCCAGA GGTAGAGTCC ACTCGGACAG   
  
  
- GTTGCAGAGC CATGAACATA CTAGCTTCCC GAACACCGTT CCTTTGTCAG AAGCCCCTGA TTGTAGATAG   
  
  
- CCCGAGAATT CATGACATTT CTCGGAGAAC CTTTTATGAA CGAGAGGATG CCGTGAGTCA CCCAATGAGA   
  
  
- TAGCGTTCGG TATCGTCGTT CCGAACCACC CGGAGGATTT CACGCTGAAT GTCCGTAACT ACTAGGACAA   
  
  
- AGATTCATAT GAACACTACG ATCGAACCTC CGATAACCCT TTGCTAATCG CAGAGATAAA CTTTTCAAAT   
  
  
- TTTATGAGCA GCTCAAGTTA CGTAACGGGC AAATACCTGG ACTACAGTCC ACCCTTTACG ACCTACACTC   
  
  
- CGGGTCCCTC CGAAACCGGC AATTAACAGG TAATGTCGAG GTAGTGTGAG GACTGCTCTC ACAGCTACAC   
  
  
- TCGTTGGGAT CCCTATCCGA AGAGTCTTAC CACTTTAACG AGCCAGGATT CCATACGTGA AACCAACTCG   
  
  
- TTCTTAGTTT GTGGTTGTGA TGTGCAAAGA ACTGGGCCAA GTATCTCTGG GAACTGATGA TGAGCTAGTC   
  
  
- GGTACAAAC

+     MYB

| Site Name | Organism | Position | Strand | Matrix score. | sequence | function |
| --- | --- | --- | --- | --- | --- | --- |
| MYB | Arabidopsis thaliana | 1844 | - | 6 | CAACAG |  |
| MYB | Arabidopsis thaliana | 2656 | - | 6 | CAACCA |  |

>HU08G00019.1   
+ +Up\_Stream \_Len000AGTTGT AGCAAGCACA TTAAAGTTGA TTATCATTTT GTCCGTGAAA CGGTGAGCAA   
  
  
+ ACGTCATCTC CAGGTCAAAT TCATTCGTAC TCAATCACAA GTTGCTGACA TTTTTACCAA AGGCTAATCG   
  
  
+ TCGCAAAAGT TTCTTTAGTT TAAGGCCAAT CTCTCCGTTG TTTCACCCCA TACAGATTGA GGGGTTGTAA   
  
  
+ TAGTGTATAT ATATAGCCTT GTGTTAGCGG GGCTTGGGCA TTAGACTTTT ACTCTTGTAA ACCCTAATGT   
  
  
+ GTTATTATAA ATATAGATCT AGCCACCCAT GTTGAGGTAG GCACTCTAAT TCTGACACAG GTAGGATAAG   
  
  
+ TTTTCATAGT TTATGTTGTG AATCTTGTCT TACTTTGTGG TTCCTATTGA CAAAAGTTTG AATCCGAGCA   
  
  
+ TGAGAGTTCA TCAATCTCTT TTTCTTTCCT ATTTTATTTC TTGTGGACTT AATTGGATTT TCTGGTAGAG   
  
  
+ GAAAGTTCCT TGCTGGAATT TGGTCGAAAG TGATATACAT TTTGGGATTT AATTAGCCAT AAATTTGGTT   
  
  
+ TTACACCTTG TTAGTTTGGA GTTCTGGTGT GTTATTGTAT GTGTTCTGGT GGGTTTTTCT TTTAGCTAAA   
  
  
+ TGTTGGAGCA TTTTAAGGGA TCTAGATATC TGAAGAAGTG CTTGGGAAAT GAAGCATATT TCCCTTATAG   
  
  
+ TCTGACATAA CTTTAATGAA TTGATCATGA ACAAAGGGCA ATTAGTTCAG AAAGGTTCAA CTCCTCTAGG   
  
  
+ TGTTGTGCTA AAGAGATGTG AGTTAGTAAC AATTGCTTAC TAACTATATA GGTTGTTTTA TTACTTGTAG   
  
  
+ AGGTTTTTTT TTTGGGATAT CTTTTTGGGG TTTATAAATG AGAAGGTTGA TTTGTTGTCT ACTCTTCTTT   
  
  
+ CGATTCAATA GTAATTCTCT CAGAGAAGGC TAAAATGACA TGGCATTCCA TCGCCTCTCA TGAGGCTTTT   
  
  
+ TGCATGTGCT TATGTATCTT TCATCTCTTA CTTTGGTGGG GTGGCATTGG GGAGGAAAAG GGGGTTTCGA   
  
  
+ TTCGATAGAT CGTCACTTTC GGAAATGACG TCTCTGACCA ATGGTAAGGG TAAGGTTAAT ACATGTGGAT   
  
  
+ ATTTCCAGAG TTAGTTGTGA TTGTTATTGT TGTTGTTGTA ACTTTATGTC ACGGGGAAAA CATAATAATT   
  
  
+ ATCTGTTTAT CTGTTTGTTT GTTATTTTCA AGGGGAAGGG GAGTGGGCGG ATGCTTAGTA TGTAATCATT   
  
  
+ TTATATAGGG AAAATTCATA TAGCATGAAA GTAAAACTTT CATTGCCAGC ATTCTAAGCT AGGGGTAGGG   
  
  
+ TTGTCTACAC CTTGATCTCC CTAGAAGCAT GGTGATCATT CCTCAGATTG TGTTATAGAA TAAAGAATGT   
  
  
+ GGTTCTGCTG TAATGAGACT TATGTATTAT CAAATGATGA ATCTGTTTAG AACTTCCTGT TTATTTTTTC   
  
  
+ TGCAAAATTT TAAGAGACTA GCATCTATCG GGCTCGACTC TAGAGGTTCT CTTGTGTTAC CCTTCTCAAC   
  
  
+ TCAGTTTGAT TGTGATACAG TTACTATATT GAGTGATAGT CGAGAGCATC ACAACCCGCT AAGGAGTCTA   
  
  
+ TCAACAAGAA GCCCTTCTTG TAATTCTCCC CTTGAAACTA GCAGTTATCA TCACTTGTCA TCGAATAGCC   
  
  
+ CTTTTTGTAA TTCTACCCTT GAAACTAGCA GTTATTGTCA TCAGTTCAAT GTGAGTCCCG TTAGATATTC   
  
  
+ CCATCAAGGC ATAGATTATG GAGTGAGCAT GAAGAACGCT TTGCACGAGC TAGAGACTGC TCTAATTGGT   
  
  
+ GTAGATGGCG AGAAAGCATC TGTTGCTAAT CAACCTATGG GGGGAATTCA TTAGTCCGGG ATCCCAAGTT   
  
  
+ AGAGATCGAA GTCATTGAGT GAAGATCCAC AGGGTTCACA TCCTCCTCAG CTTGATTCAT CATCTTTTTC   
  
  
+ AAGGGTGAAA AGATTCGGAG ATGAAAGCCA GAGAGAGAGA AATGCCACAA GGCAATGGAA GAACCAGCGG   
  
  
+ AACTACTAAG TTTCTCACCA GGTGATTTAA AGCAGTTGCT AATTGAATGT GCGAGGGCTT TATCAGATAA   
  
  
+ CCGAATAGAT GACTTTGAGA GTTTGGCTAA ACGGCCAAGG AAAGAGGTCT CCATCTCAGG TGAGCCTGTC   
  
  
+ CAACGTCTCG GTACTTGTAT GATCGAAGGG CTTGTGGCAA GGAAACAGTC TTCGGGGACT AACATCTATC   
  
  
+ GGGCTCTTAA GTACTGTAAA GAGCCTCTTG GAAAATACTT GCTCTCCTAC GGCACTCAGT GGGTTACTCT   
  
  
+ ATCGCAAGCC ATAGCAGCAA GGCTTGGTGG GCCTCCTAAA GTGCGACTTA CAGGCATTGA TGATCCTGTT   
  
  
+ TCTAAGTATA CTTGTGATGC TAGCTTGGAG GCTATTGGGA AACGATTAGC GTCTCTATTT GAAAAGTTTA   
  
  
+ AAATACTCGT CGAGTTCAAT GCATTGCCCG TTTATGGACC TGATGTCAGG TGGGAAATGC TGGATGTGAG   
  
  
+ GCCCAGGGAG GCTTTGGCCG TTAATTGTCC ATTACAGCTC CATCACACTC CTGACGAGAG TGTCGATGTG   
  
  
+ AGCAACCCTA GGGATAGGCT TCTCAGAATG GTGAAATTGC TCGGTCCTAA GGTATGCACT TTGGTTGAGC   
  
  
+ AAGAATCAAA CACCAACACT ACACGTTTCT TGACCCGGTT CATAGAGACC CTTGACTACT ACTCGATCAG   
  
  
+ CCATGTTTG  

- +Up\_Stream \_Len000TCAACA TCGTTCGTGT AATTTCAACT AATAGTAAAA CAGGCACTTT GCCACTCGTT   
  
  
- TGCAGTAGAG GTCCAGTTTA AGTAAGCATG AGTTAGTGTT CAACGACTGT AAAAATGGTT TCCGATTAGC   
  
  
- AGCGTTTTCA AAGAAATCAA ATTCCGGTTA GAGAGGCAAC AAAGTGGGGT ATGTCTAACT CCCCAACATT   
  
  
- ATCACATATA TATATCGGAA CACAATCGCC CCGAACCCGT AATCTGAAAA TGAGAACATT TGGGATTACA   
  
  
- CAATAATATT TATATCTAGA TCGGTGGGTA CAACTCCATC CGTGAGATTA AGACTGTGTC CATCCTATTC   
  
  
- AAAAGTATCA AATACAACAC TTAGAACAGA ATGAAACACC AAGGATAACT GTTTTCAAAC TTAGGCTCGT   
  
  
- ACTCTCAAGT AGTTAGAGAA AAAGAAAGGA TAAAATAAAG AACACCTGAA TTAACCTAAA AGACCATCTC   
  
  
- CTTTCAAGGA ACGACCTTAA ACCAGCTTTC ACTATATGTA AAACCCTAAA TTAATCGGTA TTTAAACCAA   
  
  
- AATGTGGAAC AATCAAACCT CAAGACCACA CAATAACATA CACAAGACCA CCCAAAAAGA AAATCGATTT   
  
  
- ACAACCTCGT AAAATTCCCT AGATCTATAG ACTTCTTCAC GAACCCTTTA CTTCGTATAA AGGGAATATC   
  
  
- AGACTGTATT GAAATTACTT AACTAGTACT TGTTTCCCGT TAATCAAGTC TTTCCAAGTT GAGGAGATCC   
  
  
- ACAACACGAT TTCTCTACAC TCAATCATTG TTAACGAATG ATTGATATAT CCAACAAAAT AATGAACATC   
  
  
- TCCAAAAAAA AAACCCTATA GAAAAACCCC AAATATTTAC TCTTCCAACT AAACAACAGA TGAGAAGAAA   
  
  
- GCTAAGTTAT CATTAAGAGA GTCTCTTCCG ATTTTACTGT ACCGTAAGGT AGCGGAGAGT ACTCCGAAAA   
  
  
- ACGTACACGA ATACATAGAA AGTAGAGAAT GAAACCACCC CACCGTAACC CCTCCTTTTC CCCCAAAGCT   
  
  
- AAGCTATCTA GCAGTGAAAG CCTTTACTGC AGAGACTGGT TACCATTCCC ATTCCAATTA TGTACACCTA   
  
  
- TAAAGGTCTC AATCAACACT AACAATAACA ACAACAACAT TGAAATACAG TGCCCCTTTT GTATTATTAA   
  
  
- TAGACAAATA GACAAACAAA CAATAAAAGT TCCCCTTCCC CTCACCCGCC TACGAATCAT ACATTAGTAA   
  
  
- AATATATCCC TTTTAAGTAT ATCGTACTTT CATTTTGAAA GTAACGGTCG TAAGATTCGA TCCCCATCCC   
  
  
- AACAGATGTG GAACTAGAGG GATCTTCGTA CCACTAGTAA GGAGTCTAAC ACAATATCTT ATTTCTTACA   
  
  
- CCAAGACGAC ATTACTCTGA ATACATAATA GTTTACTACT TAGACAAATC TTGAAGGACA AATAAAAAAG   
  
  
- ACGTTTTAAA ATTCTCTGAT CGTAGATAGC CCGAGCTGAG ATCTCCAAGA GAACACAATG GGAAGAGTTG   
  
  
- AGTCAAACTA ACACTATGTC AATGATATAA CTCACTATCA GCTCTCGTAG TGTTGGGCGA TTCCTCAGAT   
  
  
- AGTTGTTCTT CGGGAAGAAC ATTAAGAGGG GAACTTTGAT CGTCAATAGT AGTGAACAGT AGCTTATCGG   
  
  
- GAAAAACATT AAGATGGGAA CTTTGATCGT CAATAACAGT AGTCAAGTTA CACTCAGGGC AATCTATAAG   
  
  
- GGTAGTTCCG TATCTAATAC CTCACTCGTA CTTCTTGCGA AACGTGCTCG ATCTCTGACG AGATTAACCA   
  
  
- CATCTACCGC TCTTTCGTAG ACAACGATTA GTTGGATACC CCCCTTAAGT AATCAGGCCC TAGGGTTCAA   
  
  
- TCTCTAGCTT CAGTAACTCA CTTCTAGGTG TCCCAAGTGT AGGAGGAGTC GAACTAAGTA GTAGAAAAAG   
  
  
- TTCCCACTTT TCTAAGCCTC TACTTTCGGT CTCTCTCTCT TTACGGTGTT CCGTTACCTT CTTGGTCGCC   
  
  
- TTGATGATTC AAAGAGTGGT CCACTAAATT TCGTCAACGA TTAACTTACA CGCTCCCGAA ATAGTCTATT   
  
  
- GGCTTATCTA CTGAAACTCT CAAACCGATT TGCCGGTTCC TTTCTCCAGA GGTAGAGTCC ACTCGGACAG   
  
  
- GTTGCAGAGC CATGAACATA CTAGCTTCCC GAACACCGTT CCTTTGTCAG AAGCCCCTGA TTGTAGATAG   
  
  
- CCCGAGAATT CATGACATTT CTCGGAGAAC CTTTTATGAA CGAGAGGATG CCGTGAGTCA CCCAATGAGA   
  
  
- TAGCGTTCGG TATCGTCGTT CCGAACCACC CGGAGGATTT CACGCTGAAT GTCCGTAACT ACTAGGACAA   
  
  
- AGATTCATAT GAACACTACG ATCGAACCTC CGATAACCCT TTGCTAATCG CAGAGATAAA CTTTTCAAAT   
  
  
- TTTATGAGCA GCTCAAGTTA CGTAACGGGC AAATACCTGG ACTACAGTCC ACCCTTTACG ACCTACACTC   
  
  
- CGGGTCCCTC CGAAACCGGC AATTAACAGG TAATGTCGAG GTAGTGTGAG GACTGCTCTC ACAGCTACAC   
  
  
- TCGTTGGGAT CCCTATCCGA AGAGTCTTAC CACTTTAACG AGCCAGGATT CCATACGTGA AACCAACTCG   
  
  
- TTCTTAGTTT GTGGTTGTGA TGTGCAAAGA ACTGGGCCAA GTATCTCTGG GAACTGATGA TGAGCTAGTC   
  
  
- GGTACAAAC

+     MYB recognition site

| Site Name | Organism | Position | Strand | Matrix score. | sequence | function |
| --- | --- | --- | --- | --- | --- | --- |
| MYB recognition site | Arabidopsis thaliana | 179 | + | 6 | CCGTTG |  |

>HU08G00019.1   
+ +Up\_Stream \_Len000AGTTGT AGCAAGCACA TTAAAGTTGA TTATCATTTT GTCCGTGAAA CGGTGAGCAA   
  
  
+ ACGTCATCTC CAGGTCAAAT TCATTCGTAC TCAATCACAA GTTGCTGACA TTTTTACCAA AGGCTAATCG   
  
  
+ TCGCAAAAGT TTCTTTAGTT TAAGGCCAAT CTCTCCGTTG TTTCACCCCA TACAGATTGA GGGGTTGTAA   
  
  
+ TAGTGTATAT ATATAGCCTT GTGTTAGCGG GGCTTGGGCA TTAGACTTTT ACTCTTGTAA ACCCTAATGT   
  
  
+ GTTATTATAA ATATAGATCT AGCCACCCAT GTTGAGGTAG GCACTCTAAT TCTGACACAG GTAGGATAAG   
  
  
+ TTTTCATAGT TTATGTTGTG AATCTTGTCT TACTTTGTGG TTCCTATTGA CAAAAGTTTG AATCCGAGCA   
  
  
+ TGAGAGTTCA TCAATCTCTT TTTCTTTCCT ATTTTATTTC TTGTGGACTT AATTGGATTT TCTGGTAGAG   
  
  
+ GAAAGTTCCT TGCTGGAATT TGGTCGAAAG TGATATACAT TTTGGGATTT AATTAGCCAT AAATTTGGTT   
  
  
+ TTACACCTTG TTAGTTTGGA GTTCTGGTGT GTTATTGTAT GTGTTCTGGT GGGTTTTTCT TTTAGCTAAA   
  
  
+ TGTTGGAGCA TTTTAAGGGA TCTAGATATC TGAAGAAGTG CTTGGGAAAT GAAGCATATT TCCCTTATAG   
  
  
+ TCTGACATAA CTTTAATGAA TTGATCATGA ACAAAGGGCA ATTAGTTCAG AAAGGTTCAA CTCCTCTAGG   
  
  
+ TGTTGTGCTA AAGAGATGTG AGTTAGTAAC AATTGCTTAC TAACTATATA GGTTGTTTTA TTACTTGTAG   
  
  
+ AGGTTTTTTT TTTGGGATAT CTTTTTGGGG TTTATAAATG AGAAGGTTGA TTTGTTGTCT ACTCTTCTTT   
  
  
+ CGATTCAATA GTAATTCTCT CAGAGAAGGC TAAAATGACA TGGCATTCCA TCGCCTCTCA TGAGGCTTTT   
  
  
+ TGCATGTGCT TATGTATCTT TCATCTCTTA CTTTGGTGGG GTGGCATTGG GGAGGAAAAG GGGGTTTCGA   
  
  
+ TTCGATAGAT CGTCACTTTC GGAAATGACG TCTCTGACCA ATGGTAAGGG TAAGGTTAAT ACATGTGGAT   
  
  
+ ATTTCCAGAG TTAGTTGTGA TTGTTATTGT TGTTGTTGTA ACTTTATGTC ACGGGGAAAA CATAATAATT   
  
  
+ ATCTGTTTAT CTGTTTGTTT GTTATTTTCA AGGGGAAGGG GAGTGGGCGG ATGCTTAGTA TGTAATCATT   
  
  
+ TTATATAGGG AAAATTCATA TAGCATGAAA GTAAAACTTT CATTGCCAGC ATTCTAAGCT AGGGGTAGGG   
  
  
+ TTGTCTACAC CTTGATCTCC CTAGAAGCAT GGTGATCATT CCTCAGATTG TGTTATAGAA TAAAGAATGT   
  
  
+ GGTTCTGCTG TAATGAGACT TATGTATTAT CAAATGATGA ATCTGTTTAG AACTTCCTGT TTATTTTTTC   
  
  
+ TGCAAAATTT TAAGAGACTA GCATCTATCG GGCTCGACTC TAGAGGTTCT CTTGTGTTAC CCTTCTCAAC   
  
  
+ TCAGTTTGAT TGTGATACAG TTACTATATT GAGTGATAGT CGAGAGCATC ACAACCCGCT AAGGAGTCTA   
  
  
+ TCAACAAGAA GCCCTTCTTG TAATTCTCCC CTTGAAACTA GCAGTTATCA TCACTTGTCA TCGAATAGCC   
  
  
+ CTTTTTGTAA TTCTACCCTT GAAACTAGCA GTTATTGTCA TCAGTTCAAT GTGAGTCCCG TTAGATATTC   
  
  
+ CCATCAAGGC ATAGATTATG GAGTGAGCAT GAAGAACGCT TTGCACGAGC TAGAGACTGC TCTAATTGGT   
  
  
+ GTAGATGGCG AGAAAGCATC TGTTGCTAAT CAACCTATGG GGGGAATTCA TTAGTCCGGG ATCCCAAGTT   
  
  
+ AGAGATCGAA GTCATTGAGT GAAGATCCAC AGGGTTCACA TCCTCCTCAG CTTGATTCAT CATCTTTTTC   
  
  
+ AAGGGTGAAA AGATTCGGAG ATGAAAGCCA GAGAGAGAGA AATGCCACAA GGCAATGGAA GAACCAGCGG   
  
  
+ AACTACTAAG TTTCTCACCA GGTGATTTAA AGCAGTTGCT AATTGAATGT GCGAGGGCTT TATCAGATAA   
  
  
+ CCGAATAGAT GACTTTGAGA GTTTGGCTAA ACGGCCAAGG AAAGAGGTCT CCATCTCAGG TGAGCCTGTC   
  
  
+ CAACGTCTCG GTACTTGTAT GATCGAAGGG CTTGTGGCAA GGAAACAGTC TTCGGGGACT AACATCTATC   
  
  
+ GGGCTCTTAA GTACTGTAAA GAGCCTCTTG GAAAATACTT GCTCTCCTAC GGCACTCAGT GGGTTACTCT   
  
  
+ ATCGCAAGCC ATAGCAGCAA GGCTTGGTGG GCCTCCTAAA GTGCGACTTA CAGGCATTGA TGATCCTGTT   
  
  
+ TCTAAGTATA CTTGTGATGC TAGCTTGGAG GCTATTGGGA AACGATTAGC GTCTCTATTT GAAAAGTTTA   
  
  
+ AAATACTCGT CGAGTTCAAT GCATTGCCCG TTTATGGACC TGATGTCAGG TGGGAAATGC TGGATGTGAG   
  
  
+ GCCCAGGGAG GCTTTGGCCG TTAATTGTCC ATTACAGCTC CATCACACTC CTGACGAGAG TGTCGATGTG   
  
  
+ AGCAACCCTA GGGATAGGCT TCTCAGAATG GTGAAATTGC TCGGTCCTAA GGTATGCACT TTGGTTGAGC   
  
  
+ AAGAATCAAA CACCAACACT ACACGTTTCT TGACCCGGTT CATAGAGACC CTTGACTACT ACTCGATCAG   
  
  
+ CCATGTTTG  

- +Up\_Stream \_Len000TCAACA TCGTTCGTGT AATTTCAACT AATAGTAAAA CAGGCACTTT GCCACTCGTT   
  
  
- TGCAGTAGAG GTCCAGTTTA AGTAAGCATG AGTTAGTGTT CAACGACTGT AAAAATGGTT TCCGATTAGC   
  
  
- AGCGTTTTCA AAGAAATCAA ATTCCGGTTA GAGAGGCAAC AAAGTGGGGT ATGTCTAACT CCCCAACATT   
  
  
- ATCACATATA TATATCGGAA CACAATCGCC CCGAACCCGT AATCTGAAAA TGAGAACATT TGGGATTACA   
  
  
- CAATAATATT TATATCTAGA TCGGTGGGTA CAACTCCATC CGTGAGATTA AGACTGTGTC CATCCTATTC   
  
  
- AAAAGTATCA AATACAACAC TTAGAACAGA ATGAAACACC AAGGATAACT GTTTTCAAAC TTAGGCTCGT   
  
  
- ACTCTCAAGT AGTTAGAGAA AAAGAAAGGA TAAAATAAAG AACACCTGAA TTAACCTAAA AGACCATCTC   
  
  
- CTTTCAAGGA ACGACCTTAA ACCAGCTTTC ACTATATGTA AAACCCTAAA TTAATCGGTA TTTAAACCAA   
  
  
- AATGTGGAAC AATCAAACCT CAAGACCACA CAATAACATA CACAAGACCA CCCAAAAAGA AAATCGATTT   
  
  
- ACAACCTCGT AAAATTCCCT AGATCTATAG ACTTCTTCAC GAACCCTTTA CTTCGTATAA AGGGAATATC   
  
  
- AGACTGTATT GAAATTACTT AACTAGTACT TGTTTCCCGT TAATCAAGTC TTTCCAAGTT GAGGAGATCC   
  
  
- ACAACACGAT TTCTCTACAC TCAATCATTG TTAACGAATG ATTGATATAT CCAACAAAAT AATGAACATC   
  
  
- TCCAAAAAAA AAACCCTATA GAAAAACCCC AAATATTTAC TCTTCCAACT AAACAACAGA TGAGAAGAAA   
  
  
- GCTAAGTTAT CATTAAGAGA GTCTCTTCCG ATTTTACTGT ACCGTAAGGT AGCGGAGAGT ACTCCGAAAA   
  
  
- ACGTACACGA ATACATAGAA AGTAGAGAAT GAAACCACCC CACCGTAACC CCTCCTTTTC CCCCAAAGCT   
  
  
- AAGCTATCTA GCAGTGAAAG CCTTTACTGC AGAGACTGGT TACCATTCCC ATTCCAATTA TGTACACCTA   
  
  
- TAAAGGTCTC AATCAACACT AACAATAACA ACAACAACAT TGAAATACAG TGCCCCTTTT GTATTATTAA   
  
  
- TAGACAAATA GACAAACAAA CAATAAAAGT TCCCCTTCCC CTCACCCGCC TACGAATCAT ACATTAGTAA   
  
  
- AATATATCCC TTTTAAGTAT ATCGTACTTT CATTTTGAAA GTAACGGTCG TAAGATTCGA TCCCCATCCC   
  
  
- AACAGATGTG GAACTAGAGG GATCTTCGTA CCACTAGTAA GGAGTCTAAC ACAATATCTT ATTTCTTACA   
  
  
- CCAAGACGAC ATTACTCTGA ATACATAATA GTTTACTACT TAGACAAATC TTGAAGGACA AATAAAAAAG   
  
  
- ACGTTTTAAA ATTCTCTGAT CGTAGATAGC CCGAGCTGAG ATCTCCAAGA GAACACAATG GGAAGAGTTG   
  
  
- AGTCAAACTA ACACTATGTC AATGATATAA CTCACTATCA GCTCTCGTAG TGTTGGGCGA TTCCTCAGAT   
  
  
- AGTTGTTCTT CGGGAAGAAC ATTAAGAGGG GAACTTTGAT CGTCAATAGT AGTGAACAGT AGCTTATCGG   
  
  
- GAAAAACATT AAGATGGGAA CTTTGATCGT CAATAACAGT AGTCAAGTTA CACTCAGGGC AATCTATAAG   
  
  
- GGTAGTTCCG TATCTAATAC CTCACTCGTA CTTCTTGCGA AACGTGCTCG ATCTCTGACG AGATTAACCA   
  
  
- CATCTACCGC TCTTTCGTAG ACAACGATTA GTTGGATACC CCCCTTAAGT AATCAGGCCC TAGGGTTCAA   
  
  
- TCTCTAGCTT CAGTAACTCA CTTCTAGGTG TCCCAAGTGT AGGAGGAGTC GAACTAAGTA GTAGAAAAAG   
  
  
- TTCCCACTTT TCTAAGCCTC TACTTTCGGT CTCTCTCTCT TTACGGTGTT CCGTTACCTT CTTGGTCGCC   
  
  
- TTGATGATTC AAAGAGTGGT CCACTAAATT TCGTCAACGA TTAACTTACA CGCTCCCGAA ATAGTCTATT   
  
  
- GGCTTATCTA CTGAAACTCT CAAACCGATT TGCCGGTTCC TTTCTCCAGA GGTAGAGTCC ACTCGGACAG   
  
  
- GTTGCAGAGC CATGAACATA CTAGCTTCCC GAACACCGTT CCTTTGTCAG AAGCCCCTGA TTGTAGATAG   
  
  
- CCCGAGAATT CATGACATTT CTCGGAGAAC CTTTTATGAA CGAGAGGATG CCGTGAGTCA CCCAATGAGA   
  
  
- TAGCGTTCGG TATCGTCGTT CCGAACCACC CGGAGGATTT CACGCTGAAT GTCCGTAACT ACTAGGACAA   
  
  
- AGATTCATAT GAACACTACG ATCGAACCTC CGATAACCCT TTGCTAATCG CAGAGATAAA CTTTTCAAAT   
  
  
- TTTATGAGCA GCTCAAGTTA CGTAACGGGC AAATACCTGG ACTACAGTCC ACCCTTTACG ACCTACACTC   
  
  
- CGGGTCCCTC CGAAACCGGC AATTAACAGG TAATGTCGAG GTAGTGTGAG GACTGCTCTC ACAGCTACAC   
  
  
- TCGTTGGGAT CCCTATCCGA AGAGTCTTAC CACTTTAACG AGCCAGGATT CCATACGTGA AACCAACTCG   
  
  
- TTCTTAGTTT GTGGTTGTGA TGTGCAAAGA ACTGGGCCAA GTATCTCTGG GAACTGATGA TGAGCTAGTC   
  
  
- GGTACAAAC

+     MYC

| Site Name | Organism | Position | Strand | Matrix score. | sequence | function |
| --- | --- | --- | --- | --- | --- | --- |
| MYC | Arabidopsis thaliana | 1116 | + | 6 | CATGTG |  |
| MYC | Arabidopsis thaliana | 804 | + | 6 | CAATTG |  |
| MYC | Arabidopsis thaliana | 987 | + | 6 | CATGTG |  |
| MYC | Arabidopsis thaliana | 1435 | - | 6 | CATTTG |  |

>HU08G00019.1   
+ +Up\_Stream \_Len000AGTTGT AGCAAGCACA TTAAAGTTGA TTATCATTTT GTCCGTGAAA CGGTGAGCAA   
  
  
+ ACGTCATCTC CAGGTCAAAT TCATTCGTAC TCAATCACAA GTTGCTGACA TTTTTACCAA AGGCTAATCG   
  
  
+ TCGCAAAAGT TTCTTTAGTT TAAGGCCAAT CTCTCCGTTG TTTCACCCCA TACAGATTGA GGGGTTGTAA   
  
  
+ TAGTGTATAT ATATAGCCTT GTGTTAGCGG GGCTTGGGCA TTAGACTTTT ACTCTTGTAA ACCCTAATGT   
  
  
+ GTTATTATAA ATATAGATCT AGCCACCCAT GTTGAGGTAG GCACTCTAAT TCTGACACAG GTAGGATAAG   
  
  
+ TTTTCATAGT TTATGTTGTG AATCTTGTCT TACTTTGTGG TTCCTATTGA CAAAAGTTTG AATCCGAGCA   
  
  
+ TGAGAGTTCA TCAATCTCTT TTTCTTTCCT ATTTTATTTC TTGTGGACTT AATTGGATTT TCTGGTAGAG   
  
  
+ GAAAGTTCCT TGCTGGAATT TGGTCGAAAG TGATATACAT TTTGGGATTT AATTAGCCAT AAATTTGGTT   
  
  
+ TTACACCTTG TTAGTTTGGA GTTCTGGTGT GTTATTGTAT GTGTTCTGGT GGGTTTTTCT TTTAGCTAAA   
  
  
+ TGTTGGAGCA TTTTAAGGGA TCTAGATATC TGAAGAAGTG CTTGGGAAAT GAAGCATATT TCCCTTATAG   
  
  
+ TCTGACATAA CTTTAATGAA TTGATCATGA ACAAAGGGCA ATTAGTTCAG AAAGGTTCAA CTCCTCTAGG   
  
  
+ TGTTGTGCTA AAGAGATGTG AGTTAGTAAC AATTGCTTAC TAACTATATA GGTTGTTTTA TTACTTGTAG   
  
  
+ AGGTTTTTTT TTTGGGATAT CTTTTTGGGG TTTATAAATG AGAAGGTTGA TTTGTTGTCT ACTCTTCTTT   
  
  
+ CGATTCAATA GTAATTCTCT CAGAGAAGGC TAAAATGACA TGGCATTCCA TCGCCTCTCA TGAGGCTTTT   
  
  
+ TGCATGTGCT TATGTATCTT TCATCTCTTA CTTTGGTGGG GTGGCATTGG GGAGGAAAAG GGGGTTTCGA   
  
  
+ TTCGATAGAT CGTCACTTTC GGAAATGACG TCTCTGACCA ATGGTAAGGG TAAGGTTAAT ACATGTGGAT   
  
  
+ ATTTCCAGAG TTAGTTGTGA TTGTTATTGT TGTTGTTGTA ACTTTATGTC ACGGGGAAAA CATAATAATT   
  
  
+ ATCTGTTTAT CTGTTTGTTT GTTATTTTCA AGGGGAAGGG GAGTGGGCGG ATGCTTAGTA TGTAATCATT   
  
  
+ TTATATAGGG AAAATTCATA TAGCATGAAA GTAAAACTTT CATTGCCAGC ATTCTAAGCT AGGGGTAGGG   
  
  
+ TTGTCTACAC CTTGATCTCC CTAGAAGCAT GGTGATCATT CCTCAGATTG TGTTATAGAA TAAAGAATGT   
  
  
+ GGTTCTGCTG TAATGAGACT TATGTATTAT CAAATGATGA ATCTGTTTAG AACTTCCTGT TTATTTTTTC   
  
  
+ TGCAAAATTT TAAGAGACTA GCATCTATCG GGCTCGACTC TAGAGGTTCT CTTGTGTTAC CCTTCTCAAC   
  
  
+ TCAGTTTGAT TGTGATACAG TTACTATATT GAGTGATAGT CGAGAGCATC ACAACCCGCT AAGGAGTCTA   
  
  
+ TCAACAAGAA GCCCTTCTTG TAATTCTCCC CTTGAAACTA GCAGTTATCA TCACTTGTCA TCGAATAGCC   
  
  
+ CTTTTTGTAA TTCTACCCTT GAAACTAGCA GTTATTGTCA TCAGTTCAAT GTGAGTCCCG TTAGATATTC   
  
  
+ CCATCAAGGC ATAGATTATG GAGTGAGCAT GAAGAACGCT TTGCACGAGC TAGAGACTGC TCTAATTGGT   
  
  
+ GTAGATGGCG AGAAAGCATC TGTTGCTAAT CAACCTATGG GGGGAATTCA TTAGTCCGGG ATCCCAAGTT   
  
  
+ AGAGATCGAA GTCATTGAGT GAAGATCCAC AGGGTTCACA TCCTCCTCAG CTTGATTCAT CATCTTTTTC   
  
  
+ AAGGGTGAAA AGATTCGGAG ATGAAAGCCA GAGAGAGAGA AATGCCACAA GGCAATGGAA GAACCAGCGG   
  
  
+ AACTACTAAG TTTCTCACCA GGTGATTTAA AGCAGTTGCT AATTGAATGT GCGAGGGCTT TATCAGATAA   
  
  
+ CCGAATAGAT GACTTTGAGA GTTTGGCTAA ACGGCCAAGG AAAGAGGTCT CCATCTCAGG TGAGCCTGTC   
  
  
+ CAACGTCTCG GTACTTGTAT GATCGAAGGG CTTGTGGCAA GGAAACAGTC TTCGGGGACT AACATCTATC   
  
  
+ GGGCTCTTAA GTACTGTAAA GAGCCTCTTG GAAAATACTT GCTCTCCTAC GGCACTCAGT GGGTTACTCT   
  
  
+ ATCGCAAGCC ATAGCAGCAA GGCTTGGTGG GCCTCCTAAA GTGCGACTTA CAGGCATTGA TGATCCTGTT   
  
  
+ TCTAAGTATA CTTGTGATGC TAGCTTGGAG GCTATTGGGA AACGATTAGC GTCTCTATTT GAAAAGTTTA   
  
  
+ AAATACTCGT CGAGTTCAAT GCATTGCCCG TTTATGGACC TGATGTCAGG TGGGAAATGC TGGATGTGAG   
  
  
+ GCCCAGGGAG GCTTTGGCCG TTAATTGTCC ATTACAGCTC CATCACACTC CTGACGAGAG TGTCGATGTG   
  
  
+ AGCAACCCTA GGGATAGGCT TCTCAGAATG GTGAAATTGC TCGGTCCTAA GGTATGCACT TTGGTTGAGC   
  
  
+ AAGAATCAAA CACCAACACT ACACGTTTCT TGACCCGGTT CATAGAGACC CTTGACTACT ACTCGATCAG   
  
  
+ CCATGTTTG  

- +Up\_Stream \_Len000TCAACA TCGTTCGTGT AATTTCAACT AATAGTAAAA CAGGCACTTT GCCACTCGTT   
  
  
- TGCAGTAGAG GTCCAGTTTA AGTAAGCATG AGTTAGTGTT CAACGACTGT AAAAATGGTT TCCGATTAGC   
  
  
- AGCGTTTTCA AAGAAATCAA ATTCCGGTTA GAGAGGCAAC AAAGTGGGGT ATGTCTAACT CCCCAACATT   
  
  
- ATCACATATA TATATCGGAA CACAATCGCC CCGAACCCGT AATCTGAAAA TGAGAACATT TGGGATTACA   
  
  
- CAATAATATT TATATCTAGA TCGGTGGGTA CAACTCCATC CGTGAGATTA AGACTGTGTC CATCCTATTC   
  
  
- AAAAGTATCA AATACAACAC TTAGAACAGA ATGAAACACC AAGGATAACT GTTTTCAAAC TTAGGCTCGT   
  
  
- ACTCTCAAGT AGTTAGAGAA AAAGAAAGGA TAAAATAAAG AACACCTGAA TTAACCTAAA AGACCATCTC   
  
  
- CTTTCAAGGA ACGACCTTAA ACCAGCTTTC ACTATATGTA AAACCCTAAA TTAATCGGTA TTTAAACCAA   
  
  
- AATGTGGAAC AATCAAACCT CAAGACCACA CAATAACATA CACAAGACCA CCCAAAAAGA AAATCGATTT   
  
  
- ACAACCTCGT AAAATTCCCT AGATCTATAG ACTTCTTCAC GAACCCTTTA CTTCGTATAA AGGGAATATC   
  
  
- AGACTGTATT GAAATTACTT AACTAGTACT TGTTTCCCGT TAATCAAGTC TTTCCAAGTT GAGGAGATCC   
  
  
- ACAACACGAT TTCTCTACAC TCAATCATTG TTAACGAATG ATTGATATAT CCAACAAAAT AATGAACATC   
  
  
- TCCAAAAAAA AAACCCTATA GAAAAACCCC AAATATTTAC TCTTCCAACT AAACAACAGA TGAGAAGAAA   
  
  
- GCTAAGTTAT CATTAAGAGA GTCTCTTCCG ATTTTACTGT ACCGTAAGGT AGCGGAGAGT ACTCCGAAAA   
  
  
- ACGTACACGA ATACATAGAA AGTAGAGAAT GAAACCACCC CACCGTAACC CCTCCTTTTC CCCCAAAGCT   
  
  
- AAGCTATCTA GCAGTGAAAG CCTTTACTGC AGAGACTGGT TACCATTCCC ATTCCAATTA TGTACACCTA   
  
  
- TAAAGGTCTC AATCAACACT AACAATAACA ACAACAACAT TGAAATACAG TGCCCCTTTT GTATTATTAA   
  
  
- TAGACAAATA GACAAACAAA CAATAAAAGT TCCCCTTCCC CTCACCCGCC TACGAATCAT ACATTAGTAA   
  
  
- AATATATCCC TTTTAAGTAT ATCGTACTTT CATTTTGAAA GTAACGGTCG TAAGATTCGA TCCCCATCCC   
  
  
- AACAGATGTG GAACTAGAGG GATCTTCGTA CCACTAGTAA GGAGTCTAAC ACAATATCTT ATTTCTTACA   
  
  
- CCAAGACGAC ATTACTCTGA ATACATAATA GTTTACTACT TAGACAAATC TTGAAGGACA AATAAAAAAG   
  
  
- ACGTTTTAAA ATTCTCTGAT CGTAGATAGC CCGAGCTGAG ATCTCCAAGA GAACACAATG GGAAGAGTTG   
  
  
- AGTCAAACTA ACACTATGTC AATGATATAA CTCACTATCA GCTCTCGTAG TGTTGGGCGA TTCCTCAGAT   
  
  
- AGTTGTTCTT CGGGAAGAAC ATTAAGAGGG GAACTTTGAT CGTCAATAGT AGTGAACAGT AGCTTATCGG   
  
  
- GAAAAACATT AAGATGGGAA CTTTGATCGT CAATAACAGT AGTCAAGTTA CACTCAGGGC AATCTATAAG   
  
  
- GGTAGTTCCG TATCTAATAC CTCACTCGTA CTTCTTGCGA AACGTGCTCG ATCTCTGACG AGATTAACCA   
  
  
- CATCTACCGC TCTTTCGTAG ACAACGATTA GTTGGATACC CCCCTTAAGT AATCAGGCCC TAGGGTTCAA   
  
  
- TCTCTAGCTT CAGTAACTCA CTTCTAGGTG TCCCAAGTGT AGGAGGAGTC GAACTAAGTA GTAGAAAAAG   
  
  
- TTCCCACTTT TCTAAGCCTC TACTTTCGGT CTCTCTCTCT TTACGGTGTT CCGTTACCTT CTTGGTCGCC   
  
  
- TTGATGATTC AAAGAGTGGT CCACTAAATT TCGTCAACGA TTAACTTACA CGCTCCCGAA ATAGTCTATT   
  
  
- GGCTTATCTA CTGAAACTCT CAAACCGATT TGCCGGTTCC TTTCTCCAGA GGTAGAGTCC ACTCGGACAG   
  
  
- GTTGCAGAGC CATGAACATA CTAGCTTCCC GAACACCGTT CCTTTGTCAG AAGCCCCTGA TTGTAGATAG   
  
  
- CCCGAGAATT CATGACATTT CTCGGAGAAC CTTTTATGAA CGAGAGGATG CCGTGAGTCA CCCAATGAGA   
  
  
- TAGCGTTCGG TATCGTCGTT CCGAACCACC CGGAGGATTT CACGCTGAAT GTCCGTAACT ACTAGGACAA   
  
  
- AGATTCATAT GAACACTACG ATCGAACCTC CGATAACCCT TTGCTAATCG CAGAGATAAA CTTTTCAAAT   
  
  
- TTTATGAGCA GCTCAAGTTA CGTAACGGGC AAATACCTGG ACTACAGTCC ACCCTTTACG ACCTACACTC   
  
  
- CGGGTCCCTC CGAAACCGGC AATTAACAGG TAATGTCGAG GTAGTGTGAG GACTGCTCTC ACAGCTACAC   
  
  
- TCGTTGGGAT CCCTATCCGA AGAGTCTTAC CACTTTAACG AGCCAGGATT CCATACGTGA AACCAACTCG   
  
  
- TTCTTAGTTT GTGGTTGTGA TGTGCAAAGA ACTGGGCCAA GTATCTCTGG GAACTGATGA TGAGCTAGTC   
  
  
- GGTACAAAC

+     Myb

| Site Name | Organism | Position | Strand | Matrix score. | sequence | function |
| --- | --- | --- | --- | --- | --- | --- |
| Myb | Arabidopsis thaliana | 1562 | - | 6 | TAACTG |  |
| Myb | Arabidopsis thaliana | 1656 | - | 6 | TAACTG |  |
| Myb | Arabidopsis thaliana | 2067 | - | 6 | CAACTG |  |
| Myb | Arabidopsis thaliana | 1713 | - | 6 | TAACTG |  |

>HU08G00019.1   
+ +Up\_Stream \_Len000AGTTGT AGCAAGCACA TTAAAGTTGA TTATCATTTT GTCCGTGAAA CGGTGAGCAA   
  
  
+ ACGTCATCTC CAGGTCAAAT TCATTCGTAC TCAATCACAA GTTGCTGACA TTTTTACCAA AGGCTAATCG   
  
  
+ TCGCAAAAGT TTCTTTAGTT TAAGGCCAAT CTCTCCGTTG TTTCACCCCA TACAGATTGA GGGGTTGTAA   
  
  
+ TAGTGTATAT ATATAGCCTT GTGTTAGCGG GGCTTGGGCA TTAGACTTTT ACTCTTGTAA ACCCTAATGT   
  
  
+ GTTATTATAA ATATAGATCT AGCCACCCAT GTTGAGGTAG GCACTCTAAT TCTGACACAG GTAGGATAAG   
  
  
+ TTTTCATAGT TTATGTTGTG AATCTTGTCT TACTTTGTGG TTCCTATTGA CAAAAGTTTG AATCCGAGCA   
  
  
+ TGAGAGTTCA TCAATCTCTT TTTCTTTCCT ATTTTATTTC TTGTGGACTT AATTGGATTT TCTGGTAGAG   
  
  
+ GAAAGTTCCT TGCTGGAATT TGGTCGAAAG TGATATACAT TTTGGGATTT AATTAGCCAT AAATTTGGTT   
  
  
+ TTACACCTTG TTAGTTTGGA GTTCTGGTGT GTTATTGTAT GTGTTCTGGT GGGTTTTTCT TTTAGCTAAA   
  
  
+ TGTTGGAGCA TTTTAAGGGA TCTAGATATC TGAAGAAGTG CTTGGGAAAT GAAGCATATT TCCCTTATAG   
  
  
+ TCTGACATAA CTTTAATGAA TTGATCATGA ACAAAGGGCA ATTAGTTCAG AAAGGTTCAA CTCCTCTAGG   
  
  
+ TGTTGTGCTA AAGAGATGTG AGTTAGTAAC AATTGCTTAC TAACTATATA GGTTGTTTTA TTACTTGTAG   
  
  
+ AGGTTTTTTT TTTGGGATAT CTTTTTGGGG TTTATAAATG AGAAGGTTGA TTTGTTGTCT ACTCTTCTTT   
  
  
+ CGATTCAATA GTAATTCTCT CAGAGAAGGC TAAAATGACA TGGCATTCCA TCGCCTCTCA TGAGGCTTTT   
  
  
+ TGCATGTGCT TATGTATCTT TCATCTCTTA CTTTGGTGGG GTGGCATTGG GGAGGAAAAG GGGGTTTCGA   
  
  
+ TTCGATAGAT CGTCACTTTC GGAAATGACG TCTCTGACCA ATGGTAAGGG TAAGGTTAAT ACATGTGGAT   
  
  
+ ATTTCCAGAG TTAGTTGTGA TTGTTATTGT TGTTGTTGTA ACTTTATGTC ACGGGGAAAA CATAATAATT   
  
  
+ ATCTGTTTAT CTGTTTGTTT GTTATTTTCA AGGGGAAGGG GAGTGGGCGG ATGCTTAGTA TGTAATCATT   
  
  
+ TTATATAGGG AAAATTCATA TAGCATGAAA GTAAAACTTT CATTGCCAGC ATTCTAAGCT AGGGGTAGGG   
  
  
+ TTGTCTACAC CTTGATCTCC CTAGAAGCAT GGTGATCATT CCTCAGATTG TGTTATAGAA TAAAGAATGT   
  
  
+ GGTTCTGCTG TAATGAGACT TATGTATTAT CAAATGATGA ATCTGTTTAG AACTTCCTGT TTATTTTTTC   
  
  
+ TGCAAAATTT TAAGAGACTA GCATCTATCG GGCTCGACTC TAGAGGTTCT CTTGTGTTAC CCTTCTCAAC   
  
  
+ TCAGTTTGAT TGTGATACAG TTACTATATT GAGTGATAGT CGAGAGCATC ACAACCCGCT AAGGAGTCTA   
  
  
+ TCAACAAGAA GCCCTTCTTG TAATTCTCCC CTTGAAACTA GCAGTTATCA TCACTTGTCA TCGAATAGCC   
  
  
+ CTTTTTGTAA TTCTACCCTT GAAACTAGCA GTTATTGTCA TCAGTTCAAT GTGAGTCCCG TTAGATATTC   
  
  
+ CCATCAAGGC ATAGATTATG GAGTGAGCAT GAAGAACGCT TTGCACGAGC TAGAGACTGC TCTAATTGGT   
  
  
+ GTAGATGGCG AGAAAGCATC TGTTGCTAAT CAACCTATGG GGGGAATTCA TTAGTCCGGG ATCCCAAGTT   
  
  
+ AGAGATCGAA GTCATTGAGT GAAGATCCAC AGGGTTCACA TCCTCCTCAG CTTGATTCAT CATCTTTTTC   
  
  
+ AAGGGTGAAA AGATTCGGAG ATGAAAGCCA GAGAGAGAGA AATGCCACAA GGCAATGGAA GAACCAGCGG   
  
  
+ AACTACTAAG TTTCTCACCA GGTGATTTAA AGCAGTTGCT AATTGAATGT GCGAGGGCTT TATCAGATAA   
  
  
+ CCGAATAGAT GACTTTGAGA GTTTGGCTAA ACGGCCAAGG AAAGAGGTCT CCATCTCAGG TGAGCCTGTC   
  
  
+ CAACGTCTCG GTACTTGTAT GATCGAAGGG CTTGTGGCAA GGAAACAGTC TTCGGGGACT AACATCTATC   
  
  
+ GGGCTCTTAA GTACTGTAAA GAGCCTCTTG GAAAATACTT GCTCTCCTAC GGCACTCAGT GGGTTACTCT   
  
  
+ ATCGCAAGCC ATAGCAGCAA GGCTTGGTGG GCCTCCTAAA GTGCGACTTA CAGGCATTGA TGATCCTGTT   
  
  
+ TCTAAGTATA CTTGTGATGC TAGCTTGGAG GCTATTGGGA AACGATTAGC GTCTCTATTT GAAAAGTTTA   
  
  
+ AAATACTCGT CGAGTTCAAT GCATTGCCCG TTTATGGACC TGATGTCAGG TGGGAAATGC TGGATGTGAG   
  
  
+ GCCCAGGGAG GCTTTGGCCG TTAATTGTCC ATTACAGCTC CATCACACTC CTGACGAGAG TGTCGATGTG   
  
  
+ AGCAACCCTA GGGATAGGCT TCTCAGAATG GTGAAATTGC TCGGTCCTAA GGTATGCACT TTGGTTGAGC   
  
  
+ AAGAATCAAA CACCAACACT ACACGTTTCT TGACCCGGTT CATAGAGACC CTTGACTACT ACTCGATCAG   
  
  
+ CCATGTTTG  

- +Up\_Stream \_Len000TCAACA TCGTTCGTGT AATTTCAACT AATAGTAAAA CAGGCACTTT GCCACTCGTT   
  
  
- TGCAGTAGAG GTCCAGTTTA AGTAAGCATG AGTTAGTGTT CAACGACTGT AAAAATGGTT TCCGATTAGC   
  
  
- AGCGTTTTCA AAGAAATCAA ATTCCGGTTA GAGAGGCAAC AAAGTGGGGT ATGTCTAACT CCCCAACATT   
  
  
- ATCACATATA TATATCGGAA CACAATCGCC CCGAACCCGT AATCTGAAAA TGAGAACATT TGGGATTACA   
  
  
- CAATAATATT TATATCTAGA TCGGTGGGTA CAACTCCATC CGTGAGATTA AGACTGTGTC CATCCTATTC   
  
  
- AAAAGTATCA AATACAACAC TTAGAACAGA ATGAAACACC AAGGATAACT GTTTTCAAAC TTAGGCTCGT   
  
  
- ACTCTCAAGT AGTTAGAGAA AAAGAAAGGA TAAAATAAAG AACACCTGAA TTAACCTAAA AGACCATCTC   
  
  
- CTTTCAAGGA ACGACCTTAA ACCAGCTTTC ACTATATGTA AAACCCTAAA TTAATCGGTA TTTAAACCAA   
  
  
- AATGTGGAAC AATCAAACCT CAAGACCACA CAATAACATA CACAAGACCA CCCAAAAAGA AAATCGATTT   
  
  
- ACAACCTCGT AAAATTCCCT AGATCTATAG ACTTCTTCAC GAACCCTTTA CTTCGTATAA AGGGAATATC   
  
  
- AGACTGTATT GAAATTACTT AACTAGTACT TGTTTCCCGT TAATCAAGTC TTTCCAAGTT GAGGAGATCC   
  
  
- ACAACACGAT TTCTCTACAC TCAATCATTG TTAACGAATG ATTGATATAT CCAACAAAAT AATGAACATC   
  
  
- TCCAAAAAAA AAACCCTATA GAAAAACCCC AAATATTTAC TCTTCCAACT AAACAACAGA TGAGAAGAAA   
  
  
- GCTAAGTTAT CATTAAGAGA GTCTCTTCCG ATTTTACTGT ACCGTAAGGT AGCGGAGAGT ACTCCGAAAA   
  
  
- ACGTACACGA ATACATAGAA AGTAGAGAAT GAAACCACCC CACCGTAACC CCTCCTTTTC CCCCAAAGCT   
  
  
- AAGCTATCTA GCAGTGAAAG CCTTTACTGC AGAGACTGGT TACCATTCCC ATTCCAATTA TGTACACCTA   
  
  
- TAAAGGTCTC AATCAACACT AACAATAACA ACAACAACAT TGAAATACAG TGCCCCTTTT GTATTATTAA   
  
  
- TAGACAAATA GACAAACAAA CAATAAAAGT TCCCCTTCCC CTCACCCGCC TACGAATCAT ACATTAGTAA   
  
  
- AATATATCCC TTTTAAGTAT ATCGTACTTT CATTTTGAAA GTAACGGTCG TAAGATTCGA TCCCCATCCC   
  
  
- AACAGATGTG GAACTAGAGG GATCTTCGTA CCACTAGTAA GGAGTCTAAC ACAATATCTT ATTTCTTACA   
  
  
- CCAAGACGAC ATTACTCTGA ATACATAATA GTTTACTACT TAGACAAATC TTGAAGGACA AATAAAAAAG   
  
  
- ACGTTTTAAA ATTCTCTGAT CGTAGATAGC CCGAGCTGAG ATCTCCAAGA GAACACAATG GGAAGAGTTG   
  
  
- AGTCAAACTA ACACTATGTC AATGATATAA CTCACTATCA GCTCTCGTAG TGTTGGGCGA TTCCTCAGAT   
  
  
- AGTTGTTCTT CGGGAAGAAC ATTAAGAGGG GAACTTTGAT CGTCAATAGT AGTGAACAGT AGCTTATCGG   
  
  
- GAAAAACATT AAGATGGGAA CTTTGATCGT CAATAACAGT AGTCAAGTTA CACTCAGGGC AATCTATAAG   
  
  
- GGTAGTTCCG TATCTAATAC CTCACTCGTA CTTCTTGCGA AACGTGCTCG ATCTCTGACG AGATTAACCA   
  
  
- CATCTACCGC TCTTTCGTAG ACAACGATTA GTTGGATACC CCCCTTAAGT AATCAGGCCC TAGGGTTCAA   
  
  
- TCTCTAGCTT CAGTAACTCA CTTCTAGGTG TCCCAAGTGT AGGAGGAGTC GAACTAAGTA GTAGAAAAAG   
  
  
- TTCCCACTTT TCTAAGCCTC TACTTTCGGT CTCTCTCTCT TTACGGTGTT CCGTTACCTT CTTGGTCGCC   
  
  
- TTGATGATTC AAAGAGTGGT CCACTAAATT TCGTCAACGA TTAACTTACA CGCTCCCGAA ATAGTCTATT   
  
  
- GGCTTATCTA CTGAAACTCT CAAACCGATT TGCCGGTTCC TTTCTCCAGA GGTAGAGTCC ACTCGGACAG   
  
  
- GTTGCAGAGC CATGAACATA CTAGCTTCCC GAACACCGTT CCTTTGTCAG AAGCCCCTGA TTGTAGATAG   
  
  
- CCCGAGAATT CATGACATTT CTCGGAGAAC CTTTTATGAA CGAGAGGATG CCGTGAGTCA CCCAATGAGA   
  
  
- TAGCGTTCGG TATCGTCGTT CCGAACCACC CGGAGGATTT CACGCTGAAT GTCCGTAACT ACTAGGACAA   
  
  
- AGATTCATAT GAACACTACG ATCGAACCTC CGATAACCCT TTGCTAATCG CAGAGATAAA CTTTTCAAAT   
  
  
- TTTATGAGCA GCTCAAGTTA CGTAACGGGC AAATACCTGG ACTACAGTCC ACCCTTTACG ACCTACACTC   
  
  
- CGGGTCCCTC CGAAACCGGC AATTAACAGG TAATGTCGAG GTAGTGTGAG GACTGCTCTC ACAGCTACAC   
  
  
- TCGTTGGGAT CCCTATCCGA AGAGTCTTAC CACTTTAACG AGCCAGGATT CCATACGTGA AACCAACTCG   
  
  
- TTCTTAGTTT GTGGTTGTGA TGTGCAAAGA ACTGGGCCAA GTATCTCTGG GAACTGATGA TGAGCTAGTC   
  
  
- GGTACAAAC

+     Myb-binding site

| Site Name | Organism | Position | Strand | Matrix score. | sequence | function |
| --- | --- | --- | --- | --- | --- | --- |
| Myb-binding site | Nicotiana tabacum | 1844 | - | 6 | CAACAG |  |

>HU08G00019.1   
+ +Up\_Stream \_Len000AGTTGT AGCAAGCACA TTAAAGTTGA TTATCATTTT GTCCGTGAAA CGGTGAGCAA   
  
  
+ ACGTCATCTC CAGGTCAAAT TCATTCGTAC TCAATCACAA GTTGCTGACA TTTTTACCAA AGGCTAATCG   
  
  
+ TCGCAAAAGT TTCTTTAGTT TAAGGCCAAT CTCTCCGTTG TTTCACCCCA TACAGATTGA GGGGTTGTAA   
  
  
+ TAGTGTATAT ATATAGCCTT GTGTTAGCGG GGCTTGGGCA TTAGACTTTT ACTCTTGTAA ACCCTAATGT   
  
  
+ GTTATTATAA ATATAGATCT AGCCACCCAT GTTGAGGTAG GCACTCTAAT TCTGACACAG GTAGGATAAG   
  
  
+ TTTTCATAGT TTATGTTGTG AATCTTGTCT TACTTTGTGG TTCCTATTGA CAAAAGTTTG AATCCGAGCA   
  
  
+ TGAGAGTTCA TCAATCTCTT TTTCTTTCCT ATTTTATTTC TTGTGGACTT AATTGGATTT TCTGGTAGAG   
  
  
+ GAAAGTTCCT TGCTGGAATT TGGTCGAAAG TGATATACAT TTTGGGATTT AATTAGCCAT AAATTTGGTT   
  
  
+ TTACACCTTG TTAGTTTGGA GTTCTGGTGT GTTATTGTAT GTGTTCTGGT GGGTTTTTCT TTTAGCTAAA   
  
  
+ TGTTGGAGCA TTTTAAGGGA TCTAGATATC TGAAGAAGTG CTTGGGAAAT GAAGCATATT TCCCTTATAG   
  
  
+ TCTGACATAA CTTTAATGAA TTGATCATGA ACAAAGGGCA ATTAGTTCAG AAAGGTTCAA CTCCTCTAGG   
  
  
+ TGTTGTGCTA AAGAGATGTG AGTTAGTAAC AATTGCTTAC TAACTATATA GGTTGTTTTA TTACTTGTAG   
  
  
+ AGGTTTTTTT TTTGGGATAT CTTTTTGGGG TTTATAAATG AGAAGGTTGA TTTGTTGTCT ACTCTTCTTT   
  
  
+ CGATTCAATA GTAATTCTCT CAGAGAAGGC TAAAATGACA TGGCATTCCA TCGCCTCTCA TGAGGCTTTT   
  
  
+ TGCATGTGCT TATGTATCTT TCATCTCTTA CTTTGGTGGG GTGGCATTGG GGAGGAAAAG GGGGTTTCGA   
  
  
+ TTCGATAGAT CGTCACTTTC GGAAATGACG TCTCTGACCA ATGGTAAGGG TAAGGTTAAT ACATGTGGAT   
  
  
+ ATTTCCAGAG TTAGTTGTGA TTGTTATTGT TGTTGTTGTA ACTTTATGTC ACGGGGAAAA CATAATAATT   
  
  
+ ATCTGTTTAT CTGTTTGTTT GTTATTTTCA AGGGGAAGGG GAGTGGGCGG ATGCTTAGTA TGTAATCATT   
  
  
+ TTATATAGGG AAAATTCATA TAGCATGAAA GTAAAACTTT CATTGCCAGC ATTCTAAGCT AGGGGTAGGG   
  
  
+ TTGTCTACAC CTTGATCTCC CTAGAAGCAT GGTGATCATT CCTCAGATTG TGTTATAGAA TAAAGAATGT   
  
  
+ GGTTCTGCTG TAATGAGACT TATGTATTAT CAAATGATGA ATCTGTTTAG AACTTCCTGT TTATTTTTTC   
  
  
+ TGCAAAATTT TAAGAGACTA GCATCTATCG GGCTCGACTC TAGAGGTTCT CTTGTGTTAC CCTTCTCAAC   
  
  
+ TCAGTTTGAT TGTGATACAG TTACTATATT GAGTGATAGT CGAGAGCATC ACAACCCGCT AAGGAGTCTA   
  
  
+ TCAACAAGAA GCCCTTCTTG TAATTCTCCC CTTGAAACTA GCAGTTATCA TCACTTGTCA TCGAATAGCC   
  
  
+ CTTTTTGTAA TTCTACCCTT GAAACTAGCA GTTATTGTCA TCAGTTCAAT GTGAGTCCCG TTAGATATTC   
  
  
+ CCATCAAGGC ATAGATTATG GAGTGAGCAT GAAGAACGCT TTGCACGAGC TAGAGACTGC TCTAATTGGT   
  
  
+ GTAGATGGCG AGAAAGCATC TGTTGCTAAT CAACCTATGG GGGGAATTCA TTAGTCCGGG ATCCCAAGTT   
  
  
+ AGAGATCGAA GTCATTGAGT GAAGATCCAC AGGGTTCACA TCCTCCTCAG CTTGATTCAT CATCTTTTTC   
  
  
+ AAGGGTGAAA AGATTCGGAG ATGAAAGCCA GAGAGAGAGA AATGCCACAA GGCAATGGAA GAACCAGCGG   
  
  
+ AACTACTAAG TTTCTCACCA GGTGATTTAA AGCAGTTGCT AATTGAATGT GCGAGGGCTT TATCAGATAA   
  
  
+ CCGAATAGAT GACTTTGAGA GTTTGGCTAA ACGGCCAAGG AAAGAGGTCT CCATCTCAGG TGAGCCTGTC   
  
  
+ CAACGTCTCG GTACTTGTAT GATCGAAGGG CTTGTGGCAA GGAAACAGTC TTCGGGGACT AACATCTATC   
  
  
+ GGGCTCTTAA GTACTGTAAA GAGCCTCTTG GAAAATACTT GCTCTCCTAC GGCACTCAGT GGGTTACTCT   
  
  
+ ATCGCAAGCC ATAGCAGCAA GGCTTGGTGG GCCTCCTAAA GTGCGACTTA CAGGCATTGA TGATCCTGTT   
  
  
+ TCTAAGTATA CTTGTGATGC TAGCTTGGAG GCTATTGGGA AACGATTAGC GTCTCTATTT GAAAAGTTTA   
  
  
+ AAATACTCGT CGAGTTCAAT GCATTGCCCG TTTATGGACC TGATGTCAGG TGGGAAATGC TGGATGTGAG   
  
  
+ GCCCAGGGAG GCTTTGGCCG TTAATTGTCC ATTACAGCTC CATCACACTC CTGACGAGAG TGTCGATGTG   
  
  
+ AGCAACCCTA GGGATAGGCT TCTCAGAATG GTGAAATTGC TCGGTCCTAA GGTATGCACT TTGGTTGAGC   
  
  
+ AAGAATCAAA CACCAACACT ACACGTTTCT TGACCCGGTT CATAGAGACC CTTGACTACT ACTCGATCAG   
  
  
+ CCATGTTTG  

- +Up\_Stream \_Len000TCAACA TCGTTCGTGT AATTTCAACT AATAGTAAAA CAGGCACTTT GCCACTCGTT   
  
  
- TGCAGTAGAG GTCCAGTTTA AGTAAGCATG AGTTAGTGTT CAACGACTGT AAAAATGGTT TCCGATTAGC   
  
  
- AGCGTTTTCA AAGAAATCAA ATTCCGGTTA GAGAGGCAAC AAAGTGGGGT ATGTCTAACT CCCCAACATT   
  
  
- ATCACATATA TATATCGGAA CACAATCGCC CCGAACCCGT AATCTGAAAA TGAGAACATT TGGGATTACA   
  
  
- CAATAATATT TATATCTAGA TCGGTGGGTA CAACTCCATC CGTGAGATTA AGACTGTGTC CATCCTATTC   
  
  
- AAAAGTATCA AATACAACAC TTAGAACAGA ATGAAACACC AAGGATAACT GTTTTCAAAC TTAGGCTCGT   
  
  
- ACTCTCAAGT AGTTAGAGAA AAAGAAAGGA TAAAATAAAG AACACCTGAA TTAACCTAAA AGACCATCTC   
  
  
- CTTTCAAGGA ACGACCTTAA ACCAGCTTTC ACTATATGTA AAACCCTAAA TTAATCGGTA TTTAAACCAA   
  
  
- AATGTGGAAC AATCAAACCT CAAGACCACA CAATAACATA CACAAGACCA CCCAAAAAGA AAATCGATTT   
  
  
- ACAACCTCGT AAAATTCCCT AGATCTATAG ACTTCTTCAC GAACCCTTTA CTTCGTATAA AGGGAATATC   
  
  
- AGACTGTATT GAAATTACTT AACTAGTACT TGTTTCCCGT TAATCAAGTC TTTCCAAGTT GAGGAGATCC   
  
  
- ACAACACGAT TTCTCTACAC TCAATCATTG TTAACGAATG ATTGATATAT CCAACAAAAT AATGAACATC   
  
  
- TCCAAAAAAA AAACCCTATA GAAAAACCCC AAATATTTAC TCTTCCAACT AAACAACAGA TGAGAAGAAA   
  
  
- GCTAAGTTAT CATTAAGAGA GTCTCTTCCG ATTTTACTGT ACCGTAAGGT AGCGGAGAGT ACTCCGAAAA   
  
  
- ACGTACACGA ATACATAGAA AGTAGAGAAT GAAACCACCC CACCGTAACC CCTCCTTTTC CCCCAAAGCT   
  
  
- AAGCTATCTA GCAGTGAAAG CCTTTACTGC AGAGACTGGT TACCATTCCC ATTCCAATTA TGTACACCTA   
  
  
- TAAAGGTCTC AATCAACACT AACAATAACA ACAACAACAT TGAAATACAG TGCCCCTTTT GTATTATTAA   
  
  
- TAGACAAATA GACAAACAAA CAATAAAAGT TCCCCTTCCC CTCACCCGCC TACGAATCAT ACATTAGTAA   
  
  
- AATATATCCC TTTTAAGTAT ATCGTACTTT CATTTTGAAA GTAACGGTCG TAAGATTCGA TCCCCATCCC   
  
  
- AACAGATGTG GAACTAGAGG GATCTTCGTA CCACTAGTAA GGAGTCTAAC ACAATATCTT ATTTCTTACA   
  
  
- CCAAGACGAC ATTACTCTGA ATACATAATA GTTTACTACT TAGACAAATC TTGAAGGACA AATAAAAAAG   
  
  
- ACGTTTTAAA ATTCTCTGAT CGTAGATAGC CCGAGCTGAG ATCTCCAAGA GAACACAATG GGAAGAGTTG   
  
  
- AGTCAAACTA ACACTATGTC AATGATATAA CTCACTATCA GCTCTCGTAG TGTTGGGCGA TTCCTCAGAT   
  
  
- AGTTGTTCTT CGGGAAGAAC ATTAAGAGGG GAACTTTGAT CGTCAATAGT AGTGAACAGT AGCTTATCGG   
  
  
- GAAAAACATT AAGATGGGAA CTTTGATCGT CAATAACAGT AGTCAAGTTA CACTCAGGGC AATCTATAAG   
  
  
- GGTAGTTCCG TATCTAATAC CTCACTCGTA CTTCTTGCGA AACGTGCTCG ATCTCTGACG AGATTAACCA   
  
  
- CATCTACCGC TCTTTCGTAG ACAACGATTA GTTGGATACC CCCCTTAAGT AATCAGGCCC TAGGGTTCAA   
  
  
- TCTCTAGCTT CAGTAACTCA CTTCTAGGTG TCCCAAGTGT AGGAGGAGTC GAACTAAGTA GTAGAAAAAG   
  
  
- TTCCCACTTT TCTAAGCCTC TACTTTCGGT CTCTCTCTCT TTACGGTGTT CCGTTACCTT CTTGGTCGCC   
  
  
- TTGATGATTC AAAGAGTGGT CCACTAAATT TCGTCAACGA TTAACTTACA CGCTCCCGAA ATAGTCTATT   
  
  
- GGCTTATCTA CTGAAACTCT CAAACCGATT TGCCGGTTCC TTTCTCCAGA GGTAGAGTCC ACTCGGACAG   
  
  
- GTTGCAGAGC CATGAACATA CTAGCTTCCC GAACACCGTT CCTTTGTCAG AAGCCCCTGA TTGTAGATAG   
  
  
- CCCGAGAATT CATGACATTT CTCGGAGAAC CTTTTATGAA CGAGAGGATG CCGTGAGTCA CCCAATGAGA   
  
  
- TAGCGTTCGG TATCGTCGTT CCGAACCACC CGGAGGATTT CACGCTGAAT GTCCGTAACT ACTAGGACAA   
  
  
- AGATTCATAT GAACACTACG ATCGAACCTC CGATAACCCT TTGCTAATCG CAGAGATAAA CTTTTCAAAT   
  
  
- TTTATGAGCA GCTCAAGTTA CGTAACGGGC AAATACCTGG ACTACAGTCC ACCCTTTACG ACCTACACTC   
  
  
- CGGGTCCCTC CGAAACCGGC AATTAACAGG TAATGTCGAG GTAGTGTGAG GACTGCTCTC ACAGCTACAC   
  
  
- TCGTTGGGAT CCCTATCCGA AGAGTCTTAC CACTTTAACG AGCCAGGATT CCATACGTGA AACCAACTCG   
  
  
- TTCTTAGTTT GTGGTTGTGA TGTGCAAAGA ACTGGGCCAA GTATCTCTGG GAACTGATGA TGAGCTAGTC   
  
  
- GGTACAAAC

+     Myc

| Site Name | Organism | Position | Strand | Matrix score. | sequence | function |
| --- | --- | --- | --- | --- | --- | --- |
| Myc | Arabidopsis thaliana | 1008 | + | 7 | TCTCTTA |  |
| Myc | Arabidopsis thaliana | 1485 | - | 7 | TCTCTTA |  |

>HU08G00019.1   
+ +Up\_Stream \_Len000AGTTGT AGCAAGCACA TTAAAGTTGA TTATCATTTT GTCCGTGAAA CGGTGAGCAA   
  
  
+ ACGTCATCTC CAGGTCAAAT TCATTCGTAC TCAATCACAA GTTGCTGACA TTTTTACCAA AGGCTAATCG   
  
  
+ TCGCAAAAGT TTCTTTAGTT TAAGGCCAAT CTCTCCGTTG TTTCACCCCA TACAGATTGA GGGGTTGTAA   
  
  
+ TAGTGTATAT ATATAGCCTT GTGTTAGCGG GGCTTGGGCA TTAGACTTTT ACTCTTGTAA ACCCTAATGT   
  
  
+ GTTATTATAA ATATAGATCT AGCCACCCAT GTTGAGGTAG GCACTCTAAT TCTGACACAG GTAGGATAAG   
  
  
+ TTTTCATAGT TTATGTTGTG AATCTTGTCT TACTTTGTGG TTCCTATTGA CAAAAGTTTG AATCCGAGCA   
  
  
+ TGAGAGTTCA TCAATCTCTT TTTCTTTCCT ATTTTATTTC TTGTGGACTT AATTGGATTT TCTGGTAGAG   
  
  
+ GAAAGTTCCT TGCTGGAATT TGGTCGAAAG TGATATACAT TTTGGGATTT AATTAGCCAT AAATTTGGTT   
  
  
+ TTACACCTTG TTAGTTTGGA GTTCTGGTGT GTTATTGTAT GTGTTCTGGT GGGTTTTTCT TTTAGCTAAA   
  
  
+ TGTTGGAGCA TTTTAAGGGA TCTAGATATC TGAAGAAGTG CTTGGGAAAT GAAGCATATT TCCCTTATAG   
  
  
+ TCTGACATAA CTTTAATGAA TTGATCATGA ACAAAGGGCA ATTAGTTCAG AAAGGTTCAA CTCCTCTAGG   
  
  
+ TGTTGTGCTA AAGAGATGTG AGTTAGTAAC AATTGCTTAC TAACTATATA GGTTGTTTTA TTACTTGTAG   
  
  
+ AGGTTTTTTT TTTGGGATAT CTTTTTGGGG TTTATAAATG AGAAGGTTGA TTTGTTGTCT ACTCTTCTTT   
  
  
+ CGATTCAATA GTAATTCTCT CAGAGAAGGC TAAAATGACA TGGCATTCCA TCGCCTCTCA TGAGGCTTTT   
  
  
+ TGCATGTGCT TATGTATCTT TCATCTCTTA CTTTGGTGGG GTGGCATTGG GGAGGAAAAG GGGGTTTCGA   
  
  
+ TTCGATAGAT CGTCACTTTC GGAAATGACG TCTCTGACCA ATGGTAAGGG TAAGGTTAAT ACATGTGGAT   
  
  
+ ATTTCCAGAG TTAGTTGTGA TTGTTATTGT TGTTGTTGTA ACTTTATGTC ACGGGGAAAA CATAATAATT   
  
  
+ ATCTGTTTAT CTGTTTGTTT GTTATTTTCA AGGGGAAGGG GAGTGGGCGG ATGCTTAGTA TGTAATCATT   
  
  
+ TTATATAGGG AAAATTCATA TAGCATGAAA GTAAAACTTT CATTGCCAGC ATTCTAAGCT AGGGGTAGGG   
  
  
+ TTGTCTACAC CTTGATCTCC CTAGAAGCAT GGTGATCATT CCTCAGATTG TGTTATAGAA TAAAGAATGT   
  
  
+ GGTTCTGCTG TAATGAGACT TATGTATTAT CAAATGATGA ATCTGTTTAG AACTTCCTGT TTATTTTTTC   
  
  
+ TGCAAAATTT TAAGAGACTA GCATCTATCG GGCTCGACTC TAGAGGTTCT CTTGTGTTAC CCTTCTCAAC   
  
  
+ TCAGTTTGAT TGTGATACAG TTACTATATT GAGTGATAGT CGAGAGCATC ACAACCCGCT AAGGAGTCTA   
  
  
+ TCAACAAGAA GCCCTTCTTG TAATTCTCCC CTTGAAACTA GCAGTTATCA TCACTTGTCA TCGAATAGCC   
  
  
+ CTTTTTGTAA TTCTACCCTT GAAACTAGCA GTTATTGTCA TCAGTTCAAT GTGAGTCCCG TTAGATATTC   
  
  
+ CCATCAAGGC ATAGATTATG GAGTGAGCAT GAAGAACGCT TTGCACGAGC TAGAGACTGC TCTAATTGGT   
  
  
+ GTAGATGGCG AGAAAGCATC TGTTGCTAAT CAACCTATGG GGGGAATTCA TTAGTCCGGG ATCCCAAGTT   
  
  
+ AGAGATCGAA GTCATTGAGT GAAGATCCAC AGGGTTCACA TCCTCCTCAG CTTGATTCAT CATCTTTTTC   
  
  
+ AAGGGTGAAA AGATTCGGAG ATGAAAGCCA GAGAGAGAGA AATGCCACAA GGCAATGGAA GAACCAGCGG   
  
  
+ AACTACTAAG TTTCTCACCA GGTGATTTAA AGCAGTTGCT AATTGAATGT GCGAGGGCTT TATCAGATAA   
  
  
+ CCGAATAGAT GACTTTGAGA GTTTGGCTAA ACGGCCAAGG AAAGAGGTCT CCATCTCAGG TGAGCCTGTC   
  
  
+ CAACGTCTCG GTACTTGTAT GATCGAAGGG CTTGTGGCAA GGAAACAGTC TTCGGGGACT AACATCTATC   
  
  
+ GGGCTCTTAA GTACTGTAAA GAGCCTCTTG GAAAATACTT GCTCTCCTAC GGCACTCAGT GGGTTACTCT   
  
  
+ ATCGCAAGCC ATAGCAGCAA GGCTTGGTGG GCCTCCTAAA GTGCGACTTA CAGGCATTGA TGATCCTGTT   
  
  
+ TCTAAGTATA CTTGTGATGC TAGCTTGGAG GCTATTGGGA AACGATTAGC GTCTCTATTT GAAAAGTTTA   
  
  
+ AAATACTCGT CGAGTTCAAT GCATTGCCCG TTTATGGACC TGATGTCAGG TGGGAAATGC TGGATGTGAG   
  
  
+ GCCCAGGGAG GCTTTGGCCG TTAATTGTCC ATTACAGCTC CATCACACTC CTGACGAGAG TGTCGATGTG   
  
  
+ AGCAACCCTA GGGATAGGCT TCTCAGAATG GTGAAATTGC TCGGTCCTAA GGTATGCACT TTGGTTGAGC   
  
  
+ AAGAATCAAA CACCAACACT ACACGTTTCT TGACCCGGTT CATAGAGACC CTTGACTACT ACTCGATCAG   
  
  
+ CCATGTTTG  

- +Up\_Stream \_Len000TCAACA TCGTTCGTGT AATTTCAACT AATAGTAAAA CAGGCACTTT GCCACTCGTT   
  
  
- TGCAGTAGAG GTCCAGTTTA AGTAAGCATG AGTTAGTGTT CAACGACTGT AAAAATGGTT TCCGATTAGC   
  
  
- AGCGTTTTCA AAGAAATCAA ATTCCGGTTA GAGAGGCAAC AAAGTGGGGT ATGTCTAACT CCCCAACATT   
  
  
- ATCACATATA TATATCGGAA CACAATCGCC CCGAACCCGT AATCTGAAAA TGAGAACATT TGGGATTACA   
  
  
- CAATAATATT TATATCTAGA TCGGTGGGTA CAACTCCATC CGTGAGATTA AGACTGTGTC CATCCTATTC   
  
  
- AAAAGTATCA AATACAACAC TTAGAACAGA ATGAAACACC AAGGATAACT GTTTTCAAAC TTAGGCTCGT   
  
  
- ACTCTCAAGT AGTTAGAGAA AAAGAAAGGA TAAAATAAAG AACACCTGAA TTAACCTAAA AGACCATCTC   
  
  
- CTTTCAAGGA ACGACCTTAA ACCAGCTTTC ACTATATGTA AAACCCTAAA TTAATCGGTA TTTAAACCAA   
  
  
- AATGTGGAAC AATCAAACCT CAAGACCACA CAATAACATA CACAAGACCA CCCAAAAAGA AAATCGATTT   
  
  
- ACAACCTCGT AAAATTCCCT AGATCTATAG ACTTCTTCAC GAACCCTTTA CTTCGTATAA AGGGAATATC   
  
  
- AGACTGTATT GAAATTACTT AACTAGTACT TGTTTCCCGT TAATCAAGTC TTTCCAAGTT GAGGAGATCC   
  
  
- ACAACACGAT TTCTCTACAC TCAATCATTG TTAACGAATG ATTGATATAT CCAACAAAAT AATGAACATC   
  
  
- TCCAAAAAAA AAACCCTATA GAAAAACCCC AAATATTTAC TCTTCCAACT AAACAACAGA TGAGAAGAAA   
  
  
- GCTAAGTTAT CATTAAGAGA GTCTCTTCCG ATTTTACTGT ACCGTAAGGT AGCGGAGAGT ACTCCGAAAA   
  
  
- ACGTACACGA ATACATAGAA AGTAGAGAAT GAAACCACCC CACCGTAACC CCTCCTTTTC CCCCAAAGCT   
  
  
- AAGCTATCTA GCAGTGAAAG CCTTTACTGC AGAGACTGGT TACCATTCCC ATTCCAATTA TGTACACCTA   
  
  
- TAAAGGTCTC AATCAACACT AACAATAACA ACAACAACAT TGAAATACAG TGCCCCTTTT GTATTATTAA   
  
  
- TAGACAAATA GACAAACAAA CAATAAAAGT TCCCCTTCCC CTCACCCGCC TACGAATCAT ACATTAGTAA   
  
  
- AATATATCCC TTTTAAGTAT ATCGTACTTT CATTTTGAAA GTAACGGTCG TAAGATTCGA TCCCCATCCC   
  
  
- AACAGATGTG GAACTAGAGG GATCTTCGTA CCACTAGTAA GGAGTCTAAC ACAATATCTT ATTTCTTACA   
  
  
- CCAAGACGAC ATTACTCTGA ATACATAATA GTTTACTACT TAGACAAATC TTGAAGGACA AATAAAAAAG   
  
  
- ACGTTTTAAA ATTCTCTGAT CGTAGATAGC CCGAGCTGAG ATCTCCAAGA GAACACAATG GGAAGAGTTG   
  
  
- AGTCAAACTA ACACTATGTC AATGATATAA CTCACTATCA GCTCTCGTAG TGTTGGGCGA TTCCTCAGAT   
  
  
- AGTTGTTCTT CGGGAAGAAC ATTAAGAGGG GAACTTTGAT CGTCAATAGT AGTGAACAGT AGCTTATCGG   
  
  
- GAAAAACATT AAGATGGGAA CTTTGATCGT CAATAACAGT AGTCAAGTTA CACTCAGGGC AATCTATAAG   
  
  
- GGTAGTTCCG TATCTAATAC CTCACTCGTA CTTCTTGCGA AACGTGCTCG ATCTCTGACG AGATTAACCA   
  
  
- CATCTACCGC TCTTTCGTAG ACAACGATTA GTTGGATACC CCCCTTAAGT AATCAGGCCC TAGGGTTCAA   
  
  
- TCTCTAGCTT CAGTAACTCA CTTCTAGGTG TCCCAAGTGT AGGAGGAGTC GAACTAAGTA GTAGAAAAAG   
  
  
- TTCCCACTTT TCTAAGCCTC TACTTTCGGT CTCTCTCTCT TTACGGTGTT CCGTTACCTT CTTGGTCGCC   
  
  
- TTGATGATTC AAAGAGTGGT CCACTAAATT TCGTCAACGA TTAACTTACA CGCTCCCGAA ATAGTCTATT   
  
  
- GGCTTATCTA CTGAAACTCT CAAACCGATT TGCCGGTTCC TTTCTCCAGA GGTAGAGTCC ACTCGGACAG   
  
  
- GTTGCAGAGC CATGAACATA CTAGCTTCCC GAACACCGTT CCTTTGTCAG AAGCCCCTGA TTGTAGATAG   
  
  
- CCCGAGAATT CATGACATTT CTCGGAGAAC CTTTTATGAA CGAGAGGATG CCGTGAGTCA CCCAATGAGA   
  
  
- TAGCGTTCGG TATCGTCGTT CCGAACCACC CGGAGGATTT CACGCTGAAT GTCCGTAACT ACTAGGACAA   
  
  
- AGATTCATAT GAACACTACG ATCGAACCTC CGATAACCCT TTGCTAATCG CAGAGATAAA CTTTTCAAAT   
  
  
- TTTATGAGCA GCTCAAGTTA CGTAACGGGC AAATACCTGG ACTACAGTCC ACCCTTTACG ACCTACACTC   
  
  
- CGGGTCCCTC CGAAACCGGC AATTAACAGG TAATGTCGAG GTAGTGTGAG GACTGCTCTC ACAGCTACAC   
  
  
- TCGTTGGGAT CCCTATCCGA AGAGTCTTAC CACTTTAACG AGCCAGGATT CCATACGTGA AACCAACTCG   
  
  
- TTCTTAGTTT GTGGTTGTGA TGTGCAAAGA ACTGGGCCAA GTATCTCTGG GAACTGATGA TGAGCTAGTC   
  
  
- GGTACAAAC

+     O2-site

| Site Name | Organism | Position | Strand | Matrix score. | sequence | function |
| --- | --- | --- | --- | --- | --- | --- |
| O2-site | Zea mays | 948 | + | 9 | GATGACATGG | cis-acting regulatory element involved in zein metabolism regulation |

>HU08G00019.1   
+ +Up\_Stream \_Len000AGTTGT AGCAAGCACA TTAAAGTTGA TTATCATTTT GTCCGTGAAA CGGTGAGCAA   
  
  
+ ACGTCATCTC CAGGTCAAAT TCATTCGTAC TCAATCACAA GTTGCTGACA TTTTTACCAA AGGCTAATCG   
  
  
+ TCGCAAAAGT TTCTTTAGTT TAAGGCCAAT CTCTCCGTTG TTTCACCCCA TACAGATTGA GGGGTTGTAA   
  
  
+ TAGTGTATAT ATATAGCCTT GTGTTAGCGG GGCTTGGGCA TTAGACTTTT ACTCTTGTAA ACCCTAATGT   
  
  
+ GTTATTATAA ATATAGATCT AGCCACCCAT GTTGAGGTAG GCACTCTAAT TCTGACACAG GTAGGATAAG   
  
  
+ TTTTCATAGT TTATGTTGTG AATCTTGTCT TACTTTGTGG TTCCTATTGA CAAAAGTTTG AATCCGAGCA   
  
  
+ TGAGAGTTCA TCAATCTCTT TTTCTTTCCT ATTTTATTTC TTGTGGACTT AATTGGATTT TCTGGTAGAG   
  
  
+ GAAAGTTCCT TGCTGGAATT TGGTCGAAAG TGATATACAT TTTGGGATTT AATTAGCCAT AAATTTGGTT   
  
  
+ TTACACCTTG TTAGTTTGGA GTTCTGGTGT GTTATTGTAT GTGTTCTGGT GGGTTTTTCT TTTAGCTAAA   
  
  
+ TGTTGGAGCA TTTTAAGGGA TCTAGATATC TGAAGAAGTG CTTGGGAAAT GAAGCATATT TCCCTTATAG   
  
  
+ TCTGACATAA CTTTAATGAA TTGATCATGA ACAAAGGGCA ATTAGTTCAG AAAGGTTCAA CTCCTCTAGG   
  
  
+ TGTTGTGCTA AAGAGATGTG AGTTAGTAAC AATTGCTTAC TAACTATATA GGTTGTTTTA TTACTTGTAG   
  
  
+ AGGTTTTTTT TTTGGGATAT CTTTTTGGGG TTTATAAATG AGAAGGTTGA TTTGTTGTCT ACTCTTCTTT   
  
  
+ CGATTCAATA GTAATTCTCT CAGAGAAGGC TAAAATGACA TGGCATTCCA TCGCCTCTCA TGAGGCTTTT   
  
  
+ TGCATGTGCT TATGTATCTT TCATCTCTTA CTTTGGTGGG GTGGCATTGG GGAGGAAAAG GGGGTTTCGA   
  
  
+ TTCGATAGAT CGTCACTTTC GGAAATGACG TCTCTGACCA ATGGTAAGGG TAAGGTTAAT ACATGTGGAT   
  
  
+ ATTTCCAGAG TTAGTTGTGA TTGTTATTGT TGTTGTTGTA ACTTTATGTC ACGGGGAAAA CATAATAATT   
  
  
+ ATCTGTTTAT CTGTTTGTTT GTTATTTTCA AGGGGAAGGG GAGTGGGCGG ATGCTTAGTA TGTAATCATT   
  
  
+ TTATATAGGG AAAATTCATA TAGCATGAAA GTAAAACTTT CATTGCCAGC ATTCTAAGCT AGGGGTAGGG   
  
  
+ TTGTCTACAC CTTGATCTCC CTAGAAGCAT GGTGATCATT CCTCAGATTG TGTTATAGAA TAAAGAATGT   
  
  
+ GGTTCTGCTG TAATGAGACT TATGTATTAT CAAATGATGA ATCTGTTTAG AACTTCCTGT TTATTTTTTC   
  
  
+ TGCAAAATTT TAAGAGACTA GCATCTATCG GGCTCGACTC TAGAGGTTCT CTTGTGTTAC CCTTCTCAAC   
  
  
+ TCAGTTTGAT TGTGATACAG TTACTATATT GAGTGATAGT CGAGAGCATC ACAACCCGCT AAGGAGTCTA   
  
  
+ TCAACAAGAA GCCCTTCTTG TAATTCTCCC CTTGAAACTA GCAGTTATCA TCACTTGTCA TCGAATAGCC   
  
  
+ CTTTTTGTAA TTCTACCCTT GAAACTAGCA GTTATTGTCA TCAGTTCAAT GTGAGTCCCG TTAGATATTC   
  
  
+ CCATCAAGGC ATAGATTATG GAGTGAGCAT GAAGAACGCT TTGCACGAGC TAGAGACTGC TCTAATTGGT   
  
  
+ GTAGATGGCG AGAAAGCATC TGTTGCTAAT CAACCTATGG GGGGAATTCA TTAGTCCGGG ATCCCAAGTT   
  
  
+ AGAGATCGAA GTCATTGAGT GAAGATCCAC AGGGTTCACA TCCTCCTCAG CTTGATTCAT CATCTTTTTC   
  
  
+ AAGGGTGAAA AGATTCGGAG ATGAAAGCCA GAGAGAGAGA AATGCCACAA GGCAATGGAA GAACCAGCGG   
  
  
+ AACTACTAAG TTTCTCACCA GGTGATTTAA AGCAGTTGCT AATTGAATGT GCGAGGGCTT TATCAGATAA   
  
  
+ CCGAATAGAT GACTTTGAGA GTTTGGCTAA ACGGCCAAGG AAAGAGGTCT CCATCTCAGG TGAGCCTGTC   
  
  
+ CAACGTCTCG GTACTTGTAT GATCGAAGGG CTTGTGGCAA GGAAACAGTC TTCGGGGACT AACATCTATC   
  
  
+ GGGCTCTTAA GTACTGTAAA GAGCCTCTTG GAAAATACTT GCTCTCCTAC GGCACTCAGT GGGTTACTCT   
  
  
+ ATCGCAAGCC ATAGCAGCAA GGCTTGGTGG GCCTCCTAAA GTGCGACTTA CAGGCATTGA TGATCCTGTT   
  
  
+ TCTAAGTATA CTTGTGATGC TAGCTTGGAG GCTATTGGGA AACGATTAGC GTCTCTATTT GAAAAGTTTA   
  
  
+ AAATACTCGT CGAGTTCAAT GCATTGCCCG TTTATGGACC TGATGTCAGG TGGGAAATGC TGGATGTGAG   
  
  
+ GCCCAGGGAG GCTTTGGCCG TTAATTGTCC ATTACAGCTC CATCACACTC CTGACGAGAG TGTCGATGTG   
  
  
+ AGCAACCCTA GGGATAGGCT TCTCAGAATG GTGAAATTGC TCGGTCCTAA GGTATGCACT TTGGTTGAGC   
  
  
+ AAGAATCAAA CACCAACACT ACACGTTTCT TGACCCGGTT CATAGAGACC CTTGACTACT ACTCGATCAG   
  
  
+ CCATGTTTG  

- +Up\_Stream \_Len000TCAACA TCGTTCGTGT AATTTCAACT AATAGTAAAA CAGGCACTTT GCCACTCGTT   
  
  
- TGCAGTAGAG GTCCAGTTTA AGTAAGCATG AGTTAGTGTT CAACGACTGT AAAAATGGTT TCCGATTAGC   
  
  
- AGCGTTTTCA AAGAAATCAA ATTCCGGTTA GAGAGGCAAC AAAGTGGGGT ATGTCTAACT CCCCAACATT   
  
  
- ATCACATATA TATATCGGAA CACAATCGCC CCGAACCCGT AATCTGAAAA TGAGAACATT TGGGATTACA   
  
  
- CAATAATATT TATATCTAGA TCGGTGGGTA CAACTCCATC CGTGAGATTA AGACTGTGTC CATCCTATTC   
  
  
- AAAAGTATCA AATACAACAC TTAGAACAGA ATGAAACACC AAGGATAACT GTTTTCAAAC TTAGGCTCGT   
  
  
- ACTCTCAAGT AGTTAGAGAA AAAGAAAGGA TAAAATAAAG AACACCTGAA TTAACCTAAA AGACCATCTC   
  
  
- CTTTCAAGGA ACGACCTTAA ACCAGCTTTC ACTATATGTA AAACCCTAAA TTAATCGGTA TTTAAACCAA   
  
  
- AATGTGGAAC AATCAAACCT CAAGACCACA CAATAACATA CACAAGACCA CCCAAAAAGA AAATCGATTT   
  
  
- ACAACCTCGT AAAATTCCCT AGATCTATAG ACTTCTTCAC GAACCCTTTA CTTCGTATAA AGGGAATATC   
  
  
- AGACTGTATT GAAATTACTT AACTAGTACT TGTTTCCCGT TAATCAAGTC TTTCCAAGTT GAGGAGATCC   
  
  
- ACAACACGAT TTCTCTACAC TCAATCATTG TTAACGAATG ATTGATATAT CCAACAAAAT AATGAACATC   
  
  
- TCCAAAAAAA AAACCCTATA GAAAAACCCC AAATATTTAC TCTTCCAACT AAACAACAGA TGAGAAGAAA   
  
  
- GCTAAGTTAT CATTAAGAGA GTCTCTTCCG ATTTTACTGT ACCGTAAGGT AGCGGAGAGT ACTCCGAAAA   
  
  
- ACGTACACGA ATACATAGAA AGTAGAGAAT GAAACCACCC CACCGTAACC CCTCCTTTTC CCCCAAAGCT   
  
  
- AAGCTATCTA GCAGTGAAAG CCTTTACTGC AGAGACTGGT TACCATTCCC ATTCCAATTA TGTACACCTA   
  
  
- TAAAGGTCTC AATCAACACT AACAATAACA ACAACAACAT TGAAATACAG TGCCCCTTTT GTATTATTAA   
  
  
- TAGACAAATA GACAAACAAA CAATAAAAGT TCCCCTTCCC CTCACCCGCC TACGAATCAT ACATTAGTAA   
  
  
- AATATATCCC TTTTAAGTAT ATCGTACTTT CATTTTGAAA GTAACGGTCG TAAGATTCGA TCCCCATCCC   
  
  
- AACAGATGTG GAACTAGAGG GATCTTCGTA CCACTAGTAA GGAGTCTAAC ACAATATCTT ATTTCTTACA   
  
  
- CCAAGACGAC ATTACTCTGA ATACATAATA GTTTACTACT TAGACAAATC TTGAAGGACA AATAAAAAAG   
  
  
- ACGTTTTAAA ATTCTCTGAT CGTAGATAGC CCGAGCTGAG ATCTCCAAGA GAACACAATG GGAAGAGTTG   
  
  
- AGTCAAACTA ACACTATGTC AATGATATAA CTCACTATCA GCTCTCGTAG TGTTGGGCGA TTCCTCAGAT   
  
  
- AGTTGTTCTT CGGGAAGAAC ATTAAGAGGG GAACTTTGAT CGTCAATAGT AGTGAACAGT AGCTTATCGG   
  
  
- GAAAAACATT AAGATGGGAA CTTTGATCGT CAATAACAGT AGTCAAGTTA CACTCAGGGC AATCTATAAG   
  
  
- GGTAGTTCCG TATCTAATAC CTCACTCGTA CTTCTTGCGA AACGTGCTCG ATCTCTGACG AGATTAACCA   
  
  
- CATCTACCGC TCTTTCGTAG ACAACGATTA GTTGGATACC CCCCTTAAGT AATCAGGCCC TAGGGTTCAA   
  
  
- TCTCTAGCTT CAGTAACTCA CTTCTAGGTG TCCCAAGTGT AGGAGGAGTC GAACTAAGTA GTAGAAAAAG   
  
  
- TTCCCACTTT TCTAAGCCTC TACTTTCGGT CTCTCTCTCT TTACGGTGTT CCGTTACCTT CTTGGTCGCC   
  
  
- TTGATGATTC AAAGAGTGGT CCACTAAATT TCGTCAACGA TTAACTTACA CGCTCCCGAA ATAGTCTATT   
  
  
- GGCTTATCTA CTGAAACTCT CAAACCGATT TGCCGGTTCC TTTCTCCAGA GGTAGAGTCC ACTCGGACAG   
  
  
- GTTGCAGAGC CATGAACATA CTAGCTTCCC GAACACCGTT CCTTTGTCAG AAGCCCCTGA TTGTAGATAG   
  
  
- CCCGAGAATT CATGACATTT CTCGGAGAAC CTTTTATGAA CGAGAGGATG CCGTGAGTCA CCCAATGAGA   
  
  
- TAGCGTTCGG TATCGTCGTT CCGAACCACC CGGAGGATTT CACGCTGAAT GTCCGTAACT ACTAGGACAA   
  
  
- AGATTCATAT GAACACTACG ATCGAACCTC CGATAACCCT TTGCTAATCG CAGAGATAAA CTTTTCAAAT   
  
  
- TTTATGAGCA GCTCAAGTTA CGTAACGGGC AAATACCTGG ACTACAGTCC ACCCTTTACG ACCTACACTC   
  
  
- CGGGTCCCTC CGAAACCGGC AATTAACAGG TAATGTCGAG GTAGTGTGAG GACTGCTCTC ACAGCTACAC   
  
  
- TCGTTGGGAT CCCTATCCGA AGAGTCTTAC CACTTTAACG AGCCAGGATT CCATACGTGA AACCAACTCG   
  
  
- TTCTTAGTTT GTGGTTGTGA TGTGCAAAGA ACTGGGCCAA GTATCTCTGG GAACTGATGA TGAGCTAGTC   
  
  
- GGTACAAAC

+     STRE

| Site Name | Organism | Position | Strand | Matrix score. | sequence | function |
| --- | --- | --- | --- | --- | --- | --- |
| STRE | Arabidopsis thaliana | 204 | + | 5 | AGGGG |  |
| STRE | Arabidopsis thaliana | 1642 | - | 5 | AGGGG |  |
| STRE | Arabidopsis thaliana | 1325 | + | 5 | AGGGG |  |
| STRE | Arabidopsis thaliana | 1043 | + | 5 | AGGGG |  |
| STRE | Arabidopsis thaliana | 1225 | + | 5 | AGGGG |  |
| STRE | Arabidopsis thaliana | 1231 | + | 5 | AGGGG |  |

>HU08G00019.1   
+ +Up\_Stream \_Len000AGTTGT AGCAAGCACA TTAAAGTTGA TTATCATTTT GTCCGTGAAA CGGTGAGCAA   
  
  
+ ACGTCATCTC CAGGTCAAAT TCATTCGTAC TCAATCACAA GTTGCTGACA TTTTTACCAA AGGCTAATCG   
  
  
+ TCGCAAAAGT TTCTTTAGTT TAAGGCCAAT CTCTCCGTTG TTTCACCCCA TACAGATTGA GGGGTTGTAA   
  
  
+ TAGTGTATAT ATATAGCCTT GTGTTAGCGG GGCTTGGGCA TTAGACTTTT ACTCTTGTAA ACCCTAATGT   
  
  
+ GTTATTATAA ATATAGATCT AGCCACCCAT GTTGAGGTAG GCACTCTAAT TCTGACACAG GTAGGATAAG   
  
  
+ TTTTCATAGT TTATGTTGTG AATCTTGTCT TACTTTGTGG TTCCTATTGA CAAAAGTTTG AATCCGAGCA   
  
  
+ TGAGAGTTCA TCAATCTCTT TTTCTTTCCT ATTTTATTTC TTGTGGACTT AATTGGATTT TCTGGTAGAG   
  
  
+ GAAAGTTCCT TGCTGGAATT TGGTCGAAAG TGATATACAT TTTGGGATTT AATTAGCCAT AAATTTGGTT   
  
  
+ TTACACCTTG TTAGTTTGGA GTTCTGGTGT GTTATTGTAT GTGTTCTGGT GGGTTTTTCT TTTAGCTAAA   
  
  
+ TGTTGGAGCA TTTTAAGGGA TCTAGATATC TGAAGAAGTG CTTGGGAAAT GAAGCATATT TCCCTTATAG   
  
  
+ TCTGACATAA CTTTAATGAA TTGATCATGA ACAAAGGGCA ATTAGTTCAG AAAGGTTCAA CTCCTCTAGG   
  
  
+ TGTTGTGCTA AAGAGATGTG AGTTAGTAAC AATTGCTTAC TAACTATATA GGTTGTTTTA TTACTTGTAG   
  
  
+ AGGTTTTTTT TTTGGGATAT CTTTTTGGGG TTTATAAATG AGAAGGTTGA TTTGTTGTCT ACTCTTCTTT   
  
  
+ CGATTCAATA GTAATTCTCT CAGAGAAGGC TAAAATGACA TGGCATTCCA TCGCCTCTCA TGAGGCTTTT   
  
  
+ TGCATGTGCT TATGTATCTT TCATCTCTTA CTTTGGTGGG GTGGCATTGG GGAGGAAAAG GGGGTTTCGA   
  
  
+ TTCGATAGAT CGTCACTTTC GGAAATGACG TCTCTGACCA ATGGTAAGGG TAAGGTTAAT ACATGTGGAT   
  
  
+ ATTTCCAGAG TTAGTTGTGA TTGTTATTGT TGTTGTTGTA ACTTTATGTC ACGGGGAAAA CATAATAATT   
  
  
+ ATCTGTTTAT CTGTTTGTTT GTTATTTTCA AGGGGAAGGG GAGTGGGCGG ATGCTTAGTA TGTAATCATT   
  
  
+ TTATATAGGG AAAATTCATA TAGCATGAAA GTAAAACTTT CATTGCCAGC ATTCTAAGCT AGGGGTAGGG   
  
  
+ TTGTCTACAC CTTGATCTCC CTAGAAGCAT GGTGATCATT CCTCAGATTG TGTTATAGAA TAAAGAATGT   
  
  
+ GGTTCTGCTG TAATGAGACT TATGTATTAT CAAATGATGA ATCTGTTTAG AACTTCCTGT TTATTTTTTC   
  
  
+ TGCAAAATTT TAAGAGACTA GCATCTATCG GGCTCGACTC TAGAGGTTCT CTTGTGTTAC CCTTCTCAAC   
  
  
+ TCAGTTTGAT TGTGATACAG TTACTATATT GAGTGATAGT CGAGAGCATC ACAACCCGCT AAGGAGTCTA   
  
  
+ TCAACAAGAA GCCCTTCTTG TAATTCTCCC CTTGAAACTA GCAGTTATCA TCACTTGTCA TCGAATAGCC   
  
  
+ CTTTTTGTAA TTCTACCCTT GAAACTAGCA GTTATTGTCA TCAGTTCAAT GTGAGTCCCG TTAGATATTC   
  
  
+ CCATCAAGGC ATAGATTATG GAGTGAGCAT GAAGAACGCT TTGCACGAGC TAGAGACTGC TCTAATTGGT   
  
  
+ GTAGATGGCG AGAAAGCATC TGTTGCTAAT CAACCTATGG GGGGAATTCA TTAGTCCGGG ATCCCAAGTT   
  
  
+ AGAGATCGAA GTCATTGAGT GAAGATCCAC AGGGTTCACA TCCTCCTCAG CTTGATTCAT CATCTTTTTC   
  
  
+ AAGGGTGAAA AGATTCGGAG ATGAAAGCCA GAGAGAGAGA AATGCCACAA GGCAATGGAA GAACCAGCGG   
  
  
+ AACTACTAAG TTTCTCACCA GGTGATTTAA AGCAGTTGCT AATTGAATGT GCGAGGGCTT TATCAGATAA   
  
  
+ CCGAATAGAT GACTTTGAGA GTTTGGCTAA ACGGCCAAGG AAAGAGGTCT CCATCTCAGG TGAGCCTGTC   
  
  
+ CAACGTCTCG GTACTTGTAT GATCGAAGGG CTTGTGGCAA GGAAACAGTC TTCGGGGACT AACATCTATC   
  
  
+ GGGCTCTTAA GTACTGTAAA GAGCCTCTTG GAAAATACTT GCTCTCCTAC GGCACTCAGT GGGTTACTCT   
  
  
+ ATCGCAAGCC ATAGCAGCAA GGCTTGGTGG GCCTCCTAAA GTGCGACTTA CAGGCATTGA TGATCCTGTT   
  
  
+ TCTAAGTATA CTTGTGATGC TAGCTTGGAG GCTATTGGGA AACGATTAGC GTCTCTATTT GAAAAGTTTA   
  
  
+ AAATACTCGT CGAGTTCAAT GCATTGCCCG TTTATGGACC TGATGTCAGG TGGGAAATGC TGGATGTGAG   
  
  
+ GCCCAGGGAG GCTTTGGCCG TTAATTGTCC ATTACAGCTC CATCACACTC CTGACGAGAG TGTCGATGTG   
  
  
+ AGCAACCCTA GGGATAGGCT TCTCAGAATG GTGAAATTGC TCGGTCCTAA GGTATGCACT TTGGTTGAGC   
  
  
+ AAGAATCAAA CACCAACACT ACACGTTTCT TGACCCGGTT CATAGAGACC CTTGACTACT ACTCGATCAG   
  
  
+ CCATGTTTG  

- +Up\_Stream \_Len000TCAACA TCGTTCGTGT AATTTCAACT AATAGTAAAA CAGGCACTTT GCCACTCGTT   
  
  
- TGCAGTAGAG GTCCAGTTTA AGTAAGCATG AGTTAGTGTT CAACGACTGT AAAAATGGTT TCCGATTAGC   
  
  
- AGCGTTTTCA AAGAAATCAA ATTCCGGTTA GAGAGGCAAC AAAGTGGGGT ATGTCTAACT CCCCAACATT   
  
  
- ATCACATATA TATATCGGAA CACAATCGCC CCGAACCCGT AATCTGAAAA TGAGAACATT TGGGATTACA   
  
  
- CAATAATATT TATATCTAGA TCGGTGGGTA CAACTCCATC CGTGAGATTA AGACTGTGTC CATCCTATTC   
  
  
- AAAAGTATCA AATACAACAC TTAGAACAGA ATGAAACACC AAGGATAACT GTTTTCAAAC TTAGGCTCGT   
  
  
- ACTCTCAAGT AGTTAGAGAA AAAGAAAGGA TAAAATAAAG AACACCTGAA TTAACCTAAA AGACCATCTC   
  
  
- CTTTCAAGGA ACGACCTTAA ACCAGCTTTC ACTATATGTA AAACCCTAAA TTAATCGGTA TTTAAACCAA   
  
  
- AATGTGGAAC AATCAAACCT CAAGACCACA CAATAACATA CACAAGACCA CCCAAAAAGA AAATCGATTT   
  
  
- ACAACCTCGT AAAATTCCCT AGATCTATAG ACTTCTTCAC GAACCCTTTA CTTCGTATAA AGGGAATATC   
  
  
- AGACTGTATT GAAATTACTT AACTAGTACT TGTTTCCCGT TAATCAAGTC TTTCCAAGTT GAGGAGATCC   
  
  
- ACAACACGAT TTCTCTACAC TCAATCATTG TTAACGAATG ATTGATATAT CCAACAAAAT AATGAACATC   
  
  
- TCCAAAAAAA AAACCCTATA GAAAAACCCC AAATATTTAC TCTTCCAACT AAACAACAGA TGAGAAGAAA   
  
  
- GCTAAGTTAT CATTAAGAGA GTCTCTTCCG ATTTTACTGT ACCGTAAGGT AGCGGAGAGT ACTCCGAAAA   
  
  
- ACGTACACGA ATACATAGAA AGTAGAGAAT GAAACCACCC CACCGTAACC CCTCCTTTTC CCCCAAAGCT   
  
  
- AAGCTATCTA GCAGTGAAAG CCTTTACTGC AGAGACTGGT TACCATTCCC ATTCCAATTA TGTACACCTA   
  
  
- TAAAGGTCTC AATCAACACT AACAATAACA ACAACAACAT TGAAATACAG TGCCCCTTTT GTATTATTAA   
  
  
- TAGACAAATA GACAAACAAA CAATAAAAGT TCCCCTTCCC CTCACCCGCC TACGAATCAT ACATTAGTAA   
  
  
- AATATATCCC TTTTAAGTAT ATCGTACTTT CATTTTGAAA GTAACGGTCG TAAGATTCGA TCCCCATCCC   
  
  
- AACAGATGTG GAACTAGAGG GATCTTCGTA CCACTAGTAA GGAGTCTAAC ACAATATCTT ATTTCTTACA   
  
  
- CCAAGACGAC ATTACTCTGA ATACATAATA GTTTACTACT TAGACAAATC TTGAAGGACA AATAAAAAAG   
  
  
- ACGTTTTAAA ATTCTCTGAT CGTAGATAGC CCGAGCTGAG ATCTCCAAGA GAACACAATG GGAAGAGTTG   
  
  
- AGTCAAACTA ACACTATGTC AATGATATAA CTCACTATCA GCTCTCGTAG TGTTGGGCGA TTCCTCAGAT   
  
  
- AGTTGTTCTT CGGGAAGAAC ATTAAGAGGG GAACTTTGAT CGTCAATAGT AGTGAACAGT AGCTTATCGG   
  
  
- GAAAAACATT AAGATGGGAA CTTTGATCGT CAATAACAGT AGTCAAGTTA CACTCAGGGC AATCTATAAG   
  
  
- GGTAGTTCCG TATCTAATAC CTCACTCGTA CTTCTTGCGA AACGTGCTCG ATCTCTGACG AGATTAACCA   
  
  
- CATCTACCGC TCTTTCGTAG ACAACGATTA GTTGGATACC CCCCTTAAGT AATCAGGCCC TAGGGTTCAA   
  
  
- TCTCTAGCTT CAGTAACTCA CTTCTAGGTG TCCCAAGTGT AGGAGGAGTC GAACTAAGTA GTAGAAAAAG   
  
  
- TTCCCACTTT TCTAAGCCTC TACTTTCGGT CTCTCTCTCT TTACGGTGTT CCGTTACCTT CTTGGTCGCC   
  
  
- TTGATGATTC AAAGAGTGGT CCACTAAATT TCGTCAACGA TTAACTTACA CGCTCCCGAA ATAGTCTATT   
  
  
- GGCTTATCTA CTGAAACTCT CAAACCGATT TGCCGGTTCC TTTCTCCAGA GGTAGAGTCC ACTCGGACAG   
  
  
- GTTGCAGAGC CATGAACATA CTAGCTTCCC GAACACCGTT CCTTTGTCAG AAGCCCCTGA TTGTAGATAG   
  
  
- CCCGAGAATT CATGACATTT CTCGGAGAAC CTTTTATGAA CGAGAGGATG CCGTGAGTCA CCCAATGAGA   
  
  
- TAGCGTTCGG TATCGTCGTT CCGAACCACC CGGAGGATTT CACGCTGAAT GTCCGTAACT ACTAGGACAA   
  
  
- AGATTCATAT GAACACTACG ATCGAACCTC CGATAACCCT TTGCTAATCG CAGAGATAAA CTTTTCAAAT   
  
  
- TTTATGAGCA GCTCAAGTTA CGTAACGGGC AAATACCTGG ACTACAGTCC ACCCTTTACG ACCTACACTC   
  
  
- CGGGTCCCTC CGAAACCGGC AATTAACAGG TAATGTCGAG GTAGTGTGAG GACTGCTCTC ACAGCTACAC   
  
  
- TCGTTGGGAT CCCTATCCGA AGAGTCTTAC CACTTTAACG AGCCAGGATT CCATACGTGA AACCAACTCG   
  
  
- TTCTTAGTTT GTGGTTGTGA TGTGCAAAGA ACTGGGCCAA GTATCTCTGG GAACTGATGA TGAGCTAGTC   
  
  
- GGTACAAAC

+     Sp1

| Site Name | Organism | Position | Strand | Matrix score. | sequence | function |
| --- | --- | --- | --- | --- | --- | --- |
| Sp1 | Oryza sativa | 1239 | + | 6 | GGGCGG | light responsive element |

>HU08G00019.1   
+ +Up\_Stream \_Len000AGTTGT AGCAAGCACA TTAAAGTTGA TTATCATTTT GTCCGTGAAA CGGTGAGCAA   
  
  
+ ACGTCATCTC CAGGTCAAAT TCATTCGTAC TCAATCACAA GTTGCTGACA TTTTTACCAA AGGCTAATCG   
  
  
+ TCGCAAAAGT TTCTTTAGTT TAAGGCCAAT CTCTCCGTTG TTTCACCCCA TACAGATTGA GGGGTTGTAA   
  
  
+ TAGTGTATAT ATATAGCCTT GTGTTAGCGG GGCTTGGGCA TTAGACTTTT ACTCTTGTAA ACCCTAATGT   
  
  
+ GTTATTATAA ATATAGATCT AGCCACCCAT GTTGAGGTAG GCACTCTAAT TCTGACACAG GTAGGATAAG   
  
  
+ TTTTCATAGT TTATGTTGTG AATCTTGTCT TACTTTGTGG TTCCTATTGA CAAAAGTTTG AATCCGAGCA   
  
  
+ TGAGAGTTCA TCAATCTCTT TTTCTTTCCT ATTTTATTTC TTGTGGACTT AATTGGATTT TCTGGTAGAG   
  
  
+ GAAAGTTCCT TGCTGGAATT TGGTCGAAAG TGATATACAT TTTGGGATTT AATTAGCCAT AAATTTGGTT   
  
  
+ TTACACCTTG TTAGTTTGGA GTTCTGGTGT GTTATTGTAT GTGTTCTGGT GGGTTTTTCT TTTAGCTAAA   
  
  
+ TGTTGGAGCA TTTTAAGGGA TCTAGATATC TGAAGAAGTG CTTGGGAAAT GAAGCATATT TCCCTTATAG   
  
  
+ TCTGACATAA CTTTAATGAA TTGATCATGA ACAAAGGGCA ATTAGTTCAG AAAGGTTCAA CTCCTCTAGG   
  
  
+ TGTTGTGCTA AAGAGATGTG AGTTAGTAAC AATTGCTTAC TAACTATATA GGTTGTTTTA TTACTTGTAG   
  
  
+ AGGTTTTTTT TTTGGGATAT CTTTTTGGGG TTTATAAATG AGAAGGTTGA TTTGTTGTCT ACTCTTCTTT   
  
  
+ CGATTCAATA GTAATTCTCT CAGAGAAGGC TAAAATGACA TGGCATTCCA TCGCCTCTCA TGAGGCTTTT   
  
  
+ TGCATGTGCT TATGTATCTT TCATCTCTTA CTTTGGTGGG GTGGCATTGG GGAGGAAAAG GGGGTTTCGA   
  
  
+ TTCGATAGAT CGTCACTTTC GGAAATGACG TCTCTGACCA ATGGTAAGGG TAAGGTTAAT ACATGTGGAT   
  
  
+ ATTTCCAGAG TTAGTTGTGA TTGTTATTGT TGTTGTTGTA ACTTTATGTC ACGGGGAAAA CATAATAATT   
  
  
+ ATCTGTTTAT CTGTTTGTTT GTTATTTTCA AGGGGAAGGG GAGTGGGCGG ATGCTTAGTA TGTAATCATT   
  
  
+ TTATATAGGG AAAATTCATA TAGCATGAAA GTAAAACTTT CATTGCCAGC ATTCTAAGCT AGGGGTAGGG   
  
  
+ TTGTCTACAC CTTGATCTCC CTAGAAGCAT GGTGATCATT CCTCAGATTG TGTTATAGAA TAAAGAATGT   
  
  
+ GGTTCTGCTG TAATGAGACT TATGTATTAT CAAATGATGA ATCTGTTTAG AACTTCCTGT TTATTTTTTC   
  
  
+ TGCAAAATTT TAAGAGACTA GCATCTATCG GGCTCGACTC TAGAGGTTCT CTTGTGTTAC CCTTCTCAAC   
  
  
+ TCAGTTTGAT TGTGATACAG TTACTATATT GAGTGATAGT CGAGAGCATC ACAACCCGCT AAGGAGTCTA   
  
  
+ TCAACAAGAA GCCCTTCTTG TAATTCTCCC CTTGAAACTA GCAGTTATCA TCACTTGTCA TCGAATAGCC   
  
  
+ CTTTTTGTAA TTCTACCCTT GAAACTAGCA GTTATTGTCA TCAGTTCAAT GTGAGTCCCG TTAGATATTC   
  
  
+ CCATCAAGGC ATAGATTATG GAGTGAGCAT GAAGAACGCT TTGCACGAGC TAGAGACTGC TCTAATTGGT   
  
  
+ GTAGATGGCG AGAAAGCATC TGTTGCTAAT CAACCTATGG GGGGAATTCA TTAGTCCGGG ATCCCAAGTT   
  
  
+ AGAGATCGAA GTCATTGAGT GAAGATCCAC AGGGTTCACA TCCTCCTCAG CTTGATTCAT CATCTTTTTC   
  
  
+ AAGGGTGAAA AGATTCGGAG ATGAAAGCCA GAGAGAGAGA AATGCCACAA GGCAATGGAA GAACCAGCGG   
  
  
+ AACTACTAAG TTTCTCACCA GGTGATTTAA AGCAGTTGCT AATTGAATGT GCGAGGGCTT TATCAGATAA   
  
  
+ CCGAATAGAT GACTTTGAGA GTTTGGCTAA ACGGCCAAGG AAAGAGGTCT CCATCTCAGG TGAGCCTGTC   
  
  
+ CAACGTCTCG GTACTTGTAT GATCGAAGGG CTTGTGGCAA GGAAACAGTC TTCGGGGACT AACATCTATC   
  
  
+ GGGCTCTTAA GTACTGTAAA GAGCCTCTTG GAAAATACTT GCTCTCCTAC GGCACTCAGT GGGTTACTCT   
  
  
+ ATCGCAAGCC ATAGCAGCAA GGCTTGGTGG GCCTCCTAAA GTGCGACTTA CAGGCATTGA TGATCCTGTT   
  
  
+ TCTAAGTATA CTTGTGATGC TAGCTTGGAG GCTATTGGGA AACGATTAGC GTCTCTATTT GAAAAGTTTA   
  
  
+ AAATACTCGT CGAGTTCAAT GCATTGCCCG TTTATGGACC TGATGTCAGG TGGGAAATGC TGGATGTGAG   
  
  
+ GCCCAGGGAG GCTTTGGCCG TTAATTGTCC ATTACAGCTC CATCACACTC CTGACGAGAG TGTCGATGTG   
  
  
+ AGCAACCCTA GGGATAGGCT TCTCAGAATG GTGAAATTGC TCGGTCCTAA GGTATGCACT TTGGTTGAGC   
  
  
+ AAGAATCAAA CACCAACACT ACACGTTTCT TGACCCGGTT CATAGAGACC CTTGACTACT ACTCGATCAG   
  
  
+ CCATGTTTG  

- +Up\_Stream \_Len000TCAACA TCGTTCGTGT AATTTCAACT AATAGTAAAA CAGGCACTTT GCCACTCGTT   
  
  
- TGCAGTAGAG GTCCAGTTTA AGTAAGCATG AGTTAGTGTT CAACGACTGT AAAAATGGTT TCCGATTAGC   
  
  
- AGCGTTTTCA AAGAAATCAA ATTCCGGTTA GAGAGGCAAC AAAGTGGGGT ATGTCTAACT CCCCAACATT   
  
  
- ATCACATATA TATATCGGAA CACAATCGCC CCGAACCCGT AATCTGAAAA TGAGAACATT TGGGATTACA   
  
  
- CAATAATATT TATATCTAGA TCGGTGGGTA CAACTCCATC CGTGAGATTA AGACTGTGTC CATCCTATTC   
  
  
- AAAAGTATCA AATACAACAC TTAGAACAGA ATGAAACACC AAGGATAACT GTTTTCAAAC TTAGGCTCGT   
  
  
- ACTCTCAAGT AGTTAGAGAA AAAGAAAGGA TAAAATAAAG AACACCTGAA TTAACCTAAA AGACCATCTC   
  
  
- CTTTCAAGGA ACGACCTTAA ACCAGCTTTC ACTATATGTA AAACCCTAAA TTAATCGGTA TTTAAACCAA   
  
  
- AATGTGGAAC AATCAAACCT CAAGACCACA CAATAACATA CACAAGACCA CCCAAAAAGA AAATCGATTT   
  
  
- ACAACCTCGT AAAATTCCCT AGATCTATAG ACTTCTTCAC GAACCCTTTA CTTCGTATAA AGGGAATATC   
  
  
- AGACTGTATT GAAATTACTT AACTAGTACT TGTTTCCCGT TAATCAAGTC TTTCCAAGTT GAGGAGATCC   
  
  
- ACAACACGAT TTCTCTACAC TCAATCATTG TTAACGAATG ATTGATATAT CCAACAAAAT AATGAACATC   
  
  
- TCCAAAAAAA AAACCCTATA GAAAAACCCC AAATATTTAC TCTTCCAACT AAACAACAGA TGAGAAGAAA   
  
  
- GCTAAGTTAT CATTAAGAGA GTCTCTTCCG ATTTTACTGT ACCGTAAGGT AGCGGAGAGT ACTCCGAAAA   
  
  
- ACGTACACGA ATACATAGAA AGTAGAGAAT GAAACCACCC CACCGTAACC CCTCCTTTTC CCCCAAAGCT   
  
  
- AAGCTATCTA GCAGTGAAAG CCTTTACTGC AGAGACTGGT TACCATTCCC ATTCCAATTA TGTACACCTA   
  
  
- TAAAGGTCTC AATCAACACT AACAATAACA ACAACAACAT TGAAATACAG TGCCCCTTTT GTATTATTAA   
  
  
- TAGACAAATA GACAAACAAA CAATAAAAGT TCCCCTTCCC CTCACCCGCC TACGAATCAT ACATTAGTAA   
  
  
- AATATATCCC TTTTAAGTAT ATCGTACTTT CATTTTGAAA GTAACGGTCG TAAGATTCGA TCCCCATCCC   
  
  
- AACAGATGTG GAACTAGAGG GATCTTCGTA CCACTAGTAA GGAGTCTAAC ACAATATCTT ATTTCTTACA   
  
  
- CCAAGACGAC ATTACTCTGA ATACATAATA GTTTACTACT TAGACAAATC TTGAAGGACA AATAAAAAAG   
  
  
- ACGTTTTAAA ATTCTCTGAT CGTAGATAGC CCGAGCTGAG ATCTCCAAGA GAACACAATG GGAAGAGTTG   
  
  
- AGTCAAACTA ACACTATGTC AATGATATAA CTCACTATCA GCTCTCGTAG TGTTGGGCGA TTCCTCAGAT   
  
  
- AGTTGTTCTT CGGGAAGAAC ATTAAGAGGG GAACTTTGAT CGTCAATAGT AGTGAACAGT AGCTTATCGG   
  
  
- GAAAAACATT AAGATGGGAA CTTTGATCGT CAATAACAGT AGTCAAGTTA CACTCAGGGC AATCTATAAG   
  
  
- GGTAGTTCCG TATCTAATAC CTCACTCGTA CTTCTTGCGA AACGTGCTCG ATCTCTGACG AGATTAACCA   
  
  
- CATCTACCGC TCTTTCGTAG ACAACGATTA GTTGGATACC CCCCTTAAGT AATCAGGCCC TAGGGTTCAA   
  
  
- TCTCTAGCTT CAGTAACTCA CTTCTAGGTG TCCCAAGTGT AGGAGGAGTC GAACTAAGTA GTAGAAAAAG   
  
  
- TTCCCACTTT TCTAAGCCTC TACTTTCGGT CTCTCTCTCT TTACGGTGTT CCGTTACCTT CTTGGTCGCC   
  
  
- TTGATGATTC AAAGAGTGGT CCACTAAATT TCGTCAACGA TTAACTTACA CGCTCCCGAA ATAGTCTATT   
  
  
- GGCTTATCTA CTGAAACTCT CAAACCGATT TGCCGGTTCC TTTCTCCAGA GGTAGAGTCC ACTCGGACAG   
  
  
- GTTGCAGAGC CATGAACATA CTAGCTTCCC GAACACCGTT CCTTTGTCAG AAGCCCCTGA TTGTAGATAG   
  
  
- CCCGAGAATT CATGACATTT CTCGGAGAAC CTTTTATGAA CGAGAGGATG CCGTGAGTCA CCCAATGAGA   
  
  
- TAGCGTTCGG TATCGTCGTT CCGAACCACC CGGAGGATTT CACGCTGAAT GTCCGTAACT ACTAGGACAA   
  
  
- AGATTCATAT GAACACTACG ATCGAACCTC CGATAACCCT TTGCTAATCG CAGAGATAAA CTTTTCAAAT   
  
  
- TTTATGAGCA GCTCAAGTTA CGTAACGGGC AAATACCTGG ACTACAGTCC ACCCTTTACG ACCTACACTC   
  
  
- CGGGTCCCTC CGAAACCGGC AATTAACAGG TAATGTCGAG GTAGTGTGAG GACTGCTCTC ACAGCTACAC   
  
  
- TCGTTGGGAT CCCTATCCGA AGAGTCTTAC CACTTTAACG AGCCAGGATT CCATACGTGA AACCAACTCG   
  
  
- TTCTTAGTTT GTGGTTGTGA TGTGCAAAGA ACTGGGCCAA GTATCTCTGG GAACTGATGA TGAGCTAGTC   
  
  
- GGTACAAAC

+     TATA

| Site Name | Organism | Position | Strand | Matrix score. | sequence | function |
| --- | --- | --- | --- | --- | --- | --- |
| TATA | Arabidopsis thaliana | 1262 | - | 8 | TATAAAAT |  |

>HU08G00019.1   
+ +Up\_Stream \_Len000AGTTGT AGCAAGCACA TTAAAGTTGA TTATCATTTT GTCCGTGAAA CGGTGAGCAA   
  
  
+ ACGTCATCTC CAGGTCAAAT TCATTCGTAC TCAATCACAA GTTGCTGACA TTTTTACCAA AGGCTAATCG   
  
  
+ TCGCAAAAGT TTCTTTAGTT TAAGGCCAAT CTCTCCGTTG TTTCACCCCA TACAGATTGA GGGGTTGTAA   
  
  
+ TAGTGTATAT ATATAGCCTT GTGTTAGCGG GGCTTGGGCA TTAGACTTTT ACTCTTGTAA ACCCTAATGT   
  
  
+ GTTATTATAA ATATAGATCT AGCCACCCAT GTTGAGGTAG GCACTCTAAT TCTGACACAG GTAGGATAAG   
  
  
+ TTTTCATAGT TTATGTTGTG AATCTTGTCT TACTTTGTGG TTCCTATTGA CAAAAGTTTG AATCCGAGCA   
  
  
+ TGAGAGTTCA TCAATCTCTT TTTCTTTCCT ATTTTATTTC TTGTGGACTT AATTGGATTT TCTGGTAGAG   
  
  
+ GAAAGTTCCT TGCTGGAATT TGGTCGAAAG TGATATACAT TTTGGGATTT AATTAGCCAT AAATTTGGTT   
  
  
+ TTACACCTTG TTAGTTTGGA GTTCTGGTGT GTTATTGTAT GTGTTCTGGT GGGTTTTTCT TTTAGCTAAA   
  
  
+ TGTTGGAGCA TTTTAAGGGA TCTAGATATC TGAAGAAGTG CTTGGGAAAT GAAGCATATT TCCCTTATAG   
  
  
+ TCTGACATAA CTTTAATGAA TTGATCATGA ACAAAGGGCA ATTAGTTCAG AAAGGTTCAA CTCCTCTAGG   
  
  
+ TGTTGTGCTA AAGAGATGTG AGTTAGTAAC AATTGCTTAC TAACTATATA GGTTGTTTTA TTACTTGTAG   
  
  
+ AGGTTTTTTT TTTGGGATAT CTTTTTGGGG TTTATAAATG AGAAGGTTGA TTTGTTGTCT ACTCTTCTTT   
  
  
+ CGATTCAATA GTAATTCTCT CAGAGAAGGC TAAAATGACA TGGCATTCCA TCGCCTCTCA TGAGGCTTTT   
  
  
+ TGCATGTGCT TATGTATCTT TCATCTCTTA CTTTGGTGGG GTGGCATTGG GGAGGAAAAG GGGGTTTCGA   
  
  
+ TTCGATAGAT CGTCACTTTC GGAAATGACG TCTCTGACCA ATGGTAAGGG TAAGGTTAAT ACATGTGGAT   
  
  
+ ATTTCCAGAG TTAGTTGTGA TTGTTATTGT TGTTGTTGTA ACTTTATGTC ACGGGGAAAA CATAATAATT   
  
  
+ ATCTGTTTAT CTGTTTGTTT GTTATTTTCA AGGGGAAGGG GAGTGGGCGG ATGCTTAGTA TGTAATCATT   
  
  
+ TTATATAGGG AAAATTCATA TAGCATGAAA GTAAAACTTT CATTGCCAGC ATTCTAAGCT AGGGGTAGGG   
  
  
+ TTGTCTACAC CTTGATCTCC CTAGAAGCAT GGTGATCATT CCTCAGATTG TGTTATAGAA TAAAGAATGT   
  
  
+ GGTTCTGCTG TAATGAGACT TATGTATTAT CAAATGATGA ATCTGTTTAG AACTTCCTGT TTATTTTTTC   
  
  
+ TGCAAAATTT TAAGAGACTA GCATCTATCG GGCTCGACTC TAGAGGTTCT CTTGTGTTAC CCTTCTCAAC   
  
  
+ TCAGTTTGAT TGTGATACAG TTACTATATT GAGTGATAGT CGAGAGCATC ACAACCCGCT AAGGAGTCTA   
  
  
+ TCAACAAGAA GCCCTTCTTG TAATTCTCCC CTTGAAACTA GCAGTTATCA TCACTTGTCA TCGAATAGCC   
  
  
+ CTTTTTGTAA TTCTACCCTT GAAACTAGCA GTTATTGTCA TCAGTTCAAT GTGAGTCCCG TTAGATATTC   
  
  
+ CCATCAAGGC ATAGATTATG GAGTGAGCAT GAAGAACGCT TTGCACGAGC TAGAGACTGC TCTAATTGGT   
  
  
+ GTAGATGGCG AGAAAGCATC TGTTGCTAAT CAACCTATGG GGGGAATTCA TTAGTCCGGG ATCCCAAGTT   
  
  
+ AGAGATCGAA GTCATTGAGT GAAGATCCAC AGGGTTCACA TCCTCCTCAG CTTGATTCAT CATCTTTTTC   
  
  
+ AAGGGTGAAA AGATTCGGAG ATGAAAGCCA GAGAGAGAGA AATGCCACAA GGCAATGGAA GAACCAGCGG   
  
  
+ AACTACTAAG TTTCTCACCA GGTGATTTAA AGCAGTTGCT AATTGAATGT GCGAGGGCTT TATCAGATAA   
  
  
+ CCGAATAGAT GACTTTGAGA GTTTGGCTAA ACGGCCAAGG AAAGAGGTCT CCATCTCAGG TGAGCCTGTC   
  
  
+ CAACGTCTCG GTACTTGTAT GATCGAAGGG CTTGTGGCAA GGAAACAGTC TTCGGGGACT AACATCTATC   
  
  
+ GGGCTCTTAA GTACTGTAAA GAGCCTCTTG GAAAATACTT GCTCTCCTAC GGCACTCAGT GGGTTACTCT   
  
  
+ ATCGCAAGCC ATAGCAGCAA GGCTTGGTGG GCCTCCTAAA GTGCGACTTA CAGGCATTGA TGATCCTGTT   
  
  
+ TCTAAGTATA CTTGTGATGC TAGCTTGGAG GCTATTGGGA AACGATTAGC GTCTCTATTT GAAAAGTTTA   
  
  
+ AAATACTCGT CGAGTTCAAT GCATTGCCCG TTTATGGACC TGATGTCAGG TGGGAAATGC TGGATGTGAG   
  
  
+ GCCCAGGGAG GCTTTGGCCG TTAATTGTCC ATTACAGCTC CATCACACTC CTGACGAGAG TGTCGATGTG   
  
  
+ AGCAACCCTA GGGATAGGCT TCTCAGAATG GTGAAATTGC TCGGTCCTAA GGTATGCACT TTGGTTGAGC   
  
  
+ AAGAATCAAA CACCAACACT ACACGTTTCT TGACCCGGTT CATAGAGACC CTTGACTACT ACTCGATCAG   
  
  
+ CCATGTTTG  

- +Up\_Stream \_Len000TCAACA TCGTTCGTGT AATTTCAACT AATAGTAAAA CAGGCACTTT GCCACTCGTT   
  
  
- TGCAGTAGAG GTCCAGTTTA AGTAAGCATG AGTTAGTGTT CAACGACTGT AAAAATGGTT TCCGATTAGC   
  
  
- AGCGTTTTCA AAGAAATCAA ATTCCGGTTA GAGAGGCAAC AAAGTGGGGT ATGTCTAACT CCCCAACATT   
  
  
- ATCACATATA TATATCGGAA CACAATCGCC CCGAACCCGT AATCTGAAAA TGAGAACATT TGGGATTACA   
  
  
- CAATAATATT TATATCTAGA TCGGTGGGTA CAACTCCATC CGTGAGATTA AGACTGTGTC CATCCTATTC   
  
  
- AAAAGTATCA AATACAACAC TTAGAACAGA ATGAAACACC AAGGATAACT GTTTTCAAAC TTAGGCTCGT   
  
  
- ACTCTCAAGT AGTTAGAGAA AAAGAAAGGA TAAAATAAAG AACACCTGAA TTAACCTAAA AGACCATCTC   
  
  
- CTTTCAAGGA ACGACCTTAA ACCAGCTTTC ACTATATGTA AAACCCTAAA TTAATCGGTA TTTAAACCAA   
  
  
- AATGTGGAAC AATCAAACCT CAAGACCACA CAATAACATA CACAAGACCA CCCAAAAAGA AAATCGATTT   
  
  
- ACAACCTCGT AAAATTCCCT AGATCTATAG ACTTCTTCAC GAACCCTTTA CTTCGTATAA AGGGAATATC   
  
  
- AGACTGTATT GAAATTACTT AACTAGTACT TGTTTCCCGT TAATCAAGTC TTTCCAAGTT GAGGAGATCC   
  
  
- ACAACACGAT TTCTCTACAC TCAATCATTG TTAACGAATG ATTGATATAT CCAACAAAAT AATGAACATC   
  
  
- TCCAAAAAAA AAACCCTATA GAAAAACCCC AAATATTTAC TCTTCCAACT AAACAACAGA TGAGAAGAAA   
  
  
- GCTAAGTTAT CATTAAGAGA GTCTCTTCCG ATTTTACTGT ACCGTAAGGT AGCGGAGAGT ACTCCGAAAA   
  
  
- ACGTACACGA ATACATAGAA AGTAGAGAAT GAAACCACCC CACCGTAACC CCTCCTTTTC CCCCAAAGCT   
  
  
- AAGCTATCTA GCAGTGAAAG CCTTTACTGC AGAGACTGGT TACCATTCCC ATTCCAATTA TGTACACCTA   
  
  
- TAAAGGTCTC AATCAACACT AACAATAACA ACAACAACAT TGAAATACAG TGCCCCTTTT GTATTATTAA   
  
  
- TAGACAAATA GACAAACAAA CAATAAAAGT TCCCCTTCCC CTCACCCGCC TACGAATCAT ACATTAGTAA   
  
  
- AATATATCCC TTTTAAGTAT ATCGTACTTT CATTTTGAAA GTAACGGTCG TAAGATTCGA TCCCCATCCC   
  
  
- AACAGATGTG GAACTAGAGG GATCTTCGTA CCACTAGTAA GGAGTCTAAC ACAATATCTT ATTTCTTACA   
  
  
- CCAAGACGAC ATTACTCTGA ATACATAATA GTTTACTACT TAGACAAATC TTGAAGGACA AATAAAAAAG   
  
  
- ACGTTTTAAA ATTCTCTGAT CGTAGATAGC CCGAGCTGAG ATCTCCAAGA GAACACAATG GGAAGAGTTG   
  
  
- AGTCAAACTA ACACTATGTC AATGATATAA CTCACTATCA GCTCTCGTAG TGTTGGGCGA TTCCTCAGAT   
  
  
- AGTTGTTCTT CGGGAAGAAC ATTAAGAGGG GAACTTTGAT CGTCAATAGT AGTGAACAGT AGCTTATCGG   
  
  
- GAAAAACATT AAGATGGGAA CTTTGATCGT CAATAACAGT AGTCAAGTTA CACTCAGGGC AATCTATAAG   
  
  
- GGTAGTTCCG TATCTAATAC CTCACTCGTA CTTCTTGCGA AACGTGCTCG ATCTCTGACG AGATTAACCA   
  
  
- CATCTACCGC TCTTTCGTAG ACAACGATTA GTTGGATACC CCCCTTAAGT AATCAGGCCC TAGGGTTCAA   
  
  
- TCTCTAGCTT CAGTAACTCA CTTCTAGGTG TCCCAAGTGT AGGAGGAGTC GAACTAAGTA GTAGAAAAAG   
  
  
- TTCCCACTTT TCTAAGCCTC TACTTTCGGT CTCTCTCTCT TTACGGTGTT CCGTTACCTT CTTGGTCGCC   
  
  
- TTGATGATTC AAAGAGTGGT CCACTAAATT TCGTCAACGA TTAACTTACA CGCTCCCGAA ATAGTCTATT   
  
  
- GGCTTATCTA CTGAAACTCT CAAACCGATT TGCCGGTTCC TTTCTCCAGA GGTAGAGTCC ACTCGGACAG   
  
  
- GTTGCAGAGC CATGAACATA CTAGCTTCCC GAACACCGTT CCTTTGTCAG AAGCCCCTGA TTGTAGATAG   
  
  
- CCCGAGAATT CATGACATTT CTCGGAGAAC CTTTTATGAA CGAGAGGATG CCGTGAGTCA CCCAATGAGA   
  
  
- TAGCGTTCGG TATCGTCGTT CCGAACCACC CGGAGGATTT CACGCTGAAT GTCCGTAACT ACTAGGACAA   
  
  
- AGATTCATAT GAACACTACG ATCGAACCTC CGATAACCCT TTGCTAATCG CAGAGATAAA CTTTTCAAAT   
  
  
- TTTATGAGCA GCTCAAGTTA CGTAACGGGC AAATACCTGG ACTACAGTCC ACCCTTTACG ACCTACACTC   
  
  
- CGGGTCCCTC CGAAACCGGC AATTAACAGG TAATGTCGAG GTAGTGTGAG GACTGCTCTC ACAGCTACAC   
  
  
- TCGTTGGGAT CCCTATCCGA AGAGTCTTAC CACTTTAACG AGCCAGGATT CCATACGTGA AACCAACTCG   
  
  
- TTCTTAGTTT GTGGTTGTGA TGTGCAAAGA ACTGGGCCAA GTATCTCTGG GAACTGATGA TGAGCTAGTC   
  
  
- GGTACAAAC

+     TATA-box

| Site Name | Organism | Position | Strand | Matrix score. | sequence | function |
| --- | --- | --- | --- | --- | --- | --- |
| TATA-box | Brassica napus | 288 | + | 6 | ATTATA | core promoter element around -30 of transcription start |
| TATA-box | Brassica napus | 221 | + | 6 | ATATAT | core promoter element around -30 of transcription start |
| TATA-box | Arabidopsis thaliana | 1266 | + | 6 | TATATA | core promoter element around -30 of transcription start |
| TATA-box | Arabidopsis thaliana | 1264 | - | 9 | ccTATAAAaa | core promoter element around -30 of transcription start |
| TATA-box | Arabidopsis thaliana | 877 | + | 4 | TATA | core promoter element around -30 of transcription start |
| TATA-box | Pisum sativum | 1263 | - | 7 | TATAAAA | core promoter element around -30 of transcription start |
| TATA-box | Helianthus annuus | 875 | - | 6 | TATAAA | core promoter element around -30 of transcription start |
| TATA-box | Arabidopsis thaliana | 222 | + | 6 | TATATA | core promoter element around -30 of transcription start |
| TATA-box | Arabidopsis thaliana | 224 | + | 6 | TATATA | core promoter element around -30 of transcription start |
| TATA-box | Oryza sativa | 1687 | - | 7 | TACAAAA | core promoter element around -30 of transcription start |
| TATA-box | Arabidopsis thaliana | 1265 | - | 7 | TATATAA | core promoter element around -30 of transcription start |
| TATA-box | Arabidopsis thaliana | 289 | - | 5 | TATAA | core promoter element around -30 of transcription start |
| TATA-box | Arabidopsis thaliana | 226 | + | 4 | TATA | core promoter element around -30 of transcription start |
| TATA-box | Brassica napus | 223 | + | 6 | ATATAT | core promoter element around -30 of transcription start |
| TATA-box | Arabidopsis thaliana | 1283 | + | 4 | TATA | core promoter element around -30 of transcription start |
| TATA-box | Arabidopsis thaliana | 699 | - | 5 | TATAA | core promoter element around -30 of transcription start |
| TATA-box | Arabidopsis thaliana | 876 | - | 5 | TATAA | core promoter element around -30 of transcription start |
| TATA-box | Helianthus annuus | 218 | - | 6 | TATACA | core promoter element around -30 of transcription start |
| TATA-box | Arabidopsis thaliana | 296 | + | 4 | TATA | core promoter element around -30 of transcription start |
| TATA-box | Arabidopsis thaliana | 290 | + | 4 | TATA | core promoter element around -30 of transcription start |
| TATA-box | Arabidopsis thaliana | 220 | + | 6 | TATATA | core promoter element around -30 of transcription start |
| TATA-box | Arabidopsis thaliana | 1388 | - | 4 | TATA | core promoter element around -30 of transcription start |
| TATA-box | Arabidopsis thaliana | 1387 | - | 5 | TATAA | core promoter element around -30 of transcription start |
| TATA-box | Arabidopsis thaliana | 821 | + | 4 | TATA | core promoter element around -30 of transcription start |
| TATA-box | Arabidopsis thaliana | 819 | + | 6 | TATATA | core promoter element around -30 of transcription start |
| TATA-box | Arabidopsis thaliana | 1268 | + | 4 | TATA | core promoter element around -30 of transcription start |
| TATA-box | Arabidopsis thaliana | 528 | + | 4 | TATA | core promoter element around -30 of transcription start |
| TATA-box | Arabidopsis thaliana | 2391 | - | 4 | TATA | core promoter element around -30 of transcription start |
| TATA-box | Arabidopsis thaliana | 1569 | - | 4 | TATA | core promoter element around -30 of transcription start |
| TATA-box | Arabidopsis thaliana | 700 | + | 4 | TATA | core promoter element around -30 of transcription start |

>HU08G00019.1   
+ +Up\_Stream \_Len000AGTTGT AGCAAGCACA TTAAAGTTGA TTATCATTTT GTCCGTGAAA CGGTGAGCAA   
  
  
+ ACGTCATCTC CAGGTCAAAT TCATTCGTAC TCAATCACAA GTTGCTGACA TTTTTACCAA AGGCTAATCG   
  
  
+ TCGCAAAAGT TTCTTTAGTT TAAGGCCAAT CTCTCCGTTG TTTCACCCCA TACAGATTGA GGGGTTGTAA   
  
  
+ TAGTGTATAT ATATAGCCTT GTGTTAGCGG GGCTTGGGCA TTAGACTTTT ACTCTTGTAA ACCCTAATGT   
  
  
+ GTTATTATAA ATATAGATCT AGCCACCCAT GTTGAGGTAG GCACTCTAAT TCTGACACAG GTAGGATAAG   
  
  
+ TTTTCATAGT TTATGTTGTG AATCTTGTCT TACTTTGTGG TTCCTATTGA CAAAAGTTTG AATCCGAGCA   
  
  
+ TGAGAGTTCA TCAATCTCTT TTTCTTTCCT ATTTTATTTC TTGTGGACTT AATTGGATTT TCTGGTAGAG   
  
  
+ GAAAGTTCCT TGCTGGAATT TGGTCGAAAG TGATATACAT TTTGGGATTT AATTAGCCAT AAATTTGGTT   
  
  
+ TTACACCTTG TTAGTTTGGA GTTCTGGTGT GTTATTGTAT GTGTTCTGGT GGGTTTTTCT TTTAGCTAAA   
  
  
+ TGTTGGAGCA TTTTAAGGGA TCTAGATATC TGAAGAAGTG CTTGGGAAAT GAAGCATATT TCCCTTATAG   
  
  
+ TCTGACATAA CTTTAATGAA TTGATCATGA ACAAAGGGCA ATTAGTTCAG AAAGGTTCAA CTCCTCTAGG   
  
  
+ TGTTGTGCTA AAGAGATGTG AGTTAGTAAC AATTGCTTAC TAACTATATA GGTTGTTTTA TTACTTGTAG   
  
  
+ AGGTTTTTTT TTTGGGATAT CTTTTTGGGG TTTATAAATG AGAAGGTTGA TTTGTTGTCT ACTCTTCTTT   
  
  
+ CGATTCAATA GTAATTCTCT CAGAGAAGGC TAAAATGACA TGGCATTCCA TCGCCTCTCA TGAGGCTTTT   
  
  
+ TGCATGTGCT TATGTATCTT TCATCTCTTA CTTTGGTGGG GTGGCATTGG GGAGGAAAAG GGGGTTTCGA   
  
  
+ TTCGATAGAT CGTCACTTTC GGAAATGACG TCTCTGACCA ATGGTAAGGG TAAGGTTAAT ACATGTGGAT   
  
  
+ ATTTCCAGAG TTAGTTGTGA TTGTTATTGT TGTTGTTGTA ACTTTATGTC ACGGGGAAAA CATAATAATT   
  
  
+ ATCTGTTTAT CTGTTTGTTT GTTATTTTCA AGGGGAAGGG GAGTGGGCGG ATGCTTAGTA TGTAATCATT   
  
  
+ TTATATAGGG AAAATTCATA TAGCATGAAA GTAAAACTTT CATTGCCAGC ATTCTAAGCT AGGGGTAGGG   
  
  
+ TTGTCTACAC CTTGATCTCC CTAGAAGCAT GGTGATCATT CCTCAGATTG TGTTATAGAA TAAAGAATGT   
  
  
+ GGTTCTGCTG TAATGAGACT TATGTATTAT CAAATGATGA ATCTGTTTAG AACTTCCTGT TTATTTTTTC   
  
  
+ TGCAAAATTT TAAGAGACTA GCATCTATCG GGCTCGACTC TAGAGGTTCT CTTGTGTTAC CCTTCTCAAC   
  
  
+ TCAGTTTGAT TGTGATACAG TTACTATATT GAGTGATAGT CGAGAGCATC ACAACCCGCT AAGGAGTCTA   
  
  
+ TCAACAAGAA GCCCTTCTTG TAATTCTCCC CTTGAAACTA GCAGTTATCA TCACTTGTCA TCGAATAGCC   
  
  
+ CTTTTTGTAA TTCTACCCTT GAAACTAGCA GTTATTGTCA TCAGTTCAAT GTGAGTCCCG TTAGATATTC   
  
  
+ CCATCAAGGC ATAGATTATG GAGTGAGCAT GAAGAACGCT TTGCACGAGC TAGAGACTGC TCTAATTGGT   
  
  
+ GTAGATGGCG AGAAAGCATC TGTTGCTAAT CAACCTATGG GGGGAATTCA TTAGTCCGGG ATCCCAAGTT   
  
  
+ AGAGATCGAA GTCATTGAGT GAAGATCCAC AGGGTTCACA TCCTCCTCAG CTTGATTCAT CATCTTTTTC   
  
  
+ AAGGGTGAAA AGATTCGGAG ATGAAAGCCA GAGAGAGAGA AATGCCACAA GGCAATGGAA GAACCAGCGG   
  
  
+ AACTACTAAG TTTCTCACCA GGTGATTTAA AGCAGTTGCT AATTGAATGT GCGAGGGCTT TATCAGATAA   
  
  
+ CCGAATAGAT GACTTTGAGA GTTTGGCTAA ACGGCCAAGG AAAGAGGTCT CCATCTCAGG TGAGCCTGTC   
  
  
+ CAACGTCTCG GTACTTGTAT GATCGAAGGG CTTGTGGCAA GGAAACAGTC TTCGGGGACT AACATCTATC   
  
  
+ GGGCTCTTAA GTACTGTAAA GAGCCTCTTG GAAAATACTT GCTCTCCTAC GGCACTCAGT GGGTTACTCT   
  
  
+ ATCGCAAGCC ATAGCAGCAA GGCTTGGTGG GCCTCCTAAA GTGCGACTTA CAGGCATTGA TGATCCTGTT   
  
  
+ TCTAAGTATA CTTGTGATGC TAGCTTGGAG GCTATTGGGA AACGATTAGC GTCTCTATTT GAAAAGTTTA   
  
  
+ AAATACTCGT CGAGTTCAAT GCATTGCCCG TTTATGGACC TGATGTCAGG TGGGAAATGC TGGATGTGAG   
  
  
+ GCCCAGGGAG GCTTTGGCCG TTAATTGTCC ATTACAGCTC CATCACACTC CTGACGAGAG TGTCGATGTG   
  
  
+ AGCAACCCTA GGGATAGGCT TCTCAGAATG GTGAAATTGC TCGGTCCTAA GGTATGCACT TTGGTTGAGC   
  
  
+ AAGAATCAAA CACCAACACT ACACGTTTCT TGACCCGGTT CATAGAGACC CTTGACTACT ACTCGATCAG   
  
  
+ CCATGTTTG  

- +Up\_Stream \_Len000TCAACA TCGTTCGTGT AATTTCAACT AATAGTAAAA CAGGCACTTT GCCACTCGTT   
  
  
- TGCAGTAGAG GTCCAGTTTA AGTAAGCATG AGTTAGTGTT CAACGACTGT AAAAATGGTT TCCGATTAGC   
  
  
- AGCGTTTTCA AAGAAATCAA ATTCCGGTTA GAGAGGCAAC AAAGTGGGGT ATGTCTAACT CCCCAACATT   
  
  
- ATCACATATA TATATCGGAA CACAATCGCC CCGAACCCGT AATCTGAAAA TGAGAACATT TGGGATTACA   
  
  
- CAATAATATT TATATCTAGA TCGGTGGGTA CAACTCCATC CGTGAGATTA AGACTGTGTC CATCCTATTC   
  
  
- AAAAGTATCA AATACAACAC TTAGAACAGA ATGAAACACC AAGGATAACT GTTTTCAAAC TTAGGCTCGT   
  
  
- ACTCTCAAGT AGTTAGAGAA AAAGAAAGGA TAAAATAAAG AACACCTGAA TTAACCTAAA AGACCATCTC   
  
  
- CTTTCAAGGA ACGACCTTAA ACCAGCTTTC ACTATATGTA AAACCCTAAA TTAATCGGTA TTTAAACCAA   
  
  
- AATGTGGAAC AATCAAACCT CAAGACCACA CAATAACATA CACAAGACCA CCCAAAAAGA AAATCGATTT   
  
  
- ACAACCTCGT AAAATTCCCT AGATCTATAG ACTTCTTCAC GAACCCTTTA CTTCGTATAA AGGGAATATC   
  
  
- AGACTGTATT GAAATTACTT AACTAGTACT TGTTTCCCGT TAATCAAGTC TTTCCAAGTT GAGGAGATCC   
  
  
- ACAACACGAT TTCTCTACAC TCAATCATTG TTAACGAATG ATTGATATAT CCAACAAAAT AATGAACATC   
  
  
- TCCAAAAAAA AAACCCTATA GAAAAACCCC AAATATTTAC TCTTCCAACT AAACAACAGA TGAGAAGAAA   
  
  
- GCTAAGTTAT CATTAAGAGA GTCTCTTCCG ATTTTACTGT ACCGTAAGGT AGCGGAGAGT ACTCCGAAAA   
  
  
- ACGTACACGA ATACATAGAA AGTAGAGAAT GAAACCACCC CACCGTAACC CCTCCTTTTC CCCCAAAGCT   
  
  
- AAGCTATCTA GCAGTGAAAG CCTTTACTGC AGAGACTGGT TACCATTCCC ATTCCAATTA TGTACACCTA   
  
  
- TAAAGGTCTC AATCAACACT AACAATAACA ACAACAACAT TGAAATACAG TGCCCCTTTT GTATTATTAA   
  
  
- TAGACAAATA GACAAACAAA CAATAAAAGT TCCCCTTCCC CTCACCCGCC TACGAATCAT ACATTAGTAA   
  
  
- AATATATCCC TTTTAAGTAT ATCGTACTTT CATTTTGAAA GTAACGGTCG TAAGATTCGA TCCCCATCCC   
  
  
- AACAGATGTG GAACTAGAGG GATCTTCGTA CCACTAGTAA GGAGTCTAAC ACAATATCTT ATTTCTTACA   
  
  
- CCAAGACGAC ATTACTCTGA ATACATAATA GTTTACTACT TAGACAAATC TTGAAGGACA AATAAAAAAG   
  
  
- ACGTTTTAAA ATTCTCTGAT CGTAGATAGC CCGAGCTGAG ATCTCCAAGA GAACACAATG GGAAGAGTTG   
  
  
- AGTCAAACTA ACACTATGTC AATGATATAA CTCACTATCA GCTCTCGTAG TGTTGGGCGA TTCCTCAGAT   
  
  
- AGTTGTTCTT CGGGAAGAAC ATTAAGAGGG GAACTTTGAT CGTCAATAGT AGTGAACAGT AGCTTATCGG   
  
  
- GAAAAACATT AAGATGGGAA CTTTGATCGT CAATAACAGT AGTCAAGTTA CACTCAGGGC AATCTATAAG   
  
  
- GGTAGTTCCG TATCTAATAC CTCACTCGTA CTTCTTGCGA AACGTGCTCG ATCTCTGACG AGATTAACCA   
  
  
- CATCTACCGC TCTTTCGTAG ACAACGATTA GTTGGATACC CCCCTTAAGT AATCAGGCCC TAGGGTTCAA   
  
  
- TCTCTAGCTT CAGTAACTCA CTTCTAGGTG TCCCAAGTGT AGGAGGAGTC GAACTAAGTA GTAGAAAAAG   
  
  
- TTCCCACTTT TCTAAGCCTC TACTTTCGGT CTCTCTCTCT TTACGGTGTT CCGTTACCTT CTTGGTCGCC   
  
  
- TTGATGATTC AAAGAGTGGT CCACTAAATT TCGTCAACGA TTAACTTACA CGCTCCCGAA ATAGTCTATT   
  
  
- GGCTTATCTA CTGAAACTCT CAAACCGATT TGCCGGTTCC TTTCTCCAGA GGTAGAGTCC ACTCGGACAG   
  
  
- GTTGCAGAGC CATGAACATA CTAGCTTCCC GAACACCGTT CCTTTGTCAG AAGCCCCTGA TTGTAGATAG   
  
  
- CCCGAGAATT CATGACATTT CTCGGAGAAC CTTTTATGAA CGAGAGGATG CCGTGAGTCA CCCAATGAGA   
  
  
- TAGCGTTCGG TATCGTCGTT CCGAACCACC CGGAGGATTT CACGCTGAAT GTCCGTAACT ACTAGGACAA   
  
  
- AGATTCATAT GAACACTACG ATCGAACCTC CGATAACCCT TTGCTAATCG CAGAGATAAA CTTTTCAAAT   
  
  
- TTTATGAGCA GCTCAAGTTA CGTAACGGGC AAATACCTGG ACTACAGTCC ACCCTTTACG ACCTACACTC   
  
  
- CGGGTCCCTC CGAAACCGGC AATTAACAGG TAATGTCGAG GTAGTGTGAG GACTGCTCTC ACAGCTACAC   
  
  
- TCGTTGGGAT CCCTATCCGA AGAGTCTTAC CACTTTAACG AGCCAGGATT CCATACGTGA AACCAACTCG   
  
  
- TTCTTAGTTT GTGGTTGTGA TGTGCAAAGA ACTGGGCCAA GTATCTCTGG GAACTGATGA TGAGCTAGTC   
  
  
- GGTACAAAC

+     TATC-box

| Site Name | Organism | Position | Strand | Matrix score. | sequence | function |
| --- | --- | --- | --- | --- | --- | --- |
| TATC-box | Oryza sativa | 857 | - | 7 | TATCCCA | cis-acting element involved in gibberellin-responsiveness |

>HU08G00019.1   
+ +Up\_Stream \_Len000AGTTGT AGCAAGCACA TTAAAGTTGA TTATCATTTT GTCCGTGAAA CGGTGAGCAA   
  
  
+ ACGTCATCTC CAGGTCAAAT TCATTCGTAC TCAATCACAA GTTGCTGACA TTTTTACCAA AGGCTAATCG   
  
  
+ TCGCAAAAGT TTCTTTAGTT TAAGGCCAAT CTCTCCGTTG TTTCACCCCA TACAGATTGA GGGGTTGTAA   
  
  
+ TAGTGTATAT ATATAGCCTT GTGTTAGCGG GGCTTGGGCA TTAGACTTTT ACTCTTGTAA ACCCTAATGT   
  
  
+ GTTATTATAA ATATAGATCT AGCCACCCAT GTTGAGGTAG GCACTCTAAT TCTGACACAG GTAGGATAAG   
  
  
+ TTTTCATAGT TTATGTTGTG AATCTTGTCT TACTTTGTGG TTCCTATTGA CAAAAGTTTG AATCCGAGCA   
  
  
+ TGAGAGTTCA TCAATCTCTT TTTCTTTCCT ATTTTATTTC TTGTGGACTT AATTGGATTT TCTGGTAGAG   
  
  
+ GAAAGTTCCT TGCTGGAATT TGGTCGAAAG TGATATACAT TTTGGGATTT AATTAGCCAT AAATTTGGTT   
  
  
+ TTACACCTTG TTAGTTTGGA GTTCTGGTGT GTTATTGTAT GTGTTCTGGT GGGTTTTTCT TTTAGCTAAA   
  
  
+ TGTTGGAGCA TTTTAAGGGA TCTAGATATC TGAAGAAGTG CTTGGGAAAT GAAGCATATT TCCCTTATAG   
  
  
+ TCTGACATAA CTTTAATGAA TTGATCATGA ACAAAGGGCA ATTAGTTCAG AAAGGTTCAA CTCCTCTAGG   
  
  
+ TGTTGTGCTA AAGAGATGTG AGTTAGTAAC AATTGCTTAC TAACTATATA GGTTGTTTTA TTACTTGTAG   
  
  
+ AGGTTTTTTT TTTGGGATAT CTTTTTGGGG TTTATAAATG AGAAGGTTGA TTTGTTGTCT ACTCTTCTTT   
  
  
+ CGATTCAATA GTAATTCTCT CAGAGAAGGC TAAAATGACA TGGCATTCCA TCGCCTCTCA TGAGGCTTTT   
  
  
+ TGCATGTGCT TATGTATCTT TCATCTCTTA CTTTGGTGGG GTGGCATTGG GGAGGAAAAG GGGGTTTCGA   
  
  
+ TTCGATAGAT CGTCACTTTC GGAAATGACG TCTCTGACCA ATGGTAAGGG TAAGGTTAAT ACATGTGGAT   
  
  
+ ATTTCCAGAG TTAGTTGTGA TTGTTATTGT TGTTGTTGTA ACTTTATGTC ACGGGGAAAA CATAATAATT   
  
  
+ ATCTGTTTAT CTGTTTGTTT GTTATTTTCA AGGGGAAGGG GAGTGGGCGG ATGCTTAGTA TGTAATCATT   
  
  
+ TTATATAGGG AAAATTCATA TAGCATGAAA GTAAAACTTT CATTGCCAGC ATTCTAAGCT AGGGGTAGGG   
  
  
+ TTGTCTACAC CTTGATCTCC CTAGAAGCAT GGTGATCATT CCTCAGATTG TGTTATAGAA TAAAGAATGT   
  
  
+ GGTTCTGCTG TAATGAGACT TATGTATTAT CAAATGATGA ATCTGTTTAG AACTTCCTGT TTATTTTTTC   
  
  
+ TGCAAAATTT TAAGAGACTA GCATCTATCG GGCTCGACTC TAGAGGTTCT CTTGTGTTAC CCTTCTCAAC   
  
  
+ TCAGTTTGAT TGTGATACAG TTACTATATT GAGTGATAGT CGAGAGCATC ACAACCCGCT AAGGAGTCTA   
  
  
+ TCAACAAGAA GCCCTTCTTG TAATTCTCCC CTTGAAACTA GCAGTTATCA TCACTTGTCA TCGAATAGCC   
  
  
+ CTTTTTGTAA TTCTACCCTT GAAACTAGCA GTTATTGTCA TCAGTTCAAT GTGAGTCCCG TTAGATATTC   
  
  
+ CCATCAAGGC ATAGATTATG GAGTGAGCAT GAAGAACGCT TTGCACGAGC TAGAGACTGC TCTAATTGGT   
  
  
+ GTAGATGGCG AGAAAGCATC TGTTGCTAAT CAACCTATGG GGGGAATTCA TTAGTCCGGG ATCCCAAGTT   
  
  
+ AGAGATCGAA GTCATTGAGT GAAGATCCAC AGGGTTCACA TCCTCCTCAG CTTGATTCAT CATCTTTTTC   
  
  
+ AAGGGTGAAA AGATTCGGAG ATGAAAGCCA GAGAGAGAGA AATGCCACAA GGCAATGGAA GAACCAGCGG   
  
  
+ AACTACTAAG TTTCTCACCA GGTGATTTAA AGCAGTTGCT AATTGAATGT GCGAGGGCTT TATCAGATAA   
  
  
+ CCGAATAGAT GACTTTGAGA GTTTGGCTAA ACGGCCAAGG AAAGAGGTCT CCATCTCAGG TGAGCCTGTC   
  
  
+ CAACGTCTCG GTACTTGTAT GATCGAAGGG CTTGTGGCAA GGAAACAGTC TTCGGGGACT AACATCTATC   
  
  
+ GGGCTCTTAA GTACTGTAAA GAGCCTCTTG GAAAATACTT GCTCTCCTAC GGCACTCAGT GGGTTACTCT   
  
  
+ ATCGCAAGCC ATAGCAGCAA GGCTTGGTGG GCCTCCTAAA GTGCGACTTA CAGGCATTGA TGATCCTGTT   
  
  
+ TCTAAGTATA CTTGTGATGC TAGCTTGGAG GCTATTGGGA AACGATTAGC GTCTCTATTT GAAAAGTTTA   
  
  
+ AAATACTCGT CGAGTTCAAT GCATTGCCCG TTTATGGACC TGATGTCAGG TGGGAAATGC TGGATGTGAG   
  
  
+ GCCCAGGGAG GCTTTGGCCG TTAATTGTCC ATTACAGCTC CATCACACTC CTGACGAGAG TGTCGATGTG   
  
  
+ AGCAACCCTA GGGATAGGCT TCTCAGAATG GTGAAATTGC TCGGTCCTAA GGTATGCACT TTGGTTGAGC   
  
  
+ AAGAATCAAA CACCAACACT ACACGTTTCT TGACCCGGTT CATAGAGACC CTTGACTACT ACTCGATCAG   
  
  
+ CCATGTTTG  

- +Up\_Stream \_Len000TCAACA TCGTTCGTGT AATTTCAACT AATAGTAAAA CAGGCACTTT GCCACTCGTT   
  
  
- TGCAGTAGAG GTCCAGTTTA AGTAAGCATG AGTTAGTGTT CAACGACTGT AAAAATGGTT TCCGATTAGC   
  
  
- AGCGTTTTCA AAGAAATCAA ATTCCGGTTA GAGAGGCAAC AAAGTGGGGT ATGTCTAACT CCCCAACATT   
  
  
- ATCACATATA TATATCGGAA CACAATCGCC CCGAACCCGT AATCTGAAAA TGAGAACATT TGGGATTACA   
  
  
- CAATAATATT TATATCTAGA TCGGTGGGTA CAACTCCATC CGTGAGATTA AGACTGTGTC CATCCTATTC   
  
  
- AAAAGTATCA AATACAACAC TTAGAACAGA ATGAAACACC AAGGATAACT GTTTTCAAAC TTAGGCTCGT   
  
  
- ACTCTCAAGT AGTTAGAGAA AAAGAAAGGA TAAAATAAAG AACACCTGAA TTAACCTAAA AGACCATCTC   
  
  
- CTTTCAAGGA ACGACCTTAA ACCAGCTTTC ACTATATGTA AAACCCTAAA TTAATCGGTA TTTAAACCAA   
  
  
- AATGTGGAAC AATCAAACCT CAAGACCACA CAATAACATA CACAAGACCA CCCAAAAAGA AAATCGATTT   
  
  
- ACAACCTCGT AAAATTCCCT AGATCTATAG ACTTCTTCAC GAACCCTTTA CTTCGTATAA AGGGAATATC   
  
  
- AGACTGTATT GAAATTACTT AACTAGTACT TGTTTCCCGT TAATCAAGTC TTTCCAAGTT GAGGAGATCC   
  
  
- ACAACACGAT TTCTCTACAC TCAATCATTG TTAACGAATG ATTGATATAT CCAACAAAAT AATGAACATC   
  
  
- TCCAAAAAAA AAACCCTATA GAAAAACCCC AAATATTTAC TCTTCCAACT AAACAACAGA TGAGAAGAAA   
  
  
- GCTAAGTTAT CATTAAGAGA GTCTCTTCCG ATTTTACTGT ACCGTAAGGT AGCGGAGAGT ACTCCGAAAA   
  
  
- ACGTACACGA ATACATAGAA AGTAGAGAAT GAAACCACCC CACCGTAACC CCTCCTTTTC CCCCAAAGCT   
  
  
- AAGCTATCTA GCAGTGAAAG CCTTTACTGC AGAGACTGGT TACCATTCCC ATTCCAATTA TGTACACCTA   
  
  
- TAAAGGTCTC AATCAACACT AACAATAACA ACAACAACAT TGAAATACAG TGCCCCTTTT GTATTATTAA   
  
  
- TAGACAAATA GACAAACAAA CAATAAAAGT TCCCCTTCCC CTCACCCGCC TACGAATCAT ACATTAGTAA   
  
  
- AATATATCCC TTTTAAGTAT ATCGTACTTT CATTTTGAAA GTAACGGTCG TAAGATTCGA TCCCCATCCC   
  
  
- AACAGATGTG GAACTAGAGG GATCTTCGTA CCACTAGTAA GGAGTCTAAC ACAATATCTT ATTTCTTACA   
  
  
- CCAAGACGAC ATTACTCTGA ATACATAATA GTTTACTACT TAGACAAATC TTGAAGGACA AATAAAAAAG   
  
  
- ACGTTTTAAA ATTCTCTGAT CGTAGATAGC CCGAGCTGAG ATCTCCAAGA GAACACAATG GGAAGAGTTG   
  
  
- AGTCAAACTA ACACTATGTC AATGATATAA CTCACTATCA GCTCTCGTAG TGTTGGGCGA TTCCTCAGAT   
  
  
- AGTTGTTCTT CGGGAAGAAC ATTAAGAGGG GAACTTTGAT CGTCAATAGT AGTGAACAGT AGCTTATCGG   
  
  
- GAAAAACATT AAGATGGGAA CTTTGATCGT CAATAACAGT AGTCAAGTTA CACTCAGGGC AATCTATAAG   
  
  
- GGTAGTTCCG TATCTAATAC CTCACTCGTA CTTCTTGCGA AACGTGCTCG ATCTCTGACG AGATTAACCA   
  
  
- CATCTACCGC TCTTTCGTAG ACAACGATTA GTTGGATACC CCCCTTAAGT AATCAGGCCC TAGGGTTCAA   
  
  
- TCTCTAGCTT CAGTAACTCA CTTCTAGGTG TCCCAAGTGT AGGAGGAGTC GAACTAAGTA GTAGAAAAAG   
  
  
- TTCCCACTTT TCTAAGCCTC TACTTTCGGT CTCTCTCTCT TTACGGTGTT CCGTTACCTT CTTGGTCGCC   
  
  
- TTGATGATTC AAAGAGTGGT CCACTAAATT TCGTCAACGA TTAACTTACA CGCTCCCGAA ATAGTCTATT   
  
  
- GGCTTATCTA CTGAAACTCT CAAACCGATT TGCCGGTTCC TTTCTCCAGA GGTAGAGTCC ACTCGGACAG   
  
  
- GTTGCAGAGC CATGAACATA CTAGCTTCCC GAACACCGTT CCTTTGTCAG AAGCCCCTGA TTGTAGATAG   
  
  
- CCCGAGAATT CATGACATTT CTCGGAGAAC CTTTTATGAA CGAGAGGATG CCGTGAGTCA CCCAATGAGA   
  
  
- TAGCGTTCGG TATCGTCGTT CCGAACCACC CGGAGGATTT CACGCTGAAT GTCCGTAACT ACTAGGACAA   
  
  
- AGATTCATAT GAACACTACG ATCGAACCTC CGATAACCCT TTGCTAATCG CAGAGATAAA CTTTTCAAAT   
  
  
- TTTATGAGCA GCTCAAGTTA CGTAACGGGC AAATACCTGG ACTACAGTCC ACCCTTTACG ACCTACACTC   
  
  
- CGGGTCCCTC CGAAACCGGC AATTAACAGG TAATGTCGAG GTAGTGTGAG GACTGCTCTC ACAGCTACAC   
  
  
- TCGTTGGGAT CCCTATCCGA AGAGTCTTAC CACTTTAACG AGCCAGGATT CCATACGTGA AACCAACTCG   
  
  
- TTCTTAGTTT GTGGTTGTGA TGTGCAAAGA ACTGGGCCAA GTATCTCTGG GAACTGATGA TGAGCTAGTC   
  
  
- GGTACAAAC

+     TC-rich repeats

| Site Name | Organism | Position | Strand | Matrix score. | sequence | function |
| --- | --- | --- | --- | --- | --- | --- |
| TC-rich repeats | Nicotiana tabacum | 829 | + | 9 | GTTTTCTTAC | cis-acting element involved in defense and stress responsiveness |
| TC-rich repeats | Nicotiana tabacum | 1386 | - | 9 | ATTCTCTAAC | cis-acting element involved in defense and stress responsiveness |

>HU08G00019.1   
+ +Up\_Stream \_Len000AGTTGT AGCAAGCACA TTAAAGTTGA TTATCATTTT GTCCGTGAAA CGGTGAGCAA   
  
  
+ ACGTCATCTC CAGGTCAAAT TCATTCGTAC TCAATCACAA GTTGCTGACA TTTTTACCAA AGGCTAATCG   
  
  
+ TCGCAAAAGT TTCTTTAGTT TAAGGCCAAT CTCTCCGTTG TTTCACCCCA TACAGATTGA GGGGTTGTAA   
  
  
+ TAGTGTATAT ATATAGCCTT GTGTTAGCGG GGCTTGGGCA TTAGACTTTT ACTCTTGTAA ACCCTAATGT   
  
  
+ GTTATTATAA ATATAGATCT AGCCACCCAT GTTGAGGTAG GCACTCTAAT TCTGACACAG GTAGGATAAG   
  
  
+ TTTTCATAGT TTATGTTGTG AATCTTGTCT TACTTTGTGG TTCCTATTGA CAAAAGTTTG AATCCGAGCA   
  
  
+ TGAGAGTTCA TCAATCTCTT TTTCTTTCCT ATTTTATTTC TTGTGGACTT AATTGGATTT TCTGGTAGAG   
  
  
+ GAAAGTTCCT TGCTGGAATT TGGTCGAAAG TGATATACAT TTTGGGATTT AATTAGCCAT AAATTTGGTT   
  
  
+ TTACACCTTG TTAGTTTGGA GTTCTGGTGT GTTATTGTAT GTGTTCTGGT GGGTTTTTCT TTTAGCTAAA   
  
  
+ TGTTGGAGCA TTTTAAGGGA TCTAGATATC TGAAGAAGTG CTTGGGAAAT GAAGCATATT TCCCTTATAG   
  
  
+ TCTGACATAA CTTTAATGAA TTGATCATGA ACAAAGGGCA ATTAGTTCAG AAAGGTTCAA CTCCTCTAGG   
  
  
+ TGTTGTGCTA AAGAGATGTG AGTTAGTAAC AATTGCTTAC TAACTATATA GGTTGTTTTA TTACTTGTAG   
  
  
+ AGGTTTTTTT TTTGGGATAT CTTTTTGGGG TTTATAAATG AGAAGGTTGA TTTGTTGTCT ACTCTTCTTT   
  
  
+ CGATTCAATA GTAATTCTCT CAGAGAAGGC TAAAATGACA TGGCATTCCA TCGCCTCTCA TGAGGCTTTT   
  
  
+ TGCATGTGCT TATGTATCTT TCATCTCTTA CTTTGGTGGG GTGGCATTGG GGAGGAAAAG GGGGTTTCGA   
  
  
+ TTCGATAGAT CGTCACTTTC GGAAATGACG TCTCTGACCA ATGGTAAGGG TAAGGTTAAT ACATGTGGAT   
  
  
+ ATTTCCAGAG TTAGTTGTGA TTGTTATTGT TGTTGTTGTA ACTTTATGTC ACGGGGAAAA CATAATAATT   
  
  
+ ATCTGTTTAT CTGTTTGTTT GTTATTTTCA AGGGGAAGGG GAGTGGGCGG ATGCTTAGTA TGTAATCATT   
  
  
+ TTATATAGGG AAAATTCATA TAGCATGAAA GTAAAACTTT CATTGCCAGC ATTCTAAGCT AGGGGTAGGG   
  
  
+ TTGTCTACAC CTTGATCTCC CTAGAAGCAT GGTGATCATT CCTCAGATTG TGTTATAGAA TAAAGAATGT   
  
  
+ GGTTCTGCTG TAATGAGACT TATGTATTAT CAAATGATGA ATCTGTTTAG AACTTCCTGT TTATTTTTTC   
  
  
+ TGCAAAATTT TAAGAGACTA GCATCTATCG GGCTCGACTC TAGAGGTTCT CTTGTGTTAC CCTTCTCAAC   
  
  
+ TCAGTTTGAT TGTGATACAG TTACTATATT GAGTGATAGT CGAGAGCATC ACAACCCGCT AAGGAGTCTA   
  
  
+ TCAACAAGAA GCCCTTCTTG TAATTCTCCC CTTGAAACTA GCAGTTATCA TCACTTGTCA TCGAATAGCC   
  
  
+ CTTTTTGTAA TTCTACCCTT GAAACTAGCA GTTATTGTCA TCAGTTCAAT GTGAGTCCCG TTAGATATTC   
  
  
+ CCATCAAGGC ATAGATTATG GAGTGAGCAT GAAGAACGCT TTGCACGAGC TAGAGACTGC TCTAATTGGT   
  
  
+ GTAGATGGCG AGAAAGCATC TGTTGCTAAT CAACCTATGG GGGGAATTCA TTAGTCCGGG ATCCCAAGTT   
  
  
+ AGAGATCGAA GTCATTGAGT GAAGATCCAC AGGGTTCACA TCCTCCTCAG CTTGATTCAT CATCTTTTTC   
  
  
+ AAGGGTGAAA AGATTCGGAG ATGAAAGCCA GAGAGAGAGA AATGCCACAA GGCAATGGAA GAACCAGCGG   
  
  
+ AACTACTAAG TTTCTCACCA GGTGATTTAA AGCAGTTGCT AATTGAATGT GCGAGGGCTT TATCAGATAA   
  
  
+ CCGAATAGAT GACTTTGAGA GTTTGGCTAA ACGGCCAAGG AAAGAGGTCT CCATCTCAGG TGAGCCTGTC   
  
  
+ CAACGTCTCG GTACTTGTAT GATCGAAGGG CTTGTGGCAA GGAAACAGTC TTCGGGGACT AACATCTATC   
  
  
+ GGGCTCTTAA GTACTGTAAA GAGCCTCTTG GAAAATACTT GCTCTCCTAC GGCACTCAGT GGGTTACTCT   
  
  
+ ATCGCAAGCC ATAGCAGCAA GGCTTGGTGG GCCTCCTAAA GTGCGACTTA CAGGCATTGA TGATCCTGTT   
  
  
+ TCTAAGTATA CTTGTGATGC TAGCTTGGAG GCTATTGGGA AACGATTAGC GTCTCTATTT GAAAAGTTTA   
  
  
+ AAATACTCGT CGAGTTCAAT GCATTGCCCG TTTATGGACC TGATGTCAGG TGGGAAATGC TGGATGTGAG   
  
  
+ GCCCAGGGAG GCTTTGGCCG TTAATTGTCC ATTACAGCTC CATCACACTC CTGACGAGAG TGTCGATGTG   
  
  
+ AGCAACCCTA GGGATAGGCT TCTCAGAATG GTGAAATTGC TCGGTCCTAA GGTATGCACT TTGGTTGAGC   
  
  
+ AAGAATCAAA CACCAACACT ACACGTTTCT TGACCCGGTT CATAGAGACC CTTGACTACT ACTCGATCAG   
  
  
+ CCATGTTTG  

- +Up\_Stream \_Len000TCAACA TCGTTCGTGT AATTTCAACT AATAGTAAAA CAGGCACTTT GCCACTCGTT   
  
  
- TGCAGTAGAG GTCCAGTTTA AGTAAGCATG AGTTAGTGTT CAACGACTGT AAAAATGGTT TCCGATTAGC   
  
  
- AGCGTTTTCA AAGAAATCAA ATTCCGGTTA GAGAGGCAAC AAAGTGGGGT ATGTCTAACT CCCCAACATT   
  
  
- ATCACATATA TATATCGGAA CACAATCGCC CCGAACCCGT AATCTGAAAA TGAGAACATT TGGGATTACA   
  
  
- CAATAATATT TATATCTAGA TCGGTGGGTA CAACTCCATC CGTGAGATTA AGACTGTGTC CATCCTATTC   
  
  
- AAAAGTATCA AATACAACAC TTAGAACAGA ATGAAACACC AAGGATAACT GTTTTCAAAC TTAGGCTCGT   
  
  
- ACTCTCAAGT AGTTAGAGAA AAAGAAAGGA TAAAATAAAG AACACCTGAA TTAACCTAAA AGACCATCTC   
  
  
- CTTTCAAGGA ACGACCTTAA ACCAGCTTTC ACTATATGTA AAACCCTAAA TTAATCGGTA TTTAAACCAA   
  
  
- AATGTGGAAC AATCAAACCT CAAGACCACA CAATAACATA CACAAGACCA CCCAAAAAGA AAATCGATTT   
  
  
- ACAACCTCGT AAAATTCCCT AGATCTATAG ACTTCTTCAC GAACCCTTTA CTTCGTATAA AGGGAATATC   
  
  
- AGACTGTATT GAAATTACTT AACTAGTACT TGTTTCCCGT TAATCAAGTC TTTCCAAGTT GAGGAGATCC   
  
  
- ACAACACGAT TTCTCTACAC TCAATCATTG TTAACGAATG ATTGATATAT CCAACAAAAT AATGAACATC   
  
  
- TCCAAAAAAA AAACCCTATA GAAAAACCCC AAATATTTAC TCTTCCAACT AAACAACAGA TGAGAAGAAA   
  
  
- GCTAAGTTAT CATTAAGAGA GTCTCTTCCG ATTTTACTGT ACCGTAAGGT AGCGGAGAGT ACTCCGAAAA   
  
  
- ACGTACACGA ATACATAGAA AGTAGAGAAT GAAACCACCC CACCGTAACC CCTCCTTTTC CCCCAAAGCT   
  
  
- AAGCTATCTA GCAGTGAAAG CCTTTACTGC AGAGACTGGT TACCATTCCC ATTCCAATTA TGTACACCTA   
  
  
- TAAAGGTCTC AATCAACACT AACAATAACA ACAACAACAT TGAAATACAG TGCCCCTTTT GTATTATTAA   
  
  
- TAGACAAATA GACAAACAAA CAATAAAAGT TCCCCTTCCC CTCACCCGCC TACGAATCAT ACATTAGTAA   
  
  
- AATATATCCC TTTTAAGTAT ATCGTACTTT CATTTTGAAA GTAACGGTCG TAAGATTCGA TCCCCATCCC   
  
  
- AACAGATGTG GAACTAGAGG GATCTTCGTA CCACTAGTAA GGAGTCTAAC ACAATATCTT ATTTCTTACA   
  
  
- CCAAGACGAC ATTACTCTGA ATACATAATA GTTTACTACT TAGACAAATC TTGAAGGACA AATAAAAAAG   
  
  
- ACGTTTTAAA ATTCTCTGAT CGTAGATAGC CCGAGCTGAG ATCTCCAAGA GAACACAATG GGAAGAGTTG   
  
  
- AGTCAAACTA ACACTATGTC AATGATATAA CTCACTATCA GCTCTCGTAG TGTTGGGCGA TTCCTCAGAT   
  
  
- AGTTGTTCTT CGGGAAGAAC ATTAAGAGGG GAACTTTGAT CGTCAATAGT AGTGAACAGT AGCTTATCGG   
  
  
- GAAAAACATT AAGATGGGAA CTTTGATCGT CAATAACAGT AGTCAAGTTA CACTCAGGGC AATCTATAAG   
  
  
- GGTAGTTCCG TATCTAATAC CTCACTCGTA CTTCTTGCGA AACGTGCTCG ATCTCTGACG AGATTAACCA   
  
  
- CATCTACCGC TCTTTCGTAG ACAACGATTA GTTGGATACC CCCCTTAAGT AATCAGGCCC TAGGGTTCAA   
  
  
- TCTCTAGCTT CAGTAACTCA CTTCTAGGTG TCCCAAGTGT AGGAGGAGTC GAACTAAGTA GTAGAAAAAG   
  
  
- TTCCCACTTT TCTAAGCCTC TACTTTCGGT CTCTCTCTCT TTACGGTGTT CCGTTACCTT CTTGGTCGCC   
  
  
- TTGATGATTC AAAGAGTGGT CCACTAAATT TCGTCAACGA TTAACTTACA CGCTCCCGAA ATAGTCTATT   
  
  
- GGCTTATCTA CTGAAACTCT CAAACCGATT TGCCGGTTCC TTTCTCCAGA GGTAGAGTCC ACTCGGACAG   
  
  
- GTTGCAGAGC CATGAACATA CTAGCTTCCC GAACACCGTT CCTTTGTCAG AAGCCCCTGA TTGTAGATAG   
  
  
- CCCGAGAATT CATGACATTT CTCGGAGAAC CTTTTATGAA CGAGAGGATG CCGTGAGTCA CCCAATGAGA   
  
  
- TAGCGTTCGG TATCGTCGTT CCGAACCACC CGGAGGATTT CACGCTGAAT GTCCGTAACT ACTAGGACAA   
  
  
- AGATTCATAT GAACACTACG ATCGAACCTC CGATAACCCT TTGCTAATCG CAGAGATAAA CTTTTCAAAT   
  
  
- TTTATGAGCA GCTCAAGTTA CGTAACGGGC AAATACCTGG ACTACAGTCC ACCCTTTACG ACCTACACTC   
  
  
- CGGGTCCCTC CGAAACCGGC AATTAACAGG TAATGTCGAG GTAGTGTGAG GACTGCTCTC ACAGCTACAC   
  
  
- TCGTTGGGAT CCCTATCCGA AGAGTCTTAC CACTTTAACG AGCCAGGATT CCATACGTGA AACCAACTCG   
  
  
- TTCTTAGTTT GTGGTTGTGA TGTGCAAAGA ACTGGGCCAA GTATCTCTGG GAACTGATGA TGAGCTAGTC   
  
  
- GGTACAAAC

+     TCA-element

| Site Name | Organism | Position | Strand | Matrix score. | sequence | function |
| --- | --- | --- | --- | --- | --- | --- |
| TCA-element | Nicotiana tabacum | 1954 | + | 9 | CCATCTTTTT | cis-acting element involved in salicylic acid responsiveness |

>HU08G00019.1   
+ +Up\_Stream \_Len000AGTTGT AGCAAGCACA TTAAAGTTGA TTATCATTTT GTCCGTGAAA CGGTGAGCAA   
  
  
+ ACGTCATCTC CAGGTCAAAT TCATTCGTAC TCAATCACAA GTTGCTGACA TTTTTACCAA AGGCTAATCG   
  
  
+ TCGCAAAAGT TTCTTTAGTT TAAGGCCAAT CTCTCCGTTG TTTCACCCCA TACAGATTGA GGGGTTGTAA   
  
  
+ TAGTGTATAT ATATAGCCTT GTGTTAGCGG GGCTTGGGCA TTAGACTTTT ACTCTTGTAA ACCCTAATGT   
  
  
+ GTTATTATAA ATATAGATCT AGCCACCCAT GTTGAGGTAG GCACTCTAAT TCTGACACAG GTAGGATAAG   
  
  
+ TTTTCATAGT TTATGTTGTG AATCTTGTCT TACTTTGTGG TTCCTATTGA CAAAAGTTTG AATCCGAGCA   
  
  
+ TGAGAGTTCA TCAATCTCTT TTTCTTTCCT ATTTTATTTC TTGTGGACTT AATTGGATTT TCTGGTAGAG   
  
  
+ GAAAGTTCCT TGCTGGAATT TGGTCGAAAG TGATATACAT TTTGGGATTT AATTAGCCAT AAATTTGGTT   
  
  
+ TTACACCTTG TTAGTTTGGA GTTCTGGTGT GTTATTGTAT GTGTTCTGGT GGGTTTTTCT TTTAGCTAAA   
  
  
+ TGTTGGAGCA TTTTAAGGGA TCTAGATATC TGAAGAAGTG CTTGGGAAAT GAAGCATATT TCCCTTATAG   
  
  
+ TCTGACATAA CTTTAATGAA TTGATCATGA ACAAAGGGCA ATTAGTTCAG AAAGGTTCAA CTCCTCTAGG   
  
  
+ TGTTGTGCTA AAGAGATGTG AGTTAGTAAC AATTGCTTAC TAACTATATA GGTTGTTTTA TTACTTGTAG   
  
  
+ AGGTTTTTTT TTTGGGATAT CTTTTTGGGG TTTATAAATG AGAAGGTTGA TTTGTTGTCT ACTCTTCTTT   
  
  
+ CGATTCAATA GTAATTCTCT CAGAGAAGGC TAAAATGACA TGGCATTCCA TCGCCTCTCA TGAGGCTTTT   
  
  
+ TGCATGTGCT TATGTATCTT TCATCTCTTA CTTTGGTGGG GTGGCATTGG GGAGGAAAAG GGGGTTTCGA   
  
  
+ TTCGATAGAT CGTCACTTTC GGAAATGACG TCTCTGACCA ATGGTAAGGG TAAGGTTAAT ACATGTGGAT   
  
  
+ ATTTCCAGAG TTAGTTGTGA TTGTTATTGT TGTTGTTGTA ACTTTATGTC ACGGGGAAAA CATAATAATT   
  
  
+ ATCTGTTTAT CTGTTTGTTT GTTATTTTCA AGGGGAAGGG GAGTGGGCGG ATGCTTAGTA TGTAATCATT   
  
  
+ TTATATAGGG AAAATTCATA TAGCATGAAA GTAAAACTTT CATTGCCAGC ATTCTAAGCT AGGGGTAGGG   
  
  
+ TTGTCTACAC CTTGATCTCC CTAGAAGCAT GGTGATCATT CCTCAGATTG TGTTATAGAA TAAAGAATGT   
  
  
+ GGTTCTGCTG TAATGAGACT TATGTATTAT CAAATGATGA ATCTGTTTAG AACTTCCTGT TTATTTTTTC   
  
  
+ TGCAAAATTT TAAGAGACTA GCATCTATCG GGCTCGACTC TAGAGGTTCT CTTGTGTTAC CCTTCTCAAC   
  
  
+ TCAGTTTGAT TGTGATACAG TTACTATATT GAGTGATAGT CGAGAGCATC ACAACCCGCT AAGGAGTCTA   
  
  
+ TCAACAAGAA GCCCTTCTTG TAATTCTCCC CTTGAAACTA GCAGTTATCA TCACTTGTCA TCGAATAGCC   
  
  
+ CTTTTTGTAA TTCTACCCTT GAAACTAGCA GTTATTGTCA TCAGTTCAAT GTGAGTCCCG TTAGATATTC   
  
  
+ CCATCAAGGC ATAGATTATG GAGTGAGCAT GAAGAACGCT TTGCACGAGC TAGAGACTGC TCTAATTGGT   
  
  
+ GTAGATGGCG AGAAAGCATC TGTTGCTAAT CAACCTATGG GGGGAATTCA TTAGTCCGGG ATCCCAAGTT   
  
  
+ AGAGATCGAA GTCATTGAGT GAAGATCCAC AGGGTTCACA TCCTCCTCAG CTTGATTCAT CATCTTTTTC   
  
  
+ AAGGGTGAAA AGATTCGGAG ATGAAAGCCA GAGAGAGAGA AATGCCACAA GGCAATGGAA GAACCAGCGG   
  
  
+ AACTACTAAG TTTCTCACCA GGTGATTTAA AGCAGTTGCT AATTGAATGT GCGAGGGCTT TATCAGATAA   
  
  
+ CCGAATAGAT GACTTTGAGA GTTTGGCTAA ACGGCCAAGG AAAGAGGTCT CCATCTCAGG TGAGCCTGTC   
  
  
+ CAACGTCTCG GTACTTGTAT GATCGAAGGG CTTGTGGCAA GGAAACAGTC TTCGGGGACT AACATCTATC   
  
  
+ GGGCTCTTAA GTACTGTAAA GAGCCTCTTG GAAAATACTT GCTCTCCTAC GGCACTCAGT GGGTTACTCT   
  
  
+ ATCGCAAGCC ATAGCAGCAA GGCTTGGTGG GCCTCCTAAA GTGCGACTTA CAGGCATTGA TGATCCTGTT   
  
  
+ TCTAAGTATA CTTGTGATGC TAGCTTGGAG GCTATTGGGA AACGATTAGC GTCTCTATTT GAAAAGTTTA   
  
  
+ AAATACTCGT CGAGTTCAAT GCATTGCCCG TTTATGGACC TGATGTCAGG TGGGAAATGC TGGATGTGAG   
  
  
+ GCCCAGGGAG GCTTTGGCCG TTAATTGTCC ATTACAGCTC CATCACACTC CTGACGAGAG TGTCGATGTG   
  
  
+ AGCAACCCTA GGGATAGGCT TCTCAGAATG GTGAAATTGC TCGGTCCTAA GGTATGCACT TTGGTTGAGC   
  
  
+ AAGAATCAAA CACCAACACT ACACGTTTCT TGACCCGGTT CATAGAGACC CTTGACTACT ACTCGATCAG   
  
  
+ CCATGTTTG  

- +Up\_Stream \_Len000TCAACA TCGTTCGTGT AATTTCAACT AATAGTAAAA CAGGCACTTT GCCACTCGTT   
  
  
- TGCAGTAGAG GTCCAGTTTA AGTAAGCATG AGTTAGTGTT CAACGACTGT AAAAATGGTT TCCGATTAGC   
  
  
- AGCGTTTTCA AAGAAATCAA ATTCCGGTTA GAGAGGCAAC AAAGTGGGGT ATGTCTAACT CCCCAACATT   
  
  
- ATCACATATA TATATCGGAA CACAATCGCC CCGAACCCGT AATCTGAAAA TGAGAACATT TGGGATTACA   
  
  
- CAATAATATT TATATCTAGA TCGGTGGGTA CAACTCCATC CGTGAGATTA AGACTGTGTC CATCCTATTC   
  
  
- AAAAGTATCA AATACAACAC TTAGAACAGA ATGAAACACC AAGGATAACT GTTTTCAAAC TTAGGCTCGT   
  
  
- ACTCTCAAGT AGTTAGAGAA AAAGAAAGGA TAAAATAAAG AACACCTGAA TTAACCTAAA AGACCATCTC   
  
  
- CTTTCAAGGA ACGACCTTAA ACCAGCTTTC ACTATATGTA AAACCCTAAA TTAATCGGTA TTTAAACCAA   
  
  
- AATGTGGAAC AATCAAACCT CAAGACCACA CAATAACATA CACAAGACCA CCCAAAAAGA AAATCGATTT   
  
  
- ACAACCTCGT AAAATTCCCT AGATCTATAG ACTTCTTCAC GAACCCTTTA CTTCGTATAA AGGGAATATC   
  
  
- AGACTGTATT GAAATTACTT AACTAGTACT TGTTTCCCGT TAATCAAGTC TTTCCAAGTT GAGGAGATCC   
  
  
- ACAACACGAT TTCTCTACAC TCAATCATTG TTAACGAATG ATTGATATAT CCAACAAAAT AATGAACATC   
  
  
- TCCAAAAAAA AAACCCTATA GAAAAACCCC AAATATTTAC TCTTCCAACT AAACAACAGA TGAGAAGAAA   
  
  
- GCTAAGTTAT CATTAAGAGA GTCTCTTCCG ATTTTACTGT ACCGTAAGGT AGCGGAGAGT ACTCCGAAAA   
  
  
- ACGTACACGA ATACATAGAA AGTAGAGAAT GAAACCACCC CACCGTAACC CCTCCTTTTC CCCCAAAGCT   
  
  
- AAGCTATCTA GCAGTGAAAG CCTTTACTGC AGAGACTGGT TACCATTCCC ATTCCAATTA TGTACACCTA   
  
  
- TAAAGGTCTC AATCAACACT AACAATAACA ACAACAACAT TGAAATACAG TGCCCCTTTT GTATTATTAA   
  
  
- TAGACAAATA GACAAACAAA CAATAAAAGT TCCCCTTCCC CTCACCCGCC TACGAATCAT ACATTAGTAA   
  
  
- AATATATCCC TTTTAAGTAT ATCGTACTTT CATTTTGAAA GTAACGGTCG TAAGATTCGA TCCCCATCCC   
  
  
- AACAGATGTG GAACTAGAGG GATCTTCGTA CCACTAGTAA GGAGTCTAAC ACAATATCTT ATTTCTTACA   
  
  
- CCAAGACGAC ATTACTCTGA ATACATAATA GTTTACTACT TAGACAAATC TTGAAGGACA AATAAAAAAG   
  
  
- ACGTTTTAAA ATTCTCTGAT CGTAGATAGC CCGAGCTGAG ATCTCCAAGA GAACACAATG GGAAGAGTTG   
  
  
- AGTCAAACTA ACACTATGTC AATGATATAA CTCACTATCA GCTCTCGTAG TGTTGGGCGA TTCCTCAGAT   
  
  
- AGTTGTTCTT CGGGAAGAAC ATTAAGAGGG GAACTTTGAT CGTCAATAGT AGTGAACAGT AGCTTATCGG   
  
  
- GAAAAACATT AAGATGGGAA CTTTGATCGT CAATAACAGT AGTCAAGTTA CACTCAGGGC AATCTATAAG   
  
  
- GGTAGTTCCG TATCTAATAC CTCACTCGTA CTTCTTGCGA AACGTGCTCG ATCTCTGACG AGATTAACCA   
  
  
- CATCTACCGC TCTTTCGTAG ACAACGATTA GTTGGATACC CCCCTTAAGT AATCAGGCCC TAGGGTTCAA   
  
  
- TCTCTAGCTT CAGTAACTCA CTTCTAGGTG TCCCAAGTGT AGGAGGAGTC GAACTAAGTA GTAGAAAAAG   
  
  
- TTCCCACTTT TCTAAGCCTC TACTTTCGGT CTCTCTCTCT TTACGGTGTT CCGTTACCTT CTTGGTCGCC   
  
  
- TTGATGATTC AAAGAGTGGT CCACTAAATT TCGTCAACGA TTAACTTACA CGCTCCCGAA ATAGTCTATT   
  
  
- GGCTTATCTA CTGAAACTCT CAAACCGATT TGCCGGTTCC TTTCTCCAGA GGTAGAGTCC ACTCGGACAG   
  
  
- GTTGCAGAGC CATGAACATA CTAGCTTCCC GAACACCGTT CCTTTGTCAG AAGCCCCTGA TTGTAGATAG   
  
  
- CCCGAGAATT CATGACATTT CTCGGAGAAC CTTTTATGAA CGAGAGGATG CCGTGAGTCA CCCAATGAGA   
  
  
- TAGCGTTCGG TATCGTCGTT CCGAACCACC CGGAGGATTT CACGCTGAAT GTCCGTAACT ACTAGGACAA   
  
  
- AGATTCATAT GAACACTACG ATCGAACCTC CGATAACCCT TTGCTAATCG CAGAGATAAA CTTTTCAAAT   
  
  
- TTTATGAGCA GCTCAAGTTA CGTAACGGGC AAATACCTGG ACTACAGTCC ACCCTTTACG ACCTACACTC   
  
  
- CGGGTCCCTC CGAAACCGGC AATTAACAGG TAATGTCGAG GTAGTGTGAG GACTGCTCTC ACAGCTACAC   
  
  
- TCGTTGGGAT CCCTATCCGA AGAGTCTTAC CACTTTAACG AGCCAGGATT CCATACGTGA AACCAACTCG   
  
  
- TTCTTAGTTT GTGGTTGTGA TGTGCAAAGA ACTGGGCCAA GTATCTCTGG GAACTGATGA TGAGCTAGTC   
  
  
- GGTACAAAC

+     TCCC-motif

| Site Name | Organism | Position | Strand | Matrix score. | sequence | function |
| --- | --- | --- | --- | --- | --- | --- |
| TCCC-motif | Spinacia oleracea | 1350 | + | 7 | TCTCCCT | part of a light responsive element |

>HU08G00019.1   
+ +Up\_Stream \_Len000AGTTGT AGCAAGCACA TTAAAGTTGA TTATCATTTT GTCCGTGAAA CGGTGAGCAA   
  
  
+ ACGTCATCTC CAGGTCAAAT TCATTCGTAC TCAATCACAA GTTGCTGACA TTTTTACCAA AGGCTAATCG   
  
  
+ TCGCAAAAGT TTCTTTAGTT TAAGGCCAAT CTCTCCGTTG TTTCACCCCA TACAGATTGA GGGGTTGTAA   
  
  
+ TAGTGTATAT ATATAGCCTT GTGTTAGCGG GGCTTGGGCA TTAGACTTTT ACTCTTGTAA ACCCTAATGT   
  
  
+ GTTATTATAA ATATAGATCT AGCCACCCAT GTTGAGGTAG GCACTCTAAT TCTGACACAG GTAGGATAAG   
  
  
+ TTTTCATAGT TTATGTTGTG AATCTTGTCT TACTTTGTGG TTCCTATTGA CAAAAGTTTG AATCCGAGCA   
  
  
+ TGAGAGTTCA TCAATCTCTT TTTCTTTCCT ATTTTATTTC TTGTGGACTT AATTGGATTT TCTGGTAGAG   
  
  
+ GAAAGTTCCT TGCTGGAATT TGGTCGAAAG TGATATACAT TTTGGGATTT AATTAGCCAT AAATTTGGTT   
  
  
+ TTACACCTTG TTAGTTTGGA GTTCTGGTGT GTTATTGTAT GTGTTCTGGT GGGTTTTTCT TTTAGCTAAA   
  
  
+ TGTTGGAGCA TTTTAAGGGA TCTAGATATC TGAAGAAGTG CTTGGGAAAT GAAGCATATT TCCCTTATAG   
  
  
+ TCTGACATAA CTTTAATGAA TTGATCATGA ACAAAGGGCA ATTAGTTCAG AAAGGTTCAA CTCCTCTAGG   
  
  
+ TGTTGTGCTA AAGAGATGTG AGTTAGTAAC AATTGCTTAC TAACTATATA GGTTGTTTTA TTACTTGTAG   
  
  
+ AGGTTTTTTT TTTGGGATAT CTTTTTGGGG TTTATAAATG AGAAGGTTGA TTTGTTGTCT ACTCTTCTTT   
  
  
+ CGATTCAATA GTAATTCTCT CAGAGAAGGC TAAAATGACA TGGCATTCCA TCGCCTCTCA TGAGGCTTTT   
  
  
+ TGCATGTGCT TATGTATCTT TCATCTCTTA CTTTGGTGGG GTGGCATTGG GGAGGAAAAG GGGGTTTCGA   
  
  
+ TTCGATAGAT CGTCACTTTC GGAAATGACG TCTCTGACCA ATGGTAAGGG TAAGGTTAAT ACATGTGGAT   
  
  
+ ATTTCCAGAG TTAGTTGTGA TTGTTATTGT TGTTGTTGTA ACTTTATGTC ACGGGGAAAA CATAATAATT   
  
  
+ ATCTGTTTAT CTGTTTGTTT GTTATTTTCA AGGGGAAGGG GAGTGGGCGG ATGCTTAGTA TGTAATCATT   
  
  
+ TTATATAGGG AAAATTCATA TAGCATGAAA GTAAAACTTT CATTGCCAGC ATTCTAAGCT AGGGGTAGGG   
  
  
+ TTGTCTACAC CTTGATCTCC CTAGAAGCAT GGTGATCATT CCTCAGATTG TGTTATAGAA TAAAGAATGT   
  
  
+ GGTTCTGCTG TAATGAGACT TATGTATTAT CAAATGATGA ATCTGTTTAG AACTTCCTGT TTATTTTTTC   
  
  
+ TGCAAAATTT TAAGAGACTA GCATCTATCG GGCTCGACTC TAGAGGTTCT CTTGTGTTAC CCTTCTCAAC   
  
  
+ TCAGTTTGAT TGTGATACAG TTACTATATT GAGTGATAGT CGAGAGCATC ACAACCCGCT AAGGAGTCTA   
  
  
+ TCAACAAGAA GCCCTTCTTG TAATTCTCCC CTTGAAACTA GCAGTTATCA TCACTTGTCA TCGAATAGCC   
  
  
+ CTTTTTGTAA TTCTACCCTT GAAACTAGCA GTTATTGTCA TCAGTTCAAT GTGAGTCCCG TTAGATATTC   
  
  
+ CCATCAAGGC ATAGATTATG GAGTGAGCAT GAAGAACGCT TTGCACGAGC TAGAGACTGC TCTAATTGGT   
  
  
+ GTAGATGGCG AGAAAGCATC TGTTGCTAAT CAACCTATGG GGGGAATTCA TTAGTCCGGG ATCCCAAGTT   
  
  
+ AGAGATCGAA GTCATTGAGT GAAGATCCAC AGGGTTCACA TCCTCCTCAG CTTGATTCAT CATCTTTTTC   
  
  
+ AAGGGTGAAA AGATTCGGAG ATGAAAGCCA GAGAGAGAGA AATGCCACAA GGCAATGGAA GAACCAGCGG   
  
  
+ AACTACTAAG TTTCTCACCA GGTGATTTAA AGCAGTTGCT AATTGAATGT GCGAGGGCTT TATCAGATAA   
  
  
+ CCGAATAGAT GACTTTGAGA GTTTGGCTAA ACGGCCAAGG AAAGAGGTCT CCATCTCAGG TGAGCCTGTC   
  
  
+ CAACGTCTCG GTACTTGTAT GATCGAAGGG CTTGTGGCAA GGAAACAGTC TTCGGGGACT AACATCTATC   
  
  
+ GGGCTCTTAA GTACTGTAAA GAGCCTCTTG GAAAATACTT GCTCTCCTAC GGCACTCAGT GGGTTACTCT   
  
  
+ ATCGCAAGCC ATAGCAGCAA GGCTTGGTGG GCCTCCTAAA GTGCGACTTA CAGGCATTGA TGATCCTGTT   
  
  
+ TCTAAGTATA CTTGTGATGC TAGCTTGGAG GCTATTGGGA AACGATTAGC GTCTCTATTT GAAAAGTTTA   
  
  
+ AAATACTCGT CGAGTTCAAT GCATTGCCCG TTTATGGACC TGATGTCAGG TGGGAAATGC TGGATGTGAG   
  
  
+ GCCCAGGGAG GCTTTGGCCG TTAATTGTCC ATTACAGCTC CATCACACTC CTGACGAGAG TGTCGATGTG   
  
  
+ AGCAACCCTA GGGATAGGCT TCTCAGAATG GTGAAATTGC TCGGTCCTAA GGTATGCACT TTGGTTGAGC   
  
  
+ AAGAATCAAA CACCAACACT ACACGTTTCT TGACCCGGTT CATAGAGACC CTTGACTACT ACTCGATCAG   
  
  
+ CCATGTTTG  

- +Up\_Stream \_Len000TCAACA TCGTTCGTGT AATTTCAACT AATAGTAAAA CAGGCACTTT GCCACTCGTT   
  
  
- TGCAGTAGAG GTCCAGTTTA AGTAAGCATG AGTTAGTGTT CAACGACTGT AAAAATGGTT TCCGATTAGC   
  
  
- AGCGTTTTCA AAGAAATCAA ATTCCGGTTA GAGAGGCAAC AAAGTGGGGT ATGTCTAACT CCCCAACATT   
  
  
- ATCACATATA TATATCGGAA CACAATCGCC CCGAACCCGT AATCTGAAAA TGAGAACATT TGGGATTACA   
  
  
- CAATAATATT TATATCTAGA TCGGTGGGTA CAACTCCATC CGTGAGATTA AGACTGTGTC CATCCTATTC   
  
  
- AAAAGTATCA AATACAACAC TTAGAACAGA ATGAAACACC AAGGATAACT GTTTTCAAAC TTAGGCTCGT   
  
  
- ACTCTCAAGT AGTTAGAGAA AAAGAAAGGA TAAAATAAAG AACACCTGAA TTAACCTAAA AGACCATCTC   
  
  
- CTTTCAAGGA ACGACCTTAA ACCAGCTTTC ACTATATGTA AAACCCTAAA TTAATCGGTA TTTAAACCAA   
  
  
- AATGTGGAAC AATCAAACCT CAAGACCACA CAATAACATA CACAAGACCA CCCAAAAAGA AAATCGATTT   
  
  
- ACAACCTCGT AAAATTCCCT AGATCTATAG ACTTCTTCAC GAACCCTTTA CTTCGTATAA AGGGAATATC   
  
  
- AGACTGTATT GAAATTACTT AACTAGTACT TGTTTCCCGT TAATCAAGTC TTTCCAAGTT GAGGAGATCC   
  
  
- ACAACACGAT TTCTCTACAC TCAATCATTG TTAACGAATG ATTGATATAT CCAACAAAAT AATGAACATC   
  
  
- TCCAAAAAAA AAACCCTATA GAAAAACCCC AAATATTTAC TCTTCCAACT AAACAACAGA TGAGAAGAAA   
  
  
- GCTAAGTTAT CATTAAGAGA GTCTCTTCCG ATTTTACTGT ACCGTAAGGT AGCGGAGAGT ACTCCGAAAA   
  
  
- ACGTACACGA ATACATAGAA AGTAGAGAAT GAAACCACCC CACCGTAACC CCTCCTTTTC CCCCAAAGCT   
  
  
- AAGCTATCTA GCAGTGAAAG CCTTTACTGC AGAGACTGGT TACCATTCCC ATTCCAATTA TGTACACCTA   
  
  
- TAAAGGTCTC AATCAACACT AACAATAACA ACAACAACAT TGAAATACAG TGCCCCTTTT GTATTATTAA   
  
  
- TAGACAAATA GACAAACAAA CAATAAAAGT TCCCCTTCCC CTCACCCGCC TACGAATCAT ACATTAGTAA   
  
  
- AATATATCCC TTTTAAGTAT ATCGTACTTT CATTTTGAAA GTAACGGTCG TAAGATTCGA TCCCCATCCC   
  
  
- AACAGATGTG GAACTAGAGG GATCTTCGTA CCACTAGTAA GGAGTCTAAC ACAATATCTT ATTTCTTACA   
  
  
- CCAAGACGAC ATTACTCTGA ATACATAATA GTTTACTACT TAGACAAATC TTGAAGGACA AATAAAAAAG   
  
  
- ACGTTTTAAA ATTCTCTGAT CGTAGATAGC CCGAGCTGAG ATCTCCAAGA GAACACAATG GGAAGAGTTG   
  
  
- AGTCAAACTA ACACTATGTC AATGATATAA CTCACTATCA GCTCTCGTAG TGTTGGGCGA TTCCTCAGAT   
  
  
- AGTTGTTCTT CGGGAAGAAC ATTAAGAGGG GAACTTTGAT CGTCAATAGT AGTGAACAGT AGCTTATCGG   
  
  
- GAAAAACATT AAGATGGGAA CTTTGATCGT CAATAACAGT AGTCAAGTTA CACTCAGGGC AATCTATAAG   
  
  
- GGTAGTTCCG TATCTAATAC CTCACTCGTA CTTCTTGCGA AACGTGCTCG ATCTCTGACG AGATTAACCA   
  
  
- CATCTACCGC TCTTTCGTAG ACAACGATTA GTTGGATACC CCCCTTAAGT AATCAGGCCC TAGGGTTCAA   
  
  
- TCTCTAGCTT CAGTAACTCA CTTCTAGGTG TCCCAAGTGT AGGAGGAGTC GAACTAAGTA GTAGAAAAAG   
  
  
- TTCCCACTTT TCTAAGCCTC TACTTTCGGT CTCTCTCTCT TTACGGTGTT CCGTTACCTT CTTGGTCGCC   
  
  
- TTGATGATTC AAAGAGTGGT CCACTAAATT TCGTCAACGA TTAACTTACA CGCTCCCGAA ATAGTCTATT   
  
  
- GGCTTATCTA CTGAAACTCT CAAACCGATT TGCCGGTTCC TTTCTCCAGA GGTAGAGTCC ACTCGGACAG   
  
  
- GTTGCAGAGC CATGAACATA CTAGCTTCCC GAACACCGTT CCTTTGTCAG AAGCCCCTGA TTGTAGATAG   
  
  
- CCCGAGAATT CATGACATTT CTCGGAGAAC CTTTTATGAA CGAGAGGATG CCGTGAGTCA CCCAATGAGA   
  
  
- TAGCGTTCGG TATCGTCGTT CCGAACCACC CGGAGGATTT CACGCTGAAT GTCCGTAACT ACTAGGACAA   
  
  
- AGATTCATAT GAACACTACG ATCGAACCTC CGATAACCCT TTGCTAATCG CAGAGATAAA CTTTTCAAAT   
  
  
- TTTATGAGCA GCTCAAGTTA CGTAACGGGC AAATACCTGG ACTACAGTCC ACCCTTTACG ACCTACACTC   
  
  
- CGGGTCCCTC CGAAACCGGC AATTAACAGG TAATGTCGAG GTAGTGTGAG GACTGCTCTC ACAGCTACAC   
  
  
- TCGTTGGGAT CCCTATCCGA AGAGTCTTAC CACTTTAACG AGCCAGGATT CCATACGTGA AACCAACTCG   
  
  
- TTCTTAGTTT GTGGTTGTGA TGTGCAAAGA ACTGGGCCAA GTATCTCTGG GAACTGATGA TGAGCTAGTC   
  
  
- GGTACAAAC

+     TCT-motif

| Site Name | Organism | Position | Strand | Matrix score. | sequence | function |
| --- | --- | --- | --- | --- | --- | --- |
| TCT-motif | Arabidopsis thaliana | 1010 | + | 6 | TCTTAC | part of a light responsive element |
| TCT-motif | Arabidopsis thaliana | 382 | + | 6 | TCTTAC | part of a light responsive element |

>HU08G00019.1   
+ +Up\_Stream \_Len000AGTTGT AGCAAGCACA TTAAAGTTGA TTATCATTTT GTCCGTGAAA CGGTGAGCAA   
  
  
+ ACGTCATCTC CAGGTCAAAT TCATTCGTAC TCAATCACAA GTTGCTGACA TTTTTACCAA AGGCTAATCG   
  
  
+ TCGCAAAAGT TTCTTTAGTT TAAGGCCAAT CTCTCCGTTG TTTCACCCCA TACAGATTGA GGGGTTGTAA   
  
  
+ TAGTGTATAT ATATAGCCTT GTGTTAGCGG GGCTTGGGCA TTAGACTTTT ACTCTTGTAA ACCCTAATGT   
  
  
+ GTTATTATAA ATATAGATCT AGCCACCCAT GTTGAGGTAG GCACTCTAAT TCTGACACAG GTAGGATAAG   
  
  
+ TTTTCATAGT TTATGTTGTG AATCTTGTCT TACTTTGTGG TTCCTATTGA CAAAAGTTTG AATCCGAGCA   
  
  
+ TGAGAGTTCA TCAATCTCTT TTTCTTTCCT ATTTTATTTC TTGTGGACTT AATTGGATTT TCTGGTAGAG   
  
  
+ GAAAGTTCCT TGCTGGAATT TGGTCGAAAG TGATATACAT TTTGGGATTT AATTAGCCAT AAATTTGGTT   
  
  
+ TTACACCTTG TTAGTTTGGA GTTCTGGTGT GTTATTGTAT GTGTTCTGGT GGGTTTTTCT TTTAGCTAAA   
  
  
+ TGTTGGAGCA TTTTAAGGGA TCTAGATATC TGAAGAAGTG CTTGGGAAAT GAAGCATATT TCCCTTATAG   
  
  
+ TCTGACATAA CTTTAATGAA TTGATCATGA ACAAAGGGCA ATTAGTTCAG AAAGGTTCAA CTCCTCTAGG   
  
  
+ TGTTGTGCTA AAGAGATGTG AGTTAGTAAC AATTGCTTAC TAACTATATA GGTTGTTTTA TTACTTGTAG   
  
  
+ AGGTTTTTTT TTTGGGATAT CTTTTTGGGG TTTATAAATG AGAAGGTTGA TTTGTTGTCT ACTCTTCTTT   
  
  
+ CGATTCAATA GTAATTCTCT CAGAGAAGGC TAAAATGACA TGGCATTCCA TCGCCTCTCA TGAGGCTTTT   
  
  
+ TGCATGTGCT TATGTATCTT TCATCTCTTA CTTTGGTGGG GTGGCATTGG GGAGGAAAAG GGGGTTTCGA   
  
  
+ TTCGATAGAT CGTCACTTTC GGAAATGACG TCTCTGACCA ATGGTAAGGG TAAGGTTAAT ACATGTGGAT   
  
  
+ ATTTCCAGAG TTAGTTGTGA TTGTTATTGT TGTTGTTGTA ACTTTATGTC ACGGGGAAAA CATAATAATT   
  
  
+ ATCTGTTTAT CTGTTTGTTT GTTATTTTCA AGGGGAAGGG GAGTGGGCGG ATGCTTAGTA TGTAATCATT   
  
  
+ TTATATAGGG AAAATTCATA TAGCATGAAA GTAAAACTTT CATTGCCAGC ATTCTAAGCT AGGGGTAGGG   
  
  
+ TTGTCTACAC CTTGATCTCC CTAGAAGCAT GGTGATCATT CCTCAGATTG TGTTATAGAA TAAAGAATGT   
  
  
+ GGTTCTGCTG TAATGAGACT TATGTATTAT CAAATGATGA ATCTGTTTAG AACTTCCTGT TTATTTTTTC   
  
  
+ TGCAAAATTT TAAGAGACTA GCATCTATCG GGCTCGACTC TAGAGGTTCT CTTGTGTTAC CCTTCTCAAC   
  
  
+ TCAGTTTGAT TGTGATACAG TTACTATATT GAGTGATAGT CGAGAGCATC ACAACCCGCT AAGGAGTCTA   
  
  
+ TCAACAAGAA GCCCTTCTTG TAATTCTCCC CTTGAAACTA GCAGTTATCA TCACTTGTCA TCGAATAGCC   
  
  
+ CTTTTTGTAA TTCTACCCTT GAAACTAGCA GTTATTGTCA TCAGTTCAAT GTGAGTCCCG TTAGATATTC   
  
  
+ CCATCAAGGC ATAGATTATG GAGTGAGCAT GAAGAACGCT TTGCACGAGC TAGAGACTGC TCTAATTGGT   
  
  
+ GTAGATGGCG AGAAAGCATC TGTTGCTAAT CAACCTATGG GGGGAATTCA TTAGTCCGGG ATCCCAAGTT   
  
  
+ AGAGATCGAA GTCATTGAGT GAAGATCCAC AGGGTTCACA TCCTCCTCAG CTTGATTCAT CATCTTTTTC   
  
  
+ AAGGGTGAAA AGATTCGGAG ATGAAAGCCA GAGAGAGAGA AATGCCACAA GGCAATGGAA GAACCAGCGG   
  
  
+ AACTACTAAG TTTCTCACCA GGTGATTTAA AGCAGTTGCT AATTGAATGT GCGAGGGCTT TATCAGATAA   
  
  
+ CCGAATAGAT GACTTTGAGA GTTTGGCTAA ACGGCCAAGG AAAGAGGTCT CCATCTCAGG TGAGCCTGTC   
  
  
+ CAACGTCTCG GTACTTGTAT GATCGAAGGG CTTGTGGCAA GGAAACAGTC TTCGGGGACT AACATCTATC   
  
  
+ GGGCTCTTAA GTACTGTAAA GAGCCTCTTG GAAAATACTT GCTCTCCTAC GGCACTCAGT GGGTTACTCT   
  
  
+ ATCGCAAGCC ATAGCAGCAA GGCTTGGTGG GCCTCCTAAA GTGCGACTTA CAGGCATTGA TGATCCTGTT   
  
  
+ TCTAAGTATA CTTGTGATGC TAGCTTGGAG GCTATTGGGA AACGATTAGC GTCTCTATTT GAAAAGTTTA   
  
  
+ AAATACTCGT CGAGTTCAAT GCATTGCCCG TTTATGGACC TGATGTCAGG TGGGAAATGC TGGATGTGAG   
  
  
+ GCCCAGGGAG GCTTTGGCCG TTAATTGTCC ATTACAGCTC CATCACACTC CTGACGAGAG TGTCGATGTG   
  
  
+ AGCAACCCTA GGGATAGGCT TCTCAGAATG GTGAAATTGC TCGGTCCTAA GGTATGCACT TTGGTTGAGC   
  
  
+ AAGAATCAAA CACCAACACT ACACGTTTCT TGACCCGGTT CATAGAGACC CTTGACTACT ACTCGATCAG   
  
  
+ CCATGTTTG  

- +Up\_Stream \_Len000TCAACA TCGTTCGTGT AATTTCAACT AATAGTAAAA CAGGCACTTT GCCACTCGTT   
  
  
- TGCAGTAGAG GTCCAGTTTA AGTAAGCATG AGTTAGTGTT CAACGACTGT AAAAATGGTT TCCGATTAGC   
  
  
- AGCGTTTTCA AAGAAATCAA ATTCCGGTTA GAGAGGCAAC AAAGTGGGGT ATGTCTAACT CCCCAACATT   
  
  
- ATCACATATA TATATCGGAA CACAATCGCC CCGAACCCGT AATCTGAAAA TGAGAACATT TGGGATTACA   
  
  
- CAATAATATT TATATCTAGA TCGGTGGGTA CAACTCCATC CGTGAGATTA AGACTGTGTC CATCCTATTC   
  
  
- AAAAGTATCA AATACAACAC TTAGAACAGA ATGAAACACC AAGGATAACT GTTTTCAAAC TTAGGCTCGT   
  
  
- ACTCTCAAGT AGTTAGAGAA AAAGAAAGGA TAAAATAAAG AACACCTGAA TTAACCTAAA AGACCATCTC   
  
  
- CTTTCAAGGA ACGACCTTAA ACCAGCTTTC ACTATATGTA AAACCCTAAA TTAATCGGTA TTTAAACCAA   
  
  
- AATGTGGAAC AATCAAACCT CAAGACCACA CAATAACATA CACAAGACCA CCCAAAAAGA AAATCGATTT   
  
  
- ACAACCTCGT AAAATTCCCT AGATCTATAG ACTTCTTCAC GAACCCTTTA CTTCGTATAA AGGGAATATC   
  
  
- AGACTGTATT GAAATTACTT AACTAGTACT TGTTTCCCGT TAATCAAGTC TTTCCAAGTT GAGGAGATCC   
  
  
- ACAACACGAT TTCTCTACAC TCAATCATTG TTAACGAATG ATTGATATAT CCAACAAAAT AATGAACATC   
  
  
- TCCAAAAAAA AAACCCTATA GAAAAACCCC AAATATTTAC TCTTCCAACT AAACAACAGA TGAGAAGAAA   
  
  
- GCTAAGTTAT CATTAAGAGA GTCTCTTCCG ATTTTACTGT ACCGTAAGGT AGCGGAGAGT ACTCCGAAAA   
  
  
- ACGTACACGA ATACATAGAA AGTAGAGAAT GAAACCACCC CACCGTAACC CCTCCTTTTC CCCCAAAGCT   
  
  
- AAGCTATCTA GCAGTGAAAG CCTTTACTGC AGAGACTGGT TACCATTCCC ATTCCAATTA TGTACACCTA   
  
  
- TAAAGGTCTC AATCAACACT AACAATAACA ACAACAACAT TGAAATACAG TGCCCCTTTT GTATTATTAA   
  
  
- TAGACAAATA GACAAACAAA CAATAAAAGT TCCCCTTCCC CTCACCCGCC TACGAATCAT ACATTAGTAA   
  
  
- AATATATCCC TTTTAAGTAT ATCGTACTTT CATTTTGAAA GTAACGGTCG TAAGATTCGA TCCCCATCCC   
  
  
- AACAGATGTG GAACTAGAGG GATCTTCGTA CCACTAGTAA GGAGTCTAAC ACAATATCTT ATTTCTTACA   
  
  
- CCAAGACGAC ATTACTCTGA ATACATAATA GTTTACTACT TAGACAAATC TTGAAGGACA AATAAAAAAG   
  
  
- ACGTTTTAAA ATTCTCTGAT CGTAGATAGC CCGAGCTGAG ATCTCCAAGA GAACACAATG GGAAGAGTTG   
  
  
- AGTCAAACTA ACACTATGTC AATGATATAA CTCACTATCA GCTCTCGTAG TGTTGGGCGA TTCCTCAGAT   
  
  
- AGTTGTTCTT CGGGAAGAAC ATTAAGAGGG GAACTTTGAT CGTCAATAGT AGTGAACAGT AGCTTATCGG   
  
  
- GAAAAACATT AAGATGGGAA CTTTGATCGT CAATAACAGT AGTCAAGTTA CACTCAGGGC AATCTATAAG   
  
  
- GGTAGTTCCG TATCTAATAC CTCACTCGTA CTTCTTGCGA AACGTGCTCG ATCTCTGACG AGATTAACCA   
  
  
- CATCTACCGC TCTTTCGTAG ACAACGATTA GTTGGATACC CCCCTTAAGT AATCAGGCCC TAGGGTTCAA   
  
  
- TCTCTAGCTT CAGTAACTCA CTTCTAGGTG TCCCAAGTGT AGGAGGAGTC GAACTAAGTA GTAGAAAAAG   
  
  
- TTCCCACTTT TCTAAGCCTC TACTTTCGGT CTCTCTCTCT TTACGGTGTT CCGTTACCTT CTTGGTCGCC   
  
  
- TTGATGATTC AAAGAGTGGT CCACTAAATT TCGTCAACGA TTAACTTACA CGCTCCCGAA ATAGTCTATT   
  
  
- GGCTTATCTA CTGAAACTCT CAAACCGATT TGCCGGTTCC TTTCTCCAGA GGTAGAGTCC ACTCGGACAG   
  
  
- GTTGCAGAGC CATGAACATA CTAGCTTCCC GAACACCGTT CCTTTGTCAG AAGCCCCTGA TTGTAGATAG   
  
  
- CCCGAGAATT CATGACATTT CTCGGAGAAC CTTTTATGAA CGAGAGGATG CCGTGAGTCA CCCAATGAGA   
  
  
- TAGCGTTCGG TATCGTCGTT CCGAACCACC CGGAGGATTT CACGCTGAAT GTCCGTAACT ACTAGGACAA   
  
  
- AGATTCATAT GAACACTACG ATCGAACCTC CGATAACCCT TTGCTAATCG CAGAGATAAA CTTTTCAAAT   
  
  
- TTTATGAGCA GCTCAAGTTA CGTAACGGGC AAATACCTGG ACTACAGTCC ACCCTTTACG ACCTACACTC   
  
  
- CGGGTCCCTC CGAAACCGGC AATTAACAGG TAATGTCGAG GTAGTGTGAG GACTGCTCTC ACAGCTACAC   
  
  
- TCGTTGGGAT CCCTATCCGA AGAGTCTTAC CACTTTAACG AGCCAGGATT CCATACGTGA AACCAACTCG   
  
  
- TTCTTAGTTT GTGGTTGTGA TGTGCAAAGA ACTGGGCCAA GTATCTCTGG GAACTGATGA TGAGCTAGTC   
  
  
- GGTACAAAC

+     TGACG-motif

| Site Name | Organism | Position | Strand | Matrix score. | sequence | function |
| --- | --- | --- | --- | --- | --- | --- |
| TGACG-motif | Hordeum vulgare | 1080 | + | 5 | TGACG | cis-acting regulatory element involved in the MeJA-responsiveness |
| TGACG-motif | Hordeum vulgare | 2576 | + | 5 | TGACG | cis-acting regulatory element involved in the MeJA-responsiveness |
| TGACG-motif | Hordeum vulgare | 76 | - | 5 | TGACG | cis-acting regulatory element involved in the MeJA-responsiveness |
| TGACG-motif | Hordeum vulgare | 1065 | - | 5 | TGACG | cis-acting regulatory element involved in the MeJA-responsiveness |

>HU08G00019.1   
+ +Up\_Stream \_Len000AGTTGT AGCAAGCACA TTAAAGTTGA TTATCATTTT GTCCGTGAAA CGGTGAGCAA   
  
  
+ ACGTCATCTC CAGGTCAAAT TCATTCGTAC TCAATCACAA GTTGCTGACA TTTTTACCAA AGGCTAATCG   
  
  
+ TCGCAAAAGT TTCTTTAGTT TAAGGCCAAT CTCTCCGTTG TTTCACCCCA TACAGATTGA GGGGTTGTAA   
  
  
+ TAGTGTATAT ATATAGCCTT GTGTTAGCGG GGCTTGGGCA TTAGACTTTT ACTCTTGTAA ACCCTAATGT   
  
  
+ GTTATTATAA ATATAGATCT AGCCACCCAT GTTGAGGTAG GCACTCTAAT TCTGACACAG GTAGGATAAG   
  
  
+ TTTTCATAGT TTATGTTGTG AATCTTGTCT TACTTTGTGG TTCCTATTGA CAAAAGTTTG AATCCGAGCA   
  
  
+ TGAGAGTTCA TCAATCTCTT TTTCTTTCCT ATTTTATTTC TTGTGGACTT AATTGGATTT TCTGGTAGAG   
  
  
+ GAAAGTTCCT TGCTGGAATT TGGTCGAAAG TGATATACAT TTTGGGATTT AATTAGCCAT AAATTTGGTT   
  
  
+ TTACACCTTG TTAGTTTGGA GTTCTGGTGT GTTATTGTAT GTGTTCTGGT GGGTTTTTCT TTTAGCTAAA   
  
  
+ TGTTGGAGCA TTTTAAGGGA TCTAGATATC TGAAGAAGTG CTTGGGAAAT GAAGCATATT TCCCTTATAG   
  
  
+ TCTGACATAA CTTTAATGAA TTGATCATGA ACAAAGGGCA ATTAGTTCAG AAAGGTTCAA CTCCTCTAGG   
  
  
+ TGTTGTGCTA AAGAGATGTG AGTTAGTAAC AATTGCTTAC TAACTATATA GGTTGTTTTA TTACTTGTAG   
  
  
+ AGGTTTTTTT TTTGGGATAT CTTTTTGGGG TTTATAAATG AGAAGGTTGA TTTGTTGTCT ACTCTTCTTT   
  
  
+ CGATTCAATA GTAATTCTCT CAGAGAAGGC TAAAATGACA TGGCATTCCA TCGCCTCTCA TGAGGCTTTT   
  
  
+ TGCATGTGCT TATGTATCTT TCATCTCTTA CTTTGGTGGG GTGGCATTGG GGAGGAAAAG GGGGTTTCGA   
  
  
+ TTCGATAGAT CGTCACTTTC GGAAATGACG TCTCTGACCA ATGGTAAGGG TAAGGTTAAT ACATGTGGAT   
  
  
+ ATTTCCAGAG TTAGTTGTGA TTGTTATTGT TGTTGTTGTA ACTTTATGTC ACGGGGAAAA CATAATAATT   
  
  
+ ATCTGTTTAT CTGTTTGTTT GTTATTTTCA AGGGGAAGGG GAGTGGGCGG ATGCTTAGTA TGTAATCATT   
  
  
+ TTATATAGGG AAAATTCATA TAGCATGAAA GTAAAACTTT CATTGCCAGC ATTCTAAGCT AGGGGTAGGG   
  
  
+ TTGTCTACAC CTTGATCTCC CTAGAAGCAT GGTGATCATT CCTCAGATTG TGTTATAGAA TAAAGAATGT   
  
  
+ GGTTCTGCTG TAATGAGACT TATGTATTAT CAAATGATGA ATCTGTTTAG AACTTCCTGT TTATTTTTTC   
  
  
+ TGCAAAATTT TAAGAGACTA GCATCTATCG GGCTCGACTC TAGAGGTTCT CTTGTGTTAC CCTTCTCAAC   
  
  
+ TCAGTTTGAT TGTGATACAG TTACTATATT GAGTGATAGT CGAGAGCATC ACAACCCGCT AAGGAGTCTA   
  
  
+ TCAACAAGAA GCCCTTCTTG TAATTCTCCC CTTGAAACTA GCAGTTATCA TCACTTGTCA TCGAATAGCC   
  
  
+ CTTTTTGTAA TTCTACCCTT GAAACTAGCA GTTATTGTCA TCAGTTCAAT GTGAGTCCCG TTAGATATTC   
  
  
+ CCATCAAGGC ATAGATTATG GAGTGAGCAT GAAGAACGCT TTGCACGAGC TAGAGACTGC TCTAATTGGT   
  
  
+ GTAGATGGCG AGAAAGCATC TGTTGCTAAT CAACCTATGG GGGGAATTCA TTAGTCCGGG ATCCCAAGTT   
  
  
+ AGAGATCGAA GTCATTGAGT GAAGATCCAC AGGGTTCACA TCCTCCTCAG CTTGATTCAT CATCTTTTTC   
  
  
+ AAGGGTGAAA AGATTCGGAG ATGAAAGCCA GAGAGAGAGA AATGCCACAA GGCAATGGAA GAACCAGCGG   
  
  
+ AACTACTAAG TTTCTCACCA GGTGATTTAA AGCAGTTGCT AATTGAATGT GCGAGGGCTT TATCAGATAA   
  
  
+ CCGAATAGAT GACTTTGAGA GTTTGGCTAA ACGGCCAAGG AAAGAGGTCT CCATCTCAGG TGAGCCTGTC   
  
  
+ CAACGTCTCG GTACTTGTAT GATCGAAGGG CTTGTGGCAA GGAAACAGTC TTCGGGGACT AACATCTATC   
  
  
+ GGGCTCTTAA GTACTGTAAA GAGCCTCTTG GAAAATACTT GCTCTCCTAC GGCACTCAGT GGGTTACTCT   
  
  
+ ATCGCAAGCC ATAGCAGCAA GGCTTGGTGG GCCTCCTAAA GTGCGACTTA CAGGCATTGA TGATCCTGTT   
  
  
+ TCTAAGTATA CTTGTGATGC TAGCTTGGAG GCTATTGGGA AACGATTAGC GTCTCTATTT GAAAAGTTTA   
  
  
+ AAATACTCGT CGAGTTCAAT GCATTGCCCG TTTATGGACC TGATGTCAGG TGGGAAATGC TGGATGTGAG   
  
  
+ GCCCAGGGAG GCTTTGGCCG TTAATTGTCC ATTACAGCTC CATCACACTC CTGACGAGAG TGTCGATGTG   
  
  
+ AGCAACCCTA GGGATAGGCT TCTCAGAATG GTGAAATTGC TCGGTCCTAA GGTATGCACT TTGGTTGAGC   
  
  
+ AAGAATCAAA CACCAACACT ACACGTTTCT TGACCCGGTT CATAGAGACC CTTGACTACT ACTCGATCAG   
  
  
+ CCATGTTTG  

- +Up\_Stream \_Len000TCAACA TCGTTCGTGT AATTTCAACT AATAGTAAAA CAGGCACTTT GCCACTCGTT   
  
  
- TGCAGTAGAG GTCCAGTTTA AGTAAGCATG AGTTAGTGTT CAACGACTGT AAAAATGGTT TCCGATTAGC   
  
  
- AGCGTTTTCA AAGAAATCAA ATTCCGGTTA GAGAGGCAAC AAAGTGGGGT ATGTCTAACT CCCCAACATT   
  
  
- ATCACATATA TATATCGGAA CACAATCGCC CCGAACCCGT AATCTGAAAA TGAGAACATT TGGGATTACA   
  
  
- CAATAATATT TATATCTAGA TCGGTGGGTA CAACTCCATC CGTGAGATTA AGACTGTGTC CATCCTATTC   
  
  
- AAAAGTATCA AATACAACAC TTAGAACAGA ATGAAACACC AAGGATAACT GTTTTCAAAC TTAGGCTCGT   
  
  
- ACTCTCAAGT AGTTAGAGAA AAAGAAAGGA TAAAATAAAG AACACCTGAA TTAACCTAAA AGACCATCTC   
  
  
- CTTTCAAGGA ACGACCTTAA ACCAGCTTTC ACTATATGTA AAACCCTAAA TTAATCGGTA TTTAAACCAA   
  
  
- AATGTGGAAC AATCAAACCT CAAGACCACA CAATAACATA CACAAGACCA CCCAAAAAGA AAATCGATTT   
  
  
- ACAACCTCGT AAAATTCCCT AGATCTATAG ACTTCTTCAC GAACCCTTTA CTTCGTATAA AGGGAATATC   
  
  
- AGACTGTATT GAAATTACTT AACTAGTACT TGTTTCCCGT TAATCAAGTC TTTCCAAGTT GAGGAGATCC   
  
  
- ACAACACGAT TTCTCTACAC TCAATCATTG TTAACGAATG ATTGATATAT CCAACAAAAT AATGAACATC   
  
  
- TCCAAAAAAA AAACCCTATA GAAAAACCCC AAATATTTAC TCTTCCAACT AAACAACAGA TGAGAAGAAA   
  
  
- GCTAAGTTAT CATTAAGAGA GTCTCTTCCG ATTTTACTGT ACCGTAAGGT AGCGGAGAGT ACTCCGAAAA   
  
  
- ACGTACACGA ATACATAGAA AGTAGAGAAT GAAACCACCC CACCGTAACC CCTCCTTTTC CCCCAAAGCT   
  
  
- AAGCTATCTA GCAGTGAAAG CCTTTACTGC AGAGACTGGT TACCATTCCC ATTCCAATTA TGTACACCTA   
  
  
- TAAAGGTCTC AATCAACACT AACAATAACA ACAACAACAT TGAAATACAG TGCCCCTTTT GTATTATTAA   
  
  
- TAGACAAATA GACAAACAAA CAATAAAAGT TCCCCTTCCC CTCACCCGCC TACGAATCAT ACATTAGTAA   
  
  
- AATATATCCC TTTTAAGTAT ATCGTACTTT CATTTTGAAA GTAACGGTCG TAAGATTCGA TCCCCATCCC   
  
  
- AACAGATGTG GAACTAGAGG GATCTTCGTA CCACTAGTAA GGAGTCTAAC ACAATATCTT ATTTCTTACA   
  
  
- CCAAGACGAC ATTACTCTGA ATACATAATA GTTTACTACT TAGACAAATC TTGAAGGACA AATAAAAAAG   
  
  
- ACGTTTTAAA ATTCTCTGAT CGTAGATAGC CCGAGCTGAG ATCTCCAAGA GAACACAATG GGAAGAGTTG   
  
  
- AGTCAAACTA ACACTATGTC AATGATATAA CTCACTATCA GCTCTCGTAG TGTTGGGCGA TTCCTCAGAT   
  
  
- AGTTGTTCTT CGGGAAGAAC ATTAAGAGGG GAACTTTGAT CGTCAATAGT AGTGAACAGT AGCTTATCGG   
  
  
- GAAAAACATT AAGATGGGAA CTTTGATCGT CAATAACAGT AGTCAAGTTA CACTCAGGGC AATCTATAAG   
  
  
- GGTAGTTCCG TATCTAATAC CTCACTCGTA CTTCTTGCGA AACGTGCTCG ATCTCTGACG AGATTAACCA   
  
  
- CATCTACCGC TCTTTCGTAG ACAACGATTA GTTGGATACC CCCCTTAAGT AATCAGGCCC TAGGGTTCAA   
  
  
- TCTCTAGCTT CAGTAACTCA CTTCTAGGTG TCCCAAGTGT AGGAGGAGTC GAACTAAGTA GTAGAAAAAG   
  
  
- TTCCCACTTT TCTAAGCCTC TACTTTCGGT CTCTCTCTCT TTACGGTGTT CCGTTACCTT CTTGGTCGCC   
  
  
- TTGATGATTC AAAGAGTGGT CCACTAAATT TCGTCAACGA TTAACTTACA CGCTCCCGAA ATAGTCTATT   
  
  
- GGCTTATCTA CTGAAACTCT CAAACCGATT TGCCGGTTCC TTTCTCCAGA GGTAGAGTCC ACTCGGACAG   
  
  
- GTTGCAGAGC CATGAACATA CTAGCTTCCC GAACACCGTT CCTTTGTCAG AAGCCCCTGA TTGTAGATAG   
  
  
- CCCGAGAATT CATGACATTT CTCGGAGAAC CTTTTATGAA CGAGAGGATG CCGTGAGTCA CCCAATGAGA   
  
  
- TAGCGTTCGG TATCGTCGTT CCGAACCACC CGGAGGATTT CACGCTGAAT GTCCGTAACT ACTAGGACAA   
  
  
- AGATTCATAT GAACACTACG ATCGAACCTC CGATAACCCT TTGCTAATCG CAGAGATAAA CTTTTCAAAT   
  
  
- TTTATGAGCA GCTCAAGTTA CGTAACGGGC AAATACCTGG ACTACAGTCC ACCCTTTACG ACCTACACTC   
  
  
- CGGGTCCCTC CGAAACCGGC AATTAACAGG TAATGTCGAG GTAGTGTGAG GACTGCTCTC ACAGCTACAC   
  
  
- TCGTTGGGAT CCCTATCCGA AGAGTCTTAC CACTTTAACG AGCCAGGATT CCATACGTGA AACCAACTCG   
  
  
- TTCTTAGTTT GTGGTTGTGA TGTGCAAAGA ACTGGGCCAA GTATCTCTGG GAACTGATGA TGAGCTAGTC   
  
  
- GGTACAAAC

+     Unnamed\_\_4

| Site Name | Organism | Position | Strand | Matrix score. | sequence | function |
| --- | --- | --- | --- | --- | --- | --- |
| Unnamed\_\_4 | Petroselinum hortense | 2531 | - | 4 | CTCC |  |
| Unnamed\_\_4 | Petroselinum hortense | 2411 | - | 4 | CTCC |  |
| Unnamed\_\_4 | Petroselinum hortense | 2572 | + | 4 | CTCC |  |
| Unnamed\_\_4 | Petroselinum hortense | 2562 | + | 4 | CTCC |  |
| Unnamed\_\_4 | Petroselinum hortense | 1640 | + | 4 | CTCC |  |
| Unnamed\_\_4 | Petroselinum hortense | 1607 | - | 4 | CTCC |  |
| Unnamed\_\_4 | Petroselinum hortense | 2347 | + | 4 | CTCC |  |
| Unnamed\_\_4 | Petroselinum hortense | 1774 | - | 4 | CTCC |  |
| Unnamed\_\_4 | Petroselinum hortense | 1234 | - | 4 | CTCC |  |
| Unnamed\_\_4 | Petroselinum hortense | 582 | - | 4 | CTCC |  |
| Unnamed\_\_4 | Petroselinum hortense | 639 | - | 4 | CTCC |  |
| Unnamed\_\_4 | Petroselinum hortense | 82 | + | 4 | CTCC |  |
| Unnamed\_\_4 | Petroselinum hortense | 1981 | - | 4 | CTCC |  |
| Unnamed\_\_4 | Petroselinum hortense | 765 | + | 4 | CTCC |  |
| Unnamed\_\_4 | Petroselinum hortense | 2288 | + | 4 | CTCC |  |
| Unnamed\_\_4 | Petroselinum hortense | 177 | + | 4 | CTCC |  |
| Unnamed\_\_4 | Petroselinum hortense | 1937 | + | 4 | CTCC |  |
| Unnamed\_\_4 | Petroselinum hortense | 1351 | + | 4 | CTCC |  |
| Unnamed\_\_4 | Petroselinum hortense | 2153 | + | 4 | CTCC |  |
| Unnamed\_\_4 | Petroselinum hortense | 1035 | - | 4 | CTCC |  |

>HU08G00019.1   
+ +Up\_Stream \_Len000AGTTGT AGCAAGCACA TTAAAGTTGA TTATCATTTT GTCCGTGAAA CGGTGAGCAA   
  
  
+ ACGTCATCTC CAGGTCAAAT TCATTCGTAC TCAATCACAA GTTGCTGACA TTTTTACCAA AGGCTAATCG   
  
  
+ TCGCAAAAGT TTCTTTAGTT TAAGGCCAAT CTCTCCGTTG TTTCACCCCA TACAGATTGA GGGGTTGTAA   
  
  
+ TAGTGTATAT ATATAGCCTT GTGTTAGCGG GGCTTGGGCA TTAGACTTTT ACTCTTGTAA ACCCTAATGT   
  
  
+ GTTATTATAA ATATAGATCT AGCCACCCAT GTTGAGGTAG GCACTCTAAT TCTGACACAG GTAGGATAAG   
  
  
+ TTTTCATAGT TTATGTTGTG AATCTTGTCT TACTTTGTGG TTCCTATTGA CAAAAGTTTG AATCCGAGCA   
  
  
+ TGAGAGTTCA TCAATCTCTT TTTCTTTCCT ATTTTATTTC TTGTGGACTT AATTGGATTT TCTGGTAGAG   
  
  
+ GAAAGTTCCT TGCTGGAATT TGGTCGAAAG TGATATACAT TTTGGGATTT AATTAGCCAT AAATTTGGTT   
  
  
+ TTACACCTTG TTAGTTTGGA GTTCTGGTGT GTTATTGTAT GTGTTCTGGT GGGTTTTTCT TTTAGCTAAA   
  
  
+ TGTTGGAGCA TTTTAAGGGA TCTAGATATC TGAAGAAGTG CTTGGGAAAT GAAGCATATT TCCCTTATAG   
  
  
+ TCTGACATAA CTTTAATGAA TTGATCATGA ACAAAGGGCA ATTAGTTCAG AAAGGTTCAA CTCCTCTAGG   
  
  
+ TGTTGTGCTA AAGAGATGTG AGTTAGTAAC AATTGCTTAC TAACTATATA GGTTGTTTTA TTACTTGTAG   
  
  
+ AGGTTTTTTT TTTGGGATAT CTTTTTGGGG TTTATAAATG AGAAGGTTGA TTTGTTGTCT ACTCTTCTTT   
  
  
+ CGATTCAATA GTAATTCTCT CAGAGAAGGC TAAAATGACA TGGCATTCCA TCGCCTCTCA TGAGGCTTTT   
  
  
+ TGCATGTGCT TATGTATCTT TCATCTCTTA CTTTGGTGGG GTGGCATTGG GGAGGAAAAG GGGGTTTCGA   
  
  
+ TTCGATAGAT CGTCACTTTC GGAAATGACG TCTCTGACCA ATGGTAAGGG TAAGGTTAAT ACATGTGGAT   
  
  
+ ATTTCCAGAG TTAGTTGTGA TTGTTATTGT TGTTGTTGTA ACTTTATGTC ACGGGGAAAA CATAATAATT   
  
  
+ ATCTGTTTAT CTGTTTGTTT GTTATTTTCA AGGGGAAGGG GAGTGGGCGG ATGCTTAGTA TGTAATCATT   
  
  
+ TTATATAGGG AAAATTCATA TAGCATGAAA GTAAAACTTT CATTGCCAGC ATTCTAAGCT AGGGGTAGGG   
  
  
+ TTGTCTACAC CTTGATCTCC CTAGAAGCAT GGTGATCATT CCTCAGATTG TGTTATAGAA TAAAGAATGT   
  
  
+ GGTTCTGCTG TAATGAGACT TATGTATTAT CAAATGATGA ATCTGTTTAG AACTTCCTGT TTATTTTTTC   
  
  
+ TGCAAAATTT TAAGAGACTA GCATCTATCG GGCTCGACTC TAGAGGTTCT CTTGTGTTAC CCTTCTCAAC   
  
  
+ TCAGTTTGAT TGTGATACAG TTACTATATT GAGTGATAGT CGAGAGCATC ACAACCCGCT AAGGAGTCTA   
  
  
+ TCAACAAGAA GCCCTTCTTG TAATTCTCCC CTTGAAACTA GCAGTTATCA TCACTTGTCA TCGAATAGCC   
  
  
+ CTTTTTGTAA TTCTACCCTT GAAACTAGCA GTTATTGTCA TCAGTTCAAT GTGAGTCCCG TTAGATATTC   
  
  
+ CCATCAAGGC ATAGATTATG GAGTGAGCAT GAAGAACGCT TTGCACGAGC TAGAGACTGC TCTAATTGGT   
  
  
+ GTAGATGGCG AGAAAGCATC TGTTGCTAAT CAACCTATGG GGGGAATTCA TTAGTCCGGG ATCCCAAGTT   
  
  
+ AGAGATCGAA GTCATTGAGT GAAGATCCAC AGGGTTCACA TCCTCCTCAG CTTGATTCAT CATCTTTTTC   
  
  
+ AAGGGTGAAA AGATTCGGAG ATGAAAGCCA GAGAGAGAGA AATGCCACAA GGCAATGGAA GAACCAGCGG   
  
  
+ AACTACTAAG TTTCTCACCA GGTGATTTAA AGCAGTTGCT AATTGAATGT GCGAGGGCTT TATCAGATAA   
  
  
+ CCGAATAGAT GACTTTGAGA GTTTGGCTAA ACGGCCAAGG AAAGAGGTCT CCATCTCAGG TGAGCCTGTC   
  
  
+ CAACGTCTCG GTACTTGTAT GATCGAAGGG CTTGTGGCAA GGAAACAGTC TTCGGGGACT AACATCTATC   
  
  
+ GGGCTCTTAA GTACTGTAAA GAGCCTCTTG GAAAATACTT GCTCTCCTAC GGCACTCAGT GGGTTACTCT   
  
  
+ ATCGCAAGCC ATAGCAGCAA GGCTTGGTGG GCCTCCTAAA GTGCGACTTA CAGGCATTGA TGATCCTGTT   
  
  
+ TCTAAGTATA CTTGTGATGC TAGCTTGGAG GCTATTGGGA AACGATTAGC GTCTCTATTT GAAAAGTTTA   
  
  
+ AAATACTCGT CGAGTTCAAT GCATTGCCCG TTTATGGACC TGATGTCAGG TGGGAAATGC TGGATGTGAG   
  
  
+ GCCCAGGGAG GCTTTGGCCG TTAATTGTCC ATTACAGCTC CATCACACTC CTGACGAGAG TGTCGATGTG   
  
  
+ AGCAACCCTA GGGATAGGCT TCTCAGAATG GTGAAATTGC TCGGTCCTAA GGTATGCACT TTGGTTGAGC   
  
  
+ AAGAATCAAA CACCAACACT ACACGTTTCT TGACCCGGTT CATAGAGACC CTTGACTACT ACTCGATCAG   
  
  
+ CCATGTTTG  

- +Up\_Stream \_Len000TCAACA TCGTTCGTGT AATTTCAACT AATAGTAAAA CAGGCACTTT GCCACTCGTT   
  
  
- TGCAGTAGAG GTCCAGTTTA AGTAAGCATG AGTTAGTGTT CAACGACTGT AAAAATGGTT TCCGATTAGC   
  
  
- AGCGTTTTCA AAGAAATCAA ATTCCGGTTA GAGAGGCAAC AAAGTGGGGT ATGTCTAACT CCCCAACATT   
  
  
- ATCACATATA TATATCGGAA CACAATCGCC CCGAACCCGT AATCTGAAAA TGAGAACATT TGGGATTACA   
  
  
- CAATAATATT TATATCTAGA TCGGTGGGTA CAACTCCATC CGTGAGATTA AGACTGTGTC CATCCTATTC   
  
  
- AAAAGTATCA AATACAACAC TTAGAACAGA ATGAAACACC AAGGATAACT GTTTTCAAAC TTAGGCTCGT   
  
  
- ACTCTCAAGT AGTTAGAGAA AAAGAAAGGA TAAAATAAAG AACACCTGAA TTAACCTAAA AGACCATCTC   
  
  
- CTTTCAAGGA ACGACCTTAA ACCAGCTTTC ACTATATGTA AAACCCTAAA TTAATCGGTA TTTAAACCAA   
  
  
- AATGTGGAAC AATCAAACCT CAAGACCACA CAATAACATA CACAAGACCA CCCAAAAAGA AAATCGATTT   
  
  
- ACAACCTCGT AAAATTCCCT AGATCTATAG ACTTCTTCAC GAACCCTTTA CTTCGTATAA AGGGAATATC   
  
  
- AGACTGTATT GAAATTACTT AACTAGTACT TGTTTCCCGT TAATCAAGTC TTTCCAAGTT GAGGAGATCC   
  
  
- ACAACACGAT TTCTCTACAC TCAATCATTG TTAACGAATG ATTGATATAT CCAACAAAAT AATGAACATC   
  
  
- TCCAAAAAAA AAACCCTATA GAAAAACCCC AAATATTTAC TCTTCCAACT AAACAACAGA TGAGAAGAAA   
  
  
- GCTAAGTTAT CATTAAGAGA GTCTCTTCCG ATTTTACTGT ACCGTAAGGT AGCGGAGAGT ACTCCGAAAA   
  
  
- ACGTACACGA ATACATAGAA AGTAGAGAAT GAAACCACCC CACCGTAACC CCTCCTTTTC CCCCAAAGCT   
  
  
- AAGCTATCTA GCAGTGAAAG CCTTTACTGC AGAGACTGGT TACCATTCCC ATTCCAATTA TGTACACCTA   
  
  
- TAAAGGTCTC AATCAACACT AACAATAACA ACAACAACAT TGAAATACAG TGCCCCTTTT GTATTATTAA   
  
  
- TAGACAAATA GACAAACAAA CAATAAAAGT TCCCCTTCCC CTCACCCGCC TACGAATCAT ACATTAGTAA   
  
  
- AATATATCCC TTTTAAGTAT ATCGTACTTT CATTTTGAAA GTAACGGTCG TAAGATTCGA TCCCCATCCC   
  
  
- AACAGATGTG GAACTAGAGG GATCTTCGTA CCACTAGTAA GGAGTCTAAC ACAATATCTT ATTTCTTACA   
  
  
- CCAAGACGAC ATTACTCTGA ATACATAATA GTTTACTACT TAGACAAATC TTGAAGGACA AATAAAAAAG   
  
  
- ACGTTTTAAA ATTCTCTGAT CGTAGATAGC CCGAGCTGAG ATCTCCAAGA GAACACAATG GGAAGAGTTG   
  
  
- AGTCAAACTA ACACTATGTC AATGATATAA CTCACTATCA GCTCTCGTAG TGTTGGGCGA TTCCTCAGAT   
  
  
- AGTTGTTCTT CGGGAAGAAC ATTAAGAGGG GAACTTTGAT CGTCAATAGT AGTGAACAGT AGCTTATCGG   
  
  
- GAAAAACATT AAGATGGGAA CTTTGATCGT CAATAACAGT AGTCAAGTTA CACTCAGGGC AATCTATAAG   
  
  
- GGTAGTTCCG TATCTAATAC CTCACTCGTA CTTCTTGCGA AACGTGCTCG ATCTCTGACG AGATTAACCA   
  
  
- CATCTACCGC TCTTTCGTAG ACAACGATTA GTTGGATACC CCCCTTAAGT AATCAGGCCC TAGGGTTCAA   
  
  
- TCTCTAGCTT CAGTAACTCA CTTCTAGGTG TCCCAAGTGT AGGAGGAGTC GAACTAAGTA GTAGAAAAAG   
  
  
- TTCCCACTTT TCTAAGCCTC TACTTTCGGT CTCTCTCTCT TTACGGTGTT CCGTTACCTT CTTGGTCGCC   
  
  
- TTGATGATTC AAAGAGTGGT CCACTAAATT TCGTCAACGA TTAACTTACA CGCTCCCGAA ATAGTCTATT   
  
  
- GGCTTATCTA CTGAAACTCT CAAACCGATT TGCCGGTTCC TTTCTCCAGA GGTAGAGTCC ACTCGGACAG   
  
  
- GTTGCAGAGC CATGAACATA CTAGCTTCCC GAACACCGTT CCTTTGTCAG AAGCCCCTGA TTGTAGATAG   
  
  
- CCCGAGAATT CATGACATTT CTCGGAGAAC CTTTTATGAA CGAGAGGATG CCGTGAGTCA CCCAATGAGA   
  
  
- TAGCGTTCGG TATCGTCGTT CCGAACCACC CGGAGGATTT CACGCTGAAT GTCCGTAACT ACTAGGACAA   
  
  
- AGATTCATAT GAACACTACG ATCGAACCTC CGATAACCCT TTGCTAATCG CAGAGATAAA CTTTTCAAAT   
  
  
- TTTATGAGCA GCTCAAGTTA CGTAACGGGC AAATACCTGG ACTACAGTCC ACCCTTTACG ACCTACACTC   
  
  
- CGGGTCCCTC CGAAACCGGC AATTAACAGG TAATGTCGAG GTAGTGTGAG GACTGCTCTC ACAGCTACAC   
  
  
- TCGTTGGGAT CCCTATCCGA AGAGTCTTAC CACTTTAACG AGCCAGGATT CCATACGTGA AACCAACTCG   
  
  
- TTCTTAGTTT GTGGTTGTGA TGTGCAAAGA ACTGGGCCAA GTATCTCTGG GAACTGATGA TGAGCTAGTC   
  
  
- GGTACAAAC

+     W box

| Site Name | Organism | Position | Strand | Matrix score. | sequence | function |
| --- | --- | --- | --- | --- | --- | --- |
| W box | Arabidopsis thaliana | 2694 | + | 6 | TTGACC |  |
| W box | Arabidopsis thaliana | 87 | - | 6 | TTGACC |  |

>HU08G00019.1   
+ +Up\_Stream \_Len000AGTTGT AGCAAGCACA TTAAAGTTGA TTATCATTTT GTCCGTGAAA CGGTGAGCAA   
  
  
+ ACGTCATCTC CAGGTCAAAT TCATTCGTAC TCAATCACAA GTTGCTGACA TTTTTACCAA AGGCTAATCG   
  
  
+ TCGCAAAAGT TTCTTTAGTT TAAGGCCAAT CTCTCCGTTG TTTCACCCCA TACAGATTGA GGGGTTGTAA   
  
  
+ TAGTGTATAT ATATAGCCTT GTGTTAGCGG GGCTTGGGCA TTAGACTTTT ACTCTTGTAA ACCCTAATGT   
  
  
+ GTTATTATAA ATATAGATCT AGCCACCCAT GTTGAGGTAG GCACTCTAAT TCTGACACAG GTAGGATAAG   
  
  
+ TTTTCATAGT TTATGTTGTG AATCTTGTCT TACTTTGTGG TTCCTATTGA CAAAAGTTTG AATCCGAGCA   
  
  
+ TGAGAGTTCA TCAATCTCTT TTTCTTTCCT ATTTTATTTC TTGTGGACTT AATTGGATTT TCTGGTAGAG   
  
  
+ GAAAGTTCCT TGCTGGAATT TGGTCGAAAG TGATATACAT TTTGGGATTT AATTAGCCAT AAATTTGGTT   
  
  
+ TTACACCTTG TTAGTTTGGA GTTCTGGTGT GTTATTGTAT GTGTTCTGGT GGGTTTTTCT TTTAGCTAAA   
  
  
+ TGTTGGAGCA TTTTAAGGGA TCTAGATATC TGAAGAAGTG CTTGGGAAAT GAAGCATATT TCCCTTATAG   
  
  
+ TCTGACATAA CTTTAATGAA TTGATCATGA ACAAAGGGCA ATTAGTTCAG AAAGGTTCAA CTCCTCTAGG   
  
  
+ TGTTGTGCTA AAGAGATGTG AGTTAGTAAC AATTGCTTAC TAACTATATA GGTTGTTTTA TTACTTGTAG   
  
  
+ AGGTTTTTTT TTTGGGATAT CTTTTTGGGG TTTATAAATG AGAAGGTTGA TTTGTTGTCT ACTCTTCTTT   
  
  
+ CGATTCAATA GTAATTCTCT CAGAGAAGGC TAAAATGACA TGGCATTCCA TCGCCTCTCA TGAGGCTTTT   
  
  
+ TGCATGTGCT TATGTATCTT TCATCTCTTA CTTTGGTGGG GTGGCATTGG GGAGGAAAAG GGGGTTTCGA   
  
  
+ TTCGATAGAT CGTCACTTTC GGAAATGACG TCTCTGACCA ATGGTAAGGG TAAGGTTAAT ACATGTGGAT   
  
  
+ ATTTCCAGAG TTAGTTGTGA TTGTTATTGT TGTTGTTGTA ACTTTATGTC ACGGGGAAAA CATAATAATT   
  
  
+ ATCTGTTTAT CTGTTTGTTT GTTATTTTCA AGGGGAAGGG GAGTGGGCGG ATGCTTAGTA TGTAATCATT   
  
  
+ TTATATAGGG AAAATTCATA TAGCATGAAA GTAAAACTTT CATTGCCAGC ATTCTAAGCT AGGGGTAGGG   
  
  
+ TTGTCTACAC CTTGATCTCC CTAGAAGCAT GGTGATCATT CCTCAGATTG TGTTATAGAA TAAAGAATGT   
  
  
+ GGTTCTGCTG TAATGAGACT TATGTATTAT CAAATGATGA ATCTGTTTAG AACTTCCTGT TTATTTTTTC   
  
  
+ TGCAAAATTT TAAGAGACTA GCATCTATCG GGCTCGACTC TAGAGGTTCT CTTGTGTTAC CCTTCTCAAC   
  
  
+ TCAGTTTGAT TGTGATACAG TTACTATATT GAGTGATAGT CGAGAGCATC ACAACCCGCT AAGGAGTCTA   
  
  
+ TCAACAAGAA GCCCTTCTTG TAATTCTCCC CTTGAAACTA GCAGTTATCA TCACTTGTCA TCGAATAGCC   
  
  
+ CTTTTTGTAA TTCTACCCTT GAAACTAGCA GTTATTGTCA TCAGTTCAAT GTGAGTCCCG TTAGATATTC   
  
  
+ CCATCAAGGC ATAGATTATG GAGTGAGCAT GAAGAACGCT TTGCACGAGC TAGAGACTGC TCTAATTGGT   
  
  
+ GTAGATGGCG AGAAAGCATC TGTTGCTAAT CAACCTATGG GGGGAATTCA TTAGTCCGGG ATCCCAAGTT   
  
  
+ AGAGATCGAA GTCATTGAGT GAAGATCCAC AGGGTTCACA TCCTCCTCAG CTTGATTCAT CATCTTTTTC   
  
  
+ AAGGGTGAAA AGATTCGGAG ATGAAAGCCA GAGAGAGAGA AATGCCACAA GGCAATGGAA GAACCAGCGG   
  
  
+ AACTACTAAG TTTCTCACCA GGTGATTTAA AGCAGTTGCT AATTGAATGT GCGAGGGCTT TATCAGATAA   
  
  
+ CCGAATAGAT GACTTTGAGA GTTTGGCTAA ACGGCCAAGG AAAGAGGTCT CCATCTCAGG TGAGCCTGTC   
  
  
+ CAACGTCTCG GTACTTGTAT GATCGAAGGG CTTGTGGCAA GGAAACAGTC TTCGGGGACT AACATCTATC   
  
  
+ GGGCTCTTAA GTACTGTAAA GAGCCTCTTG GAAAATACTT GCTCTCCTAC GGCACTCAGT GGGTTACTCT   
  
  
+ ATCGCAAGCC ATAGCAGCAA GGCTTGGTGG GCCTCCTAAA GTGCGACTTA CAGGCATTGA TGATCCTGTT   
  
  
+ TCTAAGTATA CTTGTGATGC TAGCTTGGAG GCTATTGGGA AACGATTAGC GTCTCTATTT GAAAAGTTTA   
  
  
+ AAATACTCGT CGAGTTCAAT GCATTGCCCG TTTATGGACC TGATGTCAGG TGGGAAATGC TGGATGTGAG   
  
  
+ GCCCAGGGAG GCTTTGGCCG TTAATTGTCC ATTACAGCTC CATCACACTC CTGACGAGAG TGTCGATGTG   
  
  
+ AGCAACCCTA GGGATAGGCT TCTCAGAATG GTGAAATTGC TCGGTCCTAA GGTATGCACT TTGGTTGAGC   
  
  
+ AAGAATCAAA CACCAACACT ACACGTTTCT TGACCCGGTT CATAGAGACC CTTGACTACT ACTCGATCAG   
  
  
+ CCATGTTTG  

- +Up\_Stream \_Len000TCAACA TCGTTCGTGT AATTTCAACT AATAGTAAAA CAGGCACTTT GCCACTCGTT   
  
  
- TGCAGTAGAG GTCCAGTTTA AGTAAGCATG AGTTAGTGTT CAACGACTGT AAAAATGGTT TCCGATTAGC   
  
  
- AGCGTTTTCA AAGAAATCAA ATTCCGGTTA GAGAGGCAAC AAAGTGGGGT ATGTCTAACT CCCCAACATT   
  
  
- ATCACATATA TATATCGGAA CACAATCGCC CCGAACCCGT AATCTGAAAA TGAGAACATT TGGGATTACA   
  
  
- CAATAATATT TATATCTAGA TCGGTGGGTA CAACTCCATC CGTGAGATTA AGACTGTGTC CATCCTATTC   
  
  
- AAAAGTATCA AATACAACAC TTAGAACAGA ATGAAACACC AAGGATAACT GTTTTCAAAC TTAGGCTCGT   
  
  
- ACTCTCAAGT AGTTAGAGAA AAAGAAAGGA TAAAATAAAG AACACCTGAA TTAACCTAAA AGACCATCTC   
  
  
- CTTTCAAGGA ACGACCTTAA ACCAGCTTTC ACTATATGTA AAACCCTAAA TTAATCGGTA TTTAAACCAA   
  
  
- AATGTGGAAC AATCAAACCT CAAGACCACA CAATAACATA CACAAGACCA CCCAAAAAGA AAATCGATTT   
  
  
- ACAACCTCGT AAAATTCCCT AGATCTATAG ACTTCTTCAC GAACCCTTTA CTTCGTATAA AGGGAATATC   
  
  
- AGACTGTATT GAAATTACTT AACTAGTACT TGTTTCCCGT TAATCAAGTC TTTCCAAGTT GAGGAGATCC   
  
  
- ACAACACGAT TTCTCTACAC TCAATCATTG TTAACGAATG ATTGATATAT CCAACAAAAT AATGAACATC   
  
  
- TCCAAAAAAA AAACCCTATA GAAAAACCCC AAATATTTAC TCTTCCAACT AAACAACAGA TGAGAAGAAA   
  
  
- GCTAAGTTAT CATTAAGAGA GTCTCTTCCG ATTTTACTGT ACCGTAAGGT AGCGGAGAGT ACTCCGAAAA   
  
  
- ACGTACACGA ATACATAGAA AGTAGAGAAT GAAACCACCC CACCGTAACC CCTCCTTTTC CCCCAAAGCT   
  
  
- AAGCTATCTA GCAGTGAAAG CCTTTACTGC AGAGACTGGT TACCATTCCC ATTCCAATTA TGTACACCTA   
  
  
- TAAAGGTCTC AATCAACACT AACAATAACA ACAACAACAT TGAAATACAG TGCCCCTTTT GTATTATTAA   
  
  
- TAGACAAATA GACAAACAAA CAATAAAAGT TCCCCTTCCC CTCACCCGCC TACGAATCAT ACATTAGTAA   
  
  
- AATATATCCC TTTTAAGTAT ATCGTACTTT CATTTTGAAA GTAACGGTCG TAAGATTCGA TCCCCATCCC   
  
  
- AACAGATGTG GAACTAGAGG GATCTTCGTA CCACTAGTAA GGAGTCTAAC ACAATATCTT ATTTCTTACA   
  
  
- CCAAGACGAC ATTACTCTGA ATACATAATA GTTTACTACT TAGACAAATC TTGAAGGACA AATAAAAAAG   
  
  
- ACGTTTTAAA ATTCTCTGAT CGTAGATAGC CCGAGCTGAG ATCTCCAAGA GAACACAATG GGAAGAGTTG   
  
  
- AGTCAAACTA ACACTATGTC AATGATATAA CTCACTATCA GCTCTCGTAG TGTTGGGCGA TTCCTCAGAT   
  
  
- AGTTGTTCTT CGGGAAGAAC ATTAAGAGGG GAACTTTGAT CGTCAATAGT AGTGAACAGT AGCTTATCGG   
  
  
- GAAAAACATT AAGATGGGAA CTTTGATCGT CAATAACAGT AGTCAAGTTA CACTCAGGGC AATCTATAAG   
  
  
- GGTAGTTCCG TATCTAATAC CTCACTCGTA CTTCTTGCGA AACGTGCTCG ATCTCTGACG AGATTAACCA   
  
  
- CATCTACCGC TCTTTCGTAG ACAACGATTA GTTGGATACC CCCCTTAAGT AATCAGGCCC TAGGGTTCAA   
  
  
- TCTCTAGCTT CAGTAACTCA CTTCTAGGTG TCCCAAGTGT AGGAGGAGTC GAACTAAGTA GTAGAAAAAG   
  
  
- TTCCCACTTT TCTAAGCCTC TACTTTCGGT CTCTCTCTCT TTACGGTGTT CCGTTACCTT CTTGGTCGCC   
  
  
- TTGATGATTC AAAGAGTGGT CCACTAAATT TCGTCAACGA TTAACTTACA CGCTCCCGAA ATAGTCTATT   
  
  
- GGCTTATCTA CTGAAACTCT CAAACCGATT TGCCGGTTCC TTTCTCCAGA GGTAGAGTCC ACTCGGACAG   
  
  
- GTTGCAGAGC CATGAACATA CTAGCTTCCC GAACACCGTT CCTTTGTCAG AAGCCCCTGA TTGTAGATAG   
  
  
- CCCGAGAATT CATGACATTT CTCGGAGAAC CTTTTATGAA CGAGAGGATG CCGTGAGTCA CCCAATGAGA   
  
  
- TAGCGTTCGG TATCGTCGTT CCGAACCACC CGGAGGATTT CACGCTGAAT GTCCGTAACT ACTAGGACAA   
  
  
- AGATTCATAT GAACACTACG ATCGAACCTC CGATAACCCT TTGCTAATCG CAGAGATAAA CTTTTCAAAT   
  
  
- TTTATGAGCA GCTCAAGTTA CGTAACGGGC AAATACCTGG ACTACAGTCC ACCCTTTACG ACCTACACTC   
  
  
- CGGGTCCCTC CGAAACCGGC AATTAACAGG TAATGTCGAG GTAGTGTGAG GACTGCTCTC ACAGCTACAC   
  
  
- TCGTTGGGAT CCCTATCCGA AGAGTCTTAC CACTTTAACG AGCCAGGATT CCATACGTGA AACCAACTCG   
  
  
- TTCTTAGTTT GTGGTTGTGA TGTGCAAAGA ACTGGGCCAA GTATCTCTGG GAACTGATGA TGAGCTAGTC   
  
  
- GGTACAAAC

+     WRE3

| Site Name | Organism | Position | Strand | Matrix score. | sequence | function |
| --- | --- | --- | --- | --- | --- | --- |
| WRE3 | Pisum sativum | 2502 | - | 6 | CCACCT |  |

>HU08G00019.1   
+ +Up\_Stream \_Len000AGTTGT AGCAAGCACA TTAAAGTTGA TTATCATTTT GTCCGTGAAA CGGTGAGCAA   
  
  
+ ACGTCATCTC CAGGTCAAAT TCATTCGTAC TCAATCACAA GTTGCTGACA TTTTTACCAA AGGCTAATCG   
  
  
+ TCGCAAAAGT TTCTTTAGTT TAAGGCCAAT CTCTCCGTTG TTTCACCCCA TACAGATTGA GGGGTTGTAA   
  
  
+ TAGTGTATAT ATATAGCCTT GTGTTAGCGG GGCTTGGGCA TTAGACTTTT ACTCTTGTAA ACCCTAATGT   
  
  
+ GTTATTATAA ATATAGATCT AGCCACCCAT GTTGAGGTAG GCACTCTAAT TCTGACACAG GTAGGATAAG   
  
  
+ TTTTCATAGT TTATGTTGTG AATCTTGTCT TACTTTGTGG TTCCTATTGA CAAAAGTTTG AATCCGAGCA   
  
  
+ TGAGAGTTCA TCAATCTCTT TTTCTTTCCT ATTTTATTTC TTGTGGACTT AATTGGATTT TCTGGTAGAG   
  
  
+ GAAAGTTCCT TGCTGGAATT TGGTCGAAAG TGATATACAT TTTGGGATTT AATTAGCCAT AAATTTGGTT   
  
  
+ TTACACCTTG TTAGTTTGGA GTTCTGGTGT GTTATTGTAT GTGTTCTGGT GGGTTTTTCT TTTAGCTAAA   
  
  
+ TGTTGGAGCA TTTTAAGGGA TCTAGATATC TGAAGAAGTG CTTGGGAAAT GAAGCATATT TCCCTTATAG   
  
  
+ TCTGACATAA CTTTAATGAA TTGATCATGA ACAAAGGGCA ATTAGTTCAG AAAGGTTCAA CTCCTCTAGG   
  
  
+ TGTTGTGCTA AAGAGATGTG AGTTAGTAAC AATTGCTTAC TAACTATATA GGTTGTTTTA TTACTTGTAG   
  
  
+ AGGTTTTTTT TTTGGGATAT CTTTTTGGGG TTTATAAATG AGAAGGTTGA TTTGTTGTCT ACTCTTCTTT   
  
  
+ CGATTCAATA GTAATTCTCT CAGAGAAGGC TAAAATGACA TGGCATTCCA TCGCCTCTCA TGAGGCTTTT   
  
  
+ TGCATGTGCT TATGTATCTT TCATCTCTTA CTTTGGTGGG GTGGCATTGG GGAGGAAAAG GGGGTTTCGA   
  
  
+ TTCGATAGAT CGTCACTTTC GGAAATGACG TCTCTGACCA ATGGTAAGGG TAAGGTTAAT ACATGTGGAT   
  
  
+ ATTTCCAGAG TTAGTTGTGA TTGTTATTGT TGTTGTTGTA ACTTTATGTC ACGGGGAAAA CATAATAATT   
  
  
+ ATCTGTTTAT CTGTTTGTTT GTTATTTTCA AGGGGAAGGG GAGTGGGCGG ATGCTTAGTA TGTAATCATT   
  
  
+ TTATATAGGG AAAATTCATA TAGCATGAAA GTAAAACTTT CATTGCCAGC ATTCTAAGCT AGGGGTAGGG   
  
  
+ TTGTCTACAC CTTGATCTCC CTAGAAGCAT GGTGATCATT CCTCAGATTG TGTTATAGAA TAAAGAATGT   
  
  
+ GGTTCTGCTG TAATGAGACT TATGTATTAT CAAATGATGA ATCTGTTTAG AACTTCCTGT TTATTTTTTC   
  
  
+ TGCAAAATTT TAAGAGACTA GCATCTATCG GGCTCGACTC TAGAGGTTCT CTTGTGTTAC CCTTCTCAAC   
  
  
+ TCAGTTTGAT TGTGATACAG TTACTATATT GAGTGATAGT CGAGAGCATC ACAACCCGCT AAGGAGTCTA   
  
  
+ TCAACAAGAA GCCCTTCTTG TAATTCTCCC CTTGAAACTA GCAGTTATCA TCACTTGTCA TCGAATAGCC   
  
  
+ CTTTTTGTAA TTCTACCCTT GAAACTAGCA GTTATTGTCA TCAGTTCAAT GTGAGTCCCG TTAGATATTC   
  
  
+ CCATCAAGGC ATAGATTATG GAGTGAGCAT GAAGAACGCT TTGCACGAGC TAGAGACTGC TCTAATTGGT   
  
  
+ GTAGATGGCG AGAAAGCATC TGTTGCTAAT CAACCTATGG GGGGAATTCA TTAGTCCGGG ATCCCAAGTT   
  
  
+ AGAGATCGAA GTCATTGAGT GAAGATCCAC AGGGTTCACA TCCTCCTCAG CTTGATTCAT CATCTTTTTC   
  
  
+ AAGGGTGAAA AGATTCGGAG ATGAAAGCCA GAGAGAGAGA AATGCCACAA GGCAATGGAA GAACCAGCGG   
  
  
+ AACTACTAAG TTTCTCACCA GGTGATTTAA AGCAGTTGCT AATTGAATGT GCGAGGGCTT TATCAGATAA   
  
  
+ CCGAATAGAT GACTTTGAGA GTTTGGCTAA ACGGCCAAGG AAAGAGGTCT CCATCTCAGG TGAGCCTGTC   
  
  
+ CAACGTCTCG GTACTTGTAT GATCGAAGGG CTTGTGGCAA GGAAACAGTC TTCGGGGACT AACATCTATC   
  
  
+ GGGCTCTTAA GTACTGTAAA GAGCCTCTTG GAAAATACTT GCTCTCCTAC GGCACTCAGT GGGTTACTCT   
  
  
+ ATCGCAAGCC ATAGCAGCAA GGCTTGGTGG GCCTCCTAAA GTGCGACTTA CAGGCATTGA TGATCCTGTT   
  
  
+ TCTAAGTATA CTTGTGATGC TAGCTTGGAG GCTATTGGGA AACGATTAGC GTCTCTATTT GAAAAGTTTA   
  
  
+ AAATACTCGT CGAGTTCAAT GCATTGCCCG TTTATGGACC TGATGTCAGG TGGGAAATGC TGGATGTGAG   
  
  
+ GCCCAGGGAG GCTTTGGCCG TTAATTGTCC ATTACAGCTC CATCACACTC CTGACGAGAG TGTCGATGTG   
  
  
+ AGCAACCCTA GGGATAGGCT TCTCAGAATG GTGAAATTGC TCGGTCCTAA GGTATGCACT TTGGTTGAGC   
  
  
+ AAGAATCAAA CACCAACACT ACACGTTTCT TGACCCGGTT CATAGAGACC CTTGACTACT ACTCGATCAG   
  
  
+ CCATGTTTG  

- +Up\_Stream \_Len000TCAACA TCGTTCGTGT AATTTCAACT AATAGTAAAA CAGGCACTTT GCCACTCGTT   
  
  
- TGCAGTAGAG GTCCAGTTTA AGTAAGCATG AGTTAGTGTT CAACGACTGT AAAAATGGTT TCCGATTAGC   
  
  
- AGCGTTTTCA AAGAAATCAA ATTCCGGTTA GAGAGGCAAC AAAGTGGGGT ATGTCTAACT CCCCAACATT   
  
  
- ATCACATATA TATATCGGAA CACAATCGCC CCGAACCCGT AATCTGAAAA TGAGAACATT TGGGATTACA   
  
  
- CAATAATATT TATATCTAGA TCGGTGGGTA CAACTCCATC CGTGAGATTA AGACTGTGTC CATCCTATTC   
  
  
- AAAAGTATCA AATACAACAC TTAGAACAGA ATGAAACACC AAGGATAACT GTTTTCAAAC TTAGGCTCGT   
  
  
- ACTCTCAAGT AGTTAGAGAA AAAGAAAGGA TAAAATAAAG AACACCTGAA TTAACCTAAA AGACCATCTC   
  
  
- CTTTCAAGGA ACGACCTTAA ACCAGCTTTC ACTATATGTA AAACCCTAAA TTAATCGGTA TTTAAACCAA   
  
  
- AATGTGGAAC AATCAAACCT CAAGACCACA CAATAACATA CACAAGACCA CCCAAAAAGA AAATCGATTT   
  
  
- ACAACCTCGT AAAATTCCCT AGATCTATAG ACTTCTTCAC GAACCCTTTA CTTCGTATAA AGGGAATATC   
  
  
- AGACTGTATT GAAATTACTT AACTAGTACT TGTTTCCCGT TAATCAAGTC TTTCCAAGTT GAGGAGATCC   
  
  
- ACAACACGAT TTCTCTACAC TCAATCATTG TTAACGAATG ATTGATATAT CCAACAAAAT AATGAACATC   
  
  
- TCCAAAAAAA AAACCCTATA GAAAAACCCC AAATATTTAC TCTTCCAACT AAACAACAGA TGAGAAGAAA   
  
  
- GCTAAGTTAT CATTAAGAGA GTCTCTTCCG ATTTTACTGT ACCGTAAGGT AGCGGAGAGT ACTCCGAAAA   
  
  
- ACGTACACGA ATACATAGAA AGTAGAGAAT GAAACCACCC CACCGTAACC CCTCCTTTTC CCCCAAAGCT   
  
  
- AAGCTATCTA GCAGTGAAAG CCTTTACTGC AGAGACTGGT TACCATTCCC ATTCCAATTA TGTACACCTA   
  
  
- TAAAGGTCTC AATCAACACT AACAATAACA ACAACAACAT TGAAATACAG TGCCCCTTTT GTATTATTAA   
  
  
- TAGACAAATA GACAAACAAA CAATAAAAGT TCCCCTTCCC CTCACCCGCC TACGAATCAT ACATTAGTAA   
  
  
- AATATATCCC TTTTAAGTAT ATCGTACTTT CATTTTGAAA GTAACGGTCG TAAGATTCGA TCCCCATCCC   
  
  
- AACAGATGTG GAACTAGAGG GATCTTCGTA CCACTAGTAA GGAGTCTAAC ACAATATCTT ATTTCTTACA   
  
  
- CCAAGACGAC ATTACTCTGA ATACATAATA GTTTACTACT TAGACAAATC TTGAAGGACA AATAAAAAAG   
  
  
- ACGTTTTAAA ATTCTCTGAT CGTAGATAGC CCGAGCTGAG ATCTCCAAGA GAACACAATG GGAAGAGTTG   
  
  
- AGTCAAACTA ACACTATGTC AATGATATAA CTCACTATCA GCTCTCGTAG TGTTGGGCGA TTCCTCAGAT   
  
  
- AGTTGTTCTT CGGGAAGAAC ATTAAGAGGG GAACTTTGAT CGTCAATAGT AGTGAACAGT AGCTTATCGG   
  
  
- GAAAAACATT AAGATGGGAA CTTTGATCGT CAATAACAGT AGTCAAGTTA CACTCAGGGC AATCTATAAG   
  
  
- GGTAGTTCCG TATCTAATAC CTCACTCGTA CTTCTTGCGA AACGTGCTCG ATCTCTGACG AGATTAACCA   
  
  
- CATCTACCGC TCTTTCGTAG ACAACGATTA GTTGGATACC CCCCTTAAGT AATCAGGCCC TAGGGTTCAA   
  
  
- TCTCTAGCTT CAGTAACTCA CTTCTAGGTG TCCCAAGTGT AGGAGGAGTC GAACTAAGTA GTAGAAAAAG   
  
  
- TTCCCACTTT TCTAAGCCTC TACTTTCGGT CTCTCTCTCT TTACGGTGTT CCGTTACCTT CTTGGTCGCC   
  
  
- TTGATGATTC AAAGAGTGGT CCACTAAATT TCGTCAACGA TTAACTTACA CGCTCCCGAA ATAGTCTATT   
  
  
- GGCTTATCTA CTGAAACTCT CAAACCGATT TGCCGGTTCC TTTCTCCAGA GGTAGAGTCC ACTCGGACAG   
  
  
- GTTGCAGAGC CATGAACATA CTAGCTTCCC GAACACCGTT CCTTTGTCAG AAGCCCCTGA TTGTAGATAG   
  
  
- CCCGAGAATT CATGACATTT CTCGGAGAAC CTTTTATGAA CGAGAGGATG CCGTGAGTCA CCCAATGAGA   
  
  
- TAGCGTTCGG TATCGTCGTT CCGAACCACC CGGAGGATTT CACGCTGAAT GTCCGTAACT ACTAGGACAA   
  
  
- AGATTCATAT GAACACTACG ATCGAACCTC CGATAACCCT TTGCTAATCG CAGAGATAAA CTTTTCAAAT   
  
  
- TTTATGAGCA GCTCAAGTTA CGTAACGGGC AAATACCTGG ACTACAGTCC ACCCTTTACG ACCTACACTC   
  
  
- CGGGTCCCTC CGAAACCGGC AATTAACAGG TAATGTCGAG GTAGTGTGAG GACTGCTCTC ACAGCTACAC   
  
  
- TCGTTGGGAT CCCTATCCGA AGAGTCTTAC CACTTTAACG AGCCAGGATT CCATACGTGA AACCAACTCG   
  
  
- TTCTTAGTTT GTGGTTGTGA TGTGCAAAGA ACTGGGCCAA GTATCTCTGG GAACTGATGA TGAGCTAGTC   
  
  
- GGTACAAAC

+     as-1

| Site Name | Organism | Position | Strand | Matrix score. | sequence | function |
| --- | --- | --- | --- | --- | --- | --- |
| as-1 | Arabidopsis thaliana | 1065 | - | 5 | TGACG |  |
| as-1 | Arabidopsis thaliana | 76 | - | 5 | TGACG |  |
| as-1 | Arabidopsis thaliana | 2576 | + | 5 | TGACG |  |
| as-1 | Arabidopsis thaliana | 1080 | + | 5 | TGACG |  |

>HU08G00019.1   
+ +Up\_Stream \_Len000AGTTGT AGCAAGCACA TTAAAGTTGA TTATCATTTT GTCCGTGAAA CGGTGAGCAA   
  
  
+ ACGTCATCTC CAGGTCAAAT TCATTCGTAC TCAATCACAA GTTGCTGACA TTTTTACCAA AGGCTAATCG   
  
  
+ TCGCAAAAGT TTCTTTAGTT TAAGGCCAAT CTCTCCGTTG TTTCACCCCA TACAGATTGA GGGGTTGTAA   
  
  
+ TAGTGTATAT ATATAGCCTT GTGTTAGCGG GGCTTGGGCA TTAGACTTTT ACTCTTGTAA ACCCTAATGT   
  
  
+ GTTATTATAA ATATAGATCT AGCCACCCAT GTTGAGGTAG GCACTCTAAT TCTGACACAG GTAGGATAAG   
  
  
+ TTTTCATAGT TTATGTTGTG AATCTTGTCT TACTTTGTGG TTCCTATTGA CAAAAGTTTG AATCCGAGCA   
  
  
+ TGAGAGTTCA TCAATCTCTT TTTCTTTCCT ATTTTATTTC TTGTGGACTT AATTGGATTT TCTGGTAGAG   
  
  
+ GAAAGTTCCT TGCTGGAATT TGGTCGAAAG TGATATACAT TTTGGGATTT AATTAGCCAT AAATTTGGTT   
  
  
+ TTACACCTTG TTAGTTTGGA GTTCTGGTGT GTTATTGTAT GTGTTCTGGT GGGTTTTTCT TTTAGCTAAA   
  
  
+ TGTTGGAGCA TTTTAAGGGA TCTAGATATC TGAAGAAGTG CTTGGGAAAT GAAGCATATT TCCCTTATAG   
  
  
+ TCTGACATAA CTTTAATGAA TTGATCATGA ACAAAGGGCA ATTAGTTCAG AAAGGTTCAA CTCCTCTAGG   
  
  
+ TGTTGTGCTA AAGAGATGTG AGTTAGTAAC AATTGCTTAC TAACTATATA GGTTGTTTTA TTACTTGTAG   
  
  
+ AGGTTTTTTT TTTGGGATAT CTTTTTGGGG TTTATAAATG AGAAGGTTGA TTTGTTGTCT ACTCTTCTTT   
  
  
+ CGATTCAATA GTAATTCTCT CAGAGAAGGC TAAAATGACA TGGCATTCCA TCGCCTCTCA TGAGGCTTTT   
  
  
+ TGCATGTGCT TATGTATCTT TCATCTCTTA CTTTGGTGGG GTGGCATTGG GGAGGAAAAG GGGGTTTCGA   
  
  
+ TTCGATAGAT CGTCACTTTC GGAAATGACG TCTCTGACCA ATGGTAAGGG TAAGGTTAAT ACATGTGGAT   
  
  
+ ATTTCCAGAG TTAGTTGTGA TTGTTATTGT TGTTGTTGTA ACTTTATGTC ACGGGGAAAA CATAATAATT   
  
  
+ ATCTGTTTAT CTGTTTGTTT GTTATTTTCA AGGGGAAGGG GAGTGGGCGG ATGCTTAGTA TGTAATCATT   
  
  
+ TTATATAGGG AAAATTCATA TAGCATGAAA GTAAAACTTT CATTGCCAGC ATTCTAAGCT AGGGGTAGGG   
  
  
+ TTGTCTACAC CTTGATCTCC CTAGAAGCAT GGTGATCATT CCTCAGATTG TGTTATAGAA TAAAGAATGT   
  
  
+ GGTTCTGCTG TAATGAGACT TATGTATTAT CAAATGATGA ATCTGTTTAG AACTTCCTGT TTATTTTTTC   
  
  
+ TGCAAAATTT TAAGAGACTA GCATCTATCG GGCTCGACTC TAGAGGTTCT CTTGTGTTAC CCTTCTCAAC   
  
  
+ TCAGTTTGAT TGTGATACAG TTACTATATT GAGTGATAGT CGAGAGCATC ACAACCCGCT AAGGAGTCTA   
  
  
+ TCAACAAGAA GCCCTTCTTG TAATTCTCCC CTTGAAACTA GCAGTTATCA TCACTTGTCA TCGAATAGCC   
  
  
+ CTTTTTGTAA TTCTACCCTT GAAACTAGCA GTTATTGTCA TCAGTTCAAT GTGAGTCCCG TTAGATATTC   
  
  
+ CCATCAAGGC ATAGATTATG GAGTGAGCAT GAAGAACGCT TTGCACGAGC TAGAGACTGC TCTAATTGGT   
  
  
+ GTAGATGGCG AGAAAGCATC TGTTGCTAAT CAACCTATGG GGGGAATTCA TTAGTCCGGG ATCCCAAGTT   
  
  
+ AGAGATCGAA GTCATTGAGT GAAGATCCAC AGGGTTCACA TCCTCCTCAG CTTGATTCAT CATCTTTTTC   
  
  
+ AAGGGTGAAA AGATTCGGAG ATGAAAGCCA GAGAGAGAGA AATGCCACAA GGCAATGGAA GAACCAGCGG   
  
  
+ AACTACTAAG TTTCTCACCA GGTGATTTAA AGCAGTTGCT AATTGAATGT GCGAGGGCTT TATCAGATAA   
  
  
+ CCGAATAGAT GACTTTGAGA GTTTGGCTAA ACGGCCAAGG AAAGAGGTCT CCATCTCAGG TGAGCCTGTC   
  
  
+ CAACGTCTCG GTACTTGTAT GATCGAAGGG CTTGTGGCAA GGAAACAGTC TTCGGGGACT AACATCTATC   
  
  
+ GGGCTCTTAA GTACTGTAAA GAGCCTCTTG GAAAATACTT GCTCTCCTAC GGCACTCAGT GGGTTACTCT   
  
  
+ ATCGCAAGCC ATAGCAGCAA GGCTTGGTGG GCCTCCTAAA GTGCGACTTA CAGGCATTGA TGATCCTGTT   
  
  
+ TCTAAGTATA CTTGTGATGC TAGCTTGGAG GCTATTGGGA AACGATTAGC GTCTCTATTT GAAAAGTTTA   
  
  
+ AAATACTCGT CGAGTTCAAT GCATTGCCCG TTTATGGACC TGATGTCAGG TGGGAAATGC TGGATGTGAG   
  
  
+ GCCCAGGGAG GCTTTGGCCG TTAATTGTCC ATTACAGCTC CATCACACTC CTGACGAGAG TGTCGATGTG   
  
  
+ AGCAACCCTA GGGATAGGCT TCTCAGAATG GTGAAATTGC TCGGTCCTAA GGTATGCACT TTGGTTGAGC   
  
  
+ AAGAATCAAA CACCAACACT ACACGTTTCT TGACCCGGTT CATAGAGACC CTTGACTACT ACTCGATCAG   
  
  
+ CCATGTTTG  

- +Up\_Stream \_Len000TCAACA TCGTTCGTGT AATTTCAACT AATAGTAAAA CAGGCACTTT GCCACTCGTT   
  
  
- TGCAGTAGAG GTCCAGTTTA AGTAAGCATG AGTTAGTGTT CAACGACTGT AAAAATGGTT TCCGATTAGC   
  
  
- AGCGTTTTCA AAGAAATCAA ATTCCGGTTA GAGAGGCAAC AAAGTGGGGT ATGTCTAACT CCCCAACATT   
  
  
- ATCACATATA TATATCGGAA CACAATCGCC CCGAACCCGT AATCTGAAAA TGAGAACATT TGGGATTACA   
  
  
- CAATAATATT TATATCTAGA TCGGTGGGTA CAACTCCATC CGTGAGATTA AGACTGTGTC CATCCTATTC   
  
  
- AAAAGTATCA AATACAACAC TTAGAACAGA ATGAAACACC AAGGATAACT GTTTTCAAAC TTAGGCTCGT   
  
  
- ACTCTCAAGT AGTTAGAGAA AAAGAAAGGA TAAAATAAAG AACACCTGAA TTAACCTAAA AGACCATCTC   
  
  
- CTTTCAAGGA ACGACCTTAA ACCAGCTTTC ACTATATGTA AAACCCTAAA TTAATCGGTA TTTAAACCAA   
  
  
- AATGTGGAAC AATCAAACCT CAAGACCACA CAATAACATA CACAAGACCA CCCAAAAAGA AAATCGATTT   
  
  
- ACAACCTCGT AAAATTCCCT AGATCTATAG ACTTCTTCAC GAACCCTTTA CTTCGTATAA AGGGAATATC   
  
  
- AGACTGTATT GAAATTACTT AACTAGTACT TGTTTCCCGT TAATCAAGTC TTTCCAAGTT GAGGAGATCC   
  
  
- ACAACACGAT TTCTCTACAC TCAATCATTG TTAACGAATG ATTGATATAT CCAACAAAAT AATGAACATC   
  
  
- TCCAAAAAAA AAACCCTATA GAAAAACCCC AAATATTTAC TCTTCCAACT AAACAACAGA TGAGAAGAAA   
  
  
- GCTAAGTTAT CATTAAGAGA GTCTCTTCCG ATTTTACTGT ACCGTAAGGT AGCGGAGAGT ACTCCGAAAA   
  
  
- ACGTACACGA ATACATAGAA AGTAGAGAAT GAAACCACCC CACCGTAACC CCTCCTTTTC CCCCAAAGCT   
  
  
- AAGCTATCTA GCAGTGAAAG CCTTTACTGC AGAGACTGGT TACCATTCCC ATTCCAATTA TGTACACCTA   
  
  
- TAAAGGTCTC AATCAACACT AACAATAACA ACAACAACAT TGAAATACAG TGCCCCTTTT GTATTATTAA   
  
  
- TAGACAAATA GACAAACAAA CAATAAAAGT TCCCCTTCCC CTCACCCGCC TACGAATCAT ACATTAGTAA   
  
  
- AATATATCCC TTTTAAGTAT ATCGTACTTT CATTTTGAAA GTAACGGTCG TAAGATTCGA TCCCCATCCC   
  
  
- AACAGATGTG GAACTAGAGG GATCTTCGTA CCACTAGTAA GGAGTCTAAC ACAATATCTT ATTTCTTACA   
  
  
- CCAAGACGAC ATTACTCTGA ATACATAATA GTTTACTACT TAGACAAATC TTGAAGGACA AATAAAAAAG   
  
  
- ACGTTTTAAA ATTCTCTGAT CGTAGATAGC CCGAGCTGAG ATCTCCAAGA GAACACAATG GGAAGAGTTG   
  
  
- AGTCAAACTA ACACTATGTC AATGATATAA CTCACTATCA GCTCTCGTAG TGTTGGGCGA TTCCTCAGAT   
  
  
- AGTTGTTCTT CGGGAAGAAC ATTAAGAGGG GAACTTTGAT CGTCAATAGT AGTGAACAGT AGCTTATCGG   
  
  
- GAAAAACATT AAGATGGGAA CTTTGATCGT CAATAACAGT AGTCAAGTTA CACTCAGGGC AATCTATAAG   
  
  
- GGTAGTTCCG TATCTAATAC CTCACTCGTA CTTCTTGCGA AACGTGCTCG ATCTCTGACG AGATTAACCA   
  
  
- CATCTACCGC TCTTTCGTAG ACAACGATTA GTTGGATACC CCCCTTAAGT AATCAGGCCC TAGGGTTCAA   
  
  
- TCTCTAGCTT CAGTAACTCA CTTCTAGGTG TCCCAAGTGT AGGAGGAGTC GAACTAAGTA GTAGAAAAAG   
  
  
- TTCCCACTTT TCTAAGCCTC TACTTTCGGT CTCTCTCTCT TTACGGTGTT CCGTTACCTT CTTGGTCGCC   
  
  
- TTGATGATTC AAAGAGTGGT CCACTAAATT TCGTCAACGA TTAACTTACA CGCTCCCGAA ATAGTCTATT   
  
  
- GGCTTATCTA CTGAAACTCT CAAACCGATT TGCCGGTTCC TTTCTCCAGA GGTAGAGTCC ACTCGGACAG   
  
  
- GTTGCAGAGC CATGAACATA CTAGCTTCCC GAACACCGTT CCTTTGTCAG AAGCCCCTGA TTGTAGATAG   
  
  
- CCCGAGAATT CATGACATTT CTCGGAGAAC CTTTTATGAA CGAGAGGATG CCGTGAGTCA CCCAATGAGA   
  
  
- TAGCGTTCGG TATCGTCGTT CCGAACCACC CGGAGGATTT CACGCTGAAT GTCCGTAACT ACTAGGACAA   
  
  
- AGATTCATAT GAACACTACG ATCGAACCTC CGATAACCCT TTGCTAATCG CAGAGATAAA CTTTTCAAAT   
  
  
- TTTATGAGCA GCTCAAGTTA CGTAACGGGC AAATACCTGG ACTACAGTCC ACCCTTTACG ACCTACACTC   
  
  
- CGGGTCCCTC CGAAACCGGC AATTAACAGG TAATGTCGAG GTAGTGTGAG GACTGCTCTC ACAGCTACAC   
  
  
- TCGTTGGGAT CCCTATCCGA AGAGTCTTAC CACTTTAACG AGCCAGGATT CCATACGTGA AACCAACTCG   
  
  
- TTCTTAGTTT GTGGTTGTGA TGTGCAAAGA ACTGGGCCAA GTATCTCTGG GAACTGATGA TGAGCTAGTC   
  
  
- GGTACAAAC

+     box S

| Site Name | Organism | Position | Strand | Matrix score. | sequence | function |
| --- | --- | --- | --- | --- | --- | --- |
| box S | Arabidopsis thaliana | 305 | + | 7 | AGCCACC |  |

>HU08G00019.1   
+ +Up\_Stream \_Len000AGTTGT AGCAAGCACA TTAAAGTTGA TTATCATTTT GTCCGTGAAA CGGTGAGCAA   
  
  
+ ACGTCATCTC CAGGTCAAAT TCATTCGTAC TCAATCACAA GTTGCTGACA TTTTTACCAA AGGCTAATCG   
  
  
+ TCGCAAAAGT TTCTTTAGTT TAAGGCCAAT CTCTCCGTTG TTTCACCCCA TACAGATTGA GGGGTTGTAA   
  
  
+ TAGTGTATAT ATATAGCCTT GTGTTAGCGG GGCTTGGGCA TTAGACTTTT ACTCTTGTAA ACCCTAATGT   
  
  
+ GTTATTATAA ATATAGATCT AGCCACCCAT GTTGAGGTAG GCACTCTAAT TCTGACACAG GTAGGATAAG   
  
  
+ TTTTCATAGT TTATGTTGTG AATCTTGTCT TACTTTGTGG TTCCTATTGA CAAAAGTTTG AATCCGAGCA   
  
  
+ TGAGAGTTCA TCAATCTCTT TTTCTTTCCT ATTTTATTTC TTGTGGACTT AATTGGATTT TCTGGTAGAG   
  
  
+ GAAAGTTCCT TGCTGGAATT TGGTCGAAAG TGATATACAT TTTGGGATTT AATTAGCCAT AAATTTGGTT   
  
  
+ TTACACCTTG TTAGTTTGGA GTTCTGGTGT GTTATTGTAT GTGTTCTGGT GGGTTTTTCT TTTAGCTAAA   
  
  
+ TGTTGGAGCA TTTTAAGGGA TCTAGATATC TGAAGAAGTG CTTGGGAAAT GAAGCATATT TCCCTTATAG   
  
  
+ TCTGACATAA CTTTAATGAA TTGATCATGA ACAAAGGGCA ATTAGTTCAG AAAGGTTCAA CTCCTCTAGG   
  
  
+ TGTTGTGCTA AAGAGATGTG AGTTAGTAAC AATTGCTTAC TAACTATATA GGTTGTTTTA TTACTTGTAG   
  
  
+ AGGTTTTTTT TTTGGGATAT CTTTTTGGGG TTTATAAATG AGAAGGTTGA TTTGTTGTCT ACTCTTCTTT   
  
  
+ CGATTCAATA GTAATTCTCT CAGAGAAGGC TAAAATGACA TGGCATTCCA TCGCCTCTCA TGAGGCTTTT   
  
  
+ TGCATGTGCT TATGTATCTT TCATCTCTTA CTTTGGTGGG GTGGCATTGG GGAGGAAAAG GGGGTTTCGA   
  
  
+ TTCGATAGAT CGTCACTTTC GGAAATGACG TCTCTGACCA ATGGTAAGGG TAAGGTTAAT ACATGTGGAT   
  
  
+ ATTTCCAGAG TTAGTTGTGA TTGTTATTGT TGTTGTTGTA ACTTTATGTC ACGGGGAAAA CATAATAATT   
  
  
+ ATCTGTTTAT CTGTTTGTTT GTTATTTTCA AGGGGAAGGG GAGTGGGCGG ATGCTTAGTA TGTAATCATT   
  
  
+ TTATATAGGG AAAATTCATA TAGCATGAAA GTAAAACTTT CATTGCCAGC ATTCTAAGCT AGGGGTAGGG   
  
  
+ TTGTCTACAC CTTGATCTCC CTAGAAGCAT GGTGATCATT CCTCAGATTG TGTTATAGAA TAAAGAATGT   
  
  
+ GGTTCTGCTG TAATGAGACT TATGTATTAT CAAATGATGA ATCTGTTTAG AACTTCCTGT TTATTTTTTC   
  
  
+ TGCAAAATTT TAAGAGACTA GCATCTATCG GGCTCGACTC TAGAGGTTCT CTTGTGTTAC CCTTCTCAAC   
  
  
+ TCAGTTTGAT TGTGATACAG TTACTATATT GAGTGATAGT CGAGAGCATC ACAACCCGCT AAGGAGTCTA   
  
  
+ TCAACAAGAA GCCCTTCTTG TAATTCTCCC CTTGAAACTA GCAGTTATCA TCACTTGTCA TCGAATAGCC   
  
  
+ CTTTTTGTAA TTCTACCCTT GAAACTAGCA GTTATTGTCA TCAGTTCAAT GTGAGTCCCG TTAGATATTC   
  
  
+ CCATCAAGGC ATAGATTATG GAGTGAGCAT GAAGAACGCT TTGCACGAGC TAGAGACTGC TCTAATTGGT   
  
  
+ GTAGATGGCG AGAAAGCATC TGTTGCTAAT CAACCTATGG GGGGAATTCA TTAGTCCGGG ATCCCAAGTT   
  
  
+ AGAGATCGAA GTCATTGAGT GAAGATCCAC AGGGTTCACA TCCTCCTCAG CTTGATTCAT CATCTTTTTC   
  
  
+ AAGGGTGAAA AGATTCGGAG ATGAAAGCCA GAGAGAGAGA AATGCCACAA GGCAATGGAA GAACCAGCGG   
  
  
+ AACTACTAAG TTTCTCACCA GGTGATTTAA AGCAGTTGCT AATTGAATGT GCGAGGGCTT TATCAGATAA   
  
  
+ CCGAATAGAT GACTTTGAGA GTTTGGCTAA ACGGCCAAGG AAAGAGGTCT CCATCTCAGG TGAGCCTGTC   
  
  
+ CAACGTCTCG GTACTTGTAT GATCGAAGGG CTTGTGGCAA GGAAACAGTC TTCGGGGACT AACATCTATC   
  
  
+ GGGCTCTTAA GTACTGTAAA GAGCCTCTTG GAAAATACTT GCTCTCCTAC GGCACTCAGT GGGTTACTCT   
  
  
+ ATCGCAAGCC ATAGCAGCAA GGCTTGGTGG GCCTCCTAAA GTGCGACTTA CAGGCATTGA TGATCCTGTT   
  
  
+ TCTAAGTATA CTTGTGATGC TAGCTTGGAG GCTATTGGGA AACGATTAGC GTCTCTATTT GAAAAGTTTA   
  
  
+ AAATACTCGT CGAGTTCAAT GCATTGCCCG TTTATGGACC TGATGTCAGG TGGGAAATGC TGGATGTGAG   
  
  
+ GCCCAGGGAG GCTTTGGCCG TTAATTGTCC ATTACAGCTC CATCACACTC CTGACGAGAG TGTCGATGTG   
  
  
+ AGCAACCCTA GGGATAGGCT TCTCAGAATG GTGAAATTGC TCGGTCCTAA GGTATGCACT TTGGTTGAGC   
  
  
+ AAGAATCAAA CACCAACACT ACACGTTTCT TGACCCGGTT CATAGAGACC CTTGACTACT ACTCGATCAG   
  
  
+ CCATGTTTG  

- +Up\_Stream \_Len000TCAACA TCGTTCGTGT AATTTCAACT AATAGTAAAA CAGGCACTTT GCCACTCGTT   
  
  
- TGCAGTAGAG GTCCAGTTTA AGTAAGCATG AGTTAGTGTT CAACGACTGT AAAAATGGTT TCCGATTAGC   
  
  
- AGCGTTTTCA AAGAAATCAA ATTCCGGTTA GAGAGGCAAC AAAGTGGGGT ATGTCTAACT CCCCAACATT   
  
  
- ATCACATATA TATATCGGAA CACAATCGCC CCGAACCCGT AATCTGAAAA TGAGAACATT TGGGATTACA   
  
  
- CAATAATATT TATATCTAGA TCGGTGGGTA CAACTCCATC CGTGAGATTA AGACTGTGTC CATCCTATTC   
  
  
- AAAAGTATCA AATACAACAC TTAGAACAGA ATGAAACACC AAGGATAACT GTTTTCAAAC TTAGGCTCGT   
  
  
- ACTCTCAAGT AGTTAGAGAA AAAGAAAGGA TAAAATAAAG AACACCTGAA TTAACCTAAA AGACCATCTC   
  
  
- CTTTCAAGGA ACGACCTTAA ACCAGCTTTC ACTATATGTA AAACCCTAAA TTAATCGGTA TTTAAACCAA   
  
  
- AATGTGGAAC AATCAAACCT CAAGACCACA CAATAACATA CACAAGACCA CCCAAAAAGA AAATCGATTT   
  
  
- ACAACCTCGT AAAATTCCCT AGATCTATAG ACTTCTTCAC GAACCCTTTA CTTCGTATAA AGGGAATATC   
  
  
- AGACTGTATT GAAATTACTT AACTAGTACT TGTTTCCCGT TAATCAAGTC TTTCCAAGTT GAGGAGATCC   
  
  
- ACAACACGAT TTCTCTACAC TCAATCATTG TTAACGAATG ATTGATATAT CCAACAAAAT AATGAACATC   
  
  
- TCCAAAAAAA AAACCCTATA GAAAAACCCC AAATATTTAC TCTTCCAACT AAACAACAGA TGAGAAGAAA   
  
  
- GCTAAGTTAT CATTAAGAGA GTCTCTTCCG ATTTTACTGT ACCGTAAGGT AGCGGAGAGT ACTCCGAAAA   
  
  
- ACGTACACGA ATACATAGAA AGTAGAGAAT GAAACCACCC CACCGTAACC CCTCCTTTTC CCCCAAAGCT   
  
  
- AAGCTATCTA GCAGTGAAAG CCTTTACTGC AGAGACTGGT TACCATTCCC ATTCCAATTA TGTACACCTA   
  
  
- TAAAGGTCTC AATCAACACT AACAATAACA ACAACAACAT TGAAATACAG TGCCCCTTTT GTATTATTAA   
  
  
- TAGACAAATA GACAAACAAA CAATAAAAGT TCCCCTTCCC CTCACCCGCC TACGAATCAT ACATTAGTAA   
  
  
- AATATATCCC TTTTAAGTAT ATCGTACTTT CATTTTGAAA GTAACGGTCG TAAGATTCGA TCCCCATCCC   
  
  
- AACAGATGTG GAACTAGAGG GATCTTCGTA CCACTAGTAA GGAGTCTAAC ACAATATCTT ATTTCTTACA   
  
  
- CCAAGACGAC ATTACTCTGA ATACATAATA GTTTACTACT TAGACAAATC TTGAAGGACA AATAAAAAAG   
  
  
- ACGTTTTAAA ATTCTCTGAT CGTAGATAGC CCGAGCTGAG ATCTCCAAGA GAACACAATG GGAAGAGTTG   
  
  
- AGTCAAACTA ACACTATGTC AATGATATAA CTCACTATCA GCTCTCGTAG TGTTGGGCGA TTCCTCAGAT   
  
  
- AGTTGTTCTT CGGGAAGAAC ATTAAGAGGG GAACTTTGAT CGTCAATAGT AGTGAACAGT AGCTTATCGG   
  
  
- GAAAAACATT AAGATGGGAA CTTTGATCGT CAATAACAGT AGTCAAGTTA CACTCAGGGC AATCTATAAG   
  
  
- GGTAGTTCCG TATCTAATAC CTCACTCGTA CTTCTTGCGA AACGTGCTCG ATCTCTGACG AGATTAACCA   
  
  
- CATCTACCGC TCTTTCGTAG ACAACGATTA GTTGGATACC CCCCTTAAGT AATCAGGCCC TAGGGTTCAA   
  
  
- TCTCTAGCTT CAGTAACTCA CTTCTAGGTG TCCCAAGTGT AGGAGGAGTC GAACTAAGTA GTAGAAAAAG   
  
  
- TTCCCACTTT TCTAAGCCTC TACTTTCGGT CTCTCTCTCT TTACGGTGTT CCGTTACCTT CTTGGTCGCC   
  
  
- TTGATGATTC AAAGAGTGGT CCACTAAATT TCGTCAACGA TTAACTTACA CGCTCCCGAA ATAGTCTATT   
  
  
- GGCTTATCTA CTGAAACTCT CAAACCGATT TGCCGGTTCC TTTCTCCAGA GGTAGAGTCC ACTCGGACAG   
  
  
- GTTGCAGAGC CATGAACATA CTAGCTTCCC GAACACCGTT CCTTTGTCAG AAGCCCCTGA TTGTAGATAG   
  
  
- CCCGAGAATT CATGACATTT CTCGGAGAAC CTTTTATGAA CGAGAGGATG CCGTGAGTCA CCCAATGAGA   
  
  
- TAGCGTTCGG TATCGTCGTT CCGAACCACC CGGAGGATTT CACGCTGAAT GTCCGTAACT ACTAGGACAA   
  
  
- AGATTCATAT GAACACTACG ATCGAACCTC CGATAACCCT TTGCTAATCG CAGAGATAAA CTTTTCAAAT   
  
  
- TTTATGAGCA GCTCAAGTTA CGTAACGGGC AAATACCTGG ACTACAGTCC ACCCTTTACG ACCTACACTC   
  
  
- CGGGTCCCTC CGAAACCGGC AATTAACAGG TAATGTCGAG GTAGTGTGAG GACTGCTCTC ACAGCTACAC   
  
  
- TCGTTGGGAT CCCTATCCGA AGAGTCTTAC CACTTTAACG AGCCAGGATT CCATACGTGA AACCAACTCG   
  
  
- TTCTTAGTTT GTGGTTGTGA TGTGCAAAGA ACTGGGCCAA GTATCTCTGG GAACTGATGA TGAGCTAGTC   
  
  
- GGTACAAAC

+     circadian

| Site Name | Organism | Position | Strand | Matrix score. | sequence | function |
| --- | --- | --- | --- | --- | --- | --- |
| circadian | Lycopersicon esculentum | 860 | - | 9 | CAAAGATATC | cis-acting regulatory element involved in circadian control |

>HU08G00019.1   
+ +Up\_Stream \_Len000AGTTGT AGCAAGCACA TTAAAGTTGA TTATCATTTT GTCCGTGAAA CGGTGAGCAA   
  
  
+ ACGTCATCTC CAGGTCAAAT TCATTCGTAC TCAATCACAA GTTGCTGACA TTTTTACCAA AGGCTAATCG   
  
  
+ TCGCAAAAGT TTCTTTAGTT TAAGGCCAAT CTCTCCGTTG TTTCACCCCA TACAGATTGA GGGGTTGTAA   
  
  
+ TAGTGTATAT ATATAGCCTT GTGTTAGCGG GGCTTGGGCA TTAGACTTTT ACTCTTGTAA ACCCTAATGT   
  
  
+ GTTATTATAA ATATAGATCT AGCCACCCAT GTTGAGGTAG GCACTCTAAT TCTGACACAG GTAGGATAAG   
  
  
+ TTTTCATAGT TTATGTTGTG AATCTTGTCT TACTTTGTGG TTCCTATTGA CAAAAGTTTG AATCCGAGCA   
  
  
+ TGAGAGTTCA TCAATCTCTT TTTCTTTCCT ATTTTATTTC TTGTGGACTT AATTGGATTT TCTGGTAGAG   
  
  
+ GAAAGTTCCT TGCTGGAATT TGGTCGAAAG TGATATACAT TTTGGGATTT AATTAGCCAT AAATTTGGTT   
  
  
+ TTACACCTTG TTAGTTTGGA GTTCTGGTGT GTTATTGTAT GTGTTCTGGT GGGTTTTTCT TTTAGCTAAA   
  
  
+ TGTTGGAGCA TTTTAAGGGA TCTAGATATC TGAAGAAGTG CTTGGGAAAT GAAGCATATT TCCCTTATAG   
  
  
+ TCTGACATAA CTTTAATGAA TTGATCATGA ACAAAGGGCA ATTAGTTCAG AAAGGTTCAA CTCCTCTAGG   
  
  
+ TGTTGTGCTA AAGAGATGTG AGTTAGTAAC AATTGCTTAC TAACTATATA GGTTGTTTTA TTACTTGTAG   
  
  
+ AGGTTTTTTT TTTGGGATAT CTTTTTGGGG TTTATAAATG AGAAGGTTGA TTTGTTGTCT ACTCTTCTTT   
  
  
+ CGATTCAATA GTAATTCTCT CAGAGAAGGC TAAAATGACA TGGCATTCCA TCGCCTCTCA TGAGGCTTTT   
  
  
+ TGCATGTGCT TATGTATCTT TCATCTCTTA CTTTGGTGGG GTGGCATTGG GGAGGAAAAG GGGGTTTCGA   
  
  
+ TTCGATAGAT CGTCACTTTC GGAAATGACG TCTCTGACCA ATGGTAAGGG TAAGGTTAAT ACATGTGGAT   
  
  
+ ATTTCCAGAG TTAGTTGTGA TTGTTATTGT TGTTGTTGTA ACTTTATGTC ACGGGGAAAA CATAATAATT   
  
  
+ ATCTGTTTAT CTGTTTGTTT GTTATTTTCA AGGGGAAGGG GAGTGGGCGG ATGCTTAGTA TGTAATCATT   
  
  
+ TTATATAGGG AAAATTCATA TAGCATGAAA GTAAAACTTT CATTGCCAGC ATTCTAAGCT AGGGGTAGGG   
  
  
+ TTGTCTACAC CTTGATCTCC CTAGAAGCAT GGTGATCATT CCTCAGATTG TGTTATAGAA TAAAGAATGT   
  
  
+ GGTTCTGCTG TAATGAGACT TATGTATTAT CAAATGATGA ATCTGTTTAG AACTTCCTGT TTATTTTTTC   
  
  
+ TGCAAAATTT TAAGAGACTA GCATCTATCG GGCTCGACTC TAGAGGTTCT CTTGTGTTAC CCTTCTCAAC   
  
  
+ TCAGTTTGAT TGTGATACAG TTACTATATT GAGTGATAGT CGAGAGCATC ACAACCCGCT AAGGAGTCTA   
  
  
+ TCAACAAGAA GCCCTTCTTG TAATTCTCCC CTTGAAACTA GCAGTTATCA TCACTTGTCA TCGAATAGCC   
  
  
+ CTTTTTGTAA TTCTACCCTT GAAACTAGCA GTTATTGTCA TCAGTTCAAT GTGAGTCCCG TTAGATATTC   
  
  
+ CCATCAAGGC ATAGATTATG GAGTGAGCAT GAAGAACGCT TTGCACGAGC TAGAGACTGC TCTAATTGGT   
  
  
+ GTAGATGGCG AGAAAGCATC TGTTGCTAAT CAACCTATGG GGGGAATTCA TTAGTCCGGG ATCCCAAGTT   
  
  
+ AGAGATCGAA GTCATTGAGT GAAGATCCAC AGGGTTCACA TCCTCCTCAG CTTGATTCAT CATCTTTTTC   
  
  
+ AAGGGTGAAA AGATTCGGAG ATGAAAGCCA GAGAGAGAGA AATGCCACAA GGCAATGGAA GAACCAGCGG   
  
  
+ AACTACTAAG TTTCTCACCA GGTGATTTAA AGCAGTTGCT AATTGAATGT GCGAGGGCTT TATCAGATAA   
  
  
+ CCGAATAGAT GACTTTGAGA GTTTGGCTAA ACGGCCAAGG AAAGAGGTCT CCATCTCAGG TGAGCCTGTC   
  
  
+ CAACGTCTCG GTACTTGTAT GATCGAAGGG CTTGTGGCAA GGAAACAGTC TTCGGGGACT AACATCTATC   
  
  
+ GGGCTCTTAA GTACTGTAAA GAGCCTCTTG GAAAATACTT GCTCTCCTAC GGCACTCAGT GGGTTACTCT   
  
  
+ ATCGCAAGCC ATAGCAGCAA GGCTTGGTGG GCCTCCTAAA GTGCGACTTA CAGGCATTGA TGATCCTGTT   
  
  
+ TCTAAGTATA CTTGTGATGC TAGCTTGGAG GCTATTGGGA AACGATTAGC GTCTCTATTT GAAAAGTTTA   
  
  
+ AAATACTCGT CGAGTTCAAT GCATTGCCCG TTTATGGACC TGATGTCAGG TGGGAAATGC TGGATGTGAG   
  
  
+ GCCCAGGGAG GCTTTGGCCG TTAATTGTCC ATTACAGCTC CATCACACTC CTGACGAGAG TGTCGATGTG   
  
  
+ AGCAACCCTA GGGATAGGCT TCTCAGAATG GTGAAATTGC TCGGTCCTAA GGTATGCACT TTGGTTGAGC   
  
  
+ AAGAATCAAA CACCAACACT ACACGTTTCT TGACCCGGTT CATAGAGACC CTTGACTACT ACTCGATCAG   
  
  
+ CCATGTTTG  

- +Up\_Stream \_Len000TCAACA TCGTTCGTGT AATTTCAACT AATAGTAAAA CAGGCACTTT GCCACTCGTT   
  
  
- TGCAGTAGAG GTCCAGTTTA AGTAAGCATG AGTTAGTGTT CAACGACTGT AAAAATGGTT TCCGATTAGC   
  
  
- AGCGTTTTCA AAGAAATCAA ATTCCGGTTA GAGAGGCAAC AAAGTGGGGT ATGTCTAACT CCCCAACATT   
  
  
- ATCACATATA TATATCGGAA CACAATCGCC CCGAACCCGT AATCTGAAAA TGAGAACATT TGGGATTACA   
  
  
- CAATAATATT TATATCTAGA TCGGTGGGTA CAACTCCATC CGTGAGATTA AGACTGTGTC CATCCTATTC   
  
  
- AAAAGTATCA AATACAACAC TTAGAACAGA ATGAAACACC AAGGATAACT GTTTTCAAAC TTAGGCTCGT   
  
  
- ACTCTCAAGT AGTTAGAGAA AAAGAAAGGA TAAAATAAAG AACACCTGAA TTAACCTAAA AGACCATCTC   
  
  
- CTTTCAAGGA ACGACCTTAA ACCAGCTTTC ACTATATGTA AAACCCTAAA TTAATCGGTA TTTAAACCAA   
  
  
- AATGTGGAAC AATCAAACCT CAAGACCACA CAATAACATA CACAAGACCA CCCAAAAAGA AAATCGATTT   
  
  
- ACAACCTCGT AAAATTCCCT AGATCTATAG ACTTCTTCAC GAACCCTTTA CTTCGTATAA AGGGAATATC   
  
  
- AGACTGTATT GAAATTACTT AACTAGTACT TGTTTCCCGT TAATCAAGTC TTTCCAAGTT GAGGAGATCC   
  
  
- ACAACACGAT TTCTCTACAC TCAATCATTG TTAACGAATG ATTGATATAT CCAACAAAAT AATGAACATC   
  
  
- TCCAAAAAAA AAACCCTATA GAAAAACCCC AAATATTTAC TCTTCCAACT AAACAACAGA TGAGAAGAAA   
  
  
- GCTAAGTTAT CATTAAGAGA GTCTCTTCCG ATTTTACTGT ACCGTAAGGT AGCGGAGAGT ACTCCGAAAA   
  
  
- ACGTACACGA ATACATAGAA AGTAGAGAAT GAAACCACCC CACCGTAACC CCTCCTTTTC CCCCAAAGCT   
  
  
- AAGCTATCTA GCAGTGAAAG CCTTTACTGC AGAGACTGGT TACCATTCCC ATTCCAATTA TGTACACCTA   
  
  
- TAAAGGTCTC AATCAACACT AACAATAACA ACAACAACAT TGAAATACAG TGCCCCTTTT GTATTATTAA   
  
  
- TAGACAAATA GACAAACAAA CAATAAAAGT TCCCCTTCCC CTCACCCGCC TACGAATCAT ACATTAGTAA   
  
  
- AATATATCCC TTTTAAGTAT ATCGTACTTT CATTTTGAAA GTAACGGTCG TAAGATTCGA TCCCCATCCC   
  
  
- AACAGATGTG GAACTAGAGG GATCTTCGTA CCACTAGTAA GGAGTCTAAC ACAATATCTT ATTTCTTACA   
  
  
- CCAAGACGAC ATTACTCTGA ATACATAATA GTTTACTACT TAGACAAATC TTGAAGGACA AATAAAAAAG   
  
  
- ACGTTTTAAA ATTCTCTGAT CGTAGATAGC CCGAGCTGAG ATCTCCAAGA GAACACAATG GGAAGAGTTG   
  
  
- AGTCAAACTA ACACTATGTC AATGATATAA CTCACTATCA GCTCTCGTAG TGTTGGGCGA TTCCTCAGAT   
  
  
- AGTTGTTCTT CGGGAAGAAC ATTAAGAGGG GAACTTTGAT CGTCAATAGT AGTGAACAGT AGCTTATCGG   
  
  
- GAAAAACATT AAGATGGGAA CTTTGATCGT CAATAACAGT AGTCAAGTTA CACTCAGGGC AATCTATAAG   
  
  
- GGTAGTTCCG TATCTAATAC CTCACTCGTA CTTCTTGCGA AACGTGCTCG ATCTCTGACG AGATTAACCA   
  
  
- CATCTACCGC TCTTTCGTAG ACAACGATTA GTTGGATACC CCCCTTAAGT AATCAGGCCC TAGGGTTCAA   
  
  
- TCTCTAGCTT CAGTAACTCA CTTCTAGGTG TCCCAAGTGT AGGAGGAGTC GAACTAAGTA GTAGAAAAAG   
  
  
- TTCCCACTTT TCTAAGCCTC TACTTTCGGT CTCTCTCTCT TTACGGTGTT CCGTTACCTT CTTGGTCGCC   
  
  
- TTGATGATTC AAAGAGTGGT CCACTAAATT TCGTCAACGA TTAACTTACA CGCTCCCGAA ATAGTCTATT   
  
  
- GGCTTATCTA CTGAAACTCT CAAACCGATT TGCCGGTTCC TTTCTCCAGA GGTAGAGTCC ACTCGGACAG   
  
  
- GTTGCAGAGC CATGAACATA CTAGCTTCCC GAACACCGTT CCTTTGTCAG AAGCCCCTGA TTGTAGATAG   
  
  
- CCCGAGAATT CATGACATTT CTCGGAGAAC CTTTTATGAA CGAGAGGATG CCGTGAGTCA CCCAATGAGA   
  
  
- TAGCGTTCGG TATCGTCGTT CCGAACCACC CGGAGGATTT CACGCTGAAT GTCCGTAACT ACTAGGACAA   
  
  
- AGATTCATAT GAACACTACG ATCGAACCTC CGATAACCCT TTGCTAATCG CAGAGATAAA CTTTTCAAAT   
  
  
- TTTATGAGCA GCTCAAGTTA CGTAACGGGC AAATACCTGG ACTACAGTCC ACCCTTTACG ACCTACACTC   
  
  
- CGGGTCCCTC CGAAACCGGC AATTAACAGG TAATGTCGAG GTAGTGTGAG GACTGCTCTC ACAGCTACAC   
  
  
- TCGTTGGGAT CCCTATCCGA AGAGTCTTAC CACTTTAACG AGCCAGGATT CCATACGTGA AACCAACTCG   
  
  
- TTCTTAGTTT GTGGTTGTGA TGTGCAAAGA ACTGGGCCAA GTATCTCTGG GAACTGATGA TGAGCTAGTC   
  
  
- GGTACAAAC
