# Supplementary material for: Genome-Wide Identification and Expression Pattern of the GRAS Gene Family in Pitaya (Selenicereus undatus L.)
Source: Biology (Basel). 2022 Dec 21;12(1):11. doi: 10.3390/biology12010011 (PMC9854919; doi:10.3390/biology12010011)
Supplement: Supplementary file 1 [file biology-12-00011-s001.zip › Supplementary file S5/HU08G00229.1_plantcare.html]

Content-Type: text/html; charset=ISO-8859-1


PlantCARE


Webmaster Firefox specific output  
To save the result:
click on the frame with the right mouse button and save the source code as a text file with extension .html  
REFERENCE:PlantCARE: a database of plant cis-acting regulatory elements and a portal to tools for in silico analysis of promoter sequences.  
Lescot, M., Déhais, P., Moreau, Y., De Moor, B., Rouzé ,P.,and Rombauts, S.  
Nucleic Acids Res., Database issue(2002), 30(1):325-327.   


---

>HU08G00229.1   
+ -Up\_Stream \_Len000AAATTT ATATGATTAT GGGAGCAAAT ATTAATCGGT CTAAAACAAA AAAGAATCGG   
  
  
+ CCGAACAAAA AAAAGGGCCC AGAACTAGTC CTTTTAATGG AGTCCCAAAA TGTGGTATAA ATTAGTCTTG   
  
  
+ TTCACTCATT TCTCACTCCT CTCACTCATT CTAACCCTAA ATCTCAATCC TCACTCTCTG TCCTCTCTAT   
  
  
+ CTGCGTCTCG AGCTTCTCTT TCCTCTGGGC TTCCTTGGAC TTTTTCGACG CCATGACAAT GGAACACTGA   
  
  
+ GCTCCACCGT TGCCCCTCTC CACAACTGTC TTCTAACGGT GGTTCTCCGT AGCCACCGTA CCTCAAAAAC   
  
  
+ CAAAGCGATG CACAACTACC ACCCCACCAC TGCTGAAAAG TGCCCACGAG TCTCGATCCG CGCAAGCCTG   
  
  
+ ATGAGAATCG ATACCACCAC CATGTAGATA AGCTGAAAAC GGGCATAAGG AAGCGACCCA CGCGCCCAAC   
  
  
+ ACGCCATTGA GGCAGTGACA CGTGGGTTGA TGCGCTTCCA AAGATGGAAT TTTCACATTT TTCTTTTCAA   
  
  
+ GGTTGCGTAG ATCGGTGTGT GTGGATGGCG ATCAAGGAAG AGTTAAAATG AACTAGTCAT GCGTCGAATT   
  
  
+ GTCCAAAAAA AATTTTCTCT TTCAGTCGAG TCTTAAAGGG GCAGTCATCA AGAGCATGTT TGAAAAACAT   
  
  
+ TATGGCCATA ACCACTTAGT AGTAATTCAA ATAATCATGG AAACATATTC AACATGAATT CAATATGACT   
  
  
+ AAAAATTCCA TCTTTTAGTT TGTTCTGGTC AACACAGAGC ATGCCTTTTT AATTGCATAA TAACGATATT   
  
  
+ TAACCAACCA ATCATGTTTC TCACATATTA ATATAGGCAA GGGTAGATTA ACAGTCATAA TCGCATAGTA   
  
  
+ TGCAAGAATT TATTTGCAAT CTGGGCATAC TGTTTTATAA AGCACAAGTG TTCATGACTT AAAATCGATA   
  
  
+ AAGTCTTAAT CTTTTGTCGA TCCTTGAGTG TGCAGACAAT ATAATTGTGA ATGTATGCTA TTAACTTTAA   
  
  
+ TAATTACGAA AATTAAGCAA CTAATCATAT AGTCATGTTC TCAAAAACAA GCATGCGTAT TATTCAGATC   
  
  
+ ATTAAATGCA TTAAATGGGA CTAATCCGAT CAGTCAAGCG CTGATACGAC TATTGAATAA ACAGGACTAA   
  
  
+ CCTAGAATGC GGAATTAACT GTCGAGAATT GTCCACATCG GTAGTGATGC TTGGCGGCTA CCAGAGTCAC   
  
  
+ CTTAGGGTGT TCACATCCTA GACCCGAAAA CCTGCTAATG ATGGAAGGGC AGCCGTGGGA GGAGGAGAGG   
  
  
+ AGAGTGTGGA TTTTTCTATT TACAAAAAAG TGTATAAGTG CTAATCCCCC GAAACCTCCT TTTATAGGTT   
  
  
+ AGGGGGGCAA CTAGGGTTAT CGCCCTAGGT GACGCTTGTG GCCGTCGGCC TAATTAAATA GGCCCGACCA   
  
  
+ ACAGCTGTGA CTGGAGCGAT AGCTGAAGCA TGCAGAAATG AGGATAACAT ACATATTATA GATTTCCACA   
  
  
+ TTGCATAGGG CACTCAGTGG GTTACTGTAT CGCAAGCCAT AGCAGCAAGG CTTGGTGGGC CTCCTAAAGT   
  
  
+ GCGACTTACA GGCATTGATG ATCCTGTTTC TAAGTATACT TGTGATGCTA GCTTGGAGGC TGGTGGGAAA   
  
  
+ CGATTAGCGT CTCTATTTGA AAAGTTTAAA ATACTTGTCG AGTTCAATGC ATTGCCCGTT TATGGACCCA   
  
  
+ ATGTCAGGTG GGAAATGCTG GATGTGAGGC CCAGGGAGGC TTTGGTCGTT AATTGTCCAT TACAGCTCCA   
  
  
+ TTACACTCCT GACGAGAGTG TCGATGTGAG GAACCCTAGG GATAGGCTTC TCAGAATGGT GAAATTGCTC   
  
  
+ GGTCCTAAGG TAGCCACTTT GGTTGAGCAA GAATCAAACA CCAACACTAC ACCTTTCTTG ACCCGGTTCA   
  
  
+ TAGAGACCCT TGACTACTAC TCGATTAACC ATGTTTGAGT CTATAGACGT GACCATGCCG AGAGACCGGA   
  
  
+ AGGAGAGGAT CAATGTTGAG CAGCATTGTT TAGCTAAGGA CATTGTGAAT ATCATAGCTT GCGAGGGCAA   
  
  
+ GGAGATGGTG GAGCGTCATG AGCTTTTTGG GAAATGGCAG TCGAGGTTTA CCATGGTAGG GTTCAGGCAG   
  
  
+ TACCCGTTGA GCTCATACAT AAACTCTGTG ATAAGAAGCC TACTCCGGTG TTACTCCAAG CATTATACTC   
  
  
+ TGATAGAGAA GGATAGTGCC ATGCTTCTGG GTTGGAGGGG CCGAATGCTG ATTTCAGCTT CGGCATG  

- -Up\_Stream \_Len000TTTAAA TATACTAATA CCCTCGTTTA TAATTAGCCA GATTTTGTTT TTTCTTAGCC   
  
  
- GGCTTGTTTT TTTTCCCGGG TCTTGATCAG GAAAATTACC TCAGGGTTTT ACACCATATT TAATCAGAAC   
  
  
- AAGTGAGTAA AGAGTGAGGA GAGTGAGTAA GATTGGGATT TAGAGTTAGG AGTGAGAGAC AGGAGAGATA   
  
  
- GACGCAGAGC TCGAAGAGAA AGGAGACCCG AAGGAACCTG AAAAAGCTGC GGTACTGTTA CCTTGTGACT   
  
  
- CGAGGTGGCA ACGGGGAGAG GTGTTGACAG AAGATTGCCA CCAAGAGGCA TCGGTGGCAT GGAGTTTTTG   
  
  
- GTTTCGCTAC GTGTTGATGG TGGGGTGGTG ACGACTTTTC ACGGGTGCTC AGAGCTAGGC GCGTTCGGAC   
  
  
- TACTCTTAGC TATGGTGGTG GTACATCTAT TCGACTTTTG CCCGTATTCC TTCGCTGGGT GCGCGGGTTG   
  
  
- TGCGGTAACT CCGTCACTGT GCACCCAACT ACGCGAAGGT TTCTACCTTA AAAGTGTAAA AAGAAAAGTT   
  
  
- CCAACGCATC TAGCCACACA CACCTACCGC TAGTTCCTTC TCAATTTTAC TTGATCAGTA CGCAGCTTAA   
  
  
- CAGGTTTTTT TTAAAAGAGA AAGTCAGCTC AGAATTTCCC CGTCAGTAGT TCTCGTACAA ACTTTTTGTA   
  
  
- ATACCGGTAT TGGTGAATCA TCATTAAGTT TATTAGTACC TTTGTATAAG TTGTACTTAA GTTATACTGA   
  
  
- TTTTTAAGGT AGAAAATCAA ACAAGACCAG TTGTGTCTCG TACGGAAAAA TTAACGTATT ATTGCTATAA   
  
  
- ATTGGTTGGT TAGTACAAAG AGTGTATAAT TATATCCGTT CCCATCTAAT TGTCAGTATT AGCGTATCAT   
  
  
- ACGTTCTTAA ATAAACGTTA GACCCGTATG ACAAAATATT TCGTGTTCAC AAGTACTGAA TTTTAGCTAT   
  
  
- TTCAGAATTA GAAAACAGCT AGGAACTCAC ACGTCTGTTA TATTAACACT TACATACGAT AATTGAAATT   
  
  
- ATTAATGCTT TTAATTCGTT GATTAGTATA TCAGTACAAG AGTTTTTGTT CGTACGCATA ATAAGTCTAG   
  
  
- TAATTTACGT AATTTACCCT GATTAGGCTA GTCAGTTCGC GACTATGCTG ATAACTTATT TGTCCTGATT   
  
  
- GGATCTTACG CCTTAATTGA CAGCTCTTAA CAGGTGTAGC CATCACTACG AACCGCCGAT GGTCTCAGTG   
  
  
- GAATCCCACA AGTGTAGGAT CTGGGCTTTT GGACGATTAC TACCTTCCCG TCGGCACCCT CCTCCTCTCC   
  
  
- TCTCACACCT AAAAAGATAA ATGTTTTTTC ACATATTCAC GATTAGGGGG CTTTGGAGGA AAATATCCAA   
  
  
- TCCCCCCGTT GATCCCAATA GCGGGATCCA CTGCGAACAC CGGCAGCCGG ATTAATTTAT CCGGGCTGGT   
  
  
- TGTCGACACT GACCTCGCTA TCGACTTCGT ACGTCTTTAC TCCTATTGTA TGTATAATAT CTAAAGGTGT   
  
  
- AACGTATCCC GTGAGTCACC CAATGACATA GCGTTCGGTA TCGTCGTTCC GAACCACCCG GAGGATTTCA   
  
  
- CGCTGAATGT CCGTAACTAC TAGGACAAAG ATTCATATGA ACACTACGAT CGAACCTCCG ACCACCCTTT   
  
  
- GCTAATCGCA GAGATAAACT TTTCAAATTT TATGAACAGC TCAAGTTACG TAACGGGCAA ATACCTGGGT   
  
  
- TACAGTCCAC CCTTTACGAC CTACACTCCG GGTCCCTCCG AAACCAGCAA TTAACAGGTA ATGTCGAGGT   
  
  
- AATGTGAGGA CTGCTCTCAC AGCTACACTC CTTGGGATCC CTATCCGAAG AGTCTTACCA CTTTAACGAG   
  
  
- CCAGGATTCC ATCGGTGAAA CCAACTCGTT CTTAGTTTGT GGTTGTGATG TGGAAAGAAC TGGGCCAAGT   
  
  
- ATCTCTGGGA ACTGATGATG AGCTAATTGG TACAAACTCA GATATCTGCA CTGGTACGGC TCTCTGGCCT   
  
  
- TCCTCTCCTA GTTACAACTC GTCGTAACAA ATCGATTCCT GTAACACTTA TAGTATCGAA CGCTCCCGTT   
  
  
- CCTCTACCAC CTCGCAGTAC TCGAAAAACC CTTTACCGTC AGCTCCAAAT GGTACCATCC CAAGTCCGTC   
  
  
- ATGGGCAACT CGAGTATGTA TTTGAGACAC TATTCTTCGG ATGAGGCCAC AATGAGGTTC GTAATATGAG   
  
  
- ACTATCTCTT CCTATCACGG TACGAAGACC CAACCTCCCC GGCTTACGAC TAAAGTCGAA GCCGTAC

  
  
Motifs Found  

+   

| Site Name | Organism | Position | Strand | Matrix score. | sequence | function |
| --- | --- | --- | --- | --- | --- | --- |
|  | organism | 2072 | - | 4 | motif\_sequence | short\_function |
|  | organism | 213 | + | 4 | motif\_sequence | short\_function |
|  | organism | 2105 | - | 4 | motif\_sequence | short\_function |
|  | organism | 887 | - | 4 | motif\_sequence | short\_function |
|  | organism | 1333 | - | 4 | motif\_sequence | short\_function |
|  | organism | 1328 | - | 4 | motif\_sequence | short\_function |
|  | organism | 2036 | - | 4 | motif\_sequence | short\_function |
|  | organism | 601 | - | 4 | motif\_sequence | short\_function |
|  | organism | 571 | - | 4 | motif\_sequence | short\_function |
|  | organism | 202 | + | 4 | motif\_sequence | short\_function |
|  | organism | 300 | + | 4 | motif\_sequence | short\_function |
|  | organism | 448 | - | 4 | motif\_sequence | short\_function |
|  | organism | 1089 | + | 4 | motif\_sequence | short\_function |

>HU08G00229.1   
+ -Up\_Stream \_Len000AAATTT ATATGATTAT GGGAGCAAAT ATTAATCGGT CTAAAACAAA AAAGAATCGG   
  
  
+ CCGAACAAAA AAAAGGGCCC AGAACTAGTC CTTTTAATGG AGTCCCAAAA TGTGGTATAA ATTAGTCTTG   
  
  
+ TTCACTCATT TCTCACTCCT CTCACTCATT CTAACCCTAA ATCTCAATCC TCACTCTCTG TCCTCTCTAT   
  
  
+ CTGCGTCTCG AGCTTCTCTT TCCTCTGGGC TTCCTTGGAC TTTTTCGACG CCATGACAAT GGAACACTGA   
  
  
+ GCTCCACCGT TGCCCCTCTC CACAACTGTC TTCTAACGGT GGTTCTCCGT AGCCACCGTA CCTCAAAAAC   
  
  
+ CAAAGCGATG CACAACTACC ACCCCACCAC TGCTGAAAAG TGCCCACGAG TCTCGATCCG CGCAAGCCTG   
  
  
+ ATGAGAATCG ATACCACCAC CATGTAGATA AGCTGAAAAC GGGCATAAGG AAGCGACCCA CGCGCCCAAC   
  
  
+ ACGCCATTGA GGCAGTGACA CGTGGGTTGA TGCGCTTCCA AAGATGGAAT TTTCACATTT TTCTTTTCAA   
  
  
+ GGTTGCGTAG ATCGGTGTGT GTGGATGGCG ATCAAGGAAG AGTTAAAATG AACTAGTCAT GCGTCGAATT   
  
  
+ GTCCAAAAAA AATTTTCTCT TTCAGTCGAG TCTTAAAGGG GCAGTCATCA AGAGCATGTT TGAAAAACAT   
  
  
+ TATGGCCATA ACCACTTAGT AGTAATTCAA ATAATCATGG AAACATATTC AACATGAATT CAATATGACT   
  
  
+ AAAAATTCCA TCTTTTAGTT TGTTCTGGTC AACACAGAGC ATGCCTTTTT AATTGCATAA TAACGATATT   
  
  
+ TAACCAACCA ATCATGTTTC TCACATATTA ATATAGGCAA GGGTAGATTA ACAGTCATAA TCGCATAGTA   
  
  
+ TGCAAGAATT TATTTGCAAT CTGGGCATAC TGTTTTATAA AGCACAAGTG TTCATGACTT AAAATCGATA   
  
  
+ AAGTCTTAAT CTTTTGTCGA TCCTTGAGTG TGCAGACAAT ATAATTGTGA ATGTATGCTA TTAACTTTAA   
  
  
+ TAATTACGAA AATTAAGCAA CTAATCATAT AGTCATGTTC TCAAAAACAA GCATGCGTAT TATTCAGATC   
  
  
+ ATTAAATGCA TTAAATGGGA CTAATCCGAT CAGTCAAGCG CTGATACGAC TATTGAATAA ACAGGACTAA   
  
  
+ CCTAGAATGC GGAATTAACT GTCGAGAATT GTCCACATCG GTAGTGATGC TTGGCGGCTA CCAGAGTCAC   
  
  
+ CTTAGGGTGT TCACATCCTA GACCCGAAAA CCTGCTAATG ATGGAAGGGC AGCCGTGGGA GGAGGAGAGG   
  
  
+ AGAGTGTGGA TTTTTCTATT TACAAAAAAG TGTATAAGTG CTAATCCCCC GAAACCTCCT TTTATAGGTT   
  
  
+ AGGGGGGCAA CTAGGGTTAT CGCCCTAGGT GACGCTTGTG GCCGTCGGCC TAATTAAATA GGCCCGACCA   
  
  
+ ACAGCTGTGA CTGGAGCGAT AGCTGAAGCA TGCAGAAATG AGGATAACAT ACATATTATA GATTTCCACA   
  
  
+ TTGCATAGGG CACTCAGTGG GTTACTGTAT CGCAAGCCAT AGCAGCAAGG CTTGGTGGGC CTCCTAAAGT   
  
  
+ GCGACTTACA GGCATTGATG ATCCTGTTTC TAAGTATACT TGTGATGCTA GCTTGGAGGC TGGTGGGAAA   
  
  
+ CGATTAGCGT CTCTATTTGA AAAGTTTAAA ATACTTGTCG AGTTCAATGC ATTGCCCGTT TATGGACCCA   
  
  
+ ATGTCAGGTG GGAAATGCTG GATGTGAGGC CCAGGGAGGC TTTGGTCGTT AATTGTCCAT TACAGCTCCA   
  
  
+ TTACACTCCT GACGAGAGTG TCGATGTGAG GAACCCTAGG GATAGGCTTC TCAGAATGGT GAAATTGCTC   
  
  
+ GGTCCTAAGG TAGCCACTTT GGTTGAGCAA GAATCAAACA CCAACACTAC ACCTTTCTTG ACCCGGTTCA   
  
  
+ TAGAGACCCT TGACTACTAC TCGATTAACC ATGTTTGAGT CTATAGACGT GACCATGCCG AGAGACCGGA   
  
  
+ AGGAGAGGAT CAATGTTGAG CAGCATTGTT TAGCTAAGGA CATTGTGAAT ATCATAGCTT GCGAGGGCAA   
  
  
+ GGAGATGGTG GAGCGTCATG AGCTTTTTGG GAAATGGCAG TCGAGGTTTA CCATGGTAGG GTTCAGGCAG   
  
  
+ TACCCGTTGA GCTCATACAT AAACTCTGTG ATAAGAAGCC TACTCCGGTG TTACTCCAAG CATTATACTC   
  
  
+ TGATAGAGAA GGATAGTGCC ATGCTTCTGG GTTGGAGGGG CCGAATGCTG ATTTCAGCTT CGGCATG  

- -Up\_Stream \_Len000TTTAAA TATACTAATA CCCTCGTTTA TAATTAGCCA GATTTTGTTT TTTCTTAGCC   
  
  
- GGCTTGTTTT TTTTCCCGGG TCTTGATCAG GAAAATTACC TCAGGGTTTT ACACCATATT TAATCAGAAC   
  
  
- AAGTGAGTAA AGAGTGAGGA GAGTGAGTAA GATTGGGATT TAGAGTTAGG AGTGAGAGAC AGGAGAGATA   
  
  
- GACGCAGAGC TCGAAGAGAA AGGAGACCCG AAGGAACCTG AAAAAGCTGC GGTACTGTTA CCTTGTGACT   
  
  
- CGAGGTGGCA ACGGGGAGAG GTGTTGACAG AAGATTGCCA CCAAGAGGCA TCGGTGGCAT GGAGTTTTTG   
  
  
- GTTTCGCTAC GTGTTGATGG TGGGGTGGTG ACGACTTTTC ACGGGTGCTC AGAGCTAGGC GCGTTCGGAC   
  
  
- TACTCTTAGC TATGGTGGTG GTACATCTAT TCGACTTTTG CCCGTATTCC TTCGCTGGGT GCGCGGGTTG   
  
  
- TGCGGTAACT CCGTCACTGT GCACCCAACT ACGCGAAGGT TTCTACCTTA AAAGTGTAAA AAGAAAAGTT   
  
  
- CCAACGCATC TAGCCACACA CACCTACCGC TAGTTCCTTC TCAATTTTAC TTGATCAGTA CGCAGCTTAA   
  
  
- CAGGTTTTTT TTAAAAGAGA AAGTCAGCTC AGAATTTCCC CGTCAGTAGT TCTCGTACAA ACTTTTTGTA   
  
  
- ATACCGGTAT TGGTGAATCA TCATTAAGTT TATTAGTACC TTTGTATAAG TTGTACTTAA GTTATACTGA   
  
  
- TTTTTAAGGT AGAAAATCAA ACAAGACCAG TTGTGTCTCG TACGGAAAAA TTAACGTATT ATTGCTATAA   
  
  
- ATTGGTTGGT TAGTACAAAG AGTGTATAAT TATATCCGTT CCCATCTAAT TGTCAGTATT AGCGTATCAT   
  
  
- ACGTTCTTAA ATAAACGTTA GACCCGTATG ACAAAATATT TCGTGTTCAC AAGTACTGAA TTTTAGCTAT   
  
  
- TTCAGAATTA GAAAACAGCT AGGAACTCAC ACGTCTGTTA TATTAACACT TACATACGAT AATTGAAATT   
  
  
- ATTAATGCTT TTAATTCGTT GATTAGTATA TCAGTACAAG AGTTTTTGTT CGTACGCATA ATAAGTCTAG   
  
  
- TAATTTACGT AATTTACCCT GATTAGGCTA GTCAGTTCGC GACTATGCTG ATAACTTATT TGTCCTGATT   
  
  
- GGATCTTACG CCTTAATTGA CAGCTCTTAA CAGGTGTAGC CATCACTACG AACCGCCGAT GGTCTCAGTG   
  
  
- GAATCCCACA AGTGTAGGAT CTGGGCTTTT GGACGATTAC TACCTTCCCG TCGGCACCCT CCTCCTCTCC   
  
  
- TCTCACACCT AAAAAGATAA ATGTTTTTTC ACATATTCAC GATTAGGGGG CTTTGGAGGA AAATATCCAA   
  
  
- TCCCCCCGTT GATCCCAATA GCGGGATCCA CTGCGAACAC CGGCAGCCGG ATTAATTTAT CCGGGCTGGT   
  
  
- TGTCGACACT GACCTCGCTA TCGACTTCGT ACGTCTTTAC TCCTATTGTA TGTATAATAT CTAAAGGTGT   
  
  
- AACGTATCCC GTGAGTCACC CAATGACATA GCGTTCGGTA TCGTCGTTCC GAACCACCCG GAGGATTTCA   
  
  
- CGCTGAATGT CCGTAACTAC TAGGACAAAG ATTCATATGA ACACTACGAT CGAACCTCCG ACCACCCTTT   
  
  
- GCTAATCGCA GAGATAAACT TTTCAAATTT TATGAACAGC TCAAGTTACG TAACGGGCAA ATACCTGGGT   
  
  
- TACAGTCCAC CCTTTACGAC CTACACTCCG GGTCCCTCCG AAACCAGCAA TTAACAGGTA ATGTCGAGGT   
  
  
- AATGTGAGGA CTGCTCTCAC AGCTACACTC CTTGGGATCC CTATCCGAAG AGTCTTACCA CTTTAACGAG   
  
  
- CCAGGATTCC ATCGGTGAAA CCAACTCGTT CTTAGTTTGT GGTTGTGATG TGGAAAGAAC TGGGCCAAGT   
  
  
- ATCTCTGGGA ACTGATGATG AGCTAATTGG TACAAACTCA GATATCTGCA CTGGTACGGC TCTCTGGCCT   
  
  
- TCCTCTCCTA GTTACAACTC GTCGTAACAA ATCGATTCCT GTAACACTTA TAGTATCGAA CGCTCCCGTT   
  
  
- CCTCTACCAC CTCGCAGTAC TCGAAAAACC CTTTACCGTC AGCTCCAAAT GGTACCATCC CAAGTCCGTC   
  
  
- ATGGGCAACT CGAGTATGTA TTTGAGACAC TATTCTTCGG ATGAGGCCAC AATGAGGTTC GTAATATGAG   
  
  
- ACTATCTCTT CCTATCACGG TACGAAGACC CAACCTCCCC GGCTTACGAC TAAAGTCGAA GCCGTAC

+     AAGAA-motif

| Site Name | Organism | Position | Strand | Matrix score. | sequence | function |
| --- | --- | --- | --- | --- | --- | --- |
| AAGAA-motif | Avena sativa | 1948 | - | 9 | gGTAAAGAAA |  |

>HU08G00229.1   
+ -Up\_Stream \_Len000AAATTT ATATGATTAT GGGAGCAAAT ATTAATCGGT CTAAAACAAA AAAGAATCGG   
  
  
+ CCGAACAAAA AAAAGGGCCC AGAACTAGTC CTTTTAATGG AGTCCCAAAA TGTGGTATAA ATTAGTCTTG   
  
  
+ TTCACTCATT TCTCACTCCT CTCACTCATT CTAACCCTAA ATCTCAATCC TCACTCTCTG TCCTCTCTAT   
  
  
+ CTGCGTCTCG AGCTTCTCTT TCCTCTGGGC TTCCTTGGAC TTTTTCGACG CCATGACAAT GGAACACTGA   
  
  
+ GCTCCACCGT TGCCCCTCTC CACAACTGTC TTCTAACGGT GGTTCTCCGT AGCCACCGTA CCTCAAAAAC   
  
  
+ CAAAGCGATG CACAACTACC ACCCCACCAC TGCTGAAAAG TGCCCACGAG TCTCGATCCG CGCAAGCCTG   
  
  
+ ATGAGAATCG ATACCACCAC CATGTAGATA AGCTGAAAAC GGGCATAAGG AAGCGACCCA CGCGCCCAAC   
  
  
+ ACGCCATTGA GGCAGTGACA CGTGGGTTGA TGCGCTTCCA AAGATGGAAT TTTCACATTT TTCTTTTCAA   
  
  
+ GGTTGCGTAG ATCGGTGTGT GTGGATGGCG ATCAAGGAAG AGTTAAAATG AACTAGTCAT GCGTCGAATT   
  
  
+ GTCCAAAAAA AATTTTCTCT TTCAGTCGAG TCTTAAAGGG GCAGTCATCA AGAGCATGTT TGAAAAACAT   
  
  
+ TATGGCCATA ACCACTTAGT AGTAATTCAA ATAATCATGG AAACATATTC AACATGAATT CAATATGACT   
  
  
+ AAAAATTCCA TCTTTTAGTT TGTTCTGGTC AACACAGAGC ATGCCTTTTT AATTGCATAA TAACGATATT   
  
  
+ TAACCAACCA ATCATGTTTC TCACATATTA ATATAGGCAA GGGTAGATTA ACAGTCATAA TCGCATAGTA   
  
  
+ TGCAAGAATT TATTTGCAAT CTGGGCATAC TGTTTTATAA AGCACAAGTG TTCATGACTT AAAATCGATA   
  
  
+ AAGTCTTAAT CTTTTGTCGA TCCTTGAGTG TGCAGACAAT ATAATTGTGA ATGTATGCTA TTAACTTTAA   
  
  
+ TAATTACGAA AATTAAGCAA CTAATCATAT AGTCATGTTC TCAAAAACAA GCATGCGTAT TATTCAGATC   
  
  
+ ATTAAATGCA TTAAATGGGA CTAATCCGAT CAGTCAAGCG CTGATACGAC TATTGAATAA ACAGGACTAA   
  
  
+ CCTAGAATGC GGAATTAACT GTCGAGAATT GTCCACATCG GTAGTGATGC TTGGCGGCTA CCAGAGTCAC   
  
  
+ CTTAGGGTGT TCACATCCTA GACCCGAAAA CCTGCTAATG ATGGAAGGGC AGCCGTGGGA GGAGGAGAGG   
  
  
+ AGAGTGTGGA TTTTTCTATT TACAAAAAAG TGTATAAGTG CTAATCCCCC GAAACCTCCT TTTATAGGTT   
  
  
+ AGGGGGGCAA CTAGGGTTAT CGCCCTAGGT GACGCTTGTG GCCGTCGGCC TAATTAAATA GGCCCGACCA   
  
  
+ ACAGCTGTGA CTGGAGCGAT AGCTGAAGCA TGCAGAAATG AGGATAACAT ACATATTATA GATTTCCACA   
  
  
+ TTGCATAGGG CACTCAGTGG GTTACTGTAT CGCAAGCCAT AGCAGCAAGG CTTGGTGGGC CTCCTAAAGT   
  
  
+ GCGACTTACA GGCATTGATG ATCCTGTTTC TAAGTATACT TGTGATGCTA GCTTGGAGGC TGGTGGGAAA   
  
  
+ CGATTAGCGT CTCTATTTGA AAAGTTTAAA ATACTTGTCG AGTTCAATGC ATTGCCCGTT TATGGACCCA   
  
  
+ ATGTCAGGTG GGAAATGCTG GATGTGAGGC CCAGGGAGGC TTTGGTCGTT AATTGTCCAT TACAGCTCCA   
  
  
+ TTACACTCCT GACGAGAGTG TCGATGTGAG GAACCCTAGG GATAGGCTTC TCAGAATGGT GAAATTGCTC   
  
  
+ GGTCCTAAGG TAGCCACTTT GGTTGAGCAA GAATCAAACA CCAACACTAC ACCTTTCTTG ACCCGGTTCA   
  
  
+ TAGAGACCCT TGACTACTAC TCGATTAACC ATGTTTGAGT CTATAGACGT GACCATGCCG AGAGACCGGA   
  
  
+ AGGAGAGGAT CAATGTTGAG CAGCATTGTT TAGCTAAGGA CATTGTGAAT ATCATAGCTT GCGAGGGCAA   
  
  
+ GGAGATGGTG GAGCGTCATG AGCTTTTTGG GAAATGGCAG TCGAGGTTTA CCATGGTAGG GTTCAGGCAG   
  
  
+ TACCCGTTGA GCTCATACAT AAACTCTGTG ATAAGAAGCC TACTCCGGTG TTACTCCAAG CATTATACTC   
  
  
+ TGATAGAGAA GGATAGTGCC ATGCTTCTGG GTTGGAGGGG CCGAATGCTG ATTTCAGCTT CGGCATG  

- -Up\_Stream \_Len000TTTAAA TATACTAATA CCCTCGTTTA TAATTAGCCA GATTTTGTTT TTTCTTAGCC   
  
  
- GGCTTGTTTT TTTTCCCGGG TCTTGATCAG GAAAATTACC TCAGGGTTTT ACACCATATT TAATCAGAAC   
  
  
- AAGTGAGTAA AGAGTGAGGA GAGTGAGTAA GATTGGGATT TAGAGTTAGG AGTGAGAGAC AGGAGAGATA   
  
  
- GACGCAGAGC TCGAAGAGAA AGGAGACCCG AAGGAACCTG AAAAAGCTGC GGTACTGTTA CCTTGTGACT   
  
  
- CGAGGTGGCA ACGGGGAGAG GTGTTGACAG AAGATTGCCA CCAAGAGGCA TCGGTGGCAT GGAGTTTTTG   
  
  
- GTTTCGCTAC GTGTTGATGG TGGGGTGGTG ACGACTTTTC ACGGGTGCTC AGAGCTAGGC GCGTTCGGAC   
  
  
- TACTCTTAGC TATGGTGGTG GTACATCTAT TCGACTTTTG CCCGTATTCC TTCGCTGGGT GCGCGGGTTG   
  
  
- TGCGGTAACT CCGTCACTGT GCACCCAACT ACGCGAAGGT TTCTACCTTA AAAGTGTAAA AAGAAAAGTT   
  
  
- CCAACGCATC TAGCCACACA CACCTACCGC TAGTTCCTTC TCAATTTTAC TTGATCAGTA CGCAGCTTAA   
  
  
- CAGGTTTTTT TTAAAAGAGA AAGTCAGCTC AGAATTTCCC CGTCAGTAGT TCTCGTACAA ACTTTTTGTA   
  
  
- ATACCGGTAT TGGTGAATCA TCATTAAGTT TATTAGTACC TTTGTATAAG TTGTACTTAA GTTATACTGA   
  
  
- TTTTTAAGGT AGAAAATCAA ACAAGACCAG TTGTGTCTCG TACGGAAAAA TTAACGTATT ATTGCTATAA   
  
  
- ATTGGTTGGT TAGTACAAAG AGTGTATAAT TATATCCGTT CCCATCTAAT TGTCAGTATT AGCGTATCAT   
  
  
- ACGTTCTTAA ATAAACGTTA GACCCGTATG ACAAAATATT TCGTGTTCAC AAGTACTGAA TTTTAGCTAT   
  
  
- TTCAGAATTA GAAAACAGCT AGGAACTCAC ACGTCTGTTA TATTAACACT TACATACGAT AATTGAAATT   
  
  
- ATTAATGCTT TTAATTCGTT GATTAGTATA TCAGTACAAG AGTTTTTGTT CGTACGCATA ATAAGTCTAG   
  
  
- TAATTTACGT AATTTACCCT GATTAGGCTA GTCAGTTCGC GACTATGCTG ATAACTTATT TGTCCTGATT   
  
  
- GGATCTTACG CCTTAATTGA CAGCTCTTAA CAGGTGTAGC CATCACTACG AACCGCCGAT GGTCTCAGTG   
  
  
- GAATCCCACA AGTGTAGGAT CTGGGCTTTT GGACGATTAC TACCTTCCCG TCGGCACCCT CCTCCTCTCC   
  
  
- TCTCACACCT AAAAAGATAA ATGTTTTTTC ACATATTCAC GATTAGGGGG CTTTGGAGGA AAATATCCAA   
  
  
- TCCCCCCGTT GATCCCAATA GCGGGATCCA CTGCGAACAC CGGCAGCCGG ATTAATTTAT CCGGGCTGGT   
  
  
- TGTCGACACT GACCTCGCTA TCGACTTCGT ACGTCTTTAC TCCTATTGTA TGTATAATAT CTAAAGGTGT   
  
  
- AACGTATCCC GTGAGTCACC CAATGACATA GCGTTCGGTA TCGTCGTTCC GAACCACCCG GAGGATTTCA   
  
  
- CGCTGAATGT CCGTAACTAC TAGGACAAAG ATTCATATGA ACACTACGAT CGAACCTCCG ACCACCCTTT   
  
  
- GCTAATCGCA GAGATAAACT TTTCAAATTT TATGAACAGC TCAAGTTACG TAACGGGCAA ATACCTGGGT   
  
  
- TACAGTCCAC CCTTTACGAC CTACACTCCG GGTCCCTCCG AAACCAGCAA TTAACAGGTA ATGTCGAGGT   
  
  
- AATGTGAGGA CTGCTCTCAC AGCTACACTC CTTGGGATCC CTATCCGAAG AGTCTTACCA CTTTAACGAG   
  
  
- CCAGGATTCC ATCGGTGAAA CCAACTCGTT CTTAGTTTGT GGTTGTGATG TGGAAAGAAC TGGGCCAAGT   
  
  
- ATCTCTGGGA ACTGATGATG AGCTAATTGG TACAAACTCA GATATCTGCA CTGGTACGGC TCTCTGGCCT   
  
  
- TCCTCTCCTA GTTACAACTC GTCGTAACAA ATCGATTCCT GTAACACTTA TAGTATCGAA CGCTCCCGTT   
  
  
- CCTCTACCAC CTCGCAGTAC TCGAAAAACC CTTTACCGTC AGCTCCAAAT GGTACCATCC CAAGTCCGTC   
  
  
- ATGGGCAACT CGAGTATGTA TTTGAGACAC TATTCTTCGG ATGAGGCCAC AATGAGGTTC GTAATATGAG   
  
  
- ACTATCTCTT CCTATCACGG TACGAAGACC CAACCTCCCC GGCTTACGAC TAAAGTCGAA GCCGTAC

+     ABRE

| Site Name | Organism | Position | Strand | Matrix score. | sequence | function |
| --- | --- | --- | --- | --- | --- | --- |
| ABRE | Arabidopsis thaliana | 2011 | + | 5 | ACGTG | cis-acting element involved in the abscisic acid responsiveness |
| ABRE | Arabidopsis thaliana | 514 | + | 5 | ACGTG | cis-acting element involved in the abscisic acid responsiveness |
| ABRE | Arabidopsis thaliana | 513 | + | 6 | CACGTG | cis-acting element involved in the abscisic acid responsiveness |
| ABRE | Hordeum vulgare | 511 | - | 9 | CGCACGTGTC | cis-acting element involved in the abscisic acid responsiveness |

>HU08G00229.1   
+ -Up\_Stream \_Len000AAATTT ATATGATTAT GGGAGCAAAT ATTAATCGGT CTAAAACAAA AAAGAATCGG   
  
  
+ CCGAACAAAA AAAAGGGCCC AGAACTAGTC CTTTTAATGG AGTCCCAAAA TGTGGTATAA ATTAGTCTTG   
  
  
+ TTCACTCATT TCTCACTCCT CTCACTCATT CTAACCCTAA ATCTCAATCC TCACTCTCTG TCCTCTCTAT   
  
  
+ CTGCGTCTCG AGCTTCTCTT TCCTCTGGGC TTCCTTGGAC TTTTTCGACG CCATGACAAT GGAACACTGA   
  
  
+ GCTCCACCGT TGCCCCTCTC CACAACTGTC TTCTAACGGT GGTTCTCCGT AGCCACCGTA CCTCAAAAAC   
  
  
+ CAAAGCGATG CACAACTACC ACCCCACCAC TGCTGAAAAG TGCCCACGAG TCTCGATCCG CGCAAGCCTG   
  
  
+ ATGAGAATCG ATACCACCAC CATGTAGATA AGCTGAAAAC GGGCATAAGG AAGCGACCCA CGCGCCCAAC   
  
  
+ ACGCCATTGA GGCAGTGACA CGTGGGTTGA TGCGCTTCCA AAGATGGAAT TTTCACATTT TTCTTTTCAA   
  
  
+ GGTTGCGTAG ATCGGTGTGT GTGGATGGCG ATCAAGGAAG AGTTAAAATG AACTAGTCAT GCGTCGAATT   
  
  
+ GTCCAAAAAA AATTTTCTCT TTCAGTCGAG TCTTAAAGGG GCAGTCATCA AGAGCATGTT TGAAAAACAT   
  
  
+ TATGGCCATA ACCACTTAGT AGTAATTCAA ATAATCATGG AAACATATTC AACATGAATT CAATATGACT   
  
  
+ AAAAATTCCA TCTTTTAGTT TGTTCTGGTC AACACAGAGC ATGCCTTTTT AATTGCATAA TAACGATATT   
  
  
+ TAACCAACCA ATCATGTTTC TCACATATTA ATATAGGCAA GGGTAGATTA ACAGTCATAA TCGCATAGTA   
  
  
+ TGCAAGAATT TATTTGCAAT CTGGGCATAC TGTTTTATAA AGCACAAGTG TTCATGACTT AAAATCGATA   
  
  
+ AAGTCTTAAT CTTTTGTCGA TCCTTGAGTG TGCAGACAAT ATAATTGTGA ATGTATGCTA TTAACTTTAA   
  
  
+ TAATTACGAA AATTAAGCAA CTAATCATAT AGTCATGTTC TCAAAAACAA GCATGCGTAT TATTCAGATC   
  
  
+ ATTAAATGCA TTAAATGGGA CTAATCCGAT CAGTCAAGCG CTGATACGAC TATTGAATAA ACAGGACTAA   
  
  
+ CCTAGAATGC GGAATTAACT GTCGAGAATT GTCCACATCG GTAGTGATGC TTGGCGGCTA CCAGAGTCAC   
  
  
+ CTTAGGGTGT TCACATCCTA GACCCGAAAA CCTGCTAATG ATGGAAGGGC AGCCGTGGGA GGAGGAGAGG   
  
  
+ AGAGTGTGGA TTTTTCTATT TACAAAAAAG TGTATAAGTG CTAATCCCCC GAAACCTCCT TTTATAGGTT   
  
  
+ AGGGGGGCAA CTAGGGTTAT CGCCCTAGGT GACGCTTGTG GCCGTCGGCC TAATTAAATA GGCCCGACCA   
  
  
+ ACAGCTGTGA CTGGAGCGAT AGCTGAAGCA TGCAGAAATG AGGATAACAT ACATATTATA GATTTCCACA   
  
  
+ TTGCATAGGG CACTCAGTGG GTTACTGTAT CGCAAGCCAT AGCAGCAAGG CTTGGTGGGC CTCCTAAAGT   
  
  
+ GCGACTTACA GGCATTGATG ATCCTGTTTC TAAGTATACT TGTGATGCTA GCTTGGAGGC TGGTGGGAAA   
  
  
+ CGATTAGCGT CTCTATTTGA AAAGTTTAAA ATACTTGTCG AGTTCAATGC ATTGCCCGTT TATGGACCCA   
  
  
+ ATGTCAGGTG GGAAATGCTG GATGTGAGGC CCAGGGAGGC TTTGGTCGTT AATTGTCCAT TACAGCTCCA   
  
  
+ TTACACTCCT GACGAGAGTG TCGATGTGAG GAACCCTAGG GATAGGCTTC TCAGAATGGT GAAATTGCTC   
  
  
+ GGTCCTAAGG TAGCCACTTT GGTTGAGCAA GAATCAAACA CCAACACTAC ACCTTTCTTG ACCCGGTTCA   
  
  
+ TAGAGACCCT TGACTACTAC TCGATTAACC ATGTTTGAGT CTATAGACGT GACCATGCCG AGAGACCGGA   
  
  
+ AGGAGAGGAT CAATGTTGAG CAGCATTGTT TAGCTAAGGA CATTGTGAAT ATCATAGCTT GCGAGGGCAA   
  
  
+ GGAGATGGTG GAGCGTCATG AGCTTTTTGG GAAATGGCAG TCGAGGTTTA CCATGGTAGG GTTCAGGCAG   
  
  
+ TACCCGTTGA GCTCATACAT AAACTCTGTG ATAAGAAGCC TACTCCGGTG TTACTCCAAG CATTATACTC   
  
  
+ TGATAGAGAA GGATAGTGCC ATGCTTCTGG GTTGGAGGGG CCGAATGCTG ATTTCAGCTT CGGCATG  

- -Up\_Stream \_Len000TTTAAA TATACTAATA CCCTCGTTTA TAATTAGCCA GATTTTGTTT TTTCTTAGCC   
  
  
- GGCTTGTTTT TTTTCCCGGG TCTTGATCAG GAAAATTACC TCAGGGTTTT ACACCATATT TAATCAGAAC   
  
  
- AAGTGAGTAA AGAGTGAGGA GAGTGAGTAA GATTGGGATT TAGAGTTAGG AGTGAGAGAC AGGAGAGATA   
  
  
- GACGCAGAGC TCGAAGAGAA AGGAGACCCG AAGGAACCTG AAAAAGCTGC GGTACTGTTA CCTTGTGACT   
  
  
- CGAGGTGGCA ACGGGGAGAG GTGTTGACAG AAGATTGCCA CCAAGAGGCA TCGGTGGCAT GGAGTTTTTG   
  
  
- GTTTCGCTAC GTGTTGATGG TGGGGTGGTG ACGACTTTTC ACGGGTGCTC AGAGCTAGGC GCGTTCGGAC   
  
  
- TACTCTTAGC TATGGTGGTG GTACATCTAT TCGACTTTTG CCCGTATTCC TTCGCTGGGT GCGCGGGTTG   
  
  
- TGCGGTAACT CCGTCACTGT GCACCCAACT ACGCGAAGGT TTCTACCTTA AAAGTGTAAA AAGAAAAGTT   
  
  
- CCAACGCATC TAGCCACACA CACCTACCGC TAGTTCCTTC TCAATTTTAC TTGATCAGTA CGCAGCTTAA   
  
  
- CAGGTTTTTT TTAAAAGAGA AAGTCAGCTC AGAATTTCCC CGTCAGTAGT TCTCGTACAA ACTTTTTGTA   
  
  
- ATACCGGTAT TGGTGAATCA TCATTAAGTT TATTAGTACC TTTGTATAAG TTGTACTTAA GTTATACTGA   
  
  
- TTTTTAAGGT AGAAAATCAA ACAAGACCAG TTGTGTCTCG TACGGAAAAA TTAACGTATT ATTGCTATAA   
  
  
- ATTGGTTGGT TAGTACAAAG AGTGTATAAT TATATCCGTT CCCATCTAAT TGTCAGTATT AGCGTATCAT   
  
  
- ACGTTCTTAA ATAAACGTTA GACCCGTATG ACAAAATATT TCGTGTTCAC AAGTACTGAA TTTTAGCTAT   
  
  
- TTCAGAATTA GAAAACAGCT AGGAACTCAC ACGTCTGTTA TATTAACACT TACATACGAT AATTGAAATT   
  
  
- ATTAATGCTT TTAATTCGTT GATTAGTATA TCAGTACAAG AGTTTTTGTT CGTACGCATA ATAAGTCTAG   
  
  
- TAATTTACGT AATTTACCCT GATTAGGCTA GTCAGTTCGC GACTATGCTG ATAACTTATT TGTCCTGATT   
  
  
- GGATCTTACG CCTTAATTGA CAGCTCTTAA CAGGTGTAGC CATCACTACG AACCGCCGAT GGTCTCAGTG   
  
  
- GAATCCCACA AGTGTAGGAT CTGGGCTTTT GGACGATTAC TACCTTCCCG TCGGCACCCT CCTCCTCTCC   
  
  
- TCTCACACCT AAAAAGATAA ATGTTTTTTC ACATATTCAC GATTAGGGGG CTTTGGAGGA AAATATCCAA   
  
  
- TCCCCCCGTT GATCCCAATA GCGGGATCCA CTGCGAACAC CGGCAGCCGG ATTAATTTAT CCGGGCTGGT   
  
  
- TGTCGACACT GACCTCGCTA TCGACTTCGT ACGTCTTTAC TCCTATTGTA TGTATAATAT CTAAAGGTGT   
  
  
- AACGTATCCC GTGAGTCACC CAATGACATA GCGTTCGGTA TCGTCGTTCC GAACCACCCG GAGGATTTCA   
  
  
- CGCTGAATGT CCGTAACTAC TAGGACAAAG ATTCATATGA ACACTACGAT CGAACCTCCG ACCACCCTTT   
  
  
- GCTAATCGCA GAGATAAACT TTTCAAATTT TATGAACAGC TCAAGTTACG TAACGGGCAA ATACCTGGGT   
  
  
- TACAGTCCAC CCTTTACGAC CTACACTCCG GGTCCCTCCG AAACCAGCAA TTAACAGGTA ATGTCGAGGT   
  
  
- AATGTGAGGA CTGCTCTCAC AGCTACACTC CTTGGGATCC CTATCCGAAG AGTCTTACCA CTTTAACGAG   
  
  
- CCAGGATTCC ATCGGTGAAA CCAACTCGTT CTTAGTTTGT GGTTGTGATG TGGAAAGAAC TGGGCCAAGT   
  
  
- ATCTCTGGGA ACTGATGATG AGCTAATTGG TACAAACTCA GATATCTGCA CTGGTACGGC TCTCTGGCCT   
  
  
- TCCTCTCCTA GTTACAACTC GTCGTAACAA ATCGATTCCT GTAACACTTA TAGTATCGAA CGCTCCCGTT   
  
  
- CCTCTACCAC CTCGCAGTAC TCGAAAAACC CTTTACCGTC AGCTCCAAAT GGTACCATCC CAAGTCCGTC   
  
  
- ATGGGCAACT CGAGTATGTA TTTGAGACAC TATTCTTCGG ATGAGGCCAC AATGAGGTTC GTAATATGAG   
  
  
- ACTATCTCTT CCTATCACGG TACGAAGACC CAACCTCCCC GGCTTACGAC TAAAGTCGAA GCCGTAC

+     ARE

| Site Name | Organism | Position | Strand | Matrix score. | sequence | function |
| --- | --- | --- | --- | --- | --- | --- |
| ARE | Zea mays | 351 | + | 6 | AAACCA | cis-acting regulatory element essential for the anaerobic induction |

>HU08G00229.1   
+ -Up\_Stream \_Len000AAATTT ATATGATTAT GGGAGCAAAT ATTAATCGGT CTAAAACAAA AAAGAATCGG   
  
  
+ CCGAACAAAA AAAAGGGCCC AGAACTAGTC CTTTTAATGG AGTCCCAAAA TGTGGTATAA ATTAGTCTTG   
  
  
+ TTCACTCATT TCTCACTCCT CTCACTCATT CTAACCCTAA ATCTCAATCC TCACTCTCTG TCCTCTCTAT   
  
  
+ CTGCGTCTCG AGCTTCTCTT TCCTCTGGGC TTCCTTGGAC TTTTTCGACG CCATGACAAT GGAACACTGA   
  
  
+ GCTCCACCGT TGCCCCTCTC CACAACTGTC TTCTAACGGT GGTTCTCCGT AGCCACCGTA CCTCAAAAAC   
  
  
+ CAAAGCGATG CACAACTACC ACCCCACCAC TGCTGAAAAG TGCCCACGAG TCTCGATCCG CGCAAGCCTG   
  
  
+ ATGAGAATCG ATACCACCAC CATGTAGATA AGCTGAAAAC GGGCATAAGG AAGCGACCCA CGCGCCCAAC   
  
  
+ ACGCCATTGA GGCAGTGACA CGTGGGTTGA TGCGCTTCCA AAGATGGAAT TTTCACATTT TTCTTTTCAA   
  
  
+ GGTTGCGTAG ATCGGTGTGT GTGGATGGCG ATCAAGGAAG AGTTAAAATG AACTAGTCAT GCGTCGAATT   
  
  
+ GTCCAAAAAA AATTTTCTCT TTCAGTCGAG TCTTAAAGGG GCAGTCATCA AGAGCATGTT TGAAAAACAT   
  
  
+ TATGGCCATA ACCACTTAGT AGTAATTCAA ATAATCATGG AAACATATTC AACATGAATT CAATATGACT   
  
  
+ AAAAATTCCA TCTTTTAGTT TGTTCTGGTC AACACAGAGC ATGCCTTTTT AATTGCATAA TAACGATATT   
  
  
+ TAACCAACCA ATCATGTTTC TCACATATTA ATATAGGCAA GGGTAGATTA ACAGTCATAA TCGCATAGTA   
  
  
+ TGCAAGAATT TATTTGCAAT CTGGGCATAC TGTTTTATAA AGCACAAGTG TTCATGACTT AAAATCGATA   
  
  
+ AAGTCTTAAT CTTTTGTCGA TCCTTGAGTG TGCAGACAAT ATAATTGTGA ATGTATGCTA TTAACTTTAA   
  
  
+ TAATTACGAA AATTAAGCAA CTAATCATAT AGTCATGTTC TCAAAAACAA GCATGCGTAT TATTCAGATC   
  
  
+ ATTAAATGCA TTAAATGGGA CTAATCCGAT CAGTCAAGCG CTGATACGAC TATTGAATAA ACAGGACTAA   
  
  
+ CCTAGAATGC GGAATTAACT GTCGAGAATT GTCCACATCG GTAGTGATGC TTGGCGGCTA CCAGAGTCAC   
  
  
+ CTTAGGGTGT TCACATCCTA GACCCGAAAA CCTGCTAATG ATGGAAGGGC AGCCGTGGGA GGAGGAGAGG   
  
  
+ AGAGTGTGGA TTTTTCTATT TACAAAAAAG TGTATAAGTG CTAATCCCCC GAAACCTCCT TTTATAGGTT   
  
  
+ AGGGGGGCAA CTAGGGTTAT CGCCCTAGGT GACGCTTGTG GCCGTCGGCC TAATTAAATA GGCCCGACCA   
  
  
+ ACAGCTGTGA CTGGAGCGAT AGCTGAAGCA TGCAGAAATG AGGATAACAT ACATATTATA GATTTCCACA   
  
  
+ TTGCATAGGG CACTCAGTGG GTTACTGTAT CGCAAGCCAT AGCAGCAAGG CTTGGTGGGC CTCCTAAAGT   
  
  
+ GCGACTTACA GGCATTGATG ATCCTGTTTC TAAGTATACT TGTGATGCTA GCTTGGAGGC TGGTGGGAAA   
  
  
+ CGATTAGCGT CTCTATTTGA AAAGTTTAAA ATACTTGTCG AGTTCAATGC ATTGCCCGTT TATGGACCCA   
  
  
+ ATGTCAGGTG GGAAATGCTG GATGTGAGGC CCAGGGAGGC TTTGGTCGTT AATTGTCCAT TACAGCTCCA   
  
  
+ TTACACTCCT GACGAGAGTG TCGATGTGAG GAACCCTAGG GATAGGCTTC TCAGAATGGT GAAATTGCTC   
  
  
+ GGTCCTAAGG TAGCCACTTT GGTTGAGCAA GAATCAAACA CCAACACTAC ACCTTTCTTG ACCCGGTTCA   
  
  
+ TAGAGACCCT TGACTACTAC TCGATTAACC ATGTTTGAGT CTATAGACGT GACCATGCCG AGAGACCGGA   
  
  
+ AGGAGAGGAT CAATGTTGAG CAGCATTGTT TAGCTAAGGA CATTGTGAAT ATCATAGCTT GCGAGGGCAA   
  
  
+ GGAGATGGTG GAGCGTCATG AGCTTTTTGG GAAATGGCAG TCGAGGTTTA CCATGGTAGG GTTCAGGCAG   
  
  
+ TACCCGTTGA GCTCATACAT AAACTCTGTG ATAAGAAGCC TACTCCGGTG TTACTCCAAG CATTATACTC   
  
  
+ TGATAGAGAA GGATAGTGCC ATGCTTCTGG GTTGGAGGGG CCGAATGCTG ATTTCAGCTT CGGCATG  

- -Up\_Stream \_Len000TTTAAA TATACTAATA CCCTCGTTTA TAATTAGCCA GATTTTGTTT TTTCTTAGCC   
  
  
- GGCTTGTTTT TTTTCCCGGG TCTTGATCAG GAAAATTACC TCAGGGTTTT ACACCATATT TAATCAGAAC   
  
  
- AAGTGAGTAA AGAGTGAGGA GAGTGAGTAA GATTGGGATT TAGAGTTAGG AGTGAGAGAC AGGAGAGATA   
  
  
- GACGCAGAGC TCGAAGAGAA AGGAGACCCG AAGGAACCTG AAAAAGCTGC GGTACTGTTA CCTTGTGACT   
  
  
- CGAGGTGGCA ACGGGGAGAG GTGTTGACAG AAGATTGCCA CCAAGAGGCA TCGGTGGCAT GGAGTTTTTG   
  
  
- GTTTCGCTAC GTGTTGATGG TGGGGTGGTG ACGACTTTTC ACGGGTGCTC AGAGCTAGGC GCGTTCGGAC   
  
  
- TACTCTTAGC TATGGTGGTG GTACATCTAT TCGACTTTTG CCCGTATTCC TTCGCTGGGT GCGCGGGTTG   
  
  
- TGCGGTAACT CCGTCACTGT GCACCCAACT ACGCGAAGGT TTCTACCTTA AAAGTGTAAA AAGAAAAGTT   
  
  
- CCAACGCATC TAGCCACACA CACCTACCGC TAGTTCCTTC TCAATTTTAC TTGATCAGTA CGCAGCTTAA   
  
  
- CAGGTTTTTT TTAAAAGAGA AAGTCAGCTC AGAATTTCCC CGTCAGTAGT TCTCGTACAA ACTTTTTGTA   
  
  
- ATACCGGTAT TGGTGAATCA TCATTAAGTT TATTAGTACC TTTGTATAAG TTGTACTTAA GTTATACTGA   
  
  
- TTTTTAAGGT AGAAAATCAA ACAAGACCAG TTGTGTCTCG TACGGAAAAA TTAACGTATT ATTGCTATAA   
  
  
- ATTGGTTGGT TAGTACAAAG AGTGTATAAT TATATCCGTT CCCATCTAAT TGTCAGTATT AGCGTATCAT   
  
  
- ACGTTCTTAA ATAAACGTTA GACCCGTATG ACAAAATATT TCGTGTTCAC AAGTACTGAA TTTTAGCTAT   
  
  
- TTCAGAATTA GAAAACAGCT AGGAACTCAC ACGTCTGTTA TATTAACACT TACATACGAT AATTGAAATT   
  
  
- ATTAATGCTT TTAATTCGTT GATTAGTATA TCAGTACAAG AGTTTTTGTT CGTACGCATA ATAAGTCTAG   
  
  
- TAATTTACGT AATTTACCCT GATTAGGCTA GTCAGTTCGC GACTATGCTG ATAACTTATT TGTCCTGATT   
  
  
- GGATCTTACG CCTTAATTGA CAGCTCTTAA CAGGTGTAGC CATCACTACG AACCGCCGAT GGTCTCAGTG   
  
  
- GAATCCCACA AGTGTAGGAT CTGGGCTTTT GGACGATTAC TACCTTCCCG TCGGCACCCT CCTCCTCTCC   
  
  
- TCTCACACCT AAAAAGATAA ATGTTTTTTC ACATATTCAC GATTAGGGGG CTTTGGAGGA AAATATCCAA   
  
  
- TCCCCCCGTT GATCCCAATA GCGGGATCCA CTGCGAACAC CGGCAGCCGG ATTAATTTAT CCGGGCTGGT   
  
  
- TGTCGACACT GACCTCGCTA TCGACTTCGT ACGTCTTTAC TCCTATTGTA TGTATAATAT CTAAAGGTGT   
  
  
- AACGTATCCC GTGAGTCACC CAATGACATA GCGTTCGGTA TCGTCGTTCC GAACCACCCG GAGGATTTCA   
  
  
- CGCTGAATGT CCGTAACTAC TAGGACAAAG ATTCATATGA ACACTACGAT CGAACCTCCG ACCACCCTTT   
  
  
- GCTAATCGCA GAGATAAACT TTTCAAATTT TATGAACAGC TCAAGTTACG TAACGGGCAA ATACCTGGGT   
  
  
- TACAGTCCAC CCTTTACGAC CTACACTCCG GGTCCCTCCG AAACCAGCAA TTAACAGGTA ATGTCGAGGT   
  
  
- AATGTGAGGA CTGCTCTCAC AGCTACACTC CTTGGGATCC CTATCCGAAG AGTCTTACCA CTTTAACGAG   
  
  
- CCAGGATTCC ATCGGTGAAA CCAACTCGTT CTTAGTTTGT GGTTGTGATG TGGAAAGAAC TGGGCCAAGT   
  
  
- ATCTCTGGGA ACTGATGATG AGCTAATTGG TACAAACTCA GATATCTGCA CTGGTACGGC TCTCTGGCCT   
  
  
- TCCTCTCCTA GTTACAACTC GTCGTAACAA ATCGATTCCT GTAACACTTA TAGTATCGAA CGCTCCCGTT   
  
  
- CCTCTACCAC CTCGCAGTAC TCGAAAAACC CTTTACCGTC AGCTCCAAAT GGTACCATCC CAAGTCCGTC   
  
  
- ATGGGCAACT CGAGTATGTA TTTGAGACAC TATTCTTCGG ATGAGGCCAC AATGAGGTTC GTAATATGAG   
  
  
- ACTATCTCTT CCTATCACGG TACGAAGACC CAACCTCCCC GGCTTACGAC TAAAGTCGAA GCCGTAC

+     AT-rich sequence

| Site Name | Organism | Position | Strand | Matrix score. | sequence | function |
| --- | --- | --- | --- | --- | --- | --- |
| AT-rich sequence | Pisum sativum | 1711 | + | 9 | TAAAATACT | element for maximal elicitor-mediated activation (2copies) |

>HU08G00229.1   
+ -Up\_Stream \_Len000AAATTT ATATGATTAT GGGAGCAAAT ATTAATCGGT CTAAAACAAA AAAGAATCGG   
  
  
+ CCGAACAAAA AAAAGGGCCC AGAACTAGTC CTTTTAATGG AGTCCCAAAA TGTGGTATAA ATTAGTCTTG   
  
  
+ TTCACTCATT TCTCACTCCT CTCACTCATT CTAACCCTAA ATCTCAATCC TCACTCTCTG TCCTCTCTAT   
  
  
+ CTGCGTCTCG AGCTTCTCTT TCCTCTGGGC TTCCTTGGAC TTTTTCGACG CCATGACAAT GGAACACTGA   
  
  
+ GCTCCACCGT TGCCCCTCTC CACAACTGTC TTCTAACGGT GGTTCTCCGT AGCCACCGTA CCTCAAAAAC   
  
  
+ CAAAGCGATG CACAACTACC ACCCCACCAC TGCTGAAAAG TGCCCACGAG TCTCGATCCG CGCAAGCCTG   
  
  
+ ATGAGAATCG ATACCACCAC CATGTAGATA AGCTGAAAAC GGGCATAAGG AAGCGACCCA CGCGCCCAAC   
  
  
+ ACGCCATTGA GGCAGTGACA CGTGGGTTGA TGCGCTTCCA AAGATGGAAT TTTCACATTT TTCTTTTCAA   
  
  
+ GGTTGCGTAG ATCGGTGTGT GTGGATGGCG ATCAAGGAAG AGTTAAAATG AACTAGTCAT GCGTCGAATT   
  
  
+ GTCCAAAAAA AATTTTCTCT TTCAGTCGAG TCTTAAAGGG GCAGTCATCA AGAGCATGTT TGAAAAACAT   
  
  
+ TATGGCCATA ACCACTTAGT AGTAATTCAA ATAATCATGG AAACATATTC AACATGAATT CAATATGACT   
  
  
+ AAAAATTCCA TCTTTTAGTT TGTTCTGGTC AACACAGAGC ATGCCTTTTT AATTGCATAA TAACGATATT   
  
  
+ TAACCAACCA ATCATGTTTC TCACATATTA ATATAGGCAA GGGTAGATTA ACAGTCATAA TCGCATAGTA   
  
  
+ TGCAAGAATT TATTTGCAAT CTGGGCATAC TGTTTTATAA AGCACAAGTG TTCATGACTT AAAATCGATA   
  
  
+ AAGTCTTAAT CTTTTGTCGA TCCTTGAGTG TGCAGACAAT ATAATTGTGA ATGTATGCTA TTAACTTTAA   
  
  
+ TAATTACGAA AATTAAGCAA CTAATCATAT AGTCATGTTC TCAAAAACAA GCATGCGTAT TATTCAGATC   
  
  
+ ATTAAATGCA TTAAATGGGA CTAATCCGAT CAGTCAAGCG CTGATACGAC TATTGAATAA ACAGGACTAA   
  
  
+ CCTAGAATGC GGAATTAACT GTCGAGAATT GTCCACATCG GTAGTGATGC TTGGCGGCTA CCAGAGTCAC   
  
  
+ CTTAGGGTGT TCACATCCTA GACCCGAAAA CCTGCTAATG ATGGAAGGGC AGCCGTGGGA GGAGGAGAGG   
  
  
+ AGAGTGTGGA TTTTTCTATT TACAAAAAAG TGTATAAGTG CTAATCCCCC GAAACCTCCT TTTATAGGTT   
  
  
+ AGGGGGGCAA CTAGGGTTAT CGCCCTAGGT GACGCTTGTG GCCGTCGGCC TAATTAAATA GGCCCGACCA   
  
  
+ ACAGCTGTGA CTGGAGCGAT AGCTGAAGCA TGCAGAAATG AGGATAACAT ACATATTATA GATTTCCACA   
  
  
+ TTGCATAGGG CACTCAGTGG GTTACTGTAT CGCAAGCCAT AGCAGCAAGG CTTGGTGGGC CTCCTAAAGT   
  
  
+ GCGACTTACA GGCATTGATG ATCCTGTTTC TAAGTATACT TGTGATGCTA GCTTGGAGGC TGGTGGGAAA   
  
  
+ CGATTAGCGT CTCTATTTGA AAAGTTTAAA ATACTTGTCG AGTTCAATGC ATTGCCCGTT TATGGACCCA   
  
  
+ ATGTCAGGTG GGAAATGCTG GATGTGAGGC CCAGGGAGGC TTTGGTCGTT AATTGTCCAT TACAGCTCCA   
  
  
+ TTACACTCCT GACGAGAGTG TCGATGTGAG GAACCCTAGG GATAGGCTTC TCAGAATGGT GAAATTGCTC   
  
  
+ GGTCCTAAGG TAGCCACTTT GGTTGAGCAA GAATCAAACA CCAACACTAC ACCTTTCTTG ACCCGGTTCA   
  
  
+ TAGAGACCCT TGACTACTAC TCGATTAACC ATGTTTGAGT CTATAGACGT GACCATGCCG AGAGACCGGA   
  
  
+ AGGAGAGGAT CAATGTTGAG CAGCATTGTT TAGCTAAGGA CATTGTGAAT ATCATAGCTT GCGAGGGCAA   
  
  
+ GGAGATGGTG GAGCGTCATG AGCTTTTTGG GAAATGGCAG TCGAGGTTTA CCATGGTAGG GTTCAGGCAG   
  
  
+ TACCCGTTGA GCTCATACAT AAACTCTGTG ATAAGAAGCC TACTCCGGTG TTACTCCAAG CATTATACTC   
  
  
+ TGATAGAGAA GGATAGTGCC ATGCTTCTGG GTTGGAGGGG CCGAATGCTG ATTTCAGCTT CGGCATG  

- -Up\_Stream \_Len000TTTAAA TATACTAATA CCCTCGTTTA TAATTAGCCA GATTTTGTTT TTTCTTAGCC   
  
  
- GGCTTGTTTT TTTTCCCGGG TCTTGATCAG GAAAATTACC TCAGGGTTTT ACACCATATT TAATCAGAAC   
  
  
- AAGTGAGTAA AGAGTGAGGA GAGTGAGTAA GATTGGGATT TAGAGTTAGG AGTGAGAGAC AGGAGAGATA   
  
  
- GACGCAGAGC TCGAAGAGAA AGGAGACCCG AAGGAACCTG AAAAAGCTGC GGTACTGTTA CCTTGTGACT   
  
  
- CGAGGTGGCA ACGGGGAGAG GTGTTGACAG AAGATTGCCA CCAAGAGGCA TCGGTGGCAT GGAGTTTTTG   
  
  
- GTTTCGCTAC GTGTTGATGG TGGGGTGGTG ACGACTTTTC ACGGGTGCTC AGAGCTAGGC GCGTTCGGAC   
  
  
- TACTCTTAGC TATGGTGGTG GTACATCTAT TCGACTTTTG CCCGTATTCC TTCGCTGGGT GCGCGGGTTG   
  
  
- TGCGGTAACT CCGTCACTGT GCACCCAACT ACGCGAAGGT TTCTACCTTA AAAGTGTAAA AAGAAAAGTT   
  
  
- CCAACGCATC TAGCCACACA CACCTACCGC TAGTTCCTTC TCAATTTTAC TTGATCAGTA CGCAGCTTAA   
  
  
- CAGGTTTTTT TTAAAAGAGA AAGTCAGCTC AGAATTTCCC CGTCAGTAGT TCTCGTACAA ACTTTTTGTA   
  
  
- ATACCGGTAT TGGTGAATCA TCATTAAGTT TATTAGTACC TTTGTATAAG TTGTACTTAA GTTATACTGA   
  
  
- TTTTTAAGGT AGAAAATCAA ACAAGACCAG TTGTGTCTCG TACGGAAAAA TTAACGTATT ATTGCTATAA   
  
  
- ATTGGTTGGT TAGTACAAAG AGTGTATAAT TATATCCGTT CCCATCTAAT TGTCAGTATT AGCGTATCAT   
  
  
- ACGTTCTTAA ATAAACGTTA GACCCGTATG ACAAAATATT TCGTGTTCAC AAGTACTGAA TTTTAGCTAT   
  
  
- TTCAGAATTA GAAAACAGCT AGGAACTCAC ACGTCTGTTA TATTAACACT TACATACGAT AATTGAAATT   
  
  
- ATTAATGCTT TTAATTCGTT GATTAGTATA TCAGTACAAG AGTTTTTGTT CGTACGCATA ATAAGTCTAG   
  
  
- TAATTTACGT AATTTACCCT GATTAGGCTA GTCAGTTCGC GACTATGCTG ATAACTTATT TGTCCTGATT   
  
  
- GGATCTTACG CCTTAATTGA CAGCTCTTAA CAGGTGTAGC CATCACTACG AACCGCCGAT GGTCTCAGTG   
  
  
- GAATCCCACA AGTGTAGGAT CTGGGCTTTT GGACGATTAC TACCTTCCCG TCGGCACCCT CCTCCTCTCC   
  
  
- TCTCACACCT AAAAAGATAA ATGTTTTTTC ACATATTCAC GATTAGGGGG CTTTGGAGGA AAATATCCAA   
  
  
- TCCCCCCGTT GATCCCAATA GCGGGATCCA CTGCGAACAC CGGCAGCCGG ATTAATTTAT CCGGGCTGGT   
  
  
- TGTCGACACT GACCTCGCTA TCGACTTCGT ACGTCTTTAC TCCTATTGTA TGTATAATAT CTAAAGGTGT   
  
  
- AACGTATCCC GTGAGTCACC CAATGACATA GCGTTCGGTA TCGTCGTTCC GAACCACCCG GAGGATTTCA   
  
  
- CGCTGAATGT CCGTAACTAC TAGGACAAAG ATTCATATGA ACACTACGAT CGAACCTCCG ACCACCCTTT   
  
  
- GCTAATCGCA GAGATAAACT TTTCAAATTT TATGAACAGC TCAAGTTACG TAACGGGCAA ATACCTGGGT   
  
  
- TACAGTCCAC CCTTTACGAC CTACACTCCG GGTCCCTCCG AAACCAGCAA TTAACAGGTA ATGTCGAGGT   
  
  
- AATGTGAGGA CTGCTCTCAC AGCTACACTC CTTGGGATCC CTATCCGAAG AGTCTTACCA CTTTAACGAG   
  
  
- CCAGGATTCC ATCGGTGAAA CCAACTCGTT CTTAGTTTGT GGTTGTGATG TGGAAAGAAC TGGGCCAAGT   
  
  
- ATCTCTGGGA ACTGATGATG AGCTAATTGG TACAAACTCA GATATCTGCA CTGGTACGGC TCTCTGGCCT   
  
  
- TCCTCTCCTA GTTACAACTC GTCGTAACAA ATCGATTCCT GTAACACTTA TAGTATCGAA CGCTCCCGTT   
  
  
- CCTCTACCAC CTCGCAGTAC TCGAAAAACC CTTTACCGTC AGCTCCAAAT GGTACCATCC CAAGTCCGTC   
  
  
- ATGGGCAACT CGAGTATGTA TTTGAGACAC TATTCTTCGG ATGAGGCCAC AATGAGGTTC GTAATATGAG   
  
  
- ACTATCTCTT CCTATCACGG TACGAAGACC CAACCTCCCC GGCTTACGAC TAAAGTCGAA GCCGTAC

+     AuxRR-core

| Site Name | Organism | Position | Strand | Matrix score. | sequence | function |
| --- | --- | --- | --- | --- | --- | --- |
| AuxRR-core | Nicotiana tabacum | 1746 | - | 7 | GGTCCAT | cis-acting regulatory element involved in auxin responsiveness |

>HU08G00229.1   
+ -Up\_Stream \_Len000AAATTT ATATGATTAT GGGAGCAAAT ATTAATCGGT CTAAAACAAA AAAGAATCGG   
  
  
+ CCGAACAAAA AAAAGGGCCC AGAACTAGTC CTTTTAATGG AGTCCCAAAA TGTGGTATAA ATTAGTCTTG   
  
  
+ TTCACTCATT TCTCACTCCT CTCACTCATT CTAACCCTAA ATCTCAATCC TCACTCTCTG TCCTCTCTAT   
  
  
+ CTGCGTCTCG AGCTTCTCTT TCCTCTGGGC TTCCTTGGAC TTTTTCGACG CCATGACAAT GGAACACTGA   
  
  
+ GCTCCACCGT TGCCCCTCTC CACAACTGTC TTCTAACGGT GGTTCTCCGT AGCCACCGTA CCTCAAAAAC   
  
  
+ CAAAGCGATG CACAACTACC ACCCCACCAC TGCTGAAAAG TGCCCACGAG TCTCGATCCG CGCAAGCCTG   
  
  
+ ATGAGAATCG ATACCACCAC CATGTAGATA AGCTGAAAAC GGGCATAAGG AAGCGACCCA CGCGCCCAAC   
  
  
+ ACGCCATTGA GGCAGTGACA CGTGGGTTGA TGCGCTTCCA AAGATGGAAT TTTCACATTT TTCTTTTCAA   
  
  
+ GGTTGCGTAG ATCGGTGTGT GTGGATGGCG ATCAAGGAAG AGTTAAAATG AACTAGTCAT GCGTCGAATT   
  
  
+ GTCCAAAAAA AATTTTCTCT TTCAGTCGAG TCTTAAAGGG GCAGTCATCA AGAGCATGTT TGAAAAACAT   
  
  
+ TATGGCCATA ACCACTTAGT AGTAATTCAA ATAATCATGG AAACATATTC AACATGAATT CAATATGACT   
  
  
+ AAAAATTCCA TCTTTTAGTT TGTTCTGGTC AACACAGAGC ATGCCTTTTT AATTGCATAA TAACGATATT   
  
  
+ TAACCAACCA ATCATGTTTC TCACATATTA ATATAGGCAA GGGTAGATTA ACAGTCATAA TCGCATAGTA   
  
  
+ TGCAAGAATT TATTTGCAAT CTGGGCATAC TGTTTTATAA AGCACAAGTG TTCATGACTT AAAATCGATA   
  
  
+ AAGTCTTAAT CTTTTGTCGA TCCTTGAGTG TGCAGACAAT ATAATTGTGA ATGTATGCTA TTAACTTTAA   
  
  
+ TAATTACGAA AATTAAGCAA CTAATCATAT AGTCATGTTC TCAAAAACAA GCATGCGTAT TATTCAGATC   
  
  
+ ATTAAATGCA TTAAATGGGA CTAATCCGAT CAGTCAAGCG CTGATACGAC TATTGAATAA ACAGGACTAA   
  
  
+ CCTAGAATGC GGAATTAACT GTCGAGAATT GTCCACATCG GTAGTGATGC TTGGCGGCTA CCAGAGTCAC   
  
  
+ CTTAGGGTGT TCACATCCTA GACCCGAAAA CCTGCTAATG ATGGAAGGGC AGCCGTGGGA GGAGGAGAGG   
  
  
+ AGAGTGTGGA TTTTTCTATT TACAAAAAAG TGTATAAGTG CTAATCCCCC GAAACCTCCT TTTATAGGTT   
  
  
+ AGGGGGGCAA CTAGGGTTAT CGCCCTAGGT GACGCTTGTG GCCGTCGGCC TAATTAAATA GGCCCGACCA   
  
  
+ ACAGCTGTGA CTGGAGCGAT AGCTGAAGCA TGCAGAAATG AGGATAACAT ACATATTATA GATTTCCACA   
  
  
+ TTGCATAGGG CACTCAGTGG GTTACTGTAT CGCAAGCCAT AGCAGCAAGG CTTGGTGGGC CTCCTAAAGT   
  
  
+ GCGACTTACA GGCATTGATG ATCCTGTTTC TAAGTATACT TGTGATGCTA GCTTGGAGGC TGGTGGGAAA   
  
  
+ CGATTAGCGT CTCTATTTGA AAAGTTTAAA ATACTTGTCG AGTTCAATGC ATTGCCCGTT TATGGACCCA   
  
  
+ ATGTCAGGTG GGAAATGCTG GATGTGAGGC CCAGGGAGGC TTTGGTCGTT AATTGTCCAT TACAGCTCCA   
  
  
+ TTACACTCCT GACGAGAGTG TCGATGTGAG GAACCCTAGG GATAGGCTTC TCAGAATGGT GAAATTGCTC   
  
  
+ GGTCCTAAGG TAGCCACTTT GGTTGAGCAA GAATCAAACA CCAACACTAC ACCTTTCTTG ACCCGGTTCA   
  
  
+ TAGAGACCCT TGACTACTAC TCGATTAACC ATGTTTGAGT CTATAGACGT GACCATGCCG AGAGACCGGA   
  
  
+ AGGAGAGGAT CAATGTTGAG CAGCATTGTT TAGCTAAGGA CATTGTGAAT ATCATAGCTT GCGAGGGCAA   
  
  
+ GGAGATGGTG GAGCGTCATG AGCTTTTTGG GAAATGGCAG TCGAGGTTTA CCATGGTAGG GTTCAGGCAG   
  
  
+ TACCCGTTGA GCTCATACAT AAACTCTGTG ATAAGAAGCC TACTCCGGTG TTACTCCAAG CATTATACTC   
  
  
+ TGATAGAGAA GGATAGTGCC ATGCTTCTGG GTTGGAGGGG CCGAATGCTG ATTTCAGCTT CGGCATG  

- -Up\_Stream \_Len000TTTAAA TATACTAATA CCCTCGTTTA TAATTAGCCA GATTTTGTTT TTTCTTAGCC   
  
  
- GGCTTGTTTT TTTTCCCGGG TCTTGATCAG GAAAATTACC TCAGGGTTTT ACACCATATT TAATCAGAAC   
  
  
- AAGTGAGTAA AGAGTGAGGA GAGTGAGTAA GATTGGGATT TAGAGTTAGG AGTGAGAGAC AGGAGAGATA   
  
  
- GACGCAGAGC TCGAAGAGAA AGGAGACCCG AAGGAACCTG AAAAAGCTGC GGTACTGTTA CCTTGTGACT   
  
  
- CGAGGTGGCA ACGGGGAGAG GTGTTGACAG AAGATTGCCA CCAAGAGGCA TCGGTGGCAT GGAGTTTTTG   
  
  
- GTTTCGCTAC GTGTTGATGG TGGGGTGGTG ACGACTTTTC ACGGGTGCTC AGAGCTAGGC GCGTTCGGAC   
  
  
- TACTCTTAGC TATGGTGGTG GTACATCTAT TCGACTTTTG CCCGTATTCC TTCGCTGGGT GCGCGGGTTG   
  
  
- TGCGGTAACT CCGTCACTGT GCACCCAACT ACGCGAAGGT TTCTACCTTA AAAGTGTAAA AAGAAAAGTT   
  
  
- CCAACGCATC TAGCCACACA CACCTACCGC TAGTTCCTTC TCAATTTTAC TTGATCAGTA CGCAGCTTAA   
  
  
- CAGGTTTTTT TTAAAAGAGA AAGTCAGCTC AGAATTTCCC CGTCAGTAGT TCTCGTACAA ACTTTTTGTA   
  
  
- ATACCGGTAT TGGTGAATCA TCATTAAGTT TATTAGTACC TTTGTATAAG TTGTACTTAA GTTATACTGA   
  
  
- TTTTTAAGGT AGAAAATCAA ACAAGACCAG TTGTGTCTCG TACGGAAAAA TTAACGTATT ATTGCTATAA   
  
  
- ATTGGTTGGT TAGTACAAAG AGTGTATAAT TATATCCGTT CCCATCTAAT TGTCAGTATT AGCGTATCAT   
  
  
- ACGTTCTTAA ATAAACGTTA GACCCGTATG ACAAAATATT TCGTGTTCAC AAGTACTGAA TTTTAGCTAT   
  
  
- TTCAGAATTA GAAAACAGCT AGGAACTCAC ACGTCTGTTA TATTAACACT TACATACGAT AATTGAAATT   
  
  
- ATTAATGCTT TTAATTCGTT GATTAGTATA TCAGTACAAG AGTTTTTGTT CGTACGCATA ATAAGTCTAG   
  
  
- TAATTTACGT AATTTACCCT GATTAGGCTA GTCAGTTCGC GACTATGCTG ATAACTTATT TGTCCTGATT   
  
  
- GGATCTTACG CCTTAATTGA CAGCTCTTAA CAGGTGTAGC CATCACTACG AACCGCCGAT GGTCTCAGTG   
  
  
- GAATCCCACA AGTGTAGGAT CTGGGCTTTT GGACGATTAC TACCTTCCCG TCGGCACCCT CCTCCTCTCC   
  
  
- TCTCACACCT AAAAAGATAA ATGTTTTTTC ACATATTCAC GATTAGGGGG CTTTGGAGGA AAATATCCAA   
  
  
- TCCCCCCGTT GATCCCAATA GCGGGATCCA CTGCGAACAC CGGCAGCCGG ATTAATTTAT CCGGGCTGGT   
  
  
- TGTCGACACT GACCTCGCTA TCGACTTCGT ACGTCTTTAC TCCTATTGTA TGTATAATAT CTAAAGGTGT   
  
  
- AACGTATCCC GTGAGTCACC CAATGACATA GCGTTCGGTA TCGTCGTTCC GAACCACCCG GAGGATTTCA   
  
  
- CGCTGAATGT CCGTAACTAC TAGGACAAAG ATTCATATGA ACACTACGAT CGAACCTCCG ACCACCCTTT   
  
  
- GCTAATCGCA GAGATAAACT TTTCAAATTT TATGAACAGC TCAAGTTACG TAACGGGCAA ATACCTGGGT   
  
  
- TACAGTCCAC CCTTTACGAC CTACACTCCG GGTCCCTCCG AAACCAGCAA TTAACAGGTA ATGTCGAGGT   
  
  
- AATGTGAGGA CTGCTCTCAC AGCTACACTC CTTGGGATCC CTATCCGAAG AGTCTTACCA CTTTAACGAG   
  
  
- CCAGGATTCC ATCGGTGAAA CCAACTCGTT CTTAGTTTGT GGTTGTGATG TGGAAAGAAC TGGGCCAAGT   
  
  
- ATCTCTGGGA ACTGATGATG AGCTAATTGG TACAAACTCA GATATCTGCA CTGGTACGGC TCTCTGGCCT   
  
  
- TCCTCTCCTA GTTACAACTC GTCGTAACAA ATCGATTCCT GTAACACTTA TAGTATCGAA CGCTCCCGTT   
  
  
- CCTCTACCAC CTCGCAGTAC TCGAAAAACC CTTTACCGTC AGCTCCAAAT GGTACCATCC CAAGTCCGTC   
  
  
- ATGGGCAACT CGAGTATGTA TTTGAGACAC TATTCTTCGG ATGAGGCCAC AATGAGGTTC GTAATATGAG   
  
  
- ACTATCTCTT CCTATCACGG TACGAAGACC CAACCTCCCC GGCTTACGAC TAAAGTCGAA GCCGTAC

+     Box 4

| Site Name | Organism | Position | Strand | Matrix score. | sequence | function |
| --- | --- | --- | --- | --- | --- | --- |
| Box 4 | Petroselinum crispum | 871 | + | 6 | ATTAAT | part of a conserved DNA module involved in light responsiveness |
| Box 4 | Petroselinum crispum | 45 | + | 6 | ATTAAT | part of a conserved DNA module involved in light responsiveness |

>HU08G00229.1   
+ -Up\_Stream \_Len000AAATTT ATATGATTAT GGGAGCAAAT ATTAATCGGT CTAAAACAAA AAAGAATCGG   
  
  
+ CCGAACAAAA AAAAGGGCCC AGAACTAGTC CTTTTAATGG AGTCCCAAAA TGTGGTATAA ATTAGTCTTG   
  
  
+ TTCACTCATT TCTCACTCCT CTCACTCATT CTAACCCTAA ATCTCAATCC TCACTCTCTG TCCTCTCTAT   
  
  
+ CTGCGTCTCG AGCTTCTCTT TCCTCTGGGC TTCCTTGGAC TTTTTCGACG CCATGACAAT GGAACACTGA   
  
  
+ GCTCCACCGT TGCCCCTCTC CACAACTGTC TTCTAACGGT GGTTCTCCGT AGCCACCGTA CCTCAAAAAC   
  
  
+ CAAAGCGATG CACAACTACC ACCCCACCAC TGCTGAAAAG TGCCCACGAG TCTCGATCCG CGCAAGCCTG   
  
  
+ ATGAGAATCG ATACCACCAC CATGTAGATA AGCTGAAAAC GGGCATAAGG AAGCGACCCA CGCGCCCAAC   
  
  
+ ACGCCATTGA GGCAGTGACA CGTGGGTTGA TGCGCTTCCA AAGATGGAAT TTTCACATTT TTCTTTTCAA   
  
  
+ GGTTGCGTAG ATCGGTGTGT GTGGATGGCG ATCAAGGAAG AGTTAAAATG AACTAGTCAT GCGTCGAATT   
  
  
+ GTCCAAAAAA AATTTTCTCT TTCAGTCGAG TCTTAAAGGG GCAGTCATCA AGAGCATGTT TGAAAAACAT   
  
  
+ TATGGCCATA ACCACTTAGT AGTAATTCAA ATAATCATGG AAACATATTC AACATGAATT CAATATGACT   
  
  
+ AAAAATTCCA TCTTTTAGTT TGTTCTGGTC AACACAGAGC ATGCCTTTTT AATTGCATAA TAACGATATT   
  
  
+ TAACCAACCA ATCATGTTTC TCACATATTA ATATAGGCAA GGGTAGATTA ACAGTCATAA TCGCATAGTA   
  
  
+ TGCAAGAATT TATTTGCAAT CTGGGCATAC TGTTTTATAA AGCACAAGTG TTCATGACTT AAAATCGATA   
  
  
+ AAGTCTTAAT CTTTTGTCGA TCCTTGAGTG TGCAGACAAT ATAATTGTGA ATGTATGCTA TTAACTTTAA   
  
  
+ TAATTACGAA AATTAAGCAA CTAATCATAT AGTCATGTTC TCAAAAACAA GCATGCGTAT TATTCAGATC   
  
  
+ ATTAAATGCA TTAAATGGGA CTAATCCGAT CAGTCAAGCG CTGATACGAC TATTGAATAA ACAGGACTAA   
  
  
+ CCTAGAATGC GGAATTAACT GTCGAGAATT GTCCACATCG GTAGTGATGC TTGGCGGCTA CCAGAGTCAC   
  
  
+ CTTAGGGTGT TCACATCCTA GACCCGAAAA CCTGCTAATG ATGGAAGGGC AGCCGTGGGA GGAGGAGAGG   
  
  
+ AGAGTGTGGA TTTTTCTATT TACAAAAAAG TGTATAAGTG CTAATCCCCC GAAACCTCCT TTTATAGGTT   
  
  
+ AGGGGGGCAA CTAGGGTTAT CGCCCTAGGT GACGCTTGTG GCCGTCGGCC TAATTAAATA GGCCCGACCA   
  
  
+ ACAGCTGTGA CTGGAGCGAT AGCTGAAGCA TGCAGAAATG AGGATAACAT ACATATTATA GATTTCCACA   
  
  
+ TTGCATAGGG CACTCAGTGG GTTACTGTAT CGCAAGCCAT AGCAGCAAGG CTTGGTGGGC CTCCTAAAGT   
  
  
+ GCGACTTACA GGCATTGATG ATCCTGTTTC TAAGTATACT TGTGATGCTA GCTTGGAGGC TGGTGGGAAA   
  
  
+ CGATTAGCGT CTCTATTTGA AAAGTTTAAA ATACTTGTCG AGTTCAATGC ATTGCCCGTT TATGGACCCA   
  
  
+ ATGTCAGGTG GGAAATGCTG GATGTGAGGC CCAGGGAGGC TTTGGTCGTT AATTGTCCAT TACAGCTCCA   
  
  
+ TTACACTCCT GACGAGAGTG TCGATGTGAG GAACCCTAGG GATAGGCTTC TCAGAATGGT GAAATTGCTC   
  
  
+ GGTCCTAAGG TAGCCACTTT GGTTGAGCAA GAATCAAACA CCAACACTAC ACCTTTCTTG ACCCGGTTCA   
  
  
+ TAGAGACCCT TGACTACTAC TCGATTAACC ATGTTTGAGT CTATAGACGT GACCATGCCG AGAGACCGGA   
  
  
+ AGGAGAGGAT CAATGTTGAG CAGCATTGTT TAGCTAAGGA CATTGTGAAT ATCATAGCTT GCGAGGGCAA   
  
  
+ GGAGATGGTG GAGCGTCATG AGCTTTTTGG GAAATGGCAG TCGAGGTTTA CCATGGTAGG GTTCAGGCAG   
  
  
+ TACCCGTTGA GCTCATACAT AAACTCTGTG ATAAGAAGCC TACTCCGGTG TTACTCCAAG CATTATACTC   
  
  
+ TGATAGAGAA GGATAGTGCC ATGCTTCTGG GTTGGAGGGG CCGAATGCTG ATTTCAGCTT CGGCATG  

- -Up\_Stream \_Len000TTTAAA TATACTAATA CCCTCGTTTA TAATTAGCCA GATTTTGTTT TTTCTTAGCC   
  
  
- GGCTTGTTTT TTTTCCCGGG TCTTGATCAG GAAAATTACC TCAGGGTTTT ACACCATATT TAATCAGAAC   
  
  
- AAGTGAGTAA AGAGTGAGGA GAGTGAGTAA GATTGGGATT TAGAGTTAGG AGTGAGAGAC AGGAGAGATA   
  
  
- GACGCAGAGC TCGAAGAGAA AGGAGACCCG AAGGAACCTG AAAAAGCTGC GGTACTGTTA CCTTGTGACT   
  
  
- CGAGGTGGCA ACGGGGAGAG GTGTTGACAG AAGATTGCCA CCAAGAGGCA TCGGTGGCAT GGAGTTTTTG   
  
  
- GTTTCGCTAC GTGTTGATGG TGGGGTGGTG ACGACTTTTC ACGGGTGCTC AGAGCTAGGC GCGTTCGGAC   
  
  
- TACTCTTAGC TATGGTGGTG GTACATCTAT TCGACTTTTG CCCGTATTCC TTCGCTGGGT GCGCGGGTTG   
  
  
- TGCGGTAACT CCGTCACTGT GCACCCAACT ACGCGAAGGT TTCTACCTTA AAAGTGTAAA AAGAAAAGTT   
  
  
- CCAACGCATC TAGCCACACA CACCTACCGC TAGTTCCTTC TCAATTTTAC TTGATCAGTA CGCAGCTTAA   
  
  
- CAGGTTTTTT TTAAAAGAGA AAGTCAGCTC AGAATTTCCC CGTCAGTAGT TCTCGTACAA ACTTTTTGTA   
  
  
- ATACCGGTAT TGGTGAATCA TCATTAAGTT TATTAGTACC TTTGTATAAG TTGTACTTAA GTTATACTGA   
  
  
- TTTTTAAGGT AGAAAATCAA ACAAGACCAG TTGTGTCTCG TACGGAAAAA TTAACGTATT ATTGCTATAA   
  
  
- ATTGGTTGGT TAGTACAAAG AGTGTATAAT TATATCCGTT CCCATCTAAT TGTCAGTATT AGCGTATCAT   
  
  
- ACGTTCTTAA ATAAACGTTA GACCCGTATG ACAAAATATT TCGTGTTCAC AAGTACTGAA TTTTAGCTAT   
  
  
- TTCAGAATTA GAAAACAGCT AGGAACTCAC ACGTCTGTTA TATTAACACT TACATACGAT AATTGAAATT   
  
  
- ATTAATGCTT TTAATTCGTT GATTAGTATA TCAGTACAAG AGTTTTTGTT CGTACGCATA ATAAGTCTAG   
  
  
- TAATTTACGT AATTTACCCT GATTAGGCTA GTCAGTTCGC GACTATGCTG ATAACTTATT TGTCCTGATT   
  
  
- GGATCTTACG CCTTAATTGA CAGCTCTTAA CAGGTGTAGC CATCACTACG AACCGCCGAT GGTCTCAGTG   
  
  
- GAATCCCACA AGTGTAGGAT CTGGGCTTTT GGACGATTAC TACCTTCCCG TCGGCACCCT CCTCCTCTCC   
  
  
- TCTCACACCT AAAAAGATAA ATGTTTTTTC ACATATTCAC GATTAGGGGG CTTTGGAGGA AAATATCCAA   
  
  
- TCCCCCCGTT GATCCCAATA GCGGGATCCA CTGCGAACAC CGGCAGCCGG ATTAATTTAT CCGGGCTGGT   
  
  
- TGTCGACACT GACCTCGCTA TCGACTTCGT ACGTCTTTAC TCCTATTGTA TGTATAATAT CTAAAGGTGT   
  
  
- AACGTATCCC GTGAGTCACC CAATGACATA GCGTTCGGTA TCGTCGTTCC GAACCACCCG GAGGATTTCA   
  
  
- CGCTGAATGT CCGTAACTAC TAGGACAAAG ATTCATATGA ACACTACGAT CGAACCTCCG ACCACCCTTT   
  
  
- GCTAATCGCA GAGATAAACT TTTCAAATTT TATGAACAGC TCAAGTTACG TAACGGGCAA ATACCTGGGT   
  
  
- TACAGTCCAC CCTTTACGAC CTACACTCCG GGTCCCTCCG AAACCAGCAA TTAACAGGTA ATGTCGAGGT   
  
  
- AATGTGAGGA CTGCTCTCAC AGCTACACTC CTTGGGATCC CTATCCGAAG AGTCTTACCA CTTTAACGAG   
  
  
- CCAGGATTCC ATCGGTGAAA CCAACTCGTT CTTAGTTTGT GGTTGTGATG TGGAAAGAAC TGGGCCAAGT   
  
  
- ATCTCTGGGA ACTGATGATG AGCTAATTGG TACAAACTCA GATATCTGCA CTGGTACGGC TCTCTGGCCT   
  
  
- TCCTCTCCTA GTTACAACTC GTCGTAACAA ATCGATTCCT GTAACACTTA TAGTATCGAA CGCTCCCGTT   
  
  
- CCTCTACCAC CTCGCAGTAC TCGAAAAACC CTTTACCGTC AGCTCCAAAT GGTACCATCC CAAGTCCGTC   
  
  
- ATGGGCAACT CGAGTATGTA TTTGAGACAC TATTCTTCGG ATGAGGCCAC AATGAGGTTC GTAATATGAG   
  
  
- ACTATCTCTT CCTATCACGG TACGAAGACC CAACCTCCCC GGCTTACGAC TAAAGTCGAA GCCGTAC

+     CAAT-box

| Site Name | Organism | Position | Strand | Matrix score. | sequence | function |
| --- | --- | --- | --- | --- | --- | --- |
| CAAT-box | Pisum sativum | 926 | - | 5 | CAAAT | common cis-acting element in promoter and enhancer regions |
| CAAT-box | Arabidopsis thaliana | 852 | + | 5 | CCAAT | common cis-acting element in promoter and enhancer regions |
| CAAT-box | Nicotiana glutinosa | 1028 | - | 4 | CAAT |  |
| CAAT-box | Nicotiana glutinosa | 826 | - | 4 | CAAT |  |
| CAAT-box | Nicotiana glutinosa | 632 | - | 4 | CAAT |  |
| CAAT-box | Nicotiana glutinosa | 1806 | - | 4 | CAAT |  |
| CAAT-box | Nicotiana glutinosa | 2059 | - | 4 | CAAT |  |
| CAAT-box | Nicotiana glutinosa | 1628 | - | 4 | CAAT |  |
| CAAT-box | Nicotiana glutinosa | 1176 | - | 4 | CAAT |  |
| CAAT-box | Pisum sativum | 40 | + | 5 | CAAAT | common cis-acting element in promoter and enhancer regions |
| CAAT-box | Arabidopsis thaliana | 1752 | + | 5 | CCAAT | common cis-acting element in promoter and enhancer regions |
| CAAT-box | Nicotiana glutinosa | 1735 | - | 4 | CAAT |  |
| CAAT-box | Nicotiana glutinosa | 1729 | + | 4 | CAAT |  |
| CAAT-box | Nicotiana glutinosa | 500 | - | 4 | CAAT |  |
| CAAT-box | Nicotiana glutinosa | 1222 | - | 4 | CAAT |  |
| CAAT-box | Nicotiana glutinosa | 271 | + | 4 | CAAT |  |
| CAAT-box | Nicotiana glutinosa | 853 | + | 4 | CAAT |  |
| CAAT-box | Pisum sativum | 732 | + | 5 | CAAAT | common cis-acting element in promoter and enhancer regions |
| CAAT-box | Nicotiana glutinosa | 1888 | - | 4 | CAAT |  |
| CAAT-box | Nicotiana glutinosa | 1021 | + | 4 | CAAT |  |
| CAAT-box | Nicotiana glutinosa | 1544 | - | 4 | CAAT |  |
| CAAT-box | Nicotiana glutinosa | 189 | + | 4 | CAAT |  |
| CAAT-box | Nicotiana glutinosa | 1753 | + | 4 | CAAT |  |
| CAAT-box | Nicotiana glutinosa | 931 | + | 4 | CAAT |  |
| CAAT-box | Nicotiana glutinosa | 2076 | - | 4 | CAAT |  |
| CAAT-box | Nicotiana glutinosa | 765 | + | 4 | CAAT |  |
| CAAT-box | Pisum sativum | 1699 | - | 5 | CAAAT | common cis-acting element in promoter and enhancer regions |
| CAAT-box | Nicotiana glutinosa | 2045 | + | 4 | CAAT |  |

>HU08G00229.1   
+ -Up\_Stream \_Len000AAATTT ATATGATTAT GGGAGCAAAT ATTAATCGGT CTAAAACAAA AAAGAATCGG   
  
  
+ CCGAACAAAA AAAAGGGCCC AGAACTAGTC CTTTTAATGG AGTCCCAAAA TGTGGTATAA ATTAGTCTTG   
  
  
+ TTCACTCATT TCTCACTCCT CTCACTCATT CTAACCCTAA ATCTCAATCC TCACTCTCTG TCCTCTCTAT   
  
  
+ CTGCGTCTCG AGCTTCTCTT TCCTCTGGGC TTCCTTGGAC TTTTTCGACG CCATGACAAT GGAACACTGA   
  
  
+ GCTCCACCGT TGCCCCTCTC CACAACTGTC TTCTAACGGT GGTTCTCCGT AGCCACCGTA CCTCAAAAAC   
  
  
+ CAAAGCGATG CACAACTACC ACCCCACCAC TGCTGAAAAG TGCCCACGAG TCTCGATCCG CGCAAGCCTG   
  
  
+ ATGAGAATCG ATACCACCAC CATGTAGATA AGCTGAAAAC GGGCATAAGG AAGCGACCCA CGCGCCCAAC   
  
  
+ ACGCCATTGA GGCAGTGACA CGTGGGTTGA TGCGCTTCCA AAGATGGAAT TTTCACATTT TTCTTTTCAA   
  
  
+ GGTTGCGTAG ATCGGTGTGT GTGGATGGCG ATCAAGGAAG AGTTAAAATG AACTAGTCAT GCGTCGAATT   
  
  
+ GTCCAAAAAA AATTTTCTCT TTCAGTCGAG TCTTAAAGGG GCAGTCATCA AGAGCATGTT TGAAAAACAT   
  
  
+ TATGGCCATA ACCACTTAGT AGTAATTCAA ATAATCATGG AAACATATTC AACATGAATT CAATATGACT   
  
  
+ AAAAATTCCA TCTTTTAGTT TGTTCTGGTC AACACAGAGC ATGCCTTTTT AATTGCATAA TAACGATATT   
  
  
+ TAACCAACCA ATCATGTTTC TCACATATTA ATATAGGCAA GGGTAGATTA ACAGTCATAA TCGCATAGTA   
  
  
+ TGCAAGAATT TATTTGCAAT CTGGGCATAC TGTTTTATAA AGCACAAGTG TTCATGACTT AAAATCGATA   
  
  
+ AAGTCTTAAT CTTTTGTCGA TCCTTGAGTG TGCAGACAAT ATAATTGTGA ATGTATGCTA TTAACTTTAA   
  
  
+ TAATTACGAA AATTAAGCAA CTAATCATAT AGTCATGTTC TCAAAAACAA GCATGCGTAT TATTCAGATC   
  
  
+ ATTAAATGCA TTAAATGGGA CTAATCCGAT CAGTCAAGCG CTGATACGAC TATTGAATAA ACAGGACTAA   
  
  
+ CCTAGAATGC GGAATTAACT GTCGAGAATT GTCCACATCG GTAGTGATGC TTGGCGGCTA CCAGAGTCAC   
  
  
+ CTTAGGGTGT TCACATCCTA GACCCGAAAA CCTGCTAATG ATGGAAGGGC AGCCGTGGGA GGAGGAGAGG   
  
  
+ AGAGTGTGGA TTTTTCTATT TACAAAAAAG TGTATAAGTG CTAATCCCCC GAAACCTCCT TTTATAGGTT   
  
  
+ AGGGGGGCAA CTAGGGTTAT CGCCCTAGGT GACGCTTGTG GCCGTCGGCC TAATTAAATA GGCCCGACCA   
  
  
+ ACAGCTGTGA CTGGAGCGAT AGCTGAAGCA TGCAGAAATG AGGATAACAT ACATATTATA GATTTCCACA   
  
  
+ TTGCATAGGG CACTCAGTGG GTTACTGTAT CGCAAGCCAT AGCAGCAAGG CTTGGTGGGC CTCCTAAAGT   
  
  
+ GCGACTTACA GGCATTGATG ATCCTGTTTC TAAGTATACT TGTGATGCTA GCTTGGAGGC TGGTGGGAAA   
  
  
+ CGATTAGCGT CTCTATTTGA AAAGTTTAAA ATACTTGTCG AGTTCAATGC ATTGCCCGTT TATGGACCCA   
  
  
+ ATGTCAGGTG GGAAATGCTG GATGTGAGGC CCAGGGAGGC TTTGGTCGTT AATTGTCCAT TACAGCTCCA   
  
  
+ TTACACTCCT GACGAGAGTG TCGATGTGAG GAACCCTAGG GATAGGCTTC TCAGAATGGT GAAATTGCTC   
  
  
+ GGTCCTAAGG TAGCCACTTT GGTTGAGCAA GAATCAAACA CCAACACTAC ACCTTTCTTG ACCCGGTTCA   
  
  
+ TAGAGACCCT TGACTACTAC TCGATTAACC ATGTTTGAGT CTATAGACGT GACCATGCCG AGAGACCGGA   
  
  
+ AGGAGAGGAT CAATGTTGAG CAGCATTGTT TAGCTAAGGA CATTGTGAAT ATCATAGCTT GCGAGGGCAA   
  
  
+ GGAGATGGTG GAGCGTCATG AGCTTTTTGG GAAATGGCAG TCGAGGTTTA CCATGGTAGG GTTCAGGCAG   
  
  
+ TACCCGTTGA GCTCATACAT AAACTCTGTG ATAAGAAGCC TACTCCGGTG TTACTCCAAG CATTATACTC   
  
  
+ TGATAGAGAA GGATAGTGCC ATGCTTCTGG GTTGGAGGGG CCGAATGCTG ATTTCAGCTT CGGCATG  

- -Up\_Stream \_Len000TTTAAA TATACTAATA CCCTCGTTTA TAATTAGCCA GATTTTGTTT TTTCTTAGCC   
  
  
- GGCTTGTTTT TTTTCCCGGG TCTTGATCAG GAAAATTACC TCAGGGTTTT ACACCATATT TAATCAGAAC   
  
  
- AAGTGAGTAA AGAGTGAGGA GAGTGAGTAA GATTGGGATT TAGAGTTAGG AGTGAGAGAC AGGAGAGATA   
  
  
- GACGCAGAGC TCGAAGAGAA AGGAGACCCG AAGGAACCTG AAAAAGCTGC GGTACTGTTA CCTTGTGACT   
  
  
- CGAGGTGGCA ACGGGGAGAG GTGTTGACAG AAGATTGCCA CCAAGAGGCA TCGGTGGCAT GGAGTTTTTG   
  
  
- GTTTCGCTAC GTGTTGATGG TGGGGTGGTG ACGACTTTTC ACGGGTGCTC AGAGCTAGGC GCGTTCGGAC   
  
  
- TACTCTTAGC TATGGTGGTG GTACATCTAT TCGACTTTTG CCCGTATTCC TTCGCTGGGT GCGCGGGTTG   
  
  
- TGCGGTAACT CCGTCACTGT GCACCCAACT ACGCGAAGGT TTCTACCTTA AAAGTGTAAA AAGAAAAGTT   
  
  
- CCAACGCATC TAGCCACACA CACCTACCGC TAGTTCCTTC TCAATTTTAC TTGATCAGTA CGCAGCTTAA   
  
  
- CAGGTTTTTT TTAAAAGAGA AAGTCAGCTC AGAATTTCCC CGTCAGTAGT TCTCGTACAA ACTTTTTGTA   
  
  
- ATACCGGTAT TGGTGAATCA TCATTAAGTT TATTAGTACC TTTGTATAAG TTGTACTTAA GTTATACTGA   
  
  
- TTTTTAAGGT AGAAAATCAA ACAAGACCAG TTGTGTCTCG TACGGAAAAA TTAACGTATT ATTGCTATAA   
  
  
- ATTGGTTGGT TAGTACAAAG AGTGTATAAT TATATCCGTT CCCATCTAAT TGTCAGTATT AGCGTATCAT   
  
  
- ACGTTCTTAA ATAAACGTTA GACCCGTATG ACAAAATATT TCGTGTTCAC AAGTACTGAA TTTTAGCTAT   
  
  
- TTCAGAATTA GAAAACAGCT AGGAACTCAC ACGTCTGTTA TATTAACACT TACATACGAT AATTGAAATT   
  
  
- ATTAATGCTT TTAATTCGTT GATTAGTATA TCAGTACAAG AGTTTTTGTT CGTACGCATA ATAAGTCTAG   
  
  
- TAATTTACGT AATTTACCCT GATTAGGCTA GTCAGTTCGC GACTATGCTG ATAACTTATT TGTCCTGATT   
  
  
- GGATCTTACG CCTTAATTGA CAGCTCTTAA CAGGTGTAGC CATCACTACG AACCGCCGAT GGTCTCAGTG   
  
  
- GAATCCCACA AGTGTAGGAT CTGGGCTTTT GGACGATTAC TACCTTCCCG TCGGCACCCT CCTCCTCTCC   
  
  
- TCTCACACCT AAAAAGATAA ATGTTTTTTC ACATATTCAC GATTAGGGGG CTTTGGAGGA AAATATCCAA   
  
  
- TCCCCCCGTT GATCCCAATA GCGGGATCCA CTGCGAACAC CGGCAGCCGG ATTAATTTAT CCGGGCTGGT   
  
  
- TGTCGACACT GACCTCGCTA TCGACTTCGT ACGTCTTTAC TCCTATTGTA TGTATAATAT CTAAAGGTGT   
  
  
- AACGTATCCC GTGAGTCACC CAATGACATA GCGTTCGGTA TCGTCGTTCC GAACCACCCG GAGGATTTCA   
  
  
- CGCTGAATGT CCGTAACTAC TAGGACAAAG ATTCATATGA ACACTACGAT CGAACCTCCG ACCACCCTTT   
  
  
- GCTAATCGCA GAGATAAACT TTTCAAATTT TATGAACAGC TCAAGTTACG TAACGGGCAA ATACCTGGGT   
  
  
- TACAGTCCAC CCTTTACGAC CTACACTCCG GGTCCCTCCG AAACCAGCAA TTAACAGGTA ATGTCGAGGT   
  
  
- AATGTGAGGA CTGCTCTCAC AGCTACACTC CTTGGGATCC CTATCCGAAG AGTCTTACCA CTTTAACGAG   
  
  
- CCAGGATTCC ATCGGTGAAA CCAACTCGTT CTTAGTTTGT GGTTGTGATG TGGAAAGAAC TGGGCCAAGT   
  
  
- ATCTCTGGGA ACTGATGATG AGCTAATTGG TACAAACTCA GATATCTGCA CTGGTACGGC TCTCTGGCCT   
  
  
- TCCTCTCCTA GTTACAACTC GTCGTAACAA ATCGATTCCT GTAACACTTA TAGTATCGAA CGCTCCCGTT   
  
  
- CCTCTACCAC CTCGCAGTAC TCGAAAAACC CTTTACCGTC AGCTCCAAAT GGTACCATCC CAAGTCCGTC   
  
  
- ATGGGCAACT CGAGTATGTA TTTGAGACAC TATTCTTCGG ATGAGGCCAC AATGAGGTTC GTAATATGAG   
  
  
- ACTATCTCTT CCTATCACGG TACGAAGACC CAACCTCCCC GGCTTACGAC TAAAGTCGAA GCCGTAC

+     CAT-box

| Site Name | Organism | Position | Strand | Matrix score. | sequence | function |
| --- | --- | --- | --- | --- | --- | --- |
| CAT-box | Arabidopsis thaliana | 1907 | + | 6 | GCCACT | cis-acting regulatory element related to meristem expression |

>HU08G00229.1   
+ -Up\_Stream \_Len000AAATTT ATATGATTAT GGGAGCAAAT ATTAATCGGT CTAAAACAAA AAAGAATCGG   
  
  
+ CCGAACAAAA AAAAGGGCCC AGAACTAGTC CTTTTAATGG AGTCCCAAAA TGTGGTATAA ATTAGTCTTG   
  
  
+ TTCACTCATT TCTCACTCCT CTCACTCATT CTAACCCTAA ATCTCAATCC TCACTCTCTG TCCTCTCTAT   
  
  
+ CTGCGTCTCG AGCTTCTCTT TCCTCTGGGC TTCCTTGGAC TTTTTCGACG CCATGACAAT GGAACACTGA   
  
  
+ GCTCCACCGT TGCCCCTCTC CACAACTGTC TTCTAACGGT GGTTCTCCGT AGCCACCGTA CCTCAAAAAC   
  
  
+ CAAAGCGATG CACAACTACC ACCCCACCAC TGCTGAAAAG TGCCCACGAG TCTCGATCCG CGCAAGCCTG   
  
  
+ ATGAGAATCG ATACCACCAC CATGTAGATA AGCTGAAAAC GGGCATAAGG AAGCGACCCA CGCGCCCAAC   
  
  
+ ACGCCATTGA GGCAGTGACA CGTGGGTTGA TGCGCTTCCA AAGATGGAAT TTTCACATTT TTCTTTTCAA   
  
  
+ GGTTGCGTAG ATCGGTGTGT GTGGATGGCG ATCAAGGAAG AGTTAAAATG AACTAGTCAT GCGTCGAATT   
  
  
+ GTCCAAAAAA AATTTTCTCT TTCAGTCGAG TCTTAAAGGG GCAGTCATCA AGAGCATGTT TGAAAAACAT   
  
  
+ TATGGCCATA ACCACTTAGT AGTAATTCAA ATAATCATGG AAACATATTC AACATGAATT CAATATGACT   
  
  
+ AAAAATTCCA TCTTTTAGTT TGTTCTGGTC AACACAGAGC ATGCCTTTTT AATTGCATAA TAACGATATT   
  
  
+ TAACCAACCA ATCATGTTTC TCACATATTA ATATAGGCAA GGGTAGATTA ACAGTCATAA TCGCATAGTA   
  
  
+ TGCAAGAATT TATTTGCAAT CTGGGCATAC TGTTTTATAA AGCACAAGTG TTCATGACTT AAAATCGATA   
  
  
+ AAGTCTTAAT CTTTTGTCGA TCCTTGAGTG TGCAGACAAT ATAATTGTGA ATGTATGCTA TTAACTTTAA   
  
  
+ TAATTACGAA AATTAAGCAA CTAATCATAT AGTCATGTTC TCAAAAACAA GCATGCGTAT TATTCAGATC   
  
  
+ ATTAAATGCA TTAAATGGGA CTAATCCGAT CAGTCAAGCG CTGATACGAC TATTGAATAA ACAGGACTAA   
  
  
+ CCTAGAATGC GGAATTAACT GTCGAGAATT GTCCACATCG GTAGTGATGC TTGGCGGCTA CCAGAGTCAC   
  
  
+ CTTAGGGTGT TCACATCCTA GACCCGAAAA CCTGCTAATG ATGGAAGGGC AGCCGTGGGA GGAGGAGAGG   
  
  
+ AGAGTGTGGA TTTTTCTATT TACAAAAAAG TGTATAAGTG CTAATCCCCC GAAACCTCCT TTTATAGGTT   
  
  
+ AGGGGGGCAA CTAGGGTTAT CGCCCTAGGT GACGCTTGTG GCCGTCGGCC TAATTAAATA GGCCCGACCA   
  
  
+ ACAGCTGTGA CTGGAGCGAT AGCTGAAGCA TGCAGAAATG AGGATAACAT ACATATTATA GATTTCCACA   
  
  
+ TTGCATAGGG CACTCAGTGG GTTACTGTAT CGCAAGCCAT AGCAGCAAGG CTTGGTGGGC CTCCTAAAGT   
  
  
+ GCGACTTACA GGCATTGATG ATCCTGTTTC TAAGTATACT TGTGATGCTA GCTTGGAGGC TGGTGGGAAA   
  
  
+ CGATTAGCGT CTCTATTTGA AAAGTTTAAA ATACTTGTCG AGTTCAATGC ATTGCCCGTT TATGGACCCA   
  
  
+ ATGTCAGGTG GGAAATGCTG GATGTGAGGC CCAGGGAGGC TTTGGTCGTT AATTGTCCAT TACAGCTCCA   
  
  
+ TTACACTCCT GACGAGAGTG TCGATGTGAG GAACCCTAGG GATAGGCTTC TCAGAATGGT GAAATTGCTC   
  
  
+ GGTCCTAAGG TAGCCACTTT GGTTGAGCAA GAATCAAACA CCAACACTAC ACCTTTCTTG ACCCGGTTCA   
  
  
+ TAGAGACCCT TGACTACTAC TCGATTAACC ATGTTTGAGT CTATAGACGT GACCATGCCG AGAGACCGGA   
  
  
+ AGGAGAGGAT CAATGTTGAG CAGCATTGTT TAGCTAAGGA CATTGTGAAT ATCATAGCTT GCGAGGGCAA   
  
  
+ GGAGATGGTG GAGCGTCATG AGCTTTTTGG GAAATGGCAG TCGAGGTTTA CCATGGTAGG GTTCAGGCAG   
  
  
+ TACCCGTTGA GCTCATACAT AAACTCTGTG ATAAGAAGCC TACTCCGGTG TTACTCCAAG CATTATACTC   
  
  
+ TGATAGAGAA GGATAGTGCC ATGCTTCTGG GTTGGAGGGG CCGAATGCTG ATTTCAGCTT CGGCATG  

- -Up\_Stream \_Len000TTTAAA TATACTAATA CCCTCGTTTA TAATTAGCCA GATTTTGTTT TTTCTTAGCC   
  
  
- GGCTTGTTTT TTTTCCCGGG TCTTGATCAG GAAAATTACC TCAGGGTTTT ACACCATATT TAATCAGAAC   
  
  
- AAGTGAGTAA AGAGTGAGGA GAGTGAGTAA GATTGGGATT TAGAGTTAGG AGTGAGAGAC AGGAGAGATA   
  
  
- GACGCAGAGC TCGAAGAGAA AGGAGACCCG AAGGAACCTG AAAAAGCTGC GGTACTGTTA CCTTGTGACT   
  
  
- CGAGGTGGCA ACGGGGAGAG GTGTTGACAG AAGATTGCCA CCAAGAGGCA TCGGTGGCAT GGAGTTTTTG   
  
  
- GTTTCGCTAC GTGTTGATGG TGGGGTGGTG ACGACTTTTC ACGGGTGCTC AGAGCTAGGC GCGTTCGGAC   
  
  
- TACTCTTAGC TATGGTGGTG GTACATCTAT TCGACTTTTG CCCGTATTCC TTCGCTGGGT GCGCGGGTTG   
  
  
- TGCGGTAACT CCGTCACTGT GCACCCAACT ACGCGAAGGT TTCTACCTTA AAAGTGTAAA AAGAAAAGTT   
  
  
- CCAACGCATC TAGCCACACA CACCTACCGC TAGTTCCTTC TCAATTTTAC TTGATCAGTA CGCAGCTTAA   
  
  
- CAGGTTTTTT TTAAAAGAGA AAGTCAGCTC AGAATTTCCC CGTCAGTAGT TCTCGTACAA ACTTTTTGTA   
  
  
- ATACCGGTAT TGGTGAATCA TCATTAAGTT TATTAGTACC TTTGTATAAG TTGTACTTAA GTTATACTGA   
  
  
- TTTTTAAGGT AGAAAATCAA ACAAGACCAG TTGTGTCTCG TACGGAAAAA TTAACGTATT ATTGCTATAA   
  
  
- ATTGGTTGGT TAGTACAAAG AGTGTATAAT TATATCCGTT CCCATCTAAT TGTCAGTATT AGCGTATCAT   
  
  
- ACGTTCTTAA ATAAACGTTA GACCCGTATG ACAAAATATT TCGTGTTCAC AAGTACTGAA TTTTAGCTAT   
  
  
- TTCAGAATTA GAAAACAGCT AGGAACTCAC ACGTCTGTTA TATTAACACT TACATACGAT AATTGAAATT   
  
  
- ATTAATGCTT TTAATTCGTT GATTAGTATA TCAGTACAAG AGTTTTTGTT CGTACGCATA ATAAGTCTAG   
  
  
- TAATTTACGT AATTTACCCT GATTAGGCTA GTCAGTTCGC GACTATGCTG ATAACTTATT TGTCCTGATT   
  
  
- GGATCTTACG CCTTAATTGA CAGCTCTTAA CAGGTGTAGC CATCACTACG AACCGCCGAT GGTCTCAGTG   
  
  
- GAATCCCACA AGTGTAGGAT CTGGGCTTTT GGACGATTAC TACCTTCCCG TCGGCACCCT CCTCCTCTCC   
  
  
- TCTCACACCT AAAAAGATAA ATGTTTTTTC ACATATTCAC GATTAGGGGG CTTTGGAGGA AAATATCCAA   
  
  
- TCCCCCCGTT GATCCCAATA GCGGGATCCA CTGCGAACAC CGGCAGCCGG ATTAATTTAT CCGGGCTGGT   
  
  
- TGTCGACACT GACCTCGCTA TCGACTTCGT ACGTCTTTAC TCCTATTGTA TGTATAATAT CTAAAGGTGT   
  
  
- AACGTATCCC GTGAGTCACC CAATGACATA GCGTTCGGTA TCGTCGTTCC GAACCACCCG GAGGATTTCA   
  
  
- CGCTGAATGT CCGTAACTAC TAGGACAAAG ATTCATATGA ACACTACGAT CGAACCTCCG ACCACCCTTT   
  
  
- GCTAATCGCA GAGATAAACT TTTCAAATTT TATGAACAGC TCAAGTTACG TAACGGGCAA ATACCTGGGT   
  
  
- TACAGTCCAC CCTTTACGAC CTACACTCCG GGTCCCTCCG AAACCAGCAA TTAACAGGTA ATGTCGAGGT   
  
  
- AATGTGAGGA CTGCTCTCAC AGCTACACTC CTTGGGATCC CTATCCGAAG AGTCTTACCA CTTTAACGAG   
  
  
- CCAGGATTCC ATCGGTGAAA CCAACTCGTT CTTAGTTTGT GGTTGTGATG TGGAAAGAAC TGGGCCAAGT   
  
  
- ATCTCTGGGA ACTGATGATG AGCTAATTGG TACAAACTCA GATATCTGCA CTGGTACGGC TCTCTGGCCT   
  
  
- TCCTCTCCTA GTTACAACTC GTCGTAACAA ATCGATTCCT GTAACACTTA TAGTATCGAA CGCTCCCGTT   
  
  
- CCTCTACCAC CTCGCAGTAC TCGAAAAACC CTTTACCGTC AGCTCCAAAT GGTACCATCC CAAGTCCGTC   
  
  
- ATGGGCAACT CGAGTATGTA TTTGAGACAC TATTCTTCGG ATGAGGCCAC AATGAGGTTC GTAATATGAG   
  
  
- ACTATCTCTT CCTATCACGG TACGAAGACC CAACCTCCCC GGCTTACGAC TAAAGTCGAA GCCGTAC

+     CCAAT-box

| Site Name | Organism | Position | Strand | Matrix score. | sequence | function |
| --- | --- | --- | --- | --- | --- | --- |
| CCAAT-box | Hordeum vulgare | 291 | - | 6 | CAACGG | MYBHv1 binding site |
| CCAAT-box | Hordeum vulgare | 2178 | - | 6 | CAACGG | MYBHv1 binding site |

>HU08G00229.1   
+ -Up\_Stream \_Len000AAATTT ATATGATTAT GGGAGCAAAT ATTAATCGGT CTAAAACAAA AAAGAATCGG   
  
  
+ CCGAACAAAA AAAAGGGCCC AGAACTAGTC CTTTTAATGG AGTCCCAAAA TGTGGTATAA ATTAGTCTTG   
  
  
+ TTCACTCATT TCTCACTCCT CTCACTCATT CTAACCCTAA ATCTCAATCC TCACTCTCTG TCCTCTCTAT   
  
  
+ CTGCGTCTCG AGCTTCTCTT TCCTCTGGGC TTCCTTGGAC TTTTTCGACG CCATGACAAT GGAACACTGA   
  
  
+ GCTCCACCGT TGCCCCTCTC CACAACTGTC TTCTAACGGT GGTTCTCCGT AGCCACCGTA CCTCAAAAAC   
  
  
+ CAAAGCGATG CACAACTACC ACCCCACCAC TGCTGAAAAG TGCCCACGAG TCTCGATCCG CGCAAGCCTG   
  
  
+ ATGAGAATCG ATACCACCAC CATGTAGATA AGCTGAAAAC GGGCATAAGG AAGCGACCCA CGCGCCCAAC   
  
  
+ ACGCCATTGA GGCAGTGACA CGTGGGTTGA TGCGCTTCCA AAGATGGAAT TTTCACATTT TTCTTTTCAA   
  
  
+ GGTTGCGTAG ATCGGTGTGT GTGGATGGCG ATCAAGGAAG AGTTAAAATG AACTAGTCAT GCGTCGAATT   
  
  
+ GTCCAAAAAA AATTTTCTCT TTCAGTCGAG TCTTAAAGGG GCAGTCATCA AGAGCATGTT TGAAAAACAT   
  
  
+ TATGGCCATA ACCACTTAGT AGTAATTCAA ATAATCATGG AAACATATTC AACATGAATT CAATATGACT   
  
  
+ AAAAATTCCA TCTTTTAGTT TGTTCTGGTC AACACAGAGC ATGCCTTTTT AATTGCATAA TAACGATATT   
  
  
+ TAACCAACCA ATCATGTTTC TCACATATTA ATATAGGCAA GGGTAGATTA ACAGTCATAA TCGCATAGTA   
  
  
+ TGCAAGAATT TATTTGCAAT CTGGGCATAC TGTTTTATAA AGCACAAGTG TTCATGACTT AAAATCGATA   
  
  
+ AAGTCTTAAT CTTTTGTCGA TCCTTGAGTG TGCAGACAAT ATAATTGTGA ATGTATGCTA TTAACTTTAA   
  
  
+ TAATTACGAA AATTAAGCAA CTAATCATAT AGTCATGTTC TCAAAAACAA GCATGCGTAT TATTCAGATC   
  
  
+ ATTAAATGCA TTAAATGGGA CTAATCCGAT CAGTCAAGCG CTGATACGAC TATTGAATAA ACAGGACTAA   
  
  
+ CCTAGAATGC GGAATTAACT GTCGAGAATT GTCCACATCG GTAGTGATGC TTGGCGGCTA CCAGAGTCAC   
  
  
+ CTTAGGGTGT TCACATCCTA GACCCGAAAA CCTGCTAATG ATGGAAGGGC AGCCGTGGGA GGAGGAGAGG   
  
  
+ AGAGTGTGGA TTTTTCTATT TACAAAAAAG TGTATAAGTG CTAATCCCCC GAAACCTCCT TTTATAGGTT   
  
  
+ AGGGGGGCAA CTAGGGTTAT CGCCCTAGGT GACGCTTGTG GCCGTCGGCC TAATTAAATA GGCCCGACCA   
  
  
+ ACAGCTGTGA CTGGAGCGAT AGCTGAAGCA TGCAGAAATG AGGATAACAT ACATATTATA GATTTCCACA   
  
  
+ TTGCATAGGG CACTCAGTGG GTTACTGTAT CGCAAGCCAT AGCAGCAAGG CTTGGTGGGC CTCCTAAAGT   
  
  
+ GCGACTTACA GGCATTGATG ATCCTGTTTC TAAGTATACT TGTGATGCTA GCTTGGAGGC TGGTGGGAAA   
  
  
+ CGATTAGCGT CTCTATTTGA AAAGTTTAAA ATACTTGTCG AGTTCAATGC ATTGCCCGTT TATGGACCCA   
  
  
+ ATGTCAGGTG GGAAATGCTG GATGTGAGGC CCAGGGAGGC TTTGGTCGTT AATTGTCCAT TACAGCTCCA   
  
  
+ TTACACTCCT GACGAGAGTG TCGATGTGAG GAACCCTAGG GATAGGCTTC TCAGAATGGT GAAATTGCTC   
  
  
+ GGTCCTAAGG TAGCCACTTT GGTTGAGCAA GAATCAAACA CCAACACTAC ACCTTTCTTG ACCCGGTTCA   
  
  
+ TAGAGACCCT TGACTACTAC TCGATTAACC ATGTTTGAGT CTATAGACGT GACCATGCCG AGAGACCGGA   
  
  
+ AGGAGAGGAT CAATGTTGAG CAGCATTGTT TAGCTAAGGA CATTGTGAAT ATCATAGCTT GCGAGGGCAA   
  
  
+ GGAGATGGTG GAGCGTCATG AGCTTTTTGG GAAATGGCAG TCGAGGTTTA CCATGGTAGG GTTCAGGCAG   
  
  
+ TACCCGTTGA GCTCATACAT AAACTCTGTG ATAAGAAGCC TACTCCGGTG TTACTCCAAG CATTATACTC   
  
  
+ TGATAGAGAA GGATAGTGCC ATGCTTCTGG GTTGGAGGGG CCGAATGCTG ATTTCAGCTT CGGCATG  

- -Up\_Stream \_Len000TTTAAA TATACTAATA CCCTCGTTTA TAATTAGCCA GATTTTGTTT TTTCTTAGCC   
  
  
- GGCTTGTTTT TTTTCCCGGG TCTTGATCAG GAAAATTACC TCAGGGTTTT ACACCATATT TAATCAGAAC   
  
  
- AAGTGAGTAA AGAGTGAGGA GAGTGAGTAA GATTGGGATT TAGAGTTAGG AGTGAGAGAC AGGAGAGATA   
  
  
- GACGCAGAGC TCGAAGAGAA AGGAGACCCG AAGGAACCTG AAAAAGCTGC GGTACTGTTA CCTTGTGACT   
  
  
- CGAGGTGGCA ACGGGGAGAG GTGTTGACAG AAGATTGCCA CCAAGAGGCA TCGGTGGCAT GGAGTTTTTG   
  
  
- GTTTCGCTAC GTGTTGATGG TGGGGTGGTG ACGACTTTTC ACGGGTGCTC AGAGCTAGGC GCGTTCGGAC   
  
  
- TACTCTTAGC TATGGTGGTG GTACATCTAT TCGACTTTTG CCCGTATTCC TTCGCTGGGT GCGCGGGTTG   
  
  
- TGCGGTAACT CCGTCACTGT GCACCCAACT ACGCGAAGGT TTCTACCTTA AAAGTGTAAA AAGAAAAGTT   
  
  
- CCAACGCATC TAGCCACACA CACCTACCGC TAGTTCCTTC TCAATTTTAC TTGATCAGTA CGCAGCTTAA   
  
  
- CAGGTTTTTT TTAAAAGAGA AAGTCAGCTC AGAATTTCCC CGTCAGTAGT TCTCGTACAA ACTTTTTGTA   
  
  
- ATACCGGTAT TGGTGAATCA TCATTAAGTT TATTAGTACC TTTGTATAAG TTGTACTTAA GTTATACTGA   
  
  
- TTTTTAAGGT AGAAAATCAA ACAAGACCAG TTGTGTCTCG TACGGAAAAA TTAACGTATT ATTGCTATAA   
  
  
- ATTGGTTGGT TAGTACAAAG AGTGTATAAT TATATCCGTT CCCATCTAAT TGTCAGTATT AGCGTATCAT   
  
  
- ACGTTCTTAA ATAAACGTTA GACCCGTATG ACAAAATATT TCGTGTTCAC AAGTACTGAA TTTTAGCTAT   
  
  
- TTCAGAATTA GAAAACAGCT AGGAACTCAC ACGTCTGTTA TATTAACACT TACATACGAT AATTGAAATT   
  
  
- ATTAATGCTT TTAATTCGTT GATTAGTATA TCAGTACAAG AGTTTTTGTT CGTACGCATA ATAAGTCTAG   
  
  
- TAATTTACGT AATTTACCCT GATTAGGCTA GTCAGTTCGC GACTATGCTG ATAACTTATT TGTCCTGATT   
  
  
- GGATCTTACG CCTTAATTGA CAGCTCTTAA CAGGTGTAGC CATCACTACG AACCGCCGAT GGTCTCAGTG   
  
  
- GAATCCCACA AGTGTAGGAT CTGGGCTTTT GGACGATTAC TACCTTCCCG TCGGCACCCT CCTCCTCTCC   
  
  
- TCTCACACCT AAAAAGATAA ATGTTTTTTC ACATATTCAC GATTAGGGGG CTTTGGAGGA AAATATCCAA   
  
  
- TCCCCCCGTT GATCCCAATA GCGGGATCCA CTGCGAACAC CGGCAGCCGG ATTAATTTAT CCGGGCTGGT   
  
  
- TGTCGACACT GACCTCGCTA TCGACTTCGT ACGTCTTTAC TCCTATTGTA TGTATAATAT CTAAAGGTGT   
  
  
- AACGTATCCC GTGAGTCACC CAATGACATA GCGTTCGGTA TCGTCGTTCC GAACCACCCG GAGGATTTCA   
  
  
- CGCTGAATGT CCGTAACTAC TAGGACAAAG ATTCATATGA ACACTACGAT CGAACCTCCG ACCACCCTTT   
  
  
- GCTAATCGCA GAGATAAACT TTTCAAATTT TATGAACAGC TCAAGTTACG TAACGGGCAA ATACCTGGGT   
  
  
- TACAGTCCAC CCTTTACGAC CTACACTCCG GGTCCCTCCG AAACCAGCAA TTAACAGGTA ATGTCGAGGT   
  
  
- AATGTGAGGA CTGCTCTCAC AGCTACACTC CTTGGGATCC CTATCCGAAG AGTCTTACCA CTTTAACGAG   
  
  
- CCAGGATTCC ATCGGTGAAA CCAACTCGTT CTTAGTTTGT GGTTGTGATG TGGAAAGAAC TGGGCCAAGT   
  
  
- ATCTCTGGGA ACTGATGATG AGCTAATTGG TACAAACTCA GATATCTGCA CTGGTACGGC TCTCTGGCCT   
  
  
- TCCTCTCCTA GTTACAACTC GTCGTAACAA ATCGATTCCT GTAACACTTA TAGTATCGAA CGCTCCCGTT   
  
  
- CCTCTACCAC CTCGCAGTAC TCGAAAAACC CTTTACCGTC AGCTCCAAAT GGTACCATCC CAAGTCCGTC   
  
  
- ATGGGCAACT CGAGTATGTA TTTGAGACAC TATTCTTCGG ATGAGGCCAC AATGAGGTTC GTAATATGAG   
  
  
- ACTATCTCTT CCTATCACGG TACGAAGACC CAACCTCCCC GGCTTACGAC TAAAGTCGAA GCCGTAC

+     CGTCA-motif

| Site Name | Organism | Position | Strand | Matrix score. | sequence | function |
| --- | --- | --- | --- | --- | --- | --- |
| CGTCA-motif | Hordeum vulgare | 2118 | + | 5 | CGTCA | cis-acting regulatory element involved in the MeJA-responsiveness |
| CGTCA-motif | Hordeum vulgare | 1434 | - | 5 | CGTCA | cis-acting regulatory element involved in the MeJA-responsiveness |
| CGTCA-motif | Hordeum vulgare | 1834 | - | 5 | CGTCA | cis-acting regulatory element involved in the MeJA-responsiveness |

>HU08G00229.1   
+ -Up\_Stream \_Len000AAATTT ATATGATTAT GGGAGCAAAT ATTAATCGGT CTAAAACAAA AAAGAATCGG   
  
  
+ CCGAACAAAA AAAAGGGCCC AGAACTAGTC CTTTTAATGG AGTCCCAAAA TGTGGTATAA ATTAGTCTTG   
  
  
+ TTCACTCATT TCTCACTCCT CTCACTCATT CTAACCCTAA ATCTCAATCC TCACTCTCTG TCCTCTCTAT   
  
  
+ CTGCGTCTCG AGCTTCTCTT TCCTCTGGGC TTCCTTGGAC TTTTTCGACG CCATGACAAT GGAACACTGA   
  
  
+ GCTCCACCGT TGCCCCTCTC CACAACTGTC TTCTAACGGT GGTTCTCCGT AGCCACCGTA CCTCAAAAAC   
  
  
+ CAAAGCGATG CACAACTACC ACCCCACCAC TGCTGAAAAG TGCCCACGAG TCTCGATCCG CGCAAGCCTG   
  
  
+ ATGAGAATCG ATACCACCAC CATGTAGATA AGCTGAAAAC GGGCATAAGG AAGCGACCCA CGCGCCCAAC   
  
  
+ ACGCCATTGA GGCAGTGACA CGTGGGTTGA TGCGCTTCCA AAGATGGAAT TTTCACATTT TTCTTTTCAA   
  
  
+ GGTTGCGTAG ATCGGTGTGT GTGGATGGCG ATCAAGGAAG AGTTAAAATG AACTAGTCAT GCGTCGAATT   
  
  
+ GTCCAAAAAA AATTTTCTCT TTCAGTCGAG TCTTAAAGGG GCAGTCATCA AGAGCATGTT TGAAAAACAT   
  
  
+ TATGGCCATA ACCACTTAGT AGTAATTCAA ATAATCATGG AAACATATTC AACATGAATT CAATATGACT   
  
  
+ AAAAATTCCA TCTTTTAGTT TGTTCTGGTC AACACAGAGC ATGCCTTTTT AATTGCATAA TAACGATATT   
  
  
+ TAACCAACCA ATCATGTTTC TCACATATTA ATATAGGCAA GGGTAGATTA ACAGTCATAA TCGCATAGTA   
  
  
+ TGCAAGAATT TATTTGCAAT CTGGGCATAC TGTTTTATAA AGCACAAGTG TTCATGACTT AAAATCGATA   
  
  
+ AAGTCTTAAT CTTTTGTCGA TCCTTGAGTG TGCAGACAAT ATAATTGTGA ATGTATGCTA TTAACTTTAA   
  
  
+ TAATTACGAA AATTAAGCAA CTAATCATAT AGTCATGTTC TCAAAAACAA GCATGCGTAT TATTCAGATC   
  
  
+ ATTAAATGCA TTAAATGGGA CTAATCCGAT CAGTCAAGCG CTGATACGAC TATTGAATAA ACAGGACTAA   
  
  
+ CCTAGAATGC GGAATTAACT GTCGAGAATT GTCCACATCG GTAGTGATGC TTGGCGGCTA CCAGAGTCAC   
  
  
+ CTTAGGGTGT TCACATCCTA GACCCGAAAA CCTGCTAATG ATGGAAGGGC AGCCGTGGGA GGAGGAGAGG   
  
  
+ AGAGTGTGGA TTTTTCTATT TACAAAAAAG TGTATAAGTG CTAATCCCCC GAAACCTCCT TTTATAGGTT   
  
  
+ AGGGGGGCAA CTAGGGTTAT CGCCCTAGGT GACGCTTGTG GCCGTCGGCC TAATTAAATA GGCCCGACCA   
  
  
+ ACAGCTGTGA CTGGAGCGAT AGCTGAAGCA TGCAGAAATG AGGATAACAT ACATATTATA GATTTCCACA   
  
  
+ TTGCATAGGG CACTCAGTGG GTTACTGTAT CGCAAGCCAT AGCAGCAAGG CTTGGTGGGC CTCCTAAAGT   
  
  
+ GCGACTTACA GGCATTGATG ATCCTGTTTC TAAGTATACT TGTGATGCTA GCTTGGAGGC TGGTGGGAAA   
  
  
+ CGATTAGCGT CTCTATTTGA AAAGTTTAAA ATACTTGTCG AGTTCAATGC ATTGCCCGTT TATGGACCCA   
  
  
+ ATGTCAGGTG GGAAATGCTG GATGTGAGGC CCAGGGAGGC TTTGGTCGTT AATTGTCCAT TACAGCTCCA   
  
  
+ TTACACTCCT GACGAGAGTG TCGATGTGAG GAACCCTAGG GATAGGCTTC TCAGAATGGT GAAATTGCTC   
  
  
+ GGTCCTAAGG TAGCCACTTT GGTTGAGCAA GAATCAAACA CCAACACTAC ACCTTTCTTG ACCCGGTTCA   
  
  
+ TAGAGACCCT TGACTACTAC TCGATTAACC ATGTTTGAGT CTATAGACGT GACCATGCCG AGAGACCGGA   
  
  
+ AGGAGAGGAT CAATGTTGAG CAGCATTGTT TAGCTAAGGA CATTGTGAAT ATCATAGCTT GCGAGGGCAA   
  
  
+ GGAGATGGTG GAGCGTCATG AGCTTTTTGG GAAATGGCAG TCGAGGTTTA CCATGGTAGG GTTCAGGCAG   
  
  
+ TACCCGTTGA GCTCATACAT AAACTCTGTG ATAAGAAGCC TACTCCGGTG TTACTCCAAG CATTATACTC   
  
  
+ TGATAGAGAA GGATAGTGCC ATGCTTCTGG GTTGGAGGGG CCGAATGCTG ATTTCAGCTT CGGCATG  

- -Up\_Stream \_Len000TTTAAA TATACTAATA CCCTCGTTTA TAATTAGCCA GATTTTGTTT TTTCTTAGCC   
  
  
- GGCTTGTTTT TTTTCCCGGG TCTTGATCAG GAAAATTACC TCAGGGTTTT ACACCATATT TAATCAGAAC   
  
  
- AAGTGAGTAA AGAGTGAGGA GAGTGAGTAA GATTGGGATT TAGAGTTAGG AGTGAGAGAC AGGAGAGATA   
  
  
- GACGCAGAGC TCGAAGAGAA AGGAGACCCG AAGGAACCTG AAAAAGCTGC GGTACTGTTA CCTTGTGACT   
  
  
- CGAGGTGGCA ACGGGGAGAG GTGTTGACAG AAGATTGCCA CCAAGAGGCA TCGGTGGCAT GGAGTTTTTG   
  
  
- GTTTCGCTAC GTGTTGATGG TGGGGTGGTG ACGACTTTTC ACGGGTGCTC AGAGCTAGGC GCGTTCGGAC   
  
  
- TACTCTTAGC TATGGTGGTG GTACATCTAT TCGACTTTTG CCCGTATTCC TTCGCTGGGT GCGCGGGTTG   
  
  
- TGCGGTAACT CCGTCACTGT GCACCCAACT ACGCGAAGGT TTCTACCTTA AAAGTGTAAA AAGAAAAGTT   
  
  
- CCAACGCATC TAGCCACACA CACCTACCGC TAGTTCCTTC TCAATTTTAC TTGATCAGTA CGCAGCTTAA   
  
  
- CAGGTTTTTT TTAAAAGAGA AAGTCAGCTC AGAATTTCCC CGTCAGTAGT TCTCGTACAA ACTTTTTGTA   
  
  
- ATACCGGTAT TGGTGAATCA TCATTAAGTT TATTAGTACC TTTGTATAAG TTGTACTTAA GTTATACTGA   
  
  
- TTTTTAAGGT AGAAAATCAA ACAAGACCAG TTGTGTCTCG TACGGAAAAA TTAACGTATT ATTGCTATAA   
  
  
- ATTGGTTGGT TAGTACAAAG AGTGTATAAT TATATCCGTT CCCATCTAAT TGTCAGTATT AGCGTATCAT   
  
  
- ACGTTCTTAA ATAAACGTTA GACCCGTATG ACAAAATATT TCGTGTTCAC AAGTACTGAA TTTTAGCTAT   
  
  
- TTCAGAATTA GAAAACAGCT AGGAACTCAC ACGTCTGTTA TATTAACACT TACATACGAT AATTGAAATT   
  
  
- ATTAATGCTT TTAATTCGTT GATTAGTATA TCAGTACAAG AGTTTTTGTT CGTACGCATA ATAAGTCTAG   
  
  
- TAATTTACGT AATTTACCCT GATTAGGCTA GTCAGTTCGC GACTATGCTG ATAACTTATT TGTCCTGATT   
  
  
- GGATCTTACG CCTTAATTGA CAGCTCTTAA CAGGTGTAGC CATCACTACG AACCGCCGAT GGTCTCAGTG   
  
  
- GAATCCCACA AGTGTAGGAT CTGGGCTTTT GGACGATTAC TACCTTCCCG TCGGCACCCT CCTCCTCTCC   
  
  
- TCTCACACCT AAAAAGATAA ATGTTTTTTC ACATATTCAC GATTAGGGGG CTTTGGAGGA AAATATCCAA   
  
  
- TCCCCCCGTT GATCCCAATA GCGGGATCCA CTGCGAACAC CGGCAGCCGG ATTAATTTAT CCGGGCTGGT   
  
  
- TGTCGACACT GACCTCGCTA TCGACTTCGT ACGTCTTTAC TCCTATTGTA TGTATAATAT CTAAAGGTGT   
  
  
- AACGTATCCC GTGAGTCACC CAATGACATA GCGTTCGGTA TCGTCGTTCC GAACCACCCG GAGGATTTCA   
  
  
- CGCTGAATGT CCGTAACTAC TAGGACAAAG ATTCATATGA ACACTACGAT CGAACCTCCG ACCACCCTTT   
  
  
- GCTAATCGCA GAGATAAACT TTTCAAATTT TATGAACAGC TCAAGTTACG TAACGGGCAA ATACCTGGGT   
  
  
- TACAGTCCAC CCTTTACGAC CTACACTCCG GGTCCCTCCG AAACCAGCAA TTAACAGGTA ATGTCGAGGT   
  
  
- AATGTGAGGA CTGCTCTCAC AGCTACACTC CTTGGGATCC CTATCCGAAG AGTCTTACCA CTTTAACGAG   
  
  
- CCAGGATTCC ATCGGTGAAA CCAACTCGTT CTTAGTTTGT GGTTGTGATG TGGAAAGAAC TGGGCCAAGT   
  
  
- ATCTCTGGGA ACTGATGATG AGCTAATTGG TACAAACTCA GATATCTGCA CTGGTACGGC TCTCTGGCCT   
  
  
- TCCTCTCCTA GTTACAACTC GTCGTAACAA ATCGATTCCT GTAACACTTA TAGTATCGAA CGCTCCCGTT   
  
  
- CCTCTACCAC CTCGCAGTAC TCGAAAAACC CTTTACCGTC AGCTCCAAAT GGTACCATCC CAAGTCCGTC   
  
  
- ATGGGCAACT CGAGTATGTA TTTGAGACAC TATTCTTCGG ATGAGGCCAC AATGAGGTTC GTAATATGAG   
  
  
- ACTATCTCTT CCTATCACGG TACGAAGACC CAACCTCCCC GGCTTACGAC TAAAGTCGAA GCCGTAC

+     DRE core

| Site Name | Organism | Position | Strand | Matrix score. | sequence | function |
| --- | --- | --- | --- | --- | --- | --- |
| DRE core | Arabidopsis thaliana | 1448 | - | 6 | GCCGAC |  |

>HU08G00229.1   
+ -Up\_Stream \_Len000AAATTT ATATGATTAT GGGAGCAAAT ATTAATCGGT CTAAAACAAA AAAGAATCGG   
  
  
+ CCGAACAAAA AAAAGGGCCC AGAACTAGTC CTTTTAATGG AGTCCCAAAA TGTGGTATAA ATTAGTCTTG   
  
  
+ TTCACTCATT TCTCACTCCT CTCACTCATT CTAACCCTAA ATCTCAATCC TCACTCTCTG TCCTCTCTAT   
  
  
+ CTGCGTCTCG AGCTTCTCTT TCCTCTGGGC TTCCTTGGAC TTTTTCGACG CCATGACAAT GGAACACTGA   
  
  
+ GCTCCACCGT TGCCCCTCTC CACAACTGTC TTCTAACGGT GGTTCTCCGT AGCCACCGTA CCTCAAAAAC   
  
  
+ CAAAGCGATG CACAACTACC ACCCCACCAC TGCTGAAAAG TGCCCACGAG TCTCGATCCG CGCAAGCCTG   
  
  
+ ATGAGAATCG ATACCACCAC CATGTAGATA AGCTGAAAAC GGGCATAAGG AAGCGACCCA CGCGCCCAAC   
  
  
+ ACGCCATTGA GGCAGTGACA CGTGGGTTGA TGCGCTTCCA AAGATGGAAT TTTCACATTT TTCTTTTCAA   
  
  
+ GGTTGCGTAG ATCGGTGTGT GTGGATGGCG ATCAAGGAAG AGTTAAAATG AACTAGTCAT GCGTCGAATT   
  
  
+ GTCCAAAAAA AATTTTCTCT TTCAGTCGAG TCTTAAAGGG GCAGTCATCA AGAGCATGTT TGAAAAACAT   
  
  
+ TATGGCCATA ACCACTTAGT AGTAATTCAA ATAATCATGG AAACATATTC AACATGAATT CAATATGACT   
  
  
+ AAAAATTCCA TCTTTTAGTT TGTTCTGGTC AACACAGAGC ATGCCTTTTT AATTGCATAA TAACGATATT   
  
  
+ TAACCAACCA ATCATGTTTC TCACATATTA ATATAGGCAA GGGTAGATTA ACAGTCATAA TCGCATAGTA   
  
  
+ TGCAAGAATT TATTTGCAAT CTGGGCATAC TGTTTTATAA AGCACAAGTG TTCATGACTT AAAATCGATA   
  
  
+ AAGTCTTAAT CTTTTGTCGA TCCTTGAGTG TGCAGACAAT ATAATTGTGA ATGTATGCTA TTAACTTTAA   
  
  
+ TAATTACGAA AATTAAGCAA CTAATCATAT AGTCATGTTC TCAAAAACAA GCATGCGTAT TATTCAGATC   
  
  
+ ATTAAATGCA TTAAATGGGA CTAATCCGAT CAGTCAAGCG CTGATACGAC TATTGAATAA ACAGGACTAA   
  
  
+ CCTAGAATGC GGAATTAACT GTCGAGAATT GTCCACATCG GTAGTGATGC TTGGCGGCTA CCAGAGTCAC   
  
  
+ CTTAGGGTGT TCACATCCTA GACCCGAAAA CCTGCTAATG ATGGAAGGGC AGCCGTGGGA GGAGGAGAGG   
  
  
+ AGAGTGTGGA TTTTTCTATT TACAAAAAAG TGTATAAGTG CTAATCCCCC GAAACCTCCT TTTATAGGTT   
  
  
+ AGGGGGGCAA CTAGGGTTAT CGCCCTAGGT GACGCTTGTG GCCGTCGGCC TAATTAAATA GGCCCGACCA   
  
  
+ ACAGCTGTGA CTGGAGCGAT AGCTGAAGCA TGCAGAAATG AGGATAACAT ACATATTATA GATTTCCACA   
  
  
+ TTGCATAGGG CACTCAGTGG GTTACTGTAT CGCAAGCCAT AGCAGCAAGG CTTGGTGGGC CTCCTAAAGT   
  
  
+ GCGACTTACA GGCATTGATG ATCCTGTTTC TAAGTATACT TGTGATGCTA GCTTGGAGGC TGGTGGGAAA   
  
  
+ CGATTAGCGT CTCTATTTGA AAAGTTTAAA ATACTTGTCG AGTTCAATGC ATTGCCCGTT TATGGACCCA   
  
  
+ ATGTCAGGTG GGAAATGCTG GATGTGAGGC CCAGGGAGGC TTTGGTCGTT AATTGTCCAT TACAGCTCCA   
  
  
+ TTACACTCCT GACGAGAGTG TCGATGTGAG GAACCCTAGG GATAGGCTTC TCAGAATGGT GAAATTGCTC   
  
  
+ GGTCCTAAGG TAGCCACTTT GGTTGAGCAA GAATCAAACA CCAACACTAC ACCTTTCTTG ACCCGGTTCA   
  
  
+ TAGAGACCCT TGACTACTAC TCGATTAACC ATGTTTGAGT CTATAGACGT GACCATGCCG AGAGACCGGA   
  
  
+ AGGAGAGGAT CAATGTTGAG CAGCATTGTT TAGCTAAGGA CATTGTGAAT ATCATAGCTT GCGAGGGCAA   
  
  
+ GGAGATGGTG GAGCGTCATG AGCTTTTTGG GAAATGGCAG TCGAGGTTTA CCATGGTAGG GTTCAGGCAG   
  
  
+ TACCCGTTGA GCTCATACAT AAACTCTGTG ATAAGAAGCC TACTCCGGTG TTACTCCAAG CATTATACTC   
  
  
+ TGATAGAGAA GGATAGTGCC ATGCTTCTGG GTTGGAGGGG CCGAATGCTG ATTTCAGCTT CGGCATG  

- -Up\_Stream \_Len000TTTAAA TATACTAATA CCCTCGTTTA TAATTAGCCA GATTTTGTTT TTTCTTAGCC   
  
  
- GGCTTGTTTT TTTTCCCGGG TCTTGATCAG GAAAATTACC TCAGGGTTTT ACACCATATT TAATCAGAAC   
  
  
- AAGTGAGTAA AGAGTGAGGA GAGTGAGTAA GATTGGGATT TAGAGTTAGG AGTGAGAGAC AGGAGAGATA   
  
  
- GACGCAGAGC TCGAAGAGAA AGGAGACCCG AAGGAACCTG AAAAAGCTGC GGTACTGTTA CCTTGTGACT   
  
  
- CGAGGTGGCA ACGGGGAGAG GTGTTGACAG AAGATTGCCA CCAAGAGGCA TCGGTGGCAT GGAGTTTTTG   
  
  
- GTTTCGCTAC GTGTTGATGG TGGGGTGGTG ACGACTTTTC ACGGGTGCTC AGAGCTAGGC GCGTTCGGAC   
  
  
- TACTCTTAGC TATGGTGGTG GTACATCTAT TCGACTTTTG CCCGTATTCC TTCGCTGGGT GCGCGGGTTG   
  
  
- TGCGGTAACT CCGTCACTGT GCACCCAACT ACGCGAAGGT TTCTACCTTA AAAGTGTAAA AAGAAAAGTT   
  
  
- CCAACGCATC TAGCCACACA CACCTACCGC TAGTTCCTTC TCAATTTTAC TTGATCAGTA CGCAGCTTAA   
  
  
- CAGGTTTTTT TTAAAAGAGA AAGTCAGCTC AGAATTTCCC CGTCAGTAGT TCTCGTACAA ACTTTTTGTA   
  
  
- ATACCGGTAT TGGTGAATCA TCATTAAGTT TATTAGTACC TTTGTATAAG TTGTACTTAA GTTATACTGA   
  
  
- TTTTTAAGGT AGAAAATCAA ACAAGACCAG TTGTGTCTCG TACGGAAAAA TTAACGTATT ATTGCTATAA   
  
  
- ATTGGTTGGT TAGTACAAAG AGTGTATAAT TATATCCGTT CCCATCTAAT TGTCAGTATT AGCGTATCAT   
  
  
- ACGTTCTTAA ATAAACGTTA GACCCGTATG ACAAAATATT TCGTGTTCAC AAGTACTGAA TTTTAGCTAT   
  
  
- TTCAGAATTA GAAAACAGCT AGGAACTCAC ACGTCTGTTA TATTAACACT TACATACGAT AATTGAAATT   
  
  
- ATTAATGCTT TTAATTCGTT GATTAGTATA TCAGTACAAG AGTTTTTGTT CGTACGCATA ATAAGTCTAG   
  
  
- TAATTTACGT AATTTACCCT GATTAGGCTA GTCAGTTCGC GACTATGCTG ATAACTTATT TGTCCTGATT   
  
  
- GGATCTTACG CCTTAATTGA CAGCTCTTAA CAGGTGTAGC CATCACTACG AACCGCCGAT GGTCTCAGTG   
  
  
- GAATCCCACA AGTGTAGGAT CTGGGCTTTT GGACGATTAC TACCTTCCCG TCGGCACCCT CCTCCTCTCC   
  
  
- TCTCACACCT AAAAAGATAA ATGTTTTTTC ACATATTCAC GATTAGGGGG CTTTGGAGGA AAATATCCAA   
  
  
- TCCCCCCGTT GATCCCAATA GCGGGATCCA CTGCGAACAC CGGCAGCCGG ATTAATTTAT CCGGGCTGGT   
  
  
- TGTCGACACT GACCTCGCTA TCGACTTCGT ACGTCTTTAC TCCTATTGTA TGTATAATAT CTAAAGGTGT   
  
  
- AACGTATCCC GTGAGTCACC CAATGACATA GCGTTCGGTA TCGTCGTTCC GAACCACCCG GAGGATTTCA   
  
  
- CGCTGAATGT CCGTAACTAC TAGGACAAAG ATTCATATGA ACACTACGAT CGAACCTCCG ACCACCCTTT   
  
  
- GCTAATCGCA GAGATAAACT TTTCAAATTT TATGAACAGC TCAAGTTACG TAACGGGCAA ATACCTGGGT   
  
  
- TACAGTCCAC CCTTTACGAC CTACACTCCG GGTCCCTCCG AAACCAGCAA TTAACAGGTA ATGTCGAGGT   
  
  
- AATGTGAGGA CTGCTCTCAC AGCTACACTC CTTGGGATCC CTATCCGAAG AGTCTTACCA CTTTAACGAG   
  
  
- CCAGGATTCC ATCGGTGAAA CCAACTCGTT CTTAGTTTGT GGTTGTGATG TGGAAAGAAC TGGGCCAAGT   
  
  
- ATCTCTGGGA ACTGATGATG AGCTAATTGG TACAAACTCA GATATCTGCA CTGGTACGGC TCTCTGGCCT   
  
  
- TCCTCTCCTA GTTACAACTC GTCGTAACAA ATCGATTCCT GTAACACTTA TAGTATCGAA CGCTCCCGTT   
  
  
- CCTCTACCAC CTCGCAGTAC TCGAAAAACC CTTTACCGTC AGCTCCAAAT GGTACCATCC CAAGTCCGTC   
  
  
- ATGGGCAACT CGAGTATGTA TTTGAGACAC TATTCTTCGG ATGAGGCCAC AATGAGGTTC GTAATATGAG   
  
  
- ACTATCTCTT CCTATCACGG TACGAAGACC CAACCTCCCC GGCTTACGAC TAAAGTCGAA GCCGTAC

+     ERE

| Site Name | Organism | Position | Strand | Matrix score. | sequence | function |
| --- | --- | --- | --- | --- | --- | --- |
| ERE | Nicotiana glutinos | 1709 | - | 8 | ATTTTAAA |  |

>HU08G00229.1   
+ -Up\_Stream \_Len000AAATTT ATATGATTAT GGGAGCAAAT ATTAATCGGT CTAAAACAAA AAAGAATCGG   
  
  
+ CCGAACAAAA AAAAGGGCCC AGAACTAGTC CTTTTAATGG AGTCCCAAAA TGTGGTATAA ATTAGTCTTG   
  
  
+ TTCACTCATT TCTCACTCCT CTCACTCATT CTAACCCTAA ATCTCAATCC TCACTCTCTG TCCTCTCTAT   
  
  
+ CTGCGTCTCG AGCTTCTCTT TCCTCTGGGC TTCCTTGGAC TTTTTCGACG CCATGACAAT GGAACACTGA   
  
  
+ GCTCCACCGT TGCCCCTCTC CACAACTGTC TTCTAACGGT GGTTCTCCGT AGCCACCGTA CCTCAAAAAC   
  
  
+ CAAAGCGATG CACAACTACC ACCCCACCAC TGCTGAAAAG TGCCCACGAG TCTCGATCCG CGCAAGCCTG   
  
  
+ ATGAGAATCG ATACCACCAC CATGTAGATA AGCTGAAAAC GGGCATAAGG AAGCGACCCA CGCGCCCAAC   
  
  
+ ACGCCATTGA GGCAGTGACA CGTGGGTTGA TGCGCTTCCA AAGATGGAAT TTTCACATTT TTCTTTTCAA   
  
  
+ GGTTGCGTAG ATCGGTGTGT GTGGATGGCG ATCAAGGAAG AGTTAAAATG AACTAGTCAT GCGTCGAATT   
  
  
+ GTCCAAAAAA AATTTTCTCT TTCAGTCGAG TCTTAAAGGG GCAGTCATCA AGAGCATGTT TGAAAAACAT   
  
  
+ TATGGCCATA ACCACTTAGT AGTAATTCAA ATAATCATGG AAACATATTC AACATGAATT CAATATGACT   
  
  
+ AAAAATTCCA TCTTTTAGTT TGTTCTGGTC AACACAGAGC ATGCCTTTTT AATTGCATAA TAACGATATT   
  
  
+ TAACCAACCA ATCATGTTTC TCACATATTA ATATAGGCAA GGGTAGATTA ACAGTCATAA TCGCATAGTA   
  
  
+ TGCAAGAATT TATTTGCAAT CTGGGCATAC TGTTTTATAA AGCACAAGTG TTCATGACTT AAAATCGATA   
  
  
+ AAGTCTTAAT CTTTTGTCGA TCCTTGAGTG TGCAGACAAT ATAATTGTGA ATGTATGCTA TTAACTTTAA   
  
  
+ TAATTACGAA AATTAAGCAA CTAATCATAT AGTCATGTTC TCAAAAACAA GCATGCGTAT TATTCAGATC   
  
  
+ ATTAAATGCA TTAAATGGGA CTAATCCGAT CAGTCAAGCG CTGATACGAC TATTGAATAA ACAGGACTAA   
  
  
+ CCTAGAATGC GGAATTAACT GTCGAGAATT GTCCACATCG GTAGTGATGC TTGGCGGCTA CCAGAGTCAC   
  
  
+ CTTAGGGTGT TCACATCCTA GACCCGAAAA CCTGCTAATG ATGGAAGGGC AGCCGTGGGA GGAGGAGAGG   
  
  
+ AGAGTGTGGA TTTTTCTATT TACAAAAAAG TGTATAAGTG CTAATCCCCC GAAACCTCCT TTTATAGGTT   
  
  
+ AGGGGGGCAA CTAGGGTTAT CGCCCTAGGT GACGCTTGTG GCCGTCGGCC TAATTAAATA GGCCCGACCA   
  
  
+ ACAGCTGTGA CTGGAGCGAT AGCTGAAGCA TGCAGAAATG AGGATAACAT ACATATTATA GATTTCCACA   
  
  
+ TTGCATAGGG CACTCAGTGG GTTACTGTAT CGCAAGCCAT AGCAGCAAGG CTTGGTGGGC CTCCTAAAGT   
  
  
+ GCGACTTACA GGCATTGATG ATCCTGTTTC TAAGTATACT TGTGATGCTA GCTTGGAGGC TGGTGGGAAA   
  
  
+ CGATTAGCGT CTCTATTTGA AAAGTTTAAA ATACTTGTCG AGTTCAATGC ATTGCCCGTT TATGGACCCA   
  
  
+ ATGTCAGGTG GGAAATGCTG GATGTGAGGC CCAGGGAGGC TTTGGTCGTT AATTGTCCAT TACAGCTCCA   
  
  
+ TTACACTCCT GACGAGAGTG TCGATGTGAG GAACCCTAGG GATAGGCTTC TCAGAATGGT GAAATTGCTC   
  
  
+ GGTCCTAAGG TAGCCACTTT GGTTGAGCAA GAATCAAACA CCAACACTAC ACCTTTCTTG ACCCGGTTCA   
  
  
+ TAGAGACCCT TGACTACTAC TCGATTAACC ATGTTTGAGT CTATAGACGT GACCATGCCG AGAGACCGGA   
  
  
+ AGGAGAGGAT CAATGTTGAG CAGCATTGTT TAGCTAAGGA CATTGTGAAT ATCATAGCTT GCGAGGGCAA   
  
  
+ GGAGATGGTG GAGCGTCATG AGCTTTTTGG GAAATGGCAG TCGAGGTTTA CCATGGTAGG GTTCAGGCAG   
  
  
+ TACCCGTTGA GCTCATACAT AAACTCTGTG ATAAGAAGCC TACTCCGGTG TTACTCCAAG CATTATACTC   
  
  
+ TGATAGAGAA GGATAGTGCC ATGCTTCTGG GTTGGAGGGG CCGAATGCTG ATTTCAGCTT CGGCATG  

- -Up\_Stream \_Len000TTTAAA TATACTAATA CCCTCGTTTA TAATTAGCCA GATTTTGTTT TTTCTTAGCC   
  
  
- GGCTTGTTTT TTTTCCCGGG TCTTGATCAG GAAAATTACC TCAGGGTTTT ACACCATATT TAATCAGAAC   
  
  
- AAGTGAGTAA AGAGTGAGGA GAGTGAGTAA GATTGGGATT TAGAGTTAGG AGTGAGAGAC AGGAGAGATA   
  
  
- GACGCAGAGC TCGAAGAGAA AGGAGACCCG AAGGAACCTG AAAAAGCTGC GGTACTGTTA CCTTGTGACT   
  
  
- CGAGGTGGCA ACGGGGAGAG GTGTTGACAG AAGATTGCCA CCAAGAGGCA TCGGTGGCAT GGAGTTTTTG   
  
  
- GTTTCGCTAC GTGTTGATGG TGGGGTGGTG ACGACTTTTC ACGGGTGCTC AGAGCTAGGC GCGTTCGGAC   
  
  
- TACTCTTAGC TATGGTGGTG GTACATCTAT TCGACTTTTG CCCGTATTCC TTCGCTGGGT GCGCGGGTTG   
  
  
- TGCGGTAACT CCGTCACTGT GCACCCAACT ACGCGAAGGT TTCTACCTTA AAAGTGTAAA AAGAAAAGTT   
  
  
- CCAACGCATC TAGCCACACA CACCTACCGC TAGTTCCTTC TCAATTTTAC TTGATCAGTA CGCAGCTTAA   
  
  
- CAGGTTTTTT TTAAAAGAGA AAGTCAGCTC AGAATTTCCC CGTCAGTAGT TCTCGTACAA ACTTTTTGTA   
  
  
- ATACCGGTAT TGGTGAATCA TCATTAAGTT TATTAGTACC TTTGTATAAG TTGTACTTAA GTTATACTGA   
  
  
- TTTTTAAGGT AGAAAATCAA ACAAGACCAG TTGTGTCTCG TACGGAAAAA TTAACGTATT ATTGCTATAA   
  
  
- ATTGGTTGGT TAGTACAAAG AGTGTATAAT TATATCCGTT CCCATCTAAT TGTCAGTATT AGCGTATCAT   
  
  
- ACGTTCTTAA ATAAACGTTA GACCCGTATG ACAAAATATT TCGTGTTCAC AAGTACTGAA TTTTAGCTAT   
  
  
- TTCAGAATTA GAAAACAGCT AGGAACTCAC ACGTCTGTTA TATTAACACT TACATACGAT AATTGAAATT   
  
  
- ATTAATGCTT TTAATTCGTT GATTAGTATA TCAGTACAAG AGTTTTTGTT CGTACGCATA ATAAGTCTAG   
  
  
- TAATTTACGT AATTTACCCT GATTAGGCTA GTCAGTTCGC GACTATGCTG ATAACTTATT TGTCCTGATT   
  
  
- GGATCTTACG CCTTAATTGA CAGCTCTTAA CAGGTGTAGC CATCACTACG AACCGCCGAT GGTCTCAGTG   
  
  
- GAATCCCACA AGTGTAGGAT CTGGGCTTTT GGACGATTAC TACCTTCCCG TCGGCACCCT CCTCCTCTCC   
  
  
- TCTCACACCT AAAAAGATAA ATGTTTTTTC ACATATTCAC GATTAGGGGG CTTTGGAGGA AAATATCCAA   
  
  
- TCCCCCCGTT GATCCCAATA GCGGGATCCA CTGCGAACAC CGGCAGCCGG ATTAATTTAT CCGGGCTGGT   
  
  
- TGTCGACACT GACCTCGCTA TCGACTTCGT ACGTCTTTAC TCCTATTGTA TGTATAATAT CTAAAGGTGT   
  
  
- AACGTATCCC GTGAGTCACC CAATGACATA GCGTTCGGTA TCGTCGTTCC GAACCACCCG GAGGATTTCA   
  
  
- CGCTGAATGT CCGTAACTAC TAGGACAAAG ATTCATATGA ACACTACGAT CGAACCTCCG ACCACCCTTT   
  
  
- GCTAATCGCA GAGATAAACT TTTCAAATTT TATGAACAGC TCAAGTTACG TAACGGGCAA ATACCTGGGT   
  
  
- TACAGTCCAC CCTTTACGAC CTACACTCCG GGTCCCTCCG AAACCAGCAA TTAACAGGTA ATGTCGAGGT   
  
  
- AATGTGAGGA CTGCTCTCAC AGCTACACTC CTTGGGATCC CTATCCGAAG AGTCTTACCA CTTTAACGAG   
  
  
- CCAGGATTCC ATCGGTGAAA CCAACTCGTT CTTAGTTTGT GGTTGTGATG TGGAAAGAAC TGGGCCAAGT   
  
  
- ATCTCTGGGA ACTGATGATG AGCTAATTGG TACAAACTCA GATATCTGCA CTGGTACGGC TCTCTGGCCT   
  
  
- TCCTCTCCTA GTTACAACTC GTCGTAACAA ATCGATTCCT GTAACACTTA TAGTATCGAA CGCTCCCGTT   
  
  
- CCTCTACCAC CTCGCAGTAC TCGAAAAACC CTTTACCGTC AGCTCCAAAT GGTACCATCC CAAGTCCGTC   
  
  
- ATGGGCAACT CGAGTATGTA TTTGAGACAC TATTCTTCGG ATGAGGCCAC AATGAGGTTC GTAATATGAG   
  
  
- ACTATCTCTT CCTATCACGG TACGAAGACC CAACCTCCCC GGCTTACGAC TAAAGTCGAA GCCGTAC

+     G-Box

| Site Name | Organism | Position | Strand | Matrix score. | sequence | function |
| --- | --- | --- | --- | --- | --- | --- |
| G-Box | Triticum aestivum | 1539 | + | 10 | TCCACATGGCA | cis-acting regulatory element involved in light responsiveness |
| G-Box | Pisum sativum | 513 | + | 6 | CACGTG | cis-acting regulatory element involved in light responsiveness |

>HU08G00229.1   
+ -Up\_Stream \_Len000AAATTT ATATGATTAT GGGAGCAAAT ATTAATCGGT CTAAAACAAA AAAGAATCGG   
  
  
+ CCGAACAAAA AAAAGGGCCC AGAACTAGTC CTTTTAATGG AGTCCCAAAA TGTGGTATAA ATTAGTCTTG   
  
  
+ TTCACTCATT TCTCACTCCT CTCACTCATT CTAACCCTAA ATCTCAATCC TCACTCTCTG TCCTCTCTAT   
  
  
+ CTGCGTCTCG AGCTTCTCTT TCCTCTGGGC TTCCTTGGAC TTTTTCGACG CCATGACAAT GGAACACTGA   
  
  
+ GCTCCACCGT TGCCCCTCTC CACAACTGTC TTCTAACGGT GGTTCTCCGT AGCCACCGTA CCTCAAAAAC   
  
  
+ CAAAGCGATG CACAACTACC ACCCCACCAC TGCTGAAAAG TGCCCACGAG TCTCGATCCG CGCAAGCCTG   
  
  
+ ATGAGAATCG ATACCACCAC CATGTAGATA AGCTGAAAAC GGGCATAAGG AAGCGACCCA CGCGCCCAAC   
  
  
+ ACGCCATTGA GGCAGTGACA CGTGGGTTGA TGCGCTTCCA AAGATGGAAT TTTCACATTT TTCTTTTCAA   
  
  
+ GGTTGCGTAG ATCGGTGTGT GTGGATGGCG ATCAAGGAAG AGTTAAAATG AACTAGTCAT GCGTCGAATT   
  
  
+ GTCCAAAAAA AATTTTCTCT TTCAGTCGAG TCTTAAAGGG GCAGTCATCA AGAGCATGTT TGAAAAACAT   
  
  
+ TATGGCCATA ACCACTTAGT AGTAATTCAA ATAATCATGG AAACATATTC AACATGAATT CAATATGACT   
  
  
+ AAAAATTCCA TCTTTTAGTT TGTTCTGGTC AACACAGAGC ATGCCTTTTT AATTGCATAA TAACGATATT   
  
  
+ TAACCAACCA ATCATGTTTC TCACATATTA ATATAGGCAA GGGTAGATTA ACAGTCATAA TCGCATAGTA   
  
  
+ TGCAAGAATT TATTTGCAAT CTGGGCATAC TGTTTTATAA AGCACAAGTG TTCATGACTT AAAATCGATA   
  
  
+ AAGTCTTAAT CTTTTGTCGA TCCTTGAGTG TGCAGACAAT ATAATTGTGA ATGTATGCTA TTAACTTTAA   
  
  
+ TAATTACGAA AATTAAGCAA CTAATCATAT AGTCATGTTC TCAAAAACAA GCATGCGTAT TATTCAGATC   
  
  
+ ATTAAATGCA TTAAATGGGA CTAATCCGAT CAGTCAAGCG CTGATACGAC TATTGAATAA ACAGGACTAA   
  
  
+ CCTAGAATGC GGAATTAACT GTCGAGAATT GTCCACATCG GTAGTGATGC TTGGCGGCTA CCAGAGTCAC   
  
  
+ CTTAGGGTGT TCACATCCTA GACCCGAAAA CCTGCTAATG ATGGAAGGGC AGCCGTGGGA GGAGGAGAGG   
  
  
+ AGAGTGTGGA TTTTTCTATT TACAAAAAAG TGTATAAGTG CTAATCCCCC GAAACCTCCT TTTATAGGTT   
  
  
+ AGGGGGGCAA CTAGGGTTAT CGCCCTAGGT GACGCTTGTG GCCGTCGGCC TAATTAAATA GGCCCGACCA   
  
  
+ ACAGCTGTGA CTGGAGCGAT AGCTGAAGCA TGCAGAAATG AGGATAACAT ACATATTATA GATTTCCACA   
  
  
+ TTGCATAGGG CACTCAGTGG GTTACTGTAT CGCAAGCCAT AGCAGCAAGG CTTGGTGGGC CTCCTAAAGT   
  
  
+ GCGACTTACA GGCATTGATG ATCCTGTTTC TAAGTATACT TGTGATGCTA GCTTGGAGGC TGGTGGGAAA   
  
  
+ CGATTAGCGT CTCTATTTGA AAAGTTTAAA ATACTTGTCG AGTTCAATGC ATTGCCCGTT TATGGACCCA   
  
  
+ ATGTCAGGTG GGAAATGCTG GATGTGAGGC CCAGGGAGGC TTTGGTCGTT AATTGTCCAT TACAGCTCCA   
  
  
+ TTACACTCCT GACGAGAGTG TCGATGTGAG GAACCCTAGG GATAGGCTTC TCAGAATGGT GAAATTGCTC   
  
  
+ GGTCCTAAGG TAGCCACTTT GGTTGAGCAA GAATCAAACA CCAACACTAC ACCTTTCTTG ACCCGGTTCA   
  
  
+ TAGAGACCCT TGACTACTAC TCGATTAACC ATGTTTGAGT CTATAGACGT GACCATGCCG AGAGACCGGA   
  
  
+ AGGAGAGGAT CAATGTTGAG CAGCATTGTT TAGCTAAGGA CATTGTGAAT ATCATAGCTT GCGAGGGCAA   
  
  
+ GGAGATGGTG GAGCGTCATG AGCTTTTTGG GAAATGGCAG TCGAGGTTTA CCATGGTAGG GTTCAGGCAG   
  
  
+ TACCCGTTGA GCTCATACAT AAACTCTGTG ATAAGAAGCC TACTCCGGTG TTACTCCAAG CATTATACTC   
  
  
+ TGATAGAGAA GGATAGTGCC ATGCTTCTGG GTTGGAGGGG CCGAATGCTG ATTTCAGCTT CGGCATG  

- -Up\_Stream \_Len000TTTAAA TATACTAATA CCCTCGTTTA TAATTAGCCA GATTTTGTTT TTTCTTAGCC   
  
  
- GGCTTGTTTT TTTTCCCGGG TCTTGATCAG GAAAATTACC TCAGGGTTTT ACACCATATT TAATCAGAAC   
  
  
- AAGTGAGTAA AGAGTGAGGA GAGTGAGTAA GATTGGGATT TAGAGTTAGG AGTGAGAGAC AGGAGAGATA   
  
  
- GACGCAGAGC TCGAAGAGAA AGGAGACCCG AAGGAACCTG AAAAAGCTGC GGTACTGTTA CCTTGTGACT   
  
  
- CGAGGTGGCA ACGGGGAGAG GTGTTGACAG AAGATTGCCA CCAAGAGGCA TCGGTGGCAT GGAGTTTTTG   
  
  
- GTTTCGCTAC GTGTTGATGG TGGGGTGGTG ACGACTTTTC ACGGGTGCTC AGAGCTAGGC GCGTTCGGAC   
  
  
- TACTCTTAGC TATGGTGGTG GTACATCTAT TCGACTTTTG CCCGTATTCC TTCGCTGGGT GCGCGGGTTG   
  
  
- TGCGGTAACT CCGTCACTGT GCACCCAACT ACGCGAAGGT TTCTACCTTA AAAGTGTAAA AAGAAAAGTT   
  
  
- CCAACGCATC TAGCCACACA CACCTACCGC TAGTTCCTTC TCAATTTTAC TTGATCAGTA CGCAGCTTAA   
  
  
- CAGGTTTTTT TTAAAAGAGA AAGTCAGCTC AGAATTTCCC CGTCAGTAGT TCTCGTACAA ACTTTTTGTA   
  
  
- ATACCGGTAT TGGTGAATCA TCATTAAGTT TATTAGTACC TTTGTATAAG TTGTACTTAA GTTATACTGA   
  
  
- TTTTTAAGGT AGAAAATCAA ACAAGACCAG TTGTGTCTCG TACGGAAAAA TTAACGTATT ATTGCTATAA   
  
  
- ATTGGTTGGT TAGTACAAAG AGTGTATAAT TATATCCGTT CCCATCTAAT TGTCAGTATT AGCGTATCAT   
  
  
- ACGTTCTTAA ATAAACGTTA GACCCGTATG ACAAAATATT TCGTGTTCAC AAGTACTGAA TTTTAGCTAT   
  
  
- TTCAGAATTA GAAAACAGCT AGGAACTCAC ACGTCTGTTA TATTAACACT TACATACGAT AATTGAAATT   
  
  
- ATTAATGCTT TTAATTCGTT GATTAGTATA TCAGTACAAG AGTTTTTGTT CGTACGCATA ATAAGTCTAG   
  
  
- TAATTTACGT AATTTACCCT GATTAGGCTA GTCAGTTCGC GACTATGCTG ATAACTTATT TGTCCTGATT   
  
  
- GGATCTTACG CCTTAATTGA CAGCTCTTAA CAGGTGTAGC CATCACTACG AACCGCCGAT GGTCTCAGTG   
  
  
- GAATCCCACA AGTGTAGGAT CTGGGCTTTT GGACGATTAC TACCTTCCCG TCGGCACCCT CCTCCTCTCC   
  
  
- TCTCACACCT AAAAAGATAA ATGTTTTTTC ACATATTCAC GATTAGGGGG CTTTGGAGGA AAATATCCAA   
  
  
- TCCCCCCGTT GATCCCAATA GCGGGATCCA CTGCGAACAC CGGCAGCCGG ATTAATTTAT CCGGGCTGGT   
  
  
- TGTCGACACT GACCTCGCTA TCGACTTCGT ACGTCTTTAC TCCTATTGTA TGTATAATAT CTAAAGGTGT   
  
  
- AACGTATCCC GTGAGTCACC CAATGACATA GCGTTCGGTA TCGTCGTTCC GAACCACCCG GAGGATTTCA   
  
  
- CGCTGAATGT CCGTAACTAC TAGGACAAAG ATTCATATGA ACACTACGAT CGAACCTCCG ACCACCCTTT   
  
  
- GCTAATCGCA GAGATAAACT TTTCAAATTT TATGAACAGC TCAAGTTACG TAACGGGCAA ATACCTGGGT   
  
  
- TACAGTCCAC CCTTTACGAC CTACACTCCG GGTCCCTCCG AAACCAGCAA TTAACAGGTA ATGTCGAGGT   
  
  
- AATGTGAGGA CTGCTCTCAC AGCTACACTC CTTGGGATCC CTATCCGAAG AGTCTTACCA CTTTAACGAG   
  
  
- CCAGGATTCC ATCGGTGAAA CCAACTCGTT CTTAGTTTGT GGTTGTGATG TGGAAAGAAC TGGGCCAAGT   
  
  
- ATCTCTGGGA ACTGATGATG AGCTAATTGG TACAAACTCA GATATCTGCA CTGGTACGGC TCTCTGGCCT   
  
  
- TCCTCTCCTA GTTACAACTC GTCGTAACAA ATCGATTCCT GTAACACTTA TAGTATCGAA CGCTCCCGTT   
  
  
- CCTCTACCAC CTCGCAGTAC TCGAAAAACC CTTTACCGTC AGCTCCAAAT GGTACCATCC CAAGTCCGTC   
  
  
- ATGGGCAACT CGAGTATGTA TTTGAGACAC TATTCTTCGG ATGAGGCCAC AATGAGGTTC GTAATATGAG   
  
  
- ACTATCTCTT CCTATCACGG TACGAAGACC CAACCTCCCC GGCTTACGAC TAAAGTCGAA GCCGTAC

+     G-box

| Site Name | Organism | Position | Strand | Matrix score. | sequence | function |
| --- | --- | --- | --- | --- | --- | --- |
| G-box | Glycine max | 508 | - | 12 | tcCACGTGTCACT | cis-acting regulatory element involved in light responsiveness |
| G-box | Zea mays | 2010 | - | 6 | CACGTC | cis-acting regulatory element involved in light responsiveness |
| G-box | Arabidopsis thaliana | 513 | + | 6 | CACGTG | cis-acting regulatory element involved in light responsiveness |

>HU08G00229.1   
+ -Up\_Stream \_Len000AAATTT ATATGATTAT GGGAGCAAAT ATTAATCGGT CTAAAACAAA AAAGAATCGG   
  
  
+ CCGAACAAAA AAAAGGGCCC AGAACTAGTC CTTTTAATGG AGTCCCAAAA TGTGGTATAA ATTAGTCTTG   
  
  
+ TTCACTCATT TCTCACTCCT CTCACTCATT CTAACCCTAA ATCTCAATCC TCACTCTCTG TCCTCTCTAT   
  
  
+ CTGCGTCTCG AGCTTCTCTT TCCTCTGGGC TTCCTTGGAC TTTTTCGACG CCATGACAAT GGAACACTGA   
  
  
+ GCTCCACCGT TGCCCCTCTC CACAACTGTC TTCTAACGGT GGTTCTCCGT AGCCACCGTA CCTCAAAAAC   
  
  
+ CAAAGCGATG CACAACTACC ACCCCACCAC TGCTGAAAAG TGCCCACGAG TCTCGATCCG CGCAAGCCTG   
  
  
+ ATGAGAATCG ATACCACCAC CATGTAGATA AGCTGAAAAC GGGCATAAGG AAGCGACCCA CGCGCCCAAC   
  
  
+ ACGCCATTGA GGCAGTGACA CGTGGGTTGA TGCGCTTCCA AAGATGGAAT TTTCACATTT TTCTTTTCAA   
  
  
+ GGTTGCGTAG ATCGGTGTGT GTGGATGGCG ATCAAGGAAG AGTTAAAATG AACTAGTCAT GCGTCGAATT   
  
  
+ GTCCAAAAAA AATTTTCTCT TTCAGTCGAG TCTTAAAGGG GCAGTCATCA AGAGCATGTT TGAAAAACAT   
  
  
+ TATGGCCATA ACCACTTAGT AGTAATTCAA ATAATCATGG AAACATATTC AACATGAATT CAATATGACT   
  
  
+ AAAAATTCCA TCTTTTAGTT TGTTCTGGTC AACACAGAGC ATGCCTTTTT AATTGCATAA TAACGATATT   
  
  
+ TAACCAACCA ATCATGTTTC TCACATATTA ATATAGGCAA GGGTAGATTA ACAGTCATAA TCGCATAGTA   
  
  
+ TGCAAGAATT TATTTGCAAT CTGGGCATAC TGTTTTATAA AGCACAAGTG TTCATGACTT AAAATCGATA   
  
  
+ AAGTCTTAAT CTTTTGTCGA TCCTTGAGTG TGCAGACAAT ATAATTGTGA ATGTATGCTA TTAACTTTAA   
  
  
+ TAATTACGAA AATTAAGCAA CTAATCATAT AGTCATGTTC TCAAAAACAA GCATGCGTAT TATTCAGATC   
  
  
+ ATTAAATGCA TTAAATGGGA CTAATCCGAT CAGTCAAGCG CTGATACGAC TATTGAATAA ACAGGACTAA   
  
  
+ CCTAGAATGC GGAATTAACT GTCGAGAATT GTCCACATCG GTAGTGATGC TTGGCGGCTA CCAGAGTCAC   
  
  
+ CTTAGGGTGT TCACATCCTA GACCCGAAAA CCTGCTAATG ATGGAAGGGC AGCCGTGGGA GGAGGAGAGG   
  
  
+ AGAGTGTGGA TTTTTCTATT TACAAAAAAG TGTATAAGTG CTAATCCCCC GAAACCTCCT TTTATAGGTT   
  
  
+ AGGGGGGCAA CTAGGGTTAT CGCCCTAGGT GACGCTTGTG GCCGTCGGCC TAATTAAATA GGCCCGACCA   
  
  
+ ACAGCTGTGA CTGGAGCGAT AGCTGAAGCA TGCAGAAATG AGGATAACAT ACATATTATA GATTTCCACA   
  
  
+ TTGCATAGGG CACTCAGTGG GTTACTGTAT CGCAAGCCAT AGCAGCAAGG CTTGGTGGGC CTCCTAAAGT   
  
  
+ GCGACTTACA GGCATTGATG ATCCTGTTTC TAAGTATACT TGTGATGCTA GCTTGGAGGC TGGTGGGAAA   
  
  
+ CGATTAGCGT CTCTATTTGA AAAGTTTAAA ATACTTGTCG AGTTCAATGC ATTGCCCGTT TATGGACCCA   
  
  
+ ATGTCAGGTG GGAAATGCTG GATGTGAGGC CCAGGGAGGC TTTGGTCGTT AATTGTCCAT TACAGCTCCA   
  
  
+ TTACACTCCT GACGAGAGTG TCGATGTGAG GAACCCTAGG GATAGGCTTC TCAGAATGGT GAAATTGCTC   
  
  
+ GGTCCTAAGG TAGCCACTTT GGTTGAGCAA GAATCAAACA CCAACACTAC ACCTTTCTTG ACCCGGTTCA   
  
  
+ TAGAGACCCT TGACTACTAC TCGATTAACC ATGTTTGAGT CTATAGACGT GACCATGCCG AGAGACCGGA   
  
  
+ AGGAGAGGAT CAATGTTGAG CAGCATTGTT TAGCTAAGGA CATTGTGAAT ATCATAGCTT GCGAGGGCAA   
  
  
+ GGAGATGGTG GAGCGTCATG AGCTTTTTGG GAAATGGCAG TCGAGGTTTA CCATGGTAGG GTTCAGGCAG   
  
  
+ TACCCGTTGA GCTCATACAT AAACTCTGTG ATAAGAAGCC TACTCCGGTG TTACTCCAAG CATTATACTC   
  
  
+ TGATAGAGAA GGATAGTGCC ATGCTTCTGG GTTGGAGGGG CCGAATGCTG ATTTCAGCTT CGGCATG  

- -Up\_Stream \_Len000TTTAAA TATACTAATA CCCTCGTTTA TAATTAGCCA GATTTTGTTT TTTCTTAGCC   
  
  
- GGCTTGTTTT TTTTCCCGGG TCTTGATCAG GAAAATTACC TCAGGGTTTT ACACCATATT TAATCAGAAC   
  
  
- AAGTGAGTAA AGAGTGAGGA GAGTGAGTAA GATTGGGATT TAGAGTTAGG AGTGAGAGAC AGGAGAGATA   
  
  
- GACGCAGAGC TCGAAGAGAA AGGAGACCCG AAGGAACCTG AAAAAGCTGC GGTACTGTTA CCTTGTGACT   
  
  
- CGAGGTGGCA ACGGGGAGAG GTGTTGACAG AAGATTGCCA CCAAGAGGCA TCGGTGGCAT GGAGTTTTTG   
  
  
- GTTTCGCTAC GTGTTGATGG TGGGGTGGTG ACGACTTTTC ACGGGTGCTC AGAGCTAGGC GCGTTCGGAC   
  
  
- TACTCTTAGC TATGGTGGTG GTACATCTAT TCGACTTTTG CCCGTATTCC TTCGCTGGGT GCGCGGGTTG   
  
  
- TGCGGTAACT CCGTCACTGT GCACCCAACT ACGCGAAGGT TTCTACCTTA AAAGTGTAAA AAGAAAAGTT   
  
  
- CCAACGCATC TAGCCACACA CACCTACCGC TAGTTCCTTC TCAATTTTAC TTGATCAGTA CGCAGCTTAA   
  
  
- CAGGTTTTTT TTAAAAGAGA AAGTCAGCTC AGAATTTCCC CGTCAGTAGT TCTCGTACAA ACTTTTTGTA   
  
  
- ATACCGGTAT TGGTGAATCA TCATTAAGTT TATTAGTACC TTTGTATAAG TTGTACTTAA GTTATACTGA   
  
  
- TTTTTAAGGT AGAAAATCAA ACAAGACCAG TTGTGTCTCG TACGGAAAAA TTAACGTATT ATTGCTATAA   
  
  
- ATTGGTTGGT TAGTACAAAG AGTGTATAAT TATATCCGTT CCCATCTAAT TGTCAGTATT AGCGTATCAT   
  
  
- ACGTTCTTAA ATAAACGTTA GACCCGTATG ACAAAATATT TCGTGTTCAC AAGTACTGAA TTTTAGCTAT   
  
  
- TTCAGAATTA GAAAACAGCT AGGAACTCAC ACGTCTGTTA TATTAACACT TACATACGAT AATTGAAATT   
  
  
- ATTAATGCTT TTAATTCGTT GATTAGTATA TCAGTACAAG AGTTTTTGTT CGTACGCATA ATAAGTCTAG   
  
  
- TAATTTACGT AATTTACCCT GATTAGGCTA GTCAGTTCGC GACTATGCTG ATAACTTATT TGTCCTGATT   
  
  
- GGATCTTACG CCTTAATTGA CAGCTCTTAA CAGGTGTAGC CATCACTACG AACCGCCGAT GGTCTCAGTG   
  
  
- GAATCCCACA AGTGTAGGAT CTGGGCTTTT GGACGATTAC TACCTTCCCG TCGGCACCCT CCTCCTCTCC   
  
  
- TCTCACACCT AAAAAGATAA ATGTTTTTTC ACATATTCAC GATTAGGGGG CTTTGGAGGA AAATATCCAA   
  
  
- TCCCCCCGTT GATCCCAATA GCGGGATCCA CTGCGAACAC CGGCAGCCGG ATTAATTTAT CCGGGCTGGT   
  
  
- TGTCGACACT GACCTCGCTA TCGACTTCGT ACGTCTTTAC TCCTATTGTA TGTATAATAT CTAAAGGTGT   
  
  
- AACGTATCCC GTGAGTCACC CAATGACATA GCGTTCGGTA TCGTCGTTCC GAACCACCCG GAGGATTTCA   
  
  
- CGCTGAATGT CCGTAACTAC TAGGACAAAG ATTCATATGA ACACTACGAT CGAACCTCCG ACCACCCTTT   
  
  
- GCTAATCGCA GAGATAAACT TTTCAAATTT TATGAACAGC TCAAGTTACG TAACGGGCAA ATACCTGGGT   
  
  
- TACAGTCCAC CCTTTACGAC CTACACTCCG GGTCCCTCCG AAACCAGCAA TTAACAGGTA ATGTCGAGGT   
  
  
- AATGTGAGGA CTGCTCTCAC AGCTACACTC CTTGGGATCC CTATCCGAAG AGTCTTACCA CTTTAACGAG   
  
  
- CCAGGATTCC ATCGGTGAAA CCAACTCGTT CTTAGTTTGT GGTTGTGATG TGGAAAGAAC TGGGCCAAGT   
  
  
- ATCTCTGGGA ACTGATGATG AGCTAATTGG TACAAACTCA GATATCTGCA CTGGTACGGC TCTCTGGCCT   
  
  
- TCCTCTCCTA GTTACAACTC GTCGTAACAA ATCGATTCCT GTAACACTTA TAGTATCGAA CGCTCCCGTT   
  
  
- CCTCTACCAC CTCGCAGTAC TCGAAAAACC CTTTACCGTC AGCTCCAAAT GGTACCATCC CAAGTCCGTC   
  
  
- ATGGGCAACT CGAGTATGTA TTTGAGACAC TATTCTTCGG ATGAGGCCAC AATGAGGTTC GTAATATGAG   
  
  
- ACTATCTCTT CCTATCACGG TACGAAGACC CAACCTCCCC GGCTTACGAC TAAAGTCGAA GCCGTAC

+     GC-motif

| Site Name | Organism | Position | Strand | Matrix score. | sequence | function |
| --- | --- | --- | --- | --- | --- | --- |
| GC-motif | Zea mays | 1380 | + | 6 | CCCCCG | enhancer-like element involved in anoxic specific inducibility |

>HU08G00229.1   
+ -Up\_Stream \_Len000AAATTT ATATGATTAT GGGAGCAAAT ATTAATCGGT CTAAAACAAA AAAGAATCGG   
  
  
+ CCGAACAAAA AAAAGGGCCC AGAACTAGTC CTTTTAATGG AGTCCCAAAA TGTGGTATAA ATTAGTCTTG   
  
  
+ TTCACTCATT TCTCACTCCT CTCACTCATT CTAACCCTAA ATCTCAATCC TCACTCTCTG TCCTCTCTAT   
  
  
+ CTGCGTCTCG AGCTTCTCTT TCCTCTGGGC TTCCTTGGAC TTTTTCGACG CCATGACAAT GGAACACTGA   
  
  
+ GCTCCACCGT TGCCCCTCTC CACAACTGTC TTCTAACGGT GGTTCTCCGT AGCCACCGTA CCTCAAAAAC   
  
  
+ CAAAGCGATG CACAACTACC ACCCCACCAC TGCTGAAAAG TGCCCACGAG TCTCGATCCG CGCAAGCCTG   
  
  
+ ATGAGAATCG ATACCACCAC CATGTAGATA AGCTGAAAAC GGGCATAAGG AAGCGACCCA CGCGCCCAAC   
  
  
+ ACGCCATTGA GGCAGTGACA CGTGGGTTGA TGCGCTTCCA AAGATGGAAT TTTCACATTT TTCTTTTCAA   
  
  
+ GGTTGCGTAG ATCGGTGTGT GTGGATGGCG ATCAAGGAAG AGTTAAAATG AACTAGTCAT GCGTCGAATT   
  
  
+ GTCCAAAAAA AATTTTCTCT TTCAGTCGAG TCTTAAAGGG GCAGTCATCA AGAGCATGTT TGAAAAACAT   
  
  
+ TATGGCCATA ACCACTTAGT AGTAATTCAA ATAATCATGG AAACATATTC AACATGAATT CAATATGACT   
  
  
+ AAAAATTCCA TCTTTTAGTT TGTTCTGGTC AACACAGAGC ATGCCTTTTT AATTGCATAA TAACGATATT   
  
  
+ TAACCAACCA ATCATGTTTC TCACATATTA ATATAGGCAA GGGTAGATTA ACAGTCATAA TCGCATAGTA   
  
  
+ TGCAAGAATT TATTTGCAAT CTGGGCATAC TGTTTTATAA AGCACAAGTG TTCATGACTT AAAATCGATA   
  
  
+ AAGTCTTAAT CTTTTGTCGA TCCTTGAGTG TGCAGACAAT ATAATTGTGA ATGTATGCTA TTAACTTTAA   
  
  
+ TAATTACGAA AATTAAGCAA CTAATCATAT AGTCATGTTC TCAAAAACAA GCATGCGTAT TATTCAGATC   
  
  
+ ATTAAATGCA TTAAATGGGA CTAATCCGAT CAGTCAAGCG CTGATACGAC TATTGAATAA ACAGGACTAA   
  
  
+ CCTAGAATGC GGAATTAACT GTCGAGAATT GTCCACATCG GTAGTGATGC TTGGCGGCTA CCAGAGTCAC   
  
  
+ CTTAGGGTGT TCACATCCTA GACCCGAAAA CCTGCTAATG ATGGAAGGGC AGCCGTGGGA GGAGGAGAGG   
  
  
+ AGAGTGTGGA TTTTTCTATT TACAAAAAAG TGTATAAGTG CTAATCCCCC GAAACCTCCT TTTATAGGTT   
  
  
+ AGGGGGGCAA CTAGGGTTAT CGCCCTAGGT GACGCTTGTG GCCGTCGGCC TAATTAAATA GGCCCGACCA   
  
  
+ ACAGCTGTGA CTGGAGCGAT AGCTGAAGCA TGCAGAAATG AGGATAACAT ACATATTATA GATTTCCACA   
  
  
+ TTGCATAGGG CACTCAGTGG GTTACTGTAT CGCAAGCCAT AGCAGCAAGG CTTGGTGGGC CTCCTAAAGT   
  
  
+ GCGACTTACA GGCATTGATG ATCCTGTTTC TAAGTATACT TGTGATGCTA GCTTGGAGGC TGGTGGGAAA   
  
  
+ CGATTAGCGT CTCTATTTGA AAAGTTTAAA ATACTTGTCG AGTTCAATGC ATTGCCCGTT TATGGACCCA   
  
  
+ ATGTCAGGTG GGAAATGCTG GATGTGAGGC CCAGGGAGGC TTTGGTCGTT AATTGTCCAT TACAGCTCCA   
  
  
+ TTACACTCCT GACGAGAGTG TCGATGTGAG GAACCCTAGG GATAGGCTTC TCAGAATGGT GAAATTGCTC   
  
  
+ GGTCCTAAGG TAGCCACTTT GGTTGAGCAA GAATCAAACA CCAACACTAC ACCTTTCTTG ACCCGGTTCA   
  
  
+ TAGAGACCCT TGACTACTAC TCGATTAACC ATGTTTGAGT CTATAGACGT GACCATGCCG AGAGACCGGA   
  
  
+ AGGAGAGGAT CAATGTTGAG CAGCATTGTT TAGCTAAGGA CATTGTGAAT ATCATAGCTT GCGAGGGCAA   
  
  
+ GGAGATGGTG GAGCGTCATG AGCTTTTTGG GAAATGGCAG TCGAGGTTTA CCATGGTAGG GTTCAGGCAG   
  
  
+ TACCCGTTGA GCTCATACAT AAACTCTGTG ATAAGAAGCC TACTCCGGTG TTACTCCAAG CATTATACTC   
  
  
+ TGATAGAGAA GGATAGTGCC ATGCTTCTGG GTTGGAGGGG CCGAATGCTG ATTTCAGCTT CGGCATG  

- -Up\_Stream \_Len000TTTAAA TATACTAATA CCCTCGTTTA TAATTAGCCA GATTTTGTTT TTTCTTAGCC   
  
  
- GGCTTGTTTT TTTTCCCGGG TCTTGATCAG GAAAATTACC TCAGGGTTTT ACACCATATT TAATCAGAAC   
  
  
- AAGTGAGTAA AGAGTGAGGA GAGTGAGTAA GATTGGGATT TAGAGTTAGG AGTGAGAGAC AGGAGAGATA   
  
  
- GACGCAGAGC TCGAAGAGAA AGGAGACCCG AAGGAACCTG AAAAAGCTGC GGTACTGTTA CCTTGTGACT   
  
  
- CGAGGTGGCA ACGGGGAGAG GTGTTGACAG AAGATTGCCA CCAAGAGGCA TCGGTGGCAT GGAGTTTTTG   
  
  
- GTTTCGCTAC GTGTTGATGG TGGGGTGGTG ACGACTTTTC ACGGGTGCTC AGAGCTAGGC GCGTTCGGAC   
  
  
- TACTCTTAGC TATGGTGGTG GTACATCTAT TCGACTTTTG CCCGTATTCC TTCGCTGGGT GCGCGGGTTG   
  
  
- TGCGGTAACT CCGTCACTGT GCACCCAACT ACGCGAAGGT TTCTACCTTA AAAGTGTAAA AAGAAAAGTT   
  
  
- CCAACGCATC TAGCCACACA CACCTACCGC TAGTTCCTTC TCAATTTTAC TTGATCAGTA CGCAGCTTAA   
  
  
- CAGGTTTTTT TTAAAAGAGA AAGTCAGCTC AGAATTTCCC CGTCAGTAGT TCTCGTACAA ACTTTTTGTA   
  
  
- ATACCGGTAT TGGTGAATCA TCATTAAGTT TATTAGTACC TTTGTATAAG TTGTACTTAA GTTATACTGA   
  
  
- TTTTTAAGGT AGAAAATCAA ACAAGACCAG TTGTGTCTCG TACGGAAAAA TTAACGTATT ATTGCTATAA   
  
  
- ATTGGTTGGT TAGTACAAAG AGTGTATAAT TATATCCGTT CCCATCTAAT TGTCAGTATT AGCGTATCAT   
  
  
- ACGTTCTTAA ATAAACGTTA GACCCGTATG ACAAAATATT TCGTGTTCAC AAGTACTGAA TTTTAGCTAT   
  
  
- TTCAGAATTA GAAAACAGCT AGGAACTCAC ACGTCTGTTA TATTAACACT TACATACGAT AATTGAAATT   
  
  
- ATTAATGCTT TTAATTCGTT GATTAGTATA TCAGTACAAG AGTTTTTGTT CGTACGCATA ATAAGTCTAG   
  
  
- TAATTTACGT AATTTACCCT GATTAGGCTA GTCAGTTCGC GACTATGCTG ATAACTTATT TGTCCTGATT   
  
  
- GGATCTTACG CCTTAATTGA CAGCTCTTAA CAGGTGTAGC CATCACTACG AACCGCCGAT GGTCTCAGTG   
  
  
- GAATCCCACA AGTGTAGGAT CTGGGCTTTT GGACGATTAC TACCTTCCCG TCGGCACCCT CCTCCTCTCC   
  
  
- TCTCACACCT AAAAAGATAA ATGTTTTTTC ACATATTCAC GATTAGGGGG CTTTGGAGGA AAATATCCAA   
  
  
- TCCCCCCGTT GATCCCAATA GCGGGATCCA CTGCGAACAC CGGCAGCCGG ATTAATTTAT CCGGGCTGGT   
  
  
- TGTCGACACT GACCTCGCTA TCGACTTCGT ACGTCTTTAC TCCTATTGTA TGTATAATAT CTAAAGGTGT   
  
  
- AACGTATCCC GTGAGTCACC CAATGACATA GCGTTCGGTA TCGTCGTTCC GAACCACCCG GAGGATTTCA   
  
  
- CGCTGAATGT CCGTAACTAC TAGGACAAAG ATTCATATGA ACACTACGAT CGAACCTCCG ACCACCCTTT   
  
  
- GCTAATCGCA GAGATAAACT TTTCAAATTT TATGAACAGC TCAAGTTACG TAACGGGCAA ATACCTGGGT   
  
  
- TACAGTCCAC CCTTTACGAC CTACACTCCG GGTCCCTCCG AAACCAGCAA TTAACAGGTA ATGTCGAGGT   
  
  
- AATGTGAGGA CTGCTCTCAC AGCTACACTC CTTGGGATCC CTATCCGAAG AGTCTTACCA CTTTAACGAG   
  
  
- CCAGGATTCC ATCGGTGAAA CCAACTCGTT CTTAGTTTGT GGTTGTGATG TGGAAAGAAC TGGGCCAAGT   
  
  
- ATCTCTGGGA ACTGATGATG AGCTAATTGG TACAAACTCA GATATCTGCA CTGGTACGGC TCTCTGGCCT   
  
  
- TCCTCTCCTA GTTACAACTC GTCGTAACAA ATCGATTCCT GTAACACTTA TAGTATCGAA CGCTCCCGTT   
  
  
- CCTCTACCAC CTCGCAGTAC TCGAAAAACC CTTTACCGTC AGCTCCAAAT GGTACCATCC CAAGTCCGTC   
  
  
- ATGGGCAACT CGAGTATGTA TTTGAGACAC TATTCTTCGG ATGAGGCCAC AATGAGGTTC GTAATATGAG   
  
  
- ACTATCTCTT CCTATCACGG TACGAAGACC CAACCTCCCC GGCTTACGAC TAAAGTCGAA GCCGTAC

+     GT1-motif

| Site Name | Organism | Position | Strand | Matrix score. | sequence | function |
| --- | --- | --- | --- | --- | --- | --- |
| GT1-motif | Avena sativa | 1988 | - | 7 | GGTTAAT | light responsive element |
| GT1-motif | Arabidopsis thaliana | 844 | - | 6 | GGTTAA | light responsive element |
| GT1-motif | Arabidopsis thaliana | 1989 | - | 6 | GGTTAA | light responsive element |

>HU08G00229.1   
+ -Up\_Stream \_Len000AAATTT ATATGATTAT GGGAGCAAAT ATTAATCGGT CTAAAACAAA AAAGAATCGG   
  
  
+ CCGAACAAAA AAAAGGGCCC AGAACTAGTC CTTTTAATGG AGTCCCAAAA TGTGGTATAA ATTAGTCTTG   
  
  
+ TTCACTCATT TCTCACTCCT CTCACTCATT CTAACCCTAA ATCTCAATCC TCACTCTCTG TCCTCTCTAT   
  
  
+ CTGCGTCTCG AGCTTCTCTT TCCTCTGGGC TTCCTTGGAC TTTTTCGACG CCATGACAAT GGAACACTGA   
  
  
+ GCTCCACCGT TGCCCCTCTC CACAACTGTC TTCTAACGGT GGTTCTCCGT AGCCACCGTA CCTCAAAAAC   
  
  
+ CAAAGCGATG CACAACTACC ACCCCACCAC TGCTGAAAAG TGCCCACGAG TCTCGATCCG CGCAAGCCTG   
  
  
+ ATGAGAATCG ATACCACCAC CATGTAGATA AGCTGAAAAC GGGCATAAGG AAGCGACCCA CGCGCCCAAC   
  
  
+ ACGCCATTGA GGCAGTGACA CGTGGGTTGA TGCGCTTCCA AAGATGGAAT TTTCACATTT TTCTTTTCAA   
  
  
+ GGTTGCGTAG ATCGGTGTGT GTGGATGGCG ATCAAGGAAG AGTTAAAATG AACTAGTCAT GCGTCGAATT   
  
  
+ GTCCAAAAAA AATTTTCTCT TTCAGTCGAG TCTTAAAGGG GCAGTCATCA AGAGCATGTT TGAAAAACAT   
  
  
+ TATGGCCATA ACCACTTAGT AGTAATTCAA ATAATCATGG AAACATATTC AACATGAATT CAATATGACT   
  
  
+ AAAAATTCCA TCTTTTAGTT TGTTCTGGTC AACACAGAGC ATGCCTTTTT AATTGCATAA TAACGATATT   
  
  
+ TAACCAACCA ATCATGTTTC TCACATATTA ATATAGGCAA GGGTAGATTA ACAGTCATAA TCGCATAGTA   
  
  
+ TGCAAGAATT TATTTGCAAT CTGGGCATAC TGTTTTATAA AGCACAAGTG TTCATGACTT AAAATCGATA   
  
  
+ AAGTCTTAAT CTTTTGTCGA TCCTTGAGTG TGCAGACAAT ATAATTGTGA ATGTATGCTA TTAACTTTAA   
  
  
+ TAATTACGAA AATTAAGCAA CTAATCATAT AGTCATGTTC TCAAAAACAA GCATGCGTAT TATTCAGATC   
  
  
+ ATTAAATGCA TTAAATGGGA CTAATCCGAT CAGTCAAGCG CTGATACGAC TATTGAATAA ACAGGACTAA   
  
  
+ CCTAGAATGC GGAATTAACT GTCGAGAATT GTCCACATCG GTAGTGATGC TTGGCGGCTA CCAGAGTCAC   
  
  
+ CTTAGGGTGT TCACATCCTA GACCCGAAAA CCTGCTAATG ATGGAAGGGC AGCCGTGGGA GGAGGAGAGG   
  
  
+ AGAGTGTGGA TTTTTCTATT TACAAAAAAG TGTATAAGTG CTAATCCCCC GAAACCTCCT TTTATAGGTT   
  
  
+ AGGGGGGCAA CTAGGGTTAT CGCCCTAGGT GACGCTTGTG GCCGTCGGCC TAATTAAATA GGCCCGACCA   
  
  
+ ACAGCTGTGA CTGGAGCGAT AGCTGAAGCA TGCAGAAATG AGGATAACAT ACATATTATA GATTTCCACA   
  
  
+ TTGCATAGGG CACTCAGTGG GTTACTGTAT CGCAAGCCAT AGCAGCAAGG CTTGGTGGGC CTCCTAAAGT   
  
  
+ GCGACTTACA GGCATTGATG ATCCTGTTTC TAAGTATACT TGTGATGCTA GCTTGGAGGC TGGTGGGAAA   
  
  
+ CGATTAGCGT CTCTATTTGA AAAGTTTAAA ATACTTGTCG AGTTCAATGC ATTGCCCGTT TATGGACCCA   
  
  
+ ATGTCAGGTG GGAAATGCTG GATGTGAGGC CCAGGGAGGC TTTGGTCGTT AATTGTCCAT TACAGCTCCA   
  
  
+ TTACACTCCT GACGAGAGTG TCGATGTGAG GAACCCTAGG GATAGGCTTC TCAGAATGGT GAAATTGCTC   
  
  
+ GGTCCTAAGG TAGCCACTTT GGTTGAGCAA GAATCAAACA CCAACACTAC ACCTTTCTTG ACCCGGTTCA   
  
  
+ TAGAGACCCT TGACTACTAC TCGATTAACC ATGTTTGAGT CTATAGACGT GACCATGCCG AGAGACCGGA   
  
  
+ AGGAGAGGAT CAATGTTGAG CAGCATTGTT TAGCTAAGGA CATTGTGAAT ATCATAGCTT GCGAGGGCAA   
  
  
+ GGAGATGGTG GAGCGTCATG AGCTTTTTGG GAAATGGCAG TCGAGGTTTA CCATGGTAGG GTTCAGGCAG   
  
  
+ TACCCGTTGA GCTCATACAT AAACTCTGTG ATAAGAAGCC TACTCCGGTG TTACTCCAAG CATTATACTC   
  
  
+ TGATAGAGAA GGATAGTGCC ATGCTTCTGG GTTGGAGGGG CCGAATGCTG ATTTCAGCTT CGGCATG  

- -Up\_Stream \_Len000TTTAAA TATACTAATA CCCTCGTTTA TAATTAGCCA GATTTTGTTT TTTCTTAGCC   
  
  
- GGCTTGTTTT TTTTCCCGGG TCTTGATCAG GAAAATTACC TCAGGGTTTT ACACCATATT TAATCAGAAC   
  
  
- AAGTGAGTAA AGAGTGAGGA GAGTGAGTAA GATTGGGATT TAGAGTTAGG AGTGAGAGAC AGGAGAGATA   
  
  
- GACGCAGAGC TCGAAGAGAA AGGAGACCCG AAGGAACCTG AAAAAGCTGC GGTACTGTTA CCTTGTGACT   
  
  
- CGAGGTGGCA ACGGGGAGAG GTGTTGACAG AAGATTGCCA CCAAGAGGCA TCGGTGGCAT GGAGTTTTTG   
  
  
- GTTTCGCTAC GTGTTGATGG TGGGGTGGTG ACGACTTTTC ACGGGTGCTC AGAGCTAGGC GCGTTCGGAC   
  
  
- TACTCTTAGC TATGGTGGTG GTACATCTAT TCGACTTTTG CCCGTATTCC TTCGCTGGGT GCGCGGGTTG   
  
  
- TGCGGTAACT CCGTCACTGT GCACCCAACT ACGCGAAGGT TTCTACCTTA AAAGTGTAAA AAGAAAAGTT   
  
  
- CCAACGCATC TAGCCACACA CACCTACCGC TAGTTCCTTC TCAATTTTAC TTGATCAGTA CGCAGCTTAA   
  
  
- CAGGTTTTTT TTAAAAGAGA AAGTCAGCTC AGAATTTCCC CGTCAGTAGT TCTCGTACAA ACTTTTTGTA   
  
  
- ATACCGGTAT TGGTGAATCA TCATTAAGTT TATTAGTACC TTTGTATAAG TTGTACTTAA GTTATACTGA   
  
  
- TTTTTAAGGT AGAAAATCAA ACAAGACCAG TTGTGTCTCG TACGGAAAAA TTAACGTATT ATTGCTATAA   
  
  
- ATTGGTTGGT TAGTACAAAG AGTGTATAAT TATATCCGTT CCCATCTAAT TGTCAGTATT AGCGTATCAT   
  
  
- ACGTTCTTAA ATAAACGTTA GACCCGTATG ACAAAATATT TCGTGTTCAC AAGTACTGAA TTTTAGCTAT   
  
  
- TTCAGAATTA GAAAACAGCT AGGAACTCAC ACGTCTGTTA TATTAACACT TACATACGAT AATTGAAATT   
  
  
- ATTAATGCTT TTAATTCGTT GATTAGTATA TCAGTACAAG AGTTTTTGTT CGTACGCATA ATAAGTCTAG   
  
  
- TAATTTACGT AATTTACCCT GATTAGGCTA GTCAGTTCGC GACTATGCTG ATAACTTATT TGTCCTGATT   
  
  
- GGATCTTACG CCTTAATTGA CAGCTCTTAA CAGGTGTAGC CATCACTACG AACCGCCGAT GGTCTCAGTG   
  
  
- GAATCCCACA AGTGTAGGAT CTGGGCTTTT GGACGATTAC TACCTTCCCG TCGGCACCCT CCTCCTCTCC   
  
  
- TCTCACACCT AAAAAGATAA ATGTTTTTTC ACATATTCAC GATTAGGGGG CTTTGGAGGA AAATATCCAA   
  
  
- TCCCCCCGTT GATCCCAATA GCGGGATCCA CTGCGAACAC CGGCAGCCGG ATTAATTTAT CCGGGCTGGT   
  
  
- TGTCGACACT GACCTCGCTA TCGACTTCGT ACGTCTTTAC TCCTATTGTA TGTATAATAT CTAAAGGTGT   
  
  
- AACGTATCCC GTGAGTCACC CAATGACATA GCGTTCGGTA TCGTCGTTCC GAACCACCCG GAGGATTTCA   
  
  
- CGCTGAATGT CCGTAACTAC TAGGACAAAG ATTCATATGA ACACTACGAT CGAACCTCCG ACCACCCTTT   
  
  
- GCTAATCGCA GAGATAAACT TTTCAAATTT TATGAACAGC TCAAGTTACG TAACGGGCAA ATACCTGGGT   
  
  
- TACAGTCCAC CCTTTACGAC CTACACTCCG GGTCCCTCCG AAACCAGCAA TTAACAGGTA ATGTCGAGGT   
  
  
- AATGTGAGGA CTGCTCTCAC AGCTACACTC CTTGGGATCC CTATCCGAAG AGTCTTACCA CTTTAACGAG   
  
  
- CCAGGATTCC ATCGGTGAAA CCAACTCGTT CTTAGTTTGT GGTTGTGATG TGGAAAGAAC TGGGCCAAGT   
  
  
- ATCTCTGGGA ACTGATGATG AGCTAATTGG TACAAACTCA GATATCTGCA CTGGTACGGC TCTCTGGCCT   
  
  
- TCCTCTCCTA GTTACAACTC GTCGTAACAA ATCGATTCCT GTAACACTTA TAGTATCGAA CGCTCCCGTT   
  
  
- CCTCTACCAC CTCGCAGTAC TCGAAAAACC CTTTACCGTC AGCTCCAAAT GGTACCATCC CAAGTCCGTC   
  
  
- ATGGGCAACT CGAGTATGTA TTTGAGACAC TATTCTTCGG ATGAGGCCAC AATGAGGTTC GTAATATGAG   
  
  
- ACTATCTCTT CCTATCACGG TACGAAGACC CAACCTCCCC GGCTTACGAC TAAAGTCGAA GCCGTAC

+     LTR

| Site Name | Organism | Position | Strand | Matrix score. | sequence | function |
| --- | --- | --- | --- | --- | --- | --- |
| LTR | Hordeum vulgare | 1288 | + | 6 | CCGAAA | cis-acting element involved in low-temperature responsiveness |
| LTR | Hordeum vulgare | 1383 | + | 6 | CCGAAA | cis-acting element involved in low-temperature responsiveness |

>HU08G00229.1   
+ -Up\_Stream \_Len000AAATTT ATATGATTAT GGGAGCAAAT ATTAATCGGT CTAAAACAAA AAAGAATCGG   
  
  
+ CCGAACAAAA AAAAGGGCCC AGAACTAGTC CTTTTAATGG AGTCCCAAAA TGTGGTATAA ATTAGTCTTG   
  
  
+ TTCACTCATT TCTCACTCCT CTCACTCATT CTAACCCTAA ATCTCAATCC TCACTCTCTG TCCTCTCTAT   
  
  
+ CTGCGTCTCG AGCTTCTCTT TCCTCTGGGC TTCCTTGGAC TTTTTCGACG CCATGACAAT GGAACACTGA   
  
  
+ GCTCCACCGT TGCCCCTCTC CACAACTGTC TTCTAACGGT GGTTCTCCGT AGCCACCGTA CCTCAAAAAC   
  
  
+ CAAAGCGATG CACAACTACC ACCCCACCAC TGCTGAAAAG TGCCCACGAG TCTCGATCCG CGCAAGCCTG   
  
  
+ ATGAGAATCG ATACCACCAC CATGTAGATA AGCTGAAAAC GGGCATAAGG AAGCGACCCA CGCGCCCAAC   
  
  
+ ACGCCATTGA GGCAGTGACA CGTGGGTTGA TGCGCTTCCA AAGATGGAAT TTTCACATTT TTCTTTTCAA   
  
  
+ GGTTGCGTAG ATCGGTGTGT GTGGATGGCG ATCAAGGAAG AGTTAAAATG AACTAGTCAT GCGTCGAATT   
  
  
+ GTCCAAAAAA AATTTTCTCT TTCAGTCGAG TCTTAAAGGG GCAGTCATCA AGAGCATGTT TGAAAAACAT   
  
  
+ TATGGCCATA ACCACTTAGT AGTAATTCAA ATAATCATGG AAACATATTC AACATGAATT CAATATGACT   
  
  
+ AAAAATTCCA TCTTTTAGTT TGTTCTGGTC AACACAGAGC ATGCCTTTTT AATTGCATAA TAACGATATT   
  
  
+ TAACCAACCA ATCATGTTTC TCACATATTA ATATAGGCAA GGGTAGATTA ACAGTCATAA TCGCATAGTA   
  
  
+ TGCAAGAATT TATTTGCAAT CTGGGCATAC TGTTTTATAA AGCACAAGTG TTCATGACTT AAAATCGATA   
  
  
+ AAGTCTTAAT CTTTTGTCGA TCCTTGAGTG TGCAGACAAT ATAATTGTGA ATGTATGCTA TTAACTTTAA   
  
  
+ TAATTACGAA AATTAAGCAA CTAATCATAT AGTCATGTTC TCAAAAACAA GCATGCGTAT TATTCAGATC   
  
  
+ ATTAAATGCA TTAAATGGGA CTAATCCGAT CAGTCAAGCG CTGATACGAC TATTGAATAA ACAGGACTAA   
  
  
+ CCTAGAATGC GGAATTAACT GTCGAGAATT GTCCACATCG GTAGTGATGC TTGGCGGCTA CCAGAGTCAC   
  
  
+ CTTAGGGTGT TCACATCCTA GACCCGAAAA CCTGCTAATG ATGGAAGGGC AGCCGTGGGA GGAGGAGAGG   
  
  
+ AGAGTGTGGA TTTTTCTATT TACAAAAAAG TGTATAAGTG CTAATCCCCC GAAACCTCCT TTTATAGGTT   
  
  
+ AGGGGGGCAA CTAGGGTTAT CGCCCTAGGT GACGCTTGTG GCCGTCGGCC TAATTAAATA GGCCCGACCA   
  
  
+ ACAGCTGTGA CTGGAGCGAT AGCTGAAGCA TGCAGAAATG AGGATAACAT ACATATTATA GATTTCCACA   
  
  
+ TTGCATAGGG CACTCAGTGG GTTACTGTAT CGCAAGCCAT AGCAGCAAGG CTTGGTGGGC CTCCTAAAGT   
  
  
+ GCGACTTACA GGCATTGATG ATCCTGTTTC TAAGTATACT TGTGATGCTA GCTTGGAGGC TGGTGGGAAA   
  
  
+ CGATTAGCGT CTCTATTTGA AAAGTTTAAA ATACTTGTCG AGTTCAATGC ATTGCCCGTT TATGGACCCA   
  
  
+ ATGTCAGGTG GGAAATGCTG GATGTGAGGC CCAGGGAGGC TTTGGTCGTT AATTGTCCAT TACAGCTCCA   
  
  
+ TTACACTCCT GACGAGAGTG TCGATGTGAG GAACCCTAGG GATAGGCTTC TCAGAATGGT GAAATTGCTC   
  
  
+ GGTCCTAAGG TAGCCACTTT GGTTGAGCAA GAATCAAACA CCAACACTAC ACCTTTCTTG ACCCGGTTCA   
  
  
+ TAGAGACCCT TGACTACTAC TCGATTAACC ATGTTTGAGT CTATAGACGT GACCATGCCG AGAGACCGGA   
  
  
+ AGGAGAGGAT CAATGTTGAG CAGCATTGTT TAGCTAAGGA CATTGTGAAT ATCATAGCTT GCGAGGGCAA   
  
  
+ GGAGATGGTG GAGCGTCATG AGCTTTTTGG GAAATGGCAG TCGAGGTTTA CCATGGTAGG GTTCAGGCAG   
  
  
+ TACCCGTTGA GCTCATACAT AAACTCTGTG ATAAGAAGCC TACTCCGGTG TTACTCCAAG CATTATACTC   
  
  
+ TGATAGAGAA GGATAGTGCC ATGCTTCTGG GTTGGAGGGG CCGAATGCTG ATTTCAGCTT CGGCATG  

- -Up\_Stream \_Len000TTTAAA TATACTAATA CCCTCGTTTA TAATTAGCCA GATTTTGTTT TTTCTTAGCC   
  
  
- GGCTTGTTTT TTTTCCCGGG TCTTGATCAG GAAAATTACC TCAGGGTTTT ACACCATATT TAATCAGAAC   
  
  
- AAGTGAGTAA AGAGTGAGGA GAGTGAGTAA GATTGGGATT TAGAGTTAGG AGTGAGAGAC AGGAGAGATA   
  
  
- GACGCAGAGC TCGAAGAGAA AGGAGACCCG AAGGAACCTG AAAAAGCTGC GGTACTGTTA CCTTGTGACT   
  
  
- CGAGGTGGCA ACGGGGAGAG GTGTTGACAG AAGATTGCCA CCAAGAGGCA TCGGTGGCAT GGAGTTTTTG   
  
  
- GTTTCGCTAC GTGTTGATGG TGGGGTGGTG ACGACTTTTC ACGGGTGCTC AGAGCTAGGC GCGTTCGGAC   
  
  
- TACTCTTAGC TATGGTGGTG GTACATCTAT TCGACTTTTG CCCGTATTCC TTCGCTGGGT GCGCGGGTTG   
  
  
- TGCGGTAACT CCGTCACTGT GCACCCAACT ACGCGAAGGT TTCTACCTTA AAAGTGTAAA AAGAAAAGTT   
  
  
- CCAACGCATC TAGCCACACA CACCTACCGC TAGTTCCTTC TCAATTTTAC TTGATCAGTA CGCAGCTTAA   
  
  
- CAGGTTTTTT TTAAAAGAGA AAGTCAGCTC AGAATTTCCC CGTCAGTAGT TCTCGTACAA ACTTTTTGTA   
  
  
- ATACCGGTAT TGGTGAATCA TCATTAAGTT TATTAGTACC TTTGTATAAG TTGTACTTAA GTTATACTGA   
  
  
- TTTTTAAGGT AGAAAATCAA ACAAGACCAG TTGTGTCTCG TACGGAAAAA TTAACGTATT ATTGCTATAA   
  
  
- ATTGGTTGGT TAGTACAAAG AGTGTATAAT TATATCCGTT CCCATCTAAT TGTCAGTATT AGCGTATCAT   
  
  
- ACGTTCTTAA ATAAACGTTA GACCCGTATG ACAAAATATT TCGTGTTCAC AAGTACTGAA TTTTAGCTAT   
  
  
- TTCAGAATTA GAAAACAGCT AGGAACTCAC ACGTCTGTTA TATTAACACT TACATACGAT AATTGAAATT   
  
  
- ATTAATGCTT TTAATTCGTT GATTAGTATA TCAGTACAAG AGTTTTTGTT CGTACGCATA ATAAGTCTAG   
  
  
- TAATTTACGT AATTTACCCT GATTAGGCTA GTCAGTTCGC GACTATGCTG ATAACTTATT TGTCCTGATT   
  
  
- GGATCTTACG CCTTAATTGA CAGCTCTTAA CAGGTGTAGC CATCACTACG AACCGCCGAT GGTCTCAGTG   
  
  
- GAATCCCACA AGTGTAGGAT CTGGGCTTTT GGACGATTAC TACCTTCCCG TCGGCACCCT CCTCCTCTCC   
  
  
- TCTCACACCT AAAAAGATAA ATGTTTTTTC ACATATTCAC GATTAGGGGG CTTTGGAGGA AAATATCCAA   
  
  
- TCCCCCCGTT GATCCCAATA GCGGGATCCA CTGCGAACAC CGGCAGCCGG ATTAATTTAT CCGGGCTGGT   
  
  
- TGTCGACACT GACCTCGCTA TCGACTTCGT ACGTCTTTAC TCCTATTGTA TGTATAATAT CTAAAGGTGT   
  
  
- AACGTATCCC GTGAGTCACC CAATGACATA GCGTTCGGTA TCGTCGTTCC GAACCACCCG GAGGATTTCA   
  
  
- CGCTGAATGT CCGTAACTAC TAGGACAAAG ATTCATATGA ACACTACGAT CGAACCTCCG ACCACCCTTT   
  
  
- GCTAATCGCA GAGATAAACT TTTCAAATTT TATGAACAGC TCAAGTTACG TAACGGGCAA ATACCTGGGT   
  
  
- TACAGTCCAC CCTTTACGAC CTACACTCCG GGTCCCTCCG AAACCAGCAA TTAACAGGTA ATGTCGAGGT   
  
  
- AATGTGAGGA CTGCTCTCAC AGCTACACTC CTTGGGATCC CTATCCGAAG AGTCTTACCA CTTTAACGAG   
  
  
- CCAGGATTCC ATCGGTGAAA CCAACTCGTT CTTAGTTTGT GGTTGTGATG TGGAAAGAAC TGGGCCAAGT   
  
  
- ATCTCTGGGA ACTGATGATG AGCTAATTGG TACAAACTCA GATATCTGCA CTGGTACGGC TCTCTGGCCT   
  
  
- TCCTCTCCTA GTTACAACTC GTCGTAACAA ATCGATTCCT GTAACACTTA TAGTATCGAA CGCTCCCGTT   
  
  
- CCTCTACCAC CTCGCAGTAC TCGAAAAACC CTTTACCGTC AGCTCCAAAT GGTACCATCC CAAGTCCGTC   
  
  
- ATGGGCAACT CGAGTATGTA TTTGAGACAC TATTCTTCGG ATGAGGCCAC AATGAGGTTC GTAATATGAG   
  
  
- ACTATCTCTT CCTATCACGG TACGAAGACC CAACCTCCCC GGCTTACGAC TAAAGTCGAA GCCGTAC

+     MBS

| Site Name | Organism | Position | Strand | Matrix score. | sequence | function |
| --- | --- | --- | --- | --- | --- | --- |
| MBS | Arabidopsis thaliana | 307 | + | 6 | CAACTG | MYB binding site involved in drought-inducibility |

>HU08G00229.1   
+ -Up\_Stream \_Len000AAATTT ATATGATTAT GGGAGCAAAT ATTAATCGGT CTAAAACAAA AAAGAATCGG   
  
  
+ CCGAACAAAA AAAAGGGCCC AGAACTAGTC CTTTTAATGG AGTCCCAAAA TGTGGTATAA ATTAGTCTTG   
  
  
+ TTCACTCATT TCTCACTCCT CTCACTCATT CTAACCCTAA ATCTCAATCC TCACTCTCTG TCCTCTCTAT   
  
  
+ CTGCGTCTCG AGCTTCTCTT TCCTCTGGGC TTCCTTGGAC TTTTTCGACG CCATGACAAT GGAACACTGA   
  
  
+ GCTCCACCGT TGCCCCTCTC CACAACTGTC TTCTAACGGT GGTTCTCCGT AGCCACCGTA CCTCAAAAAC   
  
  
+ CAAAGCGATG CACAACTACC ACCCCACCAC TGCTGAAAAG TGCCCACGAG TCTCGATCCG CGCAAGCCTG   
  
  
+ ATGAGAATCG ATACCACCAC CATGTAGATA AGCTGAAAAC GGGCATAAGG AAGCGACCCA CGCGCCCAAC   
  
  
+ ACGCCATTGA GGCAGTGACA CGTGGGTTGA TGCGCTTCCA AAGATGGAAT TTTCACATTT TTCTTTTCAA   
  
  
+ GGTTGCGTAG ATCGGTGTGT GTGGATGGCG ATCAAGGAAG AGTTAAAATG AACTAGTCAT GCGTCGAATT   
  
  
+ GTCCAAAAAA AATTTTCTCT TTCAGTCGAG TCTTAAAGGG GCAGTCATCA AGAGCATGTT TGAAAAACAT   
  
  
+ TATGGCCATA ACCACTTAGT AGTAATTCAA ATAATCATGG AAACATATTC AACATGAATT CAATATGACT   
  
  
+ AAAAATTCCA TCTTTTAGTT TGTTCTGGTC AACACAGAGC ATGCCTTTTT AATTGCATAA TAACGATATT   
  
  
+ TAACCAACCA ATCATGTTTC TCACATATTA ATATAGGCAA GGGTAGATTA ACAGTCATAA TCGCATAGTA   
  
  
+ TGCAAGAATT TATTTGCAAT CTGGGCATAC TGTTTTATAA AGCACAAGTG TTCATGACTT AAAATCGATA   
  
  
+ AAGTCTTAAT CTTTTGTCGA TCCTTGAGTG TGCAGACAAT ATAATTGTGA ATGTATGCTA TTAACTTTAA   
  
  
+ TAATTACGAA AATTAAGCAA CTAATCATAT AGTCATGTTC TCAAAAACAA GCATGCGTAT TATTCAGATC   
  
  
+ ATTAAATGCA TTAAATGGGA CTAATCCGAT CAGTCAAGCG CTGATACGAC TATTGAATAA ACAGGACTAA   
  
  
+ CCTAGAATGC GGAATTAACT GTCGAGAATT GTCCACATCG GTAGTGATGC TTGGCGGCTA CCAGAGTCAC   
  
  
+ CTTAGGGTGT TCACATCCTA GACCCGAAAA CCTGCTAATG ATGGAAGGGC AGCCGTGGGA GGAGGAGAGG   
  
  
+ AGAGTGTGGA TTTTTCTATT TACAAAAAAG TGTATAAGTG CTAATCCCCC GAAACCTCCT TTTATAGGTT   
  
  
+ AGGGGGGCAA CTAGGGTTAT CGCCCTAGGT GACGCTTGTG GCCGTCGGCC TAATTAAATA GGCCCGACCA   
  
  
+ ACAGCTGTGA CTGGAGCGAT AGCTGAAGCA TGCAGAAATG AGGATAACAT ACATATTATA GATTTCCACA   
  
  
+ TTGCATAGGG CACTCAGTGG GTTACTGTAT CGCAAGCCAT AGCAGCAAGG CTTGGTGGGC CTCCTAAAGT   
  
  
+ GCGACTTACA GGCATTGATG ATCCTGTTTC TAAGTATACT TGTGATGCTA GCTTGGAGGC TGGTGGGAAA   
  
  
+ CGATTAGCGT CTCTATTTGA AAAGTTTAAA ATACTTGTCG AGTTCAATGC ATTGCCCGTT TATGGACCCA   
  
  
+ ATGTCAGGTG GGAAATGCTG GATGTGAGGC CCAGGGAGGC TTTGGTCGTT AATTGTCCAT TACAGCTCCA   
  
  
+ TTACACTCCT GACGAGAGTG TCGATGTGAG GAACCCTAGG GATAGGCTTC TCAGAATGGT GAAATTGCTC   
  
  
+ GGTCCTAAGG TAGCCACTTT GGTTGAGCAA GAATCAAACA CCAACACTAC ACCTTTCTTG ACCCGGTTCA   
  
  
+ TAGAGACCCT TGACTACTAC TCGATTAACC ATGTTTGAGT CTATAGACGT GACCATGCCG AGAGACCGGA   
  
  
+ AGGAGAGGAT CAATGTTGAG CAGCATTGTT TAGCTAAGGA CATTGTGAAT ATCATAGCTT GCGAGGGCAA   
  
  
+ GGAGATGGTG GAGCGTCATG AGCTTTTTGG GAAATGGCAG TCGAGGTTTA CCATGGTAGG GTTCAGGCAG   
  
  
+ TACCCGTTGA GCTCATACAT AAACTCTGTG ATAAGAAGCC TACTCCGGTG TTACTCCAAG CATTATACTC   
  
  
+ TGATAGAGAA GGATAGTGCC ATGCTTCTGG GTTGGAGGGG CCGAATGCTG ATTTCAGCTT CGGCATG  

- -Up\_Stream \_Len000TTTAAA TATACTAATA CCCTCGTTTA TAATTAGCCA GATTTTGTTT TTTCTTAGCC   
  
  
- GGCTTGTTTT TTTTCCCGGG TCTTGATCAG GAAAATTACC TCAGGGTTTT ACACCATATT TAATCAGAAC   
  
  
- AAGTGAGTAA AGAGTGAGGA GAGTGAGTAA GATTGGGATT TAGAGTTAGG AGTGAGAGAC AGGAGAGATA   
  
  
- GACGCAGAGC TCGAAGAGAA AGGAGACCCG AAGGAACCTG AAAAAGCTGC GGTACTGTTA CCTTGTGACT   
  
  
- CGAGGTGGCA ACGGGGAGAG GTGTTGACAG AAGATTGCCA CCAAGAGGCA TCGGTGGCAT GGAGTTTTTG   
  
  
- GTTTCGCTAC GTGTTGATGG TGGGGTGGTG ACGACTTTTC ACGGGTGCTC AGAGCTAGGC GCGTTCGGAC   
  
  
- TACTCTTAGC TATGGTGGTG GTACATCTAT TCGACTTTTG CCCGTATTCC TTCGCTGGGT GCGCGGGTTG   
  
  
- TGCGGTAACT CCGTCACTGT GCACCCAACT ACGCGAAGGT TTCTACCTTA AAAGTGTAAA AAGAAAAGTT   
  
  
- CCAACGCATC TAGCCACACA CACCTACCGC TAGTTCCTTC TCAATTTTAC TTGATCAGTA CGCAGCTTAA   
  
  
- CAGGTTTTTT TTAAAAGAGA AAGTCAGCTC AGAATTTCCC CGTCAGTAGT TCTCGTACAA ACTTTTTGTA   
  
  
- ATACCGGTAT TGGTGAATCA TCATTAAGTT TATTAGTACC TTTGTATAAG TTGTACTTAA GTTATACTGA   
  
  
- TTTTTAAGGT AGAAAATCAA ACAAGACCAG TTGTGTCTCG TACGGAAAAA TTAACGTATT ATTGCTATAA   
  
  
- ATTGGTTGGT TAGTACAAAG AGTGTATAAT TATATCCGTT CCCATCTAAT TGTCAGTATT AGCGTATCAT   
  
  
- ACGTTCTTAA ATAAACGTTA GACCCGTATG ACAAAATATT TCGTGTTCAC AAGTACTGAA TTTTAGCTAT   
  
  
- TTCAGAATTA GAAAACAGCT AGGAACTCAC ACGTCTGTTA TATTAACACT TACATACGAT AATTGAAATT   
  
  
- ATTAATGCTT TTAATTCGTT GATTAGTATA TCAGTACAAG AGTTTTTGTT CGTACGCATA ATAAGTCTAG   
  
  
- TAATTTACGT AATTTACCCT GATTAGGCTA GTCAGTTCGC GACTATGCTG ATAACTTATT TGTCCTGATT   
  
  
- GGATCTTACG CCTTAATTGA CAGCTCTTAA CAGGTGTAGC CATCACTACG AACCGCCGAT GGTCTCAGTG   
  
  
- GAATCCCACA AGTGTAGGAT CTGGGCTTTT GGACGATTAC TACCTTCCCG TCGGCACCCT CCTCCTCTCC   
  
  
- TCTCACACCT AAAAAGATAA ATGTTTTTTC ACATATTCAC GATTAGGGGG CTTTGGAGGA AAATATCCAA   
  
  
- TCCCCCCGTT GATCCCAATA GCGGGATCCA CTGCGAACAC CGGCAGCCGG ATTAATTTAT CCGGGCTGGT   
  
  
- TGTCGACACT GACCTCGCTA TCGACTTCGT ACGTCTTTAC TCCTATTGTA TGTATAATAT CTAAAGGTGT   
  
  
- AACGTATCCC GTGAGTCACC CAATGACATA GCGTTCGGTA TCGTCGTTCC GAACCACCCG GAGGATTTCA   
  
  
- CGCTGAATGT CCGTAACTAC TAGGACAAAG ATTCATATGA ACACTACGAT CGAACCTCCG ACCACCCTTT   
  
  
- GCTAATCGCA GAGATAAACT TTTCAAATTT TATGAACAGC TCAAGTTACG TAACGGGCAA ATACCTGGGT   
  
  
- TACAGTCCAC CCTTTACGAC CTACACTCCG GGTCCCTCCG AAACCAGCAA TTAACAGGTA ATGTCGAGGT   
  
  
- AATGTGAGGA CTGCTCTCAC AGCTACACTC CTTGGGATCC CTATCCGAAG AGTCTTACCA CTTTAACGAG   
  
  
- CCAGGATTCC ATCGGTGAAA CCAACTCGTT CTTAGTTTGT GGTTGTGATG TGGAAAGAAC TGGGCCAAGT   
  
  
- ATCTCTGGGA ACTGATGATG AGCTAATTGG TACAAACTCA GATATCTGCA CTGGTACGGC TCTCTGGCCT   
  
  
- TCCTCTCCTA GTTACAACTC GTCGTAACAA ATCGATTCCT GTAACACTTA TAGTATCGAA CGCTCCCGTT   
  
  
- CCTCTACCAC CTCGCAGTAC TCGAAAAACC CTTTACCGTC AGCTCCAAAT GGTACCATCC CAAGTCCGTC   
  
  
- ATGGGCAACT CGAGTATGTA TTTGAGACAC TATTCTTCGG ATGAGGCCAC AATGAGGTTC GTAATATGAG   
  
  
- ACTATCTCTT CCTATCACGG TACGAAGACC CAACCTCCCC GGCTTACGAC TAAAGTCGAA GCCGTAC

+     MYB

| Site Name | Organism | Position | Strand | Matrix score. | sequence | function |
| --- | --- | --- | --- | --- | --- | --- |
| MYB | Arabidopsis thaliana | 1914 | - | 6 | CAACCA |  |
| MYB | Arabidopsis thaliana | 1990 | + | 6 | TAACCA |  |
| MYB | Arabidopsis thaliana | 713 | + | 6 | TAACCA |  |
| MYB | Arabidopsis thaliana | 849 | + | 6 | CAACCA |  |
| MYB | Arabidopsis thaliana | 1473 | + | 6 | CAACAG |  |
| MYB | Arabidopsis thaliana | 845 | + | 6 | TAACCA |  |

>HU08G00229.1   
+ -Up\_Stream \_Len000AAATTT ATATGATTAT GGGAGCAAAT ATTAATCGGT CTAAAACAAA AAAGAATCGG   
  
  
+ CCGAACAAAA AAAAGGGCCC AGAACTAGTC CTTTTAATGG AGTCCCAAAA TGTGGTATAA ATTAGTCTTG   
  
  
+ TTCACTCATT TCTCACTCCT CTCACTCATT CTAACCCTAA ATCTCAATCC TCACTCTCTG TCCTCTCTAT   
  
  
+ CTGCGTCTCG AGCTTCTCTT TCCTCTGGGC TTCCTTGGAC TTTTTCGACG CCATGACAAT GGAACACTGA   
  
  
+ GCTCCACCGT TGCCCCTCTC CACAACTGTC TTCTAACGGT GGTTCTCCGT AGCCACCGTA CCTCAAAAAC   
  
  
+ CAAAGCGATG CACAACTACC ACCCCACCAC TGCTGAAAAG TGCCCACGAG TCTCGATCCG CGCAAGCCTG   
  
  
+ ATGAGAATCG ATACCACCAC CATGTAGATA AGCTGAAAAC GGGCATAAGG AAGCGACCCA CGCGCCCAAC   
  
  
+ ACGCCATTGA GGCAGTGACA CGTGGGTTGA TGCGCTTCCA AAGATGGAAT TTTCACATTT TTCTTTTCAA   
  
  
+ GGTTGCGTAG ATCGGTGTGT GTGGATGGCG ATCAAGGAAG AGTTAAAATG AACTAGTCAT GCGTCGAATT   
  
  
+ GTCCAAAAAA AATTTTCTCT TTCAGTCGAG TCTTAAAGGG GCAGTCATCA AGAGCATGTT TGAAAAACAT   
  
  
+ TATGGCCATA ACCACTTAGT AGTAATTCAA ATAATCATGG AAACATATTC AACATGAATT CAATATGACT   
  
  
+ AAAAATTCCA TCTTTTAGTT TGTTCTGGTC AACACAGAGC ATGCCTTTTT AATTGCATAA TAACGATATT   
  
  
+ TAACCAACCA ATCATGTTTC TCACATATTA ATATAGGCAA GGGTAGATTA ACAGTCATAA TCGCATAGTA   
  
  
+ TGCAAGAATT TATTTGCAAT CTGGGCATAC TGTTTTATAA AGCACAAGTG TTCATGACTT AAAATCGATA   
  
  
+ AAGTCTTAAT CTTTTGTCGA TCCTTGAGTG TGCAGACAAT ATAATTGTGA ATGTATGCTA TTAACTTTAA   
  
  
+ TAATTACGAA AATTAAGCAA CTAATCATAT AGTCATGTTC TCAAAAACAA GCATGCGTAT TATTCAGATC   
  
  
+ ATTAAATGCA TTAAATGGGA CTAATCCGAT CAGTCAAGCG CTGATACGAC TATTGAATAA ACAGGACTAA   
  
  
+ CCTAGAATGC GGAATTAACT GTCGAGAATT GTCCACATCG GTAGTGATGC TTGGCGGCTA CCAGAGTCAC   
  
  
+ CTTAGGGTGT TCACATCCTA GACCCGAAAA CCTGCTAATG ATGGAAGGGC AGCCGTGGGA GGAGGAGAGG   
  
  
+ AGAGTGTGGA TTTTTCTATT TACAAAAAAG TGTATAAGTG CTAATCCCCC GAAACCTCCT TTTATAGGTT   
  
  
+ AGGGGGGCAA CTAGGGTTAT CGCCCTAGGT GACGCTTGTG GCCGTCGGCC TAATTAAATA GGCCCGACCA   
  
  
+ ACAGCTGTGA CTGGAGCGAT AGCTGAAGCA TGCAGAAATG AGGATAACAT ACATATTATA GATTTCCACA   
  
  
+ TTGCATAGGG CACTCAGTGG GTTACTGTAT CGCAAGCCAT AGCAGCAAGG CTTGGTGGGC CTCCTAAAGT   
  
  
+ GCGACTTACA GGCATTGATG ATCCTGTTTC TAAGTATACT TGTGATGCTA GCTTGGAGGC TGGTGGGAAA   
  
  
+ CGATTAGCGT CTCTATTTGA AAAGTTTAAA ATACTTGTCG AGTTCAATGC ATTGCCCGTT TATGGACCCA   
  
  
+ ATGTCAGGTG GGAAATGCTG GATGTGAGGC CCAGGGAGGC TTTGGTCGTT AATTGTCCAT TACAGCTCCA   
  
  
+ TTACACTCCT GACGAGAGTG TCGATGTGAG GAACCCTAGG GATAGGCTTC TCAGAATGGT GAAATTGCTC   
  
  
+ GGTCCTAAGG TAGCCACTTT GGTTGAGCAA GAATCAAACA CCAACACTAC ACCTTTCTTG ACCCGGTTCA   
  
  
+ TAGAGACCCT TGACTACTAC TCGATTAACC ATGTTTGAGT CTATAGACGT GACCATGCCG AGAGACCGGA   
  
  
+ AGGAGAGGAT CAATGTTGAG CAGCATTGTT TAGCTAAGGA CATTGTGAAT ATCATAGCTT GCGAGGGCAA   
  
  
+ GGAGATGGTG GAGCGTCATG AGCTTTTTGG GAAATGGCAG TCGAGGTTTA CCATGGTAGG GTTCAGGCAG   
  
  
+ TACCCGTTGA GCTCATACAT AAACTCTGTG ATAAGAAGCC TACTCCGGTG TTACTCCAAG CATTATACTC   
  
  
+ TGATAGAGAA GGATAGTGCC ATGCTTCTGG GTTGGAGGGG CCGAATGCTG ATTTCAGCTT CGGCATG  

- -Up\_Stream \_Len000TTTAAA TATACTAATA CCCTCGTTTA TAATTAGCCA GATTTTGTTT TTTCTTAGCC   
  
  
- GGCTTGTTTT TTTTCCCGGG TCTTGATCAG GAAAATTACC TCAGGGTTTT ACACCATATT TAATCAGAAC   
  
  
- AAGTGAGTAA AGAGTGAGGA GAGTGAGTAA GATTGGGATT TAGAGTTAGG AGTGAGAGAC AGGAGAGATA   
  
  
- GACGCAGAGC TCGAAGAGAA AGGAGACCCG AAGGAACCTG AAAAAGCTGC GGTACTGTTA CCTTGTGACT   
  
  
- CGAGGTGGCA ACGGGGAGAG GTGTTGACAG AAGATTGCCA CCAAGAGGCA TCGGTGGCAT GGAGTTTTTG   
  
  
- GTTTCGCTAC GTGTTGATGG TGGGGTGGTG ACGACTTTTC ACGGGTGCTC AGAGCTAGGC GCGTTCGGAC   
  
  
- TACTCTTAGC TATGGTGGTG GTACATCTAT TCGACTTTTG CCCGTATTCC TTCGCTGGGT GCGCGGGTTG   
  
  
- TGCGGTAACT CCGTCACTGT GCACCCAACT ACGCGAAGGT TTCTACCTTA AAAGTGTAAA AAGAAAAGTT   
  
  
- CCAACGCATC TAGCCACACA CACCTACCGC TAGTTCCTTC TCAATTTTAC TTGATCAGTA CGCAGCTTAA   
  
  
- CAGGTTTTTT TTAAAAGAGA AAGTCAGCTC AGAATTTCCC CGTCAGTAGT TCTCGTACAA ACTTTTTGTA   
  
  
- ATACCGGTAT TGGTGAATCA TCATTAAGTT TATTAGTACC TTTGTATAAG TTGTACTTAA GTTATACTGA   
  
  
- TTTTTAAGGT AGAAAATCAA ACAAGACCAG TTGTGTCTCG TACGGAAAAA TTAACGTATT ATTGCTATAA   
  
  
- ATTGGTTGGT TAGTACAAAG AGTGTATAAT TATATCCGTT CCCATCTAAT TGTCAGTATT AGCGTATCAT   
  
  
- ACGTTCTTAA ATAAACGTTA GACCCGTATG ACAAAATATT TCGTGTTCAC AAGTACTGAA TTTTAGCTAT   
  
  
- TTCAGAATTA GAAAACAGCT AGGAACTCAC ACGTCTGTTA TATTAACACT TACATACGAT AATTGAAATT   
  
  
- ATTAATGCTT TTAATTCGTT GATTAGTATA TCAGTACAAG AGTTTTTGTT CGTACGCATA ATAAGTCTAG   
  
  
- TAATTTACGT AATTTACCCT GATTAGGCTA GTCAGTTCGC GACTATGCTG ATAACTTATT TGTCCTGATT   
  
  
- GGATCTTACG CCTTAATTGA CAGCTCTTAA CAGGTGTAGC CATCACTACG AACCGCCGAT GGTCTCAGTG   
  
  
- GAATCCCACA AGTGTAGGAT CTGGGCTTTT GGACGATTAC TACCTTCCCG TCGGCACCCT CCTCCTCTCC   
  
  
- TCTCACACCT AAAAAGATAA ATGTTTTTTC ACATATTCAC GATTAGGGGG CTTTGGAGGA AAATATCCAA   
  
  
- TCCCCCCGTT GATCCCAATA GCGGGATCCA CTGCGAACAC CGGCAGCCGG ATTAATTTAT CCGGGCTGGT   
  
  
- TGTCGACACT GACCTCGCTA TCGACTTCGT ACGTCTTTAC TCCTATTGTA TGTATAATAT CTAAAGGTGT   
  
  
- AACGTATCCC GTGAGTCACC CAATGACATA GCGTTCGGTA TCGTCGTTCC GAACCACCCG GAGGATTTCA   
  
  
- CGCTGAATGT CCGTAACTAC TAGGACAAAG ATTCATATGA ACACTACGAT CGAACCTCCG ACCACCCTTT   
  
  
- GCTAATCGCA GAGATAAACT TTTCAAATTT TATGAACAGC TCAAGTTACG TAACGGGCAA ATACCTGGGT   
  
  
- TACAGTCCAC CCTTTACGAC CTACACTCCG GGTCCCTCCG AAACCAGCAA TTAACAGGTA ATGTCGAGGT   
  
  
- AATGTGAGGA CTGCTCTCAC AGCTACACTC CTTGGGATCC CTATCCGAAG AGTCTTACCA CTTTAACGAG   
  
  
- CCAGGATTCC ATCGGTGAAA CCAACTCGTT CTTAGTTTGT GGTTGTGATG TGGAAAGAAC TGGGCCAAGT   
  
  
- ATCTCTGGGA ACTGATGATG AGCTAATTGG TACAAACTCA GATATCTGCA CTGGTACGGC TCTCTGGCCT   
  
  
- TCCTCTCCTA GTTACAACTC GTCGTAACAA ATCGATTCCT GTAACACTTA TAGTATCGAA CGCTCCCGTT   
  
  
- CCTCTACCAC CTCGCAGTAC TCGAAAAACC CTTTACCGTC AGCTCCAAAT GGTACCATCC CAAGTCCGTC   
  
  
- ATGGGCAACT CGAGTATGTA TTTGAGACAC TATTCTTCGG ATGAGGCCAC AATGAGGTTC GTAATATGAG   
  
  
- ACTATCTCTT CCTATCACGG TACGAAGACC CAACCTCCCC GGCTTACGAC TAAAGTCGAA GCCGTAC

+     MYB recognition site

| Site Name | Organism | Position | Strand | Matrix score. | sequence | function |
| --- | --- | --- | --- | --- | --- | --- |
| MYB recognition site | Arabidopsis thaliana | 291 | + | 6 | CCGTTG |  |
| MYB recognition site | Arabidopsis thaliana | 2178 | + | 6 | CCGTTG |  |

>HU08G00229.1   
+ -Up\_Stream \_Len000AAATTT ATATGATTAT GGGAGCAAAT ATTAATCGGT CTAAAACAAA AAAGAATCGG   
  
  
+ CCGAACAAAA AAAAGGGCCC AGAACTAGTC CTTTTAATGG AGTCCCAAAA TGTGGTATAA ATTAGTCTTG   
  
  
+ TTCACTCATT TCTCACTCCT CTCACTCATT CTAACCCTAA ATCTCAATCC TCACTCTCTG TCCTCTCTAT   
  
  
+ CTGCGTCTCG AGCTTCTCTT TCCTCTGGGC TTCCTTGGAC TTTTTCGACG CCATGACAAT GGAACACTGA   
  
  
+ GCTCCACCGT TGCCCCTCTC CACAACTGTC TTCTAACGGT GGTTCTCCGT AGCCACCGTA CCTCAAAAAC   
  
  
+ CAAAGCGATG CACAACTACC ACCCCACCAC TGCTGAAAAG TGCCCACGAG TCTCGATCCG CGCAAGCCTG   
  
  
+ ATGAGAATCG ATACCACCAC CATGTAGATA AGCTGAAAAC GGGCATAAGG AAGCGACCCA CGCGCCCAAC   
  
  
+ ACGCCATTGA GGCAGTGACA CGTGGGTTGA TGCGCTTCCA AAGATGGAAT TTTCACATTT TTCTTTTCAA   
  
  
+ GGTTGCGTAG ATCGGTGTGT GTGGATGGCG ATCAAGGAAG AGTTAAAATG AACTAGTCAT GCGTCGAATT   
  
  
+ GTCCAAAAAA AATTTTCTCT TTCAGTCGAG TCTTAAAGGG GCAGTCATCA AGAGCATGTT TGAAAAACAT   
  
  
+ TATGGCCATA ACCACTTAGT AGTAATTCAA ATAATCATGG AAACATATTC AACATGAATT CAATATGACT   
  
  
+ AAAAATTCCA TCTTTTAGTT TGTTCTGGTC AACACAGAGC ATGCCTTTTT AATTGCATAA TAACGATATT   
  
  
+ TAACCAACCA ATCATGTTTC TCACATATTA ATATAGGCAA GGGTAGATTA ACAGTCATAA TCGCATAGTA   
  
  
+ TGCAAGAATT TATTTGCAAT CTGGGCATAC TGTTTTATAA AGCACAAGTG TTCATGACTT AAAATCGATA   
  
  
+ AAGTCTTAAT CTTTTGTCGA TCCTTGAGTG TGCAGACAAT ATAATTGTGA ATGTATGCTA TTAACTTTAA   
  
  
+ TAATTACGAA AATTAAGCAA CTAATCATAT AGTCATGTTC TCAAAAACAA GCATGCGTAT TATTCAGATC   
  
  
+ ATTAAATGCA TTAAATGGGA CTAATCCGAT CAGTCAAGCG CTGATACGAC TATTGAATAA ACAGGACTAA   
  
  
+ CCTAGAATGC GGAATTAACT GTCGAGAATT GTCCACATCG GTAGTGATGC TTGGCGGCTA CCAGAGTCAC   
  
  
+ CTTAGGGTGT TCACATCCTA GACCCGAAAA CCTGCTAATG ATGGAAGGGC AGCCGTGGGA GGAGGAGAGG   
  
  
+ AGAGTGTGGA TTTTTCTATT TACAAAAAAG TGTATAAGTG CTAATCCCCC GAAACCTCCT TTTATAGGTT   
  
  
+ AGGGGGGCAA CTAGGGTTAT CGCCCTAGGT GACGCTTGTG GCCGTCGGCC TAATTAAATA GGCCCGACCA   
  
  
+ ACAGCTGTGA CTGGAGCGAT AGCTGAAGCA TGCAGAAATG AGGATAACAT ACATATTATA GATTTCCACA   
  
  
+ TTGCATAGGG CACTCAGTGG GTTACTGTAT CGCAAGCCAT AGCAGCAAGG CTTGGTGGGC CTCCTAAAGT   
  
  
+ GCGACTTACA GGCATTGATG ATCCTGTTTC TAAGTATACT TGTGATGCTA GCTTGGAGGC TGGTGGGAAA   
  
  
+ CGATTAGCGT CTCTATTTGA AAAGTTTAAA ATACTTGTCG AGTTCAATGC ATTGCCCGTT TATGGACCCA   
  
  
+ ATGTCAGGTG GGAAATGCTG GATGTGAGGC CCAGGGAGGC TTTGGTCGTT AATTGTCCAT TACAGCTCCA   
  
  
+ TTACACTCCT GACGAGAGTG TCGATGTGAG GAACCCTAGG GATAGGCTTC TCAGAATGGT GAAATTGCTC   
  
  
+ GGTCCTAAGG TAGCCACTTT GGTTGAGCAA GAATCAAACA CCAACACTAC ACCTTTCTTG ACCCGGTTCA   
  
  
+ TAGAGACCCT TGACTACTAC TCGATTAACC ATGTTTGAGT CTATAGACGT GACCATGCCG AGAGACCGGA   
  
  
+ AGGAGAGGAT CAATGTTGAG CAGCATTGTT TAGCTAAGGA CATTGTGAAT ATCATAGCTT GCGAGGGCAA   
  
  
+ GGAGATGGTG GAGCGTCATG AGCTTTTTGG GAAATGGCAG TCGAGGTTTA CCATGGTAGG GTTCAGGCAG   
  
  
+ TACCCGTTGA GCTCATACAT AAACTCTGTG ATAAGAAGCC TACTCCGGTG TTACTCCAAG CATTATACTC   
  
  
+ TGATAGAGAA GGATAGTGCC ATGCTTCTGG GTTGGAGGGG CCGAATGCTG ATTTCAGCTT CGGCATG  

- -Up\_Stream \_Len000TTTAAA TATACTAATA CCCTCGTTTA TAATTAGCCA GATTTTGTTT TTTCTTAGCC   
  
  
- GGCTTGTTTT TTTTCCCGGG TCTTGATCAG GAAAATTACC TCAGGGTTTT ACACCATATT TAATCAGAAC   
  
  
- AAGTGAGTAA AGAGTGAGGA GAGTGAGTAA GATTGGGATT TAGAGTTAGG AGTGAGAGAC AGGAGAGATA   
  
  
- GACGCAGAGC TCGAAGAGAA AGGAGACCCG AAGGAACCTG AAAAAGCTGC GGTACTGTTA CCTTGTGACT   
  
  
- CGAGGTGGCA ACGGGGAGAG GTGTTGACAG AAGATTGCCA CCAAGAGGCA TCGGTGGCAT GGAGTTTTTG   
  
  
- GTTTCGCTAC GTGTTGATGG TGGGGTGGTG ACGACTTTTC ACGGGTGCTC AGAGCTAGGC GCGTTCGGAC   
  
  
- TACTCTTAGC TATGGTGGTG GTACATCTAT TCGACTTTTG CCCGTATTCC TTCGCTGGGT GCGCGGGTTG   
  
  
- TGCGGTAACT CCGTCACTGT GCACCCAACT ACGCGAAGGT TTCTACCTTA AAAGTGTAAA AAGAAAAGTT   
  
  
- CCAACGCATC TAGCCACACA CACCTACCGC TAGTTCCTTC TCAATTTTAC TTGATCAGTA CGCAGCTTAA   
  
  
- CAGGTTTTTT TTAAAAGAGA AAGTCAGCTC AGAATTTCCC CGTCAGTAGT TCTCGTACAA ACTTTTTGTA   
  
  
- ATACCGGTAT TGGTGAATCA TCATTAAGTT TATTAGTACC TTTGTATAAG TTGTACTTAA GTTATACTGA   
  
  
- TTTTTAAGGT AGAAAATCAA ACAAGACCAG TTGTGTCTCG TACGGAAAAA TTAACGTATT ATTGCTATAA   
  
  
- ATTGGTTGGT TAGTACAAAG AGTGTATAAT TATATCCGTT CCCATCTAAT TGTCAGTATT AGCGTATCAT   
  
  
- ACGTTCTTAA ATAAACGTTA GACCCGTATG ACAAAATATT TCGTGTTCAC AAGTACTGAA TTTTAGCTAT   
  
  
- TTCAGAATTA GAAAACAGCT AGGAACTCAC ACGTCTGTTA TATTAACACT TACATACGAT AATTGAAATT   
  
  
- ATTAATGCTT TTAATTCGTT GATTAGTATA TCAGTACAAG AGTTTTTGTT CGTACGCATA ATAAGTCTAG   
  
  
- TAATTTACGT AATTTACCCT GATTAGGCTA GTCAGTTCGC GACTATGCTG ATAACTTATT TGTCCTGATT   
  
  
- GGATCTTACG CCTTAATTGA CAGCTCTTAA CAGGTGTAGC CATCACTACG AACCGCCGAT GGTCTCAGTG   
  
  
- GAATCCCACA AGTGTAGGAT CTGGGCTTTT GGACGATTAC TACCTTCCCG TCGGCACCCT CCTCCTCTCC   
  
  
- TCTCACACCT AAAAAGATAA ATGTTTTTTC ACATATTCAC GATTAGGGGG CTTTGGAGGA AAATATCCAA   
  
  
- TCCCCCCGTT GATCCCAATA GCGGGATCCA CTGCGAACAC CGGCAGCCGG ATTAATTTAT CCGGGCTGGT   
  
  
- TGTCGACACT GACCTCGCTA TCGACTTCGT ACGTCTTTAC TCCTATTGTA TGTATAATAT CTAAAGGTGT   
  
  
- AACGTATCCC GTGAGTCACC CAATGACATA GCGTTCGGTA TCGTCGTTCC GAACCACCCG GAGGATTTCA   
  
  
- CGCTGAATGT CCGTAACTAC TAGGACAAAG ATTCATATGA ACACTACGAT CGAACCTCCG ACCACCCTTT   
  
  
- GCTAATCGCA GAGATAAACT TTTCAAATTT TATGAACAGC TCAAGTTACG TAACGGGCAA ATACCTGGGT   
  
  
- TACAGTCCAC CCTTTACGAC CTACACTCCG GGTCCCTCCG AAACCAGCAA TTAACAGGTA ATGTCGAGGT   
  
  
- AATGTGAGGA CTGCTCTCAC AGCTACACTC CTTGGGATCC CTATCCGAAG AGTCTTACCA CTTTAACGAG   
  
  
- CCAGGATTCC ATCGGTGAAA CCAACTCGTT CTTAGTTTGT GGTTGTGATG TGGAAAGAAC TGGGCCAAGT   
  
  
- ATCTCTGGGA ACTGATGATG AGCTAATTGG TACAAACTCA GATATCTGCA CTGGTACGGC TCTCTGGCCT   
  
  
- TCCTCTCCTA GTTACAACTC GTCGTAACAA ATCGATTCCT GTAACACTTA TAGTATCGAA CGCTCCCGTT   
  
  
- CCTCTACCAC CTCGCAGTAC TCGAAAAACC CTTTACCGTC AGCTCCAAAT GGTACCATCC CAAGTCCGTC   
  
  
- ATGGGCAACT CGAGTATGTA TTTGAGACAC TATTCTTCGG ATGAGGCCAC AATGAGGTTC GTAATATGAG   
  
  
- ACTATCTCTT CCTATCACGG TACGAAGACC CAACCTCCCC GGCTTACGAC TAAAGTCGAA GCCGTAC

+     MYB-like sequence

| Site Name | Organism | Position | Strand | Matrix score. | sequence | function |
| --- | --- | --- | --- | --- | --- | --- |
| MYB-like sequence | Arabidopsis thaliana | 845 | + | 6 | TAACCA |  |
| MYB-like sequence | Arabidopsis thaliana | 713 | + | 6 | TAACCA |  |
| MYB-like sequence | Arabidopsis thaliana | 1990 | + | 6 | TAACCA |  |

>HU08G00229.1   
+ -Up\_Stream \_Len000AAATTT ATATGATTAT GGGAGCAAAT ATTAATCGGT CTAAAACAAA AAAGAATCGG   
  
  
+ CCGAACAAAA AAAAGGGCCC AGAACTAGTC CTTTTAATGG AGTCCCAAAA TGTGGTATAA ATTAGTCTTG   
  
  
+ TTCACTCATT TCTCACTCCT CTCACTCATT CTAACCCTAA ATCTCAATCC TCACTCTCTG TCCTCTCTAT   
  
  
+ CTGCGTCTCG AGCTTCTCTT TCCTCTGGGC TTCCTTGGAC TTTTTCGACG CCATGACAAT GGAACACTGA   
  
  
+ GCTCCACCGT TGCCCCTCTC CACAACTGTC TTCTAACGGT GGTTCTCCGT AGCCACCGTA CCTCAAAAAC   
  
  
+ CAAAGCGATG CACAACTACC ACCCCACCAC TGCTGAAAAG TGCCCACGAG TCTCGATCCG CGCAAGCCTG   
  
  
+ ATGAGAATCG ATACCACCAC CATGTAGATA AGCTGAAAAC GGGCATAAGG AAGCGACCCA CGCGCCCAAC   
  
  
+ ACGCCATTGA GGCAGTGACA CGTGGGTTGA TGCGCTTCCA AAGATGGAAT TTTCACATTT TTCTTTTCAA   
  
  
+ GGTTGCGTAG ATCGGTGTGT GTGGATGGCG ATCAAGGAAG AGTTAAAATG AACTAGTCAT GCGTCGAATT   
  
  
+ GTCCAAAAAA AATTTTCTCT TTCAGTCGAG TCTTAAAGGG GCAGTCATCA AGAGCATGTT TGAAAAACAT   
  
  
+ TATGGCCATA ACCACTTAGT AGTAATTCAA ATAATCATGG AAACATATTC AACATGAATT CAATATGACT   
  
  
+ AAAAATTCCA TCTTTTAGTT TGTTCTGGTC AACACAGAGC ATGCCTTTTT AATTGCATAA TAACGATATT   
  
  
+ TAACCAACCA ATCATGTTTC TCACATATTA ATATAGGCAA GGGTAGATTA ACAGTCATAA TCGCATAGTA   
  
  
+ TGCAAGAATT TATTTGCAAT CTGGGCATAC TGTTTTATAA AGCACAAGTG TTCATGACTT AAAATCGATA   
  
  
+ AAGTCTTAAT CTTTTGTCGA TCCTTGAGTG TGCAGACAAT ATAATTGTGA ATGTATGCTA TTAACTTTAA   
  
  
+ TAATTACGAA AATTAAGCAA CTAATCATAT AGTCATGTTC TCAAAAACAA GCATGCGTAT TATTCAGATC   
  
  
+ ATTAAATGCA TTAAATGGGA CTAATCCGAT CAGTCAAGCG CTGATACGAC TATTGAATAA ACAGGACTAA   
  
  
+ CCTAGAATGC GGAATTAACT GTCGAGAATT GTCCACATCG GTAGTGATGC TTGGCGGCTA CCAGAGTCAC   
  
  
+ CTTAGGGTGT TCACATCCTA GACCCGAAAA CCTGCTAATG ATGGAAGGGC AGCCGTGGGA GGAGGAGAGG   
  
  
+ AGAGTGTGGA TTTTTCTATT TACAAAAAAG TGTATAAGTG CTAATCCCCC GAAACCTCCT TTTATAGGTT   
  
  
+ AGGGGGGCAA CTAGGGTTAT CGCCCTAGGT GACGCTTGTG GCCGTCGGCC TAATTAAATA GGCCCGACCA   
  
  
+ ACAGCTGTGA CTGGAGCGAT AGCTGAAGCA TGCAGAAATG AGGATAACAT ACATATTATA GATTTCCACA   
  
  
+ TTGCATAGGG CACTCAGTGG GTTACTGTAT CGCAAGCCAT AGCAGCAAGG CTTGGTGGGC CTCCTAAAGT   
  
  
+ GCGACTTACA GGCATTGATG ATCCTGTTTC TAAGTATACT TGTGATGCTA GCTTGGAGGC TGGTGGGAAA   
  
  
+ CGATTAGCGT CTCTATTTGA AAAGTTTAAA ATACTTGTCG AGTTCAATGC ATTGCCCGTT TATGGACCCA   
  
  
+ ATGTCAGGTG GGAAATGCTG GATGTGAGGC CCAGGGAGGC TTTGGTCGTT AATTGTCCAT TACAGCTCCA   
  
  
+ TTACACTCCT GACGAGAGTG TCGATGTGAG GAACCCTAGG GATAGGCTTC TCAGAATGGT GAAATTGCTC   
  
  
+ GGTCCTAAGG TAGCCACTTT GGTTGAGCAA GAATCAAACA CCAACACTAC ACCTTTCTTG ACCCGGTTCA   
  
  
+ TAGAGACCCT TGACTACTAC TCGATTAACC ATGTTTGAGT CTATAGACGT GACCATGCCG AGAGACCGGA   
  
  
+ AGGAGAGGAT CAATGTTGAG CAGCATTGTT TAGCTAAGGA CATTGTGAAT ATCATAGCTT GCGAGGGCAA   
  
  
+ GGAGATGGTG GAGCGTCATG AGCTTTTTGG GAAATGGCAG TCGAGGTTTA CCATGGTAGG GTTCAGGCAG   
  
  
+ TACCCGTTGA GCTCATACAT AAACTCTGTG ATAAGAAGCC TACTCCGGTG TTACTCCAAG CATTATACTC   
  
  
+ TGATAGAGAA GGATAGTGCC ATGCTTCTGG GTTGGAGGGG CCGAATGCTG ATTTCAGCTT CGGCATG  

- -Up\_Stream \_Len000TTTAAA TATACTAATA CCCTCGTTTA TAATTAGCCA GATTTTGTTT TTTCTTAGCC   
  
  
- GGCTTGTTTT TTTTCCCGGG TCTTGATCAG GAAAATTACC TCAGGGTTTT ACACCATATT TAATCAGAAC   
  
  
- AAGTGAGTAA AGAGTGAGGA GAGTGAGTAA GATTGGGATT TAGAGTTAGG AGTGAGAGAC AGGAGAGATA   
  
  
- GACGCAGAGC TCGAAGAGAA AGGAGACCCG AAGGAACCTG AAAAAGCTGC GGTACTGTTA CCTTGTGACT   
  
  
- CGAGGTGGCA ACGGGGAGAG GTGTTGACAG AAGATTGCCA CCAAGAGGCA TCGGTGGCAT GGAGTTTTTG   
  
  
- GTTTCGCTAC GTGTTGATGG TGGGGTGGTG ACGACTTTTC ACGGGTGCTC AGAGCTAGGC GCGTTCGGAC   
  
  
- TACTCTTAGC TATGGTGGTG GTACATCTAT TCGACTTTTG CCCGTATTCC TTCGCTGGGT GCGCGGGTTG   
  
  
- TGCGGTAACT CCGTCACTGT GCACCCAACT ACGCGAAGGT TTCTACCTTA AAAGTGTAAA AAGAAAAGTT   
  
  
- CCAACGCATC TAGCCACACA CACCTACCGC TAGTTCCTTC TCAATTTTAC TTGATCAGTA CGCAGCTTAA   
  
  
- CAGGTTTTTT TTAAAAGAGA AAGTCAGCTC AGAATTTCCC CGTCAGTAGT TCTCGTACAA ACTTTTTGTA   
  
  
- ATACCGGTAT TGGTGAATCA TCATTAAGTT TATTAGTACC TTTGTATAAG TTGTACTTAA GTTATACTGA   
  
  
- TTTTTAAGGT AGAAAATCAA ACAAGACCAG TTGTGTCTCG TACGGAAAAA TTAACGTATT ATTGCTATAA   
  
  
- ATTGGTTGGT TAGTACAAAG AGTGTATAAT TATATCCGTT CCCATCTAAT TGTCAGTATT AGCGTATCAT   
  
  
- ACGTTCTTAA ATAAACGTTA GACCCGTATG ACAAAATATT TCGTGTTCAC AAGTACTGAA TTTTAGCTAT   
  
  
- TTCAGAATTA GAAAACAGCT AGGAACTCAC ACGTCTGTTA TATTAACACT TACATACGAT AATTGAAATT   
  
  
- ATTAATGCTT TTAATTCGTT GATTAGTATA TCAGTACAAG AGTTTTTGTT CGTACGCATA ATAAGTCTAG   
  
  
- TAATTTACGT AATTTACCCT GATTAGGCTA GTCAGTTCGC GACTATGCTG ATAACTTATT TGTCCTGATT   
  
  
- GGATCTTACG CCTTAATTGA CAGCTCTTAA CAGGTGTAGC CATCACTACG AACCGCCGAT GGTCTCAGTG   
  
  
- GAATCCCACA AGTGTAGGAT CTGGGCTTTT GGACGATTAC TACCTTCCCG TCGGCACCCT CCTCCTCTCC   
  
  
- TCTCACACCT AAAAAGATAA ATGTTTTTTC ACATATTCAC GATTAGGGGG CTTTGGAGGA AAATATCCAA   
  
  
- TCCCCCCGTT GATCCCAATA GCGGGATCCA CTGCGAACAC CGGCAGCCGG ATTAATTTAT CCGGGCTGGT   
  
  
- TGTCGACACT GACCTCGCTA TCGACTTCGT ACGTCTTTAC TCCTATTGTA TGTATAATAT CTAAAGGTGT   
  
  
- AACGTATCCC GTGAGTCACC CAATGACATA GCGTTCGGTA TCGTCGTTCC GAACCACCCG GAGGATTTCA   
  
  
- CGCTGAATGT CCGTAACTAC TAGGACAAAG ATTCATATGA ACACTACGAT CGAACCTCCG ACCACCCTTT   
  
  
- GCTAATCGCA GAGATAAACT TTTCAAATTT TATGAACAGC TCAAGTTACG TAACGGGCAA ATACCTGGGT   
  
  
- TACAGTCCAC CCTTTACGAC CTACACTCCG GGTCCCTCCG AAACCAGCAA TTAACAGGTA ATGTCGAGGT   
  
  
- AATGTGAGGA CTGCTCTCAC AGCTACACTC CTTGGGATCC CTATCCGAAG AGTCTTACCA CTTTAACGAG   
  
  
- CCAGGATTCC ATCGGTGAAA CCAACTCGTT CTTAGTTTGT GGTTGTGATG TGGAAAGAAC TGGGCCAAGT   
  
  
- ATCTCTGGGA ACTGATGATG AGCTAATTGG TACAAACTCA GATATCTGCA CTGGTACGGC TCTCTGGCCT   
  
  
- TCCTCTCCTA GTTACAACTC GTCGTAACAA ATCGATTCCT GTAACACTTA TAGTATCGAA CGCTCCCGTT   
  
  
- CCTCTACCAC CTCGCAGTAC TCGAAAAACC CTTTACCGTC AGCTCCAAAT GGTACCATCC CAAGTCCGTC   
  
  
- ATGGGCAACT CGAGTATGTA TTTGAGACAC TATTCTTCGG ATGAGGCCAC AATGAGGTTC GTAATATGAG   
  
  
- ACTATCTCTT CCTATCACGG TACGAAGACC CAACCTCCCC GGCTTACGAC TAAAGTCGAA GCCGTAC

+     Myb

| Site Name | Organism | Position | Strand | Matrix score. | sequence | function |
| --- | --- | --- | --- | --- | --- | --- |
| Myb | Arabidopsis thaliana | 1210 | + | 6 | TAACTG |  |
| Myb | Arabidopsis thaliana | 307 | + | 6 | CAACTG |  |

>HU08G00229.1   
+ -Up\_Stream \_Len000AAATTT ATATGATTAT GGGAGCAAAT ATTAATCGGT CTAAAACAAA AAAGAATCGG   
  
  
+ CCGAACAAAA AAAAGGGCCC AGAACTAGTC CTTTTAATGG AGTCCCAAAA TGTGGTATAA ATTAGTCTTG   
  
  
+ TTCACTCATT TCTCACTCCT CTCACTCATT CTAACCCTAA ATCTCAATCC TCACTCTCTG TCCTCTCTAT   
  
  
+ CTGCGTCTCG AGCTTCTCTT TCCTCTGGGC TTCCTTGGAC TTTTTCGACG CCATGACAAT GGAACACTGA   
  
  
+ GCTCCACCGT TGCCCCTCTC CACAACTGTC TTCTAACGGT GGTTCTCCGT AGCCACCGTA CCTCAAAAAC   
  
  
+ CAAAGCGATG CACAACTACC ACCCCACCAC TGCTGAAAAG TGCCCACGAG TCTCGATCCG CGCAAGCCTG   
  
  
+ ATGAGAATCG ATACCACCAC CATGTAGATA AGCTGAAAAC GGGCATAAGG AAGCGACCCA CGCGCCCAAC   
  
  
+ ACGCCATTGA GGCAGTGACA CGTGGGTTGA TGCGCTTCCA AAGATGGAAT TTTCACATTT TTCTTTTCAA   
  
  
+ GGTTGCGTAG ATCGGTGTGT GTGGATGGCG ATCAAGGAAG AGTTAAAATG AACTAGTCAT GCGTCGAATT   
  
  
+ GTCCAAAAAA AATTTTCTCT TTCAGTCGAG TCTTAAAGGG GCAGTCATCA AGAGCATGTT TGAAAAACAT   
  
  
+ TATGGCCATA ACCACTTAGT AGTAATTCAA ATAATCATGG AAACATATTC AACATGAATT CAATATGACT   
  
  
+ AAAAATTCCA TCTTTTAGTT TGTTCTGGTC AACACAGAGC ATGCCTTTTT AATTGCATAA TAACGATATT   
  
  
+ TAACCAACCA ATCATGTTTC TCACATATTA ATATAGGCAA GGGTAGATTA ACAGTCATAA TCGCATAGTA   
  
  
+ TGCAAGAATT TATTTGCAAT CTGGGCATAC TGTTTTATAA AGCACAAGTG TTCATGACTT AAAATCGATA   
  
  
+ AAGTCTTAAT CTTTTGTCGA TCCTTGAGTG TGCAGACAAT ATAATTGTGA ATGTATGCTA TTAACTTTAA   
  
  
+ TAATTACGAA AATTAAGCAA CTAATCATAT AGTCATGTTC TCAAAAACAA GCATGCGTAT TATTCAGATC   
  
  
+ ATTAAATGCA TTAAATGGGA CTAATCCGAT CAGTCAAGCG CTGATACGAC TATTGAATAA ACAGGACTAA   
  
  
+ CCTAGAATGC GGAATTAACT GTCGAGAATT GTCCACATCG GTAGTGATGC TTGGCGGCTA CCAGAGTCAC   
  
  
+ CTTAGGGTGT TCACATCCTA GACCCGAAAA CCTGCTAATG ATGGAAGGGC AGCCGTGGGA GGAGGAGAGG   
  
  
+ AGAGTGTGGA TTTTTCTATT TACAAAAAAG TGTATAAGTG CTAATCCCCC GAAACCTCCT TTTATAGGTT   
  
  
+ AGGGGGGCAA CTAGGGTTAT CGCCCTAGGT GACGCTTGTG GCCGTCGGCC TAATTAAATA GGCCCGACCA   
  
  
+ ACAGCTGTGA CTGGAGCGAT AGCTGAAGCA TGCAGAAATG AGGATAACAT ACATATTATA GATTTCCACA   
  
  
+ TTGCATAGGG CACTCAGTGG GTTACTGTAT CGCAAGCCAT AGCAGCAAGG CTTGGTGGGC CTCCTAAAGT   
  
  
+ GCGACTTACA GGCATTGATG ATCCTGTTTC TAAGTATACT TGTGATGCTA GCTTGGAGGC TGGTGGGAAA   
  
  
+ CGATTAGCGT CTCTATTTGA AAAGTTTAAA ATACTTGTCG AGTTCAATGC ATTGCCCGTT TATGGACCCA   
  
  
+ ATGTCAGGTG GGAAATGCTG GATGTGAGGC CCAGGGAGGC TTTGGTCGTT AATTGTCCAT TACAGCTCCA   
  
  
+ TTACACTCCT GACGAGAGTG TCGATGTGAG GAACCCTAGG GATAGGCTTC TCAGAATGGT GAAATTGCTC   
  
  
+ GGTCCTAAGG TAGCCACTTT GGTTGAGCAA GAATCAAACA CCAACACTAC ACCTTTCTTG ACCCGGTTCA   
  
  
+ TAGAGACCCT TGACTACTAC TCGATTAACC ATGTTTGAGT CTATAGACGT GACCATGCCG AGAGACCGGA   
  
  
+ AGGAGAGGAT CAATGTTGAG CAGCATTGTT TAGCTAAGGA CATTGTGAAT ATCATAGCTT GCGAGGGCAA   
  
  
+ GGAGATGGTG GAGCGTCATG AGCTTTTTGG GAAATGGCAG TCGAGGTTTA CCATGGTAGG GTTCAGGCAG   
  
  
+ TACCCGTTGA GCTCATACAT AAACTCTGTG ATAAGAAGCC TACTCCGGTG TTACTCCAAG CATTATACTC   
  
  
+ TGATAGAGAA GGATAGTGCC ATGCTTCTGG GTTGGAGGGG CCGAATGCTG ATTTCAGCTT CGGCATG  

- -Up\_Stream \_Len000TTTAAA TATACTAATA CCCTCGTTTA TAATTAGCCA GATTTTGTTT TTTCTTAGCC   
  
  
- GGCTTGTTTT TTTTCCCGGG TCTTGATCAG GAAAATTACC TCAGGGTTTT ACACCATATT TAATCAGAAC   
  
  
- AAGTGAGTAA AGAGTGAGGA GAGTGAGTAA GATTGGGATT TAGAGTTAGG AGTGAGAGAC AGGAGAGATA   
  
  
- GACGCAGAGC TCGAAGAGAA AGGAGACCCG AAGGAACCTG AAAAAGCTGC GGTACTGTTA CCTTGTGACT   
  
  
- CGAGGTGGCA ACGGGGAGAG GTGTTGACAG AAGATTGCCA CCAAGAGGCA TCGGTGGCAT GGAGTTTTTG   
  
  
- GTTTCGCTAC GTGTTGATGG TGGGGTGGTG ACGACTTTTC ACGGGTGCTC AGAGCTAGGC GCGTTCGGAC   
  
  
- TACTCTTAGC TATGGTGGTG GTACATCTAT TCGACTTTTG CCCGTATTCC TTCGCTGGGT GCGCGGGTTG   
  
  
- TGCGGTAACT CCGTCACTGT GCACCCAACT ACGCGAAGGT TTCTACCTTA AAAGTGTAAA AAGAAAAGTT   
  
  
- CCAACGCATC TAGCCACACA CACCTACCGC TAGTTCCTTC TCAATTTTAC TTGATCAGTA CGCAGCTTAA   
  
  
- CAGGTTTTTT TTAAAAGAGA AAGTCAGCTC AGAATTTCCC CGTCAGTAGT TCTCGTACAA ACTTTTTGTA   
  
  
- ATACCGGTAT TGGTGAATCA TCATTAAGTT TATTAGTACC TTTGTATAAG TTGTACTTAA GTTATACTGA   
  
  
- TTTTTAAGGT AGAAAATCAA ACAAGACCAG TTGTGTCTCG TACGGAAAAA TTAACGTATT ATTGCTATAA   
  
  
- ATTGGTTGGT TAGTACAAAG AGTGTATAAT TATATCCGTT CCCATCTAAT TGTCAGTATT AGCGTATCAT   
  
  
- ACGTTCTTAA ATAAACGTTA GACCCGTATG ACAAAATATT TCGTGTTCAC AAGTACTGAA TTTTAGCTAT   
  
  
- TTCAGAATTA GAAAACAGCT AGGAACTCAC ACGTCTGTTA TATTAACACT TACATACGAT AATTGAAATT   
  
  
- ATTAATGCTT TTAATTCGTT GATTAGTATA TCAGTACAAG AGTTTTTGTT CGTACGCATA ATAAGTCTAG   
  
  
- TAATTTACGT AATTTACCCT GATTAGGCTA GTCAGTTCGC GACTATGCTG ATAACTTATT TGTCCTGATT   
  
  
- GGATCTTACG CCTTAATTGA CAGCTCTTAA CAGGTGTAGC CATCACTACG AACCGCCGAT GGTCTCAGTG   
  
  
- GAATCCCACA AGTGTAGGAT CTGGGCTTTT GGACGATTAC TACCTTCCCG TCGGCACCCT CCTCCTCTCC   
  
  
- TCTCACACCT AAAAAGATAA ATGTTTTTTC ACATATTCAC GATTAGGGGG CTTTGGAGGA AAATATCCAA   
  
  
- TCCCCCCGTT GATCCCAATA GCGGGATCCA CTGCGAACAC CGGCAGCCGG ATTAATTTAT CCGGGCTGGT   
  
  
- TGTCGACACT GACCTCGCTA TCGACTTCGT ACGTCTTTAC TCCTATTGTA TGTATAATAT CTAAAGGTGT   
  
  
- AACGTATCCC GTGAGTCACC CAATGACATA GCGTTCGGTA TCGTCGTTCC GAACCACCCG GAGGATTTCA   
  
  
- CGCTGAATGT CCGTAACTAC TAGGACAAAG ATTCATATGA ACACTACGAT CGAACCTCCG ACCACCCTTT   
  
  
- GCTAATCGCA GAGATAAACT TTTCAAATTT TATGAACAGC TCAAGTTACG TAACGGGCAA ATACCTGGGT   
  
  
- TACAGTCCAC CCTTTACGAC CTACACTCCG GGTCCCTCCG AAACCAGCAA TTAACAGGTA ATGTCGAGGT   
  
  
- AATGTGAGGA CTGCTCTCAC AGCTACACTC CTTGGGATCC CTATCCGAAG AGTCTTACCA CTTTAACGAG   
  
  
- CCAGGATTCC ATCGGTGAAA CCAACTCGTT CTTAGTTTGT GGTTGTGATG TGGAAAGAAC TGGGCCAAGT   
  
  
- ATCTCTGGGA ACTGATGATG AGCTAATTGG TACAAACTCA GATATCTGCA CTGGTACGGC TCTCTGGCCT   
  
  
- TCCTCTCCTA GTTACAACTC GTCGTAACAA ATCGATTCCT GTAACACTTA TAGTATCGAA CGCTCCCGTT   
  
  
- CCTCTACCAC CTCGCAGTAC TCGAAAAACC CTTTACCGTC AGCTCCAAAT GGTACCATCC CAAGTCCGTC   
  
  
- ATGGGCAACT CGAGTATGTA TTTGAGACAC TATTCTTCGG ATGAGGCCAC AATGAGGTTC GTAATATGAG   
  
  
- ACTATCTCTT CCTATCACGG TACGAAGACC CAACCTCCCC GGCTTACGAC TAAAGTCGAA GCCGTAC

+     Myb-binding site

| Site Name | Organism | Position | Strand | Matrix score. | sequence | function |
| --- | --- | --- | --- | --- | --- | --- |
| Myb-binding site | Nicotiana tabacum | 1473 | + | 6 | CAACAG |  |

>HU08G00229.1   
+ -Up\_Stream \_Len000AAATTT ATATGATTAT GGGAGCAAAT ATTAATCGGT CTAAAACAAA AAAGAATCGG   
  
  
+ CCGAACAAAA AAAAGGGCCC AGAACTAGTC CTTTTAATGG AGTCCCAAAA TGTGGTATAA ATTAGTCTTG   
  
  
+ TTCACTCATT TCTCACTCCT CTCACTCATT CTAACCCTAA ATCTCAATCC TCACTCTCTG TCCTCTCTAT   
  
  
+ CTGCGTCTCG AGCTTCTCTT TCCTCTGGGC TTCCTTGGAC TTTTTCGACG CCATGACAAT GGAACACTGA   
  
  
+ GCTCCACCGT TGCCCCTCTC CACAACTGTC TTCTAACGGT GGTTCTCCGT AGCCACCGTA CCTCAAAAAC   
  
  
+ CAAAGCGATG CACAACTACC ACCCCACCAC TGCTGAAAAG TGCCCACGAG TCTCGATCCG CGCAAGCCTG   
  
  
+ ATGAGAATCG ATACCACCAC CATGTAGATA AGCTGAAAAC GGGCATAAGG AAGCGACCCA CGCGCCCAAC   
  
  
+ ACGCCATTGA GGCAGTGACA CGTGGGTTGA TGCGCTTCCA AAGATGGAAT TTTCACATTT TTCTTTTCAA   
  
  
+ GGTTGCGTAG ATCGGTGTGT GTGGATGGCG ATCAAGGAAG AGTTAAAATG AACTAGTCAT GCGTCGAATT   
  
  
+ GTCCAAAAAA AATTTTCTCT TTCAGTCGAG TCTTAAAGGG GCAGTCATCA AGAGCATGTT TGAAAAACAT   
  
  
+ TATGGCCATA ACCACTTAGT AGTAATTCAA ATAATCATGG AAACATATTC AACATGAATT CAATATGACT   
  
  
+ AAAAATTCCA TCTTTTAGTT TGTTCTGGTC AACACAGAGC ATGCCTTTTT AATTGCATAA TAACGATATT   
  
  
+ TAACCAACCA ATCATGTTTC TCACATATTA ATATAGGCAA GGGTAGATTA ACAGTCATAA TCGCATAGTA   
  
  
+ TGCAAGAATT TATTTGCAAT CTGGGCATAC TGTTTTATAA AGCACAAGTG TTCATGACTT AAAATCGATA   
  
  
+ AAGTCTTAAT CTTTTGTCGA TCCTTGAGTG TGCAGACAAT ATAATTGTGA ATGTATGCTA TTAACTTTAA   
  
  
+ TAATTACGAA AATTAAGCAA CTAATCATAT AGTCATGTTC TCAAAAACAA GCATGCGTAT TATTCAGATC   
  
  
+ ATTAAATGCA TTAAATGGGA CTAATCCGAT CAGTCAAGCG CTGATACGAC TATTGAATAA ACAGGACTAA   
  
  
+ CCTAGAATGC GGAATTAACT GTCGAGAATT GTCCACATCG GTAGTGATGC TTGGCGGCTA CCAGAGTCAC   
  
  
+ CTTAGGGTGT TCACATCCTA GACCCGAAAA CCTGCTAATG ATGGAAGGGC AGCCGTGGGA GGAGGAGAGG   
  
  
+ AGAGTGTGGA TTTTTCTATT TACAAAAAAG TGTATAAGTG CTAATCCCCC GAAACCTCCT TTTATAGGTT   
  
  
+ AGGGGGGCAA CTAGGGTTAT CGCCCTAGGT GACGCTTGTG GCCGTCGGCC TAATTAAATA GGCCCGACCA   
  
  
+ ACAGCTGTGA CTGGAGCGAT AGCTGAAGCA TGCAGAAATG AGGATAACAT ACATATTATA GATTTCCACA   
  
  
+ TTGCATAGGG CACTCAGTGG GTTACTGTAT CGCAAGCCAT AGCAGCAAGG CTTGGTGGGC CTCCTAAAGT   
  
  
+ GCGACTTACA GGCATTGATG ATCCTGTTTC TAAGTATACT TGTGATGCTA GCTTGGAGGC TGGTGGGAAA   
  
  
+ CGATTAGCGT CTCTATTTGA AAAGTTTAAA ATACTTGTCG AGTTCAATGC ATTGCCCGTT TATGGACCCA   
  
  
+ ATGTCAGGTG GGAAATGCTG GATGTGAGGC CCAGGGAGGC TTTGGTCGTT AATTGTCCAT TACAGCTCCA   
  
  
+ TTACACTCCT GACGAGAGTG TCGATGTGAG GAACCCTAGG GATAGGCTTC TCAGAATGGT GAAATTGCTC   
  
  
+ GGTCCTAAGG TAGCCACTTT GGTTGAGCAA GAATCAAACA CCAACACTAC ACCTTTCTTG ACCCGGTTCA   
  
  
+ TAGAGACCCT TGACTACTAC TCGATTAACC ATGTTTGAGT CTATAGACGT GACCATGCCG AGAGACCGGA   
  
  
+ AGGAGAGGAT CAATGTTGAG CAGCATTGTT TAGCTAAGGA CATTGTGAAT ATCATAGCTT GCGAGGGCAA   
  
  
+ GGAGATGGTG GAGCGTCATG AGCTTTTTGG GAAATGGCAG TCGAGGTTTA CCATGGTAGG GTTCAGGCAG   
  
  
+ TACCCGTTGA GCTCATACAT AAACTCTGTG ATAAGAAGCC TACTCCGGTG TTACTCCAAG CATTATACTC   
  
  
+ TGATAGAGAA GGATAGTGCC ATGCTTCTGG GTTGGAGGGG CCGAATGCTG ATTTCAGCTT CGGCATG  

- -Up\_Stream \_Len000TTTAAA TATACTAATA CCCTCGTTTA TAATTAGCCA GATTTTGTTT TTTCTTAGCC   
  
  
- GGCTTGTTTT TTTTCCCGGG TCTTGATCAG GAAAATTACC TCAGGGTTTT ACACCATATT TAATCAGAAC   
  
  
- AAGTGAGTAA AGAGTGAGGA GAGTGAGTAA GATTGGGATT TAGAGTTAGG AGTGAGAGAC AGGAGAGATA   
  
  
- GACGCAGAGC TCGAAGAGAA AGGAGACCCG AAGGAACCTG AAAAAGCTGC GGTACTGTTA CCTTGTGACT   
  
  
- CGAGGTGGCA ACGGGGAGAG GTGTTGACAG AAGATTGCCA CCAAGAGGCA TCGGTGGCAT GGAGTTTTTG   
  
  
- GTTTCGCTAC GTGTTGATGG TGGGGTGGTG ACGACTTTTC ACGGGTGCTC AGAGCTAGGC GCGTTCGGAC   
  
  
- TACTCTTAGC TATGGTGGTG GTACATCTAT TCGACTTTTG CCCGTATTCC TTCGCTGGGT GCGCGGGTTG   
  
  
- TGCGGTAACT CCGTCACTGT GCACCCAACT ACGCGAAGGT TTCTACCTTA AAAGTGTAAA AAGAAAAGTT   
  
  
- CCAACGCATC TAGCCACACA CACCTACCGC TAGTTCCTTC TCAATTTTAC TTGATCAGTA CGCAGCTTAA   
  
  
- CAGGTTTTTT TTAAAAGAGA AAGTCAGCTC AGAATTTCCC CGTCAGTAGT TCTCGTACAA ACTTTTTGTA   
  
  
- ATACCGGTAT TGGTGAATCA TCATTAAGTT TATTAGTACC TTTGTATAAG TTGTACTTAA GTTATACTGA   
  
  
- TTTTTAAGGT AGAAAATCAA ACAAGACCAG TTGTGTCTCG TACGGAAAAA TTAACGTATT ATTGCTATAA   
  
  
- ATTGGTTGGT TAGTACAAAG AGTGTATAAT TATATCCGTT CCCATCTAAT TGTCAGTATT AGCGTATCAT   
  
  
- ACGTTCTTAA ATAAACGTTA GACCCGTATG ACAAAATATT TCGTGTTCAC AAGTACTGAA TTTTAGCTAT   
  
  
- TTCAGAATTA GAAAACAGCT AGGAACTCAC ACGTCTGTTA TATTAACACT TACATACGAT AATTGAAATT   
  
  
- ATTAATGCTT TTAATTCGTT GATTAGTATA TCAGTACAAG AGTTTTTGTT CGTACGCATA ATAAGTCTAG   
  
  
- TAATTTACGT AATTTACCCT GATTAGGCTA GTCAGTTCGC GACTATGCTG ATAACTTATT TGTCCTGATT   
  
  
- GGATCTTACG CCTTAATTGA CAGCTCTTAA CAGGTGTAGC CATCACTACG AACCGCCGAT GGTCTCAGTG   
  
  
- GAATCCCACA AGTGTAGGAT CTGGGCTTTT GGACGATTAC TACCTTCCCG TCGGCACCCT CCTCCTCTCC   
  
  
- TCTCACACCT AAAAAGATAA ATGTTTTTTC ACATATTCAC GATTAGGGGG CTTTGGAGGA AAATATCCAA   
  
  
- TCCCCCCGTT GATCCCAATA GCGGGATCCA CTGCGAACAC CGGCAGCCGG ATTAATTTAT CCGGGCTGGT   
  
  
- TGTCGACACT GACCTCGCTA TCGACTTCGT ACGTCTTTAC TCCTATTGTA TGTATAATAT CTAAAGGTGT   
  
  
- AACGTATCCC GTGAGTCACC CAATGACATA GCGTTCGGTA TCGTCGTTCC GAACCACCCG GAGGATTTCA   
  
  
- CGCTGAATGT CCGTAACTAC TAGGACAAAG ATTCATATGA ACACTACGAT CGAACCTCCG ACCACCCTTT   
  
  
- GCTAATCGCA GAGATAAACT TTTCAAATTT TATGAACAGC TCAAGTTACG TAACGGGCAA ATACCTGGGT   
  
  
- TACAGTCCAC CCTTTACGAC CTACACTCCG GGTCCCTCCG AAACCAGCAA TTAACAGGTA ATGTCGAGGT   
  
  
- AATGTGAGGA CTGCTCTCAC AGCTACACTC CTTGGGATCC CTATCCGAAG AGTCTTACCA CTTTAACGAG   
  
  
- CCAGGATTCC ATCGGTGAAA CCAACTCGTT CTTAGTTTGT GGTTGTGATG TGGAAAGAAC TGGGCCAAGT   
  
  
- ATCTCTGGGA ACTGATGATG AGCTAATTGG TACAAACTCA GATATCTGCA CTGGTACGGC TCTCTGGCCT   
  
  
- TCCTCTCCTA GTTACAACTC GTCGTAACAA ATCGATTCCT GTAACACTTA TAGTATCGAA CGCTCCCGTT   
  
  
- CCTCTACCAC CTCGCAGTAC TCGAAAAACC CTTTACCGTC AGCTCCAAAT GGTACCATCC CAAGTCCGTC   
  
  
- ATGGGCAACT CGAGTATGTA TTTGAGACAC TATTCTTCGG ATGAGGCCAC AATGAGGTTC GTAATATGAG   
  
  
- ACTATCTCTT CCTATCACGG TACGAAGACC CAACCTCCCC GGCTTACGAC TAAAGTCGAA GCCGTAC

+     OCT

| Site Name | Organism | Position | Strand | Matrix score. | sequence | function |
| --- | --- | --- | --- | --- | --- | --- |
| OCT | Zea mays | 409 | - | 8 | CGCGGATC |  |

>HU08G00229.1   
+ -Up\_Stream \_Len000AAATTT ATATGATTAT GGGAGCAAAT ATTAATCGGT CTAAAACAAA AAAGAATCGG   
  
  
+ CCGAACAAAA AAAAGGGCCC AGAACTAGTC CTTTTAATGG AGTCCCAAAA TGTGGTATAA ATTAGTCTTG   
  
  
+ TTCACTCATT TCTCACTCCT CTCACTCATT CTAACCCTAA ATCTCAATCC TCACTCTCTG TCCTCTCTAT   
  
  
+ CTGCGTCTCG AGCTTCTCTT TCCTCTGGGC TTCCTTGGAC TTTTTCGACG CCATGACAAT GGAACACTGA   
  
  
+ GCTCCACCGT TGCCCCTCTC CACAACTGTC TTCTAACGGT GGTTCTCCGT AGCCACCGTA CCTCAAAAAC   
  
  
+ CAAAGCGATG CACAACTACC ACCCCACCAC TGCTGAAAAG TGCCCACGAG TCTCGATCCG CGCAAGCCTG   
  
  
+ ATGAGAATCG ATACCACCAC CATGTAGATA AGCTGAAAAC GGGCATAAGG AAGCGACCCA CGCGCCCAAC   
  
  
+ ACGCCATTGA GGCAGTGACA CGTGGGTTGA TGCGCTTCCA AAGATGGAAT TTTCACATTT TTCTTTTCAA   
  
  
+ GGTTGCGTAG ATCGGTGTGT GTGGATGGCG ATCAAGGAAG AGTTAAAATG AACTAGTCAT GCGTCGAATT   
  
  
+ GTCCAAAAAA AATTTTCTCT TTCAGTCGAG TCTTAAAGGG GCAGTCATCA AGAGCATGTT TGAAAAACAT   
  
  
+ TATGGCCATA ACCACTTAGT AGTAATTCAA ATAATCATGG AAACATATTC AACATGAATT CAATATGACT   
  
  
+ AAAAATTCCA TCTTTTAGTT TGTTCTGGTC AACACAGAGC ATGCCTTTTT AATTGCATAA TAACGATATT   
  
  
+ TAACCAACCA ATCATGTTTC TCACATATTA ATATAGGCAA GGGTAGATTA ACAGTCATAA TCGCATAGTA   
  
  
+ TGCAAGAATT TATTTGCAAT CTGGGCATAC TGTTTTATAA AGCACAAGTG TTCATGACTT AAAATCGATA   
  
  
+ AAGTCTTAAT CTTTTGTCGA TCCTTGAGTG TGCAGACAAT ATAATTGTGA ATGTATGCTA TTAACTTTAA   
  
  
+ TAATTACGAA AATTAAGCAA CTAATCATAT AGTCATGTTC TCAAAAACAA GCATGCGTAT TATTCAGATC   
  
  
+ ATTAAATGCA TTAAATGGGA CTAATCCGAT CAGTCAAGCG CTGATACGAC TATTGAATAA ACAGGACTAA   
  
  
+ CCTAGAATGC GGAATTAACT GTCGAGAATT GTCCACATCG GTAGTGATGC TTGGCGGCTA CCAGAGTCAC   
  
  
+ CTTAGGGTGT TCACATCCTA GACCCGAAAA CCTGCTAATG ATGGAAGGGC AGCCGTGGGA GGAGGAGAGG   
  
  
+ AGAGTGTGGA TTTTTCTATT TACAAAAAAG TGTATAAGTG CTAATCCCCC GAAACCTCCT TTTATAGGTT   
  
  
+ AGGGGGGCAA CTAGGGTTAT CGCCCTAGGT GACGCTTGTG GCCGTCGGCC TAATTAAATA GGCCCGACCA   
  
  
+ ACAGCTGTGA CTGGAGCGAT AGCTGAAGCA TGCAGAAATG AGGATAACAT ACATATTATA GATTTCCACA   
  
  
+ TTGCATAGGG CACTCAGTGG GTTACTGTAT CGCAAGCCAT AGCAGCAAGG CTTGGTGGGC CTCCTAAAGT   
  
  
+ GCGACTTACA GGCATTGATG ATCCTGTTTC TAAGTATACT TGTGATGCTA GCTTGGAGGC TGGTGGGAAA   
  
  
+ CGATTAGCGT CTCTATTTGA AAAGTTTAAA ATACTTGTCG AGTTCAATGC ATTGCCCGTT TATGGACCCA   
  
  
+ ATGTCAGGTG GGAAATGCTG GATGTGAGGC CCAGGGAGGC TTTGGTCGTT AATTGTCCAT TACAGCTCCA   
  
  
+ TTACACTCCT GACGAGAGTG TCGATGTGAG GAACCCTAGG GATAGGCTTC TCAGAATGGT GAAATTGCTC   
  
  
+ GGTCCTAAGG TAGCCACTTT GGTTGAGCAA GAATCAAACA CCAACACTAC ACCTTTCTTG ACCCGGTTCA   
  
  
+ TAGAGACCCT TGACTACTAC TCGATTAACC ATGTTTGAGT CTATAGACGT GACCATGCCG AGAGACCGGA   
  
  
+ AGGAGAGGAT CAATGTTGAG CAGCATTGTT TAGCTAAGGA CATTGTGAAT ATCATAGCTT GCGAGGGCAA   
  
  
+ GGAGATGGTG GAGCGTCATG AGCTTTTTGG GAAATGGCAG TCGAGGTTTA CCATGGTAGG GTTCAGGCAG   
  
  
+ TACCCGTTGA GCTCATACAT AAACTCTGTG ATAAGAAGCC TACTCCGGTG TTACTCCAAG CATTATACTC   
  
  
+ TGATAGAGAA GGATAGTGCC ATGCTTCTGG GTTGGAGGGG CCGAATGCTG ATTTCAGCTT CGGCATG  

- -Up\_Stream \_Len000TTTAAA TATACTAATA CCCTCGTTTA TAATTAGCCA GATTTTGTTT TTTCTTAGCC   
  
  
- GGCTTGTTTT TTTTCCCGGG TCTTGATCAG GAAAATTACC TCAGGGTTTT ACACCATATT TAATCAGAAC   
  
  
- AAGTGAGTAA AGAGTGAGGA GAGTGAGTAA GATTGGGATT TAGAGTTAGG AGTGAGAGAC AGGAGAGATA   
  
  
- GACGCAGAGC TCGAAGAGAA AGGAGACCCG AAGGAACCTG AAAAAGCTGC GGTACTGTTA CCTTGTGACT   
  
  
- CGAGGTGGCA ACGGGGAGAG GTGTTGACAG AAGATTGCCA CCAAGAGGCA TCGGTGGCAT GGAGTTTTTG   
  
  
- GTTTCGCTAC GTGTTGATGG TGGGGTGGTG ACGACTTTTC ACGGGTGCTC AGAGCTAGGC GCGTTCGGAC   
  
  
- TACTCTTAGC TATGGTGGTG GTACATCTAT TCGACTTTTG CCCGTATTCC TTCGCTGGGT GCGCGGGTTG   
  
  
- TGCGGTAACT CCGTCACTGT GCACCCAACT ACGCGAAGGT TTCTACCTTA AAAGTGTAAA AAGAAAAGTT   
  
  
- CCAACGCATC TAGCCACACA CACCTACCGC TAGTTCCTTC TCAATTTTAC TTGATCAGTA CGCAGCTTAA   
  
  
- CAGGTTTTTT TTAAAAGAGA AAGTCAGCTC AGAATTTCCC CGTCAGTAGT TCTCGTACAA ACTTTTTGTA   
  
  
- ATACCGGTAT TGGTGAATCA TCATTAAGTT TATTAGTACC TTTGTATAAG TTGTACTTAA GTTATACTGA   
  
  
- TTTTTAAGGT AGAAAATCAA ACAAGACCAG TTGTGTCTCG TACGGAAAAA TTAACGTATT ATTGCTATAA   
  
  
- ATTGGTTGGT TAGTACAAAG AGTGTATAAT TATATCCGTT CCCATCTAAT TGTCAGTATT AGCGTATCAT   
  
  
- ACGTTCTTAA ATAAACGTTA GACCCGTATG ACAAAATATT TCGTGTTCAC AAGTACTGAA TTTTAGCTAT   
  
  
- TTCAGAATTA GAAAACAGCT AGGAACTCAC ACGTCTGTTA TATTAACACT TACATACGAT AATTGAAATT   
  
  
- ATTAATGCTT TTAATTCGTT GATTAGTATA TCAGTACAAG AGTTTTTGTT CGTACGCATA ATAAGTCTAG   
  
  
- TAATTTACGT AATTTACCCT GATTAGGCTA GTCAGTTCGC GACTATGCTG ATAACTTATT TGTCCTGATT   
  
  
- GGATCTTACG CCTTAATTGA CAGCTCTTAA CAGGTGTAGC CATCACTACG AACCGCCGAT GGTCTCAGTG   
  
  
- GAATCCCACA AGTGTAGGAT CTGGGCTTTT GGACGATTAC TACCTTCCCG TCGGCACCCT CCTCCTCTCC   
  
  
- TCTCACACCT AAAAAGATAA ATGTTTTTTC ACATATTCAC GATTAGGGGG CTTTGGAGGA AAATATCCAA   
  
  
- TCCCCCCGTT GATCCCAATA GCGGGATCCA CTGCGAACAC CGGCAGCCGG ATTAATTTAT CCGGGCTGGT   
  
  
- TGTCGACACT GACCTCGCTA TCGACTTCGT ACGTCTTTAC TCCTATTGTA TGTATAATAT CTAAAGGTGT   
  
  
- AACGTATCCC GTGAGTCACC CAATGACATA GCGTTCGGTA TCGTCGTTCC GAACCACCCG GAGGATTTCA   
  
  
- CGCTGAATGT CCGTAACTAC TAGGACAAAG ATTCATATGA ACACTACGAT CGAACCTCCG ACCACCCTTT   
  
  
- GCTAATCGCA GAGATAAACT TTTCAAATTT TATGAACAGC TCAAGTTACG TAACGGGCAA ATACCTGGGT   
  
  
- TACAGTCCAC CCTTTACGAC CTACACTCCG GGTCCCTCCG AAACCAGCAA TTAACAGGTA ATGTCGAGGT   
  
  
- AATGTGAGGA CTGCTCTCAC AGCTACACTC CTTGGGATCC CTATCCGAAG AGTCTTACCA CTTTAACGAG   
  
  
- CCAGGATTCC ATCGGTGAAA CCAACTCGTT CTTAGTTTGT GGTTGTGATG TGGAAAGAAC TGGGCCAAGT   
  
  
- ATCTCTGGGA ACTGATGATG AGCTAATTGG TACAAACTCA GATATCTGCA CTGGTACGGC TCTCTGGCCT   
  
  
- TCCTCTCCTA GTTACAACTC GTCGTAACAA ATCGATTCCT GTAACACTTA TAGTATCGAA CGCTCCCGTT   
  
  
- CCTCTACCAC CTCGCAGTAC TCGAAAAACC CTTTACCGTC AGCTCCAAAT GGTACCATCC CAAGTCCGTC   
  
  
- ATGGGCAACT CGAGTATGTA TTTGAGACAC TATTCTTCGG ATGAGGCCAC AATGAGGTTC GTAATATGAG   
  
  
- ACTATCTCTT CCTATCACGG TACGAAGACC CAACCTCCCC GGCTTACGAC TAAAGTCGAA GCCGTAC

+     STRE

| Site Name | Organism | Position | Strand | Matrix score. | sequence | function |
| --- | --- | --- | --- | --- | --- | --- |
| STRE | Arabidopsis thaliana | 671 | + | 5 | AGGGG |  |
| STRE | Arabidopsis thaliana | 297 | - | 5 | AGGGG |  |
| STRE | Arabidopsis thaliana | 1405 | + | 5 | AGGGG |  |
| STRE | Arabidopsis thaliana | 2280 | + | 5 | AGGGG |  |

>HU08G00229.1   
+ -Up\_Stream \_Len000AAATTT ATATGATTAT GGGAGCAAAT ATTAATCGGT CTAAAACAAA AAAGAATCGG   
  
  
+ CCGAACAAAA AAAAGGGCCC AGAACTAGTC CTTTTAATGG AGTCCCAAAA TGTGGTATAA ATTAGTCTTG   
  
  
+ TTCACTCATT TCTCACTCCT CTCACTCATT CTAACCCTAA ATCTCAATCC TCACTCTCTG TCCTCTCTAT   
  
  
+ CTGCGTCTCG AGCTTCTCTT TCCTCTGGGC TTCCTTGGAC TTTTTCGACG CCATGACAAT GGAACACTGA   
  
  
+ GCTCCACCGT TGCCCCTCTC CACAACTGTC TTCTAACGGT GGTTCTCCGT AGCCACCGTA CCTCAAAAAC   
  
  
+ CAAAGCGATG CACAACTACC ACCCCACCAC TGCTGAAAAG TGCCCACGAG TCTCGATCCG CGCAAGCCTG   
  
  
+ ATGAGAATCG ATACCACCAC CATGTAGATA AGCTGAAAAC GGGCATAAGG AAGCGACCCA CGCGCCCAAC   
  
  
+ ACGCCATTGA GGCAGTGACA CGTGGGTTGA TGCGCTTCCA AAGATGGAAT TTTCACATTT TTCTTTTCAA   
  
  
+ GGTTGCGTAG ATCGGTGTGT GTGGATGGCG ATCAAGGAAG AGTTAAAATG AACTAGTCAT GCGTCGAATT   
  
  
+ GTCCAAAAAA AATTTTCTCT TTCAGTCGAG TCTTAAAGGG GCAGTCATCA AGAGCATGTT TGAAAAACAT   
  
  
+ TATGGCCATA ACCACTTAGT AGTAATTCAA ATAATCATGG AAACATATTC AACATGAATT CAATATGACT   
  
  
+ AAAAATTCCA TCTTTTAGTT TGTTCTGGTC AACACAGAGC ATGCCTTTTT AATTGCATAA TAACGATATT   
  
  
+ TAACCAACCA ATCATGTTTC TCACATATTA ATATAGGCAA GGGTAGATTA ACAGTCATAA TCGCATAGTA   
  
  
+ TGCAAGAATT TATTTGCAAT CTGGGCATAC TGTTTTATAA AGCACAAGTG TTCATGACTT AAAATCGATA   
  
  
+ AAGTCTTAAT CTTTTGTCGA TCCTTGAGTG TGCAGACAAT ATAATTGTGA ATGTATGCTA TTAACTTTAA   
  
  
+ TAATTACGAA AATTAAGCAA CTAATCATAT AGTCATGTTC TCAAAAACAA GCATGCGTAT TATTCAGATC   
  
  
+ ATTAAATGCA TTAAATGGGA CTAATCCGAT CAGTCAAGCG CTGATACGAC TATTGAATAA ACAGGACTAA   
  
  
+ CCTAGAATGC GGAATTAACT GTCGAGAATT GTCCACATCG GTAGTGATGC TTGGCGGCTA CCAGAGTCAC   
  
  
+ CTTAGGGTGT TCACATCCTA GACCCGAAAA CCTGCTAATG ATGGAAGGGC AGCCGTGGGA GGAGGAGAGG   
  
  
+ AGAGTGTGGA TTTTTCTATT TACAAAAAAG TGTATAAGTG CTAATCCCCC GAAACCTCCT TTTATAGGTT   
  
  
+ AGGGGGGCAA CTAGGGTTAT CGCCCTAGGT GACGCTTGTG GCCGTCGGCC TAATTAAATA GGCCCGACCA   
  
  
+ ACAGCTGTGA CTGGAGCGAT AGCTGAAGCA TGCAGAAATG AGGATAACAT ACATATTATA GATTTCCACA   
  
  
+ TTGCATAGGG CACTCAGTGG GTTACTGTAT CGCAAGCCAT AGCAGCAAGG CTTGGTGGGC CTCCTAAAGT   
  
  
+ GCGACTTACA GGCATTGATG ATCCTGTTTC TAAGTATACT TGTGATGCTA GCTTGGAGGC TGGTGGGAAA   
  
  
+ CGATTAGCGT CTCTATTTGA AAAGTTTAAA ATACTTGTCG AGTTCAATGC ATTGCCCGTT TATGGACCCA   
  
  
+ ATGTCAGGTG GGAAATGCTG GATGTGAGGC CCAGGGAGGC TTTGGTCGTT AATTGTCCAT TACAGCTCCA   
  
  
+ TTACACTCCT GACGAGAGTG TCGATGTGAG GAACCCTAGG GATAGGCTTC TCAGAATGGT GAAATTGCTC   
  
  
+ GGTCCTAAGG TAGCCACTTT GGTTGAGCAA GAATCAAACA CCAACACTAC ACCTTTCTTG ACCCGGTTCA   
  
  
+ TAGAGACCCT TGACTACTAC TCGATTAACC ATGTTTGAGT CTATAGACGT GACCATGCCG AGAGACCGGA   
  
  
+ AGGAGAGGAT CAATGTTGAG CAGCATTGTT TAGCTAAGGA CATTGTGAAT ATCATAGCTT GCGAGGGCAA   
  
  
+ GGAGATGGTG GAGCGTCATG AGCTTTTTGG GAAATGGCAG TCGAGGTTTA CCATGGTAGG GTTCAGGCAG   
  
  
+ TACCCGTTGA GCTCATACAT AAACTCTGTG ATAAGAAGCC TACTCCGGTG TTACTCCAAG CATTATACTC   
  
  
+ TGATAGAGAA GGATAGTGCC ATGCTTCTGG GTTGGAGGGG CCGAATGCTG ATTTCAGCTT CGGCATG  

- -Up\_Stream \_Len000TTTAAA TATACTAATA CCCTCGTTTA TAATTAGCCA GATTTTGTTT TTTCTTAGCC   
  
  
- GGCTTGTTTT TTTTCCCGGG TCTTGATCAG GAAAATTACC TCAGGGTTTT ACACCATATT TAATCAGAAC   
  
  
- AAGTGAGTAA AGAGTGAGGA GAGTGAGTAA GATTGGGATT TAGAGTTAGG AGTGAGAGAC AGGAGAGATA   
  
  
- GACGCAGAGC TCGAAGAGAA AGGAGACCCG AAGGAACCTG AAAAAGCTGC GGTACTGTTA CCTTGTGACT   
  
  
- CGAGGTGGCA ACGGGGAGAG GTGTTGACAG AAGATTGCCA CCAAGAGGCA TCGGTGGCAT GGAGTTTTTG   
  
  
- GTTTCGCTAC GTGTTGATGG TGGGGTGGTG ACGACTTTTC ACGGGTGCTC AGAGCTAGGC GCGTTCGGAC   
  
  
- TACTCTTAGC TATGGTGGTG GTACATCTAT TCGACTTTTG CCCGTATTCC TTCGCTGGGT GCGCGGGTTG   
  
  
- TGCGGTAACT CCGTCACTGT GCACCCAACT ACGCGAAGGT TTCTACCTTA AAAGTGTAAA AAGAAAAGTT   
  
  
- CCAACGCATC TAGCCACACA CACCTACCGC TAGTTCCTTC TCAATTTTAC TTGATCAGTA CGCAGCTTAA   
  
  
- CAGGTTTTTT TTAAAAGAGA AAGTCAGCTC AGAATTTCCC CGTCAGTAGT TCTCGTACAA ACTTTTTGTA   
  
  
- ATACCGGTAT TGGTGAATCA TCATTAAGTT TATTAGTACC TTTGTATAAG TTGTACTTAA GTTATACTGA   
  
  
- TTTTTAAGGT AGAAAATCAA ACAAGACCAG TTGTGTCTCG TACGGAAAAA TTAACGTATT ATTGCTATAA   
  
  
- ATTGGTTGGT TAGTACAAAG AGTGTATAAT TATATCCGTT CCCATCTAAT TGTCAGTATT AGCGTATCAT   
  
  
- ACGTTCTTAA ATAAACGTTA GACCCGTATG ACAAAATATT TCGTGTTCAC AAGTACTGAA TTTTAGCTAT   
  
  
- TTCAGAATTA GAAAACAGCT AGGAACTCAC ACGTCTGTTA TATTAACACT TACATACGAT AATTGAAATT   
  
  
- ATTAATGCTT TTAATTCGTT GATTAGTATA TCAGTACAAG AGTTTTTGTT CGTACGCATA ATAAGTCTAG   
  
  
- TAATTTACGT AATTTACCCT GATTAGGCTA GTCAGTTCGC GACTATGCTG ATAACTTATT TGTCCTGATT   
  
  
- GGATCTTACG CCTTAATTGA CAGCTCTTAA CAGGTGTAGC CATCACTACG AACCGCCGAT GGTCTCAGTG   
  
  
- GAATCCCACA AGTGTAGGAT CTGGGCTTTT GGACGATTAC TACCTTCCCG TCGGCACCCT CCTCCTCTCC   
  
  
- TCTCACACCT AAAAAGATAA ATGTTTTTTC ACATATTCAC GATTAGGGGG CTTTGGAGGA AAATATCCAA   
  
  
- TCCCCCCGTT GATCCCAATA GCGGGATCCA CTGCGAACAC CGGCAGCCGG ATTAATTTAT CCGGGCTGGT   
  
  
- TGTCGACACT GACCTCGCTA TCGACTTCGT ACGTCTTTAC TCCTATTGTA TGTATAATAT CTAAAGGTGT   
  
  
- AACGTATCCC GTGAGTCACC CAATGACATA GCGTTCGGTA TCGTCGTTCC GAACCACCCG GAGGATTTCA   
  
  
- CGCTGAATGT CCGTAACTAC TAGGACAAAG ATTCATATGA ACACTACGAT CGAACCTCCG ACCACCCTTT   
  
  
- GCTAATCGCA GAGATAAACT TTTCAAATTT TATGAACAGC TCAAGTTACG TAACGGGCAA ATACCTGGGT   
  
  
- TACAGTCCAC CCTTTACGAC CTACACTCCG GGTCCCTCCG AAACCAGCAA TTAACAGGTA ATGTCGAGGT   
  
  
- AATGTGAGGA CTGCTCTCAC AGCTACACTC CTTGGGATCC CTATCCGAAG AGTCTTACCA CTTTAACGAG   
  
  
- CCAGGATTCC ATCGGTGAAA CCAACTCGTT CTTAGTTTGT GGTTGTGATG TGGAAAGAAC TGGGCCAAGT   
  
  
- ATCTCTGGGA ACTGATGATG AGCTAATTGG TACAAACTCA GATATCTGCA CTGGTACGGC TCTCTGGCCT   
  
  
- TCCTCTCCTA GTTACAACTC GTCGTAACAA ATCGATTCCT GTAACACTTA TAGTATCGAA CGCTCCCGTT   
  
  
- CCTCTACCAC CTCGCAGTAC TCGAAAAACC CTTTACCGTC AGCTCCAAAT GGTACCATCC CAAGTCCGTC   
  
  
- ATGGGCAACT CGAGTATGTA TTTGAGACAC TATTCTTCGG ATGAGGCCAC AATGAGGTTC GTAATATGAG   
  
  
- ACTATCTCTT CCTATCACGG TACGAAGACC CAACCTCCCC GGCTTACGAC TAAAGTCGAA GCCGTAC

+     TATA-box

| Site Name | Organism | Position | Strand | Matrix score. | sequence | function |
| --- | --- | --- | --- | --- | --- | --- |
| TATA-box | Arabidopsis thaliana | 1367 | - | 4 | TATA | core promoter element around -30 of transcription start |
| TATA-box | Arabidopsis thaliana | 130 | + | 4 | TATA | core promoter element around -30 of transcription start |
| TATA-box | Oryza sativa | 2190 | + | 8 | TACATAAA | core promoter element around -30 of transcription start |
| TATA-box | Arabidopsis thaliana | 2006 | - | 4 | TATA | core promoter element around -30 of transcription start |
| TATA-box | Arabidopsis thaliana | 1649 | - | 4 | TATA | core promoter element around -30 of transcription start |
| TATA-box | Arabidopsis thaliana | 1530 | - | 5 | TATAA | core promoter element around -30 of transcription start |
| TATA-box | Arabidopsis thaliana | 1531 | - | 4 | TATA | core promoter element around -30 of transcription start |
| TATA-box | Helianthus annuus | 1395 | - | 6 | TATAAA | core promoter element around -30 of transcription start |
| TATA-box | Arabidopsis thaliana | 1397 | - | 4 | TATA | core promoter element around -30 of transcription start |
| TATA-box | Helianthus annuus | 1365 | - | 6 | TATACA | core promoter element around -30 of transcription start |
| TATA-box | Pisum sativum | 947 | - | 7 | TATAAAA | core promoter element around -30 of transcription start |
| TATA-box | Oryza sativa | 1355 | + | 7 | TACAAAA | core promoter element around -30 of transcription start |
| TATA-box | Arabidopsis thaliana | 949 | - | 5 | TATAA | core promoter element around -30 of transcription start |
| TATA-box | Arabidopsis thaliana | 1082 | + | 4 | TATA | core promoter element around -30 of transcription start |
| TATA-box | Helianthus annuus | 948 | - | 6 | TATAAA | core promoter element around -30 of transcription start |
| TATA-box | Arabidopsis thaliana | 2237 | - | 5 | TATAA | core promoter element around -30 of transcription start |
| TATA-box | Arabidopsis thaliana | 876 | + | 4 | TATA | core promoter element around -30 of transcription start |
| TATA-box | Arabidopsis thaliana | 2238 | - | 4 | TATA | core promoter element around -30 of transcription start |
| TATA-box | Brassica juncea | 21 | - | 7 | TATAAAT | core promoter element around -30 of transcription start |
| TATA-box | Brassica oleracea | 1023 | + | 6 | ATATAA | core promoter element around -30 of transcription start |
| TATA-box | Arabidopsis thaliana | 950 | + | 4 | TATA | core promoter element around -30 of transcription start |
| TATA-box | Pisum sativum | 1394 | - | 7 | TATAAAA | core promoter element around -30 of transcription start |
| TATA-box | Helianthus annuus | 22 | - | 6 | TATAAA | core promoter element around -30 of transcription start |
| TATA-box | Arabidopsis thaliana | 1024 | + | 4 | TATA | core promoter element around -30 of transcription start |
| TATA-box | Arabidopsis thaliana | 1393 | - | 9 | ccTATAAAaa | core promoter element around -30 of transcription start |
| TATA-box | Arabidopsis thaliana | 23 | - | 5 | TATAA | core promoter element around -30 of transcription start |
| TATA-box | Brassica napus | 2236 | + | 6 | ATTATA | core promoter element around -30 of transcription start |
| TATA-box | Arabidopsis thaliana | 24 | + | 4 | TATA | core promoter element around -30 of transcription start |
| TATA-box | Brassica napus | 1529 | + | 6 | ATTATA | core promoter element around -30 of transcription start |
| TATA-box | Arabidopsis thaliana | 1396 | - | 5 | TATAA | core promoter element around -30 of transcription start |

>HU08G00229.1   
+ -Up\_Stream \_Len000AAATTT ATATGATTAT GGGAGCAAAT ATTAATCGGT CTAAAACAAA AAAGAATCGG   
  
  
+ CCGAACAAAA AAAAGGGCCC AGAACTAGTC CTTTTAATGG AGTCCCAAAA TGTGGTATAA ATTAGTCTTG   
  
  
+ TTCACTCATT TCTCACTCCT CTCACTCATT CTAACCCTAA ATCTCAATCC TCACTCTCTG TCCTCTCTAT   
  
  
+ CTGCGTCTCG AGCTTCTCTT TCCTCTGGGC TTCCTTGGAC TTTTTCGACG CCATGACAAT GGAACACTGA   
  
  
+ GCTCCACCGT TGCCCCTCTC CACAACTGTC TTCTAACGGT GGTTCTCCGT AGCCACCGTA CCTCAAAAAC   
  
  
+ CAAAGCGATG CACAACTACC ACCCCACCAC TGCTGAAAAG TGCCCACGAG TCTCGATCCG CGCAAGCCTG   
  
  
+ ATGAGAATCG ATACCACCAC CATGTAGATA AGCTGAAAAC GGGCATAAGG AAGCGACCCA CGCGCCCAAC   
  
  
+ ACGCCATTGA GGCAGTGACA CGTGGGTTGA TGCGCTTCCA AAGATGGAAT TTTCACATTT TTCTTTTCAA   
  
  
+ GGTTGCGTAG ATCGGTGTGT GTGGATGGCG ATCAAGGAAG AGTTAAAATG AACTAGTCAT GCGTCGAATT   
  
  
+ GTCCAAAAAA AATTTTCTCT TTCAGTCGAG TCTTAAAGGG GCAGTCATCA AGAGCATGTT TGAAAAACAT   
  
  
+ TATGGCCATA ACCACTTAGT AGTAATTCAA ATAATCATGG AAACATATTC AACATGAATT CAATATGACT   
  
  
+ AAAAATTCCA TCTTTTAGTT TGTTCTGGTC AACACAGAGC ATGCCTTTTT AATTGCATAA TAACGATATT   
  
  
+ TAACCAACCA ATCATGTTTC TCACATATTA ATATAGGCAA GGGTAGATTA ACAGTCATAA TCGCATAGTA   
  
  
+ TGCAAGAATT TATTTGCAAT CTGGGCATAC TGTTTTATAA AGCACAAGTG TTCATGACTT AAAATCGATA   
  
  
+ AAGTCTTAAT CTTTTGTCGA TCCTTGAGTG TGCAGACAAT ATAATTGTGA ATGTATGCTA TTAACTTTAA   
  
  
+ TAATTACGAA AATTAAGCAA CTAATCATAT AGTCATGTTC TCAAAAACAA GCATGCGTAT TATTCAGATC   
  
  
+ ATTAAATGCA TTAAATGGGA CTAATCCGAT CAGTCAAGCG CTGATACGAC TATTGAATAA ACAGGACTAA   
  
  
+ CCTAGAATGC GGAATTAACT GTCGAGAATT GTCCACATCG GTAGTGATGC TTGGCGGCTA CCAGAGTCAC   
  
  
+ CTTAGGGTGT TCACATCCTA GACCCGAAAA CCTGCTAATG ATGGAAGGGC AGCCGTGGGA GGAGGAGAGG   
  
  
+ AGAGTGTGGA TTTTTCTATT TACAAAAAAG TGTATAAGTG CTAATCCCCC GAAACCTCCT TTTATAGGTT   
  
  
+ AGGGGGGCAA CTAGGGTTAT CGCCCTAGGT GACGCTTGTG GCCGTCGGCC TAATTAAATA GGCCCGACCA   
  
  
+ ACAGCTGTGA CTGGAGCGAT AGCTGAAGCA TGCAGAAATG AGGATAACAT ACATATTATA GATTTCCACA   
  
  
+ TTGCATAGGG CACTCAGTGG GTTACTGTAT CGCAAGCCAT AGCAGCAAGG CTTGGTGGGC CTCCTAAAGT   
  
  
+ GCGACTTACA GGCATTGATG ATCCTGTTTC TAAGTATACT TGTGATGCTA GCTTGGAGGC TGGTGGGAAA   
  
  
+ CGATTAGCGT CTCTATTTGA AAAGTTTAAA ATACTTGTCG AGTTCAATGC ATTGCCCGTT TATGGACCCA   
  
  
+ ATGTCAGGTG GGAAATGCTG GATGTGAGGC CCAGGGAGGC TTTGGTCGTT AATTGTCCAT TACAGCTCCA   
  
  
+ TTACACTCCT GACGAGAGTG TCGATGTGAG GAACCCTAGG GATAGGCTTC TCAGAATGGT GAAATTGCTC   
  
  
+ GGTCCTAAGG TAGCCACTTT GGTTGAGCAA GAATCAAACA CCAACACTAC ACCTTTCTTG ACCCGGTTCA   
  
  
+ TAGAGACCCT TGACTACTAC TCGATTAACC ATGTTTGAGT CTATAGACGT GACCATGCCG AGAGACCGGA   
  
  
+ AGGAGAGGAT CAATGTTGAG CAGCATTGTT TAGCTAAGGA CATTGTGAAT ATCATAGCTT GCGAGGGCAA   
  
  
+ GGAGATGGTG GAGCGTCATG AGCTTTTTGG GAAATGGCAG TCGAGGTTTA CCATGGTAGG GTTCAGGCAG   
  
  
+ TACCCGTTGA GCTCATACAT AAACTCTGTG ATAAGAAGCC TACTCCGGTG TTACTCCAAG CATTATACTC   
  
  
+ TGATAGAGAA GGATAGTGCC ATGCTTCTGG GTTGGAGGGG CCGAATGCTG ATTTCAGCTT CGGCATG  

- -Up\_Stream \_Len000TTTAAA TATACTAATA CCCTCGTTTA TAATTAGCCA GATTTTGTTT TTTCTTAGCC   
  
  
- GGCTTGTTTT TTTTCCCGGG TCTTGATCAG GAAAATTACC TCAGGGTTTT ACACCATATT TAATCAGAAC   
  
  
- AAGTGAGTAA AGAGTGAGGA GAGTGAGTAA GATTGGGATT TAGAGTTAGG AGTGAGAGAC AGGAGAGATA   
  
  
- GACGCAGAGC TCGAAGAGAA AGGAGACCCG AAGGAACCTG AAAAAGCTGC GGTACTGTTA CCTTGTGACT   
  
  
- CGAGGTGGCA ACGGGGAGAG GTGTTGACAG AAGATTGCCA CCAAGAGGCA TCGGTGGCAT GGAGTTTTTG   
  
  
- GTTTCGCTAC GTGTTGATGG TGGGGTGGTG ACGACTTTTC ACGGGTGCTC AGAGCTAGGC GCGTTCGGAC   
  
  
- TACTCTTAGC TATGGTGGTG GTACATCTAT TCGACTTTTG CCCGTATTCC TTCGCTGGGT GCGCGGGTTG   
  
  
- TGCGGTAACT CCGTCACTGT GCACCCAACT ACGCGAAGGT TTCTACCTTA AAAGTGTAAA AAGAAAAGTT   
  
  
- CCAACGCATC TAGCCACACA CACCTACCGC TAGTTCCTTC TCAATTTTAC TTGATCAGTA CGCAGCTTAA   
  
  
- CAGGTTTTTT TTAAAAGAGA AAGTCAGCTC AGAATTTCCC CGTCAGTAGT TCTCGTACAA ACTTTTTGTA   
  
  
- ATACCGGTAT TGGTGAATCA TCATTAAGTT TATTAGTACC TTTGTATAAG TTGTACTTAA GTTATACTGA   
  
  
- TTTTTAAGGT AGAAAATCAA ACAAGACCAG TTGTGTCTCG TACGGAAAAA TTAACGTATT ATTGCTATAA   
  
  
- ATTGGTTGGT TAGTACAAAG AGTGTATAAT TATATCCGTT CCCATCTAAT TGTCAGTATT AGCGTATCAT   
  
  
- ACGTTCTTAA ATAAACGTTA GACCCGTATG ACAAAATATT TCGTGTTCAC AAGTACTGAA TTTTAGCTAT   
  
  
- TTCAGAATTA GAAAACAGCT AGGAACTCAC ACGTCTGTTA TATTAACACT TACATACGAT AATTGAAATT   
  
  
- ATTAATGCTT TTAATTCGTT GATTAGTATA TCAGTACAAG AGTTTTTGTT CGTACGCATA ATAAGTCTAG   
  
  
- TAATTTACGT AATTTACCCT GATTAGGCTA GTCAGTTCGC GACTATGCTG ATAACTTATT TGTCCTGATT   
  
  
- GGATCTTACG CCTTAATTGA CAGCTCTTAA CAGGTGTAGC CATCACTACG AACCGCCGAT GGTCTCAGTG   
  
  
- GAATCCCACA AGTGTAGGAT CTGGGCTTTT GGACGATTAC TACCTTCCCG TCGGCACCCT CCTCCTCTCC   
  
  
- TCTCACACCT AAAAAGATAA ATGTTTTTTC ACATATTCAC GATTAGGGGG CTTTGGAGGA AAATATCCAA   
  
  
- TCCCCCCGTT GATCCCAATA GCGGGATCCA CTGCGAACAC CGGCAGCCGG ATTAATTTAT CCGGGCTGGT   
  
  
- TGTCGACACT GACCTCGCTA TCGACTTCGT ACGTCTTTAC TCCTATTGTA TGTATAATAT CTAAAGGTGT   
  
  
- AACGTATCCC GTGAGTCACC CAATGACATA GCGTTCGGTA TCGTCGTTCC GAACCACCCG GAGGATTTCA   
  
  
- CGCTGAATGT CCGTAACTAC TAGGACAAAG ATTCATATGA ACACTACGAT CGAACCTCCG ACCACCCTTT   
  
  
- GCTAATCGCA GAGATAAACT TTTCAAATTT TATGAACAGC TCAAGTTACG TAACGGGCAA ATACCTGGGT   
  
  
- TACAGTCCAC CCTTTACGAC CTACACTCCG GGTCCCTCCG AAACCAGCAA TTAACAGGTA ATGTCGAGGT   
  
  
- AATGTGAGGA CTGCTCTCAC AGCTACACTC CTTGGGATCC CTATCCGAAG AGTCTTACCA CTTTAACGAG   
  
  
- CCAGGATTCC ATCGGTGAAA CCAACTCGTT CTTAGTTTGT GGTTGTGATG TGGAAAGAAC TGGGCCAAGT   
  
  
- ATCTCTGGGA ACTGATGATG AGCTAATTGG TACAAACTCA GATATCTGCA CTGGTACGGC TCTCTGGCCT   
  
  
- TCCTCTCCTA GTTACAACTC GTCGTAACAA ATCGATTCCT GTAACACTTA TAGTATCGAA CGCTCCCGTT   
  
  
- CCTCTACCAC CTCGCAGTAC TCGAAAAACC CTTTACCGTC AGCTCCAAAT GGTACCATCC CAAGTCCGTC   
  
  
- ATGGGCAACT CGAGTATGTA TTTGAGACAC TATTCTTCGG ATGAGGCCAC AATGAGGTTC GTAATATGAG   
  
  
- ACTATCTCTT CCTATCACGG TACGAAGACC CAACCTCCCC GGCTTACGAC TAAAGTCGAA GCCGTAC

+     TCA-element

| Site Name | Organism | Position | Strand | Matrix score. | sequence | function |
| --- | --- | --- | --- | --- | --- | --- |
| TCA-element | Nicotiana tabacum | 782 | + | 9 | CCATCTTTTT | cis-acting element involved in salicylic acid responsiveness |

>HU08G00229.1   
+ -Up\_Stream \_Len000AAATTT ATATGATTAT GGGAGCAAAT ATTAATCGGT CTAAAACAAA AAAGAATCGG   
  
  
+ CCGAACAAAA AAAAGGGCCC AGAACTAGTC CTTTTAATGG AGTCCCAAAA TGTGGTATAA ATTAGTCTTG   
  
  
+ TTCACTCATT TCTCACTCCT CTCACTCATT CTAACCCTAA ATCTCAATCC TCACTCTCTG TCCTCTCTAT   
  
  
+ CTGCGTCTCG AGCTTCTCTT TCCTCTGGGC TTCCTTGGAC TTTTTCGACG CCATGACAAT GGAACACTGA   
  
  
+ GCTCCACCGT TGCCCCTCTC CACAACTGTC TTCTAACGGT GGTTCTCCGT AGCCACCGTA CCTCAAAAAC   
  
  
+ CAAAGCGATG CACAACTACC ACCCCACCAC TGCTGAAAAG TGCCCACGAG TCTCGATCCG CGCAAGCCTG   
  
  
+ ATGAGAATCG ATACCACCAC CATGTAGATA AGCTGAAAAC GGGCATAAGG AAGCGACCCA CGCGCCCAAC   
  
  
+ ACGCCATTGA GGCAGTGACA CGTGGGTTGA TGCGCTTCCA AAGATGGAAT TTTCACATTT TTCTTTTCAA   
  
  
+ GGTTGCGTAG ATCGGTGTGT GTGGATGGCG ATCAAGGAAG AGTTAAAATG AACTAGTCAT GCGTCGAATT   
  
  
+ GTCCAAAAAA AATTTTCTCT TTCAGTCGAG TCTTAAAGGG GCAGTCATCA AGAGCATGTT TGAAAAACAT   
  
  
+ TATGGCCATA ACCACTTAGT AGTAATTCAA ATAATCATGG AAACATATTC AACATGAATT CAATATGACT   
  
  
+ AAAAATTCCA TCTTTTAGTT TGTTCTGGTC AACACAGAGC ATGCCTTTTT AATTGCATAA TAACGATATT   
  
  
+ TAACCAACCA ATCATGTTTC TCACATATTA ATATAGGCAA GGGTAGATTA ACAGTCATAA TCGCATAGTA   
  
  
+ TGCAAGAATT TATTTGCAAT CTGGGCATAC TGTTTTATAA AGCACAAGTG TTCATGACTT AAAATCGATA   
  
  
+ AAGTCTTAAT CTTTTGTCGA TCCTTGAGTG TGCAGACAAT ATAATTGTGA ATGTATGCTA TTAACTTTAA   
  
  
+ TAATTACGAA AATTAAGCAA CTAATCATAT AGTCATGTTC TCAAAAACAA GCATGCGTAT TATTCAGATC   
  
  
+ ATTAAATGCA TTAAATGGGA CTAATCCGAT CAGTCAAGCG CTGATACGAC TATTGAATAA ACAGGACTAA   
  
  
+ CCTAGAATGC GGAATTAACT GTCGAGAATT GTCCACATCG GTAGTGATGC TTGGCGGCTA CCAGAGTCAC   
  
  
+ CTTAGGGTGT TCACATCCTA GACCCGAAAA CCTGCTAATG ATGGAAGGGC AGCCGTGGGA GGAGGAGAGG   
  
  
+ AGAGTGTGGA TTTTTCTATT TACAAAAAAG TGTATAAGTG CTAATCCCCC GAAACCTCCT TTTATAGGTT   
  
  
+ AGGGGGGCAA CTAGGGTTAT CGCCCTAGGT GACGCTTGTG GCCGTCGGCC TAATTAAATA GGCCCGACCA   
  
  
+ ACAGCTGTGA CTGGAGCGAT AGCTGAAGCA TGCAGAAATG AGGATAACAT ACATATTATA GATTTCCACA   
  
  
+ TTGCATAGGG CACTCAGTGG GTTACTGTAT CGCAAGCCAT AGCAGCAAGG CTTGGTGGGC CTCCTAAAGT   
  
  
+ GCGACTTACA GGCATTGATG ATCCTGTTTC TAAGTATACT TGTGATGCTA GCTTGGAGGC TGGTGGGAAA   
  
  
+ CGATTAGCGT CTCTATTTGA AAAGTTTAAA ATACTTGTCG AGTTCAATGC ATTGCCCGTT TATGGACCCA   
  
  
+ ATGTCAGGTG GGAAATGCTG GATGTGAGGC CCAGGGAGGC TTTGGTCGTT AATTGTCCAT TACAGCTCCA   
  
  
+ TTACACTCCT GACGAGAGTG TCGATGTGAG GAACCCTAGG GATAGGCTTC TCAGAATGGT GAAATTGCTC   
  
  
+ GGTCCTAAGG TAGCCACTTT GGTTGAGCAA GAATCAAACA CCAACACTAC ACCTTTCTTG ACCCGGTTCA   
  
  
+ TAGAGACCCT TGACTACTAC TCGATTAACC ATGTTTGAGT CTATAGACGT GACCATGCCG AGAGACCGGA   
  
  
+ AGGAGAGGAT CAATGTTGAG CAGCATTGTT TAGCTAAGGA CATTGTGAAT ATCATAGCTT GCGAGGGCAA   
  
  
+ GGAGATGGTG GAGCGTCATG AGCTTTTTGG GAAATGGCAG TCGAGGTTTA CCATGGTAGG GTTCAGGCAG   
  
  
+ TACCCGTTGA GCTCATACAT AAACTCTGTG ATAAGAAGCC TACTCCGGTG TTACTCCAAG CATTATACTC   
  
  
+ TGATAGAGAA GGATAGTGCC ATGCTTCTGG GTTGGAGGGG CCGAATGCTG ATTTCAGCTT CGGCATG  

- -Up\_Stream \_Len000TTTAAA TATACTAATA CCCTCGTTTA TAATTAGCCA GATTTTGTTT TTTCTTAGCC   
  
  
- GGCTTGTTTT TTTTCCCGGG TCTTGATCAG GAAAATTACC TCAGGGTTTT ACACCATATT TAATCAGAAC   
  
  
- AAGTGAGTAA AGAGTGAGGA GAGTGAGTAA GATTGGGATT TAGAGTTAGG AGTGAGAGAC AGGAGAGATA   
  
  
- GACGCAGAGC TCGAAGAGAA AGGAGACCCG AAGGAACCTG AAAAAGCTGC GGTACTGTTA CCTTGTGACT   
  
  
- CGAGGTGGCA ACGGGGAGAG GTGTTGACAG AAGATTGCCA CCAAGAGGCA TCGGTGGCAT GGAGTTTTTG   
  
  
- GTTTCGCTAC GTGTTGATGG TGGGGTGGTG ACGACTTTTC ACGGGTGCTC AGAGCTAGGC GCGTTCGGAC   
  
  
- TACTCTTAGC TATGGTGGTG GTACATCTAT TCGACTTTTG CCCGTATTCC TTCGCTGGGT GCGCGGGTTG   
  
  
- TGCGGTAACT CCGTCACTGT GCACCCAACT ACGCGAAGGT TTCTACCTTA AAAGTGTAAA AAGAAAAGTT   
  
  
- CCAACGCATC TAGCCACACA CACCTACCGC TAGTTCCTTC TCAATTTTAC TTGATCAGTA CGCAGCTTAA   
  
  
- CAGGTTTTTT TTAAAAGAGA AAGTCAGCTC AGAATTTCCC CGTCAGTAGT TCTCGTACAA ACTTTTTGTA   
  
  
- ATACCGGTAT TGGTGAATCA TCATTAAGTT TATTAGTACC TTTGTATAAG TTGTACTTAA GTTATACTGA   
  
  
- TTTTTAAGGT AGAAAATCAA ACAAGACCAG TTGTGTCTCG TACGGAAAAA TTAACGTATT ATTGCTATAA   
  
  
- ATTGGTTGGT TAGTACAAAG AGTGTATAAT TATATCCGTT CCCATCTAAT TGTCAGTATT AGCGTATCAT   
  
  
- ACGTTCTTAA ATAAACGTTA GACCCGTATG ACAAAATATT TCGTGTTCAC AAGTACTGAA TTTTAGCTAT   
  
  
- TTCAGAATTA GAAAACAGCT AGGAACTCAC ACGTCTGTTA TATTAACACT TACATACGAT AATTGAAATT   
  
  
- ATTAATGCTT TTAATTCGTT GATTAGTATA TCAGTACAAG AGTTTTTGTT CGTACGCATA ATAAGTCTAG   
  
  
- TAATTTACGT AATTTACCCT GATTAGGCTA GTCAGTTCGC GACTATGCTG ATAACTTATT TGTCCTGATT   
  
  
- GGATCTTACG CCTTAATTGA CAGCTCTTAA CAGGTGTAGC CATCACTACG AACCGCCGAT GGTCTCAGTG   
  
  
- GAATCCCACA AGTGTAGGAT CTGGGCTTTT GGACGATTAC TACCTTCCCG TCGGCACCCT CCTCCTCTCC   
  
  
- TCTCACACCT AAAAAGATAA ATGTTTTTTC ACATATTCAC GATTAGGGGG CTTTGGAGGA AAATATCCAA   
  
  
- TCCCCCCGTT GATCCCAATA GCGGGATCCA CTGCGAACAC CGGCAGCCGG ATTAATTTAT CCGGGCTGGT   
  
  
- TGTCGACACT GACCTCGCTA TCGACTTCGT ACGTCTTTAC TCCTATTGTA TGTATAATAT CTAAAGGTGT   
  
  
- AACGTATCCC GTGAGTCACC CAATGACATA GCGTTCGGTA TCGTCGTTCC GAACCACCCG GAGGATTTCA   
  
  
- CGCTGAATGT CCGTAACTAC TAGGACAAAG ATTCATATGA ACACTACGAT CGAACCTCCG ACCACCCTTT   
  
  
- GCTAATCGCA GAGATAAACT TTTCAAATTT TATGAACAGC TCAAGTTACG TAACGGGCAA ATACCTGGGT   
  
  
- TACAGTCCAC CCTTTACGAC CTACACTCCG GGTCCCTCCG AAACCAGCAA TTAACAGGTA ATGTCGAGGT   
  
  
- AATGTGAGGA CTGCTCTCAC AGCTACACTC CTTGGGATCC CTATCCGAAG AGTCTTACCA CTTTAACGAG   
  
  
- CCAGGATTCC ATCGGTGAAA CCAACTCGTT CTTAGTTTGT GGTTGTGATG TGGAAAGAAC TGGGCCAAGT   
  
  
- ATCTCTGGGA ACTGATGATG AGCTAATTGG TACAAACTCA GATATCTGCA CTGGTACGGC TCTCTGGCCT   
  
  
- TCCTCTCCTA GTTACAACTC GTCGTAACAA ATCGATTCCT GTAACACTTA TAGTATCGAA CGCTCCCGTT   
  
  
- CCTCTACCAC CTCGCAGTAC TCGAAAAACC CTTTACCGTC AGCTCCAAAT GGTACCATCC CAAGTCCGTC   
  
  
- ATGGGCAACT CGAGTATGTA TTTGAGACAC TATTCTTCGG ATGAGGCCAC AATGAGGTTC GTAATATGAG   
  
  
- ACTATCTCTT CCTATCACGG TACGAAGACC CAACCTCCCC GGCTTACGAC TAAAGTCGAA GCCGTAC

+     TGA-element

| Site Name | Organism | Position | Strand | Matrix score. | sequence | function |
| --- | --- | --- | --- | --- | --- | --- |
| TGA-element | Brassica oleracea | 1799 | - | 6 | AACGAC | auxin-responsive element |

>HU08G00229.1   
+ -Up\_Stream \_Len000AAATTT ATATGATTAT GGGAGCAAAT ATTAATCGGT CTAAAACAAA AAAGAATCGG   
  
  
+ CCGAACAAAA AAAAGGGCCC AGAACTAGTC CTTTTAATGG AGTCCCAAAA TGTGGTATAA ATTAGTCTTG   
  
  
+ TTCACTCATT TCTCACTCCT CTCACTCATT CTAACCCTAA ATCTCAATCC TCACTCTCTG TCCTCTCTAT   
  
  
+ CTGCGTCTCG AGCTTCTCTT TCCTCTGGGC TTCCTTGGAC TTTTTCGACG CCATGACAAT GGAACACTGA   
  
  
+ GCTCCACCGT TGCCCCTCTC CACAACTGTC TTCTAACGGT GGTTCTCCGT AGCCACCGTA CCTCAAAAAC   
  
  
+ CAAAGCGATG CACAACTACC ACCCCACCAC TGCTGAAAAG TGCCCACGAG TCTCGATCCG CGCAAGCCTG   
  
  
+ ATGAGAATCG ATACCACCAC CATGTAGATA AGCTGAAAAC GGGCATAAGG AAGCGACCCA CGCGCCCAAC   
  
  
+ ACGCCATTGA GGCAGTGACA CGTGGGTTGA TGCGCTTCCA AAGATGGAAT TTTCACATTT TTCTTTTCAA   
  
  
+ GGTTGCGTAG ATCGGTGTGT GTGGATGGCG ATCAAGGAAG AGTTAAAATG AACTAGTCAT GCGTCGAATT   
  
  
+ GTCCAAAAAA AATTTTCTCT TTCAGTCGAG TCTTAAAGGG GCAGTCATCA AGAGCATGTT TGAAAAACAT   
  
  
+ TATGGCCATA ACCACTTAGT AGTAATTCAA ATAATCATGG AAACATATTC AACATGAATT CAATATGACT   
  
  
+ AAAAATTCCA TCTTTTAGTT TGTTCTGGTC AACACAGAGC ATGCCTTTTT AATTGCATAA TAACGATATT   
  
  
+ TAACCAACCA ATCATGTTTC TCACATATTA ATATAGGCAA GGGTAGATTA ACAGTCATAA TCGCATAGTA   
  
  
+ TGCAAGAATT TATTTGCAAT CTGGGCATAC TGTTTTATAA AGCACAAGTG TTCATGACTT AAAATCGATA   
  
  
+ AAGTCTTAAT CTTTTGTCGA TCCTTGAGTG TGCAGACAAT ATAATTGTGA ATGTATGCTA TTAACTTTAA   
  
  
+ TAATTACGAA AATTAAGCAA CTAATCATAT AGTCATGTTC TCAAAAACAA GCATGCGTAT TATTCAGATC   
  
  
+ ATTAAATGCA TTAAATGGGA CTAATCCGAT CAGTCAAGCG CTGATACGAC TATTGAATAA ACAGGACTAA   
  
  
+ CCTAGAATGC GGAATTAACT GTCGAGAATT GTCCACATCG GTAGTGATGC TTGGCGGCTA CCAGAGTCAC   
  
  
+ CTTAGGGTGT TCACATCCTA GACCCGAAAA CCTGCTAATG ATGGAAGGGC AGCCGTGGGA GGAGGAGAGG   
  
  
+ AGAGTGTGGA TTTTTCTATT TACAAAAAAG TGTATAAGTG CTAATCCCCC GAAACCTCCT TTTATAGGTT   
  
  
+ AGGGGGGCAA CTAGGGTTAT CGCCCTAGGT GACGCTTGTG GCCGTCGGCC TAATTAAATA GGCCCGACCA   
  
  
+ ACAGCTGTGA CTGGAGCGAT AGCTGAAGCA TGCAGAAATG AGGATAACAT ACATATTATA GATTTCCACA   
  
  
+ TTGCATAGGG CACTCAGTGG GTTACTGTAT CGCAAGCCAT AGCAGCAAGG CTTGGTGGGC CTCCTAAAGT   
  
  
+ GCGACTTACA GGCATTGATG ATCCTGTTTC TAAGTATACT TGTGATGCTA GCTTGGAGGC TGGTGGGAAA   
  
  
+ CGATTAGCGT CTCTATTTGA AAAGTTTAAA ATACTTGTCG AGTTCAATGC ATTGCCCGTT TATGGACCCA   
  
  
+ ATGTCAGGTG GGAAATGCTG GATGTGAGGC CCAGGGAGGC TTTGGTCGTT AATTGTCCAT TACAGCTCCA   
  
  
+ TTACACTCCT GACGAGAGTG TCGATGTGAG GAACCCTAGG GATAGGCTTC TCAGAATGGT GAAATTGCTC   
  
  
+ GGTCCTAAGG TAGCCACTTT GGTTGAGCAA GAATCAAACA CCAACACTAC ACCTTTCTTG ACCCGGTTCA   
  
  
+ TAGAGACCCT TGACTACTAC TCGATTAACC ATGTTTGAGT CTATAGACGT GACCATGCCG AGAGACCGGA   
  
  
+ AGGAGAGGAT CAATGTTGAG CAGCATTGTT TAGCTAAGGA CATTGTGAAT ATCATAGCTT GCGAGGGCAA   
  
  
+ GGAGATGGTG GAGCGTCATG AGCTTTTTGG GAAATGGCAG TCGAGGTTTA CCATGGTAGG GTTCAGGCAG   
  
  
+ TACCCGTTGA GCTCATACAT AAACTCTGTG ATAAGAAGCC TACTCCGGTG TTACTCCAAG CATTATACTC   
  
  
+ TGATAGAGAA GGATAGTGCC ATGCTTCTGG GTTGGAGGGG CCGAATGCTG ATTTCAGCTT CGGCATG  

- -Up\_Stream \_Len000TTTAAA TATACTAATA CCCTCGTTTA TAATTAGCCA GATTTTGTTT TTTCTTAGCC   
  
  
- GGCTTGTTTT TTTTCCCGGG TCTTGATCAG GAAAATTACC TCAGGGTTTT ACACCATATT TAATCAGAAC   
  
  
- AAGTGAGTAA AGAGTGAGGA GAGTGAGTAA GATTGGGATT TAGAGTTAGG AGTGAGAGAC AGGAGAGATA   
  
  
- GACGCAGAGC TCGAAGAGAA AGGAGACCCG AAGGAACCTG AAAAAGCTGC GGTACTGTTA CCTTGTGACT   
  
  
- CGAGGTGGCA ACGGGGAGAG GTGTTGACAG AAGATTGCCA CCAAGAGGCA TCGGTGGCAT GGAGTTTTTG   
  
  
- GTTTCGCTAC GTGTTGATGG TGGGGTGGTG ACGACTTTTC ACGGGTGCTC AGAGCTAGGC GCGTTCGGAC   
  
  
- TACTCTTAGC TATGGTGGTG GTACATCTAT TCGACTTTTG CCCGTATTCC TTCGCTGGGT GCGCGGGTTG   
  
  
- TGCGGTAACT CCGTCACTGT GCACCCAACT ACGCGAAGGT TTCTACCTTA AAAGTGTAAA AAGAAAAGTT   
  
  
- CCAACGCATC TAGCCACACA CACCTACCGC TAGTTCCTTC TCAATTTTAC TTGATCAGTA CGCAGCTTAA   
  
  
- CAGGTTTTTT TTAAAAGAGA AAGTCAGCTC AGAATTTCCC CGTCAGTAGT TCTCGTACAA ACTTTTTGTA   
  
  
- ATACCGGTAT TGGTGAATCA TCATTAAGTT TATTAGTACC TTTGTATAAG TTGTACTTAA GTTATACTGA   
  
  
- TTTTTAAGGT AGAAAATCAA ACAAGACCAG TTGTGTCTCG TACGGAAAAA TTAACGTATT ATTGCTATAA   
  
  
- ATTGGTTGGT TAGTACAAAG AGTGTATAAT TATATCCGTT CCCATCTAAT TGTCAGTATT AGCGTATCAT   
  
  
- ACGTTCTTAA ATAAACGTTA GACCCGTATG ACAAAATATT TCGTGTTCAC AAGTACTGAA TTTTAGCTAT   
  
  
- TTCAGAATTA GAAAACAGCT AGGAACTCAC ACGTCTGTTA TATTAACACT TACATACGAT AATTGAAATT   
  
  
- ATTAATGCTT TTAATTCGTT GATTAGTATA TCAGTACAAG AGTTTTTGTT CGTACGCATA ATAAGTCTAG   
  
  
- TAATTTACGT AATTTACCCT GATTAGGCTA GTCAGTTCGC GACTATGCTG ATAACTTATT TGTCCTGATT   
  
  
- GGATCTTACG CCTTAATTGA CAGCTCTTAA CAGGTGTAGC CATCACTACG AACCGCCGAT GGTCTCAGTG   
  
  
- GAATCCCACA AGTGTAGGAT CTGGGCTTTT GGACGATTAC TACCTTCCCG TCGGCACCCT CCTCCTCTCC   
  
  
- TCTCACACCT AAAAAGATAA ATGTTTTTTC ACATATTCAC GATTAGGGGG CTTTGGAGGA AAATATCCAA   
  
  
- TCCCCCCGTT GATCCCAATA GCGGGATCCA CTGCGAACAC CGGCAGCCGG ATTAATTTAT CCGGGCTGGT   
  
  
- TGTCGACACT GACCTCGCTA TCGACTTCGT ACGTCTTTAC TCCTATTGTA TGTATAATAT CTAAAGGTGT   
  
  
- AACGTATCCC GTGAGTCACC CAATGACATA GCGTTCGGTA TCGTCGTTCC GAACCACCCG GAGGATTTCA   
  
  
- CGCTGAATGT CCGTAACTAC TAGGACAAAG ATTCATATGA ACACTACGAT CGAACCTCCG ACCACCCTTT   
  
  
- GCTAATCGCA GAGATAAACT TTTCAAATTT TATGAACAGC TCAAGTTACG TAACGGGCAA ATACCTGGGT   
  
  
- TACAGTCCAC CCTTTACGAC CTACACTCCG GGTCCCTCCG AAACCAGCAA TTAACAGGTA ATGTCGAGGT   
  
  
- AATGTGAGGA CTGCTCTCAC AGCTACACTC CTTGGGATCC CTATCCGAAG AGTCTTACCA CTTTAACGAG   
  
  
- CCAGGATTCC ATCGGTGAAA CCAACTCGTT CTTAGTTTGT GGTTGTGATG TGGAAAGAAC TGGGCCAAGT   
  
  
- ATCTCTGGGA ACTGATGATG AGCTAATTGG TACAAACTCA GATATCTGCA CTGGTACGGC TCTCTGGCCT   
  
  
- TCCTCTCCTA GTTACAACTC GTCGTAACAA ATCGATTCCT GTAACACTTA TAGTATCGAA CGCTCCCGTT   
  
  
- CCTCTACCAC CTCGCAGTAC TCGAAAAACC CTTTACCGTC AGCTCCAAAT GGTACCATCC CAAGTCCGTC   
  
  
- ATGGGCAACT CGAGTATGTA TTTGAGACAC TATTCTTCGG ATGAGGCCAC AATGAGGTTC GTAATATGAG   
  
  
- ACTATCTCTT CCTATCACGG TACGAAGACC CAACCTCCCC GGCTTACGAC TAAAGTCGAA GCCGTAC

+     TGACG-motif

| Site Name | Organism | Position | Strand | Matrix score. | sequence | function |
| --- | --- | --- | --- | --- | --- | --- |
| TGACG-motif | Hordeum vulgare | 1834 | + | 5 | TGACG | cis-acting regulatory element involved in the MeJA-responsiveness |
| TGACG-motif | Hordeum vulgare | 1434 | + | 5 | TGACG | cis-acting regulatory element involved in the MeJA-responsiveness |
| TGACG-motif | Hordeum vulgare | 2118 | - | 5 | TGACG | cis-acting regulatory element involved in the MeJA-responsiveness |

>HU08G00229.1   
+ -Up\_Stream \_Len000AAATTT ATATGATTAT GGGAGCAAAT ATTAATCGGT CTAAAACAAA AAAGAATCGG   
  
  
+ CCGAACAAAA AAAAGGGCCC AGAACTAGTC CTTTTAATGG AGTCCCAAAA TGTGGTATAA ATTAGTCTTG   
  
  
+ TTCACTCATT TCTCACTCCT CTCACTCATT CTAACCCTAA ATCTCAATCC TCACTCTCTG TCCTCTCTAT   
  
  
+ CTGCGTCTCG AGCTTCTCTT TCCTCTGGGC TTCCTTGGAC TTTTTCGACG CCATGACAAT GGAACACTGA   
  
  
+ GCTCCACCGT TGCCCCTCTC CACAACTGTC TTCTAACGGT GGTTCTCCGT AGCCACCGTA CCTCAAAAAC   
  
  
+ CAAAGCGATG CACAACTACC ACCCCACCAC TGCTGAAAAG TGCCCACGAG TCTCGATCCG CGCAAGCCTG   
  
  
+ ATGAGAATCG ATACCACCAC CATGTAGATA AGCTGAAAAC GGGCATAAGG AAGCGACCCA CGCGCCCAAC   
  
  
+ ACGCCATTGA GGCAGTGACA CGTGGGTTGA TGCGCTTCCA AAGATGGAAT TTTCACATTT TTCTTTTCAA   
  
  
+ GGTTGCGTAG ATCGGTGTGT GTGGATGGCG ATCAAGGAAG AGTTAAAATG AACTAGTCAT GCGTCGAATT   
  
  
+ GTCCAAAAAA AATTTTCTCT TTCAGTCGAG TCTTAAAGGG GCAGTCATCA AGAGCATGTT TGAAAAACAT   
  
  
+ TATGGCCATA ACCACTTAGT AGTAATTCAA ATAATCATGG AAACATATTC AACATGAATT CAATATGACT   
  
  
+ AAAAATTCCA TCTTTTAGTT TGTTCTGGTC AACACAGAGC ATGCCTTTTT AATTGCATAA TAACGATATT   
  
  
+ TAACCAACCA ATCATGTTTC TCACATATTA ATATAGGCAA GGGTAGATTA ACAGTCATAA TCGCATAGTA   
  
  
+ TGCAAGAATT TATTTGCAAT CTGGGCATAC TGTTTTATAA AGCACAAGTG TTCATGACTT AAAATCGATA   
  
  
+ AAGTCTTAAT CTTTTGTCGA TCCTTGAGTG TGCAGACAAT ATAATTGTGA ATGTATGCTA TTAACTTTAA   
  
  
+ TAATTACGAA AATTAAGCAA CTAATCATAT AGTCATGTTC TCAAAAACAA GCATGCGTAT TATTCAGATC   
  
  
+ ATTAAATGCA TTAAATGGGA CTAATCCGAT CAGTCAAGCG CTGATACGAC TATTGAATAA ACAGGACTAA   
  
  
+ CCTAGAATGC GGAATTAACT GTCGAGAATT GTCCACATCG GTAGTGATGC TTGGCGGCTA CCAGAGTCAC   
  
  
+ CTTAGGGTGT TCACATCCTA GACCCGAAAA CCTGCTAATG ATGGAAGGGC AGCCGTGGGA GGAGGAGAGG   
  
  
+ AGAGTGTGGA TTTTTCTATT TACAAAAAAG TGTATAAGTG CTAATCCCCC GAAACCTCCT TTTATAGGTT   
  
  
+ AGGGGGGCAA CTAGGGTTAT CGCCCTAGGT GACGCTTGTG GCCGTCGGCC TAATTAAATA GGCCCGACCA   
  
  
+ ACAGCTGTGA CTGGAGCGAT AGCTGAAGCA TGCAGAAATG AGGATAACAT ACATATTATA GATTTCCACA   
  
  
+ TTGCATAGGG CACTCAGTGG GTTACTGTAT CGCAAGCCAT AGCAGCAAGG CTTGGTGGGC CTCCTAAAGT   
  
  
+ GCGACTTACA GGCATTGATG ATCCTGTTTC TAAGTATACT TGTGATGCTA GCTTGGAGGC TGGTGGGAAA   
  
  
+ CGATTAGCGT CTCTATTTGA AAAGTTTAAA ATACTTGTCG AGTTCAATGC ATTGCCCGTT TATGGACCCA   
  
  
+ ATGTCAGGTG GGAAATGCTG GATGTGAGGC CCAGGGAGGC TTTGGTCGTT AATTGTCCAT TACAGCTCCA   
  
  
+ TTACACTCCT GACGAGAGTG TCGATGTGAG GAACCCTAGG GATAGGCTTC TCAGAATGGT GAAATTGCTC   
  
  
+ GGTCCTAAGG TAGCCACTTT GGTTGAGCAA GAATCAAACA CCAACACTAC ACCTTTCTTG ACCCGGTTCA   
  
  
+ TAGAGACCCT TGACTACTAC TCGATTAACC ATGTTTGAGT CTATAGACGT GACCATGCCG AGAGACCGGA   
  
  
+ AGGAGAGGAT CAATGTTGAG CAGCATTGTT TAGCTAAGGA CATTGTGAAT ATCATAGCTT GCGAGGGCAA   
  
  
+ GGAGATGGTG GAGCGTCATG AGCTTTTTGG GAAATGGCAG TCGAGGTTTA CCATGGTAGG GTTCAGGCAG   
  
  
+ TACCCGTTGA GCTCATACAT AAACTCTGTG ATAAGAAGCC TACTCCGGTG TTACTCCAAG CATTATACTC   
  
  
+ TGATAGAGAA GGATAGTGCC ATGCTTCTGG GTTGGAGGGG CCGAATGCTG ATTTCAGCTT CGGCATG  

- -Up\_Stream \_Len000TTTAAA TATACTAATA CCCTCGTTTA TAATTAGCCA GATTTTGTTT TTTCTTAGCC   
  
  
- GGCTTGTTTT TTTTCCCGGG TCTTGATCAG GAAAATTACC TCAGGGTTTT ACACCATATT TAATCAGAAC   
  
  
- AAGTGAGTAA AGAGTGAGGA GAGTGAGTAA GATTGGGATT TAGAGTTAGG AGTGAGAGAC AGGAGAGATA   
  
  
- GACGCAGAGC TCGAAGAGAA AGGAGACCCG AAGGAACCTG AAAAAGCTGC GGTACTGTTA CCTTGTGACT   
  
  
- CGAGGTGGCA ACGGGGAGAG GTGTTGACAG AAGATTGCCA CCAAGAGGCA TCGGTGGCAT GGAGTTTTTG   
  
  
- GTTTCGCTAC GTGTTGATGG TGGGGTGGTG ACGACTTTTC ACGGGTGCTC AGAGCTAGGC GCGTTCGGAC   
  
  
- TACTCTTAGC TATGGTGGTG GTACATCTAT TCGACTTTTG CCCGTATTCC TTCGCTGGGT GCGCGGGTTG   
  
  
- TGCGGTAACT CCGTCACTGT GCACCCAACT ACGCGAAGGT TTCTACCTTA AAAGTGTAAA AAGAAAAGTT   
  
  
- CCAACGCATC TAGCCACACA CACCTACCGC TAGTTCCTTC TCAATTTTAC TTGATCAGTA CGCAGCTTAA   
  
  
- CAGGTTTTTT TTAAAAGAGA AAGTCAGCTC AGAATTTCCC CGTCAGTAGT TCTCGTACAA ACTTTTTGTA   
  
  
- ATACCGGTAT TGGTGAATCA TCATTAAGTT TATTAGTACC TTTGTATAAG TTGTACTTAA GTTATACTGA   
  
  
- TTTTTAAGGT AGAAAATCAA ACAAGACCAG TTGTGTCTCG TACGGAAAAA TTAACGTATT ATTGCTATAA   
  
  
- ATTGGTTGGT TAGTACAAAG AGTGTATAAT TATATCCGTT CCCATCTAAT TGTCAGTATT AGCGTATCAT   
  
  
- ACGTTCTTAA ATAAACGTTA GACCCGTATG ACAAAATATT TCGTGTTCAC AAGTACTGAA TTTTAGCTAT   
  
  
- TTCAGAATTA GAAAACAGCT AGGAACTCAC ACGTCTGTTA TATTAACACT TACATACGAT AATTGAAATT   
  
  
- ATTAATGCTT TTAATTCGTT GATTAGTATA TCAGTACAAG AGTTTTTGTT CGTACGCATA ATAAGTCTAG   
  
  
- TAATTTACGT AATTTACCCT GATTAGGCTA GTCAGTTCGC GACTATGCTG ATAACTTATT TGTCCTGATT   
  
  
- GGATCTTACG CCTTAATTGA CAGCTCTTAA CAGGTGTAGC CATCACTACG AACCGCCGAT GGTCTCAGTG   
  
  
- GAATCCCACA AGTGTAGGAT CTGGGCTTTT GGACGATTAC TACCTTCCCG TCGGCACCCT CCTCCTCTCC   
  
  
- TCTCACACCT AAAAAGATAA ATGTTTTTTC ACATATTCAC GATTAGGGGG CTTTGGAGGA AAATATCCAA   
  
  
- TCCCCCCGTT GATCCCAATA GCGGGATCCA CTGCGAACAC CGGCAGCCGG ATTAATTTAT CCGGGCTGGT   
  
  
- TGTCGACACT GACCTCGCTA TCGACTTCGT ACGTCTTTAC TCCTATTGTA TGTATAATAT CTAAAGGTGT   
  
  
- AACGTATCCC GTGAGTCACC CAATGACATA GCGTTCGGTA TCGTCGTTCC GAACCACCCG GAGGATTTCA   
  
  
- CGCTGAATGT CCGTAACTAC TAGGACAAAG ATTCATATGA ACACTACGAT CGAACCTCCG ACCACCCTTT   
  
  
- GCTAATCGCA GAGATAAACT TTTCAAATTT TATGAACAGC TCAAGTTACG TAACGGGCAA ATACCTGGGT   
  
  
- TACAGTCCAC CCTTTACGAC CTACACTCCG GGTCCCTCCG AAACCAGCAA TTAACAGGTA ATGTCGAGGT   
  
  
- AATGTGAGGA CTGCTCTCAC AGCTACACTC CTTGGGATCC CTATCCGAAG AGTCTTACCA CTTTAACGAG   
  
  
- CCAGGATTCC ATCGGTGAAA CCAACTCGTT CTTAGTTTGT GGTTGTGATG TGGAAAGAAC TGGGCCAAGT   
  
  
- ATCTCTGGGA ACTGATGATG AGCTAATTGG TACAAACTCA GATATCTGCA CTGGTACGGC TCTCTGGCCT   
  
  
- TCCTCTCCTA GTTACAACTC GTCGTAACAA ATCGATTCCT GTAACACTTA TAGTATCGAA CGCTCCCGTT   
  
  
- CCTCTACCAC CTCGCAGTAC TCGAAAAACC CTTTACCGTC AGCTCCAAAT GGTACCATCC CAAGTCCGTC   
  
  
- ATGGGCAACT CGAGTATGTA TTTGAGACAC TATTCTTCGG ATGAGGCCAC AATGAGGTTC GTAATATGAG   
  
  
- ACTATCTCTT CCTATCACGG TACGAAGACC CAACCTCCCC GGCTTACGAC TAAAGTCGAA GCCGTAC

+     Unnamed\_\_1

| Site Name | Organism | Position | Strand | Matrix score. | sequence | function |
| --- | --- | --- | --- | --- | --- | --- |
| Unnamed\_\_1 | Zea mays | 398 | - | 5 | CGTGG |  |
| Unnamed\_\_1 | Zea mays | 482 | - | 5 | CGTGG |  |
| Unnamed\_\_1 | Zea mays | 515 | + | 5 | CGTGG |  |
| Unnamed\_\_1 | Zea mays | 1318 | + | 5 | CGTGG |  |

>HU08G00229.1   
+ -Up\_Stream \_Len000AAATTT ATATGATTAT GGGAGCAAAT ATTAATCGGT CTAAAACAAA AAAGAATCGG   
  
  
+ CCGAACAAAA AAAAGGGCCC AGAACTAGTC CTTTTAATGG AGTCCCAAAA TGTGGTATAA ATTAGTCTTG   
  
  
+ TTCACTCATT TCTCACTCCT CTCACTCATT CTAACCCTAA ATCTCAATCC TCACTCTCTG TCCTCTCTAT   
  
  
+ CTGCGTCTCG AGCTTCTCTT TCCTCTGGGC TTCCTTGGAC TTTTTCGACG CCATGACAAT GGAACACTGA   
  
  
+ GCTCCACCGT TGCCCCTCTC CACAACTGTC TTCTAACGGT GGTTCTCCGT AGCCACCGTA CCTCAAAAAC   
  
  
+ CAAAGCGATG CACAACTACC ACCCCACCAC TGCTGAAAAG TGCCCACGAG TCTCGATCCG CGCAAGCCTG   
  
  
+ ATGAGAATCG ATACCACCAC CATGTAGATA AGCTGAAAAC GGGCATAAGG AAGCGACCCA CGCGCCCAAC   
  
  
+ ACGCCATTGA GGCAGTGACA CGTGGGTTGA TGCGCTTCCA AAGATGGAAT TTTCACATTT TTCTTTTCAA   
  
  
+ GGTTGCGTAG ATCGGTGTGT GTGGATGGCG ATCAAGGAAG AGTTAAAATG AACTAGTCAT GCGTCGAATT   
  
  
+ GTCCAAAAAA AATTTTCTCT TTCAGTCGAG TCTTAAAGGG GCAGTCATCA AGAGCATGTT TGAAAAACAT   
  
  
+ TATGGCCATA ACCACTTAGT AGTAATTCAA ATAATCATGG AAACATATTC AACATGAATT CAATATGACT   
  
  
+ AAAAATTCCA TCTTTTAGTT TGTTCTGGTC AACACAGAGC ATGCCTTTTT AATTGCATAA TAACGATATT   
  
  
+ TAACCAACCA ATCATGTTTC TCACATATTA ATATAGGCAA GGGTAGATTA ACAGTCATAA TCGCATAGTA   
  
  
+ TGCAAGAATT TATTTGCAAT CTGGGCATAC TGTTTTATAA AGCACAAGTG TTCATGACTT AAAATCGATA   
  
  
+ AAGTCTTAAT CTTTTGTCGA TCCTTGAGTG TGCAGACAAT ATAATTGTGA ATGTATGCTA TTAACTTTAA   
  
  
+ TAATTACGAA AATTAAGCAA CTAATCATAT AGTCATGTTC TCAAAAACAA GCATGCGTAT TATTCAGATC   
  
  
+ ATTAAATGCA TTAAATGGGA CTAATCCGAT CAGTCAAGCG CTGATACGAC TATTGAATAA ACAGGACTAA   
  
  
+ CCTAGAATGC GGAATTAACT GTCGAGAATT GTCCACATCG GTAGTGATGC TTGGCGGCTA CCAGAGTCAC   
  
  
+ CTTAGGGTGT TCACATCCTA GACCCGAAAA CCTGCTAATG ATGGAAGGGC AGCCGTGGGA GGAGGAGAGG   
  
  
+ AGAGTGTGGA TTTTTCTATT TACAAAAAAG TGTATAAGTG CTAATCCCCC GAAACCTCCT TTTATAGGTT   
  
  
+ AGGGGGGCAA CTAGGGTTAT CGCCCTAGGT GACGCTTGTG GCCGTCGGCC TAATTAAATA GGCCCGACCA   
  
  
+ ACAGCTGTGA CTGGAGCGAT AGCTGAAGCA TGCAGAAATG AGGATAACAT ACATATTATA GATTTCCACA   
  
  
+ TTGCATAGGG CACTCAGTGG GTTACTGTAT CGCAAGCCAT AGCAGCAAGG CTTGGTGGGC CTCCTAAAGT   
  
  
+ GCGACTTACA GGCATTGATG ATCCTGTTTC TAAGTATACT TGTGATGCTA GCTTGGAGGC TGGTGGGAAA   
  
  
+ CGATTAGCGT CTCTATTTGA AAAGTTTAAA ATACTTGTCG AGTTCAATGC ATTGCCCGTT TATGGACCCA   
  
  
+ ATGTCAGGTG GGAAATGCTG GATGTGAGGC CCAGGGAGGC TTTGGTCGTT AATTGTCCAT TACAGCTCCA   
  
  
+ TTACACTCCT GACGAGAGTG TCGATGTGAG GAACCCTAGG GATAGGCTTC TCAGAATGGT GAAATTGCTC   
  
  
+ GGTCCTAAGG TAGCCACTTT GGTTGAGCAA GAATCAAACA CCAACACTAC ACCTTTCTTG ACCCGGTTCA   
  
  
+ TAGAGACCCT TGACTACTAC TCGATTAACC ATGTTTGAGT CTATAGACGT GACCATGCCG AGAGACCGGA   
  
  
+ AGGAGAGGAT CAATGTTGAG CAGCATTGTT TAGCTAAGGA CATTGTGAAT ATCATAGCTT GCGAGGGCAA   
  
  
+ GGAGATGGTG GAGCGTCATG AGCTTTTTGG GAAATGGCAG TCGAGGTTTA CCATGGTAGG GTTCAGGCAG   
  
  
+ TACCCGTTGA GCTCATACAT AAACTCTGTG ATAAGAAGCC TACTCCGGTG TTACTCCAAG CATTATACTC   
  
  
+ TGATAGAGAA GGATAGTGCC ATGCTTCTGG GTTGGAGGGG CCGAATGCTG ATTTCAGCTT CGGCATG  

- -Up\_Stream \_Len000TTTAAA TATACTAATA CCCTCGTTTA TAATTAGCCA GATTTTGTTT TTTCTTAGCC   
  
  
- GGCTTGTTTT TTTTCCCGGG TCTTGATCAG GAAAATTACC TCAGGGTTTT ACACCATATT TAATCAGAAC   
  
  
- AAGTGAGTAA AGAGTGAGGA GAGTGAGTAA GATTGGGATT TAGAGTTAGG AGTGAGAGAC AGGAGAGATA   
  
  
- GACGCAGAGC TCGAAGAGAA AGGAGACCCG AAGGAACCTG AAAAAGCTGC GGTACTGTTA CCTTGTGACT   
  
  
- CGAGGTGGCA ACGGGGAGAG GTGTTGACAG AAGATTGCCA CCAAGAGGCA TCGGTGGCAT GGAGTTTTTG   
  
  
- GTTTCGCTAC GTGTTGATGG TGGGGTGGTG ACGACTTTTC ACGGGTGCTC AGAGCTAGGC GCGTTCGGAC   
  
  
- TACTCTTAGC TATGGTGGTG GTACATCTAT TCGACTTTTG CCCGTATTCC TTCGCTGGGT GCGCGGGTTG   
  
  
- TGCGGTAACT CCGTCACTGT GCACCCAACT ACGCGAAGGT TTCTACCTTA AAAGTGTAAA AAGAAAAGTT   
  
  
- CCAACGCATC TAGCCACACA CACCTACCGC TAGTTCCTTC TCAATTTTAC TTGATCAGTA CGCAGCTTAA   
  
  
- CAGGTTTTTT TTAAAAGAGA AAGTCAGCTC AGAATTTCCC CGTCAGTAGT TCTCGTACAA ACTTTTTGTA   
  
  
- ATACCGGTAT TGGTGAATCA TCATTAAGTT TATTAGTACC TTTGTATAAG TTGTACTTAA GTTATACTGA   
  
  
- TTTTTAAGGT AGAAAATCAA ACAAGACCAG TTGTGTCTCG TACGGAAAAA TTAACGTATT ATTGCTATAA   
  
  
- ATTGGTTGGT TAGTACAAAG AGTGTATAAT TATATCCGTT CCCATCTAAT TGTCAGTATT AGCGTATCAT   
  
  
- ACGTTCTTAA ATAAACGTTA GACCCGTATG ACAAAATATT TCGTGTTCAC AAGTACTGAA TTTTAGCTAT   
  
  
- TTCAGAATTA GAAAACAGCT AGGAACTCAC ACGTCTGTTA TATTAACACT TACATACGAT AATTGAAATT   
  
  
- ATTAATGCTT TTAATTCGTT GATTAGTATA TCAGTACAAG AGTTTTTGTT CGTACGCATA ATAAGTCTAG   
  
  
- TAATTTACGT AATTTACCCT GATTAGGCTA GTCAGTTCGC GACTATGCTG ATAACTTATT TGTCCTGATT   
  
  
- GGATCTTACG CCTTAATTGA CAGCTCTTAA CAGGTGTAGC CATCACTACG AACCGCCGAT GGTCTCAGTG   
  
  
- GAATCCCACA AGTGTAGGAT CTGGGCTTTT GGACGATTAC TACCTTCCCG TCGGCACCCT CCTCCTCTCC   
  
  
- TCTCACACCT AAAAAGATAA ATGTTTTTTC ACATATTCAC GATTAGGGGG CTTTGGAGGA AAATATCCAA   
  
  
- TCCCCCCGTT GATCCCAATA GCGGGATCCA CTGCGAACAC CGGCAGCCGG ATTAATTTAT CCGGGCTGGT   
  
  
- TGTCGACACT GACCTCGCTA TCGACTTCGT ACGTCTTTAC TCCTATTGTA TGTATAATAT CTAAAGGTGT   
  
  
- AACGTATCCC GTGAGTCACC CAATGACATA GCGTTCGGTA TCGTCGTTCC GAACCACCCG GAGGATTTCA   
  
  
- CGCTGAATGT CCGTAACTAC TAGGACAAAG ATTCATATGA ACACTACGAT CGAACCTCCG ACCACCCTTT   
  
  
- GCTAATCGCA GAGATAAACT TTTCAAATTT TATGAACAGC TCAAGTTACG TAACGGGCAA ATACCTGGGT   
  
  
- TACAGTCCAC CCTTTACGAC CTACACTCCG GGTCCCTCCG AAACCAGCAA TTAACAGGTA ATGTCGAGGT   
  
  
- AATGTGAGGA CTGCTCTCAC AGCTACACTC CTTGGGATCC CTATCCGAAG AGTCTTACCA CTTTAACGAG   
  
  
- CCAGGATTCC ATCGGTGAAA CCAACTCGTT CTTAGTTTGT GGTTGTGATG TGGAAAGAAC TGGGCCAAGT   
  
  
- ATCTCTGGGA ACTGATGATG AGCTAATTGG TACAAACTCA GATATCTGCA CTGGTACGGC TCTCTGGCCT   
  
  
- TCCTCTCCTA GTTACAACTC GTCGTAACAA ATCGATTCCT GTAACACTTA TAGTATCGAA CGCTCCCGTT   
  
  
- CCTCTACCAC CTCGCAGTAC TCGAAAAACC CTTTACCGTC AGCTCCAAAT GGTACCATCC CAAGTCCGTC   
  
  
- ATGGGCAACT CGAGTATGTA TTTGAGACAC TATTCTTCGG ATGAGGCCAC AATGAGGTTC GTAATATGAG   
  
  
- ACTATCTCTT CCTATCACGG TACGAAGACC CAACCTCCCC GGCTTACGAC TAAAGTCGAA GCCGTAC

+     Unnamed\_\_16

| Site Name | Organism | Position | Strand | Matrix score. | sequence | function |
| --- | --- | --- | --- | --- | --- | --- |
| Unnamed\_\_16 | Zea mays | 1308 | - | 9 | GCTGCCCGTC |  |

>HU08G00229.1   
+ -Up\_Stream \_Len000AAATTT ATATGATTAT GGGAGCAAAT ATTAATCGGT CTAAAACAAA AAAGAATCGG   
  
  
+ CCGAACAAAA AAAAGGGCCC AGAACTAGTC CTTTTAATGG AGTCCCAAAA TGTGGTATAA ATTAGTCTTG   
  
  
+ TTCACTCATT TCTCACTCCT CTCACTCATT CTAACCCTAA ATCTCAATCC TCACTCTCTG TCCTCTCTAT   
  
  
+ CTGCGTCTCG AGCTTCTCTT TCCTCTGGGC TTCCTTGGAC TTTTTCGACG CCATGACAAT GGAACACTGA   
  
  
+ GCTCCACCGT TGCCCCTCTC CACAACTGTC TTCTAACGGT GGTTCTCCGT AGCCACCGTA CCTCAAAAAC   
  
  
+ CAAAGCGATG CACAACTACC ACCCCACCAC TGCTGAAAAG TGCCCACGAG TCTCGATCCG CGCAAGCCTG   
  
  
+ ATGAGAATCG ATACCACCAC CATGTAGATA AGCTGAAAAC GGGCATAAGG AAGCGACCCA CGCGCCCAAC   
  
  
+ ACGCCATTGA GGCAGTGACA CGTGGGTTGA TGCGCTTCCA AAGATGGAAT TTTCACATTT TTCTTTTCAA   
  
  
+ GGTTGCGTAG ATCGGTGTGT GTGGATGGCG ATCAAGGAAG AGTTAAAATG AACTAGTCAT GCGTCGAATT   
  
  
+ GTCCAAAAAA AATTTTCTCT TTCAGTCGAG TCTTAAAGGG GCAGTCATCA AGAGCATGTT TGAAAAACAT   
  
  
+ TATGGCCATA ACCACTTAGT AGTAATTCAA ATAATCATGG AAACATATTC AACATGAATT CAATATGACT   
  
  
+ AAAAATTCCA TCTTTTAGTT TGTTCTGGTC AACACAGAGC ATGCCTTTTT AATTGCATAA TAACGATATT   
  
  
+ TAACCAACCA ATCATGTTTC TCACATATTA ATATAGGCAA GGGTAGATTA ACAGTCATAA TCGCATAGTA   
  
  
+ TGCAAGAATT TATTTGCAAT CTGGGCATAC TGTTTTATAA AGCACAAGTG TTCATGACTT AAAATCGATA   
  
  
+ AAGTCTTAAT CTTTTGTCGA TCCTTGAGTG TGCAGACAAT ATAATTGTGA ATGTATGCTA TTAACTTTAA   
  
  
+ TAATTACGAA AATTAAGCAA CTAATCATAT AGTCATGTTC TCAAAAACAA GCATGCGTAT TATTCAGATC   
  
  
+ ATTAAATGCA TTAAATGGGA CTAATCCGAT CAGTCAAGCG CTGATACGAC TATTGAATAA ACAGGACTAA   
  
  
+ CCTAGAATGC GGAATTAACT GTCGAGAATT GTCCACATCG GTAGTGATGC TTGGCGGCTA CCAGAGTCAC   
  
  
+ CTTAGGGTGT TCACATCCTA GACCCGAAAA CCTGCTAATG ATGGAAGGGC AGCCGTGGGA GGAGGAGAGG   
  
  
+ AGAGTGTGGA TTTTTCTATT TACAAAAAAG TGTATAAGTG CTAATCCCCC GAAACCTCCT TTTATAGGTT   
  
  
+ AGGGGGGCAA CTAGGGTTAT CGCCCTAGGT GACGCTTGTG GCCGTCGGCC TAATTAAATA GGCCCGACCA   
  
  
+ ACAGCTGTGA CTGGAGCGAT AGCTGAAGCA TGCAGAAATG AGGATAACAT ACATATTATA GATTTCCACA   
  
  
+ TTGCATAGGG CACTCAGTGG GTTACTGTAT CGCAAGCCAT AGCAGCAAGG CTTGGTGGGC CTCCTAAAGT   
  
  
+ GCGACTTACA GGCATTGATG ATCCTGTTTC TAAGTATACT TGTGATGCTA GCTTGGAGGC TGGTGGGAAA   
  
  
+ CGATTAGCGT CTCTATTTGA AAAGTTTAAA ATACTTGTCG AGTTCAATGC ATTGCCCGTT TATGGACCCA   
  
  
+ ATGTCAGGTG GGAAATGCTG GATGTGAGGC CCAGGGAGGC TTTGGTCGTT AATTGTCCAT TACAGCTCCA   
  
  
+ TTACACTCCT GACGAGAGTG TCGATGTGAG GAACCCTAGG GATAGGCTTC TCAGAATGGT GAAATTGCTC   
  
  
+ GGTCCTAAGG TAGCCACTTT GGTTGAGCAA GAATCAAACA CCAACACTAC ACCTTTCTTG ACCCGGTTCA   
  
  
+ TAGAGACCCT TGACTACTAC TCGATTAACC ATGTTTGAGT CTATAGACGT GACCATGCCG AGAGACCGGA   
  
  
+ AGGAGAGGAT CAATGTTGAG CAGCATTGTT TAGCTAAGGA CATTGTGAAT ATCATAGCTT GCGAGGGCAA   
  
  
+ GGAGATGGTG GAGCGTCATG AGCTTTTTGG GAAATGGCAG TCGAGGTTTA CCATGGTAGG GTTCAGGCAG   
  
  
+ TACCCGTTGA GCTCATACAT AAACTCTGTG ATAAGAAGCC TACTCCGGTG TTACTCCAAG CATTATACTC   
  
  
+ TGATAGAGAA GGATAGTGCC ATGCTTCTGG GTTGGAGGGG CCGAATGCTG ATTTCAGCTT CGGCATG  

- -Up\_Stream \_Len000TTTAAA TATACTAATA CCCTCGTTTA TAATTAGCCA GATTTTGTTT TTTCTTAGCC   
  
  
- GGCTTGTTTT TTTTCCCGGG TCTTGATCAG GAAAATTACC TCAGGGTTTT ACACCATATT TAATCAGAAC   
  
  
- AAGTGAGTAA AGAGTGAGGA GAGTGAGTAA GATTGGGATT TAGAGTTAGG AGTGAGAGAC AGGAGAGATA   
  
  
- GACGCAGAGC TCGAAGAGAA AGGAGACCCG AAGGAACCTG AAAAAGCTGC GGTACTGTTA CCTTGTGACT   
  
  
- CGAGGTGGCA ACGGGGAGAG GTGTTGACAG AAGATTGCCA CCAAGAGGCA TCGGTGGCAT GGAGTTTTTG   
  
  
- GTTTCGCTAC GTGTTGATGG TGGGGTGGTG ACGACTTTTC ACGGGTGCTC AGAGCTAGGC GCGTTCGGAC   
  
  
- TACTCTTAGC TATGGTGGTG GTACATCTAT TCGACTTTTG CCCGTATTCC TTCGCTGGGT GCGCGGGTTG   
  
  
- TGCGGTAACT CCGTCACTGT GCACCCAACT ACGCGAAGGT TTCTACCTTA AAAGTGTAAA AAGAAAAGTT   
  
  
- CCAACGCATC TAGCCACACA CACCTACCGC TAGTTCCTTC TCAATTTTAC TTGATCAGTA CGCAGCTTAA   
  
  
- CAGGTTTTTT TTAAAAGAGA AAGTCAGCTC AGAATTTCCC CGTCAGTAGT TCTCGTACAA ACTTTTTGTA   
  
  
- ATACCGGTAT TGGTGAATCA TCATTAAGTT TATTAGTACC TTTGTATAAG TTGTACTTAA GTTATACTGA   
  
  
- TTTTTAAGGT AGAAAATCAA ACAAGACCAG TTGTGTCTCG TACGGAAAAA TTAACGTATT ATTGCTATAA   
  
  
- ATTGGTTGGT TAGTACAAAG AGTGTATAAT TATATCCGTT CCCATCTAAT TGTCAGTATT AGCGTATCAT   
  
  
- ACGTTCTTAA ATAAACGTTA GACCCGTATG ACAAAATATT TCGTGTTCAC AAGTACTGAA TTTTAGCTAT   
  
  
- TTCAGAATTA GAAAACAGCT AGGAACTCAC ACGTCTGTTA TATTAACACT TACATACGAT AATTGAAATT   
  
  
- ATTAATGCTT TTAATTCGTT GATTAGTATA TCAGTACAAG AGTTTTTGTT CGTACGCATA ATAAGTCTAG   
  
  
- TAATTTACGT AATTTACCCT GATTAGGCTA GTCAGTTCGC GACTATGCTG ATAACTTATT TGTCCTGATT   
  
  
- GGATCTTACG CCTTAATTGA CAGCTCTTAA CAGGTGTAGC CATCACTACG AACCGCCGAT GGTCTCAGTG   
  
  
- GAATCCCACA AGTGTAGGAT CTGGGCTTTT GGACGATTAC TACCTTCCCG TCGGCACCCT CCTCCTCTCC   
  
  
- TCTCACACCT AAAAAGATAA ATGTTTTTTC ACATATTCAC GATTAGGGGG CTTTGGAGGA AAATATCCAA   
  
  
- TCCCCCCGTT GATCCCAATA GCGGGATCCA CTGCGAACAC CGGCAGCCGG ATTAATTTAT CCGGGCTGGT   
  
  
- TGTCGACACT GACCTCGCTA TCGACTTCGT ACGTCTTTAC TCCTATTGTA TGTATAATAT CTAAAGGTGT   
  
  
- AACGTATCCC GTGAGTCACC CAATGACATA GCGTTCGGTA TCGTCGTTCC GAACCACCCG GAGGATTTCA   
  
  
- CGCTGAATGT CCGTAACTAC TAGGACAAAG ATTCATATGA ACACTACGAT CGAACCTCCG ACCACCCTTT   
  
  
- GCTAATCGCA GAGATAAACT TTTCAAATTT TATGAACAGC TCAAGTTACG TAACGGGCAA ATACCTGGGT   
  
  
- TACAGTCCAC CCTTTACGAC CTACACTCCG GGTCCCTCCG AAACCAGCAA TTAACAGGTA ATGTCGAGGT   
  
  
- AATGTGAGGA CTGCTCTCAC AGCTACACTC CTTGGGATCC CTATCCGAAG AGTCTTACCA CTTTAACGAG   
  
  
- CCAGGATTCC ATCGGTGAAA CCAACTCGTT CTTAGTTTGT GGTTGTGATG TGGAAAGAAC TGGGCCAAGT   
  
  
- ATCTCTGGGA ACTGATGATG AGCTAATTGG TACAAACTCA GATATCTGCA CTGGTACGGC TCTCTGGCCT   
  
  
- TCCTCTCCTA GTTACAACTC GTCGTAACAA ATCGATTCCT GTAACACTTA TAGTATCGAA CGCTCCCGTT   
  
  
- CCTCTACCAC CTCGCAGTAC TCGAAAAACC CTTTACCGTC AGCTCCAAAT GGTACCATCC CAAGTCCGTC   
  
  
- ATGGGCAACT CGAGTATGTA TTTGAGACAC TATTCTTCGG ATGAGGCCAC AATGAGGTTC GTAATATGAG   
  
  
- ACTATCTCTT CCTATCACGG TACGAAGACC CAACCTCCCC GGCTTACGAC TAAAGTCGAA GCCGTAC

+     Unnamed\_\_4

| Site Name | Organism | Position | Strand | Matrix score. | sequence | function |
| --- | --- | --- | --- | --- | --- | --- |
| Unnamed\_\_4 | Petroselinum hortense | 2114 | - | 4 | CTCC |  |
| Unnamed\_\_4 | Petroselinum hortense | 2105 | - | 4 | CTCC |  |
| Unnamed\_\_4 | Petroselinum hortense | 329 | + | 4 | CTCC |  |
| Unnamed\_\_4 | Petroselinum hortense | 302 | + | 4 | CTCC |  |
| Unnamed\_\_4 | Petroselinum hortense | 286 | + | 4 | CTCC |  |
| Unnamed\_\_4 | Petroselinum hortense | 160 | + | 4 | CTCC |  |
| Unnamed\_\_4 | Petroselinum hortense | 1487 | - | 4 | CTCC |  |
| Unnamed\_\_4 | Petroselinum hortense | 1322 | - | 4 | CTCC |  |
| Unnamed\_\_4 | Petroselinum hortense | 1390 | + | 4 | CTCC |  |
| Unnamed\_\_4 | Petroselinum hortense | 36 | - | 4 | CTCC |  |
| Unnamed\_\_4 | Petroselinum hortense | 113 | - | 4 | CTCC |  |
| Unnamed\_\_4 | Petroselinum hortense | 2278 | - | 4 | CTCC |  |
| Unnamed\_\_4 | Petroselinum hortense | 1328 | - | 4 | CTCC |  |
| Unnamed\_\_4 | Petroselinum hortense | 2036 | - | 4 | CTCC |  |
| Unnamed\_\_4 | Petroselinum hortense | 1325 | - | 4 | CTCC |  |
| Unnamed\_\_4 | Petroselinum hortense | 1333 | - | 4 | CTCC |  |
| Unnamed\_\_4 | Petroselinum hortense | 2217 | + | 4 | CTCC |  |
| Unnamed\_\_4 | Petroselinum hortense | 1669 | - | 4 | CTCC |  |
| Unnamed\_\_4 | Petroselinum hortense | 1605 | + | 4 | CTCC |  |
| Unnamed\_\_4 | Petroselinum hortense | 2228 | + | 4 | CTCC |  |
| Unnamed\_\_4 | Petroselinum hortense | 1830 | + | 4 | CTCC |  |
| Unnamed\_\_4 | Petroselinum hortense | 1789 | - | 4 | CTCC |  |
| Unnamed\_\_4 | Petroselinum hortense | 1820 | + | 4 | CTCC |  |

>HU08G00229.1   
+ -Up\_Stream \_Len000AAATTT ATATGATTAT GGGAGCAAAT ATTAATCGGT CTAAAACAAA AAAGAATCGG   
  
  
+ CCGAACAAAA AAAAGGGCCC AGAACTAGTC CTTTTAATGG AGTCCCAAAA TGTGGTATAA ATTAGTCTTG   
  
  
+ TTCACTCATT TCTCACTCCT CTCACTCATT CTAACCCTAA ATCTCAATCC TCACTCTCTG TCCTCTCTAT   
  
  
+ CTGCGTCTCG AGCTTCTCTT TCCTCTGGGC TTCCTTGGAC TTTTTCGACG CCATGACAAT GGAACACTGA   
  
  
+ GCTCCACCGT TGCCCCTCTC CACAACTGTC TTCTAACGGT GGTTCTCCGT AGCCACCGTA CCTCAAAAAC   
  
  
+ CAAAGCGATG CACAACTACC ACCCCACCAC TGCTGAAAAG TGCCCACGAG TCTCGATCCG CGCAAGCCTG   
  
  
+ ATGAGAATCG ATACCACCAC CATGTAGATA AGCTGAAAAC GGGCATAAGG AAGCGACCCA CGCGCCCAAC   
  
  
+ ACGCCATTGA GGCAGTGACA CGTGGGTTGA TGCGCTTCCA AAGATGGAAT TTTCACATTT TTCTTTTCAA   
  
  
+ GGTTGCGTAG ATCGGTGTGT GTGGATGGCG ATCAAGGAAG AGTTAAAATG AACTAGTCAT GCGTCGAATT   
  
  
+ GTCCAAAAAA AATTTTCTCT TTCAGTCGAG TCTTAAAGGG GCAGTCATCA AGAGCATGTT TGAAAAACAT   
  
  
+ TATGGCCATA ACCACTTAGT AGTAATTCAA ATAATCATGG AAACATATTC AACATGAATT CAATATGACT   
  
  
+ AAAAATTCCA TCTTTTAGTT TGTTCTGGTC AACACAGAGC ATGCCTTTTT AATTGCATAA TAACGATATT   
  
  
+ TAACCAACCA ATCATGTTTC TCACATATTA ATATAGGCAA GGGTAGATTA ACAGTCATAA TCGCATAGTA   
  
  
+ TGCAAGAATT TATTTGCAAT CTGGGCATAC TGTTTTATAA AGCACAAGTG TTCATGACTT AAAATCGATA   
  
  
+ AAGTCTTAAT CTTTTGTCGA TCCTTGAGTG TGCAGACAAT ATAATTGTGA ATGTATGCTA TTAACTTTAA   
  
  
+ TAATTACGAA AATTAAGCAA CTAATCATAT AGTCATGTTC TCAAAAACAA GCATGCGTAT TATTCAGATC   
  
  
+ ATTAAATGCA TTAAATGGGA CTAATCCGAT CAGTCAAGCG CTGATACGAC TATTGAATAA ACAGGACTAA   
  
  
+ CCTAGAATGC GGAATTAACT GTCGAGAATT GTCCACATCG GTAGTGATGC TTGGCGGCTA CCAGAGTCAC   
  
  
+ CTTAGGGTGT TCACATCCTA GACCCGAAAA CCTGCTAATG ATGGAAGGGC AGCCGTGGGA GGAGGAGAGG   
  
  
+ AGAGTGTGGA TTTTTCTATT TACAAAAAAG TGTATAAGTG CTAATCCCCC GAAACCTCCT TTTATAGGTT   
  
  
+ AGGGGGGCAA CTAGGGTTAT CGCCCTAGGT GACGCTTGTG GCCGTCGGCC TAATTAAATA GGCCCGACCA   
  
  
+ ACAGCTGTGA CTGGAGCGAT AGCTGAAGCA TGCAGAAATG AGGATAACAT ACATATTATA GATTTCCACA   
  
  
+ TTGCATAGGG CACTCAGTGG GTTACTGTAT CGCAAGCCAT AGCAGCAAGG CTTGGTGGGC CTCCTAAAGT   
  
  
+ GCGACTTACA GGCATTGATG ATCCTGTTTC TAAGTATACT TGTGATGCTA GCTTGGAGGC TGGTGGGAAA   
  
  
+ CGATTAGCGT CTCTATTTGA AAAGTTTAAA ATACTTGTCG AGTTCAATGC ATTGCCCGTT TATGGACCCA   
  
  
+ ATGTCAGGTG GGAAATGCTG GATGTGAGGC CCAGGGAGGC TTTGGTCGTT AATTGTCCAT TACAGCTCCA   
  
  
+ TTACACTCCT GACGAGAGTG TCGATGTGAG GAACCCTAGG GATAGGCTTC TCAGAATGGT GAAATTGCTC   
  
  
+ GGTCCTAAGG TAGCCACTTT GGTTGAGCAA GAATCAAACA CCAACACTAC ACCTTTCTTG ACCCGGTTCA   
  
  
+ TAGAGACCCT TGACTACTAC TCGATTAACC ATGTTTGAGT CTATAGACGT GACCATGCCG AGAGACCGGA   
  
  
+ AGGAGAGGAT CAATGTTGAG CAGCATTGTT TAGCTAAGGA CATTGTGAAT ATCATAGCTT GCGAGGGCAA   
  
  
+ GGAGATGGTG GAGCGTCATG AGCTTTTTGG GAAATGGCAG TCGAGGTTTA CCATGGTAGG GTTCAGGCAG   
  
  
+ TACCCGTTGA GCTCATACAT AAACTCTGTG ATAAGAAGCC TACTCCGGTG TTACTCCAAG CATTATACTC   
  
  
+ TGATAGAGAA GGATAGTGCC ATGCTTCTGG GTTGGAGGGG CCGAATGCTG ATTTCAGCTT CGGCATG  

- -Up\_Stream \_Len000TTTAAA TATACTAATA CCCTCGTTTA TAATTAGCCA GATTTTGTTT TTTCTTAGCC   
  
  
- GGCTTGTTTT TTTTCCCGGG TCTTGATCAG GAAAATTACC TCAGGGTTTT ACACCATATT TAATCAGAAC   
  
  
- AAGTGAGTAA AGAGTGAGGA GAGTGAGTAA GATTGGGATT TAGAGTTAGG AGTGAGAGAC AGGAGAGATA   
  
  
- GACGCAGAGC TCGAAGAGAA AGGAGACCCG AAGGAACCTG AAAAAGCTGC GGTACTGTTA CCTTGTGACT   
  
  
- CGAGGTGGCA ACGGGGAGAG GTGTTGACAG AAGATTGCCA CCAAGAGGCA TCGGTGGCAT GGAGTTTTTG   
  
  
- GTTTCGCTAC GTGTTGATGG TGGGGTGGTG ACGACTTTTC ACGGGTGCTC AGAGCTAGGC GCGTTCGGAC   
  
  
- TACTCTTAGC TATGGTGGTG GTACATCTAT TCGACTTTTG CCCGTATTCC TTCGCTGGGT GCGCGGGTTG   
  
  
- TGCGGTAACT CCGTCACTGT GCACCCAACT ACGCGAAGGT TTCTACCTTA AAAGTGTAAA AAGAAAAGTT   
  
  
- CCAACGCATC TAGCCACACA CACCTACCGC TAGTTCCTTC TCAATTTTAC TTGATCAGTA CGCAGCTTAA   
  
  
- CAGGTTTTTT TTAAAAGAGA AAGTCAGCTC AGAATTTCCC CGTCAGTAGT TCTCGTACAA ACTTTTTGTA   
  
  
- ATACCGGTAT TGGTGAATCA TCATTAAGTT TATTAGTACC TTTGTATAAG TTGTACTTAA GTTATACTGA   
  
  
- TTTTTAAGGT AGAAAATCAA ACAAGACCAG TTGTGTCTCG TACGGAAAAA TTAACGTATT ATTGCTATAA   
  
  
- ATTGGTTGGT TAGTACAAAG AGTGTATAAT TATATCCGTT CCCATCTAAT TGTCAGTATT AGCGTATCAT   
  
  
- ACGTTCTTAA ATAAACGTTA GACCCGTATG ACAAAATATT TCGTGTTCAC AAGTACTGAA TTTTAGCTAT   
  
  
- TTCAGAATTA GAAAACAGCT AGGAACTCAC ACGTCTGTTA TATTAACACT TACATACGAT AATTGAAATT   
  
  
- ATTAATGCTT TTAATTCGTT GATTAGTATA TCAGTACAAG AGTTTTTGTT CGTACGCATA ATAAGTCTAG   
  
  
- TAATTTACGT AATTTACCCT GATTAGGCTA GTCAGTTCGC GACTATGCTG ATAACTTATT TGTCCTGATT   
  
  
- GGATCTTACG CCTTAATTGA CAGCTCTTAA CAGGTGTAGC CATCACTACG AACCGCCGAT GGTCTCAGTG   
  
  
- GAATCCCACA AGTGTAGGAT CTGGGCTTTT GGACGATTAC TACCTTCCCG TCGGCACCCT CCTCCTCTCC   
  
  
- TCTCACACCT AAAAAGATAA ATGTTTTTTC ACATATTCAC GATTAGGGGG CTTTGGAGGA AAATATCCAA   
  
  
- TCCCCCCGTT GATCCCAATA GCGGGATCCA CTGCGAACAC CGGCAGCCGG ATTAATTTAT CCGGGCTGGT   
  
  
- TGTCGACACT GACCTCGCTA TCGACTTCGT ACGTCTTTAC TCCTATTGTA TGTATAATAT CTAAAGGTGT   
  
  
- AACGTATCCC GTGAGTCACC CAATGACATA GCGTTCGGTA TCGTCGTTCC GAACCACCCG GAGGATTTCA   
  
  
- CGCTGAATGT CCGTAACTAC TAGGACAAAG ATTCATATGA ACACTACGAT CGAACCTCCG ACCACCCTTT   
  
  
- GCTAATCGCA GAGATAAACT TTTCAAATTT TATGAACAGC TCAAGTTACG TAACGGGCAA ATACCTGGGT   
  
  
- TACAGTCCAC CCTTTACGAC CTACACTCCG GGTCCCTCCG AAACCAGCAA TTAACAGGTA ATGTCGAGGT   
  
  
- AATGTGAGGA CTGCTCTCAC AGCTACACTC CTTGGGATCC CTATCCGAAG AGTCTTACCA CTTTAACGAG   
  
  
- CCAGGATTCC ATCGGTGAAA CCAACTCGTT CTTAGTTTGT GGTTGTGATG TGGAAAGAAC TGGGCCAAGT   
  
  
- ATCTCTGGGA ACTGATGATG AGCTAATTGG TACAAACTCA GATATCTGCA CTGGTACGGC TCTCTGGCCT   
  
  
- TCCTCTCCTA GTTACAACTC GTCGTAACAA ATCGATTCCT GTAACACTTA TAGTATCGAA CGCTCCCGTT   
  
  
- CCTCTACCAC CTCGCAGTAC TCGAAAAACC CTTTACCGTC AGCTCCAAAT GGTACCATCC CAAGTCCGTC   
  
  
- ATGGGCAACT CGAGTATGTA TTTGAGACAC TATTCTTCGG ATGAGGCCAC AATGAGGTTC GTAATATGAG   
  
  
- ACTATCTCTT CCTATCACGG TACGAAGACC CAACCTCCCC GGCTTACGAC TAAAGTCGAA GCCGTAC

+     W box

| Site Name | Organism | Position | Strand | Matrix score. | sequence | function |
| --- | --- | --- | --- | --- | --- | --- |
| W box | Arabidopsis thaliana | 801 | - | 6 | TTGACC |  |
| W box | Arabidopsis thaliana | 1952 | + | 6 | TTGACC |  |

>HU08G00229.1   
+ -Up\_Stream \_Len000AAATTT ATATGATTAT GGGAGCAAAT ATTAATCGGT CTAAAACAAA AAAGAATCGG   
  
  
+ CCGAACAAAA AAAAGGGCCC AGAACTAGTC CTTTTAATGG AGTCCCAAAA TGTGGTATAA ATTAGTCTTG   
  
  
+ TTCACTCATT TCTCACTCCT CTCACTCATT CTAACCCTAA ATCTCAATCC TCACTCTCTG TCCTCTCTAT   
  
  
+ CTGCGTCTCG AGCTTCTCTT TCCTCTGGGC TTCCTTGGAC TTTTTCGACG CCATGACAAT GGAACACTGA   
  
  
+ GCTCCACCGT TGCCCCTCTC CACAACTGTC TTCTAACGGT GGTTCTCCGT AGCCACCGTA CCTCAAAAAC   
  
  
+ CAAAGCGATG CACAACTACC ACCCCACCAC TGCTGAAAAG TGCCCACGAG TCTCGATCCG CGCAAGCCTG   
  
  
+ ATGAGAATCG ATACCACCAC CATGTAGATA AGCTGAAAAC GGGCATAAGG AAGCGACCCA CGCGCCCAAC   
  
  
+ ACGCCATTGA GGCAGTGACA CGTGGGTTGA TGCGCTTCCA AAGATGGAAT TTTCACATTT TTCTTTTCAA   
  
  
+ GGTTGCGTAG ATCGGTGTGT GTGGATGGCG ATCAAGGAAG AGTTAAAATG AACTAGTCAT GCGTCGAATT   
  
  
+ GTCCAAAAAA AATTTTCTCT TTCAGTCGAG TCTTAAAGGG GCAGTCATCA AGAGCATGTT TGAAAAACAT   
  
  
+ TATGGCCATA ACCACTTAGT AGTAATTCAA ATAATCATGG AAACATATTC AACATGAATT CAATATGACT   
  
  
+ AAAAATTCCA TCTTTTAGTT TGTTCTGGTC AACACAGAGC ATGCCTTTTT AATTGCATAA TAACGATATT   
  
  
+ TAACCAACCA ATCATGTTTC TCACATATTA ATATAGGCAA GGGTAGATTA ACAGTCATAA TCGCATAGTA   
  
  
+ TGCAAGAATT TATTTGCAAT CTGGGCATAC TGTTTTATAA AGCACAAGTG TTCATGACTT AAAATCGATA   
  
  
+ AAGTCTTAAT CTTTTGTCGA TCCTTGAGTG TGCAGACAAT ATAATTGTGA ATGTATGCTA TTAACTTTAA   
  
  
+ TAATTACGAA AATTAAGCAA CTAATCATAT AGTCATGTTC TCAAAAACAA GCATGCGTAT TATTCAGATC   
  
  
+ ATTAAATGCA TTAAATGGGA CTAATCCGAT CAGTCAAGCG CTGATACGAC TATTGAATAA ACAGGACTAA   
  
  
+ CCTAGAATGC GGAATTAACT GTCGAGAATT GTCCACATCG GTAGTGATGC TTGGCGGCTA CCAGAGTCAC   
  
  
+ CTTAGGGTGT TCACATCCTA GACCCGAAAA CCTGCTAATG ATGGAAGGGC AGCCGTGGGA GGAGGAGAGG   
  
  
+ AGAGTGTGGA TTTTTCTATT TACAAAAAAG TGTATAAGTG CTAATCCCCC GAAACCTCCT TTTATAGGTT   
  
  
+ AGGGGGGCAA CTAGGGTTAT CGCCCTAGGT GACGCTTGTG GCCGTCGGCC TAATTAAATA GGCCCGACCA   
  
  
+ ACAGCTGTGA CTGGAGCGAT AGCTGAAGCA TGCAGAAATG AGGATAACAT ACATATTATA GATTTCCACA   
  
  
+ TTGCATAGGG CACTCAGTGG GTTACTGTAT CGCAAGCCAT AGCAGCAAGG CTTGGTGGGC CTCCTAAAGT   
  
  
+ GCGACTTACA GGCATTGATG ATCCTGTTTC TAAGTATACT TGTGATGCTA GCTTGGAGGC TGGTGGGAAA   
  
  
+ CGATTAGCGT CTCTATTTGA AAAGTTTAAA ATACTTGTCG AGTTCAATGC ATTGCCCGTT TATGGACCCA   
  
  
+ ATGTCAGGTG GGAAATGCTG GATGTGAGGC CCAGGGAGGC TTTGGTCGTT AATTGTCCAT TACAGCTCCA   
  
  
+ TTACACTCCT GACGAGAGTG TCGATGTGAG GAACCCTAGG GATAGGCTTC TCAGAATGGT GAAATTGCTC   
  
  
+ GGTCCTAAGG TAGCCACTTT GGTTGAGCAA GAATCAAACA CCAACACTAC ACCTTTCTTG ACCCGGTTCA   
  
  
+ TAGAGACCCT TGACTACTAC TCGATTAACC ATGTTTGAGT CTATAGACGT GACCATGCCG AGAGACCGGA   
  
  
+ AGGAGAGGAT CAATGTTGAG CAGCATTGTT TAGCTAAGGA CATTGTGAAT ATCATAGCTT GCGAGGGCAA   
  
  
+ GGAGATGGTG GAGCGTCATG AGCTTTTTGG GAAATGGCAG TCGAGGTTTA CCATGGTAGG GTTCAGGCAG   
  
  
+ TACCCGTTGA GCTCATACAT AAACTCTGTG ATAAGAAGCC TACTCCGGTG TTACTCCAAG CATTATACTC   
  
  
+ TGATAGAGAA GGATAGTGCC ATGCTTCTGG GTTGGAGGGG CCGAATGCTG ATTTCAGCTT CGGCATG  

- -Up\_Stream \_Len000TTTAAA TATACTAATA CCCTCGTTTA TAATTAGCCA GATTTTGTTT TTTCTTAGCC   
  
  
- GGCTTGTTTT TTTTCCCGGG TCTTGATCAG GAAAATTACC TCAGGGTTTT ACACCATATT TAATCAGAAC   
  
  
- AAGTGAGTAA AGAGTGAGGA GAGTGAGTAA GATTGGGATT TAGAGTTAGG AGTGAGAGAC AGGAGAGATA   
  
  
- GACGCAGAGC TCGAAGAGAA AGGAGACCCG AAGGAACCTG AAAAAGCTGC GGTACTGTTA CCTTGTGACT   
  
  
- CGAGGTGGCA ACGGGGAGAG GTGTTGACAG AAGATTGCCA CCAAGAGGCA TCGGTGGCAT GGAGTTTTTG   
  
  
- GTTTCGCTAC GTGTTGATGG TGGGGTGGTG ACGACTTTTC ACGGGTGCTC AGAGCTAGGC GCGTTCGGAC   
  
  
- TACTCTTAGC TATGGTGGTG GTACATCTAT TCGACTTTTG CCCGTATTCC TTCGCTGGGT GCGCGGGTTG   
  
  
- TGCGGTAACT CCGTCACTGT GCACCCAACT ACGCGAAGGT TTCTACCTTA AAAGTGTAAA AAGAAAAGTT   
  
  
- CCAACGCATC TAGCCACACA CACCTACCGC TAGTTCCTTC TCAATTTTAC TTGATCAGTA CGCAGCTTAA   
  
  
- CAGGTTTTTT TTAAAAGAGA AAGTCAGCTC AGAATTTCCC CGTCAGTAGT TCTCGTACAA ACTTTTTGTA   
  
  
- ATACCGGTAT TGGTGAATCA TCATTAAGTT TATTAGTACC TTTGTATAAG TTGTACTTAA GTTATACTGA   
  
  
- TTTTTAAGGT AGAAAATCAA ACAAGACCAG TTGTGTCTCG TACGGAAAAA TTAACGTATT ATTGCTATAA   
  
  
- ATTGGTTGGT TAGTACAAAG AGTGTATAAT TATATCCGTT CCCATCTAAT TGTCAGTATT AGCGTATCAT   
  
  
- ACGTTCTTAA ATAAACGTTA GACCCGTATG ACAAAATATT TCGTGTTCAC AAGTACTGAA TTTTAGCTAT   
  
  
- TTCAGAATTA GAAAACAGCT AGGAACTCAC ACGTCTGTTA TATTAACACT TACATACGAT AATTGAAATT   
  
  
- ATTAATGCTT TTAATTCGTT GATTAGTATA TCAGTACAAG AGTTTTTGTT CGTACGCATA ATAAGTCTAG   
  
  
- TAATTTACGT AATTTACCCT GATTAGGCTA GTCAGTTCGC GACTATGCTG ATAACTTATT TGTCCTGATT   
  
  
- GGATCTTACG CCTTAATTGA CAGCTCTTAA CAGGTGTAGC CATCACTACG AACCGCCGAT GGTCTCAGTG   
  
  
- GAATCCCACA AGTGTAGGAT CTGGGCTTTT GGACGATTAC TACCTTCCCG TCGGCACCCT CCTCCTCTCC   
  
  
- TCTCACACCT AAAAAGATAA ATGTTTTTTC ACATATTCAC GATTAGGGGG CTTTGGAGGA AAATATCCAA   
  
  
- TCCCCCCGTT GATCCCAATA GCGGGATCCA CTGCGAACAC CGGCAGCCGG ATTAATTTAT CCGGGCTGGT   
  
  
- TGTCGACACT GACCTCGCTA TCGACTTCGT ACGTCTTTAC TCCTATTGTA TGTATAATAT CTAAAGGTGT   
  
  
- AACGTATCCC GTGAGTCACC CAATGACATA GCGTTCGGTA TCGTCGTTCC GAACCACCCG GAGGATTTCA   
  
  
- CGCTGAATGT CCGTAACTAC TAGGACAAAG ATTCATATGA ACACTACGAT CGAACCTCCG ACCACCCTTT   
  
  
- GCTAATCGCA GAGATAAACT TTTCAAATTT TATGAACAGC TCAAGTTACG TAACGGGCAA ATACCTGGGT   
  
  
- TACAGTCCAC CCTTTACGAC CTACACTCCG GGTCCCTCCG AAACCAGCAA TTAACAGGTA ATGTCGAGGT   
  
  
- AATGTGAGGA CTGCTCTCAC AGCTACACTC CTTGGGATCC CTATCCGAAG AGTCTTACCA CTTTAACGAG   
  
  
- CCAGGATTCC ATCGGTGAAA CCAACTCGTT CTTAGTTTGT GGTTGTGATG TGGAAAGAAC TGGGCCAAGT   
  
  
- ATCTCTGGGA ACTGATGATG AGCTAATTGG TACAAACTCA GATATCTGCA CTGGTACGGC TCTCTGGCCT   
  
  
- TCCTCTCCTA GTTACAACTC GTCGTAACAA ATCGATTCCT GTAACACTTA TAGTATCGAA CGCTCCCGTT   
  
  
- CCTCTACCAC CTCGCAGTAC TCGAAAAACC CTTTACCGTC AGCTCCAAAT GGTACCATCC CAAGTCCGTC   
  
  
- ATGGGCAACT CGAGTATGTA TTTGAGACAC TATTCTTCGG ATGAGGCCAC AATGAGGTTC GTAATATGAG   
  
  
- ACTATCTCTT CCTATCACGG TACGAAGACC CAACCTCCCC GGCTTACGAC TAAAGTCGAA GCCGTAC

+     WRE3

| Site Name | Organism | Position | Strand | Matrix score. | sequence | function |
| --- | --- | --- | --- | --- | --- | --- |
| WRE3 | Pisum sativum | 1760 | - | 6 | CCACCT |  |

>HU08G00229.1   
+ -Up\_Stream \_Len000AAATTT ATATGATTAT GGGAGCAAAT ATTAATCGGT CTAAAACAAA AAAGAATCGG   
  
  
+ CCGAACAAAA AAAAGGGCCC AGAACTAGTC CTTTTAATGG AGTCCCAAAA TGTGGTATAA ATTAGTCTTG   
  
  
+ TTCACTCATT TCTCACTCCT CTCACTCATT CTAACCCTAA ATCTCAATCC TCACTCTCTG TCCTCTCTAT   
  
  
+ CTGCGTCTCG AGCTTCTCTT TCCTCTGGGC TTCCTTGGAC TTTTTCGACG CCATGACAAT GGAACACTGA   
  
  
+ GCTCCACCGT TGCCCCTCTC CACAACTGTC TTCTAACGGT GGTTCTCCGT AGCCACCGTA CCTCAAAAAC   
  
  
+ CAAAGCGATG CACAACTACC ACCCCACCAC TGCTGAAAAG TGCCCACGAG TCTCGATCCG CGCAAGCCTG   
  
  
+ ATGAGAATCG ATACCACCAC CATGTAGATA AGCTGAAAAC GGGCATAAGG AAGCGACCCA CGCGCCCAAC   
  
  
+ ACGCCATTGA GGCAGTGACA CGTGGGTTGA TGCGCTTCCA AAGATGGAAT TTTCACATTT TTCTTTTCAA   
  
  
+ GGTTGCGTAG ATCGGTGTGT GTGGATGGCG ATCAAGGAAG AGTTAAAATG AACTAGTCAT GCGTCGAATT   
  
  
+ GTCCAAAAAA AATTTTCTCT TTCAGTCGAG TCTTAAAGGG GCAGTCATCA AGAGCATGTT TGAAAAACAT   
  
  
+ TATGGCCATA ACCACTTAGT AGTAATTCAA ATAATCATGG AAACATATTC AACATGAATT CAATATGACT   
  
  
+ AAAAATTCCA TCTTTTAGTT TGTTCTGGTC AACACAGAGC ATGCCTTTTT AATTGCATAA TAACGATATT   
  
  
+ TAACCAACCA ATCATGTTTC TCACATATTA ATATAGGCAA GGGTAGATTA ACAGTCATAA TCGCATAGTA   
  
  
+ TGCAAGAATT TATTTGCAAT CTGGGCATAC TGTTTTATAA AGCACAAGTG TTCATGACTT AAAATCGATA   
  
  
+ AAGTCTTAAT CTTTTGTCGA TCCTTGAGTG TGCAGACAAT ATAATTGTGA ATGTATGCTA TTAACTTTAA   
  
  
+ TAATTACGAA AATTAAGCAA CTAATCATAT AGTCATGTTC TCAAAAACAA GCATGCGTAT TATTCAGATC   
  
  
+ ATTAAATGCA TTAAATGGGA CTAATCCGAT CAGTCAAGCG CTGATACGAC TATTGAATAA ACAGGACTAA   
  
  
+ CCTAGAATGC GGAATTAACT GTCGAGAATT GTCCACATCG GTAGTGATGC TTGGCGGCTA CCAGAGTCAC   
  
  
+ CTTAGGGTGT TCACATCCTA GACCCGAAAA CCTGCTAATG ATGGAAGGGC AGCCGTGGGA GGAGGAGAGG   
  
  
+ AGAGTGTGGA TTTTTCTATT TACAAAAAAG TGTATAAGTG CTAATCCCCC GAAACCTCCT TTTATAGGTT   
  
  
+ AGGGGGGCAA CTAGGGTTAT CGCCCTAGGT GACGCTTGTG GCCGTCGGCC TAATTAAATA GGCCCGACCA   
  
  
+ ACAGCTGTGA CTGGAGCGAT AGCTGAAGCA TGCAGAAATG AGGATAACAT ACATATTATA GATTTCCACA   
  
  
+ TTGCATAGGG CACTCAGTGG GTTACTGTAT CGCAAGCCAT AGCAGCAAGG CTTGGTGGGC CTCCTAAAGT   
  
  
+ GCGACTTACA GGCATTGATG ATCCTGTTTC TAAGTATACT TGTGATGCTA GCTTGGAGGC TGGTGGGAAA   
  
  
+ CGATTAGCGT CTCTATTTGA AAAGTTTAAA ATACTTGTCG AGTTCAATGC ATTGCCCGTT TATGGACCCA   
  
  
+ ATGTCAGGTG GGAAATGCTG GATGTGAGGC CCAGGGAGGC TTTGGTCGTT AATTGTCCAT TACAGCTCCA   
  
  
+ TTACACTCCT GACGAGAGTG TCGATGTGAG GAACCCTAGG GATAGGCTTC TCAGAATGGT GAAATTGCTC   
  
  
+ GGTCCTAAGG TAGCCACTTT GGTTGAGCAA GAATCAAACA CCAACACTAC ACCTTTCTTG ACCCGGTTCA   
  
  
+ TAGAGACCCT TGACTACTAC TCGATTAACC ATGTTTGAGT CTATAGACGT GACCATGCCG AGAGACCGGA   
  
  
+ AGGAGAGGAT CAATGTTGAG CAGCATTGTT TAGCTAAGGA CATTGTGAAT ATCATAGCTT GCGAGGGCAA   
  
  
+ GGAGATGGTG GAGCGTCATG AGCTTTTTGG GAAATGGCAG TCGAGGTTTA CCATGGTAGG GTTCAGGCAG   
  
  
+ TACCCGTTGA GCTCATACAT AAACTCTGTG ATAAGAAGCC TACTCCGGTG TTACTCCAAG CATTATACTC   
  
  
+ TGATAGAGAA GGATAGTGCC ATGCTTCTGG GTTGGAGGGG CCGAATGCTG ATTTCAGCTT CGGCATG  

- -Up\_Stream \_Len000TTTAAA TATACTAATA CCCTCGTTTA TAATTAGCCA GATTTTGTTT TTTCTTAGCC   
  
  
- GGCTTGTTTT TTTTCCCGGG TCTTGATCAG GAAAATTACC TCAGGGTTTT ACACCATATT TAATCAGAAC   
  
  
- AAGTGAGTAA AGAGTGAGGA GAGTGAGTAA GATTGGGATT TAGAGTTAGG AGTGAGAGAC AGGAGAGATA   
  
  
- GACGCAGAGC TCGAAGAGAA AGGAGACCCG AAGGAACCTG AAAAAGCTGC GGTACTGTTA CCTTGTGACT   
  
  
- CGAGGTGGCA ACGGGGAGAG GTGTTGACAG AAGATTGCCA CCAAGAGGCA TCGGTGGCAT GGAGTTTTTG   
  
  
- GTTTCGCTAC GTGTTGATGG TGGGGTGGTG ACGACTTTTC ACGGGTGCTC AGAGCTAGGC GCGTTCGGAC   
  
  
- TACTCTTAGC TATGGTGGTG GTACATCTAT TCGACTTTTG CCCGTATTCC TTCGCTGGGT GCGCGGGTTG   
  
  
- TGCGGTAACT CCGTCACTGT GCACCCAACT ACGCGAAGGT TTCTACCTTA AAAGTGTAAA AAGAAAAGTT   
  
  
- CCAACGCATC TAGCCACACA CACCTACCGC TAGTTCCTTC TCAATTTTAC TTGATCAGTA CGCAGCTTAA   
  
  
- CAGGTTTTTT TTAAAAGAGA AAGTCAGCTC AGAATTTCCC CGTCAGTAGT TCTCGTACAA ACTTTTTGTA   
  
  
- ATACCGGTAT TGGTGAATCA TCATTAAGTT TATTAGTACC TTTGTATAAG TTGTACTTAA GTTATACTGA   
  
  
- TTTTTAAGGT AGAAAATCAA ACAAGACCAG TTGTGTCTCG TACGGAAAAA TTAACGTATT ATTGCTATAA   
  
  
- ATTGGTTGGT TAGTACAAAG AGTGTATAAT TATATCCGTT CCCATCTAAT TGTCAGTATT AGCGTATCAT   
  
  
- ACGTTCTTAA ATAAACGTTA GACCCGTATG ACAAAATATT TCGTGTTCAC AAGTACTGAA TTTTAGCTAT   
  
  
- TTCAGAATTA GAAAACAGCT AGGAACTCAC ACGTCTGTTA TATTAACACT TACATACGAT AATTGAAATT   
  
  
- ATTAATGCTT TTAATTCGTT GATTAGTATA TCAGTACAAG AGTTTTTGTT CGTACGCATA ATAAGTCTAG   
  
  
- TAATTTACGT AATTTACCCT GATTAGGCTA GTCAGTTCGC GACTATGCTG ATAACTTATT TGTCCTGATT   
  
  
- GGATCTTACG CCTTAATTGA CAGCTCTTAA CAGGTGTAGC CATCACTACG AACCGCCGAT GGTCTCAGTG   
  
  
- GAATCCCACA AGTGTAGGAT CTGGGCTTTT GGACGATTAC TACCTTCCCG TCGGCACCCT CCTCCTCTCC   
  
  
- TCTCACACCT AAAAAGATAA ATGTTTTTTC ACATATTCAC GATTAGGGGG CTTTGGAGGA AAATATCCAA   
  
  
- TCCCCCCGTT GATCCCAATA GCGGGATCCA CTGCGAACAC CGGCAGCCGG ATTAATTTAT CCGGGCTGGT   
  
  
- TGTCGACACT GACCTCGCTA TCGACTTCGT ACGTCTTTAC TCCTATTGTA TGTATAATAT CTAAAGGTGT   
  
  
- AACGTATCCC GTGAGTCACC CAATGACATA GCGTTCGGTA TCGTCGTTCC GAACCACCCG GAGGATTTCA   
  
  
- CGCTGAATGT CCGTAACTAC TAGGACAAAG ATTCATATGA ACACTACGAT CGAACCTCCG ACCACCCTTT   
  
  
- GCTAATCGCA GAGATAAACT TTTCAAATTT TATGAACAGC TCAAGTTACG TAACGGGCAA ATACCTGGGT   
  
  
- TACAGTCCAC CCTTTACGAC CTACACTCCG GGTCCCTCCG AAACCAGCAA TTAACAGGTA ATGTCGAGGT   
  
  
- AATGTGAGGA CTGCTCTCAC AGCTACACTC CTTGGGATCC CTATCCGAAG AGTCTTACCA CTTTAACGAG   
  
  
- CCAGGATTCC ATCGGTGAAA CCAACTCGTT CTTAGTTTGT GGTTGTGATG TGGAAAGAAC TGGGCCAAGT   
  
  
- ATCTCTGGGA ACTGATGATG AGCTAATTGG TACAAACTCA GATATCTGCA CTGGTACGGC TCTCTGGCCT   
  
  
- TCCTCTCCTA GTTACAACTC GTCGTAACAA ATCGATTCCT GTAACACTTA TAGTATCGAA CGCTCCCGTT   
  
  
- CCTCTACCAC CTCGCAGTAC TCGAAAAACC CTTTACCGTC AGCTCCAAAT GGTACCATCC CAAGTCCGTC   
  
  
- ATGGGCAACT CGAGTATGTA TTTGAGACAC TATTCTTCGG ATGAGGCCAC AATGAGGTTC GTAATATGAG   
  
  
- ACTATCTCTT CCTATCACGG TACGAAGACC CAACCTCCCC GGCTTACGAC TAAAGTCGAA GCCGTAC

+     as-1

| Site Name | Organism | Position | Strand | Matrix score. | sequence | function |
| --- | --- | --- | --- | --- | --- | --- |
| as-1 | Arabidopsis thaliana | 1434 | + | 5 | TGACG |  |
| as-1 | Arabidopsis thaliana | 2118 | - | 5 | TGACG |  |
| as-1 | Arabidopsis thaliana | 1834 | + | 5 | TGACG |  |

>HU08G00229.1   
+ -Up\_Stream \_Len000AAATTT ATATGATTAT GGGAGCAAAT ATTAATCGGT CTAAAACAAA AAAGAATCGG   
  
  
+ CCGAACAAAA AAAAGGGCCC AGAACTAGTC CTTTTAATGG AGTCCCAAAA TGTGGTATAA ATTAGTCTTG   
  
  
+ TTCACTCATT TCTCACTCCT CTCACTCATT CTAACCCTAA ATCTCAATCC TCACTCTCTG TCCTCTCTAT   
  
  
+ CTGCGTCTCG AGCTTCTCTT TCCTCTGGGC TTCCTTGGAC TTTTTCGACG CCATGACAAT GGAACACTGA   
  
  
+ GCTCCACCGT TGCCCCTCTC CACAACTGTC TTCTAACGGT GGTTCTCCGT AGCCACCGTA CCTCAAAAAC   
  
  
+ CAAAGCGATG CACAACTACC ACCCCACCAC TGCTGAAAAG TGCCCACGAG TCTCGATCCG CGCAAGCCTG   
  
  
+ ATGAGAATCG ATACCACCAC CATGTAGATA AGCTGAAAAC GGGCATAAGG AAGCGACCCA CGCGCCCAAC   
  
  
+ ACGCCATTGA GGCAGTGACA CGTGGGTTGA TGCGCTTCCA AAGATGGAAT TTTCACATTT TTCTTTTCAA   
  
  
+ GGTTGCGTAG ATCGGTGTGT GTGGATGGCG ATCAAGGAAG AGTTAAAATG AACTAGTCAT GCGTCGAATT   
  
  
+ GTCCAAAAAA AATTTTCTCT TTCAGTCGAG TCTTAAAGGG GCAGTCATCA AGAGCATGTT TGAAAAACAT   
  
  
+ TATGGCCATA ACCACTTAGT AGTAATTCAA ATAATCATGG AAACATATTC AACATGAATT CAATATGACT   
  
  
+ AAAAATTCCA TCTTTTAGTT TGTTCTGGTC AACACAGAGC ATGCCTTTTT AATTGCATAA TAACGATATT   
  
  
+ TAACCAACCA ATCATGTTTC TCACATATTA ATATAGGCAA GGGTAGATTA ACAGTCATAA TCGCATAGTA   
  
  
+ TGCAAGAATT TATTTGCAAT CTGGGCATAC TGTTTTATAA AGCACAAGTG TTCATGACTT AAAATCGATA   
  
  
+ AAGTCTTAAT CTTTTGTCGA TCCTTGAGTG TGCAGACAAT ATAATTGTGA ATGTATGCTA TTAACTTTAA   
  
  
+ TAATTACGAA AATTAAGCAA CTAATCATAT AGTCATGTTC TCAAAAACAA GCATGCGTAT TATTCAGATC   
  
  
+ ATTAAATGCA TTAAATGGGA CTAATCCGAT CAGTCAAGCG CTGATACGAC TATTGAATAA ACAGGACTAA   
  
  
+ CCTAGAATGC GGAATTAACT GTCGAGAATT GTCCACATCG GTAGTGATGC TTGGCGGCTA CCAGAGTCAC   
  
  
+ CTTAGGGTGT TCACATCCTA GACCCGAAAA CCTGCTAATG ATGGAAGGGC AGCCGTGGGA GGAGGAGAGG   
  
  
+ AGAGTGTGGA TTTTTCTATT TACAAAAAAG TGTATAAGTG CTAATCCCCC GAAACCTCCT TTTATAGGTT   
  
  
+ AGGGGGGCAA CTAGGGTTAT CGCCCTAGGT GACGCTTGTG GCCGTCGGCC TAATTAAATA GGCCCGACCA   
  
  
+ ACAGCTGTGA CTGGAGCGAT AGCTGAAGCA TGCAGAAATG AGGATAACAT ACATATTATA GATTTCCACA   
  
  
+ TTGCATAGGG CACTCAGTGG GTTACTGTAT CGCAAGCCAT AGCAGCAAGG CTTGGTGGGC CTCCTAAAGT   
  
  
+ GCGACTTACA GGCATTGATG ATCCTGTTTC TAAGTATACT TGTGATGCTA GCTTGGAGGC TGGTGGGAAA   
  
  
+ CGATTAGCGT CTCTATTTGA AAAGTTTAAA ATACTTGTCG AGTTCAATGC ATTGCCCGTT TATGGACCCA   
  
  
+ ATGTCAGGTG GGAAATGCTG GATGTGAGGC CCAGGGAGGC TTTGGTCGTT AATTGTCCAT TACAGCTCCA   
  
  
+ TTACACTCCT GACGAGAGTG TCGATGTGAG GAACCCTAGG GATAGGCTTC TCAGAATGGT GAAATTGCTC   
  
  
+ GGTCCTAAGG TAGCCACTTT GGTTGAGCAA GAATCAAACA CCAACACTAC ACCTTTCTTG ACCCGGTTCA   
  
  
+ TAGAGACCCT TGACTACTAC TCGATTAACC ATGTTTGAGT CTATAGACGT GACCATGCCG AGAGACCGGA   
  
  
+ AGGAGAGGAT CAATGTTGAG CAGCATTGTT TAGCTAAGGA CATTGTGAAT ATCATAGCTT GCGAGGGCAA   
  
  
+ GGAGATGGTG GAGCGTCATG AGCTTTTTGG GAAATGGCAG TCGAGGTTTA CCATGGTAGG GTTCAGGCAG   
  
  
+ TACCCGTTGA GCTCATACAT AAACTCTGTG ATAAGAAGCC TACTCCGGTG TTACTCCAAG CATTATACTC   
  
  
+ TGATAGAGAA GGATAGTGCC ATGCTTCTGG GTTGGAGGGG CCGAATGCTG ATTTCAGCTT CGGCATG  

- -Up\_Stream \_Len000TTTAAA TATACTAATA CCCTCGTTTA TAATTAGCCA GATTTTGTTT TTTCTTAGCC   
  
  
- GGCTTGTTTT TTTTCCCGGG TCTTGATCAG GAAAATTACC TCAGGGTTTT ACACCATATT TAATCAGAAC   
  
  
- AAGTGAGTAA AGAGTGAGGA GAGTGAGTAA GATTGGGATT TAGAGTTAGG AGTGAGAGAC AGGAGAGATA   
  
  
- GACGCAGAGC TCGAAGAGAA AGGAGACCCG AAGGAACCTG AAAAAGCTGC GGTACTGTTA CCTTGTGACT   
  
  
- CGAGGTGGCA ACGGGGAGAG GTGTTGACAG AAGATTGCCA CCAAGAGGCA TCGGTGGCAT GGAGTTTTTG   
  
  
- GTTTCGCTAC GTGTTGATGG TGGGGTGGTG ACGACTTTTC ACGGGTGCTC AGAGCTAGGC GCGTTCGGAC   
  
  
- TACTCTTAGC TATGGTGGTG GTACATCTAT TCGACTTTTG CCCGTATTCC TTCGCTGGGT GCGCGGGTTG   
  
  
- TGCGGTAACT CCGTCACTGT GCACCCAACT ACGCGAAGGT TTCTACCTTA AAAGTGTAAA AAGAAAAGTT   
  
  
- CCAACGCATC TAGCCACACA CACCTACCGC TAGTTCCTTC TCAATTTTAC TTGATCAGTA CGCAGCTTAA   
  
  
- CAGGTTTTTT TTAAAAGAGA AAGTCAGCTC AGAATTTCCC CGTCAGTAGT TCTCGTACAA ACTTTTTGTA   
  
  
- ATACCGGTAT TGGTGAATCA TCATTAAGTT TATTAGTACC TTTGTATAAG TTGTACTTAA GTTATACTGA   
  
  
- TTTTTAAGGT AGAAAATCAA ACAAGACCAG TTGTGTCTCG TACGGAAAAA TTAACGTATT ATTGCTATAA   
  
  
- ATTGGTTGGT TAGTACAAAG AGTGTATAAT TATATCCGTT CCCATCTAAT TGTCAGTATT AGCGTATCAT   
  
  
- ACGTTCTTAA ATAAACGTTA GACCCGTATG ACAAAATATT TCGTGTTCAC AAGTACTGAA TTTTAGCTAT   
  
  
- TTCAGAATTA GAAAACAGCT AGGAACTCAC ACGTCTGTTA TATTAACACT TACATACGAT AATTGAAATT   
  
  
- ATTAATGCTT TTAATTCGTT GATTAGTATA TCAGTACAAG AGTTTTTGTT CGTACGCATA ATAAGTCTAG   
  
  
- TAATTTACGT AATTTACCCT GATTAGGCTA GTCAGTTCGC GACTATGCTG ATAACTTATT TGTCCTGATT   
  
  
- GGATCTTACG CCTTAATTGA CAGCTCTTAA CAGGTGTAGC CATCACTACG AACCGCCGAT GGTCTCAGTG   
  
  
- GAATCCCACA AGTGTAGGAT CTGGGCTTTT GGACGATTAC TACCTTCCCG TCGGCACCCT CCTCCTCTCC   
  
  
- TCTCACACCT AAAAAGATAA ATGTTTTTTC ACATATTCAC GATTAGGGGG CTTTGGAGGA AAATATCCAA   
  
  
- TCCCCCCGTT GATCCCAATA GCGGGATCCA CTGCGAACAC CGGCAGCCGG ATTAATTTAT CCGGGCTGGT   
  
  
- TGTCGACACT GACCTCGCTA TCGACTTCGT ACGTCTTTAC TCCTATTGTA TGTATAATAT CTAAAGGTGT   
  
  
- AACGTATCCC GTGAGTCACC CAATGACATA GCGTTCGGTA TCGTCGTTCC GAACCACCCG GAGGATTTCA   
  
  
- CGCTGAATGT CCGTAACTAC TAGGACAAAG ATTCATATGA ACACTACGAT CGAACCTCCG ACCACCCTTT   
  
  
- GCTAATCGCA GAGATAAACT TTTCAAATTT TATGAACAGC TCAAGTTACG TAACGGGCAA ATACCTGGGT   
  
  
- TACAGTCCAC CCTTTACGAC CTACACTCCG GGTCCCTCCG AAACCAGCAA TTAACAGGTA ATGTCGAGGT   
  
  
- AATGTGAGGA CTGCTCTCAC AGCTACACTC CTTGGGATCC CTATCCGAAG AGTCTTACCA CTTTAACGAG   
  
  
- CCAGGATTCC ATCGGTGAAA CCAACTCGTT CTTAGTTTGT GGTTGTGATG TGGAAAGAAC TGGGCCAAGT   
  
  
- ATCTCTGGGA ACTGATGATG AGCTAATTGG TACAAACTCA GATATCTGCA CTGGTACGGC TCTCTGGCCT   
  
  
- TCCTCTCCTA GTTACAACTC GTCGTAACAA ATCGATTCCT GTAACACTTA TAGTATCGAA CGCTCCCGTT   
  
  
- CCTCTACCAC CTCGCAGTAC TCGAAAAACC CTTTACCGTC AGCTCCAAAT GGTACCATCC CAAGTCCGTC   
  
  
- ATGGGCAACT CGAGTATGTA TTTGAGACAC TATTCTTCGG ATGAGGCCAC AATGAGGTTC GTAATATGAG   
  
  
- ACTATCTCTT CCTATCACGG TACGAAGACC CAACCTCCCC GGCTTACGAC TAAAGTCGAA GCCGTAC

+     box S

| Site Name | Organism | Position | Strand | Matrix score. | sequence | function |
| --- | --- | --- | --- | --- | --- | --- |
| box S | Arabidopsis thaliana | 335 | + | 7 | AGCCACC |  |

>HU08G00229.1   
+ -Up\_Stream \_Len000AAATTT ATATGATTAT GGGAGCAAAT ATTAATCGGT CTAAAACAAA AAAGAATCGG   
  
  
+ CCGAACAAAA AAAAGGGCCC AGAACTAGTC CTTTTAATGG AGTCCCAAAA TGTGGTATAA ATTAGTCTTG   
  
  
+ TTCACTCATT TCTCACTCCT CTCACTCATT CTAACCCTAA ATCTCAATCC TCACTCTCTG TCCTCTCTAT   
  
  
+ CTGCGTCTCG AGCTTCTCTT TCCTCTGGGC TTCCTTGGAC TTTTTCGACG CCATGACAAT GGAACACTGA   
  
  
+ GCTCCACCGT TGCCCCTCTC CACAACTGTC TTCTAACGGT GGTTCTCCGT AGCCACCGTA CCTCAAAAAC   
  
  
+ CAAAGCGATG CACAACTACC ACCCCACCAC TGCTGAAAAG TGCCCACGAG TCTCGATCCG CGCAAGCCTG   
  
  
+ ATGAGAATCG ATACCACCAC CATGTAGATA AGCTGAAAAC GGGCATAAGG AAGCGACCCA CGCGCCCAAC   
  
  
+ ACGCCATTGA GGCAGTGACA CGTGGGTTGA TGCGCTTCCA AAGATGGAAT TTTCACATTT TTCTTTTCAA   
  
  
+ GGTTGCGTAG ATCGGTGTGT GTGGATGGCG ATCAAGGAAG AGTTAAAATG AACTAGTCAT GCGTCGAATT   
  
  
+ GTCCAAAAAA AATTTTCTCT TTCAGTCGAG TCTTAAAGGG GCAGTCATCA AGAGCATGTT TGAAAAACAT   
  
  
+ TATGGCCATA ACCACTTAGT AGTAATTCAA ATAATCATGG AAACATATTC AACATGAATT CAATATGACT   
  
  
+ AAAAATTCCA TCTTTTAGTT TGTTCTGGTC AACACAGAGC ATGCCTTTTT AATTGCATAA TAACGATATT   
  
  
+ TAACCAACCA ATCATGTTTC TCACATATTA ATATAGGCAA GGGTAGATTA ACAGTCATAA TCGCATAGTA   
  
  
+ TGCAAGAATT TATTTGCAAT CTGGGCATAC TGTTTTATAA AGCACAAGTG TTCATGACTT AAAATCGATA   
  
  
+ AAGTCTTAAT CTTTTGTCGA TCCTTGAGTG TGCAGACAAT ATAATTGTGA ATGTATGCTA TTAACTTTAA   
  
  
+ TAATTACGAA AATTAAGCAA CTAATCATAT AGTCATGTTC TCAAAAACAA GCATGCGTAT TATTCAGATC   
  
  
+ ATTAAATGCA TTAAATGGGA CTAATCCGAT CAGTCAAGCG CTGATACGAC TATTGAATAA ACAGGACTAA   
  
  
+ CCTAGAATGC GGAATTAACT GTCGAGAATT GTCCACATCG GTAGTGATGC TTGGCGGCTA CCAGAGTCAC   
  
  
+ CTTAGGGTGT TCACATCCTA GACCCGAAAA CCTGCTAATG ATGGAAGGGC AGCCGTGGGA GGAGGAGAGG   
  
  
+ AGAGTGTGGA TTTTTCTATT TACAAAAAAG TGTATAAGTG CTAATCCCCC GAAACCTCCT TTTATAGGTT   
  
  
+ AGGGGGGCAA CTAGGGTTAT CGCCCTAGGT GACGCTTGTG GCCGTCGGCC TAATTAAATA GGCCCGACCA   
  
  
+ ACAGCTGTGA CTGGAGCGAT AGCTGAAGCA TGCAGAAATG AGGATAACAT ACATATTATA GATTTCCACA   
  
  
+ TTGCATAGGG CACTCAGTGG GTTACTGTAT CGCAAGCCAT AGCAGCAAGG CTTGGTGGGC CTCCTAAAGT   
  
  
+ GCGACTTACA GGCATTGATG ATCCTGTTTC TAAGTATACT TGTGATGCTA GCTTGGAGGC TGGTGGGAAA   
  
  
+ CGATTAGCGT CTCTATTTGA AAAGTTTAAA ATACTTGTCG AGTTCAATGC ATTGCCCGTT TATGGACCCA   
  
  
+ ATGTCAGGTG GGAAATGCTG GATGTGAGGC CCAGGGAGGC TTTGGTCGTT AATTGTCCAT TACAGCTCCA   
  
  
+ TTACACTCCT GACGAGAGTG TCGATGTGAG GAACCCTAGG GATAGGCTTC TCAGAATGGT GAAATTGCTC   
  
  
+ GGTCCTAAGG TAGCCACTTT GGTTGAGCAA GAATCAAACA CCAACACTAC ACCTTTCTTG ACCCGGTTCA   
  
  
+ TAGAGACCCT TGACTACTAC TCGATTAACC ATGTTTGAGT CTATAGACGT GACCATGCCG AGAGACCGGA   
  
  
+ AGGAGAGGAT CAATGTTGAG CAGCATTGTT TAGCTAAGGA CATTGTGAAT ATCATAGCTT GCGAGGGCAA   
  
  
+ GGAGATGGTG GAGCGTCATG AGCTTTTTGG GAAATGGCAG TCGAGGTTTA CCATGGTAGG GTTCAGGCAG   
  
  
+ TACCCGTTGA GCTCATACAT AAACTCTGTG ATAAGAAGCC TACTCCGGTG TTACTCCAAG CATTATACTC   
  
  
+ TGATAGAGAA GGATAGTGCC ATGCTTCTGG GTTGGAGGGG CCGAATGCTG ATTTCAGCTT CGGCATG  

- -Up\_Stream \_Len000TTTAAA TATACTAATA CCCTCGTTTA TAATTAGCCA GATTTTGTTT TTTCTTAGCC   
  
  
- GGCTTGTTTT TTTTCCCGGG TCTTGATCAG GAAAATTACC TCAGGGTTTT ACACCATATT TAATCAGAAC   
  
  
- AAGTGAGTAA AGAGTGAGGA GAGTGAGTAA GATTGGGATT TAGAGTTAGG AGTGAGAGAC AGGAGAGATA   
  
  
- GACGCAGAGC TCGAAGAGAA AGGAGACCCG AAGGAACCTG AAAAAGCTGC GGTACTGTTA CCTTGTGACT   
  
  
- CGAGGTGGCA ACGGGGAGAG GTGTTGACAG AAGATTGCCA CCAAGAGGCA TCGGTGGCAT GGAGTTTTTG   
  
  
- GTTTCGCTAC GTGTTGATGG TGGGGTGGTG ACGACTTTTC ACGGGTGCTC AGAGCTAGGC GCGTTCGGAC   
  
  
- TACTCTTAGC TATGGTGGTG GTACATCTAT TCGACTTTTG CCCGTATTCC TTCGCTGGGT GCGCGGGTTG   
  
  
- TGCGGTAACT CCGTCACTGT GCACCCAACT ACGCGAAGGT TTCTACCTTA AAAGTGTAAA AAGAAAAGTT   
  
  
- CCAACGCATC TAGCCACACA CACCTACCGC TAGTTCCTTC TCAATTTTAC TTGATCAGTA CGCAGCTTAA   
  
  
- CAGGTTTTTT TTAAAAGAGA AAGTCAGCTC AGAATTTCCC CGTCAGTAGT TCTCGTACAA ACTTTTTGTA   
  
  
- ATACCGGTAT TGGTGAATCA TCATTAAGTT TATTAGTACC TTTGTATAAG TTGTACTTAA GTTATACTGA   
  
  
- TTTTTAAGGT AGAAAATCAA ACAAGACCAG TTGTGTCTCG TACGGAAAAA TTAACGTATT ATTGCTATAA   
  
  
- ATTGGTTGGT TAGTACAAAG AGTGTATAAT TATATCCGTT CCCATCTAAT TGTCAGTATT AGCGTATCAT   
  
  
- ACGTTCTTAA ATAAACGTTA GACCCGTATG ACAAAATATT TCGTGTTCAC AAGTACTGAA TTTTAGCTAT   
  
  
- TTCAGAATTA GAAAACAGCT AGGAACTCAC ACGTCTGTTA TATTAACACT TACATACGAT AATTGAAATT   
  
  
- ATTAATGCTT TTAATTCGTT GATTAGTATA TCAGTACAAG AGTTTTTGTT CGTACGCATA ATAAGTCTAG   
  
  
- TAATTTACGT AATTTACCCT GATTAGGCTA GTCAGTTCGC GACTATGCTG ATAACTTATT TGTCCTGATT   
  
  
- GGATCTTACG CCTTAATTGA CAGCTCTTAA CAGGTGTAGC CATCACTACG AACCGCCGAT GGTCTCAGTG   
  
  
- GAATCCCACA AGTGTAGGAT CTGGGCTTTT GGACGATTAC TACCTTCCCG TCGGCACCCT CCTCCTCTCC   
  
  
- TCTCACACCT AAAAAGATAA ATGTTTTTTC ACATATTCAC GATTAGGGGG CTTTGGAGGA AAATATCCAA   
  
  
- TCCCCCCGTT GATCCCAATA GCGGGATCCA CTGCGAACAC CGGCAGCCGG ATTAATTTAT CCGGGCTGGT   
  
  
- TGTCGACACT GACCTCGCTA TCGACTTCGT ACGTCTTTAC TCCTATTGTA TGTATAATAT CTAAAGGTGT   
  
  
- AACGTATCCC GTGAGTCACC CAATGACATA GCGTTCGGTA TCGTCGTTCC GAACCACCCG GAGGATTTCA   
  
  
- CGCTGAATGT CCGTAACTAC TAGGACAAAG ATTCATATGA ACACTACGAT CGAACCTCCG ACCACCCTTT   
  
  
- GCTAATCGCA GAGATAAACT TTTCAAATTT TATGAACAGC TCAAGTTACG TAACGGGCAA ATACCTGGGT   
  
  
- TACAGTCCAC CCTTTACGAC CTACACTCCG GGTCCCTCCG AAACCAGCAA TTAACAGGTA ATGTCGAGGT   
  
  
- AATGTGAGGA CTGCTCTCAC AGCTACACTC CTTGGGATCC CTATCCGAAG AGTCTTACCA CTTTAACGAG   
  
  
- CCAGGATTCC ATCGGTGAAA CCAACTCGTT CTTAGTTTGT GGTTGTGATG TGGAAAGAAC TGGGCCAAGT   
  
  
- ATCTCTGGGA ACTGATGATG AGCTAATTGG TACAAACTCA GATATCTGCA CTGGTACGGC TCTCTGGCCT   
  
  
- TCCTCTCCTA GTTACAACTC GTCGTAACAA ATCGATTCCT GTAACACTTA TAGTATCGAA CGCTCCCGTT   
  
  
- CCTCTACCAC CTCGCAGTAC TCGAAAAACC CTTTACCGTC AGCTCCAAAT GGTACCATCC CAAGTCCGTC   
  
  
- ATGGGCAACT CGAGTATGTA TTTGAGACAC TATTCTTCGG ATGAGGCCAC AATGAGGTTC GTAATATGAG   
  
  
- ACTATCTCTT CCTATCACGG TACGAAGACC CAACCTCCCC GGCTTACGAC TAAAGTCGAA GCCGTAC
