# Supplementary material for: Genome-Wide Identification and Expression Pattern of the GRAS Gene Family in Pitaya (Selenicereus undatus L.)
Source: Biology (Basel). 2022 Dec 21;12(1):11. doi: 10.3390/biology12010011 (PMC9854919; doi:10.3390/biology12010011)
Supplement: Supplementary file 1 [file biology-12-00011-s001.zip › Supplementary file S5/HU08G00230.1_plantcare.html]

Content-Type: text/html; charset=ISO-8859-1


PlantCARE


Webmaster Firefox specific output  
To save the result:
click on the frame with the right mouse button and save the source code as a text file with extension .html  
REFERENCE:PlantCARE: a database of plant cis-acting regulatory elements and a portal to tools for in silico analysis of promoter sequences.  
Lescot, M., Déhais, P., Moreau, Y., De Moor, B., Rouzé ,P.,and Rombauts, S.  
Nucleic Acids Res., Database issue(2002), 30(1):325-327.   


---

>HU08G00230.1   
+ -Up\_Stream \_Len000GTTGTA ACAAGCACAT TAAAGTTGAT TATCATTTTG TCCGTGAAAC AGTGAGCAAA   
  
  
+ CGTCATCTCC AGGTCAAATT CATTCGTACT CAATCACAAG TTGCTGACAT TTTCACCAAA GGCTAATCAT   
  
  
+ CGCAAAAGTT TCTTTAGTTT AAGGCCAATC TCTCCATTAT TTCACCCTAT ACAGATTGAG GGGGTGTAAT   
  
  
+ AGTGTATATA TATAGCCTTG TGTTAGCGGG CCTTGGACAC TAGACTTTTA CTCTTGCAAA CCCTAATATA   
  
  
+ TTATTATAAA TATAAATCTA GCCACCCATG TTGAGGTAGG CACTCTAATT CTCACACAGG TAGGATAAGT   
  
  
+ TTTCATAGTT TATGTTGTGA ATGTGATCTT ACTTTGTGGT TCCCGTTGAC AAAAGTTTGA ATCCGAGCAT   
  
  
+ GAGAGTTCAT CAATCTCTTT TTCTTTCCTA TTTTATTTGT TGTGAACTTA ATTGGATTTT CTGGTAGAGG   
  
  
+ AAAGTTCCTT GCTGGAATTT GGTCAAAAGT GATATATATT TTGGGATTTA ATTATCCATA AATTTGGTTT   
  
  
+ TACACCTTGT TAGTTTGGAC TTCTGGTGTG TTATTGTATG TGTTCTGGTG GGTTTTTCTT TTAGCTGAAT   
  
  
+ GTTGGAGCAT TTTAAGGGAT CTAGATATCT GGAGAAGAGC TTGGGAAATA AAGCATATTT CCCTTATAGT   
  
  
+ CTGACATAAC TTTAATGAAT TGATCAGGAA CAAAGGCCAA TTAGTTCAAA AAGGTTCAAC TCCTCTAGGT   
  
  
+ GTTGTGCTAA AGAGATGTGA GTTAGTAAGT AATTGCTTAC TAACTATATA GGATGTTTTA TTACTTGTAG   
  
  
+ AGGTTTTTTT TTTGGGATAT CTTTATGGGG TTTATAAATA AGAAGGTTGA TTTGTTGTCT ACTCTTCTTT   
  
  
+ CGATTCAATA GTAATTCTCT CAGAGAAGGC TAAAATGACA TGGCATTCCA TCGCCTCTCA TGAGGCTTTT   
  
  
+ TGCATGTGCT TATGTATCTT TCATCTCTTG CTTTGGTGGG GTGGCATTGG GGAGGAAAAG GGGGTTTCGA   
  
  
+ TTCGATAGAT CGTCACTTTT GGAAATGACA TCTCTAGCCA ATGGCAAGGG TAAGGTTAAT ACATGTGGAT   
  
  
+ ATTTCCAGAG TTAGTTGTGA TTGTTATTGT TGTTGTTGTA ACTTTATGTC ACGGGGAAAA CATAATAACT   
  
  
+ ATCTGTTTAT CTGTTTGTTT GTTATTTTCA AGGGGAAGGG GAGTGGGCAG ATGCTTAGTA TGTAATCATT   
  
  
+ TTATATAGGG AAAATTCATA TAGCTTGAAA GTAAAACTTT CATTGCCAGC ATTCCAAGGT ATGGGTAGGG   
  
  
+ TTGTCTACAC CTTTATCTCC CTAGAAGCAT GGTGATCATT CCTCGGATTG TGTTATAGAA TAAAGAATGT   
  
  
+ GGTTCTGCTG TAATGAGACT TATGTATTAT CAAATGATGA ATCTGTTTAG AACTTCCTGT TTATTTTTTC   
  
  
+ TGCAAAATTT TAAGAGACTA ACATCTATCG GGCTCAACTC TAGAGGATCT CTTGTGTCAC CCTTCTCAAC   
  
  
+ TCAGTTTGAT TGTGATACAG TTACTATATT GAGTGATAGT CGAGAGCATC ACATCCCGTT AAGGAGTCTA   
  
  
+ TCAACAAGAA GCCCTTCTTG TAATTCTCCC CTTAAAACTA GCAGTTATCA TCACTTGTCA TCGAATAGCC   
  
  
+ CTTTTTGTAA TTCTACCCTT GAAACTAGCA GTTATCGTCA TCGGTTCAAC GCGAGTCCTG TTAGATATTC   
  
  
+ CCATCAAGGC ATAGATTATG GAGTGAGCAT GAAGAATGCT TTGCAGGAGC TAGAGACTAC TCTAATTGGT   
  
  
+ GTAGATGGTG AGGAAGTATC TATTGCTAAT CAACCTATGG GGGGAAGTCG TTAGTCCGGG ATCCCAAGTT   
  
  
+ AGAGATCGAA GTCATCAAGC GAAGATCCAC AGGGTTCACA TCCTACTCAG CTTGATTCAT CATCTTTTTC   
  
  
+ AAGGGTGAGA AGATTCGGAG ATGAAAGCCA GAGAGAGAGA AATGCCATAA GGCAATGGAA GAACCAGCGG   
  
  
+ AACTACTAAG TTTCTCACCA GGTGATTTAA AGCAGTTACT AATTGAATGT GCGAGGGCTT TATTAGATAA   
  
  
+ CCGAATAGAT GACTTTGAGA GTTTGGTTAA ACGGGCAAGG AAAGAGGTCT CCATCTCAGG TGAGCCTGTC   
  
  
+ CAACCTCTCG GTACCTGTAT GATCGAAGGG CTTGTGGCAA GGAAACAGTC TTCGGGGACT AACATCTATT   
  
  
+ GGGCTCTTAA GTGTAAAGAG CCTCTTGGAA AAGACTTGCT CTCCTACGGC ACTCAGTGGG TTACTGTATC   
  
  
+ GCAAGCCATA GCAGCAAGGC TTGGTGGGCC TCCTAAAGTG CGACTTACAG GCATTGATGA TCCTGTTTCT   
  
  
+ AAGTATACTT GTGATGCTAG CTTGGAGGCT GGTGGGAAAC GATTAGCGTC TCTATTTGAA AAGTTTAAAA   
  
  
+ TACTTGTCGA GTTCAATGCA TTGCCCGTTT ATGGACCCAA TGTCAGGTGG GAAATGCTGG ATGTGAGGCC   
  
  
+ CAGGGAGGCT TTGGTCGTTA ATTGTCCATT ACAGCTCCAT TACACTCCTG ACGAGAGTGT CGATGTGAGG   
  
  
+ AACCCTAGGG ATAGGCTTCT CAGAATGGTG AAATTGCTCG GTCCTAAGGT AGCCACTTTG GTTGAGCAAG   
  
  
+ AATCAAACAC CAACACTACA CCTTTCTTGA CCCGGTTCAT AGAGACCCTT GACTACTACT CGATTAACCA   
  
  
+ TGTTTG  

- -Up\_Stream \_Len000CAACAT TGTTCGTGTA ATTTCAACTA ATAGTAAAAC AGGCACTTTG TCACTCGTTT   
  
  
- GCAGTAGAGG TCCAGTTTAA GTAAGCATGA GTTAGTGTTC AACGACTGTA AAAGTGGTTT CCGATTAGTA   
  
  
- GCGTTTTCAA AGAAATCAAA TTCCGGTTAG AGAGGTAATA AAGTGGGATA TGTCTAACTC CCCCACATTA   
  
  
- TCACATATAT ATATCGGAAC ACAATCGCCC GGAACCTGTG ATCTGAAAAT GAGAACGTTT GGGATTATAT   
  
  
- AATAATATTT ATATTTAGAT CGGTGGGTAC AACTCCATCC GTGAGATTAA GAGTGTGTCC ATCCTATTCA   
  
  
- AAAGTATCAA ATACAACACT TACACTAGAA TGAAACACCA AGGGCAACTG TTTTCAAACT TAGGCTCGTA   
  
  
- CTCTCAAGTA GTTAGAGAAA AAGAAAGGAT AAAATAAACA ACACTTGAAT TAACCTAAAA GACCATCTCC   
  
  
- TTTCAAGGAA CGACCTTAAA CCAGTTTTCA CTATATATAA AACCCTAAAT TAATAGGTAT TTAAACCAAA   
  
  
- ATGTGGAACA ATCAAACCTG AAGACCACAC AATAACATAC ACAAGACCAC CCAAAAAGAA AATCGACTTA   
  
  
- CAACCTCGTA AAATTCCCTA GATCTATAGA CCTCTTCTCG AACCCTTTAT TTCGTATAAA GGGAATATCA   
  
  
- GACTGTATTG AAATTACTTA ACTAGTCCTT GTTTCCGGTT AATCAAGTTT TTCCAAGTTG AGGAGATCCA   
  
  
- CAACACGATT TCTCTACACT CAATCATTCA TTAACGAATG ATTGATATAT CCTACAAAAT AATGAACATC   
  
  
- TCCAAAAAAA AAACCCTATA GAAATACCCC AAATATTTAT TCTTCCAACT AAACAACAGA TGAGAAGAAA   
  
  
- GCTAAGTTAT CATTAAGAGA GTCTCTTCCG ATTTTACTGT ACCGTAAGGT AGCGGAGAGT ACTCCGAAAA   
  
  
- ACGTACACGA ATACATAGAA AGTAGAGAAC GAAACCACCC CACCGTAACC CCTCCTTTTC CCCCAAAGCT   
  
  
- AAGCTATCTA GCAGTGAAAA CCTTTACTGT AGAGATCGGT TACCGTTCCC ATTCCAATTA TGTACACCTA   
  
  
- TAAAGGTCTC AATCAACACT AACAATAACA ACAACAACAT TGAAATACAG TGCCCCTTTT GTATTATTGA   
  
  
- TAGACAAATA GACAAACAAA CAATAAAAGT TCCCCTTCCC CTCACCCGTC TACGAATCAT ACATTAGTAA   
  
  
- AATATATCCC TTTTAAGTAT ATCGAACTTT CATTTTGAAA GTAACGGTCG TAAGGTTCCA TACCCATCCC   
  
  
- AACAGATGTG GAAATAGAGG GATCTTCGTA CCACTAGTAA GGAGCCTAAC ACAATATCTT ATTTCTTACA   
  
  
- CCAAGACGAC ATTACTCTGA ATACATAATA GTTTACTACT TAGACAAATC TTGAAGGACA AATAAAAAAG   
  
  
- ACGTTTTAAA ATTCTCTGAT TGTAGATAGC CCGAGTTGAG ATCTCCTAGA GAACACAGTG GGAAGAGTTG   
  
  
- AGTCAAACTA ACACTATGTC AATGATATAA CTCACTATCA GCTCTCGTAG TGTAGGGCAA TTCCTCAGAT   
  
  
- AGTTGTTCTT CGGGAAGAAC ATTAAGAGGG GAATTTTGAT CGTCAATAGT AGTGAACAGT AGCTTATCGG   
  
  
- GAAAAACATT AAGATGGGAA CTTTGATCGT CAATAGCAGT AGCCAAGTTG CGCTCAGGAC AATCTATAAG   
  
  
- GGTAGTTCCG TATCTAATAC CTCACTCGTA CTTCTTACGA AACGTCCTCG ATCTCTGATG AGATTAACCA   
  
  
- CATCTACCAC TCCTTCATAG ATAACGATTA GTTGGATACC CCCCTTCAGC AATCAGGCCC TAGGGTTCAA   
  
  
- TCTCTAGCTT CAGTAGTTCG CTTCTAGGTG TCCCAAGTGT AGGATGAGTC GAACTAAGTA GTAGAAAAAG   
  
  
- TTCCCACTCT TCTAAGCCTC TACTTTCGGT CTCTCTCTCT TTACGGTATT CCGTTACCTT CTTGGTCGCC   
  
  
- TTGATGATTC AAAGAGTGGT CCACTAAATT TCGTCAATGA TTAACTTACA CGCTCCCGAA ATAATCTATT   
  
  
- GGCTTATCTA CTGAAACTCT CAAACCAATT TGCCCGTTCC TTTCTCCAGA GGTAGAGTCC ACTCGGACAG   
  
  
- GTTGGAGAGC CATGGACATA CTAGCTTCCC GAACACCGTT CCTTTGTCAG AAGCCCCTGA TTGTAGATAA   
  
  
- CCCGAGAATT CACATTTCTC GGAGAACCTT TTCTGAACGA GAGGATGCCG TGAGTCACCC AATGACATAG   
  
  
- CGTTCGGTAT CGTCGTTCCG AACCACCCGG AGGATTTCAC GCTGAATGTC CGTAACTACT AGGACAAAGA   
  
  
- TTCATATGAA CACTACGATC GAACCTCCGA CCACCCTTTG CTAATCGCAG AGATAAACTT TTCAAATTTT   
  
  
- ATGAACAGCT CAAGTTACGT AACGGGCAAA TACCTGGGTT ACAGTCCACC CTTTACGACC TACACTCCGG   
  
  
- GTCCCTCCGA AACCAGCAAT TAACAGGTAA TGTCGAGGTA ATGTGAGGAC TGCTCTCACA GCTACACTCC   
  
  
- TTGGGATCCC TATCCGAAGA GTCTTACCAC TTTAACGAGC CAGGATTCCA TCGGTGAAAC CAACTCGTTC   
  
  
- TTAGTTTGTG GTTGTGATGT GGAAAGAACT GGGCCAAGTA TCTCTGGGAA CTGATGATGA GCTAATTGGT   
  
  
- ACAAAC

  
  
Motifs Found  

+   

| Site Name | Organism | Position | Strand | Matrix score. | sequence | function |
| --- | --- | --- | --- | --- | --- | --- |
|  | organism | 2170 | + | 4 | motif\_sequence | short\_function |
|  | organism | 1973 | - | 4 | motif\_sequence | short\_function |
|  | organism | 906 | + | 4 | motif\_sequence | short\_function |
|  | organism | 1825 | - | 4 | motif\_sequence | short\_function |
|  | organism | 988 | + | 4 | motif\_sequence | short\_function |
|  | organism | 2081 | + | 4 | motif\_sequence | short\_function |
|  | organism | 1241 | - | 4 | motif\_sequence | short\_function |
|  | organism | 1915 | - | 4 | motif\_sequence | short\_function |
|  | organism | 1349 | + | 4 | motif\_sequence | short\_function |
|  | organism | 2283 | + | 4 | motif\_sequence | short\_function |
|  | organism | 668 | - | 4 | motif\_sequence | short\_function |
|  | organism | 1981 | - | 4 | motif\_sequence | short\_function |
|  | organism | 174 | + | 4 | motif\_sequence | short\_function |
|  | organism | 841 | - | 4 | motif\_sequence | short\_function |
|  | organism | 29 | - | 4 | motif\_sequence | short\_function |
|  | organism | 79 | + | 4 | motif\_sequence | short\_function |
|  | organism | 488 | - | 4 | motif\_sequence | short\_function |

>HU08G00230.1   
+ -Up\_Stream \_Len000GTTGTA ACAAGCACAT TAAAGTTGAT TATCATTTTG TCCGTGAAAC AGTGAGCAAA   
  
  
+ CGTCATCTCC AGGTCAAATT CATTCGTACT CAATCACAAG TTGCTGACAT TTTCACCAAA GGCTAATCAT   
  
  
+ CGCAAAAGTT TCTTTAGTTT AAGGCCAATC TCTCCATTAT TTCACCCTAT ACAGATTGAG GGGGTGTAAT   
  
  
+ AGTGTATATA TATAGCCTTG TGTTAGCGGG CCTTGGACAC TAGACTTTTA CTCTTGCAAA CCCTAATATA   
  
  
+ TTATTATAAA TATAAATCTA GCCACCCATG TTGAGGTAGG CACTCTAATT CTCACACAGG TAGGATAAGT   
  
  
+ TTTCATAGTT TATGTTGTGA ATGTGATCTT ACTTTGTGGT TCCCGTTGAC AAAAGTTTGA ATCCGAGCAT   
  
  
+ GAGAGTTCAT CAATCTCTTT TTCTTTCCTA TTTTATTTGT TGTGAACTTA ATTGGATTTT CTGGTAGAGG   
  
  
+ AAAGTTCCTT GCTGGAATTT GGTCAAAAGT GATATATATT TTGGGATTTA ATTATCCATA AATTTGGTTT   
  
  
+ TACACCTTGT TAGTTTGGAC TTCTGGTGTG TTATTGTATG TGTTCTGGTG GGTTTTTCTT TTAGCTGAAT   
  
  
+ GTTGGAGCAT TTTAAGGGAT CTAGATATCT GGAGAAGAGC TTGGGAAATA AAGCATATTT CCCTTATAGT   
  
  
+ CTGACATAAC TTTAATGAAT TGATCAGGAA CAAAGGCCAA TTAGTTCAAA AAGGTTCAAC TCCTCTAGGT   
  
  
+ GTTGTGCTAA AGAGATGTGA GTTAGTAAGT AATTGCTTAC TAACTATATA GGATGTTTTA TTACTTGTAG   
  
  
+ AGGTTTTTTT TTTGGGATAT CTTTATGGGG TTTATAAATA AGAAGGTTGA TTTGTTGTCT ACTCTTCTTT   
  
  
+ CGATTCAATA GTAATTCTCT CAGAGAAGGC TAAAATGACA TGGCATTCCA TCGCCTCTCA TGAGGCTTTT   
  
  
+ TGCATGTGCT TATGTATCTT TCATCTCTTG CTTTGGTGGG GTGGCATTGG GGAGGAAAAG GGGGTTTCGA   
  
  
+ TTCGATAGAT CGTCACTTTT GGAAATGACA TCTCTAGCCA ATGGCAAGGG TAAGGTTAAT ACATGTGGAT   
  
  
+ ATTTCCAGAG TTAGTTGTGA TTGTTATTGT TGTTGTTGTA ACTTTATGTC ACGGGGAAAA CATAATAACT   
  
  
+ ATCTGTTTAT CTGTTTGTTT GTTATTTTCA AGGGGAAGGG GAGTGGGCAG ATGCTTAGTA TGTAATCATT   
  
  
+ TTATATAGGG AAAATTCATA TAGCTTGAAA GTAAAACTTT CATTGCCAGC ATTCCAAGGT ATGGGTAGGG   
  
  
+ TTGTCTACAC CTTTATCTCC CTAGAAGCAT GGTGATCATT CCTCGGATTG TGTTATAGAA TAAAGAATGT   
  
  
+ GGTTCTGCTG TAATGAGACT TATGTATTAT CAAATGATGA ATCTGTTTAG AACTTCCTGT TTATTTTTTC   
  
  
+ TGCAAAATTT TAAGAGACTA ACATCTATCG GGCTCAACTC TAGAGGATCT CTTGTGTCAC CCTTCTCAAC   
  
  
+ TCAGTTTGAT TGTGATACAG TTACTATATT GAGTGATAGT CGAGAGCATC ACATCCCGTT AAGGAGTCTA   
  
  
+ TCAACAAGAA GCCCTTCTTG TAATTCTCCC CTTAAAACTA GCAGTTATCA TCACTTGTCA TCGAATAGCC   
  
  
+ CTTTTTGTAA TTCTACCCTT GAAACTAGCA GTTATCGTCA TCGGTTCAAC GCGAGTCCTG TTAGATATTC   
  
  
+ CCATCAAGGC ATAGATTATG GAGTGAGCAT GAAGAATGCT TTGCAGGAGC TAGAGACTAC TCTAATTGGT   
  
  
+ GTAGATGGTG AGGAAGTATC TATTGCTAAT CAACCTATGG GGGGAAGTCG TTAGTCCGGG ATCCCAAGTT   
  
  
+ AGAGATCGAA GTCATCAAGC GAAGATCCAC AGGGTTCACA TCCTACTCAG CTTGATTCAT CATCTTTTTC   
  
  
+ AAGGGTGAGA AGATTCGGAG ATGAAAGCCA GAGAGAGAGA AATGCCATAA GGCAATGGAA GAACCAGCGG   
  
  
+ AACTACTAAG TTTCTCACCA GGTGATTTAA AGCAGTTACT AATTGAATGT GCGAGGGCTT TATTAGATAA   
  
  
+ CCGAATAGAT GACTTTGAGA GTTTGGTTAA ACGGGCAAGG AAAGAGGTCT CCATCTCAGG TGAGCCTGTC   
  
  
+ CAACCTCTCG GTACCTGTAT GATCGAAGGG CTTGTGGCAA GGAAACAGTC TTCGGGGACT AACATCTATT   
  
  
+ GGGCTCTTAA GTGTAAAGAG CCTCTTGGAA AAGACTTGCT CTCCTACGGC ACTCAGTGGG TTACTGTATC   
  
  
+ GCAAGCCATA GCAGCAAGGC TTGGTGGGCC TCCTAAAGTG CGACTTACAG GCATTGATGA TCCTGTTTCT   
  
  
+ AAGTATACTT GTGATGCTAG CTTGGAGGCT GGTGGGAAAC GATTAGCGTC TCTATTTGAA AAGTTTAAAA   
  
  
+ TACTTGTCGA GTTCAATGCA TTGCCCGTTT ATGGACCCAA TGTCAGGTGG GAAATGCTGG ATGTGAGGCC   
  
  
+ CAGGGAGGCT TTGGTCGTTA ATTGTCCATT ACAGCTCCAT TACACTCCTG ACGAGAGTGT CGATGTGAGG   
  
  
+ AACCCTAGGG ATAGGCTTCT CAGAATGGTG AAATTGCTCG GTCCTAAGGT AGCCACTTTG GTTGAGCAAG   
  
  
+ AATCAAACAC CAACACTACA CCTTTCTTGA CCCGGTTCAT AGAGACCCTT GACTACTACT CGATTAACCA   
  
  
+ TGTTTG  

- -Up\_Stream \_Len000CAACAT TGTTCGTGTA ATTTCAACTA ATAGTAAAAC AGGCACTTTG TCACTCGTTT   
  
  
- GCAGTAGAGG TCCAGTTTAA GTAAGCATGA GTTAGTGTTC AACGACTGTA AAAGTGGTTT CCGATTAGTA   
  
  
- GCGTTTTCAA AGAAATCAAA TTCCGGTTAG AGAGGTAATA AAGTGGGATA TGTCTAACTC CCCCACATTA   
  
  
- TCACATATAT ATATCGGAAC ACAATCGCCC GGAACCTGTG ATCTGAAAAT GAGAACGTTT GGGATTATAT   
  
  
- AATAATATTT ATATTTAGAT CGGTGGGTAC AACTCCATCC GTGAGATTAA GAGTGTGTCC ATCCTATTCA   
  
  
- AAAGTATCAA ATACAACACT TACACTAGAA TGAAACACCA AGGGCAACTG TTTTCAAACT TAGGCTCGTA   
  
  
- CTCTCAAGTA GTTAGAGAAA AAGAAAGGAT AAAATAAACA ACACTTGAAT TAACCTAAAA GACCATCTCC   
  
  
- TTTCAAGGAA CGACCTTAAA CCAGTTTTCA CTATATATAA AACCCTAAAT TAATAGGTAT TTAAACCAAA   
  
  
- ATGTGGAACA ATCAAACCTG AAGACCACAC AATAACATAC ACAAGACCAC CCAAAAAGAA AATCGACTTA   
  
  
- CAACCTCGTA AAATTCCCTA GATCTATAGA CCTCTTCTCG AACCCTTTAT TTCGTATAAA GGGAATATCA   
  
  
- GACTGTATTG AAATTACTTA ACTAGTCCTT GTTTCCGGTT AATCAAGTTT TTCCAAGTTG AGGAGATCCA   
  
  
- CAACACGATT TCTCTACACT CAATCATTCA TTAACGAATG ATTGATATAT CCTACAAAAT AATGAACATC   
  
  
- TCCAAAAAAA AAACCCTATA GAAATACCCC AAATATTTAT TCTTCCAACT AAACAACAGA TGAGAAGAAA   
  
  
- GCTAAGTTAT CATTAAGAGA GTCTCTTCCG ATTTTACTGT ACCGTAAGGT AGCGGAGAGT ACTCCGAAAA   
  
  
- ACGTACACGA ATACATAGAA AGTAGAGAAC GAAACCACCC CACCGTAACC CCTCCTTTTC CCCCAAAGCT   
  
  
- AAGCTATCTA GCAGTGAAAA CCTTTACTGT AGAGATCGGT TACCGTTCCC ATTCCAATTA TGTACACCTA   
  
  
- TAAAGGTCTC AATCAACACT AACAATAACA ACAACAACAT TGAAATACAG TGCCCCTTTT GTATTATTGA   
  
  
- TAGACAAATA GACAAACAAA CAATAAAAGT TCCCCTTCCC CTCACCCGTC TACGAATCAT ACATTAGTAA   
  
  
- AATATATCCC TTTTAAGTAT ATCGAACTTT CATTTTGAAA GTAACGGTCG TAAGGTTCCA TACCCATCCC   
  
  
- AACAGATGTG GAAATAGAGG GATCTTCGTA CCACTAGTAA GGAGCCTAAC ACAATATCTT ATTTCTTACA   
  
  
- CCAAGACGAC ATTACTCTGA ATACATAATA GTTTACTACT TAGACAAATC TTGAAGGACA AATAAAAAAG   
  
  
- ACGTTTTAAA ATTCTCTGAT TGTAGATAGC CCGAGTTGAG ATCTCCTAGA GAACACAGTG GGAAGAGTTG   
  
  
- AGTCAAACTA ACACTATGTC AATGATATAA CTCACTATCA GCTCTCGTAG TGTAGGGCAA TTCCTCAGAT   
  
  
- AGTTGTTCTT CGGGAAGAAC ATTAAGAGGG GAATTTTGAT CGTCAATAGT AGTGAACAGT AGCTTATCGG   
  
  
- GAAAAACATT AAGATGGGAA CTTTGATCGT CAATAGCAGT AGCCAAGTTG CGCTCAGGAC AATCTATAAG   
  
  
- GGTAGTTCCG TATCTAATAC CTCACTCGTA CTTCTTACGA AACGTCCTCG ATCTCTGATG AGATTAACCA   
  
  
- CATCTACCAC TCCTTCATAG ATAACGATTA GTTGGATACC CCCCTTCAGC AATCAGGCCC TAGGGTTCAA   
  
  
- TCTCTAGCTT CAGTAGTTCG CTTCTAGGTG TCCCAAGTGT AGGATGAGTC GAACTAAGTA GTAGAAAAAG   
  
  
- TTCCCACTCT TCTAAGCCTC TACTTTCGGT CTCTCTCTCT TTACGGTATT CCGTTACCTT CTTGGTCGCC   
  
  
- TTGATGATTC AAAGAGTGGT CCACTAAATT TCGTCAATGA TTAACTTACA CGCTCCCGAA ATAATCTATT   
  
  
- GGCTTATCTA CTGAAACTCT CAAACCAATT TGCCCGTTCC TTTCTCCAGA GGTAGAGTCC ACTCGGACAG   
  
  
- GTTGGAGAGC CATGGACATA CTAGCTTCCC GAACACCGTT CCTTTGTCAG AAGCCCCTGA TTGTAGATAA   
  
  
- CCCGAGAATT CACATTTCTC GGAGAACCTT TTCTGAACGA GAGGATGCCG TGAGTCACCC AATGACATAG   
  
  
- CGTTCGGTAT CGTCGTTCCG AACCACCCGG AGGATTTCAC GCTGAATGTC CGTAACTACT AGGACAAAGA   
  
  
- TTCATATGAA CACTACGATC GAACCTCCGA CCACCCTTTG CTAATCGCAG AGATAAACTT TTCAAATTTT   
  
  
- ATGAACAGCT CAAGTTACGT AACGGGCAAA TACCTGGGTT ACAGTCCACC CTTTACGACC TACACTCCGG   
  
  
- GTCCCTCCGA AACCAGCAAT TAACAGGTAA TGTCGAGGTA ATGTGAGGAC TGCTCTCACA GCTACACTCC   
  
  
- TTGGGATCCC TATCCGAAGA GTCTTACCAC TTTAACGAGC CAGGATTCCA TCGGTGAAAC CAACTCGTTC   
  
  
- TTAGTTTGTG GTTGTGATGT GGAAAGAACT GGGCCAAGTA TCTCTGGGAA CTGATGATGA GCTAATTGGT   
  
  
- ACAAAC

+     AAGAA-motif

| Site Name | Organism | Position | Strand | Matrix score. | sequence | function |
| --- | --- | --- | --- | --- | --- | --- |
| AAGAA-motif | Avena sativa | 445 | - | 7 | GAAAGAA |  |
| AAGAA-motif | Avena sativa | 909 | - | 7 | GAAAGAA |  |
| AAGAA-motif | Avena sativa | 2687 | - | 9 | gGTAAAGAAA |  |

>HU08G00230.1   
+ -Up\_Stream \_Len000GTTGTA ACAAGCACAT TAAAGTTGAT TATCATTTTG TCCGTGAAAC AGTGAGCAAA   
  
  
+ CGTCATCTCC AGGTCAAATT CATTCGTACT CAATCACAAG TTGCTGACAT TTTCACCAAA GGCTAATCAT   
  
  
+ CGCAAAAGTT TCTTTAGTTT AAGGCCAATC TCTCCATTAT TTCACCCTAT ACAGATTGAG GGGGTGTAAT   
  
  
+ AGTGTATATA TATAGCCTTG TGTTAGCGGG CCTTGGACAC TAGACTTTTA CTCTTGCAAA CCCTAATATA   
  
  
+ TTATTATAAA TATAAATCTA GCCACCCATG TTGAGGTAGG CACTCTAATT CTCACACAGG TAGGATAAGT   
  
  
+ TTTCATAGTT TATGTTGTGA ATGTGATCTT ACTTTGTGGT TCCCGTTGAC AAAAGTTTGA ATCCGAGCAT   
  
  
+ GAGAGTTCAT CAATCTCTTT TTCTTTCCTA TTTTATTTGT TGTGAACTTA ATTGGATTTT CTGGTAGAGG   
  
  
+ AAAGTTCCTT GCTGGAATTT GGTCAAAAGT GATATATATT TTGGGATTTA ATTATCCATA AATTTGGTTT   
  
  
+ TACACCTTGT TAGTTTGGAC TTCTGGTGTG TTATTGTATG TGTTCTGGTG GGTTTTTCTT TTAGCTGAAT   
  
  
+ GTTGGAGCAT TTTAAGGGAT CTAGATATCT GGAGAAGAGC TTGGGAAATA AAGCATATTT CCCTTATAGT   
  
  
+ CTGACATAAC TTTAATGAAT TGATCAGGAA CAAAGGCCAA TTAGTTCAAA AAGGTTCAAC TCCTCTAGGT   
  
  
+ GTTGTGCTAA AGAGATGTGA GTTAGTAAGT AATTGCTTAC TAACTATATA GGATGTTTTA TTACTTGTAG   
  
  
+ AGGTTTTTTT TTTGGGATAT CTTTATGGGG TTTATAAATA AGAAGGTTGA TTTGTTGTCT ACTCTTCTTT   
  
  
+ CGATTCAATA GTAATTCTCT CAGAGAAGGC TAAAATGACA TGGCATTCCA TCGCCTCTCA TGAGGCTTTT   
  
  
+ TGCATGTGCT TATGTATCTT TCATCTCTTG CTTTGGTGGG GTGGCATTGG GGAGGAAAAG GGGGTTTCGA   
  
  
+ TTCGATAGAT CGTCACTTTT GGAAATGACA TCTCTAGCCA ATGGCAAGGG TAAGGTTAAT ACATGTGGAT   
  
  
+ ATTTCCAGAG TTAGTTGTGA TTGTTATTGT TGTTGTTGTA ACTTTATGTC ACGGGGAAAA CATAATAACT   
  
  
+ ATCTGTTTAT CTGTTTGTTT GTTATTTTCA AGGGGAAGGG GAGTGGGCAG ATGCTTAGTA TGTAATCATT   
  
  
+ TTATATAGGG AAAATTCATA TAGCTTGAAA GTAAAACTTT CATTGCCAGC ATTCCAAGGT ATGGGTAGGG   
  
  
+ TTGTCTACAC CTTTATCTCC CTAGAAGCAT GGTGATCATT CCTCGGATTG TGTTATAGAA TAAAGAATGT   
  
  
+ GGTTCTGCTG TAATGAGACT TATGTATTAT CAAATGATGA ATCTGTTTAG AACTTCCTGT TTATTTTTTC   
  
  
+ TGCAAAATTT TAAGAGACTA ACATCTATCG GGCTCAACTC TAGAGGATCT CTTGTGTCAC CCTTCTCAAC   
  
  
+ TCAGTTTGAT TGTGATACAG TTACTATATT GAGTGATAGT CGAGAGCATC ACATCCCGTT AAGGAGTCTA   
  
  
+ TCAACAAGAA GCCCTTCTTG TAATTCTCCC CTTAAAACTA GCAGTTATCA TCACTTGTCA TCGAATAGCC   
  
  
+ CTTTTTGTAA TTCTACCCTT GAAACTAGCA GTTATCGTCA TCGGTTCAAC GCGAGTCCTG TTAGATATTC   
  
  
+ CCATCAAGGC ATAGATTATG GAGTGAGCAT GAAGAATGCT TTGCAGGAGC TAGAGACTAC TCTAATTGGT   
  
  
+ GTAGATGGTG AGGAAGTATC TATTGCTAAT CAACCTATGG GGGGAAGTCG TTAGTCCGGG ATCCCAAGTT   
  
  
+ AGAGATCGAA GTCATCAAGC GAAGATCCAC AGGGTTCACA TCCTACTCAG CTTGATTCAT CATCTTTTTC   
  
  
+ AAGGGTGAGA AGATTCGGAG ATGAAAGCCA GAGAGAGAGA AATGCCATAA GGCAATGGAA GAACCAGCGG   
  
  
+ AACTACTAAG TTTCTCACCA GGTGATTTAA AGCAGTTACT AATTGAATGT GCGAGGGCTT TATTAGATAA   
  
  
+ CCGAATAGAT GACTTTGAGA GTTTGGTTAA ACGGGCAAGG AAAGAGGTCT CCATCTCAGG TGAGCCTGTC   
  
  
+ CAACCTCTCG GTACCTGTAT GATCGAAGGG CTTGTGGCAA GGAAACAGTC TTCGGGGACT AACATCTATT   
  
  
+ GGGCTCTTAA GTGTAAAGAG CCTCTTGGAA AAGACTTGCT CTCCTACGGC ACTCAGTGGG TTACTGTATC   
  
  
+ GCAAGCCATA GCAGCAAGGC TTGGTGGGCC TCCTAAAGTG CGACTTACAG GCATTGATGA TCCTGTTTCT   
  
  
+ AAGTATACTT GTGATGCTAG CTTGGAGGCT GGTGGGAAAC GATTAGCGTC TCTATTTGAA AAGTTTAAAA   
  
  
+ TACTTGTCGA GTTCAATGCA TTGCCCGTTT ATGGACCCAA TGTCAGGTGG GAAATGCTGG ATGTGAGGCC   
  
  
+ CAGGGAGGCT TTGGTCGTTA ATTGTCCATT ACAGCTCCAT TACACTCCTG ACGAGAGTGT CGATGTGAGG   
  
  
+ AACCCTAGGG ATAGGCTTCT CAGAATGGTG AAATTGCTCG GTCCTAAGGT AGCCACTTTG GTTGAGCAAG   
  
  
+ AATCAAACAC CAACACTACA CCTTTCTTGA CCCGGTTCAT AGAGACCCTT GACTACTACT CGATTAACCA   
  
  
+ TGTTTG  

- -Up\_Stream \_Len000CAACAT TGTTCGTGTA ATTTCAACTA ATAGTAAAAC AGGCACTTTG TCACTCGTTT   
  
  
- GCAGTAGAGG TCCAGTTTAA GTAAGCATGA GTTAGTGTTC AACGACTGTA AAAGTGGTTT CCGATTAGTA   
  
  
- GCGTTTTCAA AGAAATCAAA TTCCGGTTAG AGAGGTAATA AAGTGGGATA TGTCTAACTC CCCCACATTA   
  
  
- TCACATATAT ATATCGGAAC ACAATCGCCC GGAACCTGTG ATCTGAAAAT GAGAACGTTT GGGATTATAT   
  
  
- AATAATATTT ATATTTAGAT CGGTGGGTAC AACTCCATCC GTGAGATTAA GAGTGTGTCC ATCCTATTCA   
  
  
- AAAGTATCAA ATACAACACT TACACTAGAA TGAAACACCA AGGGCAACTG TTTTCAAACT TAGGCTCGTA   
  
  
- CTCTCAAGTA GTTAGAGAAA AAGAAAGGAT AAAATAAACA ACACTTGAAT TAACCTAAAA GACCATCTCC   
  
  
- TTTCAAGGAA CGACCTTAAA CCAGTTTTCA CTATATATAA AACCCTAAAT TAATAGGTAT TTAAACCAAA   
  
  
- ATGTGGAACA ATCAAACCTG AAGACCACAC AATAACATAC ACAAGACCAC CCAAAAAGAA AATCGACTTA   
  
  
- CAACCTCGTA AAATTCCCTA GATCTATAGA CCTCTTCTCG AACCCTTTAT TTCGTATAAA GGGAATATCA   
  
  
- GACTGTATTG AAATTACTTA ACTAGTCCTT GTTTCCGGTT AATCAAGTTT TTCCAAGTTG AGGAGATCCA   
  
  
- CAACACGATT TCTCTACACT CAATCATTCA TTAACGAATG ATTGATATAT CCTACAAAAT AATGAACATC   
  
  
- TCCAAAAAAA AAACCCTATA GAAATACCCC AAATATTTAT TCTTCCAACT AAACAACAGA TGAGAAGAAA   
  
  
- GCTAAGTTAT CATTAAGAGA GTCTCTTCCG ATTTTACTGT ACCGTAAGGT AGCGGAGAGT ACTCCGAAAA   
  
  
- ACGTACACGA ATACATAGAA AGTAGAGAAC GAAACCACCC CACCGTAACC CCTCCTTTTC CCCCAAAGCT   
  
  
- AAGCTATCTA GCAGTGAAAA CCTTTACTGT AGAGATCGGT TACCGTTCCC ATTCCAATTA TGTACACCTA   
  
  
- TAAAGGTCTC AATCAACACT AACAATAACA ACAACAACAT TGAAATACAG TGCCCCTTTT GTATTATTGA   
  
  
- TAGACAAATA GACAAACAAA CAATAAAAGT TCCCCTTCCC CTCACCCGTC TACGAATCAT ACATTAGTAA   
  
  
- AATATATCCC TTTTAAGTAT ATCGAACTTT CATTTTGAAA GTAACGGTCG TAAGGTTCCA TACCCATCCC   
  
  
- AACAGATGTG GAAATAGAGG GATCTTCGTA CCACTAGTAA GGAGCCTAAC ACAATATCTT ATTTCTTACA   
  
  
- CCAAGACGAC ATTACTCTGA ATACATAATA GTTTACTACT TAGACAAATC TTGAAGGACA AATAAAAAAG   
  
  
- ACGTTTTAAA ATTCTCTGAT TGTAGATAGC CCGAGTTGAG ATCTCCTAGA GAACACAGTG GGAAGAGTTG   
  
  
- AGTCAAACTA ACACTATGTC AATGATATAA CTCACTATCA GCTCTCGTAG TGTAGGGCAA TTCCTCAGAT   
  
  
- AGTTGTTCTT CGGGAAGAAC ATTAAGAGGG GAATTTTGAT CGTCAATAGT AGTGAACAGT AGCTTATCGG   
  
  
- GAAAAACATT AAGATGGGAA CTTTGATCGT CAATAGCAGT AGCCAAGTTG CGCTCAGGAC AATCTATAAG   
  
  
- GGTAGTTCCG TATCTAATAC CTCACTCGTA CTTCTTACGA AACGTCCTCG ATCTCTGATG AGATTAACCA   
  
  
- CATCTACCAC TCCTTCATAG ATAACGATTA GTTGGATACC CCCCTTCAGC AATCAGGCCC TAGGGTTCAA   
  
  
- TCTCTAGCTT CAGTAGTTCG CTTCTAGGTG TCCCAAGTGT AGGATGAGTC GAACTAAGTA GTAGAAAAAG   
  
  
- TTCCCACTCT TCTAAGCCTC TACTTTCGGT CTCTCTCTCT TTACGGTATT CCGTTACCTT CTTGGTCGCC   
  
  
- TTGATGATTC AAAGAGTGGT CCACTAAATT TCGTCAATGA TTAACTTACA CGCTCCCGAA ATAATCTATT   
  
  
- GGCTTATCTA CTGAAACTCT CAAACCAATT TGCCCGTTCC TTTCTCCAGA GGTAGAGTCC ACTCGGACAG   
  
  
- GTTGGAGAGC CATGGACATA CTAGCTTCCC GAACACCGTT CCTTTGTCAG AAGCCCCTGA TTGTAGATAA   
  
  
- CCCGAGAATT CACATTTCTC GGAGAACCTT TTCTGAACGA GAGGATGCCG TGAGTCACCC AATGACATAG   
  
  
- CGTTCGGTAT CGTCGTTCCG AACCACCCGG AGGATTTCAC GCTGAATGTC CGTAACTACT AGGACAAAGA   
  
  
- TTCATATGAA CACTACGATC GAACCTCCGA CCACCCTTTG CTAATCGCAG AGATAAACTT TTCAAATTTT   
  
  
- ATGAACAGCT CAAGTTACGT AACGGGCAAA TACCTGGGTT ACAGTCCACC CTTTACGACC TACACTCCGG   
  
  
- GTCCCTCCGA AACCAGCAAT TAACAGGTAA TGTCGAGGTA ATGTGAGGAC TGCTCTCACA GCTACACTCC   
  
  
- TTGGGATCCC TATCCGAAGA GTCTTACCAC TTTAACGAGC CAGGATTCCA TCGGTGAAAC CAACTCGTTC   
  
  
- TTAGTTTGTG GTTGTGATGT GGAAAGAACT GGGCCAAGTA TCTCTGGGAA CTGATGATGA GCTAATTGGT   
  
  
- ACAAAC

+     AE-box

| Site Name | Organism | Position | Strand | Matrix score. | sequence | function |
| --- | --- | --- | --- | --- | --- | --- |
| AE-box | Arabidopsis thaliana | 150 | - | 8 | AGAAACTT | part of a module for light response |
| AE-box | Arabidopsis thaliana | 2042 | - | 8 | AGAAACTT | part of a module for light response |

>HU08G00230.1   
+ -Up\_Stream \_Len000GTTGTA ACAAGCACAT TAAAGTTGAT TATCATTTTG TCCGTGAAAC AGTGAGCAAA   
  
  
+ CGTCATCTCC AGGTCAAATT CATTCGTACT CAATCACAAG TTGCTGACAT TTTCACCAAA GGCTAATCAT   
  
  
+ CGCAAAAGTT TCTTTAGTTT AAGGCCAATC TCTCCATTAT TTCACCCTAT ACAGATTGAG GGGGTGTAAT   
  
  
+ AGTGTATATA TATAGCCTTG TGTTAGCGGG CCTTGGACAC TAGACTTTTA CTCTTGCAAA CCCTAATATA   
  
  
+ TTATTATAAA TATAAATCTA GCCACCCATG TTGAGGTAGG CACTCTAATT CTCACACAGG TAGGATAAGT   
  
  
+ TTTCATAGTT TATGTTGTGA ATGTGATCTT ACTTTGTGGT TCCCGTTGAC AAAAGTTTGA ATCCGAGCAT   
  
  
+ GAGAGTTCAT CAATCTCTTT TTCTTTCCTA TTTTATTTGT TGTGAACTTA ATTGGATTTT CTGGTAGAGG   
  
  
+ AAAGTTCCTT GCTGGAATTT GGTCAAAAGT GATATATATT TTGGGATTTA ATTATCCATA AATTTGGTTT   
  
  
+ TACACCTTGT TAGTTTGGAC TTCTGGTGTG TTATTGTATG TGTTCTGGTG GGTTTTTCTT TTAGCTGAAT   
  
  
+ GTTGGAGCAT TTTAAGGGAT CTAGATATCT GGAGAAGAGC TTGGGAAATA AAGCATATTT CCCTTATAGT   
  
  
+ CTGACATAAC TTTAATGAAT TGATCAGGAA CAAAGGCCAA TTAGTTCAAA AAGGTTCAAC TCCTCTAGGT   
  
  
+ GTTGTGCTAA AGAGATGTGA GTTAGTAAGT AATTGCTTAC TAACTATATA GGATGTTTTA TTACTTGTAG   
  
  
+ AGGTTTTTTT TTTGGGATAT CTTTATGGGG TTTATAAATA AGAAGGTTGA TTTGTTGTCT ACTCTTCTTT   
  
  
+ CGATTCAATA GTAATTCTCT CAGAGAAGGC TAAAATGACA TGGCATTCCA TCGCCTCTCA TGAGGCTTTT   
  
  
+ TGCATGTGCT TATGTATCTT TCATCTCTTG CTTTGGTGGG GTGGCATTGG GGAGGAAAAG GGGGTTTCGA   
  
  
+ TTCGATAGAT CGTCACTTTT GGAAATGACA TCTCTAGCCA ATGGCAAGGG TAAGGTTAAT ACATGTGGAT   
  
  
+ ATTTCCAGAG TTAGTTGTGA TTGTTATTGT TGTTGTTGTA ACTTTATGTC ACGGGGAAAA CATAATAACT   
  
  
+ ATCTGTTTAT CTGTTTGTTT GTTATTTTCA AGGGGAAGGG GAGTGGGCAG ATGCTTAGTA TGTAATCATT   
  
  
+ TTATATAGGG AAAATTCATA TAGCTTGAAA GTAAAACTTT CATTGCCAGC ATTCCAAGGT ATGGGTAGGG   
  
  
+ TTGTCTACAC CTTTATCTCC CTAGAAGCAT GGTGATCATT CCTCGGATTG TGTTATAGAA TAAAGAATGT   
  
  
+ GGTTCTGCTG TAATGAGACT TATGTATTAT CAAATGATGA ATCTGTTTAG AACTTCCTGT TTATTTTTTC   
  
  
+ TGCAAAATTT TAAGAGACTA ACATCTATCG GGCTCAACTC TAGAGGATCT CTTGTGTCAC CCTTCTCAAC   
  
  
+ TCAGTTTGAT TGTGATACAG TTACTATATT GAGTGATAGT CGAGAGCATC ACATCCCGTT AAGGAGTCTA   
  
  
+ TCAACAAGAA GCCCTTCTTG TAATTCTCCC CTTAAAACTA GCAGTTATCA TCACTTGTCA TCGAATAGCC   
  
  
+ CTTTTTGTAA TTCTACCCTT GAAACTAGCA GTTATCGTCA TCGGTTCAAC GCGAGTCCTG TTAGATATTC   
  
  
+ CCATCAAGGC ATAGATTATG GAGTGAGCAT GAAGAATGCT TTGCAGGAGC TAGAGACTAC TCTAATTGGT   
  
  
+ GTAGATGGTG AGGAAGTATC TATTGCTAAT CAACCTATGG GGGGAAGTCG TTAGTCCGGG ATCCCAAGTT   
  
  
+ AGAGATCGAA GTCATCAAGC GAAGATCCAC AGGGTTCACA TCCTACTCAG CTTGATTCAT CATCTTTTTC   
  
  
+ AAGGGTGAGA AGATTCGGAG ATGAAAGCCA GAGAGAGAGA AATGCCATAA GGCAATGGAA GAACCAGCGG   
  
  
+ AACTACTAAG TTTCTCACCA GGTGATTTAA AGCAGTTACT AATTGAATGT GCGAGGGCTT TATTAGATAA   
  
  
+ CCGAATAGAT GACTTTGAGA GTTTGGTTAA ACGGGCAAGG AAAGAGGTCT CCATCTCAGG TGAGCCTGTC   
  
  
+ CAACCTCTCG GTACCTGTAT GATCGAAGGG CTTGTGGCAA GGAAACAGTC TTCGGGGACT AACATCTATT   
  
  
+ GGGCTCTTAA GTGTAAAGAG CCTCTTGGAA AAGACTTGCT CTCCTACGGC ACTCAGTGGG TTACTGTATC   
  
  
+ GCAAGCCATA GCAGCAAGGC TTGGTGGGCC TCCTAAAGTG CGACTTACAG GCATTGATGA TCCTGTTTCT   
  
  
+ AAGTATACTT GTGATGCTAG CTTGGAGGCT GGTGGGAAAC GATTAGCGTC TCTATTTGAA AAGTTTAAAA   
  
  
+ TACTTGTCGA GTTCAATGCA TTGCCCGTTT ATGGACCCAA TGTCAGGTGG GAAATGCTGG ATGTGAGGCC   
  
  
+ CAGGGAGGCT TTGGTCGTTA ATTGTCCATT ACAGCTCCAT TACACTCCTG ACGAGAGTGT CGATGTGAGG   
  
  
+ AACCCTAGGG ATAGGCTTCT CAGAATGGTG AAATTGCTCG GTCCTAAGGT AGCCACTTTG GTTGAGCAAG   
  
  
+ AATCAAACAC CAACACTACA CCTTTCTTGA CCCGGTTCAT AGAGACCCTT GACTACTACT CGATTAACCA   
  
  
+ TGTTTG  

- -Up\_Stream \_Len000CAACAT TGTTCGTGTA ATTTCAACTA ATAGTAAAAC AGGCACTTTG TCACTCGTTT   
  
  
- GCAGTAGAGG TCCAGTTTAA GTAAGCATGA GTTAGTGTTC AACGACTGTA AAAGTGGTTT CCGATTAGTA   
  
  
- GCGTTTTCAA AGAAATCAAA TTCCGGTTAG AGAGGTAATA AAGTGGGATA TGTCTAACTC CCCCACATTA   
  
  
- TCACATATAT ATATCGGAAC ACAATCGCCC GGAACCTGTG ATCTGAAAAT GAGAACGTTT GGGATTATAT   
  
  
- AATAATATTT ATATTTAGAT CGGTGGGTAC AACTCCATCC GTGAGATTAA GAGTGTGTCC ATCCTATTCA   
  
  
- AAAGTATCAA ATACAACACT TACACTAGAA TGAAACACCA AGGGCAACTG TTTTCAAACT TAGGCTCGTA   
  
  
- CTCTCAAGTA GTTAGAGAAA AAGAAAGGAT AAAATAAACA ACACTTGAAT TAACCTAAAA GACCATCTCC   
  
  
- TTTCAAGGAA CGACCTTAAA CCAGTTTTCA CTATATATAA AACCCTAAAT TAATAGGTAT TTAAACCAAA   
  
  
- ATGTGGAACA ATCAAACCTG AAGACCACAC AATAACATAC ACAAGACCAC CCAAAAAGAA AATCGACTTA   
  
  
- CAACCTCGTA AAATTCCCTA GATCTATAGA CCTCTTCTCG AACCCTTTAT TTCGTATAAA GGGAATATCA   
  
  
- GACTGTATTG AAATTACTTA ACTAGTCCTT GTTTCCGGTT AATCAAGTTT TTCCAAGTTG AGGAGATCCA   
  
  
- CAACACGATT TCTCTACACT CAATCATTCA TTAACGAATG ATTGATATAT CCTACAAAAT AATGAACATC   
  
  
- TCCAAAAAAA AAACCCTATA GAAATACCCC AAATATTTAT TCTTCCAACT AAACAACAGA TGAGAAGAAA   
  
  
- GCTAAGTTAT CATTAAGAGA GTCTCTTCCG ATTTTACTGT ACCGTAAGGT AGCGGAGAGT ACTCCGAAAA   
  
  
- ACGTACACGA ATACATAGAA AGTAGAGAAC GAAACCACCC CACCGTAACC CCTCCTTTTC CCCCAAAGCT   
  
  
- AAGCTATCTA GCAGTGAAAA CCTTTACTGT AGAGATCGGT TACCGTTCCC ATTCCAATTA TGTACACCTA   
  
  
- TAAAGGTCTC AATCAACACT AACAATAACA ACAACAACAT TGAAATACAG TGCCCCTTTT GTATTATTGA   
  
  
- TAGACAAATA GACAAACAAA CAATAAAAGT TCCCCTTCCC CTCACCCGTC TACGAATCAT ACATTAGTAA   
  
  
- AATATATCCC TTTTAAGTAT ATCGAACTTT CATTTTGAAA GTAACGGTCG TAAGGTTCCA TACCCATCCC   
  
  
- AACAGATGTG GAAATAGAGG GATCTTCGTA CCACTAGTAA GGAGCCTAAC ACAATATCTT ATTTCTTACA   
  
  
- CCAAGACGAC ATTACTCTGA ATACATAATA GTTTACTACT TAGACAAATC TTGAAGGACA AATAAAAAAG   
  
  
- ACGTTTTAAA ATTCTCTGAT TGTAGATAGC CCGAGTTGAG ATCTCCTAGA GAACACAGTG GGAAGAGTTG   
  
  
- AGTCAAACTA ACACTATGTC AATGATATAA CTCACTATCA GCTCTCGTAG TGTAGGGCAA TTCCTCAGAT   
  
  
- AGTTGTTCTT CGGGAAGAAC ATTAAGAGGG GAATTTTGAT CGTCAATAGT AGTGAACAGT AGCTTATCGG   
  
  
- GAAAAACATT AAGATGGGAA CTTTGATCGT CAATAGCAGT AGCCAAGTTG CGCTCAGGAC AATCTATAAG   
  
  
- GGTAGTTCCG TATCTAATAC CTCACTCGTA CTTCTTACGA AACGTCCTCG ATCTCTGATG AGATTAACCA   
  
  
- CATCTACCAC TCCTTCATAG ATAACGATTA GTTGGATACC CCCCTTCAGC AATCAGGCCC TAGGGTTCAA   
  
  
- TCTCTAGCTT CAGTAGTTCG CTTCTAGGTG TCCCAAGTGT AGGATGAGTC GAACTAAGTA GTAGAAAAAG   
  
  
- TTCCCACTCT TCTAAGCCTC TACTTTCGGT CTCTCTCTCT TTACGGTATT CCGTTACCTT CTTGGTCGCC   
  
  
- TTGATGATTC AAAGAGTGGT CCACTAAATT TCGTCAATGA TTAACTTACA CGCTCCCGAA ATAATCTATT   
  
  
- GGCTTATCTA CTGAAACTCT CAAACCAATT TGCCCGTTCC TTTCTCCAGA GGTAGAGTCC ACTCGGACAG   
  
  
- GTTGGAGAGC CATGGACATA CTAGCTTCCC GAACACCGTT CCTTTGTCAG AAGCCCCTGA TTGTAGATAA   
  
  
- CCCGAGAATT CACATTTCTC GGAGAACCTT TTCTGAACGA GAGGATGCCG TGAGTCACCC AATGACATAG   
  
  
- CGTTCGGTAT CGTCGTTCCG AACCACCCGG AGGATTTCAC GCTGAATGTC CGTAACTACT AGGACAAAGA   
  
  
- TTCATATGAA CACTACGATC GAACCTCCGA CCACCCTTTG CTAATCGCAG AGATAAACTT TTCAAATTTT   
  
  
- ATGAACAGCT CAAGTTACGT AACGGGCAAA TACCTGGGTT ACAGTCCACC CTTTACGACC TACACTCCGG   
  
  
- GTCCCTCCGA AACCAGCAAT TAACAGGTAA TGTCGAGGTA ATGTGAGGAC TGCTCTCACA GCTACACTCC   
  
  
- TTGGGATCCC TATCCGAAGA GTCTTACCAC TTTAACGAGC CAGGATTCCA TCGGTGAAAC CAACTCGTTC   
  
  
- TTAGTTTGTG GTTGTGATGT GGAAAGAACT GGGCCAAGTA TCTCTGGGAA CTGATGATGA GCTAATTGGT   
  
  
- ACAAAC

+     AP-1

| Site Name | Organism | Position | Strand | Matrix score. | sequence | function |
| --- | --- | --- | --- | --- | --- | --- |
| AP-1 | Arabidopsis thaliana | 792 | + | 8 | TGAGTTAG |  |

>HU08G00230.1   
+ -Up\_Stream \_Len000GTTGTA ACAAGCACAT TAAAGTTGAT TATCATTTTG TCCGTGAAAC AGTGAGCAAA   
  
  
+ CGTCATCTCC AGGTCAAATT CATTCGTACT CAATCACAAG TTGCTGACAT TTTCACCAAA GGCTAATCAT   
  
  
+ CGCAAAAGTT TCTTTAGTTT AAGGCCAATC TCTCCATTAT TTCACCCTAT ACAGATTGAG GGGGTGTAAT   
  
  
+ AGTGTATATA TATAGCCTTG TGTTAGCGGG CCTTGGACAC TAGACTTTTA CTCTTGCAAA CCCTAATATA   
  
  
+ TTATTATAAA TATAAATCTA GCCACCCATG TTGAGGTAGG CACTCTAATT CTCACACAGG TAGGATAAGT   
  
  
+ TTTCATAGTT TATGTTGTGA ATGTGATCTT ACTTTGTGGT TCCCGTTGAC AAAAGTTTGA ATCCGAGCAT   
  
  
+ GAGAGTTCAT CAATCTCTTT TTCTTTCCTA TTTTATTTGT TGTGAACTTA ATTGGATTTT CTGGTAGAGG   
  
  
+ AAAGTTCCTT GCTGGAATTT GGTCAAAAGT GATATATATT TTGGGATTTA ATTATCCATA AATTTGGTTT   
  
  
+ TACACCTTGT TAGTTTGGAC TTCTGGTGTG TTATTGTATG TGTTCTGGTG GGTTTTTCTT TTAGCTGAAT   
  
  
+ GTTGGAGCAT TTTAAGGGAT CTAGATATCT GGAGAAGAGC TTGGGAAATA AAGCATATTT CCCTTATAGT   
  
  
+ CTGACATAAC TTTAATGAAT TGATCAGGAA CAAAGGCCAA TTAGTTCAAA AAGGTTCAAC TCCTCTAGGT   
  
  
+ GTTGTGCTAA AGAGATGTGA GTTAGTAAGT AATTGCTTAC TAACTATATA GGATGTTTTA TTACTTGTAG   
  
  
+ AGGTTTTTTT TTTGGGATAT CTTTATGGGG TTTATAAATA AGAAGGTTGA TTTGTTGTCT ACTCTTCTTT   
  
  
+ CGATTCAATA GTAATTCTCT CAGAGAAGGC TAAAATGACA TGGCATTCCA TCGCCTCTCA TGAGGCTTTT   
  
  
+ TGCATGTGCT TATGTATCTT TCATCTCTTG CTTTGGTGGG GTGGCATTGG GGAGGAAAAG GGGGTTTCGA   
  
  
+ TTCGATAGAT CGTCACTTTT GGAAATGACA TCTCTAGCCA ATGGCAAGGG TAAGGTTAAT ACATGTGGAT   
  
  
+ ATTTCCAGAG TTAGTTGTGA TTGTTATTGT TGTTGTTGTA ACTTTATGTC ACGGGGAAAA CATAATAACT   
  
  
+ ATCTGTTTAT CTGTTTGTTT GTTATTTTCA AGGGGAAGGG GAGTGGGCAG ATGCTTAGTA TGTAATCATT   
  
  
+ TTATATAGGG AAAATTCATA TAGCTTGAAA GTAAAACTTT CATTGCCAGC ATTCCAAGGT ATGGGTAGGG   
  
  
+ TTGTCTACAC CTTTATCTCC CTAGAAGCAT GGTGATCATT CCTCGGATTG TGTTATAGAA TAAAGAATGT   
  
  
+ GGTTCTGCTG TAATGAGACT TATGTATTAT CAAATGATGA ATCTGTTTAG AACTTCCTGT TTATTTTTTC   
  
  
+ TGCAAAATTT TAAGAGACTA ACATCTATCG GGCTCAACTC TAGAGGATCT CTTGTGTCAC CCTTCTCAAC   
  
  
+ TCAGTTTGAT TGTGATACAG TTACTATATT GAGTGATAGT CGAGAGCATC ACATCCCGTT AAGGAGTCTA   
  
  
+ TCAACAAGAA GCCCTTCTTG TAATTCTCCC CTTAAAACTA GCAGTTATCA TCACTTGTCA TCGAATAGCC   
  
  
+ CTTTTTGTAA TTCTACCCTT GAAACTAGCA GTTATCGTCA TCGGTTCAAC GCGAGTCCTG TTAGATATTC   
  
  
+ CCATCAAGGC ATAGATTATG GAGTGAGCAT GAAGAATGCT TTGCAGGAGC TAGAGACTAC TCTAATTGGT   
  
  
+ GTAGATGGTG AGGAAGTATC TATTGCTAAT CAACCTATGG GGGGAAGTCG TTAGTCCGGG ATCCCAAGTT   
  
  
+ AGAGATCGAA GTCATCAAGC GAAGATCCAC AGGGTTCACA TCCTACTCAG CTTGATTCAT CATCTTTTTC   
  
  
+ AAGGGTGAGA AGATTCGGAG ATGAAAGCCA GAGAGAGAGA AATGCCATAA GGCAATGGAA GAACCAGCGG   
  
  
+ AACTACTAAG TTTCTCACCA GGTGATTTAA AGCAGTTACT AATTGAATGT GCGAGGGCTT TATTAGATAA   
  
  
+ CCGAATAGAT GACTTTGAGA GTTTGGTTAA ACGGGCAAGG AAAGAGGTCT CCATCTCAGG TGAGCCTGTC   
  
  
+ CAACCTCTCG GTACCTGTAT GATCGAAGGG CTTGTGGCAA GGAAACAGTC TTCGGGGACT AACATCTATT   
  
  
+ GGGCTCTTAA GTGTAAAGAG CCTCTTGGAA AAGACTTGCT CTCCTACGGC ACTCAGTGGG TTACTGTATC   
  
  
+ GCAAGCCATA GCAGCAAGGC TTGGTGGGCC TCCTAAAGTG CGACTTACAG GCATTGATGA TCCTGTTTCT   
  
  
+ AAGTATACTT GTGATGCTAG CTTGGAGGCT GGTGGGAAAC GATTAGCGTC TCTATTTGAA AAGTTTAAAA   
  
  
+ TACTTGTCGA GTTCAATGCA TTGCCCGTTT ATGGACCCAA TGTCAGGTGG GAAATGCTGG ATGTGAGGCC   
  
  
+ CAGGGAGGCT TTGGTCGTTA ATTGTCCATT ACAGCTCCAT TACACTCCTG ACGAGAGTGT CGATGTGAGG   
  
  
+ AACCCTAGGG ATAGGCTTCT CAGAATGGTG AAATTGCTCG GTCCTAAGGT AGCCACTTTG GTTGAGCAAG   
  
  
+ AATCAAACAC CAACACTACA CCTTTCTTGA CCCGGTTCAT AGAGACCCTT GACTACTACT CGATTAACCA   
  
  
+ TGTTTG  

- -Up\_Stream \_Len000CAACAT TGTTCGTGTA ATTTCAACTA ATAGTAAAAC AGGCACTTTG TCACTCGTTT   
  
  
- GCAGTAGAGG TCCAGTTTAA GTAAGCATGA GTTAGTGTTC AACGACTGTA AAAGTGGTTT CCGATTAGTA   
  
  
- GCGTTTTCAA AGAAATCAAA TTCCGGTTAG AGAGGTAATA AAGTGGGATA TGTCTAACTC CCCCACATTA   
  
  
- TCACATATAT ATATCGGAAC ACAATCGCCC GGAACCTGTG ATCTGAAAAT GAGAACGTTT GGGATTATAT   
  
  
- AATAATATTT ATATTTAGAT CGGTGGGTAC AACTCCATCC GTGAGATTAA GAGTGTGTCC ATCCTATTCA   
  
  
- AAAGTATCAA ATACAACACT TACACTAGAA TGAAACACCA AGGGCAACTG TTTTCAAACT TAGGCTCGTA   
  
  
- CTCTCAAGTA GTTAGAGAAA AAGAAAGGAT AAAATAAACA ACACTTGAAT TAACCTAAAA GACCATCTCC   
  
  
- TTTCAAGGAA CGACCTTAAA CCAGTTTTCA CTATATATAA AACCCTAAAT TAATAGGTAT TTAAACCAAA   
  
  
- ATGTGGAACA ATCAAACCTG AAGACCACAC AATAACATAC ACAAGACCAC CCAAAAAGAA AATCGACTTA   
  
  
- CAACCTCGTA AAATTCCCTA GATCTATAGA CCTCTTCTCG AACCCTTTAT TTCGTATAAA GGGAATATCA   
  
  
- GACTGTATTG AAATTACTTA ACTAGTCCTT GTTTCCGGTT AATCAAGTTT TTCCAAGTTG AGGAGATCCA   
  
  
- CAACACGATT TCTCTACACT CAATCATTCA TTAACGAATG ATTGATATAT CCTACAAAAT AATGAACATC   
  
  
- TCCAAAAAAA AAACCCTATA GAAATACCCC AAATATTTAT TCTTCCAACT AAACAACAGA TGAGAAGAAA   
  
  
- GCTAAGTTAT CATTAAGAGA GTCTCTTCCG ATTTTACTGT ACCGTAAGGT AGCGGAGAGT ACTCCGAAAA   
  
  
- ACGTACACGA ATACATAGAA AGTAGAGAAC GAAACCACCC CACCGTAACC CCTCCTTTTC CCCCAAAGCT   
  
  
- AAGCTATCTA GCAGTGAAAA CCTTTACTGT AGAGATCGGT TACCGTTCCC ATTCCAATTA TGTACACCTA   
  
  
- TAAAGGTCTC AATCAACACT AACAATAACA ACAACAACAT TGAAATACAG TGCCCCTTTT GTATTATTGA   
  
  
- TAGACAAATA GACAAACAAA CAATAAAAGT TCCCCTTCCC CTCACCCGTC TACGAATCAT ACATTAGTAA   
  
  
- AATATATCCC TTTTAAGTAT ATCGAACTTT CATTTTGAAA GTAACGGTCG TAAGGTTCCA TACCCATCCC   
  
  
- AACAGATGTG GAAATAGAGG GATCTTCGTA CCACTAGTAA GGAGCCTAAC ACAATATCTT ATTTCTTACA   
  
  
- CCAAGACGAC ATTACTCTGA ATACATAATA GTTTACTACT TAGACAAATC TTGAAGGACA AATAAAAAAG   
  
  
- ACGTTTTAAA ATTCTCTGAT TGTAGATAGC CCGAGTTGAG ATCTCCTAGA GAACACAGTG GGAAGAGTTG   
  
  
- AGTCAAACTA ACACTATGTC AATGATATAA CTCACTATCA GCTCTCGTAG TGTAGGGCAA TTCCTCAGAT   
  
  
- AGTTGTTCTT CGGGAAGAAC ATTAAGAGGG GAATTTTGAT CGTCAATAGT AGTGAACAGT AGCTTATCGG   
  
  
- GAAAAACATT AAGATGGGAA CTTTGATCGT CAATAGCAGT AGCCAAGTTG CGCTCAGGAC AATCTATAAG   
  
  
- GGTAGTTCCG TATCTAATAC CTCACTCGTA CTTCTTACGA AACGTCCTCG ATCTCTGATG AGATTAACCA   
  
  
- CATCTACCAC TCCTTCATAG ATAACGATTA GTTGGATACC CCCCTTCAGC AATCAGGCCC TAGGGTTCAA   
  
  
- TCTCTAGCTT CAGTAGTTCG CTTCTAGGTG TCCCAAGTGT AGGATGAGTC GAACTAAGTA GTAGAAAAAG   
  
  
- TTCCCACTCT TCTAAGCCTC TACTTTCGGT CTCTCTCTCT TTACGGTATT CCGTTACCTT CTTGGTCGCC   
  
  
- TTGATGATTC AAAGAGTGGT CCACTAAATT TCGTCAATGA TTAACTTACA CGCTCCCGAA ATAATCTATT   
  
  
- GGCTTATCTA CTGAAACTCT CAAACCAATT TGCCCGTTCC TTTCTCCAGA GGTAGAGTCC ACTCGGACAG   
  
  
- GTTGGAGAGC CATGGACATA CTAGCTTCCC GAACACCGTT CCTTTGTCAG AAGCCCCTGA TTGTAGATAA   
  
  
- CCCGAGAATT CACATTTCTC GGAGAACCTT TTCTGAACGA GAGGATGCCG TGAGTCACCC AATGACATAG   
  
  
- CGTTCGGTAT CGTCGTTCCG AACCACCCGG AGGATTTCAC GCTGAATGTC CGTAACTACT AGGACAAAGA   
  
  
- TTCATATGAA CACTACGATC GAACCTCCGA CCACCCTTTG CTAATCGCAG AGATAAACTT TTCAAATTTT   
  
  
- ATGAACAGCT CAAGTTACGT AACGGGCAAA TACCTGGGTT ACAGTCCACC CTTTACGACC TACACTCCGG   
  
  
- GTCCCTCCGA AACCAGCAAT TAACAGGTAA TGTCGAGGTA ATGTGAGGAC TGCTCTCACA GCTACACTCC   
  
  
- TTGGGATCCC TATCCGAAGA GTCTTACCAC TTTAACGAGC CAGGATTCCA TCGGTGAAAC CAACTCGTTC   
  
  
- TTAGTTTGTG GTTGTGATGT GGAAAGAACT GGGCCAAGTA TCTCTGGGAA CTGATGATGA GCTAATTGGT   
  
  
- ACAAAC

+     ARE

| Site Name | Organism | Position | Strand | Matrix score. | sequence | function |
| --- | --- | --- | --- | --- | --- | --- |
| ARE | Zea mays | 559 | - | 6 | AAACCA | cis-acting regulatory element essential for the anaerobic induction |

>HU08G00230.1   
+ -Up\_Stream \_Len000GTTGTA ACAAGCACAT TAAAGTTGAT TATCATTTTG TCCGTGAAAC AGTGAGCAAA   
  
  
+ CGTCATCTCC AGGTCAAATT CATTCGTACT CAATCACAAG TTGCTGACAT TTTCACCAAA GGCTAATCAT   
  
  
+ CGCAAAAGTT TCTTTAGTTT AAGGCCAATC TCTCCATTAT TTCACCCTAT ACAGATTGAG GGGGTGTAAT   
  
  
+ AGTGTATATA TATAGCCTTG TGTTAGCGGG CCTTGGACAC TAGACTTTTA CTCTTGCAAA CCCTAATATA   
  
  
+ TTATTATAAA TATAAATCTA GCCACCCATG TTGAGGTAGG CACTCTAATT CTCACACAGG TAGGATAAGT   
  
  
+ TTTCATAGTT TATGTTGTGA ATGTGATCTT ACTTTGTGGT TCCCGTTGAC AAAAGTTTGA ATCCGAGCAT   
  
  
+ GAGAGTTCAT CAATCTCTTT TTCTTTCCTA TTTTATTTGT TGTGAACTTA ATTGGATTTT CTGGTAGAGG   
  
  
+ AAAGTTCCTT GCTGGAATTT GGTCAAAAGT GATATATATT TTGGGATTTA ATTATCCATA AATTTGGTTT   
  
  
+ TACACCTTGT TAGTTTGGAC TTCTGGTGTG TTATTGTATG TGTTCTGGTG GGTTTTTCTT TTAGCTGAAT   
  
  
+ GTTGGAGCAT TTTAAGGGAT CTAGATATCT GGAGAAGAGC TTGGGAAATA AAGCATATTT CCCTTATAGT   
  
  
+ CTGACATAAC TTTAATGAAT TGATCAGGAA CAAAGGCCAA TTAGTTCAAA AAGGTTCAAC TCCTCTAGGT   
  
  
+ GTTGTGCTAA AGAGATGTGA GTTAGTAAGT AATTGCTTAC TAACTATATA GGATGTTTTA TTACTTGTAG   
  
  
+ AGGTTTTTTT TTTGGGATAT CTTTATGGGG TTTATAAATA AGAAGGTTGA TTTGTTGTCT ACTCTTCTTT   
  
  
+ CGATTCAATA GTAATTCTCT CAGAGAAGGC TAAAATGACA TGGCATTCCA TCGCCTCTCA TGAGGCTTTT   
  
  
+ TGCATGTGCT TATGTATCTT TCATCTCTTG CTTTGGTGGG GTGGCATTGG GGAGGAAAAG GGGGTTTCGA   
  
  
+ TTCGATAGAT CGTCACTTTT GGAAATGACA TCTCTAGCCA ATGGCAAGGG TAAGGTTAAT ACATGTGGAT   
  
  
+ ATTTCCAGAG TTAGTTGTGA TTGTTATTGT TGTTGTTGTA ACTTTATGTC ACGGGGAAAA CATAATAACT   
  
  
+ ATCTGTTTAT CTGTTTGTTT GTTATTTTCA AGGGGAAGGG GAGTGGGCAG ATGCTTAGTA TGTAATCATT   
  
  
+ TTATATAGGG AAAATTCATA TAGCTTGAAA GTAAAACTTT CATTGCCAGC ATTCCAAGGT ATGGGTAGGG   
  
  
+ TTGTCTACAC CTTTATCTCC CTAGAAGCAT GGTGATCATT CCTCGGATTG TGTTATAGAA TAAAGAATGT   
  
  
+ GGTTCTGCTG TAATGAGACT TATGTATTAT CAAATGATGA ATCTGTTTAG AACTTCCTGT TTATTTTTTC   
  
  
+ TGCAAAATTT TAAGAGACTA ACATCTATCG GGCTCAACTC TAGAGGATCT CTTGTGTCAC CCTTCTCAAC   
  
  
+ TCAGTTTGAT TGTGATACAG TTACTATATT GAGTGATAGT CGAGAGCATC ACATCCCGTT AAGGAGTCTA   
  
  
+ TCAACAAGAA GCCCTTCTTG TAATTCTCCC CTTAAAACTA GCAGTTATCA TCACTTGTCA TCGAATAGCC   
  
  
+ CTTTTTGTAA TTCTACCCTT GAAACTAGCA GTTATCGTCA TCGGTTCAAC GCGAGTCCTG TTAGATATTC   
  
  
+ CCATCAAGGC ATAGATTATG GAGTGAGCAT GAAGAATGCT TTGCAGGAGC TAGAGACTAC TCTAATTGGT   
  
  
+ GTAGATGGTG AGGAAGTATC TATTGCTAAT CAACCTATGG GGGGAAGTCG TTAGTCCGGG ATCCCAAGTT   
  
  
+ AGAGATCGAA GTCATCAAGC GAAGATCCAC AGGGTTCACA TCCTACTCAG CTTGATTCAT CATCTTTTTC   
  
  
+ AAGGGTGAGA AGATTCGGAG ATGAAAGCCA GAGAGAGAGA AATGCCATAA GGCAATGGAA GAACCAGCGG   
  
  
+ AACTACTAAG TTTCTCACCA GGTGATTTAA AGCAGTTACT AATTGAATGT GCGAGGGCTT TATTAGATAA   
  
  
+ CCGAATAGAT GACTTTGAGA GTTTGGTTAA ACGGGCAAGG AAAGAGGTCT CCATCTCAGG TGAGCCTGTC   
  
  
+ CAACCTCTCG GTACCTGTAT GATCGAAGGG CTTGTGGCAA GGAAACAGTC TTCGGGGACT AACATCTATT   
  
  
+ GGGCTCTTAA GTGTAAAGAG CCTCTTGGAA AAGACTTGCT CTCCTACGGC ACTCAGTGGG TTACTGTATC   
  
  
+ GCAAGCCATA GCAGCAAGGC TTGGTGGGCC TCCTAAAGTG CGACTTACAG GCATTGATGA TCCTGTTTCT   
  
  
+ AAGTATACTT GTGATGCTAG CTTGGAGGCT GGTGGGAAAC GATTAGCGTC TCTATTTGAA AAGTTTAAAA   
  
  
+ TACTTGTCGA GTTCAATGCA TTGCCCGTTT ATGGACCCAA TGTCAGGTGG GAAATGCTGG ATGTGAGGCC   
  
  
+ CAGGGAGGCT TTGGTCGTTA ATTGTCCATT ACAGCTCCAT TACACTCCTG ACGAGAGTGT CGATGTGAGG   
  
  
+ AACCCTAGGG ATAGGCTTCT CAGAATGGTG AAATTGCTCG GTCCTAAGGT AGCCACTTTG GTTGAGCAAG   
  
  
+ AATCAAACAC CAACACTACA CCTTTCTTGA CCCGGTTCAT AGAGACCCTT GACTACTACT CGATTAACCA   
  
  
+ TGTTTG  

- -Up\_Stream \_Len000CAACAT TGTTCGTGTA ATTTCAACTA ATAGTAAAAC AGGCACTTTG TCACTCGTTT   
  
  
- GCAGTAGAGG TCCAGTTTAA GTAAGCATGA GTTAGTGTTC AACGACTGTA AAAGTGGTTT CCGATTAGTA   
  
  
- GCGTTTTCAA AGAAATCAAA TTCCGGTTAG AGAGGTAATA AAGTGGGATA TGTCTAACTC CCCCACATTA   
  
  
- TCACATATAT ATATCGGAAC ACAATCGCCC GGAACCTGTG ATCTGAAAAT GAGAACGTTT GGGATTATAT   
  
  
- AATAATATTT ATATTTAGAT CGGTGGGTAC AACTCCATCC GTGAGATTAA GAGTGTGTCC ATCCTATTCA   
  
  
- AAAGTATCAA ATACAACACT TACACTAGAA TGAAACACCA AGGGCAACTG TTTTCAAACT TAGGCTCGTA   
  
  
- CTCTCAAGTA GTTAGAGAAA AAGAAAGGAT AAAATAAACA ACACTTGAAT TAACCTAAAA GACCATCTCC   
  
  
- TTTCAAGGAA CGACCTTAAA CCAGTTTTCA CTATATATAA AACCCTAAAT TAATAGGTAT TTAAACCAAA   
  
  
- ATGTGGAACA ATCAAACCTG AAGACCACAC AATAACATAC ACAAGACCAC CCAAAAAGAA AATCGACTTA   
  
  
- CAACCTCGTA AAATTCCCTA GATCTATAGA CCTCTTCTCG AACCCTTTAT TTCGTATAAA GGGAATATCA   
  
  
- GACTGTATTG AAATTACTTA ACTAGTCCTT GTTTCCGGTT AATCAAGTTT TTCCAAGTTG AGGAGATCCA   
  
  
- CAACACGATT TCTCTACACT CAATCATTCA TTAACGAATG ATTGATATAT CCTACAAAAT AATGAACATC   
  
  
- TCCAAAAAAA AAACCCTATA GAAATACCCC AAATATTTAT TCTTCCAACT AAACAACAGA TGAGAAGAAA   
  
  
- GCTAAGTTAT CATTAAGAGA GTCTCTTCCG ATTTTACTGT ACCGTAAGGT AGCGGAGAGT ACTCCGAAAA   
  
  
- ACGTACACGA ATACATAGAA AGTAGAGAAC GAAACCACCC CACCGTAACC CCTCCTTTTC CCCCAAAGCT   
  
  
- AAGCTATCTA GCAGTGAAAA CCTTTACTGT AGAGATCGGT TACCGTTCCC ATTCCAATTA TGTACACCTA   
  
  
- TAAAGGTCTC AATCAACACT AACAATAACA ACAACAACAT TGAAATACAG TGCCCCTTTT GTATTATTGA   
  
  
- TAGACAAATA GACAAACAAA CAATAAAAGT TCCCCTTCCC CTCACCCGTC TACGAATCAT ACATTAGTAA   
  
  
- AATATATCCC TTTTAAGTAT ATCGAACTTT CATTTTGAAA GTAACGGTCG TAAGGTTCCA TACCCATCCC   
  
  
- AACAGATGTG GAAATAGAGG GATCTTCGTA CCACTAGTAA GGAGCCTAAC ACAATATCTT ATTTCTTACA   
  
  
- CCAAGACGAC ATTACTCTGA ATACATAATA GTTTACTACT TAGACAAATC TTGAAGGACA AATAAAAAAG   
  
  
- ACGTTTTAAA ATTCTCTGAT TGTAGATAGC CCGAGTTGAG ATCTCCTAGA GAACACAGTG GGAAGAGTTG   
  
  
- AGTCAAACTA ACACTATGTC AATGATATAA CTCACTATCA GCTCTCGTAG TGTAGGGCAA TTCCTCAGAT   
  
  
- AGTTGTTCTT CGGGAAGAAC ATTAAGAGGG GAATTTTGAT CGTCAATAGT AGTGAACAGT AGCTTATCGG   
  
  
- GAAAAACATT AAGATGGGAA CTTTGATCGT CAATAGCAGT AGCCAAGTTG CGCTCAGGAC AATCTATAAG   
  
  
- GGTAGTTCCG TATCTAATAC CTCACTCGTA CTTCTTACGA AACGTCCTCG ATCTCTGATG AGATTAACCA   
  
  
- CATCTACCAC TCCTTCATAG ATAACGATTA GTTGGATACC CCCCTTCAGC AATCAGGCCC TAGGGTTCAA   
  
  
- TCTCTAGCTT CAGTAGTTCG CTTCTAGGTG TCCCAAGTGT AGGATGAGTC GAACTAAGTA GTAGAAAAAG   
  
  
- TTCCCACTCT TCTAAGCCTC TACTTTCGGT CTCTCTCTCT TTACGGTATT CCGTTACCTT CTTGGTCGCC   
  
  
- TTGATGATTC AAAGAGTGGT CCACTAAATT TCGTCAATGA TTAACTTACA CGCTCCCGAA ATAATCTATT   
  
  
- GGCTTATCTA CTGAAACTCT CAAACCAATT TGCCCGTTCC TTTCTCCAGA GGTAGAGTCC ACTCGGACAG   
  
  
- GTTGGAGAGC CATGGACATA CTAGCTTCCC GAACACCGTT CCTTTGTCAG AAGCCCCTGA TTGTAGATAA   
  
  
- CCCGAGAATT CACATTTCTC GGAGAACCTT TTCTGAACGA GAGGATGCCG TGAGTCACCC AATGACATAG   
  
  
- CGTTCGGTAT CGTCGTTCCG AACCACCCGG AGGATTTCAC GCTGAATGTC CGTAACTACT AGGACAAAGA   
  
  
- TTCATATGAA CACTACGATC GAACCTCCGA CCACCCTTTG CTAATCGCAG AGATAAACTT TTCAAATTTT   
  
  
- ATGAACAGCT CAAGTTACGT AACGGGCAAA TACCTGGGTT ACAGTCCACC CTTTACGACC TACACTCCGG   
  
  
- GTCCCTCCGA AACCAGCAAT TAACAGGTAA TGTCGAGGTA ATGTGAGGAC TGCTCTCACA GCTACACTCC   
  
  
- TTGGGATCCC TATCCGAAGA GTCTTACCAC TTTAACGAGC CAGGATTCCA TCGGTGAAAC CAACTCGTTC   
  
  
- TTAGTTTGTG GTTGTGATGT GGAAAGAACT GGGCCAAGTA TCTCTGGGAA CTGATGATGA GCTAATTGGT   
  
  
- ACAAAC

+     AT-rich sequence

| Site Name | Organism | Position | Strand | Matrix score. | sequence | function |
| --- | --- | --- | --- | --- | --- | --- |
| AT-rich sequence | Pisum sativum | 2450 | + | 9 | TAAAATACT | element for maximal elicitor-mediated activation (2copies) |

>HU08G00230.1   
+ -Up\_Stream \_Len000GTTGTA ACAAGCACAT TAAAGTTGAT TATCATTTTG TCCGTGAAAC AGTGAGCAAA   
  
  
+ CGTCATCTCC AGGTCAAATT CATTCGTACT CAATCACAAG TTGCTGACAT TTTCACCAAA GGCTAATCAT   
  
  
+ CGCAAAAGTT TCTTTAGTTT AAGGCCAATC TCTCCATTAT TTCACCCTAT ACAGATTGAG GGGGTGTAAT   
  
  
+ AGTGTATATA TATAGCCTTG TGTTAGCGGG CCTTGGACAC TAGACTTTTA CTCTTGCAAA CCCTAATATA   
  
  
+ TTATTATAAA TATAAATCTA GCCACCCATG TTGAGGTAGG CACTCTAATT CTCACACAGG TAGGATAAGT   
  
  
+ TTTCATAGTT TATGTTGTGA ATGTGATCTT ACTTTGTGGT TCCCGTTGAC AAAAGTTTGA ATCCGAGCAT   
  
  
+ GAGAGTTCAT CAATCTCTTT TTCTTTCCTA TTTTATTTGT TGTGAACTTA ATTGGATTTT CTGGTAGAGG   
  
  
+ AAAGTTCCTT GCTGGAATTT GGTCAAAAGT GATATATATT TTGGGATTTA ATTATCCATA AATTTGGTTT   
  
  
+ TACACCTTGT TAGTTTGGAC TTCTGGTGTG TTATTGTATG TGTTCTGGTG GGTTTTTCTT TTAGCTGAAT   
  
  
+ GTTGGAGCAT TTTAAGGGAT CTAGATATCT GGAGAAGAGC TTGGGAAATA AAGCATATTT CCCTTATAGT   
  
  
+ CTGACATAAC TTTAATGAAT TGATCAGGAA CAAAGGCCAA TTAGTTCAAA AAGGTTCAAC TCCTCTAGGT   
  
  
+ GTTGTGCTAA AGAGATGTGA GTTAGTAAGT AATTGCTTAC TAACTATATA GGATGTTTTA TTACTTGTAG   
  
  
+ AGGTTTTTTT TTTGGGATAT CTTTATGGGG TTTATAAATA AGAAGGTTGA TTTGTTGTCT ACTCTTCTTT   
  
  
+ CGATTCAATA GTAATTCTCT CAGAGAAGGC TAAAATGACA TGGCATTCCA TCGCCTCTCA TGAGGCTTTT   
  
  
+ TGCATGTGCT TATGTATCTT TCATCTCTTG CTTTGGTGGG GTGGCATTGG GGAGGAAAAG GGGGTTTCGA   
  
  
+ TTCGATAGAT CGTCACTTTT GGAAATGACA TCTCTAGCCA ATGGCAAGGG TAAGGTTAAT ACATGTGGAT   
  
  
+ ATTTCCAGAG TTAGTTGTGA TTGTTATTGT TGTTGTTGTA ACTTTATGTC ACGGGGAAAA CATAATAACT   
  
  
+ ATCTGTTTAT CTGTTTGTTT GTTATTTTCA AGGGGAAGGG GAGTGGGCAG ATGCTTAGTA TGTAATCATT   
  
  
+ TTATATAGGG AAAATTCATA TAGCTTGAAA GTAAAACTTT CATTGCCAGC ATTCCAAGGT ATGGGTAGGG   
  
  
+ TTGTCTACAC CTTTATCTCC CTAGAAGCAT GGTGATCATT CCTCGGATTG TGTTATAGAA TAAAGAATGT   
  
  
+ GGTTCTGCTG TAATGAGACT TATGTATTAT CAAATGATGA ATCTGTTTAG AACTTCCTGT TTATTTTTTC   
  
  
+ TGCAAAATTT TAAGAGACTA ACATCTATCG GGCTCAACTC TAGAGGATCT CTTGTGTCAC CCTTCTCAAC   
  
  
+ TCAGTTTGAT TGTGATACAG TTACTATATT GAGTGATAGT CGAGAGCATC ACATCCCGTT AAGGAGTCTA   
  
  
+ TCAACAAGAA GCCCTTCTTG TAATTCTCCC CTTAAAACTA GCAGTTATCA TCACTTGTCA TCGAATAGCC   
  
  
+ CTTTTTGTAA TTCTACCCTT GAAACTAGCA GTTATCGTCA TCGGTTCAAC GCGAGTCCTG TTAGATATTC   
  
  
+ CCATCAAGGC ATAGATTATG GAGTGAGCAT GAAGAATGCT TTGCAGGAGC TAGAGACTAC TCTAATTGGT   
  
  
+ GTAGATGGTG AGGAAGTATC TATTGCTAAT CAACCTATGG GGGGAAGTCG TTAGTCCGGG ATCCCAAGTT   
  
  
+ AGAGATCGAA GTCATCAAGC GAAGATCCAC AGGGTTCACA TCCTACTCAG CTTGATTCAT CATCTTTTTC   
  
  
+ AAGGGTGAGA AGATTCGGAG ATGAAAGCCA GAGAGAGAGA AATGCCATAA GGCAATGGAA GAACCAGCGG   
  
  
+ AACTACTAAG TTTCTCACCA GGTGATTTAA AGCAGTTACT AATTGAATGT GCGAGGGCTT TATTAGATAA   
  
  
+ CCGAATAGAT GACTTTGAGA GTTTGGTTAA ACGGGCAAGG AAAGAGGTCT CCATCTCAGG TGAGCCTGTC   
  
  
+ CAACCTCTCG GTACCTGTAT GATCGAAGGG CTTGTGGCAA GGAAACAGTC TTCGGGGACT AACATCTATT   
  
  
+ GGGCTCTTAA GTGTAAAGAG CCTCTTGGAA AAGACTTGCT CTCCTACGGC ACTCAGTGGG TTACTGTATC   
  
  
+ GCAAGCCATA GCAGCAAGGC TTGGTGGGCC TCCTAAAGTG CGACTTACAG GCATTGATGA TCCTGTTTCT   
  
  
+ AAGTATACTT GTGATGCTAG CTTGGAGGCT GGTGGGAAAC GATTAGCGTC TCTATTTGAA AAGTTTAAAA   
  
  
+ TACTTGTCGA GTTCAATGCA TTGCCCGTTT ATGGACCCAA TGTCAGGTGG GAAATGCTGG ATGTGAGGCC   
  
  
+ CAGGGAGGCT TTGGTCGTTA ATTGTCCATT ACAGCTCCAT TACACTCCTG ACGAGAGTGT CGATGTGAGG   
  
  
+ AACCCTAGGG ATAGGCTTCT CAGAATGGTG AAATTGCTCG GTCCTAAGGT AGCCACTTTG GTTGAGCAAG   
  
  
+ AATCAAACAC CAACACTACA CCTTTCTTGA CCCGGTTCAT AGAGACCCTT GACTACTACT CGATTAACCA   
  
  
+ TGTTTG  

- -Up\_Stream \_Len000CAACAT TGTTCGTGTA ATTTCAACTA ATAGTAAAAC AGGCACTTTG TCACTCGTTT   
  
  
- GCAGTAGAGG TCCAGTTTAA GTAAGCATGA GTTAGTGTTC AACGACTGTA AAAGTGGTTT CCGATTAGTA   
  
  
- GCGTTTTCAA AGAAATCAAA TTCCGGTTAG AGAGGTAATA AAGTGGGATA TGTCTAACTC CCCCACATTA   
  
  
- TCACATATAT ATATCGGAAC ACAATCGCCC GGAACCTGTG ATCTGAAAAT GAGAACGTTT GGGATTATAT   
  
  
- AATAATATTT ATATTTAGAT CGGTGGGTAC AACTCCATCC GTGAGATTAA GAGTGTGTCC ATCCTATTCA   
  
  
- AAAGTATCAA ATACAACACT TACACTAGAA TGAAACACCA AGGGCAACTG TTTTCAAACT TAGGCTCGTA   
  
  
- CTCTCAAGTA GTTAGAGAAA AAGAAAGGAT AAAATAAACA ACACTTGAAT TAACCTAAAA GACCATCTCC   
  
  
- TTTCAAGGAA CGACCTTAAA CCAGTTTTCA CTATATATAA AACCCTAAAT TAATAGGTAT TTAAACCAAA   
  
  
- ATGTGGAACA ATCAAACCTG AAGACCACAC AATAACATAC ACAAGACCAC CCAAAAAGAA AATCGACTTA   
  
  
- CAACCTCGTA AAATTCCCTA GATCTATAGA CCTCTTCTCG AACCCTTTAT TTCGTATAAA GGGAATATCA   
  
  
- GACTGTATTG AAATTACTTA ACTAGTCCTT GTTTCCGGTT AATCAAGTTT TTCCAAGTTG AGGAGATCCA   
  
  
- CAACACGATT TCTCTACACT CAATCATTCA TTAACGAATG ATTGATATAT CCTACAAAAT AATGAACATC   
  
  
- TCCAAAAAAA AAACCCTATA GAAATACCCC AAATATTTAT TCTTCCAACT AAACAACAGA TGAGAAGAAA   
  
  
- GCTAAGTTAT CATTAAGAGA GTCTCTTCCG ATTTTACTGT ACCGTAAGGT AGCGGAGAGT ACTCCGAAAA   
  
  
- ACGTACACGA ATACATAGAA AGTAGAGAAC GAAACCACCC CACCGTAACC CCTCCTTTTC CCCCAAAGCT   
  
  
- AAGCTATCTA GCAGTGAAAA CCTTTACTGT AGAGATCGGT TACCGTTCCC ATTCCAATTA TGTACACCTA   
  
  
- TAAAGGTCTC AATCAACACT AACAATAACA ACAACAACAT TGAAATACAG TGCCCCTTTT GTATTATTGA   
  
  
- TAGACAAATA GACAAACAAA CAATAAAAGT TCCCCTTCCC CTCACCCGTC TACGAATCAT ACATTAGTAA   
  
  
- AATATATCCC TTTTAAGTAT ATCGAACTTT CATTTTGAAA GTAACGGTCG TAAGGTTCCA TACCCATCCC   
  
  
- AACAGATGTG GAAATAGAGG GATCTTCGTA CCACTAGTAA GGAGCCTAAC ACAATATCTT ATTTCTTACA   
  
  
- CCAAGACGAC ATTACTCTGA ATACATAATA GTTTACTACT TAGACAAATC TTGAAGGACA AATAAAAAAG   
  
  
- ACGTTTTAAA ATTCTCTGAT TGTAGATAGC CCGAGTTGAG ATCTCCTAGA GAACACAGTG GGAAGAGTTG   
  
  
- AGTCAAACTA ACACTATGTC AATGATATAA CTCACTATCA GCTCTCGTAG TGTAGGGCAA TTCCTCAGAT   
  
  
- AGTTGTTCTT CGGGAAGAAC ATTAAGAGGG GAATTTTGAT CGTCAATAGT AGTGAACAGT AGCTTATCGG   
  
  
- GAAAAACATT AAGATGGGAA CTTTGATCGT CAATAGCAGT AGCCAAGTTG CGCTCAGGAC AATCTATAAG   
  
  
- GGTAGTTCCG TATCTAATAC CTCACTCGTA CTTCTTACGA AACGTCCTCG ATCTCTGATG AGATTAACCA   
  
  
- CATCTACCAC TCCTTCATAG ATAACGATTA GTTGGATACC CCCCTTCAGC AATCAGGCCC TAGGGTTCAA   
  
  
- TCTCTAGCTT CAGTAGTTCG CTTCTAGGTG TCCCAAGTGT AGGATGAGTC GAACTAAGTA GTAGAAAAAG   
  
  
- TTCCCACTCT TCTAAGCCTC TACTTTCGGT CTCTCTCTCT TTACGGTATT CCGTTACCTT CTTGGTCGCC   
  
  
- TTGATGATTC AAAGAGTGGT CCACTAAATT TCGTCAATGA TTAACTTACA CGCTCCCGAA ATAATCTATT   
  
  
- GGCTTATCTA CTGAAACTCT CAAACCAATT TGCCCGTTCC TTTCTCCAGA GGTAGAGTCC ACTCGGACAG   
  
  
- GTTGGAGAGC CATGGACATA CTAGCTTCCC GAACACCGTT CCTTTGTCAG AAGCCCCTGA TTGTAGATAA   
  
  
- CCCGAGAATT CACATTTCTC GGAGAACCTT TTCTGAACGA GAGGATGCCG TGAGTCACCC AATGACATAG   
  
  
- CGTTCGGTAT CGTCGTTCCG AACCACCCGG AGGATTTCAC GCTGAATGTC CGTAACTACT AGGACAAAGA   
  
  
- TTCATATGAA CACTACGATC GAACCTCCGA CCACCCTTTG CTAATCGCAG AGATAAACTT TTCAAATTTT   
  
  
- ATGAACAGCT CAAGTTACGT AACGGGCAAA TACCTGGGTT ACAGTCCACC CTTTACGACC TACACTCCGG   
  
  
- GTCCCTCCGA AACCAGCAAT TAACAGGTAA TGTCGAGGTA ATGTGAGGAC TGCTCTCACA GCTACACTCC   
  
  
- TTGGGATCCC TATCCGAAGA GTCTTACCAC TTTAACGAGC CAGGATTCCA TCGGTGAAAC CAACTCGTTC   
  
  
- TTAGTTTGTG GTTGTGATGT GGAAAGAACT GGGCCAAGTA TCTCTGGGAA CTGATGATGA GCTAATTGGT   
  
  
- ACAAAC

+     AT~TATA-box

| Site Name | Organism | Position | Strand | Matrix score. | sequence | function |
| --- | --- | --- | --- | --- | --- | --- |
| AT~TATA-box | Arabidopsis thaliana | 1266 | + | 6 | TATATA |  |
| AT~TATA-box | Arabidopsis thaliana | 819 | + | 6 | TATATA |  |
| AT~TATA-box | Arabidopsis thaliana | 221 | + | 6 | TATATA |  |
| AT~TATA-box | Arabidopsis thaliana | 223 | + | 6 | TATATA |  |
| AT~TATA-box | Arabidopsis thaliana | 1264 | - | 8 | TATATAAA |  |
| AT~TATA-box | Arabidopsis thaliana | 527 | + | 6 | TATATA |  |
| AT~TATA-box | Arabidopsis thaliana | 219 | + | 6 | TATATA |  |

>HU08G00230.1   
+ -Up\_Stream \_Len000GTTGTA ACAAGCACAT TAAAGTTGAT TATCATTTTG TCCGTGAAAC AGTGAGCAAA   
  
  
+ CGTCATCTCC AGGTCAAATT CATTCGTACT CAATCACAAG TTGCTGACAT TTTCACCAAA GGCTAATCAT   
  
  
+ CGCAAAAGTT TCTTTAGTTT AAGGCCAATC TCTCCATTAT TTCACCCTAT ACAGATTGAG GGGGTGTAAT   
  
  
+ AGTGTATATA TATAGCCTTG TGTTAGCGGG CCTTGGACAC TAGACTTTTA CTCTTGCAAA CCCTAATATA   
  
  
+ TTATTATAAA TATAAATCTA GCCACCCATG TTGAGGTAGG CACTCTAATT CTCACACAGG TAGGATAAGT   
  
  
+ TTTCATAGTT TATGTTGTGA ATGTGATCTT ACTTTGTGGT TCCCGTTGAC AAAAGTTTGA ATCCGAGCAT   
  
  
+ GAGAGTTCAT CAATCTCTTT TTCTTTCCTA TTTTATTTGT TGTGAACTTA ATTGGATTTT CTGGTAGAGG   
  
  
+ AAAGTTCCTT GCTGGAATTT GGTCAAAAGT GATATATATT TTGGGATTTA ATTATCCATA AATTTGGTTT   
  
  
+ TACACCTTGT TAGTTTGGAC TTCTGGTGTG TTATTGTATG TGTTCTGGTG GGTTTTTCTT TTAGCTGAAT   
  
  
+ GTTGGAGCAT TTTAAGGGAT CTAGATATCT GGAGAAGAGC TTGGGAAATA AAGCATATTT CCCTTATAGT   
  
  
+ CTGACATAAC TTTAATGAAT TGATCAGGAA CAAAGGCCAA TTAGTTCAAA AAGGTTCAAC TCCTCTAGGT   
  
  
+ GTTGTGCTAA AGAGATGTGA GTTAGTAAGT AATTGCTTAC TAACTATATA GGATGTTTTA TTACTTGTAG   
  
  
+ AGGTTTTTTT TTTGGGATAT CTTTATGGGG TTTATAAATA AGAAGGTTGA TTTGTTGTCT ACTCTTCTTT   
  
  
+ CGATTCAATA GTAATTCTCT CAGAGAAGGC TAAAATGACA TGGCATTCCA TCGCCTCTCA TGAGGCTTTT   
  
  
+ TGCATGTGCT TATGTATCTT TCATCTCTTG CTTTGGTGGG GTGGCATTGG GGAGGAAAAG GGGGTTTCGA   
  
  
+ TTCGATAGAT CGTCACTTTT GGAAATGACA TCTCTAGCCA ATGGCAAGGG TAAGGTTAAT ACATGTGGAT   
  
  
+ ATTTCCAGAG TTAGTTGTGA TTGTTATTGT TGTTGTTGTA ACTTTATGTC ACGGGGAAAA CATAATAACT   
  
  
+ ATCTGTTTAT CTGTTTGTTT GTTATTTTCA AGGGGAAGGG GAGTGGGCAG ATGCTTAGTA TGTAATCATT   
  
  
+ TTATATAGGG AAAATTCATA TAGCTTGAAA GTAAAACTTT CATTGCCAGC ATTCCAAGGT ATGGGTAGGG   
  
  
+ TTGTCTACAC CTTTATCTCC CTAGAAGCAT GGTGATCATT CCTCGGATTG TGTTATAGAA TAAAGAATGT   
  
  
+ GGTTCTGCTG TAATGAGACT TATGTATTAT CAAATGATGA ATCTGTTTAG AACTTCCTGT TTATTTTTTC   
  
  
+ TGCAAAATTT TAAGAGACTA ACATCTATCG GGCTCAACTC TAGAGGATCT CTTGTGTCAC CCTTCTCAAC   
  
  
+ TCAGTTTGAT TGTGATACAG TTACTATATT GAGTGATAGT CGAGAGCATC ACATCCCGTT AAGGAGTCTA   
  
  
+ TCAACAAGAA GCCCTTCTTG TAATTCTCCC CTTAAAACTA GCAGTTATCA TCACTTGTCA TCGAATAGCC   
  
  
+ CTTTTTGTAA TTCTACCCTT GAAACTAGCA GTTATCGTCA TCGGTTCAAC GCGAGTCCTG TTAGATATTC   
  
  
+ CCATCAAGGC ATAGATTATG GAGTGAGCAT GAAGAATGCT TTGCAGGAGC TAGAGACTAC TCTAATTGGT   
  
  
+ GTAGATGGTG AGGAAGTATC TATTGCTAAT CAACCTATGG GGGGAAGTCG TTAGTCCGGG ATCCCAAGTT   
  
  
+ AGAGATCGAA GTCATCAAGC GAAGATCCAC AGGGTTCACA TCCTACTCAG CTTGATTCAT CATCTTTTTC   
  
  
+ AAGGGTGAGA AGATTCGGAG ATGAAAGCCA GAGAGAGAGA AATGCCATAA GGCAATGGAA GAACCAGCGG   
  
  
+ AACTACTAAG TTTCTCACCA GGTGATTTAA AGCAGTTACT AATTGAATGT GCGAGGGCTT TATTAGATAA   
  
  
+ CCGAATAGAT GACTTTGAGA GTTTGGTTAA ACGGGCAAGG AAAGAGGTCT CCATCTCAGG TGAGCCTGTC   
  
  
+ CAACCTCTCG GTACCTGTAT GATCGAAGGG CTTGTGGCAA GGAAACAGTC TTCGGGGACT AACATCTATT   
  
  
+ GGGCTCTTAA GTGTAAAGAG CCTCTTGGAA AAGACTTGCT CTCCTACGGC ACTCAGTGGG TTACTGTATC   
  
  
+ GCAAGCCATA GCAGCAAGGC TTGGTGGGCC TCCTAAAGTG CGACTTACAG GCATTGATGA TCCTGTTTCT   
  
  
+ AAGTATACTT GTGATGCTAG CTTGGAGGCT GGTGGGAAAC GATTAGCGTC TCTATTTGAA AAGTTTAAAA   
  
  
+ TACTTGTCGA GTTCAATGCA TTGCCCGTTT ATGGACCCAA TGTCAGGTGG GAAATGCTGG ATGTGAGGCC   
  
  
+ CAGGGAGGCT TTGGTCGTTA ATTGTCCATT ACAGCTCCAT TACACTCCTG ACGAGAGTGT CGATGTGAGG   
  
  
+ AACCCTAGGG ATAGGCTTCT CAGAATGGTG AAATTGCTCG GTCCTAAGGT AGCCACTTTG GTTGAGCAAG   
  
  
+ AATCAAACAC CAACACTACA CCTTTCTTGA CCCGGTTCAT AGAGACCCTT GACTACTACT CGATTAACCA   
  
  
+ TGTTTG  

- -Up\_Stream \_Len000CAACAT TGTTCGTGTA ATTTCAACTA ATAGTAAAAC AGGCACTTTG TCACTCGTTT   
  
  
- GCAGTAGAGG TCCAGTTTAA GTAAGCATGA GTTAGTGTTC AACGACTGTA AAAGTGGTTT CCGATTAGTA   
  
  
- GCGTTTTCAA AGAAATCAAA TTCCGGTTAG AGAGGTAATA AAGTGGGATA TGTCTAACTC CCCCACATTA   
  
  
- TCACATATAT ATATCGGAAC ACAATCGCCC GGAACCTGTG ATCTGAAAAT GAGAACGTTT GGGATTATAT   
  
  
- AATAATATTT ATATTTAGAT CGGTGGGTAC AACTCCATCC GTGAGATTAA GAGTGTGTCC ATCCTATTCA   
  
  
- AAAGTATCAA ATACAACACT TACACTAGAA TGAAACACCA AGGGCAACTG TTTTCAAACT TAGGCTCGTA   
  
  
- CTCTCAAGTA GTTAGAGAAA AAGAAAGGAT AAAATAAACA ACACTTGAAT TAACCTAAAA GACCATCTCC   
  
  
- TTTCAAGGAA CGACCTTAAA CCAGTTTTCA CTATATATAA AACCCTAAAT TAATAGGTAT TTAAACCAAA   
  
  
- ATGTGGAACA ATCAAACCTG AAGACCACAC AATAACATAC ACAAGACCAC CCAAAAAGAA AATCGACTTA   
  
  
- CAACCTCGTA AAATTCCCTA GATCTATAGA CCTCTTCTCG AACCCTTTAT TTCGTATAAA GGGAATATCA   
  
  
- GACTGTATTG AAATTACTTA ACTAGTCCTT GTTTCCGGTT AATCAAGTTT TTCCAAGTTG AGGAGATCCA   
  
  
- CAACACGATT TCTCTACACT CAATCATTCA TTAACGAATG ATTGATATAT CCTACAAAAT AATGAACATC   
  
  
- TCCAAAAAAA AAACCCTATA GAAATACCCC AAATATTTAT TCTTCCAACT AAACAACAGA TGAGAAGAAA   
  
  
- GCTAAGTTAT CATTAAGAGA GTCTCTTCCG ATTTTACTGT ACCGTAAGGT AGCGGAGAGT ACTCCGAAAA   
  
  
- ACGTACACGA ATACATAGAA AGTAGAGAAC GAAACCACCC CACCGTAACC CCTCCTTTTC CCCCAAAGCT   
  
  
- AAGCTATCTA GCAGTGAAAA CCTTTACTGT AGAGATCGGT TACCGTTCCC ATTCCAATTA TGTACACCTA   
  
  
- TAAAGGTCTC AATCAACACT AACAATAACA ACAACAACAT TGAAATACAG TGCCCCTTTT GTATTATTGA   
  
  
- TAGACAAATA GACAAACAAA CAATAAAAGT TCCCCTTCCC CTCACCCGTC TACGAATCAT ACATTAGTAA   
  
  
- AATATATCCC TTTTAAGTAT ATCGAACTTT CATTTTGAAA GTAACGGTCG TAAGGTTCCA TACCCATCCC   
  
  
- AACAGATGTG GAAATAGAGG GATCTTCGTA CCACTAGTAA GGAGCCTAAC ACAATATCTT ATTTCTTACA   
  
  
- CCAAGACGAC ATTACTCTGA ATACATAATA GTTTACTACT TAGACAAATC TTGAAGGACA AATAAAAAAG   
  
  
- ACGTTTTAAA ATTCTCTGAT TGTAGATAGC CCGAGTTGAG ATCTCCTAGA GAACACAGTG GGAAGAGTTG   
  
  
- AGTCAAACTA ACACTATGTC AATGATATAA CTCACTATCA GCTCTCGTAG TGTAGGGCAA TTCCTCAGAT   
  
  
- AGTTGTTCTT CGGGAAGAAC ATTAAGAGGG GAATTTTGAT CGTCAATAGT AGTGAACAGT AGCTTATCGG   
  
  
- GAAAAACATT AAGATGGGAA CTTTGATCGT CAATAGCAGT AGCCAAGTTG CGCTCAGGAC AATCTATAAG   
  
  
- GGTAGTTCCG TATCTAATAC CTCACTCGTA CTTCTTACGA AACGTCCTCG ATCTCTGATG AGATTAACCA   
  
  
- CATCTACCAC TCCTTCATAG ATAACGATTA GTTGGATACC CCCCTTCAGC AATCAGGCCC TAGGGTTCAA   
  
  
- TCTCTAGCTT CAGTAGTTCG CTTCTAGGTG TCCCAAGTGT AGGATGAGTC GAACTAAGTA GTAGAAAAAG   
  
  
- TTCCCACTCT TCTAAGCCTC TACTTTCGGT CTCTCTCTCT TTACGGTATT CCGTTACCTT CTTGGTCGCC   
  
  
- TTGATGATTC AAAGAGTGGT CCACTAAATT TCGTCAATGA TTAACTTACA CGCTCCCGAA ATAATCTATT   
  
  
- GGCTTATCTA CTGAAACTCT CAAACCAATT TGCCCGTTCC TTTCTCCAGA GGTAGAGTCC ACTCGGACAG   
  
  
- GTTGGAGAGC CATGGACATA CTAGCTTCCC GAACACCGTT CCTTTGTCAG AAGCCCCTGA TTGTAGATAA   
  
  
- CCCGAGAATT CACATTTCTC GGAGAACCTT TTCTGAACGA GAGGATGCCG TGAGTCACCC AATGACATAG   
  
  
- CGTTCGGTAT CGTCGTTCCG AACCACCCGG AGGATTTCAC GCTGAATGTC CGTAACTACT AGGACAAAGA   
  
  
- TTCATATGAA CACTACGATC GAACCTCCGA CCACCCTTTG CTAATCGCAG AGATAAACTT TTCAAATTTT   
  
  
- ATGAACAGCT CAAGTTACGT AACGGGCAAA TACCTGGGTT ACAGTCCACC CTTTACGACC TACACTCCGG   
  
  
- GTCCCTCCGA AACCAGCAAT TAACAGGTAA TGTCGAGGTA ATGTGAGGAC TGCTCTCACA GCTACACTCC   
  
  
- TTGGGATCCC TATCCGAAGA GTCTTACCAC TTTAACGAGC CAGGATTCCA TCGGTGAAAC CAACTCGTTC   
  
  
- TTAGTTTGTG GTTGTGATGT GGAAAGAACT GGGCCAAGTA TCTCTGGGAA CTGATGATGA GCTAATTGGT   
  
  
- ACAAAC

+     AuxRR-core

| Site Name | Organism | Position | Strand | Matrix score. | sequence | function |
| --- | --- | --- | --- | --- | --- | --- |
| AuxRR-core | Nicotiana tabacum | 2485 | - | 7 | GGTCCAT | cis-acting regulatory element involved in auxin responsiveness |

>HU08G00230.1   
+ -Up\_Stream \_Len000GTTGTA ACAAGCACAT TAAAGTTGAT TATCATTTTG TCCGTGAAAC AGTGAGCAAA   
  
  
+ CGTCATCTCC AGGTCAAATT CATTCGTACT CAATCACAAG TTGCTGACAT TTTCACCAAA GGCTAATCAT   
  
  
+ CGCAAAAGTT TCTTTAGTTT AAGGCCAATC TCTCCATTAT TTCACCCTAT ACAGATTGAG GGGGTGTAAT   
  
  
+ AGTGTATATA TATAGCCTTG TGTTAGCGGG CCTTGGACAC TAGACTTTTA CTCTTGCAAA CCCTAATATA   
  
  
+ TTATTATAAA TATAAATCTA GCCACCCATG TTGAGGTAGG CACTCTAATT CTCACACAGG TAGGATAAGT   
  
  
+ TTTCATAGTT TATGTTGTGA ATGTGATCTT ACTTTGTGGT TCCCGTTGAC AAAAGTTTGA ATCCGAGCAT   
  
  
+ GAGAGTTCAT CAATCTCTTT TTCTTTCCTA TTTTATTTGT TGTGAACTTA ATTGGATTTT CTGGTAGAGG   
  
  
+ AAAGTTCCTT GCTGGAATTT GGTCAAAAGT GATATATATT TTGGGATTTA ATTATCCATA AATTTGGTTT   
  
  
+ TACACCTTGT TAGTTTGGAC TTCTGGTGTG TTATTGTATG TGTTCTGGTG GGTTTTTCTT TTAGCTGAAT   
  
  
+ GTTGGAGCAT TTTAAGGGAT CTAGATATCT GGAGAAGAGC TTGGGAAATA AAGCATATTT CCCTTATAGT   
  
  
+ CTGACATAAC TTTAATGAAT TGATCAGGAA CAAAGGCCAA TTAGTTCAAA AAGGTTCAAC TCCTCTAGGT   
  
  
+ GTTGTGCTAA AGAGATGTGA GTTAGTAAGT AATTGCTTAC TAACTATATA GGATGTTTTA TTACTTGTAG   
  
  
+ AGGTTTTTTT TTTGGGATAT CTTTATGGGG TTTATAAATA AGAAGGTTGA TTTGTTGTCT ACTCTTCTTT   
  
  
+ CGATTCAATA GTAATTCTCT CAGAGAAGGC TAAAATGACA TGGCATTCCA TCGCCTCTCA TGAGGCTTTT   
  
  
+ TGCATGTGCT TATGTATCTT TCATCTCTTG CTTTGGTGGG GTGGCATTGG GGAGGAAAAG GGGGTTTCGA   
  
  
+ TTCGATAGAT CGTCACTTTT GGAAATGACA TCTCTAGCCA ATGGCAAGGG TAAGGTTAAT ACATGTGGAT   
  
  
+ ATTTCCAGAG TTAGTTGTGA TTGTTATTGT TGTTGTTGTA ACTTTATGTC ACGGGGAAAA CATAATAACT   
  
  
+ ATCTGTTTAT CTGTTTGTTT GTTATTTTCA AGGGGAAGGG GAGTGGGCAG ATGCTTAGTA TGTAATCATT   
  
  
+ TTATATAGGG AAAATTCATA TAGCTTGAAA GTAAAACTTT CATTGCCAGC ATTCCAAGGT ATGGGTAGGG   
  
  
+ TTGTCTACAC CTTTATCTCC CTAGAAGCAT GGTGATCATT CCTCGGATTG TGTTATAGAA TAAAGAATGT   
  
  
+ GGTTCTGCTG TAATGAGACT TATGTATTAT CAAATGATGA ATCTGTTTAG AACTTCCTGT TTATTTTTTC   
  
  
+ TGCAAAATTT TAAGAGACTA ACATCTATCG GGCTCAACTC TAGAGGATCT CTTGTGTCAC CCTTCTCAAC   
  
  
+ TCAGTTTGAT TGTGATACAG TTACTATATT GAGTGATAGT CGAGAGCATC ACATCCCGTT AAGGAGTCTA   
  
  
+ TCAACAAGAA GCCCTTCTTG TAATTCTCCC CTTAAAACTA GCAGTTATCA TCACTTGTCA TCGAATAGCC   
  
  
+ CTTTTTGTAA TTCTACCCTT GAAACTAGCA GTTATCGTCA TCGGTTCAAC GCGAGTCCTG TTAGATATTC   
  
  
+ CCATCAAGGC ATAGATTATG GAGTGAGCAT GAAGAATGCT TTGCAGGAGC TAGAGACTAC TCTAATTGGT   
  
  
+ GTAGATGGTG AGGAAGTATC TATTGCTAAT CAACCTATGG GGGGAAGTCG TTAGTCCGGG ATCCCAAGTT   
  
  
+ AGAGATCGAA GTCATCAAGC GAAGATCCAC AGGGTTCACA TCCTACTCAG CTTGATTCAT CATCTTTTTC   
  
  
+ AAGGGTGAGA AGATTCGGAG ATGAAAGCCA GAGAGAGAGA AATGCCATAA GGCAATGGAA GAACCAGCGG   
  
  
+ AACTACTAAG TTTCTCACCA GGTGATTTAA AGCAGTTACT AATTGAATGT GCGAGGGCTT TATTAGATAA   
  
  
+ CCGAATAGAT GACTTTGAGA GTTTGGTTAA ACGGGCAAGG AAAGAGGTCT CCATCTCAGG TGAGCCTGTC   
  
  
+ CAACCTCTCG GTACCTGTAT GATCGAAGGG CTTGTGGCAA GGAAACAGTC TTCGGGGACT AACATCTATT   
  
  
+ GGGCTCTTAA GTGTAAAGAG CCTCTTGGAA AAGACTTGCT CTCCTACGGC ACTCAGTGGG TTACTGTATC   
  
  
+ GCAAGCCATA GCAGCAAGGC TTGGTGGGCC TCCTAAAGTG CGACTTACAG GCATTGATGA TCCTGTTTCT   
  
  
+ AAGTATACTT GTGATGCTAG CTTGGAGGCT GGTGGGAAAC GATTAGCGTC TCTATTTGAA AAGTTTAAAA   
  
  
+ TACTTGTCGA GTTCAATGCA TTGCCCGTTT ATGGACCCAA TGTCAGGTGG GAAATGCTGG ATGTGAGGCC   
  
  
+ CAGGGAGGCT TTGGTCGTTA ATTGTCCATT ACAGCTCCAT TACACTCCTG ACGAGAGTGT CGATGTGAGG   
  
  
+ AACCCTAGGG ATAGGCTTCT CAGAATGGTG AAATTGCTCG GTCCTAAGGT AGCCACTTTG GTTGAGCAAG   
  
  
+ AATCAAACAC CAACACTACA CCTTTCTTGA CCCGGTTCAT AGAGACCCTT GACTACTACT CGATTAACCA   
  
  
+ TGTTTG  

- -Up\_Stream \_Len000CAACAT TGTTCGTGTA ATTTCAACTA ATAGTAAAAC AGGCACTTTG TCACTCGTTT   
  
  
- GCAGTAGAGG TCCAGTTTAA GTAAGCATGA GTTAGTGTTC AACGACTGTA AAAGTGGTTT CCGATTAGTA   
  
  
- GCGTTTTCAA AGAAATCAAA TTCCGGTTAG AGAGGTAATA AAGTGGGATA TGTCTAACTC CCCCACATTA   
  
  
- TCACATATAT ATATCGGAAC ACAATCGCCC GGAACCTGTG ATCTGAAAAT GAGAACGTTT GGGATTATAT   
  
  
- AATAATATTT ATATTTAGAT CGGTGGGTAC AACTCCATCC GTGAGATTAA GAGTGTGTCC ATCCTATTCA   
  
  
- AAAGTATCAA ATACAACACT TACACTAGAA TGAAACACCA AGGGCAACTG TTTTCAAACT TAGGCTCGTA   
  
  
- CTCTCAAGTA GTTAGAGAAA AAGAAAGGAT AAAATAAACA ACACTTGAAT TAACCTAAAA GACCATCTCC   
  
  
- TTTCAAGGAA CGACCTTAAA CCAGTTTTCA CTATATATAA AACCCTAAAT TAATAGGTAT TTAAACCAAA   
  
  
- ATGTGGAACA ATCAAACCTG AAGACCACAC AATAACATAC ACAAGACCAC CCAAAAAGAA AATCGACTTA   
  
  
- CAACCTCGTA AAATTCCCTA GATCTATAGA CCTCTTCTCG AACCCTTTAT TTCGTATAAA GGGAATATCA   
  
  
- GACTGTATTG AAATTACTTA ACTAGTCCTT GTTTCCGGTT AATCAAGTTT TTCCAAGTTG AGGAGATCCA   
  
  
- CAACACGATT TCTCTACACT CAATCATTCA TTAACGAATG ATTGATATAT CCTACAAAAT AATGAACATC   
  
  
- TCCAAAAAAA AAACCCTATA GAAATACCCC AAATATTTAT TCTTCCAACT AAACAACAGA TGAGAAGAAA   
  
  
- GCTAAGTTAT CATTAAGAGA GTCTCTTCCG ATTTTACTGT ACCGTAAGGT AGCGGAGAGT ACTCCGAAAA   
  
  
- ACGTACACGA ATACATAGAA AGTAGAGAAC GAAACCACCC CACCGTAACC CCTCCTTTTC CCCCAAAGCT   
  
  
- AAGCTATCTA GCAGTGAAAA CCTTTACTGT AGAGATCGGT TACCGTTCCC ATTCCAATTA TGTACACCTA   
  
  
- TAAAGGTCTC AATCAACACT AACAATAACA ACAACAACAT TGAAATACAG TGCCCCTTTT GTATTATTGA   
  
  
- TAGACAAATA GACAAACAAA CAATAAAAGT TCCCCTTCCC CTCACCCGTC TACGAATCAT ACATTAGTAA   
  
  
- AATATATCCC TTTTAAGTAT ATCGAACTTT CATTTTGAAA GTAACGGTCG TAAGGTTCCA TACCCATCCC   
  
  
- AACAGATGTG GAAATAGAGG GATCTTCGTA CCACTAGTAA GGAGCCTAAC ACAATATCTT ATTTCTTACA   
  
  
- CCAAGACGAC ATTACTCTGA ATACATAATA GTTTACTACT TAGACAAATC TTGAAGGACA AATAAAAAAG   
  
  
- ACGTTTTAAA ATTCTCTGAT TGTAGATAGC CCGAGTTGAG ATCTCCTAGA GAACACAGTG GGAAGAGTTG   
  
  
- AGTCAAACTA ACACTATGTC AATGATATAA CTCACTATCA GCTCTCGTAG TGTAGGGCAA TTCCTCAGAT   
  
  
- AGTTGTTCTT CGGGAAGAAC ATTAAGAGGG GAATTTTGAT CGTCAATAGT AGTGAACAGT AGCTTATCGG   
  
  
- GAAAAACATT AAGATGGGAA CTTTGATCGT CAATAGCAGT AGCCAAGTTG CGCTCAGGAC AATCTATAAG   
  
  
- GGTAGTTCCG TATCTAATAC CTCACTCGTA CTTCTTACGA AACGTCCTCG ATCTCTGATG AGATTAACCA   
  
  
- CATCTACCAC TCCTTCATAG ATAACGATTA GTTGGATACC CCCCTTCAGC AATCAGGCCC TAGGGTTCAA   
  
  
- TCTCTAGCTT CAGTAGTTCG CTTCTAGGTG TCCCAAGTGT AGGATGAGTC GAACTAAGTA GTAGAAAAAG   
  
  
- TTCCCACTCT TCTAAGCCTC TACTTTCGGT CTCTCTCTCT TTACGGTATT CCGTTACCTT CTTGGTCGCC   
  
  
- TTGATGATTC AAAGAGTGGT CCACTAAATT TCGTCAATGA TTAACTTACA CGCTCCCGAA ATAATCTATT   
  
  
- GGCTTATCTA CTGAAACTCT CAAACCAATT TGCCCGTTCC TTTCTCCAGA GGTAGAGTCC ACTCGGACAG   
  
  
- GTTGGAGAGC CATGGACATA CTAGCTTCCC GAACACCGTT CCTTTGTCAG AAGCCCCTGA TTGTAGATAA   
  
  
- CCCGAGAATT CACATTTCTC GGAGAACCTT TTCTGAACGA GAGGATGCCG TGAGTCACCC AATGACATAG   
  
  
- CGTTCGGTAT CGTCGTTCCG AACCACCCGG AGGATTTCAC GCTGAATGTC CGTAACTACT AGGACAAAGA   
  
  
- TTCATATGAA CACTACGATC GAACCTCCGA CCACCCTTTG CTAATCGCAG AGATAAACTT TTCAAATTTT   
  
  
- ATGAACAGCT CAAGTTACGT AACGGGCAAA TACCTGGGTT ACAGTCCACC CTTTACGACC TACACTCCGG   
  
  
- GTCCCTCCGA AACCAGCAAT TAACAGGTAA TGTCGAGGTA ATGTGAGGAC TGCTCTCACA GCTACACTCC   
  
  
- TTGGGATCCC TATCCGAAGA GTCTTACCAC TTTAACGAGC CAGGATTCCA TCGGTGAAAC CAACTCGTTC   
  
  
- TTAGTTTGTG GTTGTGATGT GGAAAGAACT GGGCCAAGTA TCTCTGGGAA CTGATGATGA GCTAATTGGT   
  
  
- ACAAAC

+     CAAT-box

| Site Name | Organism | Position | Strand | Matrix score. | sequence | function |
| --- | --- | --- | --- | --- | --- | --- |
| CAAT-box | Arabidopsis thaliana | 1819 | - | 5 | CCAAT | common cis-acting element in promoter and enhancer regions |
| CAAT-box | Nicotiana glutinosa | 2492 | + | 4 | CAAT |  |
| CAAT-box | Pisum sativum | 1435 | + | 5 | CAAAT | common cis-acting element in promoter and enhancer regions |
| CAAT-box | Arabidopsis thaliana | 1092 | + | 5 | CCAAT | common cis-acting element in promoter and enhancer regions |
| CAAT-box | Nicotiana glutinosa | 806 | - | 4 | CAAT |  |
| CAAT-box | Nicotiana glutinosa | 2545 | - | 4 | CAAT |  |
| CAAT-box | Arabidopsis thaliana | 2491 | + | 5 | CCAAT | common cis-acting element in promoter and enhancer regions |
| CAAT-box | Nicotiana glutinosa | 597 | - | 4 | CAAT |  |
| CAAT-box | Nicotiana glutinosa | 2468 | + | 4 | CAAT |  |
| CAAT-box | Nicotiana glutinosa | 2627 | - | 4 | CAAT |  |
| CAAT-box | Pisum sativum | 459 | - | 5 | CAAAT | common cis-acting element in promoter and enhancer regions |
| CAAT-box | Nicotiana glutinosa | 199 | - | 4 | CAAT |  |
| CAAT-box | Nicotiana glutinosa | 2367 | - | 4 | CAAT |  |
| CAAT-box | Nicotiana glutinosa | 920 | + | 4 | CAAT |  |
| CAAT-box | Nicotiana glutinosa | 435 | + | 4 | CAAT |  |
| CAAT-box | Nicotiana glutinosa | 170 | + | 4 | CAAT |  |
| CAAT-box | Arabidopsis thaliana | 169 | + | 5 | CCAAT | common cis-acting element in promoter and enhancer regions |
| CAAT-box | Pisum sativum | 89 | + | 5 | CAAAT | common cis-acting element in promoter and enhancer regions |
| CAAT-box | Nicotiana glutinosa | 723 | - | 4 | CAAT |  |
| CAAT-box | Nicotiana glutinosa | 1306 | - | 4 | CAAT |  |
| CAAT-box | Arabidopsis thaliana | 1030 | - | 5 | CCAAT | common cis-acting element in promoter and enhancer regions |
| CAAT-box | Nicotiana glutinosa | 742 | + | 4 | CAAT |  |
| CAAT-box | Pisum sativum | 511 | - | 5 | CAAAT | common cis-acting element in promoter and enhancer regions |
| CAAT-box | Arabidopsis thaliana | 741 | + | 5 | CCAAT | common cis-acting element in promoter and enhancer regions |
| CAAT-box | Pisum sativum | 894 | - | 5 | CAAAT | common cis-acting element in promoter and enhancer regions |
| CAAT-box | Nicotiana glutinosa | 2474 | - | 4 | CAAT |  |
| CAAT-box | Nicotiana glutinosa | 105 | + | 4 | CAAT |  |
| CAAT-box | Arabidopsis thaliana | 475 | - | 5 | CCAAT | common cis-acting element in promoter and enhancer regions |
| CAAT-box | Nicotiana glutinosa | 2017 | + | 4 | CAAT |  |
| CAAT-box | Arabidopsis thaliana | 2242 | - | 5 | CCAAT | common cis-acting element in promoter and enhancer regions |
| CAAT-box | Nicotiana glutinosa | 2076 | - | 4 | CAAT |  |
| CAAT-box | Nicotiana glutinosa | 1093 | + | 4 | CAAT |  |
| CAAT-box | Pisum sativum | 556 | - | 5 | CAAAT | common cis-acting element in promoter and enhancer regions |
| CAAT-box | Nicotiana glutinosa | 1572 | - | 4 | CAAT |  |
| CAAT-box | Pisum sativum | 2438 | - | 5 | CAAAT | common cis-acting element in promoter and enhancer regions |
| CAAT-box | Nicotiana glutinosa | 1144 | - | 4 | CAAT |  |
| CAAT-box | Nicotiana glutinosa | 1150 | - | 4 | CAAT |  |
| CAAT-box | Nicotiana glutinosa | 1846 | - | 4 | CAAT |  |
| CAAT-box | Nicotiana glutinosa | 1553 | - | 4 | CAAT |  |
| CAAT-box | Nicotiana glutinosa | 1381 | - | 4 | CAAT |  |

>HU08G00230.1   
+ -Up\_Stream \_Len000GTTGTA ACAAGCACAT TAAAGTTGAT TATCATTTTG TCCGTGAAAC AGTGAGCAAA   
  
  
+ CGTCATCTCC AGGTCAAATT CATTCGTACT CAATCACAAG TTGCTGACAT TTTCACCAAA GGCTAATCAT   
  
  
+ CGCAAAAGTT TCTTTAGTTT AAGGCCAATC TCTCCATTAT TTCACCCTAT ACAGATTGAG GGGGTGTAAT   
  
  
+ AGTGTATATA TATAGCCTTG TGTTAGCGGG CCTTGGACAC TAGACTTTTA CTCTTGCAAA CCCTAATATA   
  
  
+ TTATTATAAA TATAAATCTA GCCACCCATG TTGAGGTAGG CACTCTAATT CTCACACAGG TAGGATAAGT   
  
  
+ TTTCATAGTT TATGTTGTGA ATGTGATCTT ACTTTGTGGT TCCCGTTGAC AAAAGTTTGA ATCCGAGCAT   
  
  
+ GAGAGTTCAT CAATCTCTTT TTCTTTCCTA TTTTATTTGT TGTGAACTTA ATTGGATTTT CTGGTAGAGG   
  
  
+ AAAGTTCCTT GCTGGAATTT GGTCAAAAGT GATATATATT TTGGGATTTA ATTATCCATA AATTTGGTTT   
  
  
+ TACACCTTGT TAGTTTGGAC TTCTGGTGTG TTATTGTATG TGTTCTGGTG GGTTTTTCTT TTAGCTGAAT   
  
  
+ GTTGGAGCAT TTTAAGGGAT CTAGATATCT GGAGAAGAGC TTGGGAAATA AAGCATATTT CCCTTATAGT   
  
  
+ CTGACATAAC TTTAATGAAT TGATCAGGAA CAAAGGCCAA TTAGTTCAAA AAGGTTCAAC TCCTCTAGGT   
  
  
+ GTTGTGCTAA AGAGATGTGA GTTAGTAAGT AATTGCTTAC TAACTATATA GGATGTTTTA TTACTTGTAG   
  
  
+ AGGTTTTTTT TTTGGGATAT CTTTATGGGG TTTATAAATA AGAAGGTTGA TTTGTTGTCT ACTCTTCTTT   
  
  
+ CGATTCAATA GTAATTCTCT CAGAGAAGGC TAAAATGACA TGGCATTCCA TCGCCTCTCA TGAGGCTTTT   
  
  
+ TGCATGTGCT TATGTATCTT TCATCTCTTG CTTTGGTGGG GTGGCATTGG GGAGGAAAAG GGGGTTTCGA   
  
  
+ TTCGATAGAT CGTCACTTTT GGAAATGACA TCTCTAGCCA ATGGCAAGGG TAAGGTTAAT ACATGTGGAT   
  
  
+ ATTTCCAGAG TTAGTTGTGA TTGTTATTGT TGTTGTTGTA ACTTTATGTC ACGGGGAAAA CATAATAACT   
  
  
+ ATCTGTTTAT CTGTTTGTTT GTTATTTTCA AGGGGAAGGG GAGTGGGCAG ATGCTTAGTA TGTAATCATT   
  
  
+ TTATATAGGG AAAATTCATA TAGCTTGAAA GTAAAACTTT CATTGCCAGC ATTCCAAGGT ATGGGTAGGG   
  
  
+ TTGTCTACAC CTTTATCTCC CTAGAAGCAT GGTGATCATT CCTCGGATTG TGTTATAGAA TAAAGAATGT   
  
  
+ GGTTCTGCTG TAATGAGACT TATGTATTAT CAAATGATGA ATCTGTTTAG AACTTCCTGT TTATTTTTTC   
  
  
+ TGCAAAATTT TAAGAGACTA ACATCTATCG GGCTCAACTC TAGAGGATCT CTTGTGTCAC CCTTCTCAAC   
  
  
+ TCAGTTTGAT TGTGATACAG TTACTATATT GAGTGATAGT CGAGAGCATC ACATCCCGTT AAGGAGTCTA   
  
  
+ TCAACAAGAA GCCCTTCTTG TAATTCTCCC CTTAAAACTA GCAGTTATCA TCACTTGTCA TCGAATAGCC   
  
  
+ CTTTTTGTAA TTCTACCCTT GAAACTAGCA GTTATCGTCA TCGGTTCAAC GCGAGTCCTG TTAGATATTC   
  
  
+ CCATCAAGGC ATAGATTATG GAGTGAGCAT GAAGAATGCT TTGCAGGAGC TAGAGACTAC TCTAATTGGT   
  
  
+ GTAGATGGTG AGGAAGTATC TATTGCTAAT CAACCTATGG GGGGAAGTCG TTAGTCCGGG ATCCCAAGTT   
  
  
+ AGAGATCGAA GTCATCAAGC GAAGATCCAC AGGGTTCACA TCCTACTCAG CTTGATTCAT CATCTTTTTC   
  
  
+ AAGGGTGAGA AGATTCGGAG ATGAAAGCCA GAGAGAGAGA AATGCCATAA GGCAATGGAA GAACCAGCGG   
  
  
+ AACTACTAAG TTTCTCACCA GGTGATTTAA AGCAGTTACT AATTGAATGT GCGAGGGCTT TATTAGATAA   
  
  
+ CCGAATAGAT GACTTTGAGA GTTTGGTTAA ACGGGCAAGG AAAGAGGTCT CCATCTCAGG TGAGCCTGTC   
  
  
+ CAACCTCTCG GTACCTGTAT GATCGAAGGG CTTGTGGCAA GGAAACAGTC TTCGGGGACT AACATCTATT   
  
  
+ GGGCTCTTAA GTGTAAAGAG CCTCTTGGAA AAGACTTGCT CTCCTACGGC ACTCAGTGGG TTACTGTATC   
  
  
+ GCAAGCCATA GCAGCAAGGC TTGGTGGGCC TCCTAAAGTG CGACTTACAG GCATTGATGA TCCTGTTTCT   
  
  
+ AAGTATACTT GTGATGCTAG CTTGGAGGCT GGTGGGAAAC GATTAGCGTC TCTATTTGAA AAGTTTAAAA   
  
  
+ TACTTGTCGA GTTCAATGCA TTGCCCGTTT ATGGACCCAA TGTCAGGTGG GAAATGCTGG ATGTGAGGCC   
  
  
+ CAGGGAGGCT TTGGTCGTTA ATTGTCCATT ACAGCTCCAT TACACTCCTG ACGAGAGTGT CGATGTGAGG   
  
  
+ AACCCTAGGG ATAGGCTTCT CAGAATGGTG AAATTGCTCG GTCCTAAGGT AGCCACTTTG GTTGAGCAAG   
  
  
+ AATCAAACAC CAACACTACA CCTTTCTTGA CCCGGTTCAT AGAGACCCTT GACTACTACT CGATTAACCA   
  
  
+ TGTTTG  

- -Up\_Stream \_Len000CAACAT TGTTCGTGTA ATTTCAACTA ATAGTAAAAC AGGCACTTTG TCACTCGTTT   
  
  
- GCAGTAGAGG TCCAGTTTAA GTAAGCATGA GTTAGTGTTC AACGACTGTA AAAGTGGTTT CCGATTAGTA   
  
  
- GCGTTTTCAA AGAAATCAAA TTCCGGTTAG AGAGGTAATA AAGTGGGATA TGTCTAACTC CCCCACATTA   
  
  
- TCACATATAT ATATCGGAAC ACAATCGCCC GGAACCTGTG ATCTGAAAAT GAGAACGTTT GGGATTATAT   
  
  
- AATAATATTT ATATTTAGAT CGGTGGGTAC AACTCCATCC GTGAGATTAA GAGTGTGTCC ATCCTATTCA   
  
  
- AAAGTATCAA ATACAACACT TACACTAGAA TGAAACACCA AGGGCAACTG TTTTCAAACT TAGGCTCGTA   
  
  
- CTCTCAAGTA GTTAGAGAAA AAGAAAGGAT AAAATAAACA ACACTTGAAT TAACCTAAAA GACCATCTCC   
  
  
- TTTCAAGGAA CGACCTTAAA CCAGTTTTCA CTATATATAA AACCCTAAAT TAATAGGTAT TTAAACCAAA   
  
  
- ATGTGGAACA ATCAAACCTG AAGACCACAC AATAACATAC ACAAGACCAC CCAAAAAGAA AATCGACTTA   
  
  
- CAACCTCGTA AAATTCCCTA GATCTATAGA CCTCTTCTCG AACCCTTTAT TTCGTATAAA GGGAATATCA   
  
  
- GACTGTATTG AAATTACTTA ACTAGTCCTT GTTTCCGGTT AATCAAGTTT TTCCAAGTTG AGGAGATCCA   
  
  
- CAACACGATT TCTCTACACT CAATCATTCA TTAACGAATG ATTGATATAT CCTACAAAAT AATGAACATC   
  
  
- TCCAAAAAAA AAACCCTATA GAAATACCCC AAATATTTAT TCTTCCAACT AAACAACAGA TGAGAAGAAA   
  
  
- GCTAAGTTAT CATTAAGAGA GTCTCTTCCG ATTTTACTGT ACCGTAAGGT AGCGGAGAGT ACTCCGAAAA   
  
  
- ACGTACACGA ATACATAGAA AGTAGAGAAC GAAACCACCC CACCGTAACC CCTCCTTTTC CCCCAAAGCT   
  
  
- AAGCTATCTA GCAGTGAAAA CCTTTACTGT AGAGATCGGT TACCGTTCCC ATTCCAATTA TGTACACCTA   
  
  
- TAAAGGTCTC AATCAACACT AACAATAACA ACAACAACAT TGAAATACAG TGCCCCTTTT GTATTATTGA   
  
  
- TAGACAAATA GACAAACAAA CAATAAAAGT TCCCCTTCCC CTCACCCGTC TACGAATCAT ACATTAGTAA   
  
  
- AATATATCCC TTTTAAGTAT ATCGAACTTT CATTTTGAAA GTAACGGTCG TAAGGTTCCA TACCCATCCC   
  
  
- AACAGATGTG GAAATAGAGG GATCTTCGTA CCACTAGTAA GGAGCCTAAC ACAATATCTT ATTTCTTACA   
  
  
- CCAAGACGAC ATTACTCTGA ATACATAATA GTTTACTACT TAGACAAATC TTGAAGGACA AATAAAAAAG   
  
  
- ACGTTTTAAA ATTCTCTGAT TGTAGATAGC CCGAGTTGAG ATCTCCTAGA GAACACAGTG GGAAGAGTTG   
  
  
- AGTCAAACTA ACACTATGTC AATGATATAA CTCACTATCA GCTCTCGTAG TGTAGGGCAA TTCCTCAGAT   
  
  
- AGTTGTTCTT CGGGAAGAAC ATTAAGAGGG GAATTTTGAT CGTCAATAGT AGTGAACAGT AGCTTATCGG   
  
  
- GAAAAACATT AAGATGGGAA CTTTGATCGT CAATAGCAGT AGCCAAGTTG CGCTCAGGAC AATCTATAAG   
  
  
- GGTAGTTCCG TATCTAATAC CTCACTCGTA CTTCTTACGA AACGTCCTCG ATCTCTGATG AGATTAACCA   
  
  
- CATCTACCAC TCCTTCATAG ATAACGATTA GTTGGATACC CCCCTTCAGC AATCAGGCCC TAGGGTTCAA   
  
  
- TCTCTAGCTT CAGTAGTTCG CTTCTAGGTG TCCCAAGTGT AGGATGAGTC GAACTAAGTA GTAGAAAAAG   
  
  
- TTCCCACTCT TCTAAGCCTC TACTTTCGGT CTCTCTCTCT TTACGGTATT CCGTTACCTT CTTGGTCGCC   
  
  
- TTGATGATTC AAAGAGTGGT CCACTAAATT TCGTCAATGA TTAACTTACA CGCTCCCGAA ATAATCTATT   
  
  
- GGCTTATCTA CTGAAACTCT CAAACCAATT TGCCCGTTCC TTTCTCCAGA GGTAGAGTCC ACTCGGACAG   
  
  
- GTTGGAGAGC CATGGACATA CTAGCTTCCC GAACACCGTT CCTTTGTCAG AAGCCCCTGA TTGTAGATAA   
  
  
- CCCGAGAATT CACATTTCTC GGAGAACCTT TTCTGAACGA GAGGATGCCG TGAGTCACCC AATGACATAG   
  
  
- CGTTCGGTAT CGTCGTTCCG AACCACCCGG AGGATTTCAC GCTGAATGTC CGTAACTACT AGGACAAAGA   
  
  
- TTCATATGAA CACTACGATC GAACCTCCGA CCACCCTTTG CTAATCGCAG AGATAAACTT TTCAAATTTT   
  
  
- ATGAACAGCT CAAGTTACGT AACGGGCAAA TACCTGGGTT ACAGTCCACC CTTTACGACC TACACTCCGG   
  
  
- GTCCCTCCGA AACCAGCAAT TAACAGGTAA TGTCGAGGTA ATGTGAGGAC TGCTCTCACA GCTACACTCC   
  
  
- TTGGGATCCC TATCCGAAGA GTCTTACCAC TTTAACGAGC CAGGATTCCA TCGGTGAAAC CAACTCGTTC   
  
  
- TTAGTTTGTG GTTGTGATGT GGAAAGAACT GGGCCAAGTA TCTCTGGGAA CTGATGATGA GCTAATTGGT   
  
  
- ACAAAC

+     CAT-box

| Site Name | Organism | Position | Strand | Matrix score. | sequence | function |
| --- | --- | --- | --- | --- | --- | --- |
| CAT-box | Arabidopsis thaliana | 2646 | + | 6 | GCCACT | cis-acting regulatory element related to meristem expression |

>HU08G00230.1   
+ -Up\_Stream \_Len000GTTGTA ACAAGCACAT TAAAGTTGAT TATCATTTTG TCCGTGAAAC AGTGAGCAAA   
  
  
+ CGTCATCTCC AGGTCAAATT CATTCGTACT CAATCACAAG TTGCTGACAT TTTCACCAAA GGCTAATCAT   
  
  
+ CGCAAAAGTT TCTTTAGTTT AAGGCCAATC TCTCCATTAT TTCACCCTAT ACAGATTGAG GGGGTGTAAT   
  
  
+ AGTGTATATA TATAGCCTTG TGTTAGCGGG CCTTGGACAC TAGACTTTTA CTCTTGCAAA CCCTAATATA   
  
  
+ TTATTATAAA TATAAATCTA GCCACCCATG TTGAGGTAGG CACTCTAATT CTCACACAGG TAGGATAAGT   
  
  
+ TTTCATAGTT TATGTTGTGA ATGTGATCTT ACTTTGTGGT TCCCGTTGAC AAAAGTTTGA ATCCGAGCAT   
  
  
+ GAGAGTTCAT CAATCTCTTT TTCTTTCCTA TTTTATTTGT TGTGAACTTA ATTGGATTTT CTGGTAGAGG   
  
  
+ AAAGTTCCTT GCTGGAATTT GGTCAAAAGT GATATATATT TTGGGATTTA ATTATCCATA AATTTGGTTT   
  
  
+ TACACCTTGT TAGTTTGGAC TTCTGGTGTG TTATTGTATG TGTTCTGGTG GGTTTTTCTT TTAGCTGAAT   
  
  
+ GTTGGAGCAT TTTAAGGGAT CTAGATATCT GGAGAAGAGC TTGGGAAATA AAGCATATTT CCCTTATAGT   
  
  
+ CTGACATAAC TTTAATGAAT TGATCAGGAA CAAAGGCCAA TTAGTTCAAA AAGGTTCAAC TCCTCTAGGT   
  
  
+ GTTGTGCTAA AGAGATGTGA GTTAGTAAGT AATTGCTTAC TAACTATATA GGATGTTTTA TTACTTGTAG   
  
  
+ AGGTTTTTTT TTTGGGATAT CTTTATGGGG TTTATAAATA AGAAGGTTGA TTTGTTGTCT ACTCTTCTTT   
  
  
+ CGATTCAATA GTAATTCTCT CAGAGAAGGC TAAAATGACA TGGCATTCCA TCGCCTCTCA TGAGGCTTTT   
  
  
+ TGCATGTGCT TATGTATCTT TCATCTCTTG CTTTGGTGGG GTGGCATTGG GGAGGAAAAG GGGGTTTCGA   
  
  
+ TTCGATAGAT CGTCACTTTT GGAAATGACA TCTCTAGCCA ATGGCAAGGG TAAGGTTAAT ACATGTGGAT   
  
  
+ ATTTCCAGAG TTAGTTGTGA TTGTTATTGT TGTTGTTGTA ACTTTATGTC ACGGGGAAAA CATAATAACT   
  
  
+ ATCTGTTTAT CTGTTTGTTT GTTATTTTCA AGGGGAAGGG GAGTGGGCAG ATGCTTAGTA TGTAATCATT   
  
  
+ TTATATAGGG AAAATTCATA TAGCTTGAAA GTAAAACTTT CATTGCCAGC ATTCCAAGGT ATGGGTAGGG   
  
  
+ TTGTCTACAC CTTTATCTCC CTAGAAGCAT GGTGATCATT CCTCGGATTG TGTTATAGAA TAAAGAATGT   
  
  
+ GGTTCTGCTG TAATGAGACT TATGTATTAT CAAATGATGA ATCTGTTTAG AACTTCCTGT TTATTTTTTC   
  
  
+ TGCAAAATTT TAAGAGACTA ACATCTATCG GGCTCAACTC TAGAGGATCT CTTGTGTCAC CCTTCTCAAC   
  
  
+ TCAGTTTGAT TGTGATACAG TTACTATATT GAGTGATAGT CGAGAGCATC ACATCCCGTT AAGGAGTCTA   
  
  
+ TCAACAAGAA GCCCTTCTTG TAATTCTCCC CTTAAAACTA GCAGTTATCA TCACTTGTCA TCGAATAGCC   
  
  
+ CTTTTTGTAA TTCTACCCTT GAAACTAGCA GTTATCGTCA TCGGTTCAAC GCGAGTCCTG TTAGATATTC   
  
  
+ CCATCAAGGC ATAGATTATG GAGTGAGCAT GAAGAATGCT TTGCAGGAGC TAGAGACTAC TCTAATTGGT   
  
  
+ GTAGATGGTG AGGAAGTATC TATTGCTAAT CAACCTATGG GGGGAAGTCG TTAGTCCGGG ATCCCAAGTT   
  
  
+ AGAGATCGAA GTCATCAAGC GAAGATCCAC AGGGTTCACA TCCTACTCAG CTTGATTCAT CATCTTTTTC   
  
  
+ AAGGGTGAGA AGATTCGGAG ATGAAAGCCA GAGAGAGAGA AATGCCATAA GGCAATGGAA GAACCAGCGG   
  
  
+ AACTACTAAG TTTCTCACCA GGTGATTTAA AGCAGTTACT AATTGAATGT GCGAGGGCTT TATTAGATAA   
  
  
+ CCGAATAGAT GACTTTGAGA GTTTGGTTAA ACGGGCAAGG AAAGAGGTCT CCATCTCAGG TGAGCCTGTC   
  
  
+ CAACCTCTCG GTACCTGTAT GATCGAAGGG CTTGTGGCAA GGAAACAGTC TTCGGGGACT AACATCTATT   
  
  
+ GGGCTCTTAA GTGTAAAGAG CCTCTTGGAA AAGACTTGCT CTCCTACGGC ACTCAGTGGG TTACTGTATC   
  
  
+ GCAAGCCATA GCAGCAAGGC TTGGTGGGCC TCCTAAAGTG CGACTTACAG GCATTGATGA TCCTGTTTCT   
  
  
+ AAGTATACTT GTGATGCTAG CTTGGAGGCT GGTGGGAAAC GATTAGCGTC TCTATTTGAA AAGTTTAAAA   
  
  
+ TACTTGTCGA GTTCAATGCA TTGCCCGTTT ATGGACCCAA TGTCAGGTGG GAAATGCTGG ATGTGAGGCC   
  
  
+ CAGGGAGGCT TTGGTCGTTA ATTGTCCATT ACAGCTCCAT TACACTCCTG ACGAGAGTGT CGATGTGAGG   
  
  
+ AACCCTAGGG ATAGGCTTCT CAGAATGGTG AAATTGCTCG GTCCTAAGGT AGCCACTTTG GTTGAGCAAG   
  
  
+ AATCAAACAC CAACACTACA CCTTTCTTGA CCCGGTTCAT AGAGACCCTT GACTACTACT CGATTAACCA   
  
  
+ TGTTTG  

- -Up\_Stream \_Len000CAACAT TGTTCGTGTA ATTTCAACTA ATAGTAAAAC AGGCACTTTG TCACTCGTTT   
  
  
- GCAGTAGAGG TCCAGTTTAA GTAAGCATGA GTTAGTGTTC AACGACTGTA AAAGTGGTTT CCGATTAGTA   
  
  
- GCGTTTTCAA AGAAATCAAA TTCCGGTTAG AGAGGTAATA AAGTGGGATA TGTCTAACTC CCCCACATTA   
  
  
- TCACATATAT ATATCGGAAC ACAATCGCCC GGAACCTGTG ATCTGAAAAT GAGAACGTTT GGGATTATAT   
  
  
- AATAATATTT ATATTTAGAT CGGTGGGTAC AACTCCATCC GTGAGATTAA GAGTGTGTCC ATCCTATTCA   
  
  
- AAAGTATCAA ATACAACACT TACACTAGAA TGAAACACCA AGGGCAACTG TTTTCAAACT TAGGCTCGTA   
  
  
- CTCTCAAGTA GTTAGAGAAA AAGAAAGGAT AAAATAAACA ACACTTGAAT TAACCTAAAA GACCATCTCC   
  
  
- TTTCAAGGAA CGACCTTAAA CCAGTTTTCA CTATATATAA AACCCTAAAT TAATAGGTAT TTAAACCAAA   
  
  
- ATGTGGAACA ATCAAACCTG AAGACCACAC AATAACATAC ACAAGACCAC CCAAAAAGAA AATCGACTTA   
  
  
- CAACCTCGTA AAATTCCCTA GATCTATAGA CCTCTTCTCG AACCCTTTAT TTCGTATAAA GGGAATATCA   
  
  
- GACTGTATTG AAATTACTTA ACTAGTCCTT GTTTCCGGTT AATCAAGTTT TTCCAAGTTG AGGAGATCCA   
  
  
- CAACACGATT TCTCTACACT CAATCATTCA TTAACGAATG ATTGATATAT CCTACAAAAT AATGAACATC   
  
  
- TCCAAAAAAA AAACCCTATA GAAATACCCC AAATATTTAT TCTTCCAACT AAACAACAGA TGAGAAGAAA   
  
  
- GCTAAGTTAT CATTAAGAGA GTCTCTTCCG ATTTTACTGT ACCGTAAGGT AGCGGAGAGT ACTCCGAAAA   
  
  
- ACGTACACGA ATACATAGAA AGTAGAGAAC GAAACCACCC CACCGTAACC CCTCCTTTTC CCCCAAAGCT   
  
  
- AAGCTATCTA GCAGTGAAAA CCTTTACTGT AGAGATCGGT TACCGTTCCC ATTCCAATTA TGTACACCTA   
  
  
- TAAAGGTCTC AATCAACACT AACAATAACA ACAACAACAT TGAAATACAG TGCCCCTTTT GTATTATTGA   
  
  
- TAGACAAATA GACAAACAAA CAATAAAAGT TCCCCTTCCC CTCACCCGTC TACGAATCAT ACATTAGTAA   
  
  
- AATATATCCC TTTTAAGTAT ATCGAACTTT CATTTTGAAA GTAACGGTCG TAAGGTTCCA TACCCATCCC   
  
  
- AACAGATGTG GAAATAGAGG GATCTTCGTA CCACTAGTAA GGAGCCTAAC ACAATATCTT ATTTCTTACA   
  
  
- CCAAGACGAC ATTACTCTGA ATACATAATA GTTTACTACT TAGACAAATC TTGAAGGACA AATAAAAAAG   
  
  
- ACGTTTTAAA ATTCTCTGAT TGTAGATAGC CCGAGTTGAG ATCTCCTAGA GAACACAGTG GGAAGAGTTG   
  
  
- AGTCAAACTA ACACTATGTC AATGATATAA CTCACTATCA GCTCTCGTAG TGTAGGGCAA TTCCTCAGAT   
  
  
- AGTTGTTCTT CGGGAAGAAC ATTAAGAGGG GAATTTTGAT CGTCAATAGT AGTGAACAGT AGCTTATCGG   
  
  
- GAAAAACATT AAGATGGGAA CTTTGATCGT CAATAGCAGT AGCCAAGTTG CGCTCAGGAC AATCTATAAG   
  
  
- GGTAGTTCCG TATCTAATAC CTCACTCGTA CTTCTTACGA AACGTCCTCG ATCTCTGATG AGATTAACCA   
  
  
- CATCTACCAC TCCTTCATAG ATAACGATTA GTTGGATACC CCCCTTCAGC AATCAGGCCC TAGGGTTCAA   
  
  
- TCTCTAGCTT CAGTAGTTCG CTTCTAGGTG TCCCAAGTGT AGGATGAGTC GAACTAAGTA GTAGAAAAAG   
  
  
- TTCCCACTCT TCTAAGCCTC TACTTTCGGT CTCTCTCTCT TTACGGTATT CCGTTACCTT CTTGGTCGCC   
  
  
- TTGATGATTC AAAGAGTGGT CCACTAAATT TCGTCAATGA TTAACTTACA CGCTCCCGAA ATAATCTATT   
  
  
- GGCTTATCTA CTGAAACTCT CAAACCAATT TGCCCGTTCC TTTCTCCAGA GGTAGAGTCC ACTCGGACAG   
  
  
- GTTGGAGAGC CATGGACATA CTAGCTTCCC GAACACCGTT CCTTTGTCAG AAGCCCCTGA TTGTAGATAA   
  
  
- CCCGAGAATT CACATTTCTC GGAGAACCTT TTCTGAACGA GAGGATGCCG TGAGTCACCC AATGACATAG   
  
  
- CGTTCGGTAT CGTCGTTCCG AACCACCCGG AGGATTTCAC GCTGAATGTC CGTAACTACT AGGACAAAGA   
  
  
- TTCATATGAA CACTACGATC GAACCTCCGA CCACCCTTTG CTAATCGCAG AGATAAACTT TTCAAATTTT   
  
  
- ATGAACAGCT CAAGTTACGT AACGGGCAAA TACCTGGGTT ACAGTCCACC CTTTACGACC TACACTCCGG   
  
  
- GTCCCTCCGA AACCAGCAAT TAACAGGTAA TGTCGAGGTA ATGTGAGGAC TGCTCTCACA GCTACACTCC   
  
  
- TTGGGATCCC TATCCGAAGA GTCTTACCAC TTTAACGAGC CAGGATTCCA TCGGTGAAAC CAACTCGTTC   
  
  
- TTAGTTTGTG GTTGTGATGT GGAAAGAACT GGGCCAAGTA TCTCTGGGAA CTGATGATGA GCTAATTGGT   
  
  
- ACAAAC

+     CCAAT-box

| Site Name | Organism | Position | Strand | Matrix score. | sequence | function |
| --- | --- | --- | --- | --- | --- | --- |
| CCAAT-box | Hordeum vulgare | 397 | - | 6 | CAACGG | MYBHv1 binding site |

>HU08G00230.1   
+ -Up\_Stream \_Len000GTTGTA ACAAGCACAT TAAAGTTGAT TATCATTTTG TCCGTGAAAC AGTGAGCAAA   
  
  
+ CGTCATCTCC AGGTCAAATT CATTCGTACT CAATCACAAG TTGCTGACAT TTTCACCAAA GGCTAATCAT   
  
  
+ CGCAAAAGTT TCTTTAGTTT AAGGCCAATC TCTCCATTAT TTCACCCTAT ACAGATTGAG GGGGTGTAAT   
  
  
+ AGTGTATATA TATAGCCTTG TGTTAGCGGG CCTTGGACAC TAGACTTTTA CTCTTGCAAA CCCTAATATA   
  
  
+ TTATTATAAA TATAAATCTA GCCACCCATG TTGAGGTAGG CACTCTAATT CTCACACAGG TAGGATAAGT   
  
  
+ TTTCATAGTT TATGTTGTGA ATGTGATCTT ACTTTGTGGT TCCCGTTGAC AAAAGTTTGA ATCCGAGCAT   
  
  
+ GAGAGTTCAT CAATCTCTTT TTCTTTCCTA TTTTATTTGT TGTGAACTTA ATTGGATTTT CTGGTAGAGG   
  
  
+ AAAGTTCCTT GCTGGAATTT GGTCAAAAGT GATATATATT TTGGGATTTA ATTATCCATA AATTTGGTTT   
  
  
+ TACACCTTGT TAGTTTGGAC TTCTGGTGTG TTATTGTATG TGTTCTGGTG GGTTTTTCTT TTAGCTGAAT   
  
  
+ GTTGGAGCAT TTTAAGGGAT CTAGATATCT GGAGAAGAGC TTGGGAAATA AAGCATATTT CCCTTATAGT   
  
  
+ CTGACATAAC TTTAATGAAT TGATCAGGAA CAAAGGCCAA TTAGTTCAAA AAGGTTCAAC TCCTCTAGGT   
  
  
+ GTTGTGCTAA AGAGATGTGA GTTAGTAAGT AATTGCTTAC TAACTATATA GGATGTTTTA TTACTTGTAG   
  
  
+ AGGTTTTTTT TTTGGGATAT CTTTATGGGG TTTATAAATA AGAAGGTTGA TTTGTTGTCT ACTCTTCTTT   
  
  
+ CGATTCAATA GTAATTCTCT CAGAGAAGGC TAAAATGACA TGGCATTCCA TCGCCTCTCA TGAGGCTTTT   
  
  
+ TGCATGTGCT TATGTATCTT TCATCTCTTG CTTTGGTGGG GTGGCATTGG GGAGGAAAAG GGGGTTTCGA   
  
  
+ TTCGATAGAT CGTCACTTTT GGAAATGACA TCTCTAGCCA ATGGCAAGGG TAAGGTTAAT ACATGTGGAT   
  
  
+ ATTTCCAGAG TTAGTTGTGA TTGTTATTGT TGTTGTTGTA ACTTTATGTC ACGGGGAAAA CATAATAACT   
  
  
+ ATCTGTTTAT CTGTTTGTTT GTTATTTTCA AGGGGAAGGG GAGTGGGCAG ATGCTTAGTA TGTAATCATT   
  
  
+ TTATATAGGG AAAATTCATA TAGCTTGAAA GTAAAACTTT CATTGCCAGC ATTCCAAGGT ATGGGTAGGG   
  
  
+ TTGTCTACAC CTTTATCTCC CTAGAAGCAT GGTGATCATT CCTCGGATTG TGTTATAGAA TAAAGAATGT   
  
  
+ GGTTCTGCTG TAATGAGACT TATGTATTAT CAAATGATGA ATCTGTTTAG AACTTCCTGT TTATTTTTTC   
  
  
+ TGCAAAATTT TAAGAGACTA ACATCTATCG GGCTCAACTC TAGAGGATCT CTTGTGTCAC CCTTCTCAAC   
  
  
+ TCAGTTTGAT TGTGATACAG TTACTATATT GAGTGATAGT CGAGAGCATC ACATCCCGTT AAGGAGTCTA   
  
  
+ TCAACAAGAA GCCCTTCTTG TAATTCTCCC CTTAAAACTA GCAGTTATCA TCACTTGTCA TCGAATAGCC   
  
  
+ CTTTTTGTAA TTCTACCCTT GAAACTAGCA GTTATCGTCA TCGGTTCAAC GCGAGTCCTG TTAGATATTC   
  
  
+ CCATCAAGGC ATAGATTATG GAGTGAGCAT GAAGAATGCT TTGCAGGAGC TAGAGACTAC TCTAATTGGT   
  
  
+ GTAGATGGTG AGGAAGTATC TATTGCTAAT CAACCTATGG GGGGAAGTCG TTAGTCCGGG ATCCCAAGTT   
  
  
+ AGAGATCGAA GTCATCAAGC GAAGATCCAC AGGGTTCACA TCCTACTCAG CTTGATTCAT CATCTTTTTC   
  
  
+ AAGGGTGAGA AGATTCGGAG ATGAAAGCCA GAGAGAGAGA AATGCCATAA GGCAATGGAA GAACCAGCGG   
  
  
+ AACTACTAAG TTTCTCACCA GGTGATTTAA AGCAGTTACT AATTGAATGT GCGAGGGCTT TATTAGATAA   
  
  
+ CCGAATAGAT GACTTTGAGA GTTTGGTTAA ACGGGCAAGG AAAGAGGTCT CCATCTCAGG TGAGCCTGTC   
  
  
+ CAACCTCTCG GTACCTGTAT GATCGAAGGG CTTGTGGCAA GGAAACAGTC TTCGGGGACT AACATCTATT   
  
  
+ GGGCTCTTAA GTGTAAAGAG CCTCTTGGAA AAGACTTGCT CTCCTACGGC ACTCAGTGGG TTACTGTATC   
  
  
+ GCAAGCCATA GCAGCAAGGC TTGGTGGGCC TCCTAAAGTG CGACTTACAG GCATTGATGA TCCTGTTTCT   
  
  
+ AAGTATACTT GTGATGCTAG CTTGGAGGCT GGTGGGAAAC GATTAGCGTC TCTATTTGAA AAGTTTAAAA   
  
  
+ TACTTGTCGA GTTCAATGCA TTGCCCGTTT ATGGACCCAA TGTCAGGTGG GAAATGCTGG ATGTGAGGCC   
  
  
+ CAGGGAGGCT TTGGTCGTTA ATTGTCCATT ACAGCTCCAT TACACTCCTG ACGAGAGTGT CGATGTGAGG   
  
  
+ AACCCTAGGG ATAGGCTTCT CAGAATGGTG AAATTGCTCG GTCCTAAGGT AGCCACTTTG GTTGAGCAAG   
  
  
+ AATCAAACAC CAACACTACA CCTTTCTTGA CCCGGTTCAT AGAGACCCTT GACTACTACT CGATTAACCA   
  
  
+ TGTTTG  

- -Up\_Stream \_Len000CAACAT TGTTCGTGTA ATTTCAACTA ATAGTAAAAC AGGCACTTTG TCACTCGTTT   
  
  
- GCAGTAGAGG TCCAGTTTAA GTAAGCATGA GTTAGTGTTC AACGACTGTA AAAGTGGTTT CCGATTAGTA   
  
  
- GCGTTTTCAA AGAAATCAAA TTCCGGTTAG AGAGGTAATA AAGTGGGATA TGTCTAACTC CCCCACATTA   
  
  
- TCACATATAT ATATCGGAAC ACAATCGCCC GGAACCTGTG ATCTGAAAAT GAGAACGTTT GGGATTATAT   
  
  
- AATAATATTT ATATTTAGAT CGGTGGGTAC AACTCCATCC GTGAGATTAA GAGTGTGTCC ATCCTATTCA   
  
  
- AAAGTATCAA ATACAACACT TACACTAGAA TGAAACACCA AGGGCAACTG TTTTCAAACT TAGGCTCGTA   
  
  
- CTCTCAAGTA GTTAGAGAAA AAGAAAGGAT AAAATAAACA ACACTTGAAT TAACCTAAAA GACCATCTCC   
  
  
- TTTCAAGGAA CGACCTTAAA CCAGTTTTCA CTATATATAA AACCCTAAAT TAATAGGTAT TTAAACCAAA   
  
  
- ATGTGGAACA ATCAAACCTG AAGACCACAC AATAACATAC ACAAGACCAC CCAAAAAGAA AATCGACTTA   
  
  
- CAACCTCGTA AAATTCCCTA GATCTATAGA CCTCTTCTCG AACCCTTTAT TTCGTATAAA GGGAATATCA   
  
  
- GACTGTATTG AAATTACTTA ACTAGTCCTT GTTTCCGGTT AATCAAGTTT TTCCAAGTTG AGGAGATCCA   
  
  
- CAACACGATT TCTCTACACT CAATCATTCA TTAACGAATG ATTGATATAT CCTACAAAAT AATGAACATC   
  
  
- TCCAAAAAAA AAACCCTATA GAAATACCCC AAATATTTAT TCTTCCAACT AAACAACAGA TGAGAAGAAA   
  
  
- GCTAAGTTAT CATTAAGAGA GTCTCTTCCG ATTTTACTGT ACCGTAAGGT AGCGGAGAGT ACTCCGAAAA   
  
  
- ACGTACACGA ATACATAGAA AGTAGAGAAC GAAACCACCC CACCGTAACC CCTCCTTTTC CCCCAAAGCT   
  
  
- AAGCTATCTA GCAGTGAAAA CCTTTACTGT AGAGATCGGT TACCGTTCCC ATTCCAATTA TGTACACCTA   
  
  
- TAAAGGTCTC AATCAACACT AACAATAACA ACAACAACAT TGAAATACAG TGCCCCTTTT GTATTATTGA   
  
  
- TAGACAAATA GACAAACAAA CAATAAAAGT TCCCCTTCCC CTCACCCGTC TACGAATCAT ACATTAGTAA   
  
  
- AATATATCCC TTTTAAGTAT ATCGAACTTT CATTTTGAAA GTAACGGTCG TAAGGTTCCA TACCCATCCC   
  
  
- AACAGATGTG GAAATAGAGG GATCTTCGTA CCACTAGTAA GGAGCCTAAC ACAATATCTT ATTTCTTACA   
  
  
- CCAAGACGAC ATTACTCTGA ATACATAATA GTTTACTACT TAGACAAATC TTGAAGGACA AATAAAAAAG   
  
  
- ACGTTTTAAA ATTCTCTGAT TGTAGATAGC CCGAGTTGAG ATCTCCTAGA GAACACAGTG GGAAGAGTTG   
  
  
- AGTCAAACTA ACACTATGTC AATGATATAA CTCACTATCA GCTCTCGTAG TGTAGGGCAA TTCCTCAGAT   
  
  
- AGTTGTTCTT CGGGAAGAAC ATTAAGAGGG GAATTTTGAT CGTCAATAGT AGTGAACAGT AGCTTATCGG   
  
  
- GAAAAACATT AAGATGGGAA CTTTGATCGT CAATAGCAGT AGCCAAGTTG CGCTCAGGAC AATCTATAAG   
  
  
- GGTAGTTCCG TATCTAATAC CTCACTCGTA CTTCTTACGA AACGTCCTCG ATCTCTGATG AGATTAACCA   
  
  
- CATCTACCAC TCCTTCATAG ATAACGATTA GTTGGATACC CCCCTTCAGC AATCAGGCCC TAGGGTTCAA   
  
  
- TCTCTAGCTT CAGTAGTTCG CTTCTAGGTG TCCCAAGTGT AGGATGAGTC GAACTAAGTA GTAGAAAAAG   
  
  
- TTCCCACTCT TCTAAGCCTC TACTTTCGGT CTCTCTCTCT TTACGGTATT CCGTTACCTT CTTGGTCGCC   
  
  
- TTGATGATTC AAAGAGTGGT CCACTAAATT TCGTCAATGA TTAACTTACA CGCTCCCGAA ATAATCTATT   
  
  
- GGCTTATCTA CTGAAACTCT CAAACCAATT TGCCCGTTCC TTTCTCCAGA GGTAGAGTCC ACTCGGACAG   
  
  
- GTTGGAGAGC CATGGACATA CTAGCTTCCC GAACACCGTT CCTTTGTCAG AAGCCCCTGA TTGTAGATAA   
  
  
- CCCGAGAATT CACATTTCTC GGAGAACCTT TTCTGAACGA GAGGATGCCG TGAGTCACCC AATGACATAG   
  
  
- CGTTCGGTAT CGTCGTTCCG AACCACCCGG AGGATTTCAC GCTGAATGTC CGTAACTACT AGGACAAAGA   
  
  
- TTCATATGAA CACTACGATC GAACCTCCGA CCACCCTTTG CTAATCGCAG AGATAAACTT TTCAAATTTT   
  
  
- ATGAACAGCT CAAGTTACGT AACGGGCAAA TACCTGGGTT ACAGTCCACC CTTTACGACC TACACTCCGG   
  
  
- GTCCCTCCGA AACCAGCAAT TAACAGGTAA TGTCGAGGTA ATGTGAGGAC TGCTCTCACA GCTACACTCC   
  
  
- TTGGGATCCC TATCCGAAGA GTCTTACCAC TTTAACGAGC CAGGATTCCA TCGGTGAAAC CAACTCGTTC   
  
  
- TTAGTTTGTG GTTGTGATGT GGAAAGAACT GGGCCAAGTA TCTCTGGGAA CTGATGATGA GCTAATTGGT   
  
  
- ACAAAC

+     CGTCA-motif

| Site Name | Organism | Position | Strand | Matrix score. | sequence | function |
| --- | --- | --- | --- | --- | --- | --- |
| CGTCA-motif | Hordeum vulgare | 75 | + | 5 | CGTCA | cis-acting regulatory element involved in the MeJA-responsiveness |
| CGTCA-motif | Hordeum vulgare | 2573 | - | 5 | CGTCA | cis-acting regulatory element involved in the MeJA-responsiveness |
| CGTCA-motif | Hordeum vulgare | 1065 | + | 5 | CGTCA | cis-acting regulatory element involved in the MeJA-responsiveness |
| CGTCA-motif | Hordeum vulgare | 1720 | + | 5 | CGTCA | cis-acting regulatory element involved in the MeJA-responsiveness |

>HU08G00230.1   
+ -Up\_Stream \_Len000GTTGTA ACAAGCACAT TAAAGTTGAT TATCATTTTG TCCGTGAAAC AGTGAGCAAA   
  
  
+ CGTCATCTCC AGGTCAAATT CATTCGTACT CAATCACAAG TTGCTGACAT TTTCACCAAA GGCTAATCAT   
  
  
+ CGCAAAAGTT TCTTTAGTTT AAGGCCAATC TCTCCATTAT TTCACCCTAT ACAGATTGAG GGGGTGTAAT   
  
  
+ AGTGTATATA TATAGCCTTG TGTTAGCGGG CCTTGGACAC TAGACTTTTA CTCTTGCAAA CCCTAATATA   
  
  
+ TTATTATAAA TATAAATCTA GCCACCCATG TTGAGGTAGG CACTCTAATT CTCACACAGG TAGGATAAGT   
  
  
+ TTTCATAGTT TATGTTGTGA ATGTGATCTT ACTTTGTGGT TCCCGTTGAC AAAAGTTTGA ATCCGAGCAT   
  
  
+ GAGAGTTCAT CAATCTCTTT TTCTTTCCTA TTTTATTTGT TGTGAACTTA ATTGGATTTT CTGGTAGAGG   
  
  
+ AAAGTTCCTT GCTGGAATTT GGTCAAAAGT GATATATATT TTGGGATTTA ATTATCCATA AATTTGGTTT   
  
  
+ TACACCTTGT TAGTTTGGAC TTCTGGTGTG TTATTGTATG TGTTCTGGTG GGTTTTTCTT TTAGCTGAAT   
  
  
+ GTTGGAGCAT TTTAAGGGAT CTAGATATCT GGAGAAGAGC TTGGGAAATA AAGCATATTT CCCTTATAGT   
  
  
+ CTGACATAAC TTTAATGAAT TGATCAGGAA CAAAGGCCAA TTAGTTCAAA AAGGTTCAAC TCCTCTAGGT   
  
  
+ GTTGTGCTAA AGAGATGTGA GTTAGTAAGT AATTGCTTAC TAACTATATA GGATGTTTTA TTACTTGTAG   
  
  
+ AGGTTTTTTT TTTGGGATAT CTTTATGGGG TTTATAAATA AGAAGGTTGA TTTGTTGTCT ACTCTTCTTT   
  
  
+ CGATTCAATA GTAATTCTCT CAGAGAAGGC TAAAATGACA TGGCATTCCA TCGCCTCTCA TGAGGCTTTT   
  
  
+ TGCATGTGCT TATGTATCTT TCATCTCTTG CTTTGGTGGG GTGGCATTGG GGAGGAAAAG GGGGTTTCGA   
  
  
+ TTCGATAGAT CGTCACTTTT GGAAATGACA TCTCTAGCCA ATGGCAAGGG TAAGGTTAAT ACATGTGGAT   
  
  
+ ATTTCCAGAG TTAGTTGTGA TTGTTATTGT TGTTGTTGTA ACTTTATGTC ACGGGGAAAA CATAATAACT   
  
  
+ ATCTGTTTAT CTGTTTGTTT GTTATTTTCA AGGGGAAGGG GAGTGGGCAG ATGCTTAGTA TGTAATCATT   
  
  
+ TTATATAGGG AAAATTCATA TAGCTTGAAA GTAAAACTTT CATTGCCAGC ATTCCAAGGT ATGGGTAGGG   
  
  
+ TTGTCTACAC CTTTATCTCC CTAGAAGCAT GGTGATCATT CCTCGGATTG TGTTATAGAA TAAAGAATGT   
  
  
+ GGTTCTGCTG TAATGAGACT TATGTATTAT CAAATGATGA ATCTGTTTAG AACTTCCTGT TTATTTTTTC   
  
  
+ TGCAAAATTT TAAGAGACTA ACATCTATCG GGCTCAACTC TAGAGGATCT CTTGTGTCAC CCTTCTCAAC   
  
  
+ TCAGTTTGAT TGTGATACAG TTACTATATT GAGTGATAGT CGAGAGCATC ACATCCCGTT AAGGAGTCTA   
  
  
+ TCAACAAGAA GCCCTTCTTG TAATTCTCCC CTTAAAACTA GCAGTTATCA TCACTTGTCA TCGAATAGCC   
  
  
+ CTTTTTGTAA TTCTACCCTT GAAACTAGCA GTTATCGTCA TCGGTTCAAC GCGAGTCCTG TTAGATATTC   
  
  
+ CCATCAAGGC ATAGATTATG GAGTGAGCAT GAAGAATGCT TTGCAGGAGC TAGAGACTAC TCTAATTGGT   
  
  
+ GTAGATGGTG AGGAAGTATC TATTGCTAAT CAACCTATGG GGGGAAGTCG TTAGTCCGGG ATCCCAAGTT   
  
  
+ AGAGATCGAA GTCATCAAGC GAAGATCCAC AGGGTTCACA TCCTACTCAG CTTGATTCAT CATCTTTTTC   
  
  
+ AAGGGTGAGA AGATTCGGAG ATGAAAGCCA GAGAGAGAGA AATGCCATAA GGCAATGGAA GAACCAGCGG   
  
  
+ AACTACTAAG TTTCTCACCA GGTGATTTAA AGCAGTTACT AATTGAATGT GCGAGGGCTT TATTAGATAA   
  
  
+ CCGAATAGAT GACTTTGAGA GTTTGGTTAA ACGGGCAAGG AAAGAGGTCT CCATCTCAGG TGAGCCTGTC   
  
  
+ CAACCTCTCG GTACCTGTAT GATCGAAGGG CTTGTGGCAA GGAAACAGTC TTCGGGGACT AACATCTATT   
  
  
+ GGGCTCTTAA GTGTAAAGAG CCTCTTGGAA AAGACTTGCT CTCCTACGGC ACTCAGTGGG TTACTGTATC   
  
  
+ GCAAGCCATA GCAGCAAGGC TTGGTGGGCC TCCTAAAGTG CGACTTACAG GCATTGATGA TCCTGTTTCT   
  
  
+ AAGTATACTT GTGATGCTAG CTTGGAGGCT GGTGGGAAAC GATTAGCGTC TCTATTTGAA AAGTTTAAAA   
  
  
+ TACTTGTCGA GTTCAATGCA TTGCCCGTTT ATGGACCCAA TGTCAGGTGG GAAATGCTGG ATGTGAGGCC   
  
  
+ CAGGGAGGCT TTGGTCGTTA ATTGTCCATT ACAGCTCCAT TACACTCCTG ACGAGAGTGT CGATGTGAGG   
  
  
+ AACCCTAGGG ATAGGCTTCT CAGAATGGTG AAATTGCTCG GTCCTAAGGT AGCCACTTTG GTTGAGCAAG   
  
  
+ AATCAAACAC CAACACTACA CCTTTCTTGA CCCGGTTCAT AGAGACCCTT GACTACTACT CGATTAACCA   
  
  
+ TGTTTG  

- -Up\_Stream \_Len000CAACAT TGTTCGTGTA ATTTCAACTA ATAGTAAAAC AGGCACTTTG TCACTCGTTT   
  
  
- GCAGTAGAGG TCCAGTTTAA GTAAGCATGA GTTAGTGTTC AACGACTGTA AAAGTGGTTT CCGATTAGTA   
  
  
- GCGTTTTCAA AGAAATCAAA TTCCGGTTAG AGAGGTAATA AAGTGGGATA TGTCTAACTC CCCCACATTA   
  
  
- TCACATATAT ATATCGGAAC ACAATCGCCC GGAACCTGTG ATCTGAAAAT GAGAACGTTT GGGATTATAT   
  
  
- AATAATATTT ATATTTAGAT CGGTGGGTAC AACTCCATCC GTGAGATTAA GAGTGTGTCC ATCCTATTCA   
  
  
- AAAGTATCAA ATACAACACT TACACTAGAA TGAAACACCA AGGGCAACTG TTTTCAAACT TAGGCTCGTA   
  
  
- CTCTCAAGTA GTTAGAGAAA AAGAAAGGAT AAAATAAACA ACACTTGAAT TAACCTAAAA GACCATCTCC   
  
  
- TTTCAAGGAA CGACCTTAAA CCAGTTTTCA CTATATATAA AACCCTAAAT TAATAGGTAT TTAAACCAAA   
  
  
- ATGTGGAACA ATCAAACCTG AAGACCACAC AATAACATAC ACAAGACCAC CCAAAAAGAA AATCGACTTA   
  
  
- CAACCTCGTA AAATTCCCTA GATCTATAGA CCTCTTCTCG AACCCTTTAT TTCGTATAAA GGGAATATCA   
  
  
- GACTGTATTG AAATTACTTA ACTAGTCCTT GTTTCCGGTT AATCAAGTTT TTCCAAGTTG AGGAGATCCA   
  
  
- CAACACGATT TCTCTACACT CAATCATTCA TTAACGAATG ATTGATATAT CCTACAAAAT AATGAACATC   
  
  
- TCCAAAAAAA AAACCCTATA GAAATACCCC AAATATTTAT TCTTCCAACT AAACAACAGA TGAGAAGAAA   
  
  
- GCTAAGTTAT CATTAAGAGA GTCTCTTCCG ATTTTACTGT ACCGTAAGGT AGCGGAGAGT ACTCCGAAAA   
  
  
- ACGTACACGA ATACATAGAA AGTAGAGAAC GAAACCACCC CACCGTAACC CCTCCTTTTC CCCCAAAGCT   
  
  
- AAGCTATCTA GCAGTGAAAA CCTTTACTGT AGAGATCGGT TACCGTTCCC ATTCCAATTA TGTACACCTA   
  
  
- TAAAGGTCTC AATCAACACT AACAATAACA ACAACAACAT TGAAATACAG TGCCCCTTTT GTATTATTGA   
  
  
- TAGACAAATA GACAAACAAA CAATAAAAGT TCCCCTTCCC CTCACCCGTC TACGAATCAT ACATTAGTAA   
  
  
- AATATATCCC TTTTAAGTAT ATCGAACTTT CATTTTGAAA GTAACGGTCG TAAGGTTCCA TACCCATCCC   
  
  
- AACAGATGTG GAAATAGAGG GATCTTCGTA CCACTAGTAA GGAGCCTAAC ACAATATCTT ATTTCTTACA   
  
  
- CCAAGACGAC ATTACTCTGA ATACATAATA GTTTACTACT TAGACAAATC TTGAAGGACA AATAAAAAAG   
  
  
- ACGTTTTAAA ATTCTCTGAT TGTAGATAGC CCGAGTTGAG ATCTCCTAGA GAACACAGTG GGAAGAGTTG   
  
  
- AGTCAAACTA ACACTATGTC AATGATATAA CTCACTATCA GCTCTCGTAG TGTAGGGCAA TTCCTCAGAT   
  
  
- AGTTGTTCTT CGGGAAGAAC ATTAAGAGGG GAATTTTGAT CGTCAATAGT AGTGAACAGT AGCTTATCGG   
  
  
- GAAAAACATT AAGATGGGAA CTTTGATCGT CAATAGCAGT AGCCAAGTTG CGCTCAGGAC AATCTATAAG   
  
  
- GGTAGTTCCG TATCTAATAC CTCACTCGTA CTTCTTACGA AACGTCCTCG ATCTCTGATG AGATTAACCA   
  
  
- CATCTACCAC TCCTTCATAG ATAACGATTA GTTGGATACC CCCCTTCAGC AATCAGGCCC TAGGGTTCAA   
  
  
- TCTCTAGCTT CAGTAGTTCG CTTCTAGGTG TCCCAAGTGT AGGATGAGTC GAACTAAGTA GTAGAAAAAG   
  
  
- TTCCCACTCT TCTAAGCCTC TACTTTCGGT CTCTCTCTCT TTACGGTATT CCGTTACCTT CTTGGTCGCC   
  
  
- TTGATGATTC AAAGAGTGGT CCACTAAATT TCGTCAATGA TTAACTTACA CGCTCCCGAA ATAATCTATT   
  
  
- GGCTTATCTA CTGAAACTCT CAAACCAATT TGCCCGTTCC TTTCTCCAGA GGTAGAGTCC ACTCGGACAG   
  
  
- GTTGGAGAGC CATGGACATA CTAGCTTCCC GAACACCGTT CCTTTGTCAG AAGCCCCTGA TTGTAGATAA   
  
  
- CCCGAGAATT CACATTTCTC GGAGAACCTT TTCTGAACGA GAGGATGCCG TGAGTCACCC AATGACATAG   
  
  
- CGTTCGGTAT CGTCGTTCCG AACCACCCGG AGGATTTCAC GCTGAATGTC CGTAACTACT AGGACAAAGA   
  
  
- TTCATATGAA CACTACGATC GAACCTCCGA CCACCCTTTG CTAATCGCAG AGATAAACTT TTCAAATTTT   
  
  
- ATGAACAGCT CAAGTTACGT AACGGGCAAA TACCTGGGTT ACAGTCCACC CTTTACGACC TACACTCCGG   
  
  
- GTCCCTCCGA AACCAGCAAT TAACAGGTAA TGTCGAGGTA ATGTGAGGAC TGCTCTCACA GCTACACTCC   
  
  
- TTGGGATCCC TATCCGAAGA GTCTTACCAC TTTAACGAGC CAGGATTCCA TCGGTGAAAC CAACTCGTTC   
  
  
- TTAGTTTGTG GTTGTGATGT GGAAAGAACT GGGCCAAGTA TCTCTGGGAA CTGATGATGA GCTAATTGGT   
  
  
- ACAAAC

+     DRE1

| Site Name | Organism | Position | Strand | Matrix score. | sequence | function |
| --- | --- | --- | --- | --- | --- | --- |
| DRE1 | Zea mays | 2180 | - | 7 | ACCGAGA |  |

>HU08G00230.1   
+ -Up\_Stream \_Len000GTTGTA ACAAGCACAT TAAAGTTGAT TATCATTTTG TCCGTGAAAC AGTGAGCAAA   
  
  
+ CGTCATCTCC AGGTCAAATT CATTCGTACT CAATCACAAG TTGCTGACAT TTTCACCAAA GGCTAATCAT   
  
  
+ CGCAAAAGTT TCTTTAGTTT AAGGCCAATC TCTCCATTAT TTCACCCTAT ACAGATTGAG GGGGTGTAAT   
  
  
+ AGTGTATATA TATAGCCTTG TGTTAGCGGG CCTTGGACAC TAGACTTTTA CTCTTGCAAA CCCTAATATA   
  
  
+ TTATTATAAA TATAAATCTA GCCACCCATG TTGAGGTAGG CACTCTAATT CTCACACAGG TAGGATAAGT   
  
  
+ TTTCATAGTT TATGTTGTGA ATGTGATCTT ACTTTGTGGT TCCCGTTGAC AAAAGTTTGA ATCCGAGCAT   
  
  
+ GAGAGTTCAT CAATCTCTTT TTCTTTCCTA TTTTATTTGT TGTGAACTTA ATTGGATTTT CTGGTAGAGG   
  
  
+ AAAGTTCCTT GCTGGAATTT GGTCAAAAGT GATATATATT TTGGGATTTA ATTATCCATA AATTTGGTTT   
  
  
+ TACACCTTGT TAGTTTGGAC TTCTGGTGTG TTATTGTATG TGTTCTGGTG GGTTTTTCTT TTAGCTGAAT   
  
  
+ GTTGGAGCAT TTTAAGGGAT CTAGATATCT GGAGAAGAGC TTGGGAAATA AAGCATATTT CCCTTATAGT   
  
  
+ CTGACATAAC TTTAATGAAT TGATCAGGAA CAAAGGCCAA TTAGTTCAAA AAGGTTCAAC TCCTCTAGGT   
  
  
+ GTTGTGCTAA AGAGATGTGA GTTAGTAAGT AATTGCTTAC TAACTATATA GGATGTTTTA TTACTTGTAG   
  
  
+ AGGTTTTTTT TTTGGGATAT CTTTATGGGG TTTATAAATA AGAAGGTTGA TTTGTTGTCT ACTCTTCTTT   
  
  
+ CGATTCAATA GTAATTCTCT CAGAGAAGGC TAAAATGACA TGGCATTCCA TCGCCTCTCA TGAGGCTTTT   
  
  
+ TGCATGTGCT TATGTATCTT TCATCTCTTG CTTTGGTGGG GTGGCATTGG GGAGGAAAAG GGGGTTTCGA   
  
  
+ TTCGATAGAT CGTCACTTTT GGAAATGACA TCTCTAGCCA ATGGCAAGGG TAAGGTTAAT ACATGTGGAT   
  
  
+ ATTTCCAGAG TTAGTTGTGA TTGTTATTGT TGTTGTTGTA ACTTTATGTC ACGGGGAAAA CATAATAACT   
  
  
+ ATCTGTTTAT CTGTTTGTTT GTTATTTTCA AGGGGAAGGG GAGTGGGCAG ATGCTTAGTA TGTAATCATT   
  
  
+ TTATATAGGG AAAATTCATA TAGCTTGAAA GTAAAACTTT CATTGCCAGC ATTCCAAGGT ATGGGTAGGG   
  
  
+ TTGTCTACAC CTTTATCTCC CTAGAAGCAT GGTGATCATT CCTCGGATTG TGTTATAGAA TAAAGAATGT   
  
  
+ GGTTCTGCTG TAATGAGACT TATGTATTAT CAAATGATGA ATCTGTTTAG AACTTCCTGT TTATTTTTTC   
  
  
+ TGCAAAATTT TAAGAGACTA ACATCTATCG GGCTCAACTC TAGAGGATCT CTTGTGTCAC CCTTCTCAAC   
  
  
+ TCAGTTTGAT TGTGATACAG TTACTATATT GAGTGATAGT CGAGAGCATC ACATCCCGTT AAGGAGTCTA   
  
  
+ TCAACAAGAA GCCCTTCTTG TAATTCTCCC CTTAAAACTA GCAGTTATCA TCACTTGTCA TCGAATAGCC   
  
  
+ CTTTTTGTAA TTCTACCCTT GAAACTAGCA GTTATCGTCA TCGGTTCAAC GCGAGTCCTG TTAGATATTC   
  
  
+ CCATCAAGGC ATAGATTATG GAGTGAGCAT GAAGAATGCT TTGCAGGAGC TAGAGACTAC TCTAATTGGT   
  
  
+ GTAGATGGTG AGGAAGTATC TATTGCTAAT CAACCTATGG GGGGAAGTCG TTAGTCCGGG ATCCCAAGTT   
  
  
+ AGAGATCGAA GTCATCAAGC GAAGATCCAC AGGGTTCACA TCCTACTCAG CTTGATTCAT CATCTTTTTC   
  
  
+ AAGGGTGAGA AGATTCGGAG ATGAAAGCCA GAGAGAGAGA AATGCCATAA GGCAATGGAA GAACCAGCGG   
  
  
+ AACTACTAAG TTTCTCACCA GGTGATTTAA AGCAGTTACT AATTGAATGT GCGAGGGCTT TATTAGATAA   
  
  
+ CCGAATAGAT GACTTTGAGA GTTTGGTTAA ACGGGCAAGG AAAGAGGTCT CCATCTCAGG TGAGCCTGTC   
  
  
+ CAACCTCTCG GTACCTGTAT GATCGAAGGG CTTGTGGCAA GGAAACAGTC TTCGGGGACT AACATCTATT   
  
  
+ GGGCTCTTAA GTGTAAAGAG CCTCTTGGAA AAGACTTGCT CTCCTACGGC ACTCAGTGGG TTACTGTATC   
  
  
+ GCAAGCCATA GCAGCAAGGC TTGGTGGGCC TCCTAAAGTG CGACTTACAG GCATTGATGA TCCTGTTTCT   
  
  
+ AAGTATACTT GTGATGCTAG CTTGGAGGCT GGTGGGAAAC GATTAGCGTC TCTATTTGAA AAGTTTAAAA   
  
  
+ TACTTGTCGA GTTCAATGCA TTGCCCGTTT ATGGACCCAA TGTCAGGTGG GAAATGCTGG ATGTGAGGCC   
  
  
+ CAGGGAGGCT TTGGTCGTTA ATTGTCCATT ACAGCTCCAT TACACTCCTG ACGAGAGTGT CGATGTGAGG   
  
  
+ AACCCTAGGG ATAGGCTTCT CAGAATGGTG AAATTGCTCG GTCCTAAGGT AGCCACTTTG GTTGAGCAAG   
  
  
+ AATCAAACAC CAACACTACA CCTTTCTTGA CCCGGTTCAT AGAGACCCTT GACTACTACT CGATTAACCA   
  
  
+ TGTTTG  

- -Up\_Stream \_Len000CAACAT TGTTCGTGTA ATTTCAACTA ATAGTAAAAC AGGCACTTTG TCACTCGTTT   
  
  
- GCAGTAGAGG TCCAGTTTAA GTAAGCATGA GTTAGTGTTC AACGACTGTA AAAGTGGTTT CCGATTAGTA   
  
  
- GCGTTTTCAA AGAAATCAAA TTCCGGTTAG AGAGGTAATA AAGTGGGATA TGTCTAACTC CCCCACATTA   
  
  
- TCACATATAT ATATCGGAAC ACAATCGCCC GGAACCTGTG ATCTGAAAAT GAGAACGTTT GGGATTATAT   
  
  
- AATAATATTT ATATTTAGAT CGGTGGGTAC AACTCCATCC GTGAGATTAA GAGTGTGTCC ATCCTATTCA   
  
  
- AAAGTATCAA ATACAACACT TACACTAGAA TGAAACACCA AGGGCAACTG TTTTCAAACT TAGGCTCGTA   
  
  
- CTCTCAAGTA GTTAGAGAAA AAGAAAGGAT AAAATAAACA ACACTTGAAT TAACCTAAAA GACCATCTCC   
  
  
- TTTCAAGGAA CGACCTTAAA CCAGTTTTCA CTATATATAA AACCCTAAAT TAATAGGTAT TTAAACCAAA   
  
  
- ATGTGGAACA ATCAAACCTG AAGACCACAC AATAACATAC ACAAGACCAC CCAAAAAGAA AATCGACTTA   
  
  
- CAACCTCGTA AAATTCCCTA GATCTATAGA CCTCTTCTCG AACCCTTTAT TTCGTATAAA GGGAATATCA   
  
  
- GACTGTATTG AAATTACTTA ACTAGTCCTT GTTTCCGGTT AATCAAGTTT TTCCAAGTTG AGGAGATCCA   
  
  
- CAACACGATT TCTCTACACT CAATCATTCA TTAACGAATG ATTGATATAT CCTACAAAAT AATGAACATC   
  
  
- TCCAAAAAAA AAACCCTATA GAAATACCCC AAATATTTAT TCTTCCAACT AAACAACAGA TGAGAAGAAA   
  
  
- GCTAAGTTAT CATTAAGAGA GTCTCTTCCG ATTTTACTGT ACCGTAAGGT AGCGGAGAGT ACTCCGAAAA   
  
  
- ACGTACACGA ATACATAGAA AGTAGAGAAC GAAACCACCC CACCGTAACC CCTCCTTTTC CCCCAAAGCT   
  
  
- AAGCTATCTA GCAGTGAAAA CCTTTACTGT AGAGATCGGT TACCGTTCCC ATTCCAATTA TGTACACCTA   
  
  
- TAAAGGTCTC AATCAACACT AACAATAACA ACAACAACAT TGAAATACAG TGCCCCTTTT GTATTATTGA   
  
  
- TAGACAAATA GACAAACAAA CAATAAAAGT TCCCCTTCCC CTCACCCGTC TACGAATCAT ACATTAGTAA   
  
  
- AATATATCCC TTTTAAGTAT ATCGAACTTT CATTTTGAAA GTAACGGTCG TAAGGTTCCA TACCCATCCC   
  
  
- AACAGATGTG GAAATAGAGG GATCTTCGTA CCACTAGTAA GGAGCCTAAC ACAATATCTT ATTTCTTACA   
  
  
- CCAAGACGAC ATTACTCTGA ATACATAATA GTTTACTACT TAGACAAATC TTGAAGGACA AATAAAAAAG   
  
  
- ACGTTTTAAA ATTCTCTGAT TGTAGATAGC CCGAGTTGAG ATCTCCTAGA GAACACAGTG GGAAGAGTTG   
  
  
- AGTCAAACTA ACACTATGTC AATGATATAA CTCACTATCA GCTCTCGTAG TGTAGGGCAA TTCCTCAGAT   
  
  
- AGTTGTTCTT CGGGAAGAAC ATTAAGAGGG GAATTTTGAT CGTCAATAGT AGTGAACAGT AGCTTATCGG   
  
  
- GAAAAACATT AAGATGGGAA CTTTGATCGT CAATAGCAGT AGCCAAGTTG CGCTCAGGAC AATCTATAAG   
  
  
- GGTAGTTCCG TATCTAATAC CTCACTCGTA CTTCTTACGA AACGTCCTCG ATCTCTGATG AGATTAACCA   
  
  
- CATCTACCAC TCCTTCATAG ATAACGATTA GTTGGATACC CCCCTTCAGC AATCAGGCCC TAGGGTTCAA   
  
  
- TCTCTAGCTT CAGTAGTTCG CTTCTAGGTG TCCCAAGTGT AGGATGAGTC GAACTAAGTA GTAGAAAAAG   
  
  
- TTCCCACTCT TCTAAGCCTC TACTTTCGGT CTCTCTCTCT TTACGGTATT CCGTTACCTT CTTGGTCGCC   
  
  
- TTGATGATTC AAAGAGTGGT CCACTAAATT TCGTCAATGA TTAACTTACA CGCTCCCGAA ATAATCTATT   
  
  
- GGCTTATCTA CTGAAACTCT CAAACCAATT TGCCCGTTCC TTTCTCCAGA GGTAGAGTCC ACTCGGACAG   
  
  
- GTTGGAGAGC CATGGACATA CTAGCTTCCC GAACACCGTT CCTTTGTCAG AAGCCCCTGA TTGTAGATAA   
  
  
- CCCGAGAATT CACATTTCTC GGAGAACCTT TTCTGAACGA GAGGATGCCG TGAGTCACCC AATGACATAG   
  
  
- CGTTCGGTAT CGTCGTTCCG AACCACCCGG AGGATTTCAC GCTGAATGTC CGTAACTACT AGGACAAAGA   
  
  
- TTCATATGAA CACTACGATC GAACCTCCGA CCACCCTTTG CTAATCGCAG AGATAAACTT TTCAAATTTT   
  
  
- ATGAACAGCT CAAGTTACGT AACGGGCAAA TACCTGGGTT ACAGTCCACC CTTTACGACC TACACTCCGG   
  
  
- GTCCCTCCGA AACCAGCAAT TAACAGGTAA TGTCGAGGTA ATGTGAGGAC TGCTCTCACA GCTACACTCC   
  
  
- TTGGGATCCC TATCCGAAGA GTCTTACCAC TTTAACGAGC CAGGATTCCA TCGGTGAAAC CAACTCGTTC   
  
  
- TTAGTTTGTG GTTGTGATGT GGAAAGAACT GGGCCAAGTA TCTCTGGGAA CTGATGATGA GCTAATTGGT   
  
  
- ACAAAC

+     ERE

| Site Name | Organism | Position | Strand | Matrix score. | sequence | function |
| --- | --- | --- | --- | --- | --- | --- |
| ERE | Nicotiana glutinos | 2448 | - | 8 | ATTTTAAA |  |

>HU08G00230.1   
+ -Up\_Stream \_Len000GTTGTA ACAAGCACAT TAAAGTTGAT TATCATTTTG TCCGTGAAAC AGTGAGCAAA   
  
  
+ CGTCATCTCC AGGTCAAATT CATTCGTACT CAATCACAAG TTGCTGACAT TTTCACCAAA GGCTAATCAT   
  
  
+ CGCAAAAGTT TCTTTAGTTT AAGGCCAATC TCTCCATTAT TTCACCCTAT ACAGATTGAG GGGGTGTAAT   
  
  
+ AGTGTATATA TATAGCCTTG TGTTAGCGGG CCTTGGACAC TAGACTTTTA CTCTTGCAAA CCCTAATATA   
  
  
+ TTATTATAAA TATAAATCTA GCCACCCATG TTGAGGTAGG CACTCTAATT CTCACACAGG TAGGATAAGT   
  
  
+ TTTCATAGTT TATGTTGTGA ATGTGATCTT ACTTTGTGGT TCCCGTTGAC AAAAGTTTGA ATCCGAGCAT   
  
  
+ GAGAGTTCAT CAATCTCTTT TTCTTTCCTA TTTTATTTGT TGTGAACTTA ATTGGATTTT CTGGTAGAGG   
  
  
+ AAAGTTCCTT GCTGGAATTT GGTCAAAAGT GATATATATT TTGGGATTTA ATTATCCATA AATTTGGTTT   
  
  
+ TACACCTTGT TAGTTTGGAC TTCTGGTGTG TTATTGTATG TGTTCTGGTG GGTTTTTCTT TTAGCTGAAT   
  
  
+ GTTGGAGCAT TTTAAGGGAT CTAGATATCT GGAGAAGAGC TTGGGAAATA AAGCATATTT CCCTTATAGT   
  
  
+ CTGACATAAC TTTAATGAAT TGATCAGGAA CAAAGGCCAA TTAGTTCAAA AAGGTTCAAC TCCTCTAGGT   
  
  
+ GTTGTGCTAA AGAGATGTGA GTTAGTAAGT AATTGCTTAC TAACTATATA GGATGTTTTA TTACTTGTAG   
  
  
+ AGGTTTTTTT TTTGGGATAT CTTTATGGGG TTTATAAATA AGAAGGTTGA TTTGTTGTCT ACTCTTCTTT   
  
  
+ CGATTCAATA GTAATTCTCT CAGAGAAGGC TAAAATGACA TGGCATTCCA TCGCCTCTCA TGAGGCTTTT   
  
  
+ TGCATGTGCT TATGTATCTT TCATCTCTTG CTTTGGTGGG GTGGCATTGG GGAGGAAAAG GGGGTTTCGA   
  
  
+ TTCGATAGAT CGTCACTTTT GGAAATGACA TCTCTAGCCA ATGGCAAGGG TAAGGTTAAT ACATGTGGAT   
  
  
+ ATTTCCAGAG TTAGTTGTGA TTGTTATTGT TGTTGTTGTA ACTTTATGTC ACGGGGAAAA CATAATAACT   
  
  
+ ATCTGTTTAT CTGTTTGTTT GTTATTTTCA AGGGGAAGGG GAGTGGGCAG ATGCTTAGTA TGTAATCATT   
  
  
+ TTATATAGGG AAAATTCATA TAGCTTGAAA GTAAAACTTT CATTGCCAGC ATTCCAAGGT ATGGGTAGGG   
  
  
+ TTGTCTACAC CTTTATCTCC CTAGAAGCAT GGTGATCATT CCTCGGATTG TGTTATAGAA TAAAGAATGT   
  
  
+ GGTTCTGCTG TAATGAGACT TATGTATTAT CAAATGATGA ATCTGTTTAG AACTTCCTGT TTATTTTTTC   
  
  
+ TGCAAAATTT TAAGAGACTA ACATCTATCG GGCTCAACTC TAGAGGATCT CTTGTGTCAC CCTTCTCAAC   
  
  
+ TCAGTTTGAT TGTGATACAG TTACTATATT GAGTGATAGT CGAGAGCATC ACATCCCGTT AAGGAGTCTA   
  
  
+ TCAACAAGAA GCCCTTCTTG TAATTCTCCC CTTAAAACTA GCAGTTATCA TCACTTGTCA TCGAATAGCC   
  
  
+ CTTTTTGTAA TTCTACCCTT GAAACTAGCA GTTATCGTCA TCGGTTCAAC GCGAGTCCTG TTAGATATTC   
  
  
+ CCATCAAGGC ATAGATTATG GAGTGAGCAT GAAGAATGCT TTGCAGGAGC TAGAGACTAC TCTAATTGGT   
  
  
+ GTAGATGGTG AGGAAGTATC TATTGCTAAT CAACCTATGG GGGGAAGTCG TTAGTCCGGG ATCCCAAGTT   
  
  
+ AGAGATCGAA GTCATCAAGC GAAGATCCAC AGGGTTCACA TCCTACTCAG CTTGATTCAT CATCTTTTTC   
  
  
+ AAGGGTGAGA AGATTCGGAG ATGAAAGCCA GAGAGAGAGA AATGCCATAA GGCAATGGAA GAACCAGCGG   
  
  
+ AACTACTAAG TTTCTCACCA GGTGATTTAA AGCAGTTACT AATTGAATGT GCGAGGGCTT TATTAGATAA   
  
  
+ CCGAATAGAT GACTTTGAGA GTTTGGTTAA ACGGGCAAGG AAAGAGGTCT CCATCTCAGG TGAGCCTGTC   
  
  
+ CAACCTCTCG GTACCTGTAT GATCGAAGGG CTTGTGGCAA GGAAACAGTC TTCGGGGACT AACATCTATT   
  
  
+ GGGCTCTTAA GTGTAAAGAG CCTCTTGGAA AAGACTTGCT CTCCTACGGC ACTCAGTGGG TTACTGTATC   
  
  
+ GCAAGCCATA GCAGCAAGGC TTGGTGGGCC TCCTAAAGTG CGACTTACAG GCATTGATGA TCCTGTTTCT   
  
  
+ AAGTATACTT GTGATGCTAG CTTGGAGGCT GGTGGGAAAC GATTAGCGTC TCTATTTGAA AAGTTTAAAA   
  
  
+ TACTTGTCGA GTTCAATGCA TTGCCCGTTT ATGGACCCAA TGTCAGGTGG GAAATGCTGG ATGTGAGGCC   
  
  
+ CAGGGAGGCT TTGGTCGTTA ATTGTCCATT ACAGCTCCAT TACACTCCTG ACGAGAGTGT CGATGTGAGG   
  
  
+ AACCCTAGGG ATAGGCTTCT CAGAATGGTG AAATTGCTCG GTCCTAAGGT AGCCACTTTG GTTGAGCAAG   
  
  
+ AATCAAACAC CAACACTACA CCTTTCTTGA CCCGGTTCAT AGAGACCCTT GACTACTACT CGATTAACCA   
  
  
+ TGTTTG  

- -Up\_Stream \_Len000CAACAT TGTTCGTGTA ATTTCAACTA ATAGTAAAAC AGGCACTTTG TCACTCGTTT   
  
  
- GCAGTAGAGG TCCAGTTTAA GTAAGCATGA GTTAGTGTTC AACGACTGTA AAAGTGGTTT CCGATTAGTA   
  
  
- GCGTTTTCAA AGAAATCAAA TTCCGGTTAG AGAGGTAATA AAGTGGGATA TGTCTAACTC CCCCACATTA   
  
  
- TCACATATAT ATATCGGAAC ACAATCGCCC GGAACCTGTG ATCTGAAAAT GAGAACGTTT GGGATTATAT   
  
  
- AATAATATTT ATATTTAGAT CGGTGGGTAC AACTCCATCC GTGAGATTAA GAGTGTGTCC ATCCTATTCA   
  
  
- AAAGTATCAA ATACAACACT TACACTAGAA TGAAACACCA AGGGCAACTG TTTTCAAACT TAGGCTCGTA   
  
  
- CTCTCAAGTA GTTAGAGAAA AAGAAAGGAT AAAATAAACA ACACTTGAAT TAACCTAAAA GACCATCTCC   
  
  
- TTTCAAGGAA CGACCTTAAA CCAGTTTTCA CTATATATAA AACCCTAAAT TAATAGGTAT TTAAACCAAA   
  
  
- ATGTGGAACA ATCAAACCTG AAGACCACAC AATAACATAC ACAAGACCAC CCAAAAAGAA AATCGACTTA   
  
  
- CAACCTCGTA AAATTCCCTA GATCTATAGA CCTCTTCTCG AACCCTTTAT TTCGTATAAA GGGAATATCA   
  
  
- GACTGTATTG AAATTACTTA ACTAGTCCTT GTTTCCGGTT AATCAAGTTT TTCCAAGTTG AGGAGATCCA   
  
  
- CAACACGATT TCTCTACACT CAATCATTCA TTAACGAATG ATTGATATAT CCTACAAAAT AATGAACATC   
  
  
- TCCAAAAAAA AAACCCTATA GAAATACCCC AAATATTTAT TCTTCCAACT AAACAACAGA TGAGAAGAAA   
  
  
- GCTAAGTTAT CATTAAGAGA GTCTCTTCCG ATTTTACTGT ACCGTAAGGT AGCGGAGAGT ACTCCGAAAA   
  
  
- ACGTACACGA ATACATAGAA AGTAGAGAAC GAAACCACCC CACCGTAACC CCTCCTTTTC CCCCAAAGCT   
  
  
- AAGCTATCTA GCAGTGAAAA CCTTTACTGT AGAGATCGGT TACCGTTCCC ATTCCAATTA TGTACACCTA   
  
  
- TAAAGGTCTC AATCAACACT AACAATAACA ACAACAACAT TGAAATACAG TGCCCCTTTT GTATTATTGA   
  
  
- TAGACAAATA GACAAACAAA CAATAAAAGT TCCCCTTCCC CTCACCCGTC TACGAATCAT ACATTAGTAA   
  
  
- AATATATCCC TTTTAAGTAT ATCGAACTTT CATTTTGAAA GTAACGGTCG TAAGGTTCCA TACCCATCCC   
  
  
- AACAGATGTG GAAATAGAGG GATCTTCGTA CCACTAGTAA GGAGCCTAAC ACAATATCTT ATTTCTTACA   
  
  
- CCAAGACGAC ATTACTCTGA ATACATAATA GTTTACTACT TAGACAAATC TTGAAGGACA AATAAAAAAG   
  
  
- ACGTTTTAAA ATTCTCTGAT TGTAGATAGC CCGAGTTGAG ATCTCCTAGA GAACACAGTG GGAAGAGTTG   
  
  
- AGTCAAACTA ACACTATGTC AATGATATAA CTCACTATCA GCTCTCGTAG TGTAGGGCAA TTCCTCAGAT   
  
  
- AGTTGTTCTT CGGGAAGAAC ATTAAGAGGG GAATTTTGAT CGTCAATAGT AGTGAACAGT AGCTTATCGG   
  
  
- GAAAAACATT AAGATGGGAA CTTTGATCGT CAATAGCAGT AGCCAAGTTG CGCTCAGGAC AATCTATAAG   
  
  
- GGTAGTTCCG TATCTAATAC CTCACTCGTA CTTCTTACGA AACGTCCTCG ATCTCTGATG AGATTAACCA   
  
  
- CATCTACCAC TCCTTCATAG ATAACGATTA GTTGGATACC CCCCTTCAGC AATCAGGCCC TAGGGTTCAA   
  
  
- TCTCTAGCTT CAGTAGTTCG CTTCTAGGTG TCCCAAGTGT AGGATGAGTC GAACTAAGTA GTAGAAAAAG   
  
  
- TTCCCACTCT TCTAAGCCTC TACTTTCGGT CTCTCTCTCT TTACGGTATT CCGTTACCTT CTTGGTCGCC   
  
  
- TTGATGATTC AAAGAGTGGT CCACTAAATT TCGTCAATGA TTAACTTACA CGCTCCCGAA ATAATCTATT   
  
  
- GGCTTATCTA CTGAAACTCT CAAACCAATT TGCCCGTTCC TTTCTCCAGA GGTAGAGTCC ACTCGGACAG   
  
  
- GTTGGAGAGC CATGGACATA CTAGCTTCCC GAACACCGTT CCTTTGTCAG AAGCCCCTGA TTGTAGATAA   
  
  
- CCCGAGAATT CACATTTCTC GGAGAACCTT TTCTGAACGA GAGGATGCCG TGAGTCACCC AATGACATAG   
  
  
- CGTTCGGTAT CGTCGTTCCG AACCACCCGG AGGATTTCAC GCTGAATGTC CGTAACTACT AGGACAAAGA   
  
  
- TTCATATGAA CACTACGATC GAACCTCCGA CCACCCTTTG CTAATCGCAG AGATAAACTT TTCAAATTTT   
  
  
- ATGAACAGCT CAAGTTACGT AACGGGCAAA TACCTGGGTT ACAGTCCACC CTTTACGACC TACACTCCGG   
  
  
- GTCCCTCCGA AACCAGCAAT TAACAGGTAA TGTCGAGGTA ATGTGAGGAC TGCTCTCACA GCTACACTCC   
  
  
- TTGGGATCCC TATCCGAAGA GTCTTACCAC TTTAACGAGC CAGGATTCCA TCGGTGAAAC CAACTCGTTC   
  
  
- TTAGTTTGTG GTTGTGATGT GGAAAGAACT GGGCCAAGTA TCTCTGGGAA CTGATGATGA GCTAATTGGT   
  
  
- ACAAAC

+     GATA-motif

| Site Name | Organism | Position | Strand | Matrix score. | sequence | function |
| --- | --- | --- | --- | --- | --- | --- |
| GATA-motif | Solanum tuberosum | 1100 | + | 9 | AAGGATAAGG | part of a light responsive element |

>HU08G00230.1   
+ -Up\_Stream \_Len000GTTGTA ACAAGCACAT TAAAGTTGAT TATCATTTTG TCCGTGAAAC AGTGAGCAAA   
  
  
+ CGTCATCTCC AGGTCAAATT CATTCGTACT CAATCACAAG TTGCTGACAT TTTCACCAAA GGCTAATCAT   
  
  
+ CGCAAAAGTT TCTTTAGTTT AAGGCCAATC TCTCCATTAT TTCACCCTAT ACAGATTGAG GGGGTGTAAT   
  
  
+ AGTGTATATA TATAGCCTTG TGTTAGCGGG CCTTGGACAC TAGACTTTTA CTCTTGCAAA CCCTAATATA   
  
  
+ TTATTATAAA TATAAATCTA GCCACCCATG TTGAGGTAGG CACTCTAATT CTCACACAGG TAGGATAAGT   
  
  
+ TTTCATAGTT TATGTTGTGA ATGTGATCTT ACTTTGTGGT TCCCGTTGAC AAAAGTTTGA ATCCGAGCAT   
  
  
+ GAGAGTTCAT CAATCTCTTT TTCTTTCCTA TTTTATTTGT TGTGAACTTA ATTGGATTTT CTGGTAGAGG   
  
  
+ AAAGTTCCTT GCTGGAATTT GGTCAAAAGT GATATATATT TTGGGATTTA ATTATCCATA AATTTGGTTT   
  
  
+ TACACCTTGT TAGTTTGGAC TTCTGGTGTG TTATTGTATG TGTTCTGGTG GGTTTTTCTT TTAGCTGAAT   
  
  
+ GTTGGAGCAT TTTAAGGGAT CTAGATATCT GGAGAAGAGC TTGGGAAATA AAGCATATTT CCCTTATAGT   
  
  
+ CTGACATAAC TTTAATGAAT TGATCAGGAA CAAAGGCCAA TTAGTTCAAA AAGGTTCAAC TCCTCTAGGT   
  
  
+ GTTGTGCTAA AGAGATGTGA GTTAGTAAGT AATTGCTTAC TAACTATATA GGATGTTTTA TTACTTGTAG   
  
  
+ AGGTTTTTTT TTTGGGATAT CTTTATGGGG TTTATAAATA AGAAGGTTGA TTTGTTGTCT ACTCTTCTTT   
  
  
+ CGATTCAATA GTAATTCTCT CAGAGAAGGC TAAAATGACA TGGCATTCCA TCGCCTCTCA TGAGGCTTTT   
  
  
+ TGCATGTGCT TATGTATCTT TCATCTCTTG CTTTGGTGGG GTGGCATTGG GGAGGAAAAG GGGGTTTCGA   
  
  
+ TTCGATAGAT CGTCACTTTT GGAAATGACA TCTCTAGCCA ATGGCAAGGG TAAGGTTAAT ACATGTGGAT   
  
  
+ ATTTCCAGAG TTAGTTGTGA TTGTTATTGT TGTTGTTGTA ACTTTATGTC ACGGGGAAAA CATAATAACT   
  
  
+ ATCTGTTTAT CTGTTTGTTT GTTATTTTCA AGGGGAAGGG GAGTGGGCAG ATGCTTAGTA TGTAATCATT   
  
  
+ TTATATAGGG AAAATTCATA TAGCTTGAAA GTAAAACTTT CATTGCCAGC ATTCCAAGGT ATGGGTAGGG   
  
  
+ TTGTCTACAC CTTTATCTCC CTAGAAGCAT GGTGATCATT CCTCGGATTG TGTTATAGAA TAAAGAATGT   
  
  
+ GGTTCTGCTG TAATGAGACT TATGTATTAT CAAATGATGA ATCTGTTTAG AACTTCCTGT TTATTTTTTC   
  
  
+ TGCAAAATTT TAAGAGACTA ACATCTATCG GGCTCAACTC TAGAGGATCT CTTGTGTCAC CCTTCTCAAC   
  
  
+ TCAGTTTGAT TGTGATACAG TTACTATATT GAGTGATAGT CGAGAGCATC ACATCCCGTT AAGGAGTCTA   
  
  
+ TCAACAAGAA GCCCTTCTTG TAATTCTCCC CTTAAAACTA GCAGTTATCA TCACTTGTCA TCGAATAGCC   
  
  
+ CTTTTTGTAA TTCTACCCTT GAAACTAGCA GTTATCGTCA TCGGTTCAAC GCGAGTCCTG TTAGATATTC   
  
  
+ CCATCAAGGC ATAGATTATG GAGTGAGCAT GAAGAATGCT TTGCAGGAGC TAGAGACTAC TCTAATTGGT   
  
  
+ GTAGATGGTG AGGAAGTATC TATTGCTAAT CAACCTATGG GGGGAAGTCG TTAGTCCGGG ATCCCAAGTT   
  
  
+ AGAGATCGAA GTCATCAAGC GAAGATCCAC AGGGTTCACA TCCTACTCAG CTTGATTCAT CATCTTTTTC   
  
  
+ AAGGGTGAGA AGATTCGGAG ATGAAAGCCA GAGAGAGAGA AATGCCATAA GGCAATGGAA GAACCAGCGG   
  
  
+ AACTACTAAG TTTCTCACCA GGTGATTTAA AGCAGTTACT AATTGAATGT GCGAGGGCTT TATTAGATAA   
  
  
+ CCGAATAGAT GACTTTGAGA GTTTGGTTAA ACGGGCAAGG AAAGAGGTCT CCATCTCAGG TGAGCCTGTC   
  
  
+ CAACCTCTCG GTACCTGTAT GATCGAAGGG CTTGTGGCAA GGAAACAGTC TTCGGGGACT AACATCTATT   
  
  
+ GGGCTCTTAA GTGTAAAGAG CCTCTTGGAA AAGACTTGCT CTCCTACGGC ACTCAGTGGG TTACTGTATC   
  
  
+ GCAAGCCATA GCAGCAAGGC TTGGTGGGCC TCCTAAAGTG CGACTTACAG GCATTGATGA TCCTGTTTCT   
  
  
+ AAGTATACTT GTGATGCTAG CTTGGAGGCT GGTGGGAAAC GATTAGCGTC TCTATTTGAA AAGTTTAAAA   
  
  
+ TACTTGTCGA GTTCAATGCA TTGCCCGTTT ATGGACCCAA TGTCAGGTGG GAAATGCTGG ATGTGAGGCC   
  
  
+ CAGGGAGGCT TTGGTCGTTA ATTGTCCATT ACAGCTCCAT TACACTCCTG ACGAGAGTGT CGATGTGAGG   
  
  
+ AACCCTAGGG ATAGGCTTCT CAGAATGGTG AAATTGCTCG GTCCTAAGGT AGCCACTTTG GTTGAGCAAG   
  
  
+ AATCAAACAC CAACACTACA CCTTTCTTGA CCCGGTTCAT AGAGACCCTT GACTACTACT CGATTAACCA   
  
  
+ TGTTTG  

- -Up\_Stream \_Len000CAACAT TGTTCGTGTA ATTTCAACTA ATAGTAAAAC AGGCACTTTG TCACTCGTTT   
  
  
- GCAGTAGAGG TCCAGTTTAA GTAAGCATGA GTTAGTGTTC AACGACTGTA AAAGTGGTTT CCGATTAGTA   
  
  
- GCGTTTTCAA AGAAATCAAA TTCCGGTTAG AGAGGTAATA AAGTGGGATA TGTCTAACTC CCCCACATTA   
  
  
- TCACATATAT ATATCGGAAC ACAATCGCCC GGAACCTGTG ATCTGAAAAT GAGAACGTTT GGGATTATAT   
  
  
- AATAATATTT ATATTTAGAT CGGTGGGTAC AACTCCATCC GTGAGATTAA GAGTGTGTCC ATCCTATTCA   
  
  
- AAAGTATCAA ATACAACACT TACACTAGAA TGAAACACCA AGGGCAACTG TTTTCAAACT TAGGCTCGTA   
  
  
- CTCTCAAGTA GTTAGAGAAA AAGAAAGGAT AAAATAAACA ACACTTGAAT TAACCTAAAA GACCATCTCC   
  
  
- TTTCAAGGAA CGACCTTAAA CCAGTTTTCA CTATATATAA AACCCTAAAT TAATAGGTAT TTAAACCAAA   
  
  
- ATGTGGAACA ATCAAACCTG AAGACCACAC AATAACATAC ACAAGACCAC CCAAAAAGAA AATCGACTTA   
  
  
- CAACCTCGTA AAATTCCCTA GATCTATAGA CCTCTTCTCG AACCCTTTAT TTCGTATAAA GGGAATATCA   
  
  
- GACTGTATTG AAATTACTTA ACTAGTCCTT GTTTCCGGTT AATCAAGTTT TTCCAAGTTG AGGAGATCCA   
  
  
- CAACACGATT TCTCTACACT CAATCATTCA TTAACGAATG ATTGATATAT CCTACAAAAT AATGAACATC   
  
  
- TCCAAAAAAA AAACCCTATA GAAATACCCC AAATATTTAT TCTTCCAACT AAACAACAGA TGAGAAGAAA   
  
  
- GCTAAGTTAT CATTAAGAGA GTCTCTTCCG ATTTTACTGT ACCGTAAGGT AGCGGAGAGT ACTCCGAAAA   
  
  
- ACGTACACGA ATACATAGAA AGTAGAGAAC GAAACCACCC CACCGTAACC CCTCCTTTTC CCCCAAAGCT   
  
  
- AAGCTATCTA GCAGTGAAAA CCTTTACTGT AGAGATCGGT TACCGTTCCC ATTCCAATTA TGTACACCTA   
  
  
- TAAAGGTCTC AATCAACACT AACAATAACA ACAACAACAT TGAAATACAG TGCCCCTTTT GTATTATTGA   
  
  
- TAGACAAATA GACAAACAAA CAATAAAAGT TCCCCTTCCC CTCACCCGTC TACGAATCAT ACATTAGTAA   
  
  
- AATATATCCC TTTTAAGTAT ATCGAACTTT CATTTTGAAA GTAACGGTCG TAAGGTTCCA TACCCATCCC   
  
  
- AACAGATGTG GAAATAGAGG GATCTTCGTA CCACTAGTAA GGAGCCTAAC ACAATATCTT ATTTCTTACA   
  
  
- CCAAGACGAC ATTACTCTGA ATACATAATA GTTTACTACT TAGACAAATC TTGAAGGACA AATAAAAAAG   
  
  
- ACGTTTTAAA ATTCTCTGAT TGTAGATAGC CCGAGTTGAG ATCTCCTAGA GAACACAGTG GGAAGAGTTG   
  
  
- AGTCAAACTA ACACTATGTC AATGATATAA CTCACTATCA GCTCTCGTAG TGTAGGGCAA TTCCTCAGAT   
  
  
- AGTTGTTCTT CGGGAAGAAC ATTAAGAGGG GAATTTTGAT CGTCAATAGT AGTGAACAGT AGCTTATCGG   
  
  
- GAAAAACATT AAGATGGGAA CTTTGATCGT CAATAGCAGT AGCCAAGTTG CGCTCAGGAC AATCTATAAG   
  
  
- GGTAGTTCCG TATCTAATAC CTCACTCGTA CTTCTTACGA AACGTCCTCG ATCTCTGATG AGATTAACCA   
  
  
- CATCTACCAC TCCTTCATAG ATAACGATTA GTTGGATACC CCCCTTCAGC AATCAGGCCC TAGGGTTCAA   
  
  
- TCTCTAGCTT CAGTAGTTCG CTTCTAGGTG TCCCAAGTGT AGGATGAGTC GAACTAAGTA GTAGAAAAAG   
  
  
- TTCCCACTCT TCTAAGCCTC TACTTTCGGT CTCTCTCTCT TTACGGTATT CCGTTACCTT CTTGGTCGCC   
  
  
- TTGATGATTC AAAGAGTGGT CCACTAAATT TCGTCAATGA TTAACTTACA CGCTCCCGAA ATAATCTATT   
  
  
- GGCTTATCTA CTGAAACTCT CAAACCAATT TGCCCGTTCC TTTCTCCAGA GGTAGAGTCC ACTCGGACAG   
  
  
- GTTGGAGAGC CATGGACATA CTAGCTTCCC GAACACCGTT CCTTTGTCAG AAGCCCCTGA TTGTAGATAA   
  
  
- CCCGAGAATT CACATTTCTC GGAGAACCTT TTCTGAACGA GAGGATGCCG TGAGTCACCC AATGACATAG   
  
  
- CGTTCGGTAT CGTCGTTCCG AACCACCCGG AGGATTTCAC GCTGAATGTC CGTAACTACT AGGACAAAGA   
  
  
- TTCATATGAA CACTACGATC GAACCTCCGA CCACCCTTTG CTAATCGCAG AGATAAACTT TTCAAATTTT   
  
  
- ATGAACAGCT CAAGTTACGT AACGGGCAAA TACCTGGGTT ACAGTCCACC CTTTACGACC TACACTCCGG   
  
  
- GTCCCTCCGA AACCAGCAAT TAACAGGTAA TGTCGAGGTA ATGTGAGGAC TGCTCTCACA GCTACACTCC   
  
  
- TTGGGATCCC TATCCGAAGA GTCTTACCAC TTTAACGAGC CAGGATTCCA TCGGTGAAAC CAACTCGTTC   
  
  
- TTAGTTTGTG GTTGTGATGT GGAAAGAACT GGGCCAAGTA TCTCTGGGAA CTGATGATGA GCTAATTGGT   
  
  
- ACAAAC

+     GT1-motif

| Site Name | Organism | Position | Strand | Matrix score. | sequence | function |
| --- | --- | --- | --- | --- | --- | --- |
| GT1-motif | Avena sativa | 1108 | + | 7 | GGTTAAT | light responsive element |
| GT1-motif | Avena sativa | 2727 | - | 7 | GGTTAAT | light responsive element |
| GT1-motif | Arabidopsis thaliana | 2129 | + | 6 | GGTTAA | light responsive element |
| GT1-motif | Arabidopsis thaliana | 2728 | - | 6 | GGTTAA | light responsive element |

>HU08G00230.1   
+ -Up\_Stream \_Len000GTTGTA ACAAGCACAT TAAAGTTGAT TATCATTTTG TCCGTGAAAC AGTGAGCAAA   
  
  
+ CGTCATCTCC AGGTCAAATT CATTCGTACT CAATCACAAG TTGCTGACAT TTTCACCAAA GGCTAATCAT   
  
  
+ CGCAAAAGTT TCTTTAGTTT AAGGCCAATC TCTCCATTAT TTCACCCTAT ACAGATTGAG GGGGTGTAAT   
  
  
+ AGTGTATATA TATAGCCTTG TGTTAGCGGG CCTTGGACAC TAGACTTTTA CTCTTGCAAA CCCTAATATA   
  
  
+ TTATTATAAA TATAAATCTA GCCACCCATG TTGAGGTAGG CACTCTAATT CTCACACAGG TAGGATAAGT   
  
  
+ TTTCATAGTT TATGTTGTGA ATGTGATCTT ACTTTGTGGT TCCCGTTGAC AAAAGTTTGA ATCCGAGCAT   
  
  
+ GAGAGTTCAT CAATCTCTTT TTCTTTCCTA TTTTATTTGT TGTGAACTTA ATTGGATTTT CTGGTAGAGG   
  
  
+ AAAGTTCCTT GCTGGAATTT GGTCAAAAGT GATATATATT TTGGGATTTA ATTATCCATA AATTTGGTTT   
  
  
+ TACACCTTGT TAGTTTGGAC TTCTGGTGTG TTATTGTATG TGTTCTGGTG GGTTTTTCTT TTAGCTGAAT   
  
  
+ GTTGGAGCAT TTTAAGGGAT CTAGATATCT GGAGAAGAGC TTGGGAAATA AAGCATATTT CCCTTATAGT   
  
  
+ CTGACATAAC TTTAATGAAT TGATCAGGAA CAAAGGCCAA TTAGTTCAAA AAGGTTCAAC TCCTCTAGGT   
  
  
+ GTTGTGCTAA AGAGATGTGA GTTAGTAAGT AATTGCTTAC TAACTATATA GGATGTTTTA TTACTTGTAG   
  
  
+ AGGTTTTTTT TTTGGGATAT CTTTATGGGG TTTATAAATA AGAAGGTTGA TTTGTTGTCT ACTCTTCTTT   
  
  
+ CGATTCAATA GTAATTCTCT CAGAGAAGGC TAAAATGACA TGGCATTCCA TCGCCTCTCA TGAGGCTTTT   
  
  
+ TGCATGTGCT TATGTATCTT TCATCTCTTG CTTTGGTGGG GTGGCATTGG GGAGGAAAAG GGGGTTTCGA   
  
  
+ TTCGATAGAT CGTCACTTTT GGAAATGACA TCTCTAGCCA ATGGCAAGGG TAAGGTTAAT ACATGTGGAT   
  
  
+ ATTTCCAGAG TTAGTTGTGA TTGTTATTGT TGTTGTTGTA ACTTTATGTC ACGGGGAAAA CATAATAACT   
  
  
+ ATCTGTTTAT CTGTTTGTTT GTTATTTTCA AGGGGAAGGG GAGTGGGCAG ATGCTTAGTA TGTAATCATT   
  
  
+ TTATATAGGG AAAATTCATA TAGCTTGAAA GTAAAACTTT CATTGCCAGC ATTCCAAGGT ATGGGTAGGG   
  
  
+ TTGTCTACAC CTTTATCTCC CTAGAAGCAT GGTGATCATT CCTCGGATTG TGTTATAGAA TAAAGAATGT   
  
  
+ GGTTCTGCTG TAATGAGACT TATGTATTAT CAAATGATGA ATCTGTTTAG AACTTCCTGT TTATTTTTTC   
  
  
+ TGCAAAATTT TAAGAGACTA ACATCTATCG GGCTCAACTC TAGAGGATCT CTTGTGTCAC CCTTCTCAAC   
  
  
+ TCAGTTTGAT TGTGATACAG TTACTATATT GAGTGATAGT CGAGAGCATC ACATCCCGTT AAGGAGTCTA   
  
  
+ TCAACAAGAA GCCCTTCTTG TAATTCTCCC CTTAAAACTA GCAGTTATCA TCACTTGTCA TCGAATAGCC   
  
  
+ CTTTTTGTAA TTCTACCCTT GAAACTAGCA GTTATCGTCA TCGGTTCAAC GCGAGTCCTG TTAGATATTC   
  
  
+ CCATCAAGGC ATAGATTATG GAGTGAGCAT GAAGAATGCT TTGCAGGAGC TAGAGACTAC TCTAATTGGT   
  
  
+ GTAGATGGTG AGGAAGTATC TATTGCTAAT CAACCTATGG GGGGAAGTCG TTAGTCCGGG ATCCCAAGTT   
  
  
+ AGAGATCGAA GTCATCAAGC GAAGATCCAC AGGGTTCACA TCCTACTCAG CTTGATTCAT CATCTTTTTC   
  
  
+ AAGGGTGAGA AGATTCGGAG ATGAAAGCCA GAGAGAGAGA AATGCCATAA GGCAATGGAA GAACCAGCGG   
  
  
+ AACTACTAAG TTTCTCACCA GGTGATTTAA AGCAGTTACT AATTGAATGT GCGAGGGCTT TATTAGATAA   
  
  
+ CCGAATAGAT GACTTTGAGA GTTTGGTTAA ACGGGCAAGG AAAGAGGTCT CCATCTCAGG TGAGCCTGTC   
  
  
+ CAACCTCTCG GTACCTGTAT GATCGAAGGG CTTGTGGCAA GGAAACAGTC TTCGGGGACT AACATCTATT   
  
  
+ GGGCTCTTAA GTGTAAAGAG CCTCTTGGAA AAGACTTGCT CTCCTACGGC ACTCAGTGGG TTACTGTATC   
  
  
+ GCAAGCCATA GCAGCAAGGC TTGGTGGGCC TCCTAAAGTG CGACTTACAG GCATTGATGA TCCTGTTTCT   
  
  
+ AAGTATACTT GTGATGCTAG CTTGGAGGCT GGTGGGAAAC GATTAGCGTC TCTATTTGAA AAGTTTAAAA   
  
  
+ TACTTGTCGA GTTCAATGCA TTGCCCGTTT ATGGACCCAA TGTCAGGTGG GAAATGCTGG ATGTGAGGCC   
  
  
+ CAGGGAGGCT TTGGTCGTTA ATTGTCCATT ACAGCTCCAT TACACTCCTG ACGAGAGTGT CGATGTGAGG   
  
  
+ AACCCTAGGG ATAGGCTTCT CAGAATGGTG AAATTGCTCG GTCCTAAGGT AGCCACTTTG GTTGAGCAAG   
  
  
+ AATCAAACAC CAACACTACA CCTTTCTTGA CCCGGTTCAT AGAGACCCTT GACTACTACT CGATTAACCA   
  
  
+ TGTTTG  

- -Up\_Stream \_Len000CAACAT TGTTCGTGTA ATTTCAACTA ATAGTAAAAC AGGCACTTTG TCACTCGTTT   
  
  
- GCAGTAGAGG TCCAGTTTAA GTAAGCATGA GTTAGTGTTC AACGACTGTA AAAGTGGTTT CCGATTAGTA   
  
  
- GCGTTTTCAA AGAAATCAAA TTCCGGTTAG AGAGGTAATA AAGTGGGATA TGTCTAACTC CCCCACATTA   
  
  
- TCACATATAT ATATCGGAAC ACAATCGCCC GGAACCTGTG ATCTGAAAAT GAGAACGTTT GGGATTATAT   
  
  
- AATAATATTT ATATTTAGAT CGGTGGGTAC AACTCCATCC GTGAGATTAA GAGTGTGTCC ATCCTATTCA   
  
  
- AAAGTATCAA ATACAACACT TACACTAGAA TGAAACACCA AGGGCAACTG TTTTCAAACT TAGGCTCGTA   
  
  
- CTCTCAAGTA GTTAGAGAAA AAGAAAGGAT AAAATAAACA ACACTTGAAT TAACCTAAAA GACCATCTCC   
  
  
- TTTCAAGGAA CGACCTTAAA CCAGTTTTCA CTATATATAA AACCCTAAAT TAATAGGTAT TTAAACCAAA   
  
  
- ATGTGGAACA ATCAAACCTG AAGACCACAC AATAACATAC ACAAGACCAC CCAAAAAGAA AATCGACTTA   
  
  
- CAACCTCGTA AAATTCCCTA GATCTATAGA CCTCTTCTCG AACCCTTTAT TTCGTATAAA GGGAATATCA   
  
  
- GACTGTATTG AAATTACTTA ACTAGTCCTT GTTTCCGGTT AATCAAGTTT TTCCAAGTTG AGGAGATCCA   
  
  
- CAACACGATT TCTCTACACT CAATCATTCA TTAACGAATG ATTGATATAT CCTACAAAAT AATGAACATC   
  
  
- TCCAAAAAAA AAACCCTATA GAAATACCCC AAATATTTAT TCTTCCAACT AAACAACAGA TGAGAAGAAA   
  
  
- GCTAAGTTAT CATTAAGAGA GTCTCTTCCG ATTTTACTGT ACCGTAAGGT AGCGGAGAGT ACTCCGAAAA   
  
  
- ACGTACACGA ATACATAGAA AGTAGAGAAC GAAACCACCC CACCGTAACC CCTCCTTTTC CCCCAAAGCT   
  
  
- AAGCTATCTA GCAGTGAAAA CCTTTACTGT AGAGATCGGT TACCGTTCCC ATTCCAATTA TGTACACCTA   
  
  
- TAAAGGTCTC AATCAACACT AACAATAACA ACAACAACAT TGAAATACAG TGCCCCTTTT GTATTATTGA   
  
  
- TAGACAAATA GACAAACAAA CAATAAAAGT TCCCCTTCCC CTCACCCGTC TACGAATCAT ACATTAGTAA   
  
  
- AATATATCCC TTTTAAGTAT ATCGAACTTT CATTTTGAAA GTAACGGTCG TAAGGTTCCA TACCCATCCC   
  
  
- AACAGATGTG GAAATAGAGG GATCTTCGTA CCACTAGTAA GGAGCCTAAC ACAATATCTT ATTTCTTACA   
  
  
- CCAAGACGAC ATTACTCTGA ATACATAATA GTTTACTACT TAGACAAATC TTGAAGGACA AATAAAAAAG   
  
  
- ACGTTTTAAA ATTCTCTGAT TGTAGATAGC CCGAGTTGAG ATCTCCTAGA GAACACAGTG GGAAGAGTTG   
  
  
- AGTCAAACTA ACACTATGTC AATGATATAA CTCACTATCA GCTCTCGTAG TGTAGGGCAA TTCCTCAGAT   
  
  
- AGTTGTTCTT CGGGAAGAAC ATTAAGAGGG GAATTTTGAT CGTCAATAGT AGTGAACAGT AGCTTATCGG   
  
  
- GAAAAACATT AAGATGGGAA CTTTGATCGT CAATAGCAGT AGCCAAGTTG CGCTCAGGAC AATCTATAAG   
  
  
- GGTAGTTCCG TATCTAATAC CTCACTCGTA CTTCTTACGA AACGTCCTCG ATCTCTGATG AGATTAACCA   
  
  
- CATCTACCAC TCCTTCATAG ATAACGATTA GTTGGATACC CCCCTTCAGC AATCAGGCCC TAGGGTTCAA   
  
  
- TCTCTAGCTT CAGTAGTTCG CTTCTAGGTG TCCCAAGTGT AGGATGAGTC GAACTAAGTA GTAGAAAAAG   
  
  
- TTCCCACTCT TCTAAGCCTC TACTTTCGGT CTCTCTCTCT TTACGGTATT CCGTTACCTT CTTGGTCGCC   
  
  
- TTGATGATTC AAAGAGTGGT CCACTAAATT TCGTCAATGA TTAACTTACA CGCTCCCGAA ATAATCTATT   
  
  
- GGCTTATCTA CTGAAACTCT CAAACCAATT TGCCCGTTCC TTTCTCCAGA GGTAGAGTCC ACTCGGACAG   
  
  
- GTTGGAGAGC CATGGACATA CTAGCTTCCC GAACACCGTT CCTTTGTCAG AAGCCCCTGA TTGTAGATAA   
  
  
- CCCGAGAATT CACATTTCTC GGAGAACCTT TTCTGAACGA GAGGATGCCG TGAGTCACCC AATGACATAG   
  
  
- CGTTCGGTAT CGTCGTTCCG AACCACCCGG AGGATTTCAC GCTGAATGTC CGTAACTACT AGGACAAAGA   
  
  
- TTCATATGAA CACTACGATC GAACCTCCGA CCACCCTTTG CTAATCGCAG AGATAAACTT TTCAAATTTT   
  
  
- ATGAACAGCT CAAGTTACGT AACGGGCAAA TACCTGGGTT ACAGTCCACC CTTTACGACC TACACTCCGG   
  
  
- GTCCCTCCGA AACCAGCAAT TAACAGGTAA TGTCGAGGTA ATGTGAGGAC TGCTCTCACA GCTACACTCC   
  
  
- TTGGGATCCC TATCCGAAGA GTCTTACCAC TTTAACGAGC CAGGATTCCA TCGGTGAAAC CAACTCGTTC   
  
  
- TTAGTTTGTG GTTGTGATGT GGAAAGAACT GGGCCAAGTA TCTCTGGGAA CTGATGATGA GCTAATTGGT   
  
  
- ACAAAC

+     I-box

| Site Name | Organism | Position | Strand | Matrix score. | sequence | function |
| --- | --- | --- | --- | --- | --- | --- |
| I-box | Pisum sativum | 2098 | + | 9 | TAGATAACC | part of a light responsive element |
| I-box | Larix laricina | 161 | + | 9 | GTATAAGGCC | part of a light responsive element |

>HU08G00230.1   
+ -Up\_Stream \_Len000GTTGTA ACAAGCACAT TAAAGTTGAT TATCATTTTG TCCGTGAAAC AGTGAGCAAA   
  
  
+ CGTCATCTCC AGGTCAAATT CATTCGTACT CAATCACAAG TTGCTGACAT TTTCACCAAA GGCTAATCAT   
  
  
+ CGCAAAAGTT TCTTTAGTTT AAGGCCAATC TCTCCATTAT TTCACCCTAT ACAGATTGAG GGGGTGTAAT   
  
  
+ AGTGTATATA TATAGCCTTG TGTTAGCGGG CCTTGGACAC TAGACTTTTA CTCTTGCAAA CCCTAATATA   
  
  
+ TTATTATAAA TATAAATCTA GCCACCCATG TTGAGGTAGG CACTCTAATT CTCACACAGG TAGGATAAGT   
  
  
+ TTTCATAGTT TATGTTGTGA ATGTGATCTT ACTTTGTGGT TCCCGTTGAC AAAAGTTTGA ATCCGAGCAT   
  
  
+ GAGAGTTCAT CAATCTCTTT TTCTTTCCTA TTTTATTTGT TGTGAACTTA ATTGGATTTT CTGGTAGAGG   
  
  
+ AAAGTTCCTT GCTGGAATTT GGTCAAAAGT GATATATATT TTGGGATTTA ATTATCCATA AATTTGGTTT   
  
  
+ TACACCTTGT TAGTTTGGAC TTCTGGTGTG TTATTGTATG TGTTCTGGTG GGTTTTTCTT TTAGCTGAAT   
  
  
+ GTTGGAGCAT TTTAAGGGAT CTAGATATCT GGAGAAGAGC TTGGGAAATA AAGCATATTT CCCTTATAGT   
  
  
+ CTGACATAAC TTTAATGAAT TGATCAGGAA CAAAGGCCAA TTAGTTCAAA AAGGTTCAAC TCCTCTAGGT   
  
  
+ GTTGTGCTAA AGAGATGTGA GTTAGTAAGT AATTGCTTAC TAACTATATA GGATGTTTTA TTACTTGTAG   
  
  
+ AGGTTTTTTT TTTGGGATAT CTTTATGGGG TTTATAAATA AGAAGGTTGA TTTGTTGTCT ACTCTTCTTT   
  
  
+ CGATTCAATA GTAATTCTCT CAGAGAAGGC TAAAATGACA TGGCATTCCA TCGCCTCTCA TGAGGCTTTT   
  
  
+ TGCATGTGCT TATGTATCTT TCATCTCTTG CTTTGGTGGG GTGGCATTGG GGAGGAAAAG GGGGTTTCGA   
  
  
+ TTCGATAGAT CGTCACTTTT GGAAATGACA TCTCTAGCCA ATGGCAAGGG TAAGGTTAAT ACATGTGGAT   
  
  
+ ATTTCCAGAG TTAGTTGTGA TTGTTATTGT TGTTGTTGTA ACTTTATGTC ACGGGGAAAA CATAATAACT   
  
  
+ ATCTGTTTAT CTGTTTGTTT GTTATTTTCA AGGGGAAGGG GAGTGGGCAG ATGCTTAGTA TGTAATCATT   
  
  
+ TTATATAGGG AAAATTCATA TAGCTTGAAA GTAAAACTTT CATTGCCAGC ATTCCAAGGT ATGGGTAGGG   
  
  
+ TTGTCTACAC CTTTATCTCC CTAGAAGCAT GGTGATCATT CCTCGGATTG TGTTATAGAA TAAAGAATGT   
  
  
+ GGTTCTGCTG TAATGAGACT TATGTATTAT CAAATGATGA ATCTGTTTAG AACTTCCTGT TTATTTTTTC   
  
  
+ TGCAAAATTT TAAGAGACTA ACATCTATCG GGCTCAACTC TAGAGGATCT CTTGTGTCAC CCTTCTCAAC   
  
  
+ TCAGTTTGAT TGTGATACAG TTACTATATT GAGTGATAGT CGAGAGCATC ACATCCCGTT AAGGAGTCTA   
  
  
+ TCAACAAGAA GCCCTTCTTG TAATTCTCCC CTTAAAACTA GCAGTTATCA TCACTTGTCA TCGAATAGCC   
  
  
+ CTTTTTGTAA TTCTACCCTT GAAACTAGCA GTTATCGTCA TCGGTTCAAC GCGAGTCCTG TTAGATATTC   
  
  
+ CCATCAAGGC ATAGATTATG GAGTGAGCAT GAAGAATGCT TTGCAGGAGC TAGAGACTAC TCTAATTGGT   
  
  
+ GTAGATGGTG AGGAAGTATC TATTGCTAAT CAACCTATGG GGGGAAGTCG TTAGTCCGGG ATCCCAAGTT   
  
  
+ AGAGATCGAA GTCATCAAGC GAAGATCCAC AGGGTTCACA TCCTACTCAG CTTGATTCAT CATCTTTTTC   
  
  
+ AAGGGTGAGA AGATTCGGAG ATGAAAGCCA GAGAGAGAGA AATGCCATAA GGCAATGGAA GAACCAGCGG   
  
  
+ AACTACTAAG TTTCTCACCA GGTGATTTAA AGCAGTTACT AATTGAATGT GCGAGGGCTT TATTAGATAA   
  
  
+ CCGAATAGAT GACTTTGAGA GTTTGGTTAA ACGGGCAAGG AAAGAGGTCT CCATCTCAGG TGAGCCTGTC   
  
  
+ CAACCTCTCG GTACCTGTAT GATCGAAGGG CTTGTGGCAA GGAAACAGTC TTCGGGGACT AACATCTATT   
  
  
+ GGGCTCTTAA GTGTAAAGAG CCTCTTGGAA AAGACTTGCT CTCCTACGGC ACTCAGTGGG TTACTGTATC   
  
  
+ GCAAGCCATA GCAGCAAGGC TTGGTGGGCC TCCTAAAGTG CGACTTACAG GCATTGATGA TCCTGTTTCT   
  
  
+ AAGTATACTT GTGATGCTAG CTTGGAGGCT GGTGGGAAAC GATTAGCGTC TCTATTTGAA AAGTTTAAAA   
  
  
+ TACTTGTCGA GTTCAATGCA TTGCCCGTTT ATGGACCCAA TGTCAGGTGG GAAATGCTGG ATGTGAGGCC   
  
  
+ CAGGGAGGCT TTGGTCGTTA ATTGTCCATT ACAGCTCCAT TACACTCCTG ACGAGAGTGT CGATGTGAGG   
  
  
+ AACCCTAGGG ATAGGCTTCT CAGAATGGTG AAATTGCTCG GTCCTAAGGT AGCCACTTTG GTTGAGCAAG   
  
  
+ AATCAAACAC CAACACTACA CCTTTCTTGA CCCGGTTCAT AGAGACCCTT GACTACTACT CGATTAACCA   
  
  
+ TGTTTG  

- -Up\_Stream \_Len000CAACAT TGTTCGTGTA ATTTCAACTA ATAGTAAAAC AGGCACTTTG TCACTCGTTT   
  
  
- GCAGTAGAGG TCCAGTTTAA GTAAGCATGA GTTAGTGTTC AACGACTGTA AAAGTGGTTT CCGATTAGTA   
  
  
- GCGTTTTCAA AGAAATCAAA TTCCGGTTAG AGAGGTAATA AAGTGGGATA TGTCTAACTC CCCCACATTA   
  
  
- TCACATATAT ATATCGGAAC ACAATCGCCC GGAACCTGTG ATCTGAAAAT GAGAACGTTT GGGATTATAT   
  
  
- AATAATATTT ATATTTAGAT CGGTGGGTAC AACTCCATCC GTGAGATTAA GAGTGTGTCC ATCCTATTCA   
  
  
- AAAGTATCAA ATACAACACT TACACTAGAA TGAAACACCA AGGGCAACTG TTTTCAAACT TAGGCTCGTA   
  
  
- CTCTCAAGTA GTTAGAGAAA AAGAAAGGAT AAAATAAACA ACACTTGAAT TAACCTAAAA GACCATCTCC   
  
  
- TTTCAAGGAA CGACCTTAAA CCAGTTTTCA CTATATATAA AACCCTAAAT TAATAGGTAT TTAAACCAAA   
  
  
- ATGTGGAACA ATCAAACCTG AAGACCACAC AATAACATAC ACAAGACCAC CCAAAAAGAA AATCGACTTA   
  
  
- CAACCTCGTA AAATTCCCTA GATCTATAGA CCTCTTCTCG AACCCTTTAT TTCGTATAAA GGGAATATCA   
  
  
- GACTGTATTG AAATTACTTA ACTAGTCCTT GTTTCCGGTT AATCAAGTTT TTCCAAGTTG AGGAGATCCA   
  
  
- CAACACGATT TCTCTACACT CAATCATTCA TTAACGAATG ATTGATATAT CCTACAAAAT AATGAACATC   
  
  
- TCCAAAAAAA AAACCCTATA GAAATACCCC AAATATTTAT TCTTCCAACT AAACAACAGA TGAGAAGAAA   
  
  
- GCTAAGTTAT CATTAAGAGA GTCTCTTCCG ATTTTACTGT ACCGTAAGGT AGCGGAGAGT ACTCCGAAAA   
  
  
- ACGTACACGA ATACATAGAA AGTAGAGAAC GAAACCACCC CACCGTAACC CCTCCTTTTC CCCCAAAGCT   
  
  
- AAGCTATCTA GCAGTGAAAA CCTTTACTGT AGAGATCGGT TACCGTTCCC ATTCCAATTA TGTACACCTA   
  
  
- TAAAGGTCTC AATCAACACT AACAATAACA ACAACAACAT TGAAATACAG TGCCCCTTTT GTATTATTGA   
  
  
- TAGACAAATA GACAAACAAA CAATAAAAGT TCCCCTTCCC CTCACCCGTC TACGAATCAT ACATTAGTAA   
  
  
- AATATATCCC TTTTAAGTAT ATCGAACTTT CATTTTGAAA GTAACGGTCG TAAGGTTCCA TACCCATCCC   
  
  
- AACAGATGTG GAAATAGAGG GATCTTCGTA CCACTAGTAA GGAGCCTAAC ACAATATCTT ATTTCTTACA   
  
  
- CCAAGACGAC ATTACTCTGA ATACATAATA GTTTACTACT TAGACAAATC TTGAAGGACA AATAAAAAAG   
  
  
- ACGTTTTAAA ATTCTCTGAT TGTAGATAGC CCGAGTTGAG ATCTCCTAGA GAACACAGTG GGAAGAGTTG   
  
  
- AGTCAAACTA ACACTATGTC AATGATATAA CTCACTATCA GCTCTCGTAG TGTAGGGCAA TTCCTCAGAT   
  
  
- AGTTGTTCTT CGGGAAGAAC ATTAAGAGGG GAATTTTGAT CGTCAATAGT AGTGAACAGT AGCTTATCGG   
  
  
- GAAAAACATT AAGATGGGAA CTTTGATCGT CAATAGCAGT AGCCAAGTTG CGCTCAGGAC AATCTATAAG   
  
  
- GGTAGTTCCG TATCTAATAC CTCACTCGTA CTTCTTACGA AACGTCCTCG ATCTCTGATG AGATTAACCA   
  
  
- CATCTACCAC TCCTTCATAG ATAACGATTA GTTGGATACC CCCCTTCAGC AATCAGGCCC TAGGGTTCAA   
  
  
- TCTCTAGCTT CAGTAGTTCG CTTCTAGGTG TCCCAAGTGT AGGATGAGTC GAACTAAGTA GTAGAAAAAG   
  
  
- TTCCCACTCT TCTAAGCCTC TACTTTCGGT CTCTCTCTCT TTACGGTATT CCGTTACCTT CTTGGTCGCC   
  
  
- TTGATGATTC AAAGAGTGGT CCACTAAATT TCGTCAATGA TTAACTTACA CGCTCCCGAA ATAATCTATT   
  
  
- GGCTTATCTA CTGAAACTCT CAAACCAATT TGCCCGTTCC TTTCTCCAGA GGTAGAGTCC ACTCGGACAG   
  
  
- GTTGGAGAGC CATGGACATA CTAGCTTCCC GAACACCGTT CCTTTGTCAG AAGCCCCTGA TTGTAGATAA   
  
  
- CCCGAGAATT CACATTTCTC GGAGAACCTT TTCTGAACGA GAGGATGCCG TGAGTCACCC AATGACATAG   
  
  
- CGTTCGGTAT CGTCGTTCCG AACCACCCGG AGGATTTCAC GCTGAATGTC CGTAACTACT AGGACAAAGA   
  
  
- TTCATATGAA CACTACGATC GAACCTCCGA CCACCCTTTG CTAATCGCAG AGATAAACTT TTCAAATTTT   
  
  
- ATGAACAGCT CAAGTTACGT AACGGGCAAA TACCTGGGTT ACAGTCCACC CTTTACGACC TACACTCCGG   
  
  
- GTCCCTCCGA AACCAGCAAT TAACAGGTAA TGTCGAGGTA ATGTGAGGAC TGCTCTCACA GCTACACTCC   
  
  
- TTGGGATCCC TATCCGAAGA GTCTTACCAC TTTAACGAGC CAGGATTCCA TCGGTGAAAC CAACTCGTTC   
  
  
- TTAGTTTGTG GTTGTGATGT GGAAAGAACT GGGCCAAGTA TCTCTGGGAA CTGATGATGA GCTAATTGGT   
  
  
- ACAAAC

+     MYB

| Site Name | Organism | Position | Strand | Matrix score. | sequence | function |
| --- | --- | --- | --- | --- | --- | --- |
| MYB | Arabidopsis thaliana | 2653 | - | 6 | CAACCA |  |
| MYB | Arabidopsis thaliana | 2729 | + | 6 | TAACCA |  |
| MYB | Arabidopsis thaliana | 2128 | - | 6 | TAACCA |  |

>HU08G00230.1   
+ -Up\_Stream \_Len000GTTGTA ACAAGCACAT TAAAGTTGAT TATCATTTTG TCCGTGAAAC AGTGAGCAAA   
  
  
+ CGTCATCTCC AGGTCAAATT CATTCGTACT CAATCACAAG TTGCTGACAT TTTCACCAAA GGCTAATCAT   
  
  
+ CGCAAAAGTT TCTTTAGTTT AAGGCCAATC TCTCCATTAT TTCACCCTAT ACAGATTGAG GGGGTGTAAT   
  
  
+ AGTGTATATA TATAGCCTTG TGTTAGCGGG CCTTGGACAC TAGACTTTTA CTCTTGCAAA CCCTAATATA   
  
  
+ TTATTATAAA TATAAATCTA GCCACCCATG TTGAGGTAGG CACTCTAATT CTCACACAGG TAGGATAAGT   
  
  
+ TTTCATAGTT TATGTTGTGA ATGTGATCTT ACTTTGTGGT TCCCGTTGAC AAAAGTTTGA ATCCGAGCAT   
  
  
+ GAGAGTTCAT CAATCTCTTT TTCTTTCCTA TTTTATTTGT TGTGAACTTA ATTGGATTTT CTGGTAGAGG   
  
  
+ AAAGTTCCTT GCTGGAATTT GGTCAAAAGT GATATATATT TTGGGATTTA ATTATCCATA AATTTGGTTT   
  
  
+ TACACCTTGT TAGTTTGGAC TTCTGGTGTG TTATTGTATG TGTTCTGGTG GGTTTTTCTT TTAGCTGAAT   
  
  
+ GTTGGAGCAT TTTAAGGGAT CTAGATATCT GGAGAAGAGC TTGGGAAATA AAGCATATTT CCCTTATAGT   
  
  
+ CTGACATAAC TTTAATGAAT TGATCAGGAA CAAAGGCCAA TTAGTTCAAA AAGGTTCAAC TCCTCTAGGT   
  
  
+ GTTGTGCTAA AGAGATGTGA GTTAGTAAGT AATTGCTTAC TAACTATATA GGATGTTTTA TTACTTGTAG   
  
  
+ AGGTTTTTTT TTTGGGATAT CTTTATGGGG TTTATAAATA AGAAGGTTGA TTTGTTGTCT ACTCTTCTTT   
  
  
+ CGATTCAATA GTAATTCTCT CAGAGAAGGC TAAAATGACA TGGCATTCCA TCGCCTCTCA TGAGGCTTTT   
  
  
+ TGCATGTGCT TATGTATCTT TCATCTCTTG CTTTGGTGGG GTGGCATTGG GGAGGAAAAG GGGGTTTCGA   
  
  
+ TTCGATAGAT CGTCACTTTT GGAAATGACA TCTCTAGCCA ATGGCAAGGG TAAGGTTAAT ACATGTGGAT   
  
  
+ ATTTCCAGAG TTAGTTGTGA TTGTTATTGT TGTTGTTGTA ACTTTATGTC ACGGGGAAAA CATAATAACT   
  
  
+ ATCTGTTTAT CTGTTTGTTT GTTATTTTCA AGGGGAAGGG GAGTGGGCAG ATGCTTAGTA TGTAATCATT   
  
  
+ TTATATAGGG AAAATTCATA TAGCTTGAAA GTAAAACTTT CATTGCCAGC ATTCCAAGGT ATGGGTAGGG   
  
  
+ TTGTCTACAC CTTTATCTCC CTAGAAGCAT GGTGATCATT CCTCGGATTG TGTTATAGAA TAAAGAATGT   
  
  
+ GGTTCTGCTG TAATGAGACT TATGTATTAT CAAATGATGA ATCTGTTTAG AACTTCCTGT TTATTTTTTC   
  
  
+ TGCAAAATTT TAAGAGACTA ACATCTATCG GGCTCAACTC TAGAGGATCT CTTGTGTCAC CCTTCTCAAC   
  
  
+ TCAGTTTGAT TGTGATACAG TTACTATATT GAGTGATAGT CGAGAGCATC ACATCCCGTT AAGGAGTCTA   
  
  
+ TCAACAAGAA GCCCTTCTTG TAATTCTCCC CTTAAAACTA GCAGTTATCA TCACTTGTCA TCGAATAGCC   
  
  
+ CTTTTTGTAA TTCTACCCTT GAAACTAGCA GTTATCGTCA TCGGTTCAAC GCGAGTCCTG TTAGATATTC   
  
  
+ CCATCAAGGC ATAGATTATG GAGTGAGCAT GAAGAATGCT TTGCAGGAGC TAGAGACTAC TCTAATTGGT   
  
  
+ GTAGATGGTG AGGAAGTATC TATTGCTAAT CAACCTATGG GGGGAAGTCG TTAGTCCGGG ATCCCAAGTT   
  
  
+ AGAGATCGAA GTCATCAAGC GAAGATCCAC AGGGTTCACA TCCTACTCAG CTTGATTCAT CATCTTTTTC   
  
  
+ AAGGGTGAGA AGATTCGGAG ATGAAAGCCA GAGAGAGAGA AATGCCATAA GGCAATGGAA GAACCAGCGG   
  
  
+ AACTACTAAG TTTCTCACCA GGTGATTTAA AGCAGTTACT AATTGAATGT GCGAGGGCTT TATTAGATAA   
  
  
+ CCGAATAGAT GACTTTGAGA GTTTGGTTAA ACGGGCAAGG AAAGAGGTCT CCATCTCAGG TGAGCCTGTC   
  
  
+ CAACCTCTCG GTACCTGTAT GATCGAAGGG CTTGTGGCAA GGAAACAGTC TTCGGGGACT AACATCTATT   
  
  
+ GGGCTCTTAA GTGTAAAGAG CCTCTTGGAA AAGACTTGCT CTCCTACGGC ACTCAGTGGG TTACTGTATC   
  
  
+ GCAAGCCATA GCAGCAAGGC TTGGTGGGCC TCCTAAAGTG CGACTTACAG GCATTGATGA TCCTGTTTCT   
  
  
+ AAGTATACTT GTGATGCTAG CTTGGAGGCT GGTGGGAAAC GATTAGCGTC TCTATTTGAA AAGTTTAAAA   
  
  
+ TACTTGTCGA GTTCAATGCA TTGCCCGTTT ATGGACCCAA TGTCAGGTGG GAAATGCTGG ATGTGAGGCC   
  
  
+ CAGGGAGGCT TTGGTCGTTA ATTGTCCATT ACAGCTCCAT TACACTCCTG ACGAGAGTGT CGATGTGAGG   
  
  
+ AACCCTAGGG ATAGGCTTCT CAGAATGGTG AAATTGCTCG GTCCTAAGGT AGCCACTTTG GTTGAGCAAG   
  
  
+ AATCAAACAC CAACACTACA CCTTTCTTGA CCCGGTTCAT AGAGACCCTT GACTACTACT CGATTAACCA   
  
  
+ TGTTTG  

- -Up\_Stream \_Len000CAACAT TGTTCGTGTA ATTTCAACTA ATAGTAAAAC AGGCACTTTG TCACTCGTTT   
  
  
- GCAGTAGAGG TCCAGTTTAA GTAAGCATGA GTTAGTGTTC AACGACTGTA AAAGTGGTTT CCGATTAGTA   
  
  
- GCGTTTTCAA AGAAATCAAA TTCCGGTTAG AGAGGTAATA AAGTGGGATA TGTCTAACTC CCCCACATTA   
  
  
- TCACATATAT ATATCGGAAC ACAATCGCCC GGAACCTGTG ATCTGAAAAT GAGAACGTTT GGGATTATAT   
  
  
- AATAATATTT ATATTTAGAT CGGTGGGTAC AACTCCATCC GTGAGATTAA GAGTGTGTCC ATCCTATTCA   
  
  
- AAAGTATCAA ATACAACACT TACACTAGAA TGAAACACCA AGGGCAACTG TTTTCAAACT TAGGCTCGTA   
  
  
- CTCTCAAGTA GTTAGAGAAA AAGAAAGGAT AAAATAAACA ACACTTGAAT TAACCTAAAA GACCATCTCC   
  
  
- TTTCAAGGAA CGACCTTAAA CCAGTTTTCA CTATATATAA AACCCTAAAT TAATAGGTAT TTAAACCAAA   
  
  
- ATGTGGAACA ATCAAACCTG AAGACCACAC AATAACATAC ACAAGACCAC CCAAAAAGAA AATCGACTTA   
  
  
- CAACCTCGTA AAATTCCCTA GATCTATAGA CCTCTTCTCG AACCCTTTAT TTCGTATAAA GGGAATATCA   
  
  
- GACTGTATTG AAATTACTTA ACTAGTCCTT GTTTCCGGTT AATCAAGTTT TTCCAAGTTG AGGAGATCCA   
  
  
- CAACACGATT TCTCTACACT CAATCATTCA TTAACGAATG ATTGATATAT CCTACAAAAT AATGAACATC   
  
  
- TCCAAAAAAA AAACCCTATA GAAATACCCC AAATATTTAT TCTTCCAACT AAACAACAGA TGAGAAGAAA   
  
  
- GCTAAGTTAT CATTAAGAGA GTCTCTTCCG ATTTTACTGT ACCGTAAGGT AGCGGAGAGT ACTCCGAAAA   
  
  
- ACGTACACGA ATACATAGAA AGTAGAGAAC GAAACCACCC CACCGTAACC CCTCCTTTTC CCCCAAAGCT   
  
  
- AAGCTATCTA GCAGTGAAAA CCTTTACTGT AGAGATCGGT TACCGTTCCC ATTCCAATTA TGTACACCTA   
  
  
- TAAAGGTCTC AATCAACACT AACAATAACA ACAACAACAT TGAAATACAG TGCCCCTTTT GTATTATTGA   
  
  
- TAGACAAATA GACAAACAAA CAATAAAAGT TCCCCTTCCC CTCACCCGTC TACGAATCAT ACATTAGTAA   
  
  
- AATATATCCC TTTTAAGTAT ATCGAACTTT CATTTTGAAA GTAACGGTCG TAAGGTTCCA TACCCATCCC   
  
  
- AACAGATGTG GAAATAGAGG GATCTTCGTA CCACTAGTAA GGAGCCTAAC ACAATATCTT ATTTCTTACA   
  
  
- CCAAGACGAC ATTACTCTGA ATACATAATA GTTTACTACT TAGACAAATC TTGAAGGACA AATAAAAAAG   
  
  
- ACGTTTTAAA ATTCTCTGAT TGTAGATAGC CCGAGTTGAG ATCTCCTAGA GAACACAGTG GGAAGAGTTG   
  
  
- AGTCAAACTA ACACTATGTC AATGATATAA CTCACTATCA GCTCTCGTAG TGTAGGGCAA TTCCTCAGAT   
  
  
- AGTTGTTCTT CGGGAAGAAC ATTAAGAGGG GAATTTTGAT CGTCAATAGT AGTGAACAGT AGCTTATCGG   
  
  
- GAAAAACATT AAGATGGGAA CTTTGATCGT CAATAGCAGT AGCCAAGTTG CGCTCAGGAC AATCTATAAG   
  
  
- GGTAGTTCCG TATCTAATAC CTCACTCGTA CTTCTTACGA AACGTCCTCG ATCTCTGATG AGATTAACCA   
  
  
- CATCTACCAC TCCTTCATAG ATAACGATTA GTTGGATACC CCCCTTCAGC AATCAGGCCC TAGGGTTCAA   
  
  
- TCTCTAGCTT CAGTAGTTCG CTTCTAGGTG TCCCAAGTGT AGGATGAGTC GAACTAAGTA GTAGAAAAAG   
  
  
- TTCCCACTCT TCTAAGCCTC TACTTTCGGT CTCTCTCTCT TTACGGTATT CCGTTACCTT CTTGGTCGCC   
  
  
- TTGATGATTC AAAGAGTGGT CCACTAAATT TCGTCAATGA TTAACTTACA CGCTCCCGAA ATAATCTATT   
  
  
- GGCTTATCTA CTGAAACTCT CAAACCAATT TGCCCGTTCC TTTCTCCAGA GGTAGAGTCC ACTCGGACAG   
  
  
- GTTGGAGAGC CATGGACATA CTAGCTTCCC GAACACCGTT CCTTTGTCAG AAGCCCCTGA TTGTAGATAA   
  
  
- CCCGAGAATT CACATTTCTC GGAGAACCTT TTCTGAACGA GAGGATGCCG TGAGTCACCC AATGACATAG   
  
  
- CGTTCGGTAT CGTCGTTCCG AACCACCCGG AGGATTTCAC GCTGAATGTC CGTAACTACT AGGACAAAGA   
  
  
- TTCATATGAA CACTACGATC GAACCTCCGA CCACCCTTTG CTAATCGCAG AGATAAACTT TTCAAATTTT   
  
  
- ATGAACAGCT CAAGTTACGT AACGGGCAAA TACCTGGGTT ACAGTCCACC CTTTACGACC TACACTCCGG   
  
  
- GTCCCTCCGA AACCAGCAAT TAACAGGTAA TGTCGAGGTA ATGTGAGGAC TGCTCTCACA GCTACACTCC   
  
  
- TTGGGATCCC TATCCGAAGA GTCTTACCAC TTTAACGAGC CAGGATTCCA TCGGTGAAAC CAACTCGTTC   
  
  
- TTAGTTTGTG GTTGTGATGT GGAAAGAACT GGGCCAAGTA TCTCTGGGAA CTGATGATGA GCTAATTGGT   
  
  
- ACAAAC

+     MYB recognition site

| Site Name | Organism | Position | Strand | Matrix score. | sequence | function |
| --- | --- | --- | --- | --- | --- | --- |
| MYB recognition site | Arabidopsis thaliana | 397 | + | 6 | CCGTTG |  |

>HU08G00230.1   
+ -Up\_Stream \_Len000GTTGTA ACAAGCACAT TAAAGTTGAT TATCATTTTG TCCGTGAAAC AGTGAGCAAA   
  
  
+ CGTCATCTCC AGGTCAAATT CATTCGTACT CAATCACAAG TTGCTGACAT TTTCACCAAA GGCTAATCAT   
  
  
+ CGCAAAAGTT TCTTTAGTTT AAGGCCAATC TCTCCATTAT TTCACCCTAT ACAGATTGAG GGGGTGTAAT   
  
  
+ AGTGTATATA TATAGCCTTG TGTTAGCGGG CCTTGGACAC TAGACTTTTA CTCTTGCAAA CCCTAATATA   
  
  
+ TTATTATAAA TATAAATCTA GCCACCCATG TTGAGGTAGG CACTCTAATT CTCACACAGG TAGGATAAGT   
  
  
+ TTTCATAGTT TATGTTGTGA ATGTGATCTT ACTTTGTGGT TCCCGTTGAC AAAAGTTTGA ATCCGAGCAT   
  
  
+ GAGAGTTCAT CAATCTCTTT TTCTTTCCTA TTTTATTTGT TGTGAACTTA ATTGGATTTT CTGGTAGAGG   
  
  
+ AAAGTTCCTT GCTGGAATTT GGTCAAAAGT GATATATATT TTGGGATTTA ATTATCCATA AATTTGGTTT   
  
  
+ TACACCTTGT TAGTTTGGAC TTCTGGTGTG TTATTGTATG TGTTCTGGTG GGTTTTTCTT TTAGCTGAAT   
  
  
+ GTTGGAGCAT TTTAAGGGAT CTAGATATCT GGAGAAGAGC TTGGGAAATA AAGCATATTT CCCTTATAGT   
  
  
+ CTGACATAAC TTTAATGAAT TGATCAGGAA CAAAGGCCAA TTAGTTCAAA AAGGTTCAAC TCCTCTAGGT   
  
  
+ GTTGTGCTAA AGAGATGTGA GTTAGTAAGT AATTGCTTAC TAACTATATA GGATGTTTTA TTACTTGTAG   
  
  
+ AGGTTTTTTT TTTGGGATAT CTTTATGGGG TTTATAAATA AGAAGGTTGA TTTGTTGTCT ACTCTTCTTT   
  
  
+ CGATTCAATA GTAATTCTCT CAGAGAAGGC TAAAATGACA TGGCATTCCA TCGCCTCTCA TGAGGCTTTT   
  
  
+ TGCATGTGCT TATGTATCTT TCATCTCTTG CTTTGGTGGG GTGGCATTGG GGAGGAAAAG GGGGTTTCGA   
  
  
+ TTCGATAGAT CGTCACTTTT GGAAATGACA TCTCTAGCCA ATGGCAAGGG TAAGGTTAAT ACATGTGGAT   
  
  
+ ATTTCCAGAG TTAGTTGTGA TTGTTATTGT TGTTGTTGTA ACTTTATGTC ACGGGGAAAA CATAATAACT   
  
  
+ ATCTGTTTAT CTGTTTGTTT GTTATTTTCA AGGGGAAGGG GAGTGGGCAG ATGCTTAGTA TGTAATCATT   
  
  
+ TTATATAGGG AAAATTCATA TAGCTTGAAA GTAAAACTTT CATTGCCAGC ATTCCAAGGT ATGGGTAGGG   
  
  
+ TTGTCTACAC CTTTATCTCC CTAGAAGCAT GGTGATCATT CCTCGGATTG TGTTATAGAA TAAAGAATGT   
  
  
+ GGTTCTGCTG TAATGAGACT TATGTATTAT CAAATGATGA ATCTGTTTAG AACTTCCTGT TTATTTTTTC   
  
  
+ TGCAAAATTT TAAGAGACTA ACATCTATCG GGCTCAACTC TAGAGGATCT CTTGTGTCAC CCTTCTCAAC   
  
  
+ TCAGTTTGAT TGTGATACAG TTACTATATT GAGTGATAGT CGAGAGCATC ACATCCCGTT AAGGAGTCTA   
  
  
+ TCAACAAGAA GCCCTTCTTG TAATTCTCCC CTTAAAACTA GCAGTTATCA TCACTTGTCA TCGAATAGCC   
  
  
+ CTTTTTGTAA TTCTACCCTT GAAACTAGCA GTTATCGTCA TCGGTTCAAC GCGAGTCCTG TTAGATATTC   
  
  
+ CCATCAAGGC ATAGATTATG GAGTGAGCAT GAAGAATGCT TTGCAGGAGC TAGAGACTAC TCTAATTGGT   
  
  
+ GTAGATGGTG AGGAAGTATC TATTGCTAAT CAACCTATGG GGGGAAGTCG TTAGTCCGGG ATCCCAAGTT   
  
  
+ AGAGATCGAA GTCATCAAGC GAAGATCCAC AGGGTTCACA TCCTACTCAG CTTGATTCAT CATCTTTTTC   
  
  
+ AAGGGTGAGA AGATTCGGAG ATGAAAGCCA GAGAGAGAGA AATGCCATAA GGCAATGGAA GAACCAGCGG   
  
  
+ AACTACTAAG TTTCTCACCA GGTGATTTAA AGCAGTTACT AATTGAATGT GCGAGGGCTT TATTAGATAA   
  
  
+ CCGAATAGAT GACTTTGAGA GTTTGGTTAA ACGGGCAAGG AAAGAGGTCT CCATCTCAGG TGAGCCTGTC   
  
  
+ CAACCTCTCG GTACCTGTAT GATCGAAGGG CTTGTGGCAA GGAAACAGTC TTCGGGGACT AACATCTATT   
  
  
+ GGGCTCTTAA GTGTAAAGAG CCTCTTGGAA AAGACTTGCT CTCCTACGGC ACTCAGTGGG TTACTGTATC   
  
  
+ GCAAGCCATA GCAGCAAGGC TTGGTGGGCC TCCTAAAGTG CGACTTACAG GCATTGATGA TCCTGTTTCT   
  
  
+ AAGTATACTT GTGATGCTAG CTTGGAGGCT GGTGGGAAAC GATTAGCGTC TCTATTTGAA AAGTTTAAAA   
  
  
+ TACTTGTCGA GTTCAATGCA TTGCCCGTTT ATGGACCCAA TGTCAGGTGG GAAATGCTGG ATGTGAGGCC   
  
  
+ CAGGGAGGCT TTGGTCGTTA ATTGTCCATT ACAGCTCCAT TACACTCCTG ACGAGAGTGT CGATGTGAGG   
  
  
+ AACCCTAGGG ATAGGCTTCT CAGAATGGTG AAATTGCTCG GTCCTAAGGT AGCCACTTTG GTTGAGCAAG   
  
  
+ AATCAAACAC CAACACTACA CCTTTCTTGA CCCGGTTCAT AGAGACCCTT GACTACTACT CGATTAACCA   
  
  
+ TGTTTG  

- -Up\_Stream \_Len000CAACAT TGTTCGTGTA ATTTCAACTA ATAGTAAAAC AGGCACTTTG TCACTCGTTT   
  
  
- GCAGTAGAGG TCCAGTTTAA GTAAGCATGA GTTAGTGTTC AACGACTGTA AAAGTGGTTT CCGATTAGTA   
  
  
- GCGTTTTCAA AGAAATCAAA TTCCGGTTAG AGAGGTAATA AAGTGGGATA TGTCTAACTC CCCCACATTA   
  
  
- TCACATATAT ATATCGGAAC ACAATCGCCC GGAACCTGTG ATCTGAAAAT GAGAACGTTT GGGATTATAT   
  
  
- AATAATATTT ATATTTAGAT CGGTGGGTAC AACTCCATCC GTGAGATTAA GAGTGTGTCC ATCCTATTCA   
  
  
- AAAGTATCAA ATACAACACT TACACTAGAA TGAAACACCA AGGGCAACTG TTTTCAAACT TAGGCTCGTA   
  
  
- CTCTCAAGTA GTTAGAGAAA AAGAAAGGAT AAAATAAACA ACACTTGAAT TAACCTAAAA GACCATCTCC   
  
  
- TTTCAAGGAA CGACCTTAAA CCAGTTTTCA CTATATATAA AACCCTAAAT TAATAGGTAT TTAAACCAAA   
  
  
- ATGTGGAACA ATCAAACCTG AAGACCACAC AATAACATAC ACAAGACCAC CCAAAAAGAA AATCGACTTA   
  
  
- CAACCTCGTA AAATTCCCTA GATCTATAGA CCTCTTCTCG AACCCTTTAT TTCGTATAAA GGGAATATCA   
  
  
- GACTGTATTG AAATTACTTA ACTAGTCCTT GTTTCCGGTT AATCAAGTTT TTCCAAGTTG AGGAGATCCA   
  
  
- CAACACGATT TCTCTACACT CAATCATTCA TTAACGAATG ATTGATATAT CCTACAAAAT AATGAACATC   
  
  
- TCCAAAAAAA AAACCCTATA GAAATACCCC AAATATTTAT TCTTCCAACT AAACAACAGA TGAGAAGAAA   
  
  
- GCTAAGTTAT CATTAAGAGA GTCTCTTCCG ATTTTACTGT ACCGTAAGGT AGCGGAGAGT ACTCCGAAAA   
  
  
- ACGTACACGA ATACATAGAA AGTAGAGAAC GAAACCACCC CACCGTAACC CCTCCTTTTC CCCCAAAGCT   
  
  
- AAGCTATCTA GCAGTGAAAA CCTTTACTGT AGAGATCGGT TACCGTTCCC ATTCCAATTA TGTACACCTA   
  
  
- TAAAGGTCTC AATCAACACT AACAATAACA ACAACAACAT TGAAATACAG TGCCCCTTTT GTATTATTGA   
  
  
- TAGACAAATA GACAAACAAA CAATAAAAGT TCCCCTTCCC CTCACCCGTC TACGAATCAT ACATTAGTAA   
  
  
- AATATATCCC TTTTAAGTAT ATCGAACTTT CATTTTGAAA GTAACGGTCG TAAGGTTCCA TACCCATCCC   
  
  
- AACAGATGTG GAAATAGAGG GATCTTCGTA CCACTAGTAA GGAGCCTAAC ACAATATCTT ATTTCTTACA   
  
  
- CCAAGACGAC ATTACTCTGA ATACATAATA GTTTACTACT TAGACAAATC TTGAAGGACA AATAAAAAAG   
  
  
- ACGTTTTAAA ATTCTCTGAT TGTAGATAGC CCGAGTTGAG ATCTCCTAGA GAACACAGTG GGAAGAGTTG   
  
  
- AGTCAAACTA ACACTATGTC AATGATATAA CTCACTATCA GCTCTCGTAG TGTAGGGCAA TTCCTCAGAT   
  
  
- AGTTGTTCTT CGGGAAGAAC ATTAAGAGGG GAATTTTGAT CGTCAATAGT AGTGAACAGT AGCTTATCGG   
  
  
- GAAAAACATT AAGATGGGAA CTTTGATCGT CAATAGCAGT AGCCAAGTTG CGCTCAGGAC AATCTATAAG   
  
  
- GGTAGTTCCG TATCTAATAC CTCACTCGTA CTTCTTACGA AACGTCCTCG ATCTCTGATG AGATTAACCA   
  
  
- CATCTACCAC TCCTTCATAG ATAACGATTA GTTGGATACC CCCCTTCAGC AATCAGGCCC TAGGGTTCAA   
  
  
- TCTCTAGCTT CAGTAGTTCG CTTCTAGGTG TCCCAAGTGT AGGATGAGTC GAACTAAGTA GTAGAAAAAG   
  
  
- TTCCCACTCT TCTAAGCCTC TACTTTCGGT CTCTCTCTCT TTACGGTATT CCGTTACCTT CTTGGTCGCC   
  
  
- TTGATGATTC AAAGAGTGGT CCACTAAATT TCGTCAATGA TTAACTTACA CGCTCCCGAA ATAATCTATT   
  
  
- GGCTTATCTA CTGAAACTCT CAAACCAATT TGCCCGTTCC TTTCTCCAGA GGTAGAGTCC ACTCGGACAG   
  
  
- GTTGGAGAGC CATGGACATA CTAGCTTCCC GAACACCGTT CCTTTGTCAG AAGCCCCTGA TTGTAGATAA   
  
  
- CCCGAGAATT CACATTTCTC GGAGAACCTT TTCTGAACGA GAGGATGCCG TGAGTCACCC AATGACATAG   
  
  
- CGTTCGGTAT CGTCGTTCCG AACCACCCGG AGGATTTCAC GCTGAATGTC CGTAACTACT AGGACAAAGA   
  
  
- TTCATATGAA CACTACGATC GAACCTCCGA CCACCCTTTG CTAATCGCAG AGATAAACTT TTCAAATTTT   
  
  
- ATGAACAGCT CAAGTTACGT AACGGGCAAA TACCTGGGTT ACAGTCCACC CTTTACGACC TACACTCCGG   
  
  
- GTCCCTCCGA AACCAGCAAT TAACAGGTAA TGTCGAGGTA ATGTGAGGAC TGCTCTCACA GCTACACTCC   
  
  
- TTGGGATCCC TATCCGAAGA GTCTTACCAC TTTAACGAGC CAGGATTCCA TCGGTGAAAC CAACTCGTTC   
  
  
- TTAGTTTGTG GTTGTGATGT GGAAAGAACT GGGCCAAGTA TCTCTGGGAA CTGATGATGA GCTAATTGGT   
  
  
- ACAAAC

+     MYB-like sequence

| Site Name | Organism | Position | Strand | Matrix score. | sequence | function |
| --- | --- | --- | --- | --- | --- | --- |
| MYB-like sequence | Arabidopsis thaliana | 2729 | + | 6 | TAACCA |  |
| MYB-like sequence | Arabidopsis thaliana | 2128 | - | 6 | TAACCA |  |

>HU08G00230.1   
+ -Up\_Stream \_Len000GTTGTA ACAAGCACAT TAAAGTTGAT TATCATTTTG TCCGTGAAAC AGTGAGCAAA   
  
  
+ CGTCATCTCC AGGTCAAATT CATTCGTACT CAATCACAAG TTGCTGACAT TTTCACCAAA GGCTAATCAT   
  
  
+ CGCAAAAGTT TCTTTAGTTT AAGGCCAATC TCTCCATTAT TTCACCCTAT ACAGATTGAG GGGGTGTAAT   
  
  
+ AGTGTATATA TATAGCCTTG TGTTAGCGGG CCTTGGACAC TAGACTTTTA CTCTTGCAAA CCCTAATATA   
  
  
+ TTATTATAAA TATAAATCTA GCCACCCATG TTGAGGTAGG CACTCTAATT CTCACACAGG TAGGATAAGT   
  
  
+ TTTCATAGTT TATGTTGTGA ATGTGATCTT ACTTTGTGGT TCCCGTTGAC AAAAGTTTGA ATCCGAGCAT   
  
  
+ GAGAGTTCAT CAATCTCTTT TTCTTTCCTA TTTTATTTGT TGTGAACTTA ATTGGATTTT CTGGTAGAGG   
  
  
+ AAAGTTCCTT GCTGGAATTT GGTCAAAAGT GATATATATT TTGGGATTTA ATTATCCATA AATTTGGTTT   
  
  
+ TACACCTTGT TAGTTTGGAC TTCTGGTGTG TTATTGTATG TGTTCTGGTG GGTTTTTCTT TTAGCTGAAT   
  
  
+ GTTGGAGCAT TTTAAGGGAT CTAGATATCT GGAGAAGAGC TTGGGAAATA AAGCATATTT CCCTTATAGT   
  
  
+ CTGACATAAC TTTAATGAAT TGATCAGGAA CAAAGGCCAA TTAGTTCAAA AAGGTTCAAC TCCTCTAGGT   
  
  
+ GTTGTGCTAA AGAGATGTGA GTTAGTAAGT AATTGCTTAC TAACTATATA GGATGTTTTA TTACTTGTAG   
  
  
+ AGGTTTTTTT TTTGGGATAT CTTTATGGGG TTTATAAATA AGAAGGTTGA TTTGTTGTCT ACTCTTCTTT   
  
  
+ CGATTCAATA GTAATTCTCT CAGAGAAGGC TAAAATGACA TGGCATTCCA TCGCCTCTCA TGAGGCTTTT   
  
  
+ TGCATGTGCT TATGTATCTT TCATCTCTTG CTTTGGTGGG GTGGCATTGG GGAGGAAAAG GGGGTTTCGA   
  
  
+ TTCGATAGAT CGTCACTTTT GGAAATGACA TCTCTAGCCA ATGGCAAGGG TAAGGTTAAT ACATGTGGAT   
  
  
+ ATTTCCAGAG TTAGTTGTGA TTGTTATTGT TGTTGTTGTA ACTTTATGTC ACGGGGAAAA CATAATAACT   
  
  
+ ATCTGTTTAT CTGTTTGTTT GTTATTTTCA AGGGGAAGGG GAGTGGGCAG ATGCTTAGTA TGTAATCATT   
  
  
+ TTATATAGGG AAAATTCATA TAGCTTGAAA GTAAAACTTT CATTGCCAGC ATTCCAAGGT ATGGGTAGGG   
  
  
+ TTGTCTACAC CTTTATCTCC CTAGAAGCAT GGTGATCATT CCTCGGATTG TGTTATAGAA TAAAGAATGT   
  
  
+ GGTTCTGCTG TAATGAGACT TATGTATTAT CAAATGATGA ATCTGTTTAG AACTTCCTGT TTATTTTTTC   
  
  
+ TGCAAAATTT TAAGAGACTA ACATCTATCG GGCTCAACTC TAGAGGATCT CTTGTGTCAC CCTTCTCAAC   
  
  
+ TCAGTTTGAT TGTGATACAG TTACTATATT GAGTGATAGT CGAGAGCATC ACATCCCGTT AAGGAGTCTA   
  
  
+ TCAACAAGAA GCCCTTCTTG TAATTCTCCC CTTAAAACTA GCAGTTATCA TCACTTGTCA TCGAATAGCC   
  
  
+ CTTTTTGTAA TTCTACCCTT GAAACTAGCA GTTATCGTCA TCGGTTCAAC GCGAGTCCTG TTAGATATTC   
  
  
+ CCATCAAGGC ATAGATTATG GAGTGAGCAT GAAGAATGCT TTGCAGGAGC TAGAGACTAC TCTAATTGGT   
  
  
+ GTAGATGGTG AGGAAGTATC TATTGCTAAT CAACCTATGG GGGGAAGTCG TTAGTCCGGG ATCCCAAGTT   
  
  
+ AGAGATCGAA GTCATCAAGC GAAGATCCAC AGGGTTCACA TCCTACTCAG CTTGATTCAT CATCTTTTTC   
  
  
+ AAGGGTGAGA AGATTCGGAG ATGAAAGCCA GAGAGAGAGA AATGCCATAA GGCAATGGAA GAACCAGCGG   
  
  
+ AACTACTAAG TTTCTCACCA GGTGATTTAA AGCAGTTACT AATTGAATGT GCGAGGGCTT TATTAGATAA   
  
  
+ CCGAATAGAT GACTTTGAGA GTTTGGTTAA ACGGGCAAGG AAAGAGGTCT CCATCTCAGG TGAGCCTGTC   
  
  
+ CAACCTCTCG GTACCTGTAT GATCGAAGGG CTTGTGGCAA GGAAACAGTC TTCGGGGACT AACATCTATT   
  
  
+ GGGCTCTTAA GTGTAAAGAG CCTCTTGGAA AAGACTTGCT CTCCTACGGC ACTCAGTGGG TTACTGTATC   
  
  
+ GCAAGCCATA GCAGCAAGGC TTGGTGGGCC TCCTAAAGTG CGACTTACAG GCATTGATGA TCCTGTTTCT   
  
  
+ AAGTATACTT GTGATGCTAG CTTGGAGGCT GGTGGGAAAC GATTAGCGTC TCTATTTGAA AAGTTTAAAA   
  
  
+ TACTTGTCGA GTTCAATGCA TTGCCCGTTT ATGGACCCAA TGTCAGGTGG GAAATGCTGG ATGTGAGGCC   
  
  
+ CAGGGAGGCT TTGGTCGTTA ATTGTCCATT ACAGCTCCAT TACACTCCTG ACGAGAGTGT CGATGTGAGG   
  
  
+ AACCCTAGGG ATAGGCTTCT CAGAATGGTG AAATTGCTCG GTCCTAAGGT AGCCACTTTG GTTGAGCAAG   
  
  
+ AATCAAACAC CAACACTACA CCTTTCTTGA CCCGGTTCAT AGAGACCCTT GACTACTACT CGATTAACCA   
  
  
+ TGTTTG  

- -Up\_Stream \_Len000CAACAT TGTTCGTGTA ATTTCAACTA ATAGTAAAAC AGGCACTTTG TCACTCGTTT   
  
  
- GCAGTAGAGG TCCAGTTTAA GTAAGCATGA GTTAGTGTTC AACGACTGTA AAAGTGGTTT CCGATTAGTA   
  
  
- GCGTTTTCAA AGAAATCAAA TTCCGGTTAG AGAGGTAATA AAGTGGGATA TGTCTAACTC CCCCACATTA   
  
  
- TCACATATAT ATATCGGAAC ACAATCGCCC GGAACCTGTG ATCTGAAAAT GAGAACGTTT GGGATTATAT   
  
  
- AATAATATTT ATATTTAGAT CGGTGGGTAC AACTCCATCC GTGAGATTAA GAGTGTGTCC ATCCTATTCA   
  
  
- AAAGTATCAA ATACAACACT TACACTAGAA TGAAACACCA AGGGCAACTG TTTTCAAACT TAGGCTCGTA   
  
  
- CTCTCAAGTA GTTAGAGAAA AAGAAAGGAT AAAATAAACA ACACTTGAAT TAACCTAAAA GACCATCTCC   
  
  
- TTTCAAGGAA CGACCTTAAA CCAGTTTTCA CTATATATAA AACCCTAAAT TAATAGGTAT TTAAACCAAA   
  
  
- ATGTGGAACA ATCAAACCTG AAGACCACAC AATAACATAC ACAAGACCAC CCAAAAAGAA AATCGACTTA   
  
  
- CAACCTCGTA AAATTCCCTA GATCTATAGA CCTCTTCTCG AACCCTTTAT TTCGTATAAA GGGAATATCA   
  
  
- GACTGTATTG AAATTACTTA ACTAGTCCTT GTTTCCGGTT AATCAAGTTT TTCCAAGTTG AGGAGATCCA   
  
  
- CAACACGATT TCTCTACACT CAATCATTCA TTAACGAATG ATTGATATAT CCTACAAAAT AATGAACATC   
  
  
- TCCAAAAAAA AAACCCTATA GAAATACCCC AAATATTTAT TCTTCCAACT AAACAACAGA TGAGAAGAAA   
  
  
- GCTAAGTTAT CATTAAGAGA GTCTCTTCCG ATTTTACTGT ACCGTAAGGT AGCGGAGAGT ACTCCGAAAA   
  
  
- ACGTACACGA ATACATAGAA AGTAGAGAAC GAAACCACCC CACCGTAACC CCTCCTTTTC CCCCAAAGCT   
  
  
- AAGCTATCTA GCAGTGAAAA CCTTTACTGT AGAGATCGGT TACCGTTCCC ATTCCAATTA TGTACACCTA   
  
  
- TAAAGGTCTC AATCAACACT AACAATAACA ACAACAACAT TGAAATACAG TGCCCCTTTT GTATTATTGA   
  
  
- TAGACAAATA GACAAACAAA CAATAAAAGT TCCCCTTCCC CTCACCCGTC TACGAATCAT ACATTAGTAA   
  
  
- AATATATCCC TTTTAAGTAT ATCGAACTTT CATTTTGAAA GTAACGGTCG TAAGGTTCCA TACCCATCCC   
  
  
- AACAGATGTG GAAATAGAGG GATCTTCGTA CCACTAGTAA GGAGCCTAAC ACAATATCTT ATTTCTTACA   
  
  
- CCAAGACGAC ATTACTCTGA ATACATAATA GTTTACTACT TAGACAAATC TTGAAGGACA AATAAAAAAG   
  
  
- ACGTTTTAAA ATTCTCTGAT TGTAGATAGC CCGAGTTGAG ATCTCCTAGA GAACACAGTG GGAAGAGTTG   
  
  
- AGTCAAACTA ACACTATGTC AATGATATAA CTCACTATCA GCTCTCGTAG TGTAGGGCAA TTCCTCAGAT   
  
  
- AGTTGTTCTT CGGGAAGAAC ATTAAGAGGG GAATTTTGAT CGTCAATAGT AGTGAACAGT AGCTTATCGG   
  
  
- GAAAAACATT AAGATGGGAA CTTTGATCGT CAATAGCAGT AGCCAAGTTG CGCTCAGGAC AATCTATAAG   
  
  
- GGTAGTTCCG TATCTAATAC CTCACTCGTA CTTCTTACGA AACGTCCTCG ATCTCTGATG AGATTAACCA   
  
  
- CATCTACCAC TCCTTCATAG ATAACGATTA GTTGGATACC CCCCTTCAGC AATCAGGCCC TAGGGTTCAA   
  
  
- TCTCTAGCTT CAGTAGTTCG CTTCTAGGTG TCCCAAGTGT AGGATGAGTC GAACTAAGTA GTAGAAAAAG   
  
  
- TTCCCACTCT TCTAAGCCTC TACTTTCGGT CTCTCTCTCT TTACGGTATT CCGTTACCTT CTTGGTCGCC   
  
  
- TTGATGATTC AAAGAGTGGT CCACTAAATT TCGTCAATGA TTAACTTACA CGCTCCCGAA ATAATCTATT   
  
  
- GGCTTATCTA CTGAAACTCT CAAACCAATT TGCCCGTTCC TTTCTCCAGA GGTAGAGTCC ACTCGGACAG   
  
  
- GTTGGAGAGC CATGGACATA CTAGCTTCCC GAACACCGTT CCTTTGTCAG AAGCCCCTGA TTGTAGATAA   
  
  
- CCCGAGAATT CACATTTCTC GGAGAACCTT TTCTGAACGA GAGGATGCCG TGAGTCACCC AATGACATAG   
  
  
- CGTTCGGTAT CGTCGTTCCG AACCACCCGG AGGATTTCAC GCTGAATGTC CGTAACTACT AGGACAAAGA   
  
  
- TTCATATGAA CACTACGATC GAACCTCCGA CCACCCTTTG CTAATCGCAG AGATAAACTT TTCAAATTTT   
  
  
- ATGAACAGCT CAAGTTACGT AACGGGCAAA TACCTGGGTT ACAGTCCACC CTTTACGACC TACACTCCGG   
  
  
- GTCCCTCCGA AACCAGCAAT TAACAGGTAA TGTCGAGGTA ATGTGAGGAC TGCTCTCACA GCTACACTCC   
  
  
- TTGGGATCCC TATCCGAAGA GTCTTACCAC TTTAACGAGC CAGGATTCCA TCGGTGAAAC CAACTCGTTC   
  
  
- TTAGTTTGTG GTTGTGATGT GGAAAGAACT GGGCCAAGTA TCTCTGGGAA CTGATGATGA GCTAATTGGT   
  
  
- ACAAAC

+     MYC

| Site Name | Organism | Position | Strand | Matrix score. | sequence | function |
| --- | --- | --- | --- | --- | --- | --- |
| MYC | Arabidopsis thaliana | 1116 | + | 6 | CATGTG |  |
| MYC | Arabidopsis thaliana | 987 | + | 6 | CATGTG |  |
| MYC | Arabidopsis thaliana | 1435 | - | 6 | CATTTG |  |

>HU08G00230.1   
+ -Up\_Stream \_Len000GTTGTA ACAAGCACAT TAAAGTTGAT TATCATTTTG TCCGTGAAAC AGTGAGCAAA   
  
  
+ CGTCATCTCC AGGTCAAATT CATTCGTACT CAATCACAAG TTGCTGACAT TTTCACCAAA GGCTAATCAT   
  
  
+ CGCAAAAGTT TCTTTAGTTT AAGGCCAATC TCTCCATTAT TTCACCCTAT ACAGATTGAG GGGGTGTAAT   
  
  
+ AGTGTATATA TATAGCCTTG TGTTAGCGGG CCTTGGACAC TAGACTTTTA CTCTTGCAAA CCCTAATATA   
  
  
+ TTATTATAAA TATAAATCTA GCCACCCATG TTGAGGTAGG CACTCTAATT CTCACACAGG TAGGATAAGT   
  
  
+ TTTCATAGTT TATGTTGTGA ATGTGATCTT ACTTTGTGGT TCCCGTTGAC AAAAGTTTGA ATCCGAGCAT   
  
  
+ GAGAGTTCAT CAATCTCTTT TTCTTTCCTA TTTTATTTGT TGTGAACTTA ATTGGATTTT CTGGTAGAGG   
  
  
+ AAAGTTCCTT GCTGGAATTT GGTCAAAAGT GATATATATT TTGGGATTTA ATTATCCATA AATTTGGTTT   
  
  
+ TACACCTTGT TAGTTTGGAC TTCTGGTGTG TTATTGTATG TGTTCTGGTG GGTTTTTCTT TTAGCTGAAT   
  
  
+ GTTGGAGCAT TTTAAGGGAT CTAGATATCT GGAGAAGAGC TTGGGAAATA AAGCATATTT CCCTTATAGT   
  
  
+ CTGACATAAC TTTAATGAAT TGATCAGGAA CAAAGGCCAA TTAGTTCAAA AAGGTTCAAC TCCTCTAGGT   
  
  
+ GTTGTGCTAA AGAGATGTGA GTTAGTAAGT AATTGCTTAC TAACTATATA GGATGTTTTA TTACTTGTAG   
  
  
+ AGGTTTTTTT TTTGGGATAT CTTTATGGGG TTTATAAATA AGAAGGTTGA TTTGTTGTCT ACTCTTCTTT   
  
  
+ CGATTCAATA GTAATTCTCT CAGAGAAGGC TAAAATGACA TGGCATTCCA TCGCCTCTCA TGAGGCTTTT   
  
  
+ TGCATGTGCT TATGTATCTT TCATCTCTTG CTTTGGTGGG GTGGCATTGG GGAGGAAAAG GGGGTTTCGA   
  
  
+ TTCGATAGAT CGTCACTTTT GGAAATGACA TCTCTAGCCA ATGGCAAGGG TAAGGTTAAT ACATGTGGAT   
  
  
+ ATTTCCAGAG TTAGTTGTGA TTGTTATTGT TGTTGTTGTA ACTTTATGTC ACGGGGAAAA CATAATAACT   
  
  
+ ATCTGTTTAT CTGTTTGTTT GTTATTTTCA AGGGGAAGGG GAGTGGGCAG ATGCTTAGTA TGTAATCATT   
  
  
+ TTATATAGGG AAAATTCATA TAGCTTGAAA GTAAAACTTT CATTGCCAGC ATTCCAAGGT ATGGGTAGGG   
  
  
+ TTGTCTACAC CTTTATCTCC CTAGAAGCAT GGTGATCATT CCTCGGATTG TGTTATAGAA TAAAGAATGT   
  
  
+ GGTTCTGCTG TAATGAGACT TATGTATTAT CAAATGATGA ATCTGTTTAG AACTTCCTGT TTATTTTTTC   
  
  
+ TGCAAAATTT TAAGAGACTA ACATCTATCG GGCTCAACTC TAGAGGATCT CTTGTGTCAC CCTTCTCAAC   
  
  
+ TCAGTTTGAT TGTGATACAG TTACTATATT GAGTGATAGT CGAGAGCATC ACATCCCGTT AAGGAGTCTA   
  
  
+ TCAACAAGAA GCCCTTCTTG TAATTCTCCC CTTAAAACTA GCAGTTATCA TCACTTGTCA TCGAATAGCC   
  
  
+ CTTTTTGTAA TTCTACCCTT GAAACTAGCA GTTATCGTCA TCGGTTCAAC GCGAGTCCTG TTAGATATTC   
  
  
+ CCATCAAGGC ATAGATTATG GAGTGAGCAT GAAGAATGCT TTGCAGGAGC TAGAGACTAC TCTAATTGGT   
  
  
+ GTAGATGGTG AGGAAGTATC TATTGCTAAT CAACCTATGG GGGGAAGTCG TTAGTCCGGG ATCCCAAGTT   
  
  
+ AGAGATCGAA GTCATCAAGC GAAGATCCAC AGGGTTCACA TCCTACTCAG CTTGATTCAT CATCTTTTTC   
  
  
+ AAGGGTGAGA AGATTCGGAG ATGAAAGCCA GAGAGAGAGA AATGCCATAA GGCAATGGAA GAACCAGCGG   
  
  
+ AACTACTAAG TTTCTCACCA GGTGATTTAA AGCAGTTACT AATTGAATGT GCGAGGGCTT TATTAGATAA   
  
  
+ CCGAATAGAT GACTTTGAGA GTTTGGTTAA ACGGGCAAGG AAAGAGGTCT CCATCTCAGG TGAGCCTGTC   
  
  
+ CAACCTCTCG GTACCTGTAT GATCGAAGGG CTTGTGGCAA GGAAACAGTC TTCGGGGACT AACATCTATT   
  
  
+ GGGCTCTTAA GTGTAAAGAG CCTCTTGGAA AAGACTTGCT CTCCTACGGC ACTCAGTGGG TTACTGTATC   
  
  
+ GCAAGCCATA GCAGCAAGGC TTGGTGGGCC TCCTAAAGTG CGACTTACAG GCATTGATGA TCCTGTTTCT   
  
  
+ AAGTATACTT GTGATGCTAG CTTGGAGGCT GGTGGGAAAC GATTAGCGTC TCTATTTGAA AAGTTTAAAA   
  
  
+ TACTTGTCGA GTTCAATGCA TTGCCCGTTT ATGGACCCAA TGTCAGGTGG GAAATGCTGG ATGTGAGGCC   
  
  
+ CAGGGAGGCT TTGGTCGTTA ATTGTCCATT ACAGCTCCAT TACACTCCTG ACGAGAGTGT CGATGTGAGG   
  
  
+ AACCCTAGGG ATAGGCTTCT CAGAATGGTG AAATTGCTCG GTCCTAAGGT AGCCACTTTG GTTGAGCAAG   
  
  
+ AATCAAACAC CAACACTACA CCTTTCTTGA CCCGGTTCAT AGAGACCCTT GACTACTACT CGATTAACCA   
  
  
+ TGTTTG  

- -Up\_Stream \_Len000CAACAT TGTTCGTGTA ATTTCAACTA ATAGTAAAAC AGGCACTTTG TCACTCGTTT   
  
  
- GCAGTAGAGG TCCAGTTTAA GTAAGCATGA GTTAGTGTTC AACGACTGTA AAAGTGGTTT CCGATTAGTA   
  
  
- GCGTTTTCAA AGAAATCAAA TTCCGGTTAG AGAGGTAATA AAGTGGGATA TGTCTAACTC CCCCACATTA   
  
  
- TCACATATAT ATATCGGAAC ACAATCGCCC GGAACCTGTG ATCTGAAAAT GAGAACGTTT GGGATTATAT   
  
  
- AATAATATTT ATATTTAGAT CGGTGGGTAC AACTCCATCC GTGAGATTAA GAGTGTGTCC ATCCTATTCA   
  
  
- AAAGTATCAA ATACAACACT TACACTAGAA TGAAACACCA AGGGCAACTG TTTTCAAACT TAGGCTCGTA   
  
  
- CTCTCAAGTA GTTAGAGAAA AAGAAAGGAT AAAATAAACA ACACTTGAAT TAACCTAAAA GACCATCTCC   
  
  
- TTTCAAGGAA CGACCTTAAA CCAGTTTTCA CTATATATAA AACCCTAAAT TAATAGGTAT TTAAACCAAA   
  
  
- ATGTGGAACA ATCAAACCTG AAGACCACAC AATAACATAC ACAAGACCAC CCAAAAAGAA AATCGACTTA   
  
  
- CAACCTCGTA AAATTCCCTA GATCTATAGA CCTCTTCTCG AACCCTTTAT TTCGTATAAA GGGAATATCA   
  
  
- GACTGTATTG AAATTACTTA ACTAGTCCTT GTTTCCGGTT AATCAAGTTT TTCCAAGTTG AGGAGATCCA   
  
  
- CAACACGATT TCTCTACACT CAATCATTCA TTAACGAATG ATTGATATAT CCTACAAAAT AATGAACATC   
  
  
- TCCAAAAAAA AAACCCTATA GAAATACCCC AAATATTTAT TCTTCCAACT AAACAACAGA TGAGAAGAAA   
  
  
- GCTAAGTTAT CATTAAGAGA GTCTCTTCCG ATTTTACTGT ACCGTAAGGT AGCGGAGAGT ACTCCGAAAA   
  
  
- ACGTACACGA ATACATAGAA AGTAGAGAAC GAAACCACCC CACCGTAACC CCTCCTTTTC CCCCAAAGCT   
  
  
- AAGCTATCTA GCAGTGAAAA CCTTTACTGT AGAGATCGGT TACCGTTCCC ATTCCAATTA TGTACACCTA   
  
  
- TAAAGGTCTC AATCAACACT AACAATAACA ACAACAACAT TGAAATACAG TGCCCCTTTT GTATTATTGA   
  
  
- TAGACAAATA GACAAACAAA CAATAAAAGT TCCCCTTCCC CTCACCCGTC TACGAATCAT ACATTAGTAA   
  
  
- AATATATCCC TTTTAAGTAT ATCGAACTTT CATTTTGAAA GTAACGGTCG TAAGGTTCCA TACCCATCCC   
  
  
- AACAGATGTG GAAATAGAGG GATCTTCGTA CCACTAGTAA GGAGCCTAAC ACAATATCTT ATTTCTTACA   
  
  
- CCAAGACGAC ATTACTCTGA ATACATAATA GTTTACTACT TAGACAAATC TTGAAGGACA AATAAAAAAG   
  
  
- ACGTTTTAAA ATTCTCTGAT TGTAGATAGC CCGAGTTGAG ATCTCCTAGA GAACACAGTG GGAAGAGTTG   
  
  
- AGTCAAACTA ACACTATGTC AATGATATAA CTCACTATCA GCTCTCGTAG TGTAGGGCAA TTCCTCAGAT   
  
  
- AGTTGTTCTT CGGGAAGAAC ATTAAGAGGG GAATTTTGAT CGTCAATAGT AGTGAACAGT AGCTTATCGG   
  
  
- GAAAAACATT AAGATGGGAA CTTTGATCGT CAATAGCAGT AGCCAAGTTG CGCTCAGGAC AATCTATAAG   
  
  
- GGTAGTTCCG TATCTAATAC CTCACTCGTA CTTCTTACGA AACGTCCTCG ATCTCTGATG AGATTAACCA   
  
  
- CATCTACCAC TCCTTCATAG ATAACGATTA GTTGGATACC CCCCTTCAGC AATCAGGCCC TAGGGTTCAA   
  
  
- TCTCTAGCTT CAGTAGTTCG CTTCTAGGTG TCCCAAGTGT AGGATGAGTC GAACTAAGTA GTAGAAAAAG   
  
  
- TTCCCACTCT TCTAAGCCTC TACTTTCGGT CTCTCTCTCT TTACGGTATT CCGTTACCTT CTTGGTCGCC   
  
  
- TTGATGATTC AAAGAGTGGT CCACTAAATT TCGTCAATGA TTAACTTACA CGCTCCCGAA ATAATCTATT   
  
  
- GGCTTATCTA CTGAAACTCT CAAACCAATT TGCCCGTTCC TTTCTCCAGA GGTAGAGTCC ACTCGGACAG   
  
  
- GTTGGAGAGC CATGGACATA CTAGCTTCCC GAACACCGTT CCTTTGTCAG AAGCCCCTGA TTGTAGATAA   
  
  
- CCCGAGAATT CACATTTCTC GGAGAACCTT TTCTGAACGA GAGGATGCCG TGAGTCACCC AATGACATAG   
  
  
- CGTTCGGTAT CGTCGTTCCG AACCACCCGG AGGATTTCAC GCTGAATGTC CGTAACTACT AGGACAAAGA   
  
  
- TTCATATGAA CACTACGATC GAACCTCCGA CCACCCTTTG CTAATCGCAG AGATAAACTT TTCAAATTTT   
  
  
- ATGAACAGCT CAAGTTACGT AACGGGCAAA TACCTGGGTT ACAGTCCACC CTTTACGACC TACACTCCGG   
  
  
- GTCCCTCCGA AACCAGCAAT TAACAGGTAA TGTCGAGGTA ATGTGAGGAC TGCTCTCACA GCTACACTCC   
  
  
- TTGGGATCCC TATCCGAAGA GTCTTACCAC TTTAACGAGC CAGGATTCCA TCGGTGAAAC CAACTCGTTC   
  
  
- TTAGTTTGTG GTTGTGATGT GGAAAGAACT GGGCCAAGTA TCTCTGGGAA CTGATGATGA GCTAATTGGT   
  
  
- ACAAAC

+     Myb

| Site Name | Organism | Position | Strand | Matrix score. | sequence | function |
| --- | --- | --- | --- | --- | --- | --- |
| Myb | Arabidopsis thaliana | 2067 | - | 6 | TAACTG |  |
| Myb | Arabidopsis thaliana | 1656 | - | 6 | TAACTG |  |
| Myb | Arabidopsis thaliana | 1562 | - | 6 | TAACTG |  |
| Myb | Arabidopsis thaliana | 1713 | - | 6 | TAACTG |  |

>HU08G00230.1   
+ -Up\_Stream \_Len000GTTGTA ACAAGCACAT TAAAGTTGAT TATCATTTTG TCCGTGAAAC AGTGAGCAAA   
  
  
+ CGTCATCTCC AGGTCAAATT CATTCGTACT CAATCACAAG TTGCTGACAT TTTCACCAAA GGCTAATCAT   
  
  
+ CGCAAAAGTT TCTTTAGTTT AAGGCCAATC TCTCCATTAT TTCACCCTAT ACAGATTGAG GGGGTGTAAT   
  
  
+ AGTGTATATA TATAGCCTTG TGTTAGCGGG CCTTGGACAC TAGACTTTTA CTCTTGCAAA CCCTAATATA   
  
  
+ TTATTATAAA TATAAATCTA GCCACCCATG TTGAGGTAGG CACTCTAATT CTCACACAGG TAGGATAAGT   
  
  
+ TTTCATAGTT TATGTTGTGA ATGTGATCTT ACTTTGTGGT TCCCGTTGAC AAAAGTTTGA ATCCGAGCAT   
  
  
+ GAGAGTTCAT CAATCTCTTT TTCTTTCCTA TTTTATTTGT TGTGAACTTA ATTGGATTTT CTGGTAGAGG   
  
  
+ AAAGTTCCTT GCTGGAATTT GGTCAAAAGT GATATATATT TTGGGATTTA ATTATCCATA AATTTGGTTT   
  
  
+ TACACCTTGT TAGTTTGGAC TTCTGGTGTG TTATTGTATG TGTTCTGGTG GGTTTTTCTT TTAGCTGAAT   
  
  
+ GTTGGAGCAT TTTAAGGGAT CTAGATATCT GGAGAAGAGC TTGGGAAATA AAGCATATTT CCCTTATAGT   
  
  
+ CTGACATAAC TTTAATGAAT TGATCAGGAA CAAAGGCCAA TTAGTTCAAA AAGGTTCAAC TCCTCTAGGT   
  
  
+ GTTGTGCTAA AGAGATGTGA GTTAGTAAGT AATTGCTTAC TAACTATATA GGATGTTTTA TTACTTGTAG   
  
  
+ AGGTTTTTTT TTTGGGATAT CTTTATGGGG TTTATAAATA AGAAGGTTGA TTTGTTGTCT ACTCTTCTTT   
  
  
+ CGATTCAATA GTAATTCTCT CAGAGAAGGC TAAAATGACA TGGCATTCCA TCGCCTCTCA TGAGGCTTTT   
  
  
+ TGCATGTGCT TATGTATCTT TCATCTCTTG CTTTGGTGGG GTGGCATTGG GGAGGAAAAG GGGGTTTCGA   
  
  
+ TTCGATAGAT CGTCACTTTT GGAAATGACA TCTCTAGCCA ATGGCAAGGG TAAGGTTAAT ACATGTGGAT   
  
  
+ ATTTCCAGAG TTAGTTGTGA TTGTTATTGT TGTTGTTGTA ACTTTATGTC ACGGGGAAAA CATAATAACT   
  
  
+ ATCTGTTTAT CTGTTTGTTT GTTATTTTCA AGGGGAAGGG GAGTGGGCAG ATGCTTAGTA TGTAATCATT   
  
  
+ TTATATAGGG AAAATTCATA TAGCTTGAAA GTAAAACTTT CATTGCCAGC ATTCCAAGGT ATGGGTAGGG   
  
  
+ TTGTCTACAC CTTTATCTCC CTAGAAGCAT GGTGATCATT CCTCGGATTG TGTTATAGAA TAAAGAATGT   
  
  
+ GGTTCTGCTG TAATGAGACT TATGTATTAT CAAATGATGA ATCTGTTTAG AACTTCCTGT TTATTTTTTC   
  
  
+ TGCAAAATTT TAAGAGACTA ACATCTATCG GGCTCAACTC TAGAGGATCT CTTGTGTCAC CCTTCTCAAC   
  
  
+ TCAGTTTGAT TGTGATACAG TTACTATATT GAGTGATAGT CGAGAGCATC ACATCCCGTT AAGGAGTCTA   
  
  
+ TCAACAAGAA GCCCTTCTTG TAATTCTCCC CTTAAAACTA GCAGTTATCA TCACTTGTCA TCGAATAGCC   
  
  
+ CTTTTTGTAA TTCTACCCTT GAAACTAGCA GTTATCGTCA TCGGTTCAAC GCGAGTCCTG TTAGATATTC   
  
  
+ CCATCAAGGC ATAGATTATG GAGTGAGCAT GAAGAATGCT TTGCAGGAGC TAGAGACTAC TCTAATTGGT   
  
  
+ GTAGATGGTG AGGAAGTATC TATTGCTAAT CAACCTATGG GGGGAAGTCG TTAGTCCGGG ATCCCAAGTT   
  
  
+ AGAGATCGAA GTCATCAAGC GAAGATCCAC AGGGTTCACA TCCTACTCAG CTTGATTCAT CATCTTTTTC   
  
  
+ AAGGGTGAGA AGATTCGGAG ATGAAAGCCA GAGAGAGAGA AATGCCATAA GGCAATGGAA GAACCAGCGG   
  
  
+ AACTACTAAG TTTCTCACCA GGTGATTTAA AGCAGTTACT AATTGAATGT GCGAGGGCTT TATTAGATAA   
  
  
+ CCGAATAGAT GACTTTGAGA GTTTGGTTAA ACGGGCAAGG AAAGAGGTCT CCATCTCAGG TGAGCCTGTC   
  
  
+ CAACCTCTCG GTACCTGTAT GATCGAAGGG CTTGTGGCAA GGAAACAGTC TTCGGGGACT AACATCTATT   
  
  
+ GGGCTCTTAA GTGTAAAGAG CCTCTTGGAA AAGACTTGCT CTCCTACGGC ACTCAGTGGG TTACTGTATC   
  
  
+ GCAAGCCATA GCAGCAAGGC TTGGTGGGCC TCCTAAAGTG CGACTTACAG GCATTGATGA TCCTGTTTCT   
  
  
+ AAGTATACTT GTGATGCTAG CTTGGAGGCT GGTGGGAAAC GATTAGCGTC TCTATTTGAA AAGTTTAAAA   
  
  
+ TACTTGTCGA GTTCAATGCA TTGCCCGTTT ATGGACCCAA TGTCAGGTGG GAAATGCTGG ATGTGAGGCC   
  
  
+ CAGGGAGGCT TTGGTCGTTA ATTGTCCATT ACAGCTCCAT TACACTCCTG ACGAGAGTGT CGATGTGAGG   
  
  
+ AACCCTAGGG ATAGGCTTCT CAGAATGGTG AAATTGCTCG GTCCTAAGGT AGCCACTTTG GTTGAGCAAG   
  
  
+ AATCAAACAC CAACACTACA CCTTTCTTGA CCCGGTTCAT AGAGACCCTT GACTACTACT CGATTAACCA   
  
  
+ TGTTTG  

- -Up\_Stream \_Len000CAACAT TGTTCGTGTA ATTTCAACTA ATAGTAAAAC AGGCACTTTG TCACTCGTTT   
  
  
- GCAGTAGAGG TCCAGTTTAA GTAAGCATGA GTTAGTGTTC AACGACTGTA AAAGTGGTTT CCGATTAGTA   
  
  
- GCGTTTTCAA AGAAATCAAA TTCCGGTTAG AGAGGTAATA AAGTGGGATA TGTCTAACTC CCCCACATTA   
  
  
- TCACATATAT ATATCGGAAC ACAATCGCCC GGAACCTGTG ATCTGAAAAT GAGAACGTTT GGGATTATAT   
  
  
- AATAATATTT ATATTTAGAT CGGTGGGTAC AACTCCATCC GTGAGATTAA GAGTGTGTCC ATCCTATTCA   
  
  
- AAAGTATCAA ATACAACACT TACACTAGAA TGAAACACCA AGGGCAACTG TTTTCAAACT TAGGCTCGTA   
  
  
- CTCTCAAGTA GTTAGAGAAA AAGAAAGGAT AAAATAAACA ACACTTGAAT TAACCTAAAA GACCATCTCC   
  
  
- TTTCAAGGAA CGACCTTAAA CCAGTTTTCA CTATATATAA AACCCTAAAT TAATAGGTAT TTAAACCAAA   
  
  
- ATGTGGAACA ATCAAACCTG AAGACCACAC AATAACATAC ACAAGACCAC CCAAAAAGAA AATCGACTTA   
  
  
- CAACCTCGTA AAATTCCCTA GATCTATAGA CCTCTTCTCG AACCCTTTAT TTCGTATAAA GGGAATATCA   
  
  
- GACTGTATTG AAATTACTTA ACTAGTCCTT GTTTCCGGTT AATCAAGTTT TTCCAAGTTG AGGAGATCCA   
  
  
- CAACACGATT TCTCTACACT CAATCATTCA TTAACGAATG ATTGATATAT CCTACAAAAT AATGAACATC   
  
  
- TCCAAAAAAA AAACCCTATA GAAATACCCC AAATATTTAT TCTTCCAACT AAACAACAGA TGAGAAGAAA   
  
  
- GCTAAGTTAT CATTAAGAGA GTCTCTTCCG ATTTTACTGT ACCGTAAGGT AGCGGAGAGT ACTCCGAAAA   
  
  
- ACGTACACGA ATACATAGAA AGTAGAGAAC GAAACCACCC CACCGTAACC CCTCCTTTTC CCCCAAAGCT   
  
  
- AAGCTATCTA GCAGTGAAAA CCTTTACTGT AGAGATCGGT TACCGTTCCC ATTCCAATTA TGTACACCTA   
  
  
- TAAAGGTCTC AATCAACACT AACAATAACA ACAACAACAT TGAAATACAG TGCCCCTTTT GTATTATTGA   
  
  
- TAGACAAATA GACAAACAAA CAATAAAAGT TCCCCTTCCC CTCACCCGTC TACGAATCAT ACATTAGTAA   
  
  
- AATATATCCC TTTTAAGTAT ATCGAACTTT CATTTTGAAA GTAACGGTCG TAAGGTTCCA TACCCATCCC   
  
  
- AACAGATGTG GAAATAGAGG GATCTTCGTA CCACTAGTAA GGAGCCTAAC ACAATATCTT ATTTCTTACA   
  
  
- CCAAGACGAC ATTACTCTGA ATACATAATA GTTTACTACT TAGACAAATC TTGAAGGACA AATAAAAAAG   
  
  
- ACGTTTTAAA ATTCTCTGAT TGTAGATAGC CCGAGTTGAG ATCTCCTAGA GAACACAGTG GGAAGAGTTG   
  
  
- AGTCAAACTA ACACTATGTC AATGATATAA CTCACTATCA GCTCTCGTAG TGTAGGGCAA TTCCTCAGAT   
  
  
- AGTTGTTCTT CGGGAAGAAC ATTAAGAGGG GAATTTTGAT CGTCAATAGT AGTGAACAGT AGCTTATCGG   
  
  
- GAAAAACATT AAGATGGGAA CTTTGATCGT CAATAGCAGT AGCCAAGTTG CGCTCAGGAC AATCTATAAG   
  
  
- GGTAGTTCCG TATCTAATAC CTCACTCGTA CTTCTTACGA AACGTCCTCG ATCTCTGATG AGATTAACCA   
  
  
- CATCTACCAC TCCTTCATAG ATAACGATTA GTTGGATACC CCCCTTCAGC AATCAGGCCC TAGGGTTCAA   
  
  
- TCTCTAGCTT CAGTAGTTCG CTTCTAGGTG TCCCAAGTGT AGGATGAGTC GAACTAAGTA GTAGAAAAAG   
  
  
- TTCCCACTCT TCTAAGCCTC TACTTTCGGT CTCTCTCTCT TTACGGTATT CCGTTACCTT CTTGGTCGCC   
  
  
- TTGATGATTC AAAGAGTGGT CCACTAAATT TCGTCAATGA TTAACTTACA CGCTCCCGAA ATAATCTATT   
  
  
- GGCTTATCTA CTGAAACTCT CAAACCAATT TGCCCGTTCC TTTCTCCAGA GGTAGAGTCC ACTCGGACAG   
  
  
- GTTGGAGAGC CATGGACATA CTAGCTTCCC GAACACCGTT CCTTTGTCAG AAGCCCCTGA TTGTAGATAA   
  
  
- CCCGAGAATT CACATTTCTC GGAGAACCTT TTCTGAACGA GAGGATGCCG TGAGTCACCC AATGACATAG   
  
  
- CGTTCGGTAT CGTCGTTCCG AACCACCCGG AGGATTTCAC GCTGAATGTC CGTAACTACT AGGACAAAGA   
  
  
- TTCATATGAA CACTACGATC GAACCTCCGA CCACCCTTTG CTAATCGCAG AGATAAACTT TTCAAATTTT   
  
  
- ATGAACAGCT CAAGTTACGT AACGGGCAAA TACCTGGGTT ACAGTCCACC CTTTACGACC TACACTCCGG   
  
  
- GTCCCTCCGA AACCAGCAAT TAACAGGTAA TGTCGAGGTA ATGTGAGGAC TGCTCTCACA GCTACACTCC   
  
  
- TTGGGATCCC TATCCGAAGA GTCTTACCAC TTTAACGAGC CAGGATTCCA TCGGTGAAAC CAACTCGTTC   
  
  
- TTAGTTTGTG GTTGTGATGT GGAAAGAACT GGGCCAAGTA TCTCTGGGAA CTGATGATGA GCTAATTGGT   
  
  
- ACAAAC

+     Myc

| Site Name | Organism | Position | Strand | Matrix score. | sequence | function |
| --- | --- | --- | --- | --- | --- | --- |
| Myc | Arabidopsis thaliana | 1485 | - | 7 | TCTCTTA |  |

>HU08G00230.1   
+ -Up\_Stream \_Len000GTTGTA ACAAGCACAT TAAAGTTGAT TATCATTTTG TCCGTGAAAC AGTGAGCAAA   
  
  
+ CGTCATCTCC AGGTCAAATT CATTCGTACT CAATCACAAG TTGCTGACAT TTTCACCAAA GGCTAATCAT   
  
  
+ CGCAAAAGTT TCTTTAGTTT AAGGCCAATC TCTCCATTAT TTCACCCTAT ACAGATTGAG GGGGTGTAAT   
  
  
+ AGTGTATATA TATAGCCTTG TGTTAGCGGG CCTTGGACAC TAGACTTTTA CTCTTGCAAA CCCTAATATA   
  
  
+ TTATTATAAA TATAAATCTA GCCACCCATG TTGAGGTAGG CACTCTAATT CTCACACAGG TAGGATAAGT   
  
  
+ TTTCATAGTT TATGTTGTGA ATGTGATCTT ACTTTGTGGT TCCCGTTGAC AAAAGTTTGA ATCCGAGCAT   
  
  
+ GAGAGTTCAT CAATCTCTTT TTCTTTCCTA TTTTATTTGT TGTGAACTTA ATTGGATTTT CTGGTAGAGG   
  
  
+ AAAGTTCCTT GCTGGAATTT GGTCAAAAGT GATATATATT TTGGGATTTA ATTATCCATA AATTTGGTTT   
  
  
+ TACACCTTGT TAGTTTGGAC TTCTGGTGTG TTATTGTATG TGTTCTGGTG GGTTTTTCTT TTAGCTGAAT   
  
  
+ GTTGGAGCAT TTTAAGGGAT CTAGATATCT GGAGAAGAGC TTGGGAAATA AAGCATATTT CCCTTATAGT   
  
  
+ CTGACATAAC TTTAATGAAT TGATCAGGAA CAAAGGCCAA TTAGTTCAAA AAGGTTCAAC TCCTCTAGGT   
  
  
+ GTTGTGCTAA AGAGATGTGA GTTAGTAAGT AATTGCTTAC TAACTATATA GGATGTTTTA TTACTTGTAG   
  
  
+ AGGTTTTTTT TTTGGGATAT CTTTATGGGG TTTATAAATA AGAAGGTTGA TTTGTTGTCT ACTCTTCTTT   
  
  
+ CGATTCAATA GTAATTCTCT CAGAGAAGGC TAAAATGACA TGGCATTCCA TCGCCTCTCA TGAGGCTTTT   
  
  
+ TGCATGTGCT TATGTATCTT TCATCTCTTG CTTTGGTGGG GTGGCATTGG GGAGGAAAAG GGGGTTTCGA   
  
  
+ TTCGATAGAT CGTCACTTTT GGAAATGACA TCTCTAGCCA ATGGCAAGGG TAAGGTTAAT ACATGTGGAT   
  
  
+ ATTTCCAGAG TTAGTTGTGA TTGTTATTGT TGTTGTTGTA ACTTTATGTC ACGGGGAAAA CATAATAACT   
  
  
+ ATCTGTTTAT CTGTTTGTTT GTTATTTTCA AGGGGAAGGG GAGTGGGCAG ATGCTTAGTA TGTAATCATT   
  
  
+ TTATATAGGG AAAATTCATA TAGCTTGAAA GTAAAACTTT CATTGCCAGC ATTCCAAGGT ATGGGTAGGG   
  
  
+ TTGTCTACAC CTTTATCTCC CTAGAAGCAT GGTGATCATT CCTCGGATTG TGTTATAGAA TAAAGAATGT   
  
  
+ GGTTCTGCTG TAATGAGACT TATGTATTAT CAAATGATGA ATCTGTTTAG AACTTCCTGT TTATTTTTTC   
  
  
+ TGCAAAATTT TAAGAGACTA ACATCTATCG GGCTCAACTC TAGAGGATCT CTTGTGTCAC CCTTCTCAAC   
  
  
+ TCAGTTTGAT TGTGATACAG TTACTATATT GAGTGATAGT CGAGAGCATC ACATCCCGTT AAGGAGTCTA   
  
  
+ TCAACAAGAA GCCCTTCTTG TAATTCTCCC CTTAAAACTA GCAGTTATCA TCACTTGTCA TCGAATAGCC   
  
  
+ CTTTTTGTAA TTCTACCCTT GAAACTAGCA GTTATCGTCA TCGGTTCAAC GCGAGTCCTG TTAGATATTC   
  
  
+ CCATCAAGGC ATAGATTATG GAGTGAGCAT GAAGAATGCT TTGCAGGAGC TAGAGACTAC TCTAATTGGT   
  
  
+ GTAGATGGTG AGGAAGTATC TATTGCTAAT CAACCTATGG GGGGAAGTCG TTAGTCCGGG ATCCCAAGTT   
  
  
+ AGAGATCGAA GTCATCAAGC GAAGATCCAC AGGGTTCACA TCCTACTCAG CTTGATTCAT CATCTTTTTC   
  
  
+ AAGGGTGAGA AGATTCGGAG ATGAAAGCCA GAGAGAGAGA AATGCCATAA GGCAATGGAA GAACCAGCGG   
  
  
+ AACTACTAAG TTTCTCACCA GGTGATTTAA AGCAGTTACT AATTGAATGT GCGAGGGCTT TATTAGATAA   
  
  
+ CCGAATAGAT GACTTTGAGA GTTTGGTTAA ACGGGCAAGG AAAGAGGTCT CCATCTCAGG TGAGCCTGTC   
  
  
+ CAACCTCTCG GTACCTGTAT GATCGAAGGG CTTGTGGCAA GGAAACAGTC TTCGGGGACT AACATCTATT   
  
  
+ GGGCTCTTAA GTGTAAAGAG CCTCTTGGAA AAGACTTGCT CTCCTACGGC ACTCAGTGGG TTACTGTATC   
  
  
+ GCAAGCCATA GCAGCAAGGC TTGGTGGGCC TCCTAAAGTG CGACTTACAG GCATTGATGA TCCTGTTTCT   
  
  
+ AAGTATACTT GTGATGCTAG CTTGGAGGCT GGTGGGAAAC GATTAGCGTC TCTATTTGAA AAGTTTAAAA   
  
  
+ TACTTGTCGA GTTCAATGCA TTGCCCGTTT ATGGACCCAA TGTCAGGTGG GAAATGCTGG ATGTGAGGCC   
  
  
+ CAGGGAGGCT TTGGTCGTTA ATTGTCCATT ACAGCTCCAT TACACTCCTG ACGAGAGTGT CGATGTGAGG   
  
  
+ AACCCTAGGG ATAGGCTTCT CAGAATGGTG AAATTGCTCG GTCCTAAGGT AGCCACTTTG GTTGAGCAAG   
  
  
+ AATCAAACAC CAACACTACA CCTTTCTTGA CCCGGTTCAT AGAGACCCTT GACTACTACT CGATTAACCA   
  
  
+ TGTTTG  

- -Up\_Stream \_Len000CAACAT TGTTCGTGTA ATTTCAACTA ATAGTAAAAC AGGCACTTTG TCACTCGTTT   
  
  
- GCAGTAGAGG TCCAGTTTAA GTAAGCATGA GTTAGTGTTC AACGACTGTA AAAGTGGTTT CCGATTAGTA   
  
  
- GCGTTTTCAA AGAAATCAAA TTCCGGTTAG AGAGGTAATA AAGTGGGATA TGTCTAACTC CCCCACATTA   
  
  
- TCACATATAT ATATCGGAAC ACAATCGCCC GGAACCTGTG ATCTGAAAAT GAGAACGTTT GGGATTATAT   
  
  
- AATAATATTT ATATTTAGAT CGGTGGGTAC AACTCCATCC GTGAGATTAA GAGTGTGTCC ATCCTATTCA   
  
  
- AAAGTATCAA ATACAACACT TACACTAGAA TGAAACACCA AGGGCAACTG TTTTCAAACT TAGGCTCGTA   
  
  
- CTCTCAAGTA GTTAGAGAAA AAGAAAGGAT AAAATAAACA ACACTTGAAT TAACCTAAAA GACCATCTCC   
  
  
- TTTCAAGGAA CGACCTTAAA CCAGTTTTCA CTATATATAA AACCCTAAAT TAATAGGTAT TTAAACCAAA   
  
  
- ATGTGGAACA ATCAAACCTG AAGACCACAC AATAACATAC ACAAGACCAC CCAAAAAGAA AATCGACTTA   
  
  
- CAACCTCGTA AAATTCCCTA GATCTATAGA CCTCTTCTCG AACCCTTTAT TTCGTATAAA GGGAATATCA   
  
  
- GACTGTATTG AAATTACTTA ACTAGTCCTT GTTTCCGGTT AATCAAGTTT TTCCAAGTTG AGGAGATCCA   
  
  
- CAACACGATT TCTCTACACT CAATCATTCA TTAACGAATG ATTGATATAT CCTACAAAAT AATGAACATC   
  
  
- TCCAAAAAAA AAACCCTATA GAAATACCCC AAATATTTAT TCTTCCAACT AAACAACAGA TGAGAAGAAA   
  
  
- GCTAAGTTAT CATTAAGAGA GTCTCTTCCG ATTTTACTGT ACCGTAAGGT AGCGGAGAGT ACTCCGAAAA   
  
  
- ACGTACACGA ATACATAGAA AGTAGAGAAC GAAACCACCC CACCGTAACC CCTCCTTTTC CCCCAAAGCT   
  
  
- AAGCTATCTA GCAGTGAAAA CCTTTACTGT AGAGATCGGT TACCGTTCCC ATTCCAATTA TGTACACCTA   
  
  
- TAAAGGTCTC AATCAACACT AACAATAACA ACAACAACAT TGAAATACAG TGCCCCTTTT GTATTATTGA   
  
  
- TAGACAAATA GACAAACAAA CAATAAAAGT TCCCCTTCCC CTCACCCGTC TACGAATCAT ACATTAGTAA   
  
  
- AATATATCCC TTTTAAGTAT ATCGAACTTT CATTTTGAAA GTAACGGTCG TAAGGTTCCA TACCCATCCC   
  
  
- AACAGATGTG GAAATAGAGG GATCTTCGTA CCACTAGTAA GGAGCCTAAC ACAATATCTT ATTTCTTACA   
  
  
- CCAAGACGAC ATTACTCTGA ATACATAATA GTTTACTACT TAGACAAATC TTGAAGGACA AATAAAAAAG   
  
  
- ACGTTTTAAA ATTCTCTGAT TGTAGATAGC CCGAGTTGAG ATCTCCTAGA GAACACAGTG GGAAGAGTTG   
  
  
- AGTCAAACTA ACACTATGTC AATGATATAA CTCACTATCA GCTCTCGTAG TGTAGGGCAA TTCCTCAGAT   
  
  
- AGTTGTTCTT CGGGAAGAAC ATTAAGAGGG GAATTTTGAT CGTCAATAGT AGTGAACAGT AGCTTATCGG   
  
  
- GAAAAACATT AAGATGGGAA CTTTGATCGT CAATAGCAGT AGCCAAGTTG CGCTCAGGAC AATCTATAAG   
  
  
- GGTAGTTCCG TATCTAATAC CTCACTCGTA CTTCTTACGA AACGTCCTCG ATCTCTGATG AGATTAACCA   
  
  
- CATCTACCAC TCCTTCATAG ATAACGATTA GTTGGATACC CCCCTTCAGC AATCAGGCCC TAGGGTTCAA   
  
  
- TCTCTAGCTT CAGTAGTTCG CTTCTAGGTG TCCCAAGTGT AGGATGAGTC GAACTAAGTA GTAGAAAAAG   
  
  
- TTCCCACTCT TCTAAGCCTC TACTTTCGGT CTCTCTCTCT TTACGGTATT CCGTTACCTT CTTGGTCGCC   
  
  
- TTGATGATTC AAAGAGTGGT CCACTAAATT TCGTCAATGA TTAACTTACA CGCTCCCGAA ATAATCTATT   
  
  
- GGCTTATCTA CTGAAACTCT CAAACCAATT TGCCCGTTCC TTTCTCCAGA GGTAGAGTCC ACTCGGACAG   
  
  
- GTTGGAGAGC CATGGACATA CTAGCTTCCC GAACACCGTT CCTTTGTCAG AAGCCCCTGA TTGTAGATAA   
  
  
- CCCGAGAATT CACATTTCTC GGAGAACCTT TTCTGAACGA GAGGATGCCG TGAGTCACCC AATGACATAG   
  
  
- CGTTCGGTAT CGTCGTTCCG AACCACCCGG AGGATTTCAC GCTGAATGTC CGTAACTACT AGGACAAAGA   
  
  
- TTCATATGAA CACTACGATC GAACCTCCGA CCACCCTTTG CTAATCGCAG AGATAAACTT TTCAAATTTT   
  
  
- ATGAACAGCT CAAGTTACGT AACGGGCAAA TACCTGGGTT ACAGTCCACC CTTTACGACC TACACTCCGG   
  
  
- GTCCCTCCGA AACCAGCAAT TAACAGGTAA TGTCGAGGTA ATGTGAGGAC TGCTCTCACA GCTACACTCC   
  
  
- TTGGGATCCC TATCCGAAGA GTCTTACCAC TTTAACGAGC CAGGATTCCA TCGGTGAAAC CAACTCGTTC   
  
  
- TTAGTTTGTG GTTGTGATGT GGAAAGAACT GGGCCAAGTA TCTCTGGGAA CTGATGATGA GCTAATTGGT   
  
  
- ACAAAC

+     O2-site

| Site Name | Organism | Position | Strand | Matrix score. | sequence | function |
| --- | --- | --- | --- | --- | --- | --- |
| O2-site | Zea mays | 948 | + | 9 | GATGACATGG | cis-acting regulatory element involved in zein metabolism regulation |

>HU08G00230.1   
+ -Up\_Stream \_Len000GTTGTA ACAAGCACAT TAAAGTTGAT TATCATTTTG TCCGTGAAAC AGTGAGCAAA   
  
  
+ CGTCATCTCC AGGTCAAATT CATTCGTACT CAATCACAAG TTGCTGACAT TTTCACCAAA GGCTAATCAT   
  
  
+ CGCAAAAGTT TCTTTAGTTT AAGGCCAATC TCTCCATTAT TTCACCCTAT ACAGATTGAG GGGGTGTAAT   
  
  
+ AGTGTATATA TATAGCCTTG TGTTAGCGGG CCTTGGACAC TAGACTTTTA CTCTTGCAAA CCCTAATATA   
  
  
+ TTATTATAAA TATAAATCTA GCCACCCATG TTGAGGTAGG CACTCTAATT CTCACACAGG TAGGATAAGT   
  
  
+ TTTCATAGTT TATGTTGTGA ATGTGATCTT ACTTTGTGGT TCCCGTTGAC AAAAGTTTGA ATCCGAGCAT   
  
  
+ GAGAGTTCAT CAATCTCTTT TTCTTTCCTA TTTTATTTGT TGTGAACTTA ATTGGATTTT CTGGTAGAGG   
  
  
+ AAAGTTCCTT GCTGGAATTT GGTCAAAAGT GATATATATT TTGGGATTTA ATTATCCATA AATTTGGTTT   
  
  
+ TACACCTTGT TAGTTTGGAC TTCTGGTGTG TTATTGTATG TGTTCTGGTG GGTTTTTCTT TTAGCTGAAT   
  
  
+ GTTGGAGCAT TTTAAGGGAT CTAGATATCT GGAGAAGAGC TTGGGAAATA AAGCATATTT CCCTTATAGT   
  
  
+ CTGACATAAC TTTAATGAAT TGATCAGGAA CAAAGGCCAA TTAGTTCAAA AAGGTTCAAC TCCTCTAGGT   
  
  
+ GTTGTGCTAA AGAGATGTGA GTTAGTAAGT AATTGCTTAC TAACTATATA GGATGTTTTA TTACTTGTAG   
  
  
+ AGGTTTTTTT TTTGGGATAT CTTTATGGGG TTTATAAATA AGAAGGTTGA TTTGTTGTCT ACTCTTCTTT   
  
  
+ CGATTCAATA GTAATTCTCT CAGAGAAGGC TAAAATGACA TGGCATTCCA TCGCCTCTCA TGAGGCTTTT   
  
  
+ TGCATGTGCT TATGTATCTT TCATCTCTTG CTTTGGTGGG GTGGCATTGG GGAGGAAAAG GGGGTTTCGA   
  
  
+ TTCGATAGAT CGTCACTTTT GGAAATGACA TCTCTAGCCA ATGGCAAGGG TAAGGTTAAT ACATGTGGAT   
  
  
+ ATTTCCAGAG TTAGTTGTGA TTGTTATTGT TGTTGTTGTA ACTTTATGTC ACGGGGAAAA CATAATAACT   
  
  
+ ATCTGTTTAT CTGTTTGTTT GTTATTTTCA AGGGGAAGGG GAGTGGGCAG ATGCTTAGTA TGTAATCATT   
  
  
+ TTATATAGGG AAAATTCATA TAGCTTGAAA GTAAAACTTT CATTGCCAGC ATTCCAAGGT ATGGGTAGGG   
  
  
+ TTGTCTACAC CTTTATCTCC CTAGAAGCAT GGTGATCATT CCTCGGATTG TGTTATAGAA TAAAGAATGT   
  
  
+ GGTTCTGCTG TAATGAGACT TATGTATTAT CAAATGATGA ATCTGTTTAG AACTTCCTGT TTATTTTTTC   
  
  
+ TGCAAAATTT TAAGAGACTA ACATCTATCG GGCTCAACTC TAGAGGATCT CTTGTGTCAC CCTTCTCAAC   
  
  
+ TCAGTTTGAT TGTGATACAG TTACTATATT GAGTGATAGT CGAGAGCATC ACATCCCGTT AAGGAGTCTA   
  
  
+ TCAACAAGAA GCCCTTCTTG TAATTCTCCC CTTAAAACTA GCAGTTATCA TCACTTGTCA TCGAATAGCC   
  
  
+ CTTTTTGTAA TTCTACCCTT GAAACTAGCA GTTATCGTCA TCGGTTCAAC GCGAGTCCTG TTAGATATTC   
  
  
+ CCATCAAGGC ATAGATTATG GAGTGAGCAT GAAGAATGCT TTGCAGGAGC TAGAGACTAC TCTAATTGGT   
  
  
+ GTAGATGGTG AGGAAGTATC TATTGCTAAT CAACCTATGG GGGGAAGTCG TTAGTCCGGG ATCCCAAGTT   
  
  
+ AGAGATCGAA GTCATCAAGC GAAGATCCAC AGGGTTCACA TCCTACTCAG CTTGATTCAT CATCTTTTTC   
  
  
+ AAGGGTGAGA AGATTCGGAG ATGAAAGCCA GAGAGAGAGA AATGCCATAA GGCAATGGAA GAACCAGCGG   
  
  
+ AACTACTAAG TTTCTCACCA GGTGATTTAA AGCAGTTACT AATTGAATGT GCGAGGGCTT TATTAGATAA   
  
  
+ CCGAATAGAT GACTTTGAGA GTTTGGTTAA ACGGGCAAGG AAAGAGGTCT CCATCTCAGG TGAGCCTGTC   
  
  
+ CAACCTCTCG GTACCTGTAT GATCGAAGGG CTTGTGGCAA GGAAACAGTC TTCGGGGACT AACATCTATT   
  
  
+ GGGCTCTTAA GTGTAAAGAG CCTCTTGGAA AAGACTTGCT CTCCTACGGC ACTCAGTGGG TTACTGTATC   
  
  
+ GCAAGCCATA GCAGCAAGGC TTGGTGGGCC TCCTAAAGTG CGACTTACAG GCATTGATGA TCCTGTTTCT   
  
  
+ AAGTATACTT GTGATGCTAG CTTGGAGGCT GGTGGGAAAC GATTAGCGTC TCTATTTGAA AAGTTTAAAA   
  
  
+ TACTTGTCGA GTTCAATGCA TTGCCCGTTT ATGGACCCAA TGTCAGGTGG GAAATGCTGG ATGTGAGGCC   
  
  
+ CAGGGAGGCT TTGGTCGTTA ATTGTCCATT ACAGCTCCAT TACACTCCTG ACGAGAGTGT CGATGTGAGG   
  
  
+ AACCCTAGGG ATAGGCTTCT CAGAATGGTG AAATTGCTCG GTCCTAAGGT AGCCACTTTG GTTGAGCAAG   
  
  
+ AATCAAACAC CAACACTACA CCTTTCTTGA CCCGGTTCAT AGAGACCCTT GACTACTACT CGATTAACCA   
  
  
+ TGTTTG  

- -Up\_Stream \_Len000CAACAT TGTTCGTGTA ATTTCAACTA ATAGTAAAAC AGGCACTTTG TCACTCGTTT   
  
  
- GCAGTAGAGG TCCAGTTTAA GTAAGCATGA GTTAGTGTTC AACGACTGTA AAAGTGGTTT CCGATTAGTA   
  
  
- GCGTTTTCAA AGAAATCAAA TTCCGGTTAG AGAGGTAATA AAGTGGGATA TGTCTAACTC CCCCACATTA   
  
  
- TCACATATAT ATATCGGAAC ACAATCGCCC GGAACCTGTG ATCTGAAAAT GAGAACGTTT GGGATTATAT   
  
  
- AATAATATTT ATATTTAGAT CGGTGGGTAC AACTCCATCC GTGAGATTAA GAGTGTGTCC ATCCTATTCA   
  
  
- AAAGTATCAA ATACAACACT TACACTAGAA TGAAACACCA AGGGCAACTG TTTTCAAACT TAGGCTCGTA   
  
  
- CTCTCAAGTA GTTAGAGAAA AAGAAAGGAT AAAATAAACA ACACTTGAAT TAACCTAAAA GACCATCTCC   
  
  
- TTTCAAGGAA CGACCTTAAA CCAGTTTTCA CTATATATAA AACCCTAAAT TAATAGGTAT TTAAACCAAA   
  
  
- ATGTGGAACA ATCAAACCTG AAGACCACAC AATAACATAC ACAAGACCAC CCAAAAAGAA AATCGACTTA   
  
  
- CAACCTCGTA AAATTCCCTA GATCTATAGA CCTCTTCTCG AACCCTTTAT TTCGTATAAA GGGAATATCA   
  
  
- GACTGTATTG AAATTACTTA ACTAGTCCTT GTTTCCGGTT AATCAAGTTT TTCCAAGTTG AGGAGATCCA   
  
  
- CAACACGATT TCTCTACACT CAATCATTCA TTAACGAATG ATTGATATAT CCTACAAAAT AATGAACATC   
  
  
- TCCAAAAAAA AAACCCTATA GAAATACCCC AAATATTTAT TCTTCCAACT AAACAACAGA TGAGAAGAAA   
  
  
- GCTAAGTTAT CATTAAGAGA GTCTCTTCCG ATTTTACTGT ACCGTAAGGT AGCGGAGAGT ACTCCGAAAA   
  
  
- ACGTACACGA ATACATAGAA AGTAGAGAAC GAAACCACCC CACCGTAACC CCTCCTTTTC CCCCAAAGCT   
  
  
- AAGCTATCTA GCAGTGAAAA CCTTTACTGT AGAGATCGGT TACCGTTCCC ATTCCAATTA TGTACACCTA   
  
  
- TAAAGGTCTC AATCAACACT AACAATAACA ACAACAACAT TGAAATACAG TGCCCCTTTT GTATTATTGA   
  
  
- TAGACAAATA GACAAACAAA CAATAAAAGT TCCCCTTCCC CTCACCCGTC TACGAATCAT ACATTAGTAA   
  
  
- AATATATCCC TTTTAAGTAT ATCGAACTTT CATTTTGAAA GTAACGGTCG TAAGGTTCCA TACCCATCCC   
  
  
- AACAGATGTG GAAATAGAGG GATCTTCGTA CCACTAGTAA GGAGCCTAAC ACAATATCTT ATTTCTTACA   
  
  
- CCAAGACGAC ATTACTCTGA ATACATAATA GTTTACTACT TAGACAAATC TTGAAGGACA AATAAAAAAG   
  
  
- ACGTTTTAAA ATTCTCTGAT TGTAGATAGC CCGAGTTGAG ATCTCCTAGA GAACACAGTG GGAAGAGTTG   
  
  
- AGTCAAACTA ACACTATGTC AATGATATAA CTCACTATCA GCTCTCGTAG TGTAGGGCAA TTCCTCAGAT   
  
  
- AGTTGTTCTT CGGGAAGAAC ATTAAGAGGG GAATTTTGAT CGTCAATAGT AGTGAACAGT AGCTTATCGG   
  
  
- GAAAAACATT AAGATGGGAA CTTTGATCGT CAATAGCAGT AGCCAAGTTG CGCTCAGGAC AATCTATAAG   
  
  
- GGTAGTTCCG TATCTAATAC CTCACTCGTA CTTCTTACGA AACGTCCTCG ATCTCTGATG AGATTAACCA   
  
  
- CATCTACCAC TCCTTCATAG ATAACGATTA GTTGGATACC CCCCTTCAGC AATCAGGCCC TAGGGTTCAA   
  
  
- TCTCTAGCTT CAGTAGTTCG CTTCTAGGTG TCCCAAGTGT AGGATGAGTC GAACTAAGTA GTAGAAAAAG   
  
  
- TTCCCACTCT TCTAAGCCTC TACTTTCGGT CTCTCTCTCT TTACGGTATT CCGTTACCTT CTTGGTCGCC   
  
  
- TTGATGATTC AAAGAGTGGT CCACTAAATT TCGTCAATGA TTAACTTACA CGCTCCCGAA ATAATCTATT   
  
  
- GGCTTATCTA CTGAAACTCT CAAACCAATT TGCCCGTTCC TTTCTCCAGA GGTAGAGTCC ACTCGGACAG   
  
  
- GTTGGAGAGC CATGGACATA CTAGCTTCCC GAACACCGTT CCTTTGTCAG AAGCCCCTGA TTGTAGATAA   
  
  
- CCCGAGAATT CACATTTCTC GGAGAACCTT TTCTGAACGA GAGGATGCCG TGAGTCACCC AATGACATAG   
  
  
- CGTTCGGTAT CGTCGTTCCG AACCACCCGG AGGATTTCAC GCTGAATGTC CGTAACTACT AGGACAAAGA   
  
  
- TTCATATGAA CACTACGATC GAACCTCCGA CCACCCTTTG CTAATCGCAG AGATAAACTT TTCAAATTTT   
  
  
- ATGAACAGCT CAAGTTACGT AACGGGCAAA TACCTGGGTT ACAGTCCACC CTTTACGACC TACACTCCGG   
  
  
- GTCCCTCCGA AACCAGCAAT TAACAGGTAA TGTCGAGGTA ATGTGAGGAC TGCTCTCACA GCTACACTCC   
  
  
- TTGGGATCCC TATCCGAAGA GTCTTACCAC TTTAACGAGC CAGGATTCCA TCGGTGAAAC CAACTCGTTC   
  
  
- TTAGTTTGTG GTTGTGATGT GGAAAGAACT GGGCCAAGTA TCTCTGGGAA CTGATGATGA GCTAATTGGT   
  
  
- ACAAAC

+     STRE

| Site Name | Organism | Position | Strand | Matrix score. | sequence | function |
| --- | --- | --- | --- | --- | --- | --- |
| STRE | Arabidopsis thaliana | 1231 | + | 5 | AGGGG |  |
| STRE | Arabidopsis thaliana | 1642 | - | 5 | AGGGG |  |
| STRE | Arabidopsis thaliana | 1225 | + | 5 | AGGGG |  |
| STRE | Arabidopsis thaliana | 203 | + | 5 | AGGGG |  |
| STRE | Arabidopsis thaliana | 1043 | + | 5 | AGGGG |  |

>HU08G00230.1   
+ -Up\_Stream \_Len000GTTGTA ACAAGCACAT TAAAGTTGAT TATCATTTTG TCCGTGAAAC AGTGAGCAAA   
  
  
+ CGTCATCTCC AGGTCAAATT CATTCGTACT CAATCACAAG TTGCTGACAT TTTCACCAAA GGCTAATCAT   
  
  
+ CGCAAAAGTT TCTTTAGTTT AAGGCCAATC TCTCCATTAT TTCACCCTAT ACAGATTGAG GGGGTGTAAT   
  
  
+ AGTGTATATA TATAGCCTTG TGTTAGCGGG CCTTGGACAC TAGACTTTTA CTCTTGCAAA CCCTAATATA   
  
  
+ TTATTATAAA TATAAATCTA GCCACCCATG TTGAGGTAGG CACTCTAATT CTCACACAGG TAGGATAAGT   
  
  
+ TTTCATAGTT TATGTTGTGA ATGTGATCTT ACTTTGTGGT TCCCGTTGAC AAAAGTTTGA ATCCGAGCAT   
  
  
+ GAGAGTTCAT CAATCTCTTT TTCTTTCCTA TTTTATTTGT TGTGAACTTA ATTGGATTTT CTGGTAGAGG   
  
  
+ AAAGTTCCTT GCTGGAATTT GGTCAAAAGT GATATATATT TTGGGATTTA ATTATCCATA AATTTGGTTT   
  
  
+ TACACCTTGT TAGTTTGGAC TTCTGGTGTG TTATTGTATG TGTTCTGGTG GGTTTTTCTT TTAGCTGAAT   
  
  
+ GTTGGAGCAT TTTAAGGGAT CTAGATATCT GGAGAAGAGC TTGGGAAATA AAGCATATTT CCCTTATAGT   
  
  
+ CTGACATAAC TTTAATGAAT TGATCAGGAA CAAAGGCCAA TTAGTTCAAA AAGGTTCAAC TCCTCTAGGT   
  
  
+ GTTGTGCTAA AGAGATGTGA GTTAGTAAGT AATTGCTTAC TAACTATATA GGATGTTTTA TTACTTGTAG   
  
  
+ AGGTTTTTTT TTTGGGATAT CTTTATGGGG TTTATAAATA AGAAGGTTGA TTTGTTGTCT ACTCTTCTTT   
  
  
+ CGATTCAATA GTAATTCTCT CAGAGAAGGC TAAAATGACA TGGCATTCCA TCGCCTCTCA TGAGGCTTTT   
  
  
+ TGCATGTGCT TATGTATCTT TCATCTCTTG CTTTGGTGGG GTGGCATTGG GGAGGAAAAG GGGGTTTCGA   
  
  
+ TTCGATAGAT CGTCACTTTT GGAAATGACA TCTCTAGCCA ATGGCAAGGG TAAGGTTAAT ACATGTGGAT   
  
  
+ ATTTCCAGAG TTAGTTGTGA TTGTTATTGT TGTTGTTGTA ACTTTATGTC ACGGGGAAAA CATAATAACT   
  
  
+ ATCTGTTTAT CTGTTTGTTT GTTATTTTCA AGGGGAAGGG GAGTGGGCAG ATGCTTAGTA TGTAATCATT   
  
  
+ TTATATAGGG AAAATTCATA TAGCTTGAAA GTAAAACTTT CATTGCCAGC ATTCCAAGGT ATGGGTAGGG   
  
  
+ TTGTCTACAC CTTTATCTCC CTAGAAGCAT GGTGATCATT CCTCGGATTG TGTTATAGAA TAAAGAATGT   
  
  
+ GGTTCTGCTG TAATGAGACT TATGTATTAT CAAATGATGA ATCTGTTTAG AACTTCCTGT TTATTTTTTC   
  
  
+ TGCAAAATTT TAAGAGACTA ACATCTATCG GGCTCAACTC TAGAGGATCT CTTGTGTCAC CCTTCTCAAC   
  
  
+ TCAGTTTGAT TGTGATACAG TTACTATATT GAGTGATAGT CGAGAGCATC ACATCCCGTT AAGGAGTCTA   
  
  
+ TCAACAAGAA GCCCTTCTTG TAATTCTCCC CTTAAAACTA GCAGTTATCA TCACTTGTCA TCGAATAGCC   
  
  
+ CTTTTTGTAA TTCTACCCTT GAAACTAGCA GTTATCGTCA TCGGTTCAAC GCGAGTCCTG TTAGATATTC   
  
  
+ CCATCAAGGC ATAGATTATG GAGTGAGCAT GAAGAATGCT TTGCAGGAGC TAGAGACTAC TCTAATTGGT   
  
  
+ GTAGATGGTG AGGAAGTATC TATTGCTAAT CAACCTATGG GGGGAAGTCG TTAGTCCGGG ATCCCAAGTT   
  
  
+ AGAGATCGAA GTCATCAAGC GAAGATCCAC AGGGTTCACA TCCTACTCAG CTTGATTCAT CATCTTTTTC   
  
  
+ AAGGGTGAGA AGATTCGGAG ATGAAAGCCA GAGAGAGAGA AATGCCATAA GGCAATGGAA GAACCAGCGG   
  
  
+ AACTACTAAG TTTCTCACCA GGTGATTTAA AGCAGTTACT AATTGAATGT GCGAGGGCTT TATTAGATAA   
  
  
+ CCGAATAGAT GACTTTGAGA GTTTGGTTAA ACGGGCAAGG AAAGAGGTCT CCATCTCAGG TGAGCCTGTC   
  
  
+ CAACCTCTCG GTACCTGTAT GATCGAAGGG CTTGTGGCAA GGAAACAGTC TTCGGGGACT AACATCTATT   
  
  
+ GGGCTCTTAA GTGTAAAGAG CCTCTTGGAA AAGACTTGCT CTCCTACGGC ACTCAGTGGG TTACTGTATC   
  
  
+ GCAAGCCATA GCAGCAAGGC TTGGTGGGCC TCCTAAAGTG CGACTTACAG GCATTGATGA TCCTGTTTCT   
  
  
+ AAGTATACTT GTGATGCTAG CTTGGAGGCT GGTGGGAAAC GATTAGCGTC TCTATTTGAA AAGTTTAAAA   
  
  
+ TACTTGTCGA GTTCAATGCA TTGCCCGTTT ATGGACCCAA TGTCAGGTGG GAAATGCTGG ATGTGAGGCC   
  
  
+ CAGGGAGGCT TTGGTCGTTA ATTGTCCATT ACAGCTCCAT TACACTCCTG ACGAGAGTGT CGATGTGAGG   
  
  
+ AACCCTAGGG ATAGGCTTCT CAGAATGGTG AAATTGCTCG GTCCTAAGGT AGCCACTTTG GTTGAGCAAG   
  
  
+ AATCAAACAC CAACACTACA CCTTTCTTGA CCCGGTTCAT AGAGACCCTT GACTACTACT CGATTAACCA   
  
  
+ TGTTTG  

- -Up\_Stream \_Len000CAACAT TGTTCGTGTA ATTTCAACTA ATAGTAAAAC AGGCACTTTG TCACTCGTTT   
  
  
- GCAGTAGAGG TCCAGTTTAA GTAAGCATGA GTTAGTGTTC AACGACTGTA AAAGTGGTTT CCGATTAGTA   
  
  
- GCGTTTTCAA AGAAATCAAA TTCCGGTTAG AGAGGTAATA AAGTGGGATA TGTCTAACTC CCCCACATTA   
  
  
- TCACATATAT ATATCGGAAC ACAATCGCCC GGAACCTGTG ATCTGAAAAT GAGAACGTTT GGGATTATAT   
  
  
- AATAATATTT ATATTTAGAT CGGTGGGTAC AACTCCATCC GTGAGATTAA GAGTGTGTCC ATCCTATTCA   
  
  
- AAAGTATCAA ATACAACACT TACACTAGAA TGAAACACCA AGGGCAACTG TTTTCAAACT TAGGCTCGTA   
  
  
- CTCTCAAGTA GTTAGAGAAA AAGAAAGGAT AAAATAAACA ACACTTGAAT TAACCTAAAA GACCATCTCC   
  
  
- TTTCAAGGAA CGACCTTAAA CCAGTTTTCA CTATATATAA AACCCTAAAT TAATAGGTAT TTAAACCAAA   
  
  
- ATGTGGAACA ATCAAACCTG AAGACCACAC AATAACATAC ACAAGACCAC CCAAAAAGAA AATCGACTTA   
  
  
- CAACCTCGTA AAATTCCCTA GATCTATAGA CCTCTTCTCG AACCCTTTAT TTCGTATAAA GGGAATATCA   
  
  
- GACTGTATTG AAATTACTTA ACTAGTCCTT GTTTCCGGTT AATCAAGTTT TTCCAAGTTG AGGAGATCCA   
  
  
- CAACACGATT TCTCTACACT CAATCATTCA TTAACGAATG ATTGATATAT CCTACAAAAT AATGAACATC   
  
  
- TCCAAAAAAA AAACCCTATA GAAATACCCC AAATATTTAT TCTTCCAACT AAACAACAGA TGAGAAGAAA   
  
  
- GCTAAGTTAT CATTAAGAGA GTCTCTTCCG ATTTTACTGT ACCGTAAGGT AGCGGAGAGT ACTCCGAAAA   
  
  
- ACGTACACGA ATACATAGAA AGTAGAGAAC GAAACCACCC CACCGTAACC CCTCCTTTTC CCCCAAAGCT   
  
  
- AAGCTATCTA GCAGTGAAAA CCTTTACTGT AGAGATCGGT TACCGTTCCC ATTCCAATTA TGTACACCTA   
  
  
- TAAAGGTCTC AATCAACACT AACAATAACA ACAACAACAT TGAAATACAG TGCCCCTTTT GTATTATTGA   
  
  
- TAGACAAATA GACAAACAAA CAATAAAAGT TCCCCTTCCC CTCACCCGTC TACGAATCAT ACATTAGTAA   
  
  
- AATATATCCC TTTTAAGTAT ATCGAACTTT CATTTTGAAA GTAACGGTCG TAAGGTTCCA TACCCATCCC   
  
  
- AACAGATGTG GAAATAGAGG GATCTTCGTA CCACTAGTAA GGAGCCTAAC ACAATATCTT ATTTCTTACA   
  
  
- CCAAGACGAC ATTACTCTGA ATACATAATA GTTTACTACT TAGACAAATC TTGAAGGACA AATAAAAAAG   
  
  
- ACGTTTTAAA ATTCTCTGAT TGTAGATAGC CCGAGTTGAG ATCTCCTAGA GAACACAGTG GGAAGAGTTG   
  
  
- AGTCAAACTA ACACTATGTC AATGATATAA CTCACTATCA GCTCTCGTAG TGTAGGGCAA TTCCTCAGAT   
  
  
- AGTTGTTCTT CGGGAAGAAC ATTAAGAGGG GAATTTTGAT CGTCAATAGT AGTGAACAGT AGCTTATCGG   
  
  
- GAAAAACATT AAGATGGGAA CTTTGATCGT CAATAGCAGT AGCCAAGTTG CGCTCAGGAC AATCTATAAG   
  
  
- GGTAGTTCCG TATCTAATAC CTCACTCGTA CTTCTTACGA AACGTCCTCG ATCTCTGATG AGATTAACCA   
  
  
- CATCTACCAC TCCTTCATAG ATAACGATTA GTTGGATACC CCCCTTCAGC AATCAGGCCC TAGGGTTCAA   
  
  
- TCTCTAGCTT CAGTAGTTCG CTTCTAGGTG TCCCAAGTGT AGGATGAGTC GAACTAAGTA GTAGAAAAAG   
  
  
- TTCCCACTCT TCTAAGCCTC TACTTTCGGT CTCTCTCTCT TTACGGTATT CCGTTACCTT CTTGGTCGCC   
  
  
- TTGATGATTC AAAGAGTGGT CCACTAAATT TCGTCAATGA TTAACTTACA CGCTCCCGAA ATAATCTATT   
  
  
- GGCTTATCTA CTGAAACTCT CAAACCAATT TGCCCGTTCC TTTCTCCAGA GGTAGAGTCC ACTCGGACAG   
  
  
- GTTGGAGAGC CATGGACATA CTAGCTTCCC GAACACCGTT CCTTTGTCAG AAGCCCCTGA TTGTAGATAA   
  
  
- CCCGAGAATT CACATTTCTC GGAGAACCTT TTCTGAACGA GAGGATGCCG TGAGTCACCC AATGACATAG   
  
  
- CGTTCGGTAT CGTCGTTCCG AACCACCCGG AGGATTTCAC GCTGAATGTC CGTAACTACT AGGACAAAGA   
  
  
- TTCATATGAA CACTACGATC GAACCTCCGA CCACCCTTTG CTAATCGCAG AGATAAACTT TTCAAATTTT   
  
  
- ATGAACAGCT CAAGTTACGT AACGGGCAAA TACCTGGGTT ACAGTCCACC CTTTACGACC TACACTCCGG   
  
  
- GTCCCTCCGA AACCAGCAAT TAACAGGTAA TGTCGAGGTA ATGTGAGGAC TGCTCTCACA GCTACACTCC   
  
  
- TTGGGATCCC TATCCGAAGA GTCTTACCAC TTTAACGAGC CAGGATTCCA TCGGTGAAAC CAACTCGTTC   
  
  
- TTAGTTTGTG GTTGTGATGT GGAAAGAACT GGGCCAAGTA TCTCTGGGAA CTGATGATGA GCTAATTGGT   
  
  
- ACAAAC

+     TATA

| Site Name | Organism | Position | Strand | Matrix score. | sequence | function |
| --- | --- | --- | --- | --- | --- | --- |
| TATA | Arabidopsis thaliana | 1262 | - | 8 | TATAAAAT |  |

>HU08G00230.1   
+ -Up\_Stream \_Len000GTTGTA ACAAGCACAT TAAAGTTGAT TATCATTTTG TCCGTGAAAC AGTGAGCAAA   
  
  
+ CGTCATCTCC AGGTCAAATT CATTCGTACT CAATCACAAG TTGCTGACAT TTTCACCAAA GGCTAATCAT   
  
  
+ CGCAAAAGTT TCTTTAGTTT AAGGCCAATC TCTCCATTAT TTCACCCTAT ACAGATTGAG GGGGTGTAAT   
  
  
+ AGTGTATATA TATAGCCTTG TGTTAGCGGG CCTTGGACAC TAGACTTTTA CTCTTGCAAA CCCTAATATA   
  
  
+ TTATTATAAA TATAAATCTA GCCACCCATG TTGAGGTAGG CACTCTAATT CTCACACAGG TAGGATAAGT   
  
  
+ TTTCATAGTT TATGTTGTGA ATGTGATCTT ACTTTGTGGT TCCCGTTGAC AAAAGTTTGA ATCCGAGCAT   
  
  
+ GAGAGTTCAT CAATCTCTTT TTCTTTCCTA TTTTATTTGT TGTGAACTTA ATTGGATTTT CTGGTAGAGG   
  
  
+ AAAGTTCCTT GCTGGAATTT GGTCAAAAGT GATATATATT TTGGGATTTA ATTATCCATA AATTTGGTTT   
  
  
+ TACACCTTGT TAGTTTGGAC TTCTGGTGTG TTATTGTATG TGTTCTGGTG GGTTTTTCTT TTAGCTGAAT   
  
  
+ GTTGGAGCAT TTTAAGGGAT CTAGATATCT GGAGAAGAGC TTGGGAAATA AAGCATATTT CCCTTATAGT   
  
  
+ CTGACATAAC TTTAATGAAT TGATCAGGAA CAAAGGCCAA TTAGTTCAAA AAGGTTCAAC TCCTCTAGGT   
  
  
+ GTTGTGCTAA AGAGATGTGA GTTAGTAAGT AATTGCTTAC TAACTATATA GGATGTTTTA TTACTTGTAG   
  
  
+ AGGTTTTTTT TTTGGGATAT CTTTATGGGG TTTATAAATA AGAAGGTTGA TTTGTTGTCT ACTCTTCTTT   
  
  
+ CGATTCAATA GTAATTCTCT CAGAGAAGGC TAAAATGACA TGGCATTCCA TCGCCTCTCA TGAGGCTTTT   
  
  
+ TGCATGTGCT TATGTATCTT TCATCTCTTG CTTTGGTGGG GTGGCATTGG GGAGGAAAAG GGGGTTTCGA   
  
  
+ TTCGATAGAT CGTCACTTTT GGAAATGACA TCTCTAGCCA ATGGCAAGGG TAAGGTTAAT ACATGTGGAT   
  
  
+ ATTTCCAGAG TTAGTTGTGA TTGTTATTGT TGTTGTTGTA ACTTTATGTC ACGGGGAAAA CATAATAACT   
  
  
+ ATCTGTTTAT CTGTTTGTTT GTTATTTTCA AGGGGAAGGG GAGTGGGCAG ATGCTTAGTA TGTAATCATT   
  
  
+ TTATATAGGG AAAATTCATA TAGCTTGAAA GTAAAACTTT CATTGCCAGC ATTCCAAGGT ATGGGTAGGG   
  
  
+ TTGTCTACAC CTTTATCTCC CTAGAAGCAT GGTGATCATT CCTCGGATTG TGTTATAGAA TAAAGAATGT   
  
  
+ GGTTCTGCTG TAATGAGACT TATGTATTAT CAAATGATGA ATCTGTTTAG AACTTCCTGT TTATTTTTTC   
  
  
+ TGCAAAATTT TAAGAGACTA ACATCTATCG GGCTCAACTC TAGAGGATCT CTTGTGTCAC CCTTCTCAAC   
  
  
+ TCAGTTTGAT TGTGATACAG TTACTATATT GAGTGATAGT CGAGAGCATC ACATCCCGTT AAGGAGTCTA   
  
  
+ TCAACAAGAA GCCCTTCTTG TAATTCTCCC CTTAAAACTA GCAGTTATCA TCACTTGTCA TCGAATAGCC   
  
  
+ CTTTTTGTAA TTCTACCCTT GAAACTAGCA GTTATCGTCA TCGGTTCAAC GCGAGTCCTG TTAGATATTC   
  
  
+ CCATCAAGGC ATAGATTATG GAGTGAGCAT GAAGAATGCT TTGCAGGAGC TAGAGACTAC TCTAATTGGT   
  
  
+ GTAGATGGTG AGGAAGTATC TATTGCTAAT CAACCTATGG GGGGAAGTCG TTAGTCCGGG ATCCCAAGTT   
  
  
+ AGAGATCGAA GTCATCAAGC GAAGATCCAC AGGGTTCACA TCCTACTCAG CTTGATTCAT CATCTTTTTC   
  
  
+ AAGGGTGAGA AGATTCGGAG ATGAAAGCCA GAGAGAGAGA AATGCCATAA GGCAATGGAA GAACCAGCGG   
  
  
+ AACTACTAAG TTTCTCACCA GGTGATTTAA AGCAGTTACT AATTGAATGT GCGAGGGCTT TATTAGATAA   
  
  
+ CCGAATAGAT GACTTTGAGA GTTTGGTTAA ACGGGCAAGG AAAGAGGTCT CCATCTCAGG TGAGCCTGTC   
  
  
+ CAACCTCTCG GTACCTGTAT GATCGAAGGG CTTGTGGCAA GGAAACAGTC TTCGGGGACT AACATCTATT   
  
  
+ GGGCTCTTAA GTGTAAAGAG CCTCTTGGAA AAGACTTGCT CTCCTACGGC ACTCAGTGGG TTACTGTATC   
  
  
+ GCAAGCCATA GCAGCAAGGC TTGGTGGGCC TCCTAAAGTG CGACTTACAG GCATTGATGA TCCTGTTTCT   
  
  
+ AAGTATACTT GTGATGCTAG CTTGGAGGCT GGTGGGAAAC GATTAGCGTC TCTATTTGAA AAGTTTAAAA   
  
  
+ TACTTGTCGA GTTCAATGCA TTGCCCGTTT ATGGACCCAA TGTCAGGTGG GAAATGCTGG ATGTGAGGCC   
  
  
+ CAGGGAGGCT TTGGTCGTTA ATTGTCCATT ACAGCTCCAT TACACTCCTG ACGAGAGTGT CGATGTGAGG   
  
  
+ AACCCTAGGG ATAGGCTTCT CAGAATGGTG AAATTGCTCG GTCCTAAGGT AGCCACTTTG GTTGAGCAAG   
  
  
+ AATCAAACAC CAACACTACA CCTTTCTTGA CCCGGTTCAT AGAGACCCTT GACTACTACT CGATTAACCA   
  
  
+ TGTTTG  

- -Up\_Stream \_Len000CAACAT TGTTCGTGTA ATTTCAACTA ATAGTAAAAC AGGCACTTTG TCACTCGTTT   
  
  
- GCAGTAGAGG TCCAGTTTAA GTAAGCATGA GTTAGTGTTC AACGACTGTA AAAGTGGTTT CCGATTAGTA   
  
  
- GCGTTTTCAA AGAAATCAAA TTCCGGTTAG AGAGGTAATA AAGTGGGATA TGTCTAACTC CCCCACATTA   
  
  
- TCACATATAT ATATCGGAAC ACAATCGCCC GGAACCTGTG ATCTGAAAAT GAGAACGTTT GGGATTATAT   
  
  
- AATAATATTT ATATTTAGAT CGGTGGGTAC AACTCCATCC GTGAGATTAA GAGTGTGTCC ATCCTATTCA   
  
  
- AAAGTATCAA ATACAACACT TACACTAGAA TGAAACACCA AGGGCAACTG TTTTCAAACT TAGGCTCGTA   
  
  
- CTCTCAAGTA GTTAGAGAAA AAGAAAGGAT AAAATAAACA ACACTTGAAT TAACCTAAAA GACCATCTCC   
  
  
- TTTCAAGGAA CGACCTTAAA CCAGTTTTCA CTATATATAA AACCCTAAAT TAATAGGTAT TTAAACCAAA   
  
  
- ATGTGGAACA ATCAAACCTG AAGACCACAC AATAACATAC ACAAGACCAC CCAAAAAGAA AATCGACTTA   
  
  
- CAACCTCGTA AAATTCCCTA GATCTATAGA CCTCTTCTCG AACCCTTTAT TTCGTATAAA GGGAATATCA   
  
  
- GACTGTATTG AAATTACTTA ACTAGTCCTT GTTTCCGGTT AATCAAGTTT TTCCAAGTTG AGGAGATCCA   
  
  
- CAACACGATT TCTCTACACT CAATCATTCA TTAACGAATG ATTGATATAT CCTACAAAAT AATGAACATC   
  
  
- TCCAAAAAAA AAACCCTATA GAAATACCCC AAATATTTAT TCTTCCAACT AAACAACAGA TGAGAAGAAA   
  
  
- GCTAAGTTAT CATTAAGAGA GTCTCTTCCG ATTTTACTGT ACCGTAAGGT AGCGGAGAGT ACTCCGAAAA   
  
  
- ACGTACACGA ATACATAGAA AGTAGAGAAC GAAACCACCC CACCGTAACC CCTCCTTTTC CCCCAAAGCT   
  
  
- AAGCTATCTA GCAGTGAAAA CCTTTACTGT AGAGATCGGT TACCGTTCCC ATTCCAATTA TGTACACCTA   
  
  
- TAAAGGTCTC AATCAACACT AACAATAACA ACAACAACAT TGAAATACAG TGCCCCTTTT GTATTATTGA   
  
  
- TAGACAAATA GACAAACAAA CAATAAAAGT TCCCCTTCCC CTCACCCGTC TACGAATCAT ACATTAGTAA   
  
  
- AATATATCCC TTTTAAGTAT ATCGAACTTT CATTTTGAAA GTAACGGTCG TAAGGTTCCA TACCCATCCC   
  
  
- AACAGATGTG GAAATAGAGG GATCTTCGTA CCACTAGTAA GGAGCCTAAC ACAATATCTT ATTTCTTACA   
  
  
- CCAAGACGAC ATTACTCTGA ATACATAATA GTTTACTACT TAGACAAATC TTGAAGGACA AATAAAAAAG   
  
  
- ACGTTTTAAA ATTCTCTGAT TGTAGATAGC CCGAGTTGAG ATCTCCTAGA GAACACAGTG GGAAGAGTTG   
  
  
- AGTCAAACTA ACACTATGTC AATGATATAA CTCACTATCA GCTCTCGTAG TGTAGGGCAA TTCCTCAGAT   
  
  
- AGTTGTTCTT CGGGAAGAAC ATTAAGAGGG GAATTTTGAT CGTCAATAGT AGTGAACAGT AGCTTATCGG   
  
  
- GAAAAACATT AAGATGGGAA CTTTGATCGT CAATAGCAGT AGCCAAGTTG CGCTCAGGAC AATCTATAAG   
  
  
- GGTAGTTCCG TATCTAATAC CTCACTCGTA CTTCTTACGA AACGTCCTCG ATCTCTGATG AGATTAACCA   
  
  
- CATCTACCAC TCCTTCATAG ATAACGATTA GTTGGATACC CCCCTTCAGC AATCAGGCCC TAGGGTTCAA   
  
  
- TCTCTAGCTT CAGTAGTTCG CTTCTAGGTG TCCCAAGTGT AGGATGAGTC GAACTAAGTA GTAGAAAAAG   
  
  
- TTCCCACTCT TCTAAGCCTC TACTTTCGGT CTCTCTCTCT TTACGGTATT CCGTTACCTT CTTGGTCGCC   
  
  
- TTGATGATTC AAAGAGTGGT CCACTAAATT TCGTCAATGA TTAACTTACA CGCTCCCGAA ATAATCTATT   
  
  
- GGCTTATCTA CTGAAACTCT CAAACCAATT TGCCCGTTCC TTTCTCCAGA GGTAGAGTCC ACTCGGACAG   
  
  
- GTTGGAGAGC CATGGACATA CTAGCTTCCC GAACACCGTT CCTTTGTCAG AAGCCCCTGA TTGTAGATAA   
  
  
- CCCGAGAATT CACATTTCTC GGAGAACCTT TTCTGAACGA GAGGATGCCG TGAGTCACCC AATGACATAG   
  
  
- CGTTCGGTAT CGTCGTTCCG AACCACCCGG AGGATTTCAC GCTGAATGTC CGTAACTACT AGGACAAAGA   
  
  
- TTCATATGAA CACTACGATC GAACCTCCGA CCACCCTTTG CTAATCGCAG AGATAAACTT TTCAAATTTT   
  
  
- ATGAACAGCT CAAGTTACGT AACGGGCAAA TACCTGGGTT ACAGTCCACC CTTTACGACC TACACTCCGG   
  
  
- GTCCCTCCGA AACCAGCAAT TAACAGGTAA TGTCGAGGTA ATGTGAGGAC TGCTCTCACA GCTACACTCC   
  
  
- TTGGGATCCC TATCCGAAGA GTCTTACCAC TTTAACGAGC CAGGATTCCA TCGGTGAAAC CAACTCGTTC   
  
  
- TTAGTTTGTG GTTGTGATGT GGAAAGAACT GGGCCAAGTA TCTCTGGGAA CTGATGATGA GCTAATTGGT   
  
  
- ACAAAC

+     TATA-box

| Site Name | Organism | Position | Strand | Matrix score. | sequence | function |
| --- | --- | --- | --- | --- | --- | --- |
| TATA-box | Arabidopsis thaliana | 1569 | - | 4 | TATA | core promoter element around -30 of transcription start |
| TATA-box | Arabidopsis thaliana | 877 | + | 4 | TATA | core promoter element around -30 of transcription start |
| TATA-box | Brassica napus | 528 | + | 6 | ATATAT | core promoter element around -30 of transcription start |
| TATA-box | Arabidopsis thaliana | 295 | + | 4 | TATA | core promoter element around -30 of transcription start |
| TATA-box | Arabidopsis thaliana | 289 | + | 4 | TATA | core promoter element around -30 of transcription start |
| TATA-box | Avena sativa | 292 | - | 12 | TATATTTATATTT | core promoter element around -30 of transcription start |
| TATA-box | Arabidopsis thaliana | 223 | + | 6 | TATATA | core promoter element around -30 of transcription start |
| TATA-box | Pisum sativum | 1263 | - | 7 | TATAAAA | core promoter element around -30 of transcription start |
| TATA-box | Helianthus annuus | 875 | - | 6 | TATAAA | core promoter element around -30 of transcription start |
| TATA-box | Arabidopsis thaliana | 819 | + | 6 | TATATA | core promoter element around -30 of transcription start |
| TATA-box | Arabidopsis thaliana | 288 | - | 5 | TATAA | core promoter element around -30 of transcription start |
| TATA-box | Arabidopsis thaliana | 2388 | - | 4 | TATA | core promoter element around -30 of transcription start |
| TATA-box | Arabidopsis thaliana | 281 | + | 4 | TATA | core promoter element around -30 of transcription start |
| TATA-box | Brassica oleracea | 294 | + | 6 | ATATAA | core promoter element around -30 of transcription start |
| TATA-box | Arabidopsis thaliana | 293 | + | 9 | taTATAAAtc | core promoter element around -30 of transcription start |
| TATA-box | Brassica napus | 222 | + | 6 | ATATAT | core promoter element around -30 of transcription start |
| TATA-box | Arabidopsis thaliana | 876 | - | 5 | TATAA | core promoter element around -30 of transcription start |
| TATA-box | Brassica napus | 280 | + | 6 | ATATAT | core promoter element around -30 of transcription start |
| TATA-box | Brassica napus | 220 | + | 6 | ATATAT | core promoter element around -30 of transcription start |
| TATA-box | Arabidopsis thaliana | 219 | + | 6 | TATATA | core promoter element around -30 of transcription start |
| TATA-box | Arabidopsis thaliana | 225 | + | 4 | TATA | core promoter element around -30 of transcription start |
| TATA-box | Arabidopsis thaliana | 221 | + | 6 | TATATA | core promoter element around -30 of transcription start |
| TATA-box | Oryza sativa | 1687 | - | 7 | TACAAAA | core promoter element around -30 of transcription start |
| TATA-box | Arabidopsis thaliana | 699 | + | 4 | TATA | core promoter element around -30 of transcription start |
| TATA-box | Arabidopsis thaliana | 1264 | - | 9 | ccTATAAAaa | core promoter element around -30 of transcription start |
| TATA-box | Arabidopsis thaliana | 1388 | - | 4 | TATA | core promoter element around -30 of transcription start |
| TATA-box | Arabidopsis thaliana | 821 | + | 4 | TATA | core promoter element around -30 of transcription start |
| TATA-box | Arabidopsis thaliana | 529 | + | 4 | TATA | core promoter element around -30 of transcription start |
| TATA-box | Arabidopsis thaliana | 1387 | - | 5 | TATAA | core promoter element around -30 of transcription start |
| TATA-box | Arabidopsis thaliana | 192 | + | 4 | TATA | core promoter element around -30 of transcription start |
| TATA-box | Brassica napus | 526 | + | 6 | ATATAT | core promoter element around -30 of transcription start |
| TATA-box | Arabidopsis thaliana | 698 | - | 5 | TATAA | core promoter element around -30 of transcription start |
| TATA-box | Helianthus annuus | 217 | - | 6 | TATACA | core promoter element around -30 of transcription start |
| TATA-box | Brassica napus | 287 | + | 6 | ATTATA | core promoter element around -30 of transcription start |
| TATA-box | Arabidopsis thaliana | 1268 | + | 4 | TATA | core promoter element around -30 of transcription start |
| TATA-box | Arabidopsis thaliana | 1283 | + | 4 | TATA | core promoter element around -30 of transcription start |
| TATA-box | Arabidopsis thaliana | 1266 | + | 6 | TATATA | core promoter element around -30 of transcription start |
| TATA-box | Arabidopsis thaliana | 527 | + | 6 | TATATA | core promoter element around -30 of transcription start |
| TATA-box | Arabidopsis thaliana | 1265 | - | 7 | TATATAA | core promoter element around -30 of transcription start |

>HU08G00230.1   
+ -Up\_Stream \_Len000GTTGTA ACAAGCACAT TAAAGTTGAT TATCATTTTG TCCGTGAAAC AGTGAGCAAA   
  
  
+ CGTCATCTCC AGGTCAAATT CATTCGTACT CAATCACAAG TTGCTGACAT TTTCACCAAA GGCTAATCAT   
  
  
+ CGCAAAAGTT TCTTTAGTTT AAGGCCAATC TCTCCATTAT TTCACCCTAT ACAGATTGAG GGGGTGTAAT   
  
  
+ AGTGTATATA TATAGCCTTG TGTTAGCGGG CCTTGGACAC TAGACTTTTA CTCTTGCAAA CCCTAATATA   
  
  
+ TTATTATAAA TATAAATCTA GCCACCCATG TTGAGGTAGG CACTCTAATT CTCACACAGG TAGGATAAGT   
  
  
+ TTTCATAGTT TATGTTGTGA ATGTGATCTT ACTTTGTGGT TCCCGTTGAC AAAAGTTTGA ATCCGAGCAT   
  
  
+ GAGAGTTCAT CAATCTCTTT TTCTTTCCTA TTTTATTTGT TGTGAACTTA ATTGGATTTT CTGGTAGAGG   
  
  
+ AAAGTTCCTT GCTGGAATTT GGTCAAAAGT GATATATATT TTGGGATTTA ATTATCCATA AATTTGGTTT   
  
  
+ TACACCTTGT TAGTTTGGAC TTCTGGTGTG TTATTGTATG TGTTCTGGTG GGTTTTTCTT TTAGCTGAAT   
  
  
+ GTTGGAGCAT TTTAAGGGAT CTAGATATCT GGAGAAGAGC TTGGGAAATA AAGCATATTT CCCTTATAGT   
  
  
+ CTGACATAAC TTTAATGAAT TGATCAGGAA CAAAGGCCAA TTAGTTCAAA AAGGTTCAAC TCCTCTAGGT   
  
  
+ GTTGTGCTAA AGAGATGTGA GTTAGTAAGT AATTGCTTAC TAACTATATA GGATGTTTTA TTACTTGTAG   
  
  
+ AGGTTTTTTT TTTGGGATAT CTTTATGGGG TTTATAAATA AGAAGGTTGA TTTGTTGTCT ACTCTTCTTT   
  
  
+ CGATTCAATA GTAATTCTCT CAGAGAAGGC TAAAATGACA TGGCATTCCA TCGCCTCTCA TGAGGCTTTT   
  
  
+ TGCATGTGCT TATGTATCTT TCATCTCTTG CTTTGGTGGG GTGGCATTGG GGAGGAAAAG GGGGTTTCGA   
  
  
+ TTCGATAGAT CGTCACTTTT GGAAATGACA TCTCTAGCCA ATGGCAAGGG TAAGGTTAAT ACATGTGGAT   
  
  
+ ATTTCCAGAG TTAGTTGTGA TTGTTATTGT TGTTGTTGTA ACTTTATGTC ACGGGGAAAA CATAATAACT   
  
  
+ ATCTGTTTAT CTGTTTGTTT GTTATTTTCA AGGGGAAGGG GAGTGGGCAG ATGCTTAGTA TGTAATCATT   
  
  
+ TTATATAGGG AAAATTCATA TAGCTTGAAA GTAAAACTTT CATTGCCAGC ATTCCAAGGT ATGGGTAGGG   
  
  
+ TTGTCTACAC CTTTATCTCC CTAGAAGCAT GGTGATCATT CCTCGGATTG TGTTATAGAA TAAAGAATGT   
  
  
+ GGTTCTGCTG TAATGAGACT TATGTATTAT CAAATGATGA ATCTGTTTAG AACTTCCTGT TTATTTTTTC   
  
  
+ TGCAAAATTT TAAGAGACTA ACATCTATCG GGCTCAACTC TAGAGGATCT CTTGTGTCAC CCTTCTCAAC   
  
  
+ TCAGTTTGAT TGTGATACAG TTACTATATT GAGTGATAGT CGAGAGCATC ACATCCCGTT AAGGAGTCTA   
  
  
+ TCAACAAGAA GCCCTTCTTG TAATTCTCCC CTTAAAACTA GCAGTTATCA TCACTTGTCA TCGAATAGCC   
  
  
+ CTTTTTGTAA TTCTACCCTT GAAACTAGCA GTTATCGTCA TCGGTTCAAC GCGAGTCCTG TTAGATATTC   
  
  
+ CCATCAAGGC ATAGATTATG GAGTGAGCAT GAAGAATGCT TTGCAGGAGC TAGAGACTAC TCTAATTGGT   
  
  
+ GTAGATGGTG AGGAAGTATC TATTGCTAAT CAACCTATGG GGGGAAGTCG TTAGTCCGGG ATCCCAAGTT   
  
  
+ AGAGATCGAA GTCATCAAGC GAAGATCCAC AGGGTTCACA TCCTACTCAG CTTGATTCAT CATCTTTTTC   
  
  
+ AAGGGTGAGA AGATTCGGAG ATGAAAGCCA GAGAGAGAGA AATGCCATAA GGCAATGGAA GAACCAGCGG   
  
  
+ AACTACTAAG TTTCTCACCA GGTGATTTAA AGCAGTTACT AATTGAATGT GCGAGGGCTT TATTAGATAA   
  
  
+ CCGAATAGAT GACTTTGAGA GTTTGGTTAA ACGGGCAAGG AAAGAGGTCT CCATCTCAGG TGAGCCTGTC   
  
  
+ CAACCTCTCG GTACCTGTAT GATCGAAGGG CTTGTGGCAA GGAAACAGTC TTCGGGGACT AACATCTATT   
  
  
+ GGGCTCTTAA GTGTAAAGAG CCTCTTGGAA AAGACTTGCT CTCCTACGGC ACTCAGTGGG TTACTGTATC   
  
  
+ GCAAGCCATA GCAGCAAGGC TTGGTGGGCC TCCTAAAGTG CGACTTACAG GCATTGATGA TCCTGTTTCT   
  
  
+ AAGTATACTT GTGATGCTAG CTTGGAGGCT GGTGGGAAAC GATTAGCGTC TCTATTTGAA AAGTTTAAAA   
  
  
+ TACTTGTCGA GTTCAATGCA TTGCCCGTTT ATGGACCCAA TGTCAGGTGG GAAATGCTGG ATGTGAGGCC   
  
  
+ CAGGGAGGCT TTGGTCGTTA ATTGTCCATT ACAGCTCCAT TACACTCCTG ACGAGAGTGT CGATGTGAGG   
  
  
+ AACCCTAGGG ATAGGCTTCT CAGAATGGTG AAATTGCTCG GTCCTAAGGT AGCCACTTTG GTTGAGCAAG   
  
  
+ AATCAAACAC CAACACTACA CCTTTCTTGA CCCGGTTCAT AGAGACCCTT GACTACTACT CGATTAACCA   
  
  
+ TGTTTG  

- -Up\_Stream \_Len000CAACAT TGTTCGTGTA ATTTCAACTA ATAGTAAAAC AGGCACTTTG TCACTCGTTT   
  
  
- GCAGTAGAGG TCCAGTTTAA GTAAGCATGA GTTAGTGTTC AACGACTGTA AAAGTGGTTT CCGATTAGTA   
  
  
- GCGTTTTCAA AGAAATCAAA TTCCGGTTAG AGAGGTAATA AAGTGGGATA TGTCTAACTC CCCCACATTA   
  
  
- TCACATATAT ATATCGGAAC ACAATCGCCC GGAACCTGTG ATCTGAAAAT GAGAACGTTT GGGATTATAT   
  
  
- AATAATATTT ATATTTAGAT CGGTGGGTAC AACTCCATCC GTGAGATTAA GAGTGTGTCC ATCCTATTCA   
  
  
- AAAGTATCAA ATACAACACT TACACTAGAA TGAAACACCA AGGGCAACTG TTTTCAAACT TAGGCTCGTA   
  
  
- CTCTCAAGTA GTTAGAGAAA AAGAAAGGAT AAAATAAACA ACACTTGAAT TAACCTAAAA GACCATCTCC   
  
  
- TTTCAAGGAA CGACCTTAAA CCAGTTTTCA CTATATATAA AACCCTAAAT TAATAGGTAT TTAAACCAAA   
  
  
- ATGTGGAACA ATCAAACCTG AAGACCACAC AATAACATAC ACAAGACCAC CCAAAAAGAA AATCGACTTA   
  
  
- CAACCTCGTA AAATTCCCTA GATCTATAGA CCTCTTCTCG AACCCTTTAT TTCGTATAAA GGGAATATCA   
  
  
- GACTGTATTG AAATTACTTA ACTAGTCCTT GTTTCCGGTT AATCAAGTTT TTCCAAGTTG AGGAGATCCA   
  
  
- CAACACGATT TCTCTACACT CAATCATTCA TTAACGAATG ATTGATATAT CCTACAAAAT AATGAACATC   
  
  
- TCCAAAAAAA AAACCCTATA GAAATACCCC AAATATTTAT TCTTCCAACT AAACAACAGA TGAGAAGAAA   
  
  
- GCTAAGTTAT CATTAAGAGA GTCTCTTCCG ATTTTACTGT ACCGTAAGGT AGCGGAGAGT ACTCCGAAAA   
  
  
- ACGTACACGA ATACATAGAA AGTAGAGAAC GAAACCACCC CACCGTAACC CCTCCTTTTC CCCCAAAGCT   
  
  
- AAGCTATCTA GCAGTGAAAA CCTTTACTGT AGAGATCGGT TACCGTTCCC ATTCCAATTA TGTACACCTA   
  
  
- TAAAGGTCTC AATCAACACT AACAATAACA ACAACAACAT TGAAATACAG TGCCCCTTTT GTATTATTGA   
  
  
- TAGACAAATA GACAAACAAA CAATAAAAGT TCCCCTTCCC CTCACCCGTC TACGAATCAT ACATTAGTAA   
  
  
- AATATATCCC TTTTAAGTAT ATCGAACTTT CATTTTGAAA GTAACGGTCG TAAGGTTCCA TACCCATCCC   
  
  
- AACAGATGTG GAAATAGAGG GATCTTCGTA CCACTAGTAA GGAGCCTAAC ACAATATCTT ATTTCTTACA   
  
  
- CCAAGACGAC ATTACTCTGA ATACATAATA GTTTACTACT TAGACAAATC TTGAAGGACA AATAAAAAAG   
  
  
- ACGTTTTAAA ATTCTCTGAT TGTAGATAGC CCGAGTTGAG ATCTCCTAGA GAACACAGTG GGAAGAGTTG   
  
  
- AGTCAAACTA ACACTATGTC AATGATATAA CTCACTATCA GCTCTCGTAG TGTAGGGCAA TTCCTCAGAT   
  
  
- AGTTGTTCTT CGGGAAGAAC ATTAAGAGGG GAATTTTGAT CGTCAATAGT AGTGAACAGT AGCTTATCGG   
  
  
- GAAAAACATT AAGATGGGAA CTTTGATCGT CAATAGCAGT AGCCAAGTTG CGCTCAGGAC AATCTATAAG   
  
  
- GGTAGTTCCG TATCTAATAC CTCACTCGTA CTTCTTACGA AACGTCCTCG ATCTCTGATG AGATTAACCA   
  
  
- CATCTACCAC TCCTTCATAG ATAACGATTA GTTGGATACC CCCCTTCAGC AATCAGGCCC TAGGGTTCAA   
  
  
- TCTCTAGCTT CAGTAGTTCG CTTCTAGGTG TCCCAAGTGT AGGATGAGTC GAACTAAGTA GTAGAAAAAG   
  
  
- TTCCCACTCT TCTAAGCCTC TACTTTCGGT CTCTCTCTCT TTACGGTATT CCGTTACCTT CTTGGTCGCC   
  
  
- TTGATGATTC AAAGAGTGGT CCACTAAATT TCGTCAATGA TTAACTTACA CGCTCCCGAA ATAATCTATT   
  
  
- GGCTTATCTA CTGAAACTCT CAAACCAATT TGCCCGTTCC TTTCTCCAGA GGTAGAGTCC ACTCGGACAG   
  
  
- GTTGGAGAGC CATGGACATA CTAGCTTCCC GAACACCGTT CCTTTGTCAG AAGCCCCTGA TTGTAGATAA   
  
  
- CCCGAGAATT CACATTTCTC GGAGAACCTT TTCTGAACGA GAGGATGCCG TGAGTCACCC AATGACATAG   
  
  
- CGTTCGGTAT CGTCGTTCCG AACCACCCGG AGGATTTCAC GCTGAATGTC CGTAACTACT AGGACAAAGA   
  
  
- TTCATATGAA CACTACGATC GAACCTCCGA CCACCCTTTG CTAATCGCAG AGATAAACTT TTCAAATTTT   
  
  
- ATGAACAGCT CAAGTTACGT AACGGGCAAA TACCTGGGTT ACAGTCCACC CTTTACGACC TACACTCCGG   
  
  
- GTCCCTCCGA AACCAGCAAT TAACAGGTAA TGTCGAGGTA ATGTGAGGAC TGCTCTCACA GCTACACTCC   
  
  
- TTGGGATCCC TATCCGAAGA GTCTTACCAC TTTAACGAGC CAGGATTCCA TCGGTGAAAC CAACTCGTTC   
  
  
- TTAGTTTGTG GTTGTGATGT GGAAAGAACT GGGCCAAGTA TCTCTGGGAA CTGATGATGA GCTAATTGGT   
  
  
- ACAAAC

+     TATC-box

| Site Name | Organism | Position | Strand | Matrix score. | sequence | function |
| --- | --- | --- | --- | --- | --- | --- |
| TATC-box | Oryza sativa | 857 | - | 7 | TATCCCA | cis-acting element involved in gibberellin-responsiveness |

>HU08G00230.1   
+ -Up\_Stream \_Len000GTTGTA ACAAGCACAT TAAAGTTGAT TATCATTTTG TCCGTGAAAC AGTGAGCAAA   
  
  
+ CGTCATCTCC AGGTCAAATT CATTCGTACT CAATCACAAG TTGCTGACAT TTTCACCAAA GGCTAATCAT   
  
  
+ CGCAAAAGTT TCTTTAGTTT AAGGCCAATC TCTCCATTAT TTCACCCTAT ACAGATTGAG GGGGTGTAAT   
  
  
+ AGTGTATATA TATAGCCTTG TGTTAGCGGG CCTTGGACAC TAGACTTTTA CTCTTGCAAA CCCTAATATA   
  
  
+ TTATTATAAA TATAAATCTA GCCACCCATG TTGAGGTAGG CACTCTAATT CTCACACAGG TAGGATAAGT   
  
  
+ TTTCATAGTT TATGTTGTGA ATGTGATCTT ACTTTGTGGT TCCCGTTGAC AAAAGTTTGA ATCCGAGCAT   
  
  
+ GAGAGTTCAT CAATCTCTTT TTCTTTCCTA TTTTATTTGT TGTGAACTTA ATTGGATTTT CTGGTAGAGG   
  
  
+ AAAGTTCCTT GCTGGAATTT GGTCAAAAGT GATATATATT TTGGGATTTA ATTATCCATA AATTTGGTTT   
  
  
+ TACACCTTGT TAGTTTGGAC TTCTGGTGTG TTATTGTATG TGTTCTGGTG GGTTTTTCTT TTAGCTGAAT   
  
  
+ GTTGGAGCAT TTTAAGGGAT CTAGATATCT GGAGAAGAGC TTGGGAAATA AAGCATATTT CCCTTATAGT   
  
  
+ CTGACATAAC TTTAATGAAT TGATCAGGAA CAAAGGCCAA TTAGTTCAAA AAGGTTCAAC TCCTCTAGGT   
  
  
+ GTTGTGCTAA AGAGATGTGA GTTAGTAAGT AATTGCTTAC TAACTATATA GGATGTTTTA TTACTTGTAG   
  
  
+ AGGTTTTTTT TTTGGGATAT CTTTATGGGG TTTATAAATA AGAAGGTTGA TTTGTTGTCT ACTCTTCTTT   
  
  
+ CGATTCAATA GTAATTCTCT CAGAGAAGGC TAAAATGACA TGGCATTCCA TCGCCTCTCA TGAGGCTTTT   
  
  
+ TGCATGTGCT TATGTATCTT TCATCTCTTG CTTTGGTGGG GTGGCATTGG GGAGGAAAAG GGGGTTTCGA   
  
  
+ TTCGATAGAT CGTCACTTTT GGAAATGACA TCTCTAGCCA ATGGCAAGGG TAAGGTTAAT ACATGTGGAT   
  
  
+ ATTTCCAGAG TTAGTTGTGA TTGTTATTGT TGTTGTTGTA ACTTTATGTC ACGGGGAAAA CATAATAACT   
  
  
+ ATCTGTTTAT CTGTTTGTTT GTTATTTTCA AGGGGAAGGG GAGTGGGCAG ATGCTTAGTA TGTAATCATT   
  
  
+ TTATATAGGG AAAATTCATA TAGCTTGAAA GTAAAACTTT CATTGCCAGC ATTCCAAGGT ATGGGTAGGG   
  
  
+ TTGTCTACAC CTTTATCTCC CTAGAAGCAT GGTGATCATT CCTCGGATTG TGTTATAGAA TAAAGAATGT   
  
  
+ GGTTCTGCTG TAATGAGACT TATGTATTAT CAAATGATGA ATCTGTTTAG AACTTCCTGT TTATTTTTTC   
  
  
+ TGCAAAATTT TAAGAGACTA ACATCTATCG GGCTCAACTC TAGAGGATCT CTTGTGTCAC CCTTCTCAAC   
  
  
+ TCAGTTTGAT TGTGATACAG TTACTATATT GAGTGATAGT CGAGAGCATC ACATCCCGTT AAGGAGTCTA   
  
  
+ TCAACAAGAA GCCCTTCTTG TAATTCTCCC CTTAAAACTA GCAGTTATCA TCACTTGTCA TCGAATAGCC   
  
  
+ CTTTTTGTAA TTCTACCCTT GAAACTAGCA GTTATCGTCA TCGGTTCAAC GCGAGTCCTG TTAGATATTC   
  
  
+ CCATCAAGGC ATAGATTATG GAGTGAGCAT GAAGAATGCT TTGCAGGAGC TAGAGACTAC TCTAATTGGT   
  
  
+ GTAGATGGTG AGGAAGTATC TATTGCTAAT CAACCTATGG GGGGAAGTCG TTAGTCCGGG ATCCCAAGTT   
  
  
+ AGAGATCGAA GTCATCAAGC GAAGATCCAC AGGGTTCACA TCCTACTCAG CTTGATTCAT CATCTTTTTC   
  
  
+ AAGGGTGAGA AGATTCGGAG ATGAAAGCCA GAGAGAGAGA AATGCCATAA GGCAATGGAA GAACCAGCGG   
  
  
+ AACTACTAAG TTTCTCACCA GGTGATTTAA AGCAGTTACT AATTGAATGT GCGAGGGCTT TATTAGATAA   
  
  
+ CCGAATAGAT GACTTTGAGA GTTTGGTTAA ACGGGCAAGG AAAGAGGTCT CCATCTCAGG TGAGCCTGTC   
  
  
+ CAACCTCTCG GTACCTGTAT GATCGAAGGG CTTGTGGCAA GGAAACAGTC TTCGGGGACT AACATCTATT   
  
  
+ GGGCTCTTAA GTGTAAAGAG CCTCTTGGAA AAGACTTGCT CTCCTACGGC ACTCAGTGGG TTACTGTATC   
  
  
+ GCAAGCCATA GCAGCAAGGC TTGGTGGGCC TCCTAAAGTG CGACTTACAG GCATTGATGA TCCTGTTTCT   
  
  
+ AAGTATACTT GTGATGCTAG CTTGGAGGCT GGTGGGAAAC GATTAGCGTC TCTATTTGAA AAGTTTAAAA   
  
  
+ TACTTGTCGA GTTCAATGCA TTGCCCGTTT ATGGACCCAA TGTCAGGTGG GAAATGCTGG ATGTGAGGCC   
  
  
+ CAGGGAGGCT TTGGTCGTTA ATTGTCCATT ACAGCTCCAT TACACTCCTG ACGAGAGTGT CGATGTGAGG   
  
  
+ AACCCTAGGG ATAGGCTTCT CAGAATGGTG AAATTGCTCG GTCCTAAGGT AGCCACTTTG GTTGAGCAAG   
  
  
+ AATCAAACAC CAACACTACA CCTTTCTTGA CCCGGTTCAT AGAGACCCTT GACTACTACT CGATTAACCA   
  
  
+ TGTTTG  

- -Up\_Stream \_Len000CAACAT TGTTCGTGTA ATTTCAACTA ATAGTAAAAC AGGCACTTTG TCACTCGTTT   
  
  
- GCAGTAGAGG TCCAGTTTAA GTAAGCATGA GTTAGTGTTC AACGACTGTA AAAGTGGTTT CCGATTAGTA   
  
  
- GCGTTTTCAA AGAAATCAAA TTCCGGTTAG AGAGGTAATA AAGTGGGATA TGTCTAACTC CCCCACATTA   
  
  
- TCACATATAT ATATCGGAAC ACAATCGCCC GGAACCTGTG ATCTGAAAAT GAGAACGTTT GGGATTATAT   
  
  
- AATAATATTT ATATTTAGAT CGGTGGGTAC AACTCCATCC GTGAGATTAA GAGTGTGTCC ATCCTATTCA   
  
  
- AAAGTATCAA ATACAACACT TACACTAGAA TGAAACACCA AGGGCAACTG TTTTCAAACT TAGGCTCGTA   
  
  
- CTCTCAAGTA GTTAGAGAAA AAGAAAGGAT AAAATAAACA ACACTTGAAT TAACCTAAAA GACCATCTCC   
  
  
- TTTCAAGGAA CGACCTTAAA CCAGTTTTCA CTATATATAA AACCCTAAAT TAATAGGTAT TTAAACCAAA   
  
  
- ATGTGGAACA ATCAAACCTG AAGACCACAC AATAACATAC ACAAGACCAC CCAAAAAGAA AATCGACTTA   
  
  
- CAACCTCGTA AAATTCCCTA GATCTATAGA CCTCTTCTCG AACCCTTTAT TTCGTATAAA GGGAATATCA   
  
  
- GACTGTATTG AAATTACTTA ACTAGTCCTT GTTTCCGGTT AATCAAGTTT TTCCAAGTTG AGGAGATCCA   
  
  
- CAACACGATT TCTCTACACT CAATCATTCA TTAACGAATG ATTGATATAT CCTACAAAAT AATGAACATC   
  
  
- TCCAAAAAAA AAACCCTATA GAAATACCCC AAATATTTAT TCTTCCAACT AAACAACAGA TGAGAAGAAA   
  
  
- GCTAAGTTAT CATTAAGAGA GTCTCTTCCG ATTTTACTGT ACCGTAAGGT AGCGGAGAGT ACTCCGAAAA   
  
  
- ACGTACACGA ATACATAGAA AGTAGAGAAC GAAACCACCC CACCGTAACC CCTCCTTTTC CCCCAAAGCT   
  
  
- AAGCTATCTA GCAGTGAAAA CCTTTACTGT AGAGATCGGT TACCGTTCCC ATTCCAATTA TGTACACCTA   
  
  
- TAAAGGTCTC AATCAACACT AACAATAACA ACAACAACAT TGAAATACAG TGCCCCTTTT GTATTATTGA   
  
  
- TAGACAAATA GACAAACAAA CAATAAAAGT TCCCCTTCCC CTCACCCGTC TACGAATCAT ACATTAGTAA   
  
  
- AATATATCCC TTTTAAGTAT ATCGAACTTT CATTTTGAAA GTAACGGTCG TAAGGTTCCA TACCCATCCC   
  
  
- AACAGATGTG GAAATAGAGG GATCTTCGTA CCACTAGTAA GGAGCCTAAC ACAATATCTT ATTTCTTACA   
  
  
- CCAAGACGAC ATTACTCTGA ATACATAATA GTTTACTACT TAGACAAATC TTGAAGGACA AATAAAAAAG   
  
  
- ACGTTTTAAA ATTCTCTGAT TGTAGATAGC CCGAGTTGAG ATCTCCTAGA GAACACAGTG GGAAGAGTTG   
  
  
- AGTCAAACTA ACACTATGTC AATGATATAA CTCACTATCA GCTCTCGTAG TGTAGGGCAA TTCCTCAGAT   
  
  
- AGTTGTTCTT CGGGAAGAAC ATTAAGAGGG GAATTTTGAT CGTCAATAGT AGTGAACAGT AGCTTATCGG   
  
  
- GAAAAACATT AAGATGGGAA CTTTGATCGT CAATAGCAGT AGCCAAGTTG CGCTCAGGAC AATCTATAAG   
  
  
- GGTAGTTCCG TATCTAATAC CTCACTCGTA CTTCTTACGA AACGTCCTCG ATCTCTGATG AGATTAACCA   
  
  
- CATCTACCAC TCCTTCATAG ATAACGATTA GTTGGATACC CCCCTTCAGC AATCAGGCCC TAGGGTTCAA   
  
  
- TCTCTAGCTT CAGTAGTTCG CTTCTAGGTG TCCCAAGTGT AGGATGAGTC GAACTAAGTA GTAGAAAAAG   
  
  
- TTCCCACTCT TCTAAGCCTC TACTTTCGGT CTCTCTCTCT TTACGGTATT CCGTTACCTT CTTGGTCGCC   
  
  
- TTGATGATTC AAAGAGTGGT CCACTAAATT TCGTCAATGA TTAACTTACA CGCTCCCGAA ATAATCTATT   
  
  
- GGCTTATCTA CTGAAACTCT CAAACCAATT TGCCCGTTCC TTTCTCCAGA GGTAGAGTCC ACTCGGACAG   
  
  
- GTTGGAGAGC CATGGACATA CTAGCTTCCC GAACACCGTT CCTTTGTCAG AAGCCCCTGA TTGTAGATAA   
  
  
- CCCGAGAATT CACATTTCTC GGAGAACCTT TTCTGAACGA GAGGATGCCG TGAGTCACCC AATGACATAG   
  
  
- CGTTCGGTAT CGTCGTTCCG AACCACCCGG AGGATTTCAC GCTGAATGTC CGTAACTACT AGGACAAAGA   
  
  
- TTCATATGAA CACTACGATC GAACCTCCGA CCACCCTTTG CTAATCGCAG AGATAAACTT TTCAAATTTT   
  
  
- ATGAACAGCT CAAGTTACGT AACGGGCAAA TACCTGGGTT ACAGTCCACC CTTTACGACC TACACTCCGG   
  
  
- GTCCCTCCGA AACCAGCAAT TAACAGGTAA TGTCGAGGTA ATGTGAGGAC TGCTCTCACA GCTACACTCC   
  
  
- TTGGGATCCC TATCCGAAGA GTCTTACCAC TTTAACGAGC CAGGATTCCA TCGGTGAAAC CAACTCGTTC   
  
  
- TTAGTTTGTG GTTGTGATGT GGAAAGAACT GGGCCAAGTA TCTCTGGGAA CTGATGATGA GCTAATTGGT   
  
  
- ACAAAC

+     TC-rich repeats

| Site Name | Organism | Position | Strand | Matrix score. | sequence | function |
| --- | --- | --- | --- | --- | --- | --- |
| TC-rich repeats | Nicotiana tabacum | 829 | + | 9 | GTTTTCTTAC | cis-acting element involved in defense and stress responsiveness |
| TC-rich repeats | Nicotiana tabacum | 1386 | - | 9 | ATTCTCTAAC | cis-acting element involved in defense and stress responsiveness |

>HU08G00230.1   
+ -Up\_Stream \_Len000GTTGTA ACAAGCACAT TAAAGTTGAT TATCATTTTG TCCGTGAAAC AGTGAGCAAA   
  
  
+ CGTCATCTCC AGGTCAAATT CATTCGTACT CAATCACAAG TTGCTGACAT TTTCACCAAA GGCTAATCAT   
  
  
+ CGCAAAAGTT TCTTTAGTTT AAGGCCAATC TCTCCATTAT TTCACCCTAT ACAGATTGAG GGGGTGTAAT   
  
  
+ AGTGTATATA TATAGCCTTG TGTTAGCGGG CCTTGGACAC TAGACTTTTA CTCTTGCAAA CCCTAATATA   
  
  
+ TTATTATAAA TATAAATCTA GCCACCCATG TTGAGGTAGG CACTCTAATT CTCACACAGG TAGGATAAGT   
  
  
+ TTTCATAGTT TATGTTGTGA ATGTGATCTT ACTTTGTGGT TCCCGTTGAC AAAAGTTTGA ATCCGAGCAT   
  
  
+ GAGAGTTCAT CAATCTCTTT TTCTTTCCTA TTTTATTTGT TGTGAACTTA ATTGGATTTT CTGGTAGAGG   
  
  
+ AAAGTTCCTT GCTGGAATTT GGTCAAAAGT GATATATATT TTGGGATTTA ATTATCCATA AATTTGGTTT   
  
  
+ TACACCTTGT TAGTTTGGAC TTCTGGTGTG TTATTGTATG TGTTCTGGTG GGTTTTTCTT TTAGCTGAAT   
  
  
+ GTTGGAGCAT TTTAAGGGAT CTAGATATCT GGAGAAGAGC TTGGGAAATA AAGCATATTT CCCTTATAGT   
  
  
+ CTGACATAAC TTTAATGAAT TGATCAGGAA CAAAGGCCAA TTAGTTCAAA AAGGTTCAAC TCCTCTAGGT   
  
  
+ GTTGTGCTAA AGAGATGTGA GTTAGTAAGT AATTGCTTAC TAACTATATA GGATGTTTTA TTACTTGTAG   
  
  
+ AGGTTTTTTT TTTGGGATAT CTTTATGGGG TTTATAAATA AGAAGGTTGA TTTGTTGTCT ACTCTTCTTT   
  
  
+ CGATTCAATA GTAATTCTCT CAGAGAAGGC TAAAATGACA TGGCATTCCA TCGCCTCTCA TGAGGCTTTT   
  
  
+ TGCATGTGCT TATGTATCTT TCATCTCTTG CTTTGGTGGG GTGGCATTGG GGAGGAAAAG GGGGTTTCGA   
  
  
+ TTCGATAGAT CGTCACTTTT GGAAATGACA TCTCTAGCCA ATGGCAAGGG TAAGGTTAAT ACATGTGGAT   
  
  
+ ATTTCCAGAG TTAGTTGTGA TTGTTATTGT TGTTGTTGTA ACTTTATGTC ACGGGGAAAA CATAATAACT   
  
  
+ ATCTGTTTAT CTGTTTGTTT GTTATTTTCA AGGGGAAGGG GAGTGGGCAG ATGCTTAGTA TGTAATCATT   
  
  
+ TTATATAGGG AAAATTCATA TAGCTTGAAA GTAAAACTTT CATTGCCAGC ATTCCAAGGT ATGGGTAGGG   
  
  
+ TTGTCTACAC CTTTATCTCC CTAGAAGCAT GGTGATCATT CCTCGGATTG TGTTATAGAA TAAAGAATGT   
  
  
+ GGTTCTGCTG TAATGAGACT TATGTATTAT CAAATGATGA ATCTGTTTAG AACTTCCTGT TTATTTTTTC   
  
  
+ TGCAAAATTT TAAGAGACTA ACATCTATCG GGCTCAACTC TAGAGGATCT CTTGTGTCAC CCTTCTCAAC   
  
  
+ TCAGTTTGAT TGTGATACAG TTACTATATT GAGTGATAGT CGAGAGCATC ACATCCCGTT AAGGAGTCTA   
  
  
+ TCAACAAGAA GCCCTTCTTG TAATTCTCCC CTTAAAACTA GCAGTTATCA TCACTTGTCA TCGAATAGCC   
  
  
+ CTTTTTGTAA TTCTACCCTT GAAACTAGCA GTTATCGTCA TCGGTTCAAC GCGAGTCCTG TTAGATATTC   
  
  
+ CCATCAAGGC ATAGATTATG GAGTGAGCAT GAAGAATGCT TTGCAGGAGC TAGAGACTAC TCTAATTGGT   
  
  
+ GTAGATGGTG AGGAAGTATC TATTGCTAAT CAACCTATGG GGGGAAGTCG TTAGTCCGGG ATCCCAAGTT   
  
  
+ AGAGATCGAA GTCATCAAGC GAAGATCCAC AGGGTTCACA TCCTACTCAG CTTGATTCAT CATCTTTTTC   
  
  
+ AAGGGTGAGA AGATTCGGAG ATGAAAGCCA GAGAGAGAGA AATGCCATAA GGCAATGGAA GAACCAGCGG   
  
  
+ AACTACTAAG TTTCTCACCA GGTGATTTAA AGCAGTTACT AATTGAATGT GCGAGGGCTT TATTAGATAA   
  
  
+ CCGAATAGAT GACTTTGAGA GTTTGGTTAA ACGGGCAAGG AAAGAGGTCT CCATCTCAGG TGAGCCTGTC   
  
  
+ CAACCTCTCG GTACCTGTAT GATCGAAGGG CTTGTGGCAA GGAAACAGTC TTCGGGGACT AACATCTATT   
  
  
+ GGGCTCTTAA GTGTAAAGAG CCTCTTGGAA AAGACTTGCT CTCCTACGGC ACTCAGTGGG TTACTGTATC   
  
  
+ GCAAGCCATA GCAGCAAGGC TTGGTGGGCC TCCTAAAGTG CGACTTACAG GCATTGATGA TCCTGTTTCT   
  
  
+ AAGTATACTT GTGATGCTAG CTTGGAGGCT GGTGGGAAAC GATTAGCGTC TCTATTTGAA AAGTTTAAAA   
  
  
+ TACTTGTCGA GTTCAATGCA TTGCCCGTTT ATGGACCCAA TGTCAGGTGG GAAATGCTGG ATGTGAGGCC   
  
  
+ CAGGGAGGCT TTGGTCGTTA ATTGTCCATT ACAGCTCCAT TACACTCCTG ACGAGAGTGT CGATGTGAGG   
  
  
+ AACCCTAGGG ATAGGCTTCT CAGAATGGTG AAATTGCTCG GTCCTAAGGT AGCCACTTTG GTTGAGCAAG   
  
  
+ AATCAAACAC CAACACTACA CCTTTCTTGA CCCGGTTCAT AGAGACCCTT GACTACTACT CGATTAACCA   
  
  
+ TGTTTG  

- -Up\_Stream \_Len000CAACAT TGTTCGTGTA ATTTCAACTA ATAGTAAAAC AGGCACTTTG TCACTCGTTT   
  
  
- GCAGTAGAGG TCCAGTTTAA GTAAGCATGA GTTAGTGTTC AACGACTGTA AAAGTGGTTT CCGATTAGTA   
  
  
- GCGTTTTCAA AGAAATCAAA TTCCGGTTAG AGAGGTAATA AAGTGGGATA TGTCTAACTC CCCCACATTA   
  
  
- TCACATATAT ATATCGGAAC ACAATCGCCC GGAACCTGTG ATCTGAAAAT GAGAACGTTT GGGATTATAT   
  
  
- AATAATATTT ATATTTAGAT CGGTGGGTAC AACTCCATCC GTGAGATTAA GAGTGTGTCC ATCCTATTCA   
  
  
- AAAGTATCAA ATACAACACT TACACTAGAA TGAAACACCA AGGGCAACTG TTTTCAAACT TAGGCTCGTA   
  
  
- CTCTCAAGTA GTTAGAGAAA AAGAAAGGAT AAAATAAACA ACACTTGAAT TAACCTAAAA GACCATCTCC   
  
  
- TTTCAAGGAA CGACCTTAAA CCAGTTTTCA CTATATATAA AACCCTAAAT TAATAGGTAT TTAAACCAAA   
  
  
- ATGTGGAACA ATCAAACCTG AAGACCACAC AATAACATAC ACAAGACCAC CCAAAAAGAA AATCGACTTA   
  
  
- CAACCTCGTA AAATTCCCTA GATCTATAGA CCTCTTCTCG AACCCTTTAT TTCGTATAAA GGGAATATCA   
  
  
- GACTGTATTG AAATTACTTA ACTAGTCCTT GTTTCCGGTT AATCAAGTTT TTCCAAGTTG AGGAGATCCA   
  
  
- CAACACGATT TCTCTACACT CAATCATTCA TTAACGAATG ATTGATATAT CCTACAAAAT AATGAACATC   
  
  
- TCCAAAAAAA AAACCCTATA GAAATACCCC AAATATTTAT TCTTCCAACT AAACAACAGA TGAGAAGAAA   
  
  
- GCTAAGTTAT CATTAAGAGA GTCTCTTCCG ATTTTACTGT ACCGTAAGGT AGCGGAGAGT ACTCCGAAAA   
  
  
- ACGTACACGA ATACATAGAA AGTAGAGAAC GAAACCACCC CACCGTAACC CCTCCTTTTC CCCCAAAGCT   
  
  
- AAGCTATCTA GCAGTGAAAA CCTTTACTGT AGAGATCGGT TACCGTTCCC ATTCCAATTA TGTACACCTA   
  
  
- TAAAGGTCTC AATCAACACT AACAATAACA ACAACAACAT TGAAATACAG TGCCCCTTTT GTATTATTGA   
  
  
- TAGACAAATA GACAAACAAA CAATAAAAGT TCCCCTTCCC CTCACCCGTC TACGAATCAT ACATTAGTAA   
  
  
- AATATATCCC TTTTAAGTAT ATCGAACTTT CATTTTGAAA GTAACGGTCG TAAGGTTCCA TACCCATCCC   
  
  
- AACAGATGTG GAAATAGAGG GATCTTCGTA CCACTAGTAA GGAGCCTAAC ACAATATCTT ATTTCTTACA   
  
  
- CCAAGACGAC ATTACTCTGA ATACATAATA GTTTACTACT TAGACAAATC TTGAAGGACA AATAAAAAAG   
  
  
- ACGTTTTAAA ATTCTCTGAT TGTAGATAGC CCGAGTTGAG ATCTCCTAGA GAACACAGTG GGAAGAGTTG   
  
  
- AGTCAAACTA ACACTATGTC AATGATATAA CTCACTATCA GCTCTCGTAG TGTAGGGCAA TTCCTCAGAT   
  
  
- AGTTGTTCTT CGGGAAGAAC ATTAAGAGGG GAATTTTGAT CGTCAATAGT AGTGAACAGT AGCTTATCGG   
  
  
- GAAAAACATT AAGATGGGAA CTTTGATCGT CAATAGCAGT AGCCAAGTTG CGCTCAGGAC AATCTATAAG   
  
  
- GGTAGTTCCG TATCTAATAC CTCACTCGTA CTTCTTACGA AACGTCCTCG ATCTCTGATG AGATTAACCA   
  
  
- CATCTACCAC TCCTTCATAG ATAACGATTA GTTGGATACC CCCCTTCAGC AATCAGGCCC TAGGGTTCAA   
  
  
- TCTCTAGCTT CAGTAGTTCG CTTCTAGGTG TCCCAAGTGT AGGATGAGTC GAACTAAGTA GTAGAAAAAG   
  
  
- TTCCCACTCT TCTAAGCCTC TACTTTCGGT CTCTCTCTCT TTACGGTATT CCGTTACCTT CTTGGTCGCC   
  
  
- TTGATGATTC AAAGAGTGGT CCACTAAATT TCGTCAATGA TTAACTTACA CGCTCCCGAA ATAATCTATT   
  
  
- GGCTTATCTA CTGAAACTCT CAAACCAATT TGCCCGTTCC TTTCTCCAGA GGTAGAGTCC ACTCGGACAG   
  
  
- GTTGGAGAGC CATGGACATA CTAGCTTCCC GAACACCGTT CCTTTGTCAG AAGCCCCTGA TTGTAGATAA   
  
  
- CCCGAGAATT CACATTTCTC GGAGAACCTT TTCTGAACGA GAGGATGCCG TGAGTCACCC AATGACATAG   
  
  
- CGTTCGGTAT CGTCGTTCCG AACCACCCGG AGGATTTCAC GCTGAATGTC CGTAACTACT AGGACAAAGA   
  
  
- TTCATATGAA CACTACGATC GAACCTCCGA CCACCCTTTG CTAATCGCAG AGATAAACTT TTCAAATTTT   
  
  
- ATGAACAGCT CAAGTTACGT AACGGGCAAA TACCTGGGTT ACAGTCCACC CTTTACGACC TACACTCCGG   
  
  
- GTCCCTCCGA AACCAGCAAT TAACAGGTAA TGTCGAGGTA ATGTGAGGAC TGCTCTCACA GCTACACTCC   
  
  
- TTGGGATCCC TATCCGAAGA GTCTTACCAC TTTAACGAGC CAGGATTCCA TCGGTGAAAC CAACTCGTTC   
  
  
- TTAGTTTGTG GTTGTGATGT GGAAAGAACT GGGCCAAGTA TCTCTGGGAA CTGATGATGA GCTAATTGGT   
  
  
- ACAAAC

+     TCA-element

| Site Name | Organism | Position | Strand | Matrix score. | sequence | function |
| --- | --- | --- | --- | --- | --- | --- |
| TCA-element | Nicotiana tabacum | 1954 | + | 9 | CCATCTTTTT | cis-acting element involved in salicylic acid responsiveness |

>HU08G00230.1   
+ -Up\_Stream \_Len000GTTGTA ACAAGCACAT TAAAGTTGAT TATCATTTTG TCCGTGAAAC AGTGAGCAAA   
  
  
+ CGTCATCTCC AGGTCAAATT CATTCGTACT CAATCACAAG TTGCTGACAT TTTCACCAAA GGCTAATCAT   
  
  
+ CGCAAAAGTT TCTTTAGTTT AAGGCCAATC TCTCCATTAT TTCACCCTAT ACAGATTGAG GGGGTGTAAT   
  
  
+ AGTGTATATA TATAGCCTTG TGTTAGCGGG CCTTGGACAC TAGACTTTTA CTCTTGCAAA CCCTAATATA   
  
  
+ TTATTATAAA TATAAATCTA GCCACCCATG TTGAGGTAGG CACTCTAATT CTCACACAGG TAGGATAAGT   
  
  
+ TTTCATAGTT TATGTTGTGA ATGTGATCTT ACTTTGTGGT TCCCGTTGAC AAAAGTTTGA ATCCGAGCAT   
  
  
+ GAGAGTTCAT CAATCTCTTT TTCTTTCCTA TTTTATTTGT TGTGAACTTA ATTGGATTTT CTGGTAGAGG   
  
  
+ AAAGTTCCTT GCTGGAATTT GGTCAAAAGT GATATATATT TTGGGATTTA ATTATCCATA AATTTGGTTT   
  
  
+ TACACCTTGT TAGTTTGGAC TTCTGGTGTG TTATTGTATG TGTTCTGGTG GGTTTTTCTT TTAGCTGAAT   
  
  
+ GTTGGAGCAT TTTAAGGGAT CTAGATATCT GGAGAAGAGC TTGGGAAATA AAGCATATTT CCCTTATAGT   
  
  
+ CTGACATAAC TTTAATGAAT TGATCAGGAA CAAAGGCCAA TTAGTTCAAA AAGGTTCAAC TCCTCTAGGT   
  
  
+ GTTGTGCTAA AGAGATGTGA GTTAGTAAGT AATTGCTTAC TAACTATATA GGATGTTTTA TTACTTGTAG   
  
  
+ AGGTTTTTTT TTTGGGATAT CTTTATGGGG TTTATAAATA AGAAGGTTGA TTTGTTGTCT ACTCTTCTTT   
  
  
+ CGATTCAATA GTAATTCTCT CAGAGAAGGC TAAAATGACA TGGCATTCCA TCGCCTCTCA TGAGGCTTTT   
  
  
+ TGCATGTGCT TATGTATCTT TCATCTCTTG CTTTGGTGGG GTGGCATTGG GGAGGAAAAG GGGGTTTCGA   
  
  
+ TTCGATAGAT CGTCACTTTT GGAAATGACA TCTCTAGCCA ATGGCAAGGG TAAGGTTAAT ACATGTGGAT   
  
  
+ ATTTCCAGAG TTAGTTGTGA TTGTTATTGT TGTTGTTGTA ACTTTATGTC ACGGGGAAAA CATAATAACT   
  
  
+ ATCTGTTTAT CTGTTTGTTT GTTATTTTCA AGGGGAAGGG GAGTGGGCAG ATGCTTAGTA TGTAATCATT   
  
  
+ TTATATAGGG AAAATTCATA TAGCTTGAAA GTAAAACTTT CATTGCCAGC ATTCCAAGGT ATGGGTAGGG   
  
  
+ TTGTCTACAC CTTTATCTCC CTAGAAGCAT GGTGATCATT CCTCGGATTG TGTTATAGAA TAAAGAATGT   
  
  
+ GGTTCTGCTG TAATGAGACT TATGTATTAT CAAATGATGA ATCTGTTTAG AACTTCCTGT TTATTTTTTC   
  
  
+ TGCAAAATTT TAAGAGACTA ACATCTATCG GGCTCAACTC TAGAGGATCT CTTGTGTCAC CCTTCTCAAC   
  
  
+ TCAGTTTGAT TGTGATACAG TTACTATATT GAGTGATAGT CGAGAGCATC ACATCCCGTT AAGGAGTCTA   
  
  
+ TCAACAAGAA GCCCTTCTTG TAATTCTCCC CTTAAAACTA GCAGTTATCA TCACTTGTCA TCGAATAGCC   
  
  
+ CTTTTTGTAA TTCTACCCTT GAAACTAGCA GTTATCGTCA TCGGTTCAAC GCGAGTCCTG TTAGATATTC   
  
  
+ CCATCAAGGC ATAGATTATG GAGTGAGCAT GAAGAATGCT TTGCAGGAGC TAGAGACTAC TCTAATTGGT   
  
  
+ GTAGATGGTG AGGAAGTATC TATTGCTAAT CAACCTATGG GGGGAAGTCG TTAGTCCGGG ATCCCAAGTT   
  
  
+ AGAGATCGAA GTCATCAAGC GAAGATCCAC AGGGTTCACA TCCTACTCAG CTTGATTCAT CATCTTTTTC   
  
  
+ AAGGGTGAGA AGATTCGGAG ATGAAAGCCA GAGAGAGAGA AATGCCATAA GGCAATGGAA GAACCAGCGG   
  
  
+ AACTACTAAG TTTCTCACCA GGTGATTTAA AGCAGTTACT AATTGAATGT GCGAGGGCTT TATTAGATAA   
  
  
+ CCGAATAGAT GACTTTGAGA GTTTGGTTAA ACGGGCAAGG AAAGAGGTCT CCATCTCAGG TGAGCCTGTC   
  
  
+ CAACCTCTCG GTACCTGTAT GATCGAAGGG CTTGTGGCAA GGAAACAGTC TTCGGGGACT AACATCTATT   
  
  
+ GGGCTCTTAA GTGTAAAGAG CCTCTTGGAA AAGACTTGCT CTCCTACGGC ACTCAGTGGG TTACTGTATC   
  
  
+ GCAAGCCATA GCAGCAAGGC TTGGTGGGCC TCCTAAAGTG CGACTTACAG GCATTGATGA TCCTGTTTCT   
  
  
+ AAGTATACTT GTGATGCTAG CTTGGAGGCT GGTGGGAAAC GATTAGCGTC TCTATTTGAA AAGTTTAAAA   
  
  
+ TACTTGTCGA GTTCAATGCA TTGCCCGTTT ATGGACCCAA TGTCAGGTGG GAAATGCTGG ATGTGAGGCC   
  
  
+ CAGGGAGGCT TTGGTCGTTA ATTGTCCATT ACAGCTCCAT TACACTCCTG ACGAGAGTGT CGATGTGAGG   
  
  
+ AACCCTAGGG ATAGGCTTCT CAGAATGGTG AAATTGCTCG GTCCTAAGGT AGCCACTTTG GTTGAGCAAG   
  
  
+ AATCAAACAC CAACACTACA CCTTTCTTGA CCCGGTTCAT AGAGACCCTT GACTACTACT CGATTAACCA   
  
  
+ TGTTTG  

- -Up\_Stream \_Len000CAACAT TGTTCGTGTA ATTTCAACTA ATAGTAAAAC AGGCACTTTG TCACTCGTTT   
  
  
- GCAGTAGAGG TCCAGTTTAA GTAAGCATGA GTTAGTGTTC AACGACTGTA AAAGTGGTTT CCGATTAGTA   
  
  
- GCGTTTTCAA AGAAATCAAA TTCCGGTTAG AGAGGTAATA AAGTGGGATA TGTCTAACTC CCCCACATTA   
  
  
- TCACATATAT ATATCGGAAC ACAATCGCCC GGAACCTGTG ATCTGAAAAT GAGAACGTTT GGGATTATAT   
  
  
- AATAATATTT ATATTTAGAT CGGTGGGTAC AACTCCATCC GTGAGATTAA GAGTGTGTCC ATCCTATTCA   
  
  
- AAAGTATCAA ATACAACACT TACACTAGAA TGAAACACCA AGGGCAACTG TTTTCAAACT TAGGCTCGTA   
  
  
- CTCTCAAGTA GTTAGAGAAA AAGAAAGGAT AAAATAAACA ACACTTGAAT TAACCTAAAA GACCATCTCC   
  
  
- TTTCAAGGAA CGACCTTAAA CCAGTTTTCA CTATATATAA AACCCTAAAT TAATAGGTAT TTAAACCAAA   
  
  
- ATGTGGAACA ATCAAACCTG AAGACCACAC AATAACATAC ACAAGACCAC CCAAAAAGAA AATCGACTTA   
  
  
- CAACCTCGTA AAATTCCCTA GATCTATAGA CCTCTTCTCG AACCCTTTAT TTCGTATAAA GGGAATATCA   
  
  
- GACTGTATTG AAATTACTTA ACTAGTCCTT GTTTCCGGTT AATCAAGTTT TTCCAAGTTG AGGAGATCCA   
  
  
- CAACACGATT TCTCTACACT CAATCATTCA TTAACGAATG ATTGATATAT CCTACAAAAT AATGAACATC   
  
  
- TCCAAAAAAA AAACCCTATA GAAATACCCC AAATATTTAT TCTTCCAACT AAACAACAGA TGAGAAGAAA   
  
  
- GCTAAGTTAT CATTAAGAGA GTCTCTTCCG ATTTTACTGT ACCGTAAGGT AGCGGAGAGT ACTCCGAAAA   
  
  
- ACGTACACGA ATACATAGAA AGTAGAGAAC GAAACCACCC CACCGTAACC CCTCCTTTTC CCCCAAAGCT   
  
  
- AAGCTATCTA GCAGTGAAAA CCTTTACTGT AGAGATCGGT TACCGTTCCC ATTCCAATTA TGTACACCTA   
  
  
- TAAAGGTCTC AATCAACACT AACAATAACA ACAACAACAT TGAAATACAG TGCCCCTTTT GTATTATTGA   
  
  
- TAGACAAATA GACAAACAAA CAATAAAAGT TCCCCTTCCC CTCACCCGTC TACGAATCAT ACATTAGTAA   
  
  
- AATATATCCC TTTTAAGTAT ATCGAACTTT CATTTTGAAA GTAACGGTCG TAAGGTTCCA TACCCATCCC   
  
  
- AACAGATGTG GAAATAGAGG GATCTTCGTA CCACTAGTAA GGAGCCTAAC ACAATATCTT ATTTCTTACA   
  
  
- CCAAGACGAC ATTACTCTGA ATACATAATA GTTTACTACT TAGACAAATC TTGAAGGACA AATAAAAAAG   
  
  
- ACGTTTTAAA ATTCTCTGAT TGTAGATAGC CCGAGTTGAG ATCTCCTAGA GAACACAGTG GGAAGAGTTG   
  
  
- AGTCAAACTA ACACTATGTC AATGATATAA CTCACTATCA GCTCTCGTAG TGTAGGGCAA TTCCTCAGAT   
  
  
- AGTTGTTCTT CGGGAAGAAC ATTAAGAGGG GAATTTTGAT CGTCAATAGT AGTGAACAGT AGCTTATCGG   
  
  
- GAAAAACATT AAGATGGGAA CTTTGATCGT CAATAGCAGT AGCCAAGTTG CGCTCAGGAC AATCTATAAG   
  
  
- GGTAGTTCCG TATCTAATAC CTCACTCGTA CTTCTTACGA AACGTCCTCG ATCTCTGATG AGATTAACCA   
  
  
- CATCTACCAC TCCTTCATAG ATAACGATTA GTTGGATACC CCCCTTCAGC AATCAGGCCC TAGGGTTCAA   
  
  
- TCTCTAGCTT CAGTAGTTCG CTTCTAGGTG TCCCAAGTGT AGGATGAGTC GAACTAAGTA GTAGAAAAAG   
  
  
- TTCCCACTCT TCTAAGCCTC TACTTTCGGT CTCTCTCTCT TTACGGTATT CCGTTACCTT CTTGGTCGCC   
  
  
- TTGATGATTC AAAGAGTGGT CCACTAAATT TCGTCAATGA TTAACTTACA CGCTCCCGAA ATAATCTATT   
  
  
- GGCTTATCTA CTGAAACTCT CAAACCAATT TGCCCGTTCC TTTCTCCAGA GGTAGAGTCC ACTCGGACAG   
  
  
- GTTGGAGAGC CATGGACATA CTAGCTTCCC GAACACCGTT CCTTTGTCAG AAGCCCCTGA TTGTAGATAA   
  
  
- CCCGAGAATT CACATTTCTC GGAGAACCTT TTCTGAACGA GAGGATGCCG TGAGTCACCC AATGACATAG   
  
  
- CGTTCGGTAT CGTCGTTCCG AACCACCCGG AGGATTTCAC GCTGAATGTC CGTAACTACT AGGACAAAGA   
  
  
- TTCATATGAA CACTACGATC GAACCTCCGA CCACCCTTTG CTAATCGCAG AGATAAACTT TTCAAATTTT   
  
  
- ATGAACAGCT CAAGTTACGT AACGGGCAAA TACCTGGGTT ACAGTCCACC CTTTACGACC TACACTCCGG   
  
  
- GTCCCTCCGA AACCAGCAAT TAACAGGTAA TGTCGAGGTA ATGTGAGGAC TGCTCTCACA GCTACACTCC   
  
  
- TTGGGATCCC TATCCGAAGA GTCTTACCAC TTTAACGAGC CAGGATTCCA TCGGTGAAAC CAACTCGTTC   
  
  
- TTAGTTTGTG GTTGTGATGT GGAAAGAACT GGGCCAAGTA TCTCTGGGAA CTGATGATGA GCTAATTGGT   
  
  
- ACAAAC

+     TCCC-motif

| Site Name | Organism | Position | Strand | Matrix score. | sequence | function |
| --- | --- | --- | --- | --- | --- | --- |
| TCCC-motif | Spinacia oleracea | 1350 | + | 7 | TCTCCCT | part of a light responsive element |

>HU08G00230.1   
+ -Up\_Stream \_Len000GTTGTA ACAAGCACAT TAAAGTTGAT TATCATTTTG TCCGTGAAAC AGTGAGCAAA   
  
  
+ CGTCATCTCC AGGTCAAATT CATTCGTACT CAATCACAAG TTGCTGACAT TTTCACCAAA GGCTAATCAT   
  
  
+ CGCAAAAGTT TCTTTAGTTT AAGGCCAATC TCTCCATTAT TTCACCCTAT ACAGATTGAG GGGGTGTAAT   
  
  
+ AGTGTATATA TATAGCCTTG TGTTAGCGGG CCTTGGACAC TAGACTTTTA CTCTTGCAAA CCCTAATATA   
  
  
+ TTATTATAAA TATAAATCTA GCCACCCATG TTGAGGTAGG CACTCTAATT CTCACACAGG TAGGATAAGT   
  
  
+ TTTCATAGTT TATGTTGTGA ATGTGATCTT ACTTTGTGGT TCCCGTTGAC AAAAGTTTGA ATCCGAGCAT   
  
  
+ GAGAGTTCAT CAATCTCTTT TTCTTTCCTA TTTTATTTGT TGTGAACTTA ATTGGATTTT CTGGTAGAGG   
  
  
+ AAAGTTCCTT GCTGGAATTT GGTCAAAAGT GATATATATT TTGGGATTTA ATTATCCATA AATTTGGTTT   
  
  
+ TACACCTTGT TAGTTTGGAC TTCTGGTGTG TTATTGTATG TGTTCTGGTG GGTTTTTCTT TTAGCTGAAT   
  
  
+ GTTGGAGCAT TTTAAGGGAT CTAGATATCT GGAGAAGAGC TTGGGAAATA AAGCATATTT CCCTTATAGT   
  
  
+ CTGACATAAC TTTAATGAAT TGATCAGGAA CAAAGGCCAA TTAGTTCAAA AAGGTTCAAC TCCTCTAGGT   
  
  
+ GTTGTGCTAA AGAGATGTGA GTTAGTAAGT AATTGCTTAC TAACTATATA GGATGTTTTA TTACTTGTAG   
  
  
+ AGGTTTTTTT TTTGGGATAT CTTTATGGGG TTTATAAATA AGAAGGTTGA TTTGTTGTCT ACTCTTCTTT   
  
  
+ CGATTCAATA GTAATTCTCT CAGAGAAGGC TAAAATGACA TGGCATTCCA TCGCCTCTCA TGAGGCTTTT   
  
  
+ TGCATGTGCT TATGTATCTT TCATCTCTTG CTTTGGTGGG GTGGCATTGG GGAGGAAAAG GGGGTTTCGA   
  
  
+ TTCGATAGAT CGTCACTTTT GGAAATGACA TCTCTAGCCA ATGGCAAGGG TAAGGTTAAT ACATGTGGAT   
  
  
+ ATTTCCAGAG TTAGTTGTGA TTGTTATTGT TGTTGTTGTA ACTTTATGTC ACGGGGAAAA CATAATAACT   
  
  
+ ATCTGTTTAT CTGTTTGTTT GTTATTTTCA AGGGGAAGGG GAGTGGGCAG ATGCTTAGTA TGTAATCATT   
  
  
+ TTATATAGGG AAAATTCATA TAGCTTGAAA GTAAAACTTT CATTGCCAGC ATTCCAAGGT ATGGGTAGGG   
  
  
+ TTGTCTACAC CTTTATCTCC CTAGAAGCAT GGTGATCATT CCTCGGATTG TGTTATAGAA TAAAGAATGT   
  
  
+ GGTTCTGCTG TAATGAGACT TATGTATTAT CAAATGATGA ATCTGTTTAG AACTTCCTGT TTATTTTTTC   
  
  
+ TGCAAAATTT TAAGAGACTA ACATCTATCG GGCTCAACTC TAGAGGATCT CTTGTGTCAC CCTTCTCAAC   
  
  
+ TCAGTTTGAT TGTGATACAG TTACTATATT GAGTGATAGT CGAGAGCATC ACATCCCGTT AAGGAGTCTA   
  
  
+ TCAACAAGAA GCCCTTCTTG TAATTCTCCC CTTAAAACTA GCAGTTATCA TCACTTGTCA TCGAATAGCC   
  
  
+ CTTTTTGTAA TTCTACCCTT GAAACTAGCA GTTATCGTCA TCGGTTCAAC GCGAGTCCTG TTAGATATTC   
  
  
+ CCATCAAGGC ATAGATTATG GAGTGAGCAT GAAGAATGCT TTGCAGGAGC TAGAGACTAC TCTAATTGGT   
  
  
+ GTAGATGGTG AGGAAGTATC TATTGCTAAT CAACCTATGG GGGGAAGTCG TTAGTCCGGG ATCCCAAGTT   
  
  
+ AGAGATCGAA GTCATCAAGC GAAGATCCAC AGGGTTCACA TCCTACTCAG CTTGATTCAT CATCTTTTTC   
  
  
+ AAGGGTGAGA AGATTCGGAG ATGAAAGCCA GAGAGAGAGA AATGCCATAA GGCAATGGAA GAACCAGCGG   
  
  
+ AACTACTAAG TTTCTCACCA GGTGATTTAA AGCAGTTACT AATTGAATGT GCGAGGGCTT TATTAGATAA   
  
  
+ CCGAATAGAT GACTTTGAGA GTTTGGTTAA ACGGGCAAGG AAAGAGGTCT CCATCTCAGG TGAGCCTGTC   
  
  
+ CAACCTCTCG GTACCTGTAT GATCGAAGGG CTTGTGGCAA GGAAACAGTC TTCGGGGACT AACATCTATT   
  
  
+ GGGCTCTTAA GTGTAAAGAG CCTCTTGGAA AAGACTTGCT CTCCTACGGC ACTCAGTGGG TTACTGTATC   
  
  
+ GCAAGCCATA GCAGCAAGGC TTGGTGGGCC TCCTAAAGTG CGACTTACAG GCATTGATGA TCCTGTTTCT   
  
  
+ AAGTATACTT GTGATGCTAG CTTGGAGGCT GGTGGGAAAC GATTAGCGTC TCTATTTGAA AAGTTTAAAA   
  
  
+ TACTTGTCGA GTTCAATGCA TTGCCCGTTT ATGGACCCAA TGTCAGGTGG GAAATGCTGG ATGTGAGGCC   
  
  
+ CAGGGAGGCT TTGGTCGTTA ATTGTCCATT ACAGCTCCAT TACACTCCTG ACGAGAGTGT CGATGTGAGG   
  
  
+ AACCCTAGGG ATAGGCTTCT CAGAATGGTG AAATTGCTCG GTCCTAAGGT AGCCACTTTG GTTGAGCAAG   
  
  
+ AATCAAACAC CAACACTACA CCTTTCTTGA CCCGGTTCAT AGAGACCCTT GACTACTACT CGATTAACCA   
  
  
+ TGTTTG  

- -Up\_Stream \_Len000CAACAT TGTTCGTGTA ATTTCAACTA ATAGTAAAAC AGGCACTTTG TCACTCGTTT   
  
  
- GCAGTAGAGG TCCAGTTTAA GTAAGCATGA GTTAGTGTTC AACGACTGTA AAAGTGGTTT CCGATTAGTA   
  
  
- GCGTTTTCAA AGAAATCAAA TTCCGGTTAG AGAGGTAATA AAGTGGGATA TGTCTAACTC CCCCACATTA   
  
  
- TCACATATAT ATATCGGAAC ACAATCGCCC GGAACCTGTG ATCTGAAAAT GAGAACGTTT GGGATTATAT   
  
  
- AATAATATTT ATATTTAGAT CGGTGGGTAC AACTCCATCC GTGAGATTAA GAGTGTGTCC ATCCTATTCA   
  
  
- AAAGTATCAA ATACAACACT TACACTAGAA TGAAACACCA AGGGCAACTG TTTTCAAACT TAGGCTCGTA   
  
  
- CTCTCAAGTA GTTAGAGAAA AAGAAAGGAT AAAATAAACA ACACTTGAAT TAACCTAAAA GACCATCTCC   
  
  
- TTTCAAGGAA CGACCTTAAA CCAGTTTTCA CTATATATAA AACCCTAAAT TAATAGGTAT TTAAACCAAA   
  
  
- ATGTGGAACA ATCAAACCTG AAGACCACAC AATAACATAC ACAAGACCAC CCAAAAAGAA AATCGACTTA   
  
  
- CAACCTCGTA AAATTCCCTA GATCTATAGA CCTCTTCTCG AACCCTTTAT TTCGTATAAA GGGAATATCA   
  
  
- GACTGTATTG AAATTACTTA ACTAGTCCTT GTTTCCGGTT AATCAAGTTT TTCCAAGTTG AGGAGATCCA   
  
  
- CAACACGATT TCTCTACACT CAATCATTCA TTAACGAATG ATTGATATAT CCTACAAAAT AATGAACATC   
  
  
- TCCAAAAAAA AAACCCTATA GAAATACCCC AAATATTTAT TCTTCCAACT AAACAACAGA TGAGAAGAAA   
  
  
- GCTAAGTTAT CATTAAGAGA GTCTCTTCCG ATTTTACTGT ACCGTAAGGT AGCGGAGAGT ACTCCGAAAA   
  
  
- ACGTACACGA ATACATAGAA AGTAGAGAAC GAAACCACCC CACCGTAACC CCTCCTTTTC CCCCAAAGCT   
  
  
- AAGCTATCTA GCAGTGAAAA CCTTTACTGT AGAGATCGGT TACCGTTCCC ATTCCAATTA TGTACACCTA   
  
  
- TAAAGGTCTC AATCAACACT AACAATAACA ACAACAACAT TGAAATACAG TGCCCCTTTT GTATTATTGA   
  
  
- TAGACAAATA GACAAACAAA CAATAAAAGT TCCCCTTCCC CTCACCCGTC TACGAATCAT ACATTAGTAA   
  
  
- AATATATCCC TTTTAAGTAT ATCGAACTTT CATTTTGAAA GTAACGGTCG TAAGGTTCCA TACCCATCCC   
  
  
- AACAGATGTG GAAATAGAGG GATCTTCGTA CCACTAGTAA GGAGCCTAAC ACAATATCTT ATTTCTTACA   
  
  
- CCAAGACGAC ATTACTCTGA ATACATAATA GTTTACTACT TAGACAAATC TTGAAGGACA AATAAAAAAG   
  
  
- ACGTTTTAAA ATTCTCTGAT TGTAGATAGC CCGAGTTGAG ATCTCCTAGA GAACACAGTG GGAAGAGTTG   
  
  
- AGTCAAACTA ACACTATGTC AATGATATAA CTCACTATCA GCTCTCGTAG TGTAGGGCAA TTCCTCAGAT   
  
  
- AGTTGTTCTT CGGGAAGAAC ATTAAGAGGG GAATTTTGAT CGTCAATAGT AGTGAACAGT AGCTTATCGG   
  
  
- GAAAAACATT AAGATGGGAA CTTTGATCGT CAATAGCAGT AGCCAAGTTG CGCTCAGGAC AATCTATAAG   
  
  
- GGTAGTTCCG TATCTAATAC CTCACTCGTA CTTCTTACGA AACGTCCTCG ATCTCTGATG AGATTAACCA   
  
  
- CATCTACCAC TCCTTCATAG ATAACGATTA GTTGGATACC CCCCTTCAGC AATCAGGCCC TAGGGTTCAA   
  
  
- TCTCTAGCTT CAGTAGTTCG CTTCTAGGTG TCCCAAGTGT AGGATGAGTC GAACTAAGTA GTAGAAAAAG   
  
  
- TTCCCACTCT TCTAAGCCTC TACTTTCGGT CTCTCTCTCT TTACGGTATT CCGTTACCTT CTTGGTCGCC   
  
  
- TTGATGATTC AAAGAGTGGT CCACTAAATT TCGTCAATGA TTAACTTACA CGCTCCCGAA ATAATCTATT   
  
  
- GGCTTATCTA CTGAAACTCT CAAACCAATT TGCCCGTTCC TTTCTCCAGA GGTAGAGTCC ACTCGGACAG   
  
  
- GTTGGAGAGC CATGGACATA CTAGCTTCCC GAACACCGTT CCTTTGTCAG AAGCCCCTGA TTGTAGATAA   
  
  
- CCCGAGAATT CACATTTCTC GGAGAACCTT TTCTGAACGA GAGGATGCCG TGAGTCACCC AATGACATAG   
  
  
- CGTTCGGTAT CGTCGTTCCG AACCACCCGG AGGATTTCAC GCTGAATGTC CGTAACTACT AGGACAAAGA   
  
  
- TTCATATGAA CACTACGATC GAACCTCCGA CCACCCTTTG CTAATCGCAG AGATAAACTT TTCAAATTTT   
  
  
- ATGAACAGCT CAAGTTACGT AACGGGCAAA TACCTGGGTT ACAGTCCACC CTTTACGACC TACACTCCGG   
  
  
- GTCCCTCCGA AACCAGCAAT TAACAGGTAA TGTCGAGGTA ATGTGAGGAC TGCTCTCACA GCTACACTCC   
  
  
- TTGGGATCCC TATCCGAAGA GTCTTACCAC TTTAACGAGC CAGGATTCCA TCGGTGAAAC CAACTCGTTC   
  
  
- TTAGTTTGTG GTTGTGATGT GGAAAGAACT GGGCCAAGTA TCTCTGGGAA CTGATGATGA GCTAATTGGT   
  
  
- ACAAAC

+     TCT-motif

| Site Name | Organism | Position | Strand | Matrix score. | sequence | function |
| --- | --- | --- | --- | --- | --- | --- |
| TCT-motif | Arabidopsis thaliana | 381 | + | 6 | TCTTAC | part of a light responsive element |

>HU08G00230.1   
+ -Up\_Stream \_Len000GTTGTA ACAAGCACAT TAAAGTTGAT TATCATTTTG TCCGTGAAAC AGTGAGCAAA   
  
  
+ CGTCATCTCC AGGTCAAATT CATTCGTACT CAATCACAAG TTGCTGACAT TTTCACCAAA GGCTAATCAT   
  
  
+ CGCAAAAGTT TCTTTAGTTT AAGGCCAATC TCTCCATTAT TTCACCCTAT ACAGATTGAG GGGGTGTAAT   
  
  
+ AGTGTATATA TATAGCCTTG TGTTAGCGGG CCTTGGACAC TAGACTTTTA CTCTTGCAAA CCCTAATATA   
  
  
+ TTATTATAAA TATAAATCTA GCCACCCATG TTGAGGTAGG CACTCTAATT CTCACACAGG TAGGATAAGT   
  
  
+ TTTCATAGTT TATGTTGTGA ATGTGATCTT ACTTTGTGGT TCCCGTTGAC AAAAGTTTGA ATCCGAGCAT   
  
  
+ GAGAGTTCAT CAATCTCTTT TTCTTTCCTA TTTTATTTGT TGTGAACTTA ATTGGATTTT CTGGTAGAGG   
  
  
+ AAAGTTCCTT GCTGGAATTT GGTCAAAAGT GATATATATT TTGGGATTTA ATTATCCATA AATTTGGTTT   
  
  
+ TACACCTTGT TAGTTTGGAC TTCTGGTGTG TTATTGTATG TGTTCTGGTG GGTTTTTCTT TTAGCTGAAT   
  
  
+ GTTGGAGCAT TTTAAGGGAT CTAGATATCT GGAGAAGAGC TTGGGAAATA AAGCATATTT CCCTTATAGT   
  
  
+ CTGACATAAC TTTAATGAAT TGATCAGGAA CAAAGGCCAA TTAGTTCAAA AAGGTTCAAC TCCTCTAGGT   
  
  
+ GTTGTGCTAA AGAGATGTGA GTTAGTAAGT AATTGCTTAC TAACTATATA GGATGTTTTA TTACTTGTAG   
  
  
+ AGGTTTTTTT TTTGGGATAT CTTTATGGGG TTTATAAATA AGAAGGTTGA TTTGTTGTCT ACTCTTCTTT   
  
  
+ CGATTCAATA GTAATTCTCT CAGAGAAGGC TAAAATGACA TGGCATTCCA TCGCCTCTCA TGAGGCTTTT   
  
  
+ TGCATGTGCT TATGTATCTT TCATCTCTTG CTTTGGTGGG GTGGCATTGG GGAGGAAAAG GGGGTTTCGA   
  
  
+ TTCGATAGAT CGTCACTTTT GGAAATGACA TCTCTAGCCA ATGGCAAGGG TAAGGTTAAT ACATGTGGAT   
  
  
+ ATTTCCAGAG TTAGTTGTGA TTGTTATTGT TGTTGTTGTA ACTTTATGTC ACGGGGAAAA CATAATAACT   
  
  
+ ATCTGTTTAT CTGTTTGTTT GTTATTTTCA AGGGGAAGGG GAGTGGGCAG ATGCTTAGTA TGTAATCATT   
  
  
+ TTATATAGGG AAAATTCATA TAGCTTGAAA GTAAAACTTT CATTGCCAGC ATTCCAAGGT ATGGGTAGGG   
  
  
+ TTGTCTACAC CTTTATCTCC CTAGAAGCAT GGTGATCATT CCTCGGATTG TGTTATAGAA TAAAGAATGT   
  
  
+ GGTTCTGCTG TAATGAGACT TATGTATTAT CAAATGATGA ATCTGTTTAG AACTTCCTGT TTATTTTTTC   
  
  
+ TGCAAAATTT TAAGAGACTA ACATCTATCG GGCTCAACTC TAGAGGATCT CTTGTGTCAC CCTTCTCAAC   
  
  
+ TCAGTTTGAT TGTGATACAG TTACTATATT GAGTGATAGT CGAGAGCATC ACATCCCGTT AAGGAGTCTA   
  
  
+ TCAACAAGAA GCCCTTCTTG TAATTCTCCC CTTAAAACTA GCAGTTATCA TCACTTGTCA TCGAATAGCC   
  
  
+ CTTTTTGTAA TTCTACCCTT GAAACTAGCA GTTATCGTCA TCGGTTCAAC GCGAGTCCTG TTAGATATTC   
  
  
+ CCATCAAGGC ATAGATTATG GAGTGAGCAT GAAGAATGCT TTGCAGGAGC TAGAGACTAC TCTAATTGGT   
  
  
+ GTAGATGGTG AGGAAGTATC TATTGCTAAT CAACCTATGG GGGGAAGTCG TTAGTCCGGG ATCCCAAGTT   
  
  
+ AGAGATCGAA GTCATCAAGC GAAGATCCAC AGGGTTCACA TCCTACTCAG CTTGATTCAT CATCTTTTTC   
  
  
+ AAGGGTGAGA AGATTCGGAG ATGAAAGCCA GAGAGAGAGA AATGCCATAA GGCAATGGAA GAACCAGCGG   
  
  
+ AACTACTAAG TTTCTCACCA GGTGATTTAA AGCAGTTACT AATTGAATGT GCGAGGGCTT TATTAGATAA   
  
  
+ CCGAATAGAT GACTTTGAGA GTTTGGTTAA ACGGGCAAGG AAAGAGGTCT CCATCTCAGG TGAGCCTGTC   
  
  
+ CAACCTCTCG GTACCTGTAT GATCGAAGGG CTTGTGGCAA GGAAACAGTC TTCGGGGACT AACATCTATT   
  
  
+ GGGCTCTTAA GTGTAAAGAG CCTCTTGGAA AAGACTTGCT CTCCTACGGC ACTCAGTGGG TTACTGTATC   
  
  
+ GCAAGCCATA GCAGCAAGGC TTGGTGGGCC TCCTAAAGTG CGACTTACAG GCATTGATGA TCCTGTTTCT   
  
  
+ AAGTATACTT GTGATGCTAG CTTGGAGGCT GGTGGGAAAC GATTAGCGTC TCTATTTGAA AAGTTTAAAA   
  
  
+ TACTTGTCGA GTTCAATGCA TTGCCCGTTT ATGGACCCAA TGTCAGGTGG GAAATGCTGG ATGTGAGGCC   
  
  
+ CAGGGAGGCT TTGGTCGTTA ATTGTCCATT ACAGCTCCAT TACACTCCTG ACGAGAGTGT CGATGTGAGG   
  
  
+ AACCCTAGGG ATAGGCTTCT CAGAATGGTG AAATTGCTCG GTCCTAAGGT AGCCACTTTG GTTGAGCAAG   
  
  
+ AATCAAACAC CAACACTACA CCTTTCTTGA CCCGGTTCAT AGAGACCCTT GACTACTACT CGATTAACCA   
  
  
+ TGTTTG  

- -Up\_Stream \_Len000CAACAT TGTTCGTGTA ATTTCAACTA ATAGTAAAAC AGGCACTTTG TCACTCGTTT   
  
  
- GCAGTAGAGG TCCAGTTTAA GTAAGCATGA GTTAGTGTTC AACGACTGTA AAAGTGGTTT CCGATTAGTA   
  
  
- GCGTTTTCAA AGAAATCAAA TTCCGGTTAG AGAGGTAATA AAGTGGGATA TGTCTAACTC CCCCACATTA   
  
  
- TCACATATAT ATATCGGAAC ACAATCGCCC GGAACCTGTG ATCTGAAAAT GAGAACGTTT GGGATTATAT   
  
  
- AATAATATTT ATATTTAGAT CGGTGGGTAC AACTCCATCC GTGAGATTAA GAGTGTGTCC ATCCTATTCA   
  
  
- AAAGTATCAA ATACAACACT TACACTAGAA TGAAACACCA AGGGCAACTG TTTTCAAACT TAGGCTCGTA   
  
  
- CTCTCAAGTA GTTAGAGAAA AAGAAAGGAT AAAATAAACA ACACTTGAAT TAACCTAAAA GACCATCTCC   
  
  
- TTTCAAGGAA CGACCTTAAA CCAGTTTTCA CTATATATAA AACCCTAAAT TAATAGGTAT TTAAACCAAA   
  
  
- ATGTGGAACA ATCAAACCTG AAGACCACAC AATAACATAC ACAAGACCAC CCAAAAAGAA AATCGACTTA   
  
  
- CAACCTCGTA AAATTCCCTA GATCTATAGA CCTCTTCTCG AACCCTTTAT TTCGTATAAA GGGAATATCA   
  
  
- GACTGTATTG AAATTACTTA ACTAGTCCTT GTTTCCGGTT AATCAAGTTT TTCCAAGTTG AGGAGATCCA   
  
  
- CAACACGATT TCTCTACACT CAATCATTCA TTAACGAATG ATTGATATAT CCTACAAAAT AATGAACATC   
  
  
- TCCAAAAAAA AAACCCTATA GAAATACCCC AAATATTTAT TCTTCCAACT AAACAACAGA TGAGAAGAAA   
  
  
- GCTAAGTTAT CATTAAGAGA GTCTCTTCCG ATTTTACTGT ACCGTAAGGT AGCGGAGAGT ACTCCGAAAA   
  
  
- ACGTACACGA ATACATAGAA AGTAGAGAAC GAAACCACCC CACCGTAACC CCTCCTTTTC CCCCAAAGCT   
  
  
- AAGCTATCTA GCAGTGAAAA CCTTTACTGT AGAGATCGGT TACCGTTCCC ATTCCAATTA TGTACACCTA   
  
  
- TAAAGGTCTC AATCAACACT AACAATAACA ACAACAACAT TGAAATACAG TGCCCCTTTT GTATTATTGA   
  
  
- TAGACAAATA GACAAACAAA CAATAAAAGT TCCCCTTCCC CTCACCCGTC TACGAATCAT ACATTAGTAA   
  
  
- AATATATCCC TTTTAAGTAT ATCGAACTTT CATTTTGAAA GTAACGGTCG TAAGGTTCCA TACCCATCCC   
  
  
- AACAGATGTG GAAATAGAGG GATCTTCGTA CCACTAGTAA GGAGCCTAAC ACAATATCTT ATTTCTTACA   
  
  
- CCAAGACGAC ATTACTCTGA ATACATAATA GTTTACTACT TAGACAAATC TTGAAGGACA AATAAAAAAG   
  
  
- ACGTTTTAAA ATTCTCTGAT TGTAGATAGC CCGAGTTGAG ATCTCCTAGA GAACACAGTG GGAAGAGTTG   
  
  
- AGTCAAACTA ACACTATGTC AATGATATAA CTCACTATCA GCTCTCGTAG TGTAGGGCAA TTCCTCAGAT   
  
  
- AGTTGTTCTT CGGGAAGAAC ATTAAGAGGG GAATTTTGAT CGTCAATAGT AGTGAACAGT AGCTTATCGG   
  
  
- GAAAAACATT AAGATGGGAA CTTTGATCGT CAATAGCAGT AGCCAAGTTG CGCTCAGGAC AATCTATAAG   
  
  
- GGTAGTTCCG TATCTAATAC CTCACTCGTA CTTCTTACGA AACGTCCTCG ATCTCTGATG AGATTAACCA   
  
  
- CATCTACCAC TCCTTCATAG ATAACGATTA GTTGGATACC CCCCTTCAGC AATCAGGCCC TAGGGTTCAA   
  
  
- TCTCTAGCTT CAGTAGTTCG CTTCTAGGTG TCCCAAGTGT AGGATGAGTC GAACTAAGTA GTAGAAAAAG   
  
  
- TTCCCACTCT TCTAAGCCTC TACTTTCGGT CTCTCTCTCT TTACGGTATT CCGTTACCTT CTTGGTCGCC   
  
  
- TTGATGATTC AAAGAGTGGT CCACTAAATT TCGTCAATGA TTAACTTACA CGCTCCCGAA ATAATCTATT   
  
  
- GGCTTATCTA CTGAAACTCT CAAACCAATT TGCCCGTTCC TTTCTCCAGA GGTAGAGTCC ACTCGGACAG   
  
  
- GTTGGAGAGC CATGGACATA CTAGCTTCCC GAACACCGTT CCTTTGTCAG AAGCCCCTGA TTGTAGATAA   
  
  
- CCCGAGAATT CACATTTCTC GGAGAACCTT TTCTGAACGA GAGGATGCCG TGAGTCACCC AATGACATAG   
  
  
- CGTTCGGTAT CGTCGTTCCG AACCACCCGG AGGATTTCAC GCTGAATGTC CGTAACTACT AGGACAAAGA   
  
  
- TTCATATGAA CACTACGATC GAACCTCCGA CCACCCTTTG CTAATCGCAG AGATAAACTT TTCAAATTTT   
  
  
- ATGAACAGCT CAAGTTACGT AACGGGCAAA TACCTGGGTT ACAGTCCACC CTTTACGACC TACACTCCGG   
  
  
- GTCCCTCCGA AACCAGCAAT TAACAGGTAA TGTCGAGGTA ATGTGAGGAC TGCTCTCACA GCTACACTCC   
  
  
- TTGGGATCCC TATCCGAAGA GTCTTACCAC TTTAACGAGC CAGGATTCCA TCGGTGAAAC CAACTCGTTC   
  
  
- TTAGTTTGTG GTTGTGATGT GGAAAGAACT GGGCCAAGTA TCTCTGGGAA CTGATGATGA GCTAATTGGT   
  
  
- ACAAAC

+     TGA-element

| Site Name | Organism | Position | Strand | Matrix score. | sequence | function |
| --- | --- | --- | --- | --- | --- | --- |
| TGA-element | Brassica oleracea | 2538 | - | 6 | AACGAC | auxin-responsive element |
| TGA-element | Brassica oleracea | 1871 | - | 6 | AACGAC | auxin-responsive element |

>HU08G00230.1   
+ -Up\_Stream \_Len000GTTGTA ACAAGCACAT TAAAGTTGAT TATCATTTTG TCCGTGAAAC AGTGAGCAAA   
  
  
+ CGTCATCTCC AGGTCAAATT CATTCGTACT CAATCACAAG TTGCTGACAT TTTCACCAAA GGCTAATCAT   
  
  
+ CGCAAAAGTT TCTTTAGTTT AAGGCCAATC TCTCCATTAT TTCACCCTAT ACAGATTGAG GGGGTGTAAT   
  
  
+ AGTGTATATA TATAGCCTTG TGTTAGCGGG CCTTGGACAC TAGACTTTTA CTCTTGCAAA CCCTAATATA   
  
  
+ TTATTATAAA TATAAATCTA GCCACCCATG TTGAGGTAGG CACTCTAATT CTCACACAGG TAGGATAAGT   
  
  
+ TTTCATAGTT TATGTTGTGA ATGTGATCTT ACTTTGTGGT TCCCGTTGAC AAAAGTTTGA ATCCGAGCAT   
  
  
+ GAGAGTTCAT CAATCTCTTT TTCTTTCCTA TTTTATTTGT TGTGAACTTA ATTGGATTTT CTGGTAGAGG   
  
  
+ AAAGTTCCTT GCTGGAATTT GGTCAAAAGT GATATATATT TTGGGATTTA ATTATCCATA AATTTGGTTT   
  
  
+ TACACCTTGT TAGTTTGGAC TTCTGGTGTG TTATTGTATG TGTTCTGGTG GGTTTTTCTT TTAGCTGAAT   
  
  
+ GTTGGAGCAT TTTAAGGGAT CTAGATATCT GGAGAAGAGC TTGGGAAATA AAGCATATTT CCCTTATAGT   
  
  
+ CTGACATAAC TTTAATGAAT TGATCAGGAA CAAAGGCCAA TTAGTTCAAA AAGGTTCAAC TCCTCTAGGT   
  
  
+ GTTGTGCTAA AGAGATGTGA GTTAGTAAGT AATTGCTTAC TAACTATATA GGATGTTTTA TTACTTGTAG   
  
  
+ AGGTTTTTTT TTTGGGATAT CTTTATGGGG TTTATAAATA AGAAGGTTGA TTTGTTGTCT ACTCTTCTTT   
  
  
+ CGATTCAATA GTAATTCTCT CAGAGAAGGC TAAAATGACA TGGCATTCCA TCGCCTCTCA TGAGGCTTTT   
  
  
+ TGCATGTGCT TATGTATCTT TCATCTCTTG CTTTGGTGGG GTGGCATTGG GGAGGAAAAG GGGGTTTCGA   
  
  
+ TTCGATAGAT CGTCACTTTT GGAAATGACA TCTCTAGCCA ATGGCAAGGG TAAGGTTAAT ACATGTGGAT   
  
  
+ ATTTCCAGAG TTAGTTGTGA TTGTTATTGT TGTTGTTGTA ACTTTATGTC ACGGGGAAAA CATAATAACT   
  
  
+ ATCTGTTTAT CTGTTTGTTT GTTATTTTCA AGGGGAAGGG GAGTGGGCAG ATGCTTAGTA TGTAATCATT   
  
  
+ TTATATAGGG AAAATTCATA TAGCTTGAAA GTAAAACTTT CATTGCCAGC ATTCCAAGGT ATGGGTAGGG   
  
  
+ TTGTCTACAC CTTTATCTCC CTAGAAGCAT GGTGATCATT CCTCGGATTG TGTTATAGAA TAAAGAATGT   
  
  
+ GGTTCTGCTG TAATGAGACT TATGTATTAT CAAATGATGA ATCTGTTTAG AACTTCCTGT TTATTTTTTC   
  
  
+ TGCAAAATTT TAAGAGACTA ACATCTATCG GGCTCAACTC TAGAGGATCT CTTGTGTCAC CCTTCTCAAC   
  
  
+ TCAGTTTGAT TGTGATACAG TTACTATATT GAGTGATAGT CGAGAGCATC ACATCCCGTT AAGGAGTCTA   
  
  
+ TCAACAAGAA GCCCTTCTTG TAATTCTCCC CTTAAAACTA GCAGTTATCA TCACTTGTCA TCGAATAGCC   
  
  
+ CTTTTTGTAA TTCTACCCTT GAAACTAGCA GTTATCGTCA TCGGTTCAAC GCGAGTCCTG TTAGATATTC   
  
  
+ CCATCAAGGC ATAGATTATG GAGTGAGCAT GAAGAATGCT TTGCAGGAGC TAGAGACTAC TCTAATTGGT   
  
  
+ GTAGATGGTG AGGAAGTATC TATTGCTAAT CAACCTATGG GGGGAAGTCG TTAGTCCGGG ATCCCAAGTT   
  
  
+ AGAGATCGAA GTCATCAAGC GAAGATCCAC AGGGTTCACA TCCTACTCAG CTTGATTCAT CATCTTTTTC   
  
  
+ AAGGGTGAGA AGATTCGGAG ATGAAAGCCA GAGAGAGAGA AATGCCATAA GGCAATGGAA GAACCAGCGG   
  
  
+ AACTACTAAG TTTCTCACCA GGTGATTTAA AGCAGTTACT AATTGAATGT GCGAGGGCTT TATTAGATAA   
  
  
+ CCGAATAGAT GACTTTGAGA GTTTGGTTAA ACGGGCAAGG AAAGAGGTCT CCATCTCAGG TGAGCCTGTC   
  
  
+ CAACCTCTCG GTACCTGTAT GATCGAAGGG CTTGTGGCAA GGAAACAGTC TTCGGGGACT AACATCTATT   
  
  
+ GGGCTCTTAA GTGTAAAGAG CCTCTTGGAA AAGACTTGCT CTCCTACGGC ACTCAGTGGG TTACTGTATC   
  
  
+ GCAAGCCATA GCAGCAAGGC TTGGTGGGCC TCCTAAAGTG CGACTTACAG GCATTGATGA TCCTGTTTCT   
  
  
+ AAGTATACTT GTGATGCTAG CTTGGAGGCT GGTGGGAAAC GATTAGCGTC TCTATTTGAA AAGTTTAAAA   
  
  
+ TACTTGTCGA GTTCAATGCA TTGCCCGTTT ATGGACCCAA TGTCAGGTGG GAAATGCTGG ATGTGAGGCC   
  
  
+ CAGGGAGGCT TTGGTCGTTA ATTGTCCATT ACAGCTCCAT TACACTCCTG ACGAGAGTGT CGATGTGAGG   
  
  
+ AACCCTAGGG ATAGGCTTCT CAGAATGGTG AAATTGCTCG GTCCTAAGGT AGCCACTTTG GTTGAGCAAG   
  
  
+ AATCAAACAC CAACACTACA CCTTTCTTGA CCCGGTTCAT AGAGACCCTT GACTACTACT CGATTAACCA   
  
  
+ TGTTTG  

- -Up\_Stream \_Len000CAACAT TGTTCGTGTA ATTTCAACTA ATAGTAAAAC AGGCACTTTG TCACTCGTTT   
  
  
- GCAGTAGAGG TCCAGTTTAA GTAAGCATGA GTTAGTGTTC AACGACTGTA AAAGTGGTTT CCGATTAGTA   
  
  
- GCGTTTTCAA AGAAATCAAA TTCCGGTTAG AGAGGTAATA AAGTGGGATA TGTCTAACTC CCCCACATTA   
  
  
- TCACATATAT ATATCGGAAC ACAATCGCCC GGAACCTGTG ATCTGAAAAT GAGAACGTTT GGGATTATAT   
  
  
- AATAATATTT ATATTTAGAT CGGTGGGTAC AACTCCATCC GTGAGATTAA GAGTGTGTCC ATCCTATTCA   
  
  
- AAAGTATCAA ATACAACACT TACACTAGAA TGAAACACCA AGGGCAACTG TTTTCAAACT TAGGCTCGTA   
  
  
- CTCTCAAGTA GTTAGAGAAA AAGAAAGGAT AAAATAAACA ACACTTGAAT TAACCTAAAA GACCATCTCC   
  
  
- TTTCAAGGAA CGACCTTAAA CCAGTTTTCA CTATATATAA AACCCTAAAT TAATAGGTAT TTAAACCAAA   
  
  
- ATGTGGAACA ATCAAACCTG AAGACCACAC AATAACATAC ACAAGACCAC CCAAAAAGAA AATCGACTTA   
  
  
- CAACCTCGTA AAATTCCCTA GATCTATAGA CCTCTTCTCG AACCCTTTAT TTCGTATAAA GGGAATATCA   
  
  
- GACTGTATTG AAATTACTTA ACTAGTCCTT GTTTCCGGTT AATCAAGTTT TTCCAAGTTG AGGAGATCCA   
  
  
- CAACACGATT TCTCTACACT CAATCATTCA TTAACGAATG ATTGATATAT CCTACAAAAT AATGAACATC   
  
  
- TCCAAAAAAA AAACCCTATA GAAATACCCC AAATATTTAT TCTTCCAACT AAACAACAGA TGAGAAGAAA   
  
  
- GCTAAGTTAT CATTAAGAGA GTCTCTTCCG ATTTTACTGT ACCGTAAGGT AGCGGAGAGT ACTCCGAAAA   
  
  
- ACGTACACGA ATACATAGAA AGTAGAGAAC GAAACCACCC CACCGTAACC CCTCCTTTTC CCCCAAAGCT   
  
  
- AAGCTATCTA GCAGTGAAAA CCTTTACTGT AGAGATCGGT TACCGTTCCC ATTCCAATTA TGTACACCTA   
  
  
- TAAAGGTCTC AATCAACACT AACAATAACA ACAACAACAT TGAAATACAG TGCCCCTTTT GTATTATTGA   
  
  
- TAGACAAATA GACAAACAAA CAATAAAAGT TCCCCTTCCC CTCACCCGTC TACGAATCAT ACATTAGTAA   
  
  
- AATATATCCC TTTTAAGTAT ATCGAACTTT CATTTTGAAA GTAACGGTCG TAAGGTTCCA TACCCATCCC   
  
  
- AACAGATGTG GAAATAGAGG GATCTTCGTA CCACTAGTAA GGAGCCTAAC ACAATATCTT ATTTCTTACA   
  
  
- CCAAGACGAC ATTACTCTGA ATACATAATA GTTTACTACT TAGACAAATC TTGAAGGACA AATAAAAAAG   
  
  
- ACGTTTTAAA ATTCTCTGAT TGTAGATAGC CCGAGTTGAG ATCTCCTAGA GAACACAGTG GGAAGAGTTG   
  
  
- AGTCAAACTA ACACTATGTC AATGATATAA CTCACTATCA GCTCTCGTAG TGTAGGGCAA TTCCTCAGAT   
  
  
- AGTTGTTCTT CGGGAAGAAC ATTAAGAGGG GAATTTTGAT CGTCAATAGT AGTGAACAGT AGCTTATCGG   
  
  
- GAAAAACATT AAGATGGGAA CTTTGATCGT CAATAGCAGT AGCCAAGTTG CGCTCAGGAC AATCTATAAG   
  
  
- GGTAGTTCCG TATCTAATAC CTCACTCGTA CTTCTTACGA AACGTCCTCG ATCTCTGATG AGATTAACCA   
  
  
- CATCTACCAC TCCTTCATAG ATAACGATTA GTTGGATACC CCCCTTCAGC AATCAGGCCC TAGGGTTCAA   
  
  
- TCTCTAGCTT CAGTAGTTCG CTTCTAGGTG TCCCAAGTGT AGGATGAGTC GAACTAAGTA GTAGAAAAAG   
  
  
- TTCCCACTCT TCTAAGCCTC TACTTTCGGT CTCTCTCTCT TTACGGTATT CCGTTACCTT CTTGGTCGCC   
  
  
- TTGATGATTC AAAGAGTGGT CCACTAAATT TCGTCAATGA TTAACTTACA CGCTCCCGAA ATAATCTATT   
  
  
- GGCTTATCTA CTGAAACTCT CAAACCAATT TGCCCGTTCC TTTCTCCAGA GGTAGAGTCC ACTCGGACAG   
  
  
- GTTGGAGAGC CATGGACATA CTAGCTTCCC GAACACCGTT CCTTTGTCAG AAGCCCCTGA TTGTAGATAA   
  
  
- CCCGAGAATT CACATTTCTC GGAGAACCTT TTCTGAACGA GAGGATGCCG TGAGTCACCC AATGACATAG   
  
  
- CGTTCGGTAT CGTCGTTCCG AACCACCCGG AGGATTTCAC GCTGAATGTC CGTAACTACT AGGACAAAGA   
  
  
- TTCATATGAA CACTACGATC GAACCTCCGA CCACCCTTTG CTAATCGCAG AGATAAACTT TTCAAATTTT   
  
  
- ATGAACAGCT CAAGTTACGT AACGGGCAAA TACCTGGGTT ACAGTCCACC CTTTACGACC TACACTCCGG   
  
  
- GTCCCTCCGA AACCAGCAAT TAACAGGTAA TGTCGAGGTA ATGTGAGGAC TGCTCTCACA GCTACACTCC   
  
  
- TTGGGATCCC TATCCGAAGA GTCTTACCAC TTTAACGAGC CAGGATTCCA TCGGTGAAAC CAACTCGTTC   
  
  
- TTAGTTTGTG GTTGTGATGT GGAAAGAACT GGGCCAAGTA TCTCTGGGAA CTGATGATGA GCTAATTGGT   
  
  
- ACAAAC

+     TGACG-motif

| Site Name | Organism | Position | Strand | Matrix score. | sequence | function |
| --- | --- | --- | --- | --- | --- | --- |
| TGACG-motif | Hordeum vulgare | 1720 | - | 5 | TGACG | cis-acting regulatory element involved in the MeJA-responsiveness |
| TGACG-motif | Hordeum vulgare | 2573 | + | 5 | TGACG | cis-acting regulatory element involved in the MeJA-responsiveness |
| TGACG-motif | Hordeum vulgare | 1065 | - | 5 | TGACG | cis-acting regulatory element involved in the MeJA-responsiveness |
| TGACG-motif | Hordeum vulgare | 75 | - | 5 | TGACG | cis-acting regulatory element involved in the MeJA-responsiveness |

>HU08G00230.1   
+ -Up\_Stream \_Len000GTTGTA ACAAGCACAT TAAAGTTGAT TATCATTTTG TCCGTGAAAC AGTGAGCAAA   
  
  
+ CGTCATCTCC AGGTCAAATT CATTCGTACT CAATCACAAG TTGCTGACAT TTTCACCAAA GGCTAATCAT   
  
  
+ CGCAAAAGTT TCTTTAGTTT AAGGCCAATC TCTCCATTAT TTCACCCTAT ACAGATTGAG GGGGTGTAAT   
  
  
+ AGTGTATATA TATAGCCTTG TGTTAGCGGG CCTTGGACAC TAGACTTTTA CTCTTGCAAA CCCTAATATA   
  
  
+ TTATTATAAA TATAAATCTA GCCACCCATG TTGAGGTAGG CACTCTAATT CTCACACAGG TAGGATAAGT   
  
  
+ TTTCATAGTT TATGTTGTGA ATGTGATCTT ACTTTGTGGT TCCCGTTGAC AAAAGTTTGA ATCCGAGCAT   
  
  
+ GAGAGTTCAT CAATCTCTTT TTCTTTCCTA TTTTATTTGT TGTGAACTTA ATTGGATTTT CTGGTAGAGG   
  
  
+ AAAGTTCCTT GCTGGAATTT GGTCAAAAGT GATATATATT TTGGGATTTA ATTATCCATA AATTTGGTTT   
  
  
+ TACACCTTGT TAGTTTGGAC TTCTGGTGTG TTATTGTATG TGTTCTGGTG GGTTTTTCTT TTAGCTGAAT   
  
  
+ GTTGGAGCAT TTTAAGGGAT CTAGATATCT GGAGAAGAGC TTGGGAAATA AAGCATATTT CCCTTATAGT   
  
  
+ CTGACATAAC TTTAATGAAT TGATCAGGAA CAAAGGCCAA TTAGTTCAAA AAGGTTCAAC TCCTCTAGGT   
  
  
+ GTTGTGCTAA AGAGATGTGA GTTAGTAAGT AATTGCTTAC TAACTATATA GGATGTTTTA TTACTTGTAG   
  
  
+ AGGTTTTTTT TTTGGGATAT CTTTATGGGG TTTATAAATA AGAAGGTTGA TTTGTTGTCT ACTCTTCTTT   
  
  
+ CGATTCAATA GTAATTCTCT CAGAGAAGGC TAAAATGACA TGGCATTCCA TCGCCTCTCA TGAGGCTTTT   
  
  
+ TGCATGTGCT TATGTATCTT TCATCTCTTG CTTTGGTGGG GTGGCATTGG GGAGGAAAAG GGGGTTTCGA   
  
  
+ TTCGATAGAT CGTCACTTTT GGAAATGACA TCTCTAGCCA ATGGCAAGGG TAAGGTTAAT ACATGTGGAT   
  
  
+ ATTTCCAGAG TTAGTTGTGA TTGTTATTGT TGTTGTTGTA ACTTTATGTC ACGGGGAAAA CATAATAACT   
  
  
+ ATCTGTTTAT CTGTTTGTTT GTTATTTTCA AGGGGAAGGG GAGTGGGCAG ATGCTTAGTA TGTAATCATT   
  
  
+ TTATATAGGG AAAATTCATA TAGCTTGAAA GTAAAACTTT CATTGCCAGC ATTCCAAGGT ATGGGTAGGG   
  
  
+ TTGTCTACAC CTTTATCTCC CTAGAAGCAT GGTGATCATT CCTCGGATTG TGTTATAGAA TAAAGAATGT   
  
  
+ GGTTCTGCTG TAATGAGACT TATGTATTAT CAAATGATGA ATCTGTTTAG AACTTCCTGT TTATTTTTTC   
  
  
+ TGCAAAATTT TAAGAGACTA ACATCTATCG GGCTCAACTC TAGAGGATCT CTTGTGTCAC CCTTCTCAAC   
  
  
+ TCAGTTTGAT TGTGATACAG TTACTATATT GAGTGATAGT CGAGAGCATC ACATCCCGTT AAGGAGTCTA   
  
  
+ TCAACAAGAA GCCCTTCTTG TAATTCTCCC CTTAAAACTA GCAGTTATCA TCACTTGTCA TCGAATAGCC   
  
  
+ CTTTTTGTAA TTCTACCCTT GAAACTAGCA GTTATCGTCA TCGGTTCAAC GCGAGTCCTG TTAGATATTC   
  
  
+ CCATCAAGGC ATAGATTATG GAGTGAGCAT GAAGAATGCT TTGCAGGAGC TAGAGACTAC TCTAATTGGT   
  
  
+ GTAGATGGTG AGGAAGTATC TATTGCTAAT CAACCTATGG GGGGAAGTCG TTAGTCCGGG ATCCCAAGTT   
  
  
+ AGAGATCGAA GTCATCAAGC GAAGATCCAC AGGGTTCACA TCCTACTCAG CTTGATTCAT CATCTTTTTC   
  
  
+ AAGGGTGAGA AGATTCGGAG ATGAAAGCCA GAGAGAGAGA AATGCCATAA GGCAATGGAA GAACCAGCGG   
  
  
+ AACTACTAAG TTTCTCACCA GGTGATTTAA AGCAGTTACT AATTGAATGT GCGAGGGCTT TATTAGATAA   
  
  
+ CCGAATAGAT GACTTTGAGA GTTTGGTTAA ACGGGCAAGG AAAGAGGTCT CCATCTCAGG TGAGCCTGTC   
  
  
+ CAACCTCTCG GTACCTGTAT GATCGAAGGG CTTGTGGCAA GGAAACAGTC TTCGGGGACT AACATCTATT   
  
  
+ GGGCTCTTAA GTGTAAAGAG CCTCTTGGAA AAGACTTGCT CTCCTACGGC ACTCAGTGGG TTACTGTATC   
  
  
+ GCAAGCCATA GCAGCAAGGC TTGGTGGGCC TCCTAAAGTG CGACTTACAG GCATTGATGA TCCTGTTTCT   
  
  
+ AAGTATACTT GTGATGCTAG CTTGGAGGCT GGTGGGAAAC GATTAGCGTC TCTATTTGAA AAGTTTAAAA   
  
  
+ TACTTGTCGA GTTCAATGCA TTGCCCGTTT ATGGACCCAA TGTCAGGTGG GAAATGCTGG ATGTGAGGCC   
  
  
+ CAGGGAGGCT TTGGTCGTTA ATTGTCCATT ACAGCTCCAT TACACTCCTG ACGAGAGTGT CGATGTGAGG   
  
  
+ AACCCTAGGG ATAGGCTTCT CAGAATGGTG AAATTGCTCG GTCCTAAGGT AGCCACTTTG GTTGAGCAAG   
  
  
+ AATCAAACAC CAACACTACA CCTTTCTTGA CCCGGTTCAT AGAGACCCTT GACTACTACT CGATTAACCA   
  
  
+ TGTTTG  

- -Up\_Stream \_Len000CAACAT TGTTCGTGTA ATTTCAACTA ATAGTAAAAC AGGCACTTTG TCACTCGTTT   
  
  
- GCAGTAGAGG TCCAGTTTAA GTAAGCATGA GTTAGTGTTC AACGACTGTA AAAGTGGTTT CCGATTAGTA   
  
  
- GCGTTTTCAA AGAAATCAAA TTCCGGTTAG AGAGGTAATA AAGTGGGATA TGTCTAACTC CCCCACATTA   
  
  
- TCACATATAT ATATCGGAAC ACAATCGCCC GGAACCTGTG ATCTGAAAAT GAGAACGTTT GGGATTATAT   
  
  
- AATAATATTT ATATTTAGAT CGGTGGGTAC AACTCCATCC GTGAGATTAA GAGTGTGTCC ATCCTATTCA   
  
  
- AAAGTATCAA ATACAACACT TACACTAGAA TGAAACACCA AGGGCAACTG TTTTCAAACT TAGGCTCGTA   
  
  
- CTCTCAAGTA GTTAGAGAAA AAGAAAGGAT AAAATAAACA ACACTTGAAT TAACCTAAAA GACCATCTCC   
  
  
- TTTCAAGGAA CGACCTTAAA CCAGTTTTCA CTATATATAA AACCCTAAAT TAATAGGTAT TTAAACCAAA   
  
  
- ATGTGGAACA ATCAAACCTG AAGACCACAC AATAACATAC ACAAGACCAC CCAAAAAGAA AATCGACTTA   
  
  
- CAACCTCGTA AAATTCCCTA GATCTATAGA CCTCTTCTCG AACCCTTTAT TTCGTATAAA GGGAATATCA   
  
  
- GACTGTATTG AAATTACTTA ACTAGTCCTT GTTTCCGGTT AATCAAGTTT TTCCAAGTTG AGGAGATCCA   
  
  
- CAACACGATT TCTCTACACT CAATCATTCA TTAACGAATG ATTGATATAT CCTACAAAAT AATGAACATC   
  
  
- TCCAAAAAAA AAACCCTATA GAAATACCCC AAATATTTAT TCTTCCAACT AAACAACAGA TGAGAAGAAA   
  
  
- GCTAAGTTAT CATTAAGAGA GTCTCTTCCG ATTTTACTGT ACCGTAAGGT AGCGGAGAGT ACTCCGAAAA   
  
  
- ACGTACACGA ATACATAGAA AGTAGAGAAC GAAACCACCC CACCGTAACC CCTCCTTTTC CCCCAAAGCT   
  
  
- AAGCTATCTA GCAGTGAAAA CCTTTACTGT AGAGATCGGT TACCGTTCCC ATTCCAATTA TGTACACCTA   
  
  
- TAAAGGTCTC AATCAACACT AACAATAACA ACAACAACAT TGAAATACAG TGCCCCTTTT GTATTATTGA   
  
  
- TAGACAAATA GACAAACAAA CAATAAAAGT TCCCCTTCCC CTCACCCGTC TACGAATCAT ACATTAGTAA   
  
  
- AATATATCCC TTTTAAGTAT ATCGAACTTT CATTTTGAAA GTAACGGTCG TAAGGTTCCA TACCCATCCC   
  
  
- AACAGATGTG GAAATAGAGG GATCTTCGTA CCACTAGTAA GGAGCCTAAC ACAATATCTT ATTTCTTACA   
  
  
- CCAAGACGAC ATTACTCTGA ATACATAATA GTTTACTACT TAGACAAATC TTGAAGGACA AATAAAAAAG   
  
  
- ACGTTTTAAA ATTCTCTGAT TGTAGATAGC CCGAGTTGAG ATCTCCTAGA GAACACAGTG GGAAGAGTTG   
  
  
- AGTCAAACTA ACACTATGTC AATGATATAA CTCACTATCA GCTCTCGTAG TGTAGGGCAA TTCCTCAGAT   
  
  
- AGTTGTTCTT CGGGAAGAAC ATTAAGAGGG GAATTTTGAT CGTCAATAGT AGTGAACAGT AGCTTATCGG   
  
  
- GAAAAACATT AAGATGGGAA CTTTGATCGT CAATAGCAGT AGCCAAGTTG CGCTCAGGAC AATCTATAAG   
  
  
- GGTAGTTCCG TATCTAATAC CTCACTCGTA CTTCTTACGA AACGTCCTCG ATCTCTGATG AGATTAACCA   
  
  
- CATCTACCAC TCCTTCATAG ATAACGATTA GTTGGATACC CCCCTTCAGC AATCAGGCCC TAGGGTTCAA   
  
  
- TCTCTAGCTT CAGTAGTTCG CTTCTAGGTG TCCCAAGTGT AGGATGAGTC GAACTAAGTA GTAGAAAAAG   
  
  
- TTCCCACTCT TCTAAGCCTC TACTTTCGGT CTCTCTCTCT TTACGGTATT CCGTTACCTT CTTGGTCGCC   
  
  
- TTGATGATTC AAAGAGTGGT CCACTAAATT TCGTCAATGA TTAACTTACA CGCTCCCGAA ATAATCTATT   
  
  
- GGCTTATCTA CTGAAACTCT CAAACCAATT TGCCCGTTCC TTTCTCCAGA GGTAGAGTCC ACTCGGACAG   
  
  
- GTTGGAGAGC CATGGACATA CTAGCTTCCC GAACACCGTT CCTTTGTCAG AAGCCCCTGA TTGTAGATAA   
  
  
- CCCGAGAATT CACATTTCTC GGAGAACCTT TTCTGAACGA GAGGATGCCG TGAGTCACCC AATGACATAG   
  
  
- CGTTCGGTAT CGTCGTTCCG AACCACCCGG AGGATTTCAC GCTGAATGTC CGTAACTACT AGGACAAAGA   
  
  
- TTCATATGAA CACTACGATC GAACCTCCGA CCACCCTTTG CTAATCGCAG AGATAAACTT TTCAAATTTT   
  
  
- ATGAACAGCT CAAGTTACGT AACGGGCAAA TACCTGGGTT ACAGTCCACC CTTTACGACC TACACTCCGG   
  
  
- GTCCCTCCGA AACCAGCAAT TAACAGGTAA TGTCGAGGTA ATGTGAGGAC TGCTCTCACA GCTACACTCC   
  
  
- TTGGGATCCC TATCCGAAGA GTCTTACCAC TTTAACGAGC CAGGATTCCA TCGGTGAAAC CAACTCGTTC   
  
  
- TTAGTTTGTG GTTGTGATGT GGAAAGAACT GGGCCAAGTA TCTCTGGGAA CTGATGATGA GCTAATTGGT   
  
  
- ACAAAC

+     Unnamed\_\_4

| Site Name | Organism | Position | Strand | Matrix score. | sequence | function |
| --- | --- | --- | --- | --- | --- | --- |
| Unnamed\_\_4 | Petroselinum hortense | 764 | + | 4 | CTCC |  |
| Unnamed\_\_4 | Petroselinum hortense | 665 | - | 4 | CTCC |  |
| Unnamed\_\_4 | Petroselinum hortense | 1035 | - | 4 | CTCC |  |
| Unnamed\_\_4 | Petroselinum hortense | 2344 | + | 4 | CTCC |  |
| Unnamed\_\_4 | Petroselinum hortense | 2528 | - | 4 | CTCC |  |
| Unnamed\_\_4 | Petroselinum hortense | 2569 | + | 4 | CTCC |  |
| Unnamed\_\_4 | Petroselinum hortense | 2285 | + | 4 | CTCC |  |
| Unnamed\_\_4 | Petroselinum hortense | 1351 | + | 4 | CTCC |  |
| Unnamed\_\_4 | Petroselinum hortense | 81 | + | 4 | CTCC |  |
| Unnamed\_\_4 | Petroselinum hortense | 176 | + | 4 | CTCC |  |
| Unnamed\_\_4 | Petroselinum hortense | 1234 | - | 4 | CTCC |  |
| Unnamed\_\_4 | Petroselinum hortense | 2408 | - | 4 | CTCC |  |
| Unnamed\_\_4 | Petroselinum hortense | 1800 | - | 4 | CTCC |  |
| Unnamed\_\_4 | Petroselinum hortense | 2559 | + | 4 | CTCC |  |
| Unnamed\_\_4 | Petroselinum hortense | 638 | - | 4 | CTCC |  |
| Unnamed\_\_4 | Petroselinum hortense | 1774 | - | 4 | CTCC |  |
| Unnamed\_\_4 | Petroselinum hortense | 1981 | - | 4 | CTCC |  |
| Unnamed\_\_4 | Petroselinum hortense | 1607 | - | 4 | CTCC |  |
| Unnamed\_\_4 | Petroselinum hortense | 1640 | + | 4 | CTCC |  |
| Unnamed\_\_4 | Petroselinum hortense | 2153 | + | 4 | CTCC |  |

>HU08G00230.1   
+ -Up\_Stream \_Len000GTTGTA ACAAGCACAT TAAAGTTGAT TATCATTTTG TCCGTGAAAC AGTGAGCAAA   
  
  
+ CGTCATCTCC AGGTCAAATT CATTCGTACT CAATCACAAG TTGCTGACAT TTTCACCAAA GGCTAATCAT   
  
  
+ CGCAAAAGTT TCTTTAGTTT AAGGCCAATC TCTCCATTAT TTCACCCTAT ACAGATTGAG GGGGTGTAAT   
  
  
+ AGTGTATATA TATAGCCTTG TGTTAGCGGG CCTTGGACAC TAGACTTTTA CTCTTGCAAA CCCTAATATA   
  
  
+ TTATTATAAA TATAAATCTA GCCACCCATG TTGAGGTAGG CACTCTAATT CTCACACAGG TAGGATAAGT   
  
  
+ TTTCATAGTT TATGTTGTGA ATGTGATCTT ACTTTGTGGT TCCCGTTGAC AAAAGTTTGA ATCCGAGCAT   
  
  
+ GAGAGTTCAT CAATCTCTTT TTCTTTCCTA TTTTATTTGT TGTGAACTTA ATTGGATTTT CTGGTAGAGG   
  
  
+ AAAGTTCCTT GCTGGAATTT GGTCAAAAGT GATATATATT TTGGGATTTA ATTATCCATA AATTTGGTTT   
  
  
+ TACACCTTGT TAGTTTGGAC TTCTGGTGTG TTATTGTATG TGTTCTGGTG GGTTTTTCTT TTAGCTGAAT   
  
  
+ GTTGGAGCAT TTTAAGGGAT CTAGATATCT GGAGAAGAGC TTGGGAAATA AAGCATATTT CCCTTATAGT   
  
  
+ CTGACATAAC TTTAATGAAT TGATCAGGAA CAAAGGCCAA TTAGTTCAAA AAGGTTCAAC TCCTCTAGGT   
  
  
+ GTTGTGCTAA AGAGATGTGA GTTAGTAAGT AATTGCTTAC TAACTATATA GGATGTTTTA TTACTTGTAG   
  
  
+ AGGTTTTTTT TTTGGGATAT CTTTATGGGG TTTATAAATA AGAAGGTTGA TTTGTTGTCT ACTCTTCTTT   
  
  
+ CGATTCAATA GTAATTCTCT CAGAGAAGGC TAAAATGACA TGGCATTCCA TCGCCTCTCA TGAGGCTTTT   
  
  
+ TGCATGTGCT TATGTATCTT TCATCTCTTG CTTTGGTGGG GTGGCATTGG GGAGGAAAAG GGGGTTTCGA   
  
  
+ TTCGATAGAT CGTCACTTTT GGAAATGACA TCTCTAGCCA ATGGCAAGGG TAAGGTTAAT ACATGTGGAT   
  
  
+ ATTTCCAGAG TTAGTTGTGA TTGTTATTGT TGTTGTTGTA ACTTTATGTC ACGGGGAAAA CATAATAACT   
  
  
+ ATCTGTTTAT CTGTTTGTTT GTTATTTTCA AGGGGAAGGG GAGTGGGCAG ATGCTTAGTA TGTAATCATT   
  
  
+ TTATATAGGG AAAATTCATA TAGCTTGAAA GTAAAACTTT CATTGCCAGC ATTCCAAGGT ATGGGTAGGG   
  
  
+ TTGTCTACAC CTTTATCTCC CTAGAAGCAT GGTGATCATT CCTCGGATTG TGTTATAGAA TAAAGAATGT   
  
  
+ GGTTCTGCTG TAATGAGACT TATGTATTAT CAAATGATGA ATCTGTTTAG AACTTCCTGT TTATTTTTTC   
  
  
+ TGCAAAATTT TAAGAGACTA ACATCTATCG GGCTCAACTC TAGAGGATCT CTTGTGTCAC CCTTCTCAAC   
  
  
+ TCAGTTTGAT TGTGATACAG TTACTATATT GAGTGATAGT CGAGAGCATC ACATCCCGTT AAGGAGTCTA   
  
  
+ TCAACAAGAA GCCCTTCTTG TAATTCTCCC CTTAAAACTA GCAGTTATCA TCACTTGTCA TCGAATAGCC   
  
  
+ CTTTTTGTAA TTCTACCCTT GAAACTAGCA GTTATCGTCA TCGGTTCAAC GCGAGTCCTG TTAGATATTC   
  
  
+ CCATCAAGGC ATAGATTATG GAGTGAGCAT GAAGAATGCT TTGCAGGAGC TAGAGACTAC TCTAATTGGT   
  
  
+ GTAGATGGTG AGGAAGTATC TATTGCTAAT CAACCTATGG GGGGAAGTCG TTAGTCCGGG ATCCCAAGTT   
  
  
+ AGAGATCGAA GTCATCAAGC GAAGATCCAC AGGGTTCACA TCCTACTCAG CTTGATTCAT CATCTTTTTC   
  
  
+ AAGGGTGAGA AGATTCGGAG ATGAAAGCCA GAGAGAGAGA AATGCCATAA GGCAATGGAA GAACCAGCGG   
  
  
+ AACTACTAAG TTTCTCACCA GGTGATTTAA AGCAGTTACT AATTGAATGT GCGAGGGCTT TATTAGATAA   
  
  
+ CCGAATAGAT GACTTTGAGA GTTTGGTTAA ACGGGCAAGG AAAGAGGTCT CCATCTCAGG TGAGCCTGTC   
  
  
+ CAACCTCTCG GTACCTGTAT GATCGAAGGG CTTGTGGCAA GGAAACAGTC TTCGGGGACT AACATCTATT   
  
  
+ GGGCTCTTAA GTGTAAAGAG CCTCTTGGAA AAGACTTGCT CTCCTACGGC ACTCAGTGGG TTACTGTATC   
  
  
+ GCAAGCCATA GCAGCAAGGC TTGGTGGGCC TCCTAAAGTG CGACTTACAG GCATTGATGA TCCTGTTTCT   
  
  
+ AAGTATACTT GTGATGCTAG CTTGGAGGCT GGTGGGAAAC GATTAGCGTC TCTATTTGAA AAGTTTAAAA   
  
  
+ TACTTGTCGA GTTCAATGCA TTGCCCGTTT ATGGACCCAA TGTCAGGTGG GAAATGCTGG ATGTGAGGCC   
  
  
+ CAGGGAGGCT TTGGTCGTTA ATTGTCCATT ACAGCTCCAT TACACTCCTG ACGAGAGTGT CGATGTGAGG   
  
  
+ AACCCTAGGG ATAGGCTTCT CAGAATGGTG AAATTGCTCG GTCCTAAGGT AGCCACTTTG GTTGAGCAAG   
  
  
+ AATCAAACAC CAACACTACA CCTTTCTTGA CCCGGTTCAT AGAGACCCTT GACTACTACT CGATTAACCA   
  
  
+ TGTTTG  

- -Up\_Stream \_Len000CAACAT TGTTCGTGTA ATTTCAACTA ATAGTAAAAC AGGCACTTTG TCACTCGTTT   
  
  
- GCAGTAGAGG TCCAGTTTAA GTAAGCATGA GTTAGTGTTC AACGACTGTA AAAGTGGTTT CCGATTAGTA   
  
  
- GCGTTTTCAA AGAAATCAAA TTCCGGTTAG AGAGGTAATA AAGTGGGATA TGTCTAACTC CCCCACATTA   
  
  
- TCACATATAT ATATCGGAAC ACAATCGCCC GGAACCTGTG ATCTGAAAAT GAGAACGTTT GGGATTATAT   
  
  
- AATAATATTT ATATTTAGAT CGGTGGGTAC AACTCCATCC GTGAGATTAA GAGTGTGTCC ATCCTATTCA   
  
  
- AAAGTATCAA ATACAACACT TACACTAGAA TGAAACACCA AGGGCAACTG TTTTCAAACT TAGGCTCGTA   
  
  
- CTCTCAAGTA GTTAGAGAAA AAGAAAGGAT AAAATAAACA ACACTTGAAT TAACCTAAAA GACCATCTCC   
  
  
- TTTCAAGGAA CGACCTTAAA CCAGTTTTCA CTATATATAA AACCCTAAAT TAATAGGTAT TTAAACCAAA   
  
  
- ATGTGGAACA ATCAAACCTG AAGACCACAC AATAACATAC ACAAGACCAC CCAAAAAGAA AATCGACTTA   
  
  
- CAACCTCGTA AAATTCCCTA GATCTATAGA CCTCTTCTCG AACCCTTTAT TTCGTATAAA GGGAATATCA   
  
  
- GACTGTATTG AAATTACTTA ACTAGTCCTT GTTTCCGGTT AATCAAGTTT TTCCAAGTTG AGGAGATCCA   
  
  
- CAACACGATT TCTCTACACT CAATCATTCA TTAACGAATG ATTGATATAT CCTACAAAAT AATGAACATC   
  
  
- TCCAAAAAAA AAACCCTATA GAAATACCCC AAATATTTAT TCTTCCAACT AAACAACAGA TGAGAAGAAA   
  
  
- GCTAAGTTAT CATTAAGAGA GTCTCTTCCG ATTTTACTGT ACCGTAAGGT AGCGGAGAGT ACTCCGAAAA   
  
  
- ACGTACACGA ATACATAGAA AGTAGAGAAC GAAACCACCC CACCGTAACC CCTCCTTTTC CCCCAAAGCT   
  
  
- AAGCTATCTA GCAGTGAAAA CCTTTACTGT AGAGATCGGT TACCGTTCCC ATTCCAATTA TGTACACCTA   
  
  
- TAAAGGTCTC AATCAACACT AACAATAACA ACAACAACAT TGAAATACAG TGCCCCTTTT GTATTATTGA   
  
  
- TAGACAAATA GACAAACAAA CAATAAAAGT TCCCCTTCCC CTCACCCGTC TACGAATCAT ACATTAGTAA   
  
  
- AATATATCCC TTTTAAGTAT ATCGAACTTT CATTTTGAAA GTAACGGTCG TAAGGTTCCA TACCCATCCC   
  
  
- AACAGATGTG GAAATAGAGG GATCTTCGTA CCACTAGTAA GGAGCCTAAC ACAATATCTT ATTTCTTACA   
  
  
- CCAAGACGAC ATTACTCTGA ATACATAATA GTTTACTACT TAGACAAATC TTGAAGGACA AATAAAAAAG   
  
  
- ACGTTTTAAA ATTCTCTGAT TGTAGATAGC CCGAGTTGAG ATCTCCTAGA GAACACAGTG GGAAGAGTTG   
  
  
- AGTCAAACTA ACACTATGTC AATGATATAA CTCACTATCA GCTCTCGTAG TGTAGGGCAA TTCCTCAGAT   
  
  
- AGTTGTTCTT CGGGAAGAAC ATTAAGAGGG GAATTTTGAT CGTCAATAGT AGTGAACAGT AGCTTATCGG   
  
  
- GAAAAACATT AAGATGGGAA CTTTGATCGT CAATAGCAGT AGCCAAGTTG CGCTCAGGAC AATCTATAAG   
  
  
- GGTAGTTCCG TATCTAATAC CTCACTCGTA CTTCTTACGA AACGTCCTCG ATCTCTGATG AGATTAACCA   
  
  
- CATCTACCAC TCCTTCATAG ATAACGATTA GTTGGATACC CCCCTTCAGC AATCAGGCCC TAGGGTTCAA   
  
  
- TCTCTAGCTT CAGTAGTTCG CTTCTAGGTG TCCCAAGTGT AGGATGAGTC GAACTAAGTA GTAGAAAAAG   
  
  
- TTCCCACTCT TCTAAGCCTC TACTTTCGGT CTCTCTCTCT TTACGGTATT CCGTTACCTT CTTGGTCGCC   
  
  
- TTGATGATTC AAAGAGTGGT CCACTAAATT TCGTCAATGA TTAACTTACA CGCTCCCGAA ATAATCTATT   
  
  
- GGCTTATCTA CTGAAACTCT CAAACCAATT TGCCCGTTCC TTTCTCCAGA GGTAGAGTCC ACTCGGACAG   
  
  
- GTTGGAGAGC CATGGACATA CTAGCTTCCC GAACACCGTT CCTTTGTCAG AAGCCCCTGA TTGTAGATAA   
  
  
- CCCGAGAATT CACATTTCTC GGAGAACCTT TTCTGAACGA GAGGATGCCG TGAGTCACCC AATGACATAG   
  
  
- CGTTCGGTAT CGTCGTTCCG AACCACCCGG AGGATTTCAC GCTGAATGTC CGTAACTACT AGGACAAAGA   
  
  
- TTCATATGAA CACTACGATC GAACCTCCGA CCACCCTTTG CTAATCGCAG AGATAAACTT TTCAAATTTT   
  
  
- ATGAACAGCT CAAGTTACGT AACGGGCAAA TACCTGGGTT ACAGTCCACC CTTTACGACC TACACTCCGG   
  
  
- GTCCCTCCGA AACCAGCAAT TAACAGGTAA TGTCGAGGTA ATGTGAGGAC TGCTCTCACA GCTACACTCC   
  
  
- TTGGGATCCC TATCCGAAGA GTCTTACCAC TTTAACGAGC CAGGATTCCA TCGGTGAAAC CAACTCGTTC   
  
  
- TTAGTTTGTG GTTGTGATGT GGAAAGAACT GGGCCAAGTA TCTCTGGGAA CTGATGATGA GCTAATTGGT   
  
  
- ACAAAC

+     W box

| Site Name | Organism | Position | Strand | Matrix score. | sequence | function |
| --- | --- | --- | --- | --- | --- | --- |
| W box | Arabidopsis thaliana | 2691 | + | 6 | TTGACC |  |
| W box | Arabidopsis thaliana | 515 | - | 6 | TTGACC |  |
| W box | Arabidopsis thaliana | 86 | - | 6 | TTGACC |  |

>HU08G00230.1   
+ -Up\_Stream \_Len000GTTGTA ACAAGCACAT TAAAGTTGAT TATCATTTTG TCCGTGAAAC AGTGAGCAAA   
  
  
+ CGTCATCTCC AGGTCAAATT CATTCGTACT CAATCACAAG TTGCTGACAT TTTCACCAAA GGCTAATCAT   
  
  
+ CGCAAAAGTT TCTTTAGTTT AAGGCCAATC TCTCCATTAT TTCACCCTAT ACAGATTGAG GGGGTGTAAT   
  
  
+ AGTGTATATA TATAGCCTTG TGTTAGCGGG CCTTGGACAC TAGACTTTTA CTCTTGCAAA CCCTAATATA   
  
  
+ TTATTATAAA TATAAATCTA GCCACCCATG TTGAGGTAGG CACTCTAATT CTCACACAGG TAGGATAAGT   
  
  
+ TTTCATAGTT TATGTTGTGA ATGTGATCTT ACTTTGTGGT TCCCGTTGAC AAAAGTTTGA ATCCGAGCAT   
  
  
+ GAGAGTTCAT CAATCTCTTT TTCTTTCCTA TTTTATTTGT TGTGAACTTA ATTGGATTTT CTGGTAGAGG   
  
  
+ AAAGTTCCTT GCTGGAATTT GGTCAAAAGT GATATATATT TTGGGATTTA ATTATCCATA AATTTGGTTT   
  
  
+ TACACCTTGT TAGTTTGGAC TTCTGGTGTG TTATTGTATG TGTTCTGGTG GGTTTTTCTT TTAGCTGAAT   
  
  
+ GTTGGAGCAT TTTAAGGGAT CTAGATATCT GGAGAAGAGC TTGGGAAATA AAGCATATTT CCCTTATAGT   
  
  
+ CTGACATAAC TTTAATGAAT TGATCAGGAA CAAAGGCCAA TTAGTTCAAA AAGGTTCAAC TCCTCTAGGT   
  
  
+ GTTGTGCTAA AGAGATGTGA GTTAGTAAGT AATTGCTTAC TAACTATATA GGATGTTTTA TTACTTGTAG   
  
  
+ AGGTTTTTTT TTTGGGATAT CTTTATGGGG TTTATAAATA AGAAGGTTGA TTTGTTGTCT ACTCTTCTTT   
  
  
+ CGATTCAATA GTAATTCTCT CAGAGAAGGC TAAAATGACA TGGCATTCCA TCGCCTCTCA TGAGGCTTTT   
  
  
+ TGCATGTGCT TATGTATCTT TCATCTCTTG CTTTGGTGGG GTGGCATTGG GGAGGAAAAG GGGGTTTCGA   
  
  
+ TTCGATAGAT CGTCACTTTT GGAAATGACA TCTCTAGCCA ATGGCAAGGG TAAGGTTAAT ACATGTGGAT   
  
  
+ ATTTCCAGAG TTAGTTGTGA TTGTTATTGT TGTTGTTGTA ACTTTATGTC ACGGGGAAAA CATAATAACT   
  
  
+ ATCTGTTTAT CTGTTTGTTT GTTATTTTCA AGGGGAAGGG GAGTGGGCAG ATGCTTAGTA TGTAATCATT   
  
  
+ TTATATAGGG AAAATTCATA TAGCTTGAAA GTAAAACTTT CATTGCCAGC ATTCCAAGGT ATGGGTAGGG   
  
  
+ TTGTCTACAC CTTTATCTCC CTAGAAGCAT GGTGATCATT CCTCGGATTG TGTTATAGAA TAAAGAATGT   
  
  
+ GGTTCTGCTG TAATGAGACT TATGTATTAT CAAATGATGA ATCTGTTTAG AACTTCCTGT TTATTTTTTC   
  
  
+ TGCAAAATTT TAAGAGACTA ACATCTATCG GGCTCAACTC TAGAGGATCT CTTGTGTCAC CCTTCTCAAC   
  
  
+ TCAGTTTGAT TGTGATACAG TTACTATATT GAGTGATAGT CGAGAGCATC ACATCCCGTT AAGGAGTCTA   
  
  
+ TCAACAAGAA GCCCTTCTTG TAATTCTCCC CTTAAAACTA GCAGTTATCA TCACTTGTCA TCGAATAGCC   
  
  
+ CTTTTTGTAA TTCTACCCTT GAAACTAGCA GTTATCGTCA TCGGTTCAAC GCGAGTCCTG TTAGATATTC   
  
  
+ CCATCAAGGC ATAGATTATG GAGTGAGCAT GAAGAATGCT TTGCAGGAGC TAGAGACTAC TCTAATTGGT   
  
  
+ GTAGATGGTG AGGAAGTATC TATTGCTAAT CAACCTATGG GGGGAAGTCG TTAGTCCGGG ATCCCAAGTT   
  
  
+ AGAGATCGAA GTCATCAAGC GAAGATCCAC AGGGTTCACA TCCTACTCAG CTTGATTCAT CATCTTTTTC   
  
  
+ AAGGGTGAGA AGATTCGGAG ATGAAAGCCA GAGAGAGAGA AATGCCATAA GGCAATGGAA GAACCAGCGG   
  
  
+ AACTACTAAG TTTCTCACCA GGTGATTTAA AGCAGTTACT AATTGAATGT GCGAGGGCTT TATTAGATAA   
  
  
+ CCGAATAGAT GACTTTGAGA GTTTGGTTAA ACGGGCAAGG AAAGAGGTCT CCATCTCAGG TGAGCCTGTC   
  
  
+ CAACCTCTCG GTACCTGTAT GATCGAAGGG CTTGTGGCAA GGAAACAGTC TTCGGGGACT AACATCTATT   
  
  
+ GGGCTCTTAA GTGTAAAGAG CCTCTTGGAA AAGACTTGCT CTCCTACGGC ACTCAGTGGG TTACTGTATC   
  
  
+ GCAAGCCATA GCAGCAAGGC TTGGTGGGCC TCCTAAAGTG CGACTTACAG GCATTGATGA TCCTGTTTCT   
  
  
+ AAGTATACTT GTGATGCTAG CTTGGAGGCT GGTGGGAAAC GATTAGCGTC TCTATTTGAA AAGTTTAAAA   
  
  
+ TACTTGTCGA GTTCAATGCA TTGCCCGTTT ATGGACCCAA TGTCAGGTGG GAAATGCTGG ATGTGAGGCC   
  
  
+ CAGGGAGGCT TTGGTCGTTA ATTGTCCATT ACAGCTCCAT TACACTCCTG ACGAGAGTGT CGATGTGAGG   
  
  
+ AACCCTAGGG ATAGGCTTCT CAGAATGGTG AAATTGCTCG GTCCTAAGGT AGCCACTTTG GTTGAGCAAG   
  
  
+ AATCAAACAC CAACACTACA CCTTTCTTGA CCCGGTTCAT AGAGACCCTT GACTACTACT CGATTAACCA   
  
  
+ TGTTTG  

- -Up\_Stream \_Len000CAACAT TGTTCGTGTA ATTTCAACTA ATAGTAAAAC AGGCACTTTG TCACTCGTTT   
  
  
- GCAGTAGAGG TCCAGTTTAA GTAAGCATGA GTTAGTGTTC AACGACTGTA AAAGTGGTTT CCGATTAGTA   
  
  
- GCGTTTTCAA AGAAATCAAA TTCCGGTTAG AGAGGTAATA AAGTGGGATA TGTCTAACTC CCCCACATTA   
  
  
- TCACATATAT ATATCGGAAC ACAATCGCCC GGAACCTGTG ATCTGAAAAT GAGAACGTTT GGGATTATAT   
  
  
- AATAATATTT ATATTTAGAT CGGTGGGTAC AACTCCATCC GTGAGATTAA GAGTGTGTCC ATCCTATTCA   
  
  
- AAAGTATCAA ATACAACACT TACACTAGAA TGAAACACCA AGGGCAACTG TTTTCAAACT TAGGCTCGTA   
  
  
- CTCTCAAGTA GTTAGAGAAA AAGAAAGGAT AAAATAAACA ACACTTGAAT TAACCTAAAA GACCATCTCC   
  
  
- TTTCAAGGAA CGACCTTAAA CCAGTTTTCA CTATATATAA AACCCTAAAT TAATAGGTAT TTAAACCAAA   
  
  
- ATGTGGAACA ATCAAACCTG AAGACCACAC AATAACATAC ACAAGACCAC CCAAAAAGAA AATCGACTTA   
  
  
- CAACCTCGTA AAATTCCCTA GATCTATAGA CCTCTTCTCG AACCCTTTAT TTCGTATAAA GGGAATATCA   
  
  
- GACTGTATTG AAATTACTTA ACTAGTCCTT GTTTCCGGTT AATCAAGTTT TTCCAAGTTG AGGAGATCCA   
  
  
- CAACACGATT TCTCTACACT CAATCATTCA TTAACGAATG ATTGATATAT CCTACAAAAT AATGAACATC   
  
  
- TCCAAAAAAA AAACCCTATA GAAATACCCC AAATATTTAT TCTTCCAACT AAACAACAGA TGAGAAGAAA   
  
  
- GCTAAGTTAT CATTAAGAGA GTCTCTTCCG ATTTTACTGT ACCGTAAGGT AGCGGAGAGT ACTCCGAAAA   
  
  
- ACGTACACGA ATACATAGAA AGTAGAGAAC GAAACCACCC CACCGTAACC CCTCCTTTTC CCCCAAAGCT   
  
  
- AAGCTATCTA GCAGTGAAAA CCTTTACTGT AGAGATCGGT TACCGTTCCC ATTCCAATTA TGTACACCTA   
  
  
- TAAAGGTCTC AATCAACACT AACAATAACA ACAACAACAT TGAAATACAG TGCCCCTTTT GTATTATTGA   
  
  
- TAGACAAATA GACAAACAAA CAATAAAAGT TCCCCTTCCC CTCACCCGTC TACGAATCAT ACATTAGTAA   
  
  
- AATATATCCC TTTTAAGTAT ATCGAACTTT CATTTTGAAA GTAACGGTCG TAAGGTTCCA TACCCATCCC   
  
  
- AACAGATGTG GAAATAGAGG GATCTTCGTA CCACTAGTAA GGAGCCTAAC ACAATATCTT ATTTCTTACA   
  
  
- CCAAGACGAC ATTACTCTGA ATACATAATA GTTTACTACT TAGACAAATC TTGAAGGACA AATAAAAAAG   
  
  
- ACGTTTTAAA ATTCTCTGAT TGTAGATAGC CCGAGTTGAG ATCTCCTAGA GAACACAGTG GGAAGAGTTG   
  
  
- AGTCAAACTA ACACTATGTC AATGATATAA CTCACTATCA GCTCTCGTAG TGTAGGGCAA TTCCTCAGAT   
  
  
- AGTTGTTCTT CGGGAAGAAC ATTAAGAGGG GAATTTTGAT CGTCAATAGT AGTGAACAGT AGCTTATCGG   
  
  
- GAAAAACATT AAGATGGGAA CTTTGATCGT CAATAGCAGT AGCCAAGTTG CGCTCAGGAC AATCTATAAG   
  
  
- GGTAGTTCCG TATCTAATAC CTCACTCGTA CTTCTTACGA AACGTCCTCG ATCTCTGATG AGATTAACCA   
  
  
- CATCTACCAC TCCTTCATAG ATAACGATTA GTTGGATACC CCCCTTCAGC AATCAGGCCC TAGGGTTCAA   
  
  
- TCTCTAGCTT CAGTAGTTCG CTTCTAGGTG TCCCAAGTGT AGGATGAGTC GAACTAAGTA GTAGAAAAAG   
  
  
- TTCCCACTCT TCTAAGCCTC TACTTTCGGT CTCTCTCTCT TTACGGTATT CCGTTACCTT CTTGGTCGCC   
  
  
- TTGATGATTC AAAGAGTGGT CCACTAAATT TCGTCAATGA TTAACTTACA CGCTCCCGAA ATAATCTATT   
  
  
- GGCTTATCTA CTGAAACTCT CAAACCAATT TGCCCGTTCC TTTCTCCAGA GGTAGAGTCC ACTCGGACAG   
  
  
- GTTGGAGAGC CATGGACATA CTAGCTTCCC GAACACCGTT CCTTTGTCAG AAGCCCCTGA TTGTAGATAA   
  
  
- CCCGAGAATT CACATTTCTC GGAGAACCTT TTCTGAACGA GAGGATGCCG TGAGTCACCC AATGACATAG   
  
  
- CGTTCGGTAT CGTCGTTCCG AACCACCCGG AGGATTTCAC GCTGAATGTC CGTAACTACT AGGACAAAGA   
  
  
- TTCATATGAA CACTACGATC GAACCTCCGA CCACCCTTTG CTAATCGCAG AGATAAACTT TTCAAATTTT   
  
  
- ATGAACAGCT CAAGTTACGT AACGGGCAAA TACCTGGGTT ACAGTCCACC CTTTACGACC TACACTCCGG   
  
  
- GTCCCTCCGA AACCAGCAAT TAACAGGTAA TGTCGAGGTA ATGTGAGGAC TGCTCTCACA GCTACACTCC   
  
  
- TTGGGATCCC TATCCGAAGA GTCTTACCAC TTTAACGAGC CAGGATTCCA TCGGTGAAAC CAACTCGTTC   
  
  
- TTAGTTTGTG GTTGTGATGT GGAAAGAACT GGGCCAAGTA TCTCTGGGAA CTGATGATGA GCTAATTGGT   
  
  
- ACAAAC

+     WRE3

| Site Name | Organism | Position | Strand | Matrix score. | sequence | function |
| --- | --- | --- | --- | --- | --- | --- |
| WRE3 | Pisum sativum | 2499 | - | 6 | CCACCT |  |

>HU08G00230.1   
+ -Up\_Stream \_Len000GTTGTA ACAAGCACAT TAAAGTTGAT TATCATTTTG TCCGTGAAAC AGTGAGCAAA   
  
  
+ CGTCATCTCC AGGTCAAATT CATTCGTACT CAATCACAAG TTGCTGACAT TTTCACCAAA GGCTAATCAT   
  
  
+ CGCAAAAGTT TCTTTAGTTT AAGGCCAATC TCTCCATTAT TTCACCCTAT ACAGATTGAG GGGGTGTAAT   
  
  
+ AGTGTATATA TATAGCCTTG TGTTAGCGGG CCTTGGACAC TAGACTTTTA CTCTTGCAAA CCCTAATATA   
  
  
+ TTATTATAAA TATAAATCTA GCCACCCATG TTGAGGTAGG CACTCTAATT CTCACACAGG TAGGATAAGT   
  
  
+ TTTCATAGTT TATGTTGTGA ATGTGATCTT ACTTTGTGGT TCCCGTTGAC AAAAGTTTGA ATCCGAGCAT   
  
  
+ GAGAGTTCAT CAATCTCTTT TTCTTTCCTA TTTTATTTGT TGTGAACTTA ATTGGATTTT CTGGTAGAGG   
  
  
+ AAAGTTCCTT GCTGGAATTT GGTCAAAAGT GATATATATT TTGGGATTTA ATTATCCATA AATTTGGTTT   
  
  
+ TACACCTTGT TAGTTTGGAC TTCTGGTGTG TTATTGTATG TGTTCTGGTG GGTTTTTCTT TTAGCTGAAT   
  
  
+ GTTGGAGCAT TTTAAGGGAT CTAGATATCT GGAGAAGAGC TTGGGAAATA AAGCATATTT CCCTTATAGT   
  
  
+ CTGACATAAC TTTAATGAAT TGATCAGGAA CAAAGGCCAA TTAGTTCAAA AAGGTTCAAC TCCTCTAGGT   
  
  
+ GTTGTGCTAA AGAGATGTGA GTTAGTAAGT AATTGCTTAC TAACTATATA GGATGTTTTA TTACTTGTAG   
  
  
+ AGGTTTTTTT TTTGGGATAT CTTTATGGGG TTTATAAATA AGAAGGTTGA TTTGTTGTCT ACTCTTCTTT   
  
  
+ CGATTCAATA GTAATTCTCT CAGAGAAGGC TAAAATGACA TGGCATTCCA TCGCCTCTCA TGAGGCTTTT   
  
  
+ TGCATGTGCT TATGTATCTT TCATCTCTTG CTTTGGTGGG GTGGCATTGG GGAGGAAAAG GGGGTTTCGA   
  
  
+ TTCGATAGAT CGTCACTTTT GGAAATGACA TCTCTAGCCA ATGGCAAGGG TAAGGTTAAT ACATGTGGAT   
  
  
+ ATTTCCAGAG TTAGTTGTGA TTGTTATTGT TGTTGTTGTA ACTTTATGTC ACGGGGAAAA CATAATAACT   
  
  
+ ATCTGTTTAT CTGTTTGTTT GTTATTTTCA AGGGGAAGGG GAGTGGGCAG ATGCTTAGTA TGTAATCATT   
  
  
+ TTATATAGGG AAAATTCATA TAGCTTGAAA GTAAAACTTT CATTGCCAGC ATTCCAAGGT ATGGGTAGGG   
  
  
+ TTGTCTACAC CTTTATCTCC CTAGAAGCAT GGTGATCATT CCTCGGATTG TGTTATAGAA TAAAGAATGT   
  
  
+ GGTTCTGCTG TAATGAGACT TATGTATTAT CAAATGATGA ATCTGTTTAG AACTTCCTGT TTATTTTTTC   
  
  
+ TGCAAAATTT TAAGAGACTA ACATCTATCG GGCTCAACTC TAGAGGATCT CTTGTGTCAC CCTTCTCAAC   
  
  
+ TCAGTTTGAT TGTGATACAG TTACTATATT GAGTGATAGT CGAGAGCATC ACATCCCGTT AAGGAGTCTA   
  
  
+ TCAACAAGAA GCCCTTCTTG TAATTCTCCC CTTAAAACTA GCAGTTATCA TCACTTGTCA TCGAATAGCC   
  
  
+ CTTTTTGTAA TTCTACCCTT GAAACTAGCA GTTATCGTCA TCGGTTCAAC GCGAGTCCTG TTAGATATTC   
  
  
+ CCATCAAGGC ATAGATTATG GAGTGAGCAT GAAGAATGCT TTGCAGGAGC TAGAGACTAC TCTAATTGGT   
  
  
+ GTAGATGGTG AGGAAGTATC TATTGCTAAT CAACCTATGG GGGGAAGTCG TTAGTCCGGG ATCCCAAGTT   
  
  
+ AGAGATCGAA GTCATCAAGC GAAGATCCAC AGGGTTCACA TCCTACTCAG CTTGATTCAT CATCTTTTTC   
  
  
+ AAGGGTGAGA AGATTCGGAG ATGAAAGCCA GAGAGAGAGA AATGCCATAA GGCAATGGAA GAACCAGCGG   
  
  
+ AACTACTAAG TTTCTCACCA GGTGATTTAA AGCAGTTACT AATTGAATGT GCGAGGGCTT TATTAGATAA   
  
  
+ CCGAATAGAT GACTTTGAGA GTTTGGTTAA ACGGGCAAGG AAAGAGGTCT CCATCTCAGG TGAGCCTGTC   
  
  
+ CAACCTCTCG GTACCTGTAT GATCGAAGGG CTTGTGGCAA GGAAACAGTC TTCGGGGACT AACATCTATT   
  
  
+ GGGCTCTTAA GTGTAAAGAG CCTCTTGGAA AAGACTTGCT CTCCTACGGC ACTCAGTGGG TTACTGTATC   
  
  
+ GCAAGCCATA GCAGCAAGGC TTGGTGGGCC TCCTAAAGTG CGACTTACAG GCATTGATGA TCCTGTTTCT   
  
  
+ AAGTATACTT GTGATGCTAG CTTGGAGGCT GGTGGGAAAC GATTAGCGTC TCTATTTGAA AAGTTTAAAA   
  
  
+ TACTTGTCGA GTTCAATGCA TTGCCCGTTT ATGGACCCAA TGTCAGGTGG GAAATGCTGG ATGTGAGGCC   
  
  
+ CAGGGAGGCT TTGGTCGTTA ATTGTCCATT ACAGCTCCAT TACACTCCTG ACGAGAGTGT CGATGTGAGG   
  
  
+ AACCCTAGGG ATAGGCTTCT CAGAATGGTG AAATTGCTCG GTCCTAAGGT AGCCACTTTG GTTGAGCAAG   
  
  
+ AATCAAACAC CAACACTACA CCTTTCTTGA CCCGGTTCAT AGAGACCCTT GACTACTACT CGATTAACCA   
  
  
+ TGTTTG  

- -Up\_Stream \_Len000CAACAT TGTTCGTGTA ATTTCAACTA ATAGTAAAAC AGGCACTTTG TCACTCGTTT   
  
  
- GCAGTAGAGG TCCAGTTTAA GTAAGCATGA GTTAGTGTTC AACGACTGTA AAAGTGGTTT CCGATTAGTA   
  
  
- GCGTTTTCAA AGAAATCAAA TTCCGGTTAG AGAGGTAATA AAGTGGGATA TGTCTAACTC CCCCACATTA   
  
  
- TCACATATAT ATATCGGAAC ACAATCGCCC GGAACCTGTG ATCTGAAAAT GAGAACGTTT GGGATTATAT   
  
  
- AATAATATTT ATATTTAGAT CGGTGGGTAC AACTCCATCC GTGAGATTAA GAGTGTGTCC ATCCTATTCA   
  
  
- AAAGTATCAA ATACAACACT TACACTAGAA TGAAACACCA AGGGCAACTG TTTTCAAACT TAGGCTCGTA   
  
  
- CTCTCAAGTA GTTAGAGAAA AAGAAAGGAT AAAATAAACA ACACTTGAAT TAACCTAAAA GACCATCTCC   
  
  
- TTTCAAGGAA CGACCTTAAA CCAGTTTTCA CTATATATAA AACCCTAAAT TAATAGGTAT TTAAACCAAA   
  
  
- ATGTGGAACA ATCAAACCTG AAGACCACAC AATAACATAC ACAAGACCAC CCAAAAAGAA AATCGACTTA   
  
  
- CAACCTCGTA AAATTCCCTA GATCTATAGA CCTCTTCTCG AACCCTTTAT TTCGTATAAA GGGAATATCA   
  
  
- GACTGTATTG AAATTACTTA ACTAGTCCTT GTTTCCGGTT AATCAAGTTT TTCCAAGTTG AGGAGATCCA   
  
  
- CAACACGATT TCTCTACACT CAATCATTCA TTAACGAATG ATTGATATAT CCTACAAAAT AATGAACATC   
  
  
- TCCAAAAAAA AAACCCTATA GAAATACCCC AAATATTTAT TCTTCCAACT AAACAACAGA TGAGAAGAAA   
  
  
- GCTAAGTTAT CATTAAGAGA GTCTCTTCCG ATTTTACTGT ACCGTAAGGT AGCGGAGAGT ACTCCGAAAA   
  
  
- ACGTACACGA ATACATAGAA AGTAGAGAAC GAAACCACCC CACCGTAACC CCTCCTTTTC CCCCAAAGCT   
  
  
- AAGCTATCTA GCAGTGAAAA CCTTTACTGT AGAGATCGGT TACCGTTCCC ATTCCAATTA TGTACACCTA   
  
  
- TAAAGGTCTC AATCAACACT AACAATAACA ACAACAACAT TGAAATACAG TGCCCCTTTT GTATTATTGA   
  
  
- TAGACAAATA GACAAACAAA CAATAAAAGT TCCCCTTCCC CTCACCCGTC TACGAATCAT ACATTAGTAA   
  
  
- AATATATCCC TTTTAAGTAT ATCGAACTTT CATTTTGAAA GTAACGGTCG TAAGGTTCCA TACCCATCCC   
  
  
- AACAGATGTG GAAATAGAGG GATCTTCGTA CCACTAGTAA GGAGCCTAAC ACAATATCTT ATTTCTTACA   
  
  
- CCAAGACGAC ATTACTCTGA ATACATAATA GTTTACTACT TAGACAAATC TTGAAGGACA AATAAAAAAG   
  
  
- ACGTTTTAAA ATTCTCTGAT TGTAGATAGC CCGAGTTGAG ATCTCCTAGA GAACACAGTG GGAAGAGTTG   
  
  
- AGTCAAACTA ACACTATGTC AATGATATAA CTCACTATCA GCTCTCGTAG TGTAGGGCAA TTCCTCAGAT   
  
  
- AGTTGTTCTT CGGGAAGAAC ATTAAGAGGG GAATTTTGAT CGTCAATAGT AGTGAACAGT AGCTTATCGG   
  
  
- GAAAAACATT AAGATGGGAA CTTTGATCGT CAATAGCAGT AGCCAAGTTG CGCTCAGGAC AATCTATAAG   
  
  
- GGTAGTTCCG TATCTAATAC CTCACTCGTA CTTCTTACGA AACGTCCTCG ATCTCTGATG AGATTAACCA   
  
  
- CATCTACCAC TCCTTCATAG ATAACGATTA GTTGGATACC CCCCTTCAGC AATCAGGCCC TAGGGTTCAA   
  
  
- TCTCTAGCTT CAGTAGTTCG CTTCTAGGTG TCCCAAGTGT AGGATGAGTC GAACTAAGTA GTAGAAAAAG   
  
  
- TTCCCACTCT TCTAAGCCTC TACTTTCGGT CTCTCTCTCT TTACGGTATT CCGTTACCTT CTTGGTCGCC   
  
  
- TTGATGATTC AAAGAGTGGT CCACTAAATT TCGTCAATGA TTAACTTACA CGCTCCCGAA ATAATCTATT   
  
  
- GGCTTATCTA CTGAAACTCT CAAACCAATT TGCCCGTTCC TTTCTCCAGA GGTAGAGTCC ACTCGGACAG   
  
  
- GTTGGAGAGC CATGGACATA CTAGCTTCCC GAACACCGTT CCTTTGTCAG AAGCCCCTGA TTGTAGATAA   
  
  
- CCCGAGAATT CACATTTCTC GGAGAACCTT TTCTGAACGA GAGGATGCCG TGAGTCACCC AATGACATAG   
  
  
- CGTTCGGTAT CGTCGTTCCG AACCACCCGG AGGATTTCAC GCTGAATGTC CGTAACTACT AGGACAAAGA   
  
  
- TTCATATGAA CACTACGATC GAACCTCCGA CCACCCTTTG CTAATCGCAG AGATAAACTT TTCAAATTTT   
  
  
- ATGAACAGCT CAAGTTACGT AACGGGCAAA TACCTGGGTT ACAGTCCACC CTTTACGACC TACACTCCGG   
  
  
- GTCCCTCCGA AACCAGCAAT TAACAGGTAA TGTCGAGGTA ATGTGAGGAC TGCTCTCACA GCTACACTCC   
  
  
- TTGGGATCCC TATCCGAAGA GTCTTACCAC TTTAACGAGC CAGGATTCCA TCGGTGAAAC CAACTCGTTC   
  
  
- TTAGTTTGTG GTTGTGATGT GGAAAGAACT GGGCCAAGTA TCTCTGGGAA CTGATGATGA GCTAATTGGT   
  
  
- ACAAAC

+     as-1

| Site Name | Organism | Position | Strand | Matrix score. | sequence | function |
| --- | --- | --- | --- | --- | --- | --- |
| as-1 | Arabidopsis thaliana | 2573 | + | 5 | TGACG |  |
| as-1 | Arabidopsis thaliana | 1720 | - | 5 | TGACG |  |
| as-1 | Arabidopsis thaliana | 1065 | - | 5 | TGACG |  |
| as-1 | Arabidopsis thaliana | 75 | - | 5 | TGACG |  |

>HU08G00230.1   
+ -Up\_Stream \_Len000GTTGTA ACAAGCACAT TAAAGTTGAT TATCATTTTG TCCGTGAAAC AGTGAGCAAA   
  
  
+ CGTCATCTCC AGGTCAAATT CATTCGTACT CAATCACAAG TTGCTGACAT TTTCACCAAA GGCTAATCAT   
  
  
+ CGCAAAAGTT TCTTTAGTTT AAGGCCAATC TCTCCATTAT TTCACCCTAT ACAGATTGAG GGGGTGTAAT   
  
  
+ AGTGTATATA TATAGCCTTG TGTTAGCGGG CCTTGGACAC TAGACTTTTA CTCTTGCAAA CCCTAATATA   
  
  
+ TTATTATAAA TATAAATCTA GCCACCCATG TTGAGGTAGG CACTCTAATT CTCACACAGG TAGGATAAGT   
  
  
+ TTTCATAGTT TATGTTGTGA ATGTGATCTT ACTTTGTGGT TCCCGTTGAC AAAAGTTTGA ATCCGAGCAT   
  
  
+ GAGAGTTCAT CAATCTCTTT TTCTTTCCTA TTTTATTTGT TGTGAACTTA ATTGGATTTT CTGGTAGAGG   
  
  
+ AAAGTTCCTT GCTGGAATTT GGTCAAAAGT GATATATATT TTGGGATTTA ATTATCCATA AATTTGGTTT   
  
  
+ TACACCTTGT TAGTTTGGAC TTCTGGTGTG TTATTGTATG TGTTCTGGTG GGTTTTTCTT TTAGCTGAAT   
  
  
+ GTTGGAGCAT TTTAAGGGAT CTAGATATCT GGAGAAGAGC TTGGGAAATA AAGCATATTT CCCTTATAGT   
  
  
+ CTGACATAAC TTTAATGAAT TGATCAGGAA CAAAGGCCAA TTAGTTCAAA AAGGTTCAAC TCCTCTAGGT   
  
  
+ GTTGTGCTAA AGAGATGTGA GTTAGTAAGT AATTGCTTAC TAACTATATA GGATGTTTTA TTACTTGTAG   
  
  
+ AGGTTTTTTT TTTGGGATAT CTTTATGGGG TTTATAAATA AGAAGGTTGA TTTGTTGTCT ACTCTTCTTT   
  
  
+ CGATTCAATA GTAATTCTCT CAGAGAAGGC TAAAATGACA TGGCATTCCA TCGCCTCTCA TGAGGCTTTT   
  
  
+ TGCATGTGCT TATGTATCTT TCATCTCTTG CTTTGGTGGG GTGGCATTGG GGAGGAAAAG GGGGTTTCGA   
  
  
+ TTCGATAGAT CGTCACTTTT GGAAATGACA TCTCTAGCCA ATGGCAAGGG TAAGGTTAAT ACATGTGGAT   
  
  
+ ATTTCCAGAG TTAGTTGTGA TTGTTATTGT TGTTGTTGTA ACTTTATGTC ACGGGGAAAA CATAATAACT   
  
  
+ ATCTGTTTAT CTGTTTGTTT GTTATTTTCA AGGGGAAGGG GAGTGGGCAG ATGCTTAGTA TGTAATCATT   
  
  
+ TTATATAGGG AAAATTCATA TAGCTTGAAA GTAAAACTTT CATTGCCAGC ATTCCAAGGT ATGGGTAGGG   
  
  
+ TTGTCTACAC CTTTATCTCC CTAGAAGCAT GGTGATCATT CCTCGGATTG TGTTATAGAA TAAAGAATGT   
  
  
+ GGTTCTGCTG TAATGAGACT TATGTATTAT CAAATGATGA ATCTGTTTAG AACTTCCTGT TTATTTTTTC   
  
  
+ TGCAAAATTT TAAGAGACTA ACATCTATCG GGCTCAACTC TAGAGGATCT CTTGTGTCAC CCTTCTCAAC   
  
  
+ TCAGTTTGAT TGTGATACAG TTACTATATT GAGTGATAGT CGAGAGCATC ACATCCCGTT AAGGAGTCTA   
  
  
+ TCAACAAGAA GCCCTTCTTG TAATTCTCCC CTTAAAACTA GCAGTTATCA TCACTTGTCA TCGAATAGCC   
  
  
+ CTTTTTGTAA TTCTACCCTT GAAACTAGCA GTTATCGTCA TCGGTTCAAC GCGAGTCCTG TTAGATATTC   
  
  
+ CCATCAAGGC ATAGATTATG GAGTGAGCAT GAAGAATGCT TTGCAGGAGC TAGAGACTAC TCTAATTGGT   
  
  
+ GTAGATGGTG AGGAAGTATC TATTGCTAAT CAACCTATGG GGGGAAGTCG TTAGTCCGGG ATCCCAAGTT   
  
  
+ AGAGATCGAA GTCATCAAGC GAAGATCCAC AGGGTTCACA TCCTACTCAG CTTGATTCAT CATCTTTTTC   
  
  
+ AAGGGTGAGA AGATTCGGAG ATGAAAGCCA GAGAGAGAGA AATGCCATAA GGCAATGGAA GAACCAGCGG   
  
  
+ AACTACTAAG TTTCTCACCA GGTGATTTAA AGCAGTTACT AATTGAATGT GCGAGGGCTT TATTAGATAA   
  
  
+ CCGAATAGAT GACTTTGAGA GTTTGGTTAA ACGGGCAAGG AAAGAGGTCT CCATCTCAGG TGAGCCTGTC   
  
  
+ CAACCTCTCG GTACCTGTAT GATCGAAGGG CTTGTGGCAA GGAAACAGTC TTCGGGGACT AACATCTATT   
  
  
+ GGGCTCTTAA GTGTAAAGAG CCTCTTGGAA AAGACTTGCT CTCCTACGGC ACTCAGTGGG TTACTGTATC   
  
  
+ GCAAGCCATA GCAGCAAGGC TTGGTGGGCC TCCTAAAGTG CGACTTACAG GCATTGATGA TCCTGTTTCT   
  
  
+ AAGTATACTT GTGATGCTAG CTTGGAGGCT GGTGGGAAAC GATTAGCGTC TCTATTTGAA AAGTTTAAAA   
  
  
+ TACTTGTCGA GTTCAATGCA TTGCCCGTTT ATGGACCCAA TGTCAGGTGG GAAATGCTGG ATGTGAGGCC   
  
  
+ CAGGGAGGCT TTGGTCGTTA ATTGTCCATT ACAGCTCCAT TACACTCCTG ACGAGAGTGT CGATGTGAGG   
  
  
+ AACCCTAGGG ATAGGCTTCT CAGAATGGTG AAATTGCTCG GTCCTAAGGT AGCCACTTTG GTTGAGCAAG   
  
  
+ AATCAAACAC CAACACTACA CCTTTCTTGA CCCGGTTCAT AGAGACCCTT GACTACTACT CGATTAACCA   
  
  
+ TGTTTG  

- -Up\_Stream \_Len000CAACAT TGTTCGTGTA ATTTCAACTA ATAGTAAAAC AGGCACTTTG TCACTCGTTT   
  
  
- GCAGTAGAGG TCCAGTTTAA GTAAGCATGA GTTAGTGTTC AACGACTGTA AAAGTGGTTT CCGATTAGTA   
  
  
- GCGTTTTCAA AGAAATCAAA TTCCGGTTAG AGAGGTAATA AAGTGGGATA TGTCTAACTC CCCCACATTA   
  
  
- TCACATATAT ATATCGGAAC ACAATCGCCC GGAACCTGTG ATCTGAAAAT GAGAACGTTT GGGATTATAT   
  
  
- AATAATATTT ATATTTAGAT CGGTGGGTAC AACTCCATCC GTGAGATTAA GAGTGTGTCC ATCCTATTCA   
  
  
- AAAGTATCAA ATACAACACT TACACTAGAA TGAAACACCA AGGGCAACTG TTTTCAAACT TAGGCTCGTA   
  
  
- CTCTCAAGTA GTTAGAGAAA AAGAAAGGAT AAAATAAACA ACACTTGAAT TAACCTAAAA GACCATCTCC   
  
  
- TTTCAAGGAA CGACCTTAAA CCAGTTTTCA CTATATATAA AACCCTAAAT TAATAGGTAT TTAAACCAAA   
  
  
- ATGTGGAACA ATCAAACCTG AAGACCACAC AATAACATAC ACAAGACCAC CCAAAAAGAA AATCGACTTA   
  
  
- CAACCTCGTA AAATTCCCTA GATCTATAGA CCTCTTCTCG AACCCTTTAT TTCGTATAAA GGGAATATCA   
  
  
- GACTGTATTG AAATTACTTA ACTAGTCCTT GTTTCCGGTT AATCAAGTTT TTCCAAGTTG AGGAGATCCA   
  
  
- CAACACGATT TCTCTACACT CAATCATTCA TTAACGAATG ATTGATATAT CCTACAAAAT AATGAACATC   
  
  
- TCCAAAAAAA AAACCCTATA GAAATACCCC AAATATTTAT TCTTCCAACT AAACAACAGA TGAGAAGAAA   
  
  
- GCTAAGTTAT CATTAAGAGA GTCTCTTCCG ATTTTACTGT ACCGTAAGGT AGCGGAGAGT ACTCCGAAAA   
  
  
- ACGTACACGA ATACATAGAA AGTAGAGAAC GAAACCACCC CACCGTAACC CCTCCTTTTC CCCCAAAGCT   
  
  
- AAGCTATCTA GCAGTGAAAA CCTTTACTGT AGAGATCGGT TACCGTTCCC ATTCCAATTA TGTACACCTA   
  
  
- TAAAGGTCTC AATCAACACT AACAATAACA ACAACAACAT TGAAATACAG TGCCCCTTTT GTATTATTGA   
  
  
- TAGACAAATA GACAAACAAA CAATAAAAGT TCCCCTTCCC CTCACCCGTC TACGAATCAT ACATTAGTAA   
  
  
- AATATATCCC TTTTAAGTAT ATCGAACTTT CATTTTGAAA GTAACGGTCG TAAGGTTCCA TACCCATCCC   
  
  
- AACAGATGTG GAAATAGAGG GATCTTCGTA CCACTAGTAA GGAGCCTAAC ACAATATCTT ATTTCTTACA   
  
  
- CCAAGACGAC ATTACTCTGA ATACATAATA GTTTACTACT TAGACAAATC TTGAAGGACA AATAAAAAAG   
  
  
- ACGTTTTAAA ATTCTCTGAT TGTAGATAGC CCGAGTTGAG ATCTCCTAGA GAACACAGTG GGAAGAGTTG   
  
  
- AGTCAAACTA ACACTATGTC AATGATATAA CTCACTATCA GCTCTCGTAG TGTAGGGCAA TTCCTCAGAT   
  
  
- AGTTGTTCTT CGGGAAGAAC ATTAAGAGGG GAATTTTGAT CGTCAATAGT AGTGAACAGT AGCTTATCGG   
  
  
- GAAAAACATT AAGATGGGAA CTTTGATCGT CAATAGCAGT AGCCAAGTTG CGCTCAGGAC AATCTATAAG   
  
  
- GGTAGTTCCG TATCTAATAC CTCACTCGTA CTTCTTACGA AACGTCCTCG ATCTCTGATG AGATTAACCA   
  
  
- CATCTACCAC TCCTTCATAG ATAACGATTA GTTGGATACC CCCCTTCAGC AATCAGGCCC TAGGGTTCAA   
  
  
- TCTCTAGCTT CAGTAGTTCG CTTCTAGGTG TCCCAAGTGT AGGATGAGTC GAACTAAGTA GTAGAAAAAG   
  
  
- TTCCCACTCT TCTAAGCCTC TACTTTCGGT CTCTCTCTCT TTACGGTATT CCGTTACCTT CTTGGTCGCC   
  
  
- TTGATGATTC AAAGAGTGGT CCACTAAATT TCGTCAATGA TTAACTTACA CGCTCCCGAA ATAATCTATT   
  
  
- GGCTTATCTA CTGAAACTCT CAAACCAATT TGCCCGTTCC TTTCTCCAGA GGTAGAGTCC ACTCGGACAG   
  
  
- GTTGGAGAGC CATGGACATA CTAGCTTCCC GAACACCGTT CCTTTGTCAG AAGCCCCTGA TTGTAGATAA   
  
  
- CCCGAGAATT CACATTTCTC GGAGAACCTT TTCTGAACGA GAGGATGCCG TGAGTCACCC AATGACATAG   
  
  
- CGTTCGGTAT CGTCGTTCCG AACCACCCGG AGGATTTCAC GCTGAATGTC CGTAACTACT AGGACAAAGA   
  
  
- TTCATATGAA CACTACGATC GAACCTCCGA CCACCCTTTG CTAATCGCAG AGATAAACTT TTCAAATTTT   
  
  
- ATGAACAGCT CAAGTTACGT AACGGGCAAA TACCTGGGTT ACAGTCCACC CTTTACGACC TACACTCCGG   
  
  
- GTCCCTCCGA AACCAGCAAT TAACAGGTAA TGTCGAGGTA ATGTGAGGAC TGCTCTCACA GCTACACTCC   
  
  
- TTGGGATCCC TATCCGAAGA GTCTTACCAC TTTAACGAGC CAGGATTCCA TCGGTGAAAC CAACTCGTTC   
  
  
- TTAGTTTGTG GTTGTGATGT GGAAAGAACT GGGCCAAGTA TCTCTGGGAA CTGATGATGA GCTAATTGGT   
  
  
- ACAAAC

+     box S

| Site Name | Organism | Position | Strand | Matrix score. | sequence | function |
| --- | --- | --- | --- | --- | --- | --- |
| box S | Arabidopsis thaliana | 304 | + | 7 | AGCCACC |  |

>HU08G00230.1   
+ -Up\_Stream \_Len000GTTGTA ACAAGCACAT TAAAGTTGAT TATCATTTTG TCCGTGAAAC AGTGAGCAAA   
  
  
+ CGTCATCTCC AGGTCAAATT CATTCGTACT CAATCACAAG TTGCTGACAT TTTCACCAAA GGCTAATCAT   
  
  
+ CGCAAAAGTT TCTTTAGTTT AAGGCCAATC TCTCCATTAT TTCACCCTAT ACAGATTGAG GGGGTGTAAT   
  
  
+ AGTGTATATA TATAGCCTTG TGTTAGCGGG CCTTGGACAC TAGACTTTTA CTCTTGCAAA CCCTAATATA   
  
  
+ TTATTATAAA TATAAATCTA GCCACCCATG TTGAGGTAGG CACTCTAATT CTCACACAGG TAGGATAAGT   
  
  
+ TTTCATAGTT TATGTTGTGA ATGTGATCTT ACTTTGTGGT TCCCGTTGAC AAAAGTTTGA ATCCGAGCAT   
  
  
+ GAGAGTTCAT CAATCTCTTT TTCTTTCCTA TTTTATTTGT TGTGAACTTA ATTGGATTTT CTGGTAGAGG   
  
  
+ AAAGTTCCTT GCTGGAATTT GGTCAAAAGT GATATATATT TTGGGATTTA ATTATCCATA AATTTGGTTT   
  
  
+ TACACCTTGT TAGTTTGGAC TTCTGGTGTG TTATTGTATG TGTTCTGGTG GGTTTTTCTT TTAGCTGAAT   
  
  
+ GTTGGAGCAT TTTAAGGGAT CTAGATATCT GGAGAAGAGC TTGGGAAATA AAGCATATTT CCCTTATAGT   
  
  
+ CTGACATAAC TTTAATGAAT TGATCAGGAA CAAAGGCCAA TTAGTTCAAA AAGGTTCAAC TCCTCTAGGT   
  
  
+ GTTGTGCTAA AGAGATGTGA GTTAGTAAGT AATTGCTTAC TAACTATATA GGATGTTTTA TTACTTGTAG   
  
  
+ AGGTTTTTTT TTTGGGATAT CTTTATGGGG TTTATAAATA AGAAGGTTGA TTTGTTGTCT ACTCTTCTTT   
  
  
+ CGATTCAATA GTAATTCTCT CAGAGAAGGC TAAAATGACA TGGCATTCCA TCGCCTCTCA TGAGGCTTTT   
  
  
+ TGCATGTGCT TATGTATCTT TCATCTCTTG CTTTGGTGGG GTGGCATTGG GGAGGAAAAG GGGGTTTCGA   
  
  
+ TTCGATAGAT CGTCACTTTT GGAAATGACA TCTCTAGCCA ATGGCAAGGG TAAGGTTAAT ACATGTGGAT   
  
  
+ ATTTCCAGAG TTAGTTGTGA TTGTTATTGT TGTTGTTGTA ACTTTATGTC ACGGGGAAAA CATAATAACT   
  
  
+ ATCTGTTTAT CTGTTTGTTT GTTATTTTCA AGGGGAAGGG GAGTGGGCAG ATGCTTAGTA TGTAATCATT   
  
  
+ TTATATAGGG AAAATTCATA TAGCTTGAAA GTAAAACTTT CATTGCCAGC ATTCCAAGGT ATGGGTAGGG   
  
  
+ TTGTCTACAC CTTTATCTCC CTAGAAGCAT GGTGATCATT CCTCGGATTG TGTTATAGAA TAAAGAATGT   
  
  
+ GGTTCTGCTG TAATGAGACT TATGTATTAT CAAATGATGA ATCTGTTTAG AACTTCCTGT TTATTTTTTC   
  
  
+ TGCAAAATTT TAAGAGACTA ACATCTATCG GGCTCAACTC TAGAGGATCT CTTGTGTCAC CCTTCTCAAC   
  
  
+ TCAGTTTGAT TGTGATACAG TTACTATATT GAGTGATAGT CGAGAGCATC ACATCCCGTT AAGGAGTCTA   
  
  
+ TCAACAAGAA GCCCTTCTTG TAATTCTCCC CTTAAAACTA GCAGTTATCA TCACTTGTCA TCGAATAGCC   
  
  
+ CTTTTTGTAA TTCTACCCTT GAAACTAGCA GTTATCGTCA TCGGTTCAAC GCGAGTCCTG TTAGATATTC   
  
  
+ CCATCAAGGC ATAGATTATG GAGTGAGCAT GAAGAATGCT TTGCAGGAGC TAGAGACTAC TCTAATTGGT   
  
  
+ GTAGATGGTG AGGAAGTATC TATTGCTAAT CAACCTATGG GGGGAAGTCG TTAGTCCGGG ATCCCAAGTT   
  
  
+ AGAGATCGAA GTCATCAAGC GAAGATCCAC AGGGTTCACA TCCTACTCAG CTTGATTCAT CATCTTTTTC   
  
  
+ AAGGGTGAGA AGATTCGGAG ATGAAAGCCA GAGAGAGAGA AATGCCATAA GGCAATGGAA GAACCAGCGG   
  
  
+ AACTACTAAG TTTCTCACCA GGTGATTTAA AGCAGTTACT AATTGAATGT GCGAGGGCTT TATTAGATAA   
  
  
+ CCGAATAGAT GACTTTGAGA GTTTGGTTAA ACGGGCAAGG AAAGAGGTCT CCATCTCAGG TGAGCCTGTC   
  
  
+ CAACCTCTCG GTACCTGTAT GATCGAAGGG CTTGTGGCAA GGAAACAGTC TTCGGGGACT AACATCTATT   
  
  
+ GGGCTCTTAA GTGTAAAGAG CCTCTTGGAA AAGACTTGCT CTCCTACGGC ACTCAGTGGG TTACTGTATC   
  
  
+ GCAAGCCATA GCAGCAAGGC TTGGTGGGCC TCCTAAAGTG CGACTTACAG GCATTGATGA TCCTGTTTCT   
  
  
+ AAGTATACTT GTGATGCTAG CTTGGAGGCT GGTGGGAAAC GATTAGCGTC TCTATTTGAA AAGTTTAAAA   
  
  
+ TACTTGTCGA GTTCAATGCA TTGCCCGTTT ATGGACCCAA TGTCAGGTGG GAAATGCTGG ATGTGAGGCC   
  
  
+ CAGGGAGGCT TTGGTCGTTA ATTGTCCATT ACAGCTCCAT TACACTCCTG ACGAGAGTGT CGATGTGAGG   
  
  
+ AACCCTAGGG ATAGGCTTCT CAGAATGGTG AAATTGCTCG GTCCTAAGGT AGCCACTTTG GTTGAGCAAG   
  
  
+ AATCAAACAC CAACACTACA CCTTTCTTGA CCCGGTTCAT AGAGACCCTT GACTACTACT CGATTAACCA   
  
  
+ TGTTTG  

- -Up\_Stream \_Len000CAACAT TGTTCGTGTA ATTTCAACTA ATAGTAAAAC AGGCACTTTG TCACTCGTTT   
  
  
- GCAGTAGAGG TCCAGTTTAA GTAAGCATGA GTTAGTGTTC AACGACTGTA AAAGTGGTTT CCGATTAGTA   
  
  
- GCGTTTTCAA AGAAATCAAA TTCCGGTTAG AGAGGTAATA AAGTGGGATA TGTCTAACTC CCCCACATTA   
  
  
- TCACATATAT ATATCGGAAC ACAATCGCCC GGAACCTGTG ATCTGAAAAT GAGAACGTTT GGGATTATAT   
  
  
- AATAATATTT ATATTTAGAT CGGTGGGTAC AACTCCATCC GTGAGATTAA GAGTGTGTCC ATCCTATTCA   
  
  
- AAAGTATCAA ATACAACACT TACACTAGAA TGAAACACCA AGGGCAACTG TTTTCAAACT TAGGCTCGTA   
  
  
- CTCTCAAGTA GTTAGAGAAA AAGAAAGGAT AAAATAAACA ACACTTGAAT TAACCTAAAA GACCATCTCC   
  
  
- TTTCAAGGAA CGACCTTAAA CCAGTTTTCA CTATATATAA AACCCTAAAT TAATAGGTAT TTAAACCAAA   
  
  
- ATGTGGAACA ATCAAACCTG AAGACCACAC AATAACATAC ACAAGACCAC CCAAAAAGAA AATCGACTTA   
  
  
- CAACCTCGTA AAATTCCCTA GATCTATAGA CCTCTTCTCG AACCCTTTAT TTCGTATAAA GGGAATATCA   
  
  
- GACTGTATTG AAATTACTTA ACTAGTCCTT GTTTCCGGTT AATCAAGTTT TTCCAAGTTG AGGAGATCCA   
  
  
- CAACACGATT TCTCTACACT CAATCATTCA TTAACGAATG ATTGATATAT CCTACAAAAT AATGAACATC   
  
  
- TCCAAAAAAA AAACCCTATA GAAATACCCC AAATATTTAT TCTTCCAACT AAACAACAGA TGAGAAGAAA   
  
  
- GCTAAGTTAT CATTAAGAGA GTCTCTTCCG ATTTTACTGT ACCGTAAGGT AGCGGAGAGT ACTCCGAAAA   
  
  
- ACGTACACGA ATACATAGAA AGTAGAGAAC GAAACCACCC CACCGTAACC CCTCCTTTTC CCCCAAAGCT   
  
  
- AAGCTATCTA GCAGTGAAAA CCTTTACTGT AGAGATCGGT TACCGTTCCC ATTCCAATTA TGTACACCTA   
  
  
- TAAAGGTCTC AATCAACACT AACAATAACA ACAACAACAT TGAAATACAG TGCCCCTTTT GTATTATTGA   
  
  
- TAGACAAATA GACAAACAAA CAATAAAAGT TCCCCTTCCC CTCACCCGTC TACGAATCAT ACATTAGTAA   
  
  
- AATATATCCC TTTTAAGTAT ATCGAACTTT CATTTTGAAA GTAACGGTCG TAAGGTTCCA TACCCATCCC   
  
  
- AACAGATGTG GAAATAGAGG GATCTTCGTA CCACTAGTAA GGAGCCTAAC ACAATATCTT ATTTCTTACA   
  
  
- CCAAGACGAC ATTACTCTGA ATACATAATA GTTTACTACT TAGACAAATC TTGAAGGACA AATAAAAAAG   
  
  
- ACGTTTTAAA ATTCTCTGAT TGTAGATAGC CCGAGTTGAG ATCTCCTAGA GAACACAGTG GGAAGAGTTG   
  
  
- AGTCAAACTA ACACTATGTC AATGATATAA CTCACTATCA GCTCTCGTAG TGTAGGGCAA TTCCTCAGAT   
  
  
- AGTTGTTCTT CGGGAAGAAC ATTAAGAGGG GAATTTTGAT CGTCAATAGT AGTGAACAGT AGCTTATCGG   
  
  
- GAAAAACATT AAGATGGGAA CTTTGATCGT CAATAGCAGT AGCCAAGTTG CGCTCAGGAC AATCTATAAG   
  
  
- GGTAGTTCCG TATCTAATAC CTCACTCGTA CTTCTTACGA AACGTCCTCG ATCTCTGATG AGATTAACCA   
  
  
- CATCTACCAC TCCTTCATAG ATAACGATTA GTTGGATACC CCCCTTCAGC AATCAGGCCC TAGGGTTCAA   
  
  
- TCTCTAGCTT CAGTAGTTCG CTTCTAGGTG TCCCAAGTGT AGGATGAGTC GAACTAAGTA GTAGAAAAAG   
  
  
- TTCCCACTCT TCTAAGCCTC TACTTTCGGT CTCTCTCTCT TTACGGTATT CCGTTACCTT CTTGGTCGCC   
  
  
- TTGATGATTC AAAGAGTGGT CCACTAAATT TCGTCAATGA TTAACTTACA CGCTCCCGAA ATAATCTATT   
  
  
- GGCTTATCTA CTGAAACTCT CAAACCAATT TGCCCGTTCC TTTCTCCAGA GGTAGAGTCC ACTCGGACAG   
  
  
- GTTGGAGAGC CATGGACATA CTAGCTTCCC GAACACCGTT CCTTTGTCAG AAGCCCCTGA TTGTAGATAA   
  
  
- CCCGAGAATT CACATTTCTC GGAGAACCTT TTCTGAACGA GAGGATGCCG TGAGTCACCC AATGACATAG   
  
  
- CGTTCGGTAT CGTCGTTCCG AACCACCCGG AGGATTTCAC GCTGAATGTC CGTAACTACT AGGACAAAGA   
  
  
- TTCATATGAA CACTACGATC GAACCTCCGA CCACCCTTTG CTAATCGCAG AGATAAACTT TTCAAATTTT   
  
  
- ATGAACAGCT CAAGTTACGT AACGGGCAAA TACCTGGGTT ACAGTCCACC CTTTACGACC TACACTCCGG   
  
  
- GTCCCTCCGA AACCAGCAAT TAACAGGTAA TGTCGAGGTA ATGTGAGGAC TGCTCTCACA GCTACACTCC   
  
  
- TTGGGATCCC TATCCGAAGA GTCTTACCAC TTTAACGAGC CAGGATTCCA TCGGTGAAAC CAACTCGTTC   
  
  
- TTAGTTTGTG GTTGTGATGT GGAAAGAACT GGGCCAAGTA TCTCTGGGAA CTGATGATGA GCTAATTGGT   
  
  
- ACAAAC

+     circadian

| Site Name | Organism | Position | Strand | Matrix score. | sequence | function |
| --- | --- | --- | --- | --- | --- | --- |
| circadian | Lycopersicon esculentum | 860 | - | 9 | CAAAGATATC | cis-acting regulatory element involved in circadian control |

>HU08G00230.1   
+ -Up\_Stream \_Len000GTTGTA ACAAGCACAT TAAAGTTGAT TATCATTTTG TCCGTGAAAC AGTGAGCAAA   
  
  
+ CGTCATCTCC AGGTCAAATT CATTCGTACT CAATCACAAG TTGCTGACAT TTTCACCAAA GGCTAATCAT   
  
  
+ CGCAAAAGTT TCTTTAGTTT AAGGCCAATC TCTCCATTAT TTCACCCTAT ACAGATTGAG GGGGTGTAAT   
  
  
+ AGTGTATATA TATAGCCTTG TGTTAGCGGG CCTTGGACAC TAGACTTTTA CTCTTGCAAA CCCTAATATA   
  
  
+ TTATTATAAA TATAAATCTA GCCACCCATG TTGAGGTAGG CACTCTAATT CTCACACAGG TAGGATAAGT   
  
  
+ TTTCATAGTT TATGTTGTGA ATGTGATCTT ACTTTGTGGT TCCCGTTGAC AAAAGTTTGA ATCCGAGCAT   
  
  
+ GAGAGTTCAT CAATCTCTTT TTCTTTCCTA TTTTATTTGT TGTGAACTTA ATTGGATTTT CTGGTAGAGG   
  
  
+ AAAGTTCCTT GCTGGAATTT GGTCAAAAGT GATATATATT TTGGGATTTA ATTATCCATA AATTTGGTTT   
  
  
+ TACACCTTGT TAGTTTGGAC TTCTGGTGTG TTATTGTATG TGTTCTGGTG GGTTTTTCTT TTAGCTGAAT   
  
  
+ GTTGGAGCAT TTTAAGGGAT CTAGATATCT GGAGAAGAGC TTGGGAAATA AAGCATATTT CCCTTATAGT   
  
  
+ CTGACATAAC TTTAATGAAT TGATCAGGAA CAAAGGCCAA TTAGTTCAAA AAGGTTCAAC TCCTCTAGGT   
  
  
+ GTTGTGCTAA AGAGATGTGA GTTAGTAAGT AATTGCTTAC TAACTATATA GGATGTTTTA TTACTTGTAG   
  
  
+ AGGTTTTTTT TTTGGGATAT CTTTATGGGG TTTATAAATA AGAAGGTTGA TTTGTTGTCT ACTCTTCTTT   
  
  
+ CGATTCAATA GTAATTCTCT CAGAGAAGGC TAAAATGACA TGGCATTCCA TCGCCTCTCA TGAGGCTTTT   
  
  
+ TGCATGTGCT TATGTATCTT TCATCTCTTG CTTTGGTGGG GTGGCATTGG GGAGGAAAAG GGGGTTTCGA   
  
  
+ TTCGATAGAT CGTCACTTTT GGAAATGACA TCTCTAGCCA ATGGCAAGGG TAAGGTTAAT ACATGTGGAT   
  
  
+ ATTTCCAGAG TTAGTTGTGA TTGTTATTGT TGTTGTTGTA ACTTTATGTC ACGGGGAAAA CATAATAACT   
  
  
+ ATCTGTTTAT CTGTTTGTTT GTTATTTTCA AGGGGAAGGG GAGTGGGCAG ATGCTTAGTA TGTAATCATT   
  
  
+ TTATATAGGG AAAATTCATA TAGCTTGAAA GTAAAACTTT CATTGCCAGC ATTCCAAGGT ATGGGTAGGG   
  
  
+ TTGTCTACAC CTTTATCTCC CTAGAAGCAT GGTGATCATT CCTCGGATTG TGTTATAGAA TAAAGAATGT   
  
  
+ GGTTCTGCTG TAATGAGACT TATGTATTAT CAAATGATGA ATCTGTTTAG AACTTCCTGT TTATTTTTTC   
  
  
+ TGCAAAATTT TAAGAGACTA ACATCTATCG GGCTCAACTC TAGAGGATCT CTTGTGTCAC CCTTCTCAAC   
  
  
+ TCAGTTTGAT TGTGATACAG TTACTATATT GAGTGATAGT CGAGAGCATC ACATCCCGTT AAGGAGTCTA   
  
  
+ TCAACAAGAA GCCCTTCTTG TAATTCTCCC CTTAAAACTA GCAGTTATCA TCACTTGTCA TCGAATAGCC   
  
  
+ CTTTTTGTAA TTCTACCCTT GAAACTAGCA GTTATCGTCA TCGGTTCAAC GCGAGTCCTG TTAGATATTC   
  
  
+ CCATCAAGGC ATAGATTATG GAGTGAGCAT GAAGAATGCT TTGCAGGAGC TAGAGACTAC TCTAATTGGT   
  
  
+ GTAGATGGTG AGGAAGTATC TATTGCTAAT CAACCTATGG GGGGAAGTCG TTAGTCCGGG ATCCCAAGTT   
  
  
+ AGAGATCGAA GTCATCAAGC GAAGATCCAC AGGGTTCACA TCCTACTCAG CTTGATTCAT CATCTTTTTC   
  
  
+ AAGGGTGAGA AGATTCGGAG ATGAAAGCCA GAGAGAGAGA AATGCCATAA GGCAATGGAA GAACCAGCGG   
  
  
+ AACTACTAAG TTTCTCACCA GGTGATTTAA AGCAGTTACT AATTGAATGT GCGAGGGCTT TATTAGATAA   
  
  
+ CCGAATAGAT GACTTTGAGA GTTTGGTTAA ACGGGCAAGG AAAGAGGTCT CCATCTCAGG TGAGCCTGTC   
  
  
+ CAACCTCTCG GTACCTGTAT GATCGAAGGG CTTGTGGCAA GGAAACAGTC TTCGGGGACT AACATCTATT   
  
  
+ GGGCTCTTAA GTGTAAAGAG CCTCTTGGAA AAGACTTGCT CTCCTACGGC ACTCAGTGGG TTACTGTATC   
  
  
+ GCAAGCCATA GCAGCAAGGC TTGGTGGGCC TCCTAAAGTG CGACTTACAG GCATTGATGA TCCTGTTTCT   
  
  
+ AAGTATACTT GTGATGCTAG CTTGGAGGCT GGTGGGAAAC GATTAGCGTC TCTATTTGAA AAGTTTAAAA   
  
  
+ TACTTGTCGA GTTCAATGCA TTGCCCGTTT ATGGACCCAA TGTCAGGTGG GAAATGCTGG ATGTGAGGCC   
  
  
+ CAGGGAGGCT TTGGTCGTTA ATTGTCCATT ACAGCTCCAT TACACTCCTG ACGAGAGTGT CGATGTGAGG   
  
  
+ AACCCTAGGG ATAGGCTTCT CAGAATGGTG AAATTGCTCG GTCCTAAGGT AGCCACTTTG GTTGAGCAAG   
  
  
+ AATCAAACAC CAACACTACA CCTTTCTTGA CCCGGTTCAT AGAGACCCTT GACTACTACT CGATTAACCA   
  
  
+ TGTTTG  

- -Up\_Stream \_Len000CAACAT TGTTCGTGTA ATTTCAACTA ATAGTAAAAC AGGCACTTTG TCACTCGTTT   
  
  
- GCAGTAGAGG TCCAGTTTAA GTAAGCATGA GTTAGTGTTC AACGACTGTA AAAGTGGTTT CCGATTAGTA   
  
  
- GCGTTTTCAA AGAAATCAAA TTCCGGTTAG AGAGGTAATA AAGTGGGATA TGTCTAACTC CCCCACATTA   
  
  
- TCACATATAT ATATCGGAAC ACAATCGCCC GGAACCTGTG ATCTGAAAAT GAGAACGTTT GGGATTATAT   
  
  
- AATAATATTT ATATTTAGAT CGGTGGGTAC AACTCCATCC GTGAGATTAA GAGTGTGTCC ATCCTATTCA   
  
  
- AAAGTATCAA ATACAACACT TACACTAGAA TGAAACACCA AGGGCAACTG TTTTCAAACT TAGGCTCGTA   
  
  
- CTCTCAAGTA GTTAGAGAAA AAGAAAGGAT AAAATAAACA ACACTTGAAT TAACCTAAAA GACCATCTCC   
  
  
- TTTCAAGGAA CGACCTTAAA CCAGTTTTCA CTATATATAA AACCCTAAAT TAATAGGTAT TTAAACCAAA   
  
  
- ATGTGGAACA ATCAAACCTG AAGACCACAC AATAACATAC ACAAGACCAC CCAAAAAGAA AATCGACTTA   
  
  
- CAACCTCGTA AAATTCCCTA GATCTATAGA CCTCTTCTCG AACCCTTTAT TTCGTATAAA GGGAATATCA   
  
  
- GACTGTATTG AAATTACTTA ACTAGTCCTT GTTTCCGGTT AATCAAGTTT TTCCAAGTTG AGGAGATCCA   
  
  
- CAACACGATT TCTCTACACT CAATCATTCA TTAACGAATG ATTGATATAT CCTACAAAAT AATGAACATC   
  
  
- TCCAAAAAAA AAACCCTATA GAAATACCCC AAATATTTAT TCTTCCAACT AAACAACAGA TGAGAAGAAA   
  
  
- GCTAAGTTAT CATTAAGAGA GTCTCTTCCG ATTTTACTGT ACCGTAAGGT AGCGGAGAGT ACTCCGAAAA   
  
  
- ACGTACACGA ATACATAGAA AGTAGAGAAC GAAACCACCC CACCGTAACC CCTCCTTTTC CCCCAAAGCT   
  
  
- AAGCTATCTA GCAGTGAAAA CCTTTACTGT AGAGATCGGT TACCGTTCCC ATTCCAATTA TGTACACCTA   
  
  
- TAAAGGTCTC AATCAACACT AACAATAACA ACAACAACAT TGAAATACAG TGCCCCTTTT GTATTATTGA   
  
  
- TAGACAAATA GACAAACAAA CAATAAAAGT TCCCCTTCCC CTCACCCGTC TACGAATCAT ACATTAGTAA   
  
  
- AATATATCCC TTTTAAGTAT ATCGAACTTT CATTTTGAAA GTAACGGTCG TAAGGTTCCA TACCCATCCC   
  
  
- AACAGATGTG GAAATAGAGG GATCTTCGTA CCACTAGTAA GGAGCCTAAC ACAATATCTT ATTTCTTACA   
  
  
- CCAAGACGAC ATTACTCTGA ATACATAATA GTTTACTACT TAGACAAATC TTGAAGGACA AATAAAAAAG   
  
  
- ACGTTTTAAA ATTCTCTGAT TGTAGATAGC CCGAGTTGAG ATCTCCTAGA GAACACAGTG GGAAGAGTTG   
  
  
- AGTCAAACTA ACACTATGTC AATGATATAA CTCACTATCA GCTCTCGTAG TGTAGGGCAA TTCCTCAGAT   
  
  
- AGTTGTTCTT CGGGAAGAAC ATTAAGAGGG GAATTTTGAT CGTCAATAGT AGTGAACAGT AGCTTATCGG   
  
  
- GAAAAACATT AAGATGGGAA CTTTGATCGT CAATAGCAGT AGCCAAGTTG CGCTCAGGAC AATCTATAAG   
  
  
- GGTAGTTCCG TATCTAATAC CTCACTCGTA CTTCTTACGA AACGTCCTCG ATCTCTGATG AGATTAACCA   
  
  
- CATCTACCAC TCCTTCATAG ATAACGATTA GTTGGATACC CCCCTTCAGC AATCAGGCCC TAGGGTTCAA   
  
  
- TCTCTAGCTT CAGTAGTTCG CTTCTAGGTG TCCCAAGTGT AGGATGAGTC GAACTAAGTA GTAGAAAAAG   
  
  
- TTCCCACTCT TCTAAGCCTC TACTTTCGGT CTCTCTCTCT TTACGGTATT CCGTTACCTT CTTGGTCGCC   
  
  
- TTGATGATTC AAAGAGTGGT CCACTAAATT TCGTCAATGA TTAACTTACA CGCTCCCGAA ATAATCTATT   
  
  
- GGCTTATCTA CTGAAACTCT CAAACCAATT TGCCCGTTCC TTTCTCCAGA GGTAGAGTCC ACTCGGACAG   
  
  
- GTTGGAGAGC CATGGACATA CTAGCTTCCC GAACACCGTT CCTTTGTCAG AAGCCCCTGA TTGTAGATAA   
  
  
- CCCGAGAATT CACATTTCTC GGAGAACCTT TTCTGAACGA GAGGATGCCG TGAGTCACCC AATGACATAG   
  
  
- CGTTCGGTAT CGTCGTTCCG AACCACCCGG AGGATTTCAC GCTGAATGTC CGTAACTACT AGGACAAAGA   
  
  
- TTCATATGAA CACTACGATC GAACCTCCGA CCACCCTTTG CTAATCGCAG AGATAAACTT TTCAAATTTT   
  
  
- ATGAACAGCT CAAGTTACGT AACGGGCAAA TACCTGGGTT ACAGTCCACC CTTTACGACC TACACTCCGG   
  
  
- GTCCCTCCGA AACCAGCAAT TAACAGGTAA TGTCGAGGTA ATGTGAGGAC TGCTCTCACA GCTACACTCC   
  
  
- TTGGGATCCC TATCCGAAGA GTCTTACCAC TTTAACGAGC CAGGATTCCA TCGGTGAAAC CAACTCGTTC   
  
  
- TTAGTTTGTG GTTGTGATGT GGAAAGAACT GGGCCAAGTA TCTCTGGGAA CTGATGATGA GCTAATTGGT   
  
  
- ACAAAC
