# Supplementary material for: Genome-Wide Identification and Expression Pattern of the GRAS Gene Family in Pitaya (Selenicereus undatus L.)
Source: Biology (Basel). 2022 Dec 21;12(1):11. doi: 10.3390/biology12010011 (PMC9854919; doi:10.3390/biology12010011)
Supplement: Supplementary file 1 [file biology-12-00011-s001.zip › Supplementary file S5/HU08G00284.1_plantcare.html]

Content-Type: text/html; charset=ISO-8859-1


PlantCARE


Webmaster Firefox specific output  
To save the result:
click on the frame with the right mouse button and save the source code as a text file with extension .html  
REFERENCE:PlantCARE: a database of plant cis-acting regulatory elements and a portal to tools for in silico analysis of promoter sequences.  
Lescot, M., Déhais, P., Moreau, Y., De Moor, B., Rouzé ,P.,and Rombauts, S.  
Nucleic Acids Res., Database issue(2002), 30(1):325-327.   


---

>HU08G00284.1   
+ -Up\_Stream \_Len000TTCTTT TCCTGTCTCT GTCTCTTCAT TGCACCATCA TTTAAGAGAT GGGACCATAA   
  
  
+ ATCATTCAAT CACTCCTTTA TGTTTAAACT TTGGAAGGAG GTTATTATTT ATGTGGTGGT GATCCCAATG   
  
  
+ CTGAATTTAG CTGTTGATGG CGAGGGCATA TATGTAATTA GGATTTCCAA TGCAGTTTAG ATTTTACTCA   
  
  
+ TTTGAGAATT CACTGAGGTT TGTTTGATCT GATTTTAGCA AATATTGTTC AGGGGTATAG ATGCTTATTC   
  
  
+ GGTTCTCAAC TGTATTAGTA TACAAGATGA GTGGTCCACT TGTTTAAGCT TTTACTATAT ACTTATGTTG   
  
  
+ TCCCTAATTT GGTCTCGCTT GATGTGTAGC TGTCAAGTAT TATTATAATC TTGTGTTTGA TATCACATTT   
  
  
+ GTATTGATTA GCTACAAAAG AACATATTAT ATGTGTATAG CAACTATCTC ATATATGCCC TCACCATAGC   
  
  
+ GTGGTTCCAA GGGTTCAAGT TCCACTTAGC CTCACCCTAG AAATGACTTC TTTTTTTTTT TAAAGTTAAA   
  
  
+ GATATGCAAT CACCATGGAA CCAGTCATCT TTTGACACGT TATTCCAGAT CAGATGACCA GCCTTTCTCC   
  
  
+ AAGGCCATTA GATTTTCTAG CCAATCCACT GTTAACTGCA AAAAGTGGTA TCCTTGGCTG TTCCTGTCAC   
  
  
+ TGTTTCTTAT TTGCTCTGTG ATATTTGTAC ATCTCTTTGC CAAGGATAAA AGTTCATAGC CGGGAATTGG   
  
  
+ TATTCAGTTG ACTCTTGCAG TAAACAGAGT TCATGTACTG GAGGATTTTG TGAGTCATGT CACTCAATAA   
  
  
+ AAACAGGTGT GCGACAGTGT TATAAAGCTG CTTGCTTAAT GCACATCTAC TCTCACTCAC CTTACCCAAA   
  
  
+ AAACTTCAAA ATTGTGTGCC TTCTATGAAT ATTCTGATCC ATTAGCATTC AACGGTTTCT GAATTCTGTG   
  
  
+ TAAGCCATCC ATACTCATTT TCAACAGAGA ACTTGGATTG GATGTATTCA ATACCCAAAA ACCTTGTCAA   
  
  
+ ATTACCCCTA GCTTAATTTC CTCTAATACC AACTGCTCTT TGGTTTTAGC GTACAATCCC ATAGATTACC   
  
  
+ CTTCTATAAT TAGTGATGAT TGGTTCATTT CTGCTGCATT ATGCCTTCTT TAGATCCATT GATAATATAT   
  
  
+ ACTCTTGCCA TGCTGCAACA TTCCACTGGG ATTAATATCT CACATTGTAT CCTCAGCAAC CAAGGAGACC   
  
  
+ CTACTATTTT CAGAGCCATT CATAGAAAGT TATTGCATAT AGTGATGATC TTCAATGGGT CTGATTTTTA   
  
  
+ GATAGCTGCT GTGAACATTT TACTCGTCCT CTGCCATGTG GATACTATGG TAACCTTTGT TTGCTCTCAA   
  
  
+ GCCTGAGCGT TCTGAGAAAT GATGAAAGCG CCCTCGAGTT ATAGGACTTT TCTGACTTTA GCTTTAGAAA   
  
  
+ AGAGAGGAAA ATCAACCACA CCCATCTGAT TATTGAGTTT CTGCTCCTTG AGACCTGGAT GGAGCCAGAA   
  
  
+ CTCACGGTCT TGTCAGCCAG AATAATCAGG AACTGCATTT CTGAGGACCC ATGAAAAGAC CCTGGTATAC   
  
  
+ GACTATTCAT GAAAACGTTT TGTCAGCCTT TCAGTTTTTG CATTTTATTC TAGCTGCTTT GATTGAAGCC   
  
  
+ CAATTTTATT AAAACTGTGC TTTTCTCTTG AATGTTAAAC TCTTGCAGTT CCTTGCTTGT TATTTTGGGT   
  
  
+ TTTATGTTTG CTTTCTACTG ATATCATCTT TTGGTCGTGC AATTTAATTG CCTACTATGT TATATGGATT   
  
  
+ TTTATGTTTA CTTTCTACTG ATATCATCTT TTGCTATTGC AATTTAATTG CCTTCTATGT AGAATTCGTC   
  
  
+ ACTTTGAAGT CTGCCAGGAA GGCAATACAG CTATATTTTT CTTGTGGATG AAGTGATAAA AGGCAAGTGT   
  
  
+ CAATTGCTTA CTGCTTTATT GCGCTGTACT TTTCCAGAGA TTTTGGATTA GTCAATGCAG AAGCCAACAA   
  
  
+ TTTATAGCGA CTGGCCACAG TTCTACAACC AATTTGATAA TCCACGTCTT CTTGAATCTT CATCTATAAT   
  
  
+ GGGTGGTGAT CAACTTTTCA GTTCTCCATC TACTGTAAGC ATATCTTGCA ACAGGAGTCC AGCTTCACTG   
  
  
+ CCTGAACTCG AATCCTCTTC ATCGGACTTC CAAAGTGACT CCCAGGAGAC TGTGAATGGC TCGCCAGTGA   
  
  
+ TTGATTCGTG CATGGTACGT GACATTGGTG ACTTAAGACA CAAACTTAGA GAGCTTGAGA CTGTTATGCT   
  
  
+ CGGACCAAGT TCAGACAGCT TGGATTCATG GTATGCCCCT TCAAGAGGTG AGTGTGAACC TCTGCCACCA   
  
  
+ GAAGAGCCTG ACAATGGGAA ACATTTGTTG GAGATGATAG CAAGGGGGAG CCTCAAAGAG GTGCTAATTG   
  
  
+ CTTGTGCCAA AGCAATATCA GACGATGATT TGTTAACAGC GGAGTGGTTG ATGTCAGAGC TACGCCATAT   
  
  
+ GGTTTCAGTT TCTGGAGAAC CAATTCAGAG GTTAGGAGCC TACATGTTGG AAGGGTTAGT TGCCCGGTTG   
  
  
+ TCCTCTTCAG GAAGTTCCAT CTACAAAGCT CTAAGGTGCA AAGAGCCTAC TAGTAGTGAA CTTCTTTCCT   
  
  
+ ATATGCATTT ACTCTATGAA GTTTGCCCTT ACTTCAAGTT TGGGTACATG TCTGCAAATG GGGCAATTGC   
  
  
+ TGAGGCCATG AAAAATGAGA GCAGAATTCA TATAATTGAT TTTCAGATAG CTCAAGGGAG TCAGTGGATT   
  
  
+ AGCCTTATCC AAGCCCTGGC TGCTCAGCCT GATGGGCCAC CACAGGTCCG TATTACCGGA GTCGATGATT   
  
  
+ CCCAATCTGA GTATGCTCGG GGAGGGGGGC TCGACATTGT AGCGAAGAGA CTATCTGGAC TAGCCCAGGC   
  
  
+ TTGCAGCCTA CCCTTTGAGT TCCACGCTGC AGCACTTAGT GGTTCTGAGA TCAGACTTCA AAACCTGGTT   
  
  
+ TTGCGGCCTG GAGAAGCCTT AGCAGTGAAC TTCCCATTCA TGCTGCACCA CATGCCGGAT GAGAGTGTGG   
  
  
+ GCCCTGAGAA TTATAGAGAC CAGTTATTAA GGCTGGTGAA GAGCTTCTTG CCCAAGGTGG TTACCCTTGT   
  
  
+ TGAGCAAGAA TCCAACACAA ACACGGCCCC ATTTCTACCC CGGTTTCTTG AAACCCTAGA CTACTATACT   
  
  
+ GCCATTTTCG AATCAATTGA TGTTACGCTC CCAAGAGATC ACAAGGAGCG GATCAATGTT GAGCAGCACT   
  
  
+ GTTTAGCAAG AGATATAGTC AACATAATAG CATGTGAGGG TGCCGAGAGG GTGGAACGCC ATGAGGTTCT   
  
  
+ TGGAAAGTGG AGATCTCGGT TCTCAATGGC TGGGTTTAAG CCGTACCCAT TGAGCCCGCT AGTGAATGCA   
  
  
+ ACTATCAGGA CTCTTCTGCA GAAGTATAGC AGGAGCTATG GACTTGAAGA AAGGGATGGA GCTCTTTATC   
  
  
+ TAGGCTGGAT GAACCGAGCA CTAGTTGCAT CTTGTGCATG GCAGTG  

- -Up\_Stream \_Len000AAGAAA AGGACAGAGA CAGAGAAGTA ACGTGGTAGT AAATTCTCTA CCCTGGTATT   
  
  
- TAGTAAGTTA GTGAGGAAAT ACAAATTTGA AACCTTCCTC CAATAATAAA TACACCACCA CTAGGGTTAC   
  
  
- GACTTAAATC GACAACTACC GCTCCCGTAT ATACATTAAT CCTAAAGGTT ACGTCAAATC TAAAATGAGT   
  
  
- AAACTCTTAA GTGACTCCAA ACAAACTAGA CTAAAATCGT TTATAACAAG TCCCCATATC TACGAATAAG   
  
  
- CCAAGAGTTG ACATAATCAT ATGTTCTACT CACCAGGTGA ACAAATTCGA AAATGATATA TGAATACAAC   
  
  
- AGGGATTAAA CCAGAGCGAA CTACACATCG ACAGTTCATA ATAATATTAG AACACAAACT ATAGTGTAAA   
  
  
- CATAACTAAT CGATGTTTTC TTGTATAATA TACACATATC GTTGATAGAG TATATACGGG AGTGGTATCG   
  
  
- CACCAAGGTT CCCAAGTTCA AGGTGAATCG GAGTGGGATC TTTACTGAAG AAAAAAAAAA ATTTCAATTT   
  
  
- CTATACGTTA GTGGTACCTT GGTCAGTAGA AAACTGTGCA ATAAGGTCTA GTCTACTGGT CGGAAAGAGG   
  
  
- TTCCGGTAAT CTAAAAGATC GGTTAGGTGA CAATTGACGT TTTTCACCAT AGGAACCGAC AAGGACAGTG   
  
  
- ACAAAGAATA AACGAGACAC TATAAACATG TAGAGAAACG GTTCCTATTT TCAAGTATCG GCCCTTAACC   
  
  
- ATAAGTCAAC TGAGAACGTC ATTTGTCTCA AGTACATGAC CTCCTAAAAC ACTCAGTACA GTGAGTTATT   
  
  
- TTTGTCCACA CGCTGTCACA ATATTTCGAC GAACGAATTA CGTGTAGATG AGAGTGAGTG GAATGGGTTT   
  
  
- TTTGAAGTTT TAACACACGG AAGATACTTA TAAGACTAGG TAATCGTAAG TTGCCAAAGA CTTAAGACAC   
  
  
- ATTCGGTAGG TATGAGTAAA AGTTGTCTCT TGAACCTAAC CTACATAAGT TATGGGTTTT TGGAACAGTT   
  
  
- TAATGGGGAT CGAATTAAAG GAGATTATGG TTGACGAGAA ACCAAAATCG CATGTTAGGG TATCTAATGG   
  
  
- GAAGATATTA ATCACTACTA ACCAAGTAAA GACGACGTAA TACGGAAGAA ATCTAGGTAA CTATTATATA   
  
  
- TGAGAACGGT ACGACGTTGT AAGGTGACCC TAATTATAGA GTGTAACATA GGAGTCGTTG GTTCCTCTGG   
  
  
- GATGATAAAA GTCTCGGTAA GTATCTTTCA ATAACGTATA TCACTACTAG AAGTTACCCA GACTAAAAAT   
  
  
- CTATCGACGA CACTTGTAAA ATGAGCAGGA GACGGTACAC CTATGATACC ATTGGAAACA AACGAGAGTT   
  
  
- CGGACTCGCA AGACTCTTTA CTACTTTCGC GGGAGCTCAA TATCCTGAAA AGACTGAAAT CGAAATCTTT   
  
  
- TCTCTCCTTT TAGTTGGTGT GGGTAGACTA ATAACTCAAA GACGAGGAAC TCTGGACCTA CCTCGGTCTT   
  
  
- GAGTGCCAGA ACAGTCGGTC TTATTAGTCC TTGACGTAAA GACTCCTGGG TACTTTTCTG GGACCATATG   
  
  
- CTGATAAGTA CTTTTGCAAA ACAGTCGGAA AGTCAAAAAC GTAAAATAAG ATCGACGAAA CTAACTTCGG   
  
  
- GTTAAAATAA TTTTGACACG AAAAGAGAAC TTACAATTTG AGAACGTCAA GGAACGAACA ATAAAACCCA   
  
  
- AAATACAAAC GAAAGATGAC TATAGTAGAA AACCAGCACG TTAAATTAAC GGATGATACA ATATACCTAA   
  
  
- AAATACAAAT GAAAGATGAC TATAGTAGAA AACGATAACG TTAAATTAAC GGAAGATACA TCTTAAGCAG   
  
  
- TGAAACTTCA GACGGTCCTT CCGTTATGTC GATATAAAAA GAACACCTAC TTCACTATTT TCCGTTCACA   
  
  
- GTTAACGAAT GACGAAATAA CGCGACATGA AAAGGTCTCT AAAACCTAAT CAGTTACGTC TTCGGTTGTT   
  
  
- AAATATCGCT GACCGGTGTC AAGATGTTGG TTAAACTATT AGGTGCAGAA GAACTTAGAA GTAGATATTA   
  
  
- CCCACCACTA GTTGAAAAGT CAAGAGGTAG ATGACATTCG TATAGAACGT TGTCCTCAGG TCGAAGTGAC   
  
  
- GGACTTGAGC TTAGGAGAAG TAGCCTGAAG GTTTCACTGA GGGTCCTCTG ACACTTACCG AGCGGTCACT   
  
  
- AACTAAGCAC GTACCATGCA CTGTAACCAC TGAATTCTGT GTTTGAATCT CTCGAACTCT GACAATACGA   
  
  
- GCCTGGTTCA AGTCTGTCGA ACCTAAGTAC CATACGGGGA AGTTCTCCAC TCACACTTGG AGACGGTGGT   
  
  
- CTTCTCGGAC TGTTACCCTT TGTAAACAAC CTCTACTATC GTTCCCCCTC GGAGTTTCTC CACGATTAAC   
  
  
- GAACACGGTT TCGTTATAGT CTGCTACTAA ACAATTGTCG CCTCACCAAC TACAGTCTCG ATGCGGTATA   
  
  
- CCAAAGTCAA AGACCTCTTG GTTAAGTCTC CAATCCTCGG ATGTACAACC TTCCCAATCA ACGGGCCAAC   
  
  
- AGGAGAAGTC CTTCAAGGTA GATGTTTCGA GATTCCACGT TTCTCGGATG ATCATCACTT GAAGAAAGGA   
  
  
- TATACGTAAA TGAGATACTT CAAACGGGAA TGAAGTTCAA ACCCATGTAC AGACGTTTAC CCCGTTAACG   
  
  
- ACTCCGGTAC TTTTTACTCT CGTCTTAAGT ATATTAACTA AAAGTCTATC GAGTTCCCTC AGTCACCTAA   
  
  
- TCGGAATAGG TTCGGGACCG ACGAGTCGGA CTACCCGGTG GTGTCCAGGC ATAATGGCCT CAGCTACTAA   
  
  
- GGGTTAGACT CATACGAGCC CCTCCCCCCG AGCTGTAACA TCGCTTCTCT GATAGACCTG ATCGGGTCCG   
  
  
- AACGTCGGAT GGGAAACTCA AGGTGCGACG TCGTGAATCA CCAAGACTCT AGTCTGAAGT TTTGGACCAA   
  
  
- AACGCCGGAC CTCTTCGGAA TCGTCACTTG AAGGGTAAGT ACGACGTGGT GTACGGCCTA CTCTCACACC   
  
  
- CGGGACTCTT AATATCTCTG GTCAATAATT CCGACCACTT CTCGAAGAAC GGGTTCCACC AATGGGAACA   
  
  
- ACTCGTTCTT AGGTTGTGTT TGTGCCGGGG TAAAGATGGG GCCAAAGAAC TTTGGGATCT GATGATATGA   
  
  
- CGGTAAAAGC TTAGTTAACT ACAATGCGAG GGTTCTCTAG TGTTCCTCGC CTAGTTACAA CTCGTCGTGA   
  
  
- CAAATCGTTC TCTATATCAG TTGTATTATC GTACACTCCC ACGGCTCTCC CACCTTGCGG TACTCCAAGA   
  
  
- ACCTTTCACC TCTAGAGCCA AGAGTTACCG ACCCAAATTC GGCATGGGTA ACTCGGGCGA TCACTTACGT   
  
  
- TGATAGTCCT GAGAAGACGT CTTCATATCG TCCTCGATAC CTGAACTTCT TTCCCTACCT CGAGAAATAG   
  
  
- ATCCGACCTA CTTGGCTCGT GATCAACGTA GAACACGTAC CGTCAC

  
  
Motifs Found  

+   

| Site Name | Organism | Position | Strand | Matrix score. | sequence | function |
| --- | --- | --- | --- | --- | --- | --- |
|  | organism | 1699 | + | 4 | motif\_sequence | short\_function |
|  | organism | 2090 | + | 4 | motif\_sequence | short\_function |
|  | organism | 1312 | + | 4 | motif\_sequence | short\_function |
|  | organism | 2613 | + | 4 | motif\_sequence | short\_function |
|  | organism | 2597 | + | 4 | motif\_sequence | short\_function |
|  | organism | 2414 | - | 4 | motif\_sequence | short\_function |
|  | organism | 2374 | + | 4 | motif\_sequence | short\_function |
|  | organism | 2132 | + | 4 | motif\_sequence | short\_function |
|  | organism | 807 | + | 4 | motif\_sequence | short\_function |
|  | organism | 2189 | + | 4 | motif\_sequence | short\_function |
|  | organism | 885 | - | 4 | motif\_sequence | short\_function |
|  | organism | 889 | + | 4 | motif\_sequence | short\_function |
|  | organism | 1988 | + | 4 | motif\_sequence | short\_function |
|  | organism | 2708 | - | 4 | motif\_sequence | short\_function |
|  | organism | 1363 | + | 4 | motif\_sequence | short\_function |
|  | organism | 1347 | - | 4 | motif\_sequence | short\_function |
|  | organism | 3445 | + | 4 | motif\_sequence | short\_function |
|  | organism | 692 | + | 4 | motif\_sequence | short\_function |
|  | organism | 2385 | - | 4 | motif\_sequence | short\_function |
|  | organism | 731 | - | 4 | motif\_sequence | short\_function |
|  | organism | 444 | - | 4 | motif\_sequence | short\_function |
|  | organism | 37 | + | 4 | motif\_sequence | short\_function |
|  | organism | 2918 | - | 4 | motif\_sequence | short\_function |
|  | organism | 3122 | - | 4 | motif\_sequence | short\_function |
|  | organism | 3373 | - | 4 | motif\_sequence | short\_function |

>HU08G00284.1   
+ -Up\_Stream \_Len000TTCTTT TCCTGTCTCT GTCTCTTCAT TGCACCATCA TTTAAGAGAT GGGACCATAA   
  
  
+ ATCATTCAAT CACTCCTTTA TGTTTAAACT TTGGAAGGAG GTTATTATTT ATGTGGTGGT GATCCCAATG   
  
  
+ CTGAATTTAG CTGTTGATGG CGAGGGCATA TATGTAATTA GGATTTCCAA TGCAGTTTAG ATTTTACTCA   
  
  
+ TTTGAGAATT CACTGAGGTT TGTTTGATCT GATTTTAGCA AATATTGTTC AGGGGTATAG ATGCTTATTC   
  
  
+ GGTTCTCAAC TGTATTAGTA TACAAGATGA GTGGTCCACT TGTTTAAGCT TTTACTATAT ACTTATGTTG   
  
  
+ TCCCTAATTT GGTCTCGCTT GATGTGTAGC TGTCAAGTAT TATTATAATC TTGTGTTTGA TATCACATTT   
  
  
+ GTATTGATTA GCTACAAAAG AACATATTAT ATGTGTATAG CAACTATCTC ATATATGCCC TCACCATAGC   
  
  
+ GTGGTTCCAA GGGTTCAAGT TCCACTTAGC CTCACCCTAG AAATGACTTC TTTTTTTTTT TAAAGTTAAA   
  
  
+ GATATGCAAT CACCATGGAA CCAGTCATCT TTTGACACGT TATTCCAGAT CAGATGACCA GCCTTTCTCC   
  
  
+ AAGGCCATTA GATTTTCTAG CCAATCCACT GTTAACTGCA AAAAGTGGTA TCCTTGGCTG TTCCTGTCAC   
  
  
+ TGTTTCTTAT TTGCTCTGTG ATATTTGTAC ATCTCTTTGC CAAGGATAAA AGTTCATAGC CGGGAATTGG   
  
  
+ TATTCAGTTG ACTCTTGCAG TAAACAGAGT TCATGTACTG GAGGATTTTG TGAGTCATGT CACTCAATAA   
  
  
+ AAACAGGTGT GCGACAGTGT TATAAAGCTG CTTGCTTAAT GCACATCTAC TCTCACTCAC CTTACCCAAA   
  
  
+ AAACTTCAAA ATTGTGTGCC TTCTATGAAT ATTCTGATCC ATTAGCATTC AACGGTTTCT GAATTCTGTG   
  
  
+ TAAGCCATCC ATACTCATTT TCAACAGAGA ACTTGGATTG GATGTATTCA ATACCCAAAA ACCTTGTCAA   
  
  
+ ATTACCCCTA GCTTAATTTC CTCTAATACC AACTGCTCTT TGGTTTTAGC GTACAATCCC ATAGATTACC   
  
  
+ CTTCTATAAT TAGTGATGAT TGGTTCATTT CTGCTGCATT ATGCCTTCTT TAGATCCATT GATAATATAT   
  
  
+ ACTCTTGCCA TGCTGCAACA TTCCACTGGG ATTAATATCT CACATTGTAT CCTCAGCAAC CAAGGAGACC   
  
  
+ CTACTATTTT CAGAGCCATT CATAGAAAGT TATTGCATAT AGTGATGATC TTCAATGGGT CTGATTTTTA   
  
  
+ GATAGCTGCT GTGAACATTT TACTCGTCCT CTGCCATGTG GATACTATGG TAACCTTTGT TTGCTCTCAA   
  
  
+ GCCTGAGCGT TCTGAGAAAT GATGAAAGCG CCCTCGAGTT ATAGGACTTT TCTGACTTTA GCTTTAGAAA   
  
  
+ AGAGAGGAAA ATCAACCACA CCCATCTGAT TATTGAGTTT CTGCTCCTTG AGACCTGGAT GGAGCCAGAA   
  
  
+ CTCACGGTCT TGTCAGCCAG AATAATCAGG AACTGCATTT CTGAGGACCC ATGAAAAGAC CCTGGTATAC   
  
  
+ GACTATTCAT GAAAACGTTT TGTCAGCCTT TCAGTTTTTG CATTTTATTC TAGCTGCTTT GATTGAAGCC   
  
  
+ CAATTTTATT AAAACTGTGC TTTTCTCTTG AATGTTAAAC TCTTGCAGTT CCTTGCTTGT TATTTTGGGT   
  
  
+ TTTATGTTTG CTTTCTACTG ATATCATCTT TTGGTCGTGC AATTTAATTG CCTACTATGT TATATGGATT   
  
  
+ TTTATGTTTA CTTTCTACTG ATATCATCTT TTGCTATTGC AATTTAATTG CCTTCTATGT AGAATTCGTC   
  
  
+ ACTTTGAAGT CTGCCAGGAA GGCAATACAG CTATATTTTT CTTGTGGATG AAGTGATAAA AGGCAAGTGT   
  
  
+ CAATTGCTTA CTGCTTTATT GCGCTGTACT TTTCCAGAGA TTTTGGATTA GTCAATGCAG AAGCCAACAA   
  
  
+ TTTATAGCGA CTGGCCACAG TTCTACAACC AATTTGATAA TCCACGTCTT CTTGAATCTT CATCTATAAT   
  
  
+ GGGTGGTGAT CAACTTTTCA GTTCTCCATC TACTGTAAGC ATATCTTGCA ACAGGAGTCC AGCTTCACTG   
  
  
+ CCTGAACTCG AATCCTCTTC ATCGGACTTC CAAAGTGACT CCCAGGAGAC TGTGAATGGC TCGCCAGTGA   
  
  
+ TTGATTCGTG CATGGTACGT GACATTGGTG ACTTAAGACA CAAACTTAGA GAGCTTGAGA CTGTTATGCT   
  
  
+ CGGACCAAGT TCAGACAGCT TGGATTCATG GTATGCCCCT TCAAGAGGTG AGTGTGAACC TCTGCCACCA   
  
  
+ GAAGAGCCTG ACAATGGGAA ACATTTGTTG GAGATGATAG CAAGGGGGAG CCTCAAAGAG GTGCTAATTG   
  
  
+ CTTGTGCCAA AGCAATATCA GACGATGATT TGTTAACAGC GGAGTGGTTG ATGTCAGAGC TACGCCATAT   
  
  
+ GGTTTCAGTT TCTGGAGAAC CAATTCAGAG GTTAGGAGCC TACATGTTGG AAGGGTTAGT TGCCCGGTTG   
  
  
+ TCCTCTTCAG GAAGTTCCAT CTACAAAGCT CTAAGGTGCA AAGAGCCTAC TAGTAGTGAA CTTCTTTCCT   
  
  
+ ATATGCATTT ACTCTATGAA GTTTGCCCTT ACTTCAAGTT TGGGTACATG TCTGCAAATG GGGCAATTGC   
  
  
+ TGAGGCCATG AAAAATGAGA GCAGAATTCA TATAATTGAT TTTCAGATAG CTCAAGGGAG TCAGTGGATT   
  
  
+ AGCCTTATCC AAGCCCTGGC TGCTCAGCCT GATGGGCCAC CACAGGTCCG TATTACCGGA GTCGATGATT   
  
  
+ CCCAATCTGA GTATGCTCGG GGAGGGGGGC TCGACATTGT AGCGAAGAGA CTATCTGGAC TAGCCCAGGC   
  
  
+ TTGCAGCCTA CCCTTTGAGT TCCACGCTGC AGCACTTAGT GGTTCTGAGA TCAGACTTCA AAACCTGGTT   
  
  
+ TTGCGGCCTG GAGAAGCCTT AGCAGTGAAC TTCCCATTCA TGCTGCACCA CATGCCGGAT GAGAGTGTGG   
  
  
+ GCCCTGAGAA TTATAGAGAC CAGTTATTAA GGCTGGTGAA GAGCTTCTTG CCCAAGGTGG TTACCCTTGT   
  
  
+ TGAGCAAGAA TCCAACACAA ACACGGCCCC ATTTCTACCC CGGTTTCTTG AAACCCTAGA CTACTATACT   
  
  
+ GCCATTTTCG AATCAATTGA TGTTACGCTC CCAAGAGATC ACAAGGAGCG GATCAATGTT GAGCAGCACT   
  
  
+ GTTTAGCAAG AGATATAGTC AACATAATAG CATGTGAGGG TGCCGAGAGG GTGGAACGCC ATGAGGTTCT   
  
  
+ TGGAAAGTGG AGATCTCGGT TCTCAATGGC TGGGTTTAAG CCGTACCCAT TGAGCCCGCT AGTGAATGCA   
  
  
+ ACTATCAGGA CTCTTCTGCA GAAGTATAGC AGGAGCTATG GACTTGAAGA AAGGGATGGA GCTCTTTATC   
  
  
+ TAGGCTGGAT GAACCGAGCA CTAGTTGCAT CTTGTGCATG GCAGTG  

- -Up\_Stream \_Len000AAGAAA AGGACAGAGA CAGAGAAGTA ACGTGGTAGT AAATTCTCTA CCCTGGTATT   
  
  
- TAGTAAGTTA GTGAGGAAAT ACAAATTTGA AACCTTCCTC CAATAATAAA TACACCACCA CTAGGGTTAC   
  
  
- GACTTAAATC GACAACTACC GCTCCCGTAT ATACATTAAT CCTAAAGGTT ACGTCAAATC TAAAATGAGT   
  
  
- AAACTCTTAA GTGACTCCAA ACAAACTAGA CTAAAATCGT TTATAACAAG TCCCCATATC TACGAATAAG   
  
  
- CCAAGAGTTG ACATAATCAT ATGTTCTACT CACCAGGTGA ACAAATTCGA AAATGATATA TGAATACAAC   
  
  
- AGGGATTAAA CCAGAGCGAA CTACACATCG ACAGTTCATA ATAATATTAG AACACAAACT ATAGTGTAAA   
  
  
- CATAACTAAT CGATGTTTTC TTGTATAATA TACACATATC GTTGATAGAG TATATACGGG AGTGGTATCG   
  
  
- CACCAAGGTT CCCAAGTTCA AGGTGAATCG GAGTGGGATC TTTACTGAAG AAAAAAAAAA ATTTCAATTT   
  
  
- CTATACGTTA GTGGTACCTT GGTCAGTAGA AAACTGTGCA ATAAGGTCTA GTCTACTGGT CGGAAAGAGG   
  
  
- TTCCGGTAAT CTAAAAGATC GGTTAGGTGA CAATTGACGT TTTTCACCAT AGGAACCGAC AAGGACAGTG   
  
  
- ACAAAGAATA AACGAGACAC TATAAACATG TAGAGAAACG GTTCCTATTT TCAAGTATCG GCCCTTAACC   
  
  
- ATAAGTCAAC TGAGAACGTC ATTTGTCTCA AGTACATGAC CTCCTAAAAC ACTCAGTACA GTGAGTTATT   
  
  
- TTTGTCCACA CGCTGTCACA ATATTTCGAC GAACGAATTA CGTGTAGATG AGAGTGAGTG GAATGGGTTT   
  
  
- TTTGAAGTTT TAACACACGG AAGATACTTA TAAGACTAGG TAATCGTAAG TTGCCAAAGA CTTAAGACAC   
  
  
- ATTCGGTAGG TATGAGTAAA AGTTGTCTCT TGAACCTAAC CTACATAAGT TATGGGTTTT TGGAACAGTT   
  
  
- TAATGGGGAT CGAATTAAAG GAGATTATGG TTGACGAGAA ACCAAAATCG CATGTTAGGG TATCTAATGG   
  
  
- GAAGATATTA ATCACTACTA ACCAAGTAAA GACGACGTAA TACGGAAGAA ATCTAGGTAA CTATTATATA   
  
  
- TGAGAACGGT ACGACGTTGT AAGGTGACCC TAATTATAGA GTGTAACATA GGAGTCGTTG GTTCCTCTGG   
  
  
- GATGATAAAA GTCTCGGTAA GTATCTTTCA ATAACGTATA TCACTACTAG AAGTTACCCA GACTAAAAAT   
  
  
- CTATCGACGA CACTTGTAAA ATGAGCAGGA GACGGTACAC CTATGATACC ATTGGAAACA AACGAGAGTT   
  
  
- CGGACTCGCA AGACTCTTTA CTACTTTCGC GGGAGCTCAA TATCCTGAAA AGACTGAAAT CGAAATCTTT   
  
  
- TCTCTCCTTT TAGTTGGTGT GGGTAGACTA ATAACTCAAA GACGAGGAAC TCTGGACCTA CCTCGGTCTT   
  
  
- GAGTGCCAGA ACAGTCGGTC TTATTAGTCC TTGACGTAAA GACTCCTGGG TACTTTTCTG GGACCATATG   
  
  
- CTGATAAGTA CTTTTGCAAA ACAGTCGGAA AGTCAAAAAC GTAAAATAAG ATCGACGAAA CTAACTTCGG   
  
  
- GTTAAAATAA TTTTGACACG AAAAGAGAAC TTACAATTTG AGAACGTCAA GGAACGAACA ATAAAACCCA   
  
  
- AAATACAAAC GAAAGATGAC TATAGTAGAA AACCAGCACG TTAAATTAAC GGATGATACA ATATACCTAA   
  
  
- AAATACAAAT GAAAGATGAC TATAGTAGAA AACGATAACG TTAAATTAAC GGAAGATACA TCTTAAGCAG   
  
  
- TGAAACTTCA GACGGTCCTT CCGTTATGTC GATATAAAAA GAACACCTAC TTCACTATTT TCCGTTCACA   
  
  
- GTTAACGAAT GACGAAATAA CGCGACATGA AAAGGTCTCT AAAACCTAAT CAGTTACGTC TTCGGTTGTT   
  
  
- AAATATCGCT GACCGGTGTC AAGATGTTGG TTAAACTATT AGGTGCAGAA GAACTTAGAA GTAGATATTA   
  
  
- CCCACCACTA GTTGAAAAGT CAAGAGGTAG ATGACATTCG TATAGAACGT TGTCCTCAGG TCGAAGTGAC   
  
  
- GGACTTGAGC TTAGGAGAAG TAGCCTGAAG GTTTCACTGA GGGTCCTCTG ACACTTACCG AGCGGTCACT   
  
  
- AACTAAGCAC GTACCATGCA CTGTAACCAC TGAATTCTGT GTTTGAATCT CTCGAACTCT GACAATACGA   
  
  
- GCCTGGTTCA AGTCTGTCGA ACCTAAGTAC CATACGGGGA AGTTCTCCAC TCACACTTGG AGACGGTGGT   
  
  
- CTTCTCGGAC TGTTACCCTT TGTAAACAAC CTCTACTATC GTTCCCCCTC GGAGTTTCTC CACGATTAAC   
  
  
- GAACACGGTT TCGTTATAGT CTGCTACTAA ACAATTGTCG CCTCACCAAC TACAGTCTCG ATGCGGTATA   
  
  
- CCAAAGTCAA AGACCTCTTG GTTAAGTCTC CAATCCTCGG ATGTACAACC TTCCCAATCA ACGGGCCAAC   
  
  
- AGGAGAAGTC CTTCAAGGTA GATGTTTCGA GATTCCACGT TTCTCGGATG ATCATCACTT GAAGAAAGGA   
  
  
- TATACGTAAA TGAGATACTT CAAACGGGAA TGAAGTTCAA ACCCATGTAC AGACGTTTAC CCCGTTAACG   
  
  
- ACTCCGGTAC TTTTTACTCT CGTCTTAAGT ATATTAACTA AAAGTCTATC GAGTTCCCTC AGTCACCTAA   
  
  
- TCGGAATAGG TTCGGGACCG ACGAGTCGGA CTACCCGGTG GTGTCCAGGC ATAATGGCCT CAGCTACTAA   
  
  
- GGGTTAGACT CATACGAGCC CCTCCCCCCG AGCTGTAACA TCGCTTCTCT GATAGACCTG ATCGGGTCCG   
  
  
- AACGTCGGAT GGGAAACTCA AGGTGCGACG TCGTGAATCA CCAAGACTCT AGTCTGAAGT TTTGGACCAA   
  
  
- AACGCCGGAC CTCTTCGGAA TCGTCACTTG AAGGGTAAGT ACGACGTGGT GTACGGCCTA CTCTCACACC   
  
  
- CGGGACTCTT AATATCTCTG GTCAATAATT CCGACCACTT CTCGAAGAAC GGGTTCCACC AATGGGAACA   
  
  
- ACTCGTTCTT AGGTTGTGTT TGTGCCGGGG TAAAGATGGG GCCAAAGAAC TTTGGGATCT GATGATATGA   
  
  
- CGGTAAAAGC TTAGTTAACT ACAATGCGAG GGTTCTCTAG TGTTCCTCGC CTAGTTACAA CTCGTCGTGA   
  
  
- CAAATCGTTC TCTATATCAG TTGTATTATC GTACACTCCC ACGGCTCTCC CACCTTGCGG TACTCCAAGA   
  
  
- ACCTTTCACC TCTAGAGCCA AGAGTTACCG ACCCAAATTC GGCATGGGTA ACTCGGGCGA TCACTTACGT   
  
  
- TGATAGTCCT GAGAAGACGT CTTCATATCG TCCTCGATAC CTGAACTTCT TTCCCTACCT CGAGAAATAG   
  
  
- ATCCGACCTA CTTGGCTCGT GATCAACGTA GAACACGTAC CGTCAC

+     3-AF1 binding site

| Site Name | Organism | Position | Strand | Matrix score. | sequence | function |
| --- | --- | --- | --- | --- | --- | --- |
| 3-AF1 binding site | Solanum tuberosum | 1473 | + | 10 | TAAGAGAGGAA | light responsive element |

>HU08G00284.1   
+ -Up\_Stream \_Len000TTCTTT TCCTGTCTCT GTCTCTTCAT TGCACCATCA TTTAAGAGAT GGGACCATAA   
  
  
+ ATCATTCAAT CACTCCTTTA TGTTTAAACT TTGGAAGGAG GTTATTATTT ATGTGGTGGT GATCCCAATG   
  
  
+ CTGAATTTAG CTGTTGATGG CGAGGGCATA TATGTAATTA GGATTTCCAA TGCAGTTTAG ATTTTACTCA   
  
  
+ TTTGAGAATT CACTGAGGTT TGTTTGATCT GATTTTAGCA AATATTGTTC AGGGGTATAG ATGCTTATTC   
  
  
+ GGTTCTCAAC TGTATTAGTA TACAAGATGA GTGGTCCACT TGTTTAAGCT TTTACTATAT ACTTATGTTG   
  
  
+ TCCCTAATTT GGTCTCGCTT GATGTGTAGC TGTCAAGTAT TATTATAATC TTGTGTTTGA TATCACATTT   
  
  
+ GTATTGATTA GCTACAAAAG AACATATTAT ATGTGTATAG CAACTATCTC ATATATGCCC TCACCATAGC   
  
  
+ GTGGTTCCAA GGGTTCAAGT TCCACTTAGC CTCACCCTAG AAATGACTTC TTTTTTTTTT TAAAGTTAAA   
  
  
+ GATATGCAAT CACCATGGAA CCAGTCATCT TTTGACACGT TATTCCAGAT CAGATGACCA GCCTTTCTCC   
  
  
+ AAGGCCATTA GATTTTCTAG CCAATCCACT GTTAACTGCA AAAAGTGGTA TCCTTGGCTG TTCCTGTCAC   
  
  
+ TGTTTCTTAT TTGCTCTGTG ATATTTGTAC ATCTCTTTGC CAAGGATAAA AGTTCATAGC CGGGAATTGG   
  
  
+ TATTCAGTTG ACTCTTGCAG TAAACAGAGT TCATGTACTG GAGGATTTTG TGAGTCATGT CACTCAATAA   
  
  
+ AAACAGGTGT GCGACAGTGT TATAAAGCTG CTTGCTTAAT GCACATCTAC TCTCACTCAC CTTACCCAAA   
  
  
+ AAACTTCAAA ATTGTGTGCC TTCTATGAAT ATTCTGATCC ATTAGCATTC AACGGTTTCT GAATTCTGTG   
  
  
+ TAAGCCATCC ATACTCATTT TCAACAGAGA ACTTGGATTG GATGTATTCA ATACCCAAAA ACCTTGTCAA   
  
  
+ ATTACCCCTA GCTTAATTTC CTCTAATACC AACTGCTCTT TGGTTTTAGC GTACAATCCC ATAGATTACC   
  
  
+ CTTCTATAAT TAGTGATGAT TGGTTCATTT CTGCTGCATT ATGCCTTCTT TAGATCCATT GATAATATAT   
  
  
+ ACTCTTGCCA TGCTGCAACA TTCCACTGGG ATTAATATCT CACATTGTAT CCTCAGCAAC CAAGGAGACC   
  
  
+ CTACTATTTT CAGAGCCATT CATAGAAAGT TATTGCATAT AGTGATGATC TTCAATGGGT CTGATTTTTA   
  
  
+ GATAGCTGCT GTGAACATTT TACTCGTCCT CTGCCATGTG GATACTATGG TAACCTTTGT TTGCTCTCAA   
  
  
+ GCCTGAGCGT TCTGAGAAAT GATGAAAGCG CCCTCGAGTT ATAGGACTTT TCTGACTTTA GCTTTAGAAA   
  
  
+ AGAGAGGAAA ATCAACCACA CCCATCTGAT TATTGAGTTT CTGCTCCTTG AGACCTGGAT GGAGCCAGAA   
  
  
+ CTCACGGTCT TGTCAGCCAG AATAATCAGG AACTGCATTT CTGAGGACCC ATGAAAAGAC CCTGGTATAC   
  
  
+ GACTATTCAT GAAAACGTTT TGTCAGCCTT TCAGTTTTTG CATTTTATTC TAGCTGCTTT GATTGAAGCC   
  
  
+ CAATTTTATT AAAACTGTGC TTTTCTCTTG AATGTTAAAC TCTTGCAGTT CCTTGCTTGT TATTTTGGGT   
  
  
+ TTTATGTTTG CTTTCTACTG ATATCATCTT TTGGTCGTGC AATTTAATTG CCTACTATGT TATATGGATT   
  
  
+ TTTATGTTTA CTTTCTACTG ATATCATCTT TTGCTATTGC AATTTAATTG CCTTCTATGT AGAATTCGTC   
  
  
+ ACTTTGAAGT CTGCCAGGAA GGCAATACAG CTATATTTTT CTTGTGGATG AAGTGATAAA AGGCAAGTGT   
  
  
+ CAATTGCTTA CTGCTTTATT GCGCTGTACT TTTCCAGAGA TTTTGGATTA GTCAATGCAG AAGCCAACAA   
  
  
+ TTTATAGCGA CTGGCCACAG TTCTACAACC AATTTGATAA TCCACGTCTT CTTGAATCTT CATCTATAAT   
  
  
+ GGGTGGTGAT CAACTTTTCA GTTCTCCATC TACTGTAAGC ATATCTTGCA ACAGGAGTCC AGCTTCACTG   
  
  
+ CCTGAACTCG AATCCTCTTC ATCGGACTTC CAAAGTGACT CCCAGGAGAC TGTGAATGGC TCGCCAGTGA   
  
  
+ TTGATTCGTG CATGGTACGT GACATTGGTG ACTTAAGACA CAAACTTAGA GAGCTTGAGA CTGTTATGCT   
  
  
+ CGGACCAAGT TCAGACAGCT TGGATTCATG GTATGCCCCT TCAAGAGGTG AGTGTGAACC TCTGCCACCA   
  
  
+ GAAGAGCCTG ACAATGGGAA ACATTTGTTG GAGATGATAG CAAGGGGGAG CCTCAAAGAG GTGCTAATTG   
  
  
+ CTTGTGCCAA AGCAATATCA GACGATGATT TGTTAACAGC GGAGTGGTTG ATGTCAGAGC TACGCCATAT   
  
  
+ GGTTTCAGTT TCTGGAGAAC CAATTCAGAG GTTAGGAGCC TACATGTTGG AAGGGTTAGT TGCCCGGTTG   
  
  
+ TCCTCTTCAG GAAGTTCCAT CTACAAAGCT CTAAGGTGCA AAGAGCCTAC TAGTAGTGAA CTTCTTTCCT   
  
  
+ ATATGCATTT ACTCTATGAA GTTTGCCCTT ACTTCAAGTT TGGGTACATG TCTGCAAATG GGGCAATTGC   
  
  
+ TGAGGCCATG AAAAATGAGA GCAGAATTCA TATAATTGAT TTTCAGATAG CTCAAGGGAG TCAGTGGATT   
  
  
+ AGCCTTATCC AAGCCCTGGC TGCTCAGCCT GATGGGCCAC CACAGGTCCG TATTACCGGA GTCGATGATT   
  
  
+ CCCAATCTGA GTATGCTCGG GGAGGGGGGC TCGACATTGT AGCGAAGAGA CTATCTGGAC TAGCCCAGGC   
  
  
+ TTGCAGCCTA CCCTTTGAGT TCCACGCTGC AGCACTTAGT GGTTCTGAGA TCAGACTTCA AAACCTGGTT   
  
  
+ TTGCGGCCTG GAGAAGCCTT AGCAGTGAAC TTCCCATTCA TGCTGCACCA CATGCCGGAT GAGAGTGTGG   
  
  
+ GCCCTGAGAA TTATAGAGAC CAGTTATTAA GGCTGGTGAA GAGCTTCTTG CCCAAGGTGG TTACCCTTGT   
  
  
+ TGAGCAAGAA TCCAACACAA ACACGGCCCC ATTTCTACCC CGGTTTCTTG AAACCCTAGA CTACTATACT   
  
  
+ GCCATTTTCG AATCAATTGA TGTTACGCTC CCAAGAGATC ACAAGGAGCG GATCAATGTT GAGCAGCACT   
  
  
+ GTTTAGCAAG AGATATAGTC AACATAATAG CATGTGAGGG TGCCGAGAGG GTGGAACGCC ATGAGGTTCT   
  
  
+ TGGAAAGTGG AGATCTCGGT TCTCAATGGC TGGGTTTAAG CCGTACCCAT TGAGCCCGCT AGTGAATGCA   
  
  
+ ACTATCAGGA CTCTTCTGCA GAAGTATAGC AGGAGCTATG GACTTGAAGA AAGGGATGGA GCTCTTTATC   
  
  
+ TAGGCTGGAT GAACCGAGCA CTAGTTGCAT CTTGTGCATG GCAGTG  

- -Up\_Stream \_Len000AAGAAA AGGACAGAGA CAGAGAAGTA ACGTGGTAGT AAATTCTCTA CCCTGGTATT   
  
  
- TAGTAAGTTA GTGAGGAAAT ACAAATTTGA AACCTTCCTC CAATAATAAA TACACCACCA CTAGGGTTAC   
  
  
- GACTTAAATC GACAACTACC GCTCCCGTAT ATACATTAAT CCTAAAGGTT ACGTCAAATC TAAAATGAGT   
  
  
- AAACTCTTAA GTGACTCCAA ACAAACTAGA CTAAAATCGT TTATAACAAG TCCCCATATC TACGAATAAG   
  
  
- CCAAGAGTTG ACATAATCAT ATGTTCTACT CACCAGGTGA ACAAATTCGA AAATGATATA TGAATACAAC   
  
  
- AGGGATTAAA CCAGAGCGAA CTACACATCG ACAGTTCATA ATAATATTAG AACACAAACT ATAGTGTAAA   
  
  
- CATAACTAAT CGATGTTTTC TTGTATAATA TACACATATC GTTGATAGAG TATATACGGG AGTGGTATCG   
  
  
- CACCAAGGTT CCCAAGTTCA AGGTGAATCG GAGTGGGATC TTTACTGAAG AAAAAAAAAA ATTTCAATTT   
  
  
- CTATACGTTA GTGGTACCTT GGTCAGTAGA AAACTGTGCA ATAAGGTCTA GTCTACTGGT CGGAAAGAGG   
  
  
- TTCCGGTAAT CTAAAAGATC GGTTAGGTGA CAATTGACGT TTTTCACCAT AGGAACCGAC AAGGACAGTG   
  
  
- ACAAAGAATA AACGAGACAC TATAAACATG TAGAGAAACG GTTCCTATTT TCAAGTATCG GCCCTTAACC   
  
  
- ATAAGTCAAC TGAGAACGTC ATTTGTCTCA AGTACATGAC CTCCTAAAAC ACTCAGTACA GTGAGTTATT   
  
  
- TTTGTCCACA CGCTGTCACA ATATTTCGAC GAACGAATTA CGTGTAGATG AGAGTGAGTG GAATGGGTTT   
  
  
- TTTGAAGTTT TAACACACGG AAGATACTTA TAAGACTAGG TAATCGTAAG TTGCCAAAGA CTTAAGACAC   
  
  
- ATTCGGTAGG TATGAGTAAA AGTTGTCTCT TGAACCTAAC CTACATAAGT TATGGGTTTT TGGAACAGTT   
  
  
- TAATGGGGAT CGAATTAAAG GAGATTATGG TTGACGAGAA ACCAAAATCG CATGTTAGGG TATCTAATGG   
  
  
- GAAGATATTA ATCACTACTA ACCAAGTAAA GACGACGTAA TACGGAAGAA ATCTAGGTAA CTATTATATA   
  
  
- TGAGAACGGT ACGACGTTGT AAGGTGACCC TAATTATAGA GTGTAACATA GGAGTCGTTG GTTCCTCTGG   
  
  
- GATGATAAAA GTCTCGGTAA GTATCTTTCA ATAACGTATA TCACTACTAG AAGTTACCCA GACTAAAAAT   
  
  
- CTATCGACGA CACTTGTAAA ATGAGCAGGA GACGGTACAC CTATGATACC ATTGGAAACA AACGAGAGTT   
  
  
- CGGACTCGCA AGACTCTTTA CTACTTTCGC GGGAGCTCAA TATCCTGAAA AGACTGAAAT CGAAATCTTT   
  
  
- TCTCTCCTTT TAGTTGGTGT GGGTAGACTA ATAACTCAAA GACGAGGAAC TCTGGACCTA CCTCGGTCTT   
  
  
- GAGTGCCAGA ACAGTCGGTC TTATTAGTCC TTGACGTAAA GACTCCTGGG TACTTTTCTG GGACCATATG   
  
  
- CTGATAAGTA CTTTTGCAAA ACAGTCGGAA AGTCAAAAAC GTAAAATAAG ATCGACGAAA CTAACTTCGG   
  
  
- GTTAAAATAA TTTTGACACG AAAAGAGAAC TTACAATTTG AGAACGTCAA GGAACGAACA ATAAAACCCA   
  
  
- AAATACAAAC GAAAGATGAC TATAGTAGAA AACCAGCACG TTAAATTAAC GGATGATACA ATATACCTAA   
  
  
- AAATACAAAT GAAAGATGAC TATAGTAGAA AACGATAACG TTAAATTAAC GGAAGATACA TCTTAAGCAG   
  
  
- TGAAACTTCA GACGGTCCTT CCGTTATGTC GATATAAAAA GAACACCTAC TTCACTATTT TCCGTTCACA   
  
  
- GTTAACGAAT GACGAAATAA CGCGACATGA AAAGGTCTCT AAAACCTAAT CAGTTACGTC TTCGGTTGTT   
  
  
- AAATATCGCT GACCGGTGTC AAGATGTTGG TTAAACTATT AGGTGCAGAA GAACTTAGAA GTAGATATTA   
  
  
- CCCACCACTA GTTGAAAAGT CAAGAGGTAG ATGACATTCG TATAGAACGT TGTCCTCAGG TCGAAGTGAC   
  
  
- GGACTTGAGC TTAGGAGAAG TAGCCTGAAG GTTTCACTGA GGGTCCTCTG ACACTTACCG AGCGGTCACT   
  
  
- AACTAAGCAC GTACCATGCA CTGTAACCAC TGAATTCTGT GTTTGAATCT CTCGAACTCT GACAATACGA   
  
  
- GCCTGGTTCA AGTCTGTCGA ACCTAAGTAC CATACGGGGA AGTTCTCCAC TCACACTTGG AGACGGTGGT   
  
  
- CTTCTCGGAC TGTTACCCTT TGTAAACAAC CTCTACTATC GTTCCCCCTC GGAGTTTCTC CACGATTAAC   
  
  
- GAACACGGTT TCGTTATAGT CTGCTACTAA ACAATTGTCG CCTCACCAAC TACAGTCTCG ATGCGGTATA   
  
  
- CCAAAGTCAA AGACCTCTTG GTTAAGTCTC CAATCCTCGG ATGTACAACC TTCCCAATCA ACGGGCCAAC   
  
  
- AGGAGAAGTC CTTCAAGGTA GATGTTTCGA GATTCCACGT TTCTCGGATG ATCATCACTT GAAGAAAGGA   
  
  
- TATACGTAAA TGAGATACTT CAAACGGGAA TGAAGTTCAA ACCCATGTAC AGACGTTTAC CCCGTTAACG   
  
  
- ACTCCGGTAC TTTTTACTCT CGTCTTAAGT ATATTAACTA AAAGTCTATC GAGTTCCCTC AGTCACCTAA   
  
  
- TCGGAATAGG TTCGGGACCG ACGAGTCGGA CTACCCGGTG GTGTCCAGGC ATAATGGCCT CAGCTACTAA   
  
  
- GGGTTAGACT CATACGAGCC CCTCCCCCCG AGCTGTAACA TCGCTTCTCT GATAGACCTG ATCGGGTCCG   
  
  
- AACGTCGGAT GGGAAACTCA AGGTGCGACG TCGTGAATCA CCAAGACTCT AGTCTGAAGT TTTGGACCAA   
  
  
- AACGCCGGAC CTCTTCGGAA TCGTCACTTG AAGGGTAAGT ACGACGTGGT GTACGGCCTA CTCTCACACC   
  
  
- CGGGACTCTT AATATCTCTG GTCAATAATT CCGACCACTT CTCGAAGAAC GGGTTCCACC AATGGGAACA   
  
  
- ACTCGTTCTT AGGTTGTGTT TGTGCCGGGG TAAAGATGGG GCCAAAGAAC TTTGGGATCT GATGATATGA   
  
  
- CGGTAAAAGC TTAGTTAACT ACAATGCGAG GGTTCTCTAG TGTTCCTCGC CTAGTTACAA CTCGTCGTGA   
  
  
- CAAATCGTTC TCTATATCAG TTGTATTATC GTACACTCCC ACGGCTCTCC CACCTTGCGG TACTCCAAGA   
  
  
- ACCTTTCACC TCTAGAGCCA AGAGTTACCG ACCCAAATTC GGCATGGGTA ACTCGGGCGA TCACTTACGT   
  
  
- TGATAGTCCT GAGAAGACGT CTTCATATCG TCCTCGATAC CTGAACTTCT TTCCCTACCT CGAGAAATAG   
  
  
- ATCCGACCTA CTTGGCTCGT GATCAACGTA GAACACGTAC CGTCAC

+     AAGAA-motif

| Site Name | Organism | Position | Strand | Matrix score. | sequence | function |
| --- | --- | --- | --- | --- | --- | --- |
| AAGAA-motif | Avena sativa | 2656 | - | 7 | GAAAGAA |  |

>HU08G00284.1   
+ -Up\_Stream \_Len000TTCTTT TCCTGTCTCT GTCTCTTCAT TGCACCATCA TTTAAGAGAT GGGACCATAA   
  
  
+ ATCATTCAAT CACTCCTTTA TGTTTAAACT TTGGAAGGAG GTTATTATTT ATGTGGTGGT GATCCCAATG   
  
  
+ CTGAATTTAG CTGTTGATGG CGAGGGCATA TATGTAATTA GGATTTCCAA TGCAGTTTAG ATTTTACTCA   
  
  
+ TTTGAGAATT CACTGAGGTT TGTTTGATCT GATTTTAGCA AATATTGTTC AGGGGTATAG ATGCTTATTC   
  
  
+ GGTTCTCAAC TGTATTAGTA TACAAGATGA GTGGTCCACT TGTTTAAGCT TTTACTATAT ACTTATGTTG   
  
  
+ TCCCTAATTT GGTCTCGCTT GATGTGTAGC TGTCAAGTAT TATTATAATC TTGTGTTTGA TATCACATTT   
  
  
+ GTATTGATTA GCTACAAAAG AACATATTAT ATGTGTATAG CAACTATCTC ATATATGCCC TCACCATAGC   
  
  
+ GTGGTTCCAA GGGTTCAAGT TCCACTTAGC CTCACCCTAG AAATGACTTC TTTTTTTTTT TAAAGTTAAA   
  
  
+ GATATGCAAT CACCATGGAA CCAGTCATCT TTTGACACGT TATTCCAGAT CAGATGACCA GCCTTTCTCC   
  
  
+ AAGGCCATTA GATTTTCTAG CCAATCCACT GTTAACTGCA AAAAGTGGTA TCCTTGGCTG TTCCTGTCAC   
  
  
+ TGTTTCTTAT TTGCTCTGTG ATATTTGTAC ATCTCTTTGC CAAGGATAAA AGTTCATAGC CGGGAATTGG   
  
  
+ TATTCAGTTG ACTCTTGCAG TAAACAGAGT TCATGTACTG GAGGATTTTG TGAGTCATGT CACTCAATAA   
  
  
+ AAACAGGTGT GCGACAGTGT TATAAAGCTG CTTGCTTAAT GCACATCTAC TCTCACTCAC CTTACCCAAA   
  
  
+ AAACTTCAAA ATTGTGTGCC TTCTATGAAT ATTCTGATCC ATTAGCATTC AACGGTTTCT GAATTCTGTG   
  
  
+ TAAGCCATCC ATACTCATTT TCAACAGAGA ACTTGGATTG GATGTATTCA ATACCCAAAA ACCTTGTCAA   
  
  
+ ATTACCCCTA GCTTAATTTC CTCTAATACC AACTGCTCTT TGGTTTTAGC GTACAATCCC ATAGATTACC   
  
  
+ CTTCTATAAT TAGTGATGAT TGGTTCATTT CTGCTGCATT ATGCCTTCTT TAGATCCATT GATAATATAT   
  
  
+ ACTCTTGCCA TGCTGCAACA TTCCACTGGG ATTAATATCT CACATTGTAT CCTCAGCAAC CAAGGAGACC   
  
  
+ CTACTATTTT CAGAGCCATT CATAGAAAGT TATTGCATAT AGTGATGATC TTCAATGGGT CTGATTTTTA   
  
  
+ GATAGCTGCT GTGAACATTT TACTCGTCCT CTGCCATGTG GATACTATGG TAACCTTTGT TTGCTCTCAA   
  
  
+ GCCTGAGCGT TCTGAGAAAT GATGAAAGCG CCCTCGAGTT ATAGGACTTT TCTGACTTTA GCTTTAGAAA   
  
  
+ AGAGAGGAAA ATCAACCACA CCCATCTGAT TATTGAGTTT CTGCTCCTTG AGACCTGGAT GGAGCCAGAA   
  
  
+ CTCACGGTCT TGTCAGCCAG AATAATCAGG AACTGCATTT CTGAGGACCC ATGAAAAGAC CCTGGTATAC   
  
  
+ GACTATTCAT GAAAACGTTT TGTCAGCCTT TCAGTTTTTG CATTTTATTC TAGCTGCTTT GATTGAAGCC   
  
  
+ CAATTTTATT AAAACTGTGC TTTTCTCTTG AATGTTAAAC TCTTGCAGTT CCTTGCTTGT TATTTTGGGT   
  
  
+ TTTATGTTTG CTTTCTACTG ATATCATCTT TTGGTCGTGC AATTTAATTG CCTACTATGT TATATGGATT   
  
  
+ TTTATGTTTA CTTTCTACTG ATATCATCTT TTGCTATTGC AATTTAATTG CCTTCTATGT AGAATTCGTC   
  
  
+ ACTTTGAAGT CTGCCAGGAA GGCAATACAG CTATATTTTT CTTGTGGATG AAGTGATAAA AGGCAAGTGT   
  
  
+ CAATTGCTTA CTGCTTTATT GCGCTGTACT TTTCCAGAGA TTTTGGATTA GTCAATGCAG AAGCCAACAA   
  
  
+ TTTATAGCGA CTGGCCACAG TTCTACAACC AATTTGATAA TCCACGTCTT CTTGAATCTT CATCTATAAT   
  
  
+ GGGTGGTGAT CAACTTTTCA GTTCTCCATC TACTGTAAGC ATATCTTGCA ACAGGAGTCC AGCTTCACTG   
  
  
+ CCTGAACTCG AATCCTCTTC ATCGGACTTC CAAAGTGACT CCCAGGAGAC TGTGAATGGC TCGCCAGTGA   
  
  
+ TTGATTCGTG CATGGTACGT GACATTGGTG ACTTAAGACA CAAACTTAGA GAGCTTGAGA CTGTTATGCT   
  
  
+ CGGACCAAGT TCAGACAGCT TGGATTCATG GTATGCCCCT TCAAGAGGTG AGTGTGAACC TCTGCCACCA   
  
  
+ GAAGAGCCTG ACAATGGGAA ACATTTGTTG GAGATGATAG CAAGGGGGAG CCTCAAAGAG GTGCTAATTG   
  
  
+ CTTGTGCCAA AGCAATATCA GACGATGATT TGTTAACAGC GGAGTGGTTG ATGTCAGAGC TACGCCATAT   
  
  
+ GGTTTCAGTT TCTGGAGAAC CAATTCAGAG GTTAGGAGCC TACATGTTGG AAGGGTTAGT TGCCCGGTTG   
  
  
+ TCCTCTTCAG GAAGTTCCAT CTACAAAGCT CTAAGGTGCA AAGAGCCTAC TAGTAGTGAA CTTCTTTCCT   
  
  
+ ATATGCATTT ACTCTATGAA GTTTGCCCTT ACTTCAAGTT TGGGTACATG TCTGCAAATG GGGCAATTGC   
  
  
+ TGAGGCCATG AAAAATGAGA GCAGAATTCA TATAATTGAT TTTCAGATAG CTCAAGGGAG TCAGTGGATT   
  
  
+ AGCCTTATCC AAGCCCTGGC TGCTCAGCCT GATGGGCCAC CACAGGTCCG TATTACCGGA GTCGATGATT   
  
  
+ CCCAATCTGA GTATGCTCGG GGAGGGGGGC TCGACATTGT AGCGAAGAGA CTATCTGGAC TAGCCCAGGC   
  
  
+ TTGCAGCCTA CCCTTTGAGT TCCACGCTGC AGCACTTAGT GGTTCTGAGA TCAGACTTCA AAACCTGGTT   
  
  
+ TTGCGGCCTG GAGAAGCCTT AGCAGTGAAC TTCCCATTCA TGCTGCACCA CATGCCGGAT GAGAGTGTGG   
  
  
+ GCCCTGAGAA TTATAGAGAC CAGTTATTAA GGCTGGTGAA GAGCTTCTTG CCCAAGGTGG TTACCCTTGT   
  
  
+ TGAGCAAGAA TCCAACACAA ACACGGCCCC ATTTCTACCC CGGTTTCTTG AAACCCTAGA CTACTATACT   
  
  
+ GCCATTTTCG AATCAATTGA TGTTACGCTC CCAAGAGATC ACAAGGAGCG GATCAATGTT GAGCAGCACT   
  
  
+ GTTTAGCAAG AGATATAGTC AACATAATAG CATGTGAGGG TGCCGAGAGG GTGGAACGCC ATGAGGTTCT   
  
  
+ TGGAAAGTGG AGATCTCGGT TCTCAATGGC TGGGTTTAAG CCGTACCCAT TGAGCCCGCT AGTGAATGCA   
  
  
+ ACTATCAGGA CTCTTCTGCA GAAGTATAGC AGGAGCTATG GACTTGAAGA AAGGGATGGA GCTCTTTATC   
  
  
+ TAGGCTGGAT GAACCGAGCA CTAGTTGCAT CTTGTGCATG GCAGTG  

- -Up\_Stream \_Len000AAGAAA AGGACAGAGA CAGAGAAGTA ACGTGGTAGT AAATTCTCTA CCCTGGTATT   
  
  
- TAGTAAGTTA GTGAGGAAAT ACAAATTTGA AACCTTCCTC CAATAATAAA TACACCACCA CTAGGGTTAC   
  
  
- GACTTAAATC GACAACTACC GCTCCCGTAT ATACATTAAT CCTAAAGGTT ACGTCAAATC TAAAATGAGT   
  
  
- AAACTCTTAA GTGACTCCAA ACAAACTAGA CTAAAATCGT TTATAACAAG TCCCCATATC TACGAATAAG   
  
  
- CCAAGAGTTG ACATAATCAT ATGTTCTACT CACCAGGTGA ACAAATTCGA AAATGATATA TGAATACAAC   
  
  
- AGGGATTAAA CCAGAGCGAA CTACACATCG ACAGTTCATA ATAATATTAG AACACAAACT ATAGTGTAAA   
  
  
- CATAACTAAT CGATGTTTTC TTGTATAATA TACACATATC GTTGATAGAG TATATACGGG AGTGGTATCG   
  
  
- CACCAAGGTT CCCAAGTTCA AGGTGAATCG GAGTGGGATC TTTACTGAAG AAAAAAAAAA ATTTCAATTT   
  
  
- CTATACGTTA GTGGTACCTT GGTCAGTAGA AAACTGTGCA ATAAGGTCTA GTCTACTGGT CGGAAAGAGG   
  
  
- TTCCGGTAAT CTAAAAGATC GGTTAGGTGA CAATTGACGT TTTTCACCAT AGGAACCGAC AAGGACAGTG   
  
  
- ACAAAGAATA AACGAGACAC TATAAACATG TAGAGAAACG GTTCCTATTT TCAAGTATCG GCCCTTAACC   
  
  
- ATAAGTCAAC TGAGAACGTC ATTTGTCTCA AGTACATGAC CTCCTAAAAC ACTCAGTACA GTGAGTTATT   
  
  
- TTTGTCCACA CGCTGTCACA ATATTTCGAC GAACGAATTA CGTGTAGATG AGAGTGAGTG GAATGGGTTT   
  
  
- TTTGAAGTTT TAACACACGG AAGATACTTA TAAGACTAGG TAATCGTAAG TTGCCAAAGA CTTAAGACAC   
  
  
- ATTCGGTAGG TATGAGTAAA AGTTGTCTCT TGAACCTAAC CTACATAAGT TATGGGTTTT TGGAACAGTT   
  
  
- TAATGGGGAT CGAATTAAAG GAGATTATGG TTGACGAGAA ACCAAAATCG CATGTTAGGG TATCTAATGG   
  
  
- GAAGATATTA ATCACTACTA ACCAAGTAAA GACGACGTAA TACGGAAGAA ATCTAGGTAA CTATTATATA   
  
  
- TGAGAACGGT ACGACGTTGT AAGGTGACCC TAATTATAGA GTGTAACATA GGAGTCGTTG GTTCCTCTGG   
  
  
- GATGATAAAA GTCTCGGTAA GTATCTTTCA ATAACGTATA TCACTACTAG AAGTTACCCA GACTAAAAAT   
  
  
- CTATCGACGA CACTTGTAAA ATGAGCAGGA GACGGTACAC CTATGATACC ATTGGAAACA AACGAGAGTT   
  
  
- CGGACTCGCA AGACTCTTTA CTACTTTCGC GGGAGCTCAA TATCCTGAAA AGACTGAAAT CGAAATCTTT   
  
  
- TCTCTCCTTT TAGTTGGTGT GGGTAGACTA ATAACTCAAA GACGAGGAAC TCTGGACCTA CCTCGGTCTT   
  
  
- GAGTGCCAGA ACAGTCGGTC TTATTAGTCC TTGACGTAAA GACTCCTGGG TACTTTTCTG GGACCATATG   
  
  
- CTGATAAGTA CTTTTGCAAA ACAGTCGGAA AGTCAAAAAC GTAAAATAAG ATCGACGAAA CTAACTTCGG   
  
  
- GTTAAAATAA TTTTGACACG AAAAGAGAAC TTACAATTTG AGAACGTCAA GGAACGAACA ATAAAACCCA   
  
  
- AAATACAAAC GAAAGATGAC TATAGTAGAA AACCAGCACG TTAAATTAAC GGATGATACA ATATACCTAA   
  
  
- AAATACAAAT GAAAGATGAC TATAGTAGAA AACGATAACG TTAAATTAAC GGAAGATACA TCTTAAGCAG   
  
  
- TGAAACTTCA GACGGTCCTT CCGTTATGTC GATATAAAAA GAACACCTAC TTCACTATTT TCCGTTCACA   
  
  
- GTTAACGAAT GACGAAATAA CGCGACATGA AAAGGTCTCT AAAACCTAAT CAGTTACGTC TTCGGTTGTT   
  
  
- AAATATCGCT GACCGGTGTC AAGATGTTGG TTAAACTATT AGGTGCAGAA GAACTTAGAA GTAGATATTA   
  
  
- CCCACCACTA GTTGAAAAGT CAAGAGGTAG ATGACATTCG TATAGAACGT TGTCCTCAGG TCGAAGTGAC   
  
  
- GGACTTGAGC TTAGGAGAAG TAGCCTGAAG GTTTCACTGA GGGTCCTCTG ACACTTACCG AGCGGTCACT   
  
  
- AACTAAGCAC GTACCATGCA CTGTAACCAC TGAATTCTGT GTTTGAATCT CTCGAACTCT GACAATACGA   
  
  
- GCCTGGTTCA AGTCTGTCGA ACCTAAGTAC CATACGGGGA AGTTCTCCAC TCACACTTGG AGACGGTGGT   
  
  
- CTTCTCGGAC TGTTACCCTT TGTAAACAAC CTCTACTATC GTTCCCCCTC GGAGTTTCTC CACGATTAAC   
  
  
- GAACACGGTT TCGTTATAGT CTGCTACTAA ACAATTGTCG CCTCACCAAC TACAGTCTCG ATGCGGTATA   
  
  
- CCAAAGTCAA AGACCTCTTG GTTAAGTCTC CAATCCTCGG ATGTACAACC TTCCCAATCA ACGGGCCAAC   
  
  
- AGGAGAAGTC CTTCAAGGTA GATGTTTCGA GATTCCACGT TTCTCGGATG ATCATCACTT GAAGAAAGGA   
  
  
- TATACGTAAA TGAGATACTT CAAACGGGAA TGAAGTTCAA ACCCATGTAC AGACGTTTAC CCCGTTAACG   
  
  
- ACTCCGGTAC TTTTTACTCT CGTCTTAAGT ATATTAACTA AAAGTCTATC GAGTTCCCTC AGTCACCTAA   
  
  
- TCGGAATAGG TTCGGGACCG ACGAGTCGGA CTACCCGGTG GTGTCCAGGC ATAATGGCCT CAGCTACTAA   
  
  
- GGGTTAGACT CATACGAGCC CCTCCCCCCG AGCTGTAACA TCGCTTCTCT GATAGACCTG ATCGGGTCCG   
  
  
- AACGTCGGAT GGGAAACTCA AGGTGCGACG TCGTGAATCA CCAAGACTCT AGTCTGAAGT TTTGGACCAA   
  
  
- AACGCCGGAC CTCTTCGGAA TCGTCACTTG AAGGGTAAGT ACGACGTGGT GTACGGCCTA CTCTCACACC   
  
  
- CGGGACTCTT AATATCTCTG GTCAATAATT CCGACCACTT CTCGAAGAAC GGGTTCCACC AATGGGAACA   
  
  
- ACTCGTTCTT AGGTTGTGTT TGTGCCGGGG TAAAGATGGG GCCAAAGAAC TTTGGGATCT GATGATATGA   
  
  
- CGGTAAAAGC TTAGTTAACT ACAATGCGAG GGTTCTCTAG TGTTCCTCGC CTAGTTACAA CTCGTCGTGA   
  
  
- CAAATCGTTC TCTATATCAG TTGTATTATC GTACACTCCC ACGGCTCTCC CACCTTGCGG TACTCCAAGA   
  
  
- ACCTTTCACC TCTAGAGCCA AGAGTTACCG ACCCAAATTC GGCATGGGTA ACTCGGGCGA TCACTTACGT   
  
  
- TGATAGTCCT GAGAAGACGT CTTCATATCG TCCTCGATAC CTGAACTTCT TTCCCTACCT CGAGAAATAG   
  
  
- ATCCGACCTA CTTGGCTCGT GATCAACGTA GAACACGTAC CGTCAC

+     ABRE

| Site Name | Organism | Position | Strand | Matrix score. | sequence | function |
| --- | --- | --- | --- | --- | --- | --- |
| ABRE | Arabidopsis thaliana | 2261 | + | 5 | ACGTG | cis-acting element involved in the abscisic acid responsiveness |
| ABRE | Arabidopsis thaliana | 2077 | - | 5 | ACGTG | cis-acting element involved in the abscisic acid responsiveness |
| ABRE | Arabidopsis thaliana | 600 | - | 5 | ACGTG | cis-acting element involved in the abscisic acid responsiveness |

>HU08G00284.1   
+ -Up\_Stream \_Len000TTCTTT TCCTGTCTCT GTCTCTTCAT TGCACCATCA TTTAAGAGAT GGGACCATAA   
  
  
+ ATCATTCAAT CACTCCTTTA TGTTTAAACT TTGGAAGGAG GTTATTATTT ATGTGGTGGT GATCCCAATG   
  
  
+ CTGAATTTAG CTGTTGATGG CGAGGGCATA TATGTAATTA GGATTTCCAA TGCAGTTTAG ATTTTACTCA   
  
  
+ TTTGAGAATT CACTGAGGTT TGTTTGATCT GATTTTAGCA AATATTGTTC AGGGGTATAG ATGCTTATTC   
  
  
+ GGTTCTCAAC TGTATTAGTA TACAAGATGA GTGGTCCACT TGTTTAAGCT TTTACTATAT ACTTATGTTG   
  
  
+ TCCCTAATTT GGTCTCGCTT GATGTGTAGC TGTCAAGTAT TATTATAATC TTGTGTTTGA TATCACATTT   
  
  
+ GTATTGATTA GCTACAAAAG AACATATTAT ATGTGTATAG CAACTATCTC ATATATGCCC TCACCATAGC   
  
  
+ GTGGTTCCAA GGGTTCAAGT TCCACTTAGC CTCACCCTAG AAATGACTTC TTTTTTTTTT TAAAGTTAAA   
  
  
+ GATATGCAAT CACCATGGAA CCAGTCATCT TTTGACACGT TATTCCAGAT CAGATGACCA GCCTTTCTCC   
  
  
+ AAGGCCATTA GATTTTCTAG CCAATCCACT GTTAACTGCA AAAAGTGGTA TCCTTGGCTG TTCCTGTCAC   
  
  
+ TGTTTCTTAT TTGCTCTGTG ATATTTGTAC ATCTCTTTGC CAAGGATAAA AGTTCATAGC CGGGAATTGG   
  
  
+ TATTCAGTTG ACTCTTGCAG TAAACAGAGT TCATGTACTG GAGGATTTTG TGAGTCATGT CACTCAATAA   
  
  
+ AAACAGGTGT GCGACAGTGT TATAAAGCTG CTTGCTTAAT GCACATCTAC TCTCACTCAC CTTACCCAAA   
  
  
+ AAACTTCAAA ATTGTGTGCC TTCTATGAAT ATTCTGATCC ATTAGCATTC AACGGTTTCT GAATTCTGTG   
  
  
+ TAAGCCATCC ATACTCATTT TCAACAGAGA ACTTGGATTG GATGTATTCA ATACCCAAAA ACCTTGTCAA   
  
  
+ ATTACCCCTA GCTTAATTTC CTCTAATACC AACTGCTCTT TGGTTTTAGC GTACAATCCC ATAGATTACC   
  
  
+ CTTCTATAAT TAGTGATGAT TGGTTCATTT CTGCTGCATT ATGCCTTCTT TAGATCCATT GATAATATAT   
  
  
+ ACTCTTGCCA TGCTGCAACA TTCCACTGGG ATTAATATCT CACATTGTAT CCTCAGCAAC CAAGGAGACC   
  
  
+ CTACTATTTT CAGAGCCATT CATAGAAAGT TATTGCATAT AGTGATGATC TTCAATGGGT CTGATTTTTA   
  
  
+ GATAGCTGCT GTGAACATTT TACTCGTCCT CTGCCATGTG GATACTATGG TAACCTTTGT TTGCTCTCAA   
  
  
+ GCCTGAGCGT TCTGAGAAAT GATGAAAGCG CCCTCGAGTT ATAGGACTTT TCTGACTTTA GCTTTAGAAA   
  
  
+ AGAGAGGAAA ATCAACCACA CCCATCTGAT TATTGAGTTT CTGCTCCTTG AGACCTGGAT GGAGCCAGAA   
  
  
+ CTCACGGTCT TGTCAGCCAG AATAATCAGG AACTGCATTT CTGAGGACCC ATGAAAAGAC CCTGGTATAC   
  
  
+ GACTATTCAT GAAAACGTTT TGTCAGCCTT TCAGTTTTTG CATTTTATTC TAGCTGCTTT GATTGAAGCC   
  
  
+ CAATTTTATT AAAACTGTGC TTTTCTCTTG AATGTTAAAC TCTTGCAGTT CCTTGCTTGT TATTTTGGGT   
  
  
+ TTTATGTTTG CTTTCTACTG ATATCATCTT TTGGTCGTGC AATTTAATTG CCTACTATGT TATATGGATT   
  
  
+ TTTATGTTTA CTTTCTACTG ATATCATCTT TTGCTATTGC AATTTAATTG CCTTCTATGT AGAATTCGTC   
  
  
+ ACTTTGAAGT CTGCCAGGAA GGCAATACAG CTATATTTTT CTTGTGGATG AAGTGATAAA AGGCAAGTGT   
  
  
+ CAATTGCTTA CTGCTTTATT GCGCTGTACT TTTCCAGAGA TTTTGGATTA GTCAATGCAG AAGCCAACAA   
  
  
+ TTTATAGCGA CTGGCCACAG TTCTACAACC AATTTGATAA TCCACGTCTT CTTGAATCTT CATCTATAAT   
  
  
+ GGGTGGTGAT CAACTTTTCA GTTCTCCATC TACTGTAAGC ATATCTTGCA ACAGGAGTCC AGCTTCACTG   
  
  
+ CCTGAACTCG AATCCTCTTC ATCGGACTTC CAAAGTGACT CCCAGGAGAC TGTGAATGGC TCGCCAGTGA   
  
  
+ TTGATTCGTG CATGGTACGT GACATTGGTG ACTTAAGACA CAAACTTAGA GAGCTTGAGA CTGTTATGCT   
  
  
+ CGGACCAAGT TCAGACAGCT TGGATTCATG GTATGCCCCT TCAAGAGGTG AGTGTGAACC TCTGCCACCA   
  
  
+ GAAGAGCCTG ACAATGGGAA ACATTTGTTG GAGATGATAG CAAGGGGGAG CCTCAAAGAG GTGCTAATTG   
  
  
+ CTTGTGCCAA AGCAATATCA GACGATGATT TGTTAACAGC GGAGTGGTTG ATGTCAGAGC TACGCCATAT   
  
  
+ GGTTTCAGTT TCTGGAGAAC CAATTCAGAG GTTAGGAGCC TACATGTTGG AAGGGTTAGT TGCCCGGTTG   
  
  
+ TCCTCTTCAG GAAGTTCCAT CTACAAAGCT CTAAGGTGCA AAGAGCCTAC TAGTAGTGAA CTTCTTTCCT   
  
  
+ ATATGCATTT ACTCTATGAA GTTTGCCCTT ACTTCAAGTT TGGGTACATG TCTGCAAATG GGGCAATTGC   
  
  
+ TGAGGCCATG AAAAATGAGA GCAGAATTCA TATAATTGAT TTTCAGATAG CTCAAGGGAG TCAGTGGATT   
  
  
+ AGCCTTATCC AAGCCCTGGC TGCTCAGCCT GATGGGCCAC CACAGGTCCG TATTACCGGA GTCGATGATT   
  
  
+ CCCAATCTGA GTATGCTCGG GGAGGGGGGC TCGACATTGT AGCGAAGAGA CTATCTGGAC TAGCCCAGGC   
  
  
+ TTGCAGCCTA CCCTTTGAGT TCCACGCTGC AGCACTTAGT GGTTCTGAGA TCAGACTTCA AAACCTGGTT   
  
  
+ TTGCGGCCTG GAGAAGCCTT AGCAGTGAAC TTCCCATTCA TGCTGCACCA CATGCCGGAT GAGAGTGTGG   
  
  
+ GCCCTGAGAA TTATAGAGAC CAGTTATTAA GGCTGGTGAA GAGCTTCTTG CCCAAGGTGG TTACCCTTGT   
  
  
+ TGAGCAAGAA TCCAACACAA ACACGGCCCC ATTTCTACCC CGGTTTCTTG AAACCCTAGA CTACTATACT   
  
  
+ GCCATTTTCG AATCAATTGA TGTTACGCTC CCAAGAGATC ACAAGGAGCG GATCAATGTT GAGCAGCACT   
  
  
+ GTTTAGCAAG AGATATAGTC AACATAATAG CATGTGAGGG TGCCGAGAGG GTGGAACGCC ATGAGGTTCT   
  
  
+ TGGAAAGTGG AGATCTCGGT TCTCAATGGC TGGGTTTAAG CCGTACCCAT TGAGCCCGCT AGTGAATGCA   
  
  
+ ACTATCAGGA CTCTTCTGCA GAAGTATAGC AGGAGCTATG GACTTGAAGA AAGGGATGGA GCTCTTTATC   
  
  
+ TAGGCTGGAT GAACCGAGCA CTAGTTGCAT CTTGTGCATG GCAGTG  

- -Up\_Stream \_Len000AAGAAA AGGACAGAGA CAGAGAAGTA ACGTGGTAGT AAATTCTCTA CCCTGGTATT   
  
  
- TAGTAAGTTA GTGAGGAAAT ACAAATTTGA AACCTTCCTC CAATAATAAA TACACCACCA CTAGGGTTAC   
  
  
- GACTTAAATC GACAACTACC GCTCCCGTAT ATACATTAAT CCTAAAGGTT ACGTCAAATC TAAAATGAGT   
  
  
- AAACTCTTAA GTGACTCCAA ACAAACTAGA CTAAAATCGT TTATAACAAG TCCCCATATC TACGAATAAG   
  
  
- CCAAGAGTTG ACATAATCAT ATGTTCTACT CACCAGGTGA ACAAATTCGA AAATGATATA TGAATACAAC   
  
  
- AGGGATTAAA CCAGAGCGAA CTACACATCG ACAGTTCATA ATAATATTAG AACACAAACT ATAGTGTAAA   
  
  
- CATAACTAAT CGATGTTTTC TTGTATAATA TACACATATC GTTGATAGAG TATATACGGG AGTGGTATCG   
  
  
- CACCAAGGTT CCCAAGTTCA AGGTGAATCG GAGTGGGATC TTTACTGAAG AAAAAAAAAA ATTTCAATTT   
  
  
- CTATACGTTA GTGGTACCTT GGTCAGTAGA AAACTGTGCA ATAAGGTCTA GTCTACTGGT CGGAAAGAGG   
  
  
- TTCCGGTAAT CTAAAAGATC GGTTAGGTGA CAATTGACGT TTTTCACCAT AGGAACCGAC AAGGACAGTG   
  
  
- ACAAAGAATA AACGAGACAC TATAAACATG TAGAGAAACG GTTCCTATTT TCAAGTATCG GCCCTTAACC   
  
  
- ATAAGTCAAC TGAGAACGTC ATTTGTCTCA AGTACATGAC CTCCTAAAAC ACTCAGTACA GTGAGTTATT   
  
  
- TTTGTCCACA CGCTGTCACA ATATTTCGAC GAACGAATTA CGTGTAGATG AGAGTGAGTG GAATGGGTTT   
  
  
- TTTGAAGTTT TAACACACGG AAGATACTTA TAAGACTAGG TAATCGTAAG TTGCCAAAGA CTTAAGACAC   
  
  
- ATTCGGTAGG TATGAGTAAA AGTTGTCTCT TGAACCTAAC CTACATAAGT TATGGGTTTT TGGAACAGTT   
  
  
- TAATGGGGAT CGAATTAAAG GAGATTATGG TTGACGAGAA ACCAAAATCG CATGTTAGGG TATCTAATGG   
  
  
- GAAGATATTA ATCACTACTA ACCAAGTAAA GACGACGTAA TACGGAAGAA ATCTAGGTAA CTATTATATA   
  
  
- TGAGAACGGT ACGACGTTGT AAGGTGACCC TAATTATAGA GTGTAACATA GGAGTCGTTG GTTCCTCTGG   
  
  
- GATGATAAAA GTCTCGGTAA GTATCTTTCA ATAACGTATA TCACTACTAG AAGTTACCCA GACTAAAAAT   
  
  
- CTATCGACGA CACTTGTAAA ATGAGCAGGA GACGGTACAC CTATGATACC ATTGGAAACA AACGAGAGTT   
  
  
- CGGACTCGCA AGACTCTTTA CTACTTTCGC GGGAGCTCAA TATCCTGAAA AGACTGAAAT CGAAATCTTT   
  
  
- TCTCTCCTTT TAGTTGGTGT GGGTAGACTA ATAACTCAAA GACGAGGAAC TCTGGACCTA CCTCGGTCTT   
  
  
- GAGTGCCAGA ACAGTCGGTC TTATTAGTCC TTGACGTAAA GACTCCTGGG TACTTTTCTG GGACCATATG   
  
  
- CTGATAAGTA CTTTTGCAAA ACAGTCGGAA AGTCAAAAAC GTAAAATAAG ATCGACGAAA CTAACTTCGG   
  
  
- GTTAAAATAA TTTTGACACG AAAAGAGAAC TTACAATTTG AGAACGTCAA GGAACGAACA ATAAAACCCA   
  
  
- AAATACAAAC GAAAGATGAC TATAGTAGAA AACCAGCACG TTAAATTAAC GGATGATACA ATATACCTAA   
  
  
- AAATACAAAT GAAAGATGAC TATAGTAGAA AACGATAACG TTAAATTAAC GGAAGATACA TCTTAAGCAG   
  
  
- TGAAACTTCA GACGGTCCTT CCGTTATGTC GATATAAAAA GAACACCTAC TTCACTATTT TCCGTTCACA   
  
  
- GTTAACGAAT GACGAAATAA CGCGACATGA AAAGGTCTCT AAAACCTAAT CAGTTACGTC TTCGGTTGTT   
  
  
- AAATATCGCT GACCGGTGTC AAGATGTTGG TTAAACTATT AGGTGCAGAA GAACTTAGAA GTAGATATTA   
  
  
- CCCACCACTA GTTGAAAAGT CAAGAGGTAG ATGACATTCG TATAGAACGT TGTCCTCAGG TCGAAGTGAC   
  
  
- GGACTTGAGC TTAGGAGAAG TAGCCTGAAG GTTTCACTGA GGGTCCTCTG ACACTTACCG AGCGGTCACT   
  
  
- AACTAAGCAC GTACCATGCA CTGTAACCAC TGAATTCTGT GTTTGAATCT CTCGAACTCT GACAATACGA   
  
  
- GCCTGGTTCA AGTCTGTCGA ACCTAAGTAC CATACGGGGA AGTTCTCCAC TCACACTTGG AGACGGTGGT   
  
  
- CTTCTCGGAC TGTTACCCTT TGTAAACAAC CTCTACTATC GTTCCCCCTC GGAGTTTCTC CACGATTAAC   
  
  
- GAACACGGTT TCGTTATAGT CTGCTACTAA ACAATTGTCG CCTCACCAAC TACAGTCTCG ATGCGGTATA   
  
  
- CCAAAGTCAA AGACCTCTTG GTTAAGTCTC CAATCCTCGG ATGTACAACC TTCCCAATCA ACGGGCCAAC   
  
  
- AGGAGAAGTC CTTCAAGGTA GATGTTTCGA GATTCCACGT TTCTCGGATG ATCATCACTT GAAGAAAGGA   
  
  
- TATACGTAAA TGAGATACTT CAAACGGGAA TGAAGTTCAA ACCCATGTAC AGACGTTTAC CCCGTTAACG   
  
  
- ACTCCGGTAC TTTTTACTCT CGTCTTAAGT ATATTAACTA AAAGTCTATC GAGTTCCCTC AGTCACCTAA   
  
  
- TCGGAATAGG TTCGGGACCG ACGAGTCGGA CTACCCGGTG GTGTCCAGGC ATAATGGCCT CAGCTACTAA   
  
  
- GGGTTAGACT CATACGAGCC CCTCCCCCCG AGCTGTAACA TCGCTTCTCT GATAGACCTG ATCGGGTCCG   
  
  
- AACGTCGGAT GGGAAACTCA AGGTGCGACG TCGTGAATCA CCAAGACTCT AGTCTGAAGT TTTGGACCAA   
  
  
- AACGCCGGAC CTCTTCGGAA TCGTCACTTG AAGGGTAAGT ACGACGTGGT GTACGGCCTA CTCTCACACC   
  
  
- CGGGACTCTT AATATCTCTG GTCAATAATT CCGACCACTT CTCGAAGAAC GGGTTCCACC AATGGGAACA   
  
  
- ACTCGTTCTT AGGTTGTGTT TGTGCCGGGG TAAAGATGGG GCCAAAGAAC TTTGGGATCT GATGATATGA   
  
  
- CGGTAAAAGC TTAGTTAACT ACAATGCGAG GGTTCTCTAG TGTTCCTCGC CTAGTTACAA CTCGTCGTGA   
  
  
- CAAATCGTTC TCTATATCAG TTGTATTATC GTACACTCCC ACGGCTCTCC CACCTTGCGG TACTCCAAGA   
  
  
- ACCTTTCACC TCTAGAGCCA AGAGTTACCG ACCCAAATTC GGCATGGGTA ACTCGGGCGA TCACTTACGT   
  
  
- TGATAGTCCT GAGAAGACGT CTTCATATCG TCCTCGATAC CTGAACTTCT TTCCCTACCT CGAGAAATAG   
  
  
- ATCCGACCTA CTTGGCTCGT GATCAACGTA GAACACGTAC CGTCAC

+     ABRE3a

| Site Name | Organism | Position | Strand | Matrix score. | sequence | function |
| --- | --- | --- | --- | --- | --- | --- |
| ABRE3a | Zea mays | 2260 | + | 6 | TACGTG |  |

>HU08G00284.1   
+ -Up\_Stream \_Len000TTCTTT TCCTGTCTCT GTCTCTTCAT TGCACCATCA TTTAAGAGAT GGGACCATAA   
  
  
+ ATCATTCAAT CACTCCTTTA TGTTTAAACT TTGGAAGGAG GTTATTATTT ATGTGGTGGT GATCCCAATG   
  
  
+ CTGAATTTAG CTGTTGATGG CGAGGGCATA TATGTAATTA GGATTTCCAA TGCAGTTTAG ATTTTACTCA   
  
  
+ TTTGAGAATT CACTGAGGTT TGTTTGATCT GATTTTAGCA AATATTGTTC AGGGGTATAG ATGCTTATTC   
  
  
+ GGTTCTCAAC TGTATTAGTA TACAAGATGA GTGGTCCACT TGTTTAAGCT TTTACTATAT ACTTATGTTG   
  
  
+ TCCCTAATTT GGTCTCGCTT GATGTGTAGC TGTCAAGTAT TATTATAATC TTGTGTTTGA TATCACATTT   
  
  
+ GTATTGATTA GCTACAAAAG AACATATTAT ATGTGTATAG CAACTATCTC ATATATGCCC TCACCATAGC   
  
  
+ GTGGTTCCAA GGGTTCAAGT TCCACTTAGC CTCACCCTAG AAATGACTTC TTTTTTTTTT TAAAGTTAAA   
  
  
+ GATATGCAAT CACCATGGAA CCAGTCATCT TTTGACACGT TATTCCAGAT CAGATGACCA GCCTTTCTCC   
  
  
+ AAGGCCATTA GATTTTCTAG CCAATCCACT GTTAACTGCA AAAAGTGGTA TCCTTGGCTG TTCCTGTCAC   
  
  
+ TGTTTCTTAT TTGCTCTGTG ATATTTGTAC ATCTCTTTGC CAAGGATAAA AGTTCATAGC CGGGAATTGG   
  
  
+ TATTCAGTTG ACTCTTGCAG TAAACAGAGT TCATGTACTG GAGGATTTTG TGAGTCATGT CACTCAATAA   
  
  
+ AAACAGGTGT GCGACAGTGT TATAAAGCTG CTTGCTTAAT GCACATCTAC TCTCACTCAC CTTACCCAAA   
  
  
+ AAACTTCAAA ATTGTGTGCC TTCTATGAAT ATTCTGATCC ATTAGCATTC AACGGTTTCT GAATTCTGTG   
  
  
+ TAAGCCATCC ATACTCATTT TCAACAGAGA ACTTGGATTG GATGTATTCA ATACCCAAAA ACCTTGTCAA   
  
  
+ ATTACCCCTA GCTTAATTTC CTCTAATACC AACTGCTCTT TGGTTTTAGC GTACAATCCC ATAGATTACC   
  
  
+ CTTCTATAAT TAGTGATGAT TGGTTCATTT CTGCTGCATT ATGCCTTCTT TAGATCCATT GATAATATAT   
  
  
+ ACTCTTGCCA TGCTGCAACA TTCCACTGGG ATTAATATCT CACATTGTAT CCTCAGCAAC CAAGGAGACC   
  
  
+ CTACTATTTT CAGAGCCATT CATAGAAAGT TATTGCATAT AGTGATGATC TTCAATGGGT CTGATTTTTA   
  
  
+ GATAGCTGCT GTGAACATTT TACTCGTCCT CTGCCATGTG GATACTATGG TAACCTTTGT TTGCTCTCAA   
  
  
+ GCCTGAGCGT TCTGAGAAAT GATGAAAGCG CCCTCGAGTT ATAGGACTTT TCTGACTTTA GCTTTAGAAA   
  
  
+ AGAGAGGAAA ATCAACCACA CCCATCTGAT TATTGAGTTT CTGCTCCTTG AGACCTGGAT GGAGCCAGAA   
  
  
+ CTCACGGTCT TGTCAGCCAG AATAATCAGG AACTGCATTT CTGAGGACCC ATGAAAAGAC CCTGGTATAC   
  
  
+ GACTATTCAT GAAAACGTTT TGTCAGCCTT TCAGTTTTTG CATTTTATTC TAGCTGCTTT GATTGAAGCC   
  
  
+ CAATTTTATT AAAACTGTGC TTTTCTCTTG AATGTTAAAC TCTTGCAGTT CCTTGCTTGT TATTTTGGGT   
  
  
+ TTTATGTTTG CTTTCTACTG ATATCATCTT TTGGTCGTGC AATTTAATTG CCTACTATGT TATATGGATT   
  
  
+ TTTATGTTTA CTTTCTACTG ATATCATCTT TTGCTATTGC AATTTAATTG CCTTCTATGT AGAATTCGTC   
  
  
+ ACTTTGAAGT CTGCCAGGAA GGCAATACAG CTATATTTTT CTTGTGGATG AAGTGATAAA AGGCAAGTGT   
  
  
+ CAATTGCTTA CTGCTTTATT GCGCTGTACT TTTCCAGAGA TTTTGGATTA GTCAATGCAG AAGCCAACAA   
  
  
+ TTTATAGCGA CTGGCCACAG TTCTACAACC AATTTGATAA TCCACGTCTT CTTGAATCTT CATCTATAAT   
  
  
+ GGGTGGTGAT CAACTTTTCA GTTCTCCATC TACTGTAAGC ATATCTTGCA ACAGGAGTCC AGCTTCACTG   
  
  
+ CCTGAACTCG AATCCTCTTC ATCGGACTTC CAAAGTGACT CCCAGGAGAC TGTGAATGGC TCGCCAGTGA   
  
  
+ TTGATTCGTG CATGGTACGT GACATTGGTG ACTTAAGACA CAAACTTAGA GAGCTTGAGA CTGTTATGCT   
  
  
+ CGGACCAAGT TCAGACAGCT TGGATTCATG GTATGCCCCT TCAAGAGGTG AGTGTGAACC TCTGCCACCA   
  
  
+ GAAGAGCCTG ACAATGGGAA ACATTTGTTG GAGATGATAG CAAGGGGGAG CCTCAAAGAG GTGCTAATTG   
  
  
+ CTTGTGCCAA AGCAATATCA GACGATGATT TGTTAACAGC GGAGTGGTTG ATGTCAGAGC TACGCCATAT   
  
  
+ GGTTTCAGTT TCTGGAGAAC CAATTCAGAG GTTAGGAGCC TACATGTTGG AAGGGTTAGT TGCCCGGTTG   
  
  
+ TCCTCTTCAG GAAGTTCCAT CTACAAAGCT CTAAGGTGCA AAGAGCCTAC TAGTAGTGAA CTTCTTTCCT   
  
  
+ ATATGCATTT ACTCTATGAA GTTTGCCCTT ACTTCAAGTT TGGGTACATG TCTGCAAATG GGGCAATTGC   
  
  
+ TGAGGCCATG AAAAATGAGA GCAGAATTCA TATAATTGAT TTTCAGATAG CTCAAGGGAG TCAGTGGATT   
  
  
+ AGCCTTATCC AAGCCCTGGC TGCTCAGCCT GATGGGCCAC CACAGGTCCG TATTACCGGA GTCGATGATT   
  
  
+ CCCAATCTGA GTATGCTCGG GGAGGGGGGC TCGACATTGT AGCGAAGAGA CTATCTGGAC TAGCCCAGGC   
  
  
+ TTGCAGCCTA CCCTTTGAGT TCCACGCTGC AGCACTTAGT GGTTCTGAGA TCAGACTTCA AAACCTGGTT   
  
  
+ TTGCGGCCTG GAGAAGCCTT AGCAGTGAAC TTCCCATTCA TGCTGCACCA CATGCCGGAT GAGAGTGTGG   
  
  
+ GCCCTGAGAA TTATAGAGAC CAGTTATTAA GGCTGGTGAA GAGCTTCTTG CCCAAGGTGG TTACCCTTGT   
  
  
+ TGAGCAAGAA TCCAACACAA ACACGGCCCC ATTTCTACCC CGGTTTCTTG AAACCCTAGA CTACTATACT   
  
  
+ GCCATTTTCG AATCAATTGA TGTTACGCTC CCAAGAGATC ACAAGGAGCG GATCAATGTT GAGCAGCACT   
  
  
+ GTTTAGCAAG AGATATAGTC AACATAATAG CATGTGAGGG TGCCGAGAGG GTGGAACGCC ATGAGGTTCT   
  
  
+ TGGAAAGTGG AGATCTCGGT TCTCAATGGC TGGGTTTAAG CCGTACCCAT TGAGCCCGCT AGTGAATGCA   
  
  
+ ACTATCAGGA CTCTTCTGCA GAAGTATAGC AGGAGCTATG GACTTGAAGA AAGGGATGGA GCTCTTTATC   
  
  
+ TAGGCTGGAT GAACCGAGCA CTAGTTGCAT CTTGTGCATG GCAGTG  

- -Up\_Stream \_Len000AAGAAA AGGACAGAGA CAGAGAAGTA ACGTGGTAGT AAATTCTCTA CCCTGGTATT   
  
  
- TAGTAAGTTA GTGAGGAAAT ACAAATTTGA AACCTTCCTC CAATAATAAA TACACCACCA CTAGGGTTAC   
  
  
- GACTTAAATC GACAACTACC GCTCCCGTAT ATACATTAAT CCTAAAGGTT ACGTCAAATC TAAAATGAGT   
  
  
- AAACTCTTAA GTGACTCCAA ACAAACTAGA CTAAAATCGT TTATAACAAG TCCCCATATC TACGAATAAG   
  
  
- CCAAGAGTTG ACATAATCAT ATGTTCTACT CACCAGGTGA ACAAATTCGA AAATGATATA TGAATACAAC   
  
  
- AGGGATTAAA CCAGAGCGAA CTACACATCG ACAGTTCATA ATAATATTAG AACACAAACT ATAGTGTAAA   
  
  
- CATAACTAAT CGATGTTTTC TTGTATAATA TACACATATC GTTGATAGAG TATATACGGG AGTGGTATCG   
  
  
- CACCAAGGTT CCCAAGTTCA AGGTGAATCG GAGTGGGATC TTTACTGAAG AAAAAAAAAA ATTTCAATTT   
  
  
- CTATACGTTA GTGGTACCTT GGTCAGTAGA AAACTGTGCA ATAAGGTCTA GTCTACTGGT CGGAAAGAGG   
  
  
- TTCCGGTAAT CTAAAAGATC GGTTAGGTGA CAATTGACGT TTTTCACCAT AGGAACCGAC AAGGACAGTG   
  
  
- ACAAAGAATA AACGAGACAC TATAAACATG TAGAGAAACG GTTCCTATTT TCAAGTATCG GCCCTTAACC   
  
  
- ATAAGTCAAC TGAGAACGTC ATTTGTCTCA AGTACATGAC CTCCTAAAAC ACTCAGTACA GTGAGTTATT   
  
  
- TTTGTCCACA CGCTGTCACA ATATTTCGAC GAACGAATTA CGTGTAGATG AGAGTGAGTG GAATGGGTTT   
  
  
- TTTGAAGTTT TAACACACGG AAGATACTTA TAAGACTAGG TAATCGTAAG TTGCCAAAGA CTTAAGACAC   
  
  
- ATTCGGTAGG TATGAGTAAA AGTTGTCTCT TGAACCTAAC CTACATAAGT TATGGGTTTT TGGAACAGTT   
  
  
- TAATGGGGAT CGAATTAAAG GAGATTATGG TTGACGAGAA ACCAAAATCG CATGTTAGGG TATCTAATGG   
  
  
- GAAGATATTA ATCACTACTA ACCAAGTAAA GACGACGTAA TACGGAAGAA ATCTAGGTAA CTATTATATA   
  
  
- TGAGAACGGT ACGACGTTGT AAGGTGACCC TAATTATAGA GTGTAACATA GGAGTCGTTG GTTCCTCTGG   
  
  
- GATGATAAAA GTCTCGGTAA GTATCTTTCA ATAACGTATA TCACTACTAG AAGTTACCCA GACTAAAAAT   
  
  
- CTATCGACGA CACTTGTAAA ATGAGCAGGA GACGGTACAC CTATGATACC ATTGGAAACA AACGAGAGTT   
  
  
- CGGACTCGCA AGACTCTTTA CTACTTTCGC GGGAGCTCAA TATCCTGAAA AGACTGAAAT CGAAATCTTT   
  
  
- TCTCTCCTTT TAGTTGGTGT GGGTAGACTA ATAACTCAAA GACGAGGAAC TCTGGACCTA CCTCGGTCTT   
  
  
- GAGTGCCAGA ACAGTCGGTC TTATTAGTCC TTGACGTAAA GACTCCTGGG TACTTTTCTG GGACCATATG   
  
  
- CTGATAAGTA CTTTTGCAAA ACAGTCGGAA AGTCAAAAAC GTAAAATAAG ATCGACGAAA CTAACTTCGG   
  
  
- GTTAAAATAA TTTTGACACG AAAAGAGAAC TTACAATTTG AGAACGTCAA GGAACGAACA ATAAAACCCA   
  
  
- AAATACAAAC GAAAGATGAC TATAGTAGAA AACCAGCACG TTAAATTAAC GGATGATACA ATATACCTAA   
  
  
- AAATACAAAT GAAAGATGAC TATAGTAGAA AACGATAACG TTAAATTAAC GGAAGATACA TCTTAAGCAG   
  
  
- TGAAACTTCA GACGGTCCTT CCGTTATGTC GATATAAAAA GAACACCTAC TTCACTATTT TCCGTTCACA   
  
  
- GTTAACGAAT GACGAAATAA CGCGACATGA AAAGGTCTCT AAAACCTAAT CAGTTACGTC TTCGGTTGTT   
  
  
- AAATATCGCT GACCGGTGTC AAGATGTTGG TTAAACTATT AGGTGCAGAA GAACTTAGAA GTAGATATTA   
  
  
- CCCACCACTA GTTGAAAAGT CAAGAGGTAG ATGACATTCG TATAGAACGT TGTCCTCAGG TCGAAGTGAC   
  
  
- GGACTTGAGC TTAGGAGAAG TAGCCTGAAG GTTTCACTGA GGGTCCTCTG ACACTTACCG AGCGGTCACT   
  
  
- AACTAAGCAC GTACCATGCA CTGTAACCAC TGAATTCTGT GTTTGAATCT CTCGAACTCT GACAATACGA   
  
  
- GCCTGGTTCA AGTCTGTCGA ACCTAAGTAC CATACGGGGA AGTTCTCCAC TCACACTTGG AGACGGTGGT   
  
  
- CTTCTCGGAC TGTTACCCTT TGTAAACAAC CTCTACTATC GTTCCCCCTC GGAGTTTCTC CACGATTAAC   
  
  
- GAACACGGTT TCGTTATAGT CTGCTACTAA ACAATTGTCG CCTCACCAAC TACAGTCTCG ATGCGGTATA   
  
  
- CCAAAGTCAA AGACCTCTTG GTTAAGTCTC CAATCCTCGG ATGTACAACC TTCCCAATCA ACGGGCCAAC   
  
  
- AGGAGAAGTC CTTCAAGGTA GATGTTTCGA GATTCCACGT TTCTCGGATG ATCATCACTT GAAGAAAGGA   
  
  
- TATACGTAAA TGAGATACTT CAAACGGGAA TGAAGTTCAA ACCCATGTAC AGACGTTTAC CCCGTTAACG   
  
  
- ACTCCGGTAC TTTTTACTCT CGTCTTAAGT ATATTAACTA AAAGTCTATC GAGTTCCCTC AGTCACCTAA   
  
  
- TCGGAATAGG TTCGGGACCG ACGAGTCGGA CTACCCGGTG GTGTCCAGGC ATAATGGCCT CAGCTACTAA   
  
  
- GGGTTAGACT CATACGAGCC CCTCCCCCCG AGCTGTAACA TCGCTTCTCT GATAGACCTG ATCGGGTCCG   
  
  
- AACGTCGGAT GGGAAACTCA AGGTGCGACG TCGTGAATCA CCAAGACTCT AGTCTGAAGT TTTGGACCAA   
  
  
- AACGCCGGAC CTCTTCGGAA TCGTCACTTG AAGGGTAAGT ACGACGTGGT GTACGGCCTA CTCTCACACC   
  
  
- CGGGACTCTT AATATCTCTG GTCAATAATT CCGACCACTT CTCGAAGAAC GGGTTCCACC AATGGGAACA   
  
  
- ACTCGTTCTT AGGTTGTGTT TGTGCCGGGG TAAAGATGGG GCCAAAGAAC TTTGGGATCT GATGATATGA   
  
  
- CGGTAAAAGC TTAGTTAACT ACAATGCGAG GGTTCTCTAG TGTTCCTCGC CTAGTTACAA CTCGTCGTGA   
  
  
- CAAATCGTTC TCTATATCAG TTGTATTATC GTACACTCCC ACGGCTCTCC CACCTTGCGG TACTCCAAGA   
  
  
- ACCTTTCACC TCTAGAGCCA AGAGTTACCG ACCCAAATTC GGCATGGGTA ACTCGGGCGA TCACTTACGT   
  
  
- TGATAGTCCT GAGAAGACGT CTTCATATCG TCCTCGATAC CTGAACTTCT TTCCCTACCT CGAGAAATAG   
  
  
- ATCCGACCTA CTTGGCTCGT GATCAACGTA GAACACGTAC CGTCAC

+     ABRE4

| Site Name | Organism | Position | Strand | Matrix score. | sequence | function |
| --- | --- | --- | --- | --- | --- | --- |
| ABRE4 | Zea mays | 2260 | - | 6 | CACGTA |  |

>HU08G00284.1   
+ -Up\_Stream \_Len000TTCTTT TCCTGTCTCT GTCTCTTCAT TGCACCATCA TTTAAGAGAT GGGACCATAA   
  
  
+ ATCATTCAAT CACTCCTTTA TGTTTAAACT TTGGAAGGAG GTTATTATTT ATGTGGTGGT GATCCCAATG   
  
  
+ CTGAATTTAG CTGTTGATGG CGAGGGCATA TATGTAATTA GGATTTCCAA TGCAGTTTAG ATTTTACTCA   
  
  
+ TTTGAGAATT CACTGAGGTT TGTTTGATCT GATTTTAGCA AATATTGTTC AGGGGTATAG ATGCTTATTC   
  
  
+ GGTTCTCAAC TGTATTAGTA TACAAGATGA GTGGTCCACT TGTTTAAGCT TTTACTATAT ACTTATGTTG   
  
  
+ TCCCTAATTT GGTCTCGCTT GATGTGTAGC TGTCAAGTAT TATTATAATC TTGTGTTTGA TATCACATTT   
  
  
+ GTATTGATTA GCTACAAAAG AACATATTAT ATGTGTATAG CAACTATCTC ATATATGCCC TCACCATAGC   
  
  
+ GTGGTTCCAA GGGTTCAAGT TCCACTTAGC CTCACCCTAG AAATGACTTC TTTTTTTTTT TAAAGTTAAA   
  
  
+ GATATGCAAT CACCATGGAA CCAGTCATCT TTTGACACGT TATTCCAGAT CAGATGACCA GCCTTTCTCC   
  
  
+ AAGGCCATTA GATTTTCTAG CCAATCCACT GTTAACTGCA AAAAGTGGTA TCCTTGGCTG TTCCTGTCAC   
  
  
+ TGTTTCTTAT TTGCTCTGTG ATATTTGTAC ATCTCTTTGC CAAGGATAAA AGTTCATAGC CGGGAATTGG   
  
  
+ TATTCAGTTG ACTCTTGCAG TAAACAGAGT TCATGTACTG GAGGATTTTG TGAGTCATGT CACTCAATAA   
  
  
+ AAACAGGTGT GCGACAGTGT TATAAAGCTG CTTGCTTAAT GCACATCTAC TCTCACTCAC CTTACCCAAA   
  
  
+ AAACTTCAAA ATTGTGTGCC TTCTATGAAT ATTCTGATCC ATTAGCATTC AACGGTTTCT GAATTCTGTG   
  
  
+ TAAGCCATCC ATACTCATTT TCAACAGAGA ACTTGGATTG GATGTATTCA ATACCCAAAA ACCTTGTCAA   
  
  
+ ATTACCCCTA GCTTAATTTC CTCTAATACC AACTGCTCTT TGGTTTTAGC GTACAATCCC ATAGATTACC   
  
  
+ CTTCTATAAT TAGTGATGAT TGGTTCATTT CTGCTGCATT ATGCCTTCTT TAGATCCATT GATAATATAT   
  
  
+ ACTCTTGCCA TGCTGCAACA TTCCACTGGG ATTAATATCT CACATTGTAT CCTCAGCAAC CAAGGAGACC   
  
  
+ CTACTATTTT CAGAGCCATT CATAGAAAGT TATTGCATAT AGTGATGATC TTCAATGGGT CTGATTTTTA   
  
  
+ GATAGCTGCT GTGAACATTT TACTCGTCCT CTGCCATGTG GATACTATGG TAACCTTTGT TTGCTCTCAA   
  
  
+ GCCTGAGCGT TCTGAGAAAT GATGAAAGCG CCCTCGAGTT ATAGGACTTT TCTGACTTTA GCTTTAGAAA   
  
  
+ AGAGAGGAAA ATCAACCACA CCCATCTGAT TATTGAGTTT CTGCTCCTTG AGACCTGGAT GGAGCCAGAA   
  
  
+ CTCACGGTCT TGTCAGCCAG AATAATCAGG AACTGCATTT CTGAGGACCC ATGAAAAGAC CCTGGTATAC   
  
  
+ GACTATTCAT GAAAACGTTT TGTCAGCCTT TCAGTTTTTG CATTTTATTC TAGCTGCTTT GATTGAAGCC   
  
  
+ CAATTTTATT AAAACTGTGC TTTTCTCTTG AATGTTAAAC TCTTGCAGTT CCTTGCTTGT TATTTTGGGT   
  
  
+ TTTATGTTTG CTTTCTACTG ATATCATCTT TTGGTCGTGC AATTTAATTG CCTACTATGT TATATGGATT   
  
  
+ TTTATGTTTA CTTTCTACTG ATATCATCTT TTGCTATTGC AATTTAATTG CCTTCTATGT AGAATTCGTC   
  
  
+ ACTTTGAAGT CTGCCAGGAA GGCAATACAG CTATATTTTT CTTGTGGATG AAGTGATAAA AGGCAAGTGT   
  
  
+ CAATTGCTTA CTGCTTTATT GCGCTGTACT TTTCCAGAGA TTTTGGATTA GTCAATGCAG AAGCCAACAA   
  
  
+ TTTATAGCGA CTGGCCACAG TTCTACAACC AATTTGATAA TCCACGTCTT CTTGAATCTT CATCTATAAT   
  
  
+ GGGTGGTGAT CAACTTTTCA GTTCTCCATC TACTGTAAGC ATATCTTGCA ACAGGAGTCC AGCTTCACTG   
  
  
+ CCTGAACTCG AATCCTCTTC ATCGGACTTC CAAAGTGACT CCCAGGAGAC TGTGAATGGC TCGCCAGTGA   
  
  
+ TTGATTCGTG CATGGTACGT GACATTGGTG ACTTAAGACA CAAACTTAGA GAGCTTGAGA CTGTTATGCT   
  
  
+ CGGACCAAGT TCAGACAGCT TGGATTCATG GTATGCCCCT TCAAGAGGTG AGTGTGAACC TCTGCCACCA   
  
  
+ GAAGAGCCTG ACAATGGGAA ACATTTGTTG GAGATGATAG CAAGGGGGAG CCTCAAAGAG GTGCTAATTG   
  
  
+ CTTGTGCCAA AGCAATATCA GACGATGATT TGTTAACAGC GGAGTGGTTG ATGTCAGAGC TACGCCATAT   
  
  
+ GGTTTCAGTT TCTGGAGAAC CAATTCAGAG GTTAGGAGCC TACATGTTGG AAGGGTTAGT TGCCCGGTTG   
  
  
+ TCCTCTTCAG GAAGTTCCAT CTACAAAGCT CTAAGGTGCA AAGAGCCTAC TAGTAGTGAA CTTCTTTCCT   
  
  
+ ATATGCATTT ACTCTATGAA GTTTGCCCTT ACTTCAAGTT TGGGTACATG TCTGCAAATG GGGCAATTGC   
  
  
+ TGAGGCCATG AAAAATGAGA GCAGAATTCA TATAATTGAT TTTCAGATAG CTCAAGGGAG TCAGTGGATT   
  
  
+ AGCCTTATCC AAGCCCTGGC TGCTCAGCCT GATGGGCCAC CACAGGTCCG TATTACCGGA GTCGATGATT   
  
  
+ CCCAATCTGA GTATGCTCGG GGAGGGGGGC TCGACATTGT AGCGAAGAGA CTATCTGGAC TAGCCCAGGC   
  
  
+ TTGCAGCCTA CCCTTTGAGT TCCACGCTGC AGCACTTAGT GGTTCTGAGA TCAGACTTCA AAACCTGGTT   
  
  
+ TTGCGGCCTG GAGAAGCCTT AGCAGTGAAC TTCCCATTCA TGCTGCACCA CATGCCGGAT GAGAGTGTGG   
  
  
+ GCCCTGAGAA TTATAGAGAC CAGTTATTAA GGCTGGTGAA GAGCTTCTTG CCCAAGGTGG TTACCCTTGT   
  
  
+ TGAGCAAGAA TCCAACACAA ACACGGCCCC ATTTCTACCC CGGTTTCTTG AAACCCTAGA CTACTATACT   
  
  
+ GCCATTTTCG AATCAATTGA TGTTACGCTC CCAAGAGATC ACAAGGAGCG GATCAATGTT GAGCAGCACT   
  
  
+ GTTTAGCAAG AGATATAGTC AACATAATAG CATGTGAGGG TGCCGAGAGG GTGGAACGCC ATGAGGTTCT   
  
  
+ TGGAAAGTGG AGATCTCGGT TCTCAATGGC TGGGTTTAAG CCGTACCCAT TGAGCCCGCT AGTGAATGCA   
  
  
+ ACTATCAGGA CTCTTCTGCA GAAGTATAGC AGGAGCTATG GACTTGAAGA AAGGGATGGA GCTCTTTATC   
  
  
+ TAGGCTGGAT GAACCGAGCA CTAGTTGCAT CTTGTGCATG GCAGTG  

- -Up\_Stream \_Len000AAGAAA AGGACAGAGA CAGAGAAGTA ACGTGGTAGT AAATTCTCTA CCCTGGTATT   
  
  
- TAGTAAGTTA GTGAGGAAAT ACAAATTTGA AACCTTCCTC CAATAATAAA TACACCACCA CTAGGGTTAC   
  
  
- GACTTAAATC GACAACTACC GCTCCCGTAT ATACATTAAT CCTAAAGGTT ACGTCAAATC TAAAATGAGT   
  
  
- AAACTCTTAA GTGACTCCAA ACAAACTAGA CTAAAATCGT TTATAACAAG TCCCCATATC TACGAATAAG   
  
  
- CCAAGAGTTG ACATAATCAT ATGTTCTACT CACCAGGTGA ACAAATTCGA AAATGATATA TGAATACAAC   
  
  
- AGGGATTAAA CCAGAGCGAA CTACACATCG ACAGTTCATA ATAATATTAG AACACAAACT ATAGTGTAAA   
  
  
- CATAACTAAT CGATGTTTTC TTGTATAATA TACACATATC GTTGATAGAG TATATACGGG AGTGGTATCG   
  
  
- CACCAAGGTT CCCAAGTTCA AGGTGAATCG GAGTGGGATC TTTACTGAAG AAAAAAAAAA ATTTCAATTT   
  
  
- CTATACGTTA GTGGTACCTT GGTCAGTAGA AAACTGTGCA ATAAGGTCTA GTCTACTGGT CGGAAAGAGG   
  
  
- TTCCGGTAAT CTAAAAGATC GGTTAGGTGA CAATTGACGT TTTTCACCAT AGGAACCGAC AAGGACAGTG   
  
  
- ACAAAGAATA AACGAGACAC TATAAACATG TAGAGAAACG GTTCCTATTT TCAAGTATCG GCCCTTAACC   
  
  
- ATAAGTCAAC TGAGAACGTC ATTTGTCTCA AGTACATGAC CTCCTAAAAC ACTCAGTACA GTGAGTTATT   
  
  
- TTTGTCCACA CGCTGTCACA ATATTTCGAC GAACGAATTA CGTGTAGATG AGAGTGAGTG GAATGGGTTT   
  
  
- TTTGAAGTTT TAACACACGG AAGATACTTA TAAGACTAGG TAATCGTAAG TTGCCAAAGA CTTAAGACAC   
  
  
- ATTCGGTAGG TATGAGTAAA AGTTGTCTCT TGAACCTAAC CTACATAAGT TATGGGTTTT TGGAACAGTT   
  
  
- TAATGGGGAT CGAATTAAAG GAGATTATGG TTGACGAGAA ACCAAAATCG CATGTTAGGG TATCTAATGG   
  
  
- GAAGATATTA ATCACTACTA ACCAAGTAAA GACGACGTAA TACGGAAGAA ATCTAGGTAA CTATTATATA   
  
  
- TGAGAACGGT ACGACGTTGT AAGGTGACCC TAATTATAGA GTGTAACATA GGAGTCGTTG GTTCCTCTGG   
  
  
- GATGATAAAA GTCTCGGTAA GTATCTTTCA ATAACGTATA TCACTACTAG AAGTTACCCA GACTAAAAAT   
  
  
- CTATCGACGA CACTTGTAAA ATGAGCAGGA GACGGTACAC CTATGATACC ATTGGAAACA AACGAGAGTT   
  
  
- CGGACTCGCA AGACTCTTTA CTACTTTCGC GGGAGCTCAA TATCCTGAAA AGACTGAAAT CGAAATCTTT   
  
  
- TCTCTCCTTT TAGTTGGTGT GGGTAGACTA ATAACTCAAA GACGAGGAAC TCTGGACCTA CCTCGGTCTT   
  
  
- GAGTGCCAGA ACAGTCGGTC TTATTAGTCC TTGACGTAAA GACTCCTGGG TACTTTTCTG GGACCATATG   
  
  
- CTGATAAGTA CTTTTGCAAA ACAGTCGGAA AGTCAAAAAC GTAAAATAAG ATCGACGAAA CTAACTTCGG   
  
  
- GTTAAAATAA TTTTGACACG AAAAGAGAAC TTACAATTTG AGAACGTCAA GGAACGAACA ATAAAACCCA   
  
  
- AAATACAAAC GAAAGATGAC TATAGTAGAA AACCAGCACG TTAAATTAAC GGATGATACA ATATACCTAA   
  
  
- AAATACAAAT GAAAGATGAC TATAGTAGAA AACGATAACG TTAAATTAAC GGAAGATACA TCTTAAGCAG   
  
  
- TGAAACTTCA GACGGTCCTT CCGTTATGTC GATATAAAAA GAACACCTAC TTCACTATTT TCCGTTCACA   
  
  
- GTTAACGAAT GACGAAATAA CGCGACATGA AAAGGTCTCT AAAACCTAAT CAGTTACGTC TTCGGTTGTT   
  
  
- AAATATCGCT GACCGGTGTC AAGATGTTGG TTAAACTATT AGGTGCAGAA GAACTTAGAA GTAGATATTA   
  
  
- CCCACCACTA GTTGAAAAGT CAAGAGGTAG ATGACATTCG TATAGAACGT TGTCCTCAGG TCGAAGTGAC   
  
  
- GGACTTGAGC TTAGGAGAAG TAGCCTGAAG GTTTCACTGA GGGTCCTCTG ACACTTACCG AGCGGTCACT   
  
  
- AACTAAGCAC GTACCATGCA CTGTAACCAC TGAATTCTGT GTTTGAATCT CTCGAACTCT GACAATACGA   
  
  
- GCCTGGTTCA AGTCTGTCGA ACCTAAGTAC CATACGGGGA AGTTCTCCAC TCACACTTGG AGACGGTGGT   
  
  
- CTTCTCGGAC TGTTACCCTT TGTAAACAAC CTCTACTATC GTTCCCCCTC GGAGTTTCTC CACGATTAAC   
  
  
- GAACACGGTT TCGTTATAGT CTGCTACTAA ACAATTGTCG CCTCACCAAC TACAGTCTCG ATGCGGTATA   
  
  
- CCAAAGTCAA AGACCTCTTG GTTAAGTCTC CAATCCTCGG ATGTACAACC TTCCCAATCA ACGGGCCAAC   
  
  
- AGGAGAAGTC CTTCAAGGTA GATGTTTCGA GATTCCACGT TTCTCGGATG ATCATCACTT GAAGAAAGGA   
  
  
- TATACGTAAA TGAGATACTT CAAACGGGAA TGAAGTTCAA ACCCATGTAC AGACGTTTAC CCCGTTAACG   
  
  
- ACTCCGGTAC TTTTTACTCT CGTCTTAAGT ATATTAACTA AAAGTCTATC GAGTTCCCTC AGTCACCTAA   
  
  
- TCGGAATAGG TTCGGGACCG ACGAGTCGGA CTACCCGGTG GTGTCCAGGC ATAATGGCCT CAGCTACTAA   
  
  
- GGGTTAGACT CATACGAGCC CCTCCCCCCG AGCTGTAACA TCGCTTCTCT GATAGACCTG ATCGGGTCCG   
  
  
- AACGTCGGAT GGGAAACTCA AGGTGCGACG TCGTGAATCA CCAAGACTCT AGTCTGAAGT TTTGGACCAA   
  
  
- AACGCCGGAC CTCTTCGGAA TCGTCACTTG AAGGGTAAGT ACGACGTGGT GTACGGCCTA CTCTCACACC   
  
  
- CGGGACTCTT AATATCTCTG GTCAATAATT CCGACCACTT CTCGAAGAAC GGGTTCCACC AATGGGAACA   
  
  
- ACTCGTTCTT AGGTTGTGTT TGTGCCGGGG TAAAGATGGG GCCAAAGAAC TTTGGGATCT GATGATATGA   
  
  
- CGGTAAAAGC TTAGTTAACT ACAATGCGAG GGTTCTCTAG TGTTCCTCGC CTAGTTACAA CTCGTCGTGA   
  
  
- CAAATCGTTC TCTATATCAG TTGTATTATC GTACACTCCC ACGGCTCTCC CACCTTGCGG TACTCCAAGA   
  
  
- ACCTTTCACC TCTAGAGCCA AGAGTTACCG ACCCAAATTC GGCATGGGTA ACTCGGGCGA TCACTTACGT   
  
  
- TGATAGTCCT GAGAAGACGT CTTCATATCG TCCTCGATAC CTGAACTTCT TTCCCTACCT CGAGAAATAG   
  
  
- ATCCGACCTA CTTGGCTCGT GATCAACGTA GAACACGTAC CGTCAC

+     ARE

| Site Name | Organism | Position | Strand | Matrix score. | sequence | function |
| --- | --- | --- | --- | --- | --- | --- |
| ARE | Zea mays | 3010 | - | 6 | AAACCA | cis-acting regulatory element essential for the anaerobic induction |
| ARE | Zea mays | 1095 | - | 6 | AAACCA | cis-acting regulatory element essential for the anaerobic induction |
| ARE | Zea mays | 2524 | - | 6 | AAACCA | cis-acting regulatory element essential for the anaerobic induction |

>HU08G00284.1   
+ -Up\_Stream \_Len000TTCTTT TCCTGTCTCT GTCTCTTCAT TGCACCATCA TTTAAGAGAT GGGACCATAA   
  
  
+ ATCATTCAAT CACTCCTTTA TGTTTAAACT TTGGAAGGAG GTTATTATTT ATGTGGTGGT GATCCCAATG   
  
  
+ CTGAATTTAG CTGTTGATGG CGAGGGCATA TATGTAATTA GGATTTCCAA TGCAGTTTAG ATTTTACTCA   
  
  
+ TTTGAGAATT CACTGAGGTT TGTTTGATCT GATTTTAGCA AATATTGTTC AGGGGTATAG ATGCTTATTC   
  
  
+ GGTTCTCAAC TGTATTAGTA TACAAGATGA GTGGTCCACT TGTTTAAGCT TTTACTATAT ACTTATGTTG   
  
  
+ TCCCTAATTT GGTCTCGCTT GATGTGTAGC TGTCAAGTAT TATTATAATC TTGTGTTTGA TATCACATTT   
  
  
+ GTATTGATTA GCTACAAAAG AACATATTAT ATGTGTATAG CAACTATCTC ATATATGCCC TCACCATAGC   
  
  
+ GTGGTTCCAA GGGTTCAAGT TCCACTTAGC CTCACCCTAG AAATGACTTC TTTTTTTTTT TAAAGTTAAA   
  
  
+ GATATGCAAT CACCATGGAA CCAGTCATCT TTTGACACGT TATTCCAGAT CAGATGACCA GCCTTTCTCC   
  
  
+ AAGGCCATTA GATTTTCTAG CCAATCCACT GTTAACTGCA AAAAGTGGTA TCCTTGGCTG TTCCTGTCAC   
  
  
+ TGTTTCTTAT TTGCTCTGTG ATATTTGTAC ATCTCTTTGC CAAGGATAAA AGTTCATAGC CGGGAATTGG   
  
  
+ TATTCAGTTG ACTCTTGCAG TAAACAGAGT TCATGTACTG GAGGATTTTG TGAGTCATGT CACTCAATAA   
  
  
+ AAACAGGTGT GCGACAGTGT TATAAAGCTG CTTGCTTAAT GCACATCTAC TCTCACTCAC CTTACCCAAA   
  
  
+ AAACTTCAAA ATTGTGTGCC TTCTATGAAT ATTCTGATCC ATTAGCATTC AACGGTTTCT GAATTCTGTG   
  
  
+ TAAGCCATCC ATACTCATTT TCAACAGAGA ACTTGGATTG GATGTATTCA ATACCCAAAA ACCTTGTCAA   
  
  
+ ATTACCCCTA GCTTAATTTC CTCTAATACC AACTGCTCTT TGGTTTTAGC GTACAATCCC ATAGATTACC   
  
  
+ CTTCTATAAT TAGTGATGAT TGGTTCATTT CTGCTGCATT ATGCCTTCTT TAGATCCATT GATAATATAT   
  
  
+ ACTCTTGCCA TGCTGCAACA TTCCACTGGG ATTAATATCT CACATTGTAT CCTCAGCAAC CAAGGAGACC   
  
  
+ CTACTATTTT CAGAGCCATT CATAGAAAGT TATTGCATAT AGTGATGATC TTCAATGGGT CTGATTTTTA   
  
  
+ GATAGCTGCT GTGAACATTT TACTCGTCCT CTGCCATGTG GATACTATGG TAACCTTTGT TTGCTCTCAA   
  
  
+ GCCTGAGCGT TCTGAGAAAT GATGAAAGCG CCCTCGAGTT ATAGGACTTT TCTGACTTTA GCTTTAGAAA   
  
  
+ AGAGAGGAAA ATCAACCACA CCCATCTGAT TATTGAGTTT CTGCTCCTTG AGACCTGGAT GGAGCCAGAA   
  
  
+ CTCACGGTCT TGTCAGCCAG AATAATCAGG AACTGCATTT CTGAGGACCC ATGAAAAGAC CCTGGTATAC   
  
  
+ GACTATTCAT GAAAACGTTT TGTCAGCCTT TCAGTTTTTG CATTTTATTC TAGCTGCTTT GATTGAAGCC   
  
  
+ CAATTTTATT AAAACTGTGC TTTTCTCTTG AATGTTAAAC TCTTGCAGTT CCTTGCTTGT TATTTTGGGT   
  
  
+ TTTATGTTTG CTTTCTACTG ATATCATCTT TTGGTCGTGC AATTTAATTG CCTACTATGT TATATGGATT   
  
  
+ TTTATGTTTA CTTTCTACTG ATATCATCTT TTGCTATTGC AATTTAATTG CCTTCTATGT AGAATTCGTC   
  
  
+ ACTTTGAAGT CTGCCAGGAA GGCAATACAG CTATATTTTT CTTGTGGATG AAGTGATAAA AGGCAAGTGT   
  
  
+ CAATTGCTTA CTGCTTTATT GCGCTGTACT TTTCCAGAGA TTTTGGATTA GTCAATGCAG AAGCCAACAA   
  
  
+ TTTATAGCGA CTGGCCACAG TTCTACAACC AATTTGATAA TCCACGTCTT CTTGAATCTT CATCTATAAT   
  
  
+ GGGTGGTGAT CAACTTTTCA GTTCTCCATC TACTGTAAGC ATATCTTGCA ACAGGAGTCC AGCTTCACTG   
  
  
+ CCTGAACTCG AATCCTCTTC ATCGGACTTC CAAAGTGACT CCCAGGAGAC TGTGAATGGC TCGCCAGTGA   
  
  
+ TTGATTCGTG CATGGTACGT GACATTGGTG ACTTAAGACA CAAACTTAGA GAGCTTGAGA CTGTTATGCT   
  
  
+ CGGACCAAGT TCAGACAGCT TGGATTCATG GTATGCCCCT TCAAGAGGTG AGTGTGAACC TCTGCCACCA   
  
  
+ GAAGAGCCTG ACAATGGGAA ACATTTGTTG GAGATGATAG CAAGGGGGAG CCTCAAAGAG GTGCTAATTG   
  
  
+ CTTGTGCCAA AGCAATATCA GACGATGATT TGTTAACAGC GGAGTGGTTG ATGTCAGAGC TACGCCATAT   
  
  
+ GGTTTCAGTT TCTGGAGAAC CAATTCAGAG GTTAGGAGCC TACATGTTGG AAGGGTTAGT TGCCCGGTTG   
  
  
+ TCCTCTTCAG GAAGTTCCAT CTACAAAGCT CTAAGGTGCA AAGAGCCTAC TAGTAGTGAA CTTCTTTCCT   
  
  
+ ATATGCATTT ACTCTATGAA GTTTGCCCTT ACTTCAAGTT TGGGTACATG TCTGCAAATG GGGCAATTGC   
  
  
+ TGAGGCCATG AAAAATGAGA GCAGAATTCA TATAATTGAT TTTCAGATAG CTCAAGGGAG TCAGTGGATT   
  
  
+ AGCCTTATCC AAGCCCTGGC TGCTCAGCCT GATGGGCCAC CACAGGTCCG TATTACCGGA GTCGATGATT   
  
  
+ CCCAATCTGA GTATGCTCGG GGAGGGGGGC TCGACATTGT AGCGAAGAGA CTATCTGGAC TAGCCCAGGC   
  
  
+ TTGCAGCCTA CCCTTTGAGT TCCACGCTGC AGCACTTAGT GGTTCTGAGA TCAGACTTCA AAACCTGGTT   
  
  
+ TTGCGGCCTG GAGAAGCCTT AGCAGTGAAC TTCCCATTCA TGCTGCACCA CATGCCGGAT GAGAGTGTGG   
  
  
+ GCCCTGAGAA TTATAGAGAC CAGTTATTAA GGCTGGTGAA GAGCTTCTTG CCCAAGGTGG TTACCCTTGT   
  
  
+ TGAGCAAGAA TCCAACACAA ACACGGCCCC ATTTCTACCC CGGTTTCTTG AAACCCTAGA CTACTATACT   
  
  
+ GCCATTTTCG AATCAATTGA TGTTACGCTC CCAAGAGATC ACAAGGAGCG GATCAATGTT GAGCAGCACT   
  
  
+ GTTTAGCAAG AGATATAGTC AACATAATAG CATGTGAGGG TGCCGAGAGG GTGGAACGCC ATGAGGTTCT   
  
  
+ TGGAAAGTGG AGATCTCGGT TCTCAATGGC TGGGTTTAAG CCGTACCCAT TGAGCCCGCT AGTGAATGCA   
  
  
+ ACTATCAGGA CTCTTCTGCA GAAGTATAGC AGGAGCTATG GACTTGAAGA AAGGGATGGA GCTCTTTATC   
  
  
+ TAGGCTGGAT GAACCGAGCA CTAGTTGCAT CTTGTGCATG GCAGTG  

- -Up\_Stream \_Len000AAGAAA AGGACAGAGA CAGAGAAGTA ACGTGGTAGT AAATTCTCTA CCCTGGTATT   
  
  
- TAGTAAGTTA GTGAGGAAAT ACAAATTTGA AACCTTCCTC CAATAATAAA TACACCACCA CTAGGGTTAC   
  
  
- GACTTAAATC GACAACTACC GCTCCCGTAT ATACATTAAT CCTAAAGGTT ACGTCAAATC TAAAATGAGT   
  
  
- AAACTCTTAA GTGACTCCAA ACAAACTAGA CTAAAATCGT TTATAACAAG TCCCCATATC TACGAATAAG   
  
  
- CCAAGAGTTG ACATAATCAT ATGTTCTACT CACCAGGTGA ACAAATTCGA AAATGATATA TGAATACAAC   
  
  
- AGGGATTAAA CCAGAGCGAA CTACACATCG ACAGTTCATA ATAATATTAG AACACAAACT ATAGTGTAAA   
  
  
- CATAACTAAT CGATGTTTTC TTGTATAATA TACACATATC GTTGATAGAG TATATACGGG AGTGGTATCG   
  
  
- CACCAAGGTT CCCAAGTTCA AGGTGAATCG GAGTGGGATC TTTACTGAAG AAAAAAAAAA ATTTCAATTT   
  
  
- CTATACGTTA GTGGTACCTT GGTCAGTAGA AAACTGTGCA ATAAGGTCTA GTCTACTGGT CGGAAAGAGG   
  
  
- TTCCGGTAAT CTAAAAGATC GGTTAGGTGA CAATTGACGT TTTTCACCAT AGGAACCGAC AAGGACAGTG   
  
  
- ACAAAGAATA AACGAGACAC TATAAACATG TAGAGAAACG GTTCCTATTT TCAAGTATCG GCCCTTAACC   
  
  
- ATAAGTCAAC TGAGAACGTC ATTTGTCTCA AGTACATGAC CTCCTAAAAC ACTCAGTACA GTGAGTTATT   
  
  
- TTTGTCCACA CGCTGTCACA ATATTTCGAC GAACGAATTA CGTGTAGATG AGAGTGAGTG GAATGGGTTT   
  
  
- TTTGAAGTTT TAACACACGG AAGATACTTA TAAGACTAGG TAATCGTAAG TTGCCAAAGA CTTAAGACAC   
  
  
- ATTCGGTAGG TATGAGTAAA AGTTGTCTCT TGAACCTAAC CTACATAAGT TATGGGTTTT TGGAACAGTT   
  
  
- TAATGGGGAT CGAATTAAAG GAGATTATGG TTGACGAGAA ACCAAAATCG CATGTTAGGG TATCTAATGG   
  
  
- GAAGATATTA ATCACTACTA ACCAAGTAAA GACGACGTAA TACGGAAGAA ATCTAGGTAA CTATTATATA   
  
  
- TGAGAACGGT ACGACGTTGT AAGGTGACCC TAATTATAGA GTGTAACATA GGAGTCGTTG GTTCCTCTGG   
  
  
- GATGATAAAA GTCTCGGTAA GTATCTTTCA ATAACGTATA TCACTACTAG AAGTTACCCA GACTAAAAAT   
  
  
- CTATCGACGA CACTTGTAAA ATGAGCAGGA GACGGTACAC CTATGATACC ATTGGAAACA AACGAGAGTT   
  
  
- CGGACTCGCA AGACTCTTTA CTACTTTCGC GGGAGCTCAA TATCCTGAAA AGACTGAAAT CGAAATCTTT   
  
  
- TCTCTCCTTT TAGTTGGTGT GGGTAGACTA ATAACTCAAA GACGAGGAAC TCTGGACCTA CCTCGGTCTT   
  
  
- GAGTGCCAGA ACAGTCGGTC TTATTAGTCC TTGACGTAAA GACTCCTGGG TACTTTTCTG GGACCATATG   
  
  
- CTGATAAGTA CTTTTGCAAA ACAGTCGGAA AGTCAAAAAC GTAAAATAAG ATCGACGAAA CTAACTTCGG   
  
  
- GTTAAAATAA TTTTGACACG AAAAGAGAAC TTACAATTTG AGAACGTCAA GGAACGAACA ATAAAACCCA   
  
  
- AAATACAAAC GAAAGATGAC TATAGTAGAA AACCAGCACG TTAAATTAAC GGATGATACA ATATACCTAA   
  
  
- AAATACAAAT GAAAGATGAC TATAGTAGAA AACGATAACG TTAAATTAAC GGAAGATACA TCTTAAGCAG   
  
  
- TGAAACTTCA GACGGTCCTT CCGTTATGTC GATATAAAAA GAACACCTAC TTCACTATTT TCCGTTCACA   
  
  
- GTTAACGAAT GACGAAATAA CGCGACATGA AAAGGTCTCT AAAACCTAAT CAGTTACGTC TTCGGTTGTT   
  
  
- AAATATCGCT GACCGGTGTC AAGATGTTGG TTAAACTATT AGGTGCAGAA GAACTTAGAA GTAGATATTA   
  
  
- CCCACCACTA GTTGAAAAGT CAAGAGGTAG ATGACATTCG TATAGAACGT TGTCCTCAGG TCGAAGTGAC   
  
  
- GGACTTGAGC TTAGGAGAAG TAGCCTGAAG GTTTCACTGA GGGTCCTCTG ACACTTACCG AGCGGTCACT   
  
  
- AACTAAGCAC GTACCATGCA CTGTAACCAC TGAATTCTGT GTTTGAATCT CTCGAACTCT GACAATACGA   
  
  
- GCCTGGTTCA AGTCTGTCGA ACCTAAGTAC CATACGGGGA AGTTCTCCAC TCACACTTGG AGACGGTGGT   
  
  
- CTTCTCGGAC TGTTACCCTT TGTAAACAAC CTCTACTATC GTTCCCCCTC GGAGTTTCTC CACGATTAAC   
  
  
- GAACACGGTT TCGTTATAGT CTGCTACTAA ACAATTGTCG CCTCACCAAC TACAGTCTCG ATGCGGTATA   
  
  
- CCAAAGTCAA AGACCTCTTG GTTAAGTCTC CAATCCTCGG ATGTACAACC TTCCCAATCA ACGGGCCAAC   
  
  
- AGGAGAAGTC CTTCAAGGTA GATGTTTCGA GATTCCACGT TTCTCGGATG ATCATCACTT GAAGAAAGGA   
  
  
- TATACGTAAA TGAGATACTT CAAACGGGAA TGAAGTTCAA ACCCATGTAC AGACGTTTAC CCCGTTAACG   
  
  
- ACTCCGGTAC TTTTTACTCT CGTCTTAAGT ATATTAACTA AAAGTCTATC GAGTTCCCTC AGTCACCTAA   
  
  
- TCGGAATAGG TTCGGGACCG ACGAGTCGGA CTACCCGGTG GTGTCCAGGC ATAATGGCCT CAGCTACTAA   
  
  
- GGGTTAGACT CATACGAGCC CCTCCCCCCG AGCTGTAACA TCGCTTCTCT GATAGACCTG ATCGGGTCCG   
  
  
- AACGTCGGAT GGGAAACTCA AGGTGCGACG TCGTGAATCA CCAAGACTCT AGTCTGAAGT TTTGGACCAA   
  
  
- AACGCCGGAC CTCTTCGGAA TCGTCACTTG AAGGGTAAGT ACGACGTGGT GTACGGCCTA CTCTCACACC   
  
  
- CGGGACTCTT AATATCTCTG GTCAATAATT CCGACCACTT CTCGAAGAAC GGGTTCCACC AATGGGAACA   
  
  
- ACTCGTTCTT AGGTTGTGTT TGTGCCGGGG TAAAGATGGG GCCAAAGAAC TTTGGGATCT GATGATATGA   
  
  
- CGGTAAAAGC TTAGTTAACT ACAATGCGAG GGTTCTCTAG TGTTCCTCGC CTAGTTACAA CTCGTCGTGA   
  
  
- CAAATCGTTC TCTATATCAG TTGTATTATC GTACACTCCC ACGGCTCTCC CACCTTGCGG TACTCCAAGA   
  
  
- ACCTTTCACC TCTAGAGCCA AGAGTTACCG ACCCAAATTC GGCATGGGTA ACTCGGGCGA TCACTTACGT   
  
  
- TGATAGTCCT GAGAAGACGT CTTCATATCG TCCTCGATAC CTGAACTTCT TTCCCTACCT CGAGAAATAG   
  
  
- ATCCGACCTA CTTGGCTCGT GATCAACGTA GAACACGTAC CGTCAC

+     AT~TATA-box

| Site Name | Organism | Position | Strand | Matrix score. | sequence | function |
| --- | --- | --- | --- | --- | --- | --- |
| AT~TATA-box | Arabidopsis thaliana | 1190 | + | 6 | TATATA |  |
| AT~TATA-box | Arabidopsis thaliana | 340 | + | 6 | TATATA |  |

>HU08G00284.1   
+ -Up\_Stream \_Len000TTCTTT TCCTGTCTCT GTCTCTTCAT TGCACCATCA TTTAAGAGAT GGGACCATAA   
  
  
+ ATCATTCAAT CACTCCTTTA TGTTTAAACT TTGGAAGGAG GTTATTATTT ATGTGGTGGT GATCCCAATG   
  
  
+ CTGAATTTAG CTGTTGATGG CGAGGGCATA TATGTAATTA GGATTTCCAA TGCAGTTTAG ATTTTACTCA   
  
  
+ TTTGAGAATT CACTGAGGTT TGTTTGATCT GATTTTAGCA AATATTGTTC AGGGGTATAG ATGCTTATTC   
  
  
+ GGTTCTCAAC TGTATTAGTA TACAAGATGA GTGGTCCACT TGTTTAAGCT TTTACTATAT ACTTATGTTG   
  
  
+ TCCCTAATTT GGTCTCGCTT GATGTGTAGC TGTCAAGTAT TATTATAATC TTGTGTTTGA TATCACATTT   
  
  
+ GTATTGATTA GCTACAAAAG AACATATTAT ATGTGTATAG CAACTATCTC ATATATGCCC TCACCATAGC   
  
  
+ GTGGTTCCAA GGGTTCAAGT TCCACTTAGC CTCACCCTAG AAATGACTTC TTTTTTTTTT TAAAGTTAAA   
  
  
+ GATATGCAAT CACCATGGAA CCAGTCATCT TTTGACACGT TATTCCAGAT CAGATGACCA GCCTTTCTCC   
  
  
+ AAGGCCATTA GATTTTCTAG CCAATCCACT GTTAACTGCA AAAAGTGGTA TCCTTGGCTG TTCCTGTCAC   
  
  
+ TGTTTCTTAT TTGCTCTGTG ATATTTGTAC ATCTCTTTGC CAAGGATAAA AGTTCATAGC CGGGAATTGG   
  
  
+ TATTCAGTTG ACTCTTGCAG TAAACAGAGT TCATGTACTG GAGGATTTTG TGAGTCATGT CACTCAATAA   
  
  
+ AAACAGGTGT GCGACAGTGT TATAAAGCTG CTTGCTTAAT GCACATCTAC TCTCACTCAC CTTACCCAAA   
  
  
+ AAACTTCAAA ATTGTGTGCC TTCTATGAAT ATTCTGATCC ATTAGCATTC AACGGTTTCT GAATTCTGTG   
  
  
+ TAAGCCATCC ATACTCATTT TCAACAGAGA ACTTGGATTG GATGTATTCA ATACCCAAAA ACCTTGTCAA   
  
  
+ ATTACCCCTA GCTTAATTTC CTCTAATACC AACTGCTCTT TGGTTTTAGC GTACAATCCC ATAGATTACC   
  
  
+ CTTCTATAAT TAGTGATGAT TGGTTCATTT CTGCTGCATT ATGCCTTCTT TAGATCCATT GATAATATAT   
  
  
+ ACTCTTGCCA TGCTGCAACA TTCCACTGGG ATTAATATCT CACATTGTAT CCTCAGCAAC CAAGGAGACC   
  
  
+ CTACTATTTT CAGAGCCATT CATAGAAAGT TATTGCATAT AGTGATGATC TTCAATGGGT CTGATTTTTA   
  
  
+ GATAGCTGCT GTGAACATTT TACTCGTCCT CTGCCATGTG GATACTATGG TAACCTTTGT TTGCTCTCAA   
  
  
+ GCCTGAGCGT TCTGAGAAAT GATGAAAGCG CCCTCGAGTT ATAGGACTTT TCTGACTTTA GCTTTAGAAA   
  
  
+ AGAGAGGAAA ATCAACCACA CCCATCTGAT TATTGAGTTT CTGCTCCTTG AGACCTGGAT GGAGCCAGAA   
  
  
+ CTCACGGTCT TGTCAGCCAG AATAATCAGG AACTGCATTT CTGAGGACCC ATGAAAAGAC CCTGGTATAC   
  
  
+ GACTATTCAT GAAAACGTTT TGTCAGCCTT TCAGTTTTTG CATTTTATTC TAGCTGCTTT GATTGAAGCC   
  
  
+ CAATTTTATT AAAACTGTGC TTTTCTCTTG AATGTTAAAC TCTTGCAGTT CCTTGCTTGT TATTTTGGGT   
  
  
+ TTTATGTTTG CTTTCTACTG ATATCATCTT TTGGTCGTGC AATTTAATTG CCTACTATGT TATATGGATT   
  
  
+ TTTATGTTTA CTTTCTACTG ATATCATCTT TTGCTATTGC AATTTAATTG CCTTCTATGT AGAATTCGTC   
  
  
+ ACTTTGAAGT CTGCCAGGAA GGCAATACAG CTATATTTTT CTTGTGGATG AAGTGATAAA AGGCAAGTGT   
  
  
+ CAATTGCTTA CTGCTTTATT GCGCTGTACT TTTCCAGAGA TTTTGGATTA GTCAATGCAG AAGCCAACAA   
  
  
+ TTTATAGCGA CTGGCCACAG TTCTACAACC AATTTGATAA TCCACGTCTT CTTGAATCTT CATCTATAAT   
  
  
+ GGGTGGTGAT CAACTTTTCA GTTCTCCATC TACTGTAAGC ATATCTTGCA ACAGGAGTCC AGCTTCACTG   
  
  
+ CCTGAACTCG AATCCTCTTC ATCGGACTTC CAAAGTGACT CCCAGGAGAC TGTGAATGGC TCGCCAGTGA   
  
  
+ TTGATTCGTG CATGGTACGT GACATTGGTG ACTTAAGACA CAAACTTAGA GAGCTTGAGA CTGTTATGCT   
  
  
+ CGGACCAAGT TCAGACAGCT TGGATTCATG GTATGCCCCT TCAAGAGGTG AGTGTGAACC TCTGCCACCA   
  
  
+ GAAGAGCCTG ACAATGGGAA ACATTTGTTG GAGATGATAG CAAGGGGGAG CCTCAAAGAG GTGCTAATTG   
  
  
+ CTTGTGCCAA AGCAATATCA GACGATGATT TGTTAACAGC GGAGTGGTTG ATGTCAGAGC TACGCCATAT   
  
  
+ GGTTTCAGTT TCTGGAGAAC CAATTCAGAG GTTAGGAGCC TACATGTTGG AAGGGTTAGT TGCCCGGTTG   
  
  
+ TCCTCTTCAG GAAGTTCCAT CTACAAAGCT CTAAGGTGCA AAGAGCCTAC TAGTAGTGAA CTTCTTTCCT   
  
  
+ ATATGCATTT ACTCTATGAA GTTTGCCCTT ACTTCAAGTT TGGGTACATG TCTGCAAATG GGGCAATTGC   
  
  
+ TGAGGCCATG AAAAATGAGA GCAGAATTCA TATAATTGAT TTTCAGATAG CTCAAGGGAG TCAGTGGATT   
  
  
+ AGCCTTATCC AAGCCCTGGC TGCTCAGCCT GATGGGCCAC CACAGGTCCG TATTACCGGA GTCGATGATT   
  
  
+ CCCAATCTGA GTATGCTCGG GGAGGGGGGC TCGACATTGT AGCGAAGAGA CTATCTGGAC TAGCCCAGGC   
  
  
+ TTGCAGCCTA CCCTTTGAGT TCCACGCTGC AGCACTTAGT GGTTCTGAGA TCAGACTTCA AAACCTGGTT   
  
  
+ TTGCGGCCTG GAGAAGCCTT AGCAGTGAAC TTCCCATTCA TGCTGCACCA CATGCCGGAT GAGAGTGTGG   
  
  
+ GCCCTGAGAA TTATAGAGAC CAGTTATTAA GGCTGGTGAA GAGCTTCTTG CCCAAGGTGG TTACCCTTGT   
  
  
+ TGAGCAAGAA TCCAACACAA ACACGGCCCC ATTTCTACCC CGGTTTCTTG AAACCCTAGA CTACTATACT   
  
  
+ GCCATTTTCG AATCAATTGA TGTTACGCTC CCAAGAGATC ACAAGGAGCG GATCAATGTT GAGCAGCACT   
  
  
+ GTTTAGCAAG AGATATAGTC AACATAATAG CATGTGAGGG TGCCGAGAGG GTGGAACGCC ATGAGGTTCT   
  
  
+ TGGAAAGTGG AGATCTCGGT TCTCAATGGC TGGGTTTAAG CCGTACCCAT TGAGCCCGCT AGTGAATGCA   
  
  
+ ACTATCAGGA CTCTTCTGCA GAAGTATAGC AGGAGCTATG GACTTGAAGA AAGGGATGGA GCTCTTTATC   
  
  
+ TAGGCTGGAT GAACCGAGCA CTAGTTGCAT CTTGTGCATG GCAGTG  

- -Up\_Stream \_Len000AAGAAA AGGACAGAGA CAGAGAAGTA ACGTGGTAGT AAATTCTCTA CCCTGGTATT   
  
  
- TAGTAAGTTA GTGAGGAAAT ACAAATTTGA AACCTTCCTC CAATAATAAA TACACCACCA CTAGGGTTAC   
  
  
- GACTTAAATC GACAACTACC GCTCCCGTAT ATACATTAAT CCTAAAGGTT ACGTCAAATC TAAAATGAGT   
  
  
- AAACTCTTAA GTGACTCCAA ACAAACTAGA CTAAAATCGT TTATAACAAG TCCCCATATC TACGAATAAG   
  
  
- CCAAGAGTTG ACATAATCAT ATGTTCTACT CACCAGGTGA ACAAATTCGA AAATGATATA TGAATACAAC   
  
  
- AGGGATTAAA CCAGAGCGAA CTACACATCG ACAGTTCATA ATAATATTAG AACACAAACT ATAGTGTAAA   
  
  
- CATAACTAAT CGATGTTTTC TTGTATAATA TACACATATC GTTGATAGAG TATATACGGG AGTGGTATCG   
  
  
- CACCAAGGTT CCCAAGTTCA AGGTGAATCG GAGTGGGATC TTTACTGAAG AAAAAAAAAA ATTTCAATTT   
  
  
- CTATACGTTA GTGGTACCTT GGTCAGTAGA AAACTGTGCA ATAAGGTCTA GTCTACTGGT CGGAAAGAGG   
  
  
- TTCCGGTAAT CTAAAAGATC GGTTAGGTGA CAATTGACGT TTTTCACCAT AGGAACCGAC AAGGACAGTG   
  
  
- ACAAAGAATA AACGAGACAC TATAAACATG TAGAGAAACG GTTCCTATTT TCAAGTATCG GCCCTTAACC   
  
  
- ATAAGTCAAC TGAGAACGTC ATTTGTCTCA AGTACATGAC CTCCTAAAAC ACTCAGTACA GTGAGTTATT   
  
  
- TTTGTCCACA CGCTGTCACA ATATTTCGAC GAACGAATTA CGTGTAGATG AGAGTGAGTG GAATGGGTTT   
  
  
- TTTGAAGTTT TAACACACGG AAGATACTTA TAAGACTAGG TAATCGTAAG TTGCCAAAGA CTTAAGACAC   
  
  
- ATTCGGTAGG TATGAGTAAA AGTTGTCTCT TGAACCTAAC CTACATAAGT TATGGGTTTT TGGAACAGTT   
  
  
- TAATGGGGAT CGAATTAAAG GAGATTATGG TTGACGAGAA ACCAAAATCG CATGTTAGGG TATCTAATGG   
  
  
- GAAGATATTA ATCACTACTA ACCAAGTAAA GACGACGTAA TACGGAAGAA ATCTAGGTAA CTATTATATA   
  
  
- TGAGAACGGT ACGACGTTGT AAGGTGACCC TAATTATAGA GTGTAACATA GGAGTCGTTG GTTCCTCTGG   
  
  
- GATGATAAAA GTCTCGGTAA GTATCTTTCA ATAACGTATA TCACTACTAG AAGTTACCCA GACTAAAAAT   
  
  
- CTATCGACGA CACTTGTAAA ATGAGCAGGA GACGGTACAC CTATGATACC ATTGGAAACA AACGAGAGTT   
  
  
- CGGACTCGCA AGACTCTTTA CTACTTTCGC GGGAGCTCAA TATCCTGAAA AGACTGAAAT CGAAATCTTT   
  
  
- TCTCTCCTTT TAGTTGGTGT GGGTAGACTA ATAACTCAAA GACGAGGAAC TCTGGACCTA CCTCGGTCTT   
  
  
- GAGTGCCAGA ACAGTCGGTC TTATTAGTCC TTGACGTAAA GACTCCTGGG TACTTTTCTG GGACCATATG   
  
  
- CTGATAAGTA CTTTTGCAAA ACAGTCGGAA AGTCAAAAAC GTAAAATAAG ATCGACGAAA CTAACTTCGG   
  
  
- GTTAAAATAA TTTTGACACG AAAAGAGAAC TTACAATTTG AGAACGTCAA GGAACGAACA ATAAAACCCA   
  
  
- AAATACAAAC GAAAGATGAC TATAGTAGAA AACCAGCACG TTAAATTAAC GGATGATACA ATATACCTAA   
  
  
- AAATACAAAT GAAAGATGAC TATAGTAGAA AACGATAACG TTAAATTAAC GGAAGATACA TCTTAAGCAG   
  
  
- TGAAACTTCA GACGGTCCTT CCGTTATGTC GATATAAAAA GAACACCTAC TTCACTATTT TCCGTTCACA   
  
  
- GTTAACGAAT GACGAAATAA CGCGACATGA AAAGGTCTCT AAAACCTAAT CAGTTACGTC TTCGGTTGTT   
  
  
- AAATATCGCT GACCGGTGTC AAGATGTTGG TTAAACTATT AGGTGCAGAA GAACTTAGAA GTAGATATTA   
  
  
- CCCACCACTA GTTGAAAAGT CAAGAGGTAG ATGACATTCG TATAGAACGT TGTCCTCAGG TCGAAGTGAC   
  
  
- GGACTTGAGC TTAGGAGAAG TAGCCTGAAG GTTTCACTGA GGGTCCTCTG ACACTTACCG AGCGGTCACT   
  
  
- AACTAAGCAC GTACCATGCA CTGTAACCAC TGAATTCTGT GTTTGAATCT CTCGAACTCT GACAATACGA   
  
  
- GCCTGGTTCA AGTCTGTCGA ACCTAAGTAC CATACGGGGA AGTTCTCCAC TCACACTTGG AGACGGTGGT   
  
  
- CTTCTCGGAC TGTTACCCTT TGTAAACAAC CTCTACTATC GTTCCCCCTC GGAGTTTCTC CACGATTAAC   
  
  
- GAACACGGTT TCGTTATAGT CTGCTACTAA ACAATTGTCG CCTCACCAAC TACAGTCTCG ATGCGGTATA   
  
  
- CCAAAGTCAA AGACCTCTTG GTTAAGTCTC CAATCCTCGG ATGTACAACC TTCCCAATCA ACGGGCCAAC   
  
  
- AGGAGAAGTC CTTCAAGGTA GATGTTTCGA GATTCCACGT TTCTCGGATG ATCATCACTT GAAGAAAGGA   
  
  
- TATACGTAAA TGAGATACTT CAAACGGGAA TGAAGTTCAA ACCCATGTAC AGACGTTTAC CCCGTTAACG   
  
  
- ACTCCGGTAC TTTTTACTCT CGTCTTAAGT ATATTAACTA AAAGTCTATC GAGTTCCCTC AGTCACCTAA   
  
  
- TCGGAATAGG TTCGGGACCG ACGAGTCGGA CTACCCGGTG GTGTCCAGGC ATAATGGCCT CAGCTACTAA   
  
  
- GGGTTAGACT CATACGAGCC CCTCCCCCCG AGCTGTAACA TCGCTTCTCT GATAGACCTG ATCGGGTCCG   
  
  
- AACGTCGGAT GGGAAACTCA AGGTGCGACG TCGTGAATCA CCAAGACTCT AGTCTGAAGT TTTGGACCAA   
  
  
- AACGCCGGAC CTCTTCGGAA TCGTCACTTG AAGGGTAAGT ACGACGTGGT GTACGGCCTA CTCTCACACC   
  
  
- CGGGACTCTT AATATCTCTG GTCAATAATT CCGACCACTT CTCGAAGAAC GGGTTCCACC AATGGGAACA   
  
  
- ACTCGTTCTT AGGTTGTGTT TGTGCCGGGG TAAAGATGGG GCCAAAGAAC TTTGGGATCT GATGATATGA   
  
  
- CGGTAAAAGC TTAGTTAACT ACAATGCGAG GGTTCTCTAG TGTTCCTCGC CTAGTTACAA CTCGTCGTGA   
  
  
- CAAATCGTTC TCTATATCAG TTGTATTATC GTACACTCCC ACGGCTCTCC CACCTTGCGG TACTCCAAGA   
  
  
- ACCTTTCACC TCTAGAGCCA AGAGTTACCG ACCCAAATTC GGCATGGGTA ACTCGGGCGA TCACTTACGT   
  
  
- TGATAGTCCT GAGAAGACGT CTTCATATCG TCCTCGATAC CTGAACTTCT TTCCCTACCT CGAGAAATAG   
  
  
- ATCCGACCTA CTTGGCTCGT GATCAACGTA GAACACGTAC CGTCAC

+     Box 4

| Site Name | Organism | Position | Strand | Matrix score. | sequence | function |
| --- | --- | --- | --- | --- | --- | --- |
| Box 4 | Petroselinum crispum | 1225 | + | 6 | ATTAAT | part of a conserved DNA module involved in light responsiveness |

>HU08G00284.1   
+ -Up\_Stream \_Len000TTCTTT TCCTGTCTCT GTCTCTTCAT TGCACCATCA TTTAAGAGAT GGGACCATAA   
  
  
+ ATCATTCAAT CACTCCTTTA TGTTTAAACT TTGGAAGGAG GTTATTATTT ATGTGGTGGT GATCCCAATG   
  
  
+ CTGAATTTAG CTGTTGATGG CGAGGGCATA TATGTAATTA GGATTTCCAA TGCAGTTTAG ATTTTACTCA   
  
  
+ TTTGAGAATT CACTGAGGTT TGTTTGATCT GATTTTAGCA AATATTGTTC AGGGGTATAG ATGCTTATTC   
  
  
+ GGTTCTCAAC TGTATTAGTA TACAAGATGA GTGGTCCACT TGTTTAAGCT TTTACTATAT ACTTATGTTG   
  
  
+ TCCCTAATTT GGTCTCGCTT GATGTGTAGC TGTCAAGTAT TATTATAATC TTGTGTTTGA TATCACATTT   
  
  
+ GTATTGATTA GCTACAAAAG AACATATTAT ATGTGTATAG CAACTATCTC ATATATGCCC TCACCATAGC   
  
  
+ GTGGTTCCAA GGGTTCAAGT TCCACTTAGC CTCACCCTAG AAATGACTTC TTTTTTTTTT TAAAGTTAAA   
  
  
+ GATATGCAAT CACCATGGAA CCAGTCATCT TTTGACACGT TATTCCAGAT CAGATGACCA GCCTTTCTCC   
  
  
+ AAGGCCATTA GATTTTCTAG CCAATCCACT GTTAACTGCA AAAAGTGGTA TCCTTGGCTG TTCCTGTCAC   
  
  
+ TGTTTCTTAT TTGCTCTGTG ATATTTGTAC ATCTCTTTGC CAAGGATAAA AGTTCATAGC CGGGAATTGG   
  
  
+ TATTCAGTTG ACTCTTGCAG TAAACAGAGT TCATGTACTG GAGGATTTTG TGAGTCATGT CACTCAATAA   
  
  
+ AAACAGGTGT GCGACAGTGT TATAAAGCTG CTTGCTTAAT GCACATCTAC TCTCACTCAC CTTACCCAAA   
  
  
+ AAACTTCAAA ATTGTGTGCC TTCTATGAAT ATTCTGATCC ATTAGCATTC AACGGTTTCT GAATTCTGTG   
  
  
+ TAAGCCATCC ATACTCATTT TCAACAGAGA ACTTGGATTG GATGTATTCA ATACCCAAAA ACCTTGTCAA   
  
  
+ ATTACCCCTA GCTTAATTTC CTCTAATACC AACTGCTCTT TGGTTTTAGC GTACAATCCC ATAGATTACC   
  
  
+ CTTCTATAAT TAGTGATGAT TGGTTCATTT CTGCTGCATT ATGCCTTCTT TAGATCCATT GATAATATAT   
  
  
+ ACTCTTGCCA TGCTGCAACA TTCCACTGGG ATTAATATCT CACATTGTAT CCTCAGCAAC CAAGGAGACC   
  
  
+ CTACTATTTT CAGAGCCATT CATAGAAAGT TATTGCATAT AGTGATGATC TTCAATGGGT CTGATTTTTA   
  
  
+ GATAGCTGCT GTGAACATTT TACTCGTCCT CTGCCATGTG GATACTATGG TAACCTTTGT TTGCTCTCAA   
  
  
+ GCCTGAGCGT TCTGAGAAAT GATGAAAGCG CCCTCGAGTT ATAGGACTTT TCTGACTTTA GCTTTAGAAA   
  
  
+ AGAGAGGAAA ATCAACCACA CCCATCTGAT TATTGAGTTT CTGCTCCTTG AGACCTGGAT GGAGCCAGAA   
  
  
+ CTCACGGTCT TGTCAGCCAG AATAATCAGG AACTGCATTT CTGAGGACCC ATGAAAAGAC CCTGGTATAC   
  
  
+ GACTATTCAT GAAAACGTTT TGTCAGCCTT TCAGTTTTTG CATTTTATTC TAGCTGCTTT GATTGAAGCC   
  
  
+ CAATTTTATT AAAACTGTGC TTTTCTCTTG AATGTTAAAC TCTTGCAGTT CCTTGCTTGT TATTTTGGGT   
  
  
+ TTTATGTTTG CTTTCTACTG ATATCATCTT TTGGTCGTGC AATTTAATTG CCTACTATGT TATATGGATT   
  
  
+ TTTATGTTTA CTTTCTACTG ATATCATCTT TTGCTATTGC AATTTAATTG CCTTCTATGT AGAATTCGTC   
  
  
+ ACTTTGAAGT CTGCCAGGAA GGCAATACAG CTATATTTTT CTTGTGGATG AAGTGATAAA AGGCAAGTGT   
  
  
+ CAATTGCTTA CTGCTTTATT GCGCTGTACT TTTCCAGAGA TTTTGGATTA GTCAATGCAG AAGCCAACAA   
  
  
+ TTTATAGCGA CTGGCCACAG TTCTACAACC AATTTGATAA TCCACGTCTT CTTGAATCTT CATCTATAAT   
  
  
+ GGGTGGTGAT CAACTTTTCA GTTCTCCATC TACTGTAAGC ATATCTTGCA ACAGGAGTCC AGCTTCACTG   
  
  
+ CCTGAACTCG AATCCTCTTC ATCGGACTTC CAAAGTGACT CCCAGGAGAC TGTGAATGGC TCGCCAGTGA   
  
  
+ TTGATTCGTG CATGGTACGT GACATTGGTG ACTTAAGACA CAAACTTAGA GAGCTTGAGA CTGTTATGCT   
  
  
+ CGGACCAAGT TCAGACAGCT TGGATTCATG GTATGCCCCT TCAAGAGGTG AGTGTGAACC TCTGCCACCA   
  
  
+ GAAGAGCCTG ACAATGGGAA ACATTTGTTG GAGATGATAG CAAGGGGGAG CCTCAAAGAG GTGCTAATTG   
  
  
+ CTTGTGCCAA AGCAATATCA GACGATGATT TGTTAACAGC GGAGTGGTTG ATGTCAGAGC TACGCCATAT   
  
  
+ GGTTTCAGTT TCTGGAGAAC CAATTCAGAG GTTAGGAGCC TACATGTTGG AAGGGTTAGT TGCCCGGTTG   
  
  
+ TCCTCTTCAG GAAGTTCCAT CTACAAAGCT CTAAGGTGCA AAGAGCCTAC TAGTAGTGAA CTTCTTTCCT   
  
  
+ ATATGCATTT ACTCTATGAA GTTTGCCCTT ACTTCAAGTT TGGGTACATG TCTGCAAATG GGGCAATTGC   
  
  
+ TGAGGCCATG AAAAATGAGA GCAGAATTCA TATAATTGAT TTTCAGATAG CTCAAGGGAG TCAGTGGATT   
  
  
+ AGCCTTATCC AAGCCCTGGC TGCTCAGCCT GATGGGCCAC CACAGGTCCG TATTACCGGA GTCGATGATT   
  
  
+ CCCAATCTGA GTATGCTCGG GGAGGGGGGC TCGACATTGT AGCGAAGAGA CTATCTGGAC TAGCCCAGGC   
  
  
+ TTGCAGCCTA CCCTTTGAGT TCCACGCTGC AGCACTTAGT GGTTCTGAGA TCAGACTTCA AAACCTGGTT   
  
  
+ TTGCGGCCTG GAGAAGCCTT AGCAGTGAAC TTCCCATTCA TGCTGCACCA CATGCCGGAT GAGAGTGTGG   
  
  
+ GCCCTGAGAA TTATAGAGAC CAGTTATTAA GGCTGGTGAA GAGCTTCTTG CCCAAGGTGG TTACCCTTGT   
  
  
+ TGAGCAAGAA TCCAACACAA ACACGGCCCC ATTTCTACCC CGGTTTCTTG AAACCCTAGA CTACTATACT   
  
  
+ GCCATTTTCG AATCAATTGA TGTTACGCTC CCAAGAGATC ACAAGGAGCG GATCAATGTT GAGCAGCACT   
  
  
+ GTTTAGCAAG AGATATAGTC AACATAATAG CATGTGAGGG TGCCGAGAGG GTGGAACGCC ATGAGGTTCT   
  
  
+ TGGAAAGTGG AGATCTCGGT TCTCAATGGC TGGGTTTAAG CCGTACCCAT TGAGCCCGCT AGTGAATGCA   
  
  
+ ACTATCAGGA CTCTTCTGCA GAAGTATAGC AGGAGCTATG GACTTGAAGA AAGGGATGGA GCTCTTTATC   
  
  
+ TAGGCTGGAT GAACCGAGCA CTAGTTGCAT CTTGTGCATG GCAGTG  

- -Up\_Stream \_Len000AAGAAA AGGACAGAGA CAGAGAAGTA ACGTGGTAGT AAATTCTCTA CCCTGGTATT   
  
  
- TAGTAAGTTA GTGAGGAAAT ACAAATTTGA AACCTTCCTC CAATAATAAA TACACCACCA CTAGGGTTAC   
  
  
- GACTTAAATC GACAACTACC GCTCCCGTAT ATACATTAAT CCTAAAGGTT ACGTCAAATC TAAAATGAGT   
  
  
- AAACTCTTAA GTGACTCCAA ACAAACTAGA CTAAAATCGT TTATAACAAG TCCCCATATC TACGAATAAG   
  
  
- CCAAGAGTTG ACATAATCAT ATGTTCTACT CACCAGGTGA ACAAATTCGA AAATGATATA TGAATACAAC   
  
  
- AGGGATTAAA CCAGAGCGAA CTACACATCG ACAGTTCATA ATAATATTAG AACACAAACT ATAGTGTAAA   
  
  
- CATAACTAAT CGATGTTTTC TTGTATAATA TACACATATC GTTGATAGAG TATATACGGG AGTGGTATCG   
  
  
- CACCAAGGTT CCCAAGTTCA AGGTGAATCG GAGTGGGATC TTTACTGAAG AAAAAAAAAA ATTTCAATTT   
  
  
- CTATACGTTA GTGGTACCTT GGTCAGTAGA AAACTGTGCA ATAAGGTCTA GTCTACTGGT CGGAAAGAGG   
  
  
- TTCCGGTAAT CTAAAAGATC GGTTAGGTGA CAATTGACGT TTTTCACCAT AGGAACCGAC AAGGACAGTG   
  
  
- ACAAAGAATA AACGAGACAC TATAAACATG TAGAGAAACG GTTCCTATTT TCAAGTATCG GCCCTTAACC   
  
  
- ATAAGTCAAC TGAGAACGTC ATTTGTCTCA AGTACATGAC CTCCTAAAAC ACTCAGTACA GTGAGTTATT   
  
  
- TTTGTCCACA CGCTGTCACA ATATTTCGAC GAACGAATTA CGTGTAGATG AGAGTGAGTG GAATGGGTTT   
  
  
- TTTGAAGTTT TAACACACGG AAGATACTTA TAAGACTAGG TAATCGTAAG TTGCCAAAGA CTTAAGACAC   
  
  
- ATTCGGTAGG TATGAGTAAA AGTTGTCTCT TGAACCTAAC CTACATAAGT TATGGGTTTT TGGAACAGTT   
  
  
- TAATGGGGAT CGAATTAAAG GAGATTATGG TTGACGAGAA ACCAAAATCG CATGTTAGGG TATCTAATGG   
  
  
- GAAGATATTA ATCACTACTA ACCAAGTAAA GACGACGTAA TACGGAAGAA ATCTAGGTAA CTATTATATA   
  
  
- TGAGAACGGT ACGACGTTGT AAGGTGACCC TAATTATAGA GTGTAACATA GGAGTCGTTG GTTCCTCTGG   
  
  
- GATGATAAAA GTCTCGGTAA GTATCTTTCA ATAACGTATA TCACTACTAG AAGTTACCCA GACTAAAAAT   
  
  
- CTATCGACGA CACTTGTAAA ATGAGCAGGA GACGGTACAC CTATGATACC ATTGGAAACA AACGAGAGTT   
  
  
- CGGACTCGCA AGACTCTTTA CTACTTTCGC GGGAGCTCAA TATCCTGAAA AGACTGAAAT CGAAATCTTT   
  
  
- TCTCTCCTTT TAGTTGGTGT GGGTAGACTA ATAACTCAAA GACGAGGAAC TCTGGACCTA CCTCGGTCTT   
  
  
- GAGTGCCAGA ACAGTCGGTC TTATTAGTCC TTGACGTAAA GACTCCTGGG TACTTTTCTG GGACCATATG   
  
  
- CTGATAAGTA CTTTTGCAAA ACAGTCGGAA AGTCAAAAAC GTAAAATAAG ATCGACGAAA CTAACTTCGG   
  
  
- GTTAAAATAA TTTTGACACG AAAAGAGAAC TTACAATTTG AGAACGTCAA GGAACGAACA ATAAAACCCA   
  
  
- AAATACAAAC GAAAGATGAC TATAGTAGAA AACCAGCACG TTAAATTAAC GGATGATACA ATATACCTAA   
  
  
- AAATACAAAT GAAAGATGAC TATAGTAGAA AACGATAACG TTAAATTAAC GGAAGATACA TCTTAAGCAG   
  
  
- TGAAACTTCA GACGGTCCTT CCGTTATGTC GATATAAAAA GAACACCTAC TTCACTATTT TCCGTTCACA   
  
  
- GTTAACGAAT GACGAAATAA CGCGACATGA AAAGGTCTCT AAAACCTAAT CAGTTACGTC TTCGGTTGTT   
  
  
- AAATATCGCT GACCGGTGTC AAGATGTTGG TTAAACTATT AGGTGCAGAA GAACTTAGAA GTAGATATTA   
  
  
- CCCACCACTA GTTGAAAAGT CAAGAGGTAG ATGACATTCG TATAGAACGT TGTCCTCAGG TCGAAGTGAC   
  
  
- GGACTTGAGC TTAGGAGAAG TAGCCTGAAG GTTTCACTGA GGGTCCTCTG ACACTTACCG AGCGGTCACT   
  
  
- AACTAAGCAC GTACCATGCA CTGTAACCAC TGAATTCTGT GTTTGAATCT CTCGAACTCT GACAATACGA   
  
  
- GCCTGGTTCA AGTCTGTCGA ACCTAAGTAC CATACGGGGA AGTTCTCCAC TCACACTTGG AGACGGTGGT   
  
  
- CTTCTCGGAC TGTTACCCTT TGTAAACAAC CTCTACTATC GTTCCCCCTC GGAGTTTCTC CACGATTAAC   
  
  
- GAACACGGTT TCGTTATAGT CTGCTACTAA ACAATTGTCG CCTCACCAAC TACAGTCTCG ATGCGGTATA   
  
  
- CCAAAGTCAA AGACCTCTTG GTTAAGTCTC CAATCCTCGG ATGTACAACC TTCCCAATCA ACGGGCCAAC   
  
  
- AGGAGAAGTC CTTCAAGGTA GATGTTTCGA GATTCCACGT TTCTCGGATG ATCATCACTT GAAGAAAGGA   
  
  
- TATACGTAAA TGAGATACTT CAAACGGGAA TGAAGTTCAA ACCCATGTAC AGACGTTTAC CCCGTTAACG   
  
  
- ACTCCGGTAC TTTTTACTCT CGTCTTAAGT ATATTAACTA AAAGTCTATC GAGTTCCCTC AGTCACCTAA   
  
  
- TCGGAATAGG TTCGGGACCG ACGAGTCGGA CTACCCGGTG GTGTCCAGGC ATAATGGCCT CAGCTACTAA   
  
  
- GGGTTAGACT CATACGAGCC CCTCCCCCCG AGCTGTAACA TCGCTTCTCT GATAGACCTG ATCGGGTCCG   
  
  
- AACGTCGGAT GGGAAACTCA AGGTGCGACG TCGTGAATCA CCAAGACTCT AGTCTGAAGT TTTGGACCAA   
  
  
- AACGCCGGAC CTCTTCGGAA TCGTCACTTG AAGGGTAAGT ACGACGTGGT GTACGGCCTA CTCTCACACC   
  
  
- CGGGACTCTT AATATCTCTG GTCAATAATT CCGACCACTT CTCGAAGAAC GGGTTCCACC AATGGGAACA   
  
  
- ACTCGTTCTT AGGTTGTGTT TGTGCCGGGG TAAAGATGGG GCCAAAGAAC TTTGGGATCT GATGATATGA   
  
  
- CGGTAAAAGC TTAGTTAACT ACAATGCGAG GGTTCTCTAG TGTTCCTCGC CTAGTTACAA CTCGTCGTGA   
  
  
- CAAATCGTTC TCTATATCAG TTGTATTATC GTACACTCCC ACGGCTCTCC CACCTTGCGG TACTCCAAGA   
  
  
- ACCTTTCACC TCTAGAGCCA AGAGTTACCG ACCCAAATTC GGCATGGGTA ACTCGGGCGA TCACTTACGT   
  
  
- TGATAGTCCT GAGAAGACGT CTTCATATCG TCCTCGATAC CTGAACTTCT TTCCCTACCT CGAGAAATAG   
  
  
- ATCCGACCTA CTTGGCTCGT GATCAACGTA GAACACGTAC CGTCAC

+     CAAT-box

| Site Name | Organism | Position | Strand | Matrix score. | sequence | function |
| --- | --- | --- | --- | --- | --- | --- |
| CAAT-box | Nicotiana glutinosa | 1685 | + | 4 | CAAT |  |
| CAAT-box | Arabidopsis thaliana | 1683 | + | 8 | CCCAATTT | common cis-acting element in promoter and enhancer regions |
| CAAT-box | Nicotiana glutinosa | 1296 | - | 4 | CAAT |  |
| CAAT-box | Nicotiana glutinosa | 3388 | + | 4 | CAAT |  |
| CAAT-box | Nicotiana glutinosa | 3278 | + | 4 | CAAT |  |
| CAAT-box | Nicotiana glutinosa | 2467 | + | 4 | CAAT |  |
| CAAT-box | Nicotiana glutinosa | 2244 | - | 4 | CAAT |  |
| CAAT-box | Pisum sativum | 2066 | - | 5 | CAAAT | common cis-acting element in promoter and enhancer regions |
| CAAT-box | Nicotiana glutinosa | 1238 | - | 4 | CAAT |  |
| CAAT-box | Nicotiana glutinosa | 2064 | + | 4 | CAAT |  |
| CAAT-box | Nicotiana glutinosa | 2769 | - | 4 | CAAT |  |
| CAAT-box | Nicotiana glutinosa | 1182 | - | 4 | CAAT |  |
| CAAT-box | Nicotiana glutinosa | 2728 | + | 4 | CAAT |  |
| CAAT-box | Pisum sativum | 2719 | + | 5 | CAAAT | common cis-acting element in promoter and enhancer regions |
| CAAT-box | Pisum sativum | 2407 | - | 5 | CAAAT | common cis-acting element in promoter and enhancer regions |
| CAAT-box | Nicotiana glutinosa | 1108 | + | 4 | CAAT |  |
| CAAT-box | Arabidopsis thaliana | 770 | - | 5 | CCAAT | common cis-acting element in promoter and enhancer regions |
| CAAT-box | Nicotiana glutinosa | 2910 | - | 4 | CAAT |  |
| CAAT-box | Nicotiana glutinosa | 1676 | - | 4 | CAAT |  |
| CAAT-box | Arabidopsis thaliana | 1021 | - | 5 | CCAAT | common cis-acting element in promoter and enhancer regions |
| CAAT-box | Nicotiana glutinosa | 656 | + | 4 | CAAT |  |
| CAAT-box | Arabidopsis thaliana | 655 | + | 5 | CCAAT | common cis-acting element in promoter and enhancer regions |
| CAAT-box | Nicotiana glutinosa | 1317 | + | 4 | CAAT |  |
| CAAT-box | Arabidopsis thaliana | 2876 | + | 5 | CCAAT | common cis-acting element in promoter and enhancer regions |
| CAAT-box | Nicotiana glutinosa | 2545 | + | 4 | CAAT |  |
| CAAT-box | Arabidopsis thaliana | 2544 | + | 5 | CCAAT | common cis-acting element in promoter and enhancer regions |
| CAAT-box | Arabidopsis thaliana | 2063 | + | 5 | CCAAT | common cis-acting element in promoter and enhancer regions |
| CAAT-box | Nicotiana glutinosa | 2032 | + | 4 | CAAT |  |
| CAAT-box | Pisum sativum | 214 | - | 5 | CAAAT | common cis-acting element in promoter and enhancer regions |
| CAAT-box | Arabidopsis thaliana | 191 | + | 5 | CCAAT | common cis-acting element in promoter and enhancer regions |
| CAAT-box | Nicotiana glutinosa | 2017 | + | 4 | CAAT |  |
| CAAT-box | Nicotiana glutinosa | 1917 | + | 4 | CAAT |  |
| CAAT-box | Arabidopsis thaliana | 1143 | - | 5 | CCAAT | common cis-acting element in promoter and enhancer regions |
| CAAT-box | Nicotiana glutinosa | 43 | - | 4 | CAAT |  |
| CAAT-box | Pisum sativum | 2482 | - | 5 | CAAAT | common cis-acting element in promoter and enhancer regions |
| CAAT-box | Nicotiana glutinosa | 1801 | - | 4 | CAAT |  |
| CAAT-box | Arabidopsis thaliana | 2268 | - | 5 | CCAAT | common cis-acting element in promoter and enhancer regions |
| CAAT-box | Nicotiana glutinosa | 3238 | + | 4 | CAAT |  |
| CAAT-box | Pisum sativum | 1052 | + | 5 | CAAAT | common cis-acting element in promoter and enhancer regions |
| CAAT-box | Nicotiana glutinosa | 1033 | + | 4 | CAAT |  |
| CAAT-box | Nicotiana glutinosa | 1506 | - | 4 | CAAT |  |
| CAAT-box | Nicotiana glutinosa | 1965 | + | 4 | CAAT |  |
| CAAT-box | Nicotiana glutinosa | 427 | - | 4 | CAAT |  |
| CAAT-box | Nicotiana glutinosa | 2877 | + | 4 | CAAT |  |
| CAAT-box | Nicotiana glutinosa | 140 | + | 4 | CAAT |  |
| CAAT-box | Nicotiana glutinosa | 2451 | - | 4 | CAAT |  |
| CAAT-box | Arabidopsis thaliana | 139 | + | 5 | CCAAT | common cis-acting element in promoter and enhancer regions |
| CAAT-box | Nicotiana glutinosa | 2396 | + | 4 | CAAT |  |
| CAAT-box | Nicotiana glutinosa | 839 | + | 4 | CAAT |  |
| CAAT-box | Nicotiana glutinosa | 925 | - | 4 | CAAT |  |
| CAAT-box | Arabidopsis thaliana | 1684 | + | 5 | CCAAT | common cis-acting element in promoter and enhancer regions |
| CAAT-box | Nicotiana glutinosa | 2730 | - | 4 | CAAT |  |
| CAAT-box | Pisum sativum | 713 | - | 5 | CAAAT | common cis-acting element in promoter and enhancer regions |
| CAAT-box | Nicotiana glutinosa | 1860 | - | 4 | CAAT |  |
| CAAT-box | Nicotiana glutinosa | 192 | + | 4 | CAAT |  |
| CAAT-box | Nicotiana glutinosa | 3413 | - | 4 | CAAT |  |
| CAAT-box | Nicotiana glutinosa | 1794 | + | 4 | CAAT |  |
| CAAT-box | Nicotiana glutinosa | 1982 | - | 4 | CAAT |  |
| CAAT-box | Pisum sativum | 727 | - | 5 | CAAAT | common cis-acting element in promoter and enhancer regions |
| CAAT-box | Nicotiana glutinosa | 258 | - | 4 | CAAT |  |
| CAAT-box | Nicotiana glutinosa | 3240 | - | 4 | CAAT |  |
| CAAT-box | Pisum sativum | 421 | - | 5 | CAAAT | common cis-acting element in promoter and enhancer regions |
| CAAT-box | Nicotiana glutinosa | 1967 | - | 4 | CAAT |  |
| CAAT-box | Nicotiana glutinosa | 81 | + | 4 | CAAT |  |
| CAAT-box | Nicotiana glutinosa | 1871 | - | 4 | CAAT |  |
| CAAT-box | Nicotiana glutinosa | 1864 | + | 4 | CAAT |  |
| CAAT-box | Pisum sativum | 253 | + | 5 | CAAAT | common cis-acting element in promoter and enhancer regions |
| CAAT-box | Nicotiana glutinosa | 571 | + | 4 | CAAT |  |
| CAAT-box | Pisum sativum | 361 | - | 5 | CAAAT | common cis-acting element in promoter and enhancer regions |

>HU08G00284.1   
+ -Up\_Stream \_Len000TTCTTT TCCTGTCTCT GTCTCTTCAT TGCACCATCA TTTAAGAGAT GGGACCATAA   
  
  
+ ATCATTCAAT CACTCCTTTA TGTTTAAACT TTGGAAGGAG GTTATTATTT ATGTGGTGGT GATCCCAATG   
  
  
+ CTGAATTTAG CTGTTGATGG CGAGGGCATA TATGTAATTA GGATTTCCAA TGCAGTTTAG ATTTTACTCA   
  
  
+ TTTGAGAATT CACTGAGGTT TGTTTGATCT GATTTTAGCA AATATTGTTC AGGGGTATAG ATGCTTATTC   
  
  
+ GGTTCTCAAC TGTATTAGTA TACAAGATGA GTGGTCCACT TGTTTAAGCT TTTACTATAT ACTTATGTTG   
  
  
+ TCCCTAATTT GGTCTCGCTT GATGTGTAGC TGTCAAGTAT TATTATAATC TTGTGTTTGA TATCACATTT   
  
  
+ GTATTGATTA GCTACAAAAG AACATATTAT ATGTGTATAG CAACTATCTC ATATATGCCC TCACCATAGC   
  
  
+ GTGGTTCCAA GGGTTCAAGT TCCACTTAGC CTCACCCTAG AAATGACTTC TTTTTTTTTT TAAAGTTAAA   
  
  
+ GATATGCAAT CACCATGGAA CCAGTCATCT TTTGACACGT TATTCCAGAT CAGATGACCA GCCTTTCTCC   
  
  
+ AAGGCCATTA GATTTTCTAG CCAATCCACT GTTAACTGCA AAAAGTGGTA TCCTTGGCTG TTCCTGTCAC   
  
  
+ TGTTTCTTAT TTGCTCTGTG ATATTTGTAC ATCTCTTTGC CAAGGATAAA AGTTCATAGC CGGGAATTGG   
  
  
+ TATTCAGTTG ACTCTTGCAG TAAACAGAGT TCATGTACTG GAGGATTTTG TGAGTCATGT CACTCAATAA   
  
  
+ AAACAGGTGT GCGACAGTGT TATAAAGCTG CTTGCTTAAT GCACATCTAC TCTCACTCAC CTTACCCAAA   
  
  
+ AAACTTCAAA ATTGTGTGCC TTCTATGAAT ATTCTGATCC ATTAGCATTC AACGGTTTCT GAATTCTGTG   
  
  
+ TAAGCCATCC ATACTCATTT TCAACAGAGA ACTTGGATTG GATGTATTCA ATACCCAAAA ACCTTGTCAA   
  
  
+ ATTACCCCTA GCTTAATTTC CTCTAATACC AACTGCTCTT TGGTTTTAGC GTACAATCCC ATAGATTACC   
  
  
+ CTTCTATAAT TAGTGATGAT TGGTTCATTT CTGCTGCATT ATGCCTTCTT TAGATCCATT GATAATATAT   
  
  
+ ACTCTTGCCA TGCTGCAACA TTCCACTGGG ATTAATATCT CACATTGTAT CCTCAGCAAC CAAGGAGACC   
  
  
+ CTACTATTTT CAGAGCCATT CATAGAAAGT TATTGCATAT AGTGATGATC TTCAATGGGT CTGATTTTTA   
  
  
+ GATAGCTGCT GTGAACATTT TACTCGTCCT CTGCCATGTG GATACTATGG TAACCTTTGT TTGCTCTCAA   
  
  
+ GCCTGAGCGT TCTGAGAAAT GATGAAAGCG CCCTCGAGTT ATAGGACTTT TCTGACTTTA GCTTTAGAAA   
  
  
+ AGAGAGGAAA ATCAACCACA CCCATCTGAT TATTGAGTTT CTGCTCCTTG AGACCTGGAT GGAGCCAGAA   
  
  
+ CTCACGGTCT TGTCAGCCAG AATAATCAGG AACTGCATTT CTGAGGACCC ATGAAAAGAC CCTGGTATAC   
  
  
+ GACTATTCAT GAAAACGTTT TGTCAGCCTT TCAGTTTTTG CATTTTATTC TAGCTGCTTT GATTGAAGCC   
  
  
+ CAATTTTATT AAAACTGTGC TTTTCTCTTG AATGTTAAAC TCTTGCAGTT CCTTGCTTGT TATTTTGGGT   
  
  
+ TTTATGTTTG CTTTCTACTG ATATCATCTT TTGGTCGTGC AATTTAATTG CCTACTATGT TATATGGATT   
  
  
+ TTTATGTTTA CTTTCTACTG ATATCATCTT TTGCTATTGC AATTTAATTG CCTTCTATGT AGAATTCGTC   
  
  
+ ACTTTGAAGT CTGCCAGGAA GGCAATACAG CTATATTTTT CTTGTGGATG AAGTGATAAA AGGCAAGTGT   
  
  
+ CAATTGCTTA CTGCTTTATT GCGCTGTACT TTTCCAGAGA TTTTGGATTA GTCAATGCAG AAGCCAACAA   
  
  
+ TTTATAGCGA CTGGCCACAG TTCTACAACC AATTTGATAA TCCACGTCTT CTTGAATCTT CATCTATAAT   
  
  
+ GGGTGGTGAT CAACTTTTCA GTTCTCCATC TACTGTAAGC ATATCTTGCA ACAGGAGTCC AGCTTCACTG   
  
  
+ CCTGAACTCG AATCCTCTTC ATCGGACTTC CAAAGTGACT CCCAGGAGAC TGTGAATGGC TCGCCAGTGA   
  
  
+ TTGATTCGTG CATGGTACGT GACATTGGTG ACTTAAGACA CAAACTTAGA GAGCTTGAGA CTGTTATGCT   
  
  
+ CGGACCAAGT TCAGACAGCT TGGATTCATG GTATGCCCCT TCAAGAGGTG AGTGTGAACC TCTGCCACCA   
  
  
+ GAAGAGCCTG ACAATGGGAA ACATTTGTTG GAGATGATAG CAAGGGGGAG CCTCAAAGAG GTGCTAATTG   
  
  
+ CTTGTGCCAA AGCAATATCA GACGATGATT TGTTAACAGC GGAGTGGTTG ATGTCAGAGC TACGCCATAT   
  
  
+ GGTTTCAGTT TCTGGAGAAC CAATTCAGAG GTTAGGAGCC TACATGTTGG AAGGGTTAGT TGCCCGGTTG   
  
  
+ TCCTCTTCAG GAAGTTCCAT CTACAAAGCT CTAAGGTGCA AAGAGCCTAC TAGTAGTGAA CTTCTTTCCT   
  
  
+ ATATGCATTT ACTCTATGAA GTTTGCCCTT ACTTCAAGTT TGGGTACATG TCTGCAAATG GGGCAATTGC   
  
  
+ TGAGGCCATG AAAAATGAGA GCAGAATTCA TATAATTGAT TTTCAGATAG CTCAAGGGAG TCAGTGGATT   
  
  
+ AGCCTTATCC AAGCCCTGGC TGCTCAGCCT GATGGGCCAC CACAGGTCCG TATTACCGGA GTCGATGATT   
  
  
+ CCCAATCTGA GTATGCTCGG GGAGGGGGGC TCGACATTGT AGCGAAGAGA CTATCTGGAC TAGCCCAGGC   
  
  
+ TTGCAGCCTA CCCTTTGAGT TCCACGCTGC AGCACTTAGT GGTTCTGAGA TCAGACTTCA AAACCTGGTT   
  
  
+ TTGCGGCCTG GAGAAGCCTT AGCAGTGAAC TTCCCATTCA TGCTGCACCA CATGCCGGAT GAGAGTGTGG   
  
  
+ GCCCTGAGAA TTATAGAGAC CAGTTATTAA GGCTGGTGAA GAGCTTCTTG CCCAAGGTGG TTACCCTTGT   
  
  
+ TGAGCAAGAA TCCAACACAA ACACGGCCCC ATTTCTACCC CGGTTTCTTG AAACCCTAGA CTACTATACT   
  
  
+ GCCATTTTCG AATCAATTGA TGTTACGCTC CCAAGAGATC ACAAGGAGCG GATCAATGTT GAGCAGCACT   
  
  
+ GTTTAGCAAG AGATATAGTC AACATAATAG CATGTGAGGG TGCCGAGAGG GTGGAACGCC ATGAGGTTCT   
  
  
+ TGGAAAGTGG AGATCTCGGT TCTCAATGGC TGGGTTTAAG CCGTACCCAT TGAGCCCGCT AGTGAATGCA   
  
  
+ ACTATCAGGA CTCTTCTGCA GAAGTATAGC AGGAGCTATG GACTTGAAGA AAGGGATGGA GCTCTTTATC   
  
  
+ TAGGCTGGAT GAACCGAGCA CTAGTTGCAT CTTGTGCATG GCAGTG  

- -Up\_Stream \_Len000AAGAAA AGGACAGAGA CAGAGAAGTA ACGTGGTAGT AAATTCTCTA CCCTGGTATT   
  
  
- TAGTAAGTTA GTGAGGAAAT ACAAATTTGA AACCTTCCTC CAATAATAAA TACACCACCA CTAGGGTTAC   
  
  
- GACTTAAATC GACAACTACC GCTCCCGTAT ATACATTAAT CCTAAAGGTT ACGTCAAATC TAAAATGAGT   
  
  
- AAACTCTTAA GTGACTCCAA ACAAACTAGA CTAAAATCGT TTATAACAAG TCCCCATATC TACGAATAAG   
  
  
- CCAAGAGTTG ACATAATCAT ATGTTCTACT CACCAGGTGA ACAAATTCGA AAATGATATA TGAATACAAC   
  
  
- AGGGATTAAA CCAGAGCGAA CTACACATCG ACAGTTCATA ATAATATTAG AACACAAACT ATAGTGTAAA   
  
  
- CATAACTAAT CGATGTTTTC TTGTATAATA TACACATATC GTTGATAGAG TATATACGGG AGTGGTATCG   
  
  
- CACCAAGGTT CCCAAGTTCA AGGTGAATCG GAGTGGGATC TTTACTGAAG AAAAAAAAAA ATTTCAATTT   
  
  
- CTATACGTTA GTGGTACCTT GGTCAGTAGA AAACTGTGCA ATAAGGTCTA GTCTACTGGT CGGAAAGAGG   
  
  
- TTCCGGTAAT CTAAAAGATC GGTTAGGTGA CAATTGACGT TTTTCACCAT AGGAACCGAC AAGGACAGTG   
  
  
- ACAAAGAATA AACGAGACAC TATAAACATG TAGAGAAACG GTTCCTATTT TCAAGTATCG GCCCTTAACC   
  
  
- ATAAGTCAAC TGAGAACGTC ATTTGTCTCA AGTACATGAC CTCCTAAAAC ACTCAGTACA GTGAGTTATT   
  
  
- TTTGTCCACA CGCTGTCACA ATATTTCGAC GAACGAATTA CGTGTAGATG AGAGTGAGTG GAATGGGTTT   
  
  
- TTTGAAGTTT TAACACACGG AAGATACTTA TAAGACTAGG TAATCGTAAG TTGCCAAAGA CTTAAGACAC   
  
  
- ATTCGGTAGG TATGAGTAAA AGTTGTCTCT TGAACCTAAC CTACATAAGT TATGGGTTTT TGGAACAGTT   
  
  
- TAATGGGGAT CGAATTAAAG GAGATTATGG TTGACGAGAA ACCAAAATCG CATGTTAGGG TATCTAATGG   
  
  
- GAAGATATTA ATCACTACTA ACCAAGTAAA GACGACGTAA TACGGAAGAA ATCTAGGTAA CTATTATATA   
  
  
- TGAGAACGGT ACGACGTTGT AAGGTGACCC TAATTATAGA GTGTAACATA GGAGTCGTTG GTTCCTCTGG   
  
  
- GATGATAAAA GTCTCGGTAA GTATCTTTCA ATAACGTATA TCACTACTAG AAGTTACCCA GACTAAAAAT   
  
  
- CTATCGACGA CACTTGTAAA ATGAGCAGGA GACGGTACAC CTATGATACC ATTGGAAACA AACGAGAGTT   
  
  
- CGGACTCGCA AGACTCTTTA CTACTTTCGC GGGAGCTCAA TATCCTGAAA AGACTGAAAT CGAAATCTTT   
  
  
- TCTCTCCTTT TAGTTGGTGT GGGTAGACTA ATAACTCAAA GACGAGGAAC TCTGGACCTA CCTCGGTCTT   
  
  
- GAGTGCCAGA ACAGTCGGTC TTATTAGTCC TTGACGTAAA GACTCCTGGG TACTTTTCTG GGACCATATG   
  
  
- CTGATAAGTA CTTTTGCAAA ACAGTCGGAA AGTCAAAAAC GTAAAATAAG ATCGACGAAA CTAACTTCGG   
  
  
- GTTAAAATAA TTTTGACACG AAAAGAGAAC TTACAATTTG AGAACGTCAA GGAACGAACA ATAAAACCCA   
  
  
- AAATACAAAC GAAAGATGAC TATAGTAGAA AACCAGCACG TTAAATTAAC GGATGATACA ATATACCTAA   
  
  
- AAATACAAAT GAAAGATGAC TATAGTAGAA AACGATAACG TTAAATTAAC GGAAGATACA TCTTAAGCAG   
  
  
- TGAAACTTCA GACGGTCCTT CCGTTATGTC GATATAAAAA GAACACCTAC TTCACTATTT TCCGTTCACA   
  
  
- GTTAACGAAT GACGAAATAA CGCGACATGA AAAGGTCTCT AAAACCTAAT CAGTTACGTC TTCGGTTGTT   
  
  
- AAATATCGCT GACCGGTGTC AAGATGTTGG TTAAACTATT AGGTGCAGAA GAACTTAGAA GTAGATATTA   
  
  
- CCCACCACTA GTTGAAAAGT CAAGAGGTAG ATGACATTCG TATAGAACGT TGTCCTCAGG TCGAAGTGAC   
  
  
- GGACTTGAGC TTAGGAGAAG TAGCCTGAAG GTTTCACTGA GGGTCCTCTG ACACTTACCG AGCGGTCACT   
  
  
- AACTAAGCAC GTACCATGCA CTGTAACCAC TGAATTCTGT GTTTGAATCT CTCGAACTCT GACAATACGA   
  
  
- GCCTGGTTCA AGTCTGTCGA ACCTAAGTAC CATACGGGGA AGTTCTCCAC TCACACTTGG AGACGGTGGT   
  
  
- CTTCTCGGAC TGTTACCCTT TGTAAACAAC CTCTACTATC GTTCCCCCTC GGAGTTTCTC CACGATTAAC   
  
  
- GAACACGGTT TCGTTATAGT CTGCTACTAA ACAATTGTCG CCTCACCAAC TACAGTCTCG ATGCGGTATA   
  
  
- CCAAAGTCAA AGACCTCTTG GTTAAGTCTC CAATCCTCGG ATGTACAACC TTCCCAATCA ACGGGCCAAC   
  
  
- AGGAGAAGTC CTTCAAGGTA GATGTTTCGA GATTCCACGT TTCTCGGATG ATCATCACTT GAAGAAAGGA   
  
  
- TATACGTAAA TGAGATACTT CAAACGGGAA TGAAGTTCAA ACCCATGTAC AGACGTTTAC CCCGTTAACG   
  
  
- ACTCCGGTAC TTTTTACTCT CGTCTTAAGT ATATTAACTA AAAGTCTATC GAGTTCCCTC AGTCACCTAA   
  
  
- TCGGAATAGG TTCGGGACCG ACGAGTCGGA CTACCCGGTG GTGTCCAGGC ATAATGGCCT CAGCTACTAA   
  
  
- GGGTTAGACT CATACGAGCC CCTCCCCCCG AGCTGTAACA TCGCTTCTCT GATAGACCTG ATCGGGTCCG   
  
  
- AACGTCGGAT GGGAAACTCA AGGTGCGACG TCGTGAATCA CCAAGACTCT AGTCTGAAGT TTTGGACCAA   
  
  
- AACGCCGGAC CTCTTCGGAA TCGTCACTTG AAGGGTAAGT ACGACGTGGT GTACGGCCTA CTCTCACACC   
  
  
- CGGGACTCTT AATATCTCTG GTCAATAATT CCGACCACTT CTCGAAGAAC GGGTTCCACC AATGGGAACA   
  
  
- ACTCGTTCTT AGGTTGTGTT TGTGCCGGGG TAAAGATGGG GCCAAAGAAC TTTGGGATCT GATGATATGA   
  
  
- CGGTAAAAGC TTAGTTAACT ACAATGCGAG GGTTCTCTAG TGTTCCTCGC CTAGTTACAA CTCGTCGTGA   
  
  
- CAAATCGTTC TCTATATCAG TTGTATTATC GTACACTCCC ACGGCTCTCC CACCTTGCGG TACTCCAAGA   
  
  
- ACCTTTCACC TCTAGAGCCA AGAGTTACCG ACCCAAATTC GGCATGGGTA ACTCGGGCGA TCACTTACGT   
  
  
- TGATAGTCCT GAGAAGACGT CTTCATATCG TCCTCGATAC CTGAACTTCT TTCCCTACCT CGAGAAATAG   
  
  
- ATCCGACCTA CTTGGCTCGT GATCAACGTA GAACACGTAC CGTCAC

+     CAG-motif

| Site Name | Organism | Position | Strand | Matrix score. | sequence | function |
| --- | --- | --- | --- | --- | --- | --- |
| CAG-motif | Arabidopsis thaliana | 1636 | - | 10 | GAAAGGCAGAC | part of a light response element |

>HU08G00284.1   
+ -Up\_Stream \_Len000TTCTTT TCCTGTCTCT GTCTCTTCAT TGCACCATCA TTTAAGAGAT GGGACCATAA   
  
  
+ ATCATTCAAT CACTCCTTTA TGTTTAAACT TTGGAAGGAG GTTATTATTT ATGTGGTGGT GATCCCAATG   
  
  
+ CTGAATTTAG CTGTTGATGG CGAGGGCATA TATGTAATTA GGATTTCCAA TGCAGTTTAG ATTTTACTCA   
  
  
+ TTTGAGAATT CACTGAGGTT TGTTTGATCT GATTTTAGCA AATATTGTTC AGGGGTATAG ATGCTTATTC   
  
  
+ GGTTCTCAAC TGTATTAGTA TACAAGATGA GTGGTCCACT TGTTTAAGCT TTTACTATAT ACTTATGTTG   
  
  
+ TCCCTAATTT GGTCTCGCTT GATGTGTAGC TGTCAAGTAT TATTATAATC TTGTGTTTGA TATCACATTT   
  
  
+ GTATTGATTA GCTACAAAAG AACATATTAT ATGTGTATAG CAACTATCTC ATATATGCCC TCACCATAGC   
  
  
+ GTGGTTCCAA GGGTTCAAGT TCCACTTAGC CTCACCCTAG AAATGACTTC TTTTTTTTTT TAAAGTTAAA   
  
  
+ GATATGCAAT CACCATGGAA CCAGTCATCT TTTGACACGT TATTCCAGAT CAGATGACCA GCCTTTCTCC   
  
  
+ AAGGCCATTA GATTTTCTAG CCAATCCACT GTTAACTGCA AAAAGTGGTA TCCTTGGCTG TTCCTGTCAC   
  
  
+ TGTTTCTTAT TTGCTCTGTG ATATTTGTAC ATCTCTTTGC CAAGGATAAA AGTTCATAGC CGGGAATTGG   
  
  
+ TATTCAGTTG ACTCTTGCAG TAAACAGAGT TCATGTACTG GAGGATTTTG TGAGTCATGT CACTCAATAA   
  
  
+ AAACAGGTGT GCGACAGTGT TATAAAGCTG CTTGCTTAAT GCACATCTAC TCTCACTCAC CTTACCCAAA   
  
  
+ AAACTTCAAA ATTGTGTGCC TTCTATGAAT ATTCTGATCC ATTAGCATTC AACGGTTTCT GAATTCTGTG   
  
  
+ TAAGCCATCC ATACTCATTT TCAACAGAGA ACTTGGATTG GATGTATTCA ATACCCAAAA ACCTTGTCAA   
  
  
+ ATTACCCCTA GCTTAATTTC CTCTAATACC AACTGCTCTT TGGTTTTAGC GTACAATCCC ATAGATTACC   
  
  
+ CTTCTATAAT TAGTGATGAT TGGTTCATTT CTGCTGCATT ATGCCTTCTT TAGATCCATT GATAATATAT   
  
  
+ ACTCTTGCCA TGCTGCAACA TTCCACTGGG ATTAATATCT CACATTGTAT CCTCAGCAAC CAAGGAGACC   
  
  
+ CTACTATTTT CAGAGCCATT CATAGAAAGT TATTGCATAT AGTGATGATC TTCAATGGGT CTGATTTTTA   
  
  
+ GATAGCTGCT GTGAACATTT TACTCGTCCT CTGCCATGTG GATACTATGG TAACCTTTGT TTGCTCTCAA   
  
  
+ GCCTGAGCGT TCTGAGAAAT GATGAAAGCG CCCTCGAGTT ATAGGACTTT TCTGACTTTA GCTTTAGAAA   
  
  
+ AGAGAGGAAA ATCAACCACA CCCATCTGAT TATTGAGTTT CTGCTCCTTG AGACCTGGAT GGAGCCAGAA   
  
  
+ CTCACGGTCT TGTCAGCCAG AATAATCAGG AACTGCATTT CTGAGGACCC ATGAAAAGAC CCTGGTATAC   
  
  
+ GACTATTCAT GAAAACGTTT TGTCAGCCTT TCAGTTTTTG CATTTTATTC TAGCTGCTTT GATTGAAGCC   
  
  
+ CAATTTTATT AAAACTGTGC TTTTCTCTTG AATGTTAAAC TCTTGCAGTT CCTTGCTTGT TATTTTGGGT   
  
  
+ TTTATGTTTG CTTTCTACTG ATATCATCTT TTGGTCGTGC AATTTAATTG CCTACTATGT TATATGGATT   
  
  
+ TTTATGTTTA CTTTCTACTG ATATCATCTT TTGCTATTGC AATTTAATTG CCTTCTATGT AGAATTCGTC   
  
  
+ ACTTTGAAGT CTGCCAGGAA GGCAATACAG CTATATTTTT CTTGTGGATG AAGTGATAAA AGGCAAGTGT   
  
  
+ CAATTGCTTA CTGCTTTATT GCGCTGTACT TTTCCAGAGA TTTTGGATTA GTCAATGCAG AAGCCAACAA   
  
  
+ TTTATAGCGA CTGGCCACAG TTCTACAACC AATTTGATAA TCCACGTCTT CTTGAATCTT CATCTATAAT   
  
  
+ GGGTGGTGAT CAACTTTTCA GTTCTCCATC TACTGTAAGC ATATCTTGCA ACAGGAGTCC AGCTTCACTG   
  
  
+ CCTGAACTCG AATCCTCTTC ATCGGACTTC CAAAGTGACT CCCAGGAGAC TGTGAATGGC TCGCCAGTGA   
  
  
+ TTGATTCGTG CATGGTACGT GACATTGGTG ACTTAAGACA CAAACTTAGA GAGCTTGAGA CTGTTATGCT   
  
  
+ CGGACCAAGT TCAGACAGCT TGGATTCATG GTATGCCCCT TCAAGAGGTG AGTGTGAACC TCTGCCACCA   
  
  
+ GAAGAGCCTG ACAATGGGAA ACATTTGTTG GAGATGATAG CAAGGGGGAG CCTCAAAGAG GTGCTAATTG   
  
  
+ CTTGTGCCAA AGCAATATCA GACGATGATT TGTTAACAGC GGAGTGGTTG ATGTCAGAGC TACGCCATAT   
  
  
+ GGTTTCAGTT TCTGGAGAAC CAATTCAGAG GTTAGGAGCC TACATGTTGG AAGGGTTAGT TGCCCGGTTG   
  
  
+ TCCTCTTCAG GAAGTTCCAT CTACAAAGCT CTAAGGTGCA AAGAGCCTAC TAGTAGTGAA CTTCTTTCCT   
  
  
+ ATATGCATTT ACTCTATGAA GTTTGCCCTT ACTTCAAGTT TGGGTACATG TCTGCAAATG GGGCAATTGC   
  
  
+ TGAGGCCATG AAAAATGAGA GCAGAATTCA TATAATTGAT TTTCAGATAG CTCAAGGGAG TCAGTGGATT   
  
  
+ AGCCTTATCC AAGCCCTGGC TGCTCAGCCT GATGGGCCAC CACAGGTCCG TATTACCGGA GTCGATGATT   
  
  
+ CCCAATCTGA GTATGCTCGG GGAGGGGGGC TCGACATTGT AGCGAAGAGA CTATCTGGAC TAGCCCAGGC   
  
  
+ TTGCAGCCTA CCCTTTGAGT TCCACGCTGC AGCACTTAGT GGTTCTGAGA TCAGACTTCA AAACCTGGTT   
  
  
+ TTGCGGCCTG GAGAAGCCTT AGCAGTGAAC TTCCCATTCA TGCTGCACCA CATGCCGGAT GAGAGTGTGG   
  
  
+ GCCCTGAGAA TTATAGAGAC CAGTTATTAA GGCTGGTGAA GAGCTTCTTG CCCAAGGTGG TTACCCTTGT   
  
  
+ TGAGCAAGAA TCCAACACAA ACACGGCCCC ATTTCTACCC CGGTTTCTTG AAACCCTAGA CTACTATACT   
  
  
+ GCCATTTTCG AATCAATTGA TGTTACGCTC CCAAGAGATC ACAAGGAGCG GATCAATGTT GAGCAGCACT   
  
  
+ GTTTAGCAAG AGATATAGTC AACATAATAG CATGTGAGGG TGCCGAGAGG GTGGAACGCC ATGAGGTTCT   
  
  
+ TGGAAAGTGG AGATCTCGGT TCTCAATGGC TGGGTTTAAG CCGTACCCAT TGAGCCCGCT AGTGAATGCA   
  
  
+ ACTATCAGGA CTCTTCTGCA GAAGTATAGC AGGAGCTATG GACTTGAAGA AAGGGATGGA GCTCTTTATC   
  
  
+ TAGGCTGGAT GAACCGAGCA CTAGTTGCAT CTTGTGCATG GCAGTG  

- -Up\_Stream \_Len000AAGAAA AGGACAGAGA CAGAGAAGTA ACGTGGTAGT AAATTCTCTA CCCTGGTATT   
  
  
- TAGTAAGTTA GTGAGGAAAT ACAAATTTGA AACCTTCCTC CAATAATAAA TACACCACCA CTAGGGTTAC   
  
  
- GACTTAAATC GACAACTACC GCTCCCGTAT ATACATTAAT CCTAAAGGTT ACGTCAAATC TAAAATGAGT   
  
  
- AAACTCTTAA GTGACTCCAA ACAAACTAGA CTAAAATCGT TTATAACAAG TCCCCATATC TACGAATAAG   
  
  
- CCAAGAGTTG ACATAATCAT ATGTTCTACT CACCAGGTGA ACAAATTCGA AAATGATATA TGAATACAAC   
  
  
- AGGGATTAAA CCAGAGCGAA CTACACATCG ACAGTTCATA ATAATATTAG AACACAAACT ATAGTGTAAA   
  
  
- CATAACTAAT CGATGTTTTC TTGTATAATA TACACATATC GTTGATAGAG TATATACGGG AGTGGTATCG   
  
  
- CACCAAGGTT CCCAAGTTCA AGGTGAATCG GAGTGGGATC TTTACTGAAG AAAAAAAAAA ATTTCAATTT   
  
  
- CTATACGTTA GTGGTACCTT GGTCAGTAGA AAACTGTGCA ATAAGGTCTA GTCTACTGGT CGGAAAGAGG   
  
  
- TTCCGGTAAT CTAAAAGATC GGTTAGGTGA CAATTGACGT TTTTCACCAT AGGAACCGAC AAGGACAGTG   
  
  
- ACAAAGAATA AACGAGACAC TATAAACATG TAGAGAAACG GTTCCTATTT TCAAGTATCG GCCCTTAACC   
  
  
- ATAAGTCAAC TGAGAACGTC ATTTGTCTCA AGTACATGAC CTCCTAAAAC ACTCAGTACA GTGAGTTATT   
  
  
- TTTGTCCACA CGCTGTCACA ATATTTCGAC GAACGAATTA CGTGTAGATG AGAGTGAGTG GAATGGGTTT   
  
  
- TTTGAAGTTT TAACACACGG AAGATACTTA TAAGACTAGG TAATCGTAAG TTGCCAAAGA CTTAAGACAC   
  
  
- ATTCGGTAGG TATGAGTAAA AGTTGTCTCT TGAACCTAAC CTACATAAGT TATGGGTTTT TGGAACAGTT   
  
  
- TAATGGGGAT CGAATTAAAG GAGATTATGG TTGACGAGAA ACCAAAATCG CATGTTAGGG TATCTAATGG   
  
  
- GAAGATATTA ATCACTACTA ACCAAGTAAA GACGACGTAA TACGGAAGAA ATCTAGGTAA CTATTATATA   
  
  
- TGAGAACGGT ACGACGTTGT AAGGTGACCC TAATTATAGA GTGTAACATA GGAGTCGTTG GTTCCTCTGG   
  
  
- GATGATAAAA GTCTCGGTAA GTATCTTTCA ATAACGTATA TCACTACTAG AAGTTACCCA GACTAAAAAT   
  
  
- CTATCGACGA CACTTGTAAA ATGAGCAGGA GACGGTACAC CTATGATACC ATTGGAAACA AACGAGAGTT   
  
  
- CGGACTCGCA AGACTCTTTA CTACTTTCGC GGGAGCTCAA TATCCTGAAA AGACTGAAAT CGAAATCTTT   
  
  
- TCTCTCCTTT TAGTTGGTGT GGGTAGACTA ATAACTCAAA GACGAGGAAC TCTGGACCTA CCTCGGTCTT   
  
  
- GAGTGCCAGA ACAGTCGGTC TTATTAGTCC TTGACGTAAA GACTCCTGGG TACTTTTCTG GGACCATATG   
  
  
- CTGATAAGTA CTTTTGCAAA ACAGTCGGAA AGTCAAAAAC GTAAAATAAG ATCGACGAAA CTAACTTCGG   
  
  
- GTTAAAATAA TTTTGACACG AAAAGAGAAC TTACAATTTG AGAACGTCAA GGAACGAACA ATAAAACCCA   
  
  
- AAATACAAAC GAAAGATGAC TATAGTAGAA AACCAGCACG TTAAATTAAC GGATGATACA ATATACCTAA   
  
  
- AAATACAAAT GAAAGATGAC TATAGTAGAA AACGATAACG TTAAATTAAC GGAAGATACA TCTTAAGCAG   
  
  
- TGAAACTTCA GACGGTCCTT CCGTTATGTC GATATAAAAA GAACACCTAC TTCACTATTT TCCGTTCACA   
  
  
- GTTAACGAAT GACGAAATAA CGCGACATGA AAAGGTCTCT AAAACCTAAT CAGTTACGTC TTCGGTTGTT   
  
  
- AAATATCGCT GACCGGTGTC AAGATGTTGG TTAAACTATT AGGTGCAGAA GAACTTAGAA GTAGATATTA   
  
  
- CCCACCACTA GTTGAAAAGT CAAGAGGTAG ATGACATTCG TATAGAACGT TGTCCTCAGG TCGAAGTGAC   
  
  
- GGACTTGAGC TTAGGAGAAG TAGCCTGAAG GTTTCACTGA GGGTCCTCTG ACACTTACCG AGCGGTCACT   
  
  
- AACTAAGCAC GTACCATGCA CTGTAACCAC TGAATTCTGT GTTTGAATCT CTCGAACTCT GACAATACGA   
  
  
- GCCTGGTTCA AGTCTGTCGA ACCTAAGTAC CATACGGGGA AGTTCTCCAC TCACACTTGG AGACGGTGGT   
  
  
- CTTCTCGGAC TGTTACCCTT TGTAAACAAC CTCTACTATC GTTCCCCCTC GGAGTTTCTC CACGATTAAC   
  
  
- GAACACGGTT TCGTTATAGT CTGCTACTAA ACAATTGTCG CCTCACCAAC TACAGTCTCG ATGCGGTATA   
  
  
- CCAAAGTCAA AGACCTCTTG GTTAAGTCTC CAATCCTCGG ATGTACAACC TTCCCAATCA ACGGGCCAAC   
  
  
- AGGAGAAGTC CTTCAAGGTA GATGTTTCGA GATTCCACGT TTCTCGGATG ATCATCACTT GAAGAAAGGA   
  
  
- TATACGTAAA TGAGATACTT CAAACGGGAA TGAAGTTCAA ACCCATGTAC AGACGTTTAC CCCGTTAACG   
  
  
- ACTCCGGTAC TTTTTACTCT CGTCTTAAGT ATATTAACTA AAAGTCTATC GAGTTCCCTC AGTCACCTAA   
  
  
- TCGGAATAGG TTCGGGACCG ACGAGTCGGA CTACCCGGTG GTGTCCAGGC ATAATGGCCT CAGCTACTAA   
  
  
- GGGTTAGACT CATACGAGCC CCTCCCCCCG AGCTGTAACA TCGCTTCTCT GATAGACCTG ATCGGGTCCG   
  
  
- AACGTCGGAT GGGAAACTCA AGGTGCGACG TCGTGAATCA CCAAGACTCT AGTCTGAAGT TTTGGACCAA   
  
  
- AACGCCGGAC CTCTTCGGAA TCGTCACTTG AAGGGTAAGT ACGACGTGGT GTACGGCCTA CTCTCACACC   
  
  
- CGGGACTCTT AATATCTCTG GTCAATAATT CCGACCACTT CTCGAAGAAC GGGTTCCACC AATGGGAACA   
  
  
- ACTCGTTCTT AGGTTGTGTT TGTGCCGGGG TAAAGATGGG GCCAAAGAAC TTTGGGATCT GATGATATGA   
  
  
- CGGTAAAAGC TTAGTTAACT ACAATGCGAG GGTTCTCTAG TGTTCCTCGC CTAGTTACAA CTCGTCGTGA   
  
  
- CAAATCGTTC TCTATATCAG TTGTATTATC GTACACTCCC ACGGCTCTCC CACCTTGCGG TACTCCAAGA   
  
  
- ACCTTTCACC TCTAGAGCCA AGAGTTACCG ACCCAAATTC GGCATGGGTA ACTCGGGCGA TCACTTACGT   
  
  
- TGATAGTCCT GAGAAGACGT CTTCATATCG TCCTCGATAC CTGAACTTCT TTCCCTACCT CGAGAAATAG   
  
  
- ATCCGACCTA CTTGGCTCGT GATCAACGTA GAACACGTAC CGTCAC

+     CCAAT-box

| Site Name | Organism | Position | Strand | Matrix score. | sequence | function |
| --- | --- | --- | --- | --- | --- | --- |
| CCAAT-box | Hordeum vulgare | 964 | + | 6 | CAACGG | MYBHv1 binding site |

>HU08G00284.1   
+ -Up\_Stream \_Len000TTCTTT TCCTGTCTCT GTCTCTTCAT TGCACCATCA TTTAAGAGAT GGGACCATAA   
  
  
+ ATCATTCAAT CACTCCTTTA TGTTTAAACT TTGGAAGGAG GTTATTATTT ATGTGGTGGT GATCCCAATG   
  
  
+ CTGAATTTAG CTGTTGATGG CGAGGGCATA TATGTAATTA GGATTTCCAA TGCAGTTTAG ATTTTACTCA   
  
  
+ TTTGAGAATT CACTGAGGTT TGTTTGATCT GATTTTAGCA AATATTGTTC AGGGGTATAG ATGCTTATTC   
  
  
+ GGTTCTCAAC TGTATTAGTA TACAAGATGA GTGGTCCACT TGTTTAAGCT TTTACTATAT ACTTATGTTG   
  
  
+ TCCCTAATTT GGTCTCGCTT GATGTGTAGC TGTCAAGTAT TATTATAATC TTGTGTTTGA TATCACATTT   
  
  
+ GTATTGATTA GCTACAAAAG AACATATTAT ATGTGTATAG CAACTATCTC ATATATGCCC TCACCATAGC   
  
  
+ GTGGTTCCAA GGGTTCAAGT TCCACTTAGC CTCACCCTAG AAATGACTTC TTTTTTTTTT TAAAGTTAAA   
  
  
+ GATATGCAAT CACCATGGAA CCAGTCATCT TTTGACACGT TATTCCAGAT CAGATGACCA GCCTTTCTCC   
  
  
+ AAGGCCATTA GATTTTCTAG CCAATCCACT GTTAACTGCA AAAAGTGGTA TCCTTGGCTG TTCCTGTCAC   
  
  
+ TGTTTCTTAT TTGCTCTGTG ATATTTGTAC ATCTCTTTGC CAAGGATAAA AGTTCATAGC CGGGAATTGG   
  
  
+ TATTCAGTTG ACTCTTGCAG TAAACAGAGT TCATGTACTG GAGGATTTTG TGAGTCATGT CACTCAATAA   
  
  
+ AAACAGGTGT GCGACAGTGT TATAAAGCTG CTTGCTTAAT GCACATCTAC TCTCACTCAC CTTACCCAAA   
  
  
+ AAACTTCAAA ATTGTGTGCC TTCTATGAAT ATTCTGATCC ATTAGCATTC AACGGTTTCT GAATTCTGTG   
  
  
+ TAAGCCATCC ATACTCATTT TCAACAGAGA ACTTGGATTG GATGTATTCA ATACCCAAAA ACCTTGTCAA   
  
  
+ ATTACCCCTA GCTTAATTTC CTCTAATACC AACTGCTCTT TGGTTTTAGC GTACAATCCC ATAGATTACC   
  
  
+ CTTCTATAAT TAGTGATGAT TGGTTCATTT CTGCTGCATT ATGCCTTCTT TAGATCCATT GATAATATAT   
  
  
+ ACTCTTGCCA TGCTGCAACA TTCCACTGGG ATTAATATCT CACATTGTAT CCTCAGCAAC CAAGGAGACC   
  
  
+ CTACTATTTT CAGAGCCATT CATAGAAAGT TATTGCATAT AGTGATGATC TTCAATGGGT CTGATTTTTA   
  
  
+ GATAGCTGCT GTGAACATTT TACTCGTCCT CTGCCATGTG GATACTATGG TAACCTTTGT TTGCTCTCAA   
  
  
+ GCCTGAGCGT TCTGAGAAAT GATGAAAGCG CCCTCGAGTT ATAGGACTTT TCTGACTTTA GCTTTAGAAA   
  
  
+ AGAGAGGAAA ATCAACCACA CCCATCTGAT TATTGAGTTT CTGCTCCTTG AGACCTGGAT GGAGCCAGAA   
  
  
+ CTCACGGTCT TGTCAGCCAG AATAATCAGG AACTGCATTT CTGAGGACCC ATGAAAAGAC CCTGGTATAC   
  
  
+ GACTATTCAT GAAAACGTTT TGTCAGCCTT TCAGTTTTTG CATTTTATTC TAGCTGCTTT GATTGAAGCC   
  
  
+ CAATTTTATT AAAACTGTGC TTTTCTCTTG AATGTTAAAC TCTTGCAGTT CCTTGCTTGT TATTTTGGGT   
  
  
+ TTTATGTTTG CTTTCTACTG ATATCATCTT TTGGTCGTGC AATTTAATTG CCTACTATGT TATATGGATT   
  
  
+ TTTATGTTTA CTTTCTACTG ATATCATCTT TTGCTATTGC AATTTAATTG CCTTCTATGT AGAATTCGTC   
  
  
+ ACTTTGAAGT CTGCCAGGAA GGCAATACAG CTATATTTTT CTTGTGGATG AAGTGATAAA AGGCAAGTGT   
  
  
+ CAATTGCTTA CTGCTTTATT GCGCTGTACT TTTCCAGAGA TTTTGGATTA GTCAATGCAG AAGCCAACAA   
  
  
+ TTTATAGCGA CTGGCCACAG TTCTACAACC AATTTGATAA TCCACGTCTT CTTGAATCTT CATCTATAAT   
  
  
+ GGGTGGTGAT CAACTTTTCA GTTCTCCATC TACTGTAAGC ATATCTTGCA ACAGGAGTCC AGCTTCACTG   
  
  
+ CCTGAACTCG AATCCTCTTC ATCGGACTTC CAAAGTGACT CCCAGGAGAC TGTGAATGGC TCGCCAGTGA   
  
  
+ TTGATTCGTG CATGGTACGT GACATTGGTG ACTTAAGACA CAAACTTAGA GAGCTTGAGA CTGTTATGCT   
  
  
+ CGGACCAAGT TCAGACAGCT TGGATTCATG GTATGCCCCT TCAAGAGGTG AGTGTGAACC TCTGCCACCA   
  
  
+ GAAGAGCCTG ACAATGGGAA ACATTTGTTG GAGATGATAG CAAGGGGGAG CCTCAAAGAG GTGCTAATTG   
  
  
+ CTTGTGCCAA AGCAATATCA GACGATGATT TGTTAACAGC GGAGTGGTTG ATGTCAGAGC TACGCCATAT   
  
  
+ GGTTTCAGTT TCTGGAGAAC CAATTCAGAG GTTAGGAGCC TACATGTTGG AAGGGTTAGT TGCCCGGTTG   
  
  
+ TCCTCTTCAG GAAGTTCCAT CTACAAAGCT CTAAGGTGCA AAGAGCCTAC TAGTAGTGAA CTTCTTTCCT   
  
  
+ ATATGCATTT ACTCTATGAA GTTTGCCCTT ACTTCAAGTT TGGGTACATG TCTGCAAATG GGGCAATTGC   
  
  
+ TGAGGCCATG AAAAATGAGA GCAGAATTCA TATAATTGAT TTTCAGATAG CTCAAGGGAG TCAGTGGATT   
  
  
+ AGCCTTATCC AAGCCCTGGC TGCTCAGCCT GATGGGCCAC CACAGGTCCG TATTACCGGA GTCGATGATT   
  
  
+ CCCAATCTGA GTATGCTCGG GGAGGGGGGC TCGACATTGT AGCGAAGAGA CTATCTGGAC TAGCCCAGGC   
  
  
+ TTGCAGCCTA CCCTTTGAGT TCCACGCTGC AGCACTTAGT GGTTCTGAGA TCAGACTTCA AAACCTGGTT   
  
  
+ TTGCGGCCTG GAGAAGCCTT AGCAGTGAAC TTCCCATTCA TGCTGCACCA CATGCCGGAT GAGAGTGTGG   
  
  
+ GCCCTGAGAA TTATAGAGAC CAGTTATTAA GGCTGGTGAA GAGCTTCTTG CCCAAGGTGG TTACCCTTGT   
  
  
+ TGAGCAAGAA TCCAACACAA ACACGGCCCC ATTTCTACCC CGGTTTCTTG AAACCCTAGA CTACTATACT   
  
  
+ GCCATTTTCG AATCAATTGA TGTTACGCTC CCAAGAGATC ACAAGGAGCG GATCAATGTT GAGCAGCACT   
  
  
+ GTTTAGCAAG AGATATAGTC AACATAATAG CATGTGAGGG TGCCGAGAGG GTGGAACGCC ATGAGGTTCT   
  
  
+ TGGAAAGTGG AGATCTCGGT TCTCAATGGC TGGGTTTAAG CCGTACCCAT TGAGCCCGCT AGTGAATGCA   
  
  
+ ACTATCAGGA CTCTTCTGCA GAAGTATAGC AGGAGCTATG GACTTGAAGA AAGGGATGGA GCTCTTTATC   
  
  
+ TAGGCTGGAT GAACCGAGCA CTAGTTGCAT CTTGTGCATG GCAGTG  

- -Up\_Stream \_Len000AAGAAA AGGACAGAGA CAGAGAAGTA ACGTGGTAGT AAATTCTCTA CCCTGGTATT   
  
  
- TAGTAAGTTA GTGAGGAAAT ACAAATTTGA AACCTTCCTC CAATAATAAA TACACCACCA CTAGGGTTAC   
  
  
- GACTTAAATC GACAACTACC GCTCCCGTAT ATACATTAAT CCTAAAGGTT ACGTCAAATC TAAAATGAGT   
  
  
- AAACTCTTAA GTGACTCCAA ACAAACTAGA CTAAAATCGT TTATAACAAG TCCCCATATC TACGAATAAG   
  
  
- CCAAGAGTTG ACATAATCAT ATGTTCTACT CACCAGGTGA ACAAATTCGA AAATGATATA TGAATACAAC   
  
  
- AGGGATTAAA CCAGAGCGAA CTACACATCG ACAGTTCATA ATAATATTAG AACACAAACT ATAGTGTAAA   
  
  
- CATAACTAAT CGATGTTTTC TTGTATAATA TACACATATC GTTGATAGAG TATATACGGG AGTGGTATCG   
  
  
- CACCAAGGTT CCCAAGTTCA AGGTGAATCG GAGTGGGATC TTTACTGAAG AAAAAAAAAA ATTTCAATTT   
  
  
- CTATACGTTA GTGGTACCTT GGTCAGTAGA AAACTGTGCA ATAAGGTCTA GTCTACTGGT CGGAAAGAGG   
  
  
- TTCCGGTAAT CTAAAAGATC GGTTAGGTGA CAATTGACGT TTTTCACCAT AGGAACCGAC AAGGACAGTG   
  
  
- ACAAAGAATA AACGAGACAC TATAAACATG TAGAGAAACG GTTCCTATTT TCAAGTATCG GCCCTTAACC   
  
  
- ATAAGTCAAC TGAGAACGTC ATTTGTCTCA AGTACATGAC CTCCTAAAAC ACTCAGTACA GTGAGTTATT   
  
  
- TTTGTCCACA CGCTGTCACA ATATTTCGAC GAACGAATTA CGTGTAGATG AGAGTGAGTG GAATGGGTTT   
  
  
- TTTGAAGTTT TAACACACGG AAGATACTTA TAAGACTAGG TAATCGTAAG TTGCCAAAGA CTTAAGACAC   
  
  
- ATTCGGTAGG TATGAGTAAA AGTTGTCTCT TGAACCTAAC CTACATAAGT TATGGGTTTT TGGAACAGTT   
  
  
- TAATGGGGAT CGAATTAAAG GAGATTATGG TTGACGAGAA ACCAAAATCG CATGTTAGGG TATCTAATGG   
  
  
- GAAGATATTA ATCACTACTA ACCAAGTAAA GACGACGTAA TACGGAAGAA ATCTAGGTAA CTATTATATA   
  
  
- TGAGAACGGT ACGACGTTGT AAGGTGACCC TAATTATAGA GTGTAACATA GGAGTCGTTG GTTCCTCTGG   
  
  
- GATGATAAAA GTCTCGGTAA GTATCTTTCA ATAACGTATA TCACTACTAG AAGTTACCCA GACTAAAAAT   
  
  
- CTATCGACGA CACTTGTAAA ATGAGCAGGA GACGGTACAC CTATGATACC ATTGGAAACA AACGAGAGTT   
  
  
- CGGACTCGCA AGACTCTTTA CTACTTTCGC GGGAGCTCAA TATCCTGAAA AGACTGAAAT CGAAATCTTT   
  
  
- TCTCTCCTTT TAGTTGGTGT GGGTAGACTA ATAACTCAAA GACGAGGAAC TCTGGACCTA CCTCGGTCTT   
  
  
- GAGTGCCAGA ACAGTCGGTC TTATTAGTCC TTGACGTAAA GACTCCTGGG TACTTTTCTG GGACCATATG   
  
  
- CTGATAAGTA CTTTTGCAAA ACAGTCGGAA AGTCAAAAAC GTAAAATAAG ATCGACGAAA CTAACTTCGG   
  
  
- GTTAAAATAA TTTTGACACG AAAAGAGAAC TTACAATTTG AGAACGTCAA GGAACGAACA ATAAAACCCA   
  
  
- AAATACAAAC GAAAGATGAC TATAGTAGAA AACCAGCACG TTAAATTAAC GGATGATACA ATATACCTAA   
  
  
- AAATACAAAT GAAAGATGAC TATAGTAGAA AACGATAACG TTAAATTAAC GGAAGATACA TCTTAAGCAG   
  
  
- TGAAACTTCA GACGGTCCTT CCGTTATGTC GATATAAAAA GAACACCTAC TTCACTATTT TCCGTTCACA   
  
  
- GTTAACGAAT GACGAAATAA CGCGACATGA AAAGGTCTCT AAAACCTAAT CAGTTACGTC TTCGGTTGTT   
  
  
- AAATATCGCT GACCGGTGTC AAGATGTTGG TTAAACTATT AGGTGCAGAA GAACTTAGAA GTAGATATTA   
  
  
- CCCACCACTA GTTGAAAAGT CAAGAGGTAG ATGACATTCG TATAGAACGT TGTCCTCAGG TCGAAGTGAC   
  
  
- GGACTTGAGC TTAGGAGAAG TAGCCTGAAG GTTTCACTGA GGGTCCTCTG ACACTTACCG AGCGGTCACT   
  
  
- AACTAAGCAC GTACCATGCA CTGTAACCAC TGAATTCTGT GTTTGAATCT CTCGAACTCT GACAATACGA   
  
  
- GCCTGGTTCA AGTCTGTCGA ACCTAAGTAC CATACGGGGA AGTTCTCCAC TCACACTTGG AGACGGTGGT   
  
  
- CTTCTCGGAC TGTTACCCTT TGTAAACAAC CTCTACTATC GTTCCCCCTC GGAGTTTCTC CACGATTAAC   
  
  
- GAACACGGTT TCGTTATAGT CTGCTACTAA ACAATTGTCG CCTCACCAAC TACAGTCTCG ATGCGGTATA   
  
  
- CCAAAGTCAA AGACCTCTTG GTTAAGTCTC CAATCCTCGG ATGTACAACC TTCCCAATCA ACGGGCCAAC   
  
  
- AGGAGAAGTC CTTCAAGGTA GATGTTTCGA GATTCCACGT TTCTCGGATG ATCATCACTT GAAGAAAGGA   
  
  
- TATACGTAAA TGAGATACTT CAAACGGGAA TGAAGTTCAA ACCCATGTAC AGACGTTTAC CCCGTTAACG   
  
  
- ACTCCGGTAC TTTTTACTCT CGTCTTAAGT ATATTAACTA AAAGTCTATC GAGTTCCCTC AGTCACCTAA   
  
  
- TCGGAATAGG TTCGGGACCG ACGAGTCGGA CTACCCGGTG GTGTCCAGGC ATAATGGCCT CAGCTACTAA   
  
  
- GGGTTAGACT CATACGAGCC CCTCCCCCCG AGCTGTAACA TCGCTTCTCT GATAGACCTG ATCGGGTCCG   
  
  
- AACGTCGGAT GGGAAACTCA AGGTGCGACG TCGTGAATCA CCAAGACTCT AGTCTGAAGT TTTGGACCAA   
  
  
- AACGCCGGAC CTCTTCGGAA TCGTCACTTG AAGGGTAAGT ACGACGTGGT GTACGGCCTA CTCTCACACC   
  
  
- CGGGACTCTT AATATCTCTG GTCAATAATT CCGACCACTT CTCGAAGAAC GGGTTCCACC AATGGGAACA   
  
  
- ACTCGTTCTT AGGTTGTGTT TGTGCCGGGG TAAAGATGGG GCCAAAGAAC TTTGGGATCT GATGATATGA   
  
  
- CGGTAAAAGC TTAGTTAACT ACAATGCGAG GGTTCTCTAG TGTTCCTCGC CTAGTTACAA CTCGTCGTGA   
  
  
- CAAATCGTTC TCTATATCAG TTGTATTATC GTACACTCCC ACGGCTCTCC CACCTTGCGG TACTCCAAGA   
  
  
- ACCTTTCACC TCTAGAGCCA AGAGTTACCG ACCCAAATTC GGCATGGGTA ACTCGGGCGA TCACTTACGT   
  
  
- TGATAGTCCT GAGAAGACGT CTTCATATCG TCCTCGATAC CTGAACTTCT TTCCCTACCT CGAGAAATAG   
  
  
- ATCCGACCTA CTTGGCTCGT GATCAACGTA GAACACGTAC CGTCAC

+     CGTCA-motif

| Site Name | Organism | Position | Strand | Matrix score. | sequence | function |
| --- | --- | --- | --- | --- | --- | --- |
| CGTCA-motif | Hordeum vulgare | 1891 | + | 5 | CGTCA | cis-acting regulatory element involved in the MeJA-responsiveness |

>HU08G00284.1   
+ -Up\_Stream \_Len000TTCTTT TCCTGTCTCT GTCTCTTCAT TGCACCATCA TTTAAGAGAT GGGACCATAA   
  
  
+ ATCATTCAAT CACTCCTTTA TGTTTAAACT TTGGAAGGAG GTTATTATTT ATGTGGTGGT GATCCCAATG   
  
  
+ CTGAATTTAG CTGTTGATGG CGAGGGCATA TATGTAATTA GGATTTCCAA TGCAGTTTAG ATTTTACTCA   
  
  
+ TTTGAGAATT CACTGAGGTT TGTTTGATCT GATTTTAGCA AATATTGTTC AGGGGTATAG ATGCTTATTC   
  
  
+ GGTTCTCAAC TGTATTAGTA TACAAGATGA GTGGTCCACT TGTTTAAGCT TTTACTATAT ACTTATGTTG   
  
  
+ TCCCTAATTT GGTCTCGCTT GATGTGTAGC TGTCAAGTAT TATTATAATC TTGTGTTTGA TATCACATTT   
  
  
+ GTATTGATTA GCTACAAAAG AACATATTAT ATGTGTATAG CAACTATCTC ATATATGCCC TCACCATAGC   
  
  
+ GTGGTTCCAA GGGTTCAAGT TCCACTTAGC CTCACCCTAG AAATGACTTC TTTTTTTTTT TAAAGTTAAA   
  
  
+ GATATGCAAT CACCATGGAA CCAGTCATCT TTTGACACGT TATTCCAGAT CAGATGACCA GCCTTTCTCC   
  
  
+ AAGGCCATTA GATTTTCTAG CCAATCCACT GTTAACTGCA AAAAGTGGTA TCCTTGGCTG TTCCTGTCAC   
  
  
+ TGTTTCTTAT TTGCTCTGTG ATATTTGTAC ATCTCTTTGC CAAGGATAAA AGTTCATAGC CGGGAATTGG   
  
  
+ TATTCAGTTG ACTCTTGCAG TAAACAGAGT TCATGTACTG GAGGATTTTG TGAGTCATGT CACTCAATAA   
  
  
+ AAACAGGTGT GCGACAGTGT TATAAAGCTG CTTGCTTAAT GCACATCTAC TCTCACTCAC CTTACCCAAA   
  
  
+ AAACTTCAAA ATTGTGTGCC TTCTATGAAT ATTCTGATCC ATTAGCATTC AACGGTTTCT GAATTCTGTG   
  
  
+ TAAGCCATCC ATACTCATTT TCAACAGAGA ACTTGGATTG GATGTATTCA ATACCCAAAA ACCTTGTCAA   
  
  
+ ATTACCCCTA GCTTAATTTC CTCTAATACC AACTGCTCTT TGGTTTTAGC GTACAATCCC ATAGATTACC   
  
  
+ CTTCTATAAT TAGTGATGAT TGGTTCATTT CTGCTGCATT ATGCCTTCTT TAGATCCATT GATAATATAT   
  
  
+ ACTCTTGCCA TGCTGCAACA TTCCACTGGG ATTAATATCT CACATTGTAT CCTCAGCAAC CAAGGAGACC   
  
  
+ CTACTATTTT CAGAGCCATT CATAGAAAGT TATTGCATAT AGTGATGATC TTCAATGGGT CTGATTTTTA   
  
  
+ GATAGCTGCT GTGAACATTT TACTCGTCCT CTGCCATGTG GATACTATGG TAACCTTTGT TTGCTCTCAA   
  
  
+ GCCTGAGCGT TCTGAGAAAT GATGAAAGCG CCCTCGAGTT ATAGGACTTT TCTGACTTTA GCTTTAGAAA   
  
  
+ AGAGAGGAAA ATCAACCACA CCCATCTGAT TATTGAGTTT CTGCTCCTTG AGACCTGGAT GGAGCCAGAA   
  
  
+ CTCACGGTCT TGTCAGCCAG AATAATCAGG AACTGCATTT CTGAGGACCC ATGAAAAGAC CCTGGTATAC   
  
  
+ GACTATTCAT GAAAACGTTT TGTCAGCCTT TCAGTTTTTG CATTTTATTC TAGCTGCTTT GATTGAAGCC   
  
  
+ CAATTTTATT AAAACTGTGC TTTTCTCTTG AATGTTAAAC TCTTGCAGTT CCTTGCTTGT TATTTTGGGT   
  
  
+ TTTATGTTTG CTTTCTACTG ATATCATCTT TTGGTCGTGC AATTTAATTG CCTACTATGT TATATGGATT   
  
  
+ TTTATGTTTA CTTTCTACTG ATATCATCTT TTGCTATTGC AATTTAATTG CCTTCTATGT AGAATTCGTC   
  
  
+ ACTTTGAAGT CTGCCAGGAA GGCAATACAG CTATATTTTT CTTGTGGATG AAGTGATAAA AGGCAAGTGT   
  
  
+ CAATTGCTTA CTGCTTTATT GCGCTGTACT TTTCCAGAGA TTTTGGATTA GTCAATGCAG AAGCCAACAA   
  
  
+ TTTATAGCGA CTGGCCACAG TTCTACAACC AATTTGATAA TCCACGTCTT CTTGAATCTT CATCTATAAT   
  
  
+ GGGTGGTGAT CAACTTTTCA GTTCTCCATC TACTGTAAGC ATATCTTGCA ACAGGAGTCC AGCTTCACTG   
  
  
+ CCTGAACTCG AATCCTCTTC ATCGGACTTC CAAAGTGACT CCCAGGAGAC TGTGAATGGC TCGCCAGTGA   
  
  
+ TTGATTCGTG CATGGTACGT GACATTGGTG ACTTAAGACA CAAACTTAGA GAGCTTGAGA CTGTTATGCT   
  
  
+ CGGACCAAGT TCAGACAGCT TGGATTCATG GTATGCCCCT TCAAGAGGTG AGTGTGAACC TCTGCCACCA   
  
  
+ GAAGAGCCTG ACAATGGGAA ACATTTGTTG GAGATGATAG CAAGGGGGAG CCTCAAAGAG GTGCTAATTG   
  
  
+ CTTGTGCCAA AGCAATATCA GACGATGATT TGTTAACAGC GGAGTGGTTG ATGTCAGAGC TACGCCATAT   
  
  
+ GGTTTCAGTT TCTGGAGAAC CAATTCAGAG GTTAGGAGCC TACATGTTGG AAGGGTTAGT TGCCCGGTTG   
  
  
+ TCCTCTTCAG GAAGTTCCAT CTACAAAGCT CTAAGGTGCA AAGAGCCTAC TAGTAGTGAA CTTCTTTCCT   
  
  
+ ATATGCATTT ACTCTATGAA GTTTGCCCTT ACTTCAAGTT TGGGTACATG TCTGCAAATG GGGCAATTGC   
  
  
+ TGAGGCCATG AAAAATGAGA GCAGAATTCA TATAATTGAT TTTCAGATAG CTCAAGGGAG TCAGTGGATT   
  
  
+ AGCCTTATCC AAGCCCTGGC TGCTCAGCCT GATGGGCCAC CACAGGTCCG TATTACCGGA GTCGATGATT   
  
  
+ CCCAATCTGA GTATGCTCGG GGAGGGGGGC TCGACATTGT AGCGAAGAGA CTATCTGGAC TAGCCCAGGC   
  
  
+ TTGCAGCCTA CCCTTTGAGT TCCACGCTGC AGCACTTAGT GGTTCTGAGA TCAGACTTCA AAACCTGGTT   
  
  
+ TTGCGGCCTG GAGAAGCCTT AGCAGTGAAC TTCCCATTCA TGCTGCACCA CATGCCGGAT GAGAGTGTGG   
  
  
+ GCCCTGAGAA TTATAGAGAC CAGTTATTAA GGCTGGTGAA GAGCTTCTTG CCCAAGGTGG TTACCCTTGT   
  
  
+ TGAGCAAGAA TCCAACACAA ACACGGCCCC ATTTCTACCC CGGTTTCTTG AAACCCTAGA CTACTATACT   
  
  
+ GCCATTTTCG AATCAATTGA TGTTACGCTC CCAAGAGATC ACAAGGAGCG GATCAATGTT GAGCAGCACT   
  
  
+ GTTTAGCAAG AGATATAGTC AACATAATAG CATGTGAGGG TGCCGAGAGG GTGGAACGCC ATGAGGTTCT   
  
  
+ TGGAAAGTGG AGATCTCGGT TCTCAATGGC TGGGTTTAAG CCGTACCCAT TGAGCCCGCT AGTGAATGCA   
  
  
+ ACTATCAGGA CTCTTCTGCA GAAGTATAGC AGGAGCTATG GACTTGAAGA AAGGGATGGA GCTCTTTATC   
  
  
+ TAGGCTGGAT GAACCGAGCA CTAGTTGCAT CTTGTGCATG GCAGTG  

- -Up\_Stream \_Len000AAGAAA AGGACAGAGA CAGAGAAGTA ACGTGGTAGT AAATTCTCTA CCCTGGTATT   
  
  
- TAGTAAGTTA GTGAGGAAAT ACAAATTTGA AACCTTCCTC CAATAATAAA TACACCACCA CTAGGGTTAC   
  
  
- GACTTAAATC GACAACTACC GCTCCCGTAT ATACATTAAT CCTAAAGGTT ACGTCAAATC TAAAATGAGT   
  
  
- AAACTCTTAA GTGACTCCAA ACAAACTAGA CTAAAATCGT TTATAACAAG TCCCCATATC TACGAATAAG   
  
  
- CCAAGAGTTG ACATAATCAT ATGTTCTACT CACCAGGTGA ACAAATTCGA AAATGATATA TGAATACAAC   
  
  
- AGGGATTAAA CCAGAGCGAA CTACACATCG ACAGTTCATA ATAATATTAG AACACAAACT ATAGTGTAAA   
  
  
- CATAACTAAT CGATGTTTTC TTGTATAATA TACACATATC GTTGATAGAG TATATACGGG AGTGGTATCG   
  
  
- CACCAAGGTT CCCAAGTTCA AGGTGAATCG GAGTGGGATC TTTACTGAAG AAAAAAAAAA ATTTCAATTT   
  
  
- CTATACGTTA GTGGTACCTT GGTCAGTAGA AAACTGTGCA ATAAGGTCTA GTCTACTGGT CGGAAAGAGG   
  
  
- TTCCGGTAAT CTAAAAGATC GGTTAGGTGA CAATTGACGT TTTTCACCAT AGGAACCGAC AAGGACAGTG   
  
  
- ACAAAGAATA AACGAGACAC TATAAACATG TAGAGAAACG GTTCCTATTT TCAAGTATCG GCCCTTAACC   
  
  
- ATAAGTCAAC TGAGAACGTC ATTTGTCTCA AGTACATGAC CTCCTAAAAC ACTCAGTACA GTGAGTTATT   
  
  
- TTTGTCCACA CGCTGTCACA ATATTTCGAC GAACGAATTA CGTGTAGATG AGAGTGAGTG GAATGGGTTT   
  
  
- TTTGAAGTTT TAACACACGG AAGATACTTA TAAGACTAGG TAATCGTAAG TTGCCAAAGA CTTAAGACAC   
  
  
- ATTCGGTAGG TATGAGTAAA AGTTGTCTCT TGAACCTAAC CTACATAAGT TATGGGTTTT TGGAACAGTT   
  
  
- TAATGGGGAT CGAATTAAAG GAGATTATGG TTGACGAGAA ACCAAAATCG CATGTTAGGG TATCTAATGG   
  
  
- GAAGATATTA ATCACTACTA ACCAAGTAAA GACGACGTAA TACGGAAGAA ATCTAGGTAA CTATTATATA   
  
  
- TGAGAACGGT ACGACGTTGT AAGGTGACCC TAATTATAGA GTGTAACATA GGAGTCGTTG GTTCCTCTGG   
  
  
- GATGATAAAA GTCTCGGTAA GTATCTTTCA ATAACGTATA TCACTACTAG AAGTTACCCA GACTAAAAAT   
  
  
- CTATCGACGA CACTTGTAAA ATGAGCAGGA GACGGTACAC CTATGATACC ATTGGAAACA AACGAGAGTT   
  
  
- CGGACTCGCA AGACTCTTTA CTACTTTCGC GGGAGCTCAA TATCCTGAAA AGACTGAAAT CGAAATCTTT   
  
  
- TCTCTCCTTT TAGTTGGTGT GGGTAGACTA ATAACTCAAA GACGAGGAAC TCTGGACCTA CCTCGGTCTT   
  
  
- GAGTGCCAGA ACAGTCGGTC TTATTAGTCC TTGACGTAAA GACTCCTGGG TACTTTTCTG GGACCATATG   
  
  
- CTGATAAGTA CTTTTGCAAA ACAGTCGGAA AGTCAAAAAC GTAAAATAAG ATCGACGAAA CTAACTTCGG   
  
  
- GTTAAAATAA TTTTGACACG AAAAGAGAAC TTACAATTTG AGAACGTCAA GGAACGAACA ATAAAACCCA   
  
  
- AAATACAAAC GAAAGATGAC TATAGTAGAA AACCAGCACG TTAAATTAAC GGATGATACA ATATACCTAA   
  
  
- AAATACAAAT GAAAGATGAC TATAGTAGAA AACGATAACG TTAAATTAAC GGAAGATACA TCTTAAGCAG   
  
  
- TGAAACTTCA GACGGTCCTT CCGTTATGTC GATATAAAAA GAACACCTAC TTCACTATTT TCCGTTCACA   
  
  
- GTTAACGAAT GACGAAATAA CGCGACATGA AAAGGTCTCT AAAACCTAAT CAGTTACGTC TTCGGTTGTT   
  
  
- AAATATCGCT GACCGGTGTC AAGATGTTGG TTAAACTATT AGGTGCAGAA GAACTTAGAA GTAGATATTA   
  
  
- CCCACCACTA GTTGAAAAGT CAAGAGGTAG ATGACATTCG TATAGAACGT TGTCCTCAGG TCGAAGTGAC   
  
  
- GGACTTGAGC TTAGGAGAAG TAGCCTGAAG GTTTCACTGA GGGTCCTCTG ACACTTACCG AGCGGTCACT   
  
  
- AACTAAGCAC GTACCATGCA CTGTAACCAC TGAATTCTGT GTTTGAATCT CTCGAACTCT GACAATACGA   
  
  
- GCCTGGTTCA AGTCTGTCGA ACCTAAGTAC CATACGGGGA AGTTCTCCAC TCACACTTGG AGACGGTGGT   
  
  
- CTTCTCGGAC TGTTACCCTT TGTAAACAAC CTCTACTATC GTTCCCCCTC GGAGTTTCTC CACGATTAAC   
  
  
- GAACACGGTT TCGTTATAGT CTGCTACTAA ACAATTGTCG CCTCACCAAC TACAGTCTCG ATGCGGTATA   
  
  
- CCAAAGTCAA AGACCTCTTG GTTAAGTCTC CAATCCTCGG ATGTACAACC TTCCCAATCA ACGGGCCAAC   
  
  
- AGGAGAAGTC CTTCAAGGTA GATGTTTCGA GATTCCACGT TTCTCGGATG ATCATCACTT GAAGAAAGGA   
  
  
- TATACGTAAA TGAGATACTT CAAACGGGAA TGAAGTTCAA ACCCATGTAC AGACGTTTAC CCCGTTAACG   
  
  
- ACTCCGGTAC TTTTTACTCT CGTCTTAAGT ATATTAACTA AAAGTCTATC GAGTTCCCTC AGTCACCTAA   
  
  
- TCGGAATAGG TTCGGGACCG ACGAGTCGGA CTACCCGGTG GTGTCCAGGC ATAATGGCCT CAGCTACTAA   
  
  
- GGGTTAGACT CATACGAGCC CCTCCCCCCG AGCTGTAACA TCGCTTCTCT GATAGACCTG ATCGGGTCCG   
  
  
- AACGTCGGAT GGGAAACTCA AGGTGCGACG TCGTGAATCA CCAAGACTCT AGTCTGAAGT TTTGGACCAA   
  
  
- AACGCCGGAC CTCTTCGGAA TCGTCACTTG AAGGGTAAGT ACGACGTGGT GTACGGCCTA CTCTCACACC   
  
  
- CGGGACTCTT AATATCTCTG GTCAATAATT CCGACCACTT CTCGAAGAAC GGGTTCCACC AATGGGAACA   
  
  
- ACTCGTTCTT AGGTTGTGTT TGTGCCGGGG TAAAGATGGG GCCAAAGAAC TTTGGGATCT GATGATATGA   
  
  
- CGGTAAAAGC TTAGTTAACT ACAATGCGAG GGTTCTCTAG TGTTCCTCGC CTAGTTACAA CTCGTCGTGA   
  
  
- CAAATCGTTC TCTATATCAG TTGTATTATC GTACACTCCC ACGGCTCTCC CACCTTGCGG TACTCCAAGA   
  
  
- ACCTTTCACC TCTAGAGCCA AGAGTTACCG ACCCAAATTC GGCATGGGTA ACTCGGGCGA TCACTTACGT   
  
  
- TGATAGTCCT GAGAAGACGT CTTCATATCG TCCTCGATAC CTGAACTTCT TTCCCTACCT CGAGAAATAG   
  
  
- ATCCGACCTA CTTGGCTCGT GATCAACGTA GAACACGTAC CGTCAC

+     DRE1

| Site Name | Organism | Position | Strand | Matrix score. | sequence | function |
| --- | --- | --- | --- | --- | --- | --- |
| DRE1 | Zea mays | 3378 | - | 7 | ACCGAGA |  |

>HU08G00284.1   
+ -Up\_Stream \_Len000TTCTTT TCCTGTCTCT GTCTCTTCAT TGCACCATCA TTTAAGAGAT GGGACCATAA   
  
  
+ ATCATTCAAT CACTCCTTTA TGTTTAAACT TTGGAAGGAG GTTATTATTT ATGTGGTGGT GATCCCAATG   
  
  
+ CTGAATTTAG CTGTTGATGG CGAGGGCATA TATGTAATTA GGATTTCCAA TGCAGTTTAG ATTTTACTCA   
  
  
+ TTTGAGAATT CACTGAGGTT TGTTTGATCT GATTTTAGCA AATATTGTTC AGGGGTATAG ATGCTTATTC   
  
  
+ GGTTCTCAAC TGTATTAGTA TACAAGATGA GTGGTCCACT TGTTTAAGCT TTTACTATAT ACTTATGTTG   
  
  
+ TCCCTAATTT GGTCTCGCTT GATGTGTAGC TGTCAAGTAT TATTATAATC TTGTGTTTGA TATCACATTT   
  
  
+ GTATTGATTA GCTACAAAAG AACATATTAT ATGTGTATAG CAACTATCTC ATATATGCCC TCACCATAGC   
  
  
+ GTGGTTCCAA GGGTTCAAGT TCCACTTAGC CTCACCCTAG AAATGACTTC TTTTTTTTTT TAAAGTTAAA   
  
  
+ GATATGCAAT CACCATGGAA CCAGTCATCT TTTGACACGT TATTCCAGAT CAGATGACCA GCCTTTCTCC   
  
  
+ AAGGCCATTA GATTTTCTAG CCAATCCACT GTTAACTGCA AAAAGTGGTA TCCTTGGCTG TTCCTGTCAC   
  
  
+ TGTTTCTTAT TTGCTCTGTG ATATTTGTAC ATCTCTTTGC CAAGGATAAA AGTTCATAGC CGGGAATTGG   
  
  
+ TATTCAGTTG ACTCTTGCAG TAAACAGAGT TCATGTACTG GAGGATTTTG TGAGTCATGT CACTCAATAA   
  
  
+ AAACAGGTGT GCGACAGTGT TATAAAGCTG CTTGCTTAAT GCACATCTAC TCTCACTCAC CTTACCCAAA   
  
  
+ AAACTTCAAA ATTGTGTGCC TTCTATGAAT ATTCTGATCC ATTAGCATTC AACGGTTTCT GAATTCTGTG   
  
  
+ TAAGCCATCC ATACTCATTT TCAACAGAGA ACTTGGATTG GATGTATTCA ATACCCAAAA ACCTTGTCAA   
  
  
+ ATTACCCCTA GCTTAATTTC CTCTAATACC AACTGCTCTT TGGTTTTAGC GTACAATCCC ATAGATTACC   
  
  
+ CTTCTATAAT TAGTGATGAT TGGTTCATTT CTGCTGCATT ATGCCTTCTT TAGATCCATT GATAATATAT   
  
  
+ ACTCTTGCCA TGCTGCAACA TTCCACTGGG ATTAATATCT CACATTGTAT CCTCAGCAAC CAAGGAGACC   
  
  
+ CTACTATTTT CAGAGCCATT CATAGAAAGT TATTGCATAT AGTGATGATC TTCAATGGGT CTGATTTTTA   
  
  
+ GATAGCTGCT GTGAACATTT TACTCGTCCT CTGCCATGTG GATACTATGG TAACCTTTGT TTGCTCTCAA   
  
  
+ GCCTGAGCGT TCTGAGAAAT GATGAAAGCG CCCTCGAGTT ATAGGACTTT TCTGACTTTA GCTTTAGAAA   
  
  
+ AGAGAGGAAA ATCAACCACA CCCATCTGAT TATTGAGTTT CTGCTCCTTG AGACCTGGAT GGAGCCAGAA   
  
  
+ CTCACGGTCT TGTCAGCCAG AATAATCAGG AACTGCATTT CTGAGGACCC ATGAAAAGAC CCTGGTATAC   
  
  
+ GACTATTCAT GAAAACGTTT TGTCAGCCTT TCAGTTTTTG CATTTTATTC TAGCTGCTTT GATTGAAGCC   
  
  
+ CAATTTTATT AAAACTGTGC TTTTCTCTTG AATGTTAAAC TCTTGCAGTT CCTTGCTTGT TATTTTGGGT   
  
  
+ TTTATGTTTG CTTTCTACTG ATATCATCTT TTGGTCGTGC AATTTAATTG CCTACTATGT TATATGGATT   
  
  
+ TTTATGTTTA CTTTCTACTG ATATCATCTT TTGCTATTGC AATTTAATTG CCTTCTATGT AGAATTCGTC   
  
  
+ ACTTTGAAGT CTGCCAGGAA GGCAATACAG CTATATTTTT CTTGTGGATG AAGTGATAAA AGGCAAGTGT   
  
  
+ CAATTGCTTA CTGCTTTATT GCGCTGTACT TTTCCAGAGA TTTTGGATTA GTCAATGCAG AAGCCAACAA   
  
  
+ TTTATAGCGA CTGGCCACAG TTCTACAACC AATTTGATAA TCCACGTCTT CTTGAATCTT CATCTATAAT   
  
  
+ GGGTGGTGAT CAACTTTTCA GTTCTCCATC TACTGTAAGC ATATCTTGCA ACAGGAGTCC AGCTTCACTG   
  
  
+ CCTGAACTCG AATCCTCTTC ATCGGACTTC CAAAGTGACT CCCAGGAGAC TGTGAATGGC TCGCCAGTGA   
  
  
+ TTGATTCGTG CATGGTACGT GACATTGGTG ACTTAAGACA CAAACTTAGA GAGCTTGAGA CTGTTATGCT   
  
  
+ CGGACCAAGT TCAGACAGCT TGGATTCATG GTATGCCCCT TCAAGAGGTG AGTGTGAACC TCTGCCACCA   
  
  
+ GAAGAGCCTG ACAATGGGAA ACATTTGTTG GAGATGATAG CAAGGGGGAG CCTCAAAGAG GTGCTAATTG   
  
  
+ CTTGTGCCAA AGCAATATCA GACGATGATT TGTTAACAGC GGAGTGGTTG ATGTCAGAGC TACGCCATAT   
  
  
+ GGTTTCAGTT TCTGGAGAAC CAATTCAGAG GTTAGGAGCC TACATGTTGG AAGGGTTAGT TGCCCGGTTG   
  
  
+ TCCTCTTCAG GAAGTTCCAT CTACAAAGCT CTAAGGTGCA AAGAGCCTAC TAGTAGTGAA CTTCTTTCCT   
  
  
+ ATATGCATTT ACTCTATGAA GTTTGCCCTT ACTTCAAGTT TGGGTACATG TCTGCAAATG GGGCAATTGC   
  
  
+ TGAGGCCATG AAAAATGAGA GCAGAATTCA TATAATTGAT TTTCAGATAG CTCAAGGGAG TCAGTGGATT   
  
  
+ AGCCTTATCC AAGCCCTGGC TGCTCAGCCT GATGGGCCAC CACAGGTCCG TATTACCGGA GTCGATGATT   
  
  
+ CCCAATCTGA GTATGCTCGG GGAGGGGGGC TCGACATTGT AGCGAAGAGA CTATCTGGAC TAGCCCAGGC   
  
  
+ TTGCAGCCTA CCCTTTGAGT TCCACGCTGC AGCACTTAGT GGTTCTGAGA TCAGACTTCA AAACCTGGTT   
  
  
+ TTGCGGCCTG GAGAAGCCTT AGCAGTGAAC TTCCCATTCA TGCTGCACCA CATGCCGGAT GAGAGTGTGG   
  
  
+ GCCCTGAGAA TTATAGAGAC CAGTTATTAA GGCTGGTGAA GAGCTTCTTG CCCAAGGTGG TTACCCTTGT   
  
  
+ TGAGCAAGAA TCCAACACAA ACACGGCCCC ATTTCTACCC CGGTTTCTTG AAACCCTAGA CTACTATACT   
  
  
+ GCCATTTTCG AATCAATTGA TGTTACGCTC CCAAGAGATC ACAAGGAGCG GATCAATGTT GAGCAGCACT   
  
  
+ GTTTAGCAAG AGATATAGTC AACATAATAG CATGTGAGGG TGCCGAGAGG GTGGAACGCC ATGAGGTTCT   
  
  
+ TGGAAAGTGG AGATCTCGGT TCTCAATGGC TGGGTTTAAG CCGTACCCAT TGAGCCCGCT AGTGAATGCA   
  
  
+ ACTATCAGGA CTCTTCTGCA GAAGTATAGC AGGAGCTATG GACTTGAAGA AAGGGATGGA GCTCTTTATC   
  
  
+ TAGGCTGGAT GAACCGAGCA CTAGTTGCAT CTTGTGCATG GCAGTG  

- -Up\_Stream \_Len000AAGAAA AGGACAGAGA CAGAGAAGTA ACGTGGTAGT AAATTCTCTA CCCTGGTATT   
  
  
- TAGTAAGTTA GTGAGGAAAT ACAAATTTGA AACCTTCCTC CAATAATAAA TACACCACCA CTAGGGTTAC   
  
  
- GACTTAAATC GACAACTACC GCTCCCGTAT ATACATTAAT CCTAAAGGTT ACGTCAAATC TAAAATGAGT   
  
  
- AAACTCTTAA GTGACTCCAA ACAAACTAGA CTAAAATCGT TTATAACAAG TCCCCATATC TACGAATAAG   
  
  
- CCAAGAGTTG ACATAATCAT ATGTTCTACT CACCAGGTGA ACAAATTCGA AAATGATATA TGAATACAAC   
  
  
- AGGGATTAAA CCAGAGCGAA CTACACATCG ACAGTTCATA ATAATATTAG AACACAAACT ATAGTGTAAA   
  
  
- CATAACTAAT CGATGTTTTC TTGTATAATA TACACATATC GTTGATAGAG TATATACGGG AGTGGTATCG   
  
  
- CACCAAGGTT CCCAAGTTCA AGGTGAATCG GAGTGGGATC TTTACTGAAG AAAAAAAAAA ATTTCAATTT   
  
  
- CTATACGTTA GTGGTACCTT GGTCAGTAGA AAACTGTGCA ATAAGGTCTA GTCTACTGGT CGGAAAGAGG   
  
  
- TTCCGGTAAT CTAAAAGATC GGTTAGGTGA CAATTGACGT TTTTCACCAT AGGAACCGAC AAGGACAGTG   
  
  
- ACAAAGAATA AACGAGACAC TATAAACATG TAGAGAAACG GTTCCTATTT TCAAGTATCG GCCCTTAACC   
  
  
- ATAAGTCAAC TGAGAACGTC ATTTGTCTCA AGTACATGAC CTCCTAAAAC ACTCAGTACA GTGAGTTATT   
  
  
- TTTGTCCACA CGCTGTCACA ATATTTCGAC GAACGAATTA CGTGTAGATG AGAGTGAGTG GAATGGGTTT   
  
  
- TTTGAAGTTT TAACACACGG AAGATACTTA TAAGACTAGG TAATCGTAAG TTGCCAAAGA CTTAAGACAC   
  
  
- ATTCGGTAGG TATGAGTAAA AGTTGTCTCT TGAACCTAAC CTACATAAGT TATGGGTTTT TGGAACAGTT   
  
  
- TAATGGGGAT CGAATTAAAG GAGATTATGG TTGACGAGAA ACCAAAATCG CATGTTAGGG TATCTAATGG   
  
  
- GAAGATATTA ATCACTACTA ACCAAGTAAA GACGACGTAA TACGGAAGAA ATCTAGGTAA CTATTATATA   
  
  
- TGAGAACGGT ACGACGTTGT AAGGTGACCC TAATTATAGA GTGTAACATA GGAGTCGTTG GTTCCTCTGG   
  
  
- GATGATAAAA GTCTCGGTAA GTATCTTTCA ATAACGTATA TCACTACTAG AAGTTACCCA GACTAAAAAT   
  
  
- CTATCGACGA CACTTGTAAA ATGAGCAGGA GACGGTACAC CTATGATACC ATTGGAAACA AACGAGAGTT   
  
  
- CGGACTCGCA AGACTCTTTA CTACTTTCGC GGGAGCTCAA TATCCTGAAA AGACTGAAAT CGAAATCTTT   
  
  
- TCTCTCCTTT TAGTTGGTGT GGGTAGACTA ATAACTCAAA GACGAGGAAC TCTGGACCTA CCTCGGTCTT   
  
  
- GAGTGCCAGA ACAGTCGGTC TTATTAGTCC TTGACGTAAA GACTCCTGGG TACTTTTCTG GGACCATATG   
  
  
- CTGATAAGTA CTTTTGCAAA ACAGTCGGAA AGTCAAAAAC GTAAAATAAG ATCGACGAAA CTAACTTCGG   
  
  
- GTTAAAATAA TTTTGACACG AAAAGAGAAC TTACAATTTG AGAACGTCAA GGAACGAACA ATAAAACCCA   
  
  
- AAATACAAAC GAAAGATGAC TATAGTAGAA AACCAGCACG TTAAATTAAC GGATGATACA ATATACCTAA   
  
  
- AAATACAAAT GAAAGATGAC TATAGTAGAA AACGATAACG TTAAATTAAC GGAAGATACA TCTTAAGCAG   
  
  
- TGAAACTTCA GACGGTCCTT CCGTTATGTC GATATAAAAA GAACACCTAC TTCACTATTT TCCGTTCACA   
  
  
- GTTAACGAAT GACGAAATAA CGCGACATGA AAAGGTCTCT AAAACCTAAT CAGTTACGTC TTCGGTTGTT   
  
  
- AAATATCGCT GACCGGTGTC AAGATGTTGG TTAAACTATT AGGTGCAGAA GAACTTAGAA GTAGATATTA   
  
  
- CCCACCACTA GTTGAAAAGT CAAGAGGTAG ATGACATTCG TATAGAACGT TGTCCTCAGG TCGAAGTGAC   
  
  
- GGACTTGAGC TTAGGAGAAG TAGCCTGAAG GTTTCACTGA GGGTCCTCTG ACACTTACCG AGCGGTCACT   
  
  
- AACTAAGCAC GTACCATGCA CTGTAACCAC TGAATTCTGT GTTTGAATCT CTCGAACTCT GACAATACGA   
  
  
- GCCTGGTTCA AGTCTGTCGA ACCTAAGTAC CATACGGGGA AGTTCTCCAC TCACACTTGG AGACGGTGGT   
  
  
- CTTCTCGGAC TGTTACCCTT TGTAAACAAC CTCTACTATC GTTCCCCCTC GGAGTTTCTC CACGATTAAC   
  
  
- GAACACGGTT TCGTTATAGT CTGCTACTAA ACAATTGTCG CCTCACCAAC TACAGTCTCG ATGCGGTATA   
  
  
- CCAAAGTCAA AGACCTCTTG GTTAAGTCTC CAATCCTCGG ATGTACAACC TTCCCAATCA ACGGGCCAAC   
  
  
- AGGAGAAGTC CTTCAAGGTA GATGTTTCGA GATTCCACGT TTCTCGGATG ATCATCACTT GAAGAAAGGA   
  
  
- TATACGTAAA TGAGATACTT CAAACGGGAA TGAAGTTCAA ACCCATGTAC AGACGTTTAC CCCGTTAACG   
  
  
- ACTCCGGTAC TTTTTACTCT CGTCTTAAGT ATATTAACTA AAAGTCTATC GAGTTCCCTC AGTCACCTAA   
  
  
- TCGGAATAGG TTCGGGACCG ACGAGTCGGA CTACCCGGTG GTGTCCAGGC ATAATGGCCT CAGCTACTAA   
  
  
- GGGTTAGACT CATACGAGCC CCTCCCCCCG AGCTGTAACA TCGCTTCTCT GATAGACCTG ATCGGGTCCG   
  
  
- AACGTCGGAT GGGAAACTCA AGGTGCGACG TCGTGAATCA CCAAGACTCT AGTCTGAAGT TTTGGACCAA   
  
  
- AACGCCGGAC CTCTTCGGAA TCGTCACTTG AAGGGTAAGT ACGACGTGGT GTACGGCCTA CTCTCACACC   
  
  
- CGGGACTCTT AATATCTCTG GTCAATAATT CCGACCACTT CTCGAAGAAC GGGTTCCACC AATGGGAACA   
  
  
- ACTCGTTCTT AGGTTGTGTT TGTGCCGGGG TAAAGATGGG GCCAAAGAAC TTTGGGATCT GATGATATGA   
  
  
- CGGTAAAAGC TTAGTTAACT ACAATGCGAG GGTTCTCTAG TGTTCCTCGC CTAGTTACAA CTCGTCGTGA   
  
  
- CAAATCGTTC TCTATATCAG TTGTATTATC GTACACTCCC ACGGCTCTCC CACCTTGCGG TACTCCAAGA   
  
  
- ACCTTTCACC TCTAGAGCCA AGAGTTACCG ACCCAAATTC GGCATGGGTA ACTCGGGCGA TCACTTACGT   
  
  
- TGATAGTCCT GAGAAGACGT CTTCATATCG TCCTCGATAC CTGAACTTCT TTCCCTACCT CGAGAAATAG   
  
  
- ATCCGACCTA CTTGGCTCGT GATCAACGTA GAACACGTAC CGTCAC

+     G-Box

| Site Name | Organism | Position | Strand | Matrix score. | sequence | function |
| --- | --- | --- | --- | --- | --- | --- |
| G-Box | Triticum aestivum | 1366 | - | 11 | TCCACATGGCA | cis-acting regulatory element involved in light responsiveness |
| G-Box | Pisum sativum | 600 | + | 6 | CACGTT | cis-acting regulatory element involved in light responsiveness |

>HU08G00284.1   
+ -Up\_Stream \_Len000TTCTTT TCCTGTCTCT GTCTCTTCAT TGCACCATCA TTTAAGAGAT GGGACCATAA   
  
  
+ ATCATTCAAT CACTCCTTTA TGTTTAAACT TTGGAAGGAG GTTATTATTT ATGTGGTGGT GATCCCAATG   
  
  
+ CTGAATTTAG CTGTTGATGG CGAGGGCATA TATGTAATTA GGATTTCCAA TGCAGTTTAG ATTTTACTCA   
  
  
+ TTTGAGAATT CACTGAGGTT TGTTTGATCT GATTTTAGCA AATATTGTTC AGGGGTATAG ATGCTTATTC   
  
  
+ GGTTCTCAAC TGTATTAGTA TACAAGATGA GTGGTCCACT TGTTTAAGCT TTTACTATAT ACTTATGTTG   
  
  
+ TCCCTAATTT GGTCTCGCTT GATGTGTAGC TGTCAAGTAT TATTATAATC TTGTGTTTGA TATCACATTT   
  
  
+ GTATTGATTA GCTACAAAAG AACATATTAT ATGTGTATAG CAACTATCTC ATATATGCCC TCACCATAGC   
  
  
+ GTGGTTCCAA GGGTTCAAGT TCCACTTAGC CTCACCCTAG AAATGACTTC TTTTTTTTTT TAAAGTTAAA   
  
  
+ GATATGCAAT CACCATGGAA CCAGTCATCT TTTGACACGT TATTCCAGAT CAGATGACCA GCCTTTCTCC   
  
  
+ AAGGCCATTA GATTTTCTAG CCAATCCACT GTTAACTGCA AAAAGTGGTA TCCTTGGCTG TTCCTGTCAC   
  
  
+ TGTTTCTTAT TTGCTCTGTG ATATTTGTAC ATCTCTTTGC CAAGGATAAA AGTTCATAGC CGGGAATTGG   
  
  
+ TATTCAGTTG ACTCTTGCAG TAAACAGAGT TCATGTACTG GAGGATTTTG TGAGTCATGT CACTCAATAA   
  
  
+ AAACAGGTGT GCGACAGTGT TATAAAGCTG CTTGCTTAAT GCACATCTAC TCTCACTCAC CTTACCCAAA   
  
  
+ AAACTTCAAA ATTGTGTGCC TTCTATGAAT ATTCTGATCC ATTAGCATTC AACGGTTTCT GAATTCTGTG   
  
  
+ TAAGCCATCC ATACTCATTT TCAACAGAGA ACTTGGATTG GATGTATTCA ATACCCAAAA ACCTTGTCAA   
  
  
+ ATTACCCCTA GCTTAATTTC CTCTAATACC AACTGCTCTT TGGTTTTAGC GTACAATCCC ATAGATTACC   
  
  
+ CTTCTATAAT TAGTGATGAT TGGTTCATTT CTGCTGCATT ATGCCTTCTT TAGATCCATT GATAATATAT   
  
  
+ ACTCTTGCCA TGCTGCAACA TTCCACTGGG ATTAATATCT CACATTGTAT CCTCAGCAAC CAAGGAGACC   
  
  
+ CTACTATTTT CAGAGCCATT CATAGAAAGT TATTGCATAT AGTGATGATC TTCAATGGGT CTGATTTTTA   
  
  
+ GATAGCTGCT GTGAACATTT TACTCGTCCT CTGCCATGTG GATACTATGG TAACCTTTGT TTGCTCTCAA   
  
  
+ GCCTGAGCGT TCTGAGAAAT GATGAAAGCG CCCTCGAGTT ATAGGACTTT TCTGACTTTA GCTTTAGAAA   
  
  
+ AGAGAGGAAA ATCAACCACA CCCATCTGAT TATTGAGTTT CTGCTCCTTG AGACCTGGAT GGAGCCAGAA   
  
  
+ CTCACGGTCT TGTCAGCCAG AATAATCAGG AACTGCATTT CTGAGGACCC ATGAAAAGAC CCTGGTATAC   
  
  
+ GACTATTCAT GAAAACGTTT TGTCAGCCTT TCAGTTTTTG CATTTTATTC TAGCTGCTTT GATTGAAGCC   
  
  
+ CAATTTTATT AAAACTGTGC TTTTCTCTTG AATGTTAAAC TCTTGCAGTT CCTTGCTTGT TATTTTGGGT   
  
  
+ TTTATGTTTG CTTTCTACTG ATATCATCTT TTGGTCGTGC AATTTAATTG CCTACTATGT TATATGGATT   
  
  
+ TTTATGTTTA CTTTCTACTG ATATCATCTT TTGCTATTGC AATTTAATTG CCTTCTATGT AGAATTCGTC   
  
  
+ ACTTTGAAGT CTGCCAGGAA GGCAATACAG CTATATTTTT CTTGTGGATG AAGTGATAAA AGGCAAGTGT   
  
  
+ CAATTGCTTA CTGCTTTATT GCGCTGTACT TTTCCAGAGA TTTTGGATTA GTCAATGCAG AAGCCAACAA   
  
  
+ TTTATAGCGA CTGGCCACAG TTCTACAACC AATTTGATAA TCCACGTCTT CTTGAATCTT CATCTATAAT   
  
  
+ GGGTGGTGAT CAACTTTTCA GTTCTCCATC TACTGTAAGC ATATCTTGCA ACAGGAGTCC AGCTTCACTG   
  
  
+ CCTGAACTCG AATCCTCTTC ATCGGACTTC CAAAGTGACT CCCAGGAGAC TGTGAATGGC TCGCCAGTGA   
  
  
+ TTGATTCGTG CATGGTACGT GACATTGGTG ACTTAAGACA CAAACTTAGA GAGCTTGAGA CTGTTATGCT   
  
  
+ CGGACCAAGT TCAGACAGCT TGGATTCATG GTATGCCCCT TCAAGAGGTG AGTGTGAACC TCTGCCACCA   
  
  
+ GAAGAGCCTG ACAATGGGAA ACATTTGTTG GAGATGATAG CAAGGGGGAG CCTCAAAGAG GTGCTAATTG   
  
  
+ CTTGTGCCAA AGCAATATCA GACGATGATT TGTTAACAGC GGAGTGGTTG ATGTCAGAGC TACGCCATAT   
  
  
+ GGTTTCAGTT TCTGGAGAAC CAATTCAGAG GTTAGGAGCC TACATGTTGG AAGGGTTAGT TGCCCGGTTG   
  
  
+ TCCTCTTCAG GAAGTTCCAT CTACAAAGCT CTAAGGTGCA AAGAGCCTAC TAGTAGTGAA CTTCTTTCCT   
  
  
+ ATATGCATTT ACTCTATGAA GTTTGCCCTT ACTTCAAGTT TGGGTACATG TCTGCAAATG GGGCAATTGC   
  
  
+ TGAGGCCATG AAAAATGAGA GCAGAATTCA TATAATTGAT TTTCAGATAG CTCAAGGGAG TCAGTGGATT   
  
  
+ AGCCTTATCC AAGCCCTGGC TGCTCAGCCT GATGGGCCAC CACAGGTCCG TATTACCGGA GTCGATGATT   
  
  
+ CCCAATCTGA GTATGCTCGG GGAGGGGGGC TCGACATTGT AGCGAAGAGA CTATCTGGAC TAGCCCAGGC   
  
  
+ TTGCAGCCTA CCCTTTGAGT TCCACGCTGC AGCACTTAGT GGTTCTGAGA TCAGACTTCA AAACCTGGTT   
  
  
+ TTGCGGCCTG GAGAAGCCTT AGCAGTGAAC TTCCCATTCA TGCTGCACCA CATGCCGGAT GAGAGTGTGG   
  
  
+ GCCCTGAGAA TTATAGAGAC CAGTTATTAA GGCTGGTGAA GAGCTTCTTG CCCAAGGTGG TTACCCTTGT   
  
  
+ TGAGCAAGAA TCCAACACAA ACACGGCCCC ATTTCTACCC CGGTTTCTTG AAACCCTAGA CTACTATACT   
  
  
+ GCCATTTTCG AATCAATTGA TGTTACGCTC CCAAGAGATC ACAAGGAGCG GATCAATGTT GAGCAGCACT   
  
  
+ GTTTAGCAAG AGATATAGTC AACATAATAG CATGTGAGGG TGCCGAGAGG GTGGAACGCC ATGAGGTTCT   
  
  
+ TGGAAAGTGG AGATCTCGGT TCTCAATGGC TGGGTTTAAG CCGTACCCAT TGAGCCCGCT AGTGAATGCA   
  
  
+ ACTATCAGGA CTCTTCTGCA GAAGTATAGC AGGAGCTATG GACTTGAAGA AAGGGATGGA GCTCTTTATC   
  
  
+ TAGGCTGGAT GAACCGAGCA CTAGTTGCAT CTTGTGCATG GCAGTG  

- -Up\_Stream \_Len000AAGAAA AGGACAGAGA CAGAGAAGTA ACGTGGTAGT AAATTCTCTA CCCTGGTATT   
  
  
- TAGTAAGTTA GTGAGGAAAT ACAAATTTGA AACCTTCCTC CAATAATAAA TACACCACCA CTAGGGTTAC   
  
  
- GACTTAAATC GACAACTACC GCTCCCGTAT ATACATTAAT CCTAAAGGTT ACGTCAAATC TAAAATGAGT   
  
  
- AAACTCTTAA GTGACTCCAA ACAAACTAGA CTAAAATCGT TTATAACAAG TCCCCATATC TACGAATAAG   
  
  
- CCAAGAGTTG ACATAATCAT ATGTTCTACT CACCAGGTGA ACAAATTCGA AAATGATATA TGAATACAAC   
  
  
- AGGGATTAAA CCAGAGCGAA CTACACATCG ACAGTTCATA ATAATATTAG AACACAAACT ATAGTGTAAA   
  
  
- CATAACTAAT CGATGTTTTC TTGTATAATA TACACATATC GTTGATAGAG TATATACGGG AGTGGTATCG   
  
  
- CACCAAGGTT CCCAAGTTCA AGGTGAATCG GAGTGGGATC TTTACTGAAG AAAAAAAAAA ATTTCAATTT   
  
  
- CTATACGTTA GTGGTACCTT GGTCAGTAGA AAACTGTGCA ATAAGGTCTA GTCTACTGGT CGGAAAGAGG   
  
  
- TTCCGGTAAT CTAAAAGATC GGTTAGGTGA CAATTGACGT TTTTCACCAT AGGAACCGAC AAGGACAGTG   
  
  
- ACAAAGAATA AACGAGACAC TATAAACATG TAGAGAAACG GTTCCTATTT TCAAGTATCG GCCCTTAACC   
  
  
- ATAAGTCAAC TGAGAACGTC ATTTGTCTCA AGTACATGAC CTCCTAAAAC ACTCAGTACA GTGAGTTATT   
  
  
- TTTGTCCACA CGCTGTCACA ATATTTCGAC GAACGAATTA CGTGTAGATG AGAGTGAGTG GAATGGGTTT   
  
  
- TTTGAAGTTT TAACACACGG AAGATACTTA TAAGACTAGG TAATCGTAAG TTGCCAAAGA CTTAAGACAC   
  
  
- ATTCGGTAGG TATGAGTAAA AGTTGTCTCT TGAACCTAAC CTACATAAGT TATGGGTTTT TGGAACAGTT   
  
  
- TAATGGGGAT CGAATTAAAG GAGATTATGG TTGACGAGAA ACCAAAATCG CATGTTAGGG TATCTAATGG   
  
  
- GAAGATATTA ATCACTACTA ACCAAGTAAA GACGACGTAA TACGGAAGAA ATCTAGGTAA CTATTATATA   
  
  
- TGAGAACGGT ACGACGTTGT AAGGTGACCC TAATTATAGA GTGTAACATA GGAGTCGTTG GTTCCTCTGG   
  
  
- GATGATAAAA GTCTCGGTAA GTATCTTTCA ATAACGTATA TCACTACTAG AAGTTACCCA GACTAAAAAT   
  
  
- CTATCGACGA CACTTGTAAA ATGAGCAGGA GACGGTACAC CTATGATACC ATTGGAAACA AACGAGAGTT   
  
  
- CGGACTCGCA AGACTCTTTA CTACTTTCGC GGGAGCTCAA TATCCTGAAA AGACTGAAAT CGAAATCTTT   
  
  
- TCTCTCCTTT TAGTTGGTGT GGGTAGACTA ATAACTCAAA GACGAGGAAC TCTGGACCTA CCTCGGTCTT   
  
  
- GAGTGCCAGA ACAGTCGGTC TTATTAGTCC TTGACGTAAA GACTCCTGGG TACTTTTCTG GGACCATATG   
  
  
- CTGATAAGTA CTTTTGCAAA ACAGTCGGAA AGTCAAAAAC GTAAAATAAG ATCGACGAAA CTAACTTCGG   
  
  
- GTTAAAATAA TTTTGACACG AAAAGAGAAC TTACAATTTG AGAACGTCAA GGAACGAACA ATAAAACCCA   
  
  
- AAATACAAAC GAAAGATGAC TATAGTAGAA AACCAGCACG TTAAATTAAC GGATGATACA ATATACCTAA   
  
  
- AAATACAAAT GAAAGATGAC TATAGTAGAA AACGATAACG TTAAATTAAC GGAAGATACA TCTTAAGCAG   
  
  
- TGAAACTTCA GACGGTCCTT CCGTTATGTC GATATAAAAA GAACACCTAC TTCACTATTT TCCGTTCACA   
  
  
- GTTAACGAAT GACGAAATAA CGCGACATGA AAAGGTCTCT AAAACCTAAT CAGTTACGTC TTCGGTTGTT   
  
  
- AAATATCGCT GACCGGTGTC AAGATGTTGG TTAAACTATT AGGTGCAGAA GAACTTAGAA GTAGATATTA   
  
  
- CCCACCACTA GTTGAAAAGT CAAGAGGTAG ATGACATTCG TATAGAACGT TGTCCTCAGG TCGAAGTGAC   
  
  
- GGACTTGAGC TTAGGAGAAG TAGCCTGAAG GTTTCACTGA GGGTCCTCTG ACACTTACCG AGCGGTCACT   
  
  
- AACTAAGCAC GTACCATGCA CTGTAACCAC TGAATTCTGT GTTTGAATCT CTCGAACTCT GACAATACGA   
  
  
- GCCTGGTTCA AGTCTGTCGA ACCTAAGTAC CATACGGGGA AGTTCTCCAC TCACACTTGG AGACGGTGGT   
  
  
- CTTCTCGGAC TGTTACCCTT TGTAAACAAC CTCTACTATC GTTCCCCCTC GGAGTTTCTC CACGATTAAC   
  
  
- GAACACGGTT TCGTTATAGT CTGCTACTAA ACAATTGTCG CCTCACCAAC TACAGTCTCG ATGCGGTATA   
  
  
- CCAAAGTCAA AGACCTCTTG GTTAAGTCTC CAATCCTCGG ATGTACAACC TTCCCAATCA ACGGGCCAAC   
  
  
- AGGAGAAGTC CTTCAAGGTA GATGTTTCGA GATTCCACGT TTCTCGGATG ATCATCACTT GAAGAAAGGA   
  
  
- TATACGTAAA TGAGATACTT CAAACGGGAA TGAAGTTCAA ACCCATGTAC AGACGTTTAC CCCGTTAACG   
  
  
- ACTCCGGTAC TTTTTACTCT CGTCTTAAGT ATATTAACTA AAAGTCTATC GAGTTCCCTC AGTCACCTAA   
  
  
- TCGGAATAGG TTCGGGACCG ACGAGTCGGA CTACCCGGTG GTGTCCAGGC ATAATGGCCT CAGCTACTAA   
  
  
- GGGTTAGACT CATACGAGCC CCTCCCCCCG AGCTGTAACA TCGCTTCTCT GATAGACCTG ATCGGGTCCG   
  
  
- AACGTCGGAT GGGAAACTCA AGGTGCGACG TCGTGAATCA CCAAGACTCT AGTCTGAAGT TTTGGACCAA   
  
  
- AACGCCGGAC CTCTTCGGAA TCGTCACTTG AAGGGTAAGT ACGACGTGGT GTACGGCCTA CTCTCACACC   
  
  
- CGGGACTCTT AATATCTCTG GTCAATAATT CCGACCACTT CTCGAAGAAC GGGTTCCACC AATGGGAACA   
  
  
- ACTCGTTCTT AGGTTGTGTT TGTGCCGGGG TAAAGATGGG GCCAAAGAAC TTTGGGATCT GATGATATGA   
  
  
- CGGTAAAAGC TTAGTTAACT ACAATGCGAG GGTTCTCTAG TGTTCCTCGC CTAGTTACAA CTCGTCGTGA   
  
  
- CAAATCGTTC TCTATATCAG TTGTATTATC GTACACTCCC ACGGCTCTCC CACCTTGCGG TACTCCAAGA   
  
  
- ACCTTTCACC TCTAGAGCCA AGAGTTACCG ACCCAAATTC GGCATGGGTA ACTCGGGCGA TCACTTACGT   
  
  
- TGATAGTCCT GAGAAGACGT CTTCATATCG TCCTCGATAC CTGAACTTCT TTCCCTACCT CGAGAAATAG   
  
  
- ATCCGACCTA CTTGGCTCGT GATCAACGTA GAACACGTAC CGTCAC

+     G-box

| Site Name | Organism | Position | Strand | Matrix score. | sequence | function |
| --- | --- | --- | --- | --- | --- | --- |
| G-box | Zea mays | 2077 | + | 6 | CACGTC | cis-acting regulatory element involved in light responsiveness |
| G-box | Arabidopsis thaliana | 1367 | + | 9 | GCCACGTGGA | cis-acting regulatory element involved in light responsiveness |
| G-box | Arabidopsis thaliana | 2260 | + | 6 | TACGTG | cis-acting regulatory element involved in light responsiveness |
| G-box | Zea mays | 1788 | - | 6 | CACGAC | cis-acting regulatory element involved in light responsiveness |

>HU08G00284.1   
+ -Up\_Stream \_Len000TTCTTT TCCTGTCTCT GTCTCTTCAT TGCACCATCA TTTAAGAGAT GGGACCATAA   
  
  
+ ATCATTCAAT CACTCCTTTA TGTTTAAACT TTGGAAGGAG GTTATTATTT ATGTGGTGGT GATCCCAATG   
  
  
+ CTGAATTTAG CTGTTGATGG CGAGGGCATA TATGTAATTA GGATTTCCAA TGCAGTTTAG ATTTTACTCA   
  
  
+ TTTGAGAATT CACTGAGGTT TGTTTGATCT GATTTTAGCA AATATTGTTC AGGGGTATAG ATGCTTATTC   
  
  
+ GGTTCTCAAC TGTATTAGTA TACAAGATGA GTGGTCCACT TGTTTAAGCT TTTACTATAT ACTTATGTTG   
  
  
+ TCCCTAATTT GGTCTCGCTT GATGTGTAGC TGTCAAGTAT TATTATAATC TTGTGTTTGA TATCACATTT   
  
  
+ GTATTGATTA GCTACAAAAG AACATATTAT ATGTGTATAG CAACTATCTC ATATATGCCC TCACCATAGC   
  
  
+ GTGGTTCCAA GGGTTCAAGT TCCACTTAGC CTCACCCTAG AAATGACTTC TTTTTTTTTT TAAAGTTAAA   
  
  
+ GATATGCAAT CACCATGGAA CCAGTCATCT TTTGACACGT TATTCCAGAT CAGATGACCA GCCTTTCTCC   
  
  
+ AAGGCCATTA GATTTTCTAG CCAATCCACT GTTAACTGCA AAAAGTGGTA TCCTTGGCTG TTCCTGTCAC   
  
  
+ TGTTTCTTAT TTGCTCTGTG ATATTTGTAC ATCTCTTTGC CAAGGATAAA AGTTCATAGC CGGGAATTGG   
  
  
+ TATTCAGTTG ACTCTTGCAG TAAACAGAGT TCATGTACTG GAGGATTTTG TGAGTCATGT CACTCAATAA   
  
  
+ AAACAGGTGT GCGACAGTGT TATAAAGCTG CTTGCTTAAT GCACATCTAC TCTCACTCAC CTTACCCAAA   
  
  
+ AAACTTCAAA ATTGTGTGCC TTCTATGAAT ATTCTGATCC ATTAGCATTC AACGGTTTCT GAATTCTGTG   
  
  
+ TAAGCCATCC ATACTCATTT TCAACAGAGA ACTTGGATTG GATGTATTCA ATACCCAAAA ACCTTGTCAA   
  
  
+ ATTACCCCTA GCTTAATTTC CTCTAATACC AACTGCTCTT TGGTTTTAGC GTACAATCCC ATAGATTACC   
  
  
+ CTTCTATAAT TAGTGATGAT TGGTTCATTT CTGCTGCATT ATGCCTTCTT TAGATCCATT GATAATATAT   
  
  
+ ACTCTTGCCA TGCTGCAACA TTCCACTGGG ATTAATATCT CACATTGTAT CCTCAGCAAC CAAGGAGACC   
  
  
+ CTACTATTTT CAGAGCCATT CATAGAAAGT TATTGCATAT AGTGATGATC TTCAATGGGT CTGATTTTTA   
  
  
+ GATAGCTGCT GTGAACATTT TACTCGTCCT CTGCCATGTG GATACTATGG TAACCTTTGT TTGCTCTCAA   
  
  
+ GCCTGAGCGT TCTGAGAAAT GATGAAAGCG CCCTCGAGTT ATAGGACTTT TCTGACTTTA GCTTTAGAAA   
  
  
+ AGAGAGGAAA ATCAACCACA CCCATCTGAT TATTGAGTTT CTGCTCCTTG AGACCTGGAT GGAGCCAGAA   
  
  
+ CTCACGGTCT TGTCAGCCAG AATAATCAGG AACTGCATTT CTGAGGACCC ATGAAAAGAC CCTGGTATAC   
  
  
+ GACTATTCAT GAAAACGTTT TGTCAGCCTT TCAGTTTTTG CATTTTATTC TAGCTGCTTT GATTGAAGCC   
  
  
+ CAATTTTATT AAAACTGTGC TTTTCTCTTG AATGTTAAAC TCTTGCAGTT CCTTGCTTGT TATTTTGGGT   
  
  
+ TTTATGTTTG CTTTCTACTG ATATCATCTT TTGGTCGTGC AATTTAATTG CCTACTATGT TATATGGATT   
  
  
+ TTTATGTTTA CTTTCTACTG ATATCATCTT TTGCTATTGC AATTTAATTG CCTTCTATGT AGAATTCGTC   
  
  
+ ACTTTGAAGT CTGCCAGGAA GGCAATACAG CTATATTTTT CTTGTGGATG AAGTGATAAA AGGCAAGTGT   
  
  
+ CAATTGCTTA CTGCTTTATT GCGCTGTACT TTTCCAGAGA TTTTGGATTA GTCAATGCAG AAGCCAACAA   
  
  
+ TTTATAGCGA CTGGCCACAG TTCTACAACC AATTTGATAA TCCACGTCTT CTTGAATCTT CATCTATAAT   
  
  
+ GGGTGGTGAT CAACTTTTCA GTTCTCCATC TACTGTAAGC ATATCTTGCA ACAGGAGTCC AGCTTCACTG   
  
  
+ CCTGAACTCG AATCCTCTTC ATCGGACTTC CAAAGTGACT CCCAGGAGAC TGTGAATGGC TCGCCAGTGA   
  
  
+ TTGATTCGTG CATGGTACGT GACATTGGTG ACTTAAGACA CAAACTTAGA GAGCTTGAGA CTGTTATGCT   
  
  
+ CGGACCAAGT TCAGACAGCT TGGATTCATG GTATGCCCCT TCAAGAGGTG AGTGTGAACC TCTGCCACCA   
  
  
+ GAAGAGCCTG ACAATGGGAA ACATTTGTTG GAGATGATAG CAAGGGGGAG CCTCAAAGAG GTGCTAATTG   
  
  
+ CTTGTGCCAA AGCAATATCA GACGATGATT TGTTAACAGC GGAGTGGTTG ATGTCAGAGC TACGCCATAT   
  
  
+ GGTTTCAGTT TCTGGAGAAC CAATTCAGAG GTTAGGAGCC TACATGTTGG AAGGGTTAGT TGCCCGGTTG   
  
  
+ TCCTCTTCAG GAAGTTCCAT CTACAAAGCT CTAAGGTGCA AAGAGCCTAC TAGTAGTGAA CTTCTTTCCT   
  
  
+ ATATGCATTT ACTCTATGAA GTTTGCCCTT ACTTCAAGTT TGGGTACATG TCTGCAAATG GGGCAATTGC   
  
  
+ TGAGGCCATG AAAAATGAGA GCAGAATTCA TATAATTGAT TTTCAGATAG CTCAAGGGAG TCAGTGGATT   
  
  
+ AGCCTTATCC AAGCCCTGGC TGCTCAGCCT GATGGGCCAC CACAGGTCCG TATTACCGGA GTCGATGATT   
  
  
+ CCCAATCTGA GTATGCTCGG GGAGGGGGGC TCGACATTGT AGCGAAGAGA CTATCTGGAC TAGCCCAGGC   
  
  
+ TTGCAGCCTA CCCTTTGAGT TCCACGCTGC AGCACTTAGT GGTTCTGAGA TCAGACTTCA AAACCTGGTT   
  
  
+ TTGCGGCCTG GAGAAGCCTT AGCAGTGAAC TTCCCATTCA TGCTGCACCA CATGCCGGAT GAGAGTGTGG   
  
  
+ GCCCTGAGAA TTATAGAGAC CAGTTATTAA GGCTGGTGAA GAGCTTCTTG CCCAAGGTGG TTACCCTTGT   
  
  
+ TGAGCAAGAA TCCAACACAA ACACGGCCCC ATTTCTACCC CGGTTTCTTG AAACCCTAGA CTACTATACT   
  
  
+ GCCATTTTCG AATCAATTGA TGTTACGCTC CCAAGAGATC ACAAGGAGCG GATCAATGTT GAGCAGCACT   
  
  
+ GTTTAGCAAG AGATATAGTC AACATAATAG CATGTGAGGG TGCCGAGAGG GTGGAACGCC ATGAGGTTCT   
  
  
+ TGGAAAGTGG AGATCTCGGT TCTCAATGGC TGGGTTTAAG CCGTACCCAT TGAGCCCGCT AGTGAATGCA   
  
  
+ ACTATCAGGA CTCTTCTGCA GAAGTATAGC AGGAGCTATG GACTTGAAGA AAGGGATGGA GCTCTTTATC   
  
  
+ TAGGCTGGAT GAACCGAGCA CTAGTTGCAT CTTGTGCATG GCAGTG  

- -Up\_Stream \_Len000AAGAAA AGGACAGAGA CAGAGAAGTA ACGTGGTAGT AAATTCTCTA CCCTGGTATT   
  
  
- TAGTAAGTTA GTGAGGAAAT ACAAATTTGA AACCTTCCTC CAATAATAAA TACACCACCA CTAGGGTTAC   
  
  
- GACTTAAATC GACAACTACC GCTCCCGTAT ATACATTAAT CCTAAAGGTT ACGTCAAATC TAAAATGAGT   
  
  
- AAACTCTTAA GTGACTCCAA ACAAACTAGA CTAAAATCGT TTATAACAAG TCCCCATATC TACGAATAAG   
  
  
- CCAAGAGTTG ACATAATCAT ATGTTCTACT CACCAGGTGA ACAAATTCGA AAATGATATA TGAATACAAC   
  
  
- AGGGATTAAA CCAGAGCGAA CTACACATCG ACAGTTCATA ATAATATTAG AACACAAACT ATAGTGTAAA   
  
  
- CATAACTAAT CGATGTTTTC TTGTATAATA TACACATATC GTTGATAGAG TATATACGGG AGTGGTATCG   
  
  
- CACCAAGGTT CCCAAGTTCA AGGTGAATCG GAGTGGGATC TTTACTGAAG AAAAAAAAAA ATTTCAATTT   
  
  
- CTATACGTTA GTGGTACCTT GGTCAGTAGA AAACTGTGCA ATAAGGTCTA GTCTACTGGT CGGAAAGAGG   
  
  
- TTCCGGTAAT CTAAAAGATC GGTTAGGTGA CAATTGACGT TTTTCACCAT AGGAACCGAC AAGGACAGTG   
  
  
- ACAAAGAATA AACGAGACAC TATAAACATG TAGAGAAACG GTTCCTATTT TCAAGTATCG GCCCTTAACC   
  
  
- ATAAGTCAAC TGAGAACGTC ATTTGTCTCA AGTACATGAC CTCCTAAAAC ACTCAGTACA GTGAGTTATT   
  
  
- TTTGTCCACA CGCTGTCACA ATATTTCGAC GAACGAATTA CGTGTAGATG AGAGTGAGTG GAATGGGTTT   
  
  
- TTTGAAGTTT TAACACACGG AAGATACTTA TAAGACTAGG TAATCGTAAG TTGCCAAAGA CTTAAGACAC   
  
  
- ATTCGGTAGG TATGAGTAAA AGTTGTCTCT TGAACCTAAC CTACATAAGT TATGGGTTTT TGGAACAGTT   
  
  
- TAATGGGGAT CGAATTAAAG GAGATTATGG TTGACGAGAA ACCAAAATCG CATGTTAGGG TATCTAATGG   
  
  
- GAAGATATTA ATCACTACTA ACCAAGTAAA GACGACGTAA TACGGAAGAA ATCTAGGTAA CTATTATATA   
  
  
- TGAGAACGGT ACGACGTTGT AAGGTGACCC TAATTATAGA GTGTAACATA GGAGTCGTTG GTTCCTCTGG   
  
  
- GATGATAAAA GTCTCGGTAA GTATCTTTCA ATAACGTATA TCACTACTAG AAGTTACCCA GACTAAAAAT   
  
  
- CTATCGACGA CACTTGTAAA ATGAGCAGGA GACGGTACAC CTATGATACC ATTGGAAACA AACGAGAGTT   
  
  
- CGGACTCGCA AGACTCTTTA CTACTTTCGC GGGAGCTCAA TATCCTGAAA AGACTGAAAT CGAAATCTTT   
  
  
- TCTCTCCTTT TAGTTGGTGT GGGTAGACTA ATAACTCAAA GACGAGGAAC TCTGGACCTA CCTCGGTCTT   
  
  
- GAGTGCCAGA ACAGTCGGTC TTATTAGTCC TTGACGTAAA GACTCCTGGG TACTTTTCTG GGACCATATG   
  
  
- CTGATAAGTA CTTTTGCAAA ACAGTCGGAA AGTCAAAAAC GTAAAATAAG ATCGACGAAA CTAACTTCGG   
  
  
- GTTAAAATAA TTTTGACACG AAAAGAGAAC TTACAATTTG AGAACGTCAA GGAACGAACA ATAAAACCCA   
  
  
- AAATACAAAC GAAAGATGAC TATAGTAGAA AACCAGCACG TTAAATTAAC GGATGATACA ATATACCTAA   
  
  
- AAATACAAAT GAAAGATGAC TATAGTAGAA AACGATAACG TTAAATTAAC GGAAGATACA TCTTAAGCAG   
  
  
- TGAAACTTCA GACGGTCCTT CCGTTATGTC GATATAAAAA GAACACCTAC TTCACTATTT TCCGTTCACA   
  
  
- GTTAACGAAT GACGAAATAA CGCGACATGA AAAGGTCTCT AAAACCTAAT CAGTTACGTC TTCGGTTGTT   
  
  
- AAATATCGCT GACCGGTGTC AAGATGTTGG TTAAACTATT AGGTGCAGAA GAACTTAGAA GTAGATATTA   
  
  
- CCCACCACTA GTTGAAAAGT CAAGAGGTAG ATGACATTCG TATAGAACGT TGTCCTCAGG TCGAAGTGAC   
  
  
- GGACTTGAGC TTAGGAGAAG TAGCCTGAAG GTTTCACTGA GGGTCCTCTG ACACTTACCG AGCGGTCACT   
  
  
- AACTAAGCAC GTACCATGCA CTGTAACCAC TGAATTCTGT GTTTGAATCT CTCGAACTCT GACAATACGA   
  
  
- GCCTGGTTCA AGTCTGTCGA ACCTAAGTAC CATACGGGGA AGTTCTCCAC TCACACTTGG AGACGGTGGT   
  
  
- CTTCTCGGAC TGTTACCCTT TGTAAACAAC CTCTACTATC GTTCCCCCTC GGAGTTTCTC CACGATTAAC   
  
  
- GAACACGGTT TCGTTATAGT CTGCTACTAA ACAATTGTCG CCTCACCAAC TACAGTCTCG ATGCGGTATA   
  
  
- CCAAAGTCAA AGACCTCTTG GTTAAGTCTC CAATCCTCGG ATGTACAACC TTCCCAATCA ACGGGCCAAC   
  
  
- AGGAGAAGTC CTTCAAGGTA GATGTTTCGA GATTCCACGT TTCTCGGATG ATCATCACTT GAAGAAAGGA   
  
  
- TATACGTAAA TGAGATACTT CAAACGGGAA TGAAGTTCAA ACCCATGTAC AGACGTTTAC CCCGTTAACG   
  
  
- ACTCCGGTAC TTTTTACTCT CGTCTTAAGT ATATTAACTA AAAGTCTATC GAGTTCCCTC AGTCACCTAA   
  
  
- TCGGAATAGG TTCGGGACCG ACGAGTCGGA CTACCCGGTG GTGTCCAGGC ATAATGGCCT CAGCTACTAA   
  
  
- GGGTTAGACT CATACGAGCC CCTCCCCCCG AGCTGTAACA TCGCTTCTCT GATAGACCTG ATCGGGTCCG   
  
  
- AACGTCGGAT GGGAAACTCA AGGTGCGACG TCGTGAATCA CCAAGACTCT AGTCTGAAGT TTTGGACCAA   
  
  
- AACGCCGGAC CTCTTCGGAA TCGTCACTTG AAGGGTAAGT ACGACGTGGT GTACGGCCTA CTCTCACACC   
  
  
- CGGGACTCTT AATATCTCTG GTCAATAATT CCGACCACTT CTCGAAGAAC GGGTTCCACC AATGGGAACA   
  
  
- ACTCGTTCTT AGGTTGTGTT TGTGCCGGGG TAAAGATGGG GCCAAAGAAC TTTGGGATCT GATGATATGA   
  
  
- CGGTAAAAGC TTAGTTAACT ACAATGCGAG GGTTCTCTAG TGTTCCTCGC CTAGTTACAA CTCGTCGTGA   
  
  
- CAAATCGTTC TCTATATCAG TTGTATTATC GTACACTCCC ACGGCTCTCC CACCTTGCGG TACTCCAAGA   
  
  
- ACCTTTCACC TCTAGAGCCA AGAGTTACCG ACCCAAATTC GGCATGGGTA ACTCGGGCGA TCACTTACGT   
  
  
- TGATAGTCCT GAGAAGACGT CTTCATATCG TCCTCGATAC CTGAACTTCT TTCCCTACCT CGAGAAATAG   
  
  
- ATCCGACCTA CTTGGCTCGT GATCAACGTA GAACACGTAC CGTCAC

+     GARE-motif

| Site Name | Organism | Position | Strand | Matrix score. | sequence | function |
| --- | --- | --- | --- | --- | --- | --- |
| GARE-motif | Brassica oleracea | 1006 | - | 7 | TCTGTTG | gibberellin-responsive element |

>HU08G00284.1   
+ -Up\_Stream \_Len000TTCTTT TCCTGTCTCT GTCTCTTCAT TGCACCATCA TTTAAGAGAT GGGACCATAA   
  
  
+ ATCATTCAAT CACTCCTTTA TGTTTAAACT TTGGAAGGAG GTTATTATTT ATGTGGTGGT GATCCCAATG   
  
  
+ CTGAATTTAG CTGTTGATGG CGAGGGCATA TATGTAATTA GGATTTCCAA TGCAGTTTAG ATTTTACTCA   
  
  
+ TTTGAGAATT CACTGAGGTT TGTTTGATCT GATTTTAGCA AATATTGTTC AGGGGTATAG ATGCTTATTC   
  
  
+ GGTTCTCAAC TGTATTAGTA TACAAGATGA GTGGTCCACT TGTTTAAGCT TTTACTATAT ACTTATGTTG   
  
  
+ TCCCTAATTT GGTCTCGCTT GATGTGTAGC TGTCAAGTAT TATTATAATC TTGTGTTTGA TATCACATTT   
  
  
+ GTATTGATTA GCTACAAAAG AACATATTAT ATGTGTATAG CAACTATCTC ATATATGCCC TCACCATAGC   
  
  
+ GTGGTTCCAA GGGTTCAAGT TCCACTTAGC CTCACCCTAG AAATGACTTC TTTTTTTTTT TAAAGTTAAA   
  
  
+ GATATGCAAT CACCATGGAA CCAGTCATCT TTTGACACGT TATTCCAGAT CAGATGACCA GCCTTTCTCC   
  
  
+ AAGGCCATTA GATTTTCTAG CCAATCCACT GTTAACTGCA AAAAGTGGTA TCCTTGGCTG TTCCTGTCAC   
  
  
+ TGTTTCTTAT TTGCTCTGTG ATATTTGTAC ATCTCTTTGC CAAGGATAAA AGTTCATAGC CGGGAATTGG   
  
  
+ TATTCAGTTG ACTCTTGCAG TAAACAGAGT TCATGTACTG GAGGATTTTG TGAGTCATGT CACTCAATAA   
  
  
+ AAACAGGTGT GCGACAGTGT TATAAAGCTG CTTGCTTAAT GCACATCTAC TCTCACTCAC CTTACCCAAA   
  
  
+ AAACTTCAAA ATTGTGTGCC TTCTATGAAT ATTCTGATCC ATTAGCATTC AACGGTTTCT GAATTCTGTG   
  
  
+ TAAGCCATCC ATACTCATTT TCAACAGAGA ACTTGGATTG GATGTATTCA ATACCCAAAA ACCTTGTCAA   
  
  
+ ATTACCCCTA GCTTAATTTC CTCTAATACC AACTGCTCTT TGGTTTTAGC GTACAATCCC ATAGATTACC   
  
  
+ CTTCTATAAT TAGTGATGAT TGGTTCATTT CTGCTGCATT ATGCCTTCTT TAGATCCATT GATAATATAT   
  
  
+ ACTCTTGCCA TGCTGCAACA TTCCACTGGG ATTAATATCT CACATTGTAT CCTCAGCAAC CAAGGAGACC   
  
  
+ CTACTATTTT CAGAGCCATT CATAGAAAGT TATTGCATAT AGTGATGATC TTCAATGGGT CTGATTTTTA   
  
  
+ GATAGCTGCT GTGAACATTT TACTCGTCCT CTGCCATGTG GATACTATGG TAACCTTTGT TTGCTCTCAA   
  
  
+ GCCTGAGCGT TCTGAGAAAT GATGAAAGCG CCCTCGAGTT ATAGGACTTT TCTGACTTTA GCTTTAGAAA   
  
  
+ AGAGAGGAAA ATCAACCACA CCCATCTGAT TATTGAGTTT CTGCTCCTTG AGACCTGGAT GGAGCCAGAA   
  
  
+ CTCACGGTCT TGTCAGCCAG AATAATCAGG AACTGCATTT CTGAGGACCC ATGAAAAGAC CCTGGTATAC   
  
  
+ GACTATTCAT GAAAACGTTT TGTCAGCCTT TCAGTTTTTG CATTTTATTC TAGCTGCTTT GATTGAAGCC   
  
  
+ CAATTTTATT AAAACTGTGC TTTTCTCTTG AATGTTAAAC TCTTGCAGTT CCTTGCTTGT TATTTTGGGT   
  
  
+ TTTATGTTTG CTTTCTACTG ATATCATCTT TTGGTCGTGC AATTTAATTG CCTACTATGT TATATGGATT   
  
  
+ TTTATGTTTA CTTTCTACTG ATATCATCTT TTGCTATTGC AATTTAATTG CCTTCTATGT AGAATTCGTC   
  
  
+ ACTTTGAAGT CTGCCAGGAA GGCAATACAG CTATATTTTT CTTGTGGATG AAGTGATAAA AGGCAAGTGT   
  
  
+ CAATTGCTTA CTGCTTTATT GCGCTGTACT TTTCCAGAGA TTTTGGATTA GTCAATGCAG AAGCCAACAA   
  
  
+ TTTATAGCGA CTGGCCACAG TTCTACAACC AATTTGATAA TCCACGTCTT CTTGAATCTT CATCTATAAT   
  
  
+ GGGTGGTGAT CAACTTTTCA GTTCTCCATC TACTGTAAGC ATATCTTGCA ACAGGAGTCC AGCTTCACTG   
  
  
+ CCTGAACTCG AATCCTCTTC ATCGGACTTC CAAAGTGACT CCCAGGAGAC TGTGAATGGC TCGCCAGTGA   
  
  
+ TTGATTCGTG CATGGTACGT GACATTGGTG ACTTAAGACA CAAACTTAGA GAGCTTGAGA CTGTTATGCT   
  
  
+ CGGACCAAGT TCAGACAGCT TGGATTCATG GTATGCCCCT TCAAGAGGTG AGTGTGAACC TCTGCCACCA   
  
  
+ GAAGAGCCTG ACAATGGGAA ACATTTGTTG GAGATGATAG CAAGGGGGAG CCTCAAAGAG GTGCTAATTG   
  
  
+ CTTGTGCCAA AGCAATATCA GACGATGATT TGTTAACAGC GGAGTGGTTG ATGTCAGAGC TACGCCATAT   
  
  
+ GGTTTCAGTT TCTGGAGAAC CAATTCAGAG GTTAGGAGCC TACATGTTGG AAGGGTTAGT TGCCCGGTTG   
  
  
+ TCCTCTTCAG GAAGTTCCAT CTACAAAGCT CTAAGGTGCA AAGAGCCTAC TAGTAGTGAA CTTCTTTCCT   
  
  
+ ATATGCATTT ACTCTATGAA GTTTGCCCTT ACTTCAAGTT TGGGTACATG TCTGCAAATG GGGCAATTGC   
  
  
+ TGAGGCCATG AAAAATGAGA GCAGAATTCA TATAATTGAT TTTCAGATAG CTCAAGGGAG TCAGTGGATT   
  
  
+ AGCCTTATCC AAGCCCTGGC TGCTCAGCCT GATGGGCCAC CACAGGTCCG TATTACCGGA GTCGATGATT   
  
  
+ CCCAATCTGA GTATGCTCGG GGAGGGGGGC TCGACATTGT AGCGAAGAGA CTATCTGGAC TAGCCCAGGC   
  
  
+ TTGCAGCCTA CCCTTTGAGT TCCACGCTGC AGCACTTAGT GGTTCTGAGA TCAGACTTCA AAACCTGGTT   
  
  
+ TTGCGGCCTG GAGAAGCCTT AGCAGTGAAC TTCCCATTCA TGCTGCACCA CATGCCGGAT GAGAGTGTGG   
  
  
+ GCCCTGAGAA TTATAGAGAC CAGTTATTAA GGCTGGTGAA GAGCTTCTTG CCCAAGGTGG TTACCCTTGT   
  
  
+ TGAGCAAGAA TCCAACACAA ACACGGCCCC ATTTCTACCC CGGTTTCTTG AAACCCTAGA CTACTATACT   
  
  
+ GCCATTTTCG AATCAATTGA TGTTACGCTC CCAAGAGATC ACAAGGAGCG GATCAATGTT GAGCAGCACT   
  
  
+ GTTTAGCAAG AGATATAGTC AACATAATAG CATGTGAGGG TGCCGAGAGG GTGGAACGCC ATGAGGTTCT   
  
  
+ TGGAAAGTGG AGATCTCGGT TCTCAATGGC TGGGTTTAAG CCGTACCCAT TGAGCCCGCT AGTGAATGCA   
  
  
+ ACTATCAGGA CTCTTCTGCA GAAGTATAGC AGGAGCTATG GACTTGAAGA AAGGGATGGA GCTCTTTATC   
  
  
+ TAGGCTGGAT GAACCGAGCA CTAGTTGCAT CTTGTGCATG GCAGTG  

- -Up\_Stream \_Len000AAGAAA AGGACAGAGA CAGAGAAGTA ACGTGGTAGT AAATTCTCTA CCCTGGTATT   
  
  
- TAGTAAGTTA GTGAGGAAAT ACAAATTTGA AACCTTCCTC CAATAATAAA TACACCACCA CTAGGGTTAC   
  
  
- GACTTAAATC GACAACTACC GCTCCCGTAT ATACATTAAT CCTAAAGGTT ACGTCAAATC TAAAATGAGT   
  
  
- AAACTCTTAA GTGACTCCAA ACAAACTAGA CTAAAATCGT TTATAACAAG TCCCCATATC TACGAATAAG   
  
  
- CCAAGAGTTG ACATAATCAT ATGTTCTACT CACCAGGTGA ACAAATTCGA AAATGATATA TGAATACAAC   
  
  
- AGGGATTAAA CCAGAGCGAA CTACACATCG ACAGTTCATA ATAATATTAG AACACAAACT ATAGTGTAAA   
  
  
- CATAACTAAT CGATGTTTTC TTGTATAATA TACACATATC GTTGATAGAG TATATACGGG AGTGGTATCG   
  
  
- CACCAAGGTT CCCAAGTTCA AGGTGAATCG GAGTGGGATC TTTACTGAAG AAAAAAAAAA ATTTCAATTT   
  
  
- CTATACGTTA GTGGTACCTT GGTCAGTAGA AAACTGTGCA ATAAGGTCTA GTCTACTGGT CGGAAAGAGG   
  
  
- TTCCGGTAAT CTAAAAGATC GGTTAGGTGA CAATTGACGT TTTTCACCAT AGGAACCGAC AAGGACAGTG   
  
  
- ACAAAGAATA AACGAGACAC TATAAACATG TAGAGAAACG GTTCCTATTT TCAAGTATCG GCCCTTAACC   
  
  
- ATAAGTCAAC TGAGAACGTC ATTTGTCTCA AGTACATGAC CTCCTAAAAC ACTCAGTACA GTGAGTTATT   
  
  
- TTTGTCCACA CGCTGTCACA ATATTTCGAC GAACGAATTA CGTGTAGATG AGAGTGAGTG GAATGGGTTT   
  
  
- TTTGAAGTTT TAACACACGG AAGATACTTA TAAGACTAGG TAATCGTAAG TTGCCAAAGA CTTAAGACAC   
  
  
- ATTCGGTAGG TATGAGTAAA AGTTGTCTCT TGAACCTAAC CTACATAAGT TATGGGTTTT TGGAACAGTT   
  
  
- TAATGGGGAT CGAATTAAAG GAGATTATGG TTGACGAGAA ACCAAAATCG CATGTTAGGG TATCTAATGG   
  
  
- GAAGATATTA ATCACTACTA ACCAAGTAAA GACGACGTAA TACGGAAGAA ATCTAGGTAA CTATTATATA   
  
  
- TGAGAACGGT ACGACGTTGT AAGGTGACCC TAATTATAGA GTGTAACATA GGAGTCGTTG GTTCCTCTGG   
  
  
- GATGATAAAA GTCTCGGTAA GTATCTTTCA ATAACGTATA TCACTACTAG AAGTTACCCA GACTAAAAAT   
  
  
- CTATCGACGA CACTTGTAAA ATGAGCAGGA GACGGTACAC CTATGATACC ATTGGAAACA AACGAGAGTT   
  
  
- CGGACTCGCA AGACTCTTTA CTACTTTCGC GGGAGCTCAA TATCCTGAAA AGACTGAAAT CGAAATCTTT   
  
  
- TCTCTCCTTT TAGTTGGTGT GGGTAGACTA ATAACTCAAA GACGAGGAAC TCTGGACCTA CCTCGGTCTT   
  
  
- GAGTGCCAGA ACAGTCGGTC TTATTAGTCC TTGACGTAAA GACTCCTGGG TACTTTTCTG GGACCATATG   
  
  
- CTGATAAGTA CTTTTGCAAA ACAGTCGGAA AGTCAAAAAC GTAAAATAAG ATCGACGAAA CTAACTTCGG   
  
  
- GTTAAAATAA TTTTGACACG AAAAGAGAAC TTACAATTTG AGAACGTCAA GGAACGAACA ATAAAACCCA   
  
  
- AAATACAAAC GAAAGATGAC TATAGTAGAA AACCAGCACG TTAAATTAAC GGATGATACA ATATACCTAA   
  
  
- AAATACAAAT GAAAGATGAC TATAGTAGAA AACGATAACG TTAAATTAAC GGAAGATACA TCTTAAGCAG   
  
  
- TGAAACTTCA GACGGTCCTT CCGTTATGTC GATATAAAAA GAACACCTAC TTCACTATTT TCCGTTCACA   
  
  
- GTTAACGAAT GACGAAATAA CGCGACATGA AAAGGTCTCT AAAACCTAAT CAGTTACGTC TTCGGTTGTT   
  
  
- AAATATCGCT GACCGGTGTC AAGATGTTGG TTAAACTATT AGGTGCAGAA GAACTTAGAA GTAGATATTA   
  
  
- CCCACCACTA GTTGAAAAGT CAAGAGGTAG ATGACATTCG TATAGAACGT TGTCCTCAGG TCGAAGTGAC   
  
  
- GGACTTGAGC TTAGGAGAAG TAGCCTGAAG GTTTCACTGA GGGTCCTCTG ACACTTACCG AGCGGTCACT   
  
  
- AACTAAGCAC GTACCATGCA CTGTAACCAC TGAATTCTGT GTTTGAATCT CTCGAACTCT GACAATACGA   
  
  
- GCCTGGTTCA AGTCTGTCGA ACCTAAGTAC CATACGGGGA AGTTCTCCAC TCACACTTGG AGACGGTGGT   
  
  
- CTTCTCGGAC TGTTACCCTT TGTAAACAAC CTCTACTATC GTTCCCCCTC GGAGTTTCTC CACGATTAAC   
  
  
- GAACACGGTT TCGTTATAGT CTGCTACTAA ACAATTGTCG CCTCACCAAC TACAGTCTCG ATGCGGTATA   
  
  
- CCAAAGTCAA AGACCTCTTG GTTAAGTCTC CAATCCTCGG ATGTACAACC TTCCCAATCA ACGGGCCAAC   
  
  
- AGGAGAAGTC CTTCAAGGTA GATGTTTCGA GATTCCACGT TTCTCGGATG ATCATCACTT GAAGAAAGGA   
  
  
- TATACGTAAA TGAGATACTT CAAACGGGAA TGAAGTTCAA ACCCATGTAC AGACGTTTAC CCCGTTAACG   
  
  
- ACTCCGGTAC TTTTTACTCT CGTCTTAAGT ATATTAACTA AAAGTCTATC GAGTTCCCTC AGTCACCTAA   
  
  
- TCGGAATAGG TTCGGGACCG ACGAGTCGGA CTACCCGGTG GTGTCCAGGC ATAATGGCCT CAGCTACTAA   
  
  
- GGGTTAGACT CATACGAGCC CCTCCCCCCG AGCTGTAACA TCGCTTCTCT GATAGACCTG ATCGGGTCCG   
  
  
- AACGTCGGAT GGGAAACTCA AGGTGCGACG TCGTGAATCA CCAAGACTCT AGTCTGAAGT TTTGGACCAA   
  
  
- AACGCCGGAC CTCTTCGGAA TCGTCACTTG AAGGGTAAGT ACGACGTGGT GTACGGCCTA CTCTCACACC   
  
  
- CGGGACTCTT AATATCTCTG GTCAATAATT CCGACCACTT CTCGAAGAAC GGGTTCCACC AATGGGAACA   
  
  
- ACTCGTTCTT AGGTTGTGTT TGTGCCGGGG TAAAGATGGG GCCAAAGAAC TTTGGGATCT GATGATATGA   
  
  
- CGGTAAAAGC TTAGTTAACT ACAATGCGAG GGTTCTCTAG TGTTCCTCGC CTAGTTACAA CTCGTCGTGA   
  
  
- CAAATCGTTC TCTATATCAG TTGTATTATC GTACACTCCC ACGGCTCTCC CACCTTGCGG TACTCCAAGA   
  
  
- ACCTTTCACC TCTAGAGCCA AGAGTTACCG ACCCAAATTC GGCATGGGTA ACTCGGGCGA TCACTTACGT   
  
  
- TGATAGTCCT GAGAAGACGT CTTCATATCG TCCTCGATAC CTGAACTTCT TTCCCTACCT CGAGAAATAG   
  
  
- ATCCGACCTA CTTGGCTCGT GATCAACGTA GAACACGTAC CGTCAC

+     GCN4\_motif

| Site Name | Organism | Position | Strand | Matrix score. | sequence | function |
| --- | --- | --- | --- | --- | --- | --- |
| GCN4\_motif | Oryza sativa | 825 | + | 7 | TGAGTCA | cis-regulatory element involved in endosperm expression |

>HU08G00284.1   
+ -Up\_Stream \_Len000TTCTTT TCCTGTCTCT GTCTCTTCAT TGCACCATCA TTTAAGAGAT GGGACCATAA   
  
  
+ ATCATTCAAT CACTCCTTTA TGTTTAAACT TTGGAAGGAG GTTATTATTT ATGTGGTGGT GATCCCAATG   
  
  
+ CTGAATTTAG CTGTTGATGG CGAGGGCATA TATGTAATTA GGATTTCCAA TGCAGTTTAG ATTTTACTCA   
  
  
+ TTTGAGAATT CACTGAGGTT TGTTTGATCT GATTTTAGCA AATATTGTTC AGGGGTATAG ATGCTTATTC   
  
  
+ GGTTCTCAAC TGTATTAGTA TACAAGATGA GTGGTCCACT TGTTTAAGCT TTTACTATAT ACTTATGTTG   
  
  
+ TCCCTAATTT GGTCTCGCTT GATGTGTAGC TGTCAAGTAT TATTATAATC TTGTGTTTGA TATCACATTT   
  
  
+ GTATTGATTA GCTACAAAAG AACATATTAT ATGTGTATAG CAACTATCTC ATATATGCCC TCACCATAGC   
  
  
+ GTGGTTCCAA GGGTTCAAGT TCCACTTAGC CTCACCCTAG AAATGACTTC TTTTTTTTTT TAAAGTTAAA   
  
  
+ GATATGCAAT CACCATGGAA CCAGTCATCT TTTGACACGT TATTCCAGAT CAGATGACCA GCCTTTCTCC   
  
  
+ AAGGCCATTA GATTTTCTAG CCAATCCACT GTTAACTGCA AAAAGTGGTA TCCTTGGCTG TTCCTGTCAC   
  
  
+ TGTTTCTTAT TTGCTCTGTG ATATTTGTAC ATCTCTTTGC CAAGGATAAA AGTTCATAGC CGGGAATTGG   
  
  
+ TATTCAGTTG ACTCTTGCAG TAAACAGAGT TCATGTACTG GAGGATTTTG TGAGTCATGT CACTCAATAA   
  
  
+ AAACAGGTGT GCGACAGTGT TATAAAGCTG CTTGCTTAAT GCACATCTAC TCTCACTCAC CTTACCCAAA   
  
  
+ AAACTTCAAA ATTGTGTGCC TTCTATGAAT ATTCTGATCC ATTAGCATTC AACGGTTTCT GAATTCTGTG   
  
  
+ TAAGCCATCC ATACTCATTT TCAACAGAGA ACTTGGATTG GATGTATTCA ATACCCAAAA ACCTTGTCAA   
  
  
+ ATTACCCCTA GCTTAATTTC CTCTAATACC AACTGCTCTT TGGTTTTAGC GTACAATCCC ATAGATTACC   
  
  
+ CTTCTATAAT TAGTGATGAT TGGTTCATTT CTGCTGCATT ATGCCTTCTT TAGATCCATT GATAATATAT   
  
  
+ ACTCTTGCCA TGCTGCAACA TTCCACTGGG ATTAATATCT CACATTGTAT CCTCAGCAAC CAAGGAGACC   
  
  
+ CTACTATTTT CAGAGCCATT CATAGAAAGT TATTGCATAT AGTGATGATC TTCAATGGGT CTGATTTTTA   
  
  
+ GATAGCTGCT GTGAACATTT TACTCGTCCT CTGCCATGTG GATACTATGG TAACCTTTGT TTGCTCTCAA   
  
  
+ GCCTGAGCGT TCTGAGAAAT GATGAAAGCG CCCTCGAGTT ATAGGACTTT TCTGACTTTA GCTTTAGAAA   
  
  
+ AGAGAGGAAA ATCAACCACA CCCATCTGAT TATTGAGTTT CTGCTCCTTG AGACCTGGAT GGAGCCAGAA   
  
  
+ CTCACGGTCT TGTCAGCCAG AATAATCAGG AACTGCATTT CTGAGGACCC ATGAAAAGAC CCTGGTATAC   
  
  
+ GACTATTCAT GAAAACGTTT TGTCAGCCTT TCAGTTTTTG CATTTTATTC TAGCTGCTTT GATTGAAGCC   
  
  
+ CAATTTTATT AAAACTGTGC TTTTCTCTTG AATGTTAAAC TCTTGCAGTT CCTTGCTTGT TATTTTGGGT   
  
  
+ TTTATGTTTG CTTTCTACTG ATATCATCTT TTGGTCGTGC AATTTAATTG CCTACTATGT TATATGGATT   
  
  
+ TTTATGTTTA CTTTCTACTG ATATCATCTT TTGCTATTGC AATTTAATTG CCTTCTATGT AGAATTCGTC   
  
  
+ ACTTTGAAGT CTGCCAGGAA GGCAATACAG CTATATTTTT CTTGTGGATG AAGTGATAAA AGGCAAGTGT   
  
  
+ CAATTGCTTA CTGCTTTATT GCGCTGTACT TTTCCAGAGA TTTTGGATTA GTCAATGCAG AAGCCAACAA   
  
  
+ TTTATAGCGA CTGGCCACAG TTCTACAACC AATTTGATAA TCCACGTCTT CTTGAATCTT CATCTATAAT   
  
  
+ GGGTGGTGAT CAACTTTTCA GTTCTCCATC TACTGTAAGC ATATCTTGCA ACAGGAGTCC AGCTTCACTG   
  
  
+ CCTGAACTCG AATCCTCTTC ATCGGACTTC CAAAGTGACT CCCAGGAGAC TGTGAATGGC TCGCCAGTGA   
  
  
+ TTGATTCGTG CATGGTACGT GACATTGGTG ACTTAAGACA CAAACTTAGA GAGCTTGAGA CTGTTATGCT   
  
  
+ CGGACCAAGT TCAGACAGCT TGGATTCATG GTATGCCCCT TCAAGAGGTG AGTGTGAACC TCTGCCACCA   
  
  
+ GAAGAGCCTG ACAATGGGAA ACATTTGTTG GAGATGATAG CAAGGGGGAG CCTCAAAGAG GTGCTAATTG   
  
  
+ CTTGTGCCAA AGCAATATCA GACGATGATT TGTTAACAGC GGAGTGGTTG ATGTCAGAGC TACGCCATAT   
  
  
+ GGTTTCAGTT TCTGGAGAAC CAATTCAGAG GTTAGGAGCC TACATGTTGG AAGGGTTAGT TGCCCGGTTG   
  
  
+ TCCTCTTCAG GAAGTTCCAT CTACAAAGCT CTAAGGTGCA AAGAGCCTAC TAGTAGTGAA CTTCTTTCCT   
  
  
+ ATATGCATTT ACTCTATGAA GTTTGCCCTT ACTTCAAGTT TGGGTACATG TCTGCAAATG GGGCAATTGC   
  
  
+ TGAGGCCATG AAAAATGAGA GCAGAATTCA TATAATTGAT TTTCAGATAG CTCAAGGGAG TCAGTGGATT   
  
  
+ AGCCTTATCC AAGCCCTGGC TGCTCAGCCT GATGGGCCAC CACAGGTCCG TATTACCGGA GTCGATGATT   
  
  
+ CCCAATCTGA GTATGCTCGG GGAGGGGGGC TCGACATTGT AGCGAAGAGA CTATCTGGAC TAGCCCAGGC   
  
  
+ TTGCAGCCTA CCCTTTGAGT TCCACGCTGC AGCACTTAGT GGTTCTGAGA TCAGACTTCA AAACCTGGTT   
  
  
+ TTGCGGCCTG GAGAAGCCTT AGCAGTGAAC TTCCCATTCA TGCTGCACCA CATGCCGGAT GAGAGTGTGG   
  
  
+ GCCCTGAGAA TTATAGAGAC CAGTTATTAA GGCTGGTGAA GAGCTTCTTG CCCAAGGTGG TTACCCTTGT   
  
  
+ TGAGCAAGAA TCCAACACAA ACACGGCCCC ATTTCTACCC CGGTTTCTTG AAACCCTAGA CTACTATACT   
  
  
+ GCCATTTTCG AATCAATTGA TGTTACGCTC CCAAGAGATC ACAAGGAGCG GATCAATGTT GAGCAGCACT   
  
  
+ GTTTAGCAAG AGATATAGTC AACATAATAG CATGTGAGGG TGCCGAGAGG GTGGAACGCC ATGAGGTTCT   
  
  
+ TGGAAAGTGG AGATCTCGGT TCTCAATGGC TGGGTTTAAG CCGTACCCAT TGAGCCCGCT AGTGAATGCA   
  
  
+ ACTATCAGGA CTCTTCTGCA GAAGTATAGC AGGAGCTATG GACTTGAAGA AAGGGATGGA GCTCTTTATC   
  
  
+ TAGGCTGGAT GAACCGAGCA CTAGTTGCAT CTTGTGCATG GCAGTG  

- -Up\_Stream \_Len000AAGAAA AGGACAGAGA CAGAGAAGTA ACGTGGTAGT AAATTCTCTA CCCTGGTATT   
  
  
- TAGTAAGTTA GTGAGGAAAT ACAAATTTGA AACCTTCCTC CAATAATAAA TACACCACCA CTAGGGTTAC   
  
  
- GACTTAAATC GACAACTACC GCTCCCGTAT ATACATTAAT CCTAAAGGTT ACGTCAAATC TAAAATGAGT   
  
  
- AAACTCTTAA GTGACTCCAA ACAAACTAGA CTAAAATCGT TTATAACAAG TCCCCATATC TACGAATAAG   
  
  
- CCAAGAGTTG ACATAATCAT ATGTTCTACT CACCAGGTGA ACAAATTCGA AAATGATATA TGAATACAAC   
  
  
- AGGGATTAAA CCAGAGCGAA CTACACATCG ACAGTTCATA ATAATATTAG AACACAAACT ATAGTGTAAA   
  
  
- CATAACTAAT CGATGTTTTC TTGTATAATA TACACATATC GTTGATAGAG TATATACGGG AGTGGTATCG   
  
  
- CACCAAGGTT CCCAAGTTCA AGGTGAATCG GAGTGGGATC TTTACTGAAG AAAAAAAAAA ATTTCAATTT   
  
  
- CTATACGTTA GTGGTACCTT GGTCAGTAGA AAACTGTGCA ATAAGGTCTA GTCTACTGGT CGGAAAGAGG   
  
  
- TTCCGGTAAT CTAAAAGATC GGTTAGGTGA CAATTGACGT TTTTCACCAT AGGAACCGAC AAGGACAGTG   
  
  
- ACAAAGAATA AACGAGACAC TATAAACATG TAGAGAAACG GTTCCTATTT TCAAGTATCG GCCCTTAACC   
  
  
- ATAAGTCAAC TGAGAACGTC ATTTGTCTCA AGTACATGAC CTCCTAAAAC ACTCAGTACA GTGAGTTATT   
  
  
- TTTGTCCACA CGCTGTCACA ATATTTCGAC GAACGAATTA CGTGTAGATG AGAGTGAGTG GAATGGGTTT   
  
  
- TTTGAAGTTT TAACACACGG AAGATACTTA TAAGACTAGG TAATCGTAAG TTGCCAAAGA CTTAAGACAC   
  
  
- ATTCGGTAGG TATGAGTAAA AGTTGTCTCT TGAACCTAAC CTACATAAGT TATGGGTTTT TGGAACAGTT   
  
  
- TAATGGGGAT CGAATTAAAG GAGATTATGG TTGACGAGAA ACCAAAATCG CATGTTAGGG TATCTAATGG   
  
  
- GAAGATATTA ATCACTACTA ACCAAGTAAA GACGACGTAA TACGGAAGAA ATCTAGGTAA CTATTATATA   
  
  
- TGAGAACGGT ACGACGTTGT AAGGTGACCC TAATTATAGA GTGTAACATA GGAGTCGTTG GTTCCTCTGG   
  
  
- GATGATAAAA GTCTCGGTAA GTATCTTTCA ATAACGTATA TCACTACTAG AAGTTACCCA GACTAAAAAT   
  
  
- CTATCGACGA CACTTGTAAA ATGAGCAGGA GACGGTACAC CTATGATACC ATTGGAAACA AACGAGAGTT   
  
  
- CGGACTCGCA AGACTCTTTA CTACTTTCGC GGGAGCTCAA TATCCTGAAA AGACTGAAAT CGAAATCTTT   
  
  
- TCTCTCCTTT TAGTTGGTGT GGGTAGACTA ATAACTCAAA GACGAGGAAC TCTGGACCTA CCTCGGTCTT   
  
  
- GAGTGCCAGA ACAGTCGGTC TTATTAGTCC TTGACGTAAA GACTCCTGGG TACTTTTCTG GGACCATATG   
  
  
- CTGATAAGTA CTTTTGCAAA ACAGTCGGAA AGTCAAAAAC GTAAAATAAG ATCGACGAAA CTAACTTCGG   
  
  
- GTTAAAATAA TTTTGACACG AAAAGAGAAC TTACAATTTG AGAACGTCAA GGAACGAACA ATAAAACCCA   
  
  
- AAATACAAAC GAAAGATGAC TATAGTAGAA AACCAGCACG TTAAATTAAC GGATGATACA ATATACCTAA   
  
  
- AAATACAAAT GAAAGATGAC TATAGTAGAA AACGATAACG TTAAATTAAC GGAAGATACA TCTTAAGCAG   
  
  
- TGAAACTTCA GACGGTCCTT CCGTTATGTC GATATAAAAA GAACACCTAC TTCACTATTT TCCGTTCACA   
  
  
- GTTAACGAAT GACGAAATAA CGCGACATGA AAAGGTCTCT AAAACCTAAT CAGTTACGTC TTCGGTTGTT   
  
  
- AAATATCGCT GACCGGTGTC AAGATGTTGG TTAAACTATT AGGTGCAGAA GAACTTAGAA GTAGATATTA   
  
  
- CCCACCACTA GTTGAAAAGT CAAGAGGTAG ATGACATTCG TATAGAACGT TGTCCTCAGG TCGAAGTGAC   
  
  
- GGACTTGAGC TTAGGAGAAG TAGCCTGAAG GTTTCACTGA GGGTCCTCTG ACACTTACCG AGCGGTCACT   
  
  
- AACTAAGCAC GTACCATGCA CTGTAACCAC TGAATTCTGT GTTTGAATCT CTCGAACTCT GACAATACGA   
  
  
- GCCTGGTTCA AGTCTGTCGA ACCTAAGTAC CATACGGGGA AGTTCTCCAC TCACACTTGG AGACGGTGGT   
  
  
- CTTCTCGGAC TGTTACCCTT TGTAAACAAC CTCTACTATC GTTCCCCCTC GGAGTTTCTC CACGATTAAC   
  
  
- GAACACGGTT TCGTTATAGT CTGCTACTAA ACAATTGTCG CCTCACCAAC TACAGTCTCG ATGCGGTATA   
  
  
- CCAAAGTCAA AGACCTCTTG GTTAAGTCTC CAATCCTCGG ATGTACAACC TTCCCAATCA ACGGGCCAAC   
  
  
- AGGAGAAGTC CTTCAAGGTA GATGTTTCGA GATTCCACGT TTCTCGGATG ATCATCACTT GAAGAAAGGA   
  
  
- TATACGTAAA TGAGATACTT CAAACGGGAA TGAAGTTCAA ACCCATGTAC AGACGTTTAC CCCGTTAACG   
  
  
- ACTCCGGTAC TTTTTACTCT CGTCTTAAGT ATATTAACTA AAAGTCTATC GAGTTCCCTC AGTCACCTAA   
  
  
- TCGGAATAGG TTCGGGACCG ACGAGTCGGA CTACCCGGTG GTGTCCAGGC ATAATGGCCT CAGCTACTAA   
  
  
- GGGTTAGACT CATACGAGCC CCTCCCCCCG AGCTGTAACA TCGCTTCTCT GATAGACCTG ATCGGGTCCG   
  
  
- AACGTCGGAT GGGAAACTCA AGGTGCGACG TCGTGAATCA CCAAGACTCT AGTCTGAAGT TTTGGACCAA   
  
  
- AACGCCGGAC CTCTTCGGAA TCGTCACTTG AAGGGTAAGT ACGACGTGGT GTACGGCCTA CTCTCACACC   
  
  
- CGGGACTCTT AATATCTCTG GTCAATAATT CCGACCACTT CTCGAAGAAC GGGTTCCACC AATGGGAACA   
  
  
- ACTCGTTCTT AGGTTGTGTT TGTGCCGGGG TAAAGATGGG GCCAAAGAAC TTTGGGATCT GATGATATGA   
  
  
- CGGTAAAAGC TTAGTTAACT ACAATGCGAG GGTTCTCTAG TGTTCCTCGC CTAGTTACAA CTCGTCGTGA   
  
  
- CAAATCGTTC TCTATATCAG TTGTATTATC GTACACTCCC ACGGCTCTCC CACCTTGCGG TACTCCAAGA   
  
  
- ACCTTTCACC TCTAGAGCCA AGAGTTACCG ACCCAAATTC GGCATGGGTA ACTCGGGCGA TCACTTACGT   
  
  
- TGATAGTCCT GAGAAGACGT CTTCATATCG TCCTCGATAC CTGAACTTCT TTCCCTACCT CGAGAAATAG   
  
  
- ATCCGACCTA CTTGGCTCGT GATCAACGTA GAACACGTAC CGTCAC

+     I-box

| Site Name | Organism | Position | Strand | Matrix score. | sequence | function |
| --- | --- | --- | --- | --- | --- | --- |
| I-box | Zea mays | 902 | - | 9 | gGATAAGGTG | part of a light responsive element |

>HU08G00284.1   
+ -Up\_Stream \_Len000TTCTTT TCCTGTCTCT GTCTCTTCAT TGCACCATCA TTTAAGAGAT GGGACCATAA   
  
  
+ ATCATTCAAT CACTCCTTTA TGTTTAAACT TTGGAAGGAG GTTATTATTT ATGTGGTGGT GATCCCAATG   
  
  
+ CTGAATTTAG CTGTTGATGG CGAGGGCATA TATGTAATTA GGATTTCCAA TGCAGTTTAG ATTTTACTCA   
  
  
+ TTTGAGAATT CACTGAGGTT TGTTTGATCT GATTTTAGCA AATATTGTTC AGGGGTATAG ATGCTTATTC   
  
  
+ GGTTCTCAAC TGTATTAGTA TACAAGATGA GTGGTCCACT TGTTTAAGCT TTTACTATAT ACTTATGTTG   
  
  
+ TCCCTAATTT GGTCTCGCTT GATGTGTAGC TGTCAAGTAT TATTATAATC TTGTGTTTGA TATCACATTT   
  
  
+ GTATTGATTA GCTACAAAAG AACATATTAT ATGTGTATAG CAACTATCTC ATATATGCCC TCACCATAGC   
  
  
+ GTGGTTCCAA GGGTTCAAGT TCCACTTAGC CTCACCCTAG AAATGACTTC TTTTTTTTTT TAAAGTTAAA   
  
  
+ GATATGCAAT CACCATGGAA CCAGTCATCT TTTGACACGT TATTCCAGAT CAGATGACCA GCCTTTCTCC   
  
  
+ AAGGCCATTA GATTTTCTAG CCAATCCACT GTTAACTGCA AAAAGTGGTA TCCTTGGCTG TTCCTGTCAC   
  
  
+ TGTTTCTTAT TTGCTCTGTG ATATTTGTAC ATCTCTTTGC CAAGGATAAA AGTTCATAGC CGGGAATTGG   
  
  
+ TATTCAGTTG ACTCTTGCAG TAAACAGAGT TCATGTACTG GAGGATTTTG TGAGTCATGT CACTCAATAA   
  
  
+ AAACAGGTGT GCGACAGTGT TATAAAGCTG CTTGCTTAAT GCACATCTAC TCTCACTCAC CTTACCCAAA   
  
  
+ AAACTTCAAA ATTGTGTGCC TTCTATGAAT ATTCTGATCC ATTAGCATTC AACGGTTTCT GAATTCTGTG   
  
  
+ TAAGCCATCC ATACTCATTT TCAACAGAGA ACTTGGATTG GATGTATTCA ATACCCAAAA ACCTTGTCAA   
  
  
+ ATTACCCCTA GCTTAATTTC CTCTAATACC AACTGCTCTT TGGTTTTAGC GTACAATCCC ATAGATTACC   
  
  
+ CTTCTATAAT TAGTGATGAT TGGTTCATTT CTGCTGCATT ATGCCTTCTT TAGATCCATT GATAATATAT   
  
  
+ ACTCTTGCCA TGCTGCAACA TTCCACTGGG ATTAATATCT CACATTGTAT CCTCAGCAAC CAAGGAGACC   
  
  
+ CTACTATTTT CAGAGCCATT CATAGAAAGT TATTGCATAT AGTGATGATC TTCAATGGGT CTGATTTTTA   
  
  
+ GATAGCTGCT GTGAACATTT TACTCGTCCT CTGCCATGTG GATACTATGG TAACCTTTGT TTGCTCTCAA   
  
  
+ GCCTGAGCGT TCTGAGAAAT GATGAAAGCG CCCTCGAGTT ATAGGACTTT TCTGACTTTA GCTTTAGAAA   
  
  
+ AGAGAGGAAA ATCAACCACA CCCATCTGAT TATTGAGTTT CTGCTCCTTG AGACCTGGAT GGAGCCAGAA   
  
  
+ CTCACGGTCT TGTCAGCCAG AATAATCAGG AACTGCATTT CTGAGGACCC ATGAAAAGAC CCTGGTATAC   
  
  
+ GACTATTCAT GAAAACGTTT TGTCAGCCTT TCAGTTTTTG CATTTTATTC TAGCTGCTTT GATTGAAGCC   
  
  
+ CAATTTTATT AAAACTGTGC TTTTCTCTTG AATGTTAAAC TCTTGCAGTT CCTTGCTTGT TATTTTGGGT   
  
  
+ TTTATGTTTG CTTTCTACTG ATATCATCTT TTGGTCGTGC AATTTAATTG CCTACTATGT TATATGGATT   
  
  
+ TTTATGTTTA CTTTCTACTG ATATCATCTT TTGCTATTGC AATTTAATTG CCTTCTATGT AGAATTCGTC   
  
  
+ ACTTTGAAGT CTGCCAGGAA GGCAATACAG CTATATTTTT CTTGTGGATG AAGTGATAAA AGGCAAGTGT   
  
  
+ CAATTGCTTA CTGCTTTATT GCGCTGTACT TTTCCAGAGA TTTTGGATTA GTCAATGCAG AAGCCAACAA   
  
  
+ TTTATAGCGA CTGGCCACAG TTCTACAACC AATTTGATAA TCCACGTCTT CTTGAATCTT CATCTATAAT   
  
  
+ GGGTGGTGAT CAACTTTTCA GTTCTCCATC TACTGTAAGC ATATCTTGCA ACAGGAGTCC AGCTTCACTG   
  
  
+ CCTGAACTCG AATCCTCTTC ATCGGACTTC CAAAGTGACT CCCAGGAGAC TGTGAATGGC TCGCCAGTGA   
  
  
+ TTGATTCGTG CATGGTACGT GACATTGGTG ACTTAAGACA CAAACTTAGA GAGCTTGAGA CTGTTATGCT   
  
  
+ CGGACCAAGT TCAGACAGCT TGGATTCATG GTATGCCCCT TCAAGAGGTG AGTGTGAACC TCTGCCACCA   
  
  
+ GAAGAGCCTG ACAATGGGAA ACATTTGTTG GAGATGATAG CAAGGGGGAG CCTCAAAGAG GTGCTAATTG   
  
  
+ CTTGTGCCAA AGCAATATCA GACGATGATT TGTTAACAGC GGAGTGGTTG ATGTCAGAGC TACGCCATAT   
  
  
+ GGTTTCAGTT TCTGGAGAAC CAATTCAGAG GTTAGGAGCC TACATGTTGG AAGGGTTAGT TGCCCGGTTG   
  
  
+ TCCTCTTCAG GAAGTTCCAT CTACAAAGCT CTAAGGTGCA AAGAGCCTAC TAGTAGTGAA CTTCTTTCCT   
  
  
+ ATATGCATTT ACTCTATGAA GTTTGCCCTT ACTTCAAGTT TGGGTACATG TCTGCAAATG GGGCAATTGC   
  
  
+ TGAGGCCATG AAAAATGAGA GCAGAATTCA TATAATTGAT TTTCAGATAG CTCAAGGGAG TCAGTGGATT   
  
  
+ AGCCTTATCC AAGCCCTGGC TGCTCAGCCT GATGGGCCAC CACAGGTCCG TATTACCGGA GTCGATGATT   
  
  
+ CCCAATCTGA GTATGCTCGG GGAGGGGGGC TCGACATTGT AGCGAAGAGA CTATCTGGAC TAGCCCAGGC   
  
  
+ TTGCAGCCTA CCCTTTGAGT TCCACGCTGC AGCACTTAGT GGTTCTGAGA TCAGACTTCA AAACCTGGTT   
  
  
+ TTGCGGCCTG GAGAAGCCTT AGCAGTGAAC TTCCCATTCA TGCTGCACCA CATGCCGGAT GAGAGTGTGG   
  
  
+ GCCCTGAGAA TTATAGAGAC CAGTTATTAA GGCTGGTGAA GAGCTTCTTG CCCAAGGTGG TTACCCTTGT   
  
  
+ TGAGCAAGAA TCCAACACAA ACACGGCCCC ATTTCTACCC CGGTTTCTTG AAACCCTAGA CTACTATACT   
  
  
+ GCCATTTTCG AATCAATTGA TGTTACGCTC CCAAGAGATC ACAAGGAGCG GATCAATGTT GAGCAGCACT   
  
  
+ GTTTAGCAAG AGATATAGTC AACATAATAG CATGTGAGGG TGCCGAGAGG GTGGAACGCC ATGAGGTTCT   
  
  
+ TGGAAAGTGG AGATCTCGGT TCTCAATGGC TGGGTTTAAG CCGTACCCAT TGAGCCCGCT AGTGAATGCA   
  
  
+ ACTATCAGGA CTCTTCTGCA GAAGTATAGC AGGAGCTATG GACTTGAAGA AAGGGATGGA GCTCTTTATC   
  
  
+ TAGGCTGGAT GAACCGAGCA CTAGTTGCAT CTTGTGCATG GCAGTG  

- -Up\_Stream \_Len000AAGAAA AGGACAGAGA CAGAGAAGTA ACGTGGTAGT AAATTCTCTA CCCTGGTATT   
  
  
- TAGTAAGTTA GTGAGGAAAT ACAAATTTGA AACCTTCCTC CAATAATAAA TACACCACCA CTAGGGTTAC   
  
  
- GACTTAAATC GACAACTACC GCTCCCGTAT ATACATTAAT CCTAAAGGTT ACGTCAAATC TAAAATGAGT   
  
  
- AAACTCTTAA GTGACTCCAA ACAAACTAGA CTAAAATCGT TTATAACAAG TCCCCATATC TACGAATAAG   
  
  
- CCAAGAGTTG ACATAATCAT ATGTTCTACT CACCAGGTGA ACAAATTCGA AAATGATATA TGAATACAAC   
  
  
- AGGGATTAAA CCAGAGCGAA CTACACATCG ACAGTTCATA ATAATATTAG AACACAAACT ATAGTGTAAA   
  
  
- CATAACTAAT CGATGTTTTC TTGTATAATA TACACATATC GTTGATAGAG TATATACGGG AGTGGTATCG   
  
  
- CACCAAGGTT CCCAAGTTCA AGGTGAATCG GAGTGGGATC TTTACTGAAG AAAAAAAAAA ATTTCAATTT   
  
  
- CTATACGTTA GTGGTACCTT GGTCAGTAGA AAACTGTGCA ATAAGGTCTA GTCTACTGGT CGGAAAGAGG   
  
  
- TTCCGGTAAT CTAAAAGATC GGTTAGGTGA CAATTGACGT TTTTCACCAT AGGAACCGAC AAGGACAGTG   
  
  
- ACAAAGAATA AACGAGACAC TATAAACATG TAGAGAAACG GTTCCTATTT TCAAGTATCG GCCCTTAACC   
  
  
- ATAAGTCAAC TGAGAACGTC ATTTGTCTCA AGTACATGAC CTCCTAAAAC ACTCAGTACA GTGAGTTATT   
  
  
- TTTGTCCACA CGCTGTCACA ATATTTCGAC GAACGAATTA CGTGTAGATG AGAGTGAGTG GAATGGGTTT   
  
  
- TTTGAAGTTT TAACACACGG AAGATACTTA TAAGACTAGG TAATCGTAAG TTGCCAAAGA CTTAAGACAC   
  
  
- ATTCGGTAGG TATGAGTAAA AGTTGTCTCT TGAACCTAAC CTACATAAGT TATGGGTTTT TGGAACAGTT   
  
  
- TAATGGGGAT CGAATTAAAG GAGATTATGG TTGACGAGAA ACCAAAATCG CATGTTAGGG TATCTAATGG   
  
  
- GAAGATATTA ATCACTACTA ACCAAGTAAA GACGACGTAA TACGGAAGAA ATCTAGGTAA CTATTATATA   
  
  
- TGAGAACGGT ACGACGTTGT AAGGTGACCC TAATTATAGA GTGTAACATA GGAGTCGTTG GTTCCTCTGG   
  
  
- GATGATAAAA GTCTCGGTAA GTATCTTTCA ATAACGTATA TCACTACTAG AAGTTACCCA GACTAAAAAT   
  
  
- CTATCGACGA CACTTGTAAA ATGAGCAGGA GACGGTACAC CTATGATACC ATTGGAAACA AACGAGAGTT   
  
  
- CGGACTCGCA AGACTCTTTA CTACTTTCGC GGGAGCTCAA TATCCTGAAA AGACTGAAAT CGAAATCTTT   
  
  
- TCTCTCCTTT TAGTTGGTGT GGGTAGACTA ATAACTCAAA GACGAGGAAC TCTGGACCTA CCTCGGTCTT   
  
  
- GAGTGCCAGA ACAGTCGGTC TTATTAGTCC TTGACGTAAA GACTCCTGGG TACTTTTCTG GGACCATATG   
  
  
- CTGATAAGTA CTTTTGCAAA ACAGTCGGAA AGTCAAAAAC GTAAAATAAG ATCGACGAAA CTAACTTCGG   
  
  
- GTTAAAATAA TTTTGACACG AAAAGAGAAC TTACAATTTG AGAACGTCAA GGAACGAACA ATAAAACCCA   
  
  
- AAATACAAAC GAAAGATGAC TATAGTAGAA AACCAGCACG TTAAATTAAC GGATGATACA ATATACCTAA   
  
  
- AAATACAAAT GAAAGATGAC TATAGTAGAA AACGATAACG TTAAATTAAC GGAAGATACA TCTTAAGCAG   
  
  
- TGAAACTTCA GACGGTCCTT CCGTTATGTC GATATAAAAA GAACACCTAC TTCACTATTT TCCGTTCACA   
  
  
- GTTAACGAAT GACGAAATAA CGCGACATGA AAAGGTCTCT AAAACCTAAT CAGTTACGTC TTCGGTTGTT   
  
  
- AAATATCGCT GACCGGTGTC AAGATGTTGG TTAAACTATT AGGTGCAGAA GAACTTAGAA GTAGATATTA   
  
  
- CCCACCACTA GTTGAAAAGT CAAGAGGTAG ATGACATTCG TATAGAACGT TGTCCTCAGG TCGAAGTGAC   
  
  
- GGACTTGAGC TTAGGAGAAG TAGCCTGAAG GTTTCACTGA GGGTCCTCTG ACACTTACCG AGCGGTCACT   
  
  
- AACTAAGCAC GTACCATGCA CTGTAACCAC TGAATTCTGT GTTTGAATCT CTCGAACTCT GACAATACGA   
  
  
- GCCTGGTTCA AGTCTGTCGA ACCTAAGTAC CATACGGGGA AGTTCTCCAC TCACACTTGG AGACGGTGGT   
  
  
- CTTCTCGGAC TGTTACCCTT TGTAAACAAC CTCTACTATC GTTCCCCCTC GGAGTTTCTC CACGATTAAC   
  
  
- GAACACGGTT TCGTTATAGT CTGCTACTAA ACAATTGTCG CCTCACCAAC TACAGTCTCG ATGCGGTATA   
  
  
- CCAAAGTCAA AGACCTCTTG GTTAAGTCTC CAATCCTCGG ATGTACAACC TTCCCAATCA ACGGGCCAAC   
  
  
- AGGAGAAGTC CTTCAAGGTA GATGTTTCGA GATTCCACGT TTCTCGGATG ATCATCACTT GAAGAAAGGA   
  
  
- TATACGTAAA TGAGATACTT CAAACGGGAA TGAAGTTCAA ACCCATGTAC AGACGTTTAC CCCGTTAACG   
  
  
- ACTCCGGTAC TTTTTACTCT CGTCTTAAGT ATATTAACTA AAAGTCTATC GAGTTCCCTC AGTCACCTAA   
  
  
- TCGGAATAGG TTCGGGACCG ACGAGTCGGA CTACCCGGTG GTGTCCAGGC ATAATGGCCT CAGCTACTAA   
  
  
- GGGTTAGACT CATACGAGCC CCTCCCCCCG AGCTGTAACA TCGCTTCTCT GATAGACCTG ATCGGGTCCG   
  
  
- AACGTCGGAT GGGAAACTCA AGGTGCGACG TCGTGAATCA CCAAGACTCT AGTCTGAAGT TTTGGACCAA   
  
  
- AACGCCGGAC CTCTTCGGAA TCGTCACTTG AAGGGTAAGT ACGACGTGGT GTACGGCCTA CTCTCACACC   
  
  
- CGGGACTCTT AATATCTCTG GTCAATAATT CCGACCACTT CTCGAAGAAC GGGTTCCACC AATGGGAACA   
  
  
- ACTCGTTCTT AGGTTGTGTT TGTGCCGGGG TAAAGATGGG GCCAAAGAAC TTTGGGATCT GATGATATGA   
  
  
- CGGTAAAAGC TTAGTTAACT ACAATGCGAG GGTTCTCTAG TGTTCCTCGC CTAGTTACAA CTCGTCGTGA   
  
  
- CAAATCGTTC TCTATATCAG TTGTATTATC GTACACTCCC ACGGCTCTCC CACCTTGCGG TACTCCAAGA   
  
  
- ACCTTTCACC TCTAGAGCCA AGAGTTACCG ACCCAAATTC GGCATGGGTA ACTCGGGCGA TCACTTACGT   
  
  
- TGATAGTCCT GAGAAGACGT CTTCATATCG TCCTCGATAC CTGAACTTCT TTCCCTACCT CGAGAAATAG   
  
  
- ATCCGACCTA CTTGGCTCGT GATCAACGTA GAACACGTAC CGTCAC

+     LAMP-element

| Site Name | Organism | Position | Strand | Matrix score. | sequence | function |
| --- | --- | --- | --- | --- | --- | --- |
| LAMP-element | Spinacia oleracea | 2807 | + | 9 | CCTTATCCA | part of a light responsive element |

>HU08G00284.1   
+ -Up\_Stream \_Len000TTCTTT TCCTGTCTCT GTCTCTTCAT TGCACCATCA TTTAAGAGAT GGGACCATAA   
  
  
+ ATCATTCAAT CACTCCTTTA TGTTTAAACT TTGGAAGGAG GTTATTATTT ATGTGGTGGT GATCCCAATG   
  
  
+ CTGAATTTAG CTGTTGATGG CGAGGGCATA TATGTAATTA GGATTTCCAA TGCAGTTTAG ATTTTACTCA   
  
  
+ TTTGAGAATT CACTGAGGTT TGTTTGATCT GATTTTAGCA AATATTGTTC AGGGGTATAG ATGCTTATTC   
  
  
+ GGTTCTCAAC TGTATTAGTA TACAAGATGA GTGGTCCACT TGTTTAAGCT TTTACTATAT ACTTATGTTG   
  
  
+ TCCCTAATTT GGTCTCGCTT GATGTGTAGC TGTCAAGTAT TATTATAATC TTGTGTTTGA TATCACATTT   
  
  
+ GTATTGATTA GCTACAAAAG AACATATTAT ATGTGTATAG CAACTATCTC ATATATGCCC TCACCATAGC   
  
  
+ GTGGTTCCAA GGGTTCAAGT TCCACTTAGC CTCACCCTAG AAATGACTTC TTTTTTTTTT TAAAGTTAAA   
  
  
+ GATATGCAAT CACCATGGAA CCAGTCATCT TTTGACACGT TATTCCAGAT CAGATGACCA GCCTTTCTCC   
  
  
+ AAGGCCATTA GATTTTCTAG CCAATCCACT GTTAACTGCA AAAAGTGGTA TCCTTGGCTG TTCCTGTCAC   
  
  
+ TGTTTCTTAT TTGCTCTGTG ATATTTGTAC ATCTCTTTGC CAAGGATAAA AGTTCATAGC CGGGAATTGG   
  
  
+ TATTCAGTTG ACTCTTGCAG TAAACAGAGT TCATGTACTG GAGGATTTTG TGAGTCATGT CACTCAATAA   
  
  
+ AAACAGGTGT GCGACAGTGT TATAAAGCTG CTTGCTTAAT GCACATCTAC TCTCACTCAC CTTACCCAAA   
  
  
+ AAACTTCAAA ATTGTGTGCC TTCTATGAAT ATTCTGATCC ATTAGCATTC AACGGTTTCT GAATTCTGTG   
  
  
+ TAAGCCATCC ATACTCATTT TCAACAGAGA ACTTGGATTG GATGTATTCA ATACCCAAAA ACCTTGTCAA   
  
  
+ ATTACCCCTA GCTTAATTTC CTCTAATACC AACTGCTCTT TGGTTTTAGC GTACAATCCC ATAGATTACC   
  
  
+ CTTCTATAAT TAGTGATGAT TGGTTCATTT CTGCTGCATT ATGCCTTCTT TAGATCCATT GATAATATAT   
  
  
+ ACTCTTGCCA TGCTGCAACA TTCCACTGGG ATTAATATCT CACATTGTAT CCTCAGCAAC CAAGGAGACC   
  
  
+ CTACTATTTT CAGAGCCATT CATAGAAAGT TATTGCATAT AGTGATGATC TTCAATGGGT CTGATTTTTA   
  
  
+ GATAGCTGCT GTGAACATTT TACTCGTCCT CTGCCATGTG GATACTATGG TAACCTTTGT TTGCTCTCAA   
  
  
+ GCCTGAGCGT TCTGAGAAAT GATGAAAGCG CCCTCGAGTT ATAGGACTTT TCTGACTTTA GCTTTAGAAA   
  
  
+ AGAGAGGAAA ATCAACCACA CCCATCTGAT TATTGAGTTT CTGCTCCTTG AGACCTGGAT GGAGCCAGAA   
  
  
+ CTCACGGTCT TGTCAGCCAG AATAATCAGG AACTGCATTT CTGAGGACCC ATGAAAAGAC CCTGGTATAC   
  
  
+ GACTATTCAT GAAAACGTTT TGTCAGCCTT TCAGTTTTTG CATTTTATTC TAGCTGCTTT GATTGAAGCC   
  
  
+ CAATTTTATT AAAACTGTGC TTTTCTCTTG AATGTTAAAC TCTTGCAGTT CCTTGCTTGT TATTTTGGGT   
  
  
+ TTTATGTTTG CTTTCTACTG ATATCATCTT TTGGTCGTGC AATTTAATTG CCTACTATGT TATATGGATT   
  
  
+ TTTATGTTTA CTTTCTACTG ATATCATCTT TTGCTATTGC AATTTAATTG CCTTCTATGT AGAATTCGTC   
  
  
+ ACTTTGAAGT CTGCCAGGAA GGCAATACAG CTATATTTTT CTTGTGGATG AAGTGATAAA AGGCAAGTGT   
  
  
+ CAATTGCTTA CTGCTTTATT GCGCTGTACT TTTCCAGAGA TTTTGGATTA GTCAATGCAG AAGCCAACAA   
  
  
+ TTTATAGCGA CTGGCCACAG TTCTACAACC AATTTGATAA TCCACGTCTT CTTGAATCTT CATCTATAAT   
  
  
+ GGGTGGTGAT CAACTTTTCA GTTCTCCATC TACTGTAAGC ATATCTTGCA ACAGGAGTCC AGCTTCACTG   
  
  
+ CCTGAACTCG AATCCTCTTC ATCGGACTTC CAAAGTGACT CCCAGGAGAC TGTGAATGGC TCGCCAGTGA   
  
  
+ TTGATTCGTG CATGGTACGT GACATTGGTG ACTTAAGACA CAAACTTAGA GAGCTTGAGA CTGTTATGCT   
  
  
+ CGGACCAAGT TCAGACAGCT TGGATTCATG GTATGCCCCT TCAAGAGGTG AGTGTGAACC TCTGCCACCA   
  
  
+ GAAGAGCCTG ACAATGGGAA ACATTTGTTG GAGATGATAG CAAGGGGGAG CCTCAAAGAG GTGCTAATTG   
  
  
+ CTTGTGCCAA AGCAATATCA GACGATGATT TGTTAACAGC GGAGTGGTTG ATGTCAGAGC TACGCCATAT   
  
  
+ GGTTTCAGTT TCTGGAGAAC CAATTCAGAG GTTAGGAGCC TACATGTTGG AAGGGTTAGT TGCCCGGTTG   
  
  
+ TCCTCTTCAG GAAGTTCCAT CTACAAAGCT CTAAGGTGCA AAGAGCCTAC TAGTAGTGAA CTTCTTTCCT   
  
  
+ ATATGCATTT ACTCTATGAA GTTTGCCCTT ACTTCAAGTT TGGGTACATG TCTGCAAATG GGGCAATTGC   
  
  
+ TGAGGCCATG AAAAATGAGA GCAGAATTCA TATAATTGAT TTTCAGATAG CTCAAGGGAG TCAGTGGATT   
  
  
+ AGCCTTATCC AAGCCCTGGC TGCTCAGCCT GATGGGCCAC CACAGGTCCG TATTACCGGA GTCGATGATT   
  
  
+ CCCAATCTGA GTATGCTCGG GGAGGGGGGC TCGACATTGT AGCGAAGAGA CTATCTGGAC TAGCCCAGGC   
  
  
+ TTGCAGCCTA CCCTTTGAGT TCCACGCTGC AGCACTTAGT GGTTCTGAGA TCAGACTTCA AAACCTGGTT   
  
  
+ TTGCGGCCTG GAGAAGCCTT AGCAGTGAAC TTCCCATTCA TGCTGCACCA CATGCCGGAT GAGAGTGTGG   
  
  
+ GCCCTGAGAA TTATAGAGAC CAGTTATTAA GGCTGGTGAA GAGCTTCTTG CCCAAGGTGG TTACCCTTGT   
  
  
+ TGAGCAAGAA TCCAACACAA ACACGGCCCC ATTTCTACCC CGGTTTCTTG AAACCCTAGA CTACTATACT   
  
  
+ GCCATTTTCG AATCAATTGA TGTTACGCTC CCAAGAGATC ACAAGGAGCG GATCAATGTT GAGCAGCACT   
  
  
+ GTTTAGCAAG AGATATAGTC AACATAATAG CATGTGAGGG TGCCGAGAGG GTGGAACGCC ATGAGGTTCT   
  
  
+ TGGAAAGTGG AGATCTCGGT TCTCAATGGC TGGGTTTAAG CCGTACCCAT TGAGCCCGCT AGTGAATGCA   
  
  
+ ACTATCAGGA CTCTTCTGCA GAAGTATAGC AGGAGCTATG GACTTGAAGA AAGGGATGGA GCTCTTTATC   
  
  
+ TAGGCTGGAT GAACCGAGCA CTAGTTGCAT CTTGTGCATG GCAGTG  

- -Up\_Stream \_Len000AAGAAA AGGACAGAGA CAGAGAAGTA ACGTGGTAGT AAATTCTCTA CCCTGGTATT   
  
  
- TAGTAAGTTA GTGAGGAAAT ACAAATTTGA AACCTTCCTC CAATAATAAA TACACCACCA CTAGGGTTAC   
  
  
- GACTTAAATC GACAACTACC GCTCCCGTAT ATACATTAAT CCTAAAGGTT ACGTCAAATC TAAAATGAGT   
  
  
- AAACTCTTAA GTGACTCCAA ACAAACTAGA CTAAAATCGT TTATAACAAG TCCCCATATC TACGAATAAG   
  
  
- CCAAGAGTTG ACATAATCAT ATGTTCTACT CACCAGGTGA ACAAATTCGA AAATGATATA TGAATACAAC   
  
  
- AGGGATTAAA CCAGAGCGAA CTACACATCG ACAGTTCATA ATAATATTAG AACACAAACT ATAGTGTAAA   
  
  
- CATAACTAAT CGATGTTTTC TTGTATAATA TACACATATC GTTGATAGAG TATATACGGG AGTGGTATCG   
  
  
- CACCAAGGTT CCCAAGTTCA AGGTGAATCG GAGTGGGATC TTTACTGAAG AAAAAAAAAA ATTTCAATTT   
  
  
- CTATACGTTA GTGGTACCTT GGTCAGTAGA AAACTGTGCA ATAAGGTCTA GTCTACTGGT CGGAAAGAGG   
  
  
- TTCCGGTAAT CTAAAAGATC GGTTAGGTGA CAATTGACGT TTTTCACCAT AGGAACCGAC AAGGACAGTG   
  
  
- ACAAAGAATA AACGAGACAC TATAAACATG TAGAGAAACG GTTCCTATTT TCAAGTATCG GCCCTTAACC   
  
  
- ATAAGTCAAC TGAGAACGTC ATTTGTCTCA AGTACATGAC CTCCTAAAAC ACTCAGTACA GTGAGTTATT   
  
  
- TTTGTCCACA CGCTGTCACA ATATTTCGAC GAACGAATTA CGTGTAGATG AGAGTGAGTG GAATGGGTTT   
  
  
- TTTGAAGTTT TAACACACGG AAGATACTTA TAAGACTAGG TAATCGTAAG TTGCCAAAGA CTTAAGACAC   
  
  
- ATTCGGTAGG TATGAGTAAA AGTTGTCTCT TGAACCTAAC CTACATAAGT TATGGGTTTT TGGAACAGTT   
  
  
- TAATGGGGAT CGAATTAAAG GAGATTATGG TTGACGAGAA ACCAAAATCG CATGTTAGGG TATCTAATGG   
  
  
- GAAGATATTA ATCACTACTA ACCAAGTAAA GACGACGTAA TACGGAAGAA ATCTAGGTAA CTATTATATA   
  
  
- TGAGAACGGT ACGACGTTGT AAGGTGACCC TAATTATAGA GTGTAACATA GGAGTCGTTG GTTCCTCTGG   
  
  
- GATGATAAAA GTCTCGGTAA GTATCTTTCA ATAACGTATA TCACTACTAG AAGTTACCCA GACTAAAAAT   
  
  
- CTATCGACGA CACTTGTAAA ATGAGCAGGA GACGGTACAC CTATGATACC ATTGGAAACA AACGAGAGTT   
  
  
- CGGACTCGCA AGACTCTTTA CTACTTTCGC GGGAGCTCAA TATCCTGAAA AGACTGAAAT CGAAATCTTT   
  
  
- TCTCTCCTTT TAGTTGGTGT GGGTAGACTA ATAACTCAAA GACGAGGAAC TCTGGACCTA CCTCGGTCTT   
  
  
- GAGTGCCAGA ACAGTCGGTC TTATTAGTCC TTGACGTAAA GACTCCTGGG TACTTTTCTG GGACCATATG   
  
  
- CTGATAAGTA CTTTTGCAAA ACAGTCGGAA AGTCAAAAAC GTAAAATAAG ATCGACGAAA CTAACTTCGG   
  
  
- GTTAAAATAA TTTTGACACG AAAAGAGAAC TTACAATTTG AGAACGTCAA GGAACGAACA ATAAAACCCA   
  
  
- AAATACAAAC GAAAGATGAC TATAGTAGAA AACCAGCACG TTAAATTAAC GGATGATACA ATATACCTAA   
  
  
- AAATACAAAT GAAAGATGAC TATAGTAGAA AACGATAACG TTAAATTAAC GGAAGATACA TCTTAAGCAG   
  
  
- TGAAACTTCA GACGGTCCTT CCGTTATGTC GATATAAAAA GAACACCTAC TTCACTATTT TCCGTTCACA   
  
  
- GTTAACGAAT GACGAAATAA CGCGACATGA AAAGGTCTCT AAAACCTAAT CAGTTACGTC TTCGGTTGTT   
  
  
- AAATATCGCT GACCGGTGTC AAGATGTTGG TTAAACTATT AGGTGCAGAA GAACTTAGAA GTAGATATTA   
  
  
- CCCACCACTA GTTGAAAAGT CAAGAGGTAG ATGACATTCG TATAGAACGT TGTCCTCAGG TCGAAGTGAC   
  
  
- GGACTTGAGC TTAGGAGAAG TAGCCTGAAG GTTTCACTGA GGGTCCTCTG ACACTTACCG AGCGGTCACT   
  
  
- AACTAAGCAC GTACCATGCA CTGTAACCAC TGAATTCTGT GTTTGAATCT CTCGAACTCT GACAATACGA   
  
  
- GCCTGGTTCA AGTCTGTCGA ACCTAAGTAC CATACGGGGA AGTTCTCCAC TCACACTTGG AGACGGTGGT   
  
  
- CTTCTCGGAC TGTTACCCTT TGTAAACAAC CTCTACTATC GTTCCCCCTC GGAGTTTCTC CACGATTAAC   
  
  
- GAACACGGTT TCGTTATAGT CTGCTACTAA ACAATTGTCG CCTCACCAAC TACAGTCTCG ATGCGGTATA   
  
  
- CCAAAGTCAA AGACCTCTTG GTTAAGTCTC CAATCCTCGG ATGTACAACC TTCCCAATCA ACGGGCCAAC   
  
  
- AGGAGAAGTC CTTCAAGGTA GATGTTTCGA GATTCCACGT TTCTCGGATG ATCATCACTT GAAGAAAGGA   
  
  
- TATACGTAAA TGAGATACTT CAAACGGGAA TGAAGTTCAA ACCCATGTAC AGACGTTTAC CCCGTTAACG   
  
  
- ACTCCGGTAC TTTTTACTCT CGTCTTAAGT ATATTAACTA AAAGTCTATC GAGTTCCCTC AGTCACCTAA   
  
  
- TCGGAATAGG TTCGGGACCG ACGAGTCGGA CTACCCGGTG GTGTCCAGGC ATAATGGCCT CAGCTACTAA   
  
  
- GGGTTAGACT CATACGAGCC CCTCCCCCCG AGCTGTAACA TCGCTTCTCT GATAGACCTG ATCGGGTCCG   
  
  
- AACGTCGGAT GGGAAACTCA AGGTGCGACG TCGTGAATCA CCAAGACTCT AGTCTGAAGT TTTGGACCAA   
  
  
- AACGCCGGAC CTCTTCGGAA TCGTCACTTG AAGGGTAAGT ACGACGTGGT GTACGGCCTA CTCTCACACC   
  
  
- CGGGACTCTT AATATCTCTG GTCAATAATT CCGACCACTT CTCGAAGAAC GGGTTCCACC AATGGGAACA   
  
  
- ACTCGTTCTT AGGTTGTGTT TGTGCCGGGG TAAAGATGGG GCCAAAGAAC TTTGGGATCT GATGATATGA   
  
  
- CGGTAAAAGC TTAGTTAACT ACAATGCGAG GGTTCTCTAG TGTTCCTCGC CTAGTTACAA CTCGTCGTGA   
  
  
- CAAATCGTTC TCTATATCAG TTGTATTATC GTACACTCCC ACGGCTCTCC CACCTTGCGG TACTCCAAGA   
  
  
- ACCTTTCACC TCTAGAGCCA AGAGTTACCG ACCCAAATTC GGCATGGGTA ACTCGGGCGA TCACTTACGT   
  
  
- TGATAGTCCT GAGAAGACGT CTTCATATCG TCCTCGATAC CTGAACTTCT TTCCCTACCT CGAGAAATAG   
  
  
- ATCCGACCTA CTTGGCTCGT GATCAACGTA GAACACGTAC CGTCAC

+     MBS

| Site Name | Organism | Position | Strand | Matrix score. | sequence | function |
| --- | --- | --- | --- | --- | --- | --- |
| MBS | Arabidopsis thaliana | 1084 | + | 6 | CAACTG | MYB binding site involved in drought-inducibility |
| MBS | Arabidopsis thaliana | 291 | + | 6 | CAACTG | MYB binding site involved in drought-inducibility |
| MBS | Arabidopsis thaliana | 779 | - | 6 | CAACTG | MYB binding site involved in drought-inducibility |

>HU08G00284.1   
+ -Up\_Stream \_Len000TTCTTT TCCTGTCTCT GTCTCTTCAT TGCACCATCA TTTAAGAGAT GGGACCATAA   
  
  
+ ATCATTCAAT CACTCCTTTA TGTTTAAACT TTGGAAGGAG GTTATTATTT ATGTGGTGGT GATCCCAATG   
  
  
+ CTGAATTTAG CTGTTGATGG CGAGGGCATA TATGTAATTA GGATTTCCAA TGCAGTTTAG ATTTTACTCA   
  
  
+ TTTGAGAATT CACTGAGGTT TGTTTGATCT GATTTTAGCA AATATTGTTC AGGGGTATAG ATGCTTATTC   
  
  
+ GGTTCTCAAC TGTATTAGTA TACAAGATGA GTGGTCCACT TGTTTAAGCT TTTACTATAT ACTTATGTTG   
  
  
+ TCCCTAATTT GGTCTCGCTT GATGTGTAGC TGTCAAGTAT TATTATAATC TTGTGTTTGA TATCACATTT   
  
  
+ GTATTGATTA GCTACAAAAG AACATATTAT ATGTGTATAG CAACTATCTC ATATATGCCC TCACCATAGC   
  
  
+ GTGGTTCCAA GGGTTCAAGT TCCACTTAGC CTCACCCTAG AAATGACTTC TTTTTTTTTT TAAAGTTAAA   
  
  
+ GATATGCAAT CACCATGGAA CCAGTCATCT TTTGACACGT TATTCCAGAT CAGATGACCA GCCTTTCTCC   
  
  
+ AAGGCCATTA GATTTTCTAG CCAATCCACT GTTAACTGCA AAAAGTGGTA TCCTTGGCTG TTCCTGTCAC   
  
  
+ TGTTTCTTAT TTGCTCTGTG ATATTTGTAC ATCTCTTTGC CAAGGATAAA AGTTCATAGC CGGGAATTGG   
  
  
+ TATTCAGTTG ACTCTTGCAG TAAACAGAGT TCATGTACTG GAGGATTTTG TGAGTCATGT CACTCAATAA   
  
  
+ AAACAGGTGT GCGACAGTGT TATAAAGCTG CTTGCTTAAT GCACATCTAC TCTCACTCAC CTTACCCAAA   
  
  
+ AAACTTCAAA ATTGTGTGCC TTCTATGAAT ATTCTGATCC ATTAGCATTC AACGGTTTCT GAATTCTGTG   
  
  
+ TAAGCCATCC ATACTCATTT TCAACAGAGA ACTTGGATTG GATGTATTCA ATACCCAAAA ACCTTGTCAA   
  
  
+ ATTACCCCTA GCTTAATTTC CTCTAATACC AACTGCTCTT TGGTTTTAGC GTACAATCCC ATAGATTACC   
  
  
+ CTTCTATAAT TAGTGATGAT TGGTTCATTT CTGCTGCATT ATGCCTTCTT TAGATCCATT GATAATATAT   
  
  
+ ACTCTTGCCA TGCTGCAACA TTCCACTGGG ATTAATATCT CACATTGTAT CCTCAGCAAC CAAGGAGACC   
  
  
+ CTACTATTTT CAGAGCCATT CATAGAAAGT TATTGCATAT AGTGATGATC TTCAATGGGT CTGATTTTTA   
  
  
+ GATAGCTGCT GTGAACATTT TACTCGTCCT CTGCCATGTG GATACTATGG TAACCTTTGT TTGCTCTCAA   
  
  
+ GCCTGAGCGT TCTGAGAAAT GATGAAAGCG CCCTCGAGTT ATAGGACTTT TCTGACTTTA GCTTTAGAAA   
  
  
+ AGAGAGGAAA ATCAACCACA CCCATCTGAT TATTGAGTTT CTGCTCCTTG AGACCTGGAT GGAGCCAGAA   
  
  
+ CTCACGGTCT TGTCAGCCAG AATAATCAGG AACTGCATTT CTGAGGACCC ATGAAAAGAC CCTGGTATAC   
  
  
+ GACTATTCAT GAAAACGTTT TGTCAGCCTT TCAGTTTTTG CATTTTATTC TAGCTGCTTT GATTGAAGCC   
  
  
+ CAATTTTATT AAAACTGTGC TTTTCTCTTG AATGTTAAAC TCTTGCAGTT CCTTGCTTGT TATTTTGGGT   
  
  
+ TTTATGTTTG CTTTCTACTG ATATCATCTT TTGGTCGTGC AATTTAATTG CCTACTATGT TATATGGATT   
  
  
+ TTTATGTTTA CTTTCTACTG ATATCATCTT TTGCTATTGC AATTTAATTG CCTTCTATGT AGAATTCGTC   
  
  
+ ACTTTGAAGT CTGCCAGGAA GGCAATACAG CTATATTTTT CTTGTGGATG AAGTGATAAA AGGCAAGTGT   
  
  
+ CAATTGCTTA CTGCTTTATT GCGCTGTACT TTTCCAGAGA TTTTGGATTA GTCAATGCAG AAGCCAACAA   
  
  
+ TTTATAGCGA CTGGCCACAG TTCTACAACC AATTTGATAA TCCACGTCTT CTTGAATCTT CATCTATAAT   
  
  
+ GGGTGGTGAT CAACTTTTCA GTTCTCCATC TACTGTAAGC ATATCTTGCA ACAGGAGTCC AGCTTCACTG   
  
  
+ CCTGAACTCG AATCCTCTTC ATCGGACTTC CAAAGTGACT CCCAGGAGAC TGTGAATGGC TCGCCAGTGA   
  
  
+ TTGATTCGTG CATGGTACGT GACATTGGTG ACTTAAGACA CAAACTTAGA GAGCTTGAGA CTGTTATGCT   
  
  
+ CGGACCAAGT TCAGACAGCT TGGATTCATG GTATGCCCCT TCAAGAGGTG AGTGTGAACC TCTGCCACCA   
  
  
+ GAAGAGCCTG ACAATGGGAA ACATTTGTTG GAGATGATAG CAAGGGGGAG CCTCAAAGAG GTGCTAATTG   
  
  
+ CTTGTGCCAA AGCAATATCA GACGATGATT TGTTAACAGC GGAGTGGTTG ATGTCAGAGC TACGCCATAT   
  
  
+ GGTTTCAGTT TCTGGAGAAC CAATTCAGAG GTTAGGAGCC TACATGTTGG AAGGGTTAGT TGCCCGGTTG   
  
  
+ TCCTCTTCAG GAAGTTCCAT CTACAAAGCT CTAAGGTGCA AAGAGCCTAC TAGTAGTGAA CTTCTTTCCT   
  
  
+ ATATGCATTT ACTCTATGAA GTTTGCCCTT ACTTCAAGTT TGGGTACATG TCTGCAAATG GGGCAATTGC   
  
  
+ TGAGGCCATG AAAAATGAGA GCAGAATTCA TATAATTGAT TTTCAGATAG CTCAAGGGAG TCAGTGGATT   
  
  
+ AGCCTTATCC AAGCCCTGGC TGCTCAGCCT GATGGGCCAC CACAGGTCCG TATTACCGGA GTCGATGATT   
  
  
+ CCCAATCTGA GTATGCTCGG GGAGGGGGGC TCGACATTGT AGCGAAGAGA CTATCTGGAC TAGCCCAGGC   
  
  
+ TTGCAGCCTA CCCTTTGAGT TCCACGCTGC AGCACTTAGT GGTTCTGAGA TCAGACTTCA AAACCTGGTT   
  
  
+ TTGCGGCCTG GAGAAGCCTT AGCAGTGAAC TTCCCATTCA TGCTGCACCA CATGCCGGAT GAGAGTGTGG   
  
  
+ GCCCTGAGAA TTATAGAGAC CAGTTATTAA GGCTGGTGAA GAGCTTCTTG CCCAAGGTGG TTACCCTTGT   
  
  
+ TGAGCAAGAA TCCAACACAA ACACGGCCCC ATTTCTACCC CGGTTTCTTG AAACCCTAGA CTACTATACT   
  
  
+ GCCATTTTCG AATCAATTGA TGTTACGCTC CCAAGAGATC ACAAGGAGCG GATCAATGTT GAGCAGCACT   
  
  
+ GTTTAGCAAG AGATATAGTC AACATAATAG CATGTGAGGG TGCCGAGAGG GTGGAACGCC ATGAGGTTCT   
  
  
+ TGGAAAGTGG AGATCTCGGT TCTCAATGGC TGGGTTTAAG CCGTACCCAT TGAGCCCGCT AGTGAATGCA   
  
  
+ ACTATCAGGA CTCTTCTGCA GAAGTATAGC AGGAGCTATG GACTTGAAGA AAGGGATGGA GCTCTTTATC   
  
  
+ TAGGCTGGAT GAACCGAGCA CTAGTTGCAT CTTGTGCATG GCAGTG  

- -Up\_Stream \_Len000AAGAAA AGGACAGAGA CAGAGAAGTA ACGTGGTAGT AAATTCTCTA CCCTGGTATT   
  
  
- TAGTAAGTTA GTGAGGAAAT ACAAATTTGA AACCTTCCTC CAATAATAAA TACACCACCA CTAGGGTTAC   
  
  
- GACTTAAATC GACAACTACC GCTCCCGTAT ATACATTAAT CCTAAAGGTT ACGTCAAATC TAAAATGAGT   
  
  
- AAACTCTTAA GTGACTCCAA ACAAACTAGA CTAAAATCGT TTATAACAAG TCCCCATATC TACGAATAAG   
  
  
- CCAAGAGTTG ACATAATCAT ATGTTCTACT CACCAGGTGA ACAAATTCGA AAATGATATA TGAATACAAC   
  
  
- AGGGATTAAA CCAGAGCGAA CTACACATCG ACAGTTCATA ATAATATTAG AACACAAACT ATAGTGTAAA   
  
  
- CATAACTAAT CGATGTTTTC TTGTATAATA TACACATATC GTTGATAGAG TATATACGGG AGTGGTATCG   
  
  
- CACCAAGGTT CCCAAGTTCA AGGTGAATCG GAGTGGGATC TTTACTGAAG AAAAAAAAAA ATTTCAATTT   
  
  
- CTATACGTTA GTGGTACCTT GGTCAGTAGA AAACTGTGCA ATAAGGTCTA GTCTACTGGT CGGAAAGAGG   
  
  
- TTCCGGTAAT CTAAAAGATC GGTTAGGTGA CAATTGACGT TTTTCACCAT AGGAACCGAC AAGGACAGTG   
  
  
- ACAAAGAATA AACGAGACAC TATAAACATG TAGAGAAACG GTTCCTATTT TCAAGTATCG GCCCTTAACC   
  
  
- ATAAGTCAAC TGAGAACGTC ATTTGTCTCA AGTACATGAC CTCCTAAAAC ACTCAGTACA GTGAGTTATT   
  
  
- TTTGTCCACA CGCTGTCACA ATATTTCGAC GAACGAATTA CGTGTAGATG AGAGTGAGTG GAATGGGTTT   
  
  
- TTTGAAGTTT TAACACACGG AAGATACTTA TAAGACTAGG TAATCGTAAG TTGCCAAAGA CTTAAGACAC   
  
  
- ATTCGGTAGG TATGAGTAAA AGTTGTCTCT TGAACCTAAC CTACATAAGT TATGGGTTTT TGGAACAGTT   
  
  
- TAATGGGGAT CGAATTAAAG GAGATTATGG TTGACGAGAA ACCAAAATCG CATGTTAGGG TATCTAATGG   
  
  
- GAAGATATTA ATCACTACTA ACCAAGTAAA GACGACGTAA TACGGAAGAA ATCTAGGTAA CTATTATATA   
  
  
- TGAGAACGGT ACGACGTTGT AAGGTGACCC TAATTATAGA GTGTAACATA GGAGTCGTTG GTTCCTCTGG   
  
  
- GATGATAAAA GTCTCGGTAA GTATCTTTCA ATAACGTATA TCACTACTAG AAGTTACCCA GACTAAAAAT   
  
  
- CTATCGACGA CACTTGTAAA ATGAGCAGGA GACGGTACAC CTATGATACC ATTGGAAACA AACGAGAGTT   
  
  
- CGGACTCGCA AGACTCTTTA CTACTTTCGC GGGAGCTCAA TATCCTGAAA AGACTGAAAT CGAAATCTTT   
  
  
- TCTCTCCTTT TAGTTGGTGT GGGTAGACTA ATAACTCAAA GACGAGGAAC TCTGGACCTA CCTCGGTCTT   
  
  
- GAGTGCCAGA ACAGTCGGTC TTATTAGTCC TTGACGTAAA GACTCCTGGG TACTTTTCTG GGACCATATG   
  
  
- CTGATAAGTA CTTTTGCAAA ACAGTCGGAA AGTCAAAAAC GTAAAATAAG ATCGACGAAA CTAACTTCGG   
  
  
- GTTAAAATAA TTTTGACACG AAAAGAGAAC TTACAATTTG AGAACGTCAA GGAACGAACA ATAAAACCCA   
  
  
- AAATACAAAC GAAAGATGAC TATAGTAGAA AACCAGCACG TTAAATTAAC GGATGATACA ATATACCTAA   
  
  
- AAATACAAAT GAAAGATGAC TATAGTAGAA AACGATAACG TTAAATTAAC GGAAGATACA TCTTAAGCAG   
  
  
- TGAAACTTCA GACGGTCCTT CCGTTATGTC GATATAAAAA GAACACCTAC TTCACTATTT TCCGTTCACA   
  
  
- GTTAACGAAT GACGAAATAA CGCGACATGA AAAGGTCTCT AAAACCTAAT CAGTTACGTC TTCGGTTGTT   
  
  
- AAATATCGCT GACCGGTGTC AAGATGTTGG TTAAACTATT AGGTGCAGAA GAACTTAGAA GTAGATATTA   
  
  
- CCCACCACTA GTTGAAAAGT CAAGAGGTAG ATGACATTCG TATAGAACGT TGTCCTCAGG TCGAAGTGAC   
  
  
- GGACTTGAGC TTAGGAGAAG TAGCCTGAAG GTTTCACTGA GGGTCCTCTG ACACTTACCG AGCGGTCACT   
  
  
- AACTAAGCAC GTACCATGCA CTGTAACCAC TGAATTCTGT GTTTGAATCT CTCGAACTCT GACAATACGA   
  
  
- GCCTGGTTCA AGTCTGTCGA ACCTAAGTAC CATACGGGGA AGTTCTCCAC TCACACTTGG AGACGGTGGT   
  
  
- CTTCTCGGAC TGTTACCCTT TGTAAACAAC CTCTACTATC GTTCCCCCTC GGAGTTTCTC CACGATTAAC   
  
  
- GAACACGGTT TCGTTATAGT CTGCTACTAA ACAATTGTCG CCTCACCAAC TACAGTCTCG ATGCGGTATA   
  
  
- CCAAAGTCAA AGACCTCTTG GTTAAGTCTC CAATCCTCGG ATGTACAACC TTCCCAATCA ACGGGCCAAC   
  
  
- AGGAGAAGTC CTTCAAGGTA GATGTTTCGA GATTCCACGT TTCTCGGATG ATCATCACTT GAAGAAAGGA   
  
  
- TATACGTAAA TGAGATACTT CAAACGGGAA TGAAGTTCAA ACCCATGTAC AGACGTTTAC CCCGTTAACG   
  
  
- ACTCCGGTAC TTTTTACTCT CGTCTTAAGT ATATTAACTA AAAGTCTATC GAGTTCCCTC AGTCACCTAA   
  
  
- TCGGAATAGG TTCGGGACCG ACGAGTCGGA CTACCCGGTG GTGTCCAGGC ATAATGGCCT CAGCTACTAA   
  
  
- GGGTTAGACT CATACGAGCC CCTCCCCCCG AGCTGTAACA TCGCTTCTCT GATAGACCTG ATCGGGTCCG   
  
  
- AACGTCGGAT GGGAAACTCA AGGTGCGACG TCGTGAATCA CCAAGACTCT AGTCTGAAGT TTTGGACCAA   
  
  
- AACGCCGGAC CTCTTCGGAA TCGTCACTTG AAGGGTAAGT ACGACGTGGT GTACGGCCTA CTCTCACACC   
  
  
- CGGGACTCTT AATATCTCTG GTCAATAATT CCGACCACTT CTCGAAGAAC GGGTTCCACC AATGGGAACA   
  
  
- ACTCGTTCTT AGGTTGTGTT TGTGCCGGGG TAAAGATGGG GCCAAAGAAC TTTGGGATCT GATGATATGA   
  
  
- CGGTAAAAGC TTAGTTAACT ACAATGCGAG GGTTCTCTAG TGTTCCTCGC CTAGTTACAA CTCGTCGTGA   
  
  
- CAAATCGTTC TCTATATCAG TTGTATTATC GTACACTCCC ACGGCTCTCC CACCTTGCGG TACTCCAAGA   
  
  
- ACCTTTCACC TCTAGAGCCA AGAGTTACCG ACCCAAATTC GGCATGGGTA ACTCGGGCGA TCACTTACGT   
  
  
- TGATAGTCCT GAGAAGACGT CTTCATATCG TCCTCGATAC CTGAACTTCT TTCCCTACCT CGAGAAATAG   
  
  
- ATCCGACCTA CTTGGCTCGT GATCAACGTA GAACACGTAC CGTCAC

+     MYB

| Site Name | Organism | Position | Strand | Matrix score. | sequence | function |
| --- | --- | --- | --- | --- | --- | --- |
| MYB | Arabidopsis thaliana | 1006 | + | 6 | CAACAG |  |
| MYB | Arabidopsis thaliana | 1251 | + | 6 | CAACCA |  |
| MYB | Arabidopsis thaliana | 1487 | + | 6 | CAACCA |  |
| MYB | Arabidopsis thaliana | 2153 | + | 6 | CAACAG |  |
| MYB | Arabidopsis thaliana | 155 | - | 6 | CAACAG |  |
| MYB | Arabidopsis thaliana | 2499 | - | 6 | CAACCA |  |
| MYB | Arabidopsis thaliana | 2060 | + | 6 | CAACCA |  |
| MYB | Arabidopsis thaliana | 3142 | - | 6 | TAACCA |  |

>HU08G00284.1   
+ -Up\_Stream \_Len000TTCTTT TCCTGTCTCT GTCTCTTCAT TGCACCATCA TTTAAGAGAT GGGACCATAA   
  
  
+ ATCATTCAAT CACTCCTTTA TGTTTAAACT TTGGAAGGAG GTTATTATTT ATGTGGTGGT GATCCCAATG   
  
  
+ CTGAATTTAG CTGTTGATGG CGAGGGCATA TATGTAATTA GGATTTCCAA TGCAGTTTAG ATTTTACTCA   
  
  
+ TTTGAGAATT CACTGAGGTT TGTTTGATCT GATTTTAGCA AATATTGTTC AGGGGTATAG ATGCTTATTC   
  
  
+ GGTTCTCAAC TGTATTAGTA TACAAGATGA GTGGTCCACT TGTTTAAGCT TTTACTATAT ACTTATGTTG   
  
  
+ TCCCTAATTT GGTCTCGCTT GATGTGTAGC TGTCAAGTAT TATTATAATC TTGTGTTTGA TATCACATTT   
  
  
+ GTATTGATTA GCTACAAAAG AACATATTAT ATGTGTATAG CAACTATCTC ATATATGCCC TCACCATAGC   
  
  
+ GTGGTTCCAA GGGTTCAAGT TCCACTTAGC CTCACCCTAG AAATGACTTC TTTTTTTTTT TAAAGTTAAA   
  
  
+ GATATGCAAT CACCATGGAA CCAGTCATCT TTTGACACGT TATTCCAGAT CAGATGACCA GCCTTTCTCC   
  
  
+ AAGGCCATTA GATTTTCTAG CCAATCCACT GTTAACTGCA AAAAGTGGTA TCCTTGGCTG TTCCTGTCAC   
  
  
+ TGTTTCTTAT TTGCTCTGTG ATATTTGTAC ATCTCTTTGC CAAGGATAAA AGTTCATAGC CGGGAATTGG   
  
  
+ TATTCAGTTG ACTCTTGCAG TAAACAGAGT TCATGTACTG GAGGATTTTG TGAGTCATGT CACTCAATAA   
  
  
+ AAACAGGTGT GCGACAGTGT TATAAAGCTG CTTGCTTAAT GCACATCTAC TCTCACTCAC CTTACCCAAA   
  
  
+ AAACTTCAAA ATTGTGTGCC TTCTATGAAT ATTCTGATCC ATTAGCATTC AACGGTTTCT GAATTCTGTG   
  
  
+ TAAGCCATCC ATACTCATTT TCAACAGAGA ACTTGGATTG GATGTATTCA ATACCCAAAA ACCTTGTCAA   
  
  
+ ATTACCCCTA GCTTAATTTC CTCTAATACC AACTGCTCTT TGGTTTTAGC GTACAATCCC ATAGATTACC   
  
  
+ CTTCTATAAT TAGTGATGAT TGGTTCATTT CTGCTGCATT ATGCCTTCTT TAGATCCATT GATAATATAT   
  
  
+ ACTCTTGCCA TGCTGCAACA TTCCACTGGG ATTAATATCT CACATTGTAT CCTCAGCAAC CAAGGAGACC   
  
  
+ CTACTATTTT CAGAGCCATT CATAGAAAGT TATTGCATAT AGTGATGATC TTCAATGGGT CTGATTTTTA   
  
  
+ GATAGCTGCT GTGAACATTT TACTCGTCCT CTGCCATGTG GATACTATGG TAACCTTTGT TTGCTCTCAA   
  
  
+ GCCTGAGCGT TCTGAGAAAT GATGAAAGCG CCCTCGAGTT ATAGGACTTT TCTGACTTTA GCTTTAGAAA   
  
  
+ AGAGAGGAAA ATCAACCACA CCCATCTGAT TATTGAGTTT CTGCTCCTTG AGACCTGGAT GGAGCCAGAA   
  
  
+ CTCACGGTCT TGTCAGCCAG AATAATCAGG AACTGCATTT CTGAGGACCC ATGAAAAGAC CCTGGTATAC   
  
  
+ GACTATTCAT GAAAACGTTT TGTCAGCCTT TCAGTTTTTG CATTTTATTC TAGCTGCTTT GATTGAAGCC   
  
  
+ CAATTTTATT AAAACTGTGC TTTTCTCTTG AATGTTAAAC TCTTGCAGTT CCTTGCTTGT TATTTTGGGT   
  
  
+ TTTATGTTTG CTTTCTACTG ATATCATCTT TTGGTCGTGC AATTTAATTG CCTACTATGT TATATGGATT   
  
  
+ TTTATGTTTA CTTTCTACTG ATATCATCTT TTGCTATTGC AATTTAATTG CCTTCTATGT AGAATTCGTC   
  
  
+ ACTTTGAAGT CTGCCAGGAA GGCAATACAG CTATATTTTT CTTGTGGATG AAGTGATAAA AGGCAAGTGT   
  
  
+ CAATTGCTTA CTGCTTTATT GCGCTGTACT TTTCCAGAGA TTTTGGATTA GTCAATGCAG AAGCCAACAA   
  
  
+ TTTATAGCGA CTGGCCACAG TTCTACAACC AATTTGATAA TCCACGTCTT CTTGAATCTT CATCTATAAT   
  
  
+ GGGTGGTGAT CAACTTTTCA GTTCTCCATC TACTGTAAGC ATATCTTGCA ACAGGAGTCC AGCTTCACTG   
  
  
+ CCTGAACTCG AATCCTCTTC ATCGGACTTC CAAAGTGACT CCCAGGAGAC TGTGAATGGC TCGCCAGTGA   
  
  
+ TTGATTCGTG CATGGTACGT GACATTGGTG ACTTAAGACA CAAACTTAGA GAGCTTGAGA CTGTTATGCT   
  
  
+ CGGACCAAGT TCAGACAGCT TGGATTCATG GTATGCCCCT TCAAGAGGTG AGTGTGAACC TCTGCCACCA   
  
  
+ GAAGAGCCTG ACAATGGGAA ACATTTGTTG GAGATGATAG CAAGGGGGAG CCTCAAAGAG GTGCTAATTG   
  
  
+ CTTGTGCCAA AGCAATATCA GACGATGATT TGTTAACAGC GGAGTGGTTG ATGTCAGAGC TACGCCATAT   
  
  
+ GGTTTCAGTT TCTGGAGAAC CAATTCAGAG GTTAGGAGCC TACATGTTGG AAGGGTTAGT TGCCCGGTTG   
  
  
+ TCCTCTTCAG GAAGTTCCAT CTACAAAGCT CTAAGGTGCA AAGAGCCTAC TAGTAGTGAA CTTCTTTCCT   
  
  
+ ATATGCATTT ACTCTATGAA GTTTGCCCTT ACTTCAAGTT TGGGTACATG TCTGCAAATG GGGCAATTGC   
  
  
+ TGAGGCCATG AAAAATGAGA GCAGAATTCA TATAATTGAT TTTCAGATAG CTCAAGGGAG TCAGTGGATT   
  
  
+ AGCCTTATCC AAGCCCTGGC TGCTCAGCCT GATGGGCCAC CACAGGTCCG TATTACCGGA GTCGATGATT   
  
  
+ CCCAATCTGA GTATGCTCGG GGAGGGGGGC TCGACATTGT AGCGAAGAGA CTATCTGGAC TAGCCCAGGC   
  
  
+ TTGCAGCCTA CCCTTTGAGT TCCACGCTGC AGCACTTAGT GGTTCTGAGA TCAGACTTCA AAACCTGGTT   
  
  
+ TTGCGGCCTG GAGAAGCCTT AGCAGTGAAC TTCCCATTCA TGCTGCACCA CATGCCGGAT GAGAGTGTGG   
  
  
+ GCCCTGAGAA TTATAGAGAC CAGTTATTAA GGCTGGTGAA GAGCTTCTTG CCCAAGGTGG TTACCCTTGT   
  
  
+ TGAGCAAGAA TCCAACACAA ACACGGCCCC ATTTCTACCC CGGTTTCTTG AAACCCTAGA CTACTATACT   
  
  
+ GCCATTTTCG AATCAATTGA TGTTACGCTC CCAAGAGATC ACAAGGAGCG GATCAATGTT GAGCAGCACT   
  
  
+ GTTTAGCAAG AGATATAGTC AACATAATAG CATGTGAGGG TGCCGAGAGG GTGGAACGCC ATGAGGTTCT   
  
  
+ TGGAAAGTGG AGATCTCGGT TCTCAATGGC TGGGTTTAAG CCGTACCCAT TGAGCCCGCT AGTGAATGCA   
  
  
+ ACTATCAGGA CTCTTCTGCA GAAGTATAGC AGGAGCTATG GACTTGAAGA AAGGGATGGA GCTCTTTATC   
  
  
+ TAGGCTGGAT GAACCGAGCA CTAGTTGCAT CTTGTGCATG GCAGTG  

- -Up\_Stream \_Len000AAGAAA AGGACAGAGA CAGAGAAGTA ACGTGGTAGT AAATTCTCTA CCCTGGTATT   
  
  
- TAGTAAGTTA GTGAGGAAAT ACAAATTTGA AACCTTCCTC CAATAATAAA TACACCACCA CTAGGGTTAC   
  
  
- GACTTAAATC GACAACTACC GCTCCCGTAT ATACATTAAT CCTAAAGGTT ACGTCAAATC TAAAATGAGT   
  
  
- AAACTCTTAA GTGACTCCAA ACAAACTAGA CTAAAATCGT TTATAACAAG TCCCCATATC TACGAATAAG   
  
  
- CCAAGAGTTG ACATAATCAT ATGTTCTACT CACCAGGTGA ACAAATTCGA AAATGATATA TGAATACAAC   
  
  
- AGGGATTAAA CCAGAGCGAA CTACACATCG ACAGTTCATA ATAATATTAG AACACAAACT ATAGTGTAAA   
  
  
- CATAACTAAT CGATGTTTTC TTGTATAATA TACACATATC GTTGATAGAG TATATACGGG AGTGGTATCG   
  
  
- CACCAAGGTT CCCAAGTTCA AGGTGAATCG GAGTGGGATC TTTACTGAAG AAAAAAAAAA ATTTCAATTT   
  
  
- CTATACGTTA GTGGTACCTT GGTCAGTAGA AAACTGTGCA ATAAGGTCTA GTCTACTGGT CGGAAAGAGG   
  
  
- TTCCGGTAAT CTAAAAGATC GGTTAGGTGA CAATTGACGT TTTTCACCAT AGGAACCGAC AAGGACAGTG   
  
  
- ACAAAGAATA AACGAGACAC TATAAACATG TAGAGAAACG GTTCCTATTT TCAAGTATCG GCCCTTAACC   
  
  
- ATAAGTCAAC TGAGAACGTC ATTTGTCTCA AGTACATGAC CTCCTAAAAC ACTCAGTACA GTGAGTTATT   
  
  
- TTTGTCCACA CGCTGTCACA ATATTTCGAC GAACGAATTA CGTGTAGATG AGAGTGAGTG GAATGGGTTT   
  
  
- TTTGAAGTTT TAACACACGG AAGATACTTA TAAGACTAGG TAATCGTAAG TTGCCAAAGA CTTAAGACAC   
  
  
- ATTCGGTAGG TATGAGTAAA AGTTGTCTCT TGAACCTAAC CTACATAAGT TATGGGTTTT TGGAACAGTT   
  
  
- TAATGGGGAT CGAATTAAAG GAGATTATGG TTGACGAGAA ACCAAAATCG CATGTTAGGG TATCTAATGG   
  
  
- GAAGATATTA ATCACTACTA ACCAAGTAAA GACGACGTAA TACGGAAGAA ATCTAGGTAA CTATTATATA   
  
  
- TGAGAACGGT ACGACGTTGT AAGGTGACCC TAATTATAGA GTGTAACATA GGAGTCGTTG GTTCCTCTGG   
  
  
- GATGATAAAA GTCTCGGTAA GTATCTTTCA ATAACGTATA TCACTACTAG AAGTTACCCA GACTAAAAAT   
  
  
- CTATCGACGA CACTTGTAAA ATGAGCAGGA GACGGTACAC CTATGATACC ATTGGAAACA AACGAGAGTT   
  
  
- CGGACTCGCA AGACTCTTTA CTACTTTCGC GGGAGCTCAA TATCCTGAAA AGACTGAAAT CGAAATCTTT   
  
  
- TCTCTCCTTT TAGTTGGTGT GGGTAGACTA ATAACTCAAA GACGAGGAAC TCTGGACCTA CCTCGGTCTT   
  
  
- GAGTGCCAGA ACAGTCGGTC TTATTAGTCC TTGACGTAAA GACTCCTGGG TACTTTTCTG GGACCATATG   
  
  
- CTGATAAGTA CTTTTGCAAA ACAGTCGGAA AGTCAAAAAC GTAAAATAAG ATCGACGAAA CTAACTTCGG   
  
  
- GTTAAAATAA TTTTGACACG AAAAGAGAAC TTACAATTTG AGAACGTCAA GGAACGAACA ATAAAACCCA   
  
  
- AAATACAAAC GAAAGATGAC TATAGTAGAA AACCAGCACG TTAAATTAAC GGATGATACA ATATACCTAA   
  
  
- AAATACAAAT GAAAGATGAC TATAGTAGAA AACGATAACG TTAAATTAAC GGAAGATACA TCTTAAGCAG   
  
  
- TGAAACTTCA GACGGTCCTT CCGTTATGTC GATATAAAAA GAACACCTAC TTCACTATTT TCCGTTCACA   
  
  
- GTTAACGAAT GACGAAATAA CGCGACATGA AAAGGTCTCT AAAACCTAAT CAGTTACGTC TTCGGTTGTT   
  
  
- AAATATCGCT GACCGGTGTC AAGATGTTGG TTAAACTATT AGGTGCAGAA GAACTTAGAA GTAGATATTA   
  
  
- CCCACCACTA GTTGAAAAGT CAAGAGGTAG ATGACATTCG TATAGAACGT TGTCCTCAGG TCGAAGTGAC   
  
  
- GGACTTGAGC TTAGGAGAAG TAGCCTGAAG GTTTCACTGA GGGTCCTCTG ACACTTACCG AGCGGTCACT   
  
  
- AACTAAGCAC GTACCATGCA CTGTAACCAC TGAATTCTGT GTTTGAATCT CTCGAACTCT GACAATACGA   
  
  
- GCCTGGTTCA AGTCTGTCGA ACCTAAGTAC CATACGGGGA AGTTCTCCAC TCACACTTGG AGACGGTGGT   
  
  
- CTTCTCGGAC TGTTACCCTT TGTAAACAAC CTCTACTATC GTTCCCCCTC GGAGTTTCTC CACGATTAAC   
  
  
- GAACACGGTT TCGTTATAGT CTGCTACTAA ACAATTGTCG CCTCACCAAC TACAGTCTCG ATGCGGTATA   
  
  
- CCAAAGTCAA AGACCTCTTG GTTAAGTCTC CAATCCTCGG ATGTACAACC TTCCCAATCA ACGGGCCAAC   
  
  
- AGGAGAAGTC CTTCAAGGTA GATGTTTCGA GATTCCACGT TTCTCGGATG ATCATCACTT GAAGAAAGGA   
  
  
- TATACGTAAA TGAGATACTT CAAACGGGAA TGAAGTTCAA ACCCATGTAC AGACGTTTAC CCCGTTAACG   
  
  
- ACTCCGGTAC TTTTTACTCT CGTCTTAAGT ATATTAACTA AAAGTCTATC GAGTTCCCTC AGTCACCTAA   
  
  
- TCGGAATAGG TTCGGGACCG ACGAGTCGGA CTACCCGGTG GTGTCCAGGC ATAATGGCCT CAGCTACTAA   
  
  
- GGGTTAGACT CATACGAGCC CCTCCCCCCG AGCTGTAACA TCGCTTCTCT GATAGACCTG ATCGGGTCCG   
  
  
- AACGTCGGAT GGGAAACTCA AGGTGCGACG TCGTGAATCA CCAAGACTCT AGTCTGAAGT TTTGGACCAA   
  
  
- AACGCCGGAC CTCTTCGGAA TCGTCACTTG AAGGGTAAGT ACGACGTGGT GTACGGCCTA CTCTCACACC   
  
  
- CGGGACTCTT AATATCTCTG GTCAATAATT CCGACCACTT CTCGAAGAAC GGGTTCCACC AATGGGAACA   
  
  
- ACTCGTTCTT AGGTTGTGTT TGTGCCGGGG TAAAGATGGG GCCAAAGAAC TTTGGGATCT GATGATATGA   
  
  
- CGGTAAAAGC TTAGTTAACT ACAATGCGAG GGTTCTCTAG TGTTCCTCGC CTAGTTACAA CTCGTCGTGA   
  
  
- CAAATCGTTC TCTATATCAG TTGTATTATC GTACACTCCC ACGGCTCTCC CACCTTGCGG TACTCCAAGA   
  
  
- ACCTTTCACC TCTAGAGCCA AGAGTTACCG ACCCAAATTC GGCATGGGTA ACTCGGGCGA TCACTTACGT   
  
  
- TGATAGTCCT GAGAAGACGT CTTCATATCG TCCTCGATAC CTGAACTTCT TTCCCTACCT CGAGAAATAG   
  
  
- ATCCGACCTA CTTGGCTCGT GATCAACGTA GAACACGTAC CGTCAC

+     MYB recognition site

| Site Name | Organism | Position | Strand | Matrix score. | sequence | function |
| --- | --- | --- | --- | --- | --- | --- |
| MYB recognition site | Arabidopsis thaliana | 964 | - | 6 | CCGTTG |  |

>HU08G00284.1   
+ -Up\_Stream \_Len000TTCTTT TCCTGTCTCT GTCTCTTCAT TGCACCATCA TTTAAGAGAT GGGACCATAA   
  
  
+ ATCATTCAAT CACTCCTTTA TGTTTAAACT TTGGAAGGAG GTTATTATTT ATGTGGTGGT GATCCCAATG   
  
  
+ CTGAATTTAG CTGTTGATGG CGAGGGCATA TATGTAATTA GGATTTCCAA TGCAGTTTAG ATTTTACTCA   
  
  
+ TTTGAGAATT CACTGAGGTT TGTTTGATCT GATTTTAGCA AATATTGTTC AGGGGTATAG ATGCTTATTC   
  
  
+ GGTTCTCAAC TGTATTAGTA TACAAGATGA GTGGTCCACT TGTTTAAGCT TTTACTATAT ACTTATGTTG   
  
  
+ TCCCTAATTT GGTCTCGCTT GATGTGTAGC TGTCAAGTAT TATTATAATC TTGTGTTTGA TATCACATTT   
  
  
+ GTATTGATTA GCTACAAAAG AACATATTAT ATGTGTATAG CAACTATCTC ATATATGCCC TCACCATAGC   
  
  
+ GTGGTTCCAA GGGTTCAAGT TCCACTTAGC CTCACCCTAG AAATGACTTC TTTTTTTTTT TAAAGTTAAA   
  
  
+ GATATGCAAT CACCATGGAA CCAGTCATCT TTTGACACGT TATTCCAGAT CAGATGACCA GCCTTTCTCC   
  
  
+ AAGGCCATTA GATTTTCTAG CCAATCCACT GTTAACTGCA AAAAGTGGTA TCCTTGGCTG TTCCTGTCAC   
  
  
+ TGTTTCTTAT TTGCTCTGTG ATATTTGTAC ATCTCTTTGC CAAGGATAAA AGTTCATAGC CGGGAATTGG   
  
  
+ TATTCAGTTG ACTCTTGCAG TAAACAGAGT TCATGTACTG GAGGATTTTG TGAGTCATGT CACTCAATAA   
  
  
+ AAACAGGTGT GCGACAGTGT TATAAAGCTG CTTGCTTAAT GCACATCTAC TCTCACTCAC CTTACCCAAA   
  
  
+ AAACTTCAAA ATTGTGTGCC TTCTATGAAT ATTCTGATCC ATTAGCATTC AACGGTTTCT GAATTCTGTG   
  
  
+ TAAGCCATCC ATACTCATTT TCAACAGAGA ACTTGGATTG GATGTATTCA ATACCCAAAA ACCTTGTCAA   
  
  
+ ATTACCCCTA GCTTAATTTC CTCTAATACC AACTGCTCTT TGGTTTTAGC GTACAATCCC ATAGATTACC   
  
  
+ CTTCTATAAT TAGTGATGAT TGGTTCATTT CTGCTGCATT ATGCCTTCTT TAGATCCATT GATAATATAT   
  
  
+ ACTCTTGCCA TGCTGCAACA TTCCACTGGG ATTAATATCT CACATTGTAT CCTCAGCAAC CAAGGAGACC   
  
  
+ CTACTATTTT CAGAGCCATT CATAGAAAGT TATTGCATAT AGTGATGATC TTCAATGGGT CTGATTTTTA   
  
  
+ GATAGCTGCT GTGAACATTT TACTCGTCCT CTGCCATGTG GATACTATGG TAACCTTTGT TTGCTCTCAA   
  
  
+ GCCTGAGCGT TCTGAGAAAT GATGAAAGCG CCCTCGAGTT ATAGGACTTT TCTGACTTTA GCTTTAGAAA   
  
  
+ AGAGAGGAAA ATCAACCACA CCCATCTGAT TATTGAGTTT CTGCTCCTTG AGACCTGGAT GGAGCCAGAA   
  
  
+ CTCACGGTCT TGTCAGCCAG AATAATCAGG AACTGCATTT CTGAGGACCC ATGAAAAGAC CCTGGTATAC   
  
  
+ GACTATTCAT GAAAACGTTT TGTCAGCCTT TCAGTTTTTG CATTTTATTC TAGCTGCTTT GATTGAAGCC   
  
  
+ CAATTTTATT AAAACTGTGC TTTTCTCTTG AATGTTAAAC TCTTGCAGTT CCTTGCTTGT TATTTTGGGT   
  
  
+ TTTATGTTTG CTTTCTACTG ATATCATCTT TTGGTCGTGC AATTTAATTG CCTACTATGT TATATGGATT   
  
  
+ TTTATGTTTA CTTTCTACTG ATATCATCTT TTGCTATTGC AATTTAATTG CCTTCTATGT AGAATTCGTC   
  
  
+ ACTTTGAAGT CTGCCAGGAA GGCAATACAG CTATATTTTT CTTGTGGATG AAGTGATAAA AGGCAAGTGT   
  
  
+ CAATTGCTTA CTGCTTTATT GCGCTGTACT TTTCCAGAGA TTTTGGATTA GTCAATGCAG AAGCCAACAA   
  
  
+ TTTATAGCGA CTGGCCACAG TTCTACAACC AATTTGATAA TCCACGTCTT CTTGAATCTT CATCTATAAT   
  
  
+ GGGTGGTGAT CAACTTTTCA GTTCTCCATC TACTGTAAGC ATATCTTGCA ACAGGAGTCC AGCTTCACTG   
  
  
+ CCTGAACTCG AATCCTCTTC ATCGGACTTC CAAAGTGACT CCCAGGAGAC TGTGAATGGC TCGCCAGTGA   
  
  
+ TTGATTCGTG CATGGTACGT GACATTGGTG ACTTAAGACA CAAACTTAGA GAGCTTGAGA CTGTTATGCT   
  
  
+ CGGACCAAGT TCAGACAGCT TGGATTCATG GTATGCCCCT TCAAGAGGTG AGTGTGAACC TCTGCCACCA   
  
  
+ GAAGAGCCTG ACAATGGGAA ACATTTGTTG GAGATGATAG CAAGGGGGAG CCTCAAAGAG GTGCTAATTG   
  
  
+ CTTGTGCCAA AGCAATATCA GACGATGATT TGTTAACAGC GGAGTGGTTG ATGTCAGAGC TACGCCATAT   
  
  
+ GGTTTCAGTT TCTGGAGAAC CAATTCAGAG GTTAGGAGCC TACATGTTGG AAGGGTTAGT TGCCCGGTTG   
  
  
+ TCCTCTTCAG GAAGTTCCAT CTACAAAGCT CTAAGGTGCA AAGAGCCTAC TAGTAGTGAA CTTCTTTCCT   
  
  
+ ATATGCATTT ACTCTATGAA GTTTGCCCTT ACTTCAAGTT TGGGTACATG TCTGCAAATG GGGCAATTGC   
  
  
+ TGAGGCCATG AAAAATGAGA GCAGAATTCA TATAATTGAT TTTCAGATAG CTCAAGGGAG TCAGTGGATT   
  
  
+ AGCCTTATCC AAGCCCTGGC TGCTCAGCCT GATGGGCCAC CACAGGTCCG TATTACCGGA GTCGATGATT   
  
  
+ CCCAATCTGA GTATGCTCGG GGAGGGGGGC TCGACATTGT AGCGAAGAGA CTATCTGGAC TAGCCCAGGC   
  
  
+ TTGCAGCCTA CCCTTTGAGT TCCACGCTGC AGCACTTAGT GGTTCTGAGA TCAGACTTCA AAACCTGGTT   
  
  
+ TTGCGGCCTG GAGAAGCCTT AGCAGTGAAC TTCCCATTCA TGCTGCACCA CATGCCGGAT GAGAGTGTGG   
  
  
+ GCCCTGAGAA TTATAGAGAC CAGTTATTAA GGCTGGTGAA GAGCTTCTTG CCCAAGGTGG TTACCCTTGT   
  
  
+ TGAGCAAGAA TCCAACACAA ACACGGCCCC ATTTCTACCC CGGTTTCTTG AAACCCTAGA CTACTATACT   
  
  
+ GCCATTTTCG AATCAATTGA TGTTACGCTC CCAAGAGATC ACAAGGAGCG GATCAATGTT GAGCAGCACT   
  
  
+ GTTTAGCAAG AGATATAGTC AACATAATAG CATGTGAGGG TGCCGAGAGG GTGGAACGCC ATGAGGTTCT   
  
  
+ TGGAAAGTGG AGATCTCGGT TCTCAATGGC TGGGTTTAAG CCGTACCCAT TGAGCCCGCT AGTGAATGCA   
  
  
+ ACTATCAGGA CTCTTCTGCA GAAGTATAGC AGGAGCTATG GACTTGAAGA AAGGGATGGA GCTCTTTATC   
  
  
+ TAGGCTGGAT GAACCGAGCA CTAGTTGCAT CTTGTGCATG GCAGTG  

- -Up\_Stream \_Len000AAGAAA AGGACAGAGA CAGAGAAGTA ACGTGGTAGT AAATTCTCTA CCCTGGTATT   
  
  
- TAGTAAGTTA GTGAGGAAAT ACAAATTTGA AACCTTCCTC CAATAATAAA TACACCACCA CTAGGGTTAC   
  
  
- GACTTAAATC GACAACTACC GCTCCCGTAT ATACATTAAT CCTAAAGGTT ACGTCAAATC TAAAATGAGT   
  
  
- AAACTCTTAA GTGACTCCAA ACAAACTAGA CTAAAATCGT TTATAACAAG TCCCCATATC TACGAATAAG   
  
  
- CCAAGAGTTG ACATAATCAT ATGTTCTACT CACCAGGTGA ACAAATTCGA AAATGATATA TGAATACAAC   
  
  
- AGGGATTAAA CCAGAGCGAA CTACACATCG ACAGTTCATA ATAATATTAG AACACAAACT ATAGTGTAAA   
  
  
- CATAACTAAT CGATGTTTTC TTGTATAATA TACACATATC GTTGATAGAG TATATACGGG AGTGGTATCG   
  
  
- CACCAAGGTT CCCAAGTTCA AGGTGAATCG GAGTGGGATC TTTACTGAAG AAAAAAAAAA ATTTCAATTT   
  
  
- CTATACGTTA GTGGTACCTT GGTCAGTAGA AAACTGTGCA ATAAGGTCTA GTCTACTGGT CGGAAAGAGG   
  
  
- TTCCGGTAAT CTAAAAGATC GGTTAGGTGA CAATTGACGT TTTTCACCAT AGGAACCGAC AAGGACAGTG   
  
  
- ACAAAGAATA AACGAGACAC TATAAACATG TAGAGAAACG GTTCCTATTT TCAAGTATCG GCCCTTAACC   
  
  
- ATAAGTCAAC TGAGAACGTC ATTTGTCTCA AGTACATGAC CTCCTAAAAC ACTCAGTACA GTGAGTTATT   
  
  
- TTTGTCCACA CGCTGTCACA ATATTTCGAC GAACGAATTA CGTGTAGATG AGAGTGAGTG GAATGGGTTT   
  
  
- TTTGAAGTTT TAACACACGG AAGATACTTA TAAGACTAGG TAATCGTAAG TTGCCAAAGA CTTAAGACAC   
  
  
- ATTCGGTAGG TATGAGTAAA AGTTGTCTCT TGAACCTAAC CTACATAAGT TATGGGTTTT TGGAACAGTT   
  
  
- TAATGGGGAT CGAATTAAAG GAGATTATGG TTGACGAGAA ACCAAAATCG CATGTTAGGG TATCTAATGG   
  
  
- GAAGATATTA ATCACTACTA ACCAAGTAAA GACGACGTAA TACGGAAGAA ATCTAGGTAA CTATTATATA   
  
  
- TGAGAACGGT ACGACGTTGT AAGGTGACCC TAATTATAGA GTGTAACATA GGAGTCGTTG GTTCCTCTGG   
  
  
- GATGATAAAA GTCTCGGTAA GTATCTTTCA ATAACGTATA TCACTACTAG AAGTTACCCA GACTAAAAAT   
  
  
- CTATCGACGA CACTTGTAAA ATGAGCAGGA GACGGTACAC CTATGATACC ATTGGAAACA AACGAGAGTT   
  
  
- CGGACTCGCA AGACTCTTTA CTACTTTCGC GGGAGCTCAA TATCCTGAAA AGACTGAAAT CGAAATCTTT   
  
  
- TCTCTCCTTT TAGTTGGTGT GGGTAGACTA ATAACTCAAA GACGAGGAAC TCTGGACCTA CCTCGGTCTT   
  
  
- GAGTGCCAGA ACAGTCGGTC TTATTAGTCC TTGACGTAAA GACTCCTGGG TACTTTTCTG GGACCATATG   
  
  
- CTGATAAGTA CTTTTGCAAA ACAGTCGGAA AGTCAAAAAC GTAAAATAAG ATCGACGAAA CTAACTTCGG   
  
  
- GTTAAAATAA TTTTGACACG AAAAGAGAAC TTACAATTTG AGAACGTCAA GGAACGAACA ATAAAACCCA   
  
  
- AAATACAAAC GAAAGATGAC TATAGTAGAA AACCAGCACG TTAAATTAAC GGATGATACA ATATACCTAA   
  
  
- AAATACAAAT GAAAGATGAC TATAGTAGAA AACGATAACG TTAAATTAAC GGAAGATACA TCTTAAGCAG   
  
  
- TGAAACTTCA GACGGTCCTT CCGTTATGTC GATATAAAAA GAACACCTAC TTCACTATTT TCCGTTCACA   
  
  
- GTTAACGAAT GACGAAATAA CGCGACATGA AAAGGTCTCT AAAACCTAAT CAGTTACGTC TTCGGTTGTT   
  
  
- AAATATCGCT GACCGGTGTC AAGATGTTGG TTAAACTATT AGGTGCAGAA GAACTTAGAA GTAGATATTA   
  
  
- CCCACCACTA GTTGAAAAGT CAAGAGGTAG ATGACATTCG TATAGAACGT TGTCCTCAGG TCGAAGTGAC   
  
  
- GGACTTGAGC TTAGGAGAAG TAGCCTGAAG GTTTCACTGA GGGTCCTCTG ACACTTACCG AGCGGTCACT   
  
  
- AACTAAGCAC GTACCATGCA CTGTAACCAC TGAATTCTGT GTTTGAATCT CTCGAACTCT GACAATACGA   
  
  
- GCCTGGTTCA AGTCTGTCGA ACCTAAGTAC CATACGGGGA AGTTCTCCAC TCACACTTGG AGACGGTGGT   
  
  
- CTTCTCGGAC TGTTACCCTT TGTAAACAAC CTCTACTATC GTTCCCCCTC GGAGTTTCTC CACGATTAAC   
  
  
- GAACACGGTT TCGTTATAGT CTGCTACTAA ACAATTGTCG CCTCACCAAC TACAGTCTCG ATGCGGTATA   
  
  
- CCAAAGTCAA AGACCTCTTG GTTAAGTCTC CAATCCTCGG ATGTACAACC TTCCCAATCA ACGGGCCAAC   
  
  
- AGGAGAAGTC CTTCAAGGTA GATGTTTCGA GATTCCACGT TTCTCGGATG ATCATCACTT GAAGAAAGGA   
  
  
- TATACGTAAA TGAGATACTT CAAACGGGAA TGAAGTTCAA ACCCATGTAC AGACGTTTAC CCCGTTAACG   
  
  
- ACTCCGGTAC TTTTTACTCT CGTCTTAAGT ATATTAACTA AAAGTCTATC GAGTTCCCTC AGTCACCTAA   
  
  
- TCGGAATAGG TTCGGGACCG ACGAGTCGGA CTACCCGGTG GTGTCCAGGC ATAATGGCCT CAGCTACTAA   
  
  
- GGGTTAGACT CATACGAGCC CCTCCCCCCG AGCTGTAACA TCGCTTCTCT GATAGACCTG ATCGGGTCCG   
  
  
- AACGTCGGAT GGGAAACTCA AGGTGCGACG TCGTGAATCA CCAAGACTCT AGTCTGAAGT TTTGGACCAA   
  
  
- AACGCCGGAC CTCTTCGGAA TCGTCACTTG AAGGGTAAGT ACGACGTGGT GTACGGCCTA CTCTCACACC   
  
  
- CGGGACTCTT AATATCTCTG GTCAATAATT CCGACCACTT CTCGAAGAAC GGGTTCCACC AATGGGAACA   
  
  
- ACTCGTTCTT AGGTTGTGTT TGTGCCGGGG TAAAGATGGG GCCAAAGAAC TTTGGGATCT GATGATATGA   
  
  
- CGGTAAAAGC TTAGTTAACT ACAATGCGAG GGTTCTCTAG TGTTCCTCGC CTAGTTACAA CTCGTCGTGA   
  
  
- CAAATCGTTC TCTATATCAG TTGTATTATC GTACACTCCC ACGGCTCTCC CACCTTGCGG TACTCCAAGA   
  
  
- ACCTTTCACC TCTAGAGCCA AGAGTTACCG ACCCAAATTC GGCATGGGTA ACTCGGGCGA TCACTTACGT   
  
  
- TGATAGTCCT GAGAAGACGT CTTCATATCG TCCTCGATAC CTGAACTTCT TTCCCTACCT CGAGAAATAG   
  
  
- ATCCGACCTA CTTGGCTCGT GATCAACGTA GAACACGTAC CGTCAC

+     MYB-like sequence

| Site Name | Organism | Position | Strand | Matrix score. | sequence | function |
| --- | --- | --- | --- | --- | --- | --- |
| MYB-like sequence | Arabidopsis thaliana | 3142 | - | 6 | TAACCA |  |

>HU08G00284.1   
+ -Up\_Stream \_Len000TTCTTT TCCTGTCTCT GTCTCTTCAT TGCACCATCA TTTAAGAGAT GGGACCATAA   
  
  
+ ATCATTCAAT CACTCCTTTA TGTTTAAACT TTGGAAGGAG GTTATTATTT ATGTGGTGGT GATCCCAATG   
  
  
+ CTGAATTTAG CTGTTGATGG CGAGGGCATA TATGTAATTA GGATTTCCAA TGCAGTTTAG ATTTTACTCA   
  
  
+ TTTGAGAATT CACTGAGGTT TGTTTGATCT GATTTTAGCA AATATTGTTC AGGGGTATAG ATGCTTATTC   
  
  
+ GGTTCTCAAC TGTATTAGTA TACAAGATGA GTGGTCCACT TGTTTAAGCT TTTACTATAT ACTTATGTTG   
  
  
+ TCCCTAATTT GGTCTCGCTT GATGTGTAGC TGTCAAGTAT TATTATAATC TTGTGTTTGA TATCACATTT   
  
  
+ GTATTGATTA GCTACAAAAG AACATATTAT ATGTGTATAG CAACTATCTC ATATATGCCC TCACCATAGC   
  
  
+ GTGGTTCCAA GGGTTCAAGT TCCACTTAGC CTCACCCTAG AAATGACTTC TTTTTTTTTT TAAAGTTAAA   
  
  
+ GATATGCAAT CACCATGGAA CCAGTCATCT TTTGACACGT TATTCCAGAT CAGATGACCA GCCTTTCTCC   
  
  
+ AAGGCCATTA GATTTTCTAG CCAATCCACT GTTAACTGCA AAAAGTGGTA TCCTTGGCTG TTCCTGTCAC   
  
  
+ TGTTTCTTAT TTGCTCTGTG ATATTTGTAC ATCTCTTTGC CAAGGATAAA AGTTCATAGC CGGGAATTGG   
  
  
+ TATTCAGTTG ACTCTTGCAG TAAACAGAGT TCATGTACTG GAGGATTTTG TGAGTCATGT CACTCAATAA   
  
  
+ AAACAGGTGT GCGACAGTGT TATAAAGCTG CTTGCTTAAT GCACATCTAC TCTCACTCAC CTTACCCAAA   
  
  
+ AAACTTCAAA ATTGTGTGCC TTCTATGAAT ATTCTGATCC ATTAGCATTC AACGGTTTCT GAATTCTGTG   
  
  
+ TAAGCCATCC ATACTCATTT TCAACAGAGA ACTTGGATTG GATGTATTCA ATACCCAAAA ACCTTGTCAA   
  
  
+ ATTACCCCTA GCTTAATTTC CTCTAATACC AACTGCTCTT TGGTTTTAGC GTACAATCCC ATAGATTACC   
  
  
+ CTTCTATAAT TAGTGATGAT TGGTTCATTT CTGCTGCATT ATGCCTTCTT TAGATCCATT GATAATATAT   
  
  
+ ACTCTTGCCA TGCTGCAACA TTCCACTGGG ATTAATATCT CACATTGTAT CCTCAGCAAC CAAGGAGACC   
  
  
+ CTACTATTTT CAGAGCCATT CATAGAAAGT TATTGCATAT AGTGATGATC TTCAATGGGT CTGATTTTTA   
  
  
+ GATAGCTGCT GTGAACATTT TACTCGTCCT CTGCCATGTG GATACTATGG TAACCTTTGT TTGCTCTCAA   
  
  
+ GCCTGAGCGT TCTGAGAAAT GATGAAAGCG CCCTCGAGTT ATAGGACTTT TCTGACTTTA GCTTTAGAAA   
  
  
+ AGAGAGGAAA ATCAACCACA CCCATCTGAT TATTGAGTTT CTGCTCCTTG AGACCTGGAT GGAGCCAGAA   
  
  
+ CTCACGGTCT TGTCAGCCAG AATAATCAGG AACTGCATTT CTGAGGACCC ATGAAAAGAC CCTGGTATAC   
  
  
+ GACTATTCAT GAAAACGTTT TGTCAGCCTT TCAGTTTTTG CATTTTATTC TAGCTGCTTT GATTGAAGCC   
  
  
+ CAATTTTATT AAAACTGTGC TTTTCTCTTG AATGTTAAAC TCTTGCAGTT CCTTGCTTGT TATTTTGGGT   
  
  
+ TTTATGTTTG CTTTCTACTG ATATCATCTT TTGGTCGTGC AATTTAATTG CCTACTATGT TATATGGATT   
  
  
+ TTTATGTTTA CTTTCTACTG ATATCATCTT TTGCTATTGC AATTTAATTG CCTTCTATGT AGAATTCGTC   
  
  
+ ACTTTGAAGT CTGCCAGGAA GGCAATACAG CTATATTTTT CTTGTGGATG AAGTGATAAA AGGCAAGTGT   
  
  
+ CAATTGCTTA CTGCTTTATT GCGCTGTACT TTTCCAGAGA TTTTGGATTA GTCAATGCAG AAGCCAACAA   
  
  
+ TTTATAGCGA CTGGCCACAG TTCTACAACC AATTTGATAA TCCACGTCTT CTTGAATCTT CATCTATAAT   
  
  
+ GGGTGGTGAT CAACTTTTCA GTTCTCCATC TACTGTAAGC ATATCTTGCA ACAGGAGTCC AGCTTCACTG   
  
  
+ CCTGAACTCG AATCCTCTTC ATCGGACTTC CAAAGTGACT CCCAGGAGAC TGTGAATGGC TCGCCAGTGA   
  
  
+ TTGATTCGTG CATGGTACGT GACATTGGTG ACTTAAGACA CAAACTTAGA GAGCTTGAGA CTGTTATGCT   
  
  
+ CGGACCAAGT TCAGACAGCT TGGATTCATG GTATGCCCCT TCAAGAGGTG AGTGTGAACC TCTGCCACCA   
  
  
+ GAAGAGCCTG ACAATGGGAA ACATTTGTTG GAGATGATAG CAAGGGGGAG CCTCAAAGAG GTGCTAATTG   
  
  
+ CTTGTGCCAA AGCAATATCA GACGATGATT TGTTAACAGC GGAGTGGTTG ATGTCAGAGC TACGCCATAT   
  
  
+ GGTTTCAGTT TCTGGAGAAC CAATTCAGAG GTTAGGAGCC TACATGTTGG AAGGGTTAGT TGCCCGGTTG   
  
  
+ TCCTCTTCAG GAAGTTCCAT CTACAAAGCT CTAAGGTGCA AAGAGCCTAC TAGTAGTGAA CTTCTTTCCT   
  
  
+ ATATGCATTT ACTCTATGAA GTTTGCCCTT ACTTCAAGTT TGGGTACATG TCTGCAAATG GGGCAATTGC   
  
  
+ TGAGGCCATG AAAAATGAGA GCAGAATTCA TATAATTGAT TTTCAGATAG CTCAAGGGAG TCAGTGGATT   
  
  
+ AGCCTTATCC AAGCCCTGGC TGCTCAGCCT GATGGGCCAC CACAGGTCCG TATTACCGGA GTCGATGATT   
  
  
+ CCCAATCTGA GTATGCTCGG GGAGGGGGGC TCGACATTGT AGCGAAGAGA CTATCTGGAC TAGCCCAGGC   
  
  
+ TTGCAGCCTA CCCTTTGAGT TCCACGCTGC AGCACTTAGT GGTTCTGAGA TCAGACTTCA AAACCTGGTT   
  
  
+ TTGCGGCCTG GAGAAGCCTT AGCAGTGAAC TTCCCATTCA TGCTGCACCA CATGCCGGAT GAGAGTGTGG   
  
  
+ GCCCTGAGAA TTATAGAGAC CAGTTATTAA GGCTGGTGAA GAGCTTCTTG CCCAAGGTGG TTACCCTTGT   
  
  
+ TGAGCAAGAA TCCAACACAA ACACGGCCCC ATTTCTACCC CGGTTTCTTG AAACCCTAGA CTACTATACT   
  
  
+ GCCATTTTCG AATCAATTGA TGTTACGCTC CCAAGAGATC ACAAGGAGCG GATCAATGTT GAGCAGCACT   
  
  
+ GTTTAGCAAG AGATATAGTC AACATAATAG CATGTGAGGG TGCCGAGAGG GTGGAACGCC ATGAGGTTCT   
  
  
+ TGGAAAGTGG AGATCTCGGT TCTCAATGGC TGGGTTTAAG CCGTACCCAT TGAGCCCGCT AGTGAATGCA   
  
  
+ ACTATCAGGA CTCTTCTGCA GAAGTATAGC AGGAGCTATG GACTTGAAGA AAGGGATGGA GCTCTTTATC   
  
  
+ TAGGCTGGAT GAACCGAGCA CTAGTTGCAT CTTGTGCATG GCAGTG  

- -Up\_Stream \_Len000AAGAAA AGGACAGAGA CAGAGAAGTA ACGTGGTAGT AAATTCTCTA CCCTGGTATT   
  
  
- TAGTAAGTTA GTGAGGAAAT ACAAATTTGA AACCTTCCTC CAATAATAAA TACACCACCA CTAGGGTTAC   
  
  
- GACTTAAATC GACAACTACC GCTCCCGTAT ATACATTAAT CCTAAAGGTT ACGTCAAATC TAAAATGAGT   
  
  
- AAACTCTTAA GTGACTCCAA ACAAACTAGA CTAAAATCGT TTATAACAAG TCCCCATATC TACGAATAAG   
  
  
- CCAAGAGTTG ACATAATCAT ATGTTCTACT CACCAGGTGA ACAAATTCGA AAATGATATA TGAATACAAC   
  
  
- AGGGATTAAA CCAGAGCGAA CTACACATCG ACAGTTCATA ATAATATTAG AACACAAACT ATAGTGTAAA   
  
  
- CATAACTAAT CGATGTTTTC TTGTATAATA TACACATATC GTTGATAGAG TATATACGGG AGTGGTATCG   
  
  
- CACCAAGGTT CCCAAGTTCA AGGTGAATCG GAGTGGGATC TTTACTGAAG AAAAAAAAAA ATTTCAATTT   
  
  
- CTATACGTTA GTGGTACCTT GGTCAGTAGA AAACTGTGCA ATAAGGTCTA GTCTACTGGT CGGAAAGAGG   
  
  
- TTCCGGTAAT CTAAAAGATC GGTTAGGTGA CAATTGACGT TTTTCACCAT AGGAACCGAC AAGGACAGTG   
  
  
- ACAAAGAATA AACGAGACAC TATAAACATG TAGAGAAACG GTTCCTATTT TCAAGTATCG GCCCTTAACC   
  
  
- ATAAGTCAAC TGAGAACGTC ATTTGTCTCA AGTACATGAC CTCCTAAAAC ACTCAGTACA GTGAGTTATT   
  
  
- TTTGTCCACA CGCTGTCACA ATATTTCGAC GAACGAATTA CGTGTAGATG AGAGTGAGTG GAATGGGTTT   
  
  
- TTTGAAGTTT TAACACACGG AAGATACTTA TAAGACTAGG TAATCGTAAG TTGCCAAAGA CTTAAGACAC   
  
  
- ATTCGGTAGG TATGAGTAAA AGTTGTCTCT TGAACCTAAC CTACATAAGT TATGGGTTTT TGGAACAGTT   
  
  
- TAATGGGGAT CGAATTAAAG GAGATTATGG TTGACGAGAA ACCAAAATCG CATGTTAGGG TATCTAATGG   
  
  
- GAAGATATTA ATCACTACTA ACCAAGTAAA GACGACGTAA TACGGAAGAA ATCTAGGTAA CTATTATATA   
  
  
- TGAGAACGGT ACGACGTTGT AAGGTGACCC TAATTATAGA GTGTAACATA GGAGTCGTTG GTTCCTCTGG   
  
  
- GATGATAAAA GTCTCGGTAA GTATCTTTCA ATAACGTATA TCACTACTAG AAGTTACCCA GACTAAAAAT   
  
  
- CTATCGACGA CACTTGTAAA ATGAGCAGGA GACGGTACAC CTATGATACC ATTGGAAACA AACGAGAGTT   
  
  
- CGGACTCGCA AGACTCTTTA CTACTTTCGC GGGAGCTCAA TATCCTGAAA AGACTGAAAT CGAAATCTTT   
  
  
- TCTCTCCTTT TAGTTGGTGT GGGTAGACTA ATAACTCAAA GACGAGGAAC TCTGGACCTA CCTCGGTCTT   
  
  
- GAGTGCCAGA ACAGTCGGTC TTATTAGTCC TTGACGTAAA GACTCCTGGG TACTTTTCTG GGACCATATG   
  
  
- CTGATAAGTA CTTTTGCAAA ACAGTCGGAA AGTCAAAAAC GTAAAATAAG ATCGACGAAA CTAACTTCGG   
  
  
- GTTAAAATAA TTTTGACACG AAAAGAGAAC TTACAATTTG AGAACGTCAA GGAACGAACA ATAAAACCCA   
  
  
- AAATACAAAC GAAAGATGAC TATAGTAGAA AACCAGCACG TTAAATTAAC GGATGATACA ATATACCTAA   
  
  
- AAATACAAAT GAAAGATGAC TATAGTAGAA AACGATAACG TTAAATTAAC GGAAGATACA TCTTAAGCAG   
  
  
- TGAAACTTCA GACGGTCCTT CCGTTATGTC GATATAAAAA GAACACCTAC TTCACTATTT TCCGTTCACA   
  
  
- GTTAACGAAT GACGAAATAA CGCGACATGA AAAGGTCTCT AAAACCTAAT CAGTTACGTC TTCGGTTGTT   
  
  
- AAATATCGCT GACCGGTGTC AAGATGTTGG TTAAACTATT AGGTGCAGAA GAACTTAGAA GTAGATATTA   
  
  
- CCCACCACTA GTTGAAAAGT CAAGAGGTAG ATGACATTCG TATAGAACGT TGTCCTCAGG TCGAAGTGAC   
  
  
- GGACTTGAGC TTAGGAGAAG TAGCCTGAAG GTTTCACTGA GGGTCCTCTG ACACTTACCG AGCGGTCACT   
  
  
- AACTAAGCAC GTACCATGCA CTGTAACCAC TGAATTCTGT GTTTGAATCT CTCGAACTCT GACAATACGA   
  
  
- GCCTGGTTCA AGTCTGTCGA ACCTAAGTAC CATACGGGGA AGTTCTCCAC TCACACTTGG AGACGGTGGT   
  
  
- CTTCTCGGAC TGTTACCCTT TGTAAACAAC CTCTACTATC GTTCCCCCTC GGAGTTTCTC CACGATTAAC   
  
  
- GAACACGGTT TCGTTATAGT CTGCTACTAA ACAATTGTCG CCTCACCAAC TACAGTCTCG ATGCGGTATA   
  
  
- CCAAAGTCAA AGACCTCTTG GTTAAGTCTC CAATCCTCGG ATGTACAACC TTCCCAATCA ACGGGCCAAC   
  
  
- AGGAGAAGTC CTTCAAGGTA GATGTTTCGA GATTCCACGT TTCTCGGATG ATCATCACTT GAAGAAAGGA   
  
  
- TATACGTAAA TGAGATACTT CAAACGGGAA TGAAGTTCAA ACCCATGTAC AGACGTTTAC CCCGTTAACG   
  
  
- ACTCCGGTAC TTTTTACTCT CGTCTTAAGT ATATTAACTA AAAGTCTATC GAGTTCCCTC AGTCACCTAA   
  
  
- TCGGAATAGG TTCGGGACCG ACGAGTCGGA CTACCCGGTG GTGTCCAGGC ATAATGGCCT CAGCTACTAA   
  
  
- GGGTTAGACT CATACGAGCC CCTCCCCCCG AGCTGTAACA TCGCTTCTCT GATAGACCTG ATCGGGTCCG   
  
  
- AACGTCGGAT GGGAAACTCA AGGTGCGACG TCGTGAATCA CCAAGACTCT AGTCTGAAGT TTTGGACCAA   
  
  
- AACGCCGGAC CTCTTCGGAA TCGTCACTTG AAGGGTAAGT ACGACGTGGT GTACGGCCTA CTCTCACACC   
  
  
- CGGGACTCTT AATATCTCTG GTCAATAATT CCGACCACTT CTCGAAGAAC GGGTTCCACC AATGGGAACA   
  
  
- ACTCGTTCTT AGGTTGTGTT TGTGCCGGGG TAAAGATGGG GCCAAAGAAC TTTGGGATCT GATGATATGA   
  
  
- CGGTAAAAGC TTAGTTAACT ACAATGCGAG GGTTCTCTAG TGTTCCTCGC CTAGTTACAA CTCGTCGTGA   
  
  
- CAAATCGTTC TCTATATCAG TTGTATTATC GTACACTCCC ACGGCTCTCC CACCTTGCGG TACTCCAAGA   
  
  
- ACCTTTCACC TCTAGAGCCA AGAGTTACCG ACCCAAATTC GGCATGGGTA ACTCGGGCGA TCACTTACGT   
  
  
- TGATAGTCCT GAGAAGACGT CTTCATATCG TCCTCGATAC CTGAACTTCT TTCCCTACCT CGAGAAATAG   
  
  
- ATCCGACCTA CTTGGCTCGT GATCAACGTA GAACACGTAC CGTCAC

+     MYC

| Site Name | Organism | Position | Strand | Matrix score. | sequence | function |
| --- | --- | --- | --- | --- | --- | --- |
| MYC | Arabidopsis thaliana | 2406 | + | 6 | CATTTG |  |
| MYC | Arabidopsis thaliana | 2719 | - | 6 | CATTTG |  |
| MYC | Arabidopsis thaliana | 3063 | - | 6 | CATGTG |  |
| MYC | Arabidopsis thaliana | 2728 | - | 6 | CAATTG |  |
| MYC | Arabidopsis thaliana | 3238 | - | 6 | CAATTG |  |
| MYC | Arabidopsis thaliana | 3325 | + | 6 | CATGTG |  |
| MYC | Arabidopsis thaliana | 213 | + | 6 | CATTTG |  |
| MYC | Arabidopsis thaliana | 420 | + | 6 | CATTTG |  |
| MYC | Arabidopsis thaliana | 1369 | + | 6 | CATGTG |  |
| MYC | Arabidopsis thaliana | 1965 | - | 6 | CAATTG |  |

>HU08G00284.1   
+ -Up\_Stream \_Len000TTCTTT TCCTGTCTCT GTCTCTTCAT TGCACCATCA TTTAAGAGAT GGGACCATAA   
  
  
+ ATCATTCAAT CACTCCTTTA TGTTTAAACT TTGGAAGGAG GTTATTATTT ATGTGGTGGT GATCCCAATG   
  
  
+ CTGAATTTAG CTGTTGATGG CGAGGGCATA TATGTAATTA GGATTTCCAA TGCAGTTTAG ATTTTACTCA   
  
  
+ TTTGAGAATT CACTGAGGTT TGTTTGATCT GATTTTAGCA AATATTGTTC AGGGGTATAG ATGCTTATTC   
  
  
+ GGTTCTCAAC TGTATTAGTA TACAAGATGA GTGGTCCACT TGTTTAAGCT TTTACTATAT ACTTATGTTG   
  
  
+ TCCCTAATTT GGTCTCGCTT GATGTGTAGC TGTCAAGTAT TATTATAATC TTGTGTTTGA TATCACATTT   
  
  
+ GTATTGATTA GCTACAAAAG AACATATTAT ATGTGTATAG CAACTATCTC ATATATGCCC TCACCATAGC   
  
  
+ GTGGTTCCAA GGGTTCAAGT TCCACTTAGC CTCACCCTAG AAATGACTTC TTTTTTTTTT TAAAGTTAAA   
  
  
+ GATATGCAAT CACCATGGAA CCAGTCATCT TTTGACACGT TATTCCAGAT CAGATGACCA GCCTTTCTCC   
  
  
+ AAGGCCATTA GATTTTCTAG CCAATCCACT GTTAACTGCA AAAAGTGGTA TCCTTGGCTG TTCCTGTCAC   
  
  
+ TGTTTCTTAT TTGCTCTGTG ATATTTGTAC ATCTCTTTGC CAAGGATAAA AGTTCATAGC CGGGAATTGG   
  
  
+ TATTCAGTTG ACTCTTGCAG TAAACAGAGT TCATGTACTG GAGGATTTTG TGAGTCATGT CACTCAATAA   
  
  
+ AAACAGGTGT GCGACAGTGT TATAAAGCTG CTTGCTTAAT GCACATCTAC TCTCACTCAC CTTACCCAAA   
  
  
+ AAACTTCAAA ATTGTGTGCC TTCTATGAAT ATTCTGATCC ATTAGCATTC AACGGTTTCT GAATTCTGTG   
  
  
+ TAAGCCATCC ATACTCATTT TCAACAGAGA ACTTGGATTG GATGTATTCA ATACCCAAAA ACCTTGTCAA   
  
  
+ ATTACCCCTA GCTTAATTTC CTCTAATACC AACTGCTCTT TGGTTTTAGC GTACAATCCC ATAGATTACC   
  
  
+ CTTCTATAAT TAGTGATGAT TGGTTCATTT CTGCTGCATT ATGCCTTCTT TAGATCCATT GATAATATAT   
  
  
+ ACTCTTGCCA TGCTGCAACA TTCCACTGGG ATTAATATCT CACATTGTAT CCTCAGCAAC CAAGGAGACC   
  
  
+ CTACTATTTT CAGAGCCATT CATAGAAAGT TATTGCATAT AGTGATGATC TTCAATGGGT CTGATTTTTA   
  
  
+ GATAGCTGCT GTGAACATTT TACTCGTCCT CTGCCATGTG GATACTATGG TAACCTTTGT TTGCTCTCAA   
  
  
+ GCCTGAGCGT TCTGAGAAAT GATGAAAGCG CCCTCGAGTT ATAGGACTTT TCTGACTTTA GCTTTAGAAA   
  
  
+ AGAGAGGAAA ATCAACCACA CCCATCTGAT TATTGAGTTT CTGCTCCTTG AGACCTGGAT GGAGCCAGAA   
  
  
+ CTCACGGTCT TGTCAGCCAG AATAATCAGG AACTGCATTT CTGAGGACCC ATGAAAAGAC CCTGGTATAC   
  
  
+ GACTATTCAT GAAAACGTTT TGTCAGCCTT TCAGTTTTTG CATTTTATTC TAGCTGCTTT GATTGAAGCC   
  
  
+ CAATTTTATT AAAACTGTGC TTTTCTCTTG AATGTTAAAC TCTTGCAGTT CCTTGCTTGT TATTTTGGGT   
  
  
+ TTTATGTTTG CTTTCTACTG ATATCATCTT TTGGTCGTGC AATTTAATTG CCTACTATGT TATATGGATT   
  
  
+ TTTATGTTTA CTTTCTACTG ATATCATCTT TTGCTATTGC AATTTAATTG CCTTCTATGT AGAATTCGTC   
  
  
+ ACTTTGAAGT CTGCCAGGAA GGCAATACAG CTATATTTTT CTTGTGGATG AAGTGATAAA AGGCAAGTGT   
  
  
+ CAATTGCTTA CTGCTTTATT GCGCTGTACT TTTCCAGAGA TTTTGGATTA GTCAATGCAG AAGCCAACAA   
  
  
+ TTTATAGCGA CTGGCCACAG TTCTACAACC AATTTGATAA TCCACGTCTT CTTGAATCTT CATCTATAAT   
  
  
+ GGGTGGTGAT CAACTTTTCA GTTCTCCATC TACTGTAAGC ATATCTTGCA ACAGGAGTCC AGCTTCACTG   
  
  
+ CCTGAACTCG AATCCTCTTC ATCGGACTTC CAAAGTGACT CCCAGGAGAC TGTGAATGGC TCGCCAGTGA   
  
  
+ TTGATTCGTG CATGGTACGT GACATTGGTG ACTTAAGACA CAAACTTAGA GAGCTTGAGA CTGTTATGCT   
  
  
+ CGGACCAAGT TCAGACAGCT TGGATTCATG GTATGCCCCT TCAAGAGGTG AGTGTGAACC TCTGCCACCA   
  
  
+ GAAGAGCCTG ACAATGGGAA ACATTTGTTG GAGATGATAG CAAGGGGGAG CCTCAAAGAG GTGCTAATTG   
  
  
+ CTTGTGCCAA AGCAATATCA GACGATGATT TGTTAACAGC GGAGTGGTTG ATGTCAGAGC TACGCCATAT   
  
  
+ GGTTTCAGTT TCTGGAGAAC CAATTCAGAG GTTAGGAGCC TACATGTTGG AAGGGTTAGT TGCCCGGTTG   
  
  
+ TCCTCTTCAG GAAGTTCCAT CTACAAAGCT CTAAGGTGCA AAGAGCCTAC TAGTAGTGAA CTTCTTTCCT   
  
  
+ ATATGCATTT ACTCTATGAA GTTTGCCCTT ACTTCAAGTT TGGGTACATG TCTGCAAATG GGGCAATTGC   
  
  
+ TGAGGCCATG AAAAATGAGA GCAGAATTCA TATAATTGAT TTTCAGATAG CTCAAGGGAG TCAGTGGATT   
  
  
+ AGCCTTATCC AAGCCCTGGC TGCTCAGCCT GATGGGCCAC CACAGGTCCG TATTACCGGA GTCGATGATT   
  
  
+ CCCAATCTGA GTATGCTCGG GGAGGGGGGC TCGACATTGT AGCGAAGAGA CTATCTGGAC TAGCCCAGGC   
  
  
+ TTGCAGCCTA CCCTTTGAGT TCCACGCTGC AGCACTTAGT GGTTCTGAGA TCAGACTTCA AAACCTGGTT   
  
  
+ TTGCGGCCTG GAGAAGCCTT AGCAGTGAAC TTCCCATTCA TGCTGCACCA CATGCCGGAT GAGAGTGTGG   
  
  
+ GCCCTGAGAA TTATAGAGAC CAGTTATTAA GGCTGGTGAA GAGCTTCTTG CCCAAGGTGG TTACCCTTGT   
  
  
+ TGAGCAAGAA TCCAACACAA ACACGGCCCC ATTTCTACCC CGGTTTCTTG AAACCCTAGA CTACTATACT   
  
  
+ GCCATTTTCG AATCAATTGA TGTTACGCTC CCAAGAGATC ACAAGGAGCG GATCAATGTT GAGCAGCACT   
  
  
+ GTTTAGCAAG AGATATAGTC AACATAATAG CATGTGAGGG TGCCGAGAGG GTGGAACGCC ATGAGGTTCT   
  
  
+ TGGAAAGTGG AGATCTCGGT TCTCAATGGC TGGGTTTAAG CCGTACCCAT TGAGCCCGCT AGTGAATGCA   
  
  
+ ACTATCAGGA CTCTTCTGCA GAAGTATAGC AGGAGCTATG GACTTGAAGA AAGGGATGGA GCTCTTTATC   
  
  
+ TAGGCTGGAT GAACCGAGCA CTAGTTGCAT CTTGTGCATG GCAGTG  

- -Up\_Stream \_Len000AAGAAA AGGACAGAGA CAGAGAAGTA ACGTGGTAGT AAATTCTCTA CCCTGGTATT   
  
  
- TAGTAAGTTA GTGAGGAAAT ACAAATTTGA AACCTTCCTC CAATAATAAA TACACCACCA CTAGGGTTAC   
  
  
- GACTTAAATC GACAACTACC GCTCCCGTAT ATACATTAAT CCTAAAGGTT ACGTCAAATC TAAAATGAGT   
  
  
- AAACTCTTAA GTGACTCCAA ACAAACTAGA CTAAAATCGT TTATAACAAG TCCCCATATC TACGAATAAG   
  
  
- CCAAGAGTTG ACATAATCAT ATGTTCTACT CACCAGGTGA ACAAATTCGA AAATGATATA TGAATACAAC   
  
  
- AGGGATTAAA CCAGAGCGAA CTACACATCG ACAGTTCATA ATAATATTAG AACACAAACT ATAGTGTAAA   
  
  
- CATAACTAAT CGATGTTTTC TTGTATAATA TACACATATC GTTGATAGAG TATATACGGG AGTGGTATCG   
  
  
- CACCAAGGTT CCCAAGTTCA AGGTGAATCG GAGTGGGATC TTTACTGAAG AAAAAAAAAA ATTTCAATTT   
  
  
- CTATACGTTA GTGGTACCTT GGTCAGTAGA AAACTGTGCA ATAAGGTCTA GTCTACTGGT CGGAAAGAGG   
  
  
- TTCCGGTAAT CTAAAAGATC GGTTAGGTGA CAATTGACGT TTTTCACCAT AGGAACCGAC AAGGACAGTG   
  
  
- ACAAAGAATA AACGAGACAC TATAAACATG TAGAGAAACG GTTCCTATTT TCAAGTATCG GCCCTTAACC   
  
  
- ATAAGTCAAC TGAGAACGTC ATTTGTCTCA AGTACATGAC CTCCTAAAAC ACTCAGTACA GTGAGTTATT   
  
  
- TTTGTCCACA CGCTGTCACA ATATTTCGAC GAACGAATTA CGTGTAGATG AGAGTGAGTG GAATGGGTTT   
  
  
- TTTGAAGTTT TAACACACGG AAGATACTTA TAAGACTAGG TAATCGTAAG TTGCCAAAGA CTTAAGACAC   
  
  
- ATTCGGTAGG TATGAGTAAA AGTTGTCTCT TGAACCTAAC CTACATAAGT TATGGGTTTT TGGAACAGTT   
  
  
- TAATGGGGAT CGAATTAAAG GAGATTATGG TTGACGAGAA ACCAAAATCG CATGTTAGGG TATCTAATGG   
  
  
- GAAGATATTA ATCACTACTA ACCAAGTAAA GACGACGTAA TACGGAAGAA ATCTAGGTAA CTATTATATA   
  
  
- TGAGAACGGT ACGACGTTGT AAGGTGACCC TAATTATAGA GTGTAACATA GGAGTCGTTG GTTCCTCTGG   
  
  
- GATGATAAAA GTCTCGGTAA GTATCTTTCA ATAACGTATA TCACTACTAG AAGTTACCCA GACTAAAAAT   
  
  
- CTATCGACGA CACTTGTAAA ATGAGCAGGA GACGGTACAC CTATGATACC ATTGGAAACA AACGAGAGTT   
  
  
- CGGACTCGCA AGACTCTTTA CTACTTTCGC GGGAGCTCAA TATCCTGAAA AGACTGAAAT CGAAATCTTT   
  
  
- TCTCTCCTTT TAGTTGGTGT GGGTAGACTA ATAACTCAAA GACGAGGAAC TCTGGACCTA CCTCGGTCTT   
  
  
- GAGTGCCAGA ACAGTCGGTC TTATTAGTCC TTGACGTAAA GACTCCTGGG TACTTTTCTG GGACCATATG   
  
  
- CTGATAAGTA CTTTTGCAAA ACAGTCGGAA AGTCAAAAAC GTAAAATAAG ATCGACGAAA CTAACTTCGG   
  
  
- GTTAAAATAA TTTTGACACG AAAAGAGAAC TTACAATTTG AGAACGTCAA GGAACGAACA ATAAAACCCA   
  
  
- AAATACAAAC GAAAGATGAC TATAGTAGAA AACCAGCACG TTAAATTAAC GGATGATACA ATATACCTAA   
  
  
- AAATACAAAT GAAAGATGAC TATAGTAGAA AACGATAACG TTAAATTAAC GGAAGATACA TCTTAAGCAG   
  
  
- TGAAACTTCA GACGGTCCTT CCGTTATGTC GATATAAAAA GAACACCTAC TTCACTATTT TCCGTTCACA   
  
  
- GTTAACGAAT GACGAAATAA CGCGACATGA AAAGGTCTCT AAAACCTAAT CAGTTACGTC TTCGGTTGTT   
  
  
- AAATATCGCT GACCGGTGTC AAGATGTTGG TTAAACTATT AGGTGCAGAA GAACTTAGAA GTAGATATTA   
  
  
- CCCACCACTA GTTGAAAAGT CAAGAGGTAG ATGACATTCG TATAGAACGT TGTCCTCAGG TCGAAGTGAC   
  
  
- GGACTTGAGC TTAGGAGAAG TAGCCTGAAG GTTTCACTGA GGGTCCTCTG ACACTTACCG AGCGGTCACT   
  
  
- AACTAAGCAC GTACCATGCA CTGTAACCAC TGAATTCTGT GTTTGAATCT CTCGAACTCT GACAATACGA   
  
  
- GCCTGGTTCA AGTCTGTCGA ACCTAAGTAC CATACGGGGA AGTTCTCCAC TCACACTTGG AGACGGTGGT   
  
  
- CTTCTCGGAC TGTTACCCTT TGTAAACAAC CTCTACTATC GTTCCCCCTC GGAGTTTCTC CACGATTAAC   
  
  
- GAACACGGTT TCGTTATAGT CTGCTACTAA ACAATTGTCG CCTCACCAAC TACAGTCTCG ATGCGGTATA   
  
  
- CCAAAGTCAA AGACCTCTTG GTTAAGTCTC CAATCCTCGG ATGTACAACC TTCCCAATCA ACGGGCCAAC   
  
  
- AGGAGAAGTC CTTCAAGGTA GATGTTTCGA GATTCCACGT TTCTCGGATG ATCATCACTT GAAGAAAGGA   
  
  
- TATACGTAAA TGAGATACTT CAAACGGGAA TGAAGTTCAA ACCCATGTAC AGACGTTTAC CCCGTTAACG   
  
  
- ACTCCGGTAC TTTTTACTCT CGTCTTAAGT ATATTAACTA AAAGTCTATC GAGTTCCCTC AGTCACCTAA   
  
  
- TCGGAATAGG TTCGGGACCG ACGAGTCGGA CTACCCGGTG GTGTCCAGGC ATAATGGCCT CAGCTACTAA   
  
  
- GGGTTAGACT CATACGAGCC CCTCCCCCCG AGCTGTAACA TCGCTTCTCT GATAGACCTG ATCGGGTCCG   
  
  
- AACGTCGGAT GGGAAACTCA AGGTGCGACG TCGTGAATCA CCAAGACTCT AGTCTGAAGT TTTGGACCAA   
  
  
- AACGCCGGAC CTCTTCGGAA TCGTCACTTG AAGGGTAAGT ACGACGTGGT GTACGGCCTA CTCTCACACC   
  
  
- CGGGACTCTT AATATCTCTG GTCAATAATT CCGACCACTT CTCGAAGAAC GGGTTCCACC AATGGGAACA   
  
  
- ACTCGTTCTT AGGTTGTGTT TGTGCCGGGG TAAAGATGGG GCCAAAGAAC TTTGGGATCT GATGATATGA   
  
  
- CGGTAAAAGC TTAGTTAACT ACAATGCGAG GGTTCTCTAG TGTTCCTCGC CTAGTTACAA CTCGTCGTGA   
  
  
- CAAATCGTTC TCTATATCAG TTGTATTATC GTACACTCCC ACGGCTCTCC CACCTTGCGG TACTCCAAGA   
  
  
- ACCTTTCACC TCTAGAGCCA AGAGTTACCG ACCCAAATTC GGCATGGGTA ACTCGGGCGA TCACTTACGT   
  
  
- TGATAGTCCT GAGAAGACGT CTTCATATCG TCCTCGATAC CTGAACTTCT TTCCCTACCT CGAGAAATAG   
  
  
- ATCCGACCTA CTTGGCTCGT GATCAACGTA GAACACGTAC CGTCAC

+     Myb

| Site Name | Organism | Position | Strand | Matrix score. | sequence | function |
| --- | --- | --- | --- | --- | --- | --- |
| Myb | Arabidopsis thaliana | 3105 | - | 6 | TAACTG |  |
| Myb | Arabidopsis thaliana | 779 | - | 6 | CAACTG |  |
| Myb | Arabidopsis thaliana | 1084 | + | 6 | CAACTG |  |
| Myb | Arabidopsis thaliana | 291 | + | 6 | CAACTG |  |
| Myb | Arabidopsis thaliana | 667 | + | 6 | TAACTG |  |

>HU08G00284.1   
+ -Up\_Stream \_Len000TTCTTT TCCTGTCTCT GTCTCTTCAT TGCACCATCA TTTAAGAGAT GGGACCATAA   
  
  
+ ATCATTCAAT CACTCCTTTA TGTTTAAACT TTGGAAGGAG GTTATTATTT ATGTGGTGGT GATCCCAATG   
  
  
+ CTGAATTTAG CTGTTGATGG CGAGGGCATA TATGTAATTA GGATTTCCAA TGCAGTTTAG ATTTTACTCA   
  
  
+ TTTGAGAATT CACTGAGGTT TGTTTGATCT GATTTTAGCA AATATTGTTC AGGGGTATAG ATGCTTATTC   
  
  
+ GGTTCTCAAC TGTATTAGTA TACAAGATGA GTGGTCCACT TGTTTAAGCT TTTACTATAT ACTTATGTTG   
  
  
+ TCCCTAATTT GGTCTCGCTT GATGTGTAGC TGTCAAGTAT TATTATAATC TTGTGTTTGA TATCACATTT   
  
  
+ GTATTGATTA GCTACAAAAG AACATATTAT ATGTGTATAG CAACTATCTC ATATATGCCC TCACCATAGC   
  
  
+ GTGGTTCCAA GGGTTCAAGT TCCACTTAGC CTCACCCTAG AAATGACTTC TTTTTTTTTT TAAAGTTAAA   
  
  
+ GATATGCAAT CACCATGGAA CCAGTCATCT TTTGACACGT TATTCCAGAT CAGATGACCA GCCTTTCTCC   
  
  
+ AAGGCCATTA GATTTTCTAG CCAATCCACT GTTAACTGCA AAAAGTGGTA TCCTTGGCTG TTCCTGTCAC   
  
  
+ TGTTTCTTAT TTGCTCTGTG ATATTTGTAC ATCTCTTTGC CAAGGATAAA AGTTCATAGC CGGGAATTGG   
  
  
+ TATTCAGTTG ACTCTTGCAG TAAACAGAGT TCATGTACTG GAGGATTTTG TGAGTCATGT CACTCAATAA   
  
  
+ AAACAGGTGT GCGACAGTGT TATAAAGCTG CTTGCTTAAT GCACATCTAC TCTCACTCAC CTTACCCAAA   
  
  
+ AAACTTCAAA ATTGTGTGCC TTCTATGAAT ATTCTGATCC ATTAGCATTC AACGGTTTCT GAATTCTGTG   
  
  
+ TAAGCCATCC ATACTCATTT TCAACAGAGA ACTTGGATTG GATGTATTCA ATACCCAAAA ACCTTGTCAA   
  
  
+ ATTACCCCTA GCTTAATTTC CTCTAATACC AACTGCTCTT TGGTTTTAGC GTACAATCCC ATAGATTACC   
  
  
+ CTTCTATAAT TAGTGATGAT TGGTTCATTT CTGCTGCATT ATGCCTTCTT TAGATCCATT GATAATATAT   
  
  
+ ACTCTTGCCA TGCTGCAACA TTCCACTGGG ATTAATATCT CACATTGTAT CCTCAGCAAC CAAGGAGACC   
  
  
+ CTACTATTTT CAGAGCCATT CATAGAAAGT TATTGCATAT AGTGATGATC TTCAATGGGT CTGATTTTTA   
  
  
+ GATAGCTGCT GTGAACATTT TACTCGTCCT CTGCCATGTG GATACTATGG TAACCTTTGT TTGCTCTCAA   
  
  
+ GCCTGAGCGT TCTGAGAAAT GATGAAAGCG CCCTCGAGTT ATAGGACTTT TCTGACTTTA GCTTTAGAAA   
  
  
+ AGAGAGGAAA ATCAACCACA CCCATCTGAT TATTGAGTTT CTGCTCCTTG AGACCTGGAT GGAGCCAGAA   
  
  
+ CTCACGGTCT TGTCAGCCAG AATAATCAGG AACTGCATTT CTGAGGACCC ATGAAAAGAC CCTGGTATAC   
  
  
+ GACTATTCAT GAAAACGTTT TGTCAGCCTT TCAGTTTTTG CATTTTATTC TAGCTGCTTT GATTGAAGCC   
  
  
+ CAATTTTATT AAAACTGTGC TTTTCTCTTG AATGTTAAAC TCTTGCAGTT CCTTGCTTGT TATTTTGGGT   
  
  
+ TTTATGTTTG CTTTCTACTG ATATCATCTT TTGGTCGTGC AATTTAATTG CCTACTATGT TATATGGATT   
  
  
+ TTTATGTTTA CTTTCTACTG ATATCATCTT TTGCTATTGC AATTTAATTG CCTTCTATGT AGAATTCGTC   
  
  
+ ACTTTGAAGT CTGCCAGGAA GGCAATACAG CTATATTTTT CTTGTGGATG AAGTGATAAA AGGCAAGTGT   
  
  
+ CAATTGCTTA CTGCTTTATT GCGCTGTACT TTTCCAGAGA TTTTGGATTA GTCAATGCAG AAGCCAACAA   
  
  
+ TTTATAGCGA CTGGCCACAG TTCTACAACC AATTTGATAA TCCACGTCTT CTTGAATCTT CATCTATAAT   
  
  
+ GGGTGGTGAT CAACTTTTCA GTTCTCCATC TACTGTAAGC ATATCTTGCA ACAGGAGTCC AGCTTCACTG   
  
  
+ CCTGAACTCG AATCCTCTTC ATCGGACTTC CAAAGTGACT CCCAGGAGAC TGTGAATGGC TCGCCAGTGA   
  
  
+ TTGATTCGTG CATGGTACGT GACATTGGTG ACTTAAGACA CAAACTTAGA GAGCTTGAGA CTGTTATGCT   
  
  
+ CGGACCAAGT TCAGACAGCT TGGATTCATG GTATGCCCCT TCAAGAGGTG AGTGTGAACC TCTGCCACCA   
  
  
+ GAAGAGCCTG ACAATGGGAA ACATTTGTTG GAGATGATAG CAAGGGGGAG CCTCAAAGAG GTGCTAATTG   
  
  
+ CTTGTGCCAA AGCAATATCA GACGATGATT TGTTAACAGC GGAGTGGTTG ATGTCAGAGC TACGCCATAT   
  
  
+ GGTTTCAGTT TCTGGAGAAC CAATTCAGAG GTTAGGAGCC TACATGTTGG AAGGGTTAGT TGCCCGGTTG   
  
  
+ TCCTCTTCAG GAAGTTCCAT CTACAAAGCT CTAAGGTGCA AAGAGCCTAC TAGTAGTGAA CTTCTTTCCT   
  
  
+ ATATGCATTT ACTCTATGAA GTTTGCCCTT ACTTCAAGTT TGGGTACATG TCTGCAAATG GGGCAATTGC   
  
  
+ TGAGGCCATG AAAAATGAGA GCAGAATTCA TATAATTGAT TTTCAGATAG CTCAAGGGAG TCAGTGGATT   
  
  
+ AGCCTTATCC AAGCCCTGGC TGCTCAGCCT GATGGGCCAC CACAGGTCCG TATTACCGGA GTCGATGATT   
  
  
+ CCCAATCTGA GTATGCTCGG GGAGGGGGGC TCGACATTGT AGCGAAGAGA CTATCTGGAC TAGCCCAGGC   
  
  
+ TTGCAGCCTA CCCTTTGAGT TCCACGCTGC AGCACTTAGT GGTTCTGAGA TCAGACTTCA AAACCTGGTT   
  
  
+ TTGCGGCCTG GAGAAGCCTT AGCAGTGAAC TTCCCATTCA TGCTGCACCA CATGCCGGAT GAGAGTGTGG   
  
  
+ GCCCTGAGAA TTATAGAGAC CAGTTATTAA GGCTGGTGAA GAGCTTCTTG CCCAAGGTGG TTACCCTTGT   
  
  
+ TGAGCAAGAA TCCAACACAA ACACGGCCCC ATTTCTACCC CGGTTTCTTG AAACCCTAGA CTACTATACT   
  
  
+ GCCATTTTCG AATCAATTGA TGTTACGCTC CCAAGAGATC ACAAGGAGCG GATCAATGTT GAGCAGCACT   
  
  
+ GTTTAGCAAG AGATATAGTC AACATAATAG CATGTGAGGG TGCCGAGAGG GTGGAACGCC ATGAGGTTCT   
  
  
+ TGGAAAGTGG AGATCTCGGT TCTCAATGGC TGGGTTTAAG CCGTACCCAT TGAGCCCGCT AGTGAATGCA   
  
  
+ ACTATCAGGA CTCTTCTGCA GAAGTATAGC AGGAGCTATG GACTTGAAGA AAGGGATGGA GCTCTTTATC   
  
  
+ TAGGCTGGAT GAACCGAGCA CTAGTTGCAT CTTGTGCATG GCAGTG  

- -Up\_Stream \_Len000AAGAAA AGGACAGAGA CAGAGAAGTA ACGTGGTAGT AAATTCTCTA CCCTGGTATT   
  
  
- TAGTAAGTTA GTGAGGAAAT ACAAATTTGA AACCTTCCTC CAATAATAAA TACACCACCA CTAGGGTTAC   
  
  
- GACTTAAATC GACAACTACC GCTCCCGTAT ATACATTAAT CCTAAAGGTT ACGTCAAATC TAAAATGAGT   
  
  
- AAACTCTTAA GTGACTCCAA ACAAACTAGA CTAAAATCGT TTATAACAAG TCCCCATATC TACGAATAAG   
  
  
- CCAAGAGTTG ACATAATCAT ATGTTCTACT CACCAGGTGA ACAAATTCGA AAATGATATA TGAATACAAC   
  
  
- AGGGATTAAA CCAGAGCGAA CTACACATCG ACAGTTCATA ATAATATTAG AACACAAACT ATAGTGTAAA   
  
  
- CATAACTAAT CGATGTTTTC TTGTATAATA TACACATATC GTTGATAGAG TATATACGGG AGTGGTATCG   
  
  
- CACCAAGGTT CCCAAGTTCA AGGTGAATCG GAGTGGGATC TTTACTGAAG AAAAAAAAAA ATTTCAATTT   
  
  
- CTATACGTTA GTGGTACCTT GGTCAGTAGA AAACTGTGCA ATAAGGTCTA GTCTACTGGT CGGAAAGAGG   
  
  
- TTCCGGTAAT CTAAAAGATC GGTTAGGTGA CAATTGACGT TTTTCACCAT AGGAACCGAC AAGGACAGTG   
  
  
- ACAAAGAATA AACGAGACAC TATAAACATG TAGAGAAACG GTTCCTATTT TCAAGTATCG GCCCTTAACC   
  
  
- ATAAGTCAAC TGAGAACGTC ATTTGTCTCA AGTACATGAC CTCCTAAAAC ACTCAGTACA GTGAGTTATT   
  
  
- TTTGTCCACA CGCTGTCACA ATATTTCGAC GAACGAATTA CGTGTAGATG AGAGTGAGTG GAATGGGTTT   
  
  
- TTTGAAGTTT TAACACACGG AAGATACTTA TAAGACTAGG TAATCGTAAG TTGCCAAAGA CTTAAGACAC   
  
  
- ATTCGGTAGG TATGAGTAAA AGTTGTCTCT TGAACCTAAC CTACATAAGT TATGGGTTTT TGGAACAGTT   
  
  
- TAATGGGGAT CGAATTAAAG GAGATTATGG TTGACGAGAA ACCAAAATCG CATGTTAGGG TATCTAATGG   
  
  
- GAAGATATTA ATCACTACTA ACCAAGTAAA GACGACGTAA TACGGAAGAA ATCTAGGTAA CTATTATATA   
  
  
- TGAGAACGGT ACGACGTTGT AAGGTGACCC TAATTATAGA GTGTAACATA GGAGTCGTTG GTTCCTCTGG   
  
  
- GATGATAAAA GTCTCGGTAA GTATCTTTCA ATAACGTATA TCACTACTAG AAGTTACCCA GACTAAAAAT   
  
  
- CTATCGACGA CACTTGTAAA ATGAGCAGGA GACGGTACAC CTATGATACC ATTGGAAACA AACGAGAGTT   
  
  
- CGGACTCGCA AGACTCTTTA CTACTTTCGC GGGAGCTCAA TATCCTGAAA AGACTGAAAT CGAAATCTTT   
  
  
- TCTCTCCTTT TAGTTGGTGT GGGTAGACTA ATAACTCAAA GACGAGGAAC TCTGGACCTA CCTCGGTCTT   
  
  
- GAGTGCCAGA ACAGTCGGTC TTATTAGTCC TTGACGTAAA GACTCCTGGG TACTTTTCTG GGACCATATG   
  
  
- CTGATAAGTA CTTTTGCAAA ACAGTCGGAA AGTCAAAAAC GTAAAATAAG ATCGACGAAA CTAACTTCGG   
  
  
- GTTAAAATAA TTTTGACACG AAAAGAGAAC TTACAATTTG AGAACGTCAA GGAACGAACA ATAAAACCCA   
  
  
- AAATACAAAC GAAAGATGAC TATAGTAGAA AACCAGCACG TTAAATTAAC GGATGATACA ATATACCTAA   
  
  
- AAATACAAAT GAAAGATGAC TATAGTAGAA AACGATAACG TTAAATTAAC GGAAGATACA TCTTAAGCAG   
  
  
- TGAAACTTCA GACGGTCCTT CCGTTATGTC GATATAAAAA GAACACCTAC TTCACTATTT TCCGTTCACA   
  
  
- GTTAACGAAT GACGAAATAA CGCGACATGA AAAGGTCTCT AAAACCTAAT CAGTTACGTC TTCGGTTGTT   
  
  
- AAATATCGCT GACCGGTGTC AAGATGTTGG TTAAACTATT AGGTGCAGAA GAACTTAGAA GTAGATATTA   
  
  
- CCCACCACTA GTTGAAAAGT CAAGAGGTAG ATGACATTCG TATAGAACGT TGTCCTCAGG TCGAAGTGAC   
  
  
- GGACTTGAGC TTAGGAGAAG TAGCCTGAAG GTTTCACTGA GGGTCCTCTG ACACTTACCG AGCGGTCACT   
  
  
- AACTAAGCAC GTACCATGCA CTGTAACCAC TGAATTCTGT GTTTGAATCT CTCGAACTCT GACAATACGA   
  
  
- GCCTGGTTCA AGTCTGTCGA ACCTAAGTAC CATACGGGGA AGTTCTCCAC TCACACTTGG AGACGGTGGT   
  
  
- CTTCTCGGAC TGTTACCCTT TGTAAACAAC CTCTACTATC GTTCCCCCTC GGAGTTTCTC CACGATTAAC   
  
  
- GAACACGGTT TCGTTATAGT CTGCTACTAA ACAATTGTCG CCTCACCAAC TACAGTCTCG ATGCGGTATA   
  
  
- CCAAAGTCAA AGACCTCTTG GTTAAGTCTC CAATCCTCGG ATGTACAACC TTCCCAATCA ACGGGCCAAC   
  
  
- AGGAGAAGTC CTTCAAGGTA GATGTTTCGA GATTCCACGT TTCTCGGATG ATCATCACTT GAAGAAAGGA   
  
  
- TATACGTAAA TGAGATACTT CAAACGGGAA TGAAGTTCAA ACCCATGTAC AGACGTTTAC CCCGTTAACG   
  
  
- ACTCCGGTAC TTTTTACTCT CGTCTTAAGT ATATTAACTA AAAGTCTATC GAGTTCCCTC AGTCACCTAA   
  
  
- TCGGAATAGG TTCGGGACCG ACGAGTCGGA CTACCCGGTG GTGTCCAGGC ATAATGGCCT CAGCTACTAA   
  
  
- GGGTTAGACT CATACGAGCC CCTCCCCCCG AGCTGTAACA TCGCTTCTCT GATAGACCTG ATCGGGTCCG   
  
  
- AACGTCGGAT GGGAAACTCA AGGTGCGACG TCGTGAATCA CCAAGACTCT AGTCTGAAGT TTTGGACCAA   
  
  
- AACGCCGGAC CTCTTCGGAA TCGTCACTTG AAGGGTAAGT ACGACGTGGT GTACGGCCTA CTCTCACACC   
  
  
- CGGGACTCTT AATATCTCTG GTCAATAATT CCGACCACTT CTCGAAGAAC GGGTTCCACC AATGGGAACA   
  
  
- ACTCGTTCTT AGGTTGTGTT TGTGCCGGGG TAAAGATGGG GCCAAAGAAC TTTGGGATCT GATGATATGA   
  
  
- CGGTAAAAGC TTAGTTAACT ACAATGCGAG GGTTCTCTAG TGTTCCTCGC CTAGTTACAA CTCGTCGTGA   
  
  
- CAAATCGTTC TCTATATCAG TTGTATTATC GTACACTCCC ACGGCTCTCC CACCTTGCGG TACTCCAAGA   
  
  
- ACCTTTCACC TCTAGAGCCA AGAGTTACCG ACCCAAATTC GGCATGGGTA ACTCGGGCGA TCACTTACGT   
  
  
- TGATAGTCCT GAGAAGACGT CTTCATATCG TCCTCGATAC CTGAACTTCT TTCCCTACCT CGAGAAATAG   
  
  
- ATCCGACCTA CTTGGCTCGT GATCAACGTA GAACACGTAC CGTCAC

+     Myb-binding site

| Site Name | Organism | Position | Strand | Matrix score. | sequence | function |
| --- | --- | --- | --- | --- | --- | --- |
| Myb-binding site | Nicotiana tabacum | 1006 | + | 6 | CAACAG |  |
| Myb-binding site | Nicotiana tabacum | 155 | - | 6 | CAACAG |  |
| Myb-binding site | Nicotiana tabacum | 2153 | + | 6 | CAACAG |  |

>HU08G00284.1   
+ -Up\_Stream \_Len000TTCTTT TCCTGTCTCT GTCTCTTCAT TGCACCATCA TTTAAGAGAT GGGACCATAA   
  
  
+ ATCATTCAAT CACTCCTTTA TGTTTAAACT TTGGAAGGAG GTTATTATTT ATGTGGTGGT GATCCCAATG   
  
  
+ CTGAATTTAG CTGTTGATGG CGAGGGCATA TATGTAATTA GGATTTCCAA TGCAGTTTAG ATTTTACTCA   
  
  
+ TTTGAGAATT CACTGAGGTT TGTTTGATCT GATTTTAGCA AATATTGTTC AGGGGTATAG ATGCTTATTC   
  
  
+ GGTTCTCAAC TGTATTAGTA TACAAGATGA GTGGTCCACT TGTTTAAGCT TTTACTATAT ACTTATGTTG   
  
  
+ TCCCTAATTT GGTCTCGCTT GATGTGTAGC TGTCAAGTAT TATTATAATC TTGTGTTTGA TATCACATTT   
  
  
+ GTATTGATTA GCTACAAAAG AACATATTAT ATGTGTATAG CAACTATCTC ATATATGCCC TCACCATAGC   
  
  
+ GTGGTTCCAA GGGTTCAAGT TCCACTTAGC CTCACCCTAG AAATGACTTC TTTTTTTTTT TAAAGTTAAA   
  
  
+ GATATGCAAT CACCATGGAA CCAGTCATCT TTTGACACGT TATTCCAGAT CAGATGACCA GCCTTTCTCC   
  
  
+ AAGGCCATTA GATTTTCTAG CCAATCCACT GTTAACTGCA AAAAGTGGTA TCCTTGGCTG TTCCTGTCAC   
  
  
+ TGTTTCTTAT TTGCTCTGTG ATATTTGTAC ATCTCTTTGC CAAGGATAAA AGTTCATAGC CGGGAATTGG   
  
  
+ TATTCAGTTG ACTCTTGCAG TAAACAGAGT TCATGTACTG GAGGATTTTG TGAGTCATGT CACTCAATAA   
  
  
+ AAACAGGTGT GCGACAGTGT TATAAAGCTG CTTGCTTAAT GCACATCTAC TCTCACTCAC CTTACCCAAA   
  
  
+ AAACTTCAAA ATTGTGTGCC TTCTATGAAT ATTCTGATCC ATTAGCATTC AACGGTTTCT GAATTCTGTG   
  
  
+ TAAGCCATCC ATACTCATTT TCAACAGAGA ACTTGGATTG GATGTATTCA ATACCCAAAA ACCTTGTCAA   
  
  
+ ATTACCCCTA GCTTAATTTC CTCTAATACC AACTGCTCTT TGGTTTTAGC GTACAATCCC ATAGATTACC   
  
  
+ CTTCTATAAT TAGTGATGAT TGGTTCATTT CTGCTGCATT ATGCCTTCTT TAGATCCATT GATAATATAT   
  
  
+ ACTCTTGCCA TGCTGCAACA TTCCACTGGG ATTAATATCT CACATTGTAT CCTCAGCAAC CAAGGAGACC   
  
  
+ CTACTATTTT CAGAGCCATT CATAGAAAGT TATTGCATAT AGTGATGATC TTCAATGGGT CTGATTTTTA   
  
  
+ GATAGCTGCT GTGAACATTT TACTCGTCCT CTGCCATGTG GATACTATGG TAACCTTTGT TTGCTCTCAA   
  
  
+ GCCTGAGCGT TCTGAGAAAT GATGAAAGCG CCCTCGAGTT ATAGGACTTT TCTGACTTTA GCTTTAGAAA   
  
  
+ AGAGAGGAAA ATCAACCACA CCCATCTGAT TATTGAGTTT CTGCTCCTTG AGACCTGGAT GGAGCCAGAA   
  
  
+ CTCACGGTCT TGTCAGCCAG AATAATCAGG AACTGCATTT CTGAGGACCC ATGAAAAGAC CCTGGTATAC   
  
  
+ GACTATTCAT GAAAACGTTT TGTCAGCCTT TCAGTTTTTG CATTTTATTC TAGCTGCTTT GATTGAAGCC   
  
  
+ CAATTTTATT AAAACTGTGC TTTTCTCTTG AATGTTAAAC TCTTGCAGTT CCTTGCTTGT TATTTTGGGT   
  
  
+ TTTATGTTTG CTTTCTACTG ATATCATCTT TTGGTCGTGC AATTTAATTG CCTACTATGT TATATGGATT   
  
  
+ TTTATGTTTA CTTTCTACTG ATATCATCTT TTGCTATTGC AATTTAATTG CCTTCTATGT AGAATTCGTC   
  
  
+ ACTTTGAAGT CTGCCAGGAA GGCAATACAG CTATATTTTT CTTGTGGATG AAGTGATAAA AGGCAAGTGT   
  
  
+ CAATTGCTTA CTGCTTTATT GCGCTGTACT TTTCCAGAGA TTTTGGATTA GTCAATGCAG AAGCCAACAA   
  
  
+ TTTATAGCGA CTGGCCACAG TTCTACAACC AATTTGATAA TCCACGTCTT CTTGAATCTT CATCTATAAT   
  
  
+ GGGTGGTGAT CAACTTTTCA GTTCTCCATC TACTGTAAGC ATATCTTGCA ACAGGAGTCC AGCTTCACTG   
  
  
+ CCTGAACTCG AATCCTCTTC ATCGGACTTC CAAAGTGACT CCCAGGAGAC TGTGAATGGC TCGCCAGTGA   
  
  
+ TTGATTCGTG CATGGTACGT GACATTGGTG ACTTAAGACA CAAACTTAGA GAGCTTGAGA CTGTTATGCT   
  
  
+ CGGACCAAGT TCAGACAGCT TGGATTCATG GTATGCCCCT TCAAGAGGTG AGTGTGAACC TCTGCCACCA   
  
  
+ GAAGAGCCTG ACAATGGGAA ACATTTGTTG GAGATGATAG CAAGGGGGAG CCTCAAAGAG GTGCTAATTG   
  
  
+ CTTGTGCCAA AGCAATATCA GACGATGATT TGTTAACAGC GGAGTGGTTG ATGTCAGAGC TACGCCATAT   
  
  
+ GGTTTCAGTT TCTGGAGAAC CAATTCAGAG GTTAGGAGCC TACATGTTGG AAGGGTTAGT TGCCCGGTTG   
  
  
+ TCCTCTTCAG GAAGTTCCAT CTACAAAGCT CTAAGGTGCA AAGAGCCTAC TAGTAGTGAA CTTCTTTCCT   
  
  
+ ATATGCATTT ACTCTATGAA GTTTGCCCTT ACTTCAAGTT TGGGTACATG TCTGCAAATG GGGCAATTGC   
  
  
+ TGAGGCCATG AAAAATGAGA GCAGAATTCA TATAATTGAT TTTCAGATAG CTCAAGGGAG TCAGTGGATT   
  
  
+ AGCCTTATCC AAGCCCTGGC TGCTCAGCCT GATGGGCCAC CACAGGTCCG TATTACCGGA GTCGATGATT   
  
  
+ CCCAATCTGA GTATGCTCGG GGAGGGGGGC TCGACATTGT AGCGAAGAGA CTATCTGGAC TAGCCCAGGC   
  
  
+ TTGCAGCCTA CCCTTTGAGT TCCACGCTGC AGCACTTAGT GGTTCTGAGA TCAGACTTCA AAACCTGGTT   
  
  
+ TTGCGGCCTG GAGAAGCCTT AGCAGTGAAC TTCCCATTCA TGCTGCACCA CATGCCGGAT GAGAGTGTGG   
  
  
+ GCCCTGAGAA TTATAGAGAC CAGTTATTAA GGCTGGTGAA GAGCTTCTTG CCCAAGGTGG TTACCCTTGT   
  
  
+ TGAGCAAGAA TCCAACACAA ACACGGCCCC ATTTCTACCC CGGTTTCTTG AAACCCTAGA CTACTATACT   
  
  
+ GCCATTTTCG AATCAATTGA TGTTACGCTC CCAAGAGATC ACAAGGAGCG GATCAATGTT GAGCAGCACT   
  
  
+ GTTTAGCAAG AGATATAGTC AACATAATAG CATGTGAGGG TGCCGAGAGG GTGGAACGCC ATGAGGTTCT   
  
  
+ TGGAAAGTGG AGATCTCGGT TCTCAATGGC TGGGTTTAAG CCGTACCCAT TGAGCCCGCT AGTGAATGCA   
  
  
+ ACTATCAGGA CTCTTCTGCA GAAGTATAGC AGGAGCTATG GACTTGAAGA AAGGGATGGA GCTCTTTATC   
  
  
+ TAGGCTGGAT GAACCGAGCA CTAGTTGCAT CTTGTGCATG GCAGTG  

- -Up\_Stream \_Len000AAGAAA AGGACAGAGA CAGAGAAGTA ACGTGGTAGT AAATTCTCTA CCCTGGTATT   
  
  
- TAGTAAGTTA GTGAGGAAAT ACAAATTTGA AACCTTCCTC CAATAATAAA TACACCACCA CTAGGGTTAC   
  
  
- GACTTAAATC GACAACTACC GCTCCCGTAT ATACATTAAT CCTAAAGGTT ACGTCAAATC TAAAATGAGT   
  
  
- AAACTCTTAA GTGACTCCAA ACAAACTAGA CTAAAATCGT TTATAACAAG TCCCCATATC TACGAATAAG   
  
  
- CCAAGAGTTG ACATAATCAT ATGTTCTACT CACCAGGTGA ACAAATTCGA AAATGATATA TGAATACAAC   
  
  
- AGGGATTAAA CCAGAGCGAA CTACACATCG ACAGTTCATA ATAATATTAG AACACAAACT ATAGTGTAAA   
  
  
- CATAACTAAT CGATGTTTTC TTGTATAATA TACACATATC GTTGATAGAG TATATACGGG AGTGGTATCG   
  
  
- CACCAAGGTT CCCAAGTTCA AGGTGAATCG GAGTGGGATC TTTACTGAAG AAAAAAAAAA ATTTCAATTT   
  
  
- CTATACGTTA GTGGTACCTT GGTCAGTAGA AAACTGTGCA ATAAGGTCTA GTCTACTGGT CGGAAAGAGG   
  
  
- TTCCGGTAAT CTAAAAGATC GGTTAGGTGA CAATTGACGT TTTTCACCAT AGGAACCGAC AAGGACAGTG   
  
  
- ACAAAGAATA AACGAGACAC TATAAACATG TAGAGAAACG GTTCCTATTT TCAAGTATCG GCCCTTAACC   
  
  
- ATAAGTCAAC TGAGAACGTC ATTTGTCTCA AGTACATGAC CTCCTAAAAC ACTCAGTACA GTGAGTTATT   
  
  
- TTTGTCCACA CGCTGTCACA ATATTTCGAC GAACGAATTA CGTGTAGATG AGAGTGAGTG GAATGGGTTT   
  
  
- TTTGAAGTTT TAACACACGG AAGATACTTA TAAGACTAGG TAATCGTAAG TTGCCAAAGA CTTAAGACAC   
  
  
- ATTCGGTAGG TATGAGTAAA AGTTGTCTCT TGAACCTAAC CTACATAAGT TATGGGTTTT TGGAACAGTT   
  
  
- TAATGGGGAT CGAATTAAAG GAGATTATGG TTGACGAGAA ACCAAAATCG CATGTTAGGG TATCTAATGG   
  
  
- GAAGATATTA ATCACTACTA ACCAAGTAAA GACGACGTAA TACGGAAGAA ATCTAGGTAA CTATTATATA   
  
  
- TGAGAACGGT ACGACGTTGT AAGGTGACCC TAATTATAGA GTGTAACATA GGAGTCGTTG GTTCCTCTGG   
  
  
- GATGATAAAA GTCTCGGTAA GTATCTTTCA ATAACGTATA TCACTACTAG AAGTTACCCA GACTAAAAAT   
  
  
- CTATCGACGA CACTTGTAAA ATGAGCAGGA GACGGTACAC CTATGATACC ATTGGAAACA AACGAGAGTT   
  
  
- CGGACTCGCA AGACTCTTTA CTACTTTCGC GGGAGCTCAA TATCCTGAAA AGACTGAAAT CGAAATCTTT   
  
  
- TCTCTCCTTT TAGTTGGTGT GGGTAGACTA ATAACTCAAA GACGAGGAAC TCTGGACCTA CCTCGGTCTT   
  
  
- GAGTGCCAGA ACAGTCGGTC TTATTAGTCC TTGACGTAAA GACTCCTGGG TACTTTTCTG GGACCATATG   
  
  
- CTGATAAGTA CTTTTGCAAA ACAGTCGGAA AGTCAAAAAC GTAAAATAAG ATCGACGAAA CTAACTTCGG   
  
  
- GTTAAAATAA TTTTGACACG AAAAGAGAAC TTACAATTTG AGAACGTCAA GGAACGAACA ATAAAACCCA   
  
  
- AAATACAAAC GAAAGATGAC TATAGTAGAA AACCAGCACG TTAAATTAAC GGATGATACA ATATACCTAA   
  
  
- AAATACAAAT GAAAGATGAC TATAGTAGAA AACGATAACG TTAAATTAAC GGAAGATACA TCTTAAGCAG   
  
  
- TGAAACTTCA GACGGTCCTT CCGTTATGTC GATATAAAAA GAACACCTAC TTCACTATTT TCCGTTCACA   
  
  
- GTTAACGAAT GACGAAATAA CGCGACATGA AAAGGTCTCT AAAACCTAAT CAGTTACGTC TTCGGTTGTT   
  
  
- AAATATCGCT GACCGGTGTC AAGATGTTGG TTAAACTATT AGGTGCAGAA GAACTTAGAA GTAGATATTA   
  
  
- CCCACCACTA GTTGAAAAGT CAAGAGGTAG ATGACATTCG TATAGAACGT TGTCCTCAGG TCGAAGTGAC   
  
  
- GGACTTGAGC TTAGGAGAAG TAGCCTGAAG GTTTCACTGA GGGTCCTCTG ACACTTACCG AGCGGTCACT   
  
  
- AACTAAGCAC GTACCATGCA CTGTAACCAC TGAATTCTGT GTTTGAATCT CTCGAACTCT GACAATACGA   
  
  
- GCCTGGTTCA AGTCTGTCGA ACCTAAGTAC CATACGGGGA AGTTCTCCAC TCACACTTGG AGACGGTGGT   
  
  
- CTTCTCGGAC TGTTACCCTT TGTAAACAAC CTCTACTATC GTTCCCCCTC GGAGTTTCTC CACGATTAAC   
  
  
- GAACACGGTT TCGTTATAGT CTGCTACTAA ACAATTGTCG CCTCACCAAC TACAGTCTCG ATGCGGTATA   
  
  
- CCAAAGTCAA AGACCTCTTG GTTAAGTCTC CAATCCTCGG ATGTACAACC TTCCCAATCA ACGGGCCAAC   
  
  
- AGGAGAAGTC CTTCAAGGTA GATGTTTCGA GATTCCACGT TTCTCGGATG ATCATCACTT GAAGAAAGGA   
  
  
- TATACGTAAA TGAGATACTT CAAACGGGAA TGAAGTTCAA ACCCATGTAC AGACGTTTAC CCCGTTAACG   
  
  
- ACTCCGGTAC TTTTTACTCT CGTCTTAAGT ATATTAACTA AAAGTCTATC GAGTTCCCTC AGTCACCTAA   
  
  
- TCGGAATAGG TTCGGGACCG ACGAGTCGGA CTACCCGGTG GTGTCCAGGC ATAATGGCCT CAGCTACTAA   
  
  
- GGGTTAGACT CATACGAGCC CCTCCCCCCG AGCTGTAACA TCGCTTCTCT GATAGACCTG ATCGGGTCCG   
  
  
- AACGTCGGAT GGGAAACTCA AGGTGCGACG TCGTGAATCA CCAAGACTCT AGTCTGAAGT TTTGGACCAA   
  
  
- AACGCCGGAC CTCTTCGGAA TCGTCACTTG AAGGGTAAGT ACGACGTGGT GTACGGCCTA CTCTCACACC   
  
  
- CGGGACTCTT AATATCTCTG GTCAATAATT CCGACCACTT CTCGAAGAAC GGGTTCCACC AATGGGAACA   
  
  
- ACTCGTTCTT AGGTTGTGTT TGTGCCGGGG TAAAGATGGG GCCAAAGAAC TTTGGGATCT GATGATATGA   
  
  
- CGGTAAAAGC TTAGTTAACT ACAATGCGAG GGTTCTCTAG TGTTCCTCGC CTAGTTACAA CTCGTCGTGA   
  
  
- CAAATCGTTC TCTATATCAG TTGTATTATC GTACACTCCC ACGGCTCTCC CACCTTGCGG TACTCCAAGA   
  
  
- ACCTTTCACC TCTAGAGCCA AGAGTTACCG ACCCAAATTC GGCATGGGTA ACTCGGGCGA TCACTTACGT   
  
  
- TGATAGTCCT GAGAAGACGT CTTCATATCG TCCTCGATAC CTGAACTTCT TTCCCTACCT CGAGAAATAG   
  
  
- ATCCGACCTA CTTGGCTCGT GATCAACGTA GAACACGTAC CGTCAC

+     Myc

| Site Name | Organism | Position | Strand | Matrix score. | sequence | function |
| --- | --- | --- | --- | --- | --- | --- |
| Myc | Arabidopsis thaliana | 57 | - | 7 | TCTCTTA |  |

>HU08G00284.1   
+ -Up\_Stream \_Len000TTCTTT TCCTGTCTCT GTCTCTTCAT TGCACCATCA TTTAAGAGAT GGGACCATAA   
  
  
+ ATCATTCAAT CACTCCTTTA TGTTTAAACT TTGGAAGGAG GTTATTATTT ATGTGGTGGT GATCCCAATG   
  
  
+ CTGAATTTAG CTGTTGATGG CGAGGGCATA TATGTAATTA GGATTTCCAA TGCAGTTTAG ATTTTACTCA   
  
  
+ TTTGAGAATT CACTGAGGTT TGTTTGATCT GATTTTAGCA AATATTGTTC AGGGGTATAG ATGCTTATTC   
  
  
+ GGTTCTCAAC TGTATTAGTA TACAAGATGA GTGGTCCACT TGTTTAAGCT TTTACTATAT ACTTATGTTG   
  
  
+ TCCCTAATTT GGTCTCGCTT GATGTGTAGC TGTCAAGTAT TATTATAATC TTGTGTTTGA TATCACATTT   
  
  
+ GTATTGATTA GCTACAAAAG AACATATTAT ATGTGTATAG CAACTATCTC ATATATGCCC TCACCATAGC   
  
  
+ GTGGTTCCAA GGGTTCAAGT TCCACTTAGC CTCACCCTAG AAATGACTTC TTTTTTTTTT TAAAGTTAAA   
  
  
+ GATATGCAAT CACCATGGAA CCAGTCATCT TTTGACACGT TATTCCAGAT CAGATGACCA GCCTTTCTCC   
  
  
+ AAGGCCATTA GATTTTCTAG CCAATCCACT GTTAACTGCA AAAAGTGGTA TCCTTGGCTG TTCCTGTCAC   
  
  
+ TGTTTCTTAT TTGCTCTGTG ATATTTGTAC ATCTCTTTGC CAAGGATAAA AGTTCATAGC CGGGAATTGG   
  
  
+ TATTCAGTTG ACTCTTGCAG TAAACAGAGT TCATGTACTG GAGGATTTTG TGAGTCATGT CACTCAATAA   
  
  
+ AAACAGGTGT GCGACAGTGT TATAAAGCTG CTTGCTTAAT GCACATCTAC TCTCACTCAC CTTACCCAAA   
  
  
+ AAACTTCAAA ATTGTGTGCC TTCTATGAAT ATTCTGATCC ATTAGCATTC AACGGTTTCT GAATTCTGTG   
  
  
+ TAAGCCATCC ATACTCATTT TCAACAGAGA ACTTGGATTG GATGTATTCA ATACCCAAAA ACCTTGTCAA   
  
  
+ ATTACCCCTA GCTTAATTTC CTCTAATACC AACTGCTCTT TGGTTTTAGC GTACAATCCC ATAGATTACC   
  
  
+ CTTCTATAAT TAGTGATGAT TGGTTCATTT CTGCTGCATT ATGCCTTCTT TAGATCCATT GATAATATAT   
  
  
+ ACTCTTGCCA TGCTGCAACA TTCCACTGGG ATTAATATCT CACATTGTAT CCTCAGCAAC CAAGGAGACC   
  
  
+ CTACTATTTT CAGAGCCATT CATAGAAAGT TATTGCATAT AGTGATGATC TTCAATGGGT CTGATTTTTA   
  
  
+ GATAGCTGCT GTGAACATTT TACTCGTCCT CTGCCATGTG GATACTATGG TAACCTTTGT TTGCTCTCAA   
  
  
+ GCCTGAGCGT TCTGAGAAAT GATGAAAGCG CCCTCGAGTT ATAGGACTTT TCTGACTTTA GCTTTAGAAA   
  
  
+ AGAGAGGAAA ATCAACCACA CCCATCTGAT TATTGAGTTT CTGCTCCTTG AGACCTGGAT GGAGCCAGAA   
  
  
+ CTCACGGTCT TGTCAGCCAG AATAATCAGG AACTGCATTT CTGAGGACCC ATGAAAAGAC CCTGGTATAC   
  
  
+ GACTATTCAT GAAAACGTTT TGTCAGCCTT TCAGTTTTTG CATTTTATTC TAGCTGCTTT GATTGAAGCC   
  
  
+ CAATTTTATT AAAACTGTGC TTTTCTCTTG AATGTTAAAC TCTTGCAGTT CCTTGCTTGT TATTTTGGGT   
  
  
+ TTTATGTTTG CTTTCTACTG ATATCATCTT TTGGTCGTGC AATTTAATTG CCTACTATGT TATATGGATT   
  
  
+ TTTATGTTTA CTTTCTACTG ATATCATCTT TTGCTATTGC AATTTAATTG CCTTCTATGT AGAATTCGTC   
  
  
+ ACTTTGAAGT CTGCCAGGAA GGCAATACAG CTATATTTTT CTTGTGGATG AAGTGATAAA AGGCAAGTGT   
  
  
+ CAATTGCTTA CTGCTTTATT GCGCTGTACT TTTCCAGAGA TTTTGGATTA GTCAATGCAG AAGCCAACAA   
  
  
+ TTTATAGCGA CTGGCCACAG TTCTACAACC AATTTGATAA TCCACGTCTT CTTGAATCTT CATCTATAAT   
  
  
+ GGGTGGTGAT CAACTTTTCA GTTCTCCATC TACTGTAAGC ATATCTTGCA ACAGGAGTCC AGCTTCACTG   
  
  
+ CCTGAACTCG AATCCTCTTC ATCGGACTTC CAAAGTGACT CCCAGGAGAC TGTGAATGGC TCGCCAGTGA   
  
  
+ TTGATTCGTG CATGGTACGT GACATTGGTG ACTTAAGACA CAAACTTAGA GAGCTTGAGA CTGTTATGCT   
  
  
+ CGGACCAAGT TCAGACAGCT TGGATTCATG GTATGCCCCT TCAAGAGGTG AGTGTGAACC TCTGCCACCA   
  
  
+ GAAGAGCCTG ACAATGGGAA ACATTTGTTG GAGATGATAG CAAGGGGGAG CCTCAAAGAG GTGCTAATTG   
  
  
+ CTTGTGCCAA AGCAATATCA GACGATGATT TGTTAACAGC GGAGTGGTTG ATGTCAGAGC TACGCCATAT   
  
  
+ GGTTTCAGTT TCTGGAGAAC CAATTCAGAG GTTAGGAGCC TACATGTTGG AAGGGTTAGT TGCCCGGTTG   
  
  
+ TCCTCTTCAG GAAGTTCCAT CTACAAAGCT CTAAGGTGCA AAGAGCCTAC TAGTAGTGAA CTTCTTTCCT   
  
  
+ ATATGCATTT ACTCTATGAA GTTTGCCCTT ACTTCAAGTT TGGGTACATG TCTGCAAATG GGGCAATTGC   
  
  
+ TGAGGCCATG AAAAATGAGA GCAGAATTCA TATAATTGAT TTTCAGATAG CTCAAGGGAG TCAGTGGATT   
  
  
+ AGCCTTATCC AAGCCCTGGC TGCTCAGCCT GATGGGCCAC CACAGGTCCG TATTACCGGA GTCGATGATT   
  
  
+ CCCAATCTGA GTATGCTCGG GGAGGGGGGC TCGACATTGT AGCGAAGAGA CTATCTGGAC TAGCCCAGGC   
  
  
+ TTGCAGCCTA CCCTTTGAGT TCCACGCTGC AGCACTTAGT GGTTCTGAGA TCAGACTTCA AAACCTGGTT   
  
  
+ TTGCGGCCTG GAGAAGCCTT AGCAGTGAAC TTCCCATTCA TGCTGCACCA CATGCCGGAT GAGAGTGTGG   
  
  
+ GCCCTGAGAA TTATAGAGAC CAGTTATTAA GGCTGGTGAA GAGCTTCTTG CCCAAGGTGG TTACCCTTGT   
  
  
+ TGAGCAAGAA TCCAACACAA ACACGGCCCC ATTTCTACCC CGGTTTCTTG AAACCCTAGA CTACTATACT   
  
  
+ GCCATTTTCG AATCAATTGA TGTTACGCTC CCAAGAGATC ACAAGGAGCG GATCAATGTT GAGCAGCACT   
  
  
+ GTTTAGCAAG AGATATAGTC AACATAATAG CATGTGAGGG TGCCGAGAGG GTGGAACGCC ATGAGGTTCT   
  
  
+ TGGAAAGTGG AGATCTCGGT TCTCAATGGC TGGGTTTAAG CCGTACCCAT TGAGCCCGCT AGTGAATGCA   
  
  
+ ACTATCAGGA CTCTTCTGCA GAAGTATAGC AGGAGCTATG GACTTGAAGA AAGGGATGGA GCTCTTTATC   
  
  
+ TAGGCTGGAT GAACCGAGCA CTAGTTGCAT CTTGTGCATG GCAGTG  

- -Up\_Stream \_Len000AAGAAA AGGACAGAGA CAGAGAAGTA ACGTGGTAGT AAATTCTCTA CCCTGGTATT   
  
  
- TAGTAAGTTA GTGAGGAAAT ACAAATTTGA AACCTTCCTC CAATAATAAA TACACCACCA CTAGGGTTAC   
  
  
- GACTTAAATC GACAACTACC GCTCCCGTAT ATACATTAAT CCTAAAGGTT ACGTCAAATC TAAAATGAGT   
  
  
- AAACTCTTAA GTGACTCCAA ACAAACTAGA CTAAAATCGT TTATAACAAG TCCCCATATC TACGAATAAG   
  
  
- CCAAGAGTTG ACATAATCAT ATGTTCTACT CACCAGGTGA ACAAATTCGA AAATGATATA TGAATACAAC   
  
  
- AGGGATTAAA CCAGAGCGAA CTACACATCG ACAGTTCATA ATAATATTAG AACACAAACT ATAGTGTAAA   
  
  
- CATAACTAAT CGATGTTTTC TTGTATAATA TACACATATC GTTGATAGAG TATATACGGG AGTGGTATCG   
  
  
- CACCAAGGTT CCCAAGTTCA AGGTGAATCG GAGTGGGATC TTTACTGAAG AAAAAAAAAA ATTTCAATTT   
  
  
- CTATACGTTA GTGGTACCTT GGTCAGTAGA AAACTGTGCA ATAAGGTCTA GTCTACTGGT CGGAAAGAGG   
  
  
- TTCCGGTAAT CTAAAAGATC GGTTAGGTGA CAATTGACGT TTTTCACCAT AGGAACCGAC AAGGACAGTG   
  
  
- ACAAAGAATA AACGAGACAC TATAAACATG TAGAGAAACG GTTCCTATTT TCAAGTATCG GCCCTTAACC   
  
  
- ATAAGTCAAC TGAGAACGTC ATTTGTCTCA AGTACATGAC CTCCTAAAAC ACTCAGTACA GTGAGTTATT   
  
  
- TTTGTCCACA CGCTGTCACA ATATTTCGAC GAACGAATTA CGTGTAGATG AGAGTGAGTG GAATGGGTTT   
  
  
- TTTGAAGTTT TAACACACGG AAGATACTTA TAAGACTAGG TAATCGTAAG TTGCCAAAGA CTTAAGACAC   
  
  
- ATTCGGTAGG TATGAGTAAA AGTTGTCTCT TGAACCTAAC CTACATAAGT TATGGGTTTT TGGAACAGTT   
  
  
- TAATGGGGAT CGAATTAAAG GAGATTATGG TTGACGAGAA ACCAAAATCG CATGTTAGGG TATCTAATGG   
  
  
- GAAGATATTA ATCACTACTA ACCAAGTAAA GACGACGTAA TACGGAAGAA ATCTAGGTAA CTATTATATA   
  
  
- TGAGAACGGT ACGACGTTGT AAGGTGACCC TAATTATAGA GTGTAACATA GGAGTCGTTG GTTCCTCTGG   
  
  
- GATGATAAAA GTCTCGGTAA GTATCTTTCA ATAACGTATA TCACTACTAG AAGTTACCCA GACTAAAAAT   
  
  
- CTATCGACGA CACTTGTAAA ATGAGCAGGA GACGGTACAC CTATGATACC ATTGGAAACA AACGAGAGTT   
  
  
- CGGACTCGCA AGACTCTTTA CTACTTTCGC GGGAGCTCAA TATCCTGAAA AGACTGAAAT CGAAATCTTT   
  
  
- TCTCTCCTTT TAGTTGGTGT GGGTAGACTA ATAACTCAAA GACGAGGAAC TCTGGACCTA CCTCGGTCTT   
  
  
- GAGTGCCAGA ACAGTCGGTC TTATTAGTCC TTGACGTAAA GACTCCTGGG TACTTTTCTG GGACCATATG   
  
  
- CTGATAAGTA CTTTTGCAAA ACAGTCGGAA AGTCAAAAAC GTAAAATAAG ATCGACGAAA CTAACTTCGG   
  
  
- GTTAAAATAA TTTTGACACG AAAAGAGAAC TTACAATTTG AGAACGTCAA GGAACGAACA ATAAAACCCA   
  
  
- AAATACAAAC GAAAGATGAC TATAGTAGAA AACCAGCACG TTAAATTAAC GGATGATACA ATATACCTAA   
  
  
- AAATACAAAT GAAAGATGAC TATAGTAGAA AACGATAACG TTAAATTAAC GGAAGATACA TCTTAAGCAG   
  
  
- TGAAACTTCA GACGGTCCTT CCGTTATGTC GATATAAAAA GAACACCTAC TTCACTATTT TCCGTTCACA   
  
  
- GTTAACGAAT GACGAAATAA CGCGACATGA AAAGGTCTCT AAAACCTAAT CAGTTACGTC TTCGGTTGTT   
  
  
- AAATATCGCT GACCGGTGTC AAGATGTTGG TTAAACTATT AGGTGCAGAA GAACTTAGAA GTAGATATTA   
  
  
- CCCACCACTA GTTGAAAAGT CAAGAGGTAG ATGACATTCG TATAGAACGT TGTCCTCAGG TCGAAGTGAC   
  
  
- GGACTTGAGC TTAGGAGAAG TAGCCTGAAG GTTTCACTGA GGGTCCTCTG ACACTTACCG AGCGGTCACT   
  
  
- AACTAAGCAC GTACCATGCA CTGTAACCAC TGAATTCTGT GTTTGAATCT CTCGAACTCT GACAATACGA   
  
  
- GCCTGGTTCA AGTCTGTCGA ACCTAAGTAC CATACGGGGA AGTTCTCCAC TCACACTTGG AGACGGTGGT   
  
  
- CTTCTCGGAC TGTTACCCTT TGTAAACAAC CTCTACTATC GTTCCCCCTC GGAGTTTCTC CACGATTAAC   
  
  
- GAACACGGTT TCGTTATAGT CTGCTACTAA ACAATTGTCG CCTCACCAAC TACAGTCTCG ATGCGGTATA   
  
  
- CCAAAGTCAA AGACCTCTTG GTTAAGTCTC CAATCCTCGG ATGTACAACC TTCCCAATCA ACGGGCCAAC   
  
  
- AGGAGAAGTC CTTCAAGGTA GATGTTTCGA GATTCCACGT TTCTCGGATG ATCATCACTT GAAGAAAGGA   
  
  
- TATACGTAAA TGAGATACTT CAAACGGGAA TGAAGTTCAA ACCCATGTAC AGACGTTTAC CCCGTTAACG   
  
  
- ACTCCGGTAC TTTTTACTCT CGTCTTAAGT ATATTAACTA AAAGTCTATC GAGTTCCCTC AGTCACCTAA   
  
  
- TCGGAATAGG TTCGGGACCG ACGAGTCGGA CTACCCGGTG GTGTCCAGGC ATAATGGCCT CAGCTACTAA   
  
  
- GGGTTAGACT CATACGAGCC CCTCCCCCCG AGCTGTAACA TCGCTTCTCT GATAGACCTG ATCGGGTCCG   
  
  
- AACGTCGGAT GGGAAACTCA AGGTGCGACG TCGTGAATCA CCAAGACTCT AGTCTGAAGT TTTGGACCAA   
  
  
- AACGCCGGAC CTCTTCGGAA TCGTCACTTG AAGGGTAAGT ACGACGTGGT GTACGGCCTA CTCTCACACC   
  
  
- CGGGACTCTT AATATCTCTG GTCAATAATT CCGACCACTT CTCGAAGAAC GGGTTCCACC AATGGGAACA   
  
  
- ACTCGTTCTT AGGTTGTGTT TGTGCCGGGG TAAAGATGGG GCCAAAGAAC TTTGGGATCT GATGATATGA   
  
  
- CGGTAAAAGC TTAGTTAACT ACAATGCGAG GGTTCTCTAG TGTTCCTCGC CTAGTTACAA CTCGTCGTGA   
  
  
- CAAATCGTTC TCTATATCAG TTGTATTATC GTACACTCCC ACGGCTCTCC CACCTTGCGG TACTCCAAGA   
  
  
- ACCTTTCACC TCTAGAGCCA AGAGTTACCG ACCCAAATTC GGCATGGGTA ACTCGGGCGA TCACTTACGT   
  
  
- TGATAGTCCT GAGAAGACGT CTTCATATCG TCCTCGATAC CTGAACTTCT TTCCCTACCT CGAGAAATAG   
  
  
- ATCCGACCTA CTTGGCTCGT GATCAACGTA GAACACGTAC CGTCAC

+     O2-site

| Site Name | Organism | Position | Strand | Matrix score. | sequence | function |
| --- | --- | --- | --- | --- | --- | --- |
| O2-site | Zea mays | 1941 | + | 8 | GATGA(C/T)(A/G)TG(A/G) | cis-acting regulatory element involved in zein metabolism regulation |

>HU08G00284.1   
+ -Up\_Stream \_Len000TTCTTT TCCTGTCTCT GTCTCTTCAT TGCACCATCA TTTAAGAGAT GGGACCATAA   
  
  
+ ATCATTCAAT CACTCCTTTA TGTTTAAACT TTGGAAGGAG GTTATTATTT ATGTGGTGGT GATCCCAATG   
  
  
+ CTGAATTTAG CTGTTGATGG CGAGGGCATA TATGTAATTA GGATTTCCAA TGCAGTTTAG ATTTTACTCA   
  
  
+ TTTGAGAATT CACTGAGGTT TGTTTGATCT GATTTTAGCA AATATTGTTC AGGGGTATAG ATGCTTATTC   
  
  
+ GGTTCTCAAC TGTATTAGTA TACAAGATGA GTGGTCCACT TGTTTAAGCT TTTACTATAT ACTTATGTTG   
  
  
+ TCCCTAATTT GGTCTCGCTT GATGTGTAGC TGTCAAGTAT TATTATAATC TTGTGTTTGA TATCACATTT   
  
  
+ GTATTGATTA GCTACAAAAG AACATATTAT ATGTGTATAG CAACTATCTC ATATATGCCC TCACCATAGC   
  
  
+ GTGGTTCCAA GGGTTCAAGT TCCACTTAGC CTCACCCTAG AAATGACTTC TTTTTTTTTT TAAAGTTAAA   
  
  
+ GATATGCAAT CACCATGGAA CCAGTCATCT TTTGACACGT TATTCCAGAT CAGATGACCA GCCTTTCTCC   
  
  
+ AAGGCCATTA GATTTTCTAG CCAATCCACT GTTAACTGCA AAAAGTGGTA TCCTTGGCTG TTCCTGTCAC   
  
  
+ TGTTTCTTAT TTGCTCTGTG ATATTTGTAC ATCTCTTTGC CAAGGATAAA AGTTCATAGC CGGGAATTGG   
  
  
+ TATTCAGTTG ACTCTTGCAG TAAACAGAGT TCATGTACTG GAGGATTTTG TGAGTCATGT CACTCAATAA   
  
  
+ AAACAGGTGT GCGACAGTGT TATAAAGCTG CTTGCTTAAT GCACATCTAC TCTCACTCAC CTTACCCAAA   
  
  
+ AAACTTCAAA ATTGTGTGCC TTCTATGAAT ATTCTGATCC ATTAGCATTC AACGGTTTCT GAATTCTGTG   
  
  
+ TAAGCCATCC ATACTCATTT TCAACAGAGA ACTTGGATTG GATGTATTCA ATACCCAAAA ACCTTGTCAA   
  
  
+ ATTACCCCTA GCTTAATTTC CTCTAATACC AACTGCTCTT TGGTTTTAGC GTACAATCCC ATAGATTACC   
  
  
+ CTTCTATAAT TAGTGATGAT TGGTTCATTT CTGCTGCATT ATGCCTTCTT TAGATCCATT GATAATATAT   
  
  
+ ACTCTTGCCA TGCTGCAACA TTCCACTGGG ATTAATATCT CACATTGTAT CCTCAGCAAC CAAGGAGACC   
  
  
+ CTACTATTTT CAGAGCCATT CATAGAAAGT TATTGCATAT AGTGATGATC TTCAATGGGT CTGATTTTTA   
  
  
+ GATAGCTGCT GTGAACATTT TACTCGTCCT CTGCCATGTG GATACTATGG TAACCTTTGT TTGCTCTCAA   
  
  
+ GCCTGAGCGT TCTGAGAAAT GATGAAAGCG CCCTCGAGTT ATAGGACTTT TCTGACTTTA GCTTTAGAAA   
  
  
+ AGAGAGGAAA ATCAACCACA CCCATCTGAT TATTGAGTTT CTGCTCCTTG AGACCTGGAT GGAGCCAGAA   
  
  
+ CTCACGGTCT TGTCAGCCAG AATAATCAGG AACTGCATTT CTGAGGACCC ATGAAAAGAC CCTGGTATAC   
  
  
+ GACTATTCAT GAAAACGTTT TGTCAGCCTT TCAGTTTTTG CATTTTATTC TAGCTGCTTT GATTGAAGCC   
  
  
+ CAATTTTATT AAAACTGTGC TTTTCTCTTG AATGTTAAAC TCTTGCAGTT CCTTGCTTGT TATTTTGGGT   
  
  
+ TTTATGTTTG CTTTCTACTG ATATCATCTT TTGGTCGTGC AATTTAATTG CCTACTATGT TATATGGATT   
  
  
+ TTTATGTTTA CTTTCTACTG ATATCATCTT TTGCTATTGC AATTTAATTG CCTTCTATGT AGAATTCGTC   
  
  
+ ACTTTGAAGT CTGCCAGGAA GGCAATACAG CTATATTTTT CTTGTGGATG AAGTGATAAA AGGCAAGTGT   
  
  
+ CAATTGCTTA CTGCTTTATT GCGCTGTACT TTTCCAGAGA TTTTGGATTA GTCAATGCAG AAGCCAACAA   
  
  
+ TTTATAGCGA CTGGCCACAG TTCTACAACC AATTTGATAA TCCACGTCTT CTTGAATCTT CATCTATAAT   
  
  
+ GGGTGGTGAT CAACTTTTCA GTTCTCCATC TACTGTAAGC ATATCTTGCA ACAGGAGTCC AGCTTCACTG   
  
  
+ CCTGAACTCG AATCCTCTTC ATCGGACTTC CAAAGTGACT CCCAGGAGAC TGTGAATGGC TCGCCAGTGA   
  
  
+ TTGATTCGTG CATGGTACGT GACATTGGTG ACTTAAGACA CAAACTTAGA GAGCTTGAGA CTGTTATGCT   
  
  
+ CGGACCAAGT TCAGACAGCT TGGATTCATG GTATGCCCCT TCAAGAGGTG AGTGTGAACC TCTGCCACCA   
  
  
+ GAAGAGCCTG ACAATGGGAA ACATTTGTTG GAGATGATAG CAAGGGGGAG CCTCAAAGAG GTGCTAATTG   
  
  
+ CTTGTGCCAA AGCAATATCA GACGATGATT TGTTAACAGC GGAGTGGTTG ATGTCAGAGC TACGCCATAT   
  
  
+ GGTTTCAGTT TCTGGAGAAC CAATTCAGAG GTTAGGAGCC TACATGTTGG AAGGGTTAGT TGCCCGGTTG   
  
  
+ TCCTCTTCAG GAAGTTCCAT CTACAAAGCT CTAAGGTGCA AAGAGCCTAC TAGTAGTGAA CTTCTTTCCT   
  
  
+ ATATGCATTT ACTCTATGAA GTTTGCCCTT ACTTCAAGTT TGGGTACATG TCTGCAAATG GGGCAATTGC   
  
  
+ TGAGGCCATG AAAAATGAGA GCAGAATTCA TATAATTGAT TTTCAGATAG CTCAAGGGAG TCAGTGGATT   
  
  
+ AGCCTTATCC AAGCCCTGGC TGCTCAGCCT GATGGGCCAC CACAGGTCCG TATTACCGGA GTCGATGATT   
  
  
+ CCCAATCTGA GTATGCTCGG GGAGGGGGGC TCGACATTGT AGCGAAGAGA CTATCTGGAC TAGCCCAGGC   
  
  
+ TTGCAGCCTA CCCTTTGAGT TCCACGCTGC AGCACTTAGT GGTTCTGAGA TCAGACTTCA AAACCTGGTT   
  
  
+ TTGCGGCCTG GAGAAGCCTT AGCAGTGAAC TTCCCATTCA TGCTGCACCA CATGCCGGAT GAGAGTGTGG   
  
  
+ GCCCTGAGAA TTATAGAGAC CAGTTATTAA GGCTGGTGAA GAGCTTCTTG CCCAAGGTGG TTACCCTTGT   
  
  
+ TGAGCAAGAA TCCAACACAA ACACGGCCCC ATTTCTACCC CGGTTTCTTG AAACCCTAGA CTACTATACT   
  
  
+ GCCATTTTCG AATCAATTGA TGTTACGCTC CCAAGAGATC ACAAGGAGCG GATCAATGTT GAGCAGCACT   
  
  
+ GTTTAGCAAG AGATATAGTC AACATAATAG CATGTGAGGG TGCCGAGAGG GTGGAACGCC ATGAGGTTCT   
  
  
+ TGGAAAGTGG AGATCTCGGT TCTCAATGGC TGGGTTTAAG CCGTACCCAT TGAGCCCGCT AGTGAATGCA   
  
  
+ ACTATCAGGA CTCTTCTGCA GAAGTATAGC AGGAGCTATG GACTTGAAGA AAGGGATGGA GCTCTTTATC   
  
  
+ TAGGCTGGAT GAACCGAGCA CTAGTTGCAT CTTGTGCATG GCAGTG  

- -Up\_Stream \_Len000AAGAAA AGGACAGAGA CAGAGAAGTA ACGTGGTAGT AAATTCTCTA CCCTGGTATT   
  
  
- TAGTAAGTTA GTGAGGAAAT ACAAATTTGA AACCTTCCTC CAATAATAAA TACACCACCA CTAGGGTTAC   
  
  
- GACTTAAATC GACAACTACC GCTCCCGTAT ATACATTAAT CCTAAAGGTT ACGTCAAATC TAAAATGAGT   
  
  
- AAACTCTTAA GTGACTCCAA ACAAACTAGA CTAAAATCGT TTATAACAAG TCCCCATATC TACGAATAAG   
  
  
- CCAAGAGTTG ACATAATCAT ATGTTCTACT CACCAGGTGA ACAAATTCGA AAATGATATA TGAATACAAC   
  
  
- AGGGATTAAA CCAGAGCGAA CTACACATCG ACAGTTCATA ATAATATTAG AACACAAACT ATAGTGTAAA   
  
  
- CATAACTAAT CGATGTTTTC TTGTATAATA TACACATATC GTTGATAGAG TATATACGGG AGTGGTATCG   
  
  
- CACCAAGGTT CCCAAGTTCA AGGTGAATCG GAGTGGGATC TTTACTGAAG AAAAAAAAAA ATTTCAATTT   
  
  
- CTATACGTTA GTGGTACCTT GGTCAGTAGA AAACTGTGCA ATAAGGTCTA GTCTACTGGT CGGAAAGAGG   
  
  
- TTCCGGTAAT CTAAAAGATC GGTTAGGTGA CAATTGACGT TTTTCACCAT AGGAACCGAC AAGGACAGTG   
  
  
- ACAAAGAATA AACGAGACAC TATAAACATG TAGAGAAACG GTTCCTATTT TCAAGTATCG GCCCTTAACC   
  
  
- ATAAGTCAAC TGAGAACGTC ATTTGTCTCA AGTACATGAC CTCCTAAAAC ACTCAGTACA GTGAGTTATT   
  
  
- TTTGTCCACA CGCTGTCACA ATATTTCGAC GAACGAATTA CGTGTAGATG AGAGTGAGTG GAATGGGTTT   
  
  
- TTTGAAGTTT TAACACACGG AAGATACTTA TAAGACTAGG TAATCGTAAG TTGCCAAAGA CTTAAGACAC   
  
  
- ATTCGGTAGG TATGAGTAAA AGTTGTCTCT TGAACCTAAC CTACATAAGT TATGGGTTTT TGGAACAGTT   
  
  
- TAATGGGGAT CGAATTAAAG GAGATTATGG TTGACGAGAA ACCAAAATCG CATGTTAGGG TATCTAATGG   
  
  
- GAAGATATTA ATCACTACTA ACCAAGTAAA GACGACGTAA TACGGAAGAA ATCTAGGTAA CTATTATATA   
  
  
- TGAGAACGGT ACGACGTTGT AAGGTGACCC TAATTATAGA GTGTAACATA GGAGTCGTTG GTTCCTCTGG   
  
  
- GATGATAAAA GTCTCGGTAA GTATCTTTCA ATAACGTATA TCACTACTAG AAGTTACCCA GACTAAAAAT   
  
  
- CTATCGACGA CACTTGTAAA ATGAGCAGGA GACGGTACAC CTATGATACC ATTGGAAACA AACGAGAGTT   
  
  
- CGGACTCGCA AGACTCTTTA CTACTTTCGC GGGAGCTCAA TATCCTGAAA AGACTGAAAT CGAAATCTTT   
  
  
- TCTCTCCTTT TAGTTGGTGT GGGTAGACTA ATAACTCAAA GACGAGGAAC TCTGGACCTA CCTCGGTCTT   
  
  
- GAGTGCCAGA ACAGTCGGTC TTATTAGTCC TTGACGTAAA GACTCCTGGG TACTTTTCTG GGACCATATG   
  
  
- CTGATAAGTA CTTTTGCAAA ACAGTCGGAA AGTCAAAAAC GTAAAATAAG ATCGACGAAA CTAACTTCGG   
  
  
- GTTAAAATAA TTTTGACACG AAAAGAGAAC TTACAATTTG AGAACGTCAA GGAACGAACA ATAAAACCCA   
  
  
- AAATACAAAC GAAAGATGAC TATAGTAGAA AACCAGCACG TTAAATTAAC GGATGATACA ATATACCTAA   
  
  
- AAATACAAAT GAAAGATGAC TATAGTAGAA AACGATAACG TTAAATTAAC GGAAGATACA TCTTAAGCAG   
  
  
- TGAAACTTCA GACGGTCCTT CCGTTATGTC GATATAAAAA GAACACCTAC TTCACTATTT TCCGTTCACA   
  
  
- GTTAACGAAT GACGAAATAA CGCGACATGA AAAGGTCTCT AAAACCTAAT CAGTTACGTC TTCGGTTGTT   
  
  
- AAATATCGCT GACCGGTGTC AAGATGTTGG TTAAACTATT AGGTGCAGAA GAACTTAGAA GTAGATATTA   
  
  
- CCCACCACTA GTTGAAAAGT CAAGAGGTAG ATGACATTCG TATAGAACGT TGTCCTCAGG TCGAAGTGAC   
  
  
- GGACTTGAGC TTAGGAGAAG TAGCCTGAAG GTTTCACTGA GGGTCCTCTG ACACTTACCG AGCGGTCACT   
  
  
- AACTAAGCAC GTACCATGCA CTGTAACCAC TGAATTCTGT GTTTGAATCT CTCGAACTCT GACAATACGA   
  
  
- GCCTGGTTCA AGTCTGTCGA ACCTAAGTAC CATACGGGGA AGTTCTCCAC TCACACTTGG AGACGGTGGT   
  
  
- CTTCTCGGAC TGTTACCCTT TGTAAACAAC CTCTACTATC GTTCCCCCTC GGAGTTTCTC CACGATTAAC   
  
  
- GAACACGGTT TCGTTATAGT CTGCTACTAA ACAATTGTCG CCTCACCAAC TACAGTCTCG ATGCGGTATA   
  
  
- CCAAAGTCAA AGACCTCTTG GTTAAGTCTC CAATCCTCGG ATGTACAACC TTCCCAATCA ACGGGCCAAC   
  
  
- AGGAGAAGTC CTTCAAGGTA GATGTTTCGA GATTCCACGT TTCTCGGATG ATCATCACTT GAAGAAAGGA   
  
  
- TATACGTAAA TGAGATACTT CAAACGGGAA TGAAGTTCAA ACCCATGTAC AGACGTTTAC CCCGTTAACG   
  
  
- ACTCCGGTAC TTTTTACTCT CGTCTTAAGT ATATTAACTA AAAGTCTATC GAGTTCCCTC AGTCACCTAA   
  
  
- TCGGAATAGG TTCGGGACCG ACGAGTCGGA CTACCCGGTG GTGTCCAGGC ATAATGGCCT CAGCTACTAA   
  
  
- GGGTTAGACT CATACGAGCC CCTCCCCCCG AGCTGTAACA TCGCTTCTCT GATAGACCTG ATCGGGTCCG   
  
  
- AACGTCGGAT GGGAAACTCA AGGTGCGACG TCGTGAATCA CCAAGACTCT AGTCTGAAGT TTTGGACCAA   
  
  
- AACGCCGGAC CTCTTCGGAA TCGTCACTTG AAGGGTAAGT ACGACGTGGT GTACGGCCTA CTCTCACACC   
  
  
- CGGGACTCTT AATATCTCTG GTCAATAATT CCGACCACTT CTCGAAGAAC GGGTTCCACC AATGGGAACA   
  
  
- ACTCGTTCTT AGGTTGTGTT TGTGCCGGGG TAAAGATGGG GCCAAAGAAC TTTGGGATCT GATGATATGA   
  
  
- CGGTAAAAGC TTAGTTAACT ACAATGCGAG GGTTCTCTAG TGTTCCTCGC CTAGTTACAA CTCGTCGTGA   
  
  
- CAAATCGTTC TCTATATCAG TTGTATTATC GTACACTCCC ACGGCTCTCC CACCTTGCGG TACTCCAAGA   
  
  
- ACCTTTCACC TCTAGAGCCA AGAGTTACCG ACCCAAATTC GGCATGGGTA ACTCGGGCGA TCACTTACGT   
  
  
- TGATAGTCCT GAGAAGACGT CTTCATATCG TCCTCGATAC CTGAACTTCT TTCCCTACCT CGAGAAATAG   
  
  
- ATCCGACCTA CTTGGCTCGT GATCAACGTA GAACACGTAC CGTCAC

+     STRE

| Site Name | Organism | Position | Strand | Matrix score. | sequence | function |
| --- | --- | --- | --- | --- | --- | --- |
| STRE | Arabidopsis thaliana | 2350 | - | 5 | AGGGG |  |
| STRE | Arabidopsis thaliana | 265 | + | 5 | AGGGG |  |
| STRE | Arabidopsis thaliana | 2897 | + | 5 | AGGGG |  |
| STRE | Arabidopsis thaliana | 1059 | - | 5 | AGGGG |  |
| STRE | Arabidopsis thaliana | 2427 | + | 5 | AGGGG |  |

>HU08G00284.1   
+ -Up\_Stream \_Len000TTCTTT TCCTGTCTCT GTCTCTTCAT TGCACCATCA TTTAAGAGAT GGGACCATAA   
  
  
+ ATCATTCAAT CACTCCTTTA TGTTTAAACT TTGGAAGGAG GTTATTATTT ATGTGGTGGT GATCCCAATG   
  
  
+ CTGAATTTAG CTGTTGATGG CGAGGGCATA TATGTAATTA GGATTTCCAA TGCAGTTTAG ATTTTACTCA   
  
  
+ TTTGAGAATT CACTGAGGTT TGTTTGATCT GATTTTAGCA AATATTGTTC AGGGGTATAG ATGCTTATTC   
  
  
+ GGTTCTCAAC TGTATTAGTA TACAAGATGA GTGGTCCACT TGTTTAAGCT TTTACTATAT ACTTATGTTG   
  
  
+ TCCCTAATTT GGTCTCGCTT GATGTGTAGC TGTCAAGTAT TATTATAATC TTGTGTTTGA TATCACATTT   
  
  
+ GTATTGATTA GCTACAAAAG AACATATTAT ATGTGTATAG CAACTATCTC ATATATGCCC TCACCATAGC   
  
  
+ GTGGTTCCAA GGGTTCAAGT TCCACTTAGC CTCACCCTAG AAATGACTTC TTTTTTTTTT TAAAGTTAAA   
  
  
+ GATATGCAAT CACCATGGAA CCAGTCATCT TTTGACACGT TATTCCAGAT CAGATGACCA GCCTTTCTCC   
  
  
+ AAGGCCATTA GATTTTCTAG CCAATCCACT GTTAACTGCA AAAAGTGGTA TCCTTGGCTG TTCCTGTCAC   
  
  
+ TGTTTCTTAT TTGCTCTGTG ATATTTGTAC ATCTCTTTGC CAAGGATAAA AGTTCATAGC CGGGAATTGG   
  
  
+ TATTCAGTTG ACTCTTGCAG TAAACAGAGT TCATGTACTG GAGGATTTTG TGAGTCATGT CACTCAATAA   
  
  
+ AAACAGGTGT GCGACAGTGT TATAAAGCTG CTTGCTTAAT GCACATCTAC TCTCACTCAC CTTACCCAAA   
  
  
+ AAACTTCAAA ATTGTGTGCC TTCTATGAAT ATTCTGATCC ATTAGCATTC AACGGTTTCT GAATTCTGTG   
  
  
+ TAAGCCATCC ATACTCATTT TCAACAGAGA ACTTGGATTG GATGTATTCA ATACCCAAAA ACCTTGTCAA   
  
  
+ ATTACCCCTA GCTTAATTTC CTCTAATACC AACTGCTCTT TGGTTTTAGC GTACAATCCC ATAGATTACC   
  
  
+ CTTCTATAAT TAGTGATGAT TGGTTCATTT CTGCTGCATT ATGCCTTCTT TAGATCCATT GATAATATAT   
  
  
+ ACTCTTGCCA TGCTGCAACA TTCCACTGGG ATTAATATCT CACATTGTAT CCTCAGCAAC CAAGGAGACC   
  
  
+ CTACTATTTT CAGAGCCATT CATAGAAAGT TATTGCATAT AGTGATGATC TTCAATGGGT CTGATTTTTA   
  
  
+ GATAGCTGCT GTGAACATTT TACTCGTCCT CTGCCATGTG GATACTATGG TAACCTTTGT TTGCTCTCAA   
  
  
+ GCCTGAGCGT TCTGAGAAAT GATGAAAGCG CCCTCGAGTT ATAGGACTTT TCTGACTTTA GCTTTAGAAA   
  
  
+ AGAGAGGAAA ATCAACCACA CCCATCTGAT TATTGAGTTT CTGCTCCTTG AGACCTGGAT GGAGCCAGAA   
  
  
+ CTCACGGTCT TGTCAGCCAG AATAATCAGG AACTGCATTT CTGAGGACCC ATGAAAAGAC CCTGGTATAC   
  
  
+ GACTATTCAT GAAAACGTTT TGTCAGCCTT TCAGTTTTTG CATTTTATTC TAGCTGCTTT GATTGAAGCC   
  
  
+ CAATTTTATT AAAACTGTGC TTTTCTCTTG AATGTTAAAC TCTTGCAGTT CCTTGCTTGT TATTTTGGGT   
  
  
+ TTTATGTTTG CTTTCTACTG ATATCATCTT TTGGTCGTGC AATTTAATTG CCTACTATGT TATATGGATT   
  
  
+ TTTATGTTTA CTTTCTACTG ATATCATCTT TTGCTATTGC AATTTAATTG CCTTCTATGT AGAATTCGTC   
  
  
+ ACTTTGAAGT CTGCCAGGAA GGCAATACAG CTATATTTTT CTTGTGGATG AAGTGATAAA AGGCAAGTGT   
  
  
+ CAATTGCTTA CTGCTTTATT GCGCTGTACT TTTCCAGAGA TTTTGGATTA GTCAATGCAG AAGCCAACAA   
  
  
+ TTTATAGCGA CTGGCCACAG TTCTACAACC AATTTGATAA TCCACGTCTT CTTGAATCTT CATCTATAAT   
  
  
+ GGGTGGTGAT CAACTTTTCA GTTCTCCATC TACTGTAAGC ATATCTTGCA ACAGGAGTCC AGCTTCACTG   
  
  
+ CCTGAACTCG AATCCTCTTC ATCGGACTTC CAAAGTGACT CCCAGGAGAC TGTGAATGGC TCGCCAGTGA   
  
  
+ TTGATTCGTG CATGGTACGT GACATTGGTG ACTTAAGACA CAAACTTAGA GAGCTTGAGA CTGTTATGCT   
  
  
+ CGGACCAAGT TCAGACAGCT TGGATTCATG GTATGCCCCT TCAAGAGGTG AGTGTGAACC TCTGCCACCA   
  
  
+ GAAGAGCCTG ACAATGGGAA ACATTTGTTG GAGATGATAG CAAGGGGGAG CCTCAAAGAG GTGCTAATTG   
  
  
+ CTTGTGCCAA AGCAATATCA GACGATGATT TGTTAACAGC GGAGTGGTTG ATGTCAGAGC TACGCCATAT   
  
  
+ GGTTTCAGTT TCTGGAGAAC CAATTCAGAG GTTAGGAGCC TACATGTTGG AAGGGTTAGT TGCCCGGTTG   
  
  
+ TCCTCTTCAG GAAGTTCCAT CTACAAAGCT CTAAGGTGCA AAGAGCCTAC TAGTAGTGAA CTTCTTTCCT   
  
  
+ ATATGCATTT ACTCTATGAA GTTTGCCCTT ACTTCAAGTT TGGGTACATG TCTGCAAATG GGGCAATTGC   
  
  
+ TGAGGCCATG AAAAATGAGA GCAGAATTCA TATAATTGAT TTTCAGATAG CTCAAGGGAG TCAGTGGATT   
  
  
+ AGCCTTATCC AAGCCCTGGC TGCTCAGCCT GATGGGCCAC CACAGGTCCG TATTACCGGA GTCGATGATT   
  
  
+ CCCAATCTGA GTATGCTCGG GGAGGGGGGC TCGACATTGT AGCGAAGAGA CTATCTGGAC TAGCCCAGGC   
  
  
+ TTGCAGCCTA CCCTTTGAGT TCCACGCTGC AGCACTTAGT GGTTCTGAGA TCAGACTTCA AAACCTGGTT   
  
  
+ TTGCGGCCTG GAGAAGCCTT AGCAGTGAAC TTCCCATTCA TGCTGCACCA CATGCCGGAT GAGAGTGTGG   
  
  
+ GCCCTGAGAA TTATAGAGAC CAGTTATTAA GGCTGGTGAA GAGCTTCTTG CCCAAGGTGG TTACCCTTGT   
  
  
+ TGAGCAAGAA TCCAACACAA ACACGGCCCC ATTTCTACCC CGGTTTCTTG AAACCCTAGA CTACTATACT   
  
  
+ GCCATTTTCG AATCAATTGA TGTTACGCTC CCAAGAGATC ACAAGGAGCG GATCAATGTT GAGCAGCACT   
  
  
+ GTTTAGCAAG AGATATAGTC AACATAATAG CATGTGAGGG TGCCGAGAGG GTGGAACGCC ATGAGGTTCT   
  
  
+ TGGAAAGTGG AGATCTCGGT TCTCAATGGC TGGGTTTAAG CCGTACCCAT TGAGCCCGCT AGTGAATGCA   
  
  
+ ACTATCAGGA CTCTTCTGCA GAAGTATAGC AGGAGCTATG GACTTGAAGA AAGGGATGGA GCTCTTTATC   
  
  
+ TAGGCTGGAT GAACCGAGCA CTAGTTGCAT CTTGTGCATG GCAGTG  

- -Up\_Stream \_Len000AAGAAA AGGACAGAGA CAGAGAAGTA ACGTGGTAGT AAATTCTCTA CCCTGGTATT   
  
  
- TAGTAAGTTA GTGAGGAAAT ACAAATTTGA AACCTTCCTC CAATAATAAA TACACCACCA CTAGGGTTAC   
  
  
- GACTTAAATC GACAACTACC GCTCCCGTAT ATACATTAAT CCTAAAGGTT ACGTCAAATC TAAAATGAGT   
  
  
- AAACTCTTAA GTGACTCCAA ACAAACTAGA CTAAAATCGT TTATAACAAG TCCCCATATC TACGAATAAG   
  
  
- CCAAGAGTTG ACATAATCAT ATGTTCTACT CACCAGGTGA ACAAATTCGA AAATGATATA TGAATACAAC   
  
  
- AGGGATTAAA CCAGAGCGAA CTACACATCG ACAGTTCATA ATAATATTAG AACACAAACT ATAGTGTAAA   
  
  
- CATAACTAAT CGATGTTTTC TTGTATAATA TACACATATC GTTGATAGAG TATATACGGG AGTGGTATCG   
  
  
- CACCAAGGTT CCCAAGTTCA AGGTGAATCG GAGTGGGATC TTTACTGAAG AAAAAAAAAA ATTTCAATTT   
  
  
- CTATACGTTA GTGGTACCTT GGTCAGTAGA AAACTGTGCA ATAAGGTCTA GTCTACTGGT CGGAAAGAGG   
  
  
- TTCCGGTAAT CTAAAAGATC GGTTAGGTGA CAATTGACGT TTTTCACCAT AGGAACCGAC AAGGACAGTG   
  
  
- ACAAAGAATA AACGAGACAC TATAAACATG TAGAGAAACG GTTCCTATTT TCAAGTATCG GCCCTTAACC   
  
  
- ATAAGTCAAC TGAGAACGTC ATTTGTCTCA AGTACATGAC CTCCTAAAAC ACTCAGTACA GTGAGTTATT   
  
  
- TTTGTCCACA CGCTGTCACA ATATTTCGAC GAACGAATTA CGTGTAGATG AGAGTGAGTG GAATGGGTTT   
  
  
- TTTGAAGTTT TAACACACGG AAGATACTTA TAAGACTAGG TAATCGTAAG TTGCCAAAGA CTTAAGACAC   
  
  
- ATTCGGTAGG TATGAGTAAA AGTTGTCTCT TGAACCTAAC CTACATAAGT TATGGGTTTT TGGAACAGTT   
  
  
- TAATGGGGAT CGAATTAAAG GAGATTATGG TTGACGAGAA ACCAAAATCG CATGTTAGGG TATCTAATGG   
  
  
- GAAGATATTA ATCACTACTA ACCAAGTAAA GACGACGTAA TACGGAAGAA ATCTAGGTAA CTATTATATA   
  
  
- TGAGAACGGT ACGACGTTGT AAGGTGACCC TAATTATAGA GTGTAACATA GGAGTCGTTG GTTCCTCTGG   
  
  
- GATGATAAAA GTCTCGGTAA GTATCTTTCA ATAACGTATA TCACTACTAG AAGTTACCCA GACTAAAAAT   
  
  
- CTATCGACGA CACTTGTAAA ATGAGCAGGA GACGGTACAC CTATGATACC ATTGGAAACA AACGAGAGTT   
  
  
- CGGACTCGCA AGACTCTTTA CTACTTTCGC GGGAGCTCAA TATCCTGAAA AGACTGAAAT CGAAATCTTT   
  
  
- TCTCTCCTTT TAGTTGGTGT GGGTAGACTA ATAACTCAAA GACGAGGAAC TCTGGACCTA CCTCGGTCTT   
  
  
- GAGTGCCAGA ACAGTCGGTC TTATTAGTCC TTGACGTAAA GACTCCTGGG TACTTTTCTG GGACCATATG   
  
  
- CTGATAAGTA CTTTTGCAAA ACAGTCGGAA AGTCAAAAAC GTAAAATAAG ATCGACGAAA CTAACTTCGG   
  
  
- GTTAAAATAA TTTTGACACG AAAAGAGAAC TTACAATTTG AGAACGTCAA GGAACGAACA ATAAAACCCA   
  
  
- AAATACAAAC GAAAGATGAC TATAGTAGAA AACCAGCACG TTAAATTAAC GGATGATACA ATATACCTAA   
  
  
- AAATACAAAT GAAAGATGAC TATAGTAGAA AACGATAACG TTAAATTAAC GGAAGATACA TCTTAAGCAG   
  
  
- TGAAACTTCA GACGGTCCTT CCGTTATGTC GATATAAAAA GAACACCTAC TTCACTATTT TCCGTTCACA   
  
  
- GTTAACGAAT GACGAAATAA CGCGACATGA AAAGGTCTCT AAAACCTAAT CAGTTACGTC TTCGGTTGTT   
  
  
- AAATATCGCT GACCGGTGTC AAGATGTTGG TTAAACTATT AGGTGCAGAA GAACTTAGAA GTAGATATTA   
  
  
- CCCACCACTA GTTGAAAAGT CAAGAGGTAG ATGACATTCG TATAGAACGT TGTCCTCAGG TCGAAGTGAC   
  
  
- GGACTTGAGC TTAGGAGAAG TAGCCTGAAG GTTTCACTGA GGGTCCTCTG ACACTTACCG AGCGGTCACT   
  
  
- AACTAAGCAC GTACCATGCA CTGTAACCAC TGAATTCTGT GTTTGAATCT CTCGAACTCT GACAATACGA   
  
  
- GCCTGGTTCA AGTCTGTCGA ACCTAAGTAC CATACGGGGA AGTTCTCCAC TCACACTTGG AGACGGTGGT   
  
  
- CTTCTCGGAC TGTTACCCTT TGTAAACAAC CTCTACTATC GTTCCCCCTC GGAGTTTCTC CACGATTAAC   
  
  
- GAACACGGTT TCGTTATAGT CTGCTACTAA ACAATTGTCG CCTCACCAAC TACAGTCTCG ATGCGGTATA   
  
  
- CCAAAGTCAA AGACCTCTTG GTTAAGTCTC CAATCCTCGG ATGTACAACC TTCCCAATCA ACGGGCCAAC   
  
  
- AGGAGAAGTC CTTCAAGGTA GATGTTTCGA GATTCCACGT TTCTCGGATG ATCATCACTT GAAGAAAGGA   
  
  
- TATACGTAAA TGAGATACTT CAAACGGGAA TGAAGTTCAA ACCCATGTAC AGACGTTTAC CCCGTTAACG   
  
  
- ACTCCGGTAC TTTTTACTCT CGTCTTAAGT ATATTAACTA AAAGTCTATC GAGTTCCCTC AGTCACCTAA   
  
  
- TCGGAATAGG TTCGGGACCG ACGAGTCGGA CTACCCGGTG GTGTCCAGGC ATAATGGCCT CAGCTACTAA   
  
  
- GGGTTAGACT CATACGAGCC CCTCCCCCCG AGCTGTAACA TCGCTTCTCT GATAGACCTG ATCGGGTCCG   
  
  
- AACGTCGGAT GGGAAACTCA AGGTGCGACG TCGTGAATCA CCAAGACTCT AGTCTGAAGT TTTGGACCAA   
  
  
- AACGCCGGAC CTCTTCGGAA TCGTCACTTG AAGGGTAAGT ACGACGTGGT GTACGGCCTA CTCTCACACC   
  
  
- CGGGACTCTT AATATCTCTG GTCAATAATT CCGACCACTT CTCGAAGAAC GGGTTCCACC AATGGGAACA   
  
  
- ACTCGTTCTT AGGTTGTGTT TGTGCCGGGG TAAAGATGGG GCCAAAGAAC TTTGGGATCT GATGATATGA   
  
  
- CGGTAAAAGC TTAGTTAACT ACAATGCGAG GGTTCTCTAG TGTTCCTCGC CTAGTTACAA CTCGTCGTGA   
  
  
- CAAATCGTTC TCTATATCAG TTGTATTATC GTACACTCCC ACGGCTCTCC CACCTTGCGG TACTCCAAGA   
  
  
- ACCTTTCACC TCTAGAGCCA AGAGTTACCG ACCCAAATTC GGCATGGGTA ACTCGGGCGA TCACTTACGT   
  
  
- TGATAGTCCT GAGAAGACGT CTTCATATCG TCCTCGATAC CTGAACTTCT TTCCCTACCT CGAGAAATAG   
  
  
- ATCCGACCTA CTTGGCTCGT GATCAACGTA GAACACGTAC CGTCAC

+     TATA-box

| Site Name | Organism | Position | Strand | Matrix score. | sequence | function |
| --- | --- | --- | --- | --- | --- | --- |
| TATA-box | Arabidopsis thaliana | 1814 | - | 5 | TATAA | core promoter element around -30 of transcription start |
| TATA-box | Arabidopsis thaliana | 1129 | + | 4 | TATA | core promoter element around -30 of transcription start |
| TATA-box | Arabidopsis thaliana | 476 | + | 4 | TATA | core promoter element around -30 of transcription start |
| TATA-box | Arabidopsis thaliana | 2037 | - | 4 | TATA | core promoter element around -30 of transcription start |
| TATA-box | Arabidopsis thaliana | 3459 | - | 4 | TATA | core promoter element around -30 of transcription start |
| TATA-box | Arabidopsis thaliana | 2099 | - | 4 | TATA | core promoter element around -30 of transcription start |
| TATA-box | Arabidopsis thaliana | 865 | + | 4 | TATA | core promoter element around -30 of transcription start |
| TATA-box | Arabidopsis thaliana | 3219 | - | 4 | TATA | core promoter element around -30 of transcription start |
| TATA-box | Arabidopsis thaliana | 864 | - | 5 | TATAA | core promoter element around -30 of transcription start |
| TATA-box | Arabidopsis thaliana | 460 | + | 4 | TATA | core promoter element around -30 of transcription start |
| TATA-box | Arabidopsis thaliana | 452 | + | 4 | TATA | core promoter element around -30 of transcription start |
| TATA-box | Helianthus annuus | 458 | - | 6 | TATACA | core promoter element around -30 of transcription start |
| TATA-box | Brassica oleracea | 2764 | + | 6 | ATATAA | core promoter element around -30 of transcription start |
| TATA-box | Brassica napus | 1189 | + | 6 | ATATAT | core promoter element around -30 of transcription start |
| TATA-box | Arabidopsis thaliana | 1610 | + | 4 | TATA | core promoter element around -30 of transcription start |
| TATA-box | Arabidopsis thaliana | 3096 | - | 4 | TATA | core promoter element around -30 of transcription start |
| TATA-box | Arabidopsis thaliana | 2765 | - | 4 | TATA | core promoter element around -30 of transcription start |
| TATA-box | Brassica napus | 475 | + | 6 | ATATAT | core promoter element around -30 of transcription start |
| TATA-box | Brassica napus | 450 | + | 6 | ATTATA | core promoter element around -30 of transcription start |
| TATA-box | Arabidopsis thaliana | 2664 | - | 4 | TATA | core promoter element around -30 of transcription start |
| TATA-box | Arabidopsis thaliana | 1192 | + | 4 | TATA | core promoter element around -30 of transcription start |
| TATA-box | Brassica juncea | 2034 | - | 7 | TATAAAT | core promoter element around -30 of transcription start |
| TATA-box | Arabidopsis thaliana | 451 | - | 5 | TATAA | core promoter element around -30 of transcription start |
| TATA-box | Arabidopsis thaliana | 1302 | + | 4 | TATA | core promoter element around -30 of transcription start |
| TATA-box | Arabidopsis thaliana | 397 | - | 5 | TATAA | core promoter element around -30 of transcription start |
| TATA-box | Arabidopsis thaliana | 3095 | - | 5 | TATAA | core promoter element around -30 of transcription start |
| TATA-box | Arabidopsis thaliana | 2036 | - | 5 | TATAA | core promoter element around -30 of transcription start |
| TATA-box | Arabidopsis thaliana | 3308 | - | 4 | TATA | core promoter element around -30 of transcription start |
| TATA-box | Brassica napus | 396 | + | 6 | ATTATA | core promoter element around -30 of transcription start |
| TATA-box | Arabidopsis thaliana | 303 | + | 4 | TATA | core promoter element around -30 of transcription start |
| TATA-box | Arabidopsis thaliana | 1444 | + | 4 | TATA | core promoter element around -30 of transcription start |
| TATA-box | Arabidopsis thaliana | 1443 | - | 5 | TATAA | core promoter element around -30 of transcription start |
| TATA-box | Brassica napus | 3094 | + | 6 | ATTATA | core promoter element around -30 of transcription start |
| TATA-box | Helianthus annuus | 2035 | - | 6 | TATAAA | core promoter element around -30 of transcription start |
| TATA-box | Arabidopsis thaliana | 1926 | - | 4 | TATA | core promoter element around -30 of transcription start |
| TATA-box | Arabidopsis thaliana | 1815 | - | 4 | TATA | core promoter element around -30 of transcription start |
| TATA-box | Arabidopsis thaliana | 398 | + | 4 | TATA | core promoter element around -30 of transcription start |
| TATA-box | Arabidopsis thaliana | 1190 | + | 6 | TATATA | core promoter element around -30 of transcription start |
| TATA-box | Arabidopsis thaliana | 342 | + | 4 | TATA | core promoter element around -30 of transcription start |
| TATA-box | Oryza sativa | 437 | + | 7 | TACAAAA | core promoter element around -30 of transcription start |
| TATA-box | Brassica napus | 172 | + | 6 | ATATAT | core promoter element around -30 of transcription start |
| TATA-box | Arabidopsis thaliana | 340 | + | 6 | TATATA | core promoter element around -30 of transcription start |
| TATA-box | Arabidopsis thaliana | 270 | + | 4 | TATA | core promoter element around -30 of transcription start |
| TATA-box | Arabidopsis thaliana | 173 | + | 4 | TATA | core promoter element around -30 of transcription start |

>HU08G00284.1   
+ -Up\_Stream \_Len000TTCTTT TCCTGTCTCT GTCTCTTCAT TGCACCATCA TTTAAGAGAT GGGACCATAA   
  
  
+ ATCATTCAAT CACTCCTTTA TGTTTAAACT TTGGAAGGAG GTTATTATTT ATGTGGTGGT GATCCCAATG   
  
  
+ CTGAATTTAG CTGTTGATGG CGAGGGCATA TATGTAATTA GGATTTCCAA TGCAGTTTAG ATTTTACTCA   
  
  
+ TTTGAGAATT CACTGAGGTT TGTTTGATCT GATTTTAGCA AATATTGTTC AGGGGTATAG ATGCTTATTC   
  
  
+ GGTTCTCAAC TGTATTAGTA TACAAGATGA GTGGTCCACT TGTTTAAGCT TTTACTATAT ACTTATGTTG   
  
  
+ TCCCTAATTT GGTCTCGCTT GATGTGTAGC TGTCAAGTAT TATTATAATC TTGTGTTTGA TATCACATTT   
  
  
+ GTATTGATTA GCTACAAAAG AACATATTAT ATGTGTATAG CAACTATCTC ATATATGCCC TCACCATAGC   
  
  
+ GTGGTTCCAA GGGTTCAAGT TCCACTTAGC CTCACCCTAG AAATGACTTC TTTTTTTTTT TAAAGTTAAA   
  
  
+ GATATGCAAT CACCATGGAA CCAGTCATCT TTTGACACGT TATTCCAGAT CAGATGACCA GCCTTTCTCC   
  
  
+ AAGGCCATTA GATTTTCTAG CCAATCCACT GTTAACTGCA AAAAGTGGTA TCCTTGGCTG TTCCTGTCAC   
  
  
+ TGTTTCTTAT TTGCTCTGTG ATATTTGTAC ATCTCTTTGC CAAGGATAAA AGTTCATAGC CGGGAATTGG   
  
  
+ TATTCAGTTG ACTCTTGCAG TAAACAGAGT TCATGTACTG GAGGATTTTG TGAGTCATGT CACTCAATAA   
  
  
+ AAACAGGTGT GCGACAGTGT TATAAAGCTG CTTGCTTAAT GCACATCTAC TCTCACTCAC CTTACCCAAA   
  
  
+ AAACTTCAAA ATTGTGTGCC TTCTATGAAT ATTCTGATCC ATTAGCATTC AACGGTTTCT GAATTCTGTG   
  
  
+ TAAGCCATCC ATACTCATTT TCAACAGAGA ACTTGGATTG GATGTATTCA ATACCCAAAA ACCTTGTCAA   
  
  
+ ATTACCCCTA GCTTAATTTC CTCTAATACC AACTGCTCTT TGGTTTTAGC GTACAATCCC ATAGATTACC   
  
  
+ CTTCTATAAT TAGTGATGAT TGGTTCATTT CTGCTGCATT ATGCCTTCTT TAGATCCATT GATAATATAT   
  
  
+ ACTCTTGCCA TGCTGCAACA TTCCACTGGG ATTAATATCT CACATTGTAT CCTCAGCAAC CAAGGAGACC   
  
  
+ CTACTATTTT CAGAGCCATT CATAGAAAGT TATTGCATAT AGTGATGATC TTCAATGGGT CTGATTTTTA   
  
  
+ GATAGCTGCT GTGAACATTT TACTCGTCCT CTGCCATGTG GATACTATGG TAACCTTTGT TTGCTCTCAA   
  
  
+ GCCTGAGCGT TCTGAGAAAT GATGAAAGCG CCCTCGAGTT ATAGGACTTT TCTGACTTTA GCTTTAGAAA   
  
  
+ AGAGAGGAAA ATCAACCACA CCCATCTGAT TATTGAGTTT CTGCTCCTTG AGACCTGGAT GGAGCCAGAA   
  
  
+ CTCACGGTCT TGTCAGCCAG AATAATCAGG AACTGCATTT CTGAGGACCC ATGAAAAGAC CCTGGTATAC   
  
  
+ GACTATTCAT GAAAACGTTT TGTCAGCCTT TCAGTTTTTG CATTTTATTC TAGCTGCTTT GATTGAAGCC   
  
  
+ CAATTTTATT AAAACTGTGC TTTTCTCTTG AATGTTAAAC TCTTGCAGTT CCTTGCTTGT TATTTTGGGT   
  
  
+ TTTATGTTTG CTTTCTACTG ATATCATCTT TTGGTCGTGC AATTTAATTG CCTACTATGT TATATGGATT   
  
  
+ TTTATGTTTA CTTTCTACTG ATATCATCTT TTGCTATTGC AATTTAATTG CCTTCTATGT AGAATTCGTC   
  
  
+ ACTTTGAAGT CTGCCAGGAA GGCAATACAG CTATATTTTT CTTGTGGATG AAGTGATAAA AGGCAAGTGT   
  
  
+ CAATTGCTTA CTGCTTTATT GCGCTGTACT TTTCCAGAGA TTTTGGATTA GTCAATGCAG AAGCCAACAA   
  
  
+ TTTATAGCGA CTGGCCACAG TTCTACAACC AATTTGATAA TCCACGTCTT CTTGAATCTT CATCTATAAT   
  
  
+ GGGTGGTGAT CAACTTTTCA GTTCTCCATC TACTGTAAGC ATATCTTGCA ACAGGAGTCC AGCTTCACTG   
  
  
+ CCTGAACTCG AATCCTCTTC ATCGGACTTC CAAAGTGACT CCCAGGAGAC TGTGAATGGC TCGCCAGTGA   
  
  
+ TTGATTCGTG CATGGTACGT GACATTGGTG ACTTAAGACA CAAACTTAGA GAGCTTGAGA CTGTTATGCT   
  
  
+ CGGACCAAGT TCAGACAGCT TGGATTCATG GTATGCCCCT TCAAGAGGTG AGTGTGAACC TCTGCCACCA   
  
  
+ GAAGAGCCTG ACAATGGGAA ACATTTGTTG GAGATGATAG CAAGGGGGAG CCTCAAAGAG GTGCTAATTG   
  
  
+ CTTGTGCCAA AGCAATATCA GACGATGATT TGTTAACAGC GGAGTGGTTG ATGTCAGAGC TACGCCATAT   
  
  
+ GGTTTCAGTT TCTGGAGAAC CAATTCAGAG GTTAGGAGCC TACATGTTGG AAGGGTTAGT TGCCCGGTTG   
  
  
+ TCCTCTTCAG GAAGTTCCAT CTACAAAGCT CTAAGGTGCA AAGAGCCTAC TAGTAGTGAA CTTCTTTCCT   
  
  
+ ATATGCATTT ACTCTATGAA GTTTGCCCTT ACTTCAAGTT TGGGTACATG TCTGCAAATG GGGCAATTGC   
  
  
+ TGAGGCCATG AAAAATGAGA GCAGAATTCA TATAATTGAT TTTCAGATAG CTCAAGGGAG TCAGTGGATT   
  
  
+ AGCCTTATCC AAGCCCTGGC TGCTCAGCCT GATGGGCCAC CACAGGTCCG TATTACCGGA GTCGATGATT   
  
  
+ CCCAATCTGA GTATGCTCGG GGAGGGGGGC TCGACATTGT AGCGAAGAGA CTATCTGGAC TAGCCCAGGC   
  
  
+ TTGCAGCCTA CCCTTTGAGT TCCACGCTGC AGCACTTAGT GGTTCTGAGA TCAGACTTCA AAACCTGGTT   
  
  
+ TTGCGGCCTG GAGAAGCCTT AGCAGTGAAC TTCCCATTCA TGCTGCACCA CATGCCGGAT GAGAGTGTGG   
  
  
+ GCCCTGAGAA TTATAGAGAC CAGTTATTAA GGCTGGTGAA GAGCTTCTTG CCCAAGGTGG TTACCCTTGT   
  
  
+ TGAGCAAGAA TCCAACACAA ACACGGCCCC ATTTCTACCC CGGTTTCTTG AAACCCTAGA CTACTATACT   
  
  
+ GCCATTTTCG AATCAATTGA TGTTACGCTC CCAAGAGATC ACAAGGAGCG GATCAATGTT GAGCAGCACT   
  
  
+ GTTTAGCAAG AGATATAGTC AACATAATAG CATGTGAGGG TGCCGAGAGG GTGGAACGCC ATGAGGTTCT   
  
  
+ TGGAAAGTGG AGATCTCGGT TCTCAATGGC TGGGTTTAAG CCGTACCCAT TGAGCCCGCT AGTGAATGCA   
  
  
+ ACTATCAGGA CTCTTCTGCA GAAGTATAGC AGGAGCTATG GACTTGAAGA AAGGGATGGA GCTCTTTATC   
  
  
+ TAGGCTGGAT GAACCGAGCA CTAGTTGCAT CTTGTGCATG GCAGTG  

- -Up\_Stream \_Len000AAGAAA AGGACAGAGA CAGAGAAGTA ACGTGGTAGT AAATTCTCTA CCCTGGTATT   
  
  
- TAGTAAGTTA GTGAGGAAAT ACAAATTTGA AACCTTCCTC CAATAATAAA TACACCACCA CTAGGGTTAC   
  
  
- GACTTAAATC GACAACTACC GCTCCCGTAT ATACATTAAT CCTAAAGGTT ACGTCAAATC TAAAATGAGT   
  
  
- AAACTCTTAA GTGACTCCAA ACAAACTAGA CTAAAATCGT TTATAACAAG TCCCCATATC TACGAATAAG   
  
  
- CCAAGAGTTG ACATAATCAT ATGTTCTACT CACCAGGTGA ACAAATTCGA AAATGATATA TGAATACAAC   
  
  
- AGGGATTAAA CCAGAGCGAA CTACACATCG ACAGTTCATA ATAATATTAG AACACAAACT ATAGTGTAAA   
  
  
- CATAACTAAT CGATGTTTTC TTGTATAATA TACACATATC GTTGATAGAG TATATACGGG AGTGGTATCG   
  
  
- CACCAAGGTT CCCAAGTTCA AGGTGAATCG GAGTGGGATC TTTACTGAAG AAAAAAAAAA ATTTCAATTT   
  
  
- CTATACGTTA GTGGTACCTT GGTCAGTAGA AAACTGTGCA ATAAGGTCTA GTCTACTGGT CGGAAAGAGG   
  
  
- TTCCGGTAAT CTAAAAGATC GGTTAGGTGA CAATTGACGT TTTTCACCAT AGGAACCGAC AAGGACAGTG   
  
  
- ACAAAGAATA AACGAGACAC TATAAACATG TAGAGAAACG GTTCCTATTT TCAAGTATCG GCCCTTAACC   
  
  
- ATAAGTCAAC TGAGAACGTC ATTTGTCTCA AGTACATGAC CTCCTAAAAC ACTCAGTACA GTGAGTTATT   
  
  
- TTTGTCCACA CGCTGTCACA ATATTTCGAC GAACGAATTA CGTGTAGATG AGAGTGAGTG GAATGGGTTT   
  
  
- TTTGAAGTTT TAACACACGG AAGATACTTA TAAGACTAGG TAATCGTAAG TTGCCAAAGA CTTAAGACAC   
  
  
- ATTCGGTAGG TATGAGTAAA AGTTGTCTCT TGAACCTAAC CTACATAAGT TATGGGTTTT TGGAACAGTT   
  
  
- TAATGGGGAT CGAATTAAAG GAGATTATGG TTGACGAGAA ACCAAAATCG CATGTTAGGG TATCTAATGG   
  
  
- GAAGATATTA ATCACTACTA ACCAAGTAAA GACGACGTAA TACGGAAGAA ATCTAGGTAA CTATTATATA   
  
  
- TGAGAACGGT ACGACGTTGT AAGGTGACCC TAATTATAGA GTGTAACATA GGAGTCGTTG GTTCCTCTGG   
  
  
- GATGATAAAA GTCTCGGTAA GTATCTTTCA ATAACGTATA TCACTACTAG AAGTTACCCA GACTAAAAAT   
  
  
- CTATCGACGA CACTTGTAAA ATGAGCAGGA GACGGTACAC CTATGATACC ATTGGAAACA AACGAGAGTT   
  
  
- CGGACTCGCA AGACTCTTTA CTACTTTCGC GGGAGCTCAA TATCCTGAAA AGACTGAAAT CGAAATCTTT   
  
  
- TCTCTCCTTT TAGTTGGTGT GGGTAGACTA ATAACTCAAA GACGAGGAAC TCTGGACCTA CCTCGGTCTT   
  
  
- GAGTGCCAGA ACAGTCGGTC TTATTAGTCC TTGACGTAAA GACTCCTGGG TACTTTTCTG GGACCATATG   
  
  
- CTGATAAGTA CTTTTGCAAA ACAGTCGGAA AGTCAAAAAC GTAAAATAAG ATCGACGAAA CTAACTTCGG   
  
  
- GTTAAAATAA TTTTGACACG AAAAGAGAAC TTACAATTTG AGAACGTCAA GGAACGAACA ATAAAACCCA   
  
  
- AAATACAAAC GAAAGATGAC TATAGTAGAA AACCAGCACG TTAAATTAAC GGATGATACA ATATACCTAA   
  
  
- AAATACAAAT GAAAGATGAC TATAGTAGAA AACGATAACG TTAAATTAAC GGAAGATACA TCTTAAGCAG   
  
  
- TGAAACTTCA GACGGTCCTT CCGTTATGTC GATATAAAAA GAACACCTAC TTCACTATTT TCCGTTCACA   
  
  
- GTTAACGAAT GACGAAATAA CGCGACATGA AAAGGTCTCT AAAACCTAAT CAGTTACGTC TTCGGTTGTT   
  
  
- AAATATCGCT GACCGGTGTC AAGATGTTGG TTAAACTATT AGGTGCAGAA GAACTTAGAA GTAGATATTA   
  
  
- CCCACCACTA GTTGAAAAGT CAAGAGGTAG ATGACATTCG TATAGAACGT TGTCCTCAGG TCGAAGTGAC   
  
  
- GGACTTGAGC TTAGGAGAAG TAGCCTGAAG GTTTCACTGA GGGTCCTCTG ACACTTACCG AGCGGTCACT   
  
  
- AACTAAGCAC GTACCATGCA CTGTAACCAC TGAATTCTGT GTTTGAATCT CTCGAACTCT GACAATACGA   
  
  
- GCCTGGTTCA AGTCTGTCGA ACCTAAGTAC CATACGGGGA AGTTCTCCAC TCACACTTGG AGACGGTGGT   
  
  
- CTTCTCGGAC TGTTACCCTT TGTAAACAAC CTCTACTATC GTTCCCCCTC GGAGTTTCTC CACGATTAAC   
  
  
- GAACACGGTT TCGTTATAGT CTGCTACTAA ACAATTGTCG CCTCACCAAC TACAGTCTCG ATGCGGTATA   
  
  
- CCAAAGTCAA AGACCTCTTG GTTAAGTCTC CAATCCTCGG ATGTACAACC TTCCCAATCA ACGGGCCAAC   
  
  
- AGGAGAAGTC CTTCAAGGTA GATGTTTCGA GATTCCACGT TTCTCGGATG ATCATCACTT GAAGAAAGGA   
  
  
- TATACGTAAA TGAGATACTT CAAACGGGAA TGAAGTTCAA ACCCATGTAC AGACGTTTAC CCCGTTAACG   
  
  
- ACTCCGGTAC TTTTTACTCT CGTCTTAAGT ATATTAACTA AAAGTCTATC GAGTTCCCTC AGTCACCTAA   
  
  
- TCGGAATAGG TTCGGGACCG ACGAGTCGGA CTACCCGGTG GTGTCCAGGC ATAATGGCCT CAGCTACTAA   
  
  
- GGGTTAGACT CATACGAGCC CCTCCCCCCG AGCTGTAACA TCGCTTCTCT GATAGACCTG ATCGGGTCCG   
  
  
- AACGTCGGAT GGGAAACTCA AGGTGCGACG TCGTGAATCA CCAAGACTCT AGTCTGAAGT TTTGGACCAA   
  
  
- AACGCCGGAC CTCTTCGGAA TCGTCACTTG AAGGGTAAGT ACGACGTGGT GTACGGCCTA CTCTCACACC   
  
  
- CGGGACTCTT AATATCTCTG GTCAATAATT CCGACCACTT CTCGAAGAAC GGGTTCCACC AATGGGAACA   
  
  
- ACTCGTTCTT AGGTTGTGTT TGTGCCGGGG TAAAGATGGG GCCAAAGAAC TTTGGGATCT GATGATATGA   
  
  
- CGGTAAAAGC TTAGTTAACT ACAATGCGAG GGTTCTCTAG TGTTCCTCGC CTAGTTACAA CTCGTCGTGA   
  
  
- CAAATCGTTC TCTATATCAG TTGTATTATC GTACACTCCC ACGGCTCTCC CACCTTGCGG TACTCCAAGA   
  
  
- ACCTTTCACC TCTAGAGCCA AGAGTTACCG ACCCAAATTC GGCATGGGTA ACTCGGGCGA TCACTTACGT   
  
  
- TGATAGTCCT GAGAAGACGT CTTCATATCG TCCTCGATAC CTGAACTTCT TTCCCTACCT CGAGAAATAG   
  
  
- ATCCGACCTA CTTGGCTCGT GATCAACGTA GAACACGTAC CGTCAC

+     TCA

| Site Name | Organism | Position | Strand | Matrix score. | sequence | function |
| --- | --- | --- | --- | --- | --- | --- |
| TCA | Pisum sativum | 2187 | + | 9 | TCATCTTCAT |  |

>HU08G00284.1   
+ -Up\_Stream \_Len000TTCTTT TCCTGTCTCT GTCTCTTCAT TGCACCATCA TTTAAGAGAT GGGACCATAA   
  
  
+ ATCATTCAAT CACTCCTTTA TGTTTAAACT TTGGAAGGAG GTTATTATTT ATGTGGTGGT GATCCCAATG   
  
  
+ CTGAATTTAG CTGTTGATGG CGAGGGCATA TATGTAATTA GGATTTCCAA TGCAGTTTAG ATTTTACTCA   
  
  
+ TTTGAGAATT CACTGAGGTT TGTTTGATCT GATTTTAGCA AATATTGTTC AGGGGTATAG ATGCTTATTC   
  
  
+ GGTTCTCAAC TGTATTAGTA TACAAGATGA GTGGTCCACT TGTTTAAGCT TTTACTATAT ACTTATGTTG   
  
  
+ TCCCTAATTT GGTCTCGCTT GATGTGTAGC TGTCAAGTAT TATTATAATC TTGTGTTTGA TATCACATTT   
  
  
+ GTATTGATTA GCTACAAAAG AACATATTAT ATGTGTATAG CAACTATCTC ATATATGCCC TCACCATAGC   
  
  
+ GTGGTTCCAA GGGTTCAAGT TCCACTTAGC CTCACCCTAG AAATGACTTC TTTTTTTTTT TAAAGTTAAA   
  
  
+ GATATGCAAT CACCATGGAA CCAGTCATCT TTTGACACGT TATTCCAGAT CAGATGACCA GCCTTTCTCC   
  
  
+ AAGGCCATTA GATTTTCTAG CCAATCCACT GTTAACTGCA AAAAGTGGTA TCCTTGGCTG TTCCTGTCAC   
  
  
+ TGTTTCTTAT TTGCTCTGTG ATATTTGTAC ATCTCTTTGC CAAGGATAAA AGTTCATAGC CGGGAATTGG   
  
  
+ TATTCAGTTG ACTCTTGCAG TAAACAGAGT TCATGTACTG GAGGATTTTG TGAGTCATGT CACTCAATAA   
  
  
+ AAACAGGTGT GCGACAGTGT TATAAAGCTG CTTGCTTAAT GCACATCTAC TCTCACTCAC CTTACCCAAA   
  
  
+ AAACTTCAAA ATTGTGTGCC TTCTATGAAT ATTCTGATCC ATTAGCATTC AACGGTTTCT GAATTCTGTG   
  
  
+ TAAGCCATCC ATACTCATTT TCAACAGAGA ACTTGGATTG GATGTATTCA ATACCCAAAA ACCTTGTCAA   
  
  
+ ATTACCCCTA GCTTAATTTC CTCTAATACC AACTGCTCTT TGGTTTTAGC GTACAATCCC ATAGATTACC   
  
  
+ CTTCTATAAT TAGTGATGAT TGGTTCATTT CTGCTGCATT ATGCCTTCTT TAGATCCATT GATAATATAT   
  
  
+ ACTCTTGCCA TGCTGCAACA TTCCACTGGG ATTAATATCT CACATTGTAT CCTCAGCAAC CAAGGAGACC   
  
  
+ CTACTATTTT CAGAGCCATT CATAGAAAGT TATTGCATAT AGTGATGATC TTCAATGGGT CTGATTTTTA   
  
  
+ GATAGCTGCT GTGAACATTT TACTCGTCCT CTGCCATGTG GATACTATGG TAACCTTTGT TTGCTCTCAA   
  
  
+ GCCTGAGCGT TCTGAGAAAT GATGAAAGCG CCCTCGAGTT ATAGGACTTT TCTGACTTTA GCTTTAGAAA   
  
  
+ AGAGAGGAAA ATCAACCACA CCCATCTGAT TATTGAGTTT CTGCTCCTTG AGACCTGGAT GGAGCCAGAA   
  
  
+ CTCACGGTCT TGTCAGCCAG AATAATCAGG AACTGCATTT CTGAGGACCC ATGAAAAGAC CCTGGTATAC   
  
  
+ GACTATTCAT GAAAACGTTT TGTCAGCCTT TCAGTTTTTG CATTTTATTC TAGCTGCTTT GATTGAAGCC   
  
  
+ CAATTTTATT AAAACTGTGC TTTTCTCTTG AATGTTAAAC TCTTGCAGTT CCTTGCTTGT TATTTTGGGT   
  
  
+ TTTATGTTTG CTTTCTACTG ATATCATCTT TTGGTCGTGC AATTTAATTG CCTACTATGT TATATGGATT   
  
  
+ TTTATGTTTA CTTTCTACTG ATATCATCTT TTGCTATTGC AATTTAATTG CCTTCTATGT AGAATTCGTC   
  
  
+ ACTTTGAAGT CTGCCAGGAA GGCAATACAG CTATATTTTT CTTGTGGATG AAGTGATAAA AGGCAAGTGT   
  
  
+ CAATTGCTTA CTGCTTTATT GCGCTGTACT TTTCCAGAGA TTTTGGATTA GTCAATGCAG AAGCCAACAA   
  
  
+ TTTATAGCGA CTGGCCACAG TTCTACAACC AATTTGATAA TCCACGTCTT CTTGAATCTT CATCTATAAT   
  
  
+ GGGTGGTGAT CAACTTTTCA GTTCTCCATC TACTGTAAGC ATATCTTGCA ACAGGAGTCC AGCTTCACTG   
  
  
+ CCTGAACTCG AATCCTCTTC ATCGGACTTC CAAAGTGACT CCCAGGAGAC TGTGAATGGC TCGCCAGTGA   
  
  
+ TTGATTCGTG CATGGTACGT GACATTGGTG ACTTAAGACA CAAACTTAGA GAGCTTGAGA CTGTTATGCT   
  
  
+ CGGACCAAGT TCAGACAGCT TGGATTCATG GTATGCCCCT TCAAGAGGTG AGTGTGAACC TCTGCCACCA   
  
  
+ GAAGAGCCTG ACAATGGGAA ACATTTGTTG GAGATGATAG CAAGGGGGAG CCTCAAAGAG GTGCTAATTG   
  
  
+ CTTGTGCCAA AGCAATATCA GACGATGATT TGTTAACAGC GGAGTGGTTG ATGTCAGAGC TACGCCATAT   
  
  
+ GGTTTCAGTT TCTGGAGAAC CAATTCAGAG GTTAGGAGCC TACATGTTGG AAGGGTTAGT TGCCCGGTTG   
  
  
+ TCCTCTTCAG GAAGTTCCAT CTACAAAGCT CTAAGGTGCA AAGAGCCTAC TAGTAGTGAA CTTCTTTCCT   
  
  
+ ATATGCATTT ACTCTATGAA GTTTGCCCTT ACTTCAAGTT TGGGTACATG TCTGCAAATG GGGCAATTGC   
  
  
+ TGAGGCCATG AAAAATGAGA GCAGAATTCA TATAATTGAT TTTCAGATAG CTCAAGGGAG TCAGTGGATT   
  
  
+ AGCCTTATCC AAGCCCTGGC TGCTCAGCCT GATGGGCCAC CACAGGTCCG TATTACCGGA GTCGATGATT   
  
  
+ CCCAATCTGA GTATGCTCGG GGAGGGGGGC TCGACATTGT AGCGAAGAGA CTATCTGGAC TAGCCCAGGC   
  
  
+ TTGCAGCCTA CCCTTTGAGT TCCACGCTGC AGCACTTAGT GGTTCTGAGA TCAGACTTCA AAACCTGGTT   
  
  
+ TTGCGGCCTG GAGAAGCCTT AGCAGTGAAC TTCCCATTCA TGCTGCACCA CATGCCGGAT GAGAGTGTGG   
  
  
+ GCCCTGAGAA TTATAGAGAC CAGTTATTAA GGCTGGTGAA GAGCTTCTTG CCCAAGGTGG TTACCCTTGT   
  
  
+ TGAGCAAGAA TCCAACACAA ACACGGCCCC ATTTCTACCC CGGTTTCTTG AAACCCTAGA CTACTATACT   
  
  
+ GCCATTTTCG AATCAATTGA TGTTACGCTC CCAAGAGATC ACAAGGAGCG GATCAATGTT GAGCAGCACT   
  
  
+ GTTTAGCAAG AGATATAGTC AACATAATAG CATGTGAGGG TGCCGAGAGG GTGGAACGCC ATGAGGTTCT   
  
  
+ TGGAAAGTGG AGATCTCGGT TCTCAATGGC TGGGTTTAAG CCGTACCCAT TGAGCCCGCT AGTGAATGCA   
  
  
+ ACTATCAGGA CTCTTCTGCA GAAGTATAGC AGGAGCTATG GACTTGAAGA AAGGGATGGA GCTCTTTATC   
  
  
+ TAGGCTGGAT GAACCGAGCA CTAGTTGCAT CTTGTGCATG GCAGTG  

- -Up\_Stream \_Len000AAGAAA AGGACAGAGA CAGAGAAGTA ACGTGGTAGT AAATTCTCTA CCCTGGTATT   
  
  
- TAGTAAGTTA GTGAGGAAAT ACAAATTTGA AACCTTCCTC CAATAATAAA TACACCACCA CTAGGGTTAC   
  
  
- GACTTAAATC GACAACTACC GCTCCCGTAT ATACATTAAT CCTAAAGGTT ACGTCAAATC TAAAATGAGT   
  
  
- AAACTCTTAA GTGACTCCAA ACAAACTAGA CTAAAATCGT TTATAACAAG TCCCCATATC TACGAATAAG   
  
  
- CCAAGAGTTG ACATAATCAT ATGTTCTACT CACCAGGTGA ACAAATTCGA AAATGATATA TGAATACAAC   
  
  
- AGGGATTAAA CCAGAGCGAA CTACACATCG ACAGTTCATA ATAATATTAG AACACAAACT ATAGTGTAAA   
  
  
- CATAACTAAT CGATGTTTTC TTGTATAATA TACACATATC GTTGATAGAG TATATACGGG AGTGGTATCG   
  
  
- CACCAAGGTT CCCAAGTTCA AGGTGAATCG GAGTGGGATC TTTACTGAAG AAAAAAAAAA ATTTCAATTT   
  
  
- CTATACGTTA GTGGTACCTT GGTCAGTAGA AAACTGTGCA ATAAGGTCTA GTCTACTGGT CGGAAAGAGG   
  
  
- TTCCGGTAAT CTAAAAGATC GGTTAGGTGA CAATTGACGT TTTTCACCAT AGGAACCGAC AAGGACAGTG   
  
  
- ACAAAGAATA AACGAGACAC TATAAACATG TAGAGAAACG GTTCCTATTT TCAAGTATCG GCCCTTAACC   
  
  
- ATAAGTCAAC TGAGAACGTC ATTTGTCTCA AGTACATGAC CTCCTAAAAC ACTCAGTACA GTGAGTTATT   
  
  
- TTTGTCCACA CGCTGTCACA ATATTTCGAC GAACGAATTA CGTGTAGATG AGAGTGAGTG GAATGGGTTT   
  
  
- TTTGAAGTTT TAACACACGG AAGATACTTA TAAGACTAGG TAATCGTAAG TTGCCAAAGA CTTAAGACAC   
  
  
- ATTCGGTAGG TATGAGTAAA AGTTGTCTCT TGAACCTAAC CTACATAAGT TATGGGTTTT TGGAACAGTT   
  
  
- TAATGGGGAT CGAATTAAAG GAGATTATGG TTGACGAGAA ACCAAAATCG CATGTTAGGG TATCTAATGG   
  
  
- GAAGATATTA ATCACTACTA ACCAAGTAAA GACGACGTAA TACGGAAGAA ATCTAGGTAA CTATTATATA   
  
  
- TGAGAACGGT ACGACGTTGT AAGGTGACCC TAATTATAGA GTGTAACATA GGAGTCGTTG GTTCCTCTGG   
  
  
- GATGATAAAA GTCTCGGTAA GTATCTTTCA ATAACGTATA TCACTACTAG AAGTTACCCA GACTAAAAAT   
  
  
- CTATCGACGA CACTTGTAAA ATGAGCAGGA GACGGTACAC CTATGATACC ATTGGAAACA AACGAGAGTT   
  
  
- CGGACTCGCA AGACTCTTTA CTACTTTCGC GGGAGCTCAA TATCCTGAAA AGACTGAAAT CGAAATCTTT   
  
  
- TCTCTCCTTT TAGTTGGTGT GGGTAGACTA ATAACTCAAA GACGAGGAAC TCTGGACCTA CCTCGGTCTT   
  
  
- GAGTGCCAGA ACAGTCGGTC TTATTAGTCC TTGACGTAAA GACTCCTGGG TACTTTTCTG GGACCATATG   
  
  
- CTGATAAGTA CTTTTGCAAA ACAGTCGGAA AGTCAAAAAC GTAAAATAAG ATCGACGAAA CTAACTTCGG   
  
  
- GTTAAAATAA TTTTGACACG AAAAGAGAAC TTACAATTTG AGAACGTCAA GGAACGAACA ATAAAACCCA   
  
  
- AAATACAAAC GAAAGATGAC TATAGTAGAA AACCAGCACG TTAAATTAAC GGATGATACA ATATACCTAA   
  
  
- AAATACAAAT GAAAGATGAC TATAGTAGAA AACGATAACG TTAAATTAAC GGAAGATACA TCTTAAGCAG   
  
  
- TGAAACTTCA GACGGTCCTT CCGTTATGTC GATATAAAAA GAACACCTAC TTCACTATTT TCCGTTCACA   
  
  
- GTTAACGAAT GACGAAATAA CGCGACATGA AAAGGTCTCT AAAACCTAAT CAGTTACGTC TTCGGTTGTT   
  
  
- AAATATCGCT GACCGGTGTC AAGATGTTGG TTAAACTATT AGGTGCAGAA GAACTTAGAA GTAGATATTA   
  
  
- CCCACCACTA GTTGAAAAGT CAAGAGGTAG ATGACATTCG TATAGAACGT TGTCCTCAGG TCGAAGTGAC   
  
  
- GGACTTGAGC TTAGGAGAAG TAGCCTGAAG GTTTCACTGA GGGTCCTCTG ACACTTACCG AGCGGTCACT   
  
  
- AACTAAGCAC GTACCATGCA CTGTAACCAC TGAATTCTGT GTTTGAATCT CTCGAACTCT GACAATACGA   
  
  
- GCCTGGTTCA AGTCTGTCGA ACCTAAGTAC CATACGGGGA AGTTCTCCAC TCACACTTGG AGACGGTGGT   
  
  
- CTTCTCGGAC TGTTACCCTT TGTAAACAAC CTCTACTATC GTTCCCCCTC GGAGTTTCTC CACGATTAAC   
  
  
- GAACACGGTT TCGTTATAGT CTGCTACTAA ACAATTGTCG CCTCACCAAC TACAGTCTCG ATGCGGTATA   
  
  
- CCAAAGTCAA AGACCTCTTG GTTAAGTCTC CAATCCTCGG ATGTACAACC TTCCCAATCA ACGGGCCAAC   
  
  
- AGGAGAAGTC CTTCAAGGTA GATGTTTCGA GATTCCACGT TTCTCGGATG ATCATCACTT GAAGAAAGGA   
  
  
- TATACGTAAA TGAGATACTT CAAACGGGAA TGAAGTTCAA ACCCATGTAC AGACGTTTAC CCCGTTAACG   
  
  
- ACTCCGGTAC TTTTTACTCT CGTCTTAAGT ATATTAACTA AAAGTCTATC GAGTTCCCTC AGTCACCTAA   
  
  
- TCGGAATAGG TTCGGGACCG ACGAGTCGGA CTACCCGGTG GTGTCCAGGC ATAATGGCCT CAGCTACTAA   
  
  
- GGGTTAGACT CATACGAGCC CCTCCCCCCG AGCTGTAACA TCGCTTCTCT GATAGACCTG ATCGGGTCCG   
  
  
- AACGTCGGAT GGGAAACTCA AGGTGCGACG TCGTGAATCA CCAAGACTCT AGTCTGAAGT TTTGGACCAA   
  
  
- AACGCCGGAC CTCTTCGGAA TCGTCACTTG AAGGGTAAGT ACGACGTGGT GTACGGCCTA CTCTCACACC   
  
  
- CGGGACTCTT AATATCTCTG GTCAATAATT CCGACCACTT CTCGAAGAAC GGGTTCCACC AATGGGAACA   
  
  
- ACTCGTTCTT AGGTTGTGTT TGTGCCGGGG TAAAGATGGG GCCAAAGAAC TTTGGGATCT GATGATATGA   
  
  
- CGGTAAAAGC TTAGTTAACT ACAATGCGAG GGTTCTCTAG TGTTCCTCGC CTAGTTACAA CTCGTCGTGA   
  
  
- CAAATCGTTC TCTATATCAG TTGTATTATC GTACACTCCC ACGGCTCTCC CACCTTGCGG TACTCCAAGA   
  
  
- ACCTTTCACC TCTAGAGCCA AGAGTTACCG ACCCAAATTC GGCATGGGTA ACTCGGGCGA TCACTTACGT   
  
  
- TGATAGTCCT GAGAAGACGT CTTCATATCG TCCTCGATAC CTGAACTTCT TTCCCTACCT CGAGAAATAG   
  
  
- ATCCGACCTA CTTGGCTCGT GATCAACGTA GAACACGTAC CGTCAC

+     TGACG-motif

| Site Name | Organism | Position | Strand | Matrix score. | sequence | function |
| --- | --- | --- | --- | --- | --- | --- |
| TGACG-motif | Hordeum vulgare | 1891 | - | 5 | TGACG | cis-acting regulatory element involved in the MeJA-responsiveness |

>HU08G00284.1   
+ -Up\_Stream \_Len000TTCTTT TCCTGTCTCT GTCTCTTCAT TGCACCATCA TTTAAGAGAT GGGACCATAA   
  
  
+ ATCATTCAAT CACTCCTTTA TGTTTAAACT TTGGAAGGAG GTTATTATTT ATGTGGTGGT GATCCCAATG   
  
  
+ CTGAATTTAG CTGTTGATGG CGAGGGCATA TATGTAATTA GGATTTCCAA TGCAGTTTAG ATTTTACTCA   
  
  
+ TTTGAGAATT CACTGAGGTT TGTTTGATCT GATTTTAGCA AATATTGTTC AGGGGTATAG ATGCTTATTC   
  
  
+ GGTTCTCAAC TGTATTAGTA TACAAGATGA GTGGTCCACT TGTTTAAGCT TTTACTATAT ACTTATGTTG   
  
  
+ TCCCTAATTT GGTCTCGCTT GATGTGTAGC TGTCAAGTAT TATTATAATC TTGTGTTTGA TATCACATTT   
  
  
+ GTATTGATTA GCTACAAAAG AACATATTAT ATGTGTATAG CAACTATCTC ATATATGCCC TCACCATAGC   
  
  
+ GTGGTTCCAA GGGTTCAAGT TCCACTTAGC CTCACCCTAG AAATGACTTC TTTTTTTTTT TAAAGTTAAA   
  
  
+ GATATGCAAT CACCATGGAA CCAGTCATCT TTTGACACGT TATTCCAGAT CAGATGACCA GCCTTTCTCC   
  
  
+ AAGGCCATTA GATTTTCTAG CCAATCCACT GTTAACTGCA AAAAGTGGTA TCCTTGGCTG TTCCTGTCAC   
  
  
+ TGTTTCTTAT TTGCTCTGTG ATATTTGTAC ATCTCTTTGC CAAGGATAAA AGTTCATAGC CGGGAATTGG   
  
  
+ TATTCAGTTG ACTCTTGCAG TAAACAGAGT TCATGTACTG GAGGATTTTG TGAGTCATGT CACTCAATAA   
  
  
+ AAACAGGTGT GCGACAGTGT TATAAAGCTG CTTGCTTAAT GCACATCTAC TCTCACTCAC CTTACCCAAA   
  
  
+ AAACTTCAAA ATTGTGTGCC TTCTATGAAT ATTCTGATCC ATTAGCATTC AACGGTTTCT GAATTCTGTG   
  
  
+ TAAGCCATCC ATACTCATTT TCAACAGAGA ACTTGGATTG GATGTATTCA ATACCCAAAA ACCTTGTCAA   
  
  
+ ATTACCCCTA GCTTAATTTC CTCTAATACC AACTGCTCTT TGGTTTTAGC GTACAATCCC ATAGATTACC   
  
  
+ CTTCTATAAT TAGTGATGAT TGGTTCATTT CTGCTGCATT ATGCCTTCTT TAGATCCATT GATAATATAT   
  
  
+ ACTCTTGCCA TGCTGCAACA TTCCACTGGG ATTAATATCT CACATTGTAT CCTCAGCAAC CAAGGAGACC   
  
  
+ CTACTATTTT CAGAGCCATT CATAGAAAGT TATTGCATAT AGTGATGATC TTCAATGGGT CTGATTTTTA   
  
  
+ GATAGCTGCT GTGAACATTT TACTCGTCCT CTGCCATGTG GATACTATGG TAACCTTTGT TTGCTCTCAA   
  
  
+ GCCTGAGCGT TCTGAGAAAT GATGAAAGCG CCCTCGAGTT ATAGGACTTT TCTGACTTTA GCTTTAGAAA   
  
  
+ AGAGAGGAAA ATCAACCACA CCCATCTGAT TATTGAGTTT CTGCTCCTTG AGACCTGGAT GGAGCCAGAA   
  
  
+ CTCACGGTCT TGTCAGCCAG AATAATCAGG AACTGCATTT CTGAGGACCC ATGAAAAGAC CCTGGTATAC   
  
  
+ GACTATTCAT GAAAACGTTT TGTCAGCCTT TCAGTTTTTG CATTTTATTC TAGCTGCTTT GATTGAAGCC   
  
  
+ CAATTTTATT AAAACTGTGC TTTTCTCTTG AATGTTAAAC TCTTGCAGTT CCTTGCTTGT TATTTTGGGT   
  
  
+ TTTATGTTTG CTTTCTACTG ATATCATCTT TTGGTCGTGC AATTTAATTG CCTACTATGT TATATGGATT   
  
  
+ TTTATGTTTA CTTTCTACTG ATATCATCTT TTGCTATTGC AATTTAATTG CCTTCTATGT AGAATTCGTC   
  
  
+ ACTTTGAAGT CTGCCAGGAA GGCAATACAG CTATATTTTT CTTGTGGATG AAGTGATAAA AGGCAAGTGT   
  
  
+ CAATTGCTTA CTGCTTTATT GCGCTGTACT TTTCCAGAGA TTTTGGATTA GTCAATGCAG AAGCCAACAA   
  
  
+ TTTATAGCGA CTGGCCACAG TTCTACAACC AATTTGATAA TCCACGTCTT CTTGAATCTT CATCTATAAT   
  
  
+ GGGTGGTGAT CAACTTTTCA GTTCTCCATC TACTGTAAGC ATATCTTGCA ACAGGAGTCC AGCTTCACTG   
  
  
+ CCTGAACTCG AATCCTCTTC ATCGGACTTC CAAAGTGACT CCCAGGAGAC TGTGAATGGC TCGCCAGTGA   
  
  
+ TTGATTCGTG CATGGTACGT GACATTGGTG ACTTAAGACA CAAACTTAGA GAGCTTGAGA CTGTTATGCT   
  
  
+ CGGACCAAGT TCAGACAGCT TGGATTCATG GTATGCCCCT TCAAGAGGTG AGTGTGAACC TCTGCCACCA   
  
  
+ GAAGAGCCTG ACAATGGGAA ACATTTGTTG GAGATGATAG CAAGGGGGAG CCTCAAAGAG GTGCTAATTG   
  
  
+ CTTGTGCCAA AGCAATATCA GACGATGATT TGTTAACAGC GGAGTGGTTG ATGTCAGAGC TACGCCATAT   
  
  
+ GGTTTCAGTT TCTGGAGAAC CAATTCAGAG GTTAGGAGCC TACATGTTGG AAGGGTTAGT TGCCCGGTTG   
  
  
+ TCCTCTTCAG GAAGTTCCAT CTACAAAGCT CTAAGGTGCA AAGAGCCTAC TAGTAGTGAA CTTCTTTCCT   
  
  
+ ATATGCATTT ACTCTATGAA GTTTGCCCTT ACTTCAAGTT TGGGTACATG TCTGCAAATG GGGCAATTGC   
  
  
+ TGAGGCCATG AAAAATGAGA GCAGAATTCA TATAATTGAT TTTCAGATAG CTCAAGGGAG TCAGTGGATT   
  
  
+ AGCCTTATCC AAGCCCTGGC TGCTCAGCCT GATGGGCCAC CACAGGTCCG TATTACCGGA GTCGATGATT   
  
  
+ CCCAATCTGA GTATGCTCGG GGAGGGGGGC TCGACATTGT AGCGAAGAGA CTATCTGGAC TAGCCCAGGC   
  
  
+ TTGCAGCCTA CCCTTTGAGT TCCACGCTGC AGCACTTAGT GGTTCTGAGA TCAGACTTCA AAACCTGGTT   
  
  
+ TTGCGGCCTG GAGAAGCCTT AGCAGTGAAC TTCCCATTCA TGCTGCACCA CATGCCGGAT GAGAGTGTGG   
  
  
+ GCCCTGAGAA TTATAGAGAC CAGTTATTAA GGCTGGTGAA GAGCTTCTTG CCCAAGGTGG TTACCCTTGT   
  
  
+ TGAGCAAGAA TCCAACACAA ACACGGCCCC ATTTCTACCC CGGTTTCTTG AAACCCTAGA CTACTATACT   
  
  
+ GCCATTTTCG AATCAATTGA TGTTACGCTC CCAAGAGATC ACAAGGAGCG GATCAATGTT GAGCAGCACT   
  
  
+ GTTTAGCAAG AGATATAGTC AACATAATAG CATGTGAGGG TGCCGAGAGG GTGGAACGCC ATGAGGTTCT   
  
  
+ TGGAAAGTGG AGATCTCGGT TCTCAATGGC TGGGTTTAAG CCGTACCCAT TGAGCCCGCT AGTGAATGCA   
  
  
+ ACTATCAGGA CTCTTCTGCA GAAGTATAGC AGGAGCTATG GACTTGAAGA AAGGGATGGA GCTCTTTATC   
  
  
+ TAGGCTGGAT GAACCGAGCA CTAGTTGCAT CTTGTGCATG GCAGTG  

- -Up\_Stream \_Len000AAGAAA AGGACAGAGA CAGAGAAGTA ACGTGGTAGT AAATTCTCTA CCCTGGTATT   
  
  
- TAGTAAGTTA GTGAGGAAAT ACAAATTTGA AACCTTCCTC CAATAATAAA TACACCACCA CTAGGGTTAC   
  
  
- GACTTAAATC GACAACTACC GCTCCCGTAT ATACATTAAT CCTAAAGGTT ACGTCAAATC TAAAATGAGT   
  
  
- AAACTCTTAA GTGACTCCAA ACAAACTAGA CTAAAATCGT TTATAACAAG TCCCCATATC TACGAATAAG   
  
  
- CCAAGAGTTG ACATAATCAT ATGTTCTACT CACCAGGTGA ACAAATTCGA AAATGATATA TGAATACAAC   
  
  
- AGGGATTAAA CCAGAGCGAA CTACACATCG ACAGTTCATA ATAATATTAG AACACAAACT ATAGTGTAAA   
  
  
- CATAACTAAT CGATGTTTTC TTGTATAATA TACACATATC GTTGATAGAG TATATACGGG AGTGGTATCG   
  
  
- CACCAAGGTT CCCAAGTTCA AGGTGAATCG GAGTGGGATC TTTACTGAAG AAAAAAAAAA ATTTCAATTT   
  
  
- CTATACGTTA GTGGTACCTT GGTCAGTAGA AAACTGTGCA ATAAGGTCTA GTCTACTGGT CGGAAAGAGG   
  
  
- TTCCGGTAAT CTAAAAGATC GGTTAGGTGA CAATTGACGT TTTTCACCAT AGGAACCGAC AAGGACAGTG   
  
  
- ACAAAGAATA AACGAGACAC TATAAACATG TAGAGAAACG GTTCCTATTT TCAAGTATCG GCCCTTAACC   
  
  
- ATAAGTCAAC TGAGAACGTC ATTTGTCTCA AGTACATGAC CTCCTAAAAC ACTCAGTACA GTGAGTTATT   
  
  
- TTTGTCCACA CGCTGTCACA ATATTTCGAC GAACGAATTA CGTGTAGATG AGAGTGAGTG GAATGGGTTT   
  
  
- TTTGAAGTTT TAACACACGG AAGATACTTA TAAGACTAGG TAATCGTAAG TTGCCAAAGA CTTAAGACAC   
  
  
- ATTCGGTAGG TATGAGTAAA AGTTGTCTCT TGAACCTAAC CTACATAAGT TATGGGTTTT TGGAACAGTT   
  
  
- TAATGGGGAT CGAATTAAAG GAGATTATGG TTGACGAGAA ACCAAAATCG CATGTTAGGG TATCTAATGG   
  
  
- GAAGATATTA ATCACTACTA ACCAAGTAAA GACGACGTAA TACGGAAGAA ATCTAGGTAA CTATTATATA   
  
  
- TGAGAACGGT ACGACGTTGT AAGGTGACCC TAATTATAGA GTGTAACATA GGAGTCGTTG GTTCCTCTGG   
  
  
- GATGATAAAA GTCTCGGTAA GTATCTTTCA ATAACGTATA TCACTACTAG AAGTTACCCA GACTAAAAAT   
  
  
- CTATCGACGA CACTTGTAAA ATGAGCAGGA GACGGTACAC CTATGATACC ATTGGAAACA AACGAGAGTT   
  
  
- CGGACTCGCA AGACTCTTTA CTACTTTCGC GGGAGCTCAA TATCCTGAAA AGACTGAAAT CGAAATCTTT   
  
  
- TCTCTCCTTT TAGTTGGTGT GGGTAGACTA ATAACTCAAA GACGAGGAAC TCTGGACCTA CCTCGGTCTT   
  
  
- GAGTGCCAGA ACAGTCGGTC TTATTAGTCC TTGACGTAAA GACTCCTGGG TACTTTTCTG GGACCATATG   
  
  
- CTGATAAGTA CTTTTGCAAA ACAGTCGGAA AGTCAAAAAC GTAAAATAAG ATCGACGAAA CTAACTTCGG   
  
  
- GTTAAAATAA TTTTGACACG AAAAGAGAAC TTACAATTTG AGAACGTCAA GGAACGAACA ATAAAACCCA   
  
  
- AAATACAAAC GAAAGATGAC TATAGTAGAA AACCAGCACG TTAAATTAAC GGATGATACA ATATACCTAA   
  
  
- AAATACAAAT GAAAGATGAC TATAGTAGAA AACGATAACG TTAAATTAAC GGAAGATACA TCTTAAGCAG   
  
  
- TGAAACTTCA GACGGTCCTT CCGTTATGTC GATATAAAAA GAACACCTAC TTCACTATTT TCCGTTCACA   
  
  
- GTTAACGAAT GACGAAATAA CGCGACATGA AAAGGTCTCT AAAACCTAAT CAGTTACGTC TTCGGTTGTT   
  
  
- AAATATCGCT GACCGGTGTC AAGATGTTGG TTAAACTATT AGGTGCAGAA GAACTTAGAA GTAGATATTA   
  
  
- CCCACCACTA GTTGAAAAGT CAAGAGGTAG ATGACATTCG TATAGAACGT TGTCCTCAGG TCGAAGTGAC   
  
  
- GGACTTGAGC TTAGGAGAAG TAGCCTGAAG GTTTCACTGA GGGTCCTCTG ACACTTACCG AGCGGTCACT   
  
  
- AACTAAGCAC GTACCATGCA CTGTAACCAC TGAATTCTGT GTTTGAATCT CTCGAACTCT GACAATACGA   
  
  
- GCCTGGTTCA AGTCTGTCGA ACCTAAGTAC CATACGGGGA AGTTCTCCAC TCACACTTGG AGACGGTGGT   
  
  
- CTTCTCGGAC TGTTACCCTT TGTAAACAAC CTCTACTATC GTTCCCCCTC GGAGTTTCTC CACGATTAAC   
  
  
- GAACACGGTT TCGTTATAGT CTGCTACTAA ACAATTGTCG CCTCACCAAC TACAGTCTCG ATGCGGTATA   
  
  
- CCAAAGTCAA AGACCTCTTG GTTAAGTCTC CAATCCTCGG ATGTACAACC TTCCCAATCA ACGGGCCAAC   
  
  
- AGGAGAAGTC CTTCAAGGTA GATGTTTCGA GATTCCACGT TTCTCGGATG ATCATCACTT GAAGAAAGGA   
  
  
- TATACGTAAA TGAGATACTT CAAACGGGAA TGAAGTTCAA ACCCATGTAC AGACGTTTAC CCCGTTAACG   
  
  
- ACTCCGGTAC TTTTTACTCT CGTCTTAAGT ATATTAACTA AAAGTCTATC GAGTTCCCTC AGTCACCTAA   
  
  
- TCGGAATAGG TTCGGGACCG ACGAGTCGGA CTACCCGGTG GTGTCCAGGC ATAATGGCCT CAGCTACTAA   
  
  
- GGGTTAGACT CATACGAGCC CCTCCCCCCG AGCTGTAACA TCGCTTCTCT GATAGACCTG ATCGGGTCCG   
  
  
- AACGTCGGAT GGGAAACTCA AGGTGCGACG TCGTGAATCA CCAAGACTCT AGTCTGAAGT TTTGGACCAA   
  
  
- AACGCCGGAC CTCTTCGGAA TCGTCACTTG AAGGGTAAGT ACGACGTGGT GTACGGCCTA CTCTCACACC   
  
  
- CGGGACTCTT AATATCTCTG GTCAATAATT CCGACCACTT CTCGAAGAAC GGGTTCCACC AATGGGAACA   
  
  
- ACTCGTTCTT AGGTTGTGTT TGTGCCGGGG TAAAGATGGG GCCAAAGAAC TTTGGGATCT GATGATATGA   
  
  
- CGGTAAAAGC TTAGTTAACT ACAATGCGAG GGTTCTCTAG TGTTCCTCGC CTAGTTACAA CTCGTCGTGA   
  
  
- CAAATCGTTC TCTATATCAG TTGTATTATC GTACACTCCC ACGGCTCTCC CACCTTGCGG TACTCCAAGA   
  
  
- ACCTTTCACC TCTAGAGCCA AGAGTTACCG ACCCAAATTC GGCATGGGTA ACTCGGGCGA TCACTTACGT   
  
  
- TGATAGTCCT GAGAAGACGT CTTCATATCG TCCTCGATAC CTGAACTTCT TTCCCTACCT CGAGAAATAG   
  
  
- ATCCGACCTA CTTGGCTCGT GATCAACGTA GAACACGTAC CGTCAC

+     Unnamed\_\_1

| Site Name | Organism | Position | Strand | Matrix score. | sequence | function |
| --- | --- | --- | --- | --- | --- | --- |
| Unnamed\_\_1 | Zea mays | 494 | + | 5 | CGTGG |  |
| Unnamed\_\_1 | Zea mays | 2076 | - | 5 | CGTGG |  |
| Unnamed\_\_1 | Zea mays | 2966 | - | 5 | CGTGG |  |

>HU08G00284.1   
+ -Up\_Stream \_Len000TTCTTT TCCTGTCTCT GTCTCTTCAT TGCACCATCA TTTAAGAGAT GGGACCATAA   
  
  
+ ATCATTCAAT CACTCCTTTA TGTTTAAACT TTGGAAGGAG GTTATTATTT ATGTGGTGGT GATCCCAATG   
  
  
+ CTGAATTTAG CTGTTGATGG CGAGGGCATA TATGTAATTA GGATTTCCAA TGCAGTTTAG ATTTTACTCA   
  
  
+ TTTGAGAATT CACTGAGGTT TGTTTGATCT GATTTTAGCA AATATTGTTC AGGGGTATAG ATGCTTATTC   
  
  
+ GGTTCTCAAC TGTATTAGTA TACAAGATGA GTGGTCCACT TGTTTAAGCT TTTACTATAT ACTTATGTTG   
  
  
+ TCCCTAATTT GGTCTCGCTT GATGTGTAGC TGTCAAGTAT TATTATAATC TTGTGTTTGA TATCACATTT   
  
  
+ GTATTGATTA GCTACAAAAG AACATATTAT ATGTGTATAG CAACTATCTC ATATATGCCC TCACCATAGC   
  
  
+ GTGGTTCCAA GGGTTCAAGT TCCACTTAGC CTCACCCTAG AAATGACTTC TTTTTTTTTT TAAAGTTAAA   
  
  
+ GATATGCAAT CACCATGGAA CCAGTCATCT TTTGACACGT TATTCCAGAT CAGATGACCA GCCTTTCTCC   
  
  
+ AAGGCCATTA GATTTTCTAG CCAATCCACT GTTAACTGCA AAAAGTGGTA TCCTTGGCTG TTCCTGTCAC   
  
  
+ TGTTTCTTAT TTGCTCTGTG ATATTTGTAC ATCTCTTTGC CAAGGATAAA AGTTCATAGC CGGGAATTGG   
  
  
+ TATTCAGTTG ACTCTTGCAG TAAACAGAGT TCATGTACTG GAGGATTTTG TGAGTCATGT CACTCAATAA   
  
  
+ AAACAGGTGT GCGACAGTGT TATAAAGCTG CTTGCTTAAT GCACATCTAC TCTCACTCAC CTTACCCAAA   
  
  
+ AAACTTCAAA ATTGTGTGCC TTCTATGAAT ATTCTGATCC ATTAGCATTC AACGGTTTCT GAATTCTGTG   
  
  
+ TAAGCCATCC ATACTCATTT TCAACAGAGA ACTTGGATTG GATGTATTCA ATACCCAAAA ACCTTGTCAA   
  
  
+ ATTACCCCTA GCTTAATTTC CTCTAATACC AACTGCTCTT TGGTTTTAGC GTACAATCCC ATAGATTACC   
  
  
+ CTTCTATAAT TAGTGATGAT TGGTTCATTT CTGCTGCATT ATGCCTTCTT TAGATCCATT GATAATATAT   
  
  
+ ACTCTTGCCA TGCTGCAACA TTCCACTGGG ATTAATATCT CACATTGTAT CCTCAGCAAC CAAGGAGACC   
  
  
+ CTACTATTTT CAGAGCCATT CATAGAAAGT TATTGCATAT AGTGATGATC TTCAATGGGT CTGATTTTTA   
  
  
+ GATAGCTGCT GTGAACATTT TACTCGTCCT CTGCCATGTG GATACTATGG TAACCTTTGT TTGCTCTCAA   
  
  
+ GCCTGAGCGT TCTGAGAAAT GATGAAAGCG CCCTCGAGTT ATAGGACTTT TCTGACTTTA GCTTTAGAAA   
  
  
+ AGAGAGGAAA ATCAACCACA CCCATCTGAT TATTGAGTTT CTGCTCCTTG AGACCTGGAT GGAGCCAGAA   
  
  
+ CTCACGGTCT TGTCAGCCAG AATAATCAGG AACTGCATTT CTGAGGACCC ATGAAAAGAC CCTGGTATAC   
  
  
+ GACTATTCAT GAAAACGTTT TGTCAGCCTT TCAGTTTTTG CATTTTATTC TAGCTGCTTT GATTGAAGCC   
  
  
+ CAATTTTATT AAAACTGTGC TTTTCTCTTG AATGTTAAAC TCTTGCAGTT CCTTGCTTGT TATTTTGGGT   
  
  
+ TTTATGTTTG CTTTCTACTG ATATCATCTT TTGGTCGTGC AATTTAATTG CCTACTATGT TATATGGATT   
  
  
+ TTTATGTTTA CTTTCTACTG ATATCATCTT TTGCTATTGC AATTTAATTG CCTTCTATGT AGAATTCGTC   
  
  
+ ACTTTGAAGT CTGCCAGGAA GGCAATACAG CTATATTTTT CTTGTGGATG AAGTGATAAA AGGCAAGTGT   
  
  
+ CAATTGCTTA CTGCTTTATT GCGCTGTACT TTTCCAGAGA TTTTGGATTA GTCAATGCAG AAGCCAACAA   
  
  
+ TTTATAGCGA CTGGCCACAG TTCTACAACC AATTTGATAA TCCACGTCTT CTTGAATCTT CATCTATAAT   
  
  
+ GGGTGGTGAT CAACTTTTCA GTTCTCCATC TACTGTAAGC ATATCTTGCA ACAGGAGTCC AGCTTCACTG   
  
  
+ CCTGAACTCG AATCCTCTTC ATCGGACTTC CAAAGTGACT CCCAGGAGAC TGTGAATGGC TCGCCAGTGA   
  
  
+ TTGATTCGTG CATGGTACGT GACATTGGTG ACTTAAGACA CAAACTTAGA GAGCTTGAGA CTGTTATGCT   
  
  
+ CGGACCAAGT TCAGACAGCT TGGATTCATG GTATGCCCCT TCAAGAGGTG AGTGTGAACC TCTGCCACCA   
  
  
+ GAAGAGCCTG ACAATGGGAA ACATTTGTTG GAGATGATAG CAAGGGGGAG CCTCAAAGAG GTGCTAATTG   
  
  
+ CTTGTGCCAA AGCAATATCA GACGATGATT TGTTAACAGC GGAGTGGTTG ATGTCAGAGC TACGCCATAT   
  
  
+ GGTTTCAGTT TCTGGAGAAC CAATTCAGAG GTTAGGAGCC TACATGTTGG AAGGGTTAGT TGCCCGGTTG   
  
  
+ TCCTCTTCAG GAAGTTCCAT CTACAAAGCT CTAAGGTGCA AAGAGCCTAC TAGTAGTGAA CTTCTTTCCT   
  
  
+ ATATGCATTT ACTCTATGAA GTTTGCCCTT ACTTCAAGTT TGGGTACATG TCTGCAAATG GGGCAATTGC   
  
  
+ TGAGGCCATG AAAAATGAGA GCAGAATTCA TATAATTGAT TTTCAGATAG CTCAAGGGAG TCAGTGGATT   
  
  
+ AGCCTTATCC AAGCCCTGGC TGCTCAGCCT GATGGGCCAC CACAGGTCCG TATTACCGGA GTCGATGATT   
  
  
+ CCCAATCTGA GTATGCTCGG GGAGGGGGGC TCGACATTGT AGCGAAGAGA CTATCTGGAC TAGCCCAGGC   
  
  
+ TTGCAGCCTA CCCTTTGAGT TCCACGCTGC AGCACTTAGT GGTTCTGAGA TCAGACTTCA AAACCTGGTT   
  
  
+ TTGCGGCCTG GAGAAGCCTT AGCAGTGAAC TTCCCATTCA TGCTGCACCA CATGCCGGAT GAGAGTGTGG   
  
  
+ GCCCTGAGAA TTATAGAGAC CAGTTATTAA GGCTGGTGAA GAGCTTCTTG CCCAAGGTGG TTACCCTTGT   
  
  
+ TGAGCAAGAA TCCAACACAA ACACGGCCCC ATTTCTACCC CGGTTTCTTG AAACCCTAGA CTACTATACT   
  
  
+ GCCATTTTCG AATCAATTGA TGTTACGCTC CCAAGAGATC ACAAGGAGCG GATCAATGTT GAGCAGCACT   
  
  
+ GTTTAGCAAG AGATATAGTC AACATAATAG CATGTGAGGG TGCCGAGAGG GTGGAACGCC ATGAGGTTCT   
  
  
+ TGGAAAGTGG AGATCTCGGT TCTCAATGGC TGGGTTTAAG CCGTACCCAT TGAGCCCGCT AGTGAATGCA   
  
  
+ ACTATCAGGA CTCTTCTGCA GAAGTATAGC AGGAGCTATG GACTTGAAGA AAGGGATGGA GCTCTTTATC   
  
  
+ TAGGCTGGAT GAACCGAGCA CTAGTTGCAT CTTGTGCATG GCAGTG  

- -Up\_Stream \_Len000AAGAAA AGGACAGAGA CAGAGAAGTA ACGTGGTAGT AAATTCTCTA CCCTGGTATT   
  
  
- TAGTAAGTTA GTGAGGAAAT ACAAATTTGA AACCTTCCTC CAATAATAAA TACACCACCA CTAGGGTTAC   
  
  
- GACTTAAATC GACAACTACC GCTCCCGTAT ATACATTAAT CCTAAAGGTT ACGTCAAATC TAAAATGAGT   
  
  
- AAACTCTTAA GTGACTCCAA ACAAACTAGA CTAAAATCGT TTATAACAAG TCCCCATATC TACGAATAAG   
  
  
- CCAAGAGTTG ACATAATCAT ATGTTCTACT CACCAGGTGA ACAAATTCGA AAATGATATA TGAATACAAC   
  
  
- AGGGATTAAA CCAGAGCGAA CTACACATCG ACAGTTCATA ATAATATTAG AACACAAACT ATAGTGTAAA   
  
  
- CATAACTAAT CGATGTTTTC TTGTATAATA TACACATATC GTTGATAGAG TATATACGGG AGTGGTATCG   
  
  
- CACCAAGGTT CCCAAGTTCA AGGTGAATCG GAGTGGGATC TTTACTGAAG AAAAAAAAAA ATTTCAATTT   
  
  
- CTATACGTTA GTGGTACCTT GGTCAGTAGA AAACTGTGCA ATAAGGTCTA GTCTACTGGT CGGAAAGAGG   
  
  
- TTCCGGTAAT CTAAAAGATC GGTTAGGTGA CAATTGACGT TTTTCACCAT AGGAACCGAC AAGGACAGTG   
  
  
- ACAAAGAATA AACGAGACAC TATAAACATG TAGAGAAACG GTTCCTATTT TCAAGTATCG GCCCTTAACC   
  
  
- ATAAGTCAAC TGAGAACGTC ATTTGTCTCA AGTACATGAC CTCCTAAAAC ACTCAGTACA GTGAGTTATT   
  
  
- TTTGTCCACA CGCTGTCACA ATATTTCGAC GAACGAATTA CGTGTAGATG AGAGTGAGTG GAATGGGTTT   
  
  
- TTTGAAGTTT TAACACACGG AAGATACTTA TAAGACTAGG TAATCGTAAG TTGCCAAAGA CTTAAGACAC   
  
  
- ATTCGGTAGG TATGAGTAAA AGTTGTCTCT TGAACCTAAC CTACATAAGT TATGGGTTTT TGGAACAGTT   
  
  
- TAATGGGGAT CGAATTAAAG GAGATTATGG TTGACGAGAA ACCAAAATCG CATGTTAGGG TATCTAATGG   
  
  
- GAAGATATTA ATCACTACTA ACCAAGTAAA GACGACGTAA TACGGAAGAA ATCTAGGTAA CTATTATATA   
  
  
- TGAGAACGGT ACGACGTTGT AAGGTGACCC TAATTATAGA GTGTAACATA GGAGTCGTTG GTTCCTCTGG   
  
  
- GATGATAAAA GTCTCGGTAA GTATCTTTCA ATAACGTATA TCACTACTAG AAGTTACCCA GACTAAAAAT   
  
  
- CTATCGACGA CACTTGTAAA ATGAGCAGGA GACGGTACAC CTATGATACC ATTGGAAACA AACGAGAGTT   
  
  
- CGGACTCGCA AGACTCTTTA CTACTTTCGC GGGAGCTCAA TATCCTGAAA AGACTGAAAT CGAAATCTTT   
  
  
- TCTCTCCTTT TAGTTGGTGT GGGTAGACTA ATAACTCAAA GACGAGGAAC TCTGGACCTA CCTCGGTCTT   
  
  
- GAGTGCCAGA ACAGTCGGTC TTATTAGTCC TTGACGTAAA GACTCCTGGG TACTTTTCTG GGACCATATG   
  
  
- CTGATAAGTA CTTTTGCAAA ACAGTCGGAA AGTCAAAAAC GTAAAATAAG ATCGACGAAA CTAACTTCGG   
  
  
- GTTAAAATAA TTTTGACACG AAAAGAGAAC TTACAATTTG AGAACGTCAA GGAACGAACA ATAAAACCCA   
  
  
- AAATACAAAC GAAAGATGAC TATAGTAGAA AACCAGCACG TTAAATTAAC GGATGATACA ATATACCTAA   
  
  
- AAATACAAAT GAAAGATGAC TATAGTAGAA AACGATAACG TTAAATTAAC GGAAGATACA TCTTAAGCAG   
  
  
- TGAAACTTCA GACGGTCCTT CCGTTATGTC GATATAAAAA GAACACCTAC TTCACTATTT TCCGTTCACA   
  
  
- GTTAACGAAT GACGAAATAA CGCGACATGA AAAGGTCTCT AAAACCTAAT CAGTTACGTC TTCGGTTGTT   
  
  
- AAATATCGCT GACCGGTGTC AAGATGTTGG TTAAACTATT AGGTGCAGAA GAACTTAGAA GTAGATATTA   
  
  
- CCCACCACTA GTTGAAAAGT CAAGAGGTAG ATGACATTCG TATAGAACGT TGTCCTCAGG TCGAAGTGAC   
  
  
- GGACTTGAGC TTAGGAGAAG TAGCCTGAAG GTTTCACTGA GGGTCCTCTG ACACTTACCG AGCGGTCACT   
  
  
- AACTAAGCAC GTACCATGCA CTGTAACCAC TGAATTCTGT GTTTGAATCT CTCGAACTCT GACAATACGA   
  
  
- GCCTGGTTCA AGTCTGTCGA ACCTAAGTAC CATACGGGGA AGTTCTCCAC TCACACTTGG AGACGGTGGT   
  
  
- CTTCTCGGAC TGTTACCCTT TGTAAACAAC CTCTACTATC GTTCCCCCTC GGAGTTTCTC CACGATTAAC   
  
  
- GAACACGGTT TCGTTATAGT CTGCTACTAA ACAATTGTCG CCTCACCAAC TACAGTCTCG ATGCGGTATA   
  
  
- CCAAAGTCAA AGACCTCTTG GTTAAGTCTC CAATCCTCGG ATGTACAACC TTCCCAATCA ACGGGCCAAC   
  
  
- AGGAGAAGTC CTTCAAGGTA GATGTTTCGA GATTCCACGT TTCTCGGATG ATCATCACTT GAAGAAAGGA   
  
  
- TATACGTAAA TGAGATACTT CAAACGGGAA TGAAGTTCAA ACCCATGTAC AGACGTTTAC CCCGTTAACG   
  
  
- ACTCCGGTAC TTTTTACTCT CGTCTTAAGT ATATTAACTA AAAGTCTATC GAGTTCCCTC AGTCACCTAA   
  
  
- TCGGAATAGG TTCGGGACCG ACGAGTCGGA CTACCCGGTG GTGTCCAGGC ATAATGGCCT CAGCTACTAA   
  
  
- GGGTTAGACT CATACGAGCC CCTCCCCCCG AGCTGTAACA TCGCTTCTCT GATAGACCTG ATCGGGTCCG   
  
  
- AACGTCGGAT GGGAAACTCA AGGTGCGACG TCGTGAATCA CCAAGACTCT AGTCTGAAGT TTTGGACCAA   
  
  
- AACGCCGGAC CTCTTCGGAA TCGTCACTTG AAGGGTAAGT ACGACGTGGT GTACGGCCTA CTCTCACACC   
  
  
- CGGGACTCTT AATATCTCTG GTCAATAATT CCGACCACTT CTCGAAGAAC GGGTTCCACC AATGGGAACA   
  
  
- ACTCGTTCTT AGGTTGTGTT TGTGCCGGGG TAAAGATGGG GCCAAAGAAC TTTGGGATCT GATGATATGA   
  
  
- CGGTAAAAGC TTAGTTAACT ACAATGCGAG GGTTCTCTAG TGTTCCTCGC CTAGTTACAA CTCGTCGTGA   
  
  
- CAAATCGTTC TCTATATCAG TTGTATTATC GTACACTCCC ACGGCTCTCC CACCTTGCGG TACTCCAAGA   
  
  
- ACCTTTCACC TCTAGAGCCA AGAGTTACCG ACCCAAATTC GGCATGGGTA ACTCGGGCGA TCACTTACGT   
  
  
- TGATAGTCCT GAGAAGACGT CTTCATATCG TCCTCGATAC CTGAACTTCT TTCCCTACCT CGAGAAATAG   
  
  
- ATCCGACCTA CTTGGCTCGT GATCAACGTA GAACACGTAC CGTCAC

+     Unnamed\_\_2

| Site Name | Organism | Position | Strand | Matrix score. | sequence | function |
| --- | --- | --- | --- | --- | --- | --- |
| Unnamed\_\_2 | Zea mays | 3192 | + | 6 | CCCCGG |  |

>HU08G00284.1   
+ -Up\_Stream \_Len000TTCTTT TCCTGTCTCT GTCTCTTCAT TGCACCATCA TTTAAGAGAT GGGACCATAA   
  
  
+ ATCATTCAAT CACTCCTTTA TGTTTAAACT TTGGAAGGAG GTTATTATTT ATGTGGTGGT GATCCCAATG   
  
  
+ CTGAATTTAG CTGTTGATGG CGAGGGCATA TATGTAATTA GGATTTCCAA TGCAGTTTAG ATTTTACTCA   
  
  
+ TTTGAGAATT CACTGAGGTT TGTTTGATCT GATTTTAGCA AATATTGTTC AGGGGTATAG ATGCTTATTC   
  
  
+ GGTTCTCAAC TGTATTAGTA TACAAGATGA GTGGTCCACT TGTTTAAGCT TTTACTATAT ACTTATGTTG   
  
  
+ TCCCTAATTT GGTCTCGCTT GATGTGTAGC TGTCAAGTAT TATTATAATC TTGTGTTTGA TATCACATTT   
  
  
+ GTATTGATTA GCTACAAAAG AACATATTAT ATGTGTATAG CAACTATCTC ATATATGCCC TCACCATAGC   
  
  
+ GTGGTTCCAA GGGTTCAAGT TCCACTTAGC CTCACCCTAG AAATGACTTC TTTTTTTTTT TAAAGTTAAA   
  
  
+ GATATGCAAT CACCATGGAA CCAGTCATCT TTTGACACGT TATTCCAGAT CAGATGACCA GCCTTTCTCC   
  
  
+ AAGGCCATTA GATTTTCTAG CCAATCCACT GTTAACTGCA AAAAGTGGTA TCCTTGGCTG TTCCTGTCAC   
  
  
+ TGTTTCTTAT TTGCTCTGTG ATATTTGTAC ATCTCTTTGC CAAGGATAAA AGTTCATAGC CGGGAATTGG   
  
  
+ TATTCAGTTG ACTCTTGCAG TAAACAGAGT TCATGTACTG GAGGATTTTG TGAGTCATGT CACTCAATAA   
  
  
+ AAACAGGTGT GCGACAGTGT TATAAAGCTG CTTGCTTAAT GCACATCTAC TCTCACTCAC CTTACCCAAA   
  
  
+ AAACTTCAAA ATTGTGTGCC TTCTATGAAT ATTCTGATCC ATTAGCATTC AACGGTTTCT GAATTCTGTG   
  
  
+ TAAGCCATCC ATACTCATTT TCAACAGAGA ACTTGGATTG GATGTATTCA ATACCCAAAA ACCTTGTCAA   
  
  
+ ATTACCCCTA GCTTAATTTC CTCTAATACC AACTGCTCTT TGGTTTTAGC GTACAATCCC ATAGATTACC   
  
  
+ CTTCTATAAT TAGTGATGAT TGGTTCATTT CTGCTGCATT ATGCCTTCTT TAGATCCATT GATAATATAT   
  
  
+ ACTCTTGCCA TGCTGCAACA TTCCACTGGG ATTAATATCT CACATTGTAT CCTCAGCAAC CAAGGAGACC   
  
  
+ CTACTATTTT CAGAGCCATT CATAGAAAGT TATTGCATAT AGTGATGATC TTCAATGGGT CTGATTTTTA   
  
  
+ GATAGCTGCT GTGAACATTT TACTCGTCCT CTGCCATGTG GATACTATGG TAACCTTTGT TTGCTCTCAA   
  
  
+ GCCTGAGCGT TCTGAGAAAT GATGAAAGCG CCCTCGAGTT ATAGGACTTT TCTGACTTTA GCTTTAGAAA   
  
  
+ AGAGAGGAAA ATCAACCACA CCCATCTGAT TATTGAGTTT CTGCTCCTTG AGACCTGGAT GGAGCCAGAA   
  
  
+ CTCACGGTCT TGTCAGCCAG AATAATCAGG AACTGCATTT CTGAGGACCC ATGAAAAGAC CCTGGTATAC   
  
  
+ GACTATTCAT GAAAACGTTT TGTCAGCCTT TCAGTTTTTG CATTTTATTC TAGCTGCTTT GATTGAAGCC   
  
  
+ CAATTTTATT AAAACTGTGC TTTTCTCTTG AATGTTAAAC TCTTGCAGTT CCTTGCTTGT TATTTTGGGT   
  
  
+ TTTATGTTTG CTTTCTACTG ATATCATCTT TTGGTCGTGC AATTTAATTG CCTACTATGT TATATGGATT   
  
  
+ TTTATGTTTA CTTTCTACTG ATATCATCTT TTGCTATTGC AATTTAATTG CCTTCTATGT AGAATTCGTC   
  
  
+ ACTTTGAAGT CTGCCAGGAA GGCAATACAG CTATATTTTT CTTGTGGATG AAGTGATAAA AGGCAAGTGT   
  
  
+ CAATTGCTTA CTGCTTTATT GCGCTGTACT TTTCCAGAGA TTTTGGATTA GTCAATGCAG AAGCCAACAA   
  
  
+ TTTATAGCGA CTGGCCACAG TTCTACAACC AATTTGATAA TCCACGTCTT CTTGAATCTT CATCTATAAT   
  
  
+ GGGTGGTGAT CAACTTTTCA GTTCTCCATC TACTGTAAGC ATATCTTGCA ACAGGAGTCC AGCTTCACTG   
  
  
+ CCTGAACTCG AATCCTCTTC ATCGGACTTC CAAAGTGACT CCCAGGAGAC TGTGAATGGC TCGCCAGTGA   
  
  
+ TTGATTCGTG CATGGTACGT GACATTGGTG ACTTAAGACA CAAACTTAGA GAGCTTGAGA CTGTTATGCT   
  
  
+ CGGACCAAGT TCAGACAGCT TGGATTCATG GTATGCCCCT TCAAGAGGTG AGTGTGAACC TCTGCCACCA   
  
  
+ GAAGAGCCTG ACAATGGGAA ACATTTGTTG GAGATGATAG CAAGGGGGAG CCTCAAAGAG GTGCTAATTG   
  
  
+ CTTGTGCCAA AGCAATATCA GACGATGATT TGTTAACAGC GGAGTGGTTG ATGTCAGAGC TACGCCATAT   
  
  
+ GGTTTCAGTT TCTGGAGAAC CAATTCAGAG GTTAGGAGCC TACATGTTGG AAGGGTTAGT TGCCCGGTTG   
  
  
+ TCCTCTTCAG GAAGTTCCAT CTACAAAGCT CTAAGGTGCA AAGAGCCTAC TAGTAGTGAA CTTCTTTCCT   
  
  
+ ATATGCATTT ACTCTATGAA GTTTGCCCTT ACTTCAAGTT TGGGTACATG TCTGCAAATG GGGCAATTGC   
  
  
+ TGAGGCCATG AAAAATGAGA GCAGAATTCA TATAATTGAT TTTCAGATAG CTCAAGGGAG TCAGTGGATT   
  
  
+ AGCCTTATCC AAGCCCTGGC TGCTCAGCCT GATGGGCCAC CACAGGTCCG TATTACCGGA GTCGATGATT   
  
  
+ CCCAATCTGA GTATGCTCGG GGAGGGGGGC TCGACATTGT AGCGAAGAGA CTATCTGGAC TAGCCCAGGC   
  
  
+ TTGCAGCCTA CCCTTTGAGT TCCACGCTGC AGCACTTAGT GGTTCTGAGA TCAGACTTCA AAACCTGGTT   
  
  
+ TTGCGGCCTG GAGAAGCCTT AGCAGTGAAC TTCCCATTCA TGCTGCACCA CATGCCGGAT GAGAGTGTGG   
  
  
+ GCCCTGAGAA TTATAGAGAC CAGTTATTAA GGCTGGTGAA GAGCTTCTTG CCCAAGGTGG TTACCCTTGT   
  
  
+ TGAGCAAGAA TCCAACACAA ACACGGCCCC ATTTCTACCC CGGTTTCTTG AAACCCTAGA CTACTATACT   
  
  
+ GCCATTTTCG AATCAATTGA TGTTACGCTC CCAAGAGATC ACAAGGAGCG GATCAATGTT GAGCAGCACT   
  
  
+ GTTTAGCAAG AGATATAGTC AACATAATAG CATGTGAGGG TGCCGAGAGG GTGGAACGCC ATGAGGTTCT   
  
  
+ TGGAAAGTGG AGATCTCGGT TCTCAATGGC TGGGTTTAAG CCGTACCCAT TGAGCCCGCT AGTGAATGCA   
  
  
+ ACTATCAGGA CTCTTCTGCA GAAGTATAGC AGGAGCTATG GACTTGAAGA AAGGGATGGA GCTCTTTATC   
  
  
+ TAGGCTGGAT GAACCGAGCA CTAGTTGCAT CTTGTGCATG GCAGTG  

- -Up\_Stream \_Len000AAGAAA AGGACAGAGA CAGAGAAGTA ACGTGGTAGT AAATTCTCTA CCCTGGTATT   
  
  
- TAGTAAGTTA GTGAGGAAAT ACAAATTTGA AACCTTCCTC CAATAATAAA TACACCACCA CTAGGGTTAC   
  
  
- GACTTAAATC GACAACTACC GCTCCCGTAT ATACATTAAT CCTAAAGGTT ACGTCAAATC TAAAATGAGT   
  
  
- AAACTCTTAA GTGACTCCAA ACAAACTAGA CTAAAATCGT TTATAACAAG TCCCCATATC TACGAATAAG   
  
  
- CCAAGAGTTG ACATAATCAT ATGTTCTACT CACCAGGTGA ACAAATTCGA AAATGATATA TGAATACAAC   
  
  
- AGGGATTAAA CCAGAGCGAA CTACACATCG ACAGTTCATA ATAATATTAG AACACAAACT ATAGTGTAAA   
  
  
- CATAACTAAT CGATGTTTTC TTGTATAATA TACACATATC GTTGATAGAG TATATACGGG AGTGGTATCG   
  
  
- CACCAAGGTT CCCAAGTTCA AGGTGAATCG GAGTGGGATC TTTACTGAAG AAAAAAAAAA ATTTCAATTT   
  
  
- CTATACGTTA GTGGTACCTT GGTCAGTAGA AAACTGTGCA ATAAGGTCTA GTCTACTGGT CGGAAAGAGG   
  
  
- TTCCGGTAAT CTAAAAGATC GGTTAGGTGA CAATTGACGT TTTTCACCAT AGGAACCGAC AAGGACAGTG   
  
  
- ACAAAGAATA AACGAGACAC TATAAACATG TAGAGAAACG GTTCCTATTT TCAAGTATCG GCCCTTAACC   
  
  
- ATAAGTCAAC TGAGAACGTC ATTTGTCTCA AGTACATGAC CTCCTAAAAC ACTCAGTACA GTGAGTTATT   
  
  
- TTTGTCCACA CGCTGTCACA ATATTTCGAC GAACGAATTA CGTGTAGATG AGAGTGAGTG GAATGGGTTT   
  
  
- TTTGAAGTTT TAACACACGG AAGATACTTA TAAGACTAGG TAATCGTAAG TTGCCAAAGA CTTAAGACAC   
  
  
- ATTCGGTAGG TATGAGTAAA AGTTGTCTCT TGAACCTAAC CTACATAAGT TATGGGTTTT TGGAACAGTT   
  
  
- TAATGGGGAT CGAATTAAAG GAGATTATGG TTGACGAGAA ACCAAAATCG CATGTTAGGG TATCTAATGG   
  
  
- GAAGATATTA ATCACTACTA ACCAAGTAAA GACGACGTAA TACGGAAGAA ATCTAGGTAA CTATTATATA   
  
  
- TGAGAACGGT ACGACGTTGT AAGGTGACCC TAATTATAGA GTGTAACATA GGAGTCGTTG GTTCCTCTGG   
  
  
- GATGATAAAA GTCTCGGTAA GTATCTTTCA ATAACGTATA TCACTACTAG AAGTTACCCA GACTAAAAAT   
  
  
- CTATCGACGA CACTTGTAAA ATGAGCAGGA GACGGTACAC CTATGATACC ATTGGAAACA AACGAGAGTT   
  
  
- CGGACTCGCA AGACTCTTTA CTACTTTCGC GGGAGCTCAA TATCCTGAAA AGACTGAAAT CGAAATCTTT   
  
  
- TCTCTCCTTT TAGTTGGTGT GGGTAGACTA ATAACTCAAA GACGAGGAAC TCTGGACCTA CCTCGGTCTT   
  
  
- GAGTGCCAGA ACAGTCGGTC TTATTAGTCC TTGACGTAAA GACTCCTGGG TACTTTTCTG GGACCATATG   
  
  
- CTGATAAGTA CTTTTGCAAA ACAGTCGGAA AGTCAAAAAC GTAAAATAAG ATCGACGAAA CTAACTTCGG   
  
  
- GTTAAAATAA TTTTGACACG AAAAGAGAAC TTACAATTTG AGAACGTCAA GGAACGAACA ATAAAACCCA   
  
  
- AAATACAAAC GAAAGATGAC TATAGTAGAA AACCAGCACG TTAAATTAAC GGATGATACA ATATACCTAA   
  
  
- AAATACAAAT GAAAGATGAC TATAGTAGAA AACGATAACG TTAAATTAAC GGAAGATACA TCTTAAGCAG   
  
  
- TGAAACTTCA GACGGTCCTT CCGTTATGTC GATATAAAAA GAACACCTAC TTCACTATTT TCCGTTCACA   
  
  
- GTTAACGAAT GACGAAATAA CGCGACATGA AAAGGTCTCT AAAACCTAAT CAGTTACGTC TTCGGTTGTT   
  
  
- AAATATCGCT GACCGGTGTC AAGATGTTGG TTAAACTATT AGGTGCAGAA GAACTTAGAA GTAGATATTA   
  
  
- CCCACCACTA GTTGAAAAGT CAAGAGGTAG ATGACATTCG TATAGAACGT TGTCCTCAGG TCGAAGTGAC   
  
  
- GGACTTGAGC TTAGGAGAAG TAGCCTGAAG GTTTCACTGA GGGTCCTCTG ACACTTACCG AGCGGTCACT   
  
  
- AACTAAGCAC GTACCATGCA CTGTAACCAC TGAATTCTGT GTTTGAATCT CTCGAACTCT GACAATACGA   
  
  
- GCCTGGTTCA AGTCTGTCGA ACCTAAGTAC CATACGGGGA AGTTCTCCAC TCACACTTGG AGACGGTGGT   
  
  
- CTTCTCGGAC TGTTACCCTT TGTAAACAAC CTCTACTATC GTTCCCCCTC GGAGTTTCTC CACGATTAAC   
  
  
- GAACACGGTT TCGTTATAGT CTGCTACTAA ACAATTGTCG CCTCACCAAC TACAGTCTCG ATGCGGTATA   
  
  
- CCAAAGTCAA AGACCTCTTG GTTAAGTCTC CAATCCTCGG ATGTACAACC TTCCCAATCA ACGGGCCAAC   
  
  
- AGGAGAAGTC CTTCAAGGTA GATGTTTCGA GATTCCACGT TTCTCGGATG ATCATCACTT GAAGAAAGGA   
  
  
- TATACGTAAA TGAGATACTT CAAACGGGAA TGAAGTTCAA ACCCATGTAC AGACGTTTAC CCCGTTAACG   
  
  
- ACTCCGGTAC TTTTTACTCT CGTCTTAAGT ATATTAACTA AAAGTCTATC GAGTTCCCTC AGTCACCTAA   
  
  
- TCGGAATAGG TTCGGGACCG ACGAGTCGGA CTACCCGGTG GTGTCCAGGC ATAATGGCCT CAGCTACTAA   
  
  
- GGGTTAGACT CATACGAGCC CCTCCCCCCG AGCTGTAACA TCGCTTCTCT GATAGACCTG ATCGGGTCCG   
  
  
- AACGTCGGAT GGGAAACTCA AGGTGCGACG TCGTGAATCA CCAAGACTCT AGTCTGAAGT TTTGGACCAA   
  
  
- AACGCCGGAC CTCTTCGGAA TCGTCACTTG AAGGGTAAGT ACGACGTGGT GTACGGCCTA CTCTCACACC   
  
  
- CGGGACTCTT AATATCTCTG GTCAATAATT CCGACCACTT CTCGAAGAAC GGGTTCCACC AATGGGAACA   
  
  
- ACTCGTTCTT AGGTTGTGTT TGTGCCGGGG TAAAGATGGG GCCAAAGAAC TTTGGGATCT GATGATATGA   
  
  
- CGGTAAAAGC TTAGTTAACT ACAATGCGAG GGTTCTCTAG TGTTCCTCGC CTAGTTACAA CTCGTCGTGA   
  
  
- CAAATCGTTC TCTATATCAG TTGTATTATC GTACACTCCC ACGGCTCTCC CACCTTGCGG TACTCCAAGA   
  
  
- ACCTTTCACC TCTAGAGCCA AGAGTTACCG ACCCAAATTC GGCATGGGTA ACTCGGGCGA TCACTTACGT   
  
  
- TGATAGTCCT GAGAAGACGT CTTCATATCG TCCTCGATAC CTGAACTTCT TTCCCTACCT CGAGAAATAG   
  
  
- ATCCGACCTA CTTGGCTCGT GATCAACGTA GAACACGTAC CGTCAC

+     Unnamed\_\_4

| Site Name | Organism | Position | Strand | Matrix score. | sequence | function |
| --- | --- | --- | --- | --- | --- | --- |
| Unnamed\_\_4 | Petroselinum hortense | 2431 | - | 4 | CTCC |  |
| Unnamed\_\_4 | Petroselinum hortense | 2128 | + | 4 | CTCC |  |
| Unnamed\_\_4 | Petroselinum hortense | 111 | - | 4 | CTCC |  |
| Unnamed\_\_4 | Petroselinum hortense | 631 | + | 4 | CTCC |  |
| Unnamed\_\_4 | Petroselinum hortense | 3492 | - | 4 | CTCC |  |
| Unnamed\_\_4 | Petroselinum hortense | 2495 | - | 4 | CTCC |  |
| Unnamed\_\_4 | Petroselinum hortense | 3466 | - | 4 | CTCC |  |
| Unnamed\_\_4 | Petroselinum hortense | 1518 | + | 4 | CTCC |  |
| Unnamed\_\_4 | Petroselinum hortense | 1258 | - | 4 | CTCC |  |
| Unnamed\_\_4 | Petroselinum hortense | 2895 | - | 4 | CTCC |  |
| Unnamed\_\_4 | Petroselinum hortense | 2862 | - | 4 | CTCC |  |
| Unnamed\_\_4 | Petroselinum hortense | 2219 | - | 4 | CTCC |  |
| Unnamed\_\_4 | Petroselinum hortense | 2158 | - | 4 | CTCC |  |
| Unnamed\_\_4 | Petroselinum hortense | 2213 | + | 4 | CTCC |  |
| Unnamed\_\_4 | Petroselinum hortense | 3373 | - | 4 | CTCC |  |
| Unnamed\_\_4 | Petroselinum hortense | 2414 | - | 4 | CTCC |  |
| Unnamed\_\_4 | Petroselinum hortense | 3252 | + | 4 | CTCC |  |
| Unnamed\_\_4 | Petroselinum hortense | 87 | + | 4 | CTCC |  |
| Unnamed\_\_4 | Petroselinum hortense | 814 | - | 4 | CTCC |  |
| Unnamed\_\_4 | Petroselinum hortense | 1535 | - | 4 | CTCC |  |
| Unnamed\_\_4 | Petroselinum hortense | 2538 | - | 4 | CTCC |  |
| Unnamed\_\_4 | Petroselinum hortense | 3024 | - | 4 | CTCC |  |
| Unnamed\_\_4 | Petroselinum hortense | 3269 | - | 4 | CTCC |  |
| Unnamed\_\_4 | Petroselinum hortense | 2559 | - | 4 | CTCC |  |
| Unnamed\_\_4 | Petroselinum hortense | 2791 | - | 4 | CTCC |  |

>HU08G00284.1   
+ -Up\_Stream \_Len000TTCTTT TCCTGTCTCT GTCTCTTCAT TGCACCATCA TTTAAGAGAT GGGACCATAA   
  
  
+ ATCATTCAAT CACTCCTTTA TGTTTAAACT TTGGAAGGAG GTTATTATTT ATGTGGTGGT GATCCCAATG   
  
  
+ CTGAATTTAG CTGTTGATGG CGAGGGCATA TATGTAATTA GGATTTCCAA TGCAGTTTAG ATTTTACTCA   
  
  
+ TTTGAGAATT CACTGAGGTT TGTTTGATCT GATTTTAGCA AATATTGTTC AGGGGTATAG ATGCTTATTC   
  
  
+ GGTTCTCAAC TGTATTAGTA TACAAGATGA GTGGTCCACT TGTTTAAGCT TTTACTATAT ACTTATGTTG   
  
  
+ TCCCTAATTT GGTCTCGCTT GATGTGTAGC TGTCAAGTAT TATTATAATC TTGTGTTTGA TATCACATTT   
  
  
+ GTATTGATTA GCTACAAAAG AACATATTAT ATGTGTATAG CAACTATCTC ATATATGCCC TCACCATAGC   
  
  
+ GTGGTTCCAA GGGTTCAAGT TCCACTTAGC CTCACCCTAG AAATGACTTC TTTTTTTTTT TAAAGTTAAA   
  
  
+ GATATGCAAT CACCATGGAA CCAGTCATCT TTTGACACGT TATTCCAGAT CAGATGACCA GCCTTTCTCC   
  
  
+ AAGGCCATTA GATTTTCTAG CCAATCCACT GTTAACTGCA AAAAGTGGTA TCCTTGGCTG TTCCTGTCAC   
  
  
+ TGTTTCTTAT TTGCTCTGTG ATATTTGTAC ATCTCTTTGC CAAGGATAAA AGTTCATAGC CGGGAATTGG   
  
  
+ TATTCAGTTG ACTCTTGCAG TAAACAGAGT TCATGTACTG GAGGATTTTG TGAGTCATGT CACTCAATAA   
  
  
+ AAACAGGTGT GCGACAGTGT TATAAAGCTG CTTGCTTAAT GCACATCTAC TCTCACTCAC CTTACCCAAA   
  
  
+ AAACTTCAAA ATTGTGTGCC TTCTATGAAT ATTCTGATCC ATTAGCATTC AACGGTTTCT GAATTCTGTG   
  
  
+ TAAGCCATCC ATACTCATTT TCAACAGAGA ACTTGGATTG GATGTATTCA ATACCCAAAA ACCTTGTCAA   
  
  
+ ATTACCCCTA GCTTAATTTC CTCTAATACC AACTGCTCTT TGGTTTTAGC GTACAATCCC ATAGATTACC   
  
  
+ CTTCTATAAT TAGTGATGAT TGGTTCATTT CTGCTGCATT ATGCCTTCTT TAGATCCATT GATAATATAT   
  
  
+ ACTCTTGCCA TGCTGCAACA TTCCACTGGG ATTAATATCT CACATTGTAT CCTCAGCAAC CAAGGAGACC   
  
  
+ CTACTATTTT CAGAGCCATT CATAGAAAGT TATTGCATAT AGTGATGATC TTCAATGGGT CTGATTTTTA   
  
  
+ GATAGCTGCT GTGAACATTT TACTCGTCCT CTGCCATGTG GATACTATGG TAACCTTTGT TTGCTCTCAA   
  
  
+ GCCTGAGCGT TCTGAGAAAT GATGAAAGCG CCCTCGAGTT ATAGGACTTT TCTGACTTTA GCTTTAGAAA   
  
  
+ AGAGAGGAAA ATCAACCACA CCCATCTGAT TATTGAGTTT CTGCTCCTTG AGACCTGGAT GGAGCCAGAA   
  
  
+ CTCACGGTCT TGTCAGCCAG AATAATCAGG AACTGCATTT CTGAGGACCC ATGAAAAGAC CCTGGTATAC   
  
  
+ GACTATTCAT GAAAACGTTT TGTCAGCCTT TCAGTTTTTG CATTTTATTC TAGCTGCTTT GATTGAAGCC   
  
  
+ CAATTTTATT AAAACTGTGC TTTTCTCTTG AATGTTAAAC TCTTGCAGTT CCTTGCTTGT TATTTTGGGT   
  
  
+ TTTATGTTTG CTTTCTACTG ATATCATCTT TTGGTCGTGC AATTTAATTG CCTACTATGT TATATGGATT   
  
  
+ TTTATGTTTA CTTTCTACTG ATATCATCTT TTGCTATTGC AATTTAATTG CCTTCTATGT AGAATTCGTC   
  
  
+ ACTTTGAAGT CTGCCAGGAA GGCAATACAG CTATATTTTT CTTGTGGATG AAGTGATAAA AGGCAAGTGT   
  
  
+ CAATTGCTTA CTGCTTTATT GCGCTGTACT TTTCCAGAGA TTTTGGATTA GTCAATGCAG AAGCCAACAA   
  
  
+ TTTATAGCGA CTGGCCACAG TTCTACAACC AATTTGATAA TCCACGTCTT CTTGAATCTT CATCTATAAT   
  
  
+ GGGTGGTGAT CAACTTTTCA GTTCTCCATC TACTGTAAGC ATATCTTGCA ACAGGAGTCC AGCTTCACTG   
  
  
+ CCTGAACTCG AATCCTCTTC ATCGGACTTC CAAAGTGACT CCCAGGAGAC TGTGAATGGC TCGCCAGTGA   
  
  
+ TTGATTCGTG CATGGTACGT GACATTGGTG ACTTAAGACA CAAACTTAGA GAGCTTGAGA CTGTTATGCT   
  
  
+ CGGACCAAGT TCAGACAGCT TGGATTCATG GTATGCCCCT TCAAGAGGTG AGTGTGAACC TCTGCCACCA   
  
  
+ GAAGAGCCTG ACAATGGGAA ACATTTGTTG GAGATGATAG CAAGGGGGAG CCTCAAAGAG GTGCTAATTG   
  
  
+ CTTGTGCCAA AGCAATATCA GACGATGATT TGTTAACAGC GGAGTGGTTG ATGTCAGAGC TACGCCATAT   
  
  
+ GGTTTCAGTT TCTGGAGAAC CAATTCAGAG GTTAGGAGCC TACATGTTGG AAGGGTTAGT TGCCCGGTTG   
  
  
+ TCCTCTTCAG GAAGTTCCAT CTACAAAGCT CTAAGGTGCA AAGAGCCTAC TAGTAGTGAA CTTCTTTCCT   
  
  
+ ATATGCATTT ACTCTATGAA GTTTGCCCTT ACTTCAAGTT TGGGTACATG TCTGCAAATG GGGCAATTGC   
  
  
+ TGAGGCCATG AAAAATGAGA GCAGAATTCA TATAATTGAT TTTCAGATAG CTCAAGGGAG TCAGTGGATT   
  
  
+ AGCCTTATCC AAGCCCTGGC TGCTCAGCCT GATGGGCCAC CACAGGTCCG TATTACCGGA GTCGATGATT   
  
  
+ CCCAATCTGA GTATGCTCGG GGAGGGGGGC TCGACATTGT AGCGAAGAGA CTATCTGGAC TAGCCCAGGC   
  
  
+ TTGCAGCCTA CCCTTTGAGT TCCACGCTGC AGCACTTAGT GGTTCTGAGA TCAGACTTCA AAACCTGGTT   
  
  
+ TTGCGGCCTG GAGAAGCCTT AGCAGTGAAC TTCCCATTCA TGCTGCACCA CATGCCGGAT GAGAGTGTGG   
  
  
+ GCCCTGAGAA TTATAGAGAC CAGTTATTAA GGCTGGTGAA GAGCTTCTTG CCCAAGGTGG TTACCCTTGT   
  
  
+ TGAGCAAGAA TCCAACACAA ACACGGCCCC ATTTCTACCC CGGTTTCTTG AAACCCTAGA CTACTATACT   
  
  
+ GCCATTTTCG AATCAATTGA TGTTACGCTC CCAAGAGATC ACAAGGAGCG GATCAATGTT GAGCAGCACT   
  
  
+ GTTTAGCAAG AGATATAGTC AACATAATAG CATGTGAGGG TGCCGAGAGG GTGGAACGCC ATGAGGTTCT   
  
  
+ TGGAAAGTGG AGATCTCGGT TCTCAATGGC TGGGTTTAAG CCGTACCCAT TGAGCCCGCT AGTGAATGCA   
  
  
+ ACTATCAGGA CTCTTCTGCA GAAGTATAGC AGGAGCTATG GACTTGAAGA AAGGGATGGA GCTCTTTATC   
  
  
+ TAGGCTGGAT GAACCGAGCA CTAGTTGCAT CTTGTGCATG GCAGTG  

- -Up\_Stream \_Len000AAGAAA AGGACAGAGA CAGAGAAGTA ACGTGGTAGT AAATTCTCTA CCCTGGTATT   
  
  
- TAGTAAGTTA GTGAGGAAAT ACAAATTTGA AACCTTCCTC CAATAATAAA TACACCACCA CTAGGGTTAC   
  
  
- GACTTAAATC GACAACTACC GCTCCCGTAT ATACATTAAT CCTAAAGGTT ACGTCAAATC TAAAATGAGT   
  
  
- AAACTCTTAA GTGACTCCAA ACAAACTAGA CTAAAATCGT TTATAACAAG TCCCCATATC TACGAATAAG   
  
  
- CCAAGAGTTG ACATAATCAT ATGTTCTACT CACCAGGTGA ACAAATTCGA AAATGATATA TGAATACAAC   
  
  
- AGGGATTAAA CCAGAGCGAA CTACACATCG ACAGTTCATA ATAATATTAG AACACAAACT ATAGTGTAAA   
  
  
- CATAACTAAT CGATGTTTTC TTGTATAATA TACACATATC GTTGATAGAG TATATACGGG AGTGGTATCG   
  
  
- CACCAAGGTT CCCAAGTTCA AGGTGAATCG GAGTGGGATC TTTACTGAAG AAAAAAAAAA ATTTCAATTT   
  
  
- CTATACGTTA GTGGTACCTT GGTCAGTAGA AAACTGTGCA ATAAGGTCTA GTCTACTGGT CGGAAAGAGG   
  
  
- TTCCGGTAAT CTAAAAGATC GGTTAGGTGA CAATTGACGT TTTTCACCAT AGGAACCGAC AAGGACAGTG   
  
  
- ACAAAGAATA AACGAGACAC TATAAACATG TAGAGAAACG GTTCCTATTT TCAAGTATCG GCCCTTAACC   
  
  
- ATAAGTCAAC TGAGAACGTC ATTTGTCTCA AGTACATGAC CTCCTAAAAC ACTCAGTACA GTGAGTTATT   
  
  
- TTTGTCCACA CGCTGTCACA ATATTTCGAC GAACGAATTA CGTGTAGATG AGAGTGAGTG GAATGGGTTT   
  
  
- TTTGAAGTTT TAACACACGG AAGATACTTA TAAGACTAGG TAATCGTAAG TTGCCAAAGA CTTAAGACAC   
  
  
- ATTCGGTAGG TATGAGTAAA AGTTGTCTCT TGAACCTAAC CTACATAAGT TATGGGTTTT TGGAACAGTT   
  
  
- TAATGGGGAT CGAATTAAAG GAGATTATGG TTGACGAGAA ACCAAAATCG CATGTTAGGG TATCTAATGG   
  
  
- GAAGATATTA ATCACTACTA ACCAAGTAAA GACGACGTAA TACGGAAGAA ATCTAGGTAA CTATTATATA   
  
  
- TGAGAACGGT ACGACGTTGT AAGGTGACCC TAATTATAGA GTGTAACATA GGAGTCGTTG GTTCCTCTGG   
  
  
- GATGATAAAA GTCTCGGTAA GTATCTTTCA ATAACGTATA TCACTACTAG AAGTTACCCA GACTAAAAAT   
  
  
- CTATCGACGA CACTTGTAAA ATGAGCAGGA GACGGTACAC CTATGATACC ATTGGAAACA AACGAGAGTT   
  
  
- CGGACTCGCA AGACTCTTTA CTACTTTCGC GGGAGCTCAA TATCCTGAAA AGACTGAAAT CGAAATCTTT   
  
  
- TCTCTCCTTT TAGTTGGTGT GGGTAGACTA ATAACTCAAA GACGAGGAAC TCTGGACCTA CCTCGGTCTT   
  
  
- GAGTGCCAGA ACAGTCGGTC TTATTAGTCC TTGACGTAAA GACTCCTGGG TACTTTTCTG GGACCATATG   
  
  
- CTGATAAGTA CTTTTGCAAA ACAGTCGGAA AGTCAAAAAC GTAAAATAAG ATCGACGAAA CTAACTTCGG   
  
  
- GTTAAAATAA TTTTGACACG AAAAGAGAAC TTACAATTTG AGAACGTCAA GGAACGAACA ATAAAACCCA   
  
  
- AAATACAAAC GAAAGATGAC TATAGTAGAA AACCAGCACG TTAAATTAAC GGATGATACA ATATACCTAA   
  
  
- AAATACAAAT GAAAGATGAC TATAGTAGAA AACGATAACG TTAAATTAAC GGAAGATACA TCTTAAGCAG   
  
  
- TGAAACTTCA GACGGTCCTT CCGTTATGTC GATATAAAAA GAACACCTAC TTCACTATTT TCCGTTCACA   
  
  
- GTTAACGAAT GACGAAATAA CGCGACATGA AAAGGTCTCT AAAACCTAAT CAGTTACGTC TTCGGTTGTT   
  
  
- AAATATCGCT GACCGGTGTC AAGATGTTGG TTAAACTATT AGGTGCAGAA GAACTTAGAA GTAGATATTA   
  
  
- CCCACCACTA GTTGAAAAGT CAAGAGGTAG ATGACATTCG TATAGAACGT TGTCCTCAGG TCGAAGTGAC   
  
  
- GGACTTGAGC TTAGGAGAAG TAGCCTGAAG GTTTCACTGA GGGTCCTCTG ACACTTACCG AGCGGTCACT   
  
  
- AACTAAGCAC GTACCATGCA CTGTAACCAC TGAATTCTGT GTTTGAATCT CTCGAACTCT GACAATACGA   
  
  
- GCCTGGTTCA AGTCTGTCGA ACCTAAGTAC CATACGGGGA AGTTCTCCAC TCACACTTGG AGACGGTGGT   
  
  
- CTTCTCGGAC TGTTACCCTT TGTAAACAAC CTCTACTATC GTTCCCCCTC GGAGTTTCTC CACGATTAAC   
  
  
- GAACACGGTT TCGTTATAGT CTGCTACTAA ACAATTGTCG CCTCACCAAC TACAGTCTCG ATGCGGTATA   
  
  
- CCAAAGTCAA AGACCTCTTG GTTAAGTCTC CAATCCTCGG ATGTACAACC TTCCCAATCA ACGGGCCAAC   
  
  
- AGGAGAAGTC CTTCAAGGTA GATGTTTCGA GATTCCACGT TTCTCGGATG ATCATCACTT GAAGAAAGGA   
  
  
- TATACGTAAA TGAGATACTT CAAACGGGAA TGAAGTTCAA ACCCATGTAC AGACGTTTAC CCCGTTAACG   
  
  
- ACTCCGGTAC TTTTTACTCT CGTCTTAAGT ATATTAACTA AAAGTCTATC GAGTTCCCTC AGTCACCTAA   
  
  
- TCGGAATAGG TTCGGGACCG ACGAGTCGGA CTACCCGGTG GTGTCCAGGC ATAATGGCCT CAGCTACTAA   
  
  
- GGGTTAGACT CATACGAGCC CCTCCCCCCG AGCTGTAACA TCGCTTCTCT GATAGACCTG ATCGGGTCCG   
  
  
- AACGTCGGAT GGGAAACTCA AGGTGCGACG TCGTGAATCA CCAAGACTCT AGTCTGAAGT TTTGGACCAA   
  
  
- AACGCCGGAC CTCTTCGGAA TCGTCACTTG AAGGGTAAGT ACGACGTGGT GTACGGCCTA CTCTCACACC   
  
  
- CGGGACTCTT AATATCTCTG GTCAATAATT CCGACCACTT CTCGAAGAAC GGGTTCCACC AATGGGAACA   
  
  
- ACTCGTTCTT AGGTTGTGTT TGTGCCGGGG TAAAGATGGG GCCAAAGAAC TTTGGGATCT GATGATATGA   
  
  
- CGGTAAAAGC TTAGTTAACT ACAATGCGAG GGTTCTCTAG TGTTCCTCGC CTAGTTACAA CTCGTCGTGA   
  
  
- CAAATCGTTC TCTATATCAG TTGTATTATC GTACACTCCC ACGGCTCTCC CACCTTGCGG TACTCCAAGA   
  
  
- ACCTTTCACC TCTAGAGCCA AGAGTTACCG ACCCAAATTC GGCATGGGTA ACTCGGGCGA TCACTTACGT   
  
  
- TGATAGTCCT GAGAAGACGT CTTCATATCG TCCTCGATAC CTGAACTTCT TTCCCTACCT CGAGAAATAG   
  
  
- ATCCGACCTA CTTGGCTCGT GATCAACGTA GAACACGTAC CGTCAC

+     WRE3

| Site Name | Organism | Position | Strand | Matrix score. | sequence | function |
| --- | --- | --- | --- | --- | --- | --- |
| WRE3 | Pisum sativum | 3139 | - | 6 | CCACCT |  |

>HU08G00284.1   
+ -Up\_Stream \_Len000TTCTTT TCCTGTCTCT GTCTCTTCAT TGCACCATCA TTTAAGAGAT GGGACCATAA   
  
  
+ ATCATTCAAT CACTCCTTTA TGTTTAAACT TTGGAAGGAG GTTATTATTT ATGTGGTGGT GATCCCAATG   
  
  
+ CTGAATTTAG CTGTTGATGG CGAGGGCATA TATGTAATTA GGATTTCCAA TGCAGTTTAG ATTTTACTCA   
  
  
+ TTTGAGAATT CACTGAGGTT TGTTTGATCT GATTTTAGCA AATATTGTTC AGGGGTATAG ATGCTTATTC   
  
  
+ GGTTCTCAAC TGTATTAGTA TACAAGATGA GTGGTCCACT TGTTTAAGCT TTTACTATAT ACTTATGTTG   
  
  
+ TCCCTAATTT GGTCTCGCTT GATGTGTAGC TGTCAAGTAT TATTATAATC TTGTGTTTGA TATCACATTT   
  
  
+ GTATTGATTA GCTACAAAAG AACATATTAT ATGTGTATAG CAACTATCTC ATATATGCCC TCACCATAGC   
  
  
+ GTGGTTCCAA GGGTTCAAGT TCCACTTAGC CTCACCCTAG AAATGACTTC TTTTTTTTTT TAAAGTTAAA   
  
  
+ GATATGCAAT CACCATGGAA CCAGTCATCT TTTGACACGT TATTCCAGAT CAGATGACCA GCCTTTCTCC   
  
  
+ AAGGCCATTA GATTTTCTAG CCAATCCACT GTTAACTGCA AAAAGTGGTA TCCTTGGCTG TTCCTGTCAC   
  
  
+ TGTTTCTTAT TTGCTCTGTG ATATTTGTAC ATCTCTTTGC CAAGGATAAA AGTTCATAGC CGGGAATTGG   
  
  
+ TATTCAGTTG ACTCTTGCAG TAAACAGAGT TCATGTACTG GAGGATTTTG TGAGTCATGT CACTCAATAA   
  
  
+ AAACAGGTGT GCGACAGTGT TATAAAGCTG CTTGCTTAAT GCACATCTAC TCTCACTCAC CTTACCCAAA   
  
  
+ AAACTTCAAA ATTGTGTGCC TTCTATGAAT ATTCTGATCC ATTAGCATTC AACGGTTTCT GAATTCTGTG   
  
  
+ TAAGCCATCC ATACTCATTT TCAACAGAGA ACTTGGATTG GATGTATTCA ATACCCAAAA ACCTTGTCAA   
  
  
+ ATTACCCCTA GCTTAATTTC CTCTAATACC AACTGCTCTT TGGTTTTAGC GTACAATCCC ATAGATTACC   
  
  
+ CTTCTATAAT TAGTGATGAT TGGTTCATTT CTGCTGCATT ATGCCTTCTT TAGATCCATT GATAATATAT   
  
  
+ ACTCTTGCCA TGCTGCAACA TTCCACTGGG ATTAATATCT CACATTGTAT CCTCAGCAAC CAAGGAGACC   
  
  
+ CTACTATTTT CAGAGCCATT CATAGAAAGT TATTGCATAT AGTGATGATC TTCAATGGGT CTGATTTTTA   
  
  
+ GATAGCTGCT GTGAACATTT TACTCGTCCT CTGCCATGTG GATACTATGG TAACCTTTGT TTGCTCTCAA   
  
  
+ GCCTGAGCGT TCTGAGAAAT GATGAAAGCG CCCTCGAGTT ATAGGACTTT TCTGACTTTA GCTTTAGAAA   
  
  
+ AGAGAGGAAA ATCAACCACA CCCATCTGAT TATTGAGTTT CTGCTCCTTG AGACCTGGAT GGAGCCAGAA   
  
  
+ CTCACGGTCT TGTCAGCCAG AATAATCAGG AACTGCATTT CTGAGGACCC ATGAAAAGAC CCTGGTATAC   
  
  
+ GACTATTCAT GAAAACGTTT TGTCAGCCTT TCAGTTTTTG CATTTTATTC TAGCTGCTTT GATTGAAGCC   
  
  
+ CAATTTTATT AAAACTGTGC TTTTCTCTTG AATGTTAAAC TCTTGCAGTT CCTTGCTTGT TATTTTGGGT   
  
  
+ TTTATGTTTG CTTTCTACTG ATATCATCTT TTGGTCGTGC AATTTAATTG CCTACTATGT TATATGGATT   
  
  
+ TTTATGTTTA CTTTCTACTG ATATCATCTT TTGCTATTGC AATTTAATTG CCTTCTATGT AGAATTCGTC   
  
  
+ ACTTTGAAGT CTGCCAGGAA GGCAATACAG CTATATTTTT CTTGTGGATG AAGTGATAAA AGGCAAGTGT   
  
  
+ CAATTGCTTA CTGCTTTATT GCGCTGTACT TTTCCAGAGA TTTTGGATTA GTCAATGCAG AAGCCAACAA   
  
  
+ TTTATAGCGA CTGGCCACAG TTCTACAACC AATTTGATAA TCCACGTCTT CTTGAATCTT CATCTATAAT   
  
  
+ GGGTGGTGAT CAACTTTTCA GTTCTCCATC TACTGTAAGC ATATCTTGCA ACAGGAGTCC AGCTTCACTG   
  
  
+ CCTGAACTCG AATCCTCTTC ATCGGACTTC CAAAGTGACT CCCAGGAGAC TGTGAATGGC TCGCCAGTGA   
  
  
+ TTGATTCGTG CATGGTACGT GACATTGGTG ACTTAAGACA CAAACTTAGA GAGCTTGAGA CTGTTATGCT   
  
  
+ CGGACCAAGT TCAGACAGCT TGGATTCATG GTATGCCCCT TCAAGAGGTG AGTGTGAACC TCTGCCACCA   
  
  
+ GAAGAGCCTG ACAATGGGAA ACATTTGTTG GAGATGATAG CAAGGGGGAG CCTCAAAGAG GTGCTAATTG   
  
  
+ CTTGTGCCAA AGCAATATCA GACGATGATT TGTTAACAGC GGAGTGGTTG ATGTCAGAGC TACGCCATAT   
  
  
+ GGTTTCAGTT TCTGGAGAAC CAATTCAGAG GTTAGGAGCC TACATGTTGG AAGGGTTAGT TGCCCGGTTG   
  
  
+ TCCTCTTCAG GAAGTTCCAT CTACAAAGCT CTAAGGTGCA AAGAGCCTAC TAGTAGTGAA CTTCTTTCCT   
  
  
+ ATATGCATTT ACTCTATGAA GTTTGCCCTT ACTTCAAGTT TGGGTACATG TCTGCAAATG GGGCAATTGC   
  
  
+ TGAGGCCATG AAAAATGAGA GCAGAATTCA TATAATTGAT TTTCAGATAG CTCAAGGGAG TCAGTGGATT   
  
  
+ AGCCTTATCC AAGCCCTGGC TGCTCAGCCT GATGGGCCAC CACAGGTCCG TATTACCGGA GTCGATGATT   
  
  
+ CCCAATCTGA GTATGCTCGG GGAGGGGGGC TCGACATTGT AGCGAAGAGA CTATCTGGAC TAGCCCAGGC   
  
  
+ TTGCAGCCTA CCCTTTGAGT TCCACGCTGC AGCACTTAGT GGTTCTGAGA TCAGACTTCA AAACCTGGTT   
  
  
+ TTGCGGCCTG GAGAAGCCTT AGCAGTGAAC TTCCCATTCA TGCTGCACCA CATGCCGGAT GAGAGTGTGG   
  
  
+ GCCCTGAGAA TTATAGAGAC CAGTTATTAA GGCTGGTGAA GAGCTTCTTG CCCAAGGTGG TTACCCTTGT   
  
  
+ TGAGCAAGAA TCCAACACAA ACACGGCCCC ATTTCTACCC CGGTTTCTTG AAACCCTAGA CTACTATACT   
  
  
+ GCCATTTTCG AATCAATTGA TGTTACGCTC CCAAGAGATC ACAAGGAGCG GATCAATGTT GAGCAGCACT   
  
  
+ GTTTAGCAAG AGATATAGTC AACATAATAG CATGTGAGGG TGCCGAGAGG GTGGAACGCC ATGAGGTTCT   
  
  
+ TGGAAAGTGG AGATCTCGGT TCTCAATGGC TGGGTTTAAG CCGTACCCAT TGAGCCCGCT AGTGAATGCA   
  
  
+ ACTATCAGGA CTCTTCTGCA GAAGTATAGC AGGAGCTATG GACTTGAAGA AAGGGATGGA GCTCTTTATC   
  
  
+ TAGGCTGGAT GAACCGAGCA CTAGTTGCAT CTTGTGCATG GCAGTG  

- -Up\_Stream \_Len000AAGAAA AGGACAGAGA CAGAGAAGTA ACGTGGTAGT AAATTCTCTA CCCTGGTATT   
  
  
- TAGTAAGTTA GTGAGGAAAT ACAAATTTGA AACCTTCCTC CAATAATAAA TACACCACCA CTAGGGTTAC   
  
  
- GACTTAAATC GACAACTACC GCTCCCGTAT ATACATTAAT CCTAAAGGTT ACGTCAAATC TAAAATGAGT   
  
  
- AAACTCTTAA GTGACTCCAA ACAAACTAGA CTAAAATCGT TTATAACAAG TCCCCATATC TACGAATAAG   
  
  
- CCAAGAGTTG ACATAATCAT ATGTTCTACT CACCAGGTGA ACAAATTCGA AAATGATATA TGAATACAAC   
  
  
- AGGGATTAAA CCAGAGCGAA CTACACATCG ACAGTTCATA ATAATATTAG AACACAAACT ATAGTGTAAA   
  
  
- CATAACTAAT CGATGTTTTC TTGTATAATA TACACATATC GTTGATAGAG TATATACGGG AGTGGTATCG   
  
  
- CACCAAGGTT CCCAAGTTCA AGGTGAATCG GAGTGGGATC TTTACTGAAG AAAAAAAAAA ATTTCAATTT   
  
  
- CTATACGTTA GTGGTACCTT GGTCAGTAGA AAACTGTGCA ATAAGGTCTA GTCTACTGGT CGGAAAGAGG   
  
  
- TTCCGGTAAT CTAAAAGATC GGTTAGGTGA CAATTGACGT TTTTCACCAT AGGAACCGAC AAGGACAGTG   
  
  
- ACAAAGAATA AACGAGACAC TATAAACATG TAGAGAAACG GTTCCTATTT TCAAGTATCG GCCCTTAACC   
  
  
- ATAAGTCAAC TGAGAACGTC ATTTGTCTCA AGTACATGAC CTCCTAAAAC ACTCAGTACA GTGAGTTATT   
  
  
- TTTGTCCACA CGCTGTCACA ATATTTCGAC GAACGAATTA CGTGTAGATG AGAGTGAGTG GAATGGGTTT   
  
  
- TTTGAAGTTT TAACACACGG AAGATACTTA TAAGACTAGG TAATCGTAAG TTGCCAAAGA CTTAAGACAC   
  
  
- ATTCGGTAGG TATGAGTAAA AGTTGTCTCT TGAACCTAAC CTACATAAGT TATGGGTTTT TGGAACAGTT   
  
  
- TAATGGGGAT CGAATTAAAG GAGATTATGG TTGACGAGAA ACCAAAATCG CATGTTAGGG TATCTAATGG   
  
  
- GAAGATATTA ATCACTACTA ACCAAGTAAA GACGACGTAA TACGGAAGAA ATCTAGGTAA CTATTATATA   
  
  
- TGAGAACGGT ACGACGTTGT AAGGTGACCC TAATTATAGA GTGTAACATA GGAGTCGTTG GTTCCTCTGG   
  
  
- GATGATAAAA GTCTCGGTAA GTATCTTTCA ATAACGTATA TCACTACTAG AAGTTACCCA GACTAAAAAT   
  
  
- CTATCGACGA CACTTGTAAA ATGAGCAGGA GACGGTACAC CTATGATACC ATTGGAAACA AACGAGAGTT   
  
  
- CGGACTCGCA AGACTCTTTA CTACTTTCGC GGGAGCTCAA TATCCTGAAA AGACTGAAAT CGAAATCTTT   
  
  
- TCTCTCCTTT TAGTTGGTGT GGGTAGACTA ATAACTCAAA GACGAGGAAC TCTGGACCTA CCTCGGTCTT   
  
  
- GAGTGCCAGA ACAGTCGGTC TTATTAGTCC TTGACGTAAA GACTCCTGGG TACTTTTCTG GGACCATATG   
  
  
- CTGATAAGTA CTTTTGCAAA ACAGTCGGAA AGTCAAAAAC GTAAAATAAG ATCGACGAAA CTAACTTCGG   
  
  
- GTTAAAATAA TTTTGACACG AAAAGAGAAC TTACAATTTG AGAACGTCAA GGAACGAACA ATAAAACCCA   
  
  
- AAATACAAAC GAAAGATGAC TATAGTAGAA AACCAGCACG TTAAATTAAC GGATGATACA ATATACCTAA   
  
  
- AAATACAAAT GAAAGATGAC TATAGTAGAA AACGATAACG TTAAATTAAC GGAAGATACA TCTTAAGCAG   
  
  
- TGAAACTTCA GACGGTCCTT CCGTTATGTC GATATAAAAA GAACACCTAC TTCACTATTT TCCGTTCACA   
  
  
- GTTAACGAAT GACGAAATAA CGCGACATGA AAAGGTCTCT AAAACCTAAT CAGTTACGTC TTCGGTTGTT   
  
  
- AAATATCGCT GACCGGTGTC AAGATGTTGG TTAAACTATT AGGTGCAGAA GAACTTAGAA GTAGATATTA   
  
  
- CCCACCACTA GTTGAAAAGT CAAGAGGTAG ATGACATTCG TATAGAACGT TGTCCTCAGG TCGAAGTGAC   
  
  
- GGACTTGAGC TTAGGAGAAG TAGCCTGAAG GTTTCACTGA GGGTCCTCTG ACACTTACCG AGCGGTCACT   
  
  
- AACTAAGCAC GTACCATGCA CTGTAACCAC TGAATTCTGT GTTTGAATCT CTCGAACTCT GACAATACGA   
  
  
- GCCTGGTTCA AGTCTGTCGA ACCTAAGTAC CATACGGGGA AGTTCTCCAC TCACACTTGG AGACGGTGGT   
  
  
- CTTCTCGGAC TGTTACCCTT TGTAAACAAC CTCTACTATC GTTCCCCCTC GGAGTTTCTC CACGATTAAC   
  
  
- GAACACGGTT TCGTTATAGT CTGCTACTAA ACAATTGTCG CCTCACCAAC TACAGTCTCG ATGCGGTATA   
  
  
- CCAAAGTCAA AGACCTCTTG GTTAAGTCTC CAATCCTCGG ATGTACAACC TTCCCAATCA ACGGGCCAAC   
  
  
- AGGAGAAGTC CTTCAAGGTA GATGTTTCGA GATTCCACGT TTCTCGGATG ATCATCACTT GAAGAAAGGA   
  
  
- TATACGTAAA TGAGATACTT CAAACGGGAA TGAAGTTCAA ACCCATGTAC AGACGTTTAC CCCGTTAACG   
  
  
- ACTCCGGTAC TTTTTACTCT CGTCTTAAGT ATATTAACTA AAAGTCTATC GAGTTCCCTC AGTCACCTAA   
  
  
- TCGGAATAGG TTCGGGACCG ACGAGTCGGA CTACCCGGTG GTGTCCAGGC ATAATGGCCT CAGCTACTAA   
  
  
- GGGTTAGACT CATACGAGCC CCTCCCCCCG AGCTGTAACA TCGCTTCTCT GATAGACCTG ATCGGGTCCG   
  
  
- AACGTCGGAT GGGAAACTCA AGGTGCGACG TCGTGAATCA CCAAGACTCT AGTCTGAAGT TTTGGACCAA   
  
  
- AACGCCGGAC CTCTTCGGAA TCGTCACTTG AAGGGTAAGT ACGACGTGGT GTACGGCCTA CTCTCACACC   
  
  
- CGGGACTCTT AATATCTCTG GTCAATAATT CCGACCACTT CTCGAAGAAC GGGTTCCACC AATGGGAACA   
  
  
- ACTCGTTCTT AGGTTGTGTT TGTGCCGGGG TAAAGATGGG GCCAAAGAAC TTTGGGATCT GATGATATGA   
  
  
- CGGTAAAAGC TTAGTTAACT ACAATGCGAG GGTTCTCTAG TGTTCCTCGC CTAGTTACAA CTCGTCGTGA   
  
  
- CAAATCGTTC TCTATATCAG TTGTATTATC GTACACTCCC ACGGCTCTCC CACCTTGCGG TACTCCAAGA   
  
  
- ACCTTTCACC TCTAGAGCCA AGAGTTACCG ACCCAAATTC GGCATGGGTA ACTCGGGCGA TCACTTACGT   
  
  
- TGATAGTCCT GAGAAGACGT CTTCATATCG TCCTCGATAC CTGAACTTCT TTCCCTACCT CGAGAAATAG   
  
  
- ATCCGACCTA CTTGGCTCGT GATCAACGTA GAACACGTAC CGTCAC

+     as-1

| Site Name | Organism | Position | Strand | Matrix score. | sequence | function |
| --- | --- | --- | --- | --- | --- | --- |
| as-1 | Arabidopsis thaliana | 1891 | - | 5 | TGACG |  |

>HU08G00284.1   
+ -Up\_Stream \_Len000TTCTTT TCCTGTCTCT GTCTCTTCAT TGCACCATCA TTTAAGAGAT GGGACCATAA   
  
  
+ ATCATTCAAT CACTCCTTTA TGTTTAAACT TTGGAAGGAG GTTATTATTT ATGTGGTGGT GATCCCAATG   
  
  
+ CTGAATTTAG CTGTTGATGG CGAGGGCATA TATGTAATTA GGATTTCCAA TGCAGTTTAG ATTTTACTCA   
  
  
+ TTTGAGAATT CACTGAGGTT TGTTTGATCT GATTTTAGCA AATATTGTTC AGGGGTATAG ATGCTTATTC   
  
  
+ GGTTCTCAAC TGTATTAGTA TACAAGATGA GTGGTCCACT TGTTTAAGCT TTTACTATAT ACTTATGTTG   
  
  
+ TCCCTAATTT GGTCTCGCTT GATGTGTAGC TGTCAAGTAT TATTATAATC TTGTGTTTGA TATCACATTT   
  
  
+ GTATTGATTA GCTACAAAAG AACATATTAT ATGTGTATAG CAACTATCTC ATATATGCCC TCACCATAGC   
  
  
+ GTGGTTCCAA GGGTTCAAGT TCCACTTAGC CTCACCCTAG AAATGACTTC TTTTTTTTTT TAAAGTTAAA   
  
  
+ GATATGCAAT CACCATGGAA CCAGTCATCT TTTGACACGT TATTCCAGAT CAGATGACCA GCCTTTCTCC   
  
  
+ AAGGCCATTA GATTTTCTAG CCAATCCACT GTTAACTGCA AAAAGTGGTA TCCTTGGCTG TTCCTGTCAC   
  
  
+ TGTTTCTTAT TTGCTCTGTG ATATTTGTAC ATCTCTTTGC CAAGGATAAA AGTTCATAGC CGGGAATTGG   
  
  
+ TATTCAGTTG ACTCTTGCAG TAAACAGAGT TCATGTACTG GAGGATTTTG TGAGTCATGT CACTCAATAA   
  
  
+ AAACAGGTGT GCGACAGTGT TATAAAGCTG CTTGCTTAAT GCACATCTAC TCTCACTCAC CTTACCCAAA   
  
  
+ AAACTTCAAA ATTGTGTGCC TTCTATGAAT ATTCTGATCC ATTAGCATTC AACGGTTTCT GAATTCTGTG   
  
  
+ TAAGCCATCC ATACTCATTT TCAACAGAGA ACTTGGATTG GATGTATTCA ATACCCAAAA ACCTTGTCAA   
  
  
+ ATTACCCCTA GCTTAATTTC CTCTAATACC AACTGCTCTT TGGTTTTAGC GTACAATCCC ATAGATTACC   
  
  
+ CTTCTATAAT TAGTGATGAT TGGTTCATTT CTGCTGCATT ATGCCTTCTT TAGATCCATT GATAATATAT   
  
  
+ ACTCTTGCCA TGCTGCAACA TTCCACTGGG ATTAATATCT CACATTGTAT CCTCAGCAAC CAAGGAGACC   
  
  
+ CTACTATTTT CAGAGCCATT CATAGAAAGT TATTGCATAT AGTGATGATC TTCAATGGGT CTGATTTTTA   
  
  
+ GATAGCTGCT GTGAACATTT TACTCGTCCT CTGCCATGTG GATACTATGG TAACCTTTGT TTGCTCTCAA   
  
  
+ GCCTGAGCGT TCTGAGAAAT GATGAAAGCG CCCTCGAGTT ATAGGACTTT TCTGACTTTA GCTTTAGAAA   
  
  
+ AGAGAGGAAA ATCAACCACA CCCATCTGAT TATTGAGTTT CTGCTCCTTG AGACCTGGAT GGAGCCAGAA   
  
  
+ CTCACGGTCT TGTCAGCCAG AATAATCAGG AACTGCATTT CTGAGGACCC ATGAAAAGAC CCTGGTATAC   
  
  
+ GACTATTCAT GAAAACGTTT TGTCAGCCTT TCAGTTTTTG CATTTTATTC TAGCTGCTTT GATTGAAGCC   
  
  
+ CAATTTTATT AAAACTGTGC TTTTCTCTTG AATGTTAAAC TCTTGCAGTT CCTTGCTTGT TATTTTGGGT   
  
  
+ TTTATGTTTG CTTTCTACTG ATATCATCTT TTGGTCGTGC AATTTAATTG CCTACTATGT TATATGGATT   
  
  
+ TTTATGTTTA CTTTCTACTG ATATCATCTT TTGCTATTGC AATTTAATTG CCTTCTATGT AGAATTCGTC   
  
  
+ ACTTTGAAGT CTGCCAGGAA GGCAATACAG CTATATTTTT CTTGTGGATG AAGTGATAAA AGGCAAGTGT   
  
  
+ CAATTGCTTA CTGCTTTATT GCGCTGTACT TTTCCAGAGA TTTTGGATTA GTCAATGCAG AAGCCAACAA   
  
  
+ TTTATAGCGA CTGGCCACAG TTCTACAACC AATTTGATAA TCCACGTCTT CTTGAATCTT CATCTATAAT   
  
  
+ GGGTGGTGAT CAACTTTTCA GTTCTCCATC TACTGTAAGC ATATCTTGCA ACAGGAGTCC AGCTTCACTG   
  
  
+ CCTGAACTCG AATCCTCTTC ATCGGACTTC CAAAGTGACT CCCAGGAGAC TGTGAATGGC TCGCCAGTGA   
  
  
+ TTGATTCGTG CATGGTACGT GACATTGGTG ACTTAAGACA CAAACTTAGA GAGCTTGAGA CTGTTATGCT   
  
  
+ CGGACCAAGT TCAGACAGCT TGGATTCATG GTATGCCCCT TCAAGAGGTG AGTGTGAACC TCTGCCACCA   
  
  
+ GAAGAGCCTG ACAATGGGAA ACATTTGTTG GAGATGATAG CAAGGGGGAG CCTCAAAGAG GTGCTAATTG   
  
  
+ CTTGTGCCAA AGCAATATCA GACGATGATT TGTTAACAGC GGAGTGGTTG ATGTCAGAGC TACGCCATAT   
  
  
+ GGTTTCAGTT TCTGGAGAAC CAATTCAGAG GTTAGGAGCC TACATGTTGG AAGGGTTAGT TGCCCGGTTG   
  
  
+ TCCTCTTCAG GAAGTTCCAT CTACAAAGCT CTAAGGTGCA AAGAGCCTAC TAGTAGTGAA CTTCTTTCCT   
  
  
+ ATATGCATTT ACTCTATGAA GTTTGCCCTT ACTTCAAGTT TGGGTACATG TCTGCAAATG GGGCAATTGC   
  
  
+ TGAGGCCATG AAAAATGAGA GCAGAATTCA TATAATTGAT TTTCAGATAG CTCAAGGGAG TCAGTGGATT   
  
  
+ AGCCTTATCC AAGCCCTGGC TGCTCAGCCT GATGGGCCAC CACAGGTCCG TATTACCGGA GTCGATGATT   
  
  
+ CCCAATCTGA GTATGCTCGG GGAGGGGGGC TCGACATTGT AGCGAAGAGA CTATCTGGAC TAGCCCAGGC   
  
  
+ TTGCAGCCTA CCCTTTGAGT TCCACGCTGC AGCACTTAGT GGTTCTGAGA TCAGACTTCA AAACCTGGTT   
  
  
+ TTGCGGCCTG GAGAAGCCTT AGCAGTGAAC TTCCCATTCA TGCTGCACCA CATGCCGGAT GAGAGTGTGG   
  
  
+ GCCCTGAGAA TTATAGAGAC CAGTTATTAA GGCTGGTGAA GAGCTTCTTG CCCAAGGTGG TTACCCTTGT   
  
  
+ TGAGCAAGAA TCCAACACAA ACACGGCCCC ATTTCTACCC CGGTTTCTTG AAACCCTAGA CTACTATACT   
  
  
+ GCCATTTTCG AATCAATTGA TGTTACGCTC CCAAGAGATC ACAAGGAGCG GATCAATGTT GAGCAGCACT   
  
  
+ GTTTAGCAAG AGATATAGTC AACATAATAG CATGTGAGGG TGCCGAGAGG GTGGAACGCC ATGAGGTTCT   
  
  
+ TGGAAAGTGG AGATCTCGGT TCTCAATGGC TGGGTTTAAG CCGTACCCAT TGAGCCCGCT AGTGAATGCA   
  
  
+ ACTATCAGGA CTCTTCTGCA GAAGTATAGC AGGAGCTATG GACTTGAAGA AAGGGATGGA GCTCTTTATC   
  
  
+ TAGGCTGGAT GAACCGAGCA CTAGTTGCAT CTTGTGCATG GCAGTG  

- -Up\_Stream \_Len000AAGAAA AGGACAGAGA CAGAGAAGTA ACGTGGTAGT AAATTCTCTA CCCTGGTATT   
  
  
- TAGTAAGTTA GTGAGGAAAT ACAAATTTGA AACCTTCCTC CAATAATAAA TACACCACCA CTAGGGTTAC   
  
  
- GACTTAAATC GACAACTACC GCTCCCGTAT ATACATTAAT CCTAAAGGTT ACGTCAAATC TAAAATGAGT   
  
  
- AAACTCTTAA GTGACTCCAA ACAAACTAGA CTAAAATCGT TTATAACAAG TCCCCATATC TACGAATAAG   
  
  
- CCAAGAGTTG ACATAATCAT ATGTTCTACT CACCAGGTGA ACAAATTCGA AAATGATATA TGAATACAAC   
  
  
- AGGGATTAAA CCAGAGCGAA CTACACATCG ACAGTTCATA ATAATATTAG AACACAAACT ATAGTGTAAA   
  
  
- CATAACTAAT CGATGTTTTC TTGTATAATA TACACATATC GTTGATAGAG TATATACGGG AGTGGTATCG   
  
  
- CACCAAGGTT CCCAAGTTCA AGGTGAATCG GAGTGGGATC TTTACTGAAG AAAAAAAAAA ATTTCAATTT   
  
  
- CTATACGTTA GTGGTACCTT GGTCAGTAGA AAACTGTGCA ATAAGGTCTA GTCTACTGGT CGGAAAGAGG   
  
  
- TTCCGGTAAT CTAAAAGATC GGTTAGGTGA CAATTGACGT TTTTCACCAT AGGAACCGAC AAGGACAGTG   
  
  
- ACAAAGAATA AACGAGACAC TATAAACATG TAGAGAAACG GTTCCTATTT TCAAGTATCG GCCCTTAACC   
  
  
- ATAAGTCAAC TGAGAACGTC ATTTGTCTCA AGTACATGAC CTCCTAAAAC ACTCAGTACA GTGAGTTATT   
  
  
- TTTGTCCACA CGCTGTCACA ATATTTCGAC GAACGAATTA CGTGTAGATG AGAGTGAGTG GAATGGGTTT   
  
  
- TTTGAAGTTT TAACACACGG AAGATACTTA TAAGACTAGG TAATCGTAAG TTGCCAAAGA CTTAAGACAC   
  
  
- ATTCGGTAGG TATGAGTAAA AGTTGTCTCT TGAACCTAAC CTACATAAGT TATGGGTTTT TGGAACAGTT   
  
  
- TAATGGGGAT CGAATTAAAG GAGATTATGG TTGACGAGAA ACCAAAATCG CATGTTAGGG TATCTAATGG   
  
  
- GAAGATATTA ATCACTACTA ACCAAGTAAA GACGACGTAA TACGGAAGAA ATCTAGGTAA CTATTATATA   
  
  
- TGAGAACGGT ACGACGTTGT AAGGTGACCC TAATTATAGA GTGTAACATA GGAGTCGTTG GTTCCTCTGG   
  
  
- GATGATAAAA GTCTCGGTAA GTATCTTTCA ATAACGTATA TCACTACTAG AAGTTACCCA GACTAAAAAT   
  
  
- CTATCGACGA CACTTGTAAA ATGAGCAGGA GACGGTACAC CTATGATACC ATTGGAAACA AACGAGAGTT   
  
  
- CGGACTCGCA AGACTCTTTA CTACTTTCGC GGGAGCTCAA TATCCTGAAA AGACTGAAAT CGAAATCTTT   
  
  
- TCTCTCCTTT TAGTTGGTGT GGGTAGACTA ATAACTCAAA GACGAGGAAC TCTGGACCTA CCTCGGTCTT   
  
  
- GAGTGCCAGA ACAGTCGGTC TTATTAGTCC TTGACGTAAA GACTCCTGGG TACTTTTCTG GGACCATATG   
  
  
- CTGATAAGTA CTTTTGCAAA ACAGTCGGAA AGTCAAAAAC GTAAAATAAG ATCGACGAAA CTAACTTCGG   
  
  
- GTTAAAATAA TTTTGACACG AAAAGAGAAC TTACAATTTG AGAACGTCAA GGAACGAACA ATAAAACCCA   
  
  
- AAATACAAAC GAAAGATGAC TATAGTAGAA AACCAGCACG TTAAATTAAC GGATGATACA ATATACCTAA   
  
  
- AAATACAAAT GAAAGATGAC TATAGTAGAA AACGATAACG TTAAATTAAC GGAAGATACA TCTTAAGCAG   
  
  
- TGAAACTTCA GACGGTCCTT CCGTTATGTC GATATAAAAA GAACACCTAC TTCACTATTT TCCGTTCACA   
  
  
- GTTAACGAAT GACGAAATAA CGCGACATGA AAAGGTCTCT AAAACCTAAT CAGTTACGTC TTCGGTTGTT   
  
  
- AAATATCGCT GACCGGTGTC AAGATGTTGG TTAAACTATT AGGTGCAGAA GAACTTAGAA GTAGATATTA   
  
  
- CCCACCACTA GTTGAAAAGT CAAGAGGTAG ATGACATTCG TATAGAACGT TGTCCTCAGG TCGAAGTGAC   
  
  
- GGACTTGAGC TTAGGAGAAG TAGCCTGAAG GTTTCACTGA GGGTCCTCTG ACACTTACCG AGCGGTCACT   
  
  
- AACTAAGCAC GTACCATGCA CTGTAACCAC TGAATTCTGT GTTTGAATCT CTCGAACTCT GACAATACGA   
  
  
- GCCTGGTTCA AGTCTGTCGA ACCTAAGTAC CATACGGGGA AGTTCTCCAC TCACACTTGG AGACGGTGGT   
  
  
- CTTCTCGGAC TGTTACCCTT TGTAAACAAC CTCTACTATC GTTCCCCCTC GGAGTTTCTC CACGATTAAC   
  
  
- GAACACGGTT TCGTTATAGT CTGCTACTAA ACAATTGTCG CCTCACCAAC TACAGTCTCG ATGCGGTATA   
  
  
- CCAAAGTCAA AGACCTCTTG GTTAAGTCTC CAATCCTCGG ATGTACAACC TTCCCAATCA ACGGGCCAAC   
  
  
- AGGAGAAGTC CTTCAAGGTA GATGTTTCGA GATTCCACGT TTCTCGGATG ATCATCACTT GAAGAAAGGA   
  
  
- TATACGTAAA TGAGATACTT CAAACGGGAA TGAAGTTCAA ACCCATGTAC AGACGTTTAC CCCGTTAACG   
  
  
- ACTCCGGTAC TTTTTACTCT CGTCTTAAGT ATATTAACTA AAAGTCTATC GAGTTCCCTC AGTCACCTAA   
  
  
- TCGGAATAGG TTCGGGACCG ACGAGTCGGA CTACCCGGTG GTGTCCAGGC ATAATGGCCT CAGCTACTAA   
  
  
- GGGTTAGACT CATACGAGCC CCTCCCCCCG AGCTGTAACA TCGCTTCTCT GATAGACCTG ATCGGGTCCG   
  
  
- AACGTCGGAT GGGAAACTCA AGGTGCGACG TCGTGAATCA CCAAGACTCT AGTCTGAAGT TTTGGACCAA   
  
  
- AACGCCGGAC CTCTTCGGAA TCGTCACTTG AAGGGTAAGT ACGACGTGGT GTACGGCCTA CTCTCACACC   
  
  
- CGGGACTCTT AATATCTCTG GTCAATAATT CCGACCACTT CTCGAAGAAC GGGTTCCACC AATGGGAACA   
  
  
- ACTCGTTCTT AGGTTGTGTT TGTGCCGGGG TAAAGATGGG GCCAAAGAAC TTTGGGATCT GATGATATGA   
  
  
- CGGTAAAAGC TTAGTTAACT ACAATGCGAG GGTTCTCTAG TGTTCCTCGC CTAGTTACAA CTCGTCGTGA   
  
  
- CAAATCGTTC TCTATATCAG TTGTATTATC GTACACTCCC ACGGCTCTCC CACCTTGCGG TACTCCAAGA   
  
  
- ACCTTTCACC TCTAGAGCCA AGAGTTACCG ACCCAAATTC GGCATGGGTA ACTCGGGCGA TCACTTACGT   
  
  
- TGATAGTCCT GAGAAGACGT CTTCATATCG TCCTCGATAC CTGAACTTCT TTCCCTACCT CGAGAAATAG   
  
  
- ATCCGACCTA CTTGGCTCGT GATCAACGTA GAACACGTAC CGTCAC
