# Supplementary material for: Genome-Wide Identification and Expression Pattern of the GRAS Gene Family in Pitaya (Selenicereus undatus L.)
Source: Biology (Basel). 2022 Dec 21;12(1):11. doi: 10.3390/biology12010011 (PMC9854919; doi:10.3390/biology12010011)
Supplement: Supplementary file 1 [file biology-12-00011-s001.zip › Supplementary file S5/HU08G00367.1_plantcare.html]

Content-Type: text/html; charset=ISO-8859-1


PlantCARE


Webmaster Firefox specific output  
To save the result:
click on the frame with the right mouse button and save the source code as a text file with extension .html  
REFERENCE:PlantCARE: a database of plant cis-acting regulatory elements and a portal to tools for in silico analysis of promoter sequences.  
Lescot, M., Déhais, P., Moreau, Y., De Moor, B., Rouzé ,P.,and Rombauts, S.  
Nucleic Acids Res., Database issue(2002), 30(1):325-327.   


---

>HU08G00367.1   
+ -Up\_Stream \_Len000ATGCGC CTTTCTGCTA ACTCATTATC TTTCCTTCTG AGAGGGGACA ACTTATATAA   
  
  
+ ATTGATAGGT ATTTTCAAAT CAATAGACCA GCTACCATAT ATGATAATTT TTATTACTTT TCTAAATCCA   
  
  
+ TCAACTATTT TCACAATCCT CCTTTTTCTA GAAAAATAAA ATTTGCTTAA TTCAAATCAC CTAAAATTAC   
  
  
+ TAAATCTATA GTCCTAAATC TACAAACTCA TTTGTGTTGC AACTCATGAT TTAAGTGGAA CACTAACCTT   
  
  
+ AGGAGATATG TTGTCAAAAG AAGAATTCGA TGACATATTT TCCAACAAAA GGCCACCTAA CTTCATGATT   
  
  
+ TAAAAAGGAG GAGTCTAAGC ACATCATGGA TGACTAATTT TTGATGTATT GTAACTACGT ATTGAGATAA   
  
  
+ CTTGAATGAA TCGTGACTAG AATATATCTA TTTTCAAAAA TAAAAATTCC CTAAAAGAAA AGGATAAAAA   
  
  
+ ACTATATAAT ACTAAAAAAT TCTAACTATA ATAACTAGGC TTATTAAATT TTTATAGTGA AAATTTATTT   
  
  
+ CTACTTATAA CAACATCACT TACTAAGTAG CTTATTTGTG TCTTCTTTTC TAAATAATAA TGGTAACTAT   
  
  
+ GTGTCTTTTG TAAATAACTT ATTATCTATT TTATAAATCA CCGTGTAAAG CACGGGTCTA TACTAGTTAA   
  
  
+ TTTAAATAGC ATTGTTAAAT CAAGTAAGAT ACAAACAAAT TTATTTACAA CTGTAAGTGG TCCATAAAAA   
  
  
+ TAATAGCCAT TACAAAAAAT AGTACTACTG TTTTATGAAA TGATTTTAAT ATTCTAAAAT TCCAATAAAA   
  
  
+ CATGTTATAA TCTATGATTA ATTTTAAAAC ATGATTACAT GTCAGCATAA TTTGTATTAT TGTACAGCAA   
  
  
+ ATGAAAGTTG GAGCTAAAAG AAAATGGGGA AATAAAGGCA GACAAGTAAA GATAAAATAT GCTATCTACT   
  
  
+ TTTGGAGGTA TCCTTAACCA CATTGTCTCG TTTGTCTTCA TTAGGGAGAA GTGGGATAAA TACCAAAATT   
  
  
+ TGTATGGTCA TTGCGTGGGG TTTGCTTCCA AACAAAATGG CTTTTTGGAA GCAAATTTTG TGAGCTTCTA   
  
  
+ TGCAAATTTC CACGCTTAAC CCCACAAGCA AACAAAAACA GACAGTGGTT TTTGTTTGTC TTTGTTAGGG   
  
  
+ CATGTTTGGA GCCAACTGTT AATGGGAATA AACTGGGGAG GCTTTTTCCT TGGCTCTTTC CCCCTGACCC   
  
  
+ CAAACCCACT TCAGCTCAAT ACGAAACCAG CCATTGAAAA AGGAAGGGGG GGGGGGGGGT ATGTGAGAAA   
  
  
+ AAGACAAAAC TTTTTCCCGG AGAGAGAAGA TACATATGCA ACAAAGGCTC TCCTTCTCAT AAATTCCATA   
  
  
+ TTCCTCTGTT TTTCTAGGAG AGAGAAATAT GCAAAAAACA AATCAGTTGG CGACAAAAGT TACTACCACG   
  
  
+ CATTGTCTAC TGCTGCGTAC CCATTTTTGG AGACTTTAAA GAACGCCCCT CCCATCTTGA AAAGTGAAAA   
  
  
+ CCCCCTCTTT GAAACTGAAT TTCAGGGGAA AAAAACCTCA CCAAACCTCT AGAGAGAGCT TTTAGAGAGG   
  
  
+ GAAATACAAC GATTAGAAGG AGCAATTTGG GAAATTTCTT TGGGAATTTG AATGGGTTTT GAGTGAATTG   
  
  
+ CAAATCCCAG AAAAGTTTTG GCAAGTACCG ATCTACAGTT CTCTCCTCTT CGTGTTTGGT AGATCCCCTG   
  
  
+ TTTCCTCTGT TTCATTTAGG GTACTTCTCA TTCATCCTCC CCACCCCCTT AATCGGATCT TCCTGTCGAG   
  
  
+ TCACTTTATG CTAATATTTT TTCAGTGGAT TTTTAGTTAA CCCTGTTCAT TTTTCCATTC TGTGTCCCAT   
  
  
+ TTCTCTCTTT TTCATTCATA AGTTGCCGGT TTATCTGTTG GGTGCAGCTT AGTCACAATA ATTTCTGTGT   
  
  
+ TAGGCTTCTT TACGGTTAAA AAAAAAGGAG GCACTCTTTT CGGTGTGATT GTTTATGGGA CCAATGATTC   
  
  
+ AAGATGATGG GTCATCAGTA ACTTCATCAC CCCTTCAATT TTTCTCCATG ATGTCTCCCA ATTTAGGTTC   
  
  
+ TTCCTACCCT TGGCTCAGAG AGCTAAAACC TGAAGAAAGA GGTCTTTACT TGATACATTT GTTGCTCTCT   
  
  
+ TGTGCAAATC ATGTCTCTAG TGGTAGCCTA GACAATGCGA ACTTAGCCCT CGAACAAATC TCTCAGCTTG   
  
  
+ CTGCCCCTGA TGGGGATACA ATGCAGCGTA TGGCTTCTTA TTTTGCTGAA GCCCTGGCTG AGAGGATCCT   
  
  
+ CAAGTCATGG CCTGGCATGT ATAGAGCCCT TCATTTTACG AAAATGCCTG TCATTTCAGA GGAAATTCTT   
  
  
+ GCTAGGAAGC TCTTCTTTGA GCTATTTCCT TTCTTGAAGC TGGCCTATTT GGTGACAAAC CAATCGATAA   
  
  
+ TCGAAGCCAT GGAGGGGGAA AAGATGGTTC ATATTATTGA TCTGAATGCA TCAGAACCTG CTCAATGGAT   
  
  
+ TGCCCTTATT CAGGCTTTGA GTGCAAGGCC TGGGGGTCCT CCTCATTTGA GAATTACCGG TGTTCATCAA   
  
  
+ CACAAAGAGG TTCTAGATCA AGTGGCTCAT AGGGTGACTC AAGAAGCTGA GAAATTGGAT TTGCCATTTC   
  
  
+ AGTTCAATCC TGTGGTTAGC AAGTTGGAAA ACCTTGATGT TGAAAAGCTG TGTGTTAAGA CTGGTGAGGC   
  
  
+ TCTAGCCATC AGTTCGGTCC TTCAACTGCA TACCCTTTTG GGTTCTGATG ATGAGCCCCT AAGGAAAAGT   
  
  
+ TCACCTTTAG CCTTGATGAA GTATGCAAAT GGGGCTAATA GGCAAAGCCC GAGTAATGAT TCGGCTTCTT   
  
  
+ CATCACCTCC TTCGCTCAAT ACTTCAACCA AGCTGGATGG TTTCCTCAGC GCTTTGTGGG GATTGTCCCC   
  
  
+ AAAGATTATG GTGATAGCTG AGCACGATTC CAATCACAAT GGTTCTGGAC TTATGGAGAG GTTGTCAGAA   
  
  
+ GCACTGTACT TCTATGCAGC GCTGTTTGAC TGCTTAGAAT CCACCCTGCC AAGAACATCT GTCGAAAGAA   
  
  
+ GGCGGGTAGA GAAGATGCTC CTAGGTGAAG AGATCAAGAA CATTATATCA TGCGAGGGAG GAGAAAGGAG   
  
  
+ AGAAAGGCAT GAGAAGATCG AGAAGTGGAT GCAGAGGCTA GACATGGCTG GATTCGGGAT CGTTCCTTTG   
  
  
+ AGCTATATGG GTATGCTGCA AGCAAGGCAA TTGCTTCAGG GCTATGGTTG TGATGGTTAT AGAGTGAAAG   
  
  
+ AGAATGGTTG TGTTGTCATC TGTTGGCAAG ATCGCCCCCT CTTTTCGGTA TCAGCATGGA GGTGTAGGAG   
  
  
+ ATG  

- -Up\_Stream \_Len000TACGCG GAAAGACGAT TGAGTAATAG AAAGGAAGAC TCTCCCCTGT TGAATATATT   
  
  
- TAACTATCCA TAAAAGTTTA GTTATCTGGT CGATGGTATA TACTATTAAA AATAATGAAA AGATTTAGGT   
  
  
- AGTTGATAAA AGTGTTAGGA GGAAAAAGAT CTTTTTATTT TAAACGAATT AAGTTTAGTG GATTTTAATG   
  
  
- ATTTAGATAT CAGGATTTAG ATGTTTGAGT AAACACAACG TTGAGTACTA AATTCACCTT GTGATTGGAA   
  
  
- TCCTCTATAC AACAGTTTTC TTCTTAAGCT ACTGTATAAA AGGTTGTTTT CCGGTGGATT GAAGTACTAA   
  
  
- ATTTTTCCTC CTCAGATTCG TGTAGTACCT ACTGATTAAA AACTACATAA CATTGATGCA TAACTCTATT   
  
  
- GAACTTACTT AGCACTGATC TTATATAGAT AAAAGTTTTT ATTTTTAAGG GATTTTCTTT TCCTATTTTT   
  
  
- TGATATATTA TGATTTTTTA AGATTGATAT TATTGATCCG AATAATTTAA AAATATCACT TTTAAATAAA   
  
  
- GATGAATATT GTTGTAGTGA ATGATTCATC GAATAAACAC AGAAGAAAAG ATTTATTATT ACCATTGATA   
  
  
- CACAGAAAAC ATTTATTGAA TAATAGATAA AATATTTAGT GGCACATTTC GTGCCCAGAT ATGATCAATT   
  
  
- AAATTTATCG TAACAATTTA GTTCATTCTA TGTTTGTTTA AATAAATGTT GACATTCACC AGGTATTTTT   
  
  
- ATTATCGGTA ATGTTTTTTA TCATGATGAC AAAATACTTT ACTAAAATTA TAAGATTTTA AGGTTATTTT   
  
  
- GTACAATATT AGATACTAAT TAAAATTTTG TACTAATGTA CAGTCGTATT AAACATAATA ACATGTCGTT   
  
  
- TACTTTCAAC CTCGATTTTC TTTTACCCCT TTATTTCCGT CTGTTCATTT CTATTTTATA CGATAGATGA   
  
  
- AAACCTCCAT AGGAATTGGT GTAACAGAGC AAACAGAAGT AATCCCTCTT CACCCTATTT ATGGTTTTAA   
  
  
- ACATACCAGT AACGCACCCC AAACGAAGGT TTGTTTTACC GAAAAACCTT CGTTTAAAAC ACTCGAAGAT   
  
  
- ACGTTTAAAG GTGCGAATTG GGGTGTTCGT TTGTTTTTGT CTGTCACCAA AAACAAACAG AAACAATCCC   
  
  
- GTACAAACCT CGGTTGACAA TTACCCTTAT TTGACCCCTC CGAAAAAGGA ACCGAGAAAG GGGGACTGGG   
  
  
- GTTTGGGTGA AGTCGAGTTA TGCTTTGGTC GGTAACTTTT TCCTTCCCCC CCCCCCCCCA TACACTCTTT   
  
  
- TTCTGTTTTG AAAAAGGGCC TCTCTCTTCT ATGTATACGT TGTTTCCGAG AGGAAGAGTA TTTAAGGTAT   
  
  
- AAGGAGACAA AAAGATCCTC TCTCTTTATA CGTTTTTTGT TTAGTCAACC GCTGTTTTCA ATGATGGTGC   
  
  
- GTAACAGATG ACGACGCATG GGTAAAAACC TCTGAAATTT CTTGCGGGGA GGGTAGAACT TTTCACTTTT   
  
  
- GGGGGAGAAA CTTTGACTTA AAGTCCCCTT TTTTTGGAGT GGTTTGGAGA TCTCTCTCGA AAATCTCTCC   
  
  
- CTTTATGTTG CTAATCTTCC TCGTTAAACC CTTTAAAGAA ACCCTTAAAC TTACCCAAAA CTCACTTAAC   
  
  
- GTTTAGGGTC TTTTCAAAAC CGTTCATGGC TAGATGTCAA GAGAGGAGAA GCACAAACCA TCTAGGGGAC   
  
  
- AAAGGAGACA AAGTAAATCC CATGAAGAGT AAGTAGGAGG GGTGGGGGAA TTAGCCTAGA AGGACAGCTC   
  
  
- AGTGAAATAC GATTATAAAA AAGTCACCTA AAAATCAATT GGGACAAGTA AAAAGGTAAG ACACAGGGTA   
  
  
- AAGAGAGAAA AAGTAAGTAT TCAACGGCCA AATAGACAAC CCACGTCGAA TCAGTGTTAT TAAAGACACA   
  
  
- ATCCGAAGAA ATGCCAATTT TTTTTTCCTC CGTGAGAAAA GCCACACTAA CAAATACCCT GGTTACTAAG   
  
  
- TTCTACTACC CAGTAGTCAT TGAAGTAGTG GGGAAGTTAA AAAGAGGTAC TACAGAGGGT TAAATCCAAG   
  
  
- AAGGATGGGA ACCGAGTCTC TCGATTTTGG ACTTCTTTCT CCAGAAATGA ACTATGTAAA CAACGAGAGA   
  
  
- ACACGTTTAG TACAGAGATC ACCATCGGAT CTGTTACGCT TGAATCGGGA GCTTGTTTAG AGAGTCGAAC   
  
  
- GACGGGGACT ACCCCTATGT TACGTCGCAT ACCGAAGAAT AAAACGACTT CGGGACCGAC TCTCCTAGGA   
  
  
- GTTCAGTACC GGACCGTACA TATCTCGGGA AGTAAAATGC TTTTACGGAC AGTAAAGTCT CCTTTAAGAA   
  
  
- CGATCCTTCG AGAAGAAACT CGATAAAGGA AAGAACTTCG ACCGGATAAA CCACTGTTTG GTTAGCTATT   
  
  
- AGCTTCGGTA CCTCCCCCTT TTCTACCAAG TATAATAACT AGACTTACGT AGTCTTGGAC GAGTTACCTA   
  
  
- ACGGGAATAA GTCCGAAACT CACGTTCCGG ACCCCCAGGA GGAGTAAACT CTTAATGGCC ACAAGTAGTT   
  
  
- GTGTTTCTCC AAGATCTAGT TCACCGAGTA TCCCACTGAG TTCTTCGACT CTTTAACCTA AACGGTAAAG   
  
  
- TCAAGTTAGG ACACCAATCG TTCAACCTTT TGGAACTACA ACTTTTCGAC ACACAATTCT GACCACTCCG   
  
  
- AGATCGGTAG TCAAGCCAGG AAGTTGACGT ATGGGAAAAC CCAAGACTAC TACTCGGGGA TTCCTTTTCA   
  
  
- AGTGGAAATC GGAACTACTT CATACGTTTA CCCCGATTAT CCGTTTCGGG CTCATTACTA AGCCGAAGAA   
  
  
- GTAGTGGAGG AAGCGAGTTA TGAAGTTGGT TCGACCTACC AAAGGAGTCG CGAAACACCC CTAACAGGGG   
  
  
- TTTCTAATAC CACTATCGAC TCGTGCTAAG GTTAGTGTTA CCAAGACCTG AATACCTCTC CAACAGTCTT   
  
  
- CGTGACATGA AGATACGTCG CGACAAACTG ACGAATCTTA GGTGGGACGG TTCTTGTAGA CAGCTTTCTT   
  
  
- CCGCCCATCT CTTCTACGAG GATCCACTTC TCTAGTTCTT GTAATATAGT ACGCTCCCTC CTCTTTCCTC   
  
  
- TCTTTCCGTA CTCTTCTAGC TCTTCACCTA CGTCTCCGAT CTGTACCGAC CTAAGCCCTA GCAAGGAAAC   
  
  
- TCGATATACC CATACGACGT TCGTTCCGTT AACGAAGTCC CGATACCAAC ACTACCAATA TCTCACTTTC   
  
  
- TCTTACCAAC ACAACAGTAG ACAACCGTTC TAGCGGGGGA GAAAAGCCAT AGTCGTACCT CCACATCCTC   
  
  
- TAC

  
  
Motifs Found  

+   

| Site Name | Organism | Position | Strand | Matrix score. | sequence | function |
| --- | --- | --- | --- | --- | --- | --- |
|  | organism | 3361 | - | 4 | motif\_sequence | short\_function |
|  | organism | 3185 | - | 4 | motif\_sequence | short\_function |
|  | organism | 3167 | - | 4 | motif\_sequence | short\_function |
|  | organism | 1867 | + | 4 | motif\_sequence | short\_function |
|  | organism | 1811 | + | 4 | motif\_sequence | short\_function |
|  | organism | 2394 | + | 4 | motif\_sequence | short\_function |
|  | organism | 906 | - | 4 | motif\_sequence | short\_function |
|  | organism | 1743 | - | 4 | motif\_sequence | short\_function |
|  | organism | 1730 | + | 4 | motif\_sequence | short\_function |
|  | organism | 1421 | - | 4 | motif\_sequence | short\_function |
|  | organism | 3122 | - | 4 | motif\_sequence | short\_function |
|  | organism | 3090 | - | 4 | motif\_sequence | short\_function |
|  | organism | 3111 | - | 4 | motif\_sequence | short\_function |
|  | organism | 978 | + | 4 | motif\_sequence | short\_function |
|  | organism | 1353 | - | 4 | motif\_sequence | short\_function |
|  | organism | 3151 | - | 4 | motif\_sequence | short\_function |
|  | organism | 1382 | + | 4 | motif\_sequence | short\_function |
|  | organism | 2999 | - | 4 | motif\_sequence | short\_function |
|  | organism | 1715 | + | 4 | motif\_sequence | short\_function |
|  | organism | 3018 | + | 4 | motif\_sequence | short\_function |
|  | organism | 232 | + | 4 | motif\_sequence | short\_function |
|  | organism | 3067 | - | 4 | motif\_sequence | short\_function |
|  | organism | 286 | - | 4 | motif\_sequence | short\_function |
|  | organism | 1360 | - | 4 | motif\_sequence | short\_function |
|  | organism | 3095 | - | 4 | motif\_sequence | short\_function |
|  | organism | 1725 | + | 4 | motif\_sequence | short\_function |
|  | organism | 373 | - | 4 | motif\_sequence | short\_function |

>HU08G00367.1   
+ -Up\_Stream \_Len000ATGCGC CTTTCTGCTA ACTCATTATC TTTCCTTCTG AGAGGGGACA ACTTATATAA   
  
  
+ ATTGATAGGT ATTTTCAAAT CAATAGACCA GCTACCATAT ATGATAATTT TTATTACTTT TCTAAATCCA   
  
  
+ TCAACTATTT TCACAATCCT CCTTTTTCTA GAAAAATAAA ATTTGCTTAA TTCAAATCAC CTAAAATTAC   
  
  
+ TAAATCTATA GTCCTAAATC TACAAACTCA TTTGTGTTGC AACTCATGAT TTAAGTGGAA CACTAACCTT   
  
  
+ AGGAGATATG TTGTCAAAAG AAGAATTCGA TGACATATTT TCCAACAAAA GGCCACCTAA CTTCATGATT   
  
  
+ TAAAAAGGAG GAGTCTAAGC ACATCATGGA TGACTAATTT TTGATGTATT GTAACTACGT ATTGAGATAA   
  
  
+ CTTGAATGAA TCGTGACTAG AATATATCTA TTTTCAAAAA TAAAAATTCC CTAAAAGAAA AGGATAAAAA   
  
  
+ ACTATATAAT ACTAAAAAAT TCTAACTATA ATAACTAGGC TTATTAAATT TTTATAGTGA AAATTTATTT   
  
  
+ CTACTTATAA CAACATCACT TACTAAGTAG CTTATTTGTG TCTTCTTTTC TAAATAATAA TGGTAACTAT   
  
  
+ GTGTCTTTTG TAAATAACTT ATTATCTATT TTATAAATCA CCGTGTAAAG CACGGGTCTA TACTAGTTAA   
  
  
+ TTTAAATAGC ATTGTTAAAT CAAGTAAGAT ACAAACAAAT TTATTTACAA CTGTAAGTGG TCCATAAAAA   
  
  
+ TAATAGCCAT TACAAAAAAT AGTACTACTG TTTTATGAAA TGATTTTAAT ATTCTAAAAT TCCAATAAAA   
  
  
+ CATGTTATAA TCTATGATTA ATTTTAAAAC ATGATTACAT GTCAGCATAA TTTGTATTAT TGTACAGCAA   
  
  
+ ATGAAAGTTG GAGCTAAAAG AAAATGGGGA AATAAAGGCA GACAAGTAAA GATAAAATAT GCTATCTACT   
  
  
+ TTTGGAGGTA TCCTTAACCA CATTGTCTCG TTTGTCTTCA TTAGGGAGAA GTGGGATAAA TACCAAAATT   
  
  
+ TGTATGGTCA TTGCGTGGGG TTTGCTTCCA AACAAAATGG CTTTTTGGAA GCAAATTTTG TGAGCTTCTA   
  
  
+ TGCAAATTTC CACGCTTAAC CCCACAAGCA AACAAAAACA GACAGTGGTT TTTGTTTGTC TTTGTTAGGG   
  
  
+ CATGTTTGGA GCCAACTGTT AATGGGAATA AACTGGGGAG GCTTTTTCCT TGGCTCTTTC CCCCTGACCC   
  
  
+ CAAACCCACT TCAGCTCAAT ACGAAACCAG CCATTGAAAA AGGAAGGGGG GGGGGGGGGT ATGTGAGAAA   
  
  
+ AAGACAAAAC TTTTTCCCGG AGAGAGAAGA TACATATGCA ACAAAGGCTC TCCTTCTCAT AAATTCCATA   
  
  
+ TTCCTCTGTT TTTCTAGGAG AGAGAAATAT GCAAAAAACA AATCAGTTGG CGACAAAAGT TACTACCACG   
  
  
+ CATTGTCTAC TGCTGCGTAC CCATTTTTGG AGACTTTAAA GAACGCCCCT CCCATCTTGA AAAGTGAAAA   
  
  
+ CCCCCTCTTT GAAACTGAAT TTCAGGGGAA AAAAACCTCA CCAAACCTCT AGAGAGAGCT TTTAGAGAGG   
  
  
+ GAAATACAAC GATTAGAAGG AGCAATTTGG GAAATTTCTT TGGGAATTTG AATGGGTTTT GAGTGAATTG   
  
  
+ CAAATCCCAG AAAAGTTTTG GCAAGTACCG ATCTACAGTT CTCTCCTCTT CGTGTTTGGT AGATCCCCTG   
  
  
+ TTTCCTCTGT TTCATTTAGG GTACTTCTCA TTCATCCTCC CCACCCCCTT AATCGGATCT TCCTGTCGAG   
  
  
+ TCACTTTATG CTAATATTTT TTCAGTGGAT TTTTAGTTAA CCCTGTTCAT TTTTCCATTC TGTGTCCCAT   
  
  
+ TTCTCTCTTT TTCATTCATA AGTTGCCGGT TTATCTGTTG GGTGCAGCTT AGTCACAATA ATTTCTGTGT   
  
  
+ TAGGCTTCTT TACGGTTAAA AAAAAAGGAG GCACTCTTTT CGGTGTGATT GTTTATGGGA CCAATGATTC   
  
  
+ AAGATGATGG GTCATCAGTA ACTTCATCAC CCCTTCAATT TTTCTCCATG ATGTCTCCCA ATTTAGGTTC   
  
  
+ TTCCTACCCT TGGCTCAGAG AGCTAAAACC TGAAGAAAGA GGTCTTTACT TGATACATTT GTTGCTCTCT   
  
  
+ TGTGCAAATC ATGTCTCTAG TGGTAGCCTA GACAATGCGA ACTTAGCCCT CGAACAAATC TCTCAGCTTG   
  
  
+ CTGCCCCTGA TGGGGATACA ATGCAGCGTA TGGCTTCTTA TTTTGCTGAA GCCCTGGCTG AGAGGATCCT   
  
  
+ CAAGTCATGG CCTGGCATGT ATAGAGCCCT TCATTTTACG AAAATGCCTG TCATTTCAGA GGAAATTCTT   
  
  
+ GCTAGGAAGC TCTTCTTTGA GCTATTTCCT TTCTTGAAGC TGGCCTATTT GGTGACAAAC CAATCGATAA   
  
  
+ TCGAAGCCAT GGAGGGGGAA AAGATGGTTC ATATTATTGA TCTGAATGCA TCAGAACCTG CTCAATGGAT   
  
  
+ TGCCCTTATT CAGGCTTTGA GTGCAAGGCC TGGGGGTCCT CCTCATTTGA GAATTACCGG TGTTCATCAA   
  
  
+ CACAAAGAGG TTCTAGATCA AGTGGCTCAT AGGGTGACTC AAGAAGCTGA GAAATTGGAT TTGCCATTTC   
  
  
+ AGTTCAATCC TGTGGTTAGC AAGTTGGAAA ACCTTGATGT TGAAAAGCTG TGTGTTAAGA CTGGTGAGGC   
  
  
+ TCTAGCCATC AGTTCGGTCC TTCAACTGCA TACCCTTTTG GGTTCTGATG ATGAGCCCCT AAGGAAAAGT   
  
  
+ TCACCTTTAG CCTTGATGAA GTATGCAAAT GGGGCTAATA GGCAAAGCCC GAGTAATGAT TCGGCTTCTT   
  
  
+ CATCACCTCC TTCGCTCAAT ACTTCAACCA AGCTGGATGG TTTCCTCAGC GCTTTGTGGG GATTGTCCCC   
  
  
+ AAAGATTATG GTGATAGCTG AGCACGATTC CAATCACAAT GGTTCTGGAC TTATGGAGAG GTTGTCAGAA   
  
  
+ GCACTGTACT TCTATGCAGC GCTGTTTGAC TGCTTAGAAT CCACCCTGCC AAGAACATCT GTCGAAAGAA   
  
  
+ GGCGGGTAGA GAAGATGCTC CTAGGTGAAG AGATCAAGAA CATTATATCA TGCGAGGGAG GAGAAAGGAG   
  
  
+ AGAAAGGCAT GAGAAGATCG AGAAGTGGAT GCAGAGGCTA GACATGGCTG GATTCGGGAT CGTTCCTTTG   
  
  
+ AGCTATATGG GTATGCTGCA AGCAAGGCAA TTGCTTCAGG GCTATGGTTG TGATGGTTAT AGAGTGAAAG   
  
  
+ AGAATGGTTG TGTTGTCATC TGTTGGCAAG ATCGCCCCCT CTTTTCGGTA TCAGCATGGA GGTGTAGGAG   
  
  
+ ATG  

- -Up\_Stream \_Len000TACGCG GAAAGACGAT TGAGTAATAG AAAGGAAGAC TCTCCCCTGT TGAATATATT   
  
  
- TAACTATCCA TAAAAGTTTA GTTATCTGGT CGATGGTATA TACTATTAAA AATAATGAAA AGATTTAGGT   
  
  
- AGTTGATAAA AGTGTTAGGA GGAAAAAGAT CTTTTTATTT TAAACGAATT AAGTTTAGTG GATTTTAATG   
  
  
- ATTTAGATAT CAGGATTTAG ATGTTTGAGT AAACACAACG TTGAGTACTA AATTCACCTT GTGATTGGAA   
  
  
- TCCTCTATAC AACAGTTTTC TTCTTAAGCT ACTGTATAAA AGGTTGTTTT CCGGTGGATT GAAGTACTAA   
  
  
- ATTTTTCCTC CTCAGATTCG TGTAGTACCT ACTGATTAAA AACTACATAA CATTGATGCA TAACTCTATT   
  
  
- GAACTTACTT AGCACTGATC TTATATAGAT AAAAGTTTTT ATTTTTAAGG GATTTTCTTT TCCTATTTTT   
  
  
- TGATATATTA TGATTTTTTA AGATTGATAT TATTGATCCG AATAATTTAA AAATATCACT TTTAAATAAA   
  
  
- GATGAATATT GTTGTAGTGA ATGATTCATC GAATAAACAC AGAAGAAAAG ATTTATTATT ACCATTGATA   
  
  
- CACAGAAAAC ATTTATTGAA TAATAGATAA AATATTTAGT GGCACATTTC GTGCCCAGAT ATGATCAATT   
  
  
- AAATTTATCG TAACAATTTA GTTCATTCTA TGTTTGTTTA AATAAATGTT GACATTCACC AGGTATTTTT   
  
  
- ATTATCGGTA ATGTTTTTTA TCATGATGAC AAAATACTTT ACTAAAATTA TAAGATTTTA AGGTTATTTT   
  
  
- GTACAATATT AGATACTAAT TAAAATTTTG TACTAATGTA CAGTCGTATT AAACATAATA ACATGTCGTT   
  
  
- TACTTTCAAC CTCGATTTTC TTTTACCCCT TTATTTCCGT CTGTTCATTT CTATTTTATA CGATAGATGA   
  
  
- AAACCTCCAT AGGAATTGGT GTAACAGAGC AAACAGAAGT AATCCCTCTT CACCCTATTT ATGGTTTTAA   
  
  
- ACATACCAGT AACGCACCCC AAACGAAGGT TTGTTTTACC GAAAAACCTT CGTTTAAAAC ACTCGAAGAT   
  
  
- ACGTTTAAAG GTGCGAATTG GGGTGTTCGT TTGTTTTTGT CTGTCACCAA AAACAAACAG AAACAATCCC   
  
  
- GTACAAACCT CGGTTGACAA TTACCCTTAT TTGACCCCTC CGAAAAAGGA ACCGAGAAAG GGGGACTGGG   
  
  
- GTTTGGGTGA AGTCGAGTTA TGCTTTGGTC GGTAACTTTT TCCTTCCCCC CCCCCCCCCA TACACTCTTT   
  
  
- TTCTGTTTTG AAAAAGGGCC TCTCTCTTCT ATGTATACGT TGTTTCCGAG AGGAAGAGTA TTTAAGGTAT   
  
  
- AAGGAGACAA AAAGATCCTC TCTCTTTATA CGTTTTTTGT TTAGTCAACC GCTGTTTTCA ATGATGGTGC   
  
  
- GTAACAGATG ACGACGCATG GGTAAAAACC TCTGAAATTT CTTGCGGGGA GGGTAGAACT TTTCACTTTT   
  
  
- GGGGGAGAAA CTTTGACTTA AAGTCCCCTT TTTTTGGAGT GGTTTGGAGA TCTCTCTCGA AAATCTCTCC   
  
  
- CTTTATGTTG CTAATCTTCC TCGTTAAACC CTTTAAAGAA ACCCTTAAAC TTACCCAAAA CTCACTTAAC   
  
  
- GTTTAGGGTC TTTTCAAAAC CGTTCATGGC TAGATGTCAA GAGAGGAGAA GCACAAACCA TCTAGGGGAC   
  
  
- AAAGGAGACA AAGTAAATCC CATGAAGAGT AAGTAGGAGG GGTGGGGGAA TTAGCCTAGA AGGACAGCTC   
  
  
- AGTGAAATAC GATTATAAAA AAGTCACCTA AAAATCAATT GGGACAAGTA AAAAGGTAAG ACACAGGGTA   
  
  
- AAGAGAGAAA AAGTAAGTAT TCAACGGCCA AATAGACAAC CCACGTCGAA TCAGTGTTAT TAAAGACACA   
  
  
- ATCCGAAGAA ATGCCAATTT TTTTTTCCTC CGTGAGAAAA GCCACACTAA CAAATACCCT GGTTACTAAG   
  
  
- TTCTACTACC CAGTAGTCAT TGAAGTAGTG GGGAAGTTAA AAAGAGGTAC TACAGAGGGT TAAATCCAAG   
  
  
- AAGGATGGGA ACCGAGTCTC TCGATTTTGG ACTTCTTTCT CCAGAAATGA ACTATGTAAA CAACGAGAGA   
  
  
- ACACGTTTAG TACAGAGATC ACCATCGGAT CTGTTACGCT TGAATCGGGA GCTTGTTTAG AGAGTCGAAC   
  
  
- GACGGGGACT ACCCCTATGT TACGTCGCAT ACCGAAGAAT AAAACGACTT CGGGACCGAC TCTCCTAGGA   
  
  
- GTTCAGTACC GGACCGTACA TATCTCGGGA AGTAAAATGC TTTTACGGAC AGTAAAGTCT CCTTTAAGAA   
  
  
- CGATCCTTCG AGAAGAAACT CGATAAAGGA AAGAACTTCG ACCGGATAAA CCACTGTTTG GTTAGCTATT   
  
  
- AGCTTCGGTA CCTCCCCCTT TTCTACCAAG TATAATAACT AGACTTACGT AGTCTTGGAC GAGTTACCTA   
  
  
- ACGGGAATAA GTCCGAAACT CACGTTCCGG ACCCCCAGGA GGAGTAAACT CTTAATGGCC ACAAGTAGTT   
  
  
- GTGTTTCTCC AAGATCTAGT TCACCGAGTA TCCCACTGAG TTCTTCGACT CTTTAACCTA AACGGTAAAG   
  
  
- TCAAGTTAGG ACACCAATCG TTCAACCTTT TGGAACTACA ACTTTTCGAC ACACAATTCT GACCACTCCG   
  
  
- AGATCGGTAG TCAAGCCAGG AAGTTGACGT ATGGGAAAAC CCAAGACTAC TACTCGGGGA TTCCTTTTCA   
  
  
- AGTGGAAATC GGAACTACTT CATACGTTTA CCCCGATTAT CCGTTTCGGG CTCATTACTA AGCCGAAGAA   
  
  
- GTAGTGGAGG AAGCGAGTTA TGAAGTTGGT TCGACCTACC AAAGGAGTCG CGAAACACCC CTAACAGGGG   
  
  
- TTTCTAATAC CACTATCGAC TCGTGCTAAG GTTAGTGTTA CCAAGACCTG AATACCTCTC CAACAGTCTT   
  
  
- CGTGACATGA AGATACGTCG CGACAAACTG ACGAATCTTA GGTGGGACGG TTCTTGTAGA CAGCTTTCTT   
  
  
- CCGCCCATCT CTTCTACGAG GATCCACTTC TCTAGTTCTT GTAATATAGT ACGCTCCCTC CTCTTTCCTC   
  
  
- TCTTTCCGTA CTCTTCTAGC TCTTCACCTA CGTCTCCGAT CTGTACCGAC CTAAGCCCTA GCAAGGAAAC   
  
  
- TCGATATACC CATACGACGT TCGTTCCGTT AACGAAGTCC CGATACCAAC ACTACCAATA TCTCACTTTC   
  
  
- TCTTACCAAC ACAACAGTAG ACAACCGTTC TAGCGGGGGA GAAAAGCCAT AGTCGTACCT CCACATCCTC   
  
  
- TACT

+     AAGAA-motif

| Site Name | Organism | Position | Strand | Matrix score. | sequence | function |
| --- | --- | --- | --- | --- | --- | --- |
| AAGAA-motif | Avena sativa | 3078 | + | 7 | GAAAGAA |  |

>HU08G00367.1   
+ -Up\_Stream \_Len000ATGCGC CTTTCTGCTA ACTCATTATC TTTCCTTCTG AGAGGGGACA ACTTATATAA   
  
  
+ ATTGATAGGT ATTTTCAAAT CAATAGACCA GCTACCATAT ATGATAATTT TTATTACTTT TCTAAATCCA   
  
  
+ TCAACTATTT TCACAATCCT CCTTTTTCTA GAAAAATAAA ATTTGCTTAA TTCAAATCAC CTAAAATTAC   
  
  
+ TAAATCTATA GTCCTAAATC TACAAACTCA TTTGTGTTGC AACTCATGAT TTAAGTGGAA CACTAACCTT   
  
  
+ AGGAGATATG TTGTCAAAAG AAGAATTCGA TGACATATTT TCCAACAAAA GGCCACCTAA CTTCATGATT   
  
  
+ TAAAAAGGAG GAGTCTAAGC ACATCATGGA TGACTAATTT TTGATGTATT GTAACTACGT ATTGAGATAA   
  
  
+ CTTGAATGAA TCGTGACTAG AATATATCTA TTTTCAAAAA TAAAAATTCC CTAAAAGAAA AGGATAAAAA   
  
  
+ ACTATATAAT ACTAAAAAAT TCTAACTATA ATAACTAGGC TTATTAAATT TTTATAGTGA AAATTTATTT   
  
  
+ CTACTTATAA CAACATCACT TACTAAGTAG CTTATTTGTG TCTTCTTTTC TAAATAATAA TGGTAACTAT   
  
  
+ GTGTCTTTTG TAAATAACTT ATTATCTATT TTATAAATCA CCGTGTAAAG CACGGGTCTA TACTAGTTAA   
  
  
+ TTTAAATAGC ATTGTTAAAT CAAGTAAGAT ACAAACAAAT TTATTTACAA CTGTAAGTGG TCCATAAAAA   
  
  
+ TAATAGCCAT TACAAAAAAT AGTACTACTG TTTTATGAAA TGATTTTAAT ATTCTAAAAT TCCAATAAAA   
  
  
+ CATGTTATAA TCTATGATTA ATTTTAAAAC ATGATTACAT GTCAGCATAA TTTGTATTAT TGTACAGCAA   
  
  
+ ATGAAAGTTG GAGCTAAAAG AAAATGGGGA AATAAAGGCA GACAAGTAAA GATAAAATAT GCTATCTACT   
  
  
+ TTTGGAGGTA TCCTTAACCA CATTGTCTCG TTTGTCTTCA TTAGGGAGAA GTGGGATAAA TACCAAAATT   
  
  
+ TGTATGGTCA TTGCGTGGGG TTTGCTTCCA AACAAAATGG CTTTTTGGAA GCAAATTTTG TGAGCTTCTA   
  
  
+ TGCAAATTTC CACGCTTAAC CCCACAAGCA AACAAAAACA GACAGTGGTT TTTGTTTGTC TTTGTTAGGG   
  
  
+ CATGTTTGGA GCCAACTGTT AATGGGAATA AACTGGGGAG GCTTTTTCCT TGGCTCTTTC CCCCTGACCC   
  
  
+ CAAACCCACT TCAGCTCAAT ACGAAACCAG CCATTGAAAA AGGAAGGGGG GGGGGGGGGT ATGTGAGAAA   
  
  
+ AAGACAAAAC TTTTTCCCGG AGAGAGAAGA TACATATGCA ACAAAGGCTC TCCTTCTCAT AAATTCCATA   
  
  
+ TTCCTCTGTT TTTCTAGGAG AGAGAAATAT GCAAAAAACA AATCAGTTGG CGACAAAAGT TACTACCACG   
  
  
+ CATTGTCTAC TGCTGCGTAC CCATTTTTGG AGACTTTAAA GAACGCCCCT CCCATCTTGA AAAGTGAAAA   
  
  
+ CCCCCTCTTT GAAACTGAAT TTCAGGGGAA AAAAACCTCA CCAAACCTCT AGAGAGAGCT TTTAGAGAGG   
  
  
+ GAAATACAAC GATTAGAAGG AGCAATTTGG GAAATTTCTT TGGGAATTTG AATGGGTTTT GAGTGAATTG   
  
  
+ CAAATCCCAG AAAAGTTTTG GCAAGTACCG ATCTACAGTT CTCTCCTCTT CGTGTTTGGT AGATCCCCTG   
  
  
+ TTTCCTCTGT TTCATTTAGG GTACTTCTCA TTCATCCTCC CCACCCCCTT AATCGGATCT TCCTGTCGAG   
  
  
+ TCACTTTATG CTAATATTTT TTCAGTGGAT TTTTAGTTAA CCCTGTTCAT TTTTCCATTC TGTGTCCCAT   
  
  
+ TTCTCTCTTT TTCATTCATA AGTTGCCGGT TTATCTGTTG GGTGCAGCTT AGTCACAATA ATTTCTGTGT   
  
  
+ TAGGCTTCTT TACGGTTAAA AAAAAAGGAG GCACTCTTTT CGGTGTGATT GTTTATGGGA CCAATGATTC   
  
  
+ AAGATGATGG GTCATCAGTA ACTTCATCAC CCCTTCAATT TTTCTCCATG ATGTCTCCCA ATTTAGGTTC   
  
  
+ TTCCTACCCT TGGCTCAGAG AGCTAAAACC TGAAGAAAGA GGTCTTTACT TGATACATTT GTTGCTCTCT   
  
  
+ TGTGCAAATC ATGTCTCTAG TGGTAGCCTA GACAATGCGA ACTTAGCCCT CGAACAAATC TCTCAGCTTG   
  
  
+ CTGCCCCTGA TGGGGATACA ATGCAGCGTA TGGCTTCTTA TTTTGCTGAA GCCCTGGCTG AGAGGATCCT   
  
  
+ CAAGTCATGG CCTGGCATGT ATAGAGCCCT TCATTTTACG AAAATGCCTG TCATTTCAGA GGAAATTCTT   
  
  
+ GCTAGGAAGC TCTTCTTTGA GCTATTTCCT TTCTTGAAGC TGGCCTATTT GGTGACAAAC CAATCGATAA   
  
  
+ TCGAAGCCAT GGAGGGGGAA AAGATGGTTC ATATTATTGA TCTGAATGCA TCAGAACCTG CTCAATGGAT   
  
  
+ TGCCCTTATT CAGGCTTTGA GTGCAAGGCC TGGGGGTCCT CCTCATTTGA GAATTACCGG TGTTCATCAA   
  
  
+ CACAAAGAGG TTCTAGATCA AGTGGCTCAT AGGGTGACTC AAGAAGCTGA GAAATTGGAT TTGCCATTTC   
  
  
+ AGTTCAATCC TGTGGTTAGC AAGTTGGAAA ACCTTGATGT TGAAAAGCTG TGTGTTAAGA CTGGTGAGGC   
  
  
+ TCTAGCCATC AGTTCGGTCC TTCAACTGCA TACCCTTTTG GGTTCTGATG ATGAGCCCCT AAGGAAAAGT   
  
  
+ TCACCTTTAG CCTTGATGAA GTATGCAAAT GGGGCTAATA GGCAAAGCCC GAGTAATGAT TCGGCTTCTT   
  
  
+ CATCACCTCC TTCGCTCAAT ACTTCAACCA AGCTGGATGG TTTCCTCAGC GCTTTGTGGG GATTGTCCCC   
  
  
+ AAAGATTATG GTGATAGCTG AGCACGATTC CAATCACAAT GGTTCTGGAC TTATGGAGAG GTTGTCAGAA   
  
  
+ GCACTGTACT TCTATGCAGC GCTGTTTGAC TGCTTAGAAT CCACCCTGCC AAGAACATCT GTCGAAAGAA   
  
  
+ GGCGGGTAGA GAAGATGCTC CTAGGTGAAG AGATCAAGAA CATTATATCA TGCGAGGGAG GAGAAAGGAG   
  
  
+ AGAAAGGCAT GAGAAGATCG AGAAGTGGAT GCAGAGGCTA GACATGGCTG GATTCGGGAT CGTTCCTTTG   
  
  
+ AGCTATATGG GTATGCTGCA AGCAAGGCAA TTGCTTCAGG GCTATGGTTG TGATGGTTAT AGAGTGAAAG   
  
  
+ AGAATGGTTG TGTTGTCATC TGTTGGCAAG ATCGCCCCCT CTTTTCGGTA TCAGCATGGA GGTGTAGGAG   
  
  
+ ATG  

- -Up\_Stream \_Len000TACGCG GAAAGACGAT TGAGTAATAG AAAGGAAGAC TCTCCCCTGT TGAATATATT   
  
  
- TAACTATCCA TAAAAGTTTA GTTATCTGGT CGATGGTATA TACTATTAAA AATAATGAAA AGATTTAGGT   
  
  
- AGTTGATAAA AGTGTTAGGA GGAAAAAGAT CTTTTTATTT TAAACGAATT AAGTTTAGTG GATTTTAATG   
  
  
- ATTTAGATAT CAGGATTTAG ATGTTTGAGT AAACACAACG TTGAGTACTA AATTCACCTT GTGATTGGAA   
  
  
- TCCTCTATAC AACAGTTTTC TTCTTAAGCT ACTGTATAAA AGGTTGTTTT CCGGTGGATT GAAGTACTAA   
  
  
- ATTTTTCCTC CTCAGATTCG TGTAGTACCT ACTGATTAAA AACTACATAA CATTGATGCA TAACTCTATT   
  
  
- GAACTTACTT AGCACTGATC TTATATAGAT AAAAGTTTTT ATTTTTAAGG GATTTTCTTT TCCTATTTTT   
  
  
- TGATATATTA TGATTTTTTA AGATTGATAT TATTGATCCG AATAATTTAA AAATATCACT TTTAAATAAA   
  
  
- GATGAATATT GTTGTAGTGA ATGATTCATC GAATAAACAC AGAAGAAAAG ATTTATTATT ACCATTGATA   
  
  
- CACAGAAAAC ATTTATTGAA TAATAGATAA AATATTTAGT GGCACATTTC GTGCCCAGAT ATGATCAATT   
  
  
- AAATTTATCG TAACAATTTA GTTCATTCTA TGTTTGTTTA AATAAATGTT GACATTCACC AGGTATTTTT   
  
  
- ATTATCGGTA ATGTTTTTTA TCATGATGAC AAAATACTTT ACTAAAATTA TAAGATTTTA AGGTTATTTT   
  
  
- GTACAATATT AGATACTAAT TAAAATTTTG TACTAATGTA CAGTCGTATT AAACATAATA ACATGTCGTT   
  
  
- TACTTTCAAC CTCGATTTTC TTTTACCCCT TTATTTCCGT CTGTTCATTT CTATTTTATA CGATAGATGA   
  
  
- AAACCTCCAT AGGAATTGGT GTAACAGAGC AAACAGAAGT AATCCCTCTT CACCCTATTT ATGGTTTTAA   
  
  
- ACATACCAGT AACGCACCCC AAACGAAGGT TTGTTTTACC GAAAAACCTT CGTTTAAAAC ACTCGAAGAT   
  
  
- ACGTTTAAAG GTGCGAATTG GGGTGTTCGT TTGTTTTTGT CTGTCACCAA AAACAAACAG AAACAATCCC   
  
  
- GTACAAACCT CGGTTGACAA TTACCCTTAT TTGACCCCTC CGAAAAAGGA ACCGAGAAAG GGGGACTGGG   
  
  
- GTTTGGGTGA AGTCGAGTTA TGCTTTGGTC GGTAACTTTT TCCTTCCCCC CCCCCCCCCA TACACTCTTT   
  
  
- TTCTGTTTTG AAAAAGGGCC TCTCTCTTCT ATGTATACGT TGTTTCCGAG AGGAAGAGTA TTTAAGGTAT   
  
  
- AAGGAGACAA AAAGATCCTC TCTCTTTATA CGTTTTTTGT TTAGTCAACC GCTGTTTTCA ATGATGGTGC   
  
  
- GTAACAGATG ACGACGCATG GGTAAAAACC TCTGAAATTT CTTGCGGGGA GGGTAGAACT TTTCACTTTT   
  
  
- GGGGGAGAAA CTTTGACTTA AAGTCCCCTT TTTTTGGAGT GGTTTGGAGA TCTCTCTCGA AAATCTCTCC   
  
  
- CTTTATGTTG CTAATCTTCC TCGTTAAACC CTTTAAAGAA ACCCTTAAAC TTACCCAAAA CTCACTTAAC   
  
  
- GTTTAGGGTC TTTTCAAAAC CGTTCATGGC TAGATGTCAA GAGAGGAGAA GCACAAACCA TCTAGGGGAC   
  
  
- AAAGGAGACA AAGTAAATCC CATGAAGAGT AAGTAGGAGG GGTGGGGGAA TTAGCCTAGA AGGACAGCTC   
  
  
- AGTGAAATAC GATTATAAAA AAGTCACCTA AAAATCAATT GGGACAAGTA AAAAGGTAAG ACACAGGGTA   
  
  
- AAGAGAGAAA AAGTAAGTAT TCAACGGCCA AATAGACAAC CCACGTCGAA TCAGTGTTAT TAAAGACACA   
  
  
- ATCCGAAGAA ATGCCAATTT TTTTTTCCTC CGTGAGAAAA GCCACACTAA CAAATACCCT GGTTACTAAG   
  
  
- TTCTACTACC CAGTAGTCAT TGAAGTAGTG GGGAAGTTAA AAAGAGGTAC TACAGAGGGT TAAATCCAAG   
  
  
- AAGGATGGGA ACCGAGTCTC TCGATTTTGG ACTTCTTTCT CCAGAAATGA ACTATGTAAA CAACGAGAGA   
  
  
- ACACGTTTAG TACAGAGATC ACCATCGGAT CTGTTACGCT TGAATCGGGA GCTTGTTTAG AGAGTCGAAC   
  
  
- GACGGGGACT ACCCCTATGT TACGTCGCAT ACCGAAGAAT AAAACGACTT CGGGACCGAC TCTCCTAGGA   
  
  
- GTTCAGTACC GGACCGTACA TATCTCGGGA AGTAAAATGC TTTTACGGAC AGTAAAGTCT CCTTTAAGAA   
  
  
- CGATCCTTCG AGAAGAAACT CGATAAAGGA AAGAACTTCG ACCGGATAAA CCACTGTTTG GTTAGCTATT   
  
  
- AGCTTCGGTA CCTCCCCCTT TTCTACCAAG TATAATAACT AGACTTACGT AGTCTTGGAC GAGTTACCTA   
  
  
- ACGGGAATAA GTCCGAAACT CACGTTCCGG ACCCCCAGGA GGAGTAAACT CTTAATGGCC ACAAGTAGTT   
  
  
- GTGTTTCTCC AAGATCTAGT TCACCGAGTA TCCCACTGAG TTCTTCGACT CTTTAACCTA AACGGTAAAG   
  
  
- TCAAGTTAGG ACACCAATCG TTCAACCTTT TGGAACTACA ACTTTTCGAC ACACAATTCT GACCACTCCG   
  
  
- AGATCGGTAG TCAAGCCAGG AAGTTGACGT ATGGGAAAAC CCAAGACTAC TACTCGGGGA TTCCTTTTCA   
  
  
- AGTGGAAATC GGAACTACTT CATACGTTTA CCCCGATTAT CCGTTTCGGG CTCATTACTA AGCCGAAGAA   
  
  
- GTAGTGGAGG AAGCGAGTTA TGAAGTTGGT TCGACCTACC AAAGGAGTCG CGAAACACCC CTAACAGGGG   
  
  
- TTTCTAATAC CACTATCGAC TCGTGCTAAG GTTAGTGTTA CCAAGACCTG AATACCTCTC CAACAGTCTT   
  
  
- CGTGACATGA AGATACGTCG CGACAAACTG ACGAATCTTA GGTGGGACGG TTCTTGTAGA CAGCTTTCTT   
  
  
- CCGCCCATCT CTTCTACGAG GATCCACTTC TCTAGTTCTT GTAATATAGT ACGCTCCCTC CTCTTTCCTC   
  
  
- TCTTTCCGTA CTCTTCTAGC TCTTCACCTA CGTCTCCGAT CTGTACCGAC CTAAGCCCTA GCAAGGAAAC   
  
  
- TCGATATACC CATACGACGT TCGTTCCGTT AACGAAGTCC CGATACCAAC ACTACCAATA TCTCACTTTC   
  
  
- TCTTACCAAC ACAACAGTAG ACAACCGTTC TAGCGGGGGA GAAAAGCCAT AGTCGTACCT CCACATCCTC   
  
  
- TAC

+     ACA-motif

| Site Name | Organism | Position | Strand | Matrix score. | sequence | function |
| --- | --- | --- | --- | --- | --- | --- |
| ACA-motif | Arabidopsis thaliana | 3267 | - | 12 | AATCACAACCATA | part of gapA in (gapA-CMA1) involved with light responsiveness |

>HU08G00367.1   
+ -Up\_Stream \_Len000ATGCGC CTTTCTGCTA ACTCATTATC TTTCCTTCTG AGAGGGGACA ACTTATATAA   
  
  
+ ATTGATAGGT ATTTTCAAAT CAATAGACCA GCTACCATAT ATGATAATTT TTATTACTTT TCTAAATCCA   
  
  
+ TCAACTATTT TCACAATCCT CCTTTTTCTA GAAAAATAAA ATTTGCTTAA TTCAAATCAC CTAAAATTAC   
  
  
+ TAAATCTATA GTCCTAAATC TACAAACTCA TTTGTGTTGC AACTCATGAT TTAAGTGGAA CACTAACCTT   
  
  
+ AGGAGATATG TTGTCAAAAG AAGAATTCGA TGACATATTT TCCAACAAAA GGCCACCTAA CTTCATGATT   
  
  
+ TAAAAAGGAG GAGTCTAAGC ACATCATGGA TGACTAATTT TTGATGTATT GTAACTACGT ATTGAGATAA   
  
  
+ CTTGAATGAA TCGTGACTAG AATATATCTA TTTTCAAAAA TAAAAATTCC CTAAAAGAAA AGGATAAAAA   
  
  
+ ACTATATAAT ACTAAAAAAT TCTAACTATA ATAACTAGGC TTATTAAATT TTTATAGTGA AAATTTATTT   
  
  
+ CTACTTATAA CAACATCACT TACTAAGTAG CTTATTTGTG TCTTCTTTTC TAAATAATAA TGGTAACTAT   
  
  
+ GTGTCTTTTG TAAATAACTT ATTATCTATT TTATAAATCA CCGTGTAAAG CACGGGTCTA TACTAGTTAA   
  
  
+ TTTAAATAGC ATTGTTAAAT CAAGTAAGAT ACAAACAAAT TTATTTACAA CTGTAAGTGG TCCATAAAAA   
  
  
+ TAATAGCCAT TACAAAAAAT AGTACTACTG TTTTATGAAA TGATTTTAAT ATTCTAAAAT TCCAATAAAA   
  
  
+ CATGTTATAA TCTATGATTA ATTTTAAAAC ATGATTACAT GTCAGCATAA TTTGTATTAT TGTACAGCAA   
  
  
+ ATGAAAGTTG GAGCTAAAAG AAAATGGGGA AATAAAGGCA GACAAGTAAA GATAAAATAT GCTATCTACT   
  
  
+ TTTGGAGGTA TCCTTAACCA CATTGTCTCG TTTGTCTTCA TTAGGGAGAA GTGGGATAAA TACCAAAATT   
  
  
+ TGTATGGTCA TTGCGTGGGG TTTGCTTCCA AACAAAATGG CTTTTTGGAA GCAAATTTTG TGAGCTTCTA   
  
  
+ TGCAAATTTC CACGCTTAAC CCCACAAGCA AACAAAAACA GACAGTGGTT TTTGTTTGTC TTTGTTAGGG   
  
  
+ CATGTTTGGA GCCAACTGTT AATGGGAATA AACTGGGGAG GCTTTTTCCT TGGCTCTTTC CCCCTGACCC   
  
  
+ CAAACCCACT TCAGCTCAAT ACGAAACCAG CCATTGAAAA AGGAAGGGGG GGGGGGGGGT ATGTGAGAAA   
  
  
+ AAGACAAAAC TTTTTCCCGG AGAGAGAAGA TACATATGCA ACAAAGGCTC TCCTTCTCAT AAATTCCATA   
  
  
+ TTCCTCTGTT TTTCTAGGAG AGAGAAATAT GCAAAAAACA AATCAGTTGG CGACAAAAGT TACTACCACG   
  
  
+ CATTGTCTAC TGCTGCGTAC CCATTTTTGG AGACTTTAAA GAACGCCCCT CCCATCTTGA AAAGTGAAAA   
  
  
+ CCCCCTCTTT GAAACTGAAT TTCAGGGGAA AAAAACCTCA CCAAACCTCT AGAGAGAGCT TTTAGAGAGG   
  
  
+ GAAATACAAC GATTAGAAGG AGCAATTTGG GAAATTTCTT TGGGAATTTG AATGGGTTTT GAGTGAATTG   
  
  
+ CAAATCCCAG AAAAGTTTTG GCAAGTACCG ATCTACAGTT CTCTCCTCTT CGTGTTTGGT AGATCCCCTG   
  
  
+ TTTCCTCTGT TTCATTTAGG GTACTTCTCA TTCATCCTCC CCACCCCCTT AATCGGATCT TCCTGTCGAG   
  
  
+ TCACTTTATG CTAATATTTT TTCAGTGGAT TTTTAGTTAA CCCTGTTCAT TTTTCCATTC TGTGTCCCAT   
  
  
+ TTCTCTCTTT TTCATTCATA AGTTGCCGGT TTATCTGTTG GGTGCAGCTT AGTCACAATA ATTTCTGTGT   
  
  
+ TAGGCTTCTT TACGGTTAAA AAAAAAGGAG GCACTCTTTT CGGTGTGATT GTTTATGGGA CCAATGATTC   
  
  
+ AAGATGATGG GTCATCAGTA ACTTCATCAC CCCTTCAATT TTTCTCCATG ATGTCTCCCA ATTTAGGTTC   
  
  
+ TTCCTACCCT TGGCTCAGAG AGCTAAAACC TGAAGAAAGA GGTCTTTACT TGATACATTT GTTGCTCTCT   
  
  
+ TGTGCAAATC ATGTCTCTAG TGGTAGCCTA GACAATGCGA ACTTAGCCCT CGAACAAATC TCTCAGCTTG   
  
  
+ CTGCCCCTGA TGGGGATACA ATGCAGCGTA TGGCTTCTTA TTTTGCTGAA GCCCTGGCTG AGAGGATCCT   
  
  
+ CAAGTCATGG CCTGGCATGT ATAGAGCCCT TCATTTTACG AAAATGCCTG TCATTTCAGA GGAAATTCTT   
  
  
+ GCTAGGAAGC TCTTCTTTGA GCTATTTCCT TTCTTGAAGC TGGCCTATTT GGTGACAAAC CAATCGATAA   
  
  
+ TCGAAGCCAT GGAGGGGGAA AAGATGGTTC ATATTATTGA TCTGAATGCA TCAGAACCTG CTCAATGGAT   
  
  
+ TGCCCTTATT CAGGCTTTGA GTGCAAGGCC TGGGGGTCCT CCTCATTTGA GAATTACCGG TGTTCATCAA   
  
  
+ CACAAAGAGG TTCTAGATCA AGTGGCTCAT AGGGTGACTC AAGAAGCTGA GAAATTGGAT TTGCCATTTC   
  
  
+ AGTTCAATCC TGTGGTTAGC AAGTTGGAAA ACCTTGATGT TGAAAAGCTG TGTGTTAAGA CTGGTGAGGC   
  
  
+ TCTAGCCATC AGTTCGGTCC TTCAACTGCA TACCCTTTTG GGTTCTGATG ATGAGCCCCT AAGGAAAAGT   
  
  
+ TCACCTTTAG CCTTGATGAA GTATGCAAAT GGGGCTAATA GGCAAAGCCC GAGTAATGAT TCGGCTTCTT   
  
  
+ CATCACCTCC TTCGCTCAAT ACTTCAACCA AGCTGGATGG TTTCCTCAGC GCTTTGTGGG GATTGTCCCC   
  
  
+ AAAGATTATG GTGATAGCTG AGCACGATTC CAATCACAAT GGTTCTGGAC TTATGGAGAG GTTGTCAGAA   
  
  
+ GCACTGTACT TCTATGCAGC GCTGTTTGAC TGCTTAGAAT CCACCCTGCC AAGAACATCT GTCGAAAGAA   
  
  
+ GGCGGGTAGA GAAGATGCTC CTAGGTGAAG AGATCAAGAA CATTATATCA TGCGAGGGAG GAGAAAGGAG   
  
  
+ AGAAAGGCAT GAGAAGATCG AGAAGTGGAT GCAGAGGCTA GACATGGCTG GATTCGGGAT CGTTCCTTTG   
  
  
+ AGCTATATGG GTATGCTGCA AGCAAGGCAA TTGCTTCAGG GCTATGGTTG TGATGGTTAT AGAGTGAAAG   
  
  
+ AGAATGGTTG TGTTGTCATC TGTTGGCAAG ATCGCCCCCT CTTTTCGGTA TCAGCATGGA GGTGTAGGAG   
  
  
+ ATG  

- -Up\_Stream \_Len000TACGCG GAAAGACGAT TGAGTAATAG AAAGGAAGAC TCTCCCCTGT TGAATATATT   
  
  
- TAACTATCCA TAAAAGTTTA GTTATCTGGT CGATGGTATA TACTATTAAA AATAATGAAA AGATTTAGGT   
  
  
- AGTTGATAAA AGTGTTAGGA GGAAAAAGAT CTTTTTATTT TAAACGAATT AAGTTTAGTG GATTTTAATG   
  
  
- ATTTAGATAT CAGGATTTAG ATGTTTGAGT AAACACAACG TTGAGTACTA AATTCACCTT GTGATTGGAA   
  
  
- TCCTCTATAC AACAGTTTTC TTCTTAAGCT ACTGTATAAA AGGTTGTTTT CCGGTGGATT GAAGTACTAA   
  
  
- ATTTTTCCTC CTCAGATTCG TGTAGTACCT ACTGATTAAA AACTACATAA CATTGATGCA TAACTCTATT   
  
  
- GAACTTACTT AGCACTGATC TTATATAGAT AAAAGTTTTT ATTTTTAAGG GATTTTCTTT TCCTATTTTT   
  
  
- TGATATATTA TGATTTTTTA AGATTGATAT TATTGATCCG AATAATTTAA AAATATCACT TTTAAATAAA   
  
  
- GATGAATATT GTTGTAGTGA ATGATTCATC GAATAAACAC AGAAGAAAAG ATTTATTATT ACCATTGATA   
  
  
- CACAGAAAAC ATTTATTGAA TAATAGATAA AATATTTAGT GGCACATTTC GTGCCCAGAT ATGATCAATT   
  
  
- AAATTTATCG TAACAATTTA GTTCATTCTA TGTTTGTTTA AATAAATGTT GACATTCACC AGGTATTTTT   
  
  
- ATTATCGGTA ATGTTTTTTA TCATGATGAC AAAATACTTT ACTAAAATTA TAAGATTTTA AGGTTATTTT   
  
  
- GTACAATATT AGATACTAAT TAAAATTTTG TACTAATGTA CAGTCGTATT AAACATAATA ACATGTCGTT   
  
  
- TACTTTCAAC CTCGATTTTC TTTTACCCCT TTATTTCCGT CTGTTCATTT CTATTTTATA CGATAGATGA   
  
  
- AAACCTCCAT AGGAATTGGT GTAACAGAGC AAACAGAAGT AATCCCTCTT CACCCTATTT ATGGTTTTAA   
  
  
- ACATACCAGT AACGCACCCC AAACGAAGGT TTGTTTTACC GAAAAACCTT CGTTTAAAAC ACTCGAAGAT   
  
  
- ACGTTTAAAG GTGCGAATTG GGGTGTTCGT TTGTTTTTGT CTGTCACCAA AAACAAACAG AAACAATCCC   
  
  
- GTACAAACCT CGGTTGACAA TTACCCTTAT TTGACCCCTC CGAAAAAGGA ACCGAGAAAG GGGGACTGGG   
  
  
- GTTTGGGTGA AGTCGAGTTA TGCTTTGGTC GGTAACTTTT TCCTTCCCCC CCCCCCCCCA TACACTCTTT   
  
  
- TTCTGTTTTG AAAAAGGGCC TCTCTCTTCT ATGTATACGT TGTTTCCGAG AGGAAGAGTA TTTAAGGTAT   
  
  
- AAGGAGACAA AAAGATCCTC TCTCTTTATA CGTTTTTTGT TTAGTCAACC GCTGTTTTCA ATGATGGTGC   
  
  
- GTAACAGATG ACGACGCATG GGTAAAAACC TCTGAAATTT CTTGCGGGGA GGGTAGAACT TTTCACTTTT   
  
  
- GGGGGAGAAA CTTTGACTTA AAGTCCCCTT TTTTTGGAGT GGTTTGGAGA TCTCTCTCGA AAATCTCTCC   
  
  
- CTTTATGTTG CTAATCTTCC TCGTTAAACC CTTTAAAGAA ACCCTTAAAC TTACCCAAAA CTCACTTAAC   
  
  
- GTTTAGGGTC TTTTCAAAAC CGTTCATGGC TAGATGTCAA GAGAGGAGAA GCACAAACCA TCTAGGGGAC   
  
  
- AAAGGAGACA AAGTAAATCC CATGAAGAGT AAGTAGGAGG GGTGGGGGAA TTAGCCTAGA AGGACAGCTC   
  
  
- AGTGAAATAC GATTATAAAA AAGTCACCTA AAAATCAATT GGGACAAGTA AAAAGGTAAG ACACAGGGTA   
  
  
- AAGAGAGAAA AAGTAAGTAT TCAACGGCCA AATAGACAAC CCACGTCGAA TCAGTGTTAT TAAAGACACA   
  
  
- ATCCGAAGAA ATGCCAATTT TTTTTTCCTC CGTGAGAAAA GCCACACTAA CAAATACCCT GGTTACTAAG   
  
  
- TTCTACTACC CAGTAGTCAT TGAAGTAGTG GGGAAGTTAA AAAGAGGTAC TACAGAGGGT TAAATCCAAG   
  
  
- AAGGATGGGA ACCGAGTCTC TCGATTTTGG ACTTCTTTCT CCAGAAATGA ACTATGTAAA CAACGAGAGA   
  
  
- ACACGTTTAG TACAGAGATC ACCATCGGAT CTGTTACGCT TGAATCGGGA GCTTGTTTAG AGAGTCGAAC   
  
  
- GACGGGGACT ACCCCTATGT TACGTCGCAT ACCGAAGAAT AAAACGACTT CGGGACCGAC TCTCCTAGGA   
  
  
- GTTCAGTACC GGACCGTACA TATCTCGGGA AGTAAAATGC TTTTACGGAC AGTAAAGTCT CCTTTAAGAA   
  
  
- CGATCCTTCG AGAAGAAACT CGATAAAGGA AAGAACTTCG ACCGGATAAA CCACTGTTTG GTTAGCTATT   
  
  
- AGCTTCGGTA CCTCCCCCTT TTCTACCAAG TATAATAACT AGACTTACGT AGTCTTGGAC GAGTTACCTA   
  
  
- ACGGGAATAA GTCCGAAACT CACGTTCCGG ACCCCCAGGA GGAGTAAACT CTTAATGGCC ACAAGTAGTT   
  
  
- GTGTTTCTCC AAGATCTAGT TCACCGAGTA TCCCACTGAG TTCTTCGACT CTTTAACCTA AACGGTAAAG   
  
  
- TCAAGTTAGG ACACCAATCG TTCAACCTTT TGGAACTACA ACTTTTCGAC ACACAATTCT GACCACTCCG   
  
  
- AGATCGGTAG TCAAGCCAGG AAGTTGACGT ATGGGAAAAC CCAAGACTAC TACTCGGGGA TTCCTTTTCA   
  
  
- AGTGGAAATC GGAACTACTT CATACGTTTA CCCCGATTAT CCGTTTCGGG CTCATTACTA AGCCGAAGAA   
  
  
- GTAGTGGAGG AAGCGAGTTA TGAAGTTGGT TCGACCTACC AAAGGAGTCG CGAAACACCC CTAACAGGGG   
  
  
- TTTCTAATAC CACTATCGAC TCGTGCTAAG GTTAGTGTTA CCAAGACCTG AATACCTCTC CAACAGTCTT   
  
  
- CGTGACATGA AGATACGTCG CGACAAACTG ACGAATCTTA GGTGGGACGG TTCTTGTAGA CAGCTTTCTT   
  
  
- CCGCCCATCT CTTCTACGAG GATCCACTTC TCTAGTTCTT GTAATATAGT ACGCTCCCTC CTCTTTCCTC   
  
  
- TCTTTCCGTA CTCTTCTAGC TCTTCACCTA CGTCTCCGAT CTGTACCGAC CTAAGCCCTA GCAAGGAAAC   
  
  
- TCGATATACC CATACGACGT TCGTTCCGTT AACGAAGTCC CGATACCAAC ACTACCAATA TCTCACTTTC   
  
  
- TCTTACCAAC ACAACAGTAG ACAACCGTTC TAGCGGGGGA GAAAAGCCAT AGTCGTACCT CCACATCCTC   
  
  
- TAC

+     AP-1

| Site Name | Organism | Position | Strand | Matrix score. | sequence | function |
| --- | --- | --- | --- | --- | --- | --- |
| AP-1 | Arabidopsis thaliana | 32 | - | 8 | TGAGTTAG |  |

>HU08G00367.1   
+ -Up\_Stream \_Len000ATGCGC CTTTCTGCTA ACTCATTATC TTTCCTTCTG AGAGGGGACA ACTTATATAA   
  
  
+ ATTGATAGGT ATTTTCAAAT CAATAGACCA GCTACCATAT ATGATAATTT TTATTACTTT TCTAAATCCA   
  
  
+ TCAACTATTT TCACAATCCT CCTTTTTCTA GAAAAATAAA ATTTGCTTAA TTCAAATCAC CTAAAATTAC   
  
  
+ TAAATCTATA GTCCTAAATC TACAAACTCA TTTGTGTTGC AACTCATGAT TTAAGTGGAA CACTAACCTT   
  
  
+ AGGAGATATG TTGTCAAAAG AAGAATTCGA TGACATATTT TCCAACAAAA GGCCACCTAA CTTCATGATT   
  
  
+ TAAAAAGGAG GAGTCTAAGC ACATCATGGA TGACTAATTT TTGATGTATT GTAACTACGT ATTGAGATAA   
  
  
+ CTTGAATGAA TCGTGACTAG AATATATCTA TTTTCAAAAA TAAAAATTCC CTAAAAGAAA AGGATAAAAA   
  
  
+ ACTATATAAT ACTAAAAAAT TCTAACTATA ATAACTAGGC TTATTAAATT TTTATAGTGA AAATTTATTT   
  
  
+ CTACTTATAA CAACATCACT TACTAAGTAG CTTATTTGTG TCTTCTTTTC TAAATAATAA TGGTAACTAT   
  
  
+ GTGTCTTTTG TAAATAACTT ATTATCTATT TTATAAATCA CCGTGTAAAG CACGGGTCTA TACTAGTTAA   
  
  
+ TTTAAATAGC ATTGTTAAAT CAAGTAAGAT ACAAACAAAT TTATTTACAA CTGTAAGTGG TCCATAAAAA   
  
  
+ TAATAGCCAT TACAAAAAAT AGTACTACTG TTTTATGAAA TGATTTTAAT ATTCTAAAAT TCCAATAAAA   
  
  
+ CATGTTATAA TCTATGATTA ATTTTAAAAC ATGATTACAT GTCAGCATAA TTTGTATTAT TGTACAGCAA   
  
  
+ ATGAAAGTTG GAGCTAAAAG AAAATGGGGA AATAAAGGCA GACAAGTAAA GATAAAATAT GCTATCTACT   
  
  
+ TTTGGAGGTA TCCTTAACCA CATTGTCTCG TTTGTCTTCA TTAGGGAGAA GTGGGATAAA TACCAAAATT   
  
  
+ TGTATGGTCA TTGCGTGGGG TTTGCTTCCA AACAAAATGG CTTTTTGGAA GCAAATTTTG TGAGCTTCTA   
  
  
+ TGCAAATTTC CACGCTTAAC CCCACAAGCA AACAAAAACA GACAGTGGTT TTTGTTTGTC TTTGTTAGGG   
  
  
+ CATGTTTGGA GCCAACTGTT AATGGGAATA AACTGGGGAG GCTTTTTCCT TGGCTCTTTC CCCCTGACCC   
  
  
+ CAAACCCACT TCAGCTCAAT ACGAAACCAG CCATTGAAAA AGGAAGGGGG GGGGGGGGGT ATGTGAGAAA   
  
  
+ AAGACAAAAC TTTTTCCCGG AGAGAGAAGA TACATATGCA ACAAAGGCTC TCCTTCTCAT AAATTCCATA   
  
  
+ TTCCTCTGTT TTTCTAGGAG AGAGAAATAT GCAAAAAACA AATCAGTTGG CGACAAAAGT TACTACCACG   
  
  
+ CATTGTCTAC TGCTGCGTAC CCATTTTTGG AGACTTTAAA GAACGCCCCT CCCATCTTGA AAAGTGAAAA   
  
  
+ CCCCCTCTTT GAAACTGAAT TTCAGGGGAA AAAAACCTCA CCAAACCTCT AGAGAGAGCT TTTAGAGAGG   
  
  
+ GAAATACAAC GATTAGAAGG AGCAATTTGG GAAATTTCTT TGGGAATTTG AATGGGTTTT GAGTGAATTG   
  
  
+ CAAATCCCAG AAAAGTTTTG GCAAGTACCG ATCTACAGTT CTCTCCTCTT CGTGTTTGGT AGATCCCCTG   
  
  
+ TTTCCTCTGT TTCATTTAGG GTACTTCTCA TTCATCCTCC CCACCCCCTT AATCGGATCT TCCTGTCGAG   
  
  
+ TCACTTTATG CTAATATTTT TTCAGTGGAT TTTTAGTTAA CCCTGTTCAT TTTTCCATTC TGTGTCCCAT   
  
  
+ TTCTCTCTTT TTCATTCATA AGTTGCCGGT TTATCTGTTG GGTGCAGCTT AGTCACAATA ATTTCTGTGT   
  
  
+ TAGGCTTCTT TACGGTTAAA AAAAAAGGAG GCACTCTTTT CGGTGTGATT GTTTATGGGA CCAATGATTC   
  
  
+ AAGATGATGG GTCATCAGTA ACTTCATCAC CCCTTCAATT TTTCTCCATG ATGTCTCCCA ATTTAGGTTC   
  
  
+ TTCCTACCCT TGGCTCAGAG AGCTAAAACC TGAAGAAAGA GGTCTTTACT TGATACATTT GTTGCTCTCT   
  
  
+ TGTGCAAATC ATGTCTCTAG TGGTAGCCTA GACAATGCGA ACTTAGCCCT CGAACAAATC TCTCAGCTTG   
  
  
+ CTGCCCCTGA TGGGGATACA ATGCAGCGTA TGGCTTCTTA TTTTGCTGAA GCCCTGGCTG AGAGGATCCT   
  
  
+ CAAGTCATGG CCTGGCATGT ATAGAGCCCT TCATTTTACG AAAATGCCTG TCATTTCAGA GGAAATTCTT   
  
  
+ GCTAGGAAGC TCTTCTTTGA GCTATTTCCT TTCTTGAAGC TGGCCTATTT GGTGACAAAC CAATCGATAA   
  
  
+ TCGAAGCCAT GGAGGGGGAA AAGATGGTTC ATATTATTGA TCTGAATGCA TCAGAACCTG CTCAATGGAT   
  
  
+ TGCCCTTATT CAGGCTTTGA GTGCAAGGCC TGGGGGTCCT CCTCATTTGA GAATTACCGG TGTTCATCAA   
  
  
+ CACAAAGAGG TTCTAGATCA AGTGGCTCAT AGGGTGACTC AAGAAGCTGA GAAATTGGAT TTGCCATTTC   
  
  
+ AGTTCAATCC TGTGGTTAGC AAGTTGGAAA ACCTTGATGT TGAAAAGCTG TGTGTTAAGA CTGGTGAGGC   
  
  
+ TCTAGCCATC AGTTCGGTCC TTCAACTGCA TACCCTTTTG GGTTCTGATG ATGAGCCCCT AAGGAAAAGT   
  
  
+ TCACCTTTAG CCTTGATGAA GTATGCAAAT GGGGCTAATA GGCAAAGCCC GAGTAATGAT TCGGCTTCTT   
  
  
+ CATCACCTCC TTCGCTCAAT ACTTCAACCA AGCTGGATGG TTTCCTCAGC GCTTTGTGGG GATTGTCCCC   
  
  
+ AAAGATTATG GTGATAGCTG AGCACGATTC CAATCACAAT GGTTCTGGAC TTATGGAGAG GTTGTCAGAA   
  
  
+ GCACTGTACT TCTATGCAGC GCTGTTTGAC TGCTTAGAAT CCACCCTGCC AAGAACATCT GTCGAAAGAA   
  
  
+ GGCGGGTAGA GAAGATGCTC CTAGGTGAAG AGATCAAGAA CATTATATCA TGCGAGGGAG GAGAAAGGAG   
  
  
+ AGAAAGGCAT GAGAAGATCG AGAAGTGGAT GCAGAGGCTA GACATGGCTG GATTCGGGAT CGTTCCTTTG   
  
  
+ AGCTATATGG GTATGCTGCA AGCAAGGCAA TTGCTTCAGG GCTATGGTTG TGATGGTTAT AGAGTGAAAG   
  
  
+ AGAATGGTTG TGTTGTCATC TGTTGGCAAG ATCGCCCCCT CTTTTCGGTA TCAGCATGGA GGTGTAGGAG   
  
  
+ ATG  

- -Up\_Stream \_Len000TACGCG GAAAGACGAT TGAGTAATAG AAAGGAAGAC TCTCCCCTGT TGAATATATT   
  
  
- TAACTATCCA TAAAAGTTTA GTTATCTGGT CGATGGTATA TACTATTAAA AATAATGAAA AGATTTAGGT   
  
  
- AGTTGATAAA AGTGTTAGGA GGAAAAAGAT CTTTTTATTT TAAACGAATT AAGTTTAGTG GATTTTAATG   
  
  
- ATTTAGATAT CAGGATTTAG ATGTTTGAGT AAACACAACG TTGAGTACTA AATTCACCTT GTGATTGGAA   
  
  
- TCCTCTATAC AACAGTTTTC TTCTTAAGCT ACTGTATAAA AGGTTGTTTT CCGGTGGATT GAAGTACTAA   
  
  
- ATTTTTCCTC CTCAGATTCG TGTAGTACCT ACTGATTAAA AACTACATAA CATTGATGCA TAACTCTATT   
  
  
- GAACTTACTT AGCACTGATC TTATATAGAT AAAAGTTTTT ATTTTTAAGG GATTTTCTTT TCCTATTTTT   
  
  
- TGATATATTA TGATTTTTTA AGATTGATAT TATTGATCCG AATAATTTAA AAATATCACT TTTAAATAAA   
  
  
- GATGAATATT GTTGTAGTGA ATGATTCATC GAATAAACAC AGAAGAAAAG ATTTATTATT ACCATTGATA   
  
  
- CACAGAAAAC ATTTATTGAA TAATAGATAA AATATTTAGT GGCACATTTC GTGCCCAGAT ATGATCAATT   
  
  
- AAATTTATCG TAACAATTTA GTTCATTCTA TGTTTGTTTA AATAAATGTT GACATTCACC AGGTATTTTT   
  
  
- ATTATCGGTA ATGTTTTTTA TCATGATGAC AAAATACTTT ACTAAAATTA TAAGATTTTA AGGTTATTTT   
  
  
- GTACAATATT AGATACTAAT TAAAATTTTG TACTAATGTA CAGTCGTATT AAACATAATA ACATGTCGTT   
  
  
- TACTTTCAAC CTCGATTTTC TTTTACCCCT TTATTTCCGT CTGTTCATTT CTATTTTATA CGATAGATGA   
  
  
- AAACCTCCAT AGGAATTGGT GTAACAGAGC AAACAGAAGT AATCCCTCTT CACCCTATTT ATGGTTTTAA   
  
  
- ACATACCAGT AACGCACCCC AAACGAAGGT TTGTTTTACC GAAAAACCTT CGTTTAAAAC ACTCGAAGAT   
  
  
- ACGTTTAAAG GTGCGAATTG GGGTGTTCGT TTGTTTTTGT CTGTCACCAA AAACAAACAG AAACAATCCC   
  
  
- GTACAAACCT CGGTTGACAA TTACCCTTAT TTGACCCCTC CGAAAAAGGA ACCGAGAAAG GGGGACTGGG   
  
  
- GTTTGGGTGA AGTCGAGTTA TGCTTTGGTC GGTAACTTTT TCCTTCCCCC CCCCCCCCCA TACACTCTTT   
  
  
- TTCTGTTTTG AAAAAGGGCC TCTCTCTTCT ATGTATACGT TGTTTCCGAG AGGAAGAGTA TTTAAGGTAT   
  
  
- AAGGAGACAA AAAGATCCTC TCTCTTTATA CGTTTTTTGT TTAGTCAACC GCTGTTTTCA ATGATGGTGC   
  
  
- GTAACAGATG ACGACGCATG GGTAAAAACC TCTGAAATTT CTTGCGGGGA GGGTAGAACT TTTCACTTTT   
  
  
- GGGGGAGAAA CTTTGACTTA AAGTCCCCTT TTTTTGGAGT GGTTTGGAGA TCTCTCTCGA AAATCTCTCC   
  
  
- CTTTATGTTG CTAATCTTCC TCGTTAAACC CTTTAAAGAA ACCCTTAAAC TTACCCAAAA CTCACTTAAC   
  
  
- GTTTAGGGTC TTTTCAAAAC CGTTCATGGC TAGATGTCAA GAGAGGAGAA GCACAAACCA TCTAGGGGAC   
  
  
- AAAGGAGACA AAGTAAATCC CATGAAGAGT AAGTAGGAGG GGTGGGGGAA TTAGCCTAGA AGGACAGCTC   
  
  
- AGTGAAATAC GATTATAAAA AAGTCACCTA AAAATCAATT GGGACAAGTA AAAAGGTAAG ACACAGGGTA   
  
  
- AAGAGAGAAA AAGTAAGTAT TCAACGGCCA AATAGACAAC CCACGTCGAA TCAGTGTTAT TAAAGACACA   
  
  
- ATCCGAAGAA ATGCCAATTT TTTTTTCCTC CGTGAGAAAA GCCACACTAA CAAATACCCT GGTTACTAAG   
  
  
- TTCTACTACC CAGTAGTCAT TGAAGTAGTG GGGAAGTTAA AAAGAGGTAC TACAGAGGGT TAAATCCAAG   
  
  
- AAGGATGGGA ACCGAGTCTC TCGATTTTGG ACTTCTTTCT CCAGAAATGA ACTATGTAAA CAACGAGAGA   
  
  
- ACACGTTTAG TACAGAGATC ACCATCGGAT CTGTTACGCT TGAATCGGGA GCTTGTTTAG AGAGTCGAAC   
  
  
- GACGGGGACT ACCCCTATGT TACGTCGCAT ACCGAAGAAT AAAACGACTT CGGGACCGAC TCTCCTAGGA   
  
  
- GTTCAGTACC GGACCGTACA TATCTCGGGA AGTAAAATGC TTTTACGGAC AGTAAAGTCT CCTTTAAGAA   
  
  
- CGATCCTTCG AGAAGAAACT CGATAAAGGA AAGAACTTCG ACCGGATAAA CCACTGTTTG GTTAGCTATT   
  
  
- AGCTTCGGTA CCTCCCCCTT TTCTACCAAG TATAATAACT AGACTTACGT AGTCTTGGAC GAGTTACCTA   
  
  
- ACGGGAATAA GTCCGAAACT CACGTTCCGG ACCCCCAGGA GGAGTAAACT CTTAATGGCC ACAAGTAGTT   
  
  
- GTGTTTCTCC AAGATCTAGT TCACCGAGTA TCCCACTGAG TTCTTCGACT CTTTAACCTA AACGGTAAAG   
  
  
- TCAAGTTAGG ACACCAATCG TTCAACCTTT TGGAACTACA ACTTTTCGAC ACACAATTCT GACCACTCCG   
  
  
- AGATCGGTAG TCAAGCCAGG AAGTTGACGT ATGGGAAAAC CCAAGACTAC TACTCGGGGA TTCCTTTTCA   
  
  
- AGTGGAAATC GGAACTACTT CATACGTTTA CCCCGATTAT CCGTTTCGGG CTCATTACTA AGCCGAAGAA   
  
  
- GTAGTGGAGG AAGCGAGTTA TGAAGTTGGT TCGACCTACC AAAGGAGTCG CGAAACACCC CTAACAGGGG   
  
  
- TTTCTAATAC CACTATCGAC TCGTGCTAAG GTTAGTGTTA CCAAGACCTG AATACCTCTC CAACAGTCTT   
  
  
- CGTGACATGA AGATACGTCG CGACAAACTG ACGAATCTTA GGTGGGACGG TTCTTGTAGA CAGCTTTCTT   
  
  
- CCGCCCATCT CTTCTACGAG GATCCACTTC TCTAGTTCTT GTAATATAGT ACGCTCCCTC CTCTTTCCTC   
  
  
- TCTTTCCGTA CTCTTCTAGC TCTTCACCTA CGTCTCCGAT CTGTACCGAC CTAAGCCCTA GCAAGGAAAC   
  
  
- TCGATATACC CATACGACGT TCGTTCCGTT AACGAAGTCC CGATACCAAC ACTACCAATA TCTCACTTTC   
  
  
- TCTTACCAAC ACAACAGTAG ACAACCGTTC TAGCGGGGGA GAAAAGCCAT AGTCGTACCT CCACATCCTC   
  
  
- TAC

+     ARE

| Site Name | Organism | Position | Strand | Matrix score. | sequence | function |
| --- | --- | --- | --- | --- | --- | --- |
| ARE | Zea mays | 2441 | + | 6 | AAACCA | cis-acting regulatory element essential for the anaerobic induction |
| ARE | Zea mays | 1288 | + | 6 | AAACCA | cis-acting regulatory element essential for the anaerobic induction |
| ARE | Zea mays | 1170 | - | 6 | AAACCA | cis-acting regulatory element essential for the anaerobic induction |
| ARE | Zea mays | 2912 | - | 6 | AAACCA | cis-acting regulatory element essential for the anaerobic induction |

>HU08G00367.1   
+ -Up\_Stream \_Len000ATGCGC CTTTCTGCTA ACTCATTATC TTTCCTTCTG AGAGGGGACA ACTTATATAA   
  
  
+ ATTGATAGGT ATTTTCAAAT CAATAGACCA GCTACCATAT ATGATAATTT TTATTACTTT TCTAAATCCA   
  
  
+ TCAACTATTT TCACAATCCT CCTTTTTCTA GAAAAATAAA ATTTGCTTAA TTCAAATCAC CTAAAATTAC   
  
  
+ TAAATCTATA GTCCTAAATC TACAAACTCA TTTGTGTTGC AACTCATGAT TTAAGTGGAA CACTAACCTT   
  
  
+ AGGAGATATG TTGTCAAAAG AAGAATTCGA TGACATATTT TCCAACAAAA GGCCACCTAA CTTCATGATT   
  
  
+ TAAAAAGGAG GAGTCTAAGC ACATCATGGA TGACTAATTT TTGATGTATT GTAACTACGT ATTGAGATAA   
  
  
+ CTTGAATGAA TCGTGACTAG AATATATCTA TTTTCAAAAA TAAAAATTCC CTAAAAGAAA AGGATAAAAA   
  
  
+ ACTATATAAT ACTAAAAAAT TCTAACTATA ATAACTAGGC TTATTAAATT TTTATAGTGA AAATTTATTT   
  
  
+ CTACTTATAA CAACATCACT TACTAAGTAG CTTATTTGTG TCTTCTTTTC TAAATAATAA TGGTAACTAT   
  
  
+ GTGTCTTTTG TAAATAACTT ATTATCTATT TTATAAATCA CCGTGTAAAG CACGGGTCTA TACTAGTTAA   
  
  
+ TTTAAATAGC ATTGTTAAAT CAAGTAAGAT ACAAACAAAT TTATTTACAA CTGTAAGTGG TCCATAAAAA   
  
  
+ TAATAGCCAT TACAAAAAAT AGTACTACTG TTTTATGAAA TGATTTTAAT ATTCTAAAAT TCCAATAAAA   
  
  
+ CATGTTATAA TCTATGATTA ATTTTAAAAC ATGATTACAT GTCAGCATAA TTTGTATTAT TGTACAGCAA   
  
  
+ ATGAAAGTTG GAGCTAAAAG AAAATGGGGA AATAAAGGCA GACAAGTAAA GATAAAATAT GCTATCTACT   
  
  
+ TTTGGAGGTA TCCTTAACCA CATTGTCTCG TTTGTCTTCA TTAGGGAGAA GTGGGATAAA TACCAAAATT   
  
  
+ TGTATGGTCA TTGCGTGGGG TTTGCTTCCA AACAAAATGG CTTTTTGGAA GCAAATTTTG TGAGCTTCTA   
  
  
+ TGCAAATTTC CACGCTTAAC CCCACAAGCA AACAAAAACA GACAGTGGTT TTTGTTTGTC TTTGTTAGGG   
  
  
+ CATGTTTGGA GCCAACTGTT AATGGGAATA AACTGGGGAG GCTTTTTCCT TGGCTCTTTC CCCCTGACCC   
  
  
+ CAAACCCACT TCAGCTCAAT ACGAAACCAG CCATTGAAAA AGGAAGGGGG GGGGGGGGGT ATGTGAGAAA   
  
  
+ AAGACAAAAC TTTTTCCCGG AGAGAGAAGA TACATATGCA ACAAAGGCTC TCCTTCTCAT AAATTCCATA   
  
  
+ TTCCTCTGTT TTTCTAGGAG AGAGAAATAT GCAAAAAACA AATCAGTTGG CGACAAAAGT TACTACCACG   
  
  
+ CATTGTCTAC TGCTGCGTAC CCATTTTTGG AGACTTTAAA GAACGCCCCT CCCATCTTGA AAAGTGAAAA   
  
  
+ CCCCCTCTTT GAAACTGAAT TTCAGGGGAA AAAAACCTCA CCAAACCTCT AGAGAGAGCT TTTAGAGAGG   
  
  
+ GAAATACAAC GATTAGAAGG AGCAATTTGG GAAATTTCTT TGGGAATTTG AATGGGTTTT GAGTGAATTG   
  
  
+ CAAATCCCAG AAAAGTTTTG GCAAGTACCG ATCTACAGTT CTCTCCTCTT CGTGTTTGGT AGATCCCCTG   
  
  
+ TTTCCTCTGT TTCATTTAGG GTACTTCTCA TTCATCCTCC CCACCCCCTT AATCGGATCT TCCTGTCGAG   
  
  
+ TCACTTTATG CTAATATTTT TTCAGTGGAT TTTTAGTTAA CCCTGTTCAT TTTTCCATTC TGTGTCCCAT   
  
  
+ TTCTCTCTTT TTCATTCATA AGTTGCCGGT TTATCTGTTG GGTGCAGCTT AGTCACAATA ATTTCTGTGT   
  
  
+ TAGGCTTCTT TACGGTTAAA AAAAAAGGAG GCACTCTTTT CGGTGTGATT GTTTATGGGA CCAATGATTC   
  
  
+ AAGATGATGG GTCATCAGTA ACTTCATCAC CCCTTCAATT TTTCTCCATG ATGTCTCCCA ATTTAGGTTC   
  
  
+ TTCCTACCCT TGGCTCAGAG AGCTAAAACC TGAAGAAAGA GGTCTTTACT TGATACATTT GTTGCTCTCT   
  
  
+ TGTGCAAATC ATGTCTCTAG TGGTAGCCTA GACAATGCGA ACTTAGCCCT CGAACAAATC TCTCAGCTTG   
  
  
+ CTGCCCCTGA TGGGGATACA ATGCAGCGTA TGGCTTCTTA TTTTGCTGAA GCCCTGGCTG AGAGGATCCT   
  
  
+ CAAGTCATGG CCTGGCATGT ATAGAGCCCT TCATTTTACG AAAATGCCTG TCATTTCAGA GGAAATTCTT   
  
  
+ GCTAGGAAGC TCTTCTTTGA GCTATTTCCT TTCTTGAAGC TGGCCTATTT GGTGACAAAC CAATCGATAA   
  
  
+ TCGAAGCCAT GGAGGGGGAA AAGATGGTTC ATATTATTGA TCTGAATGCA TCAGAACCTG CTCAATGGAT   
  
  
+ TGCCCTTATT CAGGCTTTGA GTGCAAGGCC TGGGGGTCCT CCTCATTTGA GAATTACCGG TGTTCATCAA   
  
  
+ CACAAAGAGG TTCTAGATCA AGTGGCTCAT AGGGTGACTC AAGAAGCTGA GAAATTGGAT TTGCCATTTC   
  
  
+ AGTTCAATCC TGTGGTTAGC AAGTTGGAAA ACCTTGATGT TGAAAAGCTG TGTGTTAAGA CTGGTGAGGC   
  
  
+ TCTAGCCATC AGTTCGGTCC TTCAACTGCA TACCCTTTTG GGTTCTGATG ATGAGCCCCT AAGGAAAAGT   
  
  
+ TCACCTTTAG CCTTGATGAA GTATGCAAAT GGGGCTAATA GGCAAAGCCC GAGTAATGAT TCGGCTTCTT   
  
  
+ CATCACCTCC TTCGCTCAAT ACTTCAACCA AGCTGGATGG TTTCCTCAGC GCTTTGTGGG GATTGTCCCC   
  
  
+ AAAGATTATG GTGATAGCTG AGCACGATTC CAATCACAAT GGTTCTGGAC TTATGGAGAG GTTGTCAGAA   
  
  
+ GCACTGTACT TCTATGCAGC GCTGTTTGAC TGCTTAGAAT CCACCCTGCC AAGAACATCT GTCGAAAGAA   
  
  
+ GGCGGGTAGA GAAGATGCTC CTAGGTGAAG AGATCAAGAA CATTATATCA TGCGAGGGAG GAGAAAGGAG   
  
  
+ AGAAAGGCAT GAGAAGATCG AGAAGTGGAT GCAGAGGCTA GACATGGCTG GATTCGGGAT CGTTCCTTTG   
  
  
+ AGCTATATGG GTATGCTGCA AGCAAGGCAA TTGCTTCAGG GCTATGGTTG TGATGGTTAT AGAGTGAAAG   
  
  
+ AGAATGGTTG TGTTGTCATC TGTTGGCAAG ATCGCCCCCT CTTTTCGGTA TCAGCATGGA GGTGTAGGAG   
  
  
+ ATG  

- -Up\_Stream \_Len000TACGCG GAAAGACGAT TGAGTAATAG AAAGGAAGAC TCTCCCCTGT TGAATATATT   
  
  
- TAACTATCCA TAAAAGTTTA GTTATCTGGT CGATGGTATA TACTATTAAA AATAATGAAA AGATTTAGGT   
  
  
- AGTTGATAAA AGTGTTAGGA GGAAAAAGAT CTTTTTATTT TAAACGAATT AAGTTTAGTG GATTTTAATG   
  
  
- ATTTAGATAT CAGGATTTAG ATGTTTGAGT AAACACAACG TTGAGTACTA AATTCACCTT GTGATTGGAA   
  
  
- TCCTCTATAC AACAGTTTTC TTCTTAAGCT ACTGTATAAA AGGTTGTTTT CCGGTGGATT GAAGTACTAA   
  
  
- ATTTTTCCTC CTCAGATTCG TGTAGTACCT ACTGATTAAA AACTACATAA CATTGATGCA TAACTCTATT   
  
  
- GAACTTACTT AGCACTGATC TTATATAGAT AAAAGTTTTT ATTTTTAAGG GATTTTCTTT TCCTATTTTT   
  
  
- TGATATATTA TGATTTTTTA AGATTGATAT TATTGATCCG AATAATTTAA AAATATCACT TTTAAATAAA   
  
  
- GATGAATATT GTTGTAGTGA ATGATTCATC GAATAAACAC AGAAGAAAAG ATTTATTATT ACCATTGATA   
  
  
- CACAGAAAAC ATTTATTGAA TAATAGATAA AATATTTAGT GGCACATTTC GTGCCCAGAT ATGATCAATT   
  
  
- AAATTTATCG TAACAATTTA GTTCATTCTA TGTTTGTTTA AATAAATGTT GACATTCACC AGGTATTTTT   
  
  
- ATTATCGGTA ATGTTTTTTA TCATGATGAC AAAATACTTT ACTAAAATTA TAAGATTTTA AGGTTATTTT   
  
  
- GTACAATATT AGATACTAAT TAAAATTTTG TACTAATGTA CAGTCGTATT AAACATAATA ACATGTCGTT   
  
  
- TACTTTCAAC CTCGATTTTC TTTTACCCCT TTATTTCCGT CTGTTCATTT CTATTTTATA CGATAGATGA   
  
  
- AAACCTCCAT AGGAATTGGT GTAACAGAGC AAACAGAAGT AATCCCTCTT CACCCTATTT ATGGTTTTAA   
  
  
- ACATACCAGT AACGCACCCC AAACGAAGGT TTGTTTTACC GAAAAACCTT CGTTTAAAAC ACTCGAAGAT   
  
  
- ACGTTTAAAG GTGCGAATTG GGGTGTTCGT TTGTTTTTGT CTGTCACCAA AAACAAACAG AAACAATCCC   
  
  
- GTACAAACCT CGGTTGACAA TTACCCTTAT TTGACCCCTC CGAAAAAGGA ACCGAGAAAG GGGGACTGGG   
  
  
- GTTTGGGTGA AGTCGAGTTA TGCTTTGGTC GGTAACTTTT TCCTTCCCCC CCCCCCCCCA TACACTCTTT   
  
  
- TTCTGTTTTG AAAAAGGGCC TCTCTCTTCT ATGTATACGT TGTTTCCGAG AGGAAGAGTA TTTAAGGTAT   
  
  
- AAGGAGACAA AAAGATCCTC TCTCTTTATA CGTTTTTTGT TTAGTCAACC GCTGTTTTCA ATGATGGTGC   
  
  
- GTAACAGATG ACGACGCATG GGTAAAAACC TCTGAAATTT CTTGCGGGGA GGGTAGAACT TTTCACTTTT   
  
  
- GGGGGAGAAA CTTTGACTTA AAGTCCCCTT TTTTTGGAGT GGTTTGGAGA TCTCTCTCGA AAATCTCTCC   
  
  
- CTTTATGTTG CTAATCTTCC TCGTTAAACC CTTTAAAGAA ACCCTTAAAC TTACCCAAAA CTCACTTAAC   
  
  
- GTTTAGGGTC TTTTCAAAAC CGTTCATGGC TAGATGTCAA GAGAGGAGAA GCACAAACCA TCTAGGGGAC   
  
  
- AAAGGAGACA AAGTAAATCC CATGAAGAGT AAGTAGGAGG GGTGGGGGAA TTAGCCTAGA AGGACAGCTC   
  
  
- AGTGAAATAC GATTATAAAA AAGTCACCTA AAAATCAATT GGGACAAGTA AAAAGGTAAG ACACAGGGTA   
  
  
- AAGAGAGAAA AAGTAAGTAT TCAACGGCCA AATAGACAAC CCACGTCGAA TCAGTGTTAT TAAAGACACA   
  
  
- ATCCGAAGAA ATGCCAATTT TTTTTTCCTC CGTGAGAAAA GCCACACTAA CAAATACCCT GGTTACTAAG   
  
  
- TTCTACTACC CAGTAGTCAT TGAAGTAGTG GGGAAGTTAA AAAGAGGTAC TACAGAGGGT TAAATCCAAG   
  
  
- AAGGATGGGA ACCGAGTCTC TCGATTTTGG ACTTCTTTCT CCAGAAATGA ACTATGTAAA CAACGAGAGA   
  
  
- ACACGTTTAG TACAGAGATC ACCATCGGAT CTGTTACGCT TGAATCGGGA GCTTGTTTAG AGAGTCGAAC   
  
  
- GACGGGGACT ACCCCTATGT TACGTCGCAT ACCGAAGAAT AAAACGACTT CGGGACCGAC TCTCCTAGGA   
  
  
- GTTCAGTACC GGACCGTACA TATCTCGGGA AGTAAAATGC TTTTACGGAC AGTAAAGTCT CCTTTAAGAA   
  
  
- CGATCCTTCG AGAAGAAACT CGATAAAGGA AAGAACTTCG ACCGGATAAA CCACTGTTTG GTTAGCTATT   
  
  
- AGCTTCGGTA CCTCCCCCTT TTCTACCAAG TATAATAACT AGACTTACGT AGTCTTGGAC GAGTTACCTA   
  
  
- ACGGGAATAA GTCCGAAACT CACGTTCCGG ACCCCCAGGA GGAGTAAACT CTTAATGGCC ACAAGTAGTT   
  
  
- GTGTTTCTCC AAGATCTAGT TCACCGAGTA TCCCACTGAG TTCTTCGACT CTTTAACCTA AACGGTAAAG   
  
  
- TCAAGTTAGG ACACCAATCG TTCAACCTTT TGGAACTACA ACTTTTCGAC ACACAATTCT GACCACTCCG   
  
  
- AGATCGGTAG TCAAGCCAGG AAGTTGACGT ATGGGAAAAC CCAAGACTAC TACTCGGGGA TTCCTTTTCA   
  
  
- AGTGGAAATC GGAACTACTT CATACGTTTA CCCCGATTAT CCGTTTCGGG CTCATTACTA AGCCGAAGAA   
  
  
- GTAGTGGAGG AAGCGAGTTA TGAAGTTGGT TCGACCTACC AAAGGAGTCG CGAAACACCC CTAACAGGGG   
  
  
- TTTCTAATAC CACTATCGAC TCGTGCTAAG GTTAGTGTTA CCAAGACCTG AATACCTCTC CAACAGTCTT   
  
  
- CGTGACATGA AGATACGTCG CGACAAACTG ACGAATCTTA GGTGGGACGG TTCTTGTAGA CAGCTTTCTT   
  
  
- CCGCCCATCT CTTCTACGAG GATCCACTTC TCTAGTTCTT GTAATATAGT ACGCTCCCTC CTCTTTCCTC   
  
  
- TCTTTCCGTA CTCTTCTAGC TCTTCACCTA CGTCTCCGAT CTGTACCGAC CTAAGCCCTA GCAAGGAAAC   
  
  
- TCGATATACC CATACGACGT TCGTTCCGTT AACGAAGTCC CGATACCAAC ACTACCAATA TCTCACTTTC   
  
  
- TCTTACCAAC ACAACAGTAG ACAACCGTTC TAGCGGGGGA GAAAAGCCAT AGTCGTACCT CCACATCCTC   
  
  
- TAC

+     AT~TATA-box

| Site Name | Organism | Position | Strand | Matrix score. | sequence | function |
| --- | --- | --- | --- | --- | --- | --- |
| AT~TATA-box | Arabidopsis thaliana | 497 | + | 6 | TATATA |  |
| AT~TATA-box | Arabidopsis thaliana | 68 | + | 6 | TATATA |  |

>HU08G00367.1   
+ -Up\_Stream \_Len000ATGCGC CTTTCTGCTA ACTCATTATC TTTCCTTCTG AGAGGGGACA ACTTATATAA   
  
  
+ ATTGATAGGT ATTTTCAAAT CAATAGACCA GCTACCATAT ATGATAATTT TTATTACTTT TCTAAATCCA   
  
  
+ TCAACTATTT TCACAATCCT CCTTTTTCTA GAAAAATAAA ATTTGCTTAA TTCAAATCAC CTAAAATTAC   
  
  
+ TAAATCTATA GTCCTAAATC TACAAACTCA TTTGTGTTGC AACTCATGAT TTAAGTGGAA CACTAACCTT   
  
  
+ AGGAGATATG TTGTCAAAAG AAGAATTCGA TGACATATTT TCCAACAAAA GGCCACCTAA CTTCATGATT   
  
  
+ TAAAAAGGAG GAGTCTAAGC ACATCATGGA TGACTAATTT TTGATGTATT GTAACTACGT ATTGAGATAA   
  
  
+ CTTGAATGAA TCGTGACTAG AATATATCTA TTTTCAAAAA TAAAAATTCC CTAAAAGAAA AGGATAAAAA   
  
  
+ ACTATATAAT ACTAAAAAAT TCTAACTATA ATAACTAGGC TTATTAAATT TTTATAGTGA AAATTTATTT   
  
  
+ CTACTTATAA CAACATCACT TACTAAGTAG CTTATTTGTG TCTTCTTTTC TAAATAATAA TGGTAACTAT   
  
  
+ GTGTCTTTTG TAAATAACTT ATTATCTATT TTATAAATCA CCGTGTAAAG CACGGGTCTA TACTAGTTAA   
  
  
+ TTTAAATAGC ATTGTTAAAT CAAGTAAGAT ACAAACAAAT TTATTTACAA CTGTAAGTGG TCCATAAAAA   
  
  
+ TAATAGCCAT TACAAAAAAT AGTACTACTG TTTTATGAAA TGATTTTAAT ATTCTAAAAT TCCAATAAAA   
  
  
+ CATGTTATAA TCTATGATTA ATTTTAAAAC ATGATTACAT GTCAGCATAA TTTGTATTAT TGTACAGCAA   
  
  
+ ATGAAAGTTG GAGCTAAAAG AAAATGGGGA AATAAAGGCA GACAAGTAAA GATAAAATAT GCTATCTACT   
  
  
+ TTTGGAGGTA TCCTTAACCA CATTGTCTCG TTTGTCTTCA TTAGGGAGAA GTGGGATAAA TACCAAAATT   
  
  
+ TGTATGGTCA TTGCGTGGGG TTTGCTTCCA AACAAAATGG CTTTTTGGAA GCAAATTTTG TGAGCTTCTA   
  
  
+ TGCAAATTTC CACGCTTAAC CCCACAAGCA AACAAAAACA GACAGTGGTT TTTGTTTGTC TTTGTTAGGG   
  
  
+ CATGTTTGGA GCCAACTGTT AATGGGAATA AACTGGGGAG GCTTTTTCCT TGGCTCTTTC CCCCTGACCC   
  
  
+ CAAACCCACT TCAGCTCAAT ACGAAACCAG CCATTGAAAA AGGAAGGGGG GGGGGGGGGT ATGTGAGAAA   
  
  
+ AAGACAAAAC TTTTTCCCGG AGAGAGAAGA TACATATGCA ACAAAGGCTC TCCTTCTCAT AAATTCCATA   
  
  
+ TTCCTCTGTT TTTCTAGGAG AGAGAAATAT GCAAAAAACA AATCAGTTGG CGACAAAAGT TACTACCACG   
  
  
+ CATTGTCTAC TGCTGCGTAC CCATTTTTGG AGACTTTAAA GAACGCCCCT CCCATCTTGA AAAGTGAAAA   
  
  
+ CCCCCTCTTT GAAACTGAAT TTCAGGGGAA AAAAACCTCA CCAAACCTCT AGAGAGAGCT TTTAGAGAGG   
  
  
+ GAAATACAAC GATTAGAAGG AGCAATTTGG GAAATTTCTT TGGGAATTTG AATGGGTTTT GAGTGAATTG   
  
  
+ CAAATCCCAG AAAAGTTTTG GCAAGTACCG ATCTACAGTT CTCTCCTCTT CGTGTTTGGT AGATCCCCTG   
  
  
+ TTTCCTCTGT TTCATTTAGG GTACTTCTCA TTCATCCTCC CCACCCCCTT AATCGGATCT TCCTGTCGAG   
  
  
+ TCACTTTATG CTAATATTTT TTCAGTGGAT TTTTAGTTAA CCCTGTTCAT TTTTCCATTC TGTGTCCCAT   
  
  
+ TTCTCTCTTT TTCATTCATA AGTTGCCGGT TTATCTGTTG GGTGCAGCTT AGTCACAATA ATTTCTGTGT   
  
  
+ TAGGCTTCTT TACGGTTAAA AAAAAAGGAG GCACTCTTTT CGGTGTGATT GTTTATGGGA CCAATGATTC   
  
  
+ AAGATGATGG GTCATCAGTA ACTTCATCAC CCCTTCAATT TTTCTCCATG ATGTCTCCCA ATTTAGGTTC   
  
  
+ TTCCTACCCT TGGCTCAGAG AGCTAAAACC TGAAGAAAGA GGTCTTTACT TGATACATTT GTTGCTCTCT   
  
  
+ TGTGCAAATC ATGTCTCTAG TGGTAGCCTA GACAATGCGA ACTTAGCCCT CGAACAAATC TCTCAGCTTG   
  
  
+ CTGCCCCTGA TGGGGATACA ATGCAGCGTA TGGCTTCTTA TTTTGCTGAA GCCCTGGCTG AGAGGATCCT   
  
  
+ CAAGTCATGG CCTGGCATGT ATAGAGCCCT TCATTTTACG AAAATGCCTG TCATTTCAGA GGAAATTCTT   
  
  
+ GCTAGGAAGC TCTTCTTTGA GCTATTTCCT TTCTTGAAGC TGGCCTATTT GGTGACAAAC CAATCGATAA   
  
  
+ TCGAAGCCAT GGAGGGGGAA AAGATGGTTC ATATTATTGA TCTGAATGCA TCAGAACCTG CTCAATGGAT   
  
  
+ TGCCCTTATT CAGGCTTTGA GTGCAAGGCC TGGGGGTCCT CCTCATTTGA GAATTACCGG TGTTCATCAA   
  
  
+ CACAAAGAGG TTCTAGATCA AGTGGCTCAT AGGGTGACTC AAGAAGCTGA GAAATTGGAT TTGCCATTTC   
  
  
+ AGTTCAATCC TGTGGTTAGC AAGTTGGAAA ACCTTGATGT TGAAAAGCTG TGTGTTAAGA CTGGTGAGGC   
  
  
+ TCTAGCCATC AGTTCGGTCC TTCAACTGCA TACCCTTTTG GGTTCTGATG ATGAGCCCCT AAGGAAAAGT   
  
  
+ TCACCTTTAG CCTTGATGAA GTATGCAAAT GGGGCTAATA GGCAAAGCCC GAGTAATGAT TCGGCTTCTT   
  
  
+ CATCACCTCC TTCGCTCAAT ACTTCAACCA AGCTGGATGG TTTCCTCAGC GCTTTGTGGG GATTGTCCCC   
  
  
+ AAAGATTATG GTGATAGCTG AGCACGATTC CAATCACAAT GGTTCTGGAC TTATGGAGAG GTTGTCAGAA   
  
  
+ GCACTGTACT TCTATGCAGC GCTGTTTGAC TGCTTAGAAT CCACCCTGCC AAGAACATCT GTCGAAAGAA   
  
  
+ GGCGGGTAGA GAAGATGCTC CTAGGTGAAG AGATCAAGAA CATTATATCA TGCGAGGGAG GAGAAAGGAG   
  
  
+ AGAAAGGCAT GAGAAGATCG AGAAGTGGAT GCAGAGGCTA GACATGGCTG GATTCGGGAT CGTTCCTTTG   
  
  
+ AGCTATATGG GTATGCTGCA AGCAAGGCAA TTGCTTCAGG GCTATGGTTG TGATGGTTAT AGAGTGAAAG   
  
  
+ AGAATGGTTG TGTTGTCATC TGTTGGCAAG ATCGCCCCCT CTTTTCGGTA TCAGCATGGA GGTGTAGGAG   
  
  
+ ATG  

- -Up\_Stream \_Len000TACGCG GAAAGACGAT TGAGTAATAG AAAGGAAGAC TCTCCCCTGT TGAATATATT   
  
  
- TAACTATCCA TAAAAGTTTA GTTATCTGGT CGATGGTATA TACTATTAAA AATAATGAAA AGATTTAGGT   
  
  
- AGTTGATAAA AGTGTTAGGA GGAAAAAGAT CTTTTTATTT TAAACGAATT AAGTTTAGTG GATTTTAATG   
  
  
- ATTTAGATAT CAGGATTTAG ATGTTTGAGT AAACACAACG TTGAGTACTA AATTCACCTT GTGATTGGAA   
  
  
- TCCTCTATAC AACAGTTTTC TTCTTAAGCT ACTGTATAAA AGGTTGTTTT CCGGTGGATT GAAGTACTAA   
  
  
- ATTTTTCCTC CTCAGATTCG TGTAGTACCT ACTGATTAAA AACTACATAA CATTGATGCA TAACTCTATT   
  
  
- GAACTTACTT AGCACTGATC TTATATAGAT AAAAGTTTTT ATTTTTAAGG GATTTTCTTT TCCTATTTTT   
  
  
- TGATATATTA TGATTTTTTA AGATTGATAT TATTGATCCG AATAATTTAA AAATATCACT TTTAAATAAA   
  
  
- GATGAATATT GTTGTAGTGA ATGATTCATC GAATAAACAC AGAAGAAAAG ATTTATTATT ACCATTGATA   
  
  
- CACAGAAAAC ATTTATTGAA TAATAGATAA AATATTTAGT GGCACATTTC GTGCCCAGAT ATGATCAATT   
  
  
- AAATTTATCG TAACAATTTA GTTCATTCTA TGTTTGTTTA AATAAATGTT GACATTCACC AGGTATTTTT   
  
  
- ATTATCGGTA ATGTTTTTTA TCATGATGAC AAAATACTTT ACTAAAATTA TAAGATTTTA AGGTTATTTT   
  
  
- GTACAATATT AGATACTAAT TAAAATTTTG TACTAATGTA CAGTCGTATT AAACATAATA ACATGTCGTT   
  
  
- TACTTTCAAC CTCGATTTTC TTTTACCCCT TTATTTCCGT CTGTTCATTT CTATTTTATA CGATAGATGA   
  
  
- AAACCTCCAT AGGAATTGGT GTAACAGAGC AAACAGAAGT AATCCCTCTT CACCCTATTT ATGGTTTTAA   
  
  
- ACATACCAGT AACGCACCCC AAACGAAGGT TTGTTTTACC GAAAAACCTT CGTTTAAAAC ACTCGAAGAT   
  
  
- ACGTTTAAAG GTGCGAATTG GGGTGTTCGT TTGTTTTTGT CTGTCACCAA AAACAAACAG AAACAATCCC   
  
  
- GTACAAACCT CGGTTGACAA TTACCCTTAT TTGACCCCTC CGAAAAAGGA ACCGAGAAAG GGGGACTGGG   
  
  
- GTTTGGGTGA AGTCGAGTTA TGCTTTGGTC GGTAACTTTT TCCTTCCCCC CCCCCCCCCA TACACTCTTT   
  
  
- TTCTGTTTTG AAAAAGGGCC TCTCTCTTCT ATGTATACGT TGTTTCCGAG AGGAAGAGTA TTTAAGGTAT   
  
  
- AAGGAGACAA AAAGATCCTC TCTCTTTATA CGTTTTTTGT TTAGTCAACC GCTGTTTTCA ATGATGGTGC   
  
  
- GTAACAGATG ACGACGCATG GGTAAAAACC TCTGAAATTT CTTGCGGGGA GGGTAGAACT TTTCACTTTT   
  
  
- GGGGGAGAAA CTTTGACTTA AAGTCCCCTT TTTTTGGAGT GGTTTGGAGA TCTCTCTCGA AAATCTCTCC   
  
  
- CTTTATGTTG CTAATCTTCC TCGTTAAACC CTTTAAAGAA ACCCTTAAAC TTACCCAAAA CTCACTTAAC   
  
  
- GTTTAGGGTC TTTTCAAAAC CGTTCATGGC TAGATGTCAA GAGAGGAGAA GCACAAACCA TCTAGGGGAC   
  
  
- AAAGGAGACA AAGTAAATCC CATGAAGAGT AAGTAGGAGG GGTGGGGGAA TTAGCCTAGA AGGACAGCTC   
  
  
- AGTGAAATAC GATTATAAAA AAGTCACCTA AAAATCAATT GGGACAAGTA AAAAGGTAAG ACACAGGGTA   
  
  
- AAGAGAGAAA AAGTAAGTAT TCAACGGCCA AATAGACAAC CCACGTCGAA TCAGTGTTAT TAAAGACACA   
  
  
- ATCCGAAGAA ATGCCAATTT TTTTTTCCTC CGTGAGAAAA GCCACACTAA CAAATACCCT GGTTACTAAG   
  
  
- TTCTACTACC CAGTAGTCAT TGAAGTAGTG GGGAAGTTAA AAAGAGGTAC TACAGAGGGT TAAATCCAAG   
  
  
- AAGGATGGGA ACCGAGTCTC TCGATTTTGG ACTTCTTTCT CCAGAAATGA ACTATGTAAA CAACGAGAGA   
  
  
- ACACGTTTAG TACAGAGATC ACCATCGGAT CTGTTACGCT TGAATCGGGA GCTTGTTTAG AGAGTCGAAC   
  
  
- GACGGGGACT ACCCCTATGT TACGTCGCAT ACCGAAGAAT AAAACGACTT CGGGACCGAC TCTCCTAGGA   
  
  
- GTTCAGTACC GGACCGTACA TATCTCGGGA AGTAAAATGC TTTTACGGAC AGTAAAGTCT CCTTTAAGAA   
  
  
- CGATCCTTCG AGAAGAAACT CGATAAAGGA AAGAACTTCG ACCGGATAAA CCACTGTTTG GTTAGCTATT   
  
  
- AGCTTCGGTA CCTCCCCCTT TTCTACCAAG TATAATAACT AGACTTACGT AGTCTTGGAC GAGTTACCTA   
  
  
- ACGGGAATAA GTCCGAAACT CACGTTCCGG ACCCCCAGGA GGAGTAAACT CTTAATGGCC ACAAGTAGTT   
  
  
- GTGTTTCTCC AAGATCTAGT TCACCGAGTA TCCCACTGAG TTCTTCGACT CTTTAACCTA AACGGTAAAG   
  
  
- TCAAGTTAGG ACACCAATCG TTCAACCTTT TGGAACTACA ACTTTTCGAC ACACAATTCT GACCACTCCG   
  
  
- AGATCGGTAG TCAAGCCAGG AAGTTGACGT ATGGGAAAAC CCAAGACTAC TACTCGGGGA TTCCTTTTCA   
  
  
- AGTGGAAATC GGAACTACTT CATACGTTTA CCCCGATTAT CCGTTTCGGG CTCATTACTA AGCCGAAGAA   
  
  
- GTAGTGGAGG AAGCGAGTTA TGAAGTTGGT TCGACCTACC AAAGGAGTCG CGAAACACCC CTAACAGGGG   
  
  
- TTTCTAATAC CACTATCGAC TCGTGCTAAG GTTAGTGTTA CCAAGACCTG AATACCTCTC CAACAGTCTT   
  
  
- CGTGACATGA AGATACGTCG CGACAAACTG ACGAATCTTA GGTGGGACGG TTCTTGTAGA CAGCTTTCTT   
  
  
- CCGCCCATCT CTTCTACGAG GATCCACTTC TCTAGTTCTT GTAATATAGT ACGCTCCCTC CTCTTTCCTC   
  
  
- TCTTTCCGTA CTCTTCTAGC TCTTCACCTA CGTCTCCGAT CTGTACCGAC CTAAGCCCTA GCAAGGAAAC   
  
  
- TCGATATACC CATACGACGT TCGTTCCGTT AACGAAGTCC CGATACCAAC ACTACCAATA TCTCACTTTC   
  
  
- TCTTACCAAC ACAACAGTAG ACAACCGTTC TAGCGGGGGA GAAAAGCCAT AGTCGTACCT CCACATCCTC   
  
  
- TAC

+     AuxRR-core

| Site Name | Organism | Position | Strand | Matrix score. | sequence | function |
| --- | --- | --- | --- | --- | --- | --- |
| AuxRR-core | Nicotiana tabacum | 763 | + | 7 | GGTCCAT | cis-acting regulatory element involved in auxin responsiveness |

>HU08G00367.1   
+ -Up\_Stream \_Len000ATGCGC CTTTCTGCTA ACTCATTATC TTTCCTTCTG AGAGGGGACA ACTTATATAA   
  
  
+ ATTGATAGGT ATTTTCAAAT CAATAGACCA GCTACCATAT ATGATAATTT TTATTACTTT TCTAAATCCA   
  
  
+ TCAACTATTT TCACAATCCT CCTTTTTCTA GAAAAATAAA ATTTGCTTAA TTCAAATCAC CTAAAATTAC   
  
  
+ TAAATCTATA GTCCTAAATC TACAAACTCA TTTGTGTTGC AACTCATGAT TTAAGTGGAA CACTAACCTT   
  
  
+ AGGAGATATG TTGTCAAAAG AAGAATTCGA TGACATATTT TCCAACAAAA GGCCACCTAA CTTCATGATT   
  
  
+ TAAAAAGGAG GAGTCTAAGC ACATCATGGA TGACTAATTT TTGATGTATT GTAACTACGT ATTGAGATAA   
  
  
+ CTTGAATGAA TCGTGACTAG AATATATCTA TTTTCAAAAA TAAAAATTCC CTAAAAGAAA AGGATAAAAA   
  
  
+ ACTATATAAT ACTAAAAAAT TCTAACTATA ATAACTAGGC TTATTAAATT TTTATAGTGA AAATTTATTT   
  
  
+ CTACTTATAA CAACATCACT TACTAAGTAG CTTATTTGTG TCTTCTTTTC TAAATAATAA TGGTAACTAT   
  
  
+ GTGTCTTTTG TAAATAACTT ATTATCTATT TTATAAATCA CCGTGTAAAG CACGGGTCTA TACTAGTTAA   
  
  
+ TTTAAATAGC ATTGTTAAAT CAAGTAAGAT ACAAACAAAT TTATTTACAA CTGTAAGTGG TCCATAAAAA   
  
  
+ TAATAGCCAT TACAAAAAAT AGTACTACTG TTTTATGAAA TGATTTTAAT ATTCTAAAAT TCCAATAAAA   
  
  
+ CATGTTATAA TCTATGATTA ATTTTAAAAC ATGATTACAT GTCAGCATAA TTTGTATTAT TGTACAGCAA   
  
  
+ ATGAAAGTTG GAGCTAAAAG AAAATGGGGA AATAAAGGCA GACAAGTAAA GATAAAATAT GCTATCTACT   
  
  
+ TTTGGAGGTA TCCTTAACCA CATTGTCTCG TTTGTCTTCA TTAGGGAGAA GTGGGATAAA TACCAAAATT   
  
  
+ TGTATGGTCA TTGCGTGGGG TTTGCTTCCA AACAAAATGG CTTTTTGGAA GCAAATTTTG TGAGCTTCTA   
  
  
+ TGCAAATTTC CACGCTTAAC CCCACAAGCA AACAAAAACA GACAGTGGTT TTTGTTTGTC TTTGTTAGGG   
  
  
+ CATGTTTGGA GCCAACTGTT AATGGGAATA AACTGGGGAG GCTTTTTCCT TGGCTCTTTC CCCCTGACCC   
  
  
+ CAAACCCACT TCAGCTCAAT ACGAAACCAG CCATTGAAAA AGGAAGGGGG GGGGGGGGGT ATGTGAGAAA   
  
  
+ AAGACAAAAC TTTTTCCCGG AGAGAGAAGA TACATATGCA ACAAAGGCTC TCCTTCTCAT AAATTCCATA   
  
  
+ TTCCTCTGTT TTTCTAGGAG AGAGAAATAT GCAAAAAACA AATCAGTTGG CGACAAAAGT TACTACCACG   
  
  
+ CATTGTCTAC TGCTGCGTAC CCATTTTTGG AGACTTTAAA GAACGCCCCT CCCATCTTGA AAAGTGAAAA   
  
  
+ CCCCCTCTTT GAAACTGAAT TTCAGGGGAA AAAAACCTCA CCAAACCTCT AGAGAGAGCT TTTAGAGAGG   
  
  
+ GAAATACAAC GATTAGAAGG AGCAATTTGG GAAATTTCTT TGGGAATTTG AATGGGTTTT GAGTGAATTG   
  
  
+ CAAATCCCAG AAAAGTTTTG GCAAGTACCG ATCTACAGTT CTCTCCTCTT CGTGTTTGGT AGATCCCCTG   
  
  
+ TTTCCTCTGT TTCATTTAGG GTACTTCTCA TTCATCCTCC CCACCCCCTT AATCGGATCT TCCTGTCGAG   
  
  
+ TCACTTTATG CTAATATTTT TTCAGTGGAT TTTTAGTTAA CCCTGTTCAT TTTTCCATTC TGTGTCCCAT   
  
  
+ TTCTCTCTTT TTCATTCATA AGTTGCCGGT TTATCTGTTG GGTGCAGCTT AGTCACAATA ATTTCTGTGT   
  
  
+ TAGGCTTCTT TACGGTTAAA AAAAAAGGAG GCACTCTTTT CGGTGTGATT GTTTATGGGA CCAATGATTC   
  
  
+ AAGATGATGG GTCATCAGTA ACTTCATCAC CCCTTCAATT TTTCTCCATG ATGTCTCCCA ATTTAGGTTC   
  
  
+ TTCCTACCCT TGGCTCAGAG AGCTAAAACC TGAAGAAAGA GGTCTTTACT TGATACATTT GTTGCTCTCT   
  
  
+ TGTGCAAATC ATGTCTCTAG TGGTAGCCTA GACAATGCGA ACTTAGCCCT CGAACAAATC TCTCAGCTTG   
  
  
+ CTGCCCCTGA TGGGGATACA ATGCAGCGTA TGGCTTCTTA TTTTGCTGAA GCCCTGGCTG AGAGGATCCT   
  
  
+ CAAGTCATGG CCTGGCATGT ATAGAGCCCT TCATTTTACG AAAATGCCTG TCATTTCAGA GGAAATTCTT   
  
  
+ GCTAGGAAGC TCTTCTTTGA GCTATTTCCT TTCTTGAAGC TGGCCTATTT GGTGACAAAC CAATCGATAA   
  
  
+ TCGAAGCCAT GGAGGGGGAA AAGATGGTTC ATATTATTGA TCTGAATGCA TCAGAACCTG CTCAATGGAT   
  
  
+ TGCCCTTATT CAGGCTTTGA GTGCAAGGCC TGGGGGTCCT CCTCATTTGA GAATTACCGG TGTTCATCAA   
  
  
+ CACAAAGAGG TTCTAGATCA AGTGGCTCAT AGGGTGACTC AAGAAGCTGA GAAATTGGAT TTGCCATTTC   
  
  
+ AGTTCAATCC TGTGGTTAGC AAGTTGGAAA ACCTTGATGT TGAAAAGCTG TGTGTTAAGA CTGGTGAGGC   
  
  
+ TCTAGCCATC AGTTCGGTCC TTCAACTGCA TACCCTTTTG GGTTCTGATG ATGAGCCCCT AAGGAAAAGT   
  
  
+ TCACCTTTAG CCTTGATGAA GTATGCAAAT GGGGCTAATA GGCAAAGCCC GAGTAATGAT TCGGCTTCTT   
  
  
+ CATCACCTCC TTCGCTCAAT ACTTCAACCA AGCTGGATGG TTTCCTCAGC GCTTTGTGGG GATTGTCCCC   
  
  
+ AAAGATTATG GTGATAGCTG AGCACGATTC CAATCACAAT GGTTCTGGAC TTATGGAGAG GTTGTCAGAA   
  
  
+ GCACTGTACT TCTATGCAGC GCTGTTTGAC TGCTTAGAAT CCACCCTGCC AAGAACATCT GTCGAAAGAA   
  
  
+ GGCGGGTAGA GAAGATGCTC CTAGGTGAAG AGATCAAGAA CATTATATCA TGCGAGGGAG GAGAAAGGAG   
  
  
+ AGAAAGGCAT GAGAAGATCG AGAAGTGGAT GCAGAGGCTA GACATGGCTG GATTCGGGAT CGTTCCTTTG   
  
  
+ AGCTATATGG GTATGCTGCA AGCAAGGCAA TTGCTTCAGG GCTATGGTTG TGATGGTTAT AGAGTGAAAG   
  
  
+ AGAATGGTTG TGTTGTCATC TGTTGGCAAG ATCGCCCCCT CTTTTCGGTA TCAGCATGGA GGTGTAGGAG   
  
  
+ ATG  

- -Up\_Stream \_Len000TACGCG GAAAGACGAT TGAGTAATAG AAAGGAAGAC TCTCCCCTGT TGAATATATT   
  
  
- TAACTATCCA TAAAAGTTTA GTTATCTGGT CGATGGTATA TACTATTAAA AATAATGAAA AGATTTAGGT   
  
  
- AGTTGATAAA AGTGTTAGGA GGAAAAAGAT CTTTTTATTT TAAACGAATT AAGTTTAGTG GATTTTAATG   
  
  
- ATTTAGATAT CAGGATTTAG ATGTTTGAGT AAACACAACG TTGAGTACTA AATTCACCTT GTGATTGGAA   
  
  
- TCCTCTATAC AACAGTTTTC TTCTTAAGCT ACTGTATAAA AGGTTGTTTT CCGGTGGATT GAAGTACTAA   
  
  
- ATTTTTCCTC CTCAGATTCG TGTAGTACCT ACTGATTAAA AACTACATAA CATTGATGCA TAACTCTATT   
  
  
- GAACTTACTT AGCACTGATC TTATATAGAT AAAAGTTTTT ATTTTTAAGG GATTTTCTTT TCCTATTTTT   
  
  
- TGATATATTA TGATTTTTTA AGATTGATAT TATTGATCCG AATAATTTAA AAATATCACT TTTAAATAAA   
  
  
- GATGAATATT GTTGTAGTGA ATGATTCATC GAATAAACAC AGAAGAAAAG ATTTATTATT ACCATTGATA   
  
  
- CACAGAAAAC ATTTATTGAA TAATAGATAA AATATTTAGT GGCACATTTC GTGCCCAGAT ATGATCAATT   
  
  
- AAATTTATCG TAACAATTTA GTTCATTCTA TGTTTGTTTA AATAAATGTT GACATTCACC AGGTATTTTT   
  
  
- ATTATCGGTA ATGTTTTTTA TCATGATGAC AAAATACTTT ACTAAAATTA TAAGATTTTA AGGTTATTTT   
  
  
- GTACAATATT AGATACTAAT TAAAATTTTG TACTAATGTA CAGTCGTATT AAACATAATA ACATGTCGTT   
  
  
- TACTTTCAAC CTCGATTTTC TTTTACCCCT TTATTTCCGT CTGTTCATTT CTATTTTATA CGATAGATGA   
  
  
- AAACCTCCAT AGGAATTGGT GTAACAGAGC AAACAGAAGT AATCCCTCTT CACCCTATTT ATGGTTTTAA   
  
  
- ACATACCAGT AACGCACCCC AAACGAAGGT TTGTTTTACC GAAAAACCTT CGTTTAAAAC ACTCGAAGAT   
  
  
- ACGTTTAAAG GTGCGAATTG GGGTGTTCGT TTGTTTTTGT CTGTCACCAA AAACAAACAG AAACAATCCC   
  
  
- GTACAAACCT CGGTTGACAA TTACCCTTAT TTGACCCCTC CGAAAAAGGA ACCGAGAAAG GGGGACTGGG   
  
  
- GTTTGGGTGA AGTCGAGTTA TGCTTTGGTC GGTAACTTTT TCCTTCCCCC CCCCCCCCCA TACACTCTTT   
  
  
- TTCTGTTTTG AAAAAGGGCC TCTCTCTTCT ATGTATACGT TGTTTCCGAG AGGAAGAGTA TTTAAGGTAT   
  
  
- AAGGAGACAA AAAGATCCTC TCTCTTTATA CGTTTTTTGT TTAGTCAACC GCTGTTTTCA ATGATGGTGC   
  
  
- GTAACAGATG ACGACGCATG GGTAAAAACC TCTGAAATTT CTTGCGGGGA GGGTAGAACT TTTCACTTTT   
  
  
- GGGGGAGAAA CTTTGACTTA AAGTCCCCTT TTTTTGGAGT GGTTTGGAGA TCTCTCTCGA AAATCTCTCC   
  
  
- CTTTATGTTG CTAATCTTCC TCGTTAAACC CTTTAAAGAA ACCCTTAAAC TTACCCAAAA CTCACTTAAC   
  
  
- GTTTAGGGTC TTTTCAAAAC CGTTCATGGC TAGATGTCAA GAGAGGAGAA GCACAAACCA TCTAGGGGAC   
  
  
- AAAGGAGACA AAGTAAATCC CATGAAGAGT AAGTAGGAGG GGTGGGGGAA TTAGCCTAGA AGGACAGCTC   
  
  
- AGTGAAATAC GATTATAAAA AAGTCACCTA AAAATCAATT GGGACAAGTA AAAAGGTAAG ACACAGGGTA   
  
  
- AAGAGAGAAA AAGTAAGTAT TCAACGGCCA AATAGACAAC CCACGTCGAA TCAGTGTTAT TAAAGACACA   
  
  
- ATCCGAAGAA ATGCCAATTT TTTTTTCCTC CGTGAGAAAA GCCACACTAA CAAATACCCT GGTTACTAAG   
  
  
- TTCTACTACC CAGTAGTCAT TGAAGTAGTG GGGAAGTTAA AAAGAGGTAC TACAGAGGGT TAAATCCAAG   
  
  
- AAGGATGGGA ACCGAGTCTC TCGATTTTGG ACTTCTTTCT CCAGAAATGA ACTATGTAAA CAACGAGAGA   
  
  
- ACACGTTTAG TACAGAGATC ACCATCGGAT CTGTTACGCT TGAATCGGGA GCTTGTTTAG AGAGTCGAAC   
  
  
- GACGGGGACT ACCCCTATGT TACGTCGCAT ACCGAAGAAT AAAACGACTT CGGGACCGAC TCTCCTAGGA   
  
  
- GTTCAGTACC GGACCGTACA TATCTCGGGA AGTAAAATGC TTTTACGGAC AGTAAAGTCT CCTTTAAGAA   
  
  
- CGATCCTTCG AGAAGAAACT CGATAAAGGA AAGAACTTCG ACCGGATAAA CCACTGTTTG GTTAGCTATT   
  
  
- AGCTTCGGTA CCTCCCCCTT TTCTACCAAG TATAATAACT AGACTTACGT AGTCTTGGAC GAGTTACCTA   
  
  
- ACGGGAATAA GTCCGAAACT CACGTTCCGG ACCCCCAGGA GGAGTAAACT CTTAATGGCC ACAAGTAGTT   
  
  
- GTGTTTCTCC AAGATCTAGT TCACCGAGTA TCCCACTGAG TTCTTCGACT CTTTAACCTA AACGGTAAAG   
  
  
- TCAAGTTAGG ACACCAATCG TTCAACCTTT TGGAACTACA ACTTTTCGAC ACACAATTCT GACCACTCCG   
  
  
- AGATCGGTAG TCAAGCCAGG AAGTTGACGT ATGGGAAAAC CCAAGACTAC TACTCGGGGA TTCCTTTTCA   
  
  
- AGTGGAAATC GGAACTACTT CATACGTTTA CCCCGATTAT CCGTTTCGGG CTCATTACTA AGCCGAAGAA   
  
  
- GTAGTGGAGG AAGCGAGTTA TGAAGTTGGT TCGACCTACC AAAGGAGTCG CGAAACACCC CTAACAGGGG   
  
  
- TTTCTAATAC CACTATCGAC TCGTGCTAAG GTTAGTGTTA CCAAGACCTG AATACCTCTC CAACAGTCTT   
  
  
- CGTGACATGA AGATACGTCG CGACAAACTG ACGAATCTTA GGTGGGACGG TTCTTGTAGA CAGCTTTCTT   
  
  
- CCGCCCATCT CTTCTACGAG GATCCACTTC TCTAGTTCTT GTAATATAGT ACGCTCCCTC CTCTTTCCTC   
  
  
- TCTTTCCGTA CTCTTCTAGC TCTTCACCTA CGTCTCCGAT CTGTACCGAC CTAAGCCCTA GCAAGGAAAC   
  
  
- TCGATATACC CATACGACGT TCGTTCCGTT AACGAAGTCC CGATACCAAC ACTACCAATA TCTCACTTTC   
  
  
- TCTTACCAAC ACAACAGTAG ACAACCGTTC TAGCGGGGGA GAAAAGCCAT AGTCGTACCT CCACATCCTC   
  
  
- TAC

+     Box 4

| Site Name | Organism | Position | Strand | Matrix score. | sequence | function |
| --- | --- | --- | --- | --- | --- | --- |
| Box 4 | Petroselinum crispum | 861 | + | 6 | ATTAAT | part of a conserved DNA module involved in light responsiveness |

>HU08G00367.1   
+ -Up\_Stream \_Len000ATGCGC CTTTCTGCTA ACTCATTATC TTTCCTTCTG AGAGGGGACA ACTTATATAA   
  
  
+ ATTGATAGGT ATTTTCAAAT CAATAGACCA GCTACCATAT ATGATAATTT TTATTACTTT TCTAAATCCA   
  
  
+ TCAACTATTT TCACAATCCT CCTTTTTCTA GAAAAATAAA ATTTGCTTAA TTCAAATCAC CTAAAATTAC   
  
  
+ TAAATCTATA GTCCTAAATC TACAAACTCA TTTGTGTTGC AACTCATGAT TTAAGTGGAA CACTAACCTT   
  
  
+ AGGAGATATG TTGTCAAAAG AAGAATTCGA TGACATATTT TCCAACAAAA GGCCACCTAA CTTCATGATT   
  
  
+ TAAAAAGGAG GAGTCTAAGC ACATCATGGA TGACTAATTT TTGATGTATT GTAACTACGT ATTGAGATAA   
  
  
+ CTTGAATGAA TCGTGACTAG AATATATCTA TTTTCAAAAA TAAAAATTCC CTAAAAGAAA AGGATAAAAA   
  
  
+ ACTATATAAT ACTAAAAAAT TCTAACTATA ATAACTAGGC TTATTAAATT TTTATAGTGA AAATTTATTT   
  
  
+ CTACTTATAA CAACATCACT TACTAAGTAG CTTATTTGTG TCTTCTTTTC TAAATAATAA TGGTAACTAT   
  
  
+ GTGTCTTTTG TAAATAACTT ATTATCTATT TTATAAATCA CCGTGTAAAG CACGGGTCTA TACTAGTTAA   
  
  
+ TTTAAATAGC ATTGTTAAAT CAAGTAAGAT ACAAACAAAT TTATTTACAA CTGTAAGTGG TCCATAAAAA   
  
  
+ TAATAGCCAT TACAAAAAAT AGTACTACTG TTTTATGAAA TGATTTTAAT ATTCTAAAAT TCCAATAAAA   
  
  
+ CATGTTATAA TCTATGATTA ATTTTAAAAC ATGATTACAT GTCAGCATAA TTTGTATTAT TGTACAGCAA   
  
  
+ ATGAAAGTTG GAGCTAAAAG AAAATGGGGA AATAAAGGCA GACAAGTAAA GATAAAATAT GCTATCTACT   
  
  
+ TTTGGAGGTA TCCTTAACCA CATTGTCTCG TTTGTCTTCA TTAGGGAGAA GTGGGATAAA TACCAAAATT   
  
  
+ TGTATGGTCA TTGCGTGGGG TTTGCTTCCA AACAAAATGG CTTTTTGGAA GCAAATTTTG TGAGCTTCTA   
  
  
+ TGCAAATTTC CACGCTTAAC CCCACAAGCA AACAAAAACA GACAGTGGTT TTTGTTTGTC TTTGTTAGGG   
  
  
+ CATGTTTGGA GCCAACTGTT AATGGGAATA AACTGGGGAG GCTTTTTCCT TGGCTCTTTC CCCCTGACCC   
  
  
+ CAAACCCACT TCAGCTCAAT ACGAAACCAG CCATTGAAAA AGGAAGGGGG GGGGGGGGGT ATGTGAGAAA   
  
  
+ AAGACAAAAC TTTTTCCCGG AGAGAGAAGA TACATATGCA ACAAAGGCTC TCCTTCTCAT AAATTCCATA   
  
  
+ TTCCTCTGTT TTTCTAGGAG AGAGAAATAT GCAAAAAACA AATCAGTTGG CGACAAAAGT TACTACCACG   
  
  
+ CATTGTCTAC TGCTGCGTAC CCATTTTTGG AGACTTTAAA GAACGCCCCT CCCATCTTGA AAAGTGAAAA   
  
  
+ CCCCCTCTTT GAAACTGAAT TTCAGGGGAA AAAAACCTCA CCAAACCTCT AGAGAGAGCT TTTAGAGAGG   
  
  
+ GAAATACAAC GATTAGAAGG AGCAATTTGG GAAATTTCTT TGGGAATTTG AATGGGTTTT GAGTGAATTG   
  
  
+ CAAATCCCAG AAAAGTTTTG GCAAGTACCG ATCTACAGTT CTCTCCTCTT CGTGTTTGGT AGATCCCCTG   
  
  
+ TTTCCTCTGT TTCATTTAGG GTACTTCTCA TTCATCCTCC CCACCCCCTT AATCGGATCT TCCTGTCGAG   
  
  
+ TCACTTTATG CTAATATTTT TTCAGTGGAT TTTTAGTTAA CCCTGTTCAT TTTTCCATTC TGTGTCCCAT   
  
  
+ TTCTCTCTTT TTCATTCATA AGTTGCCGGT TTATCTGTTG GGTGCAGCTT AGTCACAATA ATTTCTGTGT   
  
  
+ TAGGCTTCTT TACGGTTAAA AAAAAAGGAG GCACTCTTTT CGGTGTGATT GTTTATGGGA CCAATGATTC   
  
  
+ AAGATGATGG GTCATCAGTA ACTTCATCAC CCCTTCAATT TTTCTCCATG ATGTCTCCCA ATTTAGGTTC   
  
  
+ TTCCTACCCT TGGCTCAGAG AGCTAAAACC TGAAGAAAGA GGTCTTTACT TGATACATTT GTTGCTCTCT   
  
  
+ TGTGCAAATC ATGTCTCTAG TGGTAGCCTA GACAATGCGA ACTTAGCCCT CGAACAAATC TCTCAGCTTG   
  
  
+ CTGCCCCTGA TGGGGATACA ATGCAGCGTA TGGCTTCTTA TTTTGCTGAA GCCCTGGCTG AGAGGATCCT   
  
  
+ CAAGTCATGG CCTGGCATGT ATAGAGCCCT TCATTTTACG AAAATGCCTG TCATTTCAGA GGAAATTCTT   
  
  
+ GCTAGGAAGC TCTTCTTTGA GCTATTTCCT TTCTTGAAGC TGGCCTATTT GGTGACAAAC CAATCGATAA   
  
  
+ TCGAAGCCAT GGAGGGGGAA AAGATGGTTC ATATTATTGA TCTGAATGCA TCAGAACCTG CTCAATGGAT   
  
  
+ TGCCCTTATT CAGGCTTTGA GTGCAAGGCC TGGGGGTCCT CCTCATTTGA GAATTACCGG TGTTCATCAA   
  
  
+ CACAAAGAGG TTCTAGATCA AGTGGCTCAT AGGGTGACTC AAGAAGCTGA GAAATTGGAT TTGCCATTTC   
  
  
+ AGTTCAATCC TGTGGTTAGC AAGTTGGAAA ACCTTGATGT TGAAAAGCTG TGTGTTAAGA CTGGTGAGGC   
  
  
+ TCTAGCCATC AGTTCGGTCC TTCAACTGCA TACCCTTTTG GGTTCTGATG ATGAGCCCCT AAGGAAAAGT   
  
  
+ TCACCTTTAG CCTTGATGAA GTATGCAAAT GGGGCTAATA GGCAAAGCCC GAGTAATGAT TCGGCTTCTT   
  
  
+ CATCACCTCC TTCGCTCAAT ACTTCAACCA AGCTGGATGG TTTCCTCAGC GCTTTGTGGG GATTGTCCCC   
  
  
+ AAAGATTATG GTGATAGCTG AGCACGATTC CAATCACAAT GGTTCTGGAC TTATGGAGAG GTTGTCAGAA   
  
  
+ GCACTGTACT TCTATGCAGC GCTGTTTGAC TGCTTAGAAT CCACCCTGCC AAGAACATCT GTCGAAAGAA   
  
  
+ GGCGGGTAGA GAAGATGCTC CTAGGTGAAG AGATCAAGAA CATTATATCA TGCGAGGGAG GAGAAAGGAG   
  
  
+ AGAAAGGCAT GAGAAGATCG AGAAGTGGAT GCAGAGGCTA GACATGGCTG GATTCGGGAT CGTTCCTTTG   
  
  
+ AGCTATATGG GTATGCTGCA AGCAAGGCAA TTGCTTCAGG GCTATGGTTG TGATGGTTAT AGAGTGAAAG   
  
  
+ AGAATGGTTG TGTTGTCATC TGTTGGCAAG ATCGCCCCCT CTTTTCGGTA TCAGCATGGA GGTGTAGGAG   
  
  
+ ATG  

- -Up\_Stream \_Len000TACGCG GAAAGACGAT TGAGTAATAG AAAGGAAGAC TCTCCCCTGT TGAATATATT   
  
  
- TAACTATCCA TAAAAGTTTA GTTATCTGGT CGATGGTATA TACTATTAAA AATAATGAAA AGATTTAGGT   
  
  
- AGTTGATAAA AGTGTTAGGA GGAAAAAGAT CTTTTTATTT TAAACGAATT AAGTTTAGTG GATTTTAATG   
  
  
- ATTTAGATAT CAGGATTTAG ATGTTTGAGT AAACACAACG TTGAGTACTA AATTCACCTT GTGATTGGAA   
  
  
- TCCTCTATAC AACAGTTTTC TTCTTAAGCT ACTGTATAAA AGGTTGTTTT CCGGTGGATT GAAGTACTAA   
  
  
- ATTTTTCCTC CTCAGATTCG TGTAGTACCT ACTGATTAAA AACTACATAA CATTGATGCA TAACTCTATT   
  
  
- GAACTTACTT AGCACTGATC TTATATAGAT AAAAGTTTTT ATTTTTAAGG GATTTTCTTT TCCTATTTTT   
  
  
- TGATATATTA TGATTTTTTA AGATTGATAT TATTGATCCG AATAATTTAA AAATATCACT TTTAAATAAA   
  
  
- GATGAATATT GTTGTAGTGA ATGATTCATC GAATAAACAC AGAAGAAAAG ATTTATTATT ACCATTGATA   
  
  
- CACAGAAAAC ATTTATTGAA TAATAGATAA AATATTTAGT GGCACATTTC GTGCCCAGAT ATGATCAATT   
  
  
- AAATTTATCG TAACAATTTA GTTCATTCTA TGTTTGTTTA AATAAATGTT GACATTCACC AGGTATTTTT   
  
  
- ATTATCGGTA ATGTTTTTTA TCATGATGAC AAAATACTTT ACTAAAATTA TAAGATTTTA AGGTTATTTT   
  
  
- GTACAATATT AGATACTAAT TAAAATTTTG TACTAATGTA CAGTCGTATT AAACATAATA ACATGTCGTT   
  
  
- TACTTTCAAC CTCGATTTTC TTTTACCCCT TTATTTCCGT CTGTTCATTT CTATTTTATA CGATAGATGA   
  
  
- AAACCTCCAT AGGAATTGGT GTAACAGAGC AAACAGAAGT AATCCCTCTT CACCCTATTT ATGGTTTTAA   
  
  
- ACATACCAGT AACGCACCCC AAACGAAGGT TTGTTTTACC GAAAAACCTT CGTTTAAAAC ACTCGAAGAT   
  
  
- ACGTTTAAAG GTGCGAATTG GGGTGTTCGT TTGTTTTTGT CTGTCACCAA AAACAAACAG AAACAATCCC   
  
  
- GTACAAACCT CGGTTGACAA TTACCCTTAT TTGACCCCTC CGAAAAAGGA ACCGAGAAAG GGGGACTGGG   
  
  
- GTTTGGGTGA AGTCGAGTTA TGCTTTGGTC GGTAACTTTT TCCTTCCCCC CCCCCCCCCA TACACTCTTT   
  
  
- TTCTGTTTTG AAAAAGGGCC TCTCTCTTCT ATGTATACGT TGTTTCCGAG AGGAAGAGTA TTTAAGGTAT   
  
  
- AAGGAGACAA AAAGATCCTC TCTCTTTATA CGTTTTTTGT TTAGTCAACC GCTGTTTTCA ATGATGGTGC   
  
  
- GTAACAGATG ACGACGCATG GGTAAAAACC TCTGAAATTT CTTGCGGGGA GGGTAGAACT TTTCACTTTT   
  
  
- GGGGGAGAAA CTTTGACTTA AAGTCCCCTT TTTTTGGAGT GGTTTGGAGA TCTCTCTCGA AAATCTCTCC   
  
  
- CTTTATGTTG CTAATCTTCC TCGTTAAACC CTTTAAAGAA ACCCTTAAAC TTACCCAAAA CTCACTTAAC   
  
  
- GTTTAGGGTC TTTTCAAAAC CGTTCATGGC TAGATGTCAA GAGAGGAGAA GCACAAACCA TCTAGGGGAC   
  
  
- AAAGGAGACA AAGTAAATCC CATGAAGAGT AAGTAGGAGG GGTGGGGGAA TTAGCCTAGA AGGACAGCTC   
  
  
- AGTGAAATAC GATTATAAAA AAGTCACCTA AAAATCAATT GGGACAAGTA AAAAGGTAAG ACACAGGGTA   
  
  
- AAGAGAGAAA AAGTAAGTAT TCAACGGCCA AATAGACAAC CCACGTCGAA TCAGTGTTAT TAAAGACACA   
  
  
- ATCCGAAGAA ATGCCAATTT TTTTTTCCTC CGTGAGAAAA GCCACACTAA CAAATACCCT GGTTACTAAG   
  
  
- TTCTACTACC CAGTAGTCAT TGAAGTAGTG GGGAAGTTAA AAAGAGGTAC TACAGAGGGT TAAATCCAAG   
  
  
- AAGGATGGGA ACCGAGTCTC TCGATTTTGG ACTTCTTTCT CCAGAAATGA ACTATGTAAA CAACGAGAGA   
  
  
- ACACGTTTAG TACAGAGATC ACCATCGGAT CTGTTACGCT TGAATCGGGA GCTTGTTTAG AGAGTCGAAC   
  
  
- GACGGGGACT ACCCCTATGT TACGTCGCAT ACCGAAGAAT AAAACGACTT CGGGACCGAC TCTCCTAGGA   
  
  
- GTTCAGTACC GGACCGTACA TATCTCGGGA AGTAAAATGC TTTTACGGAC AGTAAAGTCT CCTTTAAGAA   
  
  
- CGATCCTTCG AGAAGAAACT CGATAAAGGA AAGAACTTCG ACCGGATAAA CCACTGTTTG GTTAGCTATT   
  
  
- AGCTTCGGTA CCTCCCCCTT TTCTACCAAG TATAATAACT AGACTTACGT AGTCTTGGAC GAGTTACCTA   
  
  
- ACGGGAATAA GTCCGAAACT CACGTTCCGG ACCCCCAGGA GGAGTAAACT CTTAATGGCC ACAAGTAGTT   
  
  
- GTGTTTCTCC AAGATCTAGT TCACCGAGTA TCCCACTGAG TTCTTCGACT CTTTAACCTA AACGGTAAAG   
  
  
- TCAAGTTAGG ACACCAATCG TTCAACCTTT TGGAACTACA ACTTTTCGAC ACACAATTCT GACCACTCCG   
  
  
- AGATCGGTAG TCAAGCCAGG AAGTTGACGT ATGGGAAAAC CCAAGACTAC TACTCGGGGA TTCCTTTTCA   
  
  
- AGTGGAAATC GGAACTACTT CATACGTTTA CCCCGATTAT CCGTTTCGGG CTCATTACTA AGCCGAAGAA   
  
  
- GTAGTGGAGG AAGCGAGTTA TGAAGTTGGT TCGACCTACC AAAGGAGTCG CGAAACACCC CTAACAGGGG   
  
  
- TTTCTAATAC CACTATCGAC TCGTGCTAAG GTTAGTGTTA CCAAGACCTG AATACCTCTC CAACAGTCTT   
  
  
- CGTGACATGA AGATACGTCG CGACAAACTG ACGAATCTTA GGTGGGACGG TTCTTGTAGA CAGCTTTCTT   
  
  
- CCGCCCATCT CTTCTACGAG GATCCACTTC TCTAGTTCTT GTAATATAGT ACGCTCCCTC CTCTTTCCTC   
  
  
- TCTTTCCGTA CTCTTCTAGC TCTTCACCTA CGTCTCCGAT CTGTACCGAC CTAAGCCCTA GCAAGGAAAC   
  
  
- TCGATATACC CATACGACGT TCGTTCCGTT AACGAAGTCC CGATACCAAC ACTACCAATA TCTCACTTTC   
  
  
- TCTTACCAAC ACAACAGTAG ACAACCGTTC TAGCGGGGGA GAAAAGCCAT AGTCGTACCT CCACATCCTC   
  
  
- TAC

+     CAAT-box

| Site Name | Organism | Position | Strand | Matrix score. | sequence | function |
| --- | --- | --- | --- | --- | --- | --- |
| CAAT-box | Petunia hybrida | 3316 | - | 7 | TGCCAAC | common cis-acting element in promoter and enhancer regions |
| CAAT-box | Nicotiana glutinosa | 3252 | + | 4 | CAAT |  |
| CAAT-box | Nicotiana glutinosa | 2445 | + | 4 | CAAT |  |
| CAAT-box | Pisum sativum | 2830 | + | 5 | CAAAT | common cis-acting element in promoter and enhancer regions |
| CAAT-box | Pisum sativum | 2179 | + | 5 | CAAAT | common cis-acting element in promoter and enhancer regions |
| CAAT-box | Pisum sativum | 1660 | - | 5 | CAAAT | common cis-acting element in promoter and enhancer regions |
| CAAT-box | Nicotiana glutinosa | 3254 | - | 4 | CAAT |  |
| CAAT-box | Arabidopsis thaliana | 2092 | + | 5 | CCAAT | common cis-acting element in promoter and enhancer regions |
| CAAT-box | Nicotiana glutinosa | 2891 | + | 4 | CAAT |  |
| CAAT-box | Nicotiana glutinosa | 2936 | - | 4 | CAAT |  |
| CAAT-box | Nicotiana glutinosa | 1281 | + | 4 | CAAT |  |
| CAAT-box | Nicotiana glutinosa | 715 | - | 4 | CAAT |  |
| CAAT-box | Pisum sativum | 598 | - | 5 | CAAAT | common cis-acting element in promoter and enhancer regions |
| CAAT-box | Nicotiana glutinosa | 415 | - | 4 | CAAT |  |
| CAAT-box | Nicotiana glutinosa | 903 | - | 4 | CAAT |  |
| CAAT-box | Pisum sativum | 894 | - | 5 | CAAAT | common cis-acting element in promoter and enhancer regions |
| CAAT-box | Pisum sativum | 2653 | - | 5 | CAAAT | common cis-acting element in promoter and enhancer regions |
| CAAT-box | Arabidopsis thaliana | 2648 | - | 5 | CCAAT | common cis-acting element in promoter and enhancer regions |
| CAAT-box | Nicotiana glutinosa | 837 | + | 4 | CAAT |  |
| CAAT-box | Pisum sativum | 1639 | - | 5 | CAAAT | common cis-acting element in promoter and enhancer regions |
| CAAT-box | Nicotiana glutinosa | 2975 | + | 4 | CAAT |  |
| CAAT-box | Nicotiana glutinosa | 2263 | + | 4 | CAAT |  |
| CAAT-box | Pisum sativum | 740 | + | 5 | CAAAT | common cis-acting element in promoter and enhancer regions |
| CAAT-box | Pisum sativum | 1685 | + | 5 | CAAAT | common cis-acting element in promoter and enhancer regions |
| CAAT-box | Pisum sativum | 1443 | + | 5 | CAAAT | common cis-acting element in promoter and enhancer regions |
| CAAT-box | Nicotiana glutinosa | 1950 | + | 4 | CAAT |  |
| CAAT-box | Nicotiana glutinosa | 2669 | + | 4 | CAAT |  |
| CAAT-box | Pisum sativum | 2569 | - | 5 | CAAAT | common cis-acting element in promoter and enhancer regions |
| CAAT-box | Pisum sativum | 2229 | + | 5 | CAAAT | common cis-acting element in promoter and enhancer regions |
| CAAT-box | Nicotiana glutinosa | 2490 | - | 4 | CAAT |  |
| CAAT-box | Pisum sativum | 1052 | - | 5 | CAAAT | common cis-acting element in promoter and enhancer regions |
| CAAT-box | Pisum sativum | 1106 | + | 5 | CAAAT | common cis-acting element in promoter and enhancer regions |
| CAAT-box | Pisum sativum | 185 | - | 5 | CAAAT | common cis-acting element in promoter and enhancer regions |
| CAAT-box | Nicotiana glutinosa | 95 | + | 4 | CAAT |  |
| CAAT-box | Arabidopsis thaliana | 2974 | + | 5 | CCAAT | common cis-acting element in promoter and enhancer regions |
| CAAT-box | Pisum sativum | 912 | + | 5 | CAAAT | common cis-acting element in promoter and enhancer regions |
| CAAT-box | Nicotiana glutinosa | 158 | + | 4 | CAAT |  |
| CAAT-box | Pisum sativum | 2431 | - | 5 | CAAAT | common cis-acting element in promoter and enhancer regions |
| CAAT-box | Nicotiana glutinosa | 2523 | - | 4 | CAAT |  |
| CAAT-box | Nicotiana glutinosa | 2981 | + | 4 | CAAT |  |
| CAAT-box | Nicotiana glutinosa | 1681 | - | 4 | CAAT |  |
| CAAT-box | Nicotiana glutinosa | 1637 | + | 4 | CAAT |  |
| CAAT-box | Nicotiana glutinosa | 1006 | - | 4 | CAAT |  |
| CAAT-box | Arabidopsis thaliana | 836 | + | 5 | CCAAT | common cis-acting element in promoter and enhancer regions |
| CAAT-box | Nicotiana glutinosa | 2207 | + | 4 | CAAT |  |
| CAAT-box | Nicotiana glutinosa | 2517 | + | 4 | CAAT |  |
| CAAT-box | Nicotiana glutinosa | 2026 | + | 4 | CAAT |  |
| CAAT-box | Arabidopsis thaliana | 2091 | + | 8 | CCCAATTT | common cis-acting element in promoter and enhancer regions |
| CAAT-box | Pisum sativum | 197 | + | 5 | CAAAT | common cis-acting element in promoter and enhancer regions |
| CAAT-box | Nicotiana glutinosa | 1297 | - | 4 | CAAT |  |
| CAAT-box | Arabidopsis thaliana | 2444 | + | 5 | CCAAT | common cis-acting element in promoter and enhancer regions |
| CAAT-box | Nicotiana glutinosa | 1476 | - | 4 | CAAT |  |
| CAAT-box | Nicotiana glutinosa | 1064 | - | 4 | CAAT |  |
| CAAT-box | Nicotiana glutinosa | 2093 | + | 4 | CAAT |  |
| CAAT-box | Nicotiana glutinosa | 75 | - | 4 | CAAT |  |
| CAAT-box | Nicotiana glutinosa | 402 | - | 4 | CAAT |  |
| CAAT-box | Pisum sativum | 2161 | - | 5 | CAAAT | common cis-acting element in promoter and enhancer regions |
| CAAT-box | Pisum sativum | 244 | - | 5 | CAAAT | common cis-acting element in promoter and enhancer regions |
| CAAT-box | Nicotiana glutinosa | 2012 | - | 4 | CAAT |  |
| CAAT-box | Nicotiana glutinosa | 2070 | + | 4 | CAAT |  |
| CAAT-box | Pisum sativum | 1127 | + | 5 | CAAAT | common cis-acting element in promoter and enhancer regions |
| CAAT-box | Pisum sativum | 90 | + | 5 | CAAAT | common cis-acting element in promoter and enhancer regions |
| CAAT-box | Arabidopsis thaliana | 2025 | + | 5 | CCAAT | common cis-acting element in promoter and enhancer regions |

>HU08G00367.1   
+ -Up\_Stream \_Len000ATGCGC CTTTCTGCTA ACTCATTATC TTTCCTTCTG AGAGGGGACA ACTTATATAA   
  
  
+ ATTGATAGGT ATTTTCAAAT CAATAGACCA GCTACCATAT ATGATAATTT TTATTACTTT TCTAAATCCA   
  
  
+ TCAACTATTT TCACAATCCT CCTTTTTCTA GAAAAATAAA ATTTGCTTAA TTCAAATCAC CTAAAATTAC   
  
  
+ TAAATCTATA GTCCTAAATC TACAAACTCA TTTGTGTTGC AACTCATGAT TTAAGTGGAA CACTAACCTT   
  
  
+ AGGAGATATG TTGTCAAAAG AAGAATTCGA TGACATATTT TCCAACAAAA GGCCACCTAA CTTCATGATT   
  
  
+ TAAAAAGGAG GAGTCTAAGC ACATCATGGA TGACTAATTT TTGATGTATT GTAACTACGT ATTGAGATAA   
  
  
+ CTTGAATGAA TCGTGACTAG AATATATCTA TTTTCAAAAA TAAAAATTCC CTAAAAGAAA AGGATAAAAA   
  
  
+ ACTATATAAT ACTAAAAAAT TCTAACTATA ATAACTAGGC TTATTAAATT TTTATAGTGA AAATTTATTT   
  
  
+ CTACTTATAA CAACATCACT TACTAAGTAG CTTATTTGTG TCTTCTTTTC TAAATAATAA TGGTAACTAT   
  
  
+ GTGTCTTTTG TAAATAACTT ATTATCTATT TTATAAATCA CCGTGTAAAG CACGGGTCTA TACTAGTTAA   
  
  
+ TTTAAATAGC ATTGTTAAAT CAAGTAAGAT ACAAACAAAT TTATTTACAA CTGTAAGTGG TCCATAAAAA   
  
  
+ TAATAGCCAT TACAAAAAAT AGTACTACTG TTTTATGAAA TGATTTTAAT ATTCTAAAAT TCCAATAAAA   
  
  
+ CATGTTATAA TCTATGATTA ATTTTAAAAC ATGATTACAT GTCAGCATAA TTTGTATTAT TGTACAGCAA   
  
  
+ ATGAAAGTTG GAGCTAAAAG AAAATGGGGA AATAAAGGCA GACAAGTAAA GATAAAATAT GCTATCTACT   
  
  
+ TTTGGAGGTA TCCTTAACCA CATTGTCTCG TTTGTCTTCA TTAGGGAGAA GTGGGATAAA TACCAAAATT   
  
  
+ TGTATGGTCA TTGCGTGGGG TTTGCTTCCA AACAAAATGG CTTTTTGGAA GCAAATTTTG TGAGCTTCTA   
  
  
+ TGCAAATTTC CACGCTTAAC CCCACAAGCA AACAAAAACA GACAGTGGTT TTTGTTTGTC TTTGTTAGGG   
  
  
+ CATGTTTGGA GCCAACTGTT AATGGGAATA AACTGGGGAG GCTTTTTCCT TGGCTCTTTC CCCCTGACCC   
  
  
+ CAAACCCACT TCAGCTCAAT ACGAAACCAG CCATTGAAAA AGGAAGGGGG GGGGGGGGGT ATGTGAGAAA   
  
  
+ AAGACAAAAC TTTTTCCCGG AGAGAGAAGA TACATATGCA ACAAAGGCTC TCCTTCTCAT AAATTCCATA   
  
  
+ TTCCTCTGTT TTTCTAGGAG AGAGAAATAT GCAAAAAACA AATCAGTTGG CGACAAAAGT TACTACCACG   
  
  
+ CATTGTCTAC TGCTGCGTAC CCATTTTTGG AGACTTTAAA GAACGCCCCT CCCATCTTGA AAAGTGAAAA   
  
  
+ CCCCCTCTTT GAAACTGAAT TTCAGGGGAA AAAAACCTCA CCAAACCTCT AGAGAGAGCT TTTAGAGAGG   
  
  
+ GAAATACAAC GATTAGAAGG AGCAATTTGG GAAATTTCTT TGGGAATTTG AATGGGTTTT GAGTGAATTG   
  
  
+ CAAATCCCAG AAAAGTTTTG GCAAGTACCG ATCTACAGTT CTCTCCTCTT CGTGTTTGGT AGATCCCCTG   
  
  
+ TTTCCTCTGT TTCATTTAGG GTACTTCTCA TTCATCCTCC CCACCCCCTT AATCGGATCT TCCTGTCGAG   
  
  
+ TCACTTTATG CTAATATTTT TTCAGTGGAT TTTTAGTTAA CCCTGTTCAT TTTTCCATTC TGTGTCCCAT   
  
  
+ TTCTCTCTTT TTCATTCATA AGTTGCCGGT TTATCTGTTG GGTGCAGCTT AGTCACAATA ATTTCTGTGT   
  
  
+ TAGGCTTCTT TACGGTTAAA AAAAAAGGAG GCACTCTTTT CGGTGTGATT GTTTATGGGA CCAATGATTC   
  
  
+ AAGATGATGG GTCATCAGTA ACTTCATCAC CCCTTCAATT TTTCTCCATG ATGTCTCCCA ATTTAGGTTC   
  
  
+ TTCCTACCCT TGGCTCAGAG AGCTAAAACC TGAAGAAAGA GGTCTTTACT TGATACATTT GTTGCTCTCT   
  
  
+ TGTGCAAATC ATGTCTCTAG TGGTAGCCTA GACAATGCGA ACTTAGCCCT CGAACAAATC TCTCAGCTTG   
  
  
+ CTGCCCCTGA TGGGGATACA ATGCAGCGTA TGGCTTCTTA TTTTGCTGAA GCCCTGGCTG AGAGGATCCT   
  
  
+ CAAGTCATGG CCTGGCATGT ATAGAGCCCT TCATTTTACG AAAATGCCTG TCATTTCAGA GGAAATTCTT   
  
  
+ GCTAGGAAGC TCTTCTTTGA GCTATTTCCT TTCTTGAAGC TGGCCTATTT GGTGACAAAC CAATCGATAA   
  
  
+ TCGAAGCCAT GGAGGGGGAA AAGATGGTTC ATATTATTGA TCTGAATGCA TCAGAACCTG CTCAATGGAT   
  
  
+ TGCCCTTATT CAGGCTTTGA GTGCAAGGCC TGGGGGTCCT CCTCATTTGA GAATTACCGG TGTTCATCAA   
  
  
+ CACAAAGAGG TTCTAGATCA AGTGGCTCAT AGGGTGACTC AAGAAGCTGA GAAATTGGAT TTGCCATTTC   
  
  
+ AGTTCAATCC TGTGGTTAGC AAGTTGGAAA ACCTTGATGT TGAAAAGCTG TGTGTTAAGA CTGGTGAGGC   
  
  
+ TCTAGCCATC AGTTCGGTCC TTCAACTGCA TACCCTTTTG GGTTCTGATG ATGAGCCCCT AAGGAAAAGT   
  
  
+ TCACCTTTAG CCTTGATGAA GTATGCAAAT GGGGCTAATA GGCAAAGCCC GAGTAATGAT TCGGCTTCTT   
  
  
+ CATCACCTCC TTCGCTCAAT ACTTCAACCA AGCTGGATGG TTTCCTCAGC GCTTTGTGGG GATTGTCCCC   
  
  
+ AAAGATTATG GTGATAGCTG AGCACGATTC CAATCACAAT GGTTCTGGAC TTATGGAGAG GTTGTCAGAA   
  
  
+ GCACTGTACT TCTATGCAGC GCTGTTTGAC TGCTTAGAAT CCACCCTGCC AAGAACATCT GTCGAAAGAA   
  
  
+ GGCGGGTAGA GAAGATGCTC CTAGGTGAAG AGATCAAGAA CATTATATCA TGCGAGGGAG GAGAAAGGAG   
  
  
+ AGAAAGGCAT GAGAAGATCG AGAAGTGGAT GCAGAGGCTA GACATGGCTG GATTCGGGAT CGTTCCTTTG   
  
  
+ AGCTATATGG GTATGCTGCA AGCAAGGCAA TTGCTTCAGG GCTATGGTTG TGATGGTTAT AGAGTGAAAG   
  
  
+ AGAATGGTTG TGTTGTCATC TGTTGGCAAG ATCGCCCCCT CTTTTCGGTA TCAGCATGGA GGTGTAGGAG   
  
  
+ ATG  

- -Up\_Stream \_Len000TACGCG GAAAGACGAT TGAGTAATAG AAAGGAAGAC TCTCCCCTGT TGAATATATT   
  
  
- TAACTATCCA TAAAAGTTTA GTTATCTGGT CGATGGTATA TACTATTAAA AATAATGAAA AGATTTAGGT   
  
  
- AGTTGATAAA AGTGTTAGGA GGAAAAAGAT CTTTTTATTT TAAACGAATT AAGTTTAGTG GATTTTAATG   
  
  
- ATTTAGATAT CAGGATTTAG ATGTTTGAGT AAACACAACG TTGAGTACTA AATTCACCTT GTGATTGGAA   
  
  
- TCCTCTATAC AACAGTTTTC TTCTTAAGCT ACTGTATAAA AGGTTGTTTT CCGGTGGATT GAAGTACTAA   
  
  
- ATTTTTCCTC CTCAGATTCG TGTAGTACCT ACTGATTAAA AACTACATAA CATTGATGCA TAACTCTATT   
  
  
- GAACTTACTT AGCACTGATC TTATATAGAT AAAAGTTTTT ATTTTTAAGG GATTTTCTTT TCCTATTTTT   
  
  
- TGATATATTA TGATTTTTTA AGATTGATAT TATTGATCCG AATAATTTAA AAATATCACT TTTAAATAAA   
  
  
- GATGAATATT GTTGTAGTGA ATGATTCATC GAATAAACAC AGAAGAAAAG ATTTATTATT ACCATTGATA   
  
  
- CACAGAAAAC ATTTATTGAA TAATAGATAA AATATTTAGT GGCACATTTC GTGCCCAGAT ATGATCAATT   
  
  
- AAATTTATCG TAACAATTTA GTTCATTCTA TGTTTGTTTA AATAAATGTT GACATTCACC AGGTATTTTT   
  
  
- ATTATCGGTA ATGTTTTTTA TCATGATGAC AAAATACTTT ACTAAAATTA TAAGATTTTA AGGTTATTTT   
  
  
- GTACAATATT AGATACTAAT TAAAATTTTG TACTAATGTA CAGTCGTATT AAACATAATA ACATGTCGTT   
  
  
- TACTTTCAAC CTCGATTTTC TTTTACCCCT TTATTTCCGT CTGTTCATTT CTATTTTATA CGATAGATGA   
  
  
- AAACCTCCAT AGGAATTGGT GTAACAGAGC AAACAGAAGT AATCCCTCTT CACCCTATTT ATGGTTTTAA   
  
  
- ACATACCAGT AACGCACCCC AAACGAAGGT TTGTTTTACC GAAAAACCTT CGTTTAAAAC ACTCGAAGAT   
  
  
- ACGTTTAAAG GTGCGAATTG GGGTGTTCGT TTGTTTTTGT CTGTCACCAA AAACAAACAG AAACAATCCC   
  
  
- GTACAAACCT CGGTTGACAA TTACCCTTAT TTGACCCCTC CGAAAAAGGA ACCGAGAAAG GGGGACTGGG   
  
  
- GTTTGGGTGA AGTCGAGTTA TGCTTTGGTC GGTAACTTTT TCCTTCCCCC CCCCCCCCCA TACACTCTTT   
  
  
- TTCTGTTTTG AAAAAGGGCC TCTCTCTTCT ATGTATACGT TGTTTCCGAG AGGAAGAGTA TTTAAGGTAT   
  
  
- AAGGAGACAA AAAGATCCTC TCTCTTTATA CGTTTTTTGT TTAGTCAACC GCTGTTTTCA ATGATGGTGC   
  
  
- GTAACAGATG ACGACGCATG GGTAAAAACC TCTGAAATTT CTTGCGGGGA GGGTAGAACT TTTCACTTTT   
  
  
- GGGGGAGAAA CTTTGACTTA AAGTCCCCTT TTTTTGGAGT GGTTTGGAGA TCTCTCTCGA AAATCTCTCC   
  
  
- CTTTATGTTG CTAATCTTCC TCGTTAAACC CTTTAAAGAA ACCCTTAAAC TTACCCAAAA CTCACTTAAC   
  
  
- GTTTAGGGTC TTTTCAAAAC CGTTCATGGC TAGATGTCAA GAGAGGAGAA GCACAAACCA TCTAGGGGAC   
  
  
- AAAGGAGACA AAGTAAATCC CATGAAGAGT AAGTAGGAGG GGTGGGGGAA TTAGCCTAGA AGGACAGCTC   
  
  
- AGTGAAATAC GATTATAAAA AAGTCACCTA AAAATCAATT GGGACAAGTA AAAAGGTAAG ACACAGGGTA   
  
  
- AAGAGAGAAA AAGTAAGTAT TCAACGGCCA AATAGACAAC CCACGTCGAA TCAGTGTTAT TAAAGACACA   
  
  
- ATCCGAAGAA ATGCCAATTT TTTTTTCCTC CGTGAGAAAA GCCACACTAA CAAATACCCT GGTTACTAAG   
  
  
- TTCTACTACC CAGTAGTCAT TGAAGTAGTG GGGAAGTTAA AAAGAGGTAC TACAGAGGGT TAAATCCAAG   
  
  
- AAGGATGGGA ACCGAGTCTC TCGATTTTGG ACTTCTTTCT CCAGAAATGA ACTATGTAAA CAACGAGAGA   
  
  
- ACACGTTTAG TACAGAGATC ACCATCGGAT CTGTTACGCT TGAATCGGGA GCTTGTTTAG AGAGTCGAAC   
  
  
- GACGGGGACT ACCCCTATGT TACGTCGCAT ACCGAAGAAT AAAACGACTT CGGGACCGAC TCTCCTAGGA   
  
  
- GTTCAGTACC GGACCGTACA TATCTCGGGA AGTAAAATGC TTTTACGGAC AGTAAAGTCT CCTTTAAGAA   
  
  
- CGATCCTTCG AGAAGAAACT CGATAAAGGA AAGAACTTCG ACCGGATAAA CCACTGTTTG GTTAGCTATT   
  
  
- AGCTTCGGTA CCTCCCCCTT TTCTACCAAG TATAATAACT AGACTTACGT AGTCTTGGAC GAGTTACCTA   
  
  
- ACGGGAATAA GTCCGAAACT CACGTTCCGG ACCCCCAGGA GGAGTAAACT CTTAATGGCC ACAAGTAGTT   
  
  
- GTGTTTCTCC AAGATCTAGT TCACCGAGTA TCCCACTGAG TTCTTCGACT CTTTAACCTA AACGGTAAAG   
  
  
- TCAAGTTAGG ACACCAATCG TTCAACCTTT TGGAACTACA ACTTTTCGAC ACACAATTCT GACCACTCCG   
  
  
- AGATCGGTAG TCAAGCCAGG AAGTTGACGT ATGGGAAAAC CCAAGACTAC TACTCGGGGA TTCCTTTTCA   
  
  
- AGTGGAAATC GGAACTACTT CATACGTTTA CCCCGATTAT CCGTTTCGGG CTCATTACTA AGCCGAAGAA   
  
  
- GTAGTGGAGG AAGCGAGTTA TGAAGTTGGT TCGACCTACC AAAGGAGTCG CGAAACACCC CTAACAGGGG   
  
  
- TTTCTAATAC CACTATCGAC TCGTGCTAAG GTTAGTGTTA CCAAGACCTG AATACCTCTC CAACAGTCTT   
  
  
- CGTGACATGA AGATACGTCG CGACAAACTG ACGAATCTTA GGTGGGACGG TTCTTGTAGA CAGCTTTCTT   
  
  
- CCGCCCATCT CTTCTACGAG GATCCACTTC TCTAGTTCTT GTAATATAGT ACGCTCCCTC CTCTTTCCTC   
  
  
- TCTTTCCGTA CTCTTCTAGC TCTTCACCTA CGTCTCCGAT CTGTACCGAC CTAAGCCCTA GCAAGGAAAC   
  
  
- TCGATATACC CATACGACGT TCGTTCCGTT AACGAAGTCC CGATACCAAC ACTACCAATA TCTCACTTTC   
  
  
- TCTTACCAAC ACAACAGTAG ACAACCGTTC TAGCGGGGGA GAAAAGCCAT AGTCGTACCT CCACATCCTC   
  
  
- TAC

+     CAG-motif

| Site Name | Organism | Position | Strand | Matrix score. | sequence | function |
| --- | --- | --- | --- | --- | --- | --- |
| CAG-motif | Arabidopsis thaliana | 947 | + | 10 | GAAAGGCAGAC | part of a light response element |

>HU08G00367.1   
+ -Up\_Stream \_Len000ATGCGC CTTTCTGCTA ACTCATTATC TTTCCTTCTG AGAGGGGACA ACTTATATAA   
  
  
+ ATTGATAGGT ATTTTCAAAT CAATAGACCA GCTACCATAT ATGATAATTT TTATTACTTT TCTAAATCCA   
  
  
+ TCAACTATTT TCACAATCCT CCTTTTTCTA GAAAAATAAA ATTTGCTTAA TTCAAATCAC CTAAAATTAC   
  
  
+ TAAATCTATA GTCCTAAATC TACAAACTCA TTTGTGTTGC AACTCATGAT TTAAGTGGAA CACTAACCTT   
  
  
+ AGGAGATATG TTGTCAAAAG AAGAATTCGA TGACATATTT TCCAACAAAA GGCCACCTAA CTTCATGATT   
  
  
+ TAAAAAGGAG GAGTCTAAGC ACATCATGGA TGACTAATTT TTGATGTATT GTAACTACGT ATTGAGATAA   
  
  
+ CTTGAATGAA TCGTGACTAG AATATATCTA TTTTCAAAAA TAAAAATTCC CTAAAAGAAA AGGATAAAAA   
  
  
+ ACTATATAAT ACTAAAAAAT TCTAACTATA ATAACTAGGC TTATTAAATT TTTATAGTGA AAATTTATTT   
  
  
+ CTACTTATAA CAACATCACT TACTAAGTAG CTTATTTGTG TCTTCTTTTC TAAATAATAA TGGTAACTAT   
  
  
+ GTGTCTTTTG TAAATAACTT ATTATCTATT TTATAAATCA CCGTGTAAAG CACGGGTCTA TACTAGTTAA   
  
  
+ TTTAAATAGC ATTGTTAAAT CAAGTAAGAT ACAAACAAAT TTATTTACAA CTGTAAGTGG TCCATAAAAA   
  
  
+ TAATAGCCAT TACAAAAAAT AGTACTACTG TTTTATGAAA TGATTTTAAT ATTCTAAAAT TCCAATAAAA   
  
  
+ CATGTTATAA TCTATGATTA ATTTTAAAAC ATGATTACAT GTCAGCATAA TTTGTATTAT TGTACAGCAA   
  
  
+ ATGAAAGTTG GAGCTAAAAG AAAATGGGGA AATAAAGGCA GACAAGTAAA GATAAAATAT GCTATCTACT   
  
  
+ TTTGGAGGTA TCCTTAACCA CATTGTCTCG TTTGTCTTCA TTAGGGAGAA GTGGGATAAA TACCAAAATT   
  
  
+ TGTATGGTCA TTGCGTGGGG TTTGCTTCCA AACAAAATGG CTTTTTGGAA GCAAATTTTG TGAGCTTCTA   
  
  
+ TGCAAATTTC CACGCTTAAC CCCACAAGCA AACAAAAACA GACAGTGGTT TTTGTTTGTC TTTGTTAGGG   
  
  
+ CATGTTTGGA GCCAACTGTT AATGGGAATA AACTGGGGAG GCTTTTTCCT TGGCTCTTTC CCCCTGACCC   
  
  
+ CAAACCCACT TCAGCTCAAT ACGAAACCAG CCATTGAAAA AGGAAGGGGG GGGGGGGGGT ATGTGAGAAA   
  
  
+ AAGACAAAAC TTTTTCCCGG AGAGAGAAGA TACATATGCA ACAAAGGCTC TCCTTCTCAT AAATTCCATA   
  
  
+ TTCCTCTGTT TTTCTAGGAG AGAGAAATAT GCAAAAAACA AATCAGTTGG CGACAAAAGT TACTACCACG   
  
  
+ CATTGTCTAC TGCTGCGTAC CCATTTTTGG AGACTTTAAA GAACGCCCCT CCCATCTTGA AAAGTGAAAA   
  
  
+ CCCCCTCTTT GAAACTGAAT TTCAGGGGAA AAAAACCTCA CCAAACCTCT AGAGAGAGCT TTTAGAGAGG   
  
  
+ GAAATACAAC GATTAGAAGG AGCAATTTGG GAAATTTCTT TGGGAATTTG AATGGGTTTT GAGTGAATTG   
  
  
+ CAAATCCCAG AAAAGTTTTG GCAAGTACCG ATCTACAGTT CTCTCCTCTT CGTGTTTGGT AGATCCCCTG   
  
  
+ TTTCCTCTGT TTCATTTAGG GTACTTCTCA TTCATCCTCC CCACCCCCTT AATCGGATCT TCCTGTCGAG   
  
  
+ TCACTTTATG CTAATATTTT TTCAGTGGAT TTTTAGTTAA CCCTGTTCAT TTTTCCATTC TGTGTCCCAT   
  
  
+ TTCTCTCTTT TTCATTCATA AGTTGCCGGT TTATCTGTTG GGTGCAGCTT AGTCACAATA ATTTCTGTGT   
  
  
+ TAGGCTTCTT TACGGTTAAA AAAAAAGGAG GCACTCTTTT CGGTGTGATT GTTTATGGGA CCAATGATTC   
  
  
+ AAGATGATGG GTCATCAGTA ACTTCATCAC CCCTTCAATT TTTCTCCATG ATGTCTCCCA ATTTAGGTTC   
  
  
+ TTCCTACCCT TGGCTCAGAG AGCTAAAACC TGAAGAAAGA GGTCTTTACT TGATACATTT GTTGCTCTCT   
  
  
+ TGTGCAAATC ATGTCTCTAG TGGTAGCCTA GACAATGCGA ACTTAGCCCT CGAACAAATC TCTCAGCTTG   
  
  
+ CTGCCCCTGA TGGGGATACA ATGCAGCGTA TGGCTTCTTA TTTTGCTGAA GCCCTGGCTG AGAGGATCCT   
  
  
+ CAAGTCATGG CCTGGCATGT ATAGAGCCCT TCATTTTACG AAAATGCCTG TCATTTCAGA GGAAATTCTT   
  
  
+ GCTAGGAAGC TCTTCTTTGA GCTATTTCCT TTCTTGAAGC TGGCCTATTT GGTGACAAAC CAATCGATAA   
  
  
+ TCGAAGCCAT GGAGGGGGAA AAGATGGTTC ATATTATTGA TCTGAATGCA TCAGAACCTG CTCAATGGAT   
  
  
+ TGCCCTTATT CAGGCTTTGA GTGCAAGGCC TGGGGGTCCT CCTCATTTGA GAATTACCGG TGTTCATCAA   
  
  
+ CACAAAGAGG TTCTAGATCA AGTGGCTCAT AGGGTGACTC AAGAAGCTGA GAAATTGGAT TTGCCATTTC   
  
  
+ AGTTCAATCC TGTGGTTAGC AAGTTGGAAA ACCTTGATGT TGAAAAGCTG TGTGTTAAGA CTGGTGAGGC   
  
  
+ TCTAGCCATC AGTTCGGTCC TTCAACTGCA TACCCTTTTG GGTTCTGATG ATGAGCCCCT AAGGAAAAGT   
  
  
+ TCACCTTTAG CCTTGATGAA GTATGCAAAT GGGGCTAATA GGCAAAGCCC GAGTAATGAT TCGGCTTCTT   
  
  
+ CATCACCTCC TTCGCTCAAT ACTTCAACCA AGCTGGATGG TTTCCTCAGC GCTTTGTGGG GATTGTCCCC   
  
  
+ AAAGATTATG GTGATAGCTG AGCACGATTC CAATCACAAT GGTTCTGGAC TTATGGAGAG GTTGTCAGAA   
  
  
+ GCACTGTACT TCTATGCAGC GCTGTTTGAC TGCTTAGAAT CCACCCTGCC AAGAACATCT GTCGAAAGAA   
  
  
+ GGCGGGTAGA GAAGATGCTC CTAGGTGAAG AGATCAAGAA CATTATATCA TGCGAGGGAG GAGAAAGGAG   
  
  
+ AGAAAGGCAT GAGAAGATCG AGAAGTGGAT GCAGAGGCTA GACATGGCTG GATTCGGGAT CGTTCCTTTG   
  
  
+ AGCTATATGG GTATGCTGCA AGCAAGGCAA TTGCTTCAGG GCTATGGTTG TGATGGTTAT AGAGTGAAAG   
  
  
+ AGAATGGTTG TGTTGTCATC TGTTGGCAAG ATCGCCCCCT CTTTTCGGTA TCAGCATGGA GGTGTAGGAG   
  
  
+ ATG  

- -Up\_Stream \_Len000TACGCG GAAAGACGAT TGAGTAATAG AAAGGAAGAC TCTCCCCTGT TGAATATATT   
  
  
- TAACTATCCA TAAAAGTTTA GTTATCTGGT CGATGGTATA TACTATTAAA AATAATGAAA AGATTTAGGT   
  
  
- AGTTGATAAA AGTGTTAGGA GGAAAAAGAT CTTTTTATTT TAAACGAATT AAGTTTAGTG GATTTTAATG   
  
  
- ATTTAGATAT CAGGATTTAG ATGTTTGAGT AAACACAACG TTGAGTACTA AATTCACCTT GTGATTGGAA   
  
  
- TCCTCTATAC AACAGTTTTC TTCTTAAGCT ACTGTATAAA AGGTTGTTTT CCGGTGGATT GAAGTACTAA   
  
  
- ATTTTTCCTC CTCAGATTCG TGTAGTACCT ACTGATTAAA AACTACATAA CATTGATGCA TAACTCTATT   
  
  
- GAACTTACTT AGCACTGATC TTATATAGAT AAAAGTTTTT ATTTTTAAGG GATTTTCTTT TCCTATTTTT   
  
  
- TGATATATTA TGATTTTTTA AGATTGATAT TATTGATCCG AATAATTTAA AAATATCACT TTTAAATAAA   
  
  
- GATGAATATT GTTGTAGTGA ATGATTCATC GAATAAACAC AGAAGAAAAG ATTTATTATT ACCATTGATA   
  
  
- CACAGAAAAC ATTTATTGAA TAATAGATAA AATATTTAGT GGCACATTTC GTGCCCAGAT ATGATCAATT   
  
  
- AAATTTATCG TAACAATTTA GTTCATTCTA TGTTTGTTTA AATAAATGTT GACATTCACC AGGTATTTTT   
  
  
- ATTATCGGTA ATGTTTTTTA TCATGATGAC AAAATACTTT ACTAAAATTA TAAGATTTTA AGGTTATTTT   
  
  
- GTACAATATT AGATACTAAT TAAAATTTTG TACTAATGTA CAGTCGTATT AAACATAATA ACATGTCGTT   
  
  
- TACTTTCAAC CTCGATTTTC TTTTACCCCT TTATTTCCGT CTGTTCATTT CTATTTTATA CGATAGATGA   
  
  
- AAACCTCCAT AGGAATTGGT GTAACAGAGC AAACAGAAGT AATCCCTCTT CACCCTATTT ATGGTTTTAA   
  
  
- ACATACCAGT AACGCACCCC AAACGAAGGT TTGTTTTACC GAAAAACCTT CGTTTAAAAC ACTCGAAGAT   
  
  
- ACGTTTAAAG GTGCGAATTG GGGTGTTCGT TTGTTTTTGT CTGTCACCAA AAACAAACAG AAACAATCCC   
  
  
- GTACAAACCT CGGTTGACAA TTACCCTTAT TTGACCCCTC CGAAAAAGGA ACCGAGAAAG GGGGACTGGG   
  
  
- GTTTGGGTGA AGTCGAGTTA TGCTTTGGTC GGTAACTTTT TCCTTCCCCC CCCCCCCCCA TACACTCTTT   
  
  
- TTCTGTTTTG AAAAAGGGCC TCTCTCTTCT ATGTATACGT TGTTTCCGAG AGGAAGAGTA TTTAAGGTAT   
  
  
- AAGGAGACAA AAAGATCCTC TCTCTTTATA CGTTTTTTGT TTAGTCAACC GCTGTTTTCA ATGATGGTGC   
  
  
- GTAACAGATG ACGACGCATG GGTAAAAACC TCTGAAATTT CTTGCGGGGA GGGTAGAACT TTTCACTTTT   
  
  
- GGGGGAGAAA CTTTGACTTA AAGTCCCCTT TTTTTGGAGT GGTTTGGAGA TCTCTCTCGA AAATCTCTCC   
  
  
- CTTTATGTTG CTAATCTTCC TCGTTAAACC CTTTAAAGAA ACCCTTAAAC TTACCCAAAA CTCACTTAAC   
  
  
- GTTTAGGGTC TTTTCAAAAC CGTTCATGGC TAGATGTCAA GAGAGGAGAA GCACAAACCA TCTAGGGGAC   
  
  
- AAAGGAGACA AAGTAAATCC CATGAAGAGT AAGTAGGAGG GGTGGGGGAA TTAGCCTAGA AGGACAGCTC   
  
  
- AGTGAAATAC GATTATAAAA AAGTCACCTA AAAATCAATT GGGACAAGTA AAAAGGTAAG ACACAGGGTA   
  
  
- AAGAGAGAAA AAGTAAGTAT TCAACGGCCA AATAGACAAC CCACGTCGAA TCAGTGTTAT TAAAGACACA   
  
  
- ATCCGAAGAA ATGCCAATTT TTTTTTCCTC CGTGAGAAAA GCCACACTAA CAAATACCCT GGTTACTAAG   
  
  
- TTCTACTACC CAGTAGTCAT TGAAGTAGTG GGGAAGTTAA AAAGAGGTAC TACAGAGGGT TAAATCCAAG   
  
  
- AAGGATGGGA ACCGAGTCTC TCGATTTTGG ACTTCTTTCT CCAGAAATGA ACTATGTAAA CAACGAGAGA   
  
  
- ACACGTTTAG TACAGAGATC ACCATCGGAT CTGTTACGCT TGAATCGGGA GCTTGTTTAG AGAGTCGAAC   
  
  
- GACGGGGACT ACCCCTATGT TACGTCGCAT ACCGAAGAAT AAAACGACTT CGGGACCGAC TCTCCTAGGA   
  
  
- GTTCAGTACC GGACCGTACA TATCTCGGGA AGTAAAATGC TTTTACGGAC AGTAAAGTCT CCTTTAAGAA   
  
  
- CGATCCTTCG AGAAGAAACT CGATAAAGGA AAGAACTTCG ACCGGATAAA CCACTGTTTG GTTAGCTATT   
  
  
- AGCTTCGGTA CCTCCCCCTT TTCTACCAAG TATAATAACT AGACTTACGT AGTCTTGGAC GAGTTACCTA   
  
  
- ACGGGAATAA GTCCGAAACT CACGTTCCGG ACCCCCAGGA GGAGTAAACT CTTAATGGCC ACAAGTAGTT   
  
  
- GTGTTTCTCC AAGATCTAGT TCACCGAGTA TCCCACTGAG TTCTTCGACT CTTTAACCTA AACGGTAAAG   
  
  
- TCAAGTTAGG ACACCAATCG TTCAACCTTT TGGAACTACA ACTTTTCGAC ACACAATTCT GACCACTCCG   
  
  
- AGATCGGTAG TCAAGCCAGG AAGTTGACGT ATGGGAAAAC CCAAGACTAC TACTCGGGGA TTCCTTTTCA   
  
  
- AGTGGAAATC GGAACTACTT CATACGTTTA CCCCGATTAT CCGTTTCGGG CTCATTACTA AGCCGAAGAA   
  
  
- GTAGTGGAGG AAGCGAGTTA TGAAGTTGGT TCGACCTACC AAAGGAGTCG CGAAACACCC CTAACAGGGG   
  
  
- TTTCTAATAC CACTATCGAC TCGTGCTAAG GTTAGTGTTA CCAAGACCTG AATACCTCTC CAACAGTCTT   
  
  
- CGTGACATGA AGATACGTCG CGACAAACTG ACGAATCTTA GGTGGGACGG TTCTTGTAGA CAGCTTTCTT   
  
  
- CCGCCCATCT CTTCTACGAG GATCCACTTC TCTAGTTCTT GTAATATAGT ACGCTCCCTC CTCTTTCCTC   
  
  
- TCTTTCCGTA CTCTTCTAGC TCTTCACCTA CGTCTCCGAT CTGTACCGAC CTAAGCCCTA GCAAGGAAAC   
  
  
- TCGATATACC CATACGACGT TCGTTCCGTT AACGAAGTCC CGATACCAAC ACTACCAATA TCTCACTTTC   
  
  
- TCTTACCAAC ACAACAGTAG ACAACCGTTC TAGCGGGGGA GAAAAGCCAT AGTCGTACCT CCACATCCTC   
  
  
- TAC

+     CAT-box

| Site Name | Organism | Position | Strand | Matrix score. | sequence | function |
| --- | --- | --- | --- | --- | --- | --- |
| CAT-box | Arabidopsis thaliana | 2615 | - | 6 | GCCACT | cis-acting regulatory element related to meristem expression |

>HU08G00367.1   
+ -Up\_Stream \_Len000ATGCGC CTTTCTGCTA ACTCATTATC TTTCCTTCTG AGAGGGGACA ACTTATATAA   
  
  
+ ATTGATAGGT ATTTTCAAAT CAATAGACCA GCTACCATAT ATGATAATTT TTATTACTTT TCTAAATCCA   
  
  
+ TCAACTATTT TCACAATCCT CCTTTTTCTA GAAAAATAAA ATTTGCTTAA TTCAAATCAC CTAAAATTAC   
  
  
+ TAAATCTATA GTCCTAAATC TACAAACTCA TTTGTGTTGC AACTCATGAT TTAAGTGGAA CACTAACCTT   
  
  
+ AGGAGATATG TTGTCAAAAG AAGAATTCGA TGACATATTT TCCAACAAAA GGCCACCTAA CTTCATGATT   
  
  
+ TAAAAAGGAG GAGTCTAAGC ACATCATGGA TGACTAATTT TTGATGTATT GTAACTACGT ATTGAGATAA   
  
  
+ CTTGAATGAA TCGTGACTAG AATATATCTA TTTTCAAAAA TAAAAATTCC CTAAAAGAAA AGGATAAAAA   
  
  
+ ACTATATAAT ACTAAAAAAT TCTAACTATA ATAACTAGGC TTATTAAATT TTTATAGTGA AAATTTATTT   
  
  
+ CTACTTATAA CAACATCACT TACTAAGTAG CTTATTTGTG TCTTCTTTTC TAAATAATAA TGGTAACTAT   
  
  
+ GTGTCTTTTG TAAATAACTT ATTATCTATT TTATAAATCA CCGTGTAAAG CACGGGTCTA TACTAGTTAA   
  
  
+ TTTAAATAGC ATTGTTAAAT CAAGTAAGAT ACAAACAAAT TTATTTACAA CTGTAAGTGG TCCATAAAAA   
  
  
+ TAATAGCCAT TACAAAAAAT AGTACTACTG TTTTATGAAA TGATTTTAAT ATTCTAAAAT TCCAATAAAA   
  
  
+ CATGTTATAA TCTATGATTA ATTTTAAAAC ATGATTACAT GTCAGCATAA TTTGTATTAT TGTACAGCAA   
  
  
+ ATGAAAGTTG GAGCTAAAAG AAAATGGGGA AATAAAGGCA GACAAGTAAA GATAAAATAT GCTATCTACT   
  
  
+ TTTGGAGGTA TCCTTAACCA CATTGTCTCG TTTGTCTTCA TTAGGGAGAA GTGGGATAAA TACCAAAATT   
  
  
+ TGTATGGTCA TTGCGTGGGG TTTGCTTCCA AACAAAATGG CTTTTTGGAA GCAAATTTTG TGAGCTTCTA   
  
  
+ TGCAAATTTC CACGCTTAAC CCCACAAGCA AACAAAAACA GACAGTGGTT TTTGTTTGTC TTTGTTAGGG   
  
  
+ CATGTTTGGA GCCAACTGTT AATGGGAATA AACTGGGGAG GCTTTTTCCT TGGCTCTTTC CCCCTGACCC   
  
  
+ CAAACCCACT TCAGCTCAAT ACGAAACCAG CCATTGAAAA AGGAAGGGGG GGGGGGGGGT ATGTGAGAAA   
  
  
+ AAGACAAAAC TTTTTCCCGG AGAGAGAAGA TACATATGCA ACAAAGGCTC TCCTTCTCAT AAATTCCATA   
  
  
+ TTCCTCTGTT TTTCTAGGAG AGAGAAATAT GCAAAAAACA AATCAGTTGG CGACAAAAGT TACTACCACG   
  
  
+ CATTGTCTAC TGCTGCGTAC CCATTTTTGG AGACTTTAAA GAACGCCCCT CCCATCTTGA AAAGTGAAAA   
  
  
+ CCCCCTCTTT GAAACTGAAT TTCAGGGGAA AAAAACCTCA CCAAACCTCT AGAGAGAGCT TTTAGAGAGG   
  
  
+ GAAATACAAC GATTAGAAGG AGCAATTTGG GAAATTTCTT TGGGAATTTG AATGGGTTTT GAGTGAATTG   
  
  
+ CAAATCCCAG AAAAGTTTTG GCAAGTACCG ATCTACAGTT CTCTCCTCTT CGTGTTTGGT AGATCCCCTG   
  
  
+ TTTCCTCTGT TTCATTTAGG GTACTTCTCA TTCATCCTCC CCACCCCCTT AATCGGATCT TCCTGTCGAG   
  
  
+ TCACTTTATG CTAATATTTT TTCAGTGGAT TTTTAGTTAA CCCTGTTCAT TTTTCCATTC TGTGTCCCAT   
  
  
+ TTCTCTCTTT TTCATTCATA AGTTGCCGGT TTATCTGTTG GGTGCAGCTT AGTCACAATA ATTTCTGTGT   
  
  
+ TAGGCTTCTT TACGGTTAAA AAAAAAGGAG GCACTCTTTT CGGTGTGATT GTTTATGGGA CCAATGATTC   
  
  
+ AAGATGATGG GTCATCAGTA ACTTCATCAC CCCTTCAATT TTTCTCCATG ATGTCTCCCA ATTTAGGTTC   
  
  
+ TTCCTACCCT TGGCTCAGAG AGCTAAAACC TGAAGAAAGA GGTCTTTACT TGATACATTT GTTGCTCTCT   
  
  
+ TGTGCAAATC ATGTCTCTAG TGGTAGCCTA GACAATGCGA ACTTAGCCCT CGAACAAATC TCTCAGCTTG   
  
  
+ CTGCCCCTGA TGGGGATACA ATGCAGCGTA TGGCTTCTTA TTTTGCTGAA GCCCTGGCTG AGAGGATCCT   
  
  
+ CAAGTCATGG CCTGGCATGT ATAGAGCCCT TCATTTTACG AAAATGCCTG TCATTTCAGA GGAAATTCTT   
  
  
+ GCTAGGAAGC TCTTCTTTGA GCTATTTCCT TTCTTGAAGC TGGCCTATTT GGTGACAAAC CAATCGATAA   
  
  
+ TCGAAGCCAT GGAGGGGGAA AAGATGGTTC ATATTATTGA TCTGAATGCA TCAGAACCTG CTCAATGGAT   
  
  
+ TGCCCTTATT CAGGCTTTGA GTGCAAGGCC TGGGGGTCCT CCTCATTTGA GAATTACCGG TGTTCATCAA   
  
  
+ CACAAAGAGG TTCTAGATCA AGTGGCTCAT AGGGTGACTC AAGAAGCTGA GAAATTGGAT TTGCCATTTC   
  
  
+ AGTTCAATCC TGTGGTTAGC AAGTTGGAAA ACCTTGATGT TGAAAAGCTG TGTGTTAAGA CTGGTGAGGC   
  
  
+ TCTAGCCATC AGTTCGGTCC TTCAACTGCA TACCCTTTTG GGTTCTGATG ATGAGCCCCT AAGGAAAAGT   
  
  
+ TCACCTTTAG CCTTGATGAA GTATGCAAAT GGGGCTAATA GGCAAAGCCC GAGTAATGAT TCGGCTTCTT   
  
  
+ CATCACCTCC TTCGCTCAAT ACTTCAACCA AGCTGGATGG TTTCCTCAGC GCTTTGTGGG GATTGTCCCC   
  
  
+ AAAGATTATG GTGATAGCTG AGCACGATTC CAATCACAAT GGTTCTGGAC TTATGGAGAG GTTGTCAGAA   
  
  
+ GCACTGTACT TCTATGCAGC GCTGTTTGAC TGCTTAGAAT CCACCCTGCC AAGAACATCT GTCGAAAGAA   
  
  
+ GGCGGGTAGA GAAGATGCTC CTAGGTGAAG AGATCAAGAA CATTATATCA TGCGAGGGAG GAGAAAGGAG   
  
  
+ AGAAAGGCAT GAGAAGATCG AGAAGTGGAT GCAGAGGCTA GACATGGCTG GATTCGGGAT CGTTCCTTTG   
  
  
+ AGCTATATGG GTATGCTGCA AGCAAGGCAA TTGCTTCAGG GCTATGGTTG TGATGGTTAT AGAGTGAAAG   
  
  
+ AGAATGGTTG TGTTGTCATC TGTTGGCAAG ATCGCCCCCT CTTTTCGGTA TCAGCATGGA GGTGTAGGAG   
  
  
+ ATG  

- -Up\_Stream \_Len000TACGCG GAAAGACGAT TGAGTAATAG AAAGGAAGAC TCTCCCCTGT TGAATATATT   
  
  
- TAACTATCCA TAAAAGTTTA GTTATCTGGT CGATGGTATA TACTATTAAA AATAATGAAA AGATTTAGGT   
  
  
- AGTTGATAAA AGTGTTAGGA GGAAAAAGAT CTTTTTATTT TAAACGAATT AAGTTTAGTG GATTTTAATG   
  
  
- ATTTAGATAT CAGGATTTAG ATGTTTGAGT AAACACAACG TTGAGTACTA AATTCACCTT GTGATTGGAA   
  
  
- TCCTCTATAC AACAGTTTTC TTCTTAAGCT ACTGTATAAA AGGTTGTTTT CCGGTGGATT GAAGTACTAA   
  
  
- ATTTTTCCTC CTCAGATTCG TGTAGTACCT ACTGATTAAA AACTACATAA CATTGATGCA TAACTCTATT   
  
  
- GAACTTACTT AGCACTGATC TTATATAGAT AAAAGTTTTT ATTTTTAAGG GATTTTCTTT TCCTATTTTT   
  
  
- TGATATATTA TGATTTTTTA AGATTGATAT TATTGATCCG AATAATTTAA AAATATCACT TTTAAATAAA   
  
  
- GATGAATATT GTTGTAGTGA ATGATTCATC GAATAAACAC AGAAGAAAAG ATTTATTATT ACCATTGATA   
  
  
- CACAGAAAAC ATTTATTGAA TAATAGATAA AATATTTAGT GGCACATTTC GTGCCCAGAT ATGATCAATT   
  
  
- AAATTTATCG TAACAATTTA GTTCATTCTA TGTTTGTTTA AATAAATGTT GACATTCACC AGGTATTTTT   
  
  
- ATTATCGGTA ATGTTTTTTA TCATGATGAC AAAATACTTT ACTAAAATTA TAAGATTTTA AGGTTATTTT   
  
  
- GTACAATATT AGATACTAAT TAAAATTTTG TACTAATGTA CAGTCGTATT AAACATAATA ACATGTCGTT   
  
  
- TACTTTCAAC CTCGATTTTC TTTTACCCCT TTATTTCCGT CTGTTCATTT CTATTTTATA CGATAGATGA   
  
  
- AAACCTCCAT AGGAATTGGT GTAACAGAGC AAACAGAAGT AATCCCTCTT CACCCTATTT ATGGTTTTAA   
  
  
- ACATACCAGT AACGCACCCC AAACGAAGGT TTGTTTTACC GAAAAACCTT CGTTTAAAAC ACTCGAAGAT   
  
  
- ACGTTTAAAG GTGCGAATTG GGGTGTTCGT TTGTTTTTGT CTGTCACCAA AAACAAACAG AAACAATCCC   
  
  
- GTACAAACCT CGGTTGACAA TTACCCTTAT TTGACCCCTC CGAAAAAGGA ACCGAGAAAG GGGGACTGGG   
  
  
- GTTTGGGTGA AGTCGAGTTA TGCTTTGGTC GGTAACTTTT TCCTTCCCCC CCCCCCCCCA TACACTCTTT   
  
  
- TTCTGTTTTG AAAAAGGGCC TCTCTCTTCT ATGTATACGT TGTTTCCGAG AGGAAGAGTA TTTAAGGTAT   
  
  
- AAGGAGACAA AAAGATCCTC TCTCTTTATA CGTTTTTTGT TTAGTCAACC GCTGTTTTCA ATGATGGTGC   
  
  
- GTAACAGATG ACGACGCATG GGTAAAAACC TCTGAAATTT CTTGCGGGGA GGGTAGAACT TTTCACTTTT   
  
  
- GGGGGAGAAA CTTTGACTTA AAGTCCCCTT TTTTTGGAGT GGTTTGGAGA TCTCTCTCGA AAATCTCTCC   
  
  
- CTTTATGTTG CTAATCTTCC TCGTTAAACC CTTTAAAGAA ACCCTTAAAC TTACCCAAAA CTCACTTAAC   
  
  
- GTTTAGGGTC TTTTCAAAAC CGTTCATGGC TAGATGTCAA GAGAGGAGAA GCACAAACCA TCTAGGGGAC   
  
  
- AAAGGAGACA AAGTAAATCC CATGAAGAGT AAGTAGGAGG GGTGGGGGAA TTAGCCTAGA AGGACAGCTC   
  
  
- AGTGAAATAC GATTATAAAA AAGTCACCTA AAAATCAATT GGGACAAGTA AAAAGGTAAG ACACAGGGTA   
  
  
- AAGAGAGAAA AAGTAAGTAT TCAACGGCCA AATAGACAAC CCACGTCGAA TCAGTGTTAT TAAAGACACA   
  
  
- ATCCGAAGAA ATGCCAATTT TTTTTTCCTC CGTGAGAAAA GCCACACTAA CAAATACCCT GGTTACTAAG   
  
  
- TTCTACTACC CAGTAGTCAT TGAAGTAGTG GGGAAGTTAA AAAGAGGTAC TACAGAGGGT TAAATCCAAG   
  
  
- AAGGATGGGA ACCGAGTCTC TCGATTTTGG ACTTCTTTCT CCAGAAATGA ACTATGTAAA CAACGAGAGA   
  
  
- ACACGTTTAG TACAGAGATC ACCATCGGAT CTGTTACGCT TGAATCGGGA GCTTGTTTAG AGAGTCGAAC   
  
  
- GACGGGGACT ACCCCTATGT TACGTCGCAT ACCGAAGAAT AAAACGACTT CGGGACCGAC TCTCCTAGGA   
  
  
- GTTCAGTACC GGACCGTACA TATCTCGGGA AGTAAAATGC TTTTACGGAC AGTAAAGTCT CCTTTAAGAA   
  
  
- CGATCCTTCG AGAAGAAACT CGATAAAGGA AAGAACTTCG ACCGGATAAA CCACTGTTTG GTTAGCTATT   
  
  
- AGCTTCGGTA CCTCCCCCTT TTCTACCAAG TATAATAACT AGACTTACGT AGTCTTGGAC GAGTTACCTA   
  
  
- ACGGGAATAA GTCCGAAACT CACGTTCCGG ACCCCCAGGA GGAGTAAACT CTTAATGGCC ACAAGTAGTT   
  
  
- GTGTTTCTCC AAGATCTAGT TCACCGAGTA TCCCACTGAG TTCTTCGACT CTTTAACCTA AACGGTAAAG   
  
  
- TCAAGTTAGG ACACCAATCG TTCAACCTTT TGGAACTACA ACTTTTCGAC ACACAATTCT GACCACTCCG   
  
  
- AGATCGGTAG TCAAGCCAGG AAGTTGACGT ATGGGAAAAC CCAAGACTAC TACTCGGGGA TTCCTTTTCA   
  
  
- AGTGGAAATC GGAACTACTT CATACGTTTA CCCCGATTAT CCGTTTCGGG CTCATTACTA AGCCGAAGAA   
  
  
- GTAGTGGAGG AAGCGAGTTA TGAAGTTGGT TCGACCTACC AAAGGAGTCG CGAAACACCC CTAACAGGGG   
  
  
- TTTCTAATAC CACTATCGAC TCGTGCTAAG GTTAGTGTTA CCAAGACCTG AATACCTCTC CAACAGTCTT   
  
  
- CGTGACATGA AGATACGTCG CGACAAACTG ACGAATCTTA GGTGGGACGG TTCTTGTAGA CAGCTTTCTT   
  
  
- CCGCCCATCT CTTCTACGAG GATCCACTTC TCTAGTTCTT GTAATATAGT ACGCTCCCTC CTCTTTCCTC   
  
  
- TCTTTCCGTA CTCTTCTAGC TCTTCACCTA CGTCTCCGAT CTGTACCGAC CTAAGCCCTA GCAAGGAAAC   
  
  
- TCGATATACC CATACGACGT TCGTTCCGTT AACGAAGTCC CGATACCAAC ACTACCAATA TCTCACTTTC   
  
  
- TCTTACCAAC ACAACAGTAG ACAACCGTTC TAGCGGGGGA GAAAAGCCAT AGTCGTACCT CCACATCCTC   
  
  
- TAC

+     ERE

| Site Name | Organism | Position | Strand | Matrix score. | sequence | function |
| --- | --- | --- | --- | --- | --- | --- |
| ERE | Nicotiana glutinos | 808 | - | 8 | ATTTCATA |  |
| ERE | Nicotiana glutinos | 865 | + | 8 | ATTTTAAA |  |

>HU08G00367.1   
+ -Up\_Stream \_Len000ATGCGC CTTTCTGCTA ACTCATTATC TTTCCTTCTG AGAGGGGACA ACTTATATAA   
  
  
+ ATTGATAGGT ATTTTCAAAT CAATAGACCA GCTACCATAT ATGATAATTT TTATTACTTT TCTAAATCCA   
  
  
+ TCAACTATTT TCACAATCCT CCTTTTTCTA GAAAAATAAA ATTTGCTTAA TTCAAATCAC CTAAAATTAC   
  
  
+ TAAATCTATA GTCCTAAATC TACAAACTCA TTTGTGTTGC AACTCATGAT TTAAGTGGAA CACTAACCTT   
  
  
+ AGGAGATATG TTGTCAAAAG AAGAATTCGA TGACATATTT TCCAACAAAA GGCCACCTAA CTTCATGATT   
  
  
+ TAAAAAGGAG GAGTCTAAGC ACATCATGGA TGACTAATTT TTGATGTATT GTAACTACGT ATTGAGATAA   
  
  
+ CTTGAATGAA TCGTGACTAG AATATATCTA TTTTCAAAAA TAAAAATTCC CTAAAAGAAA AGGATAAAAA   
  
  
+ ACTATATAAT ACTAAAAAAT TCTAACTATA ATAACTAGGC TTATTAAATT TTTATAGTGA AAATTTATTT   
  
  
+ CTACTTATAA CAACATCACT TACTAAGTAG CTTATTTGTG TCTTCTTTTC TAAATAATAA TGGTAACTAT   
  
  
+ GTGTCTTTTG TAAATAACTT ATTATCTATT TTATAAATCA CCGTGTAAAG CACGGGTCTA TACTAGTTAA   
  
  
+ TTTAAATAGC ATTGTTAAAT CAAGTAAGAT ACAAACAAAT TTATTTACAA CTGTAAGTGG TCCATAAAAA   
  
  
+ TAATAGCCAT TACAAAAAAT AGTACTACTG TTTTATGAAA TGATTTTAAT ATTCTAAAAT TCCAATAAAA   
  
  
+ CATGTTATAA TCTATGATTA ATTTTAAAAC ATGATTACAT GTCAGCATAA TTTGTATTAT TGTACAGCAA   
  
  
+ ATGAAAGTTG GAGCTAAAAG AAAATGGGGA AATAAAGGCA GACAAGTAAA GATAAAATAT GCTATCTACT   
  
  
+ TTTGGAGGTA TCCTTAACCA CATTGTCTCG TTTGTCTTCA TTAGGGAGAA GTGGGATAAA TACCAAAATT   
  
  
+ TGTATGGTCA TTGCGTGGGG TTTGCTTCCA AACAAAATGG CTTTTTGGAA GCAAATTTTG TGAGCTTCTA   
  
  
+ TGCAAATTTC CACGCTTAAC CCCACAAGCA AACAAAAACA GACAGTGGTT TTTGTTTGTC TTTGTTAGGG   
  
  
+ CATGTTTGGA GCCAACTGTT AATGGGAATA AACTGGGGAG GCTTTTTCCT TGGCTCTTTC CCCCTGACCC   
  
  
+ CAAACCCACT TCAGCTCAAT ACGAAACCAG CCATTGAAAA AGGAAGGGGG GGGGGGGGGT ATGTGAGAAA   
  
  
+ AAGACAAAAC TTTTTCCCGG AGAGAGAAGA TACATATGCA ACAAAGGCTC TCCTTCTCAT AAATTCCATA   
  
  
+ TTCCTCTGTT TTTCTAGGAG AGAGAAATAT GCAAAAAACA AATCAGTTGG CGACAAAAGT TACTACCACG   
  
  
+ CATTGTCTAC TGCTGCGTAC CCATTTTTGG AGACTTTAAA GAACGCCCCT CCCATCTTGA AAAGTGAAAA   
  
  
+ CCCCCTCTTT GAAACTGAAT TTCAGGGGAA AAAAACCTCA CCAAACCTCT AGAGAGAGCT TTTAGAGAGG   
  
  
+ GAAATACAAC GATTAGAAGG AGCAATTTGG GAAATTTCTT TGGGAATTTG AATGGGTTTT GAGTGAATTG   
  
  
+ CAAATCCCAG AAAAGTTTTG GCAAGTACCG ATCTACAGTT CTCTCCTCTT CGTGTTTGGT AGATCCCCTG   
  
  
+ TTTCCTCTGT TTCATTTAGG GTACTTCTCA TTCATCCTCC CCACCCCCTT AATCGGATCT TCCTGTCGAG   
  
  
+ TCACTTTATG CTAATATTTT TTCAGTGGAT TTTTAGTTAA CCCTGTTCAT TTTTCCATTC TGTGTCCCAT   
  
  
+ TTCTCTCTTT TTCATTCATA AGTTGCCGGT TTATCTGTTG GGTGCAGCTT AGTCACAATA ATTTCTGTGT   
  
  
+ TAGGCTTCTT TACGGTTAAA AAAAAAGGAG GCACTCTTTT CGGTGTGATT GTTTATGGGA CCAATGATTC   
  
  
+ AAGATGATGG GTCATCAGTA ACTTCATCAC CCCTTCAATT TTTCTCCATG ATGTCTCCCA ATTTAGGTTC   
  
  
+ TTCCTACCCT TGGCTCAGAG AGCTAAAACC TGAAGAAAGA GGTCTTTACT TGATACATTT GTTGCTCTCT   
  
  
+ TGTGCAAATC ATGTCTCTAG TGGTAGCCTA GACAATGCGA ACTTAGCCCT CGAACAAATC TCTCAGCTTG   
  
  
+ CTGCCCCTGA TGGGGATACA ATGCAGCGTA TGGCTTCTTA TTTTGCTGAA GCCCTGGCTG AGAGGATCCT   
  
  
+ CAAGTCATGG CCTGGCATGT ATAGAGCCCT TCATTTTACG AAAATGCCTG TCATTTCAGA GGAAATTCTT   
  
  
+ GCTAGGAAGC TCTTCTTTGA GCTATTTCCT TTCTTGAAGC TGGCCTATTT GGTGACAAAC CAATCGATAA   
  
  
+ TCGAAGCCAT GGAGGGGGAA AAGATGGTTC ATATTATTGA TCTGAATGCA TCAGAACCTG CTCAATGGAT   
  
  
+ TGCCCTTATT CAGGCTTTGA GTGCAAGGCC TGGGGGTCCT CCTCATTTGA GAATTACCGG TGTTCATCAA   
  
  
+ CACAAAGAGG TTCTAGATCA AGTGGCTCAT AGGGTGACTC AAGAAGCTGA GAAATTGGAT TTGCCATTTC   
  
  
+ AGTTCAATCC TGTGGTTAGC AAGTTGGAAA ACCTTGATGT TGAAAAGCTG TGTGTTAAGA CTGGTGAGGC   
  
  
+ TCTAGCCATC AGTTCGGTCC TTCAACTGCA TACCCTTTTG GGTTCTGATG ATGAGCCCCT AAGGAAAAGT   
  
  
+ TCACCTTTAG CCTTGATGAA GTATGCAAAT GGGGCTAATA GGCAAAGCCC GAGTAATGAT TCGGCTTCTT   
  
  
+ CATCACCTCC TTCGCTCAAT ACTTCAACCA AGCTGGATGG TTTCCTCAGC GCTTTGTGGG GATTGTCCCC   
  
  
+ AAAGATTATG GTGATAGCTG AGCACGATTC CAATCACAAT GGTTCTGGAC TTATGGAGAG GTTGTCAGAA   
  
  
+ GCACTGTACT TCTATGCAGC GCTGTTTGAC TGCTTAGAAT CCACCCTGCC AAGAACATCT GTCGAAAGAA   
  
  
+ GGCGGGTAGA GAAGATGCTC CTAGGTGAAG AGATCAAGAA CATTATATCA TGCGAGGGAG GAGAAAGGAG   
  
  
+ AGAAAGGCAT GAGAAGATCG AGAAGTGGAT GCAGAGGCTA GACATGGCTG GATTCGGGAT CGTTCCTTTG   
  
  
+ AGCTATATGG GTATGCTGCA AGCAAGGCAA TTGCTTCAGG GCTATGGTTG TGATGGTTAT AGAGTGAAAG   
  
  
+ AGAATGGTTG TGTTGTCATC TGTTGGCAAG ATCGCCCCCT CTTTTCGGTA TCAGCATGGA GGTGTAGGAG   
  
  
+ ATG  

- -Up\_Stream \_Len000TACGCG GAAAGACGAT TGAGTAATAG AAAGGAAGAC TCTCCCCTGT TGAATATATT   
  
  
- TAACTATCCA TAAAAGTTTA GTTATCTGGT CGATGGTATA TACTATTAAA AATAATGAAA AGATTTAGGT   
  
  
- AGTTGATAAA AGTGTTAGGA GGAAAAAGAT CTTTTTATTT TAAACGAATT AAGTTTAGTG GATTTTAATG   
  
  
- ATTTAGATAT CAGGATTTAG ATGTTTGAGT AAACACAACG TTGAGTACTA AATTCACCTT GTGATTGGAA   
  
  
- TCCTCTATAC AACAGTTTTC TTCTTAAGCT ACTGTATAAA AGGTTGTTTT CCGGTGGATT GAAGTACTAA   
  
  
- ATTTTTCCTC CTCAGATTCG TGTAGTACCT ACTGATTAAA AACTACATAA CATTGATGCA TAACTCTATT   
  
  
- GAACTTACTT AGCACTGATC TTATATAGAT AAAAGTTTTT ATTTTTAAGG GATTTTCTTT TCCTATTTTT   
  
  
- TGATATATTA TGATTTTTTA AGATTGATAT TATTGATCCG AATAATTTAA AAATATCACT TTTAAATAAA   
  
  
- GATGAATATT GTTGTAGTGA ATGATTCATC GAATAAACAC AGAAGAAAAG ATTTATTATT ACCATTGATA   
  
  
- CACAGAAAAC ATTTATTGAA TAATAGATAA AATATTTAGT GGCACATTTC GTGCCCAGAT ATGATCAATT   
  
  
- AAATTTATCG TAACAATTTA GTTCATTCTA TGTTTGTTTA AATAAATGTT GACATTCACC AGGTATTTTT   
  
  
- ATTATCGGTA ATGTTTTTTA TCATGATGAC AAAATACTTT ACTAAAATTA TAAGATTTTA AGGTTATTTT   
  
  
- GTACAATATT AGATACTAAT TAAAATTTTG TACTAATGTA CAGTCGTATT AAACATAATA ACATGTCGTT   
  
  
- TACTTTCAAC CTCGATTTTC TTTTACCCCT TTATTTCCGT CTGTTCATTT CTATTTTATA CGATAGATGA   
  
  
- AAACCTCCAT AGGAATTGGT GTAACAGAGC AAACAGAAGT AATCCCTCTT CACCCTATTT ATGGTTTTAA   
  
  
- ACATACCAGT AACGCACCCC AAACGAAGGT TTGTTTTACC GAAAAACCTT CGTTTAAAAC ACTCGAAGAT   
  
  
- ACGTTTAAAG GTGCGAATTG GGGTGTTCGT TTGTTTTTGT CTGTCACCAA AAACAAACAG AAACAATCCC   
  
  
- GTACAAACCT CGGTTGACAA TTACCCTTAT TTGACCCCTC CGAAAAAGGA ACCGAGAAAG GGGGACTGGG   
  
  
- GTTTGGGTGA AGTCGAGTTA TGCTTTGGTC GGTAACTTTT TCCTTCCCCC CCCCCCCCCA TACACTCTTT   
  
  
- TTCTGTTTTG AAAAAGGGCC TCTCTCTTCT ATGTATACGT TGTTTCCGAG AGGAAGAGTA TTTAAGGTAT   
  
  
- AAGGAGACAA AAAGATCCTC TCTCTTTATA CGTTTTTTGT TTAGTCAACC GCTGTTTTCA ATGATGGTGC   
  
  
- GTAACAGATG ACGACGCATG GGTAAAAACC TCTGAAATTT CTTGCGGGGA GGGTAGAACT TTTCACTTTT   
  
  
- GGGGGAGAAA CTTTGACTTA AAGTCCCCTT TTTTTGGAGT GGTTTGGAGA TCTCTCTCGA AAATCTCTCC   
  
  
- CTTTATGTTG CTAATCTTCC TCGTTAAACC CTTTAAAGAA ACCCTTAAAC TTACCCAAAA CTCACTTAAC   
  
  
- GTTTAGGGTC TTTTCAAAAC CGTTCATGGC TAGATGTCAA GAGAGGAGAA GCACAAACCA TCTAGGGGAC   
  
  
- AAAGGAGACA AAGTAAATCC CATGAAGAGT AAGTAGGAGG GGTGGGGGAA TTAGCCTAGA AGGACAGCTC   
  
  
- AGTGAAATAC GATTATAAAA AAGTCACCTA AAAATCAATT GGGACAAGTA AAAAGGTAAG ACACAGGGTA   
  
  
- AAGAGAGAAA AAGTAAGTAT TCAACGGCCA AATAGACAAC CCACGTCGAA TCAGTGTTAT TAAAGACACA   
  
  
- ATCCGAAGAA ATGCCAATTT TTTTTTCCTC CGTGAGAAAA GCCACACTAA CAAATACCCT GGTTACTAAG   
  
  
- TTCTACTACC CAGTAGTCAT TGAAGTAGTG GGGAAGTTAA AAAGAGGTAC TACAGAGGGT TAAATCCAAG   
  
  
- AAGGATGGGA ACCGAGTCTC TCGATTTTGG ACTTCTTTCT CCAGAAATGA ACTATGTAAA CAACGAGAGA   
  
  
- ACACGTTTAG TACAGAGATC ACCATCGGAT CTGTTACGCT TGAATCGGGA GCTTGTTTAG AGAGTCGAAC   
  
  
- GACGGGGACT ACCCCTATGT TACGTCGCAT ACCGAAGAAT AAAACGACTT CGGGACCGAC TCTCCTAGGA   
  
  
- GTTCAGTACC GGACCGTACA TATCTCGGGA AGTAAAATGC TTTTACGGAC AGTAAAGTCT CCTTTAAGAA   
  
  
- CGATCCTTCG AGAAGAAACT CGATAAAGGA AAGAACTTCG ACCGGATAAA CCACTGTTTG GTTAGCTATT   
  
  
- AGCTTCGGTA CCTCCCCCTT TTCTACCAAG TATAATAACT AGACTTACGT AGTCTTGGAC GAGTTACCTA   
  
  
- ACGGGAATAA GTCCGAAACT CACGTTCCGG ACCCCCAGGA GGAGTAAACT CTTAATGGCC ACAAGTAGTT   
  
  
- GTGTTTCTCC AAGATCTAGT TCACCGAGTA TCCCACTGAG TTCTTCGACT CTTTAACCTA AACGGTAAAG   
  
  
- TCAAGTTAGG ACACCAATCG TTCAACCTTT TGGAACTACA ACTTTTCGAC ACACAATTCT GACCACTCCG   
  
  
- AGATCGGTAG TCAAGCCAGG AAGTTGACGT ATGGGAAAAC CCAAGACTAC TACTCGGGGA TTCCTTTTCA   
  
  
- AGTGGAAATC GGAACTACTT CATACGTTTA CCCCGATTAT CCGTTTCGGG CTCATTACTA AGCCGAAGAA   
  
  
- GTAGTGGAGG AAGCGAGTTA TGAAGTTGGT TCGACCTACC AAAGGAGTCG CGAAACACCC CTAACAGGGG   
  
  
- TTTCTAATAC CACTATCGAC TCGTGCTAAG GTTAGTGTTA CCAAGACCTG AATACCTCTC CAACAGTCTT   
  
  
- CGTGACATGA AGATACGTCG CGACAAACTG ACGAATCTTA GGTGGGACGG TTCTTGTAGA CAGCTTTCTT   
  
  
- CCGCCCATCT CTTCTACGAG GATCCACTTC TCTAGTTCTT GTAATATAGT ACGCTCCCTC CTCTTTCCTC   
  
  
- TCTTTCCGTA CTCTTCTAGC TCTTCACCTA CGTCTCCGAT CTGTACCGAC CTAAGCCCTA GCAAGGAAAC   
  
  
- TCGATATACC CATACGACGT TCGTTCCGTT AACGAAGTCC CGATACCAAC ACTACCAATA TCTCACTTTC   
  
  
- TCTTACCAAC ACAACAGTAG ACAACCGTTC TAGCGGGGGA GAAAAGCCAT AGTCGTACCT CCACATCCTC   
  
  
- TAC

+     GA-motif

| Site Name | Organism | Position | Strand | Matrix score. | sequence | function |
| --- | --- | --- | --- | --- | --- | --- |
| GA-motif | Arabidopsis thaliana | 656 | - | 8 | ATAGATAA | part of a light responsive element |

>HU08G00367.1   
+ -Up\_Stream \_Len000ATGCGC CTTTCTGCTA ACTCATTATC TTTCCTTCTG AGAGGGGACA ACTTATATAA   
  
  
+ ATTGATAGGT ATTTTCAAAT CAATAGACCA GCTACCATAT ATGATAATTT TTATTACTTT TCTAAATCCA   
  
  
+ TCAACTATTT TCACAATCCT CCTTTTTCTA GAAAAATAAA ATTTGCTTAA TTCAAATCAC CTAAAATTAC   
  
  
+ TAAATCTATA GTCCTAAATC TACAAACTCA TTTGTGTTGC AACTCATGAT TTAAGTGGAA CACTAACCTT   
  
  
+ AGGAGATATG TTGTCAAAAG AAGAATTCGA TGACATATTT TCCAACAAAA GGCCACCTAA CTTCATGATT   
  
  
+ TAAAAAGGAG GAGTCTAAGC ACATCATGGA TGACTAATTT TTGATGTATT GTAACTACGT ATTGAGATAA   
  
  
+ CTTGAATGAA TCGTGACTAG AATATATCTA TTTTCAAAAA TAAAAATTCC CTAAAAGAAA AGGATAAAAA   
  
  
+ ACTATATAAT ACTAAAAAAT TCTAACTATA ATAACTAGGC TTATTAAATT TTTATAGTGA AAATTTATTT   
  
  
+ CTACTTATAA CAACATCACT TACTAAGTAG CTTATTTGTG TCTTCTTTTC TAAATAATAA TGGTAACTAT   
  
  
+ GTGTCTTTTG TAAATAACTT ATTATCTATT TTATAAATCA CCGTGTAAAG CACGGGTCTA TACTAGTTAA   
  
  
+ TTTAAATAGC ATTGTTAAAT CAAGTAAGAT ACAAACAAAT TTATTTACAA CTGTAAGTGG TCCATAAAAA   
  
  
+ TAATAGCCAT TACAAAAAAT AGTACTACTG TTTTATGAAA TGATTTTAAT ATTCTAAAAT TCCAATAAAA   
  
  
+ CATGTTATAA TCTATGATTA ATTTTAAAAC ATGATTACAT GTCAGCATAA TTTGTATTAT TGTACAGCAA   
  
  
+ ATGAAAGTTG GAGCTAAAAG AAAATGGGGA AATAAAGGCA GACAAGTAAA GATAAAATAT GCTATCTACT   
  
  
+ TTTGGAGGTA TCCTTAACCA CATTGTCTCG TTTGTCTTCA TTAGGGAGAA GTGGGATAAA TACCAAAATT   
  
  
+ TGTATGGTCA TTGCGTGGGG TTTGCTTCCA AACAAAATGG CTTTTTGGAA GCAAATTTTG TGAGCTTCTA   
  
  
+ TGCAAATTTC CACGCTTAAC CCCACAAGCA AACAAAAACA GACAGTGGTT TTTGTTTGTC TTTGTTAGGG   
  
  
+ CATGTTTGGA GCCAACTGTT AATGGGAATA AACTGGGGAG GCTTTTTCCT TGGCTCTTTC CCCCTGACCC   
  
  
+ CAAACCCACT TCAGCTCAAT ACGAAACCAG CCATTGAAAA AGGAAGGGGG GGGGGGGGGT ATGTGAGAAA   
  
  
+ AAGACAAAAC TTTTTCCCGG AGAGAGAAGA TACATATGCA ACAAAGGCTC TCCTTCTCAT AAATTCCATA   
  
  
+ TTCCTCTGTT TTTCTAGGAG AGAGAAATAT GCAAAAAACA AATCAGTTGG CGACAAAAGT TACTACCACG   
  
  
+ CATTGTCTAC TGCTGCGTAC CCATTTTTGG AGACTTTAAA GAACGCCCCT CCCATCTTGA AAAGTGAAAA   
  
  
+ CCCCCTCTTT GAAACTGAAT TTCAGGGGAA AAAAACCTCA CCAAACCTCT AGAGAGAGCT TTTAGAGAGG   
  
  
+ GAAATACAAC GATTAGAAGG AGCAATTTGG GAAATTTCTT TGGGAATTTG AATGGGTTTT GAGTGAATTG   
  
  
+ CAAATCCCAG AAAAGTTTTG GCAAGTACCG ATCTACAGTT CTCTCCTCTT CGTGTTTGGT AGATCCCCTG   
  
  
+ TTTCCTCTGT TTCATTTAGG GTACTTCTCA TTCATCCTCC CCACCCCCTT AATCGGATCT TCCTGTCGAG   
  
  
+ TCACTTTATG CTAATATTTT TTCAGTGGAT TTTTAGTTAA CCCTGTTCAT TTTTCCATTC TGTGTCCCAT   
  
  
+ TTCTCTCTTT TTCATTCATA AGTTGCCGGT TTATCTGTTG GGTGCAGCTT AGTCACAATA ATTTCTGTGT   
  
  
+ TAGGCTTCTT TACGGTTAAA AAAAAAGGAG GCACTCTTTT CGGTGTGATT GTTTATGGGA CCAATGATTC   
  
  
+ AAGATGATGG GTCATCAGTA ACTTCATCAC CCCTTCAATT TTTCTCCATG ATGTCTCCCA ATTTAGGTTC   
  
  
+ TTCCTACCCT TGGCTCAGAG AGCTAAAACC TGAAGAAAGA GGTCTTTACT TGATACATTT GTTGCTCTCT   
  
  
+ TGTGCAAATC ATGTCTCTAG TGGTAGCCTA GACAATGCGA ACTTAGCCCT CGAACAAATC TCTCAGCTTG   
  
  
+ CTGCCCCTGA TGGGGATACA ATGCAGCGTA TGGCTTCTTA TTTTGCTGAA GCCCTGGCTG AGAGGATCCT   
  
  
+ CAAGTCATGG CCTGGCATGT ATAGAGCCCT TCATTTTACG AAAATGCCTG TCATTTCAGA GGAAATTCTT   
  
  
+ GCTAGGAAGC TCTTCTTTGA GCTATTTCCT TTCTTGAAGC TGGCCTATTT GGTGACAAAC CAATCGATAA   
  
  
+ TCGAAGCCAT GGAGGGGGAA AAGATGGTTC ATATTATTGA TCTGAATGCA TCAGAACCTG CTCAATGGAT   
  
  
+ TGCCCTTATT CAGGCTTTGA GTGCAAGGCC TGGGGGTCCT CCTCATTTGA GAATTACCGG TGTTCATCAA   
  
  
+ CACAAAGAGG TTCTAGATCA AGTGGCTCAT AGGGTGACTC AAGAAGCTGA GAAATTGGAT TTGCCATTTC   
  
  
+ AGTTCAATCC TGTGGTTAGC AAGTTGGAAA ACCTTGATGT TGAAAAGCTG TGTGTTAAGA CTGGTGAGGC   
  
  
+ TCTAGCCATC AGTTCGGTCC TTCAACTGCA TACCCTTTTG GGTTCTGATG ATGAGCCCCT AAGGAAAAGT   
  
  
+ TCACCTTTAG CCTTGATGAA GTATGCAAAT GGGGCTAATA GGCAAAGCCC GAGTAATGAT TCGGCTTCTT   
  
  
+ CATCACCTCC TTCGCTCAAT ACTTCAACCA AGCTGGATGG TTTCCTCAGC GCTTTGTGGG GATTGTCCCC   
  
  
+ AAAGATTATG GTGATAGCTG AGCACGATTC CAATCACAAT GGTTCTGGAC TTATGGAGAG GTTGTCAGAA   
  
  
+ GCACTGTACT TCTATGCAGC GCTGTTTGAC TGCTTAGAAT CCACCCTGCC AAGAACATCT GTCGAAAGAA   
  
  
+ GGCGGGTAGA GAAGATGCTC CTAGGTGAAG AGATCAAGAA CATTATATCA TGCGAGGGAG GAGAAAGGAG   
  
  
+ AGAAAGGCAT GAGAAGATCG AGAAGTGGAT GCAGAGGCTA GACATGGCTG GATTCGGGAT CGTTCCTTTG   
  
  
+ AGCTATATGG GTATGCTGCA AGCAAGGCAA TTGCTTCAGG GCTATGGTTG TGATGGTTAT AGAGTGAAAG   
  
  
+ AGAATGGTTG TGTTGTCATC TGTTGGCAAG ATCGCCCCCT CTTTTCGGTA TCAGCATGGA GGTGTAGGAG   
  
  
+ ATG  

- -Up\_Stream \_Len000TACGCG GAAAGACGAT TGAGTAATAG AAAGGAAGAC TCTCCCCTGT TGAATATATT   
  
  
- TAACTATCCA TAAAAGTTTA GTTATCTGGT CGATGGTATA TACTATTAAA AATAATGAAA AGATTTAGGT   
  
  
- AGTTGATAAA AGTGTTAGGA GGAAAAAGAT CTTTTTATTT TAAACGAATT AAGTTTAGTG GATTTTAATG   
  
  
- ATTTAGATAT CAGGATTTAG ATGTTTGAGT AAACACAACG TTGAGTACTA AATTCACCTT GTGATTGGAA   
  
  
- TCCTCTATAC AACAGTTTTC TTCTTAAGCT ACTGTATAAA AGGTTGTTTT CCGGTGGATT GAAGTACTAA   
  
  
- ATTTTTCCTC CTCAGATTCG TGTAGTACCT ACTGATTAAA AACTACATAA CATTGATGCA TAACTCTATT   
  
  
- GAACTTACTT AGCACTGATC TTATATAGAT AAAAGTTTTT ATTTTTAAGG GATTTTCTTT TCCTATTTTT   
  
  
- TGATATATTA TGATTTTTTA AGATTGATAT TATTGATCCG AATAATTTAA AAATATCACT TTTAAATAAA   
  
  
- GATGAATATT GTTGTAGTGA ATGATTCATC GAATAAACAC AGAAGAAAAG ATTTATTATT ACCATTGATA   
  
  
- CACAGAAAAC ATTTATTGAA TAATAGATAA AATATTTAGT GGCACATTTC GTGCCCAGAT ATGATCAATT   
  
  
- AAATTTATCG TAACAATTTA GTTCATTCTA TGTTTGTTTA AATAAATGTT GACATTCACC AGGTATTTTT   
  
  
- ATTATCGGTA ATGTTTTTTA TCATGATGAC AAAATACTTT ACTAAAATTA TAAGATTTTA AGGTTATTTT   
  
  
- GTACAATATT AGATACTAAT TAAAATTTTG TACTAATGTA CAGTCGTATT AAACATAATA ACATGTCGTT   
  
  
- TACTTTCAAC CTCGATTTTC TTTTACCCCT TTATTTCCGT CTGTTCATTT CTATTTTATA CGATAGATGA   
  
  
- AAACCTCCAT AGGAATTGGT GTAACAGAGC AAACAGAAGT AATCCCTCTT CACCCTATTT ATGGTTTTAA   
  
  
- ACATACCAGT AACGCACCCC AAACGAAGGT TTGTTTTACC GAAAAACCTT CGTTTAAAAC ACTCGAAGAT   
  
  
- ACGTTTAAAG GTGCGAATTG GGGTGTTCGT TTGTTTTTGT CTGTCACCAA AAACAAACAG AAACAATCCC   
  
  
- GTACAAACCT CGGTTGACAA TTACCCTTAT TTGACCCCTC CGAAAAAGGA ACCGAGAAAG GGGGACTGGG   
  
  
- GTTTGGGTGA AGTCGAGTTA TGCTTTGGTC GGTAACTTTT TCCTTCCCCC CCCCCCCCCA TACACTCTTT   
  
  
- TTCTGTTTTG AAAAAGGGCC TCTCTCTTCT ATGTATACGT TGTTTCCGAG AGGAAGAGTA TTTAAGGTAT   
  
  
- AAGGAGACAA AAAGATCCTC TCTCTTTATA CGTTTTTTGT TTAGTCAACC GCTGTTTTCA ATGATGGTGC   
  
  
- GTAACAGATG ACGACGCATG GGTAAAAACC TCTGAAATTT CTTGCGGGGA GGGTAGAACT TTTCACTTTT   
  
  
- GGGGGAGAAA CTTTGACTTA AAGTCCCCTT TTTTTGGAGT GGTTTGGAGA TCTCTCTCGA AAATCTCTCC   
  
  
- CTTTATGTTG CTAATCTTCC TCGTTAAACC CTTTAAAGAA ACCCTTAAAC TTACCCAAAA CTCACTTAAC   
  
  
- GTTTAGGGTC TTTTCAAAAC CGTTCATGGC TAGATGTCAA GAGAGGAGAA GCACAAACCA TCTAGGGGAC   
  
  
- AAAGGAGACA AAGTAAATCC CATGAAGAGT AAGTAGGAGG GGTGGGGGAA TTAGCCTAGA AGGACAGCTC   
  
  
- AGTGAAATAC GATTATAAAA AAGTCACCTA AAAATCAATT GGGACAAGTA AAAAGGTAAG ACACAGGGTA   
  
  
- AAGAGAGAAA AAGTAAGTAT TCAACGGCCA AATAGACAAC CCACGTCGAA TCAGTGTTAT TAAAGACACA   
  
  
- ATCCGAAGAA ATGCCAATTT TTTTTTCCTC CGTGAGAAAA GCCACACTAA CAAATACCCT GGTTACTAAG   
  
  
- TTCTACTACC CAGTAGTCAT TGAAGTAGTG GGGAAGTTAA AAAGAGGTAC TACAGAGGGT TAAATCCAAG   
  
  
- AAGGATGGGA ACCGAGTCTC TCGATTTTGG ACTTCTTTCT CCAGAAATGA ACTATGTAAA CAACGAGAGA   
  
  
- ACACGTTTAG TACAGAGATC ACCATCGGAT CTGTTACGCT TGAATCGGGA GCTTGTTTAG AGAGTCGAAC   
  
  
- GACGGGGACT ACCCCTATGT TACGTCGCAT ACCGAAGAAT AAAACGACTT CGGGACCGAC TCTCCTAGGA   
  
  
- GTTCAGTACC GGACCGTACA TATCTCGGGA AGTAAAATGC TTTTACGGAC AGTAAAGTCT CCTTTAAGAA   
  
  
- CGATCCTTCG AGAAGAAACT CGATAAAGGA AAGAACTTCG ACCGGATAAA CCACTGTTTG GTTAGCTATT   
  
  
- AGCTTCGGTA CCTCCCCCTT TTCTACCAAG TATAATAACT AGACTTACGT AGTCTTGGAC GAGTTACCTA   
  
  
- ACGGGAATAA GTCCGAAACT CACGTTCCGG ACCCCCAGGA GGAGTAAACT CTTAATGGCC ACAAGTAGTT   
  
  
- GTGTTTCTCC AAGATCTAGT TCACCGAGTA TCCCACTGAG TTCTTCGACT CTTTAACCTA AACGGTAAAG   
  
  
- TCAAGTTAGG ACACCAATCG TTCAACCTTT TGGAACTACA ACTTTTCGAC ACACAATTCT GACCACTCCG   
  
  
- AGATCGGTAG TCAAGCCAGG AAGTTGACGT ATGGGAAAAC CCAAGACTAC TACTCGGGGA TTCCTTTTCA   
  
  
- AGTGGAAATC GGAACTACTT CATACGTTTA CCCCGATTAT CCGTTTCGGG CTCATTACTA AGCCGAAGAA   
  
  
- GTAGTGGAGG AAGCGAGTTA TGAAGTTGGT TCGACCTACC AAAGGAGTCG CGAAACACCC CTAACAGGGG   
  
  
- TTTCTAATAC CACTATCGAC TCGTGCTAAG GTTAGTGTTA CCAAGACCTG AATACCTCTC CAACAGTCTT   
  
  
- CGTGACATGA AGATACGTCG CGACAAACTG ACGAATCTTA GGTGGGACGG TTCTTGTAGA CAGCTTTCTT   
  
  
- CCGCCCATCT CTTCTACGAG GATCCACTTC TCTAGTTCTT GTAATATAGT ACGCTCCCTC CTCTTTCCTC   
  
  
- TCTTTCCGTA CTCTTCTAGC TCTTCACCTA CGTCTCCGAT CTGTACCGAC CTAAGCCCTA GCAAGGAAAC   
  
  
- TCGATATACC CATACGACGT TCGTTCCGTT AACGAAGTCC CGATACCAAC ACTACCAATA TCTCACTTTC   
  
  
- TCTTACCAAC ACAACAGTAG ACAACCGTTC TAGCGGGGGA GAAAAGCCAT AGTCGTACCT CCACATCCTC   
  
  
- TAC

+     GARE-motif

| Site Name | Organism | Position | Strand | Matrix score. | sequence | function |
| --- | --- | --- | --- | --- | --- | --- |
| GARE-motif | Brassica oleracea | 1928 | + | 7 | TCTGTTG | gibberellin-responsive element |
| GARE-motif | Brassica oleracea | 3313 | + | 7 | TCTGTTG | gibberellin-responsive element |

>HU08G00367.1   
+ -Up\_Stream \_Len000ATGCGC CTTTCTGCTA ACTCATTATC TTTCCTTCTG AGAGGGGACA ACTTATATAA   
  
  
+ ATTGATAGGT ATTTTCAAAT CAATAGACCA GCTACCATAT ATGATAATTT TTATTACTTT TCTAAATCCA   
  
  
+ TCAACTATTT TCACAATCCT CCTTTTTCTA GAAAAATAAA ATTTGCTTAA TTCAAATCAC CTAAAATTAC   
  
  
+ TAAATCTATA GTCCTAAATC TACAAACTCA TTTGTGTTGC AACTCATGAT TTAAGTGGAA CACTAACCTT   
  
  
+ AGGAGATATG TTGTCAAAAG AAGAATTCGA TGACATATTT TCCAACAAAA GGCCACCTAA CTTCATGATT   
  
  
+ TAAAAAGGAG GAGTCTAAGC ACATCATGGA TGACTAATTT TTGATGTATT GTAACTACGT ATTGAGATAA   
  
  
+ CTTGAATGAA TCGTGACTAG AATATATCTA TTTTCAAAAA TAAAAATTCC CTAAAAGAAA AGGATAAAAA   
  
  
+ ACTATATAAT ACTAAAAAAT TCTAACTATA ATAACTAGGC TTATTAAATT TTTATAGTGA AAATTTATTT   
  
  
+ CTACTTATAA CAACATCACT TACTAAGTAG CTTATTTGTG TCTTCTTTTC TAAATAATAA TGGTAACTAT   
  
  
+ GTGTCTTTTG TAAATAACTT ATTATCTATT TTATAAATCA CCGTGTAAAG CACGGGTCTA TACTAGTTAA   
  
  
+ TTTAAATAGC ATTGTTAAAT CAAGTAAGAT ACAAACAAAT TTATTTACAA CTGTAAGTGG TCCATAAAAA   
  
  
+ TAATAGCCAT TACAAAAAAT AGTACTACTG TTTTATGAAA TGATTTTAAT ATTCTAAAAT TCCAATAAAA   
  
  
+ CATGTTATAA TCTATGATTA ATTTTAAAAC ATGATTACAT GTCAGCATAA TTTGTATTAT TGTACAGCAA   
  
  
+ ATGAAAGTTG GAGCTAAAAG AAAATGGGGA AATAAAGGCA GACAAGTAAA GATAAAATAT GCTATCTACT   
  
  
+ TTTGGAGGTA TCCTTAACCA CATTGTCTCG TTTGTCTTCA TTAGGGAGAA GTGGGATAAA TACCAAAATT   
  
  
+ TGTATGGTCA TTGCGTGGGG TTTGCTTCCA AACAAAATGG CTTTTTGGAA GCAAATTTTG TGAGCTTCTA   
  
  
+ TGCAAATTTC CACGCTTAAC CCCACAAGCA AACAAAAACA GACAGTGGTT TTTGTTTGTC TTTGTTAGGG   
  
  
+ CATGTTTGGA GCCAACTGTT AATGGGAATA AACTGGGGAG GCTTTTTCCT TGGCTCTTTC CCCCTGACCC   
  
  
+ CAAACCCACT TCAGCTCAAT ACGAAACCAG CCATTGAAAA AGGAAGGGGG GGGGGGGGGT ATGTGAGAAA   
  
  
+ AAGACAAAAC TTTTTCCCGG AGAGAGAAGA TACATATGCA ACAAAGGCTC TCCTTCTCAT AAATTCCATA   
  
  
+ TTCCTCTGTT TTTCTAGGAG AGAGAAATAT GCAAAAAACA AATCAGTTGG CGACAAAAGT TACTACCACG   
  
  
+ CATTGTCTAC TGCTGCGTAC CCATTTTTGG AGACTTTAAA GAACGCCCCT CCCATCTTGA AAAGTGAAAA   
  
  
+ CCCCCTCTTT GAAACTGAAT TTCAGGGGAA AAAAACCTCA CCAAACCTCT AGAGAGAGCT TTTAGAGAGG   
  
  
+ GAAATACAAC GATTAGAAGG AGCAATTTGG GAAATTTCTT TGGGAATTTG AATGGGTTTT GAGTGAATTG   
  
  
+ CAAATCCCAG AAAAGTTTTG GCAAGTACCG ATCTACAGTT CTCTCCTCTT CGTGTTTGGT AGATCCCCTG   
  
  
+ TTTCCTCTGT TTCATTTAGG GTACTTCTCA TTCATCCTCC CCACCCCCTT AATCGGATCT TCCTGTCGAG   
  
  
+ TCACTTTATG CTAATATTTT TTCAGTGGAT TTTTAGTTAA CCCTGTTCAT TTTTCCATTC TGTGTCCCAT   
  
  
+ TTCTCTCTTT TTCATTCATA AGTTGCCGGT TTATCTGTTG GGTGCAGCTT AGTCACAATA ATTTCTGTGT   
  
  
+ TAGGCTTCTT TACGGTTAAA AAAAAAGGAG GCACTCTTTT CGGTGTGATT GTTTATGGGA CCAATGATTC   
  
  
+ AAGATGATGG GTCATCAGTA ACTTCATCAC CCCTTCAATT TTTCTCCATG ATGTCTCCCA ATTTAGGTTC   
  
  
+ TTCCTACCCT TGGCTCAGAG AGCTAAAACC TGAAGAAAGA GGTCTTTACT TGATACATTT GTTGCTCTCT   
  
  
+ TGTGCAAATC ATGTCTCTAG TGGTAGCCTA GACAATGCGA ACTTAGCCCT CGAACAAATC TCTCAGCTTG   
  
  
+ CTGCCCCTGA TGGGGATACA ATGCAGCGTA TGGCTTCTTA TTTTGCTGAA GCCCTGGCTG AGAGGATCCT   
  
  
+ CAAGTCATGG CCTGGCATGT ATAGAGCCCT TCATTTTACG AAAATGCCTG TCATTTCAGA GGAAATTCTT   
  
  
+ GCTAGGAAGC TCTTCTTTGA GCTATTTCCT TTCTTGAAGC TGGCCTATTT GGTGACAAAC CAATCGATAA   
  
  
+ TCGAAGCCAT GGAGGGGGAA AAGATGGTTC ATATTATTGA TCTGAATGCA TCAGAACCTG CTCAATGGAT   
  
  
+ TGCCCTTATT CAGGCTTTGA GTGCAAGGCC TGGGGGTCCT CCTCATTTGA GAATTACCGG TGTTCATCAA   
  
  
+ CACAAAGAGG TTCTAGATCA AGTGGCTCAT AGGGTGACTC AAGAAGCTGA GAAATTGGAT TTGCCATTTC   
  
  
+ AGTTCAATCC TGTGGTTAGC AAGTTGGAAA ACCTTGATGT TGAAAAGCTG TGTGTTAAGA CTGGTGAGGC   
  
  
+ TCTAGCCATC AGTTCGGTCC TTCAACTGCA TACCCTTTTG GGTTCTGATG ATGAGCCCCT AAGGAAAAGT   
  
  
+ TCACCTTTAG CCTTGATGAA GTATGCAAAT GGGGCTAATA GGCAAAGCCC GAGTAATGAT TCGGCTTCTT   
  
  
+ CATCACCTCC TTCGCTCAAT ACTTCAACCA AGCTGGATGG TTTCCTCAGC GCTTTGTGGG GATTGTCCCC   
  
  
+ AAAGATTATG GTGATAGCTG AGCACGATTC CAATCACAAT GGTTCTGGAC TTATGGAGAG GTTGTCAGAA   
  
  
+ GCACTGTACT TCTATGCAGC GCTGTTTGAC TGCTTAGAAT CCACCCTGCC AAGAACATCT GTCGAAAGAA   
  
  
+ GGCGGGTAGA GAAGATGCTC CTAGGTGAAG AGATCAAGAA CATTATATCA TGCGAGGGAG GAGAAAGGAG   
  
  
+ AGAAAGGCAT GAGAAGATCG AGAAGTGGAT GCAGAGGCTA GACATGGCTG GATTCGGGAT CGTTCCTTTG   
  
  
+ AGCTATATGG GTATGCTGCA AGCAAGGCAA TTGCTTCAGG GCTATGGTTG TGATGGTTAT AGAGTGAAAG   
  
  
+ AGAATGGTTG TGTTGTCATC TGTTGGCAAG ATCGCCCCCT CTTTTCGGTA TCAGCATGGA GGTGTAGGAG   
  
  
+ ATG  

- -Up\_Stream \_Len000TACGCG GAAAGACGAT TGAGTAATAG AAAGGAAGAC TCTCCCCTGT TGAATATATT   
  
  
- TAACTATCCA TAAAAGTTTA GTTATCTGGT CGATGGTATA TACTATTAAA AATAATGAAA AGATTTAGGT   
  
  
- AGTTGATAAA AGTGTTAGGA GGAAAAAGAT CTTTTTATTT TAAACGAATT AAGTTTAGTG GATTTTAATG   
  
  
- ATTTAGATAT CAGGATTTAG ATGTTTGAGT AAACACAACG TTGAGTACTA AATTCACCTT GTGATTGGAA   
  
  
- TCCTCTATAC AACAGTTTTC TTCTTAAGCT ACTGTATAAA AGGTTGTTTT CCGGTGGATT GAAGTACTAA   
  
  
- ATTTTTCCTC CTCAGATTCG TGTAGTACCT ACTGATTAAA AACTACATAA CATTGATGCA TAACTCTATT   
  
  
- GAACTTACTT AGCACTGATC TTATATAGAT AAAAGTTTTT ATTTTTAAGG GATTTTCTTT TCCTATTTTT   
  
  
- TGATATATTA TGATTTTTTA AGATTGATAT TATTGATCCG AATAATTTAA AAATATCACT TTTAAATAAA   
  
  
- GATGAATATT GTTGTAGTGA ATGATTCATC GAATAAACAC AGAAGAAAAG ATTTATTATT ACCATTGATA   
  
  
- CACAGAAAAC ATTTATTGAA TAATAGATAA AATATTTAGT GGCACATTTC GTGCCCAGAT ATGATCAATT   
  
  
- AAATTTATCG TAACAATTTA GTTCATTCTA TGTTTGTTTA AATAAATGTT GACATTCACC AGGTATTTTT   
  
  
- ATTATCGGTA ATGTTTTTTA TCATGATGAC AAAATACTTT ACTAAAATTA TAAGATTTTA AGGTTATTTT   
  
  
- GTACAATATT AGATACTAAT TAAAATTTTG TACTAATGTA CAGTCGTATT AAACATAATA ACATGTCGTT   
  
  
- TACTTTCAAC CTCGATTTTC TTTTACCCCT TTATTTCCGT CTGTTCATTT CTATTTTATA CGATAGATGA   
  
  
- AAACCTCCAT AGGAATTGGT GTAACAGAGC AAACAGAAGT AATCCCTCTT CACCCTATTT ATGGTTTTAA   
  
  
- ACATACCAGT AACGCACCCC AAACGAAGGT TTGTTTTACC GAAAAACCTT CGTTTAAAAC ACTCGAAGAT   
  
  
- ACGTTTAAAG GTGCGAATTG GGGTGTTCGT TTGTTTTTGT CTGTCACCAA AAACAAACAG AAACAATCCC   
  
  
- GTACAAACCT CGGTTGACAA TTACCCTTAT TTGACCCCTC CGAAAAAGGA ACCGAGAAAG GGGGACTGGG   
  
  
- GTTTGGGTGA AGTCGAGTTA TGCTTTGGTC GGTAACTTTT TCCTTCCCCC CCCCCCCCCA TACACTCTTT   
  
  
- TTCTGTTTTG AAAAAGGGCC TCTCTCTTCT ATGTATACGT TGTTTCCGAG AGGAAGAGTA TTTAAGGTAT   
  
  
- AAGGAGACAA AAAGATCCTC TCTCTTTATA CGTTTTTTGT TTAGTCAACC GCTGTTTTCA ATGATGGTGC   
  
  
- GTAACAGATG ACGACGCATG GGTAAAAACC TCTGAAATTT CTTGCGGGGA GGGTAGAACT TTTCACTTTT   
  
  
- GGGGGAGAAA CTTTGACTTA AAGTCCCCTT TTTTTGGAGT GGTTTGGAGA TCTCTCTCGA AAATCTCTCC   
  
  
- CTTTATGTTG CTAATCTTCC TCGTTAAACC CTTTAAAGAA ACCCTTAAAC TTACCCAAAA CTCACTTAAC   
  
  
- GTTTAGGGTC TTTTCAAAAC CGTTCATGGC TAGATGTCAA GAGAGGAGAA GCACAAACCA TCTAGGGGAC   
  
  
- AAAGGAGACA AAGTAAATCC CATGAAGAGT AAGTAGGAGG GGTGGGGGAA TTAGCCTAGA AGGACAGCTC   
  
  
- AGTGAAATAC GATTATAAAA AAGTCACCTA AAAATCAATT GGGACAAGTA AAAAGGTAAG ACACAGGGTA   
  
  
- AAGAGAGAAA AAGTAAGTAT TCAACGGCCA AATAGACAAC CCACGTCGAA TCAGTGTTAT TAAAGACACA   
  
  
- ATCCGAAGAA ATGCCAATTT TTTTTTCCTC CGTGAGAAAA GCCACACTAA CAAATACCCT GGTTACTAAG   
  
  
- TTCTACTACC CAGTAGTCAT TGAAGTAGTG GGGAAGTTAA AAAGAGGTAC TACAGAGGGT TAAATCCAAG   
  
  
- AAGGATGGGA ACCGAGTCTC TCGATTTTGG ACTTCTTTCT CCAGAAATGA ACTATGTAAA CAACGAGAGA   
  
  
- ACACGTTTAG TACAGAGATC ACCATCGGAT CTGTTACGCT TGAATCGGGA GCTTGTTTAG AGAGTCGAAC   
  
  
- GACGGGGACT ACCCCTATGT TACGTCGCAT ACCGAAGAAT AAAACGACTT CGGGACCGAC TCTCCTAGGA   
  
  
- GTTCAGTACC GGACCGTACA TATCTCGGGA AGTAAAATGC TTTTACGGAC AGTAAAGTCT CCTTTAAGAA   
  
  
- CGATCCTTCG AGAAGAAACT CGATAAAGGA AAGAACTTCG ACCGGATAAA CCACTGTTTG GTTAGCTATT   
  
  
- AGCTTCGGTA CCTCCCCCTT TTCTACCAAG TATAATAACT AGACTTACGT AGTCTTGGAC GAGTTACCTA   
  
  
- ACGGGAATAA GTCCGAAACT CACGTTCCGG ACCCCCAGGA GGAGTAAACT CTTAATGGCC ACAAGTAGTT   
  
  
- GTGTTTCTCC AAGATCTAGT TCACCGAGTA TCCCACTGAG TTCTTCGACT CTTTAACCTA AACGGTAAAG   
  
  
- TCAAGTTAGG ACACCAATCG TTCAACCTTT TGGAACTACA ACTTTTCGAC ACACAATTCT GACCACTCCG   
  
  
- AGATCGGTAG TCAAGCCAGG AAGTTGACGT ATGGGAAAAC CCAAGACTAC TACTCGGGGA TTCCTTTTCA   
  
  
- AGTGGAAATC GGAACTACTT CATACGTTTA CCCCGATTAT CCGTTTCGGG CTCATTACTA AGCCGAAGAA   
  
  
- GTAGTGGAGG AAGCGAGTTA TGAAGTTGGT TCGACCTACC AAAGGAGTCG CGAAACACCC CTAACAGGGG   
  
  
- TTTCTAATAC CACTATCGAC TCGTGCTAAG GTTAGTGTTA CCAAGACCTG AATACCTCTC CAACAGTCTT   
  
  
- CGTGACATGA AGATACGTCG CGACAAACTG ACGAATCTTA GGTGGGACGG TTCTTGTAGA CAGCTTTCTT   
  
  
- CCGCCCATCT CTTCTACGAG GATCCACTTC TCTAGTTCTT GTAATATAGT ACGCTCCCTC CTCTTTCCTC   
  
  
- TCTTTCCGTA CTCTTCTAGC TCTTCACCTA CGTCTCCGAT CTGTACCGAC CTAAGCCCTA GCAAGGAAAC   
  
  
- TCGATATACC CATACGACGT TCGTTCCGTT AACGAAGTCC CGATACCAAC ACTACCAATA TCTCACTTTC   
  
  
- TCTTACCAAC ACAACAGTAG ACAACCGTTC TAGCGGGGGA GAAAAGCCAT AGTCGTACCT CCACATCCTC   
  
  
- TAC

+     GCN4\_motif

| Site Name | Organism | Position | Strand | Matrix score. | sequence | function |
| --- | --- | --- | --- | --- | --- | --- |
| GCN4\_motif | Oryza sativa | 2629 | - | 7 | TGAGTCA | cis-regulatory element involved in endosperm expression |

>HU08G00367.1   
+ -Up\_Stream \_Len000ATGCGC CTTTCTGCTA ACTCATTATC TTTCCTTCTG AGAGGGGACA ACTTATATAA   
  
  
+ ATTGATAGGT ATTTTCAAAT CAATAGACCA GCTACCATAT ATGATAATTT TTATTACTTT TCTAAATCCA   
  
  
+ TCAACTATTT TCACAATCCT CCTTTTTCTA GAAAAATAAA ATTTGCTTAA TTCAAATCAC CTAAAATTAC   
  
  
+ TAAATCTATA GTCCTAAATC TACAAACTCA TTTGTGTTGC AACTCATGAT TTAAGTGGAA CACTAACCTT   
  
  
+ AGGAGATATG TTGTCAAAAG AAGAATTCGA TGACATATTT TCCAACAAAA GGCCACCTAA CTTCATGATT   
  
  
+ TAAAAAGGAG GAGTCTAAGC ACATCATGGA TGACTAATTT TTGATGTATT GTAACTACGT ATTGAGATAA   
  
  
+ CTTGAATGAA TCGTGACTAG AATATATCTA TTTTCAAAAA TAAAAATTCC CTAAAAGAAA AGGATAAAAA   
  
  
+ ACTATATAAT ACTAAAAAAT TCTAACTATA ATAACTAGGC TTATTAAATT TTTATAGTGA AAATTTATTT   
  
  
+ CTACTTATAA CAACATCACT TACTAAGTAG CTTATTTGTG TCTTCTTTTC TAAATAATAA TGGTAACTAT   
  
  
+ GTGTCTTTTG TAAATAACTT ATTATCTATT TTATAAATCA CCGTGTAAAG CACGGGTCTA TACTAGTTAA   
  
  
+ TTTAAATAGC ATTGTTAAAT CAAGTAAGAT ACAAACAAAT TTATTTACAA CTGTAAGTGG TCCATAAAAA   
  
  
+ TAATAGCCAT TACAAAAAAT AGTACTACTG TTTTATGAAA TGATTTTAAT ATTCTAAAAT TCCAATAAAA   
  
  
+ CATGTTATAA TCTATGATTA ATTTTAAAAC ATGATTACAT GTCAGCATAA TTTGTATTAT TGTACAGCAA   
  
  
+ ATGAAAGTTG GAGCTAAAAG AAAATGGGGA AATAAAGGCA GACAAGTAAA GATAAAATAT GCTATCTACT   
  
  
+ TTTGGAGGTA TCCTTAACCA CATTGTCTCG TTTGTCTTCA TTAGGGAGAA GTGGGATAAA TACCAAAATT   
  
  
+ TGTATGGTCA TTGCGTGGGG TTTGCTTCCA AACAAAATGG CTTTTTGGAA GCAAATTTTG TGAGCTTCTA   
  
  
+ TGCAAATTTC CACGCTTAAC CCCACAAGCA AACAAAAACA GACAGTGGTT TTTGTTTGTC TTTGTTAGGG   
  
  
+ CATGTTTGGA GCCAACTGTT AATGGGAATA AACTGGGGAG GCTTTTTCCT TGGCTCTTTC CCCCTGACCC   
  
  
+ CAAACCCACT TCAGCTCAAT ACGAAACCAG CCATTGAAAA AGGAAGGGGG GGGGGGGGGT ATGTGAGAAA   
  
  
+ AAGACAAAAC TTTTTCCCGG AGAGAGAAGA TACATATGCA ACAAAGGCTC TCCTTCTCAT AAATTCCATA   
  
  
+ TTCCTCTGTT TTTCTAGGAG AGAGAAATAT GCAAAAAACA AATCAGTTGG CGACAAAAGT TACTACCACG   
  
  
+ CATTGTCTAC TGCTGCGTAC CCATTTTTGG AGACTTTAAA GAACGCCCCT CCCATCTTGA AAAGTGAAAA   
  
  
+ CCCCCTCTTT GAAACTGAAT TTCAGGGGAA AAAAACCTCA CCAAACCTCT AGAGAGAGCT TTTAGAGAGG   
  
  
+ GAAATACAAC GATTAGAAGG AGCAATTTGG GAAATTTCTT TGGGAATTTG AATGGGTTTT GAGTGAATTG   
  
  
+ CAAATCCCAG AAAAGTTTTG GCAAGTACCG ATCTACAGTT CTCTCCTCTT CGTGTTTGGT AGATCCCCTG   
  
  
+ TTTCCTCTGT TTCATTTAGG GTACTTCTCA TTCATCCTCC CCACCCCCTT AATCGGATCT TCCTGTCGAG   
  
  
+ TCACTTTATG CTAATATTTT TTCAGTGGAT TTTTAGTTAA CCCTGTTCAT TTTTCCATTC TGTGTCCCAT   
  
  
+ TTCTCTCTTT TTCATTCATA AGTTGCCGGT TTATCTGTTG GGTGCAGCTT AGTCACAATA ATTTCTGTGT   
  
  
+ TAGGCTTCTT TACGGTTAAA AAAAAAGGAG GCACTCTTTT CGGTGTGATT GTTTATGGGA CCAATGATTC   
  
  
+ AAGATGATGG GTCATCAGTA ACTTCATCAC CCCTTCAATT TTTCTCCATG ATGTCTCCCA ATTTAGGTTC   
  
  
+ TTCCTACCCT TGGCTCAGAG AGCTAAAACC TGAAGAAAGA GGTCTTTACT TGATACATTT GTTGCTCTCT   
  
  
+ TGTGCAAATC ATGTCTCTAG TGGTAGCCTA GACAATGCGA ACTTAGCCCT CGAACAAATC TCTCAGCTTG   
  
  
+ CTGCCCCTGA TGGGGATACA ATGCAGCGTA TGGCTTCTTA TTTTGCTGAA GCCCTGGCTG AGAGGATCCT   
  
  
+ CAAGTCATGG CCTGGCATGT ATAGAGCCCT TCATTTTACG AAAATGCCTG TCATTTCAGA GGAAATTCTT   
  
  
+ GCTAGGAAGC TCTTCTTTGA GCTATTTCCT TTCTTGAAGC TGGCCTATTT GGTGACAAAC CAATCGATAA   
  
  
+ TCGAAGCCAT GGAGGGGGAA AAGATGGTTC ATATTATTGA TCTGAATGCA TCAGAACCTG CTCAATGGAT   
  
  
+ TGCCCTTATT CAGGCTTTGA GTGCAAGGCC TGGGGGTCCT CCTCATTTGA GAATTACCGG TGTTCATCAA   
  
  
+ CACAAAGAGG TTCTAGATCA AGTGGCTCAT AGGGTGACTC AAGAAGCTGA GAAATTGGAT TTGCCATTTC   
  
  
+ AGTTCAATCC TGTGGTTAGC AAGTTGGAAA ACCTTGATGT TGAAAAGCTG TGTGTTAAGA CTGGTGAGGC   
  
  
+ TCTAGCCATC AGTTCGGTCC TTCAACTGCA TACCCTTTTG GGTTCTGATG ATGAGCCCCT AAGGAAAAGT   
  
  
+ TCACCTTTAG CCTTGATGAA GTATGCAAAT GGGGCTAATA GGCAAAGCCC GAGTAATGAT TCGGCTTCTT   
  
  
+ CATCACCTCC TTCGCTCAAT ACTTCAACCA AGCTGGATGG TTTCCTCAGC GCTTTGTGGG GATTGTCCCC   
  
  
+ AAAGATTATG GTGATAGCTG AGCACGATTC CAATCACAAT GGTTCTGGAC TTATGGAGAG GTTGTCAGAA   
  
  
+ GCACTGTACT TCTATGCAGC GCTGTTTGAC TGCTTAGAAT CCACCCTGCC AAGAACATCT GTCGAAAGAA   
  
  
+ GGCGGGTAGA GAAGATGCTC CTAGGTGAAG AGATCAAGAA CATTATATCA TGCGAGGGAG GAGAAAGGAG   
  
  
+ AGAAAGGCAT GAGAAGATCG AGAAGTGGAT GCAGAGGCTA GACATGGCTG GATTCGGGAT CGTTCCTTTG   
  
  
+ AGCTATATGG GTATGCTGCA AGCAAGGCAA TTGCTTCAGG GCTATGGTTG TGATGGTTAT AGAGTGAAAG   
  
  
+ AGAATGGTTG TGTTGTCATC TGTTGGCAAG ATCGCCCCCT CTTTTCGGTA TCAGCATGGA GGTGTAGGAG   
  
  
+ ATG  

- -Up\_Stream \_Len000TACGCG GAAAGACGAT TGAGTAATAG AAAGGAAGAC TCTCCCCTGT TGAATATATT   
  
  
- TAACTATCCA TAAAAGTTTA GTTATCTGGT CGATGGTATA TACTATTAAA AATAATGAAA AGATTTAGGT   
  
  
- AGTTGATAAA AGTGTTAGGA GGAAAAAGAT CTTTTTATTT TAAACGAATT AAGTTTAGTG GATTTTAATG   
  
  
- ATTTAGATAT CAGGATTTAG ATGTTTGAGT AAACACAACG TTGAGTACTA AATTCACCTT GTGATTGGAA   
  
  
- TCCTCTATAC AACAGTTTTC TTCTTAAGCT ACTGTATAAA AGGTTGTTTT CCGGTGGATT GAAGTACTAA   
  
  
- ATTTTTCCTC CTCAGATTCG TGTAGTACCT ACTGATTAAA AACTACATAA CATTGATGCA TAACTCTATT   
  
  
- GAACTTACTT AGCACTGATC TTATATAGAT AAAAGTTTTT ATTTTTAAGG GATTTTCTTT TCCTATTTTT   
  
  
- TGATATATTA TGATTTTTTA AGATTGATAT TATTGATCCG AATAATTTAA AAATATCACT TTTAAATAAA   
  
  
- GATGAATATT GTTGTAGTGA ATGATTCATC GAATAAACAC AGAAGAAAAG ATTTATTATT ACCATTGATA   
  
  
- CACAGAAAAC ATTTATTGAA TAATAGATAA AATATTTAGT GGCACATTTC GTGCCCAGAT ATGATCAATT   
  
  
- AAATTTATCG TAACAATTTA GTTCATTCTA TGTTTGTTTA AATAAATGTT GACATTCACC AGGTATTTTT   
  
  
- ATTATCGGTA ATGTTTTTTA TCATGATGAC AAAATACTTT ACTAAAATTA TAAGATTTTA AGGTTATTTT   
  
  
- GTACAATATT AGATACTAAT TAAAATTTTG TACTAATGTA CAGTCGTATT AAACATAATA ACATGTCGTT   
  
  
- TACTTTCAAC CTCGATTTTC TTTTACCCCT TTATTTCCGT CTGTTCATTT CTATTTTATA CGATAGATGA   
  
  
- AAACCTCCAT AGGAATTGGT GTAACAGAGC AAACAGAAGT AATCCCTCTT CACCCTATTT ATGGTTTTAA   
  
  
- ACATACCAGT AACGCACCCC AAACGAAGGT TTGTTTTACC GAAAAACCTT CGTTTAAAAC ACTCGAAGAT   
  
  
- ACGTTTAAAG GTGCGAATTG GGGTGTTCGT TTGTTTTTGT CTGTCACCAA AAACAAACAG AAACAATCCC   
  
  
- GTACAAACCT CGGTTGACAA TTACCCTTAT TTGACCCCTC CGAAAAAGGA ACCGAGAAAG GGGGACTGGG   
  
  
- GTTTGGGTGA AGTCGAGTTA TGCTTTGGTC GGTAACTTTT TCCTTCCCCC CCCCCCCCCA TACACTCTTT   
  
  
- TTCTGTTTTG AAAAAGGGCC TCTCTCTTCT ATGTATACGT TGTTTCCGAG AGGAAGAGTA TTTAAGGTAT   
  
  
- AAGGAGACAA AAAGATCCTC TCTCTTTATA CGTTTTTTGT TTAGTCAACC GCTGTTTTCA ATGATGGTGC   
  
  
- GTAACAGATG ACGACGCATG GGTAAAAACC TCTGAAATTT CTTGCGGGGA GGGTAGAACT TTTCACTTTT   
  
  
- GGGGGAGAAA CTTTGACTTA AAGTCCCCTT TTTTTGGAGT GGTTTGGAGA TCTCTCTCGA AAATCTCTCC   
  
  
- CTTTATGTTG CTAATCTTCC TCGTTAAACC CTTTAAAGAA ACCCTTAAAC TTACCCAAAA CTCACTTAAC   
  
  
- GTTTAGGGTC TTTTCAAAAC CGTTCATGGC TAGATGTCAA GAGAGGAGAA GCACAAACCA TCTAGGGGAC   
  
  
- AAAGGAGACA AAGTAAATCC CATGAAGAGT AAGTAGGAGG GGTGGGGGAA TTAGCCTAGA AGGACAGCTC   
  
  
- AGTGAAATAC GATTATAAAA AAGTCACCTA AAAATCAATT GGGACAAGTA AAAAGGTAAG ACACAGGGTA   
  
  
- AAGAGAGAAA AAGTAAGTAT TCAACGGCCA AATAGACAAC CCACGTCGAA TCAGTGTTAT TAAAGACACA   
  
  
- ATCCGAAGAA ATGCCAATTT TTTTTTCCTC CGTGAGAAAA GCCACACTAA CAAATACCCT GGTTACTAAG   
  
  
- TTCTACTACC CAGTAGTCAT TGAAGTAGTG GGGAAGTTAA AAAGAGGTAC TACAGAGGGT TAAATCCAAG   
  
  
- AAGGATGGGA ACCGAGTCTC TCGATTTTGG ACTTCTTTCT CCAGAAATGA ACTATGTAAA CAACGAGAGA   
  
  
- ACACGTTTAG TACAGAGATC ACCATCGGAT CTGTTACGCT TGAATCGGGA GCTTGTTTAG AGAGTCGAAC   
  
  
- GACGGGGACT ACCCCTATGT TACGTCGCAT ACCGAAGAAT AAAACGACTT CGGGACCGAC TCTCCTAGGA   
  
  
- GTTCAGTACC GGACCGTACA TATCTCGGGA AGTAAAATGC TTTTACGGAC AGTAAAGTCT CCTTTAAGAA   
  
  
- CGATCCTTCG AGAAGAAACT CGATAAAGGA AAGAACTTCG ACCGGATAAA CCACTGTTTG GTTAGCTATT   
  
  
- AGCTTCGGTA CCTCCCCCTT TTCTACCAAG TATAATAACT AGACTTACGT AGTCTTGGAC GAGTTACCTA   
  
  
- ACGGGAATAA GTCCGAAACT CACGTTCCGG ACCCCCAGGA GGAGTAAACT CTTAATGGCC ACAAGTAGTT   
  
  
- GTGTTTCTCC AAGATCTAGT TCACCGAGTA TCCCACTGAG TTCTTCGACT CTTTAACCTA AACGGTAAAG   
  
  
- TCAAGTTAGG ACACCAATCG TTCAACCTTT TGGAACTACA ACTTTTCGAC ACACAATTCT GACCACTCCG   
  
  
- AGATCGGTAG TCAAGCCAGG AAGTTGACGT ATGGGAAAAC CCAAGACTAC TACTCGGGGA TTCCTTTTCA   
  
  
- AGTGGAAATC GGAACTACTT CATACGTTTA CCCCGATTAT CCGTTTCGGG CTCATTACTA AGCCGAAGAA   
  
  
- GTAGTGGAGG AAGCGAGTTA TGAAGTTGGT TCGACCTACC AAAGGAGTCG CGAAACACCC CTAACAGGGG   
  
  
- TTTCTAATAC CACTATCGAC TCGTGCTAAG GTTAGTGTTA CCAAGACCTG AATACCTCTC CAACAGTCTT   
  
  
- CGTGACATGA AGATACGTCG CGACAAACTG ACGAATCTTA GGTGGGACGG TTCTTGTAGA CAGCTTTCTT   
  
  
- CCGCCCATCT CTTCTACGAG GATCCACTTC TCTAGTTCTT GTAATATAGT ACGCTCCCTC CTCTTTCCTC   
  
  
- TCTTTCCGTA CTCTTCTAGC TCTTCACCTA CGTCTCCGAT CTGTACCGAC CTAAGCCCTA GCAAGGAAAC   
  
  
- TCGATATACC CATACGACGT TCGTTCCGTT AACGAAGTCC CGATACCAAC ACTACCAATA TCTCACTTTC   
  
  
- TCTTACCAAC ACAACAGTAG ACAACCGTTC TAGCGGGGGA GAAAAGCCAT AGTCGTACCT CCACATCCTC   
  
  
- TAC

+     GT1-motif

| Site Name | Organism | Position | Strand | Matrix score. | sequence | function |
| --- | --- | --- | --- | --- | --- | --- |
| GT1-motif | Arabidopsis thaliana | 998 | - | 6 | GGTTAA | light responsive element |
| GT1-motif | Arabidopsis thaliana | 1861 | - | 6 | GGTTAA | light responsive element |
| GT1-motif | Arabidopsis thaliana | 1140 | - | 6 | GGTTAA | light responsive element |
| GT1-motif | Arabidopsis thaliana | 1978 | + | 6 | GGTTAA | light responsive element |

>HU08G00367.1   
+ -Up\_Stream \_Len000ATGCGC CTTTCTGCTA ACTCATTATC TTTCCTTCTG AGAGGGGACA ACTTATATAA   
  
  
+ ATTGATAGGT ATTTTCAAAT CAATAGACCA GCTACCATAT ATGATAATTT TTATTACTTT TCTAAATCCA   
  
  
+ TCAACTATTT TCACAATCCT CCTTTTTCTA GAAAAATAAA ATTTGCTTAA TTCAAATCAC CTAAAATTAC   
  
  
+ TAAATCTATA GTCCTAAATC TACAAACTCA TTTGTGTTGC AACTCATGAT TTAAGTGGAA CACTAACCTT   
  
  
+ AGGAGATATG TTGTCAAAAG AAGAATTCGA TGACATATTT TCCAACAAAA GGCCACCTAA CTTCATGATT   
  
  
+ TAAAAAGGAG GAGTCTAAGC ACATCATGGA TGACTAATTT TTGATGTATT GTAACTACGT ATTGAGATAA   
  
  
+ CTTGAATGAA TCGTGACTAG AATATATCTA TTTTCAAAAA TAAAAATTCC CTAAAAGAAA AGGATAAAAA   
  
  
+ ACTATATAAT ACTAAAAAAT TCTAACTATA ATAACTAGGC TTATTAAATT TTTATAGTGA AAATTTATTT   
  
  
+ CTACTTATAA CAACATCACT TACTAAGTAG CTTATTTGTG TCTTCTTTTC TAAATAATAA TGGTAACTAT   
  
  
+ GTGTCTTTTG TAAATAACTT ATTATCTATT TTATAAATCA CCGTGTAAAG CACGGGTCTA TACTAGTTAA   
  
  
+ TTTAAATAGC ATTGTTAAAT CAAGTAAGAT ACAAACAAAT TTATTTACAA CTGTAAGTGG TCCATAAAAA   
  
  
+ TAATAGCCAT TACAAAAAAT AGTACTACTG TTTTATGAAA TGATTTTAAT ATTCTAAAAT TCCAATAAAA   
  
  
+ CATGTTATAA TCTATGATTA ATTTTAAAAC ATGATTACAT GTCAGCATAA TTTGTATTAT TGTACAGCAA   
  
  
+ ATGAAAGTTG GAGCTAAAAG AAAATGGGGA AATAAAGGCA GACAAGTAAA GATAAAATAT GCTATCTACT   
  
  
+ TTTGGAGGTA TCCTTAACCA CATTGTCTCG TTTGTCTTCA TTAGGGAGAA GTGGGATAAA TACCAAAATT   
  
  
+ TGTATGGTCA TTGCGTGGGG TTTGCTTCCA AACAAAATGG CTTTTTGGAA GCAAATTTTG TGAGCTTCTA   
  
  
+ TGCAAATTTC CACGCTTAAC CCCACAAGCA AACAAAAACA GACAGTGGTT TTTGTTTGTC TTTGTTAGGG   
  
  
+ CATGTTTGGA GCCAACTGTT AATGGGAATA AACTGGGGAG GCTTTTTCCT TGGCTCTTTC CCCCTGACCC   
  
  
+ CAAACCCACT TCAGCTCAAT ACGAAACCAG CCATTGAAAA AGGAAGGGGG GGGGGGGGGT ATGTGAGAAA   
  
  
+ AAGACAAAAC TTTTTCCCGG AGAGAGAAGA TACATATGCA ACAAAGGCTC TCCTTCTCAT AAATTCCATA   
  
  
+ TTCCTCTGTT TTTCTAGGAG AGAGAAATAT GCAAAAAACA AATCAGTTGG CGACAAAAGT TACTACCACG   
  
  
+ CATTGTCTAC TGCTGCGTAC CCATTTTTGG AGACTTTAAA GAACGCCCCT CCCATCTTGA AAAGTGAAAA   
  
  
+ CCCCCTCTTT GAAACTGAAT TTCAGGGGAA AAAAACCTCA CCAAACCTCT AGAGAGAGCT TTTAGAGAGG   
  
  
+ GAAATACAAC GATTAGAAGG AGCAATTTGG GAAATTTCTT TGGGAATTTG AATGGGTTTT GAGTGAATTG   
  
  
+ CAAATCCCAG AAAAGTTTTG GCAAGTACCG ATCTACAGTT CTCTCCTCTT CGTGTTTGGT AGATCCCCTG   
  
  
+ TTTCCTCTGT TTCATTTAGG GTACTTCTCA TTCATCCTCC CCACCCCCTT AATCGGATCT TCCTGTCGAG   
  
  
+ TCACTTTATG CTAATATTTT TTCAGTGGAT TTTTAGTTAA CCCTGTTCAT TTTTCCATTC TGTGTCCCAT   
  
  
+ TTCTCTCTTT TTCATTCATA AGTTGCCGGT TTATCTGTTG GGTGCAGCTT AGTCACAATA ATTTCTGTGT   
  
  
+ TAGGCTTCTT TACGGTTAAA AAAAAAGGAG GCACTCTTTT CGGTGTGATT GTTTATGGGA CCAATGATTC   
  
  
+ AAGATGATGG GTCATCAGTA ACTTCATCAC CCCTTCAATT TTTCTCCATG ATGTCTCCCA ATTTAGGTTC   
  
  
+ TTCCTACCCT TGGCTCAGAG AGCTAAAACC TGAAGAAAGA GGTCTTTACT TGATACATTT GTTGCTCTCT   
  
  
+ TGTGCAAATC ATGTCTCTAG TGGTAGCCTA GACAATGCGA ACTTAGCCCT CGAACAAATC TCTCAGCTTG   
  
  
+ CTGCCCCTGA TGGGGATACA ATGCAGCGTA TGGCTTCTTA TTTTGCTGAA GCCCTGGCTG AGAGGATCCT   
  
  
+ CAAGTCATGG CCTGGCATGT ATAGAGCCCT TCATTTTACG AAAATGCCTG TCATTTCAGA GGAAATTCTT   
  
  
+ GCTAGGAAGC TCTTCTTTGA GCTATTTCCT TTCTTGAAGC TGGCCTATTT GGTGACAAAC CAATCGATAA   
  
  
+ TCGAAGCCAT GGAGGGGGAA AAGATGGTTC ATATTATTGA TCTGAATGCA TCAGAACCTG CTCAATGGAT   
  
  
+ TGCCCTTATT CAGGCTTTGA GTGCAAGGCC TGGGGGTCCT CCTCATTTGA GAATTACCGG TGTTCATCAA   
  
  
+ CACAAAGAGG TTCTAGATCA AGTGGCTCAT AGGGTGACTC AAGAAGCTGA GAAATTGGAT TTGCCATTTC   
  
  
+ AGTTCAATCC TGTGGTTAGC AAGTTGGAAA ACCTTGATGT TGAAAAGCTG TGTGTTAAGA CTGGTGAGGC   
  
  
+ TCTAGCCATC AGTTCGGTCC TTCAACTGCA TACCCTTTTG GGTTCTGATG ATGAGCCCCT AAGGAAAAGT   
  
  
+ TCACCTTTAG CCTTGATGAA GTATGCAAAT GGGGCTAATA GGCAAAGCCC GAGTAATGAT TCGGCTTCTT   
  
  
+ CATCACCTCC TTCGCTCAAT ACTTCAACCA AGCTGGATGG TTTCCTCAGC GCTTTGTGGG GATTGTCCCC   
  
  
+ AAAGATTATG GTGATAGCTG AGCACGATTC CAATCACAAT GGTTCTGGAC TTATGGAGAG GTTGTCAGAA   
  
  
+ GCACTGTACT TCTATGCAGC GCTGTTTGAC TGCTTAGAAT CCACCCTGCC AAGAACATCT GTCGAAAGAA   
  
  
+ GGCGGGTAGA GAAGATGCTC CTAGGTGAAG AGATCAAGAA CATTATATCA TGCGAGGGAG GAGAAAGGAG   
  
  
+ AGAAAGGCAT GAGAAGATCG AGAAGTGGAT GCAGAGGCTA GACATGGCTG GATTCGGGAT CGTTCCTTTG   
  
  
+ AGCTATATGG GTATGCTGCA AGCAAGGCAA TTGCTTCAGG GCTATGGTTG TGATGGTTAT AGAGTGAAAG   
  
  
+ AGAATGGTTG TGTTGTCATC TGTTGGCAAG ATCGCCCCCT CTTTTCGGTA TCAGCATGGA GGTGTAGGAG   
  
  
+ ATG  

- -Up\_Stream \_Len000TACGCG GAAAGACGAT TGAGTAATAG AAAGGAAGAC TCTCCCCTGT TGAATATATT   
  
  
- TAACTATCCA TAAAAGTTTA GTTATCTGGT CGATGGTATA TACTATTAAA AATAATGAAA AGATTTAGGT   
  
  
- AGTTGATAAA AGTGTTAGGA GGAAAAAGAT CTTTTTATTT TAAACGAATT AAGTTTAGTG GATTTTAATG   
  
  
- ATTTAGATAT CAGGATTTAG ATGTTTGAGT AAACACAACG TTGAGTACTA AATTCACCTT GTGATTGGAA   
  
  
- TCCTCTATAC AACAGTTTTC TTCTTAAGCT ACTGTATAAA AGGTTGTTTT CCGGTGGATT GAAGTACTAA   
  
  
- ATTTTTCCTC CTCAGATTCG TGTAGTACCT ACTGATTAAA AACTACATAA CATTGATGCA TAACTCTATT   
  
  
- GAACTTACTT AGCACTGATC TTATATAGAT AAAAGTTTTT ATTTTTAAGG GATTTTCTTT TCCTATTTTT   
  
  
- TGATATATTA TGATTTTTTA AGATTGATAT TATTGATCCG AATAATTTAA AAATATCACT TTTAAATAAA   
  
  
- GATGAATATT GTTGTAGTGA ATGATTCATC GAATAAACAC AGAAGAAAAG ATTTATTATT ACCATTGATA   
  
  
- CACAGAAAAC ATTTATTGAA TAATAGATAA AATATTTAGT GGCACATTTC GTGCCCAGAT ATGATCAATT   
  
  
- AAATTTATCG TAACAATTTA GTTCATTCTA TGTTTGTTTA AATAAATGTT GACATTCACC AGGTATTTTT   
  
  
- ATTATCGGTA ATGTTTTTTA TCATGATGAC AAAATACTTT ACTAAAATTA TAAGATTTTA AGGTTATTTT   
  
  
- GTACAATATT AGATACTAAT TAAAATTTTG TACTAATGTA CAGTCGTATT AAACATAATA ACATGTCGTT   
  
  
- TACTTTCAAC CTCGATTTTC TTTTACCCCT TTATTTCCGT CTGTTCATTT CTATTTTATA CGATAGATGA   
  
  
- AAACCTCCAT AGGAATTGGT GTAACAGAGC AAACAGAAGT AATCCCTCTT CACCCTATTT ATGGTTTTAA   
  
  
- ACATACCAGT AACGCACCCC AAACGAAGGT TTGTTTTACC GAAAAACCTT CGTTTAAAAC ACTCGAAGAT   
  
  
- ACGTTTAAAG GTGCGAATTG GGGTGTTCGT TTGTTTTTGT CTGTCACCAA AAACAAACAG AAACAATCCC   
  
  
- GTACAAACCT CGGTTGACAA TTACCCTTAT TTGACCCCTC CGAAAAAGGA ACCGAGAAAG GGGGACTGGG   
  
  
- GTTTGGGTGA AGTCGAGTTA TGCTTTGGTC GGTAACTTTT TCCTTCCCCC CCCCCCCCCA TACACTCTTT   
  
  
- TTCTGTTTTG AAAAAGGGCC TCTCTCTTCT ATGTATACGT TGTTTCCGAG AGGAAGAGTA TTTAAGGTAT   
  
  
- AAGGAGACAA AAAGATCCTC TCTCTTTATA CGTTTTTTGT TTAGTCAACC GCTGTTTTCA ATGATGGTGC   
  
  
- GTAACAGATG ACGACGCATG GGTAAAAACC TCTGAAATTT CTTGCGGGGA GGGTAGAACT TTTCACTTTT   
  
  
- GGGGGAGAAA CTTTGACTTA AAGTCCCCTT TTTTTGGAGT GGTTTGGAGA TCTCTCTCGA AAATCTCTCC   
  
  
- CTTTATGTTG CTAATCTTCC TCGTTAAACC CTTTAAAGAA ACCCTTAAAC TTACCCAAAA CTCACTTAAC   
  
  
- GTTTAGGGTC TTTTCAAAAC CGTTCATGGC TAGATGTCAA GAGAGGAGAA GCACAAACCA TCTAGGGGAC   
  
  
- AAAGGAGACA AAGTAAATCC CATGAAGAGT AAGTAGGAGG GGTGGGGGAA TTAGCCTAGA AGGACAGCTC   
  
  
- AGTGAAATAC GATTATAAAA AAGTCACCTA AAAATCAATT GGGACAAGTA AAAAGGTAAG ACACAGGGTA   
  
  
- AAGAGAGAAA AAGTAAGTAT TCAACGGCCA AATAGACAAC CCACGTCGAA TCAGTGTTAT TAAAGACACA   
  
  
- ATCCGAAGAA ATGCCAATTT TTTTTTCCTC CGTGAGAAAA GCCACACTAA CAAATACCCT GGTTACTAAG   
  
  
- TTCTACTACC CAGTAGTCAT TGAAGTAGTG GGGAAGTTAA AAAGAGGTAC TACAGAGGGT TAAATCCAAG   
  
  
- AAGGATGGGA ACCGAGTCTC TCGATTTTGG ACTTCTTTCT CCAGAAATGA ACTATGTAAA CAACGAGAGA   
  
  
- ACACGTTTAG TACAGAGATC ACCATCGGAT CTGTTACGCT TGAATCGGGA GCTTGTTTAG AGAGTCGAAC   
  
  
- GACGGGGACT ACCCCTATGT TACGTCGCAT ACCGAAGAAT AAAACGACTT CGGGACCGAC TCTCCTAGGA   
  
  
- GTTCAGTACC GGACCGTACA TATCTCGGGA AGTAAAATGC TTTTACGGAC AGTAAAGTCT CCTTTAAGAA   
  
  
- CGATCCTTCG AGAAGAAACT CGATAAAGGA AAGAACTTCG ACCGGATAAA CCACTGTTTG GTTAGCTATT   
  
  
- AGCTTCGGTA CCTCCCCCTT TTCTACCAAG TATAATAACT AGACTTACGT AGTCTTGGAC GAGTTACCTA   
  
  
- ACGGGAATAA GTCCGAAACT CACGTTCCGG ACCCCCAGGA GGAGTAAACT CTTAATGGCC ACAAGTAGTT   
  
  
- GTGTTTCTCC AAGATCTAGT TCACCGAGTA TCCCACTGAG TTCTTCGACT CTTTAACCTA AACGGTAAAG   
  
  
- TCAAGTTAGG ACACCAATCG TTCAACCTTT TGGAACTACA ACTTTTCGAC ACACAATTCT GACCACTCCG   
  
  
- AGATCGGTAG TCAAGCCAGG AAGTTGACGT ATGGGAAAAC CCAAGACTAC TACTCGGGGA TTCCTTTTCA   
  
  
- AGTGGAAATC GGAACTACTT CATACGTTTA CCCCGATTAT CCGTTTCGGG CTCATTACTA AGCCGAAGAA   
  
  
- GTAGTGGAGG AAGCGAGTTA TGAAGTTGGT TCGACCTACC AAAGGAGTCG CGAAACACCC CTAACAGGGG   
  
  
- TTTCTAATAC CACTATCGAC TCGTGCTAAG GTTAGTGTTA CCAAGACCTG AATACCTCTC CAACAGTCTT   
  
  
- CGTGACATGA AGATACGTCG CGACAAACTG ACGAATCTTA GGTGGGACGG TTCTTGTAGA CAGCTTTCTT   
  
  
- CCGCCCATCT CTTCTACGAG GATCCACTTC TCTAGTTCTT GTAATATAGT ACGCTCCCTC CTCTTTCCTC   
  
  
- TCTTTCCGTA CTCTTCTAGC TCTTCACCTA CGTCTCCGAT CTGTACCGAC CTAAGCCCTA GCAAGGAAAC   
  
  
- TCGATATACC CATACGACGT TCGTTCCGTT AACGAAGTCC CGATACCAAC ACTACCAATA TCTCACTTTC   
  
  
- TCTTACCAAC ACAACAGTAG ACAACCGTTC TAGCGGGGGA GAAAAGCCAT AGTCGTACCT CCACATCCTC   
  
  
- TAC

+     GTGGC-motif

| Site Name | Organism | Position | Strand | Matrix score. | sequence | function |
| --- | --- | --- | --- | --- | --- | --- |
| GTGGC-motif | Hordeum vulgare | 2268 | + | 10 | CAGCGTGTGGC | part of a light responsive element |

>HU08G00367.1   
+ -Up\_Stream \_Len000ATGCGC CTTTCTGCTA ACTCATTATC TTTCCTTCTG AGAGGGGACA ACTTATATAA   
  
  
+ ATTGATAGGT ATTTTCAAAT CAATAGACCA GCTACCATAT ATGATAATTT TTATTACTTT TCTAAATCCA   
  
  
+ TCAACTATTT TCACAATCCT CCTTTTTCTA GAAAAATAAA ATTTGCTTAA TTCAAATCAC CTAAAATTAC   
  
  
+ TAAATCTATA GTCCTAAATC TACAAACTCA TTTGTGTTGC AACTCATGAT TTAAGTGGAA CACTAACCTT   
  
  
+ AGGAGATATG TTGTCAAAAG AAGAATTCGA TGACATATTT TCCAACAAAA GGCCACCTAA CTTCATGATT   
  
  
+ TAAAAAGGAG GAGTCTAAGC ACATCATGGA TGACTAATTT TTGATGTATT GTAACTACGT ATTGAGATAA   
  
  
+ CTTGAATGAA TCGTGACTAG AATATATCTA TTTTCAAAAA TAAAAATTCC CTAAAAGAAA AGGATAAAAA   
  
  
+ ACTATATAAT ACTAAAAAAT TCTAACTATA ATAACTAGGC TTATTAAATT TTTATAGTGA AAATTTATTT   
  
  
+ CTACTTATAA CAACATCACT TACTAAGTAG CTTATTTGTG TCTTCTTTTC TAAATAATAA TGGTAACTAT   
  
  
+ GTGTCTTTTG TAAATAACTT ATTATCTATT TTATAAATCA CCGTGTAAAG CACGGGTCTA TACTAGTTAA   
  
  
+ TTTAAATAGC ATTGTTAAAT CAAGTAAGAT ACAAACAAAT TTATTTACAA CTGTAAGTGG TCCATAAAAA   
  
  
+ TAATAGCCAT TACAAAAAAT AGTACTACTG TTTTATGAAA TGATTTTAAT ATTCTAAAAT TCCAATAAAA   
  
  
+ CATGTTATAA TCTATGATTA ATTTTAAAAC ATGATTACAT GTCAGCATAA TTTGTATTAT TGTACAGCAA   
  
  
+ ATGAAAGTTG GAGCTAAAAG AAAATGGGGA AATAAAGGCA GACAAGTAAA GATAAAATAT GCTATCTACT   
  
  
+ TTTGGAGGTA TCCTTAACCA CATTGTCTCG TTTGTCTTCA TTAGGGAGAA GTGGGATAAA TACCAAAATT   
  
  
+ TGTATGGTCA TTGCGTGGGG TTTGCTTCCA AACAAAATGG CTTTTTGGAA GCAAATTTTG TGAGCTTCTA   
  
  
+ TGCAAATTTC CACGCTTAAC CCCACAAGCA AACAAAAACA GACAGTGGTT TTTGTTTGTC TTTGTTAGGG   
  
  
+ CATGTTTGGA GCCAACTGTT AATGGGAATA AACTGGGGAG GCTTTTTCCT TGGCTCTTTC CCCCTGACCC   
  
  
+ CAAACCCACT TCAGCTCAAT ACGAAACCAG CCATTGAAAA AGGAAGGGGG GGGGGGGGGT ATGTGAGAAA   
  
  
+ AAGACAAAAC TTTTTCCCGG AGAGAGAAGA TACATATGCA ACAAAGGCTC TCCTTCTCAT AAATTCCATA   
  
  
+ TTCCTCTGTT TTTCTAGGAG AGAGAAATAT GCAAAAAACA AATCAGTTGG CGACAAAAGT TACTACCACG   
  
  
+ CATTGTCTAC TGCTGCGTAC CCATTTTTGG AGACTTTAAA GAACGCCCCT CCCATCTTGA AAAGTGAAAA   
  
  
+ CCCCCTCTTT GAAACTGAAT TTCAGGGGAA AAAAACCTCA CCAAACCTCT AGAGAGAGCT TTTAGAGAGG   
  
  
+ GAAATACAAC GATTAGAAGG AGCAATTTGG GAAATTTCTT TGGGAATTTG AATGGGTTTT GAGTGAATTG   
  
  
+ CAAATCCCAG AAAAGTTTTG GCAAGTACCG ATCTACAGTT CTCTCCTCTT CGTGTTTGGT AGATCCCCTG   
  
  
+ TTTCCTCTGT TTCATTTAGG GTACTTCTCA TTCATCCTCC CCACCCCCTT AATCGGATCT TCCTGTCGAG   
  
  
+ TCACTTTATG CTAATATTTT TTCAGTGGAT TTTTAGTTAA CCCTGTTCAT TTTTCCATTC TGTGTCCCAT   
  
  
+ TTCTCTCTTT TTCATTCATA AGTTGCCGGT TTATCTGTTG GGTGCAGCTT AGTCACAATA ATTTCTGTGT   
  
  
+ TAGGCTTCTT TACGGTTAAA AAAAAAGGAG GCACTCTTTT CGGTGTGATT GTTTATGGGA CCAATGATTC   
  
  
+ AAGATGATGG GTCATCAGTA ACTTCATCAC CCCTTCAATT TTTCTCCATG ATGTCTCCCA ATTTAGGTTC   
  
  
+ TTCCTACCCT TGGCTCAGAG AGCTAAAACC TGAAGAAAGA GGTCTTTACT TGATACATTT GTTGCTCTCT   
  
  
+ TGTGCAAATC ATGTCTCTAG TGGTAGCCTA GACAATGCGA ACTTAGCCCT CGAACAAATC TCTCAGCTTG   
  
  
+ CTGCCCCTGA TGGGGATACA ATGCAGCGTA TGGCTTCTTA TTTTGCTGAA GCCCTGGCTG AGAGGATCCT   
  
  
+ CAAGTCATGG CCTGGCATGT ATAGAGCCCT TCATTTTACG AAAATGCCTG TCATTTCAGA GGAAATTCTT   
  
  
+ GCTAGGAAGC TCTTCTTTGA GCTATTTCCT TTCTTGAAGC TGGCCTATTT GGTGACAAAC CAATCGATAA   
  
  
+ TCGAAGCCAT GGAGGGGGAA AAGATGGTTC ATATTATTGA TCTGAATGCA TCAGAACCTG CTCAATGGAT   
  
  
+ TGCCCTTATT CAGGCTTTGA GTGCAAGGCC TGGGGGTCCT CCTCATTTGA GAATTACCGG TGTTCATCAA   
  
  
+ CACAAAGAGG TTCTAGATCA AGTGGCTCAT AGGGTGACTC AAGAAGCTGA GAAATTGGAT TTGCCATTTC   
  
  
+ AGTTCAATCC TGTGGTTAGC AAGTTGGAAA ACCTTGATGT TGAAAAGCTG TGTGTTAAGA CTGGTGAGGC   
  
  
+ TCTAGCCATC AGTTCGGTCC TTCAACTGCA TACCCTTTTG GGTTCTGATG ATGAGCCCCT AAGGAAAAGT   
  
  
+ TCACCTTTAG CCTTGATGAA GTATGCAAAT GGGGCTAATA GGCAAAGCCC GAGTAATGAT TCGGCTTCTT   
  
  
+ CATCACCTCC TTCGCTCAAT ACTTCAACCA AGCTGGATGG TTTCCTCAGC GCTTTGTGGG GATTGTCCCC   
  
  
+ AAAGATTATG GTGATAGCTG AGCACGATTC CAATCACAAT GGTTCTGGAC TTATGGAGAG GTTGTCAGAA   
  
  
+ GCACTGTACT TCTATGCAGC GCTGTTTGAC TGCTTAGAAT CCACCCTGCC AAGAACATCT GTCGAAAGAA   
  
  
+ GGCGGGTAGA GAAGATGCTC CTAGGTGAAG AGATCAAGAA CATTATATCA TGCGAGGGAG GAGAAAGGAG   
  
  
+ AGAAAGGCAT GAGAAGATCG AGAAGTGGAT GCAGAGGCTA GACATGGCTG GATTCGGGAT CGTTCCTTTG   
  
  
+ AGCTATATGG GTATGCTGCA AGCAAGGCAA TTGCTTCAGG GCTATGGTTG TGATGGTTAT AGAGTGAAAG   
  
  
+ AGAATGGTTG TGTTGTCATC TGTTGGCAAG ATCGCCCCCT CTTTTCGGTA TCAGCATGGA GGTGTAGGAG   
  
  
+ ATG  

- -Up\_Stream \_Len000TACGCG GAAAGACGAT TGAGTAATAG AAAGGAAGAC TCTCCCCTGT TGAATATATT   
  
  
- TAACTATCCA TAAAAGTTTA GTTATCTGGT CGATGGTATA TACTATTAAA AATAATGAAA AGATTTAGGT   
  
  
- AGTTGATAAA AGTGTTAGGA GGAAAAAGAT CTTTTTATTT TAAACGAATT AAGTTTAGTG GATTTTAATG   
  
  
- ATTTAGATAT CAGGATTTAG ATGTTTGAGT AAACACAACG TTGAGTACTA AATTCACCTT GTGATTGGAA   
  
  
- TCCTCTATAC AACAGTTTTC TTCTTAAGCT ACTGTATAAA AGGTTGTTTT CCGGTGGATT GAAGTACTAA   
  
  
- ATTTTTCCTC CTCAGATTCG TGTAGTACCT ACTGATTAAA AACTACATAA CATTGATGCA TAACTCTATT   
  
  
- GAACTTACTT AGCACTGATC TTATATAGAT AAAAGTTTTT ATTTTTAAGG GATTTTCTTT TCCTATTTTT   
  
  
- TGATATATTA TGATTTTTTA AGATTGATAT TATTGATCCG AATAATTTAA AAATATCACT TTTAAATAAA   
  
  
- GATGAATATT GTTGTAGTGA ATGATTCATC GAATAAACAC AGAAGAAAAG ATTTATTATT ACCATTGATA   
  
  
- CACAGAAAAC ATTTATTGAA TAATAGATAA AATATTTAGT GGCACATTTC GTGCCCAGAT ATGATCAATT   
  
  
- AAATTTATCG TAACAATTTA GTTCATTCTA TGTTTGTTTA AATAAATGTT GACATTCACC AGGTATTTTT   
  
  
- ATTATCGGTA ATGTTTTTTA TCATGATGAC AAAATACTTT ACTAAAATTA TAAGATTTTA AGGTTATTTT   
  
  
- GTACAATATT AGATACTAAT TAAAATTTTG TACTAATGTA CAGTCGTATT AAACATAATA ACATGTCGTT   
  
  
- TACTTTCAAC CTCGATTTTC TTTTACCCCT TTATTTCCGT CTGTTCATTT CTATTTTATA CGATAGATGA   
  
  
- AAACCTCCAT AGGAATTGGT GTAACAGAGC AAACAGAAGT AATCCCTCTT CACCCTATTT ATGGTTTTAA   
  
  
- ACATACCAGT AACGCACCCC AAACGAAGGT TTGTTTTACC GAAAAACCTT CGTTTAAAAC ACTCGAAGAT   
  
  
- ACGTTTAAAG GTGCGAATTG GGGTGTTCGT TTGTTTTTGT CTGTCACCAA AAACAAACAG AAACAATCCC   
  
  
- GTACAAACCT CGGTTGACAA TTACCCTTAT TTGACCCCTC CGAAAAAGGA ACCGAGAAAG GGGGACTGGG   
  
  
- GTTTGGGTGA AGTCGAGTTA TGCTTTGGTC GGTAACTTTT TCCTTCCCCC CCCCCCCCCA TACACTCTTT   
  
  
- TTCTGTTTTG AAAAAGGGCC TCTCTCTTCT ATGTATACGT TGTTTCCGAG AGGAAGAGTA TTTAAGGTAT   
  
  
- AAGGAGACAA AAAGATCCTC TCTCTTTATA CGTTTTTTGT TTAGTCAACC GCTGTTTTCA ATGATGGTGC   
  
  
- GTAACAGATG ACGACGCATG GGTAAAAACC TCTGAAATTT CTTGCGGGGA GGGTAGAACT TTTCACTTTT   
  
  
- GGGGGAGAAA CTTTGACTTA AAGTCCCCTT TTTTTGGAGT GGTTTGGAGA TCTCTCTCGA AAATCTCTCC   
  
  
- CTTTATGTTG CTAATCTTCC TCGTTAAACC CTTTAAAGAA ACCCTTAAAC TTACCCAAAA CTCACTTAAC   
  
  
- GTTTAGGGTC TTTTCAAAAC CGTTCATGGC TAGATGTCAA GAGAGGAGAA GCACAAACCA TCTAGGGGAC   
  
  
- AAAGGAGACA AAGTAAATCC CATGAAGAGT AAGTAGGAGG GGTGGGGGAA TTAGCCTAGA AGGACAGCTC   
  
  
- AGTGAAATAC GATTATAAAA AAGTCACCTA AAAATCAATT GGGACAAGTA AAAAGGTAAG ACACAGGGTA   
  
  
- AAGAGAGAAA AAGTAAGTAT TCAACGGCCA AATAGACAAC CCACGTCGAA TCAGTGTTAT TAAAGACACA   
  
  
- ATCCGAAGAA ATGCCAATTT TTTTTTCCTC CGTGAGAAAA GCCACACTAA CAAATACCCT GGTTACTAAG   
  
  
- TTCTACTACC CAGTAGTCAT TGAAGTAGTG GGGAAGTTAA AAAGAGGTAC TACAGAGGGT TAAATCCAAG   
  
  
- AAGGATGGGA ACCGAGTCTC TCGATTTTGG ACTTCTTTCT CCAGAAATGA ACTATGTAAA CAACGAGAGA   
  
  
- ACACGTTTAG TACAGAGATC ACCATCGGAT CTGTTACGCT TGAATCGGGA GCTTGTTTAG AGAGTCGAAC   
  
  
- GACGGGGACT ACCCCTATGT TACGTCGCAT ACCGAAGAAT AAAACGACTT CGGGACCGAC TCTCCTAGGA   
  
  
- GTTCAGTACC GGACCGTACA TATCTCGGGA AGTAAAATGC TTTTACGGAC AGTAAAGTCT CCTTTAAGAA   
  
  
- CGATCCTTCG AGAAGAAACT CGATAAAGGA AAGAACTTCG ACCGGATAAA CCACTGTTTG GTTAGCTATT   
  
  
- AGCTTCGGTA CCTCCCCCTT TTCTACCAAG TATAATAACT AGACTTACGT AGTCTTGGAC GAGTTACCTA   
  
  
- ACGGGAATAA GTCCGAAACT CACGTTCCGG ACCCCCAGGA GGAGTAAACT CTTAATGGCC ACAAGTAGTT   
  
  
- GTGTTTCTCC AAGATCTAGT TCACCGAGTA TCCCACTGAG TTCTTCGACT CTTTAACCTA AACGGTAAAG   
  
  
- TCAAGTTAGG ACACCAATCG TTCAACCTTT TGGAACTACA ACTTTTCGAC ACACAATTCT GACCACTCCG   
  
  
- AGATCGGTAG TCAAGCCAGG AAGTTGACGT ATGGGAAAAC CCAAGACTAC TACTCGGGGA TTCCTTTTCA   
  
  
- AGTGGAAATC GGAACTACTT CATACGTTTA CCCCGATTAT CCGTTTCGGG CTCATTACTA AGCCGAAGAA   
  
  
- GTAGTGGAGG AAGCGAGTTA TGAAGTTGGT TCGACCTACC AAAGGAGTCG CGAAACACCC CTAACAGGGG   
  
  
- TTTCTAATAC CACTATCGAC TCGTGCTAAG GTTAGTGTTA CCAAGACCTG AATACCTCTC CAACAGTCTT   
  
  
- CGTGACATGA AGATACGTCG CGACAAACTG ACGAATCTTA GGTGGGACGG TTCTTGTAGA CAGCTTTCTT   
  
  
- CCGCCCATCT CTTCTACGAG GATCCACTTC TCTAGTTCTT GTAATATAGT ACGCTCCCTC CTCTTTCCTC   
  
  
- TCTTTCCGTA CTCTTCTAGC TCTTCACCTA CGTCTCCGAT CTGTACCGAC CTAAGCCCTA GCAAGGAAAC   
  
  
- TCGATATACC CATACGACGT TCGTTCCGTT AACGAAGTCC CGATACCAAC ACTACCAATA TCTCACTTTC   
  
  
- TCTTACCAAC ACAACAGTAG ACAACCGTTC TAGCGGGGGA GAAAAGCCAT AGTCGTACCT CCACATCCTC   
  
  
- TAC

+     LTR

| Site Name | Organism | Position | Strand | Matrix score. | sequence | function |
| --- | --- | --- | --- | --- | --- | --- |
| LTR | Hordeum vulgare | 2002 | - | 6 | CCGAAA | cis-acting element involved in low-temperature responsiveness |
| LTR | Hordeum vulgare | 3337 | - | 6 | CCGAAA | cis-acting element involved in low-temperature responsiveness |

>HU08G00367.1   
+ -Up\_Stream \_Len000ATGCGC CTTTCTGCTA ACTCATTATC TTTCCTTCTG AGAGGGGACA ACTTATATAA   
  
  
+ ATTGATAGGT ATTTTCAAAT CAATAGACCA GCTACCATAT ATGATAATTT TTATTACTTT TCTAAATCCA   
  
  
+ TCAACTATTT TCACAATCCT CCTTTTTCTA GAAAAATAAA ATTTGCTTAA TTCAAATCAC CTAAAATTAC   
  
  
+ TAAATCTATA GTCCTAAATC TACAAACTCA TTTGTGTTGC AACTCATGAT TTAAGTGGAA CACTAACCTT   
  
  
+ AGGAGATATG TTGTCAAAAG AAGAATTCGA TGACATATTT TCCAACAAAA GGCCACCTAA CTTCATGATT   
  
  
+ TAAAAAGGAG GAGTCTAAGC ACATCATGGA TGACTAATTT TTGATGTATT GTAACTACGT ATTGAGATAA   
  
  
+ CTTGAATGAA TCGTGACTAG AATATATCTA TTTTCAAAAA TAAAAATTCC CTAAAAGAAA AGGATAAAAA   
  
  
+ ACTATATAAT ACTAAAAAAT TCTAACTATA ATAACTAGGC TTATTAAATT TTTATAGTGA AAATTTATTT   
  
  
+ CTACTTATAA CAACATCACT TACTAAGTAG CTTATTTGTG TCTTCTTTTC TAAATAATAA TGGTAACTAT   
  
  
+ GTGTCTTTTG TAAATAACTT ATTATCTATT TTATAAATCA CCGTGTAAAG CACGGGTCTA TACTAGTTAA   
  
  
+ TTTAAATAGC ATTGTTAAAT CAAGTAAGAT ACAAACAAAT TTATTTACAA CTGTAAGTGG TCCATAAAAA   
  
  
+ TAATAGCCAT TACAAAAAAT AGTACTACTG TTTTATGAAA TGATTTTAAT ATTCTAAAAT TCCAATAAAA   
  
  
+ CATGTTATAA TCTATGATTA ATTTTAAAAC ATGATTACAT GTCAGCATAA TTTGTATTAT TGTACAGCAA   
  
  
+ ATGAAAGTTG GAGCTAAAAG AAAATGGGGA AATAAAGGCA GACAAGTAAA GATAAAATAT GCTATCTACT   
  
  
+ TTTGGAGGTA TCCTTAACCA CATTGTCTCG TTTGTCTTCA TTAGGGAGAA GTGGGATAAA TACCAAAATT   
  
  
+ TGTATGGTCA TTGCGTGGGG TTTGCTTCCA AACAAAATGG CTTTTTGGAA GCAAATTTTG TGAGCTTCTA   
  
  
+ TGCAAATTTC CACGCTTAAC CCCACAAGCA AACAAAAACA GACAGTGGTT TTTGTTTGTC TTTGTTAGGG   
  
  
+ CATGTTTGGA GCCAACTGTT AATGGGAATA AACTGGGGAG GCTTTTTCCT TGGCTCTTTC CCCCTGACCC   
  
  
+ CAAACCCACT TCAGCTCAAT ACGAAACCAG CCATTGAAAA AGGAAGGGGG GGGGGGGGGT ATGTGAGAAA   
  
  
+ AAGACAAAAC TTTTTCCCGG AGAGAGAAGA TACATATGCA ACAAAGGCTC TCCTTCTCAT AAATTCCATA   
  
  
+ TTCCTCTGTT TTTCTAGGAG AGAGAAATAT GCAAAAAACA AATCAGTTGG CGACAAAAGT TACTACCACG   
  
  
+ CATTGTCTAC TGCTGCGTAC CCATTTTTGG AGACTTTAAA GAACGCCCCT CCCATCTTGA AAAGTGAAAA   
  
  
+ CCCCCTCTTT GAAACTGAAT TTCAGGGGAA AAAAACCTCA CCAAACCTCT AGAGAGAGCT TTTAGAGAGG   
  
  
+ GAAATACAAC GATTAGAAGG AGCAATTTGG GAAATTTCTT TGGGAATTTG AATGGGTTTT GAGTGAATTG   
  
  
+ CAAATCCCAG AAAAGTTTTG GCAAGTACCG ATCTACAGTT CTCTCCTCTT CGTGTTTGGT AGATCCCCTG   
  
  
+ TTTCCTCTGT TTCATTTAGG GTACTTCTCA TTCATCCTCC CCACCCCCTT AATCGGATCT TCCTGTCGAG   
  
  
+ TCACTTTATG CTAATATTTT TTCAGTGGAT TTTTAGTTAA CCCTGTTCAT TTTTCCATTC TGTGTCCCAT   
  
  
+ TTCTCTCTTT TTCATTCATA AGTTGCCGGT TTATCTGTTG GGTGCAGCTT AGTCACAATA ATTTCTGTGT   
  
  
+ TAGGCTTCTT TACGGTTAAA AAAAAAGGAG GCACTCTTTT CGGTGTGATT GTTTATGGGA CCAATGATTC   
  
  
+ AAGATGATGG GTCATCAGTA ACTTCATCAC CCCTTCAATT TTTCTCCATG ATGTCTCCCA ATTTAGGTTC   
  
  
+ TTCCTACCCT TGGCTCAGAG AGCTAAAACC TGAAGAAAGA GGTCTTTACT TGATACATTT GTTGCTCTCT   
  
  
+ TGTGCAAATC ATGTCTCTAG TGGTAGCCTA GACAATGCGA ACTTAGCCCT CGAACAAATC TCTCAGCTTG   
  
  
+ CTGCCCCTGA TGGGGATACA ATGCAGCGTA TGGCTTCTTA TTTTGCTGAA GCCCTGGCTG AGAGGATCCT   
  
  
+ CAAGTCATGG CCTGGCATGT ATAGAGCCCT TCATTTTACG AAAATGCCTG TCATTTCAGA GGAAATTCTT   
  
  
+ GCTAGGAAGC TCTTCTTTGA GCTATTTCCT TTCTTGAAGC TGGCCTATTT GGTGACAAAC CAATCGATAA   
  
  
+ TCGAAGCCAT GGAGGGGGAA AAGATGGTTC ATATTATTGA TCTGAATGCA TCAGAACCTG CTCAATGGAT   
  
  
+ TGCCCTTATT CAGGCTTTGA GTGCAAGGCC TGGGGGTCCT CCTCATTTGA GAATTACCGG TGTTCATCAA   
  
  
+ CACAAAGAGG TTCTAGATCA AGTGGCTCAT AGGGTGACTC AAGAAGCTGA GAAATTGGAT TTGCCATTTC   
  
  
+ AGTTCAATCC TGTGGTTAGC AAGTTGGAAA ACCTTGATGT TGAAAAGCTG TGTGTTAAGA CTGGTGAGGC   
  
  
+ TCTAGCCATC AGTTCGGTCC TTCAACTGCA TACCCTTTTG GGTTCTGATG ATGAGCCCCT AAGGAAAAGT   
  
  
+ TCACCTTTAG CCTTGATGAA GTATGCAAAT GGGGCTAATA GGCAAAGCCC GAGTAATGAT TCGGCTTCTT   
  
  
+ CATCACCTCC TTCGCTCAAT ACTTCAACCA AGCTGGATGG TTTCCTCAGC GCTTTGTGGG GATTGTCCCC   
  
  
+ AAAGATTATG GTGATAGCTG AGCACGATTC CAATCACAAT GGTTCTGGAC TTATGGAGAG GTTGTCAGAA   
  
  
+ GCACTGTACT TCTATGCAGC GCTGTTTGAC TGCTTAGAAT CCACCCTGCC AAGAACATCT GTCGAAAGAA   
  
  
+ GGCGGGTAGA GAAGATGCTC CTAGGTGAAG AGATCAAGAA CATTATATCA TGCGAGGGAG GAGAAAGGAG   
  
  
+ AGAAAGGCAT GAGAAGATCG AGAAGTGGAT GCAGAGGCTA GACATGGCTG GATTCGGGAT CGTTCCTTTG   
  
  
+ AGCTATATGG GTATGCTGCA AGCAAGGCAA TTGCTTCAGG GCTATGGTTG TGATGGTTAT AGAGTGAAAG   
  
  
+ AGAATGGTTG TGTTGTCATC TGTTGGCAAG ATCGCCCCCT CTTTTCGGTA TCAGCATGGA GGTGTAGGAG   
  
  
+ ATG  

- -Up\_Stream \_Len000TACGCG GAAAGACGAT TGAGTAATAG AAAGGAAGAC TCTCCCCTGT TGAATATATT   
  
  
- TAACTATCCA TAAAAGTTTA GTTATCTGGT CGATGGTATA TACTATTAAA AATAATGAAA AGATTTAGGT   
  
  
- AGTTGATAAA AGTGTTAGGA GGAAAAAGAT CTTTTTATTT TAAACGAATT AAGTTTAGTG GATTTTAATG   
  
  
- ATTTAGATAT CAGGATTTAG ATGTTTGAGT AAACACAACG TTGAGTACTA AATTCACCTT GTGATTGGAA   
  
  
- TCCTCTATAC AACAGTTTTC TTCTTAAGCT ACTGTATAAA AGGTTGTTTT CCGGTGGATT GAAGTACTAA   
  
  
- ATTTTTCCTC CTCAGATTCG TGTAGTACCT ACTGATTAAA AACTACATAA CATTGATGCA TAACTCTATT   
  
  
- GAACTTACTT AGCACTGATC TTATATAGAT AAAAGTTTTT ATTTTTAAGG GATTTTCTTT TCCTATTTTT   
  
  
- TGATATATTA TGATTTTTTA AGATTGATAT TATTGATCCG AATAATTTAA AAATATCACT TTTAAATAAA   
  
  
- GATGAATATT GTTGTAGTGA ATGATTCATC GAATAAACAC AGAAGAAAAG ATTTATTATT ACCATTGATA   
  
  
- CACAGAAAAC ATTTATTGAA TAATAGATAA AATATTTAGT GGCACATTTC GTGCCCAGAT ATGATCAATT   
  
  
- AAATTTATCG TAACAATTTA GTTCATTCTA TGTTTGTTTA AATAAATGTT GACATTCACC AGGTATTTTT   
  
  
- ATTATCGGTA ATGTTTTTTA TCATGATGAC AAAATACTTT ACTAAAATTA TAAGATTTTA AGGTTATTTT   
  
  
- GTACAATATT AGATACTAAT TAAAATTTTG TACTAATGTA CAGTCGTATT AAACATAATA ACATGTCGTT   
  
  
- TACTTTCAAC CTCGATTTTC TTTTACCCCT TTATTTCCGT CTGTTCATTT CTATTTTATA CGATAGATGA   
  
  
- AAACCTCCAT AGGAATTGGT GTAACAGAGC AAACAGAAGT AATCCCTCTT CACCCTATTT ATGGTTTTAA   
  
  
- ACATACCAGT AACGCACCCC AAACGAAGGT TTGTTTTACC GAAAAACCTT CGTTTAAAAC ACTCGAAGAT   
  
  
- ACGTTTAAAG GTGCGAATTG GGGTGTTCGT TTGTTTTTGT CTGTCACCAA AAACAAACAG AAACAATCCC   
  
  
- GTACAAACCT CGGTTGACAA TTACCCTTAT TTGACCCCTC CGAAAAAGGA ACCGAGAAAG GGGGACTGGG   
  
  
- GTTTGGGTGA AGTCGAGTTA TGCTTTGGTC GGTAACTTTT TCCTTCCCCC CCCCCCCCCA TACACTCTTT   
  
  
- TTCTGTTTTG AAAAAGGGCC TCTCTCTTCT ATGTATACGT TGTTTCCGAG AGGAAGAGTA TTTAAGGTAT   
  
  
- AAGGAGACAA AAAGATCCTC TCTCTTTATA CGTTTTTTGT TTAGTCAACC GCTGTTTTCA ATGATGGTGC   
  
  
- GTAACAGATG ACGACGCATG GGTAAAAACC TCTGAAATTT CTTGCGGGGA GGGTAGAACT TTTCACTTTT   
  
  
- GGGGGAGAAA CTTTGACTTA AAGTCCCCTT TTTTTGGAGT GGTTTGGAGA TCTCTCTCGA AAATCTCTCC   
  
  
- CTTTATGTTG CTAATCTTCC TCGTTAAACC CTTTAAAGAA ACCCTTAAAC TTACCCAAAA CTCACTTAAC   
  
  
- GTTTAGGGTC TTTTCAAAAC CGTTCATGGC TAGATGTCAA GAGAGGAGAA GCACAAACCA TCTAGGGGAC   
  
  
- AAAGGAGACA AAGTAAATCC CATGAAGAGT AAGTAGGAGG GGTGGGGGAA TTAGCCTAGA AGGACAGCTC   
  
  
- AGTGAAATAC GATTATAAAA AAGTCACCTA AAAATCAATT GGGACAAGTA AAAAGGTAAG ACACAGGGTA   
  
  
- AAGAGAGAAA AAGTAAGTAT TCAACGGCCA AATAGACAAC CCACGTCGAA TCAGTGTTAT TAAAGACACA   
  
  
- ATCCGAAGAA ATGCCAATTT TTTTTTCCTC CGTGAGAAAA GCCACACTAA CAAATACCCT GGTTACTAAG   
  
  
- TTCTACTACC CAGTAGTCAT TGAAGTAGTG GGGAAGTTAA AAAGAGGTAC TACAGAGGGT TAAATCCAAG   
  
  
- AAGGATGGGA ACCGAGTCTC TCGATTTTGG ACTTCTTTCT CCAGAAATGA ACTATGTAAA CAACGAGAGA   
  
  
- ACACGTTTAG TACAGAGATC ACCATCGGAT CTGTTACGCT TGAATCGGGA GCTTGTTTAG AGAGTCGAAC   
  
  
- GACGGGGACT ACCCCTATGT TACGTCGCAT ACCGAAGAAT AAAACGACTT CGGGACCGAC TCTCCTAGGA   
  
  
- GTTCAGTACC GGACCGTACA TATCTCGGGA AGTAAAATGC TTTTACGGAC AGTAAAGTCT CCTTTAAGAA   
  
  
- CGATCCTTCG AGAAGAAACT CGATAAAGGA AAGAACTTCG ACCGGATAAA CCACTGTTTG GTTAGCTATT   
  
  
- AGCTTCGGTA CCTCCCCCTT TTCTACCAAG TATAATAACT AGACTTACGT AGTCTTGGAC GAGTTACCTA   
  
  
- ACGGGAATAA GTCCGAAACT CACGTTCCGG ACCCCCAGGA GGAGTAAACT CTTAATGGCC ACAAGTAGTT   
  
  
- GTGTTTCTCC AAGATCTAGT TCACCGAGTA TCCCACTGAG TTCTTCGACT CTTTAACCTA AACGGTAAAG   
  
  
- TCAAGTTAGG ACACCAATCG TTCAACCTTT TGGAACTACA ACTTTTCGAC ACACAATTCT GACCACTCCG   
  
  
- AGATCGGTAG TCAAGCCAGG AAGTTGACGT ATGGGAAAAC CCAAGACTAC TACTCGGGGA TTCCTTTTCA   
  
  
- AGTGGAAATC GGAACTACTT CATACGTTTA CCCCGATTAT CCGTTTCGGG CTCATTACTA AGCCGAAGAA   
  
  
- GTAGTGGAGG AAGCGAGTTA TGAAGTTGGT TCGACCTACC AAAGGAGTCG CGAAACACCC CTAACAGGGG   
  
  
- TTTCTAATAC CACTATCGAC TCGTGCTAAG GTTAGTGTTA CCAAGACCTG AATACCTCTC CAACAGTCTT   
  
  
- CGTGACATGA AGATACGTCG CGACAAACTG ACGAATCTTA GGTGGGACGG TTCTTGTAGA CAGCTTTCTT   
  
  
- CCGCCCATCT CTTCTACGAG GATCCACTTC TCTAGTTCTT GTAATATAGT ACGCTCCCTC CTCTTTCCTC   
  
  
- TCTTTCCGTA CTCTTCTAGC TCTTCACCTA CGTCTCCGAT CTGTACCGAC CTAAGCCCTA GCAAGGAAAC   
  
  
- TCGATATACC CATACGACGT TCGTTCCGTT AACGAAGTCC CGATACCAAC ACTACCAATA TCTCACTTTC   
  
  
- TCTTACCAAC ACAACAGTAG ACAACCGTTC TAGCGGGGGA GAAAAGCCAT AGTCGTACCT CCACATCCTC   
  
  
- TAC

+     MBS

| Site Name | Organism | Position | Strand | Matrix score. | sequence | function |
| --- | --- | --- | --- | --- | --- | --- |
| MBS | Arabidopsis thaliana | 1448 | - | 6 | CAACTG | MYB binding site involved in drought-inducibility |
| MBS | Arabidopsis thaliana | 1207 | + | 6 | CAACTG | MYB binding site involved in drought-inducibility |
| MBS | Arabidopsis thaliana | 752 | + | 6 | CAACTG | MYB binding site involved in drought-inducibility |
| MBS | Arabidopsis thaliana | 2757 | + | 6 | CAACTG | MYB binding site involved in drought-inducibility |

>HU08G00367.1   
+ -Up\_Stream \_Len000ATGCGC CTTTCTGCTA ACTCATTATC TTTCCTTCTG AGAGGGGACA ACTTATATAA   
  
  
+ ATTGATAGGT ATTTTCAAAT CAATAGACCA GCTACCATAT ATGATAATTT TTATTACTTT TCTAAATCCA   
  
  
+ TCAACTATTT TCACAATCCT CCTTTTTCTA GAAAAATAAA ATTTGCTTAA TTCAAATCAC CTAAAATTAC   
  
  
+ TAAATCTATA GTCCTAAATC TACAAACTCA TTTGTGTTGC AACTCATGAT TTAAGTGGAA CACTAACCTT   
  
  
+ AGGAGATATG TTGTCAAAAG AAGAATTCGA TGACATATTT TCCAACAAAA GGCCACCTAA CTTCATGATT   
  
  
+ TAAAAAGGAG GAGTCTAAGC ACATCATGGA TGACTAATTT TTGATGTATT GTAACTACGT ATTGAGATAA   
  
  
+ CTTGAATGAA TCGTGACTAG AATATATCTA TTTTCAAAAA TAAAAATTCC CTAAAAGAAA AGGATAAAAA   
  
  
+ ACTATATAAT ACTAAAAAAT TCTAACTATA ATAACTAGGC TTATTAAATT TTTATAGTGA AAATTTATTT   
  
  
+ CTACTTATAA CAACATCACT TACTAAGTAG CTTATTTGTG TCTTCTTTTC TAAATAATAA TGGTAACTAT   
  
  
+ GTGTCTTTTG TAAATAACTT ATTATCTATT TTATAAATCA CCGTGTAAAG CACGGGTCTA TACTAGTTAA   
  
  
+ TTTAAATAGC ATTGTTAAAT CAAGTAAGAT ACAAACAAAT TTATTTACAA CTGTAAGTGG TCCATAAAAA   
  
  
+ TAATAGCCAT TACAAAAAAT AGTACTACTG TTTTATGAAA TGATTTTAAT ATTCTAAAAT TCCAATAAAA   
  
  
+ CATGTTATAA TCTATGATTA ATTTTAAAAC ATGATTACAT GTCAGCATAA TTTGTATTAT TGTACAGCAA   
  
  
+ ATGAAAGTTG GAGCTAAAAG AAAATGGGGA AATAAAGGCA GACAAGTAAA GATAAAATAT GCTATCTACT   
  
  
+ TTTGGAGGTA TCCTTAACCA CATTGTCTCG TTTGTCTTCA TTAGGGAGAA GTGGGATAAA TACCAAAATT   
  
  
+ TGTATGGTCA TTGCGTGGGG TTTGCTTCCA AACAAAATGG CTTTTTGGAA GCAAATTTTG TGAGCTTCTA   
  
  
+ TGCAAATTTC CACGCTTAAC CCCACAAGCA AACAAAAACA GACAGTGGTT TTTGTTTGTC TTTGTTAGGG   
  
  
+ CATGTTTGGA GCCAACTGTT AATGGGAATA AACTGGGGAG GCTTTTTCCT TGGCTCTTTC CCCCTGACCC   
  
  
+ CAAACCCACT TCAGCTCAAT ACGAAACCAG CCATTGAAAA AGGAAGGGGG GGGGGGGGGT ATGTGAGAAA   
  
  
+ AAGACAAAAC TTTTTCCCGG AGAGAGAAGA TACATATGCA ACAAAGGCTC TCCTTCTCAT AAATTCCATA   
  
  
+ TTCCTCTGTT TTTCTAGGAG AGAGAAATAT GCAAAAAACA AATCAGTTGG CGACAAAAGT TACTACCACG   
  
  
+ CATTGTCTAC TGCTGCGTAC CCATTTTTGG AGACTTTAAA GAACGCCCCT CCCATCTTGA AAAGTGAAAA   
  
  
+ CCCCCTCTTT GAAACTGAAT TTCAGGGGAA AAAAACCTCA CCAAACCTCT AGAGAGAGCT TTTAGAGAGG   
  
  
+ GAAATACAAC GATTAGAAGG AGCAATTTGG GAAATTTCTT TGGGAATTTG AATGGGTTTT GAGTGAATTG   
  
  
+ CAAATCCCAG AAAAGTTTTG GCAAGTACCG ATCTACAGTT CTCTCCTCTT CGTGTTTGGT AGATCCCCTG   
  
  
+ TTTCCTCTGT TTCATTTAGG GTACTTCTCA TTCATCCTCC CCACCCCCTT AATCGGATCT TCCTGTCGAG   
  
  
+ TCACTTTATG CTAATATTTT TTCAGTGGAT TTTTAGTTAA CCCTGTTCAT TTTTCCATTC TGTGTCCCAT   
  
  
+ TTCTCTCTTT TTCATTCATA AGTTGCCGGT TTATCTGTTG GGTGCAGCTT AGTCACAATA ATTTCTGTGT   
  
  
+ TAGGCTTCTT TACGGTTAAA AAAAAAGGAG GCACTCTTTT CGGTGTGATT GTTTATGGGA CCAATGATTC   
  
  
+ AAGATGATGG GTCATCAGTA ACTTCATCAC CCCTTCAATT TTTCTCCATG ATGTCTCCCA ATTTAGGTTC   
  
  
+ TTCCTACCCT TGGCTCAGAG AGCTAAAACC TGAAGAAAGA GGTCTTTACT TGATACATTT GTTGCTCTCT   
  
  
+ TGTGCAAATC ATGTCTCTAG TGGTAGCCTA GACAATGCGA ACTTAGCCCT CGAACAAATC TCTCAGCTTG   
  
  
+ CTGCCCCTGA TGGGGATACA ATGCAGCGTA TGGCTTCTTA TTTTGCTGAA GCCCTGGCTG AGAGGATCCT   
  
  
+ CAAGTCATGG CCTGGCATGT ATAGAGCCCT TCATTTTACG AAAATGCCTG TCATTTCAGA GGAAATTCTT   
  
  
+ GCTAGGAAGC TCTTCTTTGA GCTATTTCCT TTCTTGAAGC TGGCCTATTT GGTGACAAAC CAATCGATAA   
  
  
+ TCGAAGCCAT GGAGGGGGAA AAGATGGTTC ATATTATTGA TCTGAATGCA TCAGAACCTG CTCAATGGAT   
  
  
+ TGCCCTTATT CAGGCTTTGA GTGCAAGGCC TGGGGGTCCT CCTCATTTGA GAATTACCGG TGTTCATCAA   
  
  
+ CACAAAGAGG TTCTAGATCA AGTGGCTCAT AGGGTGACTC AAGAAGCTGA GAAATTGGAT TTGCCATTTC   
  
  
+ AGTTCAATCC TGTGGTTAGC AAGTTGGAAA ACCTTGATGT TGAAAAGCTG TGTGTTAAGA CTGGTGAGGC   
  
  
+ TCTAGCCATC AGTTCGGTCC TTCAACTGCA TACCCTTTTG GGTTCTGATG ATGAGCCCCT AAGGAAAAGT   
  
  
+ TCACCTTTAG CCTTGATGAA GTATGCAAAT GGGGCTAATA GGCAAAGCCC GAGTAATGAT TCGGCTTCTT   
  
  
+ CATCACCTCC TTCGCTCAAT ACTTCAACCA AGCTGGATGG TTTCCTCAGC GCTTTGTGGG GATTGTCCCC   
  
  
+ AAAGATTATG GTGATAGCTG AGCACGATTC CAATCACAAT GGTTCTGGAC TTATGGAGAG GTTGTCAGAA   
  
  
+ GCACTGTACT TCTATGCAGC GCTGTTTGAC TGCTTAGAAT CCACCCTGCC AAGAACATCT GTCGAAAGAA   
  
  
+ GGCGGGTAGA GAAGATGCTC CTAGGTGAAG AGATCAAGAA CATTATATCA TGCGAGGGAG GAGAAAGGAG   
  
  
+ AGAAAGGCAT GAGAAGATCG AGAAGTGGAT GCAGAGGCTA GACATGGCTG GATTCGGGAT CGTTCCTTTG   
  
  
+ AGCTATATGG GTATGCTGCA AGCAAGGCAA TTGCTTCAGG GCTATGGTTG TGATGGTTAT AGAGTGAAAG   
  
  
+ AGAATGGTTG TGTTGTCATC TGTTGGCAAG ATCGCCCCCT CTTTTCGGTA TCAGCATGGA GGTGTAGGAG   
  
  
+ ATG  

- -Up\_Stream \_Len000TACGCG GAAAGACGAT TGAGTAATAG AAAGGAAGAC TCTCCCCTGT TGAATATATT   
  
  
- TAACTATCCA TAAAAGTTTA GTTATCTGGT CGATGGTATA TACTATTAAA AATAATGAAA AGATTTAGGT   
  
  
- AGTTGATAAA AGTGTTAGGA GGAAAAAGAT CTTTTTATTT TAAACGAATT AAGTTTAGTG GATTTTAATG   
  
  
- ATTTAGATAT CAGGATTTAG ATGTTTGAGT AAACACAACG TTGAGTACTA AATTCACCTT GTGATTGGAA   
  
  
- TCCTCTATAC AACAGTTTTC TTCTTAAGCT ACTGTATAAA AGGTTGTTTT CCGGTGGATT GAAGTACTAA   
  
  
- ATTTTTCCTC CTCAGATTCG TGTAGTACCT ACTGATTAAA AACTACATAA CATTGATGCA TAACTCTATT   
  
  
- GAACTTACTT AGCACTGATC TTATATAGAT AAAAGTTTTT ATTTTTAAGG GATTTTCTTT TCCTATTTTT   
  
  
- TGATATATTA TGATTTTTTA AGATTGATAT TATTGATCCG AATAATTTAA AAATATCACT TTTAAATAAA   
  
  
- GATGAATATT GTTGTAGTGA ATGATTCATC GAATAAACAC AGAAGAAAAG ATTTATTATT ACCATTGATA   
  
  
- CACAGAAAAC ATTTATTGAA TAATAGATAA AATATTTAGT GGCACATTTC GTGCCCAGAT ATGATCAATT   
  
  
- AAATTTATCG TAACAATTTA GTTCATTCTA TGTTTGTTTA AATAAATGTT GACATTCACC AGGTATTTTT   
  
  
- ATTATCGGTA ATGTTTTTTA TCATGATGAC AAAATACTTT ACTAAAATTA TAAGATTTTA AGGTTATTTT   
  
  
- GTACAATATT AGATACTAAT TAAAATTTTG TACTAATGTA CAGTCGTATT AAACATAATA ACATGTCGTT   
  
  
- TACTTTCAAC CTCGATTTTC TTTTACCCCT TTATTTCCGT CTGTTCATTT CTATTTTATA CGATAGATGA   
  
  
- AAACCTCCAT AGGAATTGGT GTAACAGAGC AAACAGAAGT AATCCCTCTT CACCCTATTT ATGGTTTTAA   
  
  
- ACATACCAGT AACGCACCCC AAACGAAGGT TTGTTTTACC GAAAAACCTT CGTTTAAAAC ACTCGAAGAT   
  
  
- ACGTTTAAAG GTGCGAATTG GGGTGTTCGT TTGTTTTTGT CTGTCACCAA AAACAAACAG AAACAATCCC   
  
  
- GTACAAACCT CGGTTGACAA TTACCCTTAT TTGACCCCTC CGAAAAAGGA ACCGAGAAAG GGGGACTGGG   
  
  
- GTTTGGGTGA AGTCGAGTTA TGCTTTGGTC GGTAACTTTT TCCTTCCCCC CCCCCCCCCA TACACTCTTT   
  
  
- TTCTGTTTTG AAAAAGGGCC TCTCTCTTCT ATGTATACGT TGTTTCCGAG AGGAAGAGTA TTTAAGGTAT   
  
  
- AAGGAGACAA AAAGATCCTC TCTCTTTATA CGTTTTTTGT TTAGTCAACC GCTGTTTTCA ATGATGGTGC   
  
  
- GTAACAGATG ACGACGCATG GGTAAAAACC TCTGAAATTT CTTGCGGGGA GGGTAGAACT TTTCACTTTT   
  
  
- GGGGGAGAAA CTTTGACTTA AAGTCCCCTT TTTTTGGAGT GGTTTGGAGA TCTCTCTCGA AAATCTCTCC   
  
  
- CTTTATGTTG CTAATCTTCC TCGTTAAACC CTTTAAAGAA ACCCTTAAAC TTACCCAAAA CTCACTTAAC   
  
  
- GTTTAGGGTC TTTTCAAAAC CGTTCATGGC TAGATGTCAA GAGAGGAGAA GCACAAACCA TCTAGGGGAC   
  
  
- AAAGGAGACA AAGTAAATCC CATGAAGAGT AAGTAGGAGG GGTGGGGGAA TTAGCCTAGA AGGACAGCTC   
  
  
- AGTGAAATAC GATTATAAAA AAGTCACCTA AAAATCAATT GGGACAAGTA AAAAGGTAAG ACACAGGGTA   
  
  
- AAGAGAGAAA AAGTAAGTAT TCAACGGCCA AATAGACAAC CCACGTCGAA TCAGTGTTAT TAAAGACACA   
  
  
- ATCCGAAGAA ATGCCAATTT TTTTTTCCTC CGTGAGAAAA GCCACACTAA CAAATACCCT GGTTACTAAG   
  
  
- TTCTACTACC CAGTAGTCAT TGAAGTAGTG GGGAAGTTAA AAAGAGGTAC TACAGAGGGT TAAATCCAAG   
  
  
- AAGGATGGGA ACCGAGTCTC TCGATTTTGG ACTTCTTTCT CCAGAAATGA ACTATGTAAA CAACGAGAGA   
  
  
- ACACGTTTAG TACAGAGATC ACCATCGGAT CTGTTACGCT TGAATCGGGA GCTTGTTTAG AGAGTCGAAC   
  
  
- GACGGGGACT ACCCCTATGT TACGTCGCAT ACCGAAGAAT AAAACGACTT CGGGACCGAC TCTCCTAGGA   
  
  
- GTTCAGTACC GGACCGTACA TATCTCGGGA AGTAAAATGC TTTTACGGAC AGTAAAGTCT CCTTTAAGAA   
  
  
- CGATCCTTCG AGAAGAAACT CGATAAAGGA AAGAACTTCG ACCGGATAAA CCACTGTTTG GTTAGCTATT   
  
  
- AGCTTCGGTA CCTCCCCCTT TTCTACCAAG TATAATAACT AGACTTACGT AGTCTTGGAC GAGTTACCTA   
  
  
- ACGGGAATAA GTCCGAAACT CACGTTCCGG ACCCCCAGGA GGAGTAAACT CTTAATGGCC ACAAGTAGTT   
  
  
- GTGTTTCTCC AAGATCTAGT TCACCGAGTA TCCCACTGAG TTCTTCGACT CTTTAACCTA AACGGTAAAG   
  
  
- TCAAGTTAGG ACACCAATCG TTCAACCTTT TGGAACTACA ACTTTTCGAC ACACAATTCT GACCACTCCG   
  
  
- AGATCGGTAG TCAAGCCAGG AAGTTGACGT ATGGGAAAAC CCAAGACTAC TACTCGGGGA TTCCTTTTCA   
  
  
- AGTGGAAATC GGAACTACTT CATACGTTTA CCCCGATTAT CCGTTTCGGG CTCATTACTA AGCCGAAGAA   
  
  
- GTAGTGGAGG AAGCGAGTTA TGAAGTTGGT TCGACCTACC AAAGGAGTCG CGAAACACCC CTAACAGGGG   
  
  
- TTTCTAATAC CACTATCGAC TCGTGCTAAG GTTAGTGTTA CCAAGACCTG AATACCTCTC CAACAGTCTT   
  
  
- CGTGACATGA AGATACGTCG CGACAAACTG ACGAATCTTA GGTGGGACGG TTCTTGTAGA CAGCTTTCTT   
  
  
- CCGCCCATCT CTTCTACGAG GATCCACTTC TCTAGTTCTT GTAATATAGT ACGCTCCCTC CTCTTTCCTC   
  
  
- TCTTTCCGTA CTCTTCTAGC TCTTCACCTA CGTCTCCGAT CTGTACCGAC CTAAGCCCTA GCAAGGAAAC   
  
  
- TCGATATACC CATACGACGT TCGTTCCGTT AACGAAGTCC CGATACCAAC ACTACCAATA TCTCACTTTC   
  
  
- TCTTACCAAC ACAACAGTAG ACAACCGTTC TAGCGGGGGA GAAAAGCCAT AGTCGTACCT CCACATCCTC   
  
  
- TAC

+     MBSI

| Site Name | Organism | Position | Strand | Matrix score. | sequence | function |
| --- | --- | --- | --- | --- | --- | --- |
| MBSI | Petunia hybrida | 1971 | + | 11 | TTTTTACGGTTA | MYB binding site involved in flavonoid biosynthetic genes regulation |

>HU08G00367.1   
+ -Up\_Stream \_Len000ATGCGC CTTTCTGCTA ACTCATTATC TTTCCTTCTG AGAGGGGACA ACTTATATAA   
  
  
+ ATTGATAGGT ATTTTCAAAT CAATAGACCA GCTACCATAT ATGATAATTT TTATTACTTT TCTAAATCCA   
  
  
+ TCAACTATTT TCACAATCCT CCTTTTTCTA GAAAAATAAA ATTTGCTTAA TTCAAATCAC CTAAAATTAC   
  
  
+ TAAATCTATA GTCCTAAATC TACAAACTCA TTTGTGTTGC AACTCATGAT TTAAGTGGAA CACTAACCTT   
  
  
+ AGGAGATATG TTGTCAAAAG AAGAATTCGA TGACATATTT TCCAACAAAA GGCCACCTAA CTTCATGATT   
  
  
+ TAAAAAGGAG GAGTCTAAGC ACATCATGGA TGACTAATTT TTGATGTATT GTAACTACGT ATTGAGATAA   
  
  
+ CTTGAATGAA TCGTGACTAG AATATATCTA TTTTCAAAAA TAAAAATTCC CTAAAAGAAA AGGATAAAAA   
  
  
+ ACTATATAAT ACTAAAAAAT TCTAACTATA ATAACTAGGC TTATTAAATT TTTATAGTGA AAATTTATTT   
  
  
+ CTACTTATAA CAACATCACT TACTAAGTAG CTTATTTGTG TCTTCTTTTC TAAATAATAA TGGTAACTAT   
  
  
+ GTGTCTTTTG TAAATAACTT ATTATCTATT TTATAAATCA CCGTGTAAAG CACGGGTCTA TACTAGTTAA   
  
  
+ TTTAAATAGC ATTGTTAAAT CAAGTAAGAT ACAAACAAAT TTATTTACAA CTGTAAGTGG TCCATAAAAA   
  
  
+ TAATAGCCAT TACAAAAAAT AGTACTACTG TTTTATGAAA TGATTTTAAT ATTCTAAAAT TCCAATAAAA   
  
  
+ CATGTTATAA TCTATGATTA ATTTTAAAAC ATGATTACAT GTCAGCATAA TTTGTATTAT TGTACAGCAA   
  
  
+ ATGAAAGTTG GAGCTAAAAG AAAATGGGGA AATAAAGGCA GACAAGTAAA GATAAAATAT GCTATCTACT   
  
  
+ TTTGGAGGTA TCCTTAACCA CATTGTCTCG TTTGTCTTCA TTAGGGAGAA GTGGGATAAA TACCAAAATT   
  
  
+ TGTATGGTCA TTGCGTGGGG TTTGCTTCCA AACAAAATGG CTTTTTGGAA GCAAATTTTG TGAGCTTCTA   
  
  
+ TGCAAATTTC CACGCTTAAC CCCACAAGCA AACAAAAACA GACAGTGGTT TTTGTTTGTC TTTGTTAGGG   
  
  
+ CATGTTTGGA GCCAACTGTT AATGGGAATA AACTGGGGAG GCTTTTTCCT TGGCTCTTTC CCCCTGACCC   
  
  
+ CAAACCCACT TCAGCTCAAT ACGAAACCAG CCATTGAAAA AGGAAGGGGG GGGGGGGGGT ATGTGAGAAA   
  
  
+ AAGACAAAAC TTTTTCCCGG AGAGAGAAGA TACATATGCA ACAAAGGCTC TCCTTCTCAT AAATTCCATA   
  
  
+ TTCCTCTGTT TTTCTAGGAG AGAGAAATAT GCAAAAAACA AATCAGTTGG CGACAAAAGT TACTACCACG   
  
  
+ CATTGTCTAC TGCTGCGTAC CCATTTTTGG AGACTTTAAA GAACGCCCCT CCCATCTTGA AAAGTGAAAA   
  
  
+ CCCCCTCTTT GAAACTGAAT TTCAGGGGAA AAAAACCTCA CCAAACCTCT AGAGAGAGCT TTTAGAGAGG   
  
  
+ GAAATACAAC GATTAGAAGG AGCAATTTGG GAAATTTCTT TGGGAATTTG AATGGGTTTT GAGTGAATTG   
  
  
+ CAAATCCCAG AAAAGTTTTG GCAAGTACCG ATCTACAGTT CTCTCCTCTT CGTGTTTGGT AGATCCCCTG   
  
  
+ TTTCCTCTGT TTCATTTAGG GTACTTCTCA TTCATCCTCC CCACCCCCTT AATCGGATCT TCCTGTCGAG   
  
  
+ TCACTTTATG CTAATATTTT TTCAGTGGAT TTTTAGTTAA CCCTGTTCAT TTTTCCATTC TGTGTCCCAT   
  
  
+ TTCTCTCTTT TTCATTCATA AGTTGCCGGT TTATCTGTTG GGTGCAGCTT AGTCACAATA ATTTCTGTGT   
  
  
+ TAGGCTTCTT TACGGTTAAA AAAAAAGGAG GCACTCTTTT CGGTGTGATT GTTTATGGGA CCAATGATTC   
  
  
+ AAGATGATGG GTCATCAGTA ACTTCATCAC CCCTTCAATT TTTCTCCATG ATGTCTCCCA ATTTAGGTTC   
  
  
+ TTCCTACCCT TGGCTCAGAG AGCTAAAACC TGAAGAAAGA GGTCTTTACT TGATACATTT GTTGCTCTCT   
  
  
+ TGTGCAAATC ATGTCTCTAG TGGTAGCCTA GACAATGCGA ACTTAGCCCT CGAACAAATC TCTCAGCTTG   
  
  
+ CTGCCCCTGA TGGGGATACA ATGCAGCGTA TGGCTTCTTA TTTTGCTGAA GCCCTGGCTG AGAGGATCCT   
  
  
+ CAAGTCATGG CCTGGCATGT ATAGAGCCCT TCATTTTACG AAAATGCCTG TCATTTCAGA GGAAATTCTT   
  
  
+ GCTAGGAAGC TCTTCTTTGA GCTATTTCCT TTCTTGAAGC TGGCCTATTT GGTGACAAAC CAATCGATAA   
  
  
+ TCGAAGCCAT GGAGGGGGAA AAGATGGTTC ATATTATTGA TCTGAATGCA TCAGAACCTG CTCAATGGAT   
  
  
+ TGCCCTTATT CAGGCTTTGA GTGCAAGGCC TGGGGGTCCT CCTCATTTGA GAATTACCGG TGTTCATCAA   
  
  
+ CACAAAGAGG TTCTAGATCA AGTGGCTCAT AGGGTGACTC AAGAAGCTGA GAAATTGGAT TTGCCATTTC   
  
  
+ AGTTCAATCC TGTGGTTAGC AAGTTGGAAA ACCTTGATGT TGAAAAGCTG TGTGTTAAGA CTGGTGAGGC   
  
  
+ TCTAGCCATC AGTTCGGTCC TTCAACTGCA TACCCTTTTG GGTTCTGATG ATGAGCCCCT AAGGAAAAGT   
  
  
+ TCACCTTTAG CCTTGATGAA GTATGCAAAT GGGGCTAATA GGCAAAGCCC GAGTAATGAT TCGGCTTCTT   
  
  
+ CATCACCTCC TTCGCTCAAT ACTTCAACCA AGCTGGATGG TTTCCTCAGC GCTTTGTGGG GATTGTCCCC   
  
  
+ AAAGATTATG GTGATAGCTG AGCACGATTC CAATCACAAT GGTTCTGGAC TTATGGAGAG GTTGTCAGAA   
  
  
+ GCACTGTACT TCTATGCAGC GCTGTTTGAC TGCTTAGAAT CCACCCTGCC AAGAACATCT GTCGAAAGAA   
  
  
+ GGCGGGTAGA GAAGATGCTC CTAGGTGAAG AGATCAAGAA CATTATATCA TGCGAGGGAG GAGAAAGGAG   
  
  
+ AGAAAGGCAT GAGAAGATCG AGAAGTGGAT GCAGAGGCTA GACATGGCTG GATTCGGGAT CGTTCCTTTG   
  
  
+ AGCTATATGG GTATGCTGCA AGCAAGGCAA TTGCTTCAGG GCTATGGTTG TGATGGTTAT AGAGTGAAAG   
  
  
+ AGAATGGTTG TGTTGTCATC TGTTGGCAAG ATCGCCCCCT CTTTTCGGTA TCAGCATGGA GGTGTAGGAG   
  
  
+ ATG  

- -Up\_Stream \_Len000TACGCG GAAAGACGAT TGAGTAATAG AAAGGAAGAC TCTCCCCTGT TGAATATATT   
  
  
- TAACTATCCA TAAAAGTTTA GTTATCTGGT CGATGGTATA TACTATTAAA AATAATGAAA AGATTTAGGT   
  
  
- AGTTGATAAA AGTGTTAGGA GGAAAAAGAT CTTTTTATTT TAAACGAATT AAGTTTAGTG GATTTTAATG   
  
  
- ATTTAGATAT CAGGATTTAG ATGTTTGAGT AAACACAACG TTGAGTACTA AATTCACCTT GTGATTGGAA   
  
  
- TCCTCTATAC AACAGTTTTC TTCTTAAGCT ACTGTATAAA AGGTTGTTTT CCGGTGGATT GAAGTACTAA   
  
  
- ATTTTTCCTC CTCAGATTCG TGTAGTACCT ACTGATTAAA AACTACATAA CATTGATGCA TAACTCTATT   
  
  
- GAACTTACTT AGCACTGATC TTATATAGAT AAAAGTTTTT ATTTTTAAGG GATTTTCTTT TCCTATTTTT   
  
  
- TGATATATTA TGATTTTTTA AGATTGATAT TATTGATCCG AATAATTTAA AAATATCACT TTTAAATAAA   
  
  
- GATGAATATT GTTGTAGTGA ATGATTCATC GAATAAACAC AGAAGAAAAG ATTTATTATT ACCATTGATA   
  
  
- CACAGAAAAC ATTTATTGAA TAATAGATAA AATATTTAGT GGCACATTTC GTGCCCAGAT ATGATCAATT   
  
  
- AAATTTATCG TAACAATTTA GTTCATTCTA TGTTTGTTTA AATAAATGTT GACATTCACC AGGTATTTTT   
  
  
- ATTATCGGTA ATGTTTTTTA TCATGATGAC AAAATACTTT ACTAAAATTA TAAGATTTTA AGGTTATTTT   
  
  
- GTACAATATT AGATACTAAT TAAAATTTTG TACTAATGTA CAGTCGTATT AAACATAATA ACATGTCGTT   
  
  
- TACTTTCAAC CTCGATTTTC TTTTACCCCT TTATTTCCGT CTGTTCATTT CTATTTTATA CGATAGATGA   
  
  
- AAACCTCCAT AGGAATTGGT GTAACAGAGC AAACAGAAGT AATCCCTCTT CACCCTATTT ATGGTTTTAA   
  
  
- ACATACCAGT AACGCACCCC AAACGAAGGT TTGTTTTACC GAAAAACCTT CGTTTAAAAC ACTCGAAGAT   
  
  
- ACGTTTAAAG GTGCGAATTG GGGTGTTCGT TTGTTTTTGT CTGTCACCAA AAACAAACAG AAACAATCCC   
  
  
- GTACAAACCT CGGTTGACAA TTACCCTTAT TTGACCCCTC CGAAAAAGGA ACCGAGAAAG GGGGACTGGG   
  
  
- GTTTGGGTGA AGTCGAGTTA TGCTTTGGTC GGTAACTTTT TCCTTCCCCC CCCCCCCCCA TACACTCTTT   
  
  
- TTCTGTTTTG AAAAAGGGCC TCTCTCTTCT ATGTATACGT TGTTTCCGAG AGGAAGAGTA TTTAAGGTAT   
  
  
- AAGGAGACAA AAAGATCCTC TCTCTTTATA CGTTTTTTGT TTAGTCAACC GCTGTTTTCA ATGATGGTGC   
  
  
- GTAACAGATG ACGACGCATG GGTAAAAACC TCTGAAATTT CTTGCGGGGA GGGTAGAACT TTTCACTTTT   
  
  
- GGGGGAGAAA CTTTGACTTA AAGTCCCCTT TTTTTGGAGT GGTTTGGAGA TCTCTCTCGA AAATCTCTCC   
  
  
- CTTTATGTTG CTAATCTTCC TCGTTAAACC CTTTAAAGAA ACCCTTAAAC TTACCCAAAA CTCACTTAAC   
  
  
- GTTTAGGGTC TTTTCAAAAC CGTTCATGGC TAGATGTCAA GAGAGGAGAA GCACAAACCA TCTAGGGGAC   
  
  
- AAAGGAGACA AAGTAAATCC CATGAAGAGT AAGTAGGAGG GGTGGGGGAA TTAGCCTAGA AGGACAGCTC   
  
  
- AGTGAAATAC GATTATAAAA AAGTCACCTA AAAATCAATT GGGACAAGTA AAAAGGTAAG ACACAGGGTA   
  
  
- AAGAGAGAAA AAGTAAGTAT TCAACGGCCA AATAGACAAC CCACGTCGAA TCAGTGTTAT TAAAGACACA   
  
  
- ATCCGAAGAA ATGCCAATTT TTTTTTCCTC CGTGAGAAAA GCCACACTAA CAAATACCCT GGTTACTAAG   
  
  
- TTCTACTACC CAGTAGTCAT TGAAGTAGTG GGGAAGTTAA AAAGAGGTAC TACAGAGGGT TAAATCCAAG   
  
  
- AAGGATGGGA ACCGAGTCTC TCGATTTTGG ACTTCTTTCT CCAGAAATGA ACTATGTAAA CAACGAGAGA   
  
  
- ACACGTTTAG TACAGAGATC ACCATCGGAT CTGTTACGCT TGAATCGGGA GCTTGTTTAG AGAGTCGAAC   
  
  
- GACGGGGACT ACCCCTATGT TACGTCGCAT ACCGAAGAAT AAAACGACTT CGGGACCGAC TCTCCTAGGA   
  
  
- GTTCAGTACC GGACCGTACA TATCTCGGGA AGTAAAATGC TTTTACGGAC AGTAAAGTCT CCTTTAAGAA   
  
  
- CGATCCTTCG AGAAGAAACT CGATAAAGGA AAGAACTTCG ACCGGATAAA CCACTGTTTG GTTAGCTATT   
  
  
- AGCTTCGGTA CCTCCCCCTT TTCTACCAAG TATAATAACT AGACTTACGT AGTCTTGGAC GAGTTACCTA   
  
  
- ACGGGAATAA GTCCGAAACT CACGTTCCGG ACCCCCAGGA GGAGTAAACT CTTAATGGCC ACAAGTAGTT   
  
  
- GTGTTTCTCC AAGATCTAGT TCACCGAGTA TCCCACTGAG TTCTTCGACT CTTTAACCTA AACGGTAAAG   
  
  
- TCAAGTTAGG ACACCAATCG TTCAACCTTT TGGAACTACA ACTTTTCGAC ACACAATTCT GACCACTCCG   
  
  
- AGATCGGTAG TCAAGCCAGG AAGTTGACGT ATGGGAAAAC CCAAGACTAC TACTCGGGGA TTCCTTTTCA   
  
  
- AGTGGAAATC GGAACTACTT CATACGTTTA CCCCGATTAT CCGTTTCGGG CTCATTACTA AGCCGAAGAA   
  
  
- GTAGTGGAGG AAGCGAGTTA TGAAGTTGGT TCGACCTACC AAAGGAGTCG CGAAACACCC CTAACAGGGG   
  
  
- TTTCTAATAC CACTATCGAC TCGTGCTAAG GTTAGTGTTA CCAAGACCTG AATACCTCTC CAACAGTCTT   
  
  
- CGTGACATGA AGATACGTCG CGACAAACTG ACGAATCTTA GGTGGGACGG TTCTTGTAGA CAGCTTTCTT   
  
  
- CCGCCCATCT CTTCTACGAG GATCCACTTC TCTAGTTCTT GTAATATAGT ACGCTCCCTC CTCTTTCCTC   
  
  
- TCTTTCCGTA CTCTTCTAGC TCTTCACCTA CGTCTCCGAT CTGTACCGAC CTAAGCCCTA GCAAGGAAAC   
  
  
- TCGATATACC CATACGACGT TCGTTCCGTT AACGAAGTCC CGATACCAAC ACTACCAATA TCTCACTTTC   
  
  
- TCTTACCAAC ACAACAGTAG ACAACCGTTC TAGCGGGGGA GAAAAGCCAT AGTCGTACCT CCACATCCTC   
  
  
- TAC

+     MRE

| Site Name | Organism | Position | Strand | Matrix score. | sequence | function |
| --- | --- | --- | --- | --- | --- | --- |
| MRE | Petroselinum crispum | 2097 | - | 7 | AACCTAA | MYB binding site involved in light responsiveness |

>HU08G00367.1   
+ -Up\_Stream \_Len000ATGCGC CTTTCTGCTA ACTCATTATC TTTCCTTCTG AGAGGGGACA ACTTATATAA   
  
  
+ ATTGATAGGT ATTTTCAAAT CAATAGACCA GCTACCATAT ATGATAATTT TTATTACTTT TCTAAATCCA   
  
  
+ TCAACTATTT TCACAATCCT CCTTTTTCTA GAAAAATAAA ATTTGCTTAA TTCAAATCAC CTAAAATTAC   
  
  
+ TAAATCTATA GTCCTAAATC TACAAACTCA TTTGTGTTGC AACTCATGAT TTAAGTGGAA CACTAACCTT   
  
  
+ AGGAGATATG TTGTCAAAAG AAGAATTCGA TGACATATTT TCCAACAAAA GGCCACCTAA CTTCATGATT   
  
  
+ TAAAAAGGAG GAGTCTAAGC ACATCATGGA TGACTAATTT TTGATGTATT GTAACTACGT ATTGAGATAA   
  
  
+ CTTGAATGAA TCGTGACTAG AATATATCTA TTTTCAAAAA TAAAAATTCC CTAAAAGAAA AGGATAAAAA   
  
  
+ ACTATATAAT ACTAAAAAAT TCTAACTATA ATAACTAGGC TTATTAAATT TTTATAGTGA AAATTTATTT   
  
  
+ CTACTTATAA CAACATCACT TACTAAGTAG CTTATTTGTG TCTTCTTTTC TAAATAATAA TGGTAACTAT   
  
  
+ GTGTCTTTTG TAAATAACTT ATTATCTATT TTATAAATCA CCGTGTAAAG CACGGGTCTA TACTAGTTAA   
  
  
+ TTTAAATAGC ATTGTTAAAT CAAGTAAGAT ACAAACAAAT TTATTTACAA CTGTAAGTGG TCCATAAAAA   
  
  
+ TAATAGCCAT TACAAAAAAT AGTACTACTG TTTTATGAAA TGATTTTAAT ATTCTAAAAT TCCAATAAAA   
  
  
+ CATGTTATAA TCTATGATTA ATTTTAAAAC ATGATTACAT GTCAGCATAA TTTGTATTAT TGTACAGCAA   
  
  
+ ATGAAAGTTG GAGCTAAAAG AAAATGGGGA AATAAAGGCA GACAAGTAAA GATAAAATAT GCTATCTACT   
  
  
+ TTTGGAGGTA TCCTTAACCA CATTGTCTCG TTTGTCTTCA TTAGGGAGAA GTGGGATAAA TACCAAAATT   
  
  
+ TGTATGGTCA TTGCGTGGGG TTTGCTTCCA AACAAAATGG CTTTTTGGAA GCAAATTTTG TGAGCTTCTA   
  
  
+ TGCAAATTTC CACGCTTAAC CCCACAAGCA AACAAAAACA GACAGTGGTT TTTGTTTGTC TTTGTTAGGG   
  
  
+ CATGTTTGGA GCCAACTGTT AATGGGAATA AACTGGGGAG GCTTTTTCCT TGGCTCTTTC CCCCTGACCC   
  
  
+ CAAACCCACT TCAGCTCAAT ACGAAACCAG CCATTGAAAA AGGAAGGGGG GGGGGGGGGT ATGTGAGAAA   
  
  
+ AAGACAAAAC TTTTTCCCGG AGAGAGAAGA TACATATGCA ACAAAGGCTC TCCTTCTCAT AAATTCCATA   
  
  
+ TTCCTCTGTT TTTCTAGGAG AGAGAAATAT GCAAAAAACA AATCAGTTGG CGACAAAAGT TACTACCACG   
  
  
+ CATTGTCTAC TGCTGCGTAC CCATTTTTGG AGACTTTAAA GAACGCCCCT CCCATCTTGA AAAGTGAAAA   
  
  
+ CCCCCTCTTT GAAACTGAAT TTCAGGGGAA AAAAACCTCA CCAAACCTCT AGAGAGAGCT TTTAGAGAGG   
  
  
+ GAAATACAAC GATTAGAAGG AGCAATTTGG GAAATTTCTT TGGGAATTTG AATGGGTTTT GAGTGAATTG   
  
  
+ CAAATCCCAG AAAAGTTTTG GCAAGTACCG ATCTACAGTT CTCTCCTCTT CGTGTTTGGT AGATCCCCTG   
  
  
+ TTTCCTCTGT TTCATTTAGG GTACTTCTCA TTCATCCTCC CCACCCCCTT AATCGGATCT TCCTGTCGAG   
  
  
+ TCACTTTATG CTAATATTTT TTCAGTGGAT TTTTAGTTAA CCCTGTTCAT TTTTCCATTC TGTGTCCCAT   
  
  
+ TTCTCTCTTT TTCATTCATA AGTTGCCGGT TTATCTGTTG GGTGCAGCTT AGTCACAATA ATTTCTGTGT   
  
  
+ TAGGCTTCTT TACGGTTAAA AAAAAAGGAG GCACTCTTTT CGGTGTGATT GTTTATGGGA CCAATGATTC   
  
  
+ AAGATGATGG GTCATCAGTA ACTTCATCAC CCCTTCAATT TTTCTCCATG ATGTCTCCCA ATTTAGGTTC   
  
  
+ TTCCTACCCT TGGCTCAGAG AGCTAAAACC TGAAGAAAGA GGTCTTTACT TGATACATTT GTTGCTCTCT   
  
  
+ TGTGCAAATC ATGTCTCTAG TGGTAGCCTA GACAATGCGA ACTTAGCCCT CGAACAAATC TCTCAGCTTG   
  
  
+ CTGCCCCTGA TGGGGATACA ATGCAGCGTA TGGCTTCTTA TTTTGCTGAA GCCCTGGCTG AGAGGATCCT   
  
  
+ CAAGTCATGG CCTGGCATGT ATAGAGCCCT TCATTTTACG AAAATGCCTG TCATTTCAGA GGAAATTCTT   
  
  
+ GCTAGGAAGC TCTTCTTTGA GCTATTTCCT TTCTTGAAGC TGGCCTATTT GGTGACAAAC CAATCGATAA   
  
  
+ TCGAAGCCAT GGAGGGGGAA AAGATGGTTC ATATTATTGA TCTGAATGCA TCAGAACCTG CTCAATGGAT   
  
  
+ TGCCCTTATT CAGGCTTTGA GTGCAAGGCC TGGGGGTCCT CCTCATTTGA GAATTACCGG TGTTCATCAA   
  
  
+ CACAAAGAGG TTCTAGATCA AGTGGCTCAT AGGGTGACTC AAGAAGCTGA GAAATTGGAT TTGCCATTTC   
  
  
+ AGTTCAATCC TGTGGTTAGC AAGTTGGAAA ACCTTGATGT TGAAAAGCTG TGTGTTAAGA CTGGTGAGGC   
  
  
+ TCTAGCCATC AGTTCGGTCC TTCAACTGCA TACCCTTTTG GGTTCTGATG ATGAGCCCCT AAGGAAAAGT   
  
  
+ TCACCTTTAG CCTTGATGAA GTATGCAAAT GGGGCTAATA GGCAAAGCCC GAGTAATGAT TCGGCTTCTT   
  
  
+ CATCACCTCC TTCGCTCAAT ACTTCAACCA AGCTGGATGG TTTCCTCAGC GCTTTGTGGG GATTGTCCCC   
  
  
+ AAAGATTATG GTGATAGCTG AGCACGATTC CAATCACAAT GGTTCTGGAC TTATGGAGAG GTTGTCAGAA   
  
  
+ GCACTGTACT TCTATGCAGC GCTGTTTGAC TGCTTAGAAT CCACCCTGCC AAGAACATCT GTCGAAAGAA   
  
  
+ GGCGGGTAGA GAAGATGCTC CTAGGTGAAG AGATCAAGAA CATTATATCA TGCGAGGGAG GAGAAAGGAG   
  
  
+ AGAAAGGCAT GAGAAGATCG AGAAGTGGAT GCAGAGGCTA GACATGGCTG GATTCGGGAT CGTTCCTTTG   
  
  
+ AGCTATATGG GTATGCTGCA AGCAAGGCAA TTGCTTCAGG GCTATGGTTG TGATGGTTAT AGAGTGAAAG   
  
  
+ AGAATGGTTG TGTTGTCATC TGTTGGCAAG ATCGCCCCCT CTTTTCGGTA TCAGCATGGA GGTGTAGGAG   
  
  
+ ATG  

- -Up\_Stream \_Len000TACGCG GAAAGACGAT TGAGTAATAG AAAGGAAGAC TCTCCCCTGT TGAATATATT   
  
  
- TAACTATCCA TAAAAGTTTA GTTATCTGGT CGATGGTATA TACTATTAAA AATAATGAAA AGATTTAGGT   
  
  
- AGTTGATAAA AGTGTTAGGA GGAAAAAGAT CTTTTTATTT TAAACGAATT AAGTTTAGTG GATTTTAATG   
  
  
- ATTTAGATAT CAGGATTTAG ATGTTTGAGT AAACACAACG TTGAGTACTA AATTCACCTT GTGATTGGAA   
  
  
- TCCTCTATAC AACAGTTTTC TTCTTAAGCT ACTGTATAAA AGGTTGTTTT CCGGTGGATT GAAGTACTAA   
  
  
- ATTTTTCCTC CTCAGATTCG TGTAGTACCT ACTGATTAAA AACTACATAA CATTGATGCA TAACTCTATT   
  
  
- GAACTTACTT AGCACTGATC TTATATAGAT AAAAGTTTTT ATTTTTAAGG GATTTTCTTT TCCTATTTTT   
  
  
- TGATATATTA TGATTTTTTA AGATTGATAT TATTGATCCG AATAATTTAA AAATATCACT TTTAAATAAA   
  
  
- GATGAATATT GTTGTAGTGA ATGATTCATC GAATAAACAC AGAAGAAAAG ATTTATTATT ACCATTGATA   
  
  
- CACAGAAAAC ATTTATTGAA TAATAGATAA AATATTTAGT GGCACATTTC GTGCCCAGAT ATGATCAATT   
  
  
- AAATTTATCG TAACAATTTA GTTCATTCTA TGTTTGTTTA AATAAATGTT GACATTCACC AGGTATTTTT   
  
  
- ATTATCGGTA ATGTTTTTTA TCATGATGAC AAAATACTTT ACTAAAATTA TAAGATTTTA AGGTTATTTT   
  
  
- GTACAATATT AGATACTAAT TAAAATTTTG TACTAATGTA CAGTCGTATT AAACATAATA ACATGTCGTT   
  
  
- TACTTTCAAC CTCGATTTTC TTTTACCCCT TTATTTCCGT CTGTTCATTT CTATTTTATA CGATAGATGA   
  
  
- AAACCTCCAT AGGAATTGGT GTAACAGAGC AAACAGAAGT AATCCCTCTT CACCCTATTT ATGGTTTTAA   
  
  
- ACATACCAGT AACGCACCCC AAACGAAGGT TTGTTTTACC GAAAAACCTT CGTTTAAAAC ACTCGAAGAT   
  
  
- ACGTTTAAAG GTGCGAATTG GGGTGTTCGT TTGTTTTTGT CTGTCACCAA AAACAAACAG AAACAATCCC   
  
  
- GTACAAACCT CGGTTGACAA TTACCCTTAT TTGACCCCTC CGAAAAAGGA ACCGAGAAAG GGGGACTGGG   
  
  
- GTTTGGGTGA AGTCGAGTTA TGCTTTGGTC GGTAACTTTT TCCTTCCCCC CCCCCCCCCA TACACTCTTT   
  
  
- TTCTGTTTTG AAAAAGGGCC TCTCTCTTCT ATGTATACGT TGTTTCCGAG AGGAAGAGTA TTTAAGGTAT   
  
  
- AAGGAGACAA AAAGATCCTC TCTCTTTATA CGTTTTTTGT TTAGTCAACC GCTGTTTTCA ATGATGGTGC   
  
  
- GTAACAGATG ACGACGCATG GGTAAAAACC TCTGAAATTT CTTGCGGGGA GGGTAGAACT TTTCACTTTT   
  
  
- GGGGGAGAAA CTTTGACTTA AAGTCCCCTT TTTTTGGAGT GGTTTGGAGA TCTCTCTCGA AAATCTCTCC   
  
  
- CTTTATGTTG CTAATCTTCC TCGTTAAACC CTTTAAAGAA ACCCTTAAAC TTACCCAAAA CTCACTTAAC   
  
  
- GTTTAGGGTC TTTTCAAAAC CGTTCATGGC TAGATGTCAA GAGAGGAGAA GCACAAACCA TCTAGGGGAC   
  
  
- AAAGGAGACA AAGTAAATCC CATGAAGAGT AAGTAGGAGG GGTGGGGGAA TTAGCCTAGA AGGACAGCTC   
  
  
- AGTGAAATAC GATTATAAAA AAGTCACCTA AAAATCAATT GGGACAAGTA AAAAGGTAAG ACACAGGGTA   
  
  
- AAGAGAGAAA AAGTAAGTAT TCAACGGCCA AATAGACAAC CCACGTCGAA TCAGTGTTAT TAAAGACACA   
  
  
- ATCCGAAGAA ATGCCAATTT TTTTTTCCTC CGTGAGAAAA GCCACACTAA CAAATACCCT GGTTACTAAG   
  
  
- TTCTACTACC CAGTAGTCAT TGAAGTAGTG GGGAAGTTAA AAAGAGGTAC TACAGAGGGT TAAATCCAAG   
  
  
- AAGGATGGGA ACCGAGTCTC TCGATTTTGG ACTTCTTTCT CCAGAAATGA ACTATGTAAA CAACGAGAGA   
  
  
- ACACGTTTAG TACAGAGATC ACCATCGGAT CTGTTACGCT TGAATCGGGA GCTTGTTTAG AGAGTCGAAC   
  
  
- GACGGGGACT ACCCCTATGT TACGTCGCAT ACCGAAGAAT AAAACGACTT CGGGACCGAC TCTCCTAGGA   
  
  
- GTTCAGTACC GGACCGTACA TATCTCGGGA AGTAAAATGC TTTTACGGAC AGTAAAGTCT CCTTTAAGAA   
  
  
- CGATCCTTCG AGAAGAAACT CGATAAAGGA AAGAACTTCG ACCGGATAAA CCACTGTTTG GTTAGCTATT   
  
  
- AGCTTCGGTA CCTCCCCCTT TTCTACCAAG TATAATAACT AGACTTACGT AGTCTTGGAC GAGTTACCTA   
  
  
- ACGGGAATAA GTCCGAAACT CACGTTCCGG ACCCCCAGGA GGAGTAAACT CTTAATGGCC ACAAGTAGTT   
  
  
- GTGTTTCTCC AAGATCTAGT TCACCGAGTA TCCCACTGAG TTCTTCGACT CTTTAACCTA AACGGTAAAG   
  
  
- TCAAGTTAGG ACACCAATCG TTCAACCTTT TGGAACTACA ACTTTTCGAC ACACAATTCT GACCACTCCG   
  
  
- AGATCGGTAG TCAAGCCAGG AAGTTGACGT ATGGGAAAAC CCAAGACTAC TACTCGGGGA TTCCTTTTCA   
  
  
- AGTGGAAATC GGAACTACTT CATACGTTTA CCCCGATTAT CCGTTTCGGG CTCATTACTA AGCCGAAGAA   
  
  
- GTAGTGGAGG AAGCGAGTTA TGAAGTTGGT TCGACCTACC AAAGGAGTCG CGAAACACCC CTAACAGGGG   
  
  
- TTTCTAATAC CACTATCGAC TCGTGCTAAG GTTAGTGTTA CCAAGACCTG AATACCTCTC CAACAGTCTT   
  
  
- CGTGACATGA AGATACGTCG CGACAAACTG ACGAATCTTA GGTGGGACGG TTCTTGTAGA CAGCTTTCTT   
  
  
- CCGCCCATCT CTTCTACGAG GATCCACTTC TCTAGTTCTT GTAATATAGT ACGCTCCCTC CTCTTTCCTC   
  
  
- TCTTTCCGTA CTCTTCTAGC TCTTCACCTA CGTCTCCGAT CTGTACCGAC CTAAGCCCTA GCAAGGAAAC   
  
  
- TCGATATACC CATACGACGT TCGTTCCGTT AACGAAGTCC CGATACCAAC ACTACCAATA TCTCACTTTC   
  
  
- TCTTACCAAC ACAACAGTAG ACAACCGTTC TAGCGGGGGA GAAAAGCCAT AGTCGTACCT CCACATCCTC   
  
  
- TAC

+     MYB

| Site Name | Organism | Position | Strand | Matrix score. | sequence | function |
| --- | --- | --- | --- | --- | --- | --- |
| MYB | Arabidopsis thaliana | 3278 | - | 6 | TAACCA |  |
| MYB | Arabidopsis thaliana | 999 | + | 6 | TAACCA |  |
| MYB | Arabidopsis thaliana | 2677 | - | 6 | TAACCA |  |
| MYB | Arabidopsis thaliana | 3299 | - | 6 | CAACCA |  |
| MYB | Arabidopsis thaliana | 1929 | - | 6 | CAACAG |  |
| MYB | Arabidopsis thaliana | 3269 | - | 6 | CAACCA |  |
| MYB | Arabidopsis thaliana | 2899 | + | 6 | CAACCA |  |
| MYB | Arabidopsis thaliana | 3314 | - | 6 | CAACAG |  |

>HU08G00367.1   
+ -Up\_Stream \_Len000ATGCGC CTTTCTGCTA ACTCATTATC TTTCCTTCTG AGAGGGGACA ACTTATATAA   
  
  
+ ATTGATAGGT ATTTTCAAAT CAATAGACCA GCTACCATAT ATGATAATTT TTATTACTTT TCTAAATCCA   
  
  
+ TCAACTATTT TCACAATCCT CCTTTTTCTA GAAAAATAAA ATTTGCTTAA TTCAAATCAC CTAAAATTAC   
  
  
+ TAAATCTATA GTCCTAAATC TACAAACTCA TTTGTGTTGC AACTCATGAT TTAAGTGGAA CACTAACCTT   
  
  
+ AGGAGATATG TTGTCAAAAG AAGAATTCGA TGACATATTT TCCAACAAAA GGCCACCTAA CTTCATGATT   
  
  
+ TAAAAAGGAG GAGTCTAAGC ACATCATGGA TGACTAATTT TTGATGTATT GTAACTACGT ATTGAGATAA   
  
  
+ CTTGAATGAA TCGTGACTAG AATATATCTA TTTTCAAAAA TAAAAATTCC CTAAAAGAAA AGGATAAAAA   
  
  
+ ACTATATAAT ACTAAAAAAT TCTAACTATA ATAACTAGGC TTATTAAATT TTTATAGTGA AAATTTATTT   
  
  
+ CTACTTATAA CAACATCACT TACTAAGTAG CTTATTTGTG TCTTCTTTTC TAAATAATAA TGGTAACTAT   
  
  
+ GTGTCTTTTG TAAATAACTT ATTATCTATT TTATAAATCA CCGTGTAAAG CACGGGTCTA TACTAGTTAA   
  
  
+ TTTAAATAGC ATTGTTAAAT CAAGTAAGAT ACAAACAAAT TTATTTACAA CTGTAAGTGG TCCATAAAAA   
  
  
+ TAATAGCCAT TACAAAAAAT AGTACTACTG TTTTATGAAA TGATTTTAAT ATTCTAAAAT TCCAATAAAA   
  
  
+ CATGTTATAA TCTATGATTA ATTTTAAAAC ATGATTACAT GTCAGCATAA TTTGTATTAT TGTACAGCAA   
  
  
+ ATGAAAGTTG GAGCTAAAAG AAAATGGGGA AATAAAGGCA GACAAGTAAA GATAAAATAT GCTATCTACT   
  
  
+ TTTGGAGGTA TCCTTAACCA CATTGTCTCG TTTGTCTTCA TTAGGGAGAA GTGGGATAAA TACCAAAATT   
  
  
+ TGTATGGTCA TTGCGTGGGG TTTGCTTCCA AACAAAATGG CTTTTTGGAA GCAAATTTTG TGAGCTTCTA   
  
  
+ TGCAAATTTC CACGCTTAAC CCCACAAGCA AACAAAAACA GACAGTGGTT TTTGTTTGTC TTTGTTAGGG   
  
  
+ CATGTTTGGA GCCAACTGTT AATGGGAATA AACTGGGGAG GCTTTTTCCT TGGCTCTTTC CCCCTGACCC   
  
  
+ CAAACCCACT TCAGCTCAAT ACGAAACCAG CCATTGAAAA AGGAAGGGGG GGGGGGGGGT ATGTGAGAAA   
  
  
+ AAGACAAAAC TTTTTCCCGG AGAGAGAAGA TACATATGCA ACAAAGGCTC TCCTTCTCAT AAATTCCATA   
  
  
+ TTCCTCTGTT TTTCTAGGAG AGAGAAATAT GCAAAAAACA AATCAGTTGG CGACAAAAGT TACTACCACG   
  
  
+ CATTGTCTAC TGCTGCGTAC CCATTTTTGG AGACTTTAAA GAACGCCCCT CCCATCTTGA AAAGTGAAAA   
  
  
+ CCCCCTCTTT GAAACTGAAT TTCAGGGGAA AAAAACCTCA CCAAACCTCT AGAGAGAGCT TTTAGAGAGG   
  
  
+ GAAATACAAC GATTAGAAGG AGCAATTTGG GAAATTTCTT TGGGAATTTG AATGGGTTTT GAGTGAATTG   
  
  
+ CAAATCCCAG AAAAGTTTTG GCAAGTACCG ATCTACAGTT CTCTCCTCTT CGTGTTTGGT AGATCCCCTG   
  
  
+ TTTCCTCTGT TTCATTTAGG GTACTTCTCA TTCATCCTCC CCACCCCCTT AATCGGATCT TCCTGTCGAG   
  
  
+ TCACTTTATG CTAATATTTT TTCAGTGGAT TTTTAGTTAA CCCTGTTCAT TTTTCCATTC TGTGTCCCAT   
  
  
+ TTCTCTCTTT TTCATTCATA AGTTGCCGGT TTATCTGTTG GGTGCAGCTT AGTCACAATA ATTTCTGTGT   
  
  
+ TAGGCTTCTT TACGGTTAAA AAAAAAGGAG GCACTCTTTT CGGTGTGATT GTTTATGGGA CCAATGATTC   
  
  
+ AAGATGATGG GTCATCAGTA ACTTCATCAC CCCTTCAATT TTTCTCCATG ATGTCTCCCA ATTTAGGTTC   
  
  
+ TTCCTACCCT TGGCTCAGAG AGCTAAAACC TGAAGAAAGA GGTCTTTACT TGATACATTT GTTGCTCTCT   
  
  
+ TGTGCAAATC ATGTCTCTAG TGGTAGCCTA GACAATGCGA ACTTAGCCCT CGAACAAATC TCTCAGCTTG   
  
  
+ CTGCCCCTGA TGGGGATACA ATGCAGCGTA TGGCTTCTTA TTTTGCTGAA GCCCTGGCTG AGAGGATCCT   
  
  
+ CAAGTCATGG CCTGGCATGT ATAGAGCCCT TCATTTTACG AAAATGCCTG TCATTTCAGA GGAAATTCTT   
  
  
+ GCTAGGAAGC TCTTCTTTGA GCTATTTCCT TTCTTGAAGC TGGCCTATTT GGTGACAAAC CAATCGATAA   
  
  
+ TCGAAGCCAT GGAGGGGGAA AAGATGGTTC ATATTATTGA TCTGAATGCA TCAGAACCTG CTCAATGGAT   
  
  
+ TGCCCTTATT CAGGCTTTGA GTGCAAGGCC TGGGGGTCCT CCTCATTTGA GAATTACCGG TGTTCATCAA   
  
  
+ CACAAAGAGG TTCTAGATCA AGTGGCTCAT AGGGTGACTC AAGAAGCTGA GAAATTGGAT TTGCCATTTC   
  
  
+ AGTTCAATCC TGTGGTTAGC AAGTTGGAAA ACCTTGATGT TGAAAAGCTG TGTGTTAAGA CTGGTGAGGC   
  
  
+ TCTAGCCATC AGTTCGGTCC TTCAACTGCA TACCCTTTTG GGTTCTGATG ATGAGCCCCT AAGGAAAAGT   
  
  
+ TCACCTTTAG CCTTGATGAA GTATGCAAAT GGGGCTAATA GGCAAAGCCC GAGTAATGAT TCGGCTTCTT   
  
  
+ CATCACCTCC TTCGCTCAAT ACTTCAACCA AGCTGGATGG TTTCCTCAGC GCTTTGTGGG GATTGTCCCC   
  
  
+ AAAGATTATG GTGATAGCTG AGCACGATTC CAATCACAAT GGTTCTGGAC TTATGGAGAG GTTGTCAGAA   
  
  
+ GCACTGTACT TCTATGCAGC GCTGTTTGAC TGCTTAGAAT CCACCCTGCC AAGAACATCT GTCGAAAGAA   
  
  
+ GGCGGGTAGA GAAGATGCTC CTAGGTGAAG AGATCAAGAA CATTATATCA TGCGAGGGAG GAGAAAGGAG   
  
  
+ AGAAAGGCAT GAGAAGATCG AGAAGTGGAT GCAGAGGCTA GACATGGCTG GATTCGGGAT CGTTCCTTTG   
  
  
+ AGCTATATGG GTATGCTGCA AGCAAGGCAA TTGCTTCAGG GCTATGGTTG TGATGGTTAT AGAGTGAAAG   
  
  
+ AGAATGGTTG TGTTGTCATC TGTTGGCAAG ATCGCCCCCT CTTTTCGGTA TCAGCATGGA GGTGTAGGAG   
  
  
+ ATG  

- -Up\_Stream \_Len000TACGCG GAAAGACGAT TGAGTAATAG AAAGGAAGAC TCTCCCCTGT TGAATATATT   
  
  
- TAACTATCCA TAAAAGTTTA GTTATCTGGT CGATGGTATA TACTATTAAA AATAATGAAA AGATTTAGGT   
  
  
- AGTTGATAAA AGTGTTAGGA GGAAAAAGAT CTTTTTATTT TAAACGAATT AAGTTTAGTG GATTTTAATG   
  
  
- ATTTAGATAT CAGGATTTAG ATGTTTGAGT AAACACAACG TTGAGTACTA AATTCACCTT GTGATTGGAA   
  
  
- TCCTCTATAC AACAGTTTTC TTCTTAAGCT ACTGTATAAA AGGTTGTTTT CCGGTGGATT GAAGTACTAA   
  
  
- ATTTTTCCTC CTCAGATTCG TGTAGTACCT ACTGATTAAA AACTACATAA CATTGATGCA TAACTCTATT   
  
  
- GAACTTACTT AGCACTGATC TTATATAGAT AAAAGTTTTT ATTTTTAAGG GATTTTCTTT TCCTATTTTT   
  
  
- TGATATATTA TGATTTTTTA AGATTGATAT TATTGATCCG AATAATTTAA AAATATCACT TTTAAATAAA   
  
  
- GATGAATATT GTTGTAGTGA ATGATTCATC GAATAAACAC AGAAGAAAAG ATTTATTATT ACCATTGATA   
  
  
- CACAGAAAAC ATTTATTGAA TAATAGATAA AATATTTAGT GGCACATTTC GTGCCCAGAT ATGATCAATT   
  
  
- AAATTTATCG TAACAATTTA GTTCATTCTA TGTTTGTTTA AATAAATGTT GACATTCACC AGGTATTTTT   
  
  
- ATTATCGGTA ATGTTTTTTA TCATGATGAC AAAATACTTT ACTAAAATTA TAAGATTTTA AGGTTATTTT   
  
  
- GTACAATATT AGATACTAAT TAAAATTTTG TACTAATGTA CAGTCGTATT AAACATAATA ACATGTCGTT   
  
  
- TACTTTCAAC CTCGATTTTC TTTTACCCCT TTATTTCCGT CTGTTCATTT CTATTTTATA CGATAGATGA   
  
  
- AAACCTCCAT AGGAATTGGT GTAACAGAGC AAACAGAAGT AATCCCTCTT CACCCTATTT ATGGTTTTAA   
  
  
- ACATACCAGT AACGCACCCC AAACGAAGGT TTGTTTTACC GAAAAACCTT CGTTTAAAAC ACTCGAAGAT   
  
  
- ACGTTTAAAG GTGCGAATTG GGGTGTTCGT TTGTTTTTGT CTGTCACCAA AAACAAACAG AAACAATCCC   
  
  
- GTACAAACCT CGGTTGACAA TTACCCTTAT TTGACCCCTC CGAAAAAGGA ACCGAGAAAG GGGGACTGGG   
  
  
- GTTTGGGTGA AGTCGAGTTA TGCTTTGGTC GGTAACTTTT TCCTTCCCCC CCCCCCCCCA TACACTCTTT   
  
  
- TTCTGTTTTG AAAAAGGGCC TCTCTCTTCT ATGTATACGT TGTTTCCGAG AGGAAGAGTA TTTAAGGTAT   
  
  
- AAGGAGACAA AAAGATCCTC TCTCTTTATA CGTTTTTTGT TTAGTCAACC GCTGTTTTCA ATGATGGTGC   
  
  
- GTAACAGATG ACGACGCATG GGTAAAAACC TCTGAAATTT CTTGCGGGGA GGGTAGAACT TTTCACTTTT   
  
  
- GGGGGAGAAA CTTTGACTTA AAGTCCCCTT TTTTTGGAGT GGTTTGGAGA TCTCTCTCGA AAATCTCTCC   
  
  
- CTTTATGTTG CTAATCTTCC TCGTTAAACC CTTTAAAGAA ACCCTTAAAC TTACCCAAAA CTCACTTAAC   
  
  
- GTTTAGGGTC TTTTCAAAAC CGTTCATGGC TAGATGTCAA GAGAGGAGAA GCACAAACCA TCTAGGGGAC   
  
  
- AAAGGAGACA AAGTAAATCC CATGAAGAGT AAGTAGGAGG GGTGGGGGAA TTAGCCTAGA AGGACAGCTC   
  
  
- AGTGAAATAC GATTATAAAA AAGTCACCTA AAAATCAATT GGGACAAGTA AAAAGGTAAG ACACAGGGTA   
  
  
- AAGAGAGAAA AAGTAAGTAT TCAACGGCCA AATAGACAAC CCACGTCGAA TCAGTGTTAT TAAAGACACA   
  
  
- ATCCGAAGAA ATGCCAATTT TTTTTTCCTC CGTGAGAAAA GCCACACTAA CAAATACCCT GGTTACTAAG   
  
  
- TTCTACTACC CAGTAGTCAT TGAAGTAGTG GGGAAGTTAA AAAGAGGTAC TACAGAGGGT TAAATCCAAG   
  
  
- AAGGATGGGA ACCGAGTCTC TCGATTTTGG ACTTCTTTCT CCAGAAATGA ACTATGTAAA CAACGAGAGA   
  
  
- ACACGTTTAG TACAGAGATC ACCATCGGAT CTGTTACGCT TGAATCGGGA GCTTGTTTAG AGAGTCGAAC   
  
  
- GACGGGGACT ACCCCTATGT TACGTCGCAT ACCGAAGAAT AAAACGACTT CGGGACCGAC TCTCCTAGGA   
  
  
- GTTCAGTACC GGACCGTACA TATCTCGGGA AGTAAAATGC TTTTACGGAC AGTAAAGTCT CCTTTAAGAA   
  
  
- CGATCCTTCG AGAAGAAACT CGATAAAGGA AAGAACTTCG ACCGGATAAA CCACTGTTTG GTTAGCTATT   
  
  
- AGCTTCGGTA CCTCCCCCTT TTCTACCAAG TATAATAACT AGACTTACGT AGTCTTGGAC GAGTTACCTA   
  
  
- ACGGGAATAA GTCCGAAACT CACGTTCCGG ACCCCCAGGA GGAGTAAACT CTTAATGGCC ACAAGTAGTT   
  
  
- GTGTTTCTCC AAGATCTAGT TCACCGAGTA TCCCACTGAG TTCTTCGACT CTTTAACCTA AACGGTAAAG   
  
  
- TCAAGTTAGG ACACCAATCG TTCAACCTTT TGGAACTACA ACTTTTCGAC ACACAATTCT GACCACTCCG   
  
  
- AGATCGGTAG TCAAGCCAGG AAGTTGACGT ATGGGAAAAC CCAAGACTAC TACTCGGGGA TTCCTTTTCA   
  
  
- AGTGGAAATC GGAACTACTT CATACGTTTA CCCCGATTAT CCGTTTCGGG CTCATTACTA AGCCGAAGAA   
  
  
- GTAGTGGAGG AAGCGAGTTA TGAAGTTGGT TCGACCTACC AAAGGAGTCG CGAAACACCC CTAACAGGGG   
  
  
- TTTCTAATAC CACTATCGAC TCGTGCTAAG GTTAGTGTTA CCAAGACCTG AATACCTCTC CAACAGTCTT   
  
  
- CGTGACATGA AGATACGTCG CGACAAACTG ACGAATCTTA GGTGGGACGG TTCTTGTAGA CAGCTTTCTT   
  
  
- CCGCCCATCT CTTCTACGAG GATCCACTTC TCTAGTTCTT GTAATATAGT ACGCTCCCTC CTCTTTCCTC   
  
  
- TCTTTCCGTA CTCTTCTAGC TCTTCACCTA CGTCTCCGAT CTGTACCGAC CTAAGCCCTA GCAAGGAAAC   
  
  
- TCGATATACC CATACGACGT TCGTTCCGTT AACGAAGTCC CGATACCAAC ACTACCAATA TCTCACTTTC   
  
  
- TCTTACCAAC ACAACAGTAG ACAACCGTTC TAGCGGGGGA GAAAAGCCAT AGTCGTACCT CCACATCCTC   
  
  
- TAC

+     MYB-like sequence

| Site Name | Organism | Position | Strand | Matrix score. | sequence | function |
| --- | --- | --- | --- | --- | --- | --- |
| MYB-like sequence | Arabidopsis thaliana | 999 | + | 6 | TAACCA |  |
| MYB-like sequence | Arabidopsis thaliana | 2677 | - | 6 | TAACCA |  |
| MYB-like sequence | Arabidopsis thaliana | 3278 | - | 6 | TAACCA |  |

>HU08G00367.1   
+ -Up\_Stream \_Len000ATGCGC CTTTCTGCTA ACTCATTATC TTTCCTTCTG AGAGGGGACA ACTTATATAA   
  
  
+ ATTGATAGGT ATTTTCAAAT CAATAGACCA GCTACCATAT ATGATAATTT TTATTACTTT TCTAAATCCA   
  
  
+ TCAACTATTT TCACAATCCT CCTTTTTCTA GAAAAATAAA ATTTGCTTAA TTCAAATCAC CTAAAATTAC   
  
  
+ TAAATCTATA GTCCTAAATC TACAAACTCA TTTGTGTTGC AACTCATGAT TTAAGTGGAA CACTAACCTT   
  
  
+ AGGAGATATG TTGTCAAAAG AAGAATTCGA TGACATATTT TCCAACAAAA GGCCACCTAA CTTCATGATT   
  
  
+ TAAAAAGGAG GAGTCTAAGC ACATCATGGA TGACTAATTT TTGATGTATT GTAACTACGT ATTGAGATAA   
  
  
+ CTTGAATGAA TCGTGACTAG AATATATCTA TTTTCAAAAA TAAAAATTCC CTAAAAGAAA AGGATAAAAA   
  
  
+ ACTATATAAT ACTAAAAAAT TCTAACTATA ATAACTAGGC TTATTAAATT TTTATAGTGA AAATTTATTT   
  
  
+ CTACTTATAA CAACATCACT TACTAAGTAG CTTATTTGTG TCTTCTTTTC TAAATAATAA TGGTAACTAT   
  
  
+ GTGTCTTTTG TAAATAACTT ATTATCTATT TTATAAATCA CCGTGTAAAG CACGGGTCTA TACTAGTTAA   
  
  
+ TTTAAATAGC ATTGTTAAAT CAAGTAAGAT ACAAACAAAT TTATTTACAA CTGTAAGTGG TCCATAAAAA   
  
  
+ TAATAGCCAT TACAAAAAAT AGTACTACTG TTTTATGAAA TGATTTTAAT ATTCTAAAAT TCCAATAAAA   
  
  
+ CATGTTATAA TCTATGATTA ATTTTAAAAC ATGATTACAT GTCAGCATAA TTTGTATTAT TGTACAGCAA   
  
  
+ ATGAAAGTTG GAGCTAAAAG AAAATGGGGA AATAAAGGCA GACAAGTAAA GATAAAATAT GCTATCTACT   
  
  
+ TTTGGAGGTA TCCTTAACCA CATTGTCTCG TTTGTCTTCA TTAGGGAGAA GTGGGATAAA TACCAAAATT   
  
  
+ TGTATGGTCA TTGCGTGGGG TTTGCTTCCA AACAAAATGG CTTTTTGGAA GCAAATTTTG TGAGCTTCTA   
  
  
+ TGCAAATTTC CACGCTTAAC CCCACAAGCA AACAAAAACA GACAGTGGTT TTTGTTTGTC TTTGTTAGGG   
  
  
+ CATGTTTGGA GCCAACTGTT AATGGGAATA AACTGGGGAG GCTTTTTCCT TGGCTCTTTC CCCCTGACCC   
  
  
+ CAAACCCACT TCAGCTCAAT ACGAAACCAG CCATTGAAAA AGGAAGGGGG GGGGGGGGGT ATGTGAGAAA   
  
  
+ AAGACAAAAC TTTTTCCCGG AGAGAGAAGA TACATATGCA ACAAAGGCTC TCCTTCTCAT AAATTCCATA   
  
  
+ TTCCTCTGTT TTTCTAGGAG AGAGAAATAT GCAAAAAACA AATCAGTTGG CGACAAAAGT TACTACCACG   
  
  
+ CATTGTCTAC TGCTGCGTAC CCATTTTTGG AGACTTTAAA GAACGCCCCT CCCATCTTGA AAAGTGAAAA   
  
  
+ CCCCCTCTTT GAAACTGAAT TTCAGGGGAA AAAAACCTCA CCAAACCTCT AGAGAGAGCT TTTAGAGAGG   
  
  
+ GAAATACAAC GATTAGAAGG AGCAATTTGG GAAATTTCTT TGGGAATTTG AATGGGTTTT GAGTGAATTG   
  
  
+ CAAATCCCAG AAAAGTTTTG GCAAGTACCG ATCTACAGTT CTCTCCTCTT CGTGTTTGGT AGATCCCCTG   
  
  
+ TTTCCTCTGT TTCATTTAGG GTACTTCTCA TTCATCCTCC CCACCCCCTT AATCGGATCT TCCTGTCGAG   
  
  
+ TCACTTTATG CTAATATTTT TTCAGTGGAT TTTTAGTTAA CCCTGTTCAT TTTTCCATTC TGTGTCCCAT   
  
  
+ TTCTCTCTTT TTCATTCATA AGTTGCCGGT TTATCTGTTG GGTGCAGCTT AGTCACAATA ATTTCTGTGT   
  
  
+ TAGGCTTCTT TACGGTTAAA AAAAAAGGAG GCACTCTTTT CGGTGTGATT GTTTATGGGA CCAATGATTC   
  
  
+ AAGATGATGG GTCATCAGTA ACTTCATCAC CCCTTCAATT TTTCTCCATG ATGTCTCCCA ATTTAGGTTC   
  
  
+ TTCCTACCCT TGGCTCAGAG AGCTAAAACC TGAAGAAAGA GGTCTTTACT TGATACATTT GTTGCTCTCT   
  
  
+ TGTGCAAATC ATGTCTCTAG TGGTAGCCTA GACAATGCGA ACTTAGCCCT CGAACAAATC TCTCAGCTTG   
  
  
+ CTGCCCCTGA TGGGGATACA ATGCAGCGTA TGGCTTCTTA TTTTGCTGAA GCCCTGGCTG AGAGGATCCT   
  
  
+ CAAGTCATGG CCTGGCATGT ATAGAGCCCT TCATTTTACG AAAATGCCTG TCATTTCAGA GGAAATTCTT   
  
  
+ GCTAGGAAGC TCTTCTTTGA GCTATTTCCT TTCTTGAAGC TGGCCTATTT GGTGACAAAC CAATCGATAA   
  
  
+ TCGAAGCCAT GGAGGGGGAA AAGATGGTTC ATATTATTGA TCTGAATGCA TCAGAACCTG CTCAATGGAT   
  
  
+ TGCCCTTATT CAGGCTTTGA GTGCAAGGCC TGGGGGTCCT CCTCATTTGA GAATTACCGG TGTTCATCAA   
  
  
+ CACAAAGAGG TTCTAGATCA AGTGGCTCAT AGGGTGACTC AAGAAGCTGA GAAATTGGAT TTGCCATTTC   
  
  
+ AGTTCAATCC TGTGGTTAGC AAGTTGGAAA ACCTTGATGT TGAAAAGCTG TGTGTTAAGA CTGGTGAGGC   
  
  
+ TCTAGCCATC AGTTCGGTCC TTCAACTGCA TACCCTTTTG GGTTCTGATG ATGAGCCCCT AAGGAAAAGT   
  
  
+ TCACCTTTAG CCTTGATGAA GTATGCAAAT GGGGCTAATA GGCAAAGCCC GAGTAATGAT TCGGCTTCTT   
  
  
+ CATCACCTCC TTCGCTCAAT ACTTCAACCA AGCTGGATGG TTTCCTCAGC GCTTTGTGGG GATTGTCCCC   
  
  
+ AAAGATTATG GTGATAGCTG AGCACGATTC CAATCACAAT GGTTCTGGAC TTATGGAGAG GTTGTCAGAA   
  
  
+ GCACTGTACT TCTATGCAGC GCTGTTTGAC TGCTTAGAAT CCACCCTGCC AAGAACATCT GTCGAAAGAA   
  
  
+ GGCGGGTAGA GAAGATGCTC CTAGGTGAAG AGATCAAGAA CATTATATCA TGCGAGGGAG GAGAAAGGAG   
  
  
+ AGAAAGGCAT GAGAAGATCG AGAAGTGGAT GCAGAGGCTA GACATGGCTG GATTCGGGAT CGTTCCTTTG   
  
  
+ AGCTATATGG GTATGCTGCA AGCAAGGCAA TTGCTTCAGG GCTATGGTTG TGATGGTTAT AGAGTGAAAG   
  
  
+ AGAATGGTTG TGTTGTCATC TGTTGGCAAG ATCGCCCCCT CTTTTCGGTA TCAGCATGGA GGTGTAGGAG   
  
  
+ ATG  

- -Up\_Stream \_Len000TACGCG GAAAGACGAT TGAGTAATAG AAAGGAAGAC TCTCCCCTGT TGAATATATT   
  
  
- TAACTATCCA TAAAAGTTTA GTTATCTGGT CGATGGTATA TACTATTAAA AATAATGAAA AGATTTAGGT   
  
  
- AGTTGATAAA AGTGTTAGGA GGAAAAAGAT CTTTTTATTT TAAACGAATT AAGTTTAGTG GATTTTAATG   
  
  
- ATTTAGATAT CAGGATTTAG ATGTTTGAGT AAACACAACG TTGAGTACTA AATTCACCTT GTGATTGGAA   
  
  
- TCCTCTATAC AACAGTTTTC TTCTTAAGCT ACTGTATAAA AGGTTGTTTT CCGGTGGATT GAAGTACTAA   
  
  
- ATTTTTCCTC CTCAGATTCG TGTAGTACCT ACTGATTAAA AACTACATAA CATTGATGCA TAACTCTATT   
  
  
- GAACTTACTT AGCACTGATC TTATATAGAT AAAAGTTTTT ATTTTTAAGG GATTTTCTTT TCCTATTTTT   
  
  
- TGATATATTA TGATTTTTTA AGATTGATAT TATTGATCCG AATAATTTAA AAATATCACT TTTAAATAAA   
  
  
- GATGAATATT GTTGTAGTGA ATGATTCATC GAATAAACAC AGAAGAAAAG ATTTATTATT ACCATTGATA   
  
  
- CACAGAAAAC ATTTATTGAA TAATAGATAA AATATTTAGT GGCACATTTC GTGCCCAGAT ATGATCAATT   
  
  
- AAATTTATCG TAACAATTTA GTTCATTCTA TGTTTGTTTA AATAAATGTT GACATTCACC AGGTATTTTT   
  
  
- ATTATCGGTA ATGTTTTTTA TCATGATGAC AAAATACTTT ACTAAAATTA TAAGATTTTA AGGTTATTTT   
  
  
- GTACAATATT AGATACTAAT TAAAATTTTG TACTAATGTA CAGTCGTATT AAACATAATA ACATGTCGTT   
  
  
- TACTTTCAAC CTCGATTTTC TTTTACCCCT TTATTTCCGT CTGTTCATTT CTATTTTATA CGATAGATGA   
  
  
- AAACCTCCAT AGGAATTGGT GTAACAGAGC AAACAGAAGT AATCCCTCTT CACCCTATTT ATGGTTTTAA   
  
  
- ACATACCAGT AACGCACCCC AAACGAAGGT TTGTTTTACC GAAAAACCTT CGTTTAAAAC ACTCGAAGAT   
  
  
- ACGTTTAAAG GTGCGAATTG GGGTGTTCGT TTGTTTTTGT CTGTCACCAA AAACAAACAG AAACAATCCC   
  
  
- GTACAAACCT CGGTTGACAA TTACCCTTAT TTGACCCCTC CGAAAAAGGA ACCGAGAAAG GGGGACTGGG   
  
  
- GTTTGGGTGA AGTCGAGTTA TGCTTTGGTC GGTAACTTTT TCCTTCCCCC CCCCCCCCCA TACACTCTTT   
  
  
- TTCTGTTTTG AAAAAGGGCC TCTCTCTTCT ATGTATACGT TGTTTCCGAG AGGAAGAGTA TTTAAGGTAT   
  
  
- AAGGAGACAA AAAGATCCTC TCTCTTTATA CGTTTTTTGT TTAGTCAACC GCTGTTTTCA ATGATGGTGC   
  
  
- GTAACAGATG ACGACGCATG GGTAAAAACC TCTGAAATTT CTTGCGGGGA GGGTAGAACT TTTCACTTTT   
  
  
- GGGGGAGAAA CTTTGACTTA AAGTCCCCTT TTTTTGGAGT GGTTTGGAGA TCTCTCTCGA AAATCTCTCC   
  
  
- CTTTATGTTG CTAATCTTCC TCGTTAAACC CTTTAAAGAA ACCCTTAAAC TTACCCAAAA CTCACTTAAC   
  
  
- GTTTAGGGTC TTTTCAAAAC CGTTCATGGC TAGATGTCAA GAGAGGAGAA GCACAAACCA TCTAGGGGAC   
  
  
- AAAGGAGACA AAGTAAATCC CATGAAGAGT AAGTAGGAGG GGTGGGGGAA TTAGCCTAGA AGGACAGCTC   
  
  
- AGTGAAATAC GATTATAAAA AAGTCACCTA AAAATCAATT GGGACAAGTA AAAAGGTAAG ACACAGGGTA   
  
  
- AAGAGAGAAA AAGTAAGTAT TCAACGGCCA AATAGACAAC CCACGTCGAA TCAGTGTTAT TAAAGACACA   
  
  
- ATCCGAAGAA ATGCCAATTT TTTTTTCCTC CGTGAGAAAA GCCACACTAA CAAATACCCT GGTTACTAAG   
  
  
- TTCTACTACC CAGTAGTCAT TGAAGTAGTG GGGAAGTTAA AAAGAGGTAC TACAGAGGGT TAAATCCAAG   
  
  
- AAGGATGGGA ACCGAGTCTC TCGATTTTGG ACTTCTTTCT CCAGAAATGA ACTATGTAAA CAACGAGAGA   
  
  
- ACACGTTTAG TACAGAGATC ACCATCGGAT CTGTTACGCT TGAATCGGGA GCTTGTTTAG AGAGTCGAAC   
  
  
- GACGGGGACT ACCCCTATGT TACGTCGCAT ACCGAAGAAT AAAACGACTT CGGGACCGAC TCTCCTAGGA   
  
  
- GTTCAGTACC GGACCGTACA TATCTCGGGA AGTAAAATGC TTTTACGGAC AGTAAAGTCT CCTTTAAGAA   
  
  
- CGATCCTTCG AGAAGAAACT CGATAAAGGA AAGAACTTCG ACCGGATAAA CCACTGTTTG GTTAGCTATT   
  
  
- AGCTTCGGTA CCTCCCCCTT TTCTACCAAG TATAATAACT AGACTTACGT AGTCTTGGAC GAGTTACCTA   
  
  
- ACGGGAATAA GTCCGAAACT CACGTTCCGG ACCCCCAGGA GGAGTAAACT CTTAATGGCC ACAAGTAGTT   
  
  
- GTGTTTCTCC AAGATCTAGT TCACCGAGTA TCCCACTGAG TTCTTCGACT CTTTAACCTA AACGGTAAAG   
  
  
- TCAAGTTAGG ACACCAATCG TTCAACCTTT TGGAACTACA ACTTTTCGAC ACACAATTCT GACCACTCCG   
  
  
- AGATCGGTAG TCAAGCCAGG AAGTTGACGT ATGGGAAAAC CCAAGACTAC TACTCGGGGA TTCCTTTTCA   
  
  
- AGTGGAAATC GGAACTACTT CATACGTTTA CCCCGATTAT CCGTTTCGGG CTCATTACTA AGCCGAAGAA   
  
  
- GTAGTGGAGG AAGCGAGTTA TGAAGTTGGT TCGACCTACC AAAGGAGTCG CGAAACACCC CTAACAGGGG   
  
  
- TTTCTAATAC CACTATCGAC TCGTGCTAAG GTTAGTGTTA CCAAGACCTG AATACCTCTC CAACAGTCTT   
  
  
- CGTGACATGA AGATACGTCG CGACAAACTG ACGAATCTTA GGTGGGACGG TTCTTGTAGA CAGCTTTCTT   
  
  
- CCGCCCATCT CTTCTACGAG GATCCACTTC TCTAGTTCTT GTAATATAGT ACGCTCCCTC CTCTTTCCTC   
  
  
- TCTTTCCGTA CTCTTCTAGC TCTTCACCTA CGTCTCCGAT CTGTACCGAC CTAAGCCCTA GCAAGGAAAC   
  
  
- TCGATATACC CATACGACGT TCGTTCCGTT AACGAAGTCC CGATACCAAC ACTACCAATA TCTCACTTTC   
  
  
- TCTTACCAAC ACAACAGTAG ACAACCGTTC TAGCGGGGGA GAAAAGCCAT AGTCGTACCT CCACATCCTC   
  
  
- TAC

+     MYC

| Site Name | Organism | Position | Strand | Matrix score. | sequence | function |
| --- | --- | --- | --- | --- | --- | --- |
| MYC | Arabidopsis thaliana | 3252 | - | 6 | CAATTG |  |
| MYC | Arabidopsis thaliana | 243 | + | 6 | CATTTG |  |
| MYC | Arabidopsis thaliana | 2160 | + | 6 | CATTTG |  |
| MYC | Arabidopsis thaliana | 2830 | - | 6 | CATTTG |  |
| MYC | Arabidopsis thaliana | 2568 | + | 6 | CATTTG |  |
| MYC | Arabidopsis thaliana | 912 | - | 6 | CATTTG |  |

>HU08G00367.1   
+ -Up\_Stream \_Len000ATGCGC CTTTCTGCTA ACTCATTATC TTTCCTTCTG AGAGGGGACA ACTTATATAA   
  
  
+ ATTGATAGGT ATTTTCAAAT CAATAGACCA GCTACCATAT ATGATAATTT TTATTACTTT TCTAAATCCA   
  
  
+ TCAACTATTT TCACAATCCT CCTTTTTCTA GAAAAATAAA ATTTGCTTAA TTCAAATCAC CTAAAATTAC   
  
  
+ TAAATCTATA GTCCTAAATC TACAAACTCA TTTGTGTTGC AACTCATGAT TTAAGTGGAA CACTAACCTT   
  
  
+ AGGAGATATG TTGTCAAAAG AAGAATTCGA TGACATATTT TCCAACAAAA GGCCACCTAA CTTCATGATT   
  
  
+ TAAAAAGGAG GAGTCTAAGC ACATCATGGA TGACTAATTT TTGATGTATT GTAACTACGT ATTGAGATAA   
  
  
+ CTTGAATGAA TCGTGACTAG AATATATCTA TTTTCAAAAA TAAAAATTCC CTAAAAGAAA AGGATAAAAA   
  
  
+ ACTATATAAT ACTAAAAAAT TCTAACTATA ATAACTAGGC TTATTAAATT TTTATAGTGA AAATTTATTT   
  
  
+ CTACTTATAA CAACATCACT TACTAAGTAG CTTATTTGTG TCTTCTTTTC TAAATAATAA TGGTAACTAT   
  
  
+ GTGTCTTTTG TAAATAACTT ATTATCTATT TTATAAATCA CCGTGTAAAG CACGGGTCTA TACTAGTTAA   
  
  
+ TTTAAATAGC ATTGTTAAAT CAAGTAAGAT ACAAACAAAT TTATTTACAA CTGTAAGTGG TCCATAAAAA   
  
  
+ TAATAGCCAT TACAAAAAAT AGTACTACTG TTTTATGAAA TGATTTTAAT ATTCTAAAAT TCCAATAAAA   
  
  
+ CATGTTATAA TCTATGATTA ATTTTAAAAC ATGATTACAT GTCAGCATAA TTTGTATTAT TGTACAGCAA   
  
  
+ ATGAAAGTTG GAGCTAAAAG AAAATGGGGA AATAAAGGCA GACAAGTAAA GATAAAATAT GCTATCTACT   
  
  
+ TTTGGAGGTA TCCTTAACCA CATTGTCTCG TTTGTCTTCA TTAGGGAGAA GTGGGATAAA TACCAAAATT   
  
  
+ TGTATGGTCA TTGCGTGGGG TTTGCTTCCA AACAAAATGG CTTTTTGGAA GCAAATTTTG TGAGCTTCTA   
  
  
+ TGCAAATTTC CACGCTTAAC CCCACAAGCA AACAAAAACA GACAGTGGTT TTTGTTTGTC TTTGTTAGGG   
  
  
+ CATGTTTGGA GCCAACTGTT AATGGGAATA AACTGGGGAG GCTTTTTCCT TGGCTCTTTC CCCCTGACCC   
  
  
+ CAAACCCACT TCAGCTCAAT ACGAAACCAG CCATTGAAAA AGGAAGGGGG GGGGGGGGGT ATGTGAGAAA   
  
  
+ AAGACAAAAC TTTTTCCCGG AGAGAGAAGA TACATATGCA ACAAAGGCTC TCCTTCTCAT AAATTCCATA   
  
  
+ TTCCTCTGTT TTTCTAGGAG AGAGAAATAT GCAAAAAACA AATCAGTTGG CGACAAAAGT TACTACCACG   
  
  
+ CATTGTCTAC TGCTGCGTAC CCATTTTTGG AGACTTTAAA GAACGCCCCT CCCATCTTGA AAAGTGAAAA   
  
  
+ CCCCCTCTTT GAAACTGAAT TTCAGGGGAA AAAAACCTCA CCAAACCTCT AGAGAGAGCT TTTAGAGAGG   
  
  
+ GAAATACAAC GATTAGAAGG AGCAATTTGG GAAATTTCTT TGGGAATTTG AATGGGTTTT GAGTGAATTG   
  
  
+ CAAATCCCAG AAAAGTTTTG GCAAGTACCG ATCTACAGTT CTCTCCTCTT CGTGTTTGGT AGATCCCCTG   
  
  
+ TTTCCTCTGT TTCATTTAGG GTACTTCTCA TTCATCCTCC CCACCCCCTT AATCGGATCT TCCTGTCGAG   
  
  
+ TCACTTTATG CTAATATTTT TTCAGTGGAT TTTTAGTTAA CCCTGTTCAT TTTTCCATTC TGTGTCCCAT   
  
  
+ TTCTCTCTTT TTCATTCATA AGTTGCCGGT TTATCTGTTG GGTGCAGCTT AGTCACAATA ATTTCTGTGT   
  
  
+ TAGGCTTCTT TACGGTTAAA AAAAAAGGAG GCACTCTTTT CGGTGTGATT GTTTATGGGA CCAATGATTC   
  
  
+ AAGATGATGG GTCATCAGTA ACTTCATCAC CCCTTCAATT TTTCTCCATG ATGTCTCCCA ATTTAGGTTC   
  
  
+ TTCCTACCCT TGGCTCAGAG AGCTAAAACC TGAAGAAAGA GGTCTTTACT TGATACATTT GTTGCTCTCT   
  
  
+ TGTGCAAATC ATGTCTCTAG TGGTAGCCTA GACAATGCGA ACTTAGCCCT CGAACAAATC TCTCAGCTTG   
  
  
+ CTGCCCCTGA TGGGGATACA ATGCAGCGTA TGGCTTCTTA TTTTGCTGAA GCCCTGGCTG AGAGGATCCT   
  
  
+ CAAGTCATGG CCTGGCATGT ATAGAGCCCT TCATTTTACG AAAATGCCTG TCATTTCAGA GGAAATTCTT   
  
  
+ GCTAGGAAGC TCTTCTTTGA GCTATTTCCT TTCTTGAAGC TGGCCTATTT GGTGACAAAC CAATCGATAA   
  
  
+ TCGAAGCCAT GGAGGGGGAA AAGATGGTTC ATATTATTGA TCTGAATGCA TCAGAACCTG CTCAATGGAT   
  
  
+ TGCCCTTATT CAGGCTTTGA GTGCAAGGCC TGGGGGTCCT CCTCATTTGA GAATTACCGG TGTTCATCAA   
  
  
+ CACAAAGAGG TTCTAGATCA AGTGGCTCAT AGGGTGACTC AAGAAGCTGA GAAATTGGAT TTGCCATTTC   
  
  
+ AGTTCAATCC TGTGGTTAGC AAGTTGGAAA ACCTTGATGT TGAAAAGCTG TGTGTTAAGA CTGGTGAGGC   
  
  
+ TCTAGCCATC AGTTCGGTCC TTCAACTGCA TACCCTTTTG GGTTCTGATG ATGAGCCCCT AAGGAAAAGT   
  
  
+ TCACCTTTAG CCTTGATGAA GTATGCAAAT GGGGCTAATA GGCAAAGCCC GAGTAATGAT TCGGCTTCTT   
  
  
+ CATCACCTCC TTCGCTCAAT ACTTCAACCA AGCTGGATGG TTTCCTCAGC GCTTTGTGGG GATTGTCCCC   
  
  
+ AAAGATTATG GTGATAGCTG AGCACGATTC CAATCACAAT GGTTCTGGAC TTATGGAGAG GTTGTCAGAA   
  
  
+ GCACTGTACT TCTATGCAGC GCTGTTTGAC TGCTTAGAAT CCACCCTGCC AAGAACATCT GTCGAAAGAA   
  
  
+ GGCGGGTAGA GAAGATGCTC CTAGGTGAAG AGATCAAGAA CATTATATCA TGCGAGGGAG GAGAAAGGAG   
  
  
+ AGAAAGGCAT GAGAAGATCG AGAAGTGGAT GCAGAGGCTA GACATGGCTG GATTCGGGAT CGTTCCTTTG   
  
  
+ AGCTATATGG GTATGCTGCA AGCAAGGCAA TTGCTTCAGG GCTATGGTTG TGATGGTTAT AGAGTGAAAG   
  
  
+ AGAATGGTTG TGTTGTCATC TGTTGGCAAG ATCGCCCCCT CTTTTCGGTA TCAGCATGGA GGTGTAGGAG   
  
  
+ ATG  

- -Up\_Stream \_Len000TACGCG GAAAGACGAT TGAGTAATAG AAAGGAAGAC TCTCCCCTGT TGAATATATT   
  
  
- TAACTATCCA TAAAAGTTTA GTTATCTGGT CGATGGTATA TACTATTAAA AATAATGAAA AGATTTAGGT   
  
  
- AGTTGATAAA AGTGTTAGGA GGAAAAAGAT CTTTTTATTT TAAACGAATT AAGTTTAGTG GATTTTAATG   
  
  
- ATTTAGATAT CAGGATTTAG ATGTTTGAGT AAACACAACG TTGAGTACTA AATTCACCTT GTGATTGGAA   
  
  
- TCCTCTATAC AACAGTTTTC TTCTTAAGCT ACTGTATAAA AGGTTGTTTT CCGGTGGATT GAAGTACTAA   
  
  
- ATTTTTCCTC CTCAGATTCG TGTAGTACCT ACTGATTAAA AACTACATAA CATTGATGCA TAACTCTATT   
  
  
- GAACTTACTT AGCACTGATC TTATATAGAT AAAAGTTTTT ATTTTTAAGG GATTTTCTTT TCCTATTTTT   
  
  
- TGATATATTA TGATTTTTTA AGATTGATAT TATTGATCCG AATAATTTAA AAATATCACT TTTAAATAAA   
  
  
- GATGAATATT GTTGTAGTGA ATGATTCATC GAATAAACAC AGAAGAAAAG ATTTATTATT ACCATTGATA   
  
  
- CACAGAAAAC ATTTATTGAA TAATAGATAA AATATTTAGT GGCACATTTC GTGCCCAGAT ATGATCAATT   
  
  
- AAATTTATCG TAACAATTTA GTTCATTCTA TGTTTGTTTA AATAAATGTT GACATTCACC AGGTATTTTT   
  
  
- ATTATCGGTA ATGTTTTTTA TCATGATGAC AAAATACTTT ACTAAAATTA TAAGATTTTA AGGTTATTTT   
  
  
- GTACAATATT AGATACTAAT TAAAATTTTG TACTAATGTA CAGTCGTATT AAACATAATA ACATGTCGTT   
  
  
- TACTTTCAAC CTCGATTTTC TTTTACCCCT TTATTTCCGT CTGTTCATTT CTATTTTATA CGATAGATGA   
  
  
- AAACCTCCAT AGGAATTGGT GTAACAGAGC AAACAGAAGT AATCCCTCTT CACCCTATTT ATGGTTTTAA   
  
  
- ACATACCAGT AACGCACCCC AAACGAAGGT TTGTTTTACC GAAAAACCTT CGTTTAAAAC ACTCGAAGAT   
  
  
- ACGTTTAAAG GTGCGAATTG GGGTGTTCGT TTGTTTTTGT CTGTCACCAA AAACAAACAG AAACAATCCC   
  
  
- GTACAAACCT CGGTTGACAA TTACCCTTAT TTGACCCCTC CGAAAAAGGA ACCGAGAAAG GGGGACTGGG   
  
  
- GTTTGGGTGA AGTCGAGTTA TGCTTTGGTC GGTAACTTTT TCCTTCCCCC CCCCCCCCCA TACACTCTTT   
  
  
- TTCTGTTTTG AAAAAGGGCC TCTCTCTTCT ATGTATACGT TGTTTCCGAG AGGAAGAGTA TTTAAGGTAT   
  
  
- AAGGAGACAA AAAGATCCTC TCTCTTTATA CGTTTTTTGT TTAGTCAACC GCTGTTTTCA ATGATGGTGC   
  
  
- GTAACAGATG ACGACGCATG GGTAAAAACC TCTGAAATTT CTTGCGGGGA GGGTAGAACT TTTCACTTTT   
  
  
- GGGGGAGAAA CTTTGACTTA AAGTCCCCTT TTTTTGGAGT GGTTTGGAGA TCTCTCTCGA AAATCTCTCC   
  
  
- CTTTATGTTG CTAATCTTCC TCGTTAAACC CTTTAAAGAA ACCCTTAAAC TTACCCAAAA CTCACTTAAC   
  
  
- GTTTAGGGTC TTTTCAAAAC CGTTCATGGC TAGATGTCAA GAGAGGAGAA GCACAAACCA TCTAGGGGAC   
  
  
- AAAGGAGACA AAGTAAATCC CATGAAGAGT AAGTAGGAGG GGTGGGGGAA TTAGCCTAGA AGGACAGCTC   
  
  
- AGTGAAATAC GATTATAAAA AAGTCACCTA AAAATCAATT GGGACAAGTA AAAAGGTAAG ACACAGGGTA   
  
  
- AAGAGAGAAA AAGTAAGTAT TCAACGGCCA AATAGACAAC CCACGTCGAA TCAGTGTTAT TAAAGACACA   
  
  
- ATCCGAAGAA ATGCCAATTT TTTTTTCCTC CGTGAGAAAA GCCACACTAA CAAATACCCT GGTTACTAAG   
  
  
- TTCTACTACC CAGTAGTCAT TGAAGTAGTG GGGAAGTTAA AAAGAGGTAC TACAGAGGGT TAAATCCAAG   
  
  
- AAGGATGGGA ACCGAGTCTC TCGATTTTGG ACTTCTTTCT CCAGAAATGA ACTATGTAAA CAACGAGAGA   
  
  
- ACACGTTTAG TACAGAGATC ACCATCGGAT CTGTTACGCT TGAATCGGGA GCTTGTTTAG AGAGTCGAAC   
  
  
- GACGGGGACT ACCCCTATGT TACGTCGCAT ACCGAAGAAT AAAACGACTT CGGGACCGAC TCTCCTAGGA   
  
  
- GTTCAGTACC GGACCGTACA TATCTCGGGA AGTAAAATGC TTTTACGGAC AGTAAAGTCT CCTTTAAGAA   
  
  
- CGATCCTTCG AGAAGAAACT CGATAAAGGA AAGAACTTCG ACCGGATAAA CCACTGTTTG GTTAGCTATT   
  
  
- AGCTTCGGTA CCTCCCCCTT TTCTACCAAG TATAATAACT AGACTTACGT AGTCTTGGAC GAGTTACCTA   
  
  
- ACGGGAATAA GTCCGAAACT CACGTTCCGG ACCCCCAGGA GGAGTAAACT CTTAATGGCC ACAAGTAGTT   
  
  
- GTGTTTCTCC AAGATCTAGT TCACCGAGTA TCCCACTGAG TTCTTCGACT CTTTAACCTA AACGGTAAAG   
  
  
- TCAAGTTAGG ACACCAATCG TTCAACCTTT TGGAACTACA ACTTTTCGAC ACACAATTCT GACCACTCCG   
  
  
- AGATCGGTAG TCAAGCCAGG AAGTTGACGT ATGGGAAAAC CCAAGACTAC TACTCGGGGA TTCCTTTTCA   
  
  
- AGTGGAAATC GGAACTACTT CATACGTTTA CCCCGATTAT CCGTTTCGGG CTCATTACTA AGCCGAAGAA   
  
  
- GTAGTGGAGG AAGCGAGTTA TGAAGTTGGT TCGACCTACC AAAGGAGTCG CGAAACACCC CTAACAGGGG   
  
  
- TTTCTAATAC CACTATCGAC TCGTGCTAAG GTTAGTGTTA CCAAGACCTG AATACCTCTC CAACAGTCTT   
  
  
- CGTGACATGA AGATACGTCG CGACAAACTG ACGAATCTTA GGTGGGACGG TTCTTGTAGA CAGCTTTCTT   
  
  
- CCGCCCATCT CTTCTACGAG GATCCACTTC TCTAGTTCTT GTAATATAGT ACGCTCCCTC CTCTTTCCTC   
  
  
- TCTTTCCGTA CTCTTCTAGC TCTTCACCTA CGTCTCCGAT CTGTACCGAC CTAAGCCCTA GCAAGGAAAC   
  
  
- TCGATATACC CATACGACGT TCGTTCCGTT AACGAAGTCC CGATACCAAC ACTACCAATA TCTCACTTTC   
  
  
- TCTTACCAAC ACAACAGTAG ACAACCGTTC TAGCGGGGGA GAAAAGCCAT AGTCGTACCT CCACATCCTC   
  
  
- TAC

+     Myb

| Site Name | Organism | Position | Strand | Matrix score. | sequence | function |
| --- | --- | --- | --- | --- | --- | --- |
| Myb | Arabidopsis thaliana | 1448 | - | 6 | CAACTG |  |
| Myb | Arabidopsis thaliana | 2757 | + | 6 | CAACTG |  |
| Myb | Arabidopsis thaliana | 752 | + | 6 | CAACTG |  |
| Myb | Arabidopsis thaliana | 1207 | + | 6 | CAACTG |  |

>HU08G00367.1   
+ -Up\_Stream \_Len000ATGCGC CTTTCTGCTA ACTCATTATC TTTCCTTCTG AGAGGGGACA ACTTATATAA   
  
  
+ ATTGATAGGT ATTTTCAAAT CAATAGACCA GCTACCATAT ATGATAATTT TTATTACTTT TCTAAATCCA   
  
  
+ TCAACTATTT TCACAATCCT CCTTTTTCTA GAAAAATAAA ATTTGCTTAA TTCAAATCAC CTAAAATTAC   
  
  
+ TAAATCTATA GTCCTAAATC TACAAACTCA TTTGTGTTGC AACTCATGAT TTAAGTGGAA CACTAACCTT   
  
  
+ AGGAGATATG TTGTCAAAAG AAGAATTCGA TGACATATTT TCCAACAAAA GGCCACCTAA CTTCATGATT   
  
  
+ TAAAAAGGAG GAGTCTAAGC ACATCATGGA TGACTAATTT TTGATGTATT GTAACTACGT ATTGAGATAA   
  
  
+ CTTGAATGAA TCGTGACTAG AATATATCTA TTTTCAAAAA TAAAAATTCC CTAAAAGAAA AGGATAAAAA   
  
  
+ ACTATATAAT ACTAAAAAAT TCTAACTATA ATAACTAGGC TTATTAAATT TTTATAGTGA AAATTTATTT   
  
  
+ CTACTTATAA CAACATCACT TACTAAGTAG CTTATTTGTG TCTTCTTTTC TAAATAATAA TGGTAACTAT   
  
  
+ GTGTCTTTTG TAAATAACTT ATTATCTATT TTATAAATCA CCGTGTAAAG CACGGGTCTA TACTAGTTAA   
  
  
+ TTTAAATAGC ATTGTTAAAT CAAGTAAGAT ACAAACAAAT TTATTTACAA CTGTAAGTGG TCCATAAAAA   
  
  
+ TAATAGCCAT TACAAAAAAT AGTACTACTG TTTTATGAAA TGATTTTAAT ATTCTAAAAT TCCAATAAAA   
  
  
+ CATGTTATAA TCTATGATTA ATTTTAAAAC ATGATTACAT GTCAGCATAA TTTGTATTAT TGTACAGCAA   
  
  
+ ATGAAAGTTG GAGCTAAAAG AAAATGGGGA AATAAAGGCA GACAAGTAAA GATAAAATAT GCTATCTACT   
  
  
+ TTTGGAGGTA TCCTTAACCA CATTGTCTCG TTTGTCTTCA TTAGGGAGAA GTGGGATAAA TACCAAAATT   
  
  
+ TGTATGGTCA TTGCGTGGGG TTTGCTTCCA AACAAAATGG CTTTTTGGAA GCAAATTTTG TGAGCTTCTA   
  
  
+ TGCAAATTTC CACGCTTAAC CCCACAAGCA AACAAAAACA GACAGTGGTT TTTGTTTGTC TTTGTTAGGG   
  
  
+ CATGTTTGGA GCCAACTGTT AATGGGAATA AACTGGGGAG GCTTTTTCCT TGGCTCTTTC CCCCTGACCC   
  
  
+ CAAACCCACT TCAGCTCAAT ACGAAACCAG CCATTGAAAA AGGAAGGGGG GGGGGGGGGT ATGTGAGAAA   
  
  
+ AAGACAAAAC TTTTTCCCGG AGAGAGAAGA TACATATGCA ACAAAGGCTC TCCTTCTCAT AAATTCCATA   
  
  
+ TTCCTCTGTT TTTCTAGGAG AGAGAAATAT GCAAAAAACA AATCAGTTGG CGACAAAAGT TACTACCACG   
  
  
+ CATTGTCTAC TGCTGCGTAC CCATTTTTGG AGACTTTAAA GAACGCCCCT CCCATCTTGA AAAGTGAAAA   
  
  
+ CCCCCTCTTT GAAACTGAAT TTCAGGGGAA AAAAACCTCA CCAAACCTCT AGAGAGAGCT TTTAGAGAGG   
  
  
+ GAAATACAAC GATTAGAAGG AGCAATTTGG GAAATTTCTT TGGGAATTTG AATGGGTTTT GAGTGAATTG   
  
  
+ CAAATCCCAG AAAAGTTTTG GCAAGTACCG ATCTACAGTT CTCTCCTCTT CGTGTTTGGT AGATCCCCTG   
  
  
+ TTTCCTCTGT TTCATTTAGG GTACTTCTCA TTCATCCTCC CCACCCCCTT AATCGGATCT TCCTGTCGAG   
  
  
+ TCACTTTATG CTAATATTTT TTCAGTGGAT TTTTAGTTAA CCCTGTTCAT TTTTCCATTC TGTGTCCCAT   
  
  
+ TTCTCTCTTT TTCATTCATA AGTTGCCGGT TTATCTGTTG GGTGCAGCTT AGTCACAATA ATTTCTGTGT   
  
  
+ TAGGCTTCTT TACGGTTAAA AAAAAAGGAG GCACTCTTTT CGGTGTGATT GTTTATGGGA CCAATGATTC   
  
  
+ AAGATGATGG GTCATCAGTA ACTTCATCAC CCCTTCAATT TTTCTCCATG ATGTCTCCCA ATTTAGGTTC   
  
  
+ TTCCTACCCT TGGCTCAGAG AGCTAAAACC TGAAGAAAGA GGTCTTTACT TGATACATTT GTTGCTCTCT   
  
  
+ TGTGCAAATC ATGTCTCTAG TGGTAGCCTA GACAATGCGA ACTTAGCCCT CGAACAAATC TCTCAGCTTG   
  
  
+ CTGCCCCTGA TGGGGATACA ATGCAGCGTA TGGCTTCTTA TTTTGCTGAA GCCCTGGCTG AGAGGATCCT   
  
  
+ CAAGTCATGG CCTGGCATGT ATAGAGCCCT TCATTTTACG AAAATGCCTG TCATTTCAGA GGAAATTCTT   
  
  
+ GCTAGGAAGC TCTTCTTTGA GCTATTTCCT TTCTTGAAGC TGGCCTATTT GGTGACAAAC CAATCGATAA   
  
  
+ TCGAAGCCAT GGAGGGGGAA AAGATGGTTC ATATTATTGA TCTGAATGCA TCAGAACCTG CTCAATGGAT   
  
  
+ TGCCCTTATT CAGGCTTTGA GTGCAAGGCC TGGGGGTCCT CCTCATTTGA GAATTACCGG TGTTCATCAA   
  
  
+ CACAAAGAGG TTCTAGATCA AGTGGCTCAT AGGGTGACTC AAGAAGCTGA GAAATTGGAT TTGCCATTTC   
  
  
+ AGTTCAATCC TGTGGTTAGC AAGTTGGAAA ACCTTGATGT TGAAAAGCTG TGTGTTAAGA CTGGTGAGGC   
  
  
+ TCTAGCCATC AGTTCGGTCC TTCAACTGCA TACCCTTTTG GGTTCTGATG ATGAGCCCCT AAGGAAAAGT   
  
  
+ TCACCTTTAG CCTTGATGAA GTATGCAAAT GGGGCTAATA GGCAAAGCCC GAGTAATGAT TCGGCTTCTT   
  
  
+ CATCACCTCC TTCGCTCAAT ACTTCAACCA AGCTGGATGG TTTCCTCAGC GCTTTGTGGG GATTGTCCCC   
  
  
+ AAAGATTATG GTGATAGCTG AGCACGATTC CAATCACAAT GGTTCTGGAC TTATGGAGAG GTTGTCAGAA   
  
  
+ GCACTGTACT TCTATGCAGC GCTGTTTGAC TGCTTAGAAT CCACCCTGCC AAGAACATCT GTCGAAAGAA   
  
  
+ GGCGGGTAGA GAAGATGCTC CTAGGTGAAG AGATCAAGAA CATTATATCA TGCGAGGGAG GAGAAAGGAG   
  
  
+ AGAAAGGCAT GAGAAGATCG AGAAGTGGAT GCAGAGGCTA GACATGGCTG GATTCGGGAT CGTTCCTTTG   
  
  
+ AGCTATATGG GTATGCTGCA AGCAAGGCAA TTGCTTCAGG GCTATGGTTG TGATGGTTAT AGAGTGAAAG   
  
  
+ AGAATGGTTG TGTTGTCATC TGTTGGCAAG ATCGCCCCCT CTTTTCGGTA TCAGCATGGA GGTGTAGGAG   
  
  
+ ATG  

- -Up\_Stream \_Len000TACGCG GAAAGACGAT TGAGTAATAG AAAGGAAGAC TCTCCCCTGT TGAATATATT   
  
  
- TAACTATCCA TAAAAGTTTA GTTATCTGGT CGATGGTATA TACTATTAAA AATAATGAAA AGATTTAGGT   
  
  
- AGTTGATAAA AGTGTTAGGA GGAAAAAGAT CTTTTTATTT TAAACGAATT AAGTTTAGTG GATTTTAATG   
  
  
- ATTTAGATAT CAGGATTTAG ATGTTTGAGT AAACACAACG TTGAGTACTA AATTCACCTT GTGATTGGAA   
  
  
- TCCTCTATAC AACAGTTTTC TTCTTAAGCT ACTGTATAAA AGGTTGTTTT CCGGTGGATT GAAGTACTAA   
  
  
- ATTTTTCCTC CTCAGATTCG TGTAGTACCT ACTGATTAAA AACTACATAA CATTGATGCA TAACTCTATT   
  
  
- GAACTTACTT AGCACTGATC TTATATAGAT AAAAGTTTTT ATTTTTAAGG GATTTTCTTT TCCTATTTTT   
  
  
- TGATATATTA TGATTTTTTA AGATTGATAT TATTGATCCG AATAATTTAA AAATATCACT TTTAAATAAA   
  
  
- GATGAATATT GTTGTAGTGA ATGATTCATC GAATAAACAC AGAAGAAAAG ATTTATTATT ACCATTGATA   
  
  
- CACAGAAAAC ATTTATTGAA TAATAGATAA AATATTTAGT GGCACATTTC GTGCCCAGAT ATGATCAATT   
  
  
- AAATTTATCG TAACAATTTA GTTCATTCTA TGTTTGTTTA AATAAATGTT GACATTCACC AGGTATTTTT   
  
  
- ATTATCGGTA ATGTTTTTTA TCATGATGAC AAAATACTTT ACTAAAATTA TAAGATTTTA AGGTTATTTT   
  
  
- GTACAATATT AGATACTAAT TAAAATTTTG TACTAATGTA CAGTCGTATT AAACATAATA ACATGTCGTT   
  
  
- TACTTTCAAC CTCGATTTTC TTTTACCCCT TTATTTCCGT CTGTTCATTT CTATTTTATA CGATAGATGA   
  
  
- AAACCTCCAT AGGAATTGGT GTAACAGAGC AAACAGAAGT AATCCCTCTT CACCCTATTT ATGGTTTTAA   
  
  
- ACATACCAGT AACGCACCCC AAACGAAGGT TTGTTTTACC GAAAAACCTT CGTTTAAAAC ACTCGAAGAT   
  
  
- ACGTTTAAAG GTGCGAATTG GGGTGTTCGT TTGTTTTTGT CTGTCACCAA AAACAAACAG AAACAATCCC   
  
  
- GTACAAACCT CGGTTGACAA TTACCCTTAT TTGACCCCTC CGAAAAAGGA ACCGAGAAAG GGGGACTGGG   
  
  
- GTTTGGGTGA AGTCGAGTTA TGCTTTGGTC GGTAACTTTT TCCTTCCCCC CCCCCCCCCA TACACTCTTT   
  
  
- TTCTGTTTTG AAAAAGGGCC TCTCTCTTCT ATGTATACGT TGTTTCCGAG AGGAAGAGTA TTTAAGGTAT   
  
  
- AAGGAGACAA AAAGATCCTC TCTCTTTATA CGTTTTTTGT TTAGTCAACC GCTGTTTTCA ATGATGGTGC   
  
  
- GTAACAGATG ACGACGCATG GGTAAAAACC TCTGAAATTT CTTGCGGGGA GGGTAGAACT TTTCACTTTT   
  
  
- GGGGGAGAAA CTTTGACTTA AAGTCCCCTT TTTTTGGAGT GGTTTGGAGA TCTCTCTCGA AAATCTCTCC   
  
  
- CTTTATGTTG CTAATCTTCC TCGTTAAACC CTTTAAAGAA ACCCTTAAAC TTACCCAAAA CTCACTTAAC   
  
  
- GTTTAGGGTC TTTTCAAAAC CGTTCATGGC TAGATGTCAA GAGAGGAGAA GCACAAACCA TCTAGGGGAC   
  
  
- AAAGGAGACA AAGTAAATCC CATGAAGAGT AAGTAGGAGG GGTGGGGGAA TTAGCCTAGA AGGACAGCTC   
  
  
- AGTGAAATAC GATTATAAAA AAGTCACCTA AAAATCAATT GGGACAAGTA AAAAGGTAAG ACACAGGGTA   
  
  
- AAGAGAGAAA AAGTAAGTAT TCAACGGCCA AATAGACAAC CCACGTCGAA TCAGTGTTAT TAAAGACACA   
  
  
- ATCCGAAGAA ATGCCAATTT TTTTTTCCTC CGTGAGAAAA GCCACACTAA CAAATACCCT GGTTACTAAG   
  
  
- TTCTACTACC CAGTAGTCAT TGAAGTAGTG GGGAAGTTAA AAAGAGGTAC TACAGAGGGT TAAATCCAAG   
  
  
- AAGGATGGGA ACCGAGTCTC TCGATTTTGG ACTTCTTTCT CCAGAAATGA ACTATGTAAA CAACGAGAGA   
  
  
- ACACGTTTAG TACAGAGATC ACCATCGGAT CTGTTACGCT TGAATCGGGA GCTTGTTTAG AGAGTCGAAC   
  
  
- GACGGGGACT ACCCCTATGT TACGTCGCAT ACCGAAGAAT AAAACGACTT CGGGACCGAC TCTCCTAGGA   
  
  
- GTTCAGTACC GGACCGTACA TATCTCGGGA AGTAAAATGC TTTTACGGAC AGTAAAGTCT CCTTTAAGAA   
  
  
- CGATCCTTCG AGAAGAAACT CGATAAAGGA AAGAACTTCG ACCGGATAAA CCACTGTTTG GTTAGCTATT   
  
  
- AGCTTCGGTA CCTCCCCCTT TTCTACCAAG TATAATAACT AGACTTACGT AGTCTTGGAC GAGTTACCTA   
  
  
- ACGGGAATAA GTCCGAAACT CACGTTCCGG ACCCCCAGGA GGAGTAAACT CTTAATGGCC ACAAGTAGTT   
  
  
- GTGTTTCTCC AAGATCTAGT TCACCGAGTA TCCCACTGAG TTCTTCGACT CTTTAACCTA AACGGTAAAG   
  
  
- TCAAGTTAGG ACACCAATCG TTCAACCTTT TGGAACTACA ACTTTTCGAC ACACAATTCT GACCACTCCG   
  
  
- AGATCGGTAG TCAAGCCAGG AAGTTGACGT ATGGGAAAAC CCAAGACTAC TACTCGGGGA TTCCTTTTCA   
  
  
- AGTGGAAATC GGAACTACTT CATACGTTTA CCCCGATTAT CCGTTTCGGG CTCATTACTA AGCCGAAGAA   
  
  
- GTAGTGGAGG AAGCGAGTTA TGAAGTTGGT TCGACCTACC AAAGGAGTCG CGAAACACCC CTAACAGGGG   
  
  
- TTTCTAATAC CACTATCGAC TCGTGCTAAG GTTAGTGTTA CCAAGACCTG AATACCTCTC CAACAGTCTT   
  
  
- CGTGACATGA AGATACGTCG CGACAAACTG ACGAATCTTA GGTGGGACGG TTCTTGTAGA CAGCTTTCTT   
  
  
- CCGCCCATCT CTTCTACGAG GATCCACTTC TCTAGTTCTT GTAATATAGT ACGCTCCCTC CTCTTTCCTC   
  
  
- TCTTTCCGTA CTCTTCTAGC TCTTCACCTA CGTCTCCGAT CTGTACCGAC CTAAGCCCTA GCAAGGAAAC   
  
  
- TCGATATACC CATACGACGT TCGTTCCGTT AACGAAGTCC CGATACCAAC ACTACCAATA TCTCACTTTC   
  
  
- TCTTACCAAC ACAACAGTAG ACAACCGTTC TAGCGGGGGA GAAAAGCCAT AGTCGTACCT CCACATCCTC   
  
  
- TAC

+     Myb-binding site

| Site Name | Organism | Position | Strand | Matrix score. | sequence | function |
| --- | --- | --- | --- | --- | --- | --- |
| Myb-binding site | Nicotiana tabacum | 1929 | - | 6 | CAACAG |  |
| Myb-binding site | Nicotiana tabacum | 3314 | - | 6 | CAACAG |  |

>HU08G00367.1   
+ -Up\_Stream \_Len000ATGCGC CTTTCTGCTA ACTCATTATC TTTCCTTCTG AGAGGGGACA ACTTATATAA   
  
  
+ ATTGATAGGT ATTTTCAAAT CAATAGACCA GCTACCATAT ATGATAATTT TTATTACTTT TCTAAATCCA   
  
  
+ TCAACTATTT TCACAATCCT CCTTTTTCTA GAAAAATAAA ATTTGCTTAA TTCAAATCAC CTAAAATTAC   
  
  
+ TAAATCTATA GTCCTAAATC TACAAACTCA TTTGTGTTGC AACTCATGAT TTAAGTGGAA CACTAACCTT   
  
  
+ AGGAGATATG TTGTCAAAAG AAGAATTCGA TGACATATTT TCCAACAAAA GGCCACCTAA CTTCATGATT   
  
  
+ TAAAAAGGAG GAGTCTAAGC ACATCATGGA TGACTAATTT TTGATGTATT GTAACTACGT ATTGAGATAA   
  
  
+ CTTGAATGAA TCGTGACTAG AATATATCTA TTTTCAAAAA TAAAAATTCC CTAAAAGAAA AGGATAAAAA   
  
  
+ ACTATATAAT ACTAAAAAAT TCTAACTATA ATAACTAGGC TTATTAAATT TTTATAGTGA AAATTTATTT   
  
  
+ CTACTTATAA CAACATCACT TACTAAGTAG CTTATTTGTG TCTTCTTTTC TAAATAATAA TGGTAACTAT   
  
  
+ GTGTCTTTTG TAAATAACTT ATTATCTATT TTATAAATCA CCGTGTAAAG CACGGGTCTA TACTAGTTAA   
  
  
+ TTTAAATAGC ATTGTTAAAT CAAGTAAGAT ACAAACAAAT TTATTTACAA CTGTAAGTGG TCCATAAAAA   
  
  
+ TAATAGCCAT TACAAAAAAT AGTACTACTG TTTTATGAAA TGATTTTAAT ATTCTAAAAT TCCAATAAAA   
  
  
+ CATGTTATAA TCTATGATTA ATTTTAAAAC ATGATTACAT GTCAGCATAA TTTGTATTAT TGTACAGCAA   
  
  
+ ATGAAAGTTG GAGCTAAAAG AAAATGGGGA AATAAAGGCA GACAAGTAAA GATAAAATAT GCTATCTACT   
  
  
+ TTTGGAGGTA TCCTTAACCA CATTGTCTCG TTTGTCTTCA TTAGGGAGAA GTGGGATAAA TACCAAAATT   
  
  
+ TGTATGGTCA TTGCGTGGGG TTTGCTTCCA AACAAAATGG CTTTTTGGAA GCAAATTTTG TGAGCTTCTA   
  
  
+ TGCAAATTTC CACGCTTAAC CCCACAAGCA AACAAAAACA GACAGTGGTT TTTGTTTGTC TTTGTTAGGG   
  
  
+ CATGTTTGGA GCCAACTGTT AATGGGAATA AACTGGGGAG GCTTTTTCCT TGGCTCTTTC CCCCTGACCC   
  
  
+ CAAACCCACT TCAGCTCAAT ACGAAACCAG CCATTGAAAA AGGAAGGGGG GGGGGGGGGT ATGTGAGAAA   
  
  
+ AAGACAAAAC TTTTTCCCGG AGAGAGAAGA TACATATGCA ACAAAGGCTC TCCTTCTCAT AAATTCCATA   
  
  
+ TTCCTCTGTT TTTCTAGGAG AGAGAAATAT GCAAAAAACA AATCAGTTGG CGACAAAAGT TACTACCACG   
  
  
+ CATTGTCTAC TGCTGCGTAC CCATTTTTGG AGACTTTAAA GAACGCCCCT CCCATCTTGA AAAGTGAAAA   
  
  
+ CCCCCTCTTT GAAACTGAAT TTCAGGGGAA AAAAACCTCA CCAAACCTCT AGAGAGAGCT TTTAGAGAGG   
  
  
+ GAAATACAAC GATTAGAAGG AGCAATTTGG GAAATTTCTT TGGGAATTTG AATGGGTTTT GAGTGAATTG   
  
  
+ CAAATCCCAG AAAAGTTTTG GCAAGTACCG ATCTACAGTT CTCTCCTCTT CGTGTTTGGT AGATCCCCTG   
  
  
+ TTTCCTCTGT TTCATTTAGG GTACTTCTCA TTCATCCTCC CCACCCCCTT AATCGGATCT TCCTGTCGAG   
  
  
+ TCACTTTATG CTAATATTTT TTCAGTGGAT TTTTAGTTAA CCCTGTTCAT TTTTCCATTC TGTGTCCCAT   
  
  
+ TTCTCTCTTT TTCATTCATA AGTTGCCGGT TTATCTGTTG GGTGCAGCTT AGTCACAATA ATTTCTGTGT   
  
  
+ TAGGCTTCTT TACGGTTAAA AAAAAAGGAG GCACTCTTTT CGGTGTGATT GTTTATGGGA CCAATGATTC   
  
  
+ AAGATGATGG GTCATCAGTA ACTTCATCAC CCCTTCAATT TTTCTCCATG ATGTCTCCCA ATTTAGGTTC   
  
  
+ TTCCTACCCT TGGCTCAGAG AGCTAAAACC TGAAGAAAGA GGTCTTTACT TGATACATTT GTTGCTCTCT   
  
  
+ TGTGCAAATC ATGTCTCTAG TGGTAGCCTA GACAATGCGA ACTTAGCCCT CGAACAAATC TCTCAGCTTG   
  
  
+ CTGCCCCTGA TGGGGATACA ATGCAGCGTA TGGCTTCTTA TTTTGCTGAA GCCCTGGCTG AGAGGATCCT   
  
  
+ CAAGTCATGG CCTGGCATGT ATAGAGCCCT TCATTTTACG AAAATGCCTG TCATTTCAGA GGAAATTCTT   
  
  
+ GCTAGGAAGC TCTTCTTTGA GCTATTTCCT TTCTTGAAGC TGGCCTATTT GGTGACAAAC CAATCGATAA   
  
  
+ TCGAAGCCAT GGAGGGGGAA AAGATGGTTC ATATTATTGA TCTGAATGCA TCAGAACCTG CTCAATGGAT   
  
  
+ TGCCCTTATT CAGGCTTTGA GTGCAAGGCC TGGGGGTCCT CCTCATTTGA GAATTACCGG TGTTCATCAA   
  
  
+ CACAAAGAGG TTCTAGATCA AGTGGCTCAT AGGGTGACTC AAGAAGCTGA GAAATTGGAT TTGCCATTTC   
  
  
+ AGTTCAATCC TGTGGTTAGC AAGTTGGAAA ACCTTGATGT TGAAAAGCTG TGTGTTAAGA CTGGTGAGGC   
  
  
+ TCTAGCCATC AGTTCGGTCC TTCAACTGCA TACCCTTTTG GGTTCTGATG ATGAGCCCCT AAGGAAAAGT   
  
  
+ TCACCTTTAG CCTTGATGAA GTATGCAAAT GGGGCTAATA GGCAAAGCCC GAGTAATGAT TCGGCTTCTT   
  
  
+ CATCACCTCC TTCGCTCAAT ACTTCAACCA AGCTGGATGG TTTCCTCAGC GCTTTGTGGG GATTGTCCCC   
  
  
+ AAAGATTATG GTGATAGCTG AGCACGATTC CAATCACAAT GGTTCTGGAC TTATGGAGAG GTTGTCAGAA   
  
  
+ GCACTGTACT TCTATGCAGC GCTGTTTGAC TGCTTAGAAT CCACCCTGCC AAGAACATCT GTCGAAAGAA   
  
  
+ GGCGGGTAGA GAAGATGCTC CTAGGTGAAG AGATCAAGAA CATTATATCA TGCGAGGGAG GAGAAAGGAG   
  
  
+ AGAAAGGCAT GAGAAGATCG AGAAGTGGAT GCAGAGGCTA GACATGGCTG GATTCGGGAT CGTTCCTTTG   
  
  
+ AGCTATATGG GTATGCTGCA AGCAAGGCAA TTGCTTCAGG GCTATGGTTG TGATGGTTAT AGAGTGAAAG   
  
  
+ AGAATGGTTG TGTTGTCATC TGTTGGCAAG ATCGCCCCCT CTTTTCGGTA TCAGCATGGA GGTGTAGGAG   
  
  
+ ATG  

- -Up\_Stream \_Len000TACGCG GAAAGACGAT TGAGTAATAG AAAGGAAGAC TCTCCCCTGT TGAATATATT   
  
  
- TAACTATCCA TAAAAGTTTA GTTATCTGGT CGATGGTATA TACTATTAAA AATAATGAAA AGATTTAGGT   
  
  
- AGTTGATAAA AGTGTTAGGA GGAAAAAGAT CTTTTTATTT TAAACGAATT AAGTTTAGTG GATTTTAATG   
  
  
- ATTTAGATAT CAGGATTTAG ATGTTTGAGT AAACACAACG TTGAGTACTA AATTCACCTT GTGATTGGAA   
  
  
- TCCTCTATAC AACAGTTTTC TTCTTAAGCT ACTGTATAAA AGGTTGTTTT CCGGTGGATT GAAGTACTAA   
  
  
- ATTTTTCCTC CTCAGATTCG TGTAGTACCT ACTGATTAAA AACTACATAA CATTGATGCA TAACTCTATT   
  
  
- GAACTTACTT AGCACTGATC TTATATAGAT AAAAGTTTTT ATTTTTAAGG GATTTTCTTT TCCTATTTTT   
  
  
- TGATATATTA TGATTTTTTA AGATTGATAT TATTGATCCG AATAATTTAA AAATATCACT TTTAAATAAA   
  
  
- GATGAATATT GTTGTAGTGA ATGATTCATC GAATAAACAC AGAAGAAAAG ATTTATTATT ACCATTGATA   
  
  
- CACAGAAAAC ATTTATTGAA TAATAGATAA AATATTTAGT GGCACATTTC GTGCCCAGAT ATGATCAATT   
  
  
- AAATTTATCG TAACAATTTA GTTCATTCTA TGTTTGTTTA AATAAATGTT GACATTCACC AGGTATTTTT   
  
  
- ATTATCGGTA ATGTTTTTTA TCATGATGAC AAAATACTTT ACTAAAATTA TAAGATTTTA AGGTTATTTT   
  
  
- GTACAATATT AGATACTAAT TAAAATTTTG TACTAATGTA CAGTCGTATT AAACATAATA ACATGTCGTT   
  
  
- TACTTTCAAC CTCGATTTTC TTTTACCCCT TTATTTCCGT CTGTTCATTT CTATTTTATA CGATAGATGA   
  
  
- AAACCTCCAT AGGAATTGGT GTAACAGAGC AAACAGAAGT AATCCCTCTT CACCCTATTT ATGGTTTTAA   
  
  
- ACATACCAGT AACGCACCCC AAACGAAGGT TTGTTTTACC GAAAAACCTT CGTTTAAAAC ACTCGAAGAT   
  
  
- ACGTTTAAAG GTGCGAATTG GGGTGTTCGT TTGTTTTTGT CTGTCACCAA AAACAAACAG AAACAATCCC   
  
  
- GTACAAACCT CGGTTGACAA TTACCCTTAT TTGACCCCTC CGAAAAAGGA ACCGAGAAAG GGGGACTGGG   
  
  
- GTTTGGGTGA AGTCGAGTTA TGCTTTGGTC GGTAACTTTT TCCTTCCCCC CCCCCCCCCA TACACTCTTT   
  
  
- TTCTGTTTTG AAAAAGGGCC TCTCTCTTCT ATGTATACGT TGTTTCCGAG AGGAAGAGTA TTTAAGGTAT   
  
  
- AAGGAGACAA AAAGATCCTC TCTCTTTATA CGTTTTTTGT TTAGTCAACC GCTGTTTTCA ATGATGGTGC   
  
  
- GTAACAGATG ACGACGCATG GGTAAAAACC TCTGAAATTT CTTGCGGGGA GGGTAGAACT TTTCACTTTT   
  
  
- GGGGGAGAAA CTTTGACTTA AAGTCCCCTT TTTTTGGAGT GGTTTGGAGA TCTCTCTCGA AAATCTCTCC   
  
  
- CTTTATGTTG CTAATCTTCC TCGTTAAACC CTTTAAAGAA ACCCTTAAAC TTACCCAAAA CTCACTTAAC   
  
  
- GTTTAGGGTC TTTTCAAAAC CGTTCATGGC TAGATGTCAA GAGAGGAGAA GCACAAACCA TCTAGGGGAC   
  
  
- AAAGGAGACA AAGTAAATCC CATGAAGAGT AAGTAGGAGG GGTGGGGGAA TTAGCCTAGA AGGACAGCTC   
  
  
- AGTGAAATAC GATTATAAAA AAGTCACCTA AAAATCAATT GGGACAAGTA AAAAGGTAAG ACACAGGGTA   
  
  
- AAGAGAGAAA AAGTAAGTAT TCAACGGCCA AATAGACAAC CCACGTCGAA TCAGTGTTAT TAAAGACACA   
  
  
- ATCCGAAGAA ATGCCAATTT TTTTTTCCTC CGTGAGAAAA GCCACACTAA CAAATACCCT GGTTACTAAG   
  
  
- TTCTACTACC CAGTAGTCAT TGAAGTAGTG GGGAAGTTAA AAAGAGGTAC TACAGAGGGT TAAATCCAAG   
  
  
- AAGGATGGGA ACCGAGTCTC TCGATTTTGG ACTTCTTTCT CCAGAAATGA ACTATGTAAA CAACGAGAGA   
  
  
- ACACGTTTAG TACAGAGATC ACCATCGGAT CTGTTACGCT TGAATCGGGA GCTTGTTTAG AGAGTCGAAC   
  
  
- GACGGGGACT ACCCCTATGT TACGTCGCAT ACCGAAGAAT AAAACGACTT CGGGACCGAC TCTCCTAGGA   
  
  
- GTTCAGTACC GGACCGTACA TATCTCGGGA AGTAAAATGC TTTTACGGAC AGTAAAGTCT CCTTTAAGAA   
  
  
- CGATCCTTCG AGAAGAAACT CGATAAAGGA AAGAACTTCG ACCGGATAAA CCACTGTTTG GTTAGCTATT   
  
  
- AGCTTCGGTA CCTCCCCCTT TTCTACCAAG TATAATAACT AGACTTACGT AGTCTTGGAC GAGTTACCTA   
  
  
- ACGGGAATAA GTCCGAAACT CACGTTCCGG ACCCCCAGGA GGAGTAAACT CTTAATGGCC ACAAGTAGTT   
  
  
- GTGTTTCTCC AAGATCTAGT TCACCGAGTA TCCCACTGAG TTCTTCGACT CTTTAACCTA AACGGTAAAG   
  
  
- TCAAGTTAGG ACACCAATCG TTCAACCTTT TGGAACTACA ACTTTTCGAC ACACAATTCT GACCACTCCG   
  
  
- AGATCGGTAG TCAAGCCAGG AAGTTGACGT ATGGGAAAAC CCAAGACTAC TACTCGGGGA TTCCTTTTCA   
  
  
- AGTGGAAATC GGAACTACTT CATACGTTTA CCCCGATTAT CCGTTTCGGG CTCATTACTA AGCCGAAGAA   
  
  
- GTAGTGGAGG AAGCGAGTTA TGAAGTTGGT TCGACCTACC AAAGGAGTCG CGAAACACCC CTAACAGGGG   
  
  
- TTTCTAATAC CACTATCGAC TCGTGCTAAG GTTAGTGTTA CCAAGACCTG AATACCTCTC CAACAGTCTT   
  
  
- CGTGACATGA AGATACGTCG CGACAAACTG ACGAATCTTA GGTGGGACGG TTCTTGTAGA CAGCTTTCTT   
  
  
- CCGCCCATCT CTTCTACGAG GATCCACTTC TCTAGTTCTT GTAATATAGT ACGCTCCCTC CTCTTTCCTC   
  
  
- TCTTTCCGTA CTCTTCTAGC TCTTCACCTA CGTCTCCGAT CTGTACCGAC CTAAGCCCTA GCAAGGAAAC   
  
  
- TCGATATACC CATACGACGT TCGTTCCGTT AACGAAGTCC CGATACCAAC ACTACCAATA TCTCACTTTC   
  
  
- TCTTACCAAC ACAACAGTAG ACAACCGTTC TAGCGGGGGA GAAAAGCCAT AGTCGTACCT CCACATCCTC   
  
  
- TAC

+     P-box

| Site Name | Organism | Position | Strand | Matrix score. | sequence | function |
| --- | --- | --- | --- | --- | --- | --- |
| P-box | Oryza sativa | 330 | - | 7 | CCTTTTG | gibberellin-responsive element |
| P-box | Oryza sativa | 2768 | + | 7 | CCTTTTG | gibberellin-responsive element |

>HU08G00367.1   
+ -Up\_Stream \_Len000ATGCGC CTTTCTGCTA ACTCATTATC TTTCCTTCTG AGAGGGGACA ACTTATATAA   
  
  
+ ATTGATAGGT ATTTTCAAAT CAATAGACCA GCTACCATAT ATGATAATTT TTATTACTTT TCTAAATCCA   
  
  
+ TCAACTATTT TCACAATCCT CCTTTTTCTA GAAAAATAAA ATTTGCTTAA TTCAAATCAC CTAAAATTAC   
  
  
+ TAAATCTATA GTCCTAAATC TACAAACTCA TTTGTGTTGC AACTCATGAT TTAAGTGGAA CACTAACCTT   
  
  
+ AGGAGATATG TTGTCAAAAG AAGAATTCGA TGACATATTT TCCAACAAAA GGCCACCTAA CTTCATGATT   
  
  
+ TAAAAAGGAG GAGTCTAAGC ACATCATGGA TGACTAATTT TTGATGTATT GTAACTACGT ATTGAGATAA   
  
  
+ CTTGAATGAA TCGTGACTAG AATATATCTA TTTTCAAAAA TAAAAATTCC CTAAAAGAAA AGGATAAAAA   
  
  
+ ACTATATAAT ACTAAAAAAT TCTAACTATA ATAACTAGGC TTATTAAATT TTTATAGTGA AAATTTATTT   
  
  
+ CTACTTATAA CAACATCACT TACTAAGTAG CTTATTTGTG TCTTCTTTTC TAAATAATAA TGGTAACTAT   
  
  
+ GTGTCTTTTG TAAATAACTT ATTATCTATT TTATAAATCA CCGTGTAAAG CACGGGTCTA TACTAGTTAA   
  
  
+ TTTAAATAGC ATTGTTAAAT CAAGTAAGAT ACAAACAAAT TTATTTACAA CTGTAAGTGG TCCATAAAAA   
  
  
+ TAATAGCCAT TACAAAAAAT AGTACTACTG TTTTATGAAA TGATTTTAAT ATTCTAAAAT TCCAATAAAA   
  
  
+ CATGTTATAA TCTATGATTA ATTTTAAAAC ATGATTACAT GTCAGCATAA TTTGTATTAT TGTACAGCAA   
  
  
+ ATGAAAGTTG GAGCTAAAAG AAAATGGGGA AATAAAGGCA GACAAGTAAA GATAAAATAT GCTATCTACT   
  
  
+ TTTGGAGGTA TCCTTAACCA CATTGTCTCG TTTGTCTTCA TTAGGGAGAA GTGGGATAAA TACCAAAATT   
  
  
+ TGTATGGTCA TTGCGTGGGG TTTGCTTCCA AACAAAATGG CTTTTTGGAA GCAAATTTTG TGAGCTTCTA   
  
  
+ TGCAAATTTC CACGCTTAAC CCCACAAGCA AACAAAAACA GACAGTGGTT TTTGTTTGTC TTTGTTAGGG   
  
  
+ CATGTTTGGA GCCAACTGTT AATGGGAATA AACTGGGGAG GCTTTTTCCT TGGCTCTTTC CCCCTGACCC   
  
  
+ CAAACCCACT TCAGCTCAAT ACGAAACCAG CCATTGAAAA AGGAAGGGGG GGGGGGGGGT ATGTGAGAAA   
  
  
+ AAGACAAAAC TTTTTCCCGG AGAGAGAAGA TACATATGCA ACAAAGGCTC TCCTTCTCAT AAATTCCATA   
  
  
+ TTCCTCTGTT TTTCTAGGAG AGAGAAATAT GCAAAAAACA AATCAGTTGG CGACAAAAGT TACTACCACG   
  
  
+ CATTGTCTAC TGCTGCGTAC CCATTTTTGG AGACTTTAAA GAACGCCCCT CCCATCTTGA AAAGTGAAAA   
  
  
+ CCCCCTCTTT GAAACTGAAT TTCAGGGGAA AAAAACCTCA CCAAACCTCT AGAGAGAGCT TTTAGAGAGG   
  
  
+ GAAATACAAC GATTAGAAGG AGCAATTTGG GAAATTTCTT TGGGAATTTG AATGGGTTTT GAGTGAATTG   
  
  
+ CAAATCCCAG AAAAGTTTTG GCAAGTACCG ATCTACAGTT CTCTCCTCTT CGTGTTTGGT AGATCCCCTG   
  
  
+ TTTCCTCTGT TTCATTTAGG GTACTTCTCA TTCATCCTCC CCACCCCCTT AATCGGATCT TCCTGTCGAG   
  
  
+ TCACTTTATG CTAATATTTT TTCAGTGGAT TTTTAGTTAA CCCTGTTCAT TTTTCCATTC TGTGTCCCAT   
  
  
+ TTCTCTCTTT TTCATTCATA AGTTGCCGGT TTATCTGTTG GGTGCAGCTT AGTCACAATA ATTTCTGTGT   
  
  
+ TAGGCTTCTT TACGGTTAAA AAAAAAGGAG GCACTCTTTT CGGTGTGATT GTTTATGGGA CCAATGATTC   
  
  
+ AAGATGATGG GTCATCAGTA ACTTCATCAC CCCTTCAATT TTTCTCCATG ATGTCTCCCA ATTTAGGTTC   
  
  
+ TTCCTACCCT TGGCTCAGAG AGCTAAAACC TGAAGAAAGA GGTCTTTACT TGATACATTT GTTGCTCTCT   
  
  
+ TGTGCAAATC ATGTCTCTAG TGGTAGCCTA GACAATGCGA ACTTAGCCCT CGAACAAATC TCTCAGCTTG   
  
  
+ CTGCCCCTGA TGGGGATACA ATGCAGCGTA TGGCTTCTTA TTTTGCTGAA GCCCTGGCTG AGAGGATCCT   
  
  
+ CAAGTCATGG CCTGGCATGT ATAGAGCCCT TCATTTTACG AAAATGCCTG TCATTTCAGA GGAAATTCTT   
  
  
+ GCTAGGAAGC TCTTCTTTGA GCTATTTCCT TTCTTGAAGC TGGCCTATTT GGTGACAAAC CAATCGATAA   
  
  
+ TCGAAGCCAT GGAGGGGGAA AAGATGGTTC ATATTATTGA TCTGAATGCA TCAGAACCTG CTCAATGGAT   
  
  
+ TGCCCTTATT CAGGCTTTGA GTGCAAGGCC TGGGGGTCCT CCTCATTTGA GAATTACCGG TGTTCATCAA   
  
  
+ CACAAAGAGG TTCTAGATCA AGTGGCTCAT AGGGTGACTC AAGAAGCTGA GAAATTGGAT TTGCCATTTC   
  
  
+ AGTTCAATCC TGTGGTTAGC AAGTTGGAAA ACCTTGATGT TGAAAAGCTG TGTGTTAAGA CTGGTGAGGC   
  
  
+ TCTAGCCATC AGTTCGGTCC TTCAACTGCA TACCCTTTTG GGTTCTGATG ATGAGCCCCT AAGGAAAAGT   
  
  
+ TCACCTTTAG CCTTGATGAA GTATGCAAAT GGGGCTAATA GGCAAAGCCC GAGTAATGAT TCGGCTTCTT   
  
  
+ CATCACCTCC TTCGCTCAAT ACTTCAACCA AGCTGGATGG TTTCCTCAGC GCTTTGTGGG GATTGTCCCC   
  
  
+ AAAGATTATG GTGATAGCTG AGCACGATTC CAATCACAAT GGTTCTGGAC TTATGGAGAG GTTGTCAGAA   
  
  
+ GCACTGTACT TCTATGCAGC GCTGTTTGAC TGCTTAGAAT CCACCCTGCC AAGAACATCT GTCGAAAGAA   
  
  
+ GGCGGGTAGA GAAGATGCTC CTAGGTGAAG AGATCAAGAA CATTATATCA TGCGAGGGAG GAGAAAGGAG   
  
  
+ AGAAAGGCAT GAGAAGATCG AGAAGTGGAT GCAGAGGCTA GACATGGCTG GATTCGGGAT CGTTCCTTTG   
  
  
+ AGCTATATGG GTATGCTGCA AGCAAGGCAA TTGCTTCAGG GCTATGGTTG TGATGGTTAT AGAGTGAAAG   
  
  
+ AGAATGGTTG TGTTGTCATC TGTTGGCAAG ATCGCCCCCT CTTTTCGGTA TCAGCATGGA GGTGTAGGAG   
  
  
+ ATG  

- -Up\_Stream \_Len000TACGCG GAAAGACGAT TGAGTAATAG AAAGGAAGAC TCTCCCCTGT TGAATATATT   
  
  
- TAACTATCCA TAAAAGTTTA GTTATCTGGT CGATGGTATA TACTATTAAA AATAATGAAA AGATTTAGGT   
  
  
- AGTTGATAAA AGTGTTAGGA GGAAAAAGAT CTTTTTATTT TAAACGAATT AAGTTTAGTG GATTTTAATG   
  
  
- ATTTAGATAT CAGGATTTAG ATGTTTGAGT AAACACAACG TTGAGTACTA AATTCACCTT GTGATTGGAA   
  
  
- TCCTCTATAC AACAGTTTTC TTCTTAAGCT ACTGTATAAA AGGTTGTTTT CCGGTGGATT GAAGTACTAA   
  
  
- ATTTTTCCTC CTCAGATTCG TGTAGTACCT ACTGATTAAA AACTACATAA CATTGATGCA TAACTCTATT   
  
  
- GAACTTACTT AGCACTGATC TTATATAGAT AAAAGTTTTT ATTTTTAAGG GATTTTCTTT TCCTATTTTT   
  
  
- TGATATATTA TGATTTTTTA AGATTGATAT TATTGATCCG AATAATTTAA AAATATCACT TTTAAATAAA   
  
  
- GATGAATATT GTTGTAGTGA ATGATTCATC GAATAAACAC AGAAGAAAAG ATTTATTATT ACCATTGATA   
  
  
- CACAGAAAAC ATTTATTGAA TAATAGATAA AATATTTAGT GGCACATTTC GTGCCCAGAT ATGATCAATT   
  
  
- AAATTTATCG TAACAATTTA GTTCATTCTA TGTTTGTTTA AATAAATGTT GACATTCACC AGGTATTTTT   
  
  
- ATTATCGGTA ATGTTTTTTA TCATGATGAC AAAATACTTT ACTAAAATTA TAAGATTTTA AGGTTATTTT   
  
  
- GTACAATATT AGATACTAAT TAAAATTTTG TACTAATGTA CAGTCGTATT AAACATAATA ACATGTCGTT   
  
  
- TACTTTCAAC CTCGATTTTC TTTTACCCCT TTATTTCCGT CTGTTCATTT CTATTTTATA CGATAGATGA   
  
  
- AAACCTCCAT AGGAATTGGT GTAACAGAGC AAACAGAAGT AATCCCTCTT CACCCTATTT ATGGTTTTAA   
  
  
- ACATACCAGT AACGCACCCC AAACGAAGGT TTGTTTTACC GAAAAACCTT CGTTTAAAAC ACTCGAAGAT   
  
  
- ACGTTTAAAG GTGCGAATTG GGGTGTTCGT TTGTTTTTGT CTGTCACCAA AAACAAACAG AAACAATCCC   
  
  
- GTACAAACCT CGGTTGACAA TTACCCTTAT TTGACCCCTC CGAAAAAGGA ACCGAGAAAG GGGGACTGGG   
  
  
- GTTTGGGTGA AGTCGAGTTA TGCTTTGGTC GGTAACTTTT TCCTTCCCCC CCCCCCCCCA TACACTCTTT   
  
  
- TTCTGTTTTG AAAAAGGGCC TCTCTCTTCT ATGTATACGT TGTTTCCGAG AGGAAGAGTA TTTAAGGTAT   
  
  
- AAGGAGACAA AAAGATCCTC TCTCTTTATA CGTTTTTTGT TTAGTCAACC GCTGTTTTCA ATGATGGTGC   
  
  
- GTAACAGATG ACGACGCATG GGTAAAAACC TCTGAAATTT CTTGCGGGGA GGGTAGAACT TTTCACTTTT   
  
  
- GGGGGAGAAA CTTTGACTTA AAGTCCCCTT TTTTTGGAGT GGTTTGGAGA TCTCTCTCGA AAATCTCTCC   
  
  
- CTTTATGTTG CTAATCTTCC TCGTTAAACC CTTTAAAGAA ACCCTTAAAC TTACCCAAAA CTCACTTAAC   
  
  
- GTTTAGGGTC TTTTCAAAAC CGTTCATGGC TAGATGTCAA GAGAGGAGAA GCACAAACCA TCTAGGGGAC   
  
  
- AAAGGAGACA AAGTAAATCC CATGAAGAGT AAGTAGGAGG GGTGGGGGAA TTAGCCTAGA AGGACAGCTC   
  
  
- AGTGAAATAC GATTATAAAA AAGTCACCTA AAAATCAATT GGGACAAGTA AAAAGGTAAG ACACAGGGTA   
  
  
- AAGAGAGAAA AAGTAAGTAT TCAACGGCCA AATAGACAAC CCACGTCGAA TCAGTGTTAT TAAAGACACA   
  
  
- ATCCGAAGAA ATGCCAATTT TTTTTTCCTC CGTGAGAAAA GCCACACTAA CAAATACCCT GGTTACTAAG   
  
  
- TTCTACTACC CAGTAGTCAT TGAAGTAGTG GGGAAGTTAA AAAGAGGTAC TACAGAGGGT TAAATCCAAG   
  
  
- AAGGATGGGA ACCGAGTCTC TCGATTTTGG ACTTCTTTCT CCAGAAATGA ACTATGTAAA CAACGAGAGA   
  
  
- ACACGTTTAG TACAGAGATC ACCATCGGAT CTGTTACGCT TGAATCGGGA GCTTGTTTAG AGAGTCGAAC   
  
  
- GACGGGGACT ACCCCTATGT TACGTCGCAT ACCGAAGAAT AAAACGACTT CGGGACCGAC TCTCCTAGGA   
  
  
- GTTCAGTACC GGACCGTACA TATCTCGGGA AGTAAAATGC TTTTACGGAC AGTAAAGTCT CCTTTAAGAA   
  
  
- CGATCCTTCG AGAAGAAACT CGATAAAGGA AAGAACTTCG ACCGGATAAA CCACTGTTTG GTTAGCTATT   
  
  
- AGCTTCGGTA CCTCCCCCTT TTCTACCAAG TATAATAACT AGACTTACGT AGTCTTGGAC GAGTTACCTA   
  
  
- ACGGGAATAA GTCCGAAACT CACGTTCCGG ACCCCCAGGA GGAGTAAACT CTTAATGGCC ACAAGTAGTT   
  
  
- GTGTTTCTCC AAGATCTAGT TCACCGAGTA TCCCACTGAG TTCTTCGACT CTTTAACCTA AACGGTAAAG   
  
  
- TCAAGTTAGG ACACCAATCG TTCAACCTTT TGGAACTACA ACTTTTCGAC ACACAATTCT GACCACTCCG   
  
  
- AGATCGGTAG TCAAGCCAGG AAGTTGACGT ATGGGAAAAC CCAAGACTAC TACTCGGGGA TTCCTTTTCA   
  
  
- AGTGGAAATC GGAACTACTT CATACGTTTA CCCCGATTAT CCGTTTCGGG CTCATTACTA AGCCGAAGAA   
  
  
- GTAGTGGAGG AAGCGAGTTA TGAAGTTGGT TCGACCTACC AAAGGAGTCG CGAAACACCC CTAACAGGGG   
  
  
- TTTCTAATAC CACTATCGAC TCGTGCTAAG GTTAGTGTTA CCAAGACCTG AATACCTCTC CAACAGTCTT   
  
  
- CGTGACATGA AGATACGTCG CGACAAACTG ACGAATCTTA GGTGGGACGG TTCTTGTAGA CAGCTTTCTT   
  
  
- CCGCCCATCT CTTCTACGAG GATCCACTTC TCTAGTTCTT GTAATATAGT ACGCTCCCTC CTCTTTCCTC   
  
  
- TCTTTCCGTA CTCTTCTAGC TCTTCACCTA CGTCTCCGAT CTGTACCGAC CTAAGCCCTA GCAAGGAAAC   
  
  
- TCGATATACC CATACGACGT TCGTTCCGTT AACGAAGTCC CGATACCAAC ACTACCAATA TCTCACTTTC   
  
  
- TCTTACCAAC ACAACAGTAG ACAACCGTTC TAGCGGGGGA GAAAAGCCAT AGTCGTACCT CCACATCCTC   
  
  
- TAC

+     STRE

| Site Name | Organism | Position | Strand | Matrix score. | sequence | function |
| --- | --- | --- | --- | --- | --- | --- |
| STRE | Arabidopsis thaliana | 57 | + | 5 | AGGGG |  |
| STRE | Arabidopsis thaliana | 1546 | - | 5 | AGGGG |  |
| STRE | Arabidopsis thaliana | 3330 | - | 5 | AGGGG |  |
| STRE | Arabidopsis thaliana | 2790 | - | 5 | AGGGG |  |
| STRE | Arabidopsis thaliana | 2248 | - | 5 | AGGGG |  |
| STRE | Arabidopsis thaliana | 2467 | + | 5 | AGGGG |  |
| STRE | Arabidopsis thaliana | 2064 | - | 5 | AGGGG |  |
| STRE | Arabidopsis thaliana | 1309 | + | 5 | AGGGG |  |
| STRE | Arabidopsis thaliana | 1799 | - | 5 | AGGGG |  |
| STRE | Arabidopsis thaliana | 1255 | - | 5 | AGGGG |  |
| STRE | Arabidopsis thaliana | 1568 | + | 5 | AGGGG |  |
| STRE | Arabidopsis thaliana | 1520 | - | 5 | AGGGG |  |
| STRE | Arabidopsis thaliana | 1749 | - | 5 | AGGGG |  |

>HU08G00367.1   
+ -Up\_Stream \_Len000ATGCGC CTTTCTGCTA ACTCATTATC TTTCCTTCTG AGAGGGGACA ACTTATATAA   
  
  
+ ATTGATAGGT ATTTTCAAAT CAATAGACCA GCTACCATAT ATGATAATTT TTATTACTTT TCTAAATCCA   
  
  
+ TCAACTATTT TCACAATCCT CCTTTTTCTA GAAAAATAAA ATTTGCTTAA TTCAAATCAC CTAAAATTAC   
  
  
+ TAAATCTATA GTCCTAAATC TACAAACTCA TTTGTGTTGC AACTCATGAT TTAAGTGGAA CACTAACCTT   
  
  
+ AGGAGATATG TTGTCAAAAG AAGAATTCGA TGACATATTT TCCAACAAAA GGCCACCTAA CTTCATGATT   
  
  
+ TAAAAAGGAG GAGTCTAAGC ACATCATGGA TGACTAATTT TTGATGTATT GTAACTACGT ATTGAGATAA   
  
  
+ CTTGAATGAA TCGTGACTAG AATATATCTA TTTTCAAAAA TAAAAATTCC CTAAAAGAAA AGGATAAAAA   
  
  
+ ACTATATAAT ACTAAAAAAT TCTAACTATA ATAACTAGGC TTATTAAATT TTTATAGTGA AAATTTATTT   
  
  
+ CTACTTATAA CAACATCACT TACTAAGTAG CTTATTTGTG TCTTCTTTTC TAAATAATAA TGGTAACTAT   
  
  
+ GTGTCTTTTG TAAATAACTT ATTATCTATT TTATAAATCA CCGTGTAAAG CACGGGTCTA TACTAGTTAA   
  
  
+ TTTAAATAGC ATTGTTAAAT CAAGTAAGAT ACAAACAAAT TTATTTACAA CTGTAAGTGG TCCATAAAAA   
  
  
+ TAATAGCCAT TACAAAAAAT AGTACTACTG TTTTATGAAA TGATTTTAAT ATTCTAAAAT TCCAATAAAA   
  
  
+ CATGTTATAA TCTATGATTA ATTTTAAAAC ATGATTACAT GTCAGCATAA TTTGTATTAT TGTACAGCAA   
  
  
+ ATGAAAGTTG GAGCTAAAAG AAAATGGGGA AATAAAGGCA GACAAGTAAA GATAAAATAT GCTATCTACT   
  
  
+ TTTGGAGGTA TCCTTAACCA CATTGTCTCG TTTGTCTTCA TTAGGGAGAA GTGGGATAAA TACCAAAATT   
  
  
+ TGTATGGTCA TTGCGTGGGG TTTGCTTCCA AACAAAATGG CTTTTTGGAA GCAAATTTTG TGAGCTTCTA   
  
  
+ TGCAAATTTC CACGCTTAAC CCCACAAGCA AACAAAAACA GACAGTGGTT TTTGTTTGTC TTTGTTAGGG   
  
  
+ CATGTTTGGA GCCAACTGTT AATGGGAATA AACTGGGGAG GCTTTTTCCT TGGCTCTTTC CCCCTGACCC   
  
  
+ CAAACCCACT TCAGCTCAAT ACGAAACCAG CCATTGAAAA AGGAAGGGGG GGGGGGGGGT ATGTGAGAAA   
  
  
+ AAGACAAAAC TTTTTCCCGG AGAGAGAAGA TACATATGCA ACAAAGGCTC TCCTTCTCAT AAATTCCATA   
  
  
+ TTCCTCTGTT TTTCTAGGAG AGAGAAATAT GCAAAAAACA AATCAGTTGG CGACAAAAGT TACTACCACG   
  
  
+ CATTGTCTAC TGCTGCGTAC CCATTTTTGG AGACTTTAAA GAACGCCCCT CCCATCTTGA AAAGTGAAAA   
  
  
+ CCCCCTCTTT GAAACTGAAT TTCAGGGGAA AAAAACCTCA CCAAACCTCT AGAGAGAGCT TTTAGAGAGG   
  
  
+ GAAATACAAC GATTAGAAGG AGCAATTTGG GAAATTTCTT TGGGAATTTG AATGGGTTTT GAGTGAATTG   
  
  
+ CAAATCCCAG AAAAGTTTTG GCAAGTACCG ATCTACAGTT CTCTCCTCTT CGTGTTTGGT AGATCCCCTG   
  
  
+ TTTCCTCTGT TTCATTTAGG GTACTTCTCA TTCATCCTCC CCACCCCCTT AATCGGATCT TCCTGTCGAG   
  
  
+ TCACTTTATG CTAATATTTT TTCAGTGGAT TTTTAGTTAA CCCTGTTCAT TTTTCCATTC TGTGTCCCAT   
  
  
+ TTCTCTCTTT TTCATTCATA AGTTGCCGGT TTATCTGTTG GGTGCAGCTT AGTCACAATA ATTTCTGTGT   
  
  
+ TAGGCTTCTT TACGGTTAAA AAAAAAGGAG GCACTCTTTT CGGTGTGATT GTTTATGGGA CCAATGATTC   
  
  
+ AAGATGATGG GTCATCAGTA ACTTCATCAC CCCTTCAATT TTTCTCCATG ATGTCTCCCA ATTTAGGTTC   
  
  
+ TTCCTACCCT TGGCTCAGAG AGCTAAAACC TGAAGAAAGA GGTCTTTACT TGATACATTT GTTGCTCTCT   
  
  
+ TGTGCAAATC ATGTCTCTAG TGGTAGCCTA GACAATGCGA ACTTAGCCCT CGAACAAATC TCTCAGCTTG   
  
  
+ CTGCCCCTGA TGGGGATACA ATGCAGCGTA TGGCTTCTTA TTTTGCTGAA GCCCTGGCTG AGAGGATCCT   
  
  
+ CAAGTCATGG CCTGGCATGT ATAGAGCCCT TCATTTTACG AAAATGCCTG TCATTTCAGA GGAAATTCTT   
  
  
+ GCTAGGAAGC TCTTCTTTGA GCTATTTCCT TTCTTGAAGC TGGCCTATTT GGTGACAAAC CAATCGATAA   
  
  
+ TCGAAGCCAT GGAGGGGGAA AAGATGGTTC ATATTATTGA TCTGAATGCA TCAGAACCTG CTCAATGGAT   
  
  
+ TGCCCTTATT CAGGCTTTGA GTGCAAGGCC TGGGGGTCCT CCTCATTTGA GAATTACCGG TGTTCATCAA   
  
  
+ CACAAAGAGG TTCTAGATCA AGTGGCTCAT AGGGTGACTC AAGAAGCTGA GAAATTGGAT TTGCCATTTC   
  
  
+ AGTTCAATCC TGTGGTTAGC AAGTTGGAAA ACCTTGATGT TGAAAAGCTG TGTGTTAAGA CTGGTGAGGC   
  
  
+ TCTAGCCATC AGTTCGGTCC TTCAACTGCA TACCCTTTTG GGTTCTGATG ATGAGCCCCT AAGGAAAAGT   
  
  
+ TCACCTTTAG CCTTGATGAA GTATGCAAAT GGGGCTAATA GGCAAAGCCC GAGTAATGAT TCGGCTTCTT   
  
  
+ CATCACCTCC TTCGCTCAAT ACTTCAACCA AGCTGGATGG TTTCCTCAGC GCTTTGTGGG GATTGTCCCC   
  
  
+ AAAGATTATG GTGATAGCTG AGCACGATTC CAATCACAAT GGTTCTGGAC TTATGGAGAG GTTGTCAGAA   
  
  
+ GCACTGTACT TCTATGCAGC GCTGTTTGAC TGCTTAGAAT CCACCCTGCC AAGAACATCT GTCGAAAGAA   
  
  
+ GGCGGGTAGA GAAGATGCTC CTAGGTGAAG AGATCAAGAA CATTATATCA TGCGAGGGAG GAGAAAGGAG   
  
  
+ AGAAAGGCAT GAGAAGATCG AGAAGTGGAT GCAGAGGCTA GACATGGCTG GATTCGGGAT CGTTCCTTTG   
  
  
+ AGCTATATGG GTATGCTGCA AGCAAGGCAA TTGCTTCAGG GCTATGGTTG TGATGGTTAT AGAGTGAAAG   
  
  
+ AGAATGGTTG TGTTGTCATC TGTTGGCAAG ATCGCCCCCT CTTTTCGGTA TCAGCATGGA GGTGTAGGAG   
  
  
+ ATG  

- -Up\_Stream \_Len000TACGCG GAAAGACGAT TGAGTAATAG AAAGGAAGAC TCTCCCCTGT TGAATATATT   
  
  
- TAACTATCCA TAAAAGTTTA GTTATCTGGT CGATGGTATA TACTATTAAA AATAATGAAA AGATTTAGGT   
  
  
- AGTTGATAAA AGTGTTAGGA GGAAAAAGAT CTTTTTATTT TAAACGAATT AAGTTTAGTG GATTTTAATG   
  
  
- ATTTAGATAT CAGGATTTAG ATGTTTGAGT AAACACAACG TTGAGTACTA AATTCACCTT GTGATTGGAA   
  
  
- TCCTCTATAC AACAGTTTTC TTCTTAAGCT ACTGTATAAA AGGTTGTTTT CCGGTGGATT GAAGTACTAA   
  
  
- ATTTTTCCTC CTCAGATTCG TGTAGTACCT ACTGATTAAA AACTACATAA CATTGATGCA TAACTCTATT   
  
  
- GAACTTACTT AGCACTGATC TTATATAGAT AAAAGTTTTT ATTTTTAAGG GATTTTCTTT TCCTATTTTT   
  
  
- TGATATATTA TGATTTTTTA AGATTGATAT TATTGATCCG AATAATTTAA AAATATCACT TTTAAATAAA   
  
  
- GATGAATATT GTTGTAGTGA ATGATTCATC GAATAAACAC AGAAGAAAAG ATTTATTATT ACCATTGATA   
  
  
- CACAGAAAAC ATTTATTGAA TAATAGATAA AATATTTAGT GGCACATTTC GTGCCCAGAT ATGATCAATT   
  
  
- AAATTTATCG TAACAATTTA GTTCATTCTA TGTTTGTTTA AATAAATGTT GACATTCACC AGGTATTTTT   
  
  
- ATTATCGGTA ATGTTTTTTA TCATGATGAC AAAATACTTT ACTAAAATTA TAAGATTTTA AGGTTATTTT   
  
  
- GTACAATATT AGATACTAAT TAAAATTTTG TACTAATGTA CAGTCGTATT AAACATAATA ACATGTCGTT   
  
  
- TACTTTCAAC CTCGATTTTC TTTTACCCCT TTATTTCCGT CTGTTCATTT CTATTTTATA CGATAGATGA   
  
  
- AAACCTCCAT AGGAATTGGT GTAACAGAGC AAACAGAAGT AATCCCTCTT CACCCTATTT ATGGTTTTAA   
  
  
- ACATACCAGT AACGCACCCC AAACGAAGGT TTGTTTTACC GAAAAACCTT CGTTTAAAAC ACTCGAAGAT   
  
  
- ACGTTTAAAG GTGCGAATTG GGGTGTTCGT TTGTTTTTGT CTGTCACCAA AAACAAACAG AAACAATCCC   
  
  
- GTACAAACCT CGGTTGACAA TTACCCTTAT TTGACCCCTC CGAAAAAGGA ACCGAGAAAG GGGGACTGGG   
  
  
- GTTTGGGTGA AGTCGAGTTA TGCTTTGGTC GGTAACTTTT TCCTTCCCCC CCCCCCCCCA TACACTCTTT   
  
  
- TTCTGTTTTG AAAAAGGGCC TCTCTCTTCT ATGTATACGT TGTTTCCGAG AGGAAGAGTA TTTAAGGTAT   
  
  
- AAGGAGACAA AAAGATCCTC TCTCTTTATA CGTTTTTTGT TTAGTCAACC GCTGTTTTCA ATGATGGTGC   
  
  
- GTAACAGATG ACGACGCATG GGTAAAAACC TCTGAAATTT CTTGCGGGGA GGGTAGAACT TTTCACTTTT   
  
  
- GGGGGAGAAA CTTTGACTTA AAGTCCCCTT TTTTTGGAGT GGTTTGGAGA TCTCTCTCGA AAATCTCTCC   
  
  
- CTTTATGTTG CTAATCTTCC TCGTTAAACC CTTTAAAGAA ACCCTTAAAC TTACCCAAAA CTCACTTAAC   
  
  
- GTTTAGGGTC TTTTCAAAAC CGTTCATGGC TAGATGTCAA GAGAGGAGAA GCACAAACCA TCTAGGGGAC   
  
  
- AAAGGAGACA AAGTAAATCC CATGAAGAGT AAGTAGGAGG GGTGGGGGAA TTAGCCTAGA AGGACAGCTC   
  
  
- AGTGAAATAC GATTATAAAA AAGTCACCTA AAAATCAATT GGGACAAGTA AAAAGGTAAG ACACAGGGTA   
  
  
- AAGAGAGAAA AAGTAAGTAT TCAACGGCCA AATAGACAAC CCACGTCGAA TCAGTGTTAT TAAAGACACA   
  
  
- ATCCGAAGAA ATGCCAATTT TTTTTTCCTC CGTGAGAAAA GCCACACTAA CAAATACCCT GGTTACTAAG   
  
  
- TTCTACTACC CAGTAGTCAT TGAAGTAGTG GGGAAGTTAA AAAGAGGTAC TACAGAGGGT TAAATCCAAG   
  
  
- AAGGATGGGA ACCGAGTCTC TCGATTTTGG ACTTCTTTCT CCAGAAATGA ACTATGTAAA CAACGAGAGA   
  
  
- ACACGTTTAG TACAGAGATC ACCATCGGAT CTGTTACGCT TGAATCGGGA GCTTGTTTAG AGAGTCGAAC   
  
  
- GACGGGGACT ACCCCTATGT TACGTCGCAT ACCGAAGAAT AAAACGACTT CGGGACCGAC TCTCCTAGGA   
  
  
- GTTCAGTACC GGACCGTACA TATCTCGGGA AGTAAAATGC TTTTACGGAC AGTAAAGTCT CCTTTAAGAA   
  
  
- CGATCCTTCG AGAAGAAACT CGATAAAGGA AAGAACTTCG ACCGGATAAA CCACTGTTTG GTTAGCTATT   
  
  
- AGCTTCGGTA CCTCCCCCTT TTCTACCAAG TATAATAACT AGACTTACGT AGTCTTGGAC GAGTTACCTA   
  
  
- ACGGGAATAA GTCCGAAACT CACGTTCCGG ACCCCCAGGA GGAGTAAACT CTTAATGGCC ACAAGTAGTT   
  
  
- GTGTTTCTCC AAGATCTAGT TCACCGAGTA TCCCACTGAG TTCTTCGACT CTTTAACCTA AACGGTAAAG   
  
  
- TCAAGTTAGG ACACCAATCG TTCAACCTTT TGGAACTACA ACTTTTCGAC ACACAATTCT GACCACTCCG   
  
  
- AGATCGGTAG TCAAGCCAGG AAGTTGACGT ATGGGAAAAC CCAAGACTAC TACTCGGGGA TTCCTTTTCA   
  
  
- AGTGGAAATC GGAACTACTT CATACGTTTA CCCCGATTAT CCGTTTCGGG CTCATTACTA AGCCGAAGAA   
  
  
- GTAGTGGAGG AAGCGAGTTA TGAAGTTGGT TCGACCTACC AAAGGAGTCG CGAAACACCC CTAACAGGGG   
  
  
- TTTCTAATAC CACTATCGAC TCGTGCTAAG GTTAGTGTTA CCAAGACCTG AATACCTCTC CAACAGTCTT   
  
  
- CGTGACATGA AGATACGTCG CGACAAACTG ACGAATCTTA GGTGGGACGG TTCTTGTAGA CAGCTTTCTT   
  
  
- CCGCCCATCT CTTCTACGAG GATCCACTTC TCTAGTTCTT GTAATATAGT ACGCTCCCTC CTCTTTCCTC   
  
  
- TCTTTCCGTA CTCTTCTAGC TCTTCACCTA CGTCTCCGAT CTGTACCGAC CTAAGCCCTA GCAAGGAAAC   
  
  
- TCGATATACC CATACGACGT TCGTTCCGTT AACGAAGTCC CGATACCAAC ACTACCAATA TCTCACTTTC   
  
  
- TCTTACCAAC ACAACAGTAG ACAACCGTTC TAGCGGGGGA GAAAAGCCAT AGTCGTACCT CCACATCCTC   
  
  
- TAC

+     TATA

| Site Name | Organism | Position | Strand | Matrix score. | sequence | function |
| --- | --- | --- | --- | --- | --- | --- |
| TATA | Arabidopsis thaliana | 662 | - | 8 | TATAAAAT |  |

>HU08G00367.1   
+ -Up\_Stream \_Len000ATGCGC CTTTCTGCTA ACTCATTATC TTTCCTTCTG AGAGGGGACA ACTTATATAA   
  
  
+ ATTGATAGGT ATTTTCAAAT CAATAGACCA GCTACCATAT ATGATAATTT TTATTACTTT TCTAAATCCA   
  
  
+ TCAACTATTT TCACAATCCT CCTTTTTCTA GAAAAATAAA ATTTGCTTAA TTCAAATCAC CTAAAATTAC   
  
  
+ TAAATCTATA GTCCTAAATC TACAAACTCA TTTGTGTTGC AACTCATGAT TTAAGTGGAA CACTAACCTT   
  
  
+ AGGAGATATG TTGTCAAAAG AAGAATTCGA TGACATATTT TCCAACAAAA GGCCACCTAA CTTCATGATT   
  
  
+ TAAAAAGGAG GAGTCTAAGC ACATCATGGA TGACTAATTT TTGATGTATT GTAACTACGT ATTGAGATAA   
  
  
+ CTTGAATGAA TCGTGACTAG AATATATCTA TTTTCAAAAA TAAAAATTCC CTAAAAGAAA AGGATAAAAA   
  
  
+ ACTATATAAT ACTAAAAAAT TCTAACTATA ATAACTAGGC TTATTAAATT TTTATAGTGA AAATTTATTT   
  
  
+ CTACTTATAA CAACATCACT TACTAAGTAG CTTATTTGTG TCTTCTTTTC TAAATAATAA TGGTAACTAT   
  
  
+ GTGTCTTTTG TAAATAACTT ATTATCTATT TTATAAATCA CCGTGTAAAG CACGGGTCTA TACTAGTTAA   
  
  
+ TTTAAATAGC ATTGTTAAAT CAAGTAAGAT ACAAACAAAT TTATTTACAA CTGTAAGTGG TCCATAAAAA   
  
  
+ TAATAGCCAT TACAAAAAAT AGTACTACTG TTTTATGAAA TGATTTTAAT ATTCTAAAAT TCCAATAAAA   
  
  
+ CATGTTATAA TCTATGATTA ATTTTAAAAC ATGATTACAT GTCAGCATAA TTTGTATTAT TGTACAGCAA   
  
  
+ ATGAAAGTTG GAGCTAAAAG AAAATGGGGA AATAAAGGCA GACAAGTAAA GATAAAATAT GCTATCTACT   
  
  
+ TTTGGAGGTA TCCTTAACCA CATTGTCTCG TTTGTCTTCA TTAGGGAGAA GTGGGATAAA TACCAAAATT   
  
  
+ TGTATGGTCA TTGCGTGGGG TTTGCTTCCA AACAAAATGG CTTTTTGGAA GCAAATTTTG TGAGCTTCTA   
  
  
+ TGCAAATTTC CACGCTTAAC CCCACAAGCA AACAAAAACA GACAGTGGTT TTTGTTTGTC TTTGTTAGGG   
  
  
+ CATGTTTGGA GCCAACTGTT AATGGGAATA AACTGGGGAG GCTTTTTCCT TGGCTCTTTC CCCCTGACCC   
  
  
+ CAAACCCACT TCAGCTCAAT ACGAAACCAG CCATTGAAAA AGGAAGGGGG GGGGGGGGGT ATGTGAGAAA   
  
  
+ AAGACAAAAC TTTTTCCCGG AGAGAGAAGA TACATATGCA ACAAAGGCTC TCCTTCTCAT AAATTCCATA   
  
  
+ TTCCTCTGTT TTTCTAGGAG AGAGAAATAT GCAAAAAACA AATCAGTTGG CGACAAAAGT TACTACCACG   
  
  
+ CATTGTCTAC TGCTGCGTAC CCATTTTTGG AGACTTTAAA GAACGCCCCT CCCATCTTGA AAAGTGAAAA   
  
  
+ CCCCCTCTTT GAAACTGAAT TTCAGGGGAA AAAAACCTCA CCAAACCTCT AGAGAGAGCT TTTAGAGAGG   
  
  
+ GAAATACAAC GATTAGAAGG AGCAATTTGG GAAATTTCTT TGGGAATTTG AATGGGTTTT GAGTGAATTG   
  
  
+ CAAATCCCAG AAAAGTTTTG GCAAGTACCG ATCTACAGTT CTCTCCTCTT CGTGTTTGGT AGATCCCCTG   
  
  
+ TTTCCTCTGT TTCATTTAGG GTACTTCTCA TTCATCCTCC CCACCCCCTT AATCGGATCT TCCTGTCGAG   
  
  
+ TCACTTTATG CTAATATTTT TTCAGTGGAT TTTTAGTTAA CCCTGTTCAT TTTTCCATTC TGTGTCCCAT   
  
  
+ TTCTCTCTTT TTCATTCATA AGTTGCCGGT TTATCTGTTG GGTGCAGCTT AGTCACAATA ATTTCTGTGT   
  
  
+ TAGGCTTCTT TACGGTTAAA AAAAAAGGAG GCACTCTTTT CGGTGTGATT GTTTATGGGA CCAATGATTC   
  
  
+ AAGATGATGG GTCATCAGTA ACTTCATCAC CCCTTCAATT TTTCTCCATG ATGTCTCCCA ATTTAGGTTC   
  
  
+ TTCCTACCCT TGGCTCAGAG AGCTAAAACC TGAAGAAAGA GGTCTTTACT TGATACATTT GTTGCTCTCT   
  
  
+ TGTGCAAATC ATGTCTCTAG TGGTAGCCTA GACAATGCGA ACTTAGCCCT CGAACAAATC TCTCAGCTTG   
  
  
+ CTGCCCCTGA TGGGGATACA ATGCAGCGTA TGGCTTCTTA TTTTGCTGAA GCCCTGGCTG AGAGGATCCT   
  
  
+ CAAGTCATGG CCTGGCATGT ATAGAGCCCT TCATTTTACG AAAATGCCTG TCATTTCAGA GGAAATTCTT   
  
  
+ GCTAGGAAGC TCTTCTTTGA GCTATTTCCT TTCTTGAAGC TGGCCTATTT GGTGACAAAC CAATCGATAA   
  
  
+ TCGAAGCCAT GGAGGGGGAA AAGATGGTTC ATATTATTGA TCTGAATGCA TCAGAACCTG CTCAATGGAT   
  
  
+ TGCCCTTATT CAGGCTTTGA GTGCAAGGCC TGGGGGTCCT CCTCATTTGA GAATTACCGG TGTTCATCAA   
  
  
+ CACAAAGAGG TTCTAGATCA AGTGGCTCAT AGGGTGACTC AAGAAGCTGA GAAATTGGAT TTGCCATTTC   
  
  
+ AGTTCAATCC TGTGGTTAGC AAGTTGGAAA ACCTTGATGT TGAAAAGCTG TGTGTTAAGA CTGGTGAGGC   
  
  
+ TCTAGCCATC AGTTCGGTCC TTCAACTGCA TACCCTTTTG GGTTCTGATG ATGAGCCCCT AAGGAAAAGT   
  
  
+ TCACCTTTAG CCTTGATGAA GTATGCAAAT GGGGCTAATA GGCAAAGCCC GAGTAATGAT TCGGCTTCTT   
  
  
+ CATCACCTCC TTCGCTCAAT ACTTCAACCA AGCTGGATGG TTTCCTCAGC GCTTTGTGGG GATTGTCCCC   
  
  
+ AAAGATTATG GTGATAGCTG AGCACGATTC CAATCACAAT GGTTCTGGAC TTATGGAGAG GTTGTCAGAA   
  
  
+ GCACTGTACT TCTATGCAGC GCTGTTTGAC TGCTTAGAAT CCACCCTGCC AAGAACATCT GTCGAAAGAA   
  
  
+ GGCGGGTAGA GAAGATGCTC CTAGGTGAAG AGATCAAGAA CATTATATCA TGCGAGGGAG GAGAAAGGAG   
  
  
+ AGAAAGGCAT GAGAAGATCG AGAAGTGGAT GCAGAGGCTA GACATGGCTG GATTCGGGAT CGTTCCTTTG   
  
  
+ AGCTATATGG GTATGCTGCA AGCAAGGCAA TTGCTTCAGG GCTATGGTTG TGATGGTTAT AGAGTGAAAG   
  
  
+ AGAATGGTTG TGTTGTCATC TGTTGGCAAG ATCGCCCCCT CTTTTCGGTA TCAGCATGGA GGTGTAGGAG   
  
  
+ ATG  

- -Up\_Stream \_Len000TACGCG GAAAGACGAT TGAGTAATAG AAAGGAAGAC TCTCCCCTGT TGAATATATT   
  
  
- TAACTATCCA TAAAAGTTTA GTTATCTGGT CGATGGTATA TACTATTAAA AATAATGAAA AGATTTAGGT   
  
  
- AGTTGATAAA AGTGTTAGGA GGAAAAAGAT CTTTTTATTT TAAACGAATT AAGTTTAGTG GATTTTAATG   
  
  
- ATTTAGATAT CAGGATTTAG ATGTTTGAGT AAACACAACG TTGAGTACTA AATTCACCTT GTGATTGGAA   
  
  
- TCCTCTATAC AACAGTTTTC TTCTTAAGCT ACTGTATAAA AGGTTGTTTT CCGGTGGATT GAAGTACTAA   
  
  
- ATTTTTCCTC CTCAGATTCG TGTAGTACCT ACTGATTAAA AACTACATAA CATTGATGCA TAACTCTATT   
  
  
- GAACTTACTT AGCACTGATC TTATATAGAT AAAAGTTTTT ATTTTTAAGG GATTTTCTTT TCCTATTTTT   
  
  
- TGATATATTA TGATTTTTTA AGATTGATAT TATTGATCCG AATAATTTAA AAATATCACT TTTAAATAAA   
  
  
- GATGAATATT GTTGTAGTGA ATGATTCATC GAATAAACAC AGAAGAAAAG ATTTATTATT ACCATTGATA   
  
  
- CACAGAAAAC ATTTATTGAA TAATAGATAA AATATTTAGT GGCACATTTC GTGCCCAGAT ATGATCAATT   
  
  
- AAATTTATCG TAACAATTTA GTTCATTCTA TGTTTGTTTA AATAAATGTT GACATTCACC AGGTATTTTT   
  
  
- ATTATCGGTA ATGTTTTTTA TCATGATGAC AAAATACTTT ACTAAAATTA TAAGATTTTA AGGTTATTTT   
  
  
- GTACAATATT AGATACTAAT TAAAATTTTG TACTAATGTA CAGTCGTATT AAACATAATA ACATGTCGTT   
  
  
- TACTTTCAAC CTCGATTTTC TTTTACCCCT TTATTTCCGT CTGTTCATTT CTATTTTATA CGATAGATGA   
  
  
- AAACCTCCAT AGGAATTGGT GTAACAGAGC AAACAGAAGT AATCCCTCTT CACCCTATTT ATGGTTTTAA   
  
  
- ACATACCAGT AACGCACCCC AAACGAAGGT TTGTTTTACC GAAAAACCTT CGTTTAAAAC ACTCGAAGAT   
  
  
- ACGTTTAAAG GTGCGAATTG GGGTGTTCGT TTGTTTTTGT CTGTCACCAA AAACAAACAG AAACAATCCC   
  
  
- GTACAAACCT CGGTTGACAA TTACCCTTAT TTGACCCCTC CGAAAAAGGA ACCGAGAAAG GGGGACTGGG   
  
  
- GTTTGGGTGA AGTCGAGTTA TGCTTTGGTC GGTAACTTTT TCCTTCCCCC CCCCCCCCCA TACACTCTTT   
  
  
- TTCTGTTTTG AAAAAGGGCC TCTCTCTTCT ATGTATACGT TGTTTCCGAG AGGAAGAGTA TTTAAGGTAT   
  
  
- AAGGAGACAA AAAGATCCTC TCTCTTTATA CGTTTTTTGT TTAGTCAACC GCTGTTTTCA ATGATGGTGC   
  
  
- GTAACAGATG ACGACGCATG GGTAAAAACC TCTGAAATTT CTTGCGGGGA GGGTAGAACT TTTCACTTTT   
  
  
- GGGGGAGAAA CTTTGACTTA AAGTCCCCTT TTTTTGGAGT GGTTTGGAGA TCTCTCTCGA AAATCTCTCC   
  
  
- CTTTATGTTG CTAATCTTCC TCGTTAAACC CTTTAAAGAA ACCCTTAAAC TTACCCAAAA CTCACTTAAC   
  
  
- GTTTAGGGTC TTTTCAAAAC CGTTCATGGC TAGATGTCAA GAGAGGAGAA GCACAAACCA TCTAGGGGAC   
  
  
- AAAGGAGACA AAGTAAATCC CATGAAGAGT AAGTAGGAGG GGTGGGGGAA TTAGCCTAGA AGGACAGCTC   
  
  
- AGTGAAATAC GATTATAAAA AAGTCACCTA AAAATCAATT GGGACAAGTA AAAAGGTAAG ACACAGGGTA   
  
  
- AAGAGAGAAA AAGTAAGTAT TCAACGGCCA AATAGACAAC CCACGTCGAA TCAGTGTTAT TAAAGACACA   
  
  
- ATCCGAAGAA ATGCCAATTT TTTTTTCCTC CGTGAGAAAA GCCACACTAA CAAATACCCT GGTTACTAAG   
  
  
- TTCTACTACC CAGTAGTCAT TGAAGTAGTG GGGAAGTTAA AAAGAGGTAC TACAGAGGGT TAAATCCAAG   
  
  
- AAGGATGGGA ACCGAGTCTC TCGATTTTGG ACTTCTTTCT CCAGAAATGA ACTATGTAAA CAACGAGAGA   
  
  
- ACACGTTTAG TACAGAGATC ACCATCGGAT CTGTTACGCT TGAATCGGGA GCTTGTTTAG AGAGTCGAAC   
  
  
- GACGGGGACT ACCCCTATGT TACGTCGCAT ACCGAAGAAT AAAACGACTT CGGGACCGAC TCTCCTAGGA   
  
  
- GTTCAGTACC GGACCGTACA TATCTCGGGA AGTAAAATGC TTTTACGGAC AGTAAAGTCT CCTTTAAGAA   
  
  
- CGATCCTTCG AGAAGAAACT CGATAAAGGA AAGAACTTCG ACCGGATAAA CCACTGTTTG GTTAGCTATT   
  
  
- AGCTTCGGTA CCTCCCCCTT TTCTACCAAG TATAATAACT AGACTTACGT AGTCTTGGAC GAGTTACCTA   
  
  
- ACGGGAATAA GTCCGAAACT CACGTTCCGG ACCCCCAGGA GGAGTAAACT CTTAATGGCC ACAAGTAGTT   
  
  
- GTGTTTCTCC AAGATCTAGT TCACCGAGTA TCCCACTGAG TTCTTCGACT CTTTAACCTA AACGGTAAAG   
  
  
- TCAAGTTAGG ACACCAATCG TTCAACCTTT TGGAACTACA ACTTTTCGAC ACACAATTCT GACCACTCCG   
  
  
- AGATCGGTAG TCAAGCCAGG AAGTTGACGT ATGGGAAAAC CCAAGACTAC TACTCGGGGA TTCCTTTTCA   
  
  
- AGTGGAAATC GGAACTACTT CATACGTTTA CCCCGATTAT CCGTTTCGGG CTCATTACTA AGCCGAAGAA   
  
  
- GTAGTGGAGG AAGCGAGTTA TGAAGTTGGT TCGACCTACC AAAGGAGTCG CGAAACACCC CTAACAGGGG   
  
  
- TTTCTAATAC CACTATCGAC TCGTGCTAAG GTTAGTGTTA CCAAGACCTG AATACCTCTC CAACAGTCTT   
  
  
- CGTGACATGA AGATACGTCG CGACAAACTG ACGAATCTTA GGTGGGACGG TTCTTGTAGA CAGCTTTCTT   
  
  
- CCGCCCATCT CTTCTACGAG GATCCACTTC TCTAGTTCTT GTAATATAGT ACGCTCCCTC CTCTTTCCTC   
  
  
- TCTTTCCGTA CTCTTCTAGC TCTTCACCTA CGTCTCCGAT CTGTACCGAC CTAAGCCCTA GCAAGGAAAC   
  
  
- TCGATATACC CATACGACGT TCGTTCCGTT AACGAAGTCC CGATACCAAC ACTACCAATA TCTCACTTTC   
  
  
- TCTTACCAAC ACAACAGTAG ACAACCGTTC TAGCGGGGGA GAAAAGCCAT AGTCGTACCT CCACATCCTC   
  
  
- TAC

+     TATA-box

| Site Name | Organism | Position | Strand | Matrix score. | sequence | function |
| --- | --- | --- | --- | --- | --- | --- |
| TATA-box | Arabidopsis thaliana | 570 | + | 4 | TATA | core promoter element around -30 of transcription start |
| TATA-box | Brassica napus | 111 | + | 6 | ATATAT | core promoter element around -30 of transcription start |
| TATA-box | Helianthus annuus | 545 | - | 6 | TATAAA | core promoter element around -30 of transcription start |
| TATA-box | Arabidopsis thaliana | 112 | + | 4 | TATA | core promoter element around -30 of transcription start |
| TATA-box | Arabidopsis thaliana | 3282 | - | 4 | TATA | core promoter element around -30 of transcription start |
| TATA-box | Arabidopsis thaliana | 705 | - | 8 | TATTTAAA | core promoter element around -30 of transcription start |
| TATA-box | Arabidopsis thaliana | 3281 | - | 5 | TATAA | core promoter element around -30 of transcription start |
| TATA-box | Arabidopsis thaliana | 547 | + | 4 | TATA | core promoter element around -30 of transcription start |
| TATA-box | Arabidopsis thaliana | 2334 | - | 4 | TATA | core promoter element around -30 of transcription start |
| TATA-box | Arabidopsis thaliana | 70 | + | 4 | TATA | core promoter element around -30 of transcription start |
| TATA-box | Arabidopsis thaliana | 3228 | - | 4 | TATA | core promoter element around -30 of transcription start |
| TATA-box | Helianthus annuus | 2332 | - | 6 | TATACA | core promoter element around -30 of transcription start |
| TATA-box | Arabidopsis thaliana | 3128 | - | 4 | TATA | core promoter element around -30 of transcription start |
| TATA-box | Arabidopsis thaliana | 221 | + | 4 | TATA | core promoter element around -30 of transcription start |
| TATA-box | Brassica oleracea | 69 | + | 6 | ATATAA | core promoter element around -30 of transcription start |
| TATA-box | Arabidopsis thaliana | 68 | + | 6 | TATATA | core promoter element around -30 of transcription start |
| TATA-box | Arabidopsis thaliana | 666 | + | 4 | TATA | core promoter element around -30 of transcription start |
| TATA-box | Arabidopsis thaliana | 546 | - | 5 | TATAA | core promoter element around -30 of transcription start |
| TATA-box | Arabidopsis thaliana | 521 | + | 4 | TATA | core promoter element around -30 of transcription start |
| TATA-box | Arabidopsis thaliana | 569 | - | 5 | TATAA | core promoter element around -30 of transcription start |
| TATA-box | Brassica oleracea | 498 | + | 6 | ATATAA | core promoter element around -30 of transcription start |
| TATA-box | Arabidopsis thaliana | 849 | - | 5 | TATAA | core promoter element around -30 of transcription start |
| TATA-box | Brassica napus | 446 | + | 6 | ATATAT | core promoter element around -30 of transcription start |
| TATA-box | Oryza sativa | 785 | + | 7 | TACAAAA | core promoter element around -30 of transcription start |
| TATA-box | Arabidopsis thaliana | 67 | - | 7 | TATATAA | core promoter element around -30 of transcription start |
| TATA-box | Pisum sativum | 544 | - | 7 | TATAAAA | core promoter element around -30 of transcription start |
| TATA-box | Arabidopsis thaliana | 497 | + | 6 | TATATA | core promoter element around -30 of transcription start |
| TATA-box | Arabidopsis thaliana | 447 | + | 4 | TATA | core promoter element around -30 of transcription start |
| TATA-box | Arabidopsis thaliana | 1413 | - | 9 | ccTATAAAaa | core promoter element around -30 of transcription start |
| TATA-box | Arabidopsis thaliana | 693 | + | 4 | TATA | core promoter element around -30 of transcription start |
| TATA-box | Arabidopsis thaliana | 499 | + | 4 | TATA | core promoter element around -30 of transcription start |
| TATA-box | Arabidopsis thaliana | 3127 | - | 5 | TATAA | core promoter element around -30 of transcription start |
| TATA-box | Arabidopsis thaliana | 543 | - | 9 | ccTATAAAaa | core promoter element around -30 of transcription start |
| TATA-box | Brassica napus | 3126 | + | 6 | ATTATA | core promoter element around -30 of transcription start |
| TATA-box | Arabidopsis thaliana | 665 | - | 5 | TATAA | core promoter element around -30 of transcription start |
| TATA-box | Arabidopsis thaliana | 664 | + | 9 | taTATAAAtc | core promoter element around -30 of transcription start |
| TATA-box | Pisum sativum | 663 | - | 7 | TATAAAA | core promoter element around -30 of transcription start |
| TATA-box | Arabidopsis thaliana | 850 | + | 4 | TATA | core promoter element around -30 of transcription start |
| TATA-box | Oryza sativa | 640 | - | 7 | TACAAAA | core promoter element around -30 of transcription start |

>HU08G00367.1   
+ -Up\_Stream \_Len000ATGCGC CTTTCTGCTA ACTCATTATC TTTCCTTCTG AGAGGGGACA ACTTATATAA   
  
  
+ ATTGATAGGT ATTTTCAAAT CAATAGACCA GCTACCATAT ATGATAATTT TTATTACTTT TCTAAATCCA   
  
  
+ TCAACTATTT TCACAATCCT CCTTTTTCTA GAAAAATAAA ATTTGCTTAA TTCAAATCAC CTAAAATTAC   
  
  
+ TAAATCTATA GTCCTAAATC TACAAACTCA TTTGTGTTGC AACTCATGAT TTAAGTGGAA CACTAACCTT   
  
  
+ AGGAGATATG TTGTCAAAAG AAGAATTCGA TGACATATTT TCCAACAAAA GGCCACCTAA CTTCATGATT   
  
  
+ TAAAAAGGAG GAGTCTAAGC ACATCATGGA TGACTAATTT TTGATGTATT GTAACTACGT ATTGAGATAA   
  
  
+ CTTGAATGAA TCGTGACTAG AATATATCTA TTTTCAAAAA TAAAAATTCC CTAAAAGAAA AGGATAAAAA   
  
  
+ ACTATATAAT ACTAAAAAAT TCTAACTATA ATAACTAGGC TTATTAAATT TTTATAGTGA AAATTTATTT   
  
  
+ CTACTTATAA CAACATCACT TACTAAGTAG CTTATTTGTG TCTTCTTTTC TAAATAATAA TGGTAACTAT   
  
  
+ GTGTCTTTTG TAAATAACTT ATTATCTATT TTATAAATCA CCGTGTAAAG CACGGGTCTA TACTAGTTAA   
  
  
+ TTTAAATAGC ATTGTTAAAT CAAGTAAGAT ACAAACAAAT TTATTTACAA CTGTAAGTGG TCCATAAAAA   
  
  
+ TAATAGCCAT TACAAAAAAT AGTACTACTG TTTTATGAAA TGATTTTAAT ATTCTAAAAT TCCAATAAAA   
  
  
+ CATGTTATAA TCTATGATTA ATTTTAAAAC ATGATTACAT GTCAGCATAA TTTGTATTAT TGTACAGCAA   
  
  
+ ATGAAAGTTG GAGCTAAAAG AAAATGGGGA AATAAAGGCA GACAAGTAAA GATAAAATAT GCTATCTACT   
  
  
+ TTTGGAGGTA TCCTTAACCA CATTGTCTCG TTTGTCTTCA TTAGGGAGAA GTGGGATAAA TACCAAAATT   
  
  
+ TGTATGGTCA TTGCGTGGGG TTTGCTTCCA AACAAAATGG CTTTTTGGAA GCAAATTTTG TGAGCTTCTA   
  
  
+ TGCAAATTTC CACGCTTAAC CCCACAAGCA AACAAAAACA GACAGTGGTT TTTGTTTGTC TTTGTTAGGG   
  
  
+ CATGTTTGGA GCCAACTGTT AATGGGAATA AACTGGGGAG GCTTTTTCCT TGGCTCTTTC CCCCTGACCC   
  
  
+ CAAACCCACT TCAGCTCAAT ACGAAACCAG CCATTGAAAA AGGAAGGGGG GGGGGGGGGT ATGTGAGAAA   
  
  
+ AAGACAAAAC TTTTTCCCGG AGAGAGAAGA TACATATGCA ACAAAGGCTC TCCTTCTCAT AAATTCCATA   
  
  
+ TTCCTCTGTT TTTCTAGGAG AGAGAAATAT GCAAAAAACA AATCAGTTGG CGACAAAAGT TACTACCACG   
  
  
+ CATTGTCTAC TGCTGCGTAC CCATTTTTGG AGACTTTAAA GAACGCCCCT CCCATCTTGA AAAGTGAAAA   
  
  
+ CCCCCTCTTT GAAACTGAAT TTCAGGGGAA AAAAACCTCA CCAAACCTCT AGAGAGAGCT TTTAGAGAGG   
  
  
+ GAAATACAAC GATTAGAAGG AGCAATTTGG GAAATTTCTT TGGGAATTTG AATGGGTTTT GAGTGAATTG   
  
  
+ CAAATCCCAG AAAAGTTTTG GCAAGTACCG ATCTACAGTT CTCTCCTCTT CGTGTTTGGT AGATCCCCTG   
  
  
+ TTTCCTCTGT TTCATTTAGG GTACTTCTCA TTCATCCTCC CCACCCCCTT AATCGGATCT TCCTGTCGAG   
  
  
+ TCACTTTATG CTAATATTTT TTCAGTGGAT TTTTAGTTAA CCCTGTTCAT TTTTCCATTC TGTGTCCCAT   
  
  
+ TTCTCTCTTT TTCATTCATA AGTTGCCGGT TTATCTGTTG GGTGCAGCTT AGTCACAATA ATTTCTGTGT   
  
  
+ TAGGCTTCTT TACGGTTAAA AAAAAAGGAG GCACTCTTTT CGGTGTGATT GTTTATGGGA CCAATGATTC   
  
  
+ AAGATGATGG GTCATCAGTA ACTTCATCAC CCCTTCAATT TTTCTCCATG ATGTCTCCCA ATTTAGGTTC   
  
  
+ TTCCTACCCT TGGCTCAGAG AGCTAAAACC TGAAGAAAGA GGTCTTTACT TGATACATTT GTTGCTCTCT   
  
  
+ TGTGCAAATC ATGTCTCTAG TGGTAGCCTA GACAATGCGA ACTTAGCCCT CGAACAAATC TCTCAGCTTG   
  
  
+ CTGCCCCTGA TGGGGATACA ATGCAGCGTA TGGCTTCTTA TTTTGCTGAA GCCCTGGCTG AGAGGATCCT   
  
  
+ CAAGTCATGG CCTGGCATGT ATAGAGCCCT TCATTTTACG AAAATGCCTG TCATTTCAGA GGAAATTCTT   
  
  
+ GCTAGGAAGC TCTTCTTTGA GCTATTTCCT TTCTTGAAGC TGGCCTATTT GGTGACAAAC CAATCGATAA   
  
  
+ TCGAAGCCAT GGAGGGGGAA AAGATGGTTC ATATTATTGA TCTGAATGCA TCAGAACCTG CTCAATGGAT   
  
  
+ TGCCCTTATT CAGGCTTTGA GTGCAAGGCC TGGGGGTCCT CCTCATTTGA GAATTACCGG TGTTCATCAA   
  
  
+ CACAAAGAGG TTCTAGATCA AGTGGCTCAT AGGGTGACTC AAGAAGCTGA GAAATTGGAT TTGCCATTTC   
  
  
+ AGTTCAATCC TGTGGTTAGC AAGTTGGAAA ACCTTGATGT TGAAAAGCTG TGTGTTAAGA CTGGTGAGGC   
  
  
+ TCTAGCCATC AGTTCGGTCC TTCAACTGCA TACCCTTTTG GGTTCTGATG ATGAGCCCCT AAGGAAAAGT   
  
  
+ TCACCTTTAG CCTTGATGAA GTATGCAAAT GGGGCTAATA GGCAAAGCCC GAGTAATGAT TCGGCTTCTT   
  
  
+ CATCACCTCC TTCGCTCAAT ACTTCAACCA AGCTGGATGG TTTCCTCAGC GCTTTGTGGG GATTGTCCCC   
  
  
+ AAAGATTATG GTGATAGCTG AGCACGATTC CAATCACAAT GGTTCTGGAC TTATGGAGAG GTTGTCAGAA   
  
  
+ GCACTGTACT TCTATGCAGC GCTGTTTGAC TGCTTAGAAT CCACCCTGCC AAGAACATCT GTCGAAAGAA   
  
  
+ GGCGGGTAGA GAAGATGCTC CTAGGTGAAG AGATCAAGAA CATTATATCA TGCGAGGGAG GAGAAAGGAG   
  
  
+ AGAAAGGCAT GAGAAGATCG AGAAGTGGAT GCAGAGGCTA GACATGGCTG GATTCGGGAT CGTTCCTTTG   
  
  
+ AGCTATATGG GTATGCTGCA AGCAAGGCAA TTGCTTCAGG GCTATGGTTG TGATGGTTAT AGAGTGAAAG   
  
  
+ AGAATGGTTG TGTTGTCATC TGTTGGCAAG ATCGCCCCCT CTTTTCGGTA TCAGCATGGA GGTGTAGGAG   
  
  
+ ATG  

- -Up\_Stream \_Len000TACGCG GAAAGACGAT TGAGTAATAG AAAGGAAGAC TCTCCCCTGT TGAATATATT   
  
  
- TAACTATCCA TAAAAGTTTA GTTATCTGGT CGATGGTATA TACTATTAAA AATAATGAAA AGATTTAGGT   
  
  
- AGTTGATAAA AGTGTTAGGA GGAAAAAGAT CTTTTTATTT TAAACGAATT AAGTTTAGTG GATTTTAATG   
  
  
- ATTTAGATAT CAGGATTTAG ATGTTTGAGT AAACACAACG TTGAGTACTA AATTCACCTT GTGATTGGAA   
  
  
- TCCTCTATAC AACAGTTTTC TTCTTAAGCT ACTGTATAAA AGGTTGTTTT CCGGTGGATT GAAGTACTAA   
  
  
- ATTTTTCCTC CTCAGATTCG TGTAGTACCT ACTGATTAAA AACTACATAA CATTGATGCA TAACTCTATT   
  
  
- GAACTTACTT AGCACTGATC TTATATAGAT AAAAGTTTTT ATTTTTAAGG GATTTTCTTT TCCTATTTTT   
  
  
- TGATATATTA TGATTTTTTA AGATTGATAT TATTGATCCG AATAATTTAA AAATATCACT TTTAAATAAA   
  
  
- GATGAATATT GTTGTAGTGA ATGATTCATC GAATAAACAC AGAAGAAAAG ATTTATTATT ACCATTGATA   
  
  
- CACAGAAAAC ATTTATTGAA TAATAGATAA AATATTTAGT GGCACATTTC GTGCCCAGAT ATGATCAATT   
  
  
- AAATTTATCG TAACAATTTA GTTCATTCTA TGTTTGTTTA AATAAATGTT GACATTCACC AGGTATTTTT   
  
  
- ATTATCGGTA ATGTTTTTTA TCATGATGAC AAAATACTTT ACTAAAATTA TAAGATTTTA AGGTTATTTT   
  
  
- GTACAATATT AGATACTAAT TAAAATTTTG TACTAATGTA CAGTCGTATT AAACATAATA ACATGTCGTT   
  
  
- TACTTTCAAC CTCGATTTTC TTTTACCCCT TTATTTCCGT CTGTTCATTT CTATTTTATA CGATAGATGA   
  
  
- AAACCTCCAT AGGAATTGGT GTAACAGAGC AAACAGAAGT AATCCCTCTT CACCCTATTT ATGGTTTTAA   
  
  
- ACATACCAGT AACGCACCCC AAACGAAGGT TTGTTTTACC GAAAAACCTT CGTTTAAAAC ACTCGAAGAT   
  
  
- ACGTTTAAAG GTGCGAATTG GGGTGTTCGT TTGTTTTTGT CTGTCACCAA AAACAAACAG AAACAATCCC   
  
  
- GTACAAACCT CGGTTGACAA TTACCCTTAT TTGACCCCTC CGAAAAAGGA ACCGAGAAAG GGGGACTGGG   
  
  
- GTTTGGGTGA AGTCGAGTTA TGCTTTGGTC GGTAACTTTT TCCTTCCCCC CCCCCCCCCA TACACTCTTT   
  
  
- TTCTGTTTTG AAAAAGGGCC TCTCTCTTCT ATGTATACGT TGTTTCCGAG AGGAAGAGTA TTTAAGGTAT   
  
  
- AAGGAGACAA AAAGATCCTC TCTCTTTATA CGTTTTTTGT TTAGTCAACC GCTGTTTTCA ATGATGGTGC   
  
  
- GTAACAGATG ACGACGCATG GGTAAAAACC TCTGAAATTT CTTGCGGGGA GGGTAGAACT TTTCACTTTT   
  
  
- GGGGGAGAAA CTTTGACTTA AAGTCCCCTT TTTTTGGAGT GGTTTGGAGA TCTCTCTCGA AAATCTCTCC   
  
  
- CTTTATGTTG CTAATCTTCC TCGTTAAACC CTTTAAAGAA ACCCTTAAAC TTACCCAAAA CTCACTTAAC   
  
  
- GTTTAGGGTC TTTTCAAAAC CGTTCATGGC TAGATGTCAA GAGAGGAGAA GCACAAACCA TCTAGGGGAC   
  
  
- AAAGGAGACA AAGTAAATCC CATGAAGAGT AAGTAGGAGG GGTGGGGGAA TTAGCCTAGA AGGACAGCTC   
  
  
- AGTGAAATAC GATTATAAAA AAGTCACCTA AAAATCAATT GGGACAAGTA AAAAGGTAAG ACACAGGGTA   
  
  
- AAGAGAGAAA AAGTAAGTAT TCAACGGCCA AATAGACAAC CCACGTCGAA TCAGTGTTAT TAAAGACACA   
  
  
- ATCCGAAGAA ATGCCAATTT TTTTTTCCTC CGTGAGAAAA GCCACACTAA CAAATACCCT GGTTACTAAG   
  
  
- TTCTACTACC CAGTAGTCAT TGAAGTAGTG GGGAAGTTAA AAAGAGGTAC TACAGAGGGT TAAATCCAAG   
  
  
- AAGGATGGGA ACCGAGTCTC TCGATTTTGG ACTTCTTTCT CCAGAAATGA ACTATGTAAA CAACGAGAGA   
  
  
- ACACGTTTAG TACAGAGATC ACCATCGGAT CTGTTACGCT TGAATCGGGA GCTTGTTTAG AGAGTCGAAC   
  
  
- GACGGGGACT ACCCCTATGT TACGTCGCAT ACCGAAGAAT AAAACGACTT CGGGACCGAC TCTCCTAGGA   
  
  
- GTTCAGTACC GGACCGTACA TATCTCGGGA AGTAAAATGC TTTTACGGAC AGTAAAGTCT CCTTTAAGAA   
  
  
- CGATCCTTCG AGAAGAAACT CGATAAAGGA AAGAACTTCG ACCGGATAAA CCACTGTTTG GTTAGCTATT   
  
  
- AGCTTCGGTA CCTCCCCCTT TTCTACCAAG TATAATAACT AGACTTACGT AGTCTTGGAC GAGTTACCTA   
  
  
- ACGGGAATAA GTCCGAAACT CACGTTCCGG ACCCCCAGGA GGAGTAAACT CTTAATGGCC ACAAGTAGTT   
  
  
- GTGTTTCTCC AAGATCTAGT TCACCGAGTA TCCCACTGAG TTCTTCGACT CTTTAACCTA AACGGTAAAG   
  
  
- TCAAGTTAGG ACACCAATCG TTCAACCTTT TGGAACTACA ACTTTTCGAC ACACAATTCT GACCACTCCG   
  
  
- AGATCGGTAG TCAAGCCAGG AAGTTGACGT ATGGGAAAAC CCAAGACTAC TACTCGGGGA TTCCTTTTCA   
  
  
- AGTGGAAATC GGAACTACTT CATACGTTTA CCCCGATTAT CCGTTTCGGG CTCATTACTA AGCCGAAGAA   
  
  
- GTAGTGGAGG AAGCGAGTTA TGAAGTTGGT TCGACCTACC AAAGGAGTCG CGAAACACCC CTAACAGGGG   
  
  
- TTTCTAATAC CACTATCGAC TCGTGCTAAG GTTAGTGTTA CCAAGACCTG AATACCTCTC CAACAGTCTT   
  
  
- CGTGACATGA AGATACGTCG CGACAAACTG ACGAATCTTA GGTGGGACGG TTCTTGTAGA CAGCTTTCTT   
  
  
- CCGCCCATCT CTTCTACGAG GATCCACTTC TCTAGTTCTT GTAATATAGT ACGCTCCCTC CTCTTTCCTC   
  
  
- TCTTTCCGTA CTCTTCTAGC TCTTCACCTA CGTCTCCGAT CTGTACCGAC CTAAGCCCTA GCAAGGAAAC   
  
  
- TCGATATACC CATACGACGT TCGTTCCGTT AACGAAGTCC CGATACCAAC ACTACCAATA TCTCACTTTC   
  
  
- TCTTACCAAC ACAACAGTAG ACAACCGTTC TAGCGGGGGA GAAAAGCCAT AGTCGTACCT CCACATCCTC   
  
  
- TAC

+     TATC-box

| Site Name | Organism | Position | Strand | Matrix score. | sequence | function |
| --- | --- | --- | --- | --- | --- | --- |
| TATC-box | Oryza sativa | 1036 | - | 7 | TATCCCA | cis-acting element involved in gibberellin-responsiveness |

>HU08G00367.1   
+ -Up\_Stream \_Len000ATGCGC CTTTCTGCTA ACTCATTATC TTTCCTTCTG AGAGGGGACA ACTTATATAA   
  
  
+ ATTGATAGGT ATTTTCAAAT CAATAGACCA GCTACCATAT ATGATAATTT TTATTACTTT TCTAAATCCA   
  
  
+ TCAACTATTT TCACAATCCT CCTTTTTCTA GAAAAATAAA ATTTGCTTAA TTCAAATCAC CTAAAATTAC   
  
  
+ TAAATCTATA GTCCTAAATC TACAAACTCA TTTGTGTTGC AACTCATGAT TTAAGTGGAA CACTAACCTT   
  
  
+ AGGAGATATG TTGTCAAAAG AAGAATTCGA TGACATATTT TCCAACAAAA GGCCACCTAA CTTCATGATT   
  
  
+ TAAAAAGGAG GAGTCTAAGC ACATCATGGA TGACTAATTT TTGATGTATT GTAACTACGT ATTGAGATAA   
  
  
+ CTTGAATGAA TCGTGACTAG AATATATCTA TTTTCAAAAA TAAAAATTCC CTAAAAGAAA AGGATAAAAA   
  
  
+ ACTATATAAT ACTAAAAAAT TCTAACTATA ATAACTAGGC TTATTAAATT TTTATAGTGA AAATTTATTT   
  
  
+ CTACTTATAA CAACATCACT TACTAAGTAG CTTATTTGTG TCTTCTTTTC TAAATAATAA TGGTAACTAT   
  
  
+ GTGTCTTTTG TAAATAACTT ATTATCTATT TTATAAATCA CCGTGTAAAG CACGGGTCTA TACTAGTTAA   
  
  
+ TTTAAATAGC ATTGTTAAAT CAAGTAAGAT ACAAACAAAT TTATTTACAA CTGTAAGTGG TCCATAAAAA   
  
  
+ TAATAGCCAT TACAAAAAAT AGTACTACTG TTTTATGAAA TGATTTTAAT ATTCTAAAAT TCCAATAAAA   
  
  
+ CATGTTATAA TCTATGATTA ATTTTAAAAC ATGATTACAT GTCAGCATAA TTTGTATTAT TGTACAGCAA   
  
  
+ ATGAAAGTTG GAGCTAAAAG AAAATGGGGA AATAAAGGCA GACAAGTAAA GATAAAATAT GCTATCTACT   
  
  
+ TTTGGAGGTA TCCTTAACCA CATTGTCTCG TTTGTCTTCA TTAGGGAGAA GTGGGATAAA TACCAAAATT   
  
  
+ TGTATGGTCA TTGCGTGGGG TTTGCTTCCA AACAAAATGG CTTTTTGGAA GCAAATTTTG TGAGCTTCTA   
  
  
+ TGCAAATTTC CACGCTTAAC CCCACAAGCA AACAAAAACA GACAGTGGTT TTTGTTTGTC TTTGTTAGGG   
  
  
+ CATGTTTGGA GCCAACTGTT AATGGGAATA AACTGGGGAG GCTTTTTCCT TGGCTCTTTC CCCCTGACCC   
  
  
+ CAAACCCACT TCAGCTCAAT ACGAAACCAG CCATTGAAAA AGGAAGGGGG GGGGGGGGGT ATGTGAGAAA   
  
  
+ AAGACAAAAC TTTTTCCCGG AGAGAGAAGA TACATATGCA ACAAAGGCTC TCCTTCTCAT AAATTCCATA   
  
  
+ TTCCTCTGTT TTTCTAGGAG AGAGAAATAT GCAAAAAACA AATCAGTTGG CGACAAAAGT TACTACCACG   
  
  
+ CATTGTCTAC TGCTGCGTAC CCATTTTTGG AGACTTTAAA GAACGCCCCT CCCATCTTGA AAAGTGAAAA   
  
  
+ CCCCCTCTTT GAAACTGAAT TTCAGGGGAA AAAAACCTCA CCAAACCTCT AGAGAGAGCT TTTAGAGAGG   
  
  
+ GAAATACAAC GATTAGAAGG AGCAATTTGG GAAATTTCTT TGGGAATTTG AATGGGTTTT GAGTGAATTG   
  
  
+ CAAATCCCAG AAAAGTTTTG GCAAGTACCG ATCTACAGTT CTCTCCTCTT CGTGTTTGGT AGATCCCCTG   
  
  
+ TTTCCTCTGT TTCATTTAGG GTACTTCTCA TTCATCCTCC CCACCCCCTT AATCGGATCT TCCTGTCGAG   
  
  
+ TCACTTTATG CTAATATTTT TTCAGTGGAT TTTTAGTTAA CCCTGTTCAT TTTTCCATTC TGTGTCCCAT   
  
  
+ TTCTCTCTTT TTCATTCATA AGTTGCCGGT TTATCTGTTG GGTGCAGCTT AGTCACAATA ATTTCTGTGT   
  
  
+ TAGGCTTCTT TACGGTTAAA AAAAAAGGAG GCACTCTTTT CGGTGTGATT GTTTATGGGA CCAATGATTC   
  
  
+ AAGATGATGG GTCATCAGTA ACTTCATCAC CCCTTCAATT TTTCTCCATG ATGTCTCCCA ATTTAGGTTC   
  
  
+ TTCCTACCCT TGGCTCAGAG AGCTAAAACC TGAAGAAAGA GGTCTTTACT TGATACATTT GTTGCTCTCT   
  
  
+ TGTGCAAATC ATGTCTCTAG TGGTAGCCTA GACAATGCGA ACTTAGCCCT CGAACAAATC TCTCAGCTTG   
  
  
+ CTGCCCCTGA TGGGGATACA ATGCAGCGTA TGGCTTCTTA TTTTGCTGAA GCCCTGGCTG AGAGGATCCT   
  
  
+ CAAGTCATGG CCTGGCATGT ATAGAGCCCT TCATTTTACG AAAATGCCTG TCATTTCAGA GGAAATTCTT   
  
  
+ GCTAGGAAGC TCTTCTTTGA GCTATTTCCT TTCTTGAAGC TGGCCTATTT GGTGACAAAC CAATCGATAA   
  
  
+ TCGAAGCCAT GGAGGGGGAA AAGATGGTTC ATATTATTGA TCTGAATGCA TCAGAACCTG CTCAATGGAT   
  
  
+ TGCCCTTATT CAGGCTTTGA GTGCAAGGCC TGGGGGTCCT CCTCATTTGA GAATTACCGG TGTTCATCAA   
  
  
+ CACAAAGAGG TTCTAGATCA AGTGGCTCAT AGGGTGACTC AAGAAGCTGA GAAATTGGAT TTGCCATTTC   
  
  
+ AGTTCAATCC TGTGGTTAGC AAGTTGGAAA ACCTTGATGT TGAAAAGCTG TGTGTTAAGA CTGGTGAGGC   
  
  
+ TCTAGCCATC AGTTCGGTCC TTCAACTGCA TACCCTTTTG GGTTCTGATG ATGAGCCCCT AAGGAAAAGT   
  
  
+ TCACCTTTAG CCTTGATGAA GTATGCAAAT GGGGCTAATA GGCAAAGCCC GAGTAATGAT TCGGCTTCTT   
  
  
+ CATCACCTCC TTCGCTCAAT ACTTCAACCA AGCTGGATGG TTTCCTCAGC GCTTTGTGGG GATTGTCCCC   
  
  
+ AAAGATTATG GTGATAGCTG AGCACGATTC CAATCACAAT GGTTCTGGAC TTATGGAGAG GTTGTCAGAA   
  
  
+ GCACTGTACT TCTATGCAGC GCTGTTTGAC TGCTTAGAAT CCACCCTGCC AAGAACATCT GTCGAAAGAA   
  
  
+ GGCGGGTAGA GAAGATGCTC CTAGGTGAAG AGATCAAGAA CATTATATCA TGCGAGGGAG GAGAAAGGAG   
  
  
+ AGAAAGGCAT GAGAAGATCG AGAAGTGGAT GCAGAGGCTA GACATGGCTG GATTCGGGAT CGTTCCTTTG   
  
  
+ AGCTATATGG GTATGCTGCA AGCAAGGCAA TTGCTTCAGG GCTATGGTTG TGATGGTTAT AGAGTGAAAG   
  
  
+ AGAATGGTTG TGTTGTCATC TGTTGGCAAG ATCGCCCCCT CTTTTCGGTA TCAGCATGGA GGTGTAGGAG   
  
  
+ ATG  

- -Up\_Stream \_Len000TACGCG GAAAGACGAT TGAGTAATAG AAAGGAAGAC TCTCCCCTGT TGAATATATT   
  
  
- TAACTATCCA TAAAAGTTTA GTTATCTGGT CGATGGTATA TACTATTAAA AATAATGAAA AGATTTAGGT   
  
  
- AGTTGATAAA AGTGTTAGGA GGAAAAAGAT CTTTTTATTT TAAACGAATT AAGTTTAGTG GATTTTAATG   
  
  
- ATTTAGATAT CAGGATTTAG ATGTTTGAGT AAACACAACG TTGAGTACTA AATTCACCTT GTGATTGGAA   
  
  
- TCCTCTATAC AACAGTTTTC TTCTTAAGCT ACTGTATAAA AGGTTGTTTT CCGGTGGATT GAAGTACTAA   
  
  
- ATTTTTCCTC CTCAGATTCG TGTAGTACCT ACTGATTAAA AACTACATAA CATTGATGCA TAACTCTATT   
  
  
- GAACTTACTT AGCACTGATC TTATATAGAT AAAAGTTTTT ATTTTTAAGG GATTTTCTTT TCCTATTTTT   
  
  
- TGATATATTA TGATTTTTTA AGATTGATAT TATTGATCCG AATAATTTAA AAATATCACT TTTAAATAAA   
  
  
- GATGAATATT GTTGTAGTGA ATGATTCATC GAATAAACAC AGAAGAAAAG ATTTATTATT ACCATTGATA   
  
  
- CACAGAAAAC ATTTATTGAA TAATAGATAA AATATTTAGT GGCACATTTC GTGCCCAGAT ATGATCAATT   
  
  
- AAATTTATCG TAACAATTTA GTTCATTCTA TGTTTGTTTA AATAAATGTT GACATTCACC AGGTATTTTT   
  
  
- ATTATCGGTA ATGTTTTTTA TCATGATGAC AAAATACTTT ACTAAAATTA TAAGATTTTA AGGTTATTTT   
  
  
- GTACAATATT AGATACTAAT TAAAATTTTG TACTAATGTA CAGTCGTATT AAACATAATA ACATGTCGTT   
  
  
- TACTTTCAAC CTCGATTTTC TTTTACCCCT TTATTTCCGT CTGTTCATTT CTATTTTATA CGATAGATGA   
  
  
- AAACCTCCAT AGGAATTGGT GTAACAGAGC AAACAGAAGT AATCCCTCTT CACCCTATTT ATGGTTTTAA   
  
  
- ACATACCAGT AACGCACCCC AAACGAAGGT TTGTTTTACC GAAAAACCTT CGTTTAAAAC ACTCGAAGAT   
  
  
- ACGTTTAAAG GTGCGAATTG GGGTGTTCGT TTGTTTTTGT CTGTCACCAA AAACAAACAG AAACAATCCC   
  
  
- GTACAAACCT CGGTTGACAA TTACCCTTAT TTGACCCCTC CGAAAAAGGA ACCGAGAAAG GGGGACTGGG   
  
  
- GTTTGGGTGA AGTCGAGTTA TGCTTTGGTC GGTAACTTTT TCCTTCCCCC CCCCCCCCCA TACACTCTTT   
  
  
- TTCTGTTTTG AAAAAGGGCC TCTCTCTTCT ATGTATACGT TGTTTCCGAG AGGAAGAGTA TTTAAGGTAT   
  
  
- AAGGAGACAA AAAGATCCTC TCTCTTTATA CGTTTTTTGT TTAGTCAACC GCTGTTTTCA ATGATGGTGC   
  
  
- GTAACAGATG ACGACGCATG GGTAAAAACC TCTGAAATTT CTTGCGGGGA GGGTAGAACT TTTCACTTTT   
  
  
- GGGGGAGAAA CTTTGACTTA AAGTCCCCTT TTTTTGGAGT GGTTTGGAGA TCTCTCTCGA AAATCTCTCC   
  
  
- CTTTATGTTG CTAATCTTCC TCGTTAAACC CTTTAAAGAA ACCCTTAAAC TTACCCAAAA CTCACTTAAC   
  
  
- GTTTAGGGTC TTTTCAAAAC CGTTCATGGC TAGATGTCAA GAGAGGAGAA GCACAAACCA TCTAGGGGAC   
  
  
- AAAGGAGACA AAGTAAATCC CATGAAGAGT AAGTAGGAGG GGTGGGGGAA TTAGCCTAGA AGGACAGCTC   
  
  
- AGTGAAATAC GATTATAAAA AAGTCACCTA AAAATCAATT GGGACAAGTA AAAAGGTAAG ACACAGGGTA   
  
  
- AAGAGAGAAA AAGTAAGTAT TCAACGGCCA AATAGACAAC CCACGTCGAA TCAGTGTTAT TAAAGACACA   
  
  
- ATCCGAAGAA ATGCCAATTT TTTTTTCCTC CGTGAGAAAA GCCACACTAA CAAATACCCT GGTTACTAAG   
  
  
- TTCTACTACC CAGTAGTCAT TGAAGTAGTG GGGAAGTTAA AAAGAGGTAC TACAGAGGGT TAAATCCAAG   
  
  
- AAGGATGGGA ACCGAGTCTC TCGATTTTGG ACTTCTTTCT CCAGAAATGA ACTATGTAAA CAACGAGAGA   
  
  
- ACACGTTTAG TACAGAGATC ACCATCGGAT CTGTTACGCT TGAATCGGGA GCTTGTTTAG AGAGTCGAAC   
  
  
- GACGGGGACT ACCCCTATGT TACGTCGCAT ACCGAAGAAT AAAACGACTT CGGGACCGAC TCTCCTAGGA   
  
  
- GTTCAGTACC GGACCGTACA TATCTCGGGA AGTAAAATGC TTTTACGGAC AGTAAAGTCT CCTTTAAGAA   
  
  
- CGATCCTTCG AGAAGAAACT CGATAAAGGA AAGAACTTCG ACCGGATAAA CCACTGTTTG GTTAGCTATT   
  
  
- AGCTTCGGTA CCTCCCCCTT TTCTACCAAG TATAATAACT AGACTTACGT AGTCTTGGAC GAGTTACCTA   
  
  
- ACGGGAATAA GTCCGAAACT CACGTTCCGG ACCCCCAGGA GGAGTAAACT CTTAATGGCC ACAAGTAGTT   
  
  
- GTGTTTCTCC AAGATCTAGT TCACCGAGTA TCCCACTGAG TTCTTCGACT CTTTAACCTA AACGGTAAAG   
  
  
- TCAAGTTAGG ACACCAATCG TTCAACCTTT TGGAACTACA ACTTTTCGAC ACACAATTCT GACCACTCCG   
  
  
- AGATCGGTAG TCAAGCCAGG AAGTTGACGT ATGGGAAAAC CCAAGACTAC TACTCGGGGA TTCCTTTTCA   
  
  
- AGTGGAAATC GGAACTACTT CATACGTTTA CCCCGATTAT CCGTTTCGGG CTCATTACTA AGCCGAAGAA   
  
  
- GTAGTGGAGG AAGCGAGTTA TGAAGTTGGT TCGACCTACC AAAGGAGTCG CGAAACACCC CTAACAGGGG   
  
  
- TTTCTAATAC CACTATCGAC TCGTGCTAAG GTTAGTGTTA CCAAGACCTG AATACCTCTC CAACAGTCTT   
  
  
- CGTGACATGA AGATACGTCG CGACAAACTG ACGAATCTTA GGTGGGACGG TTCTTGTAGA CAGCTTTCTT   
  
  
- CCGCCCATCT CTTCTACGAG GATCCACTTC TCTAGTTCTT GTAATATAGT ACGCTCCCTC CTCTTTCCTC   
  
  
- TCTTTCCGTA CTCTTCTAGC TCTTCACCTA CGTCTCCGAT CTGTACCGAC CTAAGCCCTA GCAAGGAAAC   
  
  
- TCGATATACC CATACGACGT TCGTTCCGTT AACGAAGTCC CGATACCAAC ACTACCAATA TCTCACTTTC   
  
  
- TCTTACCAAC ACAACAGTAG ACAACCGTTC TAGCGGGGGA GAAAAGCCAT AGTCGTACCT CCACATCCTC   
  
  
- TAC

+     TCA-element

| Site Name | Organism | Position | Strand | Matrix score. | sequence | function |
| --- | --- | --- | --- | --- | --- | --- |
| TCA-element | Nicotiana tabacum | 2472 | - | 9 | CCATCTTTTT | cis-acting element involved in salicylic acid responsiveness |

>HU08G00367.1   
+ -Up\_Stream \_Len000ATGCGC CTTTCTGCTA ACTCATTATC TTTCCTTCTG AGAGGGGACA ACTTATATAA   
  
  
+ ATTGATAGGT ATTTTCAAAT CAATAGACCA GCTACCATAT ATGATAATTT TTATTACTTT TCTAAATCCA   
  
  
+ TCAACTATTT TCACAATCCT CCTTTTTCTA GAAAAATAAA ATTTGCTTAA TTCAAATCAC CTAAAATTAC   
  
  
+ TAAATCTATA GTCCTAAATC TACAAACTCA TTTGTGTTGC AACTCATGAT TTAAGTGGAA CACTAACCTT   
  
  
+ AGGAGATATG TTGTCAAAAG AAGAATTCGA TGACATATTT TCCAACAAAA GGCCACCTAA CTTCATGATT   
  
  
+ TAAAAAGGAG GAGTCTAAGC ACATCATGGA TGACTAATTT TTGATGTATT GTAACTACGT ATTGAGATAA   
  
  
+ CTTGAATGAA TCGTGACTAG AATATATCTA TTTTCAAAAA TAAAAATTCC CTAAAAGAAA AGGATAAAAA   
  
  
+ ACTATATAAT ACTAAAAAAT TCTAACTATA ATAACTAGGC TTATTAAATT TTTATAGTGA AAATTTATTT   
  
  
+ CTACTTATAA CAACATCACT TACTAAGTAG CTTATTTGTG TCTTCTTTTC TAAATAATAA TGGTAACTAT   
  
  
+ GTGTCTTTTG TAAATAACTT ATTATCTATT TTATAAATCA CCGTGTAAAG CACGGGTCTA TACTAGTTAA   
  
  
+ TTTAAATAGC ATTGTTAAAT CAAGTAAGAT ACAAACAAAT TTATTTACAA CTGTAAGTGG TCCATAAAAA   
  
  
+ TAATAGCCAT TACAAAAAAT AGTACTACTG TTTTATGAAA TGATTTTAAT ATTCTAAAAT TCCAATAAAA   
  
  
+ CATGTTATAA TCTATGATTA ATTTTAAAAC ATGATTACAT GTCAGCATAA TTTGTATTAT TGTACAGCAA   
  
  
+ ATGAAAGTTG GAGCTAAAAG AAAATGGGGA AATAAAGGCA GACAAGTAAA GATAAAATAT GCTATCTACT   
  
  
+ TTTGGAGGTA TCCTTAACCA CATTGTCTCG TTTGTCTTCA TTAGGGAGAA GTGGGATAAA TACCAAAATT   
  
  
+ TGTATGGTCA TTGCGTGGGG TTTGCTTCCA AACAAAATGG CTTTTTGGAA GCAAATTTTG TGAGCTTCTA   
  
  
+ TGCAAATTTC CACGCTTAAC CCCACAAGCA AACAAAAACA GACAGTGGTT TTTGTTTGTC TTTGTTAGGG   
  
  
+ CATGTTTGGA GCCAACTGTT AATGGGAATA AACTGGGGAG GCTTTTTCCT TGGCTCTTTC CCCCTGACCC   
  
  
+ CAAACCCACT TCAGCTCAAT ACGAAACCAG CCATTGAAAA AGGAAGGGGG GGGGGGGGGT ATGTGAGAAA   
  
  
+ AAGACAAAAC TTTTTCCCGG AGAGAGAAGA TACATATGCA ACAAAGGCTC TCCTTCTCAT AAATTCCATA   
  
  
+ TTCCTCTGTT TTTCTAGGAG AGAGAAATAT GCAAAAAACA AATCAGTTGG CGACAAAAGT TACTACCACG   
  
  
+ CATTGTCTAC TGCTGCGTAC CCATTTTTGG AGACTTTAAA GAACGCCCCT CCCATCTTGA AAAGTGAAAA   
  
  
+ CCCCCTCTTT GAAACTGAAT TTCAGGGGAA AAAAACCTCA CCAAACCTCT AGAGAGAGCT TTTAGAGAGG   
  
  
+ GAAATACAAC GATTAGAAGG AGCAATTTGG GAAATTTCTT TGGGAATTTG AATGGGTTTT GAGTGAATTG   
  
  
+ CAAATCCCAG AAAAGTTTTG GCAAGTACCG ATCTACAGTT CTCTCCTCTT CGTGTTTGGT AGATCCCCTG   
  
  
+ TTTCCTCTGT TTCATTTAGG GTACTTCTCA TTCATCCTCC CCACCCCCTT AATCGGATCT TCCTGTCGAG   
  
  
+ TCACTTTATG CTAATATTTT TTCAGTGGAT TTTTAGTTAA CCCTGTTCAT TTTTCCATTC TGTGTCCCAT   
  
  
+ TTCTCTCTTT TTCATTCATA AGTTGCCGGT TTATCTGTTG GGTGCAGCTT AGTCACAATA ATTTCTGTGT   
  
  
+ TAGGCTTCTT TACGGTTAAA AAAAAAGGAG GCACTCTTTT CGGTGTGATT GTTTATGGGA CCAATGATTC   
  
  
+ AAGATGATGG GTCATCAGTA ACTTCATCAC CCCTTCAATT TTTCTCCATG ATGTCTCCCA ATTTAGGTTC   
  
  
+ TTCCTACCCT TGGCTCAGAG AGCTAAAACC TGAAGAAAGA GGTCTTTACT TGATACATTT GTTGCTCTCT   
  
  
+ TGTGCAAATC ATGTCTCTAG TGGTAGCCTA GACAATGCGA ACTTAGCCCT CGAACAAATC TCTCAGCTTG   
  
  
+ CTGCCCCTGA TGGGGATACA ATGCAGCGTA TGGCTTCTTA TTTTGCTGAA GCCCTGGCTG AGAGGATCCT   
  
  
+ CAAGTCATGG CCTGGCATGT ATAGAGCCCT TCATTTTACG AAAATGCCTG TCATTTCAGA GGAAATTCTT   
  
  
+ GCTAGGAAGC TCTTCTTTGA GCTATTTCCT TTCTTGAAGC TGGCCTATTT GGTGACAAAC CAATCGATAA   
  
  
+ TCGAAGCCAT GGAGGGGGAA AAGATGGTTC ATATTATTGA TCTGAATGCA TCAGAACCTG CTCAATGGAT   
  
  
+ TGCCCTTATT CAGGCTTTGA GTGCAAGGCC TGGGGGTCCT CCTCATTTGA GAATTACCGG TGTTCATCAA   
  
  
+ CACAAAGAGG TTCTAGATCA AGTGGCTCAT AGGGTGACTC AAGAAGCTGA GAAATTGGAT TTGCCATTTC   
  
  
+ AGTTCAATCC TGTGGTTAGC AAGTTGGAAA ACCTTGATGT TGAAAAGCTG TGTGTTAAGA CTGGTGAGGC   
  
  
+ TCTAGCCATC AGTTCGGTCC TTCAACTGCA TACCCTTTTG GGTTCTGATG ATGAGCCCCT AAGGAAAAGT   
  
  
+ TCACCTTTAG CCTTGATGAA GTATGCAAAT GGGGCTAATA GGCAAAGCCC GAGTAATGAT TCGGCTTCTT   
  
  
+ CATCACCTCC TTCGCTCAAT ACTTCAACCA AGCTGGATGG TTTCCTCAGC GCTTTGTGGG GATTGTCCCC   
  
  
+ AAAGATTATG GTGATAGCTG AGCACGATTC CAATCACAAT GGTTCTGGAC TTATGGAGAG GTTGTCAGAA   
  
  
+ GCACTGTACT TCTATGCAGC GCTGTTTGAC TGCTTAGAAT CCACCCTGCC AAGAACATCT GTCGAAAGAA   
  
  
+ GGCGGGTAGA GAAGATGCTC CTAGGTGAAG AGATCAAGAA CATTATATCA TGCGAGGGAG GAGAAAGGAG   
  
  
+ AGAAAGGCAT GAGAAGATCG AGAAGTGGAT GCAGAGGCTA GACATGGCTG GATTCGGGAT CGTTCCTTTG   
  
  
+ AGCTATATGG GTATGCTGCA AGCAAGGCAA TTGCTTCAGG GCTATGGTTG TGATGGTTAT AGAGTGAAAG   
  
  
+ AGAATGGTTG TGTTGTCATC TGTTGGCAAG ATCGCCCCCT CTTTTCGGTA TCAGCATGGA GGTGTAGGAG   
  
  
+ ATG  

- -Up\_Stream \_Len000TACGCG GAAAGACGAT TGAGTAATAG AAAGGAAGAC TCTCCCCTGT TGAATATATT   
  
  
- TAACTATCCA TAAAAGTTTA GTTATCTGGT CGATGGTATA TACTATTAAA AATAATGAAA AGATTTAGGT   
  
  
- AGTTGATAAA AGTGTTAGGA GGAAAAAGAT CTTTTTATTT TAAACGAATT AAGTTTAGTG GATTTTAATG   
  
  
- ATTTAGATAT CAGGATTTAG ATGTTTGAGT AAACACAACG TTGAGTACTA AATTCACCTT GTGATTGGAA   
  
  
- TCCTCTATAC AACAGTTTTC TTCTTAAGCT ACTGTATAAA AGGTTGTTTT CCGGTGGATT GAAGTACTAA   
  
  
- ATTTTTCCTC CTCAGATTCG TGTAGTACCT ACTGATTAAA AACTACATAA CATTGATGCA TAACTCTATT   
  
  
- GAACTTACTT AGCACTGATC TTATATAGAT AAAAGTTTTT ATTTTTAAGG GATTTTCTTT TCCTATTTTT   
  
  
- TGATATATTA TGATTTTTTA AGATTGATAT TATTGATCCG AATAATTTAA AAATATCACT TTTAAATAAA   
  
  
- GATGAATATT GTTGTAGTGA ATGATTCATC GAATAAACAC AGAAGAAAAG ATTTATTATT ACCATTGATA   
  
  
- CACAGAAAAC ATTTATTGAA TAATAGATAA AATATTTAGT GGCACATTTC GTGCCCAGAT ATGATCAATT   
  
  
- AAATTTATCG TAACAATTTA GTTCATTCTA TGTTTGTTTA AATAAATGTT GACATTCACC AGGTATTTTT   
  
  
- ATTATCGGTA ATGTTTTTTA TCATGATGAC AAAATACTTT ACTAAAATTA TAAGATTTTA AGGTTATTTT   
  
  
- GTACAATATT AGATACTAAT TAAAATTTTG TACTAATGTA CAGTCGTATT AAACATAATA ACATGTCGTT   
  
  
- TACTTTCAAC CTCGATTTTC TTTTACCCCT TTATTTCCGT CTGTTCATTT CTATTTTATA CGATAGATGA   
  
  
- AAACCTCCAT AGGAATTGGT GTAACAGAGC AAACAGAAGT AATCCCTCTT CACCCTATTT ATGGTTTTAA   
  
  
- ACATACCAGT AACGCACCCC AAACGAAGGT TTGTTTTACC GAAAAACCTT CGTTTAAAAC ACTCGAAGAT   
  
  
- ACGTTTAAAG GTGCGAATTG GGGTGTTCGT TTGTTTTTGT CTGTCACCAA AAACAAACAG AAACAATCCC   
  
  
- GTACAAACCT CGGTTGACAA TTACCCTTAT TTGACCCCTC CGAAAAAGGA ACCGAGAAAG GGGGACTGGG   
  
  
- GTTTGGGTGA AGTCGAGTTA TGCTTTGGTC GGTAACTTTT TCCTTCCCCC CCCCCCCCCA TACACTCTTT   
  
  
- TTCTGTTTTG AAAAAGGGCC TCTCTCTTCT ATGTATACGT TGTTTCCGAG AGGAAGAGTA TTTAAGGTAT   
  
  
- AAGGAGACAA AAAGATCCTC TCTCTTTATA CGTTTTTTGT TTAGTCAACC GCTGTTTTCA ATGATGGTGC   
  
  
- GTAACAGATG ACGACGCATG GGTAAAAACC TCTGAAATTT CTTGCGGGGA GGGTAGAACT TTTCACTTTT   
  
  
- GGGGGAGAAA CTTTGACTTA AAGTCCCCTT TTTTTGGAGT GGTTTGGAGA TCTCTCTCGA AAATCTCTCC   
  
  
- CTTTATGTTG CTAATCTTCC TCGTTAAACC CTTTAAAGAA ACCCTTAAAC TTACCCAAAA CTCACTTAAC   
  
  
- GTTTAGGGTC TTTTCAAAAC CGTTCATGGC TAGATGTCAA GAGAGGAGAA GCACAAACCA TCTAGGGGAC   
  
  
- AAAGGAGACA AAGTAAATCC CATGAAGAGT AAGTAGGAGG GGTGGGGGAA TTAGCCTAGA AGGACAGCTC   
  
  
- AGTGAAATAC GATTATAAAA AAGTCACCTA AAAATCAATT GGGACAAGTA AAAAGGTAAG ACACAGGGTA   
  
  
- AAGAGAGAAA AAGTAAGTAT TCAACGGCCA AATAGACAAC CCACGTCGAA TCAGTGTTAT TAAAGACACA   
  
  
- ATCCGAAGAA ATGCCAATTT TTTTTTCCTC CGTGAGAAAA GCCACACTAA CAAATACCCT GGTTACTAAG   
  
  
- TTCTACTACC CAGTAGTCAT TGAAGTAGTG GGGAAGTTAA AAAGAGGTAC TACAGAGGGT TAAATCCAAG   
  
  
- AAGGATGGGA ACCGAGTCTC TCGATTTTGG ACTTCTTTCT CCAGAAATGA ACTATGTAAA CAACGAGAGA   
  
  
- ACACGTTTAG TACAGAGATC ACCATCGGAT CTGTTACGCT TGAATCGGGA GCTTGTTTAG AGAGTCGAAC   
  
  
- GACGGGGACT ACCCCTATGT TACGTCGCAT ACCGAAGAAT AAAACGACTT CGGGACCGAC TCTCCTAGGA   
  
  
- GTTCAGTACC GGACCGTACA TATCTCGGGA AGTAAAATGC TTTTACGGAC AGTAAAGTCT CCTTTAAGAA   
  
  
- CGATCCTTCG AGAAGAAACT CGATAAAGGA AAGAACTTCG ACCGGATAAA CCACTGTTTG GTTAGCTATT   
  
  
- AGCTTCGGTA CCTCCCCCTT TTCTACCAAG TATAATAACT AGACTTACGT AGTCTTGGAC GAGTTACCTA   
  
  
- ACGGGAATAA GTCCGAAACT CACGTTCCGG ACCCCCAGGA GGAGTAAACT CTTAATGGCC ACAAGTAGTT   
  
  
- GTGTTTCTCC AAGATCTAGT TCACCGAGTA TCCCACTGAG TTCTTCGACT CTTTAACCTA AACGGTAAAG   
  
  
- TCAAGTTAGG ACACCAATCG TTCAACCTTT TGGAACTACA ACTTTTCGAC ACACAATTCT GACCACTCCG   
  
  
- AGATCGGTAG TCAAGCCAGG AAGTTGACGT ATGGGAAAAC CCAAGACTAC TACTCGGGGA TTCCTTTTCA   
  
  
- AGTGGAAATC GGAACTACTT CATACGTTTA CCCCGATTAT CCGTTTCGGG CTCATTACTA AGCCGAAGAA   
  
  
- GTAGTGGAGG AAGCGAGTTA TGAAGTTGGT TCGACCTACC AAAGGAGTCG CGAAACACCC CTAACAGGGG   
  
  
- TTTCTAATAC CACTATCGAC TCGTGCTAAG GTTAGTGTTA CCAAGACCTG AATACCTCTC CAACAGTCTT   
  
  
- CGTGACATGA AGATACGTCG CGACAAACTG ACGAATCTTA GGTGGGACGG TTCTTGTAGA CAGCTTTCTT   
  
  
- CCGCCCATCT CTTCTACGAG GATCCACTTC TCTAGTTCTT GTAATATAGT ACGCTCCCTC CTCTTTCCTC   
  
  
- TCTTTCCGTA CTCTTCTAGC TCTTCACCTA CGTCTCCGAT CTGTACCGAC CTAAGCCCTA GCAAGGAAAC   
  
  
- TCGATATACC CATACGACGT TCGTTCCGTT AACGAAGTCC CGATACCAAC ACTACCAATA TCTCACTTTC   
  
  
- TCTTACCAAC ACAACAGTAG ACAACCGTTC TAGCGGGGGA GAAAAGCCAT AGTCGTACCT CCACATCCTC   
  
  
- TAC

+     TCCC-motif

| Site Name | Organism | Position | Strand | Matrix score. | sequence | function |
| --- | --- | --- | --- | --- | --- | --- |
| TCCC-motif | Spinacia oleracea | 1027 | - | 7 | TCTCCCT | part of a light responsive element |

>HU08G00367.1   
+ -Up\_Stream \_Len000ATGCGC CTTTCTGCTA ACTCATTATC TTTCCTTCTG AGAGGGGACA ACTTATATAA   
  
  
+ ATTGATAGGT ATTTTCAAAT CAATAGACCA GCTACCATAT ATGATAATTT TTATTACTTT TCTAAATCCA   
  
  
+ TCAACTATTT TCACAATCCT CCTTTTTCTA GAAAAATAAA ATTTGCTTAA TTCAAATCAC CTAAAATTAC   
  
  
+ TAAATCTATA GTCCTAAATC TACAAACTCA TTTGTGTTGC AACTCATGAT TTAAGTGGAA CACTAACCTT   
  
  
+ AGGAGATATG TTGTCAAAAG AAGAATTCGA TGACATATTT TCCAACAAAA GGCCACCTAA CTTCATGATT   
  
  
+ TAAAAAGGAG GAGTCTAAGC ACATCATGGA TGACTAATTT TTGATGTATT GTAACTACGT ATTGAGATAA   
  
  
+ CTTGAATGAA TCGTGACTAG AATATATCTA TTTTCAAAAA TAAAAATTCC CTAAAAGAAA AGGATAAAAA   
  
  
+ ACTATATAAT ACTAAAAAAT TCTAACTATA ATAACTAGGC TTATTAAATT TTTATAGTGA AAATTTATTT   
  
  
+ CTACTTATAA CAACATCACT TACTAAGTAG CTTATTTGTG TCTTCTTTTC TAAATAATAA TGGTAACTAT   
  
  
+ GTGTCTTTTG TAAATAACTT ATTATCTATT TTATAAATCA CCGTGTAAAG CACGGGTCTA TACTAGTTAA   
  
  
+ TTTAAATAGC ATTGTTAAAT CAAGTAAGAT ACAAACAAAT TTATTTACAA CTGTAAGTGG TCCATAAAAA   
  
  
+ TAATAGCCAT TACAAAAAAT AGTACTACTG TTTTATGAAA TGATTTTAAT ATTCTAAAAT TCCAATAAAA   
  
  
+ CATGTTATAA TCTATGATTA ATTTTAAAAC ATGATTACAT GTCAGCATAA TTTGTATTAT TGTACAGCAA   
  
  
+ ATGAAAGTTG GAGCTAAAAG AAAATGGGGA AATAAAGGCA GACAAGTAAA GATAAAATAT GCTATCTACT   
  
  
+ TTTGGAGGTA TCCTTAACCA CATTGTCTCG TTTGTCTTCA TTAGGGAGAA GTGGGATAAA TACCAAAATT   
  
  
+ TGTATGGTCA TTGCGTGGGG TTTGCTTCCA AACAAAATGG CTTTTTGGAA GCAAATTTTG TGAGCTTCTA   
  
  
+ TGCAAATTTC CACGCTTAAC CCCACAAGCA AACAAAAACA GACAGTGGTT TTTGTTTGTC TTTGTTAGGG   
  
  
+ CATGTTTGGA GCCAACTGTT AATGGGAATA AACTGGGGAG GCTTTTTCCT TGGCTCTTTC CCCCTGACCC   
  
  
+ CAAACCCACT TCAGCTCAAT ACGAAACCAG CCATTGAAAA AGGAAGGGGG GGGGGGGGGT ATGTGAGAAA   
  
  
+ AAGACAAAAC TTTTTCCCGG AGAGAGAAGA TACATATGCA ACAAAGGCTC TCCTTCTCAT AAATTCCATA   
  
  
+ TTCCTCTGTT TTTCTAGGAG AGAGAAATAT GCAAAAAACA AATCAGTTGG CGACAAAAGT TACTACCACG   
  
  
+ CATTGTCTAC TGCTGCGTAC CCATTTTTGG AGACTTTAAA GAACGCCCCT CCCATCTTGA AAAGTGAAAA   
  
  
+ CCCCCTCTTT GAAACTGAAT TTCAGGGGAA AAAAACCTCA CCAAACCTCT AGAGAGAGCT TTTAGAGAGG   
  
  
+ GAAATACAAC GATTAGAAGG AGCAATTTGG GAAATTTCTT TGGGAATTTG AATGGGTTTT GAGTGAATTG   
  
  
+ CAAATCCCAG AAAAGTTTTG GCAAGTACCG ATCTACAGTT CTCTCCTCTT CGTGTTTGGT AGATCCCCTG   
  
  
+ TTTCCTCTGT TTCATTTAGG GTACTTCTCA TTCATCCTCC CCACCCCCTT AATCGGATCT TCCTGTCGAG   
  
  
+ TCACTTTATG CTAATATTTT TTCAGTGGAT TTTTAGTTAA CCCTGTTCAT TTTTCCATTC TGTGTCCCAT   
  
  
+ TTCTCTCTTT TTCATTCATA AGTTGCCGGT TTATCTGTTG GGTGCAGCTT AGTCACAATA ATTTCTGTGT   
  
  
+ TAGGCTTCTT TACGGTTAAA AAAAAAGGAG GCACTCTTTT CGGTGTGATT GTTTATGGGA CCAATGATTC   
  
  
+ AAGATGATGG GTCATCAGTA ACTTCATCAC CCCTTCAATT TTTCTCCATG ATGTCTCCCA ATTTAGGTTC   
  
  
+ TTCCTACCCT TGGCTCAGAG AGCTAAAACC TGAAGAAAGA GGTCTTTACT TGATACATTT GTTGCTCTCT   
  
  
+ TGTGCAAATC ATGTCTCTAG TGGTAGCCTA GACAATGCGA ACTTAGCCCT CGAACAAATC TCTCAGCTTG   
  
  
+ CTGCCCCTGA TGGGGATACA ATGCAGCGTA TGGCTTCTTA TTTTGCTGAA GCCCTGGCTG AGAGGATCCT   
  
  
+ CAAGTCATGG CCTGGCATGT ATAGAGCCCT TCATTTTACG AAAATGCCTG TCATTTCAGA GGAAATTCTT   
  
  
+ GCTAGGAAGC TCTTCTTTGA GCTATTTCCT TTCTTGAAGC TGGCCTATTT GGTGACAAAC CAATCGATAA   
  
  
+ TCGAAGCCAT GGAGGGGGAA AAGATGGTTC ATATTATTGA TCTGAATGCA TCAGAACCTG CTCAATGGAT   
  
  
+ TGCCCTTATT CAGGCTTTGA GTGCAAGGCC TGGGGGTCCT CCTCATTTGA GAATTACCGG TGTTCATCAA   
  
  
+ CACAAAGAGG TTCTAGATCA AGTGGCTCAT AGGGTGACTC AAGAAGCTGA GAAATTGGAT TTGCCATTTC   
  
  
+ AGTTCAATCC TGTGGTTAGC AAGTTGGAAA ACCTTGATGT TGAAAAGCTG TGTGTTAAGA CTGGTGAGGC   
  
  
+ TCTAGCCATC AGTTCGGTCC TTCAACTGCA TACCCTTTTG GGTTCTGATG ATGAGCCCCT AAGGAAAAGT   
  
  
+ TCACCTTTAG CCTTGATGAA GTATGCAAAT GGGGCTAATA GGCAAAGCCC GAGTAATGAT TCGGCTTCTT   
  
  
+ CATCACCTCC TTCGCTCAAT ACTTCAACCA AGCTGGATGG TTTCCTCAGC GCTTTGTGGG GATTGTCCCC   
  
  
+ AAAGATTATG GTGATAGCTG AGCACGATTC CAATCACAAT GGTTCTGGAC TTATGGAGAG GTTGTCAGAA   
  
  
+ GCACTGTACT TCTATGCAGC GCTGTTTGAC TGCTTAGAAT CCACCCTGCC AAGAACATCT GTCGAAAGAA   
  
  
+ GGCGGGTAGA GAAGATGCTC CTAGGTGAAG AGATCAAGAA CATTATATCA TGCGAGGGAG GAGAAAGGAG   
  
  
+ AGAAAGGCAT GAGAAGATCG AGAAGTGGAT GCAGAGGCTA GACATGGCTG GATTCGGGAT CGTTCCTTTG   
  
  
+ AGCTATATGG GTATGCTGCA AGCAAGGCAA TTGCTTCAGG GCTATGGTTG TGATGGTTAT AGAGTGAAAG   
  
  
+ AGAATGGTTG TGTTGTCATC TGTTGGCAAG ATCGCCCCCT CTTTTCGGTA TCAGCATGGA GGTGTAGGAG   
  
  
+ ATG  

- -Up\_Stream \_Len000TACGCG GAAAGACGAT TGAGTAATAG AAAGGAAGAC TCTCCCCTGT TGAATATATT   
  
  
- TAACTATCCA TAAAAGTTTA GTTATCTGGT CGATGGTATA TACTATTAAA AATAATGAAA AGATTTAGGT   
  
  
- AGTTGATAAA AGTGTTAGGA GGAAAAAGAT CTTTTTATTT TAAACGAATT AAGTTTAGTG GATTTTAATG   
  
  
- ATTTAGATAT CAGGATTTAG ATGTTTGAGT AAACACAACG TTGAGTACTA AATTCACCTT GTGATTGGAA   
  
  
- TCCTCTATAC AACAGTTTTC TTCTTAAGCT ACTGTATAAA AGGTTGTTTT CCGGTGGATT GAAGTACTAA   
  
  
- ATTTTTCCTC CTCAGATTCG TGTAGTACCT ACTGATTAAA AACTACATAA CATTGATGCA TAACTCTATT   
  
  
- GAACTTACTT AGCACTGATC TTATATAGAT AAAAGTTTTT ATTTTTAAGG GATTTTCTTT TCCTATTTTT   
  
  
- TGATATATTA TGATTTTTTA AGATTGATAT TATTGATCCG AATAATTTAA AAATATCACT TTTAAATAAA   
  
  
- GATGAATATT GTTGTAGTGA ATGATTCATC GAATAAACAC AGAAGAAAAG ATTTATTATT ACCATTGATA   
  
  
- CACAGAAAAC ATTTATTGAA TAATAGATAA AATATTTAGT GGCACATTTC GTGCCCAGAT ATGATCAATT   
  
  
- AAATTTATCG TAACAATTTA GTTCATTCTA TGTTTGTTTA AATAAATGTT GACATTCACC AGGTATTTTT   
  
  
- ATTATCGGTA ATGTTTTTTA TCATGATGAC AAAATACTTT ACTAAAATTA TAAGATTTTA AGGTTATTTT   
  
  
- GTACAATATT AGATACTAAT TAAAATTTTG TACTAATGTA CAGTCGTATT AAACATAATA ACATGTCGTT   
  
  
- TACTTTCAAC CTCGATTTTC TTTTACCCCT TTATTTCCGT CTGTTCATTT CTATTTTATA CGATAGATGA   
  
  
- AAACCTCCAT AGGAATTGGT GTAACAGAGC AAACAGAAGT AATCCCTCTT CACCCTATTT ATGGTTTTAA   
  
  
- ACATACCAGT AACGCACCCC AAACGAAGGT TTGTTTTACC GAAAAACCTT CGTTTAAAAC ACTCGAAGAT   
  
  
- ACGTTTAAAG GTGCGAATTG GGGTGTTCGT TTGTTTTTGT CTGTCACCAA AAACAAACAG AAACAATCCC   
  
  
- GTACAAACCT CGGTTGACAA TTACCCTTAT TTGACCCCTC CGAAAAAGGA ACCGAGAAAG GGGGACTGGG   
  
  
- GTTTGGGTGA AGTCGAGTTA TGCTTTGGTC GGTAACTTTT TCCTTCCCCC CCCCCCCCCA TACACTCTTT   
  
  
- TTCTGTTTTG AAAAAGGGCC TCTCTCTTCT ATGTATACGT TGTTTCCGAG AGGAAGAGTA TTTAAGGTAT   
  
  
- AAGGAGACAA AAAGATCCTC TCTCTTTATA CGTTTTTTGT TTAGTCAACC GCTGTTTTCA ATGATGGTGC   
  
  
- GTAACAGATG ACGACGCATG GGTAAAAACC TCTGAAATTT CTTGCGGGGA GGGTAGAACT TTTCACTTTT   
  
  
- GGGGGAGAAA CTTTGACTTA AAGTCCCCTT TTTTTGGAGT GGTTTGGAGA TCTCTCTCGA AAATCTCTCC   
  
  
- CTTTATGTTG CTAATCTTCC TCGTTAAACC CTTTAAAGAA ACCCTTAAAC TTACCCAAAA CTCACTTAAC   
  
  
- GTTTAGGGTC TTTTCAAAAC CGTTCATGGC TAGATGTCAA GAGAGGAGAA GCACAAACCA TCTAGGGGAC   
  
  
- AAAGGAGACA AAGTAAATCC CATGAAGAGT AAGTAGGAGG GGTGGGGGAA TTAGCCTAGA AGGACAGCTC   
  
  
- AGTGAAATAC GATTATAAAA AAGTCACCTA AAAATCAATT GGGACAAGTA AAAAGGTAAG ACACAGGGTA   
  
  
- AAGAGAGAAA AAGTAAGTAT TCAACGGCCA AATAGACAAC CCACGTCGAA TCAGTGTTAT TAAAGACACA   
  
  
- ATCCGAAGAA ATGCCAATTT TTTTTTCCTC CGTGAGAAAA GCCACACTAA CAAATACCCT GGTTACTAAG   
  
  
- TTCTACTACC CAGTAGTCAT TGAAGTAGTG GGGAAGTTAA AAAGAGGTAC TACAGAGGGT TAAATCCAAG   
  
  
- AAGGATGGGA ACCGAGTCTC TCGATTTTGG ACTTCTTTCT CCAGAAATGA ACTATGTAAA CAACGAGAGA   
  
  
- ACACGTTTAG TACAGAGATC ACCATCGGAT CTGTTACGCT TGAATCGGGA GCTTGTTTAG AGAGTCGAAC   
  
  
- GACGGGGACT ACCCCTATGT TACGTCGCAT ACCGAAGAAT AAAACGACTT CGGGACCGAC TCTCCTAGGA   
  
  
- GTTCAGTACC GGACCGTACA TATCTCGGGA AGTAAAATGC TTTTACGGAC AGTAAAGTCT CCTTTAAGAA   
  
  
- CGATCCTTCG AGAAGAAACT CGATAAAGGA AAGAACTTCG ACCGGATAAA CCACTGTTTG GTTAGCTATT   
  
  
- AGCTTCGGTA CCTCCCCCTT TTCTACCAAG TATAATAACT AGACTTACGT AGTCTTGGAC GAGTTACCTA   
  
  
- ACGGGAATAA GTCCGAAACT CACGTTCCGG ACCCCCAGGA GGAGTAAACT CTTAATGGCC ACAAGTAGTT   
  
  
- GTGTTTCTCC AAGATCTAGT TCACCGAGTA TCCCACTGAG TTCTTCGACT CTTTAACCTA AACGGTAAAG   
  
  
- TCAAGTTAGG ACACCAATCG TTCAACCTTT TGGAACTACA ACTTTTCGAC ACACAATTCT GACCACTCCG   
  
  
- AGATCGGTAG TCAAGCCAGG AAGTTGACGT ATGGGAAAAC CCAAGACTAC TACTCGGGGA TTCCTTTTCA   
  
  
- AGTGGAAATC GGAACTACTT CATACGTTTA CCCCGATTAT CCGTTTCGGG CTCATTACTA AGCCGAAGAA   
  
  
- GTAGTGGAGG AAGCGAGTTA TGAAGTTGGT TCGACCTACC AAAGGAGTCG CGAAACACCC CTAACAGGGG   
  
  
- TTTCTAATAC CACTATCGAC TCGTGCTAAG GTTAGTGTTA CCAAGACCTG AATACCTCTC CAACAGTCTT   
  
  
- CGTGACATGA AGATACGTCG CGACAAACTG ACGAATCTTA GGTGGGACGG TTCTTGTAGA CAGCTTTCTT   
  
  
- CCGCCCATCT CTTCTACGAG GATCCACTTC TCTAGTTCTT GTAATATAGT ACGCTCCCTC CTCTTTCCTC   
  
  
- TCTTTCCGTA CTCTTCTAGC TCTTCACCTA CGTCTCCGAT CTGTACCGAC CTAAGCCCTA GCAAGGAAAC   
  
  
- TCGATATACC CATACGACGT TCGTTCCGTT AACGAAGTCC CGATACCAAC ACTACCAATA TCTCACTTTC   
  
  
- TCTTACCAAC ACAACAGTAG ACAACCGTTC TAGCGGGGGA GAAAAGCCAT AGTCGTACCT CCACATCCTC   
  
  
- TAC

+     TCT-motif

| Site Name | Organism | Position | Strand | Matrix score. | sequence | function |
| --- | --- | --- | --- | --- | --- | --- |
| TCT-motif | Arabidopsis thaliana | 728 | - | 6 | TCTTAC | part of a light responsive element |

>HU08G00367.1   
+ -Up\_Stream \_Len000ATGCGC CTTTCTGCTA ACTCATTATC TTTCCTTCTG AGAGGGGACA ACTTATATAA   
  
  
+ ATTGATAGGT ATTTTCAAAT CAATAGACCA GCTACCATAT ATGATAATTT TTATTACTTT TCTAAATCCA   
  
  
+ TCAACTATTT TCACAATCCT CCTTTTTCTA GAAAAATAAA ATTTGCTTAA TTCAAATCAC CTAAAATTAC   
  
  
+ TAAATCTATA GTCCTAAATC TACAAACTCA TTTGTGTTGC AACTCATGAT TTAAGTGGAA CACTAACCTT   
  
  
+ AGGAGATATG TTGTCAAAAG AAGAATTCGA TGACATATTT TCCAACAAAA GGCCACCTAA CTTCATGATT   
  
  
+ TAAAAAGGAG GAGTCTAAGC ACATCATGGA TGACTAATTT TTGATGTATT GTAACTACGT ATTGAGATAA   
  
  
+ CTTGAATGAA TCGTGACTAG AATATATCTA TTTTCAAAAA TAAAAATTCC CTAAAAGAAA AGGATAAAAA   
  
  
+ ACTATATAAT ACTAAAAAAT TCTAACTATA ATAACTAGGC TTATTAAATT TTTATAGTGA AAATTTATTT   
  
  
+ CTACTTATAA CAACATCACT TACTAAGTAG CTTATTTGTG TCTTCTTTTC TAAATAATAA TGGTAACTAT   
  
  
+ GTGTCTTTTG TAAATAACTT ATTATCTATT TTATAAATCA CCGTGTAAAG CACGGGTCTA TACTAGTTAA   
  
  
+ TTTAAATAGC ATTGTTAAAT CAAGTAAGAT ACAAACAAAT TTATTTACAA CTGTAAGTGG TCCATAAAAA   
  
  
+ TAATAGCCAT TACAAAAAAT AGTACTACTG TTTTATGAAA TGATTTTAAT ATTCTAAAAT TCCAATAAAA   
  
  
+ CATGTTATAA TCTATGATTA ATTTTAAAAC ATGATTACAT GTCAGCATAA TTTGTATTAT TGTACAGCAA   
  
  
+ ATGAAAGTTG GAGCTAAAAG AAAATGGGGA AATAAAGGCA GACAAGTAAA GATAAAATAT GCTATCTACT   
  
  
+ TTTGGAGGTA TCCTTAACCA CATTGTCTCG TTTGTCTTCA TTAGGGAGAA GTGGGATAAA TACCAAAATT   
  
  
+ TGTATGGTCA TTGCGTGGGG TTTGCTTCCA AACAAAATGG CTTTTTGGAA GCAAATTTTG TGAGCTTCTA   
  
  
+ TGCAAATTTC CACGCTTAAC CCCACAAGCA AACAAAAACA GACAGTGGTT TTTGTTTGTC TTTGTTAGGG   
  
  
+ CATGTTTGGA GCCAACTGTT AATGGGAATA AACTGGGGAG GCTTTTTCCT TGGCTCTTTC CCCCTGACCC   
  
  
+ CAAACCCACT TCAGCTCAAT ACGAAACCAG CCATTGAAAA AGGAAGGGGG GGGGGGGGGT ATGTGAGAAA   
  
  
+ AAGACAAAAC TTTTTCCCGG AGAGAGAAGA TACATATGCA ACAAAGGCTC TCCTTCTCAT AAATTCCATA   
  
  
+ TTCCTCTGTT TTTCTAGGAG AGAGAAATAT GCAAAAAACA AATCAGTTGG CGACAAAAGT TACTACCACG   
  
  
+ CATTGTCTAC TGCTGCGTAC CCATTTTTGG AGACTTTAAA GAACGCCCCT CCCATCTTGA AAAGTGAAAA   
  
  
+ CCCCCTCTTT GAAACTGAAT TTCAGGGGAA AAAAACCTCA CCAAACCTCT AGAGAGAGCT TTTAGAGAGG   
  
  
+ GAAATACAAC GATTAGAAGG AGCAATTTGG GAAATTTCTT TGGGAATTTG AATGGGTTTT GAGTGAATTG   
  
  
+ CAAATCCCAG AAAAGTTTTG GCAAGTACCG ATCTACAGTT CTCTCCTCTT CGTGTTTGGT AGATCCCCTG   
  
  
+ TTTCCTCTGT TTCATTTAGG GTACTTCTCA TTCATCCTCC CCACCCCCTT AATCGGATCT TCCTGTCGAG   
  
  
+ TCACTTTATG CTAATATTTT TTCAGTGGAT TTTTAGTTAA CCCTGTTCAT TTTTCCATTC TGTGTCCCAT   
  
  
+ TTCTCTCTTT TTCATTCATA AGTTGCCGGT TTATCTGTTG GGTGCAGCTT AGTCACAATA ATTTCTGTGT   
  
  
+ TAGGCTTCTT TACGGTTAAA AAAAAAGGAG GCACTCTTTT CGGTGTGATT GTTTATGGGA CCAATGATTC   
  
  
+ AAGATGATGG GTCATCAGTA ACTTCATCAC CCCTTCAATT TTTCTCCATG ATGTCTCCCA ATTTAGGTTC   
  
  
+ TTCCTACCCT TGGCTCAGAG AGCTAAAACC TGAAGAAAGA GGTCTTTACT TGATACATTT GTTGCTCTCT   
  
  
+ TGTGCAAATC ATGTCTCTAG TGGTAGCCTA GACAATGCGA ACTTAGCCCT CGAACAAATC TCTCAGCTTG   
  
  
+ CTGCCCCTGA TGGGGATACA ATGCAGCGTA TGGCTTCTTA TTTTGCTGAA GCCCTGGCTG AGAGGATCCT   
  
  
+ CAAGTCATGG CCTGGCATGT ATAGAGCCCT TCATTTTACG AAAATGCCTG TCATTTCAGA GGAAATTCTT   
  
  
+ GCTAGGAAGC TCTTCTTTGA GCTATTTCCT TTCTTGAAGC TGGCCTATTT GGTGACAAAC CAATCGATAA   
  
  
+ TCGAAGCCAT GGAGGGGGAA AAGATGGTTC ATATTATTGA TCTGAATGCA TCAGAACCTG CTCAATGGAT   
  
  
+ TGCCCTTATT CAGGCTTTGA GTGCAAGGCC TGGGGGTCCT CCTCATTTGA GAATTACCGG TGTTCATCAA   
  
  
+ CACAAAGAGG TTCTAGATCA AGTGGCTCAT AGGGTGACTC AAGAAGCTGA GAAATTGGAT TTGCCATTTC   
  
  
+ AGTTCAATCC TGTGGTTAGC AAGTTGGAAA ACCTTGATGT TGAAAAGCTG TGTGTTAAGA CTGGTGAGGC   
  
  
+ TCTAGCCATC AGTTCGGTCC TTCAACTGCA TACCCTTTTG GGTTCTGATG ATGAGCCCCT AAGGAAAAGT   
  
  
+ TCACCTTTAG CCTTGATGAA GTATGCAAAT GGGGCTAATA GGCAAAGCCC GAGTAATGAT TCGGCTTCTT   
  
  
+ CATCACCTCC TTCGCTCAAT ACTTCAACCA AGCTGGATGG TTTCCTCAGC GCTTTGTGGG GATTGTCCCC   
  
  
+ AAAGATTATG GTGATAGCTG AGCACGATTC CAATCACAAT GGTTCTGGAC TTATGGAGAG GTTGTCAGAA   
  
  
+ GCACTGTACT TCTATGCAGC GCTGTTTGAC TGCTTAGAAT CCACCCTGCC AAGAACATCT GTCGAAAGAA   
  
  
+ GGCGGGTAGA GAAGATGCTC CTAGGTGAAG AGATCAAGAA CATTATATCA TGCGAGGGAG GAGAAAGGAG   
  
  
+ AGAAAGGCAT GAGAAGATCG AGAAGTGGAT GCAGAGGCTA GACATGGCTG GATTCGGGAT CGTTCCTTTG   
  
  
+ AGCTATATGG GTATGCTGCA AGCAAGGCAA TTGCTTCAGG GCTATGGTTG TGATGGTTAT AGAGTGAAAG   
  
  
+ AGAATGGTTG TGTTGTCATC TGTTGGCAAG ATCGCCCCCT CTTTTCGGTA TCAGCATGGA GGTGTAGGAG   
  
  
+ ATG  

- -Up\_Stream \_Len000TACGCG GAAAGACGAT TGAGTAATAG AAAGGAAGAC TCTCCCCTGT TGAATATATT   
  
  
- TAACTATCCA TAAAAGTTTA GTTATCTGGT CGATGGTATA TACTATTAAA AATAATGAAA AGATTTAGGT   
  
  
- AGTTGATAAA AGTGTTAGGA GGAAAAAGAT CTTTTTATTT TAAACGAATT AAGTTTAGTG GATTTTAATG   
  
  
- ATTTAGATAT CAGGATTTAG ATGTTTGAGT AAACACAACG TTGAGTACTA AATTCACCTT GTGATTGGAA   
  
  
- TCCTCTATAC AACAGTTTTC TTCTTAAGCT ACTGTATAAA AGGTTGTTTT CCGGTGGATT GAAGTACTAA   
  
  
- ATTTTTCCTC CTCAGATTCG TGTAGTACCT ACTGATTAAA AACTACATAA CATTGATGCA TAACTCTATT   
  
  
- GAACTTACTT AGCACTGATC TTATATAGAT AAAAGTTTTT ATTTTTAAGG GATTTTCTTT TCCTATTTTT   
  
  
- TGATATATTA TGATTTTTTA AGATTGATAT TATTGATCCG AATAATTTAA AAATATCACT TTTAAATAAA   
  
  
- GATGAATATT GTTGTAGTGA ATGATTCATC GAATAAACAC AGAAGAAAAG ATTTATTATT ACCATTGATA   
  
  
- CACAGAAAAC ATTTATTGAA TAATAGATAA AATATTTAGT GGCACATTTC GTGCCCAGAT ATGATCAATT   
  
  
- AAATTTATCG TAACAATTTA GTTCATTCTA TGTTTGTTTA AATAAATGTT GACATTCACC AGGTATTTTT   
  
  
- ATTATCGGTA ATGTTTTTTA TCATGATGAC AAAATACTTT ACTAAAATTA TAAGATTTTA AGGTTATTTT   
  
  
- GTACAATATT AGATACTAAT TAAAATTTTG TACTAATGTA CAGTCGTATT AAACATAATA ACATGTCGTT   
  
  
- TACTTTCAAC CTCGATTTTC TTTTACCCCT TTATTTCCGT CTGTTCATTT CTATTTTATA CGATAGATGA   
  
  
- AAACCTCCAT AGGAATTGGT GTAACAGAGC AAACAGAAGT AATCCCTCTT CACCCTATTT ATGGTTTTAA   
  
  
- ACATACCAGT AACGCACCCC AAACGAAGGT TTGTTTTACC GAAAAACCTT CGTTTAAAAC ACTCGAAGAT   
  
  
- ACGTTTAAAG GTGCGAATTG GGGTGTTCGT TTGTTTTTGT CTGTCACCAA AAACAAACAG AAACAATCCC   
  
  
- GTACAAACCT CGGTTGACAA TTACCCTTAT TTGACCCCTC CGAAAAAGGA ACCGAGAAAG GGGGACTGGG   
  
  
- GTTTGGGTGA AGTCGAGTTA TGCTTTGGTC GGTAACTTTT TCCTTCCCCC CCCCCCCCCA TACACTCTTT   
  
  
- TTCTGTTTTG AAAAAGGGCC TCTCTCTTCT ATGTATACGT TGTTTCCGAG AGGAAGAGTA TTTAAGGTAT   
  
  
- AAGGAGACAA AAAGATCCTC TCTCTTTATA CGTTTTTTGT TTAGTCAACC GCTGTTTTCA ATGATGGTGC   
  
  
- GTAACAGATG ACGACGCATG GGTAAAAACC TCTGAAATTT CTTGCGGGGA GGGTAGAACT TTTCACTTTT   
  
  
- GGGGGAGAAA CTTTGACTTA AAGTCCCCTT TTTTTGGAGT GGTTTGGAGA TCTCTCTCGA AAATCTCTCC   
  
  
- CTTTATGTTG CTAATCTTCC TCGTTAAACC CTTTAAAGAA ACCCTTAAAC TTACCCAAAA CTCACTTAAC   
  
  
- GTTTAGGGTC TTTTCAAAAC CGTTCATGGC TAGATGTCAA GAGAGGAGAA GCACAAACCA TCTAGGGGAC   
  
  
- AAAGGAGACA AAGTAAATCC CATGAAGAGT AAGTAGGAGG GGTGGGGGAA TTAGCCTAGA AGGACAGCTC   
  
  
- AGTGAAATAC GATTATAAAA AAGTCACCTA AAAATCAATT GGGACAAGTA AAAAGGTAAG ACACAGGGTA   
  
  
- AAGAGAGAAA AAGTAAGTAT TCAACGGCCA AATAGACAAC CCACGTCGAA TCAGTGTTAT TAAAGACACA   
  
  
- ATCCGAAGAA ATGCCAATTT TTTTTTCCTC CGTGAGAAAA GCCACACTAA CAAATACCCT GGTTACTAAG   
  
  
- TTCTACTACC CAGTAGTCAT TGAAGTAGTG GGGAAGTTAA AAAGAGGTAC TACAGAGGGT TAAATCCAAG   
  
  
- AAGGATGGGA ACCGAGTCTC TCGATTTTGG ACTTCTTTCT CCAGAAATGA ACTATGTAAA CAACGAGAGA   
  
  
- ACACGTTTAG TACAGAGATC ACCATCGGAT CTGTTACGCT TGAATCGGGA GCTTGTTTAG AGAGTCGAAC   
  
  
- GACGGGGACT ACCCCTATGT TACGTCGCAT ACCGAAGAAT AAAACGACTT CGGGACCGAC TCTCCTAGGA   
  
  
- GTTCAGTACC GGACCGTACA TATCTCGGGA AGTAAAATGC TTTTACGGAC AGTAAAGTCT CCTTTAAGAA   
  
  
- CGATCCTTCG AGAAGAAACT CGATAAAGGA AAGAACTTCG ACCGGATAAA CCACTGTTTG GTTAGCTATT   
  
  
- AGCTTCGGTA CCTCCCCCTT TTCTACCAAG TATAATAACT AGACTTACGT AGTCTTGGAC GAGTTACCTA   
  
  
- ACGGGAATAA GTCCGAAACT CACGTTCCGG ACCCCCAGGA GGAGTAAACT CTTAATGGCC ACAAGTAGTT   
  
  
- GTGTTTCTCC AAGATCTAGT TCACCGAGTA TCCCACTGAG TTCTTCGACT CTTTAACCTA AACGGTAAAG   
  
  
- TCAAGTTAGG ACACCAATCG TTCAACCTTT TGGAACTACA ACTTTTCGAC ACACAATTCT GACCACTCCG   
  
  
- AGATCGGTAG TCAAGCCAGG AAGTTGACGT ATGGGAAAAC CCAAGACTAC TACTCGGGGA TTCCTTTTCA   
  
  
- AGTGGAAATC GGAACTACTT CATACGTTTA CCCCGATTAT CCGTTTCGGG CTCATTACTA AGCCGAAGAA   
  
  
- GTAGTGGAGG AAGCGAGTTA TGAAGTTGGT TCGACCTACC AAAGGAGTCG CGAAACACCC CTAACAGGGG   
  
  
- TTTCTAATAC CACTATCGAC TCGTGCTAAG GTTAGTGTTA CCAAGACCTG AATACCTCTC CAACAGTCTT   
  
  
- CGTGACATGA AGATACGTCG CGACAAACTG ACGAATCTTA GGTGGGACGG TTCTTGTAGA CAGCTTTCTT   
  
  
- CCGCCCATCT CTTCTACGAG GATCCACTTC TCTAGTTCTT GTAATATAGT ACGCTCCCTC CTCTTTCCTC   
  
  
- TCTTTCCGTA CTCTTCTAGC TCTTCACCTA CGTCTCCGAT CTGTACCGAC CTAAGCCCTA GCAAGGAAAC   
  
  
- TCGATATACC CATACGACGT TCGTTCCGTT AACGAAGTCC CGATACCAAC ACTACCAATA TCTCACTTTC   
  
  
- TCTTACCAAC ACAACAGTAG ACAACCGTTC TAGCGGGGGA GAAAAGCCAT AGTCGTACCT CCACATCCTC   
  
  
- TAC

+     Unnamed\_\_1

| Site Name | Organism | Position | Strand | Matrix score. | sequence | function |
| --- | --- | --- | --- | --- | --- | --- |
| Unnamed\_\_1 | Zea mays | 1068 | + | 5 | CGTGG |  |
| Unnamed\_\_1 | Zea mays | 1470 | - | 5 | CGTGG |  |
| Unnamed\_\_1 | Zea mays | 1134 | - | 5 | CGTGG |  |

>HU08G00367.1   
+ -Up\_Stream \_Len000ATGCGC CTTTCTGCTA ACTCATTATC TTTCCTTCTG AGAGGGGACA ACTTATATAA   
  
  
+ ATTGATAGGT ATTTTCAAAT CAATAGACCA GCTACCATAT ATGATAATTT TTATTACTTT TCTAAATCCA   
  
  
+ TCAACTATTT TCACAATCCT CCTTTTTCTA GAAAAATAAA ATTTGCTTAA TTCAAATCAC CTAAAATTAC   
  
  
+ TAAATCTATA GTCCTAAATC TACAAACTCA TTTGTGTTGC AACTCATGAT TTAAGTGGAA CACTAACCTT   
  
  
+ AGGAGATATG TTGTCAAAAG AAGAATTCGA TGACATATTT TCCAACAAAA GGCCACCTAA CTTCATGATT   
  
  
+ TAAAAAGGAG GAGTCTAAGC ACATCATGGA TGACTAATTT TTGATGTATT GTAACTACGT ATTGAGATAA   
  
  
+ CTTGAATGAA TCGTGACTAG AATATATCTA TTTTCAAAAA TAAAAATTCC CTAAAAGAAA AGGATAAAAA   
  
  
+ ACTATATAAT ACTAAAAAAT TCTAACTATA ATAACTAGGC TTATTAAATT TTTATAGTGA AAATTTATTT   
  
  
+ CTACTTATAA CAACATCACT TACTAAGTAG CTTATTTGTG TCTTCTTTTC TAAATAATAA TGGTAACTAT   
  
  
+ GTGTCTTTTG TAAATAACTT ATTATCTATT TTATAAATCA CCGTGTAAAG CACGGGTCTA TACTAGTTAA   
  
  
+ TTTAAATAGC ATTGTTAAAT CAAGTAAGAT ACAAACAAAT TTATTTACAA CTGTAAGTGG TCCATAAAAA   
  
  
+ TAATAGCCAT TACAAAAAAT AGTACTACTG TTTTATGAAA TGATTTTAAT ATTCTAAAAT TCCAATAAAA   
  
  
+ CATGTTATAA TCTATGATTA ATTTTAAAAC ATGATTACAT GTCAGCATAA TTTGTATTAT TGTACAGCAA   
  
  
+ ATGAAAGTTG GAGCTAAAAG AAAATGGGGA AATAAAGGCA GACAAGTAAA GATAAAATAT GCTATCTACT   
  
  
+ TTTGGAGGTA TCCTTAACCA CATTGTCTCG TTTGTCTTCA TTAGGGAGAA GTGGGATAAA TACCAAAATT   
  
  
+ TGTATGGTCA TTGCGTGGGG TTTGCTTCCA AACAAAATGG CTTTTTGGAA GCAAATTTTG TGAGCTTCTA   
  
  
+ TGCAAATTTC CACGCTTAAC CCCACAAGCA AACAAAAACA GACAGTGGTT TTTGTTTGTC TTTGTTAGGG   
  
  
+ CATGTTTGGA GCCAACTGTT AATGGGAATA AACTGGGGAG GCTTTTTCCT TGGCTCTTTC CCCCTGACCC   
  
  
+ CAAACCCACT TCAGCTCAAT ACGAAACCAG CCATTGAAAA AGGAAGGGGG GGGGGGGGGT ATGTGAGAAA   
  
  
+ AAGACAAAAC TTTTTCCCGG AGAGAGAAGA TACATATGCA ACAAAGGCTC TCCTTCTCAT AAATTCCATA   
  
  
+ TTCCTCTGTT TTTCTAGGAG AGAGAAATAT GCAAAAAACA AATCAGTTGG CGACAAAAGT TACTACCACG   
  
  
+ CATTGTCTAC TGCTGCGTAC CCATTTTTGG AGACTTTAAA GAACGCCCCT CCCATCTTGA AAAGTGAAAA   
  
  
+ CCCCCTCTTT GAAACTGAAT TTCAGGGGAA AAAAACCTCA CCAAACCTCT AGAGAGAGCT TTTAGAGAGG   
  
  
+ GAAATACAAC GATTAGAAGG AGCAATTTGG GAAATTTCTT TGGGAATTTG AATGGGTTTT GAGTGAATTG   
  
  
+ CAAATCCCAG AAAAGTTTTG GCAAGTACCG ATCTACAGTT CTCTCCTCTT CGTGTTTGGT AGATCCCCTG   
  
  
+ TTTCCTCTGT TTCATTTAGG GTACTTCTCA TTCATCCTCC CCACCCCCTT AATCGGATCT TCCTGTCGAG   
  
  
+ TCACTTTATG CTAATATTTT TTCAGTGGAT TTTTAGTTAA CCCTGTTCAT TTTTCCATTC TGTGTCCCAT   
  
  
+ TTCTCTCTTT TTCATTCATA AGTTGCCGGT TTATCTGTTG GGTGCAGCTT AGTCACAATA ATTTCTGTGT   
  
  
+ TAGGCTTCTT TACGGTTAAA AAAAAAGGAG GCACTCTTTT CGGTGTGATT GTTTATGGGA CCAATGATTC   
  
  
+ AAGATGATGG GTCATCAGTA ACTTCATCAC CCCTTCAATT TTTCTCCATG ATGTCTCCCA ATTTAGGTTC   
  
  
+ TTCCTACCCT TGGCTCAGAG AGCTAAAACC TGAAGAAAGA GGTCTTTACT TGATACATTT GTTGCTCTCT   
  
  
+ TGTGCAAATC ATGTCTCTAG TGGTAGCCTA GACAATGCGA ACTTAGCCCT CGAACAAATC TCTCAGCTTG   
  
  
+ CTGCCCCTGA TGGGGATACA ATGCAGCGTA TGGCTTCTTA TTTTGCTGAA GCCCTGGCTG AGAGGATCCT   
  
  
+ CAAGTCATGG CCTGGCATGT ATAGAGCCCT TCATTTTACG AAAATGCCTG TCATTTCAGA GGAAATTCTT   
  
  
+ GCTAGGAAGC TCTTCTTTGA GCTATTTCCT TTCTTGAAGC TGGCCTATTT GGTGACAAAC CAATCGATAA   
  
  
+ TCGAAGCCAT GGAGGGGGAA AAGATGGTTC ATATTATTGA TCTGAATGCA TCAGAACCTG CTCAATGGAT   
  
  
+ TGCCCTTATT CAGGCTTTGA GTGCAAGGCC TGGGGGTCCT CCTCATTTGA GAATTACCGG TGTTCATCAA   
  
  
+ CACAAAGAGG TTCTAGATCA AGTGGCTCAT AGGGTGACTC AAGAAGCTGA GAAATTGGAT TTGCCATTTC   
  
  
+ AGTTCAATCC TGTGGTTAGC AAGTTGGAAA ACCTTGATGT TGAAAAGCTG TGTGTTAAGA CTGGTGAGGC   
  
  
+ TCTAGCCATC AGTTCGGTCC TTCAACTGCA TACCCTTTTG GGTTCTGATG ATGAGCCCCT AAGGAAAAGT   
  
  
+ TCACCTTTAG CCTTGATGAA GTATGCAAAT GGGGCTAATA GGCAAAGCCC GAGTAATGAT TCGGCTTCTT   
  
  
+ CATCACCTCC TTCGCTCAAT ACTTCAACCA AGCTGGATGG TTTCCTCAGC GCTTTGTGGG GATTGTCCCC   
  
  
+ AAAGATTATG GTGATAGCTG AGCACGATTC CAATCACAAT GGTTCTGGAC TTATGGAGAG GTTGTCAGAA   
  
  
+ GCACTGTACT TCTATGCAGC GCTGTTTGAC TGCTTAGAAT CCACCCTGCC AAGAACATCT GTCGAAAGAA   
  
  
+ GGCGGGTAGA GAAGATGCTC CTAGGTGAAG AGATCAAGAA CATTATATCA TGCGAGGGAG GAGAAAGGAG   
  
  
+ AGAAAGGCAT GAGAAGATCG AGAAGTGGAT GCAGAGGCTA GACATGGCTG GATTCGGGAT CGTTCCTTTG   
  
  
+ AGCTATATGG GTATGCTGCA AGCAAGGCAA TTGCTTCAGG GCTATGGTTG TGATGGTTAT AGAGTGAAAG   
  
  
+ AGAATGGTTG TGTTGTCATC TGTTGGCAAG ATCGCCCCCT CTTTTCGGTA TCAGCATGGA GGTGTAGGAG   
  
  
+ ATG  

- -Up\_Stream \_Len000TACGCG GAAAGACGAT TGAGTAATAG AAAGGAAGAC TCTCCCCTGT TGAATATATT   
  
  
- TAACTATCCA TAAAAGTTTA GTTATCTGGT CGATGGTATA TACTATTAAA AATAATGAAA AGATTTAGGT   
  
  
- AGTTGATAAA AGTGTTAGGA GGAAAAAGAT CTTTTTATTT TAAACGAATT AAGTTTAGTG GATTTTAATG   
  
  
- ATTTAGATAT CAGGATTTAG ATGTTTGAGT AAACACAACG TTGAGTACTA AATTCACCTT GTGATTGGAA   
  
  
- TCCTCTATAC AACAGTTTTC TTCTTAAGCT ACTGTATAAA AGGTTGTTTT CCGGTGGATT GAAGTACTAA   
  
  
- ATTTTTCCTC CTCAGATTCG TGTAGTACCT ACTGATTAAA AACTACATAA CATTGATGCA TAACTCTATT   
  
  
- GAACTTACTT AGCACTGATC TTATATAGAT AAAAGTTTTT ATTTTTAAGG GATTTTCTTT TCCTATTTTT   
  
  
- TGATATATTA TGATTTTTTA AGATTGATAT TATTGATCCG AATAATTTAA AAATATCACT TTTAAATAAA   
  
  
- GATGAATATT GTTGTAGTGA ATGATTCATC GAATAAACAC AGAAGAAAAG ATTTATTATT ACCATTGATA   
  
  
- CACAGAAAAC ATTTATTGAA TAATAGATAA AATATTTAGT GGCACATTTC GTGCCCAGAT ATGATCAATT   
  
  
- AAATTTATCG TAACAATTTA GTTCATTCTA TGTTTGTTTA AATAAATGTT GACATTCACC AGGTATTTTT   
  
  
- ATTATCGGTA ATGTTTTTTA TCATGATGAC AAAATACTTT ACTAAAATTA TAAGATTTTA AGGTTATTTT   
  
  
- GTACAATATT AGATACTAAT TAAAATTTTG TACTAATGTA CAGTCGTATT AAACATAATA ACATGTCGTT   
  
  
- TACTTTCAAC CTCGATTTTC TTTTACCCCT TTATTTCCGT CTGTTCATTT CTATTTTATA CGATAGATGA   
  
  
- AAACCTCCAT AGGAATTGGT GTAACAGAGC AAACAGAAGT AATCCCTCTT CACCCTATTT ATGGTTTTAA   
  
  
- ACATACCAGT AACGCACCCC AAACGAAGGT TTGTTTTACC GAAAAACCTT CGTTTAAAAC ACTCGAAGAT   
  
  
- ACGTTTAAAG GTGCGAATTG GGGTGTTCGT TTGTTTTTGT CTGTCACCAA AAACAAACAG AAACAATCCC   
  
  
- GTACAAACCT CGGTTGACAA TTACCCTTAT TTGACCCCTC CGAAAAAGGA ACCGAGAAAG GGGGACTGGG   
  
  
- GTTTGGGTGA AGTCGAGTTA TGCTTTGGTC GGTAACTTTT TCCTTCCCCC CCCCCCCCCA TACACTCTTT   
  
  
- TTCTGTTTTG AAAAAGGGCC TCTCTCTTCT ATGTATACGT TGTTTCCGAG AGGAAGAGTA TTTAAGGTAT   
  
  
- AAGGAGACAA AAAGATCCTC TCTCTTTATA CGTTTTTTGT TTAGTCAACC GCTGTTTTCA ATGATGGTGC   
  
  
- GTAACAGATG ACGACGCATG GGTAAAAACC TCTGAAATTT CTTGCGGGGA GGGTAGAACT TTTCACTTTT   
  
  
- GGGGGAGAAA CTTTGACTTA AAGTCCCCTT TTTTTGGAGT GGTTTGGAGA TCTCTCTCGA AAATCTCTCC   
  
  
- CTTTATGTTG CTAATCTTCC TCGTTAAACC CTTTAAAGAA ACCCTTAAAC TTACCCAAAA CTCACTTAAC   
  
  
- GTTTAGGGTC TTTTCAAAAC CGTTCATGGC TAGATGTCAA GAGAGGAGAA GCACAAACCA TCTAGGGGAC   
  
  
- AAAGGAGACA AAGTAAATCC CATGAAGAGT AAGTAGGAGG GGTGGGGGAA TTAGCCTAGA AGGACAGCTC   
  
  
- AGTGAAATAC GATTATAAAA AAGTCACCTA AAAATCAATT GGGACAAGTA AAAAGGTAAG ACACAGGGTA   
  
  
- AAGAGAGAAA AAGTAAGTAT TCAACGGCCA AATAGACAAC CCACGTCGAA TCAGTGTTAT TAAAGACACA   
  
  
- ATCCGAAGAA ATGCCAATTT TTTTTTCCTC CGTGAGAAAA GCCACACTAA CAAATACCCT GGTTACTAAG   
  
  
- TTCTACTACC CAGTAGTCAT TGAAGTAGTG GGGAAGTTAA AAAGAGGTAC TACAGAGGGT TAAATCCAAG   
  
  
- AAGGATGGGA ACCGAGTCTC TCGATTTTGG ACTTCTTTCT CCAGAAATGA ACTATGTAAA CAACGAGAGA   
  
  
- ACACGTTTAG TACAGAGATC ACCATCGGAT CTGTTACGCT TGAATCGGGA GCTTGTTTAG AGAGTCGAAC   
  
  
- GACGGGGACT ACCCCTATGT TACGTCGCAT ACCGAAGAAT AAAACGACTT CGGGACCGAC TCTCCTAGGA   
  
  
- GTTCAGTACC GGACCGTACA TATCTCGGGA AGTAAAATGC TTTTACGGAC AGTAAAGTCT CCTTTAAGAA   
  
  
- CGATCCTTCG AGAAGAAACT CGATAAAGGA AAGAACTTCG ACCGGATAAA CCACTGTTTG GTTAGCTATT   
  
  
- AGCTTCGGTA CCTCCCCCTT TTCTACCAAG TATAATAACT AGACTTACGT AGTCTTGGAC GAGTTACCTA   
  
  
- ACGGGAATAA GTCCGAAACT CACGTTCCGG ACCCCCAGGA GGAGTAAACT CTTAATGGCC ACAAGTAGTT   
  
  
- GTGTTTCTCC AAGATCTAGT TCACCGAGTA TCCCACTGAG TTCTTCGACT CTTTAACCTA AACGGTAAAG   
  
  
- TCAAGTTAGG ACACCAATCG TTCAACCTTT TGGAACTACA ACTTTTCGAC ACACAATTCT GACCACTCCG   
  
  
- AGATCGGTAG TCAAGCCAGG AAGTTGACGT ATGGGAAAAC CCAAGACTAC TACTCGGGGA TTCCTTTTCA   
  
  
- AGTGGAAATC GGAACTACTT CATACGTTTA CCCCGATTAT CCGTTTCGGG CTCATTACTA AGCCGAAGAA   
  
  
- GTAGTGGAGG AAGCGAGTTA TGAAGTTGGT TCGACCTACC AAAGGAGTCG CGAAACACCC CTAACAGGGG   
  
  
- TTTCTAATAC CACTATCGAC TCGTGCTAAG GTTAGTGTTA CCAAGACCTG AATACCTCTC CAACAGTCTT   
  
  
- CGTGACATGA AGATACGTCG CGACAAACTG ACGAATCTTA GGTGGGACGG TTCTTGTAGA CAGCTTTCTT   
  
  
- CCGCCCATCT CTTCTACGAG GATCCACTTC TCTAGTTCTT GTAATATAGT ACGCTCCCTC CTCTTTCCTC   
  
  
- TCTTTCCGTA CTCTTCTAGC TCTTCACCTA CGTCTCCGAT CTGTACCGAC CTAAGCCCTA GCAAGGAAAC   
  
  
- TCGATATACC CATACGACGT TCGTTCCGTT AACGAAGTCC CGATACCAAC ACTACCAATA TCTCACTTTC   
  
  
- TCTTACCAAC ACAACAGTAG ACAACCGTTC TAGCGGGGGA GAAAAGCCAT AGTCGTACCT CCACATCCTC   
  
  
- TAC

+     Unnamed\_\_4

| Site Name | Organism | Position | Strand | Matrix score. | sequence | function |
| --- | --- | --- | --- | --- | --- | --- |
| Unnamed\_\_4 | Petroselinum hortense | 1727 | + | 4 | CTCC |  |
| Unnamed\_\_4 | Petroselinum hortense | 1791 | + | 4 | CTCC |  |
| Unnamed\_\_4 | Petroselinum hortense | 3361 | - | 4 | CTCC |  |
| Unnamed\_\_4 | Petroselinum hortense | 1633 | - | 4 | CTCC |  |
| Unnamed\_\_4 | Petroselinum hortense | 364 | - | 4 | CTCC |  |
| Unnamed\_\_4 | Petroselinum hortense | 2881 | + | 4 | CTCC |  |
| Unnamed\_\_4 | Petroselinum hortense | 1353 | - | 4 | CTCC |  |
| Unnamed\_\_4 | Petroselinum hortense | 1503 | - | 4 | CTCC |  |
| Unnamed\_\_4 | Petroselinum hortense | 1029 | - | 4 | CTCC |  |
| Unnamed\_\_4 | Petroselinum hortense | 2465 | - | 4 | CTCC |  |
| Unnamed\_\_4 | Petroselinum hortense | 1202 | - | 4 | CTCC |  |
| Unnamed\_\_4 | Petroselinum hortense | 924 | - | 4 | CTCC |  |
| Unnamed\_\_4 | Petroselinum hortense | 286 | - | 4 | CTCC |  |
| Unnamed\_\_4 | Petroselinum hortense | 3141 | - | 4 | CTCC |  |
| Unnamed\_\_4 | Petroselinum hortense | 1384 | + | 4 | CTCC |  |
| Unnamed\_\_4 | Petroselinum hortense | 3102 | + | 4 | CTCC |  |
| Unnamed\_\_4 | Petroselinum hortense | 988 | - | 4 | CTCC |  |
| Unnamed\_\_4 | Petroselinum hortense | 1231 | - | 4 | CTCC |  |
| Unnamed\_\_4 | Petroselinum hortense | 163 | + | 4 | CTCC |  |
| Unnamed\_\_4 | Petroselinum hortense | 3352 | - | 4 | CTCC |  |
| Unnamed\_\_4 | Petroselinum hortense | 1421 | - | 4 | CTCC |  |
| Unnamed\_\_4 | Petroselinum hortense | 2078 | + | 4 | CTCC |  |
| Unnamed\_\_4 | Petroselinum hortense | 361 | - | 4 | CTCC |  |
| Unnamed\_\_4 | Petroselinum hortense | 1991 | - | 4 | CTCC |  |
| Unnamed\_\_4 | Petroselinum hortense | 3151 | - | 4 | CTCC |  |
| Unnamed\_\_4 | Petroselinum hortense | 3144 | - | 4 | CTCC |  |
| Unnamed\_\_4 | Petroselinum hortense | 1523 | + | 4 | CTCC |  |
| Unnamed\_\_4 | Petroselinum hortense | 2563 | + | 4 | CTCC |  |
| Unnamed\_\_4 | Petroselinum hortense | 2089 | + | 4 | CTCC |  |
| Unnamed\_\_4 | Petroselinum hortense | 2999 | - | 4 | CTCC |  |

>HU08G00367.1   
+ -Up\_Stream \_Len000ATGCGC CTTTCTGCTA ACTCATTATC TTTCCTTCTG AGAGGGGACA ACTTATATAA   
  
  
+ ATTGATAGGT ATTTTCAAAT CAATAGACCA GCTACCATAT ATGATAATTT TTATTACTTT TCTAAATCCA   
  
  
+ TCAACTATTT TCACAATCCT CCTTTTTCTA GAAAAATAAA ATTTGCTTAA TTCAAATCAC CTAAAATTAC   
  
  
+ TAAATCTATA GTCCTAAATC TACAAACTCA TTTGTGTTGC AACTCATGAT TTAAGTGGAA CACTAACCTT   
  
  
+ AGGAGATATG TTGTCAAAAG AAGAATTCGA TGACATATTT TCCAACAAAA GGCCACCTAA CTTCATGATT   
  
  
+ TAAAAAGGAG GAGTCTAAGC ACATCATGGA TGACTAATTT TTGATGTATT GTAACTACGT ATTGAGATAA   
  
  
+ CTTGAATGAA TCGTGACTAG AATATATCTA TTTTCAAAAA TAAAAATTCC CTAAAAGAAA AGGATAAAAA   
  
  
+ ACTATATAAT ACTAAAAAAT TCTAACTATA ATAACTAGGC TTATTAAATT TTTATAGTGA AAATTTATTT   
  
  
+ CTACTTATAA CAACATCACT TACTAAGTAG CTTATTTGTG TCTTCTTTTC TAAATAATAA TGGTAACTAT   
  
  
+ GTGTCTTTTG TAAATAACTT ATTATCTATT TTATAAATCA CCGTGTAAAG CACGGGTCTA TACTAGTTAA   
  
  
+ TTTAAATAGC ATTGTTAAAT CAAGTAAGAT ACAAACAAAT TTATTTACAA CTGTAAGTGG TCCATAAAAA   
  
  
+ TAATAGCCAT TACAAAAAAT AGTACTACTG TTTTATGAAA TGATTTTAAT ATTCTAAAAT TCCAATAAAA   
  
  
+ CATGTTATAA TCTATGATTA ATTTTAAAAC ATGATTACAT GTCAGCATAA TTTGTATTAT TGTACAGCAA   
  
  
+ ATGAAAGTTG GAGCTAAAAG AAAATGGGGA AATAAAGGCA GACAAGTAAA GATAAAATAT GCTATCTACT   
  
  
+ TTTGGAGGTA TCCTTAACCA CATTGTCTCG TTTGTCTTCA TTAGGGAGAA GTGGGATAAA TACCAAAATT   
  
  
+ TGTATGGTCA TTGCGTGGGG TTTGCTTCCA AACAAAATGG CTTTTTGGAA GCAAATTTTG TGAGCTTCTA   
  
  
+ TGCAAATTTC CACGCTTAAC CCCACAAGCA AACAAAAACA GACAGTGGTT TTTGTTTGTC TTTGTTAGGG   
  
  
+ CATGTTTGGA GCCAACTGTT AATGGGAATA AACTGGGGAG GCTTTTTCCT TGGCTCTTTC CCCCTGACCC   
  
  
+ CAAACCCACT TCAGCTCAAT ACGAAACCAG CCATTGAAAA AGGAAGGGGG GGGGGGGGGT ATGTGAGAAA   
  
  
+ AAGACAAAAC TTTTTCCCGG AGAGAGAAGA TACATATGCA ACAAAGGCTC TCCTTCTCAT AAATTCCATA   
  
  
+ TTCCTCTGTT TTTCTAGGAG AGAGAAATAT GCAAAAAACA AATCAGTTGG CGACAAAAGT TACTACCACG   
  
  
+ CATTGTCTAC TGCTGCGTAC CCATTTTTGG AGACTTTAAA GAACGCCCCT CCCATCTTGA AAAGTGAAAA   
  
  
+ CCCCCTCTTT GAAACTGAAT TTCAGGGGAA AAAAACCTCA CCAAACCTCT AGAGAGAGCT TTTAGAGAGG   
  
  
+ GAAATACAAC GATTAGAAGG AGCAATTTGG GAAATTTCTT TGGGAATTTG AATGGGTTTT GAGTGAATTG   
  
  
+ CAAATCCCAG AAAAGTTTTG GCAAGTACCG ATCTACAGTT CTCTCCTCTT CGTGTTTGGT AGATCCCCTG   
  
  
+ TTTCCTCTGT TTCATTTAGG GTACTTCTCA TTCATCCTCC CCACCCCCTT AATCGGATCT TCCTGTCGAG   
  
  
+ TCACTTTATG CTAATATTTT TTCAGTGGAT TTTTAGTTAA CCCTGTTCAT TTTTCCATTC TGTGTCCCAT   
  
  
+ TTCTCTCTTT TTCATTCATA AGTTGCCGGT TTATCTGTTG GGTGCAGCTT AGTCACAATA ATTTCTGTGT   
  
  
+ TAGGCTTCTT TACGGTTAAA AAAAAAGGAG GCACTCTTTT CGGTGTGATT GTTTATGGGA CCAATGATTC   
  
  
+ AAGATGATGG GTCATCAGTA ACTTCATCAC CCCTTCAATT TTTCTCCATG ATGTCTCCCA ATTTAGGTTC   
  
  
+ TTCCTACCCT TGGCTCAGAG AGCTAAAACC TGAAGAAAGA GGTCTTTACT TGATACATTT GTTGCTCTCT   
  
  
+ TGTGCAAATC ATGTCTCTAG TGGTAGCCTA GACAATGCGA ACTTAGCCCT CGAACAAATC TCTCAGCTTG   
  
  
+ CTGCCCCTGA TGGGGATACA ATGCAGCGTA TGGCTTCTTA TTTTGCTGAA GCCCTGGCTG AGAGGATCCT   
  
  
+ CAAGTCATGG CCTGGCATGT ATAGAGCCCT TCATTTTACG AAAATGCCTG TCATTTCAGA GGAAATTCTT   
  
  
+ GCTAGGAAGC TCTTCTTTGA GCTATTTCCT TTCTTGAAGC TGGCCTATTT GGTGACAAAC CAATCGATAA   
  
  
+ TCGAAGCCAT GGAGGGGGAA AAGATGGTTC ATATTATTGA TCTGAATGCA TCAGAACCTG CTCAATGGAT   
  
  
+ TGCCCTTATT CAGGCTTTGA GTGCAAGGCC TGGGGGTCCT CCTCATTTGA GAATTACCGG TGTTCATCAA   
  
  
+ CACAAAGAGG TTCTAGATCA AGTGGCTCAT AGGGTGACTC AAGAAGCTGA GAAATTGGAT TTGCCATTTC   
  
  
+ AGTTCAATCC TGTGGTTAGC AAGTTGGAAA ACCTTGATGT TGAAAAGCTG TGTGTTAAGA CTGGTGAGGC   
  
  
+ TCTAGCCATC AGTTCGGTCC TTCAACTGCA TACCCTTTTG GGTTCTGATG ATGAGCCCCT AAGGAAAAGT   
  
  
+ TCACCTTTAG CCTTGATGAA GTATGCAAAT GGGGCTAATA GGCAAAGCCC GAGTAATGAT TCGGCTTCTT   
  
  
+ CATCACCTCC TTCGCTCAAT ACTTCAACCA AGCTGGATGG TTTCCTCAGC GCTTTGTGGG GATTGTCCCC   
  
  
+ AAAGATTATG GTGATAGCTG AGCACGATTC CAATCACAAT GGTTCTGGAC TTATGGAGAG GTTGTCAGAA   
  
  
+ GCACTGTACT TCTATGCAGC GCTGTTTGAC TGCTTAGAAT CCACCCTGCC AAGAACATCT GTCGAAAGAA   
  
  
+ GGCGGGTAGA GAAGATGCTC CTAGGTGAAG AGATCAAGAA CATTATATCA TGCGAGGGAG GAGAAAGGAG   
  
  
+ AGAAAGGCAT GAGAAGATCG AGAAGTGGAT GCAGAGGCTA GACATGGCTG GATTCGGGAT CGTTCCTTTG   
  
  
+ AGCTATATGG GTATGCTGCA AGCAAGGCAA TTGCTTCAGG GCTATGGTTG TGATGGTTAT AGAGTGAAAG   
  
  
+ AGAATGGTTG TGTTGTCATC TGTTGGCAAG ATCGCCCCCT CTTTTCGGTA TCAGCATGGA GGTGTAGGAG   
  
  
+ ATG  

- -Up\_Stream \_Len000TACGCG GAAAGACGAT TGAGTAATAG AAAGGAAGAC TCTCCCCTGT TGAATATATT   
  
  
- TAACTATCCA TAAAAGTTTA GTTATCTGGT CGATGGTATA TACTATTAAA AATAATGAAA AGATTTAGGT   
  
  
- AGTTGATAAA AGTGTTAGGA GGAAAAAGAT CTTTTTATTT TAAACGAATT AAGTTTAGTG GATTTTAATG   
  
  
- ATTTAGATAT CAGGATTTAG ATGTTTGAGT AAACACAACG TTGAGTACTA AATTCACCTT GTGATTGGAA   
  
  
- TCCTCTATAC AACAGTTTTC TTCTTAAGCT ACTGTATAAA AGGTTGTTTT CCGGTGGATT GAAGTACTAA   
  
  
- ATTTTTCCTC CTCAGATTCG TGTAGTACCT ACTGATTAAA AACTACATAA CATTGATGCA TAACTCTATT   
  
  
- GAACTTACTT AGCACTGATC TTATATAGAT AAAAGTTTTT ATTTTTAAGG GATTTTCTTT TCCTATTTTT   
  
  
- TGATATATTA TGATTTTTTA AGATTGATAT TATTGATCCG AATAATTTAA AAATATCACT TTTAAATAAA   
  
  
- GATGAATATT GTTGTAGTGA ATGATTCATC GAATAAACAC AGAAGAAAAG ATTTATTATT ACCATTGATA   
  
  
- CACAGAAAAC ATTTATTGAA TAATAGATAA AATATTTAGT GGCACATTTC GTGCCCAGAT ATGATCAATT   
  
  
- AAATTTATCG TAACAATTTA GTTCATTCTA TGTTTGTTTA AATAAATGTT GACATTCACC AGGTATTTTT   
  
  
- ATTATCGGTA ATGTTTTTTA TCATGATGAC AAAATACTTT ACTAAAATTA TAAGATTTTA AGGTTATTTT   
  
  
- GTACAATATT AGATACTAAT TAAAATTTTG TACTAATGTA CAGTCGTATT AAACATAATA ACATGTCGTT   
  
  
- TACTTTCAAC CTCGATTTTC TTTTACCCCT TTATTTCCGT CTGTTCATTT CTATTTTATA CGATAGATGA   
  
  
- AAACCTCCAT AGGAATTGGT GTAACAGAGC AAACAGAAGT AATCCCTCTT CACCCTATTT ATGGTTTTAA   
  
  
- ACATACCAGT AACGCACCCC AAACGAAGGT TTGTTTTACC GAAAAACCTT CGTTTAAAAC ACTCGAAGAT   
  
  
- ACGTTTAAAG GTGCGAATTG GGGTGTTCGT TTGTTTTTGT CTGTCACCAA AAACAAACAG AAACAATCCC   
  
  
- GTACAAACCT CGGTTGACAA TTACCCTTAT TTGACCCCTC CGAAAAAGGA ACCGAGAAAG GGGGACTGGG   
  
  
- GTTTGGGTGA AGTCGAGTTA TGCTTTGGTC GGTAACTTTT TCCTTCCCCC CCCCCCCCCA TACACTCTTT   
  
  
- TTCTGTTTTG AAAAAGGGCC TCTCTCTTCT ATGTATACGT TGTTTCCGAG AGGAAGAGTA TTTAAGGTAT   
  
  
- AAGGAGACAA AAAGATCCTC TCTCTTTATA CGTTTTTTGT TTAGTCAACC GCTGTTTTCA ATGATGGTGC   
  
  
- GTAACAGATG ACGACGCATG GGTAAAAACC TCTGAAATTT CTTGCGGGGA GGGTAGAACT TTTCACTTTT   
  
  
- GGGGGAGAAA CTTTGACTTA AAGTCCCCTT TTTTTGGAGT GGTTTGGAGA TCTCTCTCGA AAATCTCTCC   
  
  
- CTTTATGTTG CTAATCTTCC TCGTTAAACC CTTTAAAGAA ACCCTTAAAC TTACCCAAAA CTCACTTAAC   
  
  
- GTTTAGGGTC TTTTCAAAAC CGTTCATGGC TAGATGTCAA GAGAGGAGAA GCACAAACCA TCTAGGGGAC   
  
  
- AAAGGAGACA AAGTAAATCC CATGAAGAGT AAGTAGGAGG GGTGGGGGAA TTAGCCTAGA AGGACAGCTC   
  
  
- AGTGAAATAC GATTATAAAA AAGTCACCTA AAAATCAATT GGGACAAGTA AAAAGGTAAG ACACAGGGTA   
  
  
- AAGAGAGAAA AAGTAAGTAT TCAACGGCCA AATAGACAAC CCACGTCGAA TCAGTGTTAT TAAAGACACA   
  
  
- ATCCGAAGAA ATGCCAATTT TTTTTTCCTC CGTGAGAAAA GCCACACTAA CAAATACCCT GGTTACTAAG   
  
  
- TTCTACTACC CAGTAGTCAT TGAAGTAGTG GGGAAGTTAA AAAGAGGTAC TACAGAGGGT TAAATCCAAG   
  
  
- AAGGATGGGA ACCGAGTCTC TCGATTTTGG ACTTCTTTCT CCAGAAATGA ACTATGTAAA CAACGAGAGA   
  
  
- ACACGTTTAG TACAGAGATC ACCATCGGAT CTGTTACGCT TGAATCGGGA GCTTGTTTAG AGAGTCGAAC   
  
  
- GACGGGGACT ACCCCTATGT TACGTCGCAT ACCGAAGAAT AAAACGACTT CGGGACCGAC TCTCCTAGGA   
  
  
- GTTCAGTACC GGACCGTACA TATCTCGGGA AGTAAAATGC TTTTACGGAC AGTAAAGTCT CCTTTAAGAA   
  
  
- CGATCCTTCG AGAAGAAACT CGATAAAGGA AAGAACTTCG ACCGGATAAA CCACTGTTTG GTTAGCTATT   
  
  
- AGCTTCGGTA CCTCCCCCTT TTCTACCAAG TATAATAACT AGACTTACGT AGTCTTGGAC GAGTTACCTA   
  
  
- ACGGGAATAA GTCCGAAACT CACGTTCCGG ACCCCCAGGA GGAGTAAACT CTTAATGGCC ACAAGTAGTT   
  
  
- GTGTTTCTCC AAGATCTAGT TCACCGAGTA TCCCACTGAG TTCTTCGACT CTTTAACCTA AACGGTAAAG   
  
  
- TCAAGTTAGG ACACCAATCG TTCAACCTTT TGGAACTACA ACTTTTCGAC ACACAATTCT GACCACTCCG   
  
  
- AGATCGGTAG TCAAGCCAGG AAGTTGACGT ATGGGAAAAC CCAAGACTAC TACTCGGGGA TTCCTTTTCA   
  
  
- AGTGGAAATC GGAACTACTT CATACGTTTA CCCCGATTAT CCGTTTCGGG CTCATTACTA AGCCGAAGAA   
  
  
- GTAGTGGAGG AAGCGAGTTA TGAAGTTGGT TCGACCTACC AAAGGAGTCG CGAAACACCC CTAACAGGGG   
  
  
- TTTCTAATAC CACTATCGAC TCGTGCTAAG GTTAGTGTTA CCAAGACCTG AATACCTCTC CAACAGTCTT   
  
  
- CGTGACATGA AGATACGTCG CGACAAACTG ACGAATCTTA GGTGGGACGG TTCTTGTAGA CAGCTTTCTT   
  
  
- CCGCCCATCT CTTCTACGAG GATCCACTTC TCTAGTTCTT GTAATATAGT ACGCTCCCTC CTCTTTCCTC   
  
  
- TCTTTCCGTA CTCTTCTAGC TCTTCACCTA CGTCTCCGAT CTGTACCGAC CTAAGCCCTA GCAAGGAAAC   
  
  
- TCGATATACC CATACGACGT TCGTTCCGTT AACGAAGTCC CGATACCAAC ACTACCAATA TCTCACTTTC   
  
  
- TCTTACCAAC ACAACAGTAG ACAACCGTTC TAGCGGGGGA GAAAAGCCAT AGTCGTACCT CCACATCCTC   
  
  
- TAC

+     WRE3

| Site Name | Organism | Position | Strand | Matrix score. | sequence | function |
| --- | --- | --- | --- | --- | --- | --- |
| WRE3 | Pisum sativum | 337 | + | 6 | CCACCT |  |

>HU08G00367.1   
+ -Up\_Stream \_Len000ATGCGC CTTTCTGCTA ACTCATTATC TTTCCTTCTG AGAGGGGACA ACTTATATAA   
  
  
+ ATTGATAGGT ATTTTCAAAT CAATAGACCA GCTACCATAT ATGATAATTT TTATTACTTT TCTAAATCCA   
  
  
+ TCAACTATTT TCACAATCCT CCTTTTTCTA GAAAAATAAA ATTTGCTTAA TTCAAATCAC CTAAAATTAC   
  
  
+ TAAATCTATA GTCCTAAATC TACAAACTCA TTTGTGTTGC AACTCATGAT TTAAGTGGAA CACTAACCTT   
  
  
+ AGGAGATATG TTGTCAAAAG AAGAATTCGA TGACATATTT TCCAACAAAA GGCCACCTAA CTTCATGATT   
  
  
+ TAAAAAGGAG GAGTCTAAGC ACATCATGGA TGACTAATTT TTGATGTATT GTAACTACGT ATTGAGATAA   
  
  
+ CTTGAATGAA TCGTGACTAG AATATATCTA TTTTCAAAAA TAAAAATTCC CTAAAAGAAA AGGATAAAAA   
  
  
+ ACTATATAAT ACTAAAAAAT TCTAACTATA ATAACTAGGC TTATTAAATT TTTATAGTGA AAATTTATTT   
  
  
+ CTACTTATAA CAACATCACT TACTAAGTAG CTTATTTGTG TCTTCTTTTC TAAATAATAA TGGTAACTAT   
  
  
+ GTGTCTTTTG TAAATAACTT ATTATCTATT TTATAAATCA CCGTGTAAAG CACGGGTCTA TACTAGTTAA   
  
  
+ TTTAAATAGC ATTGTTAAAT CAAGTAAGAT ACAAACAAAT TTATTTACAA CTGTAAGTGG TCCATAAAAA   
  
  
+ TAATAGCCAT TACAAAAAAT AGTACTACTG TTTTATGAAA TGATTTTAAT ATTCTAAAAT TCCAATAAAA   
  
  
+ CATGTTATAA TCTATGATTA ATTTTAAAAC ATGATTACAT GTCAGCATAA TTTGTATTAT TGTACAGCAA   
  
  
+ ATGAAAGTTG GAGCTAAAAG AAAATGGGGA AATAAAGGCA GACAAGTAAA GATAAAATAT GCTATCTACT   
  
  
+ TTTGGAGGTA TCCTTAACCA CATTGTCTCG TTTGTCTTCA TTAGGGAGAA GTGGGATAAA TACCAAAATT   
  
  
+ TGTATGGTCA TTGCGTGGGG TTTGCTTCCA AACAAAATGG CTTTTTGGAA GCAAATTTTG TGAGCTTCTA   
  
  
+ TGCAAATTTC CACGCTTAAC CCCACAAGCA AACAAAAACA GACAGTGGTT TTTGTTTGTC TTTGTTAGGG   
  
  
+ CATGTTTGGA GCCAACTGTT AATGGGAATA AACTGGGGAG GCTTTTTCCT TGGCTCTTTC CCCCTGACCC   
  
  
+ CAAACCCACT TCAGCTCAAT ACGAAACCAG CCATTGAAAA AGGAAGGGGG GGGGGGGGGT ATGTGAGAAA   
  
  
+ AAGACAAAAC TTTTTCCCGG AGAGAGAAGA TACATATGCA ACAAAGGCTC TCCTTCTCAT AAATTCCATA   
  
  
+ TTCCTCTGTT TTTCTAGGAG AGAGAAATAT GCAAAAAACA AATCAGTTGG CGACAAAAGT TACTACCACG   
  
  
+ CATTGTCTAC TGCTGCGTAC CCATTTTTGG AGACTTTAAA GAACGCCCCT CCCATCTTGA AAAGTGAAAA   
  
  
+ CCCCCTCTTT GAAACTGAAT TTCAGGGGAA AAAAACCTCA CCAAACCTCT AGAGAGAGCT TTTAGAGAGG   
  
  
+ GAAATACAAC GATTAGAAGG AGCAATTTGG GAAATTTCTT TGGGAATTTG AATGGGTTTT GAGTGAATTG   
  
  
+ CAAATCCCAG AAAAGTTTTG GCAAGTACCG ATCTACAGTT CTCTCCTCTT CGTGTTTGGT AGATCCCCTG   
  
  
+ TTTCCTCTGT TTCATTTAGG GTACTTCTCA TTCATCCTCC CCACCCCCTT AATCGGATCT TCCTGTCGAG   
  
  
+ TCACTTTATG CTAATATTTT TTCAGTGGAT TTTTAGTTAA CCCTGTTCAT TTTTCCATTC TGTGTCCCAT   
  
  
+ TTCTCTCTTT TTCATTCATA AGTTGCCGGT TTATCTGTTG GGTGCAGCTT AGTCACAATA ATTTCTGTGT   
  
  
+ TAGGCTTCTT TACGGTTAAA AAAAAAGGAG GCACTCTTTT CGGTGTGATT GTTTATGGGA CCAATGATTC   
  
  
+ AAGATGATGG GTCATCAGTA ACTTCATCAC CCCTTCAATT TTTCTCCATG ATGTCTCCCA ATTTAGGTTC   
  
  
+ TTCCTACCCT TGGCTCAGAG AGCTAAAACC TGAAGAAAGA GGTCTTTACT TGATACATTT GTTGCTCTCT   
  
  
+ TGTGCAAATC ATGTCTCTAG TGGTAGCCTA GACAATGCGA ACTTAGCCCT CGAACAAATC TCTCAGCTTG   
  
  
+ CTGCCCCTGA TGGGGATACA ATGCAGCGTA TGGCTTCTTA TTTTGCTGAA GCCCTGGCTG AGAGGATCCT   
  
  
+ CAAGTCATGG CCTGGCATGT ATAGAGCCCT TCATTTTACG AAAATGCCTG TCATTTCAGA GGAAATTCTT   
  
  
+ GCTAGGAAGC TCTTCTTTGA GCTATTTCCT TTCTTGAAGC TGGCCTATTT GGTGACAAAC CAATCGATAA   
  
  
+ TCGAAGCCAT GGAGGGGGAA AAGATGGTTC ATATTATTGA TCTGAATGCA TCAGAACCTG CTCAATGGAT   
  
  
+ TGCCCTTATT CAGGCTTTGA GTGCAAGGCC TGGGGGTCCT CCTCATTTGA GAATTACCGG TGTTCATCAA   
  
  
+ CACAAAGAGG TTCTAGATCA AGTGGCTCAT AGGGTGACTC AAGAAGCTGA GAAATTGGAT TTGCCATTTC   
  
  
+ AGTTCAATCC TGTGGTTAGC AAGTTGGAAA ACCTTGATGT TGAAAAGCTG TGTGTTAAGA CTGGTGAGGC   
  
  
+ TCTAGCCATC AGTTCGGTCC TTCAACTGCA TACCCTTTTG GGTTCTGATG ATGAGCCCCT AAGGAAAAGT   
  
  
+ TCACCTTTAG CCTTGATGAA GTATGCAAAT GGGGCTAATA GGCAAAGCCC GAGTAATGAT TCGGCTTCTT   
  
  
+ CATCACCTCC TTCGCTCAAT ACTTCAACCA AGCTGGATGG TTTCCTCAGC GCTTTGTGGG GATTGTCCCC   
  
  
+ AAAGATTATG GTGATAGCTG AGCACGATTC CAATCACAAT GGTTCTGGAC TTATGGAGAG GTTGTCAGAA   
  
  
+ GCACTGTACT TCTATGCAGC GCTGTTTGAC TGCTTAGAAT CCACCCTGCC AAGAACATCT GTCGAAAGAA   
  
  
+ GGCGGGTAGA GAAGATGCTC CTAGGTGAAG AGATCAAGAA CATTATATCA TGCGAGGGAG GAGAAAGGAG   
  
  
+ AGAAAGGCAT GAGAAGATCG AGAAGTGGAT GCAGAGGCTA GACATGGCTG GATTCGGGAT CGTTCCTTTG   
  
  
+ AGCTATATGG GTATGCTGCA AGCAAGGCAA TTGCTTCAGG GCTATGGTTG TGATGGTTAT AGAGTGAAAG   
  
  
+ AGAATGGTTG TGTTGTCATC TGTTGGCAAG ATCGCCCCCT CTTTTCGGTA TCAGCATGGA GGTGTAGGAG   
  
  
+ ATG  

- -Up\_Stream \_Len000TACGCG GAAAGACGAT TGAGTAATAG AAAGGAAGAC TCTCCCCTGT TGAATATATT   
  
  
- TAACTATCCA TAAAAGTTTA GTTATCTGGT CGATGGTATA TACTATTAAA AATAATGAAA AGATTTAGGT   
  
  
- AGTTGATAAA AGTGTTAGGA GGAAAAAGAT CTTTTTATTT TAAACGAATT AAGTTTAGTG GATTTTAATG   
  
  
- ATTTAGATAT CAGGATTTAG ATGTTTGAGT AAACACAACG TTGAGTACTA AATTCACCTT GTGATTGGAA   
  
  
- TCCTCTATAC AACAGTTTTC TTCTTAAGCT ACTGTATAAA AGGTTGTTTT CCGGTGGATT GAAGTACTAA   
  
  
- ATTTTTCCTC CTCAGATTCG TGTAGTACCT ACTGATTAAA AACTACATAA CATTGATGCA TAACTCTATT   
  
  
- GAACTTACTT AGCACTGATC TTATATAGAT AAAAGTTTTT ATTTTTAAGG GATTTTCTTT TCCTATTTTT   
  
  
- TGATATATTA TGATTTTTTA AGATTGATAT TATTGATCCG AATAATTTAA AAATATCACT TTTAAATAAA   
  
  
- GATGAATATT GTTGTAGTGA ATGATTCATC GAATAAACAC AGAAGAAAAG ATTTATTATT ACCATTGATA   
  
  
- CACAGAAAAC ATTTATTGAA TAATAGATAA AATATTTAGT GGCACATTTC GTGCCCAGAT ATGATCAATT   
  
  
- AAATTTATCG TAACAATTTA GTTCATTCTA TGTTTGTTTA AATAAATGTT GACATTCACC AGGTATTTTT   
  
  
- ATTATCGGTA ATGTTTTTTA TCATGATGAC AAAATACTTT ACTAAAATTA TAAGATTTTA AGGTTATTTT   
  
  
- GTACAATATT AGATACTAAT TAAAATTTTG TACTAATGTA CAGTCGTATT AAACATAATA ACATGTCGTT   
  
  
- TACTTTCAAC CTCGATTTTC TTTTACCCCT TTATTTCCGT CTGTTCATTT CTATTTTATA CGATAGATGA   
  
  
- AAACCTCCAT AGGAATTGGT GTAACAGAGC AAACAGAAGT AATCCCTCTT CACCCTATTT ATGGTTTTAA   
  
  
- ACATACCAGT AACGCACCCC AAACGAAGGT TTGTTTTACC GAAAAACCTT CGTTTAAAAC ACTCGAAGAT   
  
  
- ACGTTTAAAG GTGCGAATTG GGGTGTTCGT TTGTTTTTGT CTGTCACCAA AAACAAACAG AAACAATCCC   
  
  
- GTACAAACCT CGGTTGACAA TTACCCTTAT TTGACCCCTC CGAAAAAGGA ACCGAGAAAG GGGGACTGGG   
  
  
- GTTTGGGTGA AGTCGAGTTA TGCTTTGGTC GGTAACTTTT TCCTTCCCCC CCCCCCCCCA TACACTCTTT   
  
  
- TTCTGTTTTG AAAAAGGGCC TCTCTCTTCT ATGTATACGT TGTTTCCGAG AGGAAGAGTA TTTAAGGTAT   
  
  
- AAGGAGACAA AAAGATCCTC TCTCTTTATA CGTTTTTTGT TTAGTCAACC GCTGTTTTCA ATGATGGTGC   
  
  
- GTAACAGATG ACGACGCATG GGTAAAAACC TCTGAAATTT CTTGCGGGGA GGGTAGAACT TTTCACTTTT   
  
  
- GGGGGAGAAA CTTTGACTTA AAGTCCCCTT TTTTTGGAGT GGTTTGGAGA TCTCTCTCGA AAATCTCTCC   
  
  
- CTTTATGTTG CTAATCTTCC TCGTTAAACC CTTTAAAGAA ACCCTTAAAC TTACCCAAAA CTCACTTAAC   
  
  
- GTTTAGGGTC TTTTCAAAAC CGTTCATGGC TAGATGTCAA GAGAGGAGAA GCACAAACCA TCTAGGGGAC   
  
  
- AAAGGAGACA AAGTAAATCC CATGAAGAGT AAGTAGGAGG GGTGGGGGAA TTAGCCTAGA AGGACAGCTC   
  
  
- AGTGAAATAC GATTATAAAA AAGTCACCTA AAAATCAATT GGGACAAGTA AAAAGGTAAG ACACAGGGTA   
  
  
- AAGAGAGAAA AAGTAAGTAT TCAACGGCCA AATAGACAAC CCACGTCGAA TCAGTGTTAT TAAAGACACA   
  
  
- ATCCGAAGAA ATGCCAATTT TTTTTTCCTC CGTGAGAAAA GCCACACTAA CAAATACCCT GGTTACTAAG   
  
  
- TTCTACTACC CAGTAGTCAT TGAAGTAGTG GGGAAGTTAA AAAGAGGTAC TACAGAGGGT TAAATCCAAG   
  
  
- AAGGATGGGA ACCGAGTCTC TCGATTTTGG ACTTCTTTCT CCAGAAATGA ACTATGTAAA CAACGAGAGA   
  
  
- ACACGTTTAG TACAGAGATC ACCATCGGAT CTGTTACGCT TGAATCGGGA GCTTGTTTAG AGAGTCGAAC   
  
  
- GACGGGGACT ACCCCTATGT TACGTCGCAT ACCGAAGAAT AAAACGACTT CGGGACCGAC TCTCCTAGGA   
  
  
- GTTCAGTACC GGACCGTACA TATCTCGGGA AGTAAAATGC TTTTACGGAC AGTAAAGTCT CCTTTAAGAA   
  
  
- CGATCCTTCG AGAAGAAACT CGATAAAGGA AAGAACTTCG ACCGGATAAA CCACTGTTTG GTTAGCTATT   
  
  
- AGCTTCGGTA CCTCCCCCTT TTCTACCAAG TATAATAACT AGACTTACGT AGTCTTGGAC GAGTTACCTA   
  
  
- ACGGGAATAA GTCCGAAACT CACGTTCCGG ACCCCCAGGA GGAGTAAACT CTTAATGGCC ACAAGTAGTT   
  
  
- GTGTTTCTCC AAGATCTAGT TCACCGAGTA TCCCACTGAG TTCTTCGACT CTTTAACCTA AACGGTAAAG   
  
  
- TCAAGTTAGG ACACCAATCG TTCAACCTTT TGGAACTACA ACTTTTCGAC ACACAATTCT GACCACTCCG   
  
  
- AGATCGGTAG TCAAGCCAGG AAGTTGACGT ATGGGAAAAC CCAAGACTAC TACTCGGGGA TTCCTTTTCA   
  
  
- AGTGGAAATC GGAACTACTT CATACGTTTA CCCCGATTAT CCGTTTCGGG CTCATTACTA AGCCGAAGAA   
  
  
- GTAGTGGAGG AAGCGAGTTA TGAAGTTGGT TCGACCTACC AAAGGAGTCG CGAAACACCC CTAACAGGGG   
  
  
- TTTCTAATAC CACTATCGAC TCGTGCTAAG GTTAGTGTTA CCAAGACCTG AATACCTCTC CAACAGTCTT   
  
  
- CGTGACATGA AGATACGTCG CGACAAACTG ACGAATCTTA GGTGGGACGG TTCTTGTAGA CAGCTTTCTT   
  
  
- CCGCCCATCT CTTCTACGAG GATCCACTTC TCTAGTTCTT GTAATATAGT ACGCTCCCTC CTCTTTCCTC   
  
  
- TCTTTCCGTA CTCTTCTAGC TCTTCACCTA CGTCTCCGAT CTGTACCGAC CTAAGCCCTA GCAAGGAAAC   
  
  
- TCGATATACC CATACGACGT TCGTTCCGTT AACGAAGTCC CGATACCAAC ACTACCAATA TCTCACTTTC   
  
  
- TCTTACCAAC ACAACAGTAG ACAACCGTTC TAGCGGGGGA GAAAAGCCAT AGTCGTACCT CCACATCCTC   
  
  
- TAC

+     WUN-motif

| Site Name | Organism | Position | Strand | Matrix score. | sequence | function |
| --- | --- | --- | --- | --- | --- | --- |
| WUN-motif | Nicotiana glutinosa | 1646 | + | 9 | AAATTTCTT |  |
| WUN-motif | Nicotiana glutinosa | 208 | + | 9 | AAATTACTA |  |

>HU08G00367.1   
+ -Up\_Stream \_Len000ATGCGC CTTTCTGCTA ACTCATTATC TTTCCTTCTG AGAGGGGACA ACTTATATAA   
  
  
+ ATTGATAGGT ATTTTCAAAT CAATAGACCA GCTACCATAT ATGATAATTT TTATTACTTT TCTAAATCCA   
  
  
+ TCAACTATTT TCACAATCCT CCTTTTTCTA GAAAAATAAA ATTTGCTTAA TTCAAATCAC CTAAAATTAC   
  
  
+ TAAATCTATA GTCCTAAATC TACAAACTCA TTTGTGTTGC AACTCATGAT TTAAGTGGAA CACTAACCTT   
  
  
+ AGGAGATATG TTGTCAAAAG AAGAATTCGA TGACATATTT TCCAACAAAA GGCCACCTAA CTTCATGATT   
  
  
+ TAAAAAGGAG GAGTCTAAGC ACATCATGGA TGACTAATTT TTGATGTATT GTAACTACGT ATTGAGATAA   
  
  
+ CTTGAATGAA TCGTGACTAG AATATATCTA TTTTCAAAAA TAAAAATTCC CTAAAAGAAA AGGATAAAAA   
  
  
+ ACTATATAAT ACTAAAAAAT TCTAACTATA ATAACTAGGC TTATTAAATT TTTATAGTGA AAATTTATTT   
  
  
+ CTACTTATAA CAACATCACT TACTAAGTAG CTTATTTGTG TCTTCTTTTC TAAATAATAA TGGTAACTAT   
  
  
+ GTGTCTTTTG TAAATAACTT ATTATCTATT TTATAAATCA CCGTGTAAAG CACGGGTCTA TACTAGTTAA   
  
  
+ TTTAAATAGC ATTGTTAAAT CAAGTAAGAT ACAAACAAAT TTATTTACAA CTGTAAGTGG TCCATAAAAA   
  
  
+ TAATAGCCAT TACAAAAAAT AGTACTACTG TTTTATGAAA TGATTTTAAT ATTCTAAAAT TCCAATAAAA   
  
  
+ CATGTTATAA TCTATGATTA ATTTTAAAAC ATGATTACAT GTCAGCATAA TTTGTATTAT TGTACAGCAA   
  
  
+ ATGAAAGTTG GAGCTAAAAG AAAATGGGGA AATAAAGGCA GACAAGTAAA GATAAAATAT GCTATCTACT   
  
  
+ TTTGGAGGTA TCCTTAACCA CATTGTCTCG TTTGTCTTCA TTAGGGAGAA GTGGGATAAA TACCAAAATT   
  
  
+ TGTATGGTCA TTGCGTGGGG TTTGCTTCCA AACAAAATGG CTTTTTGGAA GCAAATTTTG TGAGCTTCTA   
  
  
+ TGCAAATTTC CACGCTTAAC CCCACAAGCA AACAAAAACA GACAGTGGTT TTTGTTTGTC TTTGTTAGGG   
  
  
+ CATGTTTGGA GCCAACTGTT AATGGGAATA AACTGGGGAG GCTTTTTCCT TGGCTCTTTC CCCCTGACCC   
  
  
+ CAAACCCACT TCAGCTCAAT ACGAAACCAG CCATTGAAAA AGGAAGGGGG GGGGGGGGGT ATGTGAGAAA   
  
  
+ AAGACAAAAC TTTTTCCCGG AGAGAGAAGA TACATATGCA ACAAAGGCTC TCCTTCTCAT AAATTCCATA   
  
  
+ TTCCTCTGTT TTTCTAGGAG AGAGAAATAT GCAAAAAACA AATCAGTTGG CGACAAAAGT TACTACCACG   
  
  
+ CATTGTCTAC TGCTGCGTAC CCATTTTTGG AGACTTTAAA GAACGCCCCT CCCATCTTGA AAAGTGAAAA   
  
  
+ CCCCCTCTTT GAAACTGAAT TTCAGGGGAA AAAAACCTCA CCAAACCTCT AGAGAGAGCT TTTAGAGAGG   
  
  
+ GAAATACAAC GATTAGAAGG AGCAATTTGG GAAATTTCTT TGGGAATTTG AATGGGTTTT GAGTGAATTG   
  
  
+ CAAATCCCAG AAAAGTTTTG GCAAGTACCG ATCTACAGTT CTCTCCTCTT CGTGTTTGGT AGATCCCCTG   
  
  
+ TTTCCTCTGT TTCATTTAGG GTACTTCTCA TTCATCCTCC CCACCCCCTT AATCGGATCT TCCTGTCGAG   
  
  
+ TCACTTTATG CTAATATTTT TTCAGTGGAT TTTTAGTTAA CCCTGTTCAT TTTTCCATTC TGTGTCCCAT   
  
  
+ TTCTCTCTTT TTCATTCATA AGTTGCCGGT TTATCTGTTG GGTGCAGCTT AGTCACAATA ATTTCTGTGT   
  
  
+ TAGGCTTCTT TACGGTTAAA AAAAAAGGAG GCACTCTTTT CGGTGTGATT GTTTATGGGA CCAATGATTC   
  
  
+ AAGATGATGG GTCATCAGTA ACTTCATCAC CCCTTCAATT TTTCTCCATG ATGTCTCCCA ATTTAGGTTC   
  
  
+ TTCCTACCCT TGGCTCAGAG AGCTAAAACC TGAAGAAAGA GGTCTTTACT TGATACATTT GTTGCTCTCT   
  
  
+ TGTGCAAATC ATGTCTCTAG TGGTAGCCTA GACAATGCGA ACTTAGCCCT CGAACAAATC TCTCAGCTTG   
  
  
+ CTGCCCCTGA TGGGGATACA ATGCAGCGTA TGGCTTCTTA TTTTGCTGAA GCCCTGGCTG AGAGGATCCT   
  
  
+ CAAGTCATGG CCTGGCATGT ATAGAGCCCT TCATTTTACG AAAATGCCTG TCATTTCAGA GGAAATTCTT   
  
  
+ GCTAGGAAGC TCTTCTTTGA GCTATTTCCT TTCTTGAAGC TGGCCTATTT GGTGACAAAC CAATCGATAA   
  
  
+ TCGAAGCCAT GGAGGGGGAA AAGATGGTTC ATATTATTGA TCTGAATGCA TCAGAACCTG CTCAATGGAT   
  
  
+ TGCCCTTATT CAGGCTTTGA GTGCAAGGCC TGGGGGTCCT CCTCATTTGA GAATTACCGG TGTTCATCAA   
  
  
+ CACAAAGAGG TTCTAGATCA AGTGGCTCAT AGGGTGACTC AAGAAGCTGA GAAATTGGAT TTGCCATTTC   
  
  
+ AGTTCAATCC TGTGGTTAGC AAGTTGGAAA ACCTTGATGT TGAAAAGCTG TGTGTTAAGA CTGGTGAGGC   
  
  
+ TCTAGCCATC AGTTCGGTCC TTCAACTGCA TACCCTTTTG GGTTCTGATG ATGAGCCCCT AAGGAAAAGT   
  
  
+ TCACCTTTAG CCTTGATGAA GTATGCAAAT GGGGCTAATA GGCAAAGCCC GAGTAATGAT TCGGCTTCTT   
  
  
+ CATCACCTCC TTCGCTCAAT ACTTCAACCA AGCTGGATGG TTTCCTCAGC GCTTTGTGGG GATTGTCCCC   
  
  
+ AAAGATTATG GTGATAGCTG AGCACGATTC CAATCACAAT GGTTCTGGAC TTATGGAGAG GTTGTCAGAA   
  
  
+ GCACTGTACT TCTATGCAGC GCTGTTTGAC TGCTTAGAAT CCACCCTGCC AAGAACATCT GTCGAAAGAA   
  
  
+ GGCGGGTAGA GAAGATGCTC CTAGGTGAAG AGATCAAGAA CATTATATCA TGCGAGGGAG GAGAAAGGAG   
  
  
+ AGAAAGGCAT GAGAAGATCG AGAAGTGGAT GCAGAGGCTA GACATGGCTG GATTCGGGAT CGTTCCTTTG   
  
  
+ AGCTATATGG GTATGCTGCA AGCAAGGCAA TTGCTTCAGG GCTATGGTTG TGATGGTTAT AGAGTGAAAG   
  
  
+ AGAATGGTTG TGTTGTCATC TGTTGGCAAG ATCGCCCCCT CTTTTCGGTA TCAGCATGGA GGTGTAGGAG   
  
  
+ ATG  

- -Up\_Stream \_Len000TACGCG GAAAGACGAT TGAGTAATAG AAAGGAAGAC TCTCCCCTGT TGAATATATT   
  
  
- TAACTATCCA TAAAAGTTTA GTTATCTGGT CGATGGTATA TACTATTAAA AATAATGAAA AGATTTAGGT   
  
  
- AGTTGATAAA AGTGTTAGGA GGAAAAAGAT CTTTTTATTT TAAACGAATT AAGTTTAGTG GATTTTAATG   
  
  
- ATTTAGATAT CAGGATTTAG ATGTTTGAGT AAACACAACG TTGAGTACTA AATTCACCTT GTGATTGGAA   
  
  
- TCCTCTATAC AACAGTTTTC TTCTTAAGCT ACTGTATAAA AGGTTGTTTT CCGGTGGATT GAAGTACTAA   
  
  
- ATTTTTCCTC CTCAGATTCG TGTAGTACCT ACTGATTAAA AACTACATAA CATTGATGCA TAACTCTATT   
  
  
- GAACTTACTT AGCACTGATC TTATATAGAT AAAAGTTTTT ATTTTTAAGG GATTTTCTTT TCCTATTTTT   
  
  
- TGATATATTA TGATTTTTTA AGATTGATAT TATTGATCCG AATAATTTAA AAATATCACT TTTAAATAAA   
  
  
- GATGAATATT GTTGTAGTGA ATGATTCATC GAATAAACAC AGAAGAAAAG ATTTATTATT ACCATTGATA   
  
  
- CACAGAAAAC ATTTATTGAA TAATAGATAA AATATTTAGT GGCACATTTC GTGCCCAGAT ATGATCAATT   
  
  
- AAATTTATCG TAACAATTTA GTTCATTCTA TGTTTGTTTA AATAAATGTT GACATTCACC AGGTATTTTT   
  
  
- ATTATCGGTA ATGTTTTTTA TCATGATGAC AAAATACTTT ACTAAAATTA TAAGATTTTA AGGTTATTTT   
  
  
- GTACAATATT AGATACTAAT TAAAATTTTG TACTAATGTA CAGTCGTATT AAACATAATA ACATGTCGTT   
  
  
- TACTTTCAAC CTCGATTTTC TTTTACCCCT TTATTTCCGT CTGTTCATTT CTATTTTATA CGATAGATGA   
  
  
- AAACCTCCAT AGGAATTGGT GTAACAGAGC AAACAGAAGT AATCCCTCTT CACCCTATTT ATGGTTTTAA   
  
  
- ACATACCAGT AACGCACCCC AAACGAAGGT TTGTTTTACC GAAAAACCTT CGTTTAAAAC ACTCGAAGAT   
  
  
- ACGTTTAAAG GTGCGAATTG GGGTGTTCGT TTGTTTTTGT CTGTCACCAA AAACAAACAG AAACAATCCC   
  
  
- GTACAAACCT CGGTTGACAA TTACCCTTAT TTGACCCCTC CGAAAAAGGA ACCGAGAAAG GGGGACTGGG   
  
  
- GTTTGGGTGA AGTCGAGTTA TGCTTTGGTC GGTAACTTTT TCCTTCCCCC CCCCCCCCCA TACACTCTTT   
  
  
- TTCTGTTTTG AAAAAGGGCC TCTCTCTTCT ATGTATACGT TGTTTCCGAG AGGAAGAGTA TTTAAGGTAT   
  
  
- AAGGAGACAA AAAGATCCTC TCTCTTTATA CGTTTTTTGT TTAGTCAACC GCTGTTTTCA ATGATGGTGC   
  
  
- GTAACAGATG ACGACGCATG GGTAAAAACC TCTGAAATTT CTTGCGGGGA GGGTAGAACT TTTCACTTTT   
  
  
- GGGGGAGAAA CTTTGACTTA AAGTCCCCTT TTTTTGGAGT GGTTTGGAGA TCTCTCTCGA AAATCTCTCC   
  
  
- CTTTATGTTG CTAATCTTCC TCGTTAAACC CTTTAAAGAA ACCCTTAAAC TTACCCAAAA CTCACTTAAC   
  
  
- GTTTAGGGTC TTTTCAAAAC CGTTCATGGC TAGATGTCAA GAGAGGAGAA GCACAAACCA TCTAGGGGAC   
  
  
- AAAGGAGACA AAGTAAATCC CATGAAGAGT AAGTAGGAGG GGTGGGGGAA TTAGCCTAGA AGGACAGCTC   
  
  
- AGTGAAATAC GATTATAAAA AAGTCACCTA AAAATCAATT GGGACAAGTA AAAAGGTAAG ACACAGGGTA   
  
  
- AAGAGAGAAA AAGTAAGTAT TCAACGGCCA AATAGACAAC CCACGTCGAA TCAGTGTTAT TAAAGACACA   
  
  
- ATCCGAAGAA ATGCCAATTT TTTTTTCCTC CGTGAGAAAA GCCACACTAA CAAATACCCT GGTTACTAAG   
  
  
- TTCTACTACC CAGTAGTCAT TGAAGTAGTG GGGAAGTTAA AAAGAGGTAC TACAGAGGGT TAAATCCAAG   
  
  
- AAGGATGGGA ACCGAGTCTC TCGATTTTGG ACTTCTTTCT CCAGAAATGA ACTATGTAAA CAACGAGAGA   
  
  
- ACACGTTTAG TACAGAGATC ACCATCGGAT CTGTTACGCT TGAATCGGGA GCTTGTTTAG AGAGTCGAAC   
  
  
- GACGGGGACT ACCCCTATGT TACGTCGCAT ACCGAAGAAT AAAACGACTT CGGGACCGAC TCTCCTAGGA   
  
  
- GTTCAGTACC GGACCGTACA TATCTCGGGA AGTAAAATGC TTTTACGGAC AGTAAAGTCT CCTTTAAGAA   
  
  
- CGATCCTTCG AGAAGAAACT CGATAAAGGA AAGAACTTCG ACCGGATAAA CCACTGTTTG GTTAGCTATT   
  
  
- AGCTTCGGTA CCTCCCCCTT TTCTACCAAG TATAATAACT AGACTTACGT AGTCTTGGAC GAGTTACCTA   
  
  
- ACGGGAATAA GTCCGAAACT CACGTTCCGG ACCCCCAGGA GGAGTAAACT CTTAATGGCC ACAAGTAGTT   
  
  
- GTGTTTCTCC AAGATCTAGT TCACCGAGTA TCCCACTGAG TTCTTCGACT CTTTAACCTA AACGGTAAAG   
  
  
- TCAAGTTAGG ACACCAATCG TTCAACCTTT TGGAACTACA ACTTTTCGAC ACACAATTCT GACCACTCCG   
  
  
- AGATCGGTAG TCAAGCCAGG AAGTTGACGT ATGGGAAAAC CCAAGACTAC TACTCGGGGA TTCCTTTTCA   
  
  
- AGTGGAAATC GGAACTACTT CATACGTTTA CCCCGATTAT CCGTTTCGGG CTCATTACTA AGCCGAAGAA   
  
  
- GTAGTGGAGG AAGCGAGTTA TGAAGTTGGT TCGACCTACC AAAGGAGTCG CGAAACACCC CTAACAGGGG   
  
  
- TTTCTAATAC CACTATCGAC TCGTGCTAAG GTTAGTGTTA CCAAGACCTG AATACCTCTC CAACAGTCTT   
  
  
- CGTGACATGA AGATACGTCG CGACAAACTG ACGAATCTTA GGTGGGACGG TTCTTGTAGA CAGCTTTCTT   
  
  
- CCGCCCATCT CTTCTACGAG GATCCACTTC TCTAGTTCTT GTAATATAGT ACGCTCCCTC CTCTTTCCTC   
  
  
- TCTTTCCGTA CTCTTCTAGC TCTTCACCTA CGTCTCCGAT CTGTACCGAC CTAAGCCCTA GCAAGGAAAC   
  
  
- TCGATATACC CATACGACGT TCGTTCCGTT AACGAAGTCC CGATACCAAC ACTACCAATA TCTCACTTTC   
  
  
- TCTTACCAAC ACAACAGTAG ACAACCGTTC TAGCGGGGGA GAAAAGCCAT AGTCGTACCT CCACATCCTC   
  
  
- TAC
