# Supplementary material for: Genome-Wide Identification and Expression Pattern of the GRAS Gene Family in Pitaya (Selenicereus undatus L.)
Source: Biology (Basel). 2022 Dec 21;12(1):11. doi: 10.3390/biology12010011 (PMC9854919; doi:10.3390/biology12010011)
Supplement: Supplementary file 1 [file biology-12-00011-s001.zip › Supplementary file S5/HU08G01232.1_plantcare.html]

Content-Type: text/html; charset=ISO-8859-1


PlantCARE


Webmaster Firefox specific output  
To save the result:
click on the frame with the right mouse button and save the source code as a text file with extension .html  
REFERENCE:PlantCARE: a database of plant cis-acting regulatory elements and a portal to tools for in silico analysis of promoter sequences.  
Lescot, M., Déhais, P., Moreau, Y., De Moor, B., Rouzé ,P.,and Rombauts, S.  
Nucleic Acids Res., Database issue(2002), 30(1):325-327.   


---

>HU08G01232.1   
+ -Up\_Stream \_Len000TCTACC TTTACCTGTG TTAAAAAAAA AAATTTTGTT TCCATTACTA TTCTCTTGGC   
  
  
+ ACTATTGGTG CTGATTTTTC TTCACCAAAT CAACCTCCTT ATATTTGCCA ATTTTACTGT CCTCTTTCAT   
  
  
+ CTATAAAGTC AACTCTCCTA GTACAATTTG TCACATAGAA AATCTCTAGG CACTCATCTA AACTCTCTGT   
  
  
+ TCTGGGTAAT TGATCGACGA TCTACACCAT TATTCGTTCT AGTGCACCGT TTGGACAGCC AATCATGTAC   
  
  
+ CTTTGGAGAT CAACTGCTCA TGAGTTCTTG GTGTGAAATT GGGGAAGTAA ATTCGACTTT AGGGCACTGG   
  
  
+ TCTATCACGC CATGATTTTA CCATCTATTT TCATATATAT CCAATAAGTT TGATCTTTCG CTATTAATCA   
  
  
+ CAATGATATA AACAAGTGGT GAAGAGCAAT AATGAACCAT ACATTTTAAA CTTAAGCGTT AAGGAAGACA   
  
  
+ TGAAGTTAAA AACAATGGTA AGAGCTATGC GTATGTTTGG CATATAGCTT TTTTAAGAGT GTTTTGGCTA   
  
  
+ TAGTCGGAGT TTTTTAATTA AGATTAGCTG TTTGATCAAA TAAAAAAGCT AATTTGAGTG TTTGGCGAGA   
  
  
+ AGACTTTTTA TAAGAACTTT TTTTGGTCTA AAAAGTTAAT TTAAAAAGGC TAATTCTATG AGCTTTTCGG   
  
  
+ AAGAGTTTTT TAAATAATTA ACTTTTTGTC TCATAAGCCA TAACTTTATC AGAAACAGTT AATTTTACAA   
  
  
+ AATAATTTCT CAACAAACAA CTAATTTAAA TAATTAATAA AAATAACTAA CTCAAATATC TAATAACTAA   
  
  
+ TAACTAATAT AAATAATTAA CAGTTAACAG TTGTTTACGA AACAGAATTT ATATAAAAAG CGGTAACAGA   
  
  
+ TAAAAGATAA TCATGCGAGA TTAAAAGTCA CCAGTGACAC ACAAGTACTT AAATAAATAG TCTAAACAAT   
  
  
+ GATATTTTTT GCGAGCTTCC ATGCAAATAC CGACACTTTT ATTGCCTCAG AAGGAAACAA AAACTGAGTG   
  
  
+ AAGGGGGGGG GGGGTTCTGT TTGTCTTTGT TAGGGCACGT TTGGATTCAA GTGTAGATTG GGAATAAATT   
  
  
+ CGAGGACTTT TTCTTTGCCC TTTTTCCCTG ATTCCCAAAC CCACATCAAG TCAAGACGAA ACCAGCCATT   
  
  
+ GAAAAAAGAG GGTGTTTGTG TGAGATAAAG ACAGAAATTT TATGCCAGAG AGAGAAGATA ACAAATGCAA   
  
  
+ CAACGTCGAC AGAGGCTCTC CTTCTCACAA ATTCCATATT CCTCTGTTTT TTAAGAAAGA AAAAGAAAGT   
  
  
+ GTGAGACAAG AACAACACAA GCAAACAATT CAGTTGACGA CGAATACTGA GTGATACCAC GCACTGTGTG   
  
  
+ CAACTGCGTT TCCATTTGTG GAGTCCTTCA AAGCACGCTC ATCTTCAACC CTTCCTTCGC CACGGAATTT   
  
  
+ CAGGGGGAAA AAAACCCCCA CCTAATTTTG AGAGAGAGAG AGAACAGAGA GAACGGGCGA TACAGGGATA   
  
  
+ CAAGGTTTGA GCAATTTTAG AAAATTTCTT GGGGCCGTTG ATGGGTTTTG AGTGAATTGC AAATCCCAGA   
  
  
+ AAGATTTCTG CGGTTTTTTA TCTACGGCTC TCTCTGTGGG AATTTTTTGG TAGATTCCTC TGGTTCCCAC   
  
  
+ TTTCATATCT TCTTATTTGT TCTACCCTCT TTACATCTGA TAGTTTGTTC TAACTGCTGC TTCAGTTGGT   
  
  
+ GCTTGTTCTC TTTAGTACTT TTCTGTTTTG TTCGTTTTGT TCATGCACTT GAATTCAACA AGAGCCCTTG   
  
  
+ TCCATTTTGA GCTCTTAATT CCCATCTAAC CCTGTTTCCT TCAGAAATTG CTGTTCTGTT CATATAGTTA   
  
  
+ TATTTTTTAT GCTTTGATTT GGGTATAAGT TTGCTGTTGG TGATTCCAAA GCTTGGTACT TTTGGCCATA   
  
  
+ TTTTGTTTGA GTTGGTGTTG ATTGGTACAA ATCTTGTAAT TGGTGTGATT GTTAATGGGA CCAATGCTTC   
  
  
+ AAGATGATGG TTCATCAGTA ACTTCTTCAT CACCTCTTCA ATTTTTCTCC ATGATGTCAC CCAATTTGGG   
  
  
+ TTCCTCCTAC CCTTGGCTAA GGGACTTAAA GCCTGAAGAG AGAGGTCTTT ACTTGATACA TTTGTTGCTC   
  
  
+ ACTTGTGCAA ACCATGTCTC TAATGGTAGC CTTGACAATG CAAACCTAGC CCTTGAGCAA ATCTCCCAGC   
  
  
+ TTGCAGCCCC TGATGGCGAT ACAATCCAGC GTATTGTTGC CTACTTTGCT GAATCACTTG CTGAAAGGAT   
  
  
+ CCTTAAGTCA TGGCCTGGCC TATATAAAGC CCTTCATTTC AATAGAATGC CTGTTATTTC AGAAGAATTT   
  
  
+ CTTGCTAGGA AGCTGTTTTT TGAGTTGTTT CCCTTCTTGA AGCTGGCCTT TTTGGTGACT AACCAATCAA   
  
  
+ TAATCGAGGC CATGGAGGGG GAAAAGATGG TGCATATAAT TGATCTGAAT GCGTCAGAAC CTGCACAGTG   
  
  
+ GATTGCCCTT ATTCAAGACT TGAGTGCTCG GCCTGAGGGC CCTCCTCATT TGAGGATTAC CGGGGTTCAT   
  
  
+ CAACAGAAAG AGGTTTTAGA ACAAGTAGCT CATAGATTGA CTGAAGAAGC TGAGAAGTTG GATTTGCCAT   
  
  
+ TTCAGTTCAA TCCTGTGGTT TGCAAACTAG AGAATCTCGA CATCGGAAAA CTCCGTGTTA AGACCGGGGA   
  
  
+ GGCCTTGGCT ATTACCTCGG TCCTTCAACT GCATACCCTT TTGGCTTCTG AAGAGGAAGT CCTTAAGAAA   
  
  
+ AGTTCACCCT TGGCATTGGT AAAGCAAGCC AATGGGGCTA ATTTACAGGG CTTGTTCAAT AAAGATGGAG   
  
  
+ CTAATAATAG GCGTAGCCCA AGTAATGATT CGGCTTCATC TGCACCTTCA TCCCTCAACA CTTCAGCCAA   
  
  
+ GATGGAAGGT TTCCTTAGCG CTTTGTGGGG TTTATCCCCA AAGATTATGG TGATAACCGA GCAAGATTCC   
  
  
+ AACCACAATG GGGCAGGACT AATGGAGAGA TTGTCAGAAG CATTGTACTT CTATGCAGCA TTGTTCGATT   
  
  
+ GCTTAGAATT TACCCTCCCG AGAACCTCCG TGGAGAGAAG GAAGGTCGAG ATGCTCCTCC TTGGCAAGGA   
  
  
+ AATCAAGAAC ATCATAGCGT GTGAGGGAGG AGAAAGAATA GATAGGCATG AGAAGTTGGG GAAGTGGATT   
  
  
+ AAGAGGCTTG AGATGGCCGG GTTTGGAAGC GTTCCTTTGA GCCACATAGG CATGATCCAA GCAAGGCGGT   
  
  
+ TGTTGCAGAG CTATGGCTGT GATGGTTATA GAATAAAGGA GGAGAACGGA TGTTTTGTTA TCTGCTGGCA   
  
  
+ AGATCGCCCC CTCTTTTCAG TATCTGCTTG GAGATGTAGG AGGTG  

- -Up\_Stream \_Len000AGATGG AAATGGACAC AATTTTTTTT TTTAAAACAA AGGTAATGAT AAGAGAACCG   
  
  
- TGATAACCAC GACTAAAAAG AAGTGGTTTA GTTGGAGGAA TATAAACGGT TAAAATGACA GGAGAAAGTA   
  
  
- GATATTTCAG TTGAGAGGAT CATGTTAAAC AGTGTATCTT TTAGAGATCC GTGAGTAGAT TTGAGAGACA   
  
  
- AGACCCATTA ACTAGCTGCT AGATGTGGTA ATAAGCAAGA TCACGTGGCA AACCTGTCGG TTAGTACATG   
  
  
- GAAACCTCTA GTTGACGAGT ACTCAAGAAC CACACTTTAA CCCCTTCATT TAAGCTGAAA TCCCGTGACC   
  
  
- AGATAGTGCG GTACTAAAAT GGTAGATAAA AGTATATATA GGTTATTCAA ACTAGAAAGC GATAATTAGT   
  
  
- GTTACTATAT TTGTTCACCA CTTCTCGTTA TTACTTGGTA TGTAAAATTT GAATTCGCAA TTCCTTCTGT   
  
  
- ACTTCAATTT TTGTTACCAT TCTCGATACG CATACAAACC GTATATCGAA AAAATTCTCA CAAAACCGAT   
  
  
- ATCAGCCTCA AAAAATTAAT TCTAATCGAC AAACTAGTTT ATTTTTTCGA TTAAACTCAC AAACCGCTCT   
  
  
- TCTGAAAAAT ATTCTTGAAA AAAACCAGAT TTTTCAATTA AATTTTTCCG ATTAAGATAC TCGAAAAGCC   
  
  
- TTCTCAAAAA ATTTATTAAT TGAAAAACAG AGTATTCGGT ATTGAAATAG TCTTTGTCAA TTAAAATGTT   
  
  
- TTATTAAAGA GTTGTTTGTT GATTAAATTT ATTAATTATT TTTATTGATT GAGTTTATAG ATTATTGATT   
  
  
- ATTGATTATA TTTATTAATT GTCAATTGTC AACAAATGCT TTGTCTTAAA TATATTTTTC GCCATTGTCT   
  
  
- ATTTTCTATT AGTACGCTCT AATTTTCAGT GGTCACTGTG TGTTCATGAA TTTATTTATC AGATTTGTTA   
  
  
- CTATAAAAAA CGCTCGAAGG TACGTTTATG GCTGTGAAAA TAACGGAGTC TTCCTTTGTT TTTGACTCAC   
  
  
- TTCCCCCCCC CCCCAAGACA AACAGAAACA ATCCCGTGCA AACCTAAGTT CACATCTAAC CCTTATTTAA   
  
  
- GCTCCTGAAA AAGAAACGGG AAAAAGGGAC TAAGGGTTTG GGTGTAGTTC AGTTCTGCTT TGGTCGGTAA   
  
  
- CTTTTTTCTC CCACAAACAC ACTCTATTTC TGTCTTTAAA ATACGGTCTC TCTCTTCTAT TGTTTACGTT   
  
  
- GTTGCAGCTG TCTCCGAGAG GAAGAGTGTT TAAGGTATAA GGAGACAAAA AATTCTTTCT TTTTCTTTCA   
  
  
- CACTCTGTTC TTGTTGTGTT CGTTTGTTAA GTCAACTGCT GCTTATGACT CACTATGGTG CGTGACACAC   
  
  
- GTTGACGCAA AGGTAAACAC CTCAGGAAGT TTCGTGCGAG TAGAAGTTGG GAAGGAAGCG GTGCCTTAAA   
  
  
- GTCCCCCTTT TTTTGGGGGT GGATTAAAAC TCTCTCTCTC TCTTGTCTCT CTTGCCCGCT ATGTCCCTAT   
  
  
- GTTCCAAACT CGTTAAAATC TTTTAAAGAA CCCCGGCAAC TACCCAAAAC TCACTTAACG TTTAGGGTCT   
  
  
- TTCTAAAGAC GCCAAAAAAT AGATGCCGAG AGAGACACCC TTAAAAAACC ATCTAAGGAG ACCAAGGGTG   
  
  
- AAAGTATAGA AGAATAAACA AGATGGGAGA AATGTAGACT ATCAAACAAG ATTGACGACG AAGTCAACCA   
  
  
- CGAACAAGAG AAATCATGAA AAGACAAAAC AAGCAAAACA AGTACGTGAA CTTAAGTTGT TCTCGGGAAC   
  
  
- AGGTAAAACT CGAGAATTAA GGGTAGATTG GGACAAAGGA AGTCTTTAAC GACAAGACAA GTATATCAAT   
  
  
- ATAAAAAATA CGAAACTAAA CCCATATTCA AACGACAACC ACTAAGGTTT CGAACCATGA AAACCGGTAT   
  
  
- AAAACAAACT CAACCACAAC TAACCATGTT TAGAACATTA ACCACACTAA CAATTACCCT GGTTACGAAG   
  
  
- TTCTACTACC AAGTAGTCAT TGAAGAAGTA GTGGAGAAGT TAAAAAGAGG TACTACAGTG GGTTAAACCC   
  
  
- AAGGAGGATG GGAACCGATT CCCTGAATTT CGGACTTCTC TCTCCAGAAA TGAACTATGT AAACAACGAG   
  
  
- TGAACACGTT TGGTACAGAG ATTACCATCG GAACTGTTAC GTTTGGATCG GGAACTCGTT TAGAGGGTCG   
  
  
- AACGTCGGGG ACTACCGCTA TGTTAGGTCG CATAACAACG GATGAAACGA CTTAGTGAAC GACTTTCCTA   
  
  
- GGAATTCAGT ACCGGACCGG ATATATTTCG GGAAGTAAAG TTATCTTACG GACAATAAAG TCTTCTTAAA   
  
  
- GAACGATCCT TCGACAAAAA ACTCAACAAA GGGAAGAACT TCGACCGGAA AAACCACTGA TTGGTTAGTT   
  
  
- ATTAGCTCCG GTACCTCCCC CTTTTCTACC ACGTATATTA ACTAGACTTA CGCAGTCTTG GACGTGTCAC   
  
  
- CTAACGGGAA TAAGTTCTGA ACTCACGAGC CGGACTCCCG GGAGGAGTAA ACTCCTAATG GCCCCAAGTA   
  
  
- GTTGTCTTTC TCCAAAATCT TGTTCATCGA GTATCTAACT GACTTCTTCG ACTCTTCAAC CTAAACGGTA   
  
  
- AAGTCAAGTT AGGACACCAA ACGTTTGATC TCTTAGAGCT GTAGCCTTTT GAGGCACAAT TCTGGCCCCT   
  
  
- CCGGAACCGA TAATGGAGCC AGGAAGTTGA CGTATGGGAA AACCGAAGAC TTCTCCTTCA GGAATTCTTT   
  
  
- TCAAGTGGGA ACCGTAACCA TTTCGTTCGG TTACCCCGAT TAAATGTCCC GAACAAGTTA TTTCTACCTC   
  
  
- GATTATTATC CGCATCGGGT TCATTACTAA GCCGAAGTAG ACGTGGAAGT AGGGAGTTGT GAAGTCGGTT   
  
  
- CTACCTTCCA AAGGAATCGC GAAACACCCC AAATAGGGGT TTCTAATACC ACTATTGGCT CGTTCTAAGG   
  
  
- TTGGTGTTAC CCCGTCCTGA TTACCTCTCT AACAGTCTTC GTAACATGAA GATACGTCGT AACAAGCTAA   
  
  
- CGAATCTTAA ATGGGAGGGC TCTTGGAGGC ACCTCTCTTC CTTCCAGCTC TACGAGGAGG AACCGTTCCT   
  
  
- TTAGTTCTTG TAGTATCGCA CACTCCCTCC TCTTTCTTAT CTATCCGTAC TCTTCAACCC CTTCACCTAA   
  
  
- TTCTCCGAAC TCTACCGGCC CAAACCTTCG CAAGGAAACT CGGTGTATCC GTACTAGGTT CGTTCCGCCA   
  
  
- ACAACGTCTC GATACCGACA CTACCAATAT CTTATTTCCT CCTCTTGCCT ACAAAACAAT AGACGACCGT   
  
  
- TCTAGCGGGG GAGAAAAGTC ATAGACGAAC CTCTACATCC TCCAC

  
  
Motifs Found  

+   

| Site Name | Organism | Position | Strand | Matrix score. | sequence | function |
| --- | --- | --- | --- | --- | --- | --- |
|  | organism | 3386 | + | 4 | motif\_sequence | short\_function |
|  | organism | 1516 | - | 4 | motif\_sequence | short\_function |
|  | organism | 3299 | - | 4 | motif\_sequence | short\_function |
|  | organism | 1445 | + | 4 | motif\_sequence | short\_function |
|  | organism | 1634 | + | 4 | motif\_sequence | short\_function |
|  | organism | 704 | - | 4 | motif\_sequence | short\_function |
|  | organism | 3394 | - | 4 | motif\_sequence | short\_function |
|  | organism | 3354 | + | 4 | motif\_sequence | short\_function |
|  | organism | 1248 | - | 4 | motif\_sequence | short\_function |
|  | organism | 3116 | - | 4 | motif\_sequence | short\_function |
|  | organism | 2784 | - | 4 | motif\_sequence | short\_function |
|  | organism | 2517 | - | 4 | motif\_sequence | short\_function |
|  | organism | 445 | - | 4 | motif\_sequence | short\_function |
|  | organism | 267 | - | 4 | motif\_sequence | short\_function |
|  | organism | 2139 | - | 4 | motif\_sequence | short\_function |
|  | organism | 157 | + | 4 | motif\_sequence | short\_function |
|  | organism | 3161 | - | 4 | motif\_sequence | short\_function |
|  | organism | 2235 | + | 4 | motif\_sequence | short\_function |
|  | organism | 3038 | - | 4 | motif\_sequence | short\_function |
|  | organism | 234 | + | 4 | motif\_sequence | short\_function |
|  | organism | 279 | + | 4 | motif\_sequence | short\_function |
|  | organism | 289 | - | 4 | motif\_sequence | short\_function |
|  | organism | 1880 | + | 4 | motif\_sequence | short\_function |
|  | organism | 1875 | + | 4 | motif\_sequence | short\_function |
|  | organism | 1691 | + | 4 | motif\_sequence | short\_function |
|  | organism | 1664 | - | 4 | motif\_sequence | short\_function |
|  | organism | 131 | + | 4 | motif\_sequence | short\_function |
|  | organism | 2912 | + | 4 | motif\_sequence | short\_function |
|  | organism | 1107 | - | 4 | motif\_sequence | short\_function |
|  | organism | 2068 | + | 4 | motif\_sequence | short\_function |
|  | organism | 211 | + | 4 | motif\_sequence | short\_function |
|  | organism | 1280 | + | 4 | motif\_sequence | short\_function |

>HU08G01232.1   
+ -Up\_Stream \_Len000TCTACC TTTACCTGTG TTAAAAAAAA AAATTTTGTT TCCATTACTA TTCTCTTGGC   
  
  
+ ACTATTGGTG CTGATTTTTC TTCACCAAAT CAACCTCCTT ATATTTGCCA ATTTTACTGT CCTCTTTCAT   
  
  
+ CTATAAAGTC AACTCTCCTA GTACAATTTG TCACATAGAA AATCTCTAGG CACTCATCTA AACTCTCTGT   
  
  
+ TCTGGGTAAT TGATCGACGA TCTACACCAT TATTCGTTCT AGTGCACCGT TTGGACAGCC AATCATGTAC   
  
  
+ CTTTGGAGAT CAACTGCTCA TGAGTTCTTG GTGTGAAATT GGGGAAGTAA ATTCGACTTT AGGGCACTGG   
  
  
+ TCTATCACGC CATGATTTTA CCATCTATTT TCATATATAT CCAATAAGTT TGATCTTTCG CTATTAATCA   
  
  
+ CAATGATATA AACAAGTGGT GAAGAGCAAT AATGAACCAT ACATTTTAAA CTTAAGCGTT AAGGAAGACA   
  
  
+ TGAAGTTAAA AACAATGGTA AGAGCTATGC GTATGTTTGG CATATAGCTT TTTTAAGAGT GTTTTGGCTA   
  
  
+ TAGTCGGAGT TTTTTAATTA AGATTAGCTG TTTGATCAAA TAAAAAAGCT AATTTGAGTG TTTGGCGAGA   
  
  
+ AGACTTTTTA TAAGAACTTT TTTTGGTCTA AAAAGTTAAT TTAAAAAGGC TAATTCTATG AGCTTTTCGG   
  
  
+ AAGAGTTTTT TAAATAATTA ACTTTTTGTC TCATAAGCCA TAACTTTATC AGAAACAGTT AATTTTACAA   
  
  
+ AATAATTTCT CAACAAACAA CTAATTTAAA TAATTAATAA AAATAACTAA CTCAAATATC TAATAACTAA   
  
  
+ TAACTAATAT AAATAATTAA CAGTTAACAG TTGTTTACGA AACAGAATTT ATATAAAAAG CGGTAACAGA   
  
  
+ TAAAAGATAA TCATGCGAGA TTAAAAGTCA CCAGTGACAC ACAAGTACTT AAATAAATAG TCTAAACAAT   
  
  
+ GATATTTTTT GCGAGCTTCC ATGCAAATAC CGACACTTTT ATTGCCTCAG AAGGAAACAA AAACTGAGTG   
  
  
+ AAGGGGGGGG GGGGTTCTGT TTGTCTTTGT TAGGGCACGT TTGGATTCAA GTGTAGATTG GGAATAAATT   
  
  
+ CGAGGACTTT TTCTTTGCCC TTTTTCCCTG ATTCCCAAAC CCACATCAAG TCAAGACGAA ACCAGCCATT   
  
  
+ GAAAAAAGAG GGTGTTTGTG TGAGATAAAG ACAGAAATTT TATGCCAGAG AGAGAAGATA ACAAATGCAA   
  
  
+ CAACGTCGAC AGAGGCTCTC CTTCTCACAA ATTCCATATT CCTCTGTTTT TTAAGAAAGA AAAAGAAAGT   
  
  
+ GTGAGACAAG AACAACACAA GCAAACAATT CAGTTGACGA CGAATACTGA GTGATACCAC GCACTGTGTG   
  
  
+ CAACTGCGTT TCCATTTGTG GAGTCCTTCA AAGCACGCTC ATCTTCAACC CTTCCTTCGC CACGGAATTT   
  
  
+ CAGGGGGAAA AAAACCCCCA CCTAATTTTG AGAGAGAGAG AGAACAGAGA GAACGGGCGA TACAGGGATA   
  
  
+ CAAGGTTTGA GCAATTTTAG AAAATTTCTT GGGGCCGTTG ATGGGTTTTG AGTGAATTGC AAATCCCAGA   
  
  
+ AAGATTTCTG CGGTTTTTTA TCTACGGCTC TCTCTGTGGG AATTTTTTGG TAGATTCCTC TGGTTCCCAC   
  
  
+ TTTCATATCT TCTTATTTGT TCTACCCTCT TTACATCTGA TAGTTTGTTC TAACTGCTGC TTCAGTTGGT   
  
  
+ GCTTGTTCTC TTTAGTACTT TTCTGTTTTG TTCGTTTTGT TCATGCACTT GAATTCAACA AGAGCCCTTG   
  
  
+ TCCATTTTGA GCTCTTAATT CCCATCTAAC CCTGTTTCCT TCAGAAATTG CTGTTCTGTT CATATAGTTA   
  
  
+ TATTTTTTAT GCTTTGATTT GGGTATAAGT TTGCTGTTGG TGATTCCAAA GCTTGGTACT TTTGGCCATA   
  
  
+ TTTTGTTTGA GTTGGTGTTG ATTGGTACAA ATCTTGTAAT TGGTGTGATT GTTAATGGGA CCAATGCTTC   
  
  
+ AAGATGATGG TTCATCAGTA ACTTCTTCAT CACCTCTTCA ATTTTTCTCC ATGATGTCAC CCAATTTGGG   
  
  
+ TTCCTCCTAC CCTTGGCTAA GGGACTTAAA GCCTGAAGAG AGAGGTCTTT ACTTGATACA TTTGTTGCTC   
  
  
+ ACTTGTGCAA ACCATGTCTC TAATGGTAGC CTTGACAATG CAAACCTAGC CCTTGAGCAA ATCTCCCAGC   
  
  
+ TTGCAGCCCC TGATGGCGAT ACAATCCAGC GTATTGTTGC CTACTTTGCT GAATCACTTG CTGAAAGGAT   
  
  
+ CCTTAAGTCA TGGCCTGGCC TATATAAAGC CCTTCATTTC AATAGAATGC CTGTTATTTC AGAAGAATTT   
  
  
+ CTTGCTAGGA AGCTGTTTTT TGAGTTGTTT CCCTTCTTGA AGCTGGCCTT TTTGGTGACT AACCAATCAA   
  
  
+ TAATCGAGGC CATGGAGGGG GAAAAGATGG TGCATATAAT TGATCTGAAT GCGTCAGAAC CTGCACAGTG   
  
  
+ GATTGCCCTT ATTCAAGACT TGAGTGCTCG GCCTGAGGGC CCTCCTCATT TGAGGATTAC CGGGGTTCAT   
  
  
+ CAACAGAAAG AGGTTTTAGA ACAAGTAGCT CATAGATTGA CTGAAGAAGC TGAGAAGTTG GATTTGCCAT   
  
  
+ TTCAGTTCAA TCCTGTGGTT TGCAAACTAG AGAATCTCGA CATCGGAAAA CTCCGTGTTA AGACCGGGGA   
  
  
+ GGCCTTGGCT ATTACCTCGG TCCTTCAACT GCATACCCTT TTGGCTTCTG AAGAGGAAGT CCTTAAGAAA   
  
  
+ AGTTCACCCT TGGCATTGGT AAAGCAAGCC AATGGGGCTA ATTTACAGGG CTTGTTCAAT AAAGATGGAG   
  
  
+ CTAATAATAG GCGTAGCCCA AGTAATGATT CGGCTTCATC TGCACCTTCA TCCCTCAACA CTTCAGCCAA   
  
  
+ GATGGAAGGT TTCCTTAGCG CTTTGTGGGG TTTATCCCCA AAGATTATGG TGATAACCGA GCAAGATTCC   
  
  
+ AACCACAATG GGGCAGGACT AATGGAGAGA TTGTCAGAAG CATTGTACTT CTATGCAGCA TTGTTCGATT   
  
  
+ GCTTAGAATT TACCCTCCCG AGAACCTCCG TGGAGAGAAG GAAGGTCGAG ATGCTCCTCC TTGGCAAGGA   
  
  
+ AATCAAGAAC ATCATAGCGT GTGAGGGAGG AGAAAGAATA GATAGGCATG AGAAGTTGGG GAAGTGGATT   
  
  
+ AAGAGGCTTG AGATGGCCGG GTTTGGAAGC GTTCCTTTGA GCCACATAGG CATGATCCAA GCAAGGCGGT   
  
  
+ TGTTGCAGAG CTATGGCTGT GATGGTTATA GAATAAAGGA GGAGAACGGA TGTTTTGTTA TCTGCTGGCA   
  
  
+ AGATCGCCCC CTCTTTTCAG TATCTGCTTG GAGATGTAGG AGGTG  

- -Up\_Stream \_Len000AGATGG AAATGGACAC AATTTTTTTT TTTAAAACAA AGGTAATGAT AAGAGAACCG   
  
  
- TGATAACCAC GACTAAAAAG AAGTGGTTTA GTTGGAGGAA TATAAACGGT TAAAATGACA GGAGAAAGTA   
  
  
- GATATTTCAG TTGAGAGGAT CATGTTAAAC AGTGTATCTT TTAGAGATCC GTGAGTAGAT TTGAGAGACA   
  
  
- AGACCCATTA ACTAGCTGCT AGATGTGGTA ATAAGCAAGA TCACGTGGCA AACCTGTCGG TTAGTACATG   
  
  
- GAAACCTCTA GTTGACGAGT ACTCAAGAAC CACACTTTAA CCCCTTCATT TAAGCTGAAA TCCCGTGACC   
  
  
- AGATAGTGCG GTACTAAAAT GGTAGATAAA AGTATATATA GGTTATTCAA ACTAGAAAGC GATAATTAGT   
  
  
- GTTACTATAT TTGTTCACCA CTTCTCGTTA TTACTTGGTA TGTAAAATTT GAATTCGCAA TTCCTTCTGT   
  
  
- ACTTCAATTT TTGTTACCAT TCTCGATACG CATACAAACC GTATATCGAA AAAATTCTCA CAAAACCGAT   
  
  
- ATCAGCCTCA AAAAATTAAT TCTAATCGAC AAACTAGTTT ATTTTTTCGA TTAAACTCAC AAACCGCTCT   
  
  
- TCTGAAAAAT ATTCTTGAAA AAAACCAGAT TTTTCAATTA AATTTTTCCG ATTAAGATAC TCGAAAAGCC   
  
  
- TTCTCAAAAA ATTTATTAAT TGAAAAACAG AGTATTCGGT ATTGAAATAG TCTTTGTCAA TTAAAATGTT   
  
  
- TTATTAAAGA GTTGTTTGTT GATTAAATTT ATTAATTATT TTTATTGATT GAGTTTATAG ATTATTGATT   
  
  
- ATTGATTATA TTTATTAATT GTCAATTGTC AACAAATGCT TTGTCTTAAA TATATTTTTC GCCATTGTCT   
  
  
- ATTTTCTATT AGTACGCTCT AATTTTCAGT GGTCACTGTG TGTTCATGAA TTTATTTATC AGATTTGTTA   
  
  
- CTATAAAAAA CGCTCGAAGG TACGTTTATG GCTGTGAAAA TAACGGAGTC TTCCTTTGTT TTTGACTCAC   
  
  
- TTCCCCCCCC CCCCAAGACA AACAGAAACA ATCCCGTGCA AACCTAAGTT CACATCTAAC CCTTATTTAA   
  
  
- GCTCCTGAAA AAGAAACGGG AAAAAGGGAC TAAGGGTTTG GGTGTAGTTC AGTTCTGCTT TGGTCGGTAA   
  
  
- CTTTTTTCTC CCACAAACAC ACTCTATTTC TGTCTTTAAA ATACGGTCTC TCTCTTCTAT TGTTTACGTT   
  
  
- GTTGCAGCTG TCTCCGAGAG GAAGAGTGTT TAAGGTATAA GGAGACAAAA AATTCTTTCT TTTTCTTTCA   
  
  
- CACTCTGTTC TTGTTGTGTT CGTTTGTTAA GTCAACTGCT GCTTATGACT CACTATGGTG CGTGACACAC   
  
  
- GTTGACGCAA AGGTAAACAC CTCAGGAAGT TTCGTGCGAG TAGAAGTTGG GAAGGAAGCG GTGCCTTAAA   
  
  
- GTCCCCCTTT TTTTGGGGGT GGATTAAAAC TCTCTCTCTC TCTTGTCTCT CTTGCCCGCT ATGTCCCTAT   
  
  
- GTTCCAAACT CGTTAAAATC TTTTAAAGAA CCCCGGCAAC TACCCAAAAC TCACTTAACG TTTAGGGTCT   
  
  
- TTCTAAAGAC GCCAAAAAAT AGATGCCGAG AGAGACACCC TTAAAAAACC ATCTAAGGAG ACCAAGGGTG   
  
  
- AAAGTATAGA AGAATAAACA AGATGGGAGA AATGTAGACT ATCAAACAAG ATTGACGACG AAGTCAACCA   
  
  
- CGAACAAGAG AAATCATGAA AAGACAAAAC AAGCAAAACA AGTACGTGAA CTTAAGTTGT TCTCGGGAAC   
  
  
- AGGTAAAACT CGAGAATTAA GGGTAGATTG GGACAAAGGA AGTCTTTAAC GACAAGACAA GTATATCAAT   
  
  
- ATAAAAAATA CGAAACTAAA CCCATATTCA AACGACAACC ACTAAGGTTT CGAACCATGA AAACCGGTAT   
  
  
- AAAACAAACT CAACCACAAC TAACCATGTT TAGAACATTA ACCACACTAA CAATTACCCT GGTTACGAAG   
  
  
- TTCTACTACC AAGTAGTCAT TGAAGAAGTA GTGGAGAAGT TAAAAAGAGG TACTACAGTG GGTTAAACCC   
  
  
- AAGGAGGATG GGAACCGATT CCCTGAATTT CGGACTTCTC TCTCCAGAAA TGAACTATGT AAACAACGAG   
  
  
- TGAACACGTT TGGTACAGAG ATTACCATCG GAACTGTTAC GTTTGGATCG GGAACTCGTT TAGAGGGTCG   
  
  
- AACGTCGGGG ACTACCGCTA TGTTAGGTCG CATAACAACG GATGAAACGA CTTAGTGAAC GACTTTCCTA   
  
  
- GGAATTCAGT ACCGGACCGG ATATATTTCG GGAAGTAAAG TTATCTTACG GACAATAAAG TCTTCTTAAA   
  
  
- GAACGATCCT TCGACAAAAA ACTCAACAAA GGGAAGAACT TCGACCGGAA AAACCACTGA TTGGTTAGTT   
  
  
- ATTAGCTCCG GTACCTCCCC CTTTTCTACC ACGTATATTA ACTAGACTTA CGCAGTCTTG GACGTGTCAC   
  
  
- CTAACGGGAA TAAGTTCTGA ACTCACGAGC CGGACTCCCG GGAGGAGTAA ACTCCTAATG GCCCCAAGTA   
  
  
- GTTGTCTTTC TCCAAAATCT TGTTCATCGA GTATCTAACT GACTTCTTCG ACTCTTCAAC CTAAACGGTA   
  
  
- AAGTCAAGTT AGGACACCAA ACGTTTGATC TCTTAGAGCT GTAGCCTTTT GAGGCACAAT TCTGGCCCCT   
  
  
- CCGGAACCGA TAATGGAGCC AGGAAGTTGA CGTATGGGAA AACCGAAGAC TTCTCCTTCA GGAATTCTTT   
  
  
- TCAAGTGGGA ACCGTAACCA TTTCGTTCGG TTACCCCGAT TAAATGTCCC GAACAAGTTA TTTCTACCTC   
  
  
- GATTATTATC CGCATCGGGT TCATTACTAA GCCGAAGTAG ACGTGGAAGT AGGGAGTTGT GAAGTCGGTT   
  
  
- CTACCTTCCA AAGGAATCGC GAAACACCCC AAATAGGGGT TTCTAATACC ACTATTGGCT CGTTCTAAGG   
  
  
- TTGGTGTTAC CCCGTCCTGA TTACCTCTCT AACAGTCTTC GTAACATGAA GATACGTCGT AACAAGCTAA   
  
  
- CGAATCTTAA ATGGGAGGGC TCTTGGAGGC ACCTCTCTTC CTTCCAGCTC TACGAGGAGG AACCGTTCCT   
  
  
- TTAGTTCTTG TAGTATCGCA CACTCCCTCC TCTTTCTTAT CTATCCGTAC TCTTCAACCC CTTCACCTAA   
  
  
- TTCTCCGAAC TCTACCGGCC CAAACCTTCG CAAGGAAACT CGGTGTATCC GTACTAGGTT CGTTCCGCCA   
  
  
- ACAACGTCTC GATACCGACA CTACCAATAT CTTATTTCCT CCTCTTGCCT ACAAAACAAT AGACGACCGT   
  
  
- TCTAGCGGGG GAGAAAAGTC ATAGACGAAC CTCTACATCC TCCAC

+     AAGAA-motif

| Site Name | Organism | Position | Strand | Matrix score. | sequence | function |
| --- | --- | --- | --- | --- | --- | --- |
| AAGAA-motif | Avena sativa | 3186 | + | 7 | GAAAGAA |  |
| AAGAA-motif | Avena sativa | 1319 | + | 7 | GAAAGAA |  |
| AAGAA-motif | Avena sativa | 91 | - | 9 | gGTAAAGAAA |  |
| AAGAA-motif | Avena sativa | 2822 | + | 9 | gGTAAAGAAA |  |
| AAGAA-motif | Avena sativa | 1134 | - | 9 | gGTAAAGAAA |  |

>HU08G01232.1   
+ -Up\_Stream \_Len000TCTACC TTTACCTGTG TTAAAAAAAA AAATTTTGTT TCCATTACTA TTCTCTTGGC   
  
  
+ ACTATTGGTG CTGATTTTTC TTCACCAAAT CAACCTCCTT ATATTTGCCA ATTTTACTGT CCTCTTTCAT   
  
  
+ CTATAAAGTC AACTCTCCTA GTACAATTTG TCACATAGAA AATCTCTAGG CACTCATCTA AACTCTCTGT   
  
  
+ TCTGGGTAAT TGATCGACGA TCTACACCAT TATTCGTTCT AGTGCACCGT TTGGACAGCC AATCATGTAC   
  
  
+ CTTTGGAGAT CAACTGCTCA TGAGTTCTTG GTGTGAAATT GGGGAAGTAA ATTCGACTTT AGGGCACTGG   
  
  
+ TCTATCACGC CATGATTTTA CCATCTATTT TCATATATAT CCAATAAGTT TGATCTTTCG CTATTAATCA   
  
  
+ CAATGATATA AACAAGTGGT GAAGAGCAAT AATGAACCAT ACATTTTAAA CTTAAGCGTT AAGGAAGACA   
  
  
+ TGAAGTTAAA AACAATGGTA AGAGCTATGC GTATGTTTGG CATATAGCTT TTTTAAGAGT GTTTTGGCTA   
  
  
+ TAGTCGGAGT TTTTTAATTA AGATTAGCTG TTTGATCAAA TAAAAAAGCT AATTTGAGTG TTTGGCGAGA   
  
  
+ AGACTTTTTA TAAGAACTTT TTTTGGTCTA AAAAGTTAAT TTAAAAAGGC TAATTCTATG AGCTTTTCGG   
  
  
+ AAGAGTTTTT TAAATAATTA ACTTTTTGTC TCATAAGCCA TAACTTTATC AGAAACAGTT AATTTTACAA   
  
  
+ AATAATTTCT CAACAAACAA CTAATTTAAA TAATTAATAA AAATAACTAA CTCAAATATC TAATAACTAA   
  
  
+ TAACTAATAT AAATAATTAA CAGTTAACAG TTGTTTACGA AACAGAATTT ATATAAAAAG CGGTAACAGA   
  
  
+ TAAAAGATAA TCATGCGAGA TTAAAAGTCA CCAGTGACAC ACAAGTACTT AAATAAATAG TCTAAACAAT   
  
  
+ GATATTTTTT GCGAGCTTCC ATGCAAATAC CGACACTTTT ATTGCCTCAG AAGGAAACAA AAACTGAGTG   
  
  
+ AAGGGGGGGG GGGGTTCTGT TTGTCTTTGT TAGGGCACGT TTGGATTCAA GTGTAGATTG GGAATAAATT   
  
  
+ CGAGGACTTT TTCTTTGCCC TTTTTCCCTG ATTCCCAAAC CCACATCAAG TCAAGACGAA ACCAGCCATT   
  
  
+ GAAAAAAGAG GGTGTTTGTG TGAGATAAAG ACAGAAATTT TATGCCAGAG AGAGAAGATA ACAAATGCAA   
  
  
+ CAACGTCGAC AGAGGCTCTC CTTCTCACAA ATTCCATATT CCTCTGTTTT TTAAGAAAGA AAAAGAAAGT   
  
  
+ GTGAGACAAG AACAACACAA GCAAACAATT CAGTTGACGA CGAATACTGA GTGATACCAC GCACTGTGTG   
  
  
+ CAACTGCGTT TCCATTTGTG GAGTCCTTCA AAGCACGCTC ATCTTCAACC CTTCCTTCGC CACGGAATTT   
  
  
+ CAGGGGGAAA AAAACCCCCA CCTAATTTTG AGAGAGAGAG AGAACAGAGA GAACGGGCGA TACAGGGATA   
  
  
+ CAAGGTTTGA GCAATTTTAG AAAATTTCTT GGGGCCGTTG ATGGGTTTTG AGTGAATTGC AAATCCCAGA   
  
  
+ AAGATTTCTG CGGTTTTTTA TCTACGGCTC TCTCTGTGGG AATTTTTTGG TAGATTCCTC TGGTTCCCAC   
  
  
+ TTTCATATCT TCTTATTTGT TCTACCCTCT TTACATCTGA TAGTTTGTTC TAACTGCTGC TTCAGTTGGT   
  
  
+ GCTTGTTCTC TTTAGTACTT TTCTGTTTTG TTCGTTTTGT TCATGCACTT GAATTCAACA AGAGCCCTTG   
  
  
+ TCCATTTTGA GCTCTTAATT CCCATCTAAC CCTGTTTCCT TCAGAAATTG CTGTTCTGTT CATATAGTTA   
  
  
+ TATTTTTTAT GCTTTGATTT GGGTATAAGT TTGCTGTTGG TGATTCCAAA GCTTGGTACT TTTGGCCATA   
  
  
+ TTTTGTTTGA GTTGGTGTTG ATTGGTACAA ATCTTGTAAT TGGTGTGATT GTTAATGGGA CCAATGCTTC   
  
  
+ AAGATGATGG TTCATCAGTA ACTTCTTCAT CACCTCTTCA ATTTTTCTCC ATGATGTCAC CCAATTTGGG   
  
  
+ TTCCTCCTAC CCTTGGCTAA GGGACTTAAA GCCTGAAGAG AGAGGTCTTT ACTTGATACA TTTGTTGCTC   
  
  
+ ACTTGTGCAA ACCATGTCTC TAATGGTAGC CTTGACAATG CAAACCTAGC CCTTGAGCAA ATCTCCCAGC   
  
  
+ TTGCAGCCCC TGATGGCGAT ACAATCCAGC GTATTGTTGC CTACTTTGCT GAATCACTTG CTGAAAGGAT   
  
  
+ CCTTAAGTCA TGGCCTGGCC TATATAAAGC CCTTCATTTC AATAGAATGC CTGTTATTTC AGAAGAATTT   
  
  
+ CTTGCTAGGA AGCTGTTTTT TGAGTTGTTT CCCTTCTTGA AGCTGGCCTT TTTGGTGACT AACCAATCAA   
  
  
+ TAATCGAGGC CATGGAGGGG GAAAAGATGG TGCATATAAT TGATCTGAAT GCGTCAGAAC CTGCACAGTG   
  
  
+ GATTGCCCTT ATTCAAGACT TGAGTGCTCG GCCTGAGGGC CCTCCTCATT TGAGGATTAC CGGGGTTCAT   
  
  
+ CAACAGAAAG AGGTTTTAGA ACAAGTAGCT CATAGATTGA CTGAAGAAGC TGAGAAGTTG GATTTGCCAT   
  
  
+ TTCAGTTCAA TCCTGTGGTT TGCAAACTAG AGAATCTCGA CATCGGAAAA CTCCGTGTTA AGACCGGGGA   
  
  
+ GGCCTTGGCT ATTACCTCGG TCCTTCAACT GCATACCCTT TTGGCTTCTG AAGAGGAAGT CCTTAAGAAA   
  
  
+ AGTTCACCCT TGGCATTGGT AAAGCAAGCC AATGGGGCTA ATTTACAGGG CTTGTTCAAT AAAGATGGAG   
  
  
+ CTAATAATAG GCGTAGCCCA AGTAATGATT CGGCTTCATC TGCACCTTCA TCCCTCAACA CTTCAGCCAA   
  
  
+ GATGGAAGGT TTCCTTAGCG CTTTGTGGGG TTTATCCCCA AAGATTATGG TGATAACCGA GCAAGATTCC   
  
  
+ AACCACAATG GGGCAGGACT AATGGAGAGA TTGTCAGAAG CATTGTACTT CTATGCAGCA TTGTTCGATT   
  
  
+ GCTTAGAATT TACCCTCCCG AGAACCTCCG TGGAGAGAAG GAAGGTCGAG ATGCTCCTCC TTGGCAAGGA   
  
  
+ AATCAAGAAC ATCATAGCGT GTGAGGGAGG AGAAAGAATA GATAGGCATG AGAAGTTGGG GAAGTGGATT   
  
  
+ AAGAGGCTTG AGATGGCCGG GTTTGGAAGC GTTCCTTTGA GCCACATAGG CATGATCCAA GCAAGGCGGT   
  
  
+ TGTTGCAGAG CTATGGCTGT GATGGTTATA GAATAAAGGA GGAGAACGGA TGTTTTGTTA TCTGCTGGCA   
  
  
+ AGATCGCCCC CTCTTTTCAG TATCTGCTTG GAGATGTAGG AGGTG  

- -Up\_Stream \_Len000AGATGG AAATGGACAC AATTTTTTTT TTTAAAACAA AGGTAATGAT AAGAGAACCG   
  
  
- TGATAACCAC GACTAAAAAG AAGTGGTTTA GTTGGAGGAA TATAAACGGT TAAAATGACA GGAGAAAGTA   
  
  
- GATATTTCAG TTGAGAGGAT CATGTTAAAC AGTGTATCTT TTAGAGATCC GTGAGTAGAT TTGAGAGACA   
  
  
- AGACCCATTA ACTAGCTGCT AGATGTGGTA ATAAGCAAGA TCACGTGGCA AACCTGTCGG TTAGTACATG   
  
  
- GAAACCTCTA GTTGACGAGT ACTCAAGAAC CACACTTTAA CCCCTTCATT TAAGCTGAAA TCCCGTGACC   
  
  
- AGATAGTGCG GTACTAAAAT GGTAGATAAA AGTATATATA GGTTATTCAA ACTAGAAAGC GATAATTAGT   
  
  
- GTTACTATAT TTGTTCACCA CTTCTCGTTA TTACTTGGTA TGTAAAATTT GAATTCGCAA TTCCTTCTGT   
  
  
- ACTTCAATTT TTGTTACCAT TCTCGATACG CATACAAACC GTATATCGAA AAAATTCTCA CAAAACCGAT   
  
  
- ATCAGCCTCA AAAAATTAAT TCTAATCGAC AAACTAGTTT ATTTTTTCGA TTAAACTCAC AAACCGCTCT   
  
  
- TCTGAAAAAT ATTCTTGAAA AAAACCAGAT TTTTCAATTA AATTTTTCCG ATTAAGATAC TCGAAAAGCC   
  
  
- TTCTCAAAAA ATTTATTAAT TGAAAAACAG AGTATTCGGT ATTGAAATAG TCTTTGTCAA TTAAAATGTT   
  
  
- TTATTAAAGA GTTGTTTGTT GATTAAATTT ATTAATTATT TTTATTGATT GAGTTTATAG ATTATTGATT   
  
  
- ATTGATTATA TTTATTAATT GTCAATTGTC AACAAATGCT TTGTCTTAAA TATATTTTTC GCCATTGTCT   
  
  
- ATTTTCTATT AGTACGCTCT AATTTTCAGT GGTCACTGTG TGTTCATGAA TTTATTTATC AGATTTGTTA   
  
  
- CTATAAAAAA CGCTCGAAGG TACGTTTATG GCTGTGAAAA TAACGGAGTC TTCCTTTGTT TTTGACTCAC   
  
  
- TTCCCCCCCC CCCCAAGACA AACAGAAACA ATCCCGTGCA AACCTAAGTT CACATCTAAC CCTTATTTAA   
  
  
- GCTCCTGAAA AAGAAACGGG AAAAAGGGAC TAAGGGTTTG GGTGTAGTTC AGTTCTGCTT TGGTCGGTAA   
  
  
- CTTTTTTCTC CCACAAACAC ACTCTATTTC TGTCTTTAAA ATACGGTCTC TCTCTTCTAT TGTTTACGTT   
  
  
- GTTGCAGCTG TCTCCGAGAG GAAGAGTGTT TAAGGTATAA GGAGACAAAA AATTCTTTCT TTTTCTTTCA   
  
  
- CACTCTGTTC TTGTTGTGTT CGTTTGTTAA GTCAACTGCT GCTTATGACT CACTATGGTG CGTGACACAC   
  
  
- GTTGACGCAA AGGTAAACAC CTCAGGAAGT TTCGTGCGAG TAGAAGTTGG GAAGGAAGCG GTGCCTTAAA   
  
  
- GTCCCCCTTT TTTTGGGGGT GGATTAAAAC TCTCTCTCTC TCTTGTCTCT CTTGCCCGCT ATGTCCCTAT   
  
  
- GTTCCAAACT CGTTAAAATC TTTTAAAGAA CCCCGGCAAC TACCCAAAAC TCACTTAACG TTTAGGGTCT   
  
  
- TTCTAAAGAC GCCAAAAAAT AGATGCCGAG AGAGACACCC TTAAAAAACC ATCTAAGGAG ACCAAGGGTG   
  
  
- AAAGTATAGA AGAATAAACA AGATGGGAGA AATGTAGACT ATCAAACAAG ATTGACGACG AAGTCAACCA   
  
  
- CGAACAAGAG AAATCATGAA AAGACAAAAC AAGCAAAACA AGTACGTGAA CTTAAGTTGT TCTCGGGAAC   
  
  
- AGGTAAAACT CGAGAATTAA GGGTAGATTG GGACAAAGGA AGTCTTTAAC GACAAGACAA GTATATCAAT   
  
  
- ATAAAAAATA CGAAACTAAA CCCATATTCA AACGACAACC ACTAAGGTTT CGAACCATGA AAACCGGTAT   
  
  
- AAAACAAACT CAACCACAAC TAACCATGTT TAGAACATTA ACCACACTAA CAATTACCCT GGTTACGAAG   
  
  
- TTCTACTACC AAGTAGTCAT TGAAGAAGTA GTGGAGAAGT TAAAAAGAGG TACTACAGTG GGTTAAACCC   
  
  
- AAGGAGGATG GGAACCGATT CCCTGAATTT CGGACTTCTC TCTCCAGAAA TGAACTATGT AAACAACGAG   
  
  
- TGAACACGTT TGGTACAGAG ATTACCATCG GAACTGTTAC GTTTGGATCG GGAACTCGTT TAGAGGGTCG   
  
  
- AACGTCGGGG ACTACCGCTA TGTTAGGTCG CATAACAACG GATGAAACGA CTTAGTGAAC GACTTTCCTA   
  
  
- GGAATTCAGT ACCGGACCGG ATATATTTCG GGAAGTAAAG TTATCTTACG GACAATAAAG TCTTCTTAAA   
  
  
- GAACGATCCT TCGACAAAAA ACTCAACAAA GGGAAGAACT TCGACCGGAA AAACCACTGA TTGGTTAGTT   
  
  
- ATTAGCTCCG GTACCTCCCC CTTTTCTACC ACGTATATTA ACTAGACTTA CGCAGTCTTG GACGTGTCAC   
  
  
- CTAACGGGAA TAAGTTCTGA ACTCACGAGC CGGACTCCCG GGAGGAGTAA ACTCCTAATG GCCCCAAGTA   
  
  
- GTTGTCTTTC TCCAAAATCT TGTTCATCGA GTATCTAACT GACTTCTTCG ACTCTTCAAC CTAAACGGTA   
  
  
- AAGTCAAGTT AGGACACCAA ACGTTTGATC TCTTAGAGCT GTAGCCTTTT GAGGCACAAT TCTGGCCCCT   
  
  
- CCGGAACCGA TAATGGAGCC AGGAAGTTGA CGTATGGGAA AACCGAAGAC TTCTCCTTCA GGAATTCTTT   
  
  
- TCAAGTGGGA ACCGTAACCA TTTCGTTCGG TTACCCCGAT TAAATGTCCC GAACAAGTTA TTTCTACCTC   
  
  
- GATTATTATC CGCATCGGGT TCATTACTAA GCCGAAGTAG ACGTGGAAGT AGGGAGTTGT GAAGTCGGTT   
  
  
- CTACCTTCCA AAGGAATCGC GAAACACCCC AAATAGGGGT TTCTAATACC ACTATTGGCT CGTTCTAAGG   
  
  
- TTGGTGTTAC CCCGTCCTGA TTACCTCTCT AACAGTCTTC GTAACATGAA GATACGTCGT AACAAGCTAA   
  
  
- CGAATCTTAA ATGGGAGGGC TCTTGGAGGC ACCTCTCTTC CTTCCAGCTC TACGAGGAGG AACCGTTCCT   
  
  
- TTAGTTCTTG TAGTATCGCA CACTCCCTCC TCTTTCTTAT CTATCCGTAC TCTTCAACCC CTTCACCTAA   
  
  
- TTCTCCGAAC TCTACCGGCC CAAACCTTCG CAAGGAAACT CGGTGTATCC GTACTAGGTT CGTTCCGCCA   
  
  
- ACAACGTCTC GATACCGACA CTACCAATAT CTTATTTCCT CCTCTTGCCT ACAAAACAAT AGACGACCGT   
  
  
- TCTAGCGGGG GAGAAAAGTC ATAGACGAAC CTCTACATCC TCCAC

+     ABRE

| Site Name | Organism | Position | Strand | Matrix score. | sequence | function |
| --- | --- | --- | --- | --- | --- | --- |
| ABRE | Arabidopsis thaliana | 1090 | - | 5 | ACGTG | cis-acting element involved in the abscisic acid responsiveness |
| ABRE | Arabidopsis thaliana | 3241 | - | 7 | AACCCGG | cis-acting element involved in the abscisic acid responsiveness |

>HU08G01232.1   
+ -Up\_Stream \_Len000TCTACC TTTACCTGTG TTAAAAAAAA AAATTTTGTT TCCATTACTA TTCTCTTGGC   
  
  
+ ACTATTGGTG CTGATTTTTC TTCACCAAAT CAACCTCCTT ATATTTGCCA ATTTTACTGT CCTCTTTCAT   
  
  
+ CTATAAAGTC AACTCTCCTA GTACAATTTG TCACATAGAA AATCTCTAGG CACTCATCTA AACTCTCTGT   
  
  
+ TCTGGGTAAT TGATCGACGA TCTACACCAT TATTCGTTCT AGTGCACCGT TTGGACAGCC AATCATGTAC   
  
  
+ CTTTGGAGAT CAACTGCTCA TGAGTTCTTG GTGTGAAATT GGGGAAGTAA ATTCGACTTT AGGGCACTGG   
  
  
+ TCTATCACGC CATGATTTTA CCATCTATTT TCATATATAT CCAATAAGTT TGATCTTTCG CTATTAATCA   
  
  
+ CAATGATATA AACAAGTGGT GAAGAGCAAT AATGAACCAT ACATTTTAAA CTTAAGCGTT AAGGAAGACA   
  
  
+ TGAAGTTAAA AACAATGGTA AGAGCTATGC GTATGTTTGG CATATAGCTT TTTTAAGAGT GTTTTGGCTA   
  
  
+ TAGTCGGAGT TTTTTAATTA AGATTAGCTG TTTGATCAAA TAAAAAAGCT AATTTGAGTG TTTGGCGAGA   
  
  
+ AGACTTTTTA TAAGAACTTT TTTTGGTCTA AAAAGTTAAT TTAAAAAGGC TAATTCTATG AGCTTTTCGG   
  
  
+ AAGAGTTTTT TAAATAATTA ACTTTTTGTC TCATAAGCCA TAACTTTATC AGAAACAGTT AATTTTACAA   
  
  
+ AATAATTTCT CAACAAACAA CTAATTTAAA TAATTAATAA AAATAACTAA CTCAAATATC TAATAACTAA   
  
  
+ TAACTAATAT AAATAATTAA CAGTTAACAG TTGTTTACGA AACAGAATTT ATATAAAAAG CGGTAACAGA   
  
  
+ TAAAAGATAA TCATGCGAGA TTAAAAGTCA CCAGTGACAC ACAAGTACTT AAATAAATAG TCTAAACAAT   
  
  
+ GATATTTTTT GCGAGCTTCC ATGCAAATAC CGACACTTTT ATTGCCTCAG AAGGAAACAA AAACTGAGTG   
  
  
+ AAGGGGGGGG GGGGTTCTGT TTGTCTTTGT TAGGGCACGT TTGGATTCAA GTGTAGATTG GGAATAAATT   
  
  
+ CGAGGACTTT TTCTTTGCCC TTTTTCCCTG ATTCCCAAAC CCACATCAAG TCAAGACGAA ACCAGCCATT   
  
  
+ GAAAAAAGAG GGTGTTTGTG TGAGATAAAG ACAGAAATTT TATGCCAGAG AGAGAAGATA ACAAATGCAA   
  
  
+ CAACGTCGAC AGAGGCTCTC CTTCTCACAA ATTCCATATT CCTCTGTTTT TTAAGAAAGA AAAAGAAAGT   
  
  
+ GTGAGACAAG AACAACACAA GCAAACAATT CAGTTGACGA CGAATACTGA GTGATACCAC GCACTGTGTG   
  
  
+ CAACTGCGTT TCCATTTGTG GAGTCCTTCA AAGCACGCTC ATCTTCAACC CTTCCTTCGC CACGGAATTT   
  
  
+ CAGGGGGAAA AAAACCCCCA CCTAATTTTG AGAGAGAGAG AGAACAGAGA GAACGGGCGA TACAGGGATA   
  
  
+ CAAGGTTTGA GCAATTTTAG AAAATTTCTT GGGGCCGTTG ATGGGTTTTG AGTGAATTGC AAATCCCAGA   
  
  
+ AAGATTTCTG CGGTTTTTTA TCTACGGCTC TCTCTGTGGG AATTTTTTGG TAGATTCCTC TGGTTCCCAC   
  
  
+ TTTCATATCT TCTTATTTGT TCTACCCTCT TTACATCTGA TAGTTTGTTC TAACTGCTGC TTCAGTTGGT   
  
  
+ GCTTGTTCTC TTTAGTACTT TTCTGTTTTG TTCGTTTTGT TCATGCACTT GAATTCAACA AGAGCCCTTG   
  
  
+ TCCATTTTGA GCTCTTAATT CCCATCTAAC CCTGTTTCCT TCAGAAATTG CTGTTCTGTT CATATAGTTA   
  
  
+ TATTTTTTAT GCTTTGATTT GGGTATAAGT TTGCTGTTGG TGATTCCAAA GCTTGGTACT TTTGGCCATA   
  
  
+ TTTTGTTTGA GTTGGTGTTG ATTGGTACAA ATCTTGTAAT TGGTGTGATT GTTAATGGGA CCAATGCTTC   
  
  
+ AAGATGATGG TTCATCAGTA ACTTCTTCAT CACCTCTTCA ATTTTTCTCC ATGATGTCAC CCAATTTGGG   
  
  
+ TTCCTCCTAC CCTTGGCTAA GGGACTTAAA GCCTGAAGAG AGAGGTCTTT ACTTGATACA TTTGTTGCTC   
  
  
+ ACTTGTGCAA ACCATGTCTC TAATGGTAGC CTTGACAATG CAAACCTAGC CCTTGAGCAA ATCTCCCAGC   
  
  
+ TTGCAGCCCC TGATGGCGAT ACAATCCAGC GTATTGTTGC CTACTTTGCT GAATCACTTG CTGAAAGGAT   
  
  
+ CCTTAAGTCA TGGCCTGGCC TATATAAAGC CCTTCATTTC AATAGAATGC CTGTTATTTC AGAAGAATTT   
  
  
+ CTTGCTAGGA AGCTGTTTTT TGAGTTGTTT CCCTTCTTGA AGCTGGCCTT TTTGGTGACT AACCAATCAA   
  
  
+ TAATCGAGGC CATGGAGGGG GAAAAGATGG TGCATATAAT TGATCTGAAT GCGTCAGAAC CTGCACAGTG   
  
  
+ GATTGCCCTT ATTCAAGACT TGAGTGCTCG GCCTGAGGGC CCTCCTCATT TGAGGATTAC CGGGGTTCAT   
  
  
+ CAACAGAAAG AGGTTTTAGA ACAAGTAGCT CATAGATTGA CTGAAGAAGC TGAGAAGTTG GATTTGCCAT   
  
  
+ TTCAGTTCAA TCCTGTGGTT TGCAAACTAG AGAATCTCGA CATCGGAAAA CTCCGTGTTA AGACCGGGGA   
  
  
+ GGCCTTGGCT ATTACCTCGG TCCTTCAACT GCATACCCTT TTGGCTTCTG AAGAGGAAGT CCTTAAGAAA   
  
  
+ AGTTCACCCT TGGCATTGGT AAAGCAAGCC AATGGGGCTA ATTTACAGGG CTTGTTCAAT AAAGATGGAG   
  
  
+ CTAATAATAG GCGTAGCCCA AGTAATGATT CGGCTTCATC TGCACCTTCA TCCCTCAACA CTTCAGCCAA   
  
  
+ GATGGAAGGT TTCCTTAGCG CTTTGTGGGG TTTATCCCCA AAGATTATGG TGATAACCGA GCAAGATTCC   
  
  
+ AACCACAATG GGGCAGGACT AATGGAGAGA TTGTCAGAAG CATTGTACTT CTATGCAGCA TTGTTCGATT   
  
  
+ GCTTAGAATT TACCCTCCCG AGAACCTCCG TGGAGAGAAG GAAGGTCGAG ATGCTCCTCC TTGGCAAGGA   
  
  
+ AATCAAGAAC ATCATAGCGT GTGAGGGAGG AGAAAGAATA GATAGGCATG AGAAGTTGGG GAAGTGGATT   
  
  
+ AAGAGGCTTG AGATGGCCGG GTTTGGAAGC GTTCCTTTGA GCCACATAGG CATGATCCAA GCAAGGCGGT   
  
  
+ TGTTGCAGAG CTATGGCTGT GATGGTTATA GAATAAAGGA GGAGAACGGA TGTTTTGTTA TCTGCTGGCA   
  
  
+ AGATCGCCCC CTCTTTTCAG TATCTGCTTG GAGATGTAGG AGGTG  

- -Up\_Stream \_Len000AGATGG AAATGGACAC AATTTTTTTT TTTAAAACAA AGGTAATGAT AAGAGAACCG   
  
  
- TGATAACCAC GACTAAAAAG AAGTGGTTTA GTTGGAGGAA TATAAACGGT TAAAATGACA GGAGAAAGTA   
  
  
- GATATTTCAG TTGAGAGGAT CATGTTAAAC AGTGTATCTT TTAGAGATCC GTGAGTAGAT TTGAGAGACA   
  
  
- AGACCCATTA ACTAGCTGCT AGATGTGGTA ATAAGCAAGA TCACGTGGCA AACCTGTCGG TTAGTACATG   
  
  
- GAAACCTCTA GTTGACGAGT ACTCAAGAAC CACACTTTAA CCCCTTCATT TAAGCTGAAA TCCCGTGACC   
  
  
- AGATAGTGCG GTACTAAAAT GGTAGATAAA AGTATATATA GGTTATTCAA ACTAGAAAGC GATAATTAGT   
  
  
- GTTACTATAT TTGTTCACCA CTTCTCGTTA TTACTTGGTA TGTAAAATTT GAATTCGCAA TTCCTTCTGT   
  
  
- ACTTCAATTT TTGTTACCAT TCTCGATACG CATACAAACC GTATATCGAA AAAATTCTCA CAAAACCGAT   
  
  
- ATCAGCCTCA AAAAATTAAT TCTAATCGAC AAACTAGTTT ATTTTTTCGA TTAAACTCAC AAACCGCTCT   
  
  
- TCTGAAAAAT ATTCTTGAAA AAAACCAGAT TTTTCAATTA AATTTTTCCG ATTAAGATAC TCGAAAAGCC   
  
  
- TTCTCAAAAA ATTTATTAAT TGAAAAACAG AGTATTCGGT ATTGAAATAG TCTTTGTCAA TTAAAATGTT   
  
  
- TTATTAAAGA GTTGTTTGTT GATTAAATTT ATTAATTATT TTTATTGATT GAGTTTATAG ATTATTGATT   
  
  
- ATTGATTATA TTTATTAATT GTCAATTGTC AACAAATGCT TTGTCTTAAA TATATTTTTC GCCATTGTCT   
  
  
- ATTTTCTATT AGTACGCTCT AATTTTCAGT GGTCACTGTG TGTTCATGAA TTTATTTATC AGATTTGTTA   
  
  
- CTATAAAAAA CGCTCGAAGG TACGTTTATG GCTGTGAAAA TAACGGAGTC TTCCTTTGTT TTTGACTCAC   
  
  
- TTCCCCCCCC CCCCAAGACA AACAGAAACA ATCCCGTGCA AACCTAAGTT CACATCTAAC CCTTATTTAA   
  
  
- GCTCCTGAAA AAGAAACGGG AAAAAGGGAC TAAGGGTTTG GGTGTAGTTC AGTTCTGCTT TGGTCGGTAA   
  
  
- CTTTTTTCTC CCACAAACAC ACTCTATTTC TGTCTTTAAA ATACGGTCTC TCTCTTCTAT TGTTTACGTT   
  
  
- GTTGCAGCTG TCTCCGAGAG GAAGAGTGTT TAAGGTATAA GGAGACAAAA AATTCTTTCT TTTTCTTTCA   
  
  
- CACTCTGTTC TTGTTGTGTT CGTTTGTTAA GTCAACTGCT GCTTATGACT CACTATGGTG CGTGACACAC   
  
  
- GTTGACGCAA AGGTAAACAC CTCAGGAAGT TTCGTGCGAG TAGAAGTTGG GAAGGAAGCG GTGCCTTAAA   
  
  
- GTCCCCCTTT TTTTGGGGGT GGATTAAAAC TCTCTCTCTC TCTTGTCTCT CTTGCCCGCT ATGTCCCTAT   
  
  
- GTTCCAAACT CGTTAAAATC TTTTAAAGAA CCCCGGCAAC TACCCAAAAC TCACTTAACG TTTAGGGTCT   
  
  
- TTCTAAAGAC GCCAAAAAAT AGATGCCGAG AGAGACACCC TTAAAAAACC ATCTAAGGAG ACCAAGGGTG   
  
  
- AAAGTATAGA AGAATAAACA AGATGGGAGA AATGTAGACT ATCAAACAAG ATTGACGACG AAGTCAACCA   
  
  
- CGAACAAGAG AAATCATGAA AAGACAAAAC AAGCAAAACA AGTACGTGAA CTTAAGTTGT TCTCGGGAAC   
  
  
- AGGTAAAACT CGAGAATTAA GGGTAGATTG GGACAAAGGA AGTCTTTAAC GACAAGACAA GTATATCAAT   
  
  
- ATAAAAAATA CGAAACTAAA CCCATATTCA AACGACAACC ACTAAGGTTT CGAACCATGA AAACCGGTAT   
  
  
- AAAACAAACT CAACCACAAC TAACCATGTT TAGAACATTA ACCACACTAA CAATTACCCT GGTTACGAAG   
  
  
- TTCTACTACC AAGTAGTCAT TGAAGAAGTA GTGGAGAAGT TAAAAAGAGG TACTACAGTG GGTTAAACCC   
  
  
- AAGGAGGATG GGAACCGATT CCCTGAATTT CGGACTTCTC TCTCCAGAAA TGAACTATGT AAACAACGAG   
  
  
- TGAACACGTT TGGTACAGAG ATTACCATCG GAACTGTTAC GTTTGGATCG GGAACTCGTT TAGAGGGTCG   
  
  
- AACGTCGGGG ACTACCGCTA TGTTAGGTCG CATAACAACG GATGAAACGA CTTAGTGAAC GACTTTCCTA   
  
  
- GGAATTCAGT ACCGGACCGG ATATATTTCG GGAAGTAAAG TTATCTTACG GACAATAAAG TCTTCTTAAA   
  
  
- GAACGATCCT TCGACAAAAA ACTCAACAAA GGGAAGAACT TCGACCGGAA AAACCACTGA TTGGTTAGTT   
  
  
- ATTAGCTCCG GTACCTCCCC CTTTTCTACC ACGTATATTA ACTAGACTTA CGCAGTCTTG GACGTGTCAC   
  
  
- CTAACGGGAA TAAGTTCTGA ACTCACGAGC CGGACTCCCG GGAGGAGTAA ACTCCTAATG GCCCCAAGTA   
  
  
- GTTGTCTTTC TCCAAAATCT TGTTCATCGA GTATCTAACT GACTTCTTCG ACTCTTCAAC CTAAACGGTA   
  
  
- AAGTCAAGTT AGGACACCAA ACGTTTGATC TCTTAGAGCT GTAGCCTTTT GAGGCACAAT TCTGGCCCCT   
  
  
- CCGGAACCGA TAATGGAGCC AGGAAGTTGA CGTATGGGAA AACCGAAGAC TTCTCCTTCA GGAATTCTTT   
  
  
- TCAAGTGGGA ACCGTAACCA TTTCGTTCGG TTACCCCGAT TAAATGTCCC GAACAAGTTA TTTCTACCTC   
  
  
- GATTATTATC CGCATCGGGT TCATTACTAA GCCGAAGTAG ACGTGGAAGT AGGGAGTTGT GAAGTCGGTT   
  
  
- CTACCTTCCA AAGGAATCGC GAAACACCCC AAATAGGGGT TTCTAATACC ACTATTGGCT CGTTCTAAGG   
  
  
- TTGGTGTTAC CCCGTCCTGA TTACCTCTCT AACAGTCTTC GTAACATGAA GATACGTCGT AACAAGCTAA   
  
  
- CGAATCTTAA ATGGGAGGGC TCTTGGAGGC ACCTCTCTTC CTTCCAGCTC TACGAGGAGG AACCGTTCCT   
  
  
- TTAGTTCTTG TAGTATCGCA CACTCCCTCC TCTTTCTTAT CTATCCGTAC TCTTCAACCC CTTCACCTAA   
  
  
- TTCTCCGAAC TCTACCGGCC CAAACCTTCG CAAGGAAACT CGGTGTATCC GTACTAGGTT CGTTCCGCCA   
  
  
- ACAACGTCTC GATACCGACA CTACCAATAT CTTATTTCCT CCTCTTGCCT ACAAAACAAT AGACGACCGT   
  
  
- TCTAGCGGGG GAGAAAAGTC ATAGACGAAC CTCTACATCC TCCAC

+     AP-1

| Site Name | Organism | Position | Strand | Matrix score. | sequence | function |
| --- | --- | --- | --- | --- | --- | --- |
| AP-1 | Arabidopsis thaliana | 821 | - | 8 | TGAGTTAG |  |

>HU08G01232.1   
+ -Up\_Stream \_Len000TCTACC TTTACCTGTG TTAAAAAAAA AAATTTTGTT TCCATTACTA TTCTCTTGGC   
  
  
+ ACTATTGGTG CTGATTTTTC TTCACCAAAT CAACCTCCTT ATATTTGCCA ATTTTACTGT CCTCTTTCAT   
  
  
+ CTATAAAGTC AACTCTCCTA GTACAATTTG TCACATAGAA AATCTCTAGG CACTCATCTA AACTCTCTGT   
  
  
+ TCTGGGTAAT TGATCGACGA TCTACACCAT TATTCGTTCT AGTGCACCGT TTGGACAGCC AATCATGTAC   
  
  
+ CTTTGGAGAT CAACTGCTCA TGAGTTCTTG GTGTGAAATT GGGGAAGTAA ATTCGACTTT AGGGCACTGG   
  
  
+ TCTATCACGC CATGATTTTA CCATCTATTT TCATATATAT CCAATAAGTT TGATCTTTCG CTATTAATCA   
  
  
+ CAATGATATA AACAAGTGGT GAAGAGCAAT AATGAACCAT ACATTTTAAA CTTAAGCGTT AAGGAAGACA   
  
  
+ TGAAGTTAAA AACAATGGTA AGAGCTATGC GTATGTTTGG CATATAGCTT TTTTAAGAGT GTTTTGGCTA   
  
  
+ TAGTCGGAGT TTTTTAATTA AGATTAGCTG TTTGATCAAA TAAAAAAGCT AATTTGAGTG TTTGGCGAGA   
  
  
+ AGACTTTTTA TAAGAACTTT TTTTGGTCTA AAAAGTTAAT TTAAAAAGGC TAATTCTATG AGCTTTTCGG   
  
  
+ AAGAGTTTTT TAAATAATTA ACTTTTTGTC TCATAAGCCA TAACTTTATC AGAAACAGTT AATTTTACAA   
  
  
+ AATAATTTCT CAACAAACAA CTAATTTAAA TAATTAATAA AAATAACTAA CTCAAATATC TAATAACTAA   
  
  
+ TAACTAATAT AAATAATTAA CAGTTAACAG TTGTTTACGA AACAGAATTT ATATAAAAAG CGGTAACAGA   
  
  
+ TAAAAGATAA TCATGCGAGA TTAAAAGTCA CCAGTGACAC ACAAGTACTT AAATAAATAG TCTAAACAAT   
  
  
+ GATATTTTTT GCGAGCTTCC ATGCAAATAC CGACACTTTT ATTGCCTCAG AAGGAAACAA AAACTGAGTG   
  
  
+ AAGGGGGGGG GGGGTTCTGT TTGTCTTTGT TAGGGCACGT TTGGATTCAA GTGTAGATTG GGAATAAATT   
  
  
+ CGAGGACTTT TTCTTTGCCC TTTTTCCCTG ATTCCCAAAC CCACATCAAG TCAAGACGAA ACCAGCCATT   
  
  
+ GAAAAAAGAG GGTGTTTGTG TGAGATAAAG ACAGAAATTT TATGCCAGAG AGAGAAGATA ACAAATGCAA   
  
  
+ CAACGTCGAC AGAGGCTCTC CTTCTCACAA ATTCCATATT CCTCTGTTTT TTAAGAAAGA AAAAGAAAGT   
  
  
+ GTGAGACAAG AACAACACAA GCAAACAATT CAGTTGACGA CGAATACTGA GTGATACCAC GCACTGTGTG   
  
  
+ CAACTGCGTT TCCATTTGTG GAGTCCTTCA AAGCACGCTC ATCTTCAACC CTTCCTTCGC CACGGAATTT   
  
  
+ CAGGGGGAAA AAAACCCCCA CCTAATTTTG AGAGAGAGAG AGAACAGAGA GAACGGGCGA TACAGGGATA   
  
  
+ CAAGGTTTGA GCAATTTTAG AAAATTTCTT GGGGCCGTTG ATGGGTTTTG AGTGAATTGC AAATCCCAGA   
  
  
+ AAGATTTCTG CGGTTTTTTA TCTACGGCTC TCTCTGTGGG AATTTTTTGG TAGATTCCTC TGGTTCCCAC   
  
  
+ TTTCATATCT TCTTATTTGT TCTACCCTCT TTACATCTGA TAGTTTGTTC TAACTGCTGC TTCAGTTGGT   
  
  
+ GCTTGTTCTC TTTAGTACTT TTCTGTTTTG TTCGTTTTGT TCATGCACTT GAATTCAACA AGAGCCCTTG   
  
  
+ TCCATTTTGA GCTCTTAATT CCCATCTAAC CCTGTTTCCT TCAGAAATTG CTGTTCTGTT CATATAGTTA   
  
  
+ TATTTTTTAT GCTTTGATTT GGGTATAAGT TTGCTGTTGG TGATTCCAAA GCTTGGTACT TTTGGCCATA   
  
  
+ TTTTGTTTGA GTTGGTGTTG ATTGGTACAA ATCTTGTAAT TGGTGTGATT GTTAATGGGA CCAATGCTTC   
  
  
+ AAGATGATGG TTCATCAGTA ACTTCTTCAT CACCTCTTCA ATTTTTCTCC ATGATGTCAC CCAATTTGGG   
  
  
+ TTCCTCCTAC CCTTGGCTAA GGGACTTAAA GCCTGAAGAG AGAGGTCTTT ACTTGATACA TTTGTTGCTC   
  
  
+ ACTTGTGCAA ACCATGTCTC TAATGGTAGC CTTGACAATG CAAACCTAGC CCTTGAGCAA ATCTCCCAGC   
  
  
+ TTGCAGCCCC TGATGGCGAT ACAATCCAGC GTATTGTTGC CTACTTTGCT GAATCACTTG CTGAAAGGAT   
  
  
+ CCTTAAGTCA TGGCCTGGCC TATATAAAGC CCTTCATTTC AATAGAATGC CTGTTATTTC AGAAGAATTT   
  
  
+ CTTGCTAGGA AGCTGTTTTT TGAGTTGTTT CCCTTCTTGA AGCTGGCCTT TTTGGTGACT AACCAATCAA   
  
  
+ TAATCGAGGC CATGGAGGGG GAAAAGATGG TGCATATAAT TGATCTGAAT GCGTCAGAAC CTGCACAGTG   
  
  
+ GATTGCCCTT ATTCAAGACT TGAGTGCTCG GCCTGAGGGC CCTCCTCATT TGAGGATTAC CGGGGTTCAT   
  
  
+ CAACAGAAAG AGGTTTTAGA ACAAGTAGCT CATAGATTGA CTGAAGAAGC TGAGAAGTTG GATTTGCCAT   
  
  
+ TTCAGTTCAA TCCTGTGGTT TGCAAACTAG AGAATCTCGA CATCGGAAAA CTCCGTGTTA AGACCGGGGA   
  
  
+ GGCCTTGGCT ATTACCTCGG TCCTTCAACT GCATACCCTT TTGGCTTCTG AAGAGGAAGT CCTTAAGAAA   
  
  
+ AGTTCACCCT TGGCATTGGT AAAGCAAGCC AATGGGGCTA ATTTACAGGG CTTGTTCAAT AAAGATGGAG   
  
  
+ CTAATAATAG GCGTAGCCCA AGTAATGATT CGGCTTCATC TGCACCTTCA TCCCTCAACA CTTCAGCCAA   
  
  
+ GATGGAAGGT TTCCTTAGCG CTTTGTGGGG TTTATCCCCA AAGATTATGG TGATAACCGA GCAAGATTCC   
  
  
+ AACCACAATG GGGCAGGACT AATGGAGAGA TTGTCAGAAG CATTGTACTT CTATGCAGCA TTGTTCGATT   
  
  
+ GCTTAGAATT TACCCTCCCG AGAACCTCCG TGGAGAGAAG GAAGGTCGAG ATGCTCCTCC TTGGCAAGGA   
  
  
+ AATCAAGAAC ATCATAGCGT GTGAGGGAGG AGAAAGAATA GATAGGCATG AGAAGTTGGG GAAGTGGATT   
  
  
+ AAGAGGCTTG AGATGGCCGG GTTTGGAAGC GTTCCTTTGA GCCACATAGG CATGATCCAA GCAAGGCGGT   
  
  
+ TGTTGCAGAG CTATGGCTGT GATGGTTATA GAATAAAGGA GGAGAACGGA TGTTTTGTTA TCTGCTGGCA   
  
  
+ AGATCGCCCC CTCTTTTCAG TATCTGCTTG GAGATGTAGG AGGTG  

- -Up\_Stream \_Len000AGATGG AAATGGACAC AATTTTTTTT TTTAAAACAA AGGTAATGAT AAGAGAACCG   
  
  
- TGATAACCAC GACTAAAAAG AAGTGGTTTA GTTGGAGGAA TATAAACGGT TAAAATGACA GGAGAAAGTA   
  
  
- GATATTTCAG TTGAGAGGAT CATGTTAAAC AGTGTATCTT TTAGAGATCC GTGAGTAGAT TTGAGAGACA   
  
  
- AGACCCATTA ACTAGCTGCT AGATGTGGTA ATAAGCAAGA TCACGTGGCA AACCTGTCGG TTAGTACATG   
  
  
- GAAACCTCTA GTTGACGAGT ACTCAAGAAC CACACTTTAA CCCCTTCATT TAAGCTGAAA TCCCGTGACC   
  
  
- AGATAGTGCG GTACTAAAAT GGTAGATAAA AGTATATATA GGTTATTCAA ACTAGAAAGC GATAATTAGT   
  
  
- GTTACTATAT TTGTTCACCA CTTCTCGTTA TTACTTGGTA TGTAAAATTT GAATTCGCAA TTCCTTCTGT   
  
  
- ACTTCAATTT TTGTTACCAT TCTCGATACG CATACAAACC GTATATCGAA AAAATTCTCA CAAAACCGAT   
  
  
- ATCAGCCTCA AAAAATTAAT TCTAATCGAC AAACTAGTTT ATTTTTTCGA TTAAACTCAC AAACCGCTCT   
  
  
- TCTGAAAAAT ATTCTTGAAA AAAACCAGAT TTTTCAATTA AATTTTTCCG ATTAAGATAC TCGAAAAGCC   
  
  
- TTCTCAAAAA ATTTATTAAT TGAAAAACAG AGTATTCGGT ATTGAAATAG TCTTTGTCAA TTAAAATGTT   
  
  
- TTATTAAAGA GTTGTTTGTT GATTAAATTT ATTAATTATT TTTATTGATT GAGTTTATAG ATTATTGATT   
  
  
- ATTGATTATA TTTATTAATT GTCAATTGTC AACAAATGCT TTGTCTTAAA TATATTTTTC GCCATTGTCT   
  
  
- ATTTTCTATT AGTACGCTCT AATTTTCAGT GGTCACTGTG TGTTCATGAA TTTATTTATC AGATTTGTTA   
  
  
- CTATAAAAAA CGCTCGAAGG TACGTTTATG GCTGTGAAAA TAACGGAGTC TTCCTTTGTT TTTGACTCAC   
  
  
- TTCCCCCCCC CCCCAAGACA AACAGAAACA ATCCCGTGCA AACCTAAGTT CACATCTAAC CCTTATTTAA   
  
  
- GCTCCTGAAA AAGAAACGGG AAAAAGGGAC TAAGGGTTTG GGTGTAGTTC AGTTCTGCTT TGGTCGGTAA   
  
  
- CTTTTTTCTC CCACAAACAC ACTCTATTTC TGTCTTTAAA ATACGGTCTC TCTCTTCTAT TGTTTACGTT   
  
  
- GTTGCAGCTG TCTCCGAGAG GAAGAGTGTT TAAGGTATAA GGAGACAAAA AATTCTTTCT TTTTCTTTCA   
  
  
- CACTCTGTTC TTGTTGTGTT CGTTTGTTAA GTCAACTGCT GCTTATGACT CACTATGGTG CGTGACACAC   
  
  
- GTTGACGCAA AGGTAAACAC CTCAGGAAGT TTCGTGCGAG TAGAAGTTGG GAAGGAAGCG GTGCCTTAAA   
  
  
- GTCCCCCTTT TTTTGGGGGT GGATTAAAAC TCTCTCTCTC TCTTGTCTCT CTTGCCCGCT ATGTCCCTAT   
  
  
- GTTCCAAACT CGTTAAAATC TTTTAAAGAA CCCCGGCAAC TACCCAAAAC TCACTTAACG TTTAGGGTCT   
  
  
- TTCTAAAGAC GCCAAAAAAT AGATGCCGAG AGAGACACCC TTAAAAAACC ATCTAAGGAG ACCAAGGGTG   
  
  
- AAAGTATAGA AGAATAAACA AGATGGGAGA AATGTAGACT ATCAAACAAG ATTGACGACG AAGTCAACCA   
  
  
- CGAACAAGAG AAATCATGAA AAGACAAAAC AAGCAAAACA AGTACGTGAA CTTAAGTTGT TCTCGGGAAC   
  
  
- AGGTAAAACT CGAGAATTAA GGGTAGATTG GGACAAAGGA AGTCTTTAAC GACAAGACAA GTATATCAAT   
  
  
- ATAAAAAATA CGAAACTAAA CCCATATTCA AACGACAACC ACTAAGGTTT CGAACCATGA AAACCGGTAT   
  
  
- AAAACAAACT CAACCACAAC TAACCATGTT TAGAACATTA ACCACACTAA CAATTACCCT GGTTACGAAG   
  
  
- TTCTACTACC AAGTAGTCAT TGAAGAAGTA GTGGAGAAGT TAAAAAGAGG TACTACAGTG GGTTAAACCC   
  
  
- AAGGAGGATG GGAACCGATT CCCTGAATTT CGGACTTCTC TCTCCAGAAA TGAACTATGT AAACAACGAG   
  
  
- TGAACACGTT TGGTACAGAG ATTACCATCG GAACTGTTAC GTTTGGATCG GGAACTCGTT TAGAGGGTCG   
  
  
- AACGTCGGGG ACTACCGCTA TGTTAGGTCG CATAACAACG GATGAAACGA CTTAGTGAAC GACTTTCCTA   
  
  
- GGAATTCAGT ACCGGACCGG ATATATTTCG GGAAGTAAAG TTATCTTACG GACAATAAAG TCTTCTTAAA   
  
  
- GAACGATCCT TCGACAAAAA ACTCAACAAA GGGAAGAACT TCGACCGGAA AAACCACTGA TTGGTTAGTT   
  
  
- ATTAGCTCCG GTACCTCCCC CTTTTCTACC ACGTATATTA ACTAGACTTA CGCAGTCTTG GACGTGTCAC   
  
  
- CTAACGGGAA TAAGTTCTGA ACTCACGAGC CGGACTCCCG GGAGGAGTAA ACTCCTAATG GCCCCAAGTA   
  
  
- GTTGTCTTTC TCCAAAATCT TGTTCATCGA GTATCTAACT GACTTCTTCG ACTCTTCAAC CTAAACGGTA   
  
  
- AAGTCAAGTT AGGACACCAA ACGTTTGATC TCTTAGAGCT GTAGCCTTTT GAGGCACAAT TCTGGCCCCT   
  
  
- CCGGAACCGA TAATGGAGCC AGGAAGTTGA CGTATGGGAA AACCGAAGAC TTCTCCTTCA GGAATTCTTT   
  
  
- TCAAGTGGGA ACCGTAACCA TTTCGTTCGG TTACCCCGAT TAAATGTCCC GAACAAGTTA TTTCTACCTC   
  
  
- GATTATTATC CGCATCGGGT TCATTACTAA GCCGAAGTAG ACGTGGAAGT AGGGAGTTGT GAAGTCGGTT   
  
  
- CTACCTTCCA AAGGAATCGC GAAACACCCC AAATAGGGGT TTCTAATACC ACTATTGGCT CGTTCTAAGG   
  
  
- TTGGTGTTAC CCCGTCCTGA TTACCTCTCT AACAGTCTTC GTAACATGAA GATACGTCGT AACAAGCTAA   
  
  
- CGAATCTTAA ATGGGAGGGC TCTTGGAGGC ACCTCTCTTC CTTCCAGCTC TACGAGGAGG AACCGTTCCT   
  
  
- TTAGTTCTTG TAGTATCGCA CACTCCCTCC TCTTTCTTAT CTATCCGTAC TCTTCAACCC CTTCACCTAA   
  
  
- TTCTCCGAAC TCTACCGGCC CAAACCTTCG CAAGGAAACT CGGTGTATCC GTACTAGGTT CGTTCCGCCA   
  
  
- ACAACGTCTC GATACCGACA CTACCAATAT CTTATTTCCT CCTCTTGCCT ACAAAACAAT AGACGACCGT   
  
  
- TCTAGCGGGG GAGAAAAGTC ATAGACGAAC CTCTACATCC TCCAC

+     ARE

| Site Name | Organism | Position | Strand | Matrix score. | sequence | function |
| --- | --- | --- | --- | --- | --- | --- |
| ARE | Zea mays | 2680 | - | 6 | AAACCA | cis-acting regulatory element essential for the anaerobic induction |
| ARE | Zea mays | 2183 | + | 6 | AAACCA | cis-acting regulatory element essential for the anaerobic induction |
| ARE | Zea mays | 1183 | + | 6 | AAACCA | cis-acting regulatory element essential for the anaerobic induction |

>HU08G01232.1   
+ -Up\_Stream \_Len000TCTACC TTTACCTGTG TTAAAAAAAA AAATTTTGTT TCCATTACTA TTCTCTTGGC   
  
  
+ ACTATTGGTG CTGATTTTTC TTCACCAAAT CAACCTCCTT ATATTTGCCA ATTTTACTGT CCTCTTTCAT   
  
  
+ CTATAAAGTC AACTCTCCTA GTACAATTTG TCACATAGAA AATCTCTAGG CACTCATCTA AACTCTCTGT   
  
  
+ TCTGGGTAAT TGATCGACGA TCTACACCAT TATTCGTTCT AGTGCACCGT TTGGACAGCC AATCATGTAC   
  
  
+ CTTTGGAGAT CAACTGCTCA TGAGTTCTTG GTGTGAAATT GGGGAAGTAA ATTCGACTTT AGGGCACTGG   
  
  
+ TCTATCACGC CATGATTTTA CCATCTATTT TCATATATAT CCAATAAGTT TGATCTTTCG CTATTAATCA   
  
  
+ CAATGATATA AACAAGTGGT GAAGAGCAAT AATGAACCAT ACATTTTAAA CTTAAGCGTT AAGGAAGACA   
  
  
+ TGAAGTTAAA AACAATGGTA AGAGCTATGC GTATGTTTGG CATATAGCTT TTTTAAGAGT GTTTTGGCTA   
  
  
+ TAGTCGGAGT TTTTTAATTA AGATTAGCTG TTTGATCAAA TAAAAAAGCT AATTTGAGTG TTTGGCGAGA   
  
  
+ AGACTTTTTA TAAGAACTTT TTTTGGTCTA AAAAGTTAAT TTAAAAAGGC TAATTCTATG AGCTTTTCGG   
  
  
+ AAGAGTTTTT TAAATAATTA ACTTTTTGTC TCATAAGCCA TAACTTTATC AGAAACAGTT AATTTTACAA   
  
  
+ AATAATTTCT CAACAAACAA CTAATTTAAA TAATTAATAA AAATAACTAA CTCAAATATC TAATAACTAA   
  
  
+ TAACTAATAT AAATAATTAA CAGTTAACAG TTGTTTACGA AACAGAATTT ATATAAAAAG CGGTAACAGA   
  
  
+ TAAAAGATAA TCATGCGAGA TTAAAAGTCA CCAGTGACAC ACAAGTACTT AAATAAATAG TCTAAACAAT   
  
  
+ GATATTTTTT GCGAGCTTCC ATGCAAATAC CGACACTTTT ATTGCCTCAG AAGGAAACAA AAACTGAGTG   
  
  
+ AAGGGGGGGG GGGGTTCTGT TTGTCTTTGT TAGGGCACGT TTGGATTCAA GTGTAGATTG GGAATAAATT   
  
  
+ CGAGGACTTT TTCTTTGCCC TTTTTCCCTG ATTCCCAAAC CCACATCAAG TCAAGACGAA ACCAGCCATT   
  
  
+ GAAAAAAGAG GGTGTTTGTG TGAGATAAAG ACAGAAATTT TATGCCAGAG AGAGAAGATA ACAAATGCAA   
  
  
+ CAACGTCGAC AGAGGCTCTC CTTCTCACAA ATTCCATATT CCTCTGTTTT TTAAGAAAGA AAAAGAAAGT   
  
  
+ GTGAGACAAG AACAACACAA GCAAACAATT CAGTTGACGA CGAATACTGA GTGATACCAC GCACTGTGTG   
  
  
+ CAACTGCGTT TCCATTTGTG GAGTCCTTCA AAGCACGCTC ATCTTCAACC CTTCCTTCGC CACGGAATTT   
  
  
+ CAGGGGGAAA AAAACCCCCA CCTAATTTTG AGAGAGAGAG AGAACAGAGA GAACGGGCGA TACAGGGATA   
  
  
+ CAAGGTTTGA GCAATTTTAG AAAATTTCTT GGGGCCGTTG ATGGGTTTTG AGTGAATTGC AAATCCCAGA   
  
  
+ AAGATTTCTG CGGTTTTTTA TCTACGGCTC TCTCTGTGGG AATTTTTTGG TAGATTCCTC TGGTTCCCAC   
  
  
+ TTTCATATCT TCTTATTTGT TCTACCCTCT TTACATCTGA TAGTTTGTTC TAACTGCTGC TTCAGTTGGT   
  
  
+ GCTTGTTCTC TTTAGTACTT TTCTGTTTTG TTCGTTTTGT TCATGCACTT GAATTCAACA AGAGCCCTTG   
  
  
+ TCCATTTTGA GCTCTTAATT CCCATCTAAC CCTGTTTCCT TCAGAAATTG CTGTTCTGTT CATATAGTTA   
  
  
+ TATTTTTTAT GCTTTGATTT GGGTATAAGT TTGCTGTTGG TGATTCCAAA GCTTGGTACT TTTGGCCATA   
  
  
+ TTTTGTTTGA GTTGGTGTTG ATTGGTACAA ATCTTGTAAT TGGTGTGATT GTTAATGGGA CCAATGCTTC   
  
  
+ AAGATGATGG TTCATCAGTA ACTTCTTCAT CACCTCTTCA ATTTTTCTCC ATGATGTCAC CCAATTTGGG   
  
  
+ TTCCTCCTAC CCTTGGCTAA GGGACTTAAA GCCTGAAGAG AGAGGTCTTT ACTTGATACA TTTGTTGCTC   
  
  
+ ACTTGTGCAA ACCATGTCTC TAATGGTAGC CTTGACAATG CAAACCTAGC CCTTGAGCAA ATCTCCCAGC   
  
  
+ TTGCAGCCCC TGATGGCGAT ACAATCCAGC GTATTGTTGC CTACTTTGCT GAATCACTTG CTGAAAGGAT   
  
  
+ CCTTAAGTCA TGGCCTGGCC TATATAAAGC CCTTCATTTC AATAGAATGC CTGTTATTTC AGAAGAATTT   
  
  
+ CTTGCTAGGA AGCTGTTTTT TGAGTTGTTT CCCTTCTTGA AGCTGGCCTT TTTGGTGACT AACCAATCAA   
  
  
+ TAATCGAGGC CATGGAGGGG GAAAAGATGG TGCATATAAT TGATCTGAAT GCGTCAGAAC CTGCACAGTG   
  
  
+ GATTGCCCTT ATTCAAGACT TGAGTGCTCG GCCTGAGGGC CCTCCTCATT TGAGGATTAC CGGGGTTCAT   
  
  
+ CAACAGAAAG AGGTTTTAGA ACAAGTAGCT CATAGATTGA CTGAAGAAGC TGAGAAGTTG GATTTGCCAT   
  
  
+ TTCAGTTCAA TCCTGTGGTT TGCAAACTAG AGAATCTCGA CATCGGAAAA CTCCGTGTTA AGACCGGGGA   
  
  
+ GGCCTTGGCT ATTACCTCGG TCCTTCAACT GCATACCCTT TTGGCTTCTG AAGAGGAAGT CCTTAAGAAA   
  
  
+ AGTTCACCCT TGGCATTGGT AAAGCAAGCC AATGGGGCTA ATTTACAGGG CTTGTTCAAT AAAGATGGAG   
  
  
+ CTAATAATAG GCGTAGCCCA AGTAATGATT CGGCTTCATC TGCACCTTCA TCCCTCAACA CTTCAGCCAA   
  
  
+ GATGGAAGGT TTCCTTAGCG CTTTGTGGGG TTTATCCCCA AAGATTATGG TGATAACCGA GCAAGATTCC   
  
  
+ AACCACAATG GGGCAGGACT AATGGAGAGA TTGTCAGAAG CATTGTACTT CTATGCAGCA TTGTTCGATT   
  
  
+ GCTTAGAATT TACCCTCCCG AGAACCTCCG TGGAGAGAAG GAAGGTCGAG ATGCTCCTCC TTGGCAAGGA   
  
  
+ AATCAAGAAC ATCATAGCGT GTGAGGGAGG AGAAAGAATA GATAGGCATG AGAAGTTGGG GAAGTGGATT   
  
  
+ AAGAGGCTTG AGATGGCCGG GTTTGGAAGC GTTCCTTTGA GCCACATAGG CATGATCCAA GCAAGGCGGT   
  
  
+ TGTTGCAGAG CTATGGCTGT GATGGTTATA GAATAAAGGA GGAGAACGGA TGTTTTGTTA TCTGCTGGCA   
  
  
+ AGATCGCCCC CTCTTTTCAG TATCTGCTTG GAGATGTAGG AGGTG  

- -Up\_Stream \_Len000AGATGG AAATGGACAC AATTTTTTTT TTTAAAACAA AGGTAATGAT AAGAGAACCG   
  
  
- TGATAACCAC GACTAAAAAG AAGTGGTTTA GTTGGAGGAA TATAAACGGT TAAAATGACA GGAGAAAGTA   
  
  
- GATATTTCAG TTGAGAGGAT CATGTTAAAC AGTGTATCTT TTAGAGATCC GTGAGTAGAT TTGAGAGACA   
  
  
- AGACCCATTA ACTAGCTGCT AGATGTGGTA ATAAGCAAGA TCACGTGGCA AACCTGTCGG TTAGTACATG   
  
  
- GAAACCTCTA GTTGACGAGT ACTCAAGAAC CACACTTTAA CCCCTTCATT TAAGCTGAAA TCCCGTGACC   
  
  
- AGATAGTGCG GTACTAAAAT GGTAGATAAA AGTATATATA GGTTATTCAA ACTAGAAAGC GATAATTAGT   
  
  
- GTTACTATAT TTGTTCACCA CTTCTCGTTA TTACTTGGTA TGTAAAATTT GAATTCGCAA TTCCTTCTGT   
  
  
- ACTTCAATTT TTGTTACCAT TCTCGATACG CATACAAACC GTATATCGAA AAAATTCTCA CAAAACCGAT   
  
  
- ATCAGCCTCA AAAAATTAAT TCTAATCGAC AAACTAGTTT ATTTTTTCGA TTAAACTCAC AAACCGCTCT   
  
  
- TCTGAAAAAT ATTCTTGAAA AAAACCAGAT TTTTCAATTA AATTTTTCCG ATTAAGATAC TCGAAAAGCC   
  
  
- TTCTCAAAAA ATTTATTAAT TGAAAAACAG AGTATTCGGT ATTGAAATAG TCTTTGTCAA TTAAAATGTT   
  
  
- TTATTAAAGA GTTGTTTGTT GATTAAATTT ATTAATTATT TTTATTGATT GAGTTTATAG ATTATTGATT   
  
  
- ATTGATTATA TTTATTAATT GTCAATTGTC AACAAATGCT TTGTCTTAAA TATATTTTTC GCCATTGTCT   
  
  
- ATTTTCTATT AGTACGCTCT AATTTTCAGT GGTCACTGTG TGTTCATGAA TTTATTTATC AGATTTGTTA   
  
  
- CTATAAAAAA CGCTCGAAGG TACGTTTATG GCTGTGAAAA TAACGGAGTC TTCCTTTGTT TTTGACTCAC   
  
  
- TTCCCCCCCC CCCCAAGACA AACAGAAACA ATCCCGTGCA AACCTAAGTT CACATCTAAC CCTTATTTAA   
  
  
- GCTCCTGAAA AAGAAACGGG AAAAAGGGAC TAAGGGTTTG GGTGTAGTTC AGTTCTGCTT TGGTCGGTAA   
  
  
- CTTTTTTCTC CCACAAACAC ACTCTATTTC TGTCTTTAAA ATACGGTCTC TCTCTTCTAT TGTTTACGTT   
  
  
- GTTGCAGCTG TCTCCGAGAG GAAGAGTGTT TAAGGTATAA GGAGACAAAA AATTCTTTCT TTTTCTTTCA   
  
  
- CACTCTGTTC TTGTTGTGTT CGTTTGTTAA GTCAACTGCT GCTTATGACT CACTATGGTG CGTGACACAC   
  
  
- GTTGACGCAA AGGTAAACAC CTCAGGAAGT TTCGTGCGAG TAGAAGTTGG GAAGGAAGCG GTGCCTTAAA   
  
  
- GTCCCCCTTT TTTTGGGGGT GGATTAAAAC TCTCTCTCTC TCTTGTCTCT CTTGCCCGCT ATGTCCCTAT   
  
  
- GTTCCAAACT CGTTAAAATC TTTTAAAGAA CCCCGGCAAC TACCCAAAAC TCACTTAACG TTTAGGGTCT   
  
  
- TTCTAAAGAC GCCAAAAAAT AGATGCCGAG AGAGACACCC TTAAAAAACC ATCTAAGGAG ACCAAGGGTG   
  
  
- AAAGTATAGA AGAATAAACA AGATGGGAGA AATGTAGACT ATCAAACAAG ATTGACGACG AAGTCAACCA   
  
  
- CGAACAAGAG AAATCATGAA AAGACAAAAC AAGCAAAACA AGTACGTGAA CTTAAGTTGT TCTCGGGAAC   
  
  
- AGGTAAAACT CGAGAATTAA GGGTAGATTG GGACAAAGGA AGTCTTTAAC GACAAGACAA GTATATCAAT   
  
  
- ATAAAAAATA CGAAACTAAA CCCATATTCA AACGACAACC ACTAAGGTTT CGAACCATGA AAACCGGTAT   
  
  
- AAAACAAACT CAACCACAAC TAACCATGTT TAGAACATTA ACCACACTAA CAATTACCCT GGTTACGAAG   
  
  
- TTCTACTACC AAGTAGTCAT TGAAGAAGTA GTGGAGAAGT TAAAAAGAGG TACTACAGTG GGTTAAACCC   
  
  
- AAGGAGGATG GGAACCGATT CCCTGAATTT CGGACTTCTC TCTCCAGAAA TGAACTATGT AAACAACGAG   
  
  
- TGAACACGTT TGGTACAGAG ATTACCATCG GAACTGTTAC GTTTGGATCG GGAACTCGTT TAGAGGGTCG   
  
  
- AACGTCGGGG ACTACCGCTA TGTTAGGTCG CATAACAACG GATGAAACGA CTTAGTGAAC GACTTTCCTA   
  
  
- GGAATTCAGT ACCGGACCGG ATATATTTCG GGAAGTAAAG TTATCTTACG GACAATAAAG TCTTCTTAAA   
  
  
- GAACGATCCT TCGACAAAAA ACTCAACAAA GGGAAGAACT TCGACCGGAA AAACCACTGA TTGGTTAGTT   
  
  
- ATTAGCTCCG GTACCTCCCC CTTTTCTACC ACGTATATTA ACTAGACTTA CGCAGTCTTG GACGTGTCAC   
  
  
- CTAACGGGAA TAAGTTCTGA ACTCACGAGC CGGACTCCCG GGAGGAGTAA ACTCCTAATG GCCCCAAGTA   
  
  
- GTTGTCTTTC TCCAAAATCT TGTTCATCGA GTATCTAACT GACTTCTTCG ACTCTTCAAC CTAAACGGTA   
  
  
- AAGTCAAGTT AGGACACCAA ACGTTTGATC TCTTAGAGCT GTAGCCTTTT GAGGCACAAT TCTGGCCCCT   
  
  
- CCGGAACCGA TAATGGAGCC AGGAAGTTGA CGTATGGGAA AACCGAAGAC TTCTCCTTCA GGAATTCTTT   
  
  
- TCAAGTGGGA ACCGTAACCA TTTCGTTCGG TTACCCCGAT TAAATGTCCC GAACAAGTTA TTTCTACCTC   
  
  
- GATTATTATC CGCATCGGGT TCATTACTAA GCCGAAGTAG ACGTGGAAGT AGGGAGTTGT GAAGTCGGTT   
  
  
- CTACCTTCCA AAGGAATCGC GAAACACCCC AAATAGGGGT TTCTAATACC ACTATTGGCT CGTTCTAAGG   
  
  
- TTGGTGTTAC CCCGTCCTGA TTACCTCTCT AACAGTCTTC GTAACATGAA GATACGTCGT AACAAGCTAA   
  
  
- CGAATCTTAA ATGGGAGGGC TCTTGGAGGC ACCTCTCTTC CTTCCAGCTC TACGAGGAGG AACCGTTCCT   
  
  
- TTAGTTCTTG TAGTATCGCA CACTCCCTCC TCTTTCTTAT CTATCCGTAC TCTTCAACCC CTTCACCTAA   
  
  
- TTCTCCGAAC TCTACCGGCC CAAACCTTCG CAAGGAAACT CGGTGTATCC GTACTAGGTT CGTTCCGCCA   
  
  
- ACAACGTCTC GATACCGACA CTACCAATAT CTTATTTCCT CCTCTTGCCT ACAAAACAAT AGACGACCGT   
  
  
- TCTAGCGGGG GAGAAAAGTC ATAGACGAAC CTCTACATCC TCCAC

+     AT~TATA-box

| Site Name | Organism | Position | Strand | Matrix score. | sequence | function |
| --- | --- | --- | --- | --- | --- | --- |
| AT~TATA-box | Arabidopsis thaliana | 388 | + | 6 | TATATA |  |
| AT~TATA-box | Arabidopsis thaliana | 894 | + | 6 | TATATA |  |
| AT~TATA-box | Arabidopsis thaliana | 2335 | - | 6 | TATATA |  |
| AT~TATA-box | Arabidopsis thaliana | 892 | - | 8 | TATATAAA |  |

>HU08G01232.1   
+ -Up\_Stream \_Len000TCTACC TTTACCTGTG TTAAAAAAAA AAATTTTGTT TCCATTACTA TTCTCTTGGC   
  
  
+ ACTATTGGTG CTGATTTTTC TTCACCAAAT CAACCTCCTT ATATTTGCCA ATTTTACTGT CCTCTTTCAT   
  
  
+ CTATAAAGTC AACTCTCCTA GTACAATTTG TCACATAGAA AATCTCTAGG CACTCATCTA AACTCTCTGT   
  
  
+ TCTGGGTAAT TGATCGACGA TCTACACCAT TATTCGTTCT AGTGCACCGT TTGGACAGCC AATCATGTAC   
  
  
+ CTTTGGAGAT CAACTGCTCA TGAGTTCTTG GTGTGAAATT GGGGAAGTAA ATTCGACTTT AGGGCACTGG   
  
  
+ TCTATCACGC CATGATTTTA CCATCTATTT TCATATATAT CCAATAAGTT TGATCTTTCG CTATTAATCA   
  
  
+ CAATGATATA AACAAGTGGT GAAGAGCAAT AATGAACCAT ACATTTTAAA CTTAAGCGTT AAGGAAGACA   
  
  
+ TGAAGTTAAA AACAATGGTA AGAGCTATGC GTATGTTTGG CATATAGCTT TTTTAAGAGT GTTTTGGCTA   
  
  
+ TAGTCGGAGT TTTTTAATTA AGATTAGCTG TTTGATCAAA TAAAAAAGCT AATTTGAGTG TTTGGCGAGA   
  
  
+ AGACTTTTTA TAAGAACTTT TTTTGGTCTA AAAAGTTAAT TTAAAAAGGC TAATTCTATG AGCTTTTCGG   
  
  
+ AAGAGTTTTT TAAATAATTA ACTTTTTGTC TCATAAGCCA TAACTTTATC AGAAACAGTT AATTTTACAA   
  
  
+ AATAATTTCT CAACAAACAA CTAATTTAAA TAATTAATAA AAATAACTAA CTCAAATATC TAATAACTAA   
  
  
+ TAACTAATAT AAATAATTAA CAGTTAACAG TTGTTTACGA AACAGAATTT ATATAAAAAG CGGTAACAGA   
  
  
+ TAAAAGATAA TCATGCGAGA TTAAAAGTCA CCAGTGACAC ACAAGTACTT AAATAAATAG TCTAAACAAT   
  
  
+ GATATTTTTT GCGAGCTTCC ATGCAAATAC CGACACTTTT ATTGCCTCAG AAGGAAACAA AAACTGAGTG   
  
  
+ AAGGGGGGGG GGGGTTCTGT TTGTCTTTGT TAGGGCACGT TTGGATTCAA GTGTAGATTG GGAATAAATT   
  
  
+ CGAGGACTTT TTCTTTGCCC TTTTTCCCTG ATTCCCAAAC CCACATCAAG TCAAGACGAA ACCAGCCATT   
  
  
+ GAAAAAAGAG GGTGTTTGTG TGAGATAAAG ACAGAAATTT TATGCCAGAG AGAGAAGATA ACAAATGCAA   
  
  
+ CAACGTCGAC AGAGGCTCTC CTTCTCACAA ATTCCATATT CCTCTGTTTT TTAAGAAAGA AAAAGAAAGT   
  
  
+ GTGAGACAAG AACAACACAA GCAAACAATT CAGTTGACGA CGAATACTGA GTGATACCAC GCACTGTGTG   
  
  
+ CAACTGCGTT TCCATTTGTG GAGTCCTTCA AAGCACGCTC ATCTTCAACC CTTCCTTCGC CACGGAATTT   
  
  
+ CAGGGGGAAA AAAACCCCCA CCTAATTTTG AGAGAGAGAG AGAACAGAGA GAACGGGCGA TACAGGGATA   
  
  
+ CAAGGTTTGA GCAATTTTAG AAAATTTCTT GGGGCCGTTG ATGGGTTTTG AGTGAATTGC AAATCCCAGA   
  
  
+ AAGATTTCTG CGGTTTTTTA TCTACGGCTC TCTCTGTGGG AATTTTTTGG TAGATTCCTC TGGTTCCCAC   
  
  
+ TTTCATATCT TCTTATTTGT TCTACCCTCT TTACATCTGA TAGTTTGTTC TAACTGCTGC TTCAGTTGGT   
  
  
+ GCTTGTTCTC TTTAGTACTT TTCTGTTTTG TTCGTTTTGT TCATGCACTT GAATTCAACA AGAGCCCTTG   
  
  
+ TCCATTTTGA GCTCTTAATT CCCATCTAAC CCTGTTTCCT TCAGAAATTG CTGTTCTGTT CATATAGTTA   
  
  
+ TATTTTTTAT GCTTTGATTT GGGTATAAGT TTGCTGTTGG TGATTCCAAA GCTTGGTACT TTTGGCCATA   
  
  
+ TTTTGTTTGA GTTGGTGTTG ATTGGTACAA ATCTTGTAAT TGGTGTGATT GTTAATGGGA CCAATGCTTC   
  
  
+ AAGATGATGG TTCATCAGTA ACTTCTTCAT CACCTCTTCA ATTTTTCTCC ATGATGTCAC CCAATTTGGG   
  
  
+ TTCCTCCTAC CCTTGGCTAA GGGACTTAAA GCCTGAAGAG AGAGGTCTTT ACTTGATACA TTTGTTGCTC   
  
  
+ ACTTGTGCAA ACCATGTCTC TAATGGTAGC CTTGACAATG CAAACCTAGC CCTTGAGCAA ATCTCCCAGC   
  
  
+ TTGCAGCCCC TGATGGCGAT ACAATCCAGC GTATTGTTGC CTACTTTGCT GAATCACTTG CTGAAAGGAT   
  
  
+ CCTTAAGTCA TGGCCTGGCC TATATAAAGC CCTTCATTTC AATAGAATGC CTGTTATTTC AGAAGAATTT   
  
  
+ CTTGCTAGGA AGCTGTTTTT TGAGTTGTTT CCCTTCTTGA AGCTGGCCTT TTTGGTGACT AACCAATCAA   
  
  
+ TAATCGAGGC CATGGAGGGG GAAAAGATGG TGCATATAAT TGATCTGAAT GCGTCAGAAC CTGCACAGTG   
  
  
+ GATTGCCCTT ATTCAAGACT TGAGTGCTCG GCCTGAGGGC CCTCCTCATT TGAGGATTAC CGGGGTTCAT   
  
  
+ CAACAGAAAG AGGTTTTAGA ACAAGTAGCT CATAGATTGA CTGAAGAAGC TGAGAAGTTG GATTTGCCAT   
  
  
+ TTCAGTTCAA TCCTGTGGTT TGCAAACTAG AGAATCTCGA CATCGGAAAA CTCCGTGTTA AGACCGGGGA   
  
  
+ GGCCTTGGCT ATTACCTCGG TCCTTCAACT GCATACCCTT TTGGCTTCTG AAGAGGAAGT CCTTAAGAAA   
  
  
+ AGTTCACCCT TGGCATTGGT AAAGCAAGCC AATGGGGCTA ATTTACAGGG CTTGTTCAAT AAAGATGGAG   
  
  
+ CTAATAATAG GCGTAGCCCA AGTAATGATT CGGCTTCATC TGCACCTTCA TCCCTCAACA CTTCAGCCAA   
  
  
+ GATGGAAGGT TTCCTTAGCG CTTTGTGGGG TTTATCCCCA AAGATTATGG TGATAACCGA GCAAGATTCC   
  
  
+ AACCACAATG GGGCAGGACT AATGGAGAGA TTGTCAGAAG CATTGTACTT CTATGCAGCA TTGTTCGATT   
  
  
+ GCTTAGAATT TACCCTCCCG AGAACCTCCG TGGAGAGAAG GAAGGTCGAG ATGCTCCTCC TTGGCAAGGA   
  
  
+ AATCAAGAAC ATCATAGCGT GTGAGGGAGG AGAAAGAATA GATAGGCATG AGAAGTTGGG GAAGTGGATT   
  
  
+ AAGAGGCTTG AGATGGCCGG GTTTGGAAGC GTTCCTTTGA GCCACATAGG CATGATCCAA GCAAGGCGGT   
  
  
+ TGTTGCAGAG CTATGGCTGT GATGGTTATA GAATAAAGGA GGAGAACGGA TGTTTTGTTA TCTGCTGGCA   
  
  
+ AGATCGCCCC CTCTTTTCAG TATCTGCTTG GAGATGTAGG AGGTG  

- -Up\_Stream \_Len000AGATGG AAATGGACAC AATTTTTTTT TTTAAAACAA AGGTAATGAT AAGAGAACCG   
  
  
- TGATAACCAC GACTAAAAAG AAGTGGTTTA GTTGGAGGAA TATAAACGGT TAAAATGACA GGAGAAAGTA   
  
  
- GATATTTCAG TTGAGAGGAT CATGTTAAAC AGTGTATCTT TTAGAGATCC GTGAGTAGAT TTGAGAGACA   
  
  
- AGACCCATTA ACTAGCTGCT AGATGTGGTA ATAAGCAAGA TCACGTGGCA AACCTGTCGG TTAGTACATG   
  
  
- GAAACCTCTA GTTGACGAGT ACTCAAGAAC CACACTTTAA CCCCTTCATT TAAGCTGAAA TCCCGTGACC   
  
  
- AGATAGTGCG GTACTAAAAT GGTAGATAAA AGTATATATA GGTTATTCAA ACTAGAAAGC GATAATTAGT   
  
  
- GTTACTATAT TTGTTCACCA CTTCTCGTTA TTACTTGGTA TGTAAAATTT GAATTCGCAA TTCCTTCTGT   
  
  
- ACTTCAATTT TTGTTACCAT TCTCGATACG CATACAAACC GTATATCGAA AAAATTCTCA CAAAACCGAT   
  
  
- ATCAGCCTCA AAAAATTAAT TCTAATCGAC AAACTAGTTT ATTTTTTCGA TTAAACTCAC AAACCGCTCT   
  
  
- TCTGAAAAAT ATTCTTGAAA AAAACCAGAT TTTTCAATTA AATTTTTCCG ATTAAGATAC TCGAAAAGCC   
  
  
- TTCTCAAAAA ATTTATTAAT TGAAAAACAG AGTATTCGGT ATTGAAATAG TCTTTGTCAA TTAAAATGTT   
  
  
- TTATTAAAGA GTTGTTTGTT GATTAAATTT ATTAATTATT TTTATTGATT GAGTTTATAG ATTATTGATT   
  
  
- ATTGATTATA TTTATTAATT GTCAATTGTC AACAAATGCT TTGTCTTAAA TATATTTTTC GCCATTGTCT   
  
  
- ATTTTCTATT AGTACGCTCT AATTTTCAGT GGTCACTGTG TGTTCATGAA TTTATTTATC AGATTTGTTA   
  
  
- CTATAAAAAA CGCTCGAAGG TACGTTTATG GCTGTGAAAA TAACGGAGTC TTCCTTTGTT TTTGACTCAC   
  
  
- TTCCCCCCCC CCCCAAGACA AACAGAAACA ATCCCGTGCA AACCTAAGTT CACATCTAAC CCTTATTTAA   
  
  
- GCTCCTGAAA AAGAAACGGG AAAAAGGGAC TAAGGGTTTG GGTGTAGTTC AGTTCTGCTT TGGTCGGTAA   
  
  
- CTTTTTTCTC CCACAAACAC ACTCTATTTC TGTCTTTAAA ATACGGTCTC TCTCTTCTAT TGTTTACGTT   
  
  
- GTTGCAGCTG TCTCCGAGAG GAAGAGTGTT TAAGGTATAA GGAGACAAAA AATTCTTTCT TTTTCTTTCA   
  
  
- CACTCTGTTC TTGTTGTGTT CGTTTGTTAA GTCAACTGCT GCTTATGACT CACTATGGTG CGTGACACAC   
  
  
- GTTGACGCAA AGGTAAACAC CTCAGGAAGT TTCGTGCGAG TAGAAGTTGG GAAGGAAGCG GTGCCTTAAA   
  
  
- GTCCCCCTTT TTTTGGGGGT GGATTAAAAC TCTCTCTCTC TCTTGTCTCT CTTGCCCGCT ATGTCCCTAT   
  
  
- GTTCCAAACT CGTTAAAATC TTTTAAAGAA CCCCGGCAAC TACCCAAAAC TCACTTAACG TTTAGGGTCT   
  
  
- TTCTAAAGAC GCCAAAAAAT AGATGCCGAG AGAGACACCC TTAAAAAACC ATCTAAGGAG ACCAAGGGTG   
  
  
- AAAGTATAGA AGAATAAACA AGATGGGAGA AATGTAGACT ATCAAACAAG ATTGACGACG AAGTCAACCA   
  
  
- CGAACAAGAG AAATCATGAA AAGACAAAAC AAGCAAAACA AGTACGTGAA CTTAAGTTGT TCTCGGGAAC   
  
  
- AGGTAAAACT CGAGAATTAA GGGTAGATTG GGACAAAGGA AGTCTTTAAC GACAAGACAA GTATATCAAT   
  
  
- ATAAAAAATA CGAAACTAAA CCCATATTCA AACGACAACC ACTAAGGTTT CGAACCATGA AAACCGGTAT   
  
  
- AAAACAAACT CAACCACAAC TAACCATGTT TAGAACATTA ACCACACTAA CAATTACCCT GGTTACGAAG   
  
  
- TTCTACTACC AAGTAGTCAT TGAAGAAGTA GTGGAGAAGT TAAAAAGAGG TACTACAGTG GGTTAAACCC   
  
  
- AAGGAGGATG GGAACCGATT CCCTGAATTT CGGACTTCTC TCTCCAGAAA TGAACTATGT AAACAACGAG   
  
  
- TGAACACGTT TGGTACAGAG ATTACCATCG GAACTGTTAC GTTTGGATCG GGAACTCGTT TAGAGGGTCG   
  
  
- AACGTCGGGG ACTACCGCTA TGTTAGGTCG CATAACAACG GATGAAACGA CTTAGTGAAC GACTTTCCTA   
  
  
- GGAATTCAGT ACCGGACCGG ATATATTTCG GGAAGTAAAG TTATCTTACG GACAATAAAG TCTTCTTAAA   
  
  
- GAACGATCCT TCGACAAAAA ACTCAACAAA GGGAAGAACT TCGACCGGAA AAACCACTGA TTGGTTAGTT   
  
  
- ATTAGCTCCG GTACCTCCCC CTTTTCTACC ACGTATATTA ACTAGACTTA CGCAGTCTTG GACGTGTCAC   
  
  
- CTAACGGGAA TAAGTTCTGA ACTCACGAGC CGGACTCCCG GGAGGAGTAA ACTCCTAATG GCCCCAAGTA   
  
  
- GTTGTCTTTC TCCAAAATCT TGTTCATCGA GTATCTAACT GACTTCTTCG ACTCTTCAAC CTAAACGGTA   
  
  
- AAGTCAAGTT AGGACACCAA ACGTTTGATC TCTTAGAGCT GTAGCCTTTT GAGGCACAAT TCTGGCCCCT   
  
  
- CCGGAACCGA TAATGGAGCC AGGAAGTTGA CGTATGGGAA AACCGAAGAC TTCTCCTTCA GGAATTCTTT   
  
  
- TCAAGTGGGA ACCGTAACCA TTTCGTTCGG TTACCCCGAT TAAATGTCCC GAACAAGTTA TTTCTACCTC   
  
  
- GATTATTATC CGCATCGGGT TCATTACTAA GCCGAAGTAG ACGTGGAAGT AGGGAGTTGT GAAGTCGGTT   
  
  
- CTACCTTCCA AAGGAATCGC GAAACACCCC AAATAGGGGT TTCTAATACC ACTATTGGCT CGTTCTAAGG   
  
  
- TTGGTGTTAC CCCGTCCTGA TTACCTCTCT AACAGTCTTC GTAACATGAA GATACGTCGT AACAAGCTAA   
  
  
- CGAATCTTAA ATGGGAGGGC TCTTGGAGGC ACCTCTCTTC CTTCCAGCTC TACGAGGAGG AACCGTTCCT   
  
  
- TTAGTTCTTG TAGTATCGCA CACTCCCTCC TCTTTCTTAT CTATCCGTAC TCTTCAACCC CTTCACCTAA   
  
  
- TTCTCCGAAC TCTACCGGCC CAAACCTTCG CAAGGAAACT CGGTGTATCC GTACTAGGTT CGTTCCGCCA   
  
  
- ACAACGTCTC GATACCGACA CTACCAATAT CTTATTTCCT CCTCTTGCCT ACAAAACAAT AGACGACCGT   
  
  
- TCTAGCGGGG GAGAAAAGTC ATAGACGAAC CTCTACATCC TCCAC

+     Box 4

| Site Name | Organism | Position | Strand | Matrix score. | sequence | function |
| --- | --- | --- | --- | --- | --- | --- |
| Box 4 | Petroselinum crispum | 807 | + | 6 | ATTAAT | part of a conserved DNA module involved in light responsiveness |
| Box 4 | Petroselinum crispum | 417 | + | 6 | ATTAAT | part of a conserved DNA module involved in light responsiveness |

>HU08G01232.1   
+ -Up\_Stream \_Len000TCTACC TTTACCTGTG TTAAAAAAAA AAATTTTGTT TCCATTACTA TTCTCTTGGC   
  
  
+ ACTATTGGTG CTGATTTTTC TTCACCAAAT CAACCTCCTT ATATTTGCCA ATTTTACTGT CCTCTTTCAT   
  
  
+ CTATAAAGTC AACTCTCCTA GTACAATTTG TCACATAGAA AATCTCTAGG CACTCATCTA AACTCTCTGT   
  
  
+ TCTGGGTAAT TGATCGACGA TCTACACCAT TATTCGTTCT AGTGCACCGT TTGGACAGCC AATCATGTAC   
  
  
+ CTTTGGAGAT CAACTGCTCA TGAGTTCTTG GTGTGAAATT GGGGAAGTAA ATTCGACTTT AGGGCACTGG   
  
  
+ TCTATCACGC CATGATTTTA CCATCTATTT TCATATATAT CCAATAAGTT TGATCTTTCG CTATTAATCA   
  
  
+ CAATGATATA AACAAGTGGT GAAGAGCAAT AATGAACCAT ACATTTTAAA CTTAAGCGTT AAGGAAGACA   
  
  
+ TGAAGTTAAA AACAATGGTA AGAGCTATGC GTATGTTTGG CATATAGCTT TTTTAAGAGT GTTTTGGCTA   
  
  
+ TAGTCGGAGT TTTTTAATTA AGATTAGCTG TTTGATCAAA TAAAAAAGCT AATTTGAGTG TTTGGCGAGA   
  
  
+ AGACTTTTTA TAAGAACTTT TTTTGGTCTA AAAAGTTAAT TTAAAAAGGC TAATTCTATG AGCTTTTCGG   
  
  
+ AAGAGTTTTT TAAATAATTA ACTTTTTGTC TCATAAGCCA TAACTTTATC AGAAACAGTT AATTTTACAA   
  
  
+ AATAATTTCT CAACAAACAA CTAATTTAAA TAATTAATAA AAATAACTAA CTCAAATATC TAATAACTAA   
  
  
+ TAACTAATAT AAATAATTAA CAGTTAACAG TTGTTTACGA AACAGAATTT ATATAAAAAG CGGTAACAGA   
  
  
+ TAAAAGATAA TCATGCGAGA TTAAAAGTCA CCAGTGACAC ACAAGTACTT AAATAAATAG TCTAAACAAT   
  
  
+ GATATTTTTT GCGAGCTTCC ATGCAAATAC CGACACTTTT ATTGCCTCAG AAGGAAACAA AAACTGAGTG   
  
  
+ AAGGGGGGGG GGGGTTCTGT TTGTCTTTGT TAGGGCACGT TTGGATTCAA GTGTAGATTG GGAATAAATT   
  
  
+ CGAGGACTTT TTCTTTGCCC TTTTTCCCTG ATTCCCAAAC CCACATCAAG TCAAGACGAA ACCAGCCATT   
  
  
+ GAAAAAAGAG GGTGTTTGTG TGAGATAAAG ACAGAAATTT TATGCCAGAG AGAGAAGATA ACAAATGCAA   
  
  
+ CAACGTCGAC AGAGGCTCTC CTTCTCACAA ATTCCATATT CCTCTGTTTT TTAAGAAAGA AAAAGAAAGT   
  
  
+ GTGAGACAAG AACAACACAA GCAAACAATT CAGTTGACGA CGAATACTGA GTGATACCAC GCACTGTGTG   
  
  
+ CAACTGCGTT TCCATTTGTG GAGTCCTTCA AAGCACGCTC ATCTTCAACC CTTCCTTCGC CACGGAATTT   
  
  
+ CAGGGGGAAA AAAACCCCCA CCTAATTTTG AGAGAGAGAG AGAACAGAGA GAACGGGCGA TACAGGGATA   
  
  
+ CAAGGTTTGA GCAATTTTAG AAAATTTCTT GGGGCCGTTG ATGGGTTTTG AGTGAATTGC AAATCCCAGA   
  
  
+ AAGATTTCTG CGGTTTTTTA TCTACGGCTC TCTCTGTGGG AATTTTTTGG TAGATTCCTC TGGTTCCCAC   
  
  
+ TTTCATATCT TCTTATTTGT TCTACCCTCT TTACATCTGA TAGTTTGTTC TAACTGCTGC TTCAGTTGGT   
  
  
+ GCTTGTTCTC TTTAGTACTT TTCTGTTTTG TTCGTTTTGT TCATGCACTT GAATTCAACA AGAGCCCTTG   
  
  
+ TCCATTTTGA GCTCTTAATT CCCATCTAAC CCTGTTTCCT TCAGAAATTG CTGTTCTGTT CATATAGTTA   
  
  
+ TATTTTTTAT GCTTTGATTT GGGTATAAGT TTGCTGTTGG TGATTCCAAA GCTTGGTACT TTTGGCCATA   
  
  
+ TTTTGTTTGA GTTGGTGTTG ATTGGTACAA ATCTTGTAAT TGGTGTGATT GTTAATGGGA CCAATGCTTC   
  
  
+ AAGATGATGG TTCATCAGTA ACTTCTTCAT CACCTCTTCA ATTTTTCTCC ATGATGTCAC CCAATTTGGG   
  
  
+ TTCCTCCTAC CCTTGGCTAA GGGACTTAAA GCCTGAAGAG AGAGGTCTTT ACTTGATACA TTTGTTGCTC   
  
  
+ ACTTGTGCAA ACCATGTCTC TAATGGTAGC CTTGACAATG CAAACCTAGC CCTTGAGCAA ATCTCCCAGC   
  
  
+ TTGCAGCCCC TGATGGCGAT ACAATCCAGC GTATTGTTGC CTACTTTGCT GAATCACTTG CTGAAAGGAT   
  
  
+ CCTTAAGTCA TGGCCTGGCC TATATAAAGC CCTTCATTTC AATAGAATGC CTGTTATTTC AGAAGAATTT   
  
  
+ CTTGCTAGGA AGCTGTTTTT TGAGTTGTTT CCCTTCTTGA AGCTGGCCTT TTTGGTGACT AACCAATCAA   
  
  
+ TAATCGAGGC CATGGAGGGG GAAAAGATGG TGCATATAAT TGATCTGAAT GCGTCAGAAC CTGCACAGTG   
  
  
+ GATTGCCCTT ATTCAAGACT TGAGTGCTCG GCCTGAGGGC CCTCCTCATT TGAGGATTAC CGGGGTTCAT   
  
  
+ CAACAGAAAG AGGTTTTAGA ACAAGTAGCT CATAGATTGA CTGAAGAAGC TGAGAAGTTG GATTTGCCAT   
  
  
+ TTCAGTTCAA TCCTGTGGTT TGCAAACTAG AGAATCTCGA CATCGGAAAA CTCCGTGTTA AGACCGGGGA   
  
  
+ GGCCTTGGCT ATTACCTCGG TCCTTCAACT GCATACCCTT TTGGCTTCTG AAGAGGAAGT CCTTAAGAAA   
  
  
+ AGTTCACCCT TGGCATTGGT AAAGCAAGCC AATGGGGCTA ATTTACAGGG CTTGTTCAAT AAAGATGGAG   
  
  
+ CTAATAATAG GCGTAGCCCA AGTAATGATT CGGCTTCATC TGCACCTTCA TCCCTCAACA CTTCAGCCAA   
  
  
+ GATGGAAGGT TTCCTTAGCG CTTTGTGGGG TTTATCCCCA AAGATTATGG TGATAACCGA GCAAGATTCC   
  
  
+ AACCACAATG GGGCAGGACT AATGGAGAGA TTGTCAGAAG CATTGTACTT CTATGCAGCA TTGTTCGATT   
  
  
+ GCTTAGAATT TACCCTCCCG AGAACCTCCG TGGAGAGAAG GAAGGTCGAG ATGCTCCTCC TTGGCAAGGA   
  
  
+ AATCAAGAAC ATCATAGCGT GTGAGGGAGG AGAAAGAATA GATAGGCATG AGAAGTTGGG GAAGTGGATT   
  
  
+ AAGAGGCTTG AGATGGCCGG GTTTGGAAGC GTTCCTTTGA GCCACATAGG CATGATCCAA GCAAGGCGGT   
  
  
+ TGTTGCAGAG CTATGGCTGT GATGGTTATA GAATAAAGGA GGAGAACGGA TGTTTTGTTA TCTGCTGGCA   
  
  
+ AGATCGCCCC CTCTTTTCAG TATCTGCTTG GAGATGTAGG AGGTG  

- -Up\_Stream \_Len000AGATGG AAATGGACAC AATTTTTTTT TTTAAAACAA AGGTAATGAT AAGAGAACCG   
  
  
- TGATAACCAC GACTAAAAAG AAGTGGTTTA GTTGGAGGAA TATAAACGGT TAAAATGACA GGAGAAAGTA   
  
  
- GATATTTCAG TTGAGAGGAT CATGTTAAAC AGTGTATCTT TTAGAGATCC GTGAGTAGAT TTGAGAGACA   
  
  
- AGACCCATTA ACTAGCTGCT AGATGTGGTA ATAAGCAAGA TCACGTGGCA AACCTGTCGG TTAGTACATG   
  
  
- GAAACCTCTA GTTGACGAGT ACTCAAGAAC CACACTTTAA CCCCTTCATT TAAGCTGAAA TCCCGTGACC   
  
  
- AGATAGTGCG GTACTAAAAT GGTAGATAAA AGTATATATA GGTTATTCAA ACTAGAAAGC GATAATTAGT   
  
  
- GTTACTATAT TTGTTCACCA CTTCTCGTTA TTACTTGGTA TGTAAAATTT GAATTCGCAA TTCCTTCTGT   
  
  
- ACTTCAATTT TTGTTACCAT TCTCGATACG CATACAAACC GTATATCGAA AAAATTCTCA CAAAACCGAT   
  
  
- ATCAGCCTCA AAAAATTAAT TCTAATCGAC AAACTAGTTT ATTTTTTCGA TTAAACTCAC AAACCGCTCT   
  
  
- TCTGAAAAAT ATTCTTGAAA AAAACCAGAT TTTTCAATTA AATTTTTCCG ATTAAGATAC TCGAAAAGCC   
  
  
- TTCTCAAAAA ATTTATTAAT TGAAAAACAG AGTATTCGGT ATTGAAATAG TCTTTGTCAA TTAAAATGTT   
  
  
- TTATTAAAGA GTTGTTTGTT GATTAAATTT ATTAATTATT TTTATTGATT GAGTTTATAG ATTATTGATT   
  
  
- ATTGATTATA TTTATTAATT GTCAATTGTC AACAAATGCT TTGTCTTAAA TATATTTTTC GCCATTGTCT   
  
  
- ATTTTCTATT AGTACGCTCT AATTTTCAGT GGTCACTGTG TGTTCATGAA TTTATTTATC AGATTTGTTA   
  
  
- CTATAAAAAA CGCTCGAAGG TACGTTTATG GCTGTGAAAA TAACGGAGTC TTCCTTTGTT TTTGACTCAC   
  
  
- TTCCCCCCCC CCCCAAGACA AACAGAAACA ATCCCGTGCA AACCTAAGTT CACATCTAAC CCTTATTTAA   
  
  
- GCTCCTGAAA AAGAAACGGG AAAAAGGGAC TAAGGGTTTG GGTGTAGTTC AGTTCTGCTT TGGTCGGTAA   
  
  
- CTTTTTTCTC CCACAAACAC ACTCTATTTC TGTCTTTAAA ATACGGTCTC TCTCTTCTAT TGTTTACGTT   
  
  
- GTTGCAGCTG TCTCCGAGAG GAAGAGTGTT TAAGGTATAA GGAGACAAAA AATTCTTTCT TTTTCTTTCA   
  
  
- CACTCTGTTC TTGTTGTGTT CGTTTGTTAA GTCAACTGCT GCTTATGACT CACTATGGTG CGTGACACAC   
  
  
- GTTGACGCAA AGGTAAACAC CTCAGGAAGT TTCGTGCGAG TAGAAGTTGG GAAGGAAGCG GTGCCTTAAA   
  
  
- GTCCCCCTTT TTTTGGGGGT GGATTAAAAC TCTCTCTCTC TCTTGTCTCT CTTGCCCGCT ATGTCCCTAT   
  
  
- GTTCCAAACT CGTTAAAATC TTTTAAAGAA CCCCGGCAAC TACCCAAAAC TCACTTAACG TTTAGGGTCT   
  
  
- TTCTAAAGAC GCCAAAAAAT AGATGCCGAG AGAGACACCC TTAAAAAACC ATCTAAGGAG ACCAAGGGTG   
  
  
- AAAGTATAGA AGAATAAACA AGATGGGAGA AATGTAGACT ATCAAACAAG ATTGACGACG AAGTCAACCA   
  
  
- CGAACAAGAG AAATCATGAA AAGACAAAAC AAGCAAAACA AGTACGTGAA CTTAAGTTGT TCTCGGGAAC   
  
  
- AGGTAAAACT CGAGAATTAA GGGTAGATTG GGACAAAGGA AGTCTTTAAC GACAAGACAA GTATATCAAT   
  
  
- ATAAAAAATA CGAAACTAAA CCCATATTCA AACGACAACC ACTAAGGTTT CGAACCATGA AAACCGGTAT   
  
  
- AAAACAAACT CAACCACAAC TAACCATGTT TAGAACATTA ACCACACTAA CAATTACCCT GGTTACGAAG   
  
  
- TTCTACTACC AAGTAGTCAT TGAAGAAGTA GTGGAGAAGT TAAAAAGAGG TACTACAGTG GGTTAAACCC   
  
  
- AAGGAGGATG GGAACCGATT CCCTGAATTT CGGACTTCTC TCTCCAGAAA TGAACTATGT AAACAACGAG   
  
  
- TGAACACGTT TGGTACAGAG ATTACCATCG GAACTGTTAC GTTTGGATCG GGAACTCGTT TAGAGGGTCG   
  
  
- AACGTCGGGG ACTACCGCTA TGTTAGGTCG CATAACAACG GATGAAACGA CTTAGTGAAC GACTTTCCTA   
  
  
- GGAATTCAGT ACCGGACCGG ATATATTTCG GGAAGTAAAG TTATCTTACG GACAATAAAG TCTTCTTAAA   
  
  
- GAACGATCCT TCGACAAAAA ACTCAACAAA GGGAAGAACT TCGACCGGAA AAACCACTGA TTGGTTAGTT   
  
  
- ATTAGCTCCG GTACCTCCCC CTTTTCTACC ACGTATATTA ACTAGACTTA CGCAGTCTTG GACGTGTCAC   
  
  
- CTAACGGGAA TAAGTTCTGA ACTCACGAGC CGGACTCCCG GGAGGAGTAA ACTCCTAATG GCCCCAAGTA   
  
  
- GTTGTCTTTC TCCAAAATCT TGTTCATCGA GTATCTAACT GACTTCTTCG ACTCTTCAAC CTAAACGGTA   
  
  
- AAGTCAAGTT AGGACACCAA ACGTTTGATC TCTTAGAGCT GTAGCCTTTT GAGGCACAAT TCTGGCCCCT   
  
  
- CCGGAACCGA TAATGGAGCC AGGAAGTTGA CGTATGGGAA AACCGAAGAC TTCTCCTTCA GGAATTCTTT   
  
  
- TCAAGTGGGA ACCGTAACCA TTTCGTTCGG TTACCCCGAT TAAATGTCCC GAACAAGTTA TTTCTACCTC   
  
  
- GATTATTATC CGCATCGGGT TCATTACTAA GCCGAAGTAG ACGTGGAAGT AGGGAGTTGT GAAGTCGGTT   
  
  
- CTACCTTCCA AAGGAATCGC GAAACACCCC AAATAGGGGT TTCTAATACC ACTATTGGCT CGTTCTAAGG   
  
  
- TTGGTGTTAC CCCGTCCTGA TTACCTCTCT AACAGTCTTC GTAACATGAA GATACGTCGT AACAAGCTAA   
  
  
- CGAATCTTAA ATGGGAGGGC TCTTGGAGGC ACCTCTCTTC CTTCCAGCTC TACGAGGAGG AACCGTTCCT   
  
  
- TTAGTTCTTG TAGTATCGCA CACTCCCTCC TCTTTCTTAT CTATCCGTAC TCTTCAACCC CTTCACCTAA   
  
  
- TTCTCCGAAC TCTACCGGCC CAAACCTTCG CAAGGAAACT CGGTGTATCC GTACTAGGTT CGTTCCGCCA   
  
  
- ACAACGTCTC GATACCGACA CTACCAATAT CTTATTTCCT CCTCTTGCCT ACAAAACAAT AGACGACCGT   
  
  
- TCTAGCGGGG GAGAAAAGTC ATAGACGAAC CTCTACATCC TCCAC

+     CAAT-box

| Site Name | Organism | Position | Strand | Matrix score. | sequence | function |
| --- | --- | --- | --- | --- | --- | --- |
| CAAT-box | Pisum sativum | 2656 | - | 5 | CAAAT | common cis-acting element in promoter and enhancer regions |
| CAAT-box | Nicotiana glutinosa | 2630 | - | 4 | CAAT |  |
| CAAT-box | Pisum sativum | 1911 | - | 5 | CAAAT | common cis-acting element in promoter and enhancer regions |
| CAAT-box | Pisum sativum | 1699 | - | 5 | CAAAT | common cis-acting element in promoter and enhancer regions |
| CAAT-box | Arabidopsis thaliana | 2819 | - | 5 | CCAAT | common cis-acting element in promoter and enhancer regions |
| CAAT-box | Nicotiana glutinosa | 2266 | + | 4 | CAAT |  |
| CAAT-box | Nicotiana glutinosa | 2672 | + | 4 | CAAT |  |
| CAAT-box | Pisum sativum | 2232 | + | 5 | CAAAT | common cis-acting element in promoter and enhancer regions |
| CAAT-box | Nicotiana glutinosa | 1871 | - | 4 | CAAT |  |
| CAAT-box | Pisum sativum | 1604 | + | 5 | CAAAT | common cis-acting element in promoter and enhancer regions |
| CAAT-box | Nicotiana glutinosa | 274 | + | 4 | CAAT |  |
| CAAT-box | Nicotiana glutinosa | 3056 | - | 4 | CAAT |  |
| CAAT-box | Arabidopsis thaliana | 2095 | + | 5 | CCAAT | common cis-acting element in promoter and enhancer regions |
| CAAT-box | Nicotiana glutinosa | 1025 | - | 4 | CAAT |  |
| CAAT-box | Pisum sativum | 170 | - | 5 | CAAAT | common cis-acting element in promoter and enhancer regions |
| CAAT-box | Nicotiana glutinosa | 223 | - | 4 | CAAT |  |
| CAAT-box | Nicotiana glutinosa | 2452 | + | 4 | CAAT |  |
| CAAT-box | Nicotiana glutinosa | 3044 | - | 4 | CAAT |  |
| CAAT-box | Nicotiana glutinosa | 2493 | - | 4 | CAAT |  |
| CAAT-box | Arabidopsis thaliana | 2025 | + | 5 | CCAAT | common cis-acting element in promoter and enhancer regions |
| CAAT-box | Nicotiana glutinosa | 2012 | - | 4 | CAAT |  |
| CAAT-box | Pisum sativum | 1992 | + | 5 | CAAAT | common cis-acting element in promoter and enhancer regions |
| CAAT-box | Nicotiana glutinosa | 507 | + | 4 | CAAT |  |
| CAAT-box | Nicotiana glutinosa | 396 | + | 4 | CAAT |  |
| CAAT-box | Arabidopsis thaliana | 2833 | + | 5 | CCAAT | common cis-acting element in promoter and enhancer regions |
| CAAT-box | Nicotiana glutinosa | 1192 | - | 4 | CAAT |  |
| CAAT-box | Nicotiana glutinosa | 1556 | + | 4 | CAAT |  |
| CAAT-box | Pisum sativum | 1418 | - | 5 | CAAAT | common cis-acting element in promoter and enhancer regions |
| CAAT-box | Arabidopsis thaliana | 2447 | + | 5 | CCAAT | common cis-acting element in promoter and enhancer regions |
| CAAT-box | Nicotiana glutinosa | 123 | + | 4 | CAAT |  |
| CAAT-box | Nicotiana glutinosa | 168 | + | 4 | CAAT |  |
| CAAT-box | Nicotiana glutinosa | 2354 | + | 4 | CAAT |  |
| CAAT-box | Arabidopsis thaliana | 1111 | - | 5 | CCAAT | common cis-acting element in promoter and enhancer regions |
| CAAT-box | Nicotiana glutinosa | 2526 | - | 4 | CAAT |  |
| CAAT-box | Nicotiana glutinosa | 1360 | + | 4 | CAAT |  |
| CAAT-box | Nicotiana glutinosa | 2277 | - | 4 | CAAT |  |
| CAAT-box | Nicotiana glutinosa | 425 | + | 4 | CAAT |  |
| CAAT-box | Nicotiana glutinosa | 981 | + | 4 | CAAT |  |
| CAAT-box | Pisum sativum | 2098 | - | 5 | CAAAT | common cis-acting element in promoter and enhancer regions |
| CAAT-box | Arabidopsis thaliana | 322 | - | 5 | CCAAT | common cis-acting element in promoter and enhancer regions |
| CAAT-box | Nicotiana glutinosa | 3082 | - | 4 | CAAT |  |
| CAAT-box | Pisum sativum | 2572 | - | 5 | CAAAT | common cis-acting element in promoter and enhancer regions |
| CAAT-box | Arabidopsis thaliana | 1985 | - | 5 | CCAAT | common cis-acting element in promoter and enhancer regions |
| CAAT-box | Nicotiana glutinosa | 2073 | + | 4 | CAAT |  |
| CAAT-box | Nicotiana glutinosa | 3074 | - | 4 | CAAT |  |
| CAAT-box | Nicotiana glutinosa | 2448 | + | 4 | CAAT |  |
| CAAT-box | Nicotiana glutinosa | 3020 | + | 4 | CAAT |  |
| CAAT-box | Nicotiana glutinosa | 2096 | + | 4 | CAAT |  |
| CAAT-box | Pisum sativum | 616 | - | 5 | CAAAT | common cis-acting element in promoter and enhancer regions |
| CAAT-box | Arabidopsis thaliana | 320 | - | 8 | CCCAATTT | common cis-acting element in promoter and enhancer regions |
| CAAT-box | Nicotiana glutinosa | 2834 | + | 4 | CAAT |  |
| CAAT-box | Nicotiana glutinosa | 2861 | + | 4 | CAAT |  |
| CAAT-box | Pisum sativum | 2164 | - | 5 | CAAAT | common cis-acting element in promoter and enhancer regions |
| CAAT-box | Nicotiana glutinosa | 1600 | - | 4 | CAAT |  |
| CAAT-box | Nicotiana glutinosa | 451 | + | 4 | CAAT |  |
| CAAT-box | Pisum sativum | 100 | + | 5 | CAAAT | common cis-acting element in promoter and enhancer regions |
| CAAT-box | Nicotiana glutinosa | 2210 | + | 4 | CAAT |  |
| CAAT-box | Pisum sativum | 1008 | + | 5 | CAAAT | common cis-acting element in promoter and enhancer regions |
| CAAT-box | Pisum sativum | 1256 | + | 5 | CAAAT | common cis-acting element in promoter and enhancer regions |
| CAAT-box | Pisum sativum | 827 | + | 5 | CAAAT | common cis-acting element in promoter and enhancer regions |
| CAAT-box | Pisum sativum | 1292 | + | 5 | CAAAT | common cis-acting element in promoter and enhancer regions |
| CAAT-box | Arabidopsis thaliana | 2094 | + | 8 | CCCAATTT | common cis-acting element in promoter and enhancer regions |
| CAAT-box | Arabidopsis thaliana | 273 | + | 5 | CCAAT | common cis-acting element in promoter and enhancer regions |
| CAAT-box | Arabidopsis thaliana | 2003 | - | 5 | CCAAT | common cis-acting element in promoter and enhancer regions |
| CAAT-box | Arabidopsis thaliana | 395 | + | 5 | CCAAT | common cis-acting element in promoter and enhancer regions |
| CAAT-box | Nicotiana glutinosa | 2026 | + | 4 | CAAT |  |
| CAAT-box | Pisum sativum | 601 | + | 5 | CAAAT | common cis-acting element in promoter and enhancer regions |
| CAAT-box | Pisum sativum | 117 | - | 5 | CAAAT | common cis-acting element in promoter and enhancer regions |
| CAAT-box | Arabidopsis thaliana | 122 | + | 5 | CCAAT | common cis-acting element in promoter and enhancer regions |
| CAAT-box | Arabidopsis thaliana | 78 | - | 5 | CCAAT | common cis-acting element in promoter and enhancer regions |

>HU08G01232.1   
+ -Up\_Stream \_Len000TCTACC TTTACCTGTG TTAAAAAAAA AAATTTTGTT TCCATTACTA TTCTCTTGGC   
  
  
+ ACTATTGGTG CTGATTTTTC TTCACCAAAT CAACCTCCTT ATATTTGCCA ATTTTACTGT CCTCTTTCAT   
  
  
+ CTATAAAGTC AACTCTCCTA GTACAATTTG TCACATAGAA AATCTCTAGG CACTCATCTA AACTCTCTGT   
  
  
+ TCTGGGTAAT TGATCGACGA TCTACACCAT TATTCGTTCT AGTGCACCGT TTGGACAGCC AATCATGTAC   
  
  
+ CTTTGGAGAT CAACTGCTCA TGAGTTCTTG GTGTGAAATT GGGGAAGTAA ATTCGACTTT AGGGCACTGG   
  
  
+ TCTATCACGC CATGATTTTA CCATCTATTT TCATATATAT CCAATAAGTT TGATCTTTCG CTATTAATCA   
  
  
+ CAATGATATA AACAAGTGGT GAAGAGCAAT AATGAACCAT ACATTTTAAA CTTAAGCGTT AAGGAAGACA   
  
  
+ TGAAGTTAAA AACAATGGTA AGAGCTATGC GTATGTTTGG CATATAGCTT TTTTAAGAGT GTTTTGGCTA   
  
  
+ TAGTCGGAGT TTTTTAATTA AGATTAGCTG TTTGATCAAA TAAAAAAGCT AATTTGAGTG TTTGGCGAGA   
  
  
+ AGACTTTTTA TAAGAACTTT TTTTGGTCTA AAAAGTTAAT TTAAAAAGGC TAATTCTATG AGCTTTTCGG   
  
  
+ AAGAGTTTTT TAAATAATTA ACTTTTTGTC TCATAAGCCA TAACTTTATC AGAAACAGTT AATTTTACAA   
  
  
+ AATAATTTCT CAACAAACAA CTAATTTAAA TAATTAATAA AAATAACTAA CTCAAATATC TAATAACTAA   
  
  
+ TAACTAATAT AAATAATTAA CAGTTAACAG TTGTTTACGA AACAGAATTT ATATAAAAAG CGGTAACAGA   
  
  
+ TAAAAGATAA TCATGCGAGA TTAAAAGTCA CCAGTGACAC ACAAGTACTT AAATAAATAG TCTAAACAAT   
  
  
+ GATATTTTTT GCGAGCTTCC ATGCAAATAC CGACACTTTT ATTGCCTCAG AAGGAAACAA AAACTGAGTG   
  
  
+ AAGGGGGGGG GGGGTTCTGT TTGTCTTTGT TAGGGCACGT TTGGATTCAA GTGTAGATTG GGAATAAATT   
  
  
+ CGAGGACTTT TTCTTTGCCC TTTTTCCCTG ATTCCCAAAC CCACATCAAG TCAAGACGAA ACCAGCCATT   
  
  
+ GAAAAAAGAG GGTGTTTGTG TGAGATAAAG ACAGAAATTT TATGCCAGAG AGAGAAGATA ACAAATGCAA   
  
  
+ CAACGTCGAC AGAGGCTCTC CTTCTCACAA ATTCCATATT CCTCTGTTTT TTAAGAAAGA AAAAGAAAGT   
  
  
+ GTGAGACAAG AACAACACAA GCAAACAATT CAGTTGACGA CGAATACTGA GTGATACCAC GCACTGTGTG   
  
  
+ CAACTGCGTT TCCATTTGTG GAGTCCTTCA AAGCACGCTC ATCTTCAACC CTTCCTTCGC CACGGAATTT   
  
  
+ CAGGGGGAAA AAAACCCCCA CCTAATTTTG AGAGAGAGAG AGAACAGAGA GAACGGGCGA TACAGGGATA   
  
  
+ CAAGGTTTGA GCAATTTTAG AAAATTTCTT GGGGCCGTTG ATGGGTTTTG AGTGAATTGC AAATCCCAGA   
  
  
+ AAGATTTCTG CGGTTTTTTA TCTACGGCTC TCTCTGTGGG AATTTTTTGG TAGATTCCTC TGGTTCCCAC   
  
  
+ TTTCATATCT TCTTATTTGT TCTACCCTCT TTACATCTGA TAGTTTGTTC TAACTGCTGC TTCAGTTGGT   
  
  
+ GCTTGTTCTC TTTAGTACTT TTCTGTTTTG TTCGTTTTGT TCATGCACTT GAATTCAACA AGAGCCCTTG   
  
  
+ TCCATTTTGA GCTCTTAATT CCCATCTAAC CCTGTTTCCT TCAGAAATTG CTGTTCTGTT CATATAGTTA   
  
  
+ TATTTTTTAT GCTTTGATTT GGGTATAAGT TTGCTGTTGG TGATTCCAAA GCTTGGTACT TTTGGCCATA   
  
  
+ TTTTGTTTGA GTTGGTGTTG ATTGGTACAA ATCTTGTAAT TGGTGTGATT GTTAATGGGA CCAATGCTTC   
  
  
+ AAGATGATGG TTCATCAGTA ACTTCTTCAT CACCTCTTCA ATTTTTCTCC ATGATGTCAC CCAATTTGGG   
  
  
+ TTCCTCCTAC CCTTGGCTAA GGGACTTAAA GCCTGAAGAG AGAGGTCTTT ACTTGATACA TTTGTTGCTC   
  
  
+ ACTTGTGCAA ACCATGTCTC TAATGGTAGC CTTGACAATG CAAACCTAGC CCTTGAGCAA ATCTCCCAGC   
  
  
+ TTGCAGCCCC TGATGGCGAT ACAATCCAGC GTATTGTTGC CTACTTTGCT GAATCACTTG CTGAAAGGAT   
  
  
+ CCTTAAGTCA TGGCCTGGCC TATATAAAGC CCTTCATTTC AATAGAATGC CTGTTATTTC AGAAGAATTT   
  
  
+ CTTGCTAGGA AGCTGTTTTT TGAGTTGTTT CCCTTCTTGA AGCTGGCCTT TTTGGTGACT AACCAATCAA   
  
  
+ TAATCGAGGC CATGGAGGGG GAAAAGATGG TGCATATAAT TGATCTGAAT GCGTCAGAAC CTGCACAGTG   
  
  
+ GATTGCCCTT ATTCAAGACT TGAGTGCTCG GCCTGAGGGC CCTCCTCATT TGAGGATTAC CGGGGTTCAT   
  
  
+ CAACAGAAAG AGGTTTTAGA ACAAGTAGCT CATAGATTGA CTGAAGAAGC TGAGAAGTTG GATTTGCCAT   
  
  
+ TTCAGTTCAA TCCTGTGGTT TGCAAACTAG AGAATCTCGA CATCGGAAAA CTCCGTGTTA AGACCGGGGA   
  
  
+ GGCCTTGGCT ATTACCTCGG TCCTTCAACT GCATACCCTT TTGGCTTCTG AAGAGGAAGT CCTTAAGAAA   
  
  
+ AGTTCACCCT TGGCATTGGT AAAGCAAGCC AATGGGGCTA ATTTACAGGG CTTGTTCAAT AAAGATGGAG   
  
  
+ CTAATAATAG GCGTAGCCCA AGTAATGATT CGGCTTCATC TGCACCTTCA TCCCTCAACA CTTCAGCCAA   
  
  
+ GATGGAAGGT TTCCTTAGCG CTTTGTGGGG TTTATCCCCA AAGATTATGG TGATAACCGA GCAAGATTCC   
  
  
+ AACCACAATG GGGCAGGACT AATGGAGAGA TTGTCAGAAG CATTGTACTT CTATGCAGCA TTGTTCGATT   
  
  
+ GCTTAGAATT TACCCTCCCG AGAACCTCCG TGGAGAGAAG GAAGGTCGAG ATGCTCCTCC TTGGCAAGGA   
  
  
+ AATCAAGAAC ATCATAGCGT GTGAGGGAGG AGAAAGAATA GATAGGCATG AGAAGTTGGG GAAGTGGATT   
  
  
+ AAGAGGCTTG AGATGGCCGG GTTTGGAAGC GTTCCTTTGA GCCACATAGG CATGATCCAA GCAAGGCGGT   
  
  
+ TGTTGCAGAG CTATGGCTGT GATGGTTATA GAATAAAGGA GGAGAACGGA TGTTTTGTTA TCTGCTGGCA   
  
  
+ AGATCGCCCC CTCTTTTCAG TATCTGCTTG GAGATGTAGG AGGTG  

- -Up\_Stream \_Len000AGATGG AAATGGACAC AATTTTTTTT TTTAAAACAA AGGTAATGAT AAGAGAACCG   
  
  
- TGATAACCAC GACTAAAAAG AAGTGGTTTA GTTGGAGGAA TATAAACGGT TAAAATGACA GGAGAAAGTA   
  
  
- GATATTTCAG TTGAGAGGAT CATGTTAAAC AGTGTATCTT TTAGAGATCC GTGAGTAGAT TTGAGAGACA   
  
  
- AGACCCATTA ACTAGCTGCT AGATGTGGTA ATAAGCAAGA TCACGTGGCA AACCTGTCGG TTAGTACATG   
  
  
- GAAACCTCTA GTTGACGAGT ACTCAAGAAC CACACTTTAA CCCCTTCATT TAAGCTGAAA TCCCGTGACC   
  
  
- AGATAGTGCG GTACTAAAAT GGTAGATAAA AGTATATATA GGTTATTCAA ACTAGAAAGC GATAATTAGT   
  
  
- GTTACTATAT TTGTTCACCA CTTCTCGTTA TTACTTGGTA TGTAAAATTT GAATTCGCAA TTCCTTCTGT   
  
  
- ACTTCAATTT TTGTTACCAT TCTCGATACG CATACAAACC GTATATCGAA AAAATTCTCA CAAAACCGAT   
  
  
- ATCAGCCTCA AAAAATTAAT TCTAATCGAC AAACTAGTTT ATTTTTTCGA TTAAACTCAC AAACCGCTCT   
  
  
- TCTGAAAAAT ATTCTTGAAA AAAACCAGAT TTTTCAATTA AATTTTTCCG ATTAAGATAC TCGAAAAGCC   
  
  
- TTCTCAAAAA ATTTATTAAT TGAAAAACAG AGTATTCGGT ATTGAAATAG TCTTTGTCAA TTAAAATGTT   
  
  
- TTATTAAAGA GTTGTTTGTT GATTAAATTT ATTAATTATT TTTATTGATT GAGTTTATAG ATTATTGATT   
  
  
- ATTGATTATA TTTATTAATT GTCAATTGTC AACAAATGCT TTGTCTTAAA TATATTTTTC GCCATTGTCT   
  
  
- ATTTTCTATT AGTACGCTCT AATTTTCAGT GGTCACTGTG TGTTCATGAA TTTATTTATC AGATTTGTTA   
  
  
- CTATAAAAAA CGCTCGAAGG TACGTTTATG GCTGTGAAAA TAACGGAGTC TTCCTTTGTT TTTGACTCAC   
  
  
- TTCCCCCCCC CCCCAAGACA AACAGAAACA ATCCCGTGCA AACCTAAGTT CACATCTAAC CCTTATTTAA   
  
  
- GCTCCTGAAA AAGAAACGGG AAAAAGGGAC TAAGGGTTTG GGTGTAGTTC AGTTCTGCTT TGGTCGGTAA   
  
  
- CTTTTTTCTC CCACAAACAC ACTCTATTTC TGTCTTTAAA ATACGGTCTC TCTCTTCTAT TGTTTACGTT   
  
  
- GTTGCAGCTG TCTCCGAGAG GAAGAGTGTT TAAGGTATAA GGAGACAAAA AATTCTTTCT TTTTCTTTCA   
  
  
- CACTCTGTTC TTGTTGTGTT CGTTTGTTAA GTCAACTGCT GCTTATGACT CACTATGGTG CGTGACACAC   
  
  
- GTTGACGCAA AGGTAAACAC CTCAGGAAGT TTCGTGCGAG TAGAAGTTGG GAAGGAAGCG GTGCCTTAAA   
  
  
- GTCCCCCTTT TTTTGGGGGT GGATTAAAAC TCTCTCTCTC TCTTGTCTCT CTTGCCCGCT ATGTCCCTAT   
  
  
- GTTCCAAACT CGTTAAAATC TTTTAAAGAA CCCCGGCAAC TACCCAAAAC TCACTTAACG TTTAGGGTCT   
  
  
- TTCTAAAGAC GCCAAAAAAT AGATGCCGAG AGAGACACCC TTAAAAAACC ATCTAAGGAG ACCAAGGGTG   
  
  
- AAAGTATAGA AGAATAAACA AGATGGGAGA AATGTAGACT ATCAAACAAG ATTGACGACG AAGTCAACCA   
  
  
- CGAACAAGAG AAATCATGAA AAGACAAAAC AAGCAAAACA AGTACGTGAA CTTAAGTTGT TCTCGGGAAC   
  
  
- AGGTAAAACT CGAGAATTAA GGGTAGATTG GGACAAAGGA AGTCTTTAAC GACAAGACAA GTATATCAAT   
  
  
- ATAAAAAATA CGAAACTAAA CCCATATTCA AACGACAACC ACTAAGGTTT CGAACCATGA AAACCGGTAT   
  
  
- AAAACAAACT CAACCACAAC TAACCATGTT TAGAACATTA ACCACACTAA CAATTACCCT GGTTACGAAG   
  
  
- TTCTACTACC AAGTAGTCAT TGAAGAAGTA GTGGAGAAGT TAAAAAGAGG TACTACAGTG GGTTAAACCC   
  
  
- AAGGAGGATG GGAACCGATT CCCTGAATTT CGGACTTCTC TCTCCAGAAA TGAACTATGT AAACAACGAG   
  
  
- TGAACACGTT TGGTACAGAG ATTACCATCG GAACTGTTAC GTTTGGATCG GGAACTCGTT TAGAGGGTCG   
  
  
- AACGTCGGGG ACTACCGCTA TGTTAGGTCG CATAACAACG GATGAAACGA CTTAGTGAAC GACTTTCCTA   
  
  
- GGAATTCAGT ACCGGACCGG ATATATTTCG GGAAGTAAAG TTATCTTACG GACAATAAAG TCTTCTTAAA   
  
  
- GAACGATCCT TCGACAAAAA ACTCAACAAA GGGAAGAACT TCGACCGGAA AAACCACTGA TTGGTTAGTT   
  
  
- ATTAGCTCCG GTACCTCCCC CTTTTCTACC ACGTATATTA ACTAGACTTA CGCAGTCTTG GACGTGTCAC   
  
  
- CTAACGGGAA TAAGTTCTGA ACTCACGAGC CGGACTCCCG GGAGGAGTAA ACTCCTAATG GCCCCAAGTA   
  
  
- GTTGTCTTTC TCCAAAATCT TGTTCATCGA GTATCTAACT GACTTCTTCG ACTCTTCAAC CTAAACGGTA   
  
  
- AAGTCAAGTT AGGACACCAA ACGTTTGATC TCTTAGAGCT GTAGCCTTTT GAGGCACAAT TCTGGCCCCT   
  
  
- CCGGAACCGA TAATGGAGCC AGGAAGTTGA CGTATGGGAA AACCGAAGAC TTCTCCTTCA GGAATTCTTT   
  
  
- TCAAGTGGGA ACCGTAACCA TTTCGTTCGG TTACCCCGAT TAAATGTCCC GAACAAGTTA TTTCTACCTC   
  
  
- GATTATTATC CGCATCGGGT TCATTACTAA GCCGAAGTAG ACGTGGAAGT AGGGAGTTGT GAAGTCGGTT   
  
  
- CTACCTTCCA AAGGAATCGC GAAACACCCC AAATAGGGGT TTCTAATACC ACTATTGGCT CGTTCTAAGG   
  
  
- TTGGTGTTAC CCCGTCCTGA TTACCTCTCT AACAGTCTTC GTAACATGAA GATACGTCGT AACAAGCTAA   
  
  
- CGAATCTTAA ATGGGAGGGC TCTTGGAGGC ACCTCTCTTC CTTCCAGCTC TACGAGGAGG AACCGTTCCT   
  
  
- TTAGTTCTTG TAGTATCGCA CACTCCCTCC TCTTTCTTAT CTATCCGTAC TCTTCAACCC CTTCACCTAA   
  
  
- TTCTCCGAAC TCTACCGGCC CAAACCTTCG CAAGGAAACT CGGTGTATCC GTACTAGGTT CGTTCCGCCA   
  
  
- ACAACGTCTC GATACCGACA CTACCAATAT CTTATTTCCT CCTCTTGCCT ACAAAACAAT AGACGACCGT   
  
  
- TCTAGCGGGG GAGAAAAGTC ATAGACGAAC CTCTACATCC TCCAC

+     CCAAT-box

| Site Name | Organism | Position | Strand | Matrix score. | sequence | function |
| --- | --- | --- | --- | --- | --- | --- |
| CCAAT-box | Hordeum vulgare | 1579 | - | 6 | CAACGG | MYBHv1 binding site |

>HU08G01232.1   
+ -Up\_Stream \_Len000TCTACC TTTACCTGTG TTAAAAAAAA AAATTTTGTT TCCATTACTA TTCTCTTGGC   
  
  
+ ACTATTGGTG CTGATTTTTC TTCACCAAAT CAACCTCCTT ATATTTGCCA ATTTTACTGT CCTCTTTCAT   
  
  
+ CTATAAAGTC AACTCTCCTA GTACAATTTG TCACATAGAA AATCTCTAGG CACTCATCTA AACTCTCTGT   
  
  
+ TCTGGGTAAT TGATCGACGA TCTACACCAT TATTCGTTCT AGTGCACCGT TTGGACAGCC AATCATGTAC   
  
  
+ CTTTGGAGAT CAACTGCTCA TGAGTTCTTG GTGTGAAATT GGGGAAGTAA ATTCGACTTT AGGGCACTGG   
  
  
+ TCTATCACGC CATGATTTTA CCATCTATTT TCATATATAT CCAATAAGTT TGATCTTTCG CTATTAATCA   
  
  
+ CAATGATATA AACAAGTGGT GAAGAGCAAT AATGAACCAT ACATTTTAAA CTTAAGCGTT AAGGAAGACA   
  
  
+ TGAAGTTAAA AACAATGGTA AGAGCTATGC GTATGTTTGG CATATAGCTT TTTTAAGAGT GTTTTGGCTA   
  
  
+ TAGTCGGAGT TTTTTAATTA AGATTAGCTG TTTGATCAAA TAAAAAAGCT AATTTGAGTG TTTGGCGAGA   
  
  
+ AGACTTTTTA TAAGAACTTT TTTTGGTCTA AAAAGTTAAT TTAAAAAGGC TAATTCTATG AGCTTTTCGG   
  
  
+ AAGAGTTTTT TAAATAATTA ACTTTTTGTC TCATAAGCCA TAACTTTATC AGAAACAGTT AATTTTACAA   
  
  
+ AATAATTTCT CAACAAACAA CTAATTTAAA TAATTAATAA AAATAACTAA CTCAAATATC TAATAACTAA   
  
  
+ TAACTAATAT AAATAATTAA CAGTTAACAG TTGTTTACGA AACAGAATTT ATATAAAAAG CGGTAACAGA   
  
  
+ TAAAAGATAA TCATGCGAGA TTAAAAGTCA CCAGTGACAC ACAAGTACTT AAATAAATAG TCTAAACAAT   
  
  
+ GATATTTTTT GCGAGCTTCC ATGCAAATAC CGACACTTTT ATTGCCTCAG AAGGAAACAA AAACTGAGTG   
  
  
+ AAGGGGGGGG GGGGTTCTGT TTGTCTTTGT TAGGGCACGT TTGGATTCAA GTGTAGATTG GGAATAAATT   
  
  
+ CGAGGACTTT TTCTTTGCCC TTTTTCCCTG ATTCCCAAAC CCACATCAAG TCAAGACGAA ACCAGCCATT   
  
  
+ GAAAAAAGAG GGTGTTTGTG TGAGATAAAG ACAGAAATTT TATGCCAGAG AGAGAAGATA ACAAATGCAA   
  
  
+ CAACGTCGAC AGAGGCTCTC CTTCTCACAA ATTCCATATT CCTCTGTTTT TTAAGAAAGA AAAAGAAAGT   
  
  
+ GTGAGACAAG AACAACACAA GCAAACAATT CAGTTGACGA CGAATACTGA GTGATACCAC GCACTGTGTG   
  
  
+ CAACTGCGTT TCCATTTGTG GAGTCCTTCA AAGCACGCTC ATCTTCAACC CTTCCTTCGC CACGGAATTT   
  
  
+ CAGGGGGAAA AAAACCCCCA CCTAATTTTG AGAGAGAGAG AGAACAGAGA GAACGGGCGA TACAGGGATA   
  
  
+ CAAGGTTTGA GCAATTTTAG AAAATTTCTT GGGGCCGTTG ATGGGTTTTG AGTGAATTGC AAATCCCAGA   
  
  
+ AAGATTTCTG CGGTTTTTTA TCTACGGCTC TCTCTGTGGG AATTTTTTGG TAGATTCCTC TGGTTCCCAC   
  
  
+ TTTCATATCT TCTTATTTGT TCTACCCTCT TTACATCTGA TAGTTTGTTC TAACTGCTGC TTCAGTTGGT   
  
  
+ GCTTGTTCTC TTTAGTACTT TTCTGTTTTG TTCGTTTTGT TCATGCACTT GAATTCAACA AGAGCCCTTG   
  
  
+ TCCATTTTGA GCTCTTAATT CCCATCTAAC CCTGTTTCCT TCAGAAATTG CTGTTCTGTT CATATAGTTA   
  
  
+ TATTTTTTAT GCTTTGATTT GGGTATAAGT TTGCTGTTGG TGATTCCAAA GCTTGGTACT TTTGGCCATA   
  
  
+ TTTTGTTTGA GTTGGTGTTG ATTGGTACAA ATCTTGTAAT TGGTGTGATT GTTAATGGGA CCAATGCTTC   
  
  
+ AAGATGATGG TTCATCAGTA ACTTCTTCAT CACCTCTTCA ATTTTTCTCC ATGATGTCAC CCAATTTGGG   
  
  
+ TTCCTCCTAC CCTTGGCTAA GGGACTTAAA GCCTGAAGAG AGAGGTCTTT ACTTGATACA TTTGTTGCTC   
  
  
+ ACTTGTGCAA ACCATGTCTC TAATGGTAGC CTTGACAATG CAAACCTAGC CCTTGAGCAA ATCTCCCAGC   
  
  
+ TTGCAGCCCC TGATGGCGAT ACAATCCAGC GTATTGTTGC CTACTTTGCT GAATCACTTG CTGAAAGGAT   
  
  
+ CCTTAAGTCA TGGCCTGGCC TATATAAAGC CCTTCATTTC AATAGAATGC CTGTTATTTC AGAAGAATTT   
  
  
+ CTTGCTAGGA AGCTGTTTTT TGAGTTGTTT CCCTTCTTGA AGCTGGCCTT TTTGGTGACT AACCAATCAA   
  
  
+ TAATCGAGGC CATGGAGGGG GAAAAGATGG TGCATATAAT TGATCTGAAT GCGTCAGAAC CTGCACAGTG   
  
  
+ GATTGCCCTT ATTCAAGACT TGAGTGCTCG GCCTGAGGGC CCTCCTCATT TGAGGATTAC CGGGGTTCAT   
  
  
+ CAACAGAAAG AGGTTTTAGA ACAAGTAGCT CATAGATTGA CTGAAGAAGC TGAGAAGTTG GATTTGCCAT   
  
  
+ TTCAGTTCAA TCCTGTGGTT TGCAAACTAG AGAATCTCGA CATCGGAAAA CTCCGTGTTA AGACCGGGGA   
  
  
+ GGCCTTGGCT ATTACCTCGG TCCTTCAACT GCATACCCTT TTGGCTTCTG AAGAGGAAGT CCTTAAGAAA   
  
  
+ AGTTCACCCT TGGCATTGGT AAAGCAAGCC AATGGGGCTA ATTTACAGGG CTTGTTCAAT AAAGATGGAG   
  
  
+ CTAATAATAG GCGTAGCCCA AGTAATGATT CGGCTTCATC TGCACCTTCA TCCCTCAACA CTTCAGCCAA   
  
  
+ GATGGAAGGT TTCCTTAGCG CTTTGTGGGG TTTATCCCCA AAGATTATGG TGATAACCGA GCAAGATTCC   
  
  
+ AACCACAATG GGGCAGGACT AATGGAGAGA TTGTCAGAAG CATTGTACTT CTATGCAGCA TTGTTCGATT   
  
  
+ GCTTAGAATT TACCCTCCCG AGAACCTCCG TGGAGAGAAG GAAGGTCGAG ATGCTCCTCC TTGGCAAGGA   
  
  
+ AATCAAGAAC ATCATAGCGT GTGAGGGAGG AGAAAGAATA GATAGGCATG AGAAGTTGGG GAAGTGGATT   
  
  
+ AAGAGGCTTG AGATGGCCGG GTTTGGAAGC GTTCCTTTGA GCCACATAGG CATGATCCAA GCAAGGCGGT   
  
  
+ TGTTGCAGAG CTATGGCTGT GATGGTTATA GAATAAAGGA GGAGAACGGA TGTTTTGTTA TCTGCTGGCA   
  
  
+ AGATCGCCCC CTCTTTTCAG TATCTGCTTG GAGATGTAGG AGGTG  

- -Up\_Stream \_Len000AGATGG AAATGGACAC AATTTTTTTT TTTAAAACAA AGGTAATGAT AAGAGAACCG   
  
  
- TGATAACCAC GACTAAAAAG AAGTGGTTTA GTTGGAGGAA TATAAACGGT TAAAATGACA GGAGAAAGTA   
  
  
- GATATTTCAG TTGAGAGGAT CATGTTAAAC AGTGTATCTT TTAGAGATCC GTGAGTAGAT TTGAGAGACA   
  
  
- AGACCCATTA ACTAGCTGCT AGATGTGGTA ATAAGCAAGA TCACGTGGCA AACCTGTCGG TTAGTACATG   
  
  
- GAAACCTCTA GTTGACGAGT ACTCAAGAAC CACACTTTAA CCCCTTCATT TAAGCTGAAA TCCCGTGACC   
  
  
- AGATAGTGCG GTACTAAAAT GGTAGATAAA AGTATATATA GGTTATTCAA ACTAGAAAGC GATAATTAGT   
  
  
- GTTACTATAT TTGTTCACCA CTTCTCGTTA TTACTTGGTA TGTAAAATTT GAATTCGCAA TTCCTTCTGT   
  
  
- ACTTCAATTT TTGTTACCAT TCTCGATACG CATACAAACC GTATATCGAA AAAATTCTCA CAAAACCGAT   
  
  
- ATCAGCCTCA AAAAATTAAT TCTAATCGAC AAACTAGTTT ATTTTTTCGA TTAAACTCAC AAACCGCTCT   
  
  
- TCTGAAAAAT ATTCTTGAAA AAAACCAGAT TTTTCAATTA AATTTTTCCG ATTAAGATAC TCGAAAAGCC   
  
  
- TTCTCAAAAA ATTTATTAAT TGAAAAACAG AGTATTCGGT ATTGAAATAG TCTTTGTCAA TTAAAATGTT   
  
  
- TTATTAAAGA GTTGTTTGTT GATTAAATTT ATTAATTATT TTTATTGATT GAGTTTATAG ATTATTGATT   
  
  
- ATTGATTATA TTTATTAATT GTCAATTGTC AACAAATGCT TTGTCTTAAA TATATTTTTC GCCATTGTCT   
  
  
- ATTTTCTATT AGTACGCTCT AATTTTCAGT GGTCACTGTG TGTTCATGAA TTTATTTATC AGATTTGTTA   
  
  
- CTATAAAAAA CGCTCGAAGG TACGTTTATG GCTGTGAAAA TAACGGAGTC TTCCTTTGTT TTTGACTCAC   
  
  
- TTCCCCCCCC CCCCAAGACA AACAGAAACA ATCCCGTGCA AACCTAAGTT CACATCTAAC CCTTATTTAA   
  
  
- GCTCCTGAAA AAGAAACGGG AAAAAGGGAC TAAGGGTTTG GGTGTAGTTC AGTTCTGCTT TGGTCGGTAA   
  
  
- CTTTTTTCTC CCACAAACAC ACTCTATTTC TGTCTTTAAA ATACGGTCTC TCTCTTCTAT TGTTTACGTT   
  
  
- GTTGCAGCTG TCTCCGAGAG GAAGAGTGTT TAAGGTATAA GGAGACAAAA AATTCTTTCT TTTTCTTTCA   
  
  
- CACTCTGTTC TTGTTGTGTT CGTTTGTTAA GTCAACTGCT GCTTATGACT CACTATGGTG CGTGACACAC   
  
  
- GTTGACGCAA AGGTAAACAC CTCAGGAAGT TTCGTGCGAG TAGAAGTTGG GAAGGAAGCG GTGCCTTAAA   
  
  
- GTCCCCCTTT TTTTGGGGGT GGATTAAAAC TCTCTCTCTC TCTTGTCTCT CTTGCCCGCT ATGTCCCTAT   
  
  
- GTTCCAAACT CGTTAAAATC TTTTAAAGAA CCCCGGCAAC TACCCAAAAC TCACTTAACG TTTAGGGTCT   
  
  
- TTCTAAAGAC GCCAAAAAAT AGATGCCGAG AGAGACACCC TTAAAAAACC ATCTAAGGAG ACCAAGGGTG   
  
  
- AAAGTATAGA AGAATAAACA AGATGGGAGA AATGTAGACT ATCAAACAAG ATTGACGACG AAGTCAACCA   
  
  
- CGAACAAGAG AAATCATGAA AAGACAAAAC AAGCAAAACA AGTACGTGAA CTTAAGTTGT TCTCGGGAAC   
  
  
- AGGTAAAACT CGAGAATTAA GGGTAGATTG GGACAAAGGA AGTCTTTAAC GACAAGACAA GTATATCAAT   
  
  
- ATAAAAAATA CGAAACTAAA CCCATATTCA AACGACAACC ACTAAGGTTT CGAACCATGA AAACCGGTAT   
  
  
- AAAACAAACT CAACCACAAC TAACCATGTT TAGAACATTA ACCACACTAA CAATTACCCT GGTTACGAAG   
  
  
- TTCTACTACC AAGTAGTCAT TGAAGAAGTA GTGGAGAAGT TAAAAAGAGG TACTACAGTG GGTTAAACCC   
  
  
- AAGGAGGATG GGAACCGATT CCCTGAATTT CGGACTTCTC TCTCCAGAAA TGAACTATGT AAACAACGAG   
  
  
- TGAACACGTT TGGTACAGAG ATTACCATCG GAACTGTTAC GTTTGGATCG GGAACTCGTT TAGAGGGTCG   
  
  
- AACGTCGGGG ACTACCGCTA TGTTAGGTCG CATAACAACG GATGAAACGA CTTAGTGAAC GACTTTCCTA   
  
  
- GGAATTCAGT ACCGGACCGG ATATATTTCG GGAAGTAAAG TTATCTTACG GACAATAAAG TCTTCTTAAA   
  
  
- GAACGATCCT TCGACAAAAA ACTCAACAAA GGGAAGAACT TCGACCGGAA AAACCACTGA TTGGTTAGTT   
  
  
- ATTAGCTCCG GTACCTCCCC CTTTTCTACC ACGTATATTA ACTAGACTTA CGCAGTCTTG GACGTGTCAC   
  
  
- CTAACGGGAA TAAGTTCTGA ACTCACGAGC CGGACTCCCG GGAGGAGTAA ACTCCTAATG GCCCCAAGTA   
  
  
- GTTGTCTTTC TCCAAAATCT TGTTCATCGA GTATCTAACT GACTTCTTCG ACTCTTCAAC CTAAACGGTA   
  
  
- AAGTCAAGTT AGGACACCAA ACGTTTGATC TCTTAGAGCT GTAGCCTTTT GAGGCACAAT TCTGGCCCCT   
  
  
- CCGGAACCGA TAATGGAGCC AGGAAGTTGA CGTATGGGAA AACCGAAGAC TTCTCCTTCA GGAATTCTTT   
  
  
- TCAAGTGGGA ACCGTAACCA TTTCGTTCGG TTACCCCGAT TAAATGTCCC GAACAAGTTA TTTCTACCTC   
  
  
- GATTATTATC CGCATCGGGT TCATTACTAA GCCGAAGTAG ACGTGGAAGT AGGGAGTTGT GAAGTCGGTT   
  
  
- CTACCTTCCA AAGGAATCGC GAAACACCCC AAATAGGGGT TTCTAATACC ACTATTGGCT CGTTCTAAGG   
  
  
- TTGGTGTTAC CCCGTCCTGA TTACCTCTCT AACAGTCTTC GTAACATGAA GATACGTCGT AACAAGCTAA   
  
  
- CGAATCTTAA ATGGGAGGGC TCTTGGAGGC ACCTCTCTTC CTTCCAGCTC TACGAGGAGG AACCGTTCCT   
  
  
- TTAGTTCTTG TAGTATCGCA CACTCCCTCC TCTTTCTTAT CTATCCGTAC TCTTCAACCC CTTCACCTAA   
  
  
- TTCTCCGAAC TCTACCGGCC CAAACCTTCG CAAGGAAACT CGGTGTATCC GTACTAGGTT CGTTCCGCCA   
  
  
- ACAACGTCTC GATACCGACA CTACCAATAT CTTATTTCCT CCTCTTGCCT ACAAAACAAT AGACGACCGT   
  
  
- TCTAGCGGGG GAGAAAAGTC ATAGACGAAC CTCTACATCC TCCAC

+     CGTCA-motif

| Site Name | Organism | Position | Strand | Matrix score. | sequence | function |
| --- | --- | --- | --- | --- | --- | --- |
| CGTCA-motif | Hordeum vulgare | 2506 | + | 5 | CGTCA | cis-acting regulatory element involved in the MeJA-responsiveness |
| CGTCA-motif | Hordeum vulgare | 1369 | - | 5 | CGTCA | cis-acting regulatory element involved in the MeJA-responsiveness |

>HU08G01232.1   
+ -Up\_Stream \_Len000TCTACC TTTACCTGTG TTAAAAAAAA AAATTTTGTT TCCATTACTA TTCTCTTGGC   
  
  
+ ACTATTGGTG CTGATTTTTC TTCACCAAAT CAACCTCCTT ATATTTGCCA ATTTTACTGT CCTCTTTCAT   
  
  
+ CTATAAAGTC AACTCTCCTA GTACAATTTG TCACATAGAA AATCTCTAGG CACTCATCTA AACTCTCTGT   
  
  
+ TCTGGGTAAT TGATCGACGA TCTACACCAT TATTCGTTCT AGTGCACCGT TTGGACAGCC AATCATGTAC   
  
  
+ CTTTGGAGAT CAACTGCTCA TGAGTTCTTG GTGTGAAATT GGGGAAGTAA ATTCGACTTT AGGGCACTGG   
  
  
+ TCTATCACGC CATGATTTTA CCATCTATTT TCATATATAT CCAATAAGTT TGATCTTTCG CTATTAATCA   
  
  
+ CAATGATATA AACAAGTGGT GAAGAGCAAT AATGAACCAT ACATTTTAAA CTTAAGCGTT AAGGAAGACA   
  
  
+ TGAAGTTAAA AACAATGGTA AGAGCTATGC GTATGTTTGG CATATAGCTT TTTTAAGAGT GTTTTGGCTA   
  
  
+ TAGTCGGAGT TTTTTAATTA AGATTAGCTG TTTGATCAAA TAAAAAAGCT AATTTGAGTG TTTGGCGAGA   
  
  
+ AGACTTTTTA TAAGAACTTT TTTTGGTCTA AAAAGTTAAT TTAAAAAGGC TAATTCTATG AGCTTTTCGG   
  
  
+ AAGAGTTTTT TAAATAATTA ACTTTTTGTC TCATAAGCCA TAACTTTATC AGAAACAGTT AATTTTACAA   
  
  
+ AATAATTTCT CAACAAACAA CTAATTTAAA TAATTAATAA AAATAACTAA CTCAAATATC TAATAACTAA   
  
  
+ TAACTAATAT AAATAATTAA CAGTTAACAG TTGTTTACGA AACAGAATTT ATATAAAAAG CGGTAACAGA   
  
  
+ TAAAAGATAA TCATGCGAGA TTAAAAGTCA CCAGTGACAC ACAAGTACTT AAATAAATAG TCTAAACAAT   
  
  
+ GATATTTTTT GCGAGCTTCC ATGCAAATAC CGACACTTTT ATTGCCTCAG AAGGAAACAA AAACTGAGTG   
  
  
+ AAGGGGGGGG GGGGTTCTGT TTGTCTTTGT TAGGGCACGT TTGGATTCAA GTGTAGATTG GGAATAAATT   
  
  
+ CGAGGACTTT TTCTTTGCCC TTTTTCCCTG ATTCCCAAAC CCACATCAAG TCAAGACGAA ACCAGCCATT   
  
  
+ GAAAAAAGAG GGTGTTTGTG TGAGATAAAG ACAGAAATTT TATGCCAGAG AGAGAAGATA ACAAATGCAA   
  
  
+ CAACGTCGAC AGAGGCTCTC CTTCTCACAA ATTCCATATT CCTCTGTTTT TTAAGAAAGA AAAAGAAAGT   
  
  
+ GTGAGACAAG AACAACACAA GCAAACAATT CAGTTGACGA CGAATACTGA GTGATACCAC GCACTGTGTG   
  
  
+ CAACTGCGTT TCCATTTGTG GAGTCCTTCA AAGCACGCTC ATCTTCAACC CTTCCTTCGC CACGGAATTT   
  
  
+ CAGGGGGAAA AAAACCCCCA CCTAATTTTG AGAGAGAGAG AGAACAGAGA GAACGGGCGA TACAGGGATA   
  
  
+ CAAGGTTTGA GCAATTTTAG AAAATTTCTT GGGGCCGTTG ATGGGTTTTG AGTGAATTGC AAATCCCAGA   
  
  
+ AAGATTTCTG CGGTTTTTTA TCTACGGCTC TCTCTGTGGG AATTTTTTGG TAGATTCCTC TGGTTCCCAC   
  
  
+ TTTCATATCT TCTTATTTGT TCTACCCTCT TTACATCTGA TAGTTTGTTC TAACTGCTGC TTCAGTTGGT   
  
  
+ GCTTGTTCTC TTTAGTACTT TTCTGTTTTG TTCGTTTTGT TCATGCACTT GAATTCAACA AGAGCCCTTG   
  
  
+ TCCATTTTGA GCTCTTAATT CCCATCTAAC CCTGTTTCCT TCAGAAATTG CTGTTCTGTT CATATAGTTA   
  
  
+ TATTTTTTAT GCTTTGATTT GGGTATAAGT TTGCTGTTGG TGATTCCAAA GCTTGGTACT TTTGGCCATA   
  
  
+ TTTTGTTTGA GTTGGTGTTG ATTGGTACAA ATCTTGTAAT TGGTGTGATT GTTAATGGGA CCAATGCTTC   
  
  
+ AAGATGATGG TTCATCAGTA ACTTCTTCAT CACCTCTTCA ATTTTTCTCC ATGATGTCAC CCAATTTGGG   
  
  
+ TTCCTCCTAC CCTTGGCTAA GGGACTTAAA GCCTGAAGAG AGAGGTCTTT ACTTGATACA TTTGTTGCTC   
  
  
+ ACTTGTGCAA ACCATGTCTC TAATGGTAGC CTTGACAATG CAAACCTAGC CCTTGAGCAA ATCTCCCAGC   
  
  
+ TTGCAGCCCC TGATGGCGAT ACAATCCAGC GTATTGTTGC CTACTTTGCT GAATCACTTG CTGAAAGGAT   
  
  
+ CCTTAAGTCA TGGCCTGGCC TATATAAAGC CCTTCATTTC AATAGAATGC CTGTTATTTC AGAAGAATTT   
  
  
+ CTTGCTAGGA AGCTGTTTTT TGAGTTGTTT CCCTTCTTGA AGCTGGCCTT TTTGGTGACT AACCAATCAA   
  
  
+ TAATCGAGGC CATGGAGGGG GAAAAGATGG TGCATATAAT TGATCTGAAT GCGTCAGAAC CTGCACAGTG   
  
  
+ GATTGCCCTT ATTCAAGACT TGAGTGCTCG GCCTGAGGGC CCTCCTCATT TGAGGATTAC CGGGGTTCAT   
  
  
+ CAACAGAAAG AGGTTTTAGA ACAAGTAGCT CATAGATTGA CTGAAGAAGC TGAGAAGTTG GATTTGCCAT   
  
  
+ TTCAGTTCAA TCCTGTGGTT TGCAAACTAG AGAATCTCGA CATCGGAAAA CTCCGTGTTA AGACCGGGGA   
  
  
+ GGCCTTGGCT ATTACCTCGG TCCTTCAACT GCATACCCTT TTGGCTTCTG AAGAGGAAGT CCTTAAGAAA   
  
  
+ AGTTCACCCT TGGCATTGGT AAAGCAAGCC AATGGGGCTA ATTTACAGGG CTTGTTCAAT AAAGATGGAG   
  
  
+ CTAATAATAG GCGTAGCCCA AGTAATGATT CGGCTTCATC TGCACCTTCA TCCCTCAACA CTTCAGCCAA   
  
  
+ GATGGAAGGT TTCCTTAGCG CTTTGTGGGG TTTATCCCCA AAGATTATGG TGATAACCGA GCAAGATTCC   
  
  
+ AACCACAATG GGGCAGGACT AATGGAGAGA TTGTCAGAAG CATTGTACTT CTATGCAGCA TTGTTCGATT   
  
  
+ GCTTAGAATT TACCCTCCCG AGAACCTCCG TGGAGAGAAG GAAGGTCGAG ATGCTCCTCC TTGGCAAGGA   
  
  
+ AATCAAGAAC ATCATAGCGT GTGAGGGAGG AGAAAGAATA GATAGGCATG AGAAGTTGGG GAAGTGGATT   
  
  
+ AAGAGGCTTG AGATGGCCGG GTTTGGAAGC GTTCCTTTGA GCCACATAGG CATGATCCAA GCAAGGCGGT   
  
  
+ TGTTGCAGAG CTATGGCTGT GATGGTTATA GAATAAAGGA GGAGAACGGA TGTTTTGTTA TCTGCTGGCA   
  
  
+ AGATCGCCCC CTCTTTTCAG TATCTGCTTG GAGATGTAGG AGGTG  

- -Up\_Stream \_Len000AGATGG AAATGGACAC AATTTTTTTT TTTAAAACAA AGGTAATGAT AAGAGAACCG   
  
  
- TGATAACCAC GACTAAAAAG AAGTGGTTTA GTTGGAGGAA TATAAACGGT TAAAATGACA GGAGAAAGTA   
  
  
- GATATTTCAG TTGAGAGGAT CATGTTAAAC AGTGTATCTT TTAGAGATCC GTGAGTAGAT TTGAGAGACA   
  
  
- AGACCCATTA ACTAGCTGCT AGATGTGGTA ATAAGCAAGA TCACGTGGCA AACCTGTCGG TTAGTACATG   
  
  
- GAAACCTCTA GTTGACGAGT ACTCAAGAAC CACACTTTAA CCCCTTCATT TAAGCTGAAA TCCCGTGACC   
  
  
- AGATAGTGCG GTACTAAAAT GGTAGATAAA AGTATATATA GGTTATTCAA ACTAGAAAGC GATAATTAGT   
  
  
- GTTACTATAT TTGTTCACCA CTTCTCGTTA TTACTTGGTA TGTAAAATTT GAATTCGCAA TTCCTTCTGT   
  
  
- ACTTCAATTT TTGTTACCAT TCTCGATACG CATACAAACC GTATATCGAA AAAATTCTCA CAAAACCGAT   
  
  
- ATCAGCCTCA AAAAATTAAT TCTAATCGAC AAACTAGTTT ATTTTTTCGA TTAAACTCAC AAACCGCTCT   
  
  
- TCTGAAAAAT ATTCTTGAAA AAAACCAGAT TTTTCAATTA AATTTTTCCG ATTAAGATAC TCGAAAAGCC   
  
  
- TTCTCAAAAA ATTTATTAAT TGAAAAACAG AGTATTCGGT ATTGAAATAG TCTTTGTCAA TTAAAATGTT   
  
  
- TTATTAAAGA GTTGTTTGTT GATTAAATTT ATTAATTATT TTTATTGATT GAGTTTATAG ATTATTGATT   
  
  
- ATTGATTATA TTTATTAATT GTCAATTGTC AACAAATGCT TTGTCTTAAA TATATTTTTC GCCATTGTCT   
  
  
- ATTTTCTATT AGTACGCTCT AATTTTCAGT GGTCACTGTG TGTTCATGAA TTTATTTATC AGATTTGTTA   
  
  
- CTATAAAAAA CGCTCGAAGG TACGTTTATG GCTGTGAAAA TAACGGAGTC TTCCTTTGTT TTTGACTCAC   
  
  
- TTCCCCCCCC CCCCAAGACA AACAGAAACA ATCCCGTGCA AACCTAAGTT CACATCTAAC CCTTATTTAA   
  
  
- GCTCCTGAAA AAGAAACGGG AAAAAGGGAC TAAGGGTTTG GGTGTAGTTC AGTTCTGCTT TGGTCGGTAA   
  
  
- CTTTTTTCTC CCACAAACAC ACTCTATTTC TGTCTTTAAA ATACGGTCTC TCTCTTCTAT TGTTTACGTT   
  
  
- GTTGCAGCTG TCTCCGAGAG GAAGAGTGTT TAAGGTATAA GGAGACAAAA AATTCTTTCT TTTTCTTTCA   
  
  
- CACTCTGTTC TTGTTGTGTT CGTTTGTTAA GTCAACTGCT GCTTATGACT CACTATGGTG CGTGACACAC   
  
  
- GTTGACGCAA AGGTAAACAC CTCAGGAAGT TTCGTGCGAG TAGAAGTTGG GAAGGAAGCG GTGCCTTAAA   
  
  
- GTCCCCCTTT TTTTGGGGGT GGATTAAAAC TCTCTCTCTC TCTTGTCTCT CTTGCCCGCT ATGTCCCTAT   
  
  
- GTTCCAAACT CGTTAAAATC TTTTAAAGAA CCCCGGCAAC TACCCAAAAC TCACTTAACG TTTAGGGTCT   
  
  
- TTCTAAAGAC GCCAAAAAAT AGATGCCGAG AGAGACACCC TTAAAAAACC ATCTAAGGAG ACCAAGGGTG   
  
  
- AAAGTATAGA AGAATAAACA AGATGGGAGA AATGTAGACT ATCAAACAAG ATTGACGACG AAGTCAACCA   
  
  
- CGAACAAGAG AAATCATGAA AAGACAAAAC AAGCAAAACA AGTACGTGAA CTTAAGTTGT TCTCGGGAAC   
  
  
- AGGTAAAACT CGAGAATTAA GGGTAGATTG GGACAAAGGA AGTCTTTAAC GACAAGACAA GTATATCAAT   
  
  
- ATAAAAAATA CGAAACTAAA CCCATATTCA AACGACAACC ACTAAGGTTT CGAACCATGA AAACCGGTAT   
  
  
- AAAACAAACT CAACCACAAC TAACCATGTT TAGAACATTA ACCACACTAA CAATTACCCT GGTTACGAAG   
  
  
- TTCTACTACC AAGTAGTCAT TGAAGAAGTA GTGGAGAAGT TAAAAAGAGG TACTACAGTG GGTTAAACCC   
  
  
- AAGGAGGATG GGAACCGATT CCCTGAATTT CGGACTTCTC TCTCCAGAAA TGAACTATGT AAACAACGAG   
  
  
- TGAACACGTT TGGTACAGAG ATTACCATCG GAACTGTTAC GTTTGGATCG GGAACTCGTT TAGAGGGTCG   
  
  
- AACGTCGGGG ACTACCGCTA TGTTAGGTCG CATAACAACG GATGAAACGA CTTAGTGAAC GACTTTCCTA   
  
  
- GGAATTCAGT ACCGGACCGG ATATATTTCG GGAAGTAAAG TTATCTTACG GACAATAAAG TCTTCTTAAA   
  
  
- GAACGATCCT TCGACAAAAA ACTCAACAAA GGGAAGAACT TCGACCGGAA AAACCACTGA TTGGTTAGTT   
  
  
- ATTAGCTCCG GTACCTCCCC CTTTTCTACC ACGTATATTA ACTAGACTTA CGCAGTCTTG GACGTGTCAC   
  
  
- CTAACGGGAA TAAGTTCTGA ACTCACGAGC CGGACTCCCG GGAGGAGTAA ACTCCTAATG GCCCCAAGTA   
  
  
- GTTGTCTTTC TCCAAAATCT TGTTCATCGA GTATCTAACT GACTTCTTCG ACTCTTCAAC CTAAACGGTA   
  
  
- AAGTCAAGTT AGGACACCAA ACGTTTGATC TCTTAGAGCT GTAGCCTTTT GAGGCACAAT TCTGGCCCCT   
  
  
- CCGGAACCGA TAATGGAGCC AGGAAGTTGA CGTATGGGAA AACCGAAGAC TTCTCCTTCA GGAATTCTTT   
  
  
- TCAAGTGGGA ACCGTAACCA TTTCGTTCGG TTACCCCGAT TAAATGTCCC GAACAAGTTA TTTCTACCTC   
  
  
- GATTATTATC CGCATCGGGT TCATTACTAA GCCGAAGTAG ACGTGGAAGT AGGGAGTTGT GAAGTCGGTT   
  
  
- CTACCTTCCA AAGGAATCGC GAAACACCCC AAATAGGGGT TTCTAATACC ACTATTGGCT CGTTCTAAGG   
  
  
- TTGGTGTTAC CCCGTCCTGA TTACCTCTCT AACAGTCTTC GTAACATGAA GATACGTCGT AACAAGCTAA   
  
  
- CGAATCTTAA ATGGGAGGGC TCTTGGAGGC ACCTCTCTTC CTTCCAGCTC TACGAGGAGG AACCGTTCCT   
  
  
- TTAGTTCTTG TAGTATCGCA CACTCCCTCC TCTTTCTTAT CTATCCGTAC TCTTCAACCC CTTCACCTAA   
  
  
- TTCTCCGAAC TCTACCGGCC CAAACCTTCG CAAGGAAACT CGGTGTATCC GTACTAGGTT CGTTCCGCCA   
  
  
- ACAACGTCTC GATACCGACA CTACCAATAT CTTATTTCCT CCTCTTGCCT ACAAAACAAT AGACGACCGT   
  
  
- TCTAGCGGGG GAGAAAAGTC ATAGACGAAC CTCTACATCC TCCAC

+     ERE

| Site Name | Organism | Position | Strand | Matrix score. | sequence | function |
| --- | --- | --- | --- | --- | --- | --- |
| ERE | Nicotiana glutinos | 467 | + | 8 | ATTTTAAA |  |

>HU08G01232.1   
+ -Up\_Stream \_Len000TCTACC TTTACCTGTG TTAAAAAAAA AAATTTTGTT TCCATTACTA TTCTCTTGGC   
  
  
+ ACTATTGGTG CTGATTTTTC TTCACCAAAT CAACCTCCTT ATATTTGCCA ATTTTACTGT CCTCTTTCAT   
  
  
+ CTATAAAGTC AACTCTCCTA GTACAATTTG TCACATAGAA AATCTCTAGG CACTCATCTA AACTCTCTGT   
  
  
+ TCTGGGTAAT TGATCGACGA TCTACACCAT TATTCGTTCT AGTGCACCGT TTGGACAGCC AATCATGTAC   
  
  
+ CTTTGGAGAT CAACTGCTCA TGAGTTCTTG GTGTGAAATT GGGGAAGTAA ATTCGACTTT AGGGCACTGG   
  
  
+ TCTATCACGC CATGATTTTA CCATCTATTT TCATATATAT CCAATAAGTT TGATCTTTCG CTATTAATCA   
  
  
+ CAATGATATA AACAAGTGGT GAAGAGCAAT AATGAACCAT ACATTTTAAA CTTAAGCGTT AAGGAAGACA   
  
  
+ TGAAGTTAAA AACAATGGTA AGAGCTATGC GTATGTTTGG CATATAGCTT TTTTAAGAGT GTTTTGGCTA   
  
  
+ TAGTCGGAGT TTTTTAATTA AGATTAGCTG TTTGATCAAA TAAAAAAGCT AATTTGAGTG TTTGGCGAGA   
  
  
+ AGACTTTTTA TAAGAACTTT TTTTGGTCTA AAAAGTTAAT TTAAAAAGGC TAATTCTATG AGCTTTTCGG   
  
  
+ AAGAGTTTTT TAAATAATTA ACTTTTTGTC TCATAAGCCA TAACTTTATC AGAAACAGTT AATTTTACAA   
  
  
+ AATAATTTCT CAACAAACAA CTAATTTAAA TAATTAATAA AAATAACTAA CTCAAATATC TAATAACTAA   
  
  
+ TAACTAATAT AAATAATTAA CAGTTAACAG TTGTTTACGA AACAGAATTT ATATAAAAAG CGGTAACAGA   
  
  
+ TAAAAGATAA TCATGCGAGA TTAAAAGTCA CCAGTGACAC ACAAGTACTT AAATAAATAG TCTAAACAAT   
  
  
+ GATATTTTTT GCGAGCTTCC ATGCAAATAC CGACACTTTT ATTGCCTCAG AAGGAAACAA AAACTGAGTG   
  
  
+ AAGGGGGGGG GGGGTTCTGT TTGTCTTTGT TAGGGCACGT TTGGATTCAA GTGTAGATTG GGAATAAATT   
  
  
+ CGAGGACTTT TTCTTTGCCC TTTTTCCCTG ATTCCCAAAC CCACATCAAG TCAAGACGAA ACCAGCCATT   
  
  
+ GAAAAAAGAG GGTGTTTGTG TGAGATAAAG ACAGAAATTT TATGCCAGAG AGAGAAGATA ACAAATGCAA   
  
  
+ CAACGTCGAC AGAGGCTCTC CTTCTCACAA ATTCCATATT CCTCTGTTTT TTAAGAAAGA AAAAGAAAGT   
  
  
+ GTGAGACAAG AACAACACAA GCAAACAATT CAGTTGACGA CGAATACTGA GTGATACCAC GCACTGTGTG   
  
  
+ CAACTGCGTT TCCATTTGTG GAGTCCTTCA AAGCACGCTC ATCTTCAACC CTTCCTTCGC CACGGAATTT   
  
  
+ CAGGGGGAAA AAAACCCCCA CCTAATTTTG AGAGAGAGAG AGAACAGAGA GAACGGGCGA TACAGGGATA   
  
  
+ CAAGGTTTGA GCAATTTTAG AAAATTTCTT GGGGCCGTTG ATGGGTTTTG AGTGAATTGC AAATCCCAGA   
  
  
+ AAGATTTCTG CGGTTTTTTA TCTACGGCTC TCTCTGTGGG AATTTTTTGG TAGATTCCTC TGGTTCCCAC   
  
  
+ TTTCATATCT TCTTATTTGT TCTACCCTCT TTACATCTGA TAGTTTGTTC TAACTGCTGC TTCAGTTGGT   
  
  
+ GCTTGTTCTC TTTAGTACTT TTCTGTTTTG TTCGTTTTGT TCATGCACTT GAATTCAACA AGAGCCCTTG   
  
  
+ TCCATTTTGA GCTCTTAATT CCCATCTAAC CCTGTTTCCT TCAGAAATTG CTGTTCTGTT CATATAGTTA   
  
  
+ TATTTTTTAT GCTTTGATTT GGGTATAAGT TTGCTGTTGG TGATTCCAAA GCTTGGTACT TTTGGCCATA   
  
  
+ TTTTGTTTGA GTTGGTGTTG ATTGGTACAA ATCTTGTAAT TGGTGTGATT GTTAATGGGA CCAATGCTTC   
  
  
+ AAGATGATGG TTCATCAGTA ACTTCTTCAT CACCTCTTCA ATTTTTCTCC ATGATGTCAC CCAATTTGGG   
  
  
+ TTCCTCCTAC CCTTGGCTAA GGGACTTAAA GCCTGAAGAG AGAGGTCTTT ACTTGATACA TTTGTTGCTC   
  
  
+ ACTTGTGCAA ACCATGTCTC TAATGGTAGC CTTGACAATG CAAACCTAGC CCTTGAGCAA ATCTCCCAGC   
  
  
+ TTGCAGCCCC TGATGGCGAT ACAATCCAGC GTATTGTTGC CTACTTTGCT GAATCACTTG CTGAAAGGAT   
  
  
+ CCTTAAGTCA TGGCCTGGCC TATATAAAGC CCTTCATTTC AATAGAATGC CTGTTATTTC AGAAGAATTT   
  
  
+ CTTGCTAGGA AGCTGTTTTT TGAGTTGTTT CCCTTCTTGA AGCTGGCCTT TTTGGTGACT AACCAATCAA   
  
  
+ TAATCGAGGC CATGGAGGGG GAAAAGATGG TGCATATAAT TGATCTGAAT GCGTCAGAAC CTGCACAGTG   
  
  
+ GATTGCCCTT ATTCAAGACT TGAGTGCTCG GCCTGAGGGC CCTCCTCATT TGAGGATTAC CGGGGTTCAT   
  
  
+ CAACAGAAAG AGGTTTTAGA ACAAGTAGCT CATAGATTGA CTGAAGAAGC TGAGAAGTTG GATTTGCCAT   
  
  
+ TTCAGTTCAA TCCTGTGGTT TGCAAACTAG AGAATCTCGA CATCGGAAAA CTCCGTGTTA AGACCGGGGA   
  
  
+ GGCCTTGGCT ATTACCTCGG TCCTTCAACT GCATACCCTT TTGGCTTCTG AAGAGGAAGT CCTTAAGAAA   
  
  
+ AGTTCACCCT TGGCATTGGT AAAGCAAGCC AATGGGGCTA ATTTACAGGG CTTGTTCAAT AAAGATGGAG   
  
  
+ CTAATAATAG GCGTAGCCCA AGTAATGATT CGGCTTCATC TGCACCTTCA TCCCTCAACA CTTCAGCCAA   
  
  
+ GATGGAAGGT TTCCTTAGCG CTTTGTGGGG TTTATCCCCA AAGATTATGG TGATAACCGA GCAAGATTCC   
  
  
+ AACCACAATG GGGCAGGACT AATGGAGAGA TTGTCAGAAG CATTGTACTT CTATGCAGCA TTGTTCGATT   
  
  
+ GCTTAGAATT TACCCTCCCG AGAACCTCCG TGGAGAGAAG GAAGGTCGAG ATGCTCCTCC TTGGCAAGGA   
  
  
+ AATCAAGAAC ATCATAGCGT GTGAGGGAGG AGAAAGAATA GATAGGCATG AGAAGTTGGG GAAGTGGATT   
  
  
+ AAGAGGCTTG AGATGGCCGG GTTTGGAAGC GTTCCTTTGA GCCACATAGG CATGATCCAA GCAAGGCGGT   
  
  
+ TGTTGCAGAG CTATGGCTGT GATGGTTATA GAATAAAGGA GGAGAACGGA TGTTTTGTTA TCTGCTGGCA   
  
  
+ AGATCGCCCC CTCTTTTCAG TATCTGCTTG GAGATGTAGG AGGTG  

- -Up\_Stream \_Len000AGATGG AAATGGACAC AATTTTTTTT TTTAAAACAA AGGTAATGAT AAGAGAACCG   
  
  
- TGATAACCAC GACTAAAAAG AAGTGGTTTA GTTGGAGGAA TATAAACGGT TAAAATGACA GGAGAAAGTA   
  
  
- GATATTTCAG TTGAGAGGAT CATGTTAAAC AGTGTATCTT TTAGAGATCC GTGAGTAGAT TTGAGAGACA   
  
  
- AGACCCATTA ACTAGCTGCT AGATGTGGTA ATAAGCAAGA TCACGTGGCA AACCTGTCGG TTAGTACATG   
  
  
- GAAACCTCTA GTTGACGAGT ACTCAAGAAC CACACTTTAA CCCCTTCATT TAAGCTGAAA TCCCGTGACC   
  
  
- AGATAGTGCG GTACTAAAAT GGTAGATAAA AGTATATATA GGTTATTCAA ACTAGAAAGC GATAATTAGT   
  
  
- GTTACTATAT TTGTTCACCA CTTCTCGTTA TTACTTGGTA TGTAAAATTT GAATTCGCAA TTCCTTCTGT   
  
  
- ACTTCAATTT TTGTTACCAT TCTCGATACG CATACAAACC GTATATCGAA AAAATTCTCA CAAAACCGAT   
  
  
- ATCAGCCTCA AAAAATTAAT TCTAATCGAC AAACTAGTTT ATTTTTTCGA TTAAACTCAC AAACCGCTCT   
  
  
- TCTGAAAAAT ATTCTTGAAA AAAACCAGAT TTTTCAATTA AATTTTTCCG ATTAAGATAC TCGAAAAGCC   
  
  
- TTCTCAAAAA ATTTATTAAT TGAAAAACAG AGTATTCGGT ATTGAAATAG TCTTTGTCAA TTAAAATGTT   
  
  
- TTATTAAAGA GTTGTTTGTT GATTAAATTT ATTAATTATT TTTATTGATT GAGTTTATAG ATTATTGATT   
  
  
- ATTGATTATA TTTATTAATT GTCAATTGTC AACAAATGCT TTGTCTTAAA TATATTTTTC GCCATTGTCT   
  
  
- ATTTTCTATT AGTACGCTCT AATTTTCAGT GGTCACTGTG TGTTCATGAA TTTATTTATC AGATTTGTTA   
  
  
- CTATAAAAAA CGCTCGAAGG TACGTTTATG GCTGTGAAAA TAACGGAGTC TTCCTTTGTT TTTGACTCAC   
  
  
- TTCCCCCCCC CCCCAAGACA AACAGAAACA ATCCCGTGCA AACCTAAGTT CACATCTAAC CCTTATTTAA   
  
  
- GCTCCTGAAA AAGAAACGGG AAAAAGGGAC TAAGGGTTTG GGTGTAGTTC AGTTCTGCTT TGGTCGGTAA   
  
  
- CTTTTTTCTC CCACAAACAC ACTCTATTTC TGTCTTTAAA ATACGGTCTC TCTCTTCTAT TGTTTACGTT   
  
  
- GTTGCAGCTG TCTCCGAGAG GAAGAGTGTT TAAGGTATAA GGAGACAAAA AATTCTTTCT TTTTCTTTCA   
  
  
- CACTCTGTTC TTGTTGTGTT CGTTTGTTAA GTCAACTGCT GCTTATGACT CACTATGGTG CGTGACACAC   
  
  
- GTTGACGCAA AGGTAAACAC CTCAGGAAGT TTCGTGCGAG TAGAAGTTGG GAAGGAAGCG GTGCCTTAAA   
  
  
- GTCCCCCTTT TTTTGGGGGT GGATTAAAAC TCTCTCTCTC TCTTGTCTCT CTTGCCCGCT ATGTCCCTAT   
  
  
- GTTCCAAACT CGTTAAAATC TTTTAAAGAA CCCCGGCAAC TACCCAAAAC TCACTTAACG TTTAGGGTCT   
  
  
- TTCTAAAGAC GCCAAAAAAT AGATGCCGAG AGAGACACCC TTAAAAAACC ATCTAAGGAG ACCAAGGGTG   
  
  
- AAAGTATAGA AGAATAAACA AGATGGGAGA AATGTAGACT ATCAAACAAG ATTGACGACG AAGTCAACCA   
  
  
- CGAACAAGAG AAATCATGAA AAGACAAAAC AAGCAAAACA AGTACGTGAA CTTAAGTTGT TCTCGGGAAC   
  
  
- AGGTAAAACT CGAGAATTAA GGGTAGATTG GGACAAAGGA AGTCTTTAAC GACAAGACAA GTATATCAAT   
  
  
- ATAAAAAATA CGAAACTAAA CCCATATTCA AACGACAACC ACTAAGGTTT CGAACCATGA AAACCGGTAT   
  
  
- AAAACAAACT CAACCACAAC TAACCATGTT TAGAACATTA ACCACACTAA CAATTACCCT GGTTACGAAG   
  
  
- TTCTACTACC AAGTAGTCAT TGAAGAAGTA GTGGAGAAGT TAAAAAGAGG TACTACAGTG GGTTAAACCC   
  
  
- AAGGAGGATG GGAACCGATT CCCTGAATTT CGGACTTCTC TCTCCAGAAA TGAACTATGT AAACAACGAG   
  
  
- TGAACACGTT TGGTACAGAG ATTACCATCG GAACTGTTAC GTTTGGATCG GGAACTCGTT TAGAGGGTCG   
  
  
- AACGTCGGGG ACTACCGCTA TGTTAGGTCG CATAACAACG GATGAAACGA CTTAGTGAAC GACTTTCCTA   
  
  
- GGAATTCAGT ACCGGACCGG ATATATTTCG GGAAGTAAAG TTATCTTACG GACAATAAAG TCTTCTTAAA   
  
  
- GAACGATCCT TCGACAAAAA ACTCAACAAA GGGAAGAACT TCGACCGGAA AAACCACTGA TTGGTTAGTT   
  
  
- ATTAGCTCCG GTACCTCCCC CTTTTCTACC ACGTATATTA ACTAGACTTA CGCAGTCTTG GACGTGTCAC   
  
  
- CTAACGGGAA TAAGTTCTGA ACTCACGAGC CGGACTCCCG GGAGGAGTAA ACTCCTAATG GCCCCAAGTA   
  
  
- GTTGTCTTTC TCCAAAATCT TGTTCATCGA GTATCTAACT GACTTCTTCG ACTCTTCAAC CTAAACGGTA   
  
  
- AAGTCAAGTT AGGACACCAA ACGTTTGATC TCTTAGAGCT GTAGCCTTTT GAGGCACAAT TCTGGCCCCT   
  
  
- CCGGAACCGA TAATGGAGCC AGGAAGTTGA CGTATGGGAA AACCGAAGAC TTCTCCTTCA GGAATTCTTT   
  
  
- TCAAGTGGGA ACCGTAACCA TTTCGTTCGG TTACCCCGAT TAAATGTCCC GAACAAGTTA TTTCTACCTC   
  
  
- GATTATTATC CGCATCGGGT TCATTACTAA GCCGAAGTAG ACGTGGAAGT AGGGAGTTGT GAAGTCGGTT   
  
  
- CTACCTTCCA AAGGAATCGC GAAACACCCC AAATAGGGGT TTCTAATACC ACTATTGGCT CGTTCTAAGG   
  
  
- TTGGTGTTAC CCCGTCCTGA TTACCTCTCT AACAGTCTTC GTAACATGAA GATACGTCGT AACAAGCTAA   
  
  
- CGAATCTTAA ATGGGAGGGC TCTTGGAGGC ACCTCTCTTC CTTCCAGCTC TACGAGGAGG AACCGTTCCT   
  
  
- TTAGTTCTTG TAGTATCGCA CACTCCCTCC TCTTTCTTAT CTATCCGTAC TCTTCAACCC CTTCACCTAA   
  
  
- TTCTCCGAAC TCTACCGGCC CAAACCTTCG CAAGGAAACT CGGTGTATCC GTACTAGGTT CGTTCCGCCA   
  
  
- ACAACGTCTC GATACCGACA CTACCAATAT CTTATTTCCT CCTCTTGCCT ACAAAACAAT AGACGACCGT   
  
  
- TCTAGCGGGG GAGAAAAGTC ATAGACGAAC CTCTACATCC TCCAC

+     G-Box

| Site Name | Organism | Position | Strand | Matrix score. | sequence | function |
| --- | --- | --- | --- | --- | --- | --- |
| G-Box | Pisum sativum | 1090 | + | 6 | CACGTT | cis-acting regulatory element involved in light responsiveness |

>HU08G01232.1   
+ -Up\_Stream \_Len000TCTACC TTTACCTGTG TTAAAAAAAA AAATTTTGTT TCCATTACTA TTCTCTTGGC   
  
  
+ ACTATTGGTG CTGATTTTTC TTCACCAAAT CAACCTCCTT ATATTTGCCA ATTTTACTGT CCTCTTTCAT   
  
  
+ CTATAAAGTC AACTCTCCTA GTACAATTTG TCACATAGAA AATCTCTAGG CACTCATCTA AACTCTCTGT   
  
  
+ TCTGGGTAAT TGATCGACGA TCTACACCAT TATTCGTTCT AGTGCACCGT TTGGACAGCC AATCATGTAC   
  
  
+ CTTTGGAGAT CAACTGCTCA TGAGTTCTTG GTGTGAAATT GGGGAAGTAA ATTCGACTTT AGGGCACTGG   
  
  
+ TCTATCACGC CATGATTTTA CCATCTATTT TCATATATAT CCAATAAGTT TGATCTTTCG CTATTAATCA   
  
  
+ CAATGATATA AACAAGTGGT GAAGAGCAAT AATGAACCAT ACATTTTAAA CTTAAGCGTT AAGGAAGACA   
  
  
+ TGAAGTTAAA AACAATGGTA AGAGCTATGC GTATGTTTGG CATATAGCTT TTTTAAGAGT GTTTTGGCTA   
  
  
+ TAGTCGGAGT TTTTTAATTA AGATTAGCTG TTTGATCAAA TAAAAAAGCT AATTTGAGTG TTTGGCGAGA   
  
  
+ AGACTTTTTA TAAGAACTTT TTTTGGTCTA AAAAGTTAAT TTAAAAAGGC TAATTCTATG AGCTTTTCGG   
  
  
+ AAGAGTTTTT TAAATAATTA ACTTTTTGTC TCATAAGCCA TAACTTTATC AGAAACAGTT AATTTTACAA   
  
  
+ AATAATTTCT CAACAAACAA CTAATTTAAA TAATTAATAA AAATAACTAA CTCAAATATC TAATAACTAA   
  
  
+ TAACTAATAT AAATAATTAA CAGTTAACAG TTGTTTACGA AACAGAATTT ATATAAAAAG CGGTAACAGA   
  
  
+ TAAAAGATAA TCATGCGAGA TTAAAAGTCA CCAGTGACAC ACAAGTACTT AAATAAATAG TCTAAACAAT   
  
  
+ GATATTTTTT GCGAGCTTCC ATGCAAATAC CGACACTTTT ATTGCCTCAG AAGGAAACAA AAACTGAGTG   
  
  
+ AAGGGGGGGG GGGGTTCTGT TTGTCTTTGT TAGGGCACGT TTGGATTCAA GTGTAGATTG GGAATAAATT   
  
  
+ CGAGGACTTT TTCTTTGCCC TTTTTCCCTG ATTCCCAAAC CCACATCAAG TCAAGACGAA ACCAGCCATT   
  
  
+ GAAAAAAGAG GGTGTTTGTG TGAGATAAAG ACAGAAATTT TATGCCAGAG AGAGAAGATA ACAAATGCAA   
  
  
+ CAACGTCGAC AGAGGCTCTC CTTCTCACAA ATTCCATATT CCTCTGTTTT TTAAGAAAGA AAAAGAAAGT   
  
  
+ GTGAGACAAG AACAACACAA GCAAACAATT CAGTTGACGA CGAATACTGA GTGATACCAC GCACTGTGTG   
  
  
+ CAACTGCGTT TCCATTTGTG GAGTCCTTCA AAGCACGCTC ATCTTCAACC CTTCCTTCGC CACGGAATTT   
  
  
+ CAGGGGGAAA AAAACCCCCA CCTAATTTTG AGAGAGAGAG AGAACAGAGA GAACGGGCGA TACAGGGATA   
  
  
+ CAAGGTTTGA GCAATTTTAG AAAATTTCTT GGGGCCGTTG ATGGGTTTTG AGTGAATTGC AAATCCCAGA   
  
  
+ AAGATTTCTG CGGTTTTTTA TCTACGGCTC TCTCTGTGGG AATTTTTTGG TAGATTCCTC TGGTTCCCAC   
  
  
+ TTTCATATCT TCTTATTTGT TCTACCCTCT TTACATCTGA TAGTTTGTTC TAACTGCTGC TTCAGTTGGT   
  
  
+ GCTTGTTCTC TTTAGTACTT TTCTGTTTTG TTCGTTTTGT TCATGCACTT GAATTCAACA AGAGCCCTTG   
  
  
+ TCCATTTTGA GCTCTTAATT CCCATCTAAC CCTGTTTCCT TCAGAAATTG CTGTTCTGTT CATATAGTTA   
  
  
+ TATTTTTTAT GCTTTGATTT GGGTATAAGT TTGCTGTTGG TGATTCCAAA GCTTGGTACT TTTGGCCATA   
  
  
+ TTTTGTTTGA GTTGGTGTTG ATTGGTACAA ATCTTGTAAT TGGTGTGATT GTTAATGGGA CCAATGCTTC   
  
  
+ AAGATGATGG TTCATCAGTA ACTTCTTCAT CACCTCTTCA ATTTTTCTCC ATGATGTCAC CCAATTTGGG   
  
  
+ TTCCTCCTAC CCTTGGCTAA GGGACTTAAA GCCTGAAGAG AGAGGTCTTT ACTTGATACA TTTGTTGCTC   
  
  
+ ACTTGTGCAA ACCATGTCTC TAATGGTAGC CTTGACAATG CAAACCTAGC CCTTGAGCAA ATCTCCCAGC   
  
  
+ TTGCAGCCCC TGATGGCGAT ACAATCCAGC GTATTGTTGC CTACTTTGCT GAATCACTTG CTGAAAGGAT   
  
  
+ CCTTAAGTCA TGGCCTGGCC TATATAAAGC CCTTCATTTC AATAGAATGC CTGTTATTTC AGAAGAATTT   
  
  
+ CTTGCTAGGA AGCTGTTTTT TGAGTTGTTT CCCTTCTTGA AGCTGGCCTT TTTGGTGACT AACCAATCAA   
  
  
+ TAATCGAGGC CATGGAGGGG GAAAAGATGG TGCATATAAT TGATCTGAAT GCGTCAGAAC CTGCACAGTG   
  
  
+ GATTGCCCTT ATTCAAGACT TGAGTGCTCG GCCTGAGGGC CCTCCTCATT TGAGGATTAC CGGGGTTCAT   
  
  
+ CAACAGAAAG AGGTTTTAGA ACAAGTAGCT CATAGATTGA CTGAAGAAGC TGAGAAGTTG GATTTGCCAT   
  
  
+ TTCAGTTCAA TCCTGTGGTT TGCAAACTAG AGAATCTCGA CATCGGAAAA CTCCGTGTTA AGACCGGGGA   
  
  
+ GGCCTTGGCT ATTACCTCGG TCCTTCAACT GCATACCCTT TTGGCTTCTG AAGAGGAAGT CCTTAAGAAA   
  
  
+ AGTTCACCCT TGGCATTGGT AAAGCAAGCC AATGGGGCTA ATTTACAGGG CTTGTTCAAT AAAGATGGAG   
  
  
+ CTAATAATAG GCGTAGCCCA AGTAATGATT CGGCTTCATC TGCACCTTCA TCCCTCAACA CTTCAGCCAA   
  
  
+ GATGGAAGGT TTCCTTAGCG CTTTGTGGGG TTTATCCCCA AAGATTATGG TGATAACCGA GCAAGATTCC   
  
  
+ AACCACAATG GGGCAGGACT AATGGAGAGA TTGTCAGAAG CATTGTACTT CTATGCAGCA TTGTTCGATT   
  
  
+ GCTTAGAATT TACCCTCCCG AGAACCTCCG TGGAGAGAAG GAAGGTCGAG ATGCTCCTCC TTGGCAAGGA   
  
  
+ AATCAAGAAC ATCATAGCGT GTGAGGGAGG AGAAAGAATA GATAGGCATG AGAAGTTGGG GAAGTGGATT   
  
  
+ AAGAGGCTTG AGATGGCCGG GTTTGGAAGC GTTCCTTTGA GCCACATAGG CATGATCCAA GCAAGGCGGT   
  
  
+ TGTTGCAGAG CTATGGCTGT GATGGTTATA GAATAAAGGA GGAGAACGGA TGTTTTGTTA TCTGCTGGCA   
  
  
+ AGATCGCCCC CTCTTTTCAG TATCTGCTTG GAGATGTAGG AGGTG  

- -Up\_Stream \_Len000AGATGG AAATGGACAC AATTTTTTTT TTTAAAACAA AGGTAATGAT AAGAGAACCG   
  
  
- TGATAACCAC GACTAAAAAG AAGTGGTTTA GTTGGAGGAA TATAAACGGT TAAAATGACA GGAGAAAGTA   
  
  
- GATATTTCAG TTGAGAGGAT CATGTTAAAC AGTGTATCTT TTAGAGATCC GTGAGTAGAT TTGAGAGACA   
  
  
- AGACCCATTA ACTAGCTGCT AGATGTGGTA ATAAGCAAGA TCACGTGGCA AACCTGTCGG TTAGTACATG   
  
  
- GAAACCTCTA GTTGACGAGT ACTCAAGAAC CACACTTTAA CCCCTTCATT TAAGCTGAAA TCCCGTGACC   
  
  
- AGATAGTGCG GTACTAAAAT GGTAGATAAA AGTATATATA GGTTATTCAA ACTAGAAAGC GATAATTAGT   
  
  
- GTTACTATAT TTGTTCACCA CTTCTCGTTA TTACTTGGTA TGTAAAATTT GAATTCGCAA TTCCTTCTGT   
  
  
- ACTTCAATTT TTGTTACCAT TCTCGATACG CATACAAACC GTATATCGAA AAAATTCTCA CAAAACCGAT   
  
  
- ATCAGCCTCA AAAAATTAAT TCTAATCGAC AAACTAGTTT ATTTTTTCGA TTAAACTCAC AAACCGCTCT   
  
  
- TCTGAAAAAT ATTCTTGAAA AAAACCAGAT TTTTCAATTA AATTTTTCCG ATTAAGATAC TCGAAAAGCC   
  
  
- TTCTCAAAAA ATTTATTAAT TGAAAAACAG AGTATTCGGT ATTGAAATAG TCTTTGTCAA TTAAAATGTT   
  
  
- TTATTAAAGA GTTGTTTGTT GATTAAATTT ATTAATTATT TTTATTGATT GAGTTTATAG ATTATTGATT   
  
  
- ATTGATTATA TTTATTAATT GTCAATTGTC AACAAATGCT TTGTCTTAAA TATATTTTTC GCCATTGTCT   
  
  
- ATTTTCTATT AGTACGCTCT AATTTTCAGT GGTCACTGTG TGTTCATGAA TTTATTTATC AGATTTGTTA   
  
  
- CTATAAAAAA CGCTCGAAGG TACGTTTATG GCTGTGAAAA TAACGGAGTC TTCCTTTGTT TTTGACTCAC   
  
  
- TTCCCCCCCC CCCCAAGACA AACAGAAACA ATCCCGTGCA AACCTAAGTT CACATCTAAC CCTTATTTAA   
  
  
- GCTCCTGAAA AAGAAACGGG AAAAAGGGAC TAAGGGTTTG GGTGTAGTTC AGTTCTGCTT TGGTCGGTAA   
  
  
- CTTTTTTCTC CCACAAACAC ACTCTATTTC TGTCTTTAAA ATACGGTCTC TCTCTTCTAT TGTTTACGTT   
  
  
- GTTGCAGCTG TCTCCGAGAG GAAGAGTGTT TAAGGTATAA GGAGACAAAA AATTCTTTCT TTTTCTTTCA   
  
  
- CACTCTGTTC TTGTTGTGTT CGTTTGTTAA GTCAACTGCT GCTTATGACT CACTATGGTG CGTGACACAC   
  
  
- GTTGACGCAA AGGTAAACAC CTCAGGAAGT TTCGTGCGAG TAGAAGTTGG GAAGGAAGCG GTGCCTTAAA   
  
  
- GTCCCCCTTT TTTTGGGGGT GGATTAAAAC TCTCTCTCTC TCTTGTCTCT CTTGCCCGCT ATGTCCCTAT   
  
  
- GTTCCAAACT CGTTAAAATC TTTTAAAGAA CCCCGGCAAC TACCCAAAAC TCACTTAACG TTTAGGGTCT   
  
  
- TTCTAAAGAC GCCAAAAAAT AGATGCCGAG AGAGACACCC TTAAAAAACC ATCTAAGGAG ACCAAGGGTG   
  
  
- AAAGTATAGA AGAATAAACA AGATGGGAGA AATGTAGACT ATCAAACAAG ATTGACGACG AAGTCAACCA   
  
  
- CGAACAAGAG AAATCATGAA AAGACAAAAC AAGCAAAACA AGTACGTGAA CTTAAGTTGT TCTCGGGAAC   
  
  
- AGGTAAAACT CGAGAATTAA GGGTAGATTG GGACAAAGGA AGTCTTTAAC GACAAGACAA GTATATCAAT   
  
  
- ATAAAAAATA CGAAACTAAA CCCATATTCA AACGACAACC ACTAAGGTTT CGAACCATGA AAACCGGTAT   
  
  
- AAAACAAACT CAACCACAAC TAACCATGTT TAGAACATTA ACCACACTAA CAATTACCCT GGTTACGAAG   
  
  
- TTCTACTACC AAGTAGTCAT TGAAGAAGTA GTGGAGAAGT TAAAAAGAGG TACTACAGTG GGTTAAACCC   
  
  
- AAGGAGGATG GGAACCGATT CCCTGAATTT CGGACTTCTC TCTCCAGAAA TGAACTATGT AAACAACGAG   
  
  
- TGAACACGTT TGGTACAGAG ATTACCATCG GAACTGTTAC GTTTGGATCG GGAACTCGTT TAGAGGGTCG   
  
  
- AACGTCGGGG ACTACCGCTA TGTTAGGTCG CATAACAACG GATGAAACGA CTTAGTGAAC GACTTTCCTA   
  
  
- GGAATTCAGT ACCGGACCGG ATATATTTCG GGAAGTAAAG TTATCTTACG GACAATAAAG TCTTCTTAAA   
  
  
- GAACGATCCT TCGACAAAAA ACTCAACAAA GGGAAGAACT TCGACCGGAA AAACCACTGA TTGGTTAGTT   
  
  
- ATTAGCTCCG GTACCTCCCC CTTTTCTACC ACGTATATTA ACTAGACTTA CGCAGTCTTG GACGTGTCAC   
  
  
- CTAACGGGAA TAAGTTCTGA ACTCACGAGC CGGACTCCCG GGAGGAGTAA ACTCCTAATG GCCCCAAGTA   
  
  
- GTTGTCTTTC TCCAAAATCT TGTTCATCGA GTATCTAACT GACTTCTTCG ACTCTTCAAC CTAAACGGTA   
  
  
- AAGTCAAGTT AGGACACCAA ACGTTTGATC TCTTAGAGCT GTAGCCTTTT GAGGCACAAT TCTGGCCCCT   
  
  
- CCGGAACCGA TAATGGAGCC AGGAAGTTGA CGTATGGGAA AACCGAAGAC TTCTCCTTCA GGAATTCTTT   
  
  
- TCAAGTGGGA ACCGTAACCA TTTCGTTCGG TTACCCCGAT TAAATGTCCC GAACAAGTTA TTTCTACCTC   
  
  
- GATTATTATC CGCATCGGGT TCATTACTAA GCCGAAGTAG ACGTGGAAGT AGGGAGTTGT GAAGTCGGTT   
  
  
- CTACCTTCCA AAGGAATCGC GAAACACCCC AAATAGGGGT TTCTAATACC ACTATTGGCT CGTTCTAAGG   
  
  
- TTGGTGTTAC CCCGTCCTGA TTACCTCTCT AACAGTCTTC GTAACATGAA GATACGTCGT AACAAGCTAA   
  
  
- CGAATCTTAA ATGGGAGGGC TCTTGGAGGC ACCTCTCTTC CTTCCAGCTC TACGAGGAGG AACCGTTCCT   
  
  
- TTAGTTCTTG TAGTATCGCA CACTCCCTCC TCTTTCTTAT CTATCCGTAC TCTTCAACCC CTTCACCTAA   
  
  
- TTCTCCGAAC TCTACCGGCC CAAACCTTCG CAAGGAAACT CGGTGTATCC GTACTAGGTT CGTTCCGCCA   
  
  
- ACAACGTCTC GATACCGACA CTACCAATAT CTTATTTCCT CCTCTTGCCT ACAAAACAAT AGACGACCGT   
  
  
- TCTAGCGGGG GAGAAAAGTC ATAGACGAAC CTCTACATCC TCCAC

+     G-box

| Site Name | Organism | Position | Strand | Matrix score. | sequence | function |
| --- | --- | --- | --- | --- | --- | --- |
| G-box | Brassica oleracea | 2715 | - | 9 | TAACACGTAG | cis-acting regulatory element involved in light responsiveness |

>HU08G01232.1   
+ -Up\_Stream \_Len000TCTACC TTTACCTGTG TTAAAAAAAA AAATTTTGTT TCCATTACTA TTCTCTTGGC   
  
  
+ ACTATTGGTG CTGATTTTTC TTCACCAAAT CAACCTCCTT ATATTTGCCA ATTTTACTGT CCTCTTTCAT   
  
  
+ CTATAAAGTC AACTCTCCTA GTACAATTTG TCACATAGAA AATCTCTAGG CACTCATCTA AACTCTCTGT   
  
  
+ TCTGGGTAAT TGATCGACGA TCTACACCAT TATTCGTTCT AGTGCACCGT TTGGACAGCC AATCATGTAC   
  
  
+ CTTTGGAGAT CAACTGCTCA TGAGTTCTTG GTGTGAAATT GGGGAAGTAA ATTCGACTTT AGGGCACTGG   
  
  
+ TCTATCACGC CATGATTTTA CCATCTATTT TCATATATAT CCAATAAGTT TGATCTTTCG CTATTAATCA   
  
  
+ CAATGATATA AACAAGTGGT GAAGAGCAAT AATGAACCAT ACATTTTAAA CTTAAGCGTT AAGGAAGACA   
  
  
+ TGAAGTTAAA AACAATGGTA AGAGCTATGC GTATGTTTGG CATATAGCTT TTTTAAGAGT GTTTTGGCTA   
  
  
+ TAGTCGGAGT TTTTTAATTA AGATTAGCTG TTTGATCAAA TAAAAAAGCT AATTTGAGTG TTTGGCGAGA   
  
  
+ AGACTTTTTA TAAGAACTTT TTTTGGTCTA AAAAGTTAAT TTAAAAAGGC TAATTCTATG AGCTTTTCGG   
  
  
+ AAGAGTTTTT TAAATAATTA ACTTTTTGTC TCATAAGCCA TAACTTTATC AGAAACAGTT AATTTTACAA   
  
  
+ AATAATTTCT CAACAAACAA CTAATTTAAA TAATTAATAA AAATAACTAA CTCAAATATC TAATAACTAA   
  
  
+ TAACTAATAT AAATAATTAA CAGTTAACAG TTGTTTACGA AACAGAATTT ATATAAAAAG CGGTAACAGA   
  
  
+ TAAAAGATAA TCATGCGAGA TTAAAAGTCA CCAGTGACAC ACAAGTACTT AAATAAATAG TCTAAACAAT   
  
  
+ GATATTTTTT GCGAGCTTCC ATGCAAATAC CGACACTTTT ATTGCCTCAG AAGGAAACAA AAACTGAGTG   
  
  
+ AAGGGGGGGG GGGGTTCTGT TTGTCTTTGT TAGGGCACGT TTGGATTCAA GTGTAGATTG GGAATAAATT   
  
  
+ CGAGGACTTT TTCTTTGCCC TTTTTCCCTG ATTCCCAAAC CCACATCAAG TCAAGACGAA ACCAGCCATT   
  
  
+ GAAAAAAGAG GGTGTTTGTG TGAGATAAAG ACAGAAATTT TATGCCAGAG AGAGAAGATA ACAAATGCAA   
  
  
+ CAACGTCGAC AGAGGCTCTC CTTCTCACAA ATTCCATATT CCTCTGTTTT TTAAGAAAGA AAAAGAAAGT   
  
  
+ GTGAGACAAG AACAACACAA GCAAACAATT CAGTTGACGA CGAATACTGA GTGATACCAC GCACTGTGTG   
  
  
+ CAACTGCGTT TCCATTTGTG GAGTCCTTCA AAGCACGCTC ATCTTCAACC CTTCCTTCGC CACGGAATTT   
  
  
+ CAGGGGGAAA AAAACCCCCA CCTAATTTTG AGAGAGAGAG AGAACAGAGA GAACGGGCGA TACAGGGATA   
  
  
+ CAAGGTTTGA GCAATTTTAG AAAATTTCTT GGGGCCGTTG ATGGGTTTTG AGTGAATTGC AAATCCCAGA   
  
  
+ AAGATTTCTG CGGTTTTTTA TCTACGGCTC TCTCTGTGGG AATTTTTTGG TAGATTCCTC TGGTTCCCAC   
  
  
+ TTTCATATCT TCTTATTTGT TCTACCCTCT TTACATCTGA TAGTTTGTTC TAACTGCTGC TTCAGTTGGT   
  
  
+ GCTTGTTCTC TTTAGTACTT TTCTGTTTTG TTCGTTTTGT TCATGCACTT GAATTCAACA AGAGCCCTTG   
  
  
+ TCCATTTTGA GCTCTTAATT CCCATCTAAC CCTGTTTCCT TCAGAAATTG CTGTTCTGTT CATATAGTTA   
  
  
+ TATTTTTTAT GCTTTGATTT GGGTATAAGT TTGCTGTTGG TGATTCCAAA GCTTGGTACT TTTGGCCATA   
  
  
+ TTTTGTTTGA GTTGGTGTTG ATTGGTACAA ATCTTGTAAT TGGTGTGATT GTTAATGGGA CCAATGCTTC   
  
  
+ AAGATGATGG TTCATCAGTA ACTTCTTCAT CACCTCTTCA ATTTTTCTCC ATGATGTCAC CCAATTTGGG   
  
  
+ TTCCTCCTAC CCTTGGCTAA GGGACTTAAA GCCTGAAGAG AGAGGTCTTT ACTTGATACA TTTGTTGCTC   
  
  
+ ACTTGTGCAA ACCATGTCTC TAATGGTAGC CTTGACAATG CAAACCTAGC CCTTGAGCAA ATCTCCCAGC   
  
  
+ TTGCAGCCCC TGATGGCGAT ACAATCCAGC GTATTGTTGC CTACTTTGCT GAATCACTTG CTGAAAGGAT   
  
  
+ CCTTAAGTCA TGGCCTGGCC TATATAAAGC CCTTCATTTC AATAGAATGC CTGTTATTTC AGAAGAATTT   
  
  
+ CTTGCTAGGA AGCTGTTTTT TGAGTTGTTT CCCTTCTTGA AGCTGGCCTT TTTGGTGACT AACCAATCAA   
  
  
+ TAATCGAGGC CATGGAGGGG GAAAAGATGG TGCATATAAT TGATCTGAAT GCGTCAGAAC CTGCACAGTG   
  
  
+ GATTGCCCTT ATTCAAGACT TGAGTGCTCG GCCTGAGGGC CCTCCTCATT TGAGGATTAC CGGGGTTCAT   
  
  
+ CAACAGAAAG AGGTTTTAGA ACAAGTAGCT CATAGATTGA CTGAAGAAGC TGAGAAGTTG GATTTGCCAT   
  
  
+ TTCAGTTCAA TCCTGTGGTT TGCAAACTAG AGAATCTCGA CATCGGAAAA CTCCGTGTTA AGACCGGGGA   
  
  
+ GGCCTTGGCT ATTACCTCGG TCCTTCAACT GCATACCCTT TTGGCTTCTG AAGAGGAAGT CCTTAAGAAA   
  
  
+ AGTTCACCCT TGGCATTGGT AAAGCAAGCC AATGGGGCTA ATTTACAGGG CTTGTTCAAT AAAGATGGAG   
  
  
+ CTAATAATAG GCGTAGCCCA AGTAATGATT CGGCTTCATC TGCACCTTCA TCCCTCAACA CTTCAGCCAA   
  
  
+ GATGGAAGGT TTCCTTAGCG CTTTGTGGGG TTTATCCCCA AAGATTATGG TGATAACCGA GCAAGATTCC   
  
  
+ AACCACAATG GGGCAGGACT AATGGAGAGA TTGTCAGAAG CATTGTACTT CTATGCAGCA TTGTTCGATT   
  
  
+ GCTTAGAATT TACCCTCCCG AGAACCTCCG TGGAGAGAAG GAAGGTCGAG ATGCTCCTCC TTGGCAAGGA   
  
  
+ AATCAAGAAC ATCATAGCGT GTGAGGGAGG AGAAAGAATA GATAGGCATG AGAAGTTGGG GAAGTGGATT   
  
  
+ AAGAGGCTTG AGATGGCCGG GTTTGGAAGC GTTCCTTTGA GCCACATAGG CATGATCCAA GCAAGGCGGT   
  
  
+ TGTTGCAGAG CTATGGCTGT GATGGTTATA GAATAAAGGA GGAGAACGGA TGTTTTGTTA TCTGCTGGCA   
  
  
+ AGATCGCCCC CTCTTTTCAG TATCTGCTTG GAGATGTAGG AGGTG  

- -Up\_Stream \_Len000AGATGG AAATGGACAC AATTTTTTTT TTTAAAACAA AGGTAATGAT AAGAGAACCG   
  
  
- TGATAACCAC GACTAAAAAG AAGTGGTTTA GTTGGAGGAA TATAAACGGT TAAAATGACA GGAGAAAGTA   
  
  
- GATATTTCAG TTGAGAGGAT CATGTTAAAC AGTGTATCTT TTAGAGATCC GTGAGTAGAT TTGAGAGACA   
  
  
- AGACCCATTA ACTAGCTGCT AGATGTGGTA ATAAGCAAGA TCACGTGGCA AACCTGTCGG TTAGTACATG   
  
  
- GAAACCTCTA GTTGACGAGT ACTCAAGAAC CACACTTTAA CCCCTTCATT TAAGCTGAAA TCCCGTGACC   
  
  
- AGATAGTGCG GTACTAAAAT GGTAGATAAA AGTATATATA GGTTATTCAA ACTAGAAAGC GATAATTAGT   
  
  
- GTTACTATAT TTGTTCACCA CTTCTCGTTA TTACTTGGTA TGTAAAATTT GAATTCGCAA TTCCTTCTGT   
  
  
- ACTTCAATTT TTGTTACCAT TCTCGATACG CATACAAACC GTATATCGAA AAAATTCTCA CAAAACCGAT   
  
  
- ATCAGCCTCA AAAAATTAAT TCTAATCGAC AAACTAGTTT ATTTTTTCGA TTAAACTCAC AAACCGCTCT   
  
  
- TCTGAAAAAT ATTCTTGAAA AAAACCAGAT TTTTCAATTA AATTTTTCCG ATTAAGATAC TCGAAAAGCC   
  
  
- TTCTCAAAAA ATTTATTAAT TGAAAAACAG AGTATTCGGT ATTGAAATAG TCTTTGTCAA TTAAAATGTT   
  
  
- TTATTAAAGA GTTGTTTGTT GATTAAATTT ATTAATTATT TTTATTGATT GAGTTTATAG ATTATTGATT   
  
  
- ATTGATTATA TTTATTAATT GTCAATTGTC AACAAATGCT TTGTCTTAAA TATATTTTTC GCCATTGTCT   
  
  
- ATTTTCTATT AGTACGCTCT AATTTTCAGT GGTCACTGTG TGTTCATGAA TTTATTTATC AGATTTGTTA   
  
  
- CTATAAAAAA CGCTCGAAGG TACGTTTATG GCTGTGAAAA TAACGGAGTC TTCCTTTGTT TTTGACTCAC   
  
  
- TTCCCCCCCC CCCCAAGACA AACAGAAACA ATCCCGTGCA AACCTAAGTT CACATCTAAC CCTTATTTAA   
  
  
- GCTCCTGAAA AAGAAACGGG AAAAAGGGAC TAAGGGTTTG GGTGTAGTTC AGTTCTGCTT TGGTCGGTAA   
  
  
- CTTTTTTCTC CCACAAACAC ACTCTATTTC TGTCTTTAAA ATACGGTCTC TCTCTTCTAT TGTTTACGTT   
  
  
- GTTGCAGCTG TCTCCGAGAG GAAGAGTGTT TAAGGTATAA GGAGACAAAA AATTCTTTCT TTTTCTTTCA   
  
  
- CACTCTGTTC TTGTTGTGTT CGTTTGTTAA GTCAACTGCT GCTTATGACT CACTATGGTG CGTGACACAC   
  
  
- GTTGACGCAA AGGTAAACAC CTCAGGAAGT TTCGTGCGAG TAGAAGTTGG GAAGGAAGCG GTGCCTTAAA   
  
  
- GTCCCCCTTT TTTTGGGGGT GGATTAAAAC TCTCTCTCTC TCTTGTCTCT CTTGCCCGCT ATGTCCCTAT   
  
  
- GTTCCAAACT CGTTAAAATC TTTTAAAGAA CCCCGGCAAC TACCCAAAAC TCACTTAACG TTTAGGGTCT   
  
  
- TTCTAAAGAC GCCAAAAAAT AGATGCCGAG AGAGACACCC TTAAAAAACC ATCTAAGGAG ACCAAGGGTG   
  
  
- AAAGTATAGA AGAATAAACA AGATGGGAGA AATGTAGACT ATCAAACAAG ATTGACGACG AAGTCAACCA   
  
  
- CGAACAAGAG AAATCATGAA AAGACAAAAC AAGCAAAACA AGTACGTGAA CTTAAGTTGT TCTCGGGAAC   
  
  
- AGGTAAAACT CGAGAATTAA GGGTAGATTG GGACAAAGGA AGTCTTTAAC GACAAGACAA GTATATCAAT   
  
  
- ATAAAAAATA CGAAACTAAA CCCATATTCA AACGACAACC ACTAAGGTTT CGAACCATGA AAACCGGTAT   
  
  
- AAAACAAACT CAACCACAAC TAACCATGTT TAGAACATTA ACCACACTAA CAATTACCCT GGTTACGAAG   
  
  
- TTCTACTACC AAGTAGTCAT TGAAGAAGTA GTGGAGAAGT TAAAAAGAGG TACTACAGTG GGTTAAACCC   
  
  
- AAGGAGGATG GGAACCGATT CCCTGAATTT CGGACTTCTC TCTCCAGAAA TGAACTATGT AAACAACGAG   
  
  
- TGAACACGTT TGGTACAGAG ATTACCATCG GAACTGTTAC GTTTGGATCG GGAACTCGTT TAGAGGGTCG   
  
  
- AACGTCGGGG ACTACCGCTA TGTTAGGTCG CATAACAACG GATGAAACGA CTTAGTGAAC GACTTTCCTA   
  
  
- GGAATTCAGT ACCGGACCGG ATATATTTCG GGAAGTAAAG TTATCTTACG GACAATAAAG TCTTCTTAAA   
  
  
- GAACGATCCT TCGACAAAAA ACTCAACAAA GGGAAGAACT TCGACCGGAA AAACCACTGA TTGGTTAGTT   
  
  
- ATTAGCTCCG GTACCTCCCC CTTTTCTACC ACGTATATTA ACTAGACTTA CGCAGTCTTG GACGTGTCAC   
  
  
- CTAACGGGAA TAAGTTCTGA ACTCACGAGC CGGACTCCCG GGAGGAGTAA ACTCCTAATG GCCCCAAGTA   
  
  
- GTTGTCTTTC TCCAAAATCT TGTTCATCGA GTATCTAACT GACTTCTTCG ACTCTTCAAC CTAAACGGTA   
  
  
- AAGTCAAGTT AGGACACCAA ACGTTTGATC TCTTAGAGCT GTAGCCTTTT GAGGCACAAT TCTGGCCCCT   
  
  
- CCGGAACCGA TAATGGAGCC AGGAAGTTGA CGTATGGGAA AACCGAAGAC TTCTCCTTCA GGAATTCTTT   
  
  
- TCAAGTGGGA ACCGTAACCA TTTCGTTCGG TTACCCCGAT TAAATGTCCC GAACAAGTTA TTTCTACCTC   
  
  
- GATTATTATC CGCATCGGGT TCATTACTAA GCCGAAGTAG ACGTGGAAGT AGGGAGTTGT GAAGTCGGTT   
  
  
- CTACCTTCCA AAGGAATCGC GAAACACCCC AAATAGGGGT TTCTAATACC ACTATTGGCT CGTTCTAAGG   
  
  
- TTGGTGTTAC CCCGTCCTGA TTACCTCTCT AACAGTCTTC GTAACATGAA GATACGTCGT AACAAGCTAA   
  
  
- CGAATCTTAA ATGGGAGGGC TCTTGGAGGC ACCTCTCTTC CTTCCAGCTC TACGAGGAGG AACCGTTCCT   
  
  
- TTAGTTCTTG TAGTATCGCA CACTCCCTCC TCTTTCTTAT CTATCCGTAC TCTTCAACCC CTTCACCTAA   
  
  
- TTCTCCGAAC TCTACCGGCC CAAACCTTCG CAAGGAAACT CGGTGTATCC GTACTAGGTT CGTTCCGCCA   
  
  
- ACAACGTCTC GATACCGACA CTACCAATAT CTTATTTCCT CCTCTTGCCT ACAAAACAAT AGACGACCGT   
  
  
- TCTAGCGGGG GAGAAAAGTC ATAGACGAAC CTCTACATCC TCCAC

+     GARE-motif

| Site Name | Organism | Position | Strand | Matrix score. | sequence | function |
| --- | --- | --- | --- | --- | --- | --- |
| GARE-motif | Brassica oleracea | 2595 | - | 7 | TCTGTTG | gibberellin-responsive element |

>HU08G01232.1   
+ -Up\_Stream \_Len000TCTACC TTTACCTGTG TTAAAAAAAA AAATTTTGTT TCCATTACTA TTCTCTTGGC   
  
  
+ ACTATTGGTG CTGATTTTTC TTCACCAAAT CAACCTCCTT ATATTTGCCA ATTTTACTGT CCTCTTTCAT   
  
  
+ CTATAAAGTC AACTCTCCTA GTACAATTTG TCACATAGAA AATCTCTAGG CACTCATCTA AACTCTCTGT   
  
  
+ TCTGGGTAAT TGATCGACGA TCTACACCAT TATTCGTTCT AGTGCACCGT TTGGACAGCC AATCATGTAC   
  
  
+ CTTTGGAGAT CAACTGCTCA TGAGTTCTTG GTGTGAAATT GGGGAAGTAA ATTCGACTTT AGGGCACTGG   
  
  
+ TCTATCACGC CATGATTTTA CCATCTATTT TCATATATAT CCAATAAGTT TGATCTTTCG CTATTAATCA   
  
  
+ CAATGATATA AACAAGTGGT GAAGAGCAAT AATGAACCAT ACATTTTAAA CTTAAGCGTT AAGGAAGACA   
  
  
+ TGAAGTTAAA AACAATGGTA AGAGCTATGC GTATGTTTGG CATATAGCTT TTTTAAGAGT GTTTTGGCTA   
  
  
+ TAGTCGGAGT TTTTTAATTA AGATTAGCTG TTTGATCAAA TAAAAAAGCT AATTTGAGTG TTTGGCGAGA   
  
  
+ AGACTTTTTA TAAGAACTTT TTTTGGTCTA AAAAGTTAAT TTAAAAAGGC TAATTCTATG AGCTTTTCGG   
  
  
+ AAGAGTTTTT TAAATAATTA ACTTTTTGTC TCATAAGCCA TAACTTTATC AGAAACAGTT AATTTTACAA   
  
  
+ AATAATTTCT CAACAAACAA CTAATTTAAA TAATTAATAA AAATAACTAA CTCAAATATC TAATAACTAA   
  
  
+ TAACTAATAT AAATAATTAA CAGTTAACAG TTGTTTACGA AACAGAATTT ATATAAAAAG CGGTAACAGA   
  
  
+ TAAAAGATAA TCATGCGAGA TTAAAAGTCA CCAGTGACAC ACAAGTACTT AAATAAATAG TCTAAACAAT   
  
  
+ GATATTTTTT GCGAGCTTCC ATGCAAATAC CGACACTTTT ATTGCCTCAG AAGGAAACAA AAACTGAGTG   
  
  
+ AAGGGGGGGG GGGGTTCTGT TTGTCTTTGT TAGGGCACGT TTGGATTCAA GTGTAGATTG GGAATAAATT   
  
  
+ CGAGGACTTT TTCTTTGCCC TTTTTCCCTG ATTCCCAAAC CCACATCAAG TCAAGACGAA ACCAGCCATT   
  
  
+ GAAAAAAGAG GGTGTTTGTG TGAGATAAAG ACAGAAATTT TATGCCAGAG AGAGAAGATA ACAAATGCAA   
  
  
+ CAACGTCGAC AGAGGCTCTC CTTCTCACAA ATTCCATATT CCTCTGTTTT TTAAGAAAGA AAAAGAAAGT   
  
  
+ GTGAGACAAG AACAACACAA GCAAACAATT CAGTTGACGA CGAATACTGA GTGATACCAC GCACTGTGTG   
  
  
+ CAACTGCGTT TCCATTTGTG GAGTCCTTCA AAGCACGCTC ATCTTCAACC CTTCCTTCGC CACGGAATTT   
  
  
+ CAGGGGGAAA AAAACCCCCA CCTAATTTTG AGAGAGAGAG AGAACAGAGA GAACGGGCGA TACAGGGATA   
  
  
+ CAAGGTTTGA GCAATTTTAG AAAATTTCTT GGGGCCGTTG ATGGGTTTTG AGTGAATTGC AAATCCCAGA   
  
  
+ AAGATTTCTG CGGTTTTTTA TCTACGGCTC TCTCTGTGGG AATTTTTTGG TAGATTCCTC TGGTTCCCAC   
  
  
+ TTTCATATCT TCTTATTTGT TCTACCCTCT TTACATCTGA TAGTTTGTTC TAACTGCTGC TTCAGTTGGT   
  
  
+ GCTTGTTCTC TTTAGTACTT TTCTGTTTTG TTCGTTTTGT TCATGCACTT GAATTCAACA AGAGCCCTTG   
  
  
+ TCCATTTTGA GCTCTTAATT CCCATCTAAC CCTGTTTCCT TCAGAAATTG CTGTTCTGTT CATATAGTTA   
  
  
+ TATTTTTTAT GCTTTGATTT GGGTATAAGT TTGCTGTTGG TGATTCCAAA GCTTGGTACT TTTGGCCATA   
  
  
+ TTTTGTTTGA GTTGGTGTTG ATTGGTACAA ATCTTGTAAT TGGTGTGATT GTTAATGGGA CCAATGCTTC   
  
  
+ AAGATGATGG TTCATCAGTA ACTTCTTCAT CACCTCTTCA ATTTTTCTCC ATGATGTCAC CCAATTTGGG   
  
  
+ TTCCTCCTAC CCTTGGCTAA GGGACTTAAA GCCTGAAGAG AGAGGTCTTT ACTTGATACA TTTGTTGCTC   
  
  
+ ACTTGTGCAA ACCATGTCTC TAATGGTAGC CTTGACAATG CAAACCTAGC CCTTGAGCAA ATCTCCCAGC   
  
  
+ TTGCAGCCCC TGATGGCGAT ACAATCCAGC GTATTGTTGC CTACTTTGCT GAATCACTTG CTGAAAGGAT   
  
  
+ CCTTAAGTCA TGGCCTGGCC TATATAAAGC CCTTCATTTC AATAGAATGC CTGTTATTTC AGAAGAATTT   
  
  
+ CTTGCTAGGA AGCTGTTTTT TGAGTTGTTT CCCTTCTTGA AGCTGGCCTT TTTGGTGACT AACCAATCAA   
  
  
+ TAATCGAGGC CATGGAGGGG GAAAAGATGG TGCATATAAT TGATCTGAAT GCGTCAGAAC CTGCACAGTG   
  
  
+ GATTGCCCTT ATTCAAGACT TGAGTGCTCG GCCTGAGGGC CCTCCTCATT TGAGGATTAC CGGGGTTCAT   
  
  
+ CAACAGAAAG AGGTTTTAGA ACAAGTAGCT CATAGATTGA CTGAAGAAGC TGAGAAGTTG GATTTGCCAT   
  
  
+ TTCAGTTCAA TCCTGTGGTT TGCAAACTAG AGAATCTCGA CATCGGAAAA CTCCGTGTTA AGACCGGGGA   
  
  
+ GGCCTTGGCT ATTACCTCGG TCCTTCAACT GCATACCCTT TTGGCTTCTG AAGAGGAAGT CCTTAAGAAA   
  
  
+ AGTTCACCCT TGGCATTGGT AAAGCAAGCC AATGGGGCTA ATTTACAGGG CTTGTTCAAT AAAGATGGAG   
  
  
+ CTAATAATAG GCGTAGCCCA AGTAATGATT CGGCTTCATC TGCACCTTCA TCCCTCAACA CTTCAGCCAA   
  
  
+ GATGGAAGGT TTCCTTAGCG CTTTGTGGGG TTTATCCCCA AAGATTATGG TGATAACCGA GCAAGATTCC   
  
  
+ AACCACAATG GGGCAGGACT AATGGAGAGA TTGTCAGAAG CATTGTACTT CTATGCAGCA TTGTTCGATT   
  
  
+ GCTTAGAATT TACCCTCCCG AGAACCTCCG TGGAGAGAAG GAAGGTCGAG ATGCTCCTCC TTGGCAAGGA   
  
  
+ AATCAAGAAC ATCATAGCGT GTGAGGGAGG AGAAAGAATA GATAGGCATG AGAAGTTGGG GAAGTGGATT   
  
  
+ AAGAGGCTTG AGATGGCCGG GTTTGGAAGC GTTCCTTTGA GCCACATAGG CATGATCCAA GCAAGGCGGT   
  
  
+ TGTTGCAGAG CTATGGCTGT GATGGTTATA GAATAAAGGA GGAGAACGGA TGTTTTGTTA TCTGCTGGCA   
  
  
+ AGATCGCCCC CTCTTTTCAG TATCTGCTTG GAGATGTAGG AGGTG  

- -Up\_Stream \_Len000AGATGG AAATGGACAC AATTTTTTTT TTTAAAACAA AGGTAATGAT AAGAGAACCG   
  
  
- TGATAACCAC GACTAAAAAG AAGTGGTTTA GTTGGAGGAA TATAAACGGT TAAAATGACA GGAGAAAGTA   
  
  
- GATATTTCAG TTGAGAGGAT CATGTTAAAC AGTGTATCTT TTAGAGATCC GTGAGTAGAT TTGAGAGACA   
  
  
- AGACCCATTA ACTAGCTGCT AGATGTGGTA ATAAGCAAGA TCACGTGGCA AACCTGTCGG TTAGTACATG   
  
  
- GAAACCTCTA GTTGACGAGT ACTCAAGAAC CACACTTTAA CCCCTTCATT TAAGCTGAAA TCCCGTGACC   
  
  
- AGATAGTGCG GTACTAAAAT GGTAGATAAA AGTATATATA GGTTATTCAA ACTAGAAAGC GATAATTAGT   
  
  
- GTTACTATAT TTGTTCACCA CTTCTCGTTA TTACTTGGTA TGTAAAATTT GAATTCGCAA TTCCTTCTGT   
  
  
- ACTTCAATTT TTGTTACCAT TCTCGATACG CATACAAACC GTATATCGAA AAAATTCTCA CAAAACCGAT   
  
  
- ATCAGCCTCA AAAAATTAAT TCTAATCGAC AAACTAGTTT ATTTTTTCGA TTAAACTCAC AAACCGCTCT   
  
  
- TCTGAAAAAT ATTCTTGAAA AAAACCAGAT TTTTCAATTA AATTTTTCCG ATTAAGATAC TCGAAAAGCC   
  
  
- TTCTCAAAAA ATTTATTAAT TGAAAAACAG AGTATTCGGT ATTGAAATAG TCTTTGTCAA TTAAAATGTT   
  
  
- TTATTAAAGA GTTGTTTGTT GATTAAATTT ATTAATTATT TTTATTGATT GAGTTTATAG ATTATTGATT   
  
  
- ATTGATTATA TTTATTAATT GTCAATTGTC AACAAATGCT TTGTCTTAAA TATATTTTTC GCCATTGTCT   
  
  
- ATTTTCTATT AGTACGCTCT AATTTTCAGT GGTCACTGTG TGTTCATGAA TTTATTTATC AGATTTGTTA   
  
  
- CTATAAAAAA CGCTCGAAGG TACGTTTATG GCTGTGAAAA TAACGGAGTC TTCCTTTGTT TTTGACTCAC   
  
  
- TTCCCCCCCC CCCCAAGACA AACAGAAACA ATCCCGTGCA AACCTAAGTT CACATCTAAC CCTTATTTAA   
  
  
- GCTCCTGAAA AAGAAACGGG AAAAAGGGAC TAAGGGTTTG GGTGTAGTTC AGTTCTGCTT TGGTCGGTAA   
  
  
- CTTTTTTCTC CCACAAACAC ACTCTATTTC TGTCTTTAAA ATACGGTCTC TCTCTTCTAT TGTTTACGTT   
  
  
- GTTGCAGCTG TCTCCGAGAG GAAGAGTGTT TAAGGTATAA GGAGACAAAA AATTCTTTCT TTTTCTTTCA   
  
  
- CACTCTGTTC TTGTTGTGTT CGTTTGTTAA GTCAACTGCT GCTTATGACT CACTATGGTG CGTGACACAC   
  
  
- GTTGACGCAA AGGTAAACAC CTCAGGAAGT TTCGTGCGAG TAGAAGTTGG GAAGGAAGCG GTGCCTTAAA   
  
  
- GTCCCCCTTT TTTTGGGGGT GGATTAAAAC TCTCTCTCTC TCTTGTCTCT CTTGCCCGCT ATGTCCCTAT   
  
  
- GTTCCAAACT CGTTAAAATC TTTTAAAGAA CCCCGGCAAC TACCCAAAAC TCACTTAACG TTTAGGGTCT   
  
  
- TTCTAAAGAC GCCAAAAAAT AGATGCCGAG AGAGACACCC TTAAAAAACC ATCTAAGGAG ACCAAGGGTG   
  
  
- AAAGTATAGA AGAATAAACA AGATGGGAGA AATGTAGACT ATCAAACAAG ATTGACGACG AAGTCAACCA   
  
  
- CGAACAAGAG AAATCATGAA AAGACAAAAC AAGCAAAACA AGTACGTGAA CTTAAGTTGT TCTCGGGAAC   
  
  
- AGGTAAAACT CGAGAATTAA GGGTAGATTG GGACAAAGGA AGTCTTTAAC GACAAGACAA GTATATCAAT   
  
  
- ATAAAAAATA CGAAACTAAA CCCATATTCA AACGACAACC ACTAAGGTTT CGAACCATGA AAACCGGTAT   
  
  
- AAAACAAACT CAACCACAAC TAACCATGTT TAGAACATTA ACCACACTAA CAATTACCCT GGTTACGAAG   
  
  
- TTCTACTACC AAGTAGTCAT TGAAGAAGTA GTGGAGAAGT TAAAAAGAGG TACTACAGTG GGTTAAACCC   
  
  
- AAGGAGGATG GGAACCGATT CCCTGAATTT CGGACTTCTC TCTCCAGAAA TGAACTATGT AAACAACGAG   
  
  
- TGAACACGTT TGGTACAGAG ATTACCATCG GAACTGTTAC GTTTGGATCG GGAACTCGTT TAGAGGGTCG   
  
  
- AACGTCGGGG ACTACCGCTA TGTTAGGTCG CATAACAACG GATGAAACGA CTTAGTGAAC GACTTTCCTA   
  
  
- GGAATTCAGT ACCGGACCGG ATATATTTCG GGAAGTAAAG TTATCTTACG GACAATAAAG TCTTCTTAAA   
  
  
- GAACGATCCT TCGACAAAAA ACTCAACAAA GGGAAGAACT TCGACCGGAA AAACCACTGA TTGGTTAGTT   
  
  
- ATTAGCTCCG GTACCTCCCC CTTTTCTACC ACGTATATTA ACTAGACTTA CGCAGTCTTG GACGTGTCAC   
  
  
- CTAACGGGAA TAAGTTCTGA ACTCACGAGC CGGACTCCCG GGAGGAGTAA ACTCCTAATG GCCCCAAGTA   
  
  
- GTTGTCTTTC TCCAAAATCT TGTTCATCGA GTATCTAACT GACTTCTTCG ACTCTTCAAC CTAAACGGTA   
  
  
- AAGTCAAGTT AGGACACCAA ACGTTTGATC TCTTAGAGCT GTAGCCTTTT GAGGCACAAT TCTGGCCCCT   
  
  
- CCGGAACCGA TAATGGAGCC AGGAAGTTGA CGTATGGGAA AACCGAAGAC TTCTCCTTCA GGAATTCTTT   
  
  
- TCAAGTGGGA ACCGTAACCA TTTCGTTCGG TTACCCCGAT TAAATGTCCC GAACAAGTTA TTTCTACCTC   
  
  
- GATTATTATC CGCATCGGGT TCATTACTAA GCCGAAGTAG ACGTGGAAGT AGGGAGTTGT GAAGTCGGTT   
  
  
- CTACCTTCCA AAGGAATCGC GAAACACCCC AAATAGGGGT TTCTAATACC ACTATTGGCT CGTTCTAAGG   
  
  
- TTGGTGTTAC CCCGTCCTGA TTACCTCTCT AACAGTCTTC GTAACATGAA GATACGTCGT AACAAGCTAA   
  
  
- CGAATCTTAA ATGGGAGGGC TCTTGGAGGC ACCTCTCTTC CTTCCAGCTC TACGAGGAGG AACCGTTCCT   
  
  
- TTAGTTCTTG TAGTATCGCA CACTCCCTCC TCTTTCTTAT CTATCCGTAC TCTTCAACCC CTTCACCTAA   
  
  
- TTCTCCGAAC TCTACCGGCC CAAACCTTCG CAAGGAAACT CGGTGTATCC GTACTAGGTT CGTTCCGCCA   
  
  
- ACAACGTCTC GATACCGACA CTACCAATAT CTTATTTCCT CCTCTTGCCT ACAAAACAAT AGACGACCGT   
  
  
- TCTAGCGGGG GAGAAAAGTC ATAGACGAAC CTCTACATCC TCCAC

+     LAMP-element

| Site Name | Organism | Position | Strand | Matrix score. | sequence | function |
| --- | --- | --- | --- | --- | --- | --- |
| LAMP-element | Pisum sativum | 748 | + | 8 | CTTTATCA | part of a light responsive element |

>HU08G01232.1   
+ -Up\_Stream \_Len000TCTACC TTTACCTGTG TTAAAAAAAA AAATTTTGTT TCCATTACTA TTCTCTTGGC   
  
  
+ ACTATTGGTG CTGATTTTTC TTCACCAAAT CAACCTCCTT ATATTTGCCA ATTTTACTGT CCTCTTTCAT   
  
  
+ CTATAAAGTC AACTCTCCTA GTACAATTTG TCACATAGAA AATCTCTAGG CACTCATCTA AACTCTCTGT   
  
  
+ TCTGGGTAAT TGATCGACGA TCTACACCAT TATTCGTTCT AGTGCACCGT TTGGACAGCC AATCATGTAC   
  
  
+ CTTTGGAGAT CAACTGCTCA TGAGTTCTTG GTGTGAAATT GGGGAAGTAA ATTCGACTTT AGGGCACTGG   
  
  
+ TCTATCACGC CATGATTTTA CCATCTATTT TCATATATAT CCAATAAGTT TGATCTTTCG CTATTAATCA   
  
  
+ CAATGATATA AACAAGTGGT GAAGAGCAAT AATGAACCAT ACATTTTAAA CTTAAGCGTT AAGGAAGACA   
  
  
+ TGAAGTTAAA AACAATGGTA AGAGCTATGC GTATGTTTGG CATATAGCTT TTTTAAGAGT GTTTTGGCTA   
  
  
+ TAGTCGGAGT TTTTTAATTA AGATTAGCTG TTTGATCAAA TAAAAAAGCT AATTTGAGTG TTTGGCGAGA   
  
  
+ AGACTTTTTA TAAGAACTTT TTTTGGTCTA AAAAGTTAAT TTAAAAAGGC TAATTCTATG AGCTTTTCGG   
  
  
+ AAGAGTTTTT TAAATAATTA ACTTTTTGTC TCATAAGCCA TAACTTTATC AGAAACAGTT AATTTTACAA   
  
  
+ AATAATTTCT CAACAAACAA CTAATTTAAA TAATTAATAA AAATAACTAA CTCAAATATC TAATAACTAA   
  
  
+ TAACTAATAT AAATAATTAA CAGTTAACAG TTGTTTACGA AACAGAATTT ATATAAAAAG CGGTAACAGA   
  
  
+ TAAAAGATAA TCATGCGAGA TTAAAAGTCA CCAGTGACAC ACAAGTACTT AAATAAATAG TCTAAACAAT   
  
  
+ GATATTTTTT GCGAGCTTCC ATGCAAATAC CGACACTTTT ATTGCCTCAG AAGGAAACAA AAACTGAGTG   
  
  
+ AAGGGGGGGG GGGGTTCTGT TTGTCTTTGT TAGGGCACGT TTGGATTCAA GTGTAGATTG GGAATAAATT   
  
  
+ CGAGGACTTT TTCTTTGCCC TTTTTCCCTG ATTCCCAAAC CCACATCAAG TCAAGACGAA ACCAGCCATT   
  
  
+ GAAAAAAGAG GGTGTTTGTG TGAGATAAAG ACAGAAATTT TATGCCAGAG AGAGAAGATA ACAAATGCAA   
  
  
+ CAACGTCGAC AGAGGCTCTC CTTCTCACAA ATTCCATATT CCTCTGTTTT TTAAGAAAGA AAAAGAAAGT   
  
  
+ GTGAGACAAG AACAACACAA GCAAACAATT CAGTTGACGA CGAATACTGA GTGATACCAC GCACTGTGTG   
  
  
+ CAACTGCGTT TCCATTTGTG GAGTCCTTCA AAGCACGCTC ATCTTCAACC CTTCCTTCGC CACGGAATTT   
  
  
+ CAGGGGGAAA AAAACCCCCA CCTAATTTTG AGAGAGAGAG AGAACAGAGA GAACGGGCGA TACAGGGATA   
  
  
+ CAAGGTTTGA GCAATTTTAG AAAATTTCTT GGGGCCGTTG ATGGGTTTTG AGTGAATTGC AAATCCCAGA   
  
  
+ AAGATTTCTG CGGTTTTTTA TCTACGGCTC TCTCTGTGGG AATTTTTTGG TAGATTCCTC TGGTTCCCAC   
  
  
+ TTTCATATCT TCTTATTTGT TCTACCCTCT TTACATCTGA TAGTTTGTTC TAACTGCTGC TTCAGTTGGT   
  
  
+ GCTTGTTCTC TTTAGTACTT TTCTGTTTTG TTCGTTTTGT TCATGCACTT GAATTCAACA AGAGCCCTTG   
  
  
+ TCCATTTTGA GCTCTTAATT CCCATCTAAC CCTGTTTCCT TCAGAAATTG CTGTTCTGTT CATATAGTTA   
  
  
+ TATTTTTTAT GCTTTGATTT GGGTATAAGT TTGCTGTTGG TGATTCCAAA GCTTGGTACT TTTGGCCATA   
  
  
+ TTTTGTTTGA GTTGGTGTTG ATTGGTACAA ATCTTGTAAT TGGTGTGATT GTTAATGGGA CCAATGCTTC   
  
  
+ AAGATGATGG TTCATCAGTA ACTTCTTCAT CACCTCTTCA ATTTTTCTCC ATGATGTCAC CCAATTTGGG   
  
  
+ TTCCTCCTAC CCTTGGCTAA GGGACTTAAA GCCTGAAGAG AGAGGTCTTT ACTTGATACA TTTGTTGCTC   
  
  
+ ACTTGTGCAA ACCATGTCTC TAATGGTAGC CTTGACAATG CAAACCTAGC CCTTGAGCAA ATCTCCCAGC   
  
  
+ TTGCAGCCCC TGATGGCGAT ACAATCCAGC GTATTGTTGC CTACTTTGCT GAATCACTTG CTGAAAGGAT   
  
  
+ CCTTAAGTCA TGGCCTGGCC TATATAAAGC CCTTCATTTC AATAGAATGC CTGTTATTTC AGAAGAATTT   
  
  
+ CTTGCTAGGA AGCTGTTTTT TGAGTTGTTT CCCTTCTTGA AGCTGGCCTT TTTGGTGACT AACCAATCAA   
  
  
+ TAATCGAGGC CATGGAGGGG GAAAAGATGG TGCATATAAT TGATCTGAAT GCGTCAGAAC CTGCACAGTG   
  
  
+ GATTGCCCTT ATTCAAGACT TGAGTGCTCG GCCTGAGGGC CCTCCTCATT TGAGGATTAC CGGGGTTCAT   
  
  
+ CAACAGAAAG AGGTTTTAGA ACAAGTAGCT CATAGATTGA CTGAAGAAGC TGAGAAGTTG GATTTGCCAT   
  
  
+ TTCAGTTCAA TCCTGTGGTT TGCAAACTAG AGAATCTCGA CATCGGAAAA CTCCGTGTTA AGACCGGGGA   
  
  
+ GGCCTTGGCT ATTACCTCGG TCCTTCAACT GCATACCCTT TTGGCTTCTG AAGAGGAAGT CCTTAAGAAA   
  
  
+ AGTTCACCCT TGGCATTGGT AAAGCAAGCC AATGGGGCTA ATTTACAGGG CTTGTTCAAT AAAGATGGAG   
  
  
+ CTAATAATAG GCGTAGCCCA AGTAATGATT CGGCTTCATC TGCACCTTCA TCCCTCAACA CTTCAGCCAA   
  
  
+ GATGGAAGGT TTCCTTAGCG CTTTGTGGGG TTTATCCCCA AAGATTATGG TGATAACCGA GCAAGATTCC   
  
  
+ AACCACAATG GGGCAGGACT AATGGAGAGA TTGTCAGAAG CATTGTACTT CTATGCAGCA TTGTTCGATT   
  
  
+ GCTTAGAATT TACCCTCCCG AGAACCTCCG TGGAGAGAAG GAAGGTCGAG ATGCTCCTCC TTGGCAAGGA   
  
  
+ AATCAAGAAC ATCATAGCGT GTGAGGGAGG AGAAAGAATA GATAGGCATG AGAAGTTGGG GAAGTGGATT   
  
  
+ AAGAGGCTTG AGATGGCCGG GTTTGGAAGC GTTCCTTTGA GCCACATAGG CATGATCCAA GCAAGGCGGT   
  
  
+ TGTTGCAGAG CTATGGCTGT GATGGTTATA GAATAAAGGA GGAGAACGGA TGTTTTGTTA TCTGCTGGCA   
  
  
+ AGATCGCCCC CTCTTTTCAG TATCTGCTTG GAGATGTAGG AGGTG  

- -Up\_Stream \_Len000AGATGG AAATGGACAC AATTTTTTTT TTTAAAACAA AGGTAATGAT AAGAGAACCG   
  
  
- TGATAACCAC GACTAAAAAG AAGTGGTTTA GTTGGAGGAA TATAAACGGT TAAAATGACA GGAGAAAGTA   
  
  
- GATATTTCAG TTGAGAGGAT CATGTTAAAC AGTGTATCTT TTAGAGATCC GTGAGTAGAT TTGAGAGACA   
  
  
- AGACCCATTA ACTAGCTGCT AGATGTGGTA ATAAGCAAGA TCACGTGGCA AACCTGTCGG TTAGTACATG   
  
  
- GAAACCTCTA GTTGACGAGT ACTCAAGAAC CACACTTTAA CCCCTTCATT TAAGCTGAAA TCCCGTGACC   
  
  
- AGATAGTGCG GTACTAAAAT GGTAGATAAA AGTATATATA GGTTATTCAA ACTAGAAAGC GATAATTAGT   
  
  
- GTTACTATAT TTGTTCACCA CTTCTCGTTA TTACTTGGTA TGTAAAATTT GAATTCGCAA TTCCTTCTGT   
  
  
- ACTTCAATTT TTGTTACCAT TCTCGATACG CATACAAACC GTATATCGAA AAAATTCTCA CAAAACCGAT   
  
  
- ATCAGCCTCA AAAAATTAAT TCTAATCGAC AAACTAGTTT ATTTTTTCGA TTAAACTCAC AAACCGCTCT   
  
  
- TCTGAAAAAT ATTCTTGAAA AAAACCAGAT TTTTCAATTA AATTTTTCCG ATTAAGATAC TCGAAAAGCC   
  
  
- TTCTCAAAAA ATTTATTAAT TGAAAAACAG AGTATTCGGT ATTGAAATAG TCTTTGTCAA TTAAAATGTT   
  
  
- TTATTAAAGA GTTGTTTGTT GATTAAATTT ATTAATTATT TTTATTGATT GAGTTTATAG ATTATTGATT   
  
  
- ATTGATTATA TTTATTAATT GTCAATTGTC AACAAATGCT TTGTCTTAAA TATATTTTTC GCCATTGTCT   
  
  
- ATTTTCTATT AGTACGCTCT AATTTTCAGT GGTCACTGTG TGTTCATGAA TTTATTTATC AGATTTGTTA   
  
  
- CTATAAAAAA CGCTCGAAGG TACGTTTATG GCTGTGAAAA TAACGGAGTC TTCCTTTGTT TTTGACTCAC   
  
  
- TTCCCCCCCC CCCCAAGACA AACAGAAACA ATCCCGTGCA AACCTAAGTT CACATCTAAC CCTTATTTAA   
  
  
- GCTCCTGAAA AAGAAACGGG AAAAAGGGAC TAAGGGTTTG GGTGTAGTTC AGTTCTGCTT TGGTCGGTAA   
  
  
- CTTTTTTCTC CCACAAACAC ACTCTATTTC TGTCTTTAAA ATACGGTCTC TCTCTTCTAT TGTTTACGTT   
  
  
- GTTGCAGCTG TCTCCGAGAG GAAGAGTGTT TAAGGTATAA GGAGACAAAA AATTCTTTCT TTTTCTTTCA   
  
  
- CACTCTGTTC TTGTTGTGTT CGTTTGTTAA GTCAACTGCT GCTTATGACT CACTATGGTG CGTGACACAC   
  
  
- GTTGACGCAA AGGTAAACAC CTCAGGAAGT TTCGTGCGAG TAGAAGTTGG GAAGGAAGCG GTGCCTTAAA   
  
  
- GTCCCCCTTT TTTTGGGGGT GGATTAAAAC TCTCTCTCTC TCTTGTCTCT CTTGCCCGCT ATGTCCCTAT   
  
  
- GTTCCAAACT CGTTAAAATC TTTTAAAGAA CCCCGGCAAC TACCCAAAAC TCACTTAACG TTTAGGGTCT   
  
  
- TTCTAAAGAC GCCAAAAAAT AGATGCCGAG AGAGACACCC TTAAAAAACC ATCTAAGGAG ACCAAGGGTG   
  
  
- AAAGTATAGA AGAATAAACA AGATGGGAGA AATGTAGACT ATCAAACAAG ATTGACGACG AAGTCAACCA   
  
  
- CGAACAAGAG AAATCATGAA AAGACAAAAC AAGCAAAACA AGTACGTGAA CTTAAGTTGT TCTCGGGAAC   
  
  
- AGGTAAAACT CGAGAATTAA GGGTAGATTG GGACAAAGGA AGTCTTTAAC GACAAGACAA GTATATCAAT   
  
  
- ATAAAAAATA CGAAACTAAA CCCATATTCA AACGACAACC ACTAAGGTTT CGAACCATGA AAACCGGTAT   
  
  
- AAAACAAACT CAACCACAAC TAACCATGTT TAGAACATTA ACCACACTAA CAATTACCCT GGTTACGAAG   
  
  
- TTCTACTACC AAGTAGTCAT TGAAGAAGTA GTGGAGAAGT TAAAAAGAGG TACTACAGTG GGTTAAACCC   
  
  
- AAGGAGGATG GGAACCGATT CCCTGAATTT CGGACTTCTC TCTCCAGAAA TGAACTATGT AAACAACGAG   
  
  
- TGAACACGTT TGGTACAGAG ATTACCATCG GAACTGTTAC GTTTGGATCG GGAACTCGTT TAGAGGGTCG   
  
  
- AACGTCGGGG ACTACCGCTA TGTTAGGTCG CATAACAACG GATGAAACGA CTTAGTGAAC GACTTTCCTA   
  
  
- GGAATTCAGT ACCGGACCGG ATATATTTCG GGAAGTAAAG TTATCTTACG GACAATAAAG TCTTCTTAAA   
  
  
- GAACGATCCT TCGACAAAAA ACTCAACAAA GGGAAGAACT TCGACCGGAA AAACCACTGA TTGGTTAGTT   
  
  
- ATTAGCTCCG GTACCTCCCC CTTTTCTACC ACGTATATTA ACTAGACTTA CGCAGTCTTG GACGTGTCAC   
  
  
- CTAACGGGAA TAAGTTCTGA ACTCACGAGC CGGACTCCCG GGAGGAGTAA ACTCCTAATG GCCCCAAGTA   
  
  
- GTTGTCTTTC TCCAAAATCT TGTTCATCGA GTATCTAACT GACTTCTTCG ACTCTTCAAC CTAAACGGTA   
  
  
- AAGTCAAGTT AGGACACCAA ACGTTTGATC TCTTAGAGCT GTAGCCTTTT GAGGCACAAT TCTGGCCCCT   
  
  
- CCGGAACCGA TAATGGAGCC AGGAAGTTGA CGTATGGGAA AACCGAAGAC TTCTCCTTCA GGAATTCTTT   
  
  
- TCAAGTGGGA ACCGTAACCA TTTCGTTCGG TTACCCCGAT TAAATGTCCC GAACAAGTTA TTTCTACCTC   
  
  
- GATTATTATC CGCATCGGGT TCATTACTAA GCCGAAGTAG ACGTGGAAGT AGGGAGTTGT GAAGTCGGTT   
  
  
- CTACCTTCCA AAGGAATCGC GAAACACCCC AAATAGGGGT TTCTAATACC ACTATTGGCT CGTTCTAAGG   
  
  
- TTGGTGTTAC CCCGTCCTGA TTACCTCTCT AACAGTCTTC GTAACATGAA GATACGTCGT AACAAGCTAA   
  
  
- CGAATCTTAA ATGGGAGGGC TCTTGGAGGC ACCTCTCTTC CTTCCAGCTC TACGAGGAGG AACCGTTCCT   
  
  
- TTAGTTCTTG TAGTATCGCA CACTCCCTCC TCTTTCTTAT CTATCCGTAC TCTTCAACCC CTTCACCTAA   
  
  
- TTCTCCGAAC TCTACCGGCC CAAACCTTCG CAAGGAAACT CGGTGTATCC GTACTAGGTT CGTTCCGCCA   
  
  
- ACAACGTCTC GATACCGACA CTACCAATAT CTTATTTCCT CCTCTTGCCT ACAAAACAAT AGACGACCGT   
  
  
- TCTAGCGGGG GAGAAAAGTC ATAGACGAAC CTCTACATCC TCCAC

+     LTR

| Site Name | Organism | Position | Strand | Matrix score. | sequence | function |
| --- | --- | --- | --- | --- | --- | --- |
| LTR | Hordeum vulgare | 699 | - | 6 | CCGAAA | cis-acting element involved in low-temperature responsiveness |

>HU08G01232.1   
+ -Up\_Stream \_Len000TCTACC TTTACCTGTG TTAAAAAAAA AAATTTTGTT TCCATTACTA TTCTCTTGGC   
  
  
+ ACTATTGGTG CTGATTTTTC TTCACCAAAT CAACCTCCTT ATATTTGCCA ATTTTACTGT CCTCTTTCAT   
  
  
+ CTATAAAGTC AACTCTCCTA GTACAATTTG TCACATAGAA AATCTCTAGG CACTCATCTA AACTCTCTGT   
  
  
+ TCTGGGTAAT TGATCGACGA TCTACACCAT TATTCGTTCT AGTGCACCGT TTGGACAGCC AATCATGTAC   
  
  
+ CTTTGGAGAT CAACTGCTCA TGAGTTCTTG GTGTGAAATT GGGGAAGTAA ATTCGACTTT AGGGCACTGG   
  
  
+ TCTATCACGC CATGATTTTA CCATCTATTT TCATATATAT CCAATAAGTT TGATCTTTCG CTATTAATCA   
  
  
+ CAATGATATA AACAAGTGGT GAAGAGCAAT AATGAACCAT ACATTTTAAA CTTAAGCGTT AAGGAAGACA   
  
  
+ TGAAGTTAAA AACAATGGTA AGAGCTATGC GTATGTTTGG CATATAGCTT TTTTAAGAGT GTTTTGGCTA   
  
  
+ TAGTCGGAGT TTTTTAATTA AGATTAGCTG TTTGATCAAA TAAAAAAGCT AATTTGAGTG TTTGGCGAGA   
  
  
+ AGACTTTTTA TAAGAACTTT TTTTGGTCTA AAAAGTTAAT TTAAAAAGGC TAATTCTATG AGCTTTTCGG   
  
  
+ AAGAGTTTTT TAAATAATTA ACTTTTTGTC TCATAAGCCA TAACTTTATC AGAAACAGTT AATTTTACAA   
  
  
+ AATAATTTCT CAACAAACAA CTAATTTAAA TAATTAATAA AAATAACTAA CTCAAATATC TAATAACTAA   
  
  
+ TAACTAATAT AAATAATTAA CAGTTAACAG TTGTTTACGA AACAGAATTT ATATAAAAAG CGGTAACAGA   
  
  
+ TAAAAGATAA TCATGCGAGA TTAAAAGTCA CCAGTGACAC ACAAGTACTT AAATAAATAG TCTAAACAAT   
  
  
+ GATATTTTTT GCGAGCTTCC ATGCAAATAC CGACACTTTT ATTGCCTCAG AAGGAAACAA AAACTGAGTG   
  
  
+ AAGGGGGGGG GGGGTTCTGT TTGTCTTTGT TAGGGCACGT TTGGATTCAA GTGTAGATTG GGAATAAATT   
  
  
+ CGAGGACTTT TTCTTTGCCC TTTTTCCCTG ATTCCCAAAC CCACATCAAG TCAAGACGAA ACCAGCCATT   
  
  
+ GAAAAAAGAG GGTGTTTGTG TGAGATAAAG ACAGAAATTT TATGCCAGAG AGAGAAGATA ACAAATGCAA   
  
  
+ CAACGTCGAC AGAGGCTCTC CTTCTCACAA ATTCCATATT CCTCTGTTTT TTAAGAAAGA AAAAGAAAGT   
  
  
+ GTGAGACAAG AACAACACAA GCAAACAATT CAGTTGACGA CGAATACTGA GTGATACCAC GCACTGTGTG   
  
  
+ CAACTGCGTT TCCATTTGTG GAGTCCTTCA AAGCACGCTC ATCTTCAACC CTTCCTTCGC CACGGAATTT   
  
  
+ CAGGGGGAAA AAAACCCCCA CCTAATTTTG AGAGAGAGAG AGAACAGAGA GAACGGGCGA TACAGGGATA   
  
  
+ CAAGGTTTGA GCAATTTTAG AAAATTTCTT GGGGCCGTTG ATGGGTTTTG AGTGAATTGC AAATCCCAGA   
  
  
+ AAGATTTCTG CGGTTTTTTA TCTACGGCTC TCTCTGTGGG AATTTTTTGG TAGATTCCTC TGGTTCCCAC   
  
  
+ TTTCATATCT TCTTATTTGT TCTACCCTCT TTACATCTGA TAGTTTGTTC TAACTGCTGC TTCAGTTGGT   
  
  
+ GCTTGTTCTC TTTAGTACTT TTCTGTTTTG TTCGTTTTGT TCATGCACTT GAATTCAACA AGAGCCCTTG   
  
  
+ TCCATTTTGA GCTCTTAATT CCCATCTAAC CCTGTTTCCT TCAGAAATTG CTGTTCTGTT CATATAGTTA   
  
  
+ TATTTTTTAT GCTTTGATTT GGGTATAAGT TTGCTGTTGG TGATTCCAAA GCTTGGTACT TTTGGCCATA   
  
  
+ TTTTGTTTGA GTTGGTGTTG ATTGGTACAA ATCTTGTAAT TGGTGTGATT GTTAATGGGA CCAATGCTTC   
  
  
+ AAGATGATGG TTCATCAGTA ACTTCTTCAT CACCTCTTCA ATTTTTCTCC ATGATGTCAC CCAATTTGGG   
  
  
+ TTCCTCCTAC CCTTGGCTAA GGGACTTAAA GCCTGAAGAG AGAGGTCTTT ACTTGATACA TTTGTTGCTC   
  
  
+ ACTTGTGCAA ACCATGTCTC TAATGGTAGC CTTGACAATG CAAACCTAGC CCTTGAGCAA ATCTCCCAGC   
  
  
+ TTGCAGCCCC TGATGGCGAT ACAATCCAGC GTATTGTTGC CTACTTTGCT GAATCACTTG CTGAAAGGAT   
  
  
+ CCTTAAGTCA TGGCCTGGCC TATATAAAGC CCTTCATTTC AATAGAATGC CTGTTATTTC AGAAGAATTT   
  
  
+ CTTGCTAGGA AGCTGTTTTT TGAGTTGTTT CCCTTCTTGA AGCTGGCCTT TTTGGTGACT AACCAATCAA   
  
  
+ TAATCGAGGC CATGGAGGGG GAAAAGATGG TGCATATAAT TGATCTGAAT GCGTCAGAAC CTGCACAGTG   
  
  
+ GATTGCCCTT ATTCAAGACT TGAGTGCTCG GCCTGAGGGC CCTCCTCATT TGAGGATTAC CGGGGTTCAT   
  
  
+ CAACAGAAAG AGGTTTTAGA ACAAGTAGCT CATAGATTGA CTGAAGAAGC TGAGAAGTTG GATTTGCCAT   
  
  
+ TTCAGTTCAA TCCTGTGGTT TGCAAACTAG AGAATCTCGA CATCGGAAAA CTCCGTGTTA AGACCGGGGA   
  
  
+ GGCCTTGGCT ATTACCTCGG TCCTTCAACT GCATACCCTT TTGGCTTCTG AAGAGGAAGT CCTTAAGAAA   
  
  
+ AGTTCACCCT TGGCATTGGT AAAGCAAGCC AATGGGGCTA ATTTACAGGG CTTGTTCAAT AAAGATGGAG   
  
  
+ CTAATAATAG GCGTAGCCCA AGTAATGATT CGGCTTCATC TGCACCTTCA TCCCTCAACA CTTCAGCCAA   
  
  
+ GATGGAAGGT TTCCTTAGCG CTTTGTGGGG TTTATCCCCA AAGATTATGG TGATAACCGA GCAAGATTCC   
  
  
+ AACCACAATG GGGCAGGACT AATGGAGAGA TTGTCAGAAG CATTGTACTT CTATGCAGCA TTGTTCGATT   
  
  
+ GCTTAGAATT TACCCTCCCG AGAACCTCCG TGGAGAGAAG GAAGGTCGAG ATGCTCCTCC TTGGCAAGGA   
  
  
+ AATCAAGAAC ATCATAGCGT GTGAGGGAGG AGAAAGAATA GATAGGCATG AGAAGTTGGG GAAGTGGATT   
  
  
+ AAGAGGCTTG AGATGGCCGG GTTTGGAAGC GTTCCTTTGA GCCACATAGG CATGATCCAA GCAAGGCGGT   
  
  
+ TGTTGCAGAG CTATGGCTGT GATGGTTATA GAATAAAGGA GGAGAACGGA TGTTTTGTTA TCTGCTGGCA   
  
  
+ AGATCGCCCC CTCTTTTCAG TATCTGCTTG GAGATGTAGG AGGTG  

- -Up\_Stream \_Len000AGATGG AAATGGACAC AATTTTTTTT TTTAAAACAA AGGTAATGAT AAGAGAACCG   
  
  
- TGATAACCAC GACTAAAAAG AAGTGGTTTA GTTGGAGGAA TATAAACGGT TAAAATGACA GGAGAAAGTA   
  
  
- GATATTTCAG TTGAGAGGAT CATGTTAAAC AGTGTATCTT TTAGAGATCC GTGAGTAGAT TTGAGAGACA   
  
  
- AGACCCATTA ACTAGCTGCT AGATGTGGTA ATAAGCAAGA TCACGTGGCA AACCTGTCGG TTAGTACATG   
  
  
- GAAACCTCTA GTTGACGAGT ACTCAAGAAC CACACTTTAA CCCCTTCATT TAAGCTGAAA TCCCGTGACC   
  
  
- AGATAGTGCG GTACTAAAAT GGTAGATAAA AGTATATATA GGTTATTCAA ACTAGAAAGC GATAATTAGT   
  
  
- GTTACTATAT TTGTTCACCA CTTCTCGTTA TTACTTGGTA TGTAAAATTT GAATTCGCAA TTCCTTCTGT   
  
  
- ACTTCAATTT TTGTTACCAT TCTCGATACG CATACAAACC GTATATCGAA AAAATTCTCA CAAAACCGAT   
  
  
- ATCAGCCTCA AAAAATTAAT TCTAATCGAC AAACTAGTTT ATTTTTTCGA TTAAACTCAC AAACCGCTCT   
  
  
- TCTGAAAAAT ATTCTTGAAA AAAACCAGAT TTTTCAATTA AATTTTTCCG ATTAAGATAC TCGAAAAGCC   
  
  
- TTCTCAAAAA ATTTATTAAT TGAAAAACAG AGTATTCGGT ATTGAAATAG TCTTTGTCAA TTAAAATGTT   
  
  
- TTATTAAAGA GTTGTTTGTT GATTAAATTT ATTAATTATT TTTATTGATT GAGTTTATAG ATTATTGATT   
  
  
- ATTGATTATA TTTATTAATT GTCAATTGTC AACAAATGCT TTGTCTTAAA TATATTTTTC GCCATTGTCT   
  
  
- ATTTTCTATT AGTACGCTCT AATTTTCAGT GGTCACTGTG TGTTCATGAA TTTATTTATC AGATTTGTTA   
  
  
- CTATAAAAAA CGCTCGAAGG TACGTTTATG GCTGTGAAAA TAACGGAGTC TTCCTTTGTT TTTGACTCAC   
  
  
- TTCCCCCCCC CCCCAAGACA AACAGAAACA ATCCCGTGCA AACCTAAGTT CACATCTAAC CCTTATTTAA   
  
  
- GCTCCTGAAA AAGAAACGGG AAAAAGGGAC TAAGGGTTTG GGTGTAGTTC AGTTCTGCTT TGGTCGGTAA   
  
  
- CTTTTTTCTC CCACAAACAC ACTCTATTTC TGTCTTTAAA ATACGGTCTC TCTCTTCTAT TGTTTACGTT   
  
  
- GTTGCAGCTG TCTCCGAGAG GAAGAGTGTT TAAGGTATAA GGAGACAAAA AATTCTTTCT TTTTCTTTCA   
  
  
- CACTCTGTTC TTGTTGTGTT CGTTTGTTAA GTCAACTGCT GCTTATGACT CACTATGGTG CGTGACACAC   
  
  
- GTTGACGCAA AGGTAAACAC CTCAGGAAGT TTCGTGCGAG TAGAAGTTGG GAAGGAAGCG GTGCCTTAAA   
  
  
- GTCCCCCTTT TTTTGGGGGT GGATTAAAAC TCTCTCTCTC TCTTGTCTCT CTTGCCCGCT ATGTCCCTAT   
  
  
- GTTCCAAACT CGTTAAAATC TTTTAAAGAA CCCCGGCAAC TACCCAAAAC TCACTTAACG TTTAGGGTCT   
  
  
- TTCTAAAGAC GCCAAAAAAT AGATGCCGAG AGAGACACCC TTAAAAAACC ATCTAAGGAG ACCAAGGGTG   
  
  
- AAAGTATAGA AGAATAAACA AGATGGGAGA AATGTAGACT ATCAAACAAG ATTGACGACG AAGTCAACCA   
  
  
- CGAACAAGAG AAATCATGAA AAGACAAAAC AAGCAAAACA AGTACGTGAA CTTAAGTTGT TCTCGGGAAC   
  
  
- AGGTAAAACT CGAGAATTAA GGGTAGATTG GGACAAAGGA AGTCTTTAAC GACAAGACAA GTATATCAAT   
  
  
- ATAAAAAATA CGAAACTAAA CCCATATTCA AACGACAACC ACTAAGGTTT CGAACCATGA AAACCGGTAT   
  
  
- AAAACAAACT CAACCACAAC TAACCATGTT TAGAACATTA ACCACACTAA CAATTACCCT GGTTACGAAG   
  
  
- TTCTACTACC AAGTAGTCAT TGAAGAAGTA GTGGAGAAGT TAAAAAGAGG TACTACAGTG GGTTAAACCC   
  
  
- AAGGAGGATG GGAACCGATT CCCTGAATTT CGGACTTCTC TCTCCAGAAA TGAACTATGT AAACAACGAG   
  
  
- TGAACACGTT TGGTACAGAG ATTACCATCG GAACTGTTAC GTTTGGATCG GGAACTCGTT TAGAGGGTCG   
  
  
- AACGTCGGGG ACTACCGCTA TGTTAGGTCG CATAACAACG GATGAAACGA CTTAGTGAAC GACTTTCCTA   
  
  
- GGAATTCAGT ACCGGACCGG ATATATTTCG GGAAGTAAAG TTATCTTACG GACAATAAAG TCTTCTTAAA   
  
  
- GAACGATCCT TCGACAAAAA ACTCAACAAA GGGAAGAACT TCGACCGGAA AAACCACTGA TTGGTTAGTT   
  
  
- ATTAGCTCCG GTACCTCCCC CTTTTCTACC ACGTATATTA ACTAGACTTA CGCAGTCTTG GACGTGTCAC   
  
  
- CTAACGGGAA TAAGTTCTGA ACTCACGAGC CGGACTCCCG GGAGGAGTAA ACTCCTAATG GCCCCAAGTA   
  
  
- GTTGTCTTTC TCCAAAATCT TGTTCATCGA GTATCTAACT GACTTCTTCG ACTCTTCAAC CTAAACGGTA   
  
  
- AAGTCAAGTT AGGACACCAA ACGTTTGATC TCTTAGAGCT GTAGCCTTTT GAGGCACAAT TCTGGCCCCT   
  
  
- CCGGAACCGA TAATGGAGCC AGGAAGTTGA CGTATGGGAA AACCGAAGAC TTCTCCTTCA GGAATTCTTT   
  
  
- TCAAGTGGGA ACCGTAACCA TTTCGTTCGG TTACCCCGAT TAAATGTCCC GAACAAGTTA TTTCTACCTC   
  
  
- GATTATTATC CGCATCGGGT TCATTACTAA GCCGAAGTAG ACGTGGAAGT AGGGAGTTGT GAAGTCGGTT   
  
  
- CTACCTTCCA AAGGAATCGC GAAACACCCC AAATAGGGGT TTCTAATACC ACTATTGGCT CGTTCTAAGG   
  
  
- TTGGTGTTAC CCCGTCCTGA TTACCTCTCT AACAGTCTTC GTAACATGAA GATACGTCGT AACAAGCTAA   
  
  
- CGAATCTTAA ATGGGAGGGC TCTTGGAGGC ACCTCTCTTC CTTCCAGCTC TACGAGGAGG AACCGTTCCT   
  
  
- TTAGTTCTTG TAGTATCGCA CACTCCCTCC TCTTTCTTAT CTATCCGTAC TCTTCAACCC CTTCACCTAA   
  
  
- TTCTCCGAAC TCTACCGGCC CAAACCTTCG CAAGGAAACT CGGTGTATCC GTACTAGGTT CGTTCCGCCA   
  
  
- ACAACGTCTC GATACCGACA CTACCAATAT CTTATTTCCT CCTCTTGCCT ACAAAACAAT AGACGACCGT   
  
  
- TCTAGCGGGG GAGAAAAGTC ATAGACGAAC CTCTACATCC TCCAC

+     MBS

| Site Name | Organism | Position | Strand | Matrix score. | sequence | function |
| --- | --- | --- | --- | --- | --- | --- |
| MBS | Arabidopsis thaliana | 1365 | - | 6 | CAACTG | MYB binding site involved in drought-inducibility |
| MBS | Arabidopsis thaliana | 1747 | - | 6 | CAACTG | MYB binding site involved in drought-inducibility |
| MBS | Arabidopsis thaliana | 1405 | + | 6 | CAACTG | MYB binding site involved in drought-inducibility |
| MBS | Arabidopsis thaliana | 2760 | + | 6 | CAACTG | MYB binding site involved in drought-inducibility |
| MBS | Arabidopsis thaliana | 295 | + | 6 | CAACTG | MYB binding site involved in drought-inducibility |
| MBS | Arabidopsis thaliana | 872 | - | 6 | CAACTG | MYB binding site involved in drought-inducibility |

>HU08G01232.1   
+ -Up\_Stream \_Len000TCTACC TTTACCTGTG TTAAAAAAAA AAATTTTGTT TCCATTACTA TTCTCTTGGC   
  
  
+ ACTATTGGTG CTGATTTTTC TTCACCAAAT CAACCTCCTT ATATTTGCCA ATTTTACTGT CCTCTTTCAT   
  
  
+ CTATAAAGTC AACTCTCCTA GTACAATTTG TCACATAGAA AATCTCTAGG CACTCATCTA AACTCTCTGT   
  
  
+ TCTGGGTAAT TGATCGACGA TCTACACCAT TATTCGTTCT AGTGCACCGT TTGGACAGCC AATCATGTAC   
  
  
+ CTTTGGAGAT CAACTGCTCA TGAGTTCTTG GTGTGAAATT GGGGAAGTAA ATTCGACTTT AGGGCACTGG   
  
  
+ TCTATCACGC CATGATTTTA CCATCTATTT TCATATATAT CCAATAAGTT TGATCTTTCG CTATTAATCA   
  
  
+ CAATGATATA AACAAGTGGT GAAGAGCAAT AATGAACCAT ACATTTTAAA CTTAAGCGTT AAGGAAGACA   
  
  
+ TGAAGTTAAA AACAATGGTA AGAGCTATGC GTATGTTTGG CATATAGCTT TTTTAAGAGT GTTTTGGCTA   
  
  
+ TAGTCGGAGT TTTTTAATTA AGATTAGCTG TTTGATCAAA TAAAAAAGCT AATTTGAGTG TTTGGCGAGA   
  
  
+ AGACTTTTTA TAAGAACTTT TTTTGGTCTA AAAAGTTAAT TTAAAAAGGC TAATTCTATG AGCTTTTCGG   
  
  
+ AAGAGTTTTT TAAATAATTA ACTTTTTGTC TCATAAGCCA TAACTTTATC AGAAACAGTT AATTTTACAA   
  
  
+ AATAATTTCT CAACAAACAA CTAATTTAAA TAATTAATAA AAATAACTAA CTCAAATATC TAATAACTAA   
  
  
+ TAACTAATAT AAATAATTAA CAGTTAACAG TTGTTTACGA AACAGAATTT ATATAAAAAG CGGTAACAGA   
  
  
+ TAAAAGATAA TCATGCGAGA TTAAAAGTCA CCAGTGACAC ACAAGTACTT AAATAAATAG TCTAAACAAT   
  
  
+ GATATTTTTT GCGAGCTTCC ATGCAAATAC CGACACTTTT ATTGCCTCAG AAGGAAACAA AAACTGAGTG   
  
  
+ AAGGGGGGGG GGGGTTCTGT TTGTCTTTGT TAGGGCACGT TTGGATTCAA GTGTAGATTG GGAATAAATT   
  
  
+ CGAGGACTTT TTCTTTGCCC TTTTTCCCTG ATTCCCAAAC CCACATCAAG TCAAGACGAA ACCAGCCATT   
  
  
+ GAAAAAAGAG GGTGTTTGTG TGAGATAAAG ACAGAAATTT TATGCCAGAG AGAGAAGATA ACAAATGCAA   
  
  
+ CAACGTCGAC AGAGGCTCTC CTTCTCACAA ATTCCATATT CCTCTGTTTT TTAAGAAAGA AAAAGAAAGT   
  
  
+ GTGAGACAAG AACAACACAA GCAAACAATT CAGTTGACGA CGAATACTGA GTGATACCAC GCACTGTGTG   
  
  
+ CAACTGCGTT TCCATTTGTG GAGTCCTTCA AAGCACGCTC ATCTTCAACC CTTCCTTCGC CACGGAATTT   
  
  
+ CAGGGGGAAA AAAACCCCCA CCTAATTTTG AGAGAGAGAG AGAACAGAGA GAACGGGCGA TACAGGGATA   
  
  
+ CAAGGTTTGA GCAATTTTAG AAAATTTCTT GGGGCCGTTG ATGGGTTTTG AGTGAATTGC AAATCCCAGA   
  
  
+ AAGATTTCTG CGGTTTTTTA TCTACGGCTC TCTCTGTGGG AATTTTTTGG TAGATTCCTC TGGTTCCCAC   
  
  
+ TTTCATATCT TCTTATTTGT TCTACCCTCT TTACATCTGA TAGTTTGTTC TAACTGCTGC TTCAGTTGGT   
  
  
+ GCTTGTTCTC TTTAGTACTT TTCTGTTTTG TTCGTTTTGT TCATGCACTT GAATTCAACA AGAGCCCTTG   
  
  
+ TCCATTTTGA GCTCTTAATT CCCATCTAAC CCTGTTTCCT TCAGAAATTG CTGTTCTGTT CATATAGTTA   
  
  
+ TATTTTTTAT GCTTTGATTT GGGTATAAGT TTGCTGTTGG TGATTCCAAA GCTTGGTACT TTTGGCCATA   
  
  
+ TTTTGTTTGA GTTGGTGTTG ATTGGTACAA ATCTTGTAAT TGGTGTGATT GTTAATGGGA CCAATGCTTC   
  
  
+ AAGATGATGG TTCATCAGTA ACTTCTTCAT CACCTCTTCA ATTTTTCTCC ATGATGTCAC CCAATTTGGG   
  
  
+ TTCCTCCTAC CCTTGGCTAA GGGACTTAAA GCCTGAAGAG AGAGGTCTTT ACTTGATACA TTTGTTGCTC   
  
  
+ ACTTGTGCAA ACCATGTCTC TAATGGTAGC CTTGACAATG CAAACCTAGC CCTTGAGCAA ATCTCCCAGC   
  
  
+ TTGCAGCCCC TGATGGCGAT ACAATCCAGC GTATTGTTGC CTACTTTGCT GAATCACTTG CTGAAAGGAT   
  
  
+ CCTTAAGTCA TGGCCTGGCC TATATAAAGC CCTTCATTTC AATAGAATGC CTGTTATTTC AGAAGAATTT   
  
  
+ CTTGCTAGGA AGCTGTTTTT TGAGTTGTTT CCCTTCTTGA AGCTGGCCTT TTTGGTGACT AACCAATCAA   
  
  
+ TAATCGAGGC CATGGAGGGG GAAAAGATGG TGCATATAAT TGATCTGAAT GCGTCAGAAC CTGCACAGTG   
  
  
+ GATTGCCCTT ATTCAAGACT TGAGTGCTCG GCCTGAGGGC CCTCCTCATT TGAGGATTAC CGGGGTTCAT   
  
  
+ CAACAGAAAG AGGTTTTAGA ACAAGTAGCT CATAGATTGA CTGAAGAAGC TGAGAAGTTG GATTTGCCAT   
  
  
+ TTCAGTTCAA TCCTGTGGTT TGCAAACTAG AGAATCTCGA CATCGGAAAA CTCCGTGTTA AGACCGGGGA   
  
  
+ GGCCTTGGCT ATTACCTCGG TCCTTCAACT GCATACCCTT TTGGCTTCTG AAGAGGAAGT CCTTAAGAAA   
  
  
+ AGTTCACCCT TGGCATTGGT AAAGCAAGCC AATGGGGCTA ATTTACAGGG CTTGTTCAAT AAAGATGGAG   
  
  
+ CTAATAATAG GCGTAGCCCA AGTAATGATT CGGCTTCATC TGCACCTTCA TCCCTCAACA CTTCAGCCAA   
  
  
+ GATGGAAGGT TTCCTTAGCG CTTTGTGGGG TTTATCCCCA AAGATTATGG TGATAACCGA GCAAGATTCC   
  
  
+ AACCACAATG GGGCAGGACT AATGGAGAGA TTGTCAGAAG CATTGTACTT CTATGCAGCA TTGTTCGATT   
  
  
+ GCTTAGAATT TACCCTCCCG AGAACCTCCG TGGAGAGAAG GAAGGTCGAG ATGCTCCTCC TTGGCAAGGA   
  
  
+ AATCAAGAAC ATCATAGCGT GTGAGGGAGG AGAAAGAATA GATAGGCATG AGAAGTTGGG GAAGTGGATT   
  
  
+ AAGAGGCTTG AGATGGCCGG GTTTGGAAGC GTTCCTTTGA GCCACATAGG CATGATCCAA GCAAGGCGGT   
  
  
+ TGTTGCAGAG CTATGGCTGT GATGGTTATA GAATAAAGGA GGAGAACGGA TGTTTTGTTA TCTGCTGGCA   
  
  
+ AGATCGCCCC CTCTTTTCAG TATCTGCTTG GAGATGTAGG AGGTG  

- -Up\_Stream \_Len000AGATGG AAATGGACAC AATTTTTTTT TTTAAAACAA AGGTAATGAT AAGAGAACCG   
  
  
- TGATAACCAC GACTAAAAAG AAGTGGTTTA GTTGGAGGAA TATAAACGGT TAAAATGACA GGAGAAAGTA   
  
  
- GATATTTCAG TTGAGAGGAT CATGTTAAAC AGTGTATCTT TTAGAGATCC GTGAGTAGAT TTGAGAGACA   
  
  
- AGACCCATTA ACTAGCTGCT AGATGTGGTA ATAAGCAAGA TCACGTGGCA AACCTGTCGG TTAGTACATG   
  
  
- GAAACCTCTA GTTGACGAGT ACTCAAGAAC CACACTTTAA CCCCTTCATT TAAGCTGAAA TCCCGTGACC   
  
  
- AGATAGTGCG GTACTAAAAT GGTAGATAAA AGTATATATA GGTTATTCAA ACTAGAAAGC GATAATTAGT   
  
  
- GTTACTATAT TTGTTCACCA CTTCTCGTTA TTACTTGGTA TGTAAAATTT GAATTCGCAA TTCCTTCTGT   
  
  
- ACTTCAATTT TTGTTACCAT TCTCGATACG CATACAAACC GTATATCGAA AAAATTCTCA CAAAACCGAT   
  
  
- ATCAGCCTCA AAAAATTAAT TCTAATCGAC AAACTAGTTT ATTTTTTCGA TTAAACTCAC AAACCGCTCT   
  
  
- TCTGAAAAAT ATTCTTGAAA AAAACCAGAT TTTTCAATTA AATTTTTCCG ATTAAGATAC TCGAAAAGCC   
  
  
- TTCTCAAAAA ATTTATTAAT TGAAAAACAG AGTATTCGGT ATTGAAATAG TCTTTGTCAA TTAAAATGTT   
  
  
- TTATTAAAGA GTTGTTTGTT GATTAAATTT ATTAATTATT TTTATTGATT GAGTTTATAG ATTATTGATT   
  
  
- ATTGATTATA TTTATTAATT GTCAATTGTC AACAAATGCT TTGTCTTAAA TATATTTTTC GCCATTGTCT   
  
  
- ATTTTCTATT AGTACGCTCT AATTTTCAGT GGTCACTGTG TGTTCATGAA TTTATTTATC AGATTTGTTA   
  
  
- CTATAAAAAA CGCTCGAAGG TACGTTTATG GCTGTGAAAA TAACGGAGTC TTCCTTTGTT TTTGACTCAC   
  
  
- TTCCCCCCCC CCCCAAGACA AACAGAAACA ATCCCGTGCA AACCTAAGTT CACATCTAAC CCTTATTTAA   
  
  
- GCTCCTGAAA AAGAAACGGG AAAAAGGGAC TAAGGGTTTG GGTGTAGTTC AGTTCTGCTT TGGTCGGTAA   
  
  
- CTTTTTTCTC CCACAAACAC ACTCTATTTC TGTCTTTAAA ATACGGTCTC TCTCTTCTAT TGTTTACGTT   
  
  
- GTTGCAGCTG TCTCCGAGAG GAAGAGTGTT TAAGGTATAA GGAGACAAAA AATTCTTTCT TTTTCTTTCA   
  
  
- CACTCTGTTC TTGTTGTGTT CGTTTGTTAA GTCAACTGCT GCTTATGACT CACTATGGTG CGTGACACAC   
  
  
- GTTGACGCAA AGGTAAACAC CTCAGGAAGT TTCGTGCGAG TAGAAGTTGG GAAGGAAGCG GTGCCTTAAA   
  
  
- GTCCCCCTTT TTTTGGGGGT GGATTAAAAC TCTCTCTCTC TCTTGTCTCT CTTGCCCGCT ATGTCCCTAT   
  
  
- GTTCCAAACT CGTTAAAATC TTTTAAAGAA CCCCGGCAAC TACCCAAAAC TCACTTAACG TTTAGGGTCT   
  
  
- TTCTAAAGAC GCCAAAAAAT AGATGCCGAG AGAGACACCC TTAAAAAACC ATCTAAGGAG ACCAAGGGTG   
  
  
- AAAGTATAGA AGAATAAACA AGATGGGAGA AATGTAGACT ATCAAACAAG ATTGACGACG AAGTCAACCA   
  
  
- CGAACAAGAG AAATCATGAA AAGACAAAAC AAGCAAAACA AGTACGTGAA CTTAAGTTGT TCTCGGGAAC   
  
  
- AGGTAAAACT CGAGAATTAA GGGTAGATTG GGACAAAGGA AGTCTTTAAC GACAAGACAA GTATATCAAT   
  
  
- ATAAAAAATA CGAAACTAAA CCCATATTCA AACGACAACC ACTAAGGTTT CGAACCATGA AAACCGGTAT   
  
  
- AAAACAAACT CAACCACAAC TAACCATGTT TAGAACATTA ACCACACTAA CAATTACCCT GGTTACGAAG   
  
  
- TTCTACTACC AAGTAGTCAT TGAAGAAGTA GTGGAGAAGT TAAAAAGAGG TACTACAGTG GGTTAAACCC   
  
  
- AAGGAGGATG GGAACCGATT CCCTGAATTT CGGACTTCTC TCTCCAGAAA TGAACTATGT AAACAACGAG   
  
  
- TGAACACGTT TGGTACAGAG ATTACCATCG GAACTGTTAC GTTTGGATCG GGAACTCGTT TAGAGGGTCG   
  
  
- AACGTCGGGG ACTACCGCTA TGTTAGGTCG CATAACAACG GATGAAACGA CTTAGTGAAC GACTTTCCTA   
  
  
- GGAATTCAGT ACCGGACCGG ATATATTTCG GGAAGTAAAG TTATCTTACG GACAATAAAG TCTTCTTAAA   
  
  
- GAACGATCCT TCGACAAAAA ACTCAACAAA GGGAAGAACT TCGACCGGAA AAACCACTGA TTGGTTAGTT   
  
  
- ATTAGCTCCG GTACCTCCCC CTTTTCTACC ACGTATATTA ACTAGACTTA CGCAGTCTTG GACGTGTCAC   
  
  
- CTAACGGGAA TAAGTTCTGA ACTCACGAGC CGGACTCCCG GGAGGAGTAA ACTCCTAATG GCCCCAAGTA   
  
  
- GTTGTCTTTC TCCAAAATCT TGTTCATCGA GTATCTAACT GACTTCTTCG ACTCTTCAAC CTAAACGGTA   
  
  
- AAGTCAAGTT AGGACACCAA ACGTTTGATC TCTTAGAGCT GTAGCCTTTT GAGGCACAAT TCTGGCCCCT   
  
  
- CCGGAACCGA TAATGGAGCC AGGAAGTTGA CGTATGGGAA AACCGAAGAC TTCTCCTTCA GGAATTCTTT   
  
  
- TCAAGTGGGA ACCGTAACCA TTTCGTTCGG TTACCCCGAT TAAATGTCCC GAACAAGTTA TTTCTACCTC   
  
  
- GATTATTATC CGCATCGGGT TCATTACTAA GCCGAAGTAG ACGTGGAAGT AGGGAGTTGT GAAGTCGGTT   
  
  
- CTACCTTCCA AAGGAATCGC GAAACACCCC AAATAGGGGT TTCTAATACC ACTATTGGCT CGTTCTAAGG   
  
  
- TTGGTGTTAC CCCGTCCTGA TTACCTCTCT AACAGTCTTC GTAACATGAA GATACGTCGT AACAAGCTAA   
  
  
- CGAATCTTAA ATGGGAGGGC TCTTGGAGGC ACCTCTCTTC CTTCCAGCTC TACGAGGAGG AACCGTTCCT   
  
  
- TTAGTTCTTG TAGTATCGCA CACTCCCTCC TCTTTCTTAT CTATCCGTAC TCTTCAACCC CTTCACCTAA   
  
  
- TTCTCCGAAC TCTACCGGCC CAAACCTTCG CAAGGAAACT CGGTGTATCC GTACTAGGTT CGTTCCGCCA   
  
  
- ACAACGTCTC GATACCGACA CTACCAATAT CTTATTTCCT CCTCTTGCCT ACAAAACAAT AGACGACCGT   
  
  
- TCTAGCGGGG GAGAAAAGTC ATAGACGAAC CTCTACATCC TCCAC

+     MYB

| Site Name | Organism | Position | Strand | Matrix score. | sequence | function |
| --- | --- | --- | --- | --- | --- | --- |
| MYB | Arabidopsis thaliana | 3317 | - | 6 | TAACCA |  |
| MYB | Arabidopsis thaliana | 3014 | + | 6 | CAACCA |  |
| MYB | Arabidopsis thaliana | 1928 | - | 6 | CAACAG |  |
| MYB | Arabidopsis thaliana | 2444 | + | 6 | TAACCA |  |
| MYB | Arabidopsis thaliana | 2595 | + | 6 | CAACAG |  |

>HU08G01232.1   
+ -Up\_Stream \_Len000TCTACC TTTACCTGTG TTAAAAAAAA AAATTTTGTT TCCATTACTA TTCTCTTGGC   
  
  
+ ACTATTGGTG CTGATTTTTC TTCACCAAAT CAACCTCCTT ATATTTGCCA ATTTTACTGT CCTCTTTCAT   
  
  
+ CTATAAAGTC AACTCTCCTA GTACAATTTG TCACATAGAA AATCTCTAGG CACTCATCTA AACTCTCTGT   
  
  
+ TCTGGGTAAT TGATCGACGA TCTACACCAT TATTCGTTCT AGTGCACCGT TTGGACAGCC AATCATGTAC   
  
  
+ CTTTGGAGAT CAACTGCTCA TGAGTTCTTG GTGTGAAATT GGGGAAGTAA ATTCGACTTT AGGGCACTGG   
  
  
+ TCTATCACGC CATGATTTTA CCATCTATTT TCATATATAT CCAATAAGTT TGATCTTTCG CTATTAATCA   
  
  
+ CAATGATATA AACAAGTGGT GAAGAGCAAT AATGAACCAT ACATTTTAAA CTTAAGCGTT AAGGAAGACA   
  
  
+ TGAAGTTAAA AACAATGGTA AGAGCTATGC GTATGTTTGG CATATAGCTT TTTTAAGAGT GTTTTGGCTA   
  
  
+ TAGTCGGAGT TTTTTAATTA AGATTAGCTG TTTGATCAAA TAAAAAAGCT AATTTGAGTG TTTGGCGAGA   
  
  
+ AGACTTTTTA TAAGAACTTT TTTTGGTCTA AAAAGTTAAT TTAAAAAGGC TAATTCTATG AGCTTTTCGG   
  
  
+ AAGAGTTTTT TAAATAATTA ACTTTTTGTC TCATAAGCCA TAACTTTATC AGAAACAGTT AATTTTACAA   
  
  
+ AATAATTTCT CAACAAACAA CTAATTTAAA TAATTAATAA AAATAACTAA CTCAAATATC TAATAACTAA   
  
  
+ TAACTAATAT AAATAATTAA CAGTTAACAG TTGTTTACGA AACAGAATTT ATATAAAAAG CGGTAACAGA   
  
  
+ TAAAAGATAA TCATGCGAGA TTAAAAGTCA CCAGTGACAC ACAAGTACTT AAATAAATAG TCTAAACAAT   
  
  
+ GATATTTTTT GCGAGCTTCC ATGCAAATAC CGACACTTTT ATTGCCTCAG AAGGAAACAA AAACTGAGTG   
  
  
+ AAGGGGGGGG GGGGTTCTGT TTGTCTTTGT TAGGGCACGT TTGGATTCAA GTGTAGATTG GGAATAAATT   
  
  
+ CGAGGACTTT TTCTTTGCCC TTTTTCCCTG ATTCCCAAAC CCACATCAAG TCAAGACGAA ACCAGCCATT   
  
  
+ GAAAAAAGAG GGTGTTTGTG TGAGATAAAG ACAGAAATTT TATGCCAGAG AGAGAAGATA ACAAATGCAA   
  
  
+ CAACGTCGAC AGAGGCTCTC CTTCTCACAA ATTCCATATT CCTCTGTTTT TTAAGAAAGA AAAAGAAAGT   
  
  
+ GTGAGACAAG AACAACACAA GCAAACAATT CAGTTGACGA CGAATACTGA GTGATACCAC GCACTGTGTG   
  
  
+ CAACTGCGTT TCCATTTGTG GAGTCCTTCA AAGCACGCTC ATCTTCAACC CTTCCTTCGC CACGGAATTT   
  
  
+ CAGGGGGAAA AAAACCCCCA CCTAATTTTG AGAGAGAGAG AGAACAGAGA GAACGGGCGA TACAGGGATA   
  
  
+ CAAGGTTTGA GCAATTTTAG AAAATTTCTT GGGGCCGTTG ATGGGTTTTG AGTGAATTGC AAATCCCAGA   
  
  
+ AAGATTTCTG CGGTTTTTTA TCTACGGCTC TCTCTGTGGG AATTTTTTGG TAGATTCCTC TGGTTCCCAC   
  
  
+ TTTCATATCT TCTTATTTGT TCTACCCTCT TTACATCTGA TAGTTTGTTC TAACTGCTGC TTCAGTTGGT   
  
  
+ GCTTGTTCTC TTTAGTACTT TTCTGTTTTG TTCGTTTTGT TCATGCACTT GAATTCAACA AGAGCCCTTG   
  
  
+ TCCATTTTGA GCTCTTAATT CCCATCTAAC CCTGTTTCCT TCAGAAATTG CTGTTCTGTT CATATAGTTA   
  
  
+ TATTTTTTAT GCTTTGATTT GGGTATAAGT TTGCTGTTGG TGATTCCAAA GCTTGGTACT TTTGGCCATA   
  
  
+ TTTTGTTTGA GTTGGTGTTG ATTGGTACAA ATCTTGTAAT TGGTGTGATT GTTAATGGGA CCAATGCTTC   
  
  
+ AAGATGATGG TTCATCAGTA ACTTCTTCAT CACCTCTTCA ATTTTTCTCC ATGATGTCAC CCAATTTGGG   
  
  
+ TTCCTCCTAC CCTTGGCTAA GGGACTTAAA GCCTGAAGAG AGAGGTCTTT ACTTGATACA TTTGTTGCTC   
  
  
+ ACTTGTGCAA ACCATGTCTC TAATGGTAGC CTTGACAATG CAAACCTAGC CCTTGAGCAA ATCTCCCAGC   
  
  
+ TTGCAGCCCC TGATGGCGAT ACAATCCAGC GTATTGTTGC CTACTTTGCT GAATCACTTG CTGAAAGGAT   
  
  
+ CCTTAAGTCA TGGCCTGGCC TATATAAAGC CCTTCATTTC AATAGAATGC CTGTTATTTC AGAAGAATTT   
  
  
+ CTTGCTAGGA AGCTGTTTTT TGAGTTGTTT CCCTTCTTGA AGCTGGCCTT TTTGGTGACT AACCAATCAA   
  
  
+ TAATCGAGGC CATGGAGGGG GAAAAGATGG TGCATATAAT TGATCTGAAT GCGTCAGAAC CTGCACAGTG   
  
  
+ GATTGCCCTT ATTCAAGACT TGAGTGCTCG GCCTGAGGGC CCTCCTCATT TGAGGATTAC CGGGGTTCAT   
  
  
+ CAACAGAAAG AGGTTTTAGA ACAAGTAGCT CATAGATTGA CTGAAGAAGC TGAGAAGTTG GATTTGCCAT   
  
  
+ TTCAGTTCAA TCCTGTGGTT TGCAAACTAG AGAATCTCGA CATCGGAAAA CTCCGTGTTA AGACCGGGGA   
  
  
+ GGCCTTGGCT ATTACCTCGG TCCTTCAACT GCATACCCTT TTGGCTTCTG AAGAGGAAGT CCTTAAGAAA   
  
  
+ AGTTCACCCT TGGCATTGGT AAAGCAAGCC AATGGGGCTA ATTTACAGGG CTTGTTCAAT AAAGATGGAG   
  
  
+ CTAATAATAG GCGTAGCCCA AGTAATGATT CGGCTTCATC TGCACCTTCA TCCCTCAACA CTTCAGCCAA   
  
  
+ GATGGAAGGT TTCCTTAGCG CTTTGTGGGG TTTATCCCCA AAGATTATGG TGATAACCGA GCAAGATTCC   
  
  
+ AACCACAATG GGGCAGGACT AATGGAGAGA TTGTCAGAAG CATTGTACTT CTATGCAGCA TTGTTCGATT   
  
  
+ GCTTAGAATT TACCCTCCCG AGAACCTCCG TGGAGAGAAG GAAGGTCGAG ATGCTCCTCC TTGGCAAGGA   
  
  
+ AATCAAGAAC ATCATAGCGT GTGAGGGAGG AGAAAGAATA GATAGGCATG AGAAGTTGGG GAAGTGGATT   
  
  
+ AAGAGGCTTG AGATGGCCGG GTTTGGAAGC GTTCCTTTGA GCCACATAGG CATGATCCAA GCAAGGCGGT   
  
  
+ TGTTGCAGAG CTATGGCTGT GATGGTTATA GAATAAAGGA GGAGAACGGA TGTTTTGTTA TCTGCTGGCA   
  
  
+ AGATCGCCCC CTCTTTTCAG TATCTGCTTG GAGATGTAGG AGGTG  

- -Up\_Stream \_Len000AGATGG AAATGGACAC AATTTTTTTT TTTAAAACAA AGGTAATGAT AAGAGAACCG   
  
  
- TGATAACCAC GACTAAAAAG AAGTGGTTTA GTTGGAGGAA TATAAACGGT TAAAATGACA GGAGAAAGTA   
  
  
- GATATTTCAG TTGAGAGGAT CATGTTAAAC AGTGTATCTT TTAGAGATCC GTGAGTAGAT TTGAGAGACA   
  
  
- AGACCCATTA ACTAGCTGCT AGATGTGGTA ATAAGCAAGA TCACGTGGCA AACCTGTCGG TTAGTACATG   
  
  
- GAAACCTCTA GTTGACGAGT ACTCAAGAAC CACACTTTAA CCCCTTCATT TAAGCTGAAA TCCCGTGACC   
  
  
- AGATAGTGCG GTACTAAAAT GGTAGATAAA AGTATATATA GGTTATTCAA ACTAGAAAGC GATAATTAGT   
  
  
- GTTACTATAT TTGTTCACCA CTTCTCGTTA TTACTTGGTA TGTAAAATTT GAATTCGCAA TTCCTTCTGT   
  
  
- ACTTCAATTT TTGTTACCAT TCTCGATACG CATACAAACC GTATATCGAA AAAATTCTCA CAAAACCGAT   
  
  
- ATCAGCCTCA AAAAATTAAT TCTAATCGAC AAACTAGTTT ATTTTTTCGA TTAAACTCAC AAACCGCTCT   
  
  
- TCTGAAAAAT ATTCTTGAAA AAAACCAGAT TTTTCAATTA AATTTTTCCG ATTAAGATAC TCGAAAAGCC   
  
  
- TTCTCAAAAA ATTTATTAAT TGAAAAACAG AGTATTCGGT ATTGAAATAG TCTTTGTCAA TTAAAATGTT   
  
  
- TTATTAAAGA GTTGTTTGTT GATTAAATTT ATTAATTATT TTTATTGATT GAGTTTATAG ATTATTGATT   
  
  
- ATTGATTATA TTTATTAATT GTCAATTGTC AACAAATGCT TTGTCTTAAA TATATTTTTC GCCATTGTCT   
  
  
- ATTTTCTATT AGTACGCTCT AATTTTCAGT GGTCACTGTG TGTTCATGAA TTTATTTATC AGATTTGTTA   
  
  
- CTATAAAAAA CGCTCGAAGG TACGTTTATG GCTGTGAAAA TAACGGAGTC TTCCTTTGTT TTTGACTCAC   
  
  
- TTCCCCCCCC CCCCAAGACA AACAGAAACA ATCCCGTGCA AACCTAAGTT CACATCTAAC CCTTATTTAA   
  
  
- GCTCCTGAAA AAGAAACGGG AAAAAGGGAC TAAGGGTTTG GGTGTAGTTC AGTTCTGCTT TGGTCGGTAA   
  
  
- CTTTTTTCTC CCACAAACAC ACTCTATTTC TGTCTTTAAA ATACGGTCTC TCTCTTCTAT TGTTTACGTT   
  
  
- GTTGCAGCTG TCTCCGAGAG GAAGAGTGTT TAAGGTATAA GGAGACAAAA AATTCTTTCT TTTTCTTTCA   
  
  
- CACTCTGTTC TTGTTGTGTT CGTTTGTTAA GTCAACTGCT GCTTATGACT CACTATGGTG CGTGACACAC   
  
  
- GTTGACGCAA AGGTAAACAC CTCAGGAAGT TTCGTGCGAG TAGAAGTTGG GAAGGAAGCG GTGCCTTAAA   
  
  
- GTCCCCCTTT TTTTGGGGGT GGATTAAAAC TCTCTCTCTC TCTTGTCTCT CTTGCCCGCT ATGTCCCTAT   
  
  
- GTTCCAAACT CGTTAAAATC TTTTAAAGAA CCCCGGCAAC TACCCAAAAC TCACTTAACG TTTAGGGTCT   
  
  
- TTCTAAAGAC GCCAAAAAAT AGATGCCGAG AGAGACACCC TTAAAAAACC ATCTAAGGAG ACCAAGGGTG   
  
  
- AAAGTATAGA AGAATAAACA AGATGGGAGA AATGTAGACT ATCAAACAAG ATTGACGACG AAGTCAACCA   
  
  
- CGAACAAGAG AAATCATGAA AAGACAAAAC AAGCAAAACA AGTACGTGAA CTTAAGTTGT TCTCGGGAAC   
  
  
- AGGTAAAACT CGAGAATTAA GGGTAGATTG GGACAAAGGA AGTCTTTAAC GACAAGACAA GTATATCAAT   
  
  
- ATAAAAAATA CGAAACTAAA CCCATATTCA AACGACAACC ACTAAGGTTT CGAACCATGA AAACCGGTAT   
  
  
- AAAACAAACT CAACCACAAC TAACCATGTT TAGAACATTA ACCACACTAA CAATTACCCT GGTTACGAAG   
  
  
- TTCTACTACC AAGTAGTCAT TGAAGAAGTA GTGGAGAAGT TAAAAAGAGG TACTACAGTG GGTTAAACCC   
  
  
- AAGGAGGATG GGAACCGATT CCCTGAATTT CGGACTTCTC TCTCCAGAAA TGAACTATGT AAACAACGAG   
  
  
- TGAACACGTT TGGTACAGAG ATTACCATCG GAACTGTTAC GTTTGGATCG GGAACTCGTT TAGAGGGTCG   
  
  
- AACGTCGGGG ACTACCGCTA TGTTAGGTCG CATAACAACG GATGAAACGA CTTAGTGAAC GACTTTCCTA   
  
  
- GGAATTCAGT ACCGGACCGG ATATATTTCG GGAAGTAAAG TTATCTTACG GACAATAAAG TCTTCTTAAA   
  
  
- GAACGATCCT TCGACAAAAA ACTCAACAAA GGGAAGAACT TCGACCGGAA AAACCACTGA TTGGTTAGTT   
  
  
- ATTAGCTCCG GTACCTCCCC CTTTTCTACC ACGTATATTA ACTAGACTTA CGCAGTCTTG GACGTGTCAC   
  
  
- CTAACGGGAA TAAGTTCTGA ACTCACGAGC CGGACTCCCG GGAGGAGTAA ACTCCTAATG GCCCCAAGTA   
  
  
- GTTGTCTTTC TCCAAAATCT TGTTCATCGA GTATCTAACT GACTTCTTCG ACTCTTCAAC CTAAACGGTA   
  
  
- AAGTCAAGTT AGGACACCAA ACGTTTGATC TCTTAGAGCT GTAGCCTTTT GAGGCACAAT TCTGGCCCCT   
  
  
- CCGGAACCGA TAATGGAGCC AGGAAGTTGA CGTATGGGAA AACCGAAGAC TTCTCCTTCA GGAATTCTTT   
  
  
- TCAAGTGGGA ACCGTAACCA TTTCGTTCGG TTACCCCGAT TAAATGTCCC GAACAAGTTA TTTCTACCTC   
  
  
- GATTATTATC CGCATCGGGT TCATTACTAA GCCGAAGTAG ACGTGGAAGT AGGGAGTTGT GAAGTCGGTT   
  
  
- CTACCTTCCA AAGGAATCGC GAAACACCCC AAATAGGGGT TTCTAATACC ACTATTGGCT CGTTCTAAGG   
  
  
- TTGGTGTTAC CCCGTCCTGA TTACCTCTCT AACAGTCTTC GTAACATGAA GATACGTCGT AACAAGCTAA   
  
  
- CGAATCTTAA ATGGGAGGGC TCTTGGAGGC ACCTCTCTTC CTTCCAGCTC TACGAGGAGG AACCGTTCCT   
  
  
- TTAGTTCTTG TAGTATCGCA CACTCCCTCC TCTTTCTTAT CTATCCGTAC TCTTCAACCC CTTCACCTAA   
  
  
- TTCTCCGAAC TCTACCGGCC CAAACCTTCG CAAGGAAACT CGGTGTATCC GTACTAGGTT CGTTCCGCCA   
  
  
- ACAACGTCTC GATACCGACA CTACCAATAT CTTATTTCCT CCTCTTGCCT ACAAAACAAT AGACGACCGT   
  
  
- TCTAGCGGGG GAGAAAAGTC ATAGACGAAC CTCTACATCC TCCAC

+     MYB recognition site

| Site Name | Organism | Position | Strand | Matrix score. | sequence | function |
| --- | --- | --- | --- | --- | --- | --- |
| MYB recognition site | Arabidopsis thaliana | 1579 | + | 6 | CCGTTG |  |

>HU08G01232.1   
+ -Up\_Stream \_Len000TCTACC TTTACCTGTG TTAAAAAAAA AAATTTTGTT TCCATTACTA TTCTCTTGGC   
  
  
+ ACTATTGGTG CTGATTTTTC TTCACCAAAT CAACCTCCTT ATATTTGCCA ATTTTACTGT CCTCTTTCAT   
  
  
+ CTATAAAGTC AACTCTCCTA GTACAATTTG TCACATAGAA AATCTCTAGG CACTCATCTA AACTCTCTGT   
  
  
+ TCTGGGTAAT TGATCGACGA TCTACACCAT TATTCGTTCT AGTGCACCGT TTGGACAGCC AATCATGTAC   
  
  
+ CTTTGGAGAT CAACTGCTCA TGAGTTCTTG GTGTGAAATT GGGGAAGTAA ATTCGACTTT AGGGCACTGG   
  
  
+ TCTATCACGC CATGATTTTA CCATCTATTT TCATATATAT CCAATAAGTT TGATCTTTCG CTATTAATCA   
  
  
+ CAATGATATA AACAAGTGGT GAAGAGCAAT AATGAACCAT ACATTTTAAA CTTAAGCGTT AAGGAAGACA   
  
  
+ TGAAGTTAAA AACAATGGTA AGAGCTATGC GTATGTTTGG CATATAGCTT TTTTAAGAGT GTTTTGGCTA   
  
  
+ TAGTCGGAGT TTTTTAATTA AGATTAGCTG TTTGATCAAA TAAAAAAGCT AATTTGAGTG TTTGGCGAGA   
  
  
+ AGACTTTTTA TAAGAACTTT TTTTGGTCTA AAAAGTTAAT TTAAAAAGGC TAATTCTATG AGCTTTTCGG   
  
  
+ AAGAGTTTTT TAAATAATTA ACTTTTTGTC TCATAAGCCA TAACTTTATC AGAAACAGTT AATTTTACAA   
  
  
+ AATAATTTCT CAACAAACAA CTAATTTAAA TAATTAATAA AAATAACTAA CTCAAATATC TAATAACTAA   
  
  
+ TAACTAATAT AAATAATTAA CAGTTAACAG TTGTTTACGA AACAGAATTT ATATAAAAAG CGGTAACAGA   
  
  
+ TAAAAGATAA TCATGCGAGA TTAAAAGTCA CCAGTGACAC ACAAGTACTT AAATAAATAG TCTAAACAAT   
  
  
+ GATATTTTTT GCGAGCTTCC ATGCAAATAC CGACACTTTT ATTGCCTCAG AAGGAAACAA AAACTGAGTG   
  
  
+ AAGGGGGGGG GGGGTTCTGT TTGTCTTTGT TAGGGCACGT TTGGATTCAA GTGTAGATTG GGAATAAATT   
  
  
+ CGAGGACTTT TTCTTTGCCC TTTTTCCCTG ATTCCCAAAC CCACATCAAG TCAAGACGAA ACCAGCCATT   
  
  
+ GAAAAAAGAG GGTGTTTGTG TGAGATAAAG ACAGAAATTT TATGCCAGAG AGAGAAGATA ACAAATGCAA   
  
  
+ CAACGTCGAC AGAGGCTCTC CTTCTCACAA ATTCCATATT CCTCTGTTTT TTAAGAAAGA AAAAGAAAGT   
  
  
+ GTGAGACAAG AACAACACAA GCAAACAATT CAGTTGACGA CGAATACTGA GTGATACCAC GCACTGTGTG   
  
  
+ CAACTGCGTT TCCATTTGTG GAGTCCTTCA AAGCACGCTC ATCTTCAACC CTTCCTTCGC CACGGAATTT   
  
  
+ CAGGGGGAAA AAAACCCCCA CCTAATTTTG AGAGAGAGAG AGAACAGAGA GAACGGGCGA TACAGGGATA   
  
  
+ CAAGGTTTGA GCAATTTTAG AAAATTTCTT GGGGCCGTTG ATGGGTTTTG AGTGAATTGC AAATCCCAGA   
  
  
+ AAGATTTCTG CGGTTTTTTA TCTACGGCTC TCTCTGTGGG AATTTTTTGG TAGATTCCTC TGGTTCCCAC   
  
  
+ TTTCATATCT TCTTATTTGT TCTACCCTCT TTACATCTGA TAGTTTGTTC TAACTGCTGC TTCAGTTGGT   
  
  
+ GCTTGTTCTC TTTAGTACTT TTCTGTTTTG TTCGTTTTGT TCATGCACTT GAATTCAACA AGAGCCCTTG   
  
  
+ TCCATTTTGA GCTCTTAATT CCCATCTAAC CCTGTTTCCT TCAGAAATTG CTGTTCTGTT CATATAGTTA   
  
  
+ TATTTTTTAT GCTTTGATTT GGGTATAAGT TTGCTGTTGG TGATTCCAAA GCTTGGTACT TTTGGCCATA   
  
  
+ TTTTGTTTGA GTTGGTGTTG ATTGGTACAA ATCTTGTAAT TGGTGTGATT GTTAATGGGA CCAATGCTTC   
  
  
+ AAGATGATGG TTCATCAGTA ACTTCTTCAT CACCTCTTCA ATTTTTCTCC ATGATGTCAC CCAATTTGGG   
  
  
+ TTCCTCCTAC CCTTGGCTAA GGGACTTAAA GCCTGAAGAG AGAGGTCTTT ACTTGATACA TTTGTTGCTC   
  
  
+ ACTTGTGCAA ACCATGTCTC TAATGGTAGC CTTGACAATG CAAACCTAGC CCTTGAGCAA ATCTCCCAGC   
  
  
+ TTGCAGCCCC TGATGGCGAT ACAATCCAGC GTATTGTTGC CTACTTTGCT GAATCACTTG CTGAAAGGAT   
  
  
+ CCTTAAGTCA TGGCCTGGCC TATATAAAGC CCTTCATTTC AATAGAATGC CTGTTATTTC AGAAGAATTT   
  
  
+ CTTGCTAGGA AGCTGTTTTT TGAGTTGTTT CCCTTCTTGA AGCTGGCCTT TTTGGTGACT AACCAATCAA   
  
  
+ TAATCGAGGC CATGGAGGGG GAAAAGATGG TGCATATAAT TGATCTGAAT GCGTCAGAAC CTGCACAGTG   
  
  
+ GATTGCCCTT ATTCAAGACT TGAGTGCTCG GCCTGAGGGC CCTCCTCATT TGAGGATTAC CGGGGTTCAT   
  
  
+ CAACAGAAAG AGGTTTTAGA ACAAGTAGCT CATAGATTGA CTGAAGAAGC TGAGAAGTTG GATTTGCCAT   
  
  
+ TTCAGTTCAA TCCTGTGGTT TGCAAACTAG AGAATCTCGA CATCGGAAAA CTCCGTGTTA AGACCGGGGA   
  
  
+ GGCCTTGGCT ATTACCTCGG TCCTTCAACT GCATACCCTT TTGGCTTCTG AAGAGGAAGT CCTTAAGAAA   
  
  
+ AGTTCACCCT TGGCATTGGT AAAGCAAGCC AATGGGGCTA ATTTACAGGG CTTGTTCAAT AAAGATGGAG   
  
  
+ CTAATAATAG GCGTAGCCCA AGTAATGATT CGGCTTCATC TGCACCTTCA TCCCTCAACA CTTCAGCCAA   
  
  
+ GATGGAAGGT TTCCTTAGCG CTTTGTGGGG TTTATCCCCA AAGATTATGG TGATAACCGA GCAAGATTCC   
  
  
+ AACCACAATG GGGCAGGACT AATGGAGAGA TTGTCAGAAG CATTGTACTT CTATGCAGCA TTGTTCGATT   
  
  
+ GCTTAGAATT TACCCTCCCG AGAACCTCCG TGGAGAGAAG GAAGGTCGAG ATGCTCCTCC TTGGCAAGGA   
  
  
+ AATCAAGAAC ATCATAGCGT GTGAGGGAGG AGAAAGAATA GATAGGCATG AGAAGTTGGG GAAGTGGATT   
  
  
+ AAGAGGCTTG AGATGGCCGG GTTTGGAAGC GTTCCTTTGA GCCACATAGG CATGATCCAA GCAAGGCGGT   
  
  
+ TGTTGCAGAG CTATGGCTGT GATGGTTATA GAATAAAGGA GGAGAACGGA TGTTTTGTTA TCTGCTGGCA   
  
  
+ AGATCGCCCC CTCTTTTCAG TATCTGCTTG GAGATGTAGG AGGTG  

- -Up\_Stream \_Len000AGATGG AAATGGACAC AATTTTTTTT TTTAAAACAA AGGTAATGAT AAGAGAACCG   
  
  
- TGATAACCAC GACTAAAAAG AAGTGGTTTA GTTGGAGGAA TATAAACGGT TAAAATGACA GGAGAAAGTA   
  
  
- GATATTTCAG TTGAGAGGAT CATGTTAAAC AGTGTATCTT TTAGAGATCC GTGAGTAGAT TTGAGAGACA   
  
  
- AGACCCATTA ACTAGCTGCT AGATGTGGTA ATAAGCAAGA TCACGTGGCA AACCTGTCGG TTAGTACATG   
  
  
- GAAACCTCTA GTTGACGAGT ACTCAAGAAC CACACTTTAA CCCCTTCATT TAAGCTGAAA TCCCGTGACC   
  
  
- AGATAGTGCG GTACTAAAAT GGTAGATAAA AGTATATATA GGTTATTCAA ACTAGAAAGC GATAATTAGT   
  
  
- GTTACTATAT TTGTTCACCA CTTCTCGTTA TTACTTGGTA TGTAAAATTT GAATTCGCAA TTCCTTCTGT   
  
  
- ACTTCAATTT TTGTTACCAT TCTCGATACG CATACAAACC GTATATCGAA AAAATTCTCA CAAAACCGAT   
  
  
- ATCAGCCTCA AAAAATTAAT TCTAATCGAC AAACTAGTTT ATTTTTTCGA TTAAACTCAC AAACCGCTCT   
  
  
- TCTGAAAAAT ATTCTTGAAA AAAACCAGAT TTTTCAATTA AATTTTTCCG ATTAAGATAC TCGAAAAGCC   
  
  
- TTCTCAAAAA ATTTATTAAT TGAAAAACAG AGTATTCGGT ATTGAAATAG TCTTTGTCAA TTAAAATGTT   
  
  
- TTATTAAAGA GTTGTTTGTT GATTAAATTT ATTAATTATT TTTATTGATT GAGTTTATAG ATTATTGATT   
  
  
- ATTGATTATA TTTATTAATT GTCAATTGTC AACAAATGCT TTGTCTTAAA TATATTTTTC GCCATTGTCT   
  
  
- ATTTTCTATT AGTACGCTCT AATTTTCAGT GGTCACTGTG TGTTCATGAA TTTATTTATC AGATTTGTTA   
  
  
- CTATAAAAAA CGCTCGAAGG TACGTTTATG GCTGTGAAAA TAACGGAGTC TTCCTTTGTT TTTGACTCAC   
  
  
- TTCCCCCCCC CCCCAAGACA AACAGAAACA ATCCCGTGCA AACCTAAGTT CACATCTAAC CCTTATTTAA   
  
  
- GCTCCTGAAA AAGAAACGGG AAAAAGGGAC TAAGGGTTTG GGTGTAGTTC AGTTCTGCTT TGGTCGGTAA   
  
  
- CTTTTTTCTC CCACAAACAC ACTCTATTTC TGTCTTTAAA ATACGGTCTC TCTCTTCTAT TGTTTACGTT   
  
  
- GTTGCAGCTG TCTCCGAGAG GAAGAGTGTT TAAGGTATAA GGAGACAAAA AATTCTTTCT TTTTCTTTCA   
  
  
- CACTCTGTTC TTGTTGTGTT CGTTTGTTAA GTCAACTGCT GCTTATGACT CACTATGGTG CGTGACACAC   
  
  
- GTTGACGCAA AGGTAAACAC CTCAGGAAGT TTCGTGCGAG TAGAAGTTGG GAAGGAAGCG GTGCCTTAAA   
  
  
- GTCCCCCTTT TTTTGGGGGT GGATTAAAAC TCTCTCTCTC TCTTGTCTCT CTTGCCCGCT ATGTCCCTAT   
  
  
- GTTCCAAACT CGTTAAAATC TTTTAAAGAA CCCCGGCAAC TACCCAAAAC TCACTTAACG TTTAGGGTCT   
  
  
- TTCTAAAGAC GCCAAAAAAT AGATGCCGAG AGAGACACCC TTAAAAAACC ATCTAAGGAG ACCAAGGGTG   
  
  
- AAAGTATAGA AGAATAAACA AGATGGGAGA AATGTAGACT ATCAAACAAG ATTGACGACG AAGTCAACCA   
  
  
- CGAACAAGAG AAATCATGAA AAGACAAAAC AAGCAAAACA AGTACGTGAA CTTAAGTTGT TCTCGGGAAC   
  
  
- AGGTAAAACT CGAGAATTAA GGGTAGATTG GGACAAAGGA AGTCTTTAAC GACAAGACAA GTATATCAAT   
  
  
- ATAAAAAATA CGAAACTAAA CCCATATTCA AACGACAACC ACTAAGGTTT CGAACCATGA AAACCGGTAT   
  
  
- AAAACAAACT CAACCACAAC TAACCATGTT TAGAACATTA ACCACACTAA CAATTACCCT GGTTACGAAG   
  
  
- TTCTACTACC AAGTAGTCAT TGAAGAAGTA GTGGAGAAGT TAAAAAGAGG TACTACAGTG GGTTAAACCC   
  
  
- AAGGAGGATG GGAACCGATT CCCTGAATTT CGGACTTCTC TCTCCAGAAA TGAACTATGT AAACAACGAG   
  
  
- TGAACACGTT TGGTACAGAG ATTACCATCG GAACTGTTAC GTTTGGATCG GGAACTCGTT TAGAGGGTCG   
  
  
- AACGTCGGGG ACTACCGCTA TGTTAGGTCG CATAACAACG GATGAAACGA CTTAGTGAAC GACTTTCCTA   
  
  
- GGAATTCAGT ACCGGACCGG ATATATTTCG GGAAGTAAAG TTATCTTACG GACAATAAAG TCTTCTTAAA   
  
  
- GAACGATCCT TCGACAAAAA ACTCAACAAA GGGAAGAACT TCGACCGGAA AAACCACTGA TTGGTTAGTT   
  
  
- ATTAGCTCCG GTACCTCCCC CTTTTCTACC ACGTATATTA ACTAGACTTA CGCAGTCTTG GACGTGTCAC   
  
  
- CTAACGGGAA TAAGTTCTGA ACTCACGAGC CGGACTCCCG GGAGGAGTAA ACTCCTAATG GCCCCAAGTA   
  
  
- GTTGTCTTTC TCCAAAATCT TGTTCATCGA GTATCTAACT GACTTCTTCG ACTCTTCAAC CTAAACGGTA   
  
  
- AAGTCAAGTT AGGACACCAA ACGTTTGATC TCTTAGAGCT GTAGCCTTTT GAGGCACAAT TCTGGCCCCT   
  
  
- CCGGAACCGA TAATGGAGCC AGGAAGTTGA CGTATGGGAA AACCGAAGAC TTCTCCTTCA GGAATTCTTT   
  
  
- TCAAGTGGGA ACCGTAACCA TTTCGTTCGG TTACCCCGAT TAAATGTCCC GAACAAGTTA TTTCTACCTC   
  
  
- GATTATTATC CGCATCGGGT TCATTACTAA GCCGAAGTAG ACGTGGAAGT AGGGAGTTGT GAAGTCGGTT   
  
  
- CTACCTTCCA AAGGAATCGC GAAACACCCC AAATAGGGGT TTCTAATACC ACTATTGGCT CGTTCTAAGG   
  
  
- TTGGTGTTAC CCCGTCCTGA TTACCTCTCT AACAGTCTTC GTAACATGAA GATACGTCGT AACAAGCTAA   
  
  
- CGAATCTTAA ATGGGAGGGC TCTTGGAGGC ACCTCTCTTC CTTCCAGCTC TACGAGGAGG AACCGTTCCT   
  
  
- TTAGTTCTTG TAGTATCGCA CACTCCCTCC TCTTTCTTAT CTATCCGTAC TCTTCAACCC CTTCACCTAA   
  
  
- TTCTCCGAAC TCTACCGGCC CAAACCTTCG CAAGGAAACT CGGTGTATCC GTACTAGGTT CGTTCCGCCA   
  
  
- ACAACGTCTC GATACCGACA CTACCAATAT CTTATTTCCT CCTCTTGCCT ACAAAACAAT AGACGACCGT   
  
  
- TCTAGCGGGG GAGAAAAGTC ATAGACGAAC CTCTACATCC TCCAC

+     MYB-like sequence

| Site Name | Organism | Position | Strand | Matrix score. | sequence | function |
| --- | --- | --- | --- | --- | --- | --- |
| MYB-like sequence | Arabidopsis thaliana | 2444 | + | 6 | TAACCA |  |
| MYB-like sequence | Arabidopsis thaliana | 3317 | - | 6 | TAACCA |  |

>HU08G01232.1   
+ -Up\_Stream \_Len000TCTACC TTTACCTGTG TTAAAAAAAA AAATTTTGTT TCCATTACTA TTCTCTTGGC   
  
  
+ ACTATTGGTG CTGATTTTTC TTCACCAAAT CAACCTCCTT ATATTTGCCA ATTTTACTGT CCTCTTTCAT   
  
  
+ CTATAAAGTC AACTCTCCTA GTACAATTTG TCACATAGAA AATCTCTAGG CACTCATCTA AACTCTCTGT   
  
  
+ TCTGGGTAAT TGATCGACGA TCTACACCAT TATTCGTTCT AGTGCACCGT TTGGACAGCC AATCATGTAC   
  
  
+ CTTTGGAGAT CAACTGCTCA TGAGTTCTTG GTGTGAAATT GGGGAAGTAA ATTCGACTTT AGGGCACTGG   
  
  
+ TCTATCACGC CATGATTTTA CCATCTATTT TCATATATAT CCAATAAGTT TGATCTTTCG CTATTAATCA   
  
  
+ CAATGATATA AACAAGTGGT GAAGAGCAAT AATGAACCAT ACATTTTAAA CTTAAGCGTT AAGGAAGACA   
  
  
+ TGAAGTTAAA AACAATGGTA AGAGCTATGC GTATGTTTGG CATATAGCTT TTTTAAGAGT GTTTTGGCTA   
  
  
+ TAGTCGGAGT TTTTTAATTA AGATTAGCTG TTTGATCAAA TAAAAAAGCT AATTTGAGTG TTTGGCGAGA   
  
  
+ AGACTTTTTA TAAGAACTTT TTTTGGTCTA AAAAGTTAAT TTAAAAAGGC TAATTCTATG AGCTTTTCGG   
  
  
+ AAGAGTTTTT TAAATAATTA ACTTTTTGTC TCATAAGCCA TAACTTTATC AGAAACAGTT AATTTTACAA   
  
  
+ AATAATTTCT CAACAAACAA CTAATTTAAA TAATTAATAA AAATAACTAA CTCAAATATC TAATAACTAA   
  
  
+ TAACTAATAT AAATAATTAA CAGTTAACAG TTGTTTACGA AACAGAATTT ATATAAAAAG CGGTAACAGA   
  
  
+ TAAAAGATAA TCATGCGAGA TTAAAAGTCA CCAGTGACAC ACAAGTACTT AAATAAATAG TCTAAACAAT   
  
  
+ GATATTTTTT GCGAGCTTCC ATGCAAATAC CGACACTTTT ATTGCCTCAG AAGGAAACAA AAACTGAGTG   
  
  
+ AAGGGGGGGG GGGGTTCTGT TTGTCTTTGT TAGGGCACGT TTGGATTCAA GTGTAGATTG GGAATAAATT   
  
  
+ CGAGGACTTT TTCTTTGCCC TTTTTCCCTG ATTCCCAAAC CCACATCAAG TCAAGACGAA ACCAGCCATT   
  
  
+ GAAAAAAGAG GGTGTTTGTG TGAGATAAAG ACAGAAATTT TATGCCAGAG AGAGAAGATA ACAAATGCAA   
  
  
+ CAACGTCGAC AGAGGCTCTC CTTCTCACAA ATTCCATATT CCTCTGTTTT TTAAGAAAGA AAAAGAAAGT   
  
  
+ GTGAGACAAG AACAACACAA GCAAACAATT CAGTTGACGA CGAATACTGA GTGATACCAC GCACTGTGTG   
  
  
+ CAACTGCGTT TCCATTTGTG GAGTCCTTCA AAGCACGCTC ATCTTCAACC CTTCCTTCGC CACGGAATTT   
  
  
+ CAGGGGGAAA AAAACCCCCA CCTAATTTTG AGAGAGAGAG AGAACAGAGA GAACGGGCGA TACAGGGATA   
  
  
+ CAAGGTTTGA GCAATTTTAG AAAATTTCTT GGGGCCGTTG ATGGGTTTTG AGTGAATTGC AAATCCCAGA   
  
  
+ AAGATTTCTG CGGTTTTTTA TCTACGGCTC TCTCTGTGGG AATTTTTTGG TAGATTCCTC TGGTTCCCAC   
  
  
+ TTTCATATCT TCTTATTTGT TCTACCCTCT TTACATCTGA TAGTTTGTTC TAACTGCTGC TTCAGTTGGT   
  
  
+ GCTTGTTCTC TTTAGTACTT TTCTGTTTTG TTCGTTTTGT TCATGCACTT GAATTCAACA AGAGCCCTTG   
  
  
+ TCCATTTTGA GCTCTTAATT CCCATCTAAC CCTGTTTCCT TCAGAAATTG CTGTTCTGTT CATATAGTTA   
  
  
+ TATTTTTTAT GCTTTGATTT GGGTATAAGT TTGCTGTTGG TGATTCCAAA GCTTGGTACT TTTGGCCATA   
  
  
+ TTTTGTTTGA GTTGGTGTTG ATTGGTACAA ATCTTGTAAT TGGTGTGATT GTTAATGGGA CCAATGCTTC   
  
  
+ AAGATGATGG TTCATCAGTA ACTTCTTCAT CACCTCTTCA ATTTTTCTCC ATGATGTCAC CCAATTTGGG   
  
  
+ TTCCTCCTAC CCTTGGCTAA GGGACTTAAA GCCTGAAGAG AGAGGTCTTT ACTTGATACA TTTGTTGCTC   
  
  
+ ACTTGTGCAA ACCATGTCTC TAATGGTAGC CTTGACAATG CAAACCTAGC CCTTGAGCAA ATCTCCCAGC   
  
  
+ TTGCAGCCCC TGATGGCGAT ACAATCCAGC GTATTGTTGC CTACTTTGCT GAATCACTTG CTGAAAGGAT   
  
  
+ CCTTAAGTCA TGGCCTGGCC TATATAAAGC CCTTCATTTC AATAGAATGC CTGTTATTTC AGAAGAATTT   
  
  
+ CTTGCTAGGA AGCTGTTTTT TGAGTTGTTT CCCTTCTTGA AGCTGGCCTT TTTGGTGACT AACCAATCAA   
  
  
+ TAATCGAGGC CATGGAGGGG GAAAAGATGG TGCATATAAT TGATCTGAAT GCGTCAGAAC CTGCACAGTG   
  
  
+ GATTGCCCTT ATTCAAGACT TGAGTGCTCG GCCTGAGGGC CCTCCTCATT TGAGGATTAC CGGGGTTCAT   
  
  
+ CAACAGAAAG AGGTTTTAGA ACAAGTAGCT CATAGATTGA CTGAAGAAGC TGAGAAGTTG GATTTGCCAT   
  
  
+ TTCAGTTCAA TCCTGTGGTT TGCAAACTAG AGAATCTCGA CATCGGAAAA CTCCGTGTTA AGACCGGGGA   
  
  
+ GGCCTTGGCT ATTACCTCGG TCCTTCAACT GCATACCCTT TTGGCTTCTG AAGAGGAAGT CCTTAAGAAA   
  
  
+ AGTTCACCCT TGGCATTGGT AAAGCAAGCC AATGGGGCTA ATTTACAGGG CTTGTTCAAT AAAGATGGAG   
  
  
+ CTAATAATAG GCGTAGCCCA AGTAATGATT CGGCTTCATC TGCACCTTCA TCCCTCAACA CTTCAGCCAA   
  
  
+ GATGGAAGGT TTCCTTAGCG CTTTGTGGGG TTTATCCCCA AAGATTATGG TGATAACCGA GCAAGATTCC   
  
  
+ AACCACAATG GGGCAGGACT AATGGAGAGA TTGTCAGAAG CATTGTACTT CTATGCAGCA TTGTTCGATT   
  
  
+ GCTTAGAATT TACCCTCCCG AGAACCTCCG TGGAGAGAAG GAAGGTCGAG ATGCTCCTCC TTGGCAAGGA   
  
  
+ AATCAAGAAC ATCATAGCGT GTGAGGGAGG AGAAAGAATA GATAGGCATG AGAAGTTGGG GAAGTGGATT   
  
  
+ AAGAGGCTTG AGATGGCCGG GTTTGGAAGC GTTCCTTTGA GCCACATAGG CATGATCCAA GCAAGGCGGT   
  
  
+ TGTTGCAGAG CTATGGCTGT GATGGTTATA GAATAAAGGA GGAGAACGGA TGTTTTGTTA TCTGCTGGCA   
  
  
+ AGATCGCCCC CTCTTTTCAG TATCTGCTTG GAGATGTAGG AGGTG  

- -Up\_Stream \_Len000AGATGG AAATGGACAC AATTTTTTTT TTTAAAACAA AGGTAATGAT AAGAGAACCG   
  
  
- TGATAACCAC GACTAAAAAG AAGTGGTTTA GTTGGAGGAA TATAAACGGT TAAAATGACA GGAGAAAGTA   
  
  
- GATATTTCAG TTGAGAGGAT CATGTTAAAC AGTGTATCTT TTAGAGATCC GTGAGTAGAT TTGAGAGACA   
  
  
- AGACCCATTA ACTAGCTGCT AGATGTGGTA ATAAGCAAGA TCACGTGGCA AACCTGTCGG TTAGTACATG   
  
  
- GAAACCTCTA GTTGACGAGT ACTCAAGAAC CACACTTTAA CCCCTTCATT TAAGCTGAAA TCCCGTGACC   
  
  
- AGATAGTGCG GTACTAAAAT GGTAGATAAA AGTATATATA GGTTATTCAA ACTAGAAAGC GATAATTAGT   
  
  
- GTTACTATAT TTGTTCACCA CTTCTCGTTA TTACTTGGTA TGTAAAATTT GAATTCGCAA TTCCTTCTGT   
  
  
- ACTTCAATTT TTGTTACCAT TCTCGATACG CATACAAACC GTATATCGAA AAAATTCTCA CAAAACCGAT   
  
  
- ATCAGCCTCA AAAAATTAAT TCTAATCGAC AAACTAGTTT ATTTTTTCGA TTAAACTCAC AAACCGCTCT   
  
  
- TCTGAAAAAT ATTCTTGAAA AAAACCAGAT TTTTCAATTA AATTTTTCCG ATTAAGATAC TCGAAAAGCC   
  
  
- TTCTCAAAAA ATTTATTAAT TGAAAAACAG AGTATTCGGT ATTGAAATAG TCTTTGTCAA TTAAAATGTT   
  
  
- TTATTAAAGA GTTGTTTGTT GATTAAATTT ATTAATTATT TTTATTGATT GAGTTTATAG ATTATTGATT   
  
  
- ATTGATTATA TTTATTAATT GTCAATTGTC AACAAATGCT TTGTCTTAAA TATATTTTTC GCCATTGTCT   
  
  
- ATTTTCTATT AGTACGCTCT AATTTTCAGT GGTCACTGTG TGTTCATGAA TTTATTTATC AGATTTGTTA   
  
  
- CTATAAAAAA CGCTCGAAGG TACGTTTATG GCTGTGAAAA TAACGGAGTC TTCCTTTGTT TTTGACTCAC   
  
  
- TTCCCCCCCC CCCCAAGACA AACAGAAACA ATCCCGTGCA AACCTAAGTT CACATCTAAC CCTTATTTAA   
  
  
- GCTCCTGAAA AAGAAACGGG AAAAAGGGAC TAAGGGTTTG GGTGTAGTTC AGTTCTGCTT TGGTCGGTAA   
  
  
- CTTTTTTCTC CCACAAACAC ACTCTATTTC TGTCTTTAAA ATACGGTCTC TCTCTTCTAT TGTTTACGTT   
  
  
- GTTGCAGCTG TCTCCGAGAG GAAGAGTGTT TAAGGTATAA GGAGACAAAA AATTCTTTCT TTTTCTTTCA   
  
  
- CACTCTGTTC TTGTTGTGTT CGTTTGTTAA GTCAACTGCT GCTTATGACT CACTATGGTG CGTGACACAC   
  
  
- GTTGACGCAA AGGTAAACAC CTCAGGAAGT TTCGTGCGAG TAGAAGTTGG GAAGGAAGCG GTGCCTTAAA   
  
  
- GTCCCCCTTT TTTTGGGGGT GGATTAAAAC TCTCTCTCTC TCTTGTCTCT CTTGCCCGCT ATGTCCCTAT   
  
  
- GTTCCAAACT CGTTAAAATC TTTTAAAGAA CCCCGGCAAC TACCCAAAAC TCACTTAACG TTTAGGGTCT   
  
  
- TTCTAAAGAC GCCAAAAAAT AGATGCCGAG AGAGACACCC TTAAAAAACC ATCTAAGGAG ACCAAGGGTG   
  
  
- AAAGTATAGA AGAATAAACA AGATGGGAGA AATGTAGACT ATCAAACAAG ATTGACGACG AAGTCAACCA   
  
  
- CGAACAAGAG AAATCATGAA AAGACAAAAC AAGCAAAACA AGTACGTGAA CTTAAGTTGT TCTCGGGAAC   
  
  
- AGGTAAAACT CGAGAATTAA GGGTAGATTG GGACAAAGGA AGTCTTTAAC GACAAGACAA GTATATCAAT   
  
  
- ATAAAAAATA CGAAACTAAA CCCATATTCA AACGACAACC ACTAAGGTTT CGAACCATGA AAACCGGTAT   
  
  
- AAAACAAACT CAACCACAAC TAACCATGTT TAGAACATTA ACCACACTAA CAATTACCCT GGTTACGAAG   
  
  
- TTCTACTACC AAGTAGTCAT TGAAGAAGTA GTGGAGAAGT TAAAAAGAGG TACTACAGTG GGTTAAACCC   
  
  
- AAGGAGGATG GGAACCGATT CCCTGAATTT CGGACTTCTC TCTCCAGAAA TGAACTATGT AAACAACGAG   
  
  
- TGAACACGTT TGGTACAGAG ATTACCATCG GAACTGTTAC GTTTGGATCG GGAACTCGTT TAGAGGGTCG   
  
  
- AACGTCGGGG ACTACCGCTA TGTTAGGTCG CATAACAACG GATGAAACGA CTTAGTGAAC GACTTTCCTA   
  
  
- GGAATTCAGT ACCGGACCGG ATATATTTCG GGAAGTAAAG TTATCTTACG GACAATAAAG TCTTCTTAAA   
  
  
- GAACGATCCT TCGACAAAAA ACTCAACAAA GGGAAGAACT TCGACCGGAA AAACCACTGA TTGGTTAGTT   
  
  
- ATTAGCTCCG GTACCTCCCC CTTTTCTACC ACGTATATTA ACTAGACTTA CGCAGTCTTG GACGTGTCAC   
  
  
- CTAACGGGAA TAAGTTCTGA ACTCACGAGC CGGACTCCCG GGAGGAGTAA ACTCCTAATG GCCCCAAGTA   
  
  
- GTTGTCTTTC TCCAAAATCT TGTTCATCGA GTATCTAACT GACTTCTTCG ACTCTTCAAC CTAAACGGTA   
  
  
- AAGTCAAGTT AGGACACCAA ACGTTTGATC TCTTAGAGCT GTAGCCTTTT GAGGCACAAT TCTGGCCCCT   
  
  
- CCGGAACCGA TAATGGAGCC AGGAAGTTGA CGTATGGGAA AACCGAAGAC TTCTCCTTCA GGAATTCTTT   
  
  
- TCAAGTGGGA ACCGTAACCA TTTCGTTCGG TTACCCCGAT TAAATGTCCC GAACAAGTTA TTTCTACCTC   
  
  
- GATTATTATC CGCATCGGGT TCATTACTAA GCCGAAGTAG ACGTGGAAGT AGGGAGTTGT GAAGTCGGTT   
  
  
- CTACCTTCCA AAGGAATCGC GAAACACCCC AAATAGGGGT TTCTAATACC ACTATTGGCT CGTTCTAAGG   
  
  
- TTGGTGTTAC CCCGTCCTGA TTACCTCTCT AACAGTCTTC GTAACATGAA GATACGTCGT AACAAGCTAA   
  
  
- CGAATCTTAA ATGGGAGGGC TCTTGGAGGC ACCTCTCTTC CTTCCAGCTC TACGAGGAGG AACCGTTCCT   
  
  
- TTAGTTCTTG TAGTATCGCA CACTCCCTCC TCTTTCTTAT CTATCCGTAC TCTTCAACCC CTTCACCTAA   
  
  
- TTCTCCGAAC TCTACCGGCC CAAACCTTCG CAAGGAAACT CGGTGTATCC GTACTAGGTT CGTTCCGCCA   
  
  
- ACAACGTCTC GATACCGACA CTACCAATAT CTTATTTCCT CCTCTTGCCT ACAAAACAAT AGACGACCGT   
  
  
- TCTAGCGGGG GAGAAAAGTC ATAGACGAAC CTCTACATCC TCCAC

+     MYC

| Site Name | Organism | Position | Strand | Matrix score. | sequence | function |
| --- | --- | --- | --- | --- | --- | --- |
| MYC | Arabidopsis thaliana | 1417 | + | 6 | CATTTG |  |
| MYC | Arabidopsis thaliana | 1256 | - | 6 | CATTTG |  |
| MYC | Arabidopsis thaliana | 2571 | + | 6 | CATTTG |  |
| MYC | Arabidopsis thaliana | 2163 | + | 6 | CATTTG |  |

>HU08G01232.1   
+ -Up\_Stream \_Len000TCTACC TTTACCTGTG TTAAAAAAAA AAATTTTGTT TCCATTACTA TTCTCTTGGC   
  
  
+ ACTATTGGTG CTGATTTTTC TTCACCAAAT CAACCTCCTT ATATTTGCCA ATTTTACTGT CCTCTTTCAT   
  
  
+ CTATAAAGTC AACTCTCCTA GTACAATTTG TCACATAGAA AATCTCTAGG CACTCATCTA AACTCTCTGT   
  
  
+ TCTGGGTAAT TGATCGACGA TCTACACCAT TATTCGTTCT AGTGCACCGT TTGGACAGCC AATCATGTAC   
  
  
+ CTTTGGAGAT CAACTGCTCA TGAGTTCTTG GTGTGAAATT GGGGAAGTAA ATTCGACTTT AGGGCACTGG   
  
  
+ TCTATCACGC CATGATTTTA CCATCTATTT TCATATATAT CCAATAAGTT TGATCTTTCG CTATTAATCA   
  
  
+ CAATGATATA AACAAGTGGT GAAGAGCAAT AATGAACCAT ACATTTTAAA CTTAAGCGTT AAGGAAGACA   
  
  
+ TGAAGTTAAA AACAATGGTA AGAGCTATGC GTATGTTTGG CATATAGCTT TTTTAAGAGT GTTTTGGCTA   
  
  
+ TAGTCGGAGT TTTTTAATTA AGATTAGCTG TTTGATCAAA TAAAAAAGCT AATTTGAGTG TTTGGCGAGA   
  
  
+ AGACTTTTTA TAAGAACTTT TTTTGGTCTA AAAAGTTAAT TTAAAAAGGC TAATTCTATG AGCTTTTCGG   
  
  
+ AAGAGTTTTT TAAATAATTA ACTTTTTGTC TCATAAGCCA TAACTTTATC AGAAACAGTT AATTTTACAA   
  
  
+ AATAATTTCT CAACAAACAA CTAATTTAAA TAATTAATAA AAATAACTAA CTCAAATATC TAATAACTAA   
  
  
+ TAACTAATAT AAATAATTAA CAGTTAACAG TTGTTTACGA AACAGAATTT ATATAAAAAG CGGTAACAGA   
  
  
+ TAAAAGATAA TCATGCGAGA TTAAAAGTCA CCAGTGACAC ACAAGTACTT AAATAAATAG TCTAAACAAT   
  
  
+ GATATTTTTT GCGAGCTTCC ATGCAAATAC CGACACTTTT ATTGCCTCAG AAGGAAACAA AAACTGAGTG   
  
  
+ AAGGGGGGGG GGGGTTCTGT TTGTCTTTGT TAGGGCACGT TTGGATTCAA GTGTAGATTG GGAATAAATT   
  
  
+ CGAGGACTTT TTCTTTGCCC TTTTTCCCTG ATTCCCAAAC CCACATCAAG TCAAGACGAA ACCAGCCATT   
  
  
+ GAAAAAAGAG GGTGTTTGTG TGAGATAAAG ACAGAAATTT TATGCCAGAG AGAGAAGATA ACAAATGCAA   
  
  
+ CAACGTCGAC AGAGGCTCTC CTTCTCACAA ATTCCATATT CCTCTGTTTT TTAAGAAAGA AAAAGAAAGT   
  
  
+ GTGAGACAAG AACAACACAA GCAAACAATT CAGTTGACGA CGAATACTGA GTGATACCAC GCACTGTGTG   
  
  
+ CAACTGCGTT TCCATTTGTG GAGTCCTTCA AAGCACGCTC ATCTTCAACC CTTCCTTCGC CACGGAATTT   
  
  
+ CAGGGGGAAA AAAACCCCCA CCTAATTTTG AGAGAGAGAG AGAACAGAGA GAACGGGCGA TACAGGGATA   
  
  
+ CAAGGTTTGA GCAATTTTAG AAAATTTCTT GGGGCCGTTG ATGGGTTTTG AGTGAATTGC AAATCCCAGA   
  
  
+ AAGATTTCTG CGGTTTTTTA TCTACGGCTC TCTCTGTGGG AATTTTTTGG TAGATTCCTC TGGTTCCCAC   
  
  
+ TTTCATATCT TCTTATTTGT TCTACCCTCT TTACATCTGA TAGTTTGTTC TAACTGCTGC TTCAGTTGGT   
  
  
+ GCTTGTTCTC TTTAGTACTT TTCTGTTTTG TTCGTTTTGT TCATGCACTT GAATTCAACA AGAGCCCTTG   
  
  
+ TCCATTTTGA GCTCTTAATT CCCATCTAAC CCTGTTTCCT TCAGAAATTG CTGTTCTGTT CATATAGTTA   
  
  
+ TATTTTTTAT GCTTTGATTT GGGTATAAGT TTGCTGTTGG TGATTCCAAA GCTTGGTACT TTTGGCCATA   
  
  
+ TTTTGTTTGA GTTGGTGTTG ATTGGTACAA ATCTTGTAAT TGGTGTGATT GTTAATGGGA CCAATGCTTC   
  
  
+ AAGATGATGG TTCATCAGTA ACTTCTTCAT CACCTCTTCA ATTTTTCTCC ATGATGTCAC CCAATTTGGG   
  
  
+ TTCCTCCTAC CCTTGGCTAA GGGACTTAAA GCCTGAAGAG AGAGGTCTTT ACTTGATACA TTTGTTGCTC   
  
  
+ ACTTGTGCAA ACCATGTCTC TAATGGTAGC CTTGACAATG CAAACCTAGC CCTTGAGCAA ATCTCCCAGC   
  
  
+ TTGCAGCCCC TGATGGCGAT ACAATCCAGC GTATTGTTGC CTACTTTGCT GAATCACTTG CTGAAAGGAT   
  
  
+ CCTTAAGTCA TGGCCTGGCC TATATAAAGC CCTTCATTTC AATAGAATGC CTGTTATTTC AGAAGAATTT   
  
  
+ CTTGCTAGGA AGCTGTTTTT TGAGTTGTTT CCCTTCTTGA AGCTGGCCTT TTTGGTGACT AACCAATCAA   
  
  
+ TAATCGAGGC CATGGAGGGG GAAAAGATGG TGCATATAAT TGATCTGAAT GCGTCAGAAC CTGCACAGTG   
  
  
+ GATTGCCCTT ATTCAAGACT TGAGTGCTCG GCCTGAGGGC CCTCCTCATT TGAGGATTAC CGGGGTTCAT   
  
  
+ CAACAGAAAG AGGTTTTAGA ACAAGTAGCT CATAGATTGA CTGAAGAAGC TGAGAAGTTG GATTTGCCAT   
  
  
+ TTCAGTTCAA TCCTGTGGTT TGCAAACTAG AGAATCTCGA CATCGGAAAA CTCCGTGTTA AGACCGGGGA   
  
  
+ GGCCTTGGCT ATTACCTCGG TCCTTCAACT GCATACCCTT TTGGCTTCTG AAGAGGAAGT CCTTAAGAAA   
  
  
+ AGTTCACCCT TGGCATTGGT AAAGCAAGCC AATGGGGCTA ATTTACAGGG CTTGTTCAAT AAAGATGGAG   
  
  
+ CTAATAATAG GCGTAGCCCA AGTAATGATT CGGCTTCATC TGCACCTTCA TCCCTCAACA CTTCAGCCAA   
  
  
+ GATGGAAGGT TTCCTTAGCG CTTTGTGGGG TTTATCCCCA AAGATTATGG TGATAACCGA GCAAGATTCC   
  
  
+ AACCACAATG GGGCAGGACT AATGGAGAGA TTGTCAGAAG CATTGTACTT CTATGCAGCA TTGTTCGATT   
  
  
+ GCTTAGAATT TACCCTCCCG AGAACCTCCG TGGAGAGAAG GAAGGTCGAG ATGCTCCTCC TTGGCAAGGA   
  
  
+ AATCAAGAAC ATCATAGCGT GTGAGGGAGG AGAAAGAATA GATAGGCATG AGAAGTTGGG GAAGTGGATT   
  
  
+ AAGAGGCTTG AGATGGCCGG GTTTGGAAGC GTTCCTTTGA GCCACATAGG CATGATCCAA GCAAGGCGGT   
  
  
+ TGTTGCAGAG CTATGGCTGT GATGGTTATA GAATAAAGGA GGAGAACGGA TGTTTTGTTA TCTGCTGGCA   
  
  
+ AGATCGCCCC CTCTTTTCAG TATCTGCTTG GAGATGTAGG AGGTG  

- -Up\_Stream \_Len000AGATGG AAATGGACAC AATTTTTTTT TTTAAAACAA AGGTAATGAT AAGAGAACCG   
  
  
- TGATAACCAC GACTAAAAAG AAGTGGTTTA GTTGGAGGAA TATAAACGGT TAAAATGACA GGAGAAAGTA   
  
  
- GATATTTCAG TTGAGAGGAT CATGTTAAAC AGTGTATCTT TTAGAGATCC GTGAGTAGAT TTGAGAGACA   
  
  
- AGACCCATTA ACTAGCTGCT AGATGTGGTA ATAAGCAAGA TCACGTGGCA AACCTGTCGG TTAGTACATG   
  
  
- GAAACCTCTA GTTGACGAGT ACTCAAGAAC CACACTTTAA CCCCTTCATT TAAGCTGAAA TCCCGTGACC   
  
  
- AGATAGTGCG GTACTAAAAT GGTAGATAAA AGTATATATA GGTTATTCAA ACTAGAAAGC GATAATTAGT   
  
  
- GTTACTATAT TTGTTCACCA CTTCTCGTTA TTACTTGGTA TGTAAAATTT GAATTCGCAA TTCCTTCTGT   
  
  
- ACTTCAATTT TTGTTACCAT TCTCGATACG CATACAAACC GTATATCGAA AAAATTCTCA CAAAACCGAT   
  
  
- ATCAGCCTCA AAAAATTAAT TCTAATCGAC AAACTAGTTT ATTTTTTCGA TTAAACTCAC AAACCGCTCT   
  
  
- TCTGAAAAAT ATTCTTGAAA AAAACCAGAT TTTTCAATTA AATTTTTCCG ATTAAGATAC TCGAAAAGCC   
  
  
- TTCTCAAAAA ATTTATTAAT TGAAAAACAG AGTATTCGGT ATTGAAATAG TCTTTGTCAA TTAAAATGTT   
  
  
- TTATTAAAGA GTTGTTTGTT GATTAAATTT ATTAATTATT TTTATTGATT GAGTTTATAG ATTATTGATT   
  
  
- ATTGATTATA TTTATTAATT GTCAATTGTC AACAAATGCT TTGTCTTAAA TATATTTTTC GCCATTGTCT   
  
  
- ATTTTCTATT AGTACGCTCT AATTTTCAGT GGTCACTGTG TGTTCATGAA TTTATTTATC AGATTTGTTA   
  
  
- CTATAAAAAA CGCTCGAAGG TACGTTTATG GCTGTGAAAA TAACGGAGTC TTCCTTTGTT TTTGACTCAC   
  
  
- TTCCCCCCCC CCCCAAGACA AACAGAAACA ATCCCGTGCA AACCTAAGTT CACATCTAAC CCTTATTTAA   
  
  
- GCTCCTGAAA AAGAAACGGG AAAAAGGGAC TAAGGGTTTG GGTGTAGTTC AGTTCTGCTT TGGTCGGTAA   
  
  
- CTTTTTTCTC CCACAAACAC ACTCTATTTC TGTCTTTAAA ATACGGTCTC TCTCTTCTAT TGTTTACGTT   
  
  
- GTTGCAGCTG TCTCCGAGAG GAAGAGTGTT TAAGGTATAA GGAGACAAAA AATTCTTTCT TTTTCTTTCA   
  
  
- CACTCTGTTC TTGTTGTGTT CGTTTGTTAA GTCAACTGCT GCTTATGACT CACTATGGTG CGTGACACAC   
  
  
- GTTGACGCAA AGGTAAACAC CTCAGGAAGT TTCGTGCGAG TAGAAGTTGG GAAGGAAGCG GTGCCTTAAA   
  
  
- GTCCCCCTTT TTTTGGGGGT GGATTAAAAC TCTCTCTCTC TCTTGTCTCT CTTGCCCGCT ATGTCCCTAT   
  
  
- GTTCCAAACT CGTTAAAATC TTTTAAAGAA CCCCGGCAAC TACCCAAAAC TCACTTAACG TTTAGGGTCT   
  
  
- TTCTAAAGAC GCCAAAAAAT AGATGCCGAG AGAGACACCC TTAAAAAACC ATCTAAGGAG ACCAAGGGTG   
  
  
- AAAGTATAGA AGAATAAACA AGATGGGAGA AATGTAGACT ATCAAACAAG ATTGACGACG AAGTCAACCA   
  
  
- CGAACAAGAG AAATCATGAA AAGACAAAAC AAGCAAAACA AGTACGTGAA CTTAAGTTGT TCTCGGGAAC   
  
  
- AGGTAAAACT CGAGAATTAA GGGTAGATTG GGACAAAGGA AGTCTTTAAC GACAAGACAA GTATATCAAT   
  
  
- ATAAAAAATA CGAAACTAAA CCCATATTCA AACGACAACC ACTAAGGTTT CGAACCATGA AAACCGGTAT   
  
  
- AAAACAAACT CAACCACAAC TAACCATGTT TAGAACATTA ACCACACTAA CAATTACCCT GGTTACGAAG   
  
  
- TTCTACTACC AAGTAGTCAT TGAAGAAGTA GTGGAGAAGT TAAAAAGAGG TACTACAGTG GGTTAAACCC   
  
  
- AAGGAGGATG GGAACCGATT CCCTGAATTT CGGACTTCTC TCTCCAGAAA TGAACTATGT AAACAACGAG   
  
  
- TGAACACGTT TGGTACAGAG ATTACCATCG GAACTGTTAC GTTTGGATCG GGAACTCGTT TAGAGGGTCG   
  
  
- AACGTCGGGG ACTACCGCTA TGTTAGGTCG CATAACAACG GATGAAACGA CTTAGTGAAC GACTTTCCTA   
  
  
- GGAATTCAGT ACCGGACCGG ATATATTTCG GGAAGTAAAG TTATCTTACG GACAATAAAG TCTTCTTAAA   
  
  
- GAACGATCCT TCGACAAAAA ACTCAACAAA GGGAAGAACT TCGACCGGAA AAACCACTGA TTGGTTAGTT   
  
  
- ATTAGCTCCG GTACCTCCCC CTTTTCTACC ACGTATATTA ACTAGACTTA CGCAGTCTTG GACGTGTCAC   
  
  
- CTAACGGGAA TAAGTTCTGA ACTCACGAGC CGGACTCCCG GGAGGAGTAA ACTCCTAATG GCCCCAAGTA   
  
  
- GTTGTCTTTC TCCAAAATCT TGTTCATCGA GTATCTAACT GACTTCTTCG ACTCTTCAAC CTAAACGGTA   
  
  
- AAGTCAAGTT AGGACACCAA ACGTTTGATC TCTTAGAGCT GTAGCCTTTT GAGGCACAAT TCTGGCCCCT   
  
  
- CCGGAACCGA TAATGGAGCC AGGAAGTTGA CGTATGGGAA AACCGAAGAC TTCTCCTTCA GGAATTCTTT   
  
  
- TCAAGTGGGA ACCGTAACCA TTTCGTTCGG TTACCCCGAT TAAATGTCCC GAACAAGTTA TTTCTACCTC   
  
  
- GATTATTATC CGCATCGGGT TCATTACTAA GCCGAAGTAG ACGTGGAAGT AGGGAGTTGT GAAGTCGGTT   
  
  
- CTACCTTCCA AAGGAATCGC GAAACACCCC AAATAGGGGT TTCTAATACC ACTATTGGCT CGTTCTAAGG   
  
  
- TTGGTGTTAC CCCGTCCTGA TTACCTCTCT AACAGTCTTC GTAACATGAA GATACGTCGT AACAAGCTAA   
  
  
- CGAATCTTAA ATGGGAGGGC TCTTGGAGGC ACCTCTCTTC CTTCCAGCTC TACGAGGAGG AACCGTTCCT   
  
  
- TTAGTTCTTG TAGTATCGCA CACTCCCTCC TCTTTCTTAT CTATCCGTAC TCTTCAACCC CTTCACCTAA   
  
  
- TTCTCCGAAC TCTACCGGCC CAAACCTTCG CAAGGAAACT CGGTGTATCC GTACTAGGTT CGTTCCGCCA   
  
  
- ACAACGTCTC GATACCGACA CTACCAATAT CTTATTTCCT CCTCTTGCCT ACAAAACAAT AGACGACCGT   
  
  
- TCTAGCGGGG GAGAAAAGTC ATAGACGAAC CTCTACATCC TCCAC

+     Myb

| Site Name | Organism | Position | Strand | Matrix score. | sequence | function |
| --- | --- | --- | --- | --- | --- | --- |
| Myb | Arabidopsis thaliana | 1747 | - | 6 | CAACTG |  |
| Myb | Arabidopsis thaliana | 1405 | + | 6 | CAACTG |  |
| Myb | Arabidopsis thaliana | 1365 | - | 6 | CAACTG |  |
| Myb | Arabidopsis thaliana | 760 | - | 6 | TAACTG |  |
| Myb | Arabidopsis thaliana | 295 | + | 6 | CAACTG |  |
| Myb | Arabidopsis thaliana | 865 | - | 6 | TAACTG |  |
| Myb | Arabidopsis thaliana | 872 | - | 6 | CAACTG |  |
| Myb | Arabidopsis thaliana | 1735 | + | 6 | TAACTG |  |
| Myb | Arabidopsis thaliana | 2760 | + | 6 | CAACTG |  |

>HU08G01232.1   
+ -Up\_Stream \_Len000TCTACC TTTACCTGTG TTAAAAAAAA AAATTTTGTT TCCATTACTA TTCTCTTGGC   
  
  
+ ACTATTGGTG CTGATTTTTC TTCACCAAAT CAACCTCCTT ATATTTGCCA ATTTTACTGT CCTCTTTCAT   
  
  
+ CTATAAAGTC AACTCTCCTA GTACAATTTG TCACATAGAA AATCTCTAGG CACTCATCTA AACTCTCTGT   
  
  
+ TCTGGGTAAT TGATCGACGA TCTACACCAT TATTCGTTCT AGTGCACCGT TTGGACAGCC AATCATGTAC   
  
  
+ CTTTGGAGAT CAACTGCTCA TGAGTTCTTG GTGTGAAATT GGGGAAGTAA ATTCGACTTT AGGGCACTGG   
  
  
+ TCTATCACGC CATGATTTTA CCATCTATTT TCATATATAT CCAATAAGTT TGATCTTTCG CTATTAATCA   
  
  
+ CAATGATATA AACAAGTGGT GAAGAGCAAT AATGAACCAT ACATTTTAAA CTTAAGCGTT AAGGAAGACA   
  
  
+ TGAAGTTAAA AACAATGGTA AGAGCTATGC GTATGTTTGG CATATAGCTT TTTTAAGAGT GTTTTGGCTA   
  
  
+ TAGTCGGAGT TTTTTAATTA AGATTAGCTG TTTGATCAAA TAAAAAAGCT AATTTGAGTG TTTGGCGAGA   
  
  
+ AGACTTTTTA TAAGAACTTT TTTTGGTCTA AAAAGTTAAT TTAAAAAGGC TAATTCTATG AGCTTTTCGG   
  
  
+ AAGAGTTTTT TAAATAATTA ACTTTTTGTC TCATAAGCCA TAACTTTATC AGAAACAGTT AATTTTACAA   
  
  
+ AATAATTTCT CAACAAACAA CTAATTTAAA TAATTAATAA AAATAACTAA CTCAAATATC TAATAACTAA   
  
  
+ TAACTAATAT AAATAATTAA CAGTTAACAG TTGTTTACGA AACAGAATTT ATATAAAAAG CGGTAACAGA   
  
  
+ TAAAAGATAA TCATGCGAGA TTAAAAGTCA CCAGTGACAC ACAAGTACTT AAATAAATAG TCTAAACAAT   
  
  
+ GATATTTTTT GCGAGCTTCC ATGCAAATAC CGACACTTTT ATTGCCTCAG AAGGAAACAA AAACTGAGTG   
  
  
+ AAGGGGGGGG GGGGTTCTGT TTGTCTTTGT TAGGGCACGT TTGGATTCAA GTGTAGATTG GGAATAAATT   
  
  
+ CGAGGACTTT TTCTTTGCCC TTTTTCCCTG ATTCCCAAAC CCACATCAAG TCAAGACGAA ACCAGCCATT   
  
  
+ GAAAAAAGAG GGTGTTTGTG TGAGATAAAG ACAGAAATTT TATGCCAGAG AGAGAAGATA ACAAATGCAA   
  
  
+ CAACGTCGAC AGAGGCTCTC CTTCTCACAA ATTCCATATT CCTCTGTTTT TTAAGAAAGA AAAAGAAAGT   
  
  
+ GTGAGACAAG AACAACACAA GCAAACAATT CAGTTGACGA CGAATACTGA GTGATACCAC GCACTGTGTG   
  
  
+ CAACTGCGTT TCCATTTGTG GAGTCCTTCA AAGCACGCTC ATCTTCAACC CTTCCTTCGC CACGGAATTT   
  
  
+ CAGGGGGAAA AAAACCCCCA CCTAATTTTG AGAGAGAGAG AGAACAGAGA GAACGGGCGA TACAGGGATA   
  
  
+ CAAGGTTTGA GCAATTTTAG AAAATTTCTT GGGGCCGTTG ATGGGTTTTG AGTGAATTGC AAATCCCAGA   
  
  
+ AAGATTTCTG CGGTTTTTTA TCTACGGCTC TCTCTGTGGG AATTTTTTGG TAGATTCCTC TGGTTCCCAC   
  
  
+ TTTCATATCT TCTTATTTGT TCTACCCTCT TTACATCTGA TAGTTTGTTC TAACTGCTGC TTCAGTTGGT   
  
  
+ GCTTGTTCTC TTTAGTACTT TTCTGTTTTG TTCGTTTTGT TCATGCACTT GAATTCAACA AGAGCCCTTG   
  
  
+ TCCATTTTGA GCTCTTAATT CCCATCTAAC CCTGTTTCCT TCAGAAATTG CTGTTCTGTT CATATAGTTA   
  
  
+ TATTTTTTAT GCTTTGATTT GGGTATAAGT TTGCTGTTGG TGATTCCAAA GCTTGGTACT TTTGGCCATA   
  
  
+ TTTTGTTTGA GTTGGTGTTG ATTGGTACAA ATCTTGTAAT TGGTGTGATT GTTAATGGGA CCAATGCTTC   
  
  
+ AAGATGATGG TTCATCAGTA ACTTCTTCAT CACCTCTTCA ATTTTTCTCC ATGATGTCAC CCAATTTGGG   
  
  
+ TTCCTCCTAC CCTTGGCTAA GGGACTTAAA GCCTGAAGAG AGAGGTCTTT ACTTGATACA TTTGTTGCTC   
  
  
+ ACTTGTGCAA ACCATGTCTC TAATGGTAGC CTTGACAATG CAAACCTAGC CCTTGAGCAA ATCTCCCAGC   
  
  
+ TTGCAGCCCC TGATGGCGAT ACAATCCAGC GTATTGTTGC CTACTTTGCT GAATCACTTG CTGAAAGGAT   
  
  
+ CCTTAAGTCA TGGCCTGGCC TATATAAAGC CCTTCATTTC AATAGAATGC CTGTTATTTC AGAAGAATTT   
  
  
+ CTTGCTAGGA AGCTGTTTTT TGAGTTGTTT CCCTTCTTGA AGCTGGCCTT TTTGGTGACT AACCAATCAA   
  
  
+ TAATCGAGGC CATGGAGGGG GAAAAGATGG TGCATATAAT TGATCTGAAT GCGTCAGAAC CTGCACAGTG   
  
  
+ GATTGCCCTT ATTCAAGACT TGAGTGCTCG GCCTGAGGGC CCTCCTCATT TGAGGATTAC CGGGGTTCAT   
  
  
+ CAACAGAAAG AGGTTTTAGA ACAAGTAGCT CATAGATTGA CTGAAGAAGC TGAGAAGTTG GATTTGCCAT   
  
  
+ TTCAGTTCAA TCCTGTGGTT TGCAAACTAG AGAATCTCGA CATCGGAAAA CTCCGTGTTA AGACCGGGGA   
  
  
+ GGCCTTGGCT ATTACCTCGG TCCTTCAACT GCATACCCTT TTGGCTTCTG AAGAGGAAGT CCTTAAGAAA   
  
  
+ AGTTCACCCT TGGCATTGGT AAAGCAAGCC AATGGGGCTA ATTTACAGGG CTTGTTCAAT AAAGATGGAG   
  
  
+ CTAATAATAG GCGTAGCCCA AGTAATGATT CGGCTTCATC TGCACCTTCA TCCCTCAACA CTTCAGCCAA   
  
  
+ GATGGAAGGT TTCCTTAGCG CTTTGTGGGG TTTATCCCCA AAGATTATGG TGATAACCGA GCAAGATTCC   
  
  
+ AACCACAATG GGGCAGGACT AATGGAGAGA TTGTCAGAAG CATTGTACTT CTATGCAGCA TTGTTCGATT   
  
  
+ GCTTAGAATT TACCCTCCCG AGAACCTCCG TGGAGAGAAG GAAGGTCGAG ATGCTCCTCC TTGGCAAGGA   
  
  
+ AATCAAGAAC ATCATAGCGT GTGAGGGAGG AGAAAGAATA GATAGGCATG AGAAGTTGGG GAAGTGGATT   
  
  
+ AAGAGGCTTG AGATGGCCGG GTTTGGAAGC GTTCCTTTGA GCCACATAGG CATGATCCAA GCAAGGCGGT   
  
  
+ TGTTGCAGAG CTATGGCTGT GATGGTTATA GAATAAAGGA GGAGAACGGA TGTTTTGTTA TCTGCTGGCA   
  
  
+ AGATCGCCCC CTCTTTTCAG TATCTGCTTG GAGATGTAGG AGGTG  

- -Up\_Stream \_Len000AGATGG AAATGGACAC AATTTTTTTT TTTAAAACAA AGGTAATGAT AAGAGAACCG   
  
  
- TGATAACCAC GACTAAAAAG AAGTGGTTTA GTTGGAGGAA TATAAACGGT TAAAATGACA GGAGAAAGTA   
  
  
- GATATTTCAG TTGAGAGGAT CATGTTAAAC AGTGTATCTT TTAGAGATCC GTGAGTAGAT TTGAGAGACA   
  
  
- AGACCCATTA ACTAGCTGCT AGATGTGGTA ATAAGCAAGA TCACGTGGCA AACCTGTCGG TTAGTACATG   
  
  
- GAAACCTCTA GTTGACGAGT ACTCAAGAAC CACACTTTAA CCCCTTCATT TAAGCTGAAA TCCCGTGACC   
  
  
- AGATAGTGCG GTACTAAAAT GGTAGATAAA AGTATATATA GGTTATTCAA ACTAGAAAGC GATAATTAGT   
  
  
- GTTACTATAT TTGTTCACCA CTTCTCGTTA TTACTTGGTA TGTAAAATTT GAATTCGCAA TTCCTTCTGT   
  
  
- ACTTCAATTT TTGTTACCAT TCTCGATACG CATACAAACC GTATATCGAA AAAATTCTCA CAAAACCGAT   
  
  
- ATCAGCCTCA AAAAATTAAT TCTAATCGAC AAACTAGTTT ATTTTTTCGA TTAAACTCAC AAACCGCTCT   
  
  
- TCTGAAAAAT ATTCTTGAAA AAAACCAGAT TTTTCAATTA AATTTTTCCG ATTAAGATAC TCGAAAAGCC   
  
  
- TTCTCAAAAA ATTTATTAAT TGAAAAACAG AGTATTCGGT ATTGAAATAG TCTTTGTCAA TTAAAATGTT   
  
  
- TTATTAAAGA GTTGTTTGTT GATTAAATTT ATTAATTATT TTTATTGATT GAGTTTATAG ATTATTGATT   
  
  
- ATTGATTATA TTTATTAATT GTCAATTGTC AACAAATGCT TTGTCTTAAA TATATTTTTC GCCATTGTCT   
  
  
- ATTTTCTATT AGTACGCTCT AATTTTCAGT GGTCACTGTG TGTTCATGAA TTTATTTATC AGATTTGTTA   
  
  
- CTATAAAAAA CGCTCGAAGG TACGTTTATG GCTGTGAAAA TAACGGAGTC TTCCTTTGTT TTTGACTCAC   
  
  
- TTCCCCCCCC CCCCAAGACA AACAGAAACA ATCCCGTGCA AACCTAAGTT CACATCTAAC CCTTATTTAA   
  
  
- GCTCCTGAAA AAGAAACGGG AAAAAGGGAC TAAGGGTTTG GGTGTAGTTC AGTTCTGCTT TGGTCGGTAA   
  
  
- CTTTTTTCTC CCACAAACAC ACTCTATTTC TGTCTTTAAA ATACGGTCTC TCTCTTCTAT TGTTTACGTT   
  
  
- GTTGCAGCTG TCTCCGAGAG GAAGAGTGTT TAAGGTATAA GGAGACAAAA AATTCTTTCT TTTTCTTTCA   
  
  
- CACTCTGTTC TTGTTGTGTT CGTTTGTTAA GTCAACTGCT GCTTATGACT CACTATGGTG CGTGACACAC   
  
  
- GTTGACGCAA AGGTAAACAC CTCAGGAAGT TTCGTGCGAG TAGAAGTTGG GAAGGAAGCG GTGCCTTAAA   
  
  
- GTCCCCCTTT TTTTGGGGGT GGATTAAAAC TCTCTCTCTC TCTTGTCTCT CTTGCCCGCT ATGTCCCTAT   
  
  
- GTTCCAAACT CGTTAAAATC TTTTAAAGAA CCCCGGCAAC TACCCAAAAC TCACTTAACG TTTAGGGTCT   
  
  
- TTCTAAAGAC GCCAAAAAAT AGATGCCGAG AGAGACACCC TTAAAAAACC ATCTAAGGAG ACCAAGGGTG   
  
  
- AAAGTATAGA AGAATAAACA AGATGGGAGA AATGTAGACT ATCAAACAAG ATTGACGACG AAGTCAACCA   
  
  
- CGAACAAGAG AAATCATGAA AAGACAAAAC AAGCAAAACA AGTACGTGAA CTTAAGTTGT TCTCGGGAAC   
  
  
- AGGTAAAACT CGAGAATTAA GGGTAGATTG GGACAAAGGA AGTCTTTAAC GACAAGACAA GTATATCAAT   
  
  
- ATAAAAAATA CGAAACTAAA CCCATATTCA AACGACAACC ACTAAGGTTT CGAACCATGA AAACCGGTAT   
  
  
- AAAACAAACT CAACCACAAC TAACCATGTT TAGAACATTA ACCACACTAA CAATTACCCT GGTTACGAAG   
  
  
- TTCTACTACC AAGTAGTCAT TGAAGAAGTA GTGGAGAAGT TAAAAAGAGG TACTACAGTG GGTTAAACCC   
  
  
- AAGGAGGATG GGAACCGATT CCCTGAATTT CGGACTTCTC TCTCCAGAAA TGAACTATGT AAACAACGAG   
  
  
- TGAACACGTT TGGTACAGAG ATTACCATCG GAACTGTTAC GTTTGGATCG GGAACTCGTT TAGAGGGTCG   
  
  
- AACGTCGGGG ACTACCGCTA TGTTAGGTCG CATAACAACG GATGAAACGA CTTAGTGAAC GACTTTCCTA   
  
  
- GGAATTCAGT ACCGGACCGG ATATATTTCG GGAAGTAAAG TTATCTTACG GACAATAAAG TCTTCTTAAA   
  
  
- GAACGATCCT TCGACAAAAA ACTCAACAAA GGGAAGAACT TCGACCGGAA AAACCACTGA TTGGTTAGTT   
  
  
- ATTAGCTCCG GTACCTCCCC CTTTTCTACC ACGTATATTA ACTAGACTTA CGCAGTCTTG GACGTGTCAC   
  
  
- CTAACGGGAA TAAGTTCTGA ACTCACGAGC CGGACTCCCG GGAGGAGTAA ACTCCTAATG GCCCCAAGTA   
  
  
- GTTGTCTTTC TCCAAAATCT TGTTCATCGA GTATCTAACT GACTTCTTCG ACTCTTCAAC CTAAACGGTA   
  
  
- AAGTCAAGTT AGGACACCAA ACGTTTGATC TCTTAGAGCT GTAGCCTTTT GAGGCACAAT TCTGGCCCCT   
  
  
- CCGGAACCGA TAATGGAGCC AGGAAGTTGA CGTATGGGAA AACCGAAGAC TTCTCCTTCA GGAATTCTTT   
  
  
- TCAAGTGGGA ACCGTAACCA TTTCGTTCGG TTACCCCGAT TAAATGTCCC GAACAAGTTA TTTCTACCTC   
  
  
- GATTATTATC CGCATCGGGT TCATTACTAA GCCGAAGTAG ACGTGGAAGT AGGGAGTTGT GAAGTCGGTT   
  
  
- CTACCTTCCA AAGGAATCGC GAAACACCCC AAATAGGGGT TTCTAATACC ACTATTGGCT CGTTCTAAGG   
  
  
- TTGGTGTTAC CCCGTCCTGA TTACCTCTCT AACAGTCTTC GTAACATGAA GATACGTCGT AACAAGCTAA   
  
  
- CGAATCTTAA ATGGGAGGGC TCTTGGAGGC ACCTCTCTTC CTTCCAGCTC TACGAGGAGG AACCGTTCCT   
  
  
- TTAGTTCTTG TAGTATCGCA CACTCCCTCC TCTTTCTTAT CTATCCGTAC TCTTCAACCC CTTCACCTAA   
  
  
- TTCTCCGAAC TCTACCGGCC CAAACCTTCG CAAGGAAACT CGGTGTATCC GTACTAGGTT CGTTCCGCCA   
  
  
- ACAACGTCTC GATACCGACA CTACCAATAT CTTATTTCCT CCTCTTGCCT ACAAAACAAT AGACGACCGT   
  
  
- TCTAGCGGGG GAGAAAAGTC ATAGACGAAC CTCTACATCC TCCAC

+     Myb-binding site

| Site Name | Organism | Position | Strand | Matrix score. | sequence | function |
| --- | --- | --- | --- | --- | --- | --- |
| Myb-binding site | Nicotiana tabacum | 1928 | - | 6 | CAACAG |  |
| Myb-binding site | Nicotiana tabacum | 2595 | + | 6 | CAACAG |  |

>HU08G01232.1   
+ -Up\_Stream \_Len000TCTACC TTTACCTGTG TTAAAAAAAA AAATTTTGTT TCCATTACTA TTCTCTTGGC   
  
  
+ ACTATTGGTG CTGATTTTTC TTCACCAAAT CAACCTCCTT ATATTTGCCA ATTTTACTGT CCTCTTTCAT   
  
  
+ CTATAAAGTC AACTCTCCTA GTACAATTTG TCACATAGAA AATCTCTAGG CACTCATCTA AACTCTCTGT   
  
  
+ TCTGGGTAAT TGATCGACGA TCTACACCAT TATTCGTTCT AGTGCACCGT TTGGACAGCC AATCATGTAC   
  
  
+ CTTTGGAGAT CAACTGCTCA TGAGTTCTTG GTGTGAAATT GGGGAAGTAA ATTCGACTTT AGGGCACTGG   
  
  
+ TCTATCACGC CATGATTTTA CCATCTATTT TCATATATAT CCAATAAGTT TGATCTTTCG CTATTAATCA   
  
  
+ CAATGATATA AACAAGTGGT GAAGAGCAAT AATGAACCAT ACATTTTAAA CTTAAGCGTT AAGGAAGACA   
  
  
+ TGAAGTTAAA AACAATGGTA AGAGCTATGC GTATGTTTGG CATATAGCTT TTTTAAGAGT GTTTTGGCTA   
  
  
+ TAGTCGGAGT TTTTTAATTA AGATTAGCTG TTTGATCAAA TAAAAAAGCT AATTTGAGTG TTTGGCGAGA   
  
  
+ AGACTTTTTA TAAGAACTTT TTTTGGTCTA AAAAGTTAAT TTAAAAAGGC TAATTCTATG AGCTTTTCGG   
  
  
+ AAGAGTTTTT TAAATAATTA ACTTTTTGTC TCATAAGCCA TAACTTTATC AGAAACAGTT AATTTTACAA   
  
  
+ AATAATTTCT CAACAAACAA CTAATTTAAA TAATTAATAA AAATAACTAA CTCAAATATC TAATAACTAA   
  
  
+ TAACTAATAT AAATAATTAA CAGTTAACAG TTGTTTACGA AACAGAATTT ATATAAAAAG CGGTAACAGA   
  
  
+ TAAAAGATAA TCATGCGAGA TTAAAAGTCA CCAGTGACAC ACAAGTACTT AAATAAATAG TCTAAACAAT   
  
  
+ GATATTTTTT GCGAGCTTCC ATGCAAATAC CGACACTTTT ATTGCCTCAG AAGGAAACAA AAACTGAGTG   
  
  
+ AAGGGGGGGG GGGGTTCTGT TTGTCTTTGT TAGGGCACGT TTGGATTCAA GTGTAGATTG GGAATAAATT   
  
  
+ CGAGGACTTT TTCTTTGCCC TTTTTCCCTG ATTCCCAAAC CCACATCAAG TCAAGACGAA ACCAGCCATT   
  
  
+ GAAAAAAGAG GGTGTTTGTG TGAGATAAAG ACAGAAATTT TATGCCAGAG AGAGAAGATA ACAAATGCAA   
  
  
+ CAACGTCGAC AGAGGCTCTC CTTCTCACAA ATTCCATATT CCTCTGTTTT TTAAGAAAGA AAAAGAAAGT   
  
  
+ GTGAGACAAG AACAACACAA GCAAACAATT CAGTTGACGA CGAATACTGA GTGATACCAC GCACTGTGTG   
  
  
+ CAACTGCGTT TCCATTTGTG GAGTCCTTCA AAGCACGCTC ATCTTCAACC CTTCCTTCGC CACGGAATTT   
  
  
+ CAGGGGGAAA AAAACCCCCA CCTAATTTTG AGAGAGAGAG AGAACAGAGA GAACGGGCGA TACAGGGATA   
  
  
+ CAAGGTTTGA GCAATTTTAG AAAATTTCTT GGGGCCGTTG ATGGGTTTTG AGTGAATTGC AAATCCCAGA   
  
  
+ AAGATTTCTG CGGTTTTTTA TCTACGGCTC TCTCTGTGGG AATTTTTTGG TAGATTCCTC TGGTTCCCAC   
  
  
+ TTTCATATCT TCTTATTTGT TCTACCCTCT TTACATCTGA TAGTTTGTTC TAACTGCTGC TTCAGTTGGT   
  
  
+ GCTTGTTCTC TTTAGTACTT TTCTGTTTTG TTCGTTTTGT TCATGCACTT GAATTCAACA AGAGCCCTTG   
  
  
+ TCCATTTTGA GCTCTTAATT CCCATCTAAC CCTGTTTCCT TCAGAAATTG CTGTTCTGTT CATATAGTTA   
  
  
+ TATTTTTTAT GCTTTGATTT GGGTATAAGT TTGCTGTTGG TGATTCCAAA GCTTGGTACT TTTGGCCATA   
  
  
+ TTTTGTTTGA GTTGGTGTTG ATTGGTACAA ATCTTGTAAT TGGTGTGATT GTTAATGGGA CCAATGCTTC   
  
  
+ AAGATGATGG TTCATCAGTA ACTTCTTCAT CACCTCTTCA ATTTTTCTCC ATGATGTCAC CCAATTTGGG   
  
  
+ TTCCTCCTAC CCTTGGCTAA GGGACTTAAA GCCTGAAGAG AGAGGTCTTT ACTTGATACA TTTGTTGCTC   
  
  
+ ACTTGTGCAA ACCATGTCTC TAATGGTAGC CTTGACAATG CAAACCTAGC CCTTGAGCAA ATCTCCCAGC   
  
  
+ TTGCAGCCCC TGATGGCGAT ACAATCCAGC GTATTGTTGC CTACTTTGCT GAATCACTTG CTGAAAGGAT   
  
  
+ CCTTAAGTCA TGGCCTGGCC TATATAAAGC CCTTCATTTC AATAGAATGC CTGTTATTTC AGAAGAATTT   
  
  
+ CTTGCTAGGA AGCTGTTTTT TGAGTTGTTT CCCTTCTTGA AGCTGGCCTT TTTGGTGACT AACCAATCAA   
  
  
+ TAATCGAGGC CATGGAGGGG GAAAAGATGG TGCATATAAT TGATCTGAAT GCGTCAGAAC CTGCACAGTG   
  
  
+ GATTGCCCTT ATTCAAGACT TGAGTGCTCG GCCTGAGGGC CCTCCTCATT TGAGGATTAC CGGGGTTCAT   
  
  
+ CAACAGAAAG AGGTTTTAGA ACAAGTAGCT CATAGATTGA CTGAAGAAGC TGAGAAGTTG GATTTGCCAT   
  
  
+ TTCAGTTCAA TCCTGTGGTT TGCAAACTAG AGAATCTCGA CATCGGAAAA CTCCGTGTTA AGACCGGGGA   
  
  
+ GGCCTTGGCT ATTACCTCGG TCCTTCAACT GCATACCCTT TTGGCTTCTG AAGAGGAAGT CCTTAAGAAA   
  
  
+ AGTTCACCCT TGGCATTGGT AAAGCAAGCC AATGGGGCTA ATTTACAGGG CTTGTTCAAT AAAGATGGAG   
  
  
+ CTAATAATAG GCGTAGCCCA AGTAATGATT CGGCTTCATC TGCACCTTCA TCCCTCAACA CTTCAGCCAA   
  
  
+ GATGGAAGGT TTCCTTAGCG CTTTGTGGGG TTTATCCCCA AAGATTATGG TGATAACCGA GCAAGATTCC   
  
  
+ AACCACAATG GGGCAGGACT AATGGAGAGA TTGTCAGAAG CATTGTACTT CTATGCAGCA TTGTTCGATT   
  
  
+ GCTTAGAATT TACCCTCCCG AGAACCTCCG TGGAGAGAAG GAAGGTCGAG ATGCTCCTCC TTGGCAAGGA   
  
  
+ AATCAAGAAC ATCATAGCGT GTGAGGGAGG AGAAAGAATA GATAGGCATG AGAAGTTGGG GAAGTGGATT   
  
  
+ AAGAGGCTTG AGATGGCCGG GTTTGGAAGC GTTCCTTTGA GCCACATAGG CATGATCCAA GCAAGGCGGT   
  
  
+ TGTTGCAGAG CTATGGCTGT GATGGTTATA GAATAAAGGA GGAGAACGGA TGTTTTGTTA TCTGCTGGCA   
  
  
+ AGATCGCCCC CTCTTTTCAG TATCTGCTTG GAGATGTAGG AGGTG  

- -Up\_Stream \_Len000AGATGG AAATGGACAC AATTTTTTTT TTTAAAACAA AGGTAATGAT AAGAGAACCG   
  
  
- TGATAACCAC GACTAAAAAG AAGTGGTTTA GTTGGAGGAA TATAAACGGT TAAAATGACA GGAGAAAGTA   
  
  
- GATATTTCAG TTGAGAGGAT CATGTTAAAC AGTGTATCTT TTAGAGATCC GTGAGTAGAT TTGAGAGACA   
  
  
- AGACCCATTA ACTAGCTGCT AGATGTGGTA ATAAGCAAGA TCACGTGGCA AACCTGTCGG TTAGTACATG   
  
  
- GAAACCTCTA GTTGACGAGT ACTCAAGAAC CACACTTTAA CCCCTTCATT TAAGCTGAAA TCCCGTGACC   
  
  
- AGATAGTGCG GTACTAAAAT GGTAGATAAA AGTATATATA GGTTATTCAA ACTAGAAAGC GATAATTAGT   
  
  
- GTTACTATAT TTGTTCACCA CTTCTCGTTA TTACTTGGTA TGTAAAATTT GAATTCGCAA TTCCTTCTGT   
  
  
- ACTTCAATTT TTGTTACCAT TCTCGATACG CATACAAACC GTATATCGAA AAAATTCTCA CAAAACCGAT   
  
  
- ATCAGCCTCA AAAAATTAAT TCTAATCGAC AAACTAGTTT ATTTTTTCGA TTAAACTCAC AAACCGCTCT   
  
  
- TCTGAAAAAT ATTCTTGAAA AAAACCAGAT TTTTCAATTA AATTTTTCCG ATTAAGATAC TCGAAAAGCC   
  
  
- TTCTCAAAAA ATTTATTAAT TGAAAAACAG AGTATTCGGT ATTGAAATAG TCTTTGTCAA TTAAAATGTT   
  
  
- TTATTAAAGA GTTGTTTGTT GATTAAATTT ATTAATTATT TTTATTGATT GAGTTTATAG ATTATTGATT   
  
  
- ATTGATTATA TTTATTAATT GTCAATTGTC AACAAATGCT TTGTCTTAAA TATATTTTTC GCCATTGTCT   
  
  
- ATTTTCTATT AGTACGCTCT AATTTTCAGT GGTCACTGTG TGTTCATGAA TTTATTTATC AGATTTGTTA   
  
  
- CTATAAAAAA CGCTCGAAGG TACGTTTATG GCTGTGAAAA TAACGGAGTC TTCCTTTGTT TTTGACTCAC   
  
  
- TTCCCCCCCC CCCCAAGACA AACAGAAACA ATCCCGTGCA AACCTAAGTT CACATCTAAC CCTTATTTAA   
  
  
- GCTCCTGAAA AAGAAACGGG AAAAAGGGAC TAAGGGTTTG GGTGTAGTTC AGTTCTGCTT TGGTCGGTAA   
  
  
- CTTTTTTCTC CCACAAACAC ACTCTATTTC TGTCTTTAAA ATACGGTCTC TCTCTTCTAT TGTTTACGTT   
  
  
- GTTGCAGCTG TCTCCGAGAG GAAGAGTGTT TAAGGTATAA GGAGACAAAA AATTCTTTCT TTTTCTTTCA   
  
  
- CACTCTGTTC TTGTTGTGTT CGTTTGTTAA GTCAACTGCT GCTTATGACT CACTATGGTG CGTGACACAC   
  
  
- GTTGACGCAA AGGTAAACAC CTCAGGAAGT TTCGTGCGAG TAGAAGTTGG GAAGGAAGCG GTGCCTTAAA   
  
  
- GTCCCCCTTT TTTTGGGGGT GGATTAAAAC TCTCTCTCTC TCTTGTCTCT CTTGCCCGCT ATGTCCCTAT   
  
  
- GTTCCAAACT CGTTAAAATC TTTTAAAGAA CCCCGGCAAC TACCCAAAAC TCACTTAACG TTTAGGGTCT   
  
  
- TTCTAAAGAC GCCAAAAAAT AGATGCCGAG AGAGACACCC TTAAAAAACC ATCTAAGGAG ACCAAGGGTG   
  
  
- AAAGTATAGA AGAATAAACA AGATGGGAGA AATGTAGACT ATCAAACAAG ATTGACGACG AAGTCAACCA   
  
  
- CGAACAAGAG AAATCATGAA AAGACAAAAC AAGCAAAACA AGTACGTGAA CTTAAGTTGT TCTCGGGAAC   
  
  
- AGGTAAAACT CGAGAATTAA GGGTAGATTG GGACAAAGGA AGTCTTTAAC GACAAGACAA GTATATCAAT   
  
  
- ATAAAAAATA CGAAACTAAA CCCATATTCA AACGACAACC ACTAAGGTTT CGAACCATGA AAACCGGTAT   
  
  
- AAAACAAACT CAACCACAAC TAACCATGTT TAGAACATTA ACCACACTAA CAATTACCCT GGTTACGAAG   
  
  
- TTCTACTACC AAGTAGTCAT TGAAGAAGTA GTGGAGAAGT TAAAAAGAGG TACTACAGTG GGTTAAACCC   
  
  
- AAGGAGGATG GGAACCGATT CCCTGAATTT CGGACTTCTC TCTCCAGAAA TGAACTATGT AAACAACGAG   
  
  
- TGAACACGTT TGGTACAGAG ATTACCATCG GAACTGTTAC GTTTGGATCG GGAACTCGTT TAGAGGGTCG   
  
  
- AACGTCGGGG ACTACCGCTA TGTTAGGTCG CATAACAACG GATGAAACGA CTTAGTGAAC GACTTTCCTA   
  
  
- GGAATTCAGT ACCGGACCGG ATATATTTCG GGAAGTAAAG TTATCTTACG GACAATAAAG TCTTCTTAAA   
  
  
- GAACGATCCT TCGACAAAAA ACTCAACAAA GGGAAGAACT TCGACCGGAA AAACCACTGA TTGGTTAGTT   
  
  
- ATTAGCTCCG GTACCTCCCC CTTTTCTACC ACGTATATTA ACTAGACTTA CGCAGTCTTG GACGTGTCAC   
  
  
- CTAACGGGAA TAAGTTCTGA ACTCACGAGC CGGACTCCCG GGAGGAGTAA ACTCCTAATG GCCCCAAGTA   
  
  
- GTTGTCTTTC TCCAAAATCT TGTTCATCGA GTATCTAACT GACTTCTTCG ACTCTTCAAC CTAAACGGTA   
  
  
- AAGTCAAGTT AGGACACCAA ACGTTTGATC TCTTAGAGCT GTAGCCTTTT GAGGCACAAT TCTGGCCCCT   
  
  
- CCGGAACCGA TAATGGAGCC AGGAAGTTGA CGTATGGGAA AACCGAAGAC TTCTCCTTCA GGAATTCTTT   
  
  
- TCAAGTGGGA ACCGTAACCA TTTCGTTCGG TTACCCCGAT TAAATGTCCC GAACAAGTTA TTTCTACCTC   
  
  
- GATTATTATC CGCATCGGGT TCATTACTAA GCCGAAGTAG ACGTGGAAGT AGGGAGTTGT GAAGTCGGTT   
  
  
- CTACCTTCCA AAGGAATCGC GAAACACCCC AAATAGGGGT TTCTAATACC ACTATTGGCT CGTTCTAAGG   
  
  
- TTGGTGTTAC CCCGTCCTGA TTACCTCTCT AACAGTCTTC GTAACATGAA GATACGTCGT AACAAGCTAA   
  
  
- CGAATCTTAA ATGGGAGGGC TCTTGGAGGC ACCTCTCTTC CTTCCAGCTC TACGAGGAGG AACCGTTCCT   
  
  
- TTAGTTCTTG TAGTATCGCA CACTCCCTCC TCTTTCTTAT CTATCCGTAC TCTTCAACCC CTTCACCTAA   
  
  
- TTCTCCGAAC TCTACCGGCC CAAACCTTCG CAAGGAAACT CGGTGTATCC GTACTAGGTT CGTTCCGCCA   
  
  
- ACAACGTCTC GATACCGACA CTACCAATAT CTTATTTCCT CCTCTTGCCT ACAAAACAAT AGACGACCGT   
  
  
- TCTAGCGGGG GAGAAAAGTC ATAGACGAAC CTCTACATCC TCCAC

+     P-box

| Site Name | Organism | Position | Strand | Matrix score. | sequence | function |
| --- | --- | --- | --- | --- | --- | --- |
| P-box | Oryza sativa | 2771 | + | 7 | CCTTTTG | gibberellin-responsive element |

>HU08G01232.1   
+ -Up\_Stream \_Len000TCTACC TTTACCTGTG TTAAAAAAAA AAATTTTGTT TCCATTACTA TTCTCTTGGC   
  
  
+ ACTATTGGTG CTGATTTTTC TTCACCAAAT CAACCTCCTT ATATTTGCCA ATTTTACTGT CCTCTTTCAT   
  
  
+ CTATAAAGTC AACTCTCCTA GTACAATTTG TCACATAGAA AATCTCTAGG CACTCATCTA AACTCTCTGT   
  
  
+ TCTGGGTAAT TGATCGACGA TCTACACCAT TATTCGTTCT AGTGCACCGT TTGGACAGCC AATCATGTAC   
  
  
+ CTTTGGAGAT CAACTGCTCA TGAGTTCTTG GTGTGAAATT GGGGAAGTAA ATTCGACTTT AGGGCACTGG   
  
  
+ TCTATCACGC CATGATTTTA CCATCTATTT TCATATATAT CCAATAAGTT TGATCTTTCG CTATTAATCA   
  
  
+ CAATGATATA AACAAGTGGT GAAGAGCAAT AATGAACCAT ACATTTTAAA CTTAAGCGTT AAGGAAGACA   
  
  
+ TGAAGTTAAA AACAATGGTA AGAGCTATGC GTATGTTTGG CATATAGCTT TTTTAAGAGT GTTTTGGCTA   
  
  
+ TAGTCGGAGT TTTTTAATTA AGATTAGCTG TTTGATCAAA TAAAAAAGCT AATTTGAGTG TTTGGCGAGA   
  
  
+ AGACTTTTTA TAAGAACTTT TTTTGGTCTA AAAAGTTAAT TTAAAAAGGC TAATTCTATG AGCTTTTCGG   
  
  
+ AAGAGTTTTT TAAATAATTA ACTTTTTGTC TCATAAGCCA TAACTTTATC AGAAACAGTT AATTTTACAA   
  
  
+ AATAATTTCT CAACAAACAA CTAATTTAAA TAATTAATAA AAATAACTAA CTCAAATATC TAATAACTAA   
  
  
+ TAACTAATAT AAATAATTAA CAGTTAACAG TTGTTTACGA AACAGAATTT ATATAAAAAG CGGTAACAGA   
  
  
+ TAAAAGATAA TCATGCGAGA TTAAAAGTCA CCAGTGACAC ACAAGTACTT AAATAAATAG TCTAAACAAT   
  
  
+ GATATTTTTT GCGAGCTTCC ATGCAAATAC CGACACTTTT ATTGCCTCAG AAGGAAACAA AAACTGAGTG   
  
  
+ AAGGGGGGGG GGGGTTCTGT TTGTCTTTGT TAGGGCACGT TTGGATTCAA GTGTAGATTG GGAATAAATT   
  
  
+ CGAGGACTTT TTCTTTGCCC TTTTTCCCTG ATTCCCAAAC CCACATCAAG TCAAGACGAA ACCAGCCATT   
  
  
+ GAAAAAAGAG GGTGTTTGTG TGAGATAAAG ACAGAAATTT TATGCCAGAG AGAGAAGATA ACAAATGCAA   
  
  
+ CAACGTCGAC AGAGGCTCTC CTTCTCACAA ATTCCATATT CCTCTGTTTT TTAAGAAAGA AAAAGAAAGT   
  
  
+ GTGAGACAAG AACAACACAA GCAAACAATT CAGTTGACGA CGAATACTGA GTGATACCAC GCACTGTGTG   
  
  
+ CAACTGCGTT TCCATTTGTG GAGTCCTTCA AAGCACGCTC ATCTTCAACC CTTCCTTCGC CACGGAATTT   
  
  
+ CAGGGGGAAA AAAACCCCCA CCTAATTTTG AGAGAGAGAG AGAACAGAGA GAACGGGCGA TACAGGGATA   
  
  
+ CAAGGTTTGA GCAATTTTAG AAAATTTCTT GGGGCCGTTG ATGGGTTTTG AGTGAATTGC AAATCCCAGA   
  
  
+ AAGATTTCTG CGGTTTTTTA TCTACGGCTC TCTCTGTGGG AATTTTTTGG TAGATTCCTC TGGTTCCCAC   
  
  
+ TTTCATATCT TCTTATTTGT TCTACCCTCT TTACATCTGA TAGTTTGTTC TAACTGCTGC TTCAGTTGGT   
  
  
+ GCTTGTTCTC TTTAGTACTT TTCTGTTTTG TTCGTTTTGT TCATGCACTT GAATTCAACA AGAGCCCTTG   
  
  
+ TCCATTTTGA GCTCTTAATT CCCATCTAAC CCTGTTTCCT TCAGAAATTG CTGTTCTGTT CATATAGTTA   
  
  
+ TATTTTTTAT GCTTTGATTT GGGTATAAGT TTGCTGTTGG TGATTCCAAA GCTTGGTACT TTTGGCCATA   
  
  
+ TTTTGTTTGA GTTGGTGTTG ATTGGTACAA ATCTTGTAAT TGGTGTGATT GTTAATGGGA CCAATGCTTC   
  
  
+ AAGATGATGG TTCATCAGTA ACTTCTTCAT CACCTCTTCA ATTTTTCTCC ATGATGTCAC CCAATTTGGG   
  
  
+ TTCCTCCTAC CCTTGGCTAA GGGACTTAAA GCCTGAAGAG AGAGGTCTTT ACTTGATACA TTTGTTGCTC   
  
  
+ ACTTGTGCAA ACCATGTCTC TAATGGTAGC CTTGACAATG CAAACCTAGC CCTTGAGCAA ATCTCCCAGC   
  
  
+ TTGCAGCCCC TGATGGCGAT ACAATCCAGC GTATTGTTGC CTACTTTGCT GAATCACTTG CTGAAAGGAT   
  
  
+ CCTTAAGTCA TGGCCTGGCC TATATAAAGC CCTTCATTTC AATAGAATGC CTGTTATTTC AGAAGAATTT   
  
  
+ CTTGCTAGGA AGCTGTTTTT TGAGTTGTTT CCCTTCTTGA AGCTGGCCTT TTTGGTGACT AACCAATCAA   
  
  
+ TAATCGAGGC CATGGAGGGG GAAAAGATGG TGCATATAAT TGATCTGAAT GCGTCAGAAC CTGCACAGTG   
  
  
+ GATTGCCCTT ATTCAAGACT TGAGTGCTCG GCCTGAGGGC CCTCCTCATT TGAGGATTAC CGGGGTTCAT   
  
  
+ CAACAGAAAG AGGTTTTAGA ACAAGTAGCT CATAGATTGA CTGAAGAAGC TGAGAAGTTG GATTTGCCAT   
  
  
+ TTCAGTTCAA TCCTGTGGTT TGCAAACTAG AGAATCTCGA CATCGGAAAA CTCCGTGTTA AGACCGGGGA   
  
  
+ GGCCTTGGCT ATTACCTCGG TCCTTCAACT GCATACCCTT TTGGCTTCTG AAGAGGAAGT CCTTAAGAAA   
  
  
+ AGTTCACCCT TGGCATTGGT AAAGCAAGCC AATGGGGCTA ATTTACAGGG CTTGTTCAAT AAAGATGGAG   
  
  
+ CTAATAATAG GCGTAGCCCA AGTAATGATT CGGCTTCATC TGCACCTTCA TCCCTCAACA CTTCAGCCAA   
  
  
+ GATGGAAGGT TTCCTTAGCG CTTTGTGGGG TTTATCCCCA AAGATTATGG TGATAACCGA GCAAGATTCC   
  
  
+ AACCACAATG GGGCAGGACT AATGGAGAGA TTGTCAGAAG CATTGTACTT CTATGCAGCA TTGTTCGATT   
  
  
+ GCTTAGAATT TACCCTCCCG AGAACCTCCG TGGAGAGAAG GAAGGTCGAG ATGCTCCTCC TTGGCAAGGA   
  
  
+ AATCAAGAAC ATCATAGCGT GTGAGGGAGG AGAAAGAATA GATAGGCATG AGAAGTTGGG GAAGTGGATT   
  
  
+ AAGAGGCTTG AGATGGCCGG GTTTGGAAGC GTTCCTTTGA GCCACATAGG CATGATCCAA GCAAGGCGGT   
  
  
+ TGTTGCAGAG CTATGGCTGT GATGGTTATA GAATAAAGGA GGAGAACGGA TGTTTTGTTA TCTGCTGGCA   
  
  
+ AGATCGCCCC CTCTTTTCAG TATCTGCTTG GAGATGTAGG AGGTG  

- -Up\_Stream \_Len000AGATGG AAATGGACAC AATTTTTTTT TTTAAAACAA AGGTAATGAT AAGAGAACCG   
  
  
- TGATAACCAC GACTAAAAAG AAGTGGTTTA GTTGGAGGAA TATAAACGGT TAAAATGACA GGAGAAAGTA   
  
  
- GATATTTCAG TTGAGAGGAT CATGTTAAAC AGTGTATCTT TTAGAGATCC GTGAGTAGAT TTGAGAGACA   
  
  
- AGACCCATTA ACTAGCTGCT AGATGTGGTA ATAAGCAAGA TCACGTGGCA AACCTGTCGG TTAGTACATG   
  
  
- GAAACCTCTA GTTGACGAGT ACTCAAGAAC CACACTTTAA CCCCTTCATT TAAGCTGAAA TCCCGTGACC   
  
  
- AGATAGTGCG GTACTAAAAT GGTAGATAAA AGTATATATA GGTTATTCAA ACTAGAAAGC GATAATTAGT   
  
  
- GTTACTATAT TTGTTCACCA CTTCTCGTTA TTACTTGGTA TGTAAAATTT GAATTCGCAA TTCCTTCTGT   
  
  
- ACTTCAATTT TTGTTACCAT TCTCGATACG CATACAAACC GTATATCGAA AAAATTCTCA CAAAACCGAT   
  
  
- ATCAGCCTCA AAAAATTAAT TCTAATCGAC AAACTAGTTT ATTTTTTCGA TTAAACTCAC AAACCGCTCT   
  
  
- TCTGAAAAAT ATTCTTGAAA AAAACCAGAT TTTTCAATTA AATTTTTCCG ATTAAGATAC TCGAAAAGCC   
  
  
- TTCTCAAAAA ATTTATTAAT TGAAAAACAG AGTATTCGGT ATTGAAATAG TCTTTGTCAA TTAAAATGTT   
  
  
- TTATTAAAGA GTTGTTTGTT GATTAAATTT ATTAATTATT TTTATTGATT GAGTTTATAG ATTATTGATT   
  
  
- ATTGATTATA TTTATTAATT GTCAATTGTC AACAAATGCT TTGTCTTAAA TATATTTTTC GCCATTGTCT   
  
  
- ATTTTCTATT AGTACGCTCT AATTTTCAGT GGTCACTGTG TGTTCATGAA TTTATTTATC AGATTTGTTA   
  
  
- CTATAAAAAA CGCTCGAAGG TACGTTTATG GCTGTGAAAA TAACGGAGTC TTCCTTTGTT TTTGACTCAC   
  
  
- TTCCCCCCCC CCCCAAGACA AACAGAAACA ATCCCGTGCA AACCTAAGTT CACATCTAAC CCTTATTTAA   
  
  
- GCTCCTGAAA AAGAAACGGG AAAAAGGGAC TAAGGGTTTG GGTGTAGTTC AGTTCTGCTT TGGTCGGTAA   
  
  
- CTTTTTTCTC CCACAAACAC ACTCTATTTC TGTCTTTAAA ATACGGTCTC TCTCTTCTAT TGTTTACGTT   
  
  
- GTTGCAGCTG TCTCCGAGAG GAAGAGTGTT TAAGGTATAA GGAGACAAAA AATTCTTTCT TTTTCTTTCA   
  
  
- CACTCTGTTC TTGTTGTGTT CGTTTGTTAA GTCAACTGCT GCTTATGACT CACTATGGTG CGTGACACAC   
  
  
- GTTGACGCAA AGGTAAACAC CTCAGGAAGT TTCGTGCGAG TAGAAGTTGG GAAGGAAGCG GTGCCTTAAA   
  
  
- GTCCCCCTTT TTTTGGGGGT GGATTAAAAC TCTCTCTCTC TCTTGTCTCT CTTGCCCGCT ATGTCCCTAT   
  
  
- GTTCCAAACT CGTTAAAATC TTTTAAAGAA CCCCGGCAAC TACCCAAAAC TCACTTAACG TTTAGGGTCT   
  
  
- TTCTAAAGAC GCCAAAAAAT AGATGCCGAG AGAGACACCC TTAAAAAACC ATCTAAGGAG ACCAAGGGTG   
  
  
- AAAGTATAGA AGAATAAACA AGATGGGAGA AATGTAGACT ATCAAACAAG ATTGACGACG AAGTCAACCA   
  
  
- CGAACAAGAG AAATCATGAA AAGACAAAAC AAGCAAAACA AGTACGTGAA CTTAAGTTGT TCTCGGGAAC   
  
  
- AGGTAAAACT CGAGAATTAA GGGTAGATTG GGACAAAGGA AGTCTTTAAC GACAAGACAA GTATATCAAT   
  
  
- ATAAAAAATA CGAAACTAAA CCCATATTCA AACGACAACC ACTAAGGTTT CGAACCATGA AAACCGGTAT   
  
  
- AAAACAAACT CAACCACAAC TAACCATGTT TAGAACATTA ACCACACTAA CAATTACCCT GGTTACGAAG   
  
  
- TTCTACTACC AAGTAGTCAT TGAAGAAGTA GTGGAGAAGT TAAAAAGAGG TACTACAGTG GGTTAAACCC   
  
  
- AAGGAGGATG GGAACCGATT CCCTGAATTT CGGACTTCTC TCTCCAGAAA TGAACTATGT AAACAACGAG   
  
  
- TGAACACGTT TGGTACAGAG ATTACCATCG GAACTGTTAC GTTTGGATCG GGAACTCGTT TAGAGGGTCG   
  
  
- AACGTCGGGG ACTACCGCTA TGTTAGGTCG CATAACAACG GATGAAACGA CTTAGTGAAC GACTTTCCTA   
  
  
- GGAATTCAGT ACCGGACCGG ATATATTTCG GGAAGTAAAG TTATCTTACG GACAATAAAG TCTTCTTAAA   
  
  
- GAACGATCCT TCGACAAAAA ACTCAACAAA GGGAAGAACT TCGACCGGAA AAACCACTGA TTGGTTAGTT   
  
  
- ATTAGCTCCG GTACCTCCCC CTTTTCTACC ACGTATATTA ACTAGACTTA CGCAGTCTTG GACGTGTCAC   
  
  
- CTAACGGGAA TAAGTTCTGA ACTCACGAGC CGGACTCCCG GGAGGAGTAA ACTCCTAATG GCCCCAAGTA   
  
  
- GTTGTCTTTC TCCAAAATCT TGTTCATCGA GTATCTAACT GACTTCTTCG ACTCTTCAAC CTAAACGGTA   
  
  
- AAGTCAAGTT AGGACACCAA ACGTTTGATC TCTTAGAGCT GTAGCCTTTT GAGGCACAAT TCTGGCCCCT   
  
  
- CCGGAACCGA TAATGGAGCC AGGAAGTTGA CGTATGGGAA AACCGAAGAC TTCTCCTTCA GGAATTCTTT   
  
  
- TCAAGTGGGA ACCGTAACCA TTTCGTTCGG TTACCCCGAT TAAATGTCCC GAACAAGTTA TTTCTACCTC   
  
  
- GATTATTATC CGCATCGGGT TCATTACTAA GCCGAAGTAG ACGTGGAAGT AGGGAGTTGT GAAGTCGGTT   
  
  
- CTACCTTCCA AAGGAATCGC GAAACACCCC AAATAGGGGT TTCTAATACC ACTATTGGCT CGTTCTAAGG   
  
  
- TTGGTGTTAC CCCGTCCTGA TTACCTCTCT AACAGTCTTC GTAACATGAA GATACGTCGT AACAAGCTAA   
  
  
- CGAATCTTAA ATGGGAGGGC TCTTGGAGGC ACCTCTCTTC CTTCCAGCTC TACGAGGAGG AACCGTTCCT   
  
  
- TTAGTTCTTG TAGTATCGCA CACTCCCTCC TCTTTCTTAT CTATCCGTAC TCTTCAACCC CTTCACCTAA   
  
  
- TTCTCCGAAC TCTACCGGCC CAAACCTTCG CAAGGAAACT CGGTGTATCC GTACTAGGTT CGTTCCGCCA   
  
  
- ACAACGTCTC GATACCGACA CTACCAATAT CTTATTTCCT CCTCTTGCCT ACAAAACAAT AGACGACCGT   
  
  
- TCTAGCGGGG GAGAAAAGTC ATAGACGAAC CTCTACATCC TCCAC

+     STRE

| Site Name | Organism | Position | Strand | Matrix score. | sequence | function |
| --- | --- | --- | --- | --- | --- | --- |
| STRE | Arabidopsis thaliana | 3372 | - | 5 | AGGGG |  |
| STRE | Arabidopsis thaliana | 2470 | + | 5 | AGGGG |  |
| STRE | Arabidopsis thaliana | 2251 | - | 5 | AGGGG |  |
| STRE | Arabidopsis thaliana | 1056 | + | 5 | AGGGG |  |
| STRE | Arabidopsis thaliana | 1476 | + | 5 | AGGGG |  |

>HU08G01232.1   
+ -Up\_Stream \_Len000TCTACC TTTACCTGTG TTAAAAAAAA AAATTTTGTT TCCATTACTA TTCTCTTGGC   
  
  
+ ACTATTGGTG CTGATTTTTC TTCACCAAAT CAACCTCCTT ATATTTGCCA ATTTTACTGT CCTCTTTCAT   
  
  
+ CTATAAAGTC AACTCTCCTA GTACAATTTG TCACATAGAA AATCTCTAGG CACTCATCTA AACTCTCTGT   
  
  
+ TCTGGGTAAT TGATCGACGA TCTACACCAT TATTCGTTCT AGTGCACCGT TTGGACAGCC AATCATGTAC   
  
  
+ CTTTGGAGAT CAACTGCTCA TGAGTTCTTG GTGTGAAATT GGGGAAGTAA ATTCGACTTT AGGGCACTGG   
  
  
+ TCTATCACGC CATGATTTTA CCATCTATTT TCATATATAT CCAATAAGTT TGATCTTTCG CTATTAATCA   
  
  
+ CAATGATATA AACAAGTGGT GAAGAGCAAT AATGAACCAT ACATTTTAAA CTTAAGCGTT AAGGAAGACA   
  
  
+ TGAAGTTAAA AACAATGGTA AGAGCTATGC GTATGTTTGG CATATAGCTT TTTTAAGAGT GTTTTGGCTA   
  
  
+ TAGTCGGAGT TTTTTAATTA AGATTAGCTG TTTGATCAAA TAAAAAAGCT AATTTGAGTG TTTGGCGAGA   
  
  
+ AGACTTTTTA TAAGAACTTT TTTTGGTCTA AAAAGTTAAT TTAAAAAGGC TAATTCTATG AGCTTTTCGG   
  
  
+ AAGAGTTTTT TAAATAATTA ACTTTTTGTC TCATAAGCCA TAACTTTATC AGAAACAGTT AATTTTACAA   
  
  
+ AATAATTTCT CAACAAACAA CTAATTTAAA TAATTAATAA AAATAACTAA CTCAAATATC TAATAACTAA   
  
  
+ TAACTAATAT AAATAATTAA CAGTTAACAG TTGTTTACGA AACAGAATTT ATATAAAAAG CGGTAACAGA   
  
  
+ TAAAAGATAA TCATGCGAGA TTAAAAGTCA CCAGTGACAC ACAAGTACTT AAATAAATAG TCTAAACAAT   
  
  
+ GATATTTTTT GCGAGCTTCC ATGCAAATAC CGACACTTTT ATTGCCTCAG AAGGAAACAA AAACTGAGTG   
  
  
+ AAGGGGGGGG GGGGTTCTGT TTGTCTTTGT TAGGGCACGT TTGGATTCAA GTGTAGATTG GGAATAAATT   
  
  
+ CGAGGACTTT TTCTTTGCCC TTTTTCCCTG ATTCCCAAAC CCACATCAAG TCAAGACGAA ACCAGCCATT   
  
  
+ GAAAAAAGAG GGTGTTTGTG TGAGATAAAG ACAGAAATTT TATGCCAGAG AGAGAAGATA ACAAATGCAA   
  
  
+ CAACGTCGAC AGAGGCTCTC CTTCTCACAA ATTCCATATT CCTCTGTTTT TTAAGAAAGA AAAAGAAAGT   
  
  
+ GTGAGACAAG AACAACACAA GCAAACAATT CAGTTGACGA CGAATACTGA GTGATACCAC GCACTGTGTG   
  
  
+ CAACTGCGTT TCCATTTGTG GAGTCCTTCA AAGCACGCTC ATCTTCAACC CTTCCTTCGC CACGGAATTT   
  
  
+ CAGGGGGAAA AAAACCCCCA CCTAATTTTG AGAGAGAGAG AGAACAGAGA GAACGGGCGA TACAGGGATA   
  
  
+ CAAGGTTTGA GCAATTTTAG AAAATTTCTT GGGGCCGTTG ATGGGTTTTG AGTGAATTGC AAATCCCAGA   
  
  
+ AAGATTTCTG CGGTTTTTTA TCTACGGCTC TCTCTGTGGG AATTTTTTGG TAGATTCCTC TGGTTCCCAC   
  
  
+ TTTCATATCT TCTTATTTGT TCTACCCTCT TTACATCTGA TAGTTTGTTC TAACTGCTGC TTCAGTTGGT   
  
  
+ GCTTGTTCTC TTTAGTACTT TTCTGTTTTG TTCGTTTTGT TCATGCACTT GAATTCAACA AGAGCCCTTG   
  
  
+ TCCATTTTGA GCTCTTAATT CCCATCTAAC CCTGTTTCCT TCAGAAATTG CTGTTCTGTT CATATAGTTA   
  
  
+ TATTTTTTAT GCTTTGATTT GGGTATAAGT TTGCTGTTGG TGATTCCAAA GCTTGGTACT TTTGGCCATA   
  
  
+ TTTTGTTTGA GTTGGTGTTG ATTGGTACAA ATCTTGTAAT TGGTGTGATT GTTAATGGGA CCAATGCTTC   
  
  
+ AAGATGATGG TTCATCAGTA ACTTCTTCAT CACCTCTTCA ATTTTTCTCC ATGATGTCAC CCAATTTGGG   
  
  
+ TTCCTCCTAC CCTTGGCTAA GGGACTTAAA GCCTGAAGAG AGAGGTCTTT ACTTGATACA TTTGTTGCTC   
  
  
+ ACTTGTGCAA ACCATGTCTC TAATGGTAGC CTTGACAATG CAAACCTAGC CCTTGAGCAA ATCTCCCAGC   
  
  
+ TTGCAGCCCC TGATGGCGAT ACAATCCAGC GTATTGTTGC CTACTTTGCT GAATCACTTG CTGAAAGGAT   
  
  
+ CCTTAAGTCA TGGCCTGGCC TATATAAAGC CCTTCATTTC AATAGAATGC CTGTTATTTC AGAAGAATTT   
  
  
+ CTTGCTAGGA AGCTGTTTTT TGAGTTGTTT CCCTTCTTGA AGCTGGCCTT TTTGGTGACT AACCAATCAA   
  
  
+ TAATCGAGGC CATGGAGGGG GAAAAGATGG TGCATATAAT TGATCTGAAT GCGTCAGAAC CTGCACAGTG   
  
  
+ GATTGCCCTT ATTCAAGACT TGAGTGCTCG GCCTGAGGGC CCTCCTCATT TGAGGATTAC CGGGGTTCAT   
  
  
+ CAACAGAAAG AGGTTTTAGA ACAAGTAGCT CATAGATTGA CTGAAGAAGC TGAGAAGTTG GATTTGCCAT   
  
  
+ TTCAGTTCAA TCCTGTGGTT TGCAAACTAG AGAATCTCGA CATCGGAAAA CTCCGTGTTA AGACCGGGGA   
  
  
+ GGCCTTGGCT ATTACCTCGG TCCTTCAACT GCATACCCTT TTGGCTTCTG AAGAGGAAGT CCTTAAGAAA   
  
  
+ AGTTCACCCT TGGCATTGGT AAAGCAAGCC AATGGGGCTA ATTTACAGGG CTTGTTCAAT AAAGATGGAG   
  
  
+ CTAATAATAG GCGTAGCCCA AGTAATGATT CGGCTTCATC TGCACCTTCA TCCCTCAACA CTTCAGCCAA   
  
  
+ GATGGAAGGT TTCCTTAGCG CTTTGTGGGG TTTATCCCCA AAGATTATGG TGATAACCGA GCAAGATTCC   
  
  
+ AACCACAATG GGGCAGGACT AATGGAGAGA TTGTCAGAAG CATTGTACTT CTATGCAGCA TTGTTCGATT   
  
  
+ GCTTAGAATT TACCCTCCCG AGAACCTCCG TGGAGAGAAG GAAGGTCGAG ATGCTCCTCC TTGGCAAGGA   
  
  
+ AATCAAGAAC ATCATAGCGT GTGAGGGAGG AGAAAGAATA GATAGGCATG AGAAGTTGGG GAAGTGGATT   
  
  
+ AAGAGGCTTG AGATGGCCGG GTTTGGAAGC GTTCCTTTGA GCCACATAGG CATGATCCAA GCAAGGCGGT   
  
  
+ TGTTGCAGAG CTATGGCTGT GATGGTTATA GAATAAAGGA GGAGAACGGA TGTTTTGTTA TCTGCTGGCA   
  
  
+ AGATCGCCCC CTCTTTTCAG TATCTGCTTG GAGATGTAGG AGGTG  

- -Up\_Stream \_Len000AGATGG AAATGGACAC AATTTTTTTT TTTAAAACAA AGGTAATGAT AAGAGAACCG   
  
  
- TGATAACCAC GACTAAAAAG AAGTGGTTTA GTTGGAGGAA TATAAACGGT TAAAATGACA GGAGAAAGTA   
  
  
- GATATTTCAG TTGAGAGGAT CATGTTAAAC AGTGTATCTT TTAGAGATCC GTGAGTAGAT TTGAGAGACA   
  
  
- AGACCCATTA ACTAGCTGCT AGATGTGGTA ATAAGCAAGA TCACGTGGCA AACCTGTCGG TTAGTACATG   
  
  
- GAAACCTCTA GTTGACGAGT ACTCAAGAAC CACACTTTAA CCCCTTCATT TAAGCTGAAA TCCCGTGACC   
  
  
- AGATAGTGCG GTACTAAAAT GGTAGATAAA AGTATATATA GGTTATTCAA ACTAGAAAGC GATAATTAGT   
  
  
- GTTACTATAT TTGTTCACCA CTTCTCGTTA TTACTTGGTA TGTAAAATTT GAATTCGCAA TTCCTTCTGT   
  
  
- ACTTCAATTT TTGTTACCAT TCTCGATACG CATACAAACC GTATATCGAA AAAATTCTCA CAAAACCGAT   
  
  
- ATCAGCCTCA AAAAATTAAT TCTAATCGAC AAACTAGTTT ATTTTTTCGA TTAAACTCAC AAACCGCTCT   
  
  
- TCTGAAAAAT ATTCTTGAAA AAAACCAGAT TTTTCAATTA AATTTTTCCG ATTAAGATAC TCGAAAAGCC   
  
  
- TTCTCAAAAA ATTTATTAAT TGAAAAACAG AGTATTCGGT ATTGAAATAG TCTTTGTCAA TTAAAATGTT   
  
  
- TTATTAAAGA GTTGTTTGTT GATTAAATTT ATTAATTATT TTTATTGATT GAGTTTATAG ATTATTGATT   
  
  
- ATTGATTATA TTTATTAATT GTCAATTGTC AACAAATGCT TTGTCTTAAA TATATTTTTC GCCATTGTCT   
  
  
- ATTTTCTATT AGTACGCTCT AATTTTCAGT GGTCACTGTG TGTTCATGAA TTTATTTATC AGATTTGTTA   
  
  
- CTATAAAAAA CGCTCGAAGG TACGTTTATG GCTGTGAAAA TAACGGAGTC TTCCTTTGTT TTTGACTCAC   
  
  
- TTCCCCCCCC CCCCAAGACA AACAGAAACA ATCCCGTGCA AACCTAAGTT CACATCTAAC CCTTATTTAA   
  
  
- GCTCCTGAAA AAGAAACGGG AAAAAGGGAC TAAGGGTTTG GGTGTAGTTC AGTTCTGCTT TGGTCGGTAA   
  
  
- CTTTTTTCTC CCACAAACAC ACTCTATTTC TGTCTTTAAA ATACGGTCTC TCTCTTCTAT TGTTTACGTT   
  
  
- GTTGCAGCTG TCTCCGAGAG GAAGAGTGTT TAAGGTATAA GGAGACAAAA AATTCTTTCT TTTTCTTTCA   
  
  
- CACTCTGTTC TTGTTGTGTT CGTTTGTTAA GTCAACTGCT GCTTATGACT CACTATGGTG CGTGACACAC   
  
  
- GTTGACGCAA AGGTAAACAC CTCAGGAAGT TTCGTGCGAG TAGAAGTTGG GAAGGAAGCG GTGCCTTAAA   
  
  
- GTCCCCCTTT TTTTGGGGGT GGATTAAAAC TCTCTCTCTC TCTTGTCTCT CTTGCCCGCT ATGTCCCTAT   
  
  
- GTTCCAAACT CGTTAAAATC TTTTAAAGAA CCCCGGCAAC TACCCAAAAC TCACTTAACG TTTAGGGTCT   
  
  
- TTCTAAAGAC GCCAAAAAAT AGATGCCGAG AGAGACACCC TTAAAAAACC ATCTAAGGAG ACCAAGGGTG   
  
  
- AAAGTATAGA AGAATAAACA AGATGGGAGA AATGTAGACT ATCAAACAAG ATTGACGACG AAGTCAACCA   
  
  
- CGAACAAGAG AAATCATGAA AAGACAAAAC AAGCAAAACA AGTACGTGAA CTTAAGTTGT TCTCGGGAAC   
  
  
- AGGTAAAACT CGAGAATTAA GGGTAGATTG GGACAAAGGA AGTCTTTAAC GACAAGACAA GTATATCAAT   
  
  
- ATAAAAAATA CGAAACTAAA CCCATATTCA AACGACAACC ACTAAGGTTT CGAACCATGA AAACCGGTAT   
  
  
- AAAACAAACT CAACCACAAC TAACCATGTT TAGAACATTA ACCACACTAA CAATTACCCT GGTTACGAAG   
  
  
- TTCTACTACC AAGTAGTCAT TGAAGAAGTA GTGGAGAAGT TAAAAAGAGG TACTACAGTG GGTTAAACCC   
  
  
- AAGGAGGATG GGAACCGATT CCCTGAATTT CGGACTTCTC TCTCCAGAAA TGAACTATGT AAACAACGAG   
  
  
- TGAACACGTT TGGTACAGAG ATTACCATCG GAACTGTTAC GTTTGGATCG GGAACTCGTT TAGAGGGTCG   
  
  
- AACGTCGGGG ACTACCGCTA TGTTAGGTCG CATAACAACG GATGAAACGA CTTAGTGAAC GACTTTCCTA   
  
  
- GGAATTCAGT ACCGGACCGG ATATATTTCG GGAAGTAAAG TTATCTTACG GACAATAAAG TCTTCTTAAA   
  
  
- GAACGATCCT TCGACAAAAA ACTCAACAAA GGGAAGAACT TCGACCGGAA AAACCACTGA TTGGTTAGTT   
  
  
- ATTAGCTCCG GTACCTCCCC CTTTTCTACC ACGTATATTA ACTAGACTTA CGCAGTCTTG GACGTGTCAC   
  
  
- CTAACGGGAA TAAGTTCTGA ACTCACGAGC CGGACTCCCG GGAGGAGTAA ACTCCTAATG GCCCCAAGTA   
  
  
- GTTGTCTTTC TCCAAAATCT TGTTCATCGA GTATCTAACT GACTTCTTCG ACTCTTCAAC CTAAACGGTA   
  
  
- AAGTCAAGTT AGGACACCAA ACGTTTGATC TCTTAGAGCT GTAGCCTTTT GAGGCACAAT TCTGGCCCCT   
  
  
- CCGGAACCGA TAATGGAGCC AGGAAGTTGA CGTATGGGAA AACCGAAGAC TTCTCCTTCA GGAATTCTTT   
  
  
- TCAAGTGGGA ACCGTAACCA TTTCGTTCGG TTACCCCGAT TAAATGTCCC GAACAAGTTA TTTCTACCTC   
  
  
- GATTATTATC CGCATCGGGT TCATTACTAA GCCGAAGTAG ACGTGGAAGT AGGGAGTTGT GAAGTCGGTT   
  
  
- CTACCTTCCA AAGGAATCGC GAAACACCCC AAATAGGGGT TTCTAATACC ACTATTGGCT CGTTCTAAGG   
  
  
- TTGGTGTTAC CCCGTCCTGA TTACCTCTCT AACAGTCTTC GTAACATGAA GATACGTCGT AACAAGCTAA   
  
  
- CGAATCTTAA ATGGGAGGGC TCTTGGAGGC ACCTCTCTTC CTTCCAGCTC TACGAGGAGG AACCGTTCCT   
  
  
- TTAGTTCTTG TAGTATCGCA CACTCCCTCC TCTTTCTTAT CTATCCGTAC TCTTCAACCC CTTCACCTAA   
  
  
- TTCTCCGAAC TCTACCGGCC CAAACCTTCG CAAGGAAACT CGGTGTATCC GTACTAGGTT CGTTCCGCCA   
  
  
- ACAACGTCTC GATACCGACA CTACCAATAT CTTATTTCCT CCTCTTGCCT ACAAAACAAT AGACGACCGT   
  
  
- TCTAGCGGGG GAGAAAAGTC ATAGACGAAC CTCTACATCC TCCAC

+     TATA-box

| Site Name | Organism | Position | Strand | Matrix score. | sequence | function |
| --- | --- | --- | --- | --- | --- | --- |
| TATA-box | Arabidopsis thaliana | 2489 | - | 4 | TATA | core promoter element around -30 of transcription start |
| TATA-box | Brassica oleracea | 2488 | + | 6 | ATATAA | core promoter element around -30 of transcription start |
| TATA-box | Arabidopsis thaliana | 2337 | - | 4 | TATA | core promoter element around -30 of transcription start |
| TATA-box | Arabidopsis thaliana | 799 | - | 8 | TATTTAAA | core promoter element around -30 of transcription start |
| TATA-box | Arabidopsis thaliana | 2335 | - | 6 | TATATA | core promoter element around -30 of transcription start |
| TATA-box | Arabidopsis thaliana | 1892 | - | 5 | TATAA | core promoter element around -30 of transcription start |
| TATA-box | Arabidopsis thaliana | 2333 | + | 9 | ccTATAAAaa | core promoter element around -30 of transcription start |
| TATA-box | Avena sativa | 1887 | + | 12 | TATATTTATATTT | core promoter element around -30 of transcription start |
| TATA-box | Brassica oleracea | 895 | + | 6 | ATATAA | core promoter element around -30 of transcription start |
| TATA-box | Helianthus annuus | 892 | - | 6 | TATAAA | core promoter element around -30 of transcription start |
| TATA-box | Arabidopsis thaliana | 114 | + | 4 | TATA | core promoter element around -30 of transcription start |
| TATA-box | Arabidopsis thaliana | 3320 | - | 5 | TATAA | core promoter element around -30 of transcription start |
| TATA-box | Arabidopsis thaliana | 896 | + | 4 | TATA | core promoter element around -30 of transcription start |
| TATA-box | Brassica juncea | 891 | - | 7 | TATAAAT | core promoter element around -30 of transcription start |
| TATA-box | Arabidopsis thaliana | 852 | + | 4 | TATA | core promoter element around -30 of transcription start |
| TATA-box | Arabidopsis thaliana | 113 | - | 5 | TATAA | core promoter element around -30 of transcription start |
| TATA-box | Brassica oleracea | 2336 | + | 6 | ATATAA | core promoter element around -30 of transcription start |
| TATA-box | Arabidopsis thaliana | 390 | + | 4 | TATA | core promoter element around -30 of transcription start |
| TATA-box | Arabidopsis thaliana | 431 | + | 4 | TATA | core promoter element around -30 of transcription start |
| TATA-box | Brassica napus | 387 | + | 6 | ATATAT | core promoter element around -30 of transcription start |
| TATA-box | Arabidopsis thaliana | 146 | + | 4 | TATA | core promoter element around -30 of transcription start |
| TATA-box | Arabidopsis thaliana | 537 | + | 4 | TATA | core promoter element around -30 of transcription start |
| TATA-box | Brassica napus | 389 | + | 6 | ATATAT | core promoter element around -30 of transcription start |
| TATA-box | Brassica oleracea | 430 | + | 6 | ATATAA | core promoter element around -30 of transcription start |
| TATA-box | Arabidopsis thaliana | 643 | + | 4 | TATA | core promoter element around -30 of transcription start |
| TATA-box | Helianthus annuus | 641 | - | 6 | TATAAA | core promoter element around -30 of transcription start |
| TATA-box | Arabidopsis thaliana | 3321 | - | 4 | TATA | core promoter element around -30 of transcription start |
| TATA-box | Oryza sativa | 770 | + | 7 | TACAAAA | core promoter element around -30 of transcription start |
| TATA-box | Arabidopsis thaliana | 890 | - | 9 | taTATAAAtc | core promoter element around -30 of transcription start |
| TATA-box | Arabidopsis thaliana | 894 | + | 6 | TATATA | core promoter element around -30 of transcription start |
| TATA-box | Arabidopsis thaliana | 713 | - | 8 | TATTTAAA | core promoter element around -30 of transcription start |
| TATA-box | Arabidopsis thaliana | 388 | + | 6 | TATATA | core promoter element around -30 of transcription start |
| TATA-box | Arabidopsis thaliana | 1893 | - | 4 | TATA | core promoter element around -30 of transcription start |
| TATA-box | Brassica oleracea | 851 | + | 6 | ATATAA | core promoter element around -30 of transcription start |
| TATA-box | Arabidopsis thaliana | 893 | - | 7 | TATATAA | core promoter element around -30 of transcription start |
| TATA-box | Pisum sativum | 640 | - | 7 | TATAAAA | core promoter element around -30 of transcription start |
| TATA-box | Arabidopsis thaliana | 1918 | - | 4 | TATA | core promoter element around -30 of transcription start |
| TATA-box | Arabidopsis thaliana | 642 | - | 5 | TATAA | core promoter element around -30 of transcription start |
| TATA-box | Arabidopsis thaliana | 639 | - | 9 | ccTATAAAaa | core promoter element around -30 of transcription start |
| TATA-box | Arabidopsis thaliana | 563 | + | 4 | TATA | core promoter element around -30 of transcription start |

>HU08G01232.1   
+ -Up\_Stream \_Len000TCTACC TTTACCTGTG TTAAAAAAAA AAATTTTGTT TCCATTACTA TTCTCTTGGC   
  
  
+ ACTATTGGTG CTGATTTTTC TTCACCAAAT CAACCTCCTT ATATTTGCCA ATTTTACTGT CCTCTTTCAT   
  
  
+ CTATAAAGTC AACTCTCCTA GTACAATTTG TCACATAGAA AATCTCTAGG CACTCATCTA AACTCTCTGT   
  
  
+ TCTGGGTAAT TGATCGACGA TCTACACCAT TATTCGTTCT AGTGCACCGT TTGGACAGCC AATCATGTAC   
  
  
+ CTTTGGAGAT CAACTGCTCA TGAGTTCTTG GTGTGAAATT GGGGAAGTAA ATTCGACTTT AGGGCACTGG   
  
  
+ TCTATCACGC CATGATTTTA CCATCTATTT TCATATATAT CCAATAAGTT TGATCTTTCG CTATTAATCA   
  
  
+ CAATGATATA AACAAGTGGT GAAGAGCAAT AATGAACCAT ACATTTTAAA CTTAAGCGTT AAGGAAGACA   
  
  
+ TGAAGTTAAA AACAATGGTA AGAGCTATGC GTATGTTTGG CATATAGCTT TTTTAAGAGT GTTTTGGCTA   
  
  
+ TAGTCGGAGT TTTTTAATTA AGATTAGCTG TTTGATCAAA TAAAAAAGCT AATTTGAGTG TTTGGCGAGA   
  
  
+ AGACTTTTTA TAAGAACTTT TTTTGGTCTA AAAAGTTAAT TTAAAAAGGC TAATTCTATG AGCTTTTCGG   
  
  
+ AAGAGTTTTT TAAATAATTA ACTTTTTGTC TCATAAGCCA TAACTTTATC AGAAACAGTT AATTTTACAA   
  
  
+ AATAATTTCT CAACAAACAA CTAATTTAAA TAATTAATAA AAATAACTAA CTCAAATATC TAATAACTAA   
  
  
+ TAACTAATAT AAATAATTAA CAGTTAACAG TTGTTTACGA AACAGAATTT ATATAAAAAG CGGTAACAGA   
  
  
+ TAAAAGATAA TCATGCGAGA TTAAAAGTCA CCAGTGACAC ACAAGTACTT AAATAAATAG TCTAAACAAT   
  
  
+ GATATTTTTT GCGAGCTTCC ATGCAAATAC CGACACTTTT ATTGCCTCAG AAGGAAACAA AAACTGAGTG   
  
  
+ AAGGGGGGGG GGGGTTCTGT TTGTCTTTGT TAGGGCACGT TTGGATTCAA GTGTAGATTG GGAATAAATT   
  
  
+ CGAGGACTTT TTCTTTGCCC TTTTTCCCTG ATTCCCAAAC CCACATCAAG TCAAGACGAA ACCAGCCATT   
  
  
+ GAAAAAAGAG GGTGTTTGTG TGAGATAAAG ACAGAAATTT TATGCCAGAG AGAGAAGATA ACAAATGCAA   
  
  
+ CAACGTCGAC AGAGGCTCTC CTTCTCACAA ATTCCATATT CCTCTGTTTT TTAAGAAAGA AAAAGAAAGT   
  
  
+ GTGAGACAAG AACAACACAA GCAAACAATT CAGTTGACGA CGAATACTGA GTGATACCAC GCACTGTGTG   
  
  
+ CAACTGCGTT TCCATTTGTG GAGTCCTTCA AAGCACGCTC ATCTTCAACC CTTCCTTCGC CACGGAATTT   
  
  
+ CAGGGGGAAA AAAACCCCCA CCTAATTTTG AGAGAGAGAG AGAACAGAGA GAACGGGCGA TACAGGGATA   
  
  
+ CAAGGTTTGA GCAATTTTAG AAAATTTCTT GGGGCCGTTG ATGGGTTTTG AGTGAATTGC AAATCCCAGA   
  
  
+ AAGATTTCTG CGGTTTTTTA TCTACGGCTC TCTCTGTGGG AATTTTTTGG TAGATTCCTC TGGTTCCCAC   
  
  
+ TTTCATATCT TCTTATTTGT TCTACCCTCT TTACATCTGA TAGTTTGTTC TAACTGCTGC TTCAGTTGGT   
  
  
+ GCTTGTTCTC TTTAGTACTT TTCTGTTTTG TTCGTTTTGT TCATGCACTT GAATTCAACA AGAGCCCTTG   
  
  
+ TCCATTTTGA GCTCTTAATT CCCATCTAAC CCTGTTTCCT TCAGAAATTG CTGTTCTGTT CATATAGTTA   
  
  
+ TATTTTTTAT GCTTTGATTT GGGTATAAGT TTGCTGTTGG TGATTCCAAA GCTTGGTACT TTTGGCCATA   
  
  
+ TTTTGTTTGA GTTGGTGTTG ATTGGTACAA ATCTTGTAAT TGGTGTGATT GTTAATGGGA CCAATGCTTC   
  
  
+ AAGATGATGG TTCATCAGTA ACTTCTTCAT CACCTCTTCA ATTTTTCTCC ATGATGTCAC CCAATTTGGG   
  
  
+ TTCCTCCTAC CCTTGGCTAA GGGACTTAAA GCCTGAAGAG AGAGGTCTTT ACTTGATACA TTTGTTGCTC   
  
  
+ ACTTGTGCAA ACCATGTCTC TAATGGTAGC CTTGACAATG CAAACCTAGC CCTTGAGCAA ATCTCCCAGC   
  
  
+ TTGCAGCCCC TGATGGCGAT ACAATCCAGC GTATTGTTGC CTACTTTGCT GAATCACTTG CTGAAAGGAT   
  
  
+ CCTTAAGTCA TGGCCTGGCC TATATAAAGC CCTTCATTTC AATAGAATGC CTGTTATTTC AGAAGAATTT   
  
  
+ CTTGCTAGGA AGCTGTTTTT TGAGTTGTTT CCCTTCTTGA AGCTGGCCTT TTTGGTGACT AACCAATCAA   
  
  
+ TAATCGAGGC CATGGAGGGG GAAAAGATGG TGCATATAAT TGATCTGAAT GCGTCAGAAC CTGCACAGTG   
  
  
+ GATTGCCCTT ATTCAAGACT TGAGTGCTCG GCCTGAGGGC CCTCCTCATT TGAGGATTAC CGGGGTTCAT   
  
  
+ CAACAGAAAG AGGTTTTAGA ACAAGTAGCT CATAGATTGA CTGAAGAAGC TGAGAAGTTG GATTTGCCAT   
  
  
+ TTCAGTTCAA TCCTGTGGTT TGCAAACTAG AGAATCTCGA CATCGGAAAA CTCCGTGTTA AGACCGGGGA   
  
  
+ GGCCTTGGCT ATTACCTCGG TCCTTCAACT GCATACCCTT TTGGCTTCTG AAGAGGAAGT CCTTAAGAAA   
  
  
+ AGTTCACCCT TGGCATTGGT AAAGCAAGCC AATGGGGCTA ATTTACAGGG CTTGTTCAAT AAAGATGGAG   
  
  
+ CTAATAATAG GCGTAGCCCA AGTAATGATT CGGCTTCATC TGCACCTTCA TCCCTCAACA CTTCAGCCAA   
  
  
+ GATGGAAGGT TTCCTTAGCG CTTTGTGGGG TTTATCCCCA AAGATTATGG TGATAACCGA GCAAGATTCC   
  
  
+ AACCACAATG GGGCAGGACT AATGGAGAGA TTGTCAGAAG CATTGTACTT CTATGCAGCA TTGTTCGATT   
  
  
+ GCTTAGAATT TACCCTCCCG AGAACCTCCG TGGAGAGAAG GAAGGTCGAG ATGCTCCTCC TTGGCAAGGA   
  
  
+ AATCAAGAAC ATCATAGCGT GTGAGGGAGG AGAAAGAATA GATAGGCATG AGAAGTTGGG GAAGTGGATT   
  
  
+ AAGAGGCTTG AGATGGCCGG GTTTGGAAGC GTTCCTTTGA GCCACATAGG CATGATCCAA GCAAGGCGGT   
  
  
+ TGTTGCAGAG CTATGGCTGT GATGGTTATA GAATAAAGGA GGAGAACGGA TGTTTTGTTA TCTGCTGGCA   
  
  
+ AGATCGCCCC CTCTTTTCAG TATCTGCTTG GAGATGTAGG AGGTG  

- -Up\_Stream \_Len000AGATGG AAATGGACAC AATTTTTTTT TTTAAAACAA AGGTAATGAT AAGAGAACCG   
  
  
- TGATAACCAC GACTAAAAAG AAGTGGTTTA GTTGGAGGAA TATAAACGGT TAAAATGACA GGAGAAAGTA   
  
  
- GATATTTCAG TTGAGAGGAT CATGTTAAAC AGTGTATCTT TTAGAGATCC GTGAGTAGAT TTGAGAGACA   
  
  
- AGACCCATTA ACTAGCTGCT AGATGTGGTA ATAAGCAAGA TCACGTGGCA AACCTGTCGG TTAGTACATG   
  
  
- GAAACCTCTA GTTGACGAGT ACTCAAGAAC CACACTTTAA CCCCTTCATT TAAGCTGAAA TCCCGTGACC   
  
  
- AGATAGTGCG GTACTAAAAT GGTAGATAAA AGTATATATA GGTTATTCAA ACTAGAAAGC GATAATTAGT   
  
  
- GTTACTATAT TTGTTCACCA CTTCTCGTTA TTACTTGGTA TGTAAAATTT GAATTCGCAA TTCCTTCTGT   
  
  
- ACTTCAATTT TTGTTACCAT TCTCGATACG CATACAAACC GTATATCGAA AAAATTCTCA CAAAACCGAT   
  
  
- ATCAGCCTCA AAAAATTAAT TCTAATCGAC AAACTAGTTT ATTTTTTCGA TTAAACTCAC AAACCGCTCT   
  
  
- TCTGAAAAAT ATTCTTGAAA AAAACCAGAT TTTTCAATTA AATTTTTCCG ATTAAGATAC TCGAAAAGCC   
  
  
- TTCTCAAAAA ATTTATTAAT TGAAAAACAG AGTATTCGGT ATTGAAATAG TCTTTGTCAA TTAAAATGTT   
  
  
- TTATTAAAGA GTTGTTTGTT GATTAAATTT ATTAATTATT TTTATTGATT GAGTTTATAG ATTATTGATT   
  
  
- ATTGATTATA TTTATTAATT GTCAATTGTC AACAAATGCT TTGTCTTAAA TATATTTTTC GCCATTGTCT   
  
  
- ATTTTCTATT AGTACGCTCT AATTTTCAGT GGTCACTGTG TGTTCATGAA TTTATTTATC AGATTTGTTA   
  
  
- CTATAAAAAA CGCTCGAAGG TACGTTTATG GCTGTGAAAA TAACGGAGTC TTCCTTTGTT TTTGACTCAC   
  
  
- TTCCCCCCCC CCCCAAGACA AACAGAAACA ATCCCGTGCA AACCTAAGTT CACATCTAAC CCTTATTTAA   
  
  
- GCTCCTGAAA AAGAAACGGG AAAAAGGGAC TAAGGGTTTG GGTGTAGTTC AGTTCTGCTT TGGTCGGTAA   
  
  
- CTTTTTTCTC CCACAAACAC ACTCTATTTC TGTCTTTAAA ATACGGTCTC TCTCTTCTAT TGTTTACGTT   
  
  
- GTTGCAGCTG TCTCCGAGAG GAAGAGTGTT TAAGGTATAA GGAGACAAAA AATTCTTTCT TTTTCTTTCA   
  
  
- CACTCTGTTC TTGTTGTGTT CGTTTGTTAA GTCAACTGCT GCTTATGACT CACTATGGTG CGTGACACAC   
  
  
- GTTGACGCAA AGGTAAACAC CTCAGGAAGT TTCGTGCGAG TAGAAGTTGG GAAGGAAGCG GTGCCTTAAA   
  
  
- GTCCCCCTTT TTTTGGGGGT GGATTAAAAC TCTCTCTCTC TCTTGTCTCT CTTGCCCGCT ATGTCCCTAT   
  
  
- GTTCCAAACT CGTTAAAATC TTTTAAAGAA CCCCGGCAAC TACCCAAAAC TCACTTAACG TTTAGGGTCT   
  
  
- TTCTAAAGAC GCCAAAAAAT AGATGCCGAG AGAGACACCC TTAAAAAACC ATCTAAGGAG ACCAAGGGTG   
  
  
- AAAGTATAGA AGAATAAACA AGATGGGAGA AATGTAGACT ATCAAACAAG ATTGACGACG AAGTCAACCA   
  
  
- CGAACAAGAG AAATCATGAA AAGACAAAAC AAGCAAAACA AGTACGTGAA CTTAAGTTGT TCTCGGGAAC   
  
  
- AGGTAAAACT CGAGAATTAA GGGTAGATTG GGACAAAGGA AGTCTTTAAC GACAAGACAA GTATATCAAT   
  
  
- ATAAAAAATA CGAAACTAAA CCCATATTCA AACGACAACC ACTAAGGTTT CGAACCATGA AAACCGGTAT   
  
  
- AAAACAAACT CAACCACAAC TAACCATGTT TAGAACATTA ACCACACTAA CAATTACCCT GGTTACGAAG   
  
  
- TTCTACTACC AAGTAGTCAT TGAAGAAGTA GTGGAGAAGT TAAAAAGAGG TACTACAGTG GGTTAAACCC   
  
  
- AAGGAGGATG GGAACCGATT CCCTGAATTT CGGACTTCTC TCTCCAGAAA TGAACTATGT AAACAACGAG   
  
  
- TGAACACGTT TGGTACAGAG ATTACCATCG GAACTGTTAC GTTTGGATCG GGAACTCGTT TAGAGGGTCG   
  
  
- AACGTCGGGG ACTACCGCTA TGTTAGGTCG CATAACAACG GATGAAACGA CTTAGTGAAC GACTTTCCTA   
  
  
- GGAATTCAGT ACCGGACCGG ATATATTTCG GGAAGTAAAG TTATCTTACG GACAATAAAG TCTTCTTAAA   
  
  
- GAACGATCCT TCGACAAAAA ACTCAACAAA GGGAAGAACT TCGACCGGAA AAACCACTGA TTGGTTAGTT   
  
  
- ATTAGCTCCG GTACCTCCCC CTTTTCTACC ACGTATATTA ACTAGACTTA CGCAGTCTTG GACGTGTCAC   
  
  
- CTAACGGGAA TAAGTTCTGA ACTCACGAGC CGGACTCCCG GGAGGAGTAA ACTCCTAATG GCCCCAAGTA   
  
  
- GTTGTCTTTC TCCAAAATCT TGTTCATCGA GTATCTAACT GACTTCTTCG ACTCTTCAAC CTAAACGGTA   
  
  
- AAGTCAAGTT AGGACACCAA ACGTTTGATC TCTTAGAGCT GTAGCCTTTT GAGGCACAAT TCTGGCCCCT   
  
  
- CCGGAACCGA TAATGGAGCC AGGAAGTTGA CGTATGGGAA AACCGAAGAC TTCTCCTTCA GGAATTCTTT   
  
  
- TCAAGTGGGA ACCGTAACCA TTTCGTTCGG TTACCCCGAT TAAATGTCCC GAACAAGTTA TTTCTACCTC   
  
  
- GATTATTATC CGCATCGGGT TCATTACTAA GCCGAAGTAG ACGTGGAAGT AGGGAGTTGT GAAGTCGGTT   
  
  
- CTACCTTCCA AAGGAATCGC GAAACACCCC AAATAGGGGT TTCTAATACC ACTATTGGCT CGTTCTAAGG   
  
  
- TTGGTGTTAC CCCGTCCTGA TTACCTCTCT AACAGTCTTC GTAACATGAA GATACGTCGT AACAAGCTAA   
  
  
- CGAATCTTAA ATGGGAGGGC TCTTGGAGGC ACCTCTCTTC CTTCCAGCTC TACGAGGAGG AACCGTTCCT   
  
  
- TTAGTTCTTG TAGTATCGCA CACTCCCTCC TCTTTCTTAT CTATCCGTAC TCTTCAACCC CTTCACCTAA   
  
  
- TTCTCCGAAC TCTACCGGCC CAAACCTTCG CAAGGAAACT CGGTGTATCC GTACTAGGTT CGTTCCGCCA   
  
  
- ACAACGTCTC GATACCGACA CTACCAATAT CTTATTTCCT CCTCTTGCCT ACAAAACAAT AGACGACCGT   
  
  
- TCTAGCGGGG GAGAAAAGTC ATAGACGAAC CTCTACATCC TCCAC

+     TC-rich repeats

| Site Name | Organism | Position | Strand | Matrix score. | sequence | function |
| --- | --- | --- | --- | --- | --- | --- |
| TC-rich repeats | Nicotiana tabacum | 3319 | - | 9 | ATTCTCTAAC | cis-acting element involved in defense and stress responsiveness |

>HU08G01232.1   
+ -Up\_Stream \_Len000TCTACC TTTACCTGTG TTAAAAAAAA AAATTTTGTT TCCATTACTA TTCTCTTGGC   
  
  
+ ACTATTGGTG CTGATTTTTC TTCACCAAAT CAACCTCCTT ATATTTGCCA ATTTTACTGT CCTCTTTCAT   
  
  
+ CTATAAAGTC AACTCTCCTA GTACAATTTG TCACATAGAA AATCTCTAGG CACTCATCTA AACTCTCTGT   
  
  
+ TCTGGGTAAT TGATCGACGA TCTACACCAT TATTCGTTCT AGTGCACCGT TTGGACAGCC AATCATGTAC   
  
  
+ CTTTGGAGAT CAACTGCTCA TGAGTTCTTG GTGTGAAATT GGGGAAGTAA ATTCGACTTT AGGGCACTGG   
  
  
+ TCTATCACGC CATGATTTTA CCATCTATTT TCATATATAT CCAATAAGTT TGATCTTTCG CTATTAATCA   
  
  
+ CAATGATATA AACAAGTGGT GAAGAGCAAT AATGAACCAT ACATTTTAAA CTTAAGCGTT AAGGAAGACA   
  
  
+ TGAAGTTAAA AACAATGGTA AGAGCTATGC GTATGTTTGG CATATAGCTT TTTTAAGAGT GTTTTGGCTA   
  
  
+ TAGTCGGAGT TTTTTAATTA AGATTAGCTG TTTGATCAAA TAAAAAAGCT AATTTGAGTG TTTGGCGAGA   
  
  
+ AGACTTTTTA TAAGAACTTT TTTTGGTCTA AAAAGTTAAT TTAAAAAGGC TAATTCTATG AGCTTTTCGG   
  
  
+ AAGAGTTTTT TAAATAATTA ACTTTTTGTC TCATAAGCCA TAACTTTATC AGAAACAGTT AATTTTACAA   
  
  
+ AATAATTTCT CAACAAACAA CTAATTTAAA TAATTAATAA AAATAACTAA CTCAAATATC TAATAACTAA   
  
  
+ TAACTAATAT AAATAATTAA CAGTTAACAG TTGTTTACGA AACAGAATTT ATATAAAAAG CGGTAACAGA   
  
  
+ TAAAAGATAA TCATGCGAGA TTAAAAGTCA CCAGTGACAC ACAAGTACTT AAATAAATAG TCTAAACAAT   
  
  
+ GATATTTTTT GCGAGCTTCC ATGCAAATAC CGACACTTTT ATTGCCTCAG AAGGAAACAA AAACTGAGTG   
  
  
+ AAGGGGGGGG GGGGTTCTGT TTGTCTTTGT TAGGGCACGT TTGGATTCAA GTGTAGATTG GGAATAAATT   
  
  
+ CGAGGACTTT TTCTTTGCCC TTTTTCCCTG ATTCCCAAAC CCACATCAAG TCAAGACGAA ACCAGCCATT   
  
  
+ GAAAAAAGAG GGTGTTTGTG TGAGATAAAG ACAGAAATTT TATGCCAGAG AGAGAAGATA ACAAATGCAA   
  
  
+ CAACGTCGAC AGAGGCTCTC CTTCTCACAA ATTCCATATT CCTCTGTTTT TTAAGAAAGA AAAAGAAAGT   
  
  
+ GTGAGACAAG AACAACACAA GCAAACAATT CAGTTGACGA CGAATACTGA GTGATACCAC GCACTGTGTG   
  
  
+ CAACTGCGTT TCCATTTGTG GAGTCCTTCA AAGCACGCTC ATCTTCAACC CTTCCTTCGC CACGGAATTT   
  
  
+ CAGGGGGAAA AAAACCCCCA CCTAATTTTG AGAGAGAGAG AGAACAGAGA GAACGGGCGA TACAGGGATA   
  
  
+ CAAGGTTTGA GCAATTTTAG AAAATTTCTT GGGGCCGTTG ATGGGTTTTG AGTGAATTGC AAATCCCAGA   
  
  
+ AAGATTTCTG CGGTTTTTTA TCTACGGCTC TCTCTGTGGG AATTTTTTGG TAGATTCCTC TGGTTCCCAC   
  
  
+ TTTCATATCT TCTTATTTGT TCTACCCTCT TTACATCTGA TAGTTTGTTC TAACTGCTGC TTCAGTTGGT   
  
  
+ GCTTGTTCTC TTTAGTACTT TTCTGTTTTG TTCGTTTTGT TCATGCACTT GAATTCAACA AGAGCCCTTG   
  
  
+ TCCATTTTGA GCTCTTAATT CCCATCTAAC CCTGTTTCCT TCAGAAATTG CTGTTCTGTT CATATAGTTA   
  
  
+ TATTTTTTAT GCTTTGATTT GGGTATAAGT TTGCTGTTGG TGATTCCAAA GCTTGGTACT TTTGGCCATA   
  
  
+ TTTTGTTTGA GTTGGTGTTG ATTGGTACAA ATCTTGTAAT TGGTGTGATT GTTAATGGGA CCAATGCTTC   
  
  
+ AAGATGATGG TTCATCAGTA ACTTCTTCAT CACCTCTTCA ATTTTTCTCC ATGATGTCAC CCAATTTGGG   
  
  
+ TTCCTCCTAC CCTTGGCTAA GGGACTTAAA GCCTGAAGAG AGAGGTCTTT ACTTGATACA TTTGTTGCTC   
  
  
+ ACTTGTGCAA ACCATGTCTC TAATGGTAGC CTTGACAATG CAAACCTAGC CCTTGAGCAA ATCTCCCAGC   
  
  
+ TTGCAGCCCC TGATGGCGAT ACAATCCAGC GTATTGTTGC CTACTTTGCT GAATCACTTG CTGAAAGGAT   
  
  
+ CCTTAAGTCA TGGCCTGGCC TATATAAAGC CCTTCATTTC AATAGAATGC CTGTTATTTC AGAAGAATTT   
  
  
+ CTTGCTAGGA AGCTGTTTTT TGAGTTGTTT CCCTTCTTGA AGCTGGCCTT TTTGGTGACT AACCAATCAA   
  
  
+ TAATCGAGGC CATGGAGGGG GAAAAGATGG TGCATATAAT TGATCTGAAT GCGTCAGAAC CTGCACAGTG   
  
  
+ GATTGCCCTT ATTCAAGACT TGAGTGCTCG GCCTGAGGGC CCTCCTCATT TGAGGATTAC CGGGGTTCAT   
  
  
+ CAACAGAAAG AGGTTTTAGA ACAAGTAGCT CATAGATTGA CTGAAGAAGC TGAGAAGTTG GATTTGCCAT   
  
  
+ TTCAGTTCAA TCCTGTGGTT TGCAAACTAG AGAATCTCGA CATCGGAAAA CTCCGTGTTA AGACCGGGGA   
  
  
+ GGCCTTGGCT ATTACCTCGG TCCTTCAACT GCATACCCTT TTGGCTTCTG AAGAGGAAGT CCTTAAGAAA   
  
  
+ AGTTCACCCT TGGCATTGGT AAAGCAAGCC AATGGGGCTA ATTTACAGGG CTTGTTCAAT AAAGATGGAG   
  
  
+ CTAATAATAG GCGTAGCCCA AGTAATGATT CGGCTTCATC TGCACCTTCA TCCCTCAACA CTTCAGCCAA   
  
  
+ GATGGAAGGT TTCCTTAGCG CTTTGTGGGG TTTATCCCCA AAGATTATGG TGATAACCGA GCAAGATTCC   
  
  
+ AACCACAATG GGGCAGGACT AATGGAGAGA TTGTCAGAAG CATTGTACTT CTATGCAGCA TTGTTCGATT   
  
  
+ GCTTAGAATT TACCCTCCCG AGAACCTCCG TGGAGAGAAG GAAGGTCGAG ATGCTCCTCC TTGGCAAGGA   
  
  
+ AATCAAGAAC ATCATAGCGT GTGAGGGAGG AGAAAGAATA GATAGGCATG AGAAGTTGGG GAAGTGGATT   
  
  
+ AAGAGGCTTG AGATGGCCGG GTTTGGAAGC GTTCCTTTGA GCCACATAGG CATGATCCAA GCAAGGCGGT   
  
  
+ TGTTGCAGAG CTATGGCTGT GATGGTTATA GAATAAAGGA GGAGAACGGA TGTTTTGTTA TCTGCTGGCA   
  
  
+ AGATCGCCCC CTCTTTTCAG TATCTGCTTG GAGATGTAGG AGGTG  

- -Up\_Stream \_Len000AGATGG AAATGGACAC AATTTTTTTT TTTAAAACAA AGGTAATGAT AAGAGAACCG   
  
  
- TGATAACCAC GACTAAAAAG AAGTGGTTTA GTTGGAGGAA TATAAACGGT TAAAATGACA GGAGAAAGTA   
  
  
- GATATTTCAG TTGAGAGGAT CATGTTAAAC AGTGTATCTT TTAGAGATCC GTGAGTAGAT TTGAGAGACA   
  
  
- AGACCCATTA ACTAGCTGCT AGATGTGGTA ATAAGCAAGA TCACGTGGCA AACCTGTCGG TTAGTACATG   
  
  
- GAAACCTCTA GTTGACGAGT ACTCAAGAAC CACACTTTAA CCCCTTCATT TAAGCTGAAA TCCCGTGACC   
  
  
- AGATAGTGCG GTACTAAAAT GGTAGATAAA AGTATATATA GGTTATTCAA ACTAGAAAGC GATAATTAGT   
  
  
- GTTACTATAT TTGTTCACCA CTTCTCGTTA TTACTTGGTA TGTAAAATTT GAATTCGCAA TTCCTTCTGT   
  
  
- ACTTCAATTT TTGTTACCAT TCTCGATACG CATACAAACC GTATATCGAA AAAATTCTCA CAAAACCGAT   
  
  
- ATCAGCCTCA AAAAATTAAT TCTAATCGAC AAACTAGTTT ATTTTTTCGA TTAAACTCAC AAACCGCTCT   
  
  
- TCTGAAAAAT ATTCTTGAAA AAAACCAGAT TTTTCAATTA AATTTTTCCG ATTAAGATAC TCGAAAAGCC   
  
  
- TTCTCAAAAA ATTTATTAAT TGAAAAACAG AGTATTCGGT ATTGAAATAG TCTTTGTCAA TTAAAATGTT   
  
  
- TTATTAAAGA GTTGTTTGTT GATTAAATTT ATTAATTATT TTTATTGATT GAGTTTATAG ATTATTGATT   
  
  
- ATTGATTATA TTTATTAATT GTCAATTGTC AACAAATGCT TTGTCTTAAA TATATTTTTC GCCATTGTCT   
  
  
- ATTTTCTATT AGTACGCTCT AATTTTCAGT GGTCACTGTG TGTTCATGAA TTTATTTATC AGATTTGTTA   
  
  
- CTATAAAAAA CGCTCGAAGG TACGTTTATG GCTGTGAAAA TAACGGAGTC TTCCTTTGTT TTTGACTCAC   
  
  
- TTCCCCCCCC CCCCAAGACA AACAGAAACA ATCCCGTGCA AACCTAAGTT CACATCTAAC CCTTATTTAA   
  
  
- GCTCCTGAAA AAGAAACGGG AAAAAGGGAC TAAGGGTTTG GGTGTAGTTC AGTTCTGCTT TGGTCGGTAA   
  
  
- CTTTTTTCTC CCACAAACAC ACTCTATTTC TGTCTTTAAA ATACGGTCTC TCTCTTCTAT TGTTTACGTT   
  
  
- GTTGCAGCTG TCTCCGAGAG GAAGAGTGTT TAAGGTATAA GGAGACAAAA AATTCTTTCT TTTTCTTTCA   
  
  
- CACTCTGTTC TTGTTGTGTT CGTTTGTTAA GTCAACTGCT GCTTATGACT CACTATGGTG CGTGACACAC   
  
  
- GTTGACGCAA AGGTAAACAC CTCAGGAAGT TTCGTGCGAG TAGAAGTTGG GAAGGAAGCG GTGCCTTAAA   
  
  
- GTCCCCCTTT TTTTGGGGGT GGATTAAAAC TCTCTCTCTC TCTTGTCTCT CTTGCCCGCT ATGTCCCTAT   
  
  
- GTTCCAAACT CGTTAAAATC TTTTAAAGAA CCCCGGCAAC TACCCAAAAC TCACTTAACG TTTAGGGTCT   
  
  
- TTCTAAAGAC GCCAAAAAAT AGATGCCGAG AGAGACACCC TTAAAAAACC ATCTAAGGAG ACCAAGGGTG   
  
  
- AAAGTATAGA AGAATAAACA AGATGGGAGA AATGTAGACT ATCAAACAAG ATTGACGACG AAGTCAACCA   
  
  
- CGAACAAGAG AAATCATGAA AAGACAAAAC AAGCAAAACA AGTACGTGAA CTTAAGTTGT TCTCGGGAAC   
  
  
- AGGTAAAACT CGAGAATTAA GGGTAGATTG GGACAAAGGA AGTCTTTAAC GACAAGACAA GTATATCAAT   
  
  
- ATAAAAAATA CGAAACTAAA CCCATATTCA AACGACAACC ACTAAGGTTT CGAACCATGA AAACCGGTAT   
  
  
- AAAACAAACT CAACCACAAC TAACCATGTT TAGAACATTA ACCACACTAA CAATTACCCT GGTTACGAAG   
  
  
- TTCTACTACC AAGTAGTCAT TGAAGAAGTA GTGGAGAAGT TAAAAAGAGG TACTACAGTG GGTTAAACCC   
  
  
- AAGGAGGATG GGAACCGATT CCCTGAATTT CGGACTTCTC TCTCCAGAAA TGAACTATGT AAACAACGAG   
  
  
- TGAACACGTT TGGTACAGAG ATTACCATCG GAACTGTTAC GTTTGGATCG GGAACTCGTT TAGAGGGTCG   
  
  
- AACGTCGGGG ACTACCGCTA TGTTAGGTCG CATAACAACG GATGAAACGA CTTAGTGAAC GACTTTCCTA   
  
  
- GGAATTCAGT ACCGGACCGG ATATATTTCG GGAAGTAAAG TTATCTTACG GACAATAAAG TCTTCTTAAA   
  
  
- GAACGATCCT TCGACAAAAA ACTCAACAAA GGGAAGAACT TCGACCGGAA AAACCACTGA TTGGTTAGTT   
  
  
- ATTAGCTCCG GTACCTCCCC CTTTTCTACC ACGTATATTA ACTAGACTTA CGCAGTCTTG GACGTGTCAC   
  
  
- CTAACGGGAA TAAGTTCTGA ACTCACGAGC CGGACTCCCG GGAGGAGTAA ACTCCTAATG GCCCCAAGTA   
  
  
- GTTGTCTTTC TCCAAAATCT TGTTCATCGA GTATCTAACT GACTTCTTCG ACTCTTCAAC CTAAACGGTA   
  
  
- AAGTCAAGTT AGGACACCAA ACGTTTGATC TCTTAGAGCT GTAGCCTTTT GAGGCACAAT TCTGGCCCCT   
  
  
- CCGGAACCGA TAATGGAGCC AGGAAGTTGA CGTATGGGAA AACCGAAGAC TTCTCCTTCA GGAATTCTTT   
  
  
- TCAAGTGGGA ACCGTAACCA TTTCGTTCGG TTACCCCGAT TAAATGTCCC GAACAAGTTA TTTCTACCTC   
  
  
- GATTATTATC CGCATCGGGT TCATTACTAA GCCGAAGTAG ACGTGGAAGT AGGGAGTTGT GAAGTCGGTT   
  
  
- CTACCTTCCA AAGGAATCGC GAAACACCCC AAATAGGGGT TTCTAATACC ACTATTGGCT CGTTCTAAGG   
  
  
- TTGGTGTTAC CCCGTCCTGA TTACCTCTCT AACAGTCTTC GTAACATGAA GATACGTCGT AACAAGCTAA   
  
  
- CGAATCTTAA ATGGGAGGGC TCTTGGAGGC ACCTCTCTTC CTTCCAGCTC TACGAGGAGG AACCGTTCCT   
  
  
- TTAGTTCTTG TAGTATCGCA CACTCCCTCC TCTTTCTTAT CTATCCGTAC TCTTCAACCC CTTCACCTAA   
  
  
- TTCTCCGAAC TCTACCGGCC CAAACCTTCG CAAGGAAACT CGGTGTATCC GTACTAGGTT CGTTCCGCCA   
  
  
- ACAACGTCTC GATACCGACA CTACCAATAT CTTATTTCCT CCTCTTGCCT ACAAAACAAT AGACGACCGT   
  
  
- TCTAGCGGGG GAGAAAAGTC ATAGACGAAC CTCTACATCC TCCAC

+     TCA

| Site Name | Organism | Position | Strand | Matrix score. | sequence | function |
| --- | --- | --- | --- | --- | --- | --- |
| TCA | Pisum sativum | 1443 | + | 9 | TCATCTTCAT |  |

>HU08G01232.1   
+ -Up\_Stream \_Len000TCTACC TTTACCTGTG TTAAAAAAAA AAATTTTGTT TCCATTACTA TTCTCTTGGC   
  
  
+ ACTATTGGTG CTGATTTTTC TTCACCAAAT CAACCTCCTT ATATTTGCCA ATTTTACTGT CCTCTTTCAT   
  
  
+ CTATAAAGTC AACTCTCCTA GTACAATTTG TCACATAGAA AATCTCTAGG CACTCATCTA AACTCTCTGT   
  
  
+ TCTGGGTAAT TGATCGACGA TCTACACCAT TATTCGTTCT AGTGCACCGT TTGGACAGCC AATCATGTAC   
  
  
+ CTTTGGAGAT CAACTGCTCA TGAGTTCTTG GTGTGAAATT GGGGAAGTAA ATTCGACTTT AGGGCACTGG   
  
  
+ TCTATCACGC CATGATTTTA CCATCTATTT TCATATATAT CCAATAAGTT TGATCTTTCG CTATTAATCA   
  
  
+ CAATGATATA AACAAGTGGT GAAGAGCAAT AATGAACCAT ACATTTTAAA CTTAAGCGTT AAGGAAGACA   
  
  
+ TGAAGTTAAA AACAATGGTA AGAGCTATGC GTATGTTTGG CATATAGCTT TTTTAAGAGT GTTTTGGCTA   
  
  
+ TAGTCGGAGT TTTTTAATTA AGATTAGCTG TTTGATCAAA TAAAAAAGCT AATTTGAGTG TTTGGCGAGA   
  
  
+ AGACTTTTTA TAAGAACTTT TTTTGGTCTA AAAAGTTAAT TTAAAAAGGC TAATTCTATG AGCTTTTCGG   
  
  
+ AAGAGTTTTT TAAATAATTA ACTTTTTGTC TCATAAGCCA TAACTTTATC AGAAACAGTT AATTTTACAA   
  
  
+ AATAATTTCT CAACAAACAA CTAATTTAAA TAATTAATAA AAATAACTAA CTCAAATATC TAATAACTAA   
  
  
+ TAACTAATAT AAATAATTAA CAGTTAACAG TTGTTTACGA AACAGAATTT ATATAAAAAG CGGTAACAGA   
  
  
+ TAAAAGATAA TCATGCGAGA TTAAAAGTCA CCAGTGACAC ACAAGTACTT AAATAAATAG TCTAAACAAT   
  
  
+ GATATTTTTT GCGAGCTTCC ATGCAAATAC CGACACTTTT ATTGCCTCAG AAGGAAACAA AAACTGAGTG   
  
  
+ AAGGGGGGGG GGGGTTCTGT TTGTCTTTGT TAGGGCACGT TTGGATTCAA GTGTAGATTG GGAATAAATT   
  
  
+ CGAGGACTTT TTCTTTGCCC TTTTTCCCTG ATTCCCAAAC CCACATCAAG TCAAGACGAA ACCAGCCATT   
  
  
+ GAAAAAAGAG GGTGTTTGTG TGAGATAAAG ACAGAAATTT TATGCCAGAG AGAGAAGATA ACAAATGCAA   
  
  
+ CAACGTCGAC AGAGGCTCTC CTTCTCACAA ATTCCATATT CCTCTGTTTT TTAAGAAAGA AAAAGAAAGT   
  
  
+ GTGAGACAAG AACAACACAA GCAAACAATT CAGTTGACGA CGAATACTGA GTGATACCAC GCACTGTGTG   
  
  
+ CAACTGCGTT TCCATTTGTG GAGTCCTTCA AAGCACGCTC ATCTTCAACC CTTCCTTCGC CACGGAATTT   
  
  
+ CAGGGGGAAA AAAACCCCCA CCTAATTTTG AGAGAGAGAG AGAACAGAGA GAACGGGCGA TACAGGGATA   
  
  
+ CAAGGTTTGA GCAATTTTAG AAAATTTCTT GGGGCCGTTG ATGGGTTTTG AGTGAATTGC AAATCCCAGA   
  
  
+ AAGATTTCTG CGGTTTTTTA TCTACGGCTC TCTCTGTGGG AATTTTTTGG TAGATTCCTC TGGTTCCCAC   
  
  
+ TTTCATATCT TCTTATTTGT TCTACCCTCT TTACATCTGA TAGTTTGTTC TAACTGCTGC TTCAGTTGGT   
  
  
+ GCTTGTTCTC TTTAGTACTT TTCTGTTTTG TTCGTTTTGT TCATGCACTT GAATTCAACA AGAGCCCTTG   
  
  
+ TCCATTTTGA GCTCTTAATT CCCATCTAAC CCTGTTTCCT TCAGAAATTG CTGTTCTGTT CATATAGTTA   
  
  
+ TATTTTTTAT GCTTTGATTT GGGTATAAGT TTGCTGTTGG TGATTCCAAA GCTTGGTACT TTTGGCCATA   
  
  
+ TTTTGTTTGA GTTGGTGTTG ATTGGTACAA ATCTTGTAAT TGGTGTGATT GTTAATGGGA CCAATGCTTC   
  
  
+ AAGATGATGG TTCATCAGTA ACTTCTTCAT CACCTCTTCA ATTTTTCTCC ATGATGTCAC CCAATTTGGG   
  
  
+ TTCCTCCTAC CCTTGGCTAA GGGACTTAAA GCCTGAAGAG AGAGGTCTTT ACTTGATACA TTTGTTGCTC   
  
  
+ ACTTGTGCAA ACCATGTCTC TAATGGTAGC CTTGACAATG CAAACCTAGC CCTTGAGCAA ATCTCCCAGC   
  
  
+ TTGCAGCCCC TGATGGCGAT ACAATCCAGC GTATTGTTGC CTACTTTGCT GAATCACTTG CTGAAAGGAT   
  
  
+ CCTTAAGTCA TGGCCTGGCC TATATAAAGC CCTTCATTTC AATAGAATGC CTGTTATTTC AGAAGAATTT   
  
  
+ CTTGCTAGGA AGCTGTTTTT TGAGTTGTTT CCCTTCTTGA AGCTGGCCTT TTTGGTGACT AACCAATCAA   
  
  
+ TAATCGAGGC CATGGAGGGG GAAAAGATGG TGCATATAAT TGATCTGAAT GCGTCAGAAC CTGCACAGTG   
  
  
+ GATTGCCCTT ATTCAAGACT TGAGTGCTCG GCCTGAGGGC CCTCCTCATT TGAGGATTAC CGGGGTTCAT   
  
  
+ CAACAGAAAG AGGTTTTAGA ACAAGTAGCT CATAGATTGA CTGAAGAAGC TGAGAAGTTG GATTTGCCAT   
  
  
+ TTCAGTTCAA TCCTGTGGTT TGCAAACTAG AGAATCTCGA CATCGGAAAA CTCCGTGTTA AGACCGGGGA   
  
  
+ GGCCTTGGCT ATTACCTCGG TCCTTCAACT GCATACCCTT TTGGCTTCTG AAGAGGAAGT CCTTAAGAAA   
  
  
+ AGTTCACCCT TGGCATTGGT AAAGCAAGCC AATGGGGCTA ATTTACAGGG CTTGTTCAAT AAAGATGGAG   
  
  
+ CTAATAATAG GCGTAGCCCA AGTAATGATT CGGCTTCATC TGCACCTTCA TCCCTCAACA CTTCAGCCAA   
  
  
+ GATGGAAGGT TTCCTTAGCG CTTTGTGGGG TTTATCCCCA AAGATTATGG TGATAACCGA GCAAGATTCC   
  
  
+ AACCACAATG GGGCAGGACT AATGGAGAGA TTGTCAGAAG CATTGTACTT CTATGCAGCA TTGTTCGATT   
  
  
+ GCTTAGAATT TACCCTCCCG AGAACCTCCG TGGAGAGAAG GAAGGTCGAG ATGCTCCTCC TTGGCAAGGA   
  
  
+ AATCAAGAAC ATCATAGCGT GTGAGGGAGG AGAAAGAATA GATAGGCATG AGAAGTTGGG GAAGTGGATT   
  
  
+ AAGAGGCTTG AGATGGCCGG GTTTGGAAGC GTTCCTTTGA GCCACATAGG CATGATCCAA GCAAGGCGGT   
  
  
+ TGTTGCAGAG CTATGGCTGT GATGGTTATA GAATAAAGGA GGAGAACGGA TGTTTTGTTA TCTGCTGGCA   
  
  
+ AGATCGCCCC CTCTTTTCAG TATCTGCTTG GAGATGTAGG AGGTG  

- -Up\_Stream \_Len000AGATGG AAATGGACAC AATTTTTTTT TTTAAAACAA AGGTAATGAT AAGAGAACCG   
  
  
- TGATAACCAC GACTAAAAAG AAGTGGTTTA GTTGGAGGAA TATAAACGGT TAAAATGACA GGAGAAAGTA   
  
  
- GATATTTCAG TTGAGAGGAT CATGTTAAAC AGTGTATCTT TTAGAGATCC GTGAGTAGAT TTGAGAGACA   
  
  
- AGACCCATTA ACTAGCTGCT AGATGTGGTA ATAAGCAAGA TCACGTGGCA AACCTGTCGG TTAGTACATG   
  
  
- GAAACCTCTA GTTGACGAGT ACTCAAGAAC CACACTTTAA CCCCTTCATT TAAGCTGAAA TCCCGTGACC   
  
  
- AGATAGTGCG GTACTAAAAT GGTAGATAAA AGTATATATA GGTTATTCAA ACTAGAAAGC GATAATTAGT   
  
  
- GTTACTATAT TTGTTCACCA CTTCTCGTTA TTACTTGGTA TGTAAAATTT GAATTCGCAA TTCCTTCTGT   
  
  
- ACTTCAATTT TTGTTACCAT TCTCGATACG CATACAAACC GTATATCGAA AAAATTCTCA CAAAACCGAT   
  
  
- ATCAGCCTCA AAAAATTAAT TCTAATCGAC AAACTAGTTT ATTTTTTCGA TTAAACTCAC AAACCGCTCT   
  
  
- TCTGAAAAAT ATTCTTGAAA AAAACCAGAT TTTTCAATTA AATTTTTCCG ATTAAGATAC TCGAAAAGCC   
  
  
- TTCTCAAAAA ATTTATTAAT TGAAAAACAG AGTATTCGGT ATTGAAATAG TCTTTGTCAA TTAAAATGTT   
  
  
- TTATTAAAGA GTTGTTTGTT GATTAAATTT ATTAATTATT TTTATTGATT GAGTTTATAG ATTATTGATT   
  
  
- ATTGATTATA TTTATTAATT GTCAATTGTC AACAAATGCT TTGTCTTAAA TATATTTTTC GCCATTGTCT   
  
  
- ATTTTCTATT AGTACGCTCT AATTTTCAGT GGTCACTGTG TGTTCATGAA TTTATTTATC AGATTTGTTA   
  
  
- CTATAAAAAA CGCTCGAAGG TACGTTTATG GCTGTGAAAA TAACGGAGTC TTCCTTTGTT TTTGACTCAC   
  
  
- TTCCCCCCCC CCCCAAGACA AACAGAAACA ATCCCGTGCA AACCTAAGTT CACATCTAAC CCTTATTTAA   
  
  
- GCTCCTGAAA AAGAAACGGG AAAAAGGGAC TAAGGGTTTG GGTGTAGTTC AGTTCTGCTT TGGTCGGTAA   
  
  
- CTTTTTTCTC CCACAAACAC ACTCTATTTC TGTCTTTAAA ATACGGTCTC TCTCTTCTAT TGTTTACGTT   
  
  
- GTTGCAGCTG TCTCCGAGAG GAAGAGTGTT TAAGGTATAA GGAGACAAAA AATTCTTTCT TTTTCTTTCA   
  
  
- CACTCTGTTC TTGTTGTGTT CGTTTGTTAA GTCAACTGCT GCTTATGACT CACTATGGTG CGTGACACAC   
  
  
- GTTGACGCAA AGGTAAACAC CTCAGGAAGT TTCGTGCGAG TAGAAGTTGG GAAGGAAGCG GTGCCTTAAA   
  
  
- GTCCCCCTTT TTTTGGGGGT GGATTAAAAC TCTCTCTCTC TCTTGTCTCT CTTGCCCGCT ATGTCCCTAT   
  
  
- GTTCCAAACT CGTTAAAATC TTTTAAAGAA CCCCGGCAAC TACCCAAAAC TCACTTAACG TTTAGGGTCT   
  
  
- TTCTAAAGAC GCCAAAAAAT AGATGCCGAG AGAGACACCC TTAAAAAACC ATCTAAGGAG ACCAAGGGTG   
  
  
- AAAGTATAGA AGAATAAACA AGATGGGAGA AATGTAGACT ATCAAACAAG ATTGACGACG AAGTCAACCA   
  
  
- CGAACAAGAG AAATCATGAA AAGACAAAAC AAGCAAAACA AGTACGTGAA CTTAAGTTGT TCTCGGGAAC   
  
  
- AGGTAAAACT CGAGAATTAA GGGTAGATTG GGACAAAGGA AGTCTTTAAC GACAAGACAA GTATATCAAT   
  
  
- ATAAAAAATA CGAAACTAAA CCCATATTCA AACGACAACC ACTAAGGTTT CGAACCATGA AAACCGGTAT   
  
  
- AAAACAAACT CAACCACAAC TAACCATGTT TAGAACATTA ACCACACTAA CAATTACCCT GGTTACGAAG   
  
  
- TTCTACTACC AAGTAGTCAT TGAAGAAGTA GTGGAGAAGT TAAAAAGAGG TACTACAGTG GGTTAAACCC   
  
  
- AAGGAGGATG GGAACCGATT CCCTGAATTT CGGACTTCTC TCTCCAGAAA TGAACTATGT AAACAACGAG   
  
  
- TGAACACGTT TGGTACAGAG ATTACCATCG GAACTGTTAC GTTTGGATCG GGAACTCGTT TAGAGGGTCG   
  
  
- AACGTCGGGG ACTACCGCTA TGTTAGGTCG CATAACAACG GATGAAACGA CTTAGTGAAC GACTTTCCTA   
  
  
- GGAATTCAGT ACCGGACCGG ATATATTTCG GGAAGTAAAG TTATCTTACG GACAATAAAG TCTTCTTAAA   
  
  
- GAACGATCCT TCGACAAAAA ACTCAACAAA GGGAAGAACT TCGACCGGAA AAACCACTGA TTGGTTAGTT   
  
  
- ATTAGCTCCG GTACCTCCCC CTTTTCTACC ACGTATATTA ACTAGACTTA CGCAGTCTTG GACGTGTCAC   
  
  
- CTAACGGGAA TAAGTTCTGA ACTCACGAGC CGGACTCCCG GGAGGAGTAA ACTCCTAATG GCCCCAAGTA   
  
  
- GTTGTCTTTC TCCAAAATCT TGTTCATCGA GTATCTAACT GACTTCTTCG ACTCTTCAAC CTAAACGGTA   
  
  
- AAGTCAAGTT AGGACACCAA ACGTTTGATC TCTTAGAGCT GTAGCCTTTT GAGGCACAAT TCTGGCCCCT   
  
  
- CCGGAACCGA TAATGGAGCC AGGAAGTTGA CGTATGGGAA AACCGAAGAC TTCTCCTTCA GGAATTCTTT   
  
  
- TCAAGTGGGA ACCGTAACCA TTTCGTTCGG TTACCCCGAT TAAATGTCCC GAACAAGTTA TTTCTACCTC   
  
  
- GATTATTATC CGCATCGGGT TCATTACTAA GCCGAAGTAG ACGTGGAAGT AGGGAGTTGT GAAGTCGGTT   
  
  
- CTACCTTCCA AAGGAATCGC GAAACACCCC AAATAGGGGT TTCTAATACC ACTATTGGCT CGTTCTAAGG   
  
  
- TTGGTGTTAC CCCGTCCTGA TTACCTCTCT AACAGTCTTC GTAACATGAA GATACGTCGT AACAAGCTAA   
  
  
- CGAATCTTAA ATGGGAGGGC TCTTGGAGGC ACCTCTCTTC CTTCCAGCTC TACGAGGAGG AACCGTTCCT   
  
  
- TTAGTTCTTG TAGTATCGCA CACTCCCTCC TCTTTCTTAT CTATCCGTAC TCTTCAACCC CTTCACCTAA   
  
  
- TTCTCCGAAC TCTACCGGCC CAAACCTTCG CAAGGAAACT CGGTGTATCC GTACTAGGTT CGTTCCGCCA   
  
  
- ACAACGTCTC GATACCGACA CTACCAATAT CTTATTTCCT CCTCTTGCCT ACAAAACAAT AGACGACCGT   
  
  
- TCTAGCGGGG GAGAAAAGTC ATAGACGAAC CTCTACATCC TCCAC

+     TCA-element

| Site Name | Organism | Position | Strand | Matrix score. | sequence | function |
| --- | --- | --- | --- | --- | --- | --- |
| TCA-element | Nicotiana tabacum | 2863 | - | 9 | CCATCTTTTT | cis-acting element involved in salicylic acid responsiveness |
| TCA-element | Nicotiana tabacum | 375 | + | 9 | CCATCTTTTT | cis-acting element involved in salicylic acid responsiveness |
| TCA-element | Brassica oleracea | 2781 | + | 9 | TCAGAAGAGG | cis-acting element involved in salicylic acid responsiveness |
| TCA-element | Nicotiana tabacum | 2475 | - | 9 | CCATCTTTTT | cis-acting element involved in salicylic acid responsiveness |
| TCA-element | Nicotiana tabacum | 1197 | - | 9 | CCATCTTTTT | cis-acting element involved in salicylic acid responsiveness |

>HU08G01232.1   
+ -Up\_Stream \_Len000TCTACC TTTACCTGTG TTAAAAAAAA AAATTTTGTT TCCATTACTA TTCTCTTGGC   
  
  
+ ACTATTGGTG CTGATTTTTC TTCACCAAAT CAACCTCCTT ATATTTGCCA ATTTTACTGT CCTCTTTCAT   
  
  
+ CTATAAAGTC AACTCTCCTA GTACAATTTG TCACATAGAA AATCTCTAGG CACTCATCTA AACTCTCTGT   
  
  
+ TCTGGGTAAT TGATCGACGA TCTACACCAT TATTCGTTCT AGTGCACCGT TTGGACAGCC AATCATGTAC   
  
  
+ CTTTGGAGAT CAACTGCTCA TGAGTTCTTG GTGTGAAATT GGGGAAGTAA ATTCGACTTT AGGGCACTGG   
  
  
+ TCTATCACGC CATGATTTTA CCATCTATTT TCATATATAT CCAATAAGTT TGATCTTTCG CTATTAATCA   
  
  
+ CAATGATATA AACAAGTGGT GAAGAGCAAT AATGAACCAT ACATTTTAAA CTTAAGCGTT AAGGAAGACA   
  
  
+ TGAAGTTAAA AACAATGGTA AGAGCTATGC GTATGTTTGG CATATAGCTT TTTTAAGAGT GTTTTGGCTA   
  
  
+ TAGTCGGAGT TTTTTAATTA AGATTAGCTG TTTGATCAAA TAAAAAAGCT AATTTGAGTG TTTGGCGAGA   
  
  
+ AGACTTTTTA TAAGAACTTT TTTTGGTCTA AAAAGTTAAT TTAAAAAGGC TAATTCTATG AGCTTTTCGG   
  
  
+ AAGAGTTTTT TAAATAATTA ACTTTTTGTC TCATAAGCCA TAACTTTATC AGAAACAGTT AATTTTACAA   
  
  
+ AATAATTTCT CAACAAACAA CTAATTTAAA TAATTAATAA AAATAACTAA CTCAAATATC TAATAACTAA   
  
  
+ TAACTAATAT AAATAATTAA CAGTTAACAG TTGTTTACGA AACAGAATTT ATATAAAAAG CGGTAACAGA   
  
  
+ TAAAAGATAA TCATGCGAGA TTAAAAGTCA CCAGTGACAC ACAAGTACTT AAATAAATAG TCTAAACAAT   
  
  
+ GATATTTTTT GCGAGCTTCC ATGCAAATAC CGACACTTTT ATTGCCTCAG AAGGAAACAA AAACTGAGTG   
  
  
+ AAGGGGGGGG GGGGTTCTGT TTGTCTTTGT TAGGGCACGT TTGGATTCAA GTGTAGATTG GGAATAAATT   
  
  
+ CGAGGACTTT TTCTTTGCCC TTTTTCCCTG ATTCCCAAAC CCACATCAAG TCAAGACGAA ACCAGCCATT   
  
  
+ GAAAAAAGAG GGTGTTTGTG TGAGATAAAG ACAGAAATTT TATGCCAGAG AGAGAAGATA ACAAATGCAA   
  
  
+ CAACGTCGAC AGAGGCTCTC CTTCTCACAA ATTCCATATT CCTCTGTTTT TTAAGAAAGA AAAAGAAAGT   
  
  
+ GTGAGACAAG AACAACACAA GCAAACAATT CAGTTGACGA CGAATACTGA GTGATACCAC GCACTGTGTG   
  
  
+ CAACTGCGTT TCCATTTGTG GAGTCCTTCA AAGCACGCTC ATCTTCAACC CTTCCTTCGC CACGGAATTT   
  
  
+ CAGGGGGAAA AAAACCCCCA CCTAATTTTG AGAGAGAGAG AGAACAGAGA GAACGGGCGA TACAGGGATA   
  
  
+ CAAGGTTTGA GCAATTTTAG AAAATTTCTT GGGGCCGTTG ATGGGTTTTG AGTGAATTGC AAATCCCAGA   
  
  
+ AAGATTTCTG CGGTTTTTTA TCTACGGCTC TCTCTGTGGG AATTTTTTGG TAGATTCCTC TGGTTCCCAC   
  
  
+ TTTCATATCT TCTTATTTGT TCTACCCTCT TTACATCTGA TAGTTTGTTC TAACTGCTGC TTCAGTTGGT   
  
  
+ GCTTGTTCTC TTTAGTACTT TTCTGTTTTG TTCGTTTTGT TCATGCACTT GAATTCAACA AGAGCCCTTG   
  
  
+ TCCATTTTGA GCTCTTAATT CCCATCTAAC CCTGTTTCCT TCAGAAATTG CTGTTCTGTT CATATAGTTA   
  
  
+ TATTTTTTAT GCTTTGATTT GGGTATAAGT TTGCTGTTGG TGATTCCAAA GCTTGGTACT TTTGGCCATA   
  
  
+ TTTTGTTTGA GTTGGTGTTG ATTGGTACAA ATCTTGTAAT TGGTGTGATT GTTAATGGGA CCAATGCTTC   
  
  
+ AAGATGATGG TTCATCAGTA ACTTCTTCAT CACCTCTTCA ATTTTTCTCC ATGATGTCAC CCAATTTGGG   
  
  
+ TTCCTCCTAC CCTTGGCTAA GGGACTTAAA GCCTGAAGAG AGAGGTCTTT ACTTGATACA TTTGTTGCTC   
  
  
+ ACTTGTGCAA ACCATGTCTC TAATGGTAGC CTTGACAATG CAAACCTAGC CCTTGAGCAA ATCTCCCAGC   
  
  
+ TTGCAGCCCC TGATGGCGAT ACAATCCAGC GTATTGTTGC CTACTTTGCT GAATCACTTG CTGAAAGGAT   
  
  
+ CCTTAAGTCA TGGCCTGGCC TATATAAAGC CCTTCATTTC AATAGAATGC CTGTTATTTC AGAAGAATTT   
  
  
+ CTTGCTAGGA AGCTGTTTTT TGAGTTGTTT CCCTTCTTGA AGCTGGCCTT TTTGGTGACT AACCAATCAA   
  
  
+ TAATCGAGGC CATGGAGGGG GAAAAGATGG TGCATATAAT TGATCTGAAT GCGTCAGAAC CTGCACAGTG   
  
  
+ GATTGCCCTT ATTCAAGACT TGAGTGCTCG GCCTGAGGGC CCTCCTCATT TGAGGATTAC CGGGGTTCAT   
  
  
+ CAACAGAAAG AGGTTTTAGA ACAAGTAGCT CATAGATTGA CTGAAGAAGC TGAGAAGTTG GATTTGCCAT   
  
  
+ TTCAGTTCAA TCCTGTGGTT TGCAAACTAG AGAATCTCGA CATCGGAAAA CTCCGTGTTA AGACCGGGGA   
  
  
+ GGCCTTGGCT ATTACCTCGG TCCTTCAACT GCATACCCTT TTGGCTTCTG AAGAGGAAGT CCTTAAGAAA   
  
  
+ AGTTCACCCT TGGCATTGGT AAAGCAAGCC AATGGGGCTA ATTTACAGGG CTTGTTCAAT AAAGATGGAG   
  
  
+ CTAATAATAG GCGTAGCCCA AGTAATGATT CGGCTTCATC TGCACCTTCA TCCCTCAACA CTTCAGCCAA   
  
  
+ GATGGAAGGT TTCCTTAGCG CTTTGTGGGG TTTATCCCCA AAGATTATGG TGATAACCGA GCAAGATTCC   
  
  
+ AACCACAATG GGGCAGGACT AATGGAGAGA TTGTCAGAAG CATTGTACTT CTATGCAGCA TTGTTCGATT   
  
  
+ GCTTAGAATT TACCCTCCCG AGAACCTCCG TGGAGAGAAG GAAGGTCGAG ATGCTCCTCC TTGGCAAGGA   
  
  
+ AATCAAGAAC ATCATAGCGT GTGAGGGAGG AGAAAGAATA GATAGGCATG AGAAGTTGGG GAAGTGGATT   
  
  
+ AAGAGGCTTG AGATGGCCGG GTTTGGAAGC GTTCCTTTGA GCCACATAGG CATGATCCAA GCAAGGCGGT   
  
  
+ TGTTGCAGAG CTATGGCTGT GATGGTTATA GAATAAAGGA GGAGAACGGA TGTTTTGTTA TCTGCTGGCA   
  
  
+ AGATCGCCCC CTCTTTTCAG TATCTGCTTG GAGATGTAGG AGGTG  

- -Up\_Stream \_Len000AGATGG AAATGGACAC AATTTTTTTT TTTAAAACAA AGGTAATGAT AAGAGAACCG   
  
  
- TGATAACCAC GACTAAAAAG AAGTGGTTTA GTTGGAGGAA TATAAACGGT TAAAATGACA GGAGAAAGTA   
  
  
- GATATTTCAG TTGAGAGGAT CATGTTAAAC AGTGTATCTT TTAGAGATCC GTGAGTAGAT TTGAGAGACA   
  
  
- AGACCCATTA ACTAGCTGCT AGATGTGGTA ATAAGCAAGA TCACGTGGCA AACCTGTCGG TTAGTACATG   
  
  
- GAAACCTCTA GTTGACGAGT ACTCAAGAAC CACACTTTAA CCCCTTCATT TAAGCTGAAA TCCCGTGACC   
  
  
- AGATAGTGCG GTACTAAAAT GGTAGATAAA AGTATATATA GGTTATTCAA ACTAGAAAGC GATAATTAGT   
  
  
- GTTACTATAT TTGTTCACCA CTTCTCGTTA TTACTTGGTA TGTAAAATTT GAATTCGCAA TTCCTTCTGT   
  
  
- ACTTCAATTT TTGTTACCAT TCTCGATACG CATACAAACC GTATATCGAA AAAATTCTCA CAAAACCGAT   
  
  
- ATCAGCCTCA AAAAATTAAT TCTAATCGAC AAACTAGTTT ATTTTTTCGA TTAAACTCAC AAACCGCTCT   
  
  
- TCTGAAAAAT ATTCTTGAAA AAAACCAGAT TTTTCAATTA AATTTTTCCG ATTAAGATAC TCGAAAAGCC   
  
  
- TTCTCAAAAA ATTTATTAAT TGAAAAACAG AGTATTCGGT ATTGAAATAG TCTTTGTCAA TTAAAATGTT   
  
  
- TTATTAAAGA GTTGTTTGTT GATTAAATTT ATTAATTATT TTTATTGATT GAGTTTATAG ATTATTGATT   
  
  
- ATTGATTATA TTTATTAATT GTCAATTGTC AACAAATGCT TTGTCTTAAA TATATTTTTC GCCATTGTCT   
  
  
- ATTTTCTATT AGTACGCTCT AATTTTCAGT GGTCACTGTG TGTTCATGAA TTTATTTATC AGATTTGTTA   
  
  
- CTATAAAAAA CGCTCGAAGG TACGTTTATG GCTGTGAAAA TAACGGAGTC TTCCTTTGTT TTTGACTCAC   
  
  
- TTCCCCCCCC CCCCAAGACA AACAGAAACA ATCCCGTGCA AACCTAAGTT CACATCTAAC CCTTATTTAA   
  
  
- GCTCCTGAAA AAGAAACGGG AAAAAGGGAC TAAGGGTTTG GGTGTAGTTC AGTTCTGCTT TGGTCGGTAA   
  
  
- CTTTTTTCTC CCACAAACAC ACTCTATTTC TGTCTTTAAA ATACGGTCTC TCTCTTCTAT TGTTTACGTT   
  
  
- GTTGCAGCTG TCTCCGAGAG GAAGAGTGTT TAAGGTATAA GGAGACAAAA AATTCTTTCT TTTTCTTTCA   
  
  
- CACTCTGTTC TTGTTGTGTT CGTTTGTTAA GTCAACTGCT GCTTATGACT CACTATGGTG CGTGACACAC   
  
  
- GTTGACGCAA AGGTAAACAC CTCAGGAAGT TTCGTGCGAG TAGAAGTTGG GAAGGAAGCG GTGCCTTAAA   
  
  
- GTCCCCCTTT TTTTGGGGGT GGATTAAAAC TCTCTCTCTC TCTTGTCTCT CTTGCCCGCT ATGTCCCTAT   
  
  
- GTTCCAAACT CGTTAAAATC TTTTAAAGAA CCCCGGCAAC TACCCAAAAC TCACTTAACG TTTAGGGTCT   
  
  
- TTCTAAAGAC GCCAAAAAAT AGATGCCGAG AGAGACACCC TTAAAAAACC ATCTAAGGAG ACCAAGGGTG   
  
  
- AAAGTATAGA AGAATAAACA AGATGGGAGA AATGTAGACT ATCAAACAAG ATTGACGACG AAGTCAACCA   
  
  
- CGAACAAGAG AAATCATGAA AAGACAAAAC AAGCAAAACA AGTACGTGAA CTTAAGTTGT TCTCGGGAAC   
  
  
- AGGTAAAACT CGAGAATTAA GGGTAGATTG GGACAAAGGA AGTCTTTAAC GACAAGACAA GTATATCAAT   
  
  
- ATAAAAAATA CGAAACTAAA CCCATATTCA AACGACAACC ACTAAGGTTT CGAACCATGA AAACCGGTAT   
  
  
- AAAACAAACT CAACCACAAC TAACCATGTT TAGAACATTA ACCACACTAA CAATTACCCT GGTTACGAAG   
  
  
- TTCTACTACC AAGTAGTCAT TGAAGAAGTA GTGGAGAAGT TAAAAAGAGG TACTACAGTG GGTTAAACCC   
  
  
- AAGGAGGATG GGAACCGATT CCCTGAATTT CGGACTTCTC TCTCCAGAAA TGAACTATGT AAACAACGAG   
  
  
- TGAACACGTT TGGTACAGAG ATTACCATCG GAACTGTTAC GTTTGGATCG GGAACTCGTT TAGAGGGTCG   
  
  
- AACGTCGGGG ACTACCGCTA TGTTAGGTCG CATAACAACG GATGAAACGA CTTAGTGAAC GACTTTCCTA   
  
  
- GGAATTCAGT ACCGGACCGG ATATATTTCG GGAAGTAAAG TTATCTTACG GACAATAAAG TCTTCTTAAA   
  
  
- GAACGATCCT TCGACAAAAA ACTCAACAAA GGGAAGAACT TCGACCGGAA AAACCACTGA TTGGTTAGTT   
  
  
- ATTAGCTCCG GTACCTCCCC CTTTTCTACC ACGTATATTA ACTAGACTTA CGCAGTCTTG GACGTGTCAC   
  
  
- CTAACGGGAA TAAGTTCTGA ACTCACGAGC CGGACTCCCG GGAGGAGTAA ACTCCTAATG GCCCCAAGTA   
  
  
- GTTGTCTTTC TCCAAAATCT TGTTCATCGA GTATCTAACT GACTTCTTCG ACTCTTCAAC CTAAACGGTA   
  
  
- AAGTCAAGTT AGGACACCAA ACGTTTGATC TCTTAGAGCT GTAGCCTTTT GAGGCACAAT TCTGGCCCCT   
  
  
- CCGGAACCGA TAATGGAGCC AGGAAGTTGA CGTATGGGAA AACCGAAGAC TTCTCCTTCA GGAATTCTTT   
  
  
- TCAAGTGGGA ACCGTAACCA TTTCGTTCGG TTACCCCGAT TAAATGTCCC GAACAAGTTA TTTCTACCTC   
  
  
- GATTATTATC CGCATCGGGT TCATTACTAA GCCGAAGTAG ACGTGGAAGT AGGGAGTTGT GAAGTCGGTT   
  
  
- CTACCTTCCA AAGGAATCGC GAAACACCCC AAATAGGGGT TTCTAATACC ACTATTGGCT CGTTCTAAGG   
  
  
- TTGGTGTTAC CCCGTCCTGA TTACCTCTCT AACAGTCTTC GTAACATGAA GATACGTCGT AACAAGCTAA   
  
  
- CGAATCTTAA ATGGGAGGGC TCTTGGAGGC ACCTCTCTTC CTTCCAGCTC TACGAGGAGG AACCGTTCCT   
  
  
- TTAGTTCTTG TAGTATCGCA CACTCCCTCC TCTTTCTTAT CTATCCGTAC TCTTCAACCC CTTCACCTAA   
  
  
- TTCTCCGAAC TCTACCGGCC CAAACCTTCG CAAGGAAACT CGGTGTATCC GTACTAGGTT CGTTCCGCCA   
  
  
- ACAACGTCTC GATACCGACA CTACCAATAT CTTATTTCCT CCTCTTGCCT ACAAAACAAT AGACGACCGT   
  
  
- TCTAGCGGGG GAGAAAAGTC ATAGACGAAC CTCTACATCC TCCAC

+     TCT-motif

| Site Name | Organism | Position | Strand | Matrix score. | sequence | function |
| --- | --- | --- | --- | --- | --- | --- |
| TCT-motif | Arabidopsis thaliana | 512 | - | 6 | TCTTAC | part of a light responsive element |

>HU08G01232.1   
+ -Up\_Stream \_Len000TCTACC TTTACCTGTG TTAAAAAAAA AAATTTTGTT TCCATTACTA TTCTCTTGGC   
  
  
+ ACTATTGGTG CTGATTTTTC TTCACCAAAT CAACCTCCTT ATATTTGCCA ATTTTACTGT CCTCTTTCAT   
  
  
+ CTATAAAGTC AACTCTCCTA GTACAATTTG TCACATAGAA AATCTCTAGG CACTCATCTA AACTCTCTGT   
  
  
+ TCTGGGTAAT TGATCGACGA TCTACACCAT TATTCGTTCT AGTGCACCGT TTGGACAGCC AATCATGTAC   
  
  
+ CTTTGGAGAT CAACTGCTCA TGAGTTCTTG GTGTGAAATT GGGGAAGTAA ATTCGACTTT AGGGCACTGG   
  
  
+ TCTATCACGC CATGATTTTA CCATCTATTT TCATATATAT CCAATAAGTT TGATCTTTCG CTATTAATCA   
  
  
+ CAATGATATA AACAAGTGGT GAAGAGCAAT AATGAACCAT ACATTTTAAA CTTAAGCGTT AAGGAAGACA   
  
  
+ TGAAGTTAAA AACAATGGTA AGAGCTATGC GTATGTTTGG CATATAGCTT TTTTAAGAGT GTTTTGGCTA   
  
  
+ TAGTCGGAGT TTTTTAATTA AGATTAGCTG TTTGATCAAA TAAAAAAGCT AATTTGAGTG TTTGGCGAGA   
  
  
+ AGACTTTTTA TAAGAACTTT TTTTGGTCTA AAAAGTTAAT TTAAAAAGGC TAATTCTATG AGCTTTTCGG   
  
  
+ AAGAGTTTTT TAAATAATTA ACTTTTTGTC TCATAAGCCA TAACTTTATC AGAAACAGTT AATTTTACAA   
  
  
+ AATAATTTCT CAACAAACAA CTAATTTAAA TAATTAATAA AAATAACTAA CTCAAATATC TAATAACTAA   
  
  
+ TAACTAATAT AAATAATTAA CAGTTAACAG TTGTTTACGA AACAGAATTT ATATAAAAAG CGGTAACAGA   
  
  
+ TAAAAGATAA TCATGCGAGA TTAAAAGTCA CCAGTGACAC ACAAGTACTT AAATAAATAG TCTAAACAAT   
  
  
+ GATATTTTTT GCGAGCTTCC ATGCAAATAC CGACACTTTT ATTGCCTCAG AAGGAAACAA AAACTGAGTG   
  
  
+ AAGGGGGGGG GGGGTTCTGT TTGTCTTTGT TAGGGCACGT TTGGATTCAA GTGTAGATTG GGAATAAATT   
  
  
+ CGAGGACTTT TTCTTTGCCC TTTTTCCCTG ATTCCCAAAC CCACATCAAG TCAAGACGAA ACCAGCCATT   
  
  
+ GAAAAAAGAG GGTGTTTGTG TGAGATAAAG ACAGAAATTT TATGCCAGAG AGAGAAGATA ACAAATGCAA   
  
  
+ CAACGTCGAC AGAGGCTCTC CTTCTCACAA ATTCCATATT CCTCTGTTTT TTAAGAAAGA AAAAGAAAGT   
  
  
+ GTGAGACAAG AACAACACAA GCAAACAATT CAGTTGACGA CGAATACTGA GTGATACCAC GCACTGTGTG   
  
  
+ CAACTGCGTT TCCATTTGTG GAGTCCTTCA AAGCACGCTC ATCTTCAACC CTTCCTTCGC CACGGAATTT   
  
  
+ CAGGGGGAAA AAAACCCCCA CCTAATTTTG AGAGAGAGAG AGAACAGAGA GAACGGGCGA TACAGGGATA   
  
  
+ CAAGGTTTGA GCAATTTTAG AAAATTTCTT GGGGCCGTTG ATGGGTTTTG AGTGAATTGC AAATCCCAGA   
  
  
+ AAGATTTCTG CGGTTTTTTA TCTACGGCTC TCTCTGTGGG AATTTTTTGG TAGATTCCTC TGGTTCCCAC   
  
  
+ TTTCATATCT TCTTATTTGT TCTACCCTCT TTACATCTGA TAGTTTGTTC TAACTGCTGC TTCAGTTGGT   
  
  
+ GCTTGTTCTC TTTAGTACTT TTCTGTTTTG TTCGTTTTGT TCATGCACTT GAATTCAACA AGAGCCCTTG   
  
  
+ TCCATTTTGA GCTCTTAATT CCCATCTAAC CCTGTTTCCT TCAGAAATTG CTGTTCTGTT CATATAGTTA   
  
  
+ TATTTTTTAT GCTTTGATTT GGGTATAAGT TTGCTGTTGG TGATTCCAAA GCTTGGTACT TTTGGCCATA   
  
  
+ TTTTGTTTGA GTTGGTGTTG ATTGGTACAA ATCTTGTAAT TGGTGTGATT GTTAATGGGA CCAATGCTTC   
  
  
+ AAGATGATGG TTCATCAGTA ACTTCTTCAT CACCTCTTCA ATTTTTCTCC ATGATGTCAC CCAATTTGGG   
  
  
+ TTCCTCCTAC CCTTGGCTAA GGGACTTAAA GCCTGAAGAG AGAGGTCTTT ACTTGATACA TTTGTTGCTC   
  
  
+ ACTTGTGCAA ACCATGTCTC TAATGGTAGC CTTGACAATG CAAACCTAGC CCTTGAGCAA ATCTCCCAGC   
  
  
+ TTGCAGCCCC TGATGGCGAT ACAATCCAGC GTATTGTTGC CTACTTTGCT GAATCACTTG CTGAAAGGAT   
  
  
+ CCTTAAGTCA TGGCCTGGCC TATATAAAGC CCTTCATTTC AATAGAATGC CTGTTATTTC AGAAGAATTT   
  
  
+ CTTGCTAGGA AGCTGTTTTT TGAGTTGTTT CCCTTCTTGA AGCTGGCCTT TTTGGTGACT AACCAATCAA   
  
  
+ TAATCGAGGC CATGGAGGGG GAAAAGATGG TGCATATAAT TGATCTGAAT GCGTCAGAAC CTGCACAGTG   
  
  
+ GATTGCCCTT ATTCAAGACT TGAGTGCTCG GCCTGAGGGC CCTCCTCATT TGAGGATTAC CGGGGTTCAT   
  
  
+ CAACAGAAAG AGGTTTTAGA ACAAGTAGCT CATAGATTGA CTGAAGAAGC TGAGAAGTTG GATTTGCCAT   
  
  
+ TTCAGTTCAA TCCTGTGGTT TGCAAACTAG AGAATCTCGA CATCGGAAAA CTCCGTGTTA AGACCGGGGA   
  
  
+ GGCCTTGGCT ATTACCTCGG TCCTTCAACT GCATACCCTT TTGGCTTCTG AAGAGGAAGT CCTTAAGAAA   
  
  
+ AGTTCACCCT TGGCATTGGT AAAGCAAGCC AATGGGGCTA ATTTACAGGG CTTGTTCAAT AAAGATGGAG   
  
  
+ CTAATAATAG GCGTAGCCCA AGTAATGATT CGGCTTCATC TGCACCTTCA TCCCTCAACA CTTCAGCCAA   
  
  
+ GATGGAAGGT TTCCTTAGCG CTTTGTGGGG TTTATCCCCA AAGATTATGG TGATAACCGA GCAAGATTCC   
  
  
+ AACCACAATG GGGCAGGACT AATGGAGAGA TTGTCAGAAG CATTGTACTT CTATGCAGCA TTGTTCGATT   
  
  
+ GCTTAGAATT TACCCTCCCG AGAACCTCCG TGGAGAGAAG GAAGGTCGAG ATGCTCCTCC TTGGCAAGGA   
  
  
+ AATCAAGAAC ATCATAGCGT GTGAGGGAGG AGAAAGAATA GATAGGCATG AGAAGTTGGG GAAGTGGATT   
  
  
+ AAGAGGCTTG AGATGGCCGG GTTTGGAAGC GTTCCTTTGA GCCACATAGG CATGATCCAA GCAAGGCGGT   
  
  
+ TGTTGCAGAG CTATGGCTGT GATGGTTATA GAATAAAGGA GGAGAACGGA TGTTTTGTTA TCTGCTGGCA   
  
  
+ AGATCGCCCC CTCTTTTCAG TATCTGCTTG GAGATGTAGG AGGTG  

- -Up\_Stream \_Len000AGATGG AAATGGACAC AATTTTTTTT TTTAAAACAA AGGTAATGAT AAGAGAACCG   
  
  
- TGATAACCAC GACTAAAAAG AAGTGGTTTA GTTGGAGGAA TATAAACGGT TAAAATGACA GGAGAAAGTA   
  
  
- GATATTTCAG TTGAGAGGAT CATGTTAAAC AGTGTATCTT TTAGAGATCC GTGAGTAGAT TTGAGAGACA   
  
  
- AGACCCATTA ACTAGCTGCT AGATGTGGTA ATAAGCAAGA TCACGTGGCA AACCTGTCGG TTAGTACATG   
  
  
- GAAACCTCTA GTTGACGAGT ACTCAAGAAC CACACTTTAA CCCCTTCATT TAAGCTGAAA TCCCGTGACC   
  
  
- AGATAGTGCG GTACTAAAAT GGTAGATAAA AGTATATATA GGTTATTCAA ACTAGAAAGC GATAATTAGT   
  
  
- GTTACTATAT TTGTTCACCA CTTCTCGTTA TTACTTGGTA TGTAAAATTT GAATTCGCAA TTCCTTCTGT   
  
  
- ACTTCAATTT TTGTTACCAT TCTCGATACG CATACAAACC GTATATCGAA AAAATTCTCA CAAAACCGAT   
  
  
- ATCAGCCTCA AAAAATTAAT TCTAATCGAC AAACTAGTTT ATTTTTTCGA TTAAACTCAC AAACCGCTCT   
  
  
- TCTGAAAAAT ATTCTTGAAA AAAACCAGAT TTTTCAATTA AATTTTTCCG ATTAAGATAC TCGAAAAGCC   
  
  
- TTCTCAAAAA ATTTATTAAT TGAAAAACAG AGTATTCGGT ATTGAAATAG TCTTTGTCAA TTAAAATGTT   
  
  
- TTATTAAAGA GTTGTTTGTT GATTAAATTT ATTAATTATT TTTATTGATT GAGTTTATAG ATTATTGATT   
  
  
- ATTGATTATA TTTATTAATT GTCAATTGTC AACAAATGCT TTGTCTTAAA TATATTTTTC GCCATTGTCT   
  
  
- ATTTTCTATT AGTACGCTCT AATTTTCAGT GGTCACTGTG TGTTCATGAA TTTATTTATC AGATTTGTTA   
  
  
- CTATAAAAAA CGCTCGAAGG TACGTTTATG GCTGTGAAAA TAACGGAGTC TTCCTTTGTT TTTGACTCAC   
  
  
- TTCCCCCCCC CCCCAAGACA AACAGAAACA ATCCCGTGCA AACCTAAGTT CACATCTAAC CCTTATTTAA   
  
  
- GCTCCTGAAA AAGAAACGGG AAAAAGGGAC TAAGGGTTTG GGTGTAGTTC AGTTCTGCTT TGGTCGGTAA   
  
  
- CTTTTTTCTC CCACAAACAC ACTCTATTTC TGTCTTTAAA ATACGGTCTC TCTCTTCTAT TGTTTACGTT   
  
  
- GTTGCAGCTG TCTCCGAGAG GAAGAGTGTT TAAGGTATAA GGAGACAAAA AATTCTTTCT TTTTCTTTCA   
  
  
- CACTCTGTTC TTGTTGTGTT CGTTTGTTAA GTCAACTGCT GCTTATGACT CACTATGGTG CGTGACACAC   
  
  
- GTTGACGCAA AGGTAAACAC CTCAGGAAGT TTCGTGCGAG TAGAAGTTGG GAAGGAAGCG GTGCCTTAAA   
  
  
- GTCCCCCTTT TTTTGGGGGT GGATTAAAAC TCTCTCTCTC TCTTGTCTCT CTTGCCCGCT ATGTCCCTAT   
  
  
- GTTCCAAACT CGTTAAAATC TTTTAAAGAA CCCCGGCAAC TACCCAAAAC TCACTTAACG TTTAGGGTCT   
  
  
- TTCTAAAGAC GCCAAAAAAT AGATGCCGAG AGAGACACCC TTAAAAAACC ATCTAAGGAG ACCAAGGGTG   
  
  
- AAAGTATAGA AGAATAAACA AGATGGGAGA AATGTAGACT ATCAAACAAG ATTGACGACG AAGTCAACCA   
  
  
- CGAACAAGAG AAATCATGAA AAGACAAAAC AAGCAAAACA AGTACGTGAA CTTAAGTTGT TCTCGGGAAC   
  
  
- AGGTAAAACT CGAGAATTAA GGGTAGATTG GGACAAAGGA AGTCTTTAAC GACAAGACAA GTATATCAAT   
  
  
- ATAAAAAATA CGAAACTAAA CCCATATTCA AACGACAACC ACTAAGGTTT CGAACCATGA AAACCGGTAT   
  
  
- AAAACAAACT CAACCACAAC TAACCATGTT TAGAACATTA ACCACACTAA CAATTACCCT GGTTACGAAG   
  
  
- TTCTACTACC AAGTAGTCAT TGAAGAAGTA GTGGAGAAGT TAAAAAGAGG TACTACAGTG GGTTAAACCC   
  
  
- AAGGAGGATG GGAACCGATT CCCTGAATTT CGGACTTCTC TCTCCAGAAA TGAACTATGT AAACAACGAG   
  
  
- TGAACACGTT TGGTACAGAG ATTACCATCG GAACTGTTAC GTTTGGATCG GGAACTCGTT TAGAGGGTCG   
  
  
- AACGTCGGGG ACTACCGCTA TGTTAGGTCG CATAACAACG GATGAAACGA CTTAGTGAAC GACTTTCCTA   
  
  
- GGAATTCAGT ACCGGACCGG ATATATTTCG GGAAGTAAAG TTATCTTACG GACAATAAAG TCTTCTTAAA   
  
  
- GAACGATCCT TCGACAAAAA ACTCAACAAA GGGAAGAACT TCGACCGGAA AAACCACTGA TTGGTTAGTT   
  
  
- ATTAGCTCCG GTACCTCCCC CTTTTCTACC ACGTATATTA ACTAGACTTA CGCAGTCTTG GACGTGTCAC   
  
  
- CTAACGGGAA TAAGTTCTGA ACTCACGAGC CGGACTCCCG GGAGGAGTAA ACTCCTAATG GCCCCAAGTA   
  
  
- GTTGTCTTTC TCCAAAATCT TGTTCATCGA GTATCTAACT GACTTCTTCG ACTCTTCAAC CTAAACGGTA   
  
  
- AAGTCAAGTT AGGACACCAA ACGTTTGATC TCTTAGAGCT GTAGCCTTTT GAGGCACAAT TCTGGCCCCT   
  
  
- CCGGAACCGA TAATGGAGCC AGGAAGTTGA CGTATGGGAA AACCGAAGAC TTCTCCTTCA GGAATTCTTT   
  
  
- TCAAGTGGGA ACCGTAACCA TTTCGTTCGG TTACCCCGAT TAAATGTCCC GAACAAGTTA TTTCTACCTC   
  
  
- GATTATTATC CGCATCGGGT TCATTACTAA GCCGAAGTAG ACGTGGAAGT AGGGAGTTGT GAAGTCGGTT   
  
  
- CTACCTTCCA AAGGAATCGC GAAACACCCC AAATAGGGGT TTCTAATACC ACTATTGGCT CGTTCTAAGG   
  
  
- TTGGTGTTAC CCCGTCCTGA TTACCTCTCT AACAGTCTTC GTAACATGAA GATACGTCGT AACAAGCTAA   
  
  
- CGAATCTTAA ATGGGAGGGC TCTTGGAGGC ACCTCTCTTC CTTCCAGCTC TACGAGGAGG AACCGTTCCT   
  
  
- TTAGTTCTTG TAGTATCGCA CACTCCCTCC TCTTTCTTAT CTATCCGTAC TCTTCAACCC CTTCACCTAA   
  
  
- TTCTCCGAAC TCTACCGGCC CAAACCTTCG CAAGGAAACT CGGTGTATCC GTACTAGGTT CGTTCCGCCA   
  
  
- ACAACGTCTC GATACCGACA CTACCAATAT CTTATTTCCT CCTCTTGCCT ACAAAACAAT AGACGACCGT   
  
  
- TCTAGCGGGG GAGAAAAGTC ATAGACGAAC CTCTACATCC TCCAC

+     TGACG-motif

| Site Name | Organism | Position | Strand | Matrix score. | sequence | function |
| --- | --- | --- | --- | --- | --- | --- |
| TGACG-motif | Hordeum vulgare | 1369 | + | 5 | TGACG | cis-acting regulatory element involved in the MeJA-responsiveness |
| TGACG-motif | Hordeum vulgare | 2506 | - | 5 | TGACG | cis-acting regulatory element involved in the MeJA-responsiveness |

>HU08G01232.1   
+ -Up\_Stream \_Len000TCTACC TTTACCTGTG TTAAAAAAAA AAATTTTGTT TCCATTACTA TTCTCTTGGC   
  
  
+ ACTATTGGTG CTGATTTTTC TTCACCAAAT CAACCTCCTT ATATTTGCCA ATTTTACTGT CCTCTTTCAT   
  
  
+ CTATAAAGTC AACTCTCCTA GTACAATTTG TCACATAGAA AATCTCTAGG CACTCATCTA AACTCTCTGT   
  
  
+ TCTGGGTAAT TGATCGACGA TCTACACCAT TATTCGTTCT AGTGCACCGT TTGGACAGCC AATCATGTAC   
  
  
+ CTTTGGAGAT CAACTGCTCA TGAGTTCTTG GTGTGAAATT GGGGAAGTAA ATTCGACTTT AGGGCACTGG   
  
  
+ TCTATCACGC CATGATTTTA CCATCTATTT TCATATATAT CCAATAAGTT TGATCTTTCG CTATTAATCA   
  
  
+ CAATGATATA AACAAGTGGT GAAGAGCAAT AATGAACCAT ACATTTTAAA CTTAAGCGTT AAGGAAGACA   
  
  
+ TGAAGTTAAA AACAATGGTA AGAGCTATGC GTATGTTTGG CATATAGCTT TTTTAAGAGT GTTTTGGCTA   
  
  
+ TAGTCGGAGT TTTTTAATTA AGATTAGCTG TTTGATCAAA TAAAAAAGCT AATTTGAGTG TTTGGCGAGA   
  
  
+ AGACTTTTTA TAAGAACTTT TTTTGGTCTA AAAAGTTAAT TTAAAAAGGC TAATTCTATG AGCTTTTCGG   
  
  
+ AAGAGTTTTT TAAATAATTA ACTTTTTGTC TCATAAGCCA TAACTTTATC AGAAACAGTT AATTTTACAA   
  
  
+ AATAATTTCT CAACAAACAA CTAATTTAAA TAATTAATAA AAATAACTAA CTCAAATATC TAATAACTAA   
  
  
+ TAACTAATAT AAATAATTAA CAGTTAACAG TTGTTTACGA AACAGAATTT ATATAAAAAG CGGTAACAGA   
  
  
+ TAAAAGATAA TCATGCGAGA TTAAAAGTCA CCAGTGACAC ACAAGTACTT AAATAAATAG TCTAAACAAT   
  
  
+ GATATTTTTT GCGAGCTTCC ATGCAAATAC CGACACTTTT ATTGCCTCAG AAGGAAACAA AAACTGAGTG   
  
  
+ AAGGGGGGGG GGGGTTCTGT TTGTCTTTGT TAGGGCACGT TTGGATTCAA GTGTAGATTG GGAATAAATT   
  
  
+ CGAGGACTTT TTCTTTGCCC TTTTTCCCTG ATTCCCAAAC CCACATCAAG TCAAGACGAA ACCAGCCATT   
  
  
+ GAAAAAAGAG GGTGTTTGTG TGAGATAAAG ACAGAAATTT TATGCCAGAG AGAGAAGATA ACAAATGCAA   
  
  
+ CAACGTCGAC AGAGGCTCTC CTTCTCACAA ATTCCATATT CCTCTGTTTT TTAAGAAAGA AAAAGAAAGT   
  
  
+ GTGAGACAAG AACAACACAA GCAAACAATT CAGTTGACGA CGAATACTGA GTGATACCAC GCACTGTGTG   
  
  
+ CAACTGCGTT TCCATTTGTG GAGTCCTTCA AAGCACGCTC ATCTTCAACC CTTCCTTCGC CACGGAATTT   
  
  
+ CAGGGGGAAA AAAACCCCCA CCTAATTTTG AGAGAGAGAG AGAACAGAGA GAACGGGCGA TACAGGGATA   
  
  
+ CAAGGTTTGA GCAATTTTAG AAAATTTCTT GGGGCCGTTG ATGGGTTTTG AGTGAATTGC AAATCCCAGA   
  
  
+ AAGATTTCTG CGGTTTTTTA TCTACGGCTC TCTCTGTGGG AATTTTTTGG TAGATTCCTC TGGTTCCCAC   
  
  
+ TTTCATATCT TCTTATTTGT TCTACCCTCT TTACATCTGA TAGTTTGTTC TAACTGCTGC TTCAGTTGGT   
  
  
+ GCTTGTTCTC TTTAGTACTT TTCTGTTTTG TTCGTTTTGT TCATGCACTT GAATTCAACA AGAGCCCTTG   
  
  
+ TCCATTTTGA GCTCTTAATT CCCATCTAAC CCTGTTTCCT TCAGAAATTG CTGTTCTGTT CATATAGTTA   
  
  
+ TATTTTTTAT GCTTTGATTT GGGTATAAGT TTGCTGTTGG TGATTCCAAA GCTTGGTACT TTTGGCCATA   
  
  
+ TTTTGTTTGA GTTGGTGTTG ATTGGTACAA ATCTTGTAAT TGGTGTGATT GTTAATGGGA CCAATGCTTC   
  
  
+ AAGATGATGG TTCATCAGTA ACTTCTTCAT CACCTCTTCA ATTTTTCTCC ATGATGTCAC CCAATTTGGG   
  
  
+ TTCCTCCTAC CCTTGGCTAA GGGACTTAAA GCCTGAAGAG AGAGGTCTTT ACTTGATACA TTTGTTGCTC   
  
  
+ ACTTGTGCAA ACCATGTCTC TAATGGTAGC CTTGACAATG CAAACCTAGC CCTTGAGCAA ATCTCCCAGC   
  
  
+ TTGCAGCCCC TGATGGCGAT ACAATCCAGC GTATTGTTGC CTACTTTGCT GAATCACTTG CTGAAAGGAT   
  
  
+ CCTTAAGTCA TGGCCTGGCC TATATAAAGC CCTTCATTTC AATAGAATGC CTGTTATTTC AGAAGAATTT   
  
  
+ CTTGCTAGGA AGCTGTTTTT TGAGTTGTTT CCCTTCTTGA AGCTGGCCTT TTTGGTGACT AACCAATCAA   
  
  
+ TAATCGAGGC CATGGAGGGG GAAAAGATGG TGCATATAAT TGATCTGAAT GCGTCAGAAC CTGCACAGTG   
  
  
+ GATTGCCCTT ATTCAAGACT TGAGTGCTCG GCCTGAGGGC CCTCCTCATT TGAGGATTAC CGGGGTTCAT   
  
  
+ CAACAGAAAG AGGTTTTAGA ACAAGTAGCT CATAGATTGA CTGAAGAAGC TGAGAAGTTG GATTTGCCAT   
  
  
+ TTCAGTTCAA TCCTGTGGTT TGCAAACTAG AGAATCTCGA CATCGGAAAA CTCCGTGTTA AGACCGGGGA   
  
  
+ GGCCTTGGCT ATTACCTCGG TCCTTCAACT GCATACCCTT TTGGCTTCTG AAGAGGAAGT CCTTAAGAAA   
  
  
+ AGTTCACCCT TGGCATTGGT AAAGCAAGCC AATGGGGCTA ATTTACAGGG CTTGTTCAAT AAAGATGGAG   
  
  
+ CTAATAATAG GCGTAGCCCA AGTAATGATT CGGCTTCATC TGCACCTTCA TCCCTCAACA CTTCAGCCAA   
  
  
+ GATGGAAGGT TTCCTTAGCG CTTTGTGGGG TTTATCCCCA AAGATTATGG TGATAACCGA GCAAGATTCC   
  
  
+ AACCACAATG GGGCAGGACT AATGGAGAGA TTGTCAGAAG CATTGTACTT CTATGCAGCA TTGTTCGATT   
  
  
+ GCTTAGAATT TACCCTCCCG AGAACCTCCG TGGAGAGAAG GAAGGTCGAG ATGCTCCTCC TTGGCAAGGA   
  
  
+ AATCAAGAAC ATCATAGCGT GTGAGGGAGG AGAAAGAATA GATAGGCATG AGAAGTTGGG GAAGTGGATT   
  
  
+ AAGAGGCTTG AGATGGCCGG GTTTGGAAGC GTTCCTTTGA GCCACATAGG CATGATCCAA GCAAGGCGGT   
  
  
+ TGTTGCAGAG CTATGGCTGT GATGGTTATA GAATAAAGGA GGAGAACGGA TGTTTTGTTA TCTGCTGGCA   
  
  
+ AGATCGCCCC CTCTTTTCAG TATCTGCTTG GAGATGTAGG AGGTG  

- -Up\_Stream \_Len000AGATGG AAATGGACAC AATTTTTTTT TTTAAAACAA AGGTAATGAT AAGAGAACCG   
  
  
- TGATAACCAC GACTAAAAAG AAGTGGTTTA GTTGGAGGAA TATAAACGGT TAAAATGACA GGAGAAAGTA   
  
  
- GATATTTCAG TTGAGAGGAT CATGTTAAAC AGTGTATCTT TTAGAGATCC GTGAGTAGAT TTGAGAGACA   
  
  
- AGACCCATTA ACTAGCTGCT AGATGTGGTA ATAAGCAAGA TCACGTGGCA AACCTGTCGG TTAGTACATG   
  
  
- GAAACCTCTA GTTGACGAGT ACTCAAGAAC CACACTTTAA CCCCTTCATT TAAGCTGAAA TCCCGTGACC   
  
  
- AGATAGTGCG GTACTAAAAT GGTAGATAAA AGTATATATA GGTTATTCAA ACTAGAAAGC GATAATTAGT   
  
  
- GTTACTATAT TTGTTCACCA CTTCTCGTTA TTACTTGGTA TGTAAAATTT GAATTCGCAA TTCCTTCTGT   
  
  
- ACTTCAATTT TTGTTACCAT TCTCGATACG CATACAAACC GTATATCGAA AAAATTCTCA CAAAACCGAT   
  
  
- ATCAGCCTCA AAAAATTAAT TCTAATCGAC AAACTAGTTT ATTTTTTCGA TTAAACTCAC AAACCGCTCT   
  
  
- TCTGAAAAAT ATTCTTGAAA AAAACCAGAT TTTTCAATTA AATTTTTCCG ATTAAGATAC TCGAAAAGCC   
  
  
- TTCTCAAAAA ATTTATTAAT TGAAAAACAG AGTATTCGGT ATTGAAATAG TCTTTGTCAA TTAAAATGTT   
  
  
- TTATTAAAGA GTTGTTTGTT GATTAAATTT ATTAATTATT TTTATTGATT GAGTTTATAG ATTATTGATT   
  
  
- ATTGATTATA TTTATTAATT GTCAATTGTC AACAAATGCT TTGTCTTAAA TATATTTTTC GCCATTGTCT   
  
  
- ATTTTCTATT AGTACGCTCT AATTTTCAGT GGTCACTGTG TGTTCATGAA TTTATTTATC AGATTTGTTA   
  
  
- CTATAAAAAA CGCTCGAAGG TACGTTTATG GCTGTGAAAA TAACGGAGTC TTCCTTTGTT TTTGACTCAC   
  
  
- TTCCCCCCCC CCCCAAGACA AACAGAAACA ATCCCGTGCA AACCTAAGTT CACATCTAAC CCTTATTTAA   
  
  
- GCTCCTGAAA AAGAAACGGG AAAAAGGGAC TAAGGGTTTG GGTGTAGTTC AGTTCTGCTT TGGTCGGTAA   
  
  
- CTTTTTTCTC CCACAAACAC ACTCTATTTC TGTCTTTAAA ATACGGTCTC TCTCTTCTAT TGTTTACGTT   
  
  
- GTTGCAGCTG TCTCCGAGAG GAAGAGTGTT TAAGGTATAA GGAGACAAAA AATTCTTTCT TTTTCTTTCA   
  
  
- CACTCTGTTC TTGTTGTGTT CGTTTGTTAA GTCAACTGCT GCTTATGACT CACTATGGTG CGTGACACAC   
  
  
- GTTGACGCAA AGGTAAACAC CTCAGGAAGT TTCGTGCGAG TAGAAGTTGG GAAGGAAGCG GTGCCTTAAA   
  
  
- GTCCCCCTTT TTTTGGGGGT GGATTAAAAC TCTCTCTCTC TCTTGTCTCT CTTGCCCGCT ATGTCCCTAT   
  
  
- GTTCCAAACT CGTTAAAATC TTTTAAAGAA CCCCGGCAAC TACCCAAAAC TCACTTAACG TTTAGGGTCT   
  
  
- TTCTAAAGAC GCCAAAAAAT AGATGCCGAG AGAGACACCC TTAAAAAACC ATCTAAGGAG ACCAAGGGTG   
  
  
- AAAGTATAGA AGAATAAACA AGATGGGAGA AATGTAGACT ATCAAACAAG ATTGACGACG AAGTCAACCA   
  
  
- CGAACAAGAG AAATCATGAA AAGACAAAAC AAGCAAAACA AGTACGTGAA CTTAAGTTGT TCTCGGGAAC   
  
  
- AGGTAAAACT CGAGAATTAA GGGTAGATTG GGACAAAGGA AGTCTTTAAC GACAAGACAA GTATATCAAT   
  
  
- ATAAAAAATA CGAAACTAAA CCCATATTCA AACGACAACC ACTAAGGTTT CGAACCATGA AAACCGGTAT   
  
  
- AAAACAAACT CAACCACAAC TAACCATGTT TAGAACATTA ACCACACTAA CAATTACCCT GGTTACGAAG   
  
  
- TTCTACTACC AAGTAGTCAT TGAAGAAGTA GTGGAGAAGT TAAAAAGAGG TACTACAGTG GGTTAAACCC   
  
  
- AAGGAGGATG GGAACCGATT CCCTGAATTT CGGACTTCTC TCTCCAGAAA TGAACTATGT AAACAACGAG   
  
  
- TGAACACGTT TGGTACAGAG ATTACCATCG GAACTGTTAC GTTTGGATCG GGAACTCGTT TAGAGGGTCG   
  
  
- AACGTCGGGG ACTACCGCTA TGTTAGGTCG CATAACAACG GATGAAACGA CTTAGTGAAC GACTTTCCTA   
  
  
- GGAATTCAGT ACCGGACCGG ATATATTTCG GGAAGTAAAG TTATCTTACG GACAATAAAG TCTTCTTAAA   
  
  
- GAACGATCCT TCGACAAAAA ACTCAACAAA GGGAAGAACT TCGACCGGAA AAACCACTGA TTGGTTAGTT   
  
  
- ATTAGCTCCG GTACCTCCCC CTTTTCTACC ACGTATATTA ACTAGACTTA CGCAGTCTTG GACGTGTCAC   
  
  
- CTAACGGGAA TAAGTTCTGA ACTCACGAGC CGGACTCCCG GGAGGAGTAA ACTCCTAATG GCCCCAAGTA   
  
  
- GTTGTCTTTC TCCAAAATCT TGTTCATCGA GTATCTAACT GACTTCTTCG ACTCTTCAAC CTAAACGGTA   
  
  
- AAGTCAAGTT AGGACACCAA ACGTTTGATC TCTTAGAGCT GTAGCCTTTT GAGGCACAAT TCTGGCCCCT   
  
  
- CCGGAACCGA TAATGGAGCC AGGAAGTTGA CGTATGGGAA AACCGAAGAC TTCTCCTTCA GGAATTCTTT   
  
  
- TCAAGTGGGA ACCGTAACCA TTTCGTTCGG TTACCCCGAT TAAATGTCCC GAACAAGTTA TTTCTACCTC   
  
  
- GATTATTATC CGCATCGGGT TCATTACTAA GCCGAAGTAG ACGTGGAAGT AGGGAGTTGT GAAGTCGGTT   
  
  
- CTACCTTCCA AAGGAATCGC GAAACACCCC AAATAGGGGT TTCTAATACC ACTATTGGCT CGTTCTAAGG   
  
  
- TTGGTGTTAC CCCGTCCTGA TTACCTCTCT AACAGTCTTC GTAACATGAA GATACGTCGT AACAAGCTAA   
  
  
- CGAATCTTAA ATGGGAGGGC TCTTGGAGGC ACCTCTCTTC CTTCCAGCTC TACGAGGAGG AACCGTTCCT   
  
  
- TTAGTTCTTG TAGTATCGCA CACTCCCTCC TCTTTCTTAT CTATCCGTAC TCTTCAACCC CTTCACCTAA   
  
  
- TTCTCCGAAC TCTACCGGCC CAAACCTTCG CAAGGAAACT CGGTGTATCC GTACTAGGTT CGTTCCGCCA   
  
  
- ACAACGTCTC GATACCGACA CTACCAATAT CTTATTTCCT CCTCTTGCCT ACAAAACAAT AGACGACCGT   
  
  
- TCTAGCGGGG GAGAAAAGTC ATAGACGAAC CTCTACATCC TCCAC

+     Unnamed\_\_1

| Site Name | Organism | Position | Strand | Matrix score. | sequence | function |
| --- | --- | --- | --- | --- | --- | --- |
| Unnamed\_\_1 | Zea mays | 3113 | + | 5 | CGTGG |  |
| Unnamed\_\_1 | Zea mays | 1464 | - | 5 | CGTGG |  |
| Unnamed\_\_1 | Zea mays | 1391 | - | 5 | CGTGG |  |

>HU08G01232.1   
+ -Up\_Stream \_Len000TCTACC TTTACCTGTG TTAAAAAAAA AAATTTTGTT TCCATTACTA TTCTCTTGGC   
  
  
+ ACTATTGGTG CTGATTTTTC TTCACCAAAT CAACCTCCTT ATATTTGCCA ATTTTACTGT CCTCTTTCAT   
  
  
+ CTATAAAGTC AACTCTCCTA GTACAATTTG TCACATAGAA AATCTCTAGG CACTCATCTA AACTCTCTGT   
  
  
+ TCTGGGTAAT TGATCGACGA TCTACACCAT TATTCGTTCT AGTGCACCGT TTGGACAGCC AATCATGTAC   
  
  
+ CTTTGGAGAT CAACTGCTCA TGAGTTCTTG GTGTGAAATT GGGGAAGTAA ATTCGACTTT AGGGCACTGG   
  
  
+ TCTATCACGC CATGATTTTA CCATCTATTT TCATATATAT CCAATAAGTT TGATCTTTCG CTATTAATCA   
  
  
+ CAATGATATA AACAAGTGGT GAAGAGCAAT AATGAACCAT ACATTTTAAA CTTAAGCGTT AAGGAAGACA   
  
  
+ TGAAGTTAAA AACAATGGTA AGAGCTATGC GTATGTTTGG CATATAGCTT TTTTAAGAGT GTTTTGGCTA   
  
  
+ TAGTCGGAGT TTTTTAATTA AGATTAGCTG TTTGATCAAA TAAAAAAGCT AATTTGAGTG TTTGGCGAGA   
  
  
+ AGACTTTTTA TAAGAACTTT TTTTGGTCTA AAAAGTTAAT TTAAAAAGGC TAATTCTATG AGCTTTTCGG   
  
  
+ AAGAGTTTTT TAAATAATTA ACTTTTTGTC TCATAAGCCA TAACTTTATC AGAAACAGTT AATTTTACAA   
  
  
+ AATAATTTCT CAACAAACAA CTAATTTAAA TAATTAATAA AAATAACTAA CTCAAATATC TAATAACTAA   
  
  
+ TAACTAATAT AAATAATTAA CAGTTAACAG TTGTTTACGA AACAGAATTT ATATAAAAAG CGGTAACAGA   
  
  
+ TAAAAGATAA TCATGCGAGA TTAAAAGTCA CCAGTGACAC ACAAGTACTT AAATAAATAG TCTAAACAAT   
  
  
+ GATATTTTTT GCGAGCTTCC ATGCAAATAC CGACACTTTT ATTGCCTCAG AAGGAAACAA AAACTGAGTG   
  
  
+ AAGGGGGGGG GGGGTTCTGT TTGTCTTTGT TAGGGCACGT TTGGATTCAA GTGTAGATTG GGAATAAATT   
  
  
+ CGAGGACTTT TTCTTTGCCC TTTTTCCCTG ATTCCCAAAC CCACATCAAG TCAAGACGAA ACCAGCCATT   
  
  
+ GAAAAAAGAG GGTGTTTGTG TGAGATAAAG ACAGAAATTT TATGCCAGAG AGAGAAGATA ACAAATGCAA   
  
  
+ CAACGTCGAC AGAGGCTCTC CTTCTCACAA ATTCCATATT CCTCTGTTTT TTAAGAAAGA AAAAGAAAGT   
  
  
+ GTGAGACAAG AACAACACAA GCAAACAATT CAGTTGACGA CGAATACTGA GTGATACCAC GCACTGTGTG   
  
  
+ CAACTGCGTT TCCATTTGTG GAGTCCTTCA AAGCACGCTC ATCTTCAACC CTTCCTTCGC CACGGAATTT   
  
  
+ CAGGGGGAAA AAAACCCCCA CCTAATTTTG AGAGAGAGAG AGAACAGAGA GAACGGGCGA TACAGGGATA   
  
  
+ CAAGGTTTGA GCAATTTTAG AAAATTTCTT GGGGCCGTTG ATGGGTTTTG AGTGAATTGC AAATCCCAGA   
  
  
+ AAGATTTCTG CGGTTTTTTA TCTACGGCTC TCTCTGTGGG AATTTTTTGG TAGATTCCTC TGGTTCCCAC   
  
  
+ TTTCATATCT TCTTATTTGT TCTACCCTCT TTACATCTGA TAGTTTGTTC TAACTGCTGC TTCAGTTGGT   
  
  
+ GCTTGTTCTC TTTAGTACTT TTCTGTTTTG TTCGTTTTGT TCATGCACTT GAATTCAACA AGAGCCCTTG   
  
  
+ TCCATTTTGA GCTCTTAATT CCCATCTAAC CCTGTTTCCT TCAGAAATTG CTGTTCTGTT CATATAGTTA   
  
  
+ TATTTTTTAT GCTTTGATTT GGGTATAAGT TTGCTGTTGG TGATTCCAAA GCTTGGTACT TTTGGCCATA   
  
  
+ TTTTGTTTGA GTTGGTGTTG ATTGGTACAA ATCTTGTAAT TGGTGTGATT GTTAATGGGA CCAATGCTTC   
  
  
+ AAGATGATGG TTCATCAGTA ACTTCTTCAT CACCTCTTCA ATTTTTCTCC ATGATGTCAC CCAATTTGGG   
  
  
+ TTCCTCCTAC CCTTGGCTAA GGGACTTAAA GCCTGAAGAG AGAGGTCTTT ACTTGATACA TTTGTTGCTC   
  
  
+ ACTTGTGCAA ACCATGTCTC TAATGGTAGC CTTGACAATG CAAACCTAGC CCTTGAGCAA ATCTCCCAGC   
  
  
+ TTGCAGCCCC TGATGGCGAT ACAATCCAGC GTATTGTTGC CTACTTTGCT GAATCACTTG CTGAAAGGAT   
  
  
+ CCTTAAGTCA TGGCCTGGCC TATATAAAGC CCTTCATTTC AATAGAATGC CTGTTATTTC AGAAGAATTT   
  
  
+ CTTGCTAGGA AGCTGTTTTT TGAGTTGTTT CCCTTCTTGA AGCTGGCCTT TTTGGTGACT AACCAATCAA   
  
  
+ TAATCGAGGC CATGGAGGGG GAAAAGATGG TGCATATAAT TGATCTGAAT GCGTCAGAAC CTGCACAGTG   
  
  
+ GATTGCCCTT ATTCAAGACT TGAGTGCTCG GCCTGAGGGC CCTCCTCATT TGAGGATTAC CGGGGTTCAT   
  
  
+ CAACAGAAAG AGGTTTTAGA ACAAGTAGCT CATAGATTGA CTGAAGAAGC TGAGAAGTTG GATTTGCCAT   
  
  
+ TTCAGTTCAA TCCTGTGGTT TGCAAACTAG AGAATCTCGA CATCGGAAAA CTCCGTGTTA AGACCGGGGA   
  
  
+ GGCCTTGGCT ATTACCTCGG TCCTTCAACT GCATACCCTT TTGGCTTCTG AAGAGGAAGT CCTTAAGAAA   
  
  
+ AGTTCACCCT TGGCATTGGT AAAGCAAGCC AATGGGGCTA ATTTACAGGG CTTGTTCAAT AAAGATGGAG   
  
  
+ CTAATAATAG GCGTAGCCCA AGTAATGATT CGGCTTCATC TGCACCTTCA TCCCTCAACA CTTCAGCCAA   
  
  
+ GATGGAAGGT TTCCTTAGCG CTTTGTGGGG TTTATCCCCA AAGATTATGG TGATAACCGA GCAAGATTCC   
  
  
+ AACCACAATG GGGCAGGACT AATGGAGAGA TTGTCAGAAG CATTGTACTT CTATGCAGCA TTGTTCGATT   
  
  
+ GCTTAGAATT TACCCTCCCG AGAACCTCCG TGGAGAGAAG GAAGGTCGAG ATGCTCCTCC TTGGCAAGGA   
  
  
+ AATCAAGAAC ATCATAGCGT GTGAGGGAGG AGAAAGAATA GATAGGCATG AGAAGTTGGG GAAGTGGATT   
  
  
+ AAGAGGCTTG AGATGGCCGG GTTTGGAAGC GTTCCTTTGA GCCACATAGG CATGATCCAA GCAAGGCGGT   
  
  
+ TGTTGCAGAG CTATGGCTGT GATGGTTATA GAATAAAGGA GGAGAACGGA TGTTTTGTTA TCTGCTGGCA   
  
  
+ AGATCGCCCC CTCTTTTCAG TATCTGCTTG GAGATGTAGG AGGTG  

- -Up\_Stream \_Len000AGATGG AAATGGACAC AATTTTTTTT TTTAAAACAA AGGTAATGAT AAGAGAACCG   
  
  
- TGATAACCAC GACTAAAAAG AAGTGGTTTA GTTGGAGGAA TATAAACGGT TAAAATGACA GGAGAAAGTA   
  
  
- GATATTTCAG TTGAGAGGAT CATGTTAAAC AGTGTATCTT TTAGAGATCC GTGAGTAGAT TTGAGAGACA   
  
  
- AGACCCATTA ACTAGCTGCT AGATGTGGTA ATAAGCAAGA TCACGTGGCA AACCTGTCGG TTAGTACATG   
  
  
- GAAACCTCTA GTTGACGAGT ACTCAAGAAC CACACTTTAA CCCCTTCATT TAAGCTGAAA TCCCGTGACC   
  
  
- AGATAGTGCG GTACTAAAAT GGTAGATAAA AGTATATATA GGTTATTCAA ACTAGAAAGC GATAATTAGT   
  
  
- GTTACTATAT TTGTTCACCA CTTCTCGTTA TTACTTGGTA TGTAAAATTT GAATTCGCAA TTCCTTCTGT   
  
  
- ACTTCAATTT TTGTTACCAT TCTCGATACG CATACAAACC GTATATCGAA AAAATTCTCA CAAAACCGAT   
  
  
- ATCAGCCTCA AAAAATTAAT TCTAATCGAC AAACTAGTTT ATTTTTTCGA TTAAACTCAC AAACCGCTCT   
  
  
- TCTGAAAAAT ATTCTTGAAA AAAACCAGAT TTTTCAATTA AATTTTTCCG ATTAAGATAC TCGAAAAGCC   
  
  
- TTCTCAAAAA ATTTATTAAT TGAAAAACAG AGTATTCGGT ATTGAAATAG TCTTTGTCAA TTAAAATGTT   
  
  
- TTATTAAAGA GTTGTTTGTT GATTAAATTT ATTAATTATT TTTATTGATT GAGTTTATAG ATTATTGATT   
  
  
- ATTGATTATA TTTATTAATT GTCAATTGTC AACAAATGCT TTGTCTTAAA TATATTTTTC GCCATTGTCT   
  
  
- ATTTTCTATT AGTACGCTCT AATTTTCAGT GGTCACTGTG TGTTCATGAA TTTATTTATC AGATTTGTTA   
  
  
- CTATAAAAAA CGCTCGAAGG TACGTTTATG GCTGTGAAAA TAACGGAGTC TTCCTTTGTT TTTGACTCAC   
  
  
- TTCCCCCCCC CCCCAAGACA AACAGAAACA ATCCCGTGCA AACCTAAGTT CACATCTAAC CCTTATTTAA   
  
  
- GCTCCTGAAA AAGAAACGGG AAAAAGGGAC TAAGGGTTTG GGTGTAGTTC AGTTCTGCTT TGGTCGGTAA   
  
  
- CTTTTTTCTC CCACAAACAC ACTCTATTTC TGTCTTTAAA ATACGGTCTC TCTCTTCTAT TGTTTACGTT   
  
  
- GTTGCAGCTG TCTCCGAGAG GAAGAGTGTT TAAGGTATAA GGAGACAAAA AATTCTTTCT TTTTCTTTCA   
  
  
- CACTCTGTTC TTGTTGTGTT CGTTTGTTAA GTCAACTGCT GCTTATGACT CACTATGGTG CGTGACACAC   
  
  
- GTTGACGCAA AGGTAAACAC CTCAGGAAGT TTCGTGCGAG TAGAAGTTGG GAAGGAAGCG GTGCCTTAAA   
  
  
- GTCCCCCTTT TTTTGGGGGT GGATTAAAAC TCTCTCTCTC TCTTGTCTCT CTTGCCCGCT ATGTCCCTAT   
  
  
- GTTCCAAACT CGTTAAAATC TTTTAAAGAA CCCCGGCAAC TACCCAAAAC TCACTTAACG TTTAGGGTCT   
  
  
- TTCTAAAGAC GCCAAAAAAT AGATGCCGAG AGAGACACCC TTAAAAAACC ATCTAAGGAG ACCAAGGGTG   
  
  
- AAAGTATAGA AGAATAAACA AGATGGGAGA AATGTAGACT ATCAAACAAG ATTGACGACG AAGTCAACCA   
  
  
- CGAACAAGAG AAATCATGAA AAGACAAAAC AAGCAAAACA AGTACGTGAA CTTAAGTTGT TCTCGGGAAC   
  
  
- AGGTAAAACT CGAGAATTAA GGGTAGATTG GGACAAAGGA AGTCTTTAAC GACAAGACAA GTATATCAAT   
  
  
- ATAAAAAATA CGAAACTAAA CCCATATTCA AACGACAACC ACTAAGGTTT CGAACCATGA AAACCGGTAT   
  
  
- AAAACAAACT CAACCACAAC TAACCATGTT TAGAACATTA ACCACACTAA CAATTACCCT GGTTACGAAG   
  
  
- TTCTACTACC AAGTAGTCAT TGAAGAAGTA GTGGAGAAGT TAAAAAGAGG TACTACAGTG GGTTAAACCC   
  
  
- AAGGAGGATG GGAACCGATT CCCTGAATTT CGGACTTCTC TCTCCAGAAA TGAACTATGT AAACAACGAG   
  
  
- TGAACACGTT TGGTACAGAG ATTACCATCG GAACTGTTAC GTTTGGATCG GGAACTCGTT TAGAGGGTCG   
  
  
- AACGTCGGGG ACTACCGCTA TGTTAGGTCG CATAACAACG GATGAAACGA CTTAGTGAAC GACTTTCCTA   
  
  
- GGAATTCAGT ACCGGACCGG ATATATTTCG GGAAGTAAAG TTATCTTACG GACAATAAAG TCTTCTTAAA   
  
  
- GAACGATCCT TCGACAAAAA ACTCAACAAA GGGAAGAACT TCGACCGGAA AAACCACTGA TTGGTTAGTT   
  
  
- ATTAGCTCCG GTACCTCCCC CTTTTCTACC ACGTATATTA ACTAGACTTA CGCAGTCTTG GACGTGTCAC   
  
  
- CTAACGGGAA TAAGTTCTGA ACTCACGAGC CGGACTCCCG GGAGGAGTAA ACTCCTAATG GCCCCAAGTA   
  
  
- GTTGTCTTTC TCCAAAATCT TGTTCATCGA GTATCTAACT GACTTCTTCG ACTCTTCAAC CTAAACGGTA   
  
  
- AAGTCAAGTT AGGACACCAA ACGTTTGATC TCTTAGAGCT GTAGCCTTTT GAGGCACAAT TCTGGCCCCT   
  
  
- CCGGAACCGA TAATGGAGCC AGGAAGTTGA CGTATGGGAA AACCGAAGAC TTCTCCTTCA GGAATTCTTT   
  
  
- TCAAGTGGGA ACCGTAACCA TTTCGTTCGG TTACCCCGAT TAAATGTCCC GAACAAGTTA TTTCTACCTC   
  
  
- GATTATTATC CGCATCGGGT TCATTACTAA GCCGAAGTAG ACGTGGAAGT AGGGAGTTGT GAAGTCGGTT   
  
  
- CTACCTTCCA AAGGAATCGC GAAACACCCC AAATAGGGGT TTCTAATACC ACTATTGGCT CGTTCTAAGG   
  
  
- TTGGTGTTAC CCCGTCCTGA TTACCTCTCT AACAGTCTTC GTAACATGAA GATACGTCGT AACAAGCTAA   
  
  
- CGAATCTTAA ATGGGAGGGC TCTTGGAGGC ACCTCTCTTC CTTCCAGCTC TACGAGGAGG AACCGTTCCT   
  
  
- TTAGTTCTTG TAGTATCGCA CACTCCCTCC TCTTTCTTAT CTATCCGTAC TCTTCAACCC CTTCACCTAA   
  
  
- TTCTCCGAAC TCTACCGGCC CAAACCTTCG CAAGGAAACT CGGTGTATCC GTACTAGGTT CGTTCCGCCA   
  
  
- ACAACGTCTC GATACCGACA CTACCAATAT CTTATTTCCT CCTCTTGCCT ACAAAACAAT AGACGACCGT   
  
  
- TCTAGCGGGG GAGAAAAGTC ATAGACGAAC CTCTACATCC TCCAC

+     Unnamed\_\_2

| Site Name | Organism | Position | Strand | Matrix score. | sequence | function |
| --- | --- | --- | --- | --- | --- | --- |
| Unnamed\_\_2 | Zea mays | 2584 | - | 6 | CCCCGG |  |
| Unnamed\_\_2 | Zea mays | 2728 | - | 6 | CCCCGG |  |

>HU08G01232.1   
+ -Up\_Stream \_Len000TCTACC TTTACCTGTG TTAAAAAAAA AAATTTTGTT TCCATTACTA TTCTCTTGGC   
  
  
+ ACTATTGGTG CTGATTTTTC TTCACCAAAT CAACCTCCTT ATATTTGCCA ATTTTACTGT CCTCTTTCAT   
  
  
+ CTATAAAGTC AACTCTCCTA GTACAATTTG TCACATAGAA AATCTCTAGG CACTCATCTA AACTCTCTGT   
  
  
+ TCTGGGTAAT TGATCGACGA TCTACACCAT TATTCGTTCT AGTGCACCGT TTGGACAGCC AATCATGTAC   
  
  
+ CTTTGGAGAT CAACTGCTCA TGAGTTCTTG GTGTGAAATT GGGGAAGTAA ATTCGACTTT AGGGCACTGG   
  
  
+ TCTATCACGC CATGATTTTA CCATCTATTT TCATATATAT CCAATAAGTT TGATCTTTCG CTATTAATCA   
  
  
+ CAATGATATA AACAAGTGGT GAAGAGCAAT AATGAACCAT ACATTTTAAA CTTAAGCGTT AAGGAAGACA   
  
  
+ TGAAGTTAAA AACAATGGTA AGAGCTATGC GTATGTTTGG CATATAGCTT TTTTAAGAGT GTTTTGGCTA   
  
  
+ TAGTCGGAGT TTTTTAATTA AGATTAGCTG TTTGATCAAA TAAAAAAGCT AATTTGAGTG TTTGGCGAGA   
  
  
+ AGACTTTTTA TAAGAACTTT TTTTGGTCTA AAAAGTTAAT TTAAAAAGGC TAATTCTATG AGCTTTTCGG   
  
  
+ AAGAGTTTTT TAAATAATTA ACTTTTTGTC TCATAAGCCA TAACTTTATC AGAAACAGTT AATTTTACAA   
  
  
+ AATAATTTCT CAACAAACAA CTAATTTAAA TAATTAATAA AAATAACTAA CTCAAATATC TAATAACTAA   
  
  
+ TAACTAATAT AAATAATTAA CAGTTAACAG TTGTTTACGA AACAGAATTT ATATAAAAAG CGGTAACAGA   
  
  
+ TAAAAGATAA TCATGCGAGA TTAAAAGTCA CCAGTGACAC ACAAGTACTT AAATAAATAG TCTAAACAAT   
  
  
+ GATATTTTTT GCGAGCTTCC ATGCAAATAC CGACACTTTT ATTGCCTCAG AAGGAAACAA AAACTGAGTG   
  
  
+ AAGGGGGGGG GGGGTTCTGT TTGTCTTTGT TAGGGCACGT TTGGATTCAA GTGTAGATTG GGAATAAATT   
  
  
+ CGAGGACTTT TTCTTTGCCC TTTTTCCCTG ATTCCCAAAC CCACATCAAG TCAAGACGAA ACCAGCCATT   
  
  
+ GAAAAAAGAG GGTGTTTGTG TGAGATAAAG ACAGAAATTT TATGCCAGAG AGAGAAGATA ACAAATGCAA   
  
  
+ CAACGTCGAC AGAGGCTCTC CTTCTCACAA ATTCCATATT CCTCTGTTTT TTAAGAAAGA AAAAGAAAGT   
  
  
+ GTGAGACAAG AACAACACAA GCAAACAATT CAGTTGACGA CGAATACTGA GTGATACCAC GCACTGTGTG   
  
  
+ CAACTGCGTT TCCATTTGTG GAGTCCTTCA AAGCACGCTC ATCTTCAACC CTTCCTTCGC CACGGAATTT   
  
  
+ CAGGGGGAAA AAAACCCCCA CCTAATTTTG AGAGAGAGAG AGAACAGAGA GAACGGGCGA TACAGGGATA   
  
  
+ CAAGGTTTGA GCAATTTTAG AAAATTTCTT GGGGCCGTTG ATGGGTTTTG AGTGAATTGC AAATCCCAGA   
  
  
+ AAGATTTCTG CGGTTTTTTA TCTACGGCTC TCTCTGTGGG AATTTTTTGG TAGATTCCTC TGGTTCCCAC   
  
  
+ TTTCATATCT TCTTATTTGT TCTACCCTCT TTACATCTGA TAGTTTGTTC TAACTGCTGC TTCAGTTGGT   
  
  
+ GCTTGTTCTC TTTAGTACTT TTCTGTTTTG TTCGTTTTGT TCATGCACTT GAATTCAACA AGAGCCCTTG   
  
  
+ TCCATTTTGA GCTCTTAATT CCCATCTAAC CCTGTTTCCT TCAGAAATTG CTGTTCTGTT CATATAGTTA   
  
  
+ TATTTTTTAT GCTTTGATTT GGGTATAAGT TTGCTGTTGG TGATTCCAAA GCTTGGTACT TTTGGCCATA   
  
  
+ TTTTGTTTGA GTTGGTGTTG ATTGGTACAA ATCTTGTAAT TGGTGTGATT GTTAATGGGA CCAATGCTTC   
  
  
+ AAGATGATGG TTCATCAGTA ACTTCTTCAT CACCTCTTCA ATTTTTCTCC ATGATGTCAC CCAATTTGGG   
  
  
+ TTCCTCCTAC CCTTGGCTAA GGGACTTAAA GCCTGAAGAG AGAGGTCTTT ACTTGATACA TTTGTTGCTC   
  
  
+ ACTTGTGCAA ACCATGTCTC TAATGGTAGC CTTGACAATG CAAACCTAGC CCTTGAGCAA ATCTCCCAGC   
  
  
+ TTGCAGCCCC TGATGGCGAT ACAATCCAGC GTATTGTTGC CTACTTTGCT GAATCACTTG CTGAAAGGAT   
  
  
+ CCTTAAGTCA TGGCCTGGCC TATATAAAGC CCTTCATTTC AATAGAATGC CTGTTATTTC AGAAGAATTT   
  
  
+ CTTGCTAGGA AGCTGTTTTT TGAGTTGTTT CCCTTCTTGA AGCTGGCCTT TTTGGTGACT AACCAATCAA   
  
  
+ TAATCGAGGC CATGGAGGGG GAAAAGATGG TGCATATAAT TGATCTGAAT GCGTCAGAAC CTGCACAGTG   
  
  
+ GATTGCCCTT ATTCAAGACT TGAGTGCTCG GCCTGAGGGC CCTCCTCATT TGAGGATTAC CGGGGTTCAT   
  
  
+ CAACAGAAAG AGGTTTTAGA ACAAGTAGCT CATAGATTGA CTGAAGAAGC TGAGAAGTTG GATTTGCCAT   
  
  
+ TTCAGTTCAA TCCTGTGGTT TGCAAACTAG AGAATCTCGA CATCGGAAAA CTCCGTGTTA AGACCGGGGA   
  
  
+ GGCCTTGGCT ATTACCTCGG TCCTTCAACT GCATACCCTT TTGGCTTCTG AAGAGGAAGT CCTTAAGAAA   
  
  
+ AGTTCACCCT TGGCATTGGT AAAGCAAGCC AATGGGGCTA ATTTACAGGG CTTGTTCAAT AAAGATGGAG   
  
  
+ CTAATAATAG GCGTAGCCCA AGTAATGATT CGGCTTCATC TGCACCTTCA TCCCTCAACA CTTCAGCCAA   
  
  
+ GATGGAAGGT TTCCTTAGCG CTTTGTGGGG TTTATCCCCA AAGATTATGG TGATAACCGA GCAAGATTCC   
  
  
+ AACCACAATG GGGCAGGACT AATGGAGAGA TTGTCAGAAG CATTGTACTT CTATGCAGCA TTGTTCGATT   
  
  
+ GCTTAGAATT TACCCTCCCG AGAACCTCCG TGGAGAGAAG GAAGGTCGAG ATGCTCCTCC TTGGCAAGGA   
  
  
+ AATCAAGAAC ATCATAGCGT GTGAGGGAGG AGAAAGAATA GATAGGCATG AGAAGTTGGG GAAGTGGATT   
  
  
+ AAGAGGCTTG AGATGGCCGG GTTTGGAAGC GTTCCTTTGA GCCACATAGG CATGATCCAA GCAAGGCGGT   
  
  
+ TGTTGCAGAG CTATGGCTGT GATGGTTATA GAATAAAGGA GGAGAACGGA TGTTTTGTTA TCTGCTGGCA   
  
  
+ AGATCGCCCC CTCTTTTCAG TATCTGCTTG GAGATGTAGG AGGTG  

- -Up\_Stream \_Len000AGATGG AAATGGACAC AATTTTTTTT TTTAAAACAA AGGTAATGAT AAGAGAACCG   
  
  
- TGATAACCAC GACTAAAAAG AAGTGGTTTA GTTGGAGGAA TATAAACGGT TAAAATGACA GGAGAAAGTA   
  
  
- GATATTTCAG TTGAGAGGAT CATGTTAAAC AGTGTATCTT TTAGAGATCC GTGAGTAGAT TTGAGAGACA   
  
  
- AGACCCATTA ACTAGCTGCT AGATGTGGTA ATAAGCAAGA TCACGTGGCA AACCTGTCGG TTAGTACATG   
  
  
- GAAACCTCTA GTTGACGAGT ACTCAAGAAC CACACTTTAA CCCCTTCATT TAAGCTGAAA TCCCGTGACC   
  
  
- AGATAGTGCG GTACTAAAAT GGTAGATAAA AGTATATATA GGTTATTCAA ACTAGAAAGC GATAATTAGT   
  
  
- GTTACTATAT TTGTTCACCA CTTCTCGTTA TTACTTGGTA TGTAAAATTT GAATTCGCAA TTCCTTCTGT   
  
  
- ACTTCAATTT TTGTTACCAT TCTCGATACG CATACAAACC GTATATCGAA AAAATTCTCA CAAAACCGAT   
  
  
- ATCAGCCTCA AAAAATTAAT TCTAATCGAC AAACTAGTTT ATTTTTTCGA TTAAACTCAC AAACCGCTCT   
  
  
- TCTGAAAAAT ATTCTTGAAA AAAACCAGAT TTTTCAATTA AATTTTTCCG ATTAAGATAC TCGAAAAGCC   
  
  
- TTCTCAAAAA ATTTATTAAT TGAAAAACAG AGTATTCGGT ATTGAAATAG TCTTTGTCAA TTAAAATGTT   
  
  
- TTATTAAAGA GTTGTTTGTT GATTAAATTT ATTAATTATT TTTATTGATT GAGTTTATAG ATTATTGATT   
  
  
- ATTGATTATA TTTATTAATT GTCAATTGTC AACAAATGCT TTGTCTTAAA TATATTTTTC GCCATTGTCT   
  
  
- ATTTTCTATT AGTACGCTCT AATTTTCAGT GGTCACTGTG TGTTCATGAA TTTATTTATC AGATTTGTTA   
  
  
- CTATAAAAAA CGCTCGAAGG TACGTTTATG GCTGTGAAAA TAACGGAGTC TTCCTTTGTT TTTGACTCAC   
  
  
- TTCCCCCCCC CCCCAAGACA AACAGAAACA ATCCCGTGCA AACCTAAGTT CACATCTAAC CCTTATTTAA   
  
  
- GCTCCTGAAA AAGAAACGGG AAAAAGGGAC TAAGGGTTTG GGTGTAGTTC AGTTCTGCTT TGGTCGGTAA   
  
  
- CTTTTTTCTC CCACAAACAC ACTCTATTTC TGTCTTTAAA ATACGGTCTC TCTCTTCTAT TGTTTACGTT   
  
  
- GTTGCAGCTG TCTCCGAGAG GAAGAGTGTT TAAGGTATAA GGAGACAAAA AATTCTTTCT TTTTCTTTCA   
  
  
- CACTCTGTTC TTGTTGTGTT CGTTTGTTAA GTCAACTGCT GCTTATGACT CACTATGGTG CGTGACACAC   
  
  
- GTTGACGCAA AGGTAAACAC CTCAGGAAGT TTCGTGCGAG TAGAAGTTGG GAAGGAAGCG GTGCCTTAAA   
  
  
- GTCCCCCTTT TTTTGGGGGT GGATTAAAAC TCTCTCTCTC TCTTGTCTCT CTTGCCCGCT ATGTCCCTAT   
  
  
- GTTCCAAACT CGTTAAAATC TTTTAAAGAA CCCCGGCAAC TACCCAAAAC TCACTTAACG TTTAGGGTCT   
  
  
- TTCTAAAGAC GCCAAAAAAT AGATGCCGAG AGAGACACCC TTAAAAAACC ATCTAAGGAG ACCAAGGGTG   
  
  
- AAAGTATAGA AGAATAAACA AGATGGGAGA AATGTAGACT ATCAAACAAG ATTGACGACG AAGTCAACCA   
  
  
- CGAACAAGAG AAATCATGAA AAGACAAAAC AAGCAAAACA AGTACGTGAA CTTAAGTTGT TCTCGGGAAC   
  
  
- AGGTAAAACT CGAGAATTAA GGGTAGATTG GGACAAAGGA AGTCTTTAAC GACAAGACAA GTATATCAAT   
  
  
- ATAAAAAATA CGAAACTAAA CCCATATTCA AACGACAACC ACTAAGGTTT CGAACCATGA AAACCGGTAT   
  
  
- AAAACAAACT CAACCACAAC TAACCATGTT TAGAACATTA ACCACACTAA CAATTACCCT GGTTACGAAG   
  
  
- TTCTACTACC AAGTAGTCAT TGAAGAAGTA GTGGAGAAGT TAAAAAGAGG TACTACAGTG GGTTAAACCC   
  
  
- AAGGAGGATG GGAACCGATT CCCTGAATTT CGGACTTCTC TCTCCAGAAA TGAACTATGT AAACAACGAG   
  
  
- TGAACACGTT TGGTACAGAG ATTACCATCG GAACTGTTAC GTTTGGATCG GGAACTCGTT TAGAGGGTCG   
  
  
- AACGTCGGGG ACTACCGCTA TGTTAGGTCG CATAACAACG GATGAAACGA CTTAGTGAAC GACTTTCCTA   
  
  
- GGAATTCAGT ACCGGACCGG ATATATTTCG GGAAGTAAAG TTATCTTACG GACAATAAAG TCTTCTTAAA   
  
  
- GAACGATCCT TCGACAAAAA ACTCAACAAA GGGAAGAACT TCGACCGGAA AAACCACTGA TTGGTTAGTT   
  
  
- ATTAGCTCCG GTACCTCCCC CTTTTCTACC ACGTATATTA ACTAGACTTA CGCAGTCTTG GACGTGTCAC   
  
  
- CTAACGGGAA TAAGTTCTGA ACTCACGAGC CGGACTCCCG GGAGGAGTAA ACTCCTAATG GCCCCAAGTA   
  
  
- GTTGTCTTTC TCCAAAATCT TGTTCATCGA GTATCTAACT GACTTCTTCG ACTCTTCAAC CTAAACGGTA   
  
  
- AAGTCAAGTT AGGACACCAA ACGTTTGATC TCTTAGAGCT GTAGCCTTTT GAGGCACAAT TCTGGCCCCT   
  
  
- CCGGAACCGA TAATGGAGCC AGGAAGTTGA CGTATGGGAA AACCGAAGAC TTCTCCTTCA GGAATTCTTT   
  
  
- TCAAGTGGGA ACCGTAACCA TTTCGTTCGG TTACCCCGAT TAAATGTCCC GAACAAGTTA TTTCTACCTC   
  
  
- GATTATTATC CGCATCGGGT TCATTACTAA GCCGAAGTAG ACGTGGAAGT AGGGAGTTGT GAAGTCGGTT   
  
  
- CTACCTTCCA AAGGAATCGC GAAACACCCC AAATAGGGGT TTCTAATACC ACTATTGGCT CGTTCTAAGG   
  
  
- TTGGTGTTAC CCCGTCCTGA TTACCTCTCT AACAGTCTTC GTAACATGAA GATACGTCGT AACAAGCTAA   
  
  
- CGAATCTTAA ATGGGAGGGC TCTTGGAGGC ACCTCTCTTC CTTCCAGCTC TACGAGGAGG AACCGTTCCT   
  
  
- TTAGTTCTTG TAGTATCGCA CACTCCCTCC TCTTTCTTAT CTATCCGTAC TCTTCAACCC CTTCACCTAA   
  
  
- TTCTCCGAAC TCTACCGGCC CAAACCTTCG CAAGGAAACT CGGTGTATCC GTACTAGGTT CGTTCCGCCA   
  
  
- ACAACGTCTC GATACCGACA CTACCAATAT CTTATTTCCT CCTCTTGCCT ACAAAACAAT AGACGACCGT   
  
  
- TCTAGCGGGG GAGAAAAGTC ATAGACGAAC CTCTACATCC TCCAC

+     Unnamed\_\_4

| Site Name | Organism | Position | Strand | Matrix score. | sequence | function |
| --- | --- | --- | --- | --- | --- | --- |
| Unnamed\_\_4 | Petroselinum hortense | 3394 | - | 4 | CTCC |  |
| Unnamed\_\_4 | Petroselinum hortense | 2108 | + | 4 | CTCC |  |
| Unnamed\_\_4 | Petroselinum hortense | 3403 | - | 4 | CTCC |  |
| Unnamed\_\_4 | Petroselinum hortense | 3335 | - | 4 | CTCC |  |
| Unnamed\_\_4 | Petroselinum hortense | 3141 | + | 4 | CTCC |  |
| Unnamed\_\_4 | Petroselinum hortense | 2871 | - | 4 | CTCC |  |
| Unnamed\_\_4 | Petroselinum hortense | 2081 | + | 4 | CTCC |  |
| Unnamed\_\_4 | Petroselinum hortense | 109 | + | 4 | CTCC |  |
| Unnamed\_\_4 | Petroselinum hortense | 2732 | - | 4 | CTCC |  |
| Unnamed\_\_4 | Petroselinum hortense | 3138 | + | 4 | CTCC |  |
| Unnamed\_\_4 | Petroselinum hortense | 3332 | - | 4 | CTCC |  |
| Unnamed\_\_4 | Petroselinum hortense | 2468 | - | 4 | CTCC |  |
| Unnamed\_\_4 | Petroselinum hortense | 3110 | + | 4 | CTCC |  |
| Unnamed\_\_4 | Petroselinum hortense | 3180 | - | 4 | CTCC |  |
| Unnamed\_\_4 | Petroselinum hortense | 3183 | - | 4 | CTCC |  |
| Unnamed\_\_4 | Petroselinum hortense | 289 | - | 4 | CTCC |  |
| Unnamed\_\_4 | Petroselinum hortense | 3116 | - | 4 | CTCC |  |
| Unnamed\_\_4 | Petroselinum hortense | 2237 | + | 4 | CTCC |  |
| Unnamed\_\_4 | Petroselinum hortense | 1424 | - | 4 | CTCC |  |
| Unnamed\_\_4 | Petroselinum hortense | 2715 | + | 4 | CTCC |  |
| Unnamed\_\_4 | Petroselinum hortense | 3038 | - | 4 | CTCC |  |
| Unnamed\_\_4 | Petroselinum hortense | 3099 | + | 4 | CTCC |  |
| Unnamed\_\_4 | Petroselinum hortense | 2566 | + | 4 | CTCC |  |
| Unnamed\_\_4 | Petroselinum hortense | 570 | - | 4 | CTCC |  |
| Unnamed\_\_4 | Petroselinum hortense | 1282 | + | 4 | CTCC |  |
| Unnamed\_\_4 | Petroselinum hortense | 159 | + | 4 | CTCC |  |

>HU08G01232.1   
+ -Up\_Stream \_Len000TCTACC TTTACCTGTG TTAAAAAAAA AAATTTTGTT TCCATTACTA TTCTCTTGGC   
  
  
+ ACTATTGGTG CTGATTTTTC TTCACCAAAT CAACCTCCTT ATATTTGCCA ATTTTACTGT CCTCTTTCAT   
  
  
+ CTATAAAGTC AACTCTCCTA GTACAATTTG TCACATAGAA AATCTCTAGG CACTCATCTA AACTCTCTGT   
  
  
+ TCTGGGTAAT TGATCGACGA TCTACACCAT TATTCGTTCT AGTGCACCGT TTGGACAGCC AATCATGTAC   
  
  
+ CTTTGGAGAT CAACTGCTCA TGAGTTCTTG GTGTGAAATT GGGGAAGTAA ATTCGACTTT AGGGCACTGG   
  
  
+ TCTATCACGC CATGATTTTA CCATCTATTT TCATATATAT CCAATAAGTT TGATCTTTCG CTATTAATCA   
  
  
+ CAATGATATA AACAAGTGGT GAAGAGCAAT AATGAACCAT ACATTTTAAA CTTAAGCGTT AAGGAAGACA   
  
  
+ TGAAGTTAAA AACAATGGTA AGAGCTATGC GTATGTTTGG CATATAGCTT TTTTAAGAGT GTTTTGGCTA   
  
  
+ TAGTCGGAGT TTTTTAATTA AGATTAGCTG TTTGATCAAA TAAAAAAGCT AATTTGAGTG TTTGGCGAGA   
  
  
+ AGACTTTTTA TAAGAACTTT TTTTGGTCTA AAAAGTTAAT TTAAAAAGGC TAATTCTATG AGCTTTTCGG   
  
  
+ AAGAGTTTTT TAAATAATTA ACTTTTTGTC TCATAAGCCA TAACTTTATC AGAAACAGTT AATTTTACAA   
  
  
+ AATAATTTCT CAACAAACAA CTAATTTAAA TAATTAATAA AAATAACTAA CTCAAATATC TAATAACTAA   
  
  
+ TAACTAATAT AAATAATTAA CAGTTAACAG TTGTTTACGA AACAGAATTT ATATAAAAAG CGGTAACAGA   
  
  
+ TAAAAGATAA TCATGCGAGA TTAAAAGTCA CCAGTGACAC ACAAGTACTT AAATAAATAG TCTAAACAAT   
  
  
+ GATATTTTTT GCGAGCTTCC ATGCAAATAC CGACACTTTT ATTGCCTCAG AAGGAAACAA AAACTGAGTG   
  
  
+ AAGGGGGGGG GGGGTTCTGT TTGTCTTTGT TAGGGCACGT TTGGATTCAA GTGTAGATTG GGAATAAATT   
  
  
+ CGAGGACTTT TTCTTTGCCC TTTTTCCCTG ATTCCCAAAC CCACATCAAG TCAAGACGAA ACCAGCCATT   
  
  
+ GAAAAAAGAG GGTGTTTGTG TGAGATAAAG ACAGAAATTT TATGCCAGAG AGAGAAGATA ACAAATGCAA   
  
  
+ CAACGTCGAC AGAGGCTCTC CTTCTCACAA ATTCCATATT CCTCTGTTTT TTAAGAAAGA AAAAGAAAGT   
  
  
+ GTGAGACAAG AACAACACAA GCAAACAATT CAGTTGACGA CGAATACTGA GTGATACCAC GCACTGTGTG   
  
  
+ CAACTGCGTT TCCATTTGTG GAGTCCTTCA AAGCACGCTC ATCTTCAACC CTTCCTTCGC CACGGAATTT   
  
  
+ CAGGGGGAAA AAAACCCCCA CCTAATTTTG AGAGAGAGAG AGAACAGAGA GAACGGGCGA TACAGGGATA   
  
  
+ CAAGGTTTGA GCAATTTTAG AAAATTTCTT GGGGCCGTTG ATGGGTTTTG AGTGAATTGC AAATCCCAGA   
  
  
+ AAGATTTCTG CGGTTTTTTA TCTACGGCTC TCTCTGTGGG AATTTTTTGG TAGATTCCTC TGGTTCCCAC   
  
  
+ TTTCATATCT TCTTATTTGT TCTACCCTCT TTACATCTGA TAGTTTGTTC TAACTGCTGC TTCAGTTGGT   
  
  
+ GCTTGTTCTC TTTAGTACTT TTCTGTTTTG TTCGTTTTGT TCATGCACTT GAATTCAACA AGAGCCCTTG   
  
  
+ TCCATTTTGA GCTCTTAATT CCCATCTAAC CCTGTTTCCT TCAGAAATTG CTGTTCTGTT CATATAGTTA   
  
  
+ TATTTTTTAT GCTTTGATTT GGGTATAAGT TTGCTGTTGG TGATTCCAAA GCTTGGTACT TTTGGCCATA   
  
  
+ TTTTGTTTGA GTTGGTGTTG ATTGGTACAA ATCTTGTAAT TGGTGTGATT GTTAATGGGA CCAATGCTTC   
  
  
+ AAGATGATGG TTCATCAGTA ACTTCTTCAT CACCTCTTCA ATTTTTCTCC ATGATGTCAC CCAATTTGGG   
  
  
+ TTCCTCCTAC CCTTGGCTAA GGGACTTAAA GCCTGAAGAG AGAGGTCTTT ACTTGATACA TTTGTTGCTC   
  
  
+ ACTTGTGCAA ACCATGTCTC TAATGGTAGC CTTGACAATG CAAACCTAGC CCTTGAGCAA ATCTCCCAGC   
  
  
+ TTGCAGCCCC TGATGGCGAT ACAATCCAGC GTATTGTTGC CTACTTTGCT GAATCACTTG CTGAAAGGAT   
  
  
+ CCTTAAGTCA TGGCCTGGCC TATATAAAGC CCTTCATTTC AATAGAATGC CTGTTATTTC AGAAGAATTT   
  
  
+ CTTGCTAGGA AGCTGTTTTT TGAGTTGTTT CCCTTCTTGA AGCTGGCCTT TTTGGTGACT AACCAATCAA   
  
  
+ TAATCGAGGC CATGGAGGGG GAAAAGATGG TGCATATAAT TGATCTGAAT GCGTCAGAAC CTGCACAGTG   
  
  
+ GATTGCCCTT ATTCAAGACT TGAGTGCTCG GCCTGAGGGC CCTCCTCATT TGAGGATTAC CGGGGTTCAT   
  
  
+ CAACAGAAAG AGGTTTTAGA ACAAGTAGCT CATAGATTGA CTGAAGAAGC TGAGAAGTTG GATTTGCCAT   
  
  
+ TTCAGTTCAA TCCTGTGGTT TGCAAACTAG AGAATCTCGA CATCGGAAAA CTCCGTGTTA AGACCGGGGA   
  
  
+ GGCCTTGGCT ATTACCTCGG TCCTTCAACT GCATACCCTT TTGGCTTCTG AAGAGGAAGT CCTTAAGAAA   
  
  
+ AGTTCACCCT TGGCATTGGT AAAGCAAGCC AATGGGGCTA ATTTACAGGG CTTGTTCAAT AAAGATGGAG   
  
  
+ CTAATAATAG GCGTAGCCCA AGTAATGATT CGGCTTCATC TGCACCTTCA TCCCTCAACA CTTCAGCCAA   
  
  
+ GATGGAAGGT TTCCTTAGCG CTTTGTGGGG TTTATCCCCA AAGATTATGG TGATAACCGA GCAAGATTCC   
  
  
+ AACCACAATG GGGCAGGACT AATGGAGAGA TTGTCAGAAG CATTGTACTT CTATGCAGCA TTGTTCGATT   
  
  
+ GCTTAGAATT TACCCTCCCG AGAACCTCCG TGGAGAGAAG GAAGGTCGAG ATGCTCCTCC TTGGCAAGGA   
  
  
+ AATCAAGAAC ATCATAGCGT GTGAGGGAGG AGAAAGAATA GATAGGCATG AGAAGTTGGG GAAGTGGATT   
  
  
+ AAGAGGCTTG AGATGGCCGG GTTTGGAAGC GTTCCTTTGA GCCACATAGG CATGATCCAA GCAAGGCGGT   
  
  
+ TGTTGCAGAG CTATGGCTGT GATGGTTATA GAATAAAGGA GGAGAACGGA TGTTTTGTTA TCTGCTGGCA   
  
  
+ AGATCGCCCC CTCTTTTCAG TATCTGCTTG GAGATGTAGG AGGTG  

- -Up\_Stream \_Len000AGATGG AAATGGACAC AATTTTTTTT TTTAAAACAA AGGTAATGAT AAGAGAACCG   
  
  
- TGATAACCAC GACTAAAAAG AAGTGGTTTA GTTGGAGGAA TATAAACGGT TAAAATGACA GGAGAAAGTA   
  
  
- GATATTTCAG TTGAGAGGAT CATGTTAAAC AGTGTATCTT TTAGAGATCC GTGAGTAGAT TTGAGAGACA   
  
  
- AGACCCATTA ACTAGCTGCT AGATGTGGTA ATAAGCAAGA TCACGTGGCA AACCTGTCGG TTAGTACATG   
  
  
- GAAACCTCTA GTTGACGAGT ACTCAAGAAC CACACTTTAA CCCCTTCATT TAAGCTGAAA TCCCGTGACC   
  
  
- AGATAGTGCG GTACTAAAAT GGTAGATAAA AGTATATATA GGTTATTCAA ACTAGAAAGC GATAATTAGT   
  
  
- GTTACTATAT TTGTTCACCA CTTCTCGTTA TTACTTGGTA TGTAAAATTT GAATTCGCAA TTCCTTCTGT   
  
  
- ACTTCAATTT TTGTTACCAT TCTCGATACG CATACAAACC GTATATCGAA AAAATTCTCA CAAAACCGAT   
  
  
- ATCAGCCTCA AAAAATTAAT TCTAATCGAC AAACTAGTTT ATTTTTTCGA TTAAACTCAC AAACCGCTCT   
  
  
- TCTGAAAAAT ATTCTTGAAA AAAACCAGAT TTTTCAATTA AATTTTTCCG ATTAAGATAC TCGAAAAGCC   
  
  
- TTCTCAAAAA ATTTATTAAT TGAAAAACAG AGTATTCGGT ATTGAAATAG TCTTTGTCAA TTAAAATGTT   
  
  
- TTATTAAAGA GTTGTTTGTT GATTAAATTT ATTAATTATT TTTATTGATT GAGTTTATAG ATTATTGATT   
  
  
- ATTGATTATA TTTATTAATT GTCAATTGTC AACAAATGCT TTGTCTTAAA TATATTTTTC GCCATTGTCT   
  
  
- ATTTTCTATT AGTACGCTCT AATTTTCAGT GGTCACTGTG TGTTCATGAA TTTATTTATC AGATTTGTTA   
  
  
- CTATAAAAAA CGCTCGAAGG TACGTTTATG GCTGTGAAAA TAACGGAGTC TTCCTTTGTT TTTGACTCAC   
  
  
- TTCCCCCCCC CCCCAAGACA AACAGAAACA ATCCCGTGCA AACCTAAGTT CACATCTAAC CCTTATTTAA   
  
  
- GCTCCTGAAA AAGAAACGGG AAAAAGGGAC TAAGGGTTTG GGTGTAGTTC AGTTCTGCTT TGGTCGGTAA   
  
  
- CTTTTTTCTC CCACAAACAC ACTCTATTTC TGTCTTTAAA ATACGGTCTC TCTCTTCTAT TGTTTACGTT   
  
  
- GTTGCAGCTG TCTCCGAGAG GAAGAGTGTT TAAGGTATAA GGAGACAAAA AATTCTTTCT TTTTCTTTCA   
  
  
- CACTCTGTTC TTGTTGTGTT CGTTTGTTAA GTCAACTGCT GCTTATGACT CACTATGGTG CGTGACACAC   
  
  
- GTTGACGCAA AGGTAAACAC CTCAGGAAGT TTCGTGCGAG TAGAAGTTGG GAAGGAAGCG GTGCCTTAAA   
  
  
- GTCCCCCTTT TTTTGGGGGT GGATTAAAAC TCTCTCTCTC TCTTGTCTCT CTTGCCCGCT ATGTCCCTAT   
  
  
- GTTCCAAACT CGTTAAAATC TTTTAAAGAA CCCCGGCAAC TACCCAAAAC TCACTTAACG TTTAGGGTCT   
  
  
- TTCTAAAGAC GCCAAAAAAT AGATGCCGAG AGAGACACCC TTAAAAAACC ATCTAAGGAG ACCAAGGGTG   
  
  
- AAAGTATAGA AGAATAAACA AGATGGGAGA AATGTAGACT ATCAAACAAG ATTGACGACG AAGTCAACCA   
  
  
- CGAACAAGAG AAATCATGAA AAGACAAAAC AAGCAAAACA AGTACGTGAA CTTAAGTTGT TCTCGGGAAC   
  
  
- AGGTAAAACT CGAGAATTAA GGGTAGATTG GGACAAAGGA AGTCTTTAAC GACAAGACAA GTATATCAAT   
  
  
- ATAAAAAATA CGAAACTAAA CCCATATTCA AACGACAACC ACTAAGGTTT CGAACCATGA AAACCGGTAT   
  
  
- AAAACAAACT CAACCACAAC TAACCATGTT TAGAACATTA ACCACACTAA CAATTACCCT GGTTACGAAG   
  
  
- TTCTACTACC AAGTAGTCAT TGAAGAAGTA GTGGAGAAGT TAAAAAGAGG TACTACAGTG GGTTAAACCC   
  
  
- AAGGAGGATG GGAACCGATT CCCTGAATTT CGGACTTCTC TCTCCAGAAA TGAACTATGT AAACAACGAG   
  
  
- TGAACACGTT TGGTACAGAG ATTACCATCG GAACTGTTAC GTTTGGATCG GGAACTCGTT TAGAGGGTCG   
  
  
- AACGTCGGGG ACTACCGCTA TGTTAGGTCG CATAACAACG GATGAAACGA CTTAGTGAAC GACTTTCCTA   
  
  
- GGAATTCAGT ACCGGACCGG ATATATTTCG GGAAGTAAAG TTATCTTACG GACAATAAAG TCTTCTTAAA   
  
  
- GAACGATCCT TCGACAAAAA ACTCAACAAA GGGAAGAACT TCGACCGGAA AAACCACTGA TTGGTTAGTT   
  
  
- ATTAGCTCCG GTACCTCCCC CTTTTCTACC ACGTATATTA ACTAGACTTA CGCAGTCTTG GACGTGTCAC   
  
  
- CTAACGGGAA TAAGTTCTGA ACTCACGAGC CGGACTCCCG GGAGGAGTAA ACTCCTAATG GCCCCAAGTA   
  
  
- GTTGTCTTTC TCCAAAATCT TGTTCATCGA GTATCTAACT GACTTCTTCG ACTCTTCAAC CTAAACGGTA   
  
  
- AAGTCAAGTT AGGACACCAA ACGTTTGATC TCTTAGAGCT GTAGCCTTTT GAGGCACAAT TCTGGCCCCT   
  
  
- CCGGAACCGA TAATGGAGCC AGGAAGTTGA CGTATGGGAA AACCGAAGAC TTCTCCTTCA GGAATTCTTT   
  
  
- TCAAGTGGGA ACCGTAACCA TTTCGTTCGG TTACCCCGAT TAAATGTCCC GAACAAGTTA TTTCTACCTC   
  
  
- GATTATTATC CGCATCGGGT TCATTACTAA GCCGAAGTAG ACGTGGAAGT AGGGAGTTGT GAAGTCGGTT   
  
  
- CTACCTTCCA AAGGAATCGC GAAACACCCC AAATAGGGGT TTCTAATACC ACTATTGGCT CGTTCTAAGG   
  
  
- TTGGTGTTAC CCCGTCCTGA TTACCTCTCT AACAGTCTTC GTAACATGAA GATACGTCGT AACAAGCTAA   
  
  
- CGAATCTTAA ATGGGAGGGC TCTTGGAGGC ACCTCTCTTC CTTCCAGCTC TACGAGGAGG AACCGTTCCT   
  
  
- TTAGTTCTTG TAGTATCGCA CACTCCCTCC TCTTTCTTAT CTATCCGTAC TCTTCAACCC CTTCACCTAA   
  
  
- TTCTCCGAAC TCTACCGGCC CAAACCTTCG CAAGGAAACT CGGTGTATCC GTACTAGGTT CGTTCCGCCA   
  
  
- ACAACGTCTC GATACCGACA CTACCAATAT CTTATTTCCT CCTCTTGCCT ACAAAACAAT AGACGACCGT   
  
  
- TCTAGCGGGG GAGAAAAGTC ATAGACGAAC CTCTACATCC TCCAC

+     WRE3

| Site Name | Organism | Position | Strand | Matrix score. | sequence | function |
| --- | --- | --- | --- | --- | --- | --- |
| WRE3 | Pisum sativum | 1492 | + | 6 | CCACCT |  |

>HU08G01232.1   
+ -Up\_Stream \_Len000TCTACC TTTACCTGTG TTAAAAAAAA AAATTTTGTT TCCATTACTA TTCTCTTGGC   
  
  
+ ACTATTGGTG CTGATTTTTC TTCACCAAAT CAACCTCCTT ATATTTGCCA ATTTTACTGT CCTCTTTCAT   
  
  
+ CTATAAAGTC AACTCTCCTA GTACAATTTG TCACATAGAA AATCTCTAGG CACTCATCTA AACTCTCTGT   
  
  
+ TCTGGGTAAT TGATCGACGA TCTACACCAT TATTCGTTCT AGTGCACCGT TTGGACAGCC AATCATGTAC   
  
  
+ CTTTGGAGAT CAACTGCTCA TGAGTTCTTG GTGTGAAATT GGGGAAGTAA ATTCGACTTT AGGGCACTGG   
  
  
+ TCTATCACGC CATGATTTTA CCATCTATTT TCATATATAT CCAATAAGTT TGATCTTTCG CTATTAATCA   
  
  
+ CAATGATATA AACAAGTGGT GAAGAGCAAT AATGAACCAT ACATTTTAAA CTTAAGCGTT AAGGAAGACA   
  
  
+ TGAAGTTAAA AACAATGGTA AGAGCTATGC GTATGTTTGG CATATAGCTT TTTTAAGAGT GTTTTGGCTA   
  
  
+ TAGTCGGAGT TTTTTAATTA AGATTAGCTG TTTGATCAAA TAAAAAAGCT AATTTGAGTG TTTGGCGAGA   
  
  
+ AGACTTTTTA TAAGAACTTT TTTTGGTCTA AAAAGTTAAT TTAAAAAGGC TAATTCTATG AGCTTTTCGG   
  
  
+ AAGAGTTTTT TAAATAATTA ACTTTTTGTC TCATAAGCCA TAACTTTATC AGAAACAGTT AATTTTACAA   
  
  
+ AATAATTTCT CAACAAACAA CTAATTTAAA TAATTAATAA AAATAACTAA CTCAAATATC TAATAACTAA   
  
  
+ TAACTAATAT AAATAATTAA CAGTTAACAG TTGTTTACGA AACAGAATTT ATATAAAAAG CGGTAACAGA   
  
  
+ TAAAAGATAA TCATGCGAGA TTAAAAGTCA CCAGTGACAC ACAAGTACTT AAATAAATAG TCTAAACAAT   
  
  
+ GATATTTTTT GCGAGCTTCC ATGCAAATAC CGACACTTTT ATTGCCTCAG AAGGAAACAA AAACTGAGTG   
  
  
+ AAGGGGGGGG GGGGTTCTGT TTGTCTTTGT TAGGGCACGT TTGGATTCAA GTGTAGATTG GGAATAAATT   
  
  
+ CGAGGACTTT TTCTTTGCCC TTTTTCCCTG ATTCCCAAAC CCACATCAAG TCAAGACGAA ACCAGCCATT   
  
  
+ GAAAAAAGAG GGTGTTTGTG TGAGATAAAG ACAGAAATTT TATGCCAGAG AGAGAAGATA ACAAATGCAA   
  
  
+ CAACGTCGAC AGAGGCTCTC CTTCTCACAA ATTCCATATT CCTCTGTTTT TTAAGAAAGA AAAAGAAAGT   
  
  
+ GTGAGACAAG AACAACACAA GCAAACAATT CAGTTGACGA CGAATACTGA GTGATACCAC GCACTGTGTG   
  
  
+ CAACTGCGTT TCCATTTGTG GAGTCCTTCA AAGCACGCTC ATCTTCAACC CTTCCTTCGC CACGGAATTT   
  
  
+ CAGGGGGAAA AAAACCCCCA CCTAATTTTG AGAGAGAGAG AGAACAGAGA GAACGGGCGA TACAGGGATA   
  
  
+ CAAGGTTTGA GCAATTTTAG AAAATTTCTT GGGGCCGTTG ATGGGTTTTG AGTGAATTGC AAATCCCAGA   
  
  
+ AAGATTTCTG CGGTTTTTTA TCTACGGCTC TCTCTGTGGG AATTTTTTGG TAGATTCCTC TGGTTCCCAC   
  
  
+ TTTCATATCT TCTTATTTGT TCTACCCTCT TTACATCTGA TAGTTTGTTC TAACTGCTGC TTCAGTTGGT   
  
  
+ GCTTGTTCTC TTTAGTACTT TTCTGTTTTG TTCGTTTTGT TCATGCACTT GAATTCAACA AGAGCCCTTG   
  
  
+ TCCATTTTGA GCTCTTAATT CCCATCTAAC CCTGTTTCCT TCAGAAATTG CTGTTCTGTT CATATAGTTA   
  
  
+ TATTTTTTAT GCTTTGATTT GGGTATAAGT TTGCTGTTGG TGATTCCAAA GCTTGGTACT TTTGGCCATA   
  
  
+ TTTTGTTTGA GTTGGTGTTG ATTGGTACAA ATCTTGTAAT TGGTGTGATT GTTAATGGGA CCAATGCTTC   
  
  
+ AAGATGATGG TTCATCAGTA ACTTCTTCAT CACCTCTTCA ATTTTTCTCC ATGATGTCAC CCAATTTGGG   
  
  
+ TTCCTCCTAC CCTTGGCTAA GGGACTTAAA GCCTGAAGAG AGAGGTCTTT ACTTGATACA TTTGTTGCTC   
  
  
+ ACTTGTGCAA ACCATGTCTC TAATGGTAGC CTTGACAATG CAAACCTAGC CCTTGAGCAA ATCTCCCAGC   
  
  
+ TTGCAGCCCC TGATGGCGAT ACAATCCAGC GTATTGTTGC CTACTTTGCT GAATCACTTG CTGAAAGGAT   
  
  
+ CCTTAAGTCA TGGCCTGGCC TATATAAAGC CCTTCATTTC AATAGAATGC CTGTTATTTC AGAAGAATTT   
  
  
+ CTTGCTAGGA AGCTGTTTTT TGAGTTGTTT CCCTTCTTGA AGCTGGCCTT TTTGGTGACT AACCAATCAA   
  
  
+ TAATCGAGGC CATGGAGGGG GAAAAGATGG TGCATATAAT TGATCTGAAT GCGTCAGAAC CTGCACAGTG   
  
  
+ GATTGCCCTT ATTCAAGACT TGAGTGCTCG GCCTGAGGGC CCTCCTCATT TGAGGATTAC CGGGGTTCAT   
  
  
+ CAACAGAAAG AGGTTTTAGA ACAAGTAGCT CATAGATTGA CTGAAGAAGC TGAGAAGTTG GATTTGCCAT   
  
  
+ TTCAGTTCAA TCCTGTGGTT TGCAAACTAG AGAATCTCGA CATCGGAAAA CTCCGTGTTA AGACCGGGGA   
  
  
+ GGCCTTGGCT ATTACCTCGG TCCTTCAACT GCATACCCTT TTGGCTTCTG AAGAGGAAGT CCTTAAGAAA   
  
  
+ AGTTCACCCT TGGCATTGGT AAAGCAAGCC AATGGGGCTA ATTTACAGGG CTTGTTCAAT AAAGATGGAG   
  
  
+ CTAATAATAG GCGTAGCCCA AGTAATGATT CGGCTTCATC TGCACCTTCA TCCCTCAACA CTTCAGCCAA   
  
  
+ GATGGAAGGT TTCCTTAGCG CTTTGTGGGG TTTATCCCCA AAGATTATGG TGATAACCGA GCAAGATTCC   
  
  
+ AACCACAATG GGGCAGGACT AATGGAGAGA TTGTCAGAAG CATTGTACTT CTATGCAGCA TTGTTCGATT   
  
  
+ GCTTAGAATT TACCCTCCCG AGAACCTCCG TGGAGAGAAG GAAGGTCGAG ATGCTCCTCC TTGGCAAGGA   
  
  
+ AATCAAGAAC ATCATAGCGT GTGAGGGAGG AGAAAGAATA GATAGGCATG AGAAGTTGGG GAAGTGGATT   
  
  
+ AAGAGGCTTG AGATGGCCGG GTTTGGAAGC GTTCCTTTGA GCCACATAGG CATGATCCAA GCAAGGCGGT   
  
  
+ TGTTGCAGAG CTATGGCTGT GATGGTTATA GAATAAAGGA GGAGAACGGA TGTTTTGTTA TCTGCTGGCA   
  
  
+ AGATCGCCCC CTCTTTTCAG TATCTGCTTG GAGATGTAGG AGGTG  

- -Up\_Stream \_Len000AGATGG AAATGGACAC AATTTTTTTT TTTAAAACAA AGGTAATGAT AAGAGAACCG   
  
  
- TGATAACCAC GACTAAAAAG AAGTGGTTTA GTTGGAGGAA TATAAACGGT TAAAATGACA GGAGAAAGTA   
  
  
- GATATTTCAG TTGAGAGGAT CATGTTAAAC AGTGTATCTT TTAGAGATCC GTGAGTAGAT TTGAGAGACA   
  
  
- AGACCCATTA ACTAGCTGCT AGATGTGGTA ATAAGCAAGA TCACGTGGCA AACCTGTCGG TTAGTACATG   
  
  
- GAAACCTCTA GTTGACGAGT ACTCAAGAAC CACACTTTAA CCCCTTCATT TAAGCTGAAA TCCCGTGACC   
  
  
- AGATAGTGCG GTACTAAAAT GGTAGATAAA AGTATATATA GGTTATTCAA ACTAGAAAGC GATAATTAGT   
  
  
- GTTACTATAT TTGTTCACCA CTTCTCGTTA TTACTTGGTA TGTAAAATTT GAATTCGCAA TTCCTTCTGT   
  
  
- ACTTCAATTT TTGTTACCAT TCTCGATACG CATACAAACC GTATATCGAA AAAATTCTCA CAAAACCGAT   
  
  
- ATCAGCCTCA AAAAATTAAT TCTAATCGAC AAACTAGTTT ATTTTTTCGA TTAAACTCAC AAACCGCTCT   
  
  
- TCTGAAAAAT ATTCTTGAAA AAAACCAGAT TTTTCAATTA AATTTTTCCG ATTAAGATAC TCGAAAAGCC   
  
  
- TTCTCAAAAA ATTTATTAAT TGAAAAACAG AGTATTCGGT ATTGAAATAG TCTTTGTCAA TTAAAATGTT   
  
  
- TTATTAAAGA GTTGTTTGTT GATTAAATTT ATTAATTATT TTTATTGATT GAGTTTATAG ATTATTGATT   
  
  
- ATTGATTATA TTTATTAATT GTCAATTGTC AACAAATGCT TTGTCTTAAA TATATTTTTC GCCATTGTCT   
  
  
- ATTTTCTATT AGTACGCTCT AATTTTCAGT GGTCACTGTG TGTTCATGAA TTTATTTATC AGATTTGTTA   
  
  
- CTATAAAAAA CGCTCGAAGG TACGTTTATG GCTGTGAAAA TAACGGAGTC TTCCTTTGTT TTTGACTCAC   
  
  
- TTCCCCCCCC CCCCAAGACA AACAGAAACA ATCCCGTGCA AACCTAAGTT CACATCTAAC CCTTATTTAA   
  
  
- GCTCCTGAAA AAGAAACGGG AAAAAGGGAC TAAGGGTTTG GGTGTAGTTC AGTTCTGCTT TGGTCGGTAA   
  
  
- CTTTTTTCTC CCACAAACAC ACTCTATTTC TGTCTTTAAA ATACGGTCTC TCTCTTCTAT TGTTTACGTT   
  
  
- GTTGCAGCTG TCTCCGAGAG GAAGAGTGTT TAAGGTATAA GGAGACAAAA AATTCTTTCT TTTTCTTTCA   
  
  
- CACTCTGTTC TTGTTGTGTT CGTTTGTTAA GTCAACTGCT GCTTATGACT CACTATGGTG CGTGACACAC   
  
  
- GTTGACGCAA AGGTAAACAC CTCAGGAAGT TTCGTGCGAG TAGAAGTTGG GAAGGAAGCG GTGCCTTAAA   
  
  
- GTCCCCCTTT TTTTGGGGGT GGATTAAAAC TCTCTCTCTC TCTTGTCTCT CTTGCCCGCT ATGTCCCTAT   
  
  
- GTTCCAAACT CGTTAAAATC TTTTAAAGAA CCCCGGCAAC TACCCAAAAC TCACTTAACG TTTAGGGTCT   
  
  
- TTCTAAAGAC GCCAAAAAAT AGATGCCGAG AGAGACACCC TTAAAAAACC ATCTAAGGAG ACCAAGGGTG   
  
  
- AAAGTATAGA AGAATAAACA AGATGGGAGA AATGTAGACT ATCAAACAAG ATTGACGACG AAGTCAACCA   
  
  
- CGAACAAGAG AAATCATGAA AAGACAAAAC AAGCAAAACA AGTACGTGAA CTTAAGTTGT TCTCGGGAAC   
  
  
- AGGTAAAACT CGAGAATTAA GGGTAGATTG GGACAAAGGA AGTCTTTAAC GACAAGACAA GTATATCAAT   
  
  
- ATAAAAAATA CGAAACTAAA CCCATATTCA AACGACAACC ACTAAGGTTT CGAACCATGA AAACCGGTAT   
  
  
- AAAACAAACT CAACCACAAC TAACCATGTT TAGAACATTA ACCACACTAA CAATTACCCT GGTTACGAAG   
  
  
- TTCTACTACC AAGTAGTCAT TGAAGAAGTA GTGGAGAAGT TAAAAAGAGG TACTACAGTG GGTTAAACCC   
  
  
- AAGGAGGATG GGAACCGATT CCCTGAATTT CGGACTTCTC TCTCCAGAAA TGAACTATGT AAACAACGAG   
  
  
- TGAACACGTT TGGTACAGAG ATTACCATCG GAACTGTTAC GTTTGGATCG GGAACTCGTT TAGAGGGTCG   
  
  
- AACGTCGGGG ACTACCGCTA TGTTAGGTCG CATAACAACG GATGAAACGA CTTAGTGAAC GACTTTCCTA   
  
  
- GGAATTCAGT ACCGGACCGG ATATATTTCG GGAAGTAAAG TTATCTTACG GACAATAAAG TCTTCTTAAA   
  
  
- GAACGATCCT TCGACAAAAA ACTCAACAAA GGGAAGAACT TCGACCGGAA AAACCACTGA TTGGTTAGTT   
  
  
- ATTAGCTCCG GTACCTCCCC CTTTTCTACC ACGTATATTA ACTAGACTTA CGCAGTCTTG GACGTGTCAC   
  
  
- CTAACGGGAA TAAGTTCTGA ACTCACGAGC CGGACTCCCG GGAGGAGTAA ACTCCTAATG GCCCCAAGTA   
  
  
- GTTGTCTTTC TCCAAAATCT TGTTCATCGA GTATCTAACT GACTTCTTCG ACTCTTCAAC CTAAACGGTA   
  
  
- AAGTCAAGTT AGGACACCAA ACGTTTGATC TCTTAGAGCT GTAGCCTTTT GAGGCACAAT TCTGGCCCCT   
  
  
- CCGGAACCGA TAATGGAGCC AGGAAGTTGA CGTATGGGAA AACCGAAGAC TTCTCCTTCA GGAATTCTTT   
  
  
- TCAAGTGGGA ACCGTAACCA TTTCGTTCGG TTACCCCGAT TAAATGTCCC GAACAAGTTA TTTCTACCTC   
  
  
- GATTATTATC CGCATCGGGT TCATTACTAA GCCGAAGTAG ACGTGGAAGT AGGGAGTTGT GAAGTCGGTT   
  
  
- CTACCTTCCA AAGGAATCGC GAAACACCCC AAATAGGGGT TTCTAATACC ACTATTGGCT CGTTCTAAGG   
  
  
- TTGGTGTTAC CCCGTCCTGA TTACCTCTCT AACAGTCTTC GTAACATGAA GATACGTCGT AACAAGCTAA   
  
  
- CGAATCTTAA ATGGGAGGGC TCTTGGAGGC ACCTCTCTTC CTTCCAGCTC TACGAGGAGG AACCGTTCCT   
  
  
- TTAGTTCTTG TAGTATCGCA CACTCCCTCC TCTTTCTTAT CTATCCGTAC TCTTCAACCC CTTCACCTAA   
  
  
- TTCTCCGAAC TCTACCGGCC CAAACCTTCG CAAGGAAACT CGGTGTATCC GTACTAGGTT CGTTCCGCCA   
  
  
- ACAACGTCTC GATACCGACA CTACCAATAT CTTATTTCCT CCTCTTGCCT ACAAAACAAT AGACGACCGT   
  
  
- TCTAGCGGGG GAGAAAAGTC ATAGACGAAC CTCTACATCC TCCAC

+     WUN-motif

| Site Name | Organism | Position | Strand | Matrix score. | sequence | function |
| --- | --- | --- | --- | --- | --- | --- |
| WUN-motif | Nicotiana glutinosa | 1566 | + | 9 | AAATTTCTT |  |

>HU08G01232.1   
+ -Up\_Stream \_Len000TCTACC TTTACCTGTG TTAAAAAAAA AAATTTTGTT TCCATTACTA TTCTCTTGGC   
  
  
+ ACTATTGGTG CTGATTTTTC TTCACCAAAT CAACCTCCTT ATATTTGCCA ATTTTACTGT CCTCTTTCAT   
  
  
+ CTATAAAGTC AACTCTCCTA GTACAATTTG TCACATAGAA AATCTCTAGG CACTCATCTA AACTCTCTGT   
  
  
+ TCTGGGTAAT TGATCGACGA TCTACACCAT TATTCGTTCT AGTGCACCGT TTGGACAGCC AATCATGTAC   
  
  
+ CTTTGGAGAT CAACTGCTCA TGAGTTCTTG GTGTGAAATT GGGGAAGTAA ATTCGACTTT AGGGCACTGG   
  
  
+ TCTATCACGC CATGATTTTA CCATCTATTT TCATATATAT CCAATAAGTT TGATCTTTCG CTATTAATCA   
  
  
+ CAATGATATA AACAAGTGGT GAAGAGCAAT AATGAACCAT ACATTTTAAA CTTAAGCGTT AAGGAAGACA   
  
  
+ TGAAGTTAAA AACAATGGTA AGAGCTATGC GTATGTTTGG CATATAGCTT TTTTAAGAGT GTTTTGGCTA   
  
  
+ TAGTCGGAGT TTTTTAATTA AGATTAGCTG TTTGATCAAA TAAAAAAGCT AATTTGAGTG TTTGGCGAGA   
  
  
+ AGACTTTTTA TAAGAACTTT TTTTGGTCTA AAAAGTTAAT TTAAAAAGGC TAATTCTATG AGCTTTTCGG   
  
  
+ AAGAGTTTTT TAAATAATTA ACTTTTTGTC TCATAAGCCA TAACTTTATC AGAAACAGTT AATTTTACAA   
  
  
+ AATAATTTCT CAACAAACAA CTAATTTAAA TAATTAATAA AAATAACTAA CTCAAATATC TAATAACTAA   
  
  
+ TAACTAATAT AAATAATTAA CAGTTAACAG TTGTTTACGA AACAGAATTT ATATAAAAAG CGGTAACAGA   
  
  
+ TAAAAGATAA TCATGCGAGA TTAAAAGTCA CCAGTGACAC ACAAGTACTT AAATAAATAG TCTAAACAAT   
  
  
+ GATATTTTTT GCGAGCTTCC ATGCAAATAC CGACACTTTT ATTGCCTCAG AAGGAAACAA AAACTGAGTG   
  
  
+ AAGGGGGGGG GGGGTTCTGT TTGTCTTTGT TAGGGCACGT TTGGATTCAA GTGTAGATTG GGAATAAATT   
  
  
+ CGAGGACTTT TTCTTTGCCC TTTTTCCCTG ATTCCCAAAC CCACATCAAG TCAAGACGAA ACCAGCCATT   
  
  
+ GAAAAAAGAG GGTGTTTGTG TGAGATAAAG ACAGAAATTT TATGCCAGAG AGAGAAGATA ACAAATGCAA   
  
  
+ CAACGTCGAC AGAGGCTCTC CTTCTCACAA ATTCCATATT CCTCTGTTTT TTAAGAAAGA AAAAGAAAGT   
  
  
+ GTGAGACAAG AACAACACAA GCAAACAATT CAGTTGACGA CGAATACTGA GTGATACCAC GCACTGTGTG   
  
  
+ CAACTGCGTT TCCATTTGTG GAGTCCTTCA AAGCACGCTC ATCTTCAACC CTTCCTTCGC CACGGAATTT   
  
  
+ CAGGGGGAAA AAAACCCCCA CCTAATTTTG AGAGAGAGAG AGAACAGAGA GAACGGGCGA TACAGGGATA   
  
  
+ CAAGGTTTGA GCAATTTTAG AAAATTTCTT GGGGCCGTTG ATGGGTTTTG AGTGAATTGC AAATCCCAGA   
  
  
+ AAGATTTCTG CGGTTTTTTA TCTACGGCTC TCTCTGTGGG AATTTTTTGG TAGATTCCTC TGGTTCCCAC   
  
  
+ TTTCATATCT TCTTATTTGT TCTACCCTCT TTACATCTGA TAGTTTGTTC TAACTGCTGC TTCAGTTGGT   
  
  
+ GCTTGTTCTC TTTAGTACTT TTCTGTTTTG TTCGTTTTGT TCATGCACTT GAATTCAACA AGAGCCCTTG   
  
  
+ TCCATTTTGA GCTCTTAATT CCCATCTAAC CCTGTTTCCT TCAGAAATTG CTGTTCTGTT CATATAGTTA   
  
  
+ TATTTTTTAT GCTTTGATTT GGGTATAAGT TTGCTGTTGG TGATTCCAAA GCTTGGTACT TTTGGCCATA   
  
  
+ TTTTGTTTGA GTTGGTGTTG ATTGGTACAA ATCTTGTAAT TGGTGTGATT GTTAATGGGA CCAATGCTTC   
  
  
+ AAGATGATGG TTCATCAGTA ACTTCTTCAT CACCTCTTCA ATTTTTCTCC ATGATGTCAC CCAATTTGGG   
  
  
+ TTCCTCCTAC CCTTGGCTAA GGGACTTAAA GCCTGAAGAG AGAGGTCTTT ACTTGATACA TTTGTTGCTC   
  
  
+ ACTTGTGCAA ACCATGTCTC TAATGGTAGC CTTGACAATG CAAACCTAGC CCTTGAGCAA ATCTCCCAGC   
  
  
+ TTGCAGCCCC TGATGGCGAT ACAATCCAGC GTATTGTTGC CTACTTTGCT GAATCACTTG CTGAAAGGAT   
  
  
+ CCTTAAGTCA TGGCCTGGCC TATATAAAGC CCTTCATTTC AATAGAATGC CTGTTATTTC AGAAGAATTT   
  
  
+ CTTGCTAGGA AGCTGTTTTT TGAGTTGTTT CCCTTCTTGA AGCTGGCCTT TTTGGTGACT AACCAATCAA   
  
  
+ TAATCGAGGC CATGGAGGGG GAAAAGATGG TGCATATAAT TGATCTGAAT GCGTCAGAAC CTGCACAGTG   
  
  
+ GATTGCCCTT ATTCAAGACT TGAGTGCTCG GCCTGAGGGC CCTCCTCATT TGAGGATTAC CGGGGTTCAT   
  
  
+ CAACAGAAAG AGGTTTTAGA ACAAGTAGCT CATAGATTGA CTGAAGAAGC TGAGAAGTTG GATTTGCCAT   
  
  
+ TTCAGTTCAA TCCTGTGGTT TGCAAACTAG AGAATCTCGA CATCGGAAAA CTCCGTGTTA AGACCGGGGA   
  
  
+ GGCCTTGGCT ATTACCTCGG TCCTTCAACT GCATACCCTT TTGGCTTCTG AAGAGGAAGT CCTTAAGAAA   
  
  
+ AGTTCACCCT TGGCATTGGT AAAGCAAGCC AATGGGGCTA ATTTACAGGG CTTGTTCAAT AAAGATGGAG   
  
  
+ CTAATAATAG GCGTAGCCCA AGTAATGATT CGGCTTCATC TGCACCTTCA TCCCTCAACA CTTCAGCCAA   
  
  
+ GATGGAAGGT TTCCTTAGCG CTTTGTGGGG TTTATCCCCA AAGATTATGG TGATAACCGA GCAAGATTCC   
  
  
+ AACCACAATG GGGCAGGACT AATGGAGAGA TTGTCAGAAG CATTGTACTT CTATGCAGCA TTGTTCGATT   
  
  
+ GCTTAGAATT TACCCTCCCG AGAACCTCCG TGGAGAGAAG GAAGGTCGAG ATGCTCCTCC TTGGCAAGGA   
  
  
+ AATCAAGAAC ATCATAGCGT GTGAGGGAGG AGAAAGAATA GATAGGCATG AGAAGTTGGG GAAGTGGATT   
  
  
+ AAGAGGCTTG AGATGGCCGG GTTTGGAAGC GTTCCTTTGA GCCACATAGG CATGATCCAA GCAAGGCGGT   
  
  
+ TGTTGCAGAG CTATGGCTGT GATGGTTATA GAATAAAGGA GGAGAACGGA TGTTTTGTTA TCTGCTGGCA   
  
  
+ AGATCGCCCC CTCTTTTCAG TATCTGCTTG GAGATGTAGG AGGTG  

- -Up\_Stream \_Len000AGATGG AAATGGACAC AATTTTTTTT TTTAAAACAA AGGTAATGAT AAGAGAACCG   
  
  
- TGATAACCAC GACTAAAAAG AAGTGGTTTA GTTGGAGGAA TATAAACGGT TAAAATGACA GGAGAAAGTA   
  
  
- GATATTTCAG TTGAGAGGAT CATGTTAAAC AGTGTATCTT TTAGAGATCC GTGAGTAGAT TTGAGAGACA   
  
  
- AGACCCATTA ACTAGCTGCT AGATGTGGTA ATAAGCAAGA TCACGTGGCA AACCTGTCGG TTAGTACATG   
  
  
- GAAACCTCTA GTTGACGAGT ACTCAAGAAC CACACTTTAA CCCCTTCATT TAAGCTGAAA TCCCGTGACC   
  
  
- AGATAGTGCG GTACTAAAAT GGTAGATAAA AGTATATATA GGTTATTCAA ACTAGAAAGC GATAATTAGT   
  
  
- GTTACTATAT TTGTTCACCA CTTCTCGTTA TTACTTGGTA TGTAAAATTT GAATTCGCAA TTCCTTCTGT   
  
  
- ACTTCAATTT TTGTTACCAT TCTCGATACG CATACAAACC GTATATCGAA AAAATTCTCA CAAAACCGAT   
  
  
- ATCAGCCTCA AAAAATTAAT TCTAATCGAC AAACTAGTTT ATTTTTTCGA TTAAACTCAC AAACCGCTCT   
  
  
- TCTGAAAAAT ATTCTTGAAA AAAACCAGAT TTTTCAATTA AATTTTTCCG ATTAAGATAC TCGAAAAGCC   
  
  
- TTCTCAAAAA ATTTATTAAT TGAAAAACAG AGTATTCGGT ATTGAAATAG TCTTTGTCAA TTAAAATGTT   
  
  
- TTATTAAAGA GTTGTTTGTT GATTAAATTT ATTAATTATT TTTATTGATT GAGTTTATAG ATTATTGATT   
  
  
- ATTGATTATA TTTATTAATT GTCAATTGTC AACAAATGCT TTGTCTTAAA TATATTTTTC GCCATTGTCT   
  
  
- ATTTTCTATT AGTACGCTCT AATTTTCAGT GGTCACTGTG TGTTCATGAA TTTATTTATC AGATTTGTTA   
  
  
- CTATAAAAAA CGCTCGAAGG TACGTTTATG GCTGTGAAAA TAACGGAGTC TTCCTTTGTT TTTGACTCAC   
  
  
- TTCCCCCCCC CCCCAAGACA AACAGAAACA ATCCCGTGCA AACCTAAGTT CACATCTAAC CCTTATTTAA   
  
  
- GCTCCTGAAA AAGAAACGGG AAAAAGGGAC TAAGGGTTTG GGTGTAGTTC AGTTCTGCTT TGGTCGGTAA   
  
  
- CTTTTTTCTC CCACAAACAC ACTCTATTTC TGTCTTTAAA ATACGGTCTC TCTCTTCTAT TGTTTACGTT   
  
  
- GTTGCAGCTG TCTCCGAGAG GAAGAGTGTT TAAGGTATAA GGAGACAAAA AATTCTTTCT TTTTCTTTCA   
  
  
- CACTCTGTTC TTGTTGTGTT CGTTTGTTAA GTCAACTGCT GCTTATGACT CACTATGGTG CGTGACACAC   
  
  
- GTTGACGCAA AGGTAAACAC CTCAGGAAGT TTCGTGCGAG TAGAAGTTGG GAAGGAAGCG GTGCCTTAAA   
  
  
- GTCCCCCTTT TTTTGGGGGT GGATTAAAAC TCTCTCTCTC TCTTGTCTCT CTTGCCCGCT ATGTCCCTAT   
  
  
- GTTCCAAACT CGTTAAAATC TTTTAAAGAA CCCCGGCAAC TACCCAAAAC TCACTTAACG TTTAGGGTCT   
  
  
- TTCTAAAGAC GCCAAAAAAT AGATGCCGAG AGAGACACCC TTAAAAAACC ATCTAAGGAG ACCAAGGGTG   
  
  
- AAAGTATAGA AGAATAAACA AGATGGGAGA AATGTAGACT ATCAAACAAG ATTGACGACG AAGTCAACCA   
  
  
- CGAACAAGAG AAATCATGAA AAGACAAAAC AAGCAAAACA AGTACGTGAA CTTAAGTTGT TCTCGGGAAC   
  
  
- AGGTAAAACT CGAGAATTAA GGGTAGATTG GGACAAAGGA AGTCTTTAAC GACAAGACAA GTATATCAAT   
  
  
- ATAAAAAATA CGAAACTAAA CCCATATTCA AACGACAACC ACTAAGGTTT CGAACCATGA AAACCGGTAT   
  
  
- AAAACAAACT CAACCACAAC TAACCATGTT TAGAACATTA ACCACACTAA CAATTACCCT GGTTACGAAG   
  
  
- TTCTACTACC AAGTAGTCAT TGAAGAAGTA GTGGAGAAGT TAAAAAGAGG TACTACAGTG GGTTAAACCC   
  
  
- AAGGAGGATG GGAACCGATT CCCTGAATTT CGGACTTCTC TCTCCAGAAA TGAACTATGT AAACAACGAG   
  
  
- TGAACACGTT TGGTACAGAG ATTACCATCG GAACTGTTAC GTTTGGATCG GGAACTCGTT TAGAGGGTCG   
  
  
- AACGTCGGGG ACTACCGCTA TGTTAGGTCG CATAACAACG GATGAAACGA CTTAGTGAAC GACTTTCCTA   
  
  
- GGAATTCAGT ACCGGACCGG ATATATTTCG GGAAGTAAAG TTATCTTACG GACAATAAAG TCTTCTTAAA   
  
  
- GAACGATCCT TCGACAAAAA ACTCAACAAA GGGAAGAACT TCGACCGGAA AAACCACTGA TTGGTTAGTT   
  
  
- ATTAGCTCCG GTACCTCCCC CTTTTCTACC ACGTATATTA ACTAGACTTA CGCAGTCTTG GACGTGTCAC   
  
  
- CTAACGGGAA TAAGTTCTGA ACTCACGAGC CGGACTCCCG GGAGGAGTAA ACTCCTAATG GCCCCAAGTA   
  
  
- GTTGTCTTTC TCCAAAATCT TGTTCATCGA GTATCTAACT GACTTCTTCG ACTCTTCAAC CTAAACGGTA   
  
  
- AAGTCAAGTT AGGACACCAA ACGTTTGATC TCTTAGAGCT GTAGCCTTTT GAGGCACAAT TCTGGCCCCT   
  
  
- CCGGAACCGA TAATGGAGCC AGGAAGTTGA CGTATGGGAA AACCGAAGAC TTCTCCTTCA GGAATTCTTT   
  
  
- TCAAGTGGGA ACCGTAACCA TTTCGTTCGG TTACCCCGAT TAAATGTCCC GAACAAGTTA TTTCTACCTC   
  
  
- GATTATTATC CGCATCGGGT TCATTACTAA GCCGAAGTAG ACGTGGAAGT AGGGAGTTGT GAAGTCGGTT   
  
  
- CTACCTTCCA AAGGAATCGC GAAACACCCC AAATAGGGGT TTCTAATACC ACTATTGGCT CGTTCTAAGG   
  
  
- TTGGTGTTAC CCCGTCCTGA TTACCTCTCT AACAGTCTTC GTAACATGAA GATACGTCGT AACAAGCTAA   
  
  
- CGAATCTTAA ATGGGAGGGC TCTTGGAGGC ACCTCTCTTC CTTCCAGCTC TACGAGGAGG AACCGTTCCT   
  
  
- TTAGTTCTTG TAGTATCGCA CACTCCCTCC TCTTTCTTAT CTATCCGTAC TCTTCAACCC CTTCACCTAA   
  
  
- TTCTCCGAAC TCTACCGGCC CAAACCTTCG CAAGGAAACT CGGTGTATCC GTACTAGGTT CGTTCCGCCA   
  
  
- ACAACGTCTC GATACCGACA CTACCAATAT CTTATTTCCT CCTCTTGCCT ACAAAACAAT AGACGACCGT   
  
  
- TCTAGCGGGG GAGAAAAGTC ATAGACGAAC CTCTACATCC TCCAC

+     as-1

| Site Name | Organism | Position | Strand | Matrix score. | sequence | function |
| --- | --- | --- | --- | --- | --- | --- |
| as-1 | Arabidopsis thaliana | 1369 | + | 5 | TGACG |  |
| as-1 | Arabidopsis thaliana | 2506 | - | 5 | TGACG |  |

>HU08G01232.1   
+ -Up\_Stream \_Len000TCTACC TTTACCTGTG TTAAAAAAAA AAATTTTGTT TCCATTACTA TTCTCTTGGC   
  
  
+ ACTATTGGTG CTGATTTTTC TTCACCAAAT CAACCTCCTT ATATTTGCCA ATTTTACTGT CCTCTTTCAT   
  
  
+ CTATAAAGTC AACTCTCCTA GTACAATTTG TCACATAGAA AATCTCTAGG CACTCATCTA AACTCTCTGT   
  
  
+ TCTGGGTAAT TGATCGACGA TCTACACCAT TATTCGTTCT AGTGCACCGT TTGGACAGCC AATCATGTAC   
  
  
+ CTTTGGAGAT CAACTGCTCA TGAGTTCTTG GTGTGAAATT GGGGAAGTAA ATTCGACTTT AGGGCACTGG   
  
  
+ TCTATCACGC CATGATTTTA CCATCTATTT TCATATATAT CCAATAAGTT TGATCTTTCG CTATTAATCA   
  
  
+ CAATGATATA AACAAGTGGT GAAGAGCAAT AATGAACCAT ACATTTTAAA CTTAAGCGTT AAGGAAGACA   
  
  
+ TGAAGTTAAA AACAATGGTA AGAGCTATGC GTATGTTTGG CATATAGCTT TTTTAAGAGT GTTTTGGCTA   
  
  
+ TAGTCGGAGT TTTTTAATTA AGATTAGCTG TTTGATCAAA TAAAAAAGCT AATTTGAGTG TTTGGCGAGA   
  
  
+ AGACTTTTTA TAAGAACTTT TTTTGGTCTA AAAAGTTAAT TTAAAAAGGC TAATTCTATG AGCTTTTCGG   
  
  
+ AAGAGTTTTT TAAATAATTA ACTTTTTGTC TCATAAGCCA TAACTTTATC AGAAACAGTT AATTTTACAA   
  
  
+ AATAATTTCT CAACAAACAA CTAATTTAAA TAATTAATAA AAATAACTAA CTCAAATATC TAATAACTAA   
  
  
+ TAACTAATAT AAATAATTAA CAGTTAACAG TTGTTTACGA AACAGAATTT ATATAAAAAG CGGTAACAGA   
  
  
+ TAAAAGATAA TCATGCGAGA TTAAAAGTCA CCAGTGACAC ACAAGTACTT AAATAAATAG TCTAAACAAT   
  
  
+ GATATTTTTT GCGAGCTTCC ATGCAAATAC CGACACTTTT ATTGCCTCAG AAGGAAACAA AAACTGAGTG   
  
  
+ AAGGGGGGGG GGGGTTCTGT TTGTCTTTGT TAGGGCACGT TTGGATTCAA GTGTAGATTG GGAATAAATT   
  
  
+ CGAGGACTTT TTCTTTGCCC TTTTTCCCTG ATTCCCAAAC CCACATCAAG TCAAGACGAA ACCAGCCATT   
  
  
+ GAAAAAAGAG GGTGTTTGTG TGAGATAAAG ACAGAAATTT TATGCCAGAG AGAGAAGATA ACAAATGCAA   
  
  
+ CAACGTCGAC AGAGGCTCTC CTTCTCACAA ATTCCATATT CCTCTGTTTT TTAAGAAAGA AAAAGAAAGT   
  
  
+ GTGAGACAAG AACAACACAA GCAAACAATT CAGTTGACGA CGAATACTGA GTGATACCAC GCACTGTGTG   
  
  
+ CAACTGCGTT TCCATTTGTG GAGTCCTTCA AAGCACGCTC ATCTTCAACC CTTCCTTCGC CACGGAATTT   
  
  
+ CAGGGGGAAA AAAACCCCCA CCTAATTTTG AGAGAGAGAG AGAACAGAGA GAACGGGCGA TACAGGGATA   
  
  
+ CAAGGTTTGA GCAATTTTAG AAAATTTCTT GGGGCCGTTG ATGGGTTTTG AGTGAATTGC AAATCCCAGA   
  
  
+ AAGATTTCTG CGGTTTTTTA TCTACGGCTC TCTCTGTGGG AATTTTTTGG TAGATTCCTC TGGTTCCCAC   
  
  
+ TTTCATATCT TCTTATTTGT TCTACCCTCT TTACATCTGA TAGTTTGTTC TAACTGCTGC TTCAGTTGGT   
  
  
+ GCTTGTTCTC TTTAGTACTT TTCTGTTTTG TTCGTTTTGT TCATGCACTT GAATTCAACA AGAGCCCTTG   
  
  
+ TCCATTTTGA GCTCTTAATT CCCATCTAAC CCTGTTTCCT TCAGAAATTG CTGTTCTGTT CATATAGTTA   
  
  
+ TATTTTTTAT GCTTTGATTT GGGTATAAGT TTGCTGTTGG TGATTCCAAA GCTTGGTACT TTTGGCCATA   
  
  
+ TTTTGTTTGA GTTGGTGTTG ATTGGTACAA ATCTTGTAAT TGGTGTGATT GTTAATGGGA CCAATGCTTC   
  
  
+ AAGATGATGG TTCATCAGTA ACTTCTTCAT CACCTCTTCA ATTTTTCTCC ATGATGTCAC CCAATTTGGG   
  
  
+ TTCCTCCTAC CCTTGGCTAA GGGACTTAAA GCCTGAAGAG AGAGGTCTTT ACTTGATACA TTTGTTGCTC   
  
  
+ ACTTGTGCAA ACCATGTCTC TAATGGTAGC CTTGACAATG CAAACCTAGC CCTTGAGCAA ATCTCCCAGC   
  
  
+ TTGCAGCCCC TGATGGCGAT ACAATCCAGC GTATTGTTGC CTACTTTGCT GAATCACTTG CTGAAAGGAT   
  
  
+ CCTTAAGTCA TGGCCTGGCC TATATAAAGC CCTTCATTTC AATAGAATGC CTGTTATTTC AGAAGAATTT   
  
  
+ CTTGCTAGGA AGCTGTTTTT TGAGTTGTTT CCCTTCTTGA AGCTGGCCTT TTTGGTGACT AACCAATCAA   
  
  
+ TAATCGAGGC CATGGAGGGG GAAAAGATGG TGCATATAAT TGATCTGAAT GCGTCAGAAC CTGCACAGTG   
  
  
+ GATTGCCCTT ATTCAAGACT TGAGTGCTCG GCCTGAGGGC CCTCCTCATT TGAGGATTAC CGGGGTTCAT   
  
  
+ CAACAGAAAG AGGTTTTAGA ACAAGTAGCT CATAGATTGA CTGAAGAAGC TGAGAAGTTG GATTTGCCAT   
  
  
+ TTCAGTTCAA TCCTGTGGTT TGCAAACTAG AGAATCTCGA CATCGGAAAA CTCCGTGTTA AGACCGGGGA   
  
  
+ GGCCTTGGCT ATTACCTCGG TCCTTCAACT GCATACCCTT TTGGCTTCTG AAGAGGAAGT CCTTAAGAAA   
  
  
+ AGTTCACCCT TGGCATTGGT AAAGCAAGCC AATGGGGCTA ATTTACAGGG CTTGTTCAAT AAAGATGGAG   
  
  
+ CTAATAATAG GCGTAGCCCA AGTAATGATT CGGCTTCATC TGCACCTTCA TCCCTCAACA CTTCAGCCAA   
  
  
+ GATGGAAGGT TTCCTTAGCG CTTTGTGGGG TTTATCCCCA AAGATTATGG TGATAACCGA GCAAGATTCC   
  
  
+ AACCACAATG GGGCAGGACT AATGGAGAGA TTGTCAGAAG CATTGTACTT CTATGCAGCA TTGTTCGATT   
  
  
+ GCTTAGAATT TACCCTCCCG AGAACCTCCG TGGAGAGAAG GAAGGTCGAG ATGCTCCTCC TTGGCAAGGA   
  
  
+ AATCAAGAAC ATCATAGCGT GTGAGGGAGG AGAAAGAATA GATAGGCATG AGAAGTTGGG GAAGTGGATT   
  
  
+ AAGAGGCTTG AGATGGCCGG GTTTGGAAGC GTTCCTTTGA GCCACATAGG CATGATCCAA GCAAGGCGGT   
  
  
+ TGTTGCAGAG CTATGGCTGT GATGGTTATA GAATAAAGGA GGAGAACGGA TGTTTTGTTA TCTGCTGGCA   
  
  
+ AGATCGCCCC CTCTTTTCAG TATCTGCTTG GAGATGTAGG AGGTG  

- -Up\_Stream \_Len000AGATGG AAATGGACAC AATTTTTTTT TTTAAAACAA AGGTAATGAT AAGAGAACCG   
  
  
- TGATAACCAC GACTAAAAAG AAGTGGTTTA GTTGGAGGAA TATAAACGGT TAAAATGACA GGAGAAAGTA   
  
  
- GATATTTCAG TTGAGAGGAT CATGTTAAAC AGTGTATCTT TTAGAGATCC GTGAGTAGAT TTGAGAGACA   
  
  
- AGACCCATTA ACTAGCTGCT AGATGTGGTA ATAAGCAAGA TCACGTGGCA AACCTGTCGG TTAGTACATG   
  
  
- GAAACCTCTA GTTGACGAGT ACTCAAGAAC CACACTTTAA CCCCTTCATT TAAGCTGAAA TCCCGTGACC   
  
  
- AGATAGTGCG GTACTAAAAT GGTAGATAAA AGTATATATA GGTTATTCAA ACTAGAAAGC GATAATTAGT   
  
  
- GTTACTATAT TTGTTCACCA CTTCTCGTTA TTACTTGGTA TGTAAAATTT GAATTCGCAA TTCCTTCTGT   
  
  
- ACTTCAATTT TTGTTACCAT TCTCGATACG CATACAAACC GTATATCGAA AAAATTCTCA CAAAACCGAT   
  
  
- ATCAGCCTCA AAAAATTAAT TCTAATCGAC AAACTAGTTT ATTTTTTCGA TTAAACTCAC AAACCGCTCT   
  
  
- TCTGAAAAAT ATTCTTGAAA AAAACCAGAT TTTTCAATTA AATTTTTCCG ATTAAGATAC TCGAAAAGCC   
  
  
- TTCTCAAAAA ATTTATTAAT TGAAAAACAG AGTATTCGGT ATTGAAATAG TCTTTGTCAA TTAAAATGTT   
  
  
- TTATTAAAGA GTTGTTTGTT GATTAAATTT ATTAATTATT TTTATTGATT GAGTTTATAG ATTATTGATT   
  
  
- ATTGATTATA TTTATTAATT GTCAATTGTC AACAAATGCT TTGTCTTAAA TATATTTTTC GCCATTGTCT   
  
  
- ATTTTCTATT AGTACGCTCT AATTTTCAGT GGTCACTGTG TGTTCATGAA TTTATTTATC AGATTTGTTA   
  
  
- CTATAAAAAA CGCTCGAAGG TACGTTTATG GCTGTGAAAA TAACGGAGTC TTCCTTTGTT TTTGACTCAC   
  
  
- TTCCCCCCCC CCCCAAGACA AACAGAAACA ATCCCGTGCA AACCTAAGTT CACATCTAAC CCTTATTTAA   
  
  
- GCTCCTGAAA AAGAAACGGG AAAAAGGGAC TAAGGGTTTG GGTGTAGTTC AGTTCTGCTT TGGTCGGTAA   
  
  
- CTTTTTTCTC CCACAAACAC ACTCTATTTC TGTCTTTAAA ATACGGTCTC TCTCTTCTAT TGTTTACGTT   
  
  
- GTTGCAGCTG TCTCCGAGAG GAAGAGTGTT TAAGGTATAA GGAGACAAAA AATTCTTTCT TTTTCTTTCA   
  
  
- CACTCTGTTC TTGTTGTGTT CGTTTGTTAA GTCAACTGCT GCTTATGACT CACTATGGTG CGTGACACAC   
  
  
- GTTGACGCAA AGGTAAACAC CTCAGGAAGT TTCGTGCGAG TAGAAGTTGG GAAGGAAGCG GTGCCTTAAA   
  
  
- GTCCCCCTTT TTTTGGGGGT GGATTAAAAC TCTCTCTCTC TCTTGTCTCT CTTGCCCGCT ATGTCCCTAT   
  
  
- GTTCCAAACT CGTTAAAATC TTTTAAAGAA CCCCGGCAAC TACCCAAAAC TCACTTAACG TTTAGGGTCT   
  
  
- TTCTAAAGAC GCCAAAAAAT AGATGCCGAG AGAGACACCC TTAAAAAACC ATCTAAGGAG ACCAAGGGTG   
  
  
- AAAGTATAGA AGAATAAACA AGATGGGAGA AATGTAGACT ATCAAACAAG ATTGACGACG AAGTCAACCA   
  
  
- CGAACAAGAG AAATCATGAA AAGACAAAAC AAGCAAAACA AGTACGTGAA CTTAAGTTGT TCTCGGGAAC   
  
  
- AGGTAAAACT CGAGAATTAA GGGTAGATTG GGACAAAGGA AGTCTTTAAC GACAAGACAA GTATATCAAT   
  
  
- ATAAAAAATA CGAAACTAAA CCCATATTCA AACGACAACC ACTAAGGTTT CGAACCATGA AAACCGGTAT   
  
  
- AAAACAAACT CAACCACAAC TAACCATGTT TAGAACATTA ACCACACTAA CAATTACCCT GGTTACGAAG   
  
  
- TTCTACTACC AAGTAGTCAT TGAAGAAGTA GTGGAGAAGT TAAAAAGAGG TACTACAGTG GGTTAAACCC   
  
  
- AAGGAGGATG GGAACCGATT CCCTGAATTT CGGACTTCTC TCTCCAGAAA TGAACTATGT AAACAACGAG   
  
  
- TGAACACGTT TGGTACAGAG ATTACCATCG GAACTGTTAC GTTTGGATCG GGAACTCGTT TAGAGGGTCG   
  
  
- AACGTCGGGG ACTACCGCTA TGTTAGGTCG CATAACAACG GATGAAACGA CTTAGTGAAC GACTTTCCTA   
  
  
- GGAATTCAGT ACCGGACCGG ATATATTTCG GGAAGTAAAG TTATCTTACG GACAATAAAG TCTTCTTAAA   
  
  
- GAACGATCCT TCGACAAAAA ACTCAACAAA GGGAAGAACT TCGACCGGAA AAACCACTGA TTGGTTAGTT   
  
  
- ATTAGCTCCG GTACCTCCCC CTTTTCTACC ACGTATATTA ACTAGACTTA CGCAGTCTTG GACGTGTCAC   
  
  
- CTAACGGGAA TAAGTTCTGA ACTCACGAGC CGGACTCCCG GGAGGAGTAA ACTCCTAATG GCCCCAAGTA   
  
  
- GTTGTCTTTC TCCAAAATCT TGTTCATCGA GTATCTAACT GACTTCTTCG ACTCTTCAAC CTAAACGGTA   
  
  
- AAGTCAAGTT AGGACACCAA ACGTTTGATC TCTTAGAGCT GTAGCCTTTT GAGGCACAAT TCTGGCCCCT   
  
  
- CCGGAACCGA TAATGGAGCC AGGAAGTTGA CGTATGGGAA AACCGAAGAC TTCTCCTTCA GGAATTCTTT   
  
  
- TCAAGTGGGA ACCGTAACCA TTTCGTTCGG TTACCCCGAT TAAATGTCCC GAACAAGTTA TTTCTACCTC   
  
  
- GATTATTATC CGCATCGGGT TCATTACTAA GCCGAAGTAG ACGTGGAAGT AGGGAGTTGT GAAGTCGGTT   
  
  
- CTACCTTCCA AAGGAATCGC GAAACACCCC AAATAGGGGT TTCTAATACC ACTATTGGCT CGTTCTAAGG   
  
  
- TTGGTGTTAC CCCGTCCTGA TTACCTCTCT AACAGTCTTC GTAACATGAA GATACGTCGT AACAAGCTAA   
  
  
- CGAATCTTAA ATGGGAGGGC TCTTGGAGGC ACCTCTCTTC CTTCCAGCTC TACGAGGAGG AACCGTTCCT   
  
  
- TTAGTTCTTG TAGTATCGCA CACTCCCTCC TCTTTCTTAT CTATCCGTAC TCTTCAACCC CTTCACCTAA   
  
  
- TTCTCCGAAC TCTACCGGCC CAAACCTTCG CAAGGAAACT CGGTGTATCC GTACTAGGTT CGTTCCGCCA   
  
  
- ACAACGTCTC GATACCGACA CTACCAATAT CTTATTTCCT CCTCTTGCCT ACAAAACAAT AGACGACCGT   
  
  
- TCTAGCGGGG GAGAAAAGTC ATAGACGAAC CTCTACATCC TCCAC
