# Supplementary material for: Genome-Wide Identification and Expression Pattern of the GRAS Gene Family in Pitaya (Selenicereus undatus L.)
Source: Biology (Basel). 2022 Dec 21;12(1):11. doi: 10.3390/biology12010011 (PMC9854919; doi:10.3390/biology12010011)
Supplement: Supplementary file 1 [file biology-12-00011-s001.zip › Supplementary file S5/HU08G02295.1_plantcare.html]

Content-Type: text/html; charset=ISO-8859-1


PlantCARE


Webmaster Firefox specific output  
To save the result:
click on the frame with the right mouse button and save the source code as a text file with extension .html  
REFERENCE:PlantCARE: a database of plant cis-acting regulatory elements and a portal to tools for in silico analysis of promoter sequences.  
Lescot, M., Déhais, P., Moreau, Y., De Moor, B., Rouzé ,P.,and Rombauts, S.  
Nucleic Acids Res., Database issue(2002), 30(1):325-327.   


---

>HU08G02295.1   
+ -Up\_Stream \_Len000GTTAAA AAACTCAGAA CTATTATAGA TTTTCTGTTT TCTGTTTGGA TTGAGATCCG   
  
  
+ AAGCCTTTGA GATCCCCTGT TTCTAGCTTC CAACCCCCCT CCCTGGGTTC TTTTTCTTTG GTGTGAAAGT   
  
  
+ ATGAACAAAA GCTGATGTAA GTGTGATTCA AACTCTAGCC TTGGTGAGAC TCTTGGGAAG GGCTCCTAGA   
  
  
+ GACTTTGCCA ATTGAGCTAA TTTGATGCCT ACTACTTGCT TACTTTTCTA GCTGTTGTTT AAACGATTTA   
  
  
+ TGTTCATAGT TAAGGAGCGC CATATTGCAT TACAATATCT GCTTTAGTGC TTTGTTTTTT GCATTTTCTT   
  
  
+ CTTTATTACT ATGGTTTTTG AAGAAGGCTT ACTGACTGAT ACTGGTTTCT TCGCTTAAGT TTGGTGTACA   
  
  
+ TGCTTTTCAA TCTGTGTTGC ATTTTTCTAT GATGTATAAG CCGTTTATTA CCTATTGATG TTCAACTTTT   
  
  
+ CACACTATAT ATCCTTTATG GTTTCTTCGC TTAAGTTTGG TGTACATGCT TTTCAATCTG TGTTGCATTT   
  
  
+ TTCTGTGATG TATAAGCCGT TTATTACCTA TTGATGTTCA ACTTTTCACA CTATATGTCC TTTTATTAGT   
  
  
+ TACTGATGCC TCTGTCAAAC ATTCTTAATT GCATTTAGTG TATCAACGGT CTTAAAATTT TAAGCATTGC   
  
  
+ CAACTTGAAC TTCTGAAGAT GTTTGTCAAG ATCTTCAGTC TGCAAGTTAA CTGCTGCTTG CTTGAACTTT   
  
  
+ TCAACTTTTA AGAGAGATGT CAGAGTCTAA GCATGGCTTC CTGACAAGTA CTGCATTTTA TCACTTATGA   
  
  
+ AGACTCAATT CTGGGTTTTG ACAGCTTCTA TGAACCTCTA GTGCAGGTAC AGCAGCTTGG ACATTAAGCT   
  
  
+ GCAAGTCAAT TGACTGTAGA ACGACAAGTT GTTGAAGTTT CTGCTGAAAG TTCATCACTT GTTTGATGAA   
  
  
+ TAAAGTACAG CATGGCACTT CAGATGCAAA AGTCTCGCGA TCAGTAGAGA TGATTCCATA TTTCTCATCT   
  
  
+ CATTTCCAAG TCTTTGACAA CATGTACCCA AATAATGCCA GCCATGACAC TCAGATGTCT CTCCAATCAT   
  
  
+ ACAGCGAAGG ATACTTCACT CTGGACTCAT CTCCAGCAGC AATCGGAGCG TGCAGTGTCT ATGACTACCC   
  
  
+ ATCCGTTGTC AGCACCTCTT CAAATAGAAG CCAGTTTTCT CCTCAGGGTT CCCACTCGTA CATCTCAGAC   
  
  
+ CCCCATCATT CTTCCGACAA CTATGGATCC CCAGTGAGCG GCTCTTCTGT GGTTGACGAT AATGCTGAGC   
  
  
+ TGAGGAACAG GTTCAGTGAT ATGGAGCTCC CCTTGCCACA GGACTCAGGA CACCATTATT GCTCTTTTAG   
  
  
+ CCACAGAGGA AGCCATGAAG GCTCCTATAC TTTGAGGCCA AACCAACTGA TGGATATGGC CAACATGGAG   
  
  
+ TTAAAGCAGG TGCTATACTT CTGTGCAGAA GCAATCTCAG AGAATAATCT ATCAACTGCA GAAAGACTAA   
  
  
+ TGGATGCATT GGGTAAGAGG GTGTCTGTTT TTGGTTCACC AATTGAAAGG TTGGCCGCCT ACATGTTGGA   
  
  
+ AGGGCTCAGA GCAAGGCTGG AGTTTTCTGG ATATACTATC TACAAAAAGC TCAGGTGCGA ACAGCCAACG   
  
  
+ AGCTCAGAGC TTCTTTCCTA CATGCACATC CTGTATCAAA GTTGCCCATA TTTCAAATTC GCATATATGT   
  
  
+ CCTCAAATGT TGCTATTCAA GAAGCTTTGG GGAATGAGCC GGTTATCCAC ATCATCGATT TCCAGATTGC   
  
  
+ CATGGGGACA CAATTGGTGC TCTTGATCCA GTCTCTCGCC CATCGGCCTG GCGGGCCCCC CCCTCGTTCG   
  
  
+ GATCACTGGG GTCGATGATC CACACTCGGC CTATGCTCGT GGCGGGGGGC TCGAGGTTGT GGGGCAGAGG   
  
  
+ CTAACAAAGG TGGCTGAATC ATATGGAGTC CCATTCGAGT TTCATGCTGC AGCCATGTCA GGGTGCAAGG   
  
  
+ TGAATCGCGA TGTCCTCAAG GTTCGCCCTG GGGAAGAAGC CTTGGCCGTA AACTTCCCCT ATATGTTGCA   
  
  
+ TCACATGCCC GACGAGAGCG TGAGCACCAC AAATCATCGG GACCGGCTCT TGAGGCTGGT GAAGAGGTTG   
  
  
+ TCACCAAGGA TCGTCACCTT GGTTGAGCAA GAGTCCAACA CAAACACTCC TCCCTTTCTT CAACGGTTCC   
  
  
+ GTGAAACATT GGACTACTAC ACTGCCATGT TCGAGTCCAT TGATGTGGCT CTACCTAGGG ATGACAAGAA   
  
  
+ GAGGATAAAT GCCGAGCAGC ATTGTCTTGC GCGCGATATT GTGAACATGG TCGCTTGTGA GAATGCGGAA   
  
  
+ AGGGTTGAGA GGCACGAGCC CTTCGGGAAA TGGAGGGCAA GGTTTGATAT GGCTGGGTTT CAGCAATTGC   
  
  
+ CCTCGAGCCG CTCAGTGAAT GATGCAATAA GGGGTTTGAT GCGAGAATTC CATAGGAACT ATCGGGTGCA   
  
  
+ GGATTGTCAG GGTGCCCTTT TTTTGGGCTG GAAGGAGCGC AATCTGGCAA CCTTCTCTAC ATGGTGCTGT   
  
  
+ AAAGAATG  

- -Up\_Stream \_Len000CAATTT TTTGAGTCTT GATAATATCT AAAAGACAAA AGACAAACCT AACTCTAGGC   
  
  
- TTCGGAAACT CTAGGGGACA AAGATCGAAG GTTGGGGGGA GGGACCCAAG AAAAAGAAAC CACACTTTCA   
  
  
- TACTTGTTTT CGACTACATT CACACTAAGT TTGAGATCGG AACCACTCTG AGAACCCTTC CCGAGGATCT   
  
  
- CTGAAACGGT TAACTCGATT AAACTACGGA TGATGAACGA ATGAAAAGAT CGACAACAAA TTTGCTAAAT   
  
  
- ACAAGTATCA ATTCCTCGCG GTATAACGTA ATGTTATAGA CGAAATCACG AAACAAAAAA CGTAAAAGAA   
  
  
- GAAATAATGA TACCAAAAAC TTCTTCCGAA TGACTGACTA TGACCAAAGA AGCGAATTCA AACCACATGT   
  
  
- ACGAAAAGTT AGACACAACG TAAAAAGATA CTACATATTC GGCAAATAAT GGATAACTAC AAGTTGAAAA   
  
  
- GTGTGATATA TAGGAAATAC CAAAGAAGCG AATTCAAACC ACATGTACGA AAAGTTAGAC ACAACGTAAA   
  
  
- AAGACACTAC ATATTCGGCA AATAATGGAT AACTACAAGT TGAAAAGTGT GATATACAGG AAAATAATCA   
  
  
- ATGACTACGG AGACAGTTTG TAAGAATTAA CGTAAATCAC ATAGTTGCCA GAATTTTAAA ATTCGTAACG   
  
  
- GTTGAACTTG AAGACTTCTA CAAACAGTTC TAGAAGTCAG ACGTTCAATT GACGACGAAC GAACTTGAAA   
  
  
- AGTTGAAAAT TCTCTCTACA GTCTCAGATT CGTACCGAAG GACTGTTCAT GACGTAAAAT AGTGAATACT   
  
  
- TCTGAGTTAA GACCCAAAAC TGTCGAAGAT ACTTGGAGAT CACGTCCATG TCGTCGAACC TGTAATTCGA   
  
  
- CGTTCAGTTA ACTGACATCT TGCTGTTCAA CAACTTCAAA GACGACTTTC AAGTAGTGAA CAAACTACTT   
  
  
- ATTTCATGTC GTACCGTGAA GTCTACGTTT TCAGAGCGCT AGTCATCTCT ACTAAGGTAT AAAGAGTAGA   
  
  
- GTAAAGGTTC AGAAACTGTT GTACATGGGT TTATTACGGT CGGTACTGTG AGTCTACAGA GAGGTTAGTA   
  
  
- TGTCGCTTCC TATGAAGTGA GACCTGAGTA GAGGTCGTCG TTAGCCTCGC ACGTCACAGA TACTGATGGG   
  
  
- TAGGCAACAG TCGTGGAGAA GTTTATCTTC GGTCAAAAGA GGAGTCCCAA GGGTGAGCAT GTAGAGTCTG   
  
  
- GGGGTAGTAA GAAGGCTGTT GATACCTAGG GGTCACTCGC CGAGAAGACA CCAACTGCTA TTACGACTCG   
  
  
- ACTCCTTGTC CAAGTCACTA TACCTCGAGG GGAACGGTGT CCTGAGTCCT GTGGTAATAA CGAGAAAATC   
  
  
- GGTGTCTCCT TCGGTACTTC CGAGGATATG AAACTCCGGT TTGGTTGACT ACCTATACCG GTTGTACCTC   
  
  
- AATTTCGTCC ACGATATGAA GACACGTCTT CGTTAGAGTC TCTTATTAGA TAGTTGACGT CTTTCTGATT   
  
  
- ACCTACGTAA CCCATTCTCC CACAGACAAA AACCAAGTGG TTAACTTTCC AACCGGCGGA TGTACAACCT   
  
  
- TCCCGAGTCT CGTTCCGACC TCAAAAGACC TATATGATAG ATGTTTTTCG AGTCCACGCT TGTCGGTTGC   
  
  
- TCGAGTCTCG AAGAAAGGAT GTACGTGTAG GACATAGTTT CAACGGGTAT AAAGTTTAAG CGTATATACA   
  
  
- GGAGTTTACA ACGATAAGTT CTTCGAAACC CCTTACTCGG CCAATAGGTG TAGTAGCTAA AGGTCTAACG   
  
  
- GTACCCCTGT GTTAACCACG AGAACTAGGT CAGAGAGCGG GTAGCCGGAC CGCCCGGGGG GGGAGCAAGC   
  
  
- CTAGTGACCC CAGCTACTAG GTGTGAGCCG GATACGAGCA CCGCCCCCCG AGCTCCAACA CCCCGTCTCC   
  
  
- GATTGTTTCC ACCGACTTAG TATACCTCAG GGTAAGCTCA AAGTACGACG TCGGTACAGT CCCACGTTCC   
  
  
- ACTTAGCGCT ACAGGAGTTC CAAGCGGGAC CCCTTCTTCG GAACCGGCAT TTGAAGGGGA TATACAACGT   
  
  
- AGTGTACGGG CTGCTCTCGC ACTCGTGGTG TTTAGTAGCC CTGGCCGAGA ACTCCGACCA CTTCTCCAAC   
  
  
- AGTGGTTCCT AGCAGTGGAA CCAACTCGTT CTCAGGTTGT GTTTGTGAGG AGGGAAAGAA GTTGCCAAGG   
  
  
- CACTTTGTAA CCTGATGATG TGACGGTACA AGCTCAGGTA ACTACACCGA GATGGATCCC TACTGTTCTT   
  
  
- CTCCTATTTA CGGCTCGTCG TAACAGAACG CGCGCTATAA CACTTGTACC AGCGAACACT CTTACGCCTT   
  
  
- TCCCAACTCT CCGTGCTCGG GAAGCCCTTT ACCTCCCGTT CCAAACTATA CCGACCCAAA GTCGTTAACG   
  
  
- GGAGCTCGGC GAGTCACTTA CTACGTTATT CCCCAAACTA CGCTCTTAAG GTATCCTTGA TAGCCCACGT   
  
  
- CCTAACAGTC CCACGGGAAA AAAACCCGAC CTTCCTCGCG TTAGACCGTT GGAAGAGATG TACCACGACA   
  
  
- TTTCTTAC

  
  
Motifs Found  

+   

| Site Name | Organism | Position | Strand | Matrix score. | sequence | function |
| --- | --- | --- | --- | --- | --- | --- |
|  | organism | 1252 | - | 4 | motif\_sequence | short\_function |
|  | organism | 1076 | + | 4 | motif\_sequence | short\_function |
|  | organism | 619 | + | 4 | motif\_sequence | short\_function |
|  | organism | 1958 | - | 4 | motif\_sequence | short\_function |
|  | organism | 2579 | + | 4 | motif\_sequence | short\_function |
|  | organism | 2312 | - | 4 | motif\_sequence | short\_function |
|  | organism | 420 | - | 4 | motif\_sequence | short\_function |
|  | organism | 1306 | + | 4 | motif\_sequence | short\_function |
|  | organism | 903 | - | 4 | motif\_sequence | short\_function |
|  | organism | 2357 | - | 4 | motif\_sequence | short\_function |
|  | organism | 2165 | - | 4 | motif\_sequence | short\_function |
|  | organism | 735 | + | 4 | motif\_sequence | short\_function |
|  | organism | 1339 | - | 4 | motif\_sequence | short\_function |
|  | organism | 719 | - | 4 | motif\_sequence | short\_function |
|  | organism | 989 | - | 4 | motif\_sequence | short\_function |
|  | organism | 891 | - | 4 | motif\_sequence | short\_function |
|  | organism | 1028 | - | 4 | motif\_sequence | short\_function |
|  | organism | 1153 | + | 4 | motif\_sequence | short\_function |
|  | organism | 536 | - | 4 | motif\_sequence | short\_function |
|  | organism | 598 | + | 4 | motif\_sequence | short\_function |
|  | organism | 1751 | + | 4 | motif\_sequence | short\_function |
|  | organism | 482 | + | 4 | motif\_sequence | short\_function |
|  | organism | 2271 | + | 4 | motif\_sequence | short\_function |
|  | organism | 2044 | + | 4 | motif\_sequence | short\_function |
|  | organism | 1495 | + | 4 | motif\_sequence | short\_function |
|  | organism | 1113 | + | 4 | motif\_sequence | short\_function |
|  | organism | 1708 | - | 4 | motif\_sequence | short\_function |
|  | organism | 1673 | - | 4 | motif\_sequence | short\_function |
|  | organism | 284 | + | 4 | motif\_sequence | short\_function |
|  | organism | 321 | + | 4 | motif\_sequence | short\_function |
|  | organism | 2293 | + | 4 | motif\_sequence | short\_function |
|  | organism | 1652 | + | 4 | motif\_sequence | short\_function |
|  | organism | 1210 | + | 4 | motif\_sequence | short\_function |

>HU08G02295.1   
+ -Up\_Stream \_Len000GTTAAA AAACTCAGAA CTATTATAGA TTTTCTGTTT TCTGTTTGGA TTGAGATCCG   
  
  
+ AAGCCTTTGA GATCCCCTGT TTCTAGCTTC CAACCCCCCT CCCTGGGTTC TTTTTCTTTG GTGTGAAAGT   
  
  
+ ATGAACAAAA GCTGATGTAA GTGTGATTCA AACTCTAGCC TTGGTGAGAC TCTTGGGAAG GGCTCCTAGA   
  
  
+ GACTTTGCCA ATTGAGCTAA TTTGATGCCT ACTACTTGCT TACTTTTCTA GCTGTTGTTT AAACGATTTA   
  
  
+ TGTTCATAGT TAAGGAGCGC CATATTGCAT TACAATATCT GCTTTAGTGC TTTGTTTTTT GCATTTTCTT   
  
  
+ CTTTATTACT ATGGTTTTTG AAGAAGGCTT ACTGACTGAT ACTGGTTTCT TCGCTTAAGT TTGGTGTACA   
  
  
+ TGCTTTTCAA TCTGTGTTGC ATTTTTCTAT GATGTATAAG CCGTTTATTA CCTATTGATG TTCAACTTTT   
  
  
+ CACACTATAT ATCCTTTATG GTTTCTTCGC TTAAGTTTGG TGTACATGCT TTTCAATCTG TGTTGCATTT   
  
  
+ TTCTGTGATG TATAAGCCGT TTATTACCTA TTGATGTTCA ACTTTTCACA CTATATGTCC TTTTATTAGT   
  
  
+ TACTGATGCC TCTGTCAAAC ATTCTTAATT GCATTTAGTG TATCAACGGT CTTAAAATTT TAAGCATTGC   
  
  
+ CAACTTGAAC TTCTGAAGAT GTTTGTCAAG ATCTTCAGTC TGCAAGTTAA CTGCTGCTTG CTTGAACTTT   
  
  
+ TCAACTTTTA AGAGAGATGT CAGAGTCTAA GCATGGCTTC CTGACAAGTA CTGCATTTTA TCACTTATGA   
  
  
+ AGACTCAATT CTGGGTTTTG ACAGCTTCTA TGAACCTCTA GTGCAGGTAC AGCAGCTTGG ACATTAAGCT   
  
  
+ GCAAGTCAAT TGACTGTAGA ACGACAAGTT GTTGAAGTTT CTGCTGAAAG TTCATCACTT GTTTGATGAA   
  
  
+ TAAAGTACAG CATGGCACTT CAGATGCAAA AGTCTCGCGA TCAGTAGAGA TGATTCCATA TTTCTCATCT   
  
  
+ CATTTCCAAG TCTTTGACAA CATGTACCCA AATAATGCCA GCCATGACAC TCAGATGTCT CTCCAATCAT   
  
  
+ ACAGCGAAGG ATACTTCACT CTGGACTCAT CTCCAGCAGC AATCGGAGCG TGCAGTGTCT ATGACTACCC   
  
  
+ ATCCGTTGTC AGCACCTCTT CAAATAGAAG CCAGTTTTCT CCTCAGGGTT CCCACTCGTA CATCTCAGAC   
  
  
+ CCCCATCATT CTTCCGACAA CTATGGATCC CCAGTGAGCG GCTCTTCTGT GGTTGACGAT AATGCTGAGC   
  
  
+ TGAGGAACAG GTTCAGTGAT ATGGAGCTCC CCTTGCCACA GGACTCAGGA CACCATTATT GCTCTTTTAG   
  
  
+ CCACAGAGGA AGCCATGAAG GCTCCTATAC TTTGAGGCCA AACCAACTGA TGGATATGGC CAACATGGAG   
  
  
+ TTAAAGCAGG TGCTATACTT CTGTGCAGAA GCAATCTCAG AGAATAATCT ATCAACTGCA GAAAGACTAA   
  
  
+ TGGATGCATT GGGTAAGAGG GTGTCTGTTT TTGGTTCACC AATTGAAAGG TTGGCCGCCT ACATGTTGGA   
  
  
+ AGGGCTCAGA GCAAGGCTGG AGTTTTCTGG ATATACTATC TACAAAAAGC TCAGGTGCGA ACAGCCAACG   
  
  
+ AGCTCAGAGC TTCTTTCCTA CATGCACATC CTGTATCAAA GTTGCCCATA TTTCAAATTC GCATATATGT   
  
  
+ CCTCAAATGT TGCTATTCAA GAAGCTTTGG GGAATGAGCC GGTTATCCAC ATCATCGATT TCCAGATTGC   
  
  
+ CATGGGGACA CAATTGGTGC TCTTGATCCA GTCTCTCGCC CATCGGCCTG GCGGGCCCCC CCCTCGTTCG   
  
  
+ GATCACTGGG GTCGATGATC CACACTCGGC CTATGCTCGT GGCGGGGGGC TCGAGGTTGT GGGGCAGAGG   
  
  
+ CTAACAAAGG TGGCTGAATC ATATGGAGTC CCATTCGAGT TTCATGCTGC AGCCATGTCA GGGTGCAAGG   
  
  
+ TGAATCGCGA TGTCCTCAAG GTTCGCCCTG GGGAAGAAGC CTTGGCCGTA AACTTCCCCT ATATGTTGCA   
  
  
+ TCACATGCCC GACGAGAGCG TGAGCACCAC AAATCATCGG GACCGGCTCT TGAGGCTGGT GAAGAGGTTG   
  
  
+ TCACCAAGGA TCGTCACCTT GGTTGAGCAA GAGTCCAACA CAAACACTCC TCCCTTTCTT CAACGGTTCC   
  
  
+ GTGAAACATT GGACTACTAC ACTGCCATGT TCGAGTCCAT TGATGTGGCT CTACCTAGGG ATGACAAGAA   
  
  
+ GAGGATAAAT GCCGAGCAGC ATTGTCTTGC GCGCGATATT GTGAACATGG TCGCTTGTGA GAATGCGGAA   
  
  
+ AGGGTTGAGA GGCACGAGCC CTTCGGGAAA TGGAGGGCAA GGTTTGATAT GGCTGGGTTT CAGCAATTGC   
  
  
+ CCTCGAGCCG CTCAGTGAAT GATGCAATAA GGGGTTTGAT GCGAGAATTC CATAGGAACT ATCGGGTGCA   
  
  
+ GGATTGTCAG GGTGCCCTTT TTTTGGGCTG GAAGGAGCGC AATCTGGCAA CCTTCTCTAC ATGGTGCTGT   
  
  
+ AAAGAATG  

- -Up\_Stream \_Len000CAATTT TTTGAGTCTT GATAATATCT AAAAGACAAA AGACAAACCT AACTCTAGGC   
  
  
- TTCGGAAACT CTAGGGGACA AAGATCGAAG GTTGGGGGGA GGGACCCAAG AAAAAGAAAC CACACTTTCA   
  
  
- TACTTGTTTT CGACTACATT CACACTAAGT TTGAGATCGG AACCACTCTG AGAACCCTTC CCGAGGATCT   
  
  
- CTGAAACGGT TAACTCGATT AAACTACGGA TGATGAACGA ATGAAAAGAT CGACAACAAA TTTGCTAAAT   
  
  
- ACAAGTATCA ATTCCTCGCG GTATAACGTA ATGTTATAGA CGAAATCACG AAACAAAAAA CGTAAAAGAA   
  
  
- GAAATAATGA TACCAAAAAC TTCTTCCGAA TGACTGACTA TGACCAAAGA AGCGAATTCA AACCACATGT   
  
  
- ACGAAAAGTT AGACACAACG TAAAAAGATA CTACATATTC GGCAAATAAT GGATAACTAC AAGTTGAAAA   
  
  
- GTGTGATATA TAGGAAATAC CAAAGAAGCG AATTCAAACC ACATGTACGA AAAGTTAGAC ACAACGTAAA   
  
  
- AAGACACTAC ATATTCGGCA AATAATGGAT AACTACAAGT TGAAAAGTGT GATATACAGG AAAATAATCA   
  
  
- ATGACTACGG AGACAGTTTG TAAGAATTAA CGTAAATCAC ATAGTTGCCA GAATTTTAAA ATTCGTAACG   
  
  
- GTTGAACTTG AAGACTTCTA CAAACAGTTC TAGAAGTCAG ACGTTCAATT GACGACGAAC GAACTTGAAA   
  
  
- AGTTGAAAAT TCTCTCTACA GTCTCAGATT CGTACCGAAG GACTGTTCAT GACGTAAAAT AGTGAATACT   
  
  
- TCTGAGTTAA GACCCAAAAC TGTCGAAGAT ACTTGGAGAT CACGTCCATG TCGTCGAACC TGTAATTCGA   
  
  
- CGTTCAGTTA ACTGACATCT TGCTGTTCAA CAACTTCAAA GACGACTTTC AAGTAGTGAA CAAACTACTT   
  
  
- ATTTCATGTC GTACCGTGAA GTCTACGTTT TCAGAGCGCT AGTCATCTCT ACTAAGGTAT AAAGAGTAGA   
  
  
- GTAAAGGTTC AGAAACTGTT GTACATGGGT TTATTACGGT CGGTACTGTG AGTCTACAGA GAGGTTAGTA   
  
  
- TGTCGCTTCC TATGAAGTGA GACCTGAGTA GAGGTCGTCG TTAGCCTCGC ACGTCACAGA TACTGATGGG   
  
  
- TAGGCAACAG TCGTGGAGAA GTTTATCTTC GGTCAAAAGA GGAGTCCCAA GGGTGAGCAT GTAGAGTCTG   
  
  
- GGGGTAGTAA GAAGGCTGTT GATACCTAGG GGTCACTCGC CGAGAAGACA CCAACTGCTA TTACGACTCG   
  
  
- ACTCCTTGTC CAAGTCACTA TACCTCGAGG GGAACGGTGT CCTGAGTCCT GTGGTAATAA CGAGAAAATC   
  
  
- GGTGTCTCCT TCGGTACTTC CGAGGATATG AAACTCCGGT TTGGTTGACT ACCTATACCG GTTGTACCTC   
  
  
- AATTTCGTCC ACGATATGAA GACACGTCTT CGTTAGAGTC TCTTATTAGA TAGTTGACGT CTTTCTGATT   
  
  
- ACCTACGTAA CCCATTCTCC CACAGACAAA AACCAAGTGG TTAACTTTCC AACCGGCGGA TGTACAACCT   
  
  
- TCCCGAGTCT CGTTCCGACC TCAAAAGACC TATATGATAG ATGTTTTTCG AGTCCACGCT TGTCGGTTGC   
  
  
- TCGAGTCTCG AAGAAAGGAT GTACGTGTAG GACATAGTTT CAACGGGTAT AAAGTTTAAG CGTATATACA   
  
  
- GGAGTTTACA ACGATAAGTT CTTCGAAACC CCTTACTCGG CCAATAGGTG TAGTAGCTAA AGGTCTAACG   
  
  
- GTACCCCTGT GTTAACCACG AGAACTAGGT CAGAGAGCGG GTAGCCGGAC CGCCCGGGGG GGGAGCAAGC   
  
  
- CTAGTGACCC CAGCTACTAG GTGTGAGCCG GATACGAGCA CCGCCCCCCG AGCTCCAACA CCCCGTCTCC   
  
  
- GATTGTTTCC ACCGACTTAG TATACCTCAG GGTAAGCTCA AAGTACGACG TCGGTACAGT CCCACGTTCC   
  
  
- ACTTAGCGCT ACAGGAGTTC CAAGCGGGAC CCCTTCTTCG GAACCGGCAT TTGAAGGGGA TATACAACGT   
  
  
- AGTGTACGGG CTGCTCTCGC ACTCGTGGTG TTTAGTAGCC CTGGCCGAGA ACTCCGACCA CTTCTCCAAC   
  
  
- AGTGGTTCCT AGCAGTGGAA CCAACTCGTT CTCAGGTTGT GTTTGTGAGG AGGGAAAGAA GTTGCCAAGG   
  
  
- CACTTTGTAA CCTGATGATG TGACGGTACA AGCTCAGGTA ACTACACCGA GATGGATCCC TACTGTTCTT   
  
  
- CTCCTATTTA CGGCTCGTCG TAACAGAACG CGCGCTATAA CACTTGTACC AGCGAACACT CTTACGCCTT   
  
  
- TCCCAACTCT CCGTGCTCGG GAAGCCCTTT ACCTCCCGTT CCAAACTATA CCGACCCAAA GTCGTTAACG   
  
  
- GGAGCTCGGC GAGTCACTTA CTACGTTATT CCCCAAACTA CGCTCTTAAG GTATCCTTGA TAGCCCACGT   
  
  
- CCTAACAGTC CCACGGGAAA AAAACCCGAC CTTCCTCGCG TTAGACCGTT GGAAGAGATG TACCACGACA   
  
  
- TTTCTTAC

+     AAGAA-motif

| Site Name | Organism | Position | Strand | Matrix score. | sequence | function |
| --- | --- | --- | --- | --- | --- | --- |
| AAGAA-motif | Avena sativa | 1695 | - | 7 | GAAAGAA |  |

>HU08G02295.1   
+ -Up\_Stream \_Len000GTTAAA AAACTCAGAA CTATTATAGA TTTTCTGTTT TCTGTTTGGA TTGAGATCCG   
  
  
+ AAGCCTTTGA GATCCCCTGT TTCTAGCTTC CAACCCCCCT CCCTGGGTTC TTTTTCTTTG GTGTGAAAGT   
  
  
+ ATGAACAAAA GCTGATGTAA GTGTGATTCA AACTCTAGCC TTGGTGAGAC TCTTGGGAAG GGCTCCTAGA   
  
  
+ GACTTTGCCA ATTGAGCTAA TTTGATGCCT ACTACTTGCT TACTTTTCTA GCTGTTGTTT AAACGATTTA   
  
  
+ TGTTCATAGT TAAGGAGCGC CATATTGCAT TACAATATCT GCTTTAGTGC TTTGTTTTTT GCATTTTCTT   
  
  
+ CTTTATTACT ATGGTTTTTG AAGAAGGCTT ACTGACTGAT ACTGGTTTCT TCGCTTAAGT TTGGTGTACA   
  
  
+ TGCTTTTCAA TCTGTGTTGC ATTTTTCTAT GATGTATAAG CCGTTTATTA CCTATTGATG TTCAACTTTT   
  
  
+ CACACTATAT ATCCTTTATG GTTTCTTCGC TTAAGTTTGG TGTACATGCT TTTCAATCTG TGTTGCATTT   
  
  
+ TTCTGTGATG TATAAGCCGT TTATTACCTA TTGATGTTCA ACTTTTCACA CTATATGTCC TTTTATTAGT   
  
  
+ TACTGATGCC TCTGTCAAAC ATTCTTAATT GCATTTAGTG TATCAACGGT CTTAAAATTT TAAGCATTGC   
  
  
+ CAACTTGAAC TTCTGAAGAT GTTTGTCAAG ATCTTCAGTC TGCAAGTTAA CTGCTGCTTG CTTGAACTTT   
  
  
+ TCAACTTTTA AGAGAGATGT CAGAGTCTAA GCATGGCTTC CTGACAAGTA CTGCATTTTA TCACTTATGA   
  
  
+ AGACTCAATT CTGGGTTTTG ACAGCTTCTA TGAACCTCTA GTGCAGGTAC AGCAGCTTGG ACATTAAGCT   
  
  
+ GCAAGTCAAT TGACTGTAGA ACGACAAGTT GTTGAAGTTT CTGCTGAAAG TTCATCACTT GTTTGATGAA   
  
  
+ TAAAGTACAG CATGGCACTT CAGATGCAAA AGTCTCGCGA TCAGTAGAGA TGATTCCATA TTTCTCATCT   
  
  
+ CATTTCCAAG TCTTTGACAA CATGTACCCA AATAATGCCA GCCATGACAC TCAGATGTCT CTCCAATCAT   
  
  
+ ACAGCGAAGG ATACTTCACT CTGGACTCAT CTCCAGCAGC AATCGGAGCG TGCAGTGTCT ATGACTACCC   
  
  
+ ATCCGTTGTC AGCACCTCTT CAAATAGAAG CCAGTTTTCT CCTCAGGGTT CCCACTCGTA CATCTCAGAC   
  
  
+ CCCCATCATT CTTCCGACAA CTATGGATCC CCAGTGAGCG GCTCTTCTGT GGTTGACGAT AATGCTGAGC   
  
  
+ TGAGGAACAG GTTCAGTGAT ATGGAGCTCC CCTTGCCACA GGACTCAGGA CACCATTATT GCTCTTTTAG   
  
  
+ CCACAGAGGA AGCCATGAAG GCTCCTATAC TTTGAGGCCA AACCAACTGA TGGATATGGC CAACATGGAG   
  
  
+ TTAAAGCAGG TGCTATACTT CTGTGCAGAA GCAATCTCAG AGAATAATCT ATCAACTGCA GAAAGACTAA   
  
  
+ TGGATGCATT GGGTAAGAGG GTGTCTGTTT TTGGTTCACC AATTGAAAGG TTGGCCGCCT ACATGTTGGA   
  
  
+ AGGGCTCAGA GCAAGGCTGG AGTTTTCTGG ATATACTATC TACAAAAAGC TCAGGTGCGA ACAGCCAACG   
  
  
+ AGCTCAGAGC TTCTTTCCTA CATGCACATC CTGTATCAAA GTTGCCCATA TTTCAAATTC GCATATATGT   
  
  
+ CCTCAAATGT TGCTATTCAA GAAGCTTTGG GGAATGAGCC GGTTATCCAC ATCATCGATT TCCAGATTGC   
  
  
+ CATGGGGACA CAATTGGTGC TCTTGATCCA GTCTCTCGCC CATCGGCCTG GCGGGCCCCC CCCTCGTTCG   
  
  
+ GATCACTGGG GTCGATGATC CACACTCGGC CTATGCTCGT GGCGGGGGGC TCGAGGTTGT GGGGCAGAGG   
  
  
+ CTAACAAAGG TGGCTGAATC ATATGGAGTC CCATTCGAGT TTCATGCTGC AGCCATGTCA GGGTGCAAGG   
  
  
+ TGAATCGCGA TGTCCTCAAG GTTCGCCCTG GGGAAGAAGC CTTGGCCGTA AACTTCCCCT ATATGTTGCA   
  
  
+ TCACATGCCC GACGAGAGCG TGAGCACCAC AAATCATCGG GACCGGCTCT TGAGGCTGGT GAAGAGGTTG   
  
  
+ TCACCAAGGA TCGTCACCTT GGTTGAGCAA GAGTCCAACA CAAACACTCC TCCCTTTCTT CAACGGTTCC   
  
  
+ GTGAAACATT GGACTACTAC ACTGCCATGT TCGAGTCCAT TGATGTGGCT CTACCTAGGG ATGACAAGAA   
  
  
+ GAGGATAAAT GCCGAGCAGC ATTGTCTTGC GCGCGATATT GTGAACATGG TCGCTTGTGA GAATGCGGAA   
  
  
+ AGGGTTGAGA GGCACGAGCC CTTCGGGAAA TGGAGGGCAA GGTTTGATAT GGCTGGGTTT CAGCAATTGC   
  
  
+ CCTCGAGCCG CTCAGTGAAT GATGCAATAA GGGGTTTGAT GCGAGAATTC CATAGGAACT ATCGGGTGCA   
  
  
+ GGATTGTCAG GGTGCCCTTT TTTTGGGCTG GAAGGAGCGC AATCTGGCAA CCTTCTCTAC ATGGTGCTGT   
  
  
+ AAAGAATG  

- -Up\_Stream \_Len000CAATTT TTTGAGTCTT GATAATATCT AAAAGACAAA AGACAAACCT AACTCTAGGC   
  
  
- TTCGGAAACT CTAGGGGACA AAGATCGAAG GTTGGGGGGA GGGACCCAAG AAAAAGAAAC CACACTTTCA   
  
  
- TACTTGTTTT CGACTACATT CACACTAAGT TTGAGATCGG AACCACTCTG AGAACCCTTC CCGAGGATCT   
  
  
- CTGAAACGGT TAACTCGATT AAACTACGGA TGATGAACGA ATGAAAAGAT CGACAACAAA TTTGCTAAAT   
  
  
- ACAAGTATCA ATTCCTCGCG GTATAACGTA ATGTTATAGA CGAAATCACG AAACAAAAAA CGTAAAAGAA   
  
  
- GAAATAATGA TACCAAAAAC TTCTTCCGAA TGACTGACTA TGACCAAAGA AGCGAATTCA AACCACATGT   
  
  
- ACGAAAAGTT AGACACAACG TAAAAAGATA CTACATATTC GGCAAATAAT GGATAACTAC AAGTTGAAAA   
  
  
- GTGTGATATA TAGGAAATAC CAAAGAAGCG AATTCAAACC ACATGTACGA AAAGTTAGAC ACAACGTAAA   
  
  
- AAGACACTAC ATATTCGGCA AATAATGGAT AACTACAAGT TGAAAAGTGT GATATACAGG AAAATAATCA   
  
  
- ATGACTACGG AGACAGTTTG TAAGAATTAA CGTAAATCAC ATAGTTGCCA GAATTTTAAA ATTCGTAACG   
  
  
- GTTGAACTTG AAGACTTCTA CAAACAGTTC TAGAAGTCAG ACGTTCAATT GACGACGAAC GAACTTGAAA   
  
  
- AGTTGAAAAT TCTCTCTACA GTCTCAGATT CGTACCGAAG GACTGTTCAT GACGTAAAAT AGTGAATACT   
  
  
- TCTGAGTTAA GACCCAAAAC TGTCGAAGAT ACTTGGAGAT CACGTCCATG TCGTCGAACC TGTAATTCGA   
  
  
- CGTTCAGTTA ACTGACATCT TGCTGTTCAA CAACTTCAAA GACGACTTTC AAGTAGTGAA CAAACTACTT   
  
  
- ATTTCATGTC GTACCGTGAA GTCTACGTTT TCAGAGCGCT AGTCATCTCT ACTAAGGTAT AAAGAGTAGA   
  
  
- GTAAAGGTTC AGAAACTGTT GTACATGGGT TTATTACGGT CGGTACTGTG AGTCTACAGA GAGGTTAGTA   
  
  
- TGTCGCTTCC TATGAAGTGA GACCTGAGTA GAGGTCGTCG TTAGCCTCGC ACGTCACAGA TACTGATGGG   
  
  
- TAGGCAACAG TCGTGGAGAA GTTTATCTTC GGTCAAAAGA GGAGTCCCAA GGGTGAGCAT GTAGAGTCTG   
  
  
- GGGGTAGTAA GAAGGCTGTT GATACCTAGG GGTCACTCGC CGAGAAGACA CCAACTGCTA TTACGACTCG   
  
  
- ACTCCTTGTC CAAGTCACTA TACCTCGAGG GGAACGGTGT CCTGAGTCCT GTGGTAATAA CGAGAAAATC   
  
  
- GGTGTCTCCT TCGGTACTTC CGAGGATATG AAACTCCGGT TTGGTTGACT ACCTATACCG GTTGTACCTC   
  
  
- AATTTCGTCC ACGATATGAA GACACGTCTT CGTTAGAGTC TCTTATTAGA TAGTTGACGT CTTTCTGATT   
  
  
- ACCTACGTAA CCCATTCTCC CACAGACAAA AACCAAGTGG TTAACTTTCC AACCGGCGGA TGTACAACCT   
  
  
- TCCCGAGTCT CGTTCCGACC TCAAAAGACC TATATGATAG ATGTTTTTCG AGTCCACGCT TGTCGGTTGC   
  
  
- TCGAGTCTCG AAGAAAGGAT GTACGTGTAG GACATAGTTT CAACGGGTAT AAAGTTTAAG CGTATATACA   
  
  
- GGAGTTTACA ACGATAAGTT CTTCGAAACC CCTTACTCGG CCAATAGGTG TAGTAGCTAA AGGTCTAACG   
  
  
- GTACCCCTGT GTTAACCACG AGAACTAGGT CAGAGAGCGG GTAGCCGGAC CGCCCGGGGG GGGAGCAAGC   
  
  
- CTAGTGACCC CAGCTACTAG GTGTGAGCCG GATACGAGCA CCGCCCCCCG AGCTCCAACA CCCCGTCTCC   
  
  
- GATTGTTTCC ACCGACTTAG TATACCTCAG GGTAAGCTCA AAGTACGACG TCGGTACAGT CCCACGTTCC   
  
  
- ACTTAGCGCT ACAGGAGTTC CAAGCGGGAC CCCTTCTTCG GAACCGGCAT TTGAAGGGGA TATACAACGT   
  
  
- AGTGTACGGG CTGCTCTCGC ACTCGTGGTG TTTAGTAGCC CTGGCCGAGA ACTCCGACCA CTTCTCCAAC   
  
  
- AGTGGTTCCT AGCAGTGGAA CCAACTCGTT CTCAGGTTGT GTTTGTGAGG AGGGAAAGAA GTTGCCAAGG   
  
  
- CACTTTGTAA CCTGATGATG TGACGGTACA AGCTCAGGTA ACTACACCGA GATGGATCCC TACTGTTCTT   
  
  
- CTCCTATTTA CGGCTCGTCG TAACAGAACG CGCGCTATAA CACTTGTACC AGCGAACACT CTTACGCCTT   
  
  
- TCCCAACTCT CCGTGCTCGG GAAGCCCTTT ACCTCCCGTT CCAAACTATA CCGACCCAAA GTCGTTAACG   
  
  
- GGAGCTCGGC GAGTCACTTA CTACGTTATT CCCCAAACTA CGCTCTTAAG GTATCCTTGA TAGCCCACGT   
  
  
- CCTAACAGTC CCACGGGAAA AAAACCCGAC CTTCCTCGCG TTAGACCGTT GGAAGAGATG TACCACGACA   
  
  
- TTTCTTAC

+     AE-box

| Site Name | Organism | Position | Strand | Matrix score. | sequence | function |
| --- | --- | --- | --- | --- | --- | --- |
| AE-box | Arabidopsis thaliana | 949 | - | 8 | AGAAACTT | part of a module for light response |

>HU08G02295.1   
+ -Up\_Stream \_Len000GTTAAA AAACTCAGAA CTATTATAGA TTTTCTGTTT TCTGTTTGGA TTGAGATCCG   
  
  
+ AAGCCTTTGA GATCCCCTGT TTCTAGCTTC CAACCCCCCT CCCTGGGTTC TTTTTCTTTG GTGTGAAAGT   
  
  
+ ATGAACAAAA GCTGATGTAA GTGTGATTCA AACTCTAGCC TTGGTGAGAC TCTTGGGAAG GGCTCCTAGA   
  
  
+ GACTTTGCCA ATTGAGCTAA TTTGATGCCT ACTACTTGCT TACTTTTCTA GCTGTTGTTT AAACGATTTA   
  
  
+ TGTTCATAGT TAAGGAGCGC CATATTGCAT TACAATATCT GCTTTAGTGC TTTGTTTTTT GCATTTTCTT   
  
  
+ CTTTATTACT ATGGTTTTTG AAGAAGGCTT ACTGACTGAT ACTGGTTTCT TCGCTTAAGT TTGGTGTACA   
  
  
+ TGCTTTTCAA TCTGTGTTGC ATTTTTCTAT GATGTATAAG CCGTTTATTA CCTATTGATG TTCAACTTTT   
  
  
+ CACACTATAT ATCCTTTATG GTTTCTTCGC TTAAGTTTGG TGTACATGCT TTTCAATCTG TGTTGCATTT   
  
  
+ TTCTGTGATG TATAAGCCGT TTATTACCTA TTGATGTTCA ACTTTTCACA CTATATGTCC TTTTATTAGT   
  
  
+ TACTGATGCC TCTGTCAAAC ATTCTTAATT GCATTTAGTG TATCAACGGT CTTAAAATTT TAAGCATTGC   
  
  
+ CAACTTGAAC TTCTGAAGAT GTTTGTCAAG ATCTTCAGTC TGCAAGTTAA CTGCTGCTTG CTTGAACTTT   
  
  
+ TCAACTTTTA AGAGAGATGT CAGAGTCTAA GCATGGCTTC CTGACAAGTA CTGCATTTTA TCACTTATGA   
  
  
+ AGACTCAATT CTGGGTTTTG ACAGCTTCTA TGAACCTCTA GTGCAGGTAC AGCAGCTTGG ACATTAAGCT   
  
  
+ GCAAGTCAAT TGACTGTAGA ACGACAAGTT GTTGAAGTTT CTGCTGAAAG TTCATCACTT GTTTGATGAA   
  
  
+ TAAAGTACAG CATGGCACTT CAGATGCAAA AGTCTCGCGA TCAGTAGAGA TGATTCCATA TTTCTCATCT   
  
  
+ CATTTCCAAG TCTTTGACAA CATGTACCCA AATAATGCCA GCCATGACAC TCAGATGTCT CTCCAATCAT   
  
  
+ ACAGCGAAGG ATACTTCACT CTGGACTCAT CTCCAGCAGC AATCGGAGCG TGCAGTGTCT ATGACTACCC   
  
  
+ ATCCGTTGTC AGCACCTCTT CAAATAGAAG CCAGTTTTCT CCTCAGGGTT CCCACTCGTA CATCTCAGAC   
  
  
+ CCCCATCATT CTTCCGACAA CTATGGATCC CCAGTGAGCG GCTCTTCTGT GGTTGACGAT AATGCTGAGC   
  
  
+ TGAGGAACAG GTTCAGTGAT ATGGAGCTCC CCTTGCCACA GGACTCAGGA CACCATTATT GCTCTTTTAG   
  
  
+ CCACAGAGGA AGCCATGAAG GCTCCTATAC TTTGAGGCCA AACCAACTGA TGGATATGGC CAACATGGAG   
  
  
+ TTAAAGCAGG TGCTATACTT CTGTGCAGAA GCAATCTCAG AGAATAATCT ATCAACTGCA GAAAGACTAA   
  
  
+ TGGATGCATT GGGTAAGAGG GTGTCTGTTT TTGGTTCACC AATTGAAAGG TTGGCCGCCT ACATGTTGGA   
  
  
+ AGGGCTCAGA GCAAGGCTGG AGTTTTCTGG ATATACTATC TACAAAAAGC TCAGGTGCGA ACAGCCAACG   
  
  
+ AGCTCAGAGC TTCTTTCCTA CATGCACATC CTGTATCAAA GTTGCCCATA TTTCAAATTC GCATATATGT   
  
  
+ CCTCAAATGT TGCTATTCAA GAAGCTTTGG GGAATGAGCC GGTTATCCAC ATCATCGATT TCCAGATTGC   
  
  
+ CATGGGGACA CAATTGGTGC TCTTGATCCA GTCTCTCGCC CATCGGCCTG GCGGGCCCCC CCCTCGTTCG   
  
  
+ GATCACTGGG GTCGATGATC CACACTCGGC CTATGCTCGT GGCGGGGGGC TCGAGGTTGT GGGGCAGAGG   
  
  
+ CTAACAAAGG TGGCTGAATC ATATGGAGTC CCATTCGAGT TTCATGCTGC AGCCATGTCA GGGTGCAAGG   
  
  
+ TGAATCGCGA TGTCCTCAAG GTTCGCCCTG GGGAAGAAGC CTTGGCCGTA AACTTCCCCT ATATGTTGCA   
  
  
+ TCACATGCCC GACGAGAGCG TGAGCACCAC AAATCATCGG GACCGGCTCT TGAGGCTGGT GAAGAGGTTG   
  
  
+ TCACCAAGGA TCGTCACCTT GGTTGAGCAA GAGTCCAACA CAAACACTCC TCCCTTTCTT CAACGGTTCC   
  
  
+ GTGAAACATT GGACTACTAC ACTGCCATGT TCGAGTCCAT TGATGTGGCT CTACCTAGGG ATGACAAGAA   
  
  
+ GAGGATAAAT GCCGAGCAGC ATTGTCTTGC GCGCGATATT GTGAACATGG TCGCTTGTGA GAATGCGGAA   
  
  
+ AGGGTTGAGA GGCACGAGCC CTTCGGGAAA TGGAGGGCAA GGTTTGATAT GGCTGGGTTT CAGCAATTGC   
  
  
+ CCTCGAGCCG CTCAGTGAAT GATGCAATAA GGGGTTTGAT GCGAGAATTC CATAGGAACT ATCGGGTGCA   
  
  
+ GGATTGTCAG GGTGCCCTTT TTTTGGGCTG GAAGGAGCGC AATCTGGCAA CCTTCTCTAC ATGGTGCTGT   
  
  
+ AAAGAATG  

- -Up\_Stream \_Len000CAATTT TTTGAGTCTT GATAATATCT AAAAGACAAA AGACAAACCT AACTCTAGGC   
  
  
- TTCGGAAACT CTAGGGGACA AAGATCGAAG GTTGGGGGGA GGGACCCAAG AAAAAGAAAC CACACTTTCA   
  
  
- TACTTGTTTT CGACTACATT CACACTAAGT TTGAGATCGG AACCACTCTG AGAACCCTTC CCGAGGATCT   
  
  
- CTGAAACGGT TAACTCGATT AAACTACGGA TGATGAACGA ATGAAAAGAT CGACAACAAA TTTGCTAAAT   
  
  
- ACAAGTATCA ATTCCTCGCG GTATAACGTA ATGTTATAGA CGAAATCACG AAACAAAAAA CGTAAAAGAA   
  
  
- GAAATAATGA TACCAAAAAC TTCTTCCGAA TGACTGACTA TGACCAAAGA AGCGAATTCA AACCACATGT   
  
  
- ACGAAAAGTT AGACACAACG TAAAAAGATA CTACATATTC GGCAAATAAT GGATAACTAC AAGTTGAAAA   
  
  
- GTGTGATATA TAGGAAATAC CAAAGAAGCG AATTCAAACC ACATGTACGA AAAGTTAGAC ACAACGTAAA   
  
  
- AAGACACTAC ATATTCGGCA AATAATGGAT AACTACAAGT TGAAAAGTGT GATATACAGG AAAATAATCA   
  
  
- ATGACTACGG AGACAGTTTG TAAGAATTAA CGTAAATCAC ATAGTTGCCA GAATTTTAAA ATTCGTAACG   
  
  
- GTTGAACTTG AAGACTTCTA CAAACAGTTC TAGAAGTCAG ACGTTCAATT GACGACGAAC GAACTTGAAA   
  
  
- AGTTGAAAAT TCTCTCTACA GTCTCAGATT CGTACCGAAG GACTGTTCAT GACGTAAAAT AGTGAATACT   
  
  
- TCTGAGTTAA GACCCAAAAC TGTCGAAGAT ACTTGGAGAT CACGTCCATG TCGTCGAACC TGTAATTCGA   
  
  
- CGTTCAGTTA ACTGACATCT TGCTGTTCAA CAACTTCAAA GACGACTTTC AAGTAGTGAA CAAACTACTT   
  
  
- ATTTCATGTC GTACCGTGAA GTCTACGTTT TCAGAGCGCT AGTCATCTCT ACTAAGGTAT AAAGAGTAGA   
  
  
- GTAAAGGTTC AGAAACTGTT GTACATGGGT TTATTACGGT CGGTACTGTG AGTCTACAGA GAGGTTAGTA   
  
  
- TGTCGCTTCC TATGAAGTGA GACCTGAGTA GAGGTCGTCG TTAGCCTCGC ACGTCACAGA TACTGATGGG   
  
  
- TAGGCAACAG TCGTGGAGAA GTTTATCTTC GGTCAAAAGA GGAGTCCCAA GGGTGAGCAT GTAGAGTCTG   
  
  
- GGGGTAGTAA GAAGGCTGTT GATACCTAGG GGTCACTCGC CGAGAAGACA CCAACTGCTA TTACGACTCG   
  
  
- ACTCCTTGTC CAAGTCACTA TACCTCGAGG GGAACGGTGT CCTGAGTCCT GTGGTAATAA CGAGAAAATC   
  
  
- GGTGTCTCCT TCGGTACTTC CGAGGATATG AAACTCCGGT TTGGTTGACT ACCTATACCG GTTGTACCTC   
  
  
- AATTTCGTCC ACGATATGAA GACACGTCTT CGTTAGAGTC TCTTATTAGA TAGTTGACGT CTTTCTGATT   
  
  
- ACCTACGTAA CCCATTCTCC CACAGACAAA AACCAAGTGG TTAACTTTCC AACCGGCGGA TGTACAACCT   
  
  
- TCCCGAGTCT CGTTCCGACC TCAAAAGACC TATATGATAG ATGTTTTTCG AGTCCACGCT TGTCGGTTGC   
  
  
- TCGAGTCTCG AAGAAAGGAT GTACGTGTAG GACATAGTTT CAACGGGTAT AAAGTTTAAG CGTATATACA   
  
  
- GGAGTTTACA ACGATAAGTT CTTCGAAACC CCTTACTCGG CCAATAGGTG TAGTAGCTAA AGGTCTAACG   
  
  
- GTACCCCTGT GTTAACCACG AGAACTAGGT CAGAGAGCGG GTAGCCGGAC CGCCCGGGGG GGGAGCAAGC   
  
  
- CTAGTGACCC CAGCTACTAG GTGTGAGCCG GATACGAGCA CCGCCCCCCG AGCTCCAACA CCCCGTCTCC   
  
  
- GATTGTTTCC ACCGACTTAG TATACCTCAG GGTAAGCTCA AAGTACGACG TCGGTACAGT CCCACGTTCC   
  
  
- ACTTAGCGCT ACAGGAGTTC CAAGCGGGAC CCCTTCTTCG GAACCGGCAT TTGAAGGGGA TATACAACGT   
  
  
- AGTGTACGGG CTGCTCTCGC ACTCGTGGTG TTTAGTAGCC CTGGCCGAGA ACTCCGACCA CTTCTCCAAC   
  
  
- AGTGGTTCCT AGCAGTGGAA CCAACTCGTT CTCAGGTTGT GTTTGTGAGG AGGGAAAGAA GTTGCCAAGG   
  
  
- CACTTTGTAA CCTGATGATG TGACGGTACA AGCTCAGGTA ACTACACCGA GATGGATCCC TACTGTTCTT   
  
  
- CTCCTATTTA CGGCTCGTCG TAACAGAACG CGCGCTATAA CACTTGTACC AGCGAACACT CTTACGCCTT   
  
  
- TCCCAACTCT CCGTGCTCGG GAAGCCCTTT ACCTCCCGTT CCAAACTATA CCGACCCAAA GTCGTTAACG   
  
  
- GGAGCTCGGC GAGTCACTTA CTACGTTATT CCCCAAACTA CGCTCTTAAG GTATCCTTGA TAGCCCACGT   
  
  
- CCTAACAGTC CCACGGGAAA AAAACCCGAC CTTCCTCGCG TTAGACCGTT GGAAGAGATG TACCACGACA   
  
  
- TTTCTTAC

+     ARE

| Site Name | Organism | Position | Strand | Matrix score. | sequence | function |
| --- | --- | --- | --- | --- | --- | --- |
| ARE | Zea mays | 1444 | + | 6 | AAACCA | cis-acting regulatory element essential for the anaerobic induction |
| ARE | Zea mays | 513 | - | 6 | AAACCA | cis-acting regulatory element essential for the anaerobic induction |
| ARE | Zea mays | 366 | - | 6 | AAACCA | cis-acting regulatory element essential for the anaerobic induction |
| ARE | Zea mays | 397 | - | 6 | AAACCA | cis-acting regulatory element essential for the anaerobic induction |

>HU08G02295.1   
+ -Up\_Stream \_Len000GTTAAA AAACTCAGAA CTATTATAGA TTTTCTGTTT TCTGTTTGGA TTGAGATCCG   
  
  
+ AAGCCTTTGA GATCCCCTGT TTCTAGCTTC CAACCCCCCT CCCTGGGTTC TTTTTCTTTG GTGTGAAAGT   
  
  
+ ATGAACAAAA GCTGATGTAA GTGTGATTCA AACTCTAGCC TTGGTGAGAC TCTTGGGAAG GGCTCCTAGA   
  
  
+ GACTTTGCCA ATTGAGCTAA TTTGATGCCT ACTACTTGCT TACTTTTCTA GCTGTTGTTT AAACGATTTA   
  
  
+ TGTTCATAGT TAAGGAGCGC CATATTGCAT TACAATATCT GCTTTAGTGC TTTGTTTTTT GCATTTTCTT   
  
  
+ CTTTATTACT ATGGTTTTTG AAGAAGGCTT ACTGACTGAT ACTGGTTTCT TCGCTTAAGT TTGGTGTACA   
  
  
+ TGCTTTTCAA TCTGTGTTGC ATTTTTCTAT GATGTATAAG CCGTTTATTA CCTATTGATG TTCAACTTTT   
  
  
+ CACACTATAT ATCCTTTATG GTTTCTTCGC TTAAGTTTGG TGTACATGCT TTTCAATCTG TGTTGCATTT   
  
  
+ TTCTGTGATG TATAAGCCGT TTATTACCTA TTGATGTTCA ACTTTTCACA CTATATGTCC TTTTATTAGT   
  
  
+ TACTGATGCC TCTGTCAAAC ATTCTTAATT GCATTTAGTG TATCAACGGT CTTAAAATTT TAAGCATTGC   
  
  
+ CAACTTGAAC TTCTGAAGAT GTTTGTCAAG ATCTTCAGTC TGCAAGTTAA CTGCTGCTTG CTTGAACTTT   
  
  
+ TCAACTTTTA AGAGAGATGT CAGAGTCTAA GCATGGCTTC CTGACAAGTA CTGCATTTTA TCACTTATGA   
  
  
+ AGACTCAATT CTGGGTTTTG ACAGCTTCTA TGAACCTCTA GTGCAGGTAC AGCAGCTTGG ACATTAAGCT   
  
  
+ GCAAGTCAAT TGACTGTAGA ACGACAAGTT GTTGAAGTTT CTGCTGAAAG TTCATCACTT GTTTGATGAA   
  
  
+ TAAAGTACAG CATGGCACTT CAGATGCAAA AGTCTCGCGA TCAGTAGAGA TGATTCCATA TTTCTCATCT   
  
  
+ CATTTCCAAG TCTTTGACAA CATGTACCCA AATAATGCCA GCCATGACAC TCAGATGTCT CTCCAATCAT   
  
  
+ ACAGCGAAGG ATACTTCACT CTGGACTCAT CTCCAGCAGC AATCGGAGCG TGCAGTGTCT ATGACTACCC   
  
  
+ ATCCGTTGTC AGCACCTCTT CAAATAGAAG CCAGTTTTCT CCTCAGGGTT CCCACTCGTA CATCTCAGAC   
  
  
+ CCCCATCATT CTTCCGACAA CTATGGATCC CCAGTGAGCG GCTCTTCTGT GGTTGACGAT AATGCTGAGC   
  
  
+ TGAGGAACAG GTTCAGTGAT ATGGAGCTCC CCTTGCCACA GGACTCAGGA CACCATTATT GCTCTTTTAG   
  
  
+ CCACAGAGGA AGCCATGAAG GCTCCTATAC TTTGAGGCCA AACCAACTGA TGGATATGGC CAACATGGAG   
  
  
+ TTAAAGCAGG TGCTATACTT CTGTGCAGAA GCAATCTCAG AGAATAATCT ATCAACTGCA GAAAGACTAA   
  
  
+ TGGATGCATT GGGTAAGAGG GTGTCTGTTT TTGGTTCACC AATTGAAAGG TTGGCCGCCT ACATGTTGGA   
  
  
+ AGGGCTCAGA GCAAGGCTGG AGTTTTCTGG ATATACTATC TACAAAAAGC TCAGGTGCGA ACAGCCAACG   
  
  
+ AGCTCAGAGC TTCTTTCCTA CATGCACATC CTGTATCAAA GTTGCCCATA TTTCAAATTC GCATATATGT   
  
  
+ CCTCAAATGT TGCTATTCAA GAAGCTTTGG GGAATGAGCC GGTTATCCAC ATCATCGATT TCCAGATTGC   
  
  
+ CATGGGGACA CAATTGGTGC TCTTGATCCA GTCTCTCGCC CATCGGCCTG GCGGGCCCCC CCCTCGTTCG   
  
  
+ GATCACTGGG GTCGATGATC CACACTCGGC CTATGCTCGT GGCGGGGGGC TCGAGGTTGT GGGGCAGAGG   
  
  
+ CTAACAAAGG TGGCTGAATC ATATGGAGTC CCATTCGAGT TTCATGCTGC AGCCATGTCA GGGTGCAAGG   
  
  
+ TGAATCGCGA TGTCCTCAAG GTTCGCCCTG GGGAAGAAGC CTTGGCCGTA AACTTCCCCT ATATGTTGCA   
  
  
+ TCACATGCCC GACGAGAGCG TGAGCACCAC AAATCATCGG GACCGGCTCT TGAGGCTGGT GAAGAGGTTG   
  
  
+ TCACCAAGGA TCGTCACCTT GGTTGAGCAA GAGTCCAACA CAAACACTCC TCCCTTTCTT CAACGGTTCC   
  
  
+ GTGAAACATT GGACTACTAC ACTGCCATGT TCGAGTCCAT TGATGTGGCT CTACCTAGGG ATGACAAGAA   
  
  
+ GAGGATAAAT GCCGAGCAGC ATTGTCTTGC GCGCGATATT GTGAACATGG TCGCTTGTGA GAATGCGGAA   
  
  
+ AGGGTTGAGA GGCACGAGCC CTTCGGGAAA TGGAGGGCAA GGTTTGATAT GGCTGGGTTT CAGCAATTGC   
  
  
+ CCTCGAGCCG CTCAGTGAAT GATGCAATAA GGGGTTTGAT GCGAGAATTC CATAGGAACT ATCGGGTGCA   
  
  
+ GGATTGTCAG GGTGCCCTTT TTTTGGGCTG GAAGGAGCGC AATCTGGCAA CCTTCTCTAC ATGGTGCTGT   
  
  
+ AAAGAATG  

- -Up\_Stream \_Len000CAATTT TTTGAGTCTT GATAATATCT AAAAGACAAA AGACAAACCT AACTCTAGGC   
  
  
- TTCGGAAACT CTAGGGGACA AAGATCGAAG GTTGGGGGGA GGGACCCAAG AAAAAGAAAC CACACTTTCA   
  
  
- TACTTGTTTT CGACTACATT CACACTAAGT TTGAGATCGG AACCACTCTG AGAACCCTTC CCGAGGATCT   
  
  
- CTGAAACGGT TAACTCGATT AAACTACGGA TGATGAACGA ATGAAAAGAT CGACAACAAA TTTGCTAAAT   
  
  
- ACAAGTATCA ATTCCTCGCG GTATAACGTA ATGTTATAGA CGAAATCACG AAACAAAAAA CGTAAAAGAA   
  
  
- GAAATAATGA TACCAAAAAC TTCTTCCGAA TGACTGACTA TGACCAAAGA AGCGAATTCA AACCACATGT   
  
  
- ACGAAAAGTT AGACACAACG TAAAAAGATA CTACATATTC GGCAAATAAT GGATAACTAC AAGTTGAAAA   
  
  
- GTGTGATATA TAGGAAATAC CAAAGAAGCG AATTCAAACC ACATGTACGA AAAGTTAGAC ACAACGTAAA   
  
  
- AAGACACTAC ATATTCGGCA AATAATGGAT AACTACAAGT TGAAAAGTGT GATATACAGG AAAATAATCA   
  
  
- ATGACTACGG AGACAGTTTG TAAGAATTAA CGTAAATCAC ATAGTTGCCA GAATTTTAAA ATTCGTAACG   
  
  
- GTTGAACTTG AAGACTTCTA CAAACAGTTC TAGAAGTCAG ACGTTCAATT GACGACGAAC GAACTTGAAA   
  
  
- AGTTGAAAAT TCTCTCTACA GTCTCAGATT CGTACCGAAG GACTGTTCAT GACGTAAAAT AGTGAATACT   
  
  
- TCTGAGTTAA GACCCAAAAC TGTCGAAGAT ACTTGGAGAT CACGTCCATG TCGTCGAACC TGTAATTCGA   
  
  
- CGTTCAGTTA ACTGACATCT TGCTGTTCAA CAACTTCAAA GACGACTTTC AAGTAGTGAA CAAACTACTT   
  
  
- ATTTCATGTC GTACCGTGAA GTCTACGTTT TCAGAGCGCT AGTCATCTCT ACTAAGGTAT AAAGAGTAGA   
  
  
- GTAAAGGTTC AGAAACTGTT GTACATGGGT TTATTACGGT CGGTACTGTG AGTCTACAGA GAGGTTAGTA   
  
  
- TGTCGCTTCC TATGAAGTGA GACCTGAGTA GAGGTCGTCG TTAGCCTCGC ACGTCACAGA TACTGATGGG   
  
  
- TAGGCAACAG TCGTGGAGAA GTTTATCTTC GGTCAAAAGA GGAGTCCCAA GGGTGAGCAT GTAGAGTCTG   
  
  
- GGGGTAGTAA GAAGGCTGTT GATACCTAGG GGTCACTCGC CGAGAAGACA CCAACTGCTA TTACGACTCG   
  
  
- ACTCCTTGTC CAAGTCACTA TACCTCGAGG GGAACGGTGT CCTGAGTCCT GTGGTAATAA CGAGAAAATC   
  
  
- GGTGTCTCCT TCGGTACTTC CGAGGATATG AAACTCCGGT TTGGTTGACT ACCTATACCG GTTGTACCTC   
  
  
- AATTTCGTCC ACGATATGAA GACACGTCTT CGTTAGAGTC TCTTATTAGA TAGTTGACGT CTTTCTGATT   
  
  
- ACCTACGTAA CCCATTCTCC CACAGACAAA AACCAAGTGG TTAACTTTCC AACCGGCGGA TGTACAACCT   
  
  
- TCCCGAGTCT CGTTCCGACC TCAAAAGACC TATATGATAG ATGTTTTTCG AGTCCACGCT TGTCGGTTGC   
  
  
- TCGAGTCTCG AAGAAAGGAT GTACGTGTAG GACATAGTTT CAACGGGTAT AAAGTTTAAG CGTATATACA   
  
  
- GGAGTTTACA ACGATAAGTT CTTCGAAACC CCTTACTCGG CCAATAGGTG TAGTAGCTAA AGGTCTAACG   
  
  
- GTACCCCTGT GTTAACCACG AGAACTAGGT CAGAGAGCGG GTAGCCGGAC CGCCCGGGGG GGGAGCAAGC   
  
  
- CTAGTGACCC CAGCTACTAG GTGTGAGCCG GATACGAGCA CCGCCCCCCG AGCTCCAACA CCCCGTCTCC   
  
  
- GATTGTTTCC ACCGACTTAG TATACCTCAG GGTAAGCTCA AAGTACGACG TCGGTACAGT CCCACGTTCC   
  
  
- ACTTAGCGCT ACAGGAGTTC CAAGCGGGAC CCCTTCTTCG GAACCGGCAT TTGAAGGGGA TATACAACGT   
  
  
- AGTGTACGGG CTGCTCTCGC ACTCGTGGTG TTTAGTAGCC CTGGCCGAGA ACTCCGACCA CTTCTCCAAC   
  
  
- AGTGGTTCCT AGCAGTGGAA CCAACTCGTT CTCAGGTTGT GTTTGTGAGG AGGGAAAGAA GTTGCCAAGG   
  
  
- CACTTTGTAA CCTGATGATG TGACGGTACA AGCTCAGGTA ACTACACCGA GATGGATCCC TACTGTTCTT   
  
  
- CTCCTATTTA CGGCTCGTCG TAACAGAACG CGCGCTATAA CACTTGTACC AGCGAACACT CTTACGCCTT   
  
  
- TCCCAACTCT CCGTGCTCGG GAAGCCCTTT ACCTCCCGTT CCAAACTATA CCGACCCAAA GTCGTTAACG   
  
  
- GGAGCTCGGC GAGTCACTTA CTACGTTATT CCCCAAACTA CGCTCTTAAG GTATCCTTGA TAGCCCACGT   
  
  
- CCTAACAGTC CCACGGGAAA AAAACCCGAC CTTCCTCGCG TTAGACCGTT GGAAGAGATG TACCACGACA   
  
  
- TTTCTTAC

+     AT~TATA-box

| Site Name | Organism | Position | Strand | Matrix score. | sequence | function |
| --- | --- | --- | --- | --- | --- | --- |
| AT~TATA-box | Arabidopsis thaliana | 500 | + | 6 | TATATA |  |

>HU08G02295.1   
+ -Up\_Stream \_Len000GTTAAA AAACTCAGAA CTATTATAGA TTTTCTGTTT TCTGTTTGGA TTGAGATCCG   
  
  
+ AAGCCTTTGA GATCCCCTGT TTCTAGCTTC CAACCCCCCT CCCTGGGTTC TTTTTCTTTG GTGTGAAAGT   
  
  
+ ATGAACAAAA GCTGATGTAA GTGTGATTCA AACTCTAGCC TTGGTGAGAC TCTTGGGAAG GGCTCCTAGA   
  
  
+ GACTTTGCCA ATTGAGCTAA TTTGATGCCT ACTACTTGCT TACTTTTCTA GCTGTTGTTT AAACGATTTA   
  
  
+ TGTTCATAGT TAAGGAGCGC CATATTGCAT TACAATATCT GCTTTAGTGC TTTGTTTTTT GCATTTTCTT   
  
  
+ CTTTATTACT ATGGTTTTTG AAGAAGGCTT ACTGACTGAT ACTGGTTTCT TCGCTTAAGT TTGGTGTACA   
  
  
+ TGCTTTTCAA TCTGTGTTGC ATTTTTCTAT GATGTATAAG CCGTTTATTA CCTATTGATG TTCAACTTTT   
  
  
+ CACACTATAT ATCCTTTATG GTTTCTTCGC TTAAGTTTGG TGTACATGCT TTTCAATCTG TGTTGCATTT   
  
  
+ TTCTGTGATG TATAAGCCGT TTATTACCTA TTGATGTTCA ACTTTTCACA CTATATGTCC TTTTATTAGT   
  
  
+ TACTGATGCC TCTGTCAAAC ATTCTTAATT GCATTTAGTG TATCAACGGT CTTAAAATTT TAAGCATTGC   
  
  
+ CAACTTGAAC TTCTGAAGAT GTTTGTCAAG ATCTTCAGTC TGCAAGTTAA CTGCTGCTTG CTTGAACTTT   
  
  
+ TCAACTTTTA AGAGAGATGT CAGAGTCTAA GCATGGCTTC CTGACAAGTA CTGCATTTTA TCACTTATGA   
  
  
+ AGACTCAATT CTGGGTTTTG ACAGCTTCTA TGAACCTCTA GTGCAGGTAC AGCAGCTTGG ACATTAAGCT   
  
  
+ GCAAGTCAAT TGACTGTAGA ACGACAAGTT GTTGAAGTTT CTGCTGAAAG TTCATCACTT GTTTGATGAA   
  
  
+ TAAAGTACAG CATGGCACTT CAGATGCAAA AGTCTCGCGA TCAGTAGAGA TGATTCCATA TTTCTCATCT   
  
  
+ CATTTCCAAG TCTTTGACAA CATGTACCCA AATAATGCCA GCCATGACAC TCAGATGTCT CTCCAATCAT   
  
  
+ ACAGCGAAGG ATACTTCACT CTGGACTCAT CTCCAGCAGC AATCGGAGCG TGCAGTGTCT ATGACTACCC   
  
  
+ ATCCGTTGTC AGCACCTCTT CAAATAGAAG CCAGTTTTCT CCTCAGGGTT CCCACTCGTA CATCTCAGAC   
  
  
+ CCCCATCATT CTTCCGACAA CTATGGATCC CCAGTGAGCG GCTCTTCTGT GGTTGACGAT AATGCTGAGC   
  
  
+ TGAGGAACAG GTTCAGTGAT ATGGAGCTCC CCTTGCCACA GGACTCAGGA CACCATTATT GCTCTTTTAG   
  
  
+ CCACAGAGGA AGCCATGAAG GCTCCTATAC TTTGAGGCCA AACCAACTGA TGGATATGGC CAACATGGAG   
  
  
+ TTAAAGCAGG TGCTATACTT CTGTGCAGAA GCAATCTCAG AGAATAATCT ATCAACTGCA GAAAGACTAA   
  
  
+ TGGATGCATT GGGTAAGAGG GTGTCTGTTT TTGGTTCACC AATTGAAAGG TTGGCCGCCT ACATGTTGGA   
  
  
+ AGGGCTCAGA GCAAGGCTGG AGTTTTCTGG ATATACTATC TACAAAAAGC TCAGGTGCGA ACAGCCAACG   
  
  
+ AGCTCAGAGC TTCTTTCCTA CATGCACATC CTGTATCAAA GTTGCCCATA TTTCAAATTC GCATATATGT   
  
  
+ CCTCAAATGT TGCTATTCAA GAAGCTTTGG GGAATGAGCC GGTTATCCAC ATCATCGATT TCCAGATTGC   
  
  
+ CATGGGGACA CAATTGGTGC TCTTGATCCA GTCTCTCGCC CATCGGCCTG GCGGGCCCCC CCCTCGTTCG   
  
  
+ GATCACTGGG GTCGATGATC CACACTCGGC CTATGCTCGT GGCGGGGGGC TCGAGGTTGT GGGGCAGAGG   
  
  
+ CTAACAAAGG TGGCTGAATC ATATGGAGTC CCATTCGAGT TTCATGCTGC AGCCATGTCA GGGTGCAAGG   
  
  
+ TGAATCGCGA TGTCCTCAAG GTTCGCCCTG GGGAAGAAGC CTTGGCCGTA AACTTCCCCT ATATGTTGCA   
  
  
+ TCACATGCCC GACGAGAGCG TGAGCACCAC AAATCATCGG GACCGGCTCT TGAGGCTGGT GAAGAGGTTG   
  
  
+ TCACCAAGGA TCGTCACCTT GGTTGAGCAA GAGTCCAACA CAAACACTCC TCCCTTTCTT CAACGGTTCC   
  
  
+ GTGAAACATT GGACTACTAC ACTGCCATGT TCGAGTCCAT TGATGTGGCT CTACCTAGGG ATGACAAGAA   
  
  
+ GAGGATAAAT GCCGAGCAGC ATTGTCTTGC GCGCGATATT GTGAACATGG TCGCTTGTGA GAATGCGGAA   
  
  
+ AGGGTTGAGA GGCACGAGCC CTTCGGGAAA TGGAGGGCAA GGTTTGATAT GGCTGGGTTT CAGCAATTGC   
  
  
+ CCTCGAGCCG CTCAGTGAAT GATGCAATAA GGGGTTTGAT GCGAGAATTC CATAGGAACT ATCGGGTGCA   
  
  
+ GGATTGTCAG GGTGCCCTTT TTTTGGGCTG GAAGGAGCGC AATCTGGCAA CCTTCTCTAC ATGGTGCTGT   
  
  
+ AAAGAATG  

- -Up\_Stream \_Len000CAATTT TTTGAGTCTT GATAATATCT AAAAGACAAA AGACAAACCT AACTCTAGGC   
  
  
- TTCGGAAACT CTAGGGGACA AAGATCGAAG GTTGGGGGGA GGGACCCAAG AAAAAGAAAC CACACTTTCA   
  
  
- TACTTGTTTT CGACTACATT CACACTAAGT TTGAGATCGG AACCACTCTG AGAACCCTTC CCGAGGATCT   
  
  
- CTGAAACGGT TAACTCGATT AAACTACGGA TGATGAACGA ATGAAAAGAT CGACAACAAA TTTGCTAAAT   
  
  
- ACAAGTATCA ATTCCTCGCG GTATAACGTA ATGTTATAGA CGAAATCACG AAACAAAAAA CGTAAAAGAA   
  
  
- GAAATAATGA TACCAAAAAC TTCTTCCGAA TGACTGACTA TGACCAAAGA AGCGAATTCA AACCACATGT   
  
  
- ACGAAAAGTT AGACACAACG TAAAAAGATA CTACATATTC GGCAAATAAT GGATAACTAC AAGTTGAAAA   
  
  
- GTGTGATATA TAGGAAATAC CAAAGAAGCG AATTCAAACC ACATGTACGA AAAGTTAGAC ACAACGTAAA   
  
  
- AAGACACTAC ATATTCGGCA AATAATGGAT AACTACAAGT TGAAAAGTGT GATATACAGG AAAATAATCA   
  
  
- ATGACTACGG AGACAGTTTG TAAGAATTAA CGTAAATCAC ATAGTTGCCA GAATTTTAAA ATTCGTAACG   
  
  
- GTTGAACTTG AAGACTTCTA CAAACAGTTC TAGAAGTCAG ACGTTCAATT GACGACGAAC GAACTTGAAA   
  
  
- AGTTGAAAAT TCTCTCTACA GTCTCAGATT CGTACCGAAG GACTGTTCAT GACGTAAAAT AGTGAATACT   
  
  
- TCTGAGTTAA GACCCAAAAC TGTCGAAGAT ACTTGGAGAT CACGTCCATG TCGTCGAACC TGTAATTCGA   
  
  
- CGTTCAGTTA ACTGACATCT TGCTGTTCAA CAACTTCAAA GACGACTTTC AAGTAGTGAA CAAACTACTT   
  
  
- ATTTCATGTC GTACCGTGAA GTCTACGTTT TCAGAGCGCT AGTCATCTCT ACTAAGGTAT AAAGAGTAGA   
  
  
- GTAAAGGTTC AGAAACTGTT GTACATGGGT TTATTACGGT CGGTACTGTG AGTCTACAGA GAGGTTAGTA   
  
  
- TGTCGCTTCC TATGAAGTGA GACCTGAGTA GAGGTCGTCG TTAGCCTCGC ACGTCACAGA TACTGATGGG   
  
  
- TAGGCAACAG TCGTGGAGAA GTTTATCTTC GGTCAAAAGA GGAGTCCCAA GGGTGAGCAT GTAGAGTCTG   
  
  
- GGGGTAGTAA GAAGGCTGTT GATACCTAGG GGTCACTCGC CGAGAAGACA CCAACTGCTA TTACGACTCG   
  
  
- ACTCCTTGTC CAAGTCACTA TACCTCGAGG GGAACGGTGT CCTGAGTCCT GTGGTAATAA CGAGAAAATC   
  
  
- GGTGTCTCCT TCGGTACTTC CGAGGATATG AAACTCCGGT TTGGTTGACT ACCTATACCG GTTGTACCTC   
  
  
- AATTTCGTCC ACGATATGAA GACACGTCTT CGTTAGAGTC TCTTATTAGA TAGTTGACGT CTTTCTGATT   
  
  
- ACCTACGTAA CCCATTCTCC CACAGACAAA AACCAAGTGG TTAACTTTCC AACCGGCGGA TGTACAACCT   
  
  
- TCCCGAGTCT CGTTCCGACC TCAAAAGACC TATATGATAG ATGTTTTTCG AGTCCACGCT TGTCGGTTGC   
  
  
- TCGAGTCTCG AAGAAAGGAT GTACGTGTAG GACATAGTTT CAACGGGTAT AAAGTTTAAG CGTATATACA   
  
  
- GGAGTTTACA ACGATAAGTT CTTCGAAACC CCTTACTCGG CCAATAGGTG TAGTAGCTAA AGGTCTAACG   
  
  
- GTACCCCTGT GTTAACCACG AGAACTAGGT CAGAGAGCGG GTAGCCGGAC CGCCCGGGGG GGGAGCAAGC   
  
  
- CTAGTGACCC CAGCTACTAG GTGTGAGCCG GATACGAGCA CCGCCCCCCG AGCTCCAACA CCCCGTCTCC   
  
  
- GATTGTTTCC ACCGACTTAG TATACCTCAG GGTAAGCTCA AAGTACGACG TCGGTACAGT CCCACGTTCC   
  
  
- ACTTAGCGCT ACAGGAGTTC CAAGCGGGAC CCCTTCTTCG GAACCGGCAT TTGAAGGGGA TATACAACGT   
  
  
- AGTGTACGGG CTGCTCTCGC ACTCGTGGTG TTTAGTAGCC CTGGCCGAGA ACTCCGACCA CTTCTCCAAC   
  
  
- AGTGGTTCCT AGCAGTGGAA CCAACTCGTT CTCAGGTTGT GTTTGTGAGG AGGGAAAGAA GTTGCCAAGG   
  
  
- CACTTTGTAA CCTGATGATG TGACGGTACA AGCTCAGGTA ACTACACCGA GATGGATCCC TACTGTTCTT   
  
  
- CTCCTATTTA CGGCTCGTCG TAACAGAACG CGCGCTATAA CACTTGTACC AGCGAACACT CTTACGCCTT   
  
  
- TCCCAACTCT CCGTGCTCGG GAAGCCCTTT ACCTCCCGTT CCAAACTATA CCGACCCAAA GTCGTTAACG   
  
  
- GGAGCTCGGC GAGTCACTTA CTACGTTATT CCCCAAACTA CGCTCTTAAG GTATCCTTGA TAGCCCACGT   
  
  
- CCTAACAGTC CCACGGGAAA AAAACCCGAC CTTCCTCGCG TTAGACCGTT GGAAGAGATG TACCACGACA   
  
  
- TTTCTTAC

+     CAAT-box

| Site Name | Organism | Position | Strand | Matrix score. | sequence | function |
| --- | --- | --- | --- | --- | --- | --- |
| CAAT-box | Nicotiana glutinosa | 2527 | - | 4 | CAAT |  |
| CAAT-box | Nicotiana glutinosa | 2564 | + | 4 | CAAT |  |
| CAAT-box | Pisum sativum | 2134 | + | 5 | CAAAT | common cis-acting element in promoter and enhancer regions |
| CAAT-box | Pisum sativum | 1215 | + | 5 | CAAAT | common cis-acting element in promoter and enhancer regions |
| CAAT-box | Nicotiana glutinosa | 2352 | - | 4 | CAAT |  |
| CAAT-box | Nicotiana glutinosa | 2283 | - | 4 | CAAT |  |
| CAAT-box | Nicotiana glutinosa | 1820 | - | 4 | CAAT |  |
| CAAT-box | Nicotiana glutinosa | 548 | + | 4 | CAAT |  |
| CAAT-box | Nicotiana glutinosa | 1164 | + | 4 | CAAT |  |
| CAAT-box | Nicotiana glutinosa | 921 | + | 4 | CAAT |  |
| CAAT-box | Nicotiana glutinosa | 1118 | + | 4 | CAAT |  |
| CAAT-box | Nicotiana glutinosa | 432 | + | 4 | CAAT |  |
| CAAT-box | Nicotiana glutinosa | 308 | - | 4 | CAAT |  |
| CAAT-box | Petunia hybrida | 702 | + | 7 | TGCCAAC | common cis-acting element in promoter and enhancer regions |
| CAAT-box | Nicotiana glutinosa | 2479 | + | 4 | CAAT |  |
| CAAT-box | Arabidopsis thaliana | 1837 | - | 5 | CCAAT | common cis-acting element in promoter and enhancer regions |
| CAAT-box | Pisum sativum | 1758 | + | 5 | CAAAT | common cis-acting element in promoter and enhancer regions |
| CAAT-box | Pisum sativum | 1738 | + | 5 | CAAAT | common cis-acting element in promoter and enhancer regions |
| CAAT-box | Nicotiana glutinosa | 64 | - | 4 | CAAT |  |
| CAAT-box | Nicotiana glutinosa | 2448 | + | 4 | CAAT |  |
| CAAT-box | Nicotiana glutinosa | 223 | + | 4 | CAAT |  |
| CAAT-box | Nicotiana glutinosa | 1835 | + | 4 | CAAT |  |
| CAAT-box | Nicotiana glutinosa | 225 | - | 4 | CAAT |  |
| CAAT-box | Pisum sativum | 234 | - | 5 | CAAAT | common cis-acting element in promoter and enhancer regions |
| CAAT-box | Nicotiana glutinosa | 1584 | + | 4 | CAAT |  |
| CAAT-box | Nicotiana glutinosa | 923 | - | 4 | CAAT |  |
| CAAT-box | Nicotiana glutinosa | 1506 | + | 4 | CAAT |  |
| CAAT-box | Nicotiana glutinosa | 594 | - | 4 | CAAT |  |
| CAAT-box | Nicotiana glutinosa | 478 | - | 4 | CAAT |  |
| CAAT-box | Nicotiana glutinosa | 2450 | - | 4 | CAAT |  |
| CAAT-box | Arabidopsis thaliana | 1583 | + | 5 | CCAAT | common cis-acting element in promoter and enhancer regions |
| CAAT-box | Arabidopsis thaliana | 1552 | - | 5 | CCAAT | common cis-acting element in promoter and enhancer regions |
| CAAT-box | Nicotiana glutinosa | 2335 | - | 4 | CAAT |  |
| CAAT-box | Arabidopsis thaliana | 2252 | - | 5 | CCAAT | common cis-acting element in promoter and enhancer regions |
| CAAT-box | Nicotiana glutinosa | 317 | + | 4 | CAAT |  |
| CAAT-box | Nicotiana glutinosa | 850 | + | 4 | CAAT |  |
| CAAT-box | Arabidopsis thaliana | 222 | + | 5 | CCAAT | common cis-acting element in promoter and enhancer regions |
| CAAT-box | Nicotiana glutinosa | 662 | - | 4 | CAAT |  |
| CAAT-box | Nicotiana glutinosa | 1586 | - | 4 | CAAT |  |
| CAAT-box | Pisum sativum | 1083 | + | 5 | CAAAT | common cis-acting element in promoter and enhancer regions |
| CAAT-box | Nicotiana glutinosa | 1392 | - | 4 | CAAT |  |
| CAAT-box | Nicotiana glutinosa | 700 | - | 4 | CAAT |  |
| CAAT-box | Arabidopsis thaliana | 1117 | + | 5 | CCAAT | common cis-acting element in promoter and enhancer regions |

>HU08G02295.1   
+ -Up\_Stream \_Len000GTTAAA AAACTCAGAA CTATTATAGA TTTTCTGTTT TCTGTTTGGA TTGAGATCCG   
  
  
+ AAGCCTTTGA GATCCCCTGT TTCTAGCTTC CAACCCCCCT CCCTGGGTTC TTTTTCTTTG GTGTGAAAGT   
  
  
+ ATGAACAAAA GCTGATGTAA GTGTGATTCA AACTCTAGCC TTGGTGAGAC TCTTGGGAAG GGCTCCTAGA   
  
  
+ GACTTTGCCA ATTGAGCTAA TTTGATGCCT ACTACTTGCT TACTTTTCTA GCTGTTGTTT AAACGATTTA   
  
  
+ TGTTCATAGT TAAGGAGCGC CATATTGCAT TACAATATCT GCTTTAGTGC TTTGTTTTTT GCATTTTCTT   
  
  
+ CTTTATTACT ATGGTTTTTG AAGAAGGCTT ACTGACTGAT ACTGGTTTCT TCGCTTAAGT TTGGTGTACA   
  
  
+ TGCTTTTCAA TCTGTGTTGC ATTTTTCTAT GATGTATAAG CCGTTTATTA CCTATTGATG TTCAACTTTT   
  
  
+ CACACTATAT ATCCTTTATG GTTTCTTCGC TTAAGTTTGG TGTACATGCT TTTCAATCTG TGTTGCATTT   
  
  
+ TTCTGTGATG TATAAGCCGT TTATTACCTA TTGATGTTCA ACTTTTCACA CTATATGTCC TTTTATTAGT   
  
  
+ TACTGATGCC TCTGTCAAAC ATTCTTAATT GCATTTAGTG TATCAACGGT CTTAAAATTT TAAGCATTGC   
  
  
+ CAACTTGAAC TTCTGAAGAT GTTTGTCAAG ATCTTCAGTC TGCAAGTTAA CTGCTGCTTG CTTGAACTTT   
  
  
+ TCAACTTTTA AGAGAGATGT CAGAGTCTAA GCATGGCTTC CTGACAAGTA CTGCATTTTA TCACTTATGA   
  
  
+ AGACTCAATT CTGGGTTTTG ACAGCTTCTA TGAACCTCTA GTGCAGGTAC AGCAGCTTGG ACATTAAGCT   
  
  
+ GCAAGTCAAT TGACTGTAGA ACGACAAGTT GTTGAAGTTT CTGCTGAAAG TTCATCACTT GTTTGATGAA   
  
  
+ TAAAGTACAG CATGGCACTT CAGATGCAAA AGTCTCGCGA TCAGTAGAGA TGATTCCATA TTTCTCATCT   
  
  
+ CATTTCCAAG TCTTTGACAA CATGTACCCA AATAATGCCA GCCATGACAC TCAGATGTCT CTCCAATCAT   
  
  
+ ACAGCGAAGG ATACTTCACT CTGGACTCAT CTCCAGCAGC AATCGGAGCG TGCAGTGTCT ATGACTACCC   
  
  
+ ATCCGTTGTC AGCACCTCTT CAAATAGAAG CCAGTTTTCT CCTCAGGGTT CCCACTCGTA CATCTCAGAC   
  
  
+ CCCCATCATT CTTCCGACAA CTATGGATCC CCAGTGAGCG GCTCTTCTGT GGTTGACGAT AATGCTGAGC   
  
  
+ TGAGGAACAG GTTCAGTGAT ATGGAGCTCC CCTTGCCACA GGACTCAGGA CACCATTATT GCTCTTTTAG   
  
  
+ CCACAGAGGA AGCCATGAAG GCTCCTATAC TTTGAGGCCA AACCAACTGA TGGATATGGC CAACATGGAG   
  
  
+ TTAAAGCAGG TGCTATACTT CTGTGCAGAA GCAATCTCAG AGAATAATCT ATCAACTGCA GAAAGACTAA   
  
  
+ TGGATGCATT GGGTAAGAGG GTGTCTGTTT TTGGTTCACC AATTGAAAGG TTGGCCGCCT ACATGTTGGA   
  
  
+ AGGGCTCAGA GCAAGGCTGG AGTTTTCTGG ATATACTATC TACAAAAAGC TCAGGTGCGA ACAGCCAACG   
  
  
+ AGCTCAGAGC TTCTTTCCTA CATGCACATC CTGTATCAAA GTTGCCCATA TTTCAAATTC GCATATATGT   
  
  
+ CCTCAAATGT TGCTATTCAA GAAGCTTTGG GGAATGAGCC GGTTATCCAC ATCATCGATT TCCAGATTGC   
  
  
+ CATGGGGACA CAATTGGTGC TCTTGATCCA GTCTCTCGCC CATCGGCCTG GCGGGCCCCC CCCTCGTTCG   
  
  
+ GATCACTGGG GTCGATGATC CACACTCGGC CTATGCTCGT GGCGGGGGGC TCGAGGTTGT GGGGCAGAGG   
  
  
+ CTAACAAAGG TGGCTGAATC ATATGGAGTC CCATTCGAGT TTCATGCTGC AGCCATGTCA GGGTGCAAGG   
  
  
+ TGAATCGCGA TGTCCTCAAG GTTCGCCCTG GGGAAGAAGC CTTGGCCGTA AACTTCCCCT ATATGTTGCA   
  
  
+ TCACATGCCC GACGAGAGCG TGAGCACCAC AAATCATCGG GACCGGCTCT TGAGGCTGGT GAAGAGGTTG   
  
  
+ TCACCAAGGA TCGTCACCTT GGTTGAGCAA GAGTCCAACA CAAACACTCC TCCCTTTCTT CAACGGTTCC   
  
  
+ GTGAAACATT GGACTACTAC ACTGCCATGT TCGAGTCCAT TGATGTGGCT CTACCTAGGG ATGACAAGAA   
  
  
+ GAGGATAAAT GCCGAGCAGC ATTGTCTTGC GCGCGATATT GTGAACATGG TCGCTTGTGA GAATGCGGAA   
  
  
+ AGGGTTGAGA GGCACGAGCC CTTCGGGAAA TGGAGGGCAA GGTTTGATAT GGCTGGGTTT CAGCAATTGC   
  
  
+ CCTCGAGCCG CTCAGTGAAT GATGCAATAA GGGGTTTGAT GCGAGAATTC CATAGGAACT ATCGGGTGCA   
  
  
+ GGATTGTCAG GGTGCCCTTT TTTTGGGCTG GAAGGAGCGC AATCTGGCAA CCTTCTCTAC ATGGTGCTGT   
  
  
+ AAAGAATG  

- -Up\_Stream \_Len000CAATTT TTTGAGTCTT GATAATATCT AAAAGACAAA AGACAAACCT AACTCTAGGC   
  
  
- TTCGGAAACT CTAGGGGACA AAGATCGAAG GTTGGGGGGA GGGACCCAAG AAAAAGAAAC CACACTTTCA   
  
  
- TACTTGTTTT CGACTACATT CACACTAAGT TTGAGATCGG AACCACTCTG AGAACCCTTC CCGAGGATCT   
  
  
- CTGAAACGGT TAACTCGATT AAACTACGGA TGATGAACGA ATGAAAAGAT CGACAACAAA TTTGCTAAAT   
  
  
- ACAAGTATCA ATTCCTCGCG GTATAACGTA ATGTTATAGA CGAAATCACG AAACAAAAAA CGTAAAAGAA   
  
  
- GAAATAATGA TACCAAAAAC TTCTTCCGAA TGACTGACTA TGACCAAAGA AGCGAATTCA AACCACATGT   
  
  
- ACGAAAAGTT AGACACAACG TAAAAAGATA CTACATATTC GGCAAATAAT GGATAACTAC AAGTTGAAAA   
  
  
- GTGTGATATA TAGGAAATAC CAAAGAAGCG AATTCAAACC ACATGTACGA AAAGTTAGAC ACAACGTAAA   
  
  
- AAGACACTAC ATATTCGGCA AATAATGGAT AACTACAAGT TGAAAAGTGT GATATACAGG AAAATAATCA   
  
  
- ATGACTACGG AGACAGTTTG TAAGAATTAA CGTAAATCAC ATAGTTGCCA GAATTTTAAA ATTCGTAACG   
  
  
- GTTGAACTTG AAGACTTCTA CAAACAGTTC TAGAAGTCAG ACGTTCAATT GACGACGAAC GAACTTGAAA   
  
  
- AGTTGAAAAT TCTCTCTACA GTCTCAGATT CGTACCGAAG GACTGTTCAT GACGTAAAAT AGTGAATACT   
  
  
- TCTGAGTTAA GACCCAAAAC TGTCGAAGAT ACTTGGAGAT CACGTCCATG TCGTCGAACC TGTAATTCGA   
  
  
- CGTTCAGTTA ACTGACATCT TGCTGTTCAA CAACTTCAAA GACGACTTTC AAGTAGTGAA CAAACTACTT   
  
  
- ATTTCATGTC GTACCGTGAA GTCTACGTTT TCAGAGCGCT AGTCATCTCT ACTAAGGTAT AAAGAGTAGA   
  
  
- GTAAAGGTTC AGAAACTGTT GTACATGGGT TTATTACGGT CGGTACTGTG AGTCTACAGA GAGGTTAGTA   
  
  
- TGTCGCTTCC TATGAAGTGA GACCTGAGTA GAGGTCGTCG TTAGCCTCGC ACGTCACAGA TACTGATGGG   
  
  
- TAGGCAACAG TCGTGGAGAA GTTTATCTTC GGTCAAAAGA GGAGTCCCAA GGGTGAGCAT GTAGAGTCTG   
  
  
- GGGGTAGTAA GAAGGCTGTT GATACCTAGG GGTCACTCGC CGAGAAGACA CCAACTGCTA TTACGACTCG   
  
  
- ACTCCTTGTC CAAGTCACTA TACCTCGAGG GGAACGGTGT CCTGAGTCCT GTGGTAATAA CGAGAAAATC   
  
  
- GGTGTCTCCT TCGGTACTTC CGAGGATATG AAACTCCGGT TTGGTTGACT ACCTATACCG GTTGTACCTC   
  
  
- AATTTCGTCC ACGATATGAA GACACGTCTT CGTTAGAGTC TCTTATTAGA TAGTTGACGT CTTTCTGATT   
  
  
- ACCTACGTAA CCCATTCTCC CACAGACAAA AACCAAGTGG TTAACTTTCC AACCGGCGGA TGTACAACCT   
  
  
- TCCCGAGTCT CGTTCCGACC TCAAAAGACC TATATGATAG ATGTTTTTCG AGTCCACGCT TGTCGGTTGC   
  
  
- TCGAGTCTCG AAGAAAGGAT GTACGTGTAG GACATAGTTT CAACGGGTAT AAAGTTTAAG CGTATATACA   
  
  
- GGAGTTTACA ACGATAAGTT CTTCGAAACC CCTTACTCGG CCAATAGGTG TAGTAGCTAA AGGTCTAACG   
  
  
- GTACCCCTGT GTTAACCACG AGAACTAGGT CAGAGAGCGG GTAGCCGGAC CGCCCGGGGG GGGAGCAAGC   
  
  
- CTAGTGACCC CAGCTACTAG GTGTGAGCCG GATACGAGCA CCGCCCCCCG AGCTCCAACA CCCCGTCTCC   
  
  
- GATTGTTTCC ACCGACTTAG TATACCTCAG GGTAAGCTCA AAGTACGACG TCGGTACAGT CCCACGTTCC   
  
  
- ACTTAGCGCT ACAGGAGTTC CAAGCGGGAC CCCTTCTTCG GAACCGGCAT TTGAAGGGGA TATACAACGT   
  
  
- AGTGTACGGG CTGCTCTCGC ACTCGTGGTG TTTAGTAGCC CTGGCCGAGA ACTCCGACCA CTTCTCCAAC   
  
  
- AGTGGTTCCT AGCAGTGGAA CCAACTCGTT CTCAGGTTGT GTTTGTGAGG AGGGAAAGAA GTTGCCAAGG   
  
  
- CACTTTGTAA CCTGATGATG TGACGGTACA AGCTCAGGTA ACTACACCGA GATGGATCCC TACTGTTCTT   
  
  
- CTCCTATTTA CGGCTCGTCG TAACAGAACG CGCGCTATAA CACTTGTACC AGCGAACACT CTTACGCCTT   
  
  
- TCCCAACTCT CCGTGCTCGG GAAGCCCTTT ACCTCCCGTT CCAAACTATA CCGACCCAAA GTCGTTAACG   
  
  
- GGAGCTCGGC GAGTCACTTA CTACGTTATT CCCCAAACTA CGCTCTTAAG GTATCCTTGA TAGCCCACGT   
  
  
- CCTAACAGTC CCACGGGAAA AAAACCCGAC CTTCCTCGCG TTAGACCGTT GGAAGAGATG TACCACGACA   
  
  
- TTTCTTAC

+     CCAAT-box

| Site Name | Organism | Position | Strand | Matrix score. | sequence | function |
| --- | --- | --- | --- | --- | --- | --- |
| CCAAT-box | Hordeum vulgare | 2235 | + | 6 | CAACGG | MYBHv1 binding site |
| CCAAT-box | Hordeum vulgare | 1197 | - | 6 | CAACGG | MYBHv1 binding site |
| CCAAT-box | Hordeum vulgare | 678 | + | 6 | CAACGG | MYBHv1 binding site |

>HU08G02295.1   
+ -Up\_Stream \_Len000GTTAAA AAACTCAGAA CTATTATAGA TTTTCTGTTT TCTGTTTGGA TTGAGATCCG   
  
  
+ AAGCCTTTGA GATCCCCTGT TTCTAGCTTC CAACCCCCCT CCCTGGGTTC TTTTTCTTTG GTGTGAAAGT   
  
  
+ ATGAACAAAA GCTGATGTAA GTGTGATTCA AACTCTAGCC TTGGTGAGAC TCTTGGGAAG GGCTCCTAGA   
  
  
+ GACTTTGCCA ATTGAGCTAA TTTGATGCCT ACTACTTGCT TACTTTTCTA GCTGTTGTTT AAACGATTTA   
  
  
+ TGTTCATAGT TAAGGAGCGC CATATTGCAT TACAATATCT GCTTTAGTGC TTTGTTTTTT GCATTTTCTT   
  
  
+ CTTTATTACT ATGGTTTTTG AAGAAGGCTT ACTGACTGAT ACTGGTTTCT TCGCTTAAGT TTGGTGTACA   
  
  
+ TGCTTTTCAA TCTGTGTTGC ATTTTTCTAT GATGTATAAG CCGTTTATTA CCTATTGATG TTCAACTTTT   
  
  
+ CACACTATAT ATCCTTTATG GTTTCTTCGC TTAAGTTTGG TGTACATGCT TTTCAATCTG TGTTGCATTT   
  
  
+ TTCTGTGATG TATAAGCCGT TTATTACCTA TTGATGTTCA ACTTTTCACA CTATATGTCC TTTTATTAGT   
  
  
+ TACTGATGCC TCTGTCAAAC ATTCTTAATT GCATTTAGTG TATCAACGGT CTTAAAATTT TAAGCATTGC   
  
  
+ CAACTTGAAC TTCTGAAGAT GTTTGTCAAG ATCTTCAGTC TGCAAGTTAA CTGCTGCTTG CTTGAACTTT   
  
  
+ TCAACTTTTA AGAGAGATGT CAGAGTCTAA GCATGGCTTC CTGACAAGTA CTGCATTTTA TCACTTATGA   
  
  
+ AGACTCAATT CTGGGTTTTG ACAGCTTCTA TGAACCTCTA GTGCAGGTAC AGCAGCTTGG ACATTAAGCT   
  
  
+ GCAAGTCAAT TGACTGTAGA ACGACAAGTT GTTGAAGTTT CTGCTGAAAG TTCATCACTT GTTTGATGAA   
  
  
+ TAAAGTACAG CATGGCACTT CAGATGCAAA AGTCTCGCGA TCAGTAGAGA TGATTCCATA TTTCTCATCT   
  
  
+ CATTTCCAAG TCTTTGACAA CATGTACCCA AATAATGCCA GCCATGACAC TCAGATGTCT CTCCAATCAT   
  
  
+ ACAGCGAAGG ATACTTCACT CTGGACTCAT CTCCAGCAGC AATCGGAGCG TGCAGTGTCT ATGACTACCC   
  
  
+ ATCCGTTGTC AGCACCTCTT CAAATAGAAG CCAGTTTTCT CCTCAGGGTT CCCACTCGTA CATCTCAGAC   
  
  
+ CCCCATCATT CTTCCGACAA CTATGGATCC CCAGTGAGCG GCTCTTCTGT GGTTGACGAT AATGCTGAGC   
  
  
+ TGAGGAACAG GTTCAGTGAT ATGGAGCTCC CCTTGCCACA GGACTCAGGA CACCATTATT GCTCTTTTAG   
  
  
+ CCACAGAGGA AGCCATGAAG GCTCCTATAC TTTGAGGCCA AACCAACTGA TGGATATGGC CAACATGGAG   
  
  
+ TTAAAGCAGG TGCTATACTT CTGTGCAGAA GCAATCTCAG AGAATAATCT ATCAACTGCA GAAAGACTAA   
  
  
+ TGGATGCATT GGGTAAGAGG GTGTCTGTTT TTGGTTCACC AATTGAAAGG TTGGCCGCCT ACATGTTGGA   
  
  
+ AGGGCTCAGA GCAAGGCTGG AGTTTTCTGG ATATACTATC TACAAAAAGC TCAGGTGCGA ACAGCCAACG   
  
  
+ AGCTCAGAGC TTCTTTCCTA CATGCACATC CTGTATCAAA GTTGCCCATA TTTCAAATTC GCATATATGT   
  
  
+ CCTCAAATGT TGCTATTCAA GAAGCTTTGG GGAATGAGCC GGTTATCCAC ATCATCGATT TCCAGATTGC   
  
  
+ CATGGGGACA CAATTGGTGC TCTTGATCCA GTCTCTCGCC CATCGGCCTG GCGGGCCCCC CCCTCGTTCG   
  
  
+ GATCACTGGG GTCGATGATC CACACTCGGC CTATGCTCGT GGCGGGGGGC TCGAGGTTGT GGGGCAGAGG   
  
  
+ CTAACAAAGG TGGCTGAATC ATATGGAGTC CCATTCGAGT TTCATGCTGC AGCCATGTCA GGGTGCAAGG   
  
  
+ TGAATCGCGA TGTCCTCAAG GTTCGCCCTG GGGAAGAAGC CTTGGCCGTA AACTTCCCCT ATATGTTGCA   
  
  
+ TCACATGCCC GACGAGAGCG TGAGCACCAC AAATCATCGG GACCGGCTCT TGAGGCTGGT GAAGAGGTTG   
  
  
+ TCACCAAGGA TCGTCACCTT GGTTGAGCAA GAGTCCAACA CAAACACTCC TCCCTTTCTT CAACGGTTCC   
  
  
+ GTGAAACATT GGACTACTAC ACTGCCATGT TCGAGTCCAT TGATGTGGCT CTACCTAGGG ATGACAAGAA   
  
  
+ GAGGATAAAT GCCGAGCAGC ATTGTCTTGC GCGCGATATT GTGAACATGG TCGCTTGTGA GAATGCGGAA   
  
  
+ AGGGTTGAGA GGCACGAGCC CTTCGGGAAA TGGAGGGCAA GGTTTGATAT GGCTGGGTTT CAGCAATTGC   
  
  
+ CCTCGAGCCG CTCAGTGAAT GATGCAATAA GGGGTTTGAT GCGAGAATTC CATAGGAACT ATCGGGTGCA   
  
  
+ GGATTGTCAG GGTGCCCTTT TTTTGGGCTG GAAGGAGCGC AATCTGGCAA CCTTCTCTAC ATGGTGCTGT   
  
  
+ AAAGAATG  

- -Up\_Stream \_Len000CAATTT TTTGAGTCTT GATAATATCT AAAAGACAAA AGACAAACCT AACTCTAGGC   
  
  
- TTCGGAAACT CTAGGGGACA AAGATCGAAG GTTGGGGGGA GGGACCCAAG AAAAAGAAAC CACACTTTCA   
  
  
- TACTTGTTTT CGACTACATT CACACTAAGT TTGAGATCGG AACCACTCTG AGAACCCTTC CCGAGGATCT   
  
  
- CTGAAACGGT TAACTCGATT AAACTACGGA TGATGAACGA ATGAAAAGAT CGACAACAAA TTTGCTAAAT   
  
  
- ACAAGTATCA ATTCCTCGCG GTATAACGTA ATGTTATAGA CGAAATCACG AAACAAAAAA CGTAAAAGAA   
  
  
- GAAATAATGA TACCAAAAAC TTCTTCCGAA TGACTGACTA TGACCAAAGA AGCGAATTCA AACCACATGT   
  
  
- ACGAAAAGTT AGACACAACG TAAAAAGATA CTACATATTC GGCAAATAAT GGATAACTAC AAGTTGAAAA   
  
  
- GTGTGATATA TAGGAAATAC CAAAGAAGCG AATTCAAACC ACATGTACGA AAAGTTAGAC ACAACGTAAA   
  
  
- AAGACACTAC ATATTCGGCA AATAATGGAT AACTACAAGT TGAAAAGTGT GATATACAGG AAAATAATCA   
  
  
- ATGACTACGG AGACAGTTTG TAAGAATTAA CGTAAATCAC ATAGTTGCCA GAATTTTAAA ATTCGTAACG   
  
  
- GTTGAACTTG AAGACTTCTA CAAACAGTTC TAGAAGTCAG ACGTTCAATT GACGACGAAC GAACTTGAAA   
  
  
- AGTTGAAAAT TCTCTCTACA GTCTCAGATT CGTACCGAAG GACTGTTCAT GACGTAAAAT AGTGAATACT   
  
  
- TCTGAGTTAA GACCCAAAAC TGTCGAAGAT ACTTGGAGAT CACGTCCATG TCGTCGAACC TGTAATTCGA   
  
  
- CGTTCAGTTA ACTGACATCT TGCTGTTCAA CAACTTCAAA GACGACTTTC AAGTAGTGAA CAAACTACTT   
  
  
- ATTTCATGTC GTACCGTGAA GTCTACGTTT TCAGAGCGCT AGTCATCTCT ACTAAGGTAT AAAGAGTAGA   
  
  
- GTAAAGGTTC AGAAACTGTT GTACATGGGT TTATTACGGT CGGTACTGTG AGTCTACAGA GAGGTTAGTA   
  
  
- TGTCGCTTCC TATGAAGTGA GACCTGAGTA GAGGTCGTCG TTAGCCTCGC ACGTCACAGA TACTGATGGG   
  
  
- TAGGCAACAG TCGTGGAGAA GTTTATCTTC GGTCAAAAGA GGAGTCCCAA GGGTGAGCAT GTAGAGTCTG   
  
  
- GGGGTAGTAA GAAGGCTGTT GATACCTAGG GGTCACTCGC CGAGAAGACA CCAACTGCTA TTACGACTCG   
  
  
- ACTCCTTGTC CAAGTCACTA TACCTCGAGG GGAACGGTGT CCTGAGTCCT GTGGTAATAA CGAGAAAATC   
  
  
- GGTGTCTCCT TCGGTACTTC CGAGGATATG AAACTCCGGT TTGGTTGACT ACCTATACCG GTTGTACCTC   
  
  
- AATTTCGTCC ACGATATGAA GACACGTCTT CGTTAGAGTC TCTTATTAGA TAGTTGACGT CTTTCTGATT   
  
  
- ACCTACGTAA CCCATTCTCC CACAGACAAA AACCAAGTGG TTAACTTTCC AACCGGCGGA TGTACAACCT   
  
  
- TCCCGAGTCT CGTTCCGACC TCAAAAGACC TATATGATAG ATGTTTTTCG AGTCCACGCT TGTCGGTTGC   
  
  
- TCGAGTCTCG AAGAAAGGAT GTACGTGTAG GACATAGTTT CAACGGGTAT AAAGTTTAAG CGTATATACA   
  
  
- GGAGTTTACA ACGATAAGTT CTTCGAAACC CCTTACTCGG CCAATAGGTG TAGTAGCTAA AGGTCTAACG   
  
  
- GTACCCCTGT GTTAACCACG AGAACTAGGT CAGAGAGCGG GTAGCCGGAC CGCCCGGGGG GGGAGCAAGC   
  
  
- CTAGTGACCC CAGCTACTAG GTGTGAGCCG GATACGAGCA CCGCCCCCCG AGCTCCAACA CCCCGTCTCC   
  
  
- GATTGTTTCC ACCGACTTAG TATACCTCAG GGTAAGCTCA AAGTACGACG TCGGTACAGT CCCACGTTCC   
  
  
- ACTTAGCGCT ACAGGAGTTC CAAGCGGGAC CCCTTCTTCG GAACCGGCAT TTGAAGGGGA TATACAACGT   
  
  
- AGTGTACGGG CTGCTCTCGC ACTCGTGGTG TTTAGTAGCC CTGGCCGAGA ACTCCGACCA CTTCTCCAAC   
  
  
- AGTGGTTCCT AGCAGTGGAA CCAACTCGTT CTCAGGTTGT GTTTGTGAGG AGGGAAAGAA GTTGCCAAGG   
  
  
- CACTTTGTAA CCTGATGATG TGACGGTACA AGCTCAGGTA ACTACACCGA GATGGATCCC TACTGTTCTT   
  
  
- CTCCTATTTA CGGCTCGTCG TAACAGAACG CGCGCTATAA CACTTGTACC AGCGAACACT CTTACGCCTT   
  
  
- TCCCAACTCT CCGTGCTCGG GAAGCCCTTT ACCTCCCGTT CCAAACTATA CCGACCCAAA GTCGTTAACG   
  
  
- GGAGCTCGGC GAGTCACTTA CTACGTTATT CCCCAAACTA CGCTCTTAAG GTATCCTTGA TAGCCCACGT   
  
  
- CCTAACAGTC CCACGGGAAA AAAACCCGAC CTTCCTCGCG TTAGACCGTT GGAAGAGATG TACCACGACA   
  
  
- TTTCTTAC

+     CGTCA-motif

| Site Name | Organism | Position | Strand | Matrix score. | sequence | function |
| --- | --- | --- | --- | --- | --- | --- |
| CGTCA-motif | Hordeum vulgare | 2186 | + | 5 | CGTCA | cis-acting regulatory element involved in the MeJA-responsiveness |
| CGTCA-motif | Hordeum vulgare | 1318 | - | 5 | CGTCA | cis-acting regulatory element involved in the MeJA-responsiveness |

>HU08G02295.1   
+ -Up\_Stream \_Len000GTTAAA AAACTCAGAA CTATTATAGA TTTTCTGTTT TCTGTTTGGA TTGAGATCCG   
  
  
+ AAGCCTTTGA GATCCCCTGT TTCTAGCTTC CAACCCCCCT CCCTGGGTTC TTTTTCTTTG GTGTGAAAGT   
  
  
+ ATGAACAAAA GCTGATGTAA GTGTGATTCA AACTCTAGCC TTGGTGAGAC TCTTGGGAAG GGCTCCTAGA   
  
  
+ GACTTTGCCA ATTGAGCTAA TTTGATGCCT ACTACTTGCT TACTTTTCTA GCTGTTGTTT AAACGATTTA   
  
  
+ TGTTCATAGT TAAGGAGCGC CATATTGCAT TACAATATCT GCTTTAGTGC TTTGTTTTTT GCATTTTCTT   
  
  
+ CTTTATTACT ATGGTTTTTG AAGAAGGCTT ACTGACTGAT ACTGGTTTCT TCGCTTAAGT TTGGTGTACA   
  
  
+ TGCTTTTCAA TCTGTGTTGC ATTTTTCTAT GATGTATAAG CCGTTTATTA CCTATTGATG TTCAACTTTT   
  
  
+ CACACTATAT ATCCTTTATG GTTTCTTCGC TTAAGTTTGG TGTACATGCT TTTCAATCTG TGTTGCATTT   
  
  
+ TTCTGTGATG TATAAGCCGT TTATTACCTA TTGATGTTCA ACTTTTCACA CTATATGTCC TTTTATTAGT   
  
  
+ TACTGATGCC TCTGTCAAAC ATTCTTAATT GCATTTAGTG TATCAACGGT CTTAAAATTT TAAGCATTGC   
  
  
+ CAACTTGAAC TTCTGAAGAT GTTTGTCAAG ATCTTCAGTC TGCAAGTTAA CTGCTGCTTG CTTGAACTTT   
  
  
+ TCAACTTTTA AGAGAGATGT CAGAGTCTAA GCATGGCTTC CTGACAAGTA CTGCATTTTA TCACTTATGA   
  
  
+ AGACTCAATT CTGGGTTTTG ACAGCTTCTA TGAACCTCTA GTGCAGGTAC AGCAGCTTGG ACATTAAGCT   
  
  
+ GCAAGTCAAT TGACTGTAGA ACGACAAGTT GTTGAAGTTT CTGCTGAAAG TTCATCACTT GTTTGATGAA   
  
  
+ TAAAGTACAG CATGGCACTT CAGATGCAAA AGTCTCGCGA TCAGTAGAGA TGATTCCATA TTTCTCATCT   
  
  
+ CATTTCCAAG TCTTTGACAA CATGTACCCA AATAATGCCA GCCATGACAC TCAGATGTCT CTCCAATCAT   
  
  
+ ACAGCGAAGG ATACTTCACT CTGGACTCAT CTCCAGCAGC AATCGGAGCG TGCAGTGTCT ATGACTACCC   
  
  
+ ATCCGTTGTC AGCACCTCTT CAAATAGAAG CCAGTTTTCT CCTCAGGGTT CCCACTCGTA CATCTCAGAC   
  
  
+ CCCCATCATT CTTCCGACAA CTATGGATCC CCAGTGAGCG GCTCTTCTGT GGTTGACGAT AATGCTGAGC   
  
  
+ TGAGGAACAG GTTCAGTGAT ATGGAGCTCC CCTTGCCACA GGACTCAGGA CACCATTATT GCTCTTTTAG   
  
  
+ CCACAGAGGA AGCCATGAAG GCTCCTATAC TTTGAGGCCA AACCAACTGA TGGATATGGC CAACATGGAG   
  
  
+ TTAAAGCAGG TGCTATACTT CTGTGCAGAA GCAATCTCAG AGAATAATCT ATCAACTGCA GAAAGACTAA   
  
  
+ TGGATGCATT GGGTAAGAGG GTGTCTGTTT TTGGTTCACC AATTGAAAGG TTGGCCGCCT ACATGTTGGA   
  
  
+ AGGGCTCAGA GCAAGGCTGG AGTTTTCTGG ATATACTATC TACAAAAAGC TCAGGTGCGA ACAGCCAACG   
  
  
+ AGCTCAGAGC TTCTTTCCTA CATGCACATC CTGTATCAAA GTTGCCCATA TTTCAAATTC GCATATATGT   
  
  
+ CCTCAAATGT TGCTATTCAA GAAGCTTTGG GGAATGAGCC GGTTATCCAC ATCATCGATT TCCAGATTGC   
  
  
+ CATGGGGACA CAATTGGTGC TCTTGATCCA GTCTCTCGCC CATCGGCCTG GCGGGCCCCC CCCTCGTTCG   
  
  
+ GATCACTGGG GTCGATGATC CACACTCGGC CTATGCTCGT GGCGGGGGGC TCGAGGTTGT GGGGCAGAGG   
  
  
+ CTAACAAAGG TGGCTGAATC ATATGGAGTC CCATTCGAGT TTCATGCTGC AGCCATGTCA GGGTGCAAGG   
  
  
+ TGAATCGCGA TGTCCTCAAG GTTCGCCCTG GGGAAGAAGC CTTGGCCGTA AACTTCCCCT ATATGTTGCA   
  
  
+ TCACATGCCC GACGAGAGCG TGAGCACCAC AAATCATCGG GACCGGCTCT TGAGGCTGGT GAAGAGGTTG   
  
  
+ TCACCAAGGA TCGTCACCTT GGTTGAGCAA GAGTCCAACA CAAACACTCC TCCCTTTCTT CAACGGTTCC   
  
  
+ GTGAAACATT GGACTACTAC ACTGCCATGT TCGAGTCCAT TGATGTGGCT CTACCTAGGG ATGACAAGAA   
  
  
+ GAGGATAAAT GCCGAGCAGC ATTGTCTTGC GCGCGATATT GTGAACATGG TCGCTTGTGA GAATGCGGAA   
  
  
+ AGGGTTGAGA GGCACGAGCC CTTCGGGAAA TGGAGGGCAA GGTTTGATAT GGCTGGGTTT CAGCAATTGC   
  
  
+ CCTCGAGCCG CTCAGTGAAT GATGCAATAA GGGGTTTGAT GCGAGAATTC CATAGGAACT ATCGGGTGCA   
  
  
+ GGATTGTCAG GGTGCCCTTT TTTTGGGCTG GAAGGAGCGC AATCTGGCAA CCTTCTCTAC ATGGTGCTGT   
  
  
+ AAAGAATG  

- -Up\_Stream \_Len000CAATTT TTTGAGTCTT GATAATATCT AAAAGACAAA AGACAAACCT AACTCTAGGC   
  
  
- TTCGGAAACT CTAGGGGACA AAGATCGAAG GTTGGGGGGA GGGACCCAAG AAAAAGAAAC CACACTTTCA   
  
  
- TACTTGTTTT CGACTACATT CACACTAAGT TTGAGATCGG AACCACTCTG AGAACCCTTC CCGAGGATCT   
  
  
- CTGAAACGGT TAACTCGATT AAACTACGGA TGATGAACGA ATGAAAAGAT CGACAACAAA TTTGCTAAAT   
  
  
- ACAAGTATCA ATTCCTCGCG GTATAACGTA ATGTTATAGA CGAAATCACG AAACAAAAAA CGTAAAAGAA   
  
  
- GAAATAATGA TACCAAAAAC TTCTTCCGAA TGACTGACTA TGACCAAAGA AGCGAATTCA AACCACATGT   
  
  
- ACGAAAAGTT AGACACAACG TAAAAAGATA CTACATATTC GGCAAATAAT GGATAACTAC AAGTTGAAAA   
  
  
- GTGTGATATA TAGGAAATAC CAAAGAAGCG AATTCAAACC ACATGTACGA AAAGTTAGAC ACAACGTAAA   
  
  
- AAGACACTAC ATATTCGGCA AATAATGGAT AACTACAAGT TGAAAAGTGT GATATACAGG AAAATAATCA   
  
  
- ATGACTACGG AGACAGTTTG TAAGAATTAA CGTAAATCAC ATAGTTGCCA GAATTTTAAA ATTCGTAACG   
  
  
- GTTGAACTTG AAGACTTCTA CAAACAGTTC TAGAAGTCAG ACGTTCAATT GACGACGAAC GAACTTGAAA   
  
  
- AGTTGAAAAT TCTCTCTACA GTCTCAGATT CGTACCGAAG GACTGTTCAT GACGTAAAAT AGTGAATACT   
  
  
- TCTGAGTTAA GACCCAAAAC TGTCGAAGAT ACTTGGAGAT CACGTCCATG TCGTCGAACC TGTAATTCGA   
  
  
- CGTTCAGTTA ACTGACATCT TGCTGTTCAA CAACTTCAAA GACGACTTTC AAGTAGTGAA CAAACTACTT   
  
  
- ATTTCATGTC GTACCGTGAA GTCTACGTTT TCAGAGCGCT AGTCATCTCT ACTAAGGTAT AAAGAGTAGA   
  
  
- GTAAAGGTTC AGAAACTGTT GTACATGGGT TTATTACGGT CGGTACTGTG AGTCTACAGA GAGGTTAGTA   
  
  
- TGTCGCTTCC TATGAAGTGA GACCTGAGTA GAGGTCGTCG TTAGCCTCGC ACGTCACAGA TACTGATGGG   
  
  
- TAGGCAACAG TCGTGGAGAA GTTTATCTTC GGTCAAAAGA GGAGTCCCAA GGGTGAGCAT GTAGAGTCTG   
  
  
- GGGGTAGTAA GAAGGCTGTT GATACCTAGG GGTCACTCGC CGAGAAGACA CCAACTGCTA TTACGACTCG   
  
  
- ACTCCTTGTC CAAGTCACTA TACCTCGAGG GGAACGGTGT CCTGAGTCCT GTGGTAATAA CGAGAAAATC   
  
  
- GGTGTCTCCT TCGGTACTTC CGAGGATATG AAACTCCGGT TTGGTTGACT ACCTATACCG GTTGTACCTC   
  
  
- AATTTCGTCC ACGATATGAA GACACGTCTT CGTTAGAGTC TCTTATTAGA TAGTTGACGT CTTTCTGATT   
  
  
- ACCTACGTAA CCCATTCTCC CACAGACAAA AACCAAGTGG TTAACTTTCC AACCGGCGGA TGTACAACCT   
  
  
- TCCCGAGTCT CGTTCCGACC TCAAAAGACC TATATGATAG ATGTTTTTCG AGTCCACGCT TGTCGGTTGC   
  
  
- TCGAGTCTCG AAGAAAGGAT GTACGTGTAG GACATAGTTT CAACGGGTAT AAAGTTTAAG CGTATATACA   
  
  
- GGAGTTTACA ACGATAAGTT CTTCGAAACC CCTTACTCGG CCAATAGGTG TAGTAGCTAA AGGTCTAACG   
  
  
- GTACCCCTGT GTTAACCACG AGAACTAGGT CAGAGAGCGG GTAGCCGGAC CGCCCGGGGG GGGAGCAAGC   
  
  
- CTAGTGACCC CAGCTACTAG GTGTGAGCCG GATACGAGCA CCGCCCCCCG AGCTCCAACA CCCCGTCTCC   
  
  
- GATTGTTTCC ACCGACTTAG TATACCTCAG GGTAAGCTCA AAGTACGACG TCGGTACAGT CCCACGTTCC   
  
  
- ACTTAGCGCT ACAGGAGTTC CAAGCGGGAC CCCTTCTTCG GAACCGGCAT TTGAAGGGGA TATACAACGT   
  
  
- AGTGTACGGG CTGCTCTCGC ACTCGTGGTG TTTAGTAGCC CTGGCCGAGA ACTCCGACCA CTTCTCCAAC   
  
  
- AGTGGTTCCT AGCAGTGGAA CCAACTCGTT CTCAGGTTGT GTTTGTGAGG AGGGAAAGAA GTTGCCAAGG   
  
  
- CACTTTGTAA CCTGATGATG TGACGGTACA AGCTCAGGTA ACTACACCGA GATGGATCCC TACTGTTCTT   
  
  
- CTCCTATTTA CGGCTCGTCG TAACAGAACG CGCGCTATAA CACTTGTACC AGCGAACACT CTTACGCCTT   
  
  
- TCCCAACTCT CCGTGCTCGG GAAGCCCTTT ACCTCCCGTT CCAAACTATA CCGACCCAAA GTCGTTAACG   
  
  
- GGAGCTCGGC GAGTCACTTA CTACGTTATT CCCCAAACTA CGCTCTTAAG GTATCCTTGA TAGCCCACGT   
  
  
- CCTAACAGTC CCACGGGAAA AAAACCCGAC CTTCCTCGCG TTAGACCGTT GGAAGAGATG TACCACGACA   
  
  
- TTTCTTAC

+     G-Box

| Site Name | Organism | Position | Strand | Matrix score. | sequence | function |
| --- | --- | --- | --- | --- | --- | --- |
| G-Box | Triticum aestivum | 1822 | - | 10 | TCCACATGGCA | cis-acting regulatory element involved in light responsiveness |

>HU08G02295.1   
+ -Up\_Stream \_Len000GTTAAA AAACTCAGAA CTATTATAGA TTTTCTGTTT TCTGTTTGGA TTGAGATCCG   
  
  
+ AAGCCTTTGA GATCCCCTGT TTCTAGCTTC CAACCCCCCT CCCTGGGTTC TTTTTCTTTG GTGTGAAAGT   
  
  
+ ATGAACAAAA GCTGATGTAA GTGTGATTCA AACTCTAGCC TTGGTGAGAC TCTTGGGAAG GGCTCCTAGA   
  
  
+ GACTTTGCCA ATTGAGCTAA TTTGATGCCT ACTACTTGCT TACTTTTCTA GCTGTTGTTT AAACGATTTA   
  
  
+ TGTTCATAGT TAAGGAGCGC CATATTGCAT TACAATATCT GCTTTAGTGC TTTGTTTTTT GCATTTTCTT   
  
  
+ CTTTATTACT ATGGTTTTTG AAGAAGGCTT ACTGACTGAT ACTGGTTTCT TCGCTTAAGT TTGGTGTACA   
  
  
+ TGCTTTTCAA TCTGTGTTGC ATTTTTCTAT GATGTATAAG CCGTTTATTA CCTATTGATG TTCAACTTTT   
  
  
+ CACACTATAT ATCCTTTATG GTTTCTTCGC TTAAGTTTGG TGTACATGCT TTTCAATCTG TGTTGCATTT   
  
  
+ TTCTGTGATG TATAAGCCGT TTATTACCTA TTGATGTTCA ACTTTTCACA CTATATGTCC TTTTATTAGT   
  
  
+ TACTGATGCC TCTGTCAAAC ATTCTTAATT GCATTTAGTG TATCAACGGT CTTAAAATTT TAAGCATTGC   
  
  
+ CAACTTGAAC TTCTGAAGAT GTTTGTCAAG ATCTTCAGTC TGCAAGTTAA CTGCTGCTTG CTTGAACTTT   
  
  
+ TCAACTTTTA AGAGAGATGT CAGAGTCTAA GCATGGCTTC CTGACAAGTA CTGCATTTTA TCACTTATGA   
  
  
+ AGACTCAATT CTGGGTTTTG ACAGCTTCTA TGAACCTCTA GTGCAGGTAC AGCAGCTTGG ACATTAAGCT   
  
  
+ GCAAGTCAAT TGACTGTAGA ACGACAAGTT GTTGAAGTTT CTGCTGAAAG TTCATCACTT GTTTGATGAA   
  
  
+ TAAAGTACAG CATGGCACTT CAGATGCAAA AGTCTCGCGA TCAGTAGAGA TGATTCCATA TTTCTCATCT   
  
  
+ CATTTCCAAG TCTTTGACAA CATGTACCCA AATAATGCCA GCCATGACAC TCAGATGTCT CTCCAATCAT   
  
  
+ ACAGCGAAGG ATACTTCACT CTGGACTCAT CTCCAGCAGC AATCGGAGCG TGCAGTGTCT ATGACTACCC   
  
  
+ ATCCGTTGTC AGCACCTCTT CAAATAGAAG CCAGTTTTCT CCTCAGGGTT CCCACTCGTA CATCTCAGAC   
  
  
+ CCCCATCATT CTTCCGACAA CTATGGATCC CCAGTGAGCG GCTCTTCTGT GGTTGACGAT AATGCTGAGC   
  
  
+ TGAGGAACAG GTTCAGTGAT ATGGAGCTCC CCTTGCCACA GGACTCAGGA CACCATTATT GCTCTTTTAG   
  
  
+ CCACAGAGGA AGCCATGAAG GCTCCTATAC TTTGAGGCCA AACCAACTGA TGGATATGGC CAACATGGAG   
  
  
+ TTAAAGCAGG TGCTATACTT CTGTGCAGAA GCAATCTCAG AGAATAATCT ATCAACTGCA GAAAGACTAA   
  
  
+ TGGATGCATT GGGTAAGAGG GTGTCTGTTT TTGGTTCACC AATTGAAAGG TTGGCCGCCT ACATGTTGGA   
  
  
+ AGGGCTCAGA GCAAGGCTGG AGTTTTCTGG ATATACTATC TACAAAAAGC TCAGGTGCGA ACAGCCAACG   
  
  
+ AGCTCAGAGC TTCTTTCCTA CATGCACATC CTGTATCAAA GTTGCCCATA TTTCAAATTC GCATATATGT   
  
  
+ CCTCAAATGT TGCTATTCAA GAAGCTTTGG GGAATGAGCC GGTTATCCAC ATCATCGATT TCCAGATTGC   
  
  
+ CATGGGGACA CAATTGGTGC TCTTGATCCA GTCTCTCGCC CATCGGCCTG GCGGGCCCCC CCCTCGTTCG   
  
  
+ GATCACTGGG GTCGATGATC CACACTCGGC CTATGCTCGT GGCGGGGGGC TCGAGGTTGT GGGGCAGAGG   
  
  
+ CTAACAAAGG TGGCTGAATC ATATGGAGTC CCATTCGAGT TTCATGCTGC AGCCATGTCA GGGTGCAAGG   
  
  
+ TGAATCGCGA TGTCCTCAAG GTTCGCCCTG GGGAAGAAGC CTTGGCCGTA AACTTCCCCT ATATGTTGCA   
  
  
+ TCACATGCCC GACGAGAGCG TGAGCACCAC AAATCATCGG GACCGGCTCT TGAGGCTGGT GAAGAGGTTG   
  
  
+ TCACCAAGGA TCGTCACCTT GGTTGAGCAA GAGTCCAACA CAAACACTCC TCCCTTTCTT CAACGGTTCC   
  
  
+ GTGAAACATT GGACTACTAC ACTGCCATGT TCGAGTCCAT TGATGTGGCT CTACCTAGGG ATGACAAGAA   
  
  
+ GAGGATAAAT GCCGAGCAGC ATTGTCTTGC GCGCGATATT GTGAACATGG TCGCTTGTGA GAATGCGGAA   
  
  
+ AGGGTTGAGA GGCACGAGCC CTTCGGGAAA TGGAGGGCAA GGTTTGATAT GGCTGGGTTT CAGCAATTGC   
  
  
+ CCTCGAGCCG CTCAGTGAAT GATGCAATAA GGGGTTTGAT GCGAGAATTC CATAGGAACT ATCGGGTGCA   
  
  
+ GGATTGTCAG GGTGCCCTTT TTTTGGGCTG GAAGGAGCGC AATCTGGCAA CCTTCTCTAC ATGGTGCTGT   
  
  
+ AAAGAATG  

- -Up\_Stream \_Len000CAATTT TTTGAGTCTT GATAATATCT AAAAGACAAA AGACAAACCT AACTCTAGGC   
  
  
- TTCGGAAACT CTAGGGGACA AAGATCGAAG GTTGGGGGGA GGGACCCAAG AAAAAGAAAC CACACTTTCA   
  
  
- TACTTGTTTT CGACTACATT CACACTAAGT TTGAGATCGG AACCACTCTG AGAACCCTTC CCGAGGATCT   
  
  
- CTGAAACGGT TAACTCGATT AAACTACGGA TGATGAACGA ATGAAAAGAT CGACAACAAA TTTGCTAAAT   
  
  
- ACAAGTATCA ATTCCTCGCG GTATAACGTA ATGTTATAGA CGAAATCACG AAACAAAAAA CGTAAAAGAA   
  
  
- GAAATAATGA TACCAAAAAC TTCTTCCGAA TGACTGACTA TGACCAAAGA AGCGAATTCA AACCACATGT   
  
  
- ACGAAAAGTT AGACACAACG TAAAAAGATA CTACATATTC GGCAAATAAT GGATAACTAC AAGTTGAAAA   
  
  
- GTGTGATATA TAGGAAATAC CAAAGAAGCG AATTCAAACC ACATGTACGA AAAGTTAGAC ACAACGTAAA   
  
  
- AAGACACTAC ATATTCGGCA AATAATGGAT AACTACAAGT TGAAAAGTGT GATATACAGG AAAATAATCA   
  
  
- ATGACTACGG AGACAGTTTG TAAGAATTAA CGTAAATCAC ATAGTTGCCA GAATTTTAAA ATTCGTAACG   
  
  
- GTTGAACTTG AAGACTTCTA CAAACAGTTC TAGAAGTCAG ACGTTCAATT GACGACGAAC GAACTTGAAA   
  
  
- AGTTGAAAAT TCTCTCTACA GTCTCAGATT CGTACCGAAG GACTGTTCAT GACGTAAAAT AGTGAATACT   
  
  
- TCTGAGTTAA GACCCAAAAC TGTCGAAGAT ACTTGGAGAT CACGTCCATG TCGTCGAACC TGTAATTCGA   
  
  
- CGTTCAGTTA ACTGACATCT TGCTGTTCAA CAACTTCAAA GACGACTTTC AAGTAGTGAA CAAACTACTT   
  
  
- ATTTCATGTC GTACCGTGAA GTCTACGTTT TCAGAGCGCT AGTCATCTCT ACTAAGGTAT AAAGAGTAGA   
  
  
- GTAAAGGTTC AGAAACTGTT GTACATGGGT TTATTACGGT CGGTACTGTG AGTCTACAGA GAGGTTAGTA   
  
  
- TGTCGCTTCC TATGAAGTGA GACCTGAGTA GAGGTCGTCG TTAGCCTCGC ACGTCACAGA TACTGATGGG   
  
  
- TAGGCAACAG TCGTGGAGAA GTTTATCTTC GGTCAAAAGA GGAGTCCCAA GGGTGAGCAT GTAGAGTCTG   
  
  
- GGGGTAGTAA GAAGGCTGTT GATACCTAGG GGTCACTCGC CGAGAAGACA CCAACTGCTA TTACGACTCG   
  
  
- ACTCCTTGTC CAAGTCACTA TACCTCGAGG GGAACGGTGT CCTGAGTCCT GTGGTAATAA CGAGAAAATC   
  
  
- GGTGTCTCCT TCGGTACTTC CGAGGATATG AAACTCCGGT TTGGTTGACT ACCTATACCG GTTGTACCTC   
  
  
- AATTTCGTCC ACGATATGAA GACACGTCTT CGTTAGAGTC TCTTATTAGA TAGTTGACGT CTTTCTGATT   
  
  
- ACCTACGTAA CCCATTCTCC CACAGACAAA AACCAAGTGG TTAACTTTCC AACCGGCGGA TGTACAACCT   
  
  
- TCCCGAGTCT CGTTCCGACC TCAAAAGACC TATATGATAG ATGTTTTTCG AGTCCACGCT TGTCGGTTGC   
  
  
- TCGAGTCTCG AAGAAAGGAT GTACGTGTAG GACATAGTTT CAACGGGTAT AAAGTTTAAG CGTATATACA   
  
  
- GGAGTTTACA ACGATAAGTT CTTCGAAACC CCTTACTCGG CCAATAGGTG TAGTAGCTAA AGGTCTAACG   
  
  
- GTACCCCTGT GTTAACCACG AGAACTAGGT CAGAGAGCGG GTAGCCGGAC CGCCCGGGGG GGGAGCAAGC   
  
  
- CTAGTGACCC CAGCTACTAG GTGTGAGCCG GATACGAGCA CCGCCCCCCG AGCTCCAACA CCCCGTCTCC   
  
  
- GATTGTTTCC ACCGACTTAG TATACCTCAG GGTAAGCTCA AAGTACGACG TCGGTACAGT CCCACGTTCC   
  
  
- ACTTAGCGCT ACAGGAGTTC CAAGCGGGAC CCCTTCTTCG GAACCGGCAT TTGAAGGGGA TATACAACGT   
  
  
- AGTGTACGGG CTGCTCTCGC ACTCGTGGTG TTTAGTAGCC CTGGCCGAGA ACTCCGACCA CTTCTCCAAC   
  
  
- AGTGGTTCCT AGCAGTGGAA CCAACTCGTT CTCAGGTTGT GTTTGTGAGG AGGGAAAGAA GTTGCCAAGG   
  
  
- CACTTTGTAA CCTGATGATG TGACGGTACA AGCTCAGGTA ACTACACCGA GATGGATCCC TACTGTTCTT   
  
  
- CTCCTATTTA CGGCTCGTCG TAACAGAACG CGCGCTATAA CACTTGTACC AGCGAACACT CTTACGCCTT   
  
  
- TCCCAACTCT CCGTGCTCGG GAAGCCCTTT ACCTCCCGTT CCAAACTATA CCGACCCAAA GTCGTTAACG   
  
  
- GGAGCTCGGC GAGTCACTTA CTACGTTATT CCCCAAACTA CGCTCTTAAG GTATCCTTGA TAGCCCACGT   
  
  
- CCTAACAGTC CCACGGGAAA AAAACCCGAC CTTCCTCGCG TTAGACCGTT GGAAGAGATG TACCACGACA   
  
  
- TTTCTTAC

+     GC-motif

| Site Name | Organism | Position | Strand | Matrix score. | sequence | function |
| --- | --- | --- | --- | --- | --- | --- |
| GC-motif | Zea mays | 1937 | - | 6 | CCCCCG | enhancer-like element involved in anoxic specific inducibility |

>HU08G02295.1   
+ -Up\_Stream \_Len000GTTAAA AAACTCAGAA CTATTATAGA TTTTCTGTTT TCTGTTTGGA TTGAGATCCG   
  
  
+ AAGCCTTTGA GATCCCCTGT TTCTAGCTTC CAACCCCCCT CCCTGGGTTC TTTTTCTTTG GTGTGAAAGT   
  
  
+ ATGAACAAAA GCTGATGTAA GTGTGATTCA AACTCTAGCC TTGGTGAGAC TCTTGGGAAG GGCTCCTAGA   
  
  
+ GACTTTGCCA ATTGAGCTAA TTTGATGCCT ACTACTTGCT TACTTTTCTA GCTGTTGTTT AAACGATTTA   
  
  
+ TGTTCATAGT TAAGGAGCGC CATATTGCAT TACAATATCT GCTTTAGTGC TTTGTTTTTT GCATTTTCTT   
  
  
+ CTTTATTACT ATGGTTTTTG AAGAAGGCTT ACTGACTGAT ACTGGTTTCT TCGCTTAAGT TTGGTGTACA   
  
  
+ TGCTTTTCAA TCTGTGTTGC ATTTTTCTAT GATGTATAAG CCGTTTATTA CCTATTGATG TTCAACTTTT   
  
  
+ CACACTATAT ATCCTTTATG GTTTCTTCGC TTAAGTTTGG TGTACATGCT TTTCAATCTG TGTTGCATTT   
  
  
+ TTCTGTGATG TATAAGCCGT TTATTACCTA TTGATGTTCA ACTTTTCACA CTATATGTCC TTTTATTAGT   
  
  
+ TACTGATGCC TCTGTCAAAC ATTCTTAATT GCATTTAGTG TATCAACGGT CTTAAAATTT TAAGCATTGC   
  
  
+ CAACTTGAAC TTCTGAAGAT GTTTGTCAAG ATCTTCAGTC TGCAAGTTAA CTGCTGCTTG CTTGAACTTT   
  
  
+ TCAACTTTTA AGAGAGATGT CAGAGTCTAA GCATGGCTTC CTGACAAGTA CTGCATTTTA TCACTTATGA   
  
  
+ AGACTCAATT CTGGGTTTTG ACAGCTTCTA TGAACCTCTA GTGCAGGTAC AGCAGCTTGG ACATTAAGCT   
  
  
+ GCAAGTCAAT TGACTGTAGA ACGACAAGTT GTTGAAGTTT CTGCTGAAAG TTCATCACTT GTTTGATGAA   
  
  
+ TAAAGTACAG CATGGCACTT CAGATGCAAA AGTCTCGCGA TCAGTAGAGA TGATTCCATA TTTCTCATCT   
  
  
+ CATTTCCAAG TCTTTGACAA CATGTACCCA AATAATGCCA GCCATGACAC TCAGATGTCT CTCCAATCAT   
  
  
+ ACAGCGAAGG ATACTTCACT CTGGACTCAT CTCCAGCAGC AATCGGAGCG TGCAGTGTCT ATGACTACCC   
  
  
+ ATCCGTTGTC AGCACCTCTT CAAATAGAAG CCAGTTTTCT CCTCAGGGTT CCCACTCGTA CATCTCAGAC   
  
  
+ CCCCATCATT CTTCCGACAA CTATGGATCC CCAGTGAGCG GCTCTTCTGT GGTTGACGAT AATGCTGAGC   
  
  
+ TGAGGAACAG GTTCAGTGAT ATGGAGCTCC CCTTGCCACA GGACTCAGGA CACCATTATT GCTCTTTTAG   
  
  
+ CCACAGAGGA AGCCATGAAG GCTCCTATAC TTTGAGGCCA AACCAACTGA TGGATATGGC CAACATGGAG   
  
  
+ TTAAAGCAGG TGCTATACTT CTGTGCAGAA GCAATCTCAG AGAATAATCT ATCAACTGCA GAAAGACTAA   
  
  
+ TGGATGCATT GGGTAAGAGG GTGTCTGTTT TTGGTTCACC AATTGAAAGG TTGGCCGCCT ACATGTTGGA   
  
  
+ AGGGCTCAGA GCAAGGCTGG AGTTTTCTGG ATATACTATC TACAAAAAGC TCAGGTGCGA ACAGCCAACG   
  
  
+ AGCTCAGAGC TTCTTTCCTA CATGCACATC CTGTATCAAA GTTGCCCATA TTTCAAATTC GCATATATGT   
  
  
+ CCTCAAATGT TGCTATTCAA GAAGCTTTGG GGAATGAGCC GGTTATCCAC ATCATCGATT TCCAGATTGC   
  
  
+ CATGGGGACA CAATTGGTGC TCTTGATCCA GTCTCTCGCC CATCGGCCTG GCGGGCCCCC CCCTCGTTCG   
  
  
+ GATCACTGGG GTCGATGATC CACACTCGGC CTATGCTCGT GGCGGGGGGC TCGAGGTTGT GGGGCAGAGG   
  
  
+ CTAACAAAGG TGGCTGAATC ATATGGAGTC CCATTCGAGT TTCATGCTGC AGCCATGTCA GGGTGCAAGG   
  
  
+ TGAATCGCGA TGTCCTCAAG GTTCGCCCTG GGGAAGAAGC CTTGGCCGTA AACTTCCCCT ATATGTTGCA   
  
  
+ TCACATGCCC GACGAGAGCG TGAGCACCAC AAATCATCGG GACCGGCTCT TGAGGCTGGT GAAGAGGTTG   
  
  
+ TCACCAAGGA TCGTCACCTT GGTTGAGCAA GAGTCCAACA CAAACACTCC TCCCTTTCTT CAACGGTTCC   
  
  
+ GTGAAACATT GGACTACTAC ACTGCCATGT TCGAGTCCAT TGATGTGGCT CTACCTAGGG ATGACAAGAA   
  
  
+ GAGGATAAAT GCCGAGCAGC ATTGTCTTGC GCGCGATATT GTGAACATGG TCGCTTGTGA GAATGCGGAA   
  
  
+ AGGGTTGAGA GGCACGAGCC CTTCGGGAAA TGGAGGGCAA GGTTTGATAT GGCTGGGTTT CAGCAATTGC   
  
  
+ CCTCGAGCCG CTCAGTGAAT GATGCAATAA GGGGTTTGAT GCGAGAATTC CATAGGAACT ATCGGGTGCA   
  
  
+ GGATTGTCAG GGTGCCCTTT TTTTGGGCTG GAAGGAGCGC AATCTGGCAA CCTTCTCTAC ATGGTGCTGT   
  
  
+ AAAGAATG  

- -Up\_Stream \_Len000CAATTT TTTGAGTCTT GATAATATCT AAAAGACAAA AGACAAACCT AACTCTAGGC   
  
  
- TTCGGAAACT CTAGGGGACA AAGATCGAAG GTTGGGGGGA GGGACCCAAG AAAAAGAAAC CACACTTTCA   
  
  
- TACTTGTTTT CGACTACATT CACACTAAGT TTGAGATCGG AACCACTCTG AGAACCCTTC CCGAGGATCT   
  
  
- CTGAAACGGT TAACTCGATT AAACTACGGA TGATGAACGA ATGAAAAGAT CGACAACAAA TTTGCTAAAT   
  
  
- ACAAGTATCA ATTCCTCGCG GTATAACGTA ATGTTATAGA CGAAATCACG AAACAAAAAA CGTAAAAGAA   
  
  
- GAAATAATGA TACCAAAAAC TTCTTCCGAA TGACTGACTA TGACCAAAGA AGCGAATTCA AACCACATGT   
  
  
- ACGAAAAGTT AGACACAACG TAAAAAGATA CTACATATTC GGCAAATAAT GGATAACTAC AAGTTGAAAA   
  
  
- GTGTGATATA TAGGAAATAC CAAAGAAGCG AATTCAAACC ACATGTACGA AAAGTTAGAC ACAACGTAAA   
  
  
- AAGACACTAC ATATTCGGCA AATAATGGAT AACTACAAGT TGAAAAGTGT GATATACAGG AAAATAATCA   
  
  
- ATGACTACGG AGACAGTTTG TAAGAATTAA CGTAAATCAC ATAGTTGCCA GAATTTTAAA ATTCGTAACG   
  
  
- GTTGAACTTG AAGACTTCTA CAAACAGTTC TAGAAGTCAG ACGTTCAATT GACGACGAAC GAACTTGAAA   
  
  
- AGTTGAAAAT TCTCTCTACA GTCTCAGATT CGTACCGAAG GACTGTTCAT GACGTAAAAT AGTGAATACT   
  
  
- TCTGAGTTAA GACCCAAAAC TGTCGAAGAT ACTTGGAGAT CACGTCCATG TCGTCGAACC TGTAATTCGA   
  
  
- CGTTCAGTTA ACTGACATCT TGCTGTTCAA CAACTTCAAA GACGACTTTC AAGTAGTGAA CAAACTACTT   
  
  
- ATTTCATGTC GTACCGTGAA GTCTACGTTT TCAGAGCGCT AGTCATCTCT ACTAAGGTAT AAAGAGTAGA   
  
  
- GTAAAGGTTC AGAAACTGTT GTACATGGGT TTATTACGGT CGGTACTGTG AGTCTACAGA GAGGTTAGTA   
  
  
- TGTCGCTTCC TATGAAGTGA GACCTGAGTA GAGGTCGTCG TTAGCCTCGC ACGTCACAGA TACTGATGGG   
  
  
- TAGGCAACAG TCGTGGAGAA GTTTATCTTC GGTCAAAAGA GGAGTCCCAA GGGTGAGCAT GTAGAGTCTG   
  
  
- GGGGTAGTAA GAAGGCTGTT GATACCTAGG GGTCACTCGC CGAGAAGACA CCAACTGCTA TTACGACTCG   
  
  
- ACTCCTTGTC CAAGTCACTA TACCTCGAGG GGAACGGTGT CCTGAGTCCT GTGGTAATAA CGAGAAAATC   
  
  
- GGTGTCTCCT TCGGTACTTC CGAGGATATG AAACTCCGGT TTGGTTGACT ACCTATACCG GTTGTACCTC   
  
  
- AATTTCGTCC ACGATATGAA GACACGTCTT CGTTAGAGTC TCTTATTAGA TAGTTGACGT CTTTCTGATT   
  
  
- ACCTACGTAA CCCATTCTCC CACAGACAAA AACCAAGTGG TTAACTTTCC AACCGGCGGA TGTACAACCT   
  
  
- TCCCGAGTCT CGTTCCGACC TCAAAAGACC TATATGATAG ATGTTTTTCG AGTCCACGCT TGTCGGTTGC   
  
  
- TCGAGTCTCG AAGAAAGGAT GTACGTGTAG GACATAGTTT CAACGGGTAT AAAGTTTAAG CGTATATACA   
  
  
- GGAGTTTACA ACGATAAGTT CTTCGAAACC CCTTACTCGG CCAATAGGTG TAGTAGCTAA AGGTCTAACG   
  
  
- GTACCCCTGT GTTAACCACG AGAACTAGGT CAGAGAGCGG GTAGCCGGAC CGCCCGGGGG GGGAGCAAGC   
  
  
- CTAGTGACCC CAGCTACTAG GTGTGAGCCG GATACGAGCA CCGCCCCCCG AGCTCCAACA CCCCGTCTCC   
  
  
- GATTGTTTCC ACCGACTTAG TATACCTCAG GGTAAGCTCA AAGTACGACG TCGGTACAGT CCCACGTTCC   
  
  
- ACTTAGCGCT ACAGGAGTTC CAAGCGGGAC CCCTTCTTCG GAACCGGCAT TTGAAGGGGA TATACAACGT   
  
  
- AGTGTACGGG CTGCTCTCGC ACTCGTGGTG TTTAGTAGCC CTGGCCGAGA ACTCCGACCA CTTCTCCAAC   
  
  
- AGTGGTTCCT AGCAGTGGAA CCAACTCGTT CTCAGGTTGT GTTTGTGAGG AGGGAAAGAA GTTGCCAAGG   
  
  
- CACTTTGTAA CCTGATGATG TGACGGTACA AGCTCAGGTA ACTACACCGA GATGGATCCC TACTGTTCTT   
  
  
- CTCCTATTTA CGGCTCGTCG TAACAGAACG CGCGCTATAA CACTTGTACC AGCGAACACT CTTACGCCTT   
  
  
- TCCCAACTCT CCGTGCTCGG GAAGCCCTTT ACCTCCCGTT CCAAACTATA CCGACCCAAA GTCGTTAACG   
  
  
- GGAGCTCGGC GAGTCACTTA CTACGTTATT CCCCAAACTA CGCTCTTAAG GTATCCTTGA TAGCCCACGT   
  
  
- CCTAACAGTC CCACGGGAAA AAAACCCGAC CTTCCTCGCG TTAGACCGTT GGAAGAGATG TACCACGACA   
  
  
- TTTCTTAC

+     MBS

| Site Name | Organism | Position | Strand | Matrix score. | sequence | function |
| --- | --- | --- | --- | --- | --- | --- |
| MBS | Arabidopsis thaliana | 1527 | + | 6 | CAACTG | MYB binding site involved in drought-inducibility |
| MBS | Arabidopsis thaliana | 1448 | + | 6 | CAACTG | MYB binding site involved in drought-inducibility |

>HU08G02295.1   
+ -Up\_Stream \_Len000GTTAAA AAACTCAGAA CTATTATAGA TTTTCTGTTT TCTGTTTGGA TTGAGATCCG   
  
  
+ AAGCCTTTGA GATCCCCTGT TTCTAGCTTC CAACCCCCCT CCCTGGGTTC TTTTTCTTTG GTGTGAAAGT   
  
  
+ ATGAACAAAA GCTGATGTAA GTGTGATTCA AACTCTAGCC TTGGTGAGAC TCTTGGGAAG GGCTCCTAGA   
  
  
+ GACTTTGCCA ATTGAGCTAA TTTGATGCCT ACTACTTGCT TACTTTTCTA GCTGTTGTTT AAACGATTTA   
  
  
+ TGTTCATAGT TAAGGAGCGC CATATTGCAT TACAATATCT GCTTTAGTGC TTTGTTTTTT GCATTTTCTT   
  
  
+ CTTTATTACT ATGGTTTTTG AAGAAGGCTT ACTGACTGAT ACTGGTTTCT TCGCTTAAGT TTGGTGTACA   
  
  
+ TGCTTTTCAA TCTGTGTTGC ATTTTTCTAT GATGTATAAG CCGTTTATTA CCTATTGATG TTCAACTTTT   
  
  
+ CACACTATAT ATCCTTTATG GTTTCTTCGC TTAAGTTTGG TGTACATGCT TTTCAATCTG TGTTGCATTT   
  
  
+ TTCTGTGATG TATAAGCCGT TTATTACCTA TTGATGTTCA ACTTTTCACA CTATATGTCC TTTTATTAGT   
  
  
+ TACTGATGCC TCTGTCAAAC ATTCTTAATT GCATTTAGTG TATCAACGGT CTTAAAATTT TAAGCATTGC   
  
  
+ CAACTTGAAC TTCTGAAGAT GTTTGTCAAG ATCTTCAGTC TGCAAGTTAA CTGCTGCTTG CTTGAACTTT   
  
  
+ TCAACTTTTA AGAGAGATGT CAGAGTCTAA GCATGGCTTC CTGACAAGTA CTGCATTTTA TCACTTATGA   
  
  
+ AGACTCAATT CTGGGTTTTG ACAGCTTCTA TGAACCTCTA GTGCAGGTAC AGCAGCTTGG ACATTAAGCT   
  
  
+ GCAAGTCAAT TGACTGTAGA ACGACAAGTT GTTGAAGTTT CTGCTGAAAG TTCATCACTT GTTTGATGAA   
  
  
+ TAAAGTACAG CATGGCACTT CAGATGCAAA AGTCTCGCGA TCAGTAGAGA TGATTCCATA TTTCTCATCT   
  
  
+ CATTTCCAAG TCTTTGACAA CATGTACCCA AATAATGCCA GCCATGACAC TCAGATGTCT CTCCAATCAT   
  
  
+ ACAGCGAAGG ATACTTCACT CTGGACTCAT CTCCAGCAGC AATCGGAGCG TGCAGTGTCT ATGACTACCC   
  
  
+ ATCCGTTGTC AGCACCTCTT CAAATAGAAG CCAGTTTTCT CCTCAGGGTT CCCACTCGTA CATCTCAGAC   
  
  
+ CCCCATCATT CTTCCGACAA CTATGGATCC CCAGTGAGCG GCTCTTCTGT GGTTGACGAT AATGCTGAGC   
  
  
+ TGAGGAACAG GTTCAGTGAT ATGGAGCTCC CCTTGCCACA GGACTCAGGA CACCATTATT GCTCTTTTAG   
  
  
+ CCACAGAGGA AGCCATGAAG GCTCCTATAC TTTGAGGCCA AACCAACTGA TGGATATGGC CAACATGGAG   
  
  
+ TTAAAGCAGG TGCTATACTT CTGTGCAGAA GCAATCTCAG AGAATAATCT ATCAACTGCA GAAAGACTAA   
  
  
+ TGGATGCATT GGGTAAGAGG GTGTCTGTTT TTGGTTCACC AATTGAAAGG TTGGCCGCCT ACATGTTGGA   
  
  
+ AGGGCTCAGA GCAAGGCTGG AGTTTTCTGG ATATACTATC TACAAAAAGC TCAGGTGCGA ACAGCCAACG   
  
  
+ AGCTCAGAGC TTCTTTCCTA CATGCACATC CTGTATCAAA GTTGCCCATA TTTCAAATTC GCATATATGT   
  
  
+ CCTCAAATGT TGCTATTCAA GAAGCTTTGG GGAATGAGCC GGTTATCCAC ATCATCGATT TCCAGATTGC   
  
  
+ CATGGGGACA CAATTGGTGC TCTTGATCCA GTCTCTCGCC CATCGGCCTG GCGGGCCCCC CCCTCGTTCG   
  
  
+ GATCACTGGG GTCGATGATC CACACTCGGC CTATGCTCGT GGCGGGGGGC TCGAGGTTGT GGGGCAGAGG   
  
  
+ CTAACAAAGG TGGCTGAATC ATATGGAGTC CCATTCGAGT TTCATGCTGC AGCCATGTCA GGGTGCAAGG   
  
  
+ TGAATCGCGA TGTCCTCAAG GTTCGCCCTG GGGAAGAAGC CTTGGCCGTA AACTTCCCCT ATATGTTGCA   
  
  
+ TCACATGCCC GACGAGAGCG TGAGCACCAC AAATCATCGG GACCGGCTCT TGAGGCTGGT GAAGAGGTTG   
  
  
+ TCACCAAGGA TCGTCACCTT GGTTGAGCAA GAGTCCAACA CAAACACTCC TCCCTTTCTT CAACGGTTCC   
  
  
+ GTGAAACATT GGACTACTAC ACTGCCATGT TCGAGTCCAT TGATGTGGCT CTACCTAGGG ATGACAAGAA   
  
  
+ GAGGATAAAT GCCGAGCAGC ATTGTCTTGC GCGCGATATT GTGAACATGG TCGCTTGTGA GAATGCGGAA   
  
  
+ AGGGTTGAGA GGCACGAGCC CTTCGGGAAA TGGAGGGCAA GGTTTGATAT GGCTGGGTTT CAGCAATTGC   
  
  
+ CCTCGAGCCG CTCAGTGAAT GATGCAATAA GGGGTTTGAT GCGAGAATTC CATAGGAACT ATCGGGTGCA   
  
  
+ GGATTGTCAG GGTGCCCTTT TTTTGGGCTG GAAGGAGCGC AATCTGGCAA CCTTCTCTAC ATGGTGCTGT   
  
  
+ AAAGAATG  

- -Up\_Stream \_Len000CAATTT TTTGAGTCTT GATAATATCT AAAAGACAAA AGACAAACCT AACTCTAGGC   
  
  
- TTCGGAAACT CTAGGGGACA AAGATCGAAG GTTGGGGGGA GGGACCCAAG AAAAAGAAAC CACACTTTCA   
  
  
- TACTTGTTTT CGACTACATT CACACTAAGT TTGAGATCGG AACCACTCTG AGAACCCTTC CCGAGGATCT   
  
  
- CTGAAACGGT TAACTCGATT AAACTACGGA TGATGAACGA ATGAAAAGAT CGACAACAAA TTTGCTAAAT   
  
  
- ACAAGTATCA ATTCCTCGCG GTATAACGTA ATGTTATAGA CGAAATCACG AAACAAAAAA CGTAAAAGAA   
  
  
- GAAATAATGA TACCAAAAAC TTCTTCCGAA TGACTGACTA TGACCAAAGA AGCGAATTCA AACCACATGT   
  
  
- ACGAAAAGTT AGACACAACG TAAAAAGATA CTACATATTC GGCAAATAAT GGATAACTAC AAGTTGAAAA   
  
  
- GTGTGATATA TAGGAAATAC CAAAGAAGCG AATTCAAACC ACATGTACGA AAAGTTAGAC ACAACGTAAA   
  
  
- AAGACACTAC ATATTCGGCA AATAATGGAT AACTACAAGT TGAAAAGTGT GATATACAGG AAAATAATCA   
  
  
- ATGACTACGG AGACAGTTTG TAAGAATTAA CGTAAATCAC ATAGTTGCCA GAATTTTAAA ATTCGTAACG   
  
  
- GTTGAACTTG AAGACTTCTA CAAACAGTTC TAGAAGTCAG ACGTTCAATT GACGACGAAC GAACTTGAAA   
  
  
- AGTTGAAAAT TCTCTCTACA GTCTCAGATT CGTACCGAAG GACTGTTCAT GACGTAAAAT AGTGAATACT   
  
  
- TCTGAGTTAA GACCCAAAAC TGTCGAAGAT ACTTGGAGAT CACGTCCATG TCGTCGAACC TGTAATTCGA   
  
  
- CGTTCAGTTA ACTGACATCT TGCTGTTCAA CAACTTCAAA GACGACTTTC AAGTAGTGAA CAAACTACTT   
  
  
- ATTTCATGTC GTACCGTGAA GTCTACGTTT TCAGAGCGCT AGTCATCTCT ACTAAGGTAT AAAGAGTAGA   
  
  
- GTAAAGGTTC AGAAACTGTT GTACATGGGT TTATTACGGT CGGTACTGTG AGTCTACAGA GAGGTTAGTA   
  
  
- TGTCGCTTCC TATGAAGTGA GACCTGAGTA GAGGTCGTCG TTAGCCTCGC ACGTCACAGA TACTGATGGG   
  
  
- TAGGCAACAG TCGTGGAGAA GTTTATCTTC GGTCAAAAGA GGAGTCCCAA GGGTGAGCAT GTAGAGTCTG   
  
  
- GGGGTAGTAA GAAGGCTGTT GATACCTAGG GGTCACTCGC CGAGAAGACA CCAACTGCTA TTACGACTCG   
  
  
- ACTCCTTGTC CAAGTCACTA TACCTCGAGG GGAACGGTGT CCTGAGTCCT GTGGTAATAA CGAGAAAATC   
  
  
- GGTGTCTCCT TCGGTACTTC CGAGGATATG AAACTCCGGT TTGGTTGACT ACCTATACCG GTTGTACCTC   
  
  
- AATTTCGTCC ACGATATGAA GACACGTCTT CGTTAGAGTC TCTTATTAGA TAGTTGACGT CTTTCTGATT   
  
  
- ACCTACGTAA CCCATTCTCC CACAGACAAA AACCAAGTGG TTAACTTTCC AACCGGCGGA TGTACAACCT   
  
  
- TCCCGAGTCT CGTTCCGACC TCAAAAGACC TATATGATAG ATGTTTTTCG AGTCCACGCT TGTCGGTTGC   
  
  
- TCGAGTCTCG AAGAAAGGAT GTACGTGTAG GACATAGTTT CAACGGGTAT AAAGTTTAAG CGTATATACA   
  
  
- GGAGTTTACA ACGATAAGTT CTTCGAAACC CCTTACTCGG CCAATAGGTG TAGTAGCTAA AGGTCTAACG   
  
  
- GTACCCCTGT GTTAACCACG AGAACTAGGT CAGAGAGCGG GTAGCCGGAC CGCCCGGGGG GGGAGCAAGC   
  
  
- CTAGTGACCC CAGCTACTAG GTGTGAGCCG GATACGAGCA CCGCCCCCCG AGCTCCAACA CCCCGTCTCC   
  
  
- GATTGTTTCC ACCGACTTAG TATACCTCAG GGTAAGCTCA AAGTACGACG TCGGTACAGT CCCACGTTCC   
  
  
- ACTTAGCGCT ACAGGAGTTC CAAGCGGGAC CCCTTCTTCG GAACCGGCAT TTGAAGGGGA TATACAACGT   
  
  
- AGTGTACGGG CTGCTCTCGC ACTCGTGGTG TTTAGTAGCC CTGGCCGAGA ACTCCGACCA CTTCTCCAAC   
  
  
- AGTGGTTCCT AGCAGTGGAA CCAACTCGTT CTCAGGTTGT GTTTGTGAGG AGGGAAAGAA GTTGCCAAGG   
  
  
- CACTTTGTAA CCTGATGATG TGACGGTACA AGCTCAGGTA ACTACACCGA GATGGATCCC TACTGTTCTT   
  
  
- CTCCTATTTA CGGCTCGTCG TAACAGAACG CGCGCTATAA CACTTGTACC AGCGAACACT CTTACGCCTT   
  
  
- TCCCAACTCT CCGTGCTCGG GAAGCCCTTT ACCTCCCGTT CCAAACTATA CCGACCCAAA GTCGTTAACG   
  
  
- GGAGCTCGGC GAGTCACTTA CTACGTTATT CCCCAAACTA CGCTCTTAAG GTATCCTTGA TAGCCCACGT   
  
  
- CCTAACAGTC CCACGGGAAA AAAACCCGAC CTTCCTCGCG TTAGACCGTT GGAAGAGATG TACCACGACA   
  
  
- TTTCTTAC

+     MYB

| Site Name | Organism | Position | Strand | Matrix score. | sequence | function |
| --- | --- | --- | --- | --- | --- | --- |
| MYB | Arabidopsis thaliana | 2194 | - | 6 | CAACCA |  |
| MYB | Arabidopsis thaliana | 266 | - | 6 | CAACAG |  |
| MYB | Arabidopsis thaliana | 1314 | - | 6 | CAACCA |  |

>HU08G02295.1   
+ -Up\_Stream \_Len000GTTAAA AAACTCAGAA CTATTATAGA TTTTCTGTTT TCTGTTTGGA TTGAGATCCG   
  
  
+ AAGCCTTTGA GATCCCCTGT TTCTAGCTTC CAACCCCCCT CCCTGGGTTC TTTTTCTTTG GTGTGAAAGT   
  
  
+ ATGAACAAAA GCTGATGTAA GTGTGATTCA AACTCTAGCC TTGGTGAGAC TCTTGGGAAG GGCTCCTAGA   
  
  
+ GACTTTGCCA ATTGAGCTAA TTTGATGCCT ACTACTTGCT TACTTTTCTA GCTGTTGTTT AAACGATTTA   
  
  
+ TGTTCATAGT TAAGGAGCGC CATATTGCAT TACAATATCT GCTTTAGTGC TTTGTTTTTT GCATTTTCTT   
  
  
+ CTTTATTACT ATGGTTTTTG AAGAAGGCTT ACTGACTGAT ACTGGTTTCT TCGCTTAAGT TTGGTGTACA   
  
  
+ TGCTTTTCAA TCTGTGTTGC ATTTTTCTAT GATGTATAAG CCGTTTATTA CCTATTGATG TTCAACTTTT   
  
  
+ CACACTATAT ATCCTTTATG GTTTCTTCGC TTAAGTTTGG TGTACATGCT TTTCAATCTG TGTTGCATTT   
  
  
+ TTCTGTGATG TATAAGCCGT TTATTACCTA TTGATGTTCA ACTTTTCACA CTATATGTCC TTTTATTAGT   
  
  
+ TACTGATGCC TCTGTCAAAC ATTCTTAATT GCATTTAGTG TATCAACGGT CTTAAAATTT TAAGCATTGC   
  
  
+ CAACTTGAAC TTCTGAAGAT GTTTGTCAAG ATCTTCAGTC TGCAAGTTAA CTGCTGCTTG CTTGAACTTT   
  
  
+ TCAACTTTTA AGAGAGATGT CAGAGTCTAA GCATGGCTTC CTGACAAGTA CTGCATTTTA TCACTTATGA   
  
  
+ AGACTCAATT CTGGGTTTTG ACAGCTTCTA TGAACCTCTA GTGCAGGTAC AGCAGCTTGG ACATTAAGCT   
  
  
+ GCAAGTCAAT TGACTGTAGA ACGACAAGTT GTTGAAGTTT CTGCTGAAAG TTCATCACTT GTTTGATGAA   
  
  
+ TAAAGTACAG CATGGCACTT CAGATGCAAA AGTCTCGCGA TCAGTAGAGA TGATTCCATA TTTCTCATCT   
  
  
+ CATTTCCAAG TCTTTGACAA CATGTACCCA AATAATGCCA GCCATGACAC TCAGATGTCT CTCCAATCAT   
  
  
+ ACAGCGAAGG ATACTTCACT CTGGACTCAT CTCCAGCAGC AATCGGAGCG TGCAGTGTCT ATGACTACCC   
  
  
+ ATCCGTTGTC AGCACCTCTT CAAATAGAAG CCAGTTTTCT CCTCAGGGTT CCCACTCGTA CATCTCAGAC   
  
  
+ CCCCATCATT CTTCCGACAA CTATGGATCC CCAGTGAGCG GCTCTTCTGT GGTTGACGAT AATGCTGAGC   
  
  
+ TGAGGAACAG GTTCAGTGAT ATGGAGCTCC CCTTGCCACA GGACTCAGGA CACCATTATT GCTCTTTTAG   
  
  
+ CCACAGAGGA AGCCATGAAG GCTCCTATAC TTTGAGGCCA AACCAACTGA TGGATATGGC CAACATGGAG   
  
  
+ TTAAAGCAGG TGCTATACTT CTGTGCAGAA GCAATCTCAG AGAATAATCT ATCAACTGCA GAAAGACTAA   
  
  
+ TGGATGCATT GGGTAAGAGG GTGTCTGTTT TTGGTTCACC AATTGAAAGG TTGGCCGCCT ACATGTTGGA   
  
  
+ AGGGCTCAGA GCAAGGCTGG AGTTTTCTGG ATATACTATC TACAAAAAGC TCAGGTGCGA ACAGCCAACG   
  
  
+ AGCTCAGAGC TTCTTTCCTA CATGCACATC CTGTATCAAA GTTGCCCATA TTTCAAATTC GCATATATGT   
  
  
+ CCTCAAATGT TGCTATTCAA GAAGCTTTGG GGAATGAGCC GGTTATCCAC ATCATCGATT TCCAGATTGC   
  
  
+ CATGGGGACA CAATTGGTGC TCTTGATCCA GTCTCTCGCC CATCGGCCTG GCGGGCCCCC CCCTCGTTCG   
  
  
+ GATCACTGGG GTCGATGATC CACACTCGGC CTATGCTCGT GGCGGGGGGC TCGAGGTTGT GGGGCAGAGG   
  
  
+ CTAACAAAGG TGGCTGAATC ATATGGAGTC CCATTCGAGT TTCATGCTGC AGCCATGTCA GGGTGCAAGG   
  
  
+ TGAATCGCGA TGTCCTCAAG GTTCGCCCTG GGGAAGAAGC CTTGGCCGTA AACTTCCCCT ATATGTTGCA   
  
  
+ TCACATGCCC GACGAGAGCG TGAGCACCAC AAATCATCGG GACCGGCTCT TGAGGCTGGT GAAGAGGTTG   
  
  
+ TCACCAAGGA TCGTCACCTT GGTTGAGCAA GAGTCCAACA CAAACACTCC TCCCTTTCTT CAACGGTTCC   
  
  
+ GTGAAACATT GGACTACTAC ACTGCCATGT TCGAGTCCAT TGATGTGGCT CTACCTAGGG ATGACAAGAA   
  
  
+ GAGGATAAAT GCCGAGCAGC ATTGTCTTGC GCGCGATATT GTGAACATGG TCGCTTGTGA GAATGCGGAA   
  
  
+ AGGGTTGAGA GGCACGAGCC CTTCGGGAAA TGGAGGGCAA GGTTTGATAT GGCTGGGTTT CAGCAATTGC   
  
  
+ CCTCGAGCCG CTCAGTGAAT GATGCAATAA GGGGTTTGAT GCGAGAATTC CATAGGAACT ATCGGGTGCA   
  
  
+ GGATTGTCAG GGTGCCCTTT TTTTGGGCTG GAAGGAGCGC AATCTGGCAA CCTTCTCTAC ATGGTGCTGT   
  
  
+ AAAGAATG  

- -Up\_Stream \_Len000CAATTT TTTGAGTCTT GATAATATCT AAAAGACAAA AGACAAACCT AACTCTAGGC   
  
  
- TTCGGAAACT CTAGGGGACA AAGATCGAAG GTTGGGGGGA GGGACCCAAG AAAAAGAAAC CACACTTTCA   
  
  
- TACTTGTTTT CGACTACATT CACACTAAGT TTGAGATCGG AACCACTCTG AGAACCCTTC CCGAGGATCT   
  
  
- CTGAAACGGT TAACTCGATT AAACTACGGA TGATGAACGA ATGAAAAGAT CGACAACAAA TTTGCTAAAT   
  
  
- ACAAGTATCA ATTCCTCGCG GTATAACGTA ATGTTATAGA CGAAATCACG AAACAAAAAA CGTAAAAGAA   
  
  
- GAAATAATGA TACCAAAAAC TTCTTCCGAA TGACTGACTA TGACCAAAGA AGCGAATTCA AACCACATGT   
  
  
- ACGAAAAGTT AGACACAACG TAAAAAGATA CTACATATTC GGCAAATAAT GGATAACTAC AAGTTGAAAA   
  
  
- GTGTGATATA TAGGAAATAC CAAAGAAGCG AATTCAAACC ACATGTACGA AAAGTTAGAC ACAACGTAAA   
  
  
- AAGACACTAC ATATTCGGCA AATAATGGAT AACTACAAGT TGAAAAGTGT GATATACAGG AAAATAATCA   
  
  
- ATGACTACGG AGACAGTTTG TAAGAATTAA CGTAAATCAC ATAGTTGCCA GAATTTTAAA ATTCGTAACG   
  
  
- GTTGAACTTG AAGACTTCTA CAAACAGTTC TAGAAGTCAG ACGTTCAATT GACGACGAAC GAACTTGAAA   
  
  
- AGTTGAAAAT TCTCTCTACA GTCTCAGATT CGTACCGAAG GACTGTTCAT GACGTAAAAT AGTGAATACT   
  
  
- TCTGAGTTAA GACCCAAAAC TGTCGAAGAT ACTTGGAGAT CACGTCCATG TCGTCGAACC TGTAATTCGA   
  
  
- CGTTCAGTTA ACTGACATCT TGCTGTTCAA CAACTTCAAA GACGACTTTC AAGTAGTGAA CAAACTACTT   
  
  
- ATTTCATGTC GTACCGTGAA GTCTACGTTT TCAGAGCGCT AGTCATCTCT ACTAAGGTAT AAAGAGTAGA   
  
  
- GTAAAGGTTC AGAAACTGTT GTACATGGGT TTATTACGGT CGGTACTGTG AGTCTACAGA GAGGTTAGTA   
  
  
- TGTCGCTTCC TATGAAGTGA GACCTGAGTA GAGGTCGTCG TTAGCCTCGC ACGTCACAGA TACTGATGGG   
  
  
- TAGGCAACAG TCGTGGAGAA GTTTATCTTC GGTCAAAAGA GGAGTCCCAA GGGTGAGCAT GTAGAGTCTG   
  
  
- GGGGTAGTAA GAAGGCTGTT GATACCTAGG GGTCACTCGC CGAGAAGACA CCAACTGCTA TTACGACTCG   
  
  
- ACTCCTTGTC CAAGTCACTA TACCTCGAGG GGAACGGTGT CCTGAGTCCT GTGGTAATAA CGAGAAAATC   
  
  
- GGTGTCTCCT TCGGTACTTC CGAGGATATG AAACTCCGGT TTGGTTGACT ACCTATACCG GTTGTACCTC   
  
  
- AATTTCGTCC ACGATATGAA GACACGTCTT CGTTAGAGTC TCTTATTAGA TAGTTGACGT CTTTCTGATT   
  
  
- ACCTACGTAA CCCATTCTCC CACAGACAAA AACCAAGTGG TTAACTTTCC AACCGGCGGA TGTACAACCT   
  
  
- TCCCGAGTCT CGTTCCGACC TCAAAAGACC TATATGATAG ATGTTTTTCG AGTCCACGCT TGTCGGTTGC   
  
  
- TCGAGTCTCG AAGAAAGGAT GTACGTGTAG GACATAGTTT CAACGGGTAT AAAGTTTAAG CGTATATACA   
  
  
- GGAGTTTACA ACGATAAGTT CTTCGAAACC CCTTACTCGG CCAATAGGTG TAGTAGCTAA AGGTCTAACG   
  
  
- GTACCCCTGT GTTAACCACG AGAACTAGGT CAGAGAGCGG GTAGCCGGAC CGCCCGGGGG GGGAGCAAGC   
  
  
- CTAGTGACCC CAGCTACTAG GTGTGAGCCG GATACGAGCA CCGCCCCCCG AGCTCCAACA CCCCGTCTCC   
  
  
- GATTGTTTCC ACCGACTTAG TATACCTCAG GGTAAGCTCA AAGTACGACG TCGGTACAGT CCCACGTTCC   
  
  
- ACTTAGCGCT ACAGGAGTTC CAAGCGGGAC CCCTTCTTCG GAACCGGCAT TTGAAGGGGA TATACAACGT   
  
  
- AGTGTACGGG CTGCTCTCGC ACTCGTGGTG TTTAGTAGCC CTGGCCGAGA ACTCCGACCA CTTCTCCAAC   
  
  
- AGTGGTTCCT AGCAGTGGAA CCAACTCGTT CTCAGGTTGT GTTTGTGAGG AGGGAAAGAA GTTGCCAAGG   
  
  
- CACTTTGTAA CCTGATGATG TGACGGTACA AGCTCAGGTA ACTACACCGA GATGGATCCC TACTGTTCTT   
  
  
- CTCCTATTTA CGGCTCGTCG TAACAGAACG CGCGCTATAA CACTTGTACC AGCGAACACT CTTACGCCTT   
  
  
- TCCCAACTCT CCGTGCTCGG GAAGCCCTTT ACCTCCCGTT CCAAACTATA CCGACCCAAA GTCGTTAACG   
  
  
- GGAGCTCGGC GAGTCACTTA CTACGTTATT CCCCAAACTA CGCTCTTAAG GTATCCTTGA TAGCCCACGT   
  
  
- CCTAACAGTC CCACGGGAAA AAAACCCGAC CTTCCTCGCG TTAGACCGTT GGAAGAGATG TACCACGACA   
  
  
- TTTCTTAC

+     MYB recognition site

| Site Name | Organism | Position | Strand | Matrix score. | sequence | function |
| --- | --- | --- | --- | --- | --- | --- |
| MYB recognition site | Arabidopsis thaliana | 678 | - | 6 | CCGTTG |  |
| MYB recognition site | Arabidopsis thaliana | 2235 | - | 6 | CCGTTG |  |
| MYB recognition site | Arabidopsis thaliana | 1197 | + | 6 | CCGTTG |  |

>HU08G02295.1   
+ -Up\_Stream \_Len000GTTAAA AAACTCAGAA CTATTATAGA TTTTCTGTTT TCTGTTTGGA TTGAGATCCG   
  
  
+ AAGCCTTTGA GATCCCCTGT TTCTAGCTTC CAACCCCCCT CCCTGGGTTC TTTTTCTTTG GTGTGAAAGT   
  
  
+ ATGAACAAAA GCTGATGTAA GTGTGATTCA AACTCTAGCC TTGGTGAGAC TCTTGGGAAG GGCTCCTAGA   
  
  
+ GACTTTGCCA ATTGAGCTAA TTTGATGCCT ACTACTTGCT TACTTTTCTA GCTGTTGTTT AAACGATTTA   
  
  
+ TGTTCATAGT TAAGGAGCGC CATATTGCAT TACAATATCT GCTTTAGTGC TTTGTTTTTT GCATTTTCTT   
  
  
+ CTTTATTACT ATGGTTTTTG AAGAAGGCTT ACTGACTGAT ACTGGTTTCT TCGCTTAAGT TTGGTGTACA   
  
  
+ TGCTTTTCAA TCTGTGTTGC ATTTTTCTAT GATGTATAAG CCGTTTATTA CCTATTGATG TTCAACTTTT   
  
  
+ CACACTATAT ATCCTTTATG GTTTCTTCGC TTAAGTTTGG TGTACATGCT TTTCAATCTG TGTTGCATTT   
  
  
+ TTCTGTGATG TATAAGCCGT TTATTACCTA TTGATGTTCA ACTTTTCACA CTATATGTCC TTTTATTAGT   
  
  
+ TACTGATGCC TCTGTCAAAC ATTCTTAATT GCATTTAGTG TATCAACGGT CTTAAAATTT TAAGCATTGC   
  
  
+ CAACTTGAAC TTCTGAAGAT GTTTGTCAAG ATCTTCAGTC TGCAAGTTAA CTGCTGCTTG CTTGAACTTT   
  
  
+ TCAACTTTTA AGAGAGATGT CAGAGTCTAA GCATGGCTTC CTGACAAGTA CTGCATTTTA TCACTTATGA   
  
  
+ AGACTCAATT CTGGGTTTTG ACAGCTTCTA TGAACCTCTA GTGCAGGTAC AGCAGCTTGG ACATTAAGCT   
  
  
+ GCAAGTCAAT TGACTGTAGA ACGACAAGTT GTTGAAGTTT CTGCTGAAAG TTCATCACTT GTTTGATGAA   
  
  
+ TAAAGTACAG CATGGCACTT CAGATGCAAA AGTCTCGCGA TCAGTAGAGA TGATTCCATA TTTCTCATCT   
  
  
+ CATTTCCAAG TCTTTGACAA CATGTACCCA AATAATGCCA GCCATGACAC TCAGATGTCT CTCCAATCAT   
  
  
+ ACAGCGAAGG ATACTTCACT CTGGACTCAT CTCCAGCAGC AATCGGAGCG TGCAGTGTCT ATGACTACCC   
  
  
+ ATCCGTTGTC AGCACCTCTT CAAATAGAAG CCAGTTTTCT CCTCAGGGTT CCCACTCGTA CATCTCAGAC   
  
  
+ CCCCATCATT CTTCCGACAA CTATGGATCC CCAGTGAGCG GCTCTTCTGT GGTTGACGAT AATGCTGAGC   
  
  
+ TGAGGAACAG GTTCAGTGAT ATGGAGCTCC CCTTGCCACA GGACTCAGGA CACCATTATT GCTCTTTTAG   
  
  
+ CCACAGAGGA AGCCATGAAG GCTCCTATAC TTTGAGGCCA AACCAACTGA TGGATATGGC CAACATGGAG   
  
  
+ TTAAAGCAGG TGCTATACTT CTGTGCAGAA GCAATCTCAG AGAATAATCT ATCAACTGCA GAAAGACTAA   
  
  
+ TGGATGCATT GGGTAAGAGG GTGTCTGTTT TTGGTTCACC AATTGAAAGG TTGGCCGCCT ACATGTTGGA   
  
  
+ AGGGCTCAGA GCAAGGCTGG AGTTTTCTGG ATATACTATC TACAAAAAGC TCAGGTGCGA ACAGCCAACG   
  
  
+ AGCTCAGAGC TTCTTTCCTA CATGCACATC CTGTATCAAA GTTGCCCATA TTTCAAATTC GCATATATGT   
  
  
+ CCTCAAATGT TGCTATTCAA GAAGCTTTGG GGAATGAGCC GGTTATCCAC ATCATCGATT TCCAGATTGC   
  
  
+ CATGGGGACA CAATTGGTGC TCTTGATCCA GTCTCTCGCC CATCGGCCTG GCGGGCCCCC CCCTCGTTCG   
  
  
+ GATCACTGGG GTCGATGATC CACACTCGGC CTATGCTCGT GGCGGGGGGC TCGAGGTTGT GGGGCAGAGG   
  
  
+ CTAACAAAGG TGGCTGAATC ATATGGAGTC CCATTCGAGT TTCATGCTGC AGCCATGTCA GGGTGCAAGG   
  
  
+ TGAATCGCGA TGTCCTCAAG GTTCGCCCTG GGGAAGAAGC CTTGGCCGTA AACTTCCCCT ATATGTTGCA   
  
  
+ TCACATGCCC GACGAGAGCG TGAGCACCAC AAATCATCGG GACCGGCTCT TGAGGCTGGT GAAGAGGTTG   
  
  
+ TCACCAAGGA TCGTCACCTT GGTTGAGCAA GAGTCCAACA CAAACACTCC TCCCTTTCTT CAACGGTTCC   
  
  
+ GTGAAACATT GGACTACTAC ACTGCCATGT TCGAGTCCAT TGATGTGGCT CTACCTAGGG ATGACAAGAA   
  
  
+ GAGGATAAAT GCCGAGCAGC ATTGTCTTGC GCGCGATATT GTGAACATGG TCGCTTGTGA GAATGCGGAA   
  
  
+ AGGGTTGAGA GGCACGAGCC CTTCGGGAAA TGGAGGGCAA GGTTTGATAT GGCTGGGTTT CAGCAATTGC   
  
  
+ CCTCGAGCCG CTCAGTGAAT GATGCAATAA GGGGTTTGAT GCGAGAATTC CATAGGAACT ATCGGGTGCA   
  
  
+ GGATTGTCAG GGTGCCCTTT TTTTGGGCTG GAAGGAGCGC AATCTGGCAA CCTTCTCTAC ATGGTGCTGT   
  
  
+ AAAGAATG  

- -Up\_Stream \_Len000CAATTT TTTGAGTCTT GATAATATCT AAAAGACAAA AGACAAACCT AACTCTAGGC   
  
  
- TTCGGAAACT CTAGGGGACA AAGATCGAAG GTTGGGGGGA GGGACCCAAG AAAAAGAAAC CACACTTTCA   
  
  
- TACTTGTTTT CGACTACATT CACACTAAGT TTGAGATCGG AACCACTCTG AGAACCCTTC CCGAGGATCT   
  
  
- CTGAAACGGT TAACTCGATT AAACTACGGA TGATGAACGA ATGAAAAGAT CGACAACAAA TTTGCTAAAT   
  
  
- ACAAGTATCA ATTCCTCGCG GTATAACGTA ATGTTATAGA CGAAATCACG AAACAAAAAA CGTAAAAGAA   
  
  
- GAAATAATGA TACCAAAAAC TTCTTCCGAA TGACTGACTA TGACCAAAGA AGCGAATTCA AACCACATGT   
  
  
- ACGAAAAGTT AGACACAACG TAAAAAGATA CTACATATTC GGCAAATAAT GGATAACTAC AAGTTGAAAA   
  
  
- GTGTGATATA TAGGAAATAC CAAAGAAGCG AATTCAAACC ACATGTACGA AAAGTTAGAC ACAACGTAAA   
  
  
- AAGACACTAC ATATTCGGCA AATAATGGAT AACTACAAGT TGAAAAGTGT GATATACAGG AAAATAATCA   
  
  
- ATGACTACGG AGACAGTTTG TAAGAATTAA CGTAAATCAC ATAGTTGCCA GAATTTTAAA ATTCGTAACG   
  
  
- GTTGAACTTG AAGACTTCTA CAAACAGTTC TAGAAGTCAG ACGTTCAATT GACGACGAAC GAACTTGAAA   
  
  
- AGTTGAAAAT TCTCTCTACA GTCTCAGATT CGTACCGAAG GACTGTTCAT GACGTAAAAT AGTGAATACT   
  
  
- TCTGAGTTAA GACCCAAAAC TGTCGAAGAT ACTTGGAGAT CACGTCCATG TCGTCGAACC TGTAATTCGA   
  
  
- CGTTCAGTTA ACTGACATCT TGCTGTTCAA CAACTTCAAA GACGACTTTC AAGTAGTGAA CAAACTACTT   
  
  
- ATTTCATGTC GTACCGTGAA GTCTACGTTT TCAGAGCGCT AGTCATCTCT ACTAAGGTAT AAAGAGTAGA   
  
  
- GTAAAGGTTC AGAAACTGTT GTACATGGGT TTATTACGGT CGGTACTGTG AGTCTACAGA GAGGTTAGTA   
  
  
- TGTCGCTTCC TATGAAGTGA GACCTGAGTA GAGGTCGTCG TTAGCCTCGC ACGTCACAGA TACTGATGGG   
  
  
- TAGGCAACAG TCGTGGAGAA GTTTATCTTC GGTCAAAAGA GGAGTCCCAA GGGTGAGCAT GTAGAGTCTG   
  
  
- GGGGTAGTAA GAAGGCTGTT GATACCTAGG GGTCACTCGC CGAGAAGACA CCAACTGCTA TTACGACTCG   
  
  
- ACTCCTTGTC CAAGTCACTA TACCTCGAGG GGAACGGTGT CCTGAGTCCT GTGGTAATAA CGAGAAAATC   
  
  
- GGTGTCTCCT TCGGTACTTC CGAGGATATG AAACTCCGGT TTGGTTGACT ACCTATACCG GTTGTACCTC   
  
  
- AATTTCGTCC ACGATATGAA GACACGTCTT CGTTAGAGTC TCTTATTAGA TAGTTGACGT CTTTCTGATT   
  
  
- ACCTACGTAA CCCATTCTCC CACAGACAAA AACCAAGTGG TTAACTTTCC AACCGGCGGA TGTACAACCT   
  
  
- TCCCGAGTCT CGTTCCGACC TCAAAAGACC TATATGATAG ATGTTTTTCG AGTCCACGCT TGTCGGTTGC   
  
  
- TCGAGTCTCG AAGAAAGGAT GTACGTGTAG GACATAGTTT CAACGGGTAT AAAGTTTAAG CGTATATACA   
  
  
- GGAGTTTACA ACGATAAGTT CTTCGAAACC CCTTACTCGG CCAATAGGTG TAGTAGCTAA AGGTCTAACG   
  
  
- GTACCCCTGT GTTAACCACG AGAACTAGGT CAGAGAGCGG GTAGCCGGAC CGCCCGGGGG GGGAGCAAGC   
  
  
- CTAGTGACCC CAGCTACTAG GTGTGAGCCG GATACGAGCA CCGCCCCCCG AGCTCCAACA CCCCGTCTCC   
  
  
- GATTGTTTCC ACCGACTTAG TATACCTCAG GGTAAGCTCA AAGTACGACG TCGGTACAGT CCCACGTTCC   
  
  
- ACTTAGCGCT ACAGGAGTTC CAAGCGGGAC CCCTTCTTCG GAACCGGCAT TTGAAGGGGA TATACAACGT   
  
  
- AGTGTACGGG CTGCTCTCGC ACTCGTGGTG TTTAGTAGCC CTGGCCGAGA ACTCCGACCA CTTCTCCAAC   
  
  
- AGTGGTTCCT AGCAGTGGAA CCAACTCGTT CTCAGGTTGT GTTTGTGAGG AGGGAAAGAA GTTGCCAAGG   
  
  
- CACTTTGTAA CCTGATGATG TGACGGTACA AGCTCAGGTA ACTACACCGA GATGGATCCC TACTGTTCTT   
  
  
- CTCCTATTTA CGGCTCGTCG TAACAGAACG CGCGCTATAA CACTTGTACC AGCGAACACT CTTACGCCTT   
  
  
- TCCCAACTCT CCGTGCTCGG GAAGCCCTTT ACCTCCCGTT CCAAACTATA CCGACCCAAA GTCGTTAACG   
  
  
- GGAGCTCGGC GAGTCACTTA CTACGTTATT CCCCAAACTA CGCTCTTAAG GTATCCTTGA TAGCCCACGT   
  
  
- CCTAACAGTC CCACGGGAAA AAAACCCGAC CTTCCTCGCG TTAGACCGTT GGAAGAGATG TACCACGACA   
  
  
- TTTCTTAC

+     MYC

| Site Name | Organism | Position | Strand | Matrix score. | sequence | function |
| --- | --- | --- | --- | --- | --- | --- |
| MYC | Arabidopsis thaliana | 2448 | - | 6 | CAATTG |  |
| MYC | Arabidopsis thaliana | 921 | + | 6 | CAATTG |  |
| MYC | Arabidopsis thaliana | 1835 | - | 6 | CAATTG |  |
| MYC | Arabidopsis thaliana | 1584 | - | 6 | CAATTG |  |
| MYC | Arabidopsis thaliana | 223 | + | 6 | CAATTG |  |
| MYC | Arabidopsis thaliana | 2106 | - | 6 | CATGTG |  |
| MYC | Arabidopsis thaliana | 1758 | - | 6 | CATTTG |  |

>HU08G02295.1   
+ -Up\_Stream \_Len000GTTAAA AAACTCAGAA CTATTATAGA TTTTCTGTTT TCTGTTTGGA TTGAGATCCG   
  
  
+ AAGCCTTTGA GATCCCCTGT TTCTAGCTTC CAACCCCCCT CCCTGGGTTC TTTTTCTTTG GTGTGAAAGT   
  
  
+ ATGAACAAAA GCTGATGTAA GTGTGATTCA AACTCTAGCC TTGGTGAGAC TCTTGGGAAG GGCTCCTAGA   
  
  
+ GACTTTGCCA ATTGAGCTAA TTTGATGCCT ACTACTTGCT TACTTTTCTA GCTGTTGTTT AAACGATTTA   
  
  
+ TGTTCATAGT TAAGGAGCGC CATATTGCAT TACAATATCT GCTTTAGTGC TTTGTTTTTT GCATTTTCTT   
  
  
+ CTTTATTACT ATGGTTTTTG AAGAAGGCTT ACTGACTGAT ACTGGTTTCT TCGCTTAAGT TTGGTGTACA   
  
  
+ TGCTTTTCAA TCTGTGTTGC ATTTTTCTAT GATGTATAAG CCGTTTATTA CCTATTGATG TTCAACTTTT   
  
  
+ CACACTATAT ATCCTTTATG GTTTCTTCGC TTAAGTTTGG TGTACATGCT TTTCAATCTG TGTTGCATTT   
  
  
+ TTCTGTGATG TATAAGCCGT TTATTACCTA TTGATGTTCA ACTTTTCACA CTATATGTCC TTTTATTAGT   
  
  
+ TACTGATGCC TCTGTCAAAC ATTCTTAATT GCATTTAGTG TATCAACGGT CTTAAAATTT TAAGCATTGC   
  
  
+ CAACTTGAAC TTCTGAAGAT GTTTGTCAAG ATCTTCAGTC TGCAAGTTAA CTGCTGCTTG CTTGAACTTT   
  
  
+ TCAACTTTTA AGAGAGATGT CAGAGTCTAA GCATGGCTTC CTGACAAGTA CTGCATTTTA TCACTTATGA   
  
  
+ AGACTCAATT CTGGGTTTTG ACAGCTTCTA TGAACCTCTA GTGCAGGTAC AGCAGCTTGG ACATTAAGCT   
  
  
+ GCAAGTCAAT TGACTGTAGA ACGACAAGTT GTTGAAGTTT CTGCTGAAAG TTCATCACTT GTTTGATGAA   
  
  
+ TAAAGTACAG CATGGCACTT CAGATGCAAA AGTCTCGCGA TCAGTAGAGA TGATTCCATA TTTCTCATCT   
  
  
+ CATTTCCAAG TCTTTGACAA CATGTACCCA AATAATGCCA GCCATGACAC TCAGATGTCT CTCCAATCAT   
  
  
+ ACAGCGAAGG ATACTTCACT CTGGACTCAT CTCCAGCAGC AATCGGAGCG TGCAGTGTCT ATGACTACCC   
  
  
+ ATCCGTTGTC AGCACCTCTT CAAATAGAAG CCAGTTTTCT CCTCAGGGTT CCCACTCGTA CATCTCAGAC   
  
  
+ CCCCATCATT CTTCCGACAA CTATGGATCC CCAGTGAGCG GCTCTTCTGT GGTTGACGAT AATGCTGAGC   
  
  
+ TGAGGAACAG GTTCAGTGAT ATGGAGCTCC CCTTGCCACA GGACTCAGGA CACCATTATT GCTCTTTTAG   
  
  
+ CCACAGAGGA AGCCATGAAG GCTCCTATAC TTTGAGGCCA AACCAACTGA TGGATATGGC CAACATGGAG   
  
  
+ TTAAAGCAGG TGCTATACTT CTGTGCAGAA GCAATCTCAG AGAATAATCT ATCAACTGCA GAAAGACTAA   
  
  
+ TGGATGCATT GGGTAAGAGG GTGTCTGTTT TTGGTTCACC AATTGAAAGG TTGGCCGCCT ACATGTTGGA   
  
  
+ AGGGCTCAGA GCAAGGCTGG AGTTTTCTGG ATATACTATC TACAAAAAGC TCAGGTGCGA ACAGCCAACG   
  
  
+ AGCTCAGAGC TTCTTTCCTA CATGCACATC CTGTATCAAA GTTGCCCATA TTTCAAATTC GCATATATGT   
  
  
+ CCTCAAATGT TGCTATTCAA GAAGCTTTGG GGAATGAGCC GGTTATCCAC ATCATCGATT TCCAGATTGC   
  
  
+ CATGGGGACA CAATTGGTGC TCTTGATCCA GTCTCTCGCC CATCGGCCTG GCGGGCCCCC CCCTCGTTCG   
  
  
+ GATCACTGGG GTCGATGATC CACACTCGGC CTATGCTCGT GGCGGGGGGC TCGAGGTTGT GGGGCAGAGG   
  
  
+ CTAACAAAGG TGGCTGAATC ATATGGAGTC CCATTCGAGT TTCATGCTGC AGCCATGTCA GGGTGCAAGG   
  
  
+ TGAATCGCGA TGTCCTCAAG GTTCGCCCTG GGGAAGAAGC CTTGGCCGTA AACTTCCCCT ATATGTTGCA   
  
  
+ TCACATGCCC GACGAGAGCG TGAGCACCAC AAATCATCGG GACCGGCTCT TGAGGCTGGT GAAGAGGTTG   
  
  
+ TCACCAAGGA TCGTCACCTT GGTTGAGCAA GAGTCCAACA CAAACACTCC TCCCTTTCTT CAACGGTTCC   
  
  
+ GTGAAACATT GGACTACTAC ACTGCCATGT TCGAGTCCAT TGATGTGGCT CTACCTAGGG ATGACAAGAA   
  
  
+ GAGGATAAAT GCCGAGCAGC ATTGTCTTGC GCGCGATATT GTGAACATGG TCGCTTGTGA GAATGCGGAA   
  
  
+ AGGGTTGAGA GGCACGAGCC CTTCGGGAAA TGGAGGGCAA GGTTTGATAT GGCTGGGTTT CAGCAATTGC   
  
  
+ CCTCGAGCCG CTCAGTGAAT GATGCAATAA GGGGTTTGAT GCGAGAATTC CATAGGAACT ATCGGGTGCA   
  
  
+ GGATTGTCAG GGTGCCCTTT TTTTGGGCTG GAAGGAGCGC AATCTGGCAA CCTTCTCTAC ATGGTGCTGT   
  
  
+ AAAGAATG  

- -Up\_Stream \_Len000CAATTT TTTGAGTCTT GATAATATCT AAAAGACAAA AGACAAACCT AACTCTAGGC   
  
  
- TTCGGAAACT CTAGGGGACA AAGATCGAAG GTTGGGGGGA GGGACCCAAG AAAAAGAAAC CACACTTTCA   
  
  
- TACTTGTTTT CGACTACATT CACACTAAGT TTGAGATCGG AACCACTCTG AGAACCCTTC CCGAGGATCT   
  
  
- CTGAAACGGT TAACTCGATT AAACTACGGA TGATGAACGA ATGAAAAGAT CGACAACAAA TTTGCTAAAT   
  
  
- ACAAGTATCA ATTCCTCGCG GTATAACGTA ATGTTATAGA CGAAATCACG AAACAAAAAA CGTAAAAGAA   
  
  
- GAAATAATGA TACCAAAAAC TTCTTCCGAA TGACTGACTA TGACCAAAGA AGCGAATTCA AACCACATGT   
  
  
- ACGAAAAGTT AGACACAACG TAAAAAGATA CTACATATTC GGCAAATAAT GGATAACTAC AAGTTGAAAA   
  
  
- GTGTGATATA TAGGAAATAC CAAAGAAGCG AATTCAAACC ACATGTACGA AAAGTTAGAC ACAACGTAAA   
  
  
- AAGACACTAC ATATTCGGCA AATAATGGAT AACTACAAGT TGAAAAGTGT GATATACAGG AAAATAATCA   
  
  
- ATGACTACGG AGACAGTTTG TAAGAATTAA CGTAAATCAC ATAGTTGCCA GAATTTTAAA ATTCGTAACG   
  
  
- GTTGAACTTG AAGACTTCTA CAAACAGTTC TAGAAGTCAG ACGTTCAATT GACGACGAAC GAACTTGAAA   
  
  
- AGTTGAAAAT TCTCTCTACA GTCTCAGATT CGTACCGAAG GACTGTTCAT GACGTAAAAT AGTGAATACT   
  
  
- TCTGAGTTAA GACCCAAAAC TGTCGAAGAT ACTTGGAGAT CACGTCCATG TCGTCGAACC TGTAATTCGA   
  
  
- CGTTCAGTTA ACTGACATCT TGCTGTTCAA CAACTTCAAA GACGACTTTC AAGTAGTGAA CAAACTACTT   
  
  
- ATTTCATGTC GTACCGTGAA GTCTACGTTT TCAGAGCGCT AGTCATCTCT ACTAAGGTAT AAAGAGTAGA   
  
  
- GTAAAGGTTC AGAAACTGTT GTACATGGGT TTATTACGGT CGGTACTGTG AGTCTACAGA GAGGTTAGTA   
  
  
- TGTCGCTTCC TATGAAGTGA GACCTGAGTA GAGGTCGTCG TTAGCCTCGC ACGTCACAGA TACTGATGGG   
  
  
- TAGGCAACAG TCGTGGAGAA GTTTATCTTC GGTCAAAAGA GGAGTCCCAA GGGTGAGCAT GTAGAGTCTG   
  
  
- GGGGTAGTAA GAAGGCTGTT GATACCTAGG GGTCACTCGC CGAGAAGACA CCAACTGCTA TTACGACTCG   
  
  
- ACTCCTTGTC CAAGTCACTA TACCTCGAGG GGAACGGTGT CCTGAGTCCT GTGGTAATAA CGAGAAAATC   
  
  
- GGTGTCTCCT TCGGTACTTC CGAGGATATG AAACTCCGGT TTGGTTGACT ACCTATACCG GTTGTACCTC   
  
  
- AATTTCGTCC ACGATATGAA GACACGTCTT CGTTAGAGTC TCTTATTAGA TAGTTGACGT CTTTCTGATT   
  
  
- ACCTACGTAA CCCATTCTCC CACAGACAAA AACCAAGTGG TTAACTTTCC AACCGGCGGA TGTACAACCT   
  
  
- TCCCGAGTCT CGTTCCGACC TCAAAAGACC TATATGATAG ATGTTTTTCG AGTCCACGCT TGTCGGTTGC   
  
  
- TCGAGTCTCG AAGAAAGGAT GTACGTGTAG GACATAGTTT CAACGGGTAT AAAGTTTAAG CGTATATACA   
  
  
- GGAGTTTACA ACGATAAGTT CTTCGAAACC CCTTACTCGG CCAATAGGTG TAGTAGCTAA AGGTCTAACG   
  
  
- GTACCCCTGT GTTAACCACG AGAACTAGGT CAGAGAGCGG GTAGCCGGAC CGCCCGGGGG GGGAGCAAGC   
  
  
- CTAGTGACCC CAGCTACTAG GTGTGAGCCG GATACGAGCA CCGCCCCCCG AGCTCCAACA CCCCGTCTCC   
  
  
- GATTGTTTCC ACCGACTTAG TATACCTCAG GGTAAGCTCA AAGTACGACG TCGGTACAGT CCCACGTTCC   
  
  
- ACTTAGCGCT ACAGGAGTTC CAAGCGGGAC CCCTTCTTCG GAACCGGCAT TTGAAGGGGA TATACAACGT   
  
  
- AGTGTACGGG CTGCTCTCGC ACTCGTGGTG TTTAGTAGCC CTGGCCGAGA ACTCCGACCA CTTCTCCAAC   
  
  
- AGTGGTTCCT AGCAGTGGAA CCAACTCGTT CTCAGGTTGT GTTTGTGAGG AGGGAAAGAA GTTGCCAAGG   
  
  
- CACTTTGTAA CCTGATGATG TGACGGTACA AGCTCAGGTA ACTACACCGA GATGGATCCC TACTGTTCTT   
  
  
- CTCCTATTTA CGGCTCGTCG TAACAGAACG CGCGCTATAA CACTTGTACC AGCGAACACT CTTACGCCTT   
  
  
- TCCCAACTCT CCGTGCTCGG GAAGCCCTTT ACCTCCCGTT CCAAACTATA CCGACCCAAA GTCGTTAACG   
  
  
- GGAGCTCGGC GAGTCACTTA CTACGTTATT CCCCAAACTA CGCTCTTAAG GTATCCTTGA TAGCCCACGT   
  
  
- CCTAACAGTC CCACGGGAAA AAAACCCGAC CTTCCTCGCG TTAGACCGTT GGAAGAGATG TACCACGACA   
  
  
- TTTCTTAC

+     Myb

| Site Name | Organism | Position | Strand | Matrix score. | sequence | function |
| --- | --- | --- | --- | --- | --- | --- |
| Myb | Arabidopsis thaliana | 752 | + | 6 | TAACTG |  |
| Myb | Arabidopsis thaliana | 1448 | + | 6 | CAACTG |  |
| Myb | Arabidopsis thaliana | 1527 | + | 6 | CAACTG |  |

>HU08G02295.1   
+ -Up\_Stream \_Len000GTTAAA AAACTCAGAA CTATTATAGA TTTTCTGTTT TCTGTTTGGA TTGAGATCCG   
  
  
+ AAGCCTTTGA GATCCCCTGT TTCTAGCTTC CAACCCCCCT CCCTGGGTTC TTTTTCTTTG GTGTGAAAGT   
  
  
+ ATGAACAAAA GCTGATGTAA GTGTGATTCA AACTCTAGCC TTGGTGAGAC TCTTGGGAAG GGCTCCTAGA   
  
  
+ GACTTTGCCA ATTGAGCTAA TTTGATGCCT ACTACTTGCT TACTTTTCTA GCTGTTGTTT AAACGATTTA   
  
  
+ TGTTCATAGT TAAGGAGCGC CATATTGCAT TACAATATCT GCTTTAGTGC TTTGTTTTTT GCATTTTCTT   
  
  
+ CTTTATTACT ATGGTTTTTG AAGAAGGCTT ACTGACTGAT ACTGGTTTCT TCGCTTAAGT TTGGTGTACA   
  
  
+ TGCTTTTCAA TCTGTGTTGC ATTTTTCTAT GATGTATAAG CCGTTTATTA CCTATTGATG TTCAACTTTT   
  
  
+ CACACTATAT ATCCTTTATG GTTTCTTCGC TTAAGTTTGG TGTACATGCT TTTCAATCTG TGTTGCATTT   
  
  
+ TTCTGTGATG TATAAGCCGT TTATTACCTA TTGATGTTCA ACTTTTCACA CTATATGTCC TTTTATTAGT   
  
  
+ TACTGATGCC TCTGTCAAAC ATTCTTAATT GCATTTAGTG TATCAACGGT CTTAAAATTT TAAGCATTGC   
  
  
+ CAACTTGAAC TTCTGAAGAT GTTTGTCAAG ATCTTCAGTC TGCAAGTTAA CTGCTGCTTG CTTGAACTTT   
  
  
+ TCAACTTTTA AGAGAGATGT CAGAGTCTAA GCATGGCTTC CTGACAAGTA CTGCATTTTA TCACTTATGA   
  
  
+ AGACTCAATT CTGGGTTTTG ACAGCTTCTA TGAACCTCTA GTGCAGGTAC AGCAGCTTGG ACATTAAGCT   
  
  
+ GCAAGTCAAT TGACTGTAGA ACGACAAGTT GTTGAAGTTT CTGCTGAAAG TTCATCACTT GTTTGATGAA   
  
  
+ TAAAGTACAG CATGGCACTT CAGATGCAAA AGTCTCGCGA TCAGTAGAGA TGATTCCATA TTTCTCATCT   
  
  
+ CATTTCCAAG TCTTTGACAA CATGTACCCA AATAATGCCA GCCATGACAC TCAGATGTCT CTCCAATCAT   
  
  
+ ACAGCGAAGG ATACTTCACT CTGGACTCAT CTCCAGCAGC AATCGGAGCG TGCAGTGTCT ATGACTACCC   
  
  
+ ATCCGTTGTC AGCACCTCTT CAAATAGAAG CCAGTTTTCT CCTCAGGGTT CCCACTCGTA CATCTCAGAC   
  
  
+ CCCCATCATT CTTCCGACAA CTATGGATCC CCAGTGAGCG GCTCTTCTGT GGTTGACGAT AATGCTGAGC   
  
  
+ TGAGGAACAG GTTCAGTGAT ATGGAGCTCC CCTTGCCACA GGACTCAGGA CACCATTATT GCTCTTTTAG   
  
  
+ CCACAGAGGA AGCCATGAAG GCTCCTATAC TTTGAGGCCA AACCAACTGA TGGATATGGC CAACATGGAG   
  
  
+ TTAAAGCAGG TGCTATACTT CTGTGCAGAA GCAATCTCAG AGAATAATCT ATCAACTGCA GAAAGACTAA   
  
  
+ TGGATGCATT GGGTAAGAGG GTGTCTGTTT TTGGTTCACC AATTGAAAGG TTGGCCGCCT ACATGTTGGA   
  
  
+ AGGGCTCAGA GCAAGGCTGG AGTTTTCTGG ATATACTATC TACAAAAAGC TCAGGTGCGA ACAGCCAACG   
  
  
+ AGCTCAGAGC TTCTTTCCTA CATGCACATC CTGTATCAAA GTTGCCCATA TTTCAAATTC GCATATATGT   
  
  
+ CCTCAAATGT TGCTATTCAA GAAGCTTTGG GGAATGAGCC GGTTATCCAC ATCATCGATT TCCAGATTGC   
  
  
+ CATGGGGACA CAATTGGTGC TCTTGATCCA GTCTCTCGCC CATCGGCCTG GCGGGCCCCC CCCTCGTTCG   
  
  
+ GATCACTGGG GTCGATGATC CACACTCGGC CTATGCTCGT GGCGGGGGGC TCGAGGTTGT GGGGCAGAGG   
  
  
+ CTAACAAAGG TGGCTGAATC ATATGGAGTC CCATTCGAGT TTCATGCTGC AGCCATGTCA GGGTGCAAGG   
  
  
+ TGAATCGCGA TGTCCTCAAG GTTCGCCCTG GGGAAGAAGC CTTGGCCGTA AACTTCCCCT ATATGTTGCA   
  
  
+ TCACATGCCC GACGAGAGCG TGAGCACCAC AAATCATCGG GACCGGCTCT TGAGGCTGGT GAAGAGGTTG   
  
  
+ TCACCAAGGA TCGTCACCTT GGTTGAGCAA GAGTCCAACA CAAACACTCC TCCCTTTCTT CAACGGTTCC   
  
  
+ GTGAAACATT GGACTACTAC ACTGCCATGT TCGAGTCCAT TGATGTGGCT CTACCTAGGG ATGACAAGAA   
  
  
+ GAGGATAAAT GCCGAGCAGC ATTGTCTTGC GCGCGATATT GTGAACATGG TCGCTTGTGA GAATGCGGAA   
  
  
+ AGGGTTGAGA GGCACGAGCC CTTCGGGAAA TGGAGGGCAA GGTTTGATAT GGCTGGGTTT CAGCAATTGC   
  
  
+ CCTCGAGCCG CTCAGTGAAT GATGCAATAA GGGGTTTGAT GCGAGAATTC CATAGGAACT ATCGGGTGCA   
  
  
+ GGATTGTCAG GGTGCCCTTT TTTTGGGCTG GAAGGAGCGC AATCTGGCAA CCTTCTCTAC ATGGTGCTGT   
  
  
+ AAAGAATG  

- -Up\_Stream \_Len000CAATTT TTTGAGTCTT GATAATATCT AAAAGACAAA AGACAAACCT AACTCTAGGC   
  
  
- TTCGGAAACT CTAGGGGACA AAGATCGAAG GTTGGGGGGA GGGACCCAAG AAAAAGAAAC CACACTTTCA   
  
  
- TACTTGTTTT CGACTACATT CACACTAAGT TTGAGATCGG AACCACTCTG AGAACCCTTC CCGAGGATCT   
  
  
- CTGAAACGGT TAACTCGATT AAACTACGGA TGATGAACGA ATGAAAAGAT CGACAACAAA TTTGCTAAAT   
  
  
- ACAAGTATCA ATTCCTCGCG GTATAACGTA ATGTTATAGA CGAAATCACG AAACAAAAAA CGTAAAAGAA   
  
  
- GAAATAATGA TACCAAAAAC TTCTTCCGAA TGACTGACTA TGACCAAAGA AGCGAATTCA AACCACATGT   
  
  
- ACGAAAAGTT AGACACAACG TAAAAAGATA CTACATATTC GGCAAATAAT GGATAACTAC AAGTTGAAAA   
  
  
- GTGTGATATA TAGGAAATAC CAAAGAAGCG AATTCAAACC ACATGTACGA AAAGTTAGAC ACAACGTAAA   
  
  
- AAGACACTAC ATATTCGGCA AATAATGGAT AACTACAAGT TGAAAAGTGT GATATACAGG AAAATAATCA   
  
  
- ATGACTACGG AGACAGTTTG TAAGAATTAA CGTAAATCAC ATAGTTGCCA GAATTTTAAA ATTCGTAACG   
  
  
- GTTGAACTTG AAGACTTCTA CAAACAGTTC TAGAAGTCAG ACGTTCAATT GACGACGAAC GAACTTGAAA   
  
  
- AGTTGAAAAT TCTCTCTACA GTCTCAGATT CGTACCGAAG GACTGTTCAT GACGTAAAAT AGTGAATACT   
  
  
- TCTGAGTTAA GACCCAAAAC TGTCGAAGAT ACTTGGAGAT CACGTCCATG TCGTCGAACC TGTAATTCGA   
  
  
- CGTTCAGTTA ACTGACATCT TGCTGTTCAA CAACTTCAAA GACGACTTTC AAGTAGTGAA CAAACTACTT   
  
  
- ATTTCATGTC GTACCGTGAA GTCTACGTTT TCAGAGCGCT AGTCATCTCT ACTAAGGTAT AAAGAGTAGA   
  
  
- GTAAAGGTTC AGAAACTGTT GTACATGGGT TTATTACGGT CGGTACTGTG AGTCTACAGA GAGGTTAGTA   
  
  
- TGTCGCTTCC TATGAAGTGA GACCTGAGTA GAGGTCGTCG TTAGCCTCGC ACGTCACAGA TACTGATGGG   
  
  
- TAGGCAACAG TCGTGGAGAA GTTTATCTTC GGTCAAAAGA GGAGTCCCAA GGGTGAGCAT GTAGAGTCTG   
  
  
- GGGGTAGTAA GAAGGCTGTT GATACCTAGG GGTCACTCGC CGAGAAGACA CCAACTGCTA TTACGACTCG   
  
  
- ACTCCTTGTC CAAGTCACTA TACCTCGAGG GGAACGGTGT CCTGAGTCCT GTGGTAATAA CGAGAAAATC   
  
  
- GGTGTCTCCT TCGGTACTTC CGAGGATATG AAACTCCGGT TTGGTTGACT ACCTATACCG GTTGTACCTC   
  
  
- AATTTCGTCC ACGATATGAA GACACGTCTT CGTTAGAGTC TCTTATTAGA TAGTTGACGT CTTTCTGATT   
  
  
- ACCTACGTAA CCCATTCTCC CACAGACAAA AACCAAGTGG TTAACTTTCC AACCGGCGGA TGTACAACCT   
  
  
- TCCCGAGTCT CGTTCCGACC TCAAAAGACC TATATGATAG ATGTTTTTCG AGTCCACGCT TGTCGGTTGC   
  
  
- TCGAGTCTCG AAGAAAGGAT GTACGTGTAG GACATAGTTT CAACGGGTAT AAAGTTTAAG CGTATATACA   
  
  
- GGAGTTTACA ACGATAAGTT CTTCGAAACC CCTTACTCGG CCAATAGGTG TAGTAGCTAA AGGTCTAACG   
  
  
- GTACCCCTGT GTTAACCACG AGAACTAGGT CAGAGAGCGG GTAGCCGGAC CGCCCGGGGG GGGAGCAAGC   
  
  
- CTAGTGACCC CAGCTACTAG GTGTGAGCCG GATACGAGCA CCGCCCCCCG AGCTCCAACA CCCCGTCTCC   
  
  
- GATTGTTTCC ACCGACTTAG TATACCTCAG GGTAAGCTCA AAGTACGACG TCGGTACAGT CCCACGTTCC   
  
  
- ACTTAGCGCT ACAGGAGTTC CAAGCGGGAC CCCTTCTTCG GAACCGGCAT TTGAAGGGGA TATACAACGT   
  
  
- AGTGTACGGG CTGCTCTCGC ACTCGTGGTG TTTAGTAGCC CTGGCCGAGA ACTCCGACCA CTTCTCCAAC   
  
  
- AGTGGTTCCT AGCAGTGGAA CCAACTCGTT CTCAGGTTGT GTTTGTGAGG AGGGAAAGAA GTTGCCAAGG   
  
  
- CACTTTGTAA CCTGATGATG TGACGGTACA AGCTCAGGTA ACTACACCGA GATGGATCCC TACTGTTCTT   
  
  
- CTCCTATTTA CGGCTCGTCG TAACAGAACG CGCGCTATAA CACTTGTACC AGCGAACACT CTTACGCCTT   
  
  
- TCCCAACTCT CCGTGCTCGG GAAGCCCTTT ACCTCCCGTT CCAAACTATA CCGACCCAAA GTCGTTAACG   
  
  
- GGAGCTCGGC GAGTCACTTA CTACGTTATT CCCCAAACTA CGCTCTTAAG GTATCCTTGA TAGCCCACGT   
  
  
- CCTAACAGTC CCACGGGAAA AAAACCCGAC CTTCCTCGCG TTAGACCGTT GGAAGAGATG TACCACGACA   
  
  
- TTTCTTAC

+     Myb-binding site

| Site Name | Organism | Position | Strand | Matrix score. | sequence | function |
| --- | --- | --- | --- | --- | --- | --- |
| Myb-binding site | Nicotiana tabacum | 266 | - | 6 | CAACAG |  |

>HU08G02295.1   
+ -Up\_Stream \_Len000GTTAAA AAACTCAGAA CTATTATAGA TTTTCTGTTT TCTGTTTGGA TTGAGATCCG   
  
  
+ AAGCCTTTGA GATCCCCTGT TTCTAGCTTC CAACCCCCCT CCCTGGGTTC TTTTTCTTTG GTGTGAAAGT   
  
  
+ ATGAACAAAA GCTGATGTAA GTGTGATTCA AACTCTAGCC TTGGTGAGAC TCTTGGGAAG GGCTCCTAGA   
  
  
+ GACTTTGCCA ATTGAGCTAA TTTGATGCCT ACTACTTGCT TACTTTTCTA GCTGTTGTTT AAACGATTTA   
  
  
+ TGTTCATAGT TAAGGAGCGC CATATTGCAT TACAATATCT GCTTTAGTGC TTTGTTTTTT GCATTTTCTT   
  
  
+ CTTTATTACT ATGGTTTTTG AAGAAGGCTT ACTGACTGAT ACTGGTTTCT TCGCTTAAGT TTGGTGTACA   
  
  
+ TGCTTTTCAA TCTGTGTTGC ATTTTTCTAT GATGTATAAG CCGTTTATTA CCTATTGATG TTCAACTTTT   
  
  
+ CACACTATAT ATCCTTTATG GTTTCTTCGC TTAAGTTTGG TGTACATGCT TTTCAATCTG TGTTGCATTT   
  
  
+ TTCTGTGATG TATAAGCCGT TTATTACCTA TTGATGTTCA ACTTTTCACA CTATATGTCC TTTTATTAGT   
  
  
+ TACTGATGCC TCTGTCAAAC ATTCTTAATT GCATTTAGTG TATCAACGGT CTTAAAATTT TAAGCATTGC   
  
  
+ CAACTTGAAC TTCTGAAGAT GTTTGTCAAG ATCTTCAGTC TGCAAGTTAA CTGCTGCTTG CTTGAACTTT   
  
  
+ TCAACTTTTA AGAGAGATGT CAGAGTCTAA GCATGGCTTC CTGACAAGTA CTGCATTTTA TCACTTATGA   
  
  
+ AGACTCAATT CTGGGTTTTG ACAGCTTCTA TGAACCTCTA GTGCAGGTAC AGCAGCTTGG ACATTAAGCT   
  
  
+ GCAAGTCAAT TGACTGTAGA ACGACAAGTT GTTGAAGTTT CTGCTGAAAG TTCATCACTT GTTTGATGAA   
  
  
+ TAAAGTACAG CATGGCACTT CAGATGCAAA AGTCTCGCGA TCAGTAGAGA TGATTCCATA TTTCTCATCT   
  
  
+ CATTTCCAAG TCTTTGACAA CATGTACCCA AATAATGCCA GCCATGACAC TCAGATGTCT CTCCAATCAT   
  
  
+ ACAGCGAAGG ATACTTCACT CTGGACTCAT CTCCAGCAGC AATCGGAGCG TGCAGTGTCT ATGACTACCC   
  
  
+ ATCCGTTGTC AGCACCTCTT CAAATAGAAG CCAGTTTTCT CCTCAGGGTT CCCACTCGTA CATCTCAGAC   
  
  
+ CCCCATCATT CTTCCGACAA CTATGGATCC CCAGTGAGCG GCTCTTCTGT GGTTGACGAT AATGCTGAGC   
  
  
+ TGAGGAACAG GTTCAGTGAT ATGGAGCTCC CCTTGCCACA GGACTCAGGA CACCATTATT GCTCTTTTAG   
  
  
+ CCACAGAGGA AGCCATGAAG GCTCCTATAC TTTGAGGCCA AACCAACTGA TGGATATGGC CAACATGGAG   
  
  
+ TTAAAGCAGG TGCTATACTT CTGTGCAGAA GCAATCTCAG AGAATAATCT ATCAACTGCA GAAAGACTAA   
  
  
+ TGGATGCATT GGGTAAGAGG GTGTCTGTTT TTGGTTCACC AATTGAAAGG TTGGCCGCCT ACATGTTGGA   
  
  
+ AGGGCTCAGA GCAAGGCTGG AGTTTTCTGG ATATACTATC TACAAAAAGC TCAGGTGCGA ACAGCCAACG   
  
  
+ AGCTCAGAGC TTCTTTCCTA CATGCACATC CTGTATCAAA GTTGCCCATA TTTCAAATTC GCATATATGT   
  
  
+ CCTCAAATGT TGCTATTCAA GAAGCTTTGG GGAATGAGCC GGTTATCCAC ATCATCGATT TCCAGATTGC   
  
  
+ CATGGGGACA CAATTGGTGC TCTTGATCCA GTCTCTCGCC CATCGGCCTG GCGGGCCCCC CCCTCGTTCG   
  
  
+ GATCACTGGG GTCGATGATC CACACTCGGC CTATGCTCGT GGCGGGGGGC TCGAGGTTGT GGGGCAGAGG   
  
  
+ CTAACAAAGG TGGCTGAATC ATATGGAGTC CCATTCGAGT TTCATGCTGC AGCCATGTCA GGGTGCAAGG   
  
  
+ TGAATCGCGA TGTCCTCAAG GTTCGCCCTG GGGAAGAAGC CTTGGCCGTA AACTTCCCCT ATATGTTGCA   
  
  
+ TCACATGCCC GACGAGAGCG TGAGCACCAC AAATCATCGG GACCGGCTCT TGAGGCTGGT GAAGAGGTTG   
  
  
+ TCACCAAGGA TCGTCACCTT GGTTGAGCAA GAGTCCAACA CAAACACTCC TCCCTTTCTT CAACGGTTCC   
  
  
+ GTGAAACATT GGACTACTAC ACTGCCATGT TCGAGTCCAT TGATGTGGCT CTACCTAGGG ATGACAAGAA   
  
  
+ GAGGATAAAT GCCGAGCAGC ATTGTCTTGC GCGCGATATT GTGAACATGG TCGCTTGTGA GAATGCGGAA   
  
  
+ AGGGTTGAGA GGCACGAGCC CTTCGGGAAA TGGAGGGCAA GGTTTGATAT GGCTGGGTTT CAGCAATTGC   
  
  
+ CCTCGAGCCG CTCAGTGAAT GATGCAATAA GGGGTTTGAT GCGAGAATTC CATAGGAACT ATCGGGTGCA   
  
  
+ GGATTGTCAG GGTGCCCTTT TTTTGGGCTG GAAGGAGCGC AATCTGGCAA CCTTCTCTAC ATGGTGCTGT   
  
  
+ AAAGAATG  

- -Up\_Stream \_Len000CAATTT TTTGAGTCTT GATAATATCT AAAAGACAAA AGACAAACCT AACTCTAGGC   
  
  
- TTCGGAAACT CTAGGGGACA AAGATCGAAG GTTGGGGGGA GGGACCCAAG AAAAAGAAAC CACACTTTCA   
  
  
- TACTTGTTTT CGACTACATT CACACTAAGT TTGAGATCGG AACCACTCTG AGAACCCTTC CCGAGGATCT   
  
  
- CTGAAACGGT TAACTCGATT AAACTACGGA TGATGAACGA ATGAAAAGAT CGACAACAAA TTTGCTAAAT   
  
  
- ACAAGTATCA ATTCCTCGCG GTATAACGTA ATGTTATAGA CGAAATCACG AAACAAAAAA CGTAAAAGAA   
  
  
- GAAATAATGA TACCAAAAAC TTCTTCCGAA TGACTGACTA TGACCAAAGA AGCGAATTCA AACCACATGT   
  
  
- ACGAAAAGTT AGACACAACG TAAAAAGATA CTACATATTC GGCAAATAAT GGATAACTAC AAGTTGAAAA   
  
  
- GTGTGATATA TAGGAAATAC CAAAGAAGCG AATTCAAACC ACATGTACGA AAAGTTAGAC ACAACGTAAA   
  
  
- AAGACACTAC ATATTCGGCA AATAATGGAT AACTACAAGT TGAAAAGTGT GATATACAGG AAAATAATCA   
  
  
- ATGACTACGG AGACAGTTTG TAAGAATTAA CGTAAATCAC ATAGTTGCCA GAATTTTAAA ATTCGTAACG   
  
  
- GTTGAACTTG AAGACTTCTA CAAACAGTTC TAGAAGTCAG ACGTTCAATT GACGACGAAC GAACTTGAAA   
  
  
- AGTTGAAAAT TCTCTCTACA GTCTCAGATT CGTACCGAAG GACTGTTCAT GACGTAAAAT AGTGAATACT   
  
  
- TCTGAGTTAA GACCCAAAAC TGTCGAAGAT ACTTGGAGAT CACGTCCATG TCGTCGAACC TGTAATTCGA   
  
  
- CGTTCAGTTA ACTGACATCT TGCTGTTCAA CAACTTCAAA GACGACTTTC AAGTAGTGAA CAAACTACTT   
  
  
- ATTTCATGTC GTACCGTGAA GTCTACGTTT TCAGAGCGCT AGTCATCTCT ACTAAGGTAT AAAGAGTAGA   
  
  
- GTAAAGGTTC AGAAACTGTT GTACATGGGT TTATTACGGT CGGTACTGTG AGTCTACAGA GAGGTTAGTA   
  
  
- TGTCGCTTCC TATGAAGTGA GACCTGAGTA GAGGTCGTCG TTAGCCTCGC ACGTCACAGA TACTGATGGG   
  
  
- TAGGCAACAG TCGTGGAGAA GTTTATCTTC GGTCAAAAGA GGAGTCCCAA GGGTGAGCAT GTAGAGTCTG   
  
  
- GGGGTAGTAA GAAGGCTGTT GATACCTAGG GGTCACTCGC CGAGAAGACA CCAACTGCTA TTACGACTCG   
  
  
- ACTCCTTGTC CAAGTCACTA TACCTCGAGG GGAACGGTGT CCTGAGTCCT GTGGTAATAA CGAGAAAATC   
  
  
- GGTGTCTCCT TCGGTACTTC CGAGGATATG AAACTCCGGT TTGGTTGACT ACCTATACCG GTTGTACCTC   
  
  
- AATTTCGTCC ACGATATGAA GACACGTCTT CGTTAGAGTC TCTTATTAGA TAGTTGACGT CTTTCTGATT   
  
  
- ACCTACGTAA CCCATTCTCC CACAGACAAA AACCAAGTGG TTAACTTTCC AACCGGCGGA TGTACAACCT   
  
  
- TCCCGAGTCT CGTTCCGACC TCAAAAGACC TATATGATAG ATGTTTTTCG AGTCCACGCT TGTCGGTTGC   
  
  
- TCGAGTCTCG AAGAAAGGAT GTACGTGTAG GACATAGTTT CAACGGGTAT AAAGTTTAAG CGTATATACA   
  
  
- GGAGTTTACA ACGATAAGTT CTTCGAAACC CCTTACTCGG CCAATAGGTG TAGTAGCTAA AGGTCTAACG   
  
  
- GTACCCCTGT GTTAACCACG AGAACTAGGT CAGAGAGCGG GTAGCCGGAC CGCCCGGGGG GGGAGCAAGC   
  
  
- CTAGTGACCC CAGCTACTAG GTGTGAGCCG GATACGAGCA CCGCCCCCCG AGCTCCAACA CCCCGTCTCC   
  
  
- GATTGTTTCC ACCGACTTAG TATACCTCAG GGTAAGCTCA AAGTACGACG TCGGTACAGT CCCACGTTCC   
  
  
- ACTTAGCGCT ACAGGAGTTC CAAGCGGGAC CCCTTCTTCG GAACCGGCAT TTGAAGGGGA TATACAACGT   
  
  
- AGTGTACGGG CTGCTCTCGC ACTCGTGGTG TTTAGTAGCC CTGGCCGAGA ACTCCGACCA CTTCTCCAAC   
  
  
- AGTGGTTCCT AGCAGTGGAA CCAACTCGTT CTCAGGTTGT GTTTGTGAGG AGGGAAAGAA GTTGCCAAGG   
  
  
- CACTTTGTAA CCTGATGATG TGACGGTACA AGCTCAGGTA ACTACACCGA GATGGATCCC TACTGTTCTT   
  
  
- CTCCTATTTA CGGCTCGTCG TAACAGAACG CGCGCTATAA CACTTGTACC AGCGAACACT CTTACGCCTT   
  
  
- TCCCAACTCT CCGTGCTCGG GAAGCCCTTT ACCTCCCGTT CCAAACTATA CCGACCCAAA GTCGTTAACG   
  
  
- GGAGCTCGGC GAGTCACTTA CTACGTTATT CCCCAAACTA CGCTCTTAAG GTATCCTTGA TAGCCCACGT   
  
  
- CCTAACAGTC CCACGGGAAA AAAACCCGAC CTTCCTCGCG TTAGACCGTT GGAAGAGATG TACCACGACA   
  
  
- TTTCTTAC

+     Myc

| Site Name | Organism | Position | Strand | Matrix score. | sequence | function |
| --- | --- | --- | --- | --- | --- | --- |
| Myc | Arabidopsis thaliana | 783 | - | 7 | TCTCTTA |  |

>HU08G02295.1   
+ -Up\_Stream \_Len000GTTAAA AAACTCAGAA CTATTATAGA TTTTCTGTTT TCTGTTTGGA TTGAGATCCG   
  
  
+ AAGCCTTTGA GATCCCCTGT TTCTAGCTTC CAACCCCCCT CCCTGGGTTC TTTTTCTTTG GTGTGAAAGT   
  
  
+ ATGAACAAAA GCTGATGTAA GTGTGATTCA AACTCTAGCC TTGGTGAGAC TCTTGGGAAG GGCTCCTAGA   
  
  
+ GACTTTGCCA ATTGAGCTAA TTTGATGCCT ACTACTTGCT TACTTTTCTA GCTGTTGTTT AAACGATTTA   
  
  
+ TGTTCATAGT TAAGGAGCGC CATATTGCAT TACAATATCT GCTTTAGTGC TTTGTTTTTT GCATTTTCTT   
  
  
+ CTTTATTACT ATGGTTTTTG AAGAAGGCTT ACTGACTGAT ACTGGTTTCT TCGCTTAAGT TTGGTGTACA   
  
  
+ TGCTTTTCAA TCTGTGTTGC ATTTTTCTAT GATGTATAAG CCGTTTATTA CCTATTGATG TTCAACTTTT   
  
  
+ CACACTATAT ATCCTTTATG GTTTCTTCGC TTAAGTTTGG TGTACATGCT TTTCAATCTG TGTTGCATTT   
  
  
+ TTCTGTGATG TATAAGCCGT TTATTACCTA TTGATGTTCA ACTTTTCACA CTATATGTCC TTTTATTAGT   
  
  
+ TACTGATGCC TCTGTCAAAC ATTCTTAATT GCATTTAGTG TATCAACGGT CTTAAAATTT TAAGCATTGC   
  
  
+ CAACTTGAAC TTCTGAAGAT GTTTGTCAAG ATCTTCAGTC TGCAAGTTAA CTGCTGCTTG CTTGAACTTT   
  
  
+ TCAACTTTTA AGAGAGATGT CAGAGTCTAA GCATGGCTTC CTGACAAGTA CTGCATTTTA TCACTTATGA   
  
  
+ AGACTCAATT CTGGGTTTTG ACAGCTTCTA TGAACCTCTA GTGCAGGTAC AGCAGCTTGG ACATTAAGCT   
  
  
+ GCAAGTCAAT TGACTGTAGA ACGACAAGTT GTTGAAGTTT CTGCTGAAAG TTCATCACTT GTTTGATGAA   
  
  
+ TAAAGTACAG CATGGCACTT CAGATGCAAA AGTCTCGCGA TCAGTAGAGA TGATTCCATA TTTCTCATCT   
  
  
+ CATTTCCAAG TCTTTGACAA CATGTACCCA AATAATGCCA GCCATGACAC TCAGATGTCT CTCCAATCAT   
  
  
+ ACAGCGAAGG ATACTTCACT CTGGACTCAT CTCCAGCAGC AATCGGAGCG TGCAGTGTCT ATGACTACCC   
  
  
+ ATCCGTTGTC AGCACCTCTT CAAATAGAAG CCAGTTTTCT CCTCAGGGTT CCCACTCGTA CATCTCAGAC   
  
  
+ CCCCATCATT CTTCCGACAA CTATGGATCC CCAGTGAGCG GCTCTTCTGT GGTTGACGAT AATGCTGAGC   
  
  
+ TGAGGAACAG GTTCAGTGAT ATGGAGCTCC CCTTGCCACA GGACTCAGGA CACCATTATT GCTCTTTTAG   
  
  
+ CCACAGAGGA AGCCATGAAG GCTCCTATAC TTTGAGGCCA AACCAACTGA TGGATATGGC CAACATGGAG   
  
  
+ TTAAAGCAGG TGCTATACTT CTGTGCAGAA GCAATCTCAG AGAATAATCT ATCAACTGCA GAAAGACTAA   
  
  
+ TGGATGCATT GGGTAAGAGG GTGTCTGTTT TTGGTTCACC AATTGAAAGG TTGGCCGCCT ACATGTTGGA   
  
  
+ AGGGCTCAGA GCAAGGCTGG AGTTTTCTGG ATATACTATC TACAAAAAGC TCAGGTGCGA ACAGCCAACG   
  
  
+ AGCTCAGAGC TTCTTTCCTA CATGCACATC CTGTATCAAA GTTGCCCATA TTTCAAATTC GCATATATGT   
  
  
+ CCTCAAATGT TGCTATTCAA GAAGCTTTGG GGAATGAGCC GGTTATCCAC ATCATCGATT TCCAGATTGC   
  
  
+ CATGGGGACA CAATTGGTGC TCTTGATCCA GTCTCTCGCC CATCGGCCTG GCGGGCCCCC CCCTCGTTCG   
  
  
+ GATCACTGGG GTCGATGATC CACACTCGGC CTATGCTCGT GGCGGGGGGC TCGAGGTTGT GGGGCAGAGG   
  
  
+ CTAACAAAGG TGGCTGAATC ATATGGAGTC CCATTCGAGT TTCATGCTGC AGCCATGTCA GGGTGCAAGG   
  
  
+ TGAATCGCGA TGTCCTCAAG GTTCGCCCTG GGGAAGAAGC CTTGGCCGTA AACTTCCCCT ATATGTTGCA   
  
  
+ TCACATGCCC GACGAGAGCG TGAGCACCAC AAATCATCGG GACCGGCTCT TGAGGCTGGT GAAGAGGTTG   
  
  
+ TCACCAAGGA TCGTCACCTT GGTTGAGCAA GAGTCCAACA CAAACACTCC TCCCTTTCTT CAACGGTTCC   
  
  
+ GTGAAACATT GGACTACTAC ACTGCCATGT TCGAGTCCAT TGATGTGGCT CTACCTAGGG ATGACAAGAA   
  
  
+ GAGGATAAAT GCCGAGCAGC ATTGTCTTGC GCGCGATATT GTGAACATGG TCGCTTGTGA GAATGCGGAA   
  
  
+ AGGGTTGAGA GGCACGAGCC CTTCGGGAAA TGGAGGGCAA GGTTTGATAT GGCTGGGTTT CAGCAATTGC   
  
  
+ CCTCGAGCCG CTCAGTGAAT GATGCAATAA GGGGTTTGAT GCGAGAATTC CATAGGAACT ATCGGGTGCA   
  
  
+ GGATTGTCAG GGTGCCCTTT TTTTGGGCTG GAAGGAGCGC AATCTGGCAA CCTTCTCTAC ATGGTGCTGT   
  
  
+ AAAGAATG  

- -Up\_Stream \_Len000CAATTT TTTGAGTCTT GATAATATCT AAAAGACAAA AGACAAACCT AACTCTAGGC   
  
  
- TTCGGAAACT CTAGGGGACA AAGATCGAAG GTTGGGGGGA GGGACCCAAG AAAAAGAAAC CACACTTTCA   
  
  
- TACTTGTTTT CGACTACATT CACACTAAGT TTGAGATCGG AACCACTCTG AGAACCCTTC CCGAGGATCT   
  
  
- CTGAAACGGT TAACTCGATT AAACTACGGA TGATGAACGA ATGAAAAGAT CGACAACAAA TTTGCTAAAT   
  
  
- ACAAGTATCA ATTCCTCGCG GTATAACGTA ATGTTATAGA CGAAATCACG AAACAAAAAA CGTAAAAGAA   
  
  
- GAAATAATGA TACCAAAAAC TTCTTCCGAA TGACTGACTA TGACCAAAGA AGCGAATTCA AACCACATGT   
  
  
- ACGAAAAGTT AGACACAACG TAAAAAGATA CTACATATTC GGCAAATAAT GGATAACTAC AAGTTGAAAA   
  
  
- GTGTGATATA TAGGAAATAC CAAAGAAGCG AATTCAAACC ACATGTACGA AAAGTTAGAC ACAACGTAAA   
  
  
- AAGACACTAC ATATTCGGCA AATAATGGAT AACTACAAGT TGAAAAGTGT GATATACAGG AAAATAATCA   
  
  
- ATGACTACGG AGACAGTTTG TAAGAATTAA CGTAAATCAC ATAGTTGCCA GAATTTTAAA ATTCGTAACG   
  
  
- GTTGAACTTG AAGACTTCTA CAAACAGTTC TAGAAGTCAG ACGTTCAATT GACGACGAAC GAACTTGAAA   
  
  
- AGTTGAAAAT TCTCTCTACA GTCTCAGATT CGTACCGAAG GACTGTTCAT GACGTAAAAT AGTGAATACT   
  
  
- TCTGAGTTAA GACCCAAAAC TGTCGAAGAT ACTTGGAGAT CACGTCCATG TCGTCGAACC TGTAATTCGA   
  
  
- CGTTCAGTTA ACTGACATCT TGCTGTTCAA CAACTTCAAA GACGACTTTC AAGTAGTGAA CAAACTACTT   
  
  
- ATTTCATGTC GTACCGTGAA GTCTACGTTT TCAGAGCGCT AGTCATCTCT ACTAAGGTAT AAAGAGTAGA   
  
  
- GTAAAGGTTC AGAAACTGTT GTACATGGGT TTATTACGGT CGGTACTGTG AGTCTACAGA GAGGTTAGTA   
  
  
- TGTCGCTTCC TATGAAGTGA GACCTGAGTA GAGGTCGTCG TTAGCCTCGC ACGTCACAGA TACTGATGGG   
  
  
- TAGGCAACAG TCGTGGAGAA GTTTATCTTC GGTCAAAAGA GGAGTCCCAA GGGTGAGCAT GTAGAGTCTG   
  
  
- GGGGTAGTAA GAAGGCTGTT GATACCTAGG GGTCACTCGC CGAGAAGACA CCAACTGCTA TTACGACTCG   
  
  
- ACTCCTTGTC CAAGTCACTA TACCTCGAGG GGAACGGTGT CCTGAGTCCT GTGGTAATAA CGAGAAAATC   
  
  
- GGTGTCTCCT TCGGTACTTC CGAGGATATG AAACTCCGGT TTGGTTGACT ACCTATACCG GTTGTACCTC   
  
  
- AATTTCGTCC ACGATATGAA GACACGTCTT CGTTAGAGTC TCTTATTAGA TAGTTGACGT CTTTCTGATT   
  
  
- ACCTACGTAA CCCATTCTCC CACAGACAAA AACCAAGTGG TTAACTTTCC AACCGGCGGA TGTACAACCT   
  
  
- TCCCGAGTCT CGTTCCGACC TCAAAAGACC TATATGATAG ATGTTTTTCG AGTCCACGCT TGTCGGTTGC   
  
  
- TCGAGTCTCG AAGAAAGGAT GTACGTGTAG GACATAGTTT CAACGGGTAT AAAGTTTAAG CGTATATACA   
  
  
- GGAGTTTACA ACGATAAGTT CTTCGAAACC CCTTACTCGG CCAATAGGTG TAGTAGCTAA AGGTCTAACG   
  
  
- GTACCCCTGT GTTAACCACG AGAACTAGGT CAGAGAGCGG GTAGCCGGAC CGCCCGGGGG GGGAGCAAGC   
  
  
- CTAGTGACCC CAGCTACTAG GTGTGAGCCG GATACGAGCA CCGCCCCCCG AGCTCCAACA CCCCGTCTCC   
  
  
- GATTGTTTCC ACCGACTTAG TATACCTCAG GGTAAGCTCA AAGTACGACG TCGGTACAGT CCCACGTTCC   
  
  
- ACTTAGCGCT ACAGGAGTTC CAAGCGGGAC CCCTTCTTCG GAACCGGCAT TTGAAGGGGA TATACAACGT   
  
  
- AGTGTACGGG CTGCTCTCGC ACTCGTGGTG TTTAGTAGCC CTGGCCGAGA ACTCCGACCA CTTCTCCAAC   
  
  
- AGTGGTTCCT AGCAGTGGAA CCAACTCGTT CTCAGGTTGT GTTTGTGAGG AGGGAAAGAA GTTGCCAAGG   
  
  
- CACTTTGTAA CCTGATGATG TGACGGTACA AGCTCAGGTA ACTACACCGA GATGGATCCC TACTGTTCTT   
  
  
- CTCCTATTTA CGGCTCGTCG TAACAGAACG CGCGCTATAA CACTTGTACC AGCGAACACT CTTACGCCTT   
  
  
- TCCCAACTCT CCGTGCTCGG GAAGCCCTTT ACCTCCCGTT CCAAACTATA CCGACCCAAA GTCGTTAACG   
  
  
- GGAGCTCGGC GAGTCACTTA CTACGTTATT CCCCAAACTA CGCTCTTAAG GTATCCTTGA TAGCCCACGT   
  
  
- CCTAACAGTC CCACGGGAAA AAAACCCGAC CTTCCTCGCG TTAGACCGTT GGAAGAGATG TACCACGACA   
  
  
- TTTCTTAC

+     O2-site

| Site Name | Organism | Position | Strand | Matrix score. | sequence | function |
| --- | --- | --- | --- | --- | --- | --- |
| O2-site | Zea mays | 1801 | - | 10 | GATGATGTGG | cis-acting regulatory element involved in zein metabolism regulation |

>HU08G02295.1   
+ -Up\_Stream \_Len000GTTAAA AAACTCAGAA CTATTATAGA TTTTCTGTTT TCTGTTTGGA TTGAGATCCG   
  
  
+ AAGCCTTTGA GATCCCCTGT TTCTAGCTTC CAACCCCCCT CCCTGGGTTC TTTTTCTTTG GTGTGAAAGT   
  
  
+ ATGAACAAAA GCTGATGTAA GTGTGATTCA AACTCTAGCC TTGGTGAGAC TCTTGGGAAG GGCTCCTAGA   
  
  
+ GACTTTGCCA ATTGAGCTAA TTTGATGCCT ACTACTTGCT TACTTTTCTA GCTGTTGTTT AAACGATTTA   
  
  
+ TGTTCATAGT TAAGGAGCGC CATATTGCAT TACAATATCT GCTTTAGTGC TTTGTTTTTT GCATTTTCTT   
  
  
+ CTTTATTACT ATGGTTTTTG AAGAAGGCTT ACTGACTGAT ACTGGTTTCT TCGCTTAAGT TTGGTGTACA   
  
  
+ TGCTTTTCAA TCTGTGTTGC ATTTTTCTAT GATGTATAAG CCGTTTATTA CCTATTGATG TTCAACTTTT   
  
  
+ CACACTATAT ATCCTTTATG GTTTCTTCGC TTAAGTTTGG TGTACATGCT TTTCAATCTG TGTTGCATTT   
  
  
+ TTCTGTGATG TATAAGCCGT TTATTACCTA TTGATGTTCA ACTTTTCACA CTATATGTCC TTTTATTAGT   
  
  
+ TACTGATGCC TCTGTCAAAC ATTCTTAATT GCATTTAGTG TATCAACGGT CTTAAAATTT TAAGCATTGC   
  
  
+ CAACTTGAAC TTCTGAAGAT GTTTGTCAAG ATCTTCAGTC TGCAAGTTAA CTGCTGCTTG CTTGAACTTT   
  
  
+ TCAACTTTTA AGAGAGATGT CAGAGTCTAA GCATGGCTTC CTGACAAGTA CTGCATTTTA TCACTTATGA   
  
  
+ AGACTCAATT CTGGGTTTTG ACAGCTTCTA TGAACCTCTA GTGCAGGTAC AGCAGCTTGG ACATTAAGCT   
  
  
+ GCAAGTCAAT TGACTGTAGA ACGACAAGTT GTTGAAGTTT CTGCTGAAAG TTCATCACTT GTTTGATGAA   
  
  
+ TAAAGTACAG CATGGCACTT CAGATGCAAA AGTCTCGCGA TCAGTAGAGA TGATTCCATA TTTCTCATCT   
  
  
+ CATTTCCAAG TCTTTGACAA CATGTACCCA AATAATGCCA GCCATGACAC TCAGATGTCT CTCCAATCAT   
  
  
+ ACAGCGAAGG ATACTTCACT CTGGACTCAT CTCCAGCAGC AATCGGAGCG TGCAGTGTCT ATGACTACCC   
  
  
+ ATCCGTTGTC AGCACCTCTT CAAATAGAAG CCAGTTTTCT CCTCAGGGTT CCCACTCGTA CATCTCAGAC   
  
  
+ CCCCATCATT CTTCCGACAA CTATGGATCC CCAGTGAGCG GCTCTTCTGT GGTTGACGAT AATGCTGAGC   
  
  
+ TGAGGAACAG GTTCAGTGAT ATGGAGCTCC CCTTGCCACA GGACTCAGGA CACCATTATT GCTCTTTTAG   
  
  
+ CCACAGAGGA AGCCATGAAG GCTCCTATAC TTTGAGGCCA AACCAACTGA TGGATATGGC CAACATGGAG   
  
  
+ TTAAAGCAGG TGCTATACTT CTGTGCAGAA GCAATCTCAG AGAATAATCT ATCAACTGCA GAAAGACTAA   
  
  
+ TGGATGCATT GGGTAAGAGG GTGTCTGTTT TTGGTTCACC AATTGAAAGG TTGGCCGCCT ACATGTTGGA   
  
  
+ AGGGCTCAGA GCAAGGCTGG AGTTTTCTGG ATATACTATC TACAAAAAGC TCAGGTGCGA ACAGCCAACG   
  
  
+ AGCTCAGAGC TTCTTTCCTA CATGCACATC CTGTATCAAA GTTGCCCATA TTTCAAATTC GCATATATGT   
  
  
+ CCTCAAATGT TGCTATTCAA GAAGCTTTGG GGAATGAGCC GGTTATCCAC ATCATCGATT TCCAGATTGC   
  
  
+ CATGGGGACA CAATTGGTGC TCTTGATCCA GTCTCTCGCC CATCGGCCTG GCGGGCCCCC CCCTCGTTCG   
  
  
+ GATCACTGGG GTCGATGATC CACACTCGGC CTATGCTCGT GGCGGGGGGC TCGAGGTTGT GGGGCAGAGG   
  
  
+ CTAACAAAGG TGGCTGAATC ATATGGAGTC CCATTCGAGT TTCATGCTGC AGCCATGTCA GGGTGCAAGG   
  
  
+ TGAATCGCGA TGTCCTCAAG GTTCGCCCTG GGGAAGAAGC CTTGGCCGTA AACTTCCCCT ATATGTTGCA   
  
  
+ TCACATGCCC GACGAGAGCG TGAGCACCAC AAATCATCGG GACCGGCTCT TGAGGCTGGT GAAGAGGTTG   
  
  
+ TCACCAAGGA TCGTCACCTT GGTTGAGCAA GAGTCCAACA CAAACACTCC TCCCTTTCTT CAACGGTTCC   
  
  
+ GTGAAACATT GGACTACTAC ACTGCCATGT TCGAGTCCAT TGATGTGGCT CTACCTAGGG ATGACAAGAA   
  
  
+ GAGGATAAAT GCCGAGCAGC ATTGTCTTGC GCGCGATATT GTGAACATGG TCGCTTGTGA GAATGCGGAA   
  
  
+ AGGGTTGAGA GGCACGAGCC CTTCGGGAAA TGGAGGGCAA GGTTTGATAT GGCTGGGTTT CAGCAATTGC   
  
  
+ CCTCGAGCCG CTCAGTGAAT GATGCAATAA GGGGTTTGAT GCGAGAATTC CATAGGAACT ATCGGGTGCA   
  
  
+ GGATTGTCAG GGTGCCCTTT TTTTGGGCTG GAAGGAGCGC AATCTGGCAA CCTTCTCTAC ATGGTGCTGT   
  
  
+ AAAGAATG  

- -Up\_Stream \_Len000CAATTT TTTGAGTCTT GATAATATCT AAAAGACAAA AGACAAACCT AACTCTAGGC   
  
  
- TTCGGAAACT CTAGGGGACA AAGATCGAAG GTTGGGGGGA GGGACCCAAG AAAAAGAAAC CACACTTTCA   
  
  
- TACTTGTTTT CGACTACATT CACACTAAGT TTGAGATCGG AACCACTCTG AGAACCCTTC CCGAGGATCT   
  
  
- CTGAAACGGT TAACTCGATT AAACTACGGA TGATGAACGA ATGAAAAGAT CGACAACAAA TTTGCTAAAT   
  
  
- ACAAGTATCA ATTCCTCGCG GTATAACGTA ATGTTATAGA CGAAATCACG AAACAAAAAA CGTAAAAGAA   
  
  
- GAAATAATGA TACCAAAAAC TTCTTCCGAA TGACTGACTA TGACCAAAGA AGCGAATTCA AACCACATGT   
  
  
- ACGAAAAGTT AGACACAACG TAAAAAGATA CTACATATTC GGCAAATAAT GGATAACTAC AAGTTGAAAA   
  
  
- GTGTGATATA TAGGAAATAC CAAAGAAGCG AATTCAAACC ACATGTACGA AAAGTTAGAC ACAACGTAAA   
  
  
- AAGACACTAC ATATTCGGCA AATAATGGAT AACTACAAGT TGAAAAGTGT GATATACAGG AAAATAATCA   
  
  
- ATGACTACGG AGACAGTTTG TAAGAATTAA CGTAAATCAC ATAGTTGCCA GAATTTTAAA ATTCGTAACG   
  
  
- GTTGAACTTG AAGACTTCTA CAAACAGTTC TAGAAGTCAG ACGTTCAATT GACGACGAAC GAACTTGAAA   
  
  
- AGTTGAAAAT TCTCTCTACA GTCTCAGATT CGTACCGAAG GACTGTTCAT GACGTAAAAT AGTGAATACT   
  
  
- TCTGAGTTAA GACCCAAAAC TGTCGAAGAT ACTTGGAGAT CACGTCCATG TCGTCGAACC TGTAATTCGA   
  
  
- CGTTCAGTTA ACTGACATCT TGCTGTTCAA CAACTTCAAA GACGACTTTC AAGTAGTGAA CAAACTACTT   
  
  
- ATTTCATGTC GTACCGTGAA GTCTACGTTT TCAGAGCGCT AGTCATCTCT ACTAAGGTAT AAAGAGTAGA   
  
  
- GTAAAGGTTC AGAAACTGTT GTACATGGGT TTATTACGGT CGGTACTGTG AGTCTACAGA GAGGTTAGTA   
  
  
- TGTCGCTTCC TATGAAGTGA GACCTGAGTA GAGGTCGTCG TTAGCCTCGC ACGTCACAGA TACTGATGGG   
  
  
- TAGGCAACAG TCGTGGAGAA GTTTATCTTC GGTCAAAAGA GGAGTCCCAA GGGTGAGCAT GTAGAGTCTG   
  
  
- GGGGTAGTAA GAAGGCTGTT GATACCTAGG GGTCACTCGC CGAGAAGACA CCAACTGCTA TTACGACTCG   
  
  
- ACTCCTTGTC CAAGTCACTA TACCTCGAGG GGAACGGTGT CCTGAGTCCT GTGGTAATAA CGAGAAAATC   
  
  
- GGTGTCTCCT TCGGTACTTC CGAGGATATG AAACTCCGGT TTGGTTGACT ACCTATACCG GTTGTACCTC   
  
  
- AATTTCGTCC ACGATATGAA GACACGTCTT CGTTAGAGTC TCTTATTAGA TAGTTGACGT CTTTCTGATT   
  
  
- ACCTACGTAA CCCATTCTCC CACAGACAAA AACCAAGTGG TTAACTTTCC AACCGGCGGA TGTACAACCT   
  
  
- TCCCGAGTCT CGTTCCGACC TCAAAAGACC TATATGATAG ATGTTTTTCG AGTCCACGCT TGTCGGTTGC   
  
  
- TCGAGTCTCG AAGAAAGGAT GTACGTGTAG GACATAGTTT CAACGGGTAT AAAGTTTAAG CGTATATACA   
  
  
- GGAGTTTACA ACGATAAGTT CTTCGAAACC CCTTACTCGG CCAATAGGTG TAGTAGCTAA AGGTCTAACG   
  
  
- GTACCCCTGT GTTAACCACG AGAACTAGGT CAGAGAGCGG GTAGCCGGAC CGCCCGGGGG GGGAGCAAGC   
  
  
- CTAGTGACCC CAGCTACTAG GTGTGAGCCG GATACGAGCA CCGCCCCCCG AGCTCCAACA CCCCGTCTCC   
  
  
- GATTGTTTCC ACCGACTTAG TATACCTCAG GGTAAGCTCA AAGTACGACG TCGGTACAGT CCCACGTTCC   
  
  
- ACTTAGCGCT ACAGGAGTTC CAAGCGGGAC CCCTTCTTCG GAACCGGCAT TTGAAGGGGA TATACAACGT   
  
  
- AGTGTACGGG CTGCTCTCGC ACTCGTGGTG TTTAGTAGCC CTGGCCGAGA ACTCCGACCA CTTCTCCAAC   
  
  
- AGTGGTTCCT AGCAGTGGAA CCAACTCGTT CTCAGGTTGT GTTTGTGAGG AGGGAAAGAA GTTGCCAAGG   
  
  
- CACTTTGTAA CCTGATGATG TGACGGTACA AGCTCAGGTA ACTACACCGA GATGGATCCC TACTGTTCTT   
  
  
- CTCCTATTTA CGGCTCGTCG TAACAGAACG CGCGCTATAA CACTTGTACC AGCGAACACT CTTACGCCTT   
  
  
- TCCCAACTCT CCGTGCTCGG GAAGCCCTTT ACCTCCCGTT CCAAACTATA CCGACCCAAA GTCGTTAACG   
  
  
- GGAGCTCGGC GAGTCACTTA CTACGTTATT CCCCAAACTA CGCTCTTAAG GTATCCTTGA TAGCCCACGT   
  
  
- CCTAACAGTC CCACGGGAAA AAAACCCGAC CTTCCTCGCG TTAGACCGTT GGAAGAGATG TACCACGACA   
  
  
- TTTCTTAC

+     P-box

| Site Name | Organism | Position | Strand | Matrix score. | sequence | function |
| --- | --- | --- | --- | --- | --- | --- |
| P-box | Petroselinum crispum | 2483 | - | 12 | CAACAAACCCCTT | gibberellin-responsive element and part of a light responsive element |

>HU08G02295.1   
+ -Up\_Stream \_Len000GTTAAA AAACTCAGAA CTATTATAGA TTTTCTGTTT TCTGTTTGGA TTGAGATCCG   
  
  
+ AAGCCTTTGA GATCCCCTGT TTCTAGCTTC CAACCCCCCT CCCTGGGTTC TTTTTCTTTG GTGTGAAAGT   
  
  
+ ATGAACAAAA GCTGATGTAA GTGTGATTCA AACTCTAGCC TTGGTGAGAC TCTTGGGAAG GGCTCCTAGA   
  
  
+ GACTTTGCCA ATTGAGCTAA TTTGATGCCT ACTACTTGCT TACTTTTCTA GCTGTTGTTT AAACGATTTA   
  
  
+ TGTTCATAGT TAAGGAGCGC CATATTGCAT TACAATATCT GCTTTAGTGC TTTGTTTTTT GCATTTTCTT   
  
  
+ CTTTATTACT ATGGTTTTTG AAGAAGGCTT ACTGACTGAT ACTGGTTTCT TCGCTTAAGT TTGGTGTACA   
  
  
+ TGCTTTTCAA TCTGTGTTGC ATTTTTCTAT GATGTATAAG CCGTTTATTA CCTATTGATG TTCAACTTTT   
  
  
+ CACACTATAT ATCCTTTATG GTTTCTTCGC TTAAGTTTGG TGTACATGCT TTTCAATCTG TGTTGCATTT   
  
  
+ TTCTGTGATG TATAAGCCGT TTATTACCTA TTGATGTTCA ACTTTTCACA CTATATGTCC TTTTATTAGT   
  
  
+ TACTGATGCC TCTGTCAAAC ATTCTTAATT GCATTTAGTG TATCAACGGT CTTAAAATTT TAAGCATTGC   
  
  
+ CAACTTGAAC TTCTGAAGAT GTTTGTCAAG ATCTTCAGTC TGCAAGTTAA CTGCTGCTTG CTTGAACTTT   
  
  
+ TCAACTTTTA AGAGAGATGT CAGAGTCTAA GCATGGCTTC CTGACAAGTA CTGCATTTTA TCACTTATGA   
  
  
+ AGACTCAATT CTGGGTTTTG ACAGCTTCTA TGAACCTCTA GTGCAGGTAC AGCAGCTTGG ACATTAAGCT   
  
  
+ GCAAGTCAAT TGACTGTAGA ACGACAAGTT GTTGAAGTTT CTGCTGAAAG TTCATCACTT GTTTGATGAA   
  
  
+ TAAAGTACAG CATGGCACTT CAGATGCAAA AGTCTCGCGA TCAGTAGAGA TGATTCCATA TTTCTCATCT   
  
  
+ CATTTCCAAG TCTTTGACAA CATGTACCCA AATAATGCCA GCCATGACAC TCAGATGTCT CTCCAATCAT   
  
  
+ ACAGCGAAGG ATACTTCACT CTGGACTCAT CTCCAGCAGC AATCGGAGCG TGCAGTGTCT ATGACTACCC   
  
  
+ ATCCGTTGTC AGCACCTCTT CAAATAGAAG CCAGTTTTCT CCTCAGGGTT CCCACTCGTA CATCTCAGAC   
  
  
+ CCCCATCATT CTTCCGACAA CTATGGATCC CCAGTGAGCG GCTCTTCTGT GGTTGACGAT AATGCTGAGC   
  
  
+ TGAGGAACAG GTTCAGTGAT ATGGAGCTCC CCTTGCCACA GGACTCAGGA CACCATTATT GCTCTTTTAG   
  
  
+ CCACAGAGGA AGCCATGAAG GCTCCTATAC TTTGAGGCCA AACCAACTGA TGGATATGGC CAACATGGAG   
  
  
+ TTAAAGCAGG TGCTATACTT CTGTGCAGAA GCAATCTCAG AGAATAATCT ATCAACTGCA GAAAGACTAA   
  
  
+ TGGATGCATT GGGTAAGAGG GTGTCTGTTT TTGGTTCACC AATTGAAAGG TTGGCCGCCT ACATGTTGGA   
  
  
+ AGGGCTCAGA GCAAGGCTGG AGTTTTCTGG ATATACTATC TACAAAAAGC TCAGGTGCGA ACAGCCAACG   
  
  
+ AGCTCAGAGC TTCTTTCCTA CATGCACATC CTGTATCAAA GTTGCCCATA TTTCAAATTC GCATATATGT   
  
  
+ CCTCAAATGT TGCTATTCAA GAAGCTTTGG GGAATGAGCC GGTTATCCAC ATCATCGATT TCCAGATTGC   
  
  
+ CATGGGGACA CAATTGGTGC TCTTGATCCA GTCTCTCGCC CATCGGCCTG GCGGGCCCCC CCCTCGTTCG   
  
  
+ GATCACTGGG GTCGATGATC CACACTCGGC CTATGCTCGT GGCGGGGGGC TCGAGGTTGT GGGGCAGAGG   
  
  
+ CTAACAAAGG TGGCTGAATC ATATGGAGTC CCATTCGAGT TTCATGCTGC AGCCATGTCA GGGTGCAAGG   
  
  
+ TGAATCGCGA TGTCCTCAAG GTTCGCCCTG GGGAAGAAGC CTTGGCCGTA AACTTCCCCT ATATGTTGCA   
  
  
+ TCACATGCCC GACGAGAGCG TGAGCACCAC AAATCATCGG GACCGGCTCT TGAGGCTGGT GAAGAGGTTG   
  
  
+ TCACCAAGGA TCGTCACCTT GGTTGAGCAA GAGTCCAACA CAAACACTCC TCCCTTTCTT CAACGGTTCC   
  
  
+ GTGAAACATT GGACTACTAC ACTGCCATGT TCGAGTCCAT TGATGTGGCT CTACCTAGGG ATGACAAGAA   
  
  
+ GAGGATAAAT GCCGAGCAGC ATTGTCTTGC GCGCGATATT GTGAACATGG TCGCTTGTGA GAATGCGGAA   
  
  
+ AGGGTTGAGA GGCACGAGCC CTTCGGGAAA TGGAGGGCAA GGTTTGATAT GGCTGGGTTT CAGCAATTGC   
  
  
+ CCTCGAGCCG CTCAGTGAAT GATGCAATAA GGGGTTTGAT GCGAGAATTC CATAGGAACT ATCGGGTGCA   
  
  
+ GGATTGTCAG GGTGCCCTTT TTTTGGGCTG GAAGGAGCGC AATCTGGCAA CCTTCTCTAC ATGGTGCTGT   
  
  
+ AAAGAATG  

- -Up\_Stream \_Len000CAATTT TTTGAGTCTT GATAATATCT AAAAGACAAA AGACAAACCT AACTCTAGGC   
  
  
- TTCGGAAACT CTAGGGGACA AAGATCGAAG GTTGGGGGGA GGGACCCAAG AAAAAGAAAC CACACTTTCA   
  
  
- TACTTGTTTT CGACTACATT CACACTAAGT TTGAGATCGG AACCACTCTG AGAACCCTTC CCGAGGATCT   
  
  
- CTGAAACGGT TAACTCGATT AAACTACGGA TGATGAACGA ATGAAAAGAT CGACAACAAA TTTGCTAAAT   
  
  
- ACAAGTATCA ATTCCTCGCG GTATAACGTA ATGTTATAGA CGAAATCACG AAACAAAAAA CGTAAAAGAA   
  
  
- GAAATAATGA TACCAAAAAC TTCTTCCGAA TGACTGACTA TGACCAAAGA AGCGAATTCA AACCACATGT   
  
  
- ACGAAAAGTT AGACACAACG TAAAAAGATA CTACATATTC GGCAAATAAT GGATAACTAC AAGTTGAAAA   
  
  
- GTGTGATATA TAGGAAATAC CAAAGAAGCG AATTCAAACC ACATGTACGA AAAGTTAGAC ACAACGTAAA   
  
  
- AAGACACTAC ATATTCGGCA AATAATGGAT AACTACAAGT TGAAAAGTGT GATATACAGG AAAATAATCA   
  
  
- ATGACTACGG AGACAGTTTG TAAGAATTAA CGTAAATCAC ATAGTTGCCA GAATTTTAAA ATTCGTAACG   
  
  
- GTTGAACTTG AAGACTTCTA CAAACAGTTC TAGAAGTCAG ACGTTCAATT GACGACGAAC GAACTTGAAA   
  
  
- AGTTGAAAAT TCTCTCTACA GTCTCAGATT CGTACCGAAG GACTGTTCAT GACGTAAAAT AGTGAATACT   
  
  
- TCTGAGTTAA GACCCAAAAC TGTCGAAGAT ACTTGGAGAT CACGTCCATG TCGTCGAACC TGTAATTCGA   
  
  
- CGTTCAGTTA ACTGACATCT TGCTGTTCAA CAACTTCAAA GACGACTTTC AAGTAGTGAA CAAACTACTT   
  
  
- ATTTCATGTC GTACCGTGAA GTCTACGTTT TCAGAGCGCT AGTCATCTCT ACTAAGGTAT AAAGAGTAGA   
  
  
- GTAAAGGTTC AGAAACTGTT GTACATGGGT TTATTACGGT CGGTACTGTG AGTCTACAGA GAGGTTAGTA   
  
  
- TGTCGCTTCC TATGAAGTGA GACCTGAGTA GAGGTCGTCG TTAGCCTCGC ACGTCACAGA TACTGATGGG   
  
  
- TAGGCAACAG TCGTGGAGAA GTTTATCTTC GGTCAAAAGA GGAGTCCCAA GGGTGAGCAT GTAGAGTCTG   
  
  
- GGGGTAGTAA GAAGGCTGTT GATACCTAGG GGTCACTCGC CGAGAAGACA CCAACTGCTA TTACGACTCG   
  
  
- ACTCCTTGTC CAAGTCACTA TACCTCGAGG GGAACGGTGT CCTGAGTCCT GTGGTAATAA CGAGAAAATC   
  
  
- GGTGTCTCCT TCGGTACTTC CGAGGATATG AAACTCCGGT TTGGTTGACT ACCTATACCG GTTGTACCTC   
  
  
- AATTTCGTCC ACGATATGAA GACACGTCTT CGTTAGAGTC TCTTATTAGA TAGTTGACGT CTTTCTGATT   
  
  
- ACCTACGTAA CCCATTCTCC CACAGACAAA AACCAAGTGG TTAACTTTCC AACCGGCGGA TGTACAACCT   
  
  
- TCCCGAGTCT CGTTCCGACC TCAAAAGACC TATATGATAG ATGTTTTTCG AGTCCACGCT TGTCGGTTGC   
  
  
- TCGAGTCTCG AAGAAAGGAT GTACGTGTAG GACATAGTTT CAACGGGTAT AAAGTTTAAG CGTATATACA   
  
  
- GGAGTTTACA ACGATAAGTT CTTCGAAACC CCTTACTCGG CCAATAGGTG TAGTAGCTAA AGGTCTAACG   
  
  
- GTACCCCTGT GTTAACCACG AGAACTAGGT CAGAGAGCGG GTAGCCGGAC CGCCCGGGGG GGGAGCAAGC   
  
  
- CTAGTGACCC CAGCTACTAG GTGTGAGCCG GATACGAGCA CCGCCCCCCG AGCTCCAACA CCCCGTCTCC   
  
  
- GATTGTTTCC ACCGACTTAG TATACCTCAG GGTAAGCTCA AAGTACGACG TCGGTACAGT CCCACGTTCC   
  
  
- ACTTAGCGCT ACAGGAGTTC CAAGCGGGAC CCCTTCTTCG GAACCGGCAT TTGAAGGGGA TATACAACGT   
  
  
- AGTGTACGGG CTGCTCTCGC ACTCGTGGTG TTTAGTAGCC CTGGCCGAGA ACTCCGACCA CTTCTCCAAC   
  
  
- AGTGGTTCCT AGCAGTGGAA CCAACTCGTT CTCAGGTTGT GTTTGTGAGG AGGGAAAGAA GTTGCCAAGG   
  
  
- CACTTTGTAA CCTGATGATG TGACGGTACA AGCTCAGGTA ACTACACCGA GATGGATCCC TACTGTTCTT   
  
  
- CTCCTATTTA CGGCTCGTCG TAACAGAACG CGCGCTATAA CACTTGTACC AGCGAACACT CTTACGCCTT   
  
  
- TCCCAACTCT CCGTGCTCGG GAAGCCCTTT ACCTCCCGTT CCAAACTATA CCGACCCAAA GTCGTTAACG   
  
  
- GGAGCTCGGC GAGTCACTTA CTACGTTATT CCCCAAACTA CGCTCTTAAG GTATCCTTGA TAGCCCACGT   
  
  
- CCTAACAGTC CCACGGGAAA AAAACCCGAC CTTCCTCGCG TTAGACCGTT GGAAGAGATG TACCACGACA   
  
  
- TTTCTTAC

+     STRE

| Site Name | Organism | Position | Strand | Matrix score. | sequence | function |
| --- | --- | --- | --- | --- | --- | --- |
| STRE | Arabidopsis thaliana | 1884 | - | 5 | AGGGG |  |
| STRE | Arabidopsis thaliana | 1363 | - | 5 | AGGGG |  |
| STRE | Arabidopsis thaliana | 2484 | + | 5 | AGGGG |  |
| STRE | Arabidopsis thaliana | 88 | - | 5 | AGGGG |  |
| STRE | Arabidopsis thaliana | 110 | - | 5 | AGGGG |  |
| STRE | Arabidopsis thaliana | 2090 | - | 5 | AGGGG |  |

>HU08G02295.1   
+ -Up\_Stream \_Len000GTTAAA AAACTCAGAA CTATTATAGA TTTTCTGTTT TCTGTTTGGA TTGAGATCCG   
  
  
+ AAGCCTTTGA GATCCCCTGT TTCTAGCTTC CAACCCCCCT CCCTGGGTTC TTTTTCTTTG GTGTGAAAGT   
  
  
+ ATGAACAAAA GCTGATGTAA GTGTGATTCA AACTCTAGCC TTGGTGAGAC TCTTGGGAAG GGCTCCTAGA   
  
  
+ GACTTTGCCA ATTGAGCTAA TTTGATGCCT ACTACTTGCT TACTTTTCTA GCTGTTGTTT AAACGATTTA   
  
  
+ TGTTCATAGT TAAGGAGCGC CATATTGCAT TACAATATCT GCTTTAGTGC TTTGTTTTTT GCATTTTCTT   
  
  
+ CTTTATTACT ATGGTTTTTG AAGAAGGCTT ACTGACTGAT ACTGGTTTCT TCGCTTAAGT TTGGTGTACA   
  
  
+ TGCTTTTCAA TCTGTGTTGC ATTTTTCTAT GATGTATAAG CCGTTTATTA CCTATTGATG TTCAACTTTT   
  
  
+ CACACTATAT ATCCTTTATG GTTTCTTCGC TTAAGTTTGG TGTACATGCT TTTCAATCTG TGTTGCATTT   
  
  
+ TTCTGTGATG TATAAGCCGT TTATTACCTA TTGATGTTCA ACTTTTCACA CTATATGTCC TTTTATTAGT   
  
  
+ TACTGATGCC TCTGTCAAAC ATTCTTAATT GCATTTAGTG TATCAACGGT CTTAAAATTT TAAGCATTGC   
  
  
+ CAACTTGAAC TTCTGAAGAT GTTTGTCAAG ATCTTCAGTC TGCAAGTTAA CTGCTGCTTG CTTGAACTTT   
  
  
+ TCAACTTTTA AGAGAGATGT CAGAGTCTAA GCATGGCTTC CTGACAAGTA CTGCATTTTA TCACTTATGA   
  
  
+ AGACTCAATT CTGGGTTTTG ACAGCTTCTA TGAACCTCTA GTGCAGGTAC AGCAGCTTGG ACATTAAGCT   
  
  
+ GCAAGTCAAT TGACTGTAGA ACGACAAGTT GTTGAAGTTT CTGCTGAAAG TTCATCACTT GTTTGATGAA   
  
  
+ TAAAGTACAG CATGGCACTT CAGATGCAAA AGTCTCGCGA TCAGTAGAGA TGATTCCATA TTTCTCATCT   
  
  
+ CATTTCCAAG TCTTTGACAA CATGTACCCA AATAATGCCA GCCATGACAC TCAGATGTCT CTCCAATCAT   
  
  
+ ACAGCGAAGG ATACTTCACT CTGGACTCAT CTCCAGCAGC AATCGGAGCG TGCAGTGTCT ATGACTACCC   
  
  
+ ATCCGTTGTC AGCACCTCTT CAAATAGAAG CCAGTTTTCT CCTCAGGGTT CCCACTCGTA CATCTCAGAC   
  
  
+ CCCCATCATT CTTCCGACAA CTATGGATCC CCAGTGAGCG GCTCTTCTGT GGTTGACGAT AATGCTGAGC   
  
  
+ TGAGGAACAG GTTCAGTGAT ATGGAGCTCC CCTTGCCACA GGACTCAGGA CACCATTATT GCTCTTTTAG   
  
  
+ CCACAGAGGA AGCCATGAAG GCTCCTATAC TTTGAGGCCA AACCAACTGA TGGATATGGC CAACATGGAG   
  
  
+ TTAAAGCAGG TGCTATACTT CTGTGCAGAA GCAATCTCAG AGAATAATCT ATCAACTGCA GAAAGACTAA   
  
  
+ TGGATGCATT GGGTAAGAGG GTGTCTGTTT TTGGTTCACC AATTGAAAGG TTGGCCGCCT ACATGTTGGA   
  
  
+ AGGGCTCAGA GCAAGGCTGG AGTTTTCTGG ATATACTATC TACAAAAAGC TCAGGTGCGA ACAGCCAACG   
  
  
+ AGCTCAGAGC TTCTTTCCTA CATGCACATC CTGTATCAAA GTTGCCCATA TTTCAAATTC GCATATATGT   
  
  
+ CCTCAAATGT TGCTATTCAA GAAGCTTTGG GGAATGAGCC GGTTATCCAC ATCATCGATT TCCAGATTGC   
  
  
+ CATGGGGACA CAATTGGTGC TCTTGATCCA GTCTCTCGCC CATCGGCCTG GCGGGCCCCC CCCTCGTTCG   
  
  
+ GATCACTGGG GTCGATGATC CACACTCGGC CTATGCTCGT GGCGGGGGGC TCGAGGTTGT GGGGCAGAGG   
  
  
+ CTAACAAAGG TGGCTGAATC ATATGGAGTC CCATTCGAGT TTCATGCTGC AGCCATGTCA GGGTGCAAGG   
  
  
+ TGAATCGCGA TGTCCTCAAG GTTCGCCCTG GGGAAGAAGC CTTGGCCGTA AACTTCCCCT ATATGTTGCA   
  
  
+ TCACATGCCC GACGAGAGCG TGAGCACCAC AAATCATCGG GACCGGCTCT TGAGGCTGGT GAAGAGGTTG   
  
  
+ TCACCAAGGA TCGTCACCTT GGTTGAGCAA GAGTCCAACA CAAACACTCC TCCCTTTCTT CAACGGTTCC   
  
  
+ GTGAAACATT GGACTACTAC ACTGCCATGT TCGAGTCCAT TGATGTGGCT CTACCTAGGG ATGACAAGAA   
  
  
+ GAGGATAAAT GCCGAGCAGC ATTGTCTTGC GCGCGATATT GTGAACATGG TCGCTTGTGA GAATGCGGAA   
  
  
+ AGGGTTGAGA GGCACGAGCC CTTCGGGAAA TGGAGGGCAA GGTTTGATAT GGCTGGGTTT CAGCAATTGC   
  
  
+ CCTCGAGCCG CTCAGTGAAT GATGCAATAA GGGGTTTGAT GCGAGAATTC CATAGGAACT ATCGGGTGCA   
  
  
+ GGATTGTCAG GGTGCCCTTT TTTTGGGCTG GAAGGAGCGC AATCTGGCAA CCTTCTCTAC ATGGTGCTGT   
  
  
+ AAAGAATG  

- -Up\_Stream \_Len000CAATTT TTTGAGTCTT GATAATATCT AAAAGACAAA AGACAAACCT AACTCTAGGC   
  
  
- TTCGGAAACT CTAGGGGACA AAGATCGAAG GTTGGGGGGA GGGACCCAAG AAAAAGAAAC CACACTTTCA   
  
  
- TACTTGTTTT CGACTACATT CACACTAAGT TTGAGATCGG AACCACTCTG AGAACCCTTC CCGAGGATCT   
  
  
- CTGAAACGGT TAACTCGATT AAACTACGGA TGATGAACGA ATGAAAAGAT CGACAACAAA TTTGCTAAAT   
  
  
- ACAAGTATCA ATTCCTCGCG GTATAACGTA ATGTTATAGA CGAAATCACG AAACAAAAAA CGTAAAAGAA   
  
  
- GAAATAATGA TACCAAAAAC TTCTTCCGAA TGACTGACTA TGACCAAAGA AGCGAATTCA AACCACATGT   
  
  
- ACGAAAAGTT AGACACAACG TAAAAAGATA CTACATATTC GGCAAATAAT GGATAACTAC AAGTTGAAAA   
  
  
- GTGTGATATA TAGGAAATAC CAAAGAAGCG AATTCAAACC ACATGTACGA AAAGTTAGAC ACAACGTAAA   
  
  
- AAGACACTAC ATATTCGGCA AATAATGGAT AACTACAAGT TGAAAAGTGT GATATACAGG AAAATAATCA   
  
  
- ATGACTACGG AGACAGTTTG TAAGAATTAA CGTAAATCAC ATAGTTGCCA GAATTTTAAA ATTCGTAACG   
  
  
- GTTGAACTTG AAGACTTCTA CAAACAGTTC TAGAAGTCAG ACGTTCAATT GACGACGAAC GAACTTGAAA   
  
  
- AGTTGAAAAT TCTCTCTACA GTCTCAGATT CGTACCGAAG GACTGTTCAT GACGTAAAAT AGTGAATACT   
  
  
- TCTGAGTTAA GACCCAAAAC TGTCGAAGAT ACTTGGAGAT CACGTCCATG TCGTCGAACC TGTAATTCGA   
  
  
- CGTTCAGTTA ACTGACATCT TGCTGTTCAA CAACTTCAAA GACGACTTTC AAGTAGTGAA CAAACTACTT   
  
  
- ATTTCATGTC GTACCGTGAA GTCTACGTTT TCAGAGCGCT AGTCATCTCT ACTAAGGTAT AAAGAGTAGA   
  
  
- GTAAAGGTTC AGAAACTGTT GTACATGGGT TTATTACGGT CGGTACTGTG AGTCTACAGA GAGGTTAGTA   
  
  
- TGTCGCTTCC TATGAAGTGA GACCTGAGTA GAGGTCGTCG TTAGCCTCGC ACGTCACAGA TACTGATGGG   
  
  
- TAGGCAACAG TCGTGGAGAA GTTTATCTTC GGTCAAAAGA GGAGTCCCAA GGGTGAGCAT GTAGAGTCTG   
  
  
- GGGGTAGTAA GAAGGCTGTT GATACCTAGG GGTCACTCGC CGAGAAGACA CCAACTGCTA TTACGACTCG   
  
  
- ACTCCTTGTC CAAGTCACTA TACCTCGAGG GGAACGGTGT CCTGAGTCCT GTGGTAATAA CGAGAAAATC   
  
  
- GGTGTCTCCT TCGGTACTTC CGAGGATATG AAACTCCGGT TTGGTTGACT ACCTATACCG GTTGTACCTC   
  
  
- AATTTCGTCC ACGATATGAA GACACGTCTT CGTTAGAGTC TCTTATTAGA TAGTTGACGT CTTTCTGATT   
  
  
- ACCTACGTAA CCCATTCTCC CACAGACAAA AACCAAGTGG TTAACTTTCC AACCGGCGGA TGTACAACCT   
  
  
- TCCCGAGTCT CGTTCCGACC TCAAAAGACC TATATGATAG ATGTTTTTCG AGTCCACGCT TGTCGGTTGC   
  
  
- TCGAGTCTCG AAGAAAGGAT GTACGTGTAG GACATAGTTT CAACGGGTAT AAAGTTTAAG CGTATATACA   
  
  
- GGAGTTTACA ACGATAAGTT CTTCGAAACC CCTTACTCGG CCAATAGGTG TAGTAGCTAA AGGTCTAACG   
  
  
- GTACCCCTGT GTTAACCACG AGAACTAGGT CAGAGAGCGG GTAGCCGGAC CGCCCGGGGG GGGAGCAAGC   
  
  
- CTAGTGACCC CAGCTACTAG GTGTGAGCCG GATACGAGCA CCGCCCCCCG AGCTCCAACA CCCCGTCTCC   
  
  
- GATTGTTTCC ACCGACTTAG TATACCTCAG GGTAAGCTCA AAGTACGACG TCGGTACAGT CCCACGTTCC   
  
  
- ACTTAGCGCT ACAGGAGTTC CAAGCGGGAC CCCTTCTTCG GAACCGGCAT TTGAAGGGGA TATACAACGT   
  
  
- AGTGTACGGG CTGCTCTCGC ACTCGTGGTG TTTAGTAGCC CTGGCCGAGA ACTCCGACCA CTTCTCCAAC   
  
  
- AGTGGTTCCT AGCAGTGGAA CCAACTCGTT CTCAGGTTGT GTTTGTGAGG AGGGAAAGAA GTTGCCAAGG   
  
  
- CACTTTGTAA CCTGATGATG TGACGGTACA AGCTCAGGTA ACTACACCGA GATGGATCCC TACTGTTCTT   
  
  
- CTCCTATTTA CGGCTCGTCG TAACAGAACG CGCGCTATAA CACTTGTACC AGCGAACACT CTTACGCCTT   
  
  
- TCCCAACTCT CCGTGCTCGG GAAGCCCTTT ACCTCCCGTT CCAAACTATA CCGACCCAAA GTCGTTAACG   
  
  
- GGAGCTCGGC GAGTCACTTA CTACGTTATT CCCCAAACTA CGCTCTTAAG GTATCCTTGA TAGCCCACGT   
  
  
- CCTAACAGTC CCACGGGAAA AAAACCCGAC CTTCCTCGCG TTAGACCGTT GGAAGAGATG TACCACGACA   
  
  
- TTTCTTAC

+     TATA-box

| Site Name | Organism | Position | Strand | Matrix score. | sequence | function |
| --- | --- | --- | --- | --- | --- | --- |
| TATA-box | Brassica napus | 1747 | - | 6 | ATATAT | core promoter element around -30 of transcription start |
| TATA-box | Arabidopsis thaliana | 575 | + | 4 | TATA | core promoter element around -30 of transcription start |
| TATA-box | Arabidopsis thaliana | 1488 | - | 4 | TATA | core promoter element around -30 of transcription start |
| TATA-box | Arabidopsis thaliana | 500 | + | 6 | TATATA | core promoter element around -30 of transcription start |
| TATA-box | Arabidopsis thaliana | 616 | + | 4 | TATA | core promoter element around -30 of transcription start |
| TATA-box | Arabidopsis thaliana | 459 | + | 4 | TATA | core promoter element around -30 of transcription start |
| TATA-box | Oryza sativa | 1655 | + | 7 | TACAAAA | core promoter element around -30 of transcription start |
| TATA-box | Helianthus annuus | 457 | - | 6 | TATACA | core promoter element around -30 of transcription start |
| TATA-box | Arabidopsis thaliana | 38 | - | 5 | TATAA | core promoter element around -30 of transcription start |
| TATA-box | Arabidopsis thaliana | 2094 | - | 4 | TATA | core promoter element around -30 of transcription start |
| TATA-box | Brassica napus | 37 | + | 6 | ATTATA | core promoter element around -30 of transcription start |
| TATA-box | Brassica napus | 501 | + | 6 | ATATAT | core promoter element around -30 of transcription start |
| TATA-box | Arabidopsis thaliana | 1748 | - | 4 | TATA | core promoter element around -30 of transcription start |
| TATA-box | Helianthus annuus | 573 | - | 6 | TATACA | core promoter element around -30 of transcription start |
| TATA-box | Arabidopsis thaliana | 502 | + | 4 | TATA | core promoter element around -30 of transcription start |
| TATA-box | Arabidopsis thaliana | 1430 | - | 4 | TATA | core promoter element around -30 of transcription start |
| TATA-box | Arabidopsis thaliana | 39 | + | 4 | TATA | core promoter element around -30 of transcription start |
| TATA-box | Arabidopsis thaliana | 1646 | - | 4 | TATA | core promoter element around -30 of transcription start |

>HU08G02295.1   
+ -Up\_Stream \_Len000GTTAAA AAACTCAGAA CTATTATAGA TTTTCTGTTT TCTGTTTGGA TTGAGATCCG   
  
  
+ AAGCCTTTGA GATCCCCTGT TTCTAGCTTC CAACCCCCCT CCCTGGGTTC TTTTTCTTTG GTGTGAAAGT   
  
  
+ ATGAACAAAA GCTGATGTAA GTGTGATTCA AACTCTAGCC TTGGTGAGAC TCTTGGGAAG GGCTCCTAGA   
  
  
+ GACTTTGCCA ATTGAGCTAA TTTGATGCCT ACTACTTGCT TACTTTTCTA GCTGTTGTTT AAACGATTTA   
  
  
+ TGTTCATAGT TAAGGAGCGC CATATTGCAT TACAATATCT GCTTTAGTGC TTTGTTTTTT GCATTTTCTT   
  
  
+ CTTTATTACT ATGGTTTTTG AAGAAGGCTT ACTGACTGAT ACTGGTTTCT TCGCTTAAGT TTGGTGTACA   
  
  
+ TGCTTTTCAA TCTGTGTTGC ATTTTTCTAT GATGTATAAG CCGTTTATTA CCTATTGATG TTCAACTTTT   
  
  
+ CACACTATAT ATCCTTTATG GTTTCTTCGC TTAAGTTTGG TGTACATGCT TTTCAATCTG TGTTGCATTT   
  
  
+ TTCTGTGATG TATAAGCCGT TTATTACCTA TTGATGTTCA ACTTTTCACA CTATATGTCC TTTTATTAGT   
  
  
+ TACTGATGCC TCTGTCAAAC ATTCTTAATT GCATTTAGTG TATCAACGGT CTTAAAATTT TAAGCATTGC   
  
  
+ CAACTTGAAC TTCTGAAGAT GTTTGTCAAG ATCTTCAGTC TGCAAGTTAA CTGCTGCTTG CTTGAACTTT   
  
  
+ TCAACTTTTA AGAGAGATGT CAGAGTCTAA GCATGGCTTC CTGACAAGTA CTGCATTTTA TCACTTATGA   
  
  
+ AGACTCAATT CTGGGTTTTG ACAGCTTCTA TGAACCTCTA GTGCAGGTAC AGCAGCTTGG ACATTAAGCT   
  
  
+ GCAAGTCAAT TGACTGTAGA ACGACAAGTT GTTGAAGTTT CTGCTGAAAG TTCATCACTT GTTTGATGAA   
  
  
+ TAAAGTACAG CATGGCACTT CAGATGCAAA AGTCTCGCGA TCAGTAGAGA TGATTCCATA TTTCTCATCT   
  
  
+ CATTTCCAAG TCTTTGACAA CATGTACCCA AATAATGCCA GCCATGACAC TCAGATGTCT CTCCAATCAT   
  
  
+ ACAGCGAAGG ATACTTCACT CTGGACTCAT CTCCAGCAGC AATCGGAGCG TGCAGTGTCT ATGACTACCC   
  
  
+ ATCCGTTGTC AGCACCTCTT CAAATAGAAG CCAGTTTTCT CCTCAGGGTT CCCACTCGTA CATCTCAGAC   
  
  
+ CCCCATCATT CTTCCGACAA CTATGGATCC CCAGTGAGCG GCTCTTCTGT GGTTGACGAT AATGCTGAGC   
  
  
+ TGAGGAACAG GTTCAGTGAT ATGGAGCTCC CCTTGCCACA GGACTCAGGA CACCATTATT GCTCTTTTAG   
  
  
+ CCACAGAGGA AGCCATGAAG GCTCCTATAC TTTGAGGCCA AACCAACTGA TGGATATGGC CAACATGGAG   
  
  
+ TTAAAGCAGG TGCTATACTT CTGTGCAGAA GCAATCTCAG AGAATAATCT ATCAACTGCA GAAAGACTAA   
  
  
+ TGGATGCATT GGGTAAGAGG GTGTCTGTTT TTGGTTCACC AATTGAAAGG TTGGCCGCCT ACATGTTGGA   
  
  
+ AGGGCTCAGA GCAAGGCTGG AGTTTTCTGG ATATACTATC TACAAAAAGC TCAGGTGCGA ACAGCCAACG   
  
  
+ AGCTCAGAGC TTCTTTCCTA CATGCACATC CTGTATCAAA GTTGCCCATA TTTCAAATTC GCATATATGT   
  
  
+ CCTCAAATGT TGCTATTCAA GAAGCTTTGG GGAATGAGCC GGTTATCCAC ATCATCGATT TCCAGATTGC   
  
  
+ CATGGGGACA CAATTGGTGC TCTTGATCCA GTCTCTCGCC CATCGGCCTG GCGGGCCCCC CCCTCGTTCG   
  
  
+ GATCACTGGG GTCGATGATC CACACTCGGC CTATGCTCGT GGCGGGGGGC TCGAGGTTGT GGGGCAGAGG   
  
  
+ CTAACAAAGG TGGCTGAATC ATATGGAGTC CCATTCGAGT TTCATGCTGC AGCCATGTCA GGGTGCAAGG   
  
  
+ TGAATCGCGA TGTCCTCAAG GTTCGCCCTG GGGAAGAAGC CTTGGCCGTA AACTTCCCCT ATATGTTGCA   
  
  
+ TCACATGCCC GACGAGAGCG TGAGCACCAC AAATCATCGG GACCGGCTCT TGAGGCTGGT GAAGAGGTTG   
  
  
+ TCACCAAGGA TCGTCACCTT GGTTGAGCAA GAGTCCAACA CAAACACTCC TCCCTTTCTT CAACGGTTCC   
  
  
+ GTGAAACATT GGACTACTAC ACTGCCATGT TCGAGTCCAT TGATGTGGCT CTACCTAGGG ATGACAAGAA   
  
  
+ GAGGATAAAT GCCGAGCAGC ATTGTCTTGC GCGCGATATT GTGAACATGG TCGCTTGTGA GAATGCGGAA   
  
  
+ AGGGTTGAGA GGCACGAGCC CTTCGGGAAA TGGAGGGCAA GGTTTGATAT GGCTGGGTTT CAGCAATTGC   
  
  
+ CCTCGAGCCG CTCAGTGAAT GATGCAATAA GGGGTTTGAT GCGAGAATTC CATAGGAACT ATCGGGTGCA   
  
  
+ GGATTGTCAG GGTGCCCTTT TTTTGGGCTG GAAGGAGCGC AATCTGGCAA CCTTCTCTAC ATGGTGCTGT   
  
  
+ AAAGAATG  

- -Up\_Stream \_Len000CAATTT TTTGAGTCTT GATAATATCT AAAAGACAAA AGACAAACCT AACTCTAGGC   
  
  
- TTCGGAAACT CTAGGGGACA AAGATCGAAG GTTGGGGGGA GGGACCCAAG AAAAAGAAAC CACACTTTCA   
  
  
- TACTTGTTTT CGACTACATT CACACTAAGT TTGAGATCGG AACCACTCTG AGAACCCTTC CCGAGGATCT   
  
  
- CTGAAACGGT TAACTCGATT AAACTACGGA TGATGAACGA ATGAAAAGAT CGACAACAAA TTTGCTAAAT   
  
  
- ACAAGTATCA ATTCCTCGCG GTATAACGTA ATGTTATAGA CGAAATCACG AAACAAAAAA CGTAAAAGAA   
  
  
- GAAATAATGA TACCAAAAAC TTCTTCCGAA TGACTGACTA TGACCAAAGA AGCGAATTCA AACCACATGT   
  
  
- ACGAAAAGTT AGACACAACG TAAAAAGATA CTACATATTC GGCAAATAAT GGATAACTAC AAGTTGAAAA   
  
  
- GTGTGATATA TAGGAAATAC CAAAGAAGCG AATTCAAACC ACATGTACGA AAAGTTAGAC ACAACGTAAA   
  
  
- AAGACACTAC ATATTCGGCA AATAATGGAT AACTACAAGT TGAAAAGTGT GATATACAGG AAAATAATCA   
  
  
- ATGACTACGG AGACAGTTTG TAAGAATTAA CGTAAATCAC ATAGTTGCCA GAATTTTAAA ATTCGTAACG   
  
  
- GTTGAACTTG AAGACTTCTA CAAACAGTTC TAGAAGTCAG ACGTTCAATT GACGACGAAC GAACTTGAAA   
  
  
- AGTTGAAAAT TCTCTCTACA GTCTCAGATT CGTACCGAAG GACTGTTCAT GACGTAAAAT AGTGAATACT   
  
  
- TCTGAGTTAA GACCCAAAAC TGTCGAAGAT ACTTGGAGAT CACGTCCATG TCGTCGAACC TGTAATTCGA   
  
  
- CGTTCAGTTA ACTGACATCT TGCTGTTCAA CAACTTCAAA GACGACTTTC AAGTAGTGAA CAAACTACTT   
  
  
- ATTTCATGTC GTACCGTGAA GTCTACGTTT TCAGAGCGCT AGTCATCTCT ACTAAGGTAT AAAGAGTAGA   
  
  
- GTAAAGGTTC AGAAACTGTT GTACATGGGT TTATTACGGT CGGTACTGTG AGTCTACAGA GAGGTTAGTA   
  
  
- TGTCGCTTCC TATGAAGTGA GACCTGAGTA GAGGTCGTCG TTAGCCTCGC ACGTCACAGA TACTGATGGG   
  
  
- TAGGCAACAG TCGTGGAGAA GTTTATCTTC GGTCAAAAGA GGAGTCCCAA GGGTGAGCAT GTAGAGTCTG   
  
  
- GGGGTAGTAA GAAGGCTGTT GATACCTAGG GGTCACTCGC CGAGAAGACA CCAACTGCTA TTACGACTCG   
  
  
- ACTCCTTGTC CAAGTCACTA TACCTCGAGG GGAACGGTGT CCTGAGTCCT GTGGTAATAA CGAGAAAATC   
  
  
- GGTGTCTCCT TCGGTACTTC CGAGGATATG AAACTCCGGT TTGGTTGACT ACCTATACCG GTTGTACCTC   
  
  
- AATTTCGTCC ACGATATGAA GACACGTCTT CGTTAGAGTC TCTTATTAGA TAGTTGACGT CTTTCTGATT   
  
  
- ACCTACGTAA CCCATTCTCC CACAGACAAA AACCAAGTGG TTAACTTTCC AACCGGCGGA TGTACAACCT   
  
  
- TCCCGAGTCT CGTTCCGACC TCAAAAGACC TATATGATAG ATGTTTTTCG AGTCCACGCT TGTCGGTTGC   
  
  
- TCGAGTCTCG AAGAAAGGAT GTACGTGTAG GACATAGTTT CAACGGGTAT AAAGTTTAAG CGTATATACA   
  
  
- GGAGTTTACA ACGATAAGTT CTTCGAAACC CCTTACTCGG CCAATAGGTG TAGTAGCTAA AGGTCTAACG   
  
  
- GTACCCCTGT GTTAACCACG AGAACTAGGT CAGAGAGCGG GTAGCCGGAC CGCCCGGGGG GGGAGCAAGC   
  
  
- CTAGTGACCC CAGCTACTAG GTGTGAGCCG GATACGAGCA CCGCCCCCCG AGCTCCAACA CCCCGTCTCC   
  
  
- GATTGTTTCC ACCGACTTAG TATACCTCAG GGTAAGCTCA AAGTACGACG TCGGTACAGT CCCACGTTCC   
  
  
- ACTTAGCGCT ACAGGAGTTC CAAGCGGGAC CCCTTCTTCG GAACCGGCAT TTGAAGGGGA TATACAACGT   
  
  
- AGTGTACGGG CTGCTCTCGC ACTCGTGGTG TTTAGTAGCC CTGGCCGAGA ACTCCGACCA CTTCTCCAAC   
  
  
- AGTGGTTCCT AGCAGTGGAA CCAACTCGTT CTCAGGTTGT GTTTGTGAGG AGGGAAAGAA GTTGCCAAGG   
  
  
- CACTTTGTAA CCTGATGATG TGACGGTACA AGCTCAGGTA ACTACACCGA GATGGATCCC TACTGTTCTT   
  
  
- CTCCTATTTA CGGCTCGTCG TAACAGAACG CGCGCTATAA CACTTGTACC AGCGAACACT CTTACGCCTT   
  
  
- TCCCAACTCT CCGTGCTCGG GAAGCCCTTT ACCTCCCGTT CCAAACTATA CCGACCCAAA GTCGTTAACG   
  
  
- GGAGCTCGGC GAGTCACTTA CTACGTTATT CCCCAAACTA CGCTCTTAAG GTATCCTTGA TAGCCCACGT   
  
  
- CCTAACAGTC CCACGGGAAA AAAACCCGAC CTTCCTCGCG TTAGACCGTT GGAAGAGATG TACCACGACA   
  
  
- TTTCTTAC

+     TCT-motif

| Site Name | Organism | Position | Strand | Matrix score. | sequence | function |
| --- | --- | --- | --- | --- | --- | --- |
| TCT-motif | Arabidopsis thaliana | 1557 | - | 6 | TCTTAC | part of a light responsive element |

>HU08G02295.1   
+ -Up\_Stream \_Len000GTTAAA AAACTCAGAA CTATTATAGA TTTTCTGTTT TCTGTTTGGA TTGAGATCCG   
  
  
+ AAGCCTTTGA GATCCCCTGT TTCTAGCTTC CAACCCCCCT CCCTGGGTTC TTTTTCTTTG GTGTGAAAGT   
  
  
+ ATGAACAAAA GCTGATGTAA GTGTGATTCA AACTCTAGCC TTGGTGAGAC TCTTGGGAAG GGCTCCTAGA   
  
  
+ GACTTTGCCA ATTGAGCTAA TTTGATGCCT ACTACTTGCT TACTTTTCTA GCTGTTGTTT AAACGATTTA   
  
  
+ TGTTCATAGT TAAGGAGCGC CATATTGCAT TACAATATCT GCTTTAGTGC TTTGTTTTTT GCATTTTCTT   
  
  
+ CTTTATTACT ATGGTTTTTG AAGAAGGCTT ACTGACTGAT ACTGGTTTCT TCGCTTAAGT TTGGTGTACA   
  
  
+ TGCTTTTCAA TCTGTGTTGC ATTTTTCTAT GATGTATAAG CCGTTTATTA CCTATTGATG TTCAACTTTT   
  
  
+ CACACTATAT ATCCTTTATG GTTTCTTCGC TTAAGTTTGG TGTACATGCT TTTCAATCTG TGTTGCATTT   
  
  
+ TTCTGTGATG TATAAGCCGT TTATTACCTA TTGATGTTCA ACTTTTCACA CTATATGTCC TTTTATTAGT   
  
  
+ TACTGATGCC TCTGTCAAAC ATTCTTAATT GCATTTAGTG TATCAACGGT CTTAAAATTT TAAGCATTGC   
  
  
+ CAACTTGAAC TTCTGAAGAT GTTTGTCAAG ATCTTCAGTC TGCAAGTTAA CTGCTGCTTG CTTGAACTTT   
  
  
+ TCAACTTTTA AGAGAGATGT CAGAGTCTAA GCATGGCTTC CTGACAAGTA CTGCATTTTA TCACTTATGA   
  
  
+ AGACTCAATT CTGGGTTTTG ACAGCTTCTA TGAACCTCTA GTGCAGGTAC AGCAGCTTGG ACATTAAGCT   
  
  
+ GCAAGTCAAT TGACTGTAGA ACGACAAGTT GTTGAAGTTT CTGCTGAAAG TTCATCACTT GTTTGATGAA   
  
  
+ TAAAGTACAG CATGGCACTT CAGATGCAAA AGTCTCGCGA TCAGTAGAGA TGATTCCATA TTTCTCATCT   
  
  
+ CATTTCCAAG TCTTTGACAA CATGTACCCA AATAATGCCA GCCATGACAC TCAGATGTCT CTCCAATCAT   
  
  
+ ACAGCGAAGG ATACTTCACT CTGGACTCAT CTCCAGCAGC AATCGGAGCG TGCAGTGTCT ATGACTACCC   
  
  
+ ATCCGTTGTC AGCACCTCTT CAAATAGAAG CCAGTTTTCT CCTCAGGGTT CCCACTCGTA CATCTCAGAC   
  
  
+ CCCCATCATT CTTCCGACAA CTATGGATCC CCAGTGAGCG GCTCTTCTGT GGTTGACGAT AATGCTGAGC   
  
  
+ TGAGGAACAG GTTCAGTGAT ATGGAGCTCC CCTTGCCACA GGACTCAGGA CACCATTATT GCTCTTTTAG   
  
  
+ CCACAGAGGA AGCCATGAAG GCTCCTATAC TTTGAGGCCA AACCAACTGA TGGATATGGC CAACATGGAG   
  
  
+ TTAAAGCAGG TGCTATACTT CTGTGCAGAA GCAATCTCAG AGAATAATCT ATCAACTGCA GAAAGACTAA   
  
  
+ TGGATGCATT GGGTAAGAGG GTGTCTGTTT TTGGTTCACC AATTGAAAGG TTGGCCGCCT ACATGTTGGA   
  
  
+ AGGGCTCAGA GCAAGGCTGG AGTTTTCTGG ATATACTATC TACAAAAAGC TCAGGTGCGA ACAGCCAACG   
  
  
+ AGCTCAGAGC TTCTTTCCTA CATGCACATC CTGTATCAAA GTTGCCCATA TTTCAAATTC GCATATATGT   
  
  
+ CCTCAAATGT TGCTATTCAA GAAGCTTTGG GGAATGAGCC GGTTATCCAC ATCATCGATT TCCAGATTGC   
  
  
+ CATGGGGACA CAATTGGTGC TCTTGATCCA GTCTCTCGCC CATCGGCCTG GCGGGCCCCC CCCTCGTTCG   
  
  
+ GATCACTGGG GTCGATGATC CACACTCGGC CTATGCTCGT GGCGGGGGGC TCGAGGTTGT GGGGCAGAGG   
  
  
+ CTAACAAAGG TGGCTGAATC ATATGGAGTC CCATTCGAGT TTCATGCTGC AGCCATGTCA GGGTGCAAGG   
  
  
+ TGAATCGCGA TGTCCTCAAG GTTCGCCCTG GGGAAGAAGC CTTGGCCGTA AACTTCCCCT ATATGTTGCA   
  
  
+ TCACATGCCC GACGAGAGCG TGAGCACCAC AAATCATCGG GACCGGCTCT TGAGGCTGGT GAAGAGGTTG   
  
  
+ TCACCAAGGA TCGTCACCTT GGTTGAGCAA GAGTCCAACA CAAACACTCC TCCCTTTCTT CAACGGTTCC   
  
  
+ GTGAAACATT GGACTACTAC ACTGCCATGT TCGAGTCCAT TGATGTGGCT CTACCTAGGG ATGACAAGAA   
  
  
+ GAGGATAAAT GCCGAGCAGC ATTGTCTTGC GCGCGATATT GTGAACATGG TCGCTTGTGA GAATGCGGAA   
  
  
+ AGGGTTGAGA GGCACGAGCC CTTCGGGAAA TGGAGGGCAA GGTTTGATAT GGCTGGGTTT CAGCAATTGC   
  
  
+ CCTCGAGCCG CTCAGTGAAT GATGCAATAA GGGGTTTGAT GCGAGAATTC CATAGGAACT ATCGGGTGCA   
  
  
+ GGATTGTCAG GGTGCCCTTT TTTTGGGCTG GAAGGAGCGC AATCTGGCAA CCTTCTCTAC ATGGTGCTGT   
  
  
+ AAAGAATG  

- -Up\_Stream \_Len000CAATTT TTTGAGTCTT GATAATATCT AAAAGACAAA AGACAAACCT AACTCTAGGC   
  
  
- TTCGGAAACT CTAGGGGACA AAGATCGAAG GTTGGGGGGA GGGACCCAAG AAAAAGAAAC CACACTTTCA   
  
  
- TACTTGTTTT CGACTACATT CACACTAAGT TTGAGATCGG AACCACTCTG AGAACCCTTC CCGAGGATCT   
  
  
- CTGAAACGGT TAACTCGATT AAACTACGGA TGATGAACGA ATGAAAAGAT CGACAACAAA TTTGCTAAAT   
  
  
- ACAAGTATCA ATTCCTCGCG GTATAACGTA ATGTTATAGA CGAAATCACG AAACAAAAAA CGTAAAAGAA   
  
  
- GAAATAATGA TACCAAAAAC TTCTTCCGAA TGACTGACTA TGACCAAAGA AGCGAATTCA AACCACATGT   
  
  
- ACGAAAAGTT AGACACAACG TAAAAAGATA CTACATATTC GGCAAATAAT GGATAACTAC AAGTTGAAAA   
  
  
- GTGTGATATA TAGGAAATAC CAAAGAAGCG AATTCAAACC ACATGTACGA AAAGTTAGAC ACAACGTAAA   
  
  
- AAGACACTAC ATATTCGGCA AATAATGGAT AACTACAAGT TGAAAAGTGT GATATACAGG AAAATAATCA   
  
  
- ATGACTACGG AGACAGTTTG TAAGAATTAA CGTAAATCAC ATAGTTGCCA GAATTTTAAA ATTCGTAACG   
  
  
- GTTGAACTTG AAGACTTCTA CAAACAGTTC TAGAAGTCAG ACGTTCAATT GACGACGAAC GAACTTGAAA   
  
  
- AGTTGAAAAT TCTCTCTACA GTCTCAGATT CGTACCGAAG GACTGTTCAT GACGTAAAAT AGTGAATACT   
  
  
- TCTGAGTTAA GACCCAAAAC TGTCGAAGAT ACTTGGAGAT CACGTCCATG TCGTCGAACC TGTAATTCGA   
  
  
- CGTTCAGTTA ACTGACATCT TGCTGTTCAA CAACTTCAAA GACGACTTTC AAGTAGTGAA CAAACTACTT   
  
  
- ATTTCATGTC GTACCGTGAA GTCTACGTTT TCAGAGCGCT AGTCATCTCT ACTAAGGTAT AAAGAGTAGA   
  
  
- GTAAAGGTTC AGAAACTGTT GTACATGGGT TTATTACGGT CGGTACTGTG AGTCTACAGA GAGGTTAGTA   
  
  
- TGTCGCTTCC TATGAAGTGA GACCTGAGTA GAGGTCGTCG TTAGCCTCGC ACGTCACAGA TACTGATGGG   
  
  
- TAGGCAACAG TCGTGGAGAA GTTTATCTTC GGTCAAAAGA GGAGTCCCAA GGGTGAGCAT GTAGAGTCTG   
  
  
- GGGGTAGTAA GAAGGCTGTT GATACCTAGG GGTCACTCGC CGAGAAGACA CCAACTGCTA TTACGACTCG   
  
  
- ACTCCTTGTC CAAGTCACTA TACCTCGAGG GGAACGGTGT CCTGAGTCCT GTGGTAATAA CGAGAAAATC   
  
  
- GGTGTCTCCT TCGGTACTTC CGAGGATATG AAACTCCGGT TTGGTTGACT ACCTATACCG GTTGTACCTC   
  
  
- AATTTCGTCC ACGATATGAA GACACGTCTT CGTTAGAGTC TCTTATTAGA TAGTTGACGT CTTTCTGATT   
  
  
- ACCTACGTAA CCCATTCTCC CACAGACAAA AACCAAGTGG TTAACTTTCC AACCGGCGGA TGTACAACCT   
  
  
- TCCCGAGTCT CGTTCCGACC TCAAAAGACC TATATGATAG ATGTTTTTCG AGTCCACGCT TGTCGGTTGC   
  
  
- TCGAGTCTCG AAGAAAGGAT GTACGTGTAG GACATAGTTT CAACGGGTAT AAAGTTTAAG CGTATATACA   
  
  
- GGAGTTTACA ACGATAAGTT CTTCGAAACC CCTTACTCGG CCAATAGGTG TAGTAGCTAA AGGTCTAACG   
  
  
- GTACCCCTGT GTTAACCACG AGAACTAGGT CAGAGAGCGG GTAGCCGGAC CGCCCGGGGG GGGAGCAAGC   
  
  
- CTAGTGACCC CAGCTACTAG GTGTGAGCCG GATACGAGCA CCGCCCCCCG AGCTCCAACA CCCCGTCTCC   
  
  
- GATTGTTTCC ACCGACTTAG TATACCTCAG GGTAAGCTCA AAGTACGACG TCGGTACAGT CCCACGTTCC   
  
  
- ACTTAGCGCT ACAGGAGTTC CAAGCGGGAC CCCTTCTTCG GAACCGGCAT TTGAAGGGGA TATACAACGT   
  
  
- AGTGTACGGG CTGCTCTCGC ACTCGTGGTG TTTAGTAGCC CTGGCCGAGA ACTCCGACCA CTTCTCCAAC   
  
  
- AGTGGTTCCT AGCAGTGGAA CCAACTCGTT CTCAGGTTGT GTTTGTGAGG AGGGAAAGAA GTTGCCAAGG   
  
  
- CACTTTGTAA CCTGATGATG TGACGGTACA AGCTCAGGTA ACTACACCGA GATGGATCCC TACTGTTCTT   
  
  
- CTCCTATTTA CGGCTCGTCG TAACAGAACG CGCGCTATAA CACTTGTACC AGCGAACACT CTTACGCCTT   
  
  
- TCCCAACTCT CCGTGCTCGG GAAGCCCTTT ACCTCCCGTT CCAAACTATA CCGACCCAAA GTCGTTAACG   
  
  
- GGAGCTCGGC GAGTCACTTA CTACGTTATT CCCCAAACTA CGCTCTTAAG GTATCCTTGA TAGCCCACGT   
  
  
- CCTAACAGTC CCACGGGAAA AAAACCCGAC CTTCCTCGCG TTAGACCGTT GGAAGAGATG TACCACGACA   
  
  
- TTTCTTAC

+     TGA-element

| Site Name | Organism | Position | Strand | Matrix score. | sequence | function |
| --- | --- | --- | --- | --- | --- | --- |
| TGA-element | Brassica oleracea | 934 | + | 6 | AACGAC | auxin-responsive element |

>HU08G02295.1   
+ -Up\_Stream \_Len000GTTAAA AAACTCAGAA CTATTATAGA TTTTCTGTTT TCTGTTTGGA TTGAGATCCG   
  
  
+ AAGCCTTTGA GATCCCCTGT TTCTAGCTTC CAACCCCCCT CCCTGGGTTC TTTTTCTTTG GTGTGAAAGT   
  
  
+ ATGAACAAAA GCTGATGTAA GTGTGATTCA AACTCTAGCC TTGGTGAGAC TCTTGGGAAG GGCTCCTAGA   
  
  
+ GACTTTGCCA ATTGAGCTAA TTTGATGCCT ACTACTTGCT TACTTTTCTA GCTGTTGTTT AAACGATTTA   
  
  
+ TGTTCATAGT TAAGGAGCGC CATATTGCAT TACAATATCT GCTTTAGTGC TTTGTTTTTT GCATTTTCTT   
  
  
+ CTTTATTACT ATGGTTTTTG AAGAAGGCTT ACTGACTGAT ACTGGTTTCT TCGCTTAAGT TTGGTGTACA   
  
  
+ TGCTTTTCAA TCTGTGTTGC ATTTTTCTAT GATGTATAAG CCGTTTATTA CCTATTGATG TTCAACTTTT   
  
  
+ CACACTATAT ATCCTTTATG GTTTCTTCGC TTAAGTTTGG TGTACATGCT TTTCAATCTG TGTTGCATTT   
  
  
+ TTCTGTGATG TATAAGCCGT TTATTACCTA TTGATGTTCA ACTTTTCACA CTATATGTCC TTTTATTAGT   
  
  
+ TACTGATGCC TCTGTCAAAC ATTCTTAATT GCATTTAGTG TATCAACGGT CTTAAAATTT TAAGCATTGC   
  
  
+ CAACTTGAAC TTCTGAAGAT GTTTGTCAAG ATCTTCAGTC TGCAAGTTAA CTGCTGCTTG CTTGAACTTT   
  
  
+ TCAACTTTTA AGAGAGATGT CAGAGTCTAA GCATGGCTTC CTGACAAGTA CTGCATTTTA TCACTTATGA   
  
  
+ AGACTCAATT CTGGGTTTTG ACAGCTTCTA TGAACCTCTA GTGCAGGTAC AGCAGCTTGG ACATTAAGCT   
  
  
+ GCAAGTCAAT TGACTGTAGA ACGACAAGTT GTTGAAGTTT CTGCTGAAAG TTCATCACTT GTTTGATGAA   
  
  
+ TAAAGTACAG CATGGCACTT CAGATGCAAA AGTCTCGCGA TCAGTAGAGA TGATTCCATA TTTCTCATCT   
  
  
+ CATTTCCAAG TCTTTGACAA CATGTACCCA AATAATGCCA GCCATGACAC TCAGATGTCT CTCCAATCAT   
  
  
+ ACAGCGAAGG ATACTTCACT CTGGACTCAT CTCCAGCAGC AATCGGAGCG TGCAGTGTCT ATGACTACCC   
  
  
+ ATCCGTTGTC AGCACCTCTT CAAATAGAAG CCAGTTTTCT CCTCAGGGTT CCCACTCGTA CATCTCAGAC   
  
  
+ CCCCATCATT CTTCCGACAA CTATGGATCC CCAGTGAGCG GCTCTTCTGT GGTTGACGAT AATGCTGAGC   
  
  
+ TGAGGAACAG GTTCAGTGAT ATGGAGCTCC CCTTGCCACA GGACTCAGGA CACCATTATT GCTCTTTTAG   
  
  
+ CCACAGAGGA AGCCATGAAG GCTCCTATAC TTTGAGGCCA AACCAACTGA TGGATATGGC CAACATGGAG   
  
  
+ TTAAAGCAGG TGCTATACTT CTGTGCAGAA GCAATCTCAG AGAATAATCT ATCAACTGCA GAAAGACTAA   
  
  
+ TGGATGCATT GGGTAAGAGG GTGTCTGTTT TTGGTTCACC AATTGAAAGG TTGGCCGCCT ACATGTTGGA   
  
  
+ AGGGCTCAGA GCAAGGCTGG AGTTTTCTGG ATATACTATC TACAAAAAGC TCAGGTGCGA ACAGCCAACG   
  
  
+ AGCTCAGAGC TTCTTTCCTA CATGCACATC CTGTATCAAA GTTGCCCATA TTTCAAATTC GCATATATGT   
  
  
+ CCTCAAATGT TGCTATTCAA GAAGCTTTGG GGAATGAGCC GGTTATCCAC ATCATCGATT TCCAGATTGC   
  
  
+ CATGGGGACA CAATTGGTGC TCTTGATCCA GTCTCTCGCC CATCGGCCTG GCGGGCCCCC CCCTCGTTCG   
  
  
+ GATCACTGGG GTCGATGATC CACACTCGGC CTATGCTCGT GGCGGGGGGC TCGAGGTTGT GGGGCAGAGG   
  
  
+ CTAACAAAGG TGGCTGAATC ATATGGAGTC CCATTCGAGT TTCATGCTGC AGCCATGTCA GGGTGCAAGG   
  
  
+ TGAATCGCGA TGTCCTCAAG GTTCGCCCTG GGGAAGAAGC CTTGGCCGTA AACTTCCCCT ATATGTTGCA   
  
  
+ TCACATGCCC GACGAGAGCG TGAGCACCAC AAATCATCGG GACCGGCTCT TGAGGCTGGT GAAGAGGTTG   
  
  
+ TCACCAAGGA TCGTCACCTT GGTTGAGCAA GAGTCCAACA CAAACACTCC TCCCTTTCTT CAACGGTTCC   
  
  
+ GTGAAACATT GGACTACTAC ACTGCCATGT TCGAGTCCAT TGATGTGGCT CTACCTAGGG ATGACAAGAA   
  
  
+ GAGGATAAAT GCCGAGCAGC ATTGTCTTGC GCGCGATATT GTGAACATGG TCGCTTGTGA GAATGCGGAA   
  
  
+ AGGGTTGAGA GGCACGAGCC CTTCGGGAAA TGGAGGGCAA GGTTTGATAT GGCTGGGTTT CAGCAATTGC   
  
  
+ CCTCGAGCCG CTCAGTGAAT GATGCAATAA GGGGTTTGAT GCGAGAATTC CATAGGAACT ATCGGGTGCA   
  
  
+ GGATTGTCAG GGTGCCCTTT TTTTGGGCTG GAAGGAGCGC AATCTGGCAA CCTTCTCTAC ATGGTGCTGT   
  
  
+ AAAGAATG  

- -Up\_Stream \_Len000CAATTT TTTGAGTCTT GATAATATCT AAAAGACAAA AGACAAACCT AACTCTAGGC   
  
  
- TTCGGAAACT CTAGGGGACA AAGATCGAAG GTTGGGGGGA GGGACCCAAG AAAAAGAAAC CACACTTTCA   
  
  
- TACTTGTTTT CGACTACATT CACACTAAGT TTGAGATCGG AACCACTCTG AGAACCCTTC CCGAGGATCT   
  
  
- CTGAAACGGT TAACTCGATT AAACTACGGA TGATGAACGA ATGAAAAGAT CGACAACAAA TTTGCTAAAT   
  
  
- ACAAGTATCA ATTCCTCGCG GTATAACGTA ATGTTATAGA CGAAATCACG AAACAAAAAA CGTAAAAGAA   
  
  
- GAAATAATGA TACCAAAAAC TTCTTCCGAA TGACTGACTA TGACCAAAGA AGCGAATTCA AACCACATGT   
  
  
- ACGAAAAGTT AGACACAACG TAAAAAGATA CTACATATTC GGCAAATAAT GGATAACTAC AAGTTGAAAA   
  
  
- GTGTGATATA TAGGAAATAC CAAAGAAGCG AATTCAAACC ACATGTACGA AAAGTTAGAC ACAACGTAAA   
  
  
- AAGACACTAC ATATTCGGCA AATAATGGAT AACTACAAGT TGAAAAGTGT GATATACAGG AAAATAATCA   
  
  
- ATGACTACGG AGACAGTTTG TAAGAATTAA CGTAAATCAC ATAGTTGCCA GAATTTTAAA ATTCGTAACG   
  
  
- GTTGAACTTG AAGACTTCTA CAAACAGTTC TAGAAGTCAG ACGTTCAATT GACGACGAAC GAACTTGAAA   
  
  
- AGTTGAAAAT TCTCTCTACA GTCTCAGATT CGTACCGAAG GACTGTTCAT GACGTAAAAT AGTGAATACT   
  
  
- TCTGAGTTAA GACCCAAAAC TGTCGAAGAT ACTTGGAGAT CACGTCCATG TCGTCGAACC TGTAATTCGA   
  
  
- CGTTCAGTTA ACTGACATCT TGCTGTTCAA CAACTTCAAA GACGACTTTC AAGTAGTGAA CAAACTACTT   
  
  
- ATTTCATGTC GTACCGTGAA GTCTACGTTT TCAGAGCGCT AGTCATCTCT ACTAAGGTAT AAAGAGTAGA   
  
  
- GTAAAGGTTC AGAAACTGTT GTACATGGGT TTATTACGGT CGGTACTGTG AGTCTACAGA GAGGTTAGTA   
  
  
- TGTCGCTTCC TATGAAGTGA GACCTGAGTA GAGGTCGTCG TTAGCCTCGC ACGTCACAGA TACTGATGGG   
  
  
- TAGGCAACAG TCGTGGAGAA GTTTATCTTC GGTCAAAAGA GGAGTCCCAA GGGTGAGCAT GTAGAGTCTG   
  
  
- GGGGTAGTAA GAAGGCTGTT GATACCTAGG GGTCACTCGC CGAGAAGACA CCAACTGCTA TTACGACTCG   
  
  
- ACTCCTTGTC CAAGTCACTA TACCTCGAGG GGAACGGTGT CCTGAGTCCT GTGGTAATAA CGAGAAAATC   
  
  
- GGTGTCTCCT TCGGTACTTC CGAGGATATG AAACTCCGGT TTGGTTGACT ACCTATACCG GTTGTACCTC   
  
  
- AATTTCGTCC ACGATATGAA GACACGTCTT CGTTAGAGTC TCTTATTAGA TAGTTGACGT CTTTCTGATT   
  
  
- ACCTACGTAA CCCATTCTCC CACAGACAAA AACCAAGTGG TTAACTTTCC AACCGGCGGA TGTACAACCT   
  
  
- TCCCGAGTCT CGTTCCGACC TCAAAAGACC TATATGATAG ATGTTTTTCG AGTCCACGCT TGTCGGTTGC   
  
  
- TCGAGTCTCG AAGAAAGGAT GTACGTGTAG GACATAGTTT CAACGGGTAT AAAGTTTAAG CGTATATACA   
  
  
- GGAGTTTACA ACGATAAGTT CTTCGAAACC CCTTACTCGG CCAATAGGTG TAGTAGCTAA AGGTCTAACG   
  
  
- GTACCCCTGT GTTAACCACG AGAACTAGGT CAGAGAGCGG GTAGCCGGAC CGCCCGGGGG GGGAGCAAGC   
  
  
- CTAGTGACCC CAGCTACTAG GTGTGAGCCG GATACGAGCA CCGCCCCCCG AGCTCCAACA CCCCGTCTCC   
  
  
- GATTGTTTCC ACCGACTTAG TATACCTCAG GGTAAGCTCA AAGTACGACG TCGGTACAGT CCCACGTTCC   
  
  
- ACTTAGCGCT ACAGGAGTTC CAAGCGGGAC CCCTTCTTCG GAACCGGCAT TTGAAGGGGA TATACAACGT   
  
  
- AGTGTACGGG CTGCTCTCGC ACTCGTGGTG TTTAGTAGCC CTGGCCGAGA ACTCCGACCA CTTCTCCAAC   
  
  
- AGTGGTTCCT AGCAGTGGAA CCAACTCGTT CTCAGGTTGT GTTTGTGAGG AGGGAAAGAA GTTGCCAAGG   
  
  
- CACTTTGTAA CCTGATGATG TGACGGTACA AGCTCAGGTA ACTACACCGA GATGGATCCC TACTGTTCTT   
  
  
- CTCCTATTTA CGGCTCGTCG TAACAGAACG CGCGCTATAA CACTTGTACC AGCGAACACT CTTACGCCTT   
  
  
- TCCCAACTCT CCGTGCTCGG GAAGCCCTTT ACCTCCCGTT CCAAACTATA CCGACCCAAA GTCGTTAACG   
  
  
- GGAGCTCGGC GAGTCACTTA CTACGTTATT CCCCAAACTA CGCTCTTAAG GTATCCTTGA TAGCCCACGT   
  
  
- CCTAACAGTC CCACGGGAAA AAAACCCGAC CTTCCTCGCG TTAGACCGTT GGAAGAGATG TACCACGACA   
  
  
- TTTCTTAC

+     TGACG-motif

| Site Name | Organism | Position | Strand | Matrix score. | sequence | function |
| --- | --- | --- | --- | --- | --- | --- |
| TGACG-motif | Hordeum vulgare | 1318 | + | 5 | TGACG | cis-acting regulatory element involved in the MeJA-responsiveness |
| TGACG-motif | Hordeum vulgare | 2186 | - | 5 | TGACG | cis-acting regulatory element involved in the MeJA-responsiveness |

>HU08G02295.1   
+ -Up\_Stream \_Len000GTTAAA AAACTCAGAA CTATTATAGA TTTTCTGTTT TCTGTTTGGA TTGAGATCCG   
  
  
+ AAGCCTTTGA GATCCCCTGT TTCTAGCTTC CAACCCCCCT CCCTGGGTTC TTTTTCTTTG GTGTGAAAGT   
  
  
+ ATGAACAAAA GCTGATGTAA GTGTGATTCA AACTCTAGCC TTGGTGAGAC TCTTGGGAAG GGCTCCTAGA   
  
  
+ GACTTTGCCA ATTGAGCTAA TTTGATGCCT ACTACTTGCT TACTTTTCTA GCTGTTGTTT AAACGATTTA   
  
  
+ TGTTCATAGT TAAGGAGCGC CATATTGCAT TACAATATCT GCTTTAGTGC TTTGTTTTTT GCATTTTCTT   
  
  
+ CTTTATTACT ATGGTTTTTG AAGAAGGCTT ACTGACTGAT ACTGGTTTCT TCGCTTAAGT TTGGTGTACA   
  
  
+ TGCTTTTCAA TCTGTGTTGC ATTTTTCTAT GATGTATAAG CCGTTTATTA CCTATTGATG TTCAACTTTT   
  
  
+ CACACTATAT ATCCTTTATG GTTTCTTCGC TTAAGTTTGG TGTACATGCT TTTCAATCTG TGTTGCATTT   
  
  
+ TTCTGTGATG TATAAGCCGT TTATTACCTA TTGATGTTCA ACTTTTCACA CTATATGTCC TTTTATTAGT   
  
  
+ TACTGATGCC TCTGTCAAAC ATTCTTAATT GCATTTAGTG TATCAACGGT CTTAAAATTT TAAGCATTGC   
  
  
+ CAACTTGAAC TTCTGAAGAT GTTTGTCAAG ATCTTCAGTC TGCAAGTTAA CTGCTGCTTG CTTGAACTTT   
  
  
+ TCAACTTTTA AGAGAGATGT CAGAGTCTAA GCATGGCTTC CTGACAAGTA CTGCATTTTA TCACTTATGA   
  
  
+ AGACTCAATT CTGGGTTTTG ACAGCTTCTA TGAACCTCTA GTGCAGGTAC AGCAGCTTGG ACATTAAGCT   
  
  
+ GCAAGTCAAT TGACTGTAGA ACGACAAGTT GTTGAAGTTT CTGCTGAAAG TTCATCACTT GTTTGATGAA   
  
  
+ TAAAGTACAG CATGGCACTT CAGATGCAAA AGTCTCGCGA TCAGTAGAGA TGATTCCATA TTTCTCATCT   
  
  
+ CATTTCCAAG TCTTTGACAA CATGTACCCA AATAATGCCA GCCATGACAC TCAGATGTCT CTCCAATCAT   
  
  
+ ACAGCGAAGG ATACTTCACT CTGGACTCAT CTCCAGCAGC AATCGGAGCG TGCAGTGTCT ATGACTACCC   
  
  
+ ATCCGTTGTC AGCACCTCTT CAAATAGAAG CCAGTTTTCT CCTCAGGGTT CCCACTCGTA CATCTCAGAC   
  
  
+ CCCCATCATT CTTCCGACAA CTATGGATCC CCAGTGAGCG GCTCTTCTGT GGTTGACGAT AATGCTGAGC   
  
  
+ TGAGGAACAG GTTCAGTGAT ATGGAGCTCC CCTTGCCACA GGACTCAGGA CACCATTATT GCTCTTTTAG   
  
  
+ CCACAGAGGA AGCCATGAAG GCTCCTATAC TTTGAGGCCA AACCAACTGA TGGATATGGC CAACATGGAG   
  
  
+ TTAAAGCAGG TGCTATACTT CTGTGCAGAA GCAATCTCAG AGAATAATCT ATCAACTGCA GAAAGACTAA   
  
  
+ TGGATGCATT GGGTAAGAGG GTGTCTGTTT TTGGTTCACC AATTGAAAGG TTGGCCGCCT ACATGTTGGA   
  
  
+ AGGGCTCAGA GCAAGGCTGG AGTTTTCTGG ATATACTATC TACAAAAAGC TCAGGTGCGA ACAGCCAACG   
  
  
+ AGCTCAGAGC TTCTTTCCTA CATGCACATC CTGTATCAAA GTTGCCCATA TTTCAAATTC GCATATATGT   
  
  
+ CCTCAAATGT TGCTATTCAA GAAGCTTTGG GGAATGAGCC GGTTATCCAC ATCATCGATT TCCAGATTGC   
  
  
+ CATGGGGACA CAATTGGTGC TCTTGATCCA GTCTCTCGCC CATCGGCCTG GCGGGCCCCC CCCTCGTTCG   
  
  
+ GATCACTGGG GTCGATGATC CACACTCGGC CTATGCTCGT GGCGGGGGGC TCGAGGTTGT GGGGCAGAGG   
  
  
+ CTAACAAAGG TGGCTGAATC ATATGGAGTC CCATTCGAGT TTCATGCTGC AGCCATGTCA GGGTGCAAGG   
  
  
+ TGAATCGCGA TGTCCTCAAG GTTCGCCCTG GGGAAGAAGC CTTGGCCGTA AACTTCCCCT ATATGTTGCA   
  
  
+ TCACATGCCC GACGAGAGCG TGAGCACCAC AAATCATCGG GACCGGCTCT TGAGGCTGGT GAAGAGGTTG   
  
  
+ TCACCAAGGA TCGTCACCTT GGTTGAGCAA GAGTCCAACA CAAACACTCC TCCCTTTCTT CAACGGTTCC   
  
  
+ GTGAAACATT GGACTACTAC ACTGCCATGT TCGAGTCCAT TGATGTGGCT CTACCTAGGG ATGACAAGAA   
  
  
+ GAGGATAAAT GCCGAGCAGC ATTGTCTTGC GCGCGATATT GTGAACATGG TCGCTTGTGA GAATGCGGAA   
  
  
+ AGGGTTGAGA GGCACGAGCC CTTCGGGAAA TGGAGGGCAA GGTTTGATAT GGCTGGGTTT CAGCAATTGC   
  
  
+ CCTCGAGCCG CTCAGTGAAT GATGCAATAA GGGGTTTGAT GCGAGAATTC CATAGGAACT ATCGGGTGCA   
  
  
+ GGATTGTCAG GGTGCCCTTT TTTTGGGCTG GAAGGAGCGC AATCTGGCAA CCTTCTCTAC ATGGTGCTGT   
  
  
+ AAAGAATG  

- -Up\_Stream \_Len000CAATTT TTTGAGTCTT GATAATATCT AAAAGACAAA AGACAAACCT AACTCTAGGC   
  
  
- TTCGGAAACT CTAGGGGACA AAGATCGAAG GTTGGGGGGA GGGACCCAAG AAAAAGAAAC CACACTTTCA   
  
  
- TACTTGTTTT CGACTACATT CACACTAAGT TTGAGATCGG AACCACTCTG AGAACCCTTC CCGAGGATCT   
  
  
- CTGAAACGGT TAACTCGATT AAACTACGGA TGATGAACGA ATGAAAAGAT CGACAACAAA TTTGCTAAAT   
  
  
- ACAAGTATCA ATTCCTCGCG GTATAACGTA ATGTTATAGA CGAAATCACG AAACAAAAAA CGTAAAAGAA   
  
  
- GAAATAATGA TACCAAAAAC TTCTTCCGAA TGACTGACTA TGACCAAAGA AGCGAATTCA AACCACATGT   
  
  
- ACGAAAAGTT AGACACAACG TAAAAAGATA CTACATATTC GGCAAATAAT GGATAACTAC AAGTTGAAAA   
  
  
- GTGTGATATA TAGGAAATAC CAAAGAAGCG AATTCAAACC ACATGTACGA AAAGTTAGAC ACAACGTAAA   
  
  
- AAGACACTAC ATATTCGGCA AATAATGGAT AACTACAAGT TGAAAAGTGT GATATACAGG AAAATAATCA   
  
  
- ATGACTACGG AGACAGTTTG TAAGAATTAA CGTAAATCAC ATAGTTGCCA GAATTTTAAA ATTCGTAACG   
  
  
- GTTGAACTTG AAGACTTCTA CAAACAGTTC TAGAAGTCAG ACGTTCAATT GACGACGAAC GAACTTGAAA   
  
  
- AGTTGAAAAT TCTCTCTACA GTCTCAGATT CGTACCGAAG GACTGTTCAT GACGTAAAAT AGTGAATACT   
  
  
- TCTGAGTTAA GACCCAAAAC TGTCGAAGAT ACTTGGAGAT CACGTCCATG TCGTCGAACC TGTAATTCGA   
  
  
- CGTTCAGTTA ACTGACATCT TGCTGTTCAA CAACTTCAAA GACGACTTTC AAGTAGTGAA CAAACTACTT   
  
  
- ATTTCATGTC GTACCGTGAA GTCTACGTTT TCAGAGCGCT AGTCATCTCT ACTAAGGTAT AAAGAGTAGA   
  
  
- GTAAAGGTTC AGAAACTGTT GTACATGGGT TTATTACGGT CGGTACTGTG AGTCTACAGA GAGGTTAGTA   
  
  
- TGTCGCTTCC TATGAAGTGA GACCTGAGTA GAGGTCGTCG TTAGCCTCGC ACGTCACAGA TACTGATGGG   
  
  
- TAGGCAACAG TCGTGGAGAA GTTTATCTTC GGTCAAAAGA GGAGTCCCAA GGGTGAGCAT GTAGAGTCTG   
  
  
- GGGGTAGTAA GAAGGCTGTT GATACCTAGG GGTCACTCGC CGAGAAGACA CCAACTGCTA TTACGACTCG   
  
  
- ACTCCTTGTC CAAGTCACTA TACCTCGAGG GGAACGGTGT CCTGAGTCCT GTGGTAATAA CGAGAAAATC   
  
  
- GGTGTCTCCT TCGGTACTTC CGAGGATATG AAACTCCGGT TTGGTTGACT ACCTATACCG GTTGTACCTC   
  
  
- AATTTCGTCC ACGATATGAA GACACGTCTT CGTTAGAGTC TCTTATTAGA TAGTTGACGT CTTTCTGATT   
  
  
- ACCTACGTAA CCCATTCTCC CACAGACAAA AACCAAGTGG TTAACTTTCC AACCGGCGGA TGTACAACCT   
  
  
- TCCCGAGTCT CGTTCCGACC TCAAAAGACC TATATGATAG ATGTTTTTCG AGTCCACGCT TGTCGGTTGC   
  
  
- TCGAGTCTCG AAGAAAGGAT GTACGTGTAG GACATAGTTT CAACGGGTAT AAAGTTTAAG CGTATATACA   
  
  
- GGAGTTTACA ACGATAAGTT CTTCGAAACC CCTTACTCGG CCAATAGGTG TAGTAGCTAA AGGTCTAACG   
  
  
- GTACCCCTGT GTTAACCACG AGAACTAGGT CAGAGAGCGG GTAGCCGGAC CGCCCGGGGG GGGAGCAAGC   
  
  
- CTAGTGACCC CAGCTACTAG GTGTGAGCCG GATACGAGCA CCGCCCCCCG AGCTCCAACA CCCCGTCTCC   
  
  
- GATTGTTTCC ACCGACTTAG TATACCTCAG GGTAAGCTCA AAGTACGACG TCGGTACAGT CCCACGTTCC   
  
  
- ACTTAGCGCT ACAGGAGTTC CAAGCGGGAC CCCTTCTTCG GAACCGGCAT TTGAAGGGGA TATACAACGT   
  
  
- AGTGTACGGG CTGCTCTCGC ACTCGTGGTG TTTAGTAGCC CTGGCCGAGA ACTCCGACCA CTTCTCCAAC   
  
  
- AGTGGTTCCT AGCAGTGGAA CCAACTCGTT CTCAGGTTGT GTTTGTGAGG AGGGAAAGAA GTTGCCAAGG   
  
  
- CACTTTGTAA CCTGATGATG TGACGGTACA AGCTCAGGTA ACTACACCGA GATGGATCCC TACTGTTCTT   
  
  
- CTCCTATTTA CGGCTCGTCG TAACAGAACG CGCGCTATAA CACTTGTACC AGCGAACACT CTTACGCCTT   
  
  
- TCCCAACTCT CCGTGCTCGG GAAGCCCTTT ACCTCCCGTT CCAAACTATA CCGACCCAAA GTCGTTAACG   
  
  
- GGAGCTCGGC GAGTCACTTA CTACGTTATT CCCCAAACTA CGCTCTTAAG GTATCCTTGA TAGCCCACGT   
  
  
- CCTAACAGTC CCACGGGAAA AAAACCCGAC CTTCCTCGCG TTAGACCGTT GGAAGAGATG TACCACGACA   
  
  
- TTTCTTAC

+     Unnamed\_\_1

| Site Name | Organism | Position | Strand | Matrix score. | sequence | function |
| --- | --- | --- | --- | --- | --- | --- |
| Unnamed\_\_1 | Zea mays | 1932 | + | 5 | CGTGG |  |

>HU08G02295.1   
+ -Up\_Stream \_Len000GTTAAA AAACTCAGAA CTATTATAGA TTTTCTGTTT TCTGTTTGGA TTGAGATCCG   
  
  
+ AAGCCTTTGA GATCCCCTGT TTCTAGCTTC CAACCCCCCT CCCTGGGTTC TTTTTCTTTG GTGTGAAAGT   
  
  
+ ATGAACAAAA GCTGATGTAA GTGTGATTCA AACTCTAGCC TTGGTGAGAC TCTTGGGAAG GGCTCCTAGA   
  
  
+ GACTTTGCCA ATTGAGCTAA TTTGATGCCT ACTACTTGCT TACTTTTCTA GCTGTTGTTT AAACGATTTA   
  
  
+ TGTTCATAGT TAAGGAGCGC CATATTGCAT TACAATATCT GCTTTAGTGC TTTGTTTTTT GCATTTTCTT   
  
  
+ CTTTATTACT ATGGTTTTTG AAGAAGGCTT ACTGACTGAT ACTGGTTTCT TCGCTTAAGT TTGGTGTACA   
  
  
+ TGCTTTTCAA TCTGTGTTGC ATTTTTCTAT GATGTATAAG CCGTTTATTA CCTATTGATG TTCAACTTTT   
  
  
+ CACACTATAT ATCCTTTATG GTTTCTTCGC TTAAGTTTGG TGTACATGCT TTTCAATCTG TGTTGCATTT   
  
  
+ TTCTGTGATG TATAAGCCGT TTATTACCTA TTGATGTTCA ACTTTTCACA CTATATGTCC TTTTATTAGT   
  
  
+ TACTGATGCC TCTGTCAAAC ATTCTTAATT GCATTTAGTG TATCAACGGT CTTAAAATTT TAAGCATTGC   
  
  
+ CAACTTGAAC TTCTGAAGAT GTTTGTCAAG ATCTTCAGTC TGCAAGTTAA CTGCTGCTTG CTTGAACTTT   
  
  
+ TCAACTTTTA AGAGAGATGT CAGAGTCTAA GCATGGCTTC CTGACAAGTA CTGCATTTTA TCACTTATGA   
  
  
+ AGACTCAATT CTGGGTTTTG ACAGCTTCTA TGAACCTCTA GTGCAGGTAC AGCAGCTTGG ACATTAAGCT   
  
  
+ GCAAGTCAAT TGACTGTAGA ACGACAAGTT GTTGAAGTTT CTGCTGAAAG TTCATCACTT GTTTGATGAA   
  
  
+ TAAAGTACAG CATGGCACTT CAGATGCAAA AGTCTCGCGA TCAGTAGAGA TGATTCCATA TTTCTCATCT   
  
  
+ CATTTCCAAG TCTTTGACAA CATGTACCCA AATAATGCCA GCCATGACAC TCAGATGTCT CTCCAATCAT   
  
  
+ ACAGCGAAGG ATACTTCACT CTGGACTCAT CTCCAGCAGC AATCGGAGCG TGCAGTGTCT ATGACTACCC   
  
  
+ ATCCGTTGTC AGCACCTCTT CAAATAGAAG CCAGTTTTCT CCTCAGGGTT CCCACTCGTA CATCTCAGAC   
  
  
+ CCCCATCATT CTTCCGACAA CTATGGATCC CCAGTGAGCG GCTCTTCTGT GGTTGACGAT AATGCTGAGC   
  
  
+ TGAGGAACAG GTTCAGTGAT ATGGAGCTCC CCTTGCCACA GGACTCAGGA CACCATTATT GCTCTTTTAG   
  
  
+ CCACAGAGGA AGCCATGAAG GCTCCTATAC TTTGAGGCCA AACCAACTGA TGGATATGGC CAACATGGAG   
  
  
+ TTAAAGCAGG TGCTATACTT CTGTGCAGAA GCAATCTCAG AGAATAATCT ATCAACTGCA GAAAGACTAA   
  
  
+ TGGATGCATT GGGTAAGAGG GTGTCTGTTT TTGGTTCACC AATTGAAAGG TTGGCCGCCT ACATGTTGGA   
  
  
+ AGGGCTCAGA GCAAGGCTGG AGTTTTCTGG ATATACTATC TACAAAAAGC TCAGGTGCGA ACAGCCAACG   
  
  
+ AGCTCAGAGC TTCTTTCCTA CATGCACATC CTGTATCAAA GTTGCCCATA TTTCAAATTC GCATATATGT   
  
  
+ CCTCAAATGT TGCTATTCAA GAAGCTTTGG GGAATGAGCC GGTTATCCAC ATCATCGATT TCCAGATTGC   
  
  
+ CATGGGGACA CAATTGGTGC TCTTGATCCA GTCTCTCGCC CATCGGCCTG GCGGGCCCCC CCCTCGTTCG   
  
  
+ GATCACTGGG GTCGATGATC CACACTCGGC CTATGCTCGT GGCGGGGGGC TCGAGGTTGT GGGGCAGAGG   
  
  
+ CTAACAAAGG TGGCTGAATC ATATGGAGTC CCATTCGAGT TTCATGCTGC AGCCATGTCA GGGTGCAAGG   
  
  
+ TGAATCGCGA TGTCCTCAAG GTTCGCCCTG GGGAAGAAGC CTTGGCCGTA AACTTCCCCT ATATGTTGCA   
  
  
+ TCACATGCCC GACGAGAGCG TGAGCACCAC AAATCATCGG GACCGGCTCT TGAGGCTGGT GAAGAGGTTG   
  
  
+ TCACCAAGGA TCGTCACCTT GGTTGAGCAA GAGTCCAACA CAAACACTCC TCCCTTTCTT CAACGGTTCC   
  
  
+ GTGAAACATT GGACTACTAC ACTGCCATGT TCGAGTCCAT TGATGTGGCT CTACCTAGGG ATGACAAGAA   
  
  
+ GAGGATAAAT GCCGAGCAGC ATTGTCTTGC GCGCGATATT GTGAACATGG TCGCTTGTGA GAATGCGGAA   
  
  
+ AGGGTTGAGA GGCACGAGCC CTTCGGGAAA TGGAGGGCAA GGTTTGATAT GGCTGGGTTT CAGCAATTGC   
  
  
+ CCTCGAGCCG CTCAGTGAAT GATGCAATAA GGGGTTTGAT GCGAGAATTC CATAGGAACT ATCGGGTGCA   
  
  
+ GGATTGTCAG GGTGCCCTTT TTTTGGGCTG GAAGGAGCGC AATCTGGCAA CCTTCTCTAC ATGGTGCTGT   
  
  
+ AAAGAATG  

- -Up\_Stream \_Len000CAATTT TTTGAGTCTT GATAATATCT AAAAGACAAA AGACAAACCT AACTCTAGGC   
  
  
- TTCGGAAACT CTAGGGGACA AAGATCGAAG GTTGGGGGGA GGGACCCAAG AAAAAGAAAC CACACTTTCA   
  
  
- TACTTGTTTT CGACTACATT CACACTAAGT TTGAGATCGG AACCACTCTG AGAACCCTTC CCGAGGATCT   
  
  
- CTGAAACGGT TAACTCGATT AAACTACGGA TGATGAACGA ATGAAAAGAT CGACAACAAA TTTGCTAAAT   
  
  
- ACAAGTATCA ATTCCTCGCG GTATAACGTA ATGTTATAGA CGAAATCACG AAACAAAAAA CGTAAAAGAA   
  
  
- GAAATAATGA TACCAAAAAC TTCTTCCGAA TGACTGACTA TGACCAAAGA AGCGAATTCA AACCACATGT   
  
  
- ACGAAAAGTT AGACACAACG TAAAAAGATA CTACATATTC GGCAAATAAT GGATAACTAC AAGTTGAAAA   
  
  
- GTGTGATATA TAGGAAATAC CAAAGAAGCG AATTCAAACC ACATGTACGA AAAGTTAGAC ACAACGTAAA   
  
  
- AAGACACTAC ATATTCGGCA AATAATGGAT AACTACAAGT TGAAAAGTGT GATATACAGG AAAATAATCA   
  
  
- ATGACTACGG AGACAGTTTG TAAGAATTAA CGTAAATCAC ATAGTTGCCA GAATTTTAAA ATTCGTAACG   
  
  
- GTTGAACTTG AAGACTTCTA CAAACAGTTC TAGAAGTCAG ACGTTCAATT GACGACGAAC GAACTTGAAA   
  
  
- AGTTGAAAAT TCTCTCTACA GTCTCAGATT CGTACCGAAG GACTGTTCAT GACGTAAAAT AGTGAATACT   
  
  
- TCTGAGTTAA GACCCAAAAC TGTCGAAGAT ACTTGGAGAT CACGTCCATG TCGTCGAACC TGTAATTCGA   
  
  
- CGTTCAGTTA ACTGACATCT TGCTGTTCAA CAACTTCAAA GACGACTTTC AAGTAGTGAA CAAACTACTT   
  
  
- ATTTCATGTC GTACCGTGAA GTCTACGTTT TCAGAGCGCT AGTCATCTCT ACTAAGGTAT AAAGAGTAGA   
  
  
- GTAAAGGTTC AGAAACTGTT GTACATGGGT TTATTACGGT CGGTACTGTG AGTCTACAGA GAGGTTAGTA   
  
  
- TGTCGCTTCC TATGAAGTGA GACCTGAGTA GAGGTCGTCG TTAGCCTCGC ACGTCACAGA TACTGATGGG   
  
  
- TAGGCAACAG TCGTGGAGAA GTTTATCTTC GGTCAAAAGA GGAGTCCCAA GGGTGAGCAT GTAGAGTCTG   
  
  
- GGGGTAGTAA GAAGGCTGTT GATACCTAGG GGTCACTCGC CGAGAAGACA CCAACTGCTA TTACGACTCG   
  
  
- ACTCCTTGTC CAAGTCACTA TACCTCGAGG GGAACGGTGT CCTGAGTCCT GTGGTAATAA CGAGAAAATC   
  
  
- GGTGTCTCCT TCGGTACTTC CGAGGATATG AAACTCCGGT TTGGTTGACT ACCTATACCG GTTGTACCTC   
  
  
- AATTTCGTCC ACGATATGAA GACACGTCTT CGTTAGAGTC TCTTATTAGA TAGTTGACGT CTTTCTGATT   
  
  
- ACCTACGTAA CCCATTCTCC CACAGACAAA AACCAAGTGG TTAACTTTCC AACCGGCGGA TGTACAACCT   
  
  
- TCCCGAGTCT CGTTCCGACC TCAAAAGACC TATATGATAG ATGTTTTTCG AGTCCACGCT TGTCGGTTGC   
  
  
- TCGAGTCTCG AAGAAAGGAT GTACGTGTAG GACATAGTTT CAACGGGTAT AAAGTTTAAG CGTATATACA   
  
  
- GGAGTTTACA ACGATAAGTT CTTCGAAACC CCTTACTCGG CCAATAGGTG TAGTAGCTAA AGGTCTAACG   
  
  
- GTACCCCTGT GTTAACCACG AGAACTAGGT CAGAGAGCGG GTAGCCGGAC CGCCCGGGGG GGGAGCAAGC   
  
  
- CTAGTGACCC CAGCTACTAG GTGTGAGCCG GATACGAGCA CCGCCCCCCG AGCTCCAACA CCCCGTCTCC   
  
  
- GATTGTTTCC ACCGACTTAG TATACCTCAG GGTAAGCTCA AAGTACGACG TCGGTACAGT CCCACGTTCC   
  
  
- ACTTAGCGCT ACAGGAGTTC CAAGCGGGAC CCCTTCTTCG GAACCGGCAT TTGAAGGGGA TATACAACGT   
  
  
- AGTGTACGGG CTGCTCTCGC ACTCGTGGTG TTTAGTAGCC CTGGCCGAGA ACTCCGACCA CTTCTCCAAC   
  
  
- AGTGGTTCCT AGCAGTGGAA CCAACTCGTT CTCAGGTTGT GTTTGTGAGG AGGGAAAGAA GTTGCCAAGG   
  
  
- CACTTTGTAA CCTGATGATG TGACGGTACA AGCTCAGGTA ACTACACCGA GATGGATCCC TACTGTTCTT   
  
  
- CTCCTATTTA CGGCTCGTCG TAACAGAACG CGCGCTATAA CACTTGTACC AGCGAACACT CTTACGCCTT   
  
  
- TCCCAACTCT CCGTGCTCGG GAAGCCCTTT ACCTCCCGTT CCAAACTATA CCGACCCAAA GTCGTTAACG   
  
  
- GGAGCTCGGC GAGTCACTTA CTACGTTATT CCCCAAACTA CGCTCTTAAG GTATCCTTGA TAGCCCACGT   
  
  
- CCTAACAGTC CCACGGGAAA AAAACCCGAC CTTCCTCGCG TTAGACCGTT GGAAGAGATG TACCACGACA   
  
  
- TTTCTTAC

+     Unnamed\_\_4

| Site Name | Organism | Position | Strand | Matrix score. | sequence | function |
| --- | --- | --- | --- | --- | --- | --- |
| Unnamed\_\_4 | Petroselinum hortense | 1233 | + | 4 | CTCC |  |
| Unnamed\_\_4 | Petroselinum hortense | 1169 | - | 4 | CTCC |  |
| Unnamed\_\_4 | Petroselinum hortense | 1989 | - | 4 | CTCC |  |
| Unnamed\_\_4 | Petroselinum hortense | 1357 | - | 4 | CTCC |  |
| Unnamed\_\_4 | Petroselinum hortense | 1155 | + | 4 | CTCC |  |
| Unnamed\_\_4 | Petroselinum hortense | 1361 | + | 4 | CTCC |  |
| Unnamed\_\_4 | Petroselinum hortense | 1426 | + | 4 | CTCC |  |
| Unnamed\_\_4 | Petroselinum hortense | 1115 | + | 4 | CTCC |  |
| Unnamed\_\_4 | Petroselinum hortense | 2558 | - | 4 | CTCC |  |
| Unnamed\_\_4 | Petroselinum hortense | 2224 | + | 4 | CTCC |  |
| Unnamed\_\_4 | Petroselinum hortense | 1633 | - | 4 | CTCC |  |
| Unnamed\_\_4 | Petroselinum hortense | 1471 | - | 4 | CTCC |  |
| Unnamed\_\_4 | Petroselinum hortense | 298 | - | 4 | CTCC |  |
| Unnamed\_\_4 | Petroselinum hortense | 207 | + | 4 | CTCC |  |
| Unnamed\_\_4 | Petroselinum hortense | 113 | + | 4 | CTCC |  |
| Unnamed\_\_4 | Petroselinum hortense | 2416 | - | 4 | CTCC |  |
| Unnamed\_\_4 | Petroselinum hortense | 2221 | + | 4 | CTCC |  |

>HU08G02295.1   
+ -Up\_Stream \_Len000GTTAAA AAACTCAGAA CTATTATAGA TTTTCTGTTT TCTGTTTGGA TTGAGATCCG   
  
  
+ AAGCCTTTGA GATCCCCTGT TTCTAGCTTC CAACCCCCCT CCCTGGGTTC TTTTTCTTTG GTGTGAAAGT   
  
  
+ ATGAACAAAA GCTGATGTAA GTGTGATTCA AACTCTAGCC TTGGTGAGAC TCTTGGGAAG GGCTCCTAGA   
  
  
+ GACTTTGCCA ATTGAGCTAA TTTGATGCCT ACTACTTGCT TACTTTTCTA GCTGTTGTTT AAACGATTTA   
  
  
+ TGTTCATAGT TAAGGAGCGC CATATTGCAT TACAATATCT GCTTTAGTGC TTTGTTTTTT GCATTTTCTT   
  
  
+ CTTTATTACT ATGGTTTTTG AAGAAGGCTT ACTGACTGAT ACTGGTTTCT TCGCTTAAGT TTGGTGTACA   
  
  
+ TGCTTTTCAA TCTGTGTTGC ATTTTTCTAT GATGTATAAG CCGTTTATTA CCTATTGATG TTCAACTTTT   
  
  
+ CACACTATAT ATCCTTTATG GTTTCTTCGC TTAAGTTTGG TGTACATGCT TTTCAATCTG TGTTGCATTT   
  
  
+ TTCTGTGATG TATAAGCCGT TTATTACCTA TTGATGTTCA ACTTTTCACA CTATATGTCC TTTTATTAGT   
  
  
+ TACTGATGCC TCTGTCAAAC ATTCTTAATT GCATTTAGTG TATCAACGGT CTTAAAATTT TAAGCATTGC   
  
  
+ CAACTTGAAC TTCTGAAGAT GTTTGTCAAG ATCTTCAGTC TGCAAGTTAA CTGCTGCTTG CTTGAACTTT   
  
  
+ TCAACTTTTA AGAGAGATGT CAGAGTCTAA GCATGGCTTC CTGACAAGTA CTGCATTTTA TCACTTATGA   
  
  
+ AGACTCAATT CTGGGTTTTG ACAGCTTCTA TGAACCTCTA GTGCAGGTAC AGCAGCTTGG ACATTAAGCT   
  
  
+ GCAAGTCAAT TGACTGTAGA ACGACAAGTT GTTGAAGTTT CTGCTGAAAG TTCATCACTT GTTTGATGAA   
  
  
+ TAAAGTACAG CATGGCACTT CAGATGCAAA AGTCTCGCGA TCAGTAGAGA TGATTCCATA TTTCTCATCT   
  
  
+ CATTTCCAAG TCTTTGACAA CATGTACCCA AATAATGCCA GCCATGACAC TCAGATGTCT CTCCAATCAT   
  
  
+ ACAGCGAAGG ATACTTCACT CTGGACTCAT CTCCAGCAGC AATCGGAGCG TGCAGTGTCT ATGACTACCC   
  
  
+ ATCCGTTGTC AGCACCTCTT CAAATAGAAG CCAGTTTTCT CCTCAGGGTT CCCACTCGTA CATCTCAGAC   
  
  
+ CCCCATCATT CTTCCGACAA CTATGGATCC CCAGTGAGCG GCTCTTCTGT GGTTGACGAT AATGCTGAGC   
  
  
+ TGAGGAACAG GTTCAGTGAT ATGGAGCTCC CCTTGCCACA GGACTCAGGA CACCATTATT GCTCTTTTAG   
  
  
+ CCACAGAGGA AGCCATGAAG GCTCCTATAC TTTGAGGCCA AACCAACTGA TGGATATGGC CAACATGGAG   
  
  
+ TTAAAGCAGG TGCTATACTT CTGTGCAGAA GCAATCTCAG AGAATAATCT ATCAACTGCA GAAAGACTAA   
  
  
+ TGGATGCATT GGGTAAGAGG GTGTCTGTTT TTGGTTCACC AATTGAAAGG TTGGCCGCCT ACATGTTGGA   
  
  
+ AGGGCTCAGA GCAAGGCTGG AGTTTTCTGG ATATACTATC TACAAAAAGC TCAGGTGCGA ACAGCCAACG   
  
  
+ AGCTCAGAGC TTCTTTCCTA CATGCACATC CTGTATCAAA GTTGCCCATA TTTCAAATTC GCATATATGT   
  
  
+ CCTCAAATGT TGCTATTCAA GAAGCTTTGG GGAATGAGCC GGTTATCCAC ATCATCGATT TCCAGATTGC   
  
  
+ CATGGGGACA CAATTGGTGC TCTTGATCCA GTCTCTCGCC CATCGGCCTG GCGGGCCCCC CCCTCGTTCG   
  
  
+ GATCACTGGG GTCGATGATC CACACTCGGC CTATGCTCGT GGCGGGGGGC TCGAGGTTGT GGGGCAGAGG   
  
  
+ CTAACAAAGG TGGCTGAATC ATATGGAGTC CCATTCGAGT TTCATGCTGC AGCCATGTCA GGGTGCAAGG   
  
  
+ TGAATCGCGA TGTCCTCAAG GTTCGCCCTG GGGAAGAAGC CTTGGCCGTA AACTTCCCCT ATATGTTGCA   
  
  
+ TCACATGCCC GACGAGAGCG TGAGCACCAC AAATCATCGG GACCGGCTCT TGAGGCTGGT GAAGAGGTTG   
  
  
+ TCACCAAGGA TCGTCACCTT GGTTGAGCAA GAGTCCAACA CAAACACTCC TCCCTTTCTT CAACGGTTCC   
  
  
+ GTGAAACATT GGACTACTAC ACTGCCATGT TCGAGTCCAT TGATGTGGCT CTACCTAGGG ATGACAAGAA   
  
  
+ GAGGATAAAT GCCGAGCAGC ATTGTCTTGC GCGCGATATT GTGAACATGG TCGCTTGTGA GAATGCGGAA   
  
  
+ AGGGTTGAGA GGCACGAGCC CTTCGGGAAA TGGAGGGCAA GGTTTGATAT GGCTGGGTTT CAGCAATTGC   
  
  
+ CCTCGAGCCG CTCAGTGAAT GATGCAATAA GGGGTTTGAT GCGAGAATTC CATAGGAACT ATCGGGTGCA   
  
  
+ GGATTGTCAG GGTGCCCTTT TTTTGGGCTG GAAGGAGCGC AATCTGGCAA CCTTCTCTAC ATGGTGCTGT   
  
  
+ AAAGAATG  

- -Up\_Stream \_Len000CAATTT TTTGAGTCTT GATAATATCT AAAAGACAAA AGACAAACCT AACTCTAGGC   
  
  
- TTCGGAAACT CTAGGGGACA AAGATCGAAG GTTGGGGGGA GGGACCCAAG AAAAAGAAAC CACACTTTCA   
  
  
- TACTTGTTTT CGACTACATT CACACTAAGT TTGAGATCGG AACCACTCTG AGAACCCTTC CCGAGGATCT   
  
  
- CTGAAACGGT TAACTCGATT AAACTACGGA TGATGAACGA ATGAAAAGAT CGACAACAAA TTTGCTAAAT   
  
  
- ACAAGTATCA ATTCCTCGCG GTATAACGTA ATGTTATAGA CGAAATCACG AAACAAAAAA CGTAAAAGAA   
  
  
- GAAATAATGA TACCAAAAAC TTCTTCCGAA TGACTGACTA TGACCAAAGA AGCGAATTCA AACCACATGT   
  
  
- ACGAAAAGTT AGACACAACG TAAAAAGATA CTACATATTC GGCAAATAAT GGATAACTAC AAGTTGAAAA   
  
  
- GTGTGATATA TAGGAAATAC CAAAGAAGCG AATTCAAACC ACATGTACGA AAAGTTAGAC ACAACGTAAA   
  
  
- AAGACACTAC ATATTCGGCA AATAATGGAT AACTACAAGT TGAAAAGTGT GATATACAGG AAAATAATCA   
  
  
- ATGACTACGG AGACAGTTTG TAAGAATTAA CGTAAATCAC ATAGTTGCCA GAATTTTAAA ATTCGTAACG   
  
  
- GTTGAACTTG AAGACTTCTA CAAACAGTTC TAGAAGTCAG ACGTTCAATT GACGACGAAC GAACTTGAAA   
  
  
- AGTTGAAAAT TCTCTCTACA GTCTCAGATT CGTACCGAAG GACTGTTCAT GACGTAAAAT AGTGAATACT   
  
  
- TCTGAGTTAA GACCCAAAAC TGTCGAAGAT ACTTGGAGAT CACGTCCATG TCGTCGAACC TGTAATTCGA   
  
  
- CGTTCAGTTA ACTGACATCT TGCTGTTCAA CAACTTCAAA GACGACTTTC AAGTAGTGAA CAAACTACTT   
  
  
- ATTTCATGTC GTACCGTGAA GTCTACGTTT TCAGAGCGCT AGTCATCTCT ACTAAGGTAT AAAGAGTAGA   
  
  
- GTAAAGGTTC AGAAACTGTT GTACATGGGT TTATTACGGT CGGTACTGTG AGTCTACAGA GAGGTTAGTA   
  
  
- TGTCGCTTCC TATGAAGTGA GACCTGAGTA GAGGTCGTCG TTAGCCTCGC ACGTCACAGA TACTGATGGG   
  
  
- TAGGCAACAG TCGTGGAGAA GTTTATCTTC GGTCAAAAGA GGAGTCCCAA GGGTGAGCAT GTAGAGTCTG   
  
  
- GGGGTAGTAA GAAGGCTGTT GATACCTAGG GGTCACTCGC CGAGAAGACA CCAACTGCTA TTACGACTCG   
  
  
- ACTCCTTGTC CAAGTCACTA TACCTCGAGG GGAACGGTGT CCTGAGTCCT GTGGTAATAA CGAGAAAATC   
  
  
- GGTGTCTCCT TCGGTACTTC CGAGGATATG AAACTCCGGT TTGGTTGACT ACCTATACCG GTTGTACCTC   
  
  
- AATTTCGTCC ACGATATGAA GACACGTCTT CGTTAGAGTC TCTTATTAGA TAGTTGACGT CTTTCTGATT   
  
  
- ACCTACGTAA CCCATTCTCC CACAGACAAA AACCAAGTGG TTAACTTTCC AACCGGCGGA TGTACAACCT   
  
  
- TCCCGAGTCT CGTTCCGACC TCAAAAGACC TATATGATAG ATGTTTTTCG AGTCCACGCT TGTCGGTTGC   
  
  
- TCGAGTCTCG AAGAAAGGAT GTACGTGTAG GACATAGTTT CAACGGGTAT AAAGTTTAAG CGTATATACA   
  
  
- GGAGTTTACA ACGATAAGTT CTTCGAAACC CCTTACTCGG CCAATAGGTG TAGTAGCTAA AGGTCTAACG   
  
  
- GTACCCCTGT GTTAACCACG AGAACTAGGT CAGAGAGCGG GTAGCCGGAC CGCCCGGGGG GGGAGCAAGC   
  
  
- CTAGTGACCC CAGCTACTAG GTGTGAGCCG GATACGAGCA CCGCCCCCCG AGCTCCAACA CCCCGTCTCC   
  
  
- GATTGTTTCC ACCGACTTAG TATACCTCAG GGTAAGCTCA AAGTACGACG TCGGTACAGT CCCACGTTCC   
  
  
- ACTTAGCGCT ACAGGAGTTC CAAGCGGGAC CCCTTCTTCG GAACCGGCAT TTGAAGGGGA TATACAACGT   
  
  
- AGTGTACGGG CTGCTCTCGC ACTCGTGGTG TTTAGTAGCC CTGGCCGAGA ACTCCGACCA CTTCTCCAAC   
  
  
- AGTGGTTCCT AGCAGTGGAA CCAACTCGTT CTCAGGTTGT GTTTGTGAGG AGGGAAAGAA GTTGCCAAGG   
  
  
- CACTTTGTAA CCTGATGATG TGACGGTACA AGCTCAGGTA ACTACACCGA GATGGATCCC TACTGTTCTT   
  
  
- CTCCTATTTA CGGCTCGTCG TAACAGAACG CGCGCTATAA CACTTGTACC AGCGAACACT CTTACGCCTT   
  
  
- TCCCAACTCT CCGTGCTCGG GAAGCCCTTT ACCTCCCGTT CCAAACTATA CCGACCCAAA GTCGTTAACG   
  
  
- GGAGCTCGGC GAGTCACTTA CTACGTTATT CCCCAAACTA CGCTCTTAAG GTATCCTTGA TAGCCCACGT   
  
  
- CCTAACAGTC CCACGGGAAA AAAACCCGAC CTTCCTCGCG TTAGACCGTT GGAAGAGATG TACCACGACA   
  
  
- TTTCTTAC

+     WRE3

| Site Name | Organism | Position | Strand | Matrix score. | sequence | function |
| --- | --- | --- | --- | --- | --- | --- |
| WRE3 | Pisum sativum | 1972 | - | 6 | CCACCT |  |

>HU08G02295.1   
+ -Up\_Stream \_Len000GTTAAA AAACTCAGAA CTATTATAGA TTTTCTGTTT TCTGTTTGGA TTGAGATCCG   
  
  
+ AAGCCTTTGA GATCCCCTGT TTCTAGCTTC CAACCCCCCT CCCTGGGTTC TTTTTCTTTG GTGTGAAAGT   
  
  
+ ATGAACAAAA GCTGATGTAA GTGTGATTCA AACTCTAGCC TTGGTGAGAC TCTTGGGAAG GGCTCCTAGA   
  
  
+ GACTTTGCCA ATTGAGCTAA TTTGATGCCT ACTACTTGCT TACTTTTCTA GCTGTTGTTT AAACGATTTA   
  
  
+ TGTTCATAGT TAAGGAGCGC CATATTGCAT TACAATATCT GCTTTAGTGC TTTGTTTTTT GCATTTTCTT   
  
  
+ CTTTATTACT ATGGTTTTTG AAGAAGGCTT ACTGACTGAT ACTGGTTTCT TCGCTTAAGT TTGGTGTACA   
  
  
+ TGCTTTTCAA TCTGTGTTGC ATTTTTCTAT GATGTATAAG CCGTTTATTA CCTATTGATG TTCAACTTTT   
  
  
+ CACACTATAT ATCCTTTATG GTTTCTTCGC TTAAGTTTGG TGTACATGCT TTTCAATCTG TGTTGCATTT   
  
  
+ TTCTGTGATG TATAAGCCGT TTATTACCTA TTGATGTTCA ACTTTTCACA CTATATGTCC TTTTATTAGT   
  
  
+ TACTGATGCC TCTGTCAAAC ATTCTTAATT GCATTTAGTG TATCAACGGT CTTAAAATTT TAAGCATTGC   
  
  
+ CAACTTGAAC TTCTGAAGAT GTTTGTCAAG ATCTTCAGTC TGCAAGTTAA CTGCTGCTTG CTTGAACTTT   
  
  
+ TCAACTTTTA AGAGAGATGT CAGAGTCTAA GCATGGCTTC CTGACAAGTA CTGCATTTTA TCACTTATGA   
  
  
+ AGACTCAATT CTGGGTTTTG ACAGCTTCTA TGAACCTCTA GTGCAGGTAC AGCAGCTTGG ACATTAAGCT   
  
  
+ GCAAGTCAAT TGACTGTAGA ACGACAAGTT GTTGAAGTTT CTGCTGAAAG TTCATCACTT GTTTGATGAA   
  
  
+ TAAAGTACAG CATGGCACTT CAGATGCAAA AGTCTCGCGA TCAGTAGAGA TGATTCCATA TTTCTCATCT   
  
  
+ CATTTCCAAG TCTTTGACAA CATGTACCCA AATAATGCCA GCCATGACAC TCAGATGTCT CTCCAATCAT   
  
  
+ ACAGCGAAGG ATACTTCACT CTGGACTCAT CTCCAGCAGC AATCGGAGCG TGCAGTGTCT ATGACTACCC   
  
  
+ ATCCGTTGTC AGCACCTCTT CAAATAGAAG CCAGTTTTCT CCTCAGGGTT CCCACTCGTA CATCTCAGAC   
  
  
+ CCCCATCATT CTTCCGACAA CTATGGATCC CCAGTGAGCG GCTCTTCTGT GGTTGACGAT AATGCTGAGC   
  
  
+ TGAGGAACAG GTTCAGTGAT ATGGAGCTCC CCTTGCCACA GGACTCAGGA CACCATTATT GCTCTTTTAG   
  
  
+ CCACAGAGGA AGCCATGAAG GCTCCTATAC TTTGAGGCCA AACCAACTGA TGGATATGGC CAACATGGAG   
  
  
+ TTAAAGCAGG TGCTATACTT CTGTGCAGAA GCAATCTCAG AGAATAATCT ATCAACTGCA GAAAGACTAA   
  
  
+ TGGATGCATT GGGTAAGAGG GTGTCTGTTT TTGGTTCACC AATTGAAAGG TTGGCCGCCT ACATGTTGGA   
  
  
+ AGGGCTCAGA GCAAGGCTGG AGTTTTCTGG ATATACTATC TACAAAAAGC TCAGGTGCGA ACAGCCAACG   
  
  
+ AGCTCAGAGC TTCTTTCCTA CATGCACATC CTGTATCAAA GTTGCCCATA TTTCAAATTC GCATATATGT   
  
  
+ CCTCAAATGT TGCTATTCAA GAAGCTTTGG GGAATGAGCC GGTTATCCAC ATCATCGATT TCCAGATTGC   
  
  
+ CATGGGGACA CAATTGGTGC TCTTGATCCA GTCTCTCGCC CATCGGCCTG GCGGGCCCCC CCCTCGTTCG   
  
  
+ GATCACTGGG GTCGATGATC CACACTCGGC CTATGCTCGT GGCGGGGGGC TCGAGGTTGT GGGGCAGAGG   
  
  
+ CTAACAAAGG TGGCTGAATC ATATGGAGTC CCATTCGAGT TTCATGCTGC AGCCATGTCA GGGTGCAAGG   
  
  
+ TGAATCGCGA TGTCCTCAAG GTTCGCCCTG GGGAAGAAGC CTTGGCCGTA AACTTCCCCT ATATGTTGCA   
  
  
+ TCACATGCCC GACGAGAGCG TGAGCACCAC AAATCATCGG GACCGGCTCT TGAGGCTGGT GAAGAGGTTG   
  
  
+ TCACCAAGGA TCGTCACCTT GGTTGAGCAA GAGTCCAACA CAAACACTCC TCCCTTTCTT CAACGGTTCC   
  
  
+ GTGAAACATT GGACTACTAC ACTGCCATGT TCGAGTCCAT TGATGTGGCT CTACCTAGGG ATGACAAGAA   
  
  
+ GAGGATAAAT GCCGAGCAGC ATTGTCTTGC GCGCGATATT GTGAACATGG TCGCTTGTGA GAATGCGGAA   
  
  
+ AGGGTTGAGA GGCACGAGCC CTTCGGGAAA TGGAGGGCAA GGTTTGATAT GGCTGGGTTT CAGCAATTGC   
  
  
+ CCTCGAGCCG CTCAGTGAAT GATGCAATAA GGGGTTTGAT GCGAGAATTC CATAGGAACT ATCGGGTGCA   
  
  
+ GGATTGTCAG GGTGCCCTTT TTTTGGGCTG GAAGGAGCGC AATCTGGCAA CCTTCTCTAC ATGGTGCTGT   
  
  
+ AAAGAATG  

- -Up\_Stream \_Len000CAATTT TTTGAGTCTT GATAATATCT AAAAGACAAA AGACAAACCT AACTCTAGGC   
  
  
- TTCGGAAACT CTAGGGGACA AAGATCGAAG GTTGGGGGGA GGGACCCAAG AAAAAGAAAC CACACTTTCA   
  
  
- TACTTGTTTT CGACTACATT CACACTAAGT TTGAGATCGG AACCACTCTG AGAACCCTTC CCGAGGATCT   
  
  
- CTGAAACGGT TAACTCGATT AAACTACGGA TGATGAACGA ATGAAAAGAT CGACAACAAA TTTGCTAAAT   
  
  
- ACAAGTATCA ATTCCTCGCG GTATAACGTA ATGTTATAGA CGAAATCACG AAACAAAAAA CGTAAAAGAA   
  
  
- GAAATAATGA TACCAAAAAC TTCTTCCGAA TGACTGACTA TGACCAAAGA AGCGAATTCA AACCACATGT   
  
  
- ACGAAAAGTT AGACACAACG TAAAAAGATA CTACATATTC GGCAAATAAT GGATAACTAC AAGTTGAAAA   
  
  
- GTGTGATATA TAGGAAATAC CAAAGAAGCG AATTCAAACC ACATGTACGA AAAGTTAGAC ACAACGTAAA   
  
  
- AAGACACTAC ATATTCGGCA AATAATGGAT AACTACAAGT TGAAAAGTGT GATATACAGG AAAATAATCA   
  
  
- ATGACTACGG AGACAGTTTG TAAGAATTAA CGTAAATCAC ATAGTTGCCA GAATTTTAAA ATTCGTAACG   
  
  
- GTTGAACTTG AAGACTTCTA CAAACAGTTC TAGAAGTCAG ACGTTCAATT GACGACGAAC GAACTTGAAA   
  
  
- AGTTGAAAAT TCTCTCTACA GTCTCAGATT CGTACCGAAG GACTGTTCAT GACGTAAAAT AGTGAATACT   
  
  
- TCTGAGTTAA GACCCAAAAC TGTCGAAGAT ACTTGGAGAT CACGTCCATG TCGTCGAACC TGTAATTCGA   
  
  
- CGTTCAGTTA ACTGACATCT TGCTGTTCAA CAACTTCAAA GACGACTTTC AAGTAGTGAA CAAACTACTT   
  
  
- ATTTCATGTC GTACCGTGAA GTCTACGTTT TCAGAGCGCT AGTCATCTCT ACTAAGGTAT AAAGAGTAGA   
  
  
- GTAAAGGTTC AGAAACTGTT GTACATGGGT TTATTACGGT CGGTACTGTG AGTCTACAGA GAGGTTAGTA   
  
  
- TGTCGCTTCC TATGAAGTGA GACCTGAGTA GAGGTCGTCG TTAGCCTCGC ACGTCACAGA TACTGATGGG   
  
  
- TAGGCAACAG TCGTGGAGAA GTTTATCTTC GGTCAAAAGA GGAGTCCCAA GGGTGAGCAT GTAGAGTCTG   
  
  
- GGGGTAGTAA GAAGGCTGTT GATACCTAGG GGTCACTCGC CGAGAAGACA CCAACTGCTA TTACGACTCG   
  
  
- ACTCCTTGTC CAAGTCACTA TACCTCGAGG GGAACGGTGT CCTGAGTCCT GTGGTAATAA CGAGAAAATC   
  
  
- GGTGTCTCCT TCGGTACTTC CGAGGATATG AAACTCCGGT TTGGTTGACT ACCTATACCG GTTGTACCTC   
  
  
- AATTTCGTCC ACGATATGAA GACACGTCTT CGTTAGAGTC TCTTATTAGA TAGTTGACGT CTTTCTGATT   
  
  
- ACCTACGTAA CCCATTCTCC CACAGACAAA AACCAAGTGG TTAACTTTCC AACCGGCGGA TGTACAACCT   
  
  
- TCCCGAGTCT CGTTCCGACC TCAAAAGACC TATATGATAG ATGTTTTTCG AGTCCACGCT TGTCGGTTGC   
  
  
- TCGAGTCTCG AAGAAAGGAT GTACGTGTAG GACATAGTTT CAACGGGTAT AAAGTTTAAG CGTATATACA   
  
  
- GGAGTTTACA ACGATAAGTT CTTCGAAACC CCTTACTCGG CCAATAGGTG TAGTAGCTAA AGGTCTAACG   
  
  
- GTACCCCTGT GTTAACCACG AGAACTAGGT CAGAGAGCGG GTAGCCGGAC CGCCCGGGGG GGGAGCAAGC   
  
  
- CTAGTGACCC CAGCTACTAG GTGTGAGCCG GATACGAGCA CCGCCCCCCG AGCTCCAACA CCCCGTCTCC   
  
  
- GATTGTTTCC ACCGACTTAG TATACCTCAG GGTAAGCTCA AAGTACGACG TCGGTACAGT CCCACGTTCC   
  
  
- ACTTAGCGCT ACAGGAGTTC CAAGCGGGAC CCCTTCTTCG GAACCGGCAT TTGAAGGGGA TATACAACGT   
  
  
- AGTGTACGGG CTGCTCTCGC ACTCGTGGTG TTTAGTAGCC CTGGCCGAGA ACTCCGACCA CTTCTCCAAC   
  
  
- AGTGGTTCCT AGCAGTGGAA CCAACTCGTT CTCAGGTTGT GTTTGTGAGG AGGGAAAGAA GTTGCCAAGG   
  
  
- CACTTTGTAA CCTGATGATG TGACGGTACA AGCTCAGGTA ACTACACCGA GATGGATCCC TACTGTTCTT   
  
  
- CTCCTATTTA CGGCTCGTCG TAACAGAACG CGCGCTATAA CACTTGTACC AGCGAACACT CTTACGCCTT   
  
  
- TCCCAACTCT CCGTGCTCGG GAAGCCCTTT ACCTCCCGTT CCAAACTATA CCGACCCAAA GTCGTTAACG   
  
  
- GGAGCTCGGC GAGTCACTTA CTACGTTATT CCCCAAACTA CGCTCTTAAG GTATCCTTGA TAGCCCACGT   
  
  
- CCTAACAGTC CCACGGGAAA AAAACCCGAC CTTCCTCGCG TTAGACCGTT GGAAGAGATG TACCACGACA   
  
  
- TTTCTTAC

+     as-1

| Site Name | Organism | Position | Strand | Matrix score. | sequence | function |
| --- | --- | --- | --- | --- | --- | --- |
| as-1 | Arabidopsis thaliana | 2186 | - | 5 | TGACG |  |
| as-1 | Arabidopsis thaliana | 1318 | + | 5 | TGACG |  |

>HU08G02295.1   
+ -Up\_Stream \_Len000GTTAAA AAACTCAGAA CTATTATAGA TTTTCTGTTT TCTGTTTGGA TTGAGATCCG   
  
  
+ AAGCCTTTGA GATCCCCTGT TTCTAGCTTC CAACCCCCCT CCCTGGGTTC TTTTTCTTTG GTGTGAAAGT   
  
  
+ ATGAACAAAA GCTGATGTAA GTGTGATTCA AACTCTAGCC TTGGTGAGAC TCTTGGGAAG GGCTCCTAGA   
  
  
+ GACTTTGCCA ATTGAGCTAA TTTGATGCCT ACTACTTGCT TACTTTTCTA GCTGTTGTTT AAACGATTTA   
  
  
+ TGTTCATAGT TAAGGAGCGC CATATTGCAT TACAATATCT GCTTTAGTGC TTTGTTTTTT GCATTTTCTT   
  
  
+ CTTTATTACT ATGGTTTTTG AAGAAGGCTT ACTGACTGAT ACTGGTTTCT TCGCTTAAGT TTGGTGTACA   
  
  
+ TGCTTTTCAA TCTGTGTTGC ATTTTTCTAT GATGTATAAG CCGTTTATTA CCTATTGATG TTCAACTTTT   
  
  
+ CACACTATAT ATCCTTTATG GTTTCTTCGC TTAAGTTTGG TGTACATGCT TTTCAATCTG TGTTGCATTT   
  
  
+ TTCTGTGATG TATAAGCCGT TTATTACCTA TTGATGTTCA ACTTTTCACA CTATATGTCC TTTTATTAGT   
  
  
+ TACTGATGCC TCTGTCAAAC ATTCTTAATT GCATTTAGTG TATCAACGGT CTTAAAATTT TAAGCATTGC   
  
  
+ CAACTTGAAC TTCTGAAGAT GTTTGTCAAG ATCTTCAGTC TGCAAGTTAA CTGCTGCTTG CTTGAACTTT   
  
  
+ TCAACTTTTA AGAGAGATGT CAGAGTCTAA GCATGGCTTC CTGACAAGTA CTGCATTTTA TCACTTATGA   
  
  
+ AGACTCAATT CTGGGTTTTG ACAGCTTCTA TGAACCTCTA GTGCAGGTAC AGCAGCTTGG ACATTAAGCT   
  
  
+ GCAAGTCAAT TGACTGTAGA ACGACAAGTT GTTGAAGTTT CTGCTGAAAG TTCATCACTT GTTTGATGAA   
  
  
+ TAAAGTACAG CATGGCACTT CAGATGCAAA AGTCTCGCGA TCAGTAGAGA TGATTCCATA TTTCTCATCT   
  
  
+ CATTTCCAAG TCTTTGACAA CATGTACCCA AATAATGCCA GCCATGACAC TCAGATGTCT CTCCAATCAT   
  
  
+ ACAGCGAAGG ATACTTCACT CTGGACTCAT CTCCAGCAGC AATCGGAGCG TGCAGTGTCT ATGACTACCC   
  
  
+ ATCCGTTGTC AGCACCTCTT CAAATAGAAG CCAGTTTTCT CCTCAGGGTT CCCACTCGTA CATCTCAGAC   
  
  
+ CCCCATCATT CTTCCGACAA CTATGGATCC CCAGTGAGCG GCTCTTCTGT GGTTGACGAT AATGCTGAGC   
  
  
+ TGAGGAACAG GTTCAGTGAT ATGGAGCTCC CCTTGCCACA GGACTCAGGA CACCATTATT GCTCTTTTAG   
  
  
+ CCACAGAGGA AGCCATGAAG GCTCCTATAC TTTGAGGCCA AACCAACTGA TGGATATGGC CAACATGGAG   
  
  
+ TTAAAGCAGG TGCTATACTT CTGTGCAGAA GCAATCTCAG AGAATAATCT ATCAACTGCA GAAAGACTAA   
  
  
+ TGGATGCATT GGGTAAGAGG GTGTCTGTTT TTGGTTCACC AATTGAAAGG TTGGCCGCCT ACATGTTGGA   
  
  
+ AGGGCTCAGA GCAAGGCTGG AGTTTTCTGG ATATACTATC TACAAAAAGC TCAGGTGCGA ACAGCCAACG   
  
  
+ AGCTCAGAGC TTCTTTCCTA CATGCACATC CTGTATCAAA GTTGCCCATA TTTCAAATTC GCATATATGT   
  
  
+ CCTCAAATGT TGCTATTCAA GAAGCTTTGG GGAATGAGCC GGTTATCCAC ATCATCGATT TCCAGATTGC   
  
  
+ CATGGGGACA CAATTGGTGC TCTTGATCCA GTCTCTCGCC CATCGGCCTG GCGGGCCCCC CCCTCGTTCG   
  
  
+ GATCACTGGG GTCGATGATC CACACTCGGC CTATGCTCGT GGCGGGGGGC TCGAGGTTGT GGGGCAGAGG   
  
  
+ CTAACAAAGG TGGCTGAATC ATATGGAGTC CCATTCGAGT TTCATGCTGC AGCCATGTCA GGGTGCAAGG   
  
  
+ TGAATCGCGA TGTCCTCAAG GTTCGCCCTG GGGAAGAAGC CTTGGCCGTA AACTTCCCCT ATATGTTGCA   
  
  
+ TCACATGCCC GACGAGAGCG TGAGCACCAC AAATCATCGG GACCGGCTCT TGAGGCTGGT GAAGAGGTTG   
  
  
+ TCACCAAGGA TCGTCACCTT GGTTGAGCAA GAGTCCAACA CAAACACTCC TCCCTTTCTT CAACGGTTCC   
  
  
+ GTGAAACATT GGACTACTAC ACTGCCATGT TCGAGTCCAT TGATGTGGCT CTACCTAGGG ATGACAAGAA   
  
  
+ GAGGATAAAT GCCGAGCAGC ATTGTCTTGC GCGCGATATT GTGAACATGG TCGCTTGTGA GAATGCGGAA   
  
  
+ AGGGTTGAGA GGCACGAGCC CTTCGGGAAA TGGAGGGCAA GGTTTGATAT GGCTGGGTTT CAGCAATTGC   
  
  
+ CCTCGAGCCG CTCAGTGAAT GATGCAATAA GGGGTTTGAT GCGAGAATTC CATAGGAACT ATCGGGTGCA   
  
  
+ GGATTGTCAG GGTGCCCTTT TTTTGGGCTG GAAGGAGCGC AATCTGGCAA CCTTCTCTAC ATGGTGCTGT   
  
  
+ AAAGAATG  

- -Up\_Stream \_Len000CAATTT TTTGAGTCTT GATAATATCT AAAAGACAAA AGACAAACCT AACTCTAGGC   
  
  
- TTCGGAAACT CTAGGGGACA AAGATCGAAG GTTGGGGGGA GGGACCCAAG AAAAAGAAAC CACACTTTCA   
  
  
- TACTTGTTTT CGACTACATT CACACTAAGT TTGAGATCGG AACCACTCTG AGAACCCTTC CCGAGGATCT   
  
  
- CTGAAACGGT TAACTCGATT AAACTACGGA TGATGAACGA ATGAAAAGAT CGACAACAAA TTTGCTAAAT   
  
  
- ACAAGTATCA ATTCCTCGCG GTATAACGTA ATGTTATAGA CGAAATCACG AAACAAAAAA CGTAAAAGAA   
  
  
- GAAATAATGA TACCAAAAAC TTCTTCCGAA TGACTGACTA TGACCAAAGA AGCGAATTCA AACCACATGT   
  
  
- ACGAAAAGTT AGACACAACG TAAAAAGATA CTACATATTC GGCAAATAAT GGATAACTAC AAGTTGAAAA   
  
  
- GTGTGATATA TAGGAAATAC CAAAGAAGCG AATTCAAACC ACATGTACGA AAAGTTAGAC ACAACGTAAA   
  
  
- AAGACACTAC ATATTCGGCA AATAATGGAT AACTACAAGT TGAAAAGTGT GATATACAGG AAAATAATCA   
  
  
- ATGACTACGG AGACAGTTTG TAAGAATTAA CGTAAATCAC ATAGTTGCCA GAATTTTAAA ATTCGTAACG   
  
  
- GTTGAACTTG AAGACTTCTA CAAACAGTTC TAGAAGTCAG ACGTTCAATT GACGACGAAC GAACTTGAAA   
  
  
- AGTTGAAAAT TCTCTCTACA GTCTCAGATT CGTACCGAAG GACTGTTCAT GACGTAAAAT AGTGAATACT   
  
  
- TCTGAGTTAA GACCCAAAAC TGTCGAAGAT ACTTGGAGAT CACGTCCATG TCGTCGAACC TGTAATTCGA   
  
  
- CGTTCAGTTA ACTGACATCT TGCTGTTCAA CAACTTCAAA GACGACTTTC AAGTAGTGAA CAAACTACTT   
  
  
- ATTTCATGTC GTACCGTGAA GTCTACGTTT TCAGAGCGCT AGTCATCTCT ACTAAGGTAT AAAGAGTAGA   
  
  
- GTAAAGGTTC AGAAACTGTT GTACATGGGT TTATTACGGT CGGTACTGTG AGTCTACAGA GAGGTTAGTA   
  
  
- TGTCGCTTCC TATGAAGTGA GACCTGAGTA GAGGTCGTCG TTAGCCTCGC ACGTCACAGA TACTGATGGG   
  
  
- TAGGCAACAG TCGTGGAGAA GTTTATCTTC GGTCAAAAGA GGAGTCCCAA GGGTGAGCAT GTAGAGTCTG   
  
  
- GGGGTAGTAA GAAGGCTGTT GATACCTAGG GGTCACTCGC CGAGAAGACA CCAACTGCTA TTACGACTCG   
  
  
- ACTCCTTGTC CAAGTCACTA TACCTCGAGG GGAACGGTGT CCTGAGTCCT GTGGTAATAA CGAGAAAATC   
  
  
- GGTGTCTCCT TCGGTACTTC CGAGGATATG AAACTCCGGT TTGGTTGACT ACCTATACCG GTTGTACCTC   
  
  
- AATTTCGTCC ACGATATGAA GACACGTCTT CGTTAGAGTC TCTTATTAGA TAGTTGACGT CTTTCTGATT   
  
  
- ACCTACGTAA CCCATTCTCC CACAGACAAA AACCAAGTGG TTAACTTTCC AACCGGCGGA TGTACAACCT   
  
  
- TCCCGAGTCT CGTTCCGACC TCAAAAGACC TATATGATAG ATGTTTTTCG AGTCCACGCT TGTCGGTTGC   
  
  
- TCGAGTCTCG AAGAAAGGAT GTACGTGTAG GACATAGTTT CAACGGGTAT AAAGTTTAAG CGTATATACA   
  
  
- GGAGTTTACA ACGATAAGTT CTTCGAAACC CCTTACTCGG CCAATAGGTG TAGTAGCTAA AGGTCTAACG   
  
  
- GTACCCCTGT GTTAACCACG AGAACTAGGT CAGAGAGCGG GTAGCCGGAC CGCCCGGGGG GGGAGCAAGC   
  
  
- CTAGTGACCC CAGCTACTAG GTGTGAGCCG GATACGAGCA CCGCCCCCCG AGCTCCAACA CCCCGTCTCC   
  
  
- GATTGTTTCC ACCGACTTAG TATACCTCAG GGTAAGCTCA AAGTACGACG TCGGTACAGT CCCACGTTCC   
  
  
- ACTTAGCGCT ACAGGAGTTC CAAGCGGGAC CCCTTCTTCG GAACCGGCAT TTGAAGGGGA TATACAACGT   
  
  
- AGTGTACGGG CTGCTCTCGC ACTCGTGGTG TTTAGTAGCC CTGGCCGAGA ACTCCGACCA CTTCTCCAAC   
  
  
- AGTGGTTCCT AGCAGTGGAA CCAACTCGTT CTCAGGTTGT GTTTGTGAGG AGGGAAAGAA GTTGCCAAGG   
  
  
- CACTTTGTAA CCTGATGATG TGACGGTACA AGCTCAGGTA ACTACACCGA GATGGATCCC TACTGTTCTT   
  
  
- CTCCTATTTA CGGCTCGTCG TAACAGAACG CGCGCTATAA CACTTGTACC AGCGAACACT CTTACGCCTT   
  
  
- TCCCAACTCT CCGTGCTCGG GAAGCCCTTT ACCTCCCGTT CCAAACTATA CCGACCCAAA GTCGTTAACG   
  
  
- GGAGCTCGGC GAGTCACTTA CTACGTTATT CCCCAAACTA CGCTCTTAAG GTATCCTTGA TAGCCCACGT   
  
  
- CCTAACAGTC CCACGGGAAA AAAACCCGAC CTTCCTCGCG TTAGACCGTT GGAAGAGATG TACCACGACA   
  
  
- TTTCTTAC

+     box S

| Site Name | Organism | Position | Strand | Matrix score. | sequence | function |
| --- | --- | --- | --- | --- | --- | --- |
| box S | Arabidopsis thaliana | 1973 | - | 7 | AGCCACC |  |

>HU08G02295.1   
+ -Up\_Stream \_Len000GTTAAA AAACTCAGAA CTATTATAGA TTTTCTGTTT TCTGTTTGGA TTGAGATCCG   
  
  
+ AAGCCTTTGA GATCCCCTGT TTCTAGCTTC CAACCCCCCT CCCTGGGTTC TTTTTCTTTG GTGTGAAAGT   
  
  
+ ATGAACAAAA GCTGATGTAA GTGTGATTCA AACTCTAGCC TTGGTGAGAC TCTTGGGAAG GGCTCCTAGA   
  
  
+ GACTTTGCCA ATTGAGCTAA TTTGATGCCT ACTACTTGCT TACTTTTCTA GCTGTTGTTT AAACGATTTA   
  
  
+ TGTTCATAGT TAAGGAGCGC CATATTGCAT TACAATATCT GCTTTAGTGC TTTGTTTTTT GCATTTTCTT   
  
  
+ CTTTATTACT ATGGTTTTTG AAGAAGGCTT ACTGACTGAT ACTGGTTTCT TCGCTTAAGT TTGGTGTACA   
  
  
+ TGCTTTTCAA TCTGTGTTGC ATTTTTCTAT GATGTATAAG CCGTTTATTA CCTATTGATG TTCAACTTTT   
  
  
+ CACACTATAT ATCCTTTATG GTTTCTTCGC TTAAGTTTGG TGTACATGCT TTTCAATCTG TGTTGCATTT   
  
  
+ TTCTGTGATG TATAAGCCGT TTATTACCTA TTGATGTTCA ACTTTTCACA CTATATGTCC TTTTATTAGT   
  
  
+ TACTGATGCC TCTGTCAAAC ATTCTTAATT GCATTTAGTG TATCAACGGT CTTAAAATTT TAAGCATTGC   
  
  
+ CAACTTGAAC TTCTGAAGAT GTTTGTCAAG ATCTTCAGTC TGCAAGTTAA CTGCTGCTTG CTTGAACTTT   
  
  
+ TCAACTTTTA AGAGAGATGT CAGAGTCTAA GCATGGCTTC CTGACAAGTA CTGCATTTTA TCACTTATGA   
  
  
+ AGACTCAATT CTGGGTTTTG ACAGCTTCTA TGAACCTCTA GTGCAGGTAC AGCAGCTTGG ACATTAAGCT   
  
  
+ GCAAGTCAAT TGACTGTAGA ACGACAAGTT GTTGAAGTTT CTGCTGAAAG TTCATCACTT GTTTGATGAA   
  
  
+ TAAAGTACAG CATGGCACTT CAGATGCAAA AGTCTCGCGA TCAGTAGAGA TGATTCCATA TTTCTCATCT   
  
  
+ CATTTCCAAG TCTTTGACAA CATGTACCCA AATAATGCCA GCCATGACAC TCAGATGTCT CTCCAATCAT   
  
  
+ ACAGCGAAGG ATACTTCACT CTGGACTCAT CTCCAGCAGC AATCGGAGCG TGCAGTGTCT ATGACTACCC   
  
  
+ ATCCGTTGTC AGCACCTCTT CAAATAGAAG CCAGTTTTCT CCTCAGGGTT CCCACTCGTA CATCTCAGAC   
  
  
+ CCCCATCATT CTTCCGACAA CTATGGATCC CCAGTGAGCG GCTCTTCTGT GGTTGACGAT AATGCTGAGC   
  
  
+ TGAGGAACAG GTTCAGTGAT ATGGAGCTCC CCTTGCCACA GGACTCAGGA CACCATTATT GCTCTTTTAG   
  
  
+ CCACAGAGGA AGCCATGAAG GCTCCTATAC TTTGAGGCCA AACCAACTGA TGGATATGGC CAACATGGAG   
  
  
+ TTAAAGCAGG TGCTATACTT CTGTGCAGAA GCAATCTCAG AGAATAATCT ATCAACTGCA GAAAGACTAA   
  
  
+ TGGATGCATT GGGTAAGAGG GTGTCTGTTT TTGGTTCACC AATTGAAAGG TTGGCCGCCT ACATGTTGGA   
  
  
+ AGGGCTCAGA GCAAGGCTGG AGTTTTCTGG ATATACTATC TACAAAAAGC TCAGGTGCGA ACAGCCAACG   
  
  
+ AGCTCAGAGC TTCTTTCCTA CATGCACATC CTGTATCAAA GTTGCCCATA TTTCAAATTC GCATATATGT   
  
  
+ CCTCAAATGT TGCTATTCAA GAAGCTTTGG GGAATGAGCC GGTTATCCAC ATCATCGATT TCCAGATTGC   
  
  
+ CATGGGGACA CAATTGGTGC TCTTGATCCA GTCTCTCGCC CATCGGCCTG GCGGGCCCCC CCCTCGTTCG   
  
  
+ GATCACTGGG GTCGATGATC CACACTCGGC CTATGCTCGT GGCGGGGGGC TCGAGGTTGT GGGGCAGAGG   
  
  
+ CTAACAAAGG TGGCTGAATC ATATGGAGTC CCATTCGAGT TTCATGCTGC AGCCATGTCA GGGTGCAAGG   
  
  
+ TGAATCGCGA TGTCCTCAAG GTTCGCCCTG GGGAAGAAGC CTTGGCCGTA AACTTCCCCT ATATGTTGCA   
  
  
+ TCACATGCCC GACGAGAGCG TGAGCACCAC AAATCATCGG GACCGGCTCT TGAGGCTGGT GAAGAGGTTG   
  
  
+ TCACCAAGGA TCGTCACCTT GGTTGAGCAA GAGTCCAACA CAAACACTCC TCCCTTTCTT CAACGGTTCC   
  
  
+ GTGAAACATT GGACTACTAC ACTGCCATGT TCGAGTCCAT TGATGTGGCT CTACCTAGGG ATGACAAGAA   
  
  
+ GAGGATAAAT GCCGAGCAGC ATTGTCTTGC GCGCGATATT GTGAACATGG TCGCTTGTGA GAATGCGGAA   
  
  
+ AGGGTTGAGA GGCACGAGCC CTTCGGGAAA TGGAGGGCAA GGTTTGATAT GGCTGGGTTT CAGCAATTGC   
  
  
+ CCTCGAGCCG CTCAGTGAAT GATGCAATAA GGGGTTTGAT GCGAGAATTC CATAGGAACT ATCGGGTGCA   
  
  
+ GGATTGTCAG GGTGCCCTTT TTTTGGGCTG GAAGGAGCGC AATCTGGCAA CCTTCTCTAC ATGGTGCTGT   
  
  
+ AAAGAATG  

- -Up\_Stream \_Len000CAATTT TTTGAGTCTT GATAATATCT AAAAGACAAA AGACAAACCT AACTCTAGGC   
  
  
- TTCGGAAACT CTAGGGGACA AAGATCGAAG GTTGGGGGGA GGGACCCAAG AAAAAGAAAC CACACTTTCA   
  
  
- TACTTGTTTT CGACTACATT CACACTAAGT TTGAGATCGG AACCACTCTG AGAACCCTTC CCGAGGATCT   
  
  
- CTGAAACGGT TAACTCGATT AAACTACGGA TGATGAACGA ATGAAAAGAT CGACAACAAA TTTGCTAAAT   
  
  
- ACAAGTATCA ATTCCTCGCG GTATAACGTA ATGTTATAGA CGAAATCACG AAACAAAAAA CGTAAAAGAA   
  
  
- GAAATAATGA TACCAAAAAC TTCTTCCGAA TGACTGACTA TGACCAAAGA AGCGAATTCA AACCACATGT   
  
  
- ACGAAAAGTT AGACACAACG TAAAAAGATA CTACATATTC GGCAAATAAT GGATAACTAC AAGTTGAAAA   
  
  
- GTGTGATATA TAGGAAATAC CAAAGAAGCG AATTCAAACC ACATGTACGA AAAGTTAGAC ACAACGTAAA   
  
  
- AAGACACTAC ATATTCGGCA AATAATGGAT AACTACAAGT TGAAAAGTGT GATATACAGG AAAATAATCA   
  
  
- ATGACTACGG AGACAGTTTG TAAGAATTAA CGTAAATCAC ATAGTTGCCA GAATTTTAAA ATTCGTAACG   
  
  
- GTTGAACTTG AAGACTTCTA CAAACAGTTC TAGAAGTCAG ACGTTCAATT GACGACGAAC GAACTTGAAA   
  
  
- AGTTGAAAAT TCTCTCTACA GTCTCAGATT CGTACCGAAG GACTGTTCAT GACGTAAAAT AGTGAATACT   
  
  
- TCTGAGTTAA GACCCAAAAC TGTCGAAGAT ACTTGGAGAT CACGTCCATG TCGTCGAACC TGTAATTCGA   
  
  
- CGTTCAGTTA ACTGACATCT TGCTGTTCAA CAACTTCAAA GACGACTTTC AAGTAGTGAA CAAACTACTT   
  
  
- ATTTCATGTC GTACCGTGAA GTCTACGTTT TCAGAGCGCT AGTCATCTCT ACTAAGGTAT AAAGAGTAGA   
  
  
- GTAAAGGTTC AGAAACTGTT GTACATGGGT TTATTACGGT CGGTACTGTG AGTCTACAGA GAGGTTAGTA   
  
  
- TGTCGCTTCC TATGAAGTGA GACCTGAGTA GAGGTCGTCG TTAGCCTCGC ACGTCACAGA TACTGATGGG   
  
  
- TAGGCAACAG TCGTGGAGAA GTTTATCTTC GGTCAAAAGA GGAGTCCCAA GGGTGAGCAT GTAGAGTCTG   
  
  
- GGGGTAGTAA GAAGGCTGTT GATACCTAGG GGTCACTCGC CGAGAAGACA CCAACTGCTA TTACGACTCG   
  
  
- ACTCCTTGTC CAAGTCACTA TACCTCGAGG GGAACGGTGT CCTGAGTCCT GTGGTAATAA CGAGAAAATC   
  
  
- GGTGTCTCCT TCGGTACTTC CGAGGATATG AAACTCCGGT TTGGTTGACT ACCTATACCG GTTGTACCTC   
  
  
- AATTTCGTCC ACGATATGAA GACACGTCTT CGTTAGAGTC TCTTATTAGA TAGTTGACGT CTTTCTGATT   
  
  
- ACCTACGTAA CCCATTCTCC CACAGACAAA AACCAAGTGG TTAACTTTCC AACCGGCGGA TGTACAACCT   
  
  
- TCCCGAGTCT CGTTCCGACC TCAAAAGACC TATATGATAG ATGTTTTTCG AGTCCACGCT TGTCGGTTGC   
  
  
- TCGAGTCTCG AAGAAAGGAT GTACGTGTAG GACATAGTTT CAACGGGTAT AAAGTTTAAG CGTATATACA   
  
  
- GGAGTTTACA ACGATAAGTT CTTCGAAACC CCTTACTCGG CCAATAGGTG TAGTAGCTAA AGGTCTAACG   
  
  
- GTACCCCTGT GTTAACCACG AGAACTAGGT CAGAGAGCGG GTAGCCGGAC CGCCCGGGGG GGGAGCAAGC   
  
  
- CTAGTGACCC CAGCTACTAG GTGTGAGCCG GATACGAGCA CCGCCCCCCG AGCTCCAACA CCCCGTCTCC   
  
  
- GATTGTTTCC ACCGACTTAG TATACCTCAG GGTAAGCTCA AAGTACGACG TCGGTACAGT CCCACGTTCC   
  
  
- ACTTAGCGCT ACAGGAGTTC CAAGCGGGAC CCCTTCTTCG GAACCGGCAT TTGAAGGGGA TATACAACGT   
  
  
- AGTGTACGGG CTGCTCTCGC ACTCGTGGTG TTTAGTAGCC CTGGCCGAGA ACTCCGACCA CTTCTCCAAC   
  
  
- AGTGGTTCCT AGCAGTGGAA CCAACTCGTT CTCAGGTTGT GTTTGTGAGG AGGGAAAGAA GTTGCCAAGG   
  
  
- CACTTTGTAA CCTGATGATG TGACGGTACA AGCTCAGGTA ACTACACCGA GATGGATCCC TACTGTTCTT   
  
  
- CTCCTATTTA CGGCTCGTCG TAACAGAACG CGCGCTATAA CACTTGTACC AGCGAACACT CTTACGCCTT   
  
  
- TCCCAACTCT CCGTGCTCGG GAAGCCCTTT ACCTCCCGTT CCAAACTATA CCGACCCAAA GTCGTTAACG   
  
  
- GGAGCTCGGC GAGTCACTTA CTACGTTATT CCCCAAACTA CGCTCTTAAG GTATCCTTGA TAGCCCACGT   
  
  
- CCTAACAGTC CCACGGGAAA AAAACCCGAC CTTCCTCGCG TTAGACCGTT GGAAGAGATG TACCACGACA   
  
  
- TTTCTTAC
