# Supplementary material for: Genome-Wide Identification and Expression Pattern of the GRAS Gene Family in Pitaya (Selenicereus undatus L.)
Source: Biology (Basel). 2022 Dec 21;12(1):11. doi: 10.3390/biology12010011 (PMC9854919; doi:10.3390/biology12010011)
Supplement: Supplementary file 1 [file biology-12-00011-s001.zip › Supplementary file S5/HU08G02296.1_plantcare.html]

Content-Type: text/html; charset=ISO-8859-1


PlantCARE


Webmaster Firefox specific output  
To save the result:
click on the frame with the right mouse button and save the source code as a text file with extension .html  
REFERENCE:PlantCARE: a database of plant cis-acting regulatory elements and a portal to tools for in silico analysis of promoter sequences.  
Lescot, M., Déhais, P., Moreau, Y., De Moor, B., Rouzé ,P.,and Rombauts, S.  
Nucleic Acids Res., Database issue(2002), 30(1):325-327.   


---

>HU08G02296.1   
+ -Up\_Stream \_Len000TTATTC TTACATCTTG TTGTTGTGAT TGTGTATTAA GCTTGTTGTC TCATGGTTTG   
  
  
+ CTCTTCTCTT TGGAACCTCA TGGTGGCTAG TTCCATGTTT TTGCTTTGTA GATGTAGCCG TTTGTTAAAG   
  
  
+ CAAGTTGGTT TATTTCTCCT ATGGCTTCGC TCTATTGTAA TTCAGGGGTC AGGTTGTTAT GTCAGACATT   
  
  
+ CCCATACCGC ACCTAGAAGG GAGGAGGGAG GCTCAGGAGT CTTAAAGAGT ATAGATTTGG ATTCACCTGT   
  
  
+ AGTCTATAGC TGATGTCCAC CTAATCTGGA TTTTATTCTT TCTTTCTCTC TCTTTTTTTT TCTTTTTTTG   
  
  
+ GGGGGGGGGG TGTTGTTGAG GGAGACAGGA TGAGGTCGAA TCTCTGCAGC ATAACAACCA CCATAGTCAC   
  
  
+ AATATGGAGA TTGTGGAGGC AAAGTTTGAG GACGTCCGGA TGTATTTCCT TTGGAACTGA GGAGGAGGTA   
  
  
+ GTACTTCTCT GGTAGTAGTG TTCTATATCC TTTGTTCCTT TCGTGTATTA TCCTAGCTAC CTCTCAAGCA   
  
  
+ AGTTGACATG AAATTGTAGA ATTCAAGACA ATAATAACAG CTGGTTTTTG GGTCTTTTAG TTTGCAATCC   
  
  
+ CTTGTAGCAT CTTAGTGACT TAGTGACATG AGATTTTGAC CCCAATAGAA ATAGAACAGA CAGCTGCTAA   
  
  
+ ATTCACCCAT CTCTTGTGTC AAAATCGGAC TTGTTTTGCC TCTGCTTCTC CATAAAGCAT CAAAGAGCGC   
  
  
+ TGATTGAGGC AGGGATTTTG TCAAGTGCAG TGTTGCATAT GTCATATCGT GTTGATGGTC ATGTTATCAC   
  
  
+ GGACATAAAT TTCCCAGTGT AAAGCAAAGG TGTCTGTCTT CACATAGAGT GTTCAATTCA TAGTCATGCT   
  
  
+ CTCGTCTCTG CATCATCCTG CGTTTGCTGG GGCTAAATTT GAGGGGCTTA TCACTTTGAA ATTCGGAGAA   
  
  
+ TTACTGTGAA TTTGTGTTAG TTCAGGCATA AAATACTGGA TGGGATGTTG AGATGTTCTA ATCATTATCT   
  
  
+ ATTGTTAAAA AACTCAGAAC TATTATAGAT TTTCTGTTTT CTGTTTGGAT TGAGATCCGA AGCCTTTGAG   
  
  
+ ATCCCCTGTT TCTAGCTTCC AACCCCCCTC CCTGGGTTCT TTTTCTTTGG TGTGAAAGTA TGAACAAAAG   
  
  
+ CTGATGTAAG TGTGATTCAA ACTCTAGCCT TGGTGAGACT CTTGGGAAGG GCTCCTAGAG ACTTTGCCAA   
  
  
+ TTGAGCTAAT TTGATGCCTA CTACTTGCTT ACTTTTCTAG CTGTTGTTTA AACGATTTAT GTTCATAGTT   
  
  
+ AAGGAGCGCC ATATTGCATT ACAATATCTG CTTTAGTGCT TTGTTTTTTG CATTTTCTTC TTTATTACTA   
  
  
+ TGGTTTTTGA AGAAGGCTTA CTGACTGATA CTGGTTTCTT CGCTTAAGTT TGGTGTACAT GCTTTTCAAT   
  
  
+ CTGTGTTGCA TTTTTCTATG ATGTATAAGC CGTTTATTAC CTATTGATGT TCAACTTTTC ACACTATATA   
  
  
+ TCCTTTATGG TTTCTTCGCT TAAGTTTGGT GTACATGCTT TTCAATCTGT GTTGCATTTT TCTGTGATGT   
  
  
+ ATAAGCCGTT TATTACCTAT TGATGTTCAA CTTTTCACAC TATATGTCCT TTTATTAGTT ACTGATGCCT   
  
  
+ CTGTCAAACA TTCTTAATTG CATTTAGTGT ATCAACGGTC TTAAAATTTT AAGCATTGCC AACTTGAACT   
  
  
+ TCTGAAGATG TTTGTCAAGA TCTTCAGTCT GCAAGTTAAC TGCTGCTTGC TTGAACTTTT CAACTTTTAA   
  
  
+ GAGAGATGTC AGAGTCTAAG CATGGCTTCC TGACAAGTAC TGCATTTTAT CACTTATGAA GACTCAATTC   
  
  
+ TGGGTTTTGA CAGCTTCTAT GAACCTCTAG TGCAGGTACA GCAGCTTGGA CATTAAGCTG CAAGTCAATT   
  
  
+ GACTGTAGAA CGACAAGTTG TTGAAGTTTC TGCTGAAAGT TCATCACTTG TTTGATGAAT AAAGTACAGC   
  
  
+ ATGGCACTTC AGATGCAAAA GTCTCGCGAT CAGTAGAGAT GATTCCATAT TTCTCATCTC ATTTCCAAGT   
  
  
+ CTTTGACAAC ATGTACCCAA ATAATGCCAG CCATGACACT CAGATGTCTC TCCAATCATA CAGCGAAGGA   
  
  
+ TACTTCACTC TGGACTCATC TCCAGCAGCA ATCGGAGCGT GCAGTGTCTA TGACTACCCA TCCGTTGTCA   
  
  
+ GCACCTCTTC AAATAGAAGC CAGTTTTCTC CTCAGGGTTC CCACTCGTAC ATCTCAGACC CCCATCATTC   
  
  
+ TTCCGACAAC TATGGATCCC CAGTGAGCGG CTCTTCTGTG GTTGACGATA ATGCTGAGCT GAGGAACAGG   
  
  
+ TTCAGTGATA TGGAGCTCCC CTTGCCACAG GACTCAGGAC ACCATTATTG CTCTTTTAGC CACAGAGGAA   
  
  
+ GCCATGAAGG CTCCTATACT TTGAGGCCAA ACCAACTGAT GGATATGGCC AACATGGAGT TAAAGCAGGT   
  
  
+ GCTATACTTC TGTGCAGAAG CAATCTCAGA GAATAATCTA TCAACTGCAG AAAGACTAAT GGATGCATTG   
  
  
+ GGTAAGAGGG TGTCTGTTTT TGGTTCACCA ATTGAAAGGT TGGCCGCCTA CATGTTGGAA GGGCTCAGAG   
  
  
+ CAAGGCTGGA GTTTTCTGGA TATACTATCT ACAAAAAGCT CAGGTGCGAA CAGCCAACGA GCTCAGAGCT   
  
  
+ TCTTTCCTAC ATGCACATCC TGTATCAAAG TTGCCCATAT TTCAAATTCG CATATATGTC CTCAAATGTT   
  
  
+ GCTATTCAAG AAGCTTTGGG GAATGAGCCG GTTATCCACA TCATCGATTT CCAGATTGCC ATGGGGACAC   
  
  
+ AATTGGTGCT CTTGATCCAG TCTCTCGCCC ATCGGCCTGG CGGGCCCCCC CCTCGTTCGG ATCACTGGGG   
  
  
+ TCGATG  

- -Up\_Stream \_Len000AATAAG AATGTAGAAC AACAACACTA ACACATAATT CGAACAACAG AGTACCAAAC   
  
  
- GAGAAGAGAA ACCTTGGAGT ACCACCGATC AAGGTACAAA AACGAAACAT CTACATCGGC AAACAATTTC   
  
  
- GTTCAACCAA ATAAAGAGGA TACCGAAGCG AGATAACATT AAGTCCCCAG TCCAACAATA CAGTCTGTAA   
  
  
- GGGTATGGCG TGGATCTTCC CTCCTCCCTC CGAGTCCTCA GAATTTCTCA TATCTAAACC TAAGTGGACA   
  
  
- TCAGATATCG ACTACAGGTG GATTAGACCT AAAATAAGAA AGAAAGAGAG AGAAAAAAAA AGAAAAAAAC   
  
  
- CCCCCCCCCC ACAACAACTC CCTCTGTCCT ACTCCAGCTT AGAGACGTCG TATTGTTGGT GGTATCAGTG   
  
  
- TTATACCTCT AACACCTCCG TTTCAAACTC CTGCAGGCCT ACATAAAGGA AACCTTGACT CCTCCTCCAT   
  
  
- CATGAAGAGA CCATCATCAC AAGATATAGG AAACAAGGAA AGCACATAAT AGGATCGATG GAGAGTTCGT   
  
  
- TCAACTGTAC TTTAACATCT TAAGTTCTGT TATTATTGTC GACCAAAAAC CCAGAAAATC AAACGTTAGG   
  
  
- GAACATCGTA GAATCACTGA ATCACTGTAC TCTAAAACTG GGGTTATCTT TATCTTGTCT GTCGACGATT   
  
  
- TAAGTGGGTA GAGAACACAG TTTTAGCCTG AACAAAACGG AGACGAAGAG GTATTTCGTA GTTTCTCGCG   
  
  
- ACTAACTCCG TCCCTAAAAC AGTTCACGTC ACAACGTATA CAGTATAGCA CAACTACCAG TACAATAGTG   
  
  
- CCTGTATTTA AAGGGTCACA TTTCGTTTCC ACAGACAGAA GTGTATCTCA CAAGTTAAGT ATCAGTACGA   
  
  
- GAGCAGAGAC GTAGTAGGAC GCAAACGACC CCGATTTAAA CTCCCCGAAT AGTGAAACTT TAAGCCTCTT   
  
  
- AATGACACTT AAACACAATC AAGTCCGTAT TTTATGACCT ACCCTACAAC TCTACAAGAT TAGTAATAGA   
  
  
- TAACAATTTT TTGAGTCTTG ATAATATCTA AAAGACAAAA GACAAACCTA ACTCTAGGCT TCGGAAACTC   
  
  
- TAGGGGACAA AGATCGAAGG TTGGGGGGAG GGACCCAAGA AAAAGAAACC ACACTTTCAT ACTTGTTTTC   
  
  
- GACTACATTC ACACTAAGTT TGAGATCGGA ACCACTCTGA GAACCCTTCC CGAGGATCTC TGAAACGGTT   
  
  
- AACTCGATTA AACTACGGAT GATGAACGAA TGAAAAGATC GACAACAAAT TTGCTAAATA CAAGTATCAA   
  
  
- TTCCTCGCGG TATAACGTAA TGTTATAGAC GAAATCACGA AACAAAAAAC GTAAAAGAAG AAATAATGAT   
  
  
- ACCAAAAACT TCTTCCGAAT GACTGACTAT GACCAAAGAA GCGAATTCAA ACCACATGTA CGAAAAGTTA   
  
  
- GACACAACGT AAAAAGATAC TACATATTCG GCAAATAATG GATAACTACA AGTTGAAAAG TGTGATATAT   
  
  
- AGGAAATACC AAAGAAGCGA ATTCAAACCA CATGTACGAA AAGTTAGACA CAACGTAAAA AGACACTACA   
  
  
- TATTCGGCAA ATAATGGATA ACTACAAGTT GAAAAGTGTG ATATACAGGA AAATAATCAA TGACTACGGA   
  
  
- GACAGTTTGT AAGAATTAAC GTAAATCACA TAGTTGCCAG AATTTTAAAA TTCGTAACGG TTGAACTTGA   
  
  
- AGACTTCTAC AAACAGTTCT AGAAGTCAGA CGTTCAATTG ACGACGAACG AACTTGAAAA GTTGAAAATT   
  
  
- CTCTCTACAG TCTCAGATTC GTACCGAAGG ACTGTTCATG ACGTAAAATA GTGAATACTT CTGAGTTAAG   
  
  
- ACCCAAAACT GTCGAAGATA CTTGGAGATC ACGTCCATGT CGTCGAACCT GTAATTCGAC GTTCAGTTAA   
  
  
- CTGACATCTT GCTGTTCAAC AACTTCAAAG ACGACTTTCA AGTAGTGAAC AAACTACTTA TTTCATGTCG   
  
  
- TACCGTGAAG TCTACGTTTT CAGAGCGCTA GTCATCTCTA CTAAGGTATA AAGAGTAGAG TAAAGGTTCA   
  
  
- GAAACTGTTG TACATGGGTT TATTACGGTC GGTACTGTGA GTCTACAGAG AGGTTAGTAT GTCGCTTCCT   
  
  
- ATGAAGTGAG ACCTGAGTAG AGGTCGTCGT TAGCCTCGCA CGTCACAGAT ACTGATGGGT AGGCAACAGT   
  
  
- CGTGGAGAAG TTTATCTTCG GTCAAAAGAG GAGTCCCAAG GGTGAGCATG TAGAGTCTGG GGGTAGTAAG   
  
  
- AAGGCTGTTG ATACCTAGGG GTCACTCGCC GAGAAGACAC CAACTGCTAT TACGACTCGA CTCCTTGTCC   
  
  
- AAGTCACTAT ACCTCGAGGG GAACGGTGTC CTGAGTCCTG TGGTAATAAC GAGAAAATCG GTGTCTCCTT   
  
  
- CGGTACTTCC GAGGATATGA AACTCCGGTT TGGTTGACTA CCTATACCGG TTGTACCTCA ATTTCGTCCA   
  
  
- CGATATGAAG ACACGTCTTC GTTAGAGTCT CTTATTAGAT AGTTGACGTC TTTCTGATTA CCTACGTAAC   
  
  
- CCATTCTCCC ACAGACAAAA ACCAAGTGGT TAACTTTCCA ACCGGCGGAT GTACAACCTT CCCGAGTCTC   
  
  
- GTTCCGACCT CAAAAGACCT ATATGATAGA TGTTTTTCGA GTCCACGCTT GTCGGTTGCT CGAGTCTCGA   
  
  
- AGAAAGGATG TACGTGTAGG ACATAGTTTC AACGGGTATA AAGTTTAAGC GTATATACAG GAGTTTACAA   
  
  
- CGATAAGTTC TTCGAAACCC CTTACTCGGC CAATAGGTGT AGTAGCTAAA GGTCTAACGG TACCCCTGTG   
  
  
- TTAACCACGA GAACTAGGTC AGAGAGCGGG TAGCCGGACC GCCCGGGGGG GGAGCAAGCC TAGTGACCCC   
  
  
- AGCTAC

  
  
Motifs Found  

+   

| Site Name | Organism | Position | Strand | Matrix score. | sequence | function |
| --- | --- | --- | --- | --- | --- | --- |
|  | organism | 2790 | + | 4 | motif\_sequence | short\_function |
|  | organism | 2747 | - | 4 | motif\_sequence | short\_function |
|  | organism | 2712 | - | 4 | motif\_sequence | short\_function |
|  | organism | 2691 | + | 4 | motif\_sequence | short\_function |
|  | organism | 2534 | + | 4 | motif\_sequence | short\_function |
|  | organism | 2115 | + | 4 | motif\_sequence | short\_function |
|  | organism | 2067 | - | 4 | motif\_sequence | short\_function |
|  | organism | 2028 | - | 4 | motif\_sequence | short\_function |
|  | organism | 1942 | - | 4 | motif\_sequence | short\_function |
|  | organism | 1930 | - | 4 | motif\_sequence | short\_function |
|  | organism | 1774 | + | 4 | motif\_sequence | short\_function |
|  | organism | 2345 | + | 4 | motif\_sequence | short\_function |
|  | organism | 2291 | - | 4 | motif\_sequence | short\_function |
|  | organism | 2378 | - | 4 | motif\_sequence | short\_function |
|  | organism | 2249 | + | 4 | motif\_sequence | short\_function |
|  | organism | 2192 | + | 4 | motif\_sequence | short\_function |
|  | organism | 2152 | + | 4 | motif\_sequence | short\_function |
|  | organism | 1758 | - | 4 | motif\_sequence | short\_function |
|  | organism | 1575 | - | 4 | motif\_sequence | short\_function |
|  | organism | 920 | + | 4 | motif\_sequence | short\_function |
|  | organism | 1637 | + | 4 | motif\_sequence | short\_function |
|  | organism | 1521 | + | 4 | motif\_sequence | short\_function |
|  | organism | 1658 | + | 4 | motif\_sequence | short\_function |
|  | organism | 1459 | - | 4 | motif\_sequence | short\_function |
|  | organism | 1360 | + | 4 | motif\_sequence | short\_function |
|  | organism | 1323 | + | 4 | motif\_sequence | short\_function |
|  | organism | 845 | - | 4 | motif\_sequence | short\_function |
|  | organism | 688 | - | 4 | motif\_sequence | short\_function |
|  | organism | 744 | + | 4 | motif\_sequence | short\_function |
|  | organism | 75 | + | 4 | motif\_sequence | short\_function |
|  | organism | 430 | - | 4 | motif\_sequence | short\_function |
|  | organism | 1037 | + | 4 | motif\_sequence | short\_function |
|  | organism | 122 | - | 4 | motif\_sequence | short\_function |
|  | organism | 297 | + | 4 | motif\_sequence | short\_function |
|  | organism | 396 | + | 4 | motif\_sequence | short\_function |

>HU08G02296.1   
+ -Up\_Stream \_Len000TTATTC TTACATCTTG TTGTTGTGAT TGTGTATTAA GCTTGTTGTC TCATGGTTTG   
  
  
+ CTCTTCTCTT TGGAACCTCA TGGTGGCTAG TTCCATGTTT TTGCTTTGTA GATGTAGCCG TTTGTTAAAG   
  
  
+ CAAGTTGGTT TATTTCTCCT ATGGCTTCGC TCTATTGTAA TTCAGGGGTC AGGTTGTTAT GTCAGACATT   
  
  
+ CCCATACCGC ACCTAGAAGG GAGGAGGGAG GCTCAGGAGT CTTAAAGAGT ATAGATTTGG ATTCACCTGT   
  
  
+ AGTCTATAGC TGATGTCCAC CTAATCTGGA TTTTATTCTT TCTTTCTCTC TCTTTTTTTT TCTTTTTTTG   
  
  
+ GGGGGGGGGG TGTTGTTGAG GGAGACAGGA TGAGGTCGAA TCTCTGCAGC ATAACAACCA CCATAGTCAC   
  
  
+ AATATGGAGA TTGTGGAGGC AAAGTTTGAG GACGTCCGGA TGTATTTCCT TTGGAACTGA GGAGGAGGTA   
  
  
+ GTACTTCTCT GGTAGTAGTG TTCTATATCC TTTGTTCCTT TCGTGTATTA TCCTAGCTAC CTCTCAAGCA   
  
  
+ AGTTGACATG AAATTGTAGA ATTCAAGACA ATAATAACAG CTGGTTTTTG GGTCTTTTAG TTTGCAATCC   
  
  
+ CTTGTAGCAT CTTAGTGACT TAGTGACATG AGATTTTGAC CCCAATAGAA ATAGAACAGA CAGCTGCTAA   
  
  
+ ATTCACCCAT CTCTTGTGTC AAAATCGGAC TTGTTTTGCC TCTGCTTCTC CATAAAGCAT CAAAGAGCGC   
  
  
+ TGATTGAGGC AGGGATTTTG TCAAGTGCAG TGTTGCATAT GTCATATCGT GTTGATGGTC ATGTTATCAC   
  
  
+ GGACATAAAT TTCCCAGTGT AAAGCAAAGG TGTCTGTCTT CACATAGAGT GTTCAATTCA TAGTCATGCT   
  
  
+ CTCGTCTCTG CATCATCCTG CGTTTGCTGG GGCTAAATTT GAGGGGCTTA TCACTTTGAA ATTCGGAGAA   
  
  
+ TTACTGTGAA TTTGTGTTAG TTCAGGCATA AAATACTGGA TGGGATGTTG AGATGTTCTA ATCATTATCT   
  
  
+ ATTGTTAAAA AACTCAGAAC TATTATAGAT TTTCTGTTTT CTGTTTGGAT TGAGATCCGA AGCCTTTGAG   
  
  
+ ATCCCCTGTT TCTAGCTTCC AACCCCCCTC CCTGGGTTCT TTTTCTTTGG TGTGAAAGTA TGAACAAAAG   
  
  
+ CTGATGTAAG TGTGATTCAA ACTCTAGCCT TGGTGAGACT CTTGGGAAGG GCTCCTAGAG ACTTTGCCAA   
  
  
+ TTGAGCTAAT TTGATGCCTA CTACTTGCTT ACTTTTCTAG CTGTTGTTTA AACGATTTAT GTTCATAGTT   
  
  
+ AAGGAGCGCC ATATTGCATT ACAATATCTG CTTTAGTGCT TTGTTTTTTG CATTTTCTTC TTTATTACTA   
  
  
+ TGGTTTTTGA AGAAGGCTTA CTGACTGATA CTGGTTTCTT CGCTTAAGTT TGGTGTACAT GCTTTTCAAT   
  
  
+ CTGTGTTGCA TTTTTCTATG ATGTATAAGC CGTTTATTAC CTATTGATGT TCAACTTTTC ACACTATATA   
  
  
+ TCCTTTATGG TTTCTTCGCT TAAGTTTGGT GTACATGCTT TTCAATCTGT GTTGCATTTT TCTGTGATGT   
  
  
+ ATAAGCCGTT TATTACCTAT TGATGTTCAA CTTTTCACAC TATATGTCCT TTTATTAGTT ACTGATGCCT   
  
  
+ CTGTCAAACA TTCTTAATTG CATTTAGTGT ATCAACGGTC TTAAAATTTT AAGCATTGCC AACTTGAACT   
  
  
+ TCTGAAGATG TTTGTCAAGA TCTTCAGTCT GCAAGTTAAC TGCTGCTTGC TTGAACTTTT CAACTTTTAA   
  
  
+ GAGAGATGTC AGAGTCTAAG CATGGCTTCC TGACAAGTAC TGCATTTTAT CACTTATGAA GACTCAATTC   
  
  
+ TGGGTTTTGA CAGCTTCTAT GAACCTCTAG TGCAGGTACA GCAGCTTGGA CATTAAGCTG CAAGTCAATT   
  
  
+ GACTGTAGAA CGACAAGTTG TTGAAGTTTC TGCTGAAAGT TCATCACTTG TTTGATGAAT AAAGTACAGC   
  
  
+ ATGGCACTTC AGATGCAAAA GTCTCGCGAT CAGTAGAGAT GATTCCATAT TTCTCATCTC ATTTCCAAGT   
  
  
+ CTTTGACAAC ATGTACCCAA ATAATGCCAG CCATGACACT CAGATGTCTC TCCAATCATA CAGCGAAGGA   
  
  
+ TACTTCACTC TGGACTCATC TCCAGCAGCA ATCGGAGCGT GCAGTGTCTA TGACTACCCA TCCGTTGTCA   
  
  
+ GCACCTCTTC AAATAGAAGC CAGTTTTCTC CTCAGGGTTC CCACTCGTAC ATCTCAGACC CCCATCATTC   
  
  
+ TTCCGACAAC TATGGATCCC CAGTGAGCGG CTCTTCTGTG GTTGACGATA ATGCTGAGCT GAGGAACAGG   
  
  
+ TTCAGTGATA TGGAGCTCCC CTTGCCACAG GACTCAGGAC ACCATTATTG CTCTTTTAGC CACAGAGGAA   
  
  
+ GCCATGAAGG CTCCTATACT TTGAGGCCAA ACCAACTGAT GGATATGGCC AACATGGAGT TAAAGCAGGT   
  
  
+ GCTATACTTC TGTGCAGAAG CAATCTCAGA GAATAATCTA TCAACTGCAG AAAGACTAAT GGATGCATTG   
  
  
+ GGTAAGAGGG TGTCTGTTTT TGGTTCACCA ATTGAAAGGT TGGCCGCCTA CATGTTGGAA GGGCTCAGAG   
  
  
+ CAAGGCTGGA GTTTTCTGGA TATACTATCT ACAAAAAGCT CAGGTGCGAA CAGCCAACGA GCTCAGAGCT   
  
  
+ TCTTTCCTAC ATGCACATCC TGTATCAAAG TTGCCCATAT TTCAAATTCG CATATATGTC CTCAAATGTT   
  
  
+ GCTATTCAAG AAGCTTTGGG GAATGAGCCG GTTATCCACA TCATCGATTT CCAGATTGCC ATGGGGACAC   
  
  
+ AATTGGTGCT CTTGATCCAG TCTCTCGCCC ATCGGCCTGG CGGGCCCCCC CCTCGTTCGG ATCACTGGGG   
  
  
+ TCGATG  

- -Up\_Stream \_Len000AATAAG AATGTAGAAC AACAACACTA ACACATAATT CGAACAACAG AGTACCAAAC   
  
  
- GAGAAGAGAA ACCTTGGAGT ACCACCGATC AAGGTACAAA AACGAAACAT CTACATCGGC AAACAATTTC   
  
  
- GTTCAACCAA ATAAAGAGGA TACCGAAGCG AGATAACATT AAGTCCCCAG TCCAACAATA CAGTCTGTAA   
  
  
- GGGTATGGCG TGGATCTTCC CTCCTCCCTC CGAGTCCTCA GAATTTCTCA TATCTAAACC TAAGTGGACA   
  
  
- TCAGATATCG ACTACAGGTG GATTAGACCT AAAATAAGAA AGAAAGAGAG AGAAAAAAAA AGAAAAAAAC   
  
  
- CCCCCCCCCC ACAACAACTC CCTCTGTCCT ACTCCAGCTT AGAGACGTCG TATTGTTGGT GGTATCAGTG   
  
  
- TTATACCTCT AACACCTCCG TTTCAAACTC CTGCAGGCCT ACATAAAGGA AACCTTGACT CCTCCTCCAT   
  
  
- CATGAAGAGA CCATCATCAC AAGATATAGG AAACAAGGAA AGCACATAAT AGGATCGATG GAGAGTTCGT   
  
  
- TCAACTGTAC TTTAACATCT TAAGTTCTGT TATTATTGTC GACCAAAAAC CCAGAAAATC AAACGTTAGG   
  
  
- GAACATCGTA GAATCACTGA ATCACTGTAC TCTAAAACTG GGGTTATCTT TATCTTGTCT GTCGACGATT   
  
  
- TAAGTGGGTA GAGAACACAG TTTTAGCCTG AACAAAACGG AGACGAAGAG GTATTTCGTA GTTTCTCGCG   
  
  
- ACTAACTCCG TCCCTAAAAC AGTTCACGTC ACAACGTATA CAGTATAGCA CAACTACCAG TACAATAGTG   
  
  
- CCTGTATTTA AAGGGTCACA TTTCGTTTCC ACAGACAGAA GTGTATCTCA CAAGTTAAGT ATCAGTACGA   
  
  
- GAGCAGAGAC GTAGTAGGAC GCAAACGACC CCGATTTAAA CTCCCCGAAT AGTGAAACTT TAAGCCTCTT   
  
  
- AATGACACTT AAACACAATC AAGTCCGTAT TTTATGACCT ACCCTACAAC TCTACAAGAT TAGTAATAGA   
  
  
- TAACAATTTT TTGAGTCTTG ATAATATCTA AAAGACAAAA GACAAACCTA ACTCTAGGCT TCGGAAACTC   
  
  
- TAGGGGACAA AGATCGAAGG TTGGGGGGAG GGACCCAAGA AAAAGAAACC ACACTTTCAT ACTTGTTTTC   
  
  
- GACTACATTC ACACTAAGTT TGAGATCGGA ACCACTCTGA GAACCCTTCC CGAGGATCTC TGAAACGGTT   
  
  
- AACTCGATTA AACTACGGAT GATGAACGAA TGAAAAGATC GACAACAAAT TTGCTAAATA CAAGTATCAA   
  
  
- TTCCTCGCGG TATAACGTAA TGTTATAGAC GAAATCACGA AACAAAAAAC GTAAAAGAAG AAATAATGAT   
  
  
- ACCAAAAACT TCTTCCGAAT GACTGACTAT GACCAAAGAA GCGAATTCAA ACCACATGTA CGAAAAGTTA   
  
  
- GACACAACGT AAAAAGATAC TACATATTCG GCAAATAATG GATAACTACA AGTTGAAAAG TGTGATATAT   
  
  
- AGGAAATACC AAAGAAGCGA ATTCAAACCA CATGTACGAA AAGTTAGACA CAACGTAAAA AGACACTACA   
  
  
- TATTCGGCAA ATAATGGATA ACTACAAGTT GAAAAGTGTG ATATACAGGA AAATAATCAA TGACTACGGA   
  
  
- GACAGTTTGT AAGAATTAAC GTAAATCACA TAGTTGCCAG AATTTTAAAA TTCGTAACGG TTGAACTTGA   
  
  
- AGACTTCTAC AAACAGTTCT AGAAGTCAGA CGTTCAATTG ACGACGAACG AACTTGAAAA GTTGAAAATT   
  
  
- CTCTCTACAG TCTCAGATTC GTACCGAAGG ACTGTTCATG ACGTAAAATA GTGAATACTT CTGAGTTAAG   
  
  
- ACCCAAAACT GTCGAAGATA CTTGGAGATC ACGTCCATGT CGTCGAACCT GTAATTCGAC GTTCAGTTAA   
  
  
- CTGACATCTT GCTGTTCAAC AACTTCAAAG ACGACTTTCA AGTAGTGAAC AAACTACTTA TTTCATGTCG   
  
  
- TACCGTGAAG TCTACGTTTT CAGAGCGCTA GTCATCTCTA CTAAGGTATA AAGAGTAGAG TAAAGGTTCA   
  
  
- GAAACTGTTG TACATGGGTT TATTACGGTC GGTACTGTGA GTCTACAGAG AGGTTAGTAT GTCGCTTCCT   
  
  
- ATGAAGTGAG ACCTGAGTAG AGGTCGTCGT TAGCCTCGCA CGTCACAGAT ACTGATGGGT AGGCAACAGT   
  
  
- CGTGGAGAAG TTTATCTTCG GTCAAAAGAG GAGTCCCAAG GGTGAGCATG TAGAGTCTGG GGGTAGTAAG   
  
  
- AAGGCTGTTG ATACCTAGGG GTCACTCGCC GAGAAGACAC CAACTGCTAT TACGACTCGA CTCCTTGTCC   
  
  
- AAGTCACTAT ACCTCGAGGG GAACGGTGTC CTGAGTCCTG TGGTAATAAC GAGAAAATCG GTGTCTCCTT   
  
  
- CGGTACTTCC GAGGATATGA AACTCCGGTT TGGTTGACTA CCTATACCGG TTGTACCTCA ATTTCGTCCA   
  
  
- CGATATGAAG ACACGTCTTC GTTAGAGTCT CTTATTAGAT AGTTGACGTC TTTCTGATTA CCTACGTAAC   
  
  
- CCATTCTCCC ACAGACAAAA ACCAAGTGGT TAACTTTCCA ACCGGCGGAT GTACAACCTT CCCGAGTCTC   
  
  
- GTTCCGACCT CAAAAGACCT ATATGATAGA TGTTTTTCGA GTCCACGCTT GTCGGTTGCT CGAGTCTCGA   
  
  
- AGAAAGGATG TACGTGTAGG ACATAGTTTC AACGGGTATA AAGTTTAAGC GTATATACAG GAGTTTACAA   
  
  
- CGATAAGTTC TTCGAAACCC CTTACTCGGC CAATAGGTGT AGTAGCTAAA GGTCTAACGG TACCCCTGTG   
  
  
- TTAACCACGA GAACTAGGTC AGAGAGCGGG TAGCCGGACC GCCCGGGGGG GGAGCAAGCC TAGTGACCCC   
  
  
- AGCTAC

+     AAGAA-motif

| Site Name | Organism | Position | Strand | Matrix score. | sequence | function |
| --- | --- | --- | --- | --- | --- | --- |
| AAGAA-motif | Avena sativa | 320 | - | 7 | GAAAGAA |  |
| AAGAA-motif | Avena sativa | 324 | - | 7 | GAAAGAA |  |
| AAGAA-motif | Avena sativa | 2734 | - | 7 | GAAAGAA |  |

>HU08G02296.1   
+ -Up\_Stream \_Len000TTATTC TTACATCTTG TTGTTGTGAT TGTGTATTAA GCTTGTTGTC TCATGGTTTG   
  
  
+ CTCTTCTCTT TGGAACCTCA TGGTGGCTAG TTCCATGTTT TTGCTTTGTA GATGTAGCCG TTTGTTAAAG   
  
  
+ CAAGTTGGTT TATTTCTCCT ATGGCTTCGC TCTATTGTAA TTCAGGGGTC AGGTTGTTAT GTCAGACATT   
  
  
+ CCCATACCGC ACCTAGAAGG GAGGAGGGAG GCTCAGGAGT CTTAAAGAGT ATAGATTTGG ATTCACCTGT   
  
  
+ AGTCTATAGC TGATGTCCAC CTAATCTGGA TTTTATTCTT TCTTTCTCTC TCTTTTTTTT TCTTTTTTTG   
  
  
+ GGGGGGGGGG TGTTGTTGAG GGAGACAGGA TGAGGTCGAA TCTCTGCAGC ATAACAACCA CCATAGTCAC   
  
  
+ AATATGGAGA TTGTGGAGGC AAAGTTTGAG GACGTCCGGA TGTATTTCCT TTGGAACTGA GGAGGAGGTA   
  
  
+ GTACTTCTCT GGTAGTAGTG TTCTATATCC TTTGTTCCTT TCGTGTATTA TCCTAGCTAC CTCTCAAGCA   
  
  
+ AGTTGACATG AAATTGTAGA ATTCAAGACA ATAATAACAG CTGGTTTTTG GGTCTTTTAG TTTGCAATCC   
  
  
+ CTTGTAGCAT CTTAGTGACT TAGTGACATG AGATTTTGAC CCCAATAGAA ATAGAACAGA CAGCTGCTAA   
  
  
+ ATTCACCCAT CTCTTGTGTC AAAATCGGAC TTGTTTTGCC TCTGCTTCTC CATAAAGCAT CAAAGAGCGC   
  
  
+ TGATTGAGGC AGGGATTTTG TCAAGTGCAG TGTTGCATAT GTCATATCGT GTTGATGGTC ATGTTATCAC   
  
  
+ GGACATAAAT TTCCCAGTGT AAAGCAAAGG TGTCTGTCTT CACATAGAGT GTTCAATTCA TAGTCATGCT   
  
  
+ CTCGTCTCTG CATCATCCTG CGTTTGCTGG GGCTAAATTT GAGGGGCTTA TCACTTTGAA ATTCGGAGAA   
  
  
+ TTACTGTGAA TTTGTGTTAG TTCAGGCATA AAATACTGGA TGGGATGTTG AGATGTTCTA ATCATTATCT   
  
  
+ ATTGTTAAAA AACTCAGAAC TATTATAGAT TTTCTGTTTT CTGTTTGGAT TGAGATCCGA AGCCTTTGAG   
  
  
+ ATCCCCTGTT TCTAGCTTCC AACCCCCCTC CCTGGGTTCT TTTTCTTTGG TGTGAAAGTA TGAACAAAAG   
  
  
+ CTGATGTAAG TGTGATTCAA ACTCTAGCCT TGGTGAGACT CTTGGGAAGG GCTCCTAGAG ACTTTGCCAA   
  
  
+ TTGAGCTAAT TTGATGCCTA CTACTTGCTT ACTTTTCTAG CTGTTGTTTA AACGATTTAT GTTCATAGTT   
  
  
+ AAGGAGCGCC ATATTGCATT ACAATATCTG CTTTAGTGCT TTGTTTTTTG CATTTTCTTC TTTATTACTA   
  
  
+ TGGTTTTTGA AGAAGGCTTA CTGACTGATA CTGGTTTCTT CGCTTAAGTT TGGTGTACAT GCTTTTCAAT   
  
  
+ CTGTGTTGCA TTTTTCTATG ATGTATAAGC CGTTTATTAC CTATTGATGT TCAACTTTTC ACACTATATA   
  
  
+ TCCTTTATGG TTTCTTCGCT TAAGTTTGGT GTACATGCTT TTCAATCTGT GTTGCATTTT TCTGTGATGT   
  
  
+ ATAAGCCGTT TATTACCTAT TGATGTTCAA CTTTTCACAC TATATGTCCT TTTATTAGTT ACTGATGCCT   
  
  
+ CTGTCAAACA TTCTTAATTG CATTTAGTGT ATCAACGGTC TTAAAATTTT AAGCATTGCC AACTTGAACT   
  
  
+ TCTGAAGATG TTTGTCAAGA TCTTCAGTCT GCAAGTTAAC TGCTGCTTGC TTGAACTTTT CAACTTTTAA   
  
  
+ GAGAGATGTC AGAGTCTAAG CATGGCTTCC TGACAAGTAC TGCATTTTAT CACTTATGAA GACTCAATTC   
  
  
+ TGGGTTTTGA CAGCTTCTAT GAACCTCTAG TGCAGGTACA GCAGCTTGGA CATTAAGCTG CAAGTCAATT   
  
  
+ GACTGTAGAA CGACAAGTTG TTGAAGTTTC TGCTGAAAGT TCATCACTTG TTTGATGAAT AAAGTACAGC   
  
  
+ ATGGCACTTC AGATGCAAAA GTCTCGCGAT CAGTAGAGAT GATTCCATAT TTCTCATCTC ATTTCCAAGT   
  
  
+ CTTTGACAAC ATGTACCCAA ATAATGCCAG CCATGACACT CAGATGTCTC TCCAATCATA CAGCGAAGGA   
  
  
+ TACTTCACTC TGGACTCATC TCCAGCAGCA ATCGGAGCGT GCAGTGTCTA TGACTACCCA TCCGTTGTCA   
  
  
+ GCACCTCTTC AAATAGAAGC CAGTTTTCTC CTCAGGGTTC CCACTCGTAC ATCTCAGACC CCCATCATTC   
  
  
+ TTCCGACAAC TATGGATCCC CAGTGAGCGG CTCTTCTGTG GTTGACGATA ATGCTGAGCT GAGGAACAGG   
  
  
+ TTCAGTGATA TGGAGCTCCC CTTGCCACAG GACTCAGGAC ACCATTATTG CTCTTTTAGC CACAGAGGAA   
  
  
+ GCCATGAAGG CTCCTATACT TTGAGGCCAA ACCAACTGAT GGATATGGCC AACATGGAGT TAAAGCAGGT   
  
  
+ GCTATACTTC TGTGCAGAAG CAATCTCAGA GAATAATCTA TCAACTGCAG AAAGACTAAT GGATGCATTG   
  
  
+ GGTAAGAGGG TGTCTGTTTT TGGTTCACCA ATTGAAAGGT TGGCCGCCTA CATGTTGGAA GGGCTCAGAG   
  
  
+ CAAGGCTGGA GTTTTCTGGA TATACTATCT ACAAAAAGCT CAGGTGCGAA CAGCCAACGA GCTCAGAGCT   
  
  
+ TCTTTCCTAC ATGCACATCC TGTATCAAAG TTGCCCATAT TTCAAATTCG CATATATGTC CTCAAATGTT   
  
  
+ GCTATTCAAG AAGCTTTGGG GAATGAGCCG GTTATCCACA TCATCGATTT CCAGATTGCC ATGGGGACAC   
  
  
+ AATTGGTGCT CTTGATCCAG TCTCTCGCCC ATCGGCCTGG CGGGCCCCCC CCTCGTTCGG ATCACTGGGG   
  
  
+ TCGATG  

- -Up\_Stream \_Len000AATAAG AATGTAGAAC AACAACACTA ACACATAATT CGAACAACAG AGTACCAAAC   
  
  
- GAGAAGAGAA ACCTTGGAGT ACCACCGATC AAGGTACAAA AACGAAACAT CTACATCGGC AAACAATTTC   
  
  
- GTTCAACCAA ATAAAGAGGA TACCGAAGCG AGATAACATT AAGTCCCCAG TCCAACAATA CAGTCTGTAA   
  
  
- GGGTATGGCG TGGATCTTCC CTCCTCCCTC CGAGTCCTCA GAATTTCTCA TATCTAAACC TAAGTGGACA   
  
  
- TCAGATATCG ACTACAGGTG GATTAGACCT AAAATAAGAA AGAAAGAGAG AGAAAAAAAA AGAAAAAAAC   
  
  
- CCCCCCCCCC ACAACAACTC CCTCTGTCCT ACTCCAGCTT AGAGACGTCG TATTGTTGGT GGTATCAGTG   
  
  
- TTATACCTCT AACACCTCCG TTTCAAACTC CTGCAGGCCT ACATAAAGGA AACCTTGACT CCTCCTCCAT   
  
  
- CATGAAGAGA CCATCATCAC AAGATATAGG AAACAAGGAA AGCACATAAT AGGATCGATG GAGAGTTCGT   
  
  
- TCAACTGTAC TTTAACATCT TAAGTTCTGT TATTATTGTC GACCAAAAAC CCAGAAAATC AAACGTTAGG   
  
  
- GAACATCGTA GAATCACTGA ATCACTGTAC TCTAAAACTG GGGTTATCTT TATCTTGTCT GTCGACGATT   
  
  
- TAAGTGGGTA GAGAACACAG TTTTAGCCTG AACAAAACGG AGACGAAGAG GTATTTCGTA GTTTCTCGCG   
  
  
- ACTAACTCCG TCCCTAAAAC AGTTCACGTC ACAACGTATA CAGTATAGCA CAACTACCAG TACAATAGTG   
  
  
- CCTGTATTTA AAGGGTCACA TTTCGTTTCC ACAGACAGAA GTGTATCTCA CAAGTTAAGT ATCAGTACGA   
  
  
- GAGCAGAGAC GTAGTAGGAC GCAAACGACC CCGATTTAAA CTCCCCGAAT AGTGAAACTT TAAGCCTCTT   
  
  
- AATGACACTT AAACACAATC AAGTCCGTAT TTTATGACCT ACCCTACAAC TCTACAAGAT TAGTAATAGA   
  
  
- TAACAATTTT TTGAGTCTTG ATAATATCTA AAAGACAAAA GACAAACCTA ACTCTAGGCT TCGGAAACTC   
  
  
- TAGGGGACAA AGATCGAAGG TTGGGGGGAG GGACCCAAGA AAAAGAAACC ACACTTTCAT ACTTGTTTTC   
  
  
- GACTACATTC ACACTAAGTT TGAGATCGGA ACCACTCTGA GAACCCTTCC CGAGGATCTC TGAAACGGTT   
  
  
- AACTCGATTA AACTACGGAT GATGAACGAA TGAAAAGATC GACAACAAAT TTGCTAAATA CAAGTATCAA   
  
  
- TTCCTCGCGG TATAACGTAA TGTTATAGAC GAAATCACGA AACAAAAAAC GTAAAAGAAG AAATAATGAT   
  
  
- ACCAAAAACT TCTTCCGAAT GACTGACTAT GACCAAAGAA GCGAATTCAA ACCACATGTA CGAAAAGTTA   
  
  
- GACACAACGT AAAAAGATAC TACATATTCG GCAAATAATG GATAACTACA AGTTGAAAAG TGTGATATAT   
  
  
- AGGAAATACC AAAGAAGCGA ATTCAAACCA CATGTACGAA AAGTTAGACA CAACGTAAAA AGACACTACA   
  
  
- TATTCGGCAA ATAATGGATA ACTACAAGTT GAAAAGTGTG ATATACAGGA AAATAATCAA TGACTACGGA   
  
  
- GACAGTTTGT AAGAATTAAC GTAAATCACA TAGTTGCCAG AATTTTAAAA TTCGTAACGG TTGAACTTGA   
  
  
- AGACTTCTAC AAACAGTTCT AGAAGTCAGA CGTTCAATTG ACGACGAACG AACTTGAAAA GTTGAAAATT   
  
  
- CTCTCTACAG TCTCAGATTC GTACCGAAGG ACTGTTCATG ACGTAAAATA GTGAATACTT CTGAGTTAAG   
  
  
- ACCCAAAACT GTCGAAGATA CTTGGAGATC ACGTCCATGT CGTCGAACCT GTAATTCGAC GTTCAGTTAA   
  
  
- CTGACATCTT GCTGTTCAAC AACTTCAAAG ACGACTTTCA AGTAGTGAAC AAACTACTTA TTTCATGTCG   
  
  
- TACCGTGAAG TCTACGTTTT CAGAGCGCTA GTCATCTCTA CTAAGGTATA AAGAGTAGAG TAAAGGTTCA   
  
  
- GAAACTGTTG TACATGGGTT TATTACGGTC GGTACTGTGA GTCTACAGAG AGGTTAGTAT GTCGCTTCCT   
  
  
- ATGAAGTGAG ACCTGAGTAG AGGTCGTCGT TAGCCTCGCA CGTCACAGAT ACTGATGGGT AGGCAACAGT   
  
  
- CGTGGAGAAG TTTATCTTCG GTCAAAAGAG GAGTCCCAAG GGTGAGCATG TAGAGTCTGG GGGTAGTAAG   
  
  
- AAGGCTGTTG ATACCTAGGG GTCACTCGCC GAGAAGACAC CAACTGCTAT TACGACTCGA CTCCTTGTCC   
  
  
- AAGTCACTAT ACCTCGAGGG GAACGGTGTC CTGAGTCCTG TGGTAATAAC GAGAAAATCG GTGTCTCCTT   
  
  
- CGGTACTTCC GAGGATATGA AACTCCGGTT TGGTTGACTA CCTATACCGG TTGTACCTCA ATTTCGTCCA   
  
  
- CGATATGAAG ACACGTCTTC GTTAGAGTCT CTTATTAGAT AGTTGACGTC TTTCTGATTA CCTACGTAAC   
  
  
- CCATTCTCCC ACAGACAAAA ACCAAGTGGT TAACTTTCCA ACCGGCGGAT GTACAACCTT CCCGAGTCTC   
  
  
- GTTCCGACCT CAAAAGACCT ATATGATAGA TGTTTTTCGA GTCCACGCTT GTCGGTTGCT CGAGTCTCGA   
  
  
- AGAAAGGATG TACGTGTAGG ACATAGTTTC AACGGGTATA AAGTTTAAGC GTATATACAG GAGTTTACAA   
  
  
- CGATAAGTTC TTCGAAACCC CTTACTCGGC CAATAGGTGT AGTAGCTAAA GGTCTAACGG TACCCCTGTG   
  
  
- TTAACCACGA GAACTAGGTC AGAGAGCGGG TAGCCGGACC GCCCGGGGGG GGAGCAAGCC TAGTGACCCC   
  
  
- AGCTAC

+     AE-box

| Site Name | Organism | Position | Strand | Matrix score. | sequence | function |
| --- | --- | --- | --- | --- | --- | --- |
| AE-box | Arabidopsis thaliana | 1988 | - | 8 | AGAAACTT | part of a module for light response |

>HU08G02296.1   
+ -Up\_Stream \_Len000TTATTC TTACATCTTG TTGTTGTGAT TGTGTATTAA GCTTGTTGTC TCATGGTTTG   
  
  
+ CTCTTCTCTT TGGAACCTCA TGGTGGCTAG TTCCATGTTT TTGCTTTGTA GATGTAGCCG TTTGTTAAAG   
  
  
+ CAAGTTGGTT TATTTCTCCT ATGGCTTCGC TCTATTGTAA TTCAGGGGTC AGGTTGTTAT GTCAGACATT   
  
  
+ CCCATACCGC ACCTAGAAGG GAGGAGGGAG GCTCAGGAGT CTTAAAGAGT ATAGATTTGG ATTCACCTGT   
  
  
+ AGTCTATAGC TGATGTCCAC CTAATCTGGA TTTTATTCTT TCTTTCTCTC TCTTTTTTTT TCTTTTTTTG   
  
  
+ GGGGGGGGGG TGTTGTTGAG GGAGACAGGA TGAGGTCGAA TCTCTGCAGC ATAACAACCA CCATAGTCAC   
  
  
+ AATATGGAGA TTGTGGAGGC AAAGTTTGAG GACGTCCGGA TGTATTTCCT TTGGAACTGA GGAGGAGGTA   
  
  
+ GTACTTCTCT GGTAGTAGTG TTCTATATCC TTTGTTCCTT TCGTGTATTA TCCTAGCTAC CTCTCAAGCA   
  
  
+ AGTTGACATG AAATTGTAGA ATTCAAGACA ATAATAACAG CTGGTTTTTG GGTCTTTTAG TTTGCAATCC   
  
  
+ CTTGTAGCAT CTTAGTGACT TAGTGACATG AGATTTTGAC CCCAATAGAA ATAGAACAGA CAGCTGCTAA   
  
  
+ ATTCACCCAT CTCTTGTGTC AAAATCGGAC TTGTTTTGCC TCTGCTTCTC CATAAAGCAT CAAAGAGCGC   
  
  
+ TGATTGAGGC AGGGATTTTG TCAAGTGCAG TGTTGCATAT GTCATATCGT GTTGATGGTC ATGTTATCAC   
  
  
+ GGACATAAAT TTCCCAGTGT AAAGCAAAGG TGTCTGTCTT CACATAGAGT GTTCAATTCA TAGTCATGCT   
  
  
+ CTCGTCTCTG CATCATCCTG CGTTTGCTGG GGCTAAATTT GAGGGGCTTA TCACTTTGAA ATTCGGAGAA   
  
  
+ TTACTGTGAA TTTGTGTTAG TTCAGGCATA AAATACTGGA TGGGATGTTG AGATGTTCTA ATCATTATCT   
  
  
+ ATTGTTAAAA AACTCAGAAC TATTATAGAT TTTCTGTTTT CTGTTTGGAT TGAGATCCGA AGCCTTTGAG   
  
  
+ ATCCCCTGTT TCTAGCTTCC AACCCCCCTC CCTGGGTTCT TTTTCTTTGG TGTGAAAGTA TGAACAAAAG   
  
  
+ CTGATGTAAG TGTGATTCAA ACTCTAGCCT TGGTGAGACT CTTGGGAAGG GCTCCTAGAG ACTTTGCCAA   
  
  
+ TTGAGCTAAT TTGATGCCTA CTACTTGCTT ACTTTTCTAG CTGTTGTTTA AACGATTTAT GTTCATAGTT   
  
  
+ AAGGAGCGCC ATATTGCATT ACAATATCTG CTTTAGTGCT TTGTTTTTTG CATTTTCTTC TTTATTACTA   
  
  
+ TGGTTTTTGA AGAAGGCTTA CTGACTGATA CTGGTTTCTT CGCTTAAGTT TGGTGTACAT GCTTTTCAAT   
  
  
+ CTGTGTTGCA TTTTTCTATG ATGTATAAGC CGTTTATTAC CTATTGATGT TCAACTTTTC ACACTATATA   
  
  
+ TCCTTTATGG TTTCTTCGCT TAAGTTTGGT GTACATGCTT TTCAATCTGT GTTGCATTTT TCTGTGATGT   
  
  
+ ATAAGCCGTT TATTACCTAT TGATGTTCAA CTTTTCACAC TATATGTCCT TTTATTAGTT ACTGATGCCT   
  
  
+ CTGTCAAACA TTCTTAATTG CATTTAGTGT ATCAACGGTC TTAAAATTTT AAGCATTGCC AACTTGAACT   
  
  
+ TCTGAAGATG TTTGTCAAGA TCTTCAGTCT GCAAGTTAAC TGCTGCTTGC TTGAACTTTT CAACTTTTAA   
  
  
+ GAGAGATGTC AGAGTCTAAG CATGGCTTCC TGACAAGTAC TGCATTTTAT CACTTATGAA GACTCAATTC   
  
  
+ TGGGTTTTGA CAGCTTCTAT GAACCTCTAG TGCAGGTACA GCAGCTTGGA CATTAAGCTG CAAGTCAATT   
  
  
+ GACTGTAGAA CGACAAGTTG TTGAAGTTTC TGCTGAAAGT TCATCACTTG TTTGATGAAT AAAGTACAGC   
  
  
+ ATGGCACTTC AGATGCAAAA GTCTCGCGAT CAGTAGAGAT GATTCCATAT TTCTCATCTC ATTTCCAAGT   
  
  
+ CTTTGACAAC ATGTACCCAA ATAATGCCAG CCATGACACT CAGATGTCTC TCCAATCATA CAGCGAAGGA   
  
  
+ TACTTCACTC TGGACTCATC TCCAGCAGCA ATCGGAGCGT GCAGTGTCTA TGACTACCCA TCCGTTGTCA   
  
  
+ GCACCTCTTC AAATAGAAGC CAGTTTTCTC CTCAGGGTTC CCACTCGTAC ATCTCAGACC CCCATCATTC   
  
  
+ TTCCGACAAC TATGGATCCC CAGTGAGCGG CTCTTCTGTG GTTGACGATA ATGCTGAGCT GAGGAACAGG   
  
  
+ TTCAGTGATA TGGAGCTCCC CTTGCCACAG GACTCAGGAC ACCATTATTG CTCTTTTAGC CACAGAGGAA   
  
  
+ GCCATGAAGG CTCCTATACT TTGAGGCCAA ACCAACTGAT GGATATGGCC AACATGGAGT TAAAGCAGGT   
  
  
+ GCTATACTTC TGTGCAGAAG CAATCTCAGA GAATAATCTA TCAACTGCAG AAAGACTAAT GGATGCATTG   
  
  
+ GGTAAGAGGG TGTCTGTTTT TGGTTCACCA ATTGAAAGGT TGGCCGCCTA CATGTTGGAA GGGCTCAGAG   
  
  
+ CAAGGCTGGA GTTTTCTGGA TATACTATCT ACAAAAAGCT CAGGTGCGAA CAGCCAACGA GCTCAGAGCT   
  
  
+ TCTTTCCTAC ATGCACATCC TGTATCAAAG TTGCCCATAT TTCAAATTCG CATATATGTC CTCAAATGTT   
  
  
+ GCTATTCAAG AAGCTTTGGG GAATGAGCCG GTTATCCACA TCATCGATTT CCAGATTGCC ATGGGGACAC   
  
  
+ AATTGGTGCT CTTGATCCAG TCTCTCGCCC ATCGGCCTGG CGGGCCCCCC CCTCGTTCGG ATCACTGGGG   
  
  
+ TCGATG  

- -Up\_Stream \_Len000AATAAG AATGTAGAAC AACAACACTA ACACATAATT CGAACAACAG AGTACCAAAC   
  
  
- GAGAAGAGAA ACCTTGGAGT ACCACCGATC AAGGTACAAA AACGAAACAT CTACATCGGC AAACAATTTC   
  
  
- GTTCAACCAA ATAAAGAGGA TACCGAAGCG AGATAACATT AAGTCCCCAG TCCAACAATA CAGTCTGTAA   
  
  
- GGGTATGGCG TGGATCTTCC CTCCTCCCTC CGAGTCCTCA GAATTTCTCA TATCTAAACC TAAGTGGACA   
  
  
- TCAGATATCG ACTACAGGTG GATTAGACCT AAAATAAGAA AGAAAGAGAG AGAAAAAAAA AGAAAAAAAC   
  
  
- CCCCCCCCCC ACAACAACTC CCTCTGTCCT ACTCCAGCTT AGAGACGTCG TATTGTTGGT GGTATCAGTG   
  
  
- TTATACCTCT AACACCTCCG TTTCAAACTC CTGCAGGCCT ACATAAAGGA AACCTTGACT CCTCCTCCAT   
  
  
- CATGAAGAGA CCATCATCAC AAGATATAGG AAACAAGGAA AGCACATAAT AGGATCGATG GAGAGTTCGT   
  
  
- TCAACTGTAC TTTAACATCT TAAGTTCTGT TATTATTGTC GACCAAAAAC CCAGAAAATC AAACGTTAGG   
  
  
- GAACATCGTA GAATCACTGA ATCACTGTAC TCTAAAACTG GGGTTATCTT TATCTTGTCT GTCGACGATT   
  
  
- TAAGTGGGTA GAGAACACAG TTTTAGCCTG AACAAAACGG AGACGAAGAG GTATTTCGTA GTTTCTCGCG   
  
  
- ACTAACTCCG TCCCTAAAAC AGTTCACGTC ACAACGTATA CAGTATAGCA CAACTACCAG TACAATAGTG   
  
  
- CCTGTATTTA AAGGGTCACA TTTCGTTTCC ACAGACAGAA GTGTATCTCA CAAGTTAAGT ATCAGTACGA   
  
  
- GAGCAGAGAC GTAGTAGGAC GCAAACGACC CCGATTTAAA CTCCCCGAAT AGTGAAACTT TAAGCCTCTT   
  
  
- AATGACACTT AAACACAATC AAGTCCGTAT TTTATGACCT ACCCTACAAC TCTACAAGAT TAGTAATAGA   
  
  
- TAACAATTTT TTGAGTCTTG ATAATATCTA AAAGACAAAA GACAAACCTA ACTCTAGGCT TCGGAAACTC   
  
  
- TAGGGGACAA AGATCGAAGG TTGGGGGGAG GGACCCAAGA AAAAGAAACC ACACTTTCAT ACTTGTTTTC   
  
  
- GACTACATTC ACACTAAGTT TGAGATCGGA ACCACTCTGA GAACCCTTCC CGAGGATCTC TGAAACGGTT   
  
  
- AACTCGATTA AACTACGGAT GATGAACGAA TGAAAAGATC GACAACAAAT TTGCTAAATA CAAGTATCAA   
  
  
- TTCCTCGCGG TATAACGTAA TGTTATAGAC GAAATCACGA AACAAAAAAC GTAAAAGAAG AAATAATGAT   
  
  
- ACCAAAAACT TCTTCCGAAT GACTGACTAT GACCAAAGAA GCGAATTCAA ACCACATGTA CGAAAAGTTA   
  
  
- GACACAACGT AAAAAGATAC TACATATTCG GCAAATAATG GATAACTACA AGTTGAAAAG TGTGATATAT   
  
  
- AGGAAATACC AAAGAAGCGA ATTCAAACCA CATGTACGAA AAGTTAGACA CAACGTAAAA AGACACTACA   
  
  
- TATTCGGCAA ATAATGGATA ACTACAAGTT GAAAAGTGTG ATATACAGGA AAATAATCAA TGACTACGGA   
  
  
- GACAGTTTGT AAGAATTAAC GTAAATCACA TAGTTGCCAG AATTTTAAAA TTCGTAACGG TTGAACTTGA   
  
  
- AGACTTCTAC AAACAGTTCT AGAAGTCAGA CGTTCAATTG ACGACGAACG AACTTGAAAA GTTGAAAATT   
  
  
- CTCTCTACAG TCTCAGATTC GTACCGAAGG ACTGTTCATG ACGTAAAATA GTGAATACTT CTGAGTTAAG   
  
  
- ACCCAAAACT GTCGAAGATA CTTGGAGATC ACGTCCATGT CGTCGAACCT GTAATTCGAC GTTCAGTTAA   
  
  
- CTGACATCTT GCTGTTCAAC AACTTCAAAG ACGACTTTCA AGTAGTGAAC AAACTACTTA TTTCATGTCG   
  
  
- TACCGTGAAG TCTACGTTTT CAGAGCGCTA GTCATCTCTA CTAAGGTATA AAGAGTAGAG TAAAGGTTCA   
  
  
- GAAACTGTTG TACATGGGTT TATTACGGTC GGTACTGTGA GTCTACAGAG AGGTTAGTAT GTCGCTTCCT   
  
  
- ATGAAGTGAG ACCTGAGTAG AGGTCGTCGT TAGCCTCGCA CGTCACAGAT ACTGATGGGT AGGCAACAGT   
  
  
- CGTGGAGAAG TTTATCTTCG GTCAAAAGAG GAGTCCCAAG GGTGAGCATG TAGAGTCTGG GGGTAGTAAG   
  
  
- AAGGCTGTTG ATACCTAGGG GTCACTCGCC GAGAAGACAC CAACTGCTAT TACGACTCGA CTCCTTGTCC   
  
  
- AAGTCACTAT ACCTCGAGGG GAACGGTGTC CTGAGTCCTG TGGTAATAAC GAGAAAATCG GTGTCTCCTT   
  
  
- CGGTACTTCC GAGGATATGA AACTCCGGTT TGGTTGACTA CCTATACCGG TTGTACCTCA ATTTCGTCCA   
  
  
- CGATATGAAG ACACGTCTTC GTTAGAGTCT CTTATTAGAT AGTTGACGTC TTTCTGATTA CCTACGTAAC   
  
  
- CCATTCTCCC ACAGACAAAA ACCAAGTGGT TAACTTTCCA ACCGGCGGAT GTACAACCTT CCCGAGTCTC   
  
  
- GTTCCGACCT CAAAAGACCT ATATGATAGA TGTTTTTCGA GTCCACGCTT GTCGGTTGCT CGAGTCTCGA   
  
  
- AGAAAGGATG TACGTGTAGG ACATAGTTTC AACGGGTATA AAGTTTAAGC GTATATACAG GAGTTTACAA   
  
  
- CGATAAGTTC TTCGAAACCC CTTACTCGGC CAATAGGTGT AGTAGCTAAA GGTCTAACGG TACCCCTGTG   
  
  
- TTAACCACGA GAACTAGGTC AGAGAGCGGG TAGCCGGACC GCCCGGGGGG GGAGCAAGCC TAGTGACCCC   
  
  
- AGCTAC

+     ARE

| Site Name | Organism | Position | Strand | Matrix score. | sequence | function |
| --- | --- | --- | --- | --- | --- | --- |
| ARE | Zea mays | 2483 | + | 6 | AAACCA | cis-acting regulatory element essential for the anaerobic induction |
| ARE | Zea mays | 606 | - | 6 | AAACCA | cis-acting regulatory element essential for the anaerobic induction |
| ARE | Zea mays | 1436 | - | 6 | AAACCA | cis-acting regulatory element essential for the anaerobic induction |
| ARE | Zea mays | 1552 | - | 6 | AAACCA | cis-acting regulatory element essential for the anaerobic induction |
| ARE | Zea mays | 150 | - | 6 | AAACCA | cis-acting regulatory element essential for the anaerobic induction |
| ARE | Zea mays | 68 | - | 6 | AAACCA | cis-acting regulatory element essential for the anaerobic induction |
| ARE | Zea mays | 1405 | - | 6 | AAACCA | cis-acting regulatory element essential for the anaerobic induction |

>HU08G02296.1   
+ -Up\_Stream \_Len000TTATTC TTACATCTTG TTGTTGTGAT TGTGTATTAA GCTTGTTGTC TCATGGTTTG   
  
  
+ CTCTTCTCTT TGGAACCTCA TGGTGGCTAG TTCCATGTTT TTGCTTTGTA GATGTAGCCG TTTGTTAAAG   
  
  
+ CAAGTTGGTT TATTTCTCCT ATGGCTTCGC TCTATTGTAA TTCAGGGGTC AGGTTGTTAT GTCAGACATT   
  
  
+ CCCATACCGC ACCTAGAAGG GAGGAGGGAG GCTCAGGAGT CTTAAAGAGT ATAGATTTGG ATTCACCTGT   
  
  
+ AGTCTATAGC TGATGTCCAC CTAATCTGGA TTTTATTCTT TCTTTCTCTC TCTTTTTTTT TCTTTTTTTG   
  
  
+ GGGGGGGGGG TGTTGTTGAG GGAGACAGGA TGAGGTCGAA TCTCTGCAGC ATAACAACCA CCATAGTCAC   
  
  
+ AATATGGAGA TTGTGGAGGC AAAGTTTGAG GACGTCCGGA TGTATTTCCT TTGGAACTGA GGAGGAGGTA   
  
  
+ GTACTTCTCT GGTAGTAGTG TTCTATATCC TTTGTTCCTT TCGTGTATTA TCCTAGCTAC CTCTCAAGCA   
  
  
+ AGTTGACATG AAATTGTAGA ATTCAAGACA ATAATAACAG CTGGTTTTTG GGTCTTTTAG TTTGCAATCC   
  
  
+ CTTGTAGCAT CTTAGTGACT TAGTGACATG AGATTTTGAC CCCAATAGAA ATAGAACAGA CAGCTGCTAA   
  
  
+ ATTCACCCAT CTCTTGTGTC AAAATCGGAC TTGTTTTGCC TCTGCTTCTC CATAAAGCAT CAAAGAGCGC   
  
  
+ TGATTGAGGC AGGGATTTTG TCAAGTGCAG TGTTGCATAT GTCATATCGT GTTGATGGTC ATGTTATCAC   
  
  
+ GGACATAAAT TTCCCAGTGT AAAGCAAAGG TGTCTGTCTT CACATAGAGT GTTCAATTCA TAGTCATGCT   
  
  
+ CTCGTCTCTG CATCATCCTG CGTTTGCTGG GGCTAAATTT GAGGGGCTTA TCACTTTGAA ATTCGGAGAA   
  
  
+ TTACTGTGAA TTTGTGTTAG TTCAGGCATA AAATACTGGA TGGGATGTTG AGATGTTCTA ATCATTATCT   
  
  
+ ATTGTTAAAA AACTCAGAAC TATTATAGAT TTTCTGTTTT CTGTTTGGAT TGAGATCCGA AGCCTTTGAG   
  
  
+ ATCCCCTGTT TCTAGCTTCC AACCCCCCTC CCTGGGTTCT TTTTCTTTGG TGTGAAAGTA TGAACAAAAG   
  
  
+ CTGATGTAAG TGTGATTCAA ACTCTAGCCT TGGTGAGACT CTTGGGAAGG GCTCCTAGAG ACTTTGCCAA   
  
  
+ TTGAGCTAAT TTGATGCCTA CTACTTGCTT ACTTTTCTAG CTGTTGTTTA AACGATTTAT GTTCATAGTT   
  
  
+ AAGGAGCGCC ATATTGCATT ACAATATCTG CTTTAGTGCT TTGTTTTTTG CATTTTCTTC TTTATTACTA   
  
  
+ TGGTTTTTGA AGAAGGCTTA CTGACTGATA CTGGTTTCTT CGCTTAAGTT TGGTGTACAT GCTTTTCAAT   
  
  
+ CTGTGTTGCA TTTTTCTATG ATGTATAAGC CGTTTATTAC CTATTGATGT TCAACTTTTC ACACTATATA   
  
  
+ TCCTTTATGG TTTCTTCGCT TAAGTTTGGT GTACATGCTT TTCAATCTGT GTTGCATTTT TCTGTGATGT   
  
  
+ ATAAGCCGTT TATTACCTAT TGATGTTCAA CTTTTCACAC TATATGTCCT TTTATTAGTT ACTGATGCCT   
  
  
+ CTGTCAAACA TTCTTAATTG CATTTAGTGT ATCAACGGTC TTAAAATTTT AAGCATTGCC AACTTGAACT   
  
  
+ TCTGAAGATG TTTGTCAAGA TCTTCAGTCT GCAAGTTAAC TGCTGCTTGC TTGAACTTTT CAACTTTTAA   
  
  
+ GAGAGATGTC AGAGTCTAAG CATGGCTTCC TGACAAGTAC TGCATTTTAT CACTTATGAA GACTCAATTC   
  
  
+ TGGGTTTTGA CAGCTTCTAT GAACCTCTAG TGCAGGTACA GCAGCTTGGA CATTAAGCTG CAAGTCAATT   
  
  
+ GACTGTAGAA CGACAAGTTG TTGAAGTTTC TGCTGAAAGT TCATCACTTG TTTGATGAAT AAAGTACAGC   
  
  
+ ATGGCACTTC AGATGCAAAA GTCTCGCGAT CAGTAGAGAT GATTCCATAT TTCTCATCTC ATTTCCAAGT   
  
  
+ CTTTGACAAC ATGTACCCAA ATAATGCCAG CCATGACACT CAGATGTCTC TCCAATCATA CAGCGAAGGA   
  
  
+ TACTTCACTC TGGACTCATC TCCAGCAGCA ATCGGAGCGT GCAGTGTCTA TGACTACCCA TCCGTTGTCA   
  
  
+ GCACCTCTTC AAATAGAAGC CAGTTTTCTC CTCAGGGTTC CCACTCGTAC ATCTCAGACC CCCATCATTC   
  
  
+ TTCCGACAAC TATGGATCCC CAGTGAGCGG CTCTTCTGTG GTTGACGATA ATGCTGAGCT GAGGAACAGG   
  
  
+ TTCAGTGATA TGGAGCTCCC CTTGCCACAG GACTCAGGAC ACCATTATTG CTCTTTTAGC CACAGAGGAA   
  
  
+ GCCATGAAGG CTCCTATACT TTGAGGCCAA ACCAACTGAT GGATATGGCC AACATGGAGT TAAAGCAGGT   
  
  
+ GCTATACTTC TGTGCAGAAG CAATCTCAGA GAATAATCTA TCAACTGCAG AAAGACTAAT GGATGCATTG   
  
  
+ GGTAAGAGGG TGTCTGTTTT TGGTTCACCA ATTGAAAGGT TGGCCGCCTA CATGTTGGAA GGGCTCAGAG   
  
  
+ CAAGGCTGGA GTTTTCTGGA TATACTATCT ACAAAAAGCT CAGGTGCGAA CAGCCAACGA GCTCAGAGCT   
  
  
+ TCTTTCCTAC ATGCACATCC TGTATCAAAG TTGCCCATAT TTCAAATTCG CATATATGTC CTCAAATGTT   
  
  
+ GCTATTCAAG AAGCTTTGGG GAATGAGCCG GTTATCCACA TCATCGATTT CCAGATTGCC ATGGGGACAC   
  
  
+ AATTGGTGCT CTTGATCCAG TCTCTCGCCC ATCGGCCTGG CGGGCCCCCC CCTCGTTCGG ATCACTGGGG   
  
  
+ TCGATG  

- -Up\_Stream \_Len000AATAAG AATGTAGAAC AACAACACTA ACACATAATT CGAACAACAG AGTACCAAAC   
  
  
- GAGAAGAGAA ACCTTGGAGT ACCACCGATC AAGGTACAAA AACGAAACAT CTACATCGGC AAACAATTTC   
  
  
- GTTCAACCAA ATAAAGAGGA TACCGAAGCG AGATAACATT AAGTCCCCAG TCCAACAATA CAGTCTGTAA   
  
  
- GGGTATGGCG TGGATCTTCC CTCCTCCCTC CGAGTCCTCA GAATTTCTCA TATCTAAACC TAAGTGGACA   
  
  
- TCAGATATCG ACTACAGGTG GATTAGACCT AAAATAAGAA AGAAAGAGAG AGAAAAAAAA AGAAAAAAAC   
  
  
- CCCCCCCCCC ACAACAACTC CCTCTGTCCT ACTCCAGCTT AGAGACGTCG TATTGTTGGT GGTATCAGTG   
  
  
- TTATACCTCT AACACCTCCG TTTCAAACTC CTGCAGGCCT ACATAAAGGA AACCTTGACT CCTCCTCCAT   
  
  
- CATGAAGAGA CCATCATCAC AAGATATAGG AAACAAGGAA AGCACATAAT AGGATCGATG GAGAGTTCGT   
  
  
- TCAACTGTAC TTTAACATCT TAAGTTCTGT TATTATTGTC GACCAAAAAC CCAGAAAATC AAACGTTAGG   
  
  
- GAACATCGTA GAATCACTGA ATCACTGTAC TCTAAAACTG GGGTTATCTT TATCTTGTCT GTCGACGATT   
  
  
- TAAGTGGGTA GAGAACACAG TTTTAGCCTG AACAAAACGG AGACGAAGAG GTATTTCGTA GTTTCTCGCG   
  
  
- ACTAACTCCG TCCCTAAAAC AGTTCACGTC ACAACGTATA CAGTATAGCA CAACTACCAG TACAATAGTG   
  
  
- CCTGTATTTA AAGGGTCACA TTTCGTTTCC ACAGACAGAA GTGTATCTCA CAAGTTAAGT ATCAGTACGA   
  
  
- GAGCAGAGAC GTAGTAGGAC GCAAACGACC CCGATTTAAA CTCCCCGAAT AGTGAAACTT TAAGCCTCTT   
  
  
- AATGACACTT AAACACAATC AAGTCCGTAT TTTATGACCT ACCCTACAAC TCTACAAGAT TAGTAATAGA   
  
  
- TAACAATTTT TTGAGTCTTG ATAATATCTA AAAGACAAAA GACAAACCTA ACTCTAGGCT TCGGAAACTC   
  
  
- TAGGGGACAA AGATCGAAGG TTGGGGGGAG GGACCCAAGA AAAAGAAACC ACACTTTCAT ACTTGTTTTC   
  
  
- GACTACATTC ACACTAAGTT TGAGATCGGA ACCACTCTGA GAACCCTTCC CGAGGATCTC TGAAACGGTT   
  
  
- AACTCGATTA AACTACGGAT GATGAACGAA TGAAAAGATC GACAACAAAT TTGCTAAATA CAAGTATCAA   
  
  
- TTCCTCGCGG TATAACGTAA TGTTATAGAC GAAATCACGA AACAAAAAAC GTAAAAGAAG AAATAATGAT   
  
  
- ACCAAAAACT TCTTCCGAAT GACTGACTAT GACCAAAGAA GCGAATTCAA ACCACATGTA CGAAAAGTTA   
  
  
- GACACAACGT AAAAAGATAC TACATATTCG GCAAATAATG GATAACTACA AGTTGAAAAG TGTGATATAT   
  
  
- AGGAAATACC AAAGAAGCGA ATTCAAACCA CATGTACGAA AAGTTAGACA CAACGTAAAA AGACACTACA   
  
  
- TATTCGGCAA ATAATGGATA ACTACAAGTT GAAAAGTGTG ATATACAGGA AAATAATCAA TGACTACGGA   
  
  
- GACAGTTTGT AAGAATTAAC GTAAATCACA TAGTTGCCAG AATTTTAAAA TTCGTAACGG TTGAACTTGA   
  
  
- AGACTTCTAC AAACAGTTCT AGAAGTCAGA CGTTCAATTG ACGACGAACG AACTTGAAAA GTTGAAAATT   
  
  
- CTCTCTACAG TCTCAGATTC GTACCGAAGG ACTGTTCATG ACGTAAAATA GTGAATACTT CTGAGTTAAG   
  
  
- ACCCAAAACT GTCGAAGATA CTTGGAGATC ACGTCCATGT CGTCGAACCT GTAATTCGAC GTTCAGTTAA   
  
  
- CTGACATCTT GCTGTTCAAC AACTTCAAAG ACGACTTTCA AGTAGTGAAC AAACTACTTA TTTCATGTCG   
  
  
- TACCGTGAAG TCTACGTTTT CAGAGCGCTA GTCATCTCTA CTAAGGTATA AAGAGTAGAG TAAAGGTTCA   
  
  
- GAAACTGTTG TACATGGGTT TATTACGGTC GGTACTGTGA GTCTACAGAG AGGTTAGTAT GTCGCTTCCT   
  
  
- ATGAAGTGAG ACCTGAGTAG AGGTCGTCGT TAGCCTCGCA CGTCACAGAT ACTGATGGGT AGGCAACAGT   
  
  
- CGTGGAGAAG TTTATCTTCG GTCAAAAGAG GAGTCCCAAG GGTGAGCATG TAGAGTCTGG GGGTAGTAAG   
  
  
- AAGGCTGTTG ATACCTAGGG GTCACTCGCC GAGAAGACAC CAACTGCTAT TACGACTCGA CTCCTTGTCC   
  
  
- AAGTCACTAT ACCTCGAGGG GAACGGTGTC CTGAGTCCTG TGGTAATAAC GAGAAAATCG GTGTCTCCTT   
  
  
- CGGTACTTCC GAGGATATGA AACTCCGGTT TGGTTGACTA CCTATACCGG TTGTACCTCA ATTTCGTCCA   
  
  
- CGATATGAAG ACACGTCTTC GTTAGAGTCT CTTATTAGAT AGTTGACGTC TTTCTGATTA CCTACGTAAC   
  
  
- CCATTCTCCC ACAGACAAAA ACCAAGTGGT TAACTTTCCA ACCGGCGGAT GTACAACCTT CCCGAGTCTC   
  
  
- GTTCCGACCT CAAAAGACCT ATATGATAGA TGTTTTTCGA GTCCACGCTT GTCGGTTGCT CGAGTCTCGA   
  
  
- AGAAAGGATG TACGTGTAGG ACATAGTTTC AACGGGTATA AAGTTTAAGC GTATATACAG GAGTTTACAA   
  
  
- CGATAAGTTC TTCGAAACCC CTTACTCGGC CAATAGGTGT AGTAGCTAAA GGTCTAACGG TACCCCTGTG   
  
  
- TTAACCACGA GAACTAGGTC AGAGAGCGGG TAGCCGGACC GCCCGGGGGG GGAGCAAGCC TAGTGACCCC   
  
  
- AGCTAC

+     AT-rich sequence

| Site Name | Organism | Position | Strand | Matrix score. | sequence | function |
| --- | --- | --- | --- | --- | --- | --- |
| AT-rich sequence | Pisum sativum | 1013 | + | 9 | TAAAATACT | element for maximal elicitor-mediated activation (2copies) |

>HU08G02296.1   
+ -Up\_Stream \_Len000TTATTC TTACATCTTG TTGTTGTGAT TGTGTATTAA GCTTGTTGTC TCATGGTTTG   
  
  
+ CTCTTCTCTT TGGAACCTCA TGGTGGCTAG TTCCATGTTT TTGCTTTGTA GATGTAGCCG TTTGTTAAAG   
  
  
+ CAAGTTGGTT TATTTCTCCT ATGGCTTCGC TCTATTGTAA TTCAGGGGTC AGGTTGTTAT GTCAGACATT   
  
  
+ CCCATACCGC ACCTAGAAGG GAGGAGGGAG GCTCAGGAGT CTTAAAGAGT ATAGATTTGG ATTCACCTGT   
  
  
+ AGTCTATAGC TGATGTCCAC CTAATCTGGA TTTTATTCTT TCTTTCTCTC TCTTTTTTTT TCTTTTTTTG   
  
  
+ GGGGGGGGGG TGTTGTTGAG GGAGACAGGA TGAGGTCGAA TCTCTGCAGC ATAACAACCA CCATAGTCAC   
  
  
+ AATATGGAGA TTGTGGAGGC AAAGTTTGAG GACGTCCGGA TGTATTTCCT TTGGAACTGA GGAGGAGGTA   
  
  
+ GTACTTCTCT GGTAGTAGTG TTCTATATCC TTTGTTCCTT TCGTGTATTA TCCTAGCTAC CTCTCAAGCA   
  
  
+ AGTTGACATG AAATTGTAGA ATTCAAGACA ATAATAACAG CTGGTTTTTG GGTCTTTTAG TTTGCAATCC   
  
  
+ CTTGTAGCAT CTTAGTGACT TAGTGACATG AGATTTTGAC CCCAATAGAA ATAGAACAGA CAGCTGCTAA   
  
  
+ ATTCACCCAT CTCTTGTGTC AAAATCGGAC TTGTTTTGCC TCTGCTTCTC CATAAAGCAT CAAAGAGCGC   
  
  
+ TGATTGAGGC AGGGATTTTG TCAAGTGCAG TGTTGCATAT GTCATATCGT GTTGATGGTC ATGTTATCAC   
  
  
+ GGACATAAAT TTCCCAGTGT AAAGCAAAGG TGTCTGTCTT CACATAGAGT GTTCAATTCA TAGTCATGCT   
  
  
+ CTCGTCTCTG CATCATCCTG CGTTTGCTGG GGCTAAATTT GAGGGGCTTA TCACTTTGAA ATTCGGAGAA   
  
  
+ TTACTGTGAA TTTGTGTTAG TTCAGGCATA AAATACTGGA TGGGATGTTG AGATGTTCTA ATCATTATCT   
  
  
+ ATTGTTAAAA AACTCAGAAC TATTATAGAT TTTCTGTTTT CTGTTTGGAT TGAGATCCGA AGCCTTTGAG   
  
  
+ ATCCCCTGTT TCTAGCTTCC AACCCCCCTC CCTGGGTTCT TTTTCTTTGG TGTGAAAGTA TGAACAAAAG   
  
  
+ CTGATGTAAG TGTGATTCAA ACTCTAGCCT TGGTGAGACT CTTGGGAAGG GCTCCTAGAG ACTTTGCCAA   
  
  
+ TTGAGCTAAT TTGATGCCTA CTACTTGCTT ACTTTTCTAG CTGTTGTTTA AACGATTTAT GTTCATAGTT   
  
  
+ AAGGAGCGCC ATATTGCATT ACAATATCTG CTTTAGTGCT TTGTTTTTTG CATTTTCTTC TTTATTACTA   
  
  
+ TGGTTTTTGA AGAAGGCTTA CTGACTGATA CTGGTTTCTT CGCTTAAGTT TGGTGTACAT GCTTTTCAAT   
  
  
+ CTGTGTTGCA TTTTTCTATG ATGTATAAGC CGTTTATTAC CTATTGATGT TCAACTTTTC ACACTATATA   
  
  
+ TCCTTTATGG TTTCTTCGCT TAAGTTTGGT GTACATGCTT TTCAATCTGT GTTGCATTTT TCTGTGATGT   
  
  
+ ATAAGCCGTT TATTACCTAT TGATGTTCAA CTTTTCACAC TATATGTCCT TTTATTAGTT ACTGATGCCT   
  
  
+ CTGTCAAACA TTCTTAATTG CATTTAGTGT ATCAACGGTC TTAAAATTTT AAGCATTGCC AACTTGAACT   
  
  
+ TCTGAAGATG TTTGTCAAGA TCTTCAGTCT GCAAGTTAAC TGCTGCTTGC TTGAACTTTT CAACTTTTAA   
  
  
+ GAGAGATGTC AGAGTCTAAG CATGGCTTCC TGACAAGTAC TGCATTTTAT CACTTATGAA GACTCAATTC   
  
  
+ TGGGTTTTGA CAGCTTCTAT GAACCTCTAG TGCAGGTACA GCAGCTTGGA CATTAAGCTG CAAGTCAATT   
  
  
+ GACTGTAGAA CGACAAGTTG TTGAAGTTTC TGCTGAAAGT TCATCACTTG TTTGATGAAT AAAGTACAGC   
  
  
+ ATGGCACTTC AGATGCAAAA GTCTCGCGAT CAGTAGAGAT GATTCCATAT TTCTCATCTC ATTTCCAAGT   
  
  
+ CTTTGACAAC ATGTACCCAA ATAATGCCAG CCATGACACT CAGATGTCTC TCCAATCATA CAGCGAAGGA   
  
  
+ TACTTCACTC TGGACTCATC TCCAGCAGCA ATCGGAGCGT GCAGTGTCTA TGACTACCCA TCCGTTGTCA   
  
  
+ GCACCTCTTC AAATAGAAGC CAGTTTTCTC CTCAGGGTTC CCACTCGTAC ATCTCAGACC CCCATCATTC   
  
  
+ TTCCGACAAC TATGGATCCC CAGTGAGCGG CTCTTCTGTG GTTGACGATA ATGCTGAGCT GAGGAACAGG   
  
  
+ TTCAGTGATA TGGAGCTCCC CTTGCCACAG GACTCAGGAC ACCATTATTG CTCTTTTAGC CACAGAGGAA   
  
  
+ GCCATGAAGG CTCCTATACT TTGAGGCCAA ACCAACTGAT GGATATGGCC AACATGGAGT TAAAGCAGGT   
  
  
+ GCTATACTTC TGTGCAGAAG CAATCTCAGA GAATAATCTA TCAACTGCAG AAAGACTAAT GGATGCATTG   
  
  
+ GGTAAGAGGG TGTCTGTTTT TGGTTCACCA ATTGAAAGGT TGGCCGCCTA CATGTTGGAA GGGCTCAGAG   
  
  
+ CAAGGCTGGA GTTTTCTGGA TATACTATCT ACAAAAAGCT CAGGTGCGAA CAGCCAACGA GCTCAGAGCT   
  
  
+ TCTTTCCTAC ATGCACATCC TGTATCAAAG TTGCCCATAT TTCAAATTCG CATATATGTC CTCAAATGTT   
  
  
+ GCTATTCAAG AAGCTTTGGG GAATGAGCCG GTTATCCACA TCATCGATTT CCAGATTGCC ATGGGGACAC   
  
  
+ AATTGGTGCT CTTGATCCAG TCTCTCGCCC ATCGGCCTGG CGGGCCCCCC CCTCGTTCGG ATCACTGGGG   
  
  
+ TCGATG  

- -Up\_Stream \_Len000AATAAG AATGTAGAAC AACAACACTA ACACATAATT CGAACAACAG AGTACCAAAC   
  
  
- GAGAAGAGAA ACCTTGGAGT ACCACCGATC AAGGTACAAA AACGAAACAT CTACATCGGC AAACAATTTC   
  
  
- GTTCAACCAA ATAAAGAGGA TACCGAAGCG AGATAACATT AAGTCCCCAG TCCAACAATA CAGTCTGTAA   
  
  
- GGGTATGGCG TGGATCTTCC CTCCTCCCTC CGAGTCCTCA GAATTTCTCA TATCTAAACC TAAGTGGACA   
  
  
- TCAGATATCG ACTACAGGTG GATTAGACCT AAAATAAGAA AGAAAGAGAG AGAAAAAAAA AGAAAAAAAC   
  
  
- CCCCCCCCCC ACAACAACTC CCTCTGTCCT ACTCCAGCTT AGAGACGTCG TATTGTTGGT GGTATCAGTG   
  
  
- TTATACCTCT AACACCTCCG TTTCAAACTC CTGCAGGCCT ACATAAAGGA AACCTTGACT CCTCCTCCAT   
  
  
- CATGAAGAGA CCATCATCAC AAGATATAGG AAACAAGGAA AGCACATAAT AGGATCGATG GAGAGTTCGT   
  
  
- TCAACTGTAC TTTAACATCT TAAGTTCTGT TATTATTGTC GACCAAAAAC CCAGAAAATC AAACGTTAGG   
  
  
- GAACATCGTA GAATCACTGA ATCACTGTAC TCTAAAACTG GGGTTATCTT TATCTTGTCT GTCGACGATT   
  
  
- TAAGTGGGTA GAGAACACAG TTTTAGCCTG AACAAAACGG AGACGAAGAG GTATTTCGTA GTTTCTCGCG   
  
  
- ACTAACTCCG TCCCTAAAAC AGTTCACGTC ACAACGTATA CAGTATAGCA CAACTACCAG TACAATAGTG   
  
  
- CCTGTATTTA AAGGGTCACA TTTCGTTTCC ACAGACAGAA GTGTATCTCA CAAGTTAAGT ATCAGTACGA   
  
  
- GAGCAGAGAC GTAGTAGGAC GCAAACGACC CCGATTTAAA CTCCCCGAAT AGTGAAACTT TAAGCCTCTT   
  
  
- AATGACACTT AAACACAATC AAGTCCGTAT TTTATGACCT ACCCTACAAC TCTACAAGAT TAGTAATAGA   
  
  
- TAACAATTTT TTGAGTCTTG ATAATATCTA AAAGACAAAA GACAAACCTA ACTCTAGGCT TCGGAAACTC   
  
  
- TAGGGGACAA AGATCGAAGG TTGGGGGGAG GGACCCAAGA AAAAGAAACC ACACTTTCAT ACTTGTTTTC   
  
  
- GACTACATTC ACACTAAGTT TGAGATCGGA ACCACTCTGA GAACCCTTCC CGAGGATCTC TGAAACGGTT   
  
  
- AACTCGATTA AACTACGGAT GATGAACGAA TGAAAAGATC GACAACAAAT TTGCTAAATA CAAGTATCAA   
  
  
- TTCCTCGCGG TATAACGTAA TGTTATAGAC GAAATCACGA AACAAAAAAC GTAAAAGAAG AAATAATGAT   
  
  
- ACCAAAAACT TCTTCCGAAT GACTGACTAT GACCAAAGAA GCGAATTCAA ACCACATGTA CGAAAAGTTA   
  
  
- GACACAACGT AAAAAGATAC TACATATTCG GCAAATAATG GATAACTACA AGTTGAAAAG TGTGATATAT   
  
  
- AGGAAATACC AAAGAAGCGA ATTCAAACCA CATGTACGAA AAGTTAGACA CAACGTAAAA AGACACTACA   
  
  
- TATTCGGCAA ATAATGGATA ACTACAAGTT GAAAAGTGTG ATATACAGGA AAATAATCAA TGACTACGGA   
  
  
- GACAGTTTGT AAGAATTAAC GTAAATCACA TAGTTGCCAG AATTTTAAAA TTCGTAACGG TTGAACTTGA   
  
  
- AGACTTCTAC AAACAGTTCT AGAAGTCAGA CGTTCAATTG ACGACGAACG AACTTGAAAA GTTGAAAATT   
  
  
- CTCTCTACAG TCTCAGATTC GTACCGAAGG ACTGTTCATG ACGTAAAATA GTGAATACTT CTGAGTTAAG   
  
  
- ACCCAAAACT GTCGAAGATA CTTGGAGATC ACGTCCATGT CGTCGAACCT GTAATTCGAC GTTCAGTTAA   
  
  
- CTGACATCTT GCTGTTCAAC AACTTCAAAG ACGACTTTCA AGTAGTGAAC AAACTACTTA TTTCATGTCG   
  
  
- TACCGTGAAG TCTACGTTTT CAGAGCGCTA GTCATCTCTA CTAAGGTATA AAGAGTAGAG TAAAGGTTCA   
  
  
- GAAACTGTTG TACATGGGTT TATTACGGTC GGTACTGTGA GTCTACAGAG AGGTTAGTAT GTCGCTTCCT   
  
  
- ATGAAGTGAG ACCTGAGTAG AGGTCGTCGT TAGCCTCGCA CGTCACAGAT ACTGATGGGT AGGCAACAGT   
  
  
- CGTGGAGAAG TTTATCTTCG GTCAAAAGAG GAGTCCCAAG GGTGAGCATG TAGAGTCTGG GGGTAGTAAG   
  
  
- AAGGCTGTTG ATACCTAGGG GTCACTCGCC GAGAAGACAC CAACTGCTAT TACGACTCGA CTCCTTGTCC   
  
  
- AAGTCACTAT ACCTCGAGGG GAACGGTGTC CTGAGTCCTG TGGTAATAAC GAGAAAATCG GTGTCTCCTT   
  
  
- CGGTACTTCC GAGGATATGA AACTCCGGTT TGGTTGACTA CCTATACCGG TTGTACCTCA ATTTCGTCCA   
  
  
- CGATATGAAG ACACGTCTTC GTTAGAGTCT CTTATTAGAT AGTTGACGTC TTTCTGATTA CCTACGTAAC   
  
  
- CCATTCTCCC ACAGACAAAA ACCAAGTGGT TAACTTTCCA ACCGGCGGAT GTACAACCTT CCCGAGTCTC   
  
  
- GTTCCGACCT CAAAAGACCT ATATGATAGA TGTTTTTCGA GTCCACGCTT GTCGGTTGCT CGAGTCTCGA   
  
  
- AGAAAGGATG TACGTGTAGG ACATAGTTTC AACGGGTATA AAGTTTAAGC GTATATACAG GAGTTTACAA   
  
  
- CGATAAGTTC TTCGAAACCC CTTACTCGGC CAATAGGTGT AGTAGCTAAA GGTCTAACGG TACCCCTGTG   
  
  
- TTAACCACGA GAACTAGGTC AGAGAGCGGG TAGCCGGACC GCCCGGGGGG GGAGCAAGCC TAGTGACCCC   
  
  
- AGCTAC

+     AT~TATA-box

| Site Name | Organism | Position | Strand | Matrix score. | sequence | function |
| --- | --- | --- | --- | --- | --- | --- |
| AT~TATA-box | Arabidopsis thaliana | 1539 | - | 6 | TATATA |  |

>HU08G02296.1   
+ -Up\_Stream \_Len000TTATTC TTACATCTTG TTGTTGTGAT TGTGTATTAA GCTTGTTGTC TCATGGTTTG   
  
  
+ CTCTTCTCTT TGGAACCTCA TGGTGGCTAG TTCCATGTTT TTGCTTTGTA GATGTAGCCG TTTGTTAAAG   
  
  
+ CAAGTTGGTT TATTTCTCCT ATGGCTTCGC TCTATTGTAA TTCAGGGGTC AGGTTGTTAT GTCAGACATT   
  
  
+ CCCATACCGC ACCTAGAAGG GAGGAGGGAG GCTCAGGAGT CTTAAAGAGT ATAGATTTGG ATTCACCTGT   
  
  
+ AGTCTATAGC TGATGTCCAC CTAATCTGGA TTTTATTCTT TCTTTCTCTC TCTTTTTTTT TCTTTTTTTG   
  
  
+ GGGGGGGGGG TGTTGTTGAG GGAGACAGGA TGAGGTCGAA TCTCTGCAGC ATAACAACCA CCATAGTCAC   
  
  
+ AATATGGAGA TTGTGGAGGC AAAGTTTGAG GACGTCCGGA TGTATTTCCT TTGGAACTGA GGAGGAGGTA   
  
  
+ GTACTTCTCT GGTAGTAGTG TTCTATATCC TTTGTTCCTT TCGTGTATTA TCCTAGCTAC CTCTCAAGCA   
  
  
+ AGTTGACATG AAATTGTAGA ATTCAAGACA ATAATAACAG CTGGTTTTTG GGTCTTTTAG TTTGCAATCC   
  
  
+ CTTGTAGCAT CTTAGTGACT TAGTGACATG AGATTTTGAC CCCAATAGAA ATAGAACAGA CAGCTGCTAA   
  
  
+ ATTCACCCAT CTCTTGTGTC AAAATCGGAC TTGTTTTGCC TCTGCTTCTC CATAAAGCAT CAAAGAGCGC   
  
  
+ TGATTGAGGC AGGGATTTTG TCAAGTGCAG TGTTGCATAT GTCATATCGT GTTGATGGTC ATGTTATCAC   
  
  
+ GGACATAAAT TTCCCAGTGT AAAGCAAAGG TGTCTGTCTT CACATAGAGT GTTCAATTCA TAGTCATGCT   
  
  
+ CTCGTCTCTG CATCATCCTG CGTTTGCTGG GGCTAAATTT GAGGGGCTTA TCACTTTGAA ATTCGGAGAA   
  
  
+ TTACTGTGAA TTTGTGTTAG TTCAGGCATA AAATACTGGA TGGGATGTTG AGATGTTCTA ATCATTATCT   
  
  
+ ATTGTTAAAA AACTCAGAAC TATTATAGAT TTTCTGTTTT CTGTTTGGAT TGAGATCCGA AGCCTTTGAG   
  
  
+ ATCCCCTGTT TCTAGCTTCC AACCCCCCTC CCTGGGTTCT TTTTCTTTGG TGTGAAAGTA TGAACAAAAG   
  
  
+ CTGATGTAAG TGTGATTCAA ACTCTAGCCT TGGTGAGACT CTTGGGAAGG GCTCCTAGAG ACTTTGCCAA   
  
  
+ TTGAGCTAAT TTGATGCCTA CTACTTGCTT ACTTTTCTAG CTGTTGTTTA AACGATTTAT GTTCATAGTT   
  
  
+ AAGGAGCGCC ATATTGCATT ACAATATCTG CTTTAGTGCT TTGTTTTTTG CATTTTCTTC TTTATTACTA   
  
  
+ TGGTTTTTGA AGAAGGCTTA CTGACTGATA CTGGTTTCTT CGCTTAAGTT TGGTGTACAT GCTTTTCAAT   
  
  
+ CTGTGTTGCA TTTTTCTATG ATGTATAAGC CGTTTATTAC CTATTGATGT TCAACTTTTC ACACTATATA   
  
  
+ TCCTTTATGG TTTCTTCGCT TAAGTTTGGT GTACATGCTT TTCAATCTGT GTTGCATTTT TCTGTGATGT   
  
  
+ ATAAGCCGTT TATTACCTAT TGATGTTCAA CTTTTCACAC TATATGTCCT TTTATTAGTT ACTGATGCCT   
  
  
+ CTGTCAAACA TTCTTAATTG CATTTAGTGT ATCAACGGTC TTAAAATTTT AAGCATTGCC AACTTGAACT   
  
  
+ TCTGAAGATG TTTGTCAAGA TCTTCAGTCT GCAAGTTAAC TGCTGCTTGC TTGAACTTTT CAACTTTTAA   
  
  
+ GAGAGATGTC AGAGTCTAAG CATGGCTTCC TGACAAGTAC TGCATTTTAT CACTTATGAA GACTCAATTC   
  
  
+ TGGGTTTTGA CAGCTTCTAT GAACCTCTAG TGCAGGTACA GCAGCTTGGA CATTAAGCTG CAAGTCAATT   
  
  
+ GACTGTAGAA CGACAAGTTG TTGAAGTTTC TGCTGAAAGT TCATCACTTG TTTGATGAAT AAAGTACAGC   
  
  
+ ATGGCACTTC AGATGCAAAA GTCTCGCGAT CAGTAGAGAT GATTCCATAT TTCTCATCTC ATTTCCAAGT   
  
  
+ CTTTGACAAC ATGTACCCAA ATAATGCCAG CCATGACACT CAGATGTCTC TCCAATCATA CAGCGAAGGA   
  
  
+ TACTTCACTC TGGACTCATC TCCAGCAGCA ATCGGAGCGT GCAGTGTCTA TGACTACCCA TCCGTTGTCA   
  
  
+ GCACCTCTTC AAATAGAAGC CAGTTTTCTC CTCAGGGTTC CCACTCGTAC ATCTCAGACC CCCATCATTC   
  
  
+ TTCCGACAAC TATGGATCCC CAGTGAGCGG CTCTTCTGTG GTTGACGATA ATGCTGAGCT GAGGAACAGG   
  
  
+ TTCAGTGATA TGGAGCTCCC CTTGCCACAG GACTCAGGAC ACCATTATTG CTCTTTTAGC CACAGAGGAA   
  
  
+ GCCATGAAGG CTCCTATACT TTGAGGCCAA ACCAACTGAT GGATATGGCC AACATGGAGT TAAAGCAGGT   
  
  
+ GCTATACTTC TGTGCAGAAG CAATCTCAGA GAATAATCTA TCAACTGCAG AAAGACTAAT GGATGCATTG   
  
  
+ GGTAAGAGGG TGTCTGTTTT TGGTTCACCA ATTGAAAGGT TGGCCGCCTA CATGTTGGAA GGGCTCAGAG   
  
  
+ CAAGGCTGGA GTTTTCTGGA TATACTATCT ACAAAAAGCT CAGGTGCGAA CAGCCAACGA GCTCAGAGCT   
  
  
+ TCTTTCCTAC ATGCACATCC TGTATCAAAG TTGCCCATAT TTCAAATTCG CATATATGTC CTCAAATGTT   
  
  
+ GCTATTCAAG AAGCTTTGGG GAATGAGCCG GTTATCCACA TCATCGATTT CCAGATTGCC ATGGGGACAC   
  
  
+ AATTGGTGCT CTTGATCCAG TCTCTCGCCC ATCGGCCTGG CGGGCCCCCC CCTCGTTCGG ATCACTGGGG   
  
  
+ TCGATG  

- -Up\_Stream \_Len000AATAAG AATGTAGAAC AACAACACTA ACACATAATT CGAACAACAG AGTACCAAAC   
  
  
- GAGAAGAGAA ACCTTGGAGT ACCACCGATC AAGGTACAAA AACGAAACAT CTACATCGGC AAACAATTTC   
  
  
- GTTCAACCAA ATAAAGAGGA TACCGAAGCG AGATAACATT AAGTCCCCAG TCCAACAATA CAGTCTGTAA   
  
  
- GGGTATGGCG TGGATCTTCC CTCCTCCCTC CGAGTCCTCA GAATTTCTCA TATCTAAACC TAAGTGGACA   
  
  
- TCAGATATCG ACTACAGGTG GATTAGACCT AAAATAAGAA AGAAAGAGAG AGAAAAAAAA AGAAAAAAAC   
  
  
- CCCCCCCCCC ACAACAACTC CCTCTGTCCT ACTCCAGCTT AGAGACGTCG TATTGTTGGT GGTATCAGTG   
  
  
- TTATACCTCT AACACCTCCG TTTCAAACTC CTGCAGGCCT ACATAAAGGA AACCTTGACT CCTCCTCCAT   
  
  
- CATGAAGAGA CCATCATCAC AAGATATAGG AAACAAGGAA AGCACATAAT AGGATCGATG GAGAGTTCGT   
  
  
- TCAACTGTAC TTTAACATCT TAAGTTCTGT TATTATTGTC GACCAAAAAC CCAGAAAATC AAACGTTAGG   
  
  
- GAACATCGTA GAATCACTGA ATCACTGTAC TCTAAAACTG GGGTTATCTT TATCTTGTCT GTCGACGATT   
  
  
- TAAGTGGGTA GAGAACACAG TTTTAGCCTG AACAAAACGG AGACGAAGAG GTATTTCGTA GTTTCTCGCG   
  
  
- ACTAACTCCG TCCCTAAAAC AGTTCACGTC ACAACGTATA CAGTATAGCA CAACTACCAG TACAATAGTG   
  
  
- CCTGTATTTA AAGGGTCACA TTTCGTTTCC ACAGACAGAA GTGTATCTCA CAAGTTAAGT ATCAGTACGA   
  
  
- GAGCAGAGAC GTAGTAGGAC GCAAACGACC CCGATTTAAA CTCCCCGAAT AGTGAAACTT TAAGCCTCTT   
  
  
- AATGACACTT AAACACAATC AAGTCCGTAT TTTATGACCT ACCCTACAAC TCTACAAGAT TAGTAATAGA   
  
  
- TAACAATTTT TTGAGTCTTG ATAATATCTA AAAGACAAAA GACAAACCTA ACTCTAGGCT TCGGAAACTC   
  
  
- TAGGGGACAA AGATCGAAGG TTGGGGGGAG GGACCCAAGA AAAAGAAACC ACACTTTCAT ACTTGTTTTC   
  
  
- GACTACATTC ACACTAAGTT TGAGATCGGA ACCACTCTGA GAACCCTTCC CGAGGATCTC TGAAACGGTT   
  
  
- AACTCGATTA AACTACGGAT GATGAACGAA TGAAAAGATC GACAACAAAT TTGCTAAATA CAAGTATCAA   
  
  
- TTCCTCGCGG TATAACGTAA TGTTATAGAC GAAATCACGA AACAAAAAAC GTAAAAGAAG AAATAATGAT   
  
  
- ACCAAAAACT TCTTCCGAAT GACTGACTAT GACCAAAGAA GCGAATTCAA ACCACATGTA CGAAAAGTTA   
  
  
- GACACAACGT AAAAAGATAC TACATATTCG GCAAATAATG GATAACTACA AGTTGAAAAG TGTGATATAT   
  
  
- AGGAAATACC AAAGAAGCGA ATTCAAACCA CATGTACGAA AAGTTAGACA CAACGTAAAA AGACACTACA   
  
  
- TATTCGGCAA ATAATGGATA ACTACAAGTT GAAAAGTGTG ATATACAGGA AAATAATCAA TGACTACGGA   
  
  
- GACAGTTTGT AAGAATTAAC GTAAATCACA TAGTTGCCAG AATTTTAAAA TTCGTAACGG TTGAACTTGA   
  
  
- AGACTTCTAC AAACAGTTCT AGAAGTCAGA CGTTCAATTG ACGACGAACG AACTTGAAAA GTTGAAAATT   
  
  
- CTCTCTACAG TCTCAGATTC GTACCGAAGG ACTGTTCATG ACGTAAAATA GTGAATACTT CTGAGTTAAG   
  
  
- ACCCAAAACT GTCGAAGATA CTTGGAGATC ACGTCCATGT CGTCGAACCT GTAATTCGAC GTTCAGTTAA   
  
  
- CTGACATCTT GCTGTTCAAC AACTTCAAAG ACGACTTTCA AGTAGTGAAC AAACTACTTA TTTCATGTCG   
  
  
- TACCGTGAAG TCTACGTTTT CAGAGCGCTA GTCATCTCTA CTAAGGTATA AAGAGTAGAG TAAAGGTTCA   
  
  
- GAAACTGTTG TACATGGGTT TATTACGGTC GGTACTGTGA GTCTACAGAG AGGTTAGTAT GTCGCTTCCT   
  
  
- ATGAAGTGAG ACCTGAGTAG AGGTCGTCGT TAGCCTCGCA CGTCACAGAT ACTGATGGGT AGGCAACAGT   
  
  
- CGTGGAGAAG TTTATCTTCG GTCAAAAGAG GAGTCCCAAG GGTGAGCATG TAGAGTCTGG GGGTAGTAAG   
  
  
- AAGGCTGTTG ATACCTAGGG GTCACTCGCC GAGAAGACAC CAACTGCTAT TACGACTCGA CTCCTTGTCC   
  
  
- AAGTCACTAT ACCTCGAGGG GAACGGTGTC CTGAGTCCTG TGGTAATAAC GAGAAAATCG GTGTCTCCTT   
  
  
- CGGTACTTCC GAGGATATGA AACTCCGGTT TGGTTGACTA CCTATACCGG TTGTACCTCA ATTTCGTCCA   
  
  
- CGATATGAAG ACACGTCTTC GTTAGAGTCT CTTATTAGAT AGTTGACGTC TTTCTGATTA CCTACGTAAC   
  
  
- CCATTCTCCC ACAGACAAAA ACCAAGTGGT TAACTTTCCA ACCGGCGGAT GTACAACCTT CCCGAGTCTC   
  
  
- GTTCCGACCT CAAAAGACCT ATATGATAGA TGTTTTTCGA GTCCACGCTT GTCGGTTGCT CGAGTCTCGA   
  
  
- AGAAAGGATG TACGTGTAGG ACATAGTTTC AACGGGTATA AAGTTTAAGC GTATATACAG GAGTTTACAA   
  
  
- CGATAAGTTC TTCGAAACCC CTTACTCGGC CAATAGGTGT AGTAGCTAAA GGTCTAACGG TACCCCTGTG   
  
  
- TTAACCACGA GAACTAGGTC AGAGAGCGGG TAGCCGGACC GCCCGGGGGG GGAGCAAGCC TAGTGACCCC   
  
  
- AGCTAC

+     CAAT-box

| Site Name | Organism | Position | Strand | Matrix score. | sequence | function |
| --- | --- | --- | --- | --- | --- | --- |
| CAAT-box | Arabidopsis thaliana | 676 | + | 5 | CCAAT | common cis-acting element in promoter and enhancer regions |
| CAAT-box | Arabidopsis thaliana | 2876 | - | 5 | CCAAT | common cis-acting element in promoter and enhancer regions |
| CAAT-box | Nicotiana glutinosa | 2203 | + | 4 | CAAT |  |
| CAAT-box | Nicotiana glutinosa | 2874 | + | 4 | CAAT |  |
| CAAT-box | Nicotiana glutinosa | 577 | - | 4 | CAAT |  |
| CAAT-box | Nicotiana glutinosa | 1264 | - | 4 | CAAT |  |
| CAAT-box | Nicotiana glutinosa | 1262 | + | 4 | CAAT |  |
| CAAT-box | Arabidopsis thaliana | 2622 | + | 5 | CCAAT | common cis-acting element in promoter and enhancer regions |
| CAAT-box | Nicotiana glutinosa | 2859 | - | 4 | CAAT |  |
| CAAT-box | Nicotiana glutinosa | 2625 | - | 4 | CAAT |  |
| CAAT-box | Nicotiana glutinosa | 43 | - | 4 | CAAT |  |
| CAAT-box | Nicotiana glutinosa | 2623 | + | 4 | CAAT |  |
| CAAT-box | Pisum sativum | 2777 | + | 5 | CAAAT | common cis-acting element in promoter and enhancer regions |
| CAAT-box | Pisum sativum | 2254 | + | 5 | CAAAT | common cis-acting element in promoter and enhancer regions |
| CAAT-box | Nicotiana glutinosa | 1356 | + | 4 | CAAT |  |
| CAAT-box | Pisum sativum | 2797 | + | 5 | CAAAT | common cis-acting element in promoter and enhancer regions |
| CAAT-box | Pisum sativum | 1273 | - | 5 | CAAAT | common cis-acting element in promoter and enhancer regions |
| CAAT-box | Nicotiana glutinosa | 2545 | + | 4 | CAAT |  |
| CAAT-box | Nicotiana glutinosa | 1471 | + | 4 | CAAT |  |
| CAAT-box | Nicotiana glutinosa | 2157 | + | 4 | CAAT |  |
| CAAT-box | Nicotiana glutinosa | 1962 | - | 4 | CAAT |  |
| CAAT-box | Nicotiana glutinosa | 1960 | + | 4 | CAAT |  |
| CAAT-box | Nicotiana glutinosa | 1739 | - | 4 | CAAT |  |
| CAAT-box | Nicotiana glutinosa | 1103 | - | 4 | CAAT |  |
| CAAT-box | Nicotiana glutinosa | 1055 | - | 4 | CAAT |  |
| CAAT-box | Pisum sativum | 994 | - | 5 | CAAAT | common cis-acting element in promoter and enhancer regions |
| CAAT-box | Pisum sativum | 951 | - | 5 | CAAAT | common cis-acting element in promoter and enhancer regions |
| CAAT-box | Nicotiana glutinosa | 1517 | - | 4 | CAAT |  |
| CAAT-box | Nicotiana glutinosa | 2431 | - | 4 | CAAT |  |
| CAAT-box | Nicotiana glutinosa | 593 | + | 4 | CAAT |  |
| CAAT-box | Petunia hybrida | 1741 | + | 7 | TGCCAAC | common cis-acting element in promoter and enhancer regions |
| CAAT-box | Nicotiana glutinosa | 434 | - | 4 | CAAT |  |
| CAAT-box | Pisum sativum | 269 | - | 5 | CAAAT | common cis-acting element in promoter and enhancer regions |
| CAAT-box | Nicotiana glutinosa | 629 | + | 4 | CAAT |  |
| CAAT-box | Nicotiana glutinosa | 1701 | - | 4 | CAAT |  |
| CAAT-box | Arabidopsis thaliana | 1261 | + | 5 | CCAAT | common cis-acting element in promoter and enhancer regions |
| CAAT-box | Pisum sativum | 2122 | + | 5 | CAAAT | common cis-acting element in promoter and enhancer regions |
| CAAT-box | Nicotiana glutinosa | 178 | - | 4 | CAAT |  |
| CAAT-box | Arabidopsis thaliana | 2591 | - | 5 | CCAAT | common cis-acting element in promoter and enhancer regions |
| CAAT-box | Nicotiana glutinosa | 1633 | - | 4 | CAAT |  |
| CAAT-box | Nicotiana glutinosa | 777 | - | 4 | CAAT |  |
| CAAT-box | Nicotiana glutinosa | 1587 | + | 4 | CAAT |  |
| CAAT-box | Nicotiana glutinosa | 1347 | - | 4 | CAAT |  |
| CAAT-box | Nicotiana glutinosa | 424 | + | 4 | CAAT |  |
| CAAT-box | Nicotiana glutinosa | 898 | + | 4 | CAAT |  |
| CAAT-box | Nicotiana glutinosa | 1889 | + | 4 | CAAT |  |
| CAAT-box | Nicotiana glutinosa | 677 | + | 4 | CAAT |  |
| CAAT-box | Arabidopsis thaliana | 2156 | + | 5 | CCAAT | common cis-acting element in promoter and enhancer regions |

>HU08G02296.1   
+ -Up\_Stream \_Len000TTATTC TTACATCTTG TTGTTGTGAT TGTGTATTAA GCTTGTTGTC TCATGGTTTG   
  
  
+ CTCTTCTCTT TGGAACCTCA TGGTGGCTAG TTCCATGTTT TTGCTTTGTA GATGTAGCCG TTTGTTAAAG   
  
  
+ CAAGTTGGTT TATTTCTCCT ATGGCTTCGC TCTATTGTAA TTCAGGGGTC AGGTTGTTAT GTCAGACATT   
  
  
+ CCCATACCGC ACCTAGAAGG GAGGAGGGAG GCTCAGGAGT CTTAAAGAGT ATAGATTTGG ATTCACCTGT   
  
  
+ AGTCTATAGC TGATGTCCAC CTAATCTGGA TTTTATTCTT TCTTTCTCTC TCTTTTTTTT TCTTTTTTTG   
  
  
+ GGGGGGGGGG TGTTGTTGAG GGAGACAGGA TGAGGTCGAA TCTCTGCAGC ATAACAACCA CCATAGTCAC   
  
  
+ AATATGGAGA TTGTGGAGGC AAAGTTTGAG GACGTCCGGA TGTATTTCCT TTGGAACTGA GGAGGAGGTA   
  
  
+ GTACTTCTCT GGTAGTAGTG TTCTATATCC TTTGTTCCTT TCGTGTATTA TCCTAGCTAC CTCTCAAGCA   
  
  
+ AGTTGACATG AAATTGTAGA ATTCAAGACA ATAATAACAG CTGGTTTTTG GGTCTTTTAG TTTGCAATCC   
  
  
+ CTTGTAGCAT CTTAGTGACT TAGTGACATG AGATTTTGAC CCCAATAGAA ATAGAACAGA CAGCTGCTAA   
  
  
+ ATTCACCCAT CTCTTGTGTC AAAATCGGAC TTGTTTTGCC TCTGCTTCTC CATAAAGCAT CAAAGAGCGC   
  
  
+ TGATTGAGGC AGGGATTTTG TCAAGTGCAG TGTTGCATAT GTCATATCGT GTTGATGGTC ATGTTATCAC   
  
  
+ GGACATAAAT TTCCCAGTGT AAAGCAAAGG TGTCTGTCTT CACATAGAGT GTTCAATTCA TAGTCATGCT   
  
  
+ CTCGTCTCTG CATCATCCTG CGTTTGCTGG GGCTAAATTT GAGGGGCTTA TCACTTTGAA ATTCGGAGAA   
  
  
+ TTACTGTGAA TTTGTGTTAG TTCAGGCATA AAATACTGGA TGGGATGTTG AGATGTTCTA ATCATTATCT   
  
  
+ ATTGTTAAAA AACTCAGAAC TATTATAGAT TTTCTGTTTT CTGTTTGGAT TGAGATCCGA AGCCTTTGAG   
  
  
+ ATCCCCTGTT TCTAGCTTCC AACCCCCCTC CCTGGGTTCT TTTTCTTTGG TGTGAAAGTA TGAACAAAAG   
  
  
+ CTGATGTAAG TGTGATTCAA ACTCTAGCCT TGGTGAGACT CTTGGGAAGG GCTCCTAGAG ACTTTGCCAA   
  
  
+ TTGAGCTAAT TTGATGCCTA CTACTTGCTT ACTTTTCTAG CTGTTGTTTA AACGATTTAT GTTCATAGTT   
  
  
+ AAGGAGCGCC ATATTGCATT ACAATATCTG CTTTAGTGCT TTGTTTTTTG CATTTTCTTC TTTATTACTA   
  
  
+ TGGTTTTTGA AGAAGGCTTA CTGACTGATA CTGGTTTCTT CGCTTAAGTT TGGTGTACAT GCTTTTCAAT   
  
  
+ CTGTGTTGCA TTTTTCTATG ATGTATAAGC CGTTTATTAC CTATTGATGT TCAACTTTTC ACACTATATA   
  
  
+ TCCTTTATGG TTTCTTCGCT TAAGTTTGGT GTACATGCTT TTCAATCTGT GTTGCATTTT TCTGTGATGT   
  
  
+ ATAAGCCGTT TATTACCTAT TGATGTTCAA CTTTTCACAC TATATGTCCT TTTATTAGTT ACTGATGCCT   
  
  
+ CTGTCAAACA TTCTTAATTG CATTTAGTGT ATCAACGGTC TTAAAATTTT AAGCATTGCC AACTTGAACT   
  
  
+ TCTGAAGATG TTTGTCAAGA TCTTCAGTCT GCAAGTTAAC TGCTGCTTGC TTGAACTTTT CAACTTTTAA   
  
  
+ GAGAGATGTC AGAGTCTAAG CATGGCTTCC TGACAAGTAC TGCATTTTAT CACTTATGAA GACTCAATTC   
  
  
+ TGGGTTTTGA CAGCTTCTAT GAACCTCTAG TGCAGGTACA GCAGCTTGGA CATTAAGCTG CAAGTCAATT   
  
  
+ GACTGTAGAA CGACAAGTTG TTGAAGTTTC TGCTGAAAGT TCATCACTTG TTTGATGAAT AAAGTACAGC   
  
  
+ ATGGCACTTC AGATGCAAAA GTCTCGCGAT CAGTAGAGAT GATTCCATAT TTCTCATCTC ATTTCCAAGT   
  
  
+ CTTTGACAAC ATGTACCCAA ATAATGCCAG CCATGACACT CAGATGTCTC TCCAATCATA CAGCGAAGGA   
  
  
+ TACTTCACTC TGGACTCATC TCCAGCAGCA ATCGGAGCGT GCAGTGTCTA TGACTACCCA TCCGTTGTCA   
  
  
+ GCACCTCTTC AAATAGAAGC CAGTTTTCTC CTCAGGGTTC CCACTCGTAC ATCTCAGACC CCCATCATTC   
  
  
+ TTCCGACAAC TATGGATCCC CAGTGAGCGG CTCTTCTGTG GTTGACGATA ATGCTGAGCT GAGGAACAGG   
  
  
+ TTCAGTGATA TGGAGCTCCC CTTGCCACAG GACTCAGGAC ACCATTATTG CTCTTTTAGC CACAGAGGAA   
  
  
+ GCCATGAAGG CTCCTATACT TTGAGGCCAA ACCAACTGAT GGATATGGCC AACATGGAGT TAAAGCAGGT   
  
  
+ GCTATACTTC TGTGCAGAAG CAATCTCAGA GAATAATCTA TCAACTGCAG AAAGACTAAT GGATGCATTG   
  
  
+ GGTAAGAGGG TGTCTGTTTT TGGTTCACCA ATTGAAAGGT TGGCCGCCTA CATGTTGGAA GGGCTCAGAG   
  
  
+ CAAGGCTGGA GTTTTCTGGA TATACTATCT ACAAAAAGCT CAGGTGCGAA CAGCCAACGA GCTCAGAGCT   
  
  
+ TCTTTCCTAC ATGCACATCC TGTATCAAAG TTGCCCATAT TTCAAATTCG CATATATGTC CTCAAATGTT   
  
  
+ GCTATTCAAG AAGCTTTGGG GAATGAGCCG GTTATCCACA TCATCGATTT CCAGATTGCC ATGGGGACAC   
  
  
+ AATTGGTGCT CTTGATCCAG TCTCTCGCCC ATCGGCCTGG CGGGCCCCCC CCTCGTTCGG ATCACTGGGG   
  
  
+ TCGATG  

- -Up\_Stream \_Len000AATAAG AATGTAGAAC AACAACACTA ACACATAATT CGAACAACAG AGTACCAAAC   
  
  
- GAGAAGAGAA ACCTTGGAGT ACCACCGATC AAGGTACAAA AACGAAACAT CTACATCGGC AAACAATTTC   
  
  
- GTTCAACCAA ATAAAGAGGA TACCGAAGCG AGATAACATT AAGTCCCCAG TCCAACAATA CAGTCTGTAA   
  
  
- GGGTATGGCG TGGATCTTCC CTCCTCCCTC CGAGTCCTCA GAATTTCTCA TATCTAAACC TAAGTGGACA   
  
  
- TCAGATATCG ACTACAGGTG GATTAGACCT AAAATAAGAA AGAAAGAGAG AGAAAAAAAA AGAAAAAAAC   
  
  
- CCCCCCCCCC ACAACAACTC CCTCTGTCCT ACTCCAGCTT AGAGACGTCG TATTGTTGGT GGTATCAGTG   
  
  
- TTATACCTCT AACACCTCCG TTTCAAACTC CTGCAGGCCT ACATAAAGGA AACCTTGACT CCTCCTCCAT   
  
  
- CATGAAGAGA CCATCATCAC AAGATATAGG AAACAAGGAA AGCACATAAT AGGATCGATG GAGAGTTCGT   
  
  
- TCAACTGTAC TTTAACATCT TAAGTTCTGT TATTATTGTC GACCAAAAAC CCAGAAAATC AAACGTTAGG   
  
  
- GAACATCGTA GAATCACTGA ATCACTGTAC TCTAAAACTG GGGTTATCTT TATCTTGTCT GTCGACGATT   
  
  
- TAAGTGGGTA GAGAACACAG TTTTAGCCTG AACAAAACGG AGACGAAGAG GTATTTCGTA GTTTCTCGCG   
  
  
- ACTAACTCCG TCCCTAAAAC AGTTCACGTC ACAACGTATA CAGTATAGCA CAACTACCAG TACAATAGTG   
  
  
- CCTGTATTTA AAGGGTCACA TTTCGTTTCC ACAGACAGAA GTGTATCTCA CAAGTTAAGT ATCAGTACGA   
  
  
- GAGCAGAGAC GTAGTAGGAC GCAAACGACC CCGATTTAAA CTCCCCGAAT AGTGAAACTT TAAGCCTCTT   
  
  
- AATGACACTT AAACACAATC AAGTCCGTAT TTTATGACCT ACCCTACAAC TCTACAAGAT TAGTAATAGA   
  
  
- TAACAATTTT TTGAGTCTTG ATAATATCTA AAAGACAAAA GACAAACCTA ACTCTAGGCT TCGGAAACTC   
  
  
- TAGGGGACAA AGATCGAAGG TTGGGGGGAG GGACCCAAGA AAAAGAAACC ACACTTTCAT ACTTGTTTTC   
  
  
- GACTACATTC ACACTAAGTT TGAGATCGGA ACCACTCTGA GAACCCTTCC CGAGGATCTC TGAAACGGTT   
  
  
- AACTCGATTA AACTACGGAT GATGAACGAA TGAAAAGATC GACAACAAAT TTGCTAAATA CAAGTATCAA   
  
  
- TTCCTCGCGG TATAACGTAA TGTTATAGAC GAAATCACGA AACAAAAAAC GTAAAAGAAG AAATAATGAT   
  
  
- ACCAAAAACT TCTTCCGAAT GACTGACTAT GACCAAAGAA GCGAATTCAA ACCACATGTA CGAAAAGTTA   
  
  
- GACACAACGT AAAAAGATAC TACATATTCG GCAAATAATG GATAACTACA AGTTGAAAAG TGTGATATAT   
  
  
- AGGAAATACC AAAGAAGCGA ATTCAAACCA CATGTACGAA AAGTTAGACA CAACGTAAAA AGACACTACA   
  
  
- TATTCGGCAA ATAATGGATA ACTACAAGTT GAAAAGTGTG ATATACAGGA AAATAATCAA TGACTACGGA   
  
  
- GACAGTTTGT AAGAATTAAC GTAAATCACA TAGTTGCCAG AATTTTAAAA TTCGTAACGG TTGAACTTGA   
  
  
- AGACTTCTAC AAACAGTTCT AGAAGTCAGA CGTTCAATTG ACGACGAACG AACTTGAAAA GTTGAAAATT   
  
  
- CTCTCTACAG TCTCAGATTC GTACCGAAGG ACTGTTCATG ACGTAAAATA GTGAATACTT CTGAGTTAAG   
  
  
- ACCCAAAACT GTCGAAGATA CTTGGAGATC ACGTCCATGT CGTCGAACCT GTAATTCGAC GTTCAGTTAA   
  
  
- CTGACATCTT GCTGTTCAAC AACTTCAAAG ACGACTTTCA AGTAGTGAAC AAACTACTTA TTTCATGTCG   
  
  
- TACCGTGAAG TCTACGTTTT CAGAGCGCTA GTCATCTCTA CTAAGGTATA AAGAGTAGAG TAAAGGTTCA   
  
  
- GAAACTGTTG TACATGGGTT TATTACGGTC GGTACTGTGA GTCTACAGAG AGGTTAGTAT GTCGCTTCCT   
  
  
- ATGAAGTGAG ACCTGAGTAG AGGTCGTCGT TAGCCTCGCA CGTCACAGAT ACTGATGGGT AGGCAACAGT   
  
  
- CGTGGAGAAG TTTATCTTCG GTCAAAAGAG GAGTCCCAAG GGTGAGCATG TAGAGTCTGG GGGTAGTAAG   
  
  
- AAGGCTGTTG ATACCTAGGG GTCACTCGCC GAGAAGACAC CAACTGCTAT TACGACTCGA CTCCTTGTCC   
  
  
- AAGTCACTAT ACCTCGAGGG GAACGGTGTC CTGAGTCCTG TGGTAATAAC GAGAAAATCG GTGTCTCCTT   
  
  
- CGGTACTTCC GAGGATATGA AACTCCGGTT TGGTTGACTA CCTATACCGG TTGTACCTCA ATTTCGTCCA   
  
  
- CGATATGAAG ACACGTCTTC GTTAGAGTCT CTTATTAGAT AGTTGACGTC TTTCTGATTA CCTACGTAAC   
  
  
- CCATTCTCCC ACAGACAAAA ACCAAGTGGT TAACTTTCCA ACCGGCGGAT GTACAACCTT CCCGAGTCTC   
  
  
- GTTCCGACCT CAAAAGACCT ATATGATAGA TGTTTTTCGA GTCCACGCTT GTCGGTTGCT CGAGTCTCGA   
  
  
- AGAAAGGATG TACGTGTAGG ACATAGTTTC AACGGGTATA AAGTTTAAGC GTATATACAG GAGTTTACAA   
  
  
- CGATAAGTTC TTCGAAACCC CTTACTCGGC CAATAGGTGT AGTAGCTAAA GGTCTAACGG TACCCCTGTG   
  
  
- TTAACCACGA GAACTAGGTC AGAGAGCGGG TAGCCGGACC GCCCGGGGGG GGAGCAAGCC TAGTGACCCC   
  
  
- AGCTAC

+     CCAAT-box

| Site Name | Organism | Position | Strand | Matrix score. | sequence | function |
| --- | --- | --- | --- | --- | --- | --- |
| CCAAT-box | Hordeum vulgare | 2236 | - | 6 | CAACGG | MYBHv1 binding site |
| CCAAT-box | Hordeum vulgare | 1717 | + | 6 | CAACGG | MYBHv1 binding site |

>HU08G02296.1   
+ -Up\_Stream \_Len000TTATTC TTACATCTTG TTGTTGTGAT TGTGTATTAA GCTTGTTGTC TCATGGTTTG   
  
  
+ CTCTTCTCTT TGGAACCTCA TGGTGGCTAG TTCCATGTTT TTGCTTTGTA GATGTAGCCG TTTGTTAAAG   
  
  
+ CAAGTTGGTT TATTTCTCCT ATGGCTTCGC TCTATTGTAA TTCAGGGGTC AGGTTGTTAT GTCAGACATT   
  
  
+ CCCATACCGC ACCTAGAAGG GAGGAGGGAG GCTCAGGAGT CTTAAAGAGT ATAGATTTGG ATTCACCTGT   
  
  
+ AGTCTATAGC TGATGTCCAC CTAATCTGGA TTTTATTCTT TCTTTCTCTC TCTTTTTTTT TCTTTTTTTG   
  
  
+ GGGGGGGGGG TGTTGTTGAG GGAGACAGGA TGAGGTCGAA TCTCTGCAGC ATAACAACCA CCATAGTCAC   
  
  
+ AATATGGAGA TTGTGGAGGC AAAGTTTGAG GACGTCCGGA TGTATTTCCT TTGGAACTGA GGAGGAGGTA   
  
  
+ GTACTTCTCT GGTAGTAGTG TTCTATATCC TTTGTTCCTT TCGTGTATTA TCCTAGCTAC CTCTCAAGCA   
  
  
+ AGTTGACATG AAATTGTAGA ATTCAAGACA ATAATAACAG CTGGTTTTTG GGTCTTTTAG TTTGCAATCC   
  
  
+ CTTGTAGCAT CTTAGTGACT TAGTGACATG AGATTTTGAC CCCAATAGAA ATAGAACAGA CAGCTGCTAA   
  
  
+ ATTCACCCAT CTCTTGTGTC AAAATCGGAC TTGTTTTGCC TCTGCTTCTC CATAAAGCAT CAAAGAGCGC   
  
  
+ TGATTGAGGC AGGGATTTTG TCAAGTGCAG TGTTGCATAT GTCATATCGT GTTGATGGTC ATGTTATCAC   
  
  
+ GGACATAAAT TTCCCAGTGT AAAGCAAAGG TGTCTGTCTT CACATAGAGT GTTCAATTCA TAGTCATGCT   
  
  
+ CTCGTCTCTG CATCATCCTG CGTTTGCTGG GGCTAAATTT GAGGGGCTTA TCACTTTGAA ATTCGGAGAA   
  
  
+ TTACTGTGAA TTTGTGTTAG TTCAGGCATA AAATACTGGA TGGGATGTTG AGATGTTCTA ATCATTATCT   
  
  
+ ATTGTTAAAA AACTCAGAAC TATTATAGAT TTTCTGTTTT CTGTTTGGAT TGAGATCCGA AGCCTTTGAG   
  
  
+ ATCCCCTGTT TCTAGCTTCC AACCCCCCTC CCTGGGTTCT TTTTCTTTGG TGTGAAAGTA TGAACAAAAG   
  
  
+ CTGATGTAAG TGTGATTCAA ACTCTAGCCT TGGTGAGACT CTTGGGAAGG GCTCCTAGAG ACTTTGCCAA   
  
  
+ TTGAGCTAAT TTGATGCCTA CTACTTGCTT ACTTTTCTAG CTGTTGTTTA AACGATTTAT GTTCATAGTT   
  
  
+ AAGGAGCGCC ATATTGCATT ACAATATCTG CTTTAGTGCT TTGTTTTTTG CATTTTCTTC TTTATTACTA   
  
  
+ TGGTTTTTGA AGAAGGCTTA CTGACTGATA CTGGTTTCTT CGCTTAAGTT TGGTGTACAT GCTTTTCAAT   
  
  
+ CTGTGTTGCA TTTTTCTATG ATGTATAAGC CGTTTATTAC CTATTGATGT TCAACTTTTC ACACTATATA   
  
  
+ TCCTTTATGG TTTCTTCGCT TAAGTTTGGT GTACATGCTT TTCAATCTGT GTTGCATTTT TCTGTGATGT   
  
  
+ ATAAGCCGTT TATTACCTAT TGATGTTCAA CTTTTCACAC TATATGTCCT TTTATTAGTT ACTGATGCCT   
  
  
+ CTGTCAAACA TTCTTAATTG CATTTAGTGT ATCAACGGTC TTAAAATTTT AAGCATTGCC AACTTGAACT   
  
  
+ TCTGAAGATG TTTGTCAAGA TCTTCAGTCT GCAAGTTAAC TGCTGCTTGC TTGAACTTTT CAACTTTTAA   
  
  
+ GAGAGATGTC AGAGTCTAAG CATGGCTTCC TGACAAGTAC TGCATTTTAT CACTTATGAA GACTCAATTC   
  
  
+ TGGGTTTTGA CAGCTTCTAT GAACCTCTAG TGCAGGTACA GCAGCTTGGA CATTAAGCTG CAAGTCAATT   
  
  
+ GACTGTAGAA CGACAAGTTG TTGAAGTTTC TGCTGAAAGT TCATCACTTG TTTGATGAAT AAAGTACAGC   
  
  
+ ATGGCACTTC AGATGCAAAA GTCTCGCGAT CAGTAGAGAT GATTCCATAT TTCTCATCTC ATTTCCAAGT   
  
  
+ CTTTGACAAC ATGTACCCAA ATAATGCCAG CCATGACACT CAGATGTCTC TCCAATCATA CAGCGAAGGA   
  
  
+ TACTTCACTC TGGACTCATC TCCAGCAGCA ATCGGAGCGT GCAGTGTCTA TGACTACCCA TCCGTTGTCA   
  
  
+ GCACCTCTTC AAATAGAAGC CAGTTTTCTC CTCAGGGTTC CCACTCGTAC ATCTCAGACC CCCATCATTC   
  
  
+ TTCCGACAAC TATGGATCCC CAGTGAGCGG CTCTTCTGTG GTTGACGATA ATGCTGAGCT GAGGAACAGG   
  
  
+ TTCAGTGATA TGGAGCTCCC CTTGCCACAG GACTCAGGAC ACCATTATTG CTCTTTTAGC CACAGAGGAA   
  
  
+ GCCATGAAGG CTCCTATACT TTGAGGCCAA ACCAACTGAT GGATATGGCC AACATGGAGT TAAAGCAGGT   
  
  
+ GCTATACTTC TGTGCAGAAG CAATCTCAGA GAATAATCTA TCAACTGCAG AAAGACTAAT GGATGCATTG   
  
  
+ GGTAAGAGGG TGTCTGTTTT TGGTTCACCA ATTGAAAGGT TGGCCGCCTA CATGTTGGAA GGGCTCAGAG   
  
  
+ CAAGGCTGGA GTTTTCTGGA TATACTATCT ACAAAAAGCT CAGGTGCGAA CAGCCAACGA GCTCAGAGCT   
  
  
+ TCTTTCCTAC ATGCACATCC TGTATCAAAG TTGCCCATAT TTCAAATTCG CATATATGTC CTCAAATGTT   
  
  
+ GCTATTCAAG AAGCTTTGGG GAATGAGCCG GTTATCCACA TCATCGATTT CCAGATTGCC ATGGGGACAC   
  
  
+ AATTGGTGCT CTTGATCCAG TCTCTCGCCC ATCGGCCTGG CGGGCCCCCC CCTCGTTCGG ATCACTGGGG   
  
  
+ TCGATG  

- -Up\_Stream \_Len000AATAAG AATGTAGAAC AACAACACTA ACACATAATT CGAACAACAG AGTACCAAAC   
  
  
- GAGAAGAGAA ACCTTGGAGT ACCACCGATC AAGGTACAAA AACGAAACAT CTACATCGGC AAACAATTTC   
  
  
- GTTCAACCAA ATAAAGAGGA TACCGAAGCG AGATAACATT AAGTCCCCAG TCCAACAATA CAGTCTGTAA   
  
  
- GGGTATGGCG TGGATCTTCC CTCCTCCCTC CGAGTCCTCA GAATTTCTCA TATCTAAACC TAAGTGGACA   
  
  
- TCAGATATCG ACTACAGGTG GATTAGACCT AAAATAAGAA AGAAAGAGAG AGAAAAAAAA AGAAAAAAAC   
  
  
- CCCCCCCCCC ACAACAACTC CCTCTGTCCT ACTCCAGCTT AGAGACGTCG TATTGTTGGT GGTATCAGTG   
  
  
- TTATACCTCT AACACCTCCG TTTCAAACTC CTGCAGGCCT ACATAAAGGA AACCTTGACT CCTCCTCCAT   
  
  
- CATGAAGAGA CCATCATCAC AAGATATAGG AAACAAGGAA AGCACATAAT AGGATCGATG GAGAGTTCGT   
  
  
- TCAACTGTAC TTTAACATCT TAAGTTCTGT TATTATTGTC GACCAAAAAC CCAGAAAATC AAACGTTAGG   
  
  
- GAACATCGTA GAATCACTGA ATCACTGTAC TCTAAAACTG GGGTTATCTT TATCTTGTCT GTCGACGATT   
  
  
- TAAGTGGGTA GAGAACACAG TTTTAGCCTG AACAAAACGG AGACGAAGAG GTATTTCGTA GTTTCTCGCG   
  
  
- ACTAACTCCG TCCCTAAAAC AGTTCACGTC ACAACGTATA CAGTATAGCA CAACTACCAG TACAATAGTG   
  
  
- CCTGTATTTA AAGGGTCACA TTTCGTTTCC ACAGACAGAA GTGTATCTCA CAAGTTAAGT ATCAGTACGA   
  
  
- GAGCAGAGAC GTAGTAGGAC GCAAACGACC CCGATTTAAA CTCCCCGAAT AGTGAAACTT TAAGCCTCTT   
  
  
- AATGACACTT AAACACAATC AAGTCCGTAT TTTATGACCT ACCCTACAAC TCTACAAGAT TAGTAATAGA   
  
  
- TAACAATTTT TTGAGTCTTG ATAATATCTA AAAGACAAAA GACAAACCTA ACTCTAGGCT TCGGAAACTC   
  
  
- TAGGGGACAA AGATCGAAGG TTGGGGGGAG GGACCCAAGA AAAAGAAACC ACACTTTCAT ACTTGTTTTC   
  
  
- GACTACATTC ACACTAAGTT TGAGATCGGA ACCACTCTGA GAACCCTTCC CGAGGATCTC TGAAACGGTT   
  
  
- AACTCGATTA AACTACGGAT GATGAACGAA TGAAAAGATC GACAACAAAT TTGCTAAATA CAAGTATCAA   
  
  
- TTCCTCGCGG TATAACGTAA TGTTATAGAC GAAATCACGA AACAAAAAAC GTAAAAGAAG AAATAATGAT   
  
  
- ACCAAAAACT TCTTCCGAAT GACTGACTAT GACCAAAGAA GCGAATTCAA ACCACATGTA CGAAAAGTTA   
  
  
- GACACAACGT AAAAAGATAC TACATATTCG GCAAATAATG GATAACTACA AGTTGAAAAG TGTGATATAT   
  
  
- AGGAAATACC AAAGAAGCGA ATTCAAACCA CATGTACGAA AAGTTAGACA CAACGTAAAA AGACACTACA   
  
  
- TATTCGGCAA ATAATGGATA ACTACAAGTT GAAAAGTGTG ATATACAGGA AAATAATCAA TGACTACGGA   
  
  
- GACAGTTTGT AAGAATTAAC GTAAATCACA TAGTTGCCAG AATTTTAAAA TTCGTAACGG TTGAACTTGA   
  
  
- AGACTTCTAC AAACAGTTCT AGAAGTCAGA CGTTCAATTG ACGACGAACG AACTTGAAAA GTTGAAAATT   
  
  
- CTCTCTACAG TCTCAGATTC GTACCGAAGG ACTGTTCATG ACGTAAAATA GTGAATACTT CTGAGTTAAG   
  
  
- ACCCAAAACT GTCGAAGATA CTTGGAGATC ACGTCCATGT CGTCGAACCT GTAATTCGAC GTTCAGTTAA   
  
  
- CTGACATCTT GCTGTTCAAC AACTTCAAAG ACGACTTTCA AGTAGTGAAC AAACTACTTA TTTCATGTCG   
  
  
- TACCGTGAAG TCTACGTTTT CAGAGCGCTA GTCATCTCTA CTAAGGTATA AAGAGTAGAG TAAAGGTTCA   
  
  
- GAAACTGTTG TACATGGGTT TATTACGGTC GGTACTGTGA GTCTACAGAG AGGTTAGTAT GTCGCTTCCT   
  
  
- ATGAAGTGAG ACCTGAGTAG AGGTCGTCGT TAGCCTCGCA CGTCACAGAT ACTGATGGGT AGGCAACAGT   
  
  
- CGTGGAGAAG TTTATCTTCG GTCAAAAGAG GAGTCCCAAG GGTGAGCATG TAGAGTCTGG GGGTAGTAAG   
  
  
- AAGGCTGTTG ATACCTAGGG GTCACTCGCC GAGAAGACAC CAACTGCTAT TACGACTCGA CTCCTTGTCC   
  
  
- AAGTCACTAT ACCTCGAGGG GAACGGTGTC CTGAGTCCTG TGGTAATAAC GAGAAAATCG GTGTCTCCTT   
  
  
- CGGTACTTCC GAGGATATGA AACTCCGGTT TGGTTGACTA CCTATACCGG TTGTACCTCA ATTTCGTCCA   
  
  
- CGATATGAAG ACACGTCTTC GTTAGAGTCT CTTATTAGAT AGTTGACGTC TTTCTGATTA CCTACGTAAC   
  
  
- CCATTCTCCC ACAGACAAAA ACCAAGTGGT TAACTTTCCA ACCGGCGGAT GTACAACCTT CCCGAGTCTC   
  
  
- GTTCCGACCT CAAAAGACCT ATATGATAGA TGTTTTTCGA GTCCACGCTT GTCGGTTGCT CGAGTCTCGA   
  
  
- AGAAAGGATG TACGTGTAGG ACATAGTTTC AACGGGTATA AAGTTTAAGC GTATATACAG GAGTTTACAA   
  
  
- CGATAAGTTC TTCGAAACCC CTTACTCGGC CAATAGGTGT AGTAGCTAAA GGTCTAACGG TACCCCTGTG   
  
  
- TTAACCACGA GAACTAGGTC AGAGAGCGGG TAGCCGGACC GCCCGGGGGG GGAGCAAGCC TAGTGACCCC   
  
  
- AGCTAC

+     CGTCA-motif

| Site Name | Organism | Position | Strand | Matrix score. | sequence | function |
| --- | --- | --- | --- | --- | --- | --- |
| CGTCA-motif | Hordeum vulgare | 2357 | - | 5 | CGTCA | cis-acting regulatory element involved in the MeJA-responsiveness |

>HU08G02296.1   
+ -Up\_Stream \_Len000TTATTC TTACATCTTG TTGTTGTGAT TGTGTATTAA GCTTGTTGTC TCATGGTTTG   
  
  
+ CTCTTCTCTT TGGAACCTCA TGGTGGCTAG TTCCATGTTT TTGCTTTGTA GATGTAGCCG TTTGTTAAAG   
  
  
+ CAAGTTGGTT TATTTCTCCT ATGGCTTCGC TCTATTGTAA TTCAGGGGTC AGGTTGTTAT GTCAGACATT   
  
  
+ CCCATACCGC ACCTAGAAGG GAGGAGGGAG GCTCAGGAGT CTTAAAGAGT ATAGATTTGG ATTCACCTGT   
  
  
+ AGTCTATAGC TGATGTCCAC CTAATCTGGA TTTTATTCTT TCTTTCTCTC TCTTTTTTTT TCTTTTTTTG   
  
  
+ GGGGGGGGGG TGTTGTTGAG GGAGACAGGA TGAGGTCGAA TCTCTGCAGC ATAACAACCA CCATAGTCAC   
  
  
+ AATATGGAGA TTGTGGAGGC AAAGTTTGAG GACGTCCGGA TGTATTTCCT TTGGAACTGA GGAGGAGGTA   
  
  
+ GTACTTCTCT GGTAGTAGTG TTCTATATCC TTTGTTCCTT TCGTGTATTA TCCTAGCTAC CTCTCAAGCA   
  
  
+ AGTTGACATG AAATTGTAGA ATTCAAGACA ATAATAACAG CTGGTTTTTG GGTCTTTTAG TTTGCAATCC   
  
  
+ CTTGTAGCAT CTTAGTGACT TAGTGACATG AGATTTTGAC CCCAATAGAA ATAGAACAGA CAGCTGCTAA   
  
  
+ ATTCACCCAT CTCTTGTGTC AAAATCGGAC TTGTTTTGCC TCTGCTTCTC CATAAAGCAT CAAAGAGCGC   
  
  
+ TGATTGAGGC AGGGATTTTG TCAAGTGCAG TGTTGCATAT GTCATATCGT GTTGATGGTC ATGTTATCAC   
  
  
+ GGACATAAAT TTCCCAGTGT AAAGCAAAGG TGTCTGTCTT CACATAGAGT GTTCAATTCA TAGTCATGCT   
  
  
+ CTCGTCTCTG CATCATCCTG CGTTTGCTGG GGCTAAATTT GAGGGGCTTA TCACTTTGAA ATTCGGAGAA   
  
  
+ TTACTGTGAA TTTGTGTTAG TTCAGGCATA AAATACTGGA TGGGATGTTG AGATGTTCTA ATCATTATCT   
  
  
+ ATTGTTAAAA AACTCAGAAC TATTATAGAT TTTCTGTTTT CTGTTTGGAT TGAGATCCGA AGCCTTTGAG   
  
  
+ ATCCCCTGTT TCTAGCTTCC AACCCCCCTC CCTGGGTTCT TTTTCTTTGG TGTGAAAGTA TGAACAAAAG   
  
  
+ CTGATGTAAG TGTGATTCAA ACTCTAGCCT TGGTGAGACT CTTGGGAAGG GCTCCTAGAG ACTTTGCCAA   
  
  
+ TTGAGCTAAT TTGATGCCTA CTACTTGCTT ACTTTTCTAG CTGTTGTTTA AACGATTTAT GTTCATAGTT   
  
  
+ AAGGAGCGCC ATATTGCATT ACAATATCTG CTTTAGTGCT TTGTTTTTTG CATTTTCTTC TTTATTACTA   
  
  
+ TGGTTTTTGA AGAAGGCTTA CTGACTGATA CTGGTTTCTT CGCTTAAGTT TGGTGTACAT GCTTTTCAAT   
  
  
+ CTGTGTTGCA TTTTTCTATG ATGTATAAGC CGTTTATTAC CTATTGATGT TCAACTTTTC ACACTATATA   
  
  
+ TCCTTTATGG TTTCTTCGCT TAAGTTTGGT GTACATGCTT TTCAATCTGT GTTGCATTTT TCTGTGATGT   
  
  
+ ATAAGCCGTT TATTACCTAT TGATGTTCAA CTTTTCACAC TATATGTCCT TTTATTAGTT ACTGATGCCT   
  
  
+ CTGTCAAACA TTCTTAATTG CATTTAGTGT ATCAACGGTC TTAAAATTTT AAGCATTGCC AACTTGAACT   
  
  
+ TCTGAAGATG TTTGTCAAGA TCTTCAGTCT GCAAGTTAAC TGCTGCTTGC TTGAACTTTT CAACTTTTAA   
  
  
+ GAGAGATGTC AGAGTCTAAG CATGGCTTCC TGACAAGTAC TGCATTTTAT CACTTATGAA GACTCAATTC   
  
  
+ TGGGTTTTGA CAGCTTCTAT GAACCTCTAG TGCAGGTACA GCAGCTTGGA CATTAAGCTG CAAGTCAATT   
  
  
+ GACTGTAGAA CGACAAGTTG TTGAAGTTTC TGCTGAAAGT TCATCACTTG TTTGATGAAT AAAGTACAGC   
  
  
+ ATGGCACTTC AGATGCAAAA GTCTCGCGAT CAGTAGAGAT GATTCCATAT TTCTCATCTC ATTTCCAAGT   
  
  
+ CTTTGACAAC ATGTACCCAA ATAATGCCAG CCATGACACT CAGATGTCTC TCCAATCATA CAGCGAAGGA   
  
  
+ TACTTCACTC TGGACTCATC TCCAGCAGCA ATCGGAGCGT GCAGTGTCTA TGACTACCCA TCCGTTGTCA   
  
  
+ GCACCTCTTC AAATAGAAGC CAGTTTTCTC CTCAGGGTTC CCACTCGTAC ATCTCAGACC CCCATCATTC   
  
  
+ TTCCGACAAC TATGGATCCC CAGTGAGCGG CTCTTCTGTG GTTGACGATA ATGCTGAGCT GAGGAACAGG   
  
  
+ TTCAGTGATA TGGAGCTCCC CTTGCCACAG GACTCAGGAC ACCATTATTG CTCTTTTAGC CACAGAGGAA   
  
  
+ GCCATGAAGG CTCCTATACT TTGAGGCCAA ACCAACTGAT GGATATGGCC AACATGGAGT TAAAGCAGGT   
  
  
+ GCTATACTTC TGTGCAGAAG CAATCTCAGA GAATAATCTA TCAACTGCAG AAAGACTAAT GGATGCATTG   
  
  
+ GGTAAGAGGG TGTCTGTTTT TGGTTCACCA ATTGAAAGGT TGGCCGCCTA CATGTTGGAA GGGCTCAGAG   
  
  
+ CAAGGCTGGA GTTTTCTGGA TATACTATCT ACAAAAAGCT CAGGTGCGAA CAGCCAACGA GCTCAGAGCT   
  
  
+ TCTTTCCTAC ATGCACATCC TGTATCAAAG TTGCCCATAT TTCAAATTCG CATATATGTC CTCAAATGTT   
  
  
+ GCTATTCAAG AAGCTTTGGG GAATGAGCCG GTTATCCACA TCATCGATTT CCAGATTGCC ATGGGGACAC   
  
  
+ AATTGGTGCT CTTGATCCAG TCTCTCGCCC ATCGGCCTGG CGGGCCCCCC CCTCGTTCGG ATCACTGGGG   
  
  
+ TCGATG  

- -Up\_Stream \_Len000AATAAG AATGTAGAAC AACAACACTA ACACATAATT CGAACAACAG AGTACCAAAC   
  
  
- GAGAAGAGAA ACCTTGGAGT ACCACCGATC AAGGTACAAA AACGAAACAT CTACATCGGC AAACAATTTC   
  
  
- GTTCAACCAA ATAAAGAGGA TACCGAAGCG AGATAACATT AAGTCCCCAG TCCAACAATA CAGTCTGTAA   
  
  
- GGGTATGGCG TGGATCTTCC CTCCTCCCTC CGAGTCCTCA GAATTTCTCA TATCTAAACC TAAGTGGACA   
  
  
- TCAGATATCG ACTACAGGTG GATTAGACCT AAAATAAGAA AGAAAGAGAG AGAAAAAAAA AGAAAAAAAC   
  
  
- CCCCCCCCCC ACAACAACTC CCTCTGTCCT ACTCCAGCTT AGAGACGTCG TATTGTTGGT GGTATCAGTG   
  
  
- TTATACCTCT AACACCTCCG TTTCAAACTC CTGCAGGCCT ACATAAAGGA AACCTTGACT CCTCCTCCAT   
  
  
- CATGAAGAGA CCATCATCAC AAGATATAGG AAACAAGGAA AGCACATAAT AGGATCGATG GAGAGTTCGT   
  
  
- TCAACTGTAC TTTAACATCT TAAGTTCTGT TATTATTGTC GACCAAAAAC CCAGAAAATC AAACGTTAGG   
  
  
- GAACATCGTA GAATCACTGA ATCACTGTAC TCTAAAACTG GGGTTATCTT TATCTTGTCT GTCGACGATT   
  
  
- TAAGTGGGTA GAGAACACAG TTTTAGCCTG AACAAAACGG AGACGAAGAG GTATTTCGTA GTTTCTCGCG   
  
  
- ACTAACTCCG TCCCTAAAAC AGTTCACGTC ACAACGTATA CAGTATAGCA CAACTACCAG TACAATAGTG   
  
  
- CCTGTATTTA AAGGGTCACA TTTCGTTTCC ACAGACAGAA GTGTATCTCA CAAGTTAAGT ATCAGTACGA   
  
  
- GAGCAGAGAC GTAGTAGGAC GCAAACGACC CCGATTTAAA CTCCCCGAAT AGTGAAACTT TAAGCCTCTT   
  
  
- AATGACACTT AAACACAATC AAGTCCGTAT TTTATGACCT ACCCTACAAC TCTACAAGAT TAGTAATAGA   
  
  
- TAACAATTTT TTGAGTCTTG ATAATATCTA AAAGACAAAA GACAAACCTA ACTCTAGGCT TCGGAAACTC   
  
  
- TAGGGGACAA AGATCGAAGG TTGGGGGGAG GGACCCAAGA AAAAGAAACC ACACTTTCAT ACTTGTTTTC   
  
  
- GACTACATTC ACACTAAGTT TGAGATCGGA ACCACTCTGA GAACCCTTCC CGAGGATCTC TGAAACGGTT   
  
  
- AACTCGATTA AACTACGGAT GATGAACGAA TGAAAAGATC GACAACAAAT TTGCTAAATA CAAGTATCAA   
  
  
- TTCCTCGCGG TATAACGTAA TGTTATAGAC GAAATCACGA AACAAAAAAC GTAAAAGAAG AAATAATGAT   
  
  
- ACCAAAAACT TCTTCCGAAT GACTGACTAT GACCAAAGAA GCGAATTCAA ACCACATGTA CGAAAAGTTA   
  
  
- GACACAACGT AAAAAGATAC TACATATTCG GCAAATAATG GATAACTACA AGTTGAAAAG TGTGATATAT   
  
  
- AGGAAATACC AAAGAAGCGA ATTCAAACCA CATGTACGAA AAGTTAGACA CAACGTAAAA AGACACTACA   
  
  
- TATTCGGCAA ATAATGGATA ACTACAAGTT GAAAAGTGTG ATATACAGGA AAATAATCAA TGACTACGGA   
  
  
- GACAGTTTGT AAGAATTAAC GTAAATCACA TAGTTGCCAG AATTTTAAAA TTCGTAACGG TTGAACTTGA   
  
  
- AGACTTCTAC AAACAGTTCT AGAAGTCAGA CGTTCAATTG ACGACGAACG AACTTGAAAA GTTGAAAATT   
  
  
- CTCTCTACAG TCTCAGATTC GTACCGAAGG ACTGTTCATG ACGTAAAATA GTGAATACTT CTGAGTTAAG   
  
  
- ACCCAAAACT GTCGAAGATA CTTGGAGATC ACGTCCATGT CGTCGAACCT GTAATTCGAC GTTCAGTTAA   
  
  
- CTGACATCTT GCTGTTCAAC AACTTCAAAG ACGACTTTCA AGTAGTGAAC AAACTACTTA TTTCATGTCG   
  
  
- TACCGTGAAG TCTACGTTTT CAGAGCGCTA GTCATCTCTA CTAAGGTATA AAGAGTAGAG TAAAGGTTCA   
  
  
- GAAACTGTTG TACATGGGTT TATTACGGTC GGTACTGTGA GTCTACAGAG AGGTTAGTAT GTCGCTTCCT   
  
  
- ATGAAGTGAG ACCTGAGTAG AGGTCGTCGT TAGCCTCGCA CGTCACAGAT ACTGATGGGT AGGCAACAGT   
  
  
- CGTGGAGAAG TTTATCTTCG GTCAAAAGAG GAGTCCCAAG GGTGAGCATG TAGAGTCTGG GGGTAGTAAG   
  
  
- AAGGCTGTTG ATACCTAGGG GTCACTCGCC GAGAAGACAC CAACTGCTAT TACGACTCGA CTCCTTGTCC   
  
  
- AAGTCACTAT ACCTCGAGGG GAACGGTGTC CTGAGTCCTG TGGTAATAAC GAGAAAATCG GTGTCTCCTT   
  
  
- CGGTACTTCC GAGGATATGA AACTCCGGTT TGGTTGACTA CCTATACCGG TTGTACCTCA ATTTCGTCCA   
  
  
- CGATATGAAG ACACGTCTTC GTTAGAGTCT CTTATTAGAT AGTTGACGTC TTTCTGATTA CCTACGTAAC   
  
  
- CCATTCTCCC ACAGACAAAA ACCAAGTGGT TAACTTTCCA ACCGGCGGAT GTACAACCTT CCCGAGTCTC   
  
  
- GTTCCGACCT CAAAAGACCT ATATGATAGA TGTTTTTCGA GTCCACGCTT GTCGGTTGCT CGAGTCTCGA   
  
  
- AGAAAGGATG TACGTGTAGG ACATAGTTTC AACGGGTATA AAGTTTAAGC GTATATACAG GAGTTTACAA   
  
  
- CGATAAGTTC TTCGAAACCC CTTACTCGGC CAATAGGTGT AGTAGCTAAA GGTCTAACGG TACCCCTGTG   
  
  
- TTAACCACGA GAACTAGGTC AGAGAGCGGG TAGCCGGACC GCCCGGGGGG GGAGCAAGCC TAGTGACCCC   
  
  
- AGCTAC

+     G-Box

| Site Name | Organism | Position | Strand | Matrix score. | sequence | function |
| --- | --- | --- | --- | --- | --- | --- |
| G-Box | Triticum aestivum | 2861 | - | 10 | TCCACATGGCA | cis-acting regulatory element involved in light responsiveness |

>HU08G02296.1   
+ -Up\_Stream \_Len000TTATTC TTACATCTTG TTGTTGTGAT TGTGTATTAA GCTTGTTGTC TCATGGTTTG   
  
  
+ CTCTTCTCTT TGGAACCTCA TGGTGGCTAG TTCCATGTTT TTGCTTTGTA GATGTAGCCG TTTGTTAAAG   
  
  
+ CAAGTTGGTT TATTTCTCCT ATGGCTTCGC TCTATTGTAA TTCAGGGGTC AGGTTGTTAT GTCAGACATT   
  
  
+ CCCATACCGC ACCTAGAAGG GAGGAGGGAG GCTCAGGAGT CTTAAAGAGT ATAGATTTGG ATTCACCTGT   
  
  
+ AGTCTATAGC TGATGTCCAC CTAATCTGGA TTTTATTCTT TCTTTCTCTC TCTTTTTTTT TCTTTTTTTG   
  
  
+ GGGGGGGGGG TGTTGTTGAG GGAGACAGGA TGAGGTCGAA TCTCTGCAGC ATAACAACCA CCATAGTCAC   
  
  
+ AATATGGAGA TTGTGGAGGC AAAGTTTGAG GACGTCCGGA TGTATTTCCT TTGGAACTGA GGAGGAGGTA   
  
  
+ GTACTTCTCT GGTAGTAGTG TTCTATATCC TTTGTTCCTT TCGTGTATTA TCCTAGCTAC CTCTCAAGCA   
  
  
+ AGTTGACATG AAATTGTAGA ATTCAAGACA ATAATAACAG CTGGTTTTTG GGTCTTTTAG TTTGCAATCC   
  
  
+ CTTGTAGCAT CTTAGTGACT TAGTGACATG AGATTTTGAC CCCAATAGAA ATAGAACAGA CAGCTGCTAA   
  
  
+ ATTCACCCAT CTCTTGTGTC AAAATCGGAC TTGTTTTGCC TCTGCTTCTC CATAAAGCAT CAAAGAGCGC   
  
  
+ TGATTGAGGC AGGGATTTTG TCAAGTGCAG TGTTGCATAT GTCATATCGT GTTGATGGTC ATGTTATCAC   
  
  
+ GGACATAAAT TTCCCAGTGT AAAGCAAAGG TGTCTGTCTT CACATAGAGT GTTCAATTCA TAGTCATGCT   
  
  
+ CTCGTCTCTG CATCATCCTG CGTTTGCTGG GGCTAAATTT GAGGGGCTTA TCACTTTGAA ATTCGGAGAA   
  
  
+ TTACTGTGAA TTTGTGTTAG TTCAGGCATA AAATACTGGA TGGGATGTTG AGATGTTCTA ATCATTATCT   
  
  
+ ATTGTTAAAA AACTCAGAAC TATTATAGAT TTTCTGTTTT CTGTTTGGAT TGAGATCCGA AGCCTTTGAG   
  
  
+ ATCCCCTGTT TCTAGCTTCC AACCCCCCTC CCTGGGTTCT TTTTCTTTGG TGTGAAAGTA TGAACAAAAG   
  
  
+ CTGATGTAAG TGTGATTCAA ACTCTAGCCT TGGTGAGACT CTTGGGAAGG GCTCCTAGAG ACTTTGCCAA   
  
  
+ TTGAGCTAAT TTGATGCCTA CTACTTGCTT ACTTTTCTAG CTGTTGTTTA AACGATTTAT GTTCATAGTT   
  
  
+ AAGGAGCGCC ATATTGCATT ACAATATCTG CTTTAGTGCT TTGTTTTTTG CATTTTCTTC TTTATTACTA   
  
  
+ TGGTTTTTGA AGAAGGCTTA CTGACTGATA CTGGTTTCTT CGCTTAAGTT TGGTGTACAT GCTTTTCAAT   
  
  
+ CTGTGTTGCA TTTTTCTATG ATGTATAAGC CGTTTATTAC CTATTGATGT TCAACTTTTC ACACTATATA   
  
  
+ TCCTTTATGG TTTCTTCGCT TAAGTTTGGT GTACATGCTT TTCAATCTGT GTTGCATTTT TCTGTGATGT   
  
  
+ ATAAGCCGTT TATTACCTAT TGATGTTCAA CTTTTCACAC TATATGTCCT TTTATTAGTT ACTGATGCCT   
  
  
+ CTGTCAAACA TTCTTAATTG CATTTAGTGT ATCAACGGTC TTAAAATTTT AAGCATTGCC AACTTGAACT   
  
  
+ TCTGAAGATG TTTGTCAAGA TCTTCAGTCT GCAAGTTAAC TGCTGCTTGC TTGAACTTTT CAACTTTTAA   
  
  
+ GAGAGATGTC AGAGTCTAAG CATGGCTTCC TGACAAGTAC TGCATTTTAT CACTTATGAA GACTCAATTC   
  
  
+ TGGGTTTTGA CAGCTTCTAT GAACCTCTAG TGCAGGTACA GCAGCTTGGA CATTAAGCTG CAAGTCAATT   
  
  
+ GACTGTAGAA CGACAAGTTG TTGAAGTTTC TGCTGAAAGT TCATCACTTG TTTGATGAAT AAAGTACAGC   
  
  
+ ATGGCACTTC AGATGCAAAA GTCTCGCGAT CAGTAGAGAT GATTCCATAT TTCTCATCTC ATTTCCAAGT   
  
  
+ CTTTGACAAC ATGTACCCAA ATAATGCCAG CCATGACACT CAGATGTCTC TCCAATCATA CAGCGAAGGA   
  
  
+ TACTTCACTC TGGACTCATC TCCAGCAGCA ATCGGAGCGT GCAGTGTCTA TGACTACCCA TCCGTTGTCA   
  
  
+ GCACCTCTTC AAATAGAAGC CAGTTTTCTC CTCAGGGTTC CCACTCGTAC ATCTCAGACC CCCATCATTC   
  
  
+ TTCCGACAAC TATGGATCCC CAGTGAGCGG CTCTTCTGTG GTTGACGATA ATGCTGAGCT GAGGAACAGG   
  
  
+ TTCAGTGATA TGGAGCTCCC CTTGCCACAG GACTCAGGAC ACCATTATTG CTCTTTTAGC CACAGAGGAA   
  
  
+ GCCATGAAGG CTCCTATACT TTGAGGCCAA ACCAACTGAT GGATATGGCC AACATGGAGT TAAAGCAGGT   
  
  
+ GCTATACTTC TGTGCAGAAG CAATCTCAGA GAATAATCTA TCAACTGCAG AAAGACTAAT GGATGCATTG   
  
  
+ GGTAAGAGGG TGTCTGTTTT TGGTTCACCA ATTGAAAGGT TGGCCGCCTA CATGTTGGAA GGGCTCAGAG   
  
  
+ CAAGGCTGGA GTTTTCTGGA TATACTATCT ACAAAAAGCT CAGGTGCGAA CAGCCAACGA GCTCAGAGCT   
  
  
+ TCTTTCCTAC ATGCACATCC TGTATCAAAG TTGCCCATAT TTCAAATTCG CATATATGTC CTCAAATGTT   
  
  
+ GCTATTCAAG AAGCTTTGGG GAATGAGCCG GTTATCCACA TCATCGATTT CCAGATTGCC ATGGGGACAC   
  
  
+ AATTGGTGCT CTTGATCCAG TCTCTCGCCC ATCGGCCTGG CGGGCCCCCC CCTCGTTCGG ATCACTGGGG   
  
  
+ TCGATG  

- -Up\_Stream \_Len000AATAAG AATGTAGAAC AACAACACTA ACACATAATT CGAACAACAG AGTACCAAAC   
  
  
- GAGAAGAGAA ACCTTGGAGT ACCACCGATC AAGGTACAAA AACGAAACAT CTACATCGGC AAACAATTTC   
  
  
- GTTCAACCAA ATAAAGAGGA TACCGAAGCG AGATAACATT AAGTCCCCAG TCCAACAATA CAGTCTGTAA   
  
  
- GGGTATGGCG TGGATCTTCC CTCCTCCCTC CGAGTCCTCA GAATTTCTCA TATCTAAACC TAAGTGGACA   
  
  
- TCAGATATCG ACTACAGGTG GATTAGACCT AAAATAAGAA AGAAAGAGAG AGAAAAAAAA AGAAAAAAAC   
  
  
- CCCCCCCCCC ACAACAACTC CCTCTGTCCT ACTCCAGCTT AGAGACGTCG TATTGTTGGT GGTATCAGTG   
  
  
- TTATACCTCT AACACCTCCG TTTCAAACTC CTGCAGGCCT ACATAAAGGA AACCTTGACT CCTCCTCCAT   
  
  
- CATGAAGAGA CCATCATCAC AAGATATAGG AAACAAGGAA AGCACATAAT AGGATCGATG GAGAGTTCGT   
  
  
- TCAACTGTAC TTTAACATCT TAAGTTCTGT TATTATTGTC GACCAAAAAC CCAGAAAATC AAACGTTAGG   
  
  
- GAACATCGTA GAATCACTGA ATCACTGTAC TCTAAAACTG GGGTTATCTT TATCTTGTCT GTCGACGATT   
  
  
- TAAGTGGGTA GAGAACACAG TTTTAGCCTG AACAAAACGG AGACGAAGAG GTATTTCGTA GTTTCTCGCG   
  
  
- ACTAACTCCG TCCCTAAAAC AGTTCACGTC ACAACGTATA CAGTATAGCA CAACTACCAG TACAATAGTG   
  
  
- CCTGTATTTA AAGGGTCACA TTTCGTTTCC ACAGACAGAA GTGTATCTCA CAAGTTAAGT ATCAGTACGA   
  
  
- GAGCAGAGAC GTAGTAGGAC GCAAACGACC CCGATTTAAA CTCCCCGAAT AGTGAAACTT TAAGCCTCTT   
  
  
- AATGACACTT AAACACAATC AAGTCCGTAT TTTATGACCT ACCCTACAAC TCTACAAGAT TAGTAATAGA   
  
  
- TAACAATTTT TTGAGTCTTG ATAATATCTA AAAGACAAAA GACAAACCTA ACTCTAGGCT TCGGAAACTC   
  
  
- TAGGGGACAA AGATCGAAGG TTGGGGGGAG GGACCCAAGA AAAAGAAACC ACACTTTCAT ACTTGTTTTC   
  
  
- GACTACATTC ACACTAAGTT TGAGATCGGA ACCACTCTGA GAACCCTTCC CGAGGATCTC TGAAACGGTT   
  
  
- AACTCGATTA AACTACGGAT GATGAACGAA TGAAAAGATC GACAACAAAT TTGCTAAATA CAAGTATCAA   
  
  
- TTCCTCGCGG TATAACGTAA TGTTATAGAC GAAATCACGA AACAAAAAAC GTAAAAGAAG AAATAATGAT   
  
  
- ACCAAAAACT TCTTCCGAAT GACTGACTAT GACCAAAGAA GCGAATTCAA ACCACATGTA CGAAAAGTTA   
  
  
- GACACAACGT AAAAAGATAC TACATATTCG GCAAATAATG GATAACTACA AGTTGAAAAG TGTGATATAT   
  
  
- AGGAAATACC AAAGAAGCGA ATTCAAACCA CATGTACGAA AAGTTAGACA CAACGTAAAA AGACACTACA   
  
  
- TATTCGGCAA ATAATGGATA ACTACAAGTT GAAAAGTGTG ATATACAGGA AAATAATCAA TGACTACGGA   
  
  
- GACAGTTTGT AAGAATTAAC GTAAATCACA TAGTTGCCAG AATTTTAAAA TTCGTAACGG TTGAACTTGA   
  
  
- AGACTTCTAC AAACAGTTCT AGAAGTCAGA CGTTCAATTG ACGACGAACG AACTTGAAAA GTTGAAAATT   
  
  
- CTCTCTACAG TCTCAGATTC GTACCGAAGG ACTGTTCATG ACGTAAAATA GTGAATACTT CTGAGTTAAG   
  
  
- ACCCAAAACT GTCGAAGATA CTTGGAGATC ACGTCCATGT CGTCGAACCT GTAATTCGAC GTTCAGTTAA   
  
  
- CTGACATCTT GCTGTTCAAC AACTTCAAAG ACGACTTTCA AGTAGTGAAC AAACTACTTA TTTCATGTCG   
  
  
- TACCGTGAAG TCTACGTTTT CAGAGCGCTA GTCATCTCTA CTAAGGTATA AAGAGTAGAG TAAAGGTTCA   
  
  
- GAAACTGTTG TACATGGGTT TATTACGGTC GGTACTGTGA GTCTACAGAG AGGTTAGTAT GTCGCTTCCT   
  
  
- ATGAAGTGAG ACCTGAGTAG AGGTCGTCGT TAGCCTCGCA CGTCACAGAT ACTGATGGGT AGGCAACAGT   
  
  
- CGTGGAGAAG TTTATCTTCG GTCAAAAGAG GAGTCCCAAG GGTGAGCATG TAGAGTCTGG GGGTAGTAAG   
  
  
- AAGGCTGTTG ATACCTAGGG GTCACTCGCC GAGAAGACAC CAACTGCTAT TACGACTCGA CTCCTTGTCC   
  
  
- AAGTCACTAT ACCTCGAGGG GAACGGTGTC CTGAGTCCTG TGGTAATAAC GAGAAAATCG GTGTCTCCTT   
  
  
- CGGTACTTCC GAGGATATGA AACTCCGGTT TGGTTGACTA CCTATACCGG TTGTACCTCA ATTTCGTCCA   
  
  
- CGATATGAAG ACACGTCTTC GTTAGAGTCT CTTATTAGAT AGTTGACGTC TTTCTGATTA CCTACGTAAC   
  
  
- CCATTCTCCC ACAGACAAAA ACCAAGTGGT TAACTTTCCA ACCGGCGGAT GTACAACCTT CCCGAGTCTC   
  
  
- GTTCCGACCT CAAAAGACCT ATATGATAGA TGTTTTTCGA GTCCACGCTT GTCGGTTGCT CGAGTCTCGA   
  
  
- AGAAAGGATG TACGTGTAGG ACATAGTTTC AACGGGTATA AAGTTTAAGC GTATATACAG GAGTTTACAA   
  
  
- CGATAAGTTC TTCGAAACCC CTTACTCGGC CAATAGGTGT AGTAGCTAAA GGTCTAACGG TACCCCTGTG   
  
  
- TTAACCACGA GAACTAGGTC AGAGAGCGGG TAGCCGGACC GCCCGGGGGG GGAGCAAGCC TAGTGACCCC   
  
  
- AGCTAC

+     GA-motif

| Site Name | Organism | Position | Strand | Matrix score. | sequence | function |
| --- | --- | --- | --- | --- | --- | --- |
| GA-motif | Arabidopsis thaliana | 1049 | - | 8 | ATAGATAA | part of a light responsive element |

>HU08G02296.1   
+ -Up\_Stream \_Len000TTATTC TTACATCTTG TTGTTGTGAT TGTGTATTAA GCTTGTTGTC TCATGGTTTG   
  
  
+ CTCTTCTCTT TGGAACCTCA TGGTGGCTAG TTCCATGTTT TTGCTTTGTA GATGTAGCCG TTTGTTAAAG   
  
  
+ CAAGTTGGTT TATTTCTCCT ATGGCTTCGC TCTATTGTAA TTCAGGGGTC AGGTTGTTAT GTCAGACATT   
  
  
+ CCCATACCGC ACCTAGAAGG GAGGAGGGAG GCTCAGGAGT CTTAAAGAGT ATAGATTTGG ATTCACCTGT   
  
  
+ AGTCTATAGC TGATGTCCAC CTAATCTGGA TTTTATTCTT TCTTTCTCTC TCTTTTTTTT TCTTTTTTTG   
  
  
+ GGGGGGGGGG TGTTGTTGAG GGAGACAGGA TGAGGTCGAA TCTCTGCAGC ATAACAACCA CCATAGTCAC   
  
  
+ AATATGGAGA TTGTGGAGGC AAAGTTTGAG GACGTCCGGA TGTATTTCCT TTGGAACTGA GGAGGAGGTA   
  
  
+ GTACTTCTCT GGTAGTAGTG TTCTATATCC TTTGTTCCTT TCGTGTATTA TCCTAGCTAC CTCTCAAGCA   
  
  
+ AGTTGACATG AAATTGTAGA ATTCAAGACA ATAATAACAG CTGGTTTTTG GGTCTTTTAG TTTGCAATCC   
  
  
+ CTTGTAGCAT CTTAGTGACT TAGTGACATG AGATTTTGAC CCCAATAGAA ATAGAACAGA CAGCTGCTAA   
  
  
+ ATTCACCCAT CTCTTGTGTC AAAATCGGAC TTGTTTTGCC TCTGCTTCTC CATAAAGCAT CAAAGAGCGC   
  
  
+ TGATTGAGGC AGGGATTTTG TCAAGTGCAG TGTTGCATAT GTCATATCGT GTTGATGGTC ATGTTATCAC   
  
  
+ GGACATAAAT TTCCCAGTGT AAAGCAAAGG TGTCTGTCTT CACATAGAGT GTTCAATTCA TAGTCATGCT   
  
  
+ CTCGTCTCTG CATCATCCTG CGTTTGCTGG GGCTAAATTT GAGGGGCTTA TCACTTTGAA ATTCGGAGAA   
  
  
+ TTACTGTGAA TTTGTGTTAG TTCAGGCATA AAATACTGGA TGGGATGTTG AGATGTTCTA ATCATTATCT   
  
  
+ ATTGTTAAAA AACTCAGAAC TATTATAGAT TTTCTGTTTT CTGTTTGGAT TGAGATCCGA AGCCTTTGAG   
  
  
+ ATCCCCTGTT TCTAGCTTCC AACCCCCCTC CCTGGGTTCT TTTTCTTTGG TGTGAAAGTA TGAACAAAAG   
  
  
+ CTGATGTAAG TGTGATTCAA ACTCTAGCCT TGGTGAGACT CTTGGGAAGG GCTCCTAGAG ACTTTGCCAA   
  
  
+ TTGAGCTAAT TTGATGCCTA CTACTTGCTT ACTTTTCTAG CTGTTGTTTA AACGATTTAT GTTCATAGTT   
  
  
+ AAGGAGCGCC ATATTGCATT ACAATATCTG CTTTAGTGCT TTGTTTTTTG CATTTTCTTC TTTATTACTA   
  
  
+ TGGTTTTTGA AGAAGGCTTA CTGACTGATA CTGGTTTCTT CGCTTAAGTT TGGTGTACAT GCTTTTCAAT   
  
  
+ CTGTGTTGCA TTTTTCTATG ATGTATAAGC CGTTTATTAC CTATTGATGT TCAACTTTTC ACACTATATA   
  
  
+ TCCTTTATGG TTTCTTCGCT TAAGTTTGGT GTACATGCTT TTCAATCTGT GTTGCATTTT TCTGTGATGT   
  
  
+ ATAAGCCGTT TATTACCTAT TGATGTTCAA CTTTTCACAC TATATGTCCT TTTATTAGTT ACTGATGCCT   
  
  
+ CTGTCAAACA TTCTTAATTG CATTTAGTGT ATCAACGGTC TTAAAATTTT AAGCATTGCC AACTTGAACT   
  
  
+ TCTGAAGATG TTTGTCAAGA TCTTCAGTCT GCAAGTTAAC TGCTGCTTGC TTGAACTTTT CAACTTTTAA   
  
  
+ GAGAGATGTC AGAGTCTAAG CATGGCTTCC TGACAAGTAC TGCATTTTAT CACTTATGAA GACTCAATTC   
  
  
+ TGGGTTTTGA CAGCTTCTAT GAACCTCTAG TGCAGGTACA GCAGCTTGGA CATTAAGCTG CAAGTCAATT   
  
  
+ GACTGTAGAA CGACAAGTTG TTGAAGTTTC TGCTGAAAGT TCATCACTTG TTTGATGAAT AAAGTACAGC   
  
  
+ ATGGCACTTC AGATGCAAAA GTCTCGCGAT CAGTAGAGAT GATTCCATAT TTCTCATCTC ATTTCCAAGT   
  
  
+ CTTTGACAAC ATGTACCCAA ATAATGCCAG CCATGACACT CAGATGTCTC TCCAATCATA CAGCGAAGGA   
  
  
+ TACTTCACTC TGGACTCATC TCCAGCAGCA ATCGGAGCGT GCAGTGTCTA TGACTACCCA TCCGTTGTCA   
  
  
+ GCACCTCTTC AAATAGAAGC CAGTTTTCTC CTCAGGGTTC CCACTCGTAC ATCTCAGACC CCCATCATTC   
  
  
+ TTCCGACAAC TATGGATCCC CAGTGAGCGG CTCTTCTGTG GTTGACGATA ATGCTGAGCT GAGGAACAGG   
  
  
+ TTCAGTGATA TGGAGCTCCC CTTGCCACAG GACTCAGGAC ACCATTATTG CTCTTTTAGC CACAGAGGAA   
  
  
+ GCCATGAAGG CTCCTATACT TTGAGGCCAA ACCAACTGAT GGATATGGCC AACATGGAGT TAAAGCAGGT   
  
  
+ GCTATACTTC TGTGCAGAAG CAATCTCAGA GAATAATCTA TCAACTGCAG AAAGACTAAT GGATGCATTG   
  
  
+ GGTAAGAGGG TGTCTGTTTT TGGTTCACCA ATTGAAAGGT TGGCCGCCTA CATGTTGGAA GGGCTCAGAG   
  
  
+ CAAGGCTGGA GTTTTCTGGA TATACTATCT ACAAAAAGCT CAGGTGCGAA CAGCCAACGA GCTCAGAGCT   
  
  
+ TCTTTCCTAC ATGCACATCC TGTATCAAAG TTGCCCATAT TTCAAATTCG CATATATGTC CTCAAATGTT   
  
  
+ GCTATTCAAG AAGCTTTGGG GAATGAGCCG GTTATCCACA TCATCGATTT CCAGATTGCC ATGGGGACAC   
  
  
+ AATTGGTGCT CTTGATCCAG TCTCTCGCCC ATCGGCCTGG CGGGCCCCCC CCTCGTTCGG ATCACTGGGG   
  
  
+ TCGATG  

- -Up\_Stream \_Len000AATAAG AATGTAGAAC AACAACACTA ACACATAATT CGAACAACAG AGTACCAAAC   
  
  
- GAGAAGAGAA ACCTTGGAGT ACCACCGATC AAGGTACAAA AACGAAACAT CTACATCGGC AAACAATTTC   
  
  
- GTTCAACCAA ATAAAGAGGA TACCGAAGCG AGATAACATT AAGTCCCCAG TCCAACAATA CAGTCTGTAA   
  
  
- GGGTATGGCG TGGATCTTCC CTCCTCCCTC CGAGTCCTCA GAATTTCTCA TATCTAAACC TAAGTGGACA   
  
  
- TCAGATATCG ACTACAGGTG GATTAGACCT AAAATAAGAA AGAAAGAGAG AGAAAAAAAA AGAAAAAAAC   
  
  
- CCCCCCCCCC ACAACAACTC CCTCTGTCCT ACTCCAGCTT AGAGACGTCG TATTGTTGGT GGTATCAGTG   
  
  
- TTATACCTCT AACACCTCCG TTTCAAACTC CTGCAGGCCT ACATAAAGGA AACCTTGACT CCTCCTCCAT   
  
  
- CATGAAGAGA CCATCATCAC AAGATATAGG AAACAAGGAA AGCACATAAT AGGATCGATG GAGAGTTCGT   
  
  
- TCAACTGTAC TTTAACATCT TAAGTTCTGT TATTATTGTC GACCAAAAAC CCAGAAAATC AAACGTTAGG   
  
  
- GAACATCGTA GAATCACTGA ATCACTGTAC TCTAAAACTG GGGTTATCTT TATCTTGTCT GTCGACGATT   
  
  
- TAAGTGGGTA GAGAACACAG TTTTAGCCTG AACAAAACGG AGACGAAGAG GTATTTCGTA GTTTCTCGCG   
  
  
- ACTAACTCCG TCCCTAAAAC AGTTCACGTC ACAACGTATA CAGTATAGCA CAACTACCAG TACAATAGTG   
  
  
- CCTGTATTTA AAGGGTCACA TTTCGTTTCC ACAGACAGAA GTGTATCTCA CAAGTTAAGT ATCAGTACGA   
  
  
- GAGCAGAGAC GTAGTAGGAC GCAAACGACC CCGATTTAAA CTCCCCGAAT AGTGAAACTT TAAGCCTCTT   
  
  
- AATGACACTT AAACACAATC AAGTCCGTAT TTTATGACCT ACCCTACAAC TCTACAAGAT TAGTAATAGA   
  
  
- TAACAATTTT TTGAGTCTTG ATAATATCTA AAAGACAAAA GACAAACCTA ACTCTAGGCT TCGGAAACTC   
  
  
- TAGGGGACAA AGATCGAAGG TTGGGGGGAG GGACCCAAGA AAAAGAAACC ACACTTTCAT ACTTGTTTTC   
  
  
- GACTACATTC ACACTAAGTT TGAGATCGGA ACCACTCTGA GAACCCTTCC CGAGGATCTC TGAAACGGTT   
  
  
- AACTCGATTA AACTACGGAT GATGAACGAA TGAAAAGATC GACAACAAAT TTGCTAAATA CAAGTATCAA   
  
  
- TTCCTCGCGG TATAACGTAA TGTTATAGAC GAAATCACGA AACAAAAAAC GTAAAAGAAG AAATAATGAT   
  
  
- ACCAAAAACT TCTTCCGAAT GACTGACTAT GACCAAAGAA GCGAATTCAA ACCACATGTA CGAAAAGTTA   
  
  
- GACACAACGT AAAAAGATAC TACATATTCG GCAAATAATG GATAACTACA AGTTGAAAAG TGTGATATAT   
  
  
- AGGAAATACC AAAGAAGCGA ATTCAAACCA CATGTACGAA AAGTTAGACA CAACGTAAAA AGACACTACA   
  
  
- TATTCGGCAA ATAATGGATA ACTACAAGTT GAAAAGTGTG ATATACAGGA AAATAATCAA TGACTACGGA   
  
  
- GACAGTTTGT AAGAATTAAC GTAAATCACA TAGTTGCCAG AATTTTAAAA TTCGTAACGG TTGAACTTGA   
  
  
- AGACTTCTAC AAACAGTTCT AGAAGTCAGA CGTTCAATTG ACGACGAACG AACTTGAAAA GTTGAAAATT   
  
  
- CTCTCTACAG TCTCAGATTC GTACCGAAGG ACTGTTCATG ACGTAAAATA GTGAATACTT CTGAGTTAAG   
  
  
- ACCCAAAACT GTCGAAGATA CTTGGAGATC ACGTCCATGT CGTCGAACCT GTAATTCGAC GTTCAGTTAA   
  
  
- CTGACATCTT GCTGTTCAAC AACTTCAAAG ACGACTTTCA AGTAGTGAAC AAACTACTTA TTTCATGTCG   
  
  
- TACCGTGAAG TCTACGTTTT CAGAGCGCTA GTCATCTCTA CTAAGGTATA AAGAGTAGAG TAAAGGTTCA   
  
  
- GAAACTGTTG TACATGGGTT TATTACGGTC GGTACTGTGA GTCTACAGAG AGGTTAGTAT GTCGCTTCCT   
  
  
- ATGAAGTGAG ACCTGAGTAG AGGTCGTCGT TAGCCTCGCA CGTCACAGAT ACTGATGGGT AGGCAACAGT   
  
  
- CGTGGAGAAG TTTATCTTCG GTCAAAAGAG GAGTCCCAAG GGTGAGCATG TAGAGTCTGG GGGTAGTAAG   
  
  
- AAGGCTGTTG ATACCTAGGG GTCACTCGCC GAGAAGACAC CAACTGCTAT TACGACTCGA CTCCTTGTCC   
  
  
- AAGTCACTAT ACCTCGAGGG GAACGGTGTC CTGAGTCCTG TGGTAATAAC GAGAAAATCG GTGTCTCCTT   
  
  
- CGGTACTTCC GAGGATATGA AACTCCGGTT TGGTTGACTA CCTATACCGG TTGTACCTCA ATTTCGTCCA   
  
  
- CGATATGAAG ACACGTCTTC GTTAGAGTCT CTTATTAGAT AGTTGACGTC TTTCTGATTA CCTACGTAAC   
  
  
- CCATTCTCCC ACAGACAAAA ACCAAGTGGT TAACTTTCCA ACCGGCGGAT GTACAACCTT CCCGAGTCTC   
  
  
- GTTCCGACCT CAAAAGACCT ATATGATAGA TGTTTTTCGA GTCCACGCTT GTCGGTTGCT CGAGTCTCGA   
  
  
- AGAAAGGATG TACGTGTAGG ACATAGTTTC AACGGGTATA AAGTTTAAGC GTATATACAG GAGTTTACAA   
  
  
- CGATAAGTTC TTCGAAACCC CTTACTCGGC CAATAGGTGT AGTAGCTAAA GGTCTAACGG TACCCCTGTG   
  
  
- TTAACCACGA GAACTAGGTC AGAGAGCGGG TAGCCGGACC GCCCGGGGGG GGAGCAAGCC TAGTGACCCC   
  
  
- AGCTAC

+     MBS

| Site Name | Organism | Position | Strand | Matrix score. | sequence | function |
| --- | --- | --- | --- | --- | --- | --- |
| MBS | Arabidopsis thaliana | 2487 | + | 6 | CAACTG | MYB binding site involved in drought-inducibility |
| MBS | Arabidopsis thaliana | 2566 | + | 6 | CAACTG | MYB binding site involved in drought-inducibility |

>HU08G02296.1   
+ -Up\_Stream \_Len000TTATTC TTACATCTTG TTGTTGTGAT TGTGTATTAA GCTTGTTGTC TCATGGTTTG   
  
  
+ CTCTTCTCTT TGGAACCTCA TGGTGGCTAG TTCCATGTTT TTGCTTTGTA GATGTAGCCG TTTGTTAAAG   
  
  
+ CAAGTTGGTT TATTTCTCCT ATGGCTTCGC TCTATTGTAA TTCAGGGGTC AGGTTGTTAT GTCAGACATT   
  
  
+ CCCATACCGC ACCTAGAAGG GAGGAGGGAG GCTCAGGAGT CTTAAAGAGT ATAGATTTGG ATTCACCTGT   
  
  
+ AGTCTATAGC TGATGTCCAC CTAATCTGGA TTTTATTCTT TCTTTCTCTC TCTTTTTTTT TCTTTTTTTG   
  
  
+ GGGGGGGGGG TGTTGTTGAG GGAGACAGGA TGAGGTCGAA TCTCTGCAGC ATAACAACCA CCATAGTCAC   
  
  
+ AATATGGAGA TTGTGGAGGC AAAGTTTGAG GACGTCCGGA TGTATTTCCT TTGGAACTGA GGAGGAGGTA   
  
  
+ GTACTTCTCT GGTAGTAGTG TTCTATATCC TTTGTTCCTT TCGTGTATTA TCCTAGCTAC CTCTCAAGCA   
  
  
+ AGTTGACATG AAATTGTAGA ATTCAAGACA ATAATAACAG CTGGTTTTTG GGTCTTTTAG TTTGCAATCC   
  
  
+ CTTGTAGCAT CTTAGTGACT TAGTGACATG AGATTTTGAC CCCAATAGAA ATAGAACAGA CAGCTGCTAA   
  
  
+ ATTCACCCAT CTCTTGTGTC AAAATCGGAC TTGTTTTGCC TCTGCTTCTC CATAAAGCAT CAAAGAGCGC   
  
  
+ TGATTGAGGC AGGGATTTTG TCAAGTGCAG TGTTGCATAT GTCATATCGT GTTGATGGTC ATGTTATCAC   
  
  
+ GGACATAAAT TTCCCAGTGT AAAGCAAAGG TGTCTGTCTT CACATAGAGT GTTCAATTCA TAGTCATGCT   
  
  
+ CTCGTCTCTG CATCATCCTG CGTTTGCTGG GGCTAAATTT GAGGGGCTTA TCACTTTGAA ATTCGGAGAA   
  
  
+ TTACTGTGAA TTTGTGTTAG TTCAGGCATA AAATACTGGA TGGGATGTTG AGATGTTCTA ATCATTATCT   
  
  
+ ATTGTTAAAA AACTCAGAAC TATTATAGAT TTTCTGTTTT CTGTTTGGAT TGAGATCCGA AGCCTTTGAG   
  
  
+ ATCCCCTGTT TCTAGCTTCC AACCCCCCTC CCTGGGTTCT TTTTCTTTGG TGTGAAAGTA TGAACAAAAG   
  
  
+ CTGATGTAAG TGTGATTCAA ACTCTAGCCT TGGTGAGACT CTTGGGAAGG GCTCCTAGAG ACTTTGCCAA   
  
  
+ TTGAGCTAAT TTGATGCCTA CTACTTGCTT ACTTTTCTAG CTGTTGTTTA AACGATTTAT GTTCATAGTT   
  
  
+ AAGGAGCGCC ATATTGCATT ACAATATCTG CTTTAGTGCT TTGTTTTTTG CATTTTCTTC TTTATTACTA   
  
  
+ TGGTTTTTGA AGAAGGCTTA CTGACTGATA CTGGTTTCTT CGCTTAAGTT TGGTGTACAT GCTTTTCAAT   
  
  
+ CTGTGTTGCA TTTTTCTATG ATGTATAAGC CGTTTATTAC CTATTGATGT TCAACTTTTC ACACTATATA   
  
  
+ TCCTTTATGG TTTCTTCGCT TAAGTTTGGT GTACATGCTT TTCAATCTGT GTTGCATTTT TCTGTGATGT   
  
  
+ ATAAGCCGTT TATTACCTAT TGATGTTCAA CTTTTCACAC TATATGTCCT TTTATTAGTT ACTGATGCCT   
  
  
+ CTGTCAAACA TTCTTAATTG CATTTAGTGT ATCAACGGTC TTAAAATTTT AAGCATTGCC AACTTGAACT   
  
  
+ TCTGAAGATG TTTGTCAAGA TCTTCAGTCT GCAAGTTAAC TGCTGCTTGC TTGAACTTTT CAACTTTTAA   
  
  
+ GAGAGATGTC AGAGTCTAAG CATGGCTTCC TGACAAGTAC TGCATTTTAT CACTTATGAA GACTCAATTC   
  
  
+ TGGGTTTTGA CAGCTTCTAT GAACCTCTAG TGCAGGTACA GCAGCTTGGA CATTAAGCTG CAAGTCAATT   
  
  
+ GACTGTAGAA CGACAAGTTG TTGAAGTTTC TGCTGAAAGT TCATCACTTG TTTGATGAAT AAAGTACAGC   
  
  
+ ATGGCACTTC AGATGCAAAA GTCTCGCGAT CAGTAGAGAT GATTCCATAT TTCTCATCTC ATTTCCAAGT   
  
  
+ CTTTGACAAC ATGTACCCAA ATAATGCCAG CCATGACACT CAGATGTCTC TCCAATCATA CAGCGAAGGA   
  
  
+ TACTTCACTC TGGACTCATC TCCAGCAGCA ATCGGAGCGT GCAGTGTCTA TGACTACCCA TCCGTTGTCA   
  
  
+ GCACCTCTTC AAATAGAAGC CAGTTTTCTC CTCAGGGTTC CCACTCGTAC ATCTCAGACC CCCATCATTC   
  
  
+ TTCCGACAAC TATGGATCCC CAGTGAGCGG CTCTTCTGTG GTTGACGATA ATGCTGAGCT GAGGAACAGG   
  
  
+ TTCAGTGATA TGGAGCTCCC CTTGCCACAG GACTCAGGAC ACCATTATTG CTCTTTTAGC CACAGAGGAA   
  
  
+ GCCATGAAGG CTCCTATACT TTGAGGCCAA ACCAACTGAT GGATATGGCC AACATGGAGT TAAAGCAGGT   
  
  
+ GCTATACTTC TGTGCAGAAG CAATCTCAGA GAATAATCTA TCAACTGCAG AAAGACTAAT GGATGCATTG   
  
  
+ GGTAAGAGGG TGTCTGTTTT TGGTTCACCA ATTGAAAGGT TGGCCGCCTA CATGTTGGAA GGGCTCAGAG   
  
  
+ CAAGGCTGGA GTTTTCTGGA TATACTATCT ACAAAAAGCT CAGGTGCGAA CAGCCAACGA GCTCAGAGCT   
  
  
+ TCTTTCCTAC ATGCACATCC TGTATCAAAG TTGCCCATAT TTCAAATTCG CATATATGTC CTCAAATGTT   
  
  
+ GCTATTCAAG AAGCTTTGGG GAATGAGCCG GTTATCCACA TCATCGATTT CCAGATTGCC ATGGGGACAC   
  
  
+ AATTGGTGCT CTTGATCCAG TCTCTCGCCC ATCGGCCTGG CGGGCCCCCC CCTCGTTCGG ATCACTGGGG   
  
  
+ TCGATG  

- -Up\_Stream \_Len000AATAAG AATGTAGAAC AACAACACTA ACACATAATT CGAACAACAG AGTACCAAAC   
  
  
- GAGAAGAGAA ACCTTGGAGT ACCACCGATC AAGGTACAAA AACGAAACAT CTACATCGGC AAACAATTTC   
  
  
- GTTCAACCAA ATAAAGAGGA TACCGAAGCG AGATAACATT AAGTCCCCAG TCCAACAATA CAGTCTGTAA   
  
  
- GGGTATGGCG TGGATCTTCC CTCCTCCCTC CGAGTCCTCA GAATTTCTCA TATCTAAACC TAAGTGGACA   
  
  
- TCAGATATCG ACTACAGGTG GATTAGACCT AAAATAAGAA AGAAAGAGAG AGAAAAAAAA AGAAAAAAAC   
  
  
- CCCCCCCCCC ACAACAACTC CCTCTGTCCT ACTCCAGCTT AGAGACGTCG TATTGTTGGT GGTATCAGTG   
  
  
- TTATACCTCT AACACCTCCG TTTCAAACTC CTGCAGGCCT ACATAAAGGA AACCTTGACT CCTCCTCCAT   
  
  
- CATGAAGAGA CCATCATCAC AAGATATAGG AAACAAGGAA AGCACATAAT AGGATCGATG GAGAGTTCGT   
  
  
- TCAACTGTAC TTTAACATCT TAAGTTCTGT TATTATTGTC GACCAAAAAC CCAGAAAATC AAACGTTAGG   
  
  
- GAACATCGTA GAATCACTGA ATCACTGTAC TCTAAAACTG GGGTTATCTT TATCTTGTCT GTCGACGATT   
  
  
- TAAGTGGGTA GAGAACACAG TTTTAGCCTG AACAAAACGG AGACGAAGAG GTATTTCGTA GTTTCTCGCG   
  
  
- ACTAACTCCG TCCCTAAAAC AGTTCACGTC ACAACGTATA CAGTATAGCA CAACTACCAG TACAATAGTG   
  
  
- CCTGTATTTA AAGGGTCACA TTTCGTTTCC ACAGACAGAA GTGTATCTCA CAAGTTAAGT ATCAGTACGA   
  
  
- GAGCAGAGAC GTAGTAGGAC GCAAACGACC CCGATTTAAA CTCCCCGAAT AGTGAAACTT TAAGCCTCTT   
  
  
- AATGACACTT AAACACAATC AAGTCCGTAT TTTATGACCT ACCCTACAAC TCTACAAGAT TAGTAATAGA   
  
  
- TAACAATTTT TTGAGTCTTG ATAATATCTA AAAGACAAAA GACAAACCTA ACTCTAGGCT TCGGAAACTC   
  
  
- TAGGGGACAA AGATCGAAGG TTGGGGGGAG GGACCCAAGA AAAAGAAACC ACACTTTCAT ACTTGTTTTC   
  
  
- GACTACATTC ACACTAAGTT TGAGATCGGA ACCACTCTGA GAACCCTTCC CGAGGATCTC TGAAACGGTT   
  
  
- AACTCGATTA AACTACGGAT GATGAACGAA TGAAAAGATC GACAACAAAT TTGCTAAATA CAAGTATCAA   
  
  
- TTCCTCGCGG TATAACGTAA TGTTATAGAC GAAATCACGA AACAAAAAAC GTAAAAGAAG AAATAATGAT   
  
  
- ACCAAAAACT TCTTCCGAAT GACTGACTAT GACCAAAGAA GCGAATTCAA ACCACATGTA CGAAAAGTTA   
  
  
- GACACAACGT AAAAAGATAC TACATATTCG GCAAATAATG GATAACTACA AGTTGAAAAG TGTGATATAT   
  
  
- AGGAAATACC AAAGAAGCGA ATTCAAACCA CATGTACGAA AAGTTAGACA CAACGTAAAA AGACACTACA   
  
  
- TATTCGGCAA ATAATGGATA ACTACAAGTT GAAAAGTGTG ATATACAGGA AAATAATCAA TGACTACGGA   
  
  
- GACAGTTTGT AAGAATTAAC GTAAATCACA TAGTTGCCAG AATTTTAAAA TTCGTAACGG TTGAACTTGA   
  
  
- AGACTTCTAC AAACAGTTCT AGAAGTCAGA CGTTCAATTG ACGACGAACG AACTTGAAAA GTTGAAAATT   
  
  
- CTCTCTACAG TCTCAGATTC GTACCGAAGG ACTGTTCATG ACGTAAAATA GTGAATACTT CTGAGTTAAG   
  
  
- ACCCAAAACT GTCGAAGATA CTTGGAGATC ACGTCCATGT CGTCGAACCT GTAATTCGAC GTTCAGTTAA   
  
  
- CTGACATCTT GCTGTTCAAC AACTTCAAAG ACGACTTTCA AGTAGTGAAC AAACTACTTA TTTCATGTCG   
  
  
- TACCGTGAAG TCTACGTTTT CAGAGCGCTA GTCATCTCTA CTAAGGTATA AAGAGTAGAG TAAAGGTTCA   
  
  
- GAAACTGTTG TACATGGGTT TATTACGGTC GGTACTGTGA GTCTACAGAG AGGTTAGTAT GTCGCTTCCT   
  
  
- ATGAAGTGAG ACCTGAGTAG AGGTCGTCGT TAGCCTCGCA CGTCACAGAT ACTGATGGGT AGGCAACAGT   
  
  
- CGTGGAGAAG TTTATCTTCG GTCAAAAGAG GAGTCCCAAG GGTGAGCATG TAGAGTCTGG GGGTAGTAAG   
  
  
- AAGGCTGTTG ATACCTAGGG GTCACTCGCC GAGAAGACAC CAACTGCTAT TACGACTCGA CTCCTTGTCC   
  
  
- AAGTCACTAT ACCTCGAGGG GAACGGTGTC CTGAGTCCTG TGGTAATAAC GAGAAAATCG GTGTCTCCTT   
  
  
- CGGTACTTCC GAGGATATGA AACTCCGGTT TGGTTGACTA CCTATACCGG TTGTACCTCA ATTTCGTCCA   
  
  
- CGATATGAAG ACACGTCTTC GTTAGAGTCT CTTATTAGAT AGTTGACGTC TTTCTGATTA CCTACGTAAC   
  
  
- CCATTCTCCC ACAGACAAAA ACCAAGTGGT TAACTTTCCA ACCGGCGGAT GTACAACCTT CCCGAGTCTC   
  
  
- GTTCCGACCT CAAAAGACCT ATATGATAGA TGTTTTTCGA GTCCACGCTT GTCGGTTGCT CGAGTCTCGA   
  
  
- AGAAAGGATG TACGTGTAGG ACATAGTTTC AACGGGTATA AAGTTTAAGC GTATATACAG GAGTTTACAA   
  
  
- CGATAAGTTC TTCGAAACCC CTTACTCGGC CAATAGGTGT AGTAGCTAAA GGTCTAACGG TACCCCTGTG   
  
  
- TTAACCACGA GAACTAGGTC AGAGAGCGGG TAGCCGGACC GCCCGGGGGG GGAGCAAGCC TAGTGACCCC   
  
  
- AGCTAC

+     MYB

| Site Name | Organism | Position | Strand | Matrix score. | sequence | function |
| --- | --- | --- | --- | --- | --- | --- |
| MYB | Arabidopsis thaliana | 409 | + | 6 | CAACCA |  |
| MYB | Arabidopsis thaliana | 1305 | - | 6 | CAACAG |  |
| MYB | Arabidopsis thaliana | 2353 | - | 6 | CAACCA |  |

>HU08G02296.1   
+ -Up\_Stream \_Len000TTATTC TTACATCTTG TTGTTGTGAT TGTGTATTAA GCTTGTTGTC TCATGGTTTG   
  
  
+ CTCTTCTCTT TGGAACCTCA TGGTGGCTAG TTCCATGTTT TTGCTTTGTA GATGTAGCCG TTTGTTAAAG   
  
  
+ CAAGTTGGTT TATTTCTCCT ATGGCTTCGC TCTATTGTAA TTCAGGGGTC AGGTTGTTAT GTCAGACATT   
  
  
+ CCCATACCGC ACCTAGAAGG GAGGAGGGAG GCTCAGGAGT CTTAAAGAGT ATAGATTTGG ATTCACCTGT   
  
  
+ AGTCTATAGC TGATGTCCAC CTAATCTGGA TTTTATTCTT TCTTTCTCTC TCTTTTTTTT TCTTTTTTTG   
  
  
+ GGGGGGGGGG TGTTGTTGAG GGAGACAGGA TGAGGTCGAA TCTCTGCAGC ATAACAACCA CCATAGTCAC   
  
  
+ AATATGGAGA TTGTGGAGGC AAAGTTTGAG GACGTCCGGA TGTATTTCCT TTGGAACTGA GGAGGAGGTA   
  
  
+ GTACTTCTCT GGTAGTAGTG TTCTATATCC TTTGTTCCTT TCGTGTATTA TCCTAGCTAC CTCTCAAGCA   
  
  
+ AGTTGACATG AAATTGTAGA ATTCAAGACA ATAATAACAG CTGGTTTTTG GGTCTTTTAG TTTGCAATCC   
  
  
+ CTTGTAGCAT CTTAGTGACT TAGTGACATG AGATTTTGAC CCCAATAGAA ATAGAACAGA CAGCTGCTAA   
  
  
+ ATTCACCCAT CTCTTGTGTC AAAATCGGAC TTGTTTTGCC TCTGCTTCTC CATAAAGCAT CAAAGAGCGC   
  
  
+ TGATTGAGGC AGGGATTTTG TCAAGTGCAG TGTTGCATAT GTCATATCGT GTTGATGGTC ATGTTATCAC   
  
  
+ GGACATAAAT TTCCCAGTGT AAAGCAAAGG TGTCTGTCTT CACATAGAGT GTTCAATTCA TAGTCATGCT   
  
  
+ CTCGTCTCTG CATCATCCTG CGTTTGCTGG GGCTAAATTT GAGGGGCTTA TCACTTTGAA ATTCGGAGAA   
  
  
+ TTACTGTGAA TTTGTGTTAG TTCAGGCATA AAATACTGGA TGGGATGTTG AGATGTTCTA ATCATTATCT   
  
  
+ ATTGTTAAAA AACTCAGAAC TATTATAGAT TTTCTGTTTT CTGTTTGGAT TGAGATCCGA AGCCTTTGAG   
  
  
+ ATCCCCTGTT TCTAGCTTCC AACCCCCCTC CCTGGGTTCT TTTTCTTTGG TGTGAAAGTA TGAACAAAAG   
  
  
+ CTGATGTAAG TGTGATTCAA ACTCTAGCCT TGGTGAGACT CTTGGGAAGG GCTCCTAGAG ACTTTGCCAA   
  
  
+ TTGAGCTAAT TTGATGCCTA CTACTTGCTT ACTTTTCTAG CTGTTGTTTA AACGATTTAT GTTCATAGTT   
  
  
+ AAGGAGCGCC ATATTGCATT ACAATATCTG CTTTAGTGCT TTGTTTTTTG CATTTTCTTC TTTATTACTA   
  
  
+ TGGTTTTTGA AGAAGGCTTA CTGACTGATA CTGGTTTCTT CGCTTAAGTT TGGTGTACAT GCTTTTCAAT   
  
  
+ CTGTGTTGCA TTTTTCTATG ATGTATAAGC CGTTTATTAC CTATTGATGT TCAACTTTTC ACACTATATA   
  
  
+ TCCTTTATGG TTTCTTCGCT TAAGTTTGGT GTACATGCTT TTCAATCTGT GTTGCATTTT TCTGTGATGT   
  
  
+ ATAAGCCGTT TATTACCTAT TGATGTTCAA CTTTTCACAC TATATGTCCT TTTATTAGTT ACTGATGCCT   
  
  
+ CTGTCAAACA TTCTTAATTG CATTTAGTGT ATCAACGGTC TTAAAATTTT AAGCATTGCC AACTTGAACT   
  
  
+ TCTGAAGATG TTTGTCAAGA TCTTCAGTCT GCAAGTTAAC TGCTGCTTGC TTGAACTTTT CAACTTTTAA   
  
  
+ GAGAGATGTC AGAGTCTAAG CATGGCTTCC TGACAAGTAC TGCATTTTAT CACTTATGAA GACTCAATTC   
  
  
+ TGGGTTTTGA CAGCTTCTAT GAACCTCTAG TGCAGGTACA GCAGCTTGGA CATTAAGCTG CAAGTCAATT   
  
  
+ GACTGTAGAA CGACAAGTTG TTGAAGTTTC TGCTGAAAGT TCATCACTTG TTTGATGAAT AAAGTACAGC   
  
  
+ ATGGCACTTC AGATGCAAAA GTCTCGCGAT CAGTAGAGAT GATTCCATAT TTCTCATCTC ATTTCCAAGT   
  
  
+ CTTTGACAAC ATGTACCCAA ATAATGCCAG CCATGACACT CAGATGTCTC TCCAATCATA CAGCGAAGGA   
  
  
+ TACTTCACTC TGGACTCATC TCCAGCAGCA ATCGGAGCGT GCAGTGTCTA TGACTACCCA TCCGTTGTCA   
  
  
+ GCACCTCTTC AAATAGAAGC CAGTTTTCTC CTCAGGGTTC CCACTCGTAC ATCTCAGACC CCCATCATTC   
  
  
+ TTCCGACAAC TATGGATCCC CAGTGAGCGG CTCTTCTGTG GTTGACGATA ATGCTGAGCT GAGGAACAGG   
  
  
+ TTCAGTGATA TGGAGCTCCC CTTGCCACAG GACTCAGGAC ACCATTATTG CTCTTTTAGC CACAGAGGAA   
  
  
+ GCCATGAAGG CTCCTATACT TTGAGGCCAA ACCAACTGAT GGATATGGCC AACATGGAGT TAAAGCAGGT   
  
  
+ GCTATACTTC TGTGCAGAAG CAATCTCAGA GAATAATCTA TCAACTGCAG AAAGACTAAT GGATGCATTG   
  
  
+ GGTAAGAGGG TGTCTGTTTT TGGTTCACCA ATTGAAAGGT TGGCCGCCTA CATGTTGGAA GGGCTCAGAG   
  
  
+ CAAGGCTGGA GTTTTCTGGA TATACTATCT ACAAAAAGCT CAGGTGCGAA CAGCCAACGA GCTCAGAGCT   
  
  
+ TCTTTCCTAC ATGCACATCC TGTATCAAAG TTGCCCATAT TTCAAATTCG CATATATGTC CTCAAATGTT   
  
  
+ GCTATTCAAG AAGCTTTGGG GAATGAGCCG GTTATCCACA TCATCGATTT CCAGATTGCC ATGGGGACAC   
  
  
+ AATTGGTGCT CTTGATCCAG TCTCTCGCCC ATCGGCCTGG CGGGCCCCCC CCTCGTTCGG ATCACTGGGG   
  
  
+ TCGATG  

- -Up\_Stream \_Len000AATAAG AATGTAGAAC AACAACACTA ACACATAATT CGAACAACAG AGTACCAAAC   
  
  
- GAGAAGAGAA ACCTTGGAGT ACCACCGATC AAGGTACAAA AACGAAACAT CTACATCGGC AAACAATTTC   
  
  
- GTTCAACCAA ATAAAGAGGA TACCGAAGCG AGATAACATT AAGTCCCCAG TCCAACAATA CAGTCTGTAA   
  
  
- GGGTATGGCG TGGATCTTCC CTCCTCCCTC CGAGTCCTCA GAATTTCTCA TATCTAAACC TAAGTGGACA   
  
  
- TCAGATATCG ACTACAGGTG GATTAGACCT AAAATAAGAA AGAAAGAGAG AGAAAAAAAA AGAAAAAAAC   
  
  
- CCCCCCCCCC ACAACAACTC CCTCTGTCCT ACTCCAGCTT AGAGACGTCG TATTGTTGGT GGTATCAGTG   
  
  
- TTATACCTCT AACACCTCCG TTTCAAACTC CTGCAGGCCT ACATAAAGGA AACCTTGACT CCTCCTCCAT   
  
  
- CATGAAGAGA CCATCATCAC AAGATATAGG AAACAAGGAA AGCACATAAT AGGATCGATG GAGAGTTCGT   
  
  
- TCAACTGTAC TTTAACATCT TAAGTTCTGT TATTATTGTC GACCAAAAAC CCAGAAAATC AAACGTTAGG   
  
  
- GAACATCGTA GAATCACTGA ATCACTGTAC TCTAAAACTG GGGTTATCTT TATCTTGTCT GTCGACGATT   
  
  
- TAAGTGGGTA GAGAACACAG TTTTAGCCTG AACAAAACGG AGACGAAGAG GTATTTCGTA GTTTCTCGCG   
  
  
- ACTAACTCCG TCCCTAAAAC AGTTCACGTC ACAACGTATA CAGTATAGCA CAACTACCAG TACAATAGTG   
  
  
- CCTGTATTTA AAGGGTCACA TTTCGTTTCC ACAGACAGAA GTGTATCTCA CAAGTTAAGT ATCAGTACGA   
  
  
- GAGCAGAGAC GTAGTAGGAC GCAAACGACC CCGATTTAAA CTCCCCGAAT AGTGAAACTT TAAGCCTCTT   
  
  
- AATGACACTT AAACACAATC AAGTCCGTAT TTTATGACCT ACCCTACAAC TCTACAAGAT TAGTAATAGA   
  
  
- TAACAATTTT TTGAGTCTTG ATAATATCTA AAAGACAAAA GACAAACCTA ACTCTAGGCT TCGGAAACTC   
  
  
- TAGGGGACAA AGATCGAAGG TTGGGGGGAG GGACCCAAGA AAAAGAAACC ACACTTTCAT ACTTGTTTTC   
  
  
- GACTACATTC ACACTAAGTT TGAGATCGGA ACCACTCTGA GAACCCTTCC CGAGGATCTC TGAAACGGTT   
  
  
- AACTCGATTA AACTACGGAT GATGAACGAA TGAAAAGATC GACAACAAAT TTGCTAAATA CAAGTATCAA   
  
  
- TTCCTCGCGG TATAACGTAA TGTTATAGAC GAAATCACGA AACAAAAAAC GTAAAAGAAG AAATAATGAT   
  
  
- ACCAAAAACT TCTTCCGAAT GACTGACTAT GACCAAAGAA GCGAATTCAA ACCACATGTA CGAAAAGTTA   
  
  
- GACACAACGT AAAAAGATAC TACATATTCG GCAAATAATG GATAACTACA AGTTGAAAAG TGTGATATAT   
  
  
- AGGAAATACC AAAGAAGCGA ATTCAAACCA CATGTACGAA AAGTTAGACA CAACGTAAAA AGACACTACA   
  
  
- TATTCGGCAA ATAATGGATA ACTACAAGTT GAAAAGTGTG ATATACAGGA AAATAATCAA TGACTACGGA   
  
  
- GACAGTTTGT AAGAATTAAC GTAAATCACA TAGTTGCCAG AATTTTAAAA TTCGTAACGG TTGAACTTGA   
  
  
- AGACTTCTAC AAACAGTTCT AGAAGTCAGA CGTTCAATTG ACGACGAACG AACTTGAAAA GTTGAAAATT   
  
  
- CTCTCTACAG TCTCAGATTC GTACCGAAGG ACTGTTCATG ACGTAAAATA GTGAATACTT CTGAGTTAAG   
  
  
- ACCCAAAACT GTCGAAGATA CTTGGAGATC ACGTCCATGT CGTCGAACCT GTAATTCGAC GTTCAGTTAA   
  
  
- CTGACATCTT GCTGTTCAAC AACTTCAAAG ACGACTTTCA AGTAGTGAAC AAACTACTTA TTTCATGTCG   
  
  
- TACCGTGAAG TCTACGTTTT CAGAGCGCTA GTCATCTCTA CTAAGGTATA AAGAGTAGAG TAAAGGTTCA   
  
  
- GAAACTGTTG TACATGGGTT TATTACGGTC GGTACTGTGA GTCTACAGAG AGGTTAGTAT GTCGCTTCCT   
  
  
- ATGAAGTGAG ACCTGAGTAG AGGTCGTCGT TAGCCTCGCA CGTCACAGAT ACTGATGGGT AGGCAACAGT   
  
  
- CGTGGAGAAG TTTATCTTCG GTCAAAAGAG GAGTCCCAAG GGTGAGCATG TAGAGTCTGG GGGTAGTAAG   
  
  
- AAGGCTGTTG ATACCTAGGG GTCACTCGCC GAGAAGACAC CAACTGCTAT TACGACTCGA CTCCTTGTCC   
  
  
- AAGTCACTAT ACCTCGAGGG GAACGGTGTC CTGAGTCCTG TGGTAATAAC GAGAAAATCG GTGTCTCCTT   
  
  
- CGGTACTTCC GAGGATATGA AACTCCGGTT TGGTTGACTA CCTATACCGG TTGTACCTCA ATTTCGTCCA   
  
  
- CGATATGAAG ACACGTCTTC GTTAGAGTCT CTTATTAGAT AGTTGACGTC TTTCTGATTA CCTACGTAAC   
  
  
- CCATTCTCCC ACAGACAAAA ACCAAGTGGT TAACTTTCCA ACCGGCGGAT GTACAACCTT CCCGAGTCTC   
  
  
- GTTCCGACCT CAAAAGACCT ATATGATAGA TGTTTTTCGA GTCCACGCTT GTCGGTTGCT CGAGTCTCGA   
  
  
- AGAAAGGATG TACGTGTAGG ACATAGTTTC AACGGGTATA AAGTTTAAGC GTATATACAG GAGTTTACAA   
  
  
- CGATAAGTTC TTCGAAACCC CTTACTCGGC CAATAGGTGT AGTAGCTAAA GGTCTAACGG TACCCCTGTG   
  
  
- TTAACCACGA GAACTAGGTC AGAGAGCGGG TAGCCGGACC GCCCGGGGGG GGAGCAAGCC TAGTGACCCC   
  
  
- AGCTAC

+     MYB recognition site

| Site Name | Organism | Position | Strand | Matrix score. | sequence | function |
| --- | --- | --- | --- | --- | --- | --- |
| MYB recognition site | Arabidopsis thaliana | 2236 | + | 6 | CCGTTG |  |
| MYB recognition site | Arabidopsis thaliana | 1717 | - | 6 | CCGTTG |  |

>HU08G02296.1   
+ -Up\_Stream \_Len000TTATTC TTACATCTTG TTGTTGTGAT TGTGTATTAA GCTTGTTGTC TCATGGTTTG   
  
  
+ CTCTTCTCTT TGGAACCTCA TGGTGGCTAG TTCCATGTTT TTGCTTTGTA GATGTAGCCG TTTGTTAAAG   
  
  
+ CAAGTTGGTT TATTTCTCCT ATGGCTTCGC TCTATTGTAA TTCAGGGGTC AGGTTGTTAT GTCAGACATT   
  
  
+ CCCATACCGC ACCTAGAAGG GAGGAGGGAG GCTCAGGAGT CTTAAAGAGT ATAGATTTGG ATTCACCTGT   
  
  
+ AGTCTATAGC TGATGTCCAC CTAATCTGGA TTTTATTCTT TCTTTCTCTC TCTTTTTTTT TCTTTTTTTG   
  
  
+ GGGGGGGGGG TGTTGTTGAG GGAGACAGGA TGAGGTCGAA TCTCTGCAGC ATAACAACCA CCATAGTCAC   
  
  
+ AATATGGAGA TTGTGGAGGC AAAGTTTGAG GACGTCCGGA TGTATTTCCT TTGGAACTGA GGAGGAGGTA   
  
  
+ GTACTTCTCT GGTAGTAGTG TTCTATATCC TTTGTTCCTT TCGTGTATTA TCCTAGCTAC CTCTCAAGCA   
  
  
+ AGTTGACATG AAATTGTAGA ATTCAAGACA ATAATAACAG CTGGTTTTTG GGTCTTTTAG TTTGCAATCC   
  
  
+ CTTGTAGCAT CTTAGTGACT TAGTGACATG AGATTTTGAC CCCAATAGAA ATAGAACAGA CAGCTGCTAA   
  
  
+ ATTCACCCAT CTCTTGTGTC AAAATCGGAC TTGTTTTGCC TCTGCTTCTC CATAAAGCAT CAAAGAGCGC   
  
  
+ TGATTGAGGC AGGGATTTTG TCAAGTGCAG TGTTGCATAT GTCATATCGT GTTGATGGTC ATGTTATCAC   
  
  
+ GGACATAAAT TTCCCAGTGT AAAGCAAAGG TGTCTGTCTT CACATAGAGT GTTCAATTCA TAGTCATGCT   
  
  
+ CTCGTCTCTG CATCATCCTG CGTTTGCTGG GGCTAAATTT GAGGGGCTTA TCACTTTGAA ATTCGGAGAA   
  
  
+ TTACTGTGAA TTTGTGTTAG TTCAGGCATA AAATACTGGA TGGGATGTTG AGATGTTCTA ATCATTATCT   
  
  
+ ATTGTTAAAA AACTCAGAAC TATTATAGAT TTTCTGTTTT CTGTTTGGAT TGAGATCCGA AGCCTTTGAG   
  
  
+ ATCCCCTGTT TCTAGCTTCC AACCCCCCTC CCTGGGTTCT TTTTCTTTGG TGTGAAAGTA TGAACAAAAG   
  
  
+ CTGATGTAAG TGTGATTCAA ACTCTAGCCT TGGTGAGACT CTTGGGAAGG GCTCCTAGAG ACTTTGCCAA   
  
  
+ TTGAGCTAAT TTGATGCCTA CTACTTGCTT ACTTTTCTAG CTGTTGTTTA AACGATTTAT GTTCATAGTT   
  
  
+ AAGGAGCGCC ATATTGCATT ACAATATCTG CTTTAGTGCT TTGTTTTTTG CATTTTCTTC TTTATTACTA   
  
  
+ TGGTTTTTGA AGAAGGCTTA CTGACTGATA CTGGTTTCTT CGCTTAAGTT TGGTGTACAT GCTTTTCAAT   
  
  
+ CTGTGTTGCA TTTTTCTATG ATGTATAAGC CGTTTATTAC CTATTGATGT TCAACTTTTC ACACTATATA   
  
  
+ TCCTTTATGG TTTCTTCGCT TAAGTTTGGT GTACATGCTT TTCAATCTGT GTTGCATTTT TCTGTGATGT   
  
  
+ ATAAGCCGTT TATTACCTAT TGATGTTCAA CTTTTCACAC TATATGTCCT TTTATTAGTT ACTGATGCCT   
  
  
+ CTGTCAAACA TTCTTAATTG CATTTAGTGT ATCAACGGTC TTAAAATTTT AAGCATTGCC AACTTGAACT   
  
  
+ TCTGAAGATG TTTGTCAAGA TCTTCAGTCT GCAAGTTAAC TGCTGCTTGC TTGAACTTTT CAACTTTTAA   
  
  
+ GAGAGATGTC AGAGTCTAAG CATGGCTTCC TGACAAGTAC TGCATTTTAT CACTTATGAA GACTCAATTC   
  
  
+ TGGGTTTTGA CAGCTTCTAT GAACCTCTAG TGCAGGTACA GCAGCTTGGA CATTAAGCTG CAAGTCAATT   
  
  
+ GACTGTAGAA CGACAAGTTG TTGAAGTTTC TGCTGAAAGT TCATCACTTG TTTGATGAAT AAAGTACAGC   
  
  
+ ATGGCACTTC AGATGCAAAA GTCTCGCGAT CAGTAGAGAT GATTCCATAT TTCTCATCTC ATTTCCAAGT   
  
  
+ CTTTGACAAC ATGTACCCAA ATAATGCCAG CCATGACACT CAGATGTCTC TCCAATCATA CAGCGAAGGA   
  
  
+ TACTTCACTC TGGACTCATC TCCAGCAGCA ATCGGAGCGT GCAGTGTCTA TGACTACCCA TCCGTTGTCA   
  
  
+ GCACCTCTTC AAATAGAAGC CAGTTTTCTC CTCAGGGTTC CCACTCGTAC ATCTCAGACC CCCATCATTC   
  
  
+ TTCCGACAAC TATGGATCCC CAGTGAGCGG CTCTTCTGTG GTTGACGATA ATGCTGAGCT GAGGAACAGG   
  
  
+ TTCAGTGATA TGGAGCTCCC CTTGCCACAG GACTCAGGAC ACCATTATTG CTCTTTTAGC CACAGAGGAA   
  
  
+ GCCATGAAGG CTCCTATACT TTGAGGCCAA ACCAACTGAT GGATATGGCC AACATGGAGT TAAAGCAGGT   
  
  
+ GCTATACTTC TGTGCAGAAG CAATCTCAGA GAATAATCTA TCAACTGCAG AAAGACTAAT GGATGCATTG   
  
  
+ GGTAAGAGGG TGTCTGTTTT TGGTTCACCA ATTGAAAGGT TGGCCGCCTA CATGTTGGAA GGGCTCAGAG   
  
  
+ CAAGGCTGGA GTTTTCTGGA TATACTATCT ACAAAAAGCT CAGGTGCGAA CAGCCAACGA GCTCAGAGCT   
  
  
+ TCTTTCCTAC ATGCACATCC TGTATCAAAG TTGCCCATAT TTCAAATTCG CATATATGTC CTCAAATGTT   
  
  
+ GCTATTCAAG AAGCTTTGGG GAATGAGCCG GTTATCCACA TCATCGATTT CCAGATTGCC ATGGGGACAC   
  
  
+ AATTGGTGCT CTTGATCCAG TCTCTCGCCC ATCGGCCTGG CGGGCCCCCC CCTCGTTCGG ATCACTGGGG   
  
  
+ TCGATG  

- -Up\_Stream \_Len000AATAAG AATGTAGAAC AACAACACTA ACACATAATT CGAACAACAG AGTACCAAAC   
  
  
- GAGAAGAGAA ACCTTGGAGT ACCACCGATC AAGGTACAAA AACGAAACAT CTACATCGGC AAACAATTTC   
  
  
- GTTCAACCAA ATAAAGAGGA TACCGAAGCG AGATAACATT AAGTCCCCAG TCCAACAATA CAGTCTGTAA   
  
  
- GGGTATGGCG TGGATCTTCC CTCCTCCCTC CGAGTCCTCA GAATTTCTCA TATCTAAACC TAAGTGGACA   
  
  
- TCAGATATCG ACTACAGGTG GATTAGACCT AAAATAAGAA AGAAAGAGAG AGAAAAAAAA AGAAAAAAAC   
  
  
- CCCCCCCCCC ACAACAACTC CCTCTGTCCT ACTCCAGCTT AGAGACGTCG TATTGTTGGT GGTATCAGTG   
  
  
- TTATACCTCT AACACCTCCG TTTCAAACTC CTGCAGGCCT ACATAAAGGA AACCTTGACT CCTCCTCCAT   
  
  
- CATGAAGAGA CCATCATCAC AAGATATAGG AAACAAGGAA AGCACATAAT AGGATCGATG GAGAGTTCGT   
  
  
- TCAACTGTAC TTTAACATCT TAAGTTCTGT TATTATTGTC GACCAAAAAC CCAGAAAATC AAACGTTAGG   
  
  
- GAACATCGTA GAATCACTGA ATCACTGTAC TCTAAAACTG GGGTTATCTT TATCTTGTCT GTCGACGATT   
  
  
- TAAGTGGGTA GAGAACACAG TTTTAGCCTG AACAAAACGG AGACGAAGAG GTATTTCGTA GTTTCTCGCG   
  
  
- ACTAACTCCG TCCCTAAAAC AGTTCACGTC ACAACGTATA CAGTATAGCA CAACTACCAG TACAATAGTG   
  
  
- CCTGTATTTA AAGGGTCACA TTTCGTTTCC ACAGACAGAA GTGTATCTCA CAAGTTAAGT ATCAGTACGA   
  
  
- GAGCAGAGAC GTAGTAGGAC GCAAACGACC CCGATTTAAA CTCCCCGAAT AGTGAAACTT TAAGCCTCTT   
  
  
- AATGACACTT AAACACAATC AAGTCCGTAT TTTATGACCT ACCCTACAAC TCTACAAGAT TAGTAATAGA   
  
  
- TAACAATTTT TTGAGTCTTG ATAATATCTA AAAGACAAAA GACAAACCTA ACTCTAGGCT TCGGAAACTC   
  
  
- TAGGGGACAA AGATCGAAGG TTGGGGGGAG GGACCCAAGA AAAAGAAACC ACACTTTCAT ACTTGTTTTC   
  
  
- GACTACATTC ACACTAAGTT TGAGATCGGA ACCACTCTGA GAACCCTTCC CGAGGATCTC TGAAACGGTT   
  
  
- AACTCGATTA AACTACGGAT GATGAACGAA TGAAAAGATC GACAACAAAT TTGCTAAATA CAAGTATCAA   
  
  
- TTCCTCGCGG TATAACGTAA TGTTATAGAC GAAATCACGA AACAAAAAAC GTAAAAGAAG AAATAATGAT   
  
  
- ACCAAAAACT TCTTCCGAAT GACTGACTAT GACCAAAGAA GCGAATTCAA ACCACATGTA CGAAAAGTTA   
  
  
- GACACAACGT AAAAAGATAC TACATATTCG GCAAATAATG GATAACTACA AGTTGAAAAG TGTGATATAT   
  
  
- AGGAAATACC AAAGAAGCGA ATTCAAACCA CATGTACGAA AAGTTAGACA CAACGTAAAA AGACACTACA   
  
  
- TATTCGGCAA ATAATGGATA ACTACAAGTT GAAAAGTGTG ATATACAGGA AAATAATCAA TGACTACGGA   
  
  
- GACAGTTTGT AAGAATTAAC GTAAATCACA TAGTTGCCAG AATTTTAAAA TTCGTAACGG TTGAACTTGA   
  
  
- AGACTTCTAC AAACAGTTCT AGAAGTCAGA CGTTCAATTG ACGACGAACG AACTTGAAAA GTTGAAAATT   
  
  
- CTCTCTACAG TCTCAGATTC GTACCGAAGG ACTGTTCATG ACGTAAAATA GTGAATACTT CTGAGTTAAG   
  
  
- ACCCAAAACT GTCGAAGATA CTTGGAGATC ACGTCCATGT CGTCGAACCT GTAATTCGAC GTTCAGTTAA   
  
  
- CTGACATCTT GCTGTTCAAC AACTTCAAAG ACGACTTTCA AGTAGTGAAC AAACTACTTA TTTCATGTCG   
  
  
- TACCGTGAAG TCTACGTTTT CAGAGCGCTA GTCATCTCTA CTAAGGTATA AAGAGTAGAG TAAAGGTTCA   
  
  
- GAAACTGTTG TACATGGGTT TATTACGGTC GGTACTGTGA GTCTACAGAG AGGTTAGTAT GTCGCTTCCT   
  
  
- ATGAAGTGAG ACCTGAGTAG AGGTCGTCGT TAGCCTCGCA CGTCACAGAT ACTGATGGGT AGGCAACAGT   
  
  
- CGTGGAGAAG TTTATCTTCG GTCAAAAGAG GAGTCCCAAG GGTGAGCATG TAGAGTCTGG GGGTAGTAAG   
  
  
- AAGGCTGTTG ATACCTAGGG GTCACTCGCC GAGAAGACAC CAACTGCTAT TACGACTCGA CTCCTTGTCC   
  
  
- AAGTCACTAT ACCTCGAGGG GAACGGTGTC CTGAGTCCTG TGGTAATAAC GAGAAAATCG GTGTCTCCTT   
  
  
- CGGTACTTCC GAGGATATGA AACTCCGGTT TGGTTGACTA CCTATACCGG TTGTACCTCA ATTTCGTCCA   
  
  
- CGATATGAAG ACACGTCTTC GTTAGAGTCT CTTATTAGAT AGTTGACGTC TTTCTGATTA CCTACGTAAC   
  
  
- CCATTCTCCC ACAGACAAAA ACCAAGTGGT TAACTTTCCA ACCGGCGGAT GTACAACCTT CCCGAGTCTC   
  
  
- GTTCCGACCT CAAAAGACCT ATATGATAGA TGTTTTTCGA GTCCACGCTT GTCGGTTGCT CGAGTCTCGA   
  
  
- AGAAAGGATG TACGTGTAGG ACATAGTTTC AACGGGTATA AAGTTTAAGC GTATATACAG GAGTTTACAA   
  
  
- CGATAAGTTC TTCGAAACCC CTTACTCGGC CAATAGGTGT AGTAGCTAAA GGTCTAACGG TACCCCTGTG   
  
  
- TTAACCACGA GAACTAGGTC AGAGAGCGGG TAGCCGGACC GCCCGGGGGG GGAGCAAGCC TAGTGACCCC   
  
  
- AGCTAC

+     MYC

| Site Name | Organism | Position | Strand | Matrix score. | sequence | function |
| --- | --- | --- | --- | --- | --- | --- |
| MYC | Arabidopsis thaliana | 2623 | - | 6 | CAATTG |  |
| MYC | Arabidopsis thaliana | 1262 | + | 6 | CAATTG |  |
| MYC | Arabidopsis thaliana | 2874 | - | 6 | CAATTG |  |
| MYC | Arabidopsis thaliana | 1960 | - | 6 | CAATTG |  |
| MYC | Arabidopsis thaliana | 2797 | - | 6 | CATTTG |  |

>HU08G02296.1   
+ -Up\_Stream \_Len000TTATTC TTACATCTTG TTGTTGTGAT TGTGTATTAA GCTTGTTGTC TCATGGTTTG   
  
  
+ CTCTTCTCTT TGGAACCTCA TGGTGGCTAG TTCCATGTTT TTGCTTTGTA GATGTAGCCG TTTGTTAAAG   
  
  
+ CAAGTTGGTT TATTTCTCCT ATGGCTTCGC TCTATTGTAA TTCAGGGGTC AGGTTGTTAT GTCAGACATT   
  
  
+ CCCATACCGC ACCTAGAAGG GAGGAGGGAG GCTCAGGAGT CTTAAAGAGT ATAGATTTGG ATTCACCTGT   
  
  
+ AGTCTATAGC TGATGTCCAC CTAATCTGGA TTTTATTCTT TCTTTCTCTC TCTTTTTTTT TCTTTTTTTG   
  
  
+ GGGGGGGGGG TGTTGTTGAG GGAGACAGGA TGAGGTCGAA TCTCTGCAGC ATAACAACCA CCATAGTCAC   
  
  
+ AATATGGAGA TTGTGGAGGC AAAGTTTGAG GACGTCCGGA TGTATTTCCT TTGGAACTGA GGAGGAGGTA   
  
  
+ GTACTTCTCT GGTAGTAGTG TTCTATATCC TTTGTTCCTT TCGTGTATTA TCCTAGCTAC CTCTCAAGCA   
  
  
+ AGTTGACATG AAATTGTAGA ATTCAAGACA ATAATAACAG CTGGTTTTTG GGTCTTTTAG TTTGCAATCC   
  
  
+ CTTGTAGCAT CTTAGTGACT TAGTGACATG AGATTTTGAC CCCAATAGAA ATAGAACAGA CAGCTGCTAA   
  
  
+ ATTCACCCAT CTCTTGTGTC AAAATCGGAC TTGTTTTGCC TCTGCTTCTC CATAAAGCAT CAAAGAGCGC   
  
  
+ TGATTGAGGC AGGGATTTTG TCAAGTGCAG TGTTGCATAT GTCATATCGT GTTGATGGTC ATGTTATCAC   
  
  
+ GGACATAAAT TTCCCAGTGT AAAGCAAAGG TGTCTGTCTT CACATAGAGT GTTCAATTCA TAGTCATGCT   
  
  
+ CTCGTCTCTG CATCATCCTG CGTTTGCTGG GGCTAAATTT GAGGGGCTTA TCACTTTGAA ATTCGGAGAA   
  
  
+ TTACTGTGAA TTTGTGTTAG TTCAGGCATA AAATACTGGA TGGGATGTTG AGATGTTCTA ATCATTATCT   
  
  
+ ATTGTTAAAA AACTCAGAAC TATTATAGAT TTTCTGTTTT CTGTTTGGAT TGAGATCCGA AGCCTTTGAG   
  
  
+ ATCCCCTGTT TCTAGCTTCC AACCCCCCTC CCTGGGTTCT TTTTCTTTGG TGTGAAAGTA TGAACAAAAG   
  
  
+ CTGATGTAAG TGTGATTCAA ACTCTAGCCT TGGTGAGACT CTTGGGAAGG GCTCCTAGAG ACTTTGCCAA   
  
  
+ TTGAGCTAAT TTGATGCCTA CTACTTGCTT ACTTTTCTAG CTGTTGTTTA AACGATTTAT GTTCATAGTT   
  
  
+ AAGGAGCGCC ATATTGCATT ACAATATCTG CTTTAGTGCT TTGTTTTTTG CATTTTCTTC TTTATTACTA   
  
  
+ TGGTTTTTGA AGAAGGCTTA CTGACTGATA CTGGTTTCTT CGCTTAAGTT TGGTGTACAT GCTTTTCAAT   
  
  
+ CTGTGTTGCA TTTTTCTATG ATGTATAAGC CGTTTATTAC CTATTGATGT TCAACTTTTC ACACTATATA   
  
  
+ TCCTTTATGG TTTCTTCGCT TAAGTTTGGT GTACATGCTT TTCAATCTGT GTTGCATTTT TCTGTGATGT   
  
  
+ ATAAGCCGTT TATTACCTAT TGATGTTCAA CTTTTCACAC TATATGTCCT TTTATTAGTT ACTGATGCCT   
  
  
+ CTGTCAAACA TTCTTAATTG CATTTAGTGT ATCAACGGTC TTAAAATTTT AAGCATTGCC AACTTGAACT   
  
  
+ TCTGAAGATG TTTGTCAAGA TCTTCAGTCT GCAAGTTAAC TGCTGCTTGC TTGAACTTTT CAACTTTTAA   
  
  
+ GAGAGATGTC AGAGTCTAAG CATGGCTTCC TGACAAGTAC TGCATTTTAT CACTTATGAA GACTCAATTC   
  
  
+ TGGGTTTTGA CAGCTTCTAT GAACCTCTAG TGCAGGTACA GCAGCTTGGA CATTAAGCTG CAAGTCAATT   
  
  
+ GACTGTAGAA CGACAAGTTG TTGAAGTTTC TGCTGAAAGT TCATCACTTG TTTGATGAAT AAAGTACAGC   
  
  
+ ATGGCACTTC AGATGCAAAA GTCTCGCGAT CAGTAGAGAT GATTCCATAT TTCTCATCTC ATTTCCAAGT   
  
  
+ CTTTGACAAC ATGTACCCAA ATAATGCCAG CCATGACACT CAGATGTCTC TCCAATCATA CAGCGAAGGA   
  
  
+ TACTTCACTC TGGACTCATC TCCAGCAGCA ATCGGAGCGT GCAGTGTCTA TGACTACCCA TCCGTTGTCA   
  
  
+ GCACCTCTTC AAATAGAAGC CAGTTTTCTC CTCAGGGTTC CCACTCGTAC ATCTCAGACC CCCATCATTC   
  
  
+ TTCCGACAAC TATGGATCCC CAGTGAGCGG CTCTTCTGTG GTTGACGATA ATGCTGAGCT GAGGAACAGG   
  
  
+ TTCAGTGATA TGGAGCTCCC CTTGCCACAG GACTCAGGAC ACCATTATTG CTCTTTTAGC CACAGAGGAA   
  
  
+ GCCATGAAGG CTCCTATACT TTGAGGCCAA ACCAACTGAT GGATATGGCC AACATGGAGT TAAAGCAGGT   
  
  
+ GCTATACTTC TGTGCAGAAG CAATCTCAGA GAATAATCTA TCAACTGCAG AAAGACTAAT GGATGCATTG   
  
  
+ GGTAAGAGGG TGTCTGTTTT TGGTTCACCA ATTGAAAGGT TGGCCGCCTA CATGTTGGAA GGGCTCAGAG   
  
  
+ CAAGGCTGGA GTTTTCTGGA TATACTATCT ACAAAAAGCT CAGGTGCGAA CAGCCAACGA GCTCAGAGCT   
  
  
+ TCTTTCCTAC ATGCACATCC TGTATCAAAG TTGCCCATAT TTCAAATTCG CATATATGTC CTCAAATGTT   
  
  
+ GCTATTCAAG AAGCTTTGGG GAATGAGCCG GTTATCCACA TCATCGATTT CCAGATTGCC ATGGGGACAC   
  
  
+ AATTGGTGCT CTTGATCCAG TCTCTCGCCC ATCGGCCTGG CGGGCCCCCC CCTCGTTCGG ATCACTGGGG   
  
  
+ TCGATG  

- -Up\_Stream \_Len000AATAAG AATGTAGAAC AACAACACTA ACACATAATT CGAACAACAG AGTACCAAAC   
  
  
- GAGAAGAGAA ACCTTGGAGT ACCACCGATC AAGGTACAAA AACGAAACAT CTACATCGGC AAACAATTTC   
  
  
- GTTCAACCAA ATAAAGAGGA TACCGAAGCG AGATAACATT AAGTCCCCAG TCCAACAATA CAGTCTGTAA   
  
  
- GGGTATGGCG TGGATCTTCC CTCCTCCCTC CGAGTCCTCA GAATTTCTCA TATCTAAACC TAAGTGGACA   
  
  
- TCAGATATCG ACTACAGGTG GATTAGACCT AAAATAAGAA AGAAAGAGAG AGAAAAAAAA AGAAAAAAAC   
  
  
- CCCCCCCCCC ACAACAACTC CCTCTGTCCT ACTCCAGCTT AGAGACGTCG TATTGTTGGT GGTATCAGTG   
  
  
- TTATACCTCT AACACCTCCG TTTCAAACTC CTGCAGGCCT ACATAAAGGA AACCTTGACT CCTCCTCCAT   
  
  
- CATGAAGAGA CCATCATCAC AAGATATAGG AAACAAGGAA AGCACATAAT AGGATCGATG GAGAGTTCGT   
  
  
- TCAACTGTAC TTTAACATCT TAAGTTCTGT TATTATTGTC GACCAAAAAC CCAGAAAATC AAACGTTAGG   
  
  
- GAACATCGTA GAATCACTGA ATCACTGTAC TCTAAAACTG GGGTTATCTT TATCTTGTCT GTCGACGATT   
  
  
- TAAGTGGGTA GAGAACACAG TTTTAGCCTG AACAAAACGG AGACGAAGAG GTATTTCGTA GTTTCTCGCG   
  
  
- ACTAACTCCG TCCCTAAAAC AGTTCACGTC ACAACGTATA CAGTATAGCA CAACTACCAG TACAATAGTG   
  
  
- CCTGTATTTA AAGGGTCACA TTTCGTTTCC ACAGACAGAA GTGTATCTCA CAAGTTAAGT ATCAGTACGA   
  
  
- GAGCAGAGAC GTAGTAGGAC GCAAACGACC CCGATTTAAA CTCCCCGAAT AGTGAAACTT TAAGCCTCTT   
  
  
- AATGACACTT AAACACAATC AAGTCCGTAT TTTATGACCT ACCCTACAAC TCTACAAGAT TAGTAATAGA   
  
  
- TAACAATTTT TTGAGTCTTG ATAATATCTA AAAGACAAAA GACAAACCTA ACTCTAGGCT TCGGAAACTC   
  
  
- TAGGGGACAA AGATCGAAGG TTGGGGGGAG GGACCCAAGA AAAAGAAACC ACACTTTCAT ACTTGTTTTC   
  
  
- GACTACATTC ACACTAAGTT TGAGATCGGA ACCACTCTGA GAACCCTTCC CGAGGATCTC TGAAACGGTT   
  
  
- AACTCGATTA AACTACGGAT GATGAACGAA TGAAAAGATC GACAACAAAT TTGCTAAATA CAAGTATCAA   
  
  
- TTCCTCGCGG TATAACGTAA TGTTATAGAC GAAATCACGA AACAAAAAAC GTAAAAGAAG AAATAATGAT   
  
  
- ACCAAAAACT TCTTCCGAAT GACTGACTAT GACCAAAGAA GCGAATTCAA ACCACATGTA CGAAAAGTTA   
  
  
- GACACAACGT AAAAAGATAC TACATATTCG GCAAATAATG GATAACTACA AGTTGAAAAG TGTGATATAT   
  
  
- AGGAAATACC AAAGAAGCGA ATTCAAACCA CATGTACGAA AAGTTAGACA CAACGTAAAA AGACACTACA   
  
  
- TATTCGGCAA ATAATGGATA ACTACAAGTT GAAAAGTGTG ATATACAGGA AAATAATCAA TGACTACGGA   
  
  
- GACAGTTTGT AAGAATTAAC GTAAATCACA TAGTTGCCAG AATTTTAAAA TTCGTAACGG TTGAACTTGA   
  
  
- AGACTTCTAC AAACAGTTCT AGAAGTCAGA CGTTCAATTG ACGACGAACG AACTTGAAAA GTTGAAAATT   
  
  
- CTCTCTACAG TCTCAGATTC GTACCGAAGG ACTGTTCATG ACGTAAAATA GTGAATACTT CTGAGTTAAG   
  
  
- ACCCAAAACT GTCGAAGATA CTTGGAGATC ACGTCCATGT CGTCGAACCT GTAATTCGAC GTTCAGTTAA   
  
  
- CTGACATCTT GCTGTTCAAC AACTTCAAAG ACGACTTTCA AGTAGTGAAC AAACTACTTA TTTCATGTCG   
  
  
- TACCGTGAAG TCTACGTTTT CAGAGCGCTA GTCATCTCTA CTAAGGTATA AAGAGTAGAG TAAAGGTTCA   
  
  
- GAAACTGTTG TACATGGGTT TATTACGGTC GGTACTGTGA GTCTACAGAG AGGTTAGTAT GTCGCTTCCT   
  
  
- ATGAAGTGAG ACCTGAGTAG AGGTCGTCGT TAGCCTCGCA CGTCACAGAT ACTGATGGGT AGGCAACAGT   
  
  
- CGTGGAGAAG TTTATCTTCG GTCAAAAGAG GAGTCCCAAG GGTGAGCATG TAGAGTCTGG GGGTAGTAAG   
  
  
- AAGGCTGTTG ATACCTAGGG GTCACTCGCC GAGAAGACAC CAACTGCTAT TACGACTCGA CTCCTTGTCC   
  
  
- AAGTCACTAT ACCTCGAGGG GAACGGTGTC CTGAGTCCTG TGGTAATAAC GAGAAAATCG GTGTCTCCTT   
  
  
- CGGTACTTCC GAGGATATGA AACTCCGGTT TGGTTGACTA CCTATACCGG TTGTACCTCA ATTTCGTCCA   
  
  
- CGATATGAAG ACACGTCTTC GTTAGAGTCT CTTATTAGAT AGTTGACGTC TTTCTGATTA CCTACGTAAC   
  
  
- CCATTCTCCC ACAGACAAAA ACCAAGTGGT TAACTTTCCA ACCGGCGGAT GTACAACCTT CCCGAGTCTC   
  
  
- GTTCCGACCT CAAAAGACCT ATATGATAGA TGTTTTTCGA GTCCACGCTT GTCGGTTGCT CGAGTCTCGA   
  
  
- AGAAAGGATG TACGTGTAGG ACATAGTTTC AACGGGTATA AAGTTTAAGC GTATATACAG GAGTTTACAA   
  
  
- CGATAAGTTC TTCGAAACCC CTTACTCGGC CAATAGGTGT AGTAGCTAAA GGTCTAACGG TACCCCTGTG   
  
  
- TTAACCACGA GAACTAGGTC AGAGAGCGGG TAGCCGGACC GCCCGGGGGG GGAGCAAGCC TAGTGACCCC   
  
  
- AGCTAC

+     Myb

| Site Name | Organism | Position | Strand | Matrix score. | sequence | function |
| --- | --- | --- | --- | --- | --- | --- |
| Myb | Arabidopsis thaliana | 2566 | + | 6 | CAACTG |  |
| Myb | Arabidopsis thaliana | 1791 | + | 6 | TAACTG |  |
| Myb | Arabidopsis thaliana | 2487 | + | 6 | CAACTG |  |

>HU08G02296.1   
+ -Up\_Stream \_Len000TTATTC TTACATCTTG TTGTTGTGAT TGTGTATTAA GCTTGTTGTC TCATGGTTTG   
  
  
+ CTCTTCTCTT TGGAACCTCA TGGTGGCTAG TTCCATGTTT TTGCTTTGTA GATGTAGCCG TTTGTTAAAG   
  
  
+ CAAGTTGGTT TATTTCTCCT ATGGCTTCGC TCTATTGTAA TTCAGGGGTC AGGTTGTTAT GTCAGACATT   
  
  
+ CCCATACCGC ACCTAGAAGG GAGGAGGGAG GCTCAGGAGT CTTAAAGAGT ATAGATTTGG ATTCACCTGT   
  
  
+ AGTCTATAGC TGATGTCCAC CTAATCTGGA TTTTATTCTT TCTTTCTCTC TCTTTTTTTT TCTTTTTTTG   
  
  
+ GGGGGGGGGG TGTTGTTGAG GGAGACAGGA TGAGGTCGAA TCTCTGCAGC ATAACAACCA CCATAGTCAC   
  
  
+ AATATGGAGA TTGTGGAGGC AAAGTTTGAG GACGTCCGGA TGTATTTCCT TTGGAACTGA GGAGGAGGTA   
  
  
+ GTACTTCTCT GGTAGTAGTG TTCTATATCC TTTGTTCCTT TCGTGTATTA TCCTAGCTAC CTCTCAAGCA   
  
  
+ AGTTGACATG AAATTGTAGA ATTCAAGACA ATAATAACAG CTGGTTTTTG GGTCTTTTAG TTTGCAATCC   
  
  
+ CTTGTAGCAT CTTAGTGACT TAGTGACATG AGATTTTGAC CCCAATAGAA ATAGAACAGA CAGCTGCTAA   
  
  
+ ATTCACCCAT CTCTTGTGTC AAAATCGGAC TTGTTTTGCC TCTGCTTCTC CATAAAGCAT CAAAGAGCGC   
  
  
+ TGATTGAGGC AGGGATTTTG TCAAGTGCAG TGTTGCATAT GTCATATCGT GTTGATGGTC ATGTTATCAC   
  
  
+ GGACATAAAT TTCCCAGTGT AAAGCAAAGG TGTCTGTCTT CACATAGAGT GTTCAATTCA TAGTCATGCT   
  
  
+ CTCGTCTCTG CATCATCCTG CGTTTGCTGG GGCTAAATTT GAGGGGCTTA TCACTTTGAA ATTCGGAGAA   
  
  
+ TTACTGTGAA TTTGTGTTAG TTCAGGCATA AAATACTGGA TGGGATGTTG AGATGTTCTA ATCATTATCT   
  
  
+ ATTGTTAAAA AACTCAGAAC TATTATAGAT TTTCTGTTTT CTGTTTGGAT TGAGATCCGA AGCCTTTGAG   
  
  
+ ATCCCCTGTT TCTAGCTTCC AACCCCCCTC CCTGGGTTCT TTTTCTTTGG TGTGAAAGTA TGAACAAAAG   
  
  
+ CTGATGTAAG TGTGATTCAA ACTCTAGCCT TGGTGAGACT CTTGGGAAGG GCTCCTAGAG ACTTTGCCAA   
  
  
+ TTGAGCTAAT TTGATGCCTA CTACTTGCTT ACTTTTCTAG CTGTTGTTTA AACGATTTAT GTTCATAGTT   
  
  
+ AAGGAGCGCC ATATTGCATT ACAATATCTG CTTTAGTGCT TTGTTTTTTG CATTTTCTTC TTTATTACTA   
  
  
+ TGGTTTTTGA AGAAGGCTTA CTGACTGATA CTGGTTTCTT CGCTTAAGTT TGGTGTACAT GCTTTTCAAT   
  
  
+ CTGTGTTGCA TTTTTCTATG ATGTATAAGC CGTTTATTAC CTATTGATGT TCAACTTTTC ACACTATATA   
  
  
+ TCCTTTATGG TTTCTTCGCT TAAGTTTGGT GTACATGCTT TTCAATCTGT GTTGCATTTT TCTGTGATGT   
  
  
+ ATAAGCCGTT TATTACCTAT TGATGTTCAA CTTTTCACAC TATATGTCCT TTTATTAGTT ACTGATGCCT   
  
  
+ CTGTCAAACA TTCTTAATTG CATTTAGTGT ATCAACGGTC TTAAAATTTT AAGCATTGCC AACTTGAACT   
  
  
+ TCTGAAGATG TTTGTCAAGA TCTTCAGTCT GCAAGTTAAC TGCTGCTTGC TTGAACTTTT CAACTTTTAA   
  
  
+ GAGAGATGTC AGAGTCTAAG CATGGCTTCC TGACAAGTAC TGCATTTTAT CACTTATGAA GACTCAATTC   
  
  
+ TGGGTTTTGA CAGCTTCTAT GAACCTCTAG TGCAGGTACA GCAGCTTGGA CATTAAGCTG CAAGTCAATT   
  
  
+ GACTGTAGAA CGACAAGTTG TTGAAGTTTC TGCTGAAAGT TCATCACTTG TTTGATGAAT AAAGTACAGC   
  
  
+ ATGGCACTTC AGATGCAAAA GTCTCGCGAT CAGTAGAGAT GATTCCATAT TTCTCATCTC ATTTCCAAGT   
  
  
+ CTTTGACAAC ATGTACCCAA ATAATGCCAG CCATGACACT CAGATGTCTC TCCAATCATA CAGCGAAGGA   
  
  
+ TACTTCACTC TGGACTCATC TCCAGCAGCA ATCGGAGCGT GCAGTGTCTA TGACTACCCA TCCGTTGTCA   
  
  
+ GCACCTCTTC AAATAGAAGC CAGTTTTCTC CTCAGGGTTC CCACTCGTAC ATCTCAGACC CCCATCATTC   
  
  
+ TTCCGACAAC TATGGATCCC CAGTGAGCGG CTCTTCTGTG GTTGACGATA ATGCTGAGCT GAGGAACAGG   
  
  
+ TTCAGTGATA TGGAGCTCCC CTTGCCACAG GACTCAGGAC ACCATTATTG CTCTTTTAGC CACAGAGGAA   
  
  
+ GCCATGAAGG CTCCTATACT TTGAGGCCAA ACCAACTGAT GGATATGGCC AACATGGAGT TAAAGCAGGT   
  
  
+ GCTATACTTC TGTGCAGAAG CAATCTCAGA GAATAATCTA TCAACTGCAG AAAGACTAAT GGATGCATTG   
  
  
+ GGTAAGAGGG TGTCTGTTTT TGGTTCACCA ATTGAAAGGT TGGCCGCCTA CATGTTGGAA GGGCTCAGAG   
  
  
+ CAAGGCTGGA GTTTTCTGGA TATACTATCT ACAAAAAGCT CAGGTGCGAA CAGCCAACGA GCTCAGAGCT   
  
  
+ TCTTTCCTAC ATGCACATCC TGTATCAAAG TTGCCCATAT TTCAAATTCG CATATATGTC CTCAAATGTT   
  
  
+ GCTATTCAAG AAGCTTTGGG GAATGAGCCG GTTATCCACA TCATCGATTT CCAGATTGCC ATGGGGACAC   
  
  
+ AATTGGTGCT CTTGATCCAG TCTCTCGCCC ATCGGCCTGG CGGGCCCCCC CCTCGTTCGG ATCACTGGGG   
  
  
+ TCGATG  

- -Up\_Stream \_Len000AATAAG AATGTAGAAC AACAACACTA ACACATAATT CGAACAACAG AGTACCAAAC   
  
  
- GAGAAGAGAA ACCTTGGAGT ACCACCGATC AAGGTACAAA AACGAAACAT CTACATCGGC AAACAATTTC   
  
  
- GTTCAACCAA ATAAAGAGGA TACCGAAGCG AGATAACATT AAGTCCCCAG TCCAACAATA CAGTCTGTAA   
  
  
- GGGTATGGCG TGGATCTTCC CTCCTCCCTC CGAGTCCTCA GAATTTCTCA TATCTAAACC TAAGTGGACA   
  
  
- TCAGATATCG ACTACAGGTG GATTAGACCT AAAATAAGAA AGAAAGAGAG AGAAAAAAAA AGAAAAAAAC   
  
  
- CCCCCCCCCC ACAACAACTC CCTCTGTCCT ACTCCAGCTT AGAGACGTCG TATTGTTGGT GGTATCAGTG   
  
  
- TTATACCTCT AACACCTCCG TTTCAAACTC CTGCAGGCCT ACATAAAGGA AACCTTGACT CCTCCTCCAT   
  
  
- CATGAAGAGA CCATCATCAC AAGATATAGG AAACAAGGAA AGCACATAAT AGGATCGATG GAGAGTTCGT   
  
  
- TCAACTGTAC TTTAACATCT TAAGTTCTGT TATTATTGTC GACCAAAAAC CCAGAAAATC AAACGTTAGG   
  
  
- GAACATCGTA GAATCACTGA ATCACTGTAC TCTAAAACTG GGGTTATCTT TATCTTGTCT GTCGACGATT   
  
  
- TAAGTGGGTA GAGAACACAG TTTTAGCCTG AACAAAACGG AGACGAAGAG GTATTTCGTA GTTTCTCGCG   
  
  
- ACTAACTCCG TCCCTAAAAC AGTTCACGTC ACAACGTATA CAGTATAGCA CAACTACCAG TACAATAGTG   
  
  
- CCTGTATTTA AAGGGTCACA TTTCGTTTCC ACAGACAGAA GTGTATCTCA CAAGTTAAGT ATCAGTACGA   
  
  
- GAGCAGAGAC GTAGTAGGAC GCAAACGACC CCGATTTAAA CTCCCCGAAT AGTGAAACTT TAAGCCTCTT   
  
  
- AATGACACTT AAACACAATC AAGTCCGTAT TTTATGACCT ACCCTACAAC TCTACAAGAT TAGTAATAGA   
  
  
- TAACAATTTT TTGAGTCTTG ATAATATCTA AAAGACAAAA GACAAACCTA ACTCTAGGCT TCGGAAACTC   
  
  
- TAGGGGACAA AGATCGAAGG TTGGGGGGAG GGACCCAAGA AAAAGAAACC ACACTTTCAT ACTTGTTTTC   
  
  
- GACTACATTC ACACTAAGTT TGAGATCGGA ACCACTCTGA GAACCCTTCC CGAGGATCTC TGAAACGGTT   
  
  
- AACTCGATTA AACTACGGAT GATGAACGAA TGAAAAGATC GACAACAAAT TTGCTAAATA CAAGTATCAA   
  
  
- TTCCTCGCGG TATAACGTAA TGTTATAGAC GAAATCACGA AACAAAAAAC GTAAAAGAAG AAATAATGAT   
  
  
- ACCAAAAACT TCTTCCGAAT GACTGACTAT GACCAAAGAA GCGAATTCAA ACCACATGTA CGAAAAGTTA   
  
  
- GACACAACGT AAAAAGATAC TACATATTCG GCAAATAATG GATAACTACA AGTTGAAAAG TGTGATATAT   
  
  
- AGGAAATACC AAAGAAGCGA ATTCAAACCA CATGTACGAA AAGTTAGACA CAACGTAAAA AGACACTACA   
  
  
- TATTCGGCAA ATAATGGATA ACTACAAGTT GAAAAGTGTG ATATACAGGA AAATAATCAA TGACTACGGA   
  
  
- GACAGTTTGT AAGAATTAAC GTAAATCACA TAGTTGCCAG AATTTTAAAA TTCGTAACGG TTGAACTTGA   
  
  
- AGACTTCTAC AAACAGTTCT AGAAGTCAGA CGTTCAATTG ACGACGAACG AACTTGAAAA GTTGAAAATT   
  
  
- CTCTCTACAG TCTCAGATTC GTACCGAAGG ACTGTTCATG ACGTAAAATA GTGAATACTT CTGAGTTAAG   
  
  
- ACCCAAAACT GTCGAAGATA CTTGGAGATC ACGTCCATGT CGTCGAACCT GTAATTCGAC GTTCAGTTAA   
  
  
- CTGACATCTT GCTGTTCAAC AACTTCAAAG ACGACTTTCA AGTAGTGAAC AAACTACTTA TTTCATGTCG   
  
  
- TACCGTGAAG TCTACGTTTT CAGAGCGCTA GTCATCTCTA CTAAGGTATA AAGAGTAGAG TAAAGGTTCA   
  
  
- GAAACTGTTG TACATGGGTT TATTACGGTC GGTACTGTGA GTCTACAGAG AGGTTAGTAT GTCGCTTCCT   
  
  
- ATGAAGTGAG ACCTGAGTAG AGGTCGTCGT TAGCCTCGCA CGTCACAGAT ACTGATGGGT AGGCAACAGT   
  
  
- CGTGGAGAAG TTTATCTTCG GTCAAAAGAG GAGTCCCAAG GGTGAGCATG TAGAGTCTGG GGGTAGTAAG   
  
  
- AAGGCTGTTG ATACCTAGGG GTCACTCGCC GAGAAGACAC CAACTGCTAT TACGACTCGA CTCCTTGTCC   
  
  
- AAGTCACTAT ACCTCGAGGG GAACGGTGTC CTGAGTCCTG TGGTAATAAC GAGAAAATCG GTGTCTCCTT   
  
  
- CGGTACTTCC GAGGATATGA AACTCCGGTT TGGTTGACTA CCTATACCGG TTGTACCTCA ATTTCGTCCA   
  
  
- CGATATGAAG ACACGTCTTC GTTAGAGTCT CTTATTAGAT AGTTGACGTC TTTCTGATTA CCTACGTAAC   
  
  
- CCATTCTCCC ACAGACAAAA ACCAAGTGGT TAACTTTCCA ACCGGCGGAT GTACAACCTT CCCGAGTCTC   
  
  
- GTTCCGACCT CAAAAGACCT ATATGATAGA TGTTTTTCGA GTCCACGCTT GTCGGTTGCT CGAGTCTCGA   
  
  
- AGAAAGGATG TACGTGTAGG ACATAGTTTC AACGGGTATA AAGTTTAAGC GTATATACAG GAGTTTACAA   
  
  
- CGATAAGTTC TTCGAAACCC CTTACTCGGC CAATAGGTGT AGTAGCTAAA GGTCTAACGG TACCCCTGTG   
  
  
- TTAACCACGA GAACTAGGTC AGAGAGCGGG TAGCCGGACC GCCCGGGGGG GGAGCAAGCC TAGTGACCCC   
  
  
- AGCTAC

+     Myb-binding site

| Site Name | Organism | Position | Strand | Matrix score. | sequence | function |
| --- | --- | --- | --- | --- | --- | --- |
| Myb-binding site | Nicotiana tabacum | 1305 | - | 6 | CAACAG |  |

>HU08G02296.1   
+ -Up\_Stream \_Len000TTATTC TTACATCTTG TTGTTGTGAT TGTGTATTAA GCTTGTTGTC TCATGGTTTG   
  
  
+ CTCTTCTCTT TGGAACCTCA TGGTGGCTAG TTCCATGTTT TTGCTTTGTA GATGTAGCCG TTTGTTAAAG   
  
  
+ CAAGTTGGTT TATTTCTCCT ATGGCTTCGC TCTATTGTAA TTCAGGGGTC AGGTTGTTAT GTCAGACATT   
  
  
+ CCCATACCGC ACCTAGAAGG GAGGAGGGAG GCTCAGGAGT CTTAAAGAGT ATAGATTTGG ATTCACCTGT   
  
  
+ AGTCTATAGC TGATGTCCAC CTAATCTGGA TTTTATTCTT TCTTTCTCTC TCTTTTTTTT TCTTTTTTTG   
  
  
+ GGGGGGGGGG TGTTGTTGAG GGAGACAGGA TGAGGTCGAA TCTCTGCAGC ATAACAACCA CCATAGTCAC   
  
  
+ AATATGGAGA TTGTGGAGGC AAAGTTTGAG GACGTCCGGA TGTATTTCCT TTGGAACTGA GGAGGAGGTA   
  
  
+ GTACTTCTCT GGTAGTAGTG TTCTATATCC TTTGTTCCTT TCGTGTATTA TCCTAGCTAC CTCTCAAGCA   
  
  
+ AGTTGACATG AAATTGTAGA ATTCAAGACA ATAATAACAG CTGGTTTTTG GGTCTTTTAG TTTGCAATCC   
  
  
+ CTTGTAGCAT CTTAGTGACT TAGTGACATG AGATTTTGAC CCCAATAGAA ATAGAACAGA CAGCTGCTAA   
  
  
+ ATTCACCCAT CTCTTGTGTC AAAATCGGAC TTGTTTTGCC TCTGCTTCTC CATAAAGCAT CAAAGAGCGC   
  
  
+ TGATTGAGGC AGGGATTTTG TCAAGTGCAG TGTTGCATAT GTCATATCGT GTTGATGGTC ATGTTATCAC   
  
  
+ GGACATAAAT TTCCCAGTGT AAAGCAAAGG TGTCTGTCTT CACATAGAGT GTTCAATTCA TAGTCATGCT   
  
  
+ CTCGTCTCTG CATCATCCTG CGTTTGCTGG GGCTAAATTT GAGGGGCTTA TCACTTTGAA ATTCGGAGAA   
  
  
+ TTACTGTGAA TTTGTGTTAG TTCAGGCATA AAATACTGGA TGGGATGTTG AGATGTTCTA ATCATTATCT   
  
  
+ ATTGTTAAAA AACTCAGAAC TATTATAGAT TTTCTGTTTT CTGTTTGGAT TGAGATCCGA AGCCTTTGAG   
  
  
+ ATCCCCTGTT TCTAGCTTCC AACCCCCCTC CCTGGGTTCT TTTTCTTTGG TGTGAAAGTA TGAACAAAAG   
  
  
+ CTGATGTAAG TGTGATTCAA ACTCTAGCCT TGGTGAGACT CTTGGGAAGG GCTCCTAGAG ACTTTGCCAA   
  
  
+ TTGAGCTAAT TTGATGCCTA CTACTTGCTT ACTTTTCTAG CTGTTGTTTA AACGATTTAT GTTCATAGTT   
  
  
+ AAGGAGCGCC ATATTGCATT ACAATATCTG CTTTAGTGCT TTGTTTTTTG CATTTTCTTC TTTATTACTA   
  
  
+ TGGTTTTTGA AGAAGGCTTA CTGACTGATA CTGGTTTCTT CGCTTAAGTT TGGTGTACAT GCTTTTCAAT   
  
  
+ CTGTGTTGCA TTTTTCTATG ATGTATAAGC CGTTTATTAC CTATTGATGT TCAACTTTTC ACACTATATA   
  
  
+ TCCTTTATGG TTTCTTCGCT TAAGTTTGGT GTACATGCTT TTCAATCTGT GTTGCATTTT TCTGTGATGT   
  
  
+ ATAAGCCGTT TATTACCTAT TGATGTTCAA CTTTTCACAC TATATGTCCT TTTATTAGTT ACTGATGCCT   
  
  
+ CTGTCAAACA TTCTTAATTG CATTTAGTGT ATCAACGGTC TTAAAATTTT AAGCATTGCC AACTTGAACT   
  
  
+ TCTGAAGATG TTTGTCAAGA TCTTCAGTCT GCAAGTTAAC TGCTGCTTGC TTGAACTTTT CAACTTTTAA   
  
  
+ GAGAGATGTC AGAGTCTAAG CATGGCTTCC TGACAAGTAC TGCATTTTAT CACTTATGAA GACTCAATTC   
  
  
+ TGGGTTTTGA CAGCTTCTAT GAACCTCTAG TGCAGGTACA GCAGCTTGGA CATTAAGCTG CAAGTCAATT   
  
  
+ GACTGTAGAA CGACAAGTTG TTGAAGTTTC TGCTGAAAGT TCATCACTTG TTTGATGAAT AAAGTACAGC   
  
  
+ ATGGCACTTC AGATGCAAAA GTCTCGCGAT CAGTAGAGAT GATTCCATAT TTCTCATCTC ATTTCCAAGT   
  
  
+ CTTTGACAAC ATGTACCCAA ATAATGCCAG CCATGACACT CAGATGTCTC TCCAATCATA CAGCGAAGGA   
  
  
+ TACTTCACTC TGGACTCATC TCCAGCAGCA ATCGGAGCGT GCAGTGTCTA TGACTACCCA TCCGTTGTCA   
  
  
+ GCACCTCTTC AAATAGAAGC CAGTTTTCTC CTCAGGGTTC CCACTCGTAC ATCTCAGACC CCCATCATTC   
  
  
+ TTCCGACAAC TATGGATCCC CAGTGAGCGG CTCTTCTGTG GTTGACGATA ATGCTGAGCT GAGGAACAGG   
  
  
+ TTCAGTGATA TGGAGCTCCC CTTGCCACAG GACTCAGGAC ACCATTATTG CTCTTTTAGC CACAGAGGAA   
  
  
+ GCCATGAAGG CTCCTATACT TTGAGGCCAA ACCAACTGAT GGATATGGCC AACATGGAGT TAAAGCAGGT   
  
  
+ GCTATACTTC TGTGCAGAAG CAATCTCAGA GAATAATCTA TCAACTGCAG AAAGACTAAT GGATGCATTG   
  
  
+ GGTAAGAGGG TGTCTGTTTT TGGTTCACCA ATTGAAAGGT TGGCCGCCTA CATGTTGGAA GGGCTCAGAG   
  
  
+ CAAGGCTGGA GTTTTCTGGA TATACTATCT ACAAAAAGCT CAGGTGCGAA CAGCCAACGA GCTCAGAGCT   
  
  
+ TCTTTCCTAC ATGCACATCC TGTATCAAAG TTGCCCATAT TTCAAATTCG CATATATGTC CTCAAATGTT   
  
  
+ GCTATTCAAG AAGCTTTGGG GAATGAGCCG GTTATCCACA TCATCGATTT CCAGATTGCC ATGGGGACAC   
  
  
+ AATTGGTGCT CTTGATCCAG TCTCTCGCCC ATCGGCCTGG CGGGCCCCCC CCTCGTTCGG ATCACTGGGG   
  
  
+ TCGATG  

- -Up\_Stream \_Len000AATAAG AATGTAGAAC AACAACACTA ACACATAATT CGAACAACAG AGTACCAAAC   
  
  
- GAGAAGAGAA ACCTTGGAGT ACCACCGATC AAGGTACAAA AACGAAACAT CTACATCGGC AAACAATTTC   
  
  
- GTTCAACCAA ATAAAGAGGA TACCGAAGCG AGATAACATT AAGTCCCCAG TCCAACAATA CAGTCTGTAA   
  
  
- GGGTATGGCG TGGATCTTCC CTCCTCCCTC CGAGTCCTCA GAATTTCTCA TATCTAAACC TAAGTGGACA   
  
  
- TCAGATATCG ACTACAGGTG GATTAGACCT AAAATAAGAA AGAAAGAGAG AGAAAAAAAA AGAAAAAAAC   
  
  
- CCCCCCCCCC ACAACAACTC CCTCTGTCCT ACTCCAGCTT AGAGACGTCG TATTGTTGGT GGTATCAGTG   
  
  
- TTATACCTCT AACACCTCCG TTTCAAACTC CTGCAGGCCT ACATAAAGGA AACCTTGACT CCTCCTCCAT   
  
  
- CATGAAGAGA CCATCATCAC AAGATATAGG AAACAAGGAA AGCACATAAT AGGATCGATG GAGAGTTCGT   
  
  
- TCAACTGTAC TTTAACATCT TAAGTTCTGT TATTATTGTC GACCAAAAAC CCAGAAAATC AAACGTTAGG   
  
  
- GAACATCGTA GAATCACTGA ATCACTGTAC TCTAAAACTG GGGTTATCTT TATCTTGTCT GTCGACGATT   
  
  
- TAAGTGGGTA GAGAACACAG TTTTAGCCTG AACAAAACGG AGACGAAGAG GTATTTCGTA GTTTCTCGCG   
  
  
- ACTAACTCCG TCCCTAAAAC AGTTCACGTC ACAACGTATA CAGTATAGCA CAACTACCAG TACAATAGTG   
  
  
- CCTGTATTTA AAGGGTCACA TTTCGTTTCC ACAGACAGAA GTGTATCTCA CAAGTTAAGT ATCAGTACGA   
  
  
- GAGCAGAGAC GTAGTAGGAC GCAAACGACC CCGATTTAAA CTCCCCGAAT AGTGAAACTT TAAGCCTCTT   
  
  
- AATGACACTT AAACACAATC AAGTCCGTAT TTTATGACCT ACCCTACAAC TCTACAAGAT TAGTAATAGA   
  
  
- TAACAATTTT TTGAGTCTTG ATAATATCTA AAAGACAAAA GACAAACCTA ACTCTAGGCT TCGGAAACTC   
  
  
- TAGGGGACAA AGATCGAAGG TTGGGGGGAG GGACCCAAGA AAAAGAAACC ACACTTTCAT ACTTGTTTTC   
  
  
- GACTACATTC ACACTAAGTT TGAGATCGGA ACCACTCTGA GAACCCTTCC CGAGGATCTC TGAAACGGTT   
  
  
- AACTCGATTA AACTACGGAT GATGAACGAA TGAAAAGATC GACAACAAAT TTGCTAAATA CAAGTATCAA   
  
  
- TTCCTCGCGG TATAACGTAA TGTTATAGAC GAAATCACGA AACAAAAAAC GTAAAAGAAG AAATAATGAT   
  
  
- ACCAAAAACT TCTTCCGAAT GACTGACTAT GACCAAAGAA GCGAATTCAA ACCACATGTA CGAAAAGTTA   
  
  
- GACACAACGT AAAAAGATAC TACATATTCG GCAAATAATG GATAACTACA AGTTGAAAAG TGTGATATAT   
  
  
- AGGAAATACC AAAGAAGCGA ATTCAAACCA CATGTACGAA AAGTTAGACA CAACGTAAAA AGACACTACA   
  
  
- TATTCGGCAA ATAATGGATA ACTACAAGTT GAAAAGTGTG ATATACAGGA AAATAATCAA TGACTACGGA   
  
  
- GACAGTTTGT AAGAATTAAC GTAAATCACA TAGTTGCCAG AATTTTAAAA TTCGTAACGG TTGAACTTGA   
  
  
- AGACTTCTAC AAACAGTTCT AGAAGTCAGA CGTTCAATTG ACGACGAACG AACTTGAAAA GTTGAAAATT   
  
  
- CTCTCTACAG TCTCAGATTC GTACCGAAGG ACTGTTCATG ACGTAAAATA GTGAATACTT CTGAGTTAAG   
  
  
- ACCCAAAACT GTCGAAGATA CTTGGAGATC ACGTCCATGT CGTCGAACCT GTAATTCGAC GTTCAGTTAA   
  
  
- CTGACATCTT GCTGTTCAAC AACTTCAAAG ACGACTTTCA AGTAGTGAAC AAACTACTTA TTTCATGTCG   
  
  
- TACCGTGAAG TCTACGTTTT CAGAGCGCTA GTCATCTCTA CTAAGGTATA AAGAGTAGAG TAAAGGTTCA   
  
  
- GAAACTGTTG TACATGGGTT TATTACGGTC GGTACTGTGA GTCTACAGAG AGGTTAGTAT GTCGCTTCCT   
  
  
- ATGAAGTGAG ACCTGAGTAG AGGTCGTCGT TAGCCTCGCA CGTCACAGAT ACTGATGGGT AGGCAACAGT   
  
  
- CGTGGAGAAG TTTATCTTCG GTCAAAAGAG GAGTCCCAAG GGTGAGCATG TAGAGTCTGG GGGTAGTAAG   
  
  
- AAGGCTGTTG ATACCTAGGG GTCACTCGCC GAGAAGACAC CAACTGCTAT TACGACTCGA CTCCTTGTCC   
  
  
- AAGTCACTAT ACCTCGAGGG GAACGGTGTC CTGAGTCCTG TGGTAATAAC GAGAAAATCG GTGTCTCCTT   
  
  
- CGGTACTTCC GAGGATATGA AACTCCGGTT TGGTTGACTA CCTATACCGG TTGTACCTCA ATTTCGTCCA   
  
  
- CGATATGAAG ACACGTCTTC GTTAGAGTCT CTTATTAGAT AGTTGACGTC TTTCTGATTA CCTACGTAAC   
  
  
- CCATTCTCCC ACAGACAAAA ACCAAGTGGT TAACTTTCCA ACCGGCGGAT GTACAACCTT CCCGAGTCTC   
  
  
- GTTCCGACCT CAAAAGACCT ATATGATAGA TGTTTTTCGA GTCCACGCTT GTCGGTTGCT CGAGTCTCGA   
  
  
- AGAAAGGATG TACGTGTAGG ACATAGTTTC AACGGGTATA AAGTTTAAGC GTATATACAG GAGTTTACAA   
  
  
- CGATAAGTTC TTCGAAACCC CTTACTCGGC CAATAGGTGT AGTAGCTAAA GGTCTAACGG TACCCCTGTG   
  
  
- TTAACCACGA GAACTAGGTC AGAGAGCGGG TAGCCGGACC GCCCGGGGGG GGAGCAAGCC TAGTGACCCC   
  
  
- AGCTAC

+     Myc

| Site Name | Organism | Position | Strand | Matrix score. | sequence | function |
| --- | --- | --- | --- | --- | --- | --- |
| Myc | Arabidopsis thaliana | 1822 | - | 7 | TCTCTTA |  |

>HU08G02296.1   
+ -Up\_Stream \_Len000TTATTC TTACATCTTG TTGTTGTGAT TGTGTATTAA GCTTGTTGTC TCATGGTTTG   
  
  
+ CTCTTCTCTT TGGAACCTCA TGGTGGCTAG TTCCATGTTT TTGCTTTGTA GATGTAGCCG TTTGTTAAAG   
  
  
+ CAAGTTGGTT TATTTCTCCT ATGGCTTCGC TCTATTGTAA TTCAGGGGTC AGGTTGTTAT GTCAGACATT   
  
  
+ CCCATACCGC ACCTAGAAGG GAGGAGGGAG GCTCAGGAGT CTTAAAGAGT ATAGATTTGG ATTCACCTGT   
  
  
+ AGTCTATAGC TGATGTCCAC CTAATCTGGA TTTTATTCTT TCTTTCTCTC TCTTTTTTTT TCTTTTTTTG   
  
  
+ GGGGGGGGGG TGTTGTTGAG GGAGACAGGA TGAGGTCGAA TCTCTGCAGC ATAACAACCA CCATAGTCAC   
  
  
+ AATATGGAGA TTGTGGAGGC AAAGTTTGAG GACGTCCGGA TGTATTTCCT TTGGAACTGA GGAGGAGGTA   
  
  
+ GTACTTCTCT GGTAGTAGTG TTCTATATCC TTTGTTCCTT TCGTGTATTA TCCTAGCTAC CTCTCAAGCA   
  
  
+ AGTTGACATG AAATTGTAGA ATTCAAGACA ATAATAACAG CTGGTTTTTG GGTCTTTTAG TTTGCAATCC   
  
  
+ CTTGTAGCAT CTTAGTGACT TAGTGACATG AGATTTTGAC CCCAATAGAA ATAGAACAGA CAGCTGCTAA   
  
  
+ ATTCACCCAT CTCTTGTGTC AAAATCGGAC TTGTTTTGCC TCTGCTTCTC CATAAAGCAT CAAAGAGCGC   
  
  
+ TGATTGAGGC AGGGATTTTG TCAAGTGCAG TGTTGCATAT GTCATATCGT GTTGATGGTC ATGTTATCAC   
  
  
+ GGACATAAAT TTCCCAGTGT AAAGCAAAGG TGTCTGTCTT CACATAGAGT GTTCAATTCA TAGTCATGCT   
  
  
+ CTCGTCTCTG CATCATCCTG CGTTTGCTGG GGCTAAATTT GAGGGGCTTA TCACTTTGAA ATTCGGAGAA   
  
  
+ TTACTGTGAA TTTGTGTTAG TTCAGGCATA AAATACTGGA TGGGATGTTG AGATGTTCTA ATCATTATCT   
  
  
+ ATTGTTAAAA AACTCAGAAC TATTATAGAT TTTCTGTTTT CTGTTTGGAT TGAGATCCGA AGCCTTTGAG   
  
  
+ ATCCCCTGTT TCTAGCTTCC AACCCCCCTC CCTGGGTTCT TTTTCTTTGG TGTGAAAGTA TGAACAAAAG   
  
  
+ CTGATGTAAG TGTGATTCAA ACTCTAGCCT TGGTGAGACT CTTGGGAAGG GCTCCTAGAG ACTTTGCCAA   
  
  
+ TTGAGCTAAT TTGATGCCTA CTACTTGCTT ACTTTTCTAG CTGTTGTTTA AACGATTTAT GTTCATAGTT   
  
  
+ AAGGAGCGCC ATATTGCATT ACAATATCTG CTTTAGTGCT TTGTTTTTTG CATTTTCTTC TTTATTACTA   
  
  
+ TGGTTTTTGA AGAAGGCTTA CTGACTGATA CTGGTTTCTT CGCTTAAGTT TGGTGTACAT GCTTTTCAAT   
  
  
+ CTGTGTTGCA TTTTTCTATG ATGTATAAGC CGTTTATTAC CTATTGATGT TCAACTTTTC ACACTATATA   
  
  
+ TCCTTTATGG TTTCTTCGCT TAAGTTTGGT GTACATGCTT TTCAATCTGT GTTGCATTTT TCTGTGATGT   
  
  
+ ATAAGCCGTT TATTACCTAT TGATGTTCAA CTTTTCACAC TATATGTCCT TTTATTAGTT ACTGATGCCT   
  
  
+ CTGTCAAACA TTCTTAATTG CATTTAGTGT ATCAACGGTC TTAAAATTTT AAGCATTGCC AACTTGAACT   
  
  
+ TCTGAAGATG TTTGTCAAGA TCTTCAGTCT GCAAGTTAAC TGCTGCTTGC TTGAACTTTT CAACTTTTAA   
  
  
+ GAGAGATGTC AGAGTCTAAG CATGGCTTCC TGACAAGTAC TGCATTTTAT CACTTATGAA GACTCAATTC   
  
  
+ TGGGTTTTGA CAGCTTCTAT GAACCTCTAG TGCAGGTACA GCAGCTTGGA CATTAAGCTG CAAGTCAATT   
  
  
+ GACTGTAGAA CGACAAGTTG TTGAAGTTTC TGCTGAAAGT TCATCACTTG TTTGATGAAT AAAGTACAGC   
  
  
+ ATGGCACTTC AGATGCAAAA GTCTCGCGAT CAGTAGAGAT GATTCCATAT TTCTCATCTC ATTTCCAAGT   
  
  
+ CTTTGACAAC ATGTACCCAA ATAATGCCAG CCATGACACT CAGATGTCTC TCCAATCATA CAGCGAAGGA   
  
  
+ TACTTCACTC TGGACTCATC TCCAGCAGCA ATCGGAGCGT GCAGTGTCTA TGACTACCCA TCCGTTGTCA   
  
  
+ GCACCTCTTC AAATAGAAGC CAGTTTTCTC CTCAGGGTTC CCACTCGTAC ATCTCAGACC CCCATCATTC   
  
  
+ TTCCGACAAC TATGGATCCC CAGTGAGCGG CTCTTCTGTG GTTGACGATA ATGCTGAGCT GAGGAACAGG   
  
  
+ TTCAGTGATA TGGAGCTCCC CTTGCCACAG GACTCAGGAC ACCATTATTG CTCTTTTAGC CACAGAGGAA   
  
  
+ GCCATGAAGG CTCCTATACT TTGAGGCCAA ACCAACTGAT GGATATGGCC AACATGGAGT TAAAGCAGGT   
  
  
+ GCTATACTTC TGTGCAGAAG CAATCTCAGA GAATAATCTA TCAACTGCAG AAAGACTAAT GGATGCATTG   
  
  
+ GGTAAGAGGG TGTCTGTTTT TGGTTCACCA ATTGAAAGGT TGGCCGCCTA CATGTTGGAA GGGCTCAGAG   
  
  
+ CAAGGCTGGA GTTTTCTGGA TATACTATCT ACAAAAAGCT CAGGTGCGAA CAGCCAACGA GCTCAGAGCT   
  
  
+ TCTTTCCTAC ATGCACATCC TGTATCAAAG TTGCCCATAT TTCAAATTCG CATATATGTC CTCAAATGTT   
  
  
+ GCTATTCAAG AAGCTTTGGG GAATGAGCCG GTTATCCACA TCATCGATTT CCAGATTGCC ATGGGGACAC   
  
  
+ AATTGGTGCT CTTGATCCAG TCTCTCGCCC ATCGGCCTGG CGGGCCCCCC CCTCGTTCGG ATCACTGGGG   
  
  
+ TCGATG  

- -Up\_Stream \_Len000AATAAG AATGTAGAAC AACAACACTA ACACATAATT CGAACAACAG AGTACCAAAC   
  
  
- GAGAAGAGAA ACCTTGGAGT ACCACCGATC AAGGTACAAA AACGAAACAT CTACATCGGC AAACAATTTC   
  
  
- GTTCAACCAA ATAAAGAGGA TACCGAAGCG AGATAACATT AAGTCCCCAG TCCAACAATA CAGTCTGTAA   
  
  
- GGGTATGGCG TGGATCTTCC CTCCTCCCTC CGAGTCCTCA GAATTTCTCA TATCTAAACC TAAGTGGACA   
  
  
- TCAGATATCG ACTACAGGTG GATTAGACCT AAAATAAGAA AGAAAGAGAG AGAAAAAAAA AGAAAAAAAC   
  
  
- CCCCCCCCCC ACAACAACTC CCTCTGTCCT ACTCCAGCTT AGAGACGTCG TATTGTTGGT GGTATCAGTG   
  
  
- TTATACCTCT AACACCTCCG TTTCAAACTC CTGCAGGCCT ACATAAAGGA AACCTTGACT CCTCCTCCAT   
  
  
- CATGAAGAGA CCATCATCAC AAGATATAGG AAACAAGGAA AGCACATAAT AGGATCGATG GAGAGTTCGT   
  
  
- TCAACTGTAC TTTAACATCT TAAGTTCTGT TATTATTGTC GACCAAAAAC CCAGAAAATC AAACGTTAGG   
  
  
- GAACATCGTA GAATCACTGA ATCACTGTAC TCTAAAACTG GGGTTATCTT TATCTTGTCT GTCGACGATT   
  
  
- TAAGTGGGTA GAGAACACAG TTTTAGCCTG AACAAAACGG AGACGAAGAG GTATTTCGTA GTTTCTCGCG   
  
  
- ACTAACTCCG TCCCTAAAAC AGTTCACGTC ACAACGTATA CAGTATAGCA CAACTACCAG TACAATAGTG   
  
  
- CCTGTATTTA AAGGGTCACA TTTCGTTTCC ACAGACAGAA GTGTATCTCA CAAGTTAAGT ATCAGTACGA   
  
  
- GAGCAGAGAC GTAGTAGGAC GCAAACGACC CCGATTTAAA CTCCCCGAAT AGTGAAACTT TAAGCCTCTT   
  
  
- AATGACACTT AAACACAATC AAGTCCGTAT TTTATGACCT ACCCTACAAC TCTACAAGAT TAGTAATAGA   
  
  
- TAACAATTTT TTGAGTCTTG ATAATATCTA AAAGACAAAA GACAAACCTA ACTCTAGGCT TCGGAAACTC   
  
  
- TAGGGGACAA AGATCGAAGG TTGGGGGGAG GGACCCAAGA AAAAGAAACC ACACTTTCAT ACTTGTTTTC   
  
  
- GACTACATTC ACACTAAGTT TGAGATCGGA ACCACTCTGA GAACCCTTCC CGAGGATCTC TGAAACGGTT   
  
  
- AACTCGATTA AACTACGGAT GATGAACGAA TGAAAAGATC GACAACAAAT TTGCTAAATA CAAGTATCAA   
  
  
- TTCCTCGCGG TATAACGTAA TGTTATAGAC GAAATCACGA AACAAAAAAC GTAAAAGAAG AAATAATGAT   
  
  
- ACCAAAAACT TCTTCCGAAT GACTGACTAT GACCAAAGAA GCGAATTCAA ACCACATGTA CGAAAAGTTA   
  
  
- GACACAACGT AAAAAGATAC TACATATTCG GCAAATAATG GATAACTACA AGTTGAAAAG TGTGATATAT   
  
  
- AGGAAATACC AAAGAAGCGA ATTCAAACCA CATGTACGAA AAGTTAGACA CAACGTAAAA AGACACTACA   
  
  
- TATTCGGCAA ATAATGGATA ACTACAAGTT GAAAAGTGTG ATATACAGGA AAATAATCAA TGACTACGGA   
  
  
- GACAGTTTGT AAGAATTAAC GTAAATCACA TAGTTGCCAG AATTTTAAAA TTCGTAACGG TTGAACTTGA   
  
  
- AGACTTCTAC AAACAGTTCT AGAAGTCAGA CGTTCAATTG ACGACGAACG AACTTGAAAA GTTGAAAATT   
  
  
- CTCTCTACAG TCTCAGATTC GTACCGAAGG ACTGTTCATG ACGTAAAATA GTGAATACTT CTGAGTTAAG   
  
  
- ACCCAAAACT GTCGAAGATA CTTGGAGATC ACGTCCATGT CGTCGAACCT GTAATTCGAC GTTCAGTTAA   
  
  
- CTGACATCTT GCTGTTCAAC AACTTCAAAG ACGACTTTCA AGTAGTGAAC AAACTACTTA TTTCATGTCG   
  
  
- TACCGTGAAG TCTACGTTTT CAGAGCGCTA GTCATCTCTA CTAAGGTATA AAGAGTAGAG TAAAGGTTCA   
  
  
- GAAACTGTTG TACATGGGTT TATTACGGTC GGTACTGTGA GTCTACAGAG AGGTTAGTAT GTCGCTTCCT   
  
  
- ATGAAGTGAG ACCTGAGTAG AGGTCGTCGT TAGCCTCGCA CGTCACAGAT ACTGATGGGT AGGCAACAGT   
  
  
- CGTGGAGAAG TTTATCTTCG GTCAAAAGAG GAGTCCCAAG GGTGAGCATG TAGAGTCTGG GGGTAGTAAG   
  
  
- AAGGCTGTTG ATACCTAGGG GTCACTCGCC GAGAAGACAC CAACTGCTAT TACGACTCGA CTCCTTGTCC   
  
  
- AAGTCACTAT ACCTCGAGGG GAACGGTGTC CTGAGTCCTG TGGTAATAAC GAGAAAATCG GTGTCTCCTT   
  
  
- CGGTACTTCC GAGGATATGA AACTCCGGTT TGGTTGACTA CCTATACCGG TTGTACCTCA ATTTCGTCCA   
  
  
- CGATATGAAG ACACGTCTTC GTTAGAGTCT CTTATTAGAT AGTTGACGTC TTTCTGATTA CCTACGTAAC   
  
  
- CCATTCTCCC ACAGACAAAA ACCAAGTGGT TAACTTTCCA ACCGGCGGAT GTACAACCTT CCCGAGTCTC   
  
  
- GTTCCGACCT CAAAAGACCT ATATGATAGA TGTTTTTCGA GTCCACGCTT GTCGGTTGCT CGAGTCTCGA   
  
  
- AGAAAGGATG TACGTGTAGG ACATAGTTTC AACGGGTATA AAGTTTAAGC GTATATACAG GAGTTTACAA   
  
  
- CGATAAGTTC TTCGAAACCC CTTACTCGGC CAATAGGTGT AGTAGCTAAA GGTCTAACGG TACCCCTGTG   
  
  
- TTAACCACGA GAACTAGGTC AGAGAGCGGG TAGCCGGACC GCCCGGGGGG GGAGCAAGCC TAGTGACCCC   
  
  
- AGCTAC

+     O2-site

| Site Name | Organism | Position | Strand | Matrix score. | sequence | function |
| --- | --- | --- | --- | --- | --- | --- |
| O2-site | Zea mays | 2840 | - | 10 | GATGATGTGG | cis-acting regulatory element involved in zein metabolism regulation |
| O2-site | Zea mays | 566 | + | 9 | GTTGACGTGA | cis-acting regulatory element involved in zein metabolism regulation |

>HU08G02296.1   
+ -Up\_Stream \_Len000TTATTC TTACATCTTG TTGTTGTGAT TGTGTATTAA GCTTGTTGTC TCATGGTTTG   
  
  
+ CTCTTCTCTT TGGAACCTCA TGGTGGCTAG TTCCATGTTT TTGCTTTGTA GATGTAGCCG TTTGTTAAAG   
  
  
+ CAAGTTGGTT TATTTCTCCT ATGGCTTCGC TCTATTGTAA TTCAGGGGTC AGGTTGTTAT GTCAGACATT   
  
  
+ CCCATACCGC ACCTAGAAGG GAGGAGGGAG GCTCAGGAGT CTTAAAGAGT ATAGATTTGG ATTCACCTGT   
  
  
+ AGTCTATAGC TGATGTCCAC CTAATCTGGA TTTTATTCTT TCTTTCTCTC TCTTTTTTTT TCTTTTTTTG   
  
  
+ GGGGGGGGGG TGTTGTTGAG GGAGACAGGA TGAGGTCGAA TCTCTGCAGC ATAACAACCA CCATAGTCAC   
  
  
+ AATATGGAGA TTGTGGAGGC AAAGTTTGAG GACGTCCGGA TGTATTTCCT TTGGAACTGA GGAGGAGGTA   
  
  
+ GTACTTCTCT GGTAGTAGTG TTCTATATCC TTTGTTCCTT TCGTGTATTA TCCTAGCTAC CTCTCAAGCA   
  
  
+ AGTTGACATG AAATTGTAGA ATTCAAGACA ATAATAACAG CTGGTTTTTG GGTCTTTTAG TTTGCAATCC   
  
  
+ CTTGTAGCAT CTTAGTGACT TAGTGACATG AGATTTTGAC CCCAATAGAA ATAGAACAGA CAGCTGCTAA   
  
  
+ ATTCACCCAT CTCTTGTGTC AAAATCGGAC TTGTTTTGCC TCTGCTTCTC CATAAAGCAT CAAAGAGCGC   
  
  
+ TGATTGAGGC AGGGATTTTG TCAAGTGCAG TGTTGCATAT GTCATATCGT GTTGATGGTC ATGTTATCAC   
  
  
+ GGACATAAAT TTCCCAGTGT AAAGCAAAGG TGTCTGTCTT CACATAGAGT GTTCAATTCA TAGTCATGCT   
  
  
+ CTCGTCTCTG CATCATCCTG CGTTTGCTGG GGCTAAATTT GAGGGGCTTA TCACTTTGAA ATTCGGAGAA   
  
  
+ TTACTGTGAA TTTGTGTTAG TTCAGGCATA AAATACTGGA TGGGATGTTG AGATGTTCTA ATCATTATCT   
  
  
+ ATTGTTAAAA AACTCAGAAC TATTATAGAT TTTCTGTTTT CTGTTTGGAT TGAGATCCGA AGCCTTTGAG   
  
  
+ ATCCCCTGTT TCTAGCTTCC AACCCCCCTC CCTGGGTTCT TTTTCTTTGG TGTGAAAGTA TGAACAAAAG   
  
  
+ CTGATGTAAG TGTGATTCAA ACTCTAGCCT TGGTGAGACT CTTGGGAAGG GCTCCTAGAG ACTTTGCCAA   
  
  
+ TTGAGCTAAT TTGATGCCTA CTACTTGCTT ACTTTTCTAG CTGTTGTTTA AACGATTTAT GTTCATAGTT   
  
  
+ AAGGAGCGCC ATATTGCATT ACAATATCTG CTTTAGTGCT TTGTTTTTTG CATTTTCTTC TTTATTACTA   
  
  
+ TGGTTTTTGA AGAAGGCTTA CTGACTGATA CTGGTTTCTT CGCTTAAGTT TGGTGTACAT GCTTTTCAAT   
  
  
+ CTGTGTTGCA TTTTTCTATG ATGTATAAGC CGTTTATTAC CTATTGATGT TCAACTTTTC ACACTATATA   
  
  
+ TCCTTTATGG TTTCTTCGCT TAAGTTTGGT GTACATGCTT TTCAATCTGT GTTGCATTTT TCTGTGATGT   
  
  
+ ATAAGCCGTT TATTACCTAT TGATGTTCAA CTTTTCACAC TATATGTCCT TTTATTAGTT ACTGATGCCT   
  
  
+ CTGTCAAACA TTCTTAATTG CATTTAGTGT ATCAACGGTC TTAAAATTTT AAGCATTGCC AACTTGAACT   
  
  
+ TCTGAAGATG TTTGTCAAGA TCTTCAGTCT GCAAGTTAAC TGCTGCTTGC TTGAACTTTT CAACTTTTAA   
  
  
+ GAGAGATGTC AGAGTCTAAG CATGGCTTCC TGACAAGTAC TGCATTTTAT CACTTATGAA GACTCAATTC   
  
  
+ TGGGTTTTGA CAGCTTCTAT GAACCTCTAG TGCAGGTACA GCAGCTTGGA CATTAAGCTG CAAGTCAATT   
  
  
+ GACTGTAGAA CGACAAGTTG TTGAAGTTTC TGCTGAAAGT TCATCACTTG TTTGATGAAT AAAGTACAGC   
  
  
+ ATGGCACTTC AGATGCAAAA GTCTCGCGAT CAGTAGAGAT GATTCCATAT TTCTCATCTC ATTTCCAAGT   
  
  
+ CTTTGACAAC ATGTACCCAA ATAATGCCAG CCATGACACT CAGATGTCTC TCCAATCATA CAGCGAAGGA   
  
  
+ TACTTCACTC TGGACTCATC TCCAGCAGCA ATCGGAGCGT GCAGTGTCTA TGACTACCCA TCCGTTGTCA   
  
  
+ GCACCTCTTC AAATAGAAGC CAGTTTTCTC CTCAGGGTTC CCACTCGTAC ATCTCAGACC CCCATCATTC   
  
  
+ TTCCGACAAC TATGGATCCC CAGTGAGCGG CTCTTCTGTG GTTGACGATA ATGCTGAGCT GAGGAACAGG   
  
  
+ TTCAGTGATA TGGAGCTCCC CTTGCCACAG GACTCAGGAC ACCATTATTG CTCTTTTAGC CACAGAGGAA   
  
  
+ GCCATGAAGG CTCCTATACT TTGAGGCCAA ACCAACTGAT GGATATGGCC AACATGGAGT TAAAGCAGGT   
  
  
+ GCTATACTTC TGTGCAGAAG CAATCTCAGA GAATAATCTA TCAACTGCAG AAAGACTAAT GGATGCATTG   
  
  
+ GGTAAGAGGG TGTCTGTTTT TGGTTCACCA ATTGAAAGGT TGGCCGCCTA CATGTTGGAA GGGCTCAGAG   
  
  
+ CAAGGCTGGA GTTTTCTGGA TATACTATCT ACAAAAAGCT CAGGTGCGAA CAGCCAACGA GCTCAGAGCT   
  
  
+ TCTTTCCTAC ATGCACATCC TGTATCAAAG TTGCCCATAT TTCAAATTCG CATATATGTC CTCAAATGTT   
  
  
+ GCTATTCAAG AAGCTTTGGG GAATGAGCCG GTTATCCACA TCATCGATTT CCAGATTGCC ATGGGGACAC   
  
  
+ AATTGGTGCT CTTGATCCAG TCTCTCGCCC ATCGGCCTGG CGGGCCCCCC CCTCGTTCGG ATCACTGGGG   
  
  
+ TCGATG  

- -Up\_Stream \_Len000AATAAG AATGTAGAAC AACAACACTA ACACATAATT CGAACAACAG AGTACCAAAC   
  
  
- GAGAAGAGAA ACCTTGGAGT ACCACCGATC AAGGTACAAA AACGAAACAT CTACATCGGC AAACAATTTC   
  
  
- GTTCAACCAA ATAAAGAGGA TACCGAAGCG AGATAACATT AAGTCCCCAG TCCAACAATA CAGTCTGTAA   
  
  
- GGGTATGGCG TGGATCTTCC CTCCTCCCTC CGAGTCCTCA GAATTTCTCA TATCTAAACC TAAGTGGACA   
  
  
- TCAGATATCG ACTACAGGTG GATTAGACCT AAAATAAGAA AGAAAGAGAG AGAAAAAAAA AGAAAAAAAC   
  
  
- CCCCCCCCCC ACAACAACTC CCTCTGTCCT ACTCCAGCTT AGAGACGTCG TATTGTTGGT GGTATCAGTG   
  
  
- TTATACCTCT AACACCTCCG TTTCAAACTC CTGCAGGCCT ACATAAAGGA AACCTTGACT CCTCCTCCAT   
  
  
- CATGAAGAGA CCATCATCAC AAGATATAGG AAACAAGGAA AGCACATAAT AGGATCGATG GAGAGTTCGT   
  
  
- TCAACTGTAC TTTAACATCT TAAGTTCTGT TATTATTGTC GACCAAAAAC CCAGAAAATC AAACGTTAGG   
  
  
- GAACATCGTA GAATCACTGA ATCACTGTAC TCTAAAACTG GGGTTATCTT TATCTTGTCT GTCGACGATT   
  
  
- TAAGTGGGTA GAGAACACAG TTTTAGCCTG AACAAAACGG AGACGAAGAG GTATTTCGTA GTTTCTCGCG   
  
  
- ACTAACTCCG TCCCTAAAAC AGTTCACGTC ACAACGTATA CAGTATAGCA CAACTACCAG TACAATAGTG   
  
  
- CCTGTATTTA AAGGGTCACA TTTCGTTTCC ACAGACAGAA GTGTATCTCA CAAGTTAAGT ATCAGTACGA   
  
  
- GAGCAGAGAC GTAGTAGGAC GCAAACGACC CCGATTTAAA CTCCCCGAAT AGTGAAACTT TAAGCCTCTT   
  
  
- AATGACACTT AAACACAATC AAGTCCGTAT TTTATGACCT ACCCTACAAC TCTACAAGAT TAGTAATAGA   
  
  
- TAACAATTTT TTGAGTCTTG ATAATATCTA AAAGACAAAA GACAAACCTA ACTCTAGGCT TCGGAAACTC   
  
  
- TAGGGGACAA AGATCGAAGG TTGGGGGGAG GGACCCAAGA AAAAGAAACC ACACTTTCAT ACTTGTTTTC   
  
  
- GACTACATTC ACACTAAGTT TGAGATCGGA ACCACTCTGA GAACCCTTCC CGAGGATCTC TGAAACGGTT   
  
  
- AACTCGATTA AACTACGGAT GATGAACGAA TGAAAAGATC GACAACAAAT TTGCTAAATA CAAGTATCAA   
  
  
- TTCCTCGCGG TATAACGTAA TGTTATAGAC GAAATCACGA AACAAAAAAC GTAAAAGAAG AAATAATGAT   
  
  
- ACCAAAAACT TCTTCCGAAT GACTGACTAT GACCAAAGAA GCGAATTCAA ACCACATGTA CGAAAAGTTA   
  
  
- GACACAACGT AAAAAGATAC TACATATTCG GCAAATAATG GATAACTACA AGTTGAAAAG TGTGATATAT   
  
  
- AGGAAATACC AAAGAAGCGA ATTCAAACCA CATGTACGAA AAGTTAGACA CAACGTAAAA AGACACTACA   
  
  
- TATTCGGCAA ATAATGGATA ACTACAAGTT GAAAAGTGTG ATATACAGGA AAATAATCAA TGACTACGGA   
  
  
- GACAGTTTGT AAGAATTAAC GTAAATCACA TAGTTGCCAG AATTTTAAAA TTCGTAACGG TTGAACTTGA   
  
  
- AGACTTCTAC AAACAGTTCT AGAAGTCAGA CGTTCAATTG ACGACGAACG AACTTGAAAA GTTGAAAATT   
  
  
- CTCTCTACAG TCTCAGATTC GTACCGAAGG ACTGTTCATG ACGTAAAATA GTGAATACTT CTGAGTTAAG   
  
  
- ACCCAAAACT GTCGAAGATA CTTGGAGATC ACGTCCATGT CGTCGAACCT GTAATTCGAC GTTCAGTTAA   
  
  
- CTGACATCTT GCTGTTCAAC AACTTCAAAG ACGACTTTCA AGTAGTGAAC AAACTACTTA TTTCATGTCG   
  
  
- TACCGTGAAG TCTACGTTTT CAGAGCGCTA GTCATCTCTA CTAAGGTATA AAGAGTAGAG TAAAGGTTCA   
  
  
- GAAACTGTTG TACATGGGTT TATTACGGTC GGTACTGTGA GTCTACAGAG AGGTTAGTAT GTCGCTTCCT   
  
  
- ATGAAGTGAG ACCTGAGTAG AGGTCGTCGT TAGCCTCGCA CGTCACAGAT ACTGATGGGT AGGCAACAGT   
  
  
- CGTGGAGAAG TTTATCTTCG GTCAAAAGAG GAGTCCCAAG GGTGAGCATG TAGAGTCTGG GGGTAGTAAG   
  
  
- AAGGCTGTTG ATACCTAGGG GTCACTCGCC GAGAAGACAC CAACTGCTAT TACGACTCGA CTCCTTGTCC   
  
  
- AAGTCACTAT ACCTCGAGGG GAACGGTGTC CTGAGTCCTG TGGTAATAAC GAGAAAATCG GTGTCTCCTT   
  
  
- CGGTACTTCC GAGGATATGA AACTCCGGTT TGGTTGACTA CCTATACCGG TTGTACCTCA ATTTCGTCCA   
  
  
- CGATATGAAG ACACGTCTTC GTTAGAGTCT CTTATTAGAT AGTTGACGTC TTTCTGATTA CCTACGTAAC   
  
  
- CCATTCTCCC ACAGACAAAA ACCAAGTGGT TAACTTTCCA ACCGGCGGAT GTACAACCTT CCCGAGTCTC   
  
  
- GTTCCGACCT CAAAAGACCT ATATGATAGA TGTTTTTCGA GTCCACGCTT GTCGGTTGCT CGAGTCTCGA   
  
  
- AGAAAGGATG TACGTGTAGG ACATAGTTTC AACGGGTATA AAGTTTAAGC GTATATACAG GAGTTTACAA   
  
  
- CGATAAGTTC TTCGAAACCC CTTACTCGGC CAATAGGTGT AGTAGCTAAA GGTCTAACGG TACCCCTGTG   
  
  
- TTAACCACGA GAACTAGGTC AGAGAGCGGG TAGCCGGACC GCCCGGGGGG GGAGCAAGCC TAGTGACCCC   
  
  
- AGCTAC

+     STRE

| Site Name | Organism | Position | Strand | Matrix score. | sequence | function |
| --- | --- | --- | --- | --- | --- | --- |
| STRE | Arabidopsis thaliana | 2923 | - | 5 | AGGGG |  |
| STRE | Arabidopsis thaliana | 1127 | - | 5 | AGGGG |  |
| STRE | Arabidopsis thaliana | 956 | + | 5 | AGGGG |  |
| STRE | Arabidopsis thaliana | 188 | + | 5 | AGGGG |  |
| STRE | Arabidopsis thaliana | 2402 | - | 5 | AGGGG |  |
| STRE | Arabidopsis thaliana | 1149 | - | 5 | AGGGG |  |

>HU08G02296.1   
+ -Up\_Stream \_Len000TTATTC TTACATCTTG TTGTTGTGAT TGTGTATTAA GCTTGTTGTC TCATGGTTTG   
  
  
+ CTCTTCTCTT TGGAACCTCA TGGTGGCTAG TTCCATGTTT TTGCTTTGTA GATGTAGCCG TTTGTTAAAG   
  
  
+ CAAGTTGGTT TATTTCTCCT ATGGCTTCGC TCTATTGTAA TTCAGGGGTC AGGTTGTTAT GTCAGACATT   
  
  
+ CCCATACCGC ACCTAGAAGG GAGGAGGGAG GCTCAGGAGT CTTAAAGAGT ATAGATTTGG ATTCACCTGT   
  
  
+ AGTCTATAGC TGATGTCCAC CTAATCTGGA TTTTATTCTT TCTTTCTCTC TCTTTTTTTT TCTTTTTTTG   
  
  
+ GGGGGGGGGG TGTTGTTGAG GGAGACAGGA TGAGGTCGAA TCTCTGCAGC ATAACAACCA CCATAGTCAC   
  
  
+ AATATGGAGA TTGTGGAGGC AAAGTTTGAG GACGTCCGGA TGTATTTCCT TTGGAACTGA GGAGGAGGTA   
  
  
+ GTACTTCTCT GGTAGTAGTG TTCTATATCC TTTGTTCCTT TCGTGTATTA TCCTAGCTAC CTCTCAAGCA   
  
  
+ AGTTGACATG AAATTGTAGA ATTCAAGACA ATAATAACAG CTGGTTTTTG GGTCTTTTAG TTTGCAATCC   
  
  
+ CTTGTAGCAT CTTAGTGACT TAGTGACATG AGATTTTGAC CCCAATAGAA ATAGAACAGA CAGCTGCTAA   
  
  
+ ATTCACCCAT CTCTTGTGTC AAAATCGGAC TTGTTTTGCC TCTGCTTCTC CATAAAGCAT CAAAGAGCGC   
  
  
+ TGATTGAGGC AGGGATTTTG TCAAGTGCAG TGTTGCATAT GTCATATCGT GTTGATGGTC ATGTTATCAC   
  
  
+ GGACATAAAT TTCCCAGTGT AAAGCAAAGG TGTCTGTCTT CACATAGAGT GTTCAATTCA TAGTCATGCT   
  
  
+ CTCGTCTCTG CATCATCCTG CGTTTGCTGG GGCTAAATTT GAGGGGCTTA TCACTTTGAA ATTCGGAGAA   
  
  
+ TTACTGTGAA TTTGTGTTAG TTCAGGCATA AAATACTGGA TGGGATGTTG AGATGTTCTA ATCATTATCT   
  
  
+ ATTGTTAAAA AACTCAGAAC TATTATAGAT TTTCTGTTTT CTGTTTGGAT TGAGATCCGA AGCCTTTGAG   
  
  
+ ATCCCCTGTT TCTAGCTTCC AACCCCCCTC CCTGGGTTCT TTTTCTTTGG TGTGAAAGTA TGAACAAAAG   
  
  
+ CTGATGTAAG TGTGATTCAA ACTCTAGCCT TGGTGAGACT CTTGGGAAGG GCTCCTAGAG ACTTTGCCAA   
  
  
+ TTGAGCTAAT TTGATGCCTA CTACTTGCTT ACTTTTCTAG CTGTTGTTTA AACGATTTAT GTTCATAGTT   
  
  
+ AAGGAGCGCC ATATTGCATT ACAATATCTG CTTTAGTGCT TTGTTTTTTG CATTTTCTTC TTTATTACTA   
  
  
+ TGGTTTTTGA AGAAGGCTTA CTGACTGATA CTGGTTTCTT CGCTTAAGTT TGGTGTACAT GCTTTTCAAT   
  
  
+ CTGTGTTGCA TTTTTCTATG ATGTATAAGC CGTTTATTAC CTATTGATGT TCAACTTTTC ACACTATATA   
  
  
+ TCCTTTATGG TTTCTTCGCT TAAGTTTGGT GTACATGCTT TTCAATCTGT GTTGCATTTT TCTGTGATGT   
  
  
+ ATAAGCCGTT TATTACCTAT TGATGTTCAA CTTTTCACAC TATATGTCCT TTTATTAGTT ACTGATGCCT   
  
  
+ CTGTCAAACA TTCTTAATTG CATTTAGTGT ATCAACGGTC TTAAAATTTT AAGCATTGCC AACTTGAACT   
  
  
+ TCTGAAGATG TTTGTCAAGA TCTTCAGTCT GCAAGTTAAC TGCTGCTTGC TTGAACTTTT CAACTTTTAA   
  
  
+ GAGAGATGTC AGAGTCTAAG CATGGCTTCC TGACAAGTAC TGCATTTTAT CACTTATGAA GACTCAATTC   
  
  
+ TGGGTTTTGA CAGCTTCTAT GAACCTCTAG TGCAGGTACA GCAGCTTGGA CATTAAGCTG CAAGTCAATT   
  
  
+ GACTGTAGAA CGACAAGTTG TTGAAGTTTC TGCTGAAAGT TCATCACTTG TTTGATGAAT AAAGTACAGC   
  
  
+ ATGGCACTTC AGATGCAAAA GTCTCGCGAT CAGTAGAGAT GATTCCATAT TTCTCATCTC ATTTCCAAGT   
  
  
+ CTTTGACAAC ATGTACCCAA ATAATGCCAG CCATGACACT CAGATGTCTC TCCAATCATA CAGCGAAGGA   
  
  
+ TACTTCACTC TGGACTCATC TCCAGCAGCA ATCGGAGCGT GCAGTGTCTA TGACTACCCA TCCGTTGTCA   
  
  
+ GCACCTCTTC AAATAGAAGC CAGTTTTCTC CTCAGGGTTC CCACTCGTAC ATCTCAGACC CCCATCATTC   
  
  
+ TTCCGACAAC TATGGATCCC CAGTGAGCGG CTCTTCTGTG GTTGACGATA ATGCTGAGCT GAGGAACAGG   
  
  
+ TTCAGTGATA TGGAGCTCCC CTTGCCACAG GACTCAGGAC ACCATTATTG CTCTTTTAGC CACAGAGGAA   
  
  
+ GCCATGAAGG CTCCTATACT TTGAGGCCAA ACCAACTGAT GGATATGGCC AACATGGAGT TAAAGCAGGT   
  
  
+ GCTATACTTC TGTGCAGAAG CAATCTCAGA GAATAATCTA TCAACTGCAG AAAGACTAAT GGATGCATTG   
  
  
+ GGTAAGAGGG TGTCTGTTTT TGGTTCACCA ATTGAAAGGT TGGCCGCCTA CATGTTGGAA GGGCTCAGAG   
  
  
+ CAAGGCTGGA GTTTTCTGGA TATACTATCT ACAAAAAGCT CAGGTGCGAA CAGCCAACGA GCTCAGAGCT   
  
  
+ TCTTTCCTAC ATGCACATCC TGTATCAAAG TTGCCCATAT TTCAAATTCG CATATATGTC CTCAAATGTT   
  
  
+ GCTATTCAAG AAGCTTTGGG GAATGAGCCG GTTATCCACA TCATCGATTT CCAGATTGCC ATGGGGACAC   
  
  
+ AATTGGTGCT CTTGATCCAG TCTCTCGCCC ATCGGCCTGG CGGGCCCCCC CCTCGTTCGG ATCACTGGGG   
  
  
+ TCGATG  

- -Up\_Stream \_Len000AATAAG AATGTAGAAC AACAACACTA ACACATAATT CGAACAACAG AGTACCAAAC   
  
  
- GAGAAGAGAA ACCTTGGAGT ACCACCGATC AAGGTACAAA AACGAAACAT CTACATCGGC AAACAATTTC   
  
  
- GTTCAACCAA ATAAAGAGGA TACCGAAGCG AGATAACATT AAGTCCCCAG TCCAACAATA CAGTCTGTAA   
  
  
- GGGTATGGCG TGGATCTTCC CTCCTCCCTC CGAGTCCTCA GAATTTCTCA TATCTAAACC TAAGTGGACA   
  
  
- TCAGATATCG ACTACAGGTG GATTAGACCT AAAATAAGAA AGAAAGAGAG AGAAAAAAAA AGAAAAAAAC   
  
  
- CCCCCCCCCC ACAACAACTC CCTCTGTCCT ACTCCAGCTT AGAGACGTCG TATTGTTGGT GGTATCAGTG   
  
  
- TTATACCTCT AACACCTCCG TTTCAAACTC CTGCAGGCCT ACATAAAGGA AACCTTGACT CCTCCTCCAT   
  
  
- CATGAAGAGA CCATCATCAC AAGATATAGG AAACAAGGAA AGCACATAAT AGGATCGATG GAGAGTTCGT   
  
  
- TCAACTGTAC TTTAACATCT TAAGTTCTGT TATTATTGTC GACCAAAAAC CCAGAAAATC AAACGTTAGG   
  
  
- GAACATCGTA GAATCACTGA ATCACTGTAC TCTAAAACTG GGGTTATCTT TATCTTGTCT GTCGACGATT   
  
  
- TAAGTGGGTA GAGAACACAG TTTTAGCCTG AACAAAACGG AGACGAAGAG GTATTTCGTA GTTTCTCGCG   
  
  
- ACTAACTCCG TCCCTAAAAC AGTTCACGTC ACAACGTATA CAGTATAGCA CAACTACCAG TACAATAGTG   
  
  
- CCTGTATTTA AAGGGTCACA TTTCGTTTCC ACAGACAGAA GTGTATCTCA CAAGTTAAGT ATCAGTACGA   
  
  
- GAGCAGAGAC GTAGTAGGAC GCAAACGACC CCGATTTAAA CTCCCCGAAT AGTGAAACTT TAAGCCTCTT   
  
  
- AATGACACTT AAACACAATC AAGTCCGTAT TTTATGACCT ACCCTACAAC TCTACAAGAT TAGTAATAGA   
  
  
- TAACAATTTT TTGAGTCTTG ATAATATCTA AAAGACAAAA GACAAACCTA ACTCTAGGCT TCGGAAACTC   
  
  
- TAGGGGACAA AGATCGAAGG TTGGGGGGAG GGACCCAAGA AAAAGAAACC ACACTTTCAT ACTTGTTTTC   
  
  
- GACTACATTC ACACTAAGTT TGAGATCGGA ACCACTCTGA GAACCCTTCC CGAGGATCTC TGAAACGGTT   
  
  
- AACTCGATTA AACTACGGAT GATGAACGAA TGAAAAGATC GACAACAAAT TTGCTAAATA CAAGTATCAA   
  
  
- TTCCTCGCGG TATAACGTAA TGTTATAGAC GAAATCACGA AACAAAAAAC GTAAAAGAAG AAATAATGAT   
  
  
- ACCAAAAACT TCTTCCGAAT GACTGACTAT GACCAAAGAA GCGAATTCAA ACCACATGTA CGAAAAGTTA   
  
  
- GACACAACGT AAAAAGATAC TACATATTCG GCAAATAATG GATAACTACA AGTTGAAAAG TGTGATATAT   
  
  
- AGGAAATACC AAAGAAGCGA ATTCAAACCA CATGTACGAA AAGTTAGACA CAACGTAAAA AGACACTACA   
  
  
- TATTCGGCAA ATAATGGATA ACTACAAGTT GAAAAGTGTG ATATACAGGA AAATAATCAA TGACTACGGA   
  
  
- GACAGTTTGT AAGAATTAAC GTAAATCACA TAGTTGCCAG AATTTTAAAA TTCGTAACGG TTGAACTTGA   
  
  
- AGACTTCTAC AAACAGTTCT AGAAGTCAGA CGTTCAATTG ACGACGAACG AACTTGAAAA GTTGAAAATT   
  
  
- CTCTCTACAG TCTCAGATTC GTACCGAAGG ACTGTTCATG ACGTAAAATA GTGAATACTT CTGAGTTAAG   
  
  
- ACCCAAAACT GTCGAAGATA CTTGGAGATC ACGTCCATGT CGTCGAACCT GTAATTCGAC GTTCAGTTAA   
  
  
- CTGACATCTT GCTGTTCAAC AACTTCAAAG ACGACTTTCA AGTAGTGAAC AAACTACTTA TTTCATGTCG   
  
  
- TACCGTGAAG TCTACGTTTT CAGAGCGCTA GTCATCTCTA CTAAGGTATA AAGAGTAGAG TAAAGGTTCA   
  
  
- GAAACTGTTG TACATGGGTT TATTACGGTC GGTACTGTGA GTCTACAGAG AGGTTAGTAT GTCGCTTCCT   
  
  
- ATGAAGTGAG ACCTGAGTAG AGGTCGTCGT TAGCCTCGCA CGTCACAGAT ACTGATGGGT AGGCAACAGT   
  
  
- CGTGGAGAAG TTTATCTTCG GTCAAAAGAG GAGTCCCAAG GGTGAGCATG TAGAGTCTGG GGGTAGTAAG   
  
  
- AAGGCTGTTG ATACCTAGGG GTCACTCGCC GAGAAGACAC CAACTGCTAT TACGACTCGA CTCCTTGTCC   
  
  
- AAGTCACTAT ACCTCGAGGG GAACGGTGTC CTGAGTCCTG TGGTAATAAC GAGAAAATCG GTGTCTCCTT   
  
  
- CGGTACTTCC GAGGATATGA AACTCCGGTT TGGTTGACTA CCTATACCGG TTGTACCTCA ATTTCGTCCA   
  
  
- CGATATGAAG ACACGTCTTC GTTAGAGTCT CTTATTAGAT AGTTGACGTC TTTCTGATTA CCTACGTAAC   
  
  
- CCATTCTCCC ACAGACAAAA ACCAAGTGGT TAACTTTCCA ACCGGCGGAT GTACAACCTT CCCGAGTCTC   
  
  
- GTTCCGACCT CAAAAGACCT ATATGATAGA TGTTTTTCGA GTCCACGCTT GTCGGTTGCT CGAGTCTCGA   
  
  
- AGAAAGGATG TACGTGTAGG ACATAGTTTC AACGGGTATA AAGTTTAAGC GTATATACAG GAGTTTACAA   
  
  
- CGATAAGTTC TTCGAAACCC CTTACTCGGC CAATAGGTGT AGTAGCTAAA GGTCTAACGG TACCCCTGTG   
  
  
- TTAACCACGA GAACTAGGTC AGAGAGCGGG TAGCCGGACC GCCCGGGGGG GGAGCAAGCC TAGTGACCCC   
  
  
- AGCTAC

+     TATA-box

| Site Name | Organism | Position | Strand | Matrix score. | sequence | function |
| --- | --- | --- | --- | --- | --- | --- |
| TATA-box | Brassica napus | 2786 | - | 6 | ATATAT | core promoter element around -30 of transcription start |
| TATA-box | Arabidopsis thaliana | 2787 | - | 4 | TATA | core promoter element around -30 of transcription start |
| TATA-box | Oryza sativa | 2694 | + | 7 | TACAAAA | core promoter element around -30 of transcription start |
| TATA-box | Arabidopsis thaliana | 1539 | - | 6 | TATATA | core promoter element around -30 of transcription start |
| TATA-box | Arabidopsis thaliana | 2685 | - | 4 | TATA | core promoter element around -30 of transcription start |
| TATA-box | Arabidopsis thaliana | 2527 | - | 4 | TATA | core promoter element around -30 of transcription start |
| TATA-box | Arabidopsis thaliana | 518 | + | 4 | TATA | core promoter element around -30 of transcription start |
| TATA-box | Arabidopsis thaliana | 289 | + | 4 | TATA | core promoter element around -30 of transcription start |
| TATA-box | Arabidopsis thaliana | 1614 | - | 4 | TATA | core promoter element around -30 of transcription start |
| TATA-box | Arabidopsis thaliana | 1498 | - | 4 | TATA | core promoter element around -30 of transcription start |
| TATA-box | Arabidopsis thaliana | 1655 | - | 4 | TATA | core promoter element around -30 of transcription start |
| TATA-box | Helianthus annuus | 1612 | - | 6 | TATACA | core promoter element around -30 of transcription start |
| TATA-box | Arabidopsis thaliana | 1077 | - | 5 | TATAA | core promoter element around -30 of transcription start |
| TATA-box | Arabidopsis thaliana | 1078 | + | 4 | TATA | core promoter element around -30 of transcription start |
| TATA-box | Arabidopsis thaliana | 2469 | - | 4 | TATA | core promoter element around -30 of transcription start |
| TATA-box | Arabidopsis thaliana | 1541 | - | 4 | TATA | core promoter element around -30 of transcription start |
| TATA-box | Brassica napus | 1540 | - | 6 | ATATAT | core promoter element around -30 of transcription start |
| TATA-box | Helianthus annuus | 1496 | - | 6 | TATACA | core promoter element around -30 of transcription start |
| TATA-box | Arabidopsis thaliana | 264 | + | 4 | TATA | core promoter element around -30 of transcription start |
| TATA-box | Brassica napus | 1076 | + | 6 | ATTATA | core promoter element around -30 of transcription start |

>HU08G02296.1   
+ -Up\_Stream \_Len000TTATTC TTACATCTTG TTGTTGTGAT TGTGTATTAA GCTTGTTGTC TCATGGTTTG   
  
  
+ CTCTTCTCTT TGGAACCTCA TGGTGGCTAG TTCCATGTTT TTGCTTTGTA GATGTAGCCG TTTGTTAAAG   
  
  
+ CAAGTTGGTT TATTTCTCCT ATGGCTTCGC TCTATTGTAA TTCAGGGGTC AGGTTGTTAT GTCAGACATT   
  
  
+ CCCATACCGC ACCTAGAAGG GAGGAGGGAG GCTCAGGAGT CTTAAAGAGT ATAGATTTGG ATTCACCTGT   
  
  
+ AGTCTATAGC TGATGTCCAC CTAATCTGGA TTTTATTCTT TCTTTCTCTC TCTTTTTTTT TCTTTTTTTG   
  
  
+ GGGGGGGGGG TGTTGTTGAG GGAGACAGGA TGAGGTCGAA TCTCTGCAGC ATAACAACCA CCATAGTCAC   
  
  
+ AATATGGAGA TTGTGGAGGC AAAGTTTGAG GACGTCCGGA TGTATTTCCT TTGGAACTGA GGAGGAGGTA   
  
  
+ GTACTTCTCT GGTAGTAGTG TTCTATATCC TTTGTTCCTT TCGTGTATTA TCCTAGCTAC CTCTCAAGCA   
  
  
+ AGTTGACATG AAATTGTAGA ATTCAAGACA ATAATAACAG CTGGTTTTTG GGTCTTTTAG TTTGCAATCC   
  
  
+ CTTGTAGCAT CTTAGTGACT TAGTGACATG AGATTTTGAC CCCAATAGAA ATAGAACAGA CAGCTGCTAA   
  
  
+ ATTCACCCAT CTCTTGTGTC AAAATCGGAC TTGTTTTGCC TCTGCTTCTC CATAAAGCAT CAAAGAGCGC   
  
  
+ TGATTGAGGC AGGGATTTTG TCAAGTGCAG TGTTGCATAT GTCATATCGT GTTGATGGTC ATGTTATCAC   
  
  
+ GGACATAAAT TTCCCAGTGT AAAGCAAAGG TGTCTGTCTT CACATAGAGT GTTCAATTCA TAGTCATGCT   
  
  
+ CTCGTCTCTG CATCATCCTG CGTTTGCTGG GGCTAAATTT GAGGGGCTTA TCACTTTGAA ATTCGGAGAA   
  
  
+ TTACTGTGAA TTTGTGTTAG TTCAGGCATA AAATACTGGA TGGGATGTTG AGATGTTCTA ATCATTATCT   
  
  
+ ATTGTTAAAA AACTCAGAAC TATTATAGAT TTTCTGTTTT CTGTTTGGAT TGAGATCCGA AGCCTTTGAG   
  
  
+ ATCCCCTGTT TCTAGCTTCC AACCCCCCTC CCTGGGTTCT TTTTCTTTGG TGTGAAAGTA TGAACAAAAG   
  
  
+ CTGATGTAAG TGTGATTCAA ACTCTAGCCT TGGTGAGACT CTTGGGAAGG GCTCCTAGAG ACTTTGCCAA   
  
  
+ TTGAGCTAAT TTGATGCCTA CTACTTGCTT ACTTTTCTAG CTGTTGTTTA AACGATTTAT GTTCATAGTT   
  
  
+ AAGGAGCGCC ATATTGCATT ACAATATCTG CTTTAGTGCT TTGTTTTTTG CATTTTCTTC TTTATTACTA   
  
  
+ TGGTTTTTGA AGAAGGCTTA CTGACTGATA CTGGTTTCTT CGCTTAAGTT TGGTGTACAT GCTTTTCAAT   
  
  
+ CTGTGTTGCA TTTTTCTATG ATGTATAAGC CGTTTATTAC CTATTGATGT TCAACTTTTC ACACTATATA   
  
  
+ TCCTTTATGG TTTCTTCGCT TAAGTTTGGT GTACATGCTT TTCAATCTGT GTTGCATTTT TCTGTGATGT   
  
  
+ ATAAGCCGTT TATTACCTAT TGATGTTCAA CTTTTCACAC TATATGTCCT TTTATTAGTT ACTGATGCCT   
  
  
+ CTGTCAAACA TTCTTAATTG CATTTAGTGT ATCAACGGTC TTAAAATTTT AAGCATTGCC AACTTGAACT   
  
  
+ TCTGAAGATG TTTGTCAAGA TCTTCAGTCT GCAAGTTAAC TGCTGCTTGC TTGAACTTTT CAACTTTTAA   
  
  
+ GAGAGATGTC AGAGTCTAAG CATGGCTTCC TGACAAGTAC TGCATTTTAT CACTTATGAA GACTCAATTC   
  
  
+ TGGGTTTTGA CAGCTTCTAT GAACCTCTAG TGCAGGTACA GCAGCTTGGA CATTAAGCTG CAAGTCAATT   
  
  
+ GACTGTAGAA CGACAAGTTG TTGAAGTTTC TGCTGAAAGT TCATCACTTG TTTGATGAAT AAAGTACAGC   
  
  
+ ATGGCACTTC AGATGCAAAA GTCTCGCGAT CAGTAGAGAT GATTCCATAT TTCTCATCTC ATTTCCAAGT   
  
  
+ CTTTGACAAC ATGTACCCAA ATAATGCCAG CCATGACACT CAGATGTCTC TCCAATCATA CAGCGAAGGA   
  
  
+ TACTTCACTC TGGACTCATC TCCAGCAGCA ATCGGAGCGT GCAGTGTCTA TGACTACCCA TCCGTTGTCA   
  
  
+ GCACCTCTTC AAATAGAAGC CAGTTTTCTC CTCAGGGTTC CCACTCGTAC ATCTCAGACC CCCATCATTC   
  
  
+ TTCCGACAAC TATGGATCCC CAGTGAGCGG CTCTTCTGTG GTTGACGATA ATGCTGAGCT GAGGAACAGG   
  
  
+ TTCAGTGATA TGGAGCTCCC CTTGCCACAG GACTCAGGAC ACCATTATTG CTCTTTTAGC CACAGAGGAA   
  
  
+ GCCATGAAGG CTCCTATACT TTGAGGCCAA ACCAACTGAT GGATATGGCC AACATGGAGT TAAAGCAGGT   
  
  
+ GCTATACTTC TGTGCAGAAG CAATCTCAGA GAATAATCTA TCAACTGCAG AAAGACTAAT GGATGCATTG   
  
  
+ GGTAAGAGGG TGTCTGTTTT TGGTTCACCA ATTGAAAGGT TGGCCGCCTA CATGTTGGAA GGGCTCAGAG   
  
  
+ CAAGGCTGGA GTTTTCTGGA TATACTATCT ACAAAAAGCT CAGGTGCGAA CAGCCAACGA GCTCAGAGCT   
  
  
+ TCTTTCCTAC ATGCACATCC TGTATCAAAG TTGCCCATAT TTCAAATTCG CATATATGTC CTCAAATGTT   
  
  
+ GCTATTCAAG AAGCTTTGGG GAATGAGCCG GTTATCCACA TCATCGATTT CCAGATTGCC ATGGGGACAC   
  
  
+ AATTGGTGCT CTTGATCCAG TCTCTCGCCC ATCGGCCTGG CGGGCCCCCC CCTCGTTCGG ATCACTGGGG   
  
  
+ TCGATG  

- -Up\_Stream \_Len000AATAAG AATGTAGAAC AACAACACTA ACACATAATT CGAACAACAG AGTACCAAAC   
  
  
- GAGAAGAGAA ACCTTGGAGT ACCACCGATC AAGGTACAAA AACGAAACAT CTACATCGGC AAACAATTTC   
  
  
- GTTCAACCAA ATAAAGAGGA TACCGAAGCG AGATAACATT AAGTCCCCAG TCCAACAATA CAGTCTGTAA   
  
  
- GGGTATGGCG TGGATCTTCC CTCCTCCCTC CGAGTCCTCA GAATTTCTCA TATCTAAACC TAAGTGGACA   
  
  
- TCAGATATCG ACTACAGGTG GATTAGACCT AAAATAAGAA AGAAAGAGAG AGAAAAAAAA AGAAAAAAAC   
  
  
- CCCCCCCCCC ACAACAACTC CCTCTGTCCT ACTCCAGCTT AGAGACGTCG TATTGTTGGT GGTATCAGTG   
  
  
- TTATACCTCT AACACCTCCG TTTCAAACTC CTGCAGGCCT ACATAAAGGA AACCTTGACT CCTCCTCCAT   
  
  
- CATGAAGAGA CCATCATCAC AAGATATAGG AAACAAGGAA AGCACATAAT AGGATCGATG GAGAGTTCGT   
  
  
- TCAACTGTAC TTTAACATCT TAAGTTCTGT TATTATTGTC GACCAAAAAC CCAGAAAATC AAACGTTAGG   
  
  
- GAACATCGTA GAATCACTGA ATCACTGTAC TCTAAAACTG GGGTTATCTT TATCTTGTCT GTCGACGATT   
  
  
- TAAGTGGGTA GAGAACACAG TTTTAGCCTG AACAAAACGG AGACGAAGAG GTATTTCGTA GTTTCTCGCG   
  
  
- ACTAACTCCG TCCCTAAAAC AGTTCACGTC ACAACGTATA CAGTATAGCA CAACTACCAG TACAATAGTG   
  
  
- CCTGTATTTA AAGGGTCACA TTTCGTTTCC ACAGACAGAA GTGTATCTCA CAAGTTAAGT ATCAGTACGA   
  
  
- GAGCAGAGAC GTAGTAGGAC GCAAACGACC CCGATTTAAA CTCCCCGAAT AGTGAAACTT TAAGCCTCTT   
  
  
- AATGACACTT AAACACAATC AAGTCCGTAT TTTATGACCT ACCCTACAAC TCTACAAGAT TAGTAATAGA   
  
  
- TAACAATTTT TTGAGTCTTG ATAATATCTA AAAGACAAAA GACAAACCTA ACTCTAGGCT TCGGAAACTC   
  
  
- TAGGGGACAA AGATCGAAGG TTGGGGGGAG GGACCCAAGA AAAAGAAACC ACACTTTCAT ACTTGTTTTC   
  
  
- GACTACATTC ACACTAAGTT TGAGATCGGA ACCACTCTGA GAACCCTTCC CGAGGATCTC TGAAACGGTT   
  
  
- AACTCGATTA AACTACGGAT GATGAACGAA TGAAAAGATC GACAACAAAT TTGCTAAATA CAAGTATCAA   
  
  
- TTCCTCGCGG TATAACGTAA TGTTATAGAC GAAATCACGA AACAAAAAAC GTAAAAGAAG AAATAATGAT   
  
  
- ACCAAAAACT TCTTCCGAAT GACTGACTAT GACCAAAGAA GCGAATTCAA ACCACATGTA CGAAAAGTTA   
  
  
- GACACAACGT AAAAAGATAC TACATATTCG GCAAATAATG GATAACTACA AGTTGAAAAG TGTGATATAT   
  
  
- AGGAAATACC AAAGAAGCGA ATTCAAACCA CATGTACGAA AAGTTAGACA CAACGTAAAA AGACACTACA   
  
  
- TATTCGGCAA ATAATGGATA ACTACAAGTT GAAAAGTGTG ATATACAGGA AAATAATCAA TGACTACGGA   
  
  
- GACAGTTTGT AAGAATTAAC GTAAATCACA TAGTTGCCAG AATTTTAAAA TTCGTAACGG TTGAACTTGA   
  
  
- AGACTTCTAC AAACAGTTCT AGAAGTCAGA CGTTCAATTG ACGACGAACG AACTTGAAAA GTTGAAAATT   
  
  
- CTCTCTACAG TCTCAGATTC GTACCGAAGG ACTGTTCATG ACGTAAAATA GTGAATACTT CTGAGTTAAG   
  
  
- ACCCAAAACT GTCGAAGATA CTTGGAGATC ACGTCCATGT CGTCGAACCT GTAATTCGAC GTTCAGTTAA   
  
  
- CTGACATCTT GCTGTTCAAC AACTTCAAAG ACGACTTTCA AGTAGTGAAC AAACTACTTA TTTCATGTCG   
  
  
- TACCGTGAAG TCTACGTTTT CAGAGCGCTA GTCATCTCTA CTAAGGTATA AAGAGTAGAG TAAAGGTTCA   
  
  
- GAAACTGTTG TACATGGGTT TATTACGGTC GGTACTGTGA GTCTACAGAG AGGTTAGTAT GTCGCTTCCT   
  
  
- ATGAAGTGAG ACCTGAGTAG AGGTCGTCGT TAGCCTCGCA CGTCACAGAT ACTGATGGGT AGGCAACAGT   
  
  
- CGTGGAGAAG TTTATCTTCG GTCAAAAGAG GAGTCCCAAG GGTGAGCATG TAGAGTCTGG GGGTAGTAAG   
  
  
- AAGGCTGTTG ATACCTAGGG GTCACTCGCC GAGAAGACAC CAACTGCTAT TACGACTCGA CTCCTTGTCC   
  
  
- AAGTCACTAT ACCTCGAGGG GAACGGTGTC CTGAGTCCTG TGGTAATAAC GAGAAAATCG GTGTCTCCTT   
  
  
- CGGTACTTCC GAGGATATGA AACTCCGGTT TGGTTGACTA CCTATACCGG TTGTACCTCA ATTTCGTCCA   
  
  
- CGATATGAAG ACACGTCTTC GTTAGAGTCT CTTATTAGAT AGTTGACGTC TTTCTGATTA CCTACGTAAC   
  
  
- CCATTCTCCC ACAGACAAAA ACCAAGTGGT TAACTTTCCA ACCGGCGGAT GTACAACCTT CCCGAGTCTC   
  
  
- GTTCCGACCT CAAAAGACCT ATATGATAGA TGTTTTTCGA GTCCACGCTT GTCGGTTGCT CGAGTCTCGA   
  
  
- AGAAAGGATG TACGTGTAGG ACATAGTTTC AACGGGTATA AAGTTTAAGC GTATATACAG GAGTTTACAA   
  
  
- CGATAAGTTC TTCGAAACCC CTTACTCGGC CAATAGGTGT AGTAGCTAAA GGTCTAACGG TACCCCTGTG   
  
  
- TTAACCACGA GAACTAGGTC AGAGAGCGGG TAGCCGGACC GCCCGGGGGG GGAGCAAGCC TAGTGACCCC   
  
  
- AGCTAC

+     TCA-element

| Site Name | Organism | Position | Strand | Matrix score. | sequence | function |
| --- | --- | --- | --- | --- | --- | --- |
| TCA-element | Nicotiana tabacum | 107 | + | 9 | CCATCTTTTT | cis-acting element involved in salicylic acid responsiveness |

>HU08G02296.1   
+ -Up\_Stream \_Len000TTATTC TTACATCTTG TTGTTGTGAT TGTGTATTAA GCTTGTTGTC TCATGGTTTG   
  
  
+ CTCTTCTCTT TGGAACCTCA TGGTGGCTAG TTCCATGTTT TTGCTTTGTA GATGTAGCCG TTTGTTAAAG   
  
  
+ CAAGTTGGTT TATTTCTCCT ATGGCTTCGC TCTATTGTAA TTCAGGGGTC AGGTTGTTAT GTCAGACATT   
  
  
+ CCCATACCGC ACCTAGAAGG GAGGAGGGAG GCTCAGGAGT CTTAAAGAGT ATAGATTTGG ATTCACCTGT   
  
  
+ AGTCTATAGC TGATGTCCAC CTAATCTGGA TTTTATTCTT TCTTTCTCTC TCTTTTTTTT TCTTTTTTTG   
  
  
+ GGGGGGGGGG TGTTGTTGAG GGAGACAGGA TGAGGTCGAA TCTCTGCAGC ATAACAACCA CCATAGTCAC   
  
  
+ AATATGGAGA TTGTGGAGGC AAAGTTTGAG GACGTCCGGA TGTATTTCCT TTGGAACTGA GGAGGAGGTA   
  
  
+ GTACTTCTCT GGTAGTAGTG TTCTATATCC TTTGTTCCTT TCGTGTATTA TCCTAGCTAC CTCTCAAGCA   
  
  
+ AGTTGACATG AAATTGTAGA ATTCAAGACA ATAATAACAG CTGGTTTTTG GGTCTTTTAG TTTGCAATCC   
  
  
+ CTTGTAGCAT CTTAGTGACT TAGTGACATG AGATTTTGAC CCCAATAGAA ATAGAACAGA CAGCTGCTAA   
  
  
+ ATTCACCCAT CTCTTGTGTC AAAATCGGAC TTGTTTTGCC TCTGCTTCTC CATAAAGCAT CAAAGAGCGC   
  
  
+ TGATTGAGGC AGGGATTTTG TCAAGTGCAG TGTTGCATAT GTCATATCGT GTTGATGGTC ATGTTATCAC   
  
  
+ GGACATAAAT TTCCCAGTGT AAAGCAAAGG TGTCTGTCTT CACATAGAGT GTTCAATTCA TAGTCATGCT   
  
  
+ CTCGTCTCTG CATCATCCTG CGTTTGCTGG GGCTAAATTT GAGGGGCTTA TCACTTTGAA ATTCGGAGAA   
  
  
+ TTACTGTGAA TTTGTGTTAG TTCAGGCATA AAATACTGGA TGGGATGTTG AGATGTTCTA ATCATTATCT   
  
  
+ ATTGTTAAAA AACTCAGAAC TATTATAGAT TTTCTGTTTT CTGTTTGGAT TGAGATCCGA AGCCTTTGAG   
  
  
+ ATCCCCTGTT TCTAGCTTCC AACCCCCCTC CCTGGGTTCT TTTTCTTTGG TGTGAAAGTA TGAACAAAAG   
  
  
+ CTGATGTAAG TGTGATTCAA ACTCTAGCCT TGGTGAGACT CTTGGGAAGG GCTCCTAGAG ACTTTGCCAA   
  
  
+ TTGAGCTAAT TTGATGCCTA CTACTTGCTT ACTTTTCTAG CTGTTGTTTA AACGATTTAT GTTCATAGTT   
  
  
+ AAGGAGCGCC ATATTGCATT ACAATATCTG CTTTAGTGCT TTGTTTTTTG CATTTTCTTC TTTATTACTA   
  
  
+ TGGTTTTTGA AGAAGGCTTA CTGACTGATA CTGGTTTCTT CGCTTAAGTT TGGTGTACAT GCTTTTCAAT   
  
  
+ CTGTGTTGCA TTTTTCTATG ATGTATAAGC CGTTTATTAC CTATTGATGT TCAACTTTTC ACACTATATA   
  
  
+ TCCTTTATGG TTTCTTCGCT TAAGTTTGGT GTACATGCTT TTCAATCTGT GTTGCATTTT TCTGTGATGT   
  
  
+ ATAAGCCGTT TATTACCTAT TGATGTTCAA CTTTTCACAC TATATGTCCT TTTATTAGTT ACTGATGCCT   
  
  
+ CTGTCAAACA TTCTTAATTG CATTTAGTGT ATCAACGGTC TTAAAATTTT AAGCATTGCC AACTTGAACT   
  
  
+ TCTGAAGATG TTTGTCAAGA TCTTCAGTCT GCAAGTTAAC TGCTGCTTGC TTGAACTTTT CAACTTTTAA   
  
  
+ GAGAGATGTC AGAGTCTAAG CATGGCTTCC TGACAAGTAC TGCATTTTAT CACTTATGAA GACTCAATTC   
  
  
+ TGGGTTTTGA CAGCTTCTAT GAACCTCTAG TGCAGGTACA GCAGCTTGGA CATTAAGCTG CAAGTCAATT   
  
  
+ GACTGTAGAA CGACAAGTTG TTGAAGTTTC TGCTGAAAGT TCATCACTTG TTTGATGAAT AAAGTACAGC   
  
  
+ ATGGCACTTC AGATGCAAAA GTCTCGCGAT CAGTAGAGAT GATTCCATAT TTCTCATCTC ATTTCCAAGT   
  
  
+ CTTTGACAAC ATGTACCCAA ATAATGCCAG CCATGACACT CAGATGTCTC TCCAATCATA CAGCGAAGGA   
  
  
+ TACTTCACTC TGGACTCATC TCCAGCAGCA ATCGGAGCGT GCAGTGTCTA TGACTACCCA TCCGTTGTCA   
  
  
+ GCACCTCTTC AAATAGAAGC CAGTTTTCTC CTCAGGGTTC CCACTCGTAC ATCTCAGACC CCCATCATTC   
  
  
+ TTCCGACAAC TATGGATCCC CAGTGAGCGG CTCTTCTGTG GTTGACGATA ATGCTGAGCT GAGGAACAGG   
  
  
+ TTCAGTGATA TGGAGCTCCC CTTGCCACAG GACTCAGGAC ACCATTATTG CTCTTTTAGC CACAGAGGAA   
  
  
+ GCCATGAAGG CTCCTATACT TTGAGGCCAA ACCAACTGAT GGATATGGCC AACATGGAGT TAAAGCAGGT   
  
  
+ GCTATACTTC TGTGCAGAAG CAATCTCAGA GAATAATCTA TCAACTGCAG AAAGACTAAT GGATGCATTG   
  
  
+ GGTAAGAGGG TGTCTGTTTT TGGTTCACCA ATTGAAAGGT TGGCCGCCTA CATGTTGGAA GGGCTCAGAG   
  
  
+ CAAGGCTGGA GTTTTCTGGA TATACTATCT ACAAAAAGCT CAGGTGCGAA CAGCCAACGA GCTCAGAGCT   
  
  
+ TCTTTCCTAC ATGCACATCC TGTATCAAAG TTGCCCATAT TTCAAATTCG CATATATGTC CTCAAATGTT   
  
  
+ GCTATTCAAG AAGCTTTGGG GAATGAGCCG GTTATCCACA TCATCGATTT CCAGATTGCC ATGGGGACAC   
  
  
+ AATTGGTGCT CTTGATCCAG TCTCTCGCCC ATCGGCCTGG CGGGCCCCCC CCTCGTTCGG ATCACTGGGG   
  
  
+ TCGATG  

- -Up\_Stream \_Len000AATAAG AATGTAGAAC AACAACACTA ACACATAATT CGAACAACAG AGTACCAAAC   
  
  
- GAGAAGAGAA ACCTTGGAGT ACCACCGATC AAGGTACAAA AACGAAACAT CTACATCGGC AAACAATTTC   
  
  
- GTTCAACCAA ATAAAGAGGA TACCGAAGCG AGATAACATT AAGTCCCCAG TCCAACAATA CAGTCTGTAA   
  
  
- GGGTATGGCG TGGATCTTCC CTCCTCCCTC CGAGTCCTCA GAATTTCTCA TATCTAAACC TAAGTGGACA   
  
  
- TCAGATATCG ACTACAGGTG GATTAGACCT AAAATAAGAA AGAAAGAGAG AGAAAAAAAA AGAAAAAAAC   
  
  
- CCCCCCCCCC ACAACAACTC CCTCTGTCCT ACTCCAGCTT AGAGACGTCG TATTGTTGGT GGTATCAGTG   
  
  
- TTATACCTCT AACACCTCCG TTTCAAACTC CTGCAGGCCT ACATAAAGGA AACCTTGACT CCTCCTCCAT   
  
  
- CATGAAGAGA CCATCATCAC AAGATATAGG AAACAAGGAA AGCACATAAT AGGATCGATG GAGAGTTCGT   
  
  
- TCAACTGTAC TTTAACATCT TAAGTTCTGT TATTATTGTC GACCAAAAAC CCAGAAAATC AAACGTTAGG   
  
  
- GAACATCGTA GAATCACTGA ATCACTGTAC TCTAAAACTG GGGTTATCTT TATCTTGTCT GTCGACGATT   
  
  
- TAAGTGGGTA GAGAACACAG TTTTAGCCTG AACAAAACGG AGACGAAGAG GTATTTCGTA GTTTCTCGCG   
  
  
- ACTAACTCCG TCCCTAAAAC AGTTCACGTC ACAACGTATA CAGTATAGCA CAACTACCAG TACAATAGTG   
  
  
- CCTGTATTTA AAGGGTCACA TTTCGTTTCC ACAGACAGAA GTGTATCTCA CAAGTTAAGT ATCAGTACGA   
  
  
- GAGCAGAGAC GTAGTAGGAC GCAAACGACC CCGATTTAAA CTCCCCGAAT AGTGAAACTT TAAGCCTCTT   
  
  
- AATGACACTT AAACACAATC AAGTCCGTAT TTTATGACCT ACCCTACAAC TCTACAAGAT TAGTAATAGA   
  
  
- TAACAATTTT TTGAGTCTTG ATAATATCTA AAAGACAAAA GACAAACCTA ACTCTAGGCT TCGGAAACTC   
  
  
- TAGGGGACAA AGATCGAAGG TTGGGGGGAG GGACCCAAGA AAAAGAAACC ACACTTTCAT ACTTGTTTTC   
  
  
- GACTACATTC ACACTAAGTT TGAGATCGGA ACCACTCTGA GAACCCTTCC CGAGGATCTC TGAAACGGTT   
  
  
- AACTCGATTA AACTACGGAT GATGAACGAA TGAAAAGATC GACAACAAAT TTGCTAAATA CAAGTATCAA   
  
  
- TTCCTCGCGG TATAACGTAA TGTTATAGAC GAAATCACGA AACAAAAAAC GTAAAAGAAG AAATAATGAT   
  
  
- ACCAAAAACT TCTTCCGAAT GACTGACTAT GACCAAAGAA GCGAATTCAA ACCACATGTA CGAAAAGTTA   
  
  
- GACACAACGT AAAAAGATAC TACATATTCG GCAAATAATG GATAACTACA AGTTGAAAAG TGTGATATAT   
  
  
- AGGAAATACC AAAGAAGCGA ATTCAAACCA CATGTACGAA AAGTTAGACA CAACGTAAAA AGACACTACA   
  
  
- TATTCGGCAA ATAATGGATA ACTACAAGTT GAAAAGTGTG ATATACAGGA AAATAATCAA TGACTACGGA   
  
  
- GACAGTTTGT AAGAATTAAC GTAAATCACA TAGTTGCCAG AATTTTAAAA TTCGTAACGG TTGAACTTGA   
  
  
- AGACTTCTAC AAACAGTTCT AGAAGTCAGA CGTTCAATTG ACGACGAACG AACTTGAAAA GTTGAAAATT   
  
  
- CTCTCTACAG TCTCAGATTC GTACCGAAGG ACTGTTCATG ACGTAAAATA GTGAATACTT CTGAGTTAAG   
  
  
- ACCCAAAACT GTCGAAGATA CTTGGAGATC ACGTCCATGT CGTCGAACCT GTAATTCGAC GTTCAGTTAA   
  
  
- CTGACATCTT GCTGTTCAAC AACTTCAAAG ACGACTTTCA AGTAGTGAAC AAACTACTTA TTTCATGTCG   
  
  
- TACCGTGAAG TCTACGTTTT CAGAGCGCTA GTCATCTCTA CTAAGGTATA AAGAGTAGAG TAAAGGTTCA   
  
  
- GAAACTGTTG TACATGGGTT TATTACGGTC GGTACTGTGA GTCTACAGAG AGGTTAGTAT GTCGCTTCCT   
  
  
- ATGAAGTGAG ACCTGAGTAG AGGTCGTCGT TAGCCTCGCA CGTCACAGAT ACTGATGGGT AGGCAACAGT   
  
  
- CGTGGAGAAG TTTATCTTCG GTCAAAAGAG GAGTCCCAAG GGTGAGCATG TAGAGTCTGG GGGTAGTAAG   
  
  
- AAGGCTGTTG ATACCTAGGG GTCACTCGCC GAGAAGACAC CAACTGCTAT TACGACTCGA CTCCTTGTCC   
  
  
- AAGTCACTAT ACCTCGAGGG GAACGGTGTC CTGAGTCCTG TGGTAATAAC GAGAAAATCG GTGTCTCCTT   
  
  
- CGGTACTTCC GAGGATATGA AACTCCGGTT TGGTTGACTA CCTATACCGG TTGTACCTCA ATTTCGTCCA   
  
  
- CGATATGAAG ACACGTCTTC GTTAGAGTCT CTTATTAGAT AGTTGACGTC TTTCTGATTA CCTACGTAAC   
  
  
- CCATTCTCCC ACAGACAAAA ACCAAGTGGT TAACTTTCCA ACCGGCGGAT GTACAACCTT CCCGAGTCTC   
  
  
- GTTCCGACCT CAAAAGACCT ATATGATAGA TGTTTTTCGA GTCCACGCTT GTCGGTTGCT CGAGTCTCGA   
  
  
- AGAAAGGATG TACGTGTAGG ACATAGTTTC AACGGGTATA AAGTTTAAGC GTATATACAG GAGTTTACAA   
  
  
- CGATAAGTTC TTCGAAACCC CTTACTCGGC CAATAGGTGT AGTAGCTAAA GGTCTAACGG TACCCCTGTG   
  
  
- TTAACCACGA GAACTAGGTC AGAGAGCGGG TAGCCGGACC GCCCGGGGGG GGAGCAAGCC TAGTGACCCC   
  
  
- AGCTAC

+     TCCC-motif

| Site Name | Organism | Position | Strand | Matrix score. | sequence | function |
| --- | --- | --- | --- | --- | --- | --- |
| TCCC-motif | Spinacia oleracea | 373 | - | 7 | TCTCCCT | part of a light responsive element |

>HU08G02296.1   
+ -Up\_Stream \_Len000TTATTC TTACATCTTG TTGTTGTGAT TGTGTATTAA GCTTGTTGTC TCATGGTTTG   
  
  
+ CTCTTCTCTT TGGAACCTCA TGGTGGCTAG TTCCATGTTT TTGCTTTGTA GATGTAGCCG TTTGTTAAAG   
  
  
+ CAAGTTGGTT TATTTCTCCT ATGGCTTCGC TCTATTGTAA TTCAGGGGTC AGGTTGTTAT GTCAGACATT   
  
  
+ CCCATACCGC ACCTAGAAGG GAGGAGGGAG GCTCAGGAGT CTTAAAGAGT ATAGATTTGG ATTCACCTGT   
  
  
+ AGTCTATAGC TGATGTCCAC CTAATCTGGA TTTTATTCTT TCTTTCTCTC TCTTTTTTTT TCTTTTTTTG   
  
  
+ GGGGGGGGGG TGTTGTTGAG GGAGACAGGA TGAGGTCGAA TCTCTGCAGC ATAACAACCA CCATAGTCAC   
  
  
+ AATATGGAGA TTGTGGAGGC AAAGTTTGAG GACGTCCGGA TGTATTTCCT TTGGAACTGA GGAGGAGGTA   
  
  
+ GTACTTCTCT GGTAGTAGTG TTCTATATCC TTTGTTCCTT TCGTGTATTA TCCTAGCTAC CTCTCAAGCA   
  
  
+ AGTTGACATG AAATTGTAGA ATTCAAGACA ATAATAACAG CTGGTTTTTG GGTCTTTTAG TTTGCAATCC   
  
  
+ CTTGTAGCAT CTTAGTGACT TAGTGACATG AGATTTTGAC CCCAATAGAA ATAGAACAGA CAGCTGCTAA   
  
  
+ ATTCACCCAT CTCTTGTGTC AAAATCGGAC TTGTTTTGCC TCTGCTTCTC CATAAAGCAT CAAAGAGCGC   
  
  
+ TGATTGAGGC AGGGATTTTG TCAAGTGCAG TGTTGCATAT GTCATATCGT GTTGATGGTC ATGTTATCAC   
  
  
+ GGACATAAAT TTCCCAGTGT AAAGCAAAGG TGTCTGTCTT CACATAGAGT GTTCAATTCA TAGTCATGCT   
  
  
+ CTCGTCTCTG CATCATCCTG CGTTTGCTGG GGCTAAATTT GAGGGGCTTA TCACTTTGAA ATTCGGAGAA   
  
  
+ TTACTGTGAA TTTGTGTTAG TTCAGGCATA AAATACTGGA TGGGATGTTG AGATGTTCTA ATCATTATCT   
  
  
+ ATTGTTAAAA AACTCAGAAC TATTATAGAT TTTCTGTTTT CTGTTTGGAT TGAGATCCGA AGCCTTTGAG   
  
  
+ ATCCCCTGTT TCTAGCTTCC AACCCCCCTC CCTGGGTTCT TTTTCTTTGG TGTGAAAGTA TGAACAAAAG   
  
  
+ CTGATGTAAG TGTGATTCAA ACTCTAGCCT TGGTGAGACT CTTGGGAAGG GCTCCTAGAG ACTTTGCCAA   
  
  
+ TTGAGCTAAT TTGATGCCTA CTACTTGCTT ACTTTTCTAG CTGTTGTTTA AACGATTTAT GTTCATAGTT   
  
  
+ AAGGAGCGCC ATATTGCATT ACAATATCTG CTTTAGTGCT TTGTTTTTTG CATTTTCTTC TTTATTACTA   
  
  
+ TGGTTTTTGA AGAAGGCTTA CTGACTGATA CTGGTTTCTT CGCTTAAGTT TGGTGTACAT GCTTTTCAAT   
  
  
+ CTGTGTTGCA TTTTTCTATG ATGTATAAGC CGTTTATTAC CTATTGATGT TCAACTTTTC ACACTATATA   
  
  
+ TCCTTTATGG TTTCTTCGCT TAAGTTTGGT GTACATGCTT TTCAATCTGT GTTGCATTTT TCTGTGATGT   
  
  
+ ATAAGCCGTT TATTACCTAT TGATGTTCAA CTTTTCACAC TATATGTCCT TTTATTAGTT ACTGATGCCT   
  
  
+ CTGTCAAACA TTCTTAATTG CATTTAGTGT ATCAACGGTC TTAAAATTTT AAGCATTGCC AACTTGAACT   
  
  
+ TCTGAAGATG TTTGTCAAGA TCTTCAGTCT GCAAGTTAAC TGCTGCTTGC TTGAACTTTT CAACTTTTAA   
  
  
+ GAGAGATGTC AGAGTCTAAG CATGGCTTCC TGACAAGTAC TGCATTTTAT CACTTATGAA GACTCAATTC   
  
  
+ TGGGTTTTGA CAGCTTCTAT GAACCTCTAG TGCAGGTACA GCAGCTTGGA CATTAAGCTG CAAGTCAATT   
  
  
+ GACTGTAGAA CGACAAGTTG TTGAAGTTTC TGCTGAAAGT TCATCACTTG TTTGATGAAT AAAGTACAGC   
  
  
+ ATGGCACTTC AGATGCAAAA GTCTCGCGAT CAGTAGAGAT GATTCCATAT TTCTCATCTC ATTTCCAAGT   
  
  
+ CTTTGACAAC ATGTACCCAA ATAATGCCAG CCATGACACT CAGATGTCTC TCCAATCATA CAGCGAAGGA   
  
  
+ TACTTCACTC TGGACTCATC TCCAGCAGCA ATCGGAGCGT GCAGTGTCTA TGACTACCCA TCCGTTGTCA   
  
  
+ GCACCTCTTC AAATAGAAGC CAGTTTTCTC CTCAGGGTTC CCACTCGTAC ATCTCAGACC CCCATCATTC   
  
  
+ TTCCGACAAC TATGGATCCC CAGTGAGCGG CTCTTCTGTG GTTGACGATA ATGCTGAGCT GAGGAACAGG   
  
  
+ TTCAGTGATA TGGAGCTCCC CTTGCCACAG GACTCAGGAC ACCATTATTG CTCTTTTAGC CACAGAGGAA   
  
  
+ GCCATGAAGG CTCCTATACT TTGAGGCCAA ACCAACTGAT GGATATGGCC AACATGGAGT TAAAGCAGGT   
  
  
+ GCTATACTTC TGTGCAGAAG CAATCTCAGA GAATAATCTA TCAACTGCAG AAAGACTAAT GGATGCATTG   
  
  
+ GGTAAGAGGG TGTCTGTTTT TGGTTCACCA ATTGAAAGGT TGGCCGCCTA CATGTTGGAA GGGCTCAGAG   
  
  
+ CAAGGCTGGA GTTTTCTGGA TATACTATCT ACAAAAAGCT CAGGTGCGAA CAGCCAACGA GCTCAGAGCT   
  
  
+ TCTTTCCTAC ATGCACATCC TGTATCAAAG TTGCCCATAT TTCAAATTCG CATATATGTC CTCAAATGTT   
  
  
+ GCTATTCAAG AAGCTTTGGG GAATGAGCCG GTTATCCACA TCATCGATTT CCAGATTGCC ATGGGGACAC   
  
  
+ AATTGGTGCT CTTGATCCAG TCTCTCGCCC ATCGGCCTGG CGGGCCCCCC CCTCGTTCGG ATCACTGGGG   
  
  
+ TCGATG  

- -Up\_Stream \_Len000AATAAG AATGTAGAAC AACAACACTA ACACATAATT CGAACAACAG AGTACCAAAC   
  
  
- GAGAAGAGAA ACCTTGGAGT ACCACCGATC AAGGTACAAA AACGAAACAT CTACATCGGC AAACAATTTC   
  
  
- GTTCAACCAA ATAAAGAGGA TACCGAAGCG AGATAACATT AAGTCCCCAG TCCAACAATA CAGTCTGTAA   
  
  
- GGGTATGGCG TGGATCTTCC CTCCTCCCTC CGAGTCCTCA GAATTTCTCA TATCTAAACC TAAGTGGACA   
  
  
- TCAGATATCG ACTACAGGTG GATTAGACCT AAAATAAGAA AGAAAGAGAG AGAAAAAAAA AGAAAAAAAC   
  
  
- CCCCCCCCCC ACAACAACTC CCTCTGTCCT ACTCCAGCTT AGAGACGTCG TATTGTTGGT GGTATCAGTG   
  
  
- TTATACCTCT AACACCTCCG TTTCAAACTC CTGCAGGCCT ACATAAAGGA AACCTTGACT CCTCCTCCAT   
  
  
- CATGAAGAGA CCATCATCAC AAGATATAGG AAACAAGGAA AGCACATAAT AGGATCGATG GAGAGTTCGT   
  
  
- TCAACTGTAC TTTAACATCT TAAGTTCTGT TATTATTGTC GACCAAAAAC CCAGAAAATC AAACGTTAGG   
  
  
- GAACATCGTA GAATCACTGA ATCACTGTAC TCTAAAACTG GGGTTATCTT TATCTTGTCT GTCGACGATT   
  
  
- TAAGTGGGTA GAGAACACAG TTTTAGCCTG AACAAAACGG AGACGAAGAG GTATTTCGTA GTTTCTCGCG   
  
  
- ACTAACTCCG TCCCTAAAAC AGTTCACGTC ACAACGTATA CAGTATAGCA CAACTACCAG TACAATAGTG   
  
  
- CCTGTATTTA AAGGGTCACA TTTCGTTTCC ACAGACAGAA GTGTATCTCA CAAGTTAAGT ATCAGTACGA   
  
  
- GAGCAGAGAC GTAGTAGGAC GCAAACGACC CCGATTTAAA CTCCCCGAAT AGTGAAACTT TAAGCCTCTT   
  
  
- AATGACACTT AAACACAATC AAGTCCGTAT TTTATGACCT ACCCTACAAC TCTACAAGAT TAGTAATAGA   
  
  
- TAACAATTTT TTGAGTCTTG ATAATATCTA AAAGACAAAA GACAAACCTA ACTCTAGGCT TCGGAAACTC   
  
  
- TAGGGGACAA AGATCGAAGG TTGGGGGGAG GGACCCAAGA AAAAGAAACC ACACTTTCAT ACTTGTTTTC   
  
  
- GACTACATTC ACACTAAGTT TGAGATCGGA ACCACTCTGA GAACCCTTCC CGAGGATCTC TGAAACGGTT   
  
  
- AACTCGATTA AACTACGGAT GATGAACGAA TGAAAAGATC GACAACAAAT TTGCTAAATA CAAGTATCAA   
  
  
- TTCCTCGCGG TATAACGTAA TGTTATAGAC GAAATCACGA AACAAAAAAC GTAAAAGAAG AAATAATGAT   
  
  
- ACCAAAAACT TCTTCCGAAT GACTGACTAT GACCAAAGAA GCGAATTCAA ACCACATGTA CGAAAAGTTA   
  
  
- GACACAACGT AAAAAGATAC TACATATTCG GCAAATAATG GATAACTACA AGTTGAAAAG TGTGATATAT   
  
  
- AGGAAATACC AAAGAAGCGA ATTCAAACCA CATGTACGAA AAGTTAGACA CAACGTAAAA AGACACTACA   
  
  
- TATTCGGCAA ATAATGGATA ACTACAAGTT GAAAAGTGTG ATATACAGGA AAATAATCAA TGACTACGGA   
  
  
- GACAGTTTGT AAGAATTAAC GTAAATCACA TAGTTGCCAG AATTTTAAAA TTCGTAACGG TTGAACTTGA   
  
  
- AGACTTCTAC AAACAGTTCT AGAAGTCAGA CGTTCAATTG ACGACGAACG AACTTGAAAA GTTGAAAATT   
  
  
- CTCTCTACAG TCTCAGATTC GTACCGAAGG ACTGTTCATG ACGTAAAATA GTGAATACTT CTGAGTTAAG   
  
  
- ACCCAAAACT GTCGAAGATA CTTGGAGATC ACGTCCATGT CGTCGAACCT GTAATTCGAC GTTCAGTTAA   
  
  
- CTGACATCTT GCTGTTCAAC AACTTCAAAG ACGACTTTCA AGTAGTGAAC AAACTACTTA TTTCATGTCG   
  
  
- TACCGTGAAG TCTACGTTTT CAGAGCGCTA GTCATCTCTA CTAAGGTATA AAGAGTAGAG TAAAGGTTCA   
  
  
- GAAACTGTTG TACATGGGTT TATTACGGTC GGTACTGTGA GTCTACAGAG AGGTTAGTAT GTCGCTTCCT   
  
  
- ATGAAGTGAG ACCTGAGTAG AGGTCGTCGT TAGCCTCGCA CGTCACAGAT ACTGATGGGT AGGCAACAGT   
  
  
- CGTGGAGAAG TTTATCTTCG GTCAAAAGAG GAGTCCCAAG GGTGAGCATG TAGAGTCTGG GGGTAGTAAG   
  
  
- AAGGCTGTTG ATACCTAGGG GTCACTCGCC GAGAAGACAC CAACTGCTAT TACGACTCGA CTCCTTGTCC   
  
  
- AAGTCACTAT ACCTCGAGGG GAACGGTGTC CTGAGTCCTG TGGTAATAAC GAGAAAATCG GTGTCTCCTT   
  
  
- CGGTACTTCC GAGGATATGA AACTCCGGTT TGGTTGACTA CCTATACCGG TTGTACCTCA ATTTCGTCCA   
  
  
- CGATATGAAG ACACGTCTTC GTTAGAGTCT CTTATTAGAT AGTTGACGTC TTTCTGATTA CCTACGTAAC   
  
  
- CCATTCTCCC ACAGACAAAA ACCAAGTGGT TAACTTTCCA ACCGGCGGAT GTACAACCTT CCCGAGTCTC   
  
  
- GTTCCGACCT CAAAAGACCT ATATGATAGA TGTTTTTCGA GTCCACGCTT GTCGGTTGCT CGAGTCTCGA   
  
  
- AGAAAGGATG TACGTGTAGG ACATAGTTTC AACGGGTATA AAGTTTAAGC GTATATACAG GAGTTTACAA   
  
  
- CGATAAGTTC TTCGAAACCC CTTACTCGGC CAATAGGTGT AGTAGCTAAA GGTCTAACGG TACCCCTGTG   
  
  
- TTAACCACGA GAACTAGGTC AGAGAGCGGG TAGCCGGACC GCCCGGGGGG GGAGCAAGCC TAGTGACCCC   
  
  
- AGCTAC

+     TCT-motif

| Site Name | Organism | Position | Strand | Matrix score. | sequence | function |
| --- | --- | --- | --- | --- | --- | --- |
| TCT-motif | Arabidopsis thaliana | 2596 | - | 6 | TCTTAC | part of a light responsive element |
| TCT-motif | Arabidopsis thaliana | 23 | + | 6 | TCTTAC | part of a light responsive element |

>HU08G02296.1   
+ -Up\_Stream \_Len000TTATTC TTACATCTTG TTGTTGTGAT TGTGTATTAA GCTTGTTGTC TCATGGTTTG   
  
  
+ CTCTTCTCTT TGGAACCTCA TGGTGGCTAG TTCCATGTTT TTGCTTTGTA GATGTAGCCG TTTGTTAAAG   
  
  
+ CAAGTTGGTT TATTTCTCCT ATGGCTTCGC TCTATTGTAA TTCAGGGGTC AGGTTGTTAT GTCAGACATT   
  
  
+ CCCATACCGC ACCTAGAAGG GAGGAGGGAG GCTCAGGAGT CTTAAAGAGT ATAGATTTGG ATTCACCTGT   
  
  
+ AGTCTATAGC TGATGTCCAC CTAATCTGGA TTTTATTCTT TCTTTCTCTC TCTTTTTTTT TCTTTTTTTG   
  
  
+ GGGGGGGGGG TGTTGTTGAG GGAGACAGGA TGAGGTCGAA TCTCTGCAGC ATAACAACCA CCATAGTCAC   
  
  
+ AATATGGAGA TTGTGGAGGC AAAGTTTGAG GACGTCCGGA TGTATTTCCT TTGGAACTGA GGAGGAGGTA   
  
  
+ GTACTTCTCT GGTAGTAGTG TTCTATATCC TTTGTTCCTT TCGTGTATTA TCCTAGCTAC CTCTCAAGCA   
  
  
+ AGTTGACATG AAATTGTAGA ATTCAAGACA ATAATAACAG CTGGTTTTTG GGTCTTTTAG TTTGCAATCC   
  
  
+ CTTGTAGCAT CTTAGTGACT TAGTGACATG AGATTTTGAC CCCAATAGAA ATAGAACAGA CAGCTGCTAA   
  
  
+ ATTCACCCAT CTCTTGTGTC AAAATCGGAC TTGTTTTGCC TCTGCTTCTC CATAAAGCAT CAAAGAGCGC   
  
  
+ TGATTGAGGC AGGGATTTTG TCAAGTGCAG TGTTGCATAT GTCATATCGT GTTGATGGTC ATGTTATCAC   
  
  
+ GGACATAAAT TTCCCAGTGT AAAGCAAAGG TGTCTGTCTT CACATAGAGT GTTCAATTCA TAGTCATGCT   
  
  
+ CTCGTCTCTG CATCATCCTG CGTTTGCTGG GGCTAAATTT GAGGGGCTTA TCACTTTGAA ATTCGGAGAA   
  
  
+ TTACTGTGAA TTTGTGTTAG TTCAGGCATA AAATACTGGA TGGGATGTTG AGATGTTCTA ATCATTATCT   
  
  
+ ATTGTTAAAA AACTCAGAAC TATTATAGAT TTTCTGTTTT CTGTTTGGAT TGAGATCCGA AGCCTTTGAG   
  
  
+ ATCCCCTGTT TCTAGCTTCC AACCCCCCTC CCTGGGTTCT TTTTCTTTGG TGTGAAAGTA TGAACAAAAG   
  
  
+ CTGATGTAAG TGTGATTCAA ACTCTAGCCT TGGTGAGACT CTTGGGAAGG GCTCCTAGAG ACTTTGCCAA   
  
  
+ TTGAGCTAAT TTGATGCCTA CTACTTGCTT ACTTTTCTAG CTGTTGTTTA AACGATTTAT GTTCATAGTT   
  
  
+ AAGGAGCGCC ATATTGCATT ACAATATCTG CTTTAGTGCT TTGTTTTTTG CATTTTCTTC TTTATTACTA   
  
  
+ TGGTTTTTGA AGAAGGCTTA CTGACTGATA CTGGTTTCTT CGCTTAAGTT TGGTGTACAT GCTTTTCAAT   
  
  
+ CTGTGTTGCA TTTTTCTATG ATGTATAAGC CGTTTATTAC CTATTGATGT TCAACTTTTC ACACTATATA   
  
  
+ TCCTTTATGG TTTCTTCGCT TAAGTTTGGT GTACATGCTT TTCAATCTGT GTTGCATTTT TCTGTGATGT   
  
  
+ ATAAGCCGTT TATTACCTAT TGATGTTCAA CTTTTCACAC TATATGTCCT TTTATTAGTT ACTGATGCCT   
  
  
+ CTGTCAAACA TTCTTAATTG CATTTAGTGT ATCAACGGTC TTAAAATTTT AAGCATTGCC AACTTGAACT   
  
  
+ TCTGAAGATG TTTGTCAAGA TCTTCAGTCT GCAAGTTAAC TGCTGCTTGC TTGAACTTTT CAACTTTTAA   
  
  
+ GAGAGATGTC AGAGTCTAAG CATGGCTTCC TGACAAGTAC TGCATTTTAT CACTTATGAA GACTCAATTC   
  
  
+ TGGGTTTTGA CAGCTTCTAT GAACCTCTAG TGCAGGTACA GCAGCTTGGA CATTAAGCTG CAAGTCAATT   
  
  
+ GACTGTAGAA CGACAAGTTG TTGAAGTTTC TGCTGAAAGT TCATCACTTG TTTGATGAAT AAAGTACAGC   
  
  
+ ATGGCACTTC AGATGCAAAA GTCTCGCGAT CAGTAGAGAT GATTCCATAT TTCTCATCTC ATTTCCAAGT   
  
  
+ CTTTGACAAC ATGTACCCAA ATAATGCCAG CCATGACACT CAGATGTCTC TCCAATCATA CAGCGAAGGA   
  
  
+ TACTTCACTC TGGACTCATC TCCAGCAGCA ATCGGAGCGT GCAGTGTCTA TGACTACCCA TCCGTTGTCA   
  
  
+ GCACCTCTTC AAATAGAAGC CAGTTTTCTC CTCAGGGTTC CCACTCGTAC ATCTCAGACC CCCATCATTC   
  
  
+ TTCCGACAAC TATGGATCCC CAGTGAGCGG CTCTTCTGTG GTTGACGATA ATGCTGAGCT GAGGAACAGG   
  
  
+ TTCAGTGATA TGGAGCTCCC CTTGCCACAG GACTCAGGAC ACCATTATTG CTCTTTTAGC CACAGAGGAA   
  
  
+ GCCATGAAGG CTCCTATACT TTGAGGCCAA ACCAACTGAT GGATATGGCC AACATGGAGT TAAAGCAGGT   
  
  
+ GCTATACTTC TGTGCAGAAG CAATCTCAGA GAATAATCTA TCAACTGCAG AAAGACTAAT GGATGCATTG   
  
  
+ GGTAAGAGGG TGTCTGTTTT TGGTTCACCA ATTGAAAGGT TGGCCGCCTA CATGTTGGAA GGGCTCAGAG   
  
  
+ CAAGGCTGGA GTTTTCTGGA TATACTATCT ACAAAAAGCT CAGGTGCGAA CAGCCAACGA GCTCAGAGCT   
  
  
+ TCTTTCCTAC ATGCACATCC TGTATCAAAG TTGCCCATAT TTCAAATTCG CATATATGTC CTCAAATGTT   
  
  
+ GCTATTCAAG AAGCTTTGGG GAATGAGCCG GTTATCCACA TCATCGATTT CCAGATTGCC ATGGGGACAC   
  
  
+ AATTGGTGCT CTTGATCCAG TCTCTCGCCC ATCGGCCTGG CGGGCCCCCC CCTCGTTCGG ATCACTGGGG   
  
  
+ TCGATG  

- -Up\_Stream \_Len000AATAAG AATGTAGAAC AACAACACTA ACACATAATT CGAACAACAG AGTACCAAAC   
  
  
- GAGAAGAGAA ACCTTGGAGT ACCACCGATC AAGGTACAAA AACGAAACAT CTACATCGGC AAACAATTTC   
  
  
- GTTCAACCAA ATAAAGAGGA TACCGAAGCG AGATAACATT AAGTCCCCAG TCCAACAATA CAGTCTGTAA   
  
  
- GGGTATGGCG TGGATCTTCC CTCCTCCCTC CGAGTCCTCA GAATTTCTCA TATCTAAACC TAAGTGGACA   
  
  
- TCAGATATCG ACTACAGGTG GATTAGACCT AAAATAAGAA AGAAAGAGAG AGAAAAAAAA AGAAAAAAAC   
  
  
- CCCCCCCCCC ACAACAACTC CCTCTGTCCT ACTCCAGCTT AGAGACGTCG TATTGTTGGT GGTATCAGTG   
  
  
- TTATACCTCT AACACCTCCG TTTCAAACTC CTGCAGGCCT ACATAAAGGA AACCTTGACT CCTCCTCCAT   
  
  
- CATGAAGAGA CCATCATCAC AAGATATAGG AAACAAGGAA AGCACATAAT AGGATCGATG GAGAGTTCGT   
  
  
- TCAACTGTAC TTTAACATCT TAAGTTCTGT TATTATTGTC GACCAAAAAC CCAGAAAATC AAACGTTAGG   
  
  
- GAACATCGTA GAATCACTGA ATCACTGTAC TCTAAAACTG GGGTTATCTT TATCTTGTCT GTCGACGATT   
  
  
- TAAGTGGGTA GAGAACACAG TTTTAGCCTG AACAAAACGG AGACGAAGAG GTATTTCGTA GTTTCTCGCG   
  
  
- ACTAACTCCG TCCCTAAAAC AGTTCACGTC ACAACGTATA CAGTATAGCA CAACTACCAG TACAATAGTG   
  
  
- CCTGTATTTA AAGGGTCACA TTTCGTTTCC ACAGACAGAA GTGTATCTCA CAAGTTAAGT ATCAGTACGA   
  
  
- GAGCAGAGAC GTAGTAGGAC GCAAACGACC CCGATTTAAA CTCCCCGAAT AGTGAAACTT TAAGCCTCTT   
  
  
- AATGACACTT AAACACAATC AAGTCCGTAT TTTATGACCT ACCCTACAAC TCTACAAGAT TAGTAATAGA   
  
  
- TAACAATTTT TTGAGTCTTG ATAATATCTA AAAGACAAAA GACAAACCTA ACTCTAGGCT TCGGAAACTC   
  
  
- TAGGGGACAA AGATCGAAGG TTGGGGGGAG GGACCCAAGA AAAAGAAACC ACACTTTCAT ACTTGTTTTC   
  
  
- GACTACATTC ACACTAAGTT TGAGATCGGA ACCACTCTGA GAACCCTTCC CGAGGATCTC TGAAACGGTT   
  
  
- AACTCGATTA AACTACGGAT GATGAACGAA TGAAAAGATC GACAACAAAT TTGCTAAATA CAAGTATCAA   
  
  
- TTCCTCGCGG TATAACGTAA TGTTATAGAC GAAATCACGA AACAAAAAAC GTAAAAGAAG AAATAATGAT   
  
  
- ACCAAAAACT TCTTCCGAAT GACTGACTAT GACCAAAGAA GCGAATTCAA ACCACATGTA CGAAAAGTTA   
  
  
- GACACAACGT AAAAAGATAC TACATATTCG GCAAATAATG GATAACTACA AGTTGAAAAG TGTGATATAT   
  
  
- AGGAAATACC AAAGAAGCGA ATTCAAACCA CATGTACGAA AAGTTAGACA CAACGTAAAA AGACACTACA   
  
  
- TATTCGGCAA ATAATGGATA ACTACAAGTT GAAAAGTGTG ATATACAGGA AAATAATCAA TGACTACGGA   
  
  
- GACAGTTTGT AAGAATTAAC GTAAATCACA TAGTTGCCAG AATTTTAAAA TTCGTAACGG TTGAACTTGA   
  
  
- AGACTTCTAC AAACAGTTCT AGAAGTCAGA CGTTCAATTG ACGACGAACG AACTTGAAAA GTTGAAAATT   
  
  
- CTCTCTACAG TCTCAGATTC GTACCGAAGG ACTGTTCATG ACGTAAAATA GTGAATACTT CTGAGTTAAG   
  
  
- ACCCAAAACT GTCGAAGATA CTTGGAGATC ACGTCCATGT CGTCGAACCT GTAATTCGAC GTTCAGTTAA   
  
  
- CTGACATCTT GCTGTTCAAC AACTTCAAAG ACGACTTTCA AGTAGTGAAC AAACTACTTA TTTCATGTCG   
  
  
- TACCGTGAAG TCTACGTTTT CAGAGCGCTA GTCATCTCTA CTAAGGTATA AAGAGTAGAG TAAAGGTTCA   
  
  
- GAAACTGTTG TACATGGGTT TATTACGGTC GGTACTGTGA GTCTACAGAG AGGTTAGTAT GTCGCTTCCT   
  
  
- ATGAAGTGAG ACCTGAGTAG AGGTCGTCGT TAGCCTCGCA CGTCACAGAT ACTGATGGGT AGGCAACAGT   
  
  
- CGTGGAGAAG TTTATCTTCG GTCAAAAGAG GAGTCCCAAG GGTGAGCATG TAGAGTCTGG GGGTAGTAAG   
  
  
- AAGGCTGTTG ATACCTAGGG GTCACTCGCC GAGAAGACAC CAACTGCTAT TACGACTCGA CTCCTTGTCC   
  
  
- AAGTCACTAT ACCTCGAGGG GAACGGTGTC CTGAGTCCTG TGGTAATAAC GAGAAAATCG GTGTCTCCTT   
  
  
- CGGTACTTCC GAGGATATGA AACTCCGGTT TGGTTGACTA CCTATACCGG TTGTACCTCA ATTTCGTCCA   
  
  
- CGATATGAAG ACACGTCTTC GTTAGAGTCT CTTATTAGAT AGTTGACGTC TTTCTGATTA CCTACGTAAC   
  
  
- CCATTCTCCC ACAGACAAAA ACCAAGTGGT TAACTTTCCA ACCGGCGGAT GTACAACCTT CCCGAGTCTC   
  
  
- GTTCCGACCT CAAAAGACCT ATATGATAGA TGTTTTTCGA GTCCACGCTT GTCGGTTGCT CGAGTCTCGA   
  
  
- AGAAAGGATG TACGTGTAGG ACATAGTTTC AACGGGTATA AAGTTTAAGC GTATATACAG GAGTTTACAA   
  
  
- CGATAAGTTC TTCGAAACCC CTTACTCGGC CAATAGGTGT AGTAGCTAAA GGTCTAACGG TACCCCTGTG   
  
  
- TTAACCACGA GAACTAGGTC AGAGAGCGGG TAGCCGGACC GCCCGGGGGG GGAGCAAGCC TAGTGACCCC   
  
  
- AGCTAC

+     TGA-element

| Site Name | Organism | Position | Strand | Matrix score. | sequence | function |
| --- | --- | --- | --- | --- | --- | --- |
| TGA-element | Brassica oleracea | 1973 | + | 6 | AACGAC | auxin-responsive element |

>HU08G02296.1   
+ -Up\_Stream \_Len000TTATTC TTACATCTTG TTGTTGTGAT TGTGTATTAA GCTTGTTGTC TCATGGTTTG   
  
  
+ CTCTTCTCTT TGGAACCTCA TGGTGGCTAG TTCCATGTTT TTGCTTTGTA GATGTAGCCG TTTGTTAAAG   
  
  
+ CAAGTTGGTT TATTTCTCCT ATGGCTTCGC TCTATTGTAA TTCAGGGGTC AGGTTGTTAT GTCAGACATT   
  
  
+ CCCATACCGC ACCTAGAAGG GAGGAGGGAG GCTCAGGAGT CTTAAAGAGT ATAGATTTGG ATTCACCTGT   
  
  
+ AGTCTATAGC TGATGTCCAC CTAATCTGGA TTTTATTCTT TCTTTCTCTC TCTTTTTTTT TCTTTTTTTG   
  
  
+ GGGGGGGGGG TGTTGTTGAG GGAGACAGGA TGAGGTCGAA TCTCTGCAGC ATAACAACCA CCATAGTCAC   
  
  
+ AATATGGAGA TTGTGGAGGC AAAGTTTGAG GACGTCCGGA TGTATTTCCT TTGGAACTGA GGAGGAGGTA   
  
  
+ GTACTTCTCT GGTAGTAGTG TTCTATATCC TTTGTTCCTT TCGTGTATTA TCCTAGCTAC CTCTCAAGCA   
  
  
+ AGTTGACATG AAATTGTAGA ATTCAAGACA ATAATAACAG CTGGTTTTTG GGTCTTTTAG TTTGCAATCC   
  
  
+ CTTGTAGCAT CTTAGTGACT TAGTGACATG AGATTTTGAC CCCAATAGAA ATAGAACAGA CAGCTGCTAA   
  
  
+ ATTCACCCAT CTCTTGTGTC AAAATCGGAC TTGTTTTGCC TCTGCTTCTC CATAAAGCAT CAAAGAGCGC   
  
  
+ TGATTGAGGC AGGGATTTTG TCAAGTGCAG TGTTGCATAT GTCATATCGT GTTGATGGTC ATGTTATCAC   
  
  
+ GGACATAAAT TTCCCAGTGT AAAGCAAAGG TGTCTGTCTT CACATAGAGT GTTCAATTCA TAGTCATGCT   
  
  
+ CTCGTCTCTG CATCATCCTG CGTTTGCTGG GGCTAAATTT GAGGGGCTTA TCACTTTGAA ATTCGGAGAA   
  
  
+ TTACTGTGAA TTTGTGTTAG TTCAGGCATA AAATACTGGA TGGGATGTTG AGATGTTCTA ATCATTATCT   
  
  
+ ATTGTTAAAA AACTCAGAAC TATTATAGAT TTTCTGTTTT CTGTTTGGAT TGAGATCCGA AGCCTTTGAG   
  
  
+ ATCCCCTGTT TCTAGCTTCC AACCCCCCTC CCTGGGTTCT TTTTCTTTGG TGTGAAAGTA TGAACAAAAG   
  
  
+ CTGATGTAAG TGTGATTCAA ACTCTAGCCT TGGTGAGACT CTTGGGAAGG GCTCCTAGAG ACTTTGCCAA   
  
  
+ TTGAGCTAAT TTGATGCCTA CTACTTGCTT ACTTTTCTAG CTGTTGTTTA AACGATTTAT GTTCATAGTT   
  
  
+ AAGGAGCGCC ATATTGCATT ACAATATCTG CTTTAGTGCT TTGTTTTTTG CATTTTCTTC TTTATTACTA   
  
  
+ TGGTTTTTGA AGAAGGCTTA CTGACTGATA CTGGTTTCTT CGCTTAAGTT TGGTGTACAT GCTTTTCAAT   
  
  
+ CTGTGTTGCA TTTTTCTATG ATGTATAAGC CGTTTATTAC CTATTGATGT TCAACTTTTC ACACTATATA   
  
  
+ TCCTTTATGG TTTCTTCGCT TAAGTTTGGT GTACATGCTT TTCAATCTGT GTTGCATTTT TCTGTGATGT   
  
  
+ ATAAGCCGTT TATTACCTAT TGATGTTCAA CTTTTCACAC TATATGTCCT TTTATTAGTT ACTGATGCCT   
  
  
+ CTGTCAAACA TTCTTAATTG CATTTAGTGT ATCAACGGTC TTAAAATTTT AAGCATTGCC AACTTGAACT   
  
  
+ TCTGAAGATG TTTGTCAAGA TCTTCAGTCT GCAAGTTAAC TGCTGCTTGC TTGAACTTTT CAACTTTTAA   
  
  
+ GAGAGATGTC AGAGTCTAAG CATGGCTTCC TGACAAGTAC TGCATTTTAT CACTTATGAA GACTCAATTC   
  
  
+ TGGGTTTTGA CAGCTTCTAT GAACCTCTAG TGCAGGTACA GCAGCTTGGA CATTAAGCTG CAAGTCAATT   
  
  
+ GACTGTAGAA CGACAAGTTG TTGAAGTTTC TGCTGAAAGT TCATCACTTG TTTGATGAAT AAAGTACAGC   
  
  
+ ATGGCACTTC AGATGCAAAA GTCTCGCGAT CAGTAGAGAT GATTCCATAT TTCTCATCTC ATTTCCAAGT   
  
  
+ CTTTGACAAC ATGTACCCAA ATAATGCCAG CCATGACACT CAGATGTCTC TCCAATCATA CAGCGAAGGA   
  
  
+ TACTTCACTC TGGACTCATC TCCAGCAGCA ATCGGAGCGT GCAGTGTCTA TGACTACCCA TCCGTTGTCA   
  
  
+ GCACCTCTTC AAATAGAAGC CAGTTTTCTC CTCAGGGTTC CCACTCGTAC ATCTCAGACC CCCATCATTC   
  
  
+ TTCCGACAAC TATGGATCCC CAGTGAGCGG CTCTTCTGTG GTTGACGATA ATGCTGAGCT GAGGAACAGG   
  
  
+ TTCAGTGATA TGGAGCTCCC CTTGCCACAG GACTCAGGAC ACCATTATTG CTCTTTTAGC CACAGAGGAA   
  
  
+ GCCATGAAGG CTCCTATACT TTGAGGCCAA ACCAACTGAT GGATATGGCC AACATGGAGT TAAAGCAGGT   
  
  
+ GCTATACTTC TGTGCAGAAG CAATCTCAGA GAATAATCTA TCAACTGCAG AAAGACTAAT GGATGCATTG   
  
  
+ GGTAAGAGGG TGTCTGTTTT TGGTTCACCA ATTGAAAGGT TGGCCGCCTA CATGTTGGAA GGGCTCAGAG   
  
  
+ CAAGGCTGGA GTTTTCTGGA TATACTATCT ACAAAAAGCT CAGGTGCGAA CAGCCAACGA GCTCAGAGCT   
  
  
+ TCTTTCCTAC ATGCACATCC TGTATCAAAG TTGCCCATAT TTCAAATTCG CATATATGTC CTCAAATGTT   
  
  
+ GCTATTCAAG AAGCTTTGGG GAATGAGCCG GTTATCCACA TCATCGATTT CCAGATTGCC ATGGGGACAC   
  
  
+ AATTGGTGCT CTTGATCCAG TCTCTCGCCC ATCGGCCTGG CGGGCCCCCC CCTCGTTCGG ATCACTGGGG   
  
  
+ TCGATG  

- -Up\_Stream \_Len000AATAAG AATGTAGAAC AACAACACTA ACACATAATT CGAACAACAG AGTACCAAAC   
  
  
- GAGAAGAGAA ACCTTGGAGT ACCACCGATC AAGGTACAAA AACGAAACAT CTACATCGGC AAACAATTTC   
  
  
- GTTCAACCAA ATAAAGAGGA TACCGAAGCG AGATAACATT AAGTCCCCAG TCCAACAATA CAGTCTGTAA   
  
  
- GGGTATGGCG TGGATCTTCC CTCCTCCCTC CGAGTCCTCA GAATTTCTCA TATCTAAACC TAAGTGGACA   
  
  
- TCAGATATCG ACTACAGGTG GATTAGACCT AAAATAAGAA AGAAAGAGAG AGAAAAAAAA AGAAAAAAAC   
  
  
- CCCCCCCCCC ACAACAACTC CCTCTGTCCT ACTCCAGCTT AGAGACGTCG TATTGTTGGT GGTATCAGTG   
  
  
- TTATACCTCT AACACCTCCG TTTCAAACTC CTGCAGGCCT ACATAAAGGA AACCTTGACT CCTCCTCCAT   
  
  
- CATGAAGAGA CCATCATCAC AAGATATAGG AAACAAGGAA AGCACATAAT AGGATCGATG GAGAGTTCGT   
  
  
- TCAACTGTAC TTTAACATCT TAAGTTCTGT TATTATTGTC GACCAAAAAC CCAGAAAATC AAACGTTAGG   
  
  
- GAACATCGTA GAATCACTGA ATCACTGTAC TCTAAAACTG GGGTTATCTT TATCTTGTCT GTCGACGATT   
  
  
- TAAGTGGGTA GAGAACACAG TTTTAGCCTG AACAAAACGG AGACGAAGAG GTATTTCGTA GTTTCTCGCG   
  
  
- ACTAACTCCG TCCCTAAAAC AGTTCACGTC ACAACGTATA CAGTATAGCA CAACTACCAG TACAATAGTG   
  
  
- CCTGTATTTA AAGGGTCACA TTTCGTTTCC ACAGACAGAA GTGTATCTCA CAAGTTAAGT ATCAGTACGA   
  
  
- GAGCAGAGAC GTAGTAGGAC GCAAACGACC CCGATTTAAA CTCCCCGAAT AGTGAAACTT TAAGCCTCTT   
  
  
- AATGACACTT AAACACAATC AAGTCCGTAT TTTATGACCT ACCCTACAAC TCTACAAGAT TAGTAATAGA   
  
  
- TAACAATTTT TTGAGTCTTG ATAATATCTA AAAGACAAAA GACAAACCTA ACTCTAGGCT TCGGAAACTC   
  
  
- TAGGGGACAA AGATCGAAGG TTGGGGGGAG GGACCCAAGA AAAAGAAACC ACACTTTCAT ACTTGTTTTC   
  
  
- GACTACATTC ACACTAAGTT TGAGATCGGA ACCACTCTGA GAACCCTTCC CGAGGATCTC TGAAACGGTT   
  
  
- AACTCGATTA AACTACGGAT GATGAACGAA TGAAAAGATC GACAACAAAT TTGCTAAATA CAAGTATCAA   
  
  
- TTCCTCGCGG TATAACGTAA TGTTATAGAC GAAATCACGA AACAAAAAAC GTAAAAGAAG AAATAATGAT   
  
  
- ACCAAAAACT TCTTCCGAAT GACTGACTAT GACCAAAGAA GCGAATTCAA ACCACATGTA CGAAAAGTTA   
  
  
- GACACAACGT AAAAAGATAC TACATATTCG GCAAATAATG GATAACTACA AGTTGAAAAG TGTGATATAT   
  
  
- AGGAAATACC AAAGAAGCGA ATTCAAACCA CATGTACGAA AAGTTAGACA CAACGTAAAA AGACACTACA   
  
  
- TATTCGGCAA ATAATGGATA ACTACAAGTT GAAAAGTGTG ATATACAGGA AAATAATCAA TGACTACGGA   
  
  
- GACAGTTTGT AAGAATTAAC GTAAATCACA TAGTTGCCAG AATTTTAAAA TTCGTAACGG TTGAACTTGA   
  
  
- AGACTTCTAC AAACAGTTCT AGAAGTCAGA CGTTCAATTG ACGACGAACG AACTTGAAAA GTTGAAAATT   
  
  
- CTCTCTACAG TCTCAGATTC GTACCGAAGG ACTGTTCATG ACGTAAAATA GTGAATACTT CTGAGTTAAG   
  
  
- ACCCAAAACT GTCGAAGATA CTTGGAGATC ACGTCCATGT CGTCGAACCT GTAATTCGAC GTTCAGTTAA   
  
  
- CTGACATCTT GCTGTTCAAC AACTTCAAAG ACGACTTTCA AGTAGTGAAC AAACTACTTA TTTCATGTCG   
  
  
- TACCGTGAAG TCTACGTTTT CAGAGCGCTA GTCATCTCTA CTAAGGTATA AAGAGTAGAG TAAAGGTTCA   
  
  
- GAAACTGTTG TACATGGGTT TATTACGGTC GGTACTGTGA GTCTACAGAG AGGTTAGTAT GTCGCTTCCT   
  
  
- ATGAAGTGAG ACCTGAGTAG AGGTCGTCGT TAGCCTCGCA CGTCACAGAT ACTGATGGGT AGGCAACAGT   
  
  
- CGTGGAGAAG TTTATCTTCG GTCAAAAGAG GAGTCCCAAG GGTGAGCATG TAGAGTCTGG GGGTAGTAAG   
  
  
- AAGGCTGTTG ATACCTAGGG GTCACTCGCC GAGAAGACAC CAACTGCTAT TACGACTCGA CTCCTTGTCC   
  
  
- AAGTCACTAT ACCTCGAGGG GAACGGTGTC CTGAGTCCTG TGGTAATAAC GAGAAAATCG GTGTCTCCTT   
  
  
- CGGTACTTCC GAGGATATGA AACTCCGGTT TGGTTGACTA CCTATACCGG TTGTACCTCA ATTTCGTCCA   
  
  
- CGATATGAAG ACACGTCTTC GTTAGAGTCT CTTATTAGAT AGTTGACGTC TTTCTGATTA CCTACGTAAC   
  
  
- CCATTCTCCC ACAGACAAAA ACCAAGTGGT TAACTTTCCA ACCGGCGGAT GTACAACCTT CCCGAGTCTC   
  
  
- GTTCCGACCT CAAAAGACCT ATATGATAGA TGTTTTTCGA GTCCACGCTT GTCGGTTGCT CGAGTCTCGA   
  
  
- AGAAAGGATG TACGTGTAGG ACATAGTTTC AACGGGTATA AAGTTTAAGC GTATATACAG GAGTTTACAA   
  
  
- CGATAAGTTC TTCGAAACCC CTTACTCGGC CAATAGGTGT AGTAGCTAAA GGTCTAACGG TACCCCTGTG   
  
  
- TTAACCACGA GAACTAGGTC AGAGAGCGGG TAGCCGGACC GCCCGGGGGG GGAGCAAGCC TAGTGACCCC   
  
  
- AGCTAC

+     TGACG-motif

| Site Name | Organism | Position | Strand | Matrix score. | sequence | function |
| --- | --- | --- | --- | --- | --- | --- |
| TGACG-motif | Hordeum vulgare | 2357 | + | 5 | TGACG | cis-acting regulatory element involved in the MeJA-responsiveness |

>HU08G02296.1   
+ -Up\_Stream \_Len000TTATTC TTACATCTTG TTGTTGTGAT TGTGTATTAA GCTTGTTGTC TCATGGTTTG   
  
  
+ CTCTTCTCTT TGGAACCTCA TGGTGGCTAG TTCCATGTTT TTGCTTTGTA GATGTAGCCG TTTGTTAAAG   
  
  
+ CAAGTTGGTT TATTTCTCCT ATGGCTTCGC TCTATTGTAA TTCAGGGGTC AGGTTGTTAT GTCAGACATT   
  
  
+ CCCATACCGC ACCTAGAAGG GAGGAGGGAG GCTCAGGAGT CTTAAAGAGT ATAGATTTGG ATTCACCTGT   
  
  
+ AGTCTATAGC TGATGTCCAC CTAATCTGGA TTTTATTCTT TCTTTCTCTC TCTTTTTTTT TCTTTTTTTG   
  
  
+ GGGGGGGGGG TGTTGTTGAG GGAGACAGGA TGAGGTCGAA TCTCTGCAGC ATAACAACCA CCATAGTCAC   
  
  
+ AATATGGAGA TTGTGGAGGC AAAGTTTGAG GACGTCCGGA TGTATTTCCT TTGGAACTGA GGAGGAGGTA   
  
  
+ GTACTTCTCT GGTAGTAGTG TTCTATATCC TTTGTTCCTT TCGTGTATTA TCCTAGCTAC CTCTCAAGCA   
  
  
+ AGTTGACATG AAATTGTAGA ATTCAAGACA ATAATAACAG CTGGTTTTTG GGTCTTTTAG TTTGCAATCC   
  
  
+ CTTGTAGCAT CTTAGTGACT TAGTGACATG AGATTTTGAC CCCAATAGAA ATAGAACAGA CAGCTGCTAA   
  
  
+ ATTCACCCAT CTCTTGTGTC AAAATCGGAC TTGTTTTGCC TCTGCTTCTC CATAAAGCAT CAAAGAGCGC   
  
  
+ TGATTGAGGC AGGGATTTTG TCAAGTGCAG TGTTGCATAT GTCATATCGT GTTGATGGTC ATGTTATCAC   
  
  
+ GGACATAAAT TTCCCAGTGT AAAGCAAAGG TGTCTGTCTT CACATAGAGT GTTCAATTCA TAGTCATGCT   
  
  
+ CTCGTCTCTG CATCATCCTG CGTTTGCTGG GGCTAAATTT GAGGGGCTTA TCACTTTGAA ATTCGGAGAA   
  
  
+ TTACTGTGAA TTTGTGTTAG TTCAGGCATA AAATACTGGA TGGGATGTTG AGATGTTCTA ATCATTATCT   
  
  
+ ATTGTTAAAA AACTCAGAAC TATTATAGAT TTTCTGTTTT CTGTTTGGAT TGAGATCCGA AGCCTTTGAG   
  
  
+ ATCCCCTGTT TCTAGCTTCC AACCCCCCTC CCTGGGTTCT TTTTCTTTGG TGTGAAAGTA TGAACAAAAG   
  
  
+ CTGATGTAAG TGTGATTCAA ACTCTAGCCT TGGTGAGACT CTTGGGAAGG GCTCCTAGAG ACTTTGCCAA   
  
  
+ TTGAGCTAAT TTGATGCCTA CTACTTGCTT ACTTTTCTAG CTGTTGTTTA AACGATTTAT GTTCATAGTT   
  
  
+ AAGGAGCGCC ATATTGCATT ACAATATCTG CTTTAGTGCT TTGTTTTTTG CATTTTCTTC TTTATTACTA   
  
  
+ TGGTTTTTGA AGAAGGCTTA CTGACTGATA CTGGTTTCTT CGCTTAAGTT TGGTGTACAT GCTTTTCAAT   
  
  
+ CTGTGTTGCA TTTTTCTATG ATGTATAAGC CGTTTATTAC CTATTGATGT TCAACTTTTC ACACTATATA   
  
  
+ TCCTTTATGG TTTCTTCGCT TAAGTTTGGT GTACATGCTT TTCAATCTGT GTTGCATTTT TCTGTGATGT   
  
  
+ ATAAGCCGTT TATTACCTAT TGATGTTCAA CTTTTCACAC TATATGTCCT TTTATTAGTT ACTGATGCCT   
  
  
+ CTGTCAAACA TTCTTAATTG CATTTAGTGT ATCAACGGTC TTAAAATTTT AAGCATTGCC AACTTGAACT   
  
  
+ TCTGAAGATG TTTGTCAAGA TCTTCAGTCT GCAAGTTAAC TGCTGCTTGC TTGAACTTTT CAACTTTTAA   
  
  
+ GAGAGATGTC AGAGTCTAAG CATGGCTTCC TGACAAGTAC TGCATTTTAT CACTTATGAA GACTCAATTC   
  
  
+ TGGGTTTTGA CAGCTTCTAT GAACCTCTAG TGCAGGTACA GCAGCTTGGA CATTAAGCTG CAAGTCAATT   
  
  
+ GACTGTAGAA CGACAAGTTG TTGAAGTTTC TGCTGAAAGT TCATCACTTG TTTGATGAAT AAAGTACAGC   
  
  
+ ATGGCACTTC AGATGCAAAA GTCTCGCGAT CAGTAGAGAT GATTCCATAT TTCTCATCTC ATTTCCAAGT   
  
  
+ CTTTGACAAC ATGTACCCAA ATAATGCCAG CCATGACACT CAGATGTCTC TCCAATCATA CAGCGAAGGA   
  
  
+ TACTTCACTC TGGACTCATC TCCAGCAGCA ATCGGAGCGT GCAGTGTCTA TGACTACCCA TCCGTTGTCA   
  
  
+ GCACCTCTTC AAATAGAAGC CAGTTTTCTC CTCAGGGTTC CCACTCGTAC ATCTCAGACC CCCATCATTC   
  
  
+ TTCCGACAAC TATGGATCCC CAGTGAGCGG CTCTTCTGTG GTTGACGATA ATGCTGAGCT GAGGAACAGG   
  
  
+ TTCAGTGATA TGGAGCTCCC CTTGCCACAG GACTCAGGAC ACCATTATTG CTCTTTTAGC CACAGAGGAA   
  
  
+ GCCATGAAGG CTCCTATACT TTGAGGCCAA ACCAACTGAT GGATATGGCC AACATGGAGT TAAAGCAGGT   
  
  
+ GCTATACTTC TGTGCAGAAG CAATCTCAGA GAATAATCTA TCAACTGCAG AAAGACTAAT GGATGCATTG   
  
  
+ GGTAAGAGGG TGTCTGTTTT TGGTTCACCA ATTGAAAGGT TGGCCGCCTA CATGTTGGAA GGGCTCAGAG   
  
  
+ CAAGGCTGGA GTTTTCTGGA TATACTATCT ACAAAAAGCT CAGGTGCGAA CAGCCAACGA GCTCAGAGCT   
  
  
+ TCTTTCCTAC ATGCACATCC TGTATCAAAG TTGCCCATAT TTCAAATTCG CATATATGTC CTCAAATGTT   
  
  
+ GCTATTCAAG AAGCTTTGGG GAATGAGCCG GTTATCCACA TCATCGATTT CCAGATTGCC ATGGGGACAC   
  
  
+ AATTGGTGCT CTTGATCCAG TCTCTCGCCC ATCGGCCTGG CGGGCCCCCC CCTCGTTCGG ATCACTGGGG   
  
  
+ TCGATG  

- -Up\_Stream \_Len000AATAAG AATGTAGAAC AACAACACTA ACACATAATT CGAACAACAG AGTACCAAAC   
  
  
- GAGAAGAGAA ACCTTGGAGT ACCACCGATC AAGGTACAAA AACGAAACAT CTACATCGGC AAACAATTTC   
  
  
- GTTCAACCAA ATAAAGAGGA TACCGAAGCG AGATAACATT AAGTCCCCAG TCCAACAATA CAGTCTGTAA   
  
  
- GGGTATGGCG TGGATCTTCC CTCCTCCCTC CGAGTCCTCA GAATTTCTCA TATCTAAACC TAAGTGGACA   
  
  
- TCAGATATCG ACTACAGGTG GATTAGACCT AAAATAAGAA AGAAAGAGAG AGAAAAAAAA AGAAAAAAAC   
  
  
- CCCCCCCCCC ACAACAACTC CCTCTGTCCT ACTCCAGCTT AGAGACGTCG TATTGTTGGT GGTATCAGTG   
  
  
- TTATACCTCT AACACCTCCG TTTCAAACTC CTGCAGGCCT ACATAAAGGA AACCTTGACT CCTCCTCCAT   
  
  
- CATGAAGAGA CCATCATCAC AAGATATAGG AAACAAGGAA AGCACATAAT AGGATCGATG GAGAGTTCGT   
  
  
- TCAACTGTAC TTTAACATCT TAAGTTCTGT TATTATTGTC GACCAAAAAC CCAGAAAATC AAACGTTAGG   
  
  
- GAACATCGTA GAATCACTGA ATCACTGTAC TCTAAAACTG GGGTTATCTT TATCTTGTCT GTCGACGATT   
  
  
- TAAGTGGGTA GAGAACACAG TTTTAGCCTG AACAAAACGG AGACGAAGAG GTATTTCGTA GTTTCTCGCG   
  
  
- ACTAACTCCG TCCCTAAAAC AGTTCACGTC ACAACGTATA CAGTATAGCA CAACTACCAG TACAATAGTG   
  
  
- CCTGTATTTA AAGGGTCACA TTTCGTTTCC ACAGACAGAA GTGTATCTCA CAAGTTAAGT ATCAGTACGA   
  
  
- GAGCAGAGAC GTAGTAGGAC GCAAACGACC CCGATTTAAA CTCCCCGAAT AGTGAAACTT TAAGCCTCTT   
  
  
- AATGACACTT AAACACAATC AAGTCCGTAT TTTATGACCT ACCCTACAAC TCTACAAGAT TAGTAATAGA   
  
  
- TAACAATTTT TTGAGTCTTG ATAATATCTA AAAGACAAAA GACAAACCTA ACTCTAGGCT TCGGAAACTC   
  
  
- TAGGGGACAA AGATCGAAGG TTGGGGGGAG GGACCCAAGA AAAAGAAACC ACACTTTCAT ACTTGTTTTC   
  
  
- GACTACATTC ACACTAAGTT TGAGATCGGA ACCACTCTGA GAACCCTTCC CGAGGATCTC TGAAACGGTT   
  
  
- AACTCGATTA AACTACGGAT GATGAACGAA TGAAAAGATC GACAACAAAT TTGCTAAATA CAAGTATCAA   
  
  
- TTCCTCGCGG TATAACGTAA TGTTATAGAC GAAATCACGA AACAAAAAAC GTAAAAGAAG AAATAATGAT   
  
  
- ACCAAAAACT TCTTCCGAAT GACTGACTAT GACCAAAGAA GCGAATTCAA ACCACATGTA CGAAAAGTTA   
  
  
- GACACAACGT AAAAAGATAC TACATATTCG GCAAATAATG GATAACTACA AGTTGAAAAG TGTGATATAT   
  
  
- AGGAAATACC AAAGAAGCGA ATTCAAACCA CATGTACGAA AAGTTAGACA CAACGTAAAA AGACACTACA   
  
  
- TATTCGGCAA ATAATGGATA ACTACAAGTT GAAAAGTGTG ATATACAGGA AAATAATCAA TGACTACGGA   
  
  
- GACAGTTTGT AAGAATTAAC GTAAATCACA TAGTTGCCAG AATTTTAAAA TTCGTAACGG TTGAACTTGA   
  
  
- AGACTTCTAC AAACAGTTCT AGAAGTCAGA CGTTCAATTG ACGACGAACG AACTTGAAAA GTTGAAAATT   
  
  
- CTCTCTACAG TCTCAGATTC GTACCGAAGG ACTGTTCATG ACGTAAAATA GTGAATACTT CTGAGTTAAG   
  
  
- ACCCAAAACT GTCGAAGATA CTTGGAGATC ACGTCCATGT CGTCGAACCT GTAATTCGAC GTTCAGTTAA   
  
  
- CTGACATCTT GCTGTTCAAC AACTTCAAAG ACGACTTTCA AGTAGTGAAC AAACTACTTA TTTCATGTCG   
  
  
- TACCGTGAAG TCTACGTTTT CAGAGCGCTA GTCATCTCTA CTAAGGTATA AAGAGTAGAG TAAAGGTTCA   
  
  
- GAAACTGTTG TACATGGGTT TATTACGGTC GGTACTGTGA GTCTACAGAG AGGTTAGTAT GTCGCTTCCT   
  
  
- ATGAAGTGAG ACCTGAGTAG AGGTCGTCGT TAGCCTCGCA CGTCACAGAT ACTGATGGGT AGGCAACAGT   
  
  
- CGTGGAGAAG TTTATCTTCG GTCAAAAGAG GAGTCCCAAG GGTGAGCATG TAGAGTCTGG GGGTAGTAAG   
  
  
- AAGGCTGTTG ATACCTAGGG GTCACTCGCC GAGAAGACAC CAACTGCTAT TACGACTCGA CTCCTTGTCC   
  
  
- AAGTCACTAT ACCTCGAGGG GAACGGTGTC CTGAGTCCTG TGGTAATAAC GAGAAAATCG GTGTCTCCTT   
  
  
- CGGTACTTCC GAGGATATGA AACTCCGGTT TGGTTGACTA CCTATACCGG TTGTACCTCA ATTTCGTCCA   
  
  
- CGATATGAAG ACACGTCTTC GTTAGAGTCT CTTATTAGAT AGTTGACGTC TTTCTGATTA CCTACGTAAC   
  
  
- CCATTCTCCC ACAGACAAAA ACCAAGTGGT TAACTTTCCA ACCGGCGGAT GTACAACCTT CCCGAGTCTC   
  
  
- GTTCCGACCT CAAAAGACCT ATATGATAGA TGTTTTTCGA GTCCACGCTT GTCGGTTGCT CGAGTCTCGA   
  
  
- AGAAAGGATG TACGTGTAGG ACATAGTTTC AACGGGTATA AAGTTTAAGC GTATATACAG GAGTTTACAA   
  
  
- CGATAAGTTC TTCGAAACCC CTTACTCGGC CAATAGGTGT AGTAGCTAAA GGTCTAACGG TACCCCTGTG   
  
  
- TTAACCACGA GAACTAGGTC AGAGAGCGGG TAGCCGGACC GCCCGGGGGG GGAGCAAGCC TAGTGACCCC   
  
  
- AGCTAC

+     Unnamed\_\_4

| Site Name | Organism | Position | Strand | Matrix score. | sequence | function |
| --- | --- | --- | --- | --- | --- | --- |
| Unnamed\_\_4 | Petroselinum hortense | 2510 | - | 4 | CTCC |  |
| Unnamed\_\_4 | Petroselinum hortense | 439 | - | 4 | CTCC |  |
| Unnamed\_\_4 | Petroselinum hortense | 2672 | - | 4 | CTCC |  |
| Unnamed\_\_4 | Petroselinum hortense | 2208 | - | 4 | CTCC |  |
| Unnamed\_\_4 | Petroselinum hortense | 2465 | + | 4 | CTCC |  |
| Unnamed\_\_4 | Petroselinum hortense | 2400 | + | 4 | CTCC |  |
| Unnamed\_\_4 | Petroselinum hortense | 2272 | + | 4 | CTCC |  |
| Unnamed\_\_4 | Petroselinum hortense | 2396 | - | 4 | CTCC |  |
| Unnamed\_\_4 | Petroselinum hortense | 2154 | + | 4 | CTCC |  |
| Unnamed\_\_4 | Petroselinum hortense | 1337 | - | 4 | CTCC |  |
| Unnamed\_\_4 | Petroselinum hortense | 430 | - | 4 | CTCC |  |
| Unnamed\_\_4 | Petroselinum hortense | 1246 | + | 4 | CTCC |  |
| Unnamed\_\_4 | Petroselinum hortense | 1152 | + | 4 | CTCC |  |
| Unnamed\_\_4 | Petroselinum hortense | 2194 | + | 4 | CTCC |  |
| Unnamed\_\_4 | Petroselinum hortense | 250 | - | 4 | CTCC |  |
| Unnamed\_\_4 | Petroselinum hortense | 979 | - | 4 | CTCC |  |
| Unnamed\_\_4 | Petroselinum hortense | 752 | + | 4 | CTCC |  |
| Unnamed\_\_4 | Petroselinum hortense | 488 | - | 4 | CTCC |  |
| Unnamed\_\_4 | Petroselinum hortense | 237 | - | 4 | CTCC |  |
| Unnamed\_\_4 | Petroselinum hortense | 160 | + | 4 | CTCC |  |
| Unnamed\_\_4 | Petroselinum hortense | 485 | - | 4 | CTCC |  |
| Unnamed\_\_4 | Petroselinum hortense | 375 | - | 4 | CTCC |  |
| Unnamed\_\_4 | Petroselinum hortense | 234 | - | 4 | CTCC |  |
| Unnamed\_\_4 | Petroselinum hortense | 241 | - | 4 | CTCC |  |

>HU08G02296.1   
+ -Up\_Stream \_Len000TTATTC TTACATCTTG TTGTTGTGAT TGTGTATTAA GCTTGTTGTC TCATGGTTTG   
  
  
+ CTCTTCTCTT TGGAACCTCA TGGTGGCTAG TTCCATGTTT TTGCTTTGTA GATGTAGCCG TTTGTTAAAG   
  
  
+ CAAGTTGGTT TATTTCTCCT ATGGCTTCGC TCTATTGTAA TTCAGGGGTC AGGTTGTTAT GTCAGACATT   
  
  
+ CCCATACCGC ACCTAGAAGG GAGGAGGGAG GCTCAGGAGT CTTAAAGAGT ATAGATTTGG ATTCACCTGT   
  
  
+ AGTCTATAGC TGATGTCCAC CTAATCTGGA TTTTATTCTT TCTTTCTCTC TCTTTTTTTT TCTTTTTTTG   
  
  
+ GGGGGGGGGG TGTTGTTGAG GGAGACAGGA TGAGGTCGAA TCTCTGCAGC ATAACAACCA CCATAGTCAC   
  
  
+ AATATGGAGA TTGTGGAGGC AAAGTTTGAG GACGTCCGGA TGTATTTCCT TTGGAACTGA GGAGGAGGTA   
  
  
+ GTACTTCTCT GGTAGTAGTG TTCTATATCC TTTGTTCCTT TCGTGTATTA TCCTAGCTAC CTCTCAAGCA   
  
  
+ AGTTGACATG AAATTGTAGA ATTCAAGACA ATAATAACAG CTGGTTTTTG GGTCTTTTAG TTTGCAATCC   
  
  
+ CTTGTAGCAT CTTAGTGACT TAGTGACATG AGATTTTGAC CCCAATAGAA ATAGAACAGA CAGCTGCTAA   
  
  
+ ATTCACCCAT CTCTTGTGTC AAAATCGGAC TTGTTTTGCC TCTGCTTCTC CATAAAGCAT CAAAGAGCGC   
  
  
+ TGATTGAGGC AGGGATTTTG TCAAGTGCAG TGTTGCATAT GTCATATCGT GTTGATGGTC ATGTTATCAC   
  
  
+ GGACATAAAT TTCCCAGTGT AAAGCAAAGG TGTCTGTCTT CACATAGAGT GTTCAATTCA TAGTCATGCT   
  
  
+ CTCGTCTCTG CATCATCCTG CGTTTGCTGG GGCTAAATTT GAGGGGCTTA TCACTTTGAA ATTCGGAGAA   
  
  
+ TTACTGTGAA TTTGTGTTAG TTCAGGCATA AAATACTGGA TGGGATGTTG AGATGTTCTA ATCATTATCT   
  
  
+ ATTGTTAAAA AACTCAGAAC TATTATAGAT TTTCTGTTTT CTGTTTGGAT TGAGATCCGA AGCCTTTGAG   
  
  
+ ATCCCCTGTT TCTAGCTTCC AACCCCCCTC CCTGGGTTCT TTTTCTTTGG TGTGAAAGTA TGAACAAAAG   
  
  
+ CTGATGTAAG TGTGATTCAA ACTCTAGCCT TGGTGAGACT CTTGGGAAGG GCTCCTAGAG ACTTTGCCAA   
  
  
+ TTGAGCTAAT TTGATGCCTA CTACTTGCTT ACTTTTCTAG CTGTTGTTTA AACGATTTAT GTTCATAGTT   
  
  
+ AAGGAGCGCC ATATTGCATT ACAATATCTG CTTTAGTGCT TTGTTTTTTG CATTTTCTTC TTTATTACTA   
  
  
+ TGGTTTTTGA AGAAGGCTTA CTGACTGATA CTGGTTTCTT CGCTTAAGTT TGGTGTACAT GCTTTTCAAT   
  
  
+ CTGTGTTGCA TTTTTCTATG ATGTATAAGC CGTTTATTAC CTATTGATGT TCAACTTTTC ACACTATATA   
  
  
+ TCCTTTATGG TTTCTTCGCT TAAGTTTGGT GTACATGCTT TTCAATCTGT GTTGCATTTT TCTGTGATGT   
  
  
+ ATAAGCCGTT TATTACCTAT TGATGTTCAA CTTTTCACAC TATATGTCCT TTTATTAGTT ACTGATGCCT   
  
  
+ CTGTCAAACA TTCTTAATTG CATTTAGTGT ATCAACGGTC TTAAAATTTT AAGCATTGCC AACTTGAACT   
  
  
+ TCTGAAGATG TTTGTCAAGA TCTTCAGTCT GCAAGTTAAC TGCTGCTTGC TTGAACTTTT CAACTTTTAA   
  
  
+ GAGAGATGTC AGAGTCTAAG CATGGCTTCC TGACAAGTAC TGCATTTTAT CACTTATGAA GACTCAATTC   
  
  
+ TGGGTTTTGA CAGCTTCTAT GAACCTCTAG TGCAGGTACA GCAGCTTGGA CATTAAGCTG CAAGTCAATT   
  
  
+ GACTGTAGAA CGACAAGTTG TTGAAGTTTC TGCTGAAAGT TCATCACTTG TTTGATGAAT AAAGTACAGC   
  
  
+ ATGGCACTTC AGATGCAAAA GTCTCGCGAT CAGTAGAGAT GATTCCATAT TTCTCATCTC ATTTCCAAGT   
  
  
+ CTTTGACAAC ATGTACCCAA ATAATGCCAG CCATGACACT CAGATGTCTC TCCAATCATA CAGCGAAGGA   
  
  
+ TACTTCACTC TGGACTCATC TCCAGCAGCA ATCGGAGCGT GCAGTGTCTA TGACTACCCA TCCGTTGTCA   
  
  
+ GCACCTCTTC AAATAGAAGC CAGTTTTCTC CTCAGGGTTC CCACTCGTAC ATCTCAGACC CCCATCATTC   
  
  
+ TTCCGACAAC TATGGATCCC CAGTGAGCGG CTCTTCTGTG GTTGACGATA ATGCTGAGCT GAGGAACAGG   
  
  
+ TTCAGTGATA TGGAGCTCCC CTTGCCACAG GACTCAGGAC ACCATTATTG CTCTTTTAGC CACAGAGGAA   
  
  
+ GCCATGAAGG CTCCTATACT TTGAGGCCAA ACCAACTGAT GGATATGGCC AACATGGAGT TAAAGCAGGT   
  
  
+ GCTATACTTC TGTGCAGAAG CAATCTCAGA GAATAATCTA TCAACTGCAG AAAGACTAAT GGATGCATTG   
  
  
+ GGTAAGAGGG TGTCTGTTTT TGGTTCACCA ATTGAAAGGT TGGCCGCCTA CATGTTGGAA GGGCTCAGAG   
  
  
+ CAAGGCTGGA GTTTTCTGGA TATACTATCT ACAAAAAGCT CAGGTGCGAA CAGCCAACGA GCTCAGAGCT   
  
  
+ TCTTTCCTAC ATGCACATCC TGTATCAAAG TTGCCCATAT TTCAAATTCG CATATATGTC CTCAAATGTT   
  
  
+ GCTATTCAAG AAGCTTTGGG GAATGAGCCG GTTATCCACA TCATCGATTT CCAGATTGCC ATGGGGACAC   
  
  
+ AATTGGTGCT CTTGATCCAG TCTCTCGCCC ATCGGCCTGG CGGGCCCCCC CCTCGTTCGG ATCACTGGGG   
  
  
+ TCGATG  

- -Up\_Stream \_Len000AATAAG AATGTAGAAC AACAACACTA ACACATAATT CGAACAACAG AGTACCAAAC   
  
  
- GAGAAGAGAA ACCTTGGAGT ACCACCGATC AAGGTACAAA AACGAAACAT CTACATCGGC AAACAATTTC   
  
  
- GTTCAACCAA ATAAAGAGGA TACCGAAGCG AGATAACATT AAGTCCCCAG TCCAACAATA CAGTCTGTAA   
  
  
- GGGTATGGCG TGGATCTTCC CTCCTCCCTC CGAGTCCTCA GAATTTCTCA TATCTAAACC TAAGTGGACA   
  
  
- TCAGATATCG ACTACAGGTG GATTAGACCT AAAATAAGAA AGAAAGAGAG AGAAAAAAAA AGAAAAAAAC   
  
  
- CCCCCCCCCC ACAACAACTC CCTCTGTCCT ACTCCAGCTT AGAGACGTCG TATTGTTGGT GGTATCAGTG   
  
  
- TTATACCTCT AACACCTCCG TTTCAAACTC CTGCAGGCCT ACATAAAGGA AACCTTGACT CCTCCTCCAT   
  
  
- CATGAAGAGA CCATCATCAC AAGATATAGG AAACAAGGAA AGCACATAAT AGGATCGATG GAGAGTTCGT   
  
  
- TCAACTGTAC TTTAACATCT TAAGTTCTGT TATTATTGTC GACCAAAAAC CCAGAAAATC AAACGTTAGG   
  
  
- GAACATCGTA GAATCACTGA ATCACTGTAC TCTAAAACTG GGGTTATCTT TATCTTGTCT GTCGACGATT   
  
  
- TAAGTGGGTA GAGAACACAG TTTTAGCCTG AACAAAACGG AGACGAAGAG GTATTTCGTA GTTTCTCGCG   
  
  
- ACTAACTCCG TCCCTAAAAC AGTTCACGTC ACAACGTATA CAGTATAGCA CAACTACCAG TACAATAGTG   
  
  
- CCTGTATTTA AAGGGTCACA TTTCGTTTCC ACAGACAGAA GTGTATCTCA CAAGTTAAGT ATCAGTACGA   
  
  
- GAGCAGAGAC GTAGTAGGAC GCAAACGACC CCGATTTAAA CTCCCCGAAT AGTGAAACTT TAAGCCTCTT   
  
  
- AATGACACTT AAACACAATC AAGTCCGTAT TTTATGACCT ACCCTACAAC TCTACAAGAT TAGTAATAGA   
  
  
- TAACAATTTT TTGAGTCTTG ATAATATCTA AAAGACAAAA GACAAACCTA ACTCTAGGCT TCGGAAACTC   
  
  
- TAGGGGACAA AGATCGAAGG TTGGGGGGAG GGACCCAAGA AAAAGAAACC ACACTTTCAT ACTTGTTTTC   
  
  
- GACTACATTC ACACTAAGTT TGAGATCGGA ACCACTCTGA GAACCCTTCC CGAGGATCTC TGAAACGGTT   
  
  
- AACTCGATTA AACTACGGAT GATGAACGAA TGAAAAGATC GACAACAAAT TTGCTAAATA CAAGTATCAA   
  
  
- TTCCTCGCGG TATAACGTAA TGTTATAGAC GAAATCACGA AACAAAAAAC GTAAAAGAAG AAATAATGAT   
  
  
- ACCAAAAACT TCTTCCGAAT GACTGACTAT GACCAAAGAA GCGAATTCAA ACCACATGTA CGAAAAGTTA   
  
  
- GACACAACGT AAAAAGATAC TACATATTCG GCAAATAATG GATAACTACA AGTTGAAAAG TGTGATATAT   
  
  
- AGGAAATACC AAAGAAGCGA ATTCAAACCA CATGTACGAA AAGTTAGACA CAACGTAAAA AGACACTACA   
  
  
- TATTCGGCAA ATAATGGATA ACTACAAGTT GAAAAGTGTG ATATACAGGA AAATAATCAA TGACTACGGA   
  
  
- GACAGTTTGT AAGAATTAAC GTAAATCACA TAGTTGCCAG AATTTTAAAA TTCGTAACGG TTGAACTTGA   
  
  
- AGACTTCTAC AAACAGTTCT AGAAGTCAGA CGTTCAATTG ACGACGAACG AACTTGAAAA GTTGAAAATT   
  
  
- CTCTCTACAG TCTCAGATTC GTACCGAAGG ACTGTTCATG ACGTAAAATA GTGAATACTT CTGAGTTAAG   
  
  
- ACCCAAAACT GTCGAAGATA CTTGGAGATC ACGTCCATGT CGTCGAACCT GTAATTCGAC GTTCAGTTAA   
  
  
- CTGACATCTT GCTGTTCAAC AACTTCAAAG ACGACTTTCA AGTAGTGAAC AAACTACTTA TTTCATGTCG   
  
  
- TACCGTGAAG TCTACGTTTT CAGAGCGCTA GTCATCTCTA CTAAGGTATA AAGAGTAGAG TAAAGGTTCA   
  
  
- GAAACTGTTG TACATGGGTT TATTACGGTC GGTACTGTGA GTCTACAGAG AGGTTAGTAT GTCGCTTCCT   
  
  
- ATGAAGTGAG ACCTGAGTAG AGGTCGTCGT TAGCCTCGCA CGTCACAGAT ACTGATGGGT AGGCAACAGT   
  
  
- CGTGGAGAAG TTTATCTTCG GTCAAAAGAG GAGTCCCAAG GGTGAGCATG TAGAGTCTGG GGGTAGTAAG   
  
  
- AAGGCTGTTG ATACCTAGGG GTCACTCGCC GAGAAGACAC CAACTGCTAT TACGACTCGA CTCCTTGTCC   
  
  
- AAGTCACTAT ACCTCGAGGG GAACGGTGTC CTGAGTCCTG TGGTAATAAC GAGAAAATCG GTGTCTCCTT   
  
  
- CGGTACTTCC GAGGATATGA AACTCCGGTT TGGTTGACTA CCTATACCGG TTGTACCTCA ATTTCGTCCA   
  
  
- CGATATGAAG ACACGTCTTC GTTAGAGTCT CTTATTAGAT AGTTGACGTC TTTCTGATTA CCTACGTAAC   
  
  
- CCATTCTCCC ACAGACAAAA ACCAAGTGGT TAACTTTCCA ACCGGCGGAT GTACAACCTT CCCGAGTCTC   
  
  
- GTTCCGACCT CAAAAGACCT ATATGATAGA TGTTTTTCGA GTCCACGCTT GTCGGTTGCT CGAGTCTCGA   
  
  
- AGAAAGGATG TACGTGTAGG ACATAGTTTC AACGGGTATA AAGTTTAAGC GTATATACAG GAGTTTACAA   
  
  
- CGATAAGTTC TTCGAAACCC CTTACTCGGC CAATAGGTGT AGTAGCTAAA GGTCTAACGG TACCCCTGTG   
  
  
- TTAACCACGA GAACTAGGTC AGAGAGCGGG TAGCCGGACC GCCCGGGGGG GGAGCAAGCC TAGTGACCCC   
  
  
- AGCTAC

+     W box

| Site Name | Organism | Position | Strand | Matrix score. | sequence | function |
| --- | --- | --- | --- | --- | --- | --- |
| W box | Arabidopsis thaliana | 670 | + | 6 | TTGACC |  |

>HU08G02296.1   
+ -Up\_Stream \_Len000TTATTC TTACATCTTG TTGTTGTGAT TGTGTATTAA GCTTGTTGTC TCATGGTTTG   
  
  
+ CTCTTCTCTT TGGAACCTCA TGGTGGCTAG TTCCATGTTT TTGCTTTGTA GATGTAGCCG TTTGTTAAAG   
  
  
+ CAAGTTGGTT TATTTCTCCT ATGGCTTCGC TCTATTGTAA TTCAGGGGTC AGGTTGTTAT GTCAGACATT   
  
  
+ CCCATACCGC ACCTAGAAGG GAGGAGGGAG GCTCAGGAGT CTTAAAGAGT ATAGATTTGG ATTCACCTGT   
  
  
+ AGTCTATAGC TGATGTCCAC CTAATCTGGA TTTTATTCTT TCTTTCTCTC TCTTTTTTTT TCTTTTTTTG   
  
  
+ GGGGGGGGGG TGTTGTTGAG GGAGACAGGA TGAGGTCGAA TCTCTGCAGC ATAACAACCA CCATAGTCAC   
  
  
+ AATATGGAGA TTGTGGAGGC AAAGTTTGAG GACGTCCGGA TGTATTTCCT TTGGAACTGA GGAGGAGGTA   
  
  
+ GTACTTCTCT GGTAGTAGTG TTCTATATCC TTTGTTCCTT TCGTGTATTA TCCTAGCTAC CTCTCAAGCA   
  
  
+ AGTTGACATG AAATTGTAGA ATTCAAGACA ATAATAACAG CTGGTTTTTG GGTCTTTTAG TTTGCAATCC   
  
  
+ CTTGTAGCAT CTTAGTGACT TAGTGACATG AGATTTTGAC CCCAATAGAA ATAGAACAGA CAGCTGCTAA   
  
  
+ ATTCACCCAT CTCTTGTGTC AAAATCGGAC TTGTTTTGCC TCTGCTTCTC CATAAAGCAT CAAAGAGCGC   
  
  
+ TGATTGAGGC AGGGATTTTG TCAAGTGCAG TGTTGCATAT GTCATATCGT GTTGATGGTC ATGTTATCAC   
  
  
+ GGACATAAAT TTCCCAGTGT AAAGCAAAGG TGTCTGTCTT CACATAGAGT GTTCAATTCA TAGTCATGCT   
  
  
+ CTCGTCTCTG CATCATCCTG CGTTTGCTGG GGCTAAATTT GAGGGGCTTA TCACTTTGAA ATTCGGAGAA   
  
  
+ TTACTGTGAA TTTGTGTTAG TTCAGGCATA AAATACTGGA TGGGATGTTG AGATGTTCTA ATCATTATCT   
  
  
+ ATTGTTAAAA AACTCAGAAC TATTATAGAT TTTCTGTTTT CTGTTTGGAT TGAGATCCGA AGCCTTTGAG   
  
  
+ ATCCCCTGTT TCTAGCTTCC AACCCCCCTC CCTGGGTTCT TTTTCTTTGG TGTGAAAGTA TGAACAAAAG   
  
  
+ CTGATGTAAG TGTGATTCAA ACTCTAGCCT TGGTGAGACT CTTGGGAAGG GCTCCTAGAG ACTTTGCCAA   
  
  
+ TTGAGCTAAT TTGATGCCTA CTACTTGCTT ACTTTTCTAG CTGTTGTTTA AACGATTTAT GTTCATAGTT   
  
  
+ AAGGAGCGCC ATATTGCATT ACAATATCTG CTTTAGTGCT TTGTTTTTTG CATTTTCTTC TTTATTACTA   
  
  
+ TGGTTTTTGA AGAAGGCTTA CTGACTGATA CTGGTTTCTT CGCTTAAGTT TGGTGTACAT GCTTTTCAAT   
  
  
+ CTGTGTTGCA TTTTTCTATG ATGTATAAGC CGTTTATTAC CTATTGATGT TCAACTTTTC ACACTATATA   
  
  
+ TCCTTTATGG TTTCTTCGCT TAAGTTTGGT GTACATGCTT TTCAATCTGT GTTGCATTTT TCTGTGATGT   
  
  
+ ATAAGCCGTT TATTACCTAT TGATGTTCAA CTTTTCACAC TATATGTCCT TTTATTAGTT ACTGATGCCT   
  
  
+ CTGTCAAACA TTCTTAATTG CATTTAGTGT ATCAACGGTC TTAAAATTTT AAGCATTGCC AACTTGAACT   
  
  
+ TCTGAAGATG TTTGTCAAGA TCTTCAGTCT GCAAGTTAAC TGCTGCTTGC TTGAACTTTT CAACTTTTAA   
  
  
+ GAGAGATGTC AGAGTCTAAG CATGGCTTCC TGACAAGTAC TGCATTTTAT CACTTATGAA GACTCAATTC   
  
  
+ TGGGTTTTGA CAGCTTCTAT GAACCTCTAG TGCAGGTACA GCAGCTTGGA CATTAAGCTG CAAGTCAATT   
  
  
+ GACTGTAGAA CGACAAGTTG TTGAAGTTTC TGCTGAAAGT TCATCACTTG TTTGATGAAT AAAGTACAGC   
  
  
+ ATGGCACTTC AGATGCAAAA GTCTCGCGAT CAGTAGAGAT GATTCCATAT TTCTCATCTC ATTTCCAAGT   
  
  
+ CTTTGACAAC ATGTACCCAA ATAATGCCAG CCATGACACT CAGATGTCTC TCCAATCATA CAGCGAAGGA   
  
  
+ TACTTCACTC TGGACTCATC TCCAGCAGCA ATCGGAGCGT GCAGTGTCTA TGACTACCCA TCCGTTGTCA   
  
  
+ GCACCTCTTC AAATAGAAGC CAGTTTTCTC CTCAGGGTTC CCACTCGTAC ATCTCAGACC CCCATCATTC   
  
  
+ TTCCGACAAC TATGGATCCC CAGTGAGCGG CTCTTCTGTG GTTGACGATA ATGCTGAGCT GAGGAACAGG   
  
  
+ TTCAGTGATA TGGAGCTCCC CTTGCCACAG GACTCAGGAC ACCATTATTG CTCTTTTAGC CACAGAGGAA   
  
  
+ GCCATGAAGG CTCCTATACT TTGAGGCCAA ACCAACTGAT GGATATGGCC AACATGGAGT TAAAGCAGGT   
  
  
+ GCTATACTTC TGTGCAGAAG CAATCTCAGA GAATAATCTA TCAACTGCAG AAAGACTAAT GGATGCATTG   
  
  
+ GGTAAGAGGG TGTCTGTTTT TGGTTCACCA ATTGAAAGGT TGGCCGCCTA CATGTTGGAA GGGCTCAGAG   
  
  
+ CAAGGCTGGA GTTTTCTGGA TATACTATCT ACAAAAAGCT CAGGTGCGAA CAGCCAACGA GCTCAGAGCT   
  
  
+ TCTTTCCTAC ATGCACATCC TGTATCAAAG TTGCCCATAT TTCAAATTCG CATATATGTC CTCAAATGTT   
  
  
+ GCTATTCAAG AAGCTTTGGG GAATGAGCCG GTTATCCACA TCATCGATTT CCAGATTGCC ATGGGGACAC   
  
  
+ AATTGGTGCT CTTGATCCAG TCTCTCGCCC ATCGGCCTGG CGGGCCCCCC CCTCGTTCGG ATCACTGGGG   
  
  
+ TCGATG  

- -Up\_Stream \_Len000AATAAG AATGTAGAAC AACAACACTA ACACATAATT CGAACAACAG AGTACCAAAC   
  
  
- GAGAAGAGAA ACCTTGGAGT ACCACCGATC AAGGTACAAA AACGAAACAT CTACATCGGC AAACAATTTC   
  
  
- GTTCAACCAA ATAAAGAGGA TACCGAAGCG AGATAACATT AAGTCCCCAG TCCAACAATA CAGTCTGTAA   
  
  
- GGGTATGGCG TGGATCTTCC CTCCTCCCTC CGAGTCCTCA GAATTTCTCA TATCTAAACC TAAGTGGACA   
  
  
- TCAGATATCG ACTACAGGTG GATTAGACCT AAAATAAGAA AGAAAGAGAG AGAAAAAAAA AGAAAAAAAC   
  
  
- CCCCCCCCCC ACAACAACTC CCTCTGTCCT ACTCCAGCTT AGAGACGTCG TATTGTTGGT GGTATCAGTG   
  
  
- TTATACCTCT AACACCTCCG TTTCAAACTC CTGCAGGCCT ACATAAAGGA AACCTTGACT CCTCCTCCAT   
  
  
- CATGAAGAGA CCATCATCAC AAGATATAGG AAACAAGGAA AGCACATAAT AGGATCGATG GAGAGTTCGT   
  
  
- TCAACTGTAC TTTAACATCT TAAGTTCTGT TATTATTGTC GACCAAAAAC CCAGAAAATC AAACGTTAGG   
  
  
- GAACATCGTA GAATCACTGA ATCACTGTAC TCTAAAACTG GGGTTATCTT TATCTTGTCT GTCGACGATT   
  
  
- TAAGTGGGTA GAGAACACAG TTTTAGCCTG AACAAAACGG AGACGAAGAG GTATTTCGTA GTTTCTCGCG   
  
  
- ACTAACTCCG TCCCTAAAAC AGTTCACGTC ACAACGTATA CAGTATAGCA CAACTACCAG TACAATAGTG   
  
  
- CCTGTATTTA AAGGGTCACA TTTCGTTTCC ACAGACAGAA GTGTATCTCA CAAGTTAAGT ATCAGTACGA   
  
  
- GAGCAGAGAC GTAGTAGGAC GCAAACGACC CCGATTTAAA CTCCCCGAAT AGTGAAACTT TAAGCCTCTT   
  
  
- AATGACACTT AAACACAATC AAGTCCGTAT TTTATGACCT ACCCTACAAC TCTACAAGAT TAGTAATAGA   
  
  
- TAACAATTTT TTGAGTCTTG ATAATATCTA AAAGACAAAA GACAAACCTA ACTCTAGGCT TCGGAAACTC   
  
  
- TAGGGGACAA AGATCGAAGG TTGGGGGGAG GGACCCAAGA AAAAGAAACC ACACTTTCAT ACTTGTTTTC   
  
  
- GACTACATTC ACACTAAGTT TGAGATCGGA ACCACTCTGA GAACCCTTCC CGAGGATCTC TGAAACGGTT   
  
  
- AACTCGATTA AACTACGGAT GATGAACGAA TGAAAAGATC GACAACAAAT TTGCTAAATA CAAGTATCAA   
  
  
- TTCCTCGCGG TATAACGTAA TGTTATAGAC GAAATCACGA AACAAAAAAC GTAAAAGAAG AAATAATGAT   
  
  
- ACCAAAAACT TCTTCCGAAT GACTGACTAT GACCAAAGAA GCGAATTCAA ACCACATGTA CGAAAAGTTA   
  
  
- GACACAACGT AAAAAGATAC TACATATTCG GCAAATAATG GATAACTACA AGTTGAAAAG TGTGATATAT   
  
  
- AGGAAATACC AAAGAAGCGA ATTCAAACCA CATGTACGAA AAGTTAGACA CAACGTAAAA AGACACTACA   
  
  
- TATTCGGCAA ATAATGGATA ACTACAAGTT GAAAAGTGTG ATATACAGGA AAATAATCAA TGACTACGGA   
  
  
- GACAGTTTGT AAGAATTAAC GTAAATCACA TAGTTGCCAG AATTTTAAAA TTCGTAACGG TTGAACTTGA   
  
  
- AGACTTCTAC AAACAGTTCT AGAAGTCAGA CGTTCAATTG ACGACGAACG AACTTGAAAA GTTGAAAATT   
  
  
- CTCTCTACAG TCTCAGATTC GTACCGAAGG ACTGTTCATG ACGTAAAATA GTGAATACTT CTGAGTTAAG   
  
  
- ACCCAAAACT GTCGAAGATA CTTGGAGATC ACGTCCATGT CGTCGAACCT GTAATTCGAC GTTCAGTTAA   
  
  
- CTGACATCTT GCTGTTCAAC AACTTCAAAG ACGACTTTCA AGTAGTGAAC AAACTACTTA TTTCATGTCG   
  
  
- TACCGTGAAG TCTACGTTTT CAGAGCGCTA GTCATCTCTA CTAAGGTATA AAGAGTAGAG TAAAGGTTCA   
  
  
- GAAACTGTTG TACATGGGTT TATTACGGTC GGTACTGTGA GTCTACAGAG AGGTTAGTAT GTCGCTTCCT   
  
  
- ATGAAGTGAG ACCTGAGTAG AGGTCGTCGT TAGCCTCGCA CGTCACAGAT ACTGATGGGT AGGCAACAGT   
  
  
- CGTGGAGAAG TTTATCTTCG GTCAAAAGAG GAGTCCCAAG GGTGAGCATG TAGAGTCTGG GGGTAGTAAG   
  
  
- AAGGCTGTTG ATACCTAGGG GTCACTCGCC GAGAAGACAC CAACTGCTAT TACGACTCGA CTCCTTGTCC   
  
  
- AAGTCACTAT ACCTCGAGGG GAACGGTGTC CTGAGTCCTG TGGTAATAAC GAGAAAATCG GTGTCTCCTT   
  
  
- CGGTACTTCC GAGGATATGA AACTCCGGTT TGGTTGACTA CCTATACCGG TTGTACCTCA ATTTCGTCCA   
  
  
- CGATATGAAG ACACGTCTTC GTTAGAGTCT CTTATTAGAT AGTTGACGTC TTTCTGATTA CCTACGTAAC   
  
  
- CCATTCTCCC ACAGACAAAA ACCAAGTGGT TAACTTTCCA ACCGGCGGAT GTACAACCTT CCCGAGTCTC   
  
  
- GTTCCGACCT CAAAAGACCT ATATGATAGA TGTTTTTCGA GTCCACGCTT GTCGGTTGCT CGAGTCTCGA   
  
  
- AGAAAGGATG TACGTGTAGG ACATAGTTTC AACGGGTATA AAGTTTAAGC GTATATACAG GAGTTTACAA   
  
  
- CGATAAGTTC TTCGAAACCC CTTACTCGGC CAATAGGTGT AGTAGCTAAA GGTCTAACGG TACCCCTGTG   
  
  
- TTAACCACGA GAACTAGGTC AGAGAGCGGG TAGCCGGACC GCCCGGGGGG GGAGCAAGCC TAGTGACCCC   
  
  
- AGCTAC

+     WRE3

| Site Name | Organism | Position | Strand | Matrix score. | sequence | function |
| --- | --- | --- | --- | --- | --- | --- |
| WRE3 | Pisum sativum | 301 | + | 6 | CCACCT |  |

>HU08G02296.1   
+ -Up\_Stream \_Len000TTATTC TTACATCTTG TTGTTGTGAT TGTGTATTAA GCTTGTTGTC TCATGGTTTG   
  
  
+ CTCTTCTCTT TGGAACCTCA TGGTGGCTAG TTCCATGTTT TTGCTTTGTA GATGTAGCCG TTTGTTAAAG   
  
  
+ CAAGTTGGTT TATTTCTCCT ATGGCTTCGC TCTATTGTAA TTCAGGGGTC AGGTTGTTAT GTCAGACATT   
  
  
+ CCCATACCGC ACCTAGAAGG GAGGAGGGAG GCTCAGGAGT CTTAAAGAGT ATAGATTTGG ATTCACCTGT   
  
  
+ AGTCTATAGC TGATGTCCAC CTAATCTGGA TTTTATTCTT TCTTTCTCTC TCTTTTTTTT TCTTTTTTTG   
  
  
+ GGGGGGGGGG TGTTGTTGAG GGAGACAGGA TGAGGTCGAA TCTCTGCAGC ATAACAACCA CCATAGTCAC   
  
  
+ AATATGGAGA TTGTGGAGGC AAAGTTTGAG GACGTCCGGA TGTATTTCCT TTGGAACTGA GGAGGAGGTA   
  
  
+ GTACTTCTCT GGTAGTAGTG TTCTATATCC TTTGTTCCTT TCGTGTATTA TCCTAGCTAC CTCTCAAGCA   
  
  
+ AGTTGACATG AAATTGTAGA ATTCAAGACA ATAATAACAG CTGGTTTTTG GGTCTTTTAG TTTGCAATCC   
  
  
+ CTTGTAGCAT CTTAGTGACT TAGTGACATG AGATTTTGAC CCCAATAGAA ATAGAACAGA CAGCTGCTAA   
  
  
+ ATTCACCCAT CTCTTGTGTC AAAATCGGAC TTGTTTTGCC TCTGCTTCTC CATAAAGCAT CAAAGAGCGC   
  
  
+ TGATTGAGGC AGGGATTTTG TCAAGTGCAG TGTTGCATAT GTCATATCGT GTTGATGGTC ATGTTATCAC   
  
  
+ GGACATAAAT TTCCCAGTGT AAAGCAAAGG TGTCTGTCTT CACATAGAGT GTTCAATTCA TAGTCATGCT   
  
  
+ CTCGTCTCTG CATCATCCTG CGTTTGCTGG GGCTAAATTT GAGGGGCTTA TCACTTTGAA ATTCGGAGAA   
  
  
+ TTACTGTGAA TTTGTGTTAG TTCAGGCATA AAATACTGGA TGGGATGTTG AGATGTTCTA ATCATTATCT   
  
  
+ ATTGTTAAAA AACTCAGAAC TATTATAGAT TTTCTGTTTT CTGTTTGGAT TGAGATCCGA AGCCTTTGAG   
  
  
+ ATCCCCTGTT TCTAGCTTCC AACCCCCCTC CCTGGGTTCT TTTTCTTTGG TGTGAAAGTA TGAACAAAAG   
  
  
+ CTGATGTAAG TGTGATTCAA ACTCTAGCCT TGGTGAGACT CTTGGGAAGG GCTCCTAGAG ACTTTGCCAA   
  
  
+ TTGAGCTAAT TTGATGCCTA CTACTTGCTT ACTTTTCTAG CTGTTGTTTA AACGATTTAT GTTCATAGTT   
  
  
+ AAGGAGCGCC ATATTGCATT ACAATATCTG CTTTAGTGCT TTGTTTTTTG CATTTTCTTC TTTATTACTA   
  
  
+ TGGTTTTTGA AGAAGGCTTA CTGACTGATA CTGGTTTCTT CGCTTAAGTT TGGTGTACAT GCTTTTCAAT   
  
  
+ CTGTGTTGCA TTTTTCTATG ATGTATAAGC CGTTTATTAC CTATTGATGT TCAACTTTTC ACACTATATA   
  
  
+ TCCTTTATGG TTTCTTCGCT TAAGTTTGGT GTACATGCTT TTCAATCTGT GTTGCATTTT TCTGTGATGT   
  
  
+ ATAAGCCGTT TATTACCTAT TGATGTTCAA CTTTTCACAC TATATGTCCT TTTATTAGTT ACTGATGCCT   
  
  
+ CTGTCAAACA TTCTTAATTG CATTTAGTGT ATCAACGGTC TTAAAATTTT AAGCATTGCC AACTTGAACT   
  
  
+ TCTGAAGATG TTTGTCAAGA TCTTCAGTCT GCAAGTTAAC TGCTGCTTGC TTGAACTTTT CAACTTTTAA   
  
  
+ GAGAGATGTC AGAGTCTAAG CATGGCTTCC TGACAAGTAC TGCATTTTAT CACTTATGAA GACTCAATTC   
  
  
+ TGGGTTTTGA CAGCTTCTAT GAACCTCTAG TGCAGGTACA GCAGCTTGGA CATTAAGCTG CAAGTCAATT   
  
  
+ GACTGTAGAA CGACAAGTTG TTGAAGTTTC TGCTGAAAGT TCATCACTTG TTTGATGAAT AAAGTACAGC   
  
  
+ ATGGCACTTC AGATGCAAAA GTCTCGCGAT CAGTAGAGAT GATTCCATAT TTCTCATCTC ATTTCCAAGT   
  
  
+ CTTTGACAAC ATGTACCCAA ATAATGCCAG CCATGACACT CAGATGTCTC TCCAATCATA CAGCGAAGGA   
  
  
+ TACTTCACTC TGGACTCATC TCCAGCAGCA ATCGGAGCGT GCAGTGTCTA TGACTACCCA TCCGTTGTCA   
  
  
+ GCACCTCTTC AAATAGAAGC CAGTTTTCTC CTCAGGGTTC CCACTCGTAC ATCTCAGACC CCCATCATTC   
  
  
+ TTCCGACAAC TATGGATCCC CAGTGAGCGG CTCTTCTGTG GTTGACGATA ATGCTGAGCT GAGGAACAGG   
  
  
+ TTCAGTGATA TGGAGCTCCC CTTGCCACAG GACTCAGGAC ACCATTATTG CTCTTTTAGC CACAGAGGAA   
  
  
+ GCCATGAAGG CTCCTATACT TTGAGGCCAA ACCAACTGAT GGATATGGCC AACATGGAGT TAAAGCAGGT   
  
  
+ GCTATACTTC TGTGCAGAAG CAATCTCAGA GAATAATCTA TCAACTGCAG AAAGACTAAT GGATGCATTG   
  
  
+ GGTAAGAGGG TGTCTGTTTT TGGTTCACCA ATTGAAAGGT TGGCCGCCTA CATGTTGGAA GGGCTCAGAG   
  
  
+ CAAGGCTGGA GTTTTCTGGA TATACTATCT ACAAAAAGCT CAGGTGCGAA CAGCCAACGA GCTCAGAGCT   
  
  
+ TCTTTCCTAC ATGCACATCC TGTATCAAAG TTGCCCATAT TTCAAATTCG CATATATGTC CTCAAATGTT   
  
  
+ GCTATTCAAG AAGCTTTGGG GAATGAGCCG GTTATCCACA TCATCGATTT CCAGATTGCC ATGGGGACAC   
  
  
+ AATTGGTGCT CTTGATCCAG TCTCTCGCCC ATCGGCCTGG CGGGCCCCCC CCTCGTTCGG ATCACTGGGG   
  
  
+ TCGATG  

- -Up\_Stream \_Len000AATAAG AATGTAGAAC AACAACACTA ACACATAATT CGAACAACAG AGTACCAAAC   
  
  
- GAGAAGAGAA ACCTTGGAGT ACCACCGATC AAGGTACAAA AACGAAACAT CTACATCGGC AAACAATTTC   
  
  
- GTTCAACCAA ATAAAGAGGA TACCGAAGCG AGATAACATT AAGTCCCCAG TCCAACAATA CAGTCTGTAA   
  
  
- GGGTATGGCG TGGATCTTCC CTCCTCCCTC CGAGTCCTCA GAATTTCTCA TATCTAAACC TAAGTGGACA   
  
  
- TCAGATATCG ACTACAGGTG GATTAGACCT AAAATAAGAA AGAAAGAGAG AGAAAAAAAA AGAAAAAAAC   
  
  
- CCCCCCCCCC ACAACAACTC CCTCTGTCCT ACTCCAGCTT AGAGACGTCG TATTGTTGGT GGTATCAGTG   
  
  
- TTATACCTCT AACACCTCCG TTTCAAACTC CTGCAGGCCT ACATAAAGGA AACCTTGACT CCTCCTCCAT   
  
  
- CATGAAGAGA CCATCATCAC AAGATATAGG AAACAAGGAA AGCACATAAT AGGATCGATG GAGAGTTCGT   
  
  
- TCAACTGTAC TTTAACATCT TAAGTTCTGT TATTATTGTC GACCAAAAAC CCAGAAAATC AAACGTTAGG   
  
  
- GAACATCGTA GAATCACTGA ATCACTGTAC TCTAAAACTG GGGTTATCTT TATCTTGTCT GTCGACGATT   
  
  
- TAAGTGGGTA GAGAACACAG TTTTAGCCTG AACAAAACGG AGACGAAGAG GTATTTCGTA GTTTCTCGCG   
  
  
- ACTAACTCCG TCCCTAAAAC AGTTCACGTC ACAACGTATA CAGTATAGCA CAACTACCAG TACAATAGTG   
  
  
- CCTGTATTTA AAGGGTCACA TTTCGTTTCC ACAGACAGAA GTGTATCTCA CAAGTTAAGT ATCAGTACGA   
  
  
- GAGCAGAGAC GTAGTAGGAC GCAAACGACC CCGATTTAAA CTCCCCGAAT AGTGAAACTT TAAGCCTCTT   
  
  
- AATGACACTT AAACACAATC AAGTCCGTAT TTTATGACCT ACCCTACAAC TCTACAAGAT TAGTAATAGA   
  
  
- TAACAATTTT TTGAGTCTTG ATAATATCTA AAAGACAAAA GACAAACCTA ACTCTAGGCT TCGGAAACTC   
  
  
- TAGGGGACAA AGATCGAAGG TTGGGGGGAG GGACCCAAGA AAAAGAAACC ACACTTTCAT ACTTGTTTTC   
  
  
- GACTACATTC ACACTAAGTT TGAGATCGGA ACCACTCTGA GAACCCTTCC CGAGGATCTC TGAAACGGTT   
  
  
- AACTCGATTA AACTACGGAT GATGAACGAA TGAAAAGATC GACAACAAAT TTGCTAAATA CAAGTATCAA   
  
  
- TTCCTCGCGG TATAACGTAA TGTTATAGAC GAAATCACGA AACAAAAAAC GTAAAAGAAG AAATAATGAT   
  
  
- ACCAAAAACT TCTTCCGAAT GACTGACTAT GACCAAAGAA GCGAATTCAA ACCACATGTA CGAAAAGTTA   
  
  
- GACACAACGT AAAAAGATAC TACATATTCG GCAAATAATG GATAACTACA AGTTGAAAAG TGTGATATAT   
  
  
- AGGAAATACC AAAGAAGCGA ATTCAAACCA CATGTACGAA AAGTTAGACA CAACGTAAAA AGACACTACA   
  
  
- TATTCGGCAA ATAATGGATA ACTACAAGTT GAAAAGTGTG ATATACAGGA AAATAATCAA TGACTACGGA   
  
  
- GACAGTTTGT AAGAATTAAC GTAAATCACA TAGTTGCCAG AATTTTAAAA TTCGTAACGG TTGAACTTGA   
  
  
- AGACTTCTAC AAACAGTTCT AGAAGTCAGA CGTTCAATTG ACGACGAACG AACTTGAAAA GTTGAAAATT   
  
  
- CTCTCTACAG TCTCAGATTC GTACCGAAGG ACTGTTCATG ACGTAAAATA GTGAATACTT CTGAGTTAAG   
  
  
- ACCCAAAACT GTCGAAGATA CTTGGAGATC ACGTCCATGT CGTCGAACCT GTAATTCGAC GTTCAGTTAA   
  
  
- CTGACATCTT GCTGTTCAAC AACTTCAAAG ACGACTTTCA AGTAGTGAAC AAACTACTTA TTTCATGTCG   
  
  
- TACCGTGAAG TCTACGTTTT CAGAGCGCTA GTCATCTCTA CTAAGGTATA AAGAGTAGAG TAAAGGTTCA   
  
  
- GAAACTGTTG TACATGGGTT TATTACGGTC GGTACTGTGA GTCTACAGAG AGGTTAGTAT GTCGCTTCCT   
  
  
- ATGAAGTGAG ACCTGAGTAG AGGTCGTCGT TAGCCTCGCA CGTCACAGAT ACTGATGGGT AGGCAACAGT   
  
  
- CGTGGAGAAG TTTATCTTCG GTCAAAAGAG GAGTCCCAAG GGTGAGCATG TAGAGTCTGG GGGTAGTAAG   
  
  
- AAGGCTGTTG ATACCTAGGG GTCACTCGCC GAGAAGACAC CAACTGCTAT TACGACTCGA CTCCTTGTCC   
  
  
- AAGTCACTAT ACCTCGAGGG GAACGGTGTC CTGAGTCCTG TGGTAATAAC GAGAAAATCG GTGTCTCCTT   
  
  
- CGGTACTTCC GAGGATATGA AACTCCGGTT TGGTTGACTA CCTATACCGG TTGTACCTCA ATTTCGTCCA   
  
  
- CGATATGAAG ACACGTCTTC GTTAGAGTCT CTTATTAGAT AGTTGACGTC TTTCTGATTA CCTACGTAAC   
  
  
- CCATTCTCCC ACAGACAAAA ACCAAGTGGT TAACTTTCCA ACCGGCGGAT GTACAACCTT CCCGAGTCTC   
  
  
- GTTCCGACCT CAAAAGACCT ATATGATAGA TGTTTTTCGA GTCCACGCTT GTCGGTTGCT CGAGTCTCGA   
  
  
- AGAAAGGATG TACGTGTAGG ACATAGTTTC AACGGGTATA AAGTTTAAGC GTATATACAG GAGTTTACAA   
  
  
- CGATAAGTTC TTCGAAACCC CTTACTCGGC CAATAGGTGT AGTAGCTAAA GGTCTAACGG TACCCCTGTG   
  
  
- TTAACCACGA GAACTAGGTC AGAGAGCGGG TAGCCGGACC GCCCGGGGGG GGAGCAAGCC TAGTGACCCC   
  
  
- AGCTAC

+     as-1

| Site Name | Organism | Position | Strand | Matrix score. | sequence | function |
| --- | --- | --- | --- | --- | --- | --- |
| as-1 | Arabidopsis thaliana | 2357 | + | 5 | TGACG |  |

>HU08G02296.1   
+ -Up\_Stream \_Len000TTATTC TTACATCTTG TTGTTGTGAT TGTGTATTAA GCTTGTTGTC TCATGGTTTG   
  
  
+ CTCTTCTCTT TGGAACCTCA TGGTGGCTAG TTCCATGTTT TTGCTTTGTA GATGTAGCCG TTTGTTAAAG   
  
  
+ CAAGTTGGTT TATTTCTCCT ATGGCTTCGC TCTATTGTAA TTCAGGGGTC AGGTTGTTAT GTCAGACATT   
  
  
+ CCCATACCGC ACCTAGAAGG GAGGAGGGAG GCTCAGGAGT CTTAAAGAGT ATAGATTTGG ATTCACCTGT   
  
  
+ AGTCTATAGC TGATGTCCAC CTAATCTGGA TTTTATTCTT TCTTTCTCTC TCTTTTTTTT TCTTTTTTTG   
  
  
+ GGGGGGGGGG TGTTGTTGAG GGAGACAGGA TGAGGTCGAA TCTCTGCAGC ATAACAACCA CCATAGTCAC   
  
  
+ AATATGGAGA TTGTGGAGGC AAAGTTTGAG GACGTCCGGA TGTATTTCCT TTGGAACTGA GGAGGAGGTA   
  
  
+ GTACTTCTCT GGTAGTAGTG TTCTATATCC TTTGTTCCTT TCGTGTATTA TCCTAGCTAC CTCTCAAGCA   
  
  
+ AGTTGACATG AAATTGTAGA ATTCAAGACA ATAATAACAG CTGGTTTTTG GGTCTTTTAG TTTGCAATCC   
  
  
+ CTTGTAGCAT CTTAGTGACT TAGTGACATG AGATTTTGAC CCCAATAGAA ATAGAACAGA CAGCTGCTAA   
  
  
+ ATTCACCCAT CTCTTGTGTC AAAATCGGAC TTGTTTTGCC TCTGCTTCTC CATAAAGCAT CAAAGAGCGC   
  
  
+ TGATTGAGGC AGGGATTTTG TCAAGTGCAG TGTTGCATAT GTCATATCGT GTTGATGGTC ATGTTATCAC   
  
  
+ GGACATAAAT TTCCCAGTGT AAAGCAAAGG TGTCTGTCTT CACATAGAGT GTTCAATTCA TAGTCATGCT   
  
  
+ CTCGTCTCTG CATCATCCTG CGTTTGCTGG GGCTAAATTT GAGGGGCTTA TCACTTTGAA ATTCGGAGAA   
  
  
+ TTACTGTGAA TTTGTGTTAG TTCAGGCATA AAATACTGGA TGGGATGTTG AGATGTTCTA ATCATTATCT   
  
  
+ ATTGTTAAAA AACTCAGAAC TATTATAGAT TTTCTGTTTT CTGTTTGGAT TGAGATCCGA AGCCTTTGAG   
  
  
+ ATCCCCTGTT TCTAGCTTCC AACCCCCCTC CCTGGGTTCT TTTTCTTTGG TGTGAAAGTA TGAACAAAAG   
  
  
+ CTGATGTAAG TGTGATTCAA ACTCTAGCCT TGGTGAGACT CTTGGGAAGG GCTCCTAGAG ACTTTGCCAA   
  
  
+ TTGAGCTAAT TTGATGCCTA CTACTTGCTT ACTTTTCTAG CTGTTGTTTA AACGATTTAT GTTCATAGTT   
  
  
+ AAGGAGCGCC ATATTGCATT ACAATATCTG CTTTAGTGCT TTGTTTTTTG CATTTTCTTC TTTATTACTA   
  
  
+ TGGTTTTTGA AGAAGGCTTA CTGACTGATA CTGGTTTCTT CGCTTAAGTT TGGTGTACAT GCTTTTCAAT   
  
  
+ CTGTGTTGCA TTTTTCTATG ATGTATAAGC CGTTTATTAC CTATTGATGT TCAACTTTTC ACACTATATA   
  
  
+ TCCTTTATGG TTTCTTCGCT TAAGTTTGGT GTACATGCTT TTCAATCTGT GTTGCATTTT TCTGTGATGT   
  
  
+ ATAAGCCGTT TATTACCTAT TGATGTTCAA CTTTTCACAC TATATGTCCT TTTATTAGTT ACTGATGCCT   
  
  
+ CTGTCAAACA TTCTTAATTG CATTTAGTGT ATCAACGGTC TTAAAATTTT AAGCATTGCC AACTTGAACT   
  
  
+ TCTGAAGATG TTTGTCAAGA TCTTCAGTCT GCAAGTTAAC TGCTGCTTGC TTGAACTTTT CAACTTTTAA   
  
  
+ GAGAGATGTC AGAGTCTAAG CATGGCTTCC TGACAAGTAC TGCATTTTAT CACTTATGAA GACTCAATTC   
  
  
+ TGGGTTTTGA CAGCTTCTAT GAACCTCTAG TGCAGGTACA GCAGCTTGGA CATTAAGCTG CAAGTCAATT   
  
  
+ GACTGTAGAA CGACAAGTTG TTGAAGTTTC TGCTGAAAGT TCATCACTTG TTTGATGAAT AAAGTACAGC   
  
  
+ ATGGCACTTC AGATGCAAAA GTCTCGCGAT CAGTAGAGAT GATTCCATAT TTCTCATCTC ATTTCCAAGT   
  
  
+ CTTTGACAAC ATGTACCCAA ATAATGCCAG CCATGACACT CAGATGTCTC TCCAATCATA CAGCGAAGGA   
  
  
+ TACTTCACTC TGGACTCATC TCCAGCAGCA ATCGGAGCGT GCAGTGTCTA TGACTACCCA TCCGTTGTCA   
  
  
+ GCACCTCTTC AAATAGAAGC CAGTTTTCTC CTCAGGGTTC CCACTCGTAC ATCTCAGACC CCCATCATTC   
  
  
+ TTCCGACAAC TATGGATCCC CAGTGAGCGG CTCTTCTGTG GTTGACGATA ATGCTGAGCT GAGGAACAGG   
  
  
+ TTCAGTGATA TGGAGCTCCC CTTGCCACAG GACTCAGGAC ACCATTATTG CTCTTTTAGC CACAGAGGAA   
  
  
+ GCCATGAAGG CTCCTATACT TTGAGGCCAA ACCAACTGAT GGATATGGCC AACATGGAGT TAAAGCAGGT   
  
  
+ GCTATACTTC TGTGCAGAAG CAATCTCAGA GAATAATCTA TCAACTGCAG AAAGACTAAT GGATGCATTG   
  
  
+ GGTAAGAGGG TGTCTGTTTT TGGTTCACCA ATTGAAAGGT TGGCCGCCTA CATGTTGGAA GGGCTCAGAG   
  
  
+ CAAGGCTGGA GTTTTCTGGA TATACTATCT ACAAAAAGCT CAGGTGCGAA CAGCCAACGA GCTCAGAGCT   
  
  
+ TCTTTCCTAC ATGCACATCC TGTATCAAAG TTGCCCATAT TTCAAATTCG CATATATGTC CTCAAATGTT   
  
  
+ GCTATTCAAG AAGCTTTGGG GAATGAGCCG GTTATCCACA TCATCGATTT CCAGATTGCC ATGGGGACAC   
  
  
+ AATTGGTGCT CTTGATCCAG TCTCTCGCCC ATCGGCCTGG CGGGCCCCCC CCTCGTTCGG ATCACTGGGG   
  
  
+ TCGATG  

- -Up\_Stream \_Len000AATAAG AATGTAGAAC AACAACACTA ACACATAATT CGAACAACAG AGTACCAAAC   
  
  
- GAGAAGAGAA ACCTTGGAGT ACCACCGATC AAGGTACAAA AACGAAACAT CTACATCGGC AAACAATTTC   
  
  
- GTTCAACCAA ATAAAGAGGA TACCGAAGCG AGATAACATT AAGTCCCCAG TCCAACAATA CAGTCTGTAA   
  
  
- GGGTATGGCG TGGATCTTCC CTCCTCCCTC CGAGTCCTCA GAATTTCTCA TATCTAAACC TAAGTGGACA   
  
  
- TCAGATATCG ACTACAGGTG GATTAGACCT AAAATAAGAA AGAAAGAGAG AGAAAAAAAA AGAAAAAAAC   
  
  
- CCCCCCCCCC ACAACAACTC CCTCTGTCCT ACTCCAGCTT AGAGACGTCG TATTGTTGGT GGTATCAGTG   
  
  
- TTATACCTCT AACACCTCCG TTTCAAACTC CTGCAGGCCT ACATAAAGGA AACCTTGACT CCTCCTCCAT   
  
  
- CATGAAGAGA CCATCATCAC AAGATATAGG AAACAAGGAA AGCACATAAT AGGATCGATG GAGAGTTCGT   
  
  
- TCAACTGTAC TTTAACATCT TAAGTTCTGT TATTATTGTC GACCAAAAAC CCAGAAAATC AAACGTTAGG   
  
  
- GAACATCGTA GAATCACTGA ATCACTGTAC TCTAAAACTG GGGTTATCTT TATCTTGTCT GTCGACGATT   
  
  
- TAAGTGGGTA GAGAACACAG TTTTAGCCTG AACAAAACGG AGACGAAGAG GTATTTCGTA GTTTCTCGCG   
  
  
- ACTAACTCCG TCCCTAAAAC AGTTCACGTC ACAACGTATA CAGTATAGCA CAACTACCAG TACAATAGTG   
  
  
- CCTGTATTTA AAGGGTCACA TTTCGTTTCC ACAGACAGAA GTGTATCTCA CAAGTTAAGT ATCAGTACGA   
  
  
- GAGCAGAGAC GTAGTAGGAC GCAAACGACC CCGATTTAAA CTCCCCGAAT AGTGAAACTT TAAGCCTCTT   
  
  
- AATGACACTT AAACACAATC AAGTCCGTAT TTTATGACCT ACCCTACAAC TCTACAAGAT TAGTAATAGA   
  
  
- TAACAATTTT TTGAGTCTTG ATAATATCTA AAAGACAAAA GACAAACCTA ACTCTAGGCT TCGGAAACTC   
  
  
- TAGGGGACAA AGATCGAAGG TTGGGGGGAG GGACCCAAGA AAAAGAAACC ACACTTTCAT ACTTGTTTTC   
  
  
- GACTACATTC ACACTAAGTT TGAGATCGGA ACCACTCTGA GAACCCTTCC CGAGGATCTC TGAAACGGTT   
  
  
- AACTCGATTA AACTACGGAT GATGAACGAA TGAAAAGATC GACAACAAAT TTGCTAAATA CAAGTATCAA   
  
  
- TTCCTCGCGG TATAACGTAA TGTTATAGAC GAAATCACGA AACAAAAAAC GTAAAAGAAG AAATAATGAT   
  
  
- ACCAAAAACT TCTTCCGAAT GACTGACTAT GACCAAAGAA GCGAATTCAA ACCACATGTA CGAAAAGTTA   
  
  
- GACACAACGT AAAAAGATAC TACATATTCG GCAAATAATG GATAACTACA AGTTGAAAAG TGTGATATAT   
  
  
- AGGAAATACC AAAGAAGCGA ATTCAAACCA CATGTACGAA AAGTTAGACA CAACGTAAAA AGACACTACA   
  
  
- TATTCGGCAA ATAATGGATA ACTACAAGTT GAAAAGTGTG ATATACAGGA AAATAATCAA TGACTACGGA   
  
  
- GACAGTTTGT AAGAATTAAC GTAAATCACA TAGTTGCCAG AATTTTAAAA TTCGTAACGG TTGAACTTGA   
  
  
- AGACTTCTAC AAACAGTTCT AGAAGTCAGA CGTTCAATTG ACGACGAACG AACTTGAAAA GTTGAAAATT   
  
  
- CTCTCTACAG TCTCAGATTC GTACCGAAGG ACTGTTCATG ACGTAAAATA GTGAATACTT CTGAGTTAAG   
  
  
- ACCCAAAACT GTCGAAGATA CTTGGAGATC ACGTCCATGT CGTCGAACCT GTAATTCGAC GTTCAGTTAA   
  
  
- CTGACATCTT GCTGTTCAAC AACTTCAAAG ACGACTTTCA AGTAGTGAAC AAACTACTTA TTTCATGTCG   
  
  
- TACCGTGAAG TCTACGTTTT CAGAGCGCTA GTCATCTCTA CTAAGGTATA AAGAGTAGAG TAAAGGTTCA   
  
  
- GAAACTGTTG TACATGGGTT TATTACGGTC GGTACTGTGA GTCTACAGAG AGGTTAGTAT GTCGCTTCCT   
  
  
- ATGAAGTGAG ACCTGAGTAG AGGTCGTCGT TAGCCTCGCA CGTCACAGAT ACTGATGGGT AGGCAACAGT   
  
  
- CGTGGAGAAG TTTATCTTCG GTCAAAAGAG GAGTCCCAAG GGTGAGCATG TAGAGTCTGG GGGTAGTAAG   
  
  
- AAGGCTGTTG ATACCTAGGG GTCACTCGCC GAGAAGACAC CAACTGCTAT TACGACTCGA CTCCTTGTCC   
  
  
- AAGTCACTAT ACCTCGAGGG GAACGGTGTC CTGAGTCCTG TGGTAATAAC GAGAAAATCG GTGTCTCCTT   
  
  
- CGGTACTTCC GAGGATATGA AACTCCGGTT TGGTTGACTA CCTATACCGG TTGTACCTCA ATTTCGTCCA   
  
  
- CGATATGAAG ACACGTCTTC GTTAGAGTCT CTTATTAGAT AGTTGACGTC TTTCTGATTA CCTACGTAAC   
  
  
- CCATTCTCCC ACAGACAAAA ACCAAGTGGT TAACTTTCCA ACCGGCGGAT GTACAACCTT CCCGAGTCTC   
  
  
- GTTCCGACCT CAAAAGACCT ATATGATAGA TGTTTTTCGA GTCCACGCTT GTCGGTTGCT CGAGTCTCGA   
  
  
- AGAAAGGATG TACGTGTAGG ACATAGTTTC AACGGGTATA AAGTTTAAGC GTATATACAG GAGTTTACAA   
  
  
- CGATAAGTTC TTCGAAACCC CTTACTCGGC CAATAGGTGT AGTAGCTAAA GGTCTAACGG TACCCCTGTG   
  
  
- TTAACCACGA GAACTAGGTC AGAGAGCGGG TAGCCGGACC GCCCGGGGGG GGAGCAAGCC TAGTGACCCC   
  
  
- AGCTAC

+     box S

| Site Name | Organism | Position | Strand | Matrix score. | sequence | function |
| --- | --- | --- | --- | --- | --- | --- |
| box S | Arabidopsis thaliana | 96 | - | 7 | AGCCACC |  |

>HU08G02296.1   
+ -Up\_Stream \_Len000TTATTC TTACATCTTG TTGTTGTGAT TGTGTATTAA GCTTGTTGTC TCATGGTTTG   
  
  
+ CTCTTCTCTT TGGAACCTCA TGGTGGCTAG TTCCATGTTT TTGCTTTGTA GATGTAGCCG TTTGTTAAAG   
  
  
+ CAAGTTGGTT TATTTCTCCT ATGGCTTCGC TCTATTGTAA TTCAGGGGTC AGGTTGTTAT GTCAGACATT   
  
  
+ CCCATACCGC ACCTAGAAGG GAGGAGGGAG GCTCAGGAGT CTTAAAGAGT ATAGATTTGG ATTCACCTGT   
  
  
+ AGTCTATAGC TGATGTCCAC CTAATCTGGA TTTTATTCTT TCTTTCTCTC TCTTTTTTTT TCTTTTTTTG   
  
  
+ GGGGGGGGGG TGTTGTTGAG GGAGACAGGA TGAGGTCGAA TCTCTGCAGC ATAACAACCA CCATAGTCAC   
  
  
+ AATATGGAGA TTGTGGAGGC AAAGTTTGAG GACGTCCGGA TGTATTTCCT TTGGAACTGA GGAGGAGGTA   
  
  
+ GTACTTCTCT GGTAGTAGTG TTCTATATCC TTTGTTCCTT TCGTGTATTA TCCTAGCTAC CTCTCAAGCA   
  
  
+ AGTTGACATG AAATTGTAGA ATTCAAGACA ATAATAACAG CTGGTTTTTG GGTCTTTTAG TTTGCAATCC   
  
  
+ CTTGTAGCAT CTTAGTGACT TAGTGACATG AGATTTTGAC CCCAATAGAA ATAGAACAGA CAGCTGCTAA   
  
  
+ ATTCACCCAT CTCTTGTGTC AAAATCGGAC TTGTTTTGCC TCTGCTTCTC CATAAAGCAT CAAAGAGCGC   
  
  
+ TGATTGAGGC AGGGATTTTG TCAAGTGCAG TGTTGCATAT GTCATATCGT GTTGATGGTC ATGTTATCAC   
  
  
+ GGACATAAAT TTCCCAGTGT AAAGCAAAGG TGTCTGTCTT CACATAGAGT GTTCAATTCA TAGTCATGCT   
  
  
+ CTCGTCTCTG CATCATCCTG CGTTTGCTGG GGCTAAATTT GAGGGGCTTA TCACTTTGAA ATTCGGAGAA   
  
  
+ TTACTGTGAA TTTGTGTTAG TTCAGGCATA AAATACTGGA TGGGATGTTG AGATGTTCTA ATCATTATCT   
  
  
+ ATTGTTAAAA AACTCAGAAC TATTATAGAT TTTCTGTTTT CTGTTTGGAT TGAGATCCGA AGCCTTTGAG   
  
  
+ ATCCCCTGTT TCTAGCTTCC AACCCCCCTC CCTGGGTTCT TTTTCTTTGG TGTGAAAGTA TGAACAAAAG   
  
  
+ CTGATGTAAG TGTGATTCAA ACTCTAGCCT TGGTGAGACT CTTGGGAAGG GCTCCTAGAG ACTTTGCCAA   
  
  
+ TTGAGCTAAT TTGATGCCTA CTACTTGCTT ACTTTTCTAG CTGTTGTTTA AACGATTTAT GTTCATAGTT   
  
  
+ AAGGAGCGCC ATATTGCATT ACAATATCTG CTTTAGTGCT TTGTTTTTTG CATTTTCTTC TTTATTACTA   
  
  
+ TGGTTTTTGA AGAAGGCTTA CTGACTGATA CTGGTTTCTT CGCTTAAGTT TGGTGTACAT GCTTTTCAAT   
  
  
+ CTGTGTTGCA TTTTTCTATG ATGTATAAGC CGTTTATTAC CTATTGATGT TCAACTTTTC ACACTATATA   
  
  
+ TCCTTTATGG TTTCTTCGCT TAAGTTTGGT GTACATGCTT TTCAATCTGT GTTGCATTTT TCTGTGATGT   
  
  
+ ATAAGCCGTT TATTACCTAT TGATGTTCAA CTTTTCACAC TATATGTCCT TTTATTAGTT ACTGATGCCT   
  
  
+ CTGTCAAACA TTCTTAATTG CATTTAGTGT ATCAACGGTC TTAAAATTTT AAGCATTGCC AACTTGAACT   
  
  
+ TCTGAAGATG TTTGTCAAGA TCTTCAGTCT GCAAGTTAAC TGCTGCTTGC TTGAACTTTT CAACTTTTAA   
  
  
+ GAGAGATGTC AGAGTCTAAG CATGGCTTCC TGACAAGTAC TGCATTTTAT CACTTATGAA GACTCAATTC   
  
  
+ TGGGTTTTGA CAGCTTCTAT GAACCTCTAG TGCAGGTACA GCAGCTTGGA CATTAAGCTG CAAGTCAATT   
  
  
+ GACTGTAGAA CGACAAGTTG TTGAAGTTTC TGCTGAAAGT TCATCACTTG TTTGATGAAT AAAGTACAGC   
  
  
+ ATGGCACTTC AGATGCAAAA GTCTCGCGAT CAGTAGAGAT GATTCCATAT TTCTCATCTC ATTTCCAAGT   
  
  
+ CTTTGACAAC ATGTACCCAA ATAATGCCAG CCATGACACT CAGATGTCTC TCCAATCATA CAGCGAAGGA   
  
  
+ TACTTCACTC TGGACTCATC TCCAGCAGCA ATCGGAGCGT GCAGTGTCTA TGACTACCCA TCCGTTGTCA   
  
  
+ GCACCTCTTC AAATAGAAGC CAGTTTTCTC CTCAGGGTTC CCACTCGTAC ATCTCAGACC CCCATCATTC   
  
  
+ TTCCGACAAC TATGGATCCC CAGTGAGCGG CTCTTCTGTG GTTGACGATA ATGCTGAGCT GAGGAACAGG   
  
  
+ TTCAGTGATA TGGAGCTCCC CTTGCCACAG GACTCAGGAC ACCATTATTG CTCTTTTAGC CACAGAGGAA   
  
  
+ GCCATGAAGG CTCCTATACT TTGAGGCCAA ACCAACTGAT GGATATGGCC AACATGGAGT TAAAGCAGGT   
  
  
+ GCTATACTTC TGTGCAGAAG CAATCTCAGA GAATAATCTA TCAACTGCAG AAAGACTAAT GGATGCATTG   
  
  
+ GGTAAGAGGG TGTCTGTTTT TGGTTCACCA ATTGAAAGGT TGGCCGCCTA CATGTTGGAA GGGCTCAGAG   
  
  
+ CAAGGCTGGA GTTTTCTGGA TATACTATCT ACAAAAAGCT CAGGTGCGAA CAGCCAACGA GCTCAGAGCT   
  
  
+ TCTTTCCTAC ATGCACATCC TGTATCAAAG TTGCCCATAT TTCAAATTCG CATATATGTC CTCAAATGTT   
  
  
+ GCTATTCAAG AAGCTTTGGG GAATGAGCCG GTTATCCACA TCATCGATTT CCAGATTGCC ATGGGGACAC   
  
  
+ AATTGGTGCT CTTGATCCAG TCTCTCGCCC ATCGGCCTGG CGGGCCCCCC CCTCGTTCGG ATCACTGGGG   
  
  
+ TCGATG  

- -Up\_Stream \_Len000AATAAG AATGTAGAAC AACAACACTA ACACATAATT CGAACAACAG AGTACCAAAC   
  
  
- GAGAAGAGAA ACCTTGGAGT ACCACCGATC AAGGTACAAA AACGAAACAT CTACATCGGC AAACAATTTC   
  
  
- GTTCAACCAA ATAAAGAGGA TACCGAAGCG AGATAACATT AAGTCCCCAG TCCAACAATA CAGTCTGTAA   
  
  
- GGGTATGGCG TGGATCTTCC CTCCTCCCTC CGAGTCCTCA GAATTTCTCA TATCTAAACC TAAGTGGACA   
  
  
- TCAGATATCG ACTACAGGTG GATTAGACCT AAAATAAGAA AGAAAGAGAG AGAAAAAAAA AGAAAAAAAC   
  
  
- CCCCCCCCCC ACAACAACTC CCTCTGTCCT ACTCCAGCTT AGAGACGTCG TATTGTTGGT GGTATCAGTG   
  
  
- TTATACCTCT AACACCTCCG TTTCAAACTC CTGCAGGCCT ACATAAAGGA AACCTTGACT CCTCCTCCAT   
  
  
- CATGAAGAGA CCATCATCAC AAGATATAGG AAACAAGGAA AGCACATAAT AGGATCGATG GAGAGTTCGT   
  
  
- TCAACTGTAC TTTAACATCT TAAGTTCTGT TATTATTGTC GACCAAAAAC CCAGAAAATC AAACGTTAGG   
  
  
- GAACATCGTA GAATCACTGA ATCACTGTAC TCTAAAACTG GGGTTATCTT TATCTTGTCT GTCGACGATT   
  
  
- TAAGTGGGTA GAGAACACAG TTTTAGCCTG AACAAAACGG AGACGAAGAG GTATTTCGTA GTTTCTCGCG   
  
  
- ACTAACTCCG TCCCTAAAAC AGTTCACGTC ACAACGTATA CAGTATAGCA CAACTACCAG TACAATAGTG   
  
  
- CCTGTATTTA AAGGGTCACA TTTCGTTTCC ACAGACAGAA GTGTATCTCA CAAGTTAAGT ATCAGTACGA   
  
  
- GAGCAGAGAC GTAGTAGGAC GCAAACGACC CCGATTTAAA CTCCCCGAAT AGTGAAACTT TAAGCCTCTT   
  
  
- AATGACACTT AAACACAATC AAGTCCGTAT TTTATGACCT ACCCTACAAC TCTACAAGAT TAGTAATAGA   
  
  
- TAACAATTTT TTGAGTCTTG ATAATATCTA AAAGACAAAA GACAAACCTA ACTCTAGGCT TCGGAAACTC   
  
  
- TAGGGGACAA AGATCGAAGG TTGGGGGGAG GGACCCAAGA AAAAGAAACC ACACTTTCAT ACTTGTTTTC   
  
  
- GACTACATTC ACACTAAGTT TGAGATCGGA ACCACTCTGA GAACCCTTCC CGAGGATCTC TGAAACGGTT   
  
  
- AACTCGATTA AACTACGGAT GATGAACGAA TGAAAAGATC GACAACAAAT TTGCTAAATA CAAGTATCAA   
  
  
- TTCCTCGCGG TATAACGTAA TGTTATAGAC GAAATCACGA AACAAAAAAC GTAAAAGAAG AAATAATGAT   
  
  
- ACCAAAAACT TCTTCCGAAT GACTGACTAT GACCAAAGAA GCGAATTCAA ACCACATGTA CGAAAAGTTA   
  
  
- GACACAACGT AAAAAGATAC TACATATTCG GCAAATAATG GATAACTACA AGTTGAAAAG TGTGATATAT   
  
  
- AGGAAATACC AAAGAAGCGA ATTCAAACCA CATGTACGAA AAGTTAGACA CAACGTAAAA AGACACTACA   
  
  
- TATTCGGCAA ATAATGGATA ACTACAAGTT GAAAAGTGTG ATATACAGGA AAATAATCAA TGACTACGGA   
  
  
- GACAGTTTGT AAGAATTAAC GTAAATCACA TAGTTGCCAG AATTTTAAAA TTCGTAACGG TTGAACTTGA   
  
  
- AGACTTCTAC AAACAGTTCT AGAAGTCAGA CGTTCAATTG ACGACGAACG AACTTGAAAA GTTGAAAATT   
  
  
- CTCTCTACAG TCTCAGATTC GTACCGAAGG ACTGTTCATG ACGTAAAATA GTGAATACTT CTGAGTTAAG   
  
  
- ACCCAAAACT GTCGAAGATA CTTGGAGATC ACGTCCATGT CGTCGAACCT GTAATTCGAC GTTCAGTTAA   
  
  
- CTGACATCTT GCTGTTCAAC AACTTCAAAG ACGACTTTCA AGTAGTGAAC AAACTACTTA TTTCATGTCG   
  
  
- TACCGTGAAG TCTACGTTTT CAGAGCGCTA GTCATCTCTA CTAAGGTATA AAGAGTAGAG TAAAGGTTCA   
  
  
- GAAACTGTTG TACATGGGTT TATTACGGTC GGTACTGTGA GTCTACAGAG AGGTTAGTAT GTCGCTTCCT   
  
  
- ATGAAGTGAG ACCTGAGTAG AGGTCGTCGT TAGCCTCGCA CGTCACAGAT ACTGATGGGT AGGCAACAGT   
  
  
- CGTGGAGAAG TTTATCTTCG GTCAAAAGAG GAGTCCCAAG GGTGAGCATG TAGAGTCTGG GGGTAGTAAG   
  
  
- AAGGCTGTTG ATACCTAGGG GTCACTCGCC GAGAAGACAC CAACTGCTAT TACGACTCGA CTCCTTGTCC   
  
  
- AAGTCACTAT ACCTCGAGGG GAACGGTGTC CTGAGTCCTG TGGTAATAAC GAGAAAATCG GTGTCTCCTT   
  
  
- CGGTACTTCC GAGGATATGA AACTCCGGTT TGGTTGACTA CCTATACCGG TTGTACCTCA ATTTCGTCCA   
  
  
- CGATATGAAG ACACGTCTTC GTTAGAGTCT CTTATTAGAT AGTTGACGTC TTTCTGATTA CCTACGTAAC   
  
  
- CCATTCTCCC ACAGACAAAA ACCAAGTGGT TAACTTTCCA ACCGGCGGAT GTACAACCTT CCCGAGTCTC   
  
  
- GTTCCGACCT CAAAAGACCT ATATGATAGA TGTTTTTCGA GTCCACGCTT GTCGGTTGCT CGAGTCTCGA   
  
  
- AGAAAGGATG TACGTGTAGG ACATAGTTTC AACGGGTATA AAGTTTAAGC GTATATACAG GAGTTTACAA   
  
  
- CGATAAGTTC TTCGAAACCC CTTACTCGGC CAATAGGTGT AGTAGCTAAA GGTCTAACGG TACCCCTGTG   
  
  
- TTAACCACGA GAACTAGGTC AGAGAGCGGG TAGCCGGACC GCCCGGGGGG GGAGCAAGCC TAGTGACCCC   
  
  
- AGCTAC
