# Supplementary material for: Genome-Wide Identification and Expression Pattern of the GRAS Gene Family in Pitaya (Selenicereus undatus L.)
Source: Biology (Basel). 2022 Dec 21;12(1):11. doi: 10.3390/biology12010011 (PMC9854919; doi:10.3390/biology12010011)
Supplement: Supplementary file 1 [file biology-12-00011-s001.zip › Supplementary file S5/HU10G00709.1_plantcare.html]

Content-Type: text/html; charset=ISO-8859-1


PlantCARE


Webmaster Firefox specific output  
To save the result:
click on the frame with the right mouse button and save the source code as a text file with extension .html  
REFERENCE:PlantCARE: a database of plant cis-acting regulatory elements and a portal to tools for in silico analysis of promoter sequences.  
Lescot, M., Déhais, P., Moreau, Y., De Moor, B., Rouzé ,P.,and Rombauts, S.  
Nucleic Acids Res., Database issue(2002), 30(1):325-327.   


---

>HU10G00709.1   
+ -Up\_Stream \_Len000AACCCT TTAATTTTAT CAATGTGGGA CAACACTCAT CTTCATACTC CAACAAGCTA   
  
  
+ TGATACATTC GATTATTGCC TAGTATTTCG TTTAGTAAGA TATTAGTGCC AAACTTTGTA ATAAAAATGA   
  
  
+ AACTTACTAA AAAAAATCTC GTTTTCTGTA GAGGGAAAAT TTGAAGACTT AGTGTCACAT GATGACCTAA   
  
  
+ TGGTACTTGT AGGGTCAAGA TTGAGATTGA GGGCATACTG ACGACCTGTA TAATGGTCAA GTAGGATCAA   
  
  
+ AGTCGAGAAA ACTGTCTCTG CTAACTTTCA TAATCTAACC CTTAACTCAC TCTGACCCTC AATTTAATCA   
  
  
+ GGCCTAATTC AAACTATTAA TGGTTATAAC TCTCTTTCCT TCTTTTGAGA TTTTTTTTTT CTACATGCGG   
  
  
+ TTGAAAATAA TTTTTTTAAA AAATATATAT CACTAACCAT TCATTATCAT AGTTTTAGAG TTCATATTTA   
  
  
+ TGTGGTCCTT GTTGAGTCTA ATTTAATGCT TTAGATGGCG TGAAGCACCA AATTAGTGGT AACCATAATA   
  
  
+ ATAAACTATC AAGTGTTAAG TCGAAGAAAA CGGACACTGC TCAACCAAAT CTAGTAACAT AGTGTATGAC   
  
  
+ ATGATAACTA AGTGTGCCAT GTAGTAATCA ATTCTTTCTT TTTTTTTTTT TGGTTGAGAT CACATAAATT   
  
  
+ ACGTTATCAA GGGTAGTACG ATATAATGGA TGAGGAGAAA TTAAATGTGA GATTTAGTTG ATGAATCGAA   
  
  
+ TACGTTAGTC TGAGCCATTT GGTCAAAACT GCATTGGCTA ATCAATAGAA ATTATAGAAA ATACATAACA   
  
  
+ TAATGGGATG CAATTTTCTA ATTGGGGAAA CCATGTCAAT GCAGTGATTT TGGACCATTT AGGCCTTATT   
  
  
+ CTTTTCAATT TTTTAATTGA TTATTTAGAC TCATATGAAC TTATTGTTGA CAGATTGAGC TAAAAACACT   
  
  
+ GATGTTTCAT ACATATGTAT GAATTGCACG TACGAAGGCT CTGCTCATTT CGAGAAAGAA AATTAACAGT   
  
  
+ TAGGAGCAGT ATAATTTAAC AACAAGCGAT AATTATTAGT ATGAAGTGGT ACGAATCTAT TTTAAAGGAG   
  
  
+ TACTATGAAG TACAAACAAT AACAAATAAT AAAGAGCAGT ACATTAACAT ATCTTACTAC CTAAATAAAT   
  
  
+ CGATTCTAAG AGTAAAAAAA TGTTGAAATT AACCCCATTA TAGTTTTACT TTAACAAGAA GTTTTTCTTC   
  
  
+ TTTTACCATT TACCAAGTAA GAATGATTCA GGCCGTATAG CCAAGGAAAA ATAGAAAACC ACAAAACTAA   
  
  
+ CCATGGCCCA AAAAACAACC ACCAAGAGGG GCACTAATGT GACTAACCGA CGGTTAACAT TATCATCTGT   
  
  
+ TTGATACTTG TTCATCTCTC ACTATTCGGG TAATAGTGTG AAATTACTGA TGTACCCTCT AAACTCGTCG   
  
  
+ TTTTAACTGT GTAAGAATCA GATTAACAGT CACGGACACA TGAGTAACAA ATCAATTTCG GACATGAACG   
  
  
+ AACGAACTGT GAATCCCCCC CAAAAAAAAA ATTTTTTTAA AAAAAGTTGC ATCCTTATCT TTCTGAACCA   
  
  
+ GGCCAGCTGT CTCATTCCTT GGTCCTCGCG CCCCATCACG AGCGCTGCTC ATTATTCACC CTCTCTCTCT   
  
  
+ CTCCTCTCTG ATCCACACCA CAAACACAAG GTTTGCACAG CTGCAGAGCA GTCGTACTAC TATCAGTAGT   
  
  
+ GAAAAACCAA GCAGAAATCC CCAATCCCTC TTCTCCTTCT TCCCAGATTC TTCCTCAATT CCAAGCCCAT   
  
  
+ ATCAGAAATT TAGACAACAA CAACCAAAAA AAAAAATTAA TACTGCTTGA GAAAAACAAA TATGATATGA   
  
  
+ AATTCCTCGA AAAAGGCCTG CCATAACAAA TTCCTCGTGA AAAAGCAGCT GAGAGAAGAA GAAGAAGAAA   
  
  
+ AAAATCCCGT CACAATCTCA ATCTCGATCC CAAATTGTGA AAGAGAAAGA AAAGATGAAG AGAGAACACC   
  
  
+ CCTTTTCGAA CCCTGACCAG AAACCCCCGC CGGAGATGTC GCCGGCGCCG GGGAAGTCCA AGATGTGGGA   
  
  
+ TGATGAGATG GCCCAGAACG ACGCTAAAAT GGATGAGCTG CTTGCTGTTG TGGGGTACAA GGTGAGGTCG   
  
  
+ TCGGACATGG CGGAAATTGC CCAGAAGCTT GAGCAGCTTG AAGAAGTCAT GGGTAGTGTT CAAGAAGACG   
  
  
+ GGTTATCTTA CTTGGCTTCC GAAACTGTTC ATTACAATCC TGCAGATCTG TCTACTTGGC TTGAATCTAT   
  
  
+ GCTTTCTGAG TTTAACCCTA ACCCTAATTT TGACCCTTCT CCATCGTCAA TCTCACCCAT CATCGATCCA   
  
  
+ GCTCCTCCAC TTCCTCGAAC CTCGTCGACG GTCAAATTCG AGCCCGACCC GTTTTCCGAT TCGGATCTGA   
  
  
+ AAGCAATCCC AGGTAAGGCT ATCTTGACCC CACCAAGTTC TAGCAATACT TCAAATTTGC GTGAGGCTAA   
  
  
+ GAGGTTGAAA CCCTCAAATT ACACAACCGC CCCAACGCCC ACCCCACCTC CAAAACTGCC CAATTCGGCG   
  
  
+ TCTCCACCAT CGGGCACGAC GGCGTCGAGG CCGGTGGTGC TGGTAGACTC ACAAGAAAAC GGCATCAGAT   
  
  
+ TGGTGCATGC ACTTATGGCC TGTGCCGAAG CAATTCAACT AGAAAACATG GGATTAGCTG AGGCTTTGGT   
  
  
+ TAAACAGATT AGGTATTTAG CAGCATCTCA AGCTGGACCT ATGAGGAAAG TAGCAACTTG TTTTGCAGAA   
  
  
+ GCTCTTACTT GTCGGATCTA CAAGCTATGC CCATCTGTAC CCTACGATGA ATCAGTCTCA GATGAGTTGC   
  
  
+ AGATGCACTT TTATGAGACT TGCCCATATC TTAAATTCGC CCATTTCACG GCAAATCAAG CAATTTTAGA   
  
  
+ AGCATTCAAT GGGAAGAAGA AAGTTCATGT GATTGATTTC AGCATGAAGC AAGGCATGCA ATGGCCGGCT   
  
  
+ TTGATGCAAG CCCTGGCTCT TCGACCGGAG GGTCCACCGC TTTTCCGGTT AACCGGGATT GGACCGCCCG   
  
  
+ CTCCGGACAA CTCGGACCGG CTGCAAGAGG TGGGTTGGAA GCTTGCCCAG TTCGCGGATT CGATCCGGAT   
  
  
+ TAAATTTCAG TATCGTGGGT TTGTGGCAAA CAGTTTGACC GATCTAGAAT CTTCCATGCT AGATCTTGAA   
  
  
+ CCGGACACTG AGGTGGTGGC GGTCAACTCG GTTTTCGAGC TCCACCGGCT GTTGGCTAAA CCCGGGGCGA   
  
  
+ TCGAGAAAGT GCTGGGGTTC ATGAGGGCCG TGAACCCGGT CATTGTGACG ATGGTCGAGC AGGAAGCGAA   
  
  
+ CCACAACGGA CCGGTTTTCT TGGACCGGTT CAATGAGTCG TTGCATTATT ACTCCACCTT GTTTGATTCC   
  
  
+ CTTGAGATTT GTGTTGATAA TGTAGATAAG AAGATGTCGG AGGCTTACTT GGGCCAGCAG ATCTGCAACA   
  
  
+ TGGTGGCTTG TGAAGGGTCT GACCGAGTCG AGAGGCACGA GACCCTGGCT CAGTGGCGAG CCCGGTTCGC   
  
  
+ ATCTGCCGGG TTCGACCCAG TTCATCTGGG TTCGAATGCG TTTAAGCAGG CGAGTATGTT GTTGGAGTTC   
  
  
+ TTTGCTGGTG GTGAAGGGTA CGGCGTGGAG GAGCGAGAAG GGTGTCTCAT GTTGGGATGG CATAGTAGGC   
  
  
+ CGCTTATCAC CACCTCGGCT TGGCAGCTCG CCAAGAACCC GGCTGTGAAT CGGCGATG  

- -Up\_Stream \_Len000TTGGGA AATTAAAATA GTTACACCCT GTTGTGAGTA GAAGTATGAG GTTGTTCGAT   
  
  
- ACTATGTAAG CTAATAACGG ATCATAAAGC AAATCATTCT ATAATCACGG TTTGAAACAT TATTTTTACT   
  
  
- TTGAATGATT TTTTTTAGAG CAAAAGACAT CTCCCTTTTA AACTTCTGAA TCACAGTGTA CTACTGGATT   
  
  
- ACCATGAACA TCCCAGTTCT AACTCTAACT CCCGTATGAC TGCTGGACAT ATTACCAGTT CATCCTAGTT   
  
  
- TCAGCTCTTT TGACAGAGAC GATTGAAAGT ATTAGATTGG GAATTGAGTG AGACTGGGAG TTAAATTAGT   
  
  
- CCGGATTAAG TTTGATAATT ACCAATATTG AGAGAAAGGA AGAAAACTCT AAAAAAAAAA GATGTACGCC   
  
  
- AACTTTTATT AAAAAAATTT TTTATATATA GTGATTGGTA AGTAATAGTA TCAAAATCTC AAGTATAAAT   
  
  
- ACACCAGGAA CAACTCAGAT TAAATTACGA AATCTACCGC ACTTCGTGGT TTAATCACCA TTGGTATTAT   
  
  
- TATTTGATAG TTCACAATTC AGCTTCTTTT GCCTGTGACG AGTTGGTTTA GATCATTGTA TCACATACTG   
  
  
- TACTATTGAT TCACACGGTA CATCATTAGT TAAGAAAGAA AAAAAAAAAA ACCAACTCTA GTGTATTTAA   
  
  
- TGCAATAGTT CCCATCATGC TATATTACCT ACTCCTCTTT AATTTACACT CTAAATCAAC TACTTAGCTT   
  
  
- ATGCAATCAG ACTCGGTAAA CCAGTTTTGA CGTAACCGAT TAGTTATCTT TAATATCTTT TATGTATTGT   
  
  
- ATTACCCTAC GTTAAAAGAT TAACCCCTTT GGTACAGTTA CGTCACTAAA ACCTGGTAAA TCCGGAATAA   
  
  
- GAAAAGTTAA AAAATTAACT AATAAATCTG AGTATACTTG AATAACAACT GTCTAACTCG ATTTTTGTGA   
  
  
- CTACAAAGTA TGTATACATA CTTAACGTGC ATGCTTCCGA GACGAGTAAA GCTCTTTCTT TTAATTGTCA   
  
  
- ATCCTCGTCA TATTAAATTG TTGTTCGCTA TTAATAATCA TACTTCACCA TGCTTAGATA AAATTTCCTC   
  
  
- ATGATACTTC ATGTTTGTTA TTGTTTATTA TTTCTCGTCA TGTAATTGTA TAGAATGATG GATTTATTTA   
  
  
- GCTAAGATTC TCATTTTTTT ACAACTTTAA TTGGGGTAAT ATCAAAATGA AATTGTTCTT CAAAAAGAAG   
  
  
- AAAATGGTAA ATGGTTCATT CTTACTAAGT CCGGCATATC GGTTCCTTTT TATCTTTTGG TGTTTTGATT   
  
  
- GGTACCGGGT TTTTTGTTGG TGGTTCTCCC CGTGATTACA CTGATTGGCT GCCAATTGTA ATAGTAGACA   
  
  
- AACTATGAAC AAGTAGAGAG TGATAAGCCC ATTATCACAC TTTAATGACT ACATGGGAGA TTTGAGCAGC   
  
  
- AAAATTGACA CATTCTTAGT CTAATTGTCA GTGCCTGTGT ACTCATTGTT TAGTTAAAGC CTGTACTTGC   
  
  
- TTGCTTGACA CTTAGGGGGG GTTTTTTTTT TAAAAAAATT TTTTTCAACG TAGGAATAGA AAGACTTGGT   
  
  
- CCGGTCGACA GAGTAAGGAA CCAGGAGCGC GGGGTAGTGC TCGCGACGAG TAATAAGTGG GAGAGAGAGA   
  
  
- GAGGAGAGAC TAGGTGTGGT GTTTGTGTTC CAAACGTGTC GACGTCTCGT CAGCATGATG ATAGTCATCA   
  
  
- CTTTTTGGTT CGTCTTTAGG GGTTAGGGAG AAGAGGAAGA AGGGTCTAAG AAGGAGTTAA GGTTCGGGTA   
  
  
- TAGTCTTTAA ATCTGTTGTT GTTGGTTTTT TTTTTTAATT ATGACGAACT CTTTTTGTTT ATACTATACT   
  
  
- TTAAGGAGCT TTTTCCGGAC GGTATTGTTT AAGGAGCACT TTTTCGTCGA CTCTCTTCTT CTTCTTCTTT   
  
  
- TTTTAGGGCA GTGTTAGAGT TAGAGCTAGG GTTTAACACT TTCTCTTTCT TTTCTACTTC TCTCTTGTGG   
  
  
- GGAAAAGCTT GGGACTGGTC TTTGGGGGCG GCCTCTACAG CGGCCGCGGC CCCTTCAGGT TCTACACCCT   
  
  
- ACTACTCTAC CGGGTCTTGC TGCGATTTTA CCTACTCGAC GAACGACAAC ACCCCATGTT CCACTCCAGC   
  
  
- AGCCTGTACC GCCTTTAACG GGTCTTCGAA CTCGTCGAAC TTCTTCAGTA CCCATCACAA GTTCTTCTGC   
  
  
- CCAATAGAAT GAACCGAAGG CTTTGACAAG TAATGTTAGG ACGTCTAGAC AGATGAACCG AACTTAGATA   
  
  
- CGAAAGACTC AAATTGGGAT TGGGATTAAA ACTGGGAAGA GGTAGCAGTT AGAGTGGGTA GTAGCTAGGT   
  
  
- CGAGGAGGTG AAGGAGCTTG GAGCAGCTGC CAGTTTAAGC TCGGGCTGGG CAAAAGGCTA AGCCTAGACT   
  
  
- TTCGTTAGGG TCCATTCCGA TAGAACTGGG GTGGTTCAAG ATCGTTATGA AGTTTAAACG CACTCCGATT   
  
  
- CTCCAACTTT GGGAGTTTAA TGTGTTGGCG GGGTTGCGGG TGGGGTGGAG GTTTTGACGG GTTAAGCCGC   
  
  
- AGAGGTGGTA GCCCGTGCTG CCGCAGCTCC GGCCACCACG ACCATCTGAG TGTTCTTTTG CCGTAGTCTA   
  
  
- ACCACGTACG TGAATACCGG ACACGGCTTC GTTAAGTTGA TCTTTTGTAC CCTAATCGAC TCCGAAACCA   
  
  
- ATTTGTCTAA TCCATAAATC GTCGTAGAGT TCGACCTGGA TACTCCTTTC ATCGTTGAAC AAAACGTCTT   
  
  
- CGAGAATGAA CAGCCTAGAT GTTCGATACG GGTAGACATG GGATGCTACT TAGTCAGAGT CTACTCAACG   
  
  
- TCTACGTGAA AATACTCTGA ACGGGTATAG AATTTAAGCG GGTAAAGTGC CGTTTAGTTC GTTAAAATCT   
  
  
- TCGTAAGTTA CCCTTCTTCT TTCAAGTACA CTAACTAAAG TCGTACTTCG TTCCGTACGT TACCGGCCGA   
  
  
- AACTACGTTC GGGACCGAGA AGCTGGCCTC CCAGGTGGCG AAAAGGCCAA TTGGCCCTAA CCTGGCGGGC   
  
  
- GAGGCCTGTT GAGCCTGGCC GACGTTCTCC ACCCAACCTT CGAACGGGTC AAGCGCCTAA GCTAGGCCTA   
  
  
- ATTTAAAGTC ATAGCACCCA AACACCGTTT GTCAAACTGG CTAGATCTTA GAAGGTACGA TCTAGAACTT   
  
  
- GGCCTGTGAC TCCACCACCG CCAGTTGAGC CAAAAGCTCG AGGTGGCCGA CAACCGATTT GGGCCCCGCT   
  
  
- AGCTCTTTCA CGACCCCAAG TACTCCCGGC ACTTGGGCCA GTAACACTGC TACCAGCTCG TCCTTCGCTT   
  
  
- GGTGTTGCCT GGCCAAAAGA ACCTGGCCAA GTTACTCAGC AACGTAATAA TGAGGTGGAA CAAACTAAGG   
  
  
- GAACTCTAAA CACAACTATT ACATCTATTC TTCTACAGCC TCCGAATGAA CCCGGTCGTC TAGACGTTGT   
  
  
- ACCACCGAAC ACTTCCCAGA CTGGCTCAGC TCTCCGTGCT CTGGGACCGA GTCACCGCTC GGGCCAAGCG   
  
  
- TAGACGGCCC AAGCTGGGTC AAGTAGACCC AAGCTTACGC AAATTCGTCC GCTCATACAA CAACCTCAAG   
  
  
- AAACGACCAC CACTTCCCAT GCCGCACCTC CTCGCTCTTC CCACAGAGTA CAACCCTACC GTATCATCCG   
  
  
- GCGAATAGTG GTGGAGCCGA ACCGTCGAGC GGTTCTTGGG CCGACACTTA GCCGCTAC

  
  
Motifs Found  

+   

| Site Name | Organism | Position | Strand | Matrix score. | sequence | function |
| --- | --- | --- | --- | --- | --- | --- |
|  | organism | 1683 | + | 4 | motif\_sequence | short\_function |
|  | organism | 1534 | - | 4 | motif\_sequence | short\_function |
|  | organism | 3575 | + | 4 | motif\_sequence | short\_function |
|  | organism | 3495 | + | 4 | motif\_sequence | short\_function |
|  | organism | 2873 | - | 4 | motif\_sequence | short\_function |
|  | organism | 2839 | + | 4 | motif\_sequence | short\_function |
|  | organism | 2684 | + | 4 | motif\_sequence | short\_function |
|  | organism | 1023 | + | 4 | motif\_sequence | short\_function |
|  | organism | 1454 | + | 4 | motif\_sequence | short\_function |
|  | organism | 3491 | - | 4 | motif\_sequence | short\_function |
|  | organism | 3464 | - | 4 | motif\_sequence | short\_function |
|  | organism | 3456 | - | 4 | motif\_sequence | short\_function |
|  | organism | 2820 | + | 4 | motif\_sequence | short\_function |
|  | organism | 2286 | - | 4 | motif\_sequence | short\_function |
|  | organism | 1163 | - | 4 | motif\_sequence | short\_function |
|  | organism | 2269 | + | 4 | motif\_sequence | short\_function |
|  | organism | 2177 | - | 4 | motif\_sequence | short\_function |
|  | organism | 2021 | - | 4 | motif\_sequence | short\_function |
|  | organism | 1782 | + | 4 | motif\_sequence | short\_function |
|  | organism | 300 | + | 4 | motif\_sequence | short\_function |
|  | organism | 3203 | + | 4 | motif\_sequence | short\_function |
|  | organism | 3031 | + | 4 | motif\_sequence | short\_function |
|  | organism | 53 | + | 4 | motif\_sequence | short\_function |
|  | organism | 172 | - | 4 | motif\_sequence | short\_function |
|  | organism | 2066 | - | 4 | motif\_sequence | short\_function |
|  | organism | 1727 | - | 4 | motif\_sequence | short\_function |
|  | organism | 1719 | - | 4 | motif\_sequence | short\_function |

>HU10G00709.1   
+ -Up\_Stream \_Len000AACCCT TTAATTTTAT CAATGTGGGA CAACACTCAT CTTCATACTC CAACAAGCTA   
  
  
+ TGATACATTC GATTATTGCC TAGTATTTCG TTTAGTAAGA TATTAGTGCC AAACTTTGTA ATAAAAATGA   
  
  
+ AACTTACTAA AAAAAATCTC GTTTTCTGTA GAGGGAAAAT TTGAAGACTT AGTGTCACAT GATGACCTAA   
  
  
+ TGGTACTTGT AGGGTCAAGA TTGAGATTGA GGGCATACTG ACGACCTGTA TAATGGTCAA GTAGGATCAA   
  
  
+ AGTCGAGAAA ACTGTCTCTG CTAACTTTCA TAATCTAACC CTTAACTCAC TCTGACCCTC AATTTAATCA   
  
  
+ GGCCTAATTC AAACTATTAA TGGTTATAAC TCTCTTTCCT TCTTTTGAGA TTTTTTTTTT CTACATGCGG   
  
  
+ TTGAAAATAA TTTTTTTAAA AAATATATAT CACTAACCAT TCATTATCAT AGTTTTAGAG TTCATATTTA   
  
  
+ TGTGGTCCTT GTTGAGTCTA ATTTAATGCT TTAGATGGCG TGAAGCACCA AATTAGTGGT AACCATAATA   
  
  
+ ATAAACTATC AAGTGTTAAG TCGAAGAAAA CGGACACTGC TCAACCAAAT CTAGTAACAT AGTGTATGAC   
  
  
+ ATGATAACTA AGTGTGCCAT GTAGTAATCA ATTCTTTCTT TTTTTTTTTT TGGTTGAGAT CACATAAATT   
  
  
+ ACGTTATCAA GGGTAGTACG ATATAATGGA TGAGGAGAAA TTAAATGTGA GATTTAGTTG ATGAATCGAA   
  
  
+ TACGTTAGTC TGAGCCATTT GGTCAAAACT GCATTGGCTA ATCAATAGAA ATTATAGAAA ATACATAACA   
  
  
+ TAATGGGATG CAATTTTCTA ATTGGGGAAA CCATGTCAAT GCAGTGATTT TGGACCATTT AGGCCTTATT   
  
  
+ CTTTTCAATT TTTTAATTGA TTATTTAGAC TCATATGAAC TTATTGTTGA CAGATTGAGC TAAAAACACT   
  
  
+ GATGTTTCAT ACATATGTAT GAATTGCACG TACGAAGGCT CTGCTCATTT CGAGAAAGAA AATTAACAGT   
  
  
+ TAGGAGCAGT ATAATTTAAC AACAAGCGAT AATTATTAGT ATGAAGTGGT ACGAATCTAT TTTAAAGGAG   
  
  
+ TACTATGAAG TACAAACAAT AACAAATAAT AAAGAGCAGT ACATTAACAT ATCTTACTAC CTAAATAAAT   
  
  
+ CGATTCTAAG AGTAAAAAAA TGTTGAAATT AACCCCATTA TAGTTTTACT TTAACAAGAA GTTTTTCTTC   
  
  
+ TTTTACCATT TACCAAGTAA GAATGATTCA GGCCGTATAG CCAAGGAAAA ATAGAAAACC ACAAAACTAA   
  
  
+ CCATGGCCCA AAAAACAACC ACCAAGAGGG GCACTAATGT GACTAACCGA CGGTTAACAT TATCATCTGT   
  
  
+ TTGATACTTG TTCATCTCTC ACTATTCGGG TAATAGTGTG AAATTACTGA TGTACCCTCT AAACTCGTCG   
  
  
+ TTTTAACTGT GTAAGAATCA GATTAACAGT CACGGACACA TGAGTAACAA ATCAATTTCG GACATGAACG   
  
  
+ AACGAACTGT GAATCCCCCC CAAAAAAAAA ATTTTTTTAA AAAAAGTTGC ATCCTTATCT TTCTGAACCA   
  
  
+ GGCCAGCTGT CTCATTCCTT GGTCCTCGCG CCCCATCACG AGCGCTGCTC ATTATTCACC CTCTCTCTCT   
  
  
+ CTCCTCTCTG ATCCACACCA CAAACACAAG GTTTGCACAG CTGCAGAGCA GTCGTACTAC TATCAGTAGT   
  
  
+ GAAAAACCAA GCAGAAATCC CCAATCCCTC TTCTCCTTCT TCCCAGATTC TTCCTCAATT CCAAGCCCAT   
  
  
+ ATCAGAAATT TAGACAACAA CAACCAAAAA AAAAAATTAA TACTGCTTGA GAAAAACAAA TATGATATGA   
  
  
+ AATTCCTCGA AAAAGGCCTG CCATAACAAA TTCCTCGTGA AAAAGCAGCT GAGAGAAGAA GAAGAAGAAA   
  
  
+ AAAATCCCGT CACAATCTCA ATCTCGATCC CAAATTGTGA AAGAGAAAGA AAAGATGAAG AGAGAACACC   
  
  
+ CCTTTTCGAA CCCTGACCAG AAACCCCCGC CGGAGATGTC GCCGGCGCCG GGGAAGTCCA AGATGTGGGA   
  
  
+ TGATGAGATG GCCCAGAACG ACGCTAAAAT GGATGAGCTG CTTGCTGTTG TGGGGTACAA GGTGAGGTCG   
  
  
+ TCGGACATGG CGGAAATTGC CCAGAAGCTT GAGCAGCTTG AAGAAGTCAT GGGTAGTGTT CAAGAAGACG   
  
  
+ GGTTATCTTA CTTGGCTTCC GAAACTGTTC ATTACAATCC TGCAGATCTG TCTACTTGGC TTGAATCTAT   
  
  
+ GCTTTCTGAG TTTAACCCTA ACCCTAATTT TGACCCTTCT CCATCGTCAA TCTCACCCAT CATCGATCCA   
  
  
+ GCTCCTCCAC TTCCTCGAAC CTCGTCGACG GTCAAATTCG AGCCCGACCC GTTTTCCGAT TCGGATCTGA   
  
  
+ AAGCAATCCC AGGTAAGGCT ATCTTGACCC CACCAAGTTC TAGCAATACT TCAAATTTGC GTGAGGCTAA   
  
  
+ GAGGTTGAAA CCCTCAAATT ACACAACCGC CCCAACGCCC ACCCCACCTC CAAAACTGCC CAATTCGGCG   
  
  
+ TCTCCACCAT CGGGCACGAC GGCGTCGAGG CCGGTGGTGC TGGTAGACTC ACAAGAAAAC GGCATCAGAT   
  
  
+ TGGTGCATGC ACTTATGGCC TGTGCCGAAG CAATTCAACT AGAAAACATG GGATTAGCTG AGGCTTTGGT   
  
  
+ TAAACAGATT AGGTATTTAG CAGCATCTCA AGCTGGACCT ATGAGGAAAG TAGCAACTTG TTTTGCAGAA   
  
  
+ GCTCTTACTT GTCGGATCTA CAAGCTATGC CCATCTGTAC CCTACGATGA ATCAGTCTCA GATGAGTTGC   
  
  
+ AGATGCACTT TTATGAGACT TGCCCATATC TTAAATTCGC CCATTTCACG GCAAATCAAG CAATTTTAGA   
  
  
+ AGCATTCAAT GGGAAGAAGA AAGTTCATGT GATTGATTTC AGCATGAAGC AAGGCATGCA ATGGCCGGCT   
  
  
+ TTGATGCAAG CCCTGGCTCT TCGACCGGAG GGTCCACCGC TTTTCCGGTT AACCGGGATT GGACCGCCCG   
  
  
+ CTCCGGACAA CTCGGACCGG CTGCAAGAGG TGGGTTGGAA GCTTGCCCAG TTCGCGGATT CGATCCGGAT   
  
  
+ TAAATTTCAG TATCGTGGGT TTGTGGCAAA CAGTTTGACC GATCTAGAAT CTTCCATGCT AGATCTTGAA   
  
  
+ CCGGACACTG AGGTGGTGGC GGTCAACTCG GTTTTCGAGC TCCACCGGCT GTTGGCTAAA CCCGGGGCGA   
  
  
+ TCGAGAAAGT GCTGGGGTTC ATGAGGGCCG TGAACCCGGT CATTGTGACG ATGGTCGAGC AGGAAGCGAA   
  
  
+ CCACAACGGA CCGGTTTTCT TGGACCGGTT CAATGAGTCG TTGCATTATT ACTCCACCTT GTTTGATTCC   
  
  
+ CTTGAGATTT GTGTTGATAA TGTAGATAAG AAGATGTCGG AGGCTTACTT GGGCCAGCAG ATCTGCAACA   
  
  
+ TGGTGGCTTG TGAAGGGTCT GACCGAGTCG AGAGGCACGA GACCCTGGCT CAGTGGCGAG CCCGGTTCGC   
  
  
+ ATCTGCCGGG TTCGACCCAG TTCATCTGGG TTCGAATGCG TTTAAGCAGG CGAGTATGTT GTTGGAGTTC   
  
  
+ TTTGCTGGTG GTGAAGGGTA CGGCGTGGAG GAGCGAGAAG GGTGTCTCAT GTTGGGATGG CATAGTAGGC   
  
  
+ CGCTTATCAC CACCTCGGCT TGGCAGCTCG CCAAGAACCC GGCTGTGAAT CGGCGATG  

- -Up\_Stream \_Len000TTGGGA AATTAAAATA GTTACACCCT GTTGTGAGTA GAAGTATGAG GTTGTTCGAT   
  
  
- ACTATGTAAG CTAATAACGG ATCATAAAGC AAATCATTCT ATAATCACGG TTTGAAACAT TATTTTTACT   
  
  
- TTGAATGATT TTTTTTAGAG CAAAAGACAT CTCCCTTTTA AACTTCTGAA TCACAGTGTA CTACTGGATT   
  
  
- ACCATGAACA TCCCAGTTCT AACTCTAACT CCCGTATGAC TGCTGGACAT ATTACCAGTT CATCCTAGTT   
  
  
- TCAGCTCTTT TGACAGAGAC GATTGAAAGT ATTAGATTGG GAATTGAGTG AGACTGGGAG TTAAATTAGT   
  
  
- CCGGATTAAG TTTGATAATT ACCAATATTG AGAGAAAGGA AGAAAACTCT AAAAAAAAAA GATGTACGCC   
  
  
- AACTTTTATT AAAAAAATTT TTTATATATA GTGATTGGTA AGTAATAGTA TCAAAATCTC AAGTATAAAT   
  
  
- ACACCAGGAA CAACTCAGAT TAAATTACGA AATCTACCGC ACTTCGTGGT TTAATCACCA TTGGTATTAT   
  
  
- TATTTGATAG TTCACAATTC AGCTTCTTTT GCCTGTGACG AGTTGGTTTA GATCATTGTA TCACATACTG   
  
  
- TACTATTGAT TCACACGGTA CATCATTAGT TAAGAAAGAA AAAAAAAAAA ACCAACTCTA GTGTATTTAA   
  
  
- TGCAATAGTT CCCATCATGC TATATTACCT ACTCCTCTTT AATTTACACT CTAAATCAAC TACTTAGCTT   
  
  
- ATGCAATCAG ACTCGGTAAA CCAGTTTTGA CGTAACCGAT TAGTTATCTT TAATATCTTT TATGTATTGT   
  
  
- ATTACCCTAC GTTAAAAGAT TAACCCCTTT GGTACAGTTA CGTCACTAAA ACCTGGTAAA TCCGGAATAA   
  
  
- GAAAAGTTAA AAAATTAACT AATAAATCTG AGTATACTTG AATAACAACT GTCTAACTCG ATTTTTGTGA   
  
  
- CTACAAAGTA TGTATACATA CTTAACGTGC ATGCTTCCGA GACGAGTAAA GCTCTTTCTT TTAATTGTCA   
  
  
- ATCCTCGTCA TATTAAATTG TTGTTCGCTA TTAATAATCA TACTTCACCA TGCTTAGATA AAATTTCCTC   
  
  
- ATGATACTTC ATGTTTGTTA TTGTTTATTA TTTCTCGTCA TGTAATTGTA TAGAATGATG GATTTATTTA   
  
  
- GCTAAGATTC TCATTTTTTT ACAACTTTAA TTGGGGTAAT ATCAAAATGA AATTGTTCTT CAAAAAGAAG   
  
  
- AAAATGGTAA ATGGTTCATT CTTACTAAGT CCGGCATATC GGTTCCTTTT TATCTTTTGG TGTTTTGATT   
  
  
- GGTACCGGGT TTTTTGTTGG TGGTTCTCCC CGTGATTACA CTGATTGGCT GCCAATTGTA ATAGTAGACA   
  
  
- AACTATGAAC AAGTAGAGAG TGATAAGCCC ATTATCACAC TTTAATGACT ACATGGGAGA TTTGAGCAGC   
  
  
- AAAATTGACA CATTCTTAGT CTAATTGTCA GTGCCTGTGT ACTCATTGTT TAGTTAAAGC CTGTACTTGC   
  
  
- TTGCTTGACA CTTAGGGGGG GTTTTTTTTT TAAAAAAATT TTTTTCAACG TAGGAATAGA AAGACTTGGT   
  
  
- CCGGTCGACA GAGTAAGGAA CCAGGAGCGC GGGGTAGTGC TCGCGACGAG TAATAAGTGG GAGAGAGAGA   
  
  
- GAGGAGAGAC TAGGTGTGGT GTTTGTGTTC CAAACGTGTC GACGTCTCGT CAGCATGATG ATAGTCATCA   
  
  
- CTTTTTGGTT CGTCTTTAGG GGTTAGGGAG AAGAGGAAGA AGGGTCTAAG AAGGAGTTAA GGTTCGGGTA   
  
  
- TAGTCTTTAA ATCTGTTGTT GTTGGTTTTT TTTTTTAATT ATGACGAACT CTTTTTGTTT ATACTATACT   
  
  
- TTAAGGAGCT TTTTCCGGAC GGTATTGTTT AAGGAGCACT TTTTCGTCGA CTCTCTTCTT CTTCTTCTTT   
  
  
- TTTTAGGGCA GTGTTAGAGT TAGAGCTAGG GTTTAACACT TTCTCTTTCT TTTCTACTTC TCTCTTGTGG   
  
  
- GGAAAAGCTT GGGACTGGTC TTTGGGGGCG GCCTCTACAG CGGCCGCGGC CCCTTCAGGT TCTACACCCT   
  
  
- ACTACTCTAC CGGGTCTTGC TGCGATTTTA CCTACTCGAC GAACGACAAC ACCCCATGTT CCACTCCAGC   
  
  
- AGCCTGTACC GCCTTTAACG GGTCTTCGAA CTCGTCGAAC TTCTTCAGTA CCCATCACAA GTTCTTCTGC   
  
  
- CCAATAGAAT GAACCGAAGG CTTTGACAAG TAATGTTAGG ACGTCTAGAC AGATGAACCG AACTTAGATA   
  
  
- CGAAAGACTC AAATTGGGAT TGGGATTAAA ACTGGGAAGA GGTAGCAGTT AGAGTGGGTA GTAGCTAGGT   
  
  
- CGAGGAGGTG AAGGAGCTTG GAGCAGCTGC CAGTTTAAGC TCGGGCTGGG CAAAAGGCTA AGCCTAGACT   
  
  
- TTCGTTAGGG TCCATTCCGA TAGAACTGGG GTGGTTCAAG ATCGTTATGA AGTTTAAACG CACTCCGATT   
  
  
- CTCCAACTTT GGGAGTTTAA TGTGTTGGCG GGGTTGCGGG TGGGGTGGAG GTTTTGACGG GTTAAGCCGC   
  
  
- AGAGGTGGTA GCCCGTGCTG CCGCAGCTCC GGCCACCACG ACCATCTGAG TGTTCTTTTG CCGTAGTCTA   
  
  
- ACCACGTACG TGAATACCGG ACACGGCTTC GTTAAGTTGA TCTTTTGTAC CCTAATCGAC TCCGAAACCA   
  
  
- ATTTGTCTAA TCCATAAATC GTCGTAGAGT TCGACCTGGA TACTCCTTTC ATCGTTGAAC AAAACGTCTT   
  
  
- CGAGAATGAA CAGCCTAGAT GTTCGATACG GGTAGACATG GGATGCTACT TAGTCAGAGT CTACTCAACG   
  
  
- TCTACGTGAA AATACTCTGA ACGGGTATAG AATTTAAGCG GGTAAAGTGC CGTTTAGTTC GTTAAAATCT   
  
  
- TCGTAAGTTA CCCTTCTTCT TTCAAGTACA CTAACTAAAG TCGTACTTCG TTCCGTACGT TACCGGCCGA   
  
  
- AACTACGTTC GGGACCGAGA AGCTGGCCTC CCAGGTGGCG AAAAGGCCAA TTGGCCCTAA CCTGGCGGGC   
  
  
- GAGGCCTGTT GAGCCTGGCC GACGTTCTCC ACCCAACCTT CGAACGGGTC AAGCGCCTAA GCTAGGCCTA   
  
  
- ATTTAAAGTC ATAGCACCCA AACACCGTTT GTCAAACTGG CTAGATCTTA GAAGGTACGA TCTAGAACTT   
  
  
- GGCCTGTGAC TCCACCACCG CCAGTTGAGC CAAAAGCTCG AGGTGGCCGA CAACCGATTT GGGCCCCGCT   
  
  
- AGCTCTTTCA CGACCCCAAG TACTCCCGGC ACTTGGGCCA GTAACACTGC TACCAGCTCG TCCTTCGCTT   
  
  
- GGTGTTGCCT GGCCAAAAGA ACCTGGCCAA GTTACTCAGC AACGTAATAA TGAGGTGGAA CAAACTAAGG   
  
  
- GAACTCTAAA CACAACTATT ACATCTATTC TTCTACAGCC TCCGAATGAA CCCGGTCGTC TAGACGTTGT   
  
  
- ACCACCGAAC ACTTCCCAGA CTGGCTCAGC TCTCCGTGCT CTGGGACCGA GTCACCGCTC GGGCCAAGCG   
  
  
- TAGACGGCCC AAGCTGGGTC AAGTAGACCC AAGCTTACGC AAATTCGTCC GCTCATACAA CAACCTCAAG   
  
  
- AAACGACCAC CACTTCCCAT GCCGCACCTC CTCGCTCTTC CCACAGAGTA CAACCCTACC GTATCATCCG   
  
  
- GCGAATAGTG GTGGAGCCGA ACCGTCGAGC GGTTCTTGGG CCGACACTTA GCCGCTAC

+     AAGAA-motif

| Site Name | Organism | Position | Strand | Matrix score. | sequence | function |
| --- | --- | --- | --- | --- | --- | --- |
| AAGAA-motif | Avena sativa | 1038 | + | 7 | GAAAGAA |  |
| AAGAA-motif | Avena sativa | 666 | - | 7 | GAAAGAA |  |
| AAGAA-motif | Avena sativa | 2009 | + | 7 | GAAAGAA |  |

>HU10G00709.1   
+ -Up\_Stream \_Len000AACCCT TTAATTTTAT CAATGTGGGA CAACACTCAT CTTCATACTC CAACAAGCTA   
  
  
+ TGATACATTC GATTATTGCC TAGTATTTCG TTTAGTAAGA TATTAGTGCC AAACTTTGTA ATAAAAATGA   
  
  
+ AACTTACTAA AAAAAATCTC GTTTTCTGTA GAGGGAAAAT TTGAAGACTT AGTGTCACAT GATGACCTAA   
  
  
+ TGGTACTTGT AGGGTCAAGA TTGAGATTGA GGGCATACTG ACGACCTGTA TAATGGTCAA GTAGGATCAA   
  
  
+ AGTCGAGAAA ACTGTCTCTG CTAACTTTCA TAATCTAACC CTTAACTCAC TCTGACCCTC AATTTAATCA   
  
  
+ GGCCTAATTC AAACTATTAA TGGTTATAAC TCTCTTTCCT TCTTTTGAGA TTTTTTTTTT CTACATGCGG   
  
  
+ TTGAAAATAA TTTTTTTAAA AAATATATAT CACTAACCAT TCATTATCAT AGTTTTAGAG TTCATATTTA   
  
  
+ TGTGGTCCTT GTTGAGTCTA ATTTAATGCT TTAGATGGCG TGAAGCACCA AATTAGTGGT AACCATAATA   
  
  
+ ATAAACTATC AAGTGTTAAG TCGAAGAAAA CGGACACTGC TCAACCAAAT CTAGTAACAT AGTGTATGAC   
  
  
+ ATGATAACTA AGTGTGCCAT GTAGTAATCA ATTCTTTCTT TTTTTTTTTT TGGTTGAGAT CACATAAATT   
  
  
+ ACGTTATCAA GGGTAGTACG ATATAATGGA TGAGGAGAAA TTAAATGTGA GATTTAGTTG ATGAATCGAA   
  
  
+ TACGTTAGTC TGAGCCATTT GGTCAAAACT GCATTGGCTA ATCAATAGAA ATTATAGAAA ATACATAACA   
  
  
+ TAATGGGATG CAATTTTCTA ATTGGGGAAA CCATGTCAAT GCAGTGATTT TGGACCATTT AGGCCTTATT   
  
  
+ CTTTTCAATT TTTTAATTGA TTATTTAGAC TCATATGAAC TTATTGTTGA CAGATTGAGC TAAAAACACT   
  
  
+ GATGTTTCAT ACATATGTAT GAATTGCACG TACGAAGGCT CTGCTCATTT CGAGAAAGAA AATTAACAGT   
  
  
+ TAGGAGCAGT ATAATTTAAC AACAAGCGAT AATTATTAGT ATGAAGTGGT ACGAATCTAT TTTAAAGGAG   
  
  
+ TACTATGAAG TACAAACAAT AACAAATAAT AAAGAGCAGT ACATTAACAT ATCTTACTAC CTAAATAAAT   
  
  
+ CGATTCTAAG AGTAAAAAAA TGTTGAAATT AACCCCATTA TAGTTTTACT TTAACAAGAA GTTTTTCTTC   
  
  
+ TTTTACCATT TACCAAGTAA GAATGATTCA GGCCGTATAG CCAAGGAAAA ATAGAAAACC ACAAAACTAA   
  
  
+ CCATGGCCCA AAAAACAACC ACCAAGAGGG GCACTAATGT GACTAACCGA CGGTTAACAT TATCATCTGT   
  
  
+ TTGATACTTG TTCATCTCTC ACTATTCGGG TAATAGTGTG AAATTACTGA TGTACCCTCT AAACTCGTCG   
  
  
+ TTTTAACTGT GTAAGAATCA GATTAACAGT CACGGACACA TGAGTAACAA ATCAATTTCG GACATGAACG   
  
  
+ AACGAACTGT GAATCCCCCC CAAAAAAAAA ATTTTTTTAA AAAAAGTTGC ATCCTTATCT TTCTGAACCA   
  
  
+ GGCCAGCTGT CTCATTCCTT GGTCCTCGCG CCCCATCACG AGCGCTGCTC ATTATTCACC CTCTCTCTCT   
  
  
+ CTCCTCTCTG ATCCACACCA CAAACACAAG GTTTGCACAG CTGCAGAGCA GTCGTACTAC TATCAGTAGT   
  
  
+ GAAAAACCAA GCAGAAATCC CCAATCCCTC TTCTCCTTCT TCCCAGATTC TTCCTCAATT CCAAGCCCAT   
  
  
+ ATCAGAAATT TAGACAACAA CAACCAAAAA AAAAAATTAA TACTGCTTGA GAAAAACAAA TATGATATGA   
  
  
+ AATTCCTCGA AAAAGGCCTG CCATAACAAA TTCCTCGTGA AAAAGCAGCT GAGAGAAGAA GAAGAAGAAA   
  
  
+ AAAATCCCGT CACAATCTCA ATCTCGATCC CAAATTGTGA AAGAGAAAGA AAAGATGAAG AGAGAACACC   
  
  
+ CCTTTTCGAA CCCTGACCAG AAACCCCCGC CGGAGATGTC GCCGGCGCCG GGGAAGTCCA AGATGTGGGA   
  
  
+ TGATGAGATG GCCCAGAACG ACGCTAAAAT GGATGAGCTG CTTGCTGTTG TGGGGTACAA GGTGAGGTCG   
  
  
+ TCGGACATGG CGGAAATTGC CCAGAAGCTT GAGCAGCTTG AAGAAGTCAT GGGTAGTGTT CAAGAAGACG   
  
  
+ GGTTATCTTA CTTGGCTTCC GAAACTGTTC ATTACAATCC TGCAGATCTG TCTACTTGGC TTGAATCTAT   
  
  
+ GCTTTCTGAG TTTAACCCTA ACCCTAATTT TGACCCTTCT CCATCGTCAA TCTCACCCAT CATCGATCCA   
  
  
+ GCTCCTCCAC TTCCTCGAAC CTCGTCGACG GTCAAATTCG AGCCCGACCC GTTTTCCGAT TCGGATCTGA   
  
  
+ AAGCAATCCC AGGTAAGGCT ATCTTGACCC CACCAAGTTC TAGCAATACT TCAAATTTGC GTGAGGCTAA   
  
  
+ GAGGTTGAAA CCCTCAAATT ACACAACCGC CCCAACGCCC ACCCCACCTC CAAAACTGCC CAATTCGGCG   
  
  
+ TCTCCACCAT CGGGCACGAC GGCGTCGAGG CCGGTGGTGC TGGTAGACTC ACAAGAAAAC GGCATCAGAT   
  
  
+ TGGTGCATGC ACTTATGGCC TGTGCCGAAG CAATTCAACT AGAAAACATG GGATTAGCTG AGGCTTTGGT   
  
  
+ TAAACAGATT AGGTATTTAG CAGCATCTCA AGCTGGACCT ATGAGGAAAG TAGCAACTTG TTTTGCAGAA   
  
  
+ GCTCTTACTT GTCGGATCTA CAAGCTATGC CCATCTGTAC CCTACGATGA ATCAGTCTCA GATGAGTTGC   
  
  
+ AGATGCACTT TTATGAGACT TGCCCATATC TTAAATTCGC CCATTTCACG GCAAATCAAG CAATTTTAGA   
  
  
+ AGCATTCAAT GGGAAGAAGA AAGTTCATGT GATTGATTTC AGCATGAAGC AAGGCATGCA ATGGCCGGCT   
  
  
+ TTGATGCAAG CCCTGGCTCT TCGACCGGAG GGTCCACCGC TTTTCCGGTT AACCGGGATT GGACCGCCCG   
  
  
+ CTCCGGACAA CTCGGACCGG CTGCAAGAGG TGGGTTGGAA GCTTGCCCAG TTCGCGGATT CGATCCGGAT   
  
  
+ TAAATTTCAG TATCGTGGGT TTGTGGCAAA CAGTTTGACC GATCTAGAAT CTTCCATGCT AGATCTTGAA   
  
  
+ CCGGACACTG AGGTGGTGGC GGTCAACTCG GTTTTCGAGC TCCACCGGCT GTTGGCTAAA CCCGGGGCGA   
  
  
+ TCGAGAAAGT GCTGGGGTTC ATGAGGGCCG TGAACCCGGT CATTGTGACG ATGGTCGAGC AGGAAGCGAA   
  
  
+ CCACAACGGA CCGGTTTTCT TGGACCGGTT CAATGAGTCG TTGCATTATT ACTCCACCTT GTTTGATTCC   
  
  
+ CTTGAGATTT GTGTTGATAA TGTAGATAAG AAGATGTCGG AGGCTTACTT GGGCCAGCAG ATCTGCAACA   
  
  
+ TGGTGGCTTG TGAAGGGTCT GACCGAGTCG AGAGGCACGA GACCCTGGCT CAGTGGCGAG CCCGGTTCGC   
  
  
+ ATCTGCCGGG TTCGACCCAG TTCATCTGGG TTCGAATGCG TTTAAGCAGG CGAGTATGTT GTTGGAGTTC   
  
  
+ TTTGCTGGTG GTGAAGGGTA CGGCGTGGAG GAGCGAGAAG GGTGTCTCAT GTTGGGATGG CATAGTAGGC   
  
  
+ CGCTTATCAC CACCTCGGCT TGGCAGCTCG CCAAGAACCC GGCTGTGAAT CGGCGATG  

- -Up\_Stream \_Len000TTGGGA AATTAAAATA GTTACACCCT GTTGTGAGTA GAAGTATGAG GTTGTTCGAT   
  
  
- ACTATGTAAG CTAATAACGG ATCATAAAGC AAATCATTCT ATAATCACGG TTTGAAACAT TATTTTTACT   
  
  
- TTGAATGATT TTTTTTAGAG CAAAAGACAT CTCCCTTTTA AACTTCTGAA TCACAGTGTA CTACTGGATT   
  
  
- ACCATGAACA TCCCAGTTCT AACTCTAACT CCCGTATGAC TGCTGGACAT ATTACCAGTT CATCCTAGTT   
  
  
- TCAGCTCTTT TGACAGAGAC GATTGAAAGT ATTAGATTGG GAATTGAGTG AGACTGGGAG TTAAATTAGT   
  
  
- CCGGATTAAG TTTGATAATT ACCAATATTG AGAGAAAGGA AGAAAACTCT AAAAAAAAAA GATGTACGCC   
  
  
- AACTTTTATT AAAAAAATTT TTTATATATA GTGATTGGTA AGTAATAGTA TCAAAATCTC AAGTATAAAT   
  
  
- ACACCAGGAA CAACTCAGAT TAAATTACGA AATCTACCGC ACTTCGTGGT TTAATCACCA TTGGTATTAT   
  
  
- TATTTGATAG TTCACAATTC AGCTTCTTTT GCCTGTGACG AGTTGGTTTA GATCATTGTA TCACATACTG   
  
  
- TACTATTGAT TCACACGGTA CATCATTAGT TAAGAAAGAA AAAAAAAAAA ACCAACTCTA GTGTATTTAA   
  
  
- TGCAATAGTT CCCATCATGC TATATTACCT ACTCCTCTTT AATTTACACT CTAAATCAAC TACTTAGCTT   
  
  
- ATGCAATCAG ACTCGGTAAA CCAGTTTTGA CGTAACCGAT TAGTTATCTT TAATATCTTT TATGTATTGT   
  
  
- ATTACCCTAC GTTAAAAGAT TAACCCCTTT GGTACAGTTA CGTCACTAAA ACCTGGTAAA TCCGGAATAA   
  
  
- GAAAAGTTAA AAAATTAACT AATAAATCTG AGTATACTTG AATAACAACT GTCTAACTCG ATTTTTGTGA   
  
  
- CTACAAAGTA TGTATACATA CTTAACGTGC ATGCTTCCGA GACGAGTAAA GCTCTTTCTT TTAATTGTCA   
  
  
- ATCCTCGTCA TATTAAATTG TTGTTCGCTA TTAATAATCA TACTTCACCA TGCTTAGATA AAATTTCCTC   
  
  
- ATGATACTTC ATGTTTGTTA TTGTTTATTA TTTCTCGTCA TGTAATTGTA TAGAATGATG GATTTATTTA   
  
  
- GCTAAGATTC TCATTTTTTT ACAACTTTAA TTGGGGTAAT ATCAAAATGA AATTGTTCTT CAAAAAGAAG   
  
  
- AAAATGGTAA ATGGTTCATT CTTACTAAGT CCGGCATATC GGTTCCTTTT TATCTTTTGG TGTTTTGATT   
  
  
- GGTACCGGGT TTTTTGTTGG TGGTTCTCCC CGTGATTACA CTGATTGGCT GCCAATTGTA ATAGTAGACA   
  
  
- AACTATGAAC AAGTAGAGAG TGATAAGCCC ATTATCACAC TTTAATGACT ACATGGGAGA TTTGAGCAGC   
  
  
- AAAATTGACA CATTCTTAGT CTAATTGTCA GTGCCTGTGT ACTCATTGTT TAGTTAAAGC CTGTACTTGC   
  
  
- TTGCTTGACA CTTAGGGGGG GTTTTTTTTT TAAAAAAATT TTTTTCAACG TAGGAATAGA AAGACTTGGT   
  
  
- CCGGTCGACA GAGTAAGGAA CCAGGAGCGC GGGGTAGTGC TCGCGACGAG TAATAAGTGG GAGAGAGAGA   
  
  
- GAGGAGAGAC TAGGTGTGGT GTTTGTGTTC CAAACGTGTC GACGTCTCGT CAGCATGATG ATAGTCATCA   
  
  
- CTTTTTGGTT CGTCTTTAGG GGTTAGGGAG AAGAGGAAGA AGGGTCTAAG AAGGAGTTAA GGTTCGGGTA   
  
  
- TAGTCTTTAA ATCTGTTGTT GTTGGTTTTT TTTTTTAATT ATGACGAACT CTTTTTGTTT ATACTATACT   
  
  
- TTAAGGAGCT TTTTCCGGAC GGTATTGTTT AAGGAGCACT TTTTCGTCGA CTCTCTTCTT CTTCTTCTTT   
  
  
- TTTTAGGGCA GTGTTAGAGT TAGAGCTAGG GTTTAACACT TTCTCTTTCT TTTCTACTTC TCTCTTGTGG   
  
  
- GGAAAAGCTT GGGACTGGTC TTTGGGGGCG GCCTCTACAG CGGCCGCGGC CCCTTCAGGT TCTACACCCT   
  
  
- ACTACTCTAC CGGGTCTTGC TGCGATTTTA CCTACTCGAC GAACGACAAC ACCCCATGTT CCACTCCAGC   
  
  
- AGCCTGTACC GCCTTTAACG GGTCTTCGAA CTCGTCGAAC TTCTTCAGTA CCCATCACAA GTTCTTCTGC   
  
  
- CCAATAGAAT GAACCGAAGG CTTTGACAAG TAATGTTAGG ACGTCTAGAC AGATGAACCG AACTTAGATA   
  
  
- CGAAAGACTC AAATTGGGAT TGGGATTAAA ACTGGGAAGA GGTAGCAGTT AGAGTGGGTA GTAGCTAGGT   
  
  
- CGAGGAGGTG AAGGAGCTTG GAGCAGCTGC CAGTTTAAGC TCGGGCTGGG CAAAAGGCTA AGCCTAGACT   
  
  
- TTCGTTAGGG TCCATTCCGA TAGAACTGGG GTGGTTCAAG ATCGTTATGA AGTTTAAACG CACTCCGATT   
  
  
- CTCCAACTTT GGGAGTTTAA TGTGTTGGCG GGGTTGCGGG TGGGGTGGAG GTTTTGACGG GTTAAGCCGC   
  
  
- AGAGGTGGTA GCCCGTGCTG CCGCAGCTCC GGCCACCACG ACCATCTGAG TGTTCTTTTG CCGTAGTCTA   
  
  
- ACCACGTACG TGAATACCGG ACACGGCTTC GTTAAGTTGA TCTTTTGTAC CCTAATCGAC TCCGAAACCA   
  
  
- ATTTGTCTAA TCCATAAATC GTCGTAGAGT TCGACCTGGA TACTCCTTTC ATCGTTGAAC AAAACGTCTT   
  
  
- CGAGAATGAA CAGCCTAGAT GTTCGATACG GGTAGACATG GGATGCTACT TAGTCAGAGT CTACTCAACG   
  
  
- TCTACGTGAA AATACTCTGA ACGGGTATAG AATTTAAGCG GGTAAAGTGC CGTTTAGTTC GTTAAAATCT   
  
  
- TCGTAAGTTA CCCTTCTTCT TTCAAGTACA CTAACTAAAG TCGTACTTCG TTCCGTACGT TACCGGCCGA   
  
  
- AACTACGTTC GGGACCGAGA AGCTGGCCTC CCAGGTGGCG AAAAGGCCAA TTGGCCCTAA CCTGGCGGGC   
  
  
- GAGGCCTGTT GAGCCTGGCC GACGTTCTCC ACCCAACCTT CGAACGGGTC AAGCGCCTAA GCTAGGCCTA   
  
  
- ATTTAAAGTC ATAGCACCCA AACACCGTTT GTCAAACTGG CTAGATCTTA GAAGGTACGA TCTAGAACTT   
  
  
- GGCCTGTGAC TCCACCACCG CCAGTTGAGC CAAAAGCTCG AGGTGGCCGA CAACCGATTT GGGCCCCGCT   
  
  
- AGCTCTTTCA CGACCCCAAG TACTCCCGGC ACTTGGGCCA GTAACACTGC TACCAGCTCG TCCTTCGCTT   
  
  
- GGTGTTGCCT GGCCAAAAGA ACCTGGCCAA GTTACTCAGC AACGTAATAA TGAGGTGGAA CAAACTAAGG   
  
  
- GAACTCTAAA CACAACTATT ACATCTATTC TTCTACAGCC TCCGAATGAA CCCGGTCGTC TAGACGTTGT   
  
  
- ACCACCGAAC ACTTCCCAGA CTGGCTCAGC TCTCCGTGCT CTGGGACCGA GTCACCGCTC GGGCCAAGCG   
  
  
- TAGACGGCCC AAGCTGGGTC AAGTAGACCC AAGCTTACGC AAATTCGTCC GCTCATACAA CAACCTCAAG   
  
  
- AAACGACCAC CACTTCCCAT GCCGCACCTC CTCGCTCTTC CCACAGAGTA CAACCCTACC GTATCATCCG   
  
  
- GCGAATAGTG GTGGAGCCGA ACCGTCGAGC GGTTCTTGGG CCGACACTTA GCCGCTAC

+     ABRE

| Site Name | Organism | Position | Strand | Matrix score. | sequence | function |
| --- | --- | --- | --- | --- | --- | --- |
| ABRE | Arabidopsis thaliana | 3750 | + | 7 | AACCCGG | cis-acting element involved in the abscisic acid responsiveness |
| ABRE | Arabidopsis thaliana | 3327 | + | 7 | AACCCGG | cis-acting element involved in the abscisic acid responsiveness |
| ABRE | Arabidopsis thaliana | 1011 | - | 5 | ACGTG | cis-acting element involved in the abscisic acid responsiveness |
| ABRE | Arabidopsis thaliana | 3580 | - | 7 | AACCCGG | cis-acting element involved in the abscisic acid responsiveness |
| ABRE | Hordeum vulgare | 1009 | - | 10 | CGTACGTGCA | cis-acting element involved in the abscisic acid responsiveness |
| ABRE | Arabidopsis thaliana | 3283 | + | 7 | AACCCGG | cis-acting element involved in the abscisic acid responsiveness |

>HU10G00709.1   
+ -Up\_Stream \_Len000AACCCT TTAATTTTAT CAATGTGGGA CAACACTCAT CTTCATACTC CAACAAGCTA   
  
  
+ TGATACATTC GATTATTGCC TAGTATTTCG TTTAGTAAGA TATTAGTGCC AAACTTTGTA ATAAAAATGA   
  
  
+ AACTTACTAA AAAAAATCTC GTTTTCTGTA GAGGGAAAAT TTGAAGACTT AGTGTCACAT GATGACCTAA   
  
  
+ TGGTACTTGT AGGGTCAAGA TTGAGATTGA GGGCATACTG ACGACCTGTA TAATGGTCAA GTAGGATCAA   
  
  
+ AGTCGAGAAA ACTGTCTCTG CTAACTTTCA TAATCTAACC CTTAACTCAC TCTGACCCTC AATTTAATCA   
  
  
+ GGCCTAATTC AAACTATTAA TGGTTATAAC TCTCTTTCCT TCTTTTGAGA TTTTTTTTTT CTACATGCGG   
  
  
+ TTGAAAATAA TTTTTTTAAA AAATATATAT CACTAACCAT TCATTATCAT AGTTTTAGAG TTCATATTTA   
  
  
+ TGTGGTCCTT GTTGAGTCTA ATTTAATGCT TTAGATGGCG TGAAGCACCA AATTAGTGGT AACCATAATA   
  
  
+ ATAAACTATC AAGTGTTAAG TCGAAGAAAA CGGACACTGC TCAACCAAAT CTAGTAACAT AGTGTATGAC   
  
  
+ ATGATAACTA AGTGTGCCAT GTAGTAATCA ATTCTTTCTT TTTTTTTTTT TGGTTGAGAT CACATAAATT   
  
  
+ ACGTTATCAA GGGTAGTACG ATATAATGGA TGAGGAGAAA TTAAATGTGA GATTTAGTTG ATGAATCGAA   
  
  
+ TACGTTAGTC TGAGCCATTT GGTCAAAACT GCATTGGCTA ATCAATAGAA ATTATAGAAA ATACATAACA   
  
  
+ TAATGGGATG CAATTTTCTA ATTGGGGAAA CCATGTCAAT GCAGTGATTT TGGACCATTT AGGCCTTATT   
  
  
+ CTTTTCAATT TTTTAATTGA TTATTTAGAC TCATATGAAC TTATTGTTGA CAGATTGAGC TAAAAACACT   
  
  
+ GATGTTTCAT ACATATGTAT GAATTGCACG TACGAAGGCT CTGCTCATTT CGAGAAAGAA AATTAACAGT   
  
  
+ TAGGAGCAGT ATAATTTAAC AACAAGCGAT AATTATTAGT ATGAAGTGGT ACGAATCTAT TTTAAAGGAG   
  
  
+ TACTATGAAG TACAAACAAT AACAAATAAT AAAGAGCAGT ACATTAACAT ATCTTACTAC CTAAATAAAT   
  
  
+ CGATTCTAAG AGTAAAAAAA TGTTGAAATT AACCCCATTA TAGTTTTACT TTAACAAGAA GTTTTTCTTC   
  
  
+ TTTTACCATT TACCAAGTAA GAATGATTCA GGCCGTATAG CCAAGGAAAA ATAGAAAACC ACAAAACTAA   
  
  
+ CCATGGCCCA AAAAACAACC ACCAAGAGGG GCACTAATGT GACTAACCGA CGGTTAACAT TATCATCTGT   
  
  
+ TTGATACTTG TTCATCTCTC ACTATTCGGG TAATAGTGTG AAATTACTGA TGTACCCTCT AAACTCGTCG   
  
  
+ TTTTAACTGT GTAAGAATCA GATTAACAGT CACGGACACA TGAGTAACAA ATCAATTTCG GACATGAACG   
  
  
+ AACGAACTGT GAATCCCCCC CAAAAAAAAA ATTTTTTTAA AAAAAGTTGC ATCCTTATCT TTCTGAACCA   
  
  
+ GGCCAGCTGT CTCATTCCTT GGTCCTCGCG CCCCATCACG AGCGCTGCTC ATTATTCACC CTCTCTCTCT   
  
  
+ CTCCTCTCTG ATCCACACCA CAAACACAAG GTTTGCACAG CTGCAGAGCA GTCGTACTAC TATCAGTAGT   
  
  
+ GAAAAACCAA GCAGAAATCC CCAATCCCTC TTCTCCTTCT TCCCAGATTC TTCCTCAATT CCAAGCCCAT   
  
  
+ ATCAGAAATT TAGACAACAA CAACCAAAAA AAAAAATTAA TACTGCTTGA GAAAAACAAA TATGATATGA   
  
  
+ AATTCCTCGA AAAAGGCCTG CCATAACAAA TTCCTCGTGA AAAAGCAGCT GAGAGAAGAA GAAGAAGAAA   
  
  
+ AAAATCCCGT CACAATCTCA ATCTCGATCC CAAATTGTGA AAGAGAAAGA AAAGATGAAG AGAGAACACC   
  
  
+ CCTTTTCGAA CCCTGACCAG AAACCCCCGC CGGAGATGTC GCCGGCGCCG GGGAAGTCCA AGATGTGGGA   
  
  
+ TGATGAGATG GCCCAGAACG ACGCTAAAAT GGATGAGCTG CTTGCTGTTG TGGGGTACAA GGTGAGGTCG   
  
  
+ TCGGACATGG CGGAAATTGC CCAGAAGCTT GAGCAGCTTG AAGAAGTCAT GGGTAGTGTT CAAGAAGACG   
  
  
+ GGTTATCTTA CTTGGCTTCC GAAACTGTTC ATTACAATCC TGCAGATCTG TCTACTTGGC TTGAATCTAT   
  
  
+ GCTTTCTGAG TTTAACCCTA ACCCTAATTT TGACCCTTCT CCATCGTCAA TCTCACCCAT CATCGATCCA   
  
  
+ GCTCCTCCAC TTCCTCGAAC CTCGTCGACG GTCAAATTCG AGCCCGACCC GTTTTCCGAT TCGGATCTGA   
  
  
+ AAGCAATCCC AGGTAAGGCT ATCTTGACCC CACCAAGTTC TAGCAATACT TCAAATTTGC GTGAGGCTAA   
  
  
+ GAGGTTGAAA CCCTCAAATT ACACAACCGC CCCAACGCCC ACCCCACCTC CAAAACTGCC CAATTCGGCG   
  
  
+ TCTCCACCAT CGGGCACGAC GGCGTCGAGG CCGGTGGTGC TGGTAGACTC ACAAGAAAAC GGCATCAGAT   
  
  
+ TGGTGCATGC ACTTATGGCC TGTGCCGAAG CAATTCAACT AGAAAACATG GGATTAGCTG AGGCTTTGGT   
  
  
+ TAAACAGATT AGGTATTTAG CAGCATCTCA AGCTGGACCT ATGAGGAAAG TAGCAACTTG TTTTGCAGAA   
  
  
+ GCTCTTACTT GTCGGATCTA CAAGCTATGC CCATCTGTAC CCTACGATGA ATCAGTCTCA GATGAGTTGC   
  
  
+ AGATGCACTT TTATGAGACT TGCCCATATC TTAAATTCGC CCATTTCACG GCAAATCAAG CAATTTTAGA   
  
  
+ AGCATTCAAT GGGAAGAAGA AAGTTCATGT GATTGATTTC AGCATGAAGC AAGGCATGCA ATGGCCGGCT   
  
  
+ TTGATGCAAG CCCTGGCTCT TCGACCGGAG GGTCCACCGC TTTTCCGGTT AACCGGGATT GGACCGCCCG   
  
  
+ CTCCGGACAA CTCGGACCGG CTGCAAGAGG TGGGTTGGAA GCTTGCCCAG TTCGCGGATT CGATCCGGAT   
  
  
+ TAAATTTCAG TATCGTGGGT TTGTGGCAAA CAGTTTGACC GATCTAGAAT CTTCCATGCT AGATCTTGAA   
  
  
+ CCGGACACTG AGGTGGTGGC GGTCAACTCG GTTTTCGAGC TCCACCGGCT GTTGGCTAAA CCCGGGGCGA   
  
  
+ TCGAGAAAGT GCTGGGGTTC ATGAGGGCCG TGAACCCGGT CATTGTGACG ATGGTCGAGC AGGAAGCGAA   
  
  
+ CCACAACGGA CCGGTTTTCT TGGACCGGTT CAATGAGTCG TTGCATTATT ACTCCACCTT GTTTGATTCC   
  
  
+ CTTGAGATTT GTGTTGATAA TGTAGATAAG AAGATGTCGG AGGCTTACTT GGGCCAGCAG ATCTGCAACA   
  
  
+ TGGTGGCTTG TGAAGGGTCT GACCGAGTCG AGAGGCACGA GACCCTGGCT CAGTGGCGAG CCCGGTTCGC   
  
  
+ ATCTGCCGGG TTCGACCCAG TTCATCTGGG TTCGAATGCG TTTAAGCAGG CGAGTATGTT GTTGGAGTTC   
  
  
+ TTTGCTGGTG GTGAAGGGTA CGGCGTGGAG GAGCGAGAAG GGTGTCTCAT GTTGGGATGG CATAGTAGGC   
  
  
+ CGCTTATCAC CACCTCGGCT TGGCAGCTCG CCAAGAACCC GGCTGTGAAT CGGCGATG  

- -Up\_Stream \_Len000TTGGGA AATTAAAATA GTTACACCCT GTTGTGAGTA GAAGTATGAG GTTGTTCGAT   
  
  
- ACTATGTAAG CTAATAACGG ATCATAAAGC AAATCATTCT ATAATCACGG TTTGAAACAT TATTTTTACT   
  
  
- TTGAATGATT TTTTTTAGAG CAAAAGACAT CTCCCTTTTA AACTTCTGAA TCACAGTGTA CTACTGGATT   
  
  
- ACCATGAACA TCCCAGTTCT AACTCTAACT CCCGTATGAC TGCTGGACAT ATTACCAGTT CATCCTAGTT   
  
  
- TCAGCTCTTT TGACAGAGAC GATTGAAAGT ATTAGATTGG GAATTGAGTG AGACTGGGAG TTAAATTAGT   
  
  
- CCGGATTAAG TTTGATAATT ACCAATATTG AGAGAAAGGA AGAAAACTCT AAAAAAAAAA GATGTACGCC   
  
  
- AACTTTTATT AAAAAAATTT TTTATATATA GTGATTGGTA AGTAATAGTA TCAAAATCTC AAGTATAAAT   
  
  
- ACACCAGGAA CAACTCAGAT TAAATTACGA AATCTACCGC ACTTCGTGGT TTAATCACCA TTGGTATTAT   
  
  
- TATTTGATAG TTCACAATTC AGCTTCTTTT GCCTGTGACG AGTTGGTTTA GATCATTGTA TCACATACTG   
  
  
- TACTATTGAT TCACACGGTA CATCATTAGT TAAGAAAGAA AAAAAAAAAA ACCAACTCTA GTGTATTTAA   
  
  
- TGCAATAGTT CCCATCATGC TATATTACCT ACTCCTCTTT AATTTACACT CTAAATCAAC TACTTAGCTT   
  
  
- ATGCAATCAG ACTCGGTAAA CCAGTTTTGA CGTAACCGAT TAGTTATCTT TAATATCTTT TATGTATTGT   
  
  
- ATTACCCTAC GTTAAAAGAT TAACCCCTTT GGTACAGTTA CGTCACTAAA ACCTGGTAAA TCCGGAATAA   
  
  
- GAAAAGTTAA AAAATTAACT AATAAATCTG AGTATACTTG AATAACAACT GTCTAACTCG ATTTTTGTGA   
  
  
- CTACAAAGTA TGTATACATA CTTAACGTGC ATGCTTCCGA GACGAGTAAA GCTCTTTCTT TTAATTGTCA   
  
  
- ATCCTCGTCA TATTAAATTG TTGTTCGCTA TTAATAATCA TACTTCACCA TGCTTAGATA AAATTTCCTC   
  
  
- ATGATACTTC ATGTTTGTTA TTGTTTATTA TTTCTCGTCA TGTAATTGTA TAGAATGATG GATTTATTTA   
  
  
- GCTAAGATTC TCATTTTTTT ACAACTTTAA TTGGGGTAAT ATCAAAATGA AATTGTTCTT CAAAAAGAAG   
  
  
- AAAATGGTAA ATGGTTCATT CTTACTAAGT CCGGCATATC GGTTCCTTTT TATCTTTTGG TGTTTTGATT   
  
  
- GGTACCGGGT TTTTTGTTGG TGGTTCTCCC CGTGATTACA CTGATTGGCT GCCAATTGTA ATAGTAGACA   
  
  
- AACTATGAAC AAGTAGAGAG TGATAAGCCC ATTATCACAC TTTAATGACT ACATGGGAGA TTTGAGCAGC   
  
  
- AAAATTGACA CATTCTTAGT CTAATTGTCA GTGCCTGTGT ACTCATTGTT TAGTTAAAGC CTGTACTTGC   
  
  
- TTGCTTGACA CTTAGGGGGG GTTTTTTTTT TAAAAAAATT TTTTTCAACG TAGGAATAGA AAGACTTGGT   
  
  
- CCGGTCGACA GAGTAAGGAA CCAGGAGCGC GGGGTAGTGC TCGCGACGAG TAATAAGTGG GAGAGAGAGA   
  
  
- GAGGAGAGAC TAGGTGTGGT GTTTGTGTTC CAAACGTGTC GACGTCTCGT CAGCATGATG ATAGTCATCA   
  
  
- CTTTTTGGTT CGTCTTTAGG GGTTAGGGAG AAGAGGAAGA AGGGTCTAAG AAGGAGTTAA GGTTCGGGTA   
  
  
- TAGTCTTTAA ATCTGTTGTT GTTGGTTTTT TTTTTTAATT ATGACGAACT CTTTTTGTTT ATACTATACT   
  
  
- TTAAGGAGCT TTTTCCGGAC GGTATTGTTT AAGGAGCACT TTTTCGTCGA CTCTCTTCTT CTTCTTCTTT   
  
  
- TTTTAGGGCA GTGTTAGAGT TAGAGCTAGG GTTTAACACT TTCTCTTTCT TTTCTACTTC TCTCTTGTGG   
  
  
- GGAAAAGCTT GGGACTGGTC TTTGGGGGCG GCCTCTACAG CGGCCGCGGC CCCTTCAGGT TCTACACCCT   
  
  
- ACTACTCTAC CGGGTCTTGC TGCGATTTTA CCTACTCGAC GAACGACAAC ACCCCATGTT CCACTCCAGC   
  
  
- AGCCTGTACC GCCTTTAACG GGTCTTCGAA CTCGTCGAAC TTCTTCAGTA CCCATCACAA GTTCTTCTGC   
  
  
- CCAATAGAAT GAACCGAAGG CTTTGACAAG TAATGTTAGG ACGTCTAGAC AGATGAACCG AACTTAGATA   
  
  
- CGAAAGACTC AAATTGGGAT TGGGATTAAA ACTGGGAAGA GGTAGCAGTT AGAGTGGGTA GTAGCTAGGT   
  
  
- CGAGGAGGTG AAGGAGCTTG GAGCAGCTGC CAGTTTAAGC TCGGGCTGGG CAAAAGGCTA AGCCTAGACT   
  
  
- TTCGTTAGGG TCCATTCCGA TAGAACTGGG GTGGTTCAAG ATCGTTATGA AGTTTAAACG CACTCCGATT   
  
  
- CTCCAACTTT GGGAGTTTAA TGTGTTGGCG GGGTTGCGGG TGGGGTGGAG GTTTTGACGG GTTAAGCCGC   
  
  
- AGAGGTGGTA GCCCGTGCTG CCGCAGCTCC GGCCACCACG ACCATCTGAG TGTTCTTTTG CCGTAGTCTA   
  
  
- ACCACGTACG TGAATACCGG ACACGGCTTC GTTAAGTTGA TCTTTTGTAC CCTAATCGAC TCCGAAACCA   
  
  
- ATTTGTCTAA TCCATAAATC GTCGTAGAGT TCGACCTGGA TACTCCTTTC ATCGTTGAAC AAAACGTCTT   
  
  
- CGAGAATGAA CAGCCTAGAT GTTCGATACG GGTAGACATG GGATGCTACT TAGTCAGAGT CTACTCAACG   
  
  
- TCTACGTGAA AATACTCTGA ACGGGTATAG AATTTAAGCG GGTAAAGTGC CGTTTAGTTC GTTAAAATCT   
  
  
- TCGTAAGTTA CCCTTCTTCT TTCAAGTACA CTAACTAAAG TCGTACTTCG TTCCGTACGT TACCGGCCGA   
  
  
- AACTACGTTC GGGACCGAGA AGCTGGCCTC CCAGGTGGCG AAAAGGCCAA TTGGCCCTAA CCTGGCGGGC   
  
  
- GAGGCCTGTT GAGCCTGGCC GACGTTCTCC ACCCAACCTT CGAACGGGTC AAGCGCCTAA GCTAGGCCTA   
  
  
- ATTTAAAGTC ATAGCACCCA AACACCGTTT GTCAAACTGG CTAGATCTTA GAAGGTACGA TCTAGAACTT   
  
  
- GGCCTGTGAC TCCACCACCG CCAGTTGAGC CAAAAGCTCG AGGTGGCCGA CAACCGATTT GGGCCCCGCT   
  
  
- AGCTCTTTCA CGACCCCAAG TACTCCCGGC ACTTGGGCCA GTAACACTGC TACCAGCTCG TCCTTCGCTT   
  
  
- GGTGTTGCCT GGCCAAAAGA ACCTGGCCAA GTTACTCAGC AACGTAATAA TGAGGTGGAA CAAACTAAGG   
  
  
- GAACTCTAAA CACAACTATT ACATCTATTC TTCTACAGCC TCCGAATGAA CCCGGTCGTC TAGACGTTGT   
  
  
- ACCACCGAAC ACTTCCCAGA CTGGCTCAGC TCTCCGTGCT CTGGGACCGA GTCACCGCTC GGGCCAAGCG   
  
  
- TAGACGGCCC AAGCTGGGTC AAGTAGACCC AAGCTTACGC AAATTCGTCC GCTCATACAA CAACCTCAAG   
  
  
- AAACGACCAC CACTTCCCAT GCCGCACCTC CTCGCTCTTC CCACAGAGTA CAACCCTACC GTATCATCCG   
  
  
- GCGAATAGTG GTGGAGCCGA ACCGTCGAGC GGTTCTTGGG CCGACACTTA GCCGCTAC

+     ABRE3a

| Site Name | Organism | Position | Strand | Matrix score. | sequence | function |
| --- | --- | --- | --- | --- | --- | --- |
| ABRE3a | Zea mays | 1011 | - | 6 | TACGTG |  |

>HU10G00709.1   
+ -Up\_Stream \_Len000AACCCT TTAATTTTAT CAATGTGGGA CAACACTCAT CTTCATACTC CAACAAGCTA   
  
  
+ TGATACATTC GATTATTGCC TAGTATTTCG TTTAGTAAGA TATTAGTGCC AAACTTTGTA ATAAAAATGA   
  
  
+ AACTTACTAA AAAAAATCTC GTTTTCTGTA GAGGGAAAAT TTGAAGACTT AGTGTCACAT GATGACCTAA   
  
  
+ TGGTACTTGT AGGGTCAAGA TTGAGATTGA GGGCATACTG ACGACCTGTA TAATGGTCAA GTAGGATCAA   
  
  
+ AGTCGAGAAA ACTGTCTCTG CTAACTTTCA TAATCTAACC CTTAACTCAC TCTGACCCTC AATTTAATCA   
  
  
+ GGCCTAATTC AAACTATTAA TGGTTATAAC TCTCTTTCCT TCTTTTGAGA TTTTTTTTTT CTACATGCGG   
  
  
+ TTGAAAATAA TTTTTTTAAA AAATATATAT CACTAACCAT TCATTATCAT AGTTTTAGAG TTCATATTTA   
  
  
+ TGTGGTCCTT GTTGAGTCTA ATTTAATGCT TTAGATGGCG TGAAGCACCA AATTAGTGGT AACCATAATA   
  
  
+ ATAAACTATC AAGTGTTAAG TCGAAGAAAA CGGACACTGC TCAACCAAAT CTAGTAACAT AGTGTATGAC   
  
  
+ ATGATAACTA AGTGTGCCAT GTAGTAATCA ATTCTTTCTT TTTTTTTTTT TGGTTGAGAT CACATAAATT   
  
  
+ ACGTTATCAA GGGTAGTACG ATATAATGGA TGAGGAGAAA TTAAATGTGA GATTTAGTTG ATGAATCGAA   
  
  
+ TACGTTAGTC TGAGCCATTT GGTCAAAACT GCATTGGCTA ATCAATAGAA ATTATAGAAA ATACATAACA   
  
  
+ TAATGGGATG CAATTTTCTA ATTGGGGAAA CCATGTCAAT GCAGTGATTT TGGACCATTT AGGCCTTATT   
  
  
+ CTTTTCAATT TTTTAATTGA TTATTTAGAC TCATATGAAC TTATTGTTGA CAGATTGAGC TAAAAACACT   
  
  
+ GATGTTTCAT ACATATGTAT GAATTGCACG TACGAAGGCT CTGCTCATTT CGAGAAAGAA AATTAACAGT   
  
  
+ TAGGAGCAGT ATAATTTAAC AACAAGCGAT AATTATTAGT ATGAAGTGGT ACGAATCTAT TTTAAAGGAG   
  
  
+ TACTATGAAG TACAAACAAT AACAAATAAT AAAGAGCAGT ACATTAACAT ATCTTACTAC CTAAATAAAT   
  
  
+ CGATTCTAAG AGTAAAAAAA TGTTGAAATT AACCCCATTA TAGTTTTACT TTAACAAGAA GTTTTTCTTC   
  
  
+ TTTTACCATT TACCAAGTAA GAATGATTCA GGCCGTATAG CCAAGGAAAA ATAGAAAACC ACAAAACTAA   
  
  
+ CCATGGCCCA AAAAACAACC ACCAAGAGGG GCACTAATGT GACTAACCGA CGGTTAACAT TATCATCTGT   
  
  
+ TTGATACTTG TTCATCTCTC ACTATTCGGG TAATAGTGTG AAATTACTGA TGTACCCTCT AAACTCGTCG   
  
  
+ TTTTAACTGT GTAAGAATCA GATTAACAGT CACGGACACA TGAGTAACAA ATCAATTTCG GACATGAACG   
  
  
+ AACGAACTGT GAATCCCCCC CAAAAAAAAA ATTTTTTTAA AAAAAGTTGC ATCCTTATCT TTCTGAACCA   
  
  
+ GGCCAGCTGT CTCATTCCTT GGTCCTCGCG CCCCATCACG AGCGCTGCTC ATTATTCACC CTCTCTCTCT   
  
  
+ CTCCTCTCTG ATCCACACCA CAAACACAAG GTTTGCACAG CTGCAGAGCA GTCGTACTAC TATCAGTAGT   
  
  
+ GAAAAACCAA GCAGAAATCC CCAATCCCTC TTCTCCTTCT TCCCAGATTC TTCCTCAATT CCAAGCCCAT   
  
  
+ ATCAGAAATT TAGACAACAA CAACCAAAAA AAAAAATTAA TACTGCTTGA GAAAAACAAA TATGATATGA   
  
  
+ AATTCCTCGA AAAAGGCCTG CCATAACAAA TTCCTCGTGA AAAAGCAGCT GAGAGAAGAA GAAGAAGAAA   
  
  
+ AAAATCCCGT CACAATCTCA ATCTCGATCC CAAATTGTGA AAGAGAAAGA AAAGATGAAG AGAGAACACC   
  
  
+ CCTTTTCGAA CCCTGACCAG AAACCCCCGC CGGAGATGTC GCCGGCGCCG GGGAAGTCCA AGATGTGGGA   
  
  
+ TGATGAGATG GCCCAGAACG ACGCTAAAAT GGATGAGCTG CTTGCTGTTG TGGGGTACAA GGTGAGGTCG   
  
  
+ TCGGACATGG CGGAAATTGC CCAGAAGCTT GAGCAGCTTG AAGAAGTCAT GGGTAGTGTT CAAGAAGACG   
  
  
+ GGTTATCTTA CTTGGCTTCC GAAACTGTTC ATTACAATCC TGCAGATCTG TCTACTTGGC TTGAATCTAT   
  
  
+ GCTTTCTGAG TTTAACCCTA ACCCTAATTT TGACCCTTCT CCATCGTCAA TCTCACCCAT CATCGATCCA   
  
  
+ GCTCCTCCAC TTCCTCGAAC CTCGTCGACG GTCAAATTCG AGCCCGACCC GTTTTCCGAT TCGGATCTGA   
  
  
+ AAGCAATCCC AGGTAAGGCT ATCTTGACCC CACCAAGTTC TAGCAATACT TCAAATTTGC GTGAGGCTAA   
  
  
+ GAGGTTGAAA CCCTCAAATT ACACAACCGC CCCAACGCCC ACCCCACCTC CAAAACTGCC CAATTCGGCG   
  
  
+ TCTCCACCAT CGGGCACGAC GGCGTCGAGG CCGGTGGTGC TGGTAGACTC ACAAGAAAAC GGCATCAGAT   
  
  
+ TGGTGCATGC ACTTATGGCC TGTGCCGAAG CAATTCAACT AGAAAACATG GGATTAGCTG AGGCTTTGGT   
  
  
+ TAAACAGATT AGGTATTTAG CAGCATCTCA AGCTGGACCT ATGAGGAAAG TAGCAACTTG TTTTGCAGAA   
  
  
+ GCTCTTACTT GTCGGATCTA CAAGCTATGC CCATCTGTAC CCTACGATGA ATCAGTCTCA GATGAGTTGC   
  
  
+ AGATGCACTT TTATGAGACT TGCCCATATC TTAAATTCGC CCATTTCACG GCAAATCAAG CAATTTTAGA   
  
  
+ AGCATTCAAT GGGAAGAAGA AAGTTCATGT GATTGATTTC AGCATGAAGC AAGGCATGCA ATGGCCGGCT   
  
  
+ TTGATGCAAG CCCTGGCTCT TCGACCGGAG GGTCCACCGC TTTTCCGGTT AACCGGGATT GGACCGCCCG   
  
  
+ CTCCGGACAA CTCGGACCGG CTGCAAGAGG TGGGTTGGAA GCTTGCCCAG TTCGCGGATT CGATCCGGAT   
  
  
+ TAAATTTCAG TATCGTGGGT TTGTGGCAAA CAGTTTGACC GATCTAGAAT CTTCCATGCT AGATCTTGAA   
  
  
+ CCGGACACTG AGGTGGTGGC GGTCAACTCG GTTTTCGAGC TCCACCGGCT GTTGGCTAAA CCCGGGGCGA   
  
  
+ TCGAGAAAGT GCTGGGGTTC ATGAGGGCCG TGAACCCGGT CATTGTGACG ATGGTCGAGC AGGAAGCGAA   
  
  
+ CCACAACGGA CCGGTTTTCT TGGACCGGTT CAATGAGTCG TTGCATTATT ACTCCACCTT GTTTGATTCC   
  
  
+ CTTGAGATTT GTGTTGATAA TGTAGATAAG AAGATGTCGG AGGCTTACTT GGGCCAGCAG ATCTGCAACA   
  
  
+ TGGTGGCTTG TGAAGGGTCT GACCGAGTCG AGAGGCACGA GACCCTGGCT CAGTGGCGAG CCCGGTTCGC   
  
  
+ ATCTGCCGGG TTCGACCCAG TTCATCTGGG TTCGAATGCG TTTAAGCAGG CGAGTATGTT GTTGGAGTTC   
  
  
+ TTTGCTGGTG GTGAAGGGTA CGGCGTGGAG GAGCGAGAAG GGTGTCTCAT GTTGGGATGG CATAGTAGGC   
  
  
+ CGCTTATCAC CACCTCGGCT TGGCAGCTCG CCAAGAACCC GGCTGTGAAT CGGCGATG  

- -Up\_Stream \_Len000TTGGGA AATTAAAATA GTTACACCCT GTTGTGAGTA GAAGTATGAG GTTGTTCGAT   
  
  
- ACTATGTAAG CTAATAACGG ATCATAAAGC AAATCATTCT ATAATCACGG TTTGAAACAT TATTTTTACT   
  
  
- TTGAATGATT TTTTTTAGAG CAAAAGACAT CTCCCTTTTA AACTTCTGAA TCACAGTGTA CTACTGGATT   
  
  
- ACCATGAACA TCCCAGTTCT AACTCTAACT CCCGTATGAC TGCTGGACAT ATTACCAGTT CATCCTAGTT   
  
  
- TCAGCTCTTT TGACAGAGAC GATTGAAAGT ATTAGATTGG GAATTGAGTG AGACTGGGAG TTAAATTAGT   
  
  
- CCGGATTAAG TTTGATAATT ACCAATATTG AGAGAAAGGA AGAAAACTCT AAAAAAAAAA GATGTACGCC   
  
  
- AACTTTTATT AAAAAAATTT TTTATATATA GTGATTGGTA AGTAATAGTA TCAAAATCTC AAGTATAAAT   
  
  
- ACACCAGGAA CAACTCAGAT TAAATTACGA AATCTACCGC ACTTCGTGGT TTAATCACCA TTGGTATTAT   
  
  
- TATTTGATAG TTCACAATTC AGCTTCTTTT GCCTGTGACG AGTTGGTTTA GATCATTGTA TCACATACTG   
  
  
- TACTATTGAT TCACACGGTA CATCATTAGT TAAGAAAGAA AAAAAAAAAA ACCAACTCTA GTGTATTTAA   
  
  
- TGCAATAGTT CCCATCATGC TATATTACCT ACTCCTCTTT AATTTACACT CTAAATCAAC TACTTAGCTT   
  
  
- ATGCAATCAG ACTCGGTAAA CCAGTTTTGA CGTAACCGAT TAGTTATCTT TAATATCTTT TATGTATTGT   
  
  
- ATTACCCTAC GTTAAAAGAT TAACCCCTTT GGTACAGTTA CGTCACTAAA ACCTGGTAAA TCCGGAATAA   
  
  
- GAAAAGTTAA AAAATTAACT AATAAATCTG AGTATACTTG AATAACAACT GTCTAACTCG ATTTTTGTGA   
  
  
- CTACAAAGTA TGTATACATA CTTAACGTGC ATGCTTCCGA GACGAGTAAA GCTCTTTCTT TTAATTGTCA   
  
  
- ATCCTCGTCA TATTAAATTG TTGTTCGCTA TTAATAATCA TACTTCACCA TGCTTAGATA AAATTTCCTC   
  
  
- ATGATACTTC ATGTTTGTTA TTGTTTATTA TTTCTCGTCA TGTAATTGTA TAGAATGATG GATTTATTTA   
  
  
- GCTAAGATTC TCATTTTTTT ACAACTTTAA TTGGGGTAAT ATCAAAATGA AATTGTTCTT CAAAAAGAAG   
  
  
- AAAATGGTAA ATGGTTCATT CTTACTAAGT CCGGCATATC GGTTCCTTTT TATCTTTTGG TGTTTTGATT   
  
  
- GGTACCGGGT TTTTTGTTGG TGGTTCTCCC CGTGATTACA CTGATTGGCT GCCAATTGTA ATAGTAGACA   
  
  
- AACTATGAAC AAGTAGAGAG TGATAAGCCC ATTATCACAC TTTAATGACT ACATGGGAGA TTTGAGCAGC   
  
  
- AAAATTGACA CATTCTTAGT CTAATTGTCA GTGCCTGTGT ACTCATTGTT TAGTTAAAGC CTGTACTTGC   
  
  
- TTGCTTGACA CTTAGGGGGG GTTTTTTTTT TAAAAAAATT TTTTTCAACG TAGGAATAGA AAGACTTGGT   
  
  
- CCGGTCGACA GAGTAAGGAA CCAGGAGCGC GGGGTAGTGC TCGCGACGAG TAATAAGTGG GAGAGAGAGA   
  
  
- GAGGAGAGAC TAGGTGTGGT GTTTGTGTTC CAAACGTGTC GACGTCTCGT CAGCATGATG ATAGTCATCA   
  
  
- CTTTTTGGTT CGTCTTTAGG GGTTAGGGAG AAGAGGAAGA AGGGTCTAAG AAGGAGTTAA GGTTCGGGTA   
  
  
- TAGTCTTTAA ATCTGTTGTT GTTGGTTTTT TTTTTTAATT ATGACGAACT CTTTTTGTTT ATACTATACT   
  
  
- TTAAGGAGCT TTTTCCGGAC GGTATTGTTT AAGGAGCACT TTTTCGTCGA CTCTCTTCTT CTTCTTCTTT   
  
  
- TTTTAGGGCA GTGTTAGAGT TAGAGCTAGG GTTTAACACT TTCTCTTTCT TTTCTACTTC TCTCTTGTGG   
  
  
- GGAAAAGCTT GGGACTGGTC TTTGGGGGCG GCCTCTACAG CGGCCGCGGC CCCTTCAGGT TCTACACCCT   
  
  
- ACTACTCTAC CGGGTCTTGC TGCGATTTTA CCTACTCGAC GAACGACAAC ACCCCATGTT CCACTCCAGC   
  
  
- AGCCTGTACC GCCTTTAACG GGTCTTCGAA CTCGTCGAAC TTCTTCAGTA CCCATCACAA GTTCTTCTGC   
  
  
- CCAATAGAAT GAACCGAAGG CTTTGACAAG TAATGTTAGG ACGTCTAGAC AGATGAACCG AACTTAGATA   
  
  
- CGAAAGACTC AAATTGGGAT TGGGATTAAA ACTGGGAAGA GGTAGCAGTT AGAGTGGGTA GTAGCTAGGT   
  
  
- CGAGGAGGTG AAGGAGCTTG GAGCAGCTGC CAGTTTAAGC TCGGGCTGGG CAAAAGGCTA AGCCTAGACT   
  
  
- TTCGTTAGGG TCCATTCCGA TAGAACTGGG GTGGTTCAAG ATCGTTATGA AGTTTAAACG CACTCCGATT   
  
  
- CTCCAACTTT GGGAGTTTAA TGTGTTGGCG GGGTTGCGGG TGGGGTGGAG GTTTTGACGG GTTAAGCCGC   
  
  
- AGAGGTGGTA GCCCGTGCTG CCGCAGCTCC GGCCACCACG ACCATCTGAG TGTTCTTTTG CCGTAGTCTA   
  
  
- ACCACGTACG TGAATACCGG ACACGGCTTC GTTAAGTTGA TCTTTTGTAC CCTAATCGAC TCCGAAACCA   
  
  
- ATTTGTCTAA TCCATAAATC GTCGTAGAGT TCGACCTGGA TACTCCTTTC ATCGTTGAAC AAAACGTCTT   
  
  
- CGAGAATGAA CAGCCTAGAT GTTCGATACG GGTAGACATG GGATGCTACT TAGTCAGAGT CTACTCAACG   
  
  
- TCTACGTGAA AATACTCTGA ACGGGTATAG AATTTAAGCG GGTAAAGTGC CGTTTAGTTC GTTAAAATCT   
  
  
- TCGTAAGTTA CCCTTCTTCT TTCAAGTACA CTAACTAAAG TCGTACTTCG TTCCGTACGT TACCGGCCGA   
  
  
- AACTACGTTC GGGACCGAGA AGCTGGCCTC CCAGGTGGCG AAAAGGCCAA TTGGCCCTAA CCTGGCGGGC   
  
  
- GAGGCCTGTT GAGCCTGGCC GACGTTCTCC ACCCAACCTT CGAACGGGTC AAGCGCCTAA GCTAGGCCTA   
  
  
- ATTTAAAGTC ATAGCACCCA AACACCGTTT GTCAAACTGG CTAGATCTTA GAAGGTACGA TCTAGAACTT   
  
  
- GGCCTGTGAC TCCACCACCG CCAGTTGAGC CAAAAGCTCG AGGTGGCCGA CAACCGATTT GGGCCCCGCT   
  
  
- AGCTCTTTCA CGACCCCAAG TACTCCCGGC ACTTGGGCCA GTAACACTGC TACCAGCTCG TCCTTCGCTT   
  
  
- GGTGTTGCCT GGCCAAAAGA ACCTGGCCAA GTTACTCAGC AACGTAATAA TGAGGTGGAA CAAACTAAGG   
  
  
- GAACTCTAAA CACAACTATT ACATCTATTC TTCTACAGCC TCCGAATGAA CCCGGTCGTC TAGACGTTGT   
  
  
- ACCACCGAAC ACTTCCCAGA CTGGCTCAGC TCTCCGTGCT CTGGGACCGA GTCACCGCTC GGGCCAAGCG   
  
  
- TAGACGGCCC AAGCTGGGTC AAGTAGACCC AAGCTTACGC AAATTCGTCC GCTCATACAA CAACCTCAAG   
  
  
- AAACGACCAC CACTTCCCAT GCCGCACCTC CTCGCTCTTC CCACAGAGTA CAACCCTACC GTATCATCCG   
  
  
- GCGAATAGTG GTGGAGCCGA ACCGTCGAGC GGTTCTTGGG CCGACACTTA GCCGCTAC

+     ABRE4

| Site Name | Organism | Position | Strand | Matrix score. | sequence | function |
| --- | --- | --- | --- | --- | --- | --- |
| ABRE4 | Zea mays | 1011 | + | 6 | CACGTA |  |

>HU10G00709.1   
+ -Up\_Stream \_Len000AACCCT TTAATTTTAT CAATGTGGGA CAACACTCAT CTTCATACTC CAACAAGCTA   
  
  
+ TGATACATTC GATTATTGCC TAGTATTTCG TTTAGTAAGA TATTAGTGCC AAACTTTGTA ATAAAAATGA   
  
  
+ AACTTACTAA AAAAAATCTC GTTTTCTGTA GAGGGAAAAT TTGAAGACTT AGTGTCACAT GATGACCTAA   
  
  
+ TGGTACTTGT AGGGTCAAGA TTGAGATTGA GGGCATACTG ACGACCTGTA TAATGGTCAA GTAGGATCAA   
  
  
+ AGTCGAGAAA ACTGTCTCTG CTAACTTTCA TAATCTAACC CTTAACTCAC TCTGACCCTC AATTTAATCA   
  
  
+ GGCCTAATTC AAACTATTAA TGGTTATAAC TCTCTTTCCT TCTTTTGAGA TTTTTTTTTT CTACATGCGG   
  
  
+ TTGAAAATAA TTTTTTTAAA AAATATATAT CACTAACCAT TCATTATCAT AGTTTTAGAG TTCATATTTA   
  
  
+ TGTGGTCCTT GTTGAGTCTA ATTTAATGCT TTAGATGGCG TGAAGCACCA AATTAGTGGT AACCATAATA   
  
  
+ ATAAACTATC AAGTGTTAAG TCGAAGAAAA CGGACACTGC TCAACCAAAT CTAGTAACAT AGTGTATGAC   
  
  
+ ATGATAACTA AGTGTGCCAT GTAGTAATCA ATTCTTTCTT TTTTTTTTTT TGGTTGAGAT CACATAAATT   
  
  
+ ACGTTATCAA GGGTAGTACG ATATAATGGA TGAGGAGAAA TTAAATGTGA GATTTAGTTG ATGAATCGAA   
  
  
+ TACGTTAGTC TGAGCCATTT GGTCAAAACT GCATTGGCTA ATCAATAGAA ATTATAGAAA ATACATAACA   
  
  
+ TAATGGGATG CAATTTTCTA ATTGGGGAAA CCATGTCAAT GCAGTGATTT TGGACCATTT AGGCCTTATT   
  
  
+ CTTTTCAATT TTTTAATTGA TTATTTAGAC TCATATGAAC TTATTGTTGA CAGATTGAGC TAAAAACACT   
  
  
+ GATGTTTCAT ACATATGTAT GAATTGCACG TACGAAGGCT CTGCTCATTT CGAGAAAGAA AATTAACAGT   
  
  
+ TAGGAGCAGT ATAATTTAAC AACAAGCGAT AATTATTAGT ATGAAGTGGT ACGAATCTAT TTTAAAGGAG   
  
  
+ TACTATGAAG TACAAACAAT AACAAATAAT AAAGAGCAGT ACATTAACAT ATCTTACTAC CTAAATAAAT   
  
  
+ CGATTCTAAG AGTAAAAAAA TGTTGAAATT AACCCCATTA TAGTTTTACT TTAACAAGAA GTTTTTCTTC   
  
  
+ TTTTACCATT TACCAAGTAA GAATGATTCA GGCCGTATAG CCAAGGAAAA ATAGAAAACC ACAAAACTAA   
  
  
+ CCATGGCCCA AAAAACAACC ACCAAGAGGG GCACTAATGT GACTAACCGA CGGTTAACAT TATCATCTGT   
  
  
+ TTGATACTTG TTCATCTCTC ACTATTCGGG TAATAGTGTG AAATTACTGA TGTACCCTCT AAACTCGTCG   
  
  
+ TTTTAACTGT GTAAGAATCA GATTAACAGT CACGGACACA TGAGTAACAA ATCAATTTCG GACATGAACG   
  
  
+ AACGAACTGT GAATCCCCCC CAAAAAAAAA ATTTTTTTAA AAAAAGTTGC ATCCTTATCT TTCTGAACCA   
  
  
+ GGCCAGCTGT CTCATTCCTT GGTCCTCGCG CCCCATCACG AGCGCTGCTC ATTATTCACC CTCTCTCTCT   
  
  
+ CTCCTCTCTG ATCCACACCA CAAACACAAG GTTTGCACAG CTGCAGAGCA GTCGTACTAC TATCAGTAGT   
  
  
+ GAAAAACCAA GCAGAAATCC CCAATCCCTC TTCTCCTTCT TCCCAGATTC TTCCTCAATT CCAAGCCCAT   
  
  
+ ATCAGAAATT TAGACAACAA CAACCAAAAA AAAAAATTAA TACTGCTTGA GAAAAACAAA TATGATATGA   
  
  
+ AATTCCTCGA AAAAGGCCTG CCATAACAAA TTCCTCGTGA AAAAGCAGCT GAGAGAAGAA GAAGAAGAAA   
  
  
+ AAAATCCCGT CACAATCTCA ATCTCGATCC CAAATTGTGA AAGAGAAAGA AAAGATGAAG AGAGAACACC   
  
  
+ CCTTTTCGAA CCCTGACCAG AAACCCCCGC CGGAGATGTC GCCGGCGCCG GGGAAGTCCA AGATGTGGGA   
  
  
+ TGATGAGATG GCCCAGAACG ACGCTAAAAT GGATGAGCTG CTTGCTGTTG TGGGGTACAA GGTGAGGTCG   
  
  
+ TCGGACATGG CGGAAATTGC CCAGAAGCTT GAGCAGCTTG AAGAAGTCAT GGGTAGTGTT CAAGAAGACG   
  
  
+ GGTTATCTTA CTTGGCTTCC GAAACTGTTC ATTACAATCC TGCAGATCTG TCTACTTGGC TTGAATCTAT   
  
  
+ GCTTTCTGAG TTTAACCCTA ACCCTAATTT TGACCCTTCT CCATCGTCAA TCTCACCCAT CATCGATCCA   
  
  
+ GCTCCTCCAC TTCCTCGAAC CTCGTCGACG GTCAAATTCG AGCCCGACCC GTTTTCCGAT TCGGATCTGA   
  
  
+ AAGCAATCCC AGGTAAGGCT ATCTTGACCC CACCAAGTTC TAGCAATACT TCAAATTTGC GTGAGGCTAA   
  
  
+ GAGGTTGAAA CCCTCAAATT ACACAACCGC CCCAACGCCC ACCCCACCTC CAAAACTGCC CAATTCGGCG   
  
  
+ TCTCCACCAT CGGGCACGAC GGCGTCGAGG CCGGTGGTGC TGGTAGACTC ACAAGAAAAC GGCATCAGAT   
  
  
+ TGGTGCATGC ACTTATGGCC TGTGCCGAAG CAATTCAACT AGAAAACATG GGATTAGCTG AGGCTTTGGT   
  
  
+ TAAACAGATT AGGTATTTAG CAGCATCTCA AGCTGGACCT ATGAGGAAAG TAGCAACTTG TTTTGCAGAA   
  
  
+ GCTCTTACTT GTCGGATCTA CAAGCTATGC CCATCTGTAC CCTACGATGA ATCAGTCTCA GATGAGTTGC   
  
  
+ AGATGCACTT TTATGAGACT TGCCCATATC TTAAATTCGC CCATTTCACG GCAAATCAAG CAATTTTAGA   
  
  
+ AGCATTCAAT GGGAAGAAGA AAGTTCATGT GATTGATTTC AGCATGAAGC AAGGCATGCA ATGGCCGGCT   
  
  
+ TTGATGCAAG CCCTGGCTCT TCGACCGGAG GGTCCACCGC TTTTCCGGTT AACCGGGATT GGACCGCCCG   
  
  
+ CTCCGGACAA CTCGGACCGG CTGCAAGAGG TGGGTTGGAA GCTTGCCCAG TTCGCGGATT CGATCCGGAT   
  
  
+ TAAATTTCAG TATCGTGGGT TTGTGGCAAA CAGTTTGACC GATCTAGAAT CTTCCATGCT AGATCTTGAA   
  
  
+ CCGGACACTG AGGTGGTGGC GGTCAACTCG GTTTTCGAGC TCCACCGGCT GTTGGCTAAA CCCGGGGCGA   
  
  
+ TCGAGAAAGT GCTGGGGTTC ATGAGGGCCG TGAACCCGGT CATTGTGACG ATGGTCGAGC AGGAAGCGAA   
  
  
+ CCACAACGGA CCGGTTTTCT TGGACCGGTT CAATGAGTCG TTGCATTATT ACTCCACCTT GTTTGATTCC   
  
  
+ CTTGAGATTT GTGTTGATAA TGTAGATAAG AAGATGTCGG AGGCTTACTT GGGCCAGCAG ATCTGCAACA   
  
  
+ TGGTGGCTTG TGAAGGGTCT GACCGAGTCG AGAGGCACGA GACCCTGGCT CAGTGGCGAG CCCGGTTCGC   
  
  
+ ATCTGCCGGG TTCGACCCAG TTCATCTGGG TTCGAATGCG TTTAAGCAGG CGAGTATGTT GTTGGAGTTC   
  
  
+ TTTGCTGGTG GTGAAGGGTA CGGCGTGGAG GAGCGAGAAG GGTGTCTCAT GTTGGGATGG CATAGTAGGC   
  
  
+ CGCTTATCAC CACCTCGGCT TGGCAGCTCG CCAAGAACCC GGCTGTGAAT CGGCGATG  

- -Up\_Stream \_Len000TTGGGA AATTAAAATA GTTACACCCT GTTGTGAGTA GAAGTATGAG GTTGTTCGAT   
  
  
- ACTATGTAAG CTAATAACGG ATCATAAAGC AAATCATTCT ATAATCACGG TTTGAAACAT TATTTTTACT   
  
  
- TTGAATGATT TTTTTTAGAG CAAAAGACAT CTCCCTTTTA AACTTCTGAA TCACAGTGTA CTACTGGATT   
  
  
- ACCATGAACA TCCCAGTTCT AACTCTAACT CCCGTATGAC TGCTGGACAT ATTACCAGTT CATCCTAGTT   
  
  
- TCAGCTCTTT TGACAGAGAC GATTGAAAGT ATTAGATTGG GAATTGAGTG AGACTGGGAG TTAAATTAGT   
  
  
- CCGGATTAAG TTTGATAATT ACCAATATTG AGAGAAAGGA AGAAAACTCT AAAAAAAAAA GATGTACGCC   
  
  
- AACTTTTATT AAAAAAATTT TTTATATATA GTGATTGGTA AGTAATAGTA TCAAAATCTC AAGTATAAAT   
  
  
- ACACCAGGAA CAACTCAGAT TAAATTACGA AATCTACCGC ACTTCGTGGT TTAATCACCA TTGGTATTAT   
  
  
- TATTTGATAG TTCACAATTC AGCTTCTTTT GCCTGTGACG AGTTGGTTTA GATCATTGTA TCACATACTG   
  
  
- TACTATTGAT TCACACGGTA CATCATTAGT TAAGAAAGAA AAAAAAAAAA ACCAACTCTA GTGTATTTAA   
  
  
- TGCAATAGTT CCCATCATGC TATATTACCT ACTCCTCTTT AATTTACACT CTAAATCAAC TACTTAGCTT   
  
  
- ATGCAATCAG ACTCGGTAAA CCAGTTTTGA CGTAACCGAT TAGTTATCTT TAATATCTTT TATGTATTGT   
  
  
- ATTACCCTAC GTTAAAAGAT TAACCCCTTT GGTACAGTTA CGTCACTAAA ACCTGGTAAA TCCGGAATAA   
  
  
- GAAAAGTTAA AAAATTAACT AATAAATCTG AGTATACTTG AATAACAACT GTCTAACTCG ATTTTTGTGA   
  
  
- CTACAAAGTA TGTATACATA CTTAACGTGC ATGCTTCCGA GACGAGTAAA GCTCTTTCTT TTAATTGTCA   
  
  
- ATCCTCGTCA TATTAAATTG TTGTTCGCTA TTAATAATCA TACTTCACCA TGCTTAGATA AAATTTCCTC   
  
  
- ATGATACTTC ATGTTTGTTA TTGTTTATTA TTTCTCGTCA TGTAATTGTA TAGAATGATG GATTTATTTA   
  
  
- GCTAAGATTC TCATTTTTTT ACAACTTTAA TTGGGGTAAT ATCAAAATGA AATTGTTCTT CAAAAAGAAG   
  
  
- AAAATGGTAA ATGGTTCATT CTTACTAAGT CCGGCATATC GGTTCCTTTT TATCTTTTGG TGTTTTGATT   
  
  
- GGTACCGGGT TTTTTGTTGG TGGTTCTCCC CGTGATTACA CTGATTGGCT GCCAATTGTA ATAGTAGACA   
  
  
- AACTATGAAC AAGTAGAGAG TGATAAGCCC ATTATCACAC TTTAATGACT ACATGGGAGA TTTGAGCAGC   
  
  
- AAAATTGACA CATTCTTAGT CTAATTGTCA GTGCCTGTGT ACTCATTGTT TAGTTAAAGC CTGTACTTGC   
  
  
- TTGCTTGACA CTTAGGGGGG GTTTTTTTTT TAAAAAAATT TTTTTCAACG TAGGAATAGA AAGACTTGGT   
  
  
- CCGGTCGACA GAGTAAGGAA CCAGGAGCGC GGGGTAGTGC TCGCGACGAG TAATAAGTGG GAGAGAGAGA   
  
  
- GAGGAGAGAC TAGGTGTGGT GTTTGTGTTC CAAACGTGTC GACGTCTCGT CAGCATGATG ATAGTCATCA   
  
  
- CTTTTTGGTT CGTCTTTAGG GGTTAGGGAG AAGAGGAAGA AGGGTCTAAG AAGGAGTTAA GGTTCGGGTA   
  
  
- TAGTCTTTAA ATCTGTTGTT GTTGGTTTTT TTTTTTAATT ATGACGAACT CTTTTTGTTT ATACTATACT   
  
  
- TTAAGGAGCT TTTTCCGGAC GGTATTGTTT AAGGAGCACT TTTTCGTCGA CTCTCTTCTT CTTCTTCTTT   
  
  
- TTTTAGGGCA GTGTTAGAGT TAGAGCTAGG GTTTAACACT TTCTCTTTCT TTTCTACTTC TCTCTTGTGG   
  
  
- GGAAAAGCTT GGGACTGGTC TTTGGGGGCG GCCTCTACAG CGGCCGCGGC CCCTTCAGGT TCTACACCCT   
  
  
- ACTACTCTAC CGGGTCTTGC TGCGATTTTA CCTACTCGAC GAACGACAAC ACCCCATGTT CCACTCCAGC   
  
  
- AGCCTGTACC GCCTTTAACG GGTCTTCGAA CTCGTCGAAC TTCTTCAGTA CCCATCACAA GTTCTTCTGC   
  
  
- CCAATAGAAT GAACCGAAGG CTTTGACAAG TAATGTTAGG ACGTCTAGAC AGATGAACCG AACTTAGATA   
  
  
- CGAAAGACTC AAATTGGGAT TGGGATTAAA ACTGGGAAGA GGTAGCAGTT AGAGTGGGTA GTAGCTAGGT   
  
  
- CGAGGAGGTG AAGGAGCTTG GAGCAGCTGC CAGTTTAAGC TCGGGCTGGG CAAAAGGCTA AGCCTAGACT   
  
  
- TTCGTTAGGG TCCATTCCGA TAGAACTGGG GTGGTTCAAG ATCGTTATGA AGTTTAAACG CACTCCGATT   
  
  
- CTCCAACTTT GGGAGTTTAA TGTGTTGGCG GGGTTGCGGG TGGGGTGGAG GTTTTGACGG GTTAAGCCGC   
  
  
- AGAGGTGGTA GCCCGTGCTG CCGCAGCTCC GGCCACCACG ACCATCTGAG TGTTCTTTTG CCGTAGTCTA   
  
  
- ACCACGTACG TGAATACCGG ACACGGCTTC GTTAAGTTGA TCTTTTGTAC CCTAATCGAC TCCGAAACCA   
  
  
- ATTTGTCTAA TCCATAAATC GTCGTAGAGT TCGACCTGGA TACTCCTTTC ATCGTTGAAC AAAACGTCTT   
  
  
- CGAGAATGAA CAGCCTAGAT GTTCGATACG GGTAGACATG GGATGCTACT TAGTCAGAGT CTACTCAACG   
  
  
- TCTACGTGAA AATACTCTGA ACGGGTATAG AATTTAAGCG GGTAAAGTGC CGTTTAGTTC GTTAAAATCT   
  
  
- TCGTAAGTTA CCCTTCTTCT TTCAAGTACA CTAACTAAAG TCGTACTTCG TTCCGTACGT TACCGGCCGA   
  
  
- AACTACGTTC GGGACCGAGA AGCTGGCCTC CCAGGTGGCG AAAAGGCCAA TTGGCCCTAA CCTGGCGGGC   
  
  
- GAGGCCTGTT GAGCCTGGCC GACGTTCTCC ACCCAACCTT CGAACGGGTC AAGCGCCTAA GCTAGGCCTA   
  
  
- ATTTAAAGTC ATAGCACCCA AACACCGTTT GTCAAACTGG CTAGATCTTA GAAGGTACGA TCTAGAACTT   
  
  
- GGCCTGTGAC TCCACCACCG CCAGTTGAGC CAAAAGCTCG AGGTGGCCGA CAACCGATTT GGGCCCCGCT   
  
  
- AGCTCTTTCA CGACCCCAAG TACTCCCGGC ACTTGGGCCA GTAACACTGC TACCAGCTCG TCCTTCGCTT   
  
  
- GGTGTTGCCT GGCCAAAAGA ACCTGGCCAA GTTACTCAGC AACGTAATAA TGAGGTGGAA CAAACTAAGG   
  
  
- GAACTCTAAA CACAACTATT ACATCTATTC TTCTACAGCC TCCGAATGAA CCCGGTCGTC TAGACGTTGT   
  
  
- ACCACCGAAC ACTTCCCAGA CTGGCTCAGC TCTCCGTGCT CTGGGACCGA GTCACCGCTC GGGCCAAGCG   
  
  
- TAGACGGCCC AAGCTGGGTC AAGTAGACCC AAGCTTACGC AAATTCGTCC GCTCATACAA CAACCTCAAG   
  
  
- AAACGACCAC CACTTCCCAT GCCGCACCTC CTCGCTCTTC CCACAGAGTA CAACCCTACC GTATCATCCG   
  
  
- GCGAATAGTG GTGGAGCCGA ACCGTCGAGC GGTTCTTGGG CCGACACTTA GCCGCTAC

+     AC-I

| Site Name | Organism | Position | Strand | Matrix score. | sequence | function |
| --- | --- | --- | --- | --- | --- | --- |
| AC-I | Phaseolus vulgaris | 3113 | - | 8.5 | (T/C)C(T/C)(C/T)ACC(T/C)ACC |  |

>HU10G00709.1   
+ -Up\_Stream \_Len000AACCCT TTAATTTTAT CAATGTGGGA CAACACTCAT CTTCATACTC CAACAAGCTA   
  
  
+ TGATACATTC GATTATTGCC TAGTATTTCG TTTAGTAAGA TATTAGTGCC AAACTTTGTA ATAAAAATGA   
  
  
+ AACTTACTAA AAAAAATCTC GTTTTCTGTA GAGGGAAAAT TTGAAGACTT AGTGTCACAT GATGACCTAA   
  
  
+ TGGTACTTGT AGGGTCAAGA TTGAGATTGA GGGCATACTG ACGACCTGTA TAATGGTCAA GTAGGATCAA   
  
  
+ AGTCGAGAAA ACTGTCTCTG CTAACTTTCA TAATCTAACC CTTAACTCAC TCTGACCCTC AATTTAATCA   
  
  
+ GGCCTAATTC AAACTATTAA TGGTTATAAC TCTCTTTCCT TCTTTTGAGA TTTTTTTTTT CTACATGCGG   
  
  
+ TTGAAAATAA TTTTTTTAAA AAATATATAT CACTAACCAT TCATTATCAT AGTTTTAGAG TTCATATTTA   
  
  
+ TGTGGTCCTT GTTGAGTCTA ATTTAATGCT TTAGATGGCG TGAAGCACCA AATTAGTGGT AACCATAATA   
  
  
+ ATAAACTATC AAGTGTTAAG TCGAAGAAAA CGGACACTGC TCAACCAAAT CTAGTAACAT AGTGTATGAC   
  
  
+ ATGATAACTA AGTGTGCCAT GTAGTAATCA ATTCTTTCTT TTTTTTTTTT TGGTTGAGAT CACATAAATT   
  
  
+ ACGTTATCAA GGGTAGTACG ATATAATGGA TGAGGAGAAA TTAAATGTGA GATTTAGTTG ATGAATCGAA   
  
  
+ TACGTTAGTC TGAGCCATTT GGTCAAAACT GCATTGGCTA ATCAATAGAA ATTATAGAAA ATACATAACA   
  
  
+ TAATGGGATG CAATTTTCTA ATTGGGGAAA CCATGTCAAT GCAGTGATTT TGGACCATTT AGGCCTTATT   
  
  
+ CTTTTCAATT TTTTAATTGA TTATTTAGAC TCATATGAAC TTATTGTTGA CAGATTGAGC TAAAAACACT   
  
  
+ GATGTTTCAT ACATATGTAT GAATTGCACG TACGAAGGCT CTGCTCATTT CGAGAAAGAA AATTAACAGT   
  
  
+ TAGGAGCAGT ATAATTTAAC AACAAGCGAT AATTATTAGT ATGAAGTGGT ACGAATCTAT TTTAAAGGAG   
  
  
+ TACTATGAAG TACAAACAAT AACAAATAAT AAAGAGCAGT ACATTAACAT ATCTTACTAC CTAAATAAAT   
  
  
+ CGATTCTAAG AGTAAAAAAA TGTTGAAATT AACCCCATTA TAGTTTTACT TTAACAAGAA GTTTTTCTTC   
  
  
+ TTTTACCATT TACCAAGTAA GAATGATTCA GGCCGTATAG CCAAGGAAAA ATAGAAAACC ACAAAACTAA   
  
  
+ CCATGGCCCA AAAAACAACC ACCAAGAGGG GCACTAATGT GACTAACCGA CGGTTAACAT TATCATCTGT   
  
  
+ TTGATACTTG TTCATCTCTC ACTATTCGGG TAATAGTGTG AAATTACTGA TGTACCCTCT AAACTCGTCG   
  
  
+ TTTTAACTGT GTAAGAATCA GATTAACAGT CACGGACACA TGAGTAACAA ATCAATTTCG GACATGAACG   
  
  
+ AACGAACTGT GAATCCCCCC CAAAAAAAAA ATTTTTTTAA AAAAAGTTGC ATCCTTATCT TTCTGAACCA   
  
  
+ GGCCAGCTGT CTCATTCCTT GGTCCTCGCG CCCCATCACG AGCGCTGCTC ATTATTCACC CTCTCTCTCT   
  
  
+ CTCCTCTCTG ATCCACACCA CAAACACAAG GTTTGCACAG CTGCAGAGCA GTCGTACTAC TATCAGTAGT   
  
  
+ GAAAAACCAA GCAGAAATCC CCAATCCCTC TTCTCCTTCT TCCCAGATTC TTCCTCAATT CCAAGCCCAT   
  
  
+ ATCAGAAATT TAGACAACAA CAACCAAAAA AAAAAATTAA TACTGCTTGA GAAAAACAAA TATGATATGA   
  
  
+ AATTCCTCGA AAAAGGCCTG CCATAACAAA TTCCTCGTGA AAAAGCAGCT GAGAGAAGAA GAAGAAGAAA   
  
  
+ AAAATCCCGT CACAATCTCA ATCTCGATCC CAAATTGTGA AAGAGAAAGA AAAGATGAAG AGAGAACACC   
  
  
+ CCTTTTCGAA CCCTGACCAG AAACCCCCGC CGGAGATGTC GCCGGCGCCG GGGAAGTCCA AGATGTGGGA   
  
  
+ TGATGAGATG GCCCAGAACG ACGCTAAAAT GGATGAGCTG CTTGCTGTTG TGGGGTACAA GGTGAGGTCG   
  
  
+ TCGGACATGG CGGAAATTGC CCAGAAGCTT GAGCAGCTTG AAGAAGTCAT GGGTAGTGTT CAAGAAGACG   
  
  
+ GGTTATCTTA CTTGGCTTCC GAAACTGTTC ATTACAATCC TGCAGATCTG TCTACTTGGC TTGAATCTAT   
  
  
+ GCTTTCTGAG TTTAACCCTA ACCCTAATTT TGACCCTTCT CCATCGTCAA TCTCACCCAT CATCGATCCA   
  
  
+ GCTCCTCCAC TTCCTCGAAC CTCGTCGACG GTCAAATTCG AGCCCGACCC GTTTTCCGAT TCGGATCTGA   
  
  
+ AAGCAATCCC AGGTAAGGCT ATCTTGACCC CACCAAGTTC TAGCAATACT TCAAATTTGC GTGAGGCTAA   
  
  
+ GAGGTTGAAA CCCTCAAATT ACACAACCGC CCCAACGCCC ACCCCACCTC CAAAACTGCC CAATTCGGCG   
  
  
+ TCTCCACCAT CGGGCACGAC GGCGTCGAGG CCGGTGGTGC TGGTAGACTC ACAAGAAAAC GGCATCAGAT   
  
  
+ TGGTGCATGC ACTTATGGCC TGTGCCGAAG CAATTCAACT AGAAAACATG GGATTAGCTG AGGCTTTGGT   
  
  
+ TAAACAGATT AGGTATTTAG CAGCATCTCA AGCTGGACCT ATGAGGAAAG TAGCAACTTG TTTTGCAGAA   
  
  
+ GCTCTTACTT GTCGGATCTA CAAGCTATGC CCATCTGTAC CCTACGATGA ATCAGTCTCA GATGAGTTGC   
  
  
+ AGATGCACTT TTATGAGACT TGCCCATATC TTAAATTCGC CCATTTCACG GCAAATCAAG CAATTTTAGA   
  
  
+ AGCATTCAAT GGGAAGAAGA AAGTTCATGT GATTGATTTC AGCATGAAGC AAGGCATGCA ATGGCCGGCT   
  
  
+ TTGATGCAAG CCCTGGCTCT TCGACCGGAG GGTCCACCGC TTTTCCGGTT AACCGGGATT GGACCGCCCG   
  
  
+ CTCCGGACAA CTCGGACCGG CTGCAAGAGG TGGGTTGGAA GCTTGCCCAG TTCGCGGATT CGATCCGGAT   
  
  
+ TAAATTTCAG TATCGTGGGT TTGTGGCAAA CAGTTTGACC GATCTAGAAT CTTCCATGCT AGATCTTGAA   
  
  
+ CCGGACACTG AGGTGGTGGC GGTCAACTCG GTTTTCGAGC TCCACCGGCT GTTGGCTAAA CCCGGGGCGA   
  
  
+ TCGAGAAAGT GCTGGGGTTC ATGAGGGCCG TGAACCCGGT CATTGTGACG ATGGTCGAGC AGGAAGCGAA   
  
  
+ CCACAACGGA CCGGTTTTCT TGGACCGGTT CAATGAGTCG TTGCATTATT ACTCCACCTT GTTTGATTCC   
  
  
+ CTTGAGATTT GTGTTGATAA TGTAGATAAG AAGATGTCGG AGGCTTACTT GGGCCAGCAG ATCTGCAACA   
  
  
+ TGGTGGCTTG TGAAGGGTCT GACCGAGTCG AGAGGCACGA GACCCTGGCT CAGTGGCGAG CCCGGTTCGC   
  
  
+ ATCTGCCGGG TTCGACCCAG TTCATCTGGG TTCGAATGCG TTTAAGCAGG CGAGTATGTT GTTGGAGTTC   
  
  
+ TTTGCTGGTG GTGAAGGGTA CGGCGTGGAG GAGCGAGAAG GGTGTCTCAT GTTGGGATGG CATAGTAGGC   
  
  
+ CGCTTATCAC CACCTCGGCT TGGCAGCTCG CCAAGAACCC GGCTGTGAAT CGGCGATG  

- -Up\_Stream \_Len000TTGGGA AATTAAAATA GTTACACCCT GTTGTGAGTA GAAGTATGAG GTTGTTCGAT   
  
  
- ACTATGTAAG CTAATAACGG ATCATAAAGC AAATCATTCT ATAATCACGG TTTGAAACAT TATTTTTACT   
  
  
- TTGAATGATT TTTTTTAGAG CAAAAGACAT CTCCCTTTTA AACTTCTGAA TCACAGTGTA CTACTGGATT   
  
  
- ACCATGAACA TCCCAGTTCT AACTCTAACT CCCGTATGAC TGCTGGACAT ATTACCAGTT CATCCTAGTT   
  
  
- TCAGCTCTTT TGACAGAGAC GATTGAAAGT ATTAGATTGG GAATTGAGTG AGACTGGGAG TTAAATTAGT   
  
  
- CCGGATTAAG TTTGATAATT ACCAATATTG AGAGAAAGGA AGAAAACTCT AAAAAAAAAA GATGTACGCC   
  
  
- AACTTTTATT AAAAAAATTT TTTATATATA GTGATTGGTA AGTAATAGTA TCAAAATCTC AAGTATAAAT   
  
  
- ACACCAGGAA CAACTCAGAT TAAATTACGA AATCTACCGC ACTTCGTGGT TTAATCACCA TTGGTATTAT   
  
  
- TATTTGATAG TTCACAATTC AGCTTCTTTT GCCTGTGACG AGTTGGTTTA GATCATTGTA TCACATACTG   
  
  
- TACTATTGAT TCACACGGTA CATCATTAGT TAAGAAAGAA AAAAAAAAAA ACCAACTCTA GTGTATTTAA   
  
  
- TGCAATAGTT CCCATCATGC TATATTACCT ACTCCTCTTT AATTTACACT CTAAATCAAC TACTTAGCTT   
  
  
- ATGCAATCAG ACTCGGTAAA CCAGTTTTGA CGTAACCGAT TAGTTATCTT TAATATCTTT TATGTATTGT   
  
  
- ATTACCCTAC GTTAAAAGAT TAACCCCTTT GGTACAGTTA CGTCACTAAA ACCTGGTAAA TCCGGAATAA   
  
  
- GAAAAGTTAA AAAATTAACT AATAAATCTG AGTATACTTG AATAACAACT GTCTAACTCG ATTTTTGTGA   
  
  
- CTACAAAGTA TGTATACATA CTTAACGTGC ATGCTTCCGA GACGAGTAAA GCTCTTTCTT TTAATTGTCA   
  
  
- ATCCTCGTCA TATTAAATTG TTGTTCGCTA TTAATAATCA TACTTCACCA TGCTTAGATA AAATTTCCTC   
  
  
- ATGATACTTC ATGTTTGTTA TTGTTTATTA TTTCTCGTCA TGTAATTGTA TAGAATGATG GATTTATTTA   
  
  
- GCTAAGATTC TCATTTTTTT ACAACTTTAA TTGGGGTAAT ATCAAAATGA AATTGTTCTT CAAAAAGAAG   
  
  
- AAAATGGTAA ATGGTTCATT CTTACTAAGT CCGGCATATC GGTTCCTTTT TATCTTTTGG TGTTTTGATT   
  
  
- GGTACCGGGT TTTTTGTTGG TGGTTCTCCC CGTGATTACA CTGATTGGCT GCCAATTGTA ATAGTAGACA   
  
  
- AACTATGAAC AAGTAGAGAG TGATAAGCCC ATTATCACAC TTTAATGACT ACATGGGAGA TTTGAGCAGC   
  
  
- AAAATTGACA CATTCTTAGT CTAATTGTCA GTGCCTGTGT ACTCATTGTT TAGTTAAAGC CTGTACTTGC   
  
  
- TTGCTTGACA CTTAGGGGGG GTTTTTTTTT TAAAAAAATT TTTTTCAACG TAGGAATAGA AAGACTTGGT   
  
  
- CCGGTCGACA GAGTAAGGAA CCAGGAGCGC GGGGTAGTGC TCGCGACGAG TAATAAGTGG GAGAGAGAGA   
  
  
- GAGGAGAGAC TAGGTGTGGT GTTTGTGTTC CAAACGTGTC GACGTCTCGT CAGCATGATG ATAGTCATCA   
  
  
- CTTTTTGGTT CGTCTTTAGG GGTTAGGGAG AAGAGGAAGA AGGGTCTAAG AAGGAGTTAA GGTTCGGGTA   
  
  
- TAGTCTTTAA ATCTGTTGTT GTTGGTTTTT TTTTTTAATT ATGACGAACT CTTTTTGTTT ATACTATACT   
  
  
- TTAAGGAGCT TTTTCCGGAC GGTATTGTTT AAGGAGCACT TTTTCGTCGA CTCTCTTCTT CTTCTTCTTT   
  
  
- TTTTAGGGCA GTGTTAGAGT TAGAGCTAGG GTTTAACACT TTCTCTTTCT TTTCTACTTC TCTCTTGTGG   
  
  
- GGAAAAGCTT GGGACTGGTC TTTGGGGGCG GCCTCTACAG CGGCCGCGGC CCCTTCAGGT TCTACACCCT   
  
  
- ACTACTCTAC CGGGTCTTGC TGCGATTTTA CCTACTCGAC GAACGACAAC ACCCCATGTT CCACTCCAGC   
  
  
- AGCCTGTACC GCCTTTAACG GGTCTTCGAA CTCGTCGAAC TTCTTCAGTA CCCATCACAA GTTCTTCTGC   
  
  
- CCAATAGAAT GAACCGAAGG CTTTGACAAG TAATGTTAGG ACGTCTAGAC AGATGAACCG AACTTAGATA   
  
  
- CGAAAGACTC AAATTGGGAT TGGGATTAAA ACTGGGAAGA GGTAGCAGTT AGAGTGGGTA GTAGCTAGGT   
  
  
- CGAGGAGGTG AAGGAGCTTG GAGCAGCTGC CAGTTTAAGC TCGGGCTGGG CAAAAGGCTA AGCCTAGACT   
  
  
- TTCGTTAGGG TCCATTCCGA TAGAACTGGG GTGGTTCAAG ATCGTTATGA AGTTTAAACG CACTCCGATT   
  
  
- CTCCAACTTT GGGAGTTTAA TGTGTTGGCG GGGTTGCGGG TGGGGTGGAG GTTTTGACGG GTTAAGCCGC   
  
  
- AGAGGTGGTA GCCCGTGCTG CCGCAGCTCC GGCCACCACG ACCATCTGAG TGTTCTTTTG CCGTAGTCTA   
  
  
- ACCACGTACG TGAATACCGG ACACGGCTTC GTTAAGTTGA TCTTTTGTAC CCTAATCGAC TCCGAAACCA   
  
  
- ATTTGTCTAA TCCATAAATC GTCGTAGAGT TCGACCTGGA TACTCCTTTC ATCGTTGAAC AAAACGTCTT   
  
  
- CGAGAATGAA CAGCCTAGAT GTTCGATACG GGTAGACATG GGATGCTACT TAGTCAGAGT CTACTCAACG   
  
  
- TCTACGTGAA AATACTCTGA ACGGGTATAG AATTTAAGCG GGTAAAGTGC CGTTTAGTTC GTTAAAATCT   
  
  
- TCGTAAGTTA CCCTTCTTCT TTCAAGTACA CTAACTAAAG TCGTACTTCG TTCCGTACGT TACCGGCCGA   
  
  
- AACTACGTTC GGGACCGAGA AGCTGGCCTC CCAGGTGGCG AAAAGGCCAA TTGGCCCTAA CCTGGCGGGC   
  
  
- GAGGCCTGTT GAGCCTGGCC GACGTTCTCC ACCCAACCTT CGAACGGGTC AAGCGCCTAA GCTAGGCCTA   
  
  
- ATTTAAAGTC ATAGCACCCA AACACCGTTT GTCAAACTGG CTAGATCTTA GAAGGTACGA TCTAGAACTT   
  
  
- GGCCTGTGAC TCCACCACCG CCAGTTGAGC CAAAAGCTCG AGGTGGCCGA CAACCGATTT GGGCCCCGCT   
  
  
- AGCTCTTTCA CGACCCCAAG TACTCCCGGC ACTTGGGCCA GTAACACTGC TACCAGCTCG TCCTTCGCTT   
  
  
- GGTGTTGCCT GGCCAAAAGA ACCTGGCCAA GTTACTCAGC AACGTAATAA TGAGGTGGAA CAAACTAAGG   
  
  
- GAACTCTAAA CACAACTATT ACATCTATTC TTCTACAGCC TCCGAATGAA CCCGGTCGTC TAGACGTTGT   
  
  
- ACCACCGAAC ACTTCCCAGA CTGGCTCAGC TCTCCGTGCT CTGGGACCGA GTCACCGCTC GGGCCAAGCG   
  
  
- TAGACGGCCC AAGCTGGGTC AAGTAGACCC AAGCTTACGC AAATTCGTCC GCTCATACAA CAACCTCAAG   
  
  
- AAACGACCAC CACTTCCCAT GCCGCACCTC CTCGCTCTTC CCACAGAGTA CAACCCTACC GTATCATCCG   
  
  
- GCGAATAGTG GTGGAGCCGA ACCGTCGAGC GGTTCTTGGG CCGACACTTA GCCGCTAC

+     ACE

| Site Name | Organism | Position | Strand | Matrix score. | sequence | function |
| --- | --- | --- | --- | --- | --- | --- |
| ACE | Petroselinum crispum | 773 | - | 10 | CTAACGTATT | cis-acting element involved in light responsiveness |

>HU10G00709.1   
+ -Up\_Stream \_Len000AACCCT TTAATTTTAT CAATGTGGGA CAACACTCAT CTTCATACTC CAACAAGCTA   
  
  
+ TGATACATTC GATTATTGCC TAGTATTTCG TTTAGTAAGA TATTAGTGCC AAACTTTGTA ATAAAAATGA   
  
  
+ AACTTACTAA AAAAAATCTC GTTTTCTGTA GAGGGAAAAT TTGAAGACTT AGTGTCACAT GATGACCTAA   
  
  
+ TGGTACTTGT AGGGTCAAGA TTGAGATTGA GGGCATACTG ACGACCTGTA TAATGGTCAA GTAGGATCAA   
  
  
+ AGTCGAGAAA ACTGTCTCTG CTAACTTTCA TAATCTAACC CTTAACTCAC TCTGACCCTC AATTTAATCA   
  
  
+ GGCCTAATTC AAACTATTAA TGGTTATAAC TCTCTTTCCT TCTTTTGAGA TTTTTTTTTT CTACATGCGG   
  
  
+ TTGAAAATAA TTTTTTTAAA AAATATATAT CACTAACCAT TCATTATCAT AGTTTTAGAG TTCATATTTA   
  
  
+ TGTGGTCCTT GTTGAGTCTA ATTTAATGCT TTAGATGGCG TGAAGCACCA AATTAGTGGT AACCATAATA   
  
  
+ ATAAACTATC AAGTGTTAAG TCGAAGAAAA CGGACACTGC TCAACCAAAT CTAGTAACAT AGTGTATGAC   
  
  
+ ATGATAACTA AGTGTGCCAT GTAGTAATCA ATTCTTTCTT TTTTTTTTTT TGGTTGAGAT CACATAAATT   
  
  
+ ACGTTATCAA GGGTAGTACG ATATAATGGA TGAGGAGAAA TTAAATGTGA GATTTAGTTG ATGAATCGAA   
  
  
+ TACGTTAGTC TGAGCCATTT GGTCAAAACT GCATTGGCTA ATCAATAGAA ATTATAGAAA ATACATAACA   
  
  
+ TAATGGGATG CAATTTTCTA ATTGGGGAAA CCATGTCAAT GCAGTGATTT TGGACCATTT AGGCCTTATT   
  
  
+ CTTTTCAATT TTTTAATTGA TTATTTAGAC TCATATGAAC TTATTGTTGA CAGATTGAGC TAAAAACACT   
  
  
+ GATGTTTCAT ACATATGTAT GAATTGCACG TACGAAGGCT CTGCTCATTT CGAGAAAGAA AATTAACAGT   
  
  
+ TAGGAGCAGT ATAATTTAAC AACAAGCGAT AATTATTAGT ATGAAGTGGT ACGAATCTAT TTTAAAGGAG   
  
  
+ TACTATGAAG TACAAACAAT AACAAATAAT AAAGAGCAGT ACATTAACAT ATCTTACTAC CTAAATAAAT   
  
  
+ CGATTCTAAG AGTAAAAAAA TGTTGAAATT AACCCCATTA TAGTTTTACT TTAACAAGAA GTTTTTCTTC   
  
  
+ TTTTACCATT TACCAAGTAA GAATGATTCA GGCCGTATAG CCAAGGAAAA ATAGAAAACC ACAAAACTAA   
  
  
+ CCATGGCCCA AAAAACAACC ACCAAGAGGG GCACTAATGT GACTAACCGA CGGTTAACAT TATCATCTGT   
  
  
+ TTGATACTTG TTCATCTCTC ACTATTCGGG TAATAGTGTG AAATTACTGA TGTACCCTCT AAACTCGTCG   
  
  
+ TTTTAACTGT GTAAGAATCA GATTAACAGT CACGGACACA TGAGTAACAA ATCAATTTCG GACATGAACG   
  
  
+ AACGAACTGT GAATCCCCCC CAAAAAAAAA ATTTTTTTAA AAAAAGTTGC ATCCTTATCT TTCTGAACCA   
  
  
+ GGCCAGCTGT CTCATTCCTT GGTCCTCGCG CCCCATCACG AGCGCTGCTC ATTATTCACC CTCTCTCTCT   
  
  
+ CTCCTCTCTG ATCCACACCA CAAACACAAG GTTTGCACAG CTGCAGAGCA GTCGTACTAC TATCAGTAGT   
  
  
+ GAAAAACCAA GCAGAAATCC CCAATCCCTC TTCTCCTTCT TCCCAGATTC TTCCTCAATT CCAAGCCCAT   
  
  
+ ATCAGAAATT TAGACAACAA CAACCAAAAA AAAAAATTAA TACTGCTTGA GAAAAACAAA TATGATATGA   
  
  
+ AATTCCTCGA AAAAGGCCTG CCATAACAAA TTCCTCGTGA AAAAGCAGCT GAGAGAAGAA GAAGAAGAAA   
  
  
+ AAAATCCCGT CACAATCTCA ATCTCGATCC CAAATTGTGA AAGAGAAAGA AAAGATGAAG AGAGAACACC   
  
  
+ CCTTTTCGAA CCCTGACCAG AAACCCCCGC CGGAGATGTC GCCGGCGCCG GGGAAGTCCA AGATGTGGGA   
  
  
+ TGATGAGATG GCCCAGAACG ACGCTAAAAT GGATGAGCTG CTTGCTGTTG TGGGGTACAA GGTGAGGTCG   
  
  
+ TCGGACATGG CGGAAATTGC CCAGAAGCTT GAGCAGCTTG AAGAAGTCAT GGGTAGTGTT CAAGAAGACG   
  
  
+ GGTTATCTTA CTTGGCTTCC GAAACTGTTC ATTACAATCC TGCAGATCTG TCTACTTGGC TTGAATCTAT   
  
  
+ GCTTTCTGAG TTTAACCCTA ACCCTAATTT TGACCCTTCT CCATCGTCAA TCTCACCCAT CATCGATCCA   
  
  
+ GCTCCTCCAC TTCCTCGAAC CTCGTCGACG GTCAAATTCG AGCCCGACCC GTTTTCCGAT TCGGATCTGA   
  
  
+ AAGCAATCCC AGGTAAGGCT ATCTTGACCC CACCAAGTTC TAGCAATACT TCAAATTTGC GTGAGGCTAA   
  
  
+ GAGGTTGAAA CCCTCAAATT ACACAACCGC CCCAACGCCC ACCCCACCTC CAAAACTGCC CAATTCGGCG   
  
  
+ TCTCCACCAT CGGGCACGAC GGCGTCGAGG CCGGTGGTGC TGGTAGACTC ACAAGAAAAC GGCATCAGAT   
  
  
+ TGGTGCATGC ACTTATGGCC TGTGCCGAAG CAATTCAACT AGAAAACATG GGATTAGCTG AGGCTTTGGT   
  
  
+ TAAACAGATT AGGTATTTAG CAGCATCTCA AGCTGGACCT ATGAGGAAAG TAGCAACTTG TTTTGCAGAA   
  
  
+ GCTCTTACTT GTCGGATCTA CAAGCTATGC CCATCTGTAC CCTACGATGA ATCAGTCTCA GATGAGTTGC   
  
  
+ AGATGCACTT TTATGAGACT TGCCCATATC TTAAATTCGC CCATTTCACG GCAAATCAAG CAATTTTAGA   
  
  
+ AGCATTCAAT GGGAAGAAGA AAGTTCATGT GATTGATTTC AGCATGAAGC AAGGCATGCA ATGGCCGGCT   
  
  
+ TTGATGCAAG CCCTGGCTCT TCGACCGGAG GGTCCACCGC TTTTCCGGTT AACCGGGATT GGACCGCCCG   
  
  
+ CTCCGGACAA CTCGGACCGG CTGCAAGAGG TGGGTTGGAA GCTTGCCCAG TTCGCGGATT CGATCCGGAT   
  
  
+ TAAATTTCAG TATCGTGGGT TTGTGGCAAA CAGTTTGACC GATCTAGAAT CTTCCATGCT AGATCTTGAA   
  
  
+ CCGGACACTG AGGTGGTGGC GGTCAACTCG GTTTTCGAGC TCCACCGGCT GTTGGCTAAA CCCGGGGCGA   
  
  
+ TCGAGAAAGT GCTGGGGTTC ATGAGGGCCG TGAACCCGGT CATTGTGACG ATGGTCGAGC AGGAAGCGAA   
  
  
+ CCACAACGGA CCGGTTTTCT TGGACCGGTT CAATGAGTCG TTGCATTATT ACTCCACCTT GTTTGATTCC   
  
  
+ CTTGAGATTT GTGTTGATAA TGTAGATAAG AAGATGTCGG AGGCTTACTT GGGCCAGCAG ATCTGCAACA   
  
  
+ TGGTGGCTTG TGAAGGGTCT GACCGAGTCG AGAGGCACGA GACCCTGGCT CAGTGGCGAG CCCGGTTCGC   
  
  
+ ATCTGCCGGG TTCGACCCAG TTCATCTGGG TTCGAATGCG TTTAAGCAGG CGAGTATGTT GTTGGAGTTC   
  
  
+ TTTGCTGGTG GTGAAGGGTA CGGCGTGGAG GAGCGAGAAG GGTGTCTCAT GTTGGGATGG CATAGTAGGC   
  
  
+ CGCTTATCAC CACCTCGGCT TGGCAGCTCG CCAAGAACCC GGCTGTGAAT CGGCGATG  

- -Up\_Stream \_Len000TTGGGA AATTAAAATA GTTACACCCT GTTGTGAGTA GAAGTATGAG GTTGTTCGAT   
  
  
- ACTATGTAAG CTAATAACGG ATCATAAAGC AAATCATTCT ATAATCACGG TTTGAAACAT TATTTTTACT   
  
  
- TTGAATGATT TTTTTTAGAG CAAAAGACAT CTCCCTTTTA AACTTCTGAA TCACAGTGTA CTACTGGATT   
  
  
- ACCATGAACA TCCCAGTTCT AACTCTAACT CCCGTATGAC TGCTGGACAT ATTACCAGTT CATCCTAGTT   
  
  
- TCAGCTCTTT TGACAGAGAC GATTGAAAGT ATTAGATTGG GAATTGAGTG AGACTGGGAG TTAAATTAGT   
  
  
- CCGGATTAAG TTTGATAATT ACCAATATTG AGAGAAAGGA AGAAAACTCT AAAAAAAAAA GATGTACGCC   
  
  
- AACTTTTATT AAAAAAATTT TTTATATATA GTGATTGGTA AGTAATAGTA TCAAAATCTC AAGTATAAAT   
  
  
- ACACCAGGAA CAACTCAGAT TAAATTACGA AATCTACCGC ACTTCGTGGT TTAATCACCA TTGGTATTAT   
  
  
- TATTTGATAG TTCACAATTC AGCTTCTTTT GCCTGTGACG AGTTGGTTTA GATCATTGTA TCACATACTG   
  
  
- TACTATTGAT TCACACGGTA CATCATTAGT TAAGAAAGAA AAAAAAAAAA ACCAACTCTA GTGTATTTAA   
  
  
- TGCAATAGTT CCCATCATGC TATATTACCT ACTCCTCTTT AATTTACACT CTAAATCAAC TACTTAGCTT   
  
  
- ATGCAATCAG ACTCGGTAAA CCAGTTTTGA CGTAACCGAT TAGTTATCTT TAATATCTTT TATGTATTGT   
  
  
- ATTACCCTAC GTTAAAAGAT TAACCCCTTT GGTACAGTTA CGTCACTAAA ACCTGGTAAA TCCGGAATAA   
  
  
- GAAAAGTTAA AAAATTAACT AATAAATCTG AGTATACTTG AATAACAACT GTCTAACTCG ATTTTTGTGA   
  
  
- CTACAAAGTA TGTATACATA CTTAACGTGC ATGCTTCCGA GACGAGTAAA GCTCTTTCTT TTAATTGTCA   
  
  
- ATCCTCGTCA TATTAAATTG TTGTTCGCTA TTAATAATCA TACTTCACCA TGCTTAGATA AAATTTCCTC   
  
  
- ATGATACTTC ATGTTTGTTA TTGTTTATTA TTTCTCGTCA TGTAATTGTA TAGAATGATG GATTTATTTA   
  
  
- GCTAAGATTC TCATTTTTTT ACAACTTTAA TTGGGGTAAT ATCAAAATGA AATTGTTCTT CAAAAAGAAG   
  
  
- AAAATGGTAA ATGGTTCATT CTTACTAAGT CCGGCATATC GGTTCCTTTT TATCTTTTGG TGTTTTGATT   
  
  
- GGTACCGGGT TTTTTGTTGG TGGTTCTCCC CGTGATTACA CTGATTGGCT GCCAATTGTA ATAGTAGACA   
  
  
- AACTATGAAC AAGTAGAGAG TGATAAGCCC ATTATCACAC TTTAATGACT ACATGGGAGA TTTGAGCAGC   
  
  
- AAAATTGACA CATTCTTAGT CTAATTGTCA GTGCCTGTGT ACTCATTGTT TAGTTAAAGC CTGTACTTGC   
  
  
- TTGCTTGACA CTTAGGGGGG GTTTTTTTTT TAAAAAAATT TTTTTCAACG TAGGAATAGA AAGACTTGGT   
  
  
- CCGGTCGACA GAGTAAGGAA CCAGGAGCGC GGGGTAGTGC TCGCGACGAG TAATAAGTGG GAGAGAGAGA   
  
  
- GAGGAGAGAC TAGGTGTGGT GTTTGTGTTC CAAACGTGTC GACGTCTCGT CAGCATGATG ATAGTCATCA   
  
  
- CTTTTTGGTT CGTCTTTAGG GGTTAGGGAG AAGAGGAAGA AGGGTCTAAG AAGGAGTTAA GGTTCGGGTA   
  
  
- TAGTCTTTAA ATCTGTTGTT GTTGGTTTTT TTTTTTAATT ATGACGAACT CTTTTTGTTT ATACTATACT   
  
  
- TTAAGGAGCT TTTTCCGGAC GGTATTGTTT AAGGAGCACT TTTTCGTCGA CTCTCTTCTT CTTCTTCTTT   
  
  
- TTTTAGGGCA GTGTTAGAGT TAGAGCTAGG GTTTAACACT TTCTCTTTCT TTTCTACTTC TCTCTTGTGG   
  
  
- GGAAAAGCTT GGGACTGGTC TTTGGGGGCG GCCTCTACAG CGGCCGCGGC CCCTTCAGGT TCTACACCCT   
  
  
- ACTACTCTAC CGGGTCTTGC TGCGATTTTA CCTACTCGAC GAACGACAAC ACCCCATGTT CCACTCCAGC   
  
  
- AGCCTGTACC GCCTTTAACG GGTCTTCGAA CTCGTCGAAC TTCTTCAGTA CCCATCACAA GTTCTTCTGC   
  
  
- CCAATAGAAT GAACCGAAGG CTTTGACAAG TAATGTTAGG ACGTCTAGAC AGATGAACCG AACTTAGATA   
  
  
- CGAAAGACTC AAATTGGGAT TGGGATTAAA ACTGGGAAGA GGTAGCAGTT AGAGTGGGTA GTAGCTAGGT   
  
  
- CGAGGAGGTG AAGGAGCTTG GAGCAGCTGC CAGTTTAAGC TCGGGCTGGG CAAAAGGCTA AGCCTAGACT   
  
  
- TTCGTTAGGG TCCATTCCGA TAGAACTGGG GTGGTTCAAG ATCGTTATGA AGTTTAAACG CACTCCGATT   
  
  
- CTCCAACTTT GGGAGTTTAA TGTGTTGGCG GGGTTGCGGG TGGGGTGGAG GTTTTGACGG GTTAAGCCGC   
  
  
- AGAGGTGGTA GCCCGTGCTG CCGCAGCTCC GGCCACCACG ACCATCTGAG TGTTCTTTTG CCGTAGTCTA   
  
  
- ACCACGTACG TGAATACCGG ACACGGCTTC GTTAAGTTGA TCTTTTGTAC CCTAATCGAC TCCGAAACCA   
  
  
- ATTTGTCTAA TCCATAAATC GTCGTAGAGT TCGACCTGGA TACTCCTTTC ATCGTTGAAC AAAACGTCTT   
  
  
- CGAGAATGAA CAGCCTAGAT GTTCGATACG GGTAGACATG GGATGCTACT TAGTCAGAGT CTACTCAACG   
  
  
- TCTACGTGAA AATACTCTGA ACGGGTATAG AATTTAAGCG GGTAAAGTGC CGTTTAGTTC GTTAAAATCT   
  
  
- TCGTAAGTTA CCCTTCTTCT TTCAAGTACA CTAACTAAAG TCGTACTTCG TTCCGTACGT TACCGGCCGA   
  
  
- AACTACGTTC GGGACCGAGA AGCTGGCCTC CCAGGTGGCG AAAAGGCCAA TTGGCCCTAA CCTGGCGGGC   
  
  
- GAGGCCTGTT GAGCCTGGCC GACGTTCTCC ACCCAACCTT CGAACGGGTC AAGCGCCTAA GCTAGGCCTA   
  
  
- ATTTAAAGTC ATAGCACCCA AACACCGTTT GTCAAACTGG CTAGATCTTA GAAGGTACGA TCTAGAACTT   
  
  
- GGCCTGTGAC TCCACCACCG CCAGTTGAGC CAAAAGCTCG AGGTGGCCGA CAACCGATTT GGGCCCCGCT   
  
  
- AGCTCTTTCA CGACCCCAAG TACTCCCGGC ACTTGGGCCA GTAACACTGC TACCAGCTCG TCCTTCGCTT   
  
  
- GGTGTTGCCT GGCCAAAAGA ACCTGGCCAA GTTACTCAGC AACGTAATAA TGAGGTGGAA CAAACTAAGG   
  
  
- GAACTCTAAA CACAACTATT ACATCTATTC TTCTACAGCC TCCGAATGAA CCCGGTCGTC TAGACGTTGT   
  
  
- ACCACCGAAC ACTTCCCAGA CTGGCTCAGC TCTCCGTGCT CTGGGACCGA GTCACCGCTC GGGCCAAGCG   
  
  
- TAGACGGCCC AAGCTGGGTC AAGTAGACCC AAGCTTACGC AAATTCGTCC GCTCATACAA CAACCTCAAG   
  
  
- AAACGACCAC CACTTCCCAT GCCGCACCTC CTCGCTCTTC CCACAGAGTA CAACCCTACC GTATCATCCG   
  
  
- GCGAATAGTG GTGGAGCCGA ACCGTCGAGC GGTTCTTGGG CCGACACTTA GCCGCTAC

+     ARE

| Site Name | Organism | Position | Strand | Matrix score. | sequence | function |
| --- | --- | --- | --- | --- | --- | --- |
| ARE | Zea mays | 872 | + | 6 | AAACCA | cis-acting regulatory element essential for the anaerobic induction |
| ARE | Zea mays | 1758 | + | 6 | AAACCA | cis-acting regulatory element essential for the anaerobic induction |
| ARE | Zea mays | 1320 | + | 6 | AAACCA | cis-acting regulatory element essential for the anaerobic induction |

>HU10G00709.1   
+ -Up\_Stream \_Len000AACCCT TTAATTTTAT CAATGTGGGA CAACACTCAT CTTCATACTC CAACAAGCTA   
  
  
+ TGATACATTC GATTATTGCC TAGTATTTCG TTTAGTAAGA TATTAGTGCC AAACTTTGTA ATAAAAATGA   
  
  
+ AACTTACTAA AAAAAATCTC GTTTTCTGTA GAGGGAAAAT TTGAAGACTT AGTGTCACAT GATGACCTAA   
  
  
+ TGGTACTTGT AGGGTCAAGA TTGAGATTGA GGGCATACTG ACGACCTGTA TAATGGTCAA GTAGGATCAA   
  
  
+ AGTCGAGAAA ACTGTCTCTG CTAACTTTCA TAATCTAACC CTTAACTCAC TCTGACCCTC AATTTAATCA   
  
  
+ GGCCTAATTC AAACTATTAA TGGTTATAAC TCTCTTTCCT TCTTTTGAGA TTTTTTTTTT CTACATGCGG   
  
  
+ TTGAAAATAA TTTTTTTAAA AAATATATAT CACTAACCAT TCATTATCAT AGTTTTAGAG TTCATATTTA   
  
  
+ TGTGGTCCTT GTTGAGTCTA ATTTAATGCT TTAGATGGCG TGAAGCACCA AATTAGTGGT AACCATAATA   
  
  
+ ATAAACTATC AAGTGTTAAG TCGAAGAAAA CGGACACTGC TCAACCAAAT CTAGTAACAT AGTGTATGAC   
  
  
+ ATGATAACTA AGTGTGCCAT GTAGTAATCA ATTCTTTCTT TTTTTTTTTT TGGTTGAGAT CACATAAATT   
  
  
+ ACGTTATCAA GGGTAGTACG ATATAATGGA TGAGGAGAAA TTAAATGTGA GATTTAGTTG ATGAATCGAA   
  
  
+ TACGTTAGTC TGAGCCATTT GGTCAAAACT GCATTGGCTA ATCAATAGAA ATTATAGAAA ATACATAACA   
  
  
+ TAATGGGATG CAATTTTCTA ATTGGGGAAA CCATGTCAAT GCAGTGATTT TGGACCATTT AGGCCTTATT   
  
  
+ CTTTTCAATT TTTTAATTGA TTATTTAGAC TCATATGAAC TTATTGTTGA CAGATTGAGC TAAAAACACT   
  
  
+ GATGTTTCAT ACATATGTAT GAATTGCACG TACGAAGGCT CTGCTCATTT CGAGAAAGAA AATTAACAGT   
  
  
+ TAGGAGCAGT ATAATTTAAC AACAAGCGAT AATTATTAGT ATGAAGTGGT ACGAATCTAT TTTAAAGGAG   
  
  
+ TACTATGAAG TACAAACAAT AACAAATAAT AAAGAGCAGT ACATTAACAT ATCTTACTAC CTAAATAAAT   
  
  
+ CGATTCTAAG AGTAAAAAAA TGTTGAAATT AACCCCATTA TAGTTTTACT TTAACAAGAA GTTTTTCTTC   
  
  
+ TTTTACCATT TACCAAGTAA GAATGATTCA GGCCGTATAG CCAAGGAAAA ATAGAAAACC ACAAAACTAA   
  
  
+ CCATGGCCCA AAAAACAACC ACCAAGAGGG GCACTAATGT GACTAACCGA CGGTTAACAT TATCATCTGT   
  
  
+ TTGATACTTG TTCATCTCTC ACTATTCGGG TAATAGTGTG AAATTACTGA TGTACCCTCT AAACTCGTCG   
  
  
+ TTTTAACTGT GTAAGAATCA GATTAACAGT CACGGACACA TGAGTAACAA ATCAATTTCG GACATGAACG   
  
  
+ AACGAACTGT GAATCCCCCC CAAAAAAAAA ATTTTTTTAA AAAAAGTTGC ATCCTTATCT TTCTGAACCA   
  
  
+ GGCCAGCTGT CTCATTCCTT GGTCCTCGCG CCCCATCACG AGCGCTGCTC ATTATTCACC CTCTCTCTCT   
  
  
+ CTCCTCTCTG ATCCACACCA CAAACACAAG GTTTGCACAG CTGCAGAGCA GTCGTACTAC TATCAGTAGT   
  
  
+ GAAAAACCAA GCAGAAATCC CCAATCCCTC TTCTCCTTCT TCCCAGATTC TTCCTCAATT CCAAGCCCAT   
  
  
+ ATCAGAAATT TAGACAACAA CAACCAAAAA AAAAAATTAA TACTGCTTGA GAAAAACAAA TATGATATGA   
  
  
+ AATTCCTCGA AAAAGGCCTG CCATAACAAA TTCCTCGTGA AAAAGCAGCT GAGAGAAGAA GAAGAAGAAA   
  
  
+ AAAATCCCGT CACAATCTCA ATCTCGATCC CAAATTGTGA AAGAGAAAGA AAAGATGAAG AGAGAACACC   
  
  
+ CCTTTTCGAA CCCTGACCAG AAACCCCCGC CGGAGATGTC GCCGGCGCCG GGGAAGTCCA AGATGTGGGA   
  
  
+ TGATGAGATG GCCCAGAACG ACGCTAAAAT GGATGAGCTG CTTGCTGTTG TGGGGTACAA GGTGAGGTCG   
  
  
+ TCGGACATGG CGGAAATTGC CCAGAAGCTT GAGCAGCTTG AAGAAGTCAT GGGTAGTGTT CAAGAAGACG   
  
  
+ GGTTATCTTA CTTGGCTTCC GAAACTGTTC ATTACAATCC TGCAGATCTG TCTACTTGGC TTGAATCTAT   
  
  
+ GCTTTCTGAG TTTAACCCTA ACCCTAATTT TGACCCTTCT CCATCGTCAA TCTCACCCAT CATCGATCCA   
  
  
+ GCTCCTCCAC TTCCTCGAAC CTCGTCGACG GTCAAATTCG AGCCCGACCC GTTTTCCGAT TCGGATCTGA   
  
  
+ AAGCAATCCC AGGTAAGGCT ATCTTGACCC CACCAAGTTC TAGCAATACT TCAAATTTGC GTGAGGCTAA   
  
  
+ GAGGTTGAAA CCCTCAAATT ACACAACCGC CCCAACGCCC ACCCCACCTC CAAAACTGCC CAATTCGGCG   
  
  
+ TCTCCACCAT CGGGCACGAC GGCGTCGAGG CCGGTGGTGC TGGTAGACTC ACAAGAAAAC GGCATCAGAT   
  
  
+ TGGTGCATGC ACTTATGGCC TGTGCCGAAG CAATTCAACT AGAAAACATG GGATTAGCTG AGGCTTTGGT   
  
  
+ TAAACAGATT AGGTATTTAG CAGCATCTCA AGCTGGACCT ATGAGGAAAG TAGCAACTTG TTTTGCAGAA   
  
  
+ GCTCTTACTT GTCGGATCTA CAAGCTATGC CCATCTGTAC CCTACGATGA ATCAGTCTCA GATGAGTTGC   
  
  
+ AGATGCACTT TTATGAGACT TGCCCATATC TTAAATTCGC CCATTTCACG GCAAATCAAG CAATTTTAGA   
  
  
+ AGCATTCAAT GGGAAGAAGA AAGTTCATGT GATTGATTTC AGCATGAAGC AAGGCATGCA ATGGCCGGCT   
  
  
+ TTGATGCAAG CCCTGGCTCT TCGACCGGAG GGTCCACCGC TTTTCCGGTT AACCGGGATT GGACCGCCCG   
  
  
+ CTCCGGACAA CTCGGACCGG CTGCAAGAGG TGGGTTGGAA GCTTGCCCAG TTCGCGGATT CGATCCGGAT   
  
  
+ TAAATTTCAG TATCGTGGGT TTGTGGCAAA CAGTTTGACC GATCTAGAAT CTTCCATGCT AGATCTTGAA   
  
  
+ CCGGACACTG AGGTGGTGGC GGTCAACTCG GTTTTCGAGC TCCACCGGCT GTTGGCTAAA CCCGGGGCGA   
  
  
+ TCGAGAAAGT GCTGGGGTTC ATGAGGGCCG TGAACCCGGT CATTGTGACG ATGGTCGAGC AGGAAGCGAA   
  
  
+ CCACAACGGA CCGGTTTTCT TGGACCGGTT CAATGAGTCG TTGCATTATT ACTCCACCTT GTTTGATTCC   
  
  
+ CTTGAGATTT GTGTTGATAA TGTAGATAAG AAGATGTCGG AGGCTTACTT GGGCCAGCAG ATCTGCAACA   
  
  
+ TGGTGGCTTG TGAAGGGTCT GACCGAGTCG AGAGGCACGA GACCCTGGCT CAGTGGCGAG CCCGGTTCGC   
  
  
+ ATCTGCCGGG TTCGACCCAG TTCATCTGGG TTCGAATGCG TTTAAGCAGG CGAGTATGTT GTTGGAGTTC   
  
  
+ TTTGCTGGTG GTGAAGGGTA CGGCGTGGAG GAGCGAGAAG GGTGTCTCAT GTTGGGATGG CATAGTAGGC   
  
  
+ CGCTTATCAC CACCTCGGCT TGGCAGCTCG CCAAGAACCC GGCTGTGAAT CGGCGATG  

- -Up\_Stream \_Len000TTGGGA AATTAAAATA GTTACACCCT GTTGTGAGTA GAAGTATGAG GTTGTTCGAT   
  
  
- ACTATGTAAG CTAATAACGG ATCATAAAGC AAATCATTCT ATAATCACGG TTTGAAACAT TATTTTTACT   
  
  
- TTGAATGATT TTTTTTAGAG CAAAAGACAT CTCCCTTTTA AACTTCTGAA TCACAGTGTA CTACTGGATT   
  
  
- ACCATGAACA TCCCAGTTCT AACTCTAACT CCCGTATGAC TGCTGGACAT ATTACCAGTT CATCCTAGTT   
  
  
- TCAGCTCTTT TGACAGAGAC GATTGAAAGT ATTAGATTGG GAATTGAGTG AGACTGGGAG TTAAATTAGT   
  
  
- CCGGATTAAG TTTGATAATT ACCAATATTG AGAGAAAGGA AGAAAACTCT AAAAAAAAAA GATGTACGCC   
  
  
- AACTTTTATT AAAAAAATTT TTTATATATA GTGATTGGTA AGTAATAGTA TCAAAATCTC AAGTATAAAT   
  
  
- ACACCAGGAA CAACTCAGAT TAAATTACGA AATCTACCGC ACTTCGTGGT TTAATCACCA TTGGTATTAT   
  
  
- TATTTGATAG TTCACAATTC AGCTTCTTTT GCCTGTGACG AGTTGGTTTA GATCATTGTA TCACATACTG   
  
  
- TACTATTGAT TCACACGGTA CATCATTAGT TAAGAAAGAA AAAAAAAAAA ACCAACTCTA GTGTATTTAA   
  
  
- TGCAATAGTT CCCATCATGC TATATTACCT ACTCCTCTTT AATTTACACT CTAAATCAAC TACTTAGCTT   
  
  
- ATGCAATCAG ACTCGGTAAA CCAGTTTTGA CGTAACCGAT TAGTTATCTT TAATATCTTT TATGTATTGT   
  
  
- ATTACCCTAC GTTAAAAGAT TAACCCCTTT GGTACAGTTA CGTCACTAAA ACCTGGTAAA TCCGGAATAA   
  
  
- GAAAAGTTAA AAAATTAACT AATAAATCTG AGTATACTTG AATAACAACT GTCTAACTCG ATTTTTGTGA   
  
  
- CTACAAAGTA TGTATACATA CTTAACGTGC ATGCTTCCGA GACGAGTAAA GCTCTTTCTT TTAATTGTCA   
  
  
- ATCCTCGTCA TATTAAATTG TTGTTCGCTA TTAATAATCA TACTTCACCA TGCTTAGATA AAATTTCCTC   
  
  
- ATGATACTTC ATGTTTGTTA TTGTTTATTA TTTCTCGTCA TGTAATTGTA TAGAATGATG GATTTATTTA   
  
  
- GCTAAGATTC TCATTTTTTT ACAACTTTAA TTGGGGTAAT ATCAAAATGA AATTGTTCTT CAAAAAGAAG   
  
  
- AAAATGGTAA ATGGTTCATT CTTACTAAGT CCGGCATATC GGTTCCTTTT TATCTTTTGG TGTTTTGATT   
  
  
- GGTACCGGGT TTTTTGTTGG TGGTTCTCCC CGTGATTACA CTGATTGGCT GCCAATTGTA ATAGTAGACA   
  
  
- AACTATGAAC AAGTAGAGAG TGATAAGCCC ATTATCACAC TTTAATGACT ACATGGGAGA TTTGAGCAGC   
  
  
- AAAATTGACA CATTCTTAGT CTAATTGTCA GTGCCTGTGT ACTCATTGTT TAGTTAAAGC CTGTACTTGC   
  
  
- TTGCTTGACA CTTAGGGGGG GTTTTTTTTT TAAAAAAATT TTTTTCAACG TAGGAATAGA AAGACTTGGT   
  
  
- CCGGTCGACA GAGTAAGGAA CCAGGAGCGC GGGGTAGTGC TCGCGACGAG TAATAAGTGG GAGAGAGAGA   
  
  
- GAGGAGAGAC TAGGTGTGGT GTTTGTGTTC CAAACGTGTC GACGTCTCGT CAGCATGATG ATAGTCATCA   
  
  
- CTTTTTGGTT CGTCTTTAGG GGTTAGGGAG AAGAGGAAGA AGGGTCTAAG AAGGAGTTAA GGTTCGGGTA   
  
  
- TAGTCTTTAA ATCTGTTGTT GTTGGTTTTT TTTTTTAATT ATGACGAACT CTTTTTGTTT ATACTATACT   
  
  
- TTAAGGAGCT TTTTCCGGAC GGTATTGTTT AAGGAGCACT TTTTCGTCGA CTCTCTTCTT CTTCTTCTTT   
  
  
- TTTTAGGGCA GTGTTAGAGT TAGAGCTAGG GTTTAACACT TTCTCTTTCT TTTCTACTTC TCTCTTGTGG   
  
  
- GGAAAAGCTT GGGACTGGTC TTTGGGGGCG GCCTCTACAG CGGCCGCGGC CCCTTCAGGT TCTACACCCT   
  
  
- ACTACTCTAC CGGGTCTTGC TGCGATTTTA CCTACTCGAC GAACGACAAC ACCCCATGTT CCACTCCAGC   
  
  
- AGCCTGTACC GCCTTTAACG GGTCTTCGAA CTCGTCGAAC TTCTTCAGTA CCCATCACAA GTTCTTCTGC   
  
  
- CCAATAGAAT GAACCGAAGG CTTTGACAAG TAATGTTAGG ACGTCTAGAC AGATGAACCG AACTTAGATA   
  
  
- CGAAAGACTC AAATTGGGAT TGGGATTAAA ACTGGGAAGA GGTAGCAGTT AGAGTGGGTA GTAGCTAGGT   
  
  
- CGAGGAGGTG AAGGAGCTTG GAGCAGCTGC CAGTTTAAGC TCGGGCTGGG CAAAAGGCTA AGCCTAGACT   
  
  
- TTCGTTAGGG TCCATTCCGA TAGAACTGGG GTGGTTCAAG ATCGTTATGA AGTTTAAACG CACTCCGATT   
  
  
- CTCCAACTTT GGGAGTTTAA TGTGTTGGCG GGGTTGCGGG TGGGGTGGAG GTTTTGACGG GTTAAGCCGC   
  
  
- AGAGGTGGTA GCCCGTGCTG CCGCAGCTCC GGCCACCACG ACCATCTGAG TGTTCTTTTG CCGTAGTCTA   
  
  
- ACCACGTACG TGAATACCGG ACACGGCTTC GTTAAGTTGA TCTTTTGTAC CCTAATCGAC TCCGAAACCA   
  
  
- ATTTGTCTAA TCCATAAATC GTCGTAGAGT TCGACCTGGA TACTCCTTTC ATCGTTGAAC AAAACGTCTT   
  
  
- CGAGAATGAA CAGCCTAGAT GTTCGATACG GGTAGACATG GGATGCTACT TAGTCAGAGT CTACTCAACG   
  
  
- TCTACGTGAA AATACTCTGA ACGGGTATAG AATTTAAGCG GGTAAAGTGC CGTTTAGTTC GTTAAAATCT   
  
  
- TCGTAAGTTA CCCTTCTTCT TTCAAGTACA CTAACTAAAG TCGTACTTCG TTCCGTACGT TACCGGCCGA   
  
  
- AACTACGTTC GGGACCGAGA AGCTGGCCTC CCAGGTGGCG AAAAGGCCAA TTGGCCCTAA CCTGGCGGGC   
  
  
- GAGGCCTGTT GAGCCTGGCC GACGTTCTCC ACCCAACCTT CGAACGGGTC AAGCGCCTAA GCTAGGCCTA   
  
  
- ATTTAAAGTC ATAGCACCCA AACACCGTTT GTCAAACTGG CTAGATCTTA GAAGGTACGA TCTAGAACTT   
  
  
- GGCCTGTGAC TCCACCACCG CCAGTTGAGC CAAAAGCTCG AGGTGGCCGA CAACCGATTT GGGCCCCGCT   
  
  
- AGCTCTTTCA CGACCCCAAG TACTCCCGGC ACTTGGGCCA GTAACACTGC TACCAGCTCG TCCTTCGCTT   
  
  
- GGTGTTGCCT GGCCAAAAGA ACCTGGCCAA GTTACTCAGC AACGTAATAA TGAGGTGGAA CAAACTAAGG   
  
  
- GAACTCTAAA CACAACTATT ACATCTATTC TTCTACAGCC TCCGAATGAA CCCGGTCGTC TAGACGTTGT   
  
  
- ACCACCGAAC ACTTCCCAGA CTGGCTCAGC TCTCCGTGCT CTGGGACCGA GTCACCGCTC GGGCCAAGCG   
  
  
- TAGACGGCCC AAGCTGGGTC AAGTAGACCC AAGCTTACGC AAATTCGTCC GCTCATACAA CAACCTCAAG   
  
  
- AAACGACCAC CACTTCCCAT GCCGCACCTC CTCGCTCTTC CCACAGAGTA CAACCCTACC GTATCATCCG   
  
  
- GCGAATAGTG GTGGAGCCGA ACCGTCGAGC GGTTCTTGGG CCGACACTTA GCCGCTAC

+     ATCT-motif

| Site Name | Organism | Position | Strand | Matrix score. | sequence | function |
| --- | --- | --- | --- | --- | --- | --- |
| ATCT-motif | Pisum sativum | 3151 | - | 9 | AATCTAATCC | part of a conserved DNA module involved in light responsiveness |
| ATCT-motif | Pisum sativum | 316 | + | 9 | AATCTAATCC | part of a conserved DNA module involved in light responsiveness |

>HU10G00709.1   
+ -Up\_Stream \_Len000AACCCT TTAATTTTAT CAATGTGGGA CAACACTCAT CTTCATACTC CAACAAGCTA   
  
  
+ TGATACATTC GATTATTGCC TAGTATTTCG TTTAGTAAGA TATTAGTGCC AAACTTTGTA ATAAAAATGA   
  
  
+ AACTTACTAA AAAAAATCTC GTTTTCTGTA GAGGGAAAAT TTGAAGACTT AGTGTCACAT GATGACCTAA   
  
  
+ TGGTACTTGT AGGGTCAAGA TTGAGATTGA GGGCATACTG ACGACCTGTA TAATGGTCAA GTAGGATCAA   
  
  
+ AGTCGAGAAA ACTGTCTCTG CTAACTTTCA TAATCTAACC CTTAACTCAC TCTGACCCTC AATTTAATCA   
  
  
+ GGCCTAATTC AAACTATTAA TGGTTATAAC TCTCTTTCCT TCTTTTGAGA TTTTTTTTTT CTACATGCGG   
  
  
+ TTGAAAATAA TTTTTTTAAA AAATATATAT CACTAACCAT TCATTATCAT AGTTTTAGAG TTCATATTTA   
  
  
+ TGTGGTCCTT GTTGAGTCTA ATTTAATGCT TTAGATGGCG TGAAGCACCA AATTAGTGGT AACCATAATA   
  
  
+ ATAAACTATC AAGTGTTAAG TCGAAGAAAA CGGACACTGC TCAACCAAAT CTAGTAACAT AGTGTATGAC   
  
  
+ ATGATAACTA AGTGTGCCAT GTAGTAATCA ATTCTTTCTT TTTTTTTTTT TGGTTGAGAT CACATAAATT   
  
  
+ ACGTTATCAA GGGTAGTACG ATATAATGGA TGAGGAGAAA TTAAATGTGA GATTTAGTTG ATGAATCGAA   
  
  
+ TACGTTAGTC TGAGCCATTT GGTCAAAACT GCATTGGCTA ATCAATAGAA ATTATAGAAA ATACATAACA   
  
  
+ TAATGGGATG CAATTTTCTA ATTGGGGAAA CCATGTCAAT GCAGTGATTT TGGACCATTT AGGCCTTATT   
  
  
+ CTTTTCAATT TTTTAATTGA TTATTTAGAC TCATATGAAC TTATTGTTGA CAGATTGAGC TAAAAACACT   
  
  
+ GATGTTTCAT ACATATGTAT GAATTGCACG TACGAAGGCT CTGCTCATTT CGAGAAAGAA AATTAACAGT   
  
  
+ TAGGAGCAGT ATAATTTAAC AACAAGCGAT AATTATTAGT ATGAAGTGGT ACGAATCTAT TTTAAAGGAG   
  
  
+ TACTATGAAG TACAAACAAT AACAAATAAT AAAGAGCAGT ACATTAACAT ATCTTACTAC CTAAATAAAT   
  
  
+ CGATTCTAAG AGTAAAAAAA TGTTGAAATT AACCCCATTA TAGTTTTACT TTAACAAGAA GTTTTTCTTC   
  
  
+ TTTTACCATT TACCAAGTAA GAATGATTCA GGCCGTATAG CCAAGGAAAA ATAGAAAACC ACAAAACTAA   
  
  
+ CCATGGCCCA AAAAACAACC ACCAAGAGGG GCACTAATGT GACTAACCGA CGGTTAACAT TATCATCTGT   
  
  
+ TTGATACTTG TTCATCTCTC ACTATTCGGG TAATAGTGTG AAATTACTGA TGTACCCTCT AAACTCGTCG   
  
  
+ TTTTAACTGT GTAAGAATCA GATTAACAGT CACGGACACA TGAGTAACAA ATCAATTTCG GACATGAACG   
  
  
+ AACGAACTGT GAATCCCCCC CAAAAAAAAA ATTTTTTTAA AAAAAGTTGC ATCCTTATCT TTCTGAACCA   
  
  
+ GGCCAGCTGT CTCATTCCTT GGTCCTCGCG CCCCATCACG AGCGCTGCTC ATTATTCACC CTCTCTCTCT   
  
  
+ CTCCTCTCTG ATCCACACCA CAAACACAAG GTTTGCACAG CTGCAGAGCA GTCGTACTAC TATCAGTAGT   
  
  
+ GAAAAACCAA GCAGAAATCC CCAATCCCTC TTCTCCTTCT TCCCAGATTC TTCCTCAATT CCAAGCCCAT   
  
  
+ ATCAGAAATT TAGACAACAA CAACCAAAAA AAAAAATTAA TACTGCTTGA GAAAAACAAA TATGATATGA   
  
  
+ AATTCCTCGA AAAAGGCCTG CCATAACAAA TTCCTCGTGA AAAAGCAGCT GAGAGAAGAA GAAGAAGAAA   
  
  
+ AAAATCCCGT CACAATCTCA ATCTCGATCC CAAATTGTGA AAGAGAAAGA AAAGATGAAG AGAGAACACC   
  
  
+ CCTTTTCGAA CCCTGACCAG AAACCCCCGC CGGAGATGTC GCCGGCGCCG GGGAAGTCCA AGATGTGGGA   
  
  
+ TGATGAGATG GCCCAGAACG ACGCTAAAAT GGATGAGCTG CTTGCTGTTG TGGGGTACAA GGTGAGGTCG   
  
  
+ TCGGACATGG CGGAAATTGC CCAGAAGCTT GAGCAGCTTG AAGAAGTCAT GGGTAGTGTT CAAGAAGACG   
  
  
+ GGTTATCTTA CTTGGCTTCC GAAACTGTTC ATTACAATCC TGCAGATCTG TCTACTTGGC TTGAATCTAT   
  
  
+ GCTTTCTGAG TTTAACCCTA ACCCTAATTT TGACCCTTCT CCATCGTCAA TCTCACCCAT CATCGATCCA   
  
  
+ GCTCCTCCAC TTCCTCGAAC CTCGTCGACG GTCAAATTCG AGCCCGACCC GTTTTCCGAT TCGGATCTGA   
  
  
+ AAGCAATCCC AGGTAAGGCT ATCTTGACCC CACCAAGTTC TAGCAATACT TCAAATTTGC GTGAGGCTAA   
  
  
+ GAGGTTGAAA CCCTCAAATT ACACAACCGC CCCAACGCCC ACCCCACCTC CAAAACTGCC CAATTCGGCG   
  
  
+ TCTCCACCAT CGGGCACGAC GGCGTCGAGG CCGGTGGTGC TGGTAGACTC ACAAGAAAAC GGCATCAGAT   
  
  
+ TGGTGCATGC ACTTATGGCC TGTGCCGAAG CAATTCAACT AGAAAACATG GGATTAGCTG AGGCTTTGGT   
  
  
+ TAAACAGATT AGGTATTTAG CAGCATCTCA AGCTGGACCT ATGAGGAAAG TAGCAACTTG TTTTGCAGAA   
  
  
+ GCTCTTACTT GTCGGATCTA CAAGCTATGC CCATCTGTAC CCTACGATGA ATCAGTCTCA GATGAGTTGC   
  
  
+ AGATGCACTT TTATGAGACT TGCCCATATC TTAAATTCGC CCATTTCACG GCAAATCAAG CAATTTTAGA   
  
  
+ AGCATTCAAT GGGAAGAAGA AAGTTCATGT GATTGATTTC AGCATGAAGC AAGGCATGCA ATGGCCGGCT   
  
  
+ TTGATGCAAG CCCTGGCTCT TCGACCGGAG GGTCCACCGC TTTTCCGGTT AACCGGGATT GGACCGCCCG   
  
  
+ CTCCGGACAA CTCGGACCGG CTGCAAGAGG TGGGTTGGAA GCTTGCCCAG TTCGCGGATT CGATCCGGAT   
  
  
+ TAAATTTCAG TATCGTGGGT TTGTGGCAAA CAGTTTGACC GATCTAGAAT CTTCCATGCT AGATCTTGAA   
  
  
+ CCGGACACTG AGGTGGTGGC GGTCAACTCG GTTTTCGAGC TCCACCGGCT GTTGGCTAAA CCCGGGGCGA   
  
  
+ TCGAGAAAGT GCTGGGGTTC ATGAGGGCCG TGAACCCGGT CATTGTGACG ATGGTCGAGC AGGAAGCGAA   
  
  
+ CCACAACGGA CCGGTTTTCT TGGACCGGTT CAATGAGTCG TTGCATTATT ACTCCACCTT GTTTGATTCC   
  
  
+ CTTGAGATTT GTGTTGATAA TGTAGATAAG AAGATGTCGG AGGCTTACTT GGGCCAGCAG ATCTGCAACA   
  
  
+ TGGTGGCTTG TGAAGGGTCT GACCGAGTCG AGAGGCACGA GACCCTGGCT CAGTGGCGAG CCCGGTTCGC   
  
  
+ ATCTGCCGGG TTCGACCCAG TTCATCTGGG TTCGAATGCG TTTAAGCAGG CGAGTATGTT GTTGGAGTTC   
  
  
+ TTTGCTGGTG GTGAAGGGTA CGGCGTGGAG GAGCGAGAAG GGTGTCTCAT GTTGGGATGG CATAGTAGGC   
  
  
+ CGCTTATCAC CACCTCGGCT TGGCAGCTCG CCAAGAACCC GGCTGTGAAT CGGCGATG  

- -Up\_Stream \_Len000TTGGGA AATTAAAATA GTTACACCCT GTTGTGAGTA GAAGTATGAG GTTGTTCGAT   
  
  
- ACTATGTAAG CTAATAACGG ATCATAAAGC AAATCATTCT ATAATCACGG TTTGAAACAT TATTTTTACT   
  
  
- TTGAATGATT TTTTTTAGAG CAAAAGACAT CTCCCTTTTA AACTTCTGAA TCACAGTGTA CTACTGGATT   
  
  
- ACCATGAACA TCCCAGTTCT AACTCTAACT CCCGTATGAC TGCTGGACAT ATTACCAGTT CATCCTAGTT   
  
  
- TCAGCTCTTT TGACAGAGAC GATTGAAAGT ATTAGATTGG GAATTGAGTG AGACTGGGAG TTAAATTAGT   
  
  
- CCGGATTAAG TTTGATAATT ACCAATATTG AGAGAAAGGA AGAAAACTCT AAAAAAAAAA GATGTACGCC   
  
  
- AACTTTTATT AAAAAAATTT TTTATATATA GTGATTGGTA AGTAATAGTA TCAAAATCTC AAGTATAAAT   
  
  
- ACACCAGGAA CAACTCAGAT TAAATTACGA AATCTACCGC ACTTCGTGGT TTAATCACCA TTGGTATTAT   
  
  
- TATTTGATAG TTCACAATTC AGCTTCTTTT GCCTGTGACG AGTTGGTTTA GATCATTGTA TCACATACTG   
  
  
- TACTATTGAT TCACACGGTA CATCATTAGT TAAGAAAGAA AAAAAAAAAA ACCAACTCTA GTGTATTTAA   
  
  
- TGCAATAGTT CCCATCATGC TATATTACCT ACTCCTCTTT AATTTACACT CTAAATCAAC TACTTAGCTT   
  
  
- ATGCAATCAG ACTCGGTAAA CCAGTTTTGA CGTAACCGAT TAGTTATCTT TAATATCTTT TATGTATTGT   
  
  
- ATTACCCTAC GTTAAAAGAT TAACCCCTTT GGTACAGTTA CGTCACTAAA ACCTGGTAAA TCCGGAATAA   
  
  
- GAAAAGTTAA AAAATTAACT AATAAATCTG AGTATACTTG AATAACAACT GTCTAACTCG ATTTTTGTGA   
  
  
- CTACAAAGTA TGTATACATA CTTAACGTGC ATGCTTCCGA GACGAGTAAA GCTCTTTCTT TTAATTGTCA   
  
  
- ATCCTCGTCA TATTAAATTG TTGTTCGCTA TTAATAATCA TACTTCACCA TGCTTAGATA AAATTTCCTC   
  
  
- ATGATACTTC ATGTTTGTTA TTGTTTATTA TTTCTCGTCA TGTAATTGTA TAGAATGATG GATTTATTTA   
  
  
- GCTAAGATTC TCATTTTTTT ACAACTTTAA TTGGGGTAAT ATCAAAATGA AATTGTTCTT CAAAAAGAAG   
  
  
- AAAATGGTAA ATGGTTCATT CTTACTAAGT CCGGCATATC GGTTCCTTTT TATCTTTTGG TGTTTTGATT   
  
  
- GGTACCGGGT TTTTTGTTGG TGGTTCTCCC CGTGATTACA CTGATTGGCT GCCAATTGTA ATAGTAGACA   
  
  
- AACTATGAAC AAGTAGAGAG TGATAAGCCC ATTATCACAC TTTAATGACT ACATGGGAGA TTTGAGCAGC   
  
  
- AAAATTGACA CATTCTTAGT CTAATTGTCA GTGCCTGTGT ACTCATTGTT TAGTTAAAGC CTGTACTTGC   
  
  
- TTGCTTGACA CTTAGGGGGG GTTTTTTTTT TAAAAAAATT TTTTTCAACG TAGGAATAGA AAGACTTGGT   
  
  
- CCGGTCGACA GAGTAAGGAA CCAGGAGCGC GGGGTAGTGC TCGCGACGAG TAATAAGTGG GAGAGAGAGA   
  
  
- GAGGAGAGAC TAGGTGTGGT GTTTGTGTTC CAAACGTGTC GACGTCTCGT CAGCATGATG ATAGTCATCA   
  
  
- CTTTTTGGTT CGTCTTTAGG GGTTAGGGAG AAGAGGAAGA AGGGTCTAAG AAGGAGTTAA GGTTCGGGTA   
  
  
- TAGTCTTTAA ATCTGTTGTT GTTGGTTTTT TTTTTTAATT ATGACGAACT CTTTTTGTTT ATACTATACT   
  
  
- TTAAGGAGCT TTTTCCGGAC GGTATTGTTT AAGGAGCACT TTTTCGTCGA CTCTCTTCTT CTTCTTCTTT   
  
  
- TTTTAGGGCA GTGTTAGAGT TAGAGCTAGG GTTTAACACT TTCTCTTTCT TTTCTACTTC TCTCTTGTGG   
  
  
- GGAAAAGCTT GGGACTGGTC TTTGGGGGCG GCCTCTACAG CGGCCGCGGC CCCTTCAGGT TCTACACCCT   
  
  
- ACTACTCTAC CGGGTCTTGC TGCGATTTTA CCTACTCGAC GAACGACAAC ACCCCATGTT CCACTCCAGC   
  
  
- AGCCTGTACC GCCTTTAACG GGTCTTCGAA CTCGTCGAAC TTCTTCAGTA CCCATCACAA GTTCTTCTGC   
  
  
- CCAATAGAAT GAACCGAAGG CTTTGACAAG TAATGTTAGG ACGTCTAGAC AGATGAACCG AACTTAGATA   
  
  
- CGAAAGACTC AAATTGGGAT TGGGATTAAA ACTGGGAAGA GGTAGCAGTT AGAGTGGGTA GTAGCTAGGT   
  
  
- CGAGGAGGTG AAGGAGCTTG GAGCAGCTGC CAGTTTAAGC TCGGGCTGGG CAAAAGGCTA AGCCTAGACT   
  
  
- TTCGTTAGGG TCCATTCCGA TAGAACTGGG GTGGTTCAAG ATCGTTATGA AGTTTAAACG CACTCCGATT   
  
  
- CTCCAACTTT GGGAGTTTAA TGTGTTGGCG GGGTTGCGGG TGGGGTGGAG GTTTTGACGG GTTAAGCCGC   
  
  
- AGAGGTGGTA GCCCGTGCTG CCGCAGCTCC GGCCACCACG ACCATCTGAG TGTTCTTTTG CCGTAGTCTA   
  
  
- ACCACGTACG TGAATACCGG ACACGGCTTC GTTAAGTTGA TCTTTTGTAC CCTAATCGAC TCCGAAACCA   
  
  
- ATTTGTCTAA TCCATAAATC GTCGTAGAGT TCGACCTGGA TACTCCTTTC ATCGTTGAAC AAAACGTCTT   
  
  
- CGAGAATGAA CAGCCTAGAT GTTCGATACG GGTAGACATG GGATGCTACT TAGTCAGAGT CTACTCAACG   
  
  
- TCTACGTGAA AATACTCTGA ACGGGTATAG AATTTAAGCG GGTAAAGTGC CGTTTAGTTC GTTAAAATCT   
  
  
- TCGTAAGTTA CCCTTCTTCT TTCAAGTACA CTAACTAAAG TCGTACTTCG TTCCGTACGT TACCGGCCGA   
  
  
- AACTACGTTC GGGACCGAGA AGCTGGCCTC CCAGGTGGCG AAAAGGCCAA TTGGCCCTAA CCTGGCGGGC   
  
  
- GAGGCCTGTT GAGCCTGGCC GACGTTCTCC ACCCAACCTT CGAACGGGTC AAGCGCCTAA GCTAGGCCTA   
  
  
- ATTTAAAGTC ATAGCACCCA AACACCGTTT GTCAAACTGG CTAGATCTTA GAAGGTACGA TCTAGAACTT   
  
  
- GGCCTGTGAC TCCACCACCG CCAGTTGAGC CAAAAGCTCG AGGTGGCCGA CAACCGATTT GGGCCCCGCT   
  
  
- AGCTCTTTCA CGACCCCAAG TACTCCCGGC ACTTGGGCCA GTAACACTGC TACCAGCTCG TCCTTCGCTT   
  
  
- GGTGTTGCCT GGCCAAAAGA ACCTGGCCAA GTTACTCAGC AACGTAATAA TGAGGTGGAA CAAACTAAGG   
  
  
- GAACTCTAAA CACAACTATT ACATCTATTC TTCTACAGCC TCCGAATGAA CCCGGTCGTC TAGACGTTGT   
  
  
- ACCACCGAAC ACTTCCCAGA CTGGCTCAGC TCTCCGTGCT CTGGGACCGA GTCACCGCTC GGGCCAAGCG   
  
  
- TAGACGGCCC AAGCTGGGTC AAGTAGACCC AAGCTTACGC AAATTCGTCC GCTCATACAA CAACCTCAAG   
  
  
- AAACGACCAC CACTTCCCAT GCCGCACCTC CTCGCTCTTC CCACAGAGTA CAACCCTACC GTATCATCCG   
  
  
- GCGAATAGTG GTGGAGCCGA ACCGTCGAGC GGTTCTTGGG CCGACACTTA GCCGCTAC

+     AT~TATA-box

| Site Name | Organism | Position | Strand | Matrix score. | sequence | function |
| --- | --- | --- | --- | --- | --- | --- |
| AT~TATA-box | Arabidopsis thaliana | 448 | + | 6 | TATATA |  |

>HU10G00709.1   
+ -Up\_Stream \_Len000AACCCT TTAATTTTAT CAATGTGGGA CAACACTCAT CTTCATACTC CAACAAGCTA   
  
  
+ TGATACATTC GATTATTGCC TAGTATTTCG TTTAGTAAGA TATTAGTGCC AAACTTTGTA ATAAAAATGA   
  
  
+ AACTTACTAA AAAAAATCTC GTTTTCTGTA GAGGGAAAAT TTGAAGACTT AGTGTCACAT GATGACCTAA   
  
  
+ TGGTACTTGT AGGGTCAAGA TTGAGATTGA GGGCATACTG ACGACCTGTA TAATGGTCAA GTAGGATCAA   
  
  
+ AGTCGAGAAA ACTGTCTCTG CTAACTTTCA TAATCTAACC CTTAACTCAC TCTGACCCTC AATTTAATCA   
  
  
+ GGCCTAATTC AAACTATTAA TGGTTATAAC TCTCTTTCCT TCTTTTGAGA TTTTTTTTTT CTACATGCGG   
  
  
+ TTGAAAATAA TTTTTTTAAA AAATATATAT CACTAACCAT TCATTATCAT AGTTTTAGAG TTCATATTTA   
  
  
+ TGTGGTCCTT GTTGAGTCTA ATTTAATGCT TTAGATGGCG TGAAGCACCA AATTAGTGGT AACCATAATA   
  
  
+ ATAAACTATC AAGTGTTAAG TCGAAGAAAA CGGACACTGC TCAACCAAAT CTAGTAACAT AGTGTATGAC   
  
  
+ ATGATAACTA AGTGTGCCAT GTAGTAATCA ATTCTTTCTT TTTTTTTTTT TGGTTGAGAT CACATAAATT   
  
  
+ ACGTTATCAA GGGTAGTACG ATATAATGGA TGAGGAGAAA TTAAATGTGA GATTTAGTTG ATGAATCGAA   
  
  
+ TACGTTAGTC TGAGCCATTT GGTCAAAACT GCATTGGCTA ATCAATAGAA ATTATAGAAA ATACATAACA   
  
  
+ TAATGGGATG CAATTTTCTA ATTGGGGAAA CCATGTCAAT GCAGTGATTT TGGACCATTT AGGCCTTATT   
  
  
+ CTTTTCAATT TTTTAATTGA TTATTTAGAC TCATATGAAC TTATTGTTGA CAGATTGAGC TAAAAACACT   
  
  
+ GATGTTTCAT ACATATGTAT GAATTGCACG TACGAAGGCT CTGCTCATTT CGAGAAAGAA AATTAACAGT   
  
  
+ TAGGAGCAGT ATAATTTAAC AACAAGCGAT AATTATTAGT ATGAAGTGGT ACGAATCTAT TTTAAAGGAG   
  
  
+ TACTATGAAG TACAAACAAT AACAAATAAT AAAGAGCAGT ACATTAACAT ATCTTACTAC CTAAATAAAT   
  
  
+ CGATTCTAAG AGTAAAAAAA TGTTGAAATT AACCCCATTA TAGTTTTACT TTAACAAGAA GTTTTTCTTC   
  
  
+ TTTTACCATT TACCAAGTAA GAATGATTCA GGCCGTATAG CCAAGGAAAA ATAGAAAACC ACAAAACTAA   
  
  
+ CCATGGCCCA AAAAACAACC ACCAAGAGGG GCACTAATGT GACTAACCGA CGGTTAACAT TATCATCTGT   
  
  
+ TTGATACTTG TTCATCTCTC ACTATTCGGG TAATAGTGTG AAATTACTGA TGTACCCTCT AAACTCGTCG   
  
  
+ TTTTAACTGT GTAAGAATCA GATTAACAGT CACGGACACA TGAGTAACAA ATCAATTTCG GACATGAACG   
  
  
+ AACGAACTGT GAATCCCCCC CAAAAAAAAA ATTTTTTTAA AAAAAGTTGC ATCCTTATCT TTCTGAACCA   
  
  
+ GGCCAGCTGT CTCATTCCTT GGTCCTCGCG CCCCATCACG AGCGCTGCTC ATTATTCACC CTCTCTCTCT   
  
  
+ CTCCTCTCTG ATCCACACCA CAAACACAAG GTTTGCACAG CTGCAGAGCA GTCGTACTAC TATCAGTAGT   
  
  
+ GAAAAACCAA GCAGAAATCC CCAATCCCTC TTCTCCTTCT TCCCAGATTC TTCCTCAATT CCAAGCCCAT   
  
  
+ ATCAGAAATT TAGACAACAA CAACCAAAAA AAAAAATTAA TACTGCTTGA GAAAAACAAA TATGATATGA   
  
  
+ AATTCCTCGA AAAAGGCCTG CCATAACAAA TTCCTCGTGA AAAAGCAGCT GAGAGAAGAA GAAGAAGAAA   
  
  
+ AAAATCCCGT CACAATCTCA ATCTCGATCC CAAATTGTGA AAGAGAAAGA AAAGATGAAG AGAGAACACC   
  
  
+ CCTTTTCGAA CCCTGACCAG AAACCCCCGC CGGAGATGTC GCCGGCGCCG GGGAAGTCCA AGATGTGGGA   
  
  
+ TGATGAGATG GCCCAGAACG ACGCTAAAAT GGATGAGCTG CTTGCTGTTG TGGGGTACAA GGTGAGGTCG   
  
  
+ TCGGACATGG CGGAAATTGC CCAGAAGCTT GAGCAGCTTG AAGAAGTCAT GGGTAGTGTT CAAGAAGACG   
  
  
+ GGTTATCTTA CTTGGCTTCC GAAACTGTTC ATTACAATCC TGCAGATCTG TCTACTTGGC TTGAATCTAT   
  
  
+ GCTTTCTGAG TTTAACCCTA ACCCTAATTT TGACCCTTCT CCATCGTCAA TCTCACCCAT CATCGATCCA   
  
  
+ GCTCCTCCAC TTCCTCGAAC CTCGTCGACG GTCAAATTCG AGCCCGACCC GTTTTCCGAT TCGGATCTGA   
  
  
+ AAGCAATCCC AGGTAAGGCT ATCTTGACCC CACCAAGTTC TAGCAATACT TCAAATTTGC GTGAGGCTAA   
  
  
+ GAGGTTGAAA CCCTCAAATT ACACAACCGC CCCAACGCCC ACCCCACCTC CAAAACTGCC CAATTCGGCG   
  
  
+ TCTCCACCAT CGGGCACGAC GGCGTCGAGG CCGGTGGTGC TGGTAGACTC ACAAGAAAAC GGCATCAGAT   
  
  
+ TGGTGCATGC ACTTATGGCC TGTGCCGAAG CAATTCAACT AGAAAACATG GGATTAGCTG AGGCTTTGGT   
  
  
+ TAAACAGATT AGGTATTTAG CAGCATCTCA AGCTGGACCT ATGAGGAAAG TAGCAACTTG TTTTGCAGAA   
  
  
+ GCTCTTACTT GTCGGATCTA CAAGCTATGC CCATCTGTAC CCTACGATGA ATCAGTCTCA GATGAGTTGC   
  
  
+ AGATGCACTT TTATGAGACT TGCCCATATC TTAAATTCGC CCATTTCACG GCAAATCAAG CAATTTTAGA   
  
  
+ AGCATTCAAT GGGAAGAAGA AAGTTCATGT GATTGATTTC AGCATGAAGC AAGGCATGCA ATGGCCGGCT   
  
  
+ TTGATGCAAG CCCTGGCTCT TCGACCGGAG GGTCCACCGC TTTTCCGGTT AACCGGGATT GGACCGCCCG   
  
  
+ CTCCGGACAA CTCGGACCGG CTGCAAGAGG TGGGTTGGAA GCTTGCCCAG TTCGCGGATT CGATCCGGAT   
  
  
+ TAAATTTCAG TATCGTGGGT TTGTGGCAAA CAGTTTGACC GATCTAGAAT CTTCCATGCT AGATCTTGAA   
  
  
+ CCGGACACTG AGGTGGTGGC GGTCAACTCG GTTTTCGAGC TCCACCGGCT GTTGGCTAAA CCCGGGGCGA   
  
  
+ TCGAGAAAGT GCTGGGGTTC ATGAGGGCCG TGAACCCGGT CATTGTGACG ATGGTCGAGC AGGAAGCGAA   
  
  
+ CCACAACGGA CCGGTTTTCT TGGACCGGTT CAATGAGTCG TTGCATTATT ACTCCACCTT GTTTGATTCC   
  
  
+ CTTGAGATTT GTGTTGATAA TGTAGATAAG AAGATGTCGG AGGCTTACTT GGGCCAGCAG ATCTGCAACA   
  
  
+ TGGTGGCTTG TGAAGGGTCT GACCGAGTCG AGAGGCACGA GACCCTGGCT CAGTGGCGAG CCCGGTTCGC   
  
  
+ ATCTGCCGGG TTCGACCCAG TTCATCTGGG TTCGAATGCG TTTAAGCAGG CGAGTATGTT GTTGGAGTTC   
  
  
+ TTTGCTGGTG GTGAAGGGTA CGGCGTGGAG GAGCGAGAAG GGTGTCTCAT GTTGGGATGG CATAGTAGGC   
  
  
+ CGCTTATCAC CACCTCGGCT TGGCAGCTCG CCAAGAACCC GGCTGTGAAT CGGCGATG  

- -Up\_Stream \_Len000TTGGGA AATTAAAATA GTTACACCCT GTTGTGAGTA GAAGTATGAG GTTGTTCGAT   
  
  
- ACTATGTAAG CTAATAACGG ATCATAAAGC AAATCATTCT ATAATCACGG TTTGAAACAT TATTTTTACT   
  
  
- TTGAATGATT TTTTTTAGAG CAAAAGACAT CTCCCTTTTA AACTTCTGAA TCACAGTGTA CTACTGGATT   
  
  
- ACCATGAACA TCCCAGTTCT AACTCTAACT CCCGTATGAC TGCTGGACAT ATTACCAGTT CATCCTAGTT   
  
  
- TCAGCTCTTT TGACAGAGAC GATTGAAAGT ATTAGATTGG GAATTGAGTG AGACTGGGAG TTAAATTAGT   
  
  
- CCGGATTAAG TTTGATAATT ACCAATATTG AGAGAAAGGA AGAAAACTCT AAAAAAAAAA GATGTACGCC   
  
  
- AACTTTTATT AAAAAAATTT TTTATATATA GTGATTGGTA AGTAATAGTA TCAAAATCTC AAGTATAAAT   
  
  
- ACACCAGGAA CAACTCAGAT TAAATTACGA AATCTACCGC ACTTCGTGGT TTAATCACCA TTGGTATTAT   
  
  
- TATTTGATAG TTCACAATTC AGCTTCTTTT GCCTGTGACG AGTTGGTTTA GATCATTGTA TCACATACTG   
  
  
- TACTATTGAT TCACACGGTA CATCATTAGT TAAGAAAGAA AAAAAAAAAA ACCAACTCTA GTGTATTTAA   
  
  
- TGCAATAGTT CCCATCATGC TATATTACCT ACTCCTCTTT AATTTACACT CTAAATCAAC TACTTAGCTT   
  
  
- ATGCAATCAG ACTCGGTAAA CCAGTTTTGA CGTAACCGAT TAGTTATCTT TAATATCTTT TATGTATTGT   
  
  
- ATTACCCTAC GTTAAAAGAT TAACCCCTTT GGTACAGTTA CGTCACTAAA ACCTGGTAAA TCCGGAATAA   
  
  
- GAAAAGTTAA AAAATTAACT AATAAATCTG AGTATACTTG AATAACAACT GTCTAACTCG ATTTTTGTGA   
  
  
- CTACAAAGTA TGTATACATA CTTAACGTGC ATGCTTCCGA GACGAGTAAA GCTCTTTCTT TTAATTGTCA   
  
  
- ATCCTCGTCA TATTAAATTG TTGTTCGCTA TTAATAATCA TACTTCACCA TGCTTAGATA AAATTTCCTC   
  
  
- ATGATACTTC ATGTTTGTTA TTGTTTATTA TTTCTCGTCA TGTAATTGTA TAGAATGATG GATTTATTTA   
  
  
- GCTAAGATTC TCATTTTTTT ACAACTTTAA TTGGGGTAAT ATCAAAATGA AATTGTTCTT CAAAAAGAAG   
  
  
- AAAATGGTAA ATGGTTCATT CTTACTAAGT CCGGCATATC GGTTCCTTTT TATCTTTTGG TGTTTTGATT   
  
  
- GGTACCGGGT TTTTTGTTGG TGGTTCTCCC CGTGATTACA CTGATTGGCT GCCAATTGTA ATAGTAGACA   
  
  
- AACTATGAAC AAGTAGAGAG TGATAAGCCC ATTATCACAC TTTAATGACT ACATGGGAGA TTTGAGCAGC   
  
  
- AAAATTGACA CATTCTTAGT CTAATTGTCA GTGCCTGTGT ACTCATTGTT TAGTTAAAGC CTGTACTTGC   
  
  
- TTGCTTGACA CTTAGGGGGG GTTTTTTTTT TAAAAAAATT TTTTTCAACG TAGGAATAGA AAGACTTGGT   
  
  
- CCGGTCGACA GAGTAAGGAA CCAGGAGCGC GGGGTAGTGC TCGCGACGAG TAATAAGTGG GAGAGAGAGA   
  
  
- GAGGAGAGAC TAGGTGTGGT GTTTGTGTTC CAAACGTGTC GACGTCTCGT CAGCATGATG ATAGTCATCA   
  
  
- CTTTTTGGTT CGTCTTTAGG GGTTAGGGAG AAGAGGAAGA AGGGTCTAAG AAGGAGTTAA GGTTCGGGTA   
  
  
- TAGTCTTTAA ATCTGTTGTT GTTGGTTTTT TTTTTTAATT ATGACGAACT CTTTTTGTTT ATACTATACT   
  
  
- TTAAGGAGCT TTTTCCGGAC GGTATTGTTT AAGGAGCACT TTTTCGTCGA CTCTCTTCTT CTTCTTCTTT   
  
  
- TTTTAGGGCA GTGTTAGAGT TAGAGCTAGG GTTTAACACT TTCTCTTTCT TTTCTACTTC TCTCTTGTGG   
  
  
- GGAAAAGCTT GGGACTGGTC TTTGGGGGCG GCCTCTACAG CGGCCGCGGC CCCTTCAGGT TCTACACCCT   
  
  
- ACTACTCTAC CGGGTCTTGC TGCGATTTTA CCTACTCGAC GAACGACAAC ACCCCATGTT CCACTCCAGC   
  
  
- AGCCTGTACC GCCTTTAACG GGTCTTCGAA CTCGTCGAAC TTCTTCAGTA CCCATCACAA GTTCTTCTGC   
  
  
- CCAATAGAAT GAACCGAAGG CTTTGACAAG TAATGTTAGG ACGTCTAGAC AGATGAACCG AACTTAGATA   
  
  
- CGAAAGACTC AAATTGGGAT TGGGATTAAA ACTGGGAAGA GGTAGCAGTT AGAGTGGGTA GTAGCTAGGT   
  
  
- CGAGGAGGTG AAGGAGCTTG GAGCAGCTGC CAGTTTAAGC TCGGGCTGGG CAAAAGGCTA AGCCTAGACT   
  
  
- TTCGTTAGGG TCCATTCCGA TAGAACTGGG GTGGTTCAAG ATCGTTATGA AGTTTAAACG CACTCCGATT   
  
  
- CTCCAACTTT GGGAGTTTAA TGTGTTGGCG GGGTTGCGGG TGGGGTGGAG GTTTTGACGG GTTAAGCCGC   
  
  
- AGAGGTGGTA GCCCGTGCTG CCGCAGCTCC GGCCACCACG ACCATCTGAG TGTTCTTTTG CCGTAGTCTA   
  
  
- ACCACGTACG TGAATACCGG ACACGGCTTC GTTAAGTTGA TCTTTTGTAC CCTAATCGAC TCCGAAACCA   
  
  
- ATTTGTCTAA TCCATAAATC GTCGTAGAGT TCGACCTGGA TACTCCTTTC ATCGTTGAAC AAAACGTCTT   
  
  
- CGAGAATGAA CAGCCTAGAT GTTCGATACG GGTAGACATG GGATGCTACT TAGTCAGAGT CTACTCAACG   
  
  
- TCTACGTGAA AATACTCTGA ACGGGTATAG AATTTAAGCG GGTAAAGTGC CGTTTAGTTC GTTAAAATCT   
  
  
- TCGTAAGTTA CCCTTCTTCT TTCAAGTACA CTAACTAAAG TCGTACTTCG TTCCGTACGT TACCGGCCGA   
  
  
- AACTACGTTC GGGACCGAGA AGCTGGCCTC CCAGGTGGCG AAAAGGCCAA TTGGCCCTAA CCTGGCGGGC   
  
  
- GAGGCCTGTT GAGCCTGGCC GACGTTCTCC ACCCAACCTT CGAACGGGTC AAGCGCCTAA GCTAGGCCTA   
  
  
- ATTTAAAGTC ATAGCACCCA AACACCGTTT GTCAAACTGG CTAGATCTTA GAAGGTACGA TCTAGAACTT   
  
  
- GGCCTGTGAC TCCACCACCG CCAGTTGAGC CAAAAGCTCG AGGTGGCCGA CAACCGATTT GGGCCCCGCT   
  
  
- AGCTCTTTCA CGACCCCAAG TACTCCCGGC ACTTGGGCCA GTAACACTGC TACCAGCTCG TCCTTCGCTT   
  
  
- GGTGTTGCCT GGCCAAAAGA ACCTGGCCAA GTTACTCAGC AACGTAATAA TGAGGTGGAA CAAACTAAGG   
  
  
- GAACTCTAAA CACAACTATT ACATCTATTC TTCTACAGCC TCCGAATGAA CCCGGTCGTC TAGACGTTGT   
  
  
- ACCACCGAAC ACTTCCCAGA CTGGCTCAGC TCTCCGTGCT CTGGGACCGA GTCACCGCTC GGGCCAAGCG   
  
  
- TAGACGGCCC AAGCTGGGTC AAGTAGACCC AAGCTTACGC AAATTCGTCC GCTCATACAA CAACCTCAAG   
  
  
- AAACGACCAC CACTTCCCAT GCCGCACCTC CTCGCTCTTC CCACAGAGTA CAACCCTACC GTATCATCCG   
  
  
- GCGAATAGTG GTGGAGCCGA ACCGTCGAGC GGTTCTTGGG CCGACACTTA GCCGCTAC

+     Box 4

| Site Name | Organism | Position | Strand | Matrix score. | sequence | function |
| --- | --- | --- | --- | --- | --- | --- |
| Box 4 | Petroselinum crispum | 1860 | + | 6 | ATTAAT | part of a conserved DNA module involved in light responsiveness |
| Box 4 | Petroselinum crispum | 370 | + | 6 | ATTAAT | part of a conserved DNA module involved in light responsiveness |

>HU10G00709.1   
+ -Up\_Stream \_Len000AACCCT TTAATTTTAT CAATGTGGGA CAACACTCAT CTTCATACTC CAACAAGCTA   
  
  
+ TGATACATTC GATTATTGCC TAGTATTTCG TTTAGTAAGA TATTAGTGCC AAACTTTGTA ATAAAAATGA   
  
  
+ AACTTACTAA AAAAAATCTC GTTTTCTGTA GAGGGAAAAT TTGAAGACTT AGTGTCACAT GATGACCTAA   
  
  
+ TGGTACTTGT AGGGTCAAGA TTGAGATTGA GGGCATACTG ACGACCTGTA TAATGGTCAA GTAGGATCAA   
  
  
+ AGTCGAGAAA ACTGTCTCTG CTAACTTTCA TAATCTAACC CTTAACTCAC TCTGACCCTC AATTTAATCA   
  
  
+ GGCCTAATTC AAACTATTAA TGGTTATAAC TCTCTTTCCT TCTTTTGAGA TTTTTTTTTT CTACATGCGG   
  
  
+ TTGAAAATAA TTTTTTTAAA AAATATATAT CACTAACCAT TCATTATCAT AGTTTTAGAG TTCATATTTA   
  
  
+ TGTGGTCCTT GTTGAGTCTA ATTTAATGCT TTAGATGGCG TGAAGCACCA AATTAGTGGT AACCATAATA   
  
  
+ ATAAACTATC AAGTGTTAAG TCGAAGAAAA CGGACACTGC TCAACCAAAT CTAGTAACAT AGTGTATGAC   
  
  
+ ATGATAACTA AGTGTGCCAT GTAGTAATCA ATTCTTTCTT TTTTTTTTTT TGGTTGAGAT CACATAAATT   
  
  
+ ACGTTATCAA GGGTAGTACG ATATAATGGA TGAGGAGAAA TTAAATGTGA GATTTAGTTG ATGAATCGAA   
  
  
+ TACGTTAGTC TGAGCCATTT GGTCAAAACT GCATTGGCTA ATCAATAGAA ATTATAGAAA ATACATAACA   
  
  
+ TAATGGGATG CAATTTTCTA ATTGGGGAAA CCATGTCAAT GCAGTGATTT TGGACCATTT AGGCCTTATT   
  
  
+ CTTTTCAATT TTTTAATTGA TTATTTAGAC TCATATGAAC TTATTGTTGA CAGATTGAGC TAAAAACACT   
  
  
+ GATGTTTCAT ACATATGTAT GAATTGCACG TACGAAGGCT CTGCTCATTT CGAGAAAGAA AATTAACAGT   
  
  
+ TAGGAGCAGT ATAATTTAAC AACAAGCGAT AATTATTAGT ATGAAGTGGT ACGAATCTAT TTTAAAGGAG   
  
  
+ TACTATGAAG TACAAACAAT AACAAATAAT AAAGAGCAGT ACATTAACAT ATCTTACTAC CTAAATAAAT   
  
  
+ CGATTCTAAG AGTAAAAAAA TGTTGAAATT AACCCCATTA TAGTTTTACT TTAACAAGAA GTTTTTCTTC   
  
  
+ TTTTACCATT TACCAAGTAA GAATGATTCA GGCCGTATAG CCAAGGAAAA ATAGAAAACC ACAAAACTAA   
  
  
+ CCATGGCCCA AAAAACAACC ACCAAGAGGG GCACTAATGT GACTAACCGA CGGTTAACAT TATCATCTGT   
  
  
+ TTGATACTTG TTCATCTCTC ACTATTCGGG TAATAGTGTG AAATTACTGA TGTACCCTCT AAACTCGTCG   
  
  
+ TTTTAACTGT GTAAGAATCA GATTAACAGT CACGGACACA TGAGTAACAA ATCAATTTCG GACATGAACG   
  
  
+ AACGAACTGT GAATCCCCCC CAAAAAAAAA ATTTTTTTAA AAAAAGTTGC ATCCTTATCT TTCTGAACCA   
  
  
+ GGCCAGCTGT CTCATTCCTT GGTCCTCGCG CCCCATCACG AGCGCTGCTC ATTATTCACC CTCTCTCTCT   
  
  
+ CTCCTCTCTG ATCCACACCA CAAACACAAG GTTTGCACAG CTGCAGAGCA GTCGTACTAC TATCAGTAGT   
  
  
+ GAAAAACCAA GCAGAAATCC CCAATCCCTC TTCTCCTTCT TCCCAGATTC TTCCTCAATT CCAAGCCCAT   
  
  
+ ATCAGAAATT TAGACAACAA CAACCAAAAA AAAAAATTAA TACTGCTTGA GAAAAACAAA TATGATATGA   
  
  
+ AATTCCTCGA AAAAGGCCTG CCATAACAAA TTCCTCGTGA AAAAGCAGCT GAGAGAAGAA GAAGAAGAAA   
  
  
+ AAAATCCCGT CACAATCTCA ATCTCGATCC CAAATTGTGA AAGAGAAAGA AAAGATGAAG AGAGAACACC   
  
  
+ CCTTTTCGAA CCCTGACCAG AAACCCCCGC CGGAGATGTC GCCGGCGCCG GGGAAGTCCA AGATGTGGGA   
  
  
+ TGATGAGATG GCCCAGAACG ACGCTAAAAT GGATGAGCTG CTTGCTGTTG TGGGGTACAA GGTGAGGTCG   
  
  
+ TCGGACATGG CGGAAATTGC CCAGAAGCTT GAGCAGCTTG AAGAAGTCAT GGGTAGTGTT CAAGAAGACG   
  
  
+ GGTTATCTTA CTTGGCTTCC GAAACTGTTC ATTACAATCC TGCAGATCTG TCTACTTGGC TTGAATCTAT   
  
  
+ GCTTTCTGAG TTTAACCCTA ACCCTAATTT TGACCCTTCT CCATCGTCAA TCTCACCCAT CATCGATCCA   
  
  
+ GCTCCTCCAC TTCCTCGAAC CTCGTCGACG GTCAAATTCG AGCCCGACCC GTTTTCCGAT TCGGATCTGA   
  
  
+ AAGCAATCCC AGGTAAGGCT ATCTTGACCC CACCAAGTTC TAGCAATACT TCAAATTTGC GTGAGGCTAA   
  
  
+ GAGGTTGAAA CCCTCAAATT ACACAACCGC CCCAACGCCC ACCCCACCTC CAAAACTGCC CAATTCGGCG   
  
  
+ TCTCCACCAT CGGGCACGAC GGCGTCGAGG CCGGTGGTGC TGGTAGACTC ACAAGAAAAC GGCATCAGAT   
  
  
+ TGGTGCATGC ACTTATGGCC TGTGCCGAAG CAATTCAACT AGAAAACATG GGATTAGCTG AGGCTTTGGT   
  
  
+ TAAACAGATT AGGTATTTAG CAGCATCTCA AGCTGGACCT ATGAGGAAAG TAGCAACTTG TTTTGCAGAA   
  
  
+ GCTCTTACTT GTCGGATCTA CAAGCTATGC CCATCTGTAC CCTACGATGA ATCAGTCTCA GATGAGTTGC   
  
  
+ AGATGCACTT TTATGAGACT TGCCCATATC TTAAATTCGC CCATTTCACG GCAAATCAAG CAATTTTAGA   
  
  
+ AGCATTCAAT GGGAAGAAGA AAGTTCATGT GATTGATTTC AGCATGAAGC AAGGCATGCA ATGGCCGGCT   
  
  
+ TTGATGCAAG CCCTGGCTCT TCGACCGGAG GGTCCACCGC TTTTCCGGTT AACCGGGATT GGACCGCCCG   
  
  
+ CTCCGGACAA CTCGGACCGG CTGCAAGAGG TGGGTTGGAA GCTTGCCCAG TTCGCGGATT CGATCCGGAT   
  
  
+ TAAATTTCAG TATCGTGGGT TTGTGGCAAA CAGTTTGACC GATCTAGAAT CTTCCATGCT AGATCTTGAA   
  
  
+ CCGGACACTG AGGTGGTGGC GGTCAACTCG GTTTTCGAGC TCCACCGGCT GTTGGCTAAA CCCGGGGCGA   
  
  
+ TCGAGAAAGT GCTGGGGTTC ATGAGGGCCG TGAACCCGGT CATTGTGACG ATGGTCGAGC AGGAAGCGAA   
  
  
+ CCACAACGGA CCGGTTTTCT TGGACCGGTT CAATGAGTCG TTGCATTATT ACTCCACCTT GTTTGATTCC   
  
  
+ CTTGAGATTT GTGTTGATAA TGTAGATAAG AAGATGTCGG AGGCTTACTT GGGCCAGCAG ATCTGCAACA   
  
  
+ TGGTGGCTTG TGAAGGGTCT GACCGAGTCG AGAGGCACGA GACCCTGGCT CAGTGGCGAG CCCGGTTCGC   
  
  
+ ATCTGCCGGG TTCGACCCAG TTCATCTGGG TTCGAATGCG TTTAAGCAGG CGAGTATGTT GTTGGAGTTC   
  
  
+ TTTGCTGGTG GTGAAGGGTA CGGCGTGGAG GAGCGAGAAG GGTGTCTCAT GTTGGGATGG CATAGTAGGC   
  
  
+ CGCTTATCAC CACCTCGGCT TGGCAGCTCG CCAAGAACCC GGCTGTGAAT CGGCGATG  

- -Up\_Stream \_Len000TTGGGA AATTAAAATA GTTACACCCT GTTGTGAGTA GAAGTATGAG GTTGTTCGAT   
  
  
- ACTATGTAAG CTAATAACGG ATCATAAAGC AAATCATTCT ATAATCACGG TTTGAAACAT TATTTTTACT   
  
  
- TTGAATGATT TTTTTTAGAG CAAAAGACAT CTCCCTTTTA AACTTCTGAA TCACAGTGTA CTACTGGATT   
  
  
- ACCATGAACA TCCCAGTTCT AACTCTAACT CCCGTATGAC TGCTGGACAT ATTACCAGTT CATCCTAGTT   
  
  
- TCAGCTCTTT TGACAGAGAC GATTGAAAGT ATTAGATTGG GAATTGAGTG AGACTGGGAG TTAAATTAGT   
  
  
- CCGGATTAAG TTTGATAATT ACCAATATTG AGAGAAAGGA AGAAAACTCT AAAAAAAAAA GATGTACGCC   
  
  
- AACTTTTATT AAAAAAATTT TTTATATATA GTGATTGGTA AGTAATAGTA TCAAAATCTC AAGTATAAAT   
  
  
- ACACCAGGAA CAACTCAGAT TAAATTACGA AATCTACCGC ACTTCGTGGT TTAATCACCA TTGGTATTAT   
  
  
- TATTTGATAG TTCACAATTC AGCTTCTTTT GCCTGTGACG AGTTGGTTTA GATCATTGTA TCACATACTG   
  
  
- TACTATTGAT TCACACGGTA CATCATTAGT TAAGAAAGAA AAAAAAAAAA ACCAACTCTA GTGTATTTAA   
  
  
- TGCAATAGTT CCCATCATGC TATATTACCT ACTCCTCTTT AATTTACACT CTAAATCAAC TACTTAGCTT   
  
  
- ATGCAATCAG ACTCGGTAAA CCAGTTTTGA CGTAACCGAT TAGTTATCTT TAATATCTTT TATGTATTGT   
  
  
- ATTACCCTAC GTTAAAAGAT TAACCCCTTT GGTACAGTTA CGTCACTAAA ACCTGGTAAA TCCGGAATAA   
  
  
- GAAAAGTTAA AAAATTAACT AATAAATCTG AGTATACTTG AATAACAACT GTCTAACTCG ATTTTTGTGA   
  
  
- CTACAAAGTA TGTATACATA CTTAACGTGC ATGCTTCCGA GACGAGTAAA GCTCTTTCTT TTAATTGTCA   
  
  
- ATCCTCGTCA TATTAAATTG TTGTTCGCTA TTAATAATCA TACTTCACCA TGCTTAGATA AAATTTCCTC   
  
  
- ATGATACTTC ATGTTTGTTA TTGTTTATTA TTTCTCGTCA TGTAATTGTA TAGAATGATG GATTTATTTA   
  
  
- GCTAAGATTC TCATTTTTTT ACAACTTTAA TTGGGGTAAT ATCAAAATGA AATTGTTCTT CAAAAAGAAG   
  
  
- AAAATGGTAA ATGGTTCATT CTTACTAAGT CCGGCATATC GGTTCCTTTT TATCTTTTGG TGTTTTGATT   
  
  
- GGTACCGGGT TTTTTGTTGG TGGTTCTCCC CGTGATTACA CTGATTGGCT GCCAATTGTA ATAGTAGACA   
  
  
- AACTATGAAC AAGTAGAGAG TGATAAGCCC ATTATCACAC TTTAATGACT ACATGGGAGA TTTGAGCAGC   
  
  
- AAAATTGACA CATTCTTAGT CTAATTGTCA GTGCCTGTGT ACTCATTGTT TAGTTAAAGC CTGTACTTGC   
  
  
- TTGCTTGACA CTTAGGGGGG GTTTTTTTTT TAAAAAAATT TTTTTCAACG TAGGAATAGA AAGACTTGGT   
  
  
- CCGGTCGACA GAGTAAGGAA CCAGGAGCGC GGGGTAGTGC TCGCGACGAG TAATAAGTGG GAGAGAGAGA   
  
  
- GAGGAGAGAC TAGGTGTGGT GTTTGTGTTC CAAACGTGTC GACGTCTCGT CAGCATGATG ATAGTCATCA   
  
  
- CTTTTTGGTT CGTCTTTAGG GGTTAGGGAG AAGAGGAAGA AGGGTCTAAG AAGGAGTTAA GGTTCGGGTA   
  
  
- TAGTCTTTAA ATCTGTTGTT GTTGGTTTTT TTTTTTAATT ATGACGAACT CTTTTTGTTT ATACTATACT   
  
  
- TTAAGGAGCT TTTTCCGGAC GGTATTGTTT AAGGAGCACT TTTTCGTCGA CTCTCTTCTT CTTCTTCTTT   
  
  
- TTTTAGGGCA GTGTTAGAGT TAGAGCTAGG GTTTAACACT TTCTCTTTCT TTTCTACTTC TCTCTTGTGG   
  
  
- GGAAAAGCTT GGGACTGGTC TTTGGGGGCG GCCTCTACAG CGGCCGCGGC CCCTTCAGGT TCTACACCCT   
  
  
- ACTACTCTAC CGGGTCTTGC TGCGATTTTA CCTACTCGAC GAACGACAAC ACCCCATGTT CCACTCCAGC   
  
  
- AGCCTGTACC GCCTTTAACG GGTCTTCGAA CTCGTCGAAC TTCTTCAGTA CCCATCACAA GTTCTTCTGC   
  
  
- CCAATAGAAT GAACCGAAGG CTTTGACAAG TAATGTTAGG ACGTCTAGAC AGATGAACCG AACTTAGATA   
  
  
- CGAAAGACTC AAATTGGGAT TGGGATTAAA ACTGGGAAGA GGTAGCAGTT AGAGTGGGTA GTAGCTAGGT   
  
  
- CGAGGAGGTG AAGGAGCTTG GAGCAGCTGC CAGTTTAAGC TCGGGCTGGG CAAAAGGCTA AGCCTAGACT   
  
  
- TTCGTTAGGG TCCATTCCGA TAGAACTGGG GTGGTTCAAG ATCGTTATGA AGTTTAAACG CACTCCGATT   
  
  
- CTCCAACTTT GGGAGTTTAA TGTGTTGGCG GGGTTGCGGG TGGGGTGGAG GTTTTGACGG GTTAAGCCGC   
  
  
- AGAGGTGGTA GCCCGTGCTG CCGCAGCTCC GGCCACCACG ACCATCTGAG TGTTCTTTTG CCGTAGTCTA   
  
  
- ACCACGTACG TGAATACCGG ACACGGCTTC GTTAAGTTGA TCTTTTGTAC CCTAATCGAC TCCGAAACCA   
  
  
- ATTTGTCTAA TCCATAAATC GTCGTAGAGT TCGACCTGGA TACTCCTTTC ATCGTTGAAC AAAACGTCTT   
  
  
- CGAGAATGAA CAGCCTAGAT GTTCGATACG GGTAGACATG GGATGCTACT TAGTCAGAGT CTACTCAACG   
  
  
- TCTACGTGAA AATACTCTGA ACGGGTATAG AATTTAAGCG GGTAAAGTGC CGTTTAGTTC GTTAAAATCT   
  
  
- TCGTAAGTTA CCCTTCTTCT TTCAAGTACA CTAACTAAAG TCGTACTTCG TTCCGTACGT TACCGGCCGA   
  
  
- AACTACGTTC GGGACCGAGA AGCTGGCCTC CCAGGTGGCG AAAAGGCCAA TTGGCCCTAA CCTGGCGGGC   
  
  
- GAGGCCTGTT GAGCCTGGCC GACGTTCTCC ACCCAACCTT CGAACGGGTC AAGCGCCTAA GCTAGGCCTA   
  
  
- ATTTAAAGTC ATAGCACCCA AACACCGTTT GTCAAACTGG CTAGATCTTA GAAGGTACGA TCTAGAACTT   
  
  
- GGCCTGTGAC TCCACCACCG CCAGTTGAGC CAAAAGCTCG AGGTGGCCGA CAACCGATTT GGGCCCCGCT   
  
  
- AGCTCTTTCA CGACCCCAAG TACTCCCGGC ACTTGGGCCA GTAACACTGC TACCAGCTCG TCCTTCGCTT   
  
  
- GGTGTTGCCT GGCCAAAAGA ACCTGGCCAA GTTACTCAGC AACGTAATAA TGAGGTGGAA CAAACTAAGG   
  
  
- GAACTCTAAA CACAACTATT ACATCTATTC TTCTACAGCC TCCGAATGAA CCCGGTCGTC TAGACGTTGT   
  
  
- ACCACCGAAC ACTTCCCAGA CTGGCTCAGC TCTCCGTGCT CTGGGACCGA GTCACCGCTC GGGCCAAGCG   
  
  
- TAGACGGCCC AAGCTGGGTC AAGTAGACCC AAGCTTACGC AAATTCGTCC GCTCATACAA CAACCTCAAG   
  
  
- AAACGACCAC CACTTCCCAT GCCGCACCTC CTCGCTCTTC CCACAGAGTA CAACCCTACC GTATCATCCG   
  
  
- GCGAATAGTG GTGGAGCCGA ACCGTCGAGC GGTTCTTGGG CCGACACTTA GCCGCTAC

+     CAAT-box

| Site Name | Organism | Position | Strand | Matrix score. | sequence | function |
| --- | --- | --- | --- | --- | --- | --- |
| CAAT-box | Nicotiana glutinosa | 3395 | + | 4 | CAAT |  |
| CAAT-box | Nicotiana glutinosa | 881 | + | 4 | CAAT |  |
| CAAT-box | Arabidopsis thaliana | 807 | - | 5 | CCAAT | common cis-acting element in promoter and enhancer regions |
| CAAT-box | Nicotiana glutinosa | 2585 | + | 4 | CAAT |  |
| CAAT-box | Nicotiana glutinosa | 1983 | + | 4 | CAAT |  |
| CAAT-box | Nicotiana glutinosa | 2458 | + | 4 | CAAT |  |
| CAAT-box | Nicotiana glutinosa | 3336 | - | 4 | CAAT |  |
| CAAT-box | Pisum sativum | 1522 | + | 5 | CAAAT | common cis-acting element in promoter and enhancer regions |
| CAAT-box | Pisum sativum | 1881 | + | 5 | CAAAT | common cis-acting element in promoter and enhancer regions |
| CAAT-box | Nicotiana glutinosa | 2362 | + | 4 | CAAT |  |
| CAAT-box | Nicotiana glutinosa | 2279 | + | 4 | CAAT |  |
| CAAT-box | Nicotiana glutinosa | 2190 | - | 4 | CAAT |  |
| CAAT-box | Arabidopsis thaliana | 3072 | - | 5 | CCAAT | common cis-acting element in promoter and enhancer regions |
| CAAT-box | Nicotiana glutinosa | 968 | - | 4 | CAAT |  |
| CAAT-box | Nicotiana glutinosa | 344 | + | 4 | CAAT |  |
| CAAT-box | Nicotiana glutinosa | 1998 | - | 4 | CAAT |  |
| CAAT-box | Nicotiana glutinosa | 234 | - | 4 | CAAT |  |
| CAAT-box | Nicotiana glutinosa | 920 | + | 4 | CAAT |  |
| CAAT-box | Nicotiana glutinosa | 2695 | + | 4 | CAAT |  |
| CAAT-box | Nicotiana glutinosa | 663 | + | 4 | CAAT |  |
| CAAT-box | Pisum sativum | 2539 | + | 5 | CAAAT | common cis-acting element in promoter and enhancer regions |
| CAAT-box | Arabidopsis thaliana | 2584 | + | 5 | CCAAT | common cis-acting element in promoter and enhancer regions |
| CAAT-box | Nicotiana glutinosa | 89 | - | 4 | CAAT |  |
| CAAT-box | Arabidopsis thaliana | 1775 | + | 5 | CCAAT | common cis-acting element in promoter and enhancer regions |
| CAAT-box | Pisum sativum | 1921 | + | 5 | CAAAT | common cis-acting element in promoter and enhancer regions |
| CAAT-box | Nicotiana glutinosa | 3003 | + | 4 | CAAT |  |
| CAAT-box | Nicotiana glutinosa | 35 | + | 4 | CAAT |  |
| CAAT-box | Nicotiana glutinosa | 1141 | + | 4 | CAAT |  |
| CAAT-box | Pisum sativum | 2506 | + | 5 | CAAAT | common cis-acting element in promoter and enhancer regions |
| CAAT-box | Arabidopsis thaliana | 865 | - | 5 | CCAAT | common cis-acting element in promoter and enhancer regions |
| CAAT-box | Arabidopsis thaliana | 2663 | - | 5 | CCAAT | common cis-acting element in promoter and enhancer regions |
| CAAT-box | Nicotiana glutinosa | 1527 | + | 4 | CAAT |  |
| CAAT-box | Pisum sativum | 1995 | + | 5 | CAAAT | common cis-acting element in promoter and enhancer regions |
| CAAT-box | Pisum sativum | 3441 | - | 5 | CAAAT | common cis-acting element in promoter and enhancer regions |
| CAAT-box | Nicotiana glutinosa | 1776 | + | 4 | CAAT |  |
| CAAT-box | Pisum sativum | 543 | + | 5 | CAAAT | common cis-acting element in promoter and enhancer regions |
| CAAT-box | Nicotiana glutinosa | 240 | - | 4 | CAAT |  |
| CAAT-box | Pisum sativum | 2926 | + | 5 | CAAAT | common cis-acting element in promoter and enhancer regions |
| CAAT-box | Nicotiana glutinosa | 1977 | + | 4 | CAAT |  |
| CAAT-box | Nicotiana glutinosa | 930 | - | 4 | CAAT |  |
| CAAT-box | Pisum sativum | 791 | - | 5 | CAAAT | common cis-acting element in promoter and enhancer regions |
| CAAT-box | Pisum sativum | 2509 | - | 5 | CAAAT | common cis-acting element in promoter and enhancer regions |
| CAAT-box | Nicotiana glutinosa | 957 | - | 4 | CAAT |  |
| CAAT-box | Nicotiana glutinosa | 2951 | + | 4 | CAAT |  |
| CAAT-box | Nicotiana glutinosa | 2498 | + | 4 | CAAT |  |
| CAAT-box | Pisum sativum | 610 | + | 5 | CAAAT | common cis-acting element in promoter and enhancer regions |
| CAAT-box | Nicotiana glutinosa | 1810 | + | 4 | CAAT |  |
| CAAT-box | Nicotiana glutinosa | 2976 | - | 4 | CAAT |  |
| CAAT-box | Nicotiana glutinosa | 817 | + | 4 | CAAT |  |
| CAAT-box | Pisum sativum | 2417 | + | 5 | CAAAT | common cis-acting element in promoter and enhancer regions |
| CAAT-box | Nicotiana glutinosa | 1007 | - | 4 | CAAT |  |
| CAAT-box | Nicotiana glutinosa | 855 | + | 4 | CAAT |  |
| CAAT-box | Nicotiana glutinosa | 2935 | + | 4 | CAAT |  |
| CAAT-box | Pisum sativum | 1147 | + | 5 | CAAAT | common cis-acting element in promoter and enhancer regions |
| CAAT-box | Pisum sativum | 183 | - | 5 | CAAAT | common cis-acting element in promoter and enhancer regions |

>HU10G00709.1   
+ -Up\_Stream \_Len000AACCCT TTAATTTTAT CAATGTGGGA CAACACTCAT CTTCATACTC CAACAAGCTA   
  
  
+ TGATACATTC GATTATTGCC TAGTATTTCG TTTAGTAAGA TATTAGTGCC AAACTTTGTA ATAAAAATGA   
  
  
+ AACTTACTAA AAAAAATCTC GTTTTCTGTA GAGGGAAAAT TTGAAGACTT AGTGTCACAT GATGACCTAA   
  
  
+ TGGTACTTGT AGGGTCAAGA TTGAGATTGA GGGCATACTG ACGACCTGTA TAATGGTCAA GTAGGATCAA   
  
  
+ AGTCGAGAAA ACTGTCTCTG CTAACTTTCA TAATCTAACC CTTAACTCAC TCTGACCCTC AATTTAATCA   
  
  
+ GGCCTAATTC AAACTATTAA TGGTTATAAC TCTCTTTCCT TCTTTTGAGA TTTTTTTTTT CTACATGCGG   
  
  
+ TTGAAAATAA TTTTTTTAAA AAATATATAT CACTAACCAT TCATTATCAT AGTTTTAGAG TTCATATTTA   
  
  
+ TGTGGTCCTT GTTGAGTCTA ATTTAATGCT TTAGATGGCG TGAAGCACCA AATTAGTGGT AACCATAATA   
  
  
+ ATAAACTATC AAGTGTTAAG TCGAAGAAAA CGGACACTGC TCAACCAAAT CTAGTAACAT AGTGTATGAC   
  
  
+ ATGATAACTA AGTGTGCCAT GTAGTAATCA ATTCTTTCTT TTTTTTTTTT TGGTTGAGAT CACATAAATT   
  
  
+ ACGTTATCAA GGGTAGTACG ATATAATGGA TGAGGAGAAA TTAAATGTGA GATTTAGTTG ATGAATCGAA   
  
  
+ TACGTTAGTC TGAGCCATTT GGTCAAAACT GCATTGGCTA ATCAATAGAA ATTATAGAAA ATACATAACA   
  
  
+ TAATGGGATG CAATTTTCTA ATTGGGGAAA CCATGTCAAT GCAGTGATTT TGGACCATTT AGGCCTTATT   
  
  
+ CTTTTCAATT TTTTAATTGA TTATTTAGAC TCATATGAAC TTATTGTTGA CAGATTGAGC TAAAAACACT   
  
  
+ GATGTTTCAT ACATATGTAT GAATTGCACG TACGAAGGCT CTGCTCATTT CGAGAAAGAA AATTAACAGT   
  
  
+ TAGGAGCAGT ATAATTTAAC AACAAGCGAT AATTATTAGT ATGAAGTGGT ACGAATCTAT TTTAAAGGAG   
  
  
+ TACTATGAAG TACAAACAAT AACAAATAAT AAAGAGCAGT ACATTAACAT ATCTTACTAC CTAAATAAAT   
  
  
+ CGATTCTAAG AGTAAAAAAA TGTTGAAATT AACCCCATTA TAGTTTTACT TTAACAAGAA GTTTTTCTTC   
  
  
+ TTTTACCATT TACCAAGTAA GAATGATTCA GGCCGTATAG CCAAGGAAAA ATAGAAAACC ACAAAACTAA   
  
  
+ CCATGGCCCA AAAAACAACC ACCAAGAGGG GCACTAATGT GACTAACCGA CGGTTAACAT TATCATCTGT   
  
  
+ TTGATACTTG TTCATCTCTC ACTATTCGGG TAATAGTGTG AAATTACTGA TGTACCCTCT AAACTCGTCG   
  
  
+ TTTTAACTGT GTAAGAATCA GATTAACAGT CACGGACACA TGAGTAACAA ATCAATTTCG GACATGAACG   
  
  
+ AACGAACTGT GAATCCCCCC CAAAAAAAAA ATTTTTTTAA AAAAAGTTGC ATCCTTATCT TTCTGAACCA   
  
  
+ GGCCAGCTGT CTCATTCCTT GGTCCTCGCG CCCCATCACG AGCGCTGCTC ATTATTCACC CTCTCTCTCT   
  
  
+ CTCCTCTCTG ATCCACACCA CAAACACAAG GTTTGCACAG CTGCAGAGCA GTCGTACTAC TATCAGTAGT   
  
  
+ GAAAAACCAA GCAGAAATCC CCAATCCCTC TTCTCCTTCT TCCCAGATTC TTCCTCAATT CCAAGCCCAT   
  
  
+ ATCAGAAATT TAGACAACAA CAACCAAAAA AAAAAATTAA TACTGCTTGA GAAAAACAAA TATGATATGA   
  
  
+ AATTCCTCGA AAAAGGCCTG CCATAACAAA TTCCTCGTGA AAAAGCAGCT GAGAGAAGAA GAAGAAGAAA   
  
  
+ AAAATCCCGT CACAATCTCA ATCTCGATCC CAAATTGTGA AAGAGAAAGA AAAGATGAAG AGAGAACACC   
  
  
+ CCTTTTCGAA CCCTGACCAG AAACCCCCGC CGGAGATGTC GCCGGCGCCG GGGAAGTCCA AGATGTGGGA   
  
  
+ TGATGAGATG GCCCAGAACG ACGCTAAAAT GGATGAGCTG CTTGCTGTTG TGGGGTACAA GGTGAGGTCG   
  
  
+ TCGGACATGG CGGAAATTGC CCAGAAGCTT GAGCAGCTTG AAGAAGTCAT GGGTAGTGTT CAAGAAGACG   
  
  
+ GGTTATCTTA CTTGGCTTCC GAAACTGTTC ATTACAATCC TGCAGATCTG TCTACTTGGC TTGAATCTAT   
  
  
+ GCTTTCTGAG TTTAACCCTA ACCCTAATTT TGACCCTTCT CCATCGTCAA TCTCACCCAT CATCGATCCA   
  
  
+ GCTCCTCCAC TTCCTCGAAC CTCGTCGACG GTCAAATTCG AGCCCGACCC GTTTTCCGAT TCGGATCTGA   
  
  
+ AAGCAATCCC AGGTAAGGCT ATCTTGACCC CACCAAGTTC TAGCAATACT TCAAATTTGC GTGAGGCTAA   
  
  
+ GAGGTTGAAA CCCTCAAATT ACACAACCGC CCCAACGCCC ACCCCACCTC CAAAACTGCC CAATTCGGCG   
  
  
+ TCTCCACCAT CGGGCACGAC GGCGTCGAGG CCGGTGGTGC TGGTAGACTC ACAAGAAAAC GGCATCAGAT   
  
  
+ TGGTGCATGC ACTTATGGCC TGTGCCGAAG CAATTCAACT AGAAAACATG GGATTAGCTG AGGCTTTGGT   
  
  
+ TAAACAGATT AGGTATTTAG CAGCATCTCA AGCTGGACCT ATGAGGAAAG TAGCAACTTG TTTTGCAGAA   
  
  
+ GCTCTTACTT GTCGGATCTA CAAGCTATGC CCATCTGTAC CCTACGATGA ATCAGTCTCA GATGAGTTGC   
  
  
+ AGATGCACTT TTATGAGACT TGCCCATATC TTAAATTCGC CCATTTCACG GCAAATCAAG CAATTTTAGA   
  
  
+ AGCATTCAAT GGGAAGAAGA AAGTTCATGT GATTGATTTC AGCATGAAGC AAGGCATGCA ATGGCCGGCT   
  
  
+ TTGATGCAAG CCCTGGCTCT TCGACCGGAG GGTCCACCGC TTTTCCGGTT AACCGGGATT GGACCGCCCG   
  
  
+ CTCCGGACAA CTCGGACCGG CTGCAAGAGG TGGGTTGGAA GCTTGCCCAG TTCGCGGATT CGATCCGGAT   
  
  
+ TAAATTTCAG TATCGTGGGT TTGTGGCAAA CAGTTTGACC GATCTAGAAT CTTCCATGCT AGATCTTGAA   
  
  
+ CCGGACACTG AGGTGGTGGC GGTCAACTCG GTTTTCGAGC TCCACCGGCT GTTGGCTAAA CCCGGGGCGA   
  
  
+ TCGAGAAAGT GCTGGGGTTC ATGAGGGCCG TGAACCCGGT CATTGTGACG ATGGTCGAGC AGGAAGCGAA   
  
  
+ CCACAACGGA CCGGTTTTCT TGGACCGGTT CAATGAGTCG TTGCATTATT ACTCCACCTT GTTTGATTCC   
  
  
+ CTTGAGATTT GTGTTGATAA TGTAGATAAG AAGATGTCGG AGGCTTACTT GGGCCAGCAG ATCTGCAACA   
  
  
+ TGGTGGCTTG TGAAGGGTCT GACCGAGTCG AGAGGCACGA GACCCTGGCT CAGTGGCGAG CCCGGTTCGC   
  
  
+ ATCTGCCGGG TTCGACCCAG TTCATCTGGG TTCGAATGCG TTTAAGCAGG CGAGTATGTT GTTGGAGTTC   
  
  
+ TTTGCTGGTG GTGAAGGGTA CGGCGTGGAG GAGCGAGAAG GGTGTCTCAT GTTGGGATGG CATAGTAGGC   
  
  
+ CGCTTATCAC CACCTCGGCT TGGCAGCTCG CCAAGAACCC GGCTGTGAAT CGGCGATG  

- -Up\_Stream \_Len000TTGGGA AATTAAAATA GTTACACCCT GTTGTGAGTA GAAGTATGAG GTTGTTCGAT   
  
  
- ACTATGTAAG CTAATAACGG ATCATAAAGC AAATCATTCT ATAATCACGG TTTGAAACAT TATTTTTACT   
  
  
- TTGAATGATT TTTTTTAGAG CAAAAGACAT CTCCCTTTTA AACTTCTGAA TCACAGTGTA CTACTGGATT   
  
  
- ACCATGAACA TCCCAGTTCT AACTCTAACT CCCGTATGAC TGCTGGACAT ATTACCAGTT CATCCTAGTT   
  
  
- TCAGCTCTTT TGACAGAGAC GATTGAAAGT ATTAGATTGG GAATTGAGTG AGACTGGGAG TTAAATTAGT   
  
  
- CCGGATTAAG TTTGATAATT ACCAATATTG AGAGAAAGGA AGAAAACTCT AAAAAAAAAA GATGTACGCC   
  
  
- AACTTTTATT AAAAAAATTT TTTATATATA GTGATTGGTA AGTAATAGTA TCAAAATCTC AAGTATAAAT   
  
  
- ACACCAGGAA CAACTCAGAT TAAATTACGA AATCTACCGC ACTTCGTGGT TTAATCACCA TTGGTATTAT   
  
  
- TATTTGATAG TTCACAATTC AGCTTCTTTT GCCTGTGACG AGTTGGTTTA GATCATTGTA TCACATACTG   
  
  
- TACTATTGAT TCACACGGTA CATCATTAGT TAAGAAAGAA AAAAAAAAAA ACCAACTCTA GTGTATTTAA   
  
  
- TGCAATAGTT CCCATCATGC TATATTACCT ACTCCTCTTT AATTTACACT CTAAATCAAC TACTTAGCTT   
  
  
- ATGCAATCAG ACTCGGTAAA CCAGTTTTGA CGTAACCGAT TAGTTATCTT TAATATCTTT TATGTATTGT   
  
  
- ATTACCCTAC GTTAAAAGAT TAACCCCTTT GGTACAGTTA CGTCACTAAA ACCTGGTAAA TCCGGAATAA   
  
  
- GAAAAGTTAA AAAATTAACT AATAAATCTG AGTATACTTG AATAACAACT GTCTAACTCG ATTTTTGTGA   
  
  
- CTACAAAGTA TGTATACATA CTTAACGTGC ATGCTTCCGA GACGAGTAAA GCTCTTTCTT TTAATTGTCA   
  
  
- ATCCTCGTCA TATTAAATTG TTGTTCGCTA TTAATAATCA TACTTCACCA TGCTTAGATA AAATTTCCTC   
  
  
- ATGATACTTC ATGTTTGTTA TTGTTTATTA TTTCTCGTCA TGTAATTGTA TAGAATGATG GATTTATTTA   
  
  
- GCTAAGATTC TCATTTTTTT ACAACTTTAA TTGGGGTAAT ATCAAAATGA AATTGTTCTT CAAAAAGAAG   
  
  
- AAAATGGTAA ATGGTTCATT CTTACTAAGT CCGGCATATC GGTTCCTTTT TATCTTTTGG TGTTTTGATT   
  
  
- GGTACCGGGT TTTTTGTTGG TGGTTCTCCC CGTGATTACA CTGATTGGCT GCCAATTGTA ATAGTAGACA   
  
  
- AACTATGAAC AAGTAGAGAG TGATAAGCCC ATTATCACAC TTTAATGACT ACATGGGAGA TTTGAGCAGC   
  
  
- AAAATTGACA CATTCTTAGT CTAATTGTCA GTGCCTGTGT ACTCATTGTT TAGTTAAAGC CTGTACTTGC   
  
  
- TTGCTTGACA CTTAGGGGGG GTTTTTTTTT TAAAAAAATT TTTTTCAACG TAGGAATAGA AAGACTTGGT   
  
  
- CCGGTCGACA GAGTAAGGAA CCAGGAGCGC GGGGTAGTGC TCGCGACGAG TAATAAGTGG GAGAGAGAGA   
  
  
- GAGGAGAGAC TAGGTGTGGT GTTTGTGTTC CAAACGTGTC GACGTCTCGT CAGCATGATG ATAGTCATCA   
  
  
- CTTTTTGGTT CGTCTTTAGG GGTTAGGGAG AAGAGGAAGA AGGGTCTAAG AAGGAGTTAA GGTTCGGGTA   
  
  
- TAGTCTTTAA ATCTGTTGTT GTTGGTTTTT TTTTTTAATT ATGACGAACT CTTTTTGTTT ATACTATACT   
  
  
- TTAAGGAGCT TTTTCCGGAC GGTATTGTTT AAGGAGCACT TTTTCGTCGA CTCTCTTCTT CTTCTTCTTT   
  
  
- TTTTAGGGCA GTGTTAGAGT TAGAGCTAGG GTTTAACACT TTCTCTTTCT TTTCTACTTC TCTCTTGTGG   
  
  
- GGAAAAGCTT GGGACTGGTC TTTGGGGGCG GCCTCTACAG CGGCCGCGGC CCCTTCAGGT TCTACACCCT   
  
  
- ACTACTCTAC CGGGTCTTGC TGCGATTTTA CCTACTCGAC GAACGACAAC ACCCCATGTT CCACTCCAGC   
  
  
- AGCCTGTACC GCCTTTAACG GGTCTTCGAA CTCGTCGAAC TTCTTCAGTA CCCATCACAA GTTCTTCTGC   
  
  
- CCAATAGAAT GAACCGAAGG CTTTGACAAG TAATGTTAGG ACGTCTAGAC AGATGAACCG AACTTAGATA   
  
  
- CGAAAGACTC AAATTGGGAT TGGGATTAAA ACTGGGAAGA GGTAGCAGTT AGAGTGGGTA GTAGCTAGGT   
  
  
- CGAGGAGGTG AAGGAGCTTG GAGCAGCTGC CAGTTTAAGC TCGGGCTGGG CAAAAGGCTA AGCCTAGACT   
  
  
- TTCGTTAGGG TCCATTCCGA TAGAACTGGG GTGGTTCAAG ATCGTTATGA AGTTTAAACG CACTCCGATT   
  
  
- CTCCAACTTT GGGAGTTTAA TGTGTTGGCG GGGTTGCGGG TGGGGTGGAG GTTTTGACGG GTTAAGCCGC   
  
  
- AGAGGTGGTA GCCCGTGCTG CCGCAGCTCC GGCCACCACG ACCATCTGAG TGTTCTTTTG CCGTAGTCTA   
  
  
- ACCACGTACG TGAATACCGG ACACGGCTTC GTTAAGTTGA TCTTTTGTAC CCTAATCGAC TCCGAAACCA   
  
  
- ATTTGTCTAA TCCATAAATC GTCGTAGAGT TCGACCTGGA TACTCCTTTC ATCGTTGAAC AAAACGTCTT   
  
  
- CGAGAATGAA CAGCCTAGAT GTTCGATACG GGTAGACATG GGATGCTACT TAGTCAGAGT CTACTCAACG   
  
  
- TCTACGTGAA AATACTCTGA ACGGGTATAG AATTTAAGCG GGTAAAGTGC CGTTTAGTTC GTTAAAATCT   
  
  
- TCGTAAGTTA CCCTTCTTCT TTCAAGTACA CTAACTAAAG TCGTACTTCG TTCCGTACGT TACCGGCCGA   
  
  
- AACTACGTTC GGGACCGAGA AGCTGGCCTC CCAGGTGGCG AAAAGGCCAA TTGGCCCTAA CCTGGCGGGC   
  
  
- GAGGCCTGTT GAGCCTGGCC GACGTTCTCC ACCCAACCTT CGAACGGGTC AAGCGCCTAA GCTAGGCCTA   
  
  
- ATTTAAAGTC ATAGCACCCA AACACCGTTT GTCAAACTGG CTAGATCTTA GAAGGTACGA TCTAGAACTT   
  
  
- GGCCTGTGAC TCCACCACCG CCAGTTGAGC CAAAAGCTCG AGGTGGCCGA CAACCGATTT GGGCCCCGCT   
  
  
- AGCTCTTTCA CGACCCCAAG TACTCCCGGC ACTTGGGCCA GTAACACTGC TACCAGCTCG TCCTTCGCTT   
  
  
- GGTGTTGCCT GGCCAAAAGA ACCTGGCCAA GTTACTCAGC AACGTAATAA TGAGGTGGAA CAAACTAAGG   
  
  
- GAACTCTAAA CACAACTATT ACATCTATTC TTCTACAGCC TCCGAATGAA CCCGGTCGTC TAGACGTTGT   
  
  
- ACCACCGAAC ACTTCCCAGA CTGGCTCAGC TCTCCGTGCT CTGGGACCGA GTCACCGCTC GGGCCAAGCG   
  
  
- TAGACGGCCC AAGCTGGGTC AAGTAGACCC AAGCTTACGC AAATTCGTCC GCTCATACAA CAACCTCAAG   
  
  
- AAACGACCAC CACTTCCCAT GCCGCACCTC CTCGCTCTTC CCACAGAGTA CAACCCTACC GTATCATCCG   
  
  
- GCGAATAGTG GTGGAGCCGA ACCGTCGAGC GGTTCTTGGG CCGACACTTA GCCGCTAC

+     CAT-box

| Site Name | Organism | Position | Strand | Matrix score. | sequence | function |
| --- | --- | --- | --- | --- | --- | --- |
| CAT-box | Arabidopsis thaliana | 3556 | - | 6 | GCCACT | cis-acting regulatory element related to meristem expression |

>HU10G00709.1   
+ -Up\_Stream \_Len000AACCCT TTAATTTTAT CAATGTGGGA CAACACTCAT CTTCATACTC CAACAAGCTA   
  
  
+ TGATACATTC GATTATTGCC TAGTATTTCG TTTAGTAAGA TATTAGTGCC AAACTTTGTA ATAAAAATGA   
  
  
+ AACTTACTAA AAAAAATCTC GTTTTCTGTA GAGGGAAAAT TTGAAGACTT AGTGTCACAT GATGACCTAA   
  
  
+ TGGTACTTGT AGGGTCAAGA TTGAGATTGA GGGCATACTG ACGACCTGTA TAATGGTCAA GTAGGATCAA   
  
  
+ AGTCGAGAAA ACTGTCTCTG CTAACTTTCA TAATCTAACC CTTAACTCAC TCTGACCCTC AATTTAATCA   
  
  
+ GGCCTAATTC AAACTATTAA TGGTTATAAC TCTCTTTCCT TCTTTTGAGA TTTTTTTTTT CTACATGCGG   
  
  
+ TTGAAAATAA TTTTTTTAAA AAATATATAT CACTAACCAT TCATTATCAT AGTTTTAGAG TTCATATTTA   
  
  
+ TGTGGTCCTT GTTGAGTCTA ATTTAATGCT TTAGATGGCG TGAAGCACCA AATTAGTGGT AACCATAATA   
  
  
+ ATAAACTATC AAGTGTTAAG TCGAAGAAAA CGGACACTGC TCAACCAAAT CTAGTAACAT AGTGTATGAC   
  
  
+ ATGATAACTA AGTGTGCCAT GTAGTAATCA ATTCTTTCTT TTTTTTTTTT TGGTTGAGAT CACATAAATT   
  
  
+ ACGTTATCAA GGGTAGTACG ATATAATGGA TGAGGAGAAA TTAAATGTGA GATTTAGTTG ATGAATCGAA   
  
  
+ TACGTTAGTC TGAGCCATTT GGTCAAAACT GCATTGGCTA ATCAATAGAA ATTATAGAAA ATACATAACA   
  
  
+ TAATGGGATG CAATTTTCTA ATTGGGGAAA CCATGTCAAT GCAGTGATTT TGGACCATTT AGGCCTTATT   
  
  
+ CTTTTCAATT TTTTAATTGA TTATTTAGAC TCATATGAAC TTATTGTTGA CAGATTGAGC TAAAAACACT   
  
  
+ GATGTTTCAT ACATATGTAT GAATTGCACG TACGAAGGCT CTGCTCATTT CGAGAAAGAA AATTAACAGT   
  
  
+ TAGGAGCAGT ATAATTTAAC AACAAGCGAT AATTATTAGT ATGAAGTGGT ACGAATCTAT TTTAAAGGAG   
  
  
+ TACTATGAAG TACAAACAAT AACAAATAAT AAAGAGCAGT ACATTAACAT ATCTTACTAC CTAAATAAAT   
  
  
+ CGATTCTAAG AGTAAAAAAA TGTTGAAATT AACCCCATTA TAGTTTTACT TTAACAAGAA GTTTTTCTTC   
  
  
+ TTTTACCATT TACCAAGTAA GAATGATTCA GGCCGTATAG CCAAGGAAAA ATAGAAAACC ACAAAACTAA   
  
  
+ CCATGGCCCA AAAAACAACC ACCAAGAGGG GCACTAATGT GACTAACCGA CGGTTAACAT TATCATCTGT   
  
  
+ TTGATACTTG TTCATCTCTC ACTATTCGGG TAATAGTGTG AAATTACTGA TGTACCCTCT AAACTCGTCG   
  
  
+ TTTTAACTGT GTAAGAATCA GATTAACAGT CACGGACACA TGAGTAACAA ATCAATTTCG GACATGAACG   
  
  
+ AACGAACTGT GAATCCCCCC CAAAAAAAAA ATTTTTTTAA AAAAAGTTGC ATCCTTATCT TTCTGAACCA   
  
  
+ GGCCAGCTGT CTCATTCCTT GGTCCTCGCG CCCCATCACG AGCGCTGCTC ATTATTCACC CTCTCTCTCT   
  
  
+ CTCCTCTCTG ATCCACACCA CAAACACAAG GTTTGCACAG CTGCAGAGCA GTCGTACTAC TATCAGTAGT   
  
  
+ GAAAAACCAA GCAGAAATCC CCAATCCCTC TTCTCCTTCT TCCCAGATTC TTCCTCAATT CCAAGCCCAT   
  
  
+ ATCAGAAATT TAGACAACAA CAACCAAAAA AAAAAATTAA TACTGCTTGA GAAAAACAAA TATGATATGA   
  
  
+ AATTCCTCGA AAAAGGCCTG CCATAACAAA TTCCTCGTGA AAAAGCAGCT GAGAGAAGAA GAAGAAGAAA   
  
  
+ AAAATCCCGT CACAATCTCA ATCTCGATCC CAAATTGTGA AAGAGAAAGA AAAGATGAAG AGAGAACACC   
  
  
+ CCTTTTCGAA CCCTGACCAG AAACCCCCGC CGGAGATGTC GCCGGCGCCG GGGAAGTCCA AGATGTGGGA   
  
  
+ TGATGAGATG GCCCAGAACG ACGCTAAAAT GGATGAGCTG CTTGCTGTTG TGGGGTACAA GGTGAGGTCG   
  
  
+ TCGGACATGG CGGAAATTGC CCAGAAGCTT GAGCAGCTTG AAGAAGTCAT GGGTAGTGTT CAAGAAGACG   
  
  
+ GGTTATCTTA CTTGGCTTCC GAAACTGTTC ATTACAATCC TGCAGATCTG TCTACTTGGC TTGAATCTAT   
  
  
+ GCTTTCTGAG TTTAACCCTA ACCCTAATTT TGACCCTTCT CCATCGTCAA TCTCACCCAT CATCGATCCA   
  
  
+ GCTCCTCCAC TTCCTCGAAC CTCGTCGACG GTCAAATTCG AGCCCGACCC GTTTTCCGAT TCGGATCTGA   
  
  
+ AAGCAATCCC AGGTAAGGCT ATCTTGACCC CACCAAGTTC TAGCAATACT TCAAATTTGC GTGAGGCTAA   
  
  
+ GAGGTTGAAA CCCTCAAATT ACACAACCGC CCCAACGCCC ACCCCACCTC CAAAACTGCC CAATTCGGCG   
  
  
+ TCTCCACCAT CGGGCACGAC GGCGTCGAGG CCGGTGGTGC TGGTAGACTC ACAAGAAAAC GGCATCAGAT   
  
  
+ TGGTGCATGC ACTTATGGCC TGTGCCGAAG CAATTCAACT AGAAAACATG GGATTAGCTG AGGCTTTGGT   
  
  
+ TAAACAGATT AGGTATTTAG CAGCATCTCA AGCTGGACCT ATGAGGAAAG TAGCAACTTG TTTTGCAGAA   
  
  
+ GCTCTTACTT GTCGGATCTA CAAGCTATGC CCATCTGTAC CCTACGATGA ATCAGTCTCA GATGAGTTGC   
  
  
+ AGATGCACTT TTATGAGACT TGCCCATATC TTAAATTCGC CCATTTCACG GCAAATCAAG CAATTTTAGA   
  
  
+ AGCATTCAAT GGGAAGAAGA AAGTTCATGT GATTGATTTC AGCATGAAGC AAGGCATGCA ATGGCCGGCT   
  
  
+ TTGATGCAAG CCCTGGCTCT TCGACCGGAG GGTCCACCGC TTTTCCGGTT AACCGGGATT GGACCGCCCG   
  
  
+ CTCCGGACAA CTCGGACCGG CTGCAAGAGG TGGGTTGGAA GCTTGCCCAG TTCGCGGATT CGATCCGGAT   
  
  
+ TAAATTTCAG TATCGTGGGT TTGTGGCAAA CAGTTTGACC GATCTAGAAT CTTCCATGCT AGATCTTGAA   
  
  
+ CCGGACACTG AGGTGGTGGC GGTCAACTCG GTTTTCGAGC TCCACCGGCT GTTGGCTAAA CCCGGGGCGA   
  
  
+ TCGAGAAAGT GCTGGGGTTC ATGAGGGCCG TGAACCCGGT CATTGTGACG ATGGTCGAGC AGGAAGCGAA   
  
  
+ CCACAACGGA CCGGTTTTCT TGGACCGGTT CAATGAGTCG TTGCATTATT ACTCCACCTT GTTTGATTCC   
  
  
+ CTTGAGATTT GTGTTGATAA TGTAGATAAG AAGATGTCGG AGGCTTACTT GGGCCAGCAG ATCTGCAACA   
  
  
+ TGGTGGCTTG TGAAGGGTCT GACCGAGTCG AGAGGCACGA GACCCTGGCT CAGTGGCGAG CCCGGTTCGC   
  
  
+ ATCTGCCGGG TTCGACCCAG TTCATCTGGG TTCGAATGCG TTTAAGCAGG CGAGTATGTT GTTGGAGTTC   
  
  
+ TTTGCTGGTG GTGAAGGGTA CGGCGTGGAG GAGCGAGAAG GGTGTCTCAT GTTGGGATGG CATAGTAGGC   
  
  
+ CGCTTATCAC CACCTCGGCT TGGCAGCTCG CCAAGAACCC GGCTGTGAAT CGGCGATG  

- -Up\_Stream \_Len000TTGGGA AATTAAAATA GTTACACCCT GTTGTGAGTA GAAGTATGAG GTTGTTCGAT   
  
  
- ACTATGTAAG CTAATAACGG ATCATAAAGC AAATCATTCT ATAATCACGG TTTGAAACAT TATTTTTACT   
  
  
- TTGAATGATT TTTTTTAGAG CAAAAGACAT CTCCCTTTTA AACTTCTGAA TCACAGTGTA CTACTGGATT   
  
  
- ACCATGAACA TCCCAGTTCT AACTCTAACT CCCGTATGAC TGCTGGACAT ATTACCAGTT CATCCTAGTT   
  
  
- TCAGCTCTTT TGACAGAGAC GATTGAAAGT ATTAGATTGG GAATTGAGTG AGACTGGGAG TTAAATTAGT   
  
  
- CCGGATTAAG TTTGATAATT ACCAATATTG AGAGAAAGGA AGAAAACTCT AAAAAAAAAA GATGTACGCC   
  
  
- AACTTTTATT AAAAAAATTT TTTATATATA GTGATTGGTA AGTAATAGTA TCAAAATCTC AAGTATAAAT   
  
  
- ACACCAGGAA CAACTCAGAT TAAATTACGA AATCTACCGC ACTTCGTGGT TTAATCACCA TTGGTATTAT   
  
  
- TATTTGATAG TTCACAATTC AGCTTCTTTT GCCTGTGACG AGTTGGTTTA GATCATTGTA TCACATACTG   
  
  
- TACTATTGAT TCACACGGTA CATCATTAGT TAAGAAAGAA AAAAAAAAAA ACCAACTCTA GTGTATTTAA   
  
  
- TGCAATAGTT CCCATCATGC TATATTACCT ACTCCTCTTT AATTTACACT CTAAATCAAC TACTTAGCTT   
  
  
- ATGCAATCAG ACTCGGTAAA CCAGTTTTGA CGTAACCGAT TAGTTATCTT TAATATCTTT TATGTATTGT   
  
  
- ATTACCCTAC GTTAAAAGAT TAACCCCTTT GGTACAGTTA CGTCACTAAA ACCTGGTAAA TCCGGAATAA   
  
  
- GAAAAGTTAA AAAATTAACT AATAAATCTG AGTATACTTG AATAACAACT GTCTAACTCG ATTTTTGTGA   
  
  
- CTACAAAGTA TGTATACATA CTTAACGTGC ATGCTTCCGA GACGAGTAAA GCTCTTTCTT TTAATTGTCA   
  
  
- ATCCTCGTCA TATTAAATTG TTGTTCGCTA TTAATAATCA TACTTCACCA TGCTTAGATA AAATTTCCTC   
  
  
- ATGATACTTC ATGTTTGTTA TTGTTTATTA TTTCTCGTCA TGTAATTGTA TAGAATGATG GATTTATTTA   
  
  
- GCTAAGATTC TCATTTTTTT ACAACTTTAA TTGGGGTAAT ATCAAAATGA AATTGTTCTT CAAAAAGAAG   
  
  
- AAAATGGTAA ATGGTTCATT CTTACTAAGT CCGGCATATC GGTTCCTTTT TATCTTTTGG TGTTTTGATT   
  
  
- GGTACCGGGT TTTTTGTTGG TGGTTCTCCC CGTGATTACA CTGATTGGCT GCCAATTGTA ATAGTAGACA   
  
  
- AACTATGAAC AAGTAGAGAG TGATAAGCCC ATTATCACAC TTTAATGACT ACATGGGAGA TTTGAGCAGC   
  
  
- AAAATTGACA CATTCTTAGT CTAATTGTCA GTGCCTGTGT ACTCATTGTT TAGTTAAAGC CTGTACTTGC   
  
  
- TTGCTTGACA CTTAGGGGGG GTTTTTTTTT TAAAAAAATT TTTTTCAACG TAGGAATAGA AAGACTTGGT   
  
  
- CCGGTCGACA GAGTAAGGAA CCAGGAGCGC GGGGTAGTGC TCGCGACGAG TAATAAGTGG GAGAGAGAGA   
  
  
- GAGGAGAGAC TAGGTGTGGT GTTTGTGTTC CAAACGTGTC GACGTCTCGT CAGCATGATG ATAGTCATCA   
  
  
- CTTTTTGGTT CGTCTTTAGG GGTTAGGGAG AAGAGGAAGA AGGGTCTAAG AAGGAGTTAA GGTTCGGGTA   
  
  
- TAGTCTTTAA ATCTGTTGTT GTTGGTTTTT TTTTTTAATT ATGACGAACT CTTTTTGTTT ATACTATACT   
  
  
- TTAAGGAGCT TTTTCCGGAC GGTATTGTTT AAGGAGCACT TTTTCGTCGA CTCTCTTCTT CTTCTTCTTT   
  
  
- TTTTAGGGCA GTGTTAGAGT TAGAGCTAGG GTTTAACACT TTCTCTTTCT TTTCTACTTC TCTCTTGTGG   
  
  
- GGAAAAGCTT GGGACTGGTC TTTGGGGGCG GCCTCTACAG CGGCCGCGGC CCCTTCAGGT TCTACACCCT   
  
  
- ACTACTCTAC CGGGTCTTGC TGCGATTTTA CCTACTCGAC GAACGACAAC ACCCCATGTT CCACTCCAGC   
  
  
- AGCCTGTACC GCCTTTAACG GGTCTTCGAA CTCGTCGAAC TTCTTCAGTA CCCATCACAA GTTCTTCTGC   
  
  
- CCAATAGAAT GAACCGAAGG CTTTGACAAG TAATGTTAGG ACGTCTAGAC AGATGAACCG AACTTAGATA   
  
  
- CGAAAGACTC AAATTGGGAT TGGGATTAAA ACTGGGAAGA GGTAGCAGTT AGAGTGGGTA GTAGCTAGGT   
  
  
- CGAGGAGGTG AAGGAGCTTG GAGCAGCTGC CAGTTTAAGC TCGGGCTGGG CAAAAGGCTA AGCCTAGACT   
  
  
- TTCGTTAGGG TCCATTCCGA TAGAACTGGG GTGGTTCAAG ATCGTTATGA AGTTTAAACG CACTCCGATT   
  
  
- CTCCAACTTT GGGAGTTTAA TGTGTTGGCG GGGTTGCGGG TGGGGTGGAG GTTTTGACGG GTTAAGCCGC   
  
  
- AGAGGTGGTA GCCCGTGCTG CCGCAGCTCC GGCCACCACG ACCATCTGAG TGTTCTTTTG CCGTAGTCTA   
  
  
- ACCACGTACG TGAATACCGG ACACGGCTTC GTTAAGTTGA TCTTTTGTAC CCTAATCGAC TCCGAAACCA   
  
  
- ATTTGTCTAA TCCATAAATC GTCGTAGAGT TCGACCTGGA TACTCCTTTC ATCGTTGAAC AAAACGTCTT   
  
  
- CGAGAATGAA CAGCCTAGAT GTTCGATACG GGTAGACATG GGATGCTACT TAGTCAGAGT CTACTCAACG   
  
  
- TCTACGTGAA AATACTCTGA ACGGGTATAG AATTTAAGCG GGTAAAGTGC CGTTTAGTTC GTTAAAATCT   
  
  
- TCGTAAGTTA CCCTTCTTCT TTCAAGTACA CTAACTAAAG TCGTACTTCG TTCCGTACGT TACCGGCCGA   
  
  
- AACTACGTTC GGGACCGAGA AGCTGGCCTC CCAGGTGGCG AAAAGGCCAA TTGGCCCTAA CCTGGCGGGC   
  
  
- GAGGCCTGTT GAGCCTGGCC GACGTTCTCC ACCCAACCTT CGAACGGGTC AAGCGCCTAA GCTAGGCCTA   
  
  
- ATTTAAAGTC ATAGCACCCA AACACCGTTT GTCAAACTGG CTAGATCTTA GAAGGTACGA TCTAGAACTT   
  
  
- GGCCTGTGAC TCCACCACCG CCAGTTGAGC CAAAAGCTCG AGGTGGCCGA CAACCGATTT GGGCCCCGCT   
  
  
- AGCTCTTTCA CGACCCCAAG TACTCCCGGC ACTTGGGCCA GTAACACTGC TACCAGCTCG TCCTTCGCTT   
  
  
- GGTGTTGCCT GGCCAAAAGA ACCTGGCCAA GTTACTCAGC AACGTAATAA TGAGGTGGAA CAAACTAAGG   
  
  
- GAACTCTAAA CACAACTATT ACATCTATTC TTCTACAGCC TCCGAATGAA CCCGGTCGTC TAGACGTTGT   
  
  
- ACCACCGAAC ACTTCCCAGA CTGGCTCAGC TCTCCGTGCT CTGGGACCGA GTCACCGCTC GGGCCAAGCG   
  
  
- TAGACGGCCC AAGCTGGGTC AAGTAGACCC AAGCTTACGC AAATTCGTCC GCTCATACAA CAACCTCAAG   
  
  
- AAACGACCAC CACTTCCCAT GCCGCACCTC CTCGCTCTTC CCACAGAGTA CAACCCTACC GTATCATCCG   
  
  
- GCGAATAGTG GTGGAGCCGA ACCGTCGAGC GGTTCTTGGG CCGACACTTA GCCGCTAC

+     CCAAT-box

| Site Name | Organism | Position | Strand | Matrix score. | sequence | function |
| --- | --- | --- | --- | --- | --- | --- |
| CCAAT-box | Hordeum vulgare | 3368 | + | 6 | CAACGG | MYBHv1 binding site |

>HU10G00709.1   
+ -Up\_Stream \_Len000AACCCT TTAATTTTAT CAATGTGGGA CAACACTCAT CTTCATACTC CAACAAGCTA   
  
  
+ TGATACATTC GATTATTGCC TAGTATTTCG TTTAGTAAGA TATTAGTGCC AAACTTTGTA ATAAAAATGA   
  
  
+ AACTTACTAA AAAAAATCTC GTTTTCTGTA GAGGGAAAAT TTGAAGACTT AGTGTCACAT GATGACCTAA   
  
  
+ TGGTACTTGT AGGGTCAAGA TTGAGATTGA GGGCATACTG ACGACCTGTA TAATGGTCAA GTAGGATCAA   
  
  
+ AGTCGAGAAA ACTGTCTCTG CTAACTTTCA TAATCTAACC CTTAACTCAC TCTGACCCTC AATTTAATCA   
  
  
+ GGCCTAATTC AAACTATTAA TGGTTATAAC TCTCTTTCCT TCTTTTGAGA TTTTTTTTTT CTACATGCGG   
  
  
+ TTGAAAATAA TTTTTTTAAA AAATATATAT CACTAACCAT TCATTATCAT AGTTTTAGAG TTCATATTTA   
  
  
+ TGTGGTCCTT GTTGAGTCTA ATTTAATGCT TTAGATGGCG TGAAGCACCA AATTAGTGGT AACCATAATA   
  
  
+ ATAAACTATC AAGTGTTAAG TCGAAGAAAA CGGACACTGC TCAACCAAAT CTAGTAACAT AGTGTATGAC   
  
  
+ ATGATAACTA AGTGTGCCAT GTAGTAATCA ATTCTTTCTT TTTTTTTTTT TGGTTGAGAT CACATAAATT   
  
  
+ ACGTTATCAA GGGTAGTACG ATATAATGGA TGAGGAGAAA TTAAATGTGA GATTTAGTTG ATGAATCGAA   
  
  
+ TACGTTAGTC TGAGCCATTT GGTCAAAACT GCATTGGCTA ATCAATAGAA ATTATAGAAA ATACATAACA   
  
  
+ TAATGGGATG CAATTTTCTA ATTGGGGAAA CCATGTCAAT GCAGTGATTT TGGACCATTT AGGCCTTATT   
  
  
+ CTTTTCAATT TTTTAATTGA TTATTTAGAC TCATATGAAC TTATTGTTGA CAGATTGAGC TAAAAACACT   
  
  
+ GATGTTTCAT ACATATGTAT GAATTGCACG TACGAAGGCT CTGCTCATTT CGAGAAAGAA AATTAACAGT   
  
  
+ TAGGAGCAGT ATAATTTAAC AACAAGCGAT AATTATTAGT ATGAAGTGGT ACGAATCTAT TTTAAAGGAG   
  
  
+ TACTATGAAG TACAAACAAT AACAAATAAT AAAGAGCAGT ACATTAACAT ATCTTACTAC CTAAATAAAT   
  
  
+ CGATTCTAAG AGTAAAAAAA TGTTGAAATT AACCCCATTA TAGTTTTACT TTAACAAGAA GTTTTTCTTC   
  
  
+ TTTTACCATT TACCAAGTAA GAATGATTCA GGCCGTATAG CCAAGGAAAA ATAGAAAACC ACAAAACTAA   
  
  
+ CCATGGCCCA AAAAACAACC ACCAAGAGGG GCACTAATGT GACTAACCGA CGGTTAACAT TATCATCTGT   
  
  
+ TTGATACTTG TTCATCTCTC ACTATTCGGG TAATAGTGTG AAATTACTGA TGTACCCTCT AAACTCGTCG   
  
  
+ TTTTAACTGT GTAAGAATCA GATTAACAGT CACGGACACA TGAGTAACAA ATCAATTTCG GACATGAACG   
  
  
+ AACGAACTGT GAATCCCCCC CAAAAAAAAA ATTTTTTTAA AAAAAGTTGC ATCCTTATCT TTCTGAACCA   
  
  
+ GGCCAGCTGT CTCATTCCTT GGTCCTCGCG CCCCATCACG AGCGCTGCTC ATTATTCACC CTCTCTCTCT   
  
  
+ CTCCTCTCTG ATCCACACCA CAAACACAAG GTTTGCACAG CTGCAGAGCA GTCGTACTAC TATCAGTAGT   
  
  
+ GAAAAACCAA GCAGAAATCC CCAATCCCTC TTCTCCTTCT TCCCAGATTC TTCCTCAATT CCAAGCCCAT   
  
  
+ ATCAGAAATT TAGACAACAA CAACCAAAAA AAAAAATTAA TACTGCTTGA GAAAAACAAA TATGATATGA   
  
  
+ AATTCCTCGA AAAAGGCCTG CCATAACAAA TTCCTCGTGA AAAAGCAGCT GAGAGAAGAA GAAGAAGAAA   
  
  
+ AAAATCCCGT CACAATCTCA ATCTCGATCC CAAATTGTGA AAGAGAAAGA AAAGATGAAG AGAGAACACC   
  
  
+ CCTTTTCGAA CCCTGACCAG AAACCCCCGC CGGAGATGTC GCCGGCGCCG GGGAAGTCCA AGATGTGGGA   
  
  
+ TGATGAGATG GCCCAGAACG ACGCTAAAAT GGATGAGCTG CTTGCTGTTG TGGGGTACAA GGTGAGGTCG   
  
  
+ TCGGACATGG CGGAAATTGC CCAGAAGCTT GAGCAGCTTG AAGAAGTCAT GGGTAGTGTT CAAGAAGACG   
  
  
+ GGTTATCTTA CTTGGCTTCC GAAACTGTTC ATTACAATCC TGCAGATCTG TCTACTTGGC TTGAATCTAT   
  
  
+ GCTTTCTGAG TTTAACCCTA ACCCTAATTT TGACCCTTCT CCATCGTCAA TCTCACCCAT CATCGATCCA   
  
  
+ GCTCCTCCAC TTCCTCGAAC CTCGTCGACG GTCAAATTCG AGCCCGACCC GTTTTCCGAT TCGGATCTGA   
  
  
+ AAGCAATCCC AGGTAAGGCT ATCTTGACCC CACCAAGTTC TAGCAATACT TCAAATTTGC GTGAGGCTAA   
  
  
+ GAGGTTGAAA CCCTCAAATT ACACAACCGC CCCAACGCCC ACCCCACCTC CAAAACTGCC CAATTCGGCG   
  
  
+ TCTCCACCAT CGGGCACGAC GGCGTCGAGG CCGGTGGTGC TGGTAGACTC ACAAGAAAAC GGCATCAGAT   
  
  
+ TGGTGCATGC ACTTATGGCC TGTGCCGAAG CAATTCAACT AGAAAACATG GGATTAGCTG AGGCTTTGGT   
  
  
+ TAAACAGATT AGGTATTTAG CAGCATCTCA AGCTGGACCT ATGAGGAAAG TAGCAACTTG TTTTGCAGAA   
  
  
+ GCTCTTACTT GTCGGATCTA CAAGCTATGC CCATCTGTAC CCTACGATGA ATCAGTCTCA GATGAGTTGC   
  
  
+ AGATGCACTT TTATGAGACT TGCCCATATC TTAAATTCGC CCATTTCACG GCAAATCAAG CAATTTTAGA   
  
  
+ AGCATTCAAT GGGAAGAAGA AAGTTCATGT GATTGATTTC AGCATGAAGC AAGGCATGCA ATGGCCGGCT   
  
  
+ TTGATGCAAG CCCTGGCTCT TCGACCGGAG GGTCCACCGC TTTTCCGGTT AACCGGGATT GGACCGCCCG   
  
  
+ CTCCGGACAA CTCGGACCGG CTGCAAGAGG TGGGTTGGAA GCTTGCCCAG TTCGCGGATT CGATCCGGAT   
  
  
+ TAAATTTCAG TATCGTGGGT TTGTGGCAAA CAGTTTGACC GATCTAGAAT CTTCCATGCT AGATCTTGAA   
  
  
+ CCGGACACTG AGGTGGTGGC GGTCAACTCG GTTTTCGAGC TCCACCGGCT GTTGGCTAAA CCCGGGGCGA   
  
  
+ TCGAGAAAGT GCTGGGGTTC ATGAGGGCCG TGAACCCGGT CATTGTGACG ATGGTCGAGC AGGAAGCGAA   
  
  
+ CCACAACGGA CCGGTTTTCT TGGACCGGTT CAATGAGTCG TTGCATTATT ACTCCACCTT GTTTGATTCC   
  
  
+ CTTGAGATTT GTGTTGATAA TGTAGATAAG AAGATGTCGG AGGCTTACTT GGGCCAGCAG ATCTGCAACA   
  
  
+ TGGTGGCTTG TGAAGGGTCT GACCGAGTCG AGAGGCACGA GACCCTGGCT CAGTGGCGAG CCCGGTTCGC   
  
  
+ ATCTGCCGGG TTCGACCCAG TTCATCTGGG TTCGAATGCG TTTAAGCAGG CGAGTATGTT GTTGGAGTTC   
  
  
+ TTTGCTGGTG GTGAAGGGTA CGGCGTGGAG GAGCGAGAAG GGTGTCTCAT GTTGGGATGG CATAGTAGGC   
  
  
+ CGCTTATCAC CACCTCGGCT TGGCAGCTCG CCAAGAACCC GGCTGTGAAT CGGCGATG  

- -Up\_Stream \_Len000TTGGGA AATTAAAATA GTTACACCCT GTTGTGAGTA GAAGTATGAG GTTGTTCGAT   
  
  
- ACTATGTAAG CTAATAACGG ATCATAAAGC AAATCATTCT ATAATCACGG TTTGAAACAT TATTTTTACT   
  
  
- TTGAATGATT TTTTTTAGAG CAAAAGACAT CTCCCTTTTA AACTTCTGAA TCACAGTGTA CTACTGGATT   
  
  
- ACCATGAACA TCCCAGTTCT AACTCTAACT CCCGTATGAC TGCTGGACAT ATTACCAGTT CATCCTAGTT   
  
  
- TCAGCTCTTT TGACAGAGAC GATTGAAAGT ATTAGATTGG GAATTGAGTG AGACTGGGAG TTAAATTAGT   
  
  
- CCGGATTAAG TTTGATAATT ACCAATATTG AGAGAAAGGA AGAAAACTCT AAAAAAAAAA GATGTACGCC   
  
  
- AACTTTTATT AAAAAAATTT TTTATATATA GTGATTGGTA AGTAATAGTA TCAAAATCTC AAGTATAAAT   
  
  
- ACACCAGGAA CAACTCAGAT TAAATTACGA AATCTACCGC ACTTCGTGGT TTAATCACCA TTGGTATTAT   
  
  
- TATTTGATAG TTCACAATTC AGCTTCTTTT GCCTGTGACG AGTTGGTTTA GATCATTGTA TCACATACTG   
  
  
- TACTATTGAT TCACACGGTA CATCATTAGT TAAGAAAGAA AAAAAAAAAA ACCAACTCTA GTGTATTTAA   
  
  
- TGCAATAGTT CCCATCATGC TATATTACCT ACTCCTCTTT AATTTACACT CTAAATCAAC TACTTAGCTT   
  
  
- ATGCAATCAG ACTCGGTAAA CCAGTTTTGA CGTAACCGAT TAGTTATCTT TAATATCTTT TATGTATTGT   
  
  
- ATTACCCTAC GTTAAAAGAT TAACCCCTTT GGTACAGTTA CGTCACTAAA ACCTGGTAAA TCCGGAATAA   
  
  
- GAAAAGTTAA AAAATTAACT AATAAATCTG AGTATACTTG AATAACAACT GTCTAACTCG ATTTTTGTGA   
  
  
- CTACAAAGTA TGTATACATA CTTAACGTGC ATGCTTCCGA GACGAGTAAA GCTCTTTCTT TTAATTGTCA   
  
  
- ATCCTCGTCA TATTAAATTG TTGTTCGCTA TTAATAATCA TACTTCACCA TGCTTAGATA AAATTTCCTC   
  
  
- ATGATACTTC ATGTTTGTTA TTGTTTATTA TTTCTCGTCA TGTAATTGTA TAGAATGATG GATTTATTTA   
  
  
- GCTAAGATTC TCATTTTTTT ACAACTTTAA TTGGGGTAAT ATCAAAATGA AATTGTTCTT CAAAAAGAAG   
  
  
- AAAATGGTAA ATGGTTCATT CTTACTAAGT CCGGCATATC GGTTCCTTTT TATCTTTTGG TGTTTTGATT   
  
  
- GGTACCGGGT TTTTTGTTGG TGGTTCTCCC CGTGATTACA CTGATTGGCT GCCAATTGTA ATAGTAGACA   
  
  
- AACTATGAAC AAGTAGAGAG TGATAAGCCC ATTATCACAC TTTAATGACT ACATGGGAGA TTTGAGCAGC   
  
  
- AAAATTGACA CATTCTTAGT CTAATTGTCA GTGCCTGTGT ACTCATTGTT TAGTTAAAGC CTGTACTTGC   
  
  
- TTGCTTGACA CTTAGGGGGG GTTTTTTTTT TAAAAAAATT TTTTTCAACG TAGGAATAGA AAGACTTGGT   
  
  
- CCGGTCGACA GAGTAAGGAA CCAGGAGCGC GGGGTAGTGC TCGCGACGAG TAATAAGTGG GAGAGAGAGA   
  
  
- GAGGAGAGAC TAGGTGTGGT GTTTGTGTTC CAAACGTGTC GACGTCTCGT CAGCATGATG ATAGTCATCA   
  
  
- CTTTTTGGTT CGTCTTTAGG GGTTAGGGAG AAGAGGAAGA AGGGTCTAAG AAGGAGTTAA GGTTCGGGTA   
  
  
- TAGTCTTTAA ATCTGTTGTT GTTGGTTTTT TTTTTTAATT ATGACGAACT CTTTTTGTTT ATACTATACT   
  
  
- TTAAGGAGCT TTTTCCGGAC GGTATTGTTT AAGGAGCACT TTTTCGTCGA CTCTCTTCTT CTTCTTCTTT   
  
  
- TTTTAGGGCA GTGTTAGAGT TAGAGCTAGG GTTTAACACT TTCTCTTTCT TTTCTACTTC TCTCTTGTGG   
  
  
- GGAAAAGCTT GGGACTGGTC TTTGGGGGCG GCCTCTACAG CGGCCGCGGC CCCTTCAGGT TCTACACCCT   
  
  
- ACTACTCTAC CGGGTCTTGC TGCGATTTTA CCTACTCGAC GAACGACAAC ACCCCATGTT CCACTCCAGC   
  
  
- AGCCTGTACC GCCTTTAACG GGTCTTCGAA CTCGTCGAAC TTCTTCAGTA CCCATCACAA GTTCTTCTGC   
  
  
- CCAATAGAAT GAACCGAAGG CTTTGACAAG TAATGTTAGG ACGTCTAGAC AGATGAACCG AACTTAGATA   
  
  
- CGAAAGACTC AAATTGGGAT TGGGATTAAA ACTGGGAAGA GGTAGCAGTT AGAGTGGGTA GTAGCTAGGT   
  
  
- CGAGGAGGTG AAGGAGCTTG GAGCAGCTGC CAGTTTAAGC TCGGGCTGGG CAAAAGGCTA AGCCTAGACT   
  
  
- TTCGTTAGGG TCCATTCCGA TAGAACTGGG GTGGTTCAAG ATCGTTATGA AGTTTAAACG CACTCCGATT   
  
  
- CTCCAACTTT GGGAGTTTAA TGTGTTGGCG GGGTTGCGGG TGGGGTGGAG GTTTTGACGG GTTAAGCCGC   
  
  
- AGAGGTGGTA GCCCGTGCTG CCGCAGCTCC GGCCACCACG ACCATCTGAG TGTTCTTTTG CCGTAGTCTA   
  
  
- ACCACGTACG TGAATACCGG ACACGGCTTC GTTAAGTTGA TCTTTTGTAC CCTAATCGAC TCCGAAACCA   
  
  
- ATTTGTCTAA TCCATAAATC GTCGTAGAGT TCGACCTGGA TACTCCTTTC ATCGTTGAAC AAAACGTCTT   
  
  
- CGAGAATGAA CAGCCTAGAT GTTCGATACG GGTAGACATG GGATGCTACT TAGTCAGAGT CTACTCAACG   
  
  
- TCTACGTGAA AATACTCTGA ACGGGTATAG AATTTAAGCG GGTAAAGTGC CGTTTAGTTC GTTAAAATCT   
  
  
- TCGTAAGTTA CCCTTCTTCT TTCAAGTACA CTAACTAAAG TCGTACTTCG TTCCGTACGT TACCGGCCGA   
  
  
- AACTACGTTC GGGACCGAGA AGCTGGCCTC CCAGGTGGCG AAAAGGCCAA TTGGCCCTAA CCTGGCGGGC   
  
  
- GAGGCCTGTT GAGCCTGGCC GACGTTCTCC ACCCAACCTT CGAACGGGTC AAGCGCCTAA GCTAGGCCTA   
  
  
- ATTTAAAGTC ATAGCACCCA AACACCGTTT GTCAAACTGG CTAGATCTTA GAAGGTACGA TCTAGAACTT   
  
  
- GGCCTGTGAC TCCACCACCG CCAGTTGAGC CAAAAGCTCG AGGTGGCCGA CAACCGATTT GGGCCCCGCT   
  
  
- AGCTCTTTCA CGACCCCAAG TACTCCCGGC ACTTGGGCCA GTAACACTGC TACCAGCTCG TCCTTCGCTT   
  
  
- GGTGTTGCCT GGCCAAAAGA ACCTGGCCAA GTTACTCAGC AACGTAATAA TGAGGTGGAA CAAACTAAGG   
  
  
- GAACTCTAAA CACAACTATT ACATCTATTC TTCTACAGCC TCCGAATGAA CCCGGTCGTC TAGACGTTGT   
  
  
- ACCACCGAAC ACTTCCCAGA CTGGCTCAGC TCTCCGTGCT CTGGGACCGA GTCACCGCTC GGGCCAAGCG   
  
  
- TAGACGGCCC AAGCTGGGTC AAGTAGACCC AAGCTTACGC AAATTCGTCC GCTCATACAA CAACCTCAAG   
  
  
- AAACGACCAC CACTTCCCAT GCCGCACCTC CTCGCTCTTC CCACAGAGTA CAACCCTACC GTATCATCCG   
  
  
- GCGAATAGTG GTGGAGCCGA ACCGTCGAGC GGTTCTTGGG CCGACACTTA GCCGCTAC

+     CGTCA-motif

| Site Name | Organism | Position | Strand | Matrix score. | sequence | function |
| --- | --- | --- | --- | --- | --- | --- |
| CGTCA-motif | Hordeum vulgare | 2359 | + | 5 | CGTCA | cis-acting regulatory element involved in the MeJA-responsiveness |
| CGTCA-motif | Hordeum vulgare | 1972 | + | 5 | CGTCA | cis-acting regulatory element involved in the MeJA-responsiveness |
| CGTCA-motif | Hordeum vulgare | 3340 | - | 5 | CGTCA | cis-acting regulatory element involved in the MeJA-responsiveness |
| CGTCA-motif | Hordeum vulgare | 253 | - | 5 | CGTCA | cis-acting regulatory element involved in the MeJA-responsiveness |

>HU10G00709.1   
+ -Up\_Stream \_Len000AACCCT TTAATTTTAT CAATGTGGGA CAACACTCAT CTTCATACTC CAACAAGCTA   
  
  
+ TGATACATTC GATTATTGCC TAGTATTTCG TTTAGTAAGA TATTAGTGCC AAACTTTGTA ATAAAAATGA   
  
  
+ AACTTACTAA AAAAAATCTC GTTTTCTGTA GAGGGAAAAT TTGAAGACTT AGTGTCACAT GATGACCTAA   
  
  
+ TGGTACTTGT AGGGTCAAGA TTGAGATTGA GGGCATACTG ACGACCTGTA TAATGGTCAA GTAGGATCAA   
  
  
+ AGTCGAGAAA ACTGTCTCTG CTAACTTTCA TAATCTAACC CTTAACTCAC TCTGACCCTC AATTTAATCA   
  
  
+ GGCCTAATTC AAACTATTAA TGGTTATAAC TCTCTTTCCT TCTTTTGAGA TTTTTTTTTT CTACATGCGG   
  
  
+ TTGAAAATAA TTTTTTTAAA AAATATATAT CACTAACCAT TCATTATCAT AGTTTTAGAG TTCATATTTA   
  
  
+ TGTGGTCCTT GTTGAGTCTA ATTTAATGCT TTAGATGGCG TGAAGCACCA AATTAGTGGT AACCATAATA   
  
  
+ ATAAACTATC AAGTGTTAAG TCGAAGAAAA CGGACACTGC TCAACCAAAT CTAGTAACAT AGTGTATGAC   
  
  
+ ATGATAACTA AGTGTGCCAT GTAGTAATCA ATTCTTTCTT TTTTTTTTTT TGGTTGAGAT CACATAAATT   
  
  
+ ACGTTATCAA GGGTAGTACG ATATAATGGA TGAGGAGAAA TTAAATGTGA GATTTAGTTG ATGAATCGAA   
  
  
+ TACGTTAGTC TGAGCCATTT GGTCAAAACT GCATTGGCTA ATCAATAGAA ATTATAGAAA ATACATAACA   
  
  
+ TAATGGGATG CAATTTTCTA ATTGGGGAAA CCATGTCAAT GCAGTGATTT TGGACCATTT AGGCCTTATT   
  
  
+ CTTTTCAATT TTTTAATTGA TTATTTAGAC TCATATGAAC TTATTGTTGA CAGATTGAGC TAAAAACACT   
  
  
+ GATGTTTCAT ACATATGTAT GAATTGCACG TACGAAGGCT CTGCTCATTT CGAGAAAGAA AATTAACAGT   
  
  
+ TAGGAGCAGT ATAATTTAAC AACAAGCGAT AATTATTAGT ATGAAGTGGT ACGAATCTAT TTTAAAGGAG   
  
  
+ TACTATGAAG TACAAACAAT AACAAATAAT AAAGAGCAGT ACATTAACAT ATCTTACTAC CTAAATAAAT   
  
  
+ CGATTCTAAG AGTAAAAAAA TGTTGAAATT AACCCCATTA TAGTTTTACT TTAACAAGAA GTTTTTCTTC   
  
  
+ TTTTACCATT TACCAAGTAA GAATGATTCA GGCCGTATAG CCAAGGAAAA ATAGAAAACC ACAAAACTAA   
  
  
+ CCATGGCCCA AAAAACAACC ACCAAGAGGG GCACTAATGT GACTAACCGA CGGTTAACAT TATCATCTGT   
  
  
+ TTGATACTTG TTCATCTCTC ACTATTCGGG TAATAGTGTG AAATTACTGA TGTACCCTCT AAACTCGTCG   
  
  
+ TTTTAACTGT GTAAGAATCA GATTAACAGT CACGGACACA TGAGTAACAA ATCAATTTCG GACATGAACG   
  
  
+ AACGAACTGT GAATCCCCCC CAAAAAAAAA ATTTTTTTAA AAAAAGTTGC ATCCTTATCT TTCTGAACCA   
  
  
+ GGCCAGCTGT CTCATTCCTT GGTCCTCGCG CCCCATCACG AGCGCTGCTC ATTATTCACC CTCTCTCTCT   
  
  
+ CTCCTCTCTG ATCCACACCA CAAACACAAG GTTTGCACAG CTGCAGAGCA GTCGTACTAC TATCAGTAGT   
  
  
+ GAAAAACCAA GCAGAAATCC CCAATCCCTC TTCTCCTTCT TCCCAGATTC TTCCTCAATT CCAAGCCCAT   
  
  
+ ATCAGAAATT TAGACAACAA CAACCAAAAA AAAAAATTAA TACTGCTTGA GAAAAACAAA TATGATATGA   
  
  
+ AATTCCTCGA AAAAGGCCTG CCATAACAAA TTCCTCGTGA AAAAGCAGCT GAGAGAAGAA GAAGAAGAAA   
  
  
+ AAAATCCCGT CACAATCTCA ATCTCGATCC CAAATTGTGA AAGAGAAAGA AAAGATGAAG AGAGAACACC   
  
  
+ CCTTTTCGAA CCCTGACCAG AAACCCCCGC CGGAGATGTC GCCGGCGCCG GGGAAGTCCA AGATGTGGGA   
  
  
+ TGATGAGATG GCCCAGAACG ACGCTAAAAT GGATGAGCTG CTTGCTGTTG TGGGGTACAA GGTGAGGTCG   
  
  
+ TCGGACATGG CGGAAATTGC CCAGAAGCTT GAGCAGCTTG AAGAAGTCAT GGGTAGTGTT CAAGAAGACG   
  
  
+ GGTTATCTTA CTTGGCTTCC GAAACTGTTC ATTACAATCC TGCAGATCTG TCTACTTGGC TTGAATCTAT   
  
  
+ GCTTTCTGAG TTTAACCCTA ACCCTAATTT TGACCCTTCT CCATCGTCAA TCTCACCCAT CATCGATCCA   
  
  
+ GCTCCTCCAC TTCCTCGAAC CTCGTCGACG GTCAAATTCG AGCCCGACCC GTTTTCCGAT TCGGATCTGA   
  
  
+ AAGCAATCCC AGGTAAGGCT ATCTTGACCC CACCAAGTTC TAGCAATACT TCAAATTTGC GTGAGGCTAA   
  
  
+ GAGGTTGAAA CCCTCAAATT ACACAACCGC CCCAACGCCC ACCCCACCTC CAAAACTGCC CAATTCGGCG   
  
  
+ TCTCCACCAT CGGGCACGAC GGCGTCGAGG CCGGTGGTGC TGGTAGACTC ACAAGAAAAC GGCATCAGAT   
  
  
+ TGGTGCATGC ACTTATGGCC TGTGCCGAAG CAATTCAACT AGAAAACATG GGATTAGCTG AGGCTTTGGT   
  
  
+ TAAACAGATT AGGTATTTAG CAGCATCTCA AGCTGGACCT ATGAGGAAAG TAGCAACTTG TTTTGCAGAA   
  
  
+ GCTCTTACTT GTCGGATCTA CAAGCTATGC CCATCTGTAC CCTACGATGA ATCAGTCTCA GATGAGTTGC   
  
  
+ AGATGCACTT TTATGAGACT TGCCCATATC TTAAATTCGC CCATTTCACG GCAAATCAAG CAATTTTAGA   
  
  
+ AGCATTCAAT GGGAAGAAGA AAGTTCATGT GATTGATTTC AGCATGAAGC AAGGCATGCA ATGGCCGGCT   
  
  
+ TTGATGCAAG CCCTGGCTCT TCGACCGGAG GGTCCACCGC TTTTCCGGTT AACCGGGATT GGACCGCCCG   
  
  
+ CTCCGGACAA CTCGGACCGG CTGCAAGAGG TGGGTTGGAA GCTTGCCCAG TTCGCGGATT CGATCCGGAT   
  
  
+ TAAATTTCAG TATCGTGGGT TTGTGGCAAA CAGTTTGACC GATCTAGAAT CTTCCATGCT AGATCTTGAA   
  
  
+ CCGGACACTG AGGTGGTGGC GGTCAACTCG GTTTTCGAGC TCCACCGGCT GTTGGCTAAA CCCGGGGCGA   
  
  
+ TCGAGAAAGT GCTGGGGTTC ATGAGGGCCG TGAACCCGGT CATTGTGACG ATGGTCGAGC AGGAAGCGAA   
  
  
+ CCACAACGGA CCGGTTTTCT TGGACCGGTT CAATGAGTCG TTGCATTATT ACTCCACCTT GTTTGATTCC   
  
  
+ CTTGAGATTT GTGTTGATAA TGTAGATAAG AAGATGTCGG AGGCTTACTT GGGCCAGCAG ATCTGCAACA   
  
  
+ TGGTGGCTTG TGAAGGGTCT GACCGAGTCG AGAGGCACGA GACCCTGGCT CAGTGGCGAG CCCGGTTCGC   
  
  
+ ATCTGCCGGG TTCGACCCAG TTCATCTGGG TTCGAATGCG TTTAAGCAGG CGAGTATGTT GTTGGAGTTC   
  
  
+ TTTGCTGGTG GTGAAGGGTA CGGCGTGGAG GAGCGAGAAG GGTGTCTCAT GTTGGGATGG CATAGTAGGC   
  
  
+ CGCTTATCAC CACCTCGGCT TGGCAGCTCG CCAAGAACCC GGCTGTGAAT CGGCGATG  

- -Up\_Stream \_Len000TTGGGA AATTAAAATA GTTACACCCT GTTGTGAGTA GAAGTATGAG GTTGTTCGAT   
  
  
- ACTATGTAAG CTAATAACGG ATCATAAAGC AAATCATTCT ATAATCACGG TTTGAAACAT TATTTTTACT   
  
  
- TTGAATGATT TTTTTTAGAG CAAAAGACAT CTCCCTTTTA AACTTCTGAA TCACAGTGTA CTACTGGATT   
  
  
- ACCATGAACA TCCCAGTTCT AACTCTAACT CCCGTATGAC TGCTGGACAT ATTACCAGTT CATCCTAGTT   
  
  
- TCAGCTCTTT TGACAGAGAC GATTGAAAGT ATTAGATTGG GAATTGAGTG AGACTGGGAG TTAAATTAGT   
  
  
- CCGGATTAAG TTTGATAATT ACCAATATTG AGAGAAAGGA AGAAAACTCT AAAAAAAAAA GATGTACGCC   
  
  
- AACTTTTATT AAAAAAATTT TTTATATATA GTGATTGGTA AGTAATAGTA TCAAAATCTC AAGTATAAAT   
  
  
- ACACCAGGAA CAACTCAGAT TAAATTACGA AATCTACCGC ACTTCGTGGT TTAATCACCA TTGGTATTAT   
  
  
- TATTTGATAG TTCACAATTC AGCTTCTTTT GCCTGTGACG AGTTGGTTTA GATCATTGTA TCACATACTG   
  
  
- TACTATTGAT TCACACGGTA CATCATTAGT TAAGAAAGAA AAAAAAAAAA ACCAACTCTA GTGTATTTAA   
  
  
- TGCAATAGTT CCCATCATGC TATATTACCT ACTCCTCTTT AATTTACACT CTAAATCAAC TACTTAGCTT   
  
  
- ATGCAATCAG ACTCGGTAAA CCAGTTTTGA CGTAACCGAT TAGTTATCTT TAATATCTTT TATGTATTGT   
  
  
- ATTACCCTAC GTTAAAAGAT TAACCCCTTT GGTACAGTTA CGTCACTAAA ACCTGGTAAA TCCGGAATAA   
  
  
- GAAAAGTTAA AAAATTAACT AATAAATCTG AGTATACTTG AATAACAACT GTCTAACTCG ATTTTTGTGA   
  
  
- CTACAAAGTA TGTATACATA CTTAACGTGC ATGCTTCCGA GACGAGTAAA GCTCTTTCTT TTAATTGTCA   
  
  
- ATCCTCGTCA TATTAAATTG TTGTTCGCTA TTAATAATCA TACTTCACCA TGCTTAGATA AAATTTCCTC   
  
  
- ATGATACTTC ATGTTTGTTA TTGTTTATTA TTTCTCGTCA TGTAATTGTA TAGAATGATG GATTTATTTA   
  
  
- GCTAAGATTC TCATTTTTTT ACAACTTTAA TTGGGGTAAT ATCAAAATGA AATTGTTCTT CAAAAAGAAG   
  
  
- AAAATGGTAA ATGGTTCATT CTTACTAAGT CCGGCATATC GGTTCCTTTT TATCTTTTGG TGTTTTGATT   
  
  
- GGTACCGGGT TTTTTGTTGG TGGTTCTCCC CGTGATTACA CTGATTGGCT GCCAATTGTA ATAGTAGACA   
  
  
- AACTATGAAC AAGTAGAGAG TGATAAGCCC ATTATCACAC TTTAATGACT ACATGGGAGA TTTGAGCAGC   
  
  
- AAAATTGACA CATTCTTAGT CTAATTGTCA GTGCCTGTGT ACTCATTGTT TAGTTAAAGC CTGTACTTGC   
  
  
- TTGCTTGACA CTTAGGGGGG GTTTTTTTTT TAAAAAAATT TTTTTCAACG TAGGAATAGA AAGACTTGGT   
  
  
- CCGGTCGACA GAGTAAGGAA CCAGGAGCGC GGGGTAGTGC TCGCGACGAG TAATAAGTGG GAGAGAGAGA   
  
  
- GAGGAGAGAC TAGGTGTGGT GTTTGTGTTC CAAACGTGTC GACGTCTCGT CAGCATGATG ATAGTCATCA   
  
  
- CTTTTTGGTT CGTCTTTAGG GGTTAGGGAG AAGAGGAAGA AGGGTCTAAG AAGGAGTTAA GGTTCGGGTA   
  
  
- TAGTCTTTAA ATCTGTTGTT GTTGGTTTTT TTTTTTAATT ATGACGAACT CTTTTTGTTT ATACTATACT   
  
  
- TTAAGGAGCT TTTTCCGGAC GGTATTGTTT AAGGAGCACT TTTTCGTCGA CTCTCTTCTT CTTCTTCTTT   
  
  
- TTTTAGGGCA GTGTTAGAGT TAGAGCTAGG GTTTAACACT TTCTCTTTCT TTTCTACTTC TCTCTTGTGG   
  
  
- GGAAAAGCTT GGGACTGGTC TTTGGGGGCG GCCTCTACAG CGGCCGCGGC CCCTTCAGGT TCTACACCCT   
  
  
- ACTACTCTAC CGGGTCTTGC TGCGATTTTA CCTACTCGAC GAACGACAAC ACCCCATGTT CCACTCCAGC   
  
  
- AGCCTGTACC GCCTTTAACG GGTCTTCGAA CTCGTCGAAC TTCTTCAGTA CCCATCACAA GTTCTTCTGC   
  
  
- CCAATAGAAT GAACCGAAGG CTTTGACAAG TAATGTTAGG ACGTCTAGAC AGATGAACCG AACTTAGATA   
  
  
- CGAAAGACTC AAATTGGGAT TGGGATTAAA ACTGGGAAGA GGTAGCAGTT AGAGTGGGTA GTAGCTAGGT   
  
  
- CGAGGAGGTG AAGGAGCTTG GAGCAGCTGC CAGTTTAAGC TCGGGCTGGG CAAAAGGCTA AGCCTAGACT   
  
  
- TTCGTTAGGG TCCATTCCGA TAGAACTGGG GTGGTTCAAG ATCGTTATGA AGTTTAAACG CACTCCGATT   
  
  
- CTCCAACTTT GGGAGTTTAA TGTGTTGGCG GGGTTGCGGG TGGGGTGGAG GTTTTGACGG GTTAAGCCGC   
  
  
- AGAGGTGGTA GCCCGTGCTG CCGCAGCTCC GGCCACCACG ACCATCTGAG TGTTCTTTTG CCGTAGTCTA   
  
  
- ACCACGTACG TGAATACCGG ACACGGCTTC GTTAAGTTGA TCTTTTGTAC CCTAATCGAC TCCGAAACCA   
  
  
- ATTTGTCTAA TCCATAAATC GTCGTAGAGT TCGACCTGGA TACTCCTTTC ATCGTTGAAC AAAACGTCTT   
  
  
- CGAGAATGAA CAGCCTAGAT GTTCGATACG GGTAGACATG GGATGCTACT TAGTCAGAGT CTACTCAACG   
  
  
- TCTACGTGAA AATACTCTGA ACGGGTATAG AATTTAAGCG GGTAAAGTGC CGTTTAGTTC GTTAAAATCT   
  
  
- TCGTAAGTTA CCCTTCTTCT TTCAAGTACA CTAACTAAAG TCGTACTTCG TTCCGTACGT TACCGGCCGA   
  
  
- AACTACGTTC GGGACCGAGA AGCTGGCCTC CCAGGTGGCG AAAAGGCCAA TTGGCCCTAA CCTGGCGGGC   
  
  
- GAGGCCTGTT GAGCCTGGCC GACGTTCTCC ACCCAACCTT CGAACGGGTC AAGCGCCTAA GCTAGGCCTA   
  
  
- ATTTAAAGTC ATAGCACCCA AACACCGTTT GTCAAACTGG CTAGATCTTA GAAGGTACGA TCTAGAACTT   
  
  
- GGCCTGTGAC TCCACCACCG CCAGTTGAGC CAAAAGCTCG AGGTGGCCGA CAACCGATTT GGGCCCCGCT   
  
  
- AGCTCTTTCA CGACCCCAAG TACTCCCGGC ACTTGGGCCA GTAACACTGC TACCAGCTCG TCCTTCGCTT   
  
  
- GGTGTTGCCT GGCCAAAAGA ACCTGGCCAA GTTACTCAGC AACGTAATAA TGAGGTGGAA CAAACTAAGG   
  
  
- GAACTCTAAA CACAACTATT ACATCTATTC TTCTACAGCC TCCGAATGAA CCCGGTCGTC TAGACGTTGT   
  
  
- ACCACCGAAC ACTTCCCAGA CTGGCTCAGC TCTCCGTGCT CTGGGACCGA GTCACCGCTC GGGCCAAGCG   
  
  
- TAGACGGCCC AAGCTGGGTC AAGTAGACCC AAGCTTACGC AAATTCGTCC GCTCATACAA CAACCTCAAG   
  
  
- AAACGACCAC CACTTCCCAT GCCGCACCTC CTCGCTCTTC CCACAGAGTA CAACCCTACC GTATCATCCG   
  
  
- GCGAATAGTG GTGGAGCCGA ACCGTCGAGC GGTTCTTGGG CCGACACTTA GCCGCTAC

+     ERE

| Site Name | Organism | Position | Strand | Matrix score. | sequence | function |
| --- | --- | --- | --- | --- | --- | --- |
| ERE | Nicotiana glutinos | 1890 | - | 8 | ATTTCATA |  |
| ERE | Nicotiana glutinos | 1113 | + | 8 | ATTTTAAA |  |

>HU10G00709.1   
+ -Up\_Stream \_Len000AACCCT TTAATTTTAT CAATGTGGGA CAACACTCAT CTTCATACTC CAACAAGCTA   
  
  
+ TGATACATTC GATTATTGCC TAGTATTTCG TTTAGTAAGA TATTAGTGCC AAACTTTGTA ATAAAAATGA   
  
  
+ AACTTACTAA AAAAAATCTC GTTTTCTGTA GAGGGAAAAT TTGAAGACTT AGTGTCACAT GATGACCTAA   
  
  
+ TGGTACTTGT AGGGTCAAGA TTGAGATTGA GGGCATACTG ACGACCTGTA TAATGGTCAA GTAGGATCAA   
  
  
+ AGTCGAGAAA ACTGTCTCTG CTAACTTTCA TAATCTAACC CTTAACTCAC TCTGACCCTC AATTTAATCA   
  
  
+ GGCCTAATTC AAACTATTAA TGGTTATAAC TCTCTTTCCT TCTTTTGAGA TTTTTTTTTT CTACATGCGG   
  
  
+ TTGAAAATAA TTTTTTTAAA AAATATATAT CACTAACCAT TCATTATCAT AGTTTTAGAG TTCATATTTA   
  
  
+ TGTGGTCCTT GTTGAGTCTA ATTTAATGCT TTAGATGGCG TGAAGCACCA AATTAGTGGT AACCATAATA   
  
  
+ ATAAACTATC AAGTGTTAAG TCGAAGAAAA CGGACACTGC TCAACCAAAT CTAGTAACAT AGTGTATGAC   
  
  
+ ATGATAACTA AGTGTGCCAT GTAGTAATCA ATTCTTTCTT TTTTTTTTTT TGGTTGAGAT CACATAAATT   
  
  
+ ACGTTATCAA GGGTAGTACG ATATAATGGA TGAGGAGAAA TTAAATGTGA GATTTAGTTG ATGAATCGAA   
  
  
+ TACGTTAGTC TGAGCCATTT GGTCAAAACT GCATTGGCTA ATCAATAGAA ATTATAGAAA ATACATAACA   
  
  
+ TAATGGGATG CAATTTTCTA ATTGGGGAAA CCATGTCAAT GCAGTGATTT TGGACCATTT AGGCCTTATT   
  
  
+ CTTTTCAATT TTTTAATTGA TTATTTAGAC TCATATGAAC TTATTGTTGA CAGATTGAGC TAAAAACACT   
  
  
+ GATGTTTCAT ACATATGTAT GAATTGCACG TACGAAGGCT CTGCTCATTT CGAGAAAGAA AATTAACAGT   
  
  
+ TAGGAGCAGT ATAATTTAAC AACAAGCGAT AATTATTAGT ATGAAGTGGT ACGAATCTAT TTTAAAGGAG   
  
  
+ TACTATGAAG TACAAACAAT AACAAATAAT AAAGAGCAGT ACATTAACAT ATCTTACTAC CTAAATAAAT   
  
  
+ CGATTCTAAG AGTAAAAAAA TGTTGAAATT AACCCCATTA TAGTTTTACT TTAACAAGAA GTTTTTCTTC   
  
  
+ TTTTACCATT TACCAAGTAA GAATGATTCA GGCCGTATAG CCAAGGAAAA ATAGAAAACC ACAAAACTAA   
  
  
+ CCATGGCCCA AAAAACAACC ACCAAGAGGG GCACTAATGT GACTAACCGA CGGTTAACAT TATCATCTGT   
  
  
+ TTGATACTTG TTCATCTCTC ACTATTCGGG TAATAGTGTG AAATTACTGA TGTACCCTCT AAACTCGTCG   
  
  
+ TTTTAACTGT GTAAGAATCA GATTAACAGT CACGGACACA TGAGTAACAA ATCAATTTCG GACATGAACG   
  
  
+ AACGAACTGT GAATCCCCCC CAAAAAAAAA ATTTTTTTAA AAAAAGTTGC ATCCTTATCT TTCTGAACCA   
  
  
+ GGCCAGCTGT CTCATTCCTT GGTCCTCGCG CCCCATCACG AGCGCTGCTC ATTATTCACC CTCTCTCTCT   
  
  
+ CTCCTCTCTG ATCCACACCA CAAACACAAG GTTTGCACAG CTGCAGAGCA GTCGTACTAC TATCAGTAGT   
  
  
+ GAAAAACCAA GCAGAAATCC CCAATCCCTC TTCTCCTTCT TCCCAGATTC TTCCTCAATT CCAAGCCCAT   
  
  
+ ATCAGAAATT TAGACAACAA CAACCAAAAA AAAAAATTAA TACTGCTTGA GAAAAACAAA TATGATATGA   
  
  
+ AATTCCTCGA AAAAGGCCTG CCATAACAAA TTCCTCGTGA AAAAGCAGCT GAGAGAAGAA GAAGAAGAAA   
  
  
+ AAAATCCCGT CACAATCTCA ATCTCGATCC CAAATTGTGA AAGAGAAAGA AAAGATGAAG AGAGAACACC   
  
  
+ CCTTTTCGAA CCCTGACCAG AAACCCCCGC CGGAGATGTC GCCGGCGCCG GGGAAGTCCA AGATGTGGGA   
  
  
+ TGATGAGATG GCCCAGAACG ACGCTAAAAT GGATGAGCTG CTTGCTGTTG TGGGGTACAA GGTGAGGTCG   
  
  
+ TCGGACATGG CGGAAATTGC CCAGAAGCTT GAGCAGCTTG AAGAAGTCAT GGGTAGTGTT CAAGAAGACG   
  
  
+ GGTTATCTTA CTTGGCTTCC GAAACTGTTC ATTACAATCC TGCAGATCTG TCTACTTGGC TTGAATCTAT   
  
  
+ GCTTTCTGAG TTTAACCCTA ACCCTAATTT TGACCCTTCT CCATCGTCAA TCTCACCCAT CATCGATCCA   
  
  
+ GCTCCTCCAC TTCCTCGAAC CTCGTCGACG GTCAAATTCG AGCCCGACCC GTTTTCCGAT TCGGATCTGA   
  
  
+ AAGCAATCCC AGGTAAGGCT ATCTTGACCC CACCAAGTTC TAGCAATACT TCAAATTTGC GTGAGGCTAA   
  
  
+ GAGGTTGAAA CCCTCAAATT ACACAACCGC CCCAACGCCC ACCCCACCTC CAAAACTGCC CAATTCGGCG   
  
  
+ TCTCCACCAT CGGGCACGAC GGCGTCGAGG CCGGTGGTGC TGGTAGACTC ACAAGAAAAC GGCATCAGAT   
  
  
+ TGGTGCATGC ACTTATGGCC TGTGCCGAAG CAATTCAACT AGAAAACATG GGATTAGCTG AGGCTTTGGT   
  
  
+ TAAACAGATT AGGTATTTAG CAGCATCTCA AGCTGGACCT ATGAGGAAAG TAGCAACTTG TTTTGCAGAA   
  
  
+ GCTCTTACTT GTCGGATCTA CAAGCTATGC CCATCTGTAC CCTACGATGA ATCAGTCTCA GATGAGTTGC   
  
  
+ AGATGCACTT TTATGAGACT TGCCCATATC TTAAATTCGC CCATTTCACG GCAAATCAAG CAATTTTAGA   
  
  
+ AGCATTCAAT GGGAAGAAGA AAGTTCATGT GATTGATTTC AGCATGAAGC AAGGCATGCA ATGGCCGGCT   
  
  
+ TTGATGCAAG CCCTGGCTCT TCGACCGGAG GGTCCACCGC TTTTCCGGTT AACCGGGATT GGACCGCCCG   
  
  
+ CTCCGGACAA CTCGGACCGG CTGCAAGAGG TGGGTTGGAA GCTTGCCCAG TTCGCGGATT CGATCCGGAT   
  
  
+ TAAATTTCAG TATCGTGGGT TTGTGGCAAA CAGTTTGACC GATCTAGAAT CTTCCATGCT AGATCTTGAA   
  
  
+ CCGGACACTG AGGTGGTGGC GGTCAACTCG GTTTTCGAGC TCCACCGGCT GTTGGCTAAA CCCGGGGCGA   
  
  
+ TCGAGAAAGT GCTGGGGTTC ATGAGGGCCG TGAACCCGGT CATTGTGACG ATGGTCGAGC AGGAAGCGAA   
  
  
+ CCACAACGGA CCGGTTTTCT TGGACCGGTT CAATGAGTCG TTGCATTATT ACTCCACCTT GTTTGATTCC   
  
  
+ CTTGAGATTT GTGTTGATAA TGTAGATAAG AAGATGTCGG AGGCTTACTT GGGCCAGCAG ATCTGCAACA   
  
  
+ TGGTGGCTTG TGAAGGGTCT GACCGAGTCG AGAGGCACGA GACCCTGGCT CAGTGGCGAG CCCGGTTCGC   
  
  
+ ATCTGCCGGG TTCGACCCAG TTCATCTGGG TTCGAATGCG TTTAAGCAGG CGAGTATGTT GTTGGAGTTC   
  
  
+ TTTGCTGGTG GTGAAGGGTA CGGCGTGGAG GAGCGAGAAG GGTGTCTCAT GTTGGGATGG CATAGTAGGC   
  
  
+ CGCTTATCAC CACCTCGGCT TGGCAGCTCG CCAAGAACCC GGCTGTGAAT CGGCGATG  

- -Up\_Stream \_Len000TTGGGA AATTAAAATA GTTACACCCT GTTGTGAGTA GAAGTATGAG GTTGTTCGAT   
  
  
- ACTATGTAAG CTAATAACGG ATCATAAAGC AAATCATTCT ATAATCACGG TTTGAAACAT TATTTTTACT   
  
  
- TTGAATGATT TTTTTTAGAG CAAAAGACAT CTCCCTTTTA AACTTCTGAA TCACAGTGTA CTACTGGATT   
  
  
- ACCATGAACA TCCCAGTTCT AACTCTAACT CCCGTATGAC TGCTGGACAT ATTACCAGTT CATCCTAGTT   
  
  
- TCAGCTCTTT TGACAGAGAC GATTGAAAGT ATTAGATTGG GAATTGAGTG AGACTGGGAG TTAAATTAGT   
  
  
- CCGGATTAAG TTTGATAATT ACCAATATTG AGAGAAAGGA AGAAAACTCT AAAAAAAAAA GATGTACGCC   
  
  
- AACTTTTATT AAAAAAATTT TTTATATATA GTGATTGGTA AGTAATAGTA TCAAAATCTC AAGTATAAAT   
  
  
- ACACCAGGAA CAACTCAGAT TAAATTACGA AATCTACCGC ACTTCGTGGT TTAATCACCA TTGGTATTAT   
  
  
- TATTTGATAG TTCACAATTC AGCTTCTTTT GCCTGTGACG AGTTGGTTTA GATCATTGTA TCACATACTG   
  
  
- TACTATTGAT TCACACGGTA CATCATTAGT TAAGAAAGAA AAAAAAAAAA ACCAACTCTA GTGTATTTAA   
  
  
- TGCAATAGTT CCCATCATGC TATATTACCT ACTCCTCTTT AATTTACACT CTAAATCAAC TACTTAGCTT   
  
  
- ATGCAATCAG ACTCGGTAAA CCAGTTTTGA CGTAACCGAT TAGTTATCTT TAATATCTTT TATGTATTGT   
  
  
- ATTACCCTAC GTTAAAAGAT TAACCCCTTT GGTACAGTTA CGTCACTAAA ACCTGGTAAA TCCGGAATAA   
  
  
- GAAAAGTTAA AAAATTAACT AATAAATCTG AGTATACTTG AATAACAACT GTCTAACTCG ATTTTTGTGA   
  
  
- CTACAAAGTA TGTATACATA CTTAACGTGC ATGCTTCCGA GACGAGTAAA GCTCTTTCTT TTAATTGTCA   
  
  
- ATCCTCGTCA TATTAAATTG TTGTTCGCTA TTAATAATCA TACTTCACCA TGCTTAGATA AAATTTCCTC   
  
  
- ATGATACTTC ATGTTTGTTA TTGTTTATTA TTTCTCGTCA TGTAATTGTA TAGAATGATG GATTTATTTA   
  
  
- GCTAAGATTC TCATTTTTTT ACAACTTTAA TTGGGGTAAT ATCAAAATGA AATTGTTCTT CAAAAAGAAG   
  
  
- AAAATGGTAA ATGGTTCATT CTTACTAAGT CCGGCATATC GGTTCCTTTT TATCTTTTGG TGTTTTGATT   
  
  
- GGTACCGGGT TTTTTGTTGG TGGTTCTCCC CGTGATTACA CTGATTGGCT GCCAATTGTA ATAGTAGACA   
  
  
- AACTATGAAC AAGTAGAGAG TGATAAGCCC ATTATCACAC TTTAATGACT ACATGGGAGA TTTGAGCAGC   
  
  
- AAAATTGACA CATTCTTAGT CTAATTGTCA GTGCCTGTGT ACTCATTGTT TAGTTAAAGC CTGTACTTGC   
  
  
- TTGCTTGACA CTTAGGGGGG GTTTTTTTTT TAAAAAAATT TTTTTCAACG TAGGAATAGA AAGACTTGGT   
  
  
- CCGGTCGACA GAGTAAGGAA CCAGGAGCGC GGGGTAGTGC TCGCGACGAG TAATAAGTGG GAGAGAGAGA   
  
  
- GAGGAGAGAC TAGGTGTGGT GTTTGTGTTC CAAACGTGTC GACGTCTCGT CAGCATGATG ATAGTCATCA   
  
  
- CTTTTTGGTT CGTCTTTAGG GGTTAGGGAG AAGAGGAAGA AGGGTCTAAG AAGGAGTTAA GGTTCGGGTA   
  
  
- TAGTCTTTAA ATCTGTTGTT GTTGGTTTTT TTTTTTAATT ATGACGAACT CTTTTTGTTT ATACTATACT   
  
  
- TTAAGGAGCT TTTTCCGGAC GGTATTGTTT AAGGAGCACT TTTTCGTCGA CTCTCTTCTT CTTCTTCTTT   
  
  
- TTTTAGGGCA GTGTTAGAGT TAGAGCTAGG GTTTAACACT TTCTCTTTCT TTTCTACTTC TCTCTTGTGG   
  
  
- GGAAAAGCTT GGGACTGGTC TTTGGGGGCG GCCTCTACAG CGGCCGCGGC CCCTTCAGGT TCTACACCCT   
  
  
- ACTACTCTAC CGGGTCTTGC TGCGATTTTA CCTACTCGAC GAACGACAAC ACCCCATGTT CCACTCCAGC   
  
  
- AGCCTGTACC GCCTTTAACG GGTCTTCGAA CTCGTCGAAC TTCTTCAGTA CCCATCACAA GTTCTTCTGC   
  
  
- CCAATAGAAT GAACCGAAGG CTTTGACAAG TAATGTTAGG ACGTCTAGAC AGATGAACCG AACTTAGATA   
  
  
- CGAAAGACTC AAATTGGGAT TGGGATTAAA ACTGGGAAGA GGTAGCAGTT AGAGTGGGTA GTAGCTAGGT   
  
  
- CGAGGAGGTG AAGGAGCTTG GAGCAGCTGC CAGTTTAAGC TCGGGCTGGG CAAAAGGCTA AGCCTAGACT   
  
  
- TTCGTTAGGG TCCATTCCGA TAGAACTGGG GTGGTTCAAG ATCGTTATGA AGTTTAAACG CACTCCGATT   
  
  
- CTCCAACTTT GGGAGTTTAA TGTGTTGGCG GGGTTGCGGG TGGGGTGGAG GTTTTGACGG GTTAAGCCGC   
  
  
- AGAGGTGGTA GCCCGTGCTG CCGCAGCTCC GGCCACCACG ACCATCTGAG TGTTCTTTTG CCGTAGTCTA   
  
  
- ACCACGTACG TGAATACCGG ACACGGCTTC GTTAAGTTGA TCTTTTGTAC CCTAATCGAC TCCGAAACCA   
  
  
- ATTTGTCTAA TCCATAAATC GTCGTAGAGT TCGACCTGGA TACTCCTTTC ATCGTTGAAC AAAACGTCTT   
  
  
- CGAGAATGAA CAGCCTAGAT GTTCGATACG GGTAGACATG GGATGCTACT TAGTCAGAGT CTACTCAACG   
  
  
- TCTACGTGAA AATACTCTGA ACGGGTATAG AATTTAAGCG GGTAAAGTGC CGTTTAGTTC GTTAAAATCT   
  
  
- TCGTAAGTTA CCCTTCTTCT TTCAAGTACA CTAACTAAAG TCGTACTTCG TTCCGTACGT TACCGGCCGA   
  
  
- AACTACGTTC GGGACCGAGA AGCTGGCCTC CCAGGTGGCG AAAAGGCCAA TTGGCCCTAA CCTGGCGGGC   
  
  
- GAGGCCTGTT GAGCCTGGCC GACGTTCTCC ACCCAACCTT CGAACGGGTC AAGCGCCTAA GCTAGGCCTA   
  
  
- ATTTAAAGTC ATAGCACCCA AACACCGTTT GTCAAACTGG CTAGATCTTA GAAGGTACGA TCTAGAACTT   
  
  
- GGCCTGTGAC TCCACCACCG CCAGTTGAGC CAAAAGCTCG AGGTGGCCGA CAACCGATTT GGGCCCCGCT   
  
  
- AGCTCTTTCA CGACCCCAAG TACTCCCGGC ACTTGGGCCA GTAACACTGC TACCAGCTCG TCCTTCGCTT   
  
  
- GGTGTTGCCT GGCCAAAAGA ACCTGGCCAA GTTACTCAGC AACGTAATAA TGAGGTGGAA CAAACTAAGG   
  
  
- GAACTCTAAA CACAACTATT ACATCTATTC TTCTACAGCC TCCGAATGAA CCCGGTCGTC TAGACGTTGT   
  
  
- ACCACCGAAC ACTTCCCAGA CTGGCTCAGC TCTCCGTGCT CTGGGACCGA GTCACCGCTC GGGCCAAGCG   
  
  
- TAGACGGCCC AAGCTGGGTC AAGTAGACCC AAGCTTACGC AAATTCGTCC GCTCATACAA CAACCTCAAG   
  
  
- AAACGACCAC CACTTCCCAT GCCGCACCTC CTCGCTCTTC CCACAGAGTA CAACCCTACC GTATCATCCG   
  
  
- GCGAATAGTG GTGGAGCCGA ACCGTCGAGC GGTTCTTGGG CCGACACTTA GCCGCTAC

+     G-box

| Site Name | Organism | Position | Strand | Matrix score. | sequence | function |
| --- | --- | --- | --- | --- | --- | --- |
| G-box | Arabidopsis thaliana | 1011 | - | 6 | TACGTG | cis-acting regulatory element involved in light responsiveness |
| G-box | Zea mays | 2609 | + | 6 | CACGAC | cis-acting regulatory element involved in light responsiveness |

>HU10G00709.1   
+ -Up\_Stream \_Len000AACCCT TTAATTTTAT CAATGTGGGA CAACACTCAT CTTCATACTC CAACAAGCTA   
  
  
+ TGATACATTC GATTATTGCC TAGTATTTCG TTTAGTAAGA TATTAGTGCC AAACTTTGTA ATAAAAATGA   
  
  
+ AACTTACTAA AAAAAATCTC GTTTTCTGTA GAGGGAAAAT TTGAAGACTT AGTGTCACAT GATGACCTAA   
  
  
+ TGGTACTTGT AGGGTCAAGA TTGAGATTGA GGGCATACTG ACGACCTGTA TAATGGTCAA GTAGGATCAA   
  
  
+ AGTCGAGAAA ACTGTCTCTG CTAACTTTCA TAATCTAACC CTTAACTCAC TCTGACCCTC AATTTAATCA   
  
  
+ GGCCTAATTC AAACTATTAA TGGTTATAAC TCTCTTTCCT TCTTTTGAGA TTTTTTTTTT CTACATGCGG   
  
  
+ TTGAAAATAA TTTTTTTAAA AAATATATAT CACTAACCAT TCATTATCAT AGTTTTAGAG TTCATATTTA   
  
  
+ TGTGGTCCTT GTTGAGTCTA ATTTAATGCT TTAGATGGCG TGAAGCACCA AATTAGTGGT AACCATAATA   
  
  
+ ATAAACTATC AAGTGTTAAG TCGAAGAAAA CGGACACTGC TCAACCAAAT CTAGTAACAT AGTGTATGAC   
  
  
+ ATGATAACTA AGTGTGCCAT GTAGTAATCA ATTCTTTCTT TTTTTTTTTT TGGTTGAGAT CACATAAATT   
  
  
+ ACGTTATCAA GGGTAGTACG ATATAATGGA TGAGGAGAAA TTAAATGTGA GATTTAGTTG ATGAATCGAA   
  
  
+ TACGTTAGTC TGAGCCATTT GGTCAAAACT GCATTGGCTA ATCAATAGAA ATTATAGAAA ATACATAACA   
  
  
+ TAATGGGATG CAATTTTCTA ATTGGGGAAA CCATGTCAAT GCAGTGATTT TGGACCATTT AGGCCTTATT   
  
  
+ CTTTTCAATT TTTTAATTGA TTATTTAGAC TCATATGAAC TTATTGTTGA CAGATTGAGC TAAAAACACT   
  
  
+ GATGTTTCAT ACATATGTAT GAATTGCACG TACGAAGGCT CTGCTCATTT CGAGAAAGAA AATTAACAGT   
  
  
+ TAGGAGCAGT ATAATTTAAC AACAAGCGAT AATTATTAGT ATGAAGTGGT ACGAATCTAT TTTAAAGGAG   
  
  
+ TACTATGAAG TACAAACAAT AACAAATAAT AAAGAGCAGT ACATTAACAT ATCTTACTAC CTAAATAAAT   
  
  
+ CGATTCTAAG AGTAAAAAAA TGTTGAAATT AACCCCATTA TAGTTTTACT TTAACAAGAA GTTTTTCTTC   
  
  
+ TTTTACCATT TACCAAGTAA GAATGATTCA GGCCGTATAG CCAAGGAAAA ATAGAAAACC ACAAAACTAA   
  
  
+ CCATGGCCCA AAAAACAACC ACCAAGAGGG GCACTAATGT GACTAACCGA CGGTTAACAT TATCATCTGT   
  
  
+ TTGATACTTG TTCATCTCTC ACTATTCGGG TAATAGTGTG AAATTACTGA TGTACCCTCT AAACTCGTCG   
  
  
+ TTTTAACTGT GTAAGAATCA GATTAACAGT CACGGACACA TGAGTAACAA ATCAATTTCG GACATGAACG   
  
  
+ AACGAACTGT GAATCCCCCC CAAAAAAAAA ATTTTTTTAA AAAAAGTTGC ATCCTTATCT TTCTGAACCA   
  
  
+ GGCCAGCTGT CTCATTCCTT GGTCCTCGCG CCCCATCACG AGCGCTGCTC ATTATTCACC CTCTCTCTCT   
  
  
+ CTCCTCTCTG ATCCACACCA CAAACACAAG GTTTGCACAG CTGCAGAGCA GTCGTACTAC TATCAGTAGT   
  
  
+ GAAAAACCAA GCAGAAATCC CCAATCCCTC TTCTCCTTCT TCCCAGATTC TTCCTCAATT CCAAGCCCAT   
  
  
+ ATCAGAAATT TAGACAACAA CAACCAAAAA AAAAAATTAA TACTGCTTGA GAAAAACAAA TATGATATGA   
  
  
+ AATTCCTCGA AAAAGGCCTG CCATAACAAA TTCCTCGTGA AAAAGCAGCT GAGAGAAGAA GAAGAAGAAA   
  
  
+ AAAATCCCGT CACAATCTCA ATCTCGATCC CAAATTGTGA AAGAGAAAGA AAAGATGAAG AGAGAACACC   
  
  
+ CCTTTTCGAA CCCTGACCAG AAACCCCCGC CGGAGATGTC GCCGGCGCCG GGGAAGTCCA AGATGTGGGA   
  
  
+ TGATGAGATG GCCCAGAACG ACGCTAAAAT GGATGAGCTG CTTGCTGTTG TGGGGTACAA GGTGAGGTCG   
  
  
+ TCGGACATGG CGGAAATTGC CCAGAAGCTT GAGCAGCTTG AAGAAGTCAT GGGTAGTGTT CAAGAAGACG   
  
  
+ GGTTATCTTA CTTGGCTTCC GAAACTGTTC ATTACAATCC TGCAGATCTG TCTACTTGGC TTGAATCTAT   
  
  
+ GCTTTCTGAG TTTAACCCTA ACCCTAATTT TGACCCTTCT CCATCGTCAA TCTCACCCAT CATCGATCCA   
  
  
+ GCTCCTCCAC TTCCTCGAAC CTCGTCGACG GTCAAATTCG AGCCCGACCC GTTTTCCGAT TCGGATCTGA   
  
  
+ AAGCAATCCC AGGTAAGGCT ATCTTGACCC CACCAAGTTC TAGCAATACT TCAAATTTGC GTGAGGCTAA   
  
  
+ GAGGTTGAAA CCCTCAAATT ACACAACCGC CCCAACGCCC ACCCCACCTC CAAAACTGCC CAATTCGGCG   
  
  
+ TCTCCACCAT CGGGCACGAC GGCGTCGAGG CCGGTGGTGC TGGTAGACTC ACAAGAAAAC GGCATCAGAT   
  
  
+ TGGTGCATGC ACTTATGGCC TGTGCCGAAG CAATTCAACT AGAAAACATG GGATTAGCTG AGGCTTTGGT   
  
  
+ TAAACAGATT AGGTATTTAG CAGCATCTCA AGCTGGACCT ATGAGGAAAG TAGCAACTTG TTTTGCAGAA   
  
  
+ GCTCTTACTT GTCGGATCTA CAAGCTATGC CCATCTGTAC CCTACGATGA ATCAGTCTCA GATGAGTTGC   
  
  
+ AGATGCACTT TTATGAGACT TGCCCATATC TTAAATTCGC CCATTTCACG GCAAATCAAG CAATTTTAGA   
  
  
+ AGCATTCAAT GGGAAGAAGA AAGTTCATGT GATTGATTTC AGCATGAAGC AAGGCATGCA ATGGCCGGCT   
  
  
+ TTGATGCAAG CCCTGGCTCT TCGACCGGAG GGTCCACCGC TTTTCCGGTT AACCGGGATT GGACCGCCCG   
  
  
+ CTCCGGACAA CTCGGACCGG CTGCAAGAGG TGGGTTGGAA GCTTGCCCAG TTCGCGGATT CGATCCGGAT   
  
  
+ TAAATTTCAG TATCGTGGGT TTGTGGCAAA CAGTTTGACC GATCTAGAAT CTTCCATGCT AGATCTTGAA   
  
  
+ CCGGACACTG AGGTGGTGGC GGTCAACTCG GTTTTCGAGC TCCACCGGCT GTTGGCTAAA CCCGGGGCGA   
  
  
+ TCGAGAAAGT GCTGGGGTTC ATGAGGGCCG TGAACCCGGT CATTGTGACG ATGGTCGAGC AGGAAGCGAA   
  
  
+ CCACAACGGA CCGGTTTTCT TGGACCGGTT CAATGAGTCG TTGCATTATT ACTCCACCTT GTTTGATTCC   
  
  
+ CTTGAGATTT GTGTTGATAA TGTAGATAAG AAGATGTCGG AGGCTTACTT GGGCCAGCAG ATCTGCAACA   
  
  
+ TGGTGGCTTG TGAAGGGTCT GACCGAGTCG AGAGGCACGA GACCCTGGCT CAGTGGCGAG CCCGGTTCGC   
  
  
+ ATCTGCCGGG TTCGACCCAG TTCATCTGGG TTCGAATGCG TTTAAGCAGG CGAGTATGTT GTTGGAGTTC   
  
  
+ TTTGCTGGTG GTGAAGGGTA CGGCGTGGAG GAGCGAGAAG GGTGTCTCAT GTTGGGATGG CATAGTAGGC   
  
  
+ CGCTTATCAC CACCTCGGCT TGGCAGCTCG CCAAGAACCC GGCTGTGAAT CGGCGATG  

- -Up\_Stream \_Len000TTGGGA AATTAAAATA GTTACACCCT GTTGTGAGTA GAAGTATGAG GTTGTTCGAT   
  
  
- ACTATGTAAG CTAATAACGG ATCATAAAGC AAATCATTCT ATAATCACGG TTTGAAACAT TATTTTTACT   
  
  
- TTGAATGATT TTTTTTAGAG CAAAAGACAT CTCCCTTTTA AACTTCTGAA TCACAGTGTA CTACTGGATT   
  
  
- ACCATGAACA TCCCAGTTCT AACTCTAACT CCCGTATGAC TGCTGGACAT ATTACCAGTT CATCCTAGTT   
  
  
- TCAGCTCTTT TGACAGAGAC GATTGAAAGT ATTAGATTGG GAATTGAGTG AGACTGGGAG TTAAATTAGT   
  
  
- CCGGATTAAG TTTGATAATT ACCAATATTG AGAGAAAGGA AGAAAACTCT AAAAAAAAAA GATGTACGCC   
  
  
- AACTTTTATT AAAAAAATTT TTTATATATA GTGATTGGTA AGTAATAGTA TCAAAATCTC AAGTATAAAT   
  
  
- ACACCAGGAA CAACTCAGAT TAAATTACGA AATCTACCGC ACTTCGTGGT TTAATCACCA TTGGTATTAT   
  
  
- TATTTGATAG TTCACAATTC AGCTTCTTTT GCCTGTGACG AGTTGGTTTA GATCATTGTA TCACATACTG   
  
  
- TACTATTGAT TCACACGGTA CATCATTAGT TAAGAAAGAA AAAAAAAAAA ACCAACTCTA GTGTATTTAA   
  
  
- TGCAATAGTT CCCATCATGC TATATTACCT ACTCCTCTTT AATTTACACT CTAAATCAAC TACTTAGCTT   
  
  
- ATGCAATCAG ACTCGGTAAA CCAGTTTTGA CGTAACCGAT TAGTTATCTT TAATATCTTT TATGTATTGT   
  
  
- ATTACCCTAC GTTAAAAGAT TAACCCCTTT GGTACAGTTA CGTCACTAAA ACCTGGTAAA TCCGGAATAA   
  
  
- GAAAAGTTAA AAAATTAACT AATAAATCTG AGTATACTTG AATAACAACT GTCTAACTCG ATTTTTGTGA   
  
  
- CTACAAAGTA TGTATACATA CTTAACGTGC ATGCTTCCGA GACGAGTAAA GCTCTTTCTT TTAATTGTCA   
  
  
- ATCCTCGTCA TATTAAATTG TTGTTCGCTA TTAATAATCA TACTTCACCA TGCTTAGATA AAATTTCCTC   
  
  
- ATGATACTTC ATGTTTGTTA TTGTTTATTA TTTCTCGTCA TGTAATTGTA TAGAATGATG GATTTATTTA   
  
  
- GCTAAGATTC TCATTTTTTT ACAACTTTAA TTGGGGTAAT ATCAAAATGA AATTGTTCTT CAAAAAGAAG   
  
  
- AAAATGGTAA ATGGTTCATT CTTACTAAGT CCGGCATATC GGTTCCTTTT TATCTTTTGG TGTTTTGATT   
  
  
- GGTACCGGGT TTTTTGTTGG TGGTTCTCCC CGTGATTACA CTGATTGGCT GCCAATTGTA ATAGTAGACA   
  
  
- AACTATGAAC AAGTAGAGAG TGATAAGCCC ATTATCACAC TTTAATGACT ACATGGGAGA TTTGAGCAGC   
  
  
- AAAATTGACA CATTCTTAGT CTAATTGTCA GTGCCTGTGT ACTCATTGTT TAGTTAAAGC CTGTACTTGC   
  
  
- TTGCTTGACA CTTAGGGGGG GTTTTTTTTT TAAAAAAATT TTTTTCAACG TAGGAATAGA AAGACTTGGT   
  
  
- CCGGTCGACA GAGTAAGGAA CCAGGAGCGC GGGGTAGTGC TCGCGACGAG TAATAAGTGG GAGAGAGAGA   
  
  
- GAGGAGAGAC TAGGTGTGGT GTTTGTGTTC CAAACGTGTC GACGTCTCGT CAGCATGATG ATAGTCATCA   
  
  
- CTTTTTGGTT CGTCTTTAGG GGTTAGGGAG AAGAGGAAGA AGGGTCTAAG AAGGAGTTAA GGTTCGGGTA   
  
  
- TAGTCTTTAA ATCTGTTGTT GTTGGTTTTT TTTTTTAATT ATGACGAACT CTTTTTGTTT ATACTATACT   
  
  
- TTAAGGAGCT TTTTCCGGAC GGTATTGTTT AAGGAGCACT TTTTCGTCGA CTCTCTTCTT CTTCTTCTTT   
  
  
- TTTTAGGGCA GTGTTAGAGT TAGAGCTAGG GTTTAACACT TTCTCTTTCT TTTCTACTTC TCTCTTGTGG   
  
  
- GGAAAAGCTT GGGACTGGTC TTTGGGGGCG GCCTCTACAG CGGCCGCGGC CCCTTCAGGT TCTACACCCT   
  
  
- ACTACTCTAC CGGGTCTTGC TGCGATTTTA CCTACTCGAC GAACGACAAC ACCCCATGTT CCACTCCAGC   
  
  
- AGCCTGTACC GCCTTTAACG GGTCTTCGAA CTCGTCGAAC TTCTTCAGTA CCCATCACAA GTTCTTCTGC   
  
  
- CCAATAGAAT GAACCGAAGG CTTTGACAAG TAATGTTAGG ACGTCTAGAC AGATGAACCG AACTTAGATA   
  
  
- CGAAAGACTC AAATTGGGAT TGGGATTAAA ACTGGGAAGA GGTAGCAGTT AGAGTGGGTA GTAGCTAGGT   
  
  
- CGAGGAGGTG AAGGAGCTTG GAGCAGCTGC CAGTTTAAGC TCGGGCTGGG CAAAAGGCTA AGCCTAGACT   
  
  
- TTCGTTAGGG TCCATTCCGA TAGAACTGGG GTGGTTCAAG ATCGTTATGA AGTTTAAACG CACTCCGATT   
  
  
- CTCCAACTTT GGGAGTTTAA TGTGTTGGCG GGGTTGCGGG TGGGGTGGAG GTTTTGACGG GTTAAGCCGC   
  
  
- AGAGGTGGTA GCCCGTGCTG CCGCAGCTCC GGCCACCACG ACCATCTGAG TGTTCTTTTG CCGTAGTCTA   
  
  
- ACCACGTACG TGAATACCGG ACACGGCTTC GTTAAGTTGA TCTTTTGTAC CCTAATCGAC TCCGAAACCA   
  
  
- ATTTGTCTAA TCCATAAATC GTCGTAGAGT TCGACCTGGA TACTCCTTTC ATCGTTGAAC AAAACGTCTT   
  
  
- CGAGAATGAA CAGCCTAGAT GTTCGATACG GGTAGACATG GGATGCTACT TAGTCAGAGT CTACTCAACG   
  
  
- TCTACGTGAA AATACTCTGA ACGGGTATAG AATTTAAGCG GGTAAAGTGC CGTTTAGTTC GTTAAAATCT   
  
  
- TCGTAAGTTA CCCTTCTTCT TTCAAGTACA CTAACTAAAG TCGTACTTCG TTCCGTACGT TACCGGCCGA   
  
  
- AACTACGTTC GGGACCGAGA AGCTGGCCTC CCAGGTGGCG AAAAGGCCAA TTGGCCCTAA CCTGGCGGGC   
  
  
- GAGGCCTGTT GAGCCTGGCC GACGTTCTCC ACCCAACCTT CGAACGGGTC AAGCGCCTAA GCTAGGCCTA   
  
  
- ATTTAAAGTC ATAGCACCCA AACACCGTTT GTCAAACTGG CTAGATCTTA GAAGGTACGA TCTAGAACTT   
  
  
- GGCCTGTGAC TCCACCACCG CCAGTTGAGC CAAAAGCTCG AGGTGGCCGA CAACCGATTT GGGCCCCGCT   
  
  
- AGCTCTTTCA CGACCCCAAG TACTCCCGGC ACTTGGGCCA GTAACACTGC TACCAGCTCG TCCTTCGCTT   
  
  
- GGTGTTGCCT GGCCAAAAGA ACCTGGCCAA GTTACTCAGC AACGTAATAA TGAGGTGGAA CAAACTAAGG   
  
  
- GAACTCTAAA CACAACTATT ACATCTATTC TTCTACAGCC TCCGAATGAA CCCGGTCGTC TAGACGTTGT   
  
  
- ACCACCGAAC ACTTCCCAGA CTGGCTCAGC TCTCCGTGCT CTGGGACCGA GTCACCGCTC GGGCCAAGCG   
  
  
- TAGACGGCCC AAGCTGGGTC AAGTAGACCC AAGCTTACGC AAATTCGTCC GCTCATACAA CAACCTCAAG   
  
  
- AAACGACCAC CACTTCCCAT GCCGCACCTC CTCGCTCTTC CCACAGAGTA CAACCCTACC GTATCATCCG   
  
  
- GCGAATAGTG GTGGAGCCGA ACCGTCGAGC GGTTCTTGGG CCGACACTTA GCCGCTAC

+     GATA-motif

| Site Name | Organism | Position | Strand | Matrix score. | sequence | function |
| --- | --- | --- | --- | --- | --- | --- |
| GATA-motif | Solanum tuberosum | 1597 | - | 9 | AAGGATAAGG | part of a light responsive element |
| GATA-motif | Solanum tuberosum | 908 | - | 9 | AAGGATAAGG | part of a light responsive element |

>HU10G00709.1   
+ -Up\_Stream \_Len000AACCCT TTAATTTTAT CAATGTGGGA CAACACTCAT CTTCATACTC CAACAAGCTA   
  
  
+ TGATACATTC GATTATTGCC TAGTATTTCG TTTAGTAAGA TATTAGTGCC AAACTTTGTA ATAAAAATGA   
  
  
+ AACTTACTAA AAAAAATCTC GTTTTCTGTA GAGGGAAAAT TTGAAGACTT AGTGTCACAT GATGACCTAA   
  
  
+ TGGTACTTGT AGGGTCAAGA TTGAGATTGA GGGCATACTG ACGACCTGTA TAATGGTCAA GTAGGATCAA   
  
  
+ AGTCGAGAAA ACTGTCTCTG CTAACTTTCA TAATCTAACC CTTAACTCAC TCTGACCCTC AATTTAATCA   
  
  
+ GGCCTAATTC AAACTATTAA TGGTTATAAC TCTCTTTCCT TCTTTTGAGA TTTTTTTTTT CTACATGCGG   
  
  
+ TTGAAAATAA TTTTTTTAAA AAATATATAT CACTAACCAT TCATTATCAT AGTTTTAGAG TTCATATTTA   
  
  
+ TGTGGTCCTT GTTGAGTCTA ATTTAATGCT TTAGATGGCG TGAAGCACCA AATTAGTGGT AACCATAATA   
  
  
+ ATAAACTATC AAGTGTTAAG TCGAAGAAAA CGGACACTGC TCAACCAAAT CTAGTAACAT AGTGTATGAC   
  
  
+ ATGATAACTA AGTGTGCCAT GTAGTAATCA ATTCTTTCTT TTTTTTTTTT TGGTTGAGAT CACATAAATT   
  
  
+ ACGTTATCAA GGGTAGTACG ATATAATGGA TGAGGAGAAA TTAAATGTGA GATTTAGTTG ATGAATCGAA   
  
  
+ TACGTTAGTC TGAGCCATTT GGTCAAAACT GCATTGGCTA ATCAATAGAA ATTATAGAAA ATACATAACA   
  
  
+ TAATGGGATG CAATTTTCTA ATTGGGGAAA CCATGTCAAT GCAGTGATTT TGGACCATTT AGGCCTTATT   
  
  
+ CTTTTCAATT TTTTAATTGA TTATTTAGAC TCATATGAAC TTATTGTTGA CAGATTGAGC TAAAAACACT   
  
  
+ GATGTTTCAT ACATATGTAT GAATTGCACG TACGAAGGCT CTGCTCATTT CGAGAAAGAA AATTAACAGT   
  
  
+ TAGGAGCAGT ATAATTTAAC AACAAGCGAT AATTATTAGT ATGAAGTGGT ACGAATCTAT TTTAAAGGAG   
  
  
+ TACTATGAAG TACAAACAAT AACAAATAAT AAAGAGCAGT ACATTAACAT ATCTTACTAC CTAAATAAAT   
  
  
+ CGATTCTAAG AGTAAAAAAA TGTTGAAATT AACCCCATTA TAGTTTTACT TTAACAAGAA GTTTTTCTTC   
  
  
+ TTTTACCATT TACCAAGTAA GAATGATTCA GGCCGTATAG CCAAGGAAAA ATAGAAAACC ACAAAACTAA   
  
  
+ CCATGGCCCA AAAAACAACC ACCAAGAGGG GCACTAATGT GACTAACCGA CGGTTAACAT TATCATCTGT   
  
  
+ TTGATACTTG TTCATCTCTC ACTATTCGGG TAATAGTGTG AAATTACTGA TGTACCCTCT AAACTCGTCG   
  
  
+ TTTTAACTGT GTAAGAATCA GATTAACAGT CACGGACACA TGAGTAACAA ATCAATTTCG GACATGAACG   
  
  
+ AACGAACTGT GAATCCCCCC CAAAAAAAAA ATTTTTTTAA AAAAAGTTGC ATCCTTATCT TTCTGAACCA   
  
  
+ GGCCAGCTGT CTCATTCCTT GGTCCTCGCG CCCCATCACG AGCGCTGCTC ATTATTCACC CTCTCTCTCT   
  
  
+ CTCCTCTCTG ATCCACACCA CAAACACAAG GTTTGCACAG CTGCAGAGCA GTCGTACTAC TATCAGTAGT   
  
  
+ GAAAAACCAA GCAGAAATCC CCAATCCCTC TTCTCCTTCT TCCCAGATTC TTCCTCAATT CCAAGCCCAT   
  
  
+ ATCAGAAATT TAGACAACAA CAACCAAAAA AAAAAATTAA TACTGCTTGA GAAAAACAAA TATGATATGA   
  
  
+ AATTCCTCGA AAAAGGCCTG CCATAACAAA TTCCTCGTGA AAAAGCAGCT GAGAGAAGAA GAAGAAGAAA   
  
  
+ AAAATCCCGT CACAATCTCA ATCTCGATCC CAAATTGTGA AAGAGAAAGA AAAGATGAAG AGAGAACACC   
  
  
+ CCTTTTCGAA CCCTGACCAG AAACCCCCGC CGGAGATGTC GCCGGCGCCG GGGAAGTCCA AGATGTGGGA   
  
  
+ TGATGAGATG GCCCAGAACG ACGCTAAAAT GGATGAGCTG CTTGCTGTTG TGGGGTACAA GGTGAGGTCG   
  
  
+ TCGGACATGG CGGAAATTGC CCAGAAGCTT GAGCAGCTTG AAGAAGTCAT GGGTAGTGTT CAAGAAGACG   
  
  
+ GGTTATCTTA CTTGGCTTCC GAAACTGTTC ATTACAATCC TGCAGATCTG TCTACTTGGC TTGAATCTAT   
  
  
+ GCTTTCTGAG TTTAACCCTA ACCCTAATTT TGACCCTTCT CCATCGTCAA TCTCACCCAT CATCGATCCA   
  
  
+ GCTCCTCCAC TTCCTCGAAC CTCGTCGACG GTCAAATTCG AGCCCGACCC GTTTTCCGAT TCGGATCTGA   
  
  
+ AAGCAATCCC AGGTAAGGCT ATCTTGACCC CACCAAGTTC TAGCAATACT TCAAATTTGC GTGAGGCTAA   
  
  
+ GAGGTTGAAA CCCTCAAATT ACACAACCGC CCCAACGCCC ACCCCACCTC CAAAACTGCC CAATTCGGCG   
  
  
+ TCTCCACCAT CGGGCACGAC GGCGTCGAGG CCGGTGGTGC TGGTAGACTC ACAAGAAAAC GGCATCAGAT   
  
  
+ TGGTGCATGC ACTTATGGCC TGTGCCGAAG CAATTCAACT AGAAAACATG GGATTAGCTG AGGCTTTGGT   
  
  
+ TAAACAGATT AGGTATTTAG CAGCATCTCA AGCTGGACCT ATGAGGAAAG TAGCAACTTG TTTTGCAGAA   
  
  
+ GCTCTTACTT GTCGGATCTA CAAGCTATGC CCATCTGTAC CCTACGATGA ATCAGTCTCA GATGAGTTGC   
  
  
+ AGATGCACTT TTATGAGACT TGCCCATATC TTAAATTCGC CCATTTCACG GCAAATCAAG CAATTTTAGA   
  
  
+ AGCATTCAAT GGGAAGAAGA AAGTTCATGT GATTGATTTC AGCATGAAGC AAGGCATGCA ATGGCCGGCT   
  
  
+ TTGATGCAAG CCCTGGCTCT TCGACCGGAG GGTCCACCGC TTTTCCGGTT AACCGGGATT GGACCGCCCG   
  
  
+ CTCCGGACAA CTCGGACCGG CTGCAAGAGG TGGGTTGGAA GCTTGCCCAG TTCGCGGATT CGATCCGGAT   
  
  
+ TAAATTTCAG TATCGTGGGT TTGTGGCAAA CAGTTTGACC GATCTAGAAT CTTCCATGCT AGATCTTGAA   
  
  
+ CCGGACACTG AGGTGGTGGC GGTCAACTCG GTTTTCGAGC TCCACCGGCT GTTGGCTAAA CCCGGGGCGA   
  
  
+ TCGAGAAAGT GCTGGGGTTC ATGAGGGCCG TGAACCCGGT CATTGTGACG ATGGTCGAGC AGGAAGCGAA   
  
  
+ CCACAACGGA CCGGTTTTCT TGGACCGGTT CAATGAGTCG TTGCATTATT ACTCCACCTT GTTTGATTCC   
  
  
+ CTTGAGATTT GTGTTGATAA TGTAGATAAG AAGATGTCGG AGGCTTACTT GGGCCAGCAG ATCTGCAACA   
  
  
+ TGGTGGCTTG TGAAGGGTCT GACCGAGTCG AGAGGCACGA GACCCTGGCT CAGTGGCGAG CCCGGTTCGC   
  
  
+ ATCTGCCGGG TTCGACCCAG TTCATCTGGG TTCGAATGCG TTTAAGCAGG CGAGTATGTT GTTGGAGTTC   
  
  
+ TTTGCTGGTG GTGAAGGGTA CGGCGTGGAG GAGCGAGAAG GGTGTCTCAT GTTGGGATGG CATAGTAGGC   
  
  
+ CGCTTATCAC CACCTCGGCT TGGCAGCTCG CCAAGAACCC GGCTGTGAAT CGGCGATG  

- -Up\_Stream \_Len000TTGGGA AATTAAAATA GTTACACCCT GTTGTGAGTA GAAGTATGAG GTTGTTCGAT   
  
  
- ACTATGTAAG CTAATAACGG ATCATAAAGC AAATCATTCT ATAATCACGG TTTGAAACAT TATTTTTACT   
  
  
- TTGAATGATT TTTTTTAGAG CAAAAGACAT CTCCCTTTTA AACTTCTGAA TCACAGTGTA CTACTGGATT   
  
  
- ACCATGAACA TCCCAGTTCT AACTCTAACT CCCGTATGAC TGCTGGACAT ATTACCAGTT CATCCTAGTT   
  
  
- TCAGCTCTTT TGACAGAGAC GATTGAAAGT ATTAGATTGG GAATTGAGTG AGACTGGGAG TTAAATTAGT   
  
  
- CCGGATTAAG TTTGATAATT ACCAATATTG AGAGAAAGGA AGAAAACTCT AAAAAAAAAA GATGTACGCC   
  
  
- AACTTTTATT AAAAAAATTT TTTATATATA GTGATTGGTA AGTAATAGTA TCAAAATCTC AAGTATAAAT   
  
  
- ACACCAGGAA CAACTCAGAT TAAATTACGA AATCTACCGC ACTTCGTGGT TTAATCACCA TTGGTATTAT   
  
  
- TATTTGATAG TTCACAATTC AGCTTCTTTT GCCTGTGACG AGTTGGTTTA GATCATTGTA TCACATACTG   
  
  
- TACTATTGAT TCACACGGTA CATCATTAGT TAAGAAAGAA AAAAAAAAAA ACCAACTCTA GTGTATTTAA   
  
  
- TGCAATAGTT CCCATCATGC TATATTACCT ACTCCTCTTT AATTTACACT CTAAATCAAC TACTTAGCTT   
  
  
- ATGCAATCAG ACTCGGTAAA CCAGTTTTGA CGTAACCGAT TAGTTATCTT TAATATCTTT TATGTATTGT   
  
  
- ATTACCCTAC GTTAAAAGAT TAACCCCTTT GGTACAGTTA CGTCACTAAA ACCTGGTAAA TCCGGAATAA   
  
  
- GAAAAGTTAA AAAATTAACT AATAAATCTG AGTATACTTG AATAACAACT GTCTAACTCG ATTTTTGTGA   
  
  
- CTACAAAGTA TGTATACATA CTTAACGTGC ATGCTTCCGA GACGAGTAAA GCTCTTTCTT TTAATTGTCA   
  
  
- ATCCTCGTCA TATTAAATTG TTGTTCGCTA TTAATAATCA TACTTCACCA TGCTTAGATA AAATTTCCTC   
  
  
- ATGATACTTC ATGTTTGTTA TTGTTTATTA TTTCTCGTCA TGTAATTGTA TAGAATGATG GATTTATTTA   
  
  
- GCTAAGATTC TCATTTTTTT ACAACTTTAA TTGGGGTAAT ATCAAAATGA AATTGTTCTT CAAAAAGAAG   
  
  
- AAAATGGTAA ATGGTTCATT CTTACTAAGT CCGGCATATC GGTTCCTTTT TATCTTTTGG TGTTTTGATT   
  
  
- GGTACCGGGT TTTTTGTTGG TGGTTCTCCC CGTGATTACA CTGATTGGCT GCCAATTGTA ATAGTAGACA   
  
  
- AACTATGAAC AAGTAGAGAG TGATAAGCCC ATTATCACAC TTTAATGACT ACATGGGAGA TTTGAGCAGC   
  
  
- AAAATTGACA CATTCTTAGT CTAATTGTCA GTGCCTGTGT ACTCATTGTT TAGTTAAAGC CTGTACTTGC   
  
  
- TTGCTTGACA CTTAGGGGGG GTTTTTTTTT TAAAAAAATT TTTTTCAACG TAGGAATAGA AAGACTTGGT   
  
  
- CCGGTCGACA GAGTAAGGAA CCAGGAGCGC GGGGTAGTGC TCGCGACGAG TAATAAGTGG GAGAGAGAGA   
  
  
- GAGGAGAGAC TAGGTGTGGT GTTTGTGTTC CAAACGTGTC GACGTCTCGT CAGCATGATG ATAGTCATCA   
  
  
- CTTTTTGGTT CGTCTTTAGG GGTTAGGGAG AAGAGGAAGA AGGGTCTAAG AAGGAGTTAA GGTTCGGGTA   
  
  
- TAGTCTTTAA ATCTGTTGTT GTTGGTTTTT TTTTTTAATT ATGACGAACT CTTTTTGTTT ATACTATACT   
  
  
- TTAAGGAGCT TTTTCCGGAC GGTATTGTTT AAGGAGCACT TTTTCGTCGA CTCTCTTCTT CTTCTTCTTT   
  
  
- TTTTAGGGCA GTGTTAGAGT TAGAGCTAGG GTTTAACACT TTCTCTTTCT TTTCTACTTC TCTCTTGTGG   
  
  
- GGAAAAGCTT GGGACTGGTC TTTGGGGGCG GCCTCTACAG CGGCCGCGGC CCCTTCAGGT TCTACACCCT   
  
  
- ACTACTCTAC CGGGTCTTGC TGCGATTTTA CCTACTCGAC GAACGACAAC ACCCCATGTT CCACTCCAGC   
  
  
- AGCCTGTACC GCCTTTAACG GGTCTTCGAA CTCGTCGAAC TTCTTCAGTA CCCATCACAA GTTCTTCTGC   
  
  
- CCAATAGAAT GAACCGAAGG CTTTGACAAG TAATGTTAGG ACGTCTAGAC AGATGAACCG AACTTAGATA   
  
  
- CGAAAGACTC AAATTGGGAT TGGGATTAAA ACTGGGAAGA GGTAGCAGTT AGAGTGGGTA GTAGCTAGGT   
  
  
- CGAGGAGGTG AAGGAGCTTG GAGCAGCTGC CAGTTTAAGC TCGGGCTGGG CAAAAGGCTA AGCCTAGACT   
  
  
- TTCGTTAGGG TCCATTCCGA TAGAACTGGG GTGGTTCAAG ATCGTTATGA AGTTTAAACG CACTCCGATT   
  
  
- CTCCAACTTT GGGAGTTTAA TGTGTTGGCG GGGTTGCGGG TGGGGTGGAG GTTTTGACGG GTTAAGCCGC   
  
  
- AGAGGTGGTA GCCCGTGCTG CCGCAGCTCC GGCCACCACG ACCATCTGAG TGTTCTTTTG CCGTAGTCTA   
  
  
- ACCACGTACG TGAATACCGG ACACGGCTTC GTTAAGTTGA TCTTTTGTAC CCTAATCGAC TCCGAAACCA   
  
  
- ATTTGTCTAA TCCATAAATC GTCGTAGAGT TCGACCTGGA TACTCCTTTC ATCGTTGAAC AAAACGTCTT   
  
  
- CGAGAATGAA CAGCCTAGAT GTTCGATACG GGTAGACATG GGATGCTACT TAGTCAGAGT CTACTCAACG   
  
  
- TCTACGTGAA AATACTCTGA ACGGGTATAG AATTTAAGCG GGTAAAGTGC CGTTTAGTTC GTTAAAATCT   
  
  
- TCGTAAGTTA CCCTTCTTCT TTCAAGTACA CTAACTAAAG TCGTACTTCG TTCCGTACGT TACCGGCCGA   
  
  
- AACTACGTTC GGGACCGAGA AGCTGGCCTC CCAGGTGGCG AAAAGGCCAA TTGGCCCTAA CCTGGCGGGC   
  
  
- GAGGCCTGTT GAGCCTGGCC GACGTTCTCC ACCCAACCTT CGAACGGGTC AAGCGCCTAA GCTAGGCCTA   
  
  
- ATTTAAAGTC ATAGCACCCA AACACCGTTT GTCAAACTGG CTAGATCTTA GAAGGTACGA TCTAGAACTT   
  
  
- GGCCTGTGAC TCCACCACCG CCAGTTGAGC CAAAAGCTCG AGGTGGCCGA CAACCGATTT GGGCCCCGCT   
  
  
- AGCTCTTTCA CGACCCCAAG TACTCCCGGC ACTTGGGCCA GTAACACTGC TACCAGCTCG TCCTTCGCTT   
  
  
- GGTGTTGCCT GGCCAAAAGA ACCTGGCCAA GTTACTCAGC AACGTAATAA TGAGGTGGAA CAAACTAAGG   
  
  
- GAACTCTAAA CACAACTATT ACATCTATTC TTCTACAGCC TCCGAATGAA CCCGGTCGTC TAGACGTTGT   
  
  
- ACCACCGAAC ACTTCCCAGA CTGGCTCAGC TCTCCGTGCT CTGGGACCGA GTCACCGCTC GGGCCAAGCG   
  
  
- TAGACGGCCC AAGCTGGGTC AAGTAGACCC AAGCTTACGC AAATTCGTCC GCTCATACAA CAACCTCAAG   
  
  
- AAACGACCAC CACTTCCCAT GCCGCACCTC CTCGCTCTTC CCACAGAGTA CAACCCTACC GTATCATCCG   
  
  
- GCGAATAGTG GTGGAGCCGA ACCGTCGAGC GGTTCTTGGG CCGACACTTA GCCGCTAC

+     GC-motif

| Site Name | Organism | Position | Strand | Matrix score. | sequence | function |
| --- | --- | --- | --- | --- | --- | --- |
| GC-motif | Zea mays | 2058 | + | 6 | CCCCCG | enhancer-like element involved in anoxic specific inducibility |

>HU10G00709.1   
+ -Up\_Stream \_Len000AACCCT TTAATTTTAT CAATGTGGGA CAACACTCAT CTTCATACTC CAACAAGCTA   
  
  
+ TGATACATTC GATTATTGCC TAGTATTTCG TTTAGTAAGA TATTAGTGCC AAACTTTGTA ATAAAAATGA   
  
  
+ AACTTACTAA AAAAAATCTC GTTTTCTGTA GAGGGAAAAT TTGAAGACTT AGTGTCACAT GATGACCTAA   
  
  
+ TGGTACTTGT AGGGTCAAGA TTGAGATTGA GGGCATACTG ACGACCTGTA TAATGGTCAA GTAGGATCAA   
  
  
+ AGTCGAGAAA ACTGTCTCTG CTAACTTTCA TAATCTAACC CTTAACTCAC TCTGACCCTC AATTTAATCA   
  
  
+ GGCCTAATTC AAACTATTAA TGGTTATAAC TCTCTTTCCT TCTTTTGAGA TTTTTTTTTT CTACATGCGG   
  
  
+ TTGAAAATAA TTTTTTTAAA AAATATATAT CACTAACCAT TCATTATCAT AGTTTTAGAG TTCATATTTA   
  
  
+ TGTGGTCCTT GTTGAGTCTA ATTTAATGCT TTAGATGGCG TGAAGCACCA AATTAGTGGT AACCATAATA   
  
  
+ ATAAACTATC AAGTGTTAAG TCGAAGAAAA CGGACACTGC TCAACCAAAT CTAGTAACAT AGTGTATGAC   
  
  
+ ATGATAACTA AGTGTGCCAT GTAGTAATCA ATTCTTTCTT TTTTTTTTTT TGGTTGAGAT CACATAAATT   
  
  
+ ACGTTATCAA GGGTAGTACG ATATAATGGA TGAGGAGAAA TTAAATGTGA GATTTAGTTG ATGAATCGAA   
  
  
+ TACGTTAGTC TGAGCCATTT GGTCAAAACT GCATTGGCTA ATCAATAGAA ATTATAGAAA ATACATAACA   
  
  
+ TAATGGGATG CAATTTTCTA ATTGGGGAAA CCATGTCAAT GCAGTGATTT TGGACCATTT AGGCCTTATT   
  
  
+ CTTTTCAATT TTTTAATTGA TTATTTAGAC TCATATGAAC TTATTGTTGA CAGATTGAGC TAAAAACACT   
  
  
+ GATGTTTCAT ACATATGTAT GAATTGCACG TACGAAGGCT CTGCTCATTT CGAGAAAGAA AATTAACAGT   
  
  
+ TAGGAGCAGT ATAATTTAAC AACAAGCGAT AATTATTAGT ATGAAGTGGT ACGAATCTAT TTTAAAGGAG   
  
  
+ TACTATGAAG TACAAACAAT AACAAATAAT AAAGAGCAGT ACATTAACAT ATCTTACTAC CTAAATAAAT   
  
  
+ CGATTCTAAG AGTAAAAAAA TGTTGAAATT AACCCCATTA TAGTTTTACT TTAACAAGAA GTTTTTCTTC   
  
  
+ TTTTACCATT TACCAAGTAA GAATGATTCA GGCCGTATAG CCAAGGAAAA ATAGAAAACC ACAAAACTAA   
  
  
+ CCATGGCCCA AAAAACAACC ACCAAGAGGG GCACTAATGT GACTAACCGA CGGTTAACAT TATCATCTGT   
  
  
+ TTGATACTTG TTCATCTCTC ACTATTCGGG TAATAGTGTG AAATTACTGA TGTACCCTCT AAACTCGTCG   
  
  
+ TTTTAACTGT GTAAGAATCA GATTAACAGT CACGGACACA TGAGTAACAA ATCAATTTCG GACATGAACG   
  
  
+ AACGAACTGT GAATCCCCCC CAAAAAAAAA ATTTTTTTAA AAAAAGTTGC ATCCTTATCT TTCTGAACCA   
  
  
+ GGCCAGCTGT CTCATTCCTT GGTCCTCGCG CCCCATCACG AGCGCTGCTC ATTATTCACC CTCTCTCTCT   
  
  
+ CTCCTCTCTG ATCCACACCA CAAACACAAG GTTTGCACAG CTGCAGAGCA GTCGTACTAC TATCAGTAGT   
  
  
+ GAAAAACCAA GCAGAAATCC CCAATCCCTC TTCTCCTTCT TCCCAGATTC TTCCTCAATT CCAAGCCCAT   
  
  
+ ATCAGAAATT TAGACAACAA CAACCAAAAA AAAAAATTAA TACTGCTTGA GAAAAACAAA TATGATATGA   
  
  
+ AATTCCTCGA AAAAGGCCTG CCATAACAAA TTCCTCGTGA AAAAGCAGCT GAGAGAAGAA GAAGAAGAAA   
  
  
+ AAAATCCCGT CACAATCTCA ATCTCGATCC CAAATTGTGA AAGAGAAAGA AAAGATGAAG AGAGAACACC   
  
  
+ CCTTTTCGAA CCCTGACCAG AAACCCCCGC CGGAGATGTC GCCGGCGCCG GGGAAGTCCA AGATGTGGGA   
  
  
+ TGATGAGATG GCCCAGAACG ACGCTAAAAT GGATGAGCTG CTTGCTGTTG TGGGGTACAA GGTGAGGTCG   
  
  
+ TCGGACATGG CGGAAATTGC CCAGAAGCTT GAGCAGCTTG AAGAAGTCAT GGGTAGTGTT CAAGAAGACG   
  
  
+ GGTTATCTTA CTTGGCTTCC GAAACTGTTC ATTACAATCC TGCAGATCTG TCTACTTGGC TTGAATCTAT   
  
  
+ GCTTTCTGAG TTTAACCCTA ACCCTAATTT TGACCCTTCT CCATCGTCAA TCTCACCCAT CATCGATCCA   
  
  
+ GCTCCTCCAC TTCCTCGAAC CTCGTCGACG GTCAAATTCG AGCCCGACCC GTTTTCCGAT TCGGATCTGA   
  
  
+ AAGCAATCCC AGGTAAGGCT ATCTTGACCC CACCAAGTTC TAGCAATACT TCAAATTTGC GTGAGGCTAA   
  
  
+ GAGGTTGAAA CCCTCAAATT ACACAACCGC CCCAACGCCC ACCCCACCTC CAAAACTGCC CAATTCGGCG   
  
  
+ TCTCCACCAT CGGGCACGAC GGCGTCGAGG CCGGTGGTGC TGGTAGACTC ACAAGAAAAC GGCATCAGAT   
  
  
+ TGGTGCATGC ACTTATGGCC TGTGCCGAAG CAATTCAACT AGAAAACATG GGATTAGCTG AGGCTTTGGT   
  
  
+ TAAACAGATT AGGTATTTAG CAGCATCTCA AGCTGGACCT ATGAGGAAAG TAGCAACTTG TTTTGCAGAA   
  
  
+ GCTCTTACTT GTCGGATCTA CAAGCTATGC CCATCTGTAC CCTACGATGA ATCAGTCTCA GATGAGTTGC   
  
  
+ AGATGCACTT TTATGAGACT TGCCCATATC TTAAATTCGC CCATTTCACG GCAAATCAAG CAATTTTAGA   
  
  
+ AGCATTCAAT GGGAAGAAGA AAGTTCATGT GATTGATTTC AGCATGAAGC AAGGCATGCA ATGGCCGGCT   
  
  
+ TTGATGCAAG CCCTGGCTCT TCGACCGGAG GGTCCACCGC TTTTCCGGTT AACCGGGATT GGACCGCCCG   
  
  
+ CTCCGGACAA CTCGGACCGG CTGCAAGAGG TGGGTTGGAA GCTTGCCCAG TTCGCGGATT CGATCCGGAT   
  
  
+ TAAATTTCAG TATCGTGGGT TTGTGGCAAA CAGTTTGACC GATCTAGAAT CTTCCATGCT AGATCTTGAA   
  
  
+ CCGGACACTG AGGTGGTGGC GGTCAACTCG GTTTTCGAGC TCCACCGGCT GTTGGCTAAA CCCGGGGCGA   
  
  
+ TCGAGAAAGT GCTGGGGTTC ATGAGGGCCG TGAACCCGGT CATTGTGACG ATGGTCGAGC AGGAAGCGAA   
  
  
+ CCACAACGGA CCGGTTTTCT TGGACCGGTT CAATGAGTCG TTGCATTATT ACTCCACCTT GTTTGATTCC   
  
  
+ CTTGAGATTT GTGTTGATAA TGTAGATAAG AAGATGTCGG AGGCTTACTT GGGCCAGCAG ATCTGCAACA   
  
  
+ TGGTGGCTTG TGAAGGGTCT GACCGAGTCG AGAGGCACGA GACCCTGGCT CAGTGGCGAG CCCGGTTCGC   
  
  
+ ATCTGCCGGG TTCGACCCAG TTCATCTGGG TTCGAATGCG TTTAAGCAGG CGAGTATGTT GTTGGAGTTC   
  
  
+ TTTGCTGGTG GTGAAGGGTA CGGCGTGGAG GAGCGAGAAG GGTGTCTCAT GTTGGGATGG CATAGTAGGC   
  
  
+ CGCTTATCAC CACCTCGGCT TGGCAGCTCG CCAAGAACCC GGCTGTGAAT CGGCGATG  

- -Up\_Stream \_Len000TTGGGA AATTAAAATA GTTACACCCT GTTGTGAGTA GAAGTATGAG GTTGTTCGAT   
  
  
- ACTATGTAAG CTAATAACGG ATCATAAAGC AAATCATTCT ATAATCACGG TTTGAAACAT TATTTTTACT   
  
  
- TTGAATGATT TTTTTTAGAG CAAAAGACAT CTCCCTTTTA AACTTCTGAA TCACAGTGTA CTACTGGATT   
  
  
- ACCATGAACA TCCCAGTTCT AACTCTAACT CCCGTATGAC TGCTGGACAT ATTACCAGTT CATCCTAGTT   
  
  
- TCAGCTCTTT TGACAGAGAC GATTGAAAGT ATTAGATTGG GAATTGAGTG AGACTGGGAG TTAAATTAGT   
  
  
- CCGGATTAAG TTTGATAATT ACCAATATTG AGAGAAAGGA AGAAAACTCT AAAAAAAAAA GATGTACGCC   
  
  
- AACTTTTATT AAAAAAATTT TTTATATATA GTGATTGGTA AGTAATAGTA TCAAAATCTC AAGTATAAAT   
  
  
- ACACCAGGAA CAACTCAGAT TAAATTACGA AATCTACCGC ACTTCGTGGT TTAATCACCA TTGGTATTAT   
  
  
- TATTTGATAG TTCACAATTC AGCTTCTTTT GCCTGTGACG AGTTGGTTTA GATCATTGTA TCACATACTG   
  
  
- TACTATTGAT TCACACGGTA CATCATTAGT TAAGAAAGAA AAAAAAAAAA ACCAACTCTA GTGTATTTAA   
  
  
- TGCAATAGTT CCCATCATGC TATATTACCT ACTCCTCTTT AATTTACACT CTAAATCAAC TACTTAGCTT   
  
  
- ATGCAATCAG ACTCGGTAAA CCAGTTTTGA CGTAACCGAT TAGTTATCTT TAATATCTTT TATGTATTGT   
  
  
- ATTACCCTAC GTTAAAAGAT TAACCCCTTT GGTACAGTTA CGTCACTAAA ACCTGGTAAA TCCGGAATAA   
  
  
- GAAAAGTTAA AAAATTAACT AATAAATCTG AGTATACTTG AATAACAACT GTCTAACTCG ATTTTTGTGA   
  
  
- CTACAAAGTA TGTATACATA CTTAACGTGC ATGCTTCCGA GACGAGTAAA GCTCTTTCTT TTAATTGTCA   
  
  
- ATCCTCGTCA TATTAAATTG TTGTTCGCTA TTAATAATCA TACTTCACCA TGCTTAGATA AAATTTCCTC   
  
  
- ATGATACTTC ATGTTTGTTA TTGTTTATTA TTTCTCGTCA TGTAATTGTA TAGAATGATG GATTTATTTA   
  
  
- GCTAAGATTC TCATTTTTTT ACAACTTTAA TTGGGGTAAT ATCAAAATGA AATTGTTCTT CAAAAAGAAG   
  
  
- AAAATGGTAA ATGGTTCATT CTTACTAAGT CCGGCATATC GGTTCCTTTT TATCTTTTGG TGTTTTGATT   
  
  
- GGTACCGGGT TTTTTGTTGG TGGTTCTCCC CGTGATTACA CTGATTGGCT GCCAATTGTA ATAGTAGACA   
  
  
- AACTATGAAC AAGTAGAGAG TGATAAGCCC ATTATCACAC TTTAATGACT ACATGGGAGA TTTGAGCAGC   
  
  
- AAAATTGACA CATTCTTAGT CTAATTGTCA GTGCCTGTGT ACTCATTGTT TAGTTAAAGC CTGTACTTGC   
  
  
- TTGCTTGACA CTTAGGGGGG GTTTTTTTTT TAAAAAAATT TTTTTCAACG TAGGAATAGA AAGACTTGGT   
  
  
- CCGGTCGACA GAGTAAGGAA CCAGGAGCGC GGGGTAGTGC TCGCGACGAG TAATAAGTGG GAGAGAGAGA   
  
  
- GAGGAGAGAC TAGGTGTGGT GTTTGTGTTC CAAACGTGTC GACGTCTCGT CAGCATGATG ATAGTCATCA   
  
  
- CTTTTTGGTT CGTCTTTAGG GGTTAGGGAG AAGAGGAAGA AGGGTCTAAG AAGGAGTTAA GGTTCGGGTA   
  
  
- TAGTCTTTAA ATCTGTTGTT GTTGGTTTTT TTTTTTAATT ATGACGAACT CTTTTTGTTT ATACTATACT   
  
  
- TTAAGGAGCT TTTTCCGGAC GGTATTGTTT AAGGAGCACT TTTTCGTCGA CTCTCTTCTT CTTCTTCTTT   
  
  
- TTTTAGGGCA GTGTTAGAGT TAGAGCTAGG GTTTAACACT TTCTCTTTCT TTTCTACTTC TCTCTTGTGG   
  
  
- GGAAAAGCTT GGGACTGGTC TTTGGGGGCG GCCTCTACAG CGGCCGCGGC CCCTTCAGGT TCTACACCCT   
  
  
- ACTACTCTAC CGGGTCTTGC TGCGATTTTA CCTACTCGAC GAACGACAAC ACCCCATGTT CCACTCCAGC   
  
  
- AGCCTGTACC GCCTTTAACG GGTCTTCGAA CTCGTCGAAC TTCTTCAGTA CCCATCACAA GTTCTTCTGC   
  
  
- CCAATAGAAT GAACCGAAGG CTTTGACAAG TAATGTTAGG ACGTCTAGAC AGATGAACCG AACTTAGATA   
  
  
- CGAAAGACTC AAATTGGGAT TGGGATTAAA ACTGGGAAGA GGTAGCAGTT AGAGTGGGTA GTAGCTAGGT   
  
  
- CGAGGAGGTG AAGGAGCTTG GAGCAGCTGC CAGTTTAAGC TCGGGCTGGG CAAAAGGCTA AGCCTAGACT   
  
  
- TTCGTTAGGG TCCATTCCGA TAGAACTGGG GTGGTTCAAG ATCGTTATGA AGTTTAAACG CACTCCGATT   
  
  
- CTCCAACTTT GGGAGTTTAA TGTGTTGGCG GGGTTGCGGG TGGGGTGGAG GTTTTGACGG GTTAAGCCGC   
  
  
- AGAGGTGGTA GCCCGTGCTG CCGCAGCTCC GGCCACCACG ACCATCTGAG TGTTCTTTTG CCGTAGTCTA   
  
  
- ACCACGTACG TGAATACCGG ACACGGCTTC GTTAAGTTGA TCTTTTGTAC CCTAATCGAC TCCGAAACCA   
  
  
- ATTTGTCTAA TCCATAAATC GTCGTAGAGT TCGACCTGGA TACTCCTTTC ATCGTTGAAC AAAACGTCTT   
  
  
- CGAGAATGAA CAGCCTAGAT GTTCGATACG GGTAGACATG GGATGCTACT TAGTCAGAGT CTACTCAACG   
  
  
- TCTACGTGAA AATACTCTGA ACGGGTATAG AATTTAAGCG GGTAAAGTGC CGTTTAGTTC GTTAAAATCT   
  
  
- TCGTAAGTTA CCCTTCTTCT TTCAAGTACA CTAACTAAAG TCGTACTTCG TTCCGTACGT TACCGGCCGA   
  
  
- AACTACGTTC GGGACCGAGA AGCTGGCCTC CCAGGTGGCG AAAAGGCCAA TTGGCCCTAA CCTGGCGGGC   
  
  
- GAGGCCTGTT GAGCCTGGCC GACGTTCTCC ACCCAACCTT CGAACGGGTC AAGCGCCTAA GCTAGGCCTA   
  
  
- ATTTAAAGTC ATAGCACCCA AACACCGTTT GTCAAACTGG CTAGATCTTA GAAGGTACGA TCTAGAACTT   
  
  
- GGCCTGTGAC TCCACCACCG CCAGTTGAGC CAAAAGCTCG AGGTGGCCGA CAACCGATTT GGGCCCCGCT   
  
  
- AGCTCTTTCA CGACCCCAAG TACTCCCGGC ACTTGGGCCA GTAACACTGC TACCAGCTCG TCCTTCGCTT   
  
  
- GGTGTTGCCT GGCCAAAAGA ACCTGGCCAA GTTACTCAGC AACGTAATAA TGAGGTGGAA CAAACTAAGG   
  
  
- GAACTCTAAA CACAACTATT ACATCTATTC TTCTACAGCC TCCGAATGAA CCCGGTCGTC TAGACGTTGT   
  
  
- ACCACCGAAC ACTTCCCAGA CTGGCTCAGC TCTCCGTGCT CTGGGACCGA GTCACCGCTC GGGCCAAGCG   
  
  
- TAGACGGCCC AAGCTGGGTC AAGTAGACCC AAGCTTACGC AAATTCGTCC GCTCATACAA CAACCTCAAG   
  
  
- AAACGACCAC CACTTCCCAT GCCGCACCTC CTCGCTCTTC CCACAGAGTA CAACCCTACC GTATCATCCG   
  
  
- GCGAATAGTG GTGGAGCCGA ACCGTCGAGC GGTTCTTGGG CCGACACTTA GCCGCTAC

+     GT1-motif

| Site Name | Organism | Position | Strand | Matrix score. | sequence | function |
| --- | --- | --- | --- | --- | --- | --- |
| GT1-motif | Arabidopsis thaliana | 1386 | + | 6 | GGTTAA | light responsive element |
| GT1-motif | Arabidopsis thaliana | 3061 | + | 6 | GGTTAA | light responsive element |
| GT1-motif | Arabidopsis thaliana | 1223 | - | 6 | GGTTAA | light responsive element |
| GT1-motif | Arabidopsis thaliana | 3063 | - | 6 | GGTTAA | light responsive element |
| GT1-motif | Arabidopsis thaliana | 2326 | - | 6 | GGTTAA | light responsive element |
| GT1-motif | Avena sativa | 1222 | - | 7 | GGTTAAT | light responsive element |
| GT1-motif | Arabidopsis thaliana | 2732 | + | 6 | GGTTAA | light responsive element |

>HU10G00709.1   
+ -Up\_Stream \_Len000AACCCT TTAATTTTAT CAATGTGGGA CAACACTCAT CTTCATACTC CAACAAGCTA   
  
  
+ TGATACATTC GATTATTGCC TAGTATTTCG TTTAGTAAGA TATTAGTGCC AAACTTTGTA ATAAAAATGA   
  
  
+ AACTTACTAA AAAAAATCTC GTTTTCTGTA GAGGGAAAAT TTGAAGACTT AGTGTCACAT GATGACCTAA   
  
  
+ TGGTACTTGT AGGGTCAAGA TTGAGATTGA GGGCATACTG ACGACCTGTA TAATGGTCAA GTAGGATCAA   
  
  
+ AGTCGAGAAA ACTGTCTCTG CTAACTTTCA TAATCTAACC CTTAACTCAC TCTGACCCTC AATTTAATCA   
  
  
+ GGCCTAATTC AAACTATTAA TGGTTATAAC TCTCTTTCCT TCTTTTGAGA TTTTTTTTTT CTACATGCGG   
  
  
+ TTGAAAATAA TTTTTTTAAA AAATATATAT CACTAACCAT TCATTATCAT AGTTTTAGAG TTCATATTTA   
  
  
+ TGTGGTCCTT GTTGAGTCTA ATTTAATGCT TTAGATGGCG TGAAGCACCA AATTAGTGGT AACCATAATA   
  
  
+ ATAAACTATC AAGTGTTAAG TCGAAGAAAA CGGACACTGC TCAACCAAAT CTAGTAACAT AGTGTATGAC   
  
  
+ ATGATAACTA AGTGTGCCAT GTAGTAATCA ATTCTTTCTT TTTTTTTTTT TGGTTGAGAT CACATAAATT   
  
  
+ ACGTTATCAA GGGTAGTACG ATATAATGGA TGAGGAGAAA TTAAATGTGA GATTTAGTTG ATGAATCGAA   
  
  
+ TACGTTAGTC TGAGCCATTT GGTCAAAACT GCATTGGCTA ATCAATAGAA ATTATAGAAA ATACATAACA   
  
  
+ TAATGGGATG CAATTTTCTA ATTGGGGAAA CCATGTCAAT GCAGTGATTT TGGACCATTT AGGCCTTATT   
  
  
+ CTTTTCAATT TTTTAATTGA TTATTTAGAC TCATATGAAC TTATTGTTGA CAGATTGAGC TAAAAACACT   
  
  
+ GATGTTTCAT ACATATGTAT GAATTGCACG TACGAAGGCT CTGCTCATTT CGAGAAAGAA AATTAACAGT   
  
  
+ TAGGAGCAGT ATAATTTAAC AACAAGCGAT AATTATTAGT ATGAAGTGGT ACGAATCTAT TTTAAAGGAG   
  
  
+ TACTATGAAG TACAAACAAT AACAAATAAT AAAGAGCAGT ACATTAACAT ATCTTACTAC CTAAATAAAT   
  
  
+ CGATTCTAAG AGTAAAAAAA TGTTGAAATT AACCCCATTA TAGTTTTACT TTAACAAGAA GTTTTTCTTC   
  
  
+ TTTTACCATT TACCAAGTAA GAATGATTCA GGCCGTATAG CCAAGGAAAA ATAGAAAACC ACAAAACTAA   
  
  
+ CCATGGCCCA AAAAACAACC ACCAAGAGGG GCACTAATGT GACTAACCGA CGGTTAACAT TATCATCTGT   
  
  
+ TTGATACTTG TTCATCTCTC ACTATTCGGG TAATAGTGTG AAATTACTGA TGTACCCTCT AAACTCGTCG   
  
  
+ TTTTAACTGT GTAAGAATCA GATTAACAGT CACGGACACA TGAGTAACAA ATCAATTTCG GACATGAACG   
  
  
+ AACGAACTGT GAATCCCCCC CAAAAAAAAA ATTTTTTTAA AAAAAGTTGC ATCCTTATCT TTCTGAACCA   
  
  
+ GGCCAGCTGT CTCATTCCTT GGTCCTCGCG CCCCATCACG AGCGCTGCTC ATTATTCACC CTCTCTCTCT   
  
  
+ CTCCTCTCTG ATCCACACCA CAAACACAAG GTTTGCACAG CTGCAGAGCA GTCGTACTAC TATCAGTAGT   
  
  
+ GAAAAACCAA GCAGAAATCC CCAATCCCTC TTCTCCTTCT TCCCAGATTC TTCCTCAATT CCAAGCCCAT   
  
  
+ ATCAGAAATT TAGACAACAA CAACCAAAAA AAAAAATTAA TACTGCTTGA GAAAAACAAA TATGATATGA   
  
  
+ AATTCCTCGA AAAAGGCCTG CCATAACAAA TTCCTCGTGA AAAAGCAGCT GAGAGAAGAA GAAGAAGAAA   
  
  
+ AAAATCCCGT CACAATCTCA ATCTCGATCC CAAATTGTGA AAGAGAAAGA AAAGATGAAG AGAGAACACC   
  
  
+ CCTTTTCGAA CCCTGACCAG AAACCCCCGC CGGAGATGTC GCCGGCGCCG GGGAAGTCCA AGATGTGGGA   
  
  
+ TGATGAGATG GCCCAGAACG ACGCTAAAAT GGATGAGCTG CTTGCTGTTG TGGGGTACAA GGTGAGGTCG   
  
  
+ TCGGACATGG CGGAAATTGC CCAGAAGCTT GAGCAGCTTG AAGAAGTCAT GGGTAGTGTT CAAGAAGACG   
  
  
+ GGTTATCTTA CTTGGCTTCC GAAACTGTTC ATTACAATCC TGCAGATCTG TCTACTTGGC TTGAATCTAT   
  
  
+ GCTTTCTGAG TTTAACCCTA ACCCTAATTT TGACCCTTCT CCATCGTCAA TCTCACCCAT CATCGATCCA   
  
  
+ GCTCCTCCAC TTCCTCGAAC CTCGTCGACG GTCAAATTCG AGCCCGACCC GTTTTCCGAT TCGGATCTGA   
  
  
+ AAGCAATCCC AGGTAAGGCT ATCTTGACCC CACCAAGTTC TAGCAATACT TCAAATTTGC GTGAGGCTAA   
  
  
+ GAGGTTGAAA CCCTCAAATT ACACAACCGC CCCAACGCCC ACCCCACCTC CAAAACTGCC CAATTCGGCG   
  
  
+ TCTCCACCAT CGGGCACGAC GGCGTCGAGG CCGGTGGTGC TGGTAGACTC ACAAGAAAAC GGCATCAGAT   
  
  
+ TGGTGCATGC ACTTATGGCC TGTGCCGAAG CAATTCAACT AGAAAACATG GGATTAGCTG AGGCTTTGGT   
  
  
+ TAAACAGATT AGGTATTTAG CAGCATCTCA AGCTGGACCT ATGAGGAAAG TAGCAACTTG TTTTGCAGAA   
  
  
+ GCTCTTACTT GTCGGATCTA CAAGCTATGC CCATCTGTAC CCTACGATGA ATCAGTCTCA GATGAGTTGC   
  
  
+ AGATGCACTT TTATGAGACT TGCCCATATC TTAAATTCGC CCATTTCACG GCAAATCAAG CAATTTTAGA   
  
  
+ AGCATTCAAT GGGAAGAAGA AAGTTCATGT GATTGATTTC AGCATGAAGC AAGGCATGCA ATGGCCGGCT   
  
  
+ TTGATGCAAG CCCTGGCTCT TCGACCGGAG GGTCCACCGC TTTTCCGGTT AACCGGGATT GGACCGCCCG   
  
  
+ CTCCGGACAA CTCGGACCGG CTGCAAGAGG TGGGTTGGAA GCTTGCCCAG TTCGCGGATT CGATCCGGAT   
  
  
+ TAAATTTCAG TATCGTGGGT TTGTGGCAAA CAGTTTGACC GATCTAGAAT CTTCCATGCT AGATCTTGAA   
  
  
+ CCGGACACTG AGGTGGTGGC GGTCAACTCG GTTTTCGAGC TCCACCGGCT GTTGGCTAAA CCCGGGGCGA   
  
  
+ TCGAGAAAGT GCTGGGGTTC ATGAGGGCCG TGAACCCGGT CATTGTGACG ATGGTCGAGC AGGAAGCGAA   
  
  
+ CCACAACGGA CCGGTTTTCT TGGACCGGTT CAATGAGTCG TTGCATTATT ACTCCACCTT GTTTGATTCC   
  
  
+ CTTGAGATTT GTGTTGATAA TGTAGATAAG AAGATGTCGG AGGCTTACTT GGGCCAGCAG ATCTGCAACA   
  
  
+ TGGTGGCTTG TGAAGGGTCT GACCGAGTCG AGAGGCACGA GACCCTGGCT CAGTGGCGAG CCCGGTTCGC   
  
  
+ ATCTGCCGGG TTCGACCCAG TTCATCTGGG TTCGAATGCG TTTAAGCAGG CGAGTATGTT GTTGGAGTTC   
  
  
+ TTTGCTGGTG GTGAAGGGTA CGGCGTGGAG GAGCGAGAAG GGTGTCTCAT GTTGGGATGG CATAGTAGGC   
  
  
+ CGCTTATCAC CACCTCGGCT TGGCAGCTCG CCAAGAACCC GGCTGTGAAT CGGCGATG  

- -Up\_Stream \_Len000TTGGGA AATTAAAATA GTTACACCCT GTTGTGAGTA GAAGTATGAG GTTGTTCGAT   
  
  
- ACTATGTAAG CTAATAACGG ATCATAAAGC AAATCATTCT ATAATCACGG TTTGAAACAT TATTTTTACT   
  
  
- TTGAATGATT TTTTTTAGAG CAAAAGACAT CTCCCTTTTA AACTTCTGAA TCACAGTGTA CTACTGGATT   
  
  
- ACCATGAACA TCCCAGTTCT AACTCTAACT CCCGTATGAC TGCTGGACAT ATTACCAGTT CATCCTAGTT   
  
  
- TCAGCTCTTT TGACAGAGAC GATTGAAAGT ATTAGATTGG GAATTGAGTG AGACTGGGAG TTAAATTAGT   
  
  
- CCGGATTAAG TTTGATAATT ACCAATATTG AGAGAAAGGA AGAAAACTCT AAAAAAAAAA GATGTACGCC   
  
  
- AACTTTTATT AAAAAAATTT TTTATATATA GTGATTGGTA AGTAATAGTA TCAAAATCTC AAGTATAAAT   
  
  
- ACACCAGGAA CAACTCAGAT TAAATTACGA AATCTACCGC ACTTCGTGGT TTAATCACCA TTGGTATTAT   
  
  
- TATTTGATAG TTCACAATTC AGCTTCTTTT GCCTGTGACG AGTTGGTTTA GATCATTGTA TCACATACTG   
  
  
- TACTATTGAT TCACACGGTA CATCATTAGT TAAGAAAGAA AAAAAAAAAA ACCAACTCTA GTGTATTTAA   
  
  
- TGCAATAGTT CCCATCATGC TATATTACCT ACTCCTCTTT AATTTACACT CTAAATCAAC TACTTAGCTT   
  
  
- ATGCAATCAG ACTCGGTAAA CCAGTTTTGA CGTAACCGAT TAGTTATCTT TAATATCTTT TATGTATTGT   
  
  
- ATTACCCTAC GTTAAAAGAT TAACCCCTTT GGTACAGTTA CGTCACTAAA ACCTGGTAAA TCCGGAATAA   
  
  
- GAAAAGTTAA AAAATTAACT AATAAATCTG AGTATACTTG AATAACAACT GTCTAACTCG ATTTTTGTGA   
  
  
- CTACAAAGTA TGTATACATA CTTAACGTGC ATGCTTCCGA GACGAGTAAA GCTCTTTCTT TTAATTGTCA   
  
  
- ATCCTCGTCA TATTAAATTG TTGTTCGCTA TTAATAATCA TACTTCACCA TGCTTAGATA AAATTTCCTC   
  
  
- ATGATACTTC ATGTTTGTTA TTGTTTATTA TTTCTCGTCA TGTAATTGTA TAGAATGATG GATTTATTTA   
  
  
- GCTAAGATTC TCATTTTTTT ACAACTTTAA TTGGGGTAAT ATCAAAATGA AATTGTTCTT CAAAAAGAAG   
  
  
- AAAATGGTAA ATGGTTCATT CTTACTAAGT CCGGCATATC GGTTCCTTTT TATCTTTTGG TGTTTTGATT   
  
  
- GGTACCGGGT TTTTTGTTGG TGGTTCTCCC CGTGATTACA CTGATTGGCT GCCAATTGTA ATAGTAGACA   
  
  
- AACTATGAAC AAGTAGAGAG TGATAAGCCC ATTATCACAC TTTAATGACT ACATGGGAGA TTTGAGCAGC   
  
  
- AAAATTGACA CATTCTTAGT CTAATTGTCA GTGCCTGTGT ACTCATTGTT TAGTTAAAGC CTGTACTTGC   
  
  
- TTGCTTGACA CTTAGGGGGG GTTTTTTTTT TAAAAAAATT TTTTTCAACG TAGGAATAGA AAGACTTGGT   
  
  
- CCGGTCGACA GAGTAAGGAA CCAGGAGCGC GGGGTAGTGC TCGCGACGAG TAATAAGTGG GAGAGAGAGA   
  
  
- GAGGAGAGAC TAGGTGTGGT GTTTGTGTTC CAAACGTGTC GACGTCTCGT CAGCATGATG ATAGTCATCA   
  
  
- CTTTTTGGTT CGTCTTTAGG GGTTAGGGAG AAGAGGAAGA AGGGTCTAAG AAGGAGTTAA GGTTCGGGTA   
  
  
- TAGTCTTTAA ATCTGTTGTT GTTGGTTTTT TTTTTTAATT ATGACGAACT CTTTTTGTTT ATACTATACT   
  
  
- TTAAGGAGCT TTTTCCGGAC GGTATTGTTT AAGGAGCACT TTTTCGTCGA CTCTCTTCTT CTTCTTCTTT   
  
  
- TTTTAGGGCA GTGTTAGAGT TAGAGCTAGG GTTTAACACT TTCTCTTTCT TTTCTACTTC TCTCTTGTGG   
  
  
- GGAAAAGCTT GGGACTGGTC TTTGGGGGCG GCCTCTACAG CGGCCGCGGC CCCTTCAGGT TCTACACCCT   
  
  
- ACTACTCTAC CGGGTCTTGC TGCGATTTTA CCTACTCGAC GAACGACAAC ACCCCATGTT CCACTCCAGC   
  
  
- AGCCTGTACC GCCTTTAACG GGTCTTCGAA CTCGTCGAAC TTCTTCAGTA CCCATCACAA GTTCTTCTGC   
  
  
- CCAATAGAAT GAACCGAAGG CTTTGACAAG TAATGTTAGG ACGTCTAGAC AGATGAACCG AACTTAGATA   
  
  
- CGAAAGACTC AAATTGGGAT TGGGATTAAA ACTGGGAAGA GGTAGCAGTT AGAGTGGGTA GTAGCTAGGT   
  
  
- CGAGGAGGTG AAGGAGCTTG GAGCAGCTGC CAGTTTAAGC TCGGGCTGGG CAAAAGGCTA AGCCTAGACT   
  
  
- TTCGTTAGGG TCCATTCCGA TAGAACTGGG GTGGTTCAAG ATCGTTATGA AGTTTAAACG CACTCCGATT   
  
  
- CTCCAACTTT GGGAGTTTAA TGTGTTGGCG GGGTTGCGGG TGGGGTGGAG GTTTTGACGG GTTAAGCCGC   
  
  
- AGAGGTGGTA GCCCGTGCTG CCGCAGCTCC GGCCACCACG ACCATCTGAG TGTTCTTTTG CCGTAGTCTA   
  
  
- ACCACGTACG TGAATACCGG ACACGGCTTC GTTAAGTTGA TCTTTTGTAC CCTAATCGAC TCCGAAACCA   
  
  
- ATTTGTCTAA TCCATAAATC GTCGTAGAGT TCGACCTGGA TACTCCTTTC ATCGTTGAAC AAAACGTCTT   
  
  
- CGAGAATGAA CAGCCTAGAT GTTCGATACG GGTAGACATG GGATGCTACT TAGTCAGAGT CTACTCAACG   
  
  
- TCTACGTGAA AATACTCTGA ACGGGTATAG AATTTAAGCG GGTAAAGTGC CGTTTAGTTC GTTAAAATCT   
  
  
- TCGTAAGTTA CCCTTCTTCT TTCAAGTACA CTAACTAAAG TCGTACTTCG TTCCGTACGT TACCGGCCGA   
  
  
- AACTACGTTC GGGACCGAGA AGCTGGCCTC CCAGGTGGCG AAAAGGCCAA TTGGCCCTAA CCTGGCGGGC   
  
  
- GAGGCCTGTT GAGCCTGGCC GACGTTCTCC ACCCAACCTT CGAACGGGTC AAGCGCCTAA GCTAGGCCTA   
  
  
- ATTTAAAGTC ATAGCACCCA AACACCGTTT GTCAAACTGG CTAGATCTTA GAAGGTACGA TCTAGAACTT   
  
  
- GGCCTGTGAC TCCACCACCG CCAGTTGAGC CAAAAGCTCG AGGTGGCCGA CAACCGATTT GGGCCCCGCT   
  
  
- AGCTCTTTCA CGACCCCAAG TACTCCCGGC ACTTGGGCCA GTAACACTGC TACCAGCTCG TCCTTCGCTT   
  
  
- GGTGTTGCCT GGCCAAAAGA ACCTGGCCAA GTTACTCAGC AACGTAATAA TGAGGTGGAA CAAACTAAGG   
  
  
- GAACTCTAAA CACAACTATT ACATCTATTC TTCTACAGCC TCCGAATGAA CCCGGTCGTC TAGACGTTGT   
  
  
- ACCACCGAAC ACTTCCCAGA CTGGCTCAGC TCTCCGTGCT CTGGGACCGA GTCACCGCTC GGGCCAAGCG   
  
  
- TAGACGGCCC AAGCTGGGTC AAGTAGACCC AAGCTTACGC AAATTCGTCC GCTCATACAA CAACCTCAAG   
  
  
- AAACGACCAC CACTTCCCAT GCCGCACCTC CTCGCTCTTC CCACAGAGTA CAACCCTACC GTATCATCCG   
  
  
- GCGAATAGTG GTGGAGCCGA ACCGTCGAGC GGTTCTTGGG CCGACACTTA GCCGCTAC

+     I-box

| Site Name | Organism | Position | Strand | Matrix score. | sequence | function |
| --- | --- | --- | --- | --- | --- | --- |
| I-box | Larix laricina | 906 | - | 9 | GTATAAGGCC | part of a light responsive element |
| I-box | Solanum tuberosum | 3449 | + | 9 | TGATAATGT | part of a light responsive element |
| I-box | Gossypium hirsutum | 1595 | - | 10 | AAGATAAGGCT | part of a light responsive element |
| I-box | Solanum tuberosum | 1391 | - | 9 | TGATAATGT | part of a light responsive element |
| I-box | Triticum aestivum | 1597 | - | 8 | AGATAAGG | part of a light responsive element |

>HU10G00709.1   
+ -Up\_Stream \_Len000AACCCT TTAATTTTAT CAATGTGGGA CAACACTCAT CTTCATACTC CAACAAGCTA   
  
  
+ TGATACATTC GATTATTGCC TAGTATTTCG TTTAGTAAGA TATTAGTGCC AAACTTTGTA ATAAAAATGA   
  
  
+ AACTTACTAA AAAAAATCTC GTTTTCTGTA GAGGGAAAAT TTGAAGACTT AGTGTCACAT GATGACCTAA   
  
  
+ TGGTACTTGT AGGGTCAAGA TTGAGATTGA GGGCATACTG ACGACCTGTA TAATGGTCAA GTAGGATCAA   
  
  
+ AGTCGAGAAA ACTGTCTCTG CTAACTTTCA TAATCTAACC CTTAACTCAC TCTGACCCTC AATTTAATCA   
  
  
+ GGCCTAATTC AAACTATTAA TGGTTATAAC TCTCTTTCCT TCTTTTGAGA TTTTTTTTTT CTACATGCGG   
  
  
+ TTGAAAATAA TTTTTTTAAA AAATATATAT CACTAACCAT TCATTATCAT AGTTTTAGAG TTCATATTTA   
  
  
+ TGTGGTCCTT GTTGAGTCTA ATTTAATGCT TTAGATGGCG TGAAGCACCA AATTAGTGGT AACCATAATA   
  
  
+ ATAAACTATC AAGTGTTAAG TCGAAGAAAA CGGACACTGC TCAACCAAAT CTAGTAACAT AGTGTATGAC   
  
  
+ ATGATAACTA AGTGTGCCAT GTAGTAATCA ATTCTTTCTT TTTTTTTTTT TGGTTGAGAT CACATAAATT   
  
  
+ ACGTTATCAA GGGTAGTACG ATATAATGGA TGAGGAGAAA TTAAATGTGA GATTTAGTTG ATGAATCGAA   
  
  
+ TACGTTAGTC TGAGCCATTT GGTCAAAACT GCATTGGCTA ATCAATAGAA ATTATAGAAA ATACATAACA   
  
  
+ TAATGGGATG CAATTTTCTA ATTGGGGAAA CCATGTCAAT GCAGTGATTT TGGACCATTT AGGCCTTATT   
  
  
+ CTTTTCAATT TTTTAATTGA TTATTTAGAC TCATATGAAC TTATTGTTGA CAGATTGAGC TAAAAACACT   
  
  
+ GATGTTTCAT ACATATGTAT GAATTGCACG TACGAAGGCT CTGCTCATTT CGAGAAAGAA AATTAACAGT   
  
  
+ TAGGAGCAGT ATAATTTAAC AACAAGCGAT AATTATTAGT ATGAAGTGGT ACGAATCTAT TTTAAAGGAG   
  
  
+ TACTATGAAG TACAAACAAT AACAAATAAT AAAGAGCAGT ACATTAACAT ATCTTACTAC CTAAATAAAT   
  
  
+ CGATTCTAAG AGTAAAAAAA TGTTGAAATT AACCCCATTA TAGTTTTACT TTAACAAGAA GTTTTTCTTC   
  
  
+ TTTTACCATT TACCAAGTAA GAATGATTCA GGCCGTATAG CCAAGGAAAA ATAGAAAACC ACAAAACTAA   
  
  
+ CCATGGCCCA AAAAACAACC ACCAAGAGGG GCACTAATGT GACTAACCGA CGGTTAACAT TATCATCTGT   
  
  
+ TTGATACTTG TTCATCTCTC ACTATTCGGG TAATAGTGTG AAATTACTGA TGTACCCTCT AAACTCGTCG   
  
  
+ TTTTAACTGT GTAAGAATCA GATTAACAGT CACGGACACA TGAGTAACAA ATCAATTTCG GACATGAACG   
  
  
+ AACGAACTGT GAATCCCCCC CAAAAAAAAA ATTTTTTTAA AAAAAGTTGC ATCCTTATCT TTCTGAACCA   
  
  
+ GGCCAGCTGT CTCATTCCTT GGTCCTCGCG CCCCATCACG AGCGCTGCTC ATTATTCACC CTCTCTCTCT   
  
  
+ CTCCTCTCTG ATCCACACCA CAAACACAAG GTTTGCACAG CTGCAGAGCA GTCGTACTAC TATCAGTAGT   
  
  
+ GAAAAACCAA GCAGAAATCC CCAATCCCTC TTCTCCTTCT TCCCAGATTC TTCCTCAATT CCAAGCCCAT   
  
  
+ ATCAGAAATT TAGACAACAA CAACCAAAAA AAAAAATTAA TACTGCTTGA GAAAAACAAA TATGATATGA   
  
  
+ AATTCCTCGA AAAAGGCCTG CCATAACAAA TTCCTCGTGA AAAAGCAGCT GAGAGAAGAA GAAGAAGAAA   
  
  
+ AAAATCCCGT CACAATCTCA ATCTCGATCC CAAATTGTGA AAGAGAAAGA AAAGATGAAG AGAGAACACC   
  
  
+ CCTTTTCGAA CCCTGACCAG AAACCCCCGC CGGAGATGTC GCCGGCGCCG GGGAAGTCCA AGATGTGGGA   
  
  
+ TGATGAGATG GCCCAGAACG ACGCTAAAAT GGATGAGCTG CTTGCTGTTG TGGGGTACAA GGTGAGGTCG   
  
  
+ TCGGACATGG CGGAAATTGC CCAGAAGCTT GAGCAGCTTG AAGAAGTCAT GGGTAGTGTT CAAGAAGACG   
  
  
+ GGTTATCTTA CTTGGCTTCC GAAACTGTTC ATTACAATCC TGCAGATCTG TCTACTTGGC TTGAATCTAT   
  
  
+ GCTTTCTGAG TTTAACCCTA ACCCTAATTT TGACCCTTCT CCATCGTCAA TCTCACCCAT CATCGATCCA   
  
  
+ GCTCCTCCAC TTCCTCGAAC CTCGTCGACG GTCAAATTCG AGCCCGACCC GTTTTCCGAT TCGGATCTGA   
  
  
+ AAGCAATCCC AGGTAAGGCT ATCTTGACCC CACCAAGTTC TAGCAATACT TCAAATTTGC GTGAGGCTAA   
  
  
+ GAGGTTGAAA CCCTCAAATT ACACAACCGC CCCAACGCCC ACCCCACCTC CAAAACTGCC CAATTCGGCG   
  
  
+ TCTCCACCAT CGGGCACGAC GGCGTCGAGG CCGGTGGTGC TGGTAGACTC ACAAGAAAAC GGCATCAGAT   
  
  
+ TGGTGCATGC ACTTATGGCC TGTGCCGAAG CAATTCAACT AGAAAACATG GGATTAGCTG AGGCTTTGGT   
  
  
+ TAAACAGATT AGGTATTTAG CAGCATCTCA AGCTGGACCT ATGAGGAAAG TAGCAACTTG TTTTGCAGAA   
  
  
+ GCTCTTACTT GTCGGATCTA CAAGCTATGC CCATCTGTAC CCTACGATGA ATCAGTCTCA GATGAGTTGC   
  
  
+ AGATGCACTT TTATGAGACT TGCCCATATC TTAAATTCGC CCATTTCACG GCAAATCAAG CAATTTTAGA   
  
  
+ AGCATTCAAT GGGAAGAAGA AAGTTCATGT GATTGATTTC AGCATGAAGC AAGGCATGCA ATGGCCGGCT   
  
  
+ TTGATGCAAG CCCTGGCTCT TCGACCGGAG GGTCCACCGC TTTTCCGGTT AACCGGGATT GGACCGCCCG   
  
  
+ CTCCGGACAA CTCGGACCGG CTGCAAGAGG TGGGTTGGAA GCTTGCCCAG TTCGCGGATT CGATCCGGAT   
  
  
+ TAAATTTCAG TATCGTGGGT TTGTGGCAAA CAGTTTGACC GATCTAGAAT CTTCCATGCT AGATCTTGAA   
  
  
+ CCGGACACTG AGGTGGTGGC GGTCAACTCG GTTTTCGAGC TCCACCGGCT GTTGGCTAAA CCCGGGGCGA   
  
  
+ TCGAGAAAGT GCTGGGGTTC ATGAGGGCCG TGAACCCGGT CATTGTGACG ATGGTCGAGC AGGAAGCGAA   
  
  
+ CCACAACGGA CCGGTTTTCT TGGACCGGTT CAATGAGTCG TTGCATTATT ACTCCACCTT GTTTGATTCC   
  
  
+ CTTGAGATTT GTGTTGATAA TGTAGATAAG AAGATGTCGG AGGCTTACTT GGGCCAGCAG ATCTGCAACA   
  
  
+ TGGTGGCTTG TGAAGGGTCT GACCGAGTCG AGAGGCACGA GACCCTGGCT CAGTGGCGAG CCCGGTTCGC   
  
  
+ ATCTGCCGGG TTCGACCCAG TTCATCTGGG TTCGAATGCG TTTAAGCAGG CGAGTATGTT GTTGGAGTTC   
  
  
+ TTTGCTGGTG GTGAAGGGTA CGGCGTGGAG GAGCGAGAAG GGTGTCTCAT GTTGGGATGG CATAGTAGGC   
  
  
+ CGCTTATCAC CACCTCGGCT TGGCAGCTCG CCAAGAACCC GGCTGTGAAT CGGCGATG  

- -Up\_Stream \_Len000TTGGGA AATTAAAATA GTTACACCCT GTTGTGAGTA GAAGTATGAG GTTGTTCGAT   
  
  
- ACTATGTAAG CTAATAACGG ATCATAAAGC AAATCATTCT ATAATCACGG TTTGAAACAT TATTTTTACT   
  
  
- TTGAATGATT TTTTTTAGAG CAAAAGACAT CTCCCTTTTA AACTTCTGAA TCACAGTGTA CTACTGGATT   
  
  
- ACCATGAACA TCCCAGTTCT AACTCTAACT CCCGTATGAC TGCTGGACAT ATTACCAGTT CATCCTAGTT   
  
  
- TCAGCTCTTT TGACAGAGAC GATTGAAAGT ATTAGATTGG GAATTGAGTG AGACTGGGAG TTAAATTAGT   
  
  
- CCGGATTAAG TTTGATAATT ACCAATATTG AGAGAAAGGA AGAAAACTCT AAAAAAAAAA GATGTACGCC   
  
  
- AACTTTTATT AAAAAAATTT TTTATATATA GTGATTGGTA AGTAATAGTA TCAAAATCTC AAGTATAAAT   
  
  
- ACACCAGGAA CAACTCAGAT TAAATTACGA AATCTACCGC ACTTCGTGGT TTAATCACCA TTGGTATTAT   
  
  
- TATTTGATAG TTCACAATTC AGCTTCTTTT GCCTGTGACG AGTTGGTTTA GATCATTGTA TCACATACTG   
  
  
- TACTATTGAT TCACACGGTA CATCATTAGT TAAGAAAGAA AAAAAAAAAA ACCAACTCTA GTGTATTTAA   
  
  
- TGCAATAGTT CCCATCATGC TATATTACCT ACTCCTCTTT AATTTACACT CTAAATCAAC TACTTAGCTT   
  
  
- ATGCAATCAG ACTCGGTAAA CCAGTTTTGA CGTAACCGAT TAGTTATCTT TAATATCTTT TATGTATTGT   
  
  
- ATTACCCTAC GTTAAAAGAT TAACCCCTTT GGTACAGTTA CGTCACTAAA ACCTGGTAAA TCCGGAATAA   
  
  
- GAAAAGTTAA AAAATTAACT AATAAATCTG AGTATACTTG AATAACAACT GTCTAACTCG ATTTTTGTGA   
  
  
- CTACAAAGTA TGTATACATA CTTAACGTGC ATGCTTCCGA GACGAGTAAA GCTCTTTCTT TTAATTGTCA   
  
  
- ATCCTCGTCA TATTAAATTG TTGTTCGCTA TTAATAATCA TACTTCACCA TGCTTAGATA AAATTTCCTC   
  
  
- ATGATACTTC ATGTTTGTTA TTGTTTATTA TTTCTCGTCA TGTAATTGTA TAGAATGATG GATTTATTTA   
  
  
- GCTAAGATTC TCATTTTTTT ACAACTTTAA TTGGGGTAAT ATCAAAATGA AATTGTTCTT CAAAAAGAAG   
  
  
- AAAATGGTAA ATGGTTCATT CTTACTAAGT CCGGCATATC GGTTCCTTTT TATCTTTTGG TGTTTTGATT   
  
  
- GGTACCGGGT TTTTTGTTGG TGGTTCTCCC CGTGATTACA CTGATTGGCT GCCAATTGTA ATAGTAGACA   
  
  
- AACTATGAAC AAGTAGAGAG TGATAAGCCC ATTATCACAC TTTAATGACT ACATGGGAGA TTTGAGCAGC   
  
  
- AAAATTGACA CATTCTTAGT CTAATTGTCA GTGCCTGTGT ACTCATTGTT TAGTTAAAGC CTGTACTTGC   
  
  
- TTGCTTGACA CTTAGGGGGG GTTTTTTTTT TAAAAAAATT TTTTTCAACG TAGGAATAGA AAGACTTGGT   
  
  
- CCGGTCGACA GAGTAAGGAA CCAGGAGCGC GGGGTAGTGC TCGCGACGAG TAATAAGTGG GAGAGAGAGA   
  
  
- GAGGAGAGAC TAGGTGTGGT GTTTGTGTTC CAAACGTGTC GACGTCTCGT CAGCATGATG ATAGTCATCA   
  
  
- CTTTTTGGTT CGTCTTTAGG GGTTAGGGAG AAGAGGAAGA AGGGTCTAAG AAGGAGTTAA GGTTCGGGTA   
  
  
- TAGTCTTTAA ATCTGTTGTT GTTGGTTTTT TTTTTTAATT ATGACGAACT CTTTTTGTTT ATACTATACT   
  
  
- TTAAGGAGCT TTTTCCGGAC GGTATTGTTT AAGGAGCACT TTTTCGTCGA CTCTCTTCTT CTTCTTCTTT   
  
  
- TTTTAGGGCA GTGTTAGAGT TAGAGCTAGG GTTTAACACT TTCTCTTTCT TTTCTACTTC TCTCTTGTGG   
  
  
- GGAAAAGCTT GGGACTGGTC TTTGGGGGCG GCCTCTACAG CGGCCGCGGC CCCTTCAGGT TCTACACCCT   
  
  
- ACTACTCTAC CGGGTCTTGC TGCGATTTTA CCTACTCGAC GAACGACAAC ACCCCATGTT CCACTCCAGC   
  
  
- AGCCTGTACC GCCTTTAACG GGTCTTCGAA CTCGTCGAAC TTCTTCAGTA CCCATCACAA GTTCTTCTGC   
  
  
- CCAATAGAAT GAACCGAAGG CTTTGACAAG TAATGTTAGG ACGTCTAGAC AGATGAACCG AACTTAGATA   
  
  
- CGAAAGACTC AAATTGGGAT TGGGATTAAA ACTGGGAAGA GGTAGCAGTT AGAGTGGGTA GTAGCTAGGT   
  
  
- CGAGGAGGTG AAGGAGCTTG GAGCAGCTGC CAGTTTAAGC TCGGGCTGGG CAAAAGGCTA AGCCTAGACT   
  
  
- TTCGTTAGGG TCCATTCCGA TAGAACTGGG GTGGTTCAAG ATCGTTATGA AGTTTAAACG CACTCCGATT   
  
  
- CTCCAACTTT GGGAGTTTAA TGTGTTGGCG GGGTTGCGGG TGGGGTGGAG GTTTTGACGG GTTAAGCCGC   
  
  
- AGAGGTGGTA GCCCGTGCTG CCGCAGCTCC GGCCACCACG ACCATCTGAG TGTTCTTTTG CCGTAGTCTA   
  
  
- ACCACGTACG TGAATACCGG ACACGGCTTC GTTAAGTTGA TCTTTTGTAC CCTAATCGAC TCCGAAACCA   
  
  
- ATTTGTCTAA TCCATAAATC GTCGTAGAGT TCGACCTGGA TACTCCTTTC ATCGTTGAAC AAAACGTCTT   
  
  
- CGAGAATGAA CAGCCTAGAT GTTCGATACG GGTAGACATG GGATGCTACT TAGTCAGAGT CTACTCAACG   
  
  
- TCTACGTGAA AATACTCTGA ACGGGTATAG AATTTAAGCG GGTAAAGTGC CGTTTAGTTC GTTAAAATCT   
  
  
- TCGTAAGTTA CCCTTCTTCT TTCAAGTACA CTAACTAAAG TCGTACTTCG TTCCGTACGT TACCGGCCGA   
  
  
- AACTACGTTC GGGACCGAGA AGCTGGCCTC CCAGGTGGCG AAAAGGCCAA TTGGCCCTAA CCTGGCGGGC   
  
  
- GAGGCCTGTT GAGCCTGGCC GACGTTCTCC ACCCAACCTT CGAACGGGTC AAGCGCCTAA GCTAGGCCTA   
  
  
- ATTTAAAGTC ATAGCACCCA AACACCGTTT GTCAAACTGG CTAGATCTTA GAAGGTACGA TCTAGAACTT   
  
  
- GGCCTGTGAC TCCACCACCG CCAGTTGAGC CAAAAGCTCG AGGTGGCCGA CAACCGATTT GGGCCCCGCT   
  
  
- AGCTCTTTCA CGACCCCAAG TACTCCCGGC ACTTGGGCCA GTAACACTGC TACCAGCTCG TCCTTCGCTT   
  
  
- GGTGTTGCCT GGCCAAAAGA ACCTGGCCAA GTTACTCAGC AACGTAATAA TGAGGTGGAA CAAACTAAGG   
  
  
- GAACTCTAAA CACAACTATT ACATCTATTC TTCTACAGCC TCCGAATGAA CCCGGTCGTC TAGACGTTGT   
  
  
- ACCACCGAAC ACTTCCCAGA CTGGCTCAGC TCTCCGTGCT CTGGGACCGA GTCACCGCTC GGGCCAAGCG   
  
  
- TAGACGGCCC AAGCTGGGTC AAGTAGACCC AAGCTTACGC AAATTCGTCC GCTCATACAA CAACCTCAAG   
  
  
- AAACGACCAC CACTTCCCAT GCCGCACCTC CTCGCTCTTC CCACAGAGTA CAACCCTACC GTATCATCCG   
  
  
- GCGAATAGTG GTGGAGCCGA ACCGTCGAGC GGTTCTTGGG CCGACACTTA GCCGCTAC

+     LTR

| Site Name | Organism | Position | Strand | Matrix score. | sequence | function |
| --- | --- | --- | --- | --- | --- | --- |
| LTR | Hordeum vulgare | 1530 | - | 6 | CCGAAA | cis-acting element involved in low-temperature responsiveness |
| LTR | Hordeum vulgare | 2263 | + | 6 | CCGAAA | cis-acting element involved in low-temperature responsiveness |

>HU10G00709.1   
+ -Up\_Stream \_Len000AACCCT TTAATTTTAT CAATGTGGGA CAACACTCAT CTTCATACTC CAACAAGCTA   
  
  
+ TGATACATTC GATTATTGCC TAGTATTTCG TTTAGTAAGA TATTAGTGCC AAACTTTGTA ATAAAAATGA   
  
  
+ AACTTACTAA AAAAAATCTC GTTTTCTGTA GAGGGAAAAT TTGAAGACTT AGTGTCACAT GATGACCTAA   
  
  
+ TGGTACTTGT AGGGTCAAGA TTGAGATTGA GGGCATACTG ACGACCTGTA TAATGGTCAA GTAGGATCAA   
  
  
+ AGTCGAGAAA ACTGTCTCTG CTAACTTTCA TAATCTAACC CTTAACTCAC TCTGACCCTC AATTTAATCA   
  
  
+ GGCCTAATTC AAACTATTAA TGGTTATAAC TCTCTTTCCT TCTTTTGAGA TTTTTTTTTT CTACATGCGG   
  
  
+ TTGAAAATAA TTTTTTTAAA AAATATATAT CACTAACCAT TCATTATCAT AGTTTTAGAG TTCATATTTA   
  
  
+ TGTGGTCCTT GTTGAGTCTA ATTTAATGCT TTAGATGGCG TGAAGCACCA AATTAGTGGT AACCATAATA   
  
  
+ ATAAACTATC AAGTGTTAAG TCGAAGAAAA CGGACACTGC TCAACCAAAT CTAGTAACAT AGTGTATGAC   
  
  
+ ATGATAACTA AGTGTGCCAT GTAGTAATCA ATTCTTTCTT TTTTTTTTTT TGGTTGAGAT CACATAAATT   
  
  
+ ACGTTATCAA GGGTAGTACG ATATAATGGA TGAGGAGAAA TTAAATGTGA GATTTAGTTG ATGAATCGAA   
  
  
+ TACGTTAGTC TGAGCCATTT GGTCAAAACT GCATTGGCTA ATCAATAGAA ATTATAGAAA ATACATAACA   
  
  
+ TAATGGGATG CAATTTTCTA ATTGGGGAAA CCATGTCAAT GCAGTGATTT TGGACCATTT AGGCCTTATT   
  
  
+ CTTTTCAATT TTTTAATTGA TTATTTAGAC TCATATGAAC TTATTGTTGA CAGATTGAGC TAAAAACACT   
  
  
+ GATGTTTCAT ACATATGTAT GAATTGCACG TACGAAGGCT CTGCTCATTT CGAGAAAGAA AATTAACAGT   
  
  
+ TAGGAGCAGT ATAATTTAAC AACAAGCGAT AATTATTAGT ATGAAGTGGT ACGAATCTAT TTTAAAGGAG   
  
  
+ TACTATGAAG TACAAACAAT AACAAATAAT AAAGAGCAGT ACATTAACAT ATCTTACTAC CTAAATAAAT   
  
  
+ CGATTCTAAG AGTAAAAAAA TGTTGAAATT AACCCCATTA TAGTTTTACT TTAACAAGAA GTTTTTCTTC   
  
  
+ TTTTACCATT TACCAAGTAA GAATGATTCA GGCCGTATAG CCAAGGAAAA ATAGAAAACC ACAAAACTAA   
  
  
+ CCATGGCCCA AAAAACAACC ACCAAGAGGG GCACTAATGT GACTAACCGA CGGTTAACAT TATCATCTGT   
  
  
+ TTGATACTTG TTCATCTCTC ACTATTCGGG TAATAGTGTG AAATTACTGA TGTACCCTCT AAACTCGTCG   
  
  
+ TTTTAACTGT GTAAGAATCA GATTAACAGT CACGGACACA TGAGTAACAA ATCAATTTCG GACATGAACG   
  
  
+ AACGAACTGT GAATCCCCCC CAAAAAAAAA ATTTTTTTAA AAAAAGTTGC ATCCTTATCT TTCTGAACCA   
  
  
+ GGCCAGCTGT CTCATTCCTT GGTCCTCGCG CCCCATCACG AGCGCTGCTC ATTATTCACC CTCTCTCTCT   
  
  
+ CTCCTCTCTG ATCCACACCA CAAACACAAG GTTTGCACAG CTGCAGAGCA GTCGTACTAC TATCAGTAGT   
  
  
+ GAAAAACCAA GCAGAAATCC CCAATCCCTC TTCTCCTTCT TCCCAGATTC TTCCTCAATT CCAAGCCCAT   
  
  
+ ATCAGAAATT TAGACAACAA CAACCAAAAA AAAAAATTAA TACTGCTTGA GAAAAACAAA TATGATATGA   
  
  
+ AATTCCTCGA AAAAGGCCTG CCATAACAAA TTCCTCGTGA AAAAGCAGCT GAGAGAAGAA GAAGAAGAAA   
  
  
+ AAAATCCCGT CACAATCTCA ATCTCGATCC CAAATTGTGA AAGAGAAAGA AAAGATGAAG AGAGAACACC   
  
  
+ CCTTTTCGAA CCCTGACCAG AAACCCCCGC CGGAGATGTC GCCGGCGCCG GGGAAGTCCA AGATGTGGGA   
  
  
+ TGATGAGATG GCCCAGAACG ACGCTAAAAT GGATGAGCTG CTTGCTGTTG TGGGGTACAA GGTGAGGTCG   
  
  
+ TCGGACATGG CGGAAATTGC CCAGAAGCTT GAGCAGCTTG AAGAAGTCAT GGGTAGTGTT CAAGAAGACG   
  
  
+ GGTTATCTTA CTTGGCTTCC GAAACTGTTC ATTACAATCC TGCAGATCTG TCTACTTGGC TTGAATCTAT   
  
  
+ GCTTTCTGAG TTTAACCCTA ACCCTAATTT TGACCCTTCT CCATCGTCAA TCTCACCCAT CATCGATCCA   
  
  
+ GCTCCTCCAC TTCCTCGAAC CTCGTCGACG GTCAAATTCG AGCCCGACCC GTTTTCCGAT TCGGATCTGA   
  
  
+ AAGCAATCCC AGGTAAGGCT ATCTTGACCC CACCAAGTTC TAGCAATACT TCAAATTTGC GTGAGGCTAA   
  
  
+ GAGGTTGAAA CCCTCAAATT ACACAACCGC CCCAACGCCC ACCCCACCTC CAAAACTGCC CAATTCGGCG   
  
  
+ TCTCCACCAT CGGGCACGAC GGCGTCGAGG CCGGTGGTGC TGGTAGACTC ACAAGAAAAC GGCATCAGAT   
  
  
+ TGGTGCATGC ACTTATGGCC TGTGCCGAAG CAATTCAACT AGAAAACATG GGATTAGCTG AGGCTTTGGT   
  
  
+ TAAACAGATT AGGTATTTAG CAGCATCTCA AGCTGGACCT ATGAGGAAAG TAGCAACTTG TTTTGCAGAA   
  
  
+ GCTCTTACTT GTCGGATCTA CAAGCTATGC CCATCTGTAC CCTACGATGA ATCAGTCTCA GATGAGTTGC   
  
  
+ AGATGCACTT TTATGAGACT TGCCCATATC TTAAATTCGC CCATTTCACG GCAAATCAAG CAATTTTAGA   
  
  
+ AGCATTCAAT GGGAAGAAGA AAGTTCATGT GATTGATTTC AGCATGAAGC AAGGCATGCA ATGGCCGGCT   
  
  
+ TTGATGCAAG CCCTGGCTCT TCGACCGGAG GGTCCACCGC TTTTCCGGTT AACCGGGATT GGACCGCCCG   
  
  
+ CTCCGGACAA CTCGGACCGG CTGCAAGAGG TGGGTTGGAA GCTTGCCCAG TTCGCGGATT CGATCCGGAT   
  
  
+ TAAATTTCAG TATCGTGGGT TTGTGGCAAA CAGTTTGACC GATCTAGAAT CTTCCATGCT AGATCTTGAA   
  
  
+ CCGGACACTG AGGTGGTGGC GGTCAACTCG GTTTTCGAGC TCCACCGGCT GTTGGCTAAA CCCGGGGCGA   
  
  
+ TCGAGAAAGT GCTGGGGTTC ATGAGGGCCG TGAACCCGGT CATTGTGACG ATGGTCGAGC AGGAAGCGAA   
  
  
+ CCACAACGGA CCGGTTTTCT TGGACCGGTT CAATGAGTCG TTGCATTATT ACTCCACCTT GTTTGATTCC   
  
  
+ CTTGAGATTT GTGTTGATAA TGTAGATAAG AAGATGTCGG AGGCTTACTT GGGCCAGCAG ATCTGCAACA   
  
  
+ TGGTGGCTTG TGAAGGGTCT GACCGAGTCG AGAGGCACGA GACCCTGGCT CAGTGGCGAG CCCGGTTCGC   
  
  
+ ATCTGCCGGG TTCGACCCAG TTCATCTGGG TTCGAATGCG TTTAAGCAGG CGAGTATGTT GTTGGAGTTC   
  
  
+ TTTGCTGGTG GTGAAGGGTA CGGCGTGGAG GAGCGAGAAG GGTGTCTCAT GTTGGGATGG CATAGTAGGC   
  
  
+ CGCTTATCAC CACCTCGGCT TGGCAGCTCG CCAAGAACCC GGCTGTGAAT CGGCGATG  

- -Up\_Stream \_Len000TTGGGA AATTAAAATA GTTACACCCT GTTGTGAGTA GAAGTATGAG GTTGTTCGAT   
  
  
- ACTATGTAAG CTAATAACGG ATCATAAAGC AAATCATTCT ATAATCACGG TTTGAAACAT TATTTTTACT   
  
  
- TTGAATGATT TTTTTTAGAG CAAAAGACAT CTCCCTTTTA AACTTCTGAA TCACAGTGTA CTACTGGATT   
  
  
- ACCATGAACA TCCCAGTTCT AACTCTAACT CCCGTATGAC TGCTGGACAT ATTACCAGTT CATCCTAGTT   
  
  
- TCAGCTCTTT TGACAGAGAC GATTGAAAGT ATTAGATTGG GAATTGAGTG AGACTGGGAG TTAAATTAGT   
  
  
- CCGGATTAAG TTTGATAATT ACCAATATTG AGAGAAAGGA AGAAAACTCT AAAAAAAAAA GATGTACGCC   
  
  
- AACTTTTATT AAAAAAATTT TTTATATATA GTGATTGGTA AGTAATAGTA TCAAAATCTC AAGTATAAAT   
  
  
- ACACCAGGAA CAACTCAGAT TAAATTACGA AATCTACCGC ACTTCGTGGT TTAATCACCA TTGGTATTAT   
  
  
- TATTTGATAG TTCACAATTC AGCTTCTTTT GCCTGTGACG AGTTGGTTTA GATCATTGTA TCACATACTG   
  
  
- TACTATTGAT TCACACGGTA CATCATTAGT TAAGAAAGAA AAAAAAAAAA ACCAACTCTA GTGTATTTAA   
  
  
- TGCAATAGTT CCCATCATGC TATATTACCT ACTCCTCTTT AATTTACACT CTAAATCAAC TACTTAGCTT   
  
  
- ATGCAATCAG ACTCGGTAAA CCAGTTTTGA CGTAACCGAT TAGTTATCTT TAATATCTTT TATGTATTGT   
  
  
- ATTACCCTAC GTTAAAAGAT TAACCCCTTT GGTACAGTTA CGTCACTAAA ACCTGGTAAA TCCGGAATAA   
  
  
- GAAAAGTTAA AAAATTAACT AATAAATCTG AGTATACTTG AATAACAACT GTCTAACTCG ATTTTTGTGA   
  
  
- CTACAAAGTA TGTATACATA CTTAACGTGC ATGCTTCCGA GACGAGTAAA GCTCTTTCTT TTAATTGTCA   
  
  
- ATCCTCGTCA TATTAAATTG TTGTTCGCTA TTAATAATCA TACTTCACCA TGCTTAGATA AAATTTCCTC   
  
  
- ATGATACTTC ATGTTTGTTA TTGTTTATTA TTTCTCGTCA TGTAATTGTA TAGAATGATG GATTTATTTA   
  
  
- GCTAAGATTC TCATTTTTTT ACAACTTTAA TTGGGGTAAT ATCAAAATGA AATTGTTCTT CAAAAAGAAG   
  
  
- AAAATGGTAA ATGGTTCATT CTTACTAAGT CCGGCATATC GGTTCCTTTT TATCTTTTGG TGTTTTGATT   
  
  
- GGTACCGGGT TTTTTGTTGG TGGTTCTCCC CGTGATTACA CTGATTGGCT GCCAATTGTA ATAGTAGACA   
  
  
- AACTATGAAC AAGTAGAGAG TGATAAGCCC ATTATCACAC TTTAATGACT ACATGGGAGA TTTGAGCAGC   
  
  
- AAAATTGACA CATTCTTAGT CTAATTGTCA GTGCCTGTGT ACTCATTGTT TAGTTAAAGC CTGTACTTGC   
  
  
- TTGCTTGACA CTTAGGGGGG GTTTTTTTTT TAAAAAAATT TTTTTCAACG TAGGAATAGA AAGACTTGGT   
  
  
- CCGGTCGACA GAGTAAGGAA CCAGGAGCGC GGGGTAGTGC TCGCGACGAG TAATAAGTGG GAGAGAGAGA   
  
  
- GAGGAGAGAC TAGGTGTGGT GTTTGTGTTC CAAACGTGTC GACGTCTCGT CAGCATGATG ATAGTCATCA   
  
  
- CTTTTTGGTT CGTCTTTAGG GGTTAGGGAG AAGAGGAAGA AGGGTCTAAG AAGGAGTTAA GGTTCGGGTA   
  
  
- TAGTCTTTAA ATCTGTTGTT GTTGGTTTTT TTTTTTAATT ATGACGAACT CTTTTTGTTT ATACTATACT   
  
  
- TTAAGGAGCT TTTTCCGGAC GGTATTGTTT AAGGAGCACT TTTTCGTCGA CTCTCTTCTT CTTCTTCTTT   
  
  
- TTTTAGGGCA GTGTTAGAGT TAGAGCTAGG GTTTAACACT TTCTCTTTCT TTTCTACTTC TCTCTTGTGG   
  
  
- GGAAAAGCTT GGGACTGGTC TTTGGGGGCG GCCTCTACAG CGGCCGCGGC CCCTTCAGGT TCTACACCCT   
  
  
- ACTACTCTAC CGGGTCTTGC TGCGATTTTA CCTACTCGAC GAACGACAAC ACCCCATGTT CCACTCCAGC   
  
  
- AGCCTGTACC GCCTTTAACG GGTCTTCGAA CTCGTCGAAC TTCTTCAGTA CCCATCACAA GTTCTTCTGC   
  
  
- CCAATAGAAT GAACCGAAGG CTTTGACAAG TAATGTTAGG ACGTCTAGAC AGATGAACCG AACTTAGATA   
  
  
- CGAAAGACTC AAATTGGGAT TGGGATTAAA ACTGGGAAGA GGTAGCAGTT AGAGTGGGTA GTAGCTAGGT   
  
  
- CGAGGAGGTG AAGGAGCTTG GAGCAGCTGC CAGTTTAAGC TCGGGCTGGG CAAAAGGCTA AGCCTAGACT   
  
  
- TTCGTTAGGG TCCATTCCGA TAGAACTGGG GTGGTTCAAG ATCGTTATGA AGTTTAAACG CACTCCGATT   
  
  
- CTCCAACTTT GGGAGTTTAA TGTGTTGGCG GGGTTGCGGG TGGGGTGGAG GTTTTGACGG GTTAAGCCGC   
  
  
- AGAGGTGGTA GCCCGTGCTG CCGCAGCTCC GGCCACCACG ACCATCTGAG TGTTCTTTTG CCGTAGTCTA   
  
  
- ACCACGTACG TGAATACCGG ACACGGCTTC GTTAAGTTGA TCTTTTGTAC CCTAATCGAC TCCGAAACCA   
  
  
- ATTTGTCTAA TCCATAAATC GTCGTAGAGT TCGACCTGGA TACTCCTTTC ATCGTTGAAC AAAACGTCTT   
  
  
- CGAGAATGAA CAGCCTAGAT GTTCGATACG GGTAGACATG GGATGCTACT TAGTCAGAGT CTACTCAACG   
  
  
- TCTACGTGAA AATACTCTGA ACGGGTATAG AATTTAAGCG GGTAAAGTGC CGTTTAGTTC GTTAAAATCT   
  
  
- TCGTAAGTTA CCCTTCTTCT TTCAAGTACA CTAACTAAAG TCGTACTTCG TTCCGTACGT TACCGGCCGA   
  
  
- AACTACGTTC GGGACCGAGA AGCTGGCCTC CCAGGTGGCG AAAAGGCCAA TTGGCCCTAA CCTGGCGGGC   
  
  
- GAGGCCTGTT GAGCCTGGCC GACGTTCTCC ACCCAACCTT CGAACGGGTC AAGCGCCTAA GCTAGGCCTA   
  
  
- ATTTAAAGTC ATAGCACCCA AACACCGTTT GTCAAACTGG CTAGATCTTA GAAGGTACGA TCTAGAACTT   
  
  
- GGCCTGTGAC TCCACCACCG CCAGTTGAGC CAAAAGCTCG AGGTGGCCGA CAACCGATTT GGGCCCCGCT   
  
  
- AGCTCTTTCA CGACCCCAAG TACTCCCGGC ACTTGGGCCA GTAACACTGC TACCAGCTCG TCCTTCGCTT   
  
  
- GGTGTTGCCT GGCCAAAAGA ACCTGGCCAA GTTACTCAGC AACGTAATAA TGAGGTGGAA CAAACTAAGG   
  
  
- GAACTCTAAA CACAACTATT ACATCTATTC TTCTACAGCC TCCGAATGAA CCCGGTCGTC TAGACGTTGT   
  
  
- ACCACCGAAC ACTTCCCAGA CTGGCTCAGC TCTCCGTGCT CTGGGACCGA GTCACCGCTC GGGCCAAGCG   
  
  
- TAGACGGCCC AAGCTGGGTC AAGTAGACCC AAGCTTACGC AAATTCGTCC GCTCATACAA CAACCTCAAG   
  
  
- AAACGACCAC CACTTCCCAT GCCGCACCTC CTCGCTCTTC CCACAGAGTA CAACCCTACC GTATCATCCG   
  
  
- GCGAATAGTG GTGGAGCCGA ACCGTCGAGC GGTTCTTGGG CCGACACTTA GCCGCTAC

+     MYB

| Site Name | Organism | Position | Strand | Matrix score. | sequence | function |
| --- | --- | --- | --- | --- | --- | --- |
| MYB | Arabidopsis thaliana | 685 | - | 6 | CAACCA |  |
| MYB | Arabidopsis thaliana | 554 | + | 6 | TAACCA |  |
| MYB | Arabidopsis thaliana | 2149 | - | 6 | CAACAG |  |
| MYB | Arabidopsis thaliana | 606 | + | 6 | CAACCA |  |
| MYB | Arabidopsis thaliana | 458 | + | 6 | TAACCA |  |
| MYB | Arabidopsis thaliana | 3273 | - | 6 | CAACAG |  |
| MYB | Arabidopsis thaliana | 1332 | + | 6 | TAACCA |  |
| MYB | Arabidopsis thaliana | 1845 | + | 6 | CAACCA |  |
| MYB | Arabidopsis thaliana | 375 | - | 6 | TAACCA |  |
| MYB | Arabidopsis thaliana | 2731 | - | 6 | TAACCA |  |
| MYB | Arabidopsis thaliana | 1350 | + | 6 | CAACCA |  |

>HU10G00709.1   
+ -Up\_Stream \_Len000AACCCT TTAATTTTAT CAATGTGGGA CAACACTCAT CTTCATACTC CAACAAGCTA   
  
  
+ TGATACATTC GATTATTGCC TAGTATTTCG TTTAGTAAGA TATTAGTGCC AAACTTTGTA ATAAAAATGA   
  
  
+ AACTTACTAA AAAAAATCTC GTTTTCTGTA GAGGGAAAAT TTGAAGACTT AGTGTCACAT GATGACCTAA   
  
  
+ TGGTACTTGT AGGGTCAAGA TTGAGATTGA GGGCATACTG ACGACCTGTA TAATGGTCAA GTAGGATCAA   
  
  
+ AGTCGAGAAA ACTGTCTCTG CTAACTTTCA TAATCTAACC CTTAACTCAC TCTGACCCTC AATTTAATCA   
  
  
+ GGCCTAATTC AAACTATTAA TGGTTATAAC TCTCTTTCCT TCTTTTGAGA TTTTTTTTTT CTACATGCGG   
  
  
+ TTGAAAATAA TTTTTTTAAA AAATATATAT CACTAACCAT TCATTATCAT AGTTTTAGAG TTCATATTTA   
  
  
+ TGTGGTCCTT GTTGAGTCTA ATTTAATGCT TTAGATGGCG TGAAGCACCA AATTAGTGGT AACCATAATA   
  
  
+ ATAAACTATC AAGTGTTAAG TCGAAGAAAA CGGACACTGC TCAACCAAAT CTAGTAACAT AGTGTATGAC   
  
  
+ ATGATAACTA AGTGTGCCAT GTAGTAATCA ATTCTTTCTT TTTTTTTTTT TGGTTGAGAT CACATAAATT   
  
  
+ ACGTTATCAA GGGTAGTACG ATATAATGGA TGAGGAGAAA TTAAATGTGA GATTTAGTTG ATGAATCGAA   
  
  
+ TACGTTAGTC TGAGCCATTT GGTCAAAACT GCATTGGCTA ATCAATAGAA ATTATAGAAA ATACATAACA   
  
  
+ TAATGGGATG CAATTTTCTA ATTGGGGAAA CCATGTCAAT GCAGTGATTT TGGACCATTT AGGCCTTATT   
  
  
+ CTTTTCAATT TTTTAATTGA TTATTTAGAC TCATATGAAC TTATTGTTGA CAGATTGAGC TAAAAACACT   
  
  
+ GATGTTTCAT ACATATGTAT GAATTGCACG TACGAAGGCT CTGCTCATTT CGAGAAAGAA AATTAACAGT   
  
  
+ TAGGAGCAGT ATAATTTAAC AACAAGCGAT AATTATTAGT ATGAAGTGGT ACGAATCTAT TTTAAAGGAG   
  
  
+ TACTATGAAG TACAAACAAT AACAAATAAT AAAGAGCAGT ACATTAACAT ATCTTACTAC CTAAATAAAT   
  
  
+ CGATTCTAAG AGTAAAAAAA TGTTGAAATT AACCCCATTA TAGTTTTACT TTAACAAGAA GTTTTTCTTC   
  
  
+ TTTTACCATT TACCAAGTAA GAATGATTCA GGCCGTATAG CCAAGGAAAA ATAGAAAACC ACAAAACTAA   
  
  
+ CCATGGCCCA AAAAACAACC ACCAAGAGGG GCACTAATGT GACTAACCGA CGGTTAACAT TATCATCTGT   
  
  
+ TTGATACTTG TTCATCTCTC ACTATTCGGG TAATAGTGTG AAATTACTGA TGTACCCTCT AAACTCGTCG   
  
  
+ TTTTAACTGT GTAAGAATCA GATTAACAGT CACGGACACA TGAGTAACAA ATCAATTTCG GACATGAACG   
  
  
+ AACGAACTGT GAATCCCCCC CAAAAAAAAA ATTTTTTTAA AAAAAGTTGC ATCCTTATCT TTCTGAACCA   
  
  
+ GGCCAGCTGT CTCATTCCTT GGTCCTCGCG CCCCATCACG AGCGCTGCTC ATTATTCACC CTCTCTCTCT   
  
  
+ CTCCTCTCTG ATCCACACCA CAAACACAAG GTTTGCACAG CTGCAGAGCA GTCGTACTAC TATCAGTAGT   
  
  
+ GAAAAACCAA GCAGAAATCC CCAATCCCTC TTCTCCTTCT TCCCAGATTC TTCCTCAATT CCAAGCCCAT   
  
  
+ ATCAGAAATT TAGACAACAA CAACCAAAAA AAAAAATTAA TACTGCTTGA GAAAAACAAA TATGATATGA   
  
  
+ AATTCCTCGA AAAAGGCCTG CCATAACAAA TTCCTCGTGA AAAAGCAGCT GAGAGAAGAA GAAGAAGAAA   
  
  
+ AAAATCCCGT CACAATCTCA ATCTCGATCC CAAATTGTGA AAGAGAAAGA AAAGATGAAG AGAGAACACC   
  
  
+ CCTTTTCGAA CCCTGACCAG AAACCCCCGC CGGAGATGTC GCCGGCGCCG GGGAAGTCCA AGATGTGGGA   
  
  
+ TGATGAGATG GCCCAGAACG ACGCTAAAAT GGATGAGCTG CTTGCTGTTG TGGGGTACAA GGTGAGGTCG   
  
  
+ TCGGACATGG CGGAAATTGC CCAGAAGCTT GAGCAGCTTG AAGAAGTCAT GGGTAGTGTT CAAGAAGACG   
  
  
+ GGTTATCTTA CTTGGCTTCC GAAACTGTTC ATTACAATCC TGCAGATCTG TCTACTTGGC TTGAATCTAT   
  
  
+ GCTTTCTGAG TTTAACCCTA ACCCTAATTT TGACCCTTCT CCATCGTCAA TCTCACCCAT CATCGATCCA   
  
  
+ GCTCCTCCAC TTCCTCGAAC CTCGTCGACG GTCAAATTCG AGCCCGACCC GTTTTCCGAT TCGGATCTGA   
  
  
+ AAGCAATCCC AGGTAAGGCT ATCTTGACCC CACCAAGTTC TAGCAATACT TCAAATTTGC GTGAGGCTAA   
  
  
+ GAGGTTGAAA CCCTCAAATT ACACAACCGC CCCAACGCCC ACCCCACCTC CAAAACTGCC CAATTCGGCG   
  
  
+ TCTCCACCAT CGGGCACGAC GGCGTCGAGG CCGGTGGTGC TGGTAGACTC ACAAGAAAAC GGCATCAGAT   
  
  
+ TGGTGCATGC ACTTATGGCC TGTGCCGAAG CAATTCAACT AGAAAACATG GGATTAGCTG AGGCTTTGGT   
  
  
+ TAAACAGATT AGGTATTTAG CAGCATCTCA AGCTGGACCT ATGAGGAAAG TAGCAACTTG TTTTGCAGAA   
  
  
+ GCTCTTACTT GTCGGATCTA CAAGCTATGC CCATCTGTAC CCTACGATGA ATCAGTCTCA GATGAGTTGC   
  
  
+ AGATGCACTT TTATGAGACT TGCCCATATC TTAAATTCGC CCATTTCACG GCAAATCAAG CAATTTTAGA   
  
  
+ AGCATTCAAT GGGAAGAAGA AAGTTCATGT GATTGATTTC AGCATGAAGC AAGGCATGCA ATGGCCGGCT   
  
  
+ TTGATGCAAG CCCTGGCTCT TCGACCGGAG GGTCCACCGC TTTTCCGGTT AACCGGGATT GGACCGCCCG   
  
  
+ CTCCGGACAA CTCGGACCGG CTGCAAGAGG TGGGTTGGAA GCTTGCCCAG TTCGCGGATT CGATCCGGAT   
  
  
+ TAAATTTCAG TATCGTGGGT TTGTGGCAAA CAGTTTGACC GATCTAGAAT CTTCCATGCT AGATCTTGAA   
  
  
+ CCGGACACTG AGGTGGTGGC GGTCAACTCG GTTTTCGAGC TCCACCGGCT GTTGGCTAAA CCCGGGGCGA   
  
  
+ TCGAGAAAGT GCTGGGGTTC ATGAGGGCCG TGAACCCGGT CATTGTGACG ATGGTCGAGC AGGAAGCGAA   
  
  
+ CCACAACGGA CCGGTTTTCT TGGACCGGTT CAATGAGTCG TTGCATTATT ACTCCACCTT GTTTGATTCC   
  
  
+ CTTGAGATTT GTGTTGATAA TGTAGATAAG AAGATGTCGG AGGCTTACTT GGGCCAGCAG ATCTGCAACA   
  
  
+ TGGTGGCTTG TGAAGGGTCT GACCGAGTCG AGAGGCACGA GACCCTGGCT CAGTGGCGAG CCCGGTTCGC   
  
  
+ ATCTGCCGGG TTCGACCCAG TTCATCTGGG TTCGAATGCG TTTAAGCAGG CGAGTATGTT GTTGGAGTTC   
  
  
+ TTTGCTGGTG GTGAAGGGTA CGGCGTGGAG GAGCGAGAAG GGTGTCTCAT GTTGGGATGG CATAGTAGGC   
  
  
+ CGCTTATCAC CACCTCGGCT TGGCAGCTCG CCAAGAACCC GGCTGTGAAT CGGCGATG  

- -Up\_Stream \_Len000TTGGGA AATTAAAATA GTTACACCCT GTTGTGAGTA GAAGTATGAG GTTGTTCGAT   
  
  
- ACTATGTAAG CTAATAACGG ATCATAAAGC AAATCATTCT ATAATCACGG TTTGAAACAT TATTTTTACT   
  
  
- TTGAATGATT TTTTTTAGAG CAAAAGACAT CTCCCTTTTA AACTTCTGAA TCACAGTGTA CTACTGGATT   
  
  
- ACCATGAACA TCCCAGTTCT AACTCTAACT CCCGTATGAC TGCTGGACAT ATTACCAGTT CATCCTAGTT   
  
  
- TCAGCTCTTT TGACAGAGAC GATTGAAAGT ATTAGATTGG GAATTGAGTG AGACTGGGAG TTAAATTAGT   
  
  
- CCGGATTAAG TTTGATAATT ACCAATATTG AGAGAAAGGA AGAAAACTCT AAAAAAAAAA GATGTACGCC   
  
  
- AACTTTTATT AAAAAAATTT TTTATATATA GTGATTGGTA AGTAATAGTA TCAAAATCTC AAGTATAAAT   
  
  
- ACACCAGGAA CAACTCAGAT TAAATTACGA AATCTACCGC ACTTCGTGGT TTAATCACCA TTGGTATTAT   
  
  
- TATTTGATAG TTCACAATTC AGCTTCTTTT GCCTGTGACG AGTTGGTTTA GATCATTGTA TCACATACTG   
  
  
- TACTATTGAT TCACACGGTA CATCATTAGT TAAGAAAGAA AAAAAAAAAA ACCAACTCTA GTGTATTTAA   
  
  
- TGCAATAGTT CCCATCATGC TATATTACCT ACTCCTCTTT AATTTACACT CTAAATCAAC TACTTAGCTT   
  
  
- ATGCAATCAG ACTCGGTAAA CCAGTTTTGA CGTAACCGAT TAGTTATCTT TAATATCTTT TATGTATTGT   
  
  
- ATTACCCTAC GTTAAAAGAT TAACCCCTTT GGTACAGTTA CGTCACTAAA ACCTGGTAAA TCCGGAATAA   
  
  
- GAAAAGTTAA AAAATTAACT AATAAATCTG AGTATACTTG AATAACAACT GTCTAACTCG ATTTTTGTGA   
  
  
- CTACAAAGTA TGTATACATA CTTAACGTGC ATGCTTCCGA GACGAGTAAA GCTCTTTCTT TTAATTGTCA   
  
  
- ATCCTCGTCA TATTAAATTG TTGTTCGCTA TTAATAATCA TACTTCACCA TGCTTAGATA AAATTTCCTC   
  
  
- ATGATACTTC ATGTTTGTTA TTGTTTATTA TTTCTCGTCA TGTAATTGTA TAGAATGATG GATTTATTTA   
  
  
- GCTAAGATTC TCATTTTTTT ACAACTTTAA TTGGGGTAAT ATCAAAATGA AATTGTTCTT CAAAAAGAAG   
  
  
- AAAATGGTAA ATGGTTCATT CTTACTAAGT CCGGCATATC GGTTCCTTTT TATCTTTTGG TGTTTTGATT   
  
  
- GGTACCGGGT TTTTTGTTGG TGGTTCTCCC CGTGATTACA CTGATTGGCT GCCAATTGTA ATAGTAGACA   
  
  
- AACTATGAAC AAGTAGAGAG TGATAAGCCC ATTATCACAC TTTAATGACT ACATGGGAGA TTTGAGCAGC   
  
  
- AAAATTGACA CATTCTTAGT CTAATTGTCA GTGCCTGTGT ACTCATTGTT TAGTTAAAGC CTGTACTTGC   
  
  
- TTGCTTGACA CTTAGGGGGG GTTTTTTTTT TAAAAAAATT TTTTTCAACG TAGGAATAGA AAGACTTGGT   
  
  
- CCGGTCGACA GAGTAAGGAA CCAGGAGCGC GGGGTAGTGC TCGCGACGAG TAATAAGTGG GAGAGAGAGA   
  
  
- GAGGAGAGAC TAGGTGTGGT GTTTGTGTTC CAAACGTGTC GACGTCTCGT CAGCATGATG ATAGTCATCA   
  
  
- CTTTTTGGTT CGTCTTTAGG GGTTAGGGAG AAGAGGAAGA AGGGTCTAAG AAGGAGTTAA GGTTCGGGTA   
  
  
- TAGTCTTTAA ATCTGTTGTT GTTGGTTTTT TTTTTTAATT ATGACGAACT CTTTTTGTTT ATACTATACT   
  
  
- TTAAGGAGCT TTTTCCGGAC GGTATTGTTT AAGGAGCACT TTTTCGTCGA CTCTCTTCTT CTTCTTCTTT   
  
  
- TTTTAGGGCA GTGTTAGAGT TAGAGCTAGG GTTTAACACT TTCTCTTTCT TTTCTACTTC TCTCTTGTGG   
  
  
- GGAAAAGCTT GGGACTGGTC TTTGGGGGCG GCCTCTACAG CGGCCGCGGC CCCTTCAGGT TCTACACCCT   
  
  
- ACTACTCTAC CGGGTCTTGC TGCGATTTTA CCTACTCGAC GAACGACAAC ACCCCATGTT CCACTCCAGC   
  
  
- AGCCTGTACC GCCTTTAACG GGTCTTCGAA CTCGTCGAAC TTCTTCAGTA CCCATCACAA GTTCTTCTGC   
  
  
- CCAATAGAAT GAACCGAAGG CTTTGACAAG TAATGTTAGG ACGTCTAGAC AGATGAACCG AACTTAGATA   
  
  
- CGAAAGACTC AAATTGGGAT TGGGATTAAA ACTGGGAAGA GGTAGCAGTT AGAGTGGGTA GTAGCTAGGT   
  
  
- CGAGGAGGTG AAGGAGCTTG GAGCAGCTGC CAGTTTAAGC TCGGGCTGGG CAAAAGGCTA AGCCTAGACT   
  
  
- TTCGTTAGGG TCCATTCCGA TAGAACTGGG GTGGTTCAAG ATCGTTATGA AGTTTAAACG CACTCCGATT   
  
  
- CTCCAACTTT GGGAGTTTAA TGTGTTGGCG GGGTTGCGGG TGGGGTGGAG GTTTTGACGG GTTAAGCCGC   
  
  
- AGAGGTGGTA GCCCGTGCTG CCGCAGCTCC GGCCACCACG ACCATCTGAG TGTTCTTTTG CCGTAGTCTA   
  
  
- ACCACGTACG TGAATACCGG ACACGGCTTC GTTAAGTTGA TCTTTTGTAC CCTAATCGAC TCCGAAACCA   
  
  
- ATTTGTCTAA TCCATAAATC GTCGTAGAGT TCGACCTGGA TACTCCTTTC ATCGTTGAAC AAAACGTCTT   
  
  
- CGAGAATGAA CAGCCTAGAT GTTCGATACG GGTAGACATG GGATGCTACT TAGTCAGAGT CTACTCAACG   
  
  
- TCTACGTGAA AATACTCTGA ACGGGTATAG AATTTAAGCG GGTAAAGTGC CGTTTAGTTC GTTAAAATCT   
  
  
- TCGTAAGTTA CCCTTCTTCT TTCAAGTACA CTAACTAAAG TCGTACTTCG TTCCGTACGT TACCGGCCGA   
  
  
- AACTACGTTC GGGACCGAGA AGCTGGCCTC CCAGGTGGCG AAAAGGCCAA TTGGCCCTAA CCTGGCGGGC   
  
  
- GAGGCCTGTT GAGCCTGGCC GACGTTCTCC ACCCAACCTT CGAACGGGTC AAGCGCCTAA GCTAGGCCTA   
  
  
- ATTTAAAGTC ATAGCACCCA AACACCGTTT GTCAAACTGG CTAGATCTTA GAAGGTACGA TCTAGAACTT   
  
  
- GGCCTGTGAC TCCACCACCG CCAGTTGAGC CAAAAGCTCG AGGTGGCCGA CAACCGATTT GGGCCCCGCT   
  
  
- AGCTCTTTCA CGACCCCAAG TACTCCCGGC ACTTGGGCCA GTAACACTGC TACCAGCTCG TCCTTCGCTT   
  
  
- GGTGTTGCCT GGCCAAAAGA ACCTGGCCAA GTTACTCAGC AACGTAATAA TGAGGTGGAA CAAACTAAGG   
  
  
- GAACTCTAAA CACAACTATT ACATCTATTC TTCTACAGCC TCCGAATGAA CCCGGTCGTC TAGACGTTGT   
  
  
- ACCACCGAAC ACTTCCCAGA CTGGCTCAGC TCTCCGTGCT CTGGGACCGA GTCACCGCTC GGGCCAAGCG   
  
  
- TAGACGGCCC AAGCTGGGTC AAGTAGACCC AAGCTTACGC AAATTCGTCC GCTCATACAA CAACCTCAAG   
  
  
- AAACGACCAC CACTTCCCAT GCCGCACCTC CTCGCTCTTC CCACAGAGTA CAACCCTACC GTATCATCCG   
  
  
- GCGAATAGTG GTGGAGCCGA ACCGTCGAGC GGTTCTTGGG CCGACACTTA GCCGCTAC

+     MYB recognition site

| Site Name | Organism | Position | Strand | Matrix score. | sequence | function |
| --- | --- | --- | --- | --- | --- | --- |
| MYB recognition site | Arabidopsis thaliana | 3368 | - | 6 | CCGTTG |  |

>HU10G00709.1   
+ -Up\_Stream \_Len000AACCCT TTAATTTTAT CAATGTGGGA CAACACTCAT CTTCATACTC CAACAAGCTA   
  
  
+ TGATACATTC GATTATTGCC TAGTATTTCG TTTAGTAAGA TATTAGTGCC AAACTTTGTA ATAAAAATGA   
  
  
+ AACTTACTAA AAAAAATCTC GTTTTCTGTA GAGGGAAAAT TTGAAGACTT AGTGTCACAT GATGACCTAA   
  
  
+ TGGTACTTGT AGGGTCAAGA TTGAGATTGA GGGCATACTG ACGACCTGTA TAATGGTCAA GTAGGATCAA   
  
  
+ AGTCGAGAAA ACTGTCTCTG CTAACTTTCA TAATCTAACC CTTAACTCAC TCTGACCCTC AATTTAATCA   
  
  
+ GGCCTAATTC AAACTATTAA TGGTTATAAC TCTCTTTCCT TCTTTTGAGA TTTTTTTTTT CTACATGCGG   
  
  
+ TTGAAAATAA TTTTTTTAAA AAATATATAT CACTAACCAT TCATTATCAT AGTTTTAGAG TTCATATTTA   
  
  
+ TGTGGTCCTT GTTGAGTCTA ATTTAATGCT TTAGATGGCG TGAAGCACCA AATTAGTGGT AACCATAATA   
  
  
+ ATAAACTATC AAGTGTTAAG TCGAAGAAAA CGGACACTGC TCAACCAAAT CTAGTAACAT AGTGTATGAC   
  
  
+ ATGATAACTA AGTGTGCCAT GTAGTAATCA ATTCTTTCTT TTTTTTTTTT TGGTTGAGAT CACATAAATT   
  
  
+ ACGTTATCAA GGGTAGTACG ATATAATGGA TGAGGAGAAA TTAAATGTGA GATTTAGTTG ATGAATCGAA   
  
  
+ TACGTTAGTC TGAGCCATTT GGTCAAAACT GCATTGGCTA ATCAATAGAA ATTATAGAAA ATACATAACA   
  
  
+ TAATGGGATG CAATTTTCTA ATTGGGGAAA CCATGTCAAT GCAGTGATTT TGGACCATTT AGGCCTTATT   
  
  
+ CTTTTCAATT TTTTAATTGA TTATTTAGAC TCATATGAAC TTATTGTTGA CAGATTGAGC TAAAAACACT   
  
  
+ GATGTTTCAT ACATATGTAT GAATTGCACG TACGAAGGCT CTGCTCATTT CGAGAAAGAA AATTAACAGT   
  
  
+ TAGGAGCAGT ATAATTTAAC AACAAGCGAT AATTATTAGT ATGAAGTGGT ACGAATCTAT TTTAAAGGAG   
  
  
+ TACTATGAAG TACAAACAAT AACAAATAAT AAAGAGCAGT ACATTAACAT ATCTTACTAC CTAAATAAAT   
  
  
+ CGATTCTAAG AGTAAAAAAA TGTTGAAATT AACCCCATTA TAGTTTTACT TTAACAAGAA GTTTTTCTTC   
  
  
+ TTTTACCATT TACCAAGTAA GAATGATTCA GGCCGTATAG CCAAGGAAAA ATAGAAAACC ACAAAACTAA   
  
  
+ CCATGGCCCA AAAAACAACC ACCAAGAGGG GCACTAATGT GACTAACCGA CGGTTAACAT TATCATCTGT   
  
  
+ TTGATACTTG TTCATCTCTC ACTATTCGGG TAATAGTGTG AAATTACTGA TGTACCCTCT AAACTCGTCG   
  
  
+ TTTTAACTGT GTAAGAATCA GATTAACAGT CACGGACACA TGAGTAACAA ATCAATTTCG GACATGAACG   
  
  
+ AACGAACTGT GAATCCCCCC CAAAAAAAAA ATTTTTTTAA AAAAAGTTGC ATCCTTATCT TTCTGAACCA   
  
  
+ GGCCAGCTGT CTCATTCCTT GGTCCTCGCG CCCCATCACG AGCGCTGCTC ATTATTCACC CTCTCTCTCT   
  
  
+ CTCCTCTCTG ATCCACACCA CAAACACAAG GTTTGCACAG CTGCAGAGCA GTCGTACTAC TATCAGTAGT   
  
  
+ GAAAAACCAA GCAGAAATCC CCAATCCCTC TTCTCCTTCT TCCCAGATTC TTCCTCAATT CCAAGCCCAT   
  
  
+ ATCAGAAATT TAGACAACAA CAACCAAAAA AAAAAATTAA TACTGCTTGA GAAAAACAAA TATGATATGA   
  
  
+ AATTCCTCGA AAAAGGCCTG CCATAACAAA TTCCTCGTGA AAAAGCAGCT GAGAGAAGAA GAAGAAGAAA   
  
  
+ AAAATCCCGT CACAATCTCA ATCTCGATCC CAAATTGTGA AAGAGAAAGA AAAGATGAAG AGAGAACACC   
  
  
+ CCTTTTCGAA CCCTGACCAG AAACCCCCGC CGGAGATGTC GCCGGCGCCG GGGAAGTCCA AGATGTGGGA   
  
  
+ TGATGAGATG GCCCAGAACG ACGCTAAAAT GGATGAGCTG CTTGCTGTTG TGGGGTACAA GGTGAGGTCG   
  
  
+ TCGGACATGG CGGAAATTGC CCAGAAGCTT GAGCAGCTTG AAGAAGTCAT GGGTAGTGTT CAAGAAGACG   
  
  
+ GGTTATCTTA CTTGGCTTCC GAAACTGTTC ATTACAATCC TGCAGATCTG TCTACTTGGC TTGAATCTAT   
  
  
+ GCTTTCTGAG TTTAACCCTA ACCCTAATTT TGACCCTTCT CCATCGTCAA TCTCACCCAT CATCGATCCA   
  
  
+ GCTCCTCCAC TTCCTCGAAC CTCGTCGACG GTCAAATTCG AGCCCGACCC GTTTTCCGAT TCGGATCTGA   
  
  
+ AAGCAATCCC AGGTAAGGCT ATCTTGACCC CACCAAGTTC TAGCAATACT TCAAATTTGC GTGAGGCTAA   
  
  
+ GAGGTTGAAA CCCTCAAATT ACACAACCGC CCCAACGCCC ACCCCACCTC CAAAACTGCC CAATTCGGCG   
  
  
+ TCTCCACCAT CGGGCACGAC GGCGTCGAGG CCGGTGGTGC TGGTAGACTC ACAAGAAAAC GGCATCAGAT   
  
  
+ TGGTGCATGC ACTTATGGCC TGTGCCGAAG CAATTCAACT AGAAAACATG GGATTAGCTG AGGCTTTGGT   
  
  
+ TAAACAGATT AGGTATTTAG CAGCATCTCA AGCTGGACCT ATGAGGAAAG TAGCAACTTG TTTTGCAGAA   
  
  
+ GCTCTTACTT GTCGGATCTA CAAGCTATGC CCATCTGTAC CCTACGATGA ATCAGTCTCA GATGAGTTGC   
  
  
+ AGATGCACTT TTATGAGACT TGCCCATATC TTAAATTCGC CCATTTCACG GCAAATCAAG CAATTTTAGA   
  
  
+ AGCATTCAAT GGGAAGAAGA AAGTTCATGT GATTGATTTC AGCATGAAGC AAGGCATGCA ATGGCCGGCT   
  
  
+ TTGATGCAAG CCCTGGCTCT TCGACCGGAG GGTCCACCGC TTTTCCGGTT AACCGGGATT GGACCGCCCG   
  
  
+ CTCCGGACAA CTCGGACCGG CTGCAAGAGG TGGGTTGGAA GCTTGCCCAG TTCGCGGATT CGATCCGGAT   
  
  
+ TAAATTTCAG TATCGTGGGT TTGTGGCAAA CAGTTTGACC GATCTAGAAT CTTCCATGCT AGATCTTGAA   
  
  
+ CCGGACACTG AGGTGGTGGC GGTCAACTCG GTTTTCGAGC TCCACCGGCT GTTGGCTAAA CCCGGGGCGA   
  
  
+ TCGAGAAAGT GCTGGGGTTC ATGAGGGCCG TGAACCCGGT CATTGTGACG ATGGTCGAGC AGGAAGCGAA   
  
  
+ CCACAACGGA CCGGTTTTCT TGGACCGGTT CAATGAGTCG TTGCATTATT ACTCCACCTT GTTTGATTCC   
  
  
+ CTTGAGATTT GTGTTGATAA TGTAGATAAG AAGATGTCGG AGGCTTACTT GGGCCAGCAG ATCTGCAACA   
  
  
+ TGGTGGCTTG TGAAGGGTCT GACCGAGTCG AGAGGCACGA GACCCTGGCT CAGTGGCGAG CCCGGTTCGC   
  
  
+ ATCTGCCGGG TTCGACCCAG TTCATCTGGG TTCGAATGCG TTTAAGCAGG CGAGTATGTT GTTGGAGTTC   
  
  
+ TTTGCTGGTG GTGAAGGGTA CGGCGTGGAG GAGCGAGAAG GGTGTCTCAT GTTGGGATGG CATAGTAGGC   
  
  
+ CGCTTATCAC CACCTCGGCT TGGCAGCTCG CCAAGAACCC GGCTGTGAAT CGGCGATG  

- -Up\_Stream \_Len000TTGGGA AATTAAAATA GTTACACCCT GTTGTGAGTA GAAGTATGAG GTTGTTCGAT   
  
  
- ACTATGTAAG CTAATAACGG ATCATAAAGC AAATCATTCT ATAATCACGG TTTGAAACAT TATTTTTACT   
  
  
- TTGAATGATT TTTTTTAGAG CAAAAGACAT CTCCCTTTTA AACTTCTGAA TCACAGTGTA CTACTGGATT   
  
  
- ACCATGAACA TCCCAGTTCT AACTCTAACT CCCGTATGAC TGCTGGACAT ATTACCAGTT CATCCTAGTT   
  
  
- TCAGCTCTTT TGACAGAGAC GATTGAAAGT ATTAGATTGG GAATTGAGTG AGACTGGGAG TTAAATTAGT   
  
  
- CCGGATTAAG TTTGATAATT ACCAATATTG AGAGAAAGGA AGAAAACTCT AAAAAAAAAA GATGTACGCC   
  
  
- AACTTTTATT AAAAAAATTT TTTATATATA GTGATTGGTA AGTAATAGTA TCAAAATCTC AAGTATAAAT   
  
  
- ACACCAGGAA CAACTCAGAT TAAATTACGA AATCTACCGC ACTTCGTGGT TTAATCACCA TTGGTATTAT   
  
  
- TATTTGATAG TTCACAATTC AGCTTCTTTT GCCTGTGACG AGTTGGTTTA GATCATTGTA TCACATACTG   
  
  
- TACTATTGAT TCACACGGTA CATCATTAGT TAAGAAAGAA AAAAAAAAAA ACCAACTCTA GTGTATTTAA   
  
  
- TGCAATAGTT CCCATCATGC TATATTACCT ACTCCTCTTT AATTTACACT CTAAATCAAC TACTTAGCTT   
  
  
- ATGCAATCAG ACTCGGTAAA CCAGTTTTGA CGTAACCGAT TAGTTATCTT TAATATCTTT TATGTATTGT   
  
  
- ATTACCCTAC GTTAAAAGAT TAACCCCTTT GGTACAGTTA CGTCACTAAA ACCTGGTAAA TCCGGAATAA   
  
  
- GAAAAGTTAA AAAATTAACT AATAAATCTG AGTATACTTG AATAACAACT GTCTAACTCG ATTTTTGTGA   
  
  
- CTACAAAGTA TGTATACATA CTTAACGTGC ATGCTTCCGA GACGAGTAAA GCTCTTTCTT TTAATTGTCA   
  
  
- ATCCTCGTCA TATTAAATTG TTGTTCGCTA TTAATAATCA TACTTCACCA TGCTTAGATA AAATTTCCTC   
  
  
- ATGATACTTC ATGTTTGTTA TTGTTTATTA TTTCTCGTCA TGTAATTGTA TAGAATGATG GATTTATTTA   
  
  
- GCTAAGATTC TCATTTTTTT ACAACTTTAA TTGGGGTAAT ATCAAAATGA AATTGTTCTT CAAAAAGAAG   
  
  
- AAAATGGTAA ATGGTTCATT CTTACTAAGT CCGGCATATC GGTTCCTTTT TATCTTTTGG TGTTTTGATT   
  
  
- GGTACCGGGT TTTTTGTTGG TGGTTCTCCC CGTGATTACA CTGATTGGCT GCCAATTGTA ATAGTAGACA   
  
  
- AACTATGAAC AAGTAGAGAG TGATAAGCCC ATTATCACAC TTTAATGACT ACATGGGAGA TTTGAGCAGC   
  
  
- AAAATTGACA CATTCTTAGT CTAATTGTCA GTGCCTGTGT ACTCATTGTT TAGTTAAAGC CTGTACTTGC   
  
  
- TTGCTTGACA CTTAGGGGGG GTTTTTTTTT TAAAAAAATT TTTTTCAACG TAGGAATAGA AAGACTTGGT   
  
  
- CCGGTCGACA GAGTAAGGAA CCAGGAGCGC GGGGTAGTGC TCGCGACGAG TAATAAGTGG GAGAGAGAGA   
  
  
- GAGGAGAGAC TAGGTGTGGT GTTTGTGTTC CAAACGTGTC GACGTCTCGT CAGCATGATG ATAGTCATCA   
  
  
- CTTTTTGGTT CGTCTTTAGG GGTTAGGGAG AAGAGGAAGA AGGGTCTAAG AAGGAGTTAA GGTTCGGGTA   
  
  
- TAGTCTTTAA ATCTGTTGTT GTTGGTTTTT TTTTTTAATT ATGACGAACT CTTTTTGTTT ATACTATACT   
  
  
- TTAAGGAGCT TTTTCCGGAC GGTATTGTTT AAGGAGCACT TTTTCGTCGA CTCTCTTCTT CTTCTTCTTT   
  
  
- TTTTAGGGCA GTGTTAGAGT TAGAGCTAGG GTTTAACACT TTCTCTTTCT TTTCTACTTC TCTCTTGTGG   
  
  
- GGAAAAGCTT GGGACTGGTC TTTGGGGGCG GCCTCTACAG CGGCCGCGGC CCCTTCAGGT TCTACACCCT   
  
  
- ACTACTCTAC CGGGTCTTGC TGCGATTTTA CCTACTCGAC GAACGACAAC ACCCCATGTT CCACTCCAGC   
  
  
- AGCCTGTACC GCCTTTAACG GGTCTTCGAA CTCGTCGAAC TTCTTCAGTA CCCATCACAA GTTCTTCTGC   
  
  
- CCAATAGAAT GAACCGAAGG CTTTGACAAG TAATGTTAGG ACGTCTAGAC AGATGAACCG AACTTAGATA   
  
  
- CGAAAGACTC AAATTGGGAT TGGGATTAAA ACTGGGAAGA GGTAGCAGTT AGAGTGGGTA GTAGCTAGGT   
  
  
- CGAGGAGGTG AAGGAGCTTG GAGCAGCTGC CAGTTTAAGC TCGGGCTGGG CAAAAGGCTA AGCCTAGACT   
  
  
- TTCGTTAGGG TCCATTCCGA TAGAACTGGG GTGGTTCAAG ATCGTTATGA AGTTTAAACG CACTCCGATT   
  
  
- CTCCAACTTT GGGAGTTTAA TGTGTTGGCG GGGTTGCGGG TGGGGTGGAG GTTTTGACGG GTTAAGCCGC   
  
  
- AGAGGTGGTA GCCCGTGCTG CCGCAGCTCC GGCCACCACG ACCATCTGAG TGTTCTTTTG CCGTAGTCTA   
  
  
- ACCACGTACG TGAATACCGG ACACGGCTTC GTTAAGTTGA TCTTTTGTAC CCTAATCGAC TCCGAAACCA   
  
  
- ATTTGTCTAA TCCATAAATC GTCGTAGAGT TCGACCTGGA TACTCCTTTC ATCGTTGAAC AAAACGTCTT   
  
  
- CGAGAATGAA CAGCCTAGAT GTTCGATACG GGTAGACATG GGATGCTACT TAGTCAGAGT CTACTCAACG   
  
  
- TCTACGTGAA AATACTCTGA ACGGGTATAG AATTTAAGCG GGTAAAGTGC CGTTTAGTTC GTTAAAATCT   
  
  
- TCGTAAGTTA CCCTTCTTCT TTCAAGTACA CTAACTAAAG TCGTACTTCG TTCCGTACGT TACCGGCCGA   
  
  
- AACTACGTTC GGGACCGAGA AGCTGGCCTC CCAGGTGGCG AAAAGGCCAA TTGGCCCTAA CCTGGCGGGC   
  
  
- GAGGCCTGTT GAGCCTGGCC GACGTTCTCC ACCCAACCTT CGAACGGGTC AAGCGCCTAA GCTAGGCCTA   
  
  
- ATTTAAAGTC ATAGCACCCA AACACCGTTT GTCAAACTGG CTAGATCTTA GAAGGTACGA TCTAGAACTT   
  
  
- GGCCTGTGAC TCCACCACCG CCAGTTGAGC CAAAAGCTCG AGGTGGCCGA CAACCGATTT GGGCCCCGCT   
  
  
- AGCTCTTTCA CGACCCCAAG TACTCCCGGC ACTTGGGCCA GTAACACTGC TACCAGCTCG TCCTTCGCTT   
  
  
- GGTGTTGCCT GGCCAAAAGA ACCTGGCCAA GTTACTCAGC AACGTAATAA TGAGGTGGAA CAAACTAAGG   
  
  
- GAACTCTAAA CACAACTATT ACATCTATTC TTCTACAGCC TCCGAATGAA CCCGGTCGTC TAGACGTTGT   
  
  
- ACCACCGAAC ACTTCCCAGA CTGGCTCAGC TCTCCGTGCT CTGGGACCGA GTCACCGCTC GGGCCAAGCG   
  
  
- TAGACGGCCC AAGCTGGGTC AAGTAGACCC AAGCTTACGC AAATTCGTCC GCTCATACAA CAACCTCAAG   
  
  
- AAACGACCAC CACTTCCCAT GCCGCACCTC CTCGCTCTTC CCACAGAGTA CAACCCTACC GTATCATCCG   
  
  
- GCGAATAGTG GTGGAGCCGA ACCGTCGAGC GGTTCTTGGG CCGACACTTA GCCGCTAC

+     MYB-like sequence

| Site Name | Organism | Position | Strand | Matrix score. | sequence | function |
| --- | --- | --- | --- | --- | --- | --- |
| MYB-like sequence | Arabidopsis thaliana | 2731 | - | 6 | TAACCA |  |
| MYB-like sequence | Arabidopsis thaliana | 554 | + | 6 | TAACCA |  |
| MYB-like sequence | Arabidopsis thaliana | 1332 | + | 6 | TAACCA |  |
| MYB-like sequence | Arabidopsis thaliana | 458 | + | 6 | TAACCA |  |
| MYB-like sequence | Arabidopsis thaliana | 375 | - | 6 | TAACCA |  |

>HU10G00709.1   
+ -Up\_Stream \_Len000AACCCT TTAATTTTAT CAATGTGGGA CAACACTCAT CTTCATACTC CAACAAGCTA   
  
  
+ TGATACATTC GATTATTGCC TAGTATTTCG TTTAGTAAGA TATTAGTGCC AAACTTTGTA ATAAAAATGA   
  
  
+ AACTTACTAA AAAAAATCTC GTTTTCTGTA GAGGGAAAAT TTGAAGACTT AGTGTCACAT GATGACCTAA   
  
  
+ TGGTACTTGT AGGGTCAAGA TTGAGATTGA GGGCATACTG ACGACCTGTA TAATGGTCAA GTAGGATCAA   
  
  
+ AGTCGAGAAA ACTGTCTCTG CTAACTTTCA TAATCTAACC CTTAACTCAC TCTGACCCTC AATTTAATCA   
  
  
+ GGCCTAATTC AAACTATTAA TGGTTATAAC TCTCTTTCCT TCTTTTGAGA TTTTTTTTTT CTACATGCGG   
  
  
+ TTGAAAATAA TTTTTTTAAA AAATATATAT CACTAACCAT TCATTATCAT AGTTTTAGAG TTCATATTTA   
  
  
+ TGTGGTCCTT GTTGAGTCTA ATTTAATGCT TTAGATGGCG TGAAGCACCA AATTAGTGGT AACCATAATA   
  
  
+ ATAAACTATC AAGTGTTAAG TCGAAGAAAA CGGACACTGC TCAACCAAAT CTAGTAACAT AGTGTATGAC   
  
  
+ ATGATAACTA AGTGTGCCAT GTAGTAATCA ATTCTTTCTT TTTTTTTTTT TGGTTGAGAT CACATAAATT   
  
  
+ ACGTTATCAA GGGTAGTACG ATATAATGGA TGAGGAGAAA TTAAATGTGA GATTTAGTTG ATGAATCGAA   
  
  
+ TACGTTAGTC TGAGCCATTT GGTCAAAACT GCATTGGCTA ATCAATAGAA ATTATAGAAA ATACATAACA   
  
  
+ TAATGGGATG CAATTTTCTA ATTGGGGAAA CCATGTCAAT GCAGTGATTT TGGACCATTT AGGCCTTATT   
  
  
+ CTTTTCAATT TTTTAATTGA TTATTTAGAC TCATATGAAC TTATTGTTGA CAGATTGAGC TAAAAACACT   
  
  
+ GATGTTTCAT ACATATGTAT GAATTGCACG TACGAAGGCT CTGCTCATTT CGAGAAAGAA AATTAACAGT   
  
  
+ TAGGAGCAGT ATAATTTAAC AACAAGCGAT AATTATTAGT ATGAAGTGGT ACGAATCTAT TTTAAAGGAG   
  
  
+ TACTATGAAG TACAAACAAT AACAAATAAT AAAGAGCAGT ACATTAACAT ATCTTACTAC CTAAATAAAT   
  
  
+ CGATTCTAAG AGTAAAAAAA TGTTGAAATT AACCCCATTA TAGTTTTACT TTAACAAGAA GTTTTTCTTC   
  
  
+ TTTTACCATT TACCAAGTAA GAATGATTCA GGCCGTATAG CCAAGGAAAA ATAGAAAACC ACAAAACTAA   
  
  
+ CCATGGCCCA AAAAACAACC ACCAAGAGGG GCACTAATGT GACTAACCGA CGGTTAACAT TATCATCTGT   
  
  
+ TTGATACTTG TTCATCTCTC ACTATTCGGG TAATAGTGTG AAATTACTGA TGTACCCTCT AAACTCGTCG   
  
  
+ TTTTAACTGT GTAAGAATCA GATTAACAGT CACGGACACA TGAGTAACAA ATCAATTTCG GACATGAACG   
  
  
+ AACGAACTGT GAATCCCCCC CAAAAAAAAA ATTTTTTTAA AAAAAGTTGC ATCCTTATCT TTCTGAACCA   
  
  
+ GGCCAGCTGT CTCATTCCTT GGTCCTCGCG CCCCATCACG AGCGCTGCTC ATTATTCACC CTCTCTCTCT   
  
  
+ CTCCTCTCTG ATCCACACCA CAAACACAAG GTTTGCACAG CTGCAGAGCA GTCGTACTAC TATCAGTAGT   
  
  
+ GAAAAACCAA GCAGAAATCC CCAATCCCTC TTCTCCTTCT TCCCAGATTC TTCCTCAATT CCAAGCCCAT   
  
  
+ ATCAGAAATT TAGACAACAA CAACCAAAAA AAAAAATTAA TACTGCTTGA GAAAAACAAA TATGATATGA   
  
  
+ AATTCCTCGA AAAAGGCCTG CCATAACAAA TTCCTCGTGA AAAAGCAGCT GAGAGAAGAA GAAGAAGAAA   
  
  
+ AAAATCCCGT CACAATCTCA ATCTCGATCC CAAATTGTGA AAGAGAAAGA AAAGATGAAG AGAGAACACC   
  
  
+ CCTTTTCGAA CCCTGACCAG AAACCCCCGC CGGAGATGTC GCCGGCGCCG GGGAAGTCCA AGATGTGGGA   
  
  
+ TGATGAGATG GCCCAGAACG ACGCTAAAAT GGATGAGCTG CTTGCTGTTG TGGGGTACAA GGTGAGGTCG   
  
  
+ TCGGACATGG CGGAAATTGC CCAGAAGCTT GAGCAGCTTG AAGAAGTCAT GGGTAGTGTT CAAGAAGACG   
  
  
+ GGTTATCTTA CTTGGCTTCC GAAACTGTTC ATTACAATCC TGCAGATCTG TCTACTTGGC TTGAATCTAT   
  
  
+ GCTTTCTGAG TTTAACCCTA ACCCTAATTT TGACCCTTCT CCATCGTCAA TCTCACCCAT CATCGATCCA   
  
  
+ GCTCCTCCAC TTCCTCGAAC CTCGTCGACG GTCAAATTCG AGCCCGACCC GTTTTCCGAT TCGGATCTGA   
  
  
+ AAGCAATCCC AGGTAAGGCT ATCTTGACCC CACCAAGTTC TAGCAATACT TCAAATTTGC GTGAGGCTAA   
  
  
+ GAGGTTGAAA CCCTCAAATT ACACAACCGC CCCAACGCCC ACCCCACCTC CAAAACTGCC CAATTCGGCG   
  
  
+ TCTCCACCAT CGGGCACGAC GGCGTCGAGG CCGGTGGTGC TGGTAGACTC ACAAGAAAAC GGCATCAGAT   
  
  
+ TGGTGCATGC ACTTATGGCC TGTGCCGAAG CAATTCAACT AGAAAACATG GGATTAGCTG AGGCTTTGGT   
  
  
+ TAAACAGATT AGGTATTTAG CAGCATCTCA AGCTGGACCT ATGAGGAAAG TAGCAACTTG TTTTGCAGAA   
  
  
+ GCTCTTACTT GTCGGATCTA CAAGCTATGC CCATCTGTAC CCTACGATGA ATCAGTCTCA GATGAGTTGC   
  
  
+ AGATGCACTT TTATGAGACT TGCCCATATC TTAAATTCGC CCATTTCACG GCAAATCAAG CAATTTTAGA   
  
  
+ AGCATTCAAT GGGAAGAAGA AAGTTCATGT GATTGATTTC AGCATGAAGC AAGGCATGCA ATGGCCGGCT   
  
  
+ TTGATGCAAG CCCTGGCTCT TCGACCGGAG GGTCCACCGC TTTTCCGGTT AACCGGGATT GGACCGCCCG   
  
  
+ CTCCGGACAA CTCGGACCGG CTGCAAGAGG TGGGTTGGAA GCTTGCCCAG TTCGCGGATT CGATCCGGAT   
  
  
+ TAAATTTCAG TATCGTGGGT TTGTGGCAAA CAGTTTGACC GATCTAGAAT CTTCCATGCT AGATCTTGAA   
  
  
+ CCGGACACTG AGGTGGTGGC GGTCAACTCG GTTTTCGAGC TCCACCGGCT GTTGGCTAAA CCCGGGGCGA   
  
  
+ TCGAGAAAGT GCTGGGGTTC ATGAGGGCCG TGAACCCGGT CATTGTGACG ATGGTCGAGC AGGAAGCGAA   
  
  
+ CCACAACGGA CCGGTTTTCT TGGACCGGTT CAATGAGTCG TTGCATTATT ACTCCACCTT GTTTGATTCC   
  
  
+ CTTGAGATTT GTGTTGATAA TGTAGATAAG AAGATGTCGG AGGCTTACTT GGGCCAGCAG ATCTGCAACA   
  
  
+ TGGTGGCTTG TGAAGGGTCT GACCGAGTCG AGAGGCACGA GACCCTGGCT CAGTGGCGAG CCCGGTTCGC   
  
  
+ ATCTGCCGGG TTCGACCCAG TTCATCTGGG TTCGAATGCG TTTAAGCAGG CGAGTATGTT GTTGGAGTTC   
  
  
+ TTTGCTGGTG GTGAAGGGTA CGGCGTGGAG GAGCGAGAAG GGTGTCTCAT GTTGGGATGG CATAGTAGGC   
  
  
+ CGCTTATCAC CACCTCGGCT TGGCAGCTCG CCAAGAACCC GGCTGTGAAT CGGCGATG  

- -Up\_Stream \_Len000TTGGGA AATTAAAATA GTTACACCCT GTTGTGAGTA GAAGTATGAG GTTGTTCGAT   
  
  
- ACTATGTAAG CTAATAACGG ATCATAAAGC AAATCATTCT ATAATCACGG TTTGAAACAT TATTTTTACT   
  
  
- TTGAATGATT TTTTTTAGAG CAAAAGACAT CTCCCTTTTA AACTTCTGAA TCACAGTGTA CTACTGGATT   
  
  
- ACCATGAACA TCCCAGTTCT AACTCTAACT CCCGTATGAC TGCTGGACAT ATTACCAGTT CATCCTAGTT   
  
  
- TCAGCTCTTT TGACAGAGAC GATTGAAAGT ATTAGATTGG GAATTGAGTG AGACTGGGAG TTAAATTAGT   
  
  
- CCGGATTAAG TTTGATAATT ACCAATATTG AGAGAAAGGA AGAAAACTCT AAAAAAAAAA GATGTACGCC   
  
  
- AACTTTTATT AAAAAAATTT TTTATATATA GTGATTGGTA AGTAATAGTA TCAAAATCTC AAGTATAAAT   
  
  
- ACACCAGGAA CAACTCAGAT TAAATTACGA AATCTACCGC ACTTCGTGGT TTAATCACCA TTGGTATTAT   
  
  
- TATTTGATAG TTCACAATTC AGCTTCTTTT GCCTGTGACG AGTTGGTTTA GATCATTGTA TCACATACTG   
  
  
- TACTATTGAT TCACACGGTA CATCATTAGT TAAGAAAGAA AAAAAAAAAA ACCAACTCTA GTGTATTTAA   
  
  
- TGCAATAGTT CCCATCATGC TATATTACCT ACTCCTCTTT AATTTACACT CTAAATCAAC TACTTAGCTT   
  
  
- ATGCAATCAG ACTCGGTAAA CCAGTTTTGA CGTAACCGAT TAGTTATCTT TAATATCTTT TATGTATTGT   
  
  
- ATTACCCTAC GTTAAAAGAT TAACCCCTTT GGTACAGTTA CGTCACTAAA ACCTGGTAAA TCCGGAATAA   
  
  
- GAAAAGTTAA AAAATTAACT AATAAATCTG AGTATACTTG AATAACAACT GTCTAACTCG ATTTTTGTGA   
  
  
- CTACAAAGTA TGTATACATA CTTAACGTGC ATGCTTCCGA GACGAGTAAA GCTCTTTCTT TTAATTGTCA   
  
  
- ATCCTCGTCA TATTAAATTG TTGTTCGCTA TTAATAATCA TACTTCACCA TGCTTAGATA AAATTTCCTC   
  
  
- ATGATACTTC ATGTTTGTTA TTGTTTATTA TTTCTCGTCA TGTAATTGTA TAGAATGATG GATTTATTTA   
  
  
- GCTAAGATTC TCATTTTTTT ACAACTTTAA TTGGGGTAAT ATCAAAATGA AATTGTTCTT CAAAAAGAAG   
  
  
- AAAATGGTAA ATGGTTCATT CTTACTAAGT CCGGCATATC GGTTCCTTTT TATCTTTTGG TGTTTTGATT   
  
  
- GGTACCGGGT TTTTTGTTGG TGGTTCTCCC CGTGATTACA CTGATTGGCT GCCAATTGTA ATAGTAGACA   
  
  
- AACTATGAAC AAGTAGAGAG TGATAAGCCC ATTATCACAC TTTAATGACT ACATGGGAGA TTTGAGCAGC   
  
  
- AAAATTGACA CATTCTTAGT CTAATTGTCA GTGCCTGTGT ACTCATTGTT TAGTTAAAGC CTGTACTTGC   
  
  
- TTGCTTGACA CTTAGGGGGG GTTTTTTTTT TAAAAAAATT TTTTTCAACG TAGGAATAGA AAGACTTGGT   
  
  
- CCGGTCGACA GAGTAAGGAA CCAGGAGCGC GGGGTAGTGC TCGCGACGAG TAATAAGTGG GAGAGAGAGA   
  
  
- GAGGAGAGAC TAGGTGTGGT GTTTGTGTTC CAAACGTGTC GACGTCTCGT CAGCATGATG ATAGTCATCA   
  
  
- CTTTTTGGTT CGTCTTTAGG GGTTAGGGAG AAGAGGAAGA AGGGTCTAAG AAGGAGTTAA GGTTCGGGTA   
  
  
- TAGTCTTTAA ATCTGTTGTT GTTGGTTTTT TTTTTTAATT ATGACGAACT CTTTTTGTTT ATACTATACT   
  
  
- TTAAGGAGCT TTTTCCGGAC GGTATTGTTT AAGGAGCACT TTTTCGTCGA CTCTCTTCTT CTTCTTCTTT   
  
  
- TTTTAGGGCA GTGTTAGAGT TAGAGCTAGG GTTTAACACT TTCTCTTTCT TTTCTACTTC TCTCTTGTGG   
  
  
- GGAAAAGCTT GGGACTGGTC TTTGGGGGCG GCCTCTACAG CGGCCGCGGC CCCTTCAGGT TCTACACCCT   
  
  
- ACTACTCTAC CGGGTCTTGC TGCGATTTTA CCTACTCGAC GAACGACAAC ACCCCATGTT CCACTCCAGC   
  
  
- AGCCTGTACC GCCTTTAACG GGTCTTCGAA CTCGTCGAAC TTCTTCAGTA CCCATCACAA GTTCTTCTGC   
  
  
- CCAATAGAAT GAACCGAAGG CTTTGACAAG TAATGTTAGG ACGTCTAGAC AGATGAACCG AACTTAGATA   
  
  
- CGAAAGACTC AAATTGGGAT TGGGATTAAA ACTGGGAAGA GGTAGCAGTT AGAGTGGGTA GTAGCTAGGT   
  
  
- CGAGGAGGTG AAGGAGCTTG GAGCAGCTGC CAGTTTAAGC TCGGGCTGGG CAAAAGGCTA AGCCTAGACT   
  
  
- TTCGTTAGGG TCCATTCCGA TAGAACTGGG GTGGTTCAAG ATCGTTATGA AGTTTAAACG CACTCCGATT   
  
  
- CTCCAACTTT GGGAGTTTAA TGTGTTGGCG GGGTTGCGGG TGGGGTGGAG GTTTTGACGG GTTAAGCCGC   
  
  
- AGAGGTGGTA GCCCGTGCTG CCGCAGCTCC GGCCACCACG ACCATCTGAG TGTTCTTTTG CCGTAGTCTA   
  
  
- ACCACGTACG TGAATACCGG ACACGGCTTC GTTAAGTTGA TCTTTTGTAC CCTAATCGAC TCCGAAACCA   
  
  
- ATTTGTCTAA TCCATAAATC GTCGTAGAGT TCGACCTGGA TACTCCTTTC ATCGTTGAAC AAAACGTCTT   
  
  
- CGAGAATGAA CAGCCTAGAT GTTCGATACG GGTAGACATG GGATGCTACT TAGTCAGAGT CTACTCAACG   
  
  
- TCTACGTGAA AATACTCTGA ACGGGTATAG AATTTAAGCG GGTAAAGTGC CGTTTAGTTC GTTAAAATCT   
  
  
- TCGTAAGTTA CCCTTCTTCT TTCAAGTACA CTAACTAAAG TCGTACTTCG TTCCGTACGT TACCGGCCGA   
  
  
- AACTACGTTC GGGACCGAGA AGCTGGCCTC CCAGGTGGCG AAAAGGCCAA TTGGCCCTAA CCTGGCGGGC   
  
  
- GAGGCCTGTT GAGCCTGGCC GACGTTCTCC ACCCAACCTT CGAACGGGTC AAGCGCCTAA GCTAGGCCTA   
  
  
- ATTTAAAGTC ATAGCACCCA AACACCGTTT GTCAAACTGG CTAGATCTTA GAAGGTACGA TCTAGAACTT   
  
  
- GGCCTGTGAC TCCACCACCG CCAGTTGAGC CAAAAGCTCG AGGTGGCCGA CAACCGATTT GGGCCCCGCT   
  
  
- AGCTCTTTCA CGACCCCAAG TACTCCCGGC ACTTGGGCCA GTAACACTGC TACCAGCTCG TCCTTCGCTT   
  
  
- GGTGTTGCCT GGCCAAAAGA ACCTGGCCAA GTTACTCAGC AACGTAATAA TGAGGTGGAA CAAACTAAGG   
  
  
- GAACTCTAAA CACAACTATT ACATCTATTC TTCTACAGCC TCCGAATGAA CCCGGTCGTC TAGACGTTGT   
  
  
- ACCACCGAAC ACTTCCCAGA CTGGCTCAGC TCTCCGTGCT CTGGGACCGA GTCACCGCTC GGGCCAAGCG   
  
  
- TAGACGGCCC AAGCTGGGTC AAGTAGACCC AAGCTTACGC AAATTCGTCC GCTCATACAA CAACCTCAAG   
  
  
- AAACGACCAC CACTTCCCAT GCCGCACCTC CTCGCTCTTC CCACAGAGTA CAACCCTACC GTATCATCCG   
  
  
- GCGAATAGTG GTGGAGCCGA ACCGTCGAGC GGTTCTTGGG CCGACACTTA GCCGCTAC

+     MYC

| Site Name | Organism | Position | Strand | Matrix score. | sequence | function |
| --- | --- | --- | --- | --- | --- | --- |
| MYC | Arabidopsis thaliana | 2970 | + | 6 | CATGTG |  |
| MYC | Arabidopsis thaliana | 1511 | - | 6 | CATGTG |  |
| MYC | Arabidopsis thaliana | 790 | + | 6 | CATTTG |  |
| MYC | Arabidopsis thaliana | 200 | - | 6 | CATGTG |  |

>HU10G00709.1   
+ -Up\_Stream \_Len000AACCCT TTAATTTTAT CAATGTGGGA CAACACTCAT CTTCATACTC CAACAAGCTA   
  
  
+ TGATACATTC GATTATTGCC TAGTATTTCG TTTAGTAAGA TATTAGTGCC AAACTTTGTA ATAAAAATGA   
  
  
+ AACTTACTAA AAAAAATCTC GTTTTCTGTA GAGGGAAAAT TTGAAGACTT AGTGTCACAT GATGACCTAA   
  
  
+ TGGTACTTGT AGGGTCAAGA TTGAGATTGA GGGCATACTG ACGACCTGTA TAATGGTCAA GTAGGATCAA   
  
  
+ AGTCGAGAAA ACTGTCTCTG CTAACTTTCA TAATCTAACC CTTAACTCAC TCTGACCCTC AATTTAATCA   
  
  
+ GGCCTAATTC AAACTATTAA TGGTTATAAC TCTCTTTCCT TCTTTTGAGA TTTTTTTTTT CTACATGCGG   
  
  
+ TTGAAAATAA TTTTTTTAAA AAATATATAT CACTAACCAT TCATTATCAT AGTTTTAGAG TTCATATTTA   
  
  
+ TGTGGTCCTT GTTGAGTCTA ATTTAATGCT TTAGATGGCG TGAAGCACCA AATTAGTGGT AACCATAATA   
  
  
+ ATAAACTATC AAGTGTTAAG TCGAAGAAAA CGGACACTGC TCAACCAAAT CTAGTAACAT AGTGTATGAC   
  
  
+ ATGATAACTA AGTGTGCCAT GTAGTAATCA ATTCTTTCTT TTTTTTTTTT TGGTTGAGAT CACATAAATT   
  
  
+ ACGTTATCAA GGGTAGTACG ATATAATGGA TGAGGAGAAA TTAAATGTGA GATTTAGTTG ATGAATCGAA   
  
  
+ TACGTTAGTC TGAGCCATTT GGTCAAAACT GCATTGGCTA ATCAATAGAA ATTATAGAAA ATACATAACA   
  
  
+ TAATGGGATG CAATTTTCTA ATTGGGGAAA CCATGTCAAT GCAGTGATTT TGGACCATTT AGGCCTTATT   
  
  
+ CTTTTCAATT TTTTAATTGA TTATTTAGAC TCATATGAAC TTATTGTTGA CAGATTGAGC TAAAAACACT   
  
  
+ GATGTTTCAT ACATATGTAT GAATTGCACG TACGAAGGCT CTGCTCATTT CGAGAAAGAA AATTAACAGT   
  
  
+ TAGGAGCAGT ATAATTTAAC AACAAGCGAT AATTATTAGT ATGAAGTGGT ACGAATCTAT TTTAAAGGAG   
  
  
+ TACTATGAAG TACAAACAAT AACAAATAAT AAAGAGCAGT ACATTAACAT ATCTTACTAC CTAAATAAAT   
  
  
+ CGATTCTAAG AGTAAAAAAA TGTTGAAATT AACCCCATTA TAGTTTTACT TTAACAAGAA GTTTTTCTTC   
  
  
+ TTTTACCATT TACCAAGTAA GAATGATTCA GGCCGTATAG CCAAGGAAAA ATAGAAAACC ACAAAACTAA   
  
  
+ CCATGGCCCA AAAAACAACC ACCAAGAGGG GCACTAATGT GACTAACCGA CGGTTAACAT TATCATCTGT   
  
  
+ TTGATACTTG TTCATCTCTC ACTATTCGGG TAATAGTGTG AAATTACTGA TGTACCCTCT AAACTCGTCG   
  
  
+ TTTTAACTGT GTAAGAATCA GATTAACAGT CACGGACACA TGAGTAACAA ATCAATTTCG GACATGAACG   
  
  
+ AACGAACTGT GAATCCCCCC CAAAAAAAAA ATTTTTTTAA AAAAAGTTGC ATCCTTATCT TTCTGAACCA   
  
  
+ GGCCAGCTGT CTCATTCCTT GGTCCTCGCG CCCCATCACG AGCGCTGCTC ATTATTCACC CTCTCTCTCT   
  
  
+ CTCCTCTCTG ATCCACACCA CAAACACAAG GTTTGCACAG CTGCAGAGCA GTCGTACTAC TATCAGTAGT   
  
  
+ GAAAAACCAA GCAGAAATCC CCAATCCCTC TTCTCCTTCT TCCCAGATTC TTCCTCAATT CCAAGCCCAT   
  
  
+ ATCAGAAATT TAGACAACAA CAACCAAAAA AAAAAATTAA TACTGCTTGA GAAAAACAAA TATGATATGA   
  
  
+ AATTCCTCGA AAAAGGCCTG CCATAACAAA TTCCTCGTGA AAAAGCAGCT GAGAGAAGAA GAAGAAGAAA   
  
  
+ AAAATCCCGT CACAATCTCA ATCTCGATCC CAAATTGTGA AAGAGAAAGA AAAGATGAAG AGAGAACACC   
  
  
+ CCTTTTCGAA CCCTGACCAG AAACCCCCGC CGGAGATGTC GCCGGCGCCG GGGAAGTCCA AGATGTGGGA   
  
  
+ TGATGAGATG GCCCAGAACG ACGCTAAAAT GGATGAGCTG CTTGCTGTTG TGGGGTACAA GGTGAGGTCG   
  
  
+ TCGGACATGG CGGAAATTGC CCAGAAGCTT GAGCAGCTTG AAGAAGTCAT GGGTAGTGTT CAAGAAGACG   
  
  
+ GGTTATCTTA CTTGGCTTCC GAAACTGTTC ATTACAATCC TGCAGATCTG TCTACTTGGC TTGAATCTAT   
  
  
+ GCTTTCTGAG TTTAACCCTA ACCCTAATTT TGACCCTTCT CCATCGTCAA TCTCACCCAT CATCGATCCA   
  
  
+ GCTCCTCCAC TTCCTCGAAC CTCGTCGACG GTCAAATTCG AGCCCGACCC GTTTTCCGAT TCGGATCTGA   
  
  
+ AAGCAATCCC AGGTAAGGCT ATCTTGACCC CACCAAGTTC TAGCAATACT TCAAATTTGC GTGAGGCTAA   
  
  
+ GAGGTTGAAA CCCTCAAATT ACACAACCGC CCCAACGCCC ACCCCACCTC CAAAACTGCC CAATTCGGCG   
  
  
+ TCTCCACCAT CGGGCACGAC GGCGTCGAGG CCGGTGGTGC TGGTAGACTC ACAAGAAAAC GGCATCAGAT   
  
  
+ TGGTGCATGC ACTTATGGCC TGTGCCGAAG CAATTCAACT AGAAAACATG GGATTAGCTG AGGCTTTGGT   
  
  
+ TAAACAGATT AGGTATTTAG CAGCATCTCA AGCTGGACCT ATGAGGAAAG TAGCAACTTG TTTTGCAGAA   
  
  
+ GCTCTTACTT GTCGGATCTA CAAGCTATGC CCATCTGTAC CCTACGATGA ATCAGTCTCA GATGAGTTGC   
  
  
+ AGATGCACTT TTATGAGACT TGCCCATATC TTAAATTCGC CCATTTCACG GCAAATCAAG CAATTTTAGA   
  
  
+ AGCATTCAAT GGGAAGAAGA AAGTTCATGT GATTGATTTC AGCATGAAGC AAGGCATGCA ATGGCCGGCT   
  
  
+ TTGATGCAAG CCCTGGCTCT TCGACCGGAG GGTCCACCGC TTTTCCGGTT AACCGGGATT GGACCGCCCG   
  
  
+ CTCCGGACAA CTCGGACCGG CTGCAAGAGG TGGGTTGGAA GCTTGCCCAG TTCGCGGATT CGATCCGGAT   
  
  
+ TAAATTTCAG TATCGTGGGT TTGTGGCAAA CAGTTTGACC GATCTAGAAT CTTCCATGCT AGATCTTGAA   
  
  
+ CCGGACACTG AGGTGGTGGC GGTCAACTCG GTTTTCGAGC TCCACCGGCT GTTGGCTAAA CCCGGGGCGA   
  
  
+ TCGAGAAAGT GCTGGGGTTC ATGAGGGCCG TGAACCCGGT CATTGTGACG ATGGTCGAGC AGGAAGCGAA   
  
  
+ CCACAACGGA CCGGTTTTCT TGGACCGGTT CAATGAGTCG TTGCATTATT ACTCCACCTT GTTTGATTCC   
  
  
+ CTTGAGATTT GTGTTGATAA TGTAGATAAG AAGATGTCGG AGGCTTACTT GGGCCAGCAG ATCTGCAACA   
  
  
+ TGGTGGCTTG TGAAGGGTCT GACCGAGTCG AGAGGCACGA GACCCTGGCT CAGTGGCGAG CCCGGTTCGC   
  
  
+ ATCTGCCGGG TTCGACCCAG TTCATCTGGG TTCGAATGCG TTTAAGCAGG CGAGTATGTT GTTGGAGTTC   
  
  
+ TTTGCTGGTG GTGAAGGGTA CGGCGTGGAG GAGCGAGAAG GGTGTCTCAT GTTGGGATGG CATAGTAGGC   
  
  
+ CGCTTATCAC CACCTCGGCT TGGCAGCTCG CCAAGAACCC GGCTGTGAAT CGGCGATG  

- -Up\_Stream \_Len000TTGGGA AATTAAAATA GTTACACCCT GTTGTGAGTA GAAGTATGAG GTTGTTCGAT   
  
  
- ACTATGTAAG CTAATAACGG ATCATAAAGC AAATCATTCT ATAATCACGG TTTGAAACAT TATTTTTACT   
  
  
- TTGAATGATT TTTTTTAGAG CAAAAGACAT CTCCCTTTTA AACTTCTGAA TCACAGTGTA CTACTGGATT   
  
  
- ACCATGAACA TCCCAGTTCT AACTCTAACT CCCGTATGAC TGCTGGACAT ATTACCAGTT CATCCTAGTT   
  
  
- TCAGCTCTTT TGACAGAGAC GATTGAAAGT ATTAGATTGG GAATTGAGTG AGACTGGGAG TTAAATTAGT   
  
  
- CCGGATTAAG TTTGATAATT ACCAATATTG AGAGAAAGGA AGAAAACTCT AAAAAAAAAA GATGTACGCC   
  
  
- AACTTTTATT AAAAAAATTT TTTATATATA GTGATTGGTA AGTAATAGTA TCAAAATCTC AAGTATAAAT   
  
  
- ACACCAGGAA CAACTCAGAT TAAATTACGA AATCTACCGC ACTTCGTGGT TTAATCACCA TTGGTATTAT   
  
  
- TATTTGATAG TTCACAATTC AGCTTCTTTT GCCTGTGACG AGTTGGTTTA GATCATTGTA TCACATACTG   
  
  
- TACTATTGAT TCACACGGTA CATCATTAGT TAAGAAAGAA AAAAAAAAAA ACCAACTCTA GTGTATTTAA   
  
  
- TGCAATAGTT CCCATCATGC TATATTACCT ACTCCTCTTT AATTTACACT CTAAATCAAC TACTTAGCTT   
  
  
- ATGCAATCAG ACTCGGTAAA CCAGTTTTGA CGTAACCGAT TAGTTATCTT TAATATCTTT TATGTATTGT   
  
  
- ATTACCCTAC GTTAAAAGAT TAACCCCTTT GGTACAGTTA CGTCACTAAA ACCTGGTAAA TCCGGAATAA   
  
  
- GAAAAGTTAA AAAATTAACT AATAAATCTG AGTATACTTG AATAACAACT GTCTAACTCG ATTTTTGTGA   
  
  
- CTACAAAGTA TGTATACATA CTTAACGTGC ATGCTTCCGA GACGAGTAAA GCTCTTTCTT TTAATTGTCA   
  
  
- ATCCTCGTCA TATTAAATTG TTGTTCGCTA TTAATAATCA TACTTCACCA TGCTTAGATA AAATTTCCTC   
  
  
- ATGATACTTC ATGTTTGTTA TTGTTTATTA TTTCTCGTCA TGTAATTGTA TAGAATGATG GATTTATTTA   
  
  
- GCTAAGATTC TCATTTTTTT ACAACTTTAA TTGGGGTAAT ATCAAAATGA AATTGTTCTT CAAAAAGAAG   
  
  
- AAAATGGTAA ATGGTTCATT CTTACTAAGT CCGGCATATC GGTTCCTTTT TATCTTTTGG TGTTTTGATT   
  
  
- GGTACCGGGT TTTTTGTTGG TGGTTCTCCC CGTGATTACA CTGATTGGCT GCCAATTGTA ATAGTAGACA   
  
  
- AACTATGAAC AAGTAGAGAG TGATAAGCCC ATTATCACAC TTTAATGACT ACATGGGAGA TTTGAGCAGC   
  
  
- AAAATTGACA CATTCTTAGT CTAATTGTCA GTGCCTGTGT ACTCATTGTT TAGTTAAAGC CTGTACTTGC   
  
  
- TTGCTTGACA CTTAGGGGGG GTTTTTTTTT TAAAAAAATT TTTTTCAACG TAGGAATAGA AAGACTTGGT   
  
  
- CCGGTCGACA GAGTAAGGAA CCAGGAGCGC GGGGTAGTGC TCGCGACGAG TAATAAGTGG GAGAGAGAGA   
  
  
- GAGGAGAGAC TAGGTGTGGT GTTTGTGTTC CAAACGTGTC GACGTCTCGT CAGCATGATG ATAGTCATCA   
  
  
- CTTTTTGGTT CGTCTTTAGG GGTTAGGGAG AAGAGGAAGA AGGGTCTAAG AAGGAGTTAA GGTTCGGGTA   
  
  
- TAGTCTTTAA ATCTGTTGTT GTTGGTTTTT TTTTTTAATT ATGACGAACT CTTTTTGTTT ATACTATACT   
  
  
- TTAAGGAGCT TTTTCCGGAC GGTATTGTTT AAGGAGCACT TTTTCGTCGA CTCTCTTCTT CTTCTTCTTT   
  
  
- TTTTAGGGCA GTGTTAGAGT TAGAGCTAGG GTTTAACACT TTCTCTTTCT TTTCTACTTC TCTCTTGTGG   
  
  
- GGAAAAGCTT GGGACTGGTC TTTGGGGGCG GCCTCTACAG CGGCCGCGGC CCCTTCAGGT TCTACACCCT   
  
  
- ACTACTCTAC CGGGTCTTGC TGCGATTTTA CCTACTCGAC GAACGACAAC ACCCCATGTT CCACTCCAGC   
  
  
- AGCCTGTACC GCCTTTAACG GGTCTTCGAA CTCGTCGAAC TTCTTCAGTA CCCATCACAA GTTCTTCTGC   
  
  
- CCAATAGAAT GAACCGAAGG CTTTGACAAG TAATGTTAGG ACGTCTAGAC AGATGAACCG AACTTAGATA   
  
  
- CGAAAGACTC AAATTGGGAT TGGGATTAAA ACTGGGAAGA GGTAGCAGTT AGAGTGGGTA GTAGCTAGGT   
  
  
- CGAGGAGGTG AAGGAGCTTG GAGCAGCTGC CAGTTTAAGC TCGGGCTGGG CAAAAGGCTA AGCCTAGACT   
  
  
- TTCGTTAGGG TCCATTCCGA TAGAACTGGG GTGGTTCAAG ATCGTTATGA AGTTTAAACG CACTCCGATT   
  
  
- CTCCAACTTT GGGAGTTTAA TGTGTTGGCG GGGTTGCGGG TGGGGTGGAG GTTTTGACGG GTTAAGCCGC   
  
  
- AGAGGTGGTA GCCCGTGCTG CCGCAGCTCC GGCCACCACG ACCATCTGAG TGTTCTTTTG CCGTAGTCTA   
  
  
- ACCACGTACG TGAATACCGG ACACGGCTTC GTTAAGTTGA TCTTTTGTAC CCTAATCGAC TCCGAAACCA   
  
  
- ATTTGTCTAA TCCATAAATC GTCGTAGAGT TCGACCTGGA TACTCCTTTC ATCGTTGAAC AAAACGTCTT   
  
  
- CGAGAATGAA CAGCCTAGAT GTTCGATACG GGTAGACATG GGATGCTACT TAGTCAGAGT CTACTCAACG   
  
  
- TCTACGTGAA AATACTCTGA ACGGGTATAG AATTTAAGCG GGTAAAGTGC CGTTTAGTTC GTTAAAATCT   
  
  
- TCGTAAGTTA CCCTTCTTCT TTCAAGTACA CTAACTAAAG TCGTACTTCG TTCCGTACGT TACCGGCCGA   
  
  
- AACTACGTTC GGGACCGAGA AGCTGGCCTC CCAGGTGGCG AAAAGGCCAA TTGGCCCTAA CCTGGCGGGC   
  
  
- GAGGCCTGTT GAGCCTGGCC GACGTTCTCC ACCCAACCTT CGAACGGGTC AAGCGCCTAA GCTAGGCCTA   
  
  
- ATTTAAAGTC ATAGCACCCA AACACCGTTT GTCAAACTGG CTAGATCTTA GAAGGTACGA TCTAGAACTT   
  
  
- GGCCTGTGAC TCCACCACCG CCAGTTGAGC CAAAAGCTCG AGGTGGCCGA CAACCGATTT GGGCCCCGCT   
  
  
- AGCTCTTTCA CGACCCCAAG TACTCCCGGC ACTTGGGCCA GTAACACTGC TACCAGCTCG TCCTTCGCTT   
  
  
- GGTGTTGCCT GGCCAAAAGA ACCTGGCCAA GTTACTCAGC AACGTAATAA TGAGGTGGAA CAAACTAAGG   
  
  
- GAACTCTAAA CACAACTATT ACATCTATTC TTCTACAGCC TCCGAATGAA CCCGGTCGTC TAGACGTTGT   
  
  
- ACCACCGAAC ACTTCCCAGA CTGGCTCAGC TCTCCGTGCT CTGGGACCGA GTCACCGCTC GGGCCAAGCG   
  
  
- TAGACGGCCC AAGCTGGGTC AAGTAGACCC AAGCTTACGC AAATTCGTCC GCTCATACAA CAACCTCAAG   
  
  
- AAACGACCAC CACTTCCCAT GCCGCACCTC CTCGCTCTTC CCACAGAGTA CAACCCTACC GTATCATCCG   
  
  
- GCGAATAGTG GTGGAGCCGA ACCGTCGAGC GGTTCTTGGG CCGACACTTA GCCGCTAC

+     Myb

| Site Name | Organism | Position | Strand | Matrix score. | sequence | function |
| --- | --- | --- | --- | --- | --- | --- |
| Myb | Arabidopsis thaliana | 1478 | + | 6 | TAACTG |  |
| Myb | Arabidopsis thaliana | 1051 | - | 6 | TAACTG |  |

>HU10G00709.1   
+ -Up\_Stream \_Len000AACCCT TTAATTTTAT CAATGTGGGA CAACACTCAT CTTCATACTC CAACAAGCTA   
  
  
+ TGATACATTC GATTATTGCC TAGTATTTCG TTTAGTAAGA TATTAGTGCC AAACTTTGTA ATAAAAATGA   
  
  
+ AACTTACTAA AAAAAATCTC GTTTTCTGTA GAGGGAAAAT TTGAAGACTT AGTGTCACAT GATGACCTAA   
  
  
+ TGGTACTTGT AGGGTCAAGA TTGAGATTGA GGGCATACTG ACGACCTGTA TAATGGTCAA GTAGGATCAA   
  
  
+ AGTCGAGAAA ACTGTCTCTG CTAACTTTCA TAATCTAACC CTTAACTCAC TCTGACCCTC AATTTAATCA   
  
  
+ GGCCTAATTC AAACTATTAA TGGTTATAAC TCTCTTTCCT TCTTTTGAGA TTTTTTTTTT CTACATGCGG   
  
  
+ TTGAAAATAA TTTTTTTAAA AAATATATAT CACTAACCAT TCATTATCAT AGTTTTAGAG TTCATATTTA   
  
  
+ TGTGGTCCTT GTTGAGTCTA ATTTAATGCT TTAGATGGCG TGAAGCACCA AATTAGTGGT AACCATAATA   
  
  
+ ATAAACTATC AAGTGTTAAG TCGAAGAAAA CGGACACTGC TCAACCAAAT CTAGTAACAT AGTGTATGAC   
  
  
+ ATGATAACTA AGTGTGCCAT GTAGTAATCA ATTCTTTCTT TTTTTTTTTT TGGTTGAGAT CACATAAATT   
  
  
+ ACGTTATCAA GGGTAGTACG ATATAATGGA TGAGGAGAAA TTAAATGTGA GATTTAGTTG ATGAATCGAA   
  
  
+ TACGTTAGTC TGAGCCATTT GGTCAAAACT GCATTGGCTA ATCAATAGAA ATTATAGAAA ATACATAACA   
  
  
+ TAATGGGATG CAATTTTCTA ATTGGGGAAA CCATGTCAAT GCAGTGATTT TGGACCATTT AGGCCTTATT   
  
  
+ CTTTTCAATT TTTTAATTGA TTATTTAGAC TCATATGAAC TTATTGTTGA CAGATTGAGC TAAAAACACT   
  
  
+ GATGTTTCAT ACATATGTAT GAATTGCACG TACGAAGGCT CTGCTCATTT CGAGAAAGAA AATTAACAGT   
  
  
+ TAGGAGCAGT ATAATTTAAC AACAAGCGAT AATTATTAGT ATGAAGTGGT ACGAATCTAT TTTAAAGGAG   
  
  
+ TACTATGAAG TACAAACAAT AACAAATAAT AAAGAGCAGT ACATTAACAT ATCTTACTAC CTAAATAAAT   
  
  
+ CGATTCTAAG AGTAAAAAAA TGTTGAAATT AACCCCATTA TAGTTTTACT TTAACAAGAA GTTTTTCTTC   
  
  
+ TTTTACCATT TACCAAGTAA GAATGATTCA GGCCGTATAG CCAAGGAAAA ATAGAAAACC ACAAAACTAA   
  
  
+ CCATGGCCCA AAAAACAACC ACCAAGAGGG GCACTAATGT GACTAACCGA CGGTTAACAT TATCATCTGT   
  
  
+ TTGATACTTG TTCATCTCTC ACTATTCGGG TAATAGTGTG AAATTACTGA TGTACCCTCT AAACTCGTCG   
  
  
+ TTTTAACTGT GTAAGAATCA GATTAACAGT CACGGACACA TGAGTAACAA ATCAATTTCG GACATGAACG   
  
  
+ AACGAACTGT GAATCCCCCC CAAAAAAAAA ATTTTTTTAA AAAAAGTTGC ATCCTTATCT TTCTGAACCA   
  
  
+ GGCCAGCTGT CTCATTCCTT GGTCCTCGCG CCCCATCACG AGCGCTGCTC ATTATTCACC CTCTCTCTCT   
  
  
+ CTCCTCTCTG ATCCACACCA CAAACACAAG GTTTGCACAG CTGCAGAGCA GTCGTACTAC TATCAGTAGT   
  
  
+ GAAAAACCAA GCAGAAATCC CCAATCCCTC TTCTCCTTCT TCCCAGATTC TTCCTCAATT CCAAGCCCAT   
  
  
+ ATCAGAAATT TAGACAACAA CAACCAAAAA AAAAAATTAA TACTGCTTGA GAAAAACAAA TATGATATGA   
  
  
+ AATTCCTCGA AAAAGGCCTG CCATAACAAA TTCCTCGTGA AAAAGCAGCT GAGAGAAGAA GAAGAAGAAA   
  
  
+ AAAATCCCGT CACAATCTCA ATCTCGATCC CAAATTGTGA AAGAGAAAGA AAAGATGAAG AGAGAACACC   
  
  
+ CCTTTTCGAA CCCTGACCAG AAACCCCCGC CGGAGATGTC GCCGGCGCCG GGGAAGTCCA AGATGTGGGA   
  
  
+ TGATGAGATG GCCCAGAACG ACGCTAAAAT GGATGAGCTG CTTGCTGTTG TGGGGTACAA GGTGAGGTCG   
  
  
+ TCGGACATGG CGGAAATTGC CCAGAAGCTT GAGCAGCTTG AAGAAGTCAT GGGTAGTGTT CAAGAAGACG   
  
  
+ GGTTATCTTA CTTGGCTTCC GAAACTGTTC ATTACAATCC TGCAGATCTG TCTACTTGGC TTGAATCTAT   
  
  
+ GCTTTCTGAG TTTAACCCTA ACCCTAATTT TGACCCTTCT CCATCGTCAA TCTCACCCAT CATCGATCCA   
  
  
+ GCTCCTCCAC TTCCTCGAAC CTCGTCGACG GTCAAATTCG AGCCCGACCC GTTTTCCGAT TCGGATCTGA   
  
  
+ AAGCAATCCC AGGTAAGGCT ATCTTGACCC CACCAAGTTC TAGCAATACT TCAAATTTGC GTGAGGCTAA   
  
  
+ GAGGTTGAAA CCCTCAAATT ACACAACCGC CCCAACGCCC ACCCCACCTC CAAAACTGCC CAATTCGGCG   
  
  
+ TCTCCACCAT CGGGCACGAC GGCGTCGAGG CCGGTGGTGC TGGTAGACTC ACAAGAAAAC GGCATCAGAT   
  
  
+ TGGTGCATGC ACTTATGGCC TGTGCCGAAG CAATTCAACT AGAAAACATG GGATTAGCTG AGGCTTTGGT   
  
  
+ TAAACAGATT AGGTATTTAG CAGCATCTCA AGCTGGACCT ATGAGGAAAG TAGCAACTTG TTTTGCAGAA   
  
  
+ GCTCTTACTT GTCGGATCTA CAAGCTATGC CCATCTGTAC CCTACGATGA ATCAGTCTCA GATGAGTTGC   
  
  
+ AGATGCACTT TTATGAGACT TGCCCATATC TTAAATTCGC CCATTTCACG GCAAATCAAG CAATTTTAGA   
  
  
+ AGCATTCAAT GGGAAGAAGA AAGTTCATGT GATTGATTTC AGCATGAAGC AAGGCATGCA ATGGCCGGCT   
  
  
+ TTGATGCAAG CCCTGGCTCT TCGACCGGAG GGTCCACCGC TTTTCCGGTT AACCGGGATT GGACCGCCCG   
  
  
+ CTCCGGACAA CTCGGACCGG CTGCAAGAGG TGGGTTGGAA GCTTGCCCAG TTCGCGGATT CGATCCGGAT   
  
  
+ TAAATTTCAG TATCGTGGGT TTGTGGCAAA CAGTTTGACC GATCTAGAAT CTTCCATGCT AGATCTTGAA   
  
  
+ CCGGACACTG AGGTGGTGGC GGTCAACTCG GTTTTCGAGC TCCACCGGCT GTTGGCTAAA CCCGGGGCGA   
  
  
+ TCGAGAAAGT GCTGGGGTTC ATGAGGGCCG TGAACCCGGT CATTGTGACG ATGGTCGAGC AGGAAGCGAA   
  
  
+ CCACAACGGA CCGGTTTTCT TGGACCGGTT CAATGAGTCG TTGCATTATT ACTCCACCTT GTTTGATTCC   
  
  
+ CTTGAGATTT GTGTTGATAA TGTAGATAAG AAGATGTCGG AGGCTTACTT GGGCCAGCAG ATCTGCAACA   
  
  
+ TGGTGGCTTG TGAAGGGTCT GACCGAGTCG AGAGGCACGA GACCCTGGCT CAGTGGCGAG CCCGGTTCGC   
  
  
+ ATCTGCCGGG TTCGACCCAG TTCATCTGGG TTCGAATGCG TTTAAGCAGG CGAGTATGTT GTTGGAGTTC   
  
  
+ TTTGCTGGTG GTGAAGGGTA CGGCGTGGAG GAGCGAGAAG GGTGTCTCAT GTTGGGATGG CATAGTAGGC   
  
  
+ CGCTTATCAC CACCTCGGCT TGGCAGCTCG CCAAGAACCC GGCTGTGAAT CGGCGATG  

- -Up\_Stream \_Len000TTGGGA AATTAAAATA GTTACACCCT GTTGTGAGTA GAAGTATGAG GTTGTTCGAT   
  
  
- ACTATGTAAG CTAATAACGG ATCATAAAGC AAATCATTCT ATAATCACGG TTTGAAACAT TATTTTTACT   
  
  
- TTGAATGATT TTTTTTAGAG CAAAAGACAT CTCCCTTTTA AACTTCTGAA TCACAGTGTA CTACTGGATT   
  
  
- ACCATGAACA TCCCAGTTCT AACTCTAACT CCCGTATGAC TGCTGGACAT ATTACCAGTT CATCCTAGTT   
  
  
- TCAGCTCTTT TGACAGAGAC GATTGAAAGT ATTAGATTGG GAATTGAGTG AGACTGGGAG TTAAATTAGT   
  
  
- CCGGATTAAG TTTGATAATT ACCAATATTG AGAGAAAGGA AGAAAACTCT AAAAAAAAAA GATGTACGCC   
  
  
- AACTTTTATT AAAAAAATTT TTTATATATA GTGATTGGTA AGTAATAGTA TCAAAATCTC AAGTATAAAT   
  
  
- ACACCAGGAA CAACTCAGAT TAAATTACGA AATCTACCGC ACTTCGTGGT TTAATCACCA TTGGTATTAT   
  
  
- TATTTGATAG TTCACAATTC AGCTTCTTTT GCCTGTGACG AGTTGGTTTA GATCATTGTA TCACATACTG   
  
  
- TACTATTGAT TCACACGGTA CATCATTAGT TAAGAAAGAA AAAAAAAAAA ACCAACTCTA GTGTATTTAA   
  
  
- TGCAATAGTT CCCATCATGC TATATTACCT ACTCCTCTTT AATTTACACT CTAAATCAAC TACTTAGCTT   
  
  
- ATGCAATCAG ACTCGGTAAA CCAGTTTTGA CGTAACCGAT TAGTTATCTT TAATATCTTT TATGTATTGT   
  
  
- ATTACCCTAC GTTAAAAGAT TAACCCCTTT GGTACAGTTA CGTCACTAAA ACCTGGTAAA TCCGGAATAA   
  
  
- GAAAAGTTAA AAAATTAACT AATAAATCTG AGTATACTTG AATAACAACT GTCTAACTCG ATTTTTGTGA   
  
  
- CTACAAAGTA TGTATACATA CTTAACGTGC ATGCTTCCGA GACGAGTAAA GCTCTTTCTT TTAATTGTCA   
  
  
- ATCCTCGTCA TATTAAATTG TTGTTCGCTA TTAATAATCA TACTTCACCA TGCTTAGATA AAATTTCCTC   
  
  
- ATGATACTTC ATGTTTGTTA TTGTTTATTA TTTCTCGTCA TGTAATTGTA TAGAATGATG GATTTATTTA   
  
  
- GCTAAGATTC TCATTTTTTT ACAACTTTAA TTGGGGTAAT ATCAAAATGA AATTGTTCTT CAAAAAGAAG   
  
  
- AAAATGGTAA ATGGTTCATT CTTACTAAGT CCGGCATATC GGTTCCTTTT TATCTTTTGG TGTTTTGATT   
  
  
- GGTACCGGGT TTTTTGTTGG TGGTTCTCCC CGTGATTACA CTGATTGGCT GCCAATTGTA ATAGTAGACA   
  
  
- AACTATGAAC AAGTAGAGAG TGATAAGCCC ATTATCACAC TTTAATGACT ACATGGGAGA TTTGAGCAGC   
  
  
- AAAATTGACA CATTCTTAGT CTAATTGTCA GTGCCTGTGT ACTCATTGTT TAGTTAAAGC CTGTACTTGC   
  
  
- TTGCTTGACA CTTAGGGGGG GTTTTTTTTT TAAAAAAATT TTTTTCAACG TAGGAATAGA AAGACTTGGT   
  
  
- CCGGTCGACA GAGTAAGGAA CCAGGAGCGC GGGGTAGTGC TCGCGACGAG TAATAAGTGG GAGAGAGAGA   
  
  
- GAGGAGAGAC TAGGTGTGGT GTTTGTGTTC CAAACGTGTC GACGTCTCGT CAGCATGATG ATAGTCATCA   
  
  
- CTTTTTGGTT CGTCTTTAGG GGTTAGGGAG AAGAGGAAGA AGGGTCTAAG AAGGAGTTAA GGTTCGGGTA   
  
  
- TAGTCTTTAA ATCTGTTGTT GTTGGTTTTT TTTTTTAATT ATGACGAACT CTTTTTGTTT ATACTATACT   
  
  
- TTAAGGAGCT TTTTCCGGAC GGTATTGTTT AAGGAGCACT TTTTCGTCGA CTCTCTTCTT CTTCTTCTTT   
  
  
- TTTTAGGGCA GTGTTAGAGT TAGAGCTAGG GTTTAACACT TTCTCTTTCT TTTCTACTTC TCTCTTGTGG   
  
  
- GGAAAAGCTT GGGACTGGTC TTTGGGGGCG GCCTCTACAG CGGCCGCGGC CCCTTCAGGT TCTACACCCT   
  
  
- ACTACTCTAC CGGGTCTTGC TGCGATTTTA CCTACTCGAC GAACGACAAC ACCCCATGTT CCACTCCAGC   
  
  
- AGCCTGTACC GCCTTTAACG GGTCTTCGAA CTCGTCGAAC TTCTTCAGTA CCCATCACAA GTTCTTCTGC   
  
  
- CCAATAGAAT GAACCGAAGG CTTTGACAAG TAATGTTAGG ACGTCTAGAC AGATGAACCG AACTTAGATA   
  
  
- CGAAAGACTC AAATTGGGAT TGGGATTAAA ACTGGGAAGA GGTAGCAGTT AGAGTGGGTA GTAGCTAGGT   
  
  
- CGAGGAGGTG AAGGAGCTTG GAGCAGCTGC CAGTTTAAGC TCGGGCTGGG CAAAAGGCTA AGCCTAGACT   
  
  
- TTCGTTAGGG TCCATTCCGA TAGAACTGGG GTGGTTCAAG ATCGTTATGA AGTTTAAACG CACTCCGATT   
  
  
- CTCCAACTTT GGGAGTTTAA TGTGTTGGCG GGGTTGCGGG TGGGGTGGAG GTTTTGACGG GTTAAGCCGC   
  
  
- AGAGGTGGTA GCCCGTGCTG CCGCAGCTCC GGCCACCACG ACCATCTGAG TGTTCTTTTG CCGTAGTCTA   
  
  
- ACCACGTACG TGAATACCGG ACACGGCTTC GTTAAGTTGA TCTTTTGTAC CCTAATCGAC TCCGAAACCA   
  
  
- ATTTGTCTAA TCCATAAATC GTCGTAGAGT TCGACCTGGA TACTCCTTTC ATCGTTGAAC AAAACGTCTT   
  
  
- CGAGAATGAA CAGCCTAGAT GTTCGATACG GGTAGACATG GGATGCTACT TAGTCAGAGT CTACTCAACG   
  
  
- TCTACGTGAA AATACTCTGA ACGGGTATAG AATTTAAGCG GGTAAAGTGC CGTTTAGTTC GTTAAAATCT   
  
  
- TCGTAAGTTA CCCTTCTTCT TTCAAGTACA CTAACTAAAG TCGTACTTCG TTCCGTACGT TACCGGCCGA   
  
  
- AACTACGTTC GGGACCGAGA AGCTGGCCTC CCAGGTGGCG AAAAGGCCAA TTGGCCCTAA CCTGGCGGGC   
  
  
- GAGGCCTGTT GAGCCTGGCC GACGTTCTCC ACCCAACCTT CGAACGGGTC AAGCGCCTAA GCTAGGCCTA   
  
  
- ATTTAAAGTC ATAGCACCCA AACACCGTTT GTCAAACTGG CTAGATCTTA GAAGGTACGA TCTAGAACTT   
  
  
- GGCCTGTGAC TCCACCACCG CCAGTTGAGC CAAAAGCTCG AGGTGGCCGA CAACCGATTT GGGCCCCGCT   
  
  
- AGCTCTTTCA CGACCCCAAG TACTCCCGGC ACTTGGGCCA GTAACACTGC TACCAGCTCG TCCTTCGCTT   
  
  
- GGTGTTGCCT GGCCAAAAGA ACCTGGCCAA GTTACTCAGC AACGTAATAA TGAGGTGGAA CAAACTAAGG   
  
  
- GAACTCTAAA CACAACTATT ACATCTATTC TTCTACAGCC TCCGAATGAA CCCGGTCGTC TAGACGTTGT   
  
  
- ACCACCGAAC ACTTCCCAGA CTGGCTCAGC TCTCCGTGCT CTGGGACCGA GTCACCGCTC GGGCCAAGCG   
  
  
- TAGACGGCCC AAGCTGGGTC AAGTAGACCC AAGCTTACGC AAATTCGTCC GCTCATACAA CAACCTCAAG   
  
  
- AAACGACCAC CACTTCCCAT GCCGCACCTC CTCGCTCTTC CCACAGAGTA CAACCCTACC GTATCATCCG   
  
  
- GCGAATAGTG GTGGAGCCGA ACCGTCGAGC GGTTCTTGGG CCGACACTTA GCCGCTAC

+     Myb-binding site

| Site Name | Organism | Position | Strand | Matrix score. | sequence | function |
| --- | --- | --- | --- | --- | --- | --- |
| Myb-binding site | Nicotiana tabacum | 3273 | - | 6 | CAACAG |  |
| Myb-binding site | Nicotiana tabacum | 2149 | - | 6 | CAACAG |  |

>HU10G00709.1   
+ -Up\_Stream \_Len000AACCCT TTAATTTTAT CAATGTGGGA CAACACTCAT CTTCATACTC CAACAAGCTA   
  
  
+ TGATACATTC GATTATTGCC TAGTATTTCG TTTAGTAAGA TATTAGTGCC AAACTTTGTA ATAAAAATGA   
  
  
+ AACTTACTAA AAAAAATCTC GTTTTCTGTA GAGGGAAAAT TTGAAGACTT AGTGTCACAT GATGACCTAA   
  
  
+ TGGTACTTGT AGGGTCAAGA TTGAGATTGA GGGCATACTG ACGACCTGTA TAATGGTCAA GTAGGATCAA   
  
  
+ AGTCGAGAAA ACTGTCTCTG CTAACTTTCA TAATCTAACC CTTAACTCAC TCTGACCCTC AATTTAATCA   
  
  
+ GGCCTAATTC AAACTATTAA TGGTTATAAC TCTCTTTCCT TCTTTTGAGA TTTTTTTTTT CTACATGCGG   
  
  
+ TTGAAAATAA TTTTTTTAAA AAATATATAT CACTAACCAT TCATTATCAT AGTTTTAGAG TTCATATTTA   
  
  
+ TGTGGTCCTT GTTGAGTCTA ATTTAATGCT TTAGATGGCG TGAAGCACCA AATTAGTGGT AACCATAATA   
  
  
+ ATAAACTATC AAGTGTTAAG TCGAAGAAAA CGGACACTGC TCAACCAAAT CTAGTAACAT AGTGTATGAC   
  
  
+ ATGATAACTA AGTGTGCCAT GTAGTAATCA ATTCTTTCTT TTTTTTTTTT TGGTTGAGAT CACATAAATT   
  
  
+ ACGTTATCAA GGGTAGTACG ATATAATGGA TGAGGAGAAA TTAAATGTGA GATTTAGTTG ATGAATCGAA   
  
  
+ TACGTTAGTC TGAGCCATTT GGTCAAAACT GCATTGGCTA ATCAATAGAA ATTATAGAAA ATACATAACA   
  
  
+ TAATGGGATG CAATTTTCTA ATTGGGGAAA CCATGTCAAT GCAGTGATTT TGGACCATTT AGGCCTTATT   
  
  
+ CTTTTCAATT TTTTAATTGA TTATTTAGAC TCATATGAAC TTATTGTTGA CAGATTGAGC TAAAAACACT   
  
  
+ GATGTTTCAT ACATATGTAT GAATTGCACG TACGAAGGCT CTGCTCATTT CGAGAAAGAA AATTAACAGT   
  
  
+ TAGGAGCAGT ATAATTTAAC AACAAGCGAT AATTATTAGT ATGAAGTGGT ACGAATCTAT TTTAAAGGAG   
  
  
+ TACTATGAAG TACAAACAAT AACAAATAAT AAAGAGCAGT ACATTAACAT ATCTTACTAC CTAAATAAAT   
  
  
+ CGATTCTAAG AGTAAAAAAA TGTTGAAATT AACCCCATTA TAGTTTTACT TTAACAAGAA GTTTTTCTTC   
  
  
+ TTTTACCATT TACCAAGTAA GAATGATTCA GGCCGTATAG CCAAGGAAAA ATAGAAAACC ACAAAACTAA   
  
  
+ CCATGGCCCA AAAAACAACC ACCAAGAGGG GCACTAATGT GACTAACCGA CGGTTAACAT TATCATCTGT   
  
  
+ TTGATACTTG TTCATCTCTC ACTATTCGGG TAATAGTGTG AAATTACTGA TGTACCCTCT AAACTCGTCG   
  
  
+ TTTTAACTGT GTAAGAATCA GATTAACAGT CACGGACACA TGAGTAACAA ATCAATTTCG GACATGAACG   
  
  
+ AACGAACTGT GAATCCCCCC CAAAAAAAAA ATTTTTTTAA AAAAAGTTGC ATCCTTATCT TTCTGAACCA   
  
  
+ GGCCAGCTGT CTCATTCCTT GGTCCTCGCG CCCCATCACG AGCGCTGCTC ATTATTCACC CTCTCTCTCT   
  
  
+ CTCCTCTCTG ATCCACACCA CAAACACAAG GTTTGCACAG CTGCAGAGCA GTCGTACTAC TATCAGTAGT   
  
  
+ GAAAAACCAA GCAGAAATCC CCAATCCCTC TTCTCCTTCT TCCCAGATTC TTCCTCAATT CCAAGCCCAT   
  
  
+ ATCAGAAATT TAGACAACAA CAACCAAAAA AAAAAATTAA TACTGCTTGA GAAAAACAAA TATGATATGA   
  
  
+ AATTCCTCGA AAAAGGCCTG CCATAACAAA TTCCTCGTGA AAAAGCAGCT GAGAGAAGAA GAAGAAGAAA   
  
  
+ AAAATCCCGT CACAATCTCA ATCTCGATCC CAAATTGTGA AAGAGAAAGA AAAGATGAAG AGAGAACACC   
  
  
+ CCTTTTCGAA CCCTGACCAG AAACCCCCGC CGGAGATGTC GCCGGCGCCG GGGAAGTCCA AGATGTGGGA   
  
  
+ TGATGAGATG GCCCAGAACG ACGCTAAAAT GGATGAGCTG CTTGCTGTTG TGGGGTACAA GGTGAGGTCG   
  
  
+ TCGGACATGG CGGAAATTGC CCAGAAGCTT GAGCAGCTTG AAGAAGTCAT GGGTAGTGTT CAAGAAGACG   
  
  
+ GGTTATCTTA CTTGGCTTCC GAAACTGTTC ATTACAATCC TGCAGATCTG TCTACTTGGC TTGAATCTAT   
  
  
+ GCTTTCTGAG TTTAACCCTA ACCCTAATTT TGACCCTTCT CCATCGTCAA TCTCACCCAT CATCGATCCA   
  
  
+ GCTCCTCCAC TTCCTCGAAC CTCGTCGACG GTCAAATTCG AGCCCGACCC GTTTTCCGAT TCGGATCTGA   
  
  
+ AAGCAATCCC AGGTAAGGCT ATCTTGACCC CACCAAGTTC TAGCAATACT TCAAATTTGC GTGAGGCTAA   
  
  
+ GAGGTTGAAA CCCTCAAATT ACACAACCGC CCCAACGCCC ACCCCACCTC CAAAACTGCC CAATTCGGCG   
  
  
+ TCTCCACCAT CGGGCACGAC GGCGTCGAGG CCGGTGGTGC TGGTAGACTC ACAAGAAAAC GGCATCAGAT   
  
  
+ TGGTGCATGC ACTTATGGCC TGTGCCGAAG CAATTCAACT AGAAAACATG GGATTAGCTG AGGCTTTGGT   
  
  
+ TAAACAGATT AGGTATTTAG CAGCATCTCA AGCTGGACCT ATGAGGAAAG TAGCAACTTG TTTTGCAGAA   
  
  
+ GCTCTTACTT GTCGGATCTA CAAGCTATGC CCATCTGTAC CCTACGATGA ATCAGTCTCA GATGAGTTGC   
  
  
+ AGATGCACTT TTATGAGACT TGCCCATATC TTAAATTCGC CCATTTCACG GCAAATCAAG CAATTTTAGA   
  
  
+ AGCATTCAAT GGGAAGAAGA AAGTTCATGT GATTGATTTC AGCATGAAGC AAGGCATGCA ATGGCCGGCT   
  
  
+ TTGATGCAAG CCCTGGCTCT TCGACCGGAG GGTCCACCGC TTTTCCGGTT AACCGGGATT GGACCGCCCG   
  
  
+ CTCCGGACAA CTCGGACCGG CTGCAAGAGG TGGGTTGGAA GCTTGCCCAG TTCGCGGATT CGATCCGGAT   
  
  
+ TAAATTTCAG TATCGTGGGT TTGTGGCAAA CAGTTTGACC GATCTAGAAT CTTCCATGCT AGATCTTGAA   
  
  
+ CCGGACACTG AGGTGGTGGC GGTCAACTCG GTTTTCGAGC TCCACCGGCT GTTGGCTAAA CCCGGGGCGA   
  
  
+ TCGAGAAAGT GCTGGGGTTC ATGAGGGCCG TGAACCCGGT CATTGTGACG ATGGTCGAGC AGGAAGCGAA   
  
  
+ CCACAACGGA CCGGTTTTCT TGGACCGGTT CAATGAGTCG TTGCATTATT ACTCCACCTT GTTTGATTCC   
  
  
+ CTTGAGATTT GTGTTGATAA TGTAGATAAG AAGATGTCGG AGGCTTACTT GGGCCAGCAG ATCTGCAACA   
  
  
+ TGGTGGCTTG TGAAGGGTCT GACCGAGTCG AGAGGCACGA GACCCTGGCT CAGTGGCGAG CCCGGTTCGC   
  
  
+ ATCTGCCGGG TTCGACCCAG TTCATCTGGG TTCGAATGCG TTTAAGCAGG CGAGTATGTT GTTGGAGTTC   
  
  
+ TTTGCTGGTG GTGAAGGGTA CGGCGTGGAG GAGCGAGAAG GGTGTCTCAT GTTGGGATGG CATAGTAGGC   
  
  
+ CGCTTATCAC CACCTCGGCT TGGCAGCTCG CCAAGAACCC GGCTGTGAAT CGGCGATG  

- -Up\_Stream \_Len000TTGGGA AATTAAAATA GTTACACCCT GTTGTGAGTA GAAGTATGAG GTTGTTCGAT   
  
  
- ACTATGTAAG CTAATAACGG ATCATAAAGC AAATCATTCT ATAATCACGG TTTGAAACAT TATTTTTACT   
  
  
- TTGAATGATT TTTTTTAGAG CAAAAGACAT CTCCCTTTTA AACTTCTGAA TCACAGTGTA CTACTGGATT   
  
  
- ACCATGAACA TCCCAGTTCT AACTCTAACT CCCGTATGAC TGCTGGACAT ATTACCAGTT CATCCTAGTT   
  
  
- TCAGCTCTTT TGACAGAGAC GATTGAAAGT ATTAGATTGG GAATTGAGTG AGACTGGGAG TTAAATTAGT   
  
  
- CCGGATTAAG TTTGATAATT ACCAATATTG AGAGAAAGGA AGAAAACTCT AAAAAAAAAA GATGTACGCC   
  
  
- AACTTTTATT AAAAAAATTT TTTATATATA GTGATTGGTA AGTAATAGTA TCAAAATCTC AAGTATAAAT   
  
  
- ACACCAGGAA CAACTCAGAT TAAATTACGA AATCTACCGC ACTTCGTGGT TTAATCACCA TTGGTATTAT   
  
  
- TATTTGATAG TTCACAATTC AGCTTCTTTT GCCTGTGACG AGTTGGTTTA GATCATTGTA TCACATACTG   
  
  
- TACTATTGAT TCACACGGTA CATCATTAGT TAAGAAAGAA AAAAAAAAAA ACCAACTCTA GTGTATTTAA   
  
  
- TGCAATAGTT CCCATCATGC TATATTACCT ACTCCTCTTT AATTTACACT CTAAATCAAC TACTTAGCTT   
  
  
- ATGCAATCAG ACTCGGTAAA CCAGTTTTGA CGTAACCGAT TAGTTATCTT TAATATCTTT TATGTATTGT   
  
  
- ATTACCCTAC GTTAAAAGAT TAACCCCTTT GGTACAGTTA CGTCACTAAA ACCTGGTAAA TCCGGAATAA   
  
  
- GAAAAGTTAA AAAATTAACT AATAAATCTG AGTATACTTG AATAACAACT GTCTAACTCG ATTTTTGTGA   
  
  
- CTACAAAGTA TGTATACATA CTTAACGTGC ATGCTTCCGA GACGAGTAAA GCTCTTTCTT TTAATTGTCA   
  
  
- ATCCTCGTCA TATTAAATTG TTGTTCGCTA TTAATAATCA TACTTCACCA TGCTTAGATA AAATTTCCTC   
  
  
- ATGATACTTC ATGTTTGTTA TTGTTTATTA TTTCTCGTCA TGTAATTGTA TAGAATGATG GATTTATTTA   
  
  
- GCTAAGATTC TCATTTTTTT ACAACTTTAA TTGGGGTAAT ATCAAAATGA AATTGTTCTT CAAAAAGAAG   
  
  
- AAAATGGTAA ATGGTTCATT CTTACTAAGT CCGGCATATC GGTTCCTTTT TATCTTTTGG TGTTTTGATT   
  
  
- GGTACCGGGT TTTTTGTTGG TGGTTCTCCC CGTGATTACA CTGATTGGCT GCCAATTGTA ATAGTAGACA   
  
  
- AACTATGAAC AAGTAGAGAG TGATAAGCCC ATTATCACAC TTTAATGACT ACATGGGAGA TTTGAGCAGC   
  
  
- AAAATTGACA CATTCTTAGT CTAATTGTCA GTGCCTGTGT ACTCATTGTT TAGTTAAAGC CTGTACTTGC   
  
  
- TTGCTTGACA CTTAGGGGGG GTTTTTTTTT TAAAAAAATT TTTTTCAACG TAGGAATAGA AAGACTTGGT   
  
  
- CCGGTCGACA GAGTAAGGAA CCAGGAGCGC GGGGTAGTGC TCGCGACGAG TAATAAGTGG GAGAGAGAGA   
  
  
- GAGGAGAGAC TAGGTGTGGT GTTTGTGTTC CAAACGTGTC GACGTCTCGT CAGCATGATG ATAGTCATCA   
  
  
- CTTTTTGGTT CGTCTTTAGG GGTTAGGGAG AAGAGGAAGA AGGGTCTAAG AAGGAGTTAA GGTTCGGGTA   
  
  
- TAGTCTTTAA ATCTGTTGTT GTTGGTTTTT TTTTTTAATT ATGACGAACT CTTTTTGTTT ATACTATACT   
  
  
- TTAAGGAGCT TTTTCCGGAC GGTATTGTTT AAGGAGCACT TTTTCGTCGA CTCTCTTCTT CTTCTTCTTT   
  
  
- TTTTAGGGCA GTGTTAGAGT TAGAGCTAGG GTTTAACACT TTCTCTTTCT TTTCTACTTC TCTCTTGTGG   
  
  
- GGAAAAGCTT GGGACTGGTC TTTGGGGGCG GCCTCTACAG CGGCCGCGGC CCCTTCAGGT TCTACACCCT   
  
  
- ACTACTCTAC CGGGTCTTGC TGCGATTTTA CCTACTCGAC GAACGACAAC ACCCCATGTT CCACTCCAGC   
  
  
- AGCCTGTACC GCCTTTAACG GGTCTTCGAA CTCGTCGAAC TTCTTCAGTA CCCATCACAA GTTCTTCTGC   
  
  
- CCAATAGAAT GAACCGAAGG CTTTGACAAG TAATGTTAGG ACGTCTAGAC AGATGAACCG AACTTAGATA   
  
  
- CGAAAGACTC AAATTGGGAT TGGGATTAAA ACTGGGAAGA GGTAGCAGTT AGAGTGGGTA GTAGCTAGGT   
  
  
- CGAGGAGGTG AAGGAGCTTG GAGCAGCTGC CAGTTTAAGC TCGGGCTGGG CAAAAGGCTA AGCCTAGACT   
  
  
- TTCGTTAGGG TCCATTCCGA TAGAACTGGG GTGGTTCAAG ATCGTTATGA AGTTTAAACG CACTCCGATT   
  
  
- CTCCAACTTT GGGAGTTTAA TGTGTTGGCG GGGTTGCGGG TGGGGTGGAG GTTTTGACGG GTTAAGCCGC   
  
  
- AGAGGTGGTA GCCCGTGCTG CCGCAGCTCC GGCCACCACG ACCATCTGAG TGTTCTTTTG CCGTAGTCTA   
  
  
- ACCACGTACG TGAATACCGG ACACGGCTTC GTTAAGTTGA TCTTTTGTAC CCTAATCGAC TCCGAAACCA   
  
  
- ATTTGTCTAA TCCATAAATC GTCGTAGAGT TCGACCTGGA TACTCCTTTC ATCGTTGAAC AAAACGTCTT   
  
  
- CGAGAATGAA CAGCCTAGAT GTTCGATACG GGTAGACATG GGATGCTACT TAGTCAGAGT CTACTCAACG   
  
  
- TCTACGTGAA AATACTCTGA ACGGGTATAG AATTTAAGCG GGTAAAGTGC CGTTTAGTTC GTTAAAATCT   
  
  
- TCGTAAGTTA CCCTTCTTCT TTCAAGTACA CTAACTAAAG TCGTACTTCG TTCCGTACGT TACCGGCCGA   
  
  
- AACTACGTTC GGGACCGAGA AGCTGGCCTC CCAGGTGGCG AAAAGGCCAA TTGGCCCTAA CCTGGCGGGC   
  
  
- GAGGCCTGTT GAGCCTGGCC GACGTTCTCC ACCCAACCTT CGAACGGGTC AAGCGCCTAA GCTAGGCCTA   
  
  
- ATTTAAAGTC ATAGCACCCA AACACCGTTT GTCAAACTGG CTAGATCTTA GAAGGTACGA TCTAGAACTT   
  
  
- GGCCTGTGAC TCCACCACCG CCAGTTGAGC CAAAAGCTCG AGGTGGCCGA CAACCGATTT GGGCCCCGCT   
  
  
- AGCTCTTTCA CGACCCCAAG TACTCCCGGC ACTTGGGCCA GTAACACTGC TACCAGCTCG TCCTTCGCTT   
  
  
- GGTGTTGCCT GGCCAAAAGA ACCTGGCCAA GTTACTCAGC AACGTAATAA TGAGGTGGAA CAAACTAAGG   
  
  
- GAACTCTAAA CACAACTATT ACATCTATTC TTCTACAGCC TCCGAATGAA CCCGGTCGTC TAGACGTTGT   
  
  
- ACCACCGAAC ACTTCCCAGA CTGGCTCAGC TCTCCGTGCT CTGGGACCGA GTCACCGCTC GGGCCAAGCG   
  
  
- TAGACGGCCC AAGCTGGGTC AAGTAGACCC AAGCTTACGC AAATTCGTCC GCTCATACAA CAACCTCAAG   
  
  
- AAACGACCAC CACTTCCCAT GCCGCACCTC CTCGCTCTTC CCACAGAGTA CAACCCTACC GTATCATCCG   
  
  
- GCGAATAGTG GTGGAGCCGA ACCGTCGAGC GGTTCTTGGG CCGACACTTA GCCGCTAC

+     O2-site

| Site Name | Organism | Position | Strand | Matrix score. | sequence | function |
| --- | --- | --- | --- | --- | --- | --- |
| O2-site | Zea mays | 2106 | + | 9 | GATGACATGG | cis-acting regulatory element involved in zein metabolism regulation |

>HU10G00709.1   
+ -Up\_Stream \_Len000AACCCT TTAATTTTAT CAATGTGGGA CAACACTCAT CTTCATACTC CAACAAGCTA   
  
  
+ TGATACATTC GATTATTGCC TAGTATTTCG TTTAGTAAGA TATTAGTGCC AAACTTTGTA ATAAAAATGA   
  
  
+ AACTTACTAA AAAAAATCTC GTTTTCTGTA GAGGGAAAAT TTGAAGACTT AGTGTCACAT GATGACCTAA   
  
  
+ TGGTACTTGT AGGGTCAAGA TTGAGATTGA GGGCATACTG ACGACCTGTA TAATGGTCAA GTAGGATCAA   
  
  
+ AGTCGAGAAA ACTGTCTCTG CTAACTTTCA TAATCTAACC CTTAACTCAC TCTGACCCTC AATTTAATCA   
  
  
+ GGCCTAATTC AAACTATTAA TGGTTATAAC TCTCTTTCCT TCTTTTGAGA TTTTTTTTTT CTACATGCGG   
  
  
+ TTGAAAATAA TTTTTTTAAA AAATATATAT CACTAACCAT TCATTATCAT AGTTTTAGAG TTCATATTTA   
  
  
+ TGTGGTCCTT GTTGAGTCTA ATTTAATGCT TTAGATGGCG TGAAGCACCA AATTAGTGGT AACCATAATA   
  
  
+ ATAAACTATC AAGTGTTAAG TCGAAGAAAA CGGACACTGC TCAACCAAAT CTAGTAACAT AGTGTATGAC   
  
  
+ ATGATAACTA AGTGTGCCAT GTAGTAATCA ATTCTTTCTT TTTTTTTTTT TGGTTGAGAT CACATAAATT   
  
  
+ ACGTTATCAA GGGTAGTACG ATATAATGGA TGAGGAGAAA TTAAATGTGA GATTTAGTTG ATGAATCGAA   
  
  
+ TACGTTAGTC TGAGCCATTT GGTCAAAACT GCATTGGCTA ATCAATAGAA ATTATAGAAA ATACATAACA   
  
  
+ TAATGGGATG CAATTTTCTA ATTGGGGAAA CCATGTCAAT GCAGTGATTT TGGACCATTT AGGCCTTATT   
  
  
+ CTTTTCAATT TTTTAATTGA TTATTTAGAC TCATATGAAC TTATTGTTGA CAGATTGAGC TAAAAACACT   
  
  
+ GATGTTTCAT ACATATGTAT GAATTGCACG TACGAAGGCT CTGCTCATTT CGAGAAAGAA AATTAACAGT   
  
  
+ TAGGAGCAGT ATAATTTAAC AACAAGCGAT AATTATTAGT ATGAAGTGGT ACGAATCTAT TTTAAAGGAG   
  
  
+ TACTATGAAG TACAAACAAT AACAAATAAT AAAGAGCAGT ACATTAACAT ATCTTACTAC CTAAATAAAT   
  
  
+ CGATTCTAAG AGTAAAAAAA TGTTGAAATT AACCCCATTA TAGTTTTACT TTAACAAGAA GTTTTTCTTC   
  
  
+ TTTTACCATT TACCAAGTAA GAATGATTCA GGCCGTATAG CCAAGGAAAA ATAGAAAACC ACAAAACTAA   
  
  
+ CCATGGCCCA AAAAACAACC ACCAAGAGGG GCACTAATGT GACTAACCGA CGGTTAACAT TATCATCTGT   
  
  
+ TTGATACTTG TTCATCTCTC ACTATTCGGG TAATAGTGTG AAATTACTGA TGTACCCTCT AAACTCGTCG   
  
  
+ TTTTAACTGT GTAAGAATCA GATTAACAGT CACGGACACA TGAGTAACAA ATCAATTTCG GACATGAACG   
  
  
+ AACGAACTGT GAATCCCCCC CAAAAAAAAA ATTTTTTTAA AAAAAGTTGC ATCCTTATCT TTCTGAACCA   
  
  
+ GGCCAGCTGT CTCATTCCTT GGTCCTCGCG CCCCATCACG AGCGCTGCTC ATTATTCACC CTCTCTCTCT   
  
  
+ CTCCTCTCTG ATCCACACCA CAAACACAAG GTTTGCACAG CTGCAGAGCA GTCGTACTAC TATCAGTAGT   
  
  
+ GAAAAACCAA GCAGAAATCC CCAATCCCTC TTCTCCTTCT TCCCAGATTC TTCCTCAATT CCAAGCCCAT   
  
  
+ ATCAGAAATT TAGACAACAA CAACCAAAAA AAAAAATTAA TACTGCTTGA GAAAAACAAA TATGATATGA   
  
  
+ AATTCCTCGA AAAAGGCCTG CCATAACAAA TTCCTCGTGA AAAAGCAGCT GAGAGAAGAA GAAGAAGAAA   
  
  
+ AAAATCCCGT CACAATCTCA ATCTCGATCC CAAATTGTGA AAGAGAAAGA AAAGATGAAG AGAGAACACC   
  
  
+ CCTTTTCGAA CCCTGACCAG AAACCCCCGC CGGAGATGTC GCCGGCGCCG GGGAAGTCCA AGATGTGGGA   
  
  
+ TGATGAGATG GCCCAGAACG ACGCTAAAAT GGATGAGCTG CTTGCTGTTG TGGGGTACAA GGTGAGGTCG   
  
  
+ TCGGACATGG CGGAAATTGC CCAGAAGCTT GAGCAGCTTG AAGAAGTCAT GGGTAGTGTT CAAGAAGACG   
  
  
+ GGTTATCTTA CTTGGCTTCC GAAACTGTTC ATTACAATCC TGCAGATCTG TCTACTTGGC TTGAATCTAT   
  
  
+ GCTTTCTGAG TTTAACCCTA ACCCTAATTT TGACCCTTCT CCATCGTCAA TCTCACCCAT CATCGATCCA   
  
  
+ GCTCCTCCAC TTCCTCGAAC CTCGTCGACG GTCAAATTCG AGCCCGACCC GTTTTCCGAT TCGGATCTGA   
  
  
+ AAGCAATCCC AGGTAAGGCT ATCTTGACCC CACCAAGTTC TAGCAATACT TCAAATTTGC GTGAGGCTAA   
  
  
+ GAGGTTGAAA CCCTCAAATT ACACAACCGC CCCAACGCCC ACCCCACCTC CAAAACTGCC CAATTCGGCG   
  
  
+ TCTCCACCAT CGGGCACGAC GGCGTCGAGG CCGGTGGTGC TGGTAGACTC ACAAGAAAAC GGCATCAGAT   
  
  
+ TGGTGCATGC ACTTATGGCC TGTGCCGAAG CAATTCAACT AGAAAACATG GGATTAGCTG AGGCTTTGGT   
  
  
+ TAAACAGATT AGGTATTTAG CAGCATCTCA AGCTGGACCT ATGAGGAAAG TAGCAACTTG TTTTGCAGAA   
  
  
+ GCTCTTACTT GTCGGATCTA CAAGCTATGC CCATCTGTAC CCTACGATGA ATCAGTCTCA GATGAGTTGC   
  
  
+ AGATGCACTT TTATGAGACT TGCCCATATC TTAAATTCGC CCATTTCACG GCAAATCAAG CAATTTTAGA   
  
  
+ AGCATTCAAT GGGAAGAAGA AAGTTCATGT GATTGATTTC AGCATGAAGC AAGGCATGCA ATGGCCGGCT   
  
  
+ TTGATGCAAG CCCTGGCTCT TCGACCGGAG GGTCCACCGC TTTTCCGGTT AACCGGGATT GGACCGCCCG   
  
  
+ CTCCGGACAA CTCGGACCGG CTGCAAGAGG TGGGTTGGAA GCTTGCCCAG TTCGCGGATT CGATCCGGAT   
  
  
+ TAAATTTCAG TATCGTGGGT TTGTGGCAAA CAGTTTGACC GATCTAGAAT CTTCCATGCT AGATCTTGAA   
  
  
+ CCGGACACTG AGGTGGTGGC GGTCAACTCG GTTTTCGAGC TCCACCGGCT GTTGGCTAAA CCCGGGGCGA   
  
  
+ TCGAGAAAGT GCTGGGGTTC ATGAGGGCCG TGAACCCGGT CATTGTGACG ATGGTCGAGC AGGAAGCGAA   
  
  
+ CCACAACGGA CCGGTTTTCT TGGACCGGTT CAATGAGTCG TTGCATTATT ACTCCACCTT GTTTGATTCC   
  
  
+ CTTGAGATTT GTGTTGATAA TGTAGATAAG AAGATGTCGG AGGCTTACTT GGGCCAGCAG ATCTGCAACA   
  
  
+ TGGTGGCTTG TGAAGGGTCT GACCGAGTCG AGAGGCACGA GACCCTGGCT CAGTGGCGAG CCCGGTTCGC   
  
  
+ ATCTGCCGGG TTCGACCCAG TTCATCTGGG TTCGAATGCG TTTAAGCAGG CGAGTATGTT GTTGGAGTTC   
  
  
+ TTTGCTGGTG GTGAAGGGTA CGGCGTGGAG GAGCGAGAAG GGTGTCTCAT GTTGGGATGG CATAGTAGGC   
  
  
+ CGCTTATCAC CACCTCGGCT TGGCAGCTCG CCAAGAACCC GGCTGTGAAT CGGCGATG  

- -Up\_Stream \_Len000TTGGGA AATTAAAATA GTTACACCCT GTTGTGAGTA GAAGTATGAG GTTGTTCGAT   
  
  
- ACTATGTAAG CTAATAACGG ATCATAAAGC AAATCATTCT ATAATCACGG TTTGAAACAT TATTTTTACT   
  
  
- TTGAATGATT TTTTTTAGAG CAAAAGACAT CTCCCTTTTA AACTTCTGAA TCACAGTGTA CTACTGGATT   
  
  
- ACCATGAACA TCCCAGTTCT AACTCTAACT CCCGTATGAC TGCTGGACAT ATTACCAGTT CATCCTAGTT   
  
  
- TCAGCTCTTT TGACAGAGAC GATTGAAAGT ATTAGATTGG GAATTGAGTG AGACTGGGAG TTAAATTAGT   
  
  
- CCGGATTAAG TTTGATAATT ACCAATATTG AGAGAAAGGA AGAAAACTCT AAAAAAAAAA GATGTACGCC   
  
  
- AACTTTTATT AAAAAAATTT TTTATATATA GTGATTGGTA AGTAATAGTA TCAAAATCTC AAGTATAAAT   
  
  
- ACACCAGGAA CAACTCAGAT TAAATTACGA AATCTACCGC ACTTCGTGGT TTAATCACCA TTGGTATTAT   
  
  
- TATTTGATAG TTCACAATTC AGCTTCTTTT GCCTGTGACG AGTTGGTTTA GATCATTGTA TCACATACTG   
  
  
- TACTATTGAT TCACACGGTA CATCATTAGT TAAGAAAGAA AAAAAAAAAA ACCAACTCTA GTGTATTTAA   
  
  
- TGCAATAGTT CCCATCATGC TATATTACCT ACTCCTCTTT AATTTACACT CTAAATCAAC TACTTAGCTT   
  
  
- ATGCAATCAG ACTCGGTAAA CCAGTTTTGA CGTAACCGAT TAGTTATCTT TAATATCTTT TATGTATTGT   
  
  
- ATTACCCTAC GTTAAAAGAT TAACCCCTTT GGTACAGTTA CGTCACTAAA ACCTGGTAAA TCCGGAATAA   
  
  
- GAAAAGTTAA AAAATTAACT AATAAATCTG AGTATACTTG AATAACAACT GTCTAACTCG ATTTTTGTGA   
  
  
- CTACAAAGTA TGTATACATA CTTAACGTGC ATGCTTCCGA GACGAGTAAA GCTCTTTCTT TTAATTGTCA   
  
  
- ATCCTCGTCA TATTAAATTG TTGTTCGCTA TTAATAATCA TACTTCACCA TGCTTAGATA AAATTTCCTC   
  
  
- ATGATACTTC ATGTTTGTTA TTGTTTATTA TTTCTCGTCA TGTAATTGTA TAGAATGATG GATTTATTTA   
  
  
- GCTAAGATTC TCATTTTTTT ACAACTTTAA TTGGGGTAAT ATCAAAATGA AATTGTTCTT CAAAAAGAAG   
  
  
- AAAATGGTAA ATGGTTCATT CTTACTAAGT CCGGCATATC GGTTCCTTTT TATCTTTTGG TGTTTTGATT   
  
  
- GGTACCGGGT TTTTTGTTGG TGGTTCTCCC CGTGATTACA CTGATTGGCT GCCAATTGTA ATAGTAGACA   
  
  
- AACTATGAAC AAGTAGAGAG TGATAAGCCC ATTATCACAC TTTAATGACT ACATGGGAGA TTTGAGCAGC   
  
  
- AAAATTGACA CATTCTTAGT CTAATTGTCA GTGCCTGTGT ACTCATTGTT TAGTTAAAGC CTGTACTTGC   
  
  
- TTGCTTGACA CTTAGGGGGG GTTTTTTTTT TAAAAAAATT TTTTTCAACG TAGGAATAGA AAGACTTGGT   
  
  
- CCGGTCGACA GAGTAAGGAA CCAGGAGCGC GGGGTAGTGC TCGCGACGAG TAATAAGTGG GAGAGAGAGA   
  
  
- GAGGAGAGAC TAGGTGTGGT GTTTGTGTTC CAAACGTGTC GACGTCTCGT CAGCATGATG ATAGTCATCA   
  
  
- CTTTTTGGTT CGTCTTTAGG GGTTAGGGAG AAGAGGAAGA AGGGTCTAAG AAGGAGTTAA GGTTCGGGTA   
  
  
- TAGTCTTTAA ATCTGTTGTT GTTGGTTTTT TTTTTTAATT ATGACGAACT CTTTTTGTTT ATACTATACT   
  
  
- TTAAGGAGCT TTTTCCGGAC GGTATTGTTT AAGGAGCACT TTTTCGTCGA CTCTCTTCTT CTTCTTCTTT   
  
  
- TTTTAGGGCA GTGTTAGAGT TAGAGCTAGG GTTTAACACT TTCTCTTTCT TTTCTACTTC TCTCTTGTGG   
  
  
- GGAAAAGCTT GGGACTGGTC TTTGGGGGCG GCCTCTACAG CGGCCGCGGC CCCTTCAGGT TCTACACCCT   
  
  
- ACTACTCTAC CGGGTCTTGC TGCGATTTTA CCTACTCGAC GAACGACAAC ACCCCATGTT CCACTCCAGC   
  
  
- AGCCTGTACC GCCTTTAACG GGTCTTCGAA CTCGTCGAAC TTCTTCAGTA CCCATCACAA GTTCTTCTGC   
  
  
- CCAATAGAAT GAACCGAAGG CTTTGACAAG TAATGTTAGG ACGTCTAGAC AGATGAACCG AACTTAGATA   
  
  
- CGAAAGACTC AAATTGGGAT TGGGATTAAA ACTGGGAAGA GGTAGCAGTT AGAGTGGGTA GTAGCTAGGT   
  
  
- CGAGGAGGTG AAGGAGCTTG GAGCAGCTGC CAGTTTAAGC TCGGGCTGGG CAAAAGGCTA AGCCTAGACT   
  
  
- TTCGTTAGGG TCCATTCCGA TAGAACTGGG GTGGTTCAAG ATCGTTATGA AGTTTAAACG CACTCCGATT   
  
  
- CTCCAACTTT GGGAGTTTAA TGTGTTGGCG GGGTTGCGGG TGGGGTGGAG GTTTTGACGG GTTAAGCCGC   
  
  
- AGAGGTGGTA GCCCGTGCTG CCGCAGCTCC GGCCACCACG ACCATCTGAG TGTTCTTTTG CCGTAGTCTA   
  
  
- ACCACGTACG TGAATACCGG ACACGGCTTC GTTAAGTTGA TCTTTTGTAC CCTAATCGAC TCCGAAACCA   
  
  
- ATTTGTCTAA TCCATAAATC GTCGTAGAGT TCGACCTGGA TACTCCTTTC ATCGTTGAAC AAAACGTCTT   
  
  
- CGAGAATGAA CAGCCTAGAT GTTCGATACG GGTAGACATG GGATGCTACT TAGTCAGAGT CTACTCAACG   
  
  
- TCTACGTGAA AATACTCTGA ACGGGTATAG AATTTAAGCG GGTAAAGTGC CGTTTAGTTC GTTAAAATCT   
  
  
- TCGTAAGTTA CCCTTCTTCT TTCAAGTACA CTAACTAAAG TCGTACTTCG TTCCGTACGT TACCGGCCGA   
  
  
- AACTACGTTC GGGACCGAGA AGCTGGCCTC CCAGGTGGCG AAAAGGCCAA TTGGCCCTAA CCTGGCGGGC   
  
  
- GAGGCCTGTT GAGCCTGGCC GACGTTCTCC ACCCAACCTT CGAACGGGTC AAGCGCCTAA GCTAGGCCTA   
  
  
- ATTTAAAGTC ATAGCACCCA AACACCGTTT GTCAAACTGG CTAGATCTTA GAAGGTACGA TCTAGAACTT   
  
  
- GGCCTGTGAC TCCACCACCG CCAGTTGAGC CAAAAGCTCG AGGTGGCCGA CAACCGATTT GGGCCCCGCT   
  
  
- AGCTCTTTCA CGACCCCAAG TACTCCCGGC ACTTGGGCCA GTAACACTGC TACCAGCTCG TCCTTCGCTT   
  
  
- GGTGTTGCCT GGCCAAAAGA ACCTGGCCAA GTTACTCAGC AACGTAATAA TGAGGTGGAA CAAACTAAGG   
  
  
- GAACTCTAAA CACAACTATT ACATCTATTC TTCTACAGCC TCCGAATGAA CCCGGTCGTC TAGACGTTGT   
  
  
- ACCACCGAAC ACTTCCCAGA CTGGCTCAGC TCTCCGTGCT CTGGGACCGA GTCACCGCTC GGGCCAAGCG   
  
  
- TAGACGGCCC AAGCTGGGTC AAGTAGACCC AAGCTTACGC AAATTCGTCC GCTCATACAA CAACCTCAAG   
  
  
- AAACGACCAC CACTTCCCAT GCCGCACCTC CTCGCTCTTC CCACAGAGTA CAACCCTACC GTATCATCCG   
  
  
- GCGAATAGTG GTGGAGCCGA ACCGTCGAGC GGTTCTTGGG CCGACACTTA GCCGCTAC

+     STRE

| Site Name | Organism | Position | Strand | Matrix score. | sequence | function |
| --- | --- | --- | --- | --- | --- | --- |
| STRE | Arabidopsis thaliana | 2033 | - | 5 | AGGGG |  |
| STRE | Arabidopsis thaliana | 1361 | + | 5 | AGGGG |  |

>HU10G00709.1   
+ -Up\_Stream \_Len000AACCCT TTAATTTTAT CAATGTGGGA CAACACTCAT CTTCATACTC CAACAAGCTA   
  
  
+ TGATACATTC GATTATTGCC TAGTATTTCG TTTAGTAAGA TATTAGTGCC AAACTTTGTA ATAAAAATGA   
  
  
+ AACTTACTAA AAAAAATCTC GTTTTCTGTA GAGGGAAAAT TTGAAGACTT AGTGTCACAT GATGACCTAA   
  
  
+ TGGTACTTGT AGGGTCAAGA TTGAGATTGA GGGCATACTG ACGACCTGTA TAATGGTCAA GTAGGATCAA   
  
  
+ AGTCGAGAAA ACTGTCTCTG CTAACTTTCA TAATCTAACC CTTAACTCAC TCTGACCCTC AATTTAATCA   
  
  
+ GGCCTAATTC AAACTATTAA TGGTTATAAC TCTCTTTCCT TCTTTTGAGA TTTTTTTTTT CTACATGCGG   
  
  
+ TTGAAAATAA TTTTTTTAAA AAATATATAT CACTAACCAT TCATTATCAT AGTTTTAGAG TTCATATTTA   
  
  
+ TGTGGTCCTT GTTGAGTCTA ATTTAATGCT TTAGATGGCG TGAAGCACCA AATTAGTGGT AACCATAATA   
  
  
+ ATAAACTATC AAGTGTTAAG TCGAAGAAAA CGGACACTGC TCAACCAAAT CTAGTAACAT AGTGTATGAC   
  
  
+ ATGATAACTA AGTGTGCCAT GTAGTAATCA ATTCTTTCTT TTTTTTTTTT TGGTTGAGAT CACATAAATT   
  
  
+ ACGTTATCAA GGGTAGTACG ATATAATGGA TGAGGAGAAA TTAAATGTGA GATTTAGTTG ATGAATCGAA   
  
  
+ TACGTTAGTC TGAGCCATTT GGTCAAAACT GCATTGGCTA ATCAATAGAA ATTATAGAAA ATACATAACA   
  
  
+ TAATGGGATG CAATTTTCTA ATTGGGGAAA CCATGTCAAT GCAGTGATTT TGGACCATTT AGGCCTTATT   
  
  
+ CTTTTCAATT TTTTAATTGA TTATTTAGAC TCATATGAAC TTATTGTTGA CAGATTGAGC TAAAAACACT   
  
  
+ GATGTTTCAT ACATATGTAT GAATTGCACG TACGAAGGCT CTGCTCATTT CGAGAAAGAA AATTAACAGT   
  
  
+ TAGGAGCAGT ATAATTTAAC AACAAGCGAT AATTATTAGT ATGAAGTGGT ACGAATCTAT TTTAAAGGAG   
  
  
+ TACTATGAAG TACAAACAAT AACAAATAAT AAAGAGCAGT ACATTAACAT ATCTTACTAC CTAAATAAAT   
  
  
+ CGATTCTAAG AGTAAAAAAA TGTTGAAATT AACCCCATTA TAGTTTTACT TTAACAAGAA GTTTTTCTTC   
  
  
+ TTTTACCATT TACCAAGTAA GAATGATTCA GGCCGTATAG CCAAGGAAAA ATAGAAAACC ACAAAACTAA   
  
  
+ CCATGGCCCA AAAAACAACC ACCAAGAGGG GCACTAATGT GACTAACCGA CGGTTAACAT TATCATCTGT   
  
  
+ TTGATACTTG TTCATCTCTC ACTATTCGGG TAATAGTGTG AAATTACTGA TGTACCCTCT AAACTCGTCG   
  
  
+ TTTTAACTGT GTAAGAATCA GATTAACAGT CACGGACACA TGAGTAACAA ATCAATTTCG GACATGAACG   
  
  
+ AACGAACTGT GAATCCCCCC CAAAAAAAAA ATTTTTTTAA AAAAAGTTGC ATCCTTATCT TTCTGAACCA   
  
  
+ GGCCAGCTGT CTCATTCCTT GGTCCTCGCG CCCCATCACG AGCGCTGCTC ATTATTCACC CTCTCTCTCT   
  
  
+ CTCCTCTCTG ATCCACACCA CAAACACAAG GTTTGCACAG CTGCAGAGCA GTCGTACTAC TATCAGTAGT   
  
  
+ GAAAAACCAA GCAGAAATCC CCAATCCCTC TTCTCCTTCT TCCCAGATTC TTCCTCAATT CCAAGCCCAT   
  
  
+ ATCAGAAATT TAGACAACAA CAACCAAAAA AAAAAATTAA TACTGCTTGA GAAAAACAAA TATGATATGA   
  
  
+ AATTCCTCGA AAAAGGCCTG CCATAACAAA TTCCTCGTGA AAAAGCAGCT GAGAGAAGAA GAAGAAGAAA   
  
  
+ AAAATCCCGT CACAATCTCA ATCTCGATCC CAAATTGTGA AAGAGAAAGA AAAGATGAAG AGAGAACACC   
  
  
+ CCTTTTCGAA CCCTGACCAG AAACCCCCGC CGGAGATGTC GCCGGCGCCG GGGAAGTCCA AGATGTGGGA   
  
  
+ TGATGAGATG GCCCAGAACG ACGCTAAAAT GGATGAGCTG CTTGCTGTTG TGGGGTACAA GGTGAGGTCG   
  
  
+ TCGGACATGG CGGAAATTGC CCAGAAGCTT GAGCAGCTTG AAGAAGTCAT GGGTAGTGTT CAAGAAGACG   
  
  
+ GGTTATCTTA CTTGGCTTCC GAAACTGTTC ATTACAATCC TGCAGATCTG TCTACTTGGC TTGAATCTAT   
  
  
+ GCTTTCTGAG TTTAACCCTA ACCCTAATTT TGACCCTTCT CCATCGTCAA TCTCACCCAT CATCGATCCA   
  
  
+ GCTCCTCCAC TTCCTCGAAC CTCGTCGACG GTCAAATTCG AGCCCGACCC GTTTTCCGAT TCGGATCTGA   
  
  
+ AAGCAATCCC AGGTAAGGCT ATCTTGACCC CACCAAGTTC TAGCAATACT TCAAATTTGC GTGAGGCTAA   
  
  
+ GAGGTTGAAA CCCTCAAATT ACACAACCGC CCCAACGCCC ACCCCACCTC CAAAACTGCC CAATTCGGCG   
  
  
+ TCTCCACCAT CGGGCACGAC GGCGTCGAGG CCGGTGGTGC TGGTAGACTC ACAAGAAAAC GGCATCAGAT   
  
  
+ TGGTGCATGC ACTTATGGCC TGTGCCGAAG CAATTCAACT AGAAAACATG GGATTAGCTG AGGCTTTGGT   
  
  
+ TAAACAGATT AGGTATTTAG CAGCATCTCA AGCTGGACCT ATGAGGAAAG TAGCAACTTG TTTTGCAGAA   
  
  
+ GCTCTTACTT GTCGGATCTA CAAGCTATGC CCATCTGTAC CCTACGATGA ATCAGTCTCA GATGAGTTGC   
  
  
+ AGATGCACTT TTATGAGACT TGCCCATATC TTAAATTCGC CCATTTCACG GCAAATCAAG CAATTTTAGA   
  
  
+ AGCATTCAAT GGGAAGAAGA AAGTTCATGT GATTGATTTC AGCATGAAGC AAGGCATGCA ATGGCCGGCT   
  
  
+ TTGATGCAAG CCCTGGCTCT TCGACCGGAG GGTCCACCGC TTTTCCGGTT AACCGGGATT GGACCGCCCG   
  
  
+ CTCCGGACAA CTCGGACCGG CTGCAAGAGG TGGGTTGGAA GCTTGCCCAG TTCGCGGATT CGATCCGGAT   
  
  
+ TAAATTTCAG TATCGTGGGT TTGTGGCAAA CAGTTTGACC GATCTAGAAT CTTCCATGCT AGATCTTGAA   
  
  
+ CCGGACACTG AGGTGGTGGC GGTCAACTCG GTTTTCGAGC TCCACCGGCT GTTGGCTAAA CCCGGGGCGA   
  
  
+ TCGAGAAAGT GCTGGGGTTC ATGAGGGCCG TGAACCCGGT CATTGTGACG ATGGTCGAGC AGGAAGCGAA   
  
  
+ CCACAACGGA CCGGTTTTCT TGGACCGGTT CAATGAGTCG TTGCATTATT ACTCCACCTT GTTTGATTCC   
  
  
+ CTTGAGATTT GTGTTGATAA TGTAGATAAG AAGATGTCGG AGGCTTACTT GGGCCAGCAG ATCTGCAACA   
  
  
+ TGGTGGCTTG TGAAGGGTCT GACCGAGTCG AGAGGCACGA GACCCTGGCT CAGTGGCGAG CCCGGTTCGC   
  
  
+ ATCTGCCGGG TTCGACCCAG TTCATCTGGG TTCGAATGCG TTTAAGCAGG CGAGTATGTT GTTGGAGTTC   
  
  
+ TTTGCTGGTG GTGAAGGGTA CGGCGTGGAG GAGCGAGAAG GGTGTCTCAT GTTGGGATGG CATAGTAGGC   
  
  
+ CGCTTATCAC CACCTCGGCT TGGCAGCTCG CCAAGAACCC GGCTGTGAAT CGGCGATG  

- -Up\_Stream \_Len000TTGGGA AATTAAAATA GTTACACCCT GTTGTGAGTA GAAGTATGAG GTTGTTCGAT   
  
  
- ACTATGTAAG CTAATAACGG ATCATAAAGC AAATCATTCT ATAATCACGG TTTGAAACAT TATTTTTACT   
  
  
- TTGAATGATT TTTTTTAGAG CAAAAGACAT CTCCCTTTTA AACTTCTGAA TCACAGTGTA CTACTGGATT   
  
  
- ACCATGAACA TCCCAGTTCT AACTCTAACT CCCGTATGAC TGCTGGACAT ATTACCAGTT CATCCTAGTT   
  
  
- TCAGCTCTTT TGACAGAGAC GATTGAAAGT ATTAGATTGG GAATTGAGTG AGACTGGGAG TTAAATTAGT   
  
  
- CCGGATTAAG TTTGATAATT ACCAATATTG AGAGAAAGGA AGAAAACTCT AAAAAAAAAA GATGTACGCC   
  
  
- AACTTTTATT AAAAAAATTT TTTATATATA GTGATTGGTA AGTAATAGTA TCAAAATCTC AAGTATAAAT   
  
  
- ACACCAGGAA CAACTCAGAT TAAATTACGA AATCTACCGC ACTTCGTGGT TTAATCACCA TTGGTATTAT   
  
  
- TATTTGATAG TTCACAATTC AGCTTCTTTT GCCTGTGACG AGTTGGTTTA GATCATTGTA TCACATACTG   
  
  
- TACTATTGAT TCACACGGTA CATCATTAGT TAAGAAAGAA AAAAAAAAAA ACCAACTCTA GTGTATTTAA   
  
  
- TGCAATAGTT CCCATCATGC TATATTACCT ACTCCTCTTT AATTTACACT CTAAATCAAC TACTTAGCTT   
  
  
- ATGCAATCAG ACTCGGTAAA CCAGTTTTGA CGTAACCGAT TAGTTATCTT TAATATCTTT TATGTATTGT   
  
  
- ATTACCCTAC GTTAAAAGAT TAACCCCTTT GGTACAGTTA CGTCACTAAA ACCTGGTAAA TCCGGAATAA   
  
  
- GAAAAGTTAA AAAATTAACT AATAAATCTG AGTATACTTG AATAACAACT GTCTAACTCG ATTTTTGTGA   
  
  
- CTACAAAGTA TGTATACATA CTTAACGTGC ATGCTTCCGA GACGAGTAAA GCTCTTTCTT TTAATTGTCA   
  
  
- ATCCTCGTCA TATTAAATTG TTGTTCGCTA TTAATAATCA TACTTCACCA TGCTTAGATA AAATTTCCTC   
  
  
- ATGATACTTC ATGTTTGTTA TTGTTTATTA TTTCTCGTCA TGTAATTGTA TAGAATGATG GATTTATTTA   
  
  
- GCTAAGATTC TCATTTTTTT ACAACTTTAA TTGGGGTAAT ATCAAAATGA AATTGTTCTT CAAAAAGAAG   
  
  
- AAAATGGTAA ATGGTTCATT CTTACTAAGT CCGGCATATC GGTTCCTTTT TATCTTTTGG TGTTTTGATT   
  
  
- GGTACCGGGT TTTTTGTTGG TGGTTCTCCC CGTGATTACA CTGATTGGCT GCCAATTGTA ATAGTAGACA   
  
  
- AACTATGAAC AAGTAGAGAG TGATAAGCCC ATTATCACAC TTTAATGACT ACATGGGAGA TTTGAGCAGC   
  
  
- AAAATTGACA CATTCTTAGT CTAATTGTCA GTGCCTGTGT ACTCATTGTT TAGTTAAAGC CTGTACTTGC   
  
  
- TTGCTTGACA CTTAGGGGGG GTTTTTTTTT TAAAAAAATT TTTTTCAACG TAGGAATAGA AAGACTTGGT   
  
  
- CCGGTCGACA GAGTAAGGAA CCAGGAGCGC GGGGTAGTGC TCGCGACGAG TAATAAGTGG GAGAGAGAGA   
  
  
- GAGGAGAGAC TAGGTGTGGT GTTTGTGTTC CAAACGTGTC GACGTCTCGT CAGCATGATG ATAGTCATCA   
  
  
- CTTTTTGGTT CGTCTTTAGG GGTTAGGGAG AAGAGGAAGA AGGGTCTAAG AAGGAGTTAA GGTTCGGGTA   
  
  
- TAGTCTTTAA ATCTGTTGTT GTTGGTTTTT TTTTTTAATT ATGACGAACT CTTTTTGTTT ATACTATACT   
  
  
- TTAAGGAGCT TTTTCCGGAC GGTATTGTTT AAGGAGCACT TTTTCGTCGA CTCTCTTCTT CTTCTTCTTT   
  
  
- TTTTAGGGCA GTGTTAGAGT TAGAGCTAGG GTTTAACACT TTCTCTTTCT TTTCTACTTC TCTCTTGTGG   
  
  
- GGAAAAGCTT GGGACTGGTC TTTGGGGGCG GCCTCTACAG CGGCCGCGGC CCCTTCAGGT TCTACACCCT   
  
  
- ACTACTCTAC CGGGTCTTGC TGCGATTTTA CCTACTCGAC GAACGACAAC ACCCCATGTT CCACTCCAGC   
  
  
- AGCCTGTACC GCCTTTAACG GGTCTTCGAA CTCGTCGAAC TTCTTCAGTA CCCATCACAA GTTCTTCTGC   
  
  
- CCAATAGAAT GAACCGAAGG CTTTGACAAG TAATGTTAGG ACGTCTAGAC AGATGAACCG AACTTAGATA   
  
  
- CGAAAGACTC AAATTGGGAT TGGGATTAAA ACTGGGAAGA GGTAGCAGTT AGAGTGGGTA GTAGCTAGGT   
  
  
- CGAGGAGGTG AAGGAGCTTG GAGCAGCTGC CAGTTTAAGC TCGGGCTGGG CAAAAGGCTA AGCCTAGACT   
  
  
- TTCGTTAGGG TCCATTCCGA TAGAACTGGG GTGGTTCAAG ATCGTTATGA AGTTTAAACG CACTCCGATT   
  
  
- CTCCAACTTT GGGAGTTTAA TGTGTTGGCG GGGTTGCGGG TGGGGTGGAG GTTTTGACGG GTTAAGCCGC   
  
  
- AGAGGTGGTA GCCCGTGCTG CCGCAGCTCC GGCCACCACG ACCATCTGAG TGTTCTTTTG CCGTAGTCTA   
  
  
- ACCACGTACG TGAATACCGG ACACGGCTTC GTTAAGTTGA TCTTTTGTAC CCTAATCGAC TCCGAAACCA   
  
  
- ATTTGTCTAA TCCATAAATC GTCGTAGAGT TCGACCTGGA TACTCCTTTC ATCGTTGAAC AAAACGTCTT   
  
  
- CGAGAATGAA CAGCCTAGAT GTTCGATACG GGTAGACATG GGATGCTACT TAGTCAGAGT CTACTCAACG   
  
  
- TCTACGTGAA AATACTCTGA ACGGGTATAG AATTTAAGCG GGTAAAGTGC CGTTTAGTTC GTTAAAATCT   
  
  
- TCGTAAGTTA CCCTTCTTCT TTCAAGTACA CTAACTAAAG TCGTACTTCG TTCCGTACGT TACCGGCCGA   
  
  
- AACTACGTTC GGGACCGAGA AGCTGGCCTC CCAGGTGGCG AAAAGGCCAA TTGGCCCTAA CCTGGCGGGC   
  
  
- GAGGCCTGTT GAGCCTGGCC GACGTTCTCC ACCCAACCTT CGAACGGGTC AAGCGCCTAA GCTAGGCCTA   
  
  
- ATTTAAAGTC ATAGCACCCA AACACCGTTT GTCAAACTGG CTAGATCTTA GAAGGTACGA TCTAGAACTT   
  
  
- GGCCTGTGAC TCCACCACCG CCAGTTGAGC CAAAAGCTCG AGGTGGCCGA CAACCGATTT GGGCCCCGCT   
  
  
- AGCTCTTTCA CGACCCCAAG TACTCCCGGC ACTTGGGCCA GTAACACTGC TACCAGCTCG TCCTTCGCTT   
  
  
- GGTGTTGCCT GGCCAAAAGA ACCTGGCCAA GTTACTCAGC AACGTAATAA TGAGGTGGAA CAAACTAAGG   
  
  
- GAACTCTAAA CACAACTATT ACATCTATTC TTCTACAGCC TCCGAATGAA CCCGGTCGTC TAGACGTTGT   
  
  
- ACCACCGAAC ACTTCCCAGA CTGGCTCAGC TCTCCGTGCT CTGGGACCGA GTCACCGCTC GGGCCAAGCG   
  
  
- TAGACGGCCC AAGCTGGGTC AAGTAGACCC AAGCTTACGC AAATTCGTCC GCTCATACAA CAACCTCAAG   
  
  
- AAACGACCAC CACTTCCCAT GCCGCACCTC CTCGCTCTTC CCACAGAGTA CAACCCTACC GTATCATCCG   
  
  
- GCGAATAGTG GTGGAGCCGA ACCGTCGAGC GGTTCTTGGG CCGACACTTA GCCGCTAC

+     Sp1

| Site Name | Organism | Position | Strand | Matrix score. | sequence | function |
| --- | --- | --- | --- | --- | --- | --- |
| Sp1 | Oryza sativa | 2551 | - | 6 | GGGCGG | light responsive element |
| Sp1 | Oryza sativa | 3078 | - | 6 | GGGCGG | light responsive element |

>HU10G00709.1   
+ -Up\_Stream \_Len000AACCCT TTAATTTTAT CAATGTGGGA CAACACTCAT CTTCATACTC CAACAAGCTA   
  
  
+ TGATACATTC GATTATTGCC TAGTATTTCG TTTAGTAAGA TATTAGTGCC AAACTTTGTA ATAAAAATGA   
  
  
+ AACTTACTAA AAAAAATCTC GTTTTCTGTA GAGGGAAAAT TTGAAGACTT AGTGTCACAT GATGACCTAA   
  
  
+ TGGTACTTGT AGGGTCAAGA TTGAGATTGA GGGCATACTG ACGACCTGTA TAATGGTCAA GTAGGATCAA   
  
  
+ AGTCGAGAAA ACTGTCTCTG CTAACTTTCA TAATCTAACC CTTAACTCAC TCTGACCCTC AATTTAATCA   
  
  
+ GGCCTAATTC AAACTATTAA TGGTTATAAC TCTCTTTCCT TCTTTTGAGA TTTTTTTTTT CTACATGCGG   
  
  
+ TTGAAAATAA TTTTTTTAAA AAATATATAT CACTAACCAT TCATTATCAT AGTTTTAGAG TTCATATTTA   
  
  
+ TGTGGTCCTT GTTGAGTCTA ATTTAATGCT TTAGATGGCG TGAAGCACCA AATTAGTGGT AACCATAATA   
  
  
+ ATAAACTATC AAGTGTTAAG TCGAAGAAAA CGGACACTGC TCAACCAAAT CTAGTAACAT AGTGTATGAC   
  
  
+ ATGATAACTA AGTGTGCCAT GTAGTAATCA ATTCTTTCTT TTTTTTTTTT TGGTTGAGAT CACATAAATT   
  
  
+ ACGTTATCAA GGGTAGTACG ATATAATGGA TGAGGAGAAA TTAAATGTGA GATTTAGTTG ATGAATCGAA   
  
  
+ TACGTTAGTC TGAGCCATTT GGTCAAAACT GCATTGGCTA ATCAATAGAA ATTATAGAAA ATACATAACA   
  
  
+ TAATGGGATG CAATTTTCTA ATTGGGGAAA CCATGTCAAT GCAGTGATTT TGGACCATTT AGGCCTTATT   
  
  
+ CTTTTCAATT TTTTAATTGA TTATTTAGAC TCATATGAAC TTATTGTTGA CAGATTGAGC TAAAAACACT   
  
  
+ GATGTTTCAT ACATATGTAT GAATTGCACG TACGAAGGCT CTGCTCATTT CGAGAAAGAA AATTAACAGT   
  
  
+ TAGGAGCAGT ATAATTTAAC AACAAGCGAT AATTATTAGT ATGAAGTGGT ACGAATCTAT TTTAAAGGAG   
  
  
+ TACTATGAAG TACAAACAAT AACAAATAAT AAAGAGCAGT ACATTAACAT ATCTTACTAC CTAAATAAAT   
  
  
+ CGATTCTAAG AGTAAAAAAA TGTTGAAATT AACCCCATTA TAGTTTTACT TTAACAAGAA GTTTTTCTTC   
  
  
+ TTTTACCATT TACCAAGTAA GAATGATTCA GGCCGTATAG CCAAGGAAAA ATAGAAAACC ACAAAACTAA   
  
  
+ CCATGGCCCA AAAAACAACC ACCAAGAGGG GCACTAATGT GACTAACCGA CGGTTAACAT TATCATCTGT   
  
  
+ TTGATACTTG TTCATCTCTC ACTATTCGGG TAATAGTGTG AAATTACTGA TGTACCCTCT AAACTCGTCG   
  
  
+ TTTTAACTGT GTAAGAATCA GATTAACAGT CACGGACACA TGAGTAACAA ATCAATTTCG GACATGAACG   
  
  
+ AACGAACTGT GAATCCCCCC CAAAAAAAAA ATTTTTTTAA AAAAAGTTGC ATCCTTATCT TTCTGAACCA   
  
  
+ GGCCAGCTGT CTCATTCCTT GGTCCTCGCG CCCCATCACG AGCGCTGCTC ATTATTCACC CTCTCTCTCT   
  
  
+ CTCCTCTCTG ATCCACACCA CAAACACAAG GTTTGCACAG CTGCAGAGCA GTCGTACTAC TATCAGTAGT   
  
  
+ GAAAAACCAA GCAGAAATCC CCAATCCCTC TTCTCCTTCT TCCCAGATTC TTCCTCAATT CCAAGCCCAT   
  
  
+ ATCAGAAATT TAGACAACAA CAACCAAAAA AAAAAATTAA TACTGCTTGA GAAAAACAAA TATGATATGA   
  
  
+ AATTCCTCGA AAAAGGCCTG CCATAACAAA TTCCTCGTGA AAAAGCAGCT GAGAGAAGAA GAAGAAGAAA   
  
  
+ AAAATCCCGT CACAATCTCA ATCTCGATCC CAAATTGTGA AAGAGAAAGA AAAGATGAAG AGAGAACACC   
  
  
+ CCTTTTCGAA CCCTGACCAG AAACCCCCGC CGGAGATGTC GCCGGCGCCG GGGAAGTCCA AGATGTGGGA   
  
  
+ TGATGAGATG GCCCAGAACG ACGCTAAAAT GGATGAGCTG CTTGCTGTTG TGGGGTACAA GGTGAGGTCG   
  
  
+ TCGGACATGG CGGAAATTGC CCAGAAGCTT GAGCAGCTTG AAGAAGTCAT GGGTAGTGTT CAAGAAGACG   
  
  
+ GGTTATCTTA CTTGGCTTCC GAAACTGTTC ATTACAATCC TGCAGATCTG TCTACTTGGC TTGAATCTAT   
  
  
+ GCTTTCTGAG TTTAACCCTA ACCCTAATTT TGACCCTTCT CCATCGTCAA TCTCACCCAT CATCGATCCA   
  
  
+ GCTCCTCCAC TTCCTCGAAC CTCGTCGACG GTCAAATTCG AGCCCGACCC GTTTTCCGAT TCGGATCTGA   
  
  
+ AAGCAATCCC AGGTAAGGCT ATCTTGACCC CACCAAGTTC TAGCAATACT TCAAATTTGC GTGAGGCTAA   
  
  
+ GAGGTTGAAA CCCTCAAATT ACACAACCGC CCCAACGCCC ACCCCACCTC CAAAACTGCC CAATTCGGCG   
  
  
+ TCTCCACCAT CGGGCACGAC GGCGTCGAGG CCGGTGGTGC TGGTAGACTC ACAAGAAAAC GGCATCAGAT   
  
  
+ TGGTGCATGC ACTTATGGCC TGTGCCGAAG CAATTCAACT AGAAAACATG GGATTAGCTG AGGCTTTGGT   
  
  
+ TAAACAGATT AGGTATTTAG CAGCATCTCA AGCTGGACCT ATGAGGAAAG TAGCAACTTG TTTTGCAGAA   
  
  
+ GCTCTTACTT GTCGGATCTA CAAGCTATGC CCATCTGTAC CCTACGATGA ATCAGTCTCA GATGAGTTGC   
  
  
+ AGATGCACTT TTATGAGACT TGCCCATATC TTAAATTCGC CCATTTCACG GCAAATCAAG CAATTTTAGA   
  
  
+ AGCATTCAAT GGGAAGAAGA AAGTTCATGT GATTGATTTC AGCATGAAGC AAGGCATGCA ATGGCCGGCT   
  
  
+ TTGATGCAAG CCCTGGCTCT TCGACCGGAG GGTCCACCGC TTTTCCGGTT AACCGGGATT GGACCGCCCG   
  
  
+ CTCCGGACAA CTCGGACCGG CTGCAAGAGG TGGGTTGGAA GCTTGCCCAG TTCGCGGATT CGATCCGGAT   
  
  
+ TAAATTTCAG TATCGTGGGT TTGTGGCAAA CAGTTTGACC GATCTAGAAT CTTCCATGCT AGATCTTGAA   
  
  
+ CCGGACACTG AGGTGGTGGC GGTCAACTCG GTTTTCGAGC TCCACCGGCT GTTGGCTAAA CCCGGGGCGA   
  
  
+ TCGAGAAAGT GCTGGGGTTC ATGAGGGCCG TGAACCCGGT CATTGTGACG ATGGTCGAGC AGGAAGCGAA   
  
  
+ CCACAACGGA CCGGTTTTCT TGGACCGGTT CAATGAGTCG TTGCATTATT ACTCCACCTT GTTTGATTCC   
  
  
+ CTTGAGATTT GTGTTGATAA TGTAGATAAG AAGATGTCGG AGGCTTACTT GGGCCAGCAG ATCTGCAACA   
  
  
+ TGGTGGCTTG TGAAGGGTCT GACCGAGTCG AGAGGCACGA GACCCTGGCT CAGTGGCGAG CCCGGTTCGC   
  
  
+ ATCTGCCGGG TTCGACCCAG TTCATCTGGG TTCGAATGCG TTTAAGCAGG CGAGTATGTT GTTGGAGTTC   
  
  
+ TTTGCTGGTG GTGAAGGGTA CGGCGTGGAG GAGCGAGAAG GGTGTCTCAT GTTGGGATGG CATAGTAGGC   
  
  
+ CGCTTATCAC CACCTCGGCT TGGCAGCTCG CCAAGAACCC GGCTGTGAAT CGGCGATG  

- -Up\_Stream \_Len000TTGGGA AATTAAAATA GTTACACCCT GTTGTGAGTA GAAGTATGAG GTTGTTCGAT   
  
  
- ACTATGTAAG CTAATAACGG ATCATAAAGC AAATCATTCT ATAATCACGG TTTGAAACAT TATTTTTACT   
  
  
- TTGAATGATT TTTTTTAGAG CAAAAGACAT CTCCCTTTTA AACTTCTGAA TCACAGTGTA CTACTGGATT   
  
  
- ACCATGAACA TCCCAGTTCT AACTCTAACT CCCGTATGAC TGCTGGACAT ATTACCAGTT CATCCTAGTT   
  
  
- TCAGCTCTTT TGACAGAGAC GATTGAAAGT ATTAGATTGG GAATTGAGTG AGACTGGGAG TTAAATTAGT   
  
  
- CCGGATTAAG TTTGATAATT ACCAATATTG AGAGAAAGGA AGAAAACTCT AAAAAAAAAA GATGTACGCC   
  
  
- AACTTTTATT AAAAAAATTT TTTATATATA GTGATTGGTA AGTAATAGTA TCAAAATCTC AAGTATAAAT   
  
  
- ACACCAGGAA CAACTCAGAT TAAATTACGA AATCTACCGC ACTTCGTGGT TTAATCACCA TTGGTATTAT   
  
  
- TATTTGATAG TTCACAATTC AGCTTCTTTT GCCTGTGACG AGTTGGTTTA GATCATTGTA TCACATACTG   
  
  
- TACTATTGAT TCACACGGTA CATCATTAGT TAAGAAAGAA AAAAAAAAAA ACCAACTCTA GTGTATTTAA   
  
  
- TGCAATAGTT CCCATCATGC TATATTACCT ACTCCTCTTT AATTTACACT CTAAATCAAC TACTTAGCTT   
  
  
- ATGCAATCAG ACTCGGTAAA CCAGTTTTGA CGTAACCGAT TAGTTATCTT TAATATCTTT TATGTATTGT   
  
  
- ATTACCCTAC GTTAAAAGAT TAACCCCTTT GGTACAGTTA CGTCACTAAA ACCTGGTAAA TCCGGAATAA   
  
  
- GAAAAGTTAA AAAATTAACT AATAAATCTG AGTATACTTG AATAACAACT GTCTAACTCG ATTTTTGTGA   
  
  
- CTACAAAGTA TGTATACATA CTTAACGTGC ATGCTTCCGA GACGAGTAAA GCTCTTTCTT TTAATTGTCA   
  
  
- ATCCTCGTCA TATTAAATTG TTGTTCGCTA TTAATAATCA TACTTCACCA TGCTTAGATA AAATTTCCTC   
  
  
- ATGATACTTC ATGTTTGTTA TTGTTTATTA TTTCTCGTCA TGTAATTGTA TAGAATGATG GATTTATTTA   
  
  
- GCTAAGATTC TCATTTTTTT ACAACTTTAA TTGGGGTAAT ATCAAAATGA AATTGTTCTT CAAAAAGAAG   
  
  
- AAAATGGTAA ATGGTTCATT CTTACTAAGT CCGGCATATC GGTTCCTTTT TATCTTTTGG TGTTTTGATT   
  
  
- GGTACCGGGT TTTTTGTTGG TGGTTCTCCC CGTGATTACA CTGATTGGCT GCCAATTGTA ATAGTAGACA   
  
  
- AACTATGAAC AAGTAGAGAG TGATAAGCCC ATTATCACAC TTTAATGACT ACATGGGAGA TTTGAGCAGC   
  
  
- AAAATTGACA CATTCTTAGT CTAATTGTCA GTGCCTGTGT ACTCATTGTT TAGTTAAAGC CTGTACTTGC   
  
  
- TTGCTTGACA CTTAGGGGGG GTTTTTTTTT TAAAAAAATT TTTTTCAACG TAGGAATAGA AAGACTTGGT   
  
  
- CCGGTCGACA GAGTAAGGAA CCAGGAGCGC GGGGTAGTGC TCGCGACGAG TAATAAGTGG GAGAGAGAGA   
  
  
- GAGGAGAGAC TAGGTGTGGT GTTTGTGTTC CAAACGTGTC GACGTCTCGT CAGCATGATG ATAGTCATCA   
  
  
- CTTTTTGGTT CGTCTTTAGG GGTTAGGGAG AAGAGGAAGA AGGGTCTAAG AAGGAGTTAA GGTTCGGGTA   
  
  
- TAGTCTTTAA ATCTGTTGTT GTTGGTTTTT TTTTTTAATT ATGACGAACT CTTTTTGTTT ATACTATACT   
  
  
- TTAAGGAGCT TTTTCCGGAC GGTATTGTTT AAGGAGCACT TTTTCGTCGA CTCTCTTCTT CTTCTTCTTT   
  
  
- TTTTAGGGCA GTGTTAGAGT TAGAGCTAGG GTTTAACACT TTCTCTTTCT TTTCTACTTC TCTCTTGTGG   
  
  
- GGAAAAGCTT GGGACTGGTC TTTGGGGGCG GCCTCTACAG CGGCCGCGGC CCCTTCAGGT TCTACACCCT   
  
  
- ACTACTCTAC CGGGTCTTGC TGCGATTTTA CCTACTCGAC GAACGACAAC ACCCCATGTT CCACTCCAGC   
  
  
- AGCCTGTACC GCCTTTAACG GGTCTTCGAA CTCGTCGAAC TTCTTCAGTA CCCATCACAA GTTCTTCTGC   
  
  
- CCAATAGAAT GAACCGAAGG CTTTGACAAG TAATGTTAGG ACGTCTAGAC AGATGAACCG AACTTAGATA   
  
  
- CGAAAGACTC AAATTGGGAT TGGGATTAAA ACTGGGAAGA GGTAGCAGTT AGAGTGGGTA GTAGCTAGGT   
  
  
- CGAGGAGGTG AAGGAGCTTG GAGCAGCTGC CAGTTTAAGC TCGGGCTGGG CAAAAGGCTA AGCCTAGACT   
  
  
- TTCGTTAGGG TCCATTCCGA TAGAACTGGG GTGGTTCAAG ATCGTTATGA AGTTTAAACG CACTCCGATT   
  
  
- CTCCAACTTT GGGAGTTTAA TGTGTTGGCG GGGTTGCGGG TGGGGTGGAG GTTTTGACGG GTTAAGCCGC   
  
  
- AGAGGTGGTA GCCCGTGCTG CCGCAGCTCC GGCCACCACG ACCATCTGAG TGTTCTTTTG CCGTAGTCTA   
  
  
- ACCACGTACG TGAATACCGG ACACGGCTTC GTTAAGTTGA TCTTTTGTAC CCTAATCGAC TCCGAAACCA   
  
  
- ATTTGTCTAA TCCATAAATC GTCGTAGAGT TCGACCTGGA TACTCCTTTC ATCGTTGAAC AAAACGTCTT   
  
  
- CGAGAATGAA CAGCCTAGAT GTTCGATACG GGTAGACATG GGATGCTACT TAGTCAGAGT CTACTCAACG   
  
  
- TCTACGTGAA AATACTCTGA ACGGGTATAG AATTTAAGCG GGTAAAGTGC CGTTTAGTTC GTTAAAATCT   
  
  
- TCGTAAGTTA CCCTTCTTCT TTCAAGTACA CTAACTAAAG TCGTACTTCG TTCCGTACGT TACCGGCCGA   
  
  
- AACTACGTTC GGGACCGAGA AGCTGGCCTC CCAGGTGGCG AAAAGGCCAA TTGGCCCTAA CCTGGCGGGC   
  
  
- GAGGCCTGTT GAGCCTGGCC GACGTTCTCC ACCCAACCTT CGAACGGGTC AAGCGCCTAA GCTAGGCCTA   
  
  
- ATTTAAAGTC ATAGCACCCA AACACCGTTT GTCAAACTGG CTAGATCTTA GAAGGTACGA TCTAGAACTT   
  
  
- GGCCTGTGAC TCCACCACCG CCAGTTGAGC CAAAAGCTCG AGGTGGCCGA CAACCGATTT GGGCCCCGCT   
  
  
- AGCTCTTTCA CGACCCCAAG TACTCCCGGC ACTTGGGCCA GTAACACTGC TACCAGCTCG TCCTTCGCTT   
  
  
- GGTGTTGCCT GGCCAAAAGA ACCTGGCCAA GTTACTCAGC AACGTAATAA TGAGGTGGAA CAAACTAAGG   
  
  
- GAACTCTAAA CACAACTATT ACATCTATTC TTCTACAGCC TCCGAATGAA CCCGGTCGTC TAGACGTTGT   
  
  
- ACCACCGAAC ACTTCCCAGA CTGGCTCAGC TCTCCGTGCT CTGGGACCGA GTCACCGCTC GGGCCAAGCG   
  
  
- TAGACGGCCC AAGCTGGGTC AAGTAGACCC AAGCTTACGC AAATTCGTCC GCTCATACAA CAACCTCAAG   
  
  
- AAACGACCAC CACTTCCCAT GCCGCACCTC CTCGCTCTTC CCACAGAGTA CAACCCTACC GTATCATCCG   
  
  
- GCGAATAGTG GTGGAGCCGA ACCGTCGAGC GGTTCTTGGG CCGACACTTA GCCGCTAC

+     TATA-box

| Site Name | Organism | Position | Strand | Matrix score. | sequence | function |
| --- | --- | --- | --- | --- | --- | --- |
| TATA-box | Arabidopsis thaliana | 1300 | + | 4 | TATA | core promoter element around -30 of transcription start |
| TATA-box | Arabidopsis thaliana | 1233 | + | 4 | TATA | core promoter element around -30 of transcription start |
| TATA-box | Arabidopsis thaliana | 726 | + | 4 | TATA | core promoter element around -30 of transcription start |
| TATA-box | Arabidopsis thaliana | 1064 | + | 4 | TATA | core promoter element around -30 of transcription start |
| TATA-box | Arabidopsis thaliana | 826 | - | 5 | TATAA | core promoter element around -30 of transcription start |
| TATA-box | Brassica napus | 825 | + | 6 | ATTATA | core promoter element around -30 of transcription start |
| TATA-box | Arabidopsis thaliana | 448 | + | 6 | TATATA | core promoter element around -30 of transcription start |
| TATA-box | Brassica oleracea | 725 | + | 6 | ATATAA | core promoter element around -30 of transcription start |
| TATA-box | Brassica napus | 447 | + | 6 | ATATAT | core promoter element around -30 of transcription start |
| TATA-box | Arabidopsis thaliana | 378 | - | 5 | TATAA | core promoter element around -30 of transcription start |
| TATA-box | Arabidopsis thaliana | 263 | + | 4 | TATA | core promoter element around -30 of transcription start |
| TATA-box | Arabidopsis thaliana | 379 | + | 4 | TATA | core promoter element around -30 of transcription start |
| TATA-box | Helianthus annuus | 261 | - | 6 | TATACA | core promoter element around -30 of transcription start |
| TATA-box | Arabidopsis thaliana | 450 | + | 4 | TATA | core promoter element around -30 of transcription start |
| TATA-box | Brassica napus | 449 | + | 6 | ATATAT | core promoter element around -30 of transcription start |
| TATA-box | Arabidopsis thaliana | 1232 | - | 5 | TATAA | core promoter element around -30 of transcription start |
| TATA-box | Arabidopsis thaliana | 827 | + | 4 | TATA | core promoter element around -30 of transcription start |
| TATA-box | Arabidopsis thaliana | 1186 | + | 9 | taTATAAAtc | core promoter element around -30 of transcription start |
| TATA-box | Brassica napus | 1231 | + | 6 | ATTATA | core promoter element around -30 of transcription start |

>HU10G00709.1   
+ -Up\_Stream \_Len000AACCCT TTAATTTTAT CAATGTGGGA CAACACTCAT CTTCATACTC CAACAAGCTA   
  
  
+ TGATACATTC GATTATTGCC TAGTATTTCG TTTAGTAAGA TATTAGTGCC AAACTTTGTA ATAAAAATGA   
  
  
+ AACTTACTAA AAAAAATCTC GTTTTCTGTA GAGGGAAAAT TTGAAGACTT AGTGTCACAT GATGACCTAA   
  
  
+ TGGTACTTGT AGGGTCAAGA TTGAGATTGA GGGCATACTG ACGACCTGTA TAATGGTCAA GTAGGATCAA   
  
  
+ AGTCGAGAAA ACTGTCTCTG CTAACTTTCA TAATCTAACC CTTAACTCAC TCTGACCCTC AATTTAATCA   
  
  
+ GGCCTAATTC AAACTATTAA TGGTTATAAC TCTCTTTCCT TCTTTTGAGA TTTTTTTTTT CTACATGCGG   
  
  
+ TTGAAAATAA TTTTTTTAAA AAATATATAT CACTAACCAT TCATTATCAT AGTTTTAGAG TTCATATTTA   
  
  
+ TGTGGTCCTT GTTGAGTCTA ATTTAATGCT TTAGATGGCG TGAAGCACCA AATTAGTGGT AACCATAATA   
  
  
+ ATAAACTATC AAGTGTTAAG TCGAAGAAAA CGGACACTGC TCAACCAAAT CTAGTAACAT AGTGTATGAC   
  
  
+ ATGATAACTA AGTGTGCCAT GTAGTAATCA ATTCTTTCTT TTTTTTTTTT TGGTTGAGAT CACATAAATT   
  
  
+ ACGTTATCAA GGGTAGTACG ATATAATGGA TGAGGAGAAA TTAAATGTGA GATTTAGTTG ATGAATCGAA   
  
  
+ TACGTTAGTC TGAGCCATTT GGTCAAAACT GCATTGGCTA ATCAATAGAA ATTATAGAAA ATACATAACA   
  
  
+ TAATGGGATG CAATTTTCTA ATTGGGGAAA CCATGTCAAT GCAGTGATTT TGGACCATTT AGGCCTTATT   
  
  
+ CTTTTCAATT TTTTAATTGA TTATTTAGAC TCATATGAAC TTATTGTTGA CAGATTGAGC TAAAAACACT   
  
  
+ GATGTTTCAT ACATATGTAT GAATTGCACG TACGAAGGCT CTGCTCATTT CGAGAAAGAA AATTAACAGT   
  
  
+ TAGGAGCAGT ATAATTTAAC AACAAGCGAT AATTATTAGT ATGAAGTGGT ACGAATCTAT TTTAAAGGAG   
  
  
+ TACTATGAAG TACAAACAAT AACAAATAAT AAAGAGCAGT ACATTAACAT ATCTTACTAC CTAAATAAAT   
  
  
+ CGATTCTAAG AGTAAAAAAA TGTTGAAATT AACCCCATTA TAGTTTTACT TTAACAAGAA GTTTTTCTTC   
  
  
+ TTTTACCATT TACCAAGTAA GAATGATTCA GGCCGTATAG CCAAGGAAAA ATAGAAAACC ACAAAACTAA   
  
  
+ CCATGGCCCA AAAAACAACC ACCAAGAGGG GCACTAATGT GACTAACCGA CGGTTAACAT TATCATCTGT   
  
  
+ TTGATACTTG TTCATCTCTC ACTATTCGGG TAATAGTGTG AAATTACTGA TGTACCCTCT AAACTCGTCG   
  
  
+ TTTTAACTGT GTAAGAATCA GATTAACAGT CACGGACACA TGAGTAACAA ATCAATTTCG GACATGAACG   
  
  
+ AACGAACTGT GAATCCCCCC CAAAAAAAAA ATTTTTTTAA AAAAAGTTGC ATCCTTATCT TTCTGAACCA   
  
  
+ GGCCAGCTGT CTCATTCCTT GGTCCTCGCG CCCCATCACG AGCGCTGCTC ATTATTCACC CTCTCTCTCT   
  
  
+ CTCCTCTCTG ATCCACACCA CAAACACAAG GTTTGCACAG CTGCAGAGCA GTCGTACTAC TATCAGTAGT   
  
  
+ GAAAAACCAA GCAGAAATCC CCAATCCCTC TTCTCCTTCT TCCCAGATTC TTCCTCAATT CCAAGCCCAT   
  
  
+ ATCAGAAATT TAGACAACAA CAACCAAAAA AAAAAATTAA TACTGCTTGA GAAAAACAAA TATGATATGA   
  
  
+ AATTCCTCGA AAAAGGCCTG CCATAACAAA TTCCTCGTGA AAAAGCAGCT GAGAGAAGAA GAAGAAGAAA   
  
  
+ AAAATCCCGT CACAATCTCA ATCTCGATCC CAAATTGTGA AAGAGAAAGA AAAGATGAAG AGAGAACACC   
  
  
+ CCTTTTCGAA CCCTGACCAG AAACCCCCGC CGGAGATGTC GCCGGCGCCG GGGAAGTCCA AGATGTGGGA   
  
  
+ TGATGAGATG GCCCAGAACG ACGCTAAAAT GGATGAGCTG CTTGCTGTTG TGGGGTACAA GGTGAGGTCG   
  
  
+ TCGGACATGG CGGAAATTGC CCAGAAGCTT GAGCAGCTTG AAGAAGTCAT GGGTAGTGTT CAAGAAGACG   
  
  
+ GGTTATCTTA CTTGGCTTCC GAAACTGTTC ATTACAATCC TGCAGATCTG TCTACTTGGC TTGAATCTAT   
  
  
+ GCTTTCTGAG TTTAACCCTA ACCCTAATTT TGACCCTTCT CCATCGTCAA TCTCACCCAT CATCGATCCA   
  
  
+ GCTCCTCCAC TTCCTCGAAC CTCGTCGACG GTCAAATTCG AGCCCGACCC GTTTTCCGAT TCGGATCTGA   
  
  
+ AAGCAATCCC AGGTAAGGCT ATCTTGACCC CACCAAGTTC TAGCAATACT TCAAATTTGC GTGAGGCTAA   
  
  
+ GAGGTTGAAA CCCTCAAATT ACACAACCGC CCCAACGCCC ACCCCACCTC CAAAACTGCC CAATTCGGCG   
  
  
+ TCTCCACCAT CGGGCACGAC GGCGTCGAGG CCGGTGGTGC TGGTAGACTC ACAAGAAAAC GGCATCAGAT   
  
  
+ TGGTGCATGC ACTTATGGCC TGTGCCGAAG CAATTCAACT AGAAAACATG GGATTAGCTG AGGCTTTGGT   
  
  
+ TAAACAGATT AGGTATTTAG CAGCATCTCA AGCTGGACCT ATGAGGAAAG TAGCAACTTG TTTTGCAGAA   
  
  
+ GCTCTTACTT GTCGGATCTA CAAGCTATGC CCATCTGTAC CCTACGATGA ATCAGTCTCA GATGAGTTGC   
  
  
+ AGATGCACTT TTATGAGACT TGCCCATATC TTAAATTCGC CCATTTCACG GCAAATCAAG CAATTTTAGA   
  
  
+ AGCATTCAAT GGGAAGAAGA AAGTTCATGT GATTGATTTC AGCATGAAGC AAGGCATGCA ATGGCCGGCT   
  
  
+ TTGATGCAAG CCCTGGCTCT TCGACCGGAG GGTCCACCGC TTTTCCGGTT AACCGGGATT GGACCGCCCG   
  
  
+ CTCCGGACAA CTCGGACCGG CTGCAAGAGG TGGGTTGGAA GCTTGCCCAG TTCGCGGATT CGATCCGGAT   
  
  
+ TAAATTTCAG TATCGTGGGT TTGTGGCAAA CAGTTTGACC GATCTAGAAT CTTCCATGCT AGATCTTGAA   
  
  
+ CCGGACACTG AGGTGGTGGC GGTCAACTCG GTTTTCGAGC TCCACCGGCT GTTGGCTAAA CCCGGGGCGA   
  
  
+ TCGAGAAAGT GCTGGGGTTC ATGAGGGCCG TGAACCCGGT CATTGTGACG ATGGTCGAGC AGGAAGCGAA   
  
  
+ CCACAACGGA CCGGTTTTCT TGGACCGGTT CAATGAGTCG TTGCATTATT ACTCCACCTT GTTTGATTCC   
  
  
+ CTTGAGATTT GTGTTGATAA TGTAGATAAG AAGATGTCGG AGGCTTACTT GGGCCAGCAG ATCTGCAACA   
  
  
+ TGGTGGCTTG TGAAGGGTCT GACCGAGTCG AGAGGCACGA GACCCTGGCT CAGTGGCGAG CCCGGTTCGC   
  
  
+ ATCTGCCGGG TTCGACCCAG TTCATCTGGG TTCGAATGCG TTTAAGCAGG CGAGTATGTT GTTGGAGTTC   
  
  
+ TTTGCTGGTG GTGAAGGGTA CGGCGTGGAG GAGCGAGAAG GGTGTCTCAT GTTGGGATGG CATAGTAGGC   
  
  
+ CGCTTATCAC CACCTCGGCT TGGCAGCTCG CCAAGAACCC GGCTGTGAAT CGGCGATG  

- -Up\_Stream \_Len000TTGGGA AATTAAAATA GTTACACCCT GTTGTGAGTA GAAGTATGAG GTTGTTCGAT   
  
  
- ACTATGTAAG CTAATAACGG ATCATAAAGC AAATCATTCT ATAATCACGG TTTGAAACAT TATTTTTACT   
  
  
- TTGAATGATT TTTTTTAGAG CAAAAGACAT CTCCCTTTTA AACTTCTGAA TCACAGTGTA CTACTGGATT   
  
  
- ACCATGAACA TCCCAGTTCT AACTCTAACT CCCGTATGAC TGCTGGACAT ATTACCAGTT CATCCTAGTT   
  
  
- TCAGCTCTTT TGACAGAGAC GATTGAAAGT ATTAGATTGG GAATTGAGTG AGACTGGGAG TTAAATTAGT   
  
  
- CCGGATTAAG TTTGATAATT ACCAATATTG AGAGAAAGGA AGAAAACTCT AAAAAAAAAA GATGTACGCC   
  
  
- AACTTTTATT AAAAAAATTT TTTATATATA GTGATTGGTA AGTAATAGTA TCAAAATCTC AAGTATAAAT   
  
  
- ACACCAGGAA CAACTCAGAT TAAATTACGA AATCTACCGC ACTTCGTGGT TTAATCACCA TTGGTATTAT   
  
  
- TATTTGATAG TTCACAATTC AGCTTCTTTT GCCTGTGACG AGTTGGTTTA GATCATTGTA TCACATACTG   
  
  
- TACTATTGAT TCACACGGTA CATCATTAGT TAAGAAAGAA AAAAAAAAAA ACCAACTCTA GTGTATTTAA   
  
  
- TGCAATAGTT CCCATCATGC TATATTACCT ACTCCTCTTT AATTTACACT CTAAATCAAC TACTTAGCTT   
  
  
- ATGCAATCAG ACTCGGTAAA CCAGTTTTGA CGTAACCGAT TAGTTATCTT TAATATCTTT TATGTATTGT   
  
  
- ATTACCCTAC GTTAAAAGAT TAACCCCTTT GGTACAGTTA CGTCACTAAA ACCTGGTAAA TCCGGAATAA   
  
  
- GAAAAGTTAA AAAATTAACT AATAAATCTG AGTATACTTG AATAACAACT GTCTAACTCG ATTTTTGTGA   
  
  
- CTACAAAGTA TGTATACATA CTTAACGTGC ATGCTTCCGA GACGAGTAAA GCTCTTTCTT TTAATTGTCA   
  
  
- ATCCTCGTCA TATTAAATTG TTGTTCGCTA TTAATAATCA TACTTCACCA TGCTTAGATA AAATTTCCTC   
  
  
- ATGATACTTC ATGTTTGTTA TTGTTTATTA TTTCTCGTCA TGTAATTGTA TAGAATGATG GATTTATTTA   
  
  
- GCTAAGATTC TCATTTTTTT ACAACTTTAA TTGGGGTAAT ATCAAAATGA AATTGTTCTT CAAAAAGAAG   
  
  
- AAAATGGTAA ATGGTTCATT CTTACTAAGT CCGGCATATC GGTTCCTTTT TATCTTTTGG TGTTTTGATT   
  
  
- GGTACCGGGT TTTTTGTTGG TGGTTCTCCC CGTGATTACA CTGATTGGCT GCCAATTGTA ATAGTAGACA   
  
  
- AACTATGAAC AAGTAGAGAG TGATAAGCCC ATTATCACAC TTTAATGACT ACATGGGAGA TTTGAGCAGC   
  
  
- AAAATTGACA CATTCTTAGT CTAATTGTCA GTGCCTGTGT ACTCATTGTT TAGTTAAAGC CTGTACTTGC   
  
  
- TTGCTTGACA CTTAGGGGGG GTTTTTTTTT TAAAAAAATT TTTTTCAACG TAGGAATAGA AAGACTTGGT   
  
  
- CCGGTCGACA GAGTAAGGAA CCAGGAGCGC GGGGTAGTGC TCGCGACGAG TAATAAGTGG GAGAGAGAGA   
  
  
- GAGGAGAGAC TAGGTGTGGT GTTTGTGTTC CAAACGTGTC GACGTCTCGT CAGCATGATG ATAGTCATCA   
  
  
- CTTTTTGGTT CGTCTTTAGG GGTTAGGGAG AAGAGGAAGA AGGGTCTAAG AAGGAGTTAA GGTTCGGGTA   
  
  
- TAGTCTTTAA ATCTGTTGTT GTTGGTTTTT TTTTTTAATT ATGACGAACT CTTTTTGTTT ATACTATACT   
  
  
- TTAAGGAGCT TTTTCCGGAC GGTATTGTTT AAGGAGCACT TTTTCGTCGA CTCTCTTCTT CTTCTTCTTT   
  
  
- TTTTAGGGCA GTGTTAGAGT TAGAGCTAGG GTTTAACACT TTCTCTTTCT TTTCTACTTC TCTCTTGTGG   
  
  
- GGAAAAGCTT GGGACTGGTC TTTGGGGGCG GCCTCTACAG CGGCCGCGGC CCCTTCAGGT TCTACACCCT   
  
  
- ACTACTCTAC CGGGTCTTGC TGCGATTTTA CCTACTCGAC GAACGACAAC ACCCCATGTT CCACTCCAGC   
  
  
- AGCCTGTACC GCCTTTAACG GGTCTTCGAA CTCGTCGAAC TTCTTCAGTA CCCATCACAA GTTCTTCTGC   
  
  
- CCAATAGAAT GAACCGAAGG CTTTGACAAG TAATGTTAGG ACGTCTAGAC AGATGAACCG AACTTAGATA   
  
  
- CGAAAGACTC AAATTGGGAT TGGGATTAAA ACTGGGAAGA GGTAGCAGTT AGAGTGGGTA GTAGCTAGGT   
  
  
- CGAGGAGGTG AAGGAGCTTG GAGCAGCTGC CAGTTTAAGC TCGGGCTGGG CAAAAGGCTA AGCCTAGACT   
  
  
- TTCGTTAGGG TCCATTCCGA TAGAACTGGG GTGGTTCAAG ATCGTTATGA AGTTTAAACG CACTCCGATT   
  
  
- CTCCAACTTT GGGAGTTTAA TGTGTTGGCG GGGTTGCGGG TGGGGTGGAG GTTTTGACGG GTTAAGCCGC   
  
  
- AGAGGTGGTA GCCCGTGCTG CCGCAGCTCC GGCCACCACG ACCATCTGAG TGTTCTTTTG CCGTAGTCTA   
  
  
- ACCACGTACG TGAATACCGG ACACGGCTTC GTTAAGTTGA TCTTTTGTAC CCTAATCGAC TCCGAAACCA   
  
  
- ATTTGTCTAA TCCATAAATC GTCGTAGAGT TCGACCTGGA TACTCCTTTC ATCGTTGAAC AAAACGTCTT   
  
  
- CGAGAATGAA CAGCCTAGAT GTTCGATACG GGTAGACATG GGATGCTACT TAGTCAGAGT CTACTCAACG   
  
  
- TCTACGTGAA AATACTCTGA ACGGGTATAG AATTTAAGCG GGTAAAGTGC CGTTTAGTTC GTTAAAATCT   
  
  
- TCGTAAGTTA CCCTTCTTCT TTCAAGTACA CTAACTAAAG TCGTACTTCG TTCCGTACGT TACCGGCCGA   
  
  
- AACTACGTTC GGGACCGAGA AGCTGGCCTC CCAGGTGGCG AAAAGGCCAA TTGGCCCTAA CCTGGCGGGC   
  
  
- GAGGCCTGTT GAGCCTGGCC GACGTTCTCC ACCCAACCTT CGAACGGGTC AAGCGCCTAA GCTAGGCCTA   
  
  
- ATTTAAAGTC ATAGCACCCA AACACCGTTT GTCAAACTGG CTAGATCTTA GAAGGTACGA TCTAGAACTT   
  
  
- GGCCTGTGAC TCCACCACCG CCAGTTGAGC CAAAAGCTCG AGGTGGCCGA CAACCGATTT GGGCCCCGCT   
  
  
- AGCTCTTTCA CGACCCCAAG TACTCCCGGC ACTTGGGCCA GTAACACTGC TACCAGCTCG TCCTTCGCTT   
  
  
- GGTGTTGCCT GGCCAAAAGA ACCTGGCCAA GTTACTCAGC AACGTAATAA TGAGGTGGAA CAAACTAAGG   
  
  
- GAACTCTAAA CACAACTATT ACATCTATTC TTCTACAGCC TCCGAATGAA CCCGGTCGTC TAGACGTTGT   
  
  
- ACCACCGAAC ACTTCCCAGA CTGGCTCAGC TCTCCGTGCT CTGGGACCGA GTCACCGCTC GGGCCAAGCG   
  
  
- TAGACGGCCC AAGCTGGGTC AAGTAGACCC AAGCTTACGC AAATTCGTCC GCTCATACAA CAACCTCAAG   
  
  
- AAACGACCAC CACTTCCCAT GCCGCACCTC CTCGCTCTTC CCACAGAGTA CAACCCTACC GTATCATCCG   
  
  
- GCGAATAGTG GTGGAGCCGA ACCGTCGAGC GGTTCTTGGG CCGACACTTA GCCGCTAC

+     TC-rich repeats

| Site Name | Organism | Position | Strand | Matrix score. | sequence | function |
| --- | --- | --- | --- | --- | --- | --- |
| TC-rich repeats | Nicotiana tabacum | 2246 | + | 9 | GTTTTCTTAC | cis-acting element involved in defense and stress responsiveness |

>HU10G00709.1   
+ -Up\_Stream \_Len000AACCCT TTAATTTTAT CAATGTGGGA CAACACTCAT CTTCATACTC CAACAAGCTA   
  
  
+ TGATACATTC GATTATTGCC TAGTATTTCG TTTAGTAAGA TATTAGTGCC AAACTTTGTA ATAAAAATGA   
  
  
+ AACTTACTAA AAAAAATCTC GTTTTCTGTA GAGGGAAAAT TTGAAGACTT AGTGTCACAT GATGACCTAA   
  
  
+ TGGTACTTGT AGGGTCAAGA TTGAGATTGA GGGCATACTG ACGACCTGTA TAATGGTCAA GTAGGATCAA   
  
  
+ AGTCGAGAAA ACTGTCTCTG CTAACTTTCA TAATCTAACC CTTAACTCAC TCTGACCCTC AATTTAATCA   
  
  
+ GGCCTAATTC AAACTATTAA TGGTTATAAC TCTCTTTCCT TCTTTTGAGA TTTTTTTTTT CTACATGCGG   
  
  
+ TTGAAAATAA TTTTTTTAAA AAATATATAT CACTAACCAT TCATTATCAT AGTTTTAGAG TTCATATTTA   
  
  
+ TGTGGTCCTT GTTGAGTCTA ATTTAATGCT TTAGATGGCG TGAAGCACCA AATTAGTGGT AACCATAATA   
  
  
+ ATAAACTATC AAGTGTTAAG TCGAAGAAAA CGGACACTGC TCAACCAAAT CTAGTAACAT AGTGTATGAC   
  
  
+ ATGATAACTA AGTGTGCCAT GTAGTAATCA ATTCTTTCTT TTTTTTTTTT TGGTTGAGAT CACATAAATT   
  
  
+ ACGTTATCAA GGGTAGTACG ATATAATGGA TGAGGAGAAA TTAAATGTGA GATTTAGTTG ATGAATCGAA   
  
  
+ TACGTTAGTC TGAGCCATTT GGTCAAAACT GCATTGGCTA ATCAATAGAA ATTATAGAAA ATACATAACA   
  
  
+ TAATGGGATG CAATTTTCTA ATTGGGGAAA CCATGTCAAT GCAGTGATTT TGGACCATTT AGGCCTTATT   
  
  
+ CTTTTCAATT TTTTAATTGA TTATTTAGAC TCATATGAAC TTATTGTTGA CAGATTGAGC TAAAAACACT   
  
  
+ GATGTTTCAT ACATATGTAT GAATTGCACG TACGAAGGCT CTGCTCATTT CGAGAAAGAA AATTAACAGT   
  
  
+ TAGGAGCAGT ATAATTTAAC AACAAGCGAT AATTATTAGT ATGAAGTGGT ACGAATCTAT TTTAAAGGAG   
  
  
+ TACTATGAAG TACAAACAAT AACAAATAAT AAAGAGCAGT ACATTAACAT ATCTTACTAC CTAAATAAAT   
  
  
+ CGATTCTAAG AGTAAAAAAA TGTTGAAATT AACCCCATTA TAGTTTTACT TTAACAAGAA GTTTTTCTTC   
  
  
+ TTTTACCATT TACCAAGTAA GAATGATTCA GGCCGTATAG CCAAGGAAAA ATAGAAAACC ACAAAACTAA   
  
  
+ CCATGGCCCA AAAAACAACC ACCAAGAGGG GCACTAATGT GACTAACCGA CGGTTAACAT TATCATCTGT   
  
  
+ TTGATACTTG TTCATCTCTC ACTATTCGGG TAATAGTGTG AAATTACTGA TGTACCCTCT AAACTCGTCG   
  
  
+ TTTTAACTGT GTAAGAATCA GATTAACAGT CACGGACACA TGAGTAACAA ATCAATTTCG GACATGAACG   
  
  
+ AACGAACTGT GAATCCCCCC CAAAAAAAAA ATTTTTTTAA AAAAAGTTGC ATCCTTATCT TTCTGAACCA   
  
  
+ GGCCAGCTGT CTCATTCCTT GGTCCTCGCG CCCCATCACG AGCGCTGCTC ATTATTCACC CTCTCTCTCT   
  
  
+ CTCCTCTCTG ATCCACACCA CAAACACAAG GTTTGCACAG CTGCAGAGCA GTCGTACTAC TATCAGTAGT   
  
  
+ GAAAAACCAA GCAGAAATCC CCAATCCCTC TTCTCCTTCT TCCCAGATTC TTCCTCAATT CCAAGCCCAT   
  
  
+ ATCAGAAATT TAGACAACAA CAACCAAAAA AAAAAATTAA TACTGCTTGA GAAAAACAAA TATGATATGA   
  
  
+ AATTCCTCGA AAAAGGCCTG CCATAACAAA TTCCTCGTGA AAAAGCAGCT GAGAGAAGAA GAAGAAGAAA   
  
  
+ AAAATCCCGT CACAATCTCA ATCTCGATCC CAAATTGTGA AAGAGAAAGA AAAGATGAAG AGAGAACACC   
  
  
+ CCTTTTCGAA CCCTGACCAG AAACCCCCGC CGGAGATGTC GCCGGCGCCG GGGAAGTCCA AGATGTGGGA   
  
  
+ TGATGAGATG GCCCAGAACG ACGCTAAAAT GGATGAGCTG CTTGCTGTTG TGGGGTACAA GGTGAGGTCG   
  
  
+ TCGGACATGG CGGAAATTGC CCAGAAGCTT GAGCAGCTTG AAGAAGTCAT GGGTAGTGTT CAAGAAGACG   
  
  
+ GGTTATCTTA CTTGGCTTCC GAAACTGTTC ATTACAATCC TGCAGATCTG TCTACTTGGC TTGAATCTAT   
  
  
+ GCTTTCTGAG TTTAACCCTA ACCCTAATTT TGACCCTTCT CCATCGTCAA TCTCACCCAT CATCGATCCA   
  
  
+ GCTCCTCCAC TTCCTCGAAC CTCGTCGACG GTCAAATTCG AGCCCGACCC GTTTTCCGAT TCGGATCTGA   
  
  
+ AAGCAATCCC AGGTAAGGCT ATCTTGACCC CACCAAGTTC TAGCAATACT TCAAATTTGC GTGAGGCTAA   
  
  
+ GAGGTTGAAA CCCTCAAATT ACACAACCGC CCCAACGCCC ACCCCACCTC CAAAACTGCC CAATTCGGCG   
  
  
+ TCTCCACCAT CGGGCACGAC GGCGTCGAGG CCGGTGGTGC TGGTAGACTC ACAAGAAAAC GGCATCAGAT   
  
  
+ TGGTGCATGC ACTTATGGCC TGTGCCGAAG CAATTCAACT AGAAAACATG GGATTAGCTG AGGCTTTGGT   
  
  
+ TAAACAGATT AGGTATTTAG CAGCATCTCA AGCTGGACCT ATGAGGAAAG TAGCAACTTG TTTTGCAGAA   
  
  
+ GCTCTTACTT GTCGGATCTA CAAGCTATGC CCATCTGTAC CCTACGATGA ATCAGTCTCA GATGAGTTGC   
  
  
+ AGATGCACTT TTATGAGACT TGCCCATATC TTAAATTCGC CCATTTCACG GCAAATCAAG CAATTTTAGA   
  
  
+ AGCATTCAAT GGGAAGAAGA AAGTTCATGT GATTGATTTC AGCATGAAGC AAGGCATGCA ATGGCCGGCT   
  
  
+ TTGATGCAAG CCCTGGCTCT TCGACCGGAG GGTCCACCGC TTTTCCGGTT AACCGGGATT GGACCGCCCG   
  
  
+ CTCCGGACAA CTCGGACCGG CTGCAAGAGG TGGGTTGGAA GCTTGCCCAG TTCGCGGATT CGATCCGGAT   
  
  
+ TAAATTTCAG TATCGTGGGT TTGTGGCAAA CAGTTTGACC GATCTAGAAT CTTCCATGCT AGATCTTGAA   
  
  
+ CCGGACACTG AGGTGGTGGC GGTCAACTCG GTTTTCGAGC TCCACCGGCT GTTGGCTAAA CCCGGGGCGA   
  
  
+ TCGAGAAAGT GCTGGGGTTC ATGAGGGCCG TGAACCCGGT CATTGTGACG ATGGTCGAGC AGGAAGCGAA   
  
  
+ CCACAACGGA CCGGTTTTCT TGGACCGGTT CAATGAGTCG TTGCATTATT ACTCCACCTT GTTTGATTCC   
  
  
+ CTTGAGATTT GTGTTGATAA TGTAGATAAG AAGATGTCGG AGGCTTACTT GGGCCAGCAG ATCTGCAACA   
  
  
+ TGGTGGCTTG TGAAGGGTCT GACCGAGTCG AGAGGCACGA GACCCTGGCT CAGTGGCGAG CCCGGTTCGC   
  
  
+ ATCTGCCGGG TTCGACCCAG TTCATCTGGG TTCGAATGCG TTTAAGCAGG CGAGTATGTT GTTGGAGTTC   
  
  
+ TTTGCTGGTG GTGAAGGGTA CGGCGTGGAG GAGCGAGAAG GGTGTCTCAT GTTGGGATGG CATAGTAGGC   
  
  
+ CGCTTATCAC CACCTCGGCT TGGCAGCTCG CCAAGAACCC GGCTGTGAAT CGGCGATG  

- -Up\_Stream \_Len000TTGGGA AATTAAAATA GTTACACCCT GTTGTGAGTA GAAGTATGAG GTTGTTCGAT   
  
  
- ACTATGTAAG CTAATAACGG ATCATAAAGC AAATCATTCT ATAATCACGG TTTGAAACAT TATTTTTACT   
  
  
- TTGAATGATT TTTTTTAGAG CAAAAGACAT CTCCCTTTTA AACTTCTGAA TCACAGTGTA CTACTGGATT   
  
  
- ACCATGAACA TCCCAGTTCT AACTCTAACT CCCGTATGAC TGCTGGACAT ATTACCAGTT CATCCTAGTT   
  
  
- TCAGCTCTTT TGACAGAGAC GATTGAAAGT ATTAGATTGG GAATTGAGTG AGACTGGGAG TTAAATTAGT   
  
  
- CCGGATTAAG TTTGATAATT ACCAATATTG AGAGAAAGGA AGAAAACTCT AAAAAAAAAA GATGTACGCC   
  
  
- AACTTTTATT AAAAAAATTT TTTATATATA GTGATTGGTA AGTAATAGTA TCAAAATCTC AAGTATAAAT   
  
  
- ACACCAGGAA CAACTCAGAT TAAATTACGA AATCTACCGC ACTTCGTGGT TTAATCACCA TTGGTATTAT   
  
  
- TATTTGATAG TTCACAATTC AGCTTCTTTT GCCTGTGACG AGTTGGTTTA GATCATTGTA TCACATACTG   
  
  
- TACTATTGAT TCACACGGTA CATCATTAGT TAAGAAAGAA AAAAAAAAAA ACCAACTCTA GTGTATTTAA   
  
  
- TGCAATAGTT CCCATCATGC TATATTACCT ACTCCTCTTT AATTTACACT CTAAATCAAC TACTTAGCTT   
  
  
- ATGCAATCAG ACTCGGTAAA CCAGTTTTGA CGTAACCGAT TAGTTATCTT TAATATCTTT TATGTATTGT   
  
  
- ATTACCCTAC GTTAAAAGAT TAACCCCTTT GGTACAGTTA CGTCACTAAA ACCTGGTAAA TCCGGAATAA   
  
  
- GAAAAGTTAA AAAATTAACT AATAAATCTG AGTATACTTG AATAACAACT GTCTAACTCG ATTTTTGTGA   
  
  
- CTACAAAGTA TGTATACATA CTTAACGTGC ATGCTTCCGA GACGAGTAAA GCTCTTTCTT TTAATTGTCA   
  
  
- ATCCTCGTCA TATTAAATTG TTGTTCGCTA TTAATAATCA TACTTCACCA TGCTTAGATA AAATTTCCTC   
  
  
- ATGATACTTC ATGTTTGTTA TTGTTTATTA TTTCTCGTCA TGTAATTGTA TAGAATGATG GATTTATTTA   
  
  
- GCTAAGATTC TCATTTTTTT ACAACTTTAA TTGGGGTAAT ATCAAAATGA AATTGTTCTT CAAAAAGAAG   
  
  
- AAAATGGTAA ATGGTTCATT CTTACTAAGT CCGGCATATC GGTTCCTTTT TATCTTTTGG TGTTTTGATT   
  
  
- GGTACCGGGT TTTTTGTTGG TGGTTCTCCC CGTGATTACA CTGATTGGCT GCCAATTGTA ATAGTAGACA   
  
  
- AACTATGAAC AAGTAGAGAG TGATAAGCCC ATTATCACAC TTTAATGACT ACATGGGAGA TTTGAGCAGC   
  
  
- AAAATTGACA CATTCTTAGT CTAATTGTCA GTGCCTGTGT ACTCATTGTT TAGTTAAAGC CTGTACTTGC   
  
  
- TTGCTTGACA CTTAGGGGGG GTTTTTTTTT TAAAAAAATT TTTTTCAACG TAGGAATAGA AAGACTTGGT   
  
  
- CCGGTCGACA GAGTAAGGAA CCAGGAGCGC GGGGTAGTGC TCGCGACGAG TAATAAGTGG GAGAGAGAGA   
  
  
- GAGGAGAGAC TAGGTGTGGT GTTTGTGTTC CAAACGTGTC GACGTCTCGT CAGCATGATG ATAGTCATCA   
  
  
- CTTTTTGGTT CGTCTTTAGG GGTTAGGGAG AAGAGGAAGA AGGGTCTAAG AAGGAGTTAA GGTTCGGGTA   
  
  
- TAGTCTTTAA ATCTGTTGTT GTTGGTTTTT TTTTTTAATT ATGACGAACT CTTTTTGTTT ATACTATACT   
  
  
- TTAAGGAGCT TTTTCCGGAC GGTATTGTTT AAGGAGCACT TTTTCGTCGA CTCTCTTCTT CTTCTTCTTT   
  
  
- TTTTAGGGCA GTGTTAGAGT TAGAGCTAGG GTTTAACACT TTCTCTTTCT TTTCTACTTC TCTCTTGTGG   
  
  
- GGAAAAGCTT GGGACTGGTC TTTGGGGGCG GCCTCTACAG CGGCCGCGGC CCCTTCAGGT TCTACACCCT   
  
  
- ACTACTCTAC CGGGTCTTGC TGCGATTTTA CCTACTCGAC GAACGACAAC ACCCCATGTT CCACTCCAGC   
  
  
- AGCCTGTACC GCCTTTAACG GGTCTTCGAA CTCGTCGAAC TTCTTCAGTA CCCATCACAA GTTCTTCTGC   
  
  
- CCAATAGAAT GAACCGAAGG CTTTGACAAG TAATGTTAGG ACGTCTAGAC AGATGAACCG AACTTAGATA   
  
  
- CGAAAGACTC AAATTGGGAT TGGGATTAAA ACTGGGAAGA GGTAGCAGTT AGAGTGGGTA GTAGCTAGGT   
  
  
- CGAGGAGGTG AAGGAGCTTG GAGCAGCTGC CAGTTTAAGC TCGGGCTGGG CAAAAGGCTA AGCCTAGACT   
  
  
- TTCGTTAGGG TCCATTCCGA TAGAACTGGG GTGGTTCAAG ATCGTTATGA AGTTTAAACG CACTCCGATT   
  
  
- CTCCAACTTT GGGAGTTTAA TGTGTTGGCG GGGTTGCGGG TGGGGTGGAG GTTTTGACGG GTTAAGCCGC   
  
  
- AGAGGTGGTA GCCCGTGCTG CCGCAGCTCC GGCCACCACG ACCATCTGAG TGTTCTTTTG CCGTAGTCTA   
  
  
- ACCACGTACG TGAATACCGG ACACGGCTTC GTTAAGTTGA TCTTTTGTAC CCTAATCGAC TCCGAAACCA   
  
  
- ATTTGTCTAA TCCATAAATC GTCGTAGAGT TCGACCTGGA TACTCCTTTC ATCGTTGAAC AAAACGTCTT   
  
  
- CGAGAATGAA CAGCCTAGAT GTTCGATACG GGTAGACATG GGATGCTACT TAGTCAGAGT CTACTCAACG   
  
  
- TCTACGTGAA AATACTCTGA ACGGGTATAG AATTTAAGCG GGTAAAGTGC CGTTTAGTTC GTTAAAATCT   
  
  
- TCGTAAGTTA CCCTTCTTCT TTCAAGTACA CTAACTAAAG TCGTACTTCG TTCCGTACGT TACCGGCCGA   
  
  
- AACTACGTTC GGGACCGAGA AGCTGGCCTC CCAGGTGGCG AAAAGGCCAA TTGGCCCTAA CCTGGCGGGC   
  
  
- GAGGCCTGTT GAGCCTGGCC GACGTTCTCC ACCCAACCTT CGAACGGGTC AAGCGCCTAA GCTAGGCCTA   
  
  
- ATTTAAAGTC ATAGCACCCA AACACCGTTT GTCAAACTGG CTAGATCTTA GAAGGTACGA TCTAGAACTT   
  
  
- GGCCTGTGAC TCCACCACCG CCAGTTGAGC CAAAAGCTCG AGGTGGCCGA CAACCGATTT GGGCCCCGCT   
  
  
- AGCTCTTTCA CGACCCCAAG TACTCCCGGC ACTTGGGCCA GTAACACTGC TACCAGCTCG TCCTTCGCTT   
  
  
- GGTGTTGCCT GGCCAAAAGA ACCTGGCCAA GTTACTCAGC AACGTAATAA TGAGGTGGAA CAAACTAAGG   
  
  
- GAACTCTAAA CACAACTATT ACATCTATTC TTCTACAGCC TCCGAATGAA CCCGGTCGTC TAGACGTTGT   
  
  
- ACCACCGAAC ACTTCCCAGA CTGGCTCAGC TCTCCGTGCT CTGGGACCGA GTCACCGCTC GGGCCAAGCG   
  
  
- TAGACGGCCC AAGCTGGGTC AAGTAGACCC AAGCTTACGC AAATTCGTCC GCTCATACAA CAACCTCAAG   
  
  
- AAACGACCAC CACTTCCCAT GCCGCACCTC CTCGCTCTTC CCACAGAGTA CAACCCTACC GTATCATCCG   
  
  
- GCGAATAGTG GTGGAGCCGA ACCGTCGAGC GGTTCTTGGG CCGACACTTA GCCGCTAC

+     TCA

| Site Name | Organism | Position | Strand | Matrix score. | sequence | function |
| --- | --- | --- | --- | --- | --- | --- |
| TCA | Pisum sativum | 51 | + | 10 | TCATCTTCAT |  |

>HU10G00709.1   
+ -Up\_Stream \_Len000AACCCT TTAATTTTAT CAATGTGGGA CAACACTCAT CTTCATACTC CAACAAGCTA   
  
  
+ TGATACATTC GATTATTGCC TAGTATTTCG TTTAGTAAGA TATTAGTGCC AAACTTTGTA ATAAAAATGA   
  
  
+ AACTTACTAA AAAAAATCTC GTTTTCTGTA GAGGGAAAAT TTGAAGACTT AGTGTCACAT GATGACCTAA   
  
  
+ TGGTACTTGT AGGGTCAAGA TTGAGATTGA GGGCATACTG ACGACCTGTA TAATGGTCAA GTAGGATCAA   
  
  
+ AGTCGAGAAA ACTGTCTCTG CTAACTTTCA TAATCTAACC CTTAACTCAC TCTGACCCTC AATTTAATCA   
  
  
+ GGCCTAATTC AAACTATTAA TGGTTATAAC TCTCTTTCCT TCTTTTGAGA TTTTTTTTTT CTACATGCGG   
  
  
+ TTGAAAATAA TTTTTTTAAA AAATATATAT CACTAACCAT TCATTATCAT AGTTTTAGAG TTCATATTTA   
  
  
+ TGTGGTCCTT GTTGAGTCTA ATTTAATGCT TTAGATGGCG TGAAGCACCA AATTAGTGGT AACCATAATA   
  
  
+ ATAAACTATC AAGTGTTAAG TCGAAGAAAA CGGACACTGC TCAACCAAAT CTAGTAACAT AGTGTATGAC   
  
  
+ ATGATAACTA AGTGTGCCAT GTAGTAATCA ATTCTTTCTT TTTTTTTTTT TGGTTGAGAT CACATAAATT   
  
  
+ ACGTTATCAA GGGTAGTACG ATATAATGGA TGAGGAGAAA TTAAATGTGA GATTTAGTTG ATGAATCGAA   
  
  
+ TACGTTAGTC TGAGCCATTT GGTCAAAACT GCATTGGCTA ATCAATAGAA ATTATAGAAA ATACATAACA   
  
  
+ TAATGGGATG CAATTTTCTA ATTGGGGAAA CCATGTCAAT GCAGTGATTT TGGACCATTT AGGCCTTATT   
  
  
+ CTTTTCAATT TTTTAATTGA TTATTTAGAC TCATATGAAC TTATTGTTGA CAGATTGAGC TAAAAACACT   
  
  
+ GATGTTTCAT ACATATGTAT GAATTGCACG TACGAAGGCT CTGCTCATTT CGAGAAAGAA AATTAACAGT   
  
  
+ TAGGAGCAGT ATAATTTAAC AACAAGCGAT AATTATTAGT ATGAAGTGGT ACGAATCTAT TTTAAAGGAG   
  
  
+ TACTATGAAG TACAAACAAT AACAAATAAT AAAGAGCAGT ACATTAACAT ATCTTACTAC CTAAATAAAT   
  
  
+ CGATTCTAAG AGTAAAAAAA TGTTGAAATT AACCCCATTA TAGTTTTACT TTAACAAGAA GTTTTTCTTC   
  
  
+ TTTTACCATT TACCAAGTAA GAATGATTCA GGCCGTATAG CCAAGGAAAA ATAGAAAACC ACAAAACTAA   
  
  
+ CCATGGCCCA AAAAACAACC ACCAAGAGGG GCACTAATGT GACTAACCGA CGGTTAACAT TATCATCTGT   
  
  
+ TTGATACTTG TTCATCTCTC ACTATTCGGG TAATAGTGTG AAATTACTGA TGTACCCTCT AAACTCGTCG   
  
  
+ TTTTAACTGT GTAAGAATCA GATTAACAGT CACGGACACA TGAGTAACAA ATCAATTTCG GACATGAACG   
  
  
+ AACGAACTGT GAATCCCCCC CAAAAAAAAA ATTTTTTTAA AAAAAGTTGC ATCCTTATCT TTCTGAACCA   
  
  
+ GGCCAGCTGT CTCATTCCTT GGTCCTCGCG CCCCATCACG AGCGCTGCTC ATTATTCACC CTCTCTCTCT   
  
  
+ CTCCTCTCTG ATCCACACCA CAAACACAAG GTTTGCACAG CTGCAGAGCA GTCGTACTAC TATCAGTAGT   
  
  
+ GAAAAACCAA GCAGAAATCC CCAATCCCTC TTCTCCTTCT TCCCAGATTC TTCCTCAATT CCAAGCCCAT   
  
  
+ ATCAGAAATT TAGACAACAA CAACCAAAAA AAAAAATTAA TACTGCTTGA GAAAAACAAA TATGATATGA   
  
  
+ AATTCCTCGA AAAAGGCCTG CCATAACAAA TTCCTCGTGA AAAAGCAGCT GAGAGAAGAA GAAGAAGAAA   
  
  
+ AAAATCCCGT CACAATCTCA ATCTCGATCC CAAATTGTGA AAGAGAAAGA AAAGATGAAG AGAGAACACC   
  
  
+ CCTTTTCGAA CCCTGACCAG AAACCCCCGC CGGAGATGTC GCCGGCGCCG GGGAAGTCCA AGATGTGGGA   
  
  
+ TGATGAGATG GCCCAGAACG ACGCTAAAAT GGATGAGCTG CTTGCTGTTG TGGGGTACAA GGTGAGGTCG   
  
  
+ TCGGACATGG CGGAAATTGC CCAGAAGCTT GAGCAGCTTG AAGAAGTCAT GGGTAGTGTT CAAGAAGACG   
  
  
+ GGTTATCTTA CTTGGCTTCC GAAACTGTTC ATTACAATCC TGCAGATCTG TCTACTTGGC TTGAATCTAT   
  
  
+ GCTTTCTGAG TTTAACCCTA ACCCTAATTT TGACCCTTCT CCATCGTCAA TCTCACCCAT CATCGATCCA   
  
  
+ GCTCCTCCAC TTCCTCGAAC CTCGTCGACG GTCAAATTCG AGCCCGACCC GTTTTCCGAT TCGGATCTGA   
  
  
+ AAGCAATCCC AGGTAAGGCT ATCTTGACCC CACCAAGTTC TAGCAATACT TCAAATTTGC GTGAGGCTAA   
  
  
+ GAGGTTGAAA CCCTCAAATT ACACAACCGC CCCAACGCCC ACCCCACCTC CAAAACTGCC CAATTCGGCG   
  
  
+ TCTCCACCAT CGGGCACGAC GGCGTCGAGG CCGGTGGTGC TGGTAGACTC ACAAGAAAAC GGCATCAGAT   
  
  
+ TGGTGCATGC ACTTATGGCC TGTGCCGAAG CAATTCAACT AGAAAACATG GGATTAGCTG AGGCTTTGGT   
  
  
+ TAAACAGATT AGGTATTTAG CAGCATCTCA AGCTGGACCT ATGAGGAAAG TAGCAACTTG TTTTGCAGAA   
  
  
+ GCTCTTACTT GTCGGATCTA CAAGCTATGC CCATCTGTAC CCTACGATGA ATCAGTCTCA GATGAGTTGC   
  
  
+ AGATGCACTT TTATGAGACT TGCCCATATC TTAAATTCGC CCATTTCACG GCAAATCAAG CAATTTTAGA   
  
  
+ AGCATTCAAT GGGAAGAAGA AAGTTCATGT GATTGATTTC AGCATGAAGC AAGGCATGCA ATGGCCGGCT   
  
  
+ TTGATGCAAG CCCTGGCTCT TCGACCGGAG GGTCCACCGC TTTTCCGGTT AACCGGGATT GGACCGCCCG   
  
  
+ CTCCGGACAA CTCGGACCGG CTGCAAGAGG TGGGTTGGAA GCTTGCCCAG TTCGCGGATT CGATCCGGAT   
  
  
+ TAAATTTCAG TATCGTGGGT TTGTGGCAAA CAGTTTGACC GATCTAGAAT CTTCCATGCT AGATCTTGAA   
  
  
+ CCGGACACTG AGGTGGTGGC GGTCAACTCG GTTTTCGAGC TCCACCGGCT GTTGGCTAAA CCCGGGGCGA   
  
  
+ TCGAGAAAGT GCTGGGGTTC ATGAGGGCCG TGAACCCGGT CATTGTGACG ATGGTCGAGC AGGAAGCGAA   
  
  
+ CCACAACGGA CCGGTTTTCT TGGACCGGTT CAATGAGTCG TTGCATTATT ACTCCACCTT GTTTGATTCC   
  
  
+ CTTGAGATTT GTGTTGATAA TGTAGATAAG AAGATGTCGG AGGCTTACTT GGGCCAGCAG ATCTGCAACA   
  
  
+ TGGTGGCTTG TGAAGGGTCT GACCGAGTCG AGAGGCACGA GACCCTGGCT CAGTGGCGAG CCCGGTTCGC   
  
  
+ ATCTGCCGGG TTCGACCCAG TTCATCTGGG TTCGAATGCG TTTAAGCAGG CGAGTATGTT GTTGGAGTTC   
  
  
+ TTTGCTGGTG GTGAAGGGTA CGGCGTGGAG GAGCGAGAAG GGTGTCTCAT GTTGGGATGG CATAGTAGGC   
  
  
+ CGCTTATCAC CACCTCGGCT TGGCAGCTCG CCAAGAACCC GGCTGTGAAT CGGCGATG  

- -Up\_Stream \_Len000TTGGGA AATTAAAATA GTTACACCCT GTTGTGAGTA GAAGTATGAG GTTGTTCGAT   
  
  
- ACTATGTAAG CTAATAACGG ATCATAAAGC AAATCATTCT ATAATCACGG TTTGAAACAT TATTTTTACT   
  
  
- TTGAATGATT TTTTTTAGAG CAAAAGACAT CTCCCTTTTA AACTTCTGAA TCACAGTGTA CTACTGGATT   
  
  
- ACCATGAACA TCCCAGTTCT AACTCTAACT CCCGTATGAC TGCTGGACAT ATTACCAGTT CATCCTAGTT   
  
  
- TCAGCTCTTT TGACAGAGAC GATTGAAAGT ATTAGATTGG GAATTGAGTG AGACTGGGAG TTAAATTAGT   
  
  
- CCGGATTAAG TTTGATAATT ACCAATATTG AGAGAAAGGA AGAAAACTCT AAAAAAAAAA GATGTACGCC   
  
  
- AACTTTTATT AAAAAAATTT TTTATATATA GTGATTGGTA AGTAATAGTA TCAAAATCTC AAGTATAAAT   
  
  
- ACACCAGGAA CAACTCAGAT TAAATTACGA AATCTACCGC ACTTCGTGGT TTAATCACCA TTGGTATTAT   
  
  
- TATTTGATAG TTCACAATTC AGCTTCTTTT GCCTGTGACG AGTTGGTTTA GATCATTGTA TCACATACTG   
  
  
- TACTATTGAT TCACACGGTA CATCATTAGT TAAGAAAGAA AAAAAAAAAA ACCAACTCTA GTGTATTTAA   
  
  
- TGCAATAGTT CCCATCATGC TATATTACCT ACTCCTCTTT AATTTACACT CTAAATCAAC TACTTAGCTT   
  
  
- ATGCAATCAG ACTCGGTAAA CCAGTTTTGA CGTAACCGAT TAGTTATCTT TAATATCTTT TATGTATTGT   
  
  
- ATTACCCTAC GTTAAAAGAT TAACCCCTTT GGTACAGTTA CGTCACTAAA ACCTGGTAAA TCCGGAATAA   
  
  
- GAAAAGTTAA AAAATTAACT AATAAATCTG AGTATACTTG AATAACAACT GTCTAACTCG ATTTTTGTGA   
  
  
- CTACAAAGTA TGTATACATA CTTAACGTGC ATGCTTCCGA GACGAGTAAA GCTCTTTCTT TTAATTGTCA   
  
  
- ATCCTCGTCA TATTAAATTG TTGTTCGCTA TTAATAATCA TACTTCACCA TGCTTAGATA AAATTTCCTC   
  
  
- ATGATACTTC ATGTTTGTTA TTGTTTATTA TTTCTCGTCA TGTAATTGTA TAGAATGATG GATTTATTTA   
  
  
- GCTAAGATTC TCATTTTTTT ACAACTTTAA TTGGGGTAAT ATCAAAATGA AATTGTTCTT CAAAAAGAAG   
  
  
- AAAATGGTAA ATGGTTCATT CTTACTAAGT CCGGCATATC GGTTCCTTTT TATCTTTTGG TGTTTTGATT   
  
  
- GGTACCGGGT TTTTTGTTGG TGGTTCTCCC CGTGATTACA CTGATTGGCT GCCAATTGTA ATAGTAGACA   
  
  
- AACTATGAAC AAGTAGAGAG TGATAAGCCC ATTATCACAC TTTAATGACT ACATGGGAGA TTTGAGCAGC   
  
  
- AAAATTGACA CATTCTTAGT CTAATTGTCA GTGCCTGTGT ACTCATTGTT TAGTTAAAGC CTGTACTTGC   
  
  
- TTGCTTGACA CTTAGGGGGG GTTTTTTTTT TAAAAAAATT TTTTTCAACG TAGGAATAGA AAGACTTGGT   
  
  
- CCGGTCGACA GAGTAAGGAA CCAGGAGCGC GGGGTAGTGC TCGCGACGAG TAATAAGTGG GAGAGAGAGA   
  
  
- GAGGAGAGAC TAGGTGTGGT GTTTGTGTTC CAAACGTGTC GACGTCTCGT CAGCATGATG ATAGTCATCA   
  
  
- CTTTTTGGTT CGTCTTTAGG GGTTAGGGAG AAGAGGAAGA AGGGTCTAAG AAGGAGTTAA GGTTCGGGTA   
  
  
- TAGTCTTTAA ATCTGTTGTT GTTGGTTTTT TTTTTTAATT ATGACGAACT CTTTTTGTTT ATACTATACT   
  
  
- TTAAGGAGCT TTTTCCGGAC GGTATTGTTT AAGGAGCACT TTTTCGTCGA CTCTCTTCTT CTTCTTCTTT   
  
  
- TTTTAGGGCA GTGTTAGAGT TAGAGCTAGG GTTTAACACT TTCTCTTTCT TTTCTACTTC TCTCTTGTGG   
  
  
- GGAAAAGCTT GGGACTGGTC TTTGGGGGCG GCCTCTACAG CGGCCGCGGC CCCTTCAGGT TCTACACCCT   
  
  
- ACTACTCTAC CGGGTCTTGC TGCGATTTTA CCTACTCGAC GAACGACAAC ACCCCATGTT CCACTCCAGC   
  
  
- AGCCTGTACC GCCTTTAACG GGTCTTCGAA CTCGTCGAAC TTCTTCAGTA CCCATCACAA GTTCTTCTGC   
  
  
- CCAATAGAAT GAACCGAAGG CTTTGACAAG TAATGTTAGG ACGTCTAGAC AGATGAACCG AACTTAGATA   
  
  
- CGAAAGACTC AAATTGGGAT TGGGATTAAA ACTGGGAAGA GGTAGCAGTT AGAGTGGGTA GTAGCTAGGT   
  
  
- CGAGGAGGTG AAGGAGCTTG GAGCAGCTGC CAGTTTAAGC TCGGGCTGGG CAAAAGGCTA AGCCTAGACT   
  
  
- TTCGTTAGGG TCCATTCCGA TAGAACTGGG GTGGTTCAAG ATCGTTATGA AGTTTAAACG CACTCCGATT   
  
  
- CTCCAACTTT GGGAGTTTAA TGTGTTGGCG GGGTTGCGGG TGGGGTGGAG GTTTTGACGG GTTAAGCCGC   
  
  
- AGAGGTGGTA GCCCGTGCTG CCGCAGCTCC GGCCACCACG ACCATCTGAG TGTTCTTTTG CCGTAGTCTA   
  
  
- ACCACGTACG TGAATACCGG ACACGGCTTC GTTAAGTTGA TCTTTTGTAC CCTAATCGAC TCCGAAACCA   
  
  
- ATTTGTCTAA TCCATAAATC GTCGTAGAGT TCGACCTGGA TACTCCTTTC ATCGTTGAAC AAAACGTCTT   
  
  
- CGAGAATGAA CAGCCTAGAT GTTCGATACG GGTAGACATG GGATGCTACT TAGTCAGAGT CTACTCAACG   
  
  
- TCTACGTGAA AATACTCTGA ACGGGTATAG AATTTAAGCG GGTAAAGTGC CGTTTAGTTC GTTAAAATCT   
  
  
- TCGTAAGTTA CCCTTCTTCT TTCAAGTACA CTAACTAAAG TCGTACTTCG TTCCGTACGT TACCGGCCGA   
  
  
- AACTACGTTC GGGACCGAGA AGCTGGCCTC CCAGGTGGCG AAAAGGCCAA TTGGCCCTAA CCTGGCGGGC   
  
  
- GAGGCCTGTT GAGCCTGGCC GACGTTCTCC ACCCAACCTT CGAACGGGTC AAGCGCCTAA GCTAGGCCTA   
  
  
- ATTTAAAGTC ATAGCACCCA AACACCGTTT GTCAAACTGG CTAGATCTTA GAAGGTACGA TCTAGAACTT   
  
  
- GGCCTGTGAC TCCACCACCG CCAGTTGAGC CAAAAGCTCG AGGTGGCCGA CAACCGATTT GGGCCCCGCT   
  
  
- AGCTCTTTCA CGACCCCAAG TACTCCCGGC ACTTGGGCCA GTAACACTGC TACCAGCTCG TCCTTCGCTT   
  
  
- GGTGTTGCCT GGCCAAAAGA ACCTGGCCAA GTTACTCAGC AACGTAATAA TGAGGTGGAA CAAACTAAGG   
  
  
- GAACTCTAAA CACAACTATT ACATCTATTC TTCTACAGCC TCCGAATGAA CCCGGTCGTC TAGACGTTGT   
  
  
- ACCACCGAAC ACTTCCCAGA CTGGCTCAGC TCTCCGTGCT CTGGGACCGA GTCACCGCTC GGGCCAAGCG   
  
  
- TAGACGGCCC AAGCTGGGTC AAGTAGACCC AAGCTTACGC AAATTCGTCC GCTCATACAA CAACCTCAAG   
  
  
- AAACGACCAC CACTTCCCAT GCCGCACCTC CTCGCTCTTC CCACAGAGTA CAACCCTACC GTATCATCCG   
  
  
- GCGAATAGTG GTGGAGCCGA ACCGTCGAGC GGTTCTTGGG CCGACACTTA GCCGCTAC

+     TCT-motif

| Site Name | Organism | Position | Strand | Matrix score. | sequence | function |
| --- | --- | --- | --- | --- | --- | --- |
| TCT-motif | Arabidopsis thaliana | 1485 | - | 6 | TCTTAC | part of a light responsive element |
| TCT-motif | Arabidopsis thaliana | 1281 | - | 6 | TCTTAC | part of a light responsive element |
| TCT-motif | Arabidopsis thaliana | 109 | - | 6 | TCTTAC | part of a light responsive element |
| TCT-motif | Arabidopsis thaliana | 1176 | + | 6 | TCTTAC | part of a light responsive element |
| TCT-motif | Arabidopsis thaliana | 2807 | + | 6 | TCTTAC | part of a light responsive element |
| TCT-motif | Arabidopsis thaliana | 2250 | + | 6 | TCTTAC | part of a light responsive element |

>HU10G00709.1   
+ -Up\_Stream \_Len000AACCCT TTAATTTTAT CAATGTGGGA CAACACTCAT CTTCATACTC CAACAAGCTA   
  
  
+ TGATACATTC GATTATTGCC TAGTATTTCG TTTAGTAAGA TATTAGTGCC AAACTTTGTA ATAAAAATGA   
  
  
+ AACTTACTAA AAAAAATCTC GTTTTCTGTA GAGGGAAAAT TTGAAGACTT AGTGTCACAT GATGACCTAA   
  
  
+ TGGTACTTGT AGGGTCAAGA TTGAGATTGA GGGCATACTG ACGACCTGTA TAATGGTCAA GTAGGATCAA   
  
  
+ AGTCGAGAAA ACTGTCTCTG CTAACTTTCA TAATCTAACC CTTAACTCAC TCTGACCCTC AATTTAATCA   
  
  
+ GGCCTAATTC AAACTATTAA TGGTTATAAC TCTCTTTCCT TCTTTTGAGA TTTTTTTTTT CTACATGCGG   
  
  
+ TTGAAAATAA TTTTTTTAAA AAATATATAT CACTAACCAT TCATTATCAT AGTTTTAGAG TTCATATTTA   
  
  
+ TGTGGTCCTT GTTGAGTCTA ATTTAATGCT TTAGATGGCG TGAAGCACCA AATTAGTGGT AACCATAATA   
  
  
+ ATAAACTATC AAGTGTTAAG TCGAAGAAAA CGGACACTGC TCAACCAAAT CTAGTAACAT AGTGTATGAC   
  
  
+ ATGATAACTA AGTGTGCCAT GTAGTAATCA ATTCTTTCTT TTTTTTTTTT TGGTTGAGAT CACATAAATT   
  
  
+ ACGTTATCAA GGGTAGTACG ATATAATGGA TGAGGAGAAA TTAAATGTGA GATTTAGTTG ATGAATCGAA   
  
  
+ TACGTTAGTC TGAGCCATTT GGTCAAAACT GCATTGGCTA ATCAATAGAA ATTATAGAAA ATACATAACA   
  
  
+ TAATGGGATG CAATTTTCTA ATTGGGGAAA CCATGTCAAT GCAGTGATTT TGGACCATTT AGGCCTTATT   
  
  
+ CTTTTCAATT TTTTAATTGA TTATTTAGAC TCATATGAAC TTATTGTTGA CAGATTGAGC TAAAAACACT   
  
  
+ GATGTTTCAT ACATATGTAT GAATTGCACG TACGAAGGCT CTGCTCATTT CGAGAAAGAA AATTAACAGT   
  
  
+ TAGGAGCAGT ATAATTTAAC AACAAGCGAT AATTATTAGT ATGAAGTGGT ACGAATCTAT TTTAAAGGAG   
  
  
+ TACTATGAAG TACAAACAAT AACAAATAAT AAAGAGCAGT ACATTAACAT ATCTTACTAC CTAAATAAAT   
  
  
+ CGATTCTAAG AGTAAAAAAA TGTTGAAATT AACCCCATTA TAGTTTTACT TTAACAAGAA GTTTTTCTTC   
  
  
+ TTTTACCATT TACCAAGTAA GAATGATTCA GGCCGTATAG CCAAGGAAAA ATAGAAAACC ACAAAACTAA   
  
  
+ CCATGGCCCA AAAAACAACC ACCAAGAGGG GCACTAATGT GACTAACCGA CGGTTAACAT TATCATCTGT   
  
  
+ TTGATACTTG TTCATCTCTC ACTATTCGGG TAATAGTGTG AAATTACTGA TGTACCCTCT AAACTCGTCG   
  
  
+ TTTTAACTGT GTAAGAATCA GATTAACAGT CACGGACACA TGAGTAACAA ATCAATTTCG GACATGAACG   
  
  
+ AACGAACTGT GAATCCCCCC CAAAAAAAAA ATTTTTTTAA AAAAAGTTGC ATCCTTATCT TTCTGAACCA   
  
  
+ GGCCAGCTGT CTCATTCCTT GGTCCTCGCG CCCCATCACG AGCGCTGCTC ATTATTCACC CTCTCTCTCT   
  
  
+ CTCCTCTCTG ATCCACACCA CAAACACAAG GTTTGCACAG CTGCAGAGCA GTCGTACTAC TATCAGTAGT   
  
  
+ GAAAAACCAA GCAGAAATCC CCAATCCCTC TTCTCCTTCT TCCCAGATTC TTCCTCAATT CCAAGCCCAT   
  
  
+ ATCAGAAATT TAGACAACAA CAACCAAAAA AAAAAATTAA TACTGCTTGA GAAAAACAAA TATGATATGA   
  
  
+ AATTCCTCGA AAAAGGCCTG CCATAACAAA TTCCTCGTGA AAAAGCAGCT GAGAGAAGAA GAAGAAGAAA   
  
  
+ AAAATCCCGT CACAATCTCA ATCTCGATCC CAAATTGTGA AAGAGAAAGA AAAGATGAAG AGAGAACACC   
  
  
+ CCTTTTCGAA CCCTGACCAG AAACCCCCGC CGGAGATGTC GCCGGCGCCG GGGAAGTCCA AGATGTGGGA   
  
  
+ TGATGAGATG GCCCAGAACG ACGCTAAAAT GGATGAGCTG CTTGCTGTTG TGGGGTACAA GGTGAGGTCG   
  
  
+ TCGGACATGG CGGAAATTGC CCAGAAGCTT GAGCAGCTTG AAGAAGTCAT GGGTAGTGTT CAAGAAGACG   
  
  
+ GGTTATCTTA CTTGGCTTCC GAAACTGTTC ATTACAATCC TGCAGATCTG TCTACTTGGC TTGAATCTAT   
  
  
+ GCTTTCTGAG TTTAACCCTA ACCCTAATTT TGACCCTTCT CCATCGTCAA TCTCACCCAT CATCGATCCA   
  
  
+ GCTCCTCCAC TTCCTCGAAC CTCGTCGACG GTCAAATTCG AGCCCGACCC GTTTTCCGAT TCGGATCTGA   
  
  
+ AAGCAATCCC AGGTAAGGCT ATCTTGACCC CACCAAGTTC TAGCAATACT TCAAATTTGC GTGAGGCTAA   
  
  
+ GAGGTTGAAA CCCTCAAATT ACACAACCGC CCCAACGCCC ACCCCACCTC CAAAACTGCC CAATTCGGCG   
  
  
+ TCTCCACCAT CGGGCACGAC GGCGTCGAGG CCGGTGGTGC TGGTAGACTC ACAAGAAAAC GGCATCAGAT   
  
  
+ TGGTGCATGC ACTTATGGCC TGTGCCGAAG CAATTCAACT AGAAAACATG GGATTAGCTG AGGCTTTGGT   
  
  
+ TAAACAGATT AGGTATTTAG CAGCATCTCA AGCTGGACCT ATGAGGAAAG TAGCAACTTG TTTTGCAGAA   
  
  
+ GCTCTTACTT GTCGGATCTA CAAGCTATGC CCATCTGTAC CCTACGATGA ATCAGTCTCA GATGAGTTGC   
  
  
+ AGATGCACTT TTATGAGACT TGCCCATATC TTAAATTCGC CCATTTCACG GCAAATCAAG CAATTTTAGA   
  
  
+ AGCATTCAAT GGGAAGAAGA AAGTTCATGT GATTGATTTC AGCATGAAGC AAGGCATGCA ATGGCCGGCT   
  
  
+ TTGATGCAAG CCCTGGCTCT TCGACCGGAG GGTCCACCGC TTTTCCGGTT AACCGGGATT GGACCGCCCG   
  
  
+ CTCCGGACAA CTCGGACCGG CTGCAAGAGG TGGGTTGGAA GCTTGCCCAG TTCGCGGATT CGATCCGGAT   
  
  
+ TAAATTTCAG TATCGTGGGT TTGTGGCAAA CAGTTTGACC GATCTAGAAT CTTCCATGCT AGATCTTGAA   
  
  
+ CCGGACACTG AGGTGGTGGC GGTCAACTCG GTTTTCGAGC TCCACCGGCT GTTGGCTAAA CCCGGGGCGA   
  
  
+ TCGAGAAAGT GCTGGGGTTC ATGAGGGCCG TGAACCCGGT CATTGTGACG ATGGTCGAGC AGGAAGCGAA   
  
  
+ CCACAACGGA CCGGTTTTCT TGGACCGGTT CAATGAGTCG TTGCATTATT ACTCCACCTT GTTTGATTCC   
  
  
+ CTTGAGATTT GTGTTGATAA TGTAGATAAG AAGATGTCGG AGGCTTACTT GGGCCAGCAG ATCTGCAACA   
  
  
+ TGGTGGCTTG TGAAGGGTCT GACCGAGTCG AGAGGCACGA GACCCTGGCT CAGTGGCGAG CCCGGTTCGC   
  
  
+ ATCTGCCGGG TTCGACCCAG TTCATCTGGG TTCGAATGCG TTTAAGCAGG CGAGTATGTT GTTGGAGTTC   
  
  
+ TTTGCTGGTG GTGAAGGGTA CGGCGTGGAG GAGCGAGAAG GGTGTCTCAT GTTGGGATGG CATAGTAGGC   
  
  
+ CGCTTATCAC CACCTCGGCT TGGCAGCTCG CCAAGAACCC GGCTGTGAAT CGGCGATG  

- -Up\_Stream \_Len000TTGGGA AATTAAAATA GTTACACCCT GTTGTGAGTA GAAGTATGAG GTTGTTCGAT   
  
  
- ACTATGTAAG CTAATAACGG ATCATAAAGC AAATCATTCT ATAATCACGG TTTGAAACAT TATTTTTACT   
  
  
- TTGAATGATT TTTTTTAGAG CAAAAGACAT CTCCCTTTTA AACTTCTGAA TCACAGTGTA CTACTGGATT   
  
  
- ACCATGAACA TCCCAGTTCT AACTCTAACT CCCGTATGAC TGCTGGACAT ATTACCAGTT CATCCTAGTT   
  
  
- TCAGCTCTTT TGACAGAGAC GATTGAAAGT ATTAGATTGG GAATTGAGTG AGACTGGGAG TTAAATTAGT   
  
  
- CCGGATTAAG TTTGATAATT ACCAATATTG AGAGAAAGGA AGAAAACTCT AAAAAAAAAA GATGTACGCC   
  
  
- AACTTTTATT AAAAAAATTT TTTATATATA GTGATTGGTA AGTAATAGTA TCAAAATCTC AAGTATAAAT   
  
  
- ACACCAGGAA CAACTCAGAT TAAATTACGA AATCTACCGC ACTTCGTGGT TTAATCACCA TTGGTATTAT   
  
  
- TATTTGATAG TTCACAATTC AGCTTCTTTT GCCTGTGACG AGTTGGTTTA GATCATTGTA TCACATACTG   
  
  
- TACTATTGAT TCACACGGTA CATCATTAGT TAAGAAAGAA AAAAAAAAAA ACCAACTCTA GTGTATTTAA   
  
  
- TGCAATAGTT CCCATCATGC TATATTACCT ACTCCTCTTT AATTTACACT CTAAATCAAC TACTTAGCTT   
  
  
- ATGCAATCAG ACTCGGTAAA CCAGTTTTGA CGTAACCGAT TAGTTATCTT TAATATCTTT TATGTATTGT   
  
  
- ATTACCCTAC GTTAAAAGAT TAACCCCTTT GGTACAGTTA CGTCACTAAA ACCTGGTAAA TCCGGAATAA   
  
  
- GAAAAGTTAA AAAATTAACT AATAAATCTG AGTATACTTG AATAACAACT GTCTAACTCG ATTTTTGTGA   
  
  
- CTACAAAGTA TGTATACATA CTTAACGTGC ATGCTTCCGA GACGAGTAAA GCTCTTTCTT TTAATTGTCA   
  
  
- ATCCTCGTCA TATTAAATTG TTGTTCGCTA TTAATAATCA TACTTCACCA TGCTTAGATA AAATTTCCTC   
  
  
- ATGATACTTC ATGTTTGTTA TTGTTTATTA TTTCTCGTCA TGTAATTGTA TAGAATGATG GATTTATTTA   
  
  
- GCTAAGATTC TCATTTTTTT ACAACTTTAA TTGGGGTAAT ATCAAAATGA AATTGTTCTT CAAAAAGAAG   
  
  
- AAAATGGTAA ATGGTTCATT CTTACTAAGT CCGGCATATC GGTTCCTTTT TATCTTTTGG TGTTTTGATT   
  
  
- GGTACCGGGT TTTTTGTTGG TGGTTCTCCC CGTGATTACA CTGATTGGCT GCCAATTGTA ATAGTAGACA   
  
  
- AACTATGAAC AAGTAGAGAG TGATAAGCCC ATTATCACAC TTTAATGACT ACATGGGAGA TTTGAGCAGC   
  
  
- AAAATTGACA CATTCTTAGT CTAATTGTCA GTGCCTGTGT ACTCATTGTT TAGTTAAAGC CTGTACTTGC   
  
  
- TTGCTTGACA CTTAGGGGGG GTTTTTTTTT TAAAAAAATT TTTTTCAACG TAGGAATAGA AAGACTTGGT   
  
  
- CCGGTCGACA GAGTAAGGAA CCAGGAGCGC GGGGTAGTGC TCGCGACGAG TAATAAGTGG GAGAGAGAGA   
  
  
- GAGGAGAGAC TAGGTGTGGT GTTTGTGTTC CAAACGTGTC GACGTCTCGT CAGCATGATG ATAGTCATCA   
  
  
- CTTTTTGGTT CGTCTTTAGG GGTTAGGGAG AAGAGGAAGA AGGGTCTAAG AAGGAGTTAA GGTTCGGGTA   
  
  
- TAGTCTTTAA ATCTGTTGTT GTTGGTTTTT TTTTTTAATT ATGACGAACT CTTTTTGTTT ATACTATACT   
  
  
- TTAAGGAGCT TTTTCCGGAC GGTATTGTTT AAGGAGCACT TTTTCGTCGA CTCTCTTCTT CTTCTTCTTT   
  
  
- TTTTAGGGCA GTGTTAGAGT TAGAGCTAGG GTTTAACACT TTCTCTTTCT TTTCTACTTC TCTCTTGTGG   
  
  
- GGAAAAGCTT GGGACTGGTC TTTGGGGGCG GCCTCTACAG CGGCCGCGGC CCCTTCAGGT TCTACACCCT   
  
  
- ACTACTCTAC CGGGTCTTGC TGCGATTTTA CCTACTCGAC GAACGACAAC ACCCCATGTT CCACTCCAGC   
  
  
- AGCCTGTACC GCCTTTAACG GGTCTTCGAA CTCGTCGAAC TTCTTCAGTA CCCATCACAA GTTCTTCTGC   
  
  
- CCAATAGAAT GAACCGAAGG CTTTGACAAG TAATGTTAGG ACGTCTAGAC AGATGAACCG AACTTAGATA   
  
  
- CGAAAGACTC AAATTGGGAT TGGGATTAAA ACTGGGAAGA GGTAGCAGTT AGAGTGGGTA GTAGCTAGGT   
  
  
- CGAGGAGGTG AAGGAGCTTG GAGCAGCTGC CAGTTTAAGC TCGGGCTGGG CAAAAGGCTA AGCCTAGACT   
  
  
- TTCGTTAGGG TCCATTCCGA TAGAACTGGG GTGGTTCAAG ATCGTTATGA AGTTTAAACG CACTCCGATT   
  
  
- CTCCAACTTT GGGAGTTTAA TGTGTTGGCG GGGTTGCGGG TGGGGTGGAG GTTTTGACGG GTTAAGCCGC   
  
  
- AGAGGTGGTA GCCCGTGCTG CCGCAGCTCC GGCCACCACG ACCATCTGAG TGTTCTTTTG CCGTAGTCTA   
  
  
- ACCACGTACG TGAATACCGG ACACGGCTTC GTTAAGTTGA TCTTTTGTAC CCTAATCGAC TCCGAAACCA   
  
  
- ATTTGTCTAA TCCATAAATC GTCGTAGAGT TCGACCTGGA TACTCCTTTC ATCGTTGAAC AAAACGTCTT   
  
  
- CGAGAATGAA CAGCCTAGAT GTTCGATACG GGTAGACATG GGATGCTACT TAGTCAGAGT CTACTCAACG   
  
  
- TCTACGTGAA AATACTCTGA ACGGGTATAG AATTTAAGCG GGTAAAGTGC CGTTTAGTTC GTTAAAATCT   
  
  
- TCGTAAGTTA CCCTTCTTCT TTCAAGTACA CTAACTAAAG TCGTACTTCG TTCCGTACGT TACCGGCCGA   
  
  
- AACTACGTTC GGGACCGAGA AGCTGGCCTC CCAGGTGGCG AAAAGGCCAA TTGGCCCTAA CCTGGCGGGC   
  
  
- GAGGCCTGTT GAGCCTGGCC GACGTTCTCC ACCCAACCTT CGAACGGGTC AAGCGCCTAA GCTAGGCCTA   
  
  
- ATTTAAAGTC ATAGCACCCA AACACCGTTT GTCAAACTGG CTAGATCTTA GAAGGTACGA TCTAGAACTT   
  
  
- GGCCTGTGAC TCCACCACCG CCAGTTGAGC CAAAAGCTCG AGGTGGCCGA CAACCGATTT GGGCCCCGCT   
  
  
- AGCTCTTTCA CGACCCCAAG TACTCCCGGC ACTTGGGCCA GTAACACTGC TACCAGCTCG TCCTTCGCTT   
  
  
- GGTGTTGCCT GGCCAAAAGA ACCTGGCCAA GTTACTCAGC AACGTAATAA TGAGGTGGAA CAAACTAAGG   
  
  
- GAACTCTAAA CACAACTATT ACATCTATTC TTCTACAGCC TCCGAATGAA CCCGGTCGTC TAGACGTTGT   
  
  
- ACCACCGAAC ACTTCCCAGA CTGGCTCAGC TCTCCGTGCT CTGGGACCGA GTCACCGCTC GGGCCAAGCG   
  
  
- TAGACGGCCC AAGCTGGGTC AAGTAGACCC AAGCTTACGC AAATTCGTCC GCTCATACAA CAACCTCAAG   
  
  
- AAACGACCAC CACTTCCCAT GCCGCACCTC CTCGCTCTTC CCACAGAGTA CAACCCTACC GTATCATCCG   
  
  
- GCGAATAGTG GTGGAGCCGA ACCGTCGAGC GGTTCTTGGG CCGACACTTA GCCGCTAC

+     TGA-element

| Site Name | Organism | Position | Strand | Matrix score. | sequence | function |
| --- | --- | --- | --- | --- | --- | --- |
| TGA-element | Brassica oleracea | 3401 | - | 6 | AACGAC | auxin-responsive element |
| TGA-element | Brassica oleracea | 2121 | + | 6 | AACGAC | auxin-responsive element |
| TGA-element | Brassica oleracea | 1471 | - | 6 | AACGAC | auxin-responsive element |

>HU10G00709.1   
+ -Up\_Stream \_Len000AACCCT TTAATTTTAT CAATGTGGGA CAACACTCAT CTTCATACTC CAACAAGCTA   
  
  
+ TGATACATTC GATTATTGCC TAGTATTTCG TTTAGTAAGA TATTAGTGCC AAACTTTGTA ATAAAAATGA   
  
  
+ AACTTACTAA AAAAAATCTC GTTTTCTGTA GAGGGAAAAT TTGAAGACTT AGTGTCACAT GATGACCTAA   
  
  
+ TGGTACTTGT AGGGTCAAGA TTGAGATTGA GGGCATACTG ACGACCTGTA TAATGGTCAA GTAGGATCAA   
  
  
+ AGTCGAGAAA ACTGTCTCTG CTAACTTTCA TAATCTAACC CTTAACTCAC TCTGACCCTC AATTTAATCA   
  
  
+ GGCCTAATTC AAACTATTAA TGGTTATAAC TCTCTTTCCT TCTTTTGAGA TTTTTTTTTT CTACATGCGG   
  
  
+ TTGAAAATAA TTTTTTTAAA AAATATATAT CACTAACCAT TCATTATCAT AGTTTTAGAG TTCATATTTA   
  
  
+ TGTGGTCCTT GTTGAGTCTA ATTTAATGCT TTAGATGGCG TGAAGCACCA AATTAGTGGT AACCATAATA   
  
  
+ ATAAACTATC AAGTGTTAAG TCGAAGAAAA CGGACACTGC TCAACCAAAT CTAGTAACAT AGTGTATGAC   
  
  
+ ATGATAACTA AGTGTGCCAT GTAGTAATCA ATTCTTTCTT TTTTTTTTTT TGGTTGAGAT CACATAAATT   
  
  
+ ACGTTATCAA GGGTAGTACG ATATAATGGA TGAGGAGAAA TTAAATGTGA GATTTAGTTG ATGAATCGAA   
  
  
+ TACGTTAGTC TGAGCCATTT GGTCAAAACT GCATTGGCTA ATCAATAGAA ATTATAGAAA ATACATAACA   
  
  
+ TAATGGGATG CAATTTTCTA ATTGGGGAAA CCATGTCAAT GCAGTGATTT TGGACCATTT AGGCCTTATT   
  
  
+ CTTTTCAATT TTTTAATTGA TTATTTAGAC TCATATGAAC TTATTGTTGA CAGATTGAGC TAAAAACACT   
  
  
+ GATGTTTCAT ACATATGTAT GAATTGCACG TACGAAGGCT CTGCTCATTT CGAGAAAGAA AATTAACAGT   
  
  
+ TAGGAGCAGT ATAATTTAAC AACAAGCGAT AATTATTAGT ATGAAGTGGT ACGAATCTAT TTTAAAGGAG   
  
  
+ TACTATGAAG TACAAACAAT AACAAATAAT AAAGAGCAGT ACATTAACAT ATCTTACTAC CTAAATAAAT   
  
  
+ CGATTCTAAG AGTAAAAAAA TGTTGAAATT AACCCCATTA TAGTTTTACT TTAACAAGAA GTTTTTCTTC   
  
  
+ TTTTACCATT TACCAAGTAA GAATGATTCA GGCCGTATAG CCAAGGAAAA ATAGAAAACC ACAAAACTAA   
  
  
+ CCATGGCCCA AAAAACAACC ACCAAGAGGG GCACTAATGT GACTAACCGA CGGTTAACAT TATCATCTGT   
  
  
+ TTGATACTTG TTCATCTCTC ACTATTCGGG TAATAGTGTG AAATTACTGA TGTACCCTCT AAACTCGTCG   
  
  
+ TTTTAACTGT GTAAGAATCA GATTAACAGT CACGGACACA TGAGTAACAA ATCAATTTCG GACATGAACG   
  
  
+ AACGAACTGT GAATCCCCCC CAAAAAAAAA ATTTTTTTAA AAAAAGTTGC ATCCTTATCT TTCTGAACCA   
  
  
+ GGCCAGCTGT CTCATTCCTT GGTCCTCGCG CCCCATCACG AGCGCTGCTC ATTATTCACC CTCTCTCTCT   
  
  
+ CTCCTCTCTG ATCCACACCA CAAACACAAG GTTTGCACAG CTGCAGAGCA GTCGTACTAC TATCAGTAGT   
  
  
+ GAAAAACCAA GCAGAAATCC CCAATCCCTC TTCTCCTTCT TCCCAGATTC TTCCTCAATT CCAAGCCCAT   
  
  
+ ATCAGAAATT TAGACAACAA CAACCAAAAA AAAAAATTAA TACTGCTTGA GAAAAACAAA TATGATATGA   
  
  
+ AATTCCTCGA AAAAGGCCTG CCATAACAAA TTCCTCGTGA AAAAGCAGCT GAGAGAAGAA GAAGAAGAAA   
  
  
+ AAAATCCCGT CACAATCTCA ATCTCGATCC CAAATTGTGA AAGAGAAAGA AAAGATGAAG AGAGAACACC   
  
  
+ CCTTTTCGAA CCCTGACCAG AAACCCCCGC CGGAGATGTC GCCGGCGCCG GGGAAGTCCA AGATGTGGGA   
  
  
+ TGATGAGATG GCCCAGAACG ACGCTAAAAT GGATGAGCTG CTTGCTGTTG TGGGGTACAA GGTGAGGTCG   
  
  
+ TCGGACATGG CGGAAATTGC CCAGAAGCTT GAGCAGCTTG AAGAAGTCAT GGGTAGTGTT CAAGAAGACG   
  
  
+ GGTTATCTTA CTTGGCTTCC GAAACTGTTC ATTACAATCC TGCAGATCTG TCTACTTGGC TTGAATCTAT   
  
  
+ GCTTTCTGAG TTTAACCCTA ACCCTAATTT TGACCCTTCT CCATCGTCAA TCTCACCCAT CATCGATCCA   
  
  
+ GCTCCTCCAC TTCCTCGAAC CTCGTCGACG GTCAAATTCG AGCCCGACCC GTTTTCCGAT TCGGATCTGA   
  
  
+ AAGCAATCCC AGGTAAGGCT ATCTTGACCC CACCAAGTTC TAGCAATACT TCAAATTTGC GTGAGGCTAA   
  
  
+ GAGGTTGAAA CCCTCAAATT ACACAACCGC CCCAACGCCC ACCCCACCTC CAAAACTGCC CAATTCGGCG   
  
  
+ TCTCCACCAT CGGGCACGAC GGCGTCGAGG CCGGTGGTGC TGGTAGACTC ACAAGAAAAC GGCATCAGAT   
  
  
+ TGGTGCATGC ACTTATGGCC TGTGCCGAAG CAATTCAACT AGAAAACATG GGATTAGCTG AGGCTTTGGT   
  
  
+ TAAACAGATT AGGTATTTAG CAGCATCTCA AGCTGGACCT ATGAGGAAAG TAGCAACTTG TTTTGCAGAA   
  
  
+ GCTCTTACTT GTCGGATCTA CAAGCTATGC CCATCTGTAC CCTACGATGA ATCAGTCTCA GATGAGTTGC   
  
  
+ AGATGCACTT TTATGAGACT TGCCCATATC TTAAATTCGC CCATTTCACG GCAAATCAAG CAATTTTAGA   
  
  
+ AGCATTCAAT GGGAAGAAGA AAGTTCATGT GATTGATTTC AGCATGAAGC AAGGCATGCA ATGGCCGGCT   
  
  
+ TTGATGCAAG CCCTGGCTCT TCGACCGGAG GGTCCACCGC TTTTCCGGTT AACCGGGATT GGACCGCCCG   
  
  
+ CTCCGGACAA CTCGGACCGG CTGCAAGAGG TGGGTTGGAA GCTTGCCCAG TTCGCGGATT CGATCCGGAT   
  
  
+ TAAATTTCAG TATCGTGGGT TTGTGGCAAA CAGTTTGACC GATCTAGAAT CTTCCATGCT AGATCTTGAA   
  
  
+ CCGGACACTG AGGTGGTGGC GGTCAACTCG GTTTTCGAGC TCCACCGGCT GTTGGCTAAA CCCGGGGCGA   
  
  
+ TCGAGAAAGT GCTGGGGTTC ATGAGGGCCG TGAACCCGGT CATTGTGACG ATGGTCGAGC AGGAAGCGAA   
  
  
+ CCACAACGGA CCGGTTTTCT TGGACCGGTT CAATGAGTCG TTGCATTATT ACTCCACCTT GTTTGATTCC   
  
  
+ CTTGAGATTT GTGTTGATAA TGTAGATAAG AAGATGTCGG AGGCTTACTT GGGCCAGCAG ATCTGCAACA   
  
  
+ TGGTGGCTTG TGAAGGGTCT GACCGAGTCG AGAGGCACGA GACCCTGGCT CAGTGGCGAG CCCGGTTCGC   
  
  
+ ATCTGCCGGG TTCGACCCAG TTCATCTGGG TTCGAATGCG TTTAAGCAGG CGAGTATGTT GTTGGAGTTC   
  
  
+ TTTGCTGGTG GTGAAGGGTA CGGCGTGGAG GAGCGAGAAG GGTGTCTCAT GTTGGGATGG CATAGTAGGC   
  
  
+ CGCTTATCAC CACCTCGGCT TGGCAGCTCG CCAAGAACCC GGCTGTGAAT CGGCGATG  

- -Up\_Stream \_Len000TTGGGA AATTAAAATA GTTACACCCT GTTGTGAGTA GAAGTATGAG GTTGTTCGAT   
  
  
- ACTATGTAAG CTAATAACGG ATCATAAAGC AAATCATTCT ATAATCACGG TTTGAAACAT TATTTTTACT   
  
  
- TTGAATGATT TTTTTTAGAG CAAAAGACAT CTCCCTTTTA AACTTCTGAA TCACAGTGTA CTACTGGATT   
  
  
- ACCATGAACA TCCCAGTTCT AACTCTAACT CCCGTATGAC TGCTGGACAT ATTACCAGTT CATCCTAGTT   
  
  
- TCAGCTCTTT TGACAGAGAC GATTGAAAGT ATTAGATTGG GAATTGAGTG AGACTGGGAG TTAAATTAGT   
  
  
- CCGGATTAAG TTTGATAATT ACCAATATTG AGAGAAAGGA AGAAAACTCT AAAAAAAAAA GATGTACGCC   
  
  
- AACTTTTATT AAAAAAATTT TTTATATATA GTGATTGGTA AGTAATAGTA TCAAAATCTC AAGTATAAAT   
  
  
- ACACCAGGAA CAACTCAGAT TAAATTACGA AATCTACCGC ACTTCGTGGT TTAATCACCA TTGGTATTAT   
  
  
- TATTTGATAG TTCACAATTC AGCTTCTTTT GCCTGTGACG AGTTGGTTTA GATCATTGTA TCACATACTG   
  
  
- TACTATTGAT TCACACGGTA CATCATTAGT TAAGAAAGAA AAAAAAAAAA ACCAACTCTA GTGTATTTAA   
  
  
- TGCAATAGTT CCCATCATGC TATATTACCT ACTCCTCTTT AATTTACACT CTAAATCAAC TACTTAGCTT   
  
  
- ATGCAATCAG ACTCGGTAAA CCAGTTTTGA CGTAACCGAT TAGTTATCTT TAATATCTTT TATGTATTGT   
  
  
- ATTACCCTAC GTTAAAAGAT TAACCCCTTT GGTACAGTTA CGTCACTAAA ACCTGGTAAA TCCGGAATAA   
  
  
- GAAAAGTTAA AAAATTAACT AATAAATCTG AGTATACTTG AATAACAACT GTCTAACTCG ATTTTTGTGA   
  
  
- CTACAAAGTA TGTATACATA CTTAACGTGC ATGCTTCCGA GACGAGTAAA GCTCTTTCTT TTAATTGTCA   
  
  
- ATCCTCGTCA TATTAAATTG TTGTTCGCTA TTAATAATCA TACTTCACCA TGCTTAGATA AAATTTCCTC   
  
  
- ATGATACTTC ATGTTTGTTA TTGTTTATTA TTTCTCGTCA TGTAATTGTA TAGAATGATG GATTTATTTA   
  
  
- GCTAAGATTC TCATTTTTTT ACAACTTTAA TTGGGGTAAT ATCAAAATGA AATTGTTCTT CAAAAAGAAG   
  
  
- AAAATGGTAA ATGGTTCATT CTTACTAAGT CCGGCATATC GGTTCCTTTT TATCTTTTGG TGTTTTGATT   
  
  
- GGTACCGGGT TTTTTGTTGG TGGTTCTCCC CGTGATTACA CTGATTGGCT GCCAATTGTA ATAGTAGACA   
  
  
- AACTATGAAC AAGTAGAGAG TGATAAGCCC ATTATCACAC TTTAATGACT ACATGGGAGA TTTGAGCAGC   
  
  
- AAAATTGACA CATTCTTAGT CTAATTGTCA GTGCCTGTGT ACTCATTGTT TAGTTAAAGC CTGTACTTGC   
  
  
- TTGCTTGACA CTTAGGGGGG GTTTTTTTTT TAAAAAAATT TTTTTCAACG TAGGAATAGA AAGACTTGGT   
  
  
- CCGGTCGACA GAGTAAGGAA CCAGGAGCGC GGGGTAGTGC TCGCGACGAG TAATAAGTGG GAGAGAGAGA   
  
  
- GAGGAGAGAC TAGGTGTGGT GTTTGTGTTC CAAACGTGTC GACGTCTCGT CAGCATGATG ATAGTCATCA   
  
  
- CTTTTTGGTT CGTCTTTAGG GGTTAGGGAG AAGAGGAAGA AGGGTCTAAG AAGGAGTTAA GGTTCGGGTA   
  
  
- TAGTCTTTAA ATCTGTTGTT GTTGGTTTTT TTTTTTAATT ATGACGAACT CTTTTTGTTT ATACTATACT   
  
  
- TTAAGGAGCT TTTTCCGGAC GGTATTGTTT AAGGAGCACT TTTTCGTCGA CTCTCTTCTT CTTCTTCTTT   
  
  
- TTTTAGGGCA GTGTTAGAGT TAGAGCTAGG GTTTAACACT TTCTCTTTCT TTTCTACTTC TCTCTTGTGG   
  
  
- GGAAAAGCTT GGGACTGGTC TTTGGGGGCG GCCTCTACAG CGGCCGCGGC CCCTTCAGGT TCTACACCCT   
  
  
- ACTACTCTAC CGGGTCTTGC TGCGATTTTA CCTACTCGAC GAACGACAAC ACCCCATGTT CCACTCCAGC   
  
  
- AGCCTGTACC GCCTTTAACG GGTCTTCGAA CTCGTCGAAC TTCTTCAGTA CCCATCACAA GTTCTTCTGC   
  
  
- CCAATAGAAT GAACCGAAGG CTTTGACAAG TAATGTTAGG ACGTCTAGAC AGATGAACCG AACTTAGATA   
  
  
- CGAAAGACTC AAATTGGGAT TGGGATTAAA ACTGGGAAGA GGTAGCAGTT AGAGTGGGTA GTAGCTAGGT   
  
  
- CGAGGAGGTG AAGGAGCTTG GAGCAGCTGC CAGTTTAAGC TCGGGCTGGG CAAAAGGCTA AGCCTAGACT   
  
  
- TTCGTTAGGG TCCATTCCGA TAGAACTGGG GTGGTTCAAG ATCGTTATGA AGTTTAAACG CACTCCGATT   
  
  
- CTCCAACTTT GGGAGTTTAA TGTGTTGGCG GGGTTGCGGG TGGGGTGGAG GTTTTGACGG GTTAAGCCGC   
  
  
- AGAGGTGGTA GCCCGTGCTG CCGCAGCTCC GGCCACCACG ACCATCTGAG TGTTCTTTTG CCGTAGTCTA   
  
  
- ACCACGTACG TGAATACCGG ACACGGCTTC GTTAAGTTGA TCTTTTGTAC CCTAATCGAC TCCGAAACCA   
  
  
- ATTTGTCTAA TCCATAAATC GTCGTAGAGT TCGACCTGGA TACTCCTTTC ATCGTTGAAC AAAACGTCTT   
  
  
- CGAGAATGAA CAGCCTAGAT GTTCGATACG GGTAGACATG GGATGCTACT TAGTCAGAGT CTACTCAACG   
  
  
- TCTACGTGAA AATACTCTGA ACGGGTATAG AATTTAAGCG GGTAAAGTGC CGTTTAGTTC GTTAAAATCT   
  
  
- TCGTAAGTTA CCCTTCTTCT TTCAAGTACA CTAACTAAAG TCGTACTTCG TTCCGTACGT TACCGGCCGA   
  
  
- AACTACGTTC GGGACCGAGA AGCTGGCCTC CCAGGTGGCG AAAAGGCCAA TTGGCCCTAA CCTGGCGGGC   
  
  
- GAGGCCTGTT GAGCCTGGCC GACGTTCTCC ACCCAACCTT CGAACGGGTC AAGCGCCTAA GCTAGGCCTA   
  
  
- ATTTAAAGTC ATAGCACCCA AACACCGTTT GTCAAACTGG CTAGATCTTA GAAGGTACGA TCTAGAACTT   
  
  
- GGCCTGTGAC TCCACCACCG CCAGTTGAGC CAAAAGCTCG AGGTGGCCGA CAACCGATTT GGGCCCCGCT   
  
  
- AGCTCTTTCA CGACCCCAAG TACTCCCGGC ACTTGGGCCA GTAACACTGC TACCAGCTCG TCCTTCGCTT   
  
  
- GGTGTTGCCT GGCCAAAAGA ACCTGGCCAA GTTACTCAGC AACGTAATAA TGAGGTGGAA CAAACTAAGG   
  
  
- GAACTCTAAA CACAACTATT ACATCTATTC TTCTACAGCC TCCGAATGAA CCCGGTCGTC TAGACGTTGT   
  
  
- ACCACCGAAC ACTTCCCAGA CTGGCTCAGC TCTCCGTGCT CTGGGACCGA GTCACCGCTC GGGCCAAGCG   
  
  
- TAGACGGCCC AAGCTGGGTC AAGTAGACCC AAGCTTACGC AAATTCGTCC GCTCATACAA CAACCTCAAG   
  
  
- AAACGACCAC CACTTCCCAT GCCGCACCTC CTCGCTCTTC CCACAGAGTA CAACCCTACC GTATCATCCG   
  
  
- GCGAATAGTG GTGGAGCCGA ACCGTCGAGC GGTTCTTGGG CCGACACTTA GCCGCTAC

+     TGACG-motif

| Site Name | Organism | Position | Strand | Matrix score. | sequence | function |
| --- | --- | --- | --- | --- | --- | --- |
| TGACG-motif | Hordeum vulgare | 1972 | - | 5 | TGACG | cis-acting regulatory element involved in the MeJA-responsiveness |
| TGACG-motif | Hordeum vulgare | 253 | + | 5 | TGACG | cis-acting regulatory element involved in the MeJA-responsiveness |
| TGACG-motif | Hordeum vulgare | 3340 | + | 5 | TGACG | cis-acting regulatory element involved in the MeJA-responsiveness |
| TGACG-motif | Hordeum vulgare | 2359 | - | 5 | TGACG | cis-acting regulatory element involved in the MeJA-responsiveness |

>HU10G00709.1   
+ -Up\_Stream \_Len000AACCCT TTAATTTTAT CAATGTGGGA CAACACTCAT CTTCATACTC CAACAAGCTA   
  
  
+ TGATACATTC GATTATTGCC TAGTATTTCG TTTAGTAAGA TATTAGTGCC AAACTTTGTA ATAAAAATGA   
  
  
+ AACTTACTAA AAAAAATCTC GTTTTCTGTA GAGGGAAAAT TTGAAGACTT AGTGTCACAT GATGACCTAA   
  
  
+ TGGTACTTGT AGGGTCAAGA TTGAGATTGA GGGCATACTG ACGACCTGTA TAATGGTCAA GTAGGATCAA   
  
  
+ AGTCGAGAAA ACTGTCTCTG CTAACTTTCA TAATCTAACC CTTAACTCAC TCTGACCCTC AATTTAATCA   
  
  
+ GGCCTAATTC AAACTATTAA TGGTTATAAC TCTCTTTCCT TCTTTTGAGA TTTTTTTTTT CTACATGCGG   
  
  
+ TTGAAAATAA TTTTTTTAAA AAATATATAT CACTAACCAT TCATTATCAT AGTTTTAGAG TTCATATTTA   
  
  
+ TGTGGTCCTT GTTGAGTCTA ATTTAATGCT TTAGATGGCG TGAAGCACCA AATTAGTGGT AACCATAATA   
  
  
+ ATAAACTATC AAGTGTTAAG TCGAAGAAAA CGGACACTGC TCAACCAAAT CTAGTAACAT AGTGTATGAC   
  
  
+ ATGATAACTA AGTGTGCCAT GTAGTAATCA ATTCTTTCTT TTTTTTTTTT TGGTTGAGAT CACATAAATT   
  
  
+ ACGTTATCAA GGGTAGTACG ATATAATGGA TGAGGAGAAA TTAAATGTGA GATTTAGTTG ATGAATCGAA   
  
  
+ TACGTTAGTC TGAGCCATTT GGTCAAAACT GCATTGGCTA ATCAATAGAA ATTATAGAAA ATACATAACA   
  
  
+ TAATGGGATG CAATTTTCTA ATTGGGGAAA CCATGTCAAT GCAGTGATTT TGGACCATTT AGGCCTTATT   
  
  
+ CTTTTCAATT TTTTAATTGA TTATTTAGAC TCATATGAAC TTATTGTTGA CAGATTGAGC TAAAAACACT   
  
  
+ GATGTTTCAT ACATATGTAT GAATTGCACG TACGAAGGCT CTGCTCATTT CGAGAAAGAA AATTAACAGT   
  
  
+ TAGGAGCAGT ATAATTTAAC AACAAGCGAT AATTATTAGT ATGAAGTGGT ACGAATCTAT TTTAAAGGAG   
  
  
+ TACTATGAAG TACAAACAAT AACAAATAAT AAAGAGCAGT ACATTAACAT ATCTTACTAC CTAAATAAAT   
  
  
+ CGATTCTAAG AGTAAAAAAA TGTTGAAATT AACCCCATTA TAGTTTTACT TTAACAAGAA GTTTTTCTTC   
  
  
+ TTTTACCATT TACCAAGTAA GAATGATTCA GGCCGTATAG CCAAGGAAAA ATAGAAAACC ACAAAACTAA   
  
  
+ CCATGGCCCA AAAAACAACC ACCAAGAGGG GCACTAATGT GACTAACCGA CGGTTAACAT TATCATCTGT   
  
  
+ TTGATACTTG TTCATCTCTC ACTATTCGGG TAATAGTGTG AAATTACTGA TGTACCCTCT AAACTCGTCG   
  
  
+ TTTTAACTGT GTAAGAATCA GATTAACAGT CACGGACACA TGAGTAACAA ATCAATTTCG GACATGAACG   
  
  
+ AACGAACTGT GAATCCCCCC CAAAAAAAAA ATTTTTTTAA AAAAAGTTGC ATCCTTATCT TTCTGAACCA   
  
  
+ GGCCAGCTGT CTCATTCCTT GGTCCTCGCG CCCCATCACG AGCGCTGCTC ATTATTCACC CTCTCTCTCT   
  
  
+ CTCCTCTCTG ATCCACACCA CAAACACAAG GTTTGCACAG CTGCAGAGCA GTCGTACTAC TATCAGTAGT   
  
  
+ GAAAAACCAA GCAGAAATCC CCAATCCCTC TTCTCCTTCT TCCCAGATTC TTCCTCAATT CCAAGCCCAT   
  
  
+ ATCAGAAATT TAGACAACAA CAACCAAAAA AAAAAATTAA TACTGCTTGA GAAAAACAAA TATGATATGA   
  
  
+ AATTCCTCGA AAAAGGCCTG CCATAACAAA TTCCTCGTGA AAAAGCAGCT GAGAGAAGAA GAAGAAGAAA   
  
  
+ AAAATCCCGT CACAATCTCA ATCTCGATCC CAAATTGTGA AAGAGAAAGA AAAGATGAAG AGAGAACACC   
  
  
+ CCTTTTCGAA CCCTGACCAG AAACCCCCGC CGGAGATGTC GCCGGCGCCG GGGAAGTCCA AGATGTGGGA   
  
  
+ TGATGAGATG GCCCAGAACG ACGCTAAAAT GGATGAGCTG CTTGCTGTTG TGGGGTACAA GGTGAGGTCG   
  
  
+ TCGGACATGG CGGAAATTGC CCAGAAGCTT GAGCAGCTTG AAGAAGTCAT GGGTAGTGTT CAAGAAGACG   
  
  
+ GGTTATCTTA CTTGGCTTCC GAAACTGTTC ATTACAATCC TGCAGATCTG TCTACTTGGC TTGAATCTAT   
  
  
+ GCTTTCTGAG TTTAACCCTA ACCCTAATTT TGACCCTTCT CCATCGTCAA TCTCACCCAT CATCGATCCA   
  
  
+ GCTCCTCCAC TTCCTCGAAC CTCGTCGACG GTCAAATTCG AGCCCGACCC GTTTTCCGAT TCGGATCTGA   
  
  
+ AAGCAATCCC AGGTAAGGCT ATCTTGACCC CACCAAGTTC TAGCAATACT TCAAATTTGC GTGAGGCTAA   
  
  
+ GAGGTTGAAA CCCTCAAATT ACACAACCGC CCCAACGCCC ACCCCACCTC CAAAACTGCC CAATTCGGCG   
  
  
+ TCTCCACCAT CGGGCACGAC GGCGTCGAGG CCGGTGGTGC TGGTAGACTC ACAAGAAAAC GGCATCAGAT   
  
  
+ TGGTGCATGC ACTTATGGCC TGTGCCGAAG CAATTCAACT AGAAAACATG GGATTAGCTG AGGCTTTGGT   
  
  
+ TAAACAGATT AGGTATTTAG CAGCATCTCA AGCTGGACCT ATGAGGAAAG TAGCAACTTG TTTTGCAGAA   
  
  
+ GCTCTTACTT GTCGGATCTA CAAGCTATGC CCATCTGTAC CCTACGATGA ATCAGTCTCA GATGAGTTGC   
  
  
+ AGATGCACTT TTATGAGACT TGCCCATATC TTAAATTCGC CCATTTCACG GCAAATCAAG CAATTTTAGA   
  
  
+ AGCATTCAAT GGGAAGAAGA AAGTTCATGT GATTGATTTC AGCATGAAGC AAGGCATGCA ATGGCCGGCT   
  
  
+ TTGATGCAAG CCCTGGCTCT TCGACCGGAG GGTCCACCGC TTTTCCGGTT AACCGGGATT GGACCGCCCG   
  
  
+ CTCCGGACAA CTCGGACCGG CTGCAAGAGG TGGGTTGGAA GCTTGCCCAG TTCGCGGATT CGATCCGGAT   
  
  
+ TAAATTTCAG TATCGTGGGT TTGTGGCAAA CAGTTTGACC GATCTAGAAT CTTCCATGCT AGATCTTGAA   
  
  
+ CCGGACACTG AGGTGGTGGC GGTCAACTCG GTTTTCGAGC TCCACCGGCT GTTGGCTAAA CCCGGGGCGA   
  
  
+ TCGAGAAAGT GCTGGGGTTC ATGAGGGCCG TGAACCCGGT CATTGTGACG ATGGTCGAGC AGGAAGCGAA   
  
  
+ CCACAACGGA CCGGTTTTCT TGGACCGGTT CAATGAGTCG TTGCATTATT ACTCCACCTT GTTTGATTCC   
  
  
+ CTTGAGATTT GTGTTGATAA TGTAGATAAG AAGATGTCGG AGGCTTACTT GGGCCAGCAG ATCTGCAACA   
  
  
+ TGGTGGCTTG TGAAGGGTCT GACCGAGTCG AGAGGCACGA GACCCTGGCT CAGTGGCGAG CCCGGTTCGC   
  
  
+ ATCTGCCGGG TTCGACCCAG TTCATCTGGG TTCGAATGCG TTTAAGCAGG CGAGTATGTT GTTGGAGTTC   
  
  
+ TTTGCTGGTG GTGAAGGGTA CGGCGTGGAG GAGCGAGAAG GGTGTCTCAT GTTGGGATGG CATAGTAGGC   
  
  
+ CGCTTATCAC CACCTCGGCT TGGCAGCTCG CCAAGAACCC GGCTGTGAAT CGGCGATG  

- -Up\_Stream \_Len000TTGGGA AATTAAAATA GTTACACCCT GTTGTGAGTA GAAGTATGAG GTTGTTCGAT   
  
  
- ACTATGTAAG CTAATAACGG ATCATAAAGC AAATCATTCT ATAATCACGG TTTGAAACAT TATTTTTACT   
  
  
- TTGAATGATT TTTTTTAGAG CAAAAGACAT CTCCCTTTTA AACTTCTGAA TCACAGTGTA CTACTGGATT   
  
  
- ACCATGAACA TCCCAGTTCT AACTCTAACT CCCGTATGAC TGCTGGACAT ATTACCAGTT CATCCTAGTT   
  
  
- TCAGCTCTTT TGACAGAGAC GATTGAAAGT ATTAGATTGG GAATTGAGTG AGACTGGGAG TTAAATTAGT   
  
  
- CCGGATTAAG TTTGATAATT ACCAATATTG AGAGAAAGGA AGAAAACTCT AAAAAAAAAA GATGTACGCC   
  
  
- AACTTTTATT AAAAAAATTT TTTATATATA GTGATTGGTA AGTAATAGTA TCAAAATCTC AAGTATAAAT   
  
  
- ACACCAGGAA CAACTCAGAT TAAATTACGA AATCTACCGC ACTTCGTGGT TTAATCACCA TTGGTATTAT   
  
  
- TATTTGATAG TTCACAATTC AGCTTCTTTT GCCTGTGACG AGTTGGTTTA GATCATTGTA TCACATACTG   
  
  
- TACTATTGAT TCACACGGTA CATCATTAGT TAAGAAAGAA AAAAAAAAAA ACCAACTCTA GTGTATTTAA   
  
  
- TGCAATAGTT CCCATCATGC TATATTACCT ACTCCTCTTT AATTTACACT CTAAATCAAC TACTTAGCTT   
  
  
- ATGCAATCAG ACTCGGTAAA CCAGTTTTGA CGTAACCGAT TAGTTATCTT TAATATCTTT TATGTATTGT   
  
  
- ATTACCCTAC GTTAAAAGAT TAACCCCTTT GGTACAGTTA CGTCACTAAA ACCTGGTAAA TCCGGAATAA   
  
  
- GAAAAGTTAA AAAATTAACT AATAAATCTG AGTATACTTG AATAACAACT GTCTAACTCG ATTTTTGTGA   
  
  
- CTACAAAGTA TGTATACATA CTTAACGTGC ATGCTTCCGA GACGAGTAAA GCTCTTTCTT TTAATTGTCA   
  
  
- ATCCTCGTCA TATTAAATTG TTGTTCGCTA TTAATAATCA TACTTCACCA TGCTTAGATA AAATTTCCTC   
  
  
- ATGATACTTC ATGTTTGTTA TTGTTTATTA TTTCTCGTCA TGTAATTGTA TAGAATGATG GATTTATTTA   
  
  
- GCTAAGATTC TCATTTTTTT ACAACTTTAA TTGGGGTAAT ATCAAAATGA AATTGTTCTT CAAAAAGAAG   
  
  
- AAAATGGTAA ATGGTTCATT CTTACTAAGT CCGGCATATC GGTTCCTTTT TATCTTTTGG TGTTTTGATT   
  
  
- GGTACCGGGT TTTTTGTTGG TGGTTCTCCC CGTGATTACA CTGATTGGCT GCCAATTGTA ATAGTAGACA   
  
  
- AACTATGAAC AAGTAGAGAG TGATAAGCCC ATTATCACAC TTTAATGACT ACATGGGAGA TTTGAGCAGC   
  
  
- AAAATTGACA CATTCTTAGT CTAATTGTCA GTGCCTGTGT ACTCATTGTT TAGTTAAAGC CTGTACTTGC   
  
  
- TTGCTTGACA CTTAGGGGGG GTTTTTTTTT TAAAAAAATT TTTTTCAACG TAGGAATAGA AAGACTTGGT   
  
  
- CCGGTCGACA GAGTAAGGAA CCAGGAGCGC GGGGTAGTGC TCGCGACGAG TAATAAGTGG GAGAGAGAGA   
  
  
- GAGGAGAGAC TAGGTGTGGT GTTTGTGTTC CAAACGTGTC GACGTCTCGT CAGCATGATG ATAGTCATCA   
  
  
- CTTTTTGGTT CGTCTTTAGG GGTTAGGGAG AAGAGGAAGA AGGGTCTAAG AAGGAGTTAA GGTTCGGGTA   
  
  
- TAGTCTTTAA ATCTGTTGTT GTTGGTTTTT TTTTTTAATT ATGACGAACT CTTTTTGTTT ATACTATACT   
  
  
- TTAAGGAGCT TTTTCCGGAC GGTATTGTTT AAGGAGCACT TTTTCGTCGA CTCTCTTCTT CTTCTTCTTT   
  
  
- TTTTAGGGCA GTGTTAGAGT TAGAGCTAGG GTTTAACACT TTCTCTTTCT TTTCTACTTC TCTCTTGTGG   
  
  
- GGAAAAGCTT GGGACTGGTC TTTGGGGGCG GCCTCTACAG CGGCCGCGGC CCCTTCAGGT TCTACACCCT   
  
  
- ACTACTCTAC CGGGTCTTGC TGCGATTTTA CCTACTCGAC GAACGACAAC ACCCCATGTT CCACTCCAGC   
  
  
- AGCCTGTACC GCCTTTAACG GGTCTTCGAA CTCGTCGAAC TTCTTCAGTA CCCATCACAA GTTCTTCTGC   
  
  
- CCAATAGAAT GAACCGAAGG CTTTGACAAG TAATGTTAGG ACGTCTAGAC AGATGAACCG AACTTAGATA   
  
  
- CGAAAGACTC AAATTGGGAT TGGGATTAAA ACTGGGAAGA GGTAGCAGTT AGAGTGGGTA GTAGCTAGGT   
  
  
- CGAGGAGGTG AAGGAGCTTG GAGCAGCTGC CAGTTTAAGC TCGGGCTGGG CAAAAGGCTA AGCCTAGACT   
  
  
- TTCGTTAGGG TCCATTCCGA TAGAACTGGG GTGGTTCAAG ATCGTTATGA AGTTTAAACG CACTCCGATT   
  
  
- CTCCAACTTT GGGAGTTTAA TGTGTTGGCG GGGTTGCGGG TGGGGTGGAG GTTTTGACGG GTTAAGCCGC   
  
  
- AGAGGTGGTA GCCCGTGCTG CCGCAGCTCC GGCCACCACG ACCATCTGAG TGTTCTTTTG CCGTAGTCTA   
  
  
- ACCACGTACG TGAATACCGG ACACGGCTTC GTTAAGTTGA TCTTTTGTAC CCTAATCGAC TCCGAAACCA   
  
  
- ATTTGTCTAA TCCATAAATC GTCGTAGAGT TCGACCTGGA TACTCCTTTC ATCGTTGAAC AAAACGTCTT   
  
  
- CGAGAATGAA CAGCCTAGAT GTTCGATACG GGTAGACATG GGATGCTACT TAGTCAGAGT CTACTCAACG   
  
  
- TCTACGTGAA AATACTCTGA ACGGGTATAG AATTTAAGCG GGTAAAGTGC CGTTTAGTTC GTTAAAATCT   
  
  
- TCGTAAGTTA CCCTTCTTCT TTCAAGTACA CTAACTAAAG TCGTACTTCG TTCCGTACGT TACCGGCCGA   
  
  
- AACTACGTTC GGGACCGAGA AGCTGGCCTC CCAGGTGGCG AAAAGGCCAA TTGGCCCTAA CCTGGCGGGC   
  
  
- GAGGCCTGTT GAGCCTGGCC GACGTTCTCC ACCCAACCTT CGAACGGGTC AAGCGCCTAA GCTAGGCCTA   
  
  
- ATTTAAAGTC ATAGCACCCA AACACCGTTT GTCAAACTGG CTAGATCTTA GAAGGTACGA TCTAGAACTT   
  
  
- GGCCTGTGAC TCCACCACCG CCAGTTGAGC CAAAAGCTCG AGGTGGCCGA CAACCGATTT GGGCCCCGCT   
  
  
- AGCTCTTTCA CGACCCCAAG TACTCCCGGC ACTTGGGCCA GTAACACTGC TACCAGCTCG TCCTTCGCTT   
  
  
- GGTGTTGCCT GGCCAAAAGA ACCTGGCCAA GTTACTCAGC AACGTAATAA TGAGGTGGAA CAAACTAAGG   
  
  
- GAACTCTAAA CACAACTATT ACATCTATTC TTCTACAGCC TCCGAATGAA CCCGGTCGTC TAGACGTTGT   
  
  
- ACCACCGAAC ACTTCCCAGA CTGGCTCAGC TCTCCGTGCT CTGGGACCGA GTCACCGCTC GGGCCAAGCG   
  
  
- TAGACGGCCC AAGCTGGGTC AAGTAGACCC AAGCTTACGC AAATTCGTCC GCTCATACAA CAACCTCAAG   
  
  
- AAACGACCAC CACTTCCCAT GCCGCACCTC CTCGCTCTTC CCACAGAGTA CAACCCTACC GTATCATCCG   
  
  
- GCGAATAGTG GTGGAGCCGA ACCGTCGAGC GGTTCTTGGG CCGACACTTA GCCGCTAC

+     Unnamed\_\_1

| Site Name | Organism | Position | Strand | Matrix score. | sequence | function |
| --- | --- | --- | --- | --- | --- | --- |
| Unnamed\_\_1 | Zea mays | 3168 | + | 5 | CGTGG |  |
| Unnamed\_\_1 | Zea mays | 3668 | + | 5 | CGTGG |  |

>HU10G00709.1   
+ -Up\_Stream \_Len000AACCCT TTAATTTTAT CAATGTGGGA CAACACTCAT CTTCATACTC CAACAAGCTA   
  
  
+ TGATACATTC GATTATTGCC TAGTATTTCG TTTAGTAAGA TATTAGTGCC AAACTTTGTA ATAAAAATGA   
  
  
+ AACTTACTAA AAAAAATCTC GTTTTCTGTA GAGGGAAAAT TTGAAGACTT AGTGTCACAT GATGACCTAA   
  
  
+ TGGTACTTGT AGGGTCAAGA TTGAGATTGA GGGCATACTG ACGACCTGTA TAATGGTCAA GTAGGATCAA   
  
  
+ AGTCGAGAAA ACTGTCTCTG CTAACTTTCA TAATCTAACC CTTAACTCAC TCTGACCCTC AATTTAATCA   
  
  
+ GGCCTAATTC AAACTATTAA TGGTTATAAC TCTCTTTCCT TCTTTTGAGA TTTTTTTTTT CTACATGCGG   
  
  
+ TTGAAAATAA TTTTTTTAAA AAATATATAT CACTAACCAT TCATTATCAT AGTTTTAGAG TTCATATTTA   
  
  
+ TGTGGTCCTT GTTGAGTCTA ATTTAATGCT TTAGATGGCG TGAAGCACCA AATTAGTGGT AACCATAATA   
  
  
+ ATAAACTATC AAGTGTTAAG TCGAAGAAAA CGGACACTGC TCAACCAAAT CTAGTAACAT AGTGTATGAC   
  
  
+ ATGATAACTA AGTGTGCCAT GTAGTAATCA ATTCTTTCTT TTTTTTTTTT TGGTTGAGAT CACATAAATT   
  
  
+ ACGTTATCAA GGGTAGTACG ATATAATGGA TGAGGAGAAA TTAAATGTGA GATTTAGTTG ATGAATCGAA   
  
  
+ TACGTTAGTC TGAGCCATTT GGTCAAAACT GCATTGGCTA ATCAATAGAA ATTATAGAAA ATACATAACA   
  
  
+ TAATGGGATG CAATTTTCTA ATTGGGGAAA CCATGTCAAT GCAGTGATTT TGGACCATTT AGGCCTTATT   
  
  
+ CTTTTCAATT TTTTAATTGA TTATTTAGAC TCATATGAAC TTATTGTTGA CAGATTGAGC TAAAAACACT   
  
  
+ GATGTTTCAT ACATATGTAT GAATTGCACG TACGAAGGCT CTGCTCATTT CGAGAAAGAA AATTAACAGT   
  
  
+ TAGGAGCAGT ATAATTTAAC AACAAGCGAT AATTATTAGT ATGAAGTGGT ACGAATCTAT TTTAAAGGAG   
  
  
+ TACTATGAAG TACAAACAAT AACAAATAAT AAAGAGCAGT ACATTAACAT ATCTTACTAC CTAAATAAAT   
  
  
+ CGATTCTAAG AGTAAAAAAA TGTTGAAATT AACCCCATTA TAGTTTTACT TTAACAAGAA GTTTTTCTTC   
  
  
+ TTTTACCATT TACCAAGTAA GAATGATTCA GGCCGTATAG CCAAGGAAAA ATAGAAAACC ACAAAACTAA   
  
  
+ CCATGGCCCA AAAAACAACC ACCAAGAGGG GCACTAATGT GACTAACCGA CGGTTAACAT TATCATCTGT   
  
  
+ TTGATACTTG TTCATCTCTC ACTATTCGGG TAATAGTGTG AAATTACTGA TGTACCCTCT AAACTCGTCG   
  
  
+ TTTTAACTGT GTAAGAATCA GATTAACAGT CACGGACACA TGAGTAACAA ATCAATTTCG GACATGAACG   
  
  
+ AACGAACTGT GAATCCCCCC CAAAAAAAAA ATTTTTTTAA AAAAAGTTGC ATCCTTATCT TTCTGAACCA   
  
  
+ GGCCAGCTGT CTCATTCCTT GGTCCTCGCG CCCCATCACG AGCGCTGCTC ATTATTCACC CTCTCTCTCT   
  
  
+ CTCCTCTCTG ATCCACACCA CAAACACAAG GTTTGCACAG CTGCAGAGCA GTCGTACTAC TATCAGTAGT   
  
  
+ GAAAAACCAA GCAGAAATCC CCAATCCCTC TTCTCCTTCT TCCCAGATTC TTCCTCAATT CCAAGCCCAT   
  
  
+ ATCAGAAATT TAGACAACAA CAACCAAAAA AAAAAATTAA TACTGCTTGA GAAAAACAAA TATGATATGA   
  
  
+ AATTCCTCGA AAAAGGCCTG CCATAACAAA TTCCTCGTGA AAAAGCAGCT GAGAGAAGAA GAAGAAGAAA   
  
  
+ AAAATCCCGT CACAATCTCA ATCTCGATCC CAAATTGTGA AAGAGAAAGA AAAGATGAAG AGAGAACACC   
  
  
+ CCTTTTCGAA CCCTGACCAG AAACCCCCGC CGGAGATGTC GCCGGCGCCG GGGAAGTCCA AGATGTGGGA   
  
  
+ TGATGAGATG GCCCAGAACG ACGCTAAAAT GGATGAGCTG CTTGCTGTTG TGGGGTACAA GGTGAGGTCG   
  
  
+ TCGGACATGG CGGAAATTGC CCAGAAGCTT GAGCAGCTTG AAGAAGTCAT GGGTAGTGTT CAAGAAGACG   
  
  
+ GGTTATCTTA CTTGGCTTCC GAAACTGTTC ATTACAATCC TGCAGATCTG TCTACTTGGC TTGAATCTAT   
  
  
+ GCTTTCTGAG TTTAACCCTA ACCCTAATTT TGACCCTTCT CCATCGTCAA TCTCACCCAT CATCGATCCA   
  
  
+ GCTCCTCCAC TTCCTCGAAC CTCGTCGACG GTCAAATTCG AGCCCGACCC GTTTTCCGAT TCGGATCTGA   
  
  
+ AAGCAATCCC AGGTAAGGCT ATCTTGACCC CACCAAGTTC TAGCAATACT TCAAATTTGC GTGAGGCTAA   
  
  
+ GAGGTTGAAA CCCTCAAATT ACACAACCGC CCCAACGCCC ACCCCACCTC CAAAACTGCC CAATTCGGCG   
  
  
+ TCTCCACCAT CGGGCACGAC GGCGTCGAGG CCGGTGGTGC TGGTAGACTC ACAAGAAAAC GGCATCAGAT   
  
  
+ TGGTGCATGC ACTTATGGCC TGTGCCGAAG CAATTCAACT AGAAAACATG GGATTAGCTG AGGCTTTGGT   
  
  
+ TAAACAGATT AGGTATTTAG CAGCATCTCA AGCTGGACCT ATGAGGAAAG TAGCAACTTG TTTTGCAGAA   
  
  
+ GCTCTTACTT GTCGGATCTA CAAGCTATGC CCATCTGTAC CCTACGATGA ATCAGTCTCA GATGAGTTGC   
  
  
+ AGATGCACTT TTATGAGACT TGCCCATATC TTAAATTCGC CCATTTCACG GCAAATCAAG CAATTTTAGA   
  
  
+ AGCATTCAAT GGGAAGAAGA AAGTTCATGT GATTGATTTC AGCATGAAGC AAGGCATGCA ATGGCCGGCT   
  
  
+ TTGATGCAAG CCCTGGCTCT TCGACCGGAG GGTCCACCGC TTTTCCGGTT AACCGGGATT GGACCGCCCG   
  
  
+ CTCCGGACAA CTCGGACCGG CTGCAAGAGG TGGGTTGGAA GCTTGCCCAG TTCGCGGATT CGATCCGGAT   
  
  
+ TAAATTTCAG TATCGTGGGT TTGTGGCAAA CAGTTTGACC GATCTAGAAT CTTCCATGCT AGATCTTGAA   
  
  
+ CCGGACACTG AGGTGGTGGC GGTCAACTCG GTTTTCGAGC TCCACCGGCT GTTGGCTAAA CCCGGGGCGA   
  
  
+ TCGAGAAAGT GCTGGGGTTC ATGAGGGCCG TGAACCCGGT CATTGTGACG ATGGTCGAGC AGGAAGCGAA   
  
  
+ CCACAACGGA CCGGTTTTCT TGGACCGGTT CAATGAGTCG TTGCATTATT ACTCCACCTT GTTTGATTCC   
  
  
+ CTTGAGATTT GTGTTGATAA TGTAGATAAG AAGATGTCGG AGGCTTACTT GGGCCAGCAG ATCTGCAACA   
  
  
+ TGGTGGCTTG TGAAGGGTCT GACCGAGTCG AGAGGCACGA GACCCTGGCT CAGTGGCGAG CCCGGTTCGC   
  
  
+ ATCTGCCGGG TTCGACCCAG TTCATCTGGG TTCGAATGCG TTTAAGCAGG CGAGTATGTT GTTGGAGTTC   
  
  
+ TTTGCTGGTG GTGAAGGGTA CGGCGTGGAG GAGCGAGAAG GGTGTCTCAT GTTGGGATGG CATAGTAGGC   
  
  
+ CGCTTATCAC CACCTCGGCT TGGCAGCTCG CCAAGAACCC GGCTGTGAAT CGGCGATG  

- -Up\_Stream \_Len000TTGGGA AATTAAAATA GTTACACCCT GTTGTGAGTA GAAGTATGAG GTTGTTCGAT   
  
  
- ACTATGTAAG CTAATAACGG ATCATAAAGC AAATCATTCT ATAATCACGG TTTGAAACAT TATTTTTACT   
  
  
- TTGAATGATT TTTTTTAGAG CAAAAGACAT CTCCCTTTTA AACTTCTGAA TCACAGTGTA CTACTGGATT   
  
  
- ACCATGAACA TCCCAGTTCT AACTCTAACT CCCGTATGAC TGCTGGACAT ATTACCAGTT CATCCTAGTT   
  
  
- TCAGCTCTTT TGACAGAGAC GATTGAAAGT ATTAGATTGG GAATTGAGTG AGACTGGGAG TTAAATTAGT   
  
  
- CCGGATTAAG TTTGATAATT ACCAATATTG AGAGAAAGGA AGAAAACTCT AAAAAAAAAA GATGTACGCC   
  
  
- AACTTTTATT AAAAAAATTT TTTATATATA GTGATTGGTA AGTAATAGTA TCAAAATCTC AAGTATAAAT   
  
  
- ACACCAGGAA CAACTCAGAT TAAATTACGA AATCTACCGC ACTTCGTGGT TTAATCACCA TTGGTATTAT   
  
  
- TATTTGATAG TTCACAATTC AGCTTCTTTT GCCTGTGACG AGTTGGTTTA GATCATTGTA TCACATACTG   
  
  
- TACTATTGAT TCACACGGTA CATCATTAGT TAAGAAAGAA AAAAAAAAAA ACCAACTCTA GTGTATTTAA   
  
  
- TGCAATAGTT CCCATCATGC TATATTACCT ACTCCTCTTT AATTTACACT CTAAATCAAC TACTTAGCTT   
  
  
- ATGCAATCAG ACTCGGTAAA CCAGTTTTGA CGTAACCGAT TAGTTATCTT TAATATCTTT TATGTATTGT   
  
  
- ATTACCCTAC GTTAAAAGAT TAACCCCTTT GGTACAGTTA CGTCACTAAA ACCTGGTAAA TCCGGAATAA   
  
  
- GAAAAGTTAA AAAATTAACT AATAAATCTG AGTATACTTG AATAACAACT GTCTAACTCG ATTTTTGTGA   
  
  
- CTACAAAGTA TGTATACATA CTTAACGTGC ATGCTTCCGA GACGAGTAAA GCTCTTTCTT TTAATTGTCA   
  
  
- ATCCTCGTCA TATTAAATTG TTGTTCGCTA TTAATAATCA TACTTCACCA TGCTTAGATA AAATTTCCTC   
  
  
- ATGATACTTC ATGTTTGTTA TTGTTTATTA TTTCTCGTCA TGTAATTGTA TAGAATGATG GATTTATTTA   
  
  
- GCTAAGATTC TCATTTTTTT ACAACTTTAA TTGGGGTAAT ATCAAAATGA AATTGTTCTT CAAAAAGAAG   
  
  
- AAAATGGTAA ATGGTTCATT CTTACTAAGT CCGGCATATC GGTTCCTTTT TATCTTTTGG TGTTTTGATT   
  
  
- GGTACCGGGT TTTTTGTTGG TGGTTCTCCC CGTGATTACA CTGATTGGCT GCCAATTGTA ATAGTAGACA   
  
  
- AACTATGAAC AAGTAGAGAG TGATAAGCCC ATTATCACAC TTTAATGACT ACATGGGAGA TTTGAGCAGC   
  
  
- AAAATTGACA CATTCTTAGT CTAATTGTCA GTGCCTGTGT ACTCATTGTT TAGTTAAAGC CTGTACTTGC   
  
  
- TTGCTTGACA CTTAGGGGGG GTTTTTTTTT TAAAAAAATT TTTTTCAACG TAGGAATAGA AAGACTTGGT   
  
  
- CCGGTCGACA GAGTAAGGAA CCAGGAGCGC GGGGTAGTGC TCGCGACGAG TAATAAGTGG GAGAGAGAGA   
  
  
- GAGGAGAGAC TAGGTGTGGT GTTTGTGTTC CAAACGTGTC GACGTCTCGT CAGCATGATG ATAGTCATCA   
  
  
- CTTTTTGGTT CGTCTTTAGG GGTTAGGGAG AAGAGGAAGA AGGGTCTAAG AAGGAGTTAA GGTTCGGGTA   
  
  
- TAGTCTTTAA ATCTGTTGTT GTTGGTTTTT TTTTTTAATT ATGACGAACT CTTTTTGTTT ATACTATACT   
  
  
- TTAAGGAGCT TTTTCCGGAC GGTATTGTTT AAGGAGCACT TTTTCGTCGA CTCTCTTCTT CTTCTTCTTT   
  
  
- TTTTAGGGCA GTGTTAGAGT TAGAGCTAGG GTTTAACACT TTCTCTTTCT TTTCTACTTC TCTCTTGTGG   
  
  
- GGAAAAGCTT GGGACTGGTC TTTGGGGGCG GCCTCTACAG CGGCCGCGGC CCCTTCAGGT TCTACACCCT   
  
  
- ACTACTCTAC CGGGTCTTGC TGCGATTTTA CCTACTCGAC GAACGACAAC ACCCCATGTT CCACTCCAGC   
  
  
- AGCCTGTACC GCCTTTAACG GGTCTTCGAA CTCGTCGAAC TTCTTCAGTA CCCATCACAA GTTCTTCTGC   
  
  
- CCAATAGAAT GAACCGAAGG CTTTGACAAG TAATGTTAGG ACGTCTAGAC AGATGAACCG AACTTAGATA   
  
  
- CGAAAGACTC AAATTGGGAT TGGGATTAAA ACTGGGAAGA GGTAGCAGTT AGAGTGGGTA GTAGCTAGGT   
  
  
- CGAGGAGGTG AAGGAGCTTG GAGCAGCTGC CAGTTTAAGC TCGGGCTGGG CAAAAGGCTA AGCCTAGACT   
  
  
- TTCGTTAGGG TCCATTCCGA TAGAACTGGG GTGGTTCAAG ATCGTTATGA AGTTTAAACG CACTCCGATT   
  
  
- CTCCAACTTT GGGAGTTTAA TGTGTTGGCG GGGTTGCGGG TGGGGTGGAG GTTTTGACGG GTTAAGCCGC   
  
  
- AGAGGTGGTA GCCCGTGCTG CCGCAGCTCC GGCCACCACG ACCATCTGAG TGTTCTTTTG CCGTAGTCTA   
  
  
- ACCACGTACG TGAATACCGG ACACGGCTTC GTTAAGTTGA TCTTTTGTAC CCTAATCGAC TCCGAAACCA   
  
  
- ATTTGTCTAA TCCATAAATC GTCGTAGAGT TCGACCTGGA TACTCCTTTC ATCGTTGAAC AAAACGTCTT   
  
  
- CGAGAATGAA CAGCCTAGAT GTTCGATACG GGTAGACATG GGATGCTACT TAGTCAGAGT CTACTCAACG   
  
  
- TCTACGTGAA AATACTCTGA ACGGGTATAG AATTTAAGCG GGTAAAGTGC CGTTTAGTTC GTTAAAATCT   
  
  
- TCGTAAGTTA CCCTTCTTCT TTCAAGTACA CTAACTAAAG TCGTACTTCG TTCCGTACGT TACCGGCCGA   
  
  
- AACTACGTTC GGGACCGAGA AGCTGGCCTC CCAGGTGGCG AAAAGGCCAA TTGGCCCTAA CCTGGCGGGC   
  
  
- GAGGCCTGTT GAGCCTGGCC GACGTTCTCC ACCCAACCTT CGAACGGGTC AAGCGCCTAA GCTAGGCCTA   
  
  
- ATTTAAAGTC ATAGCACCCA AACACCGTTT GTCAAACTGG CTAGATCTTA GAAGGTACGA TCTAGAACTT   
  
  
- GGCCTGTGAC TCCACCACCG CCAGTTGAGC CAAAAGCTCG AGGTGGCCGA CAACCGATTT GGGCCCCGCT   
  
  
- AGCTCTTTCA CGACCCCAAG TACTCCCGGC ACTTGGGCCA GTAACACTGC TACCAGCTCG TCCTTCGCTT   
  
  
- GGTGTTGCCT GGCCAAAAGA ACCTGGCCAA GTTACTCAGC AACGTAATAA TGAGGTGGAA CAAACTAAGG   
  
  
- GAACTCTAAA CACAACTATT ACATCTATTC TTCTACAGCC TCCGAATGAA CCCGGTCGTC TAGACGTTGT   
  
  
- ACCACCGAAC ACTTCCCAGA CTGGCTCAGC TCTCCGTGCT CTGGGACCGA GTCACCGCTC GGGCCAAGCG   
  
  
- TAGACGGCCC AAGCTGGGTC AAGTAGACCC AAGCTTACGC AAATTCGTCC GCTCATACAA CAACCTCAAG   
  
  
- AAACGACCAC CACTTCCCAT GCCGCACCTC CTCGCTCTTC CCACAGAGTA CAACCCTACC GTATCATCCG   
  
  
- GCGAATAGTG GTGGAGCCGA ACCGTCGAGC GGTTCTTGGG CCGACACTTA GCCGCTAC

+     Unnamed\_\_2

| Site Name | Organism | Position | Strand | Matrix score. | sequence | function |
| --- | --- | --- | --- | --- | --- | --- |
| Unnamed\_\_2 | Zea mays | 2082 | - | 6 | CCCCGG |  |
| Unnamed\_\_2 | Zea mays | 3286 | - | 6 | CCCCGG |  |

>HU10G00709.1   
+ -Up\_Stream \_Len000AACCCT TTAATTTTAT CAATGTGGGA CAACACTCAT CTTCATACTC CAACAAGCTA   
  
  
+ TGATACATTC GATTATTGCC TAGTATTTCG TTTAGTAAGA TATTAGTGCC AAACTTTGTA ATAAAAATGA   
  
  
+ AACTTACTAA AAAAAATCTC GTTTTCTGTA GAGGGAAAAT TTGAAGACTT AGTGTCACAT GATGACCTAA   
  
  
+ TGGTACTTGT AGGGTCAAGA TTGAGATTGA GGGCATACTG ACGACCTGTA TAATGGTCAA GTAGGATCAA   
  
  
+ AGTCGAGAAA ACTGTCTCTG CTAACTTTCA TAATCTAACC CTTAACTCAC TCTGACCCTC AATTTAATCA   
  
  
+ GGCCTAATTC AAACTATTAA TGGTTATAAC TCTCTTTCCT TCTTTTGAGA TTTTTTTTTT CTACATGCGG   
  
  
+ TTGAAAATAA TTTTTTTAAA AAATATATAT CACTAACCAT TCATTATCAT AGTTTTAGAG TTCATATTTA   
  
  
+ TGTGGTCCTT GTTGAGTCTA ATTTAATGCT TTAGATGGCG TGAAGCACCA AATTAGTGGT AACCATAATA   
  
  
+ ATAAACTATC AAGTGTTAAG TCGAAGAAAA CGGACACTGC TCAACCAAAT CTAGTAACAT AGTGTATGAC   
  
  
+ ATGATAACTA AGTGTGCCAT GTAGTAATCA ATTCTTTCTT TTTTTTTTTT TGGTTGAGAT CACATAAATT   
  
  
+ ACGTTATCAA GGGTAGTACG ATATAATGGA TGAGGAGAAA TTAAATGTGA GATTTAGTTG ATGAATCGAA   
  
  
+ TACGTTAGTC TGAGCCATTT GGTCAAAACT GCATTGGCTA ATCAATAGAA ATTATAGAAA ATACATAACA   
  
  
+ TAATGGGATG CAATTTTCTA ATTGGGGAAA CCATGTCAAT GCAGTGATTT TGGACCATTT AGGCCTTATT   
  
  
+ CTTTTCAATT TTTTAATTGA TTATTTAGAC TCATATGAAC TTATTGTTGA CAGATTGAGC TAAAAACACT   
  
  
+ GATGTTTCAT ACATATGTAT GAATTGCACG TACGAAGGCT CTGCTCATTT CGAGAAAGAA AATTAACAGT   
  
  
+ TAGGAGCAGT ATAATTTAAC AACAAGCGAT AATTATTAGT ATGAAGTGGT ACGAATCTAT TTTAAAGGAG   
  
  
+ TACTATGAAG TACAAACAAT AACAAATAAT AAAGAGCAGT ACATTAACAT ATCTTACTAC CTAAATAAAT   
  
  
+ CGATTCTAAG AGTAAAAAAA TGTTGAAATT AACCCCATTA TAGTTTTACT TTAACAAGAA GTTTTTCTTC   
  
  
+ TTTTACCATT TACCAAGTAA GAATGATTCA GGCCGTATAG CCAAGGAAAA ATAGAAAACC ACAAAACTAA   
  
  
+ CCATGGCCCA AAAAACAACC ACCAAGAGGG GCACTAATGT GACTAACCGA CGGTTAACAT TATCATCTGT   
  
  
+ TTGATACTTG TTCATCTCTC ACTATTCGGG TAATAGTGTG AAATTACTGA TGTACCCTCT AAACTCGTCG   
  
  
+ TTTTAACTGT GTAAGAATCA GATTAACAGT CACGGACACA TGAGTAACAA ATCAATTTCG GACATGAACG   
  
  
+ AACGAACTGT GAATCCCCCC CAAAAAAAAA ATTTTTTTAA AAAAAGTTGC ATCCTTATCT TTCTGAACCA   
  
  
+ GGCCAGCTGT CTCATTCCTT GGTCCTCGCG CCCCATCACG AGCGCTGCTC ATTATTCACC CTCTCTCTCT   
  
  
+ CTCCTCTCTG ATCCACACCA CAAACACAAG GTTTGCACAG CTGCAGAGCA GTCGTACTAC TATCAGTAGT   
  
  
+ GAAAAACCAA GCAGAAATCC CCAATCCCTC TTCTCCTTCT TCCCAGATTC TTCCTCAATT CCAAGCCCAT   
  
  
+ ATCAGAAATT TAGACAACAA CAACCAAAAA AAAAAATTAA TACTGCTTGA GAAAAACAAA TATGATATGA   
  
  
+ AATTCCTCGA AAAAGGCCTG CCATAACAAA TTCCTCGTGA AAAAGCAGCT GAGAGAAGAA GAAGAAGAAA   
  
  
+ AAAATCCCGT CACAATCTCA ATCTCGATCC CAAATTGTGA AAGAGAAAGA AAAGATGAAG AGAGAACACC   
  
  
+ CCTTTTCGAA CCCTGACCAG AAACCCCCGC CGGAGATGTC GCCGGCGCCG GGGAAGTCCA AGATGTGGGA   
  
  
+ TGATGAGATG GCCCAGAACG ACGCTAAAAT GGATGAGCTG CTTGCTGTTG TGGGGTACAA GGTGAGGTCG   
  
  
+ TCGGACATGG CGGAAATTGC CCAGAAGCTT GAGCAGCTTG AAGAAGTCAT GGGTAGTGTT CAAGAAGACG   
  
  
+ GGTTATCTTA CTTGGCTTCC GAAACTGTTC ATTACAATCC TGCAGATCTG TCTACTTGGC TTGAATCTAT   
  
  
+ GCTTTCTGAG TTTAACCCTA ACCCTAATTT TGACCCTTCT CCATCGTCAA TCTCACCCAT CATCGATCCA   
  
  
+ GCTCCTCCAC TTCCTCGAAC CTCGTCGACG GTCAAATTCG AGCCCGACCC GTTTTCCGAT TCGGATCTGA   
  
  
+ AAGCAATCCC AGGTAAGGCT ATCTTGACCC CACCAAGTTC TAGCAATACT TCAAATTTGC GTGAGGCTAA   
  
  
+ GAGGTTGAAA CCCTCAAATT ACACAACCGC CCCAACGCCC ACCCCACCTC CAAAACTGCC CAATTCGGCG   
  
  
+ TCTCCACCAT CGGGCACGAC GGCGTCGAGG CCGGTGGTGC TGGTAGACTC ACAAGAAAAC GGCATCAGAT   
  
  
+ TGGTGCATGC ACTTATGGCC TGTGCCGAAG CAATTCAACT AGAAAACATG GGATTAGCTG AGGCTTTGGT   
  
  
+ TAAACAGATT AGGTATTTAG CAGCATCTCA AGCTGGACCT ATGAGGAAAG TAGCAACTTG TTTTGCAGAA   
  
  
+ GCTCTTACTT GTCGGATCTA CAAGCTATGC CCATCTGTAC CCTACGATGA ATCAGTCTCA GATGAGTTGC   
  
  
+ AGATGCACTT TTATGAGACT TGCCCATATC TTAAATTCGC CCATTTCACG GCAAATCAAG CAATTTTAGA   
  
  
+ AGCATTCAAT GGGAAGAAGA AAGTTCATGT GATTGATTTC AGCATGAAGC AAGGCATGCA ATGGCCGGCT   
  
  
+ TTGATGCAAG CCCTGGCTCT TCGACCGGAG GGTCCACCGC TTTTCCGGTT AACCGGGATT GGACCGCCCG   
  
  
+ CTCCGGACAA CTCGGACCGG CTGCAAGAGG TGGGTTGGAA GCTTGCCCAG TTCGCGGATT CGATCCGGAT   
  
  
+ TAAATTTCAG TATCGTGGGT TTGTGGCAAA CAGTTTGACC GATCTAGAAT CTTCCATGCT AGATCTTGAA   
  
  
+ CCGGACACTG AGGTGGTGGC GGTCAACTCG GTTTTCGAGC TCCACCGGCT GTTGGCTAAA CCCGGGGCGA   
  
  
+ TCGAGAAAGT GCTGGGGTTC ATGAGGGCCG TGAACCCGGT CATTGTGACG ATGGTCGAGC AGGAAGCGAA   
  
  
+ CCACAACGGA CCGGTTTTCT TGGACCGGTT CAATGAGTCG TTGCATTATT ACTCCACCTT GTTTGATTCC   
  
  
+ CTTGAGATTT GTGTTGATAA TGTAGATAAG AAGATGTCGG AGGCTTACTT GGGCCAGCAG ATCTGCAACA   
  
  
+ TGGTGGCTTG TGAAGGGTCT GACCGAGTCG AGAGGCACGA GACCCTGGCT CAGTGGCGAG CCCGGTTCGC   
  
  
+ ATCTGCCGGG TTCGACCCAG TTCATCTGGG TTCGAATGCG TTTAAGCAGG CGAGTATGTT GTTGGAGTTC   
  
  
+ TTTGCTGGTG GTGAAGGGTA CGGCGTGGAG GAGCGAGAAG GGTGTCTCAT GTTGGGATGG CATAGTAGGC   
  
  
+ CGCTTATCAC CACCTCGGCT TGGCAGCTCG CCAAGAACCC GGCTGTGAAT CGGCGATG  

- -Up\_Stream \_Len000TTGGGA AATTAAAATA GTTACACCCT GTTGTGAGTA GAAGTATGAG GTTGTTCGAT   
  
  
- ACTATGTAAG CTAATAACGG ATCATAAAGC AAATCATTCT ATAATCACGG TTTGAAACAT TATTTTTACT   
  
  
- TTGAATGATT TTTTTTAGAG CAAAAGACAT CTCCCTTTTA AACTTCTGAA TCACAGTGTA CTACTGGATT   
  
  
- ACCATGAACA TCCCAGTTCT AACTCTAACT CCCGTATGAC TGCTGGACAT ATTACCAGTT CATCCTAGTT   
  
  
- TCAGCTCTTT TGACAGAGAC GATTGAAAGT ATTAGATTGG GAATTGAGTG AGACTGGGAG TTAAATTAGT   
  
  
- CCGGATTAAG TTTGATAATT ACCAATATTG AGAGAAAGGA AGAAAACTCT AAAAAAAAAA GATGTACGCC   
  
  
- AACTTTTATT AAAAAAATTT TTTATATATA GTGATTGGTA AGTAATAGTA TCAAAATCTC AAGTATAAAT   
  
  
- ACACCAGGAA CAACTCAGAT TAAATTACGA AATCTACCGC ACTTCGTGGT TTAATCACCA TTGGTATTAT   
  
  
- TATTTGATAG TTCACAATTC AGCTTCTTTT GCCTGTGACG AGTTGGTTTA GATCATTGTA TCACATACTG   
  
  
- TACTATTGAT TCACACGGTA CATCATTAGT TAAGAAAGAA AAAAAAAAAA ACCAACTCTA GTGTATTTAA   
  
  
- TGCAATAGTT CCCATCATGC TATATTACCT ACTCCTCTTT AATTTACACT CTAAATCAAC TACTTAGCTT   
  
  
- ATGCAATCAG ACTCGGTAAA CCAGTTTTGA CGTAACCGAT TAGTTATCTT TAATATCTTT TATGTATTGT   
  
  
- ATTACCCTAC GTTAAAAGAT TAACCCCTTT GGTACAGTTA CGTCACTAAA ACCTGGTAAA TCCGGAATAA   
  
  
- GAAAAGTTAA AAAATTAACT AATAAATCTG AGTATACTTG AATAACAACT GTCTAACTCG ATTTTTGTGA   
  
  
- CTACAAAGTA TGTATACATA CTTAACGTGC ATGCTTCCGA GACGAGTAAA GCTCTTTCTT TTAATTGTCA   
  
  
- ATCCTCGTCA TATTAAATTG TTGTTCGCTA TTAATAATCA TACTTCACCA TGCTTAGATA AAATTTCCTC   
  
  
- ATGATACTTC ATGTTTGTTA TTGTTTATTA TTTCTCGTCA TGTAATTGTA TAGAATGATG GATTTATTTA   
  
  
- GCTAAGATTC TCATTTTTTT ACAACTTTAA TTGGGGTAAT ATCAAAATGA AATTGTTCTT CAAAAAGAAG   
  
  
- AAAATGGTAA ATGGTTCATT CTTACTAAGT CCGGCATATC GGTTCCTTTT TATCTTTTGG TGTTTTGATT   
  
  
- GGTACCGGGT TTTTTGTTGG TGGTTCTCCC CGTGATTACA CTGATTGGCT GCCAATTGTA ATAGTAGACA   
  
  
- AACTATGAAC AAGTAGAGAG TGATAAGCCC ATTATCACAC TTTAATGACT ACATGGGAGA TTTGAGCAGC   
  
  
- AAAATTGACA CATTCTTAGT CTAATTGTCA GTGCCTGTGT ACTCATTGTT TAGTTAAAGC CTGTACTTGC   
  
  
- TTGCTTGACA CTTAGGGGGG GTTTTTTTTT TAAAAAAATT TTTTTCAACG TAGGAATAGA AAGACTTGGT
[truncated: 72,623 more chars]
