# Supplementary material for: Genome-Wide Identification and Expression Pattern of the GRAS Gene Family in Pitaya (Selenicereus undatus L.)
Source: Biology (Basel). 2022 Dec 21;12(1):11. doi: 10.3390/biology12010011 (PMC9854919; doi:10.3390/biology12010011)
Supplement: Supplementary file 1 [file biology-12-00011-s001.zip › Supplementary file S5/HU11G00778.1_plantcare.html]

Content-Type: text/html; charset=ISO-8859-1


PlantCARE


Webmaster Firefox specific output  
To save the result:
click on the frame with the right mouse button and save the source code as a text file with extension .html  
REFERENCE:PlantCARE: a database of plant cis-acting regulatory elements and a portal to tools for in silico analysis of promoter sequences.  
Lescot, M., Déhais, P., Moreau, Y., De Moor, B., Rouzé ,P.,and Rombauts, S.  
Nucleic Acids Res., Database issue(2002), 30(1):325-327.   


---

>HU11G00778.1   
+ -Up\_Stream \_Len000AAAAAA GAAAGAAATG GAGAAACCAA GCAATATGGA TAAAAAAAAA ACAAAGTAAT   
  
  
+ AAAGGAAGGA TGAGGCTCCA CATTCCACTC GCCCGCGCCA CTCATAGGAG ATGAATAGTA AAATGTGAGG   
  
  
+ GGTTAAATGA TTTGAAAAAA ATCTAGTAAG GGCCCACAAC AGTATTGGAA CCCAGCTTGC CAAGGAAATG   
  
  
+ TCAGAGGGGC TAGCAATGGA CCAGTGCAAG AAGGCCAAGC CACATTTGGT TAGGGAATTT CATCCCATCC   
  
  
+ GGCGCTAAAA GCATCAAAAT TACTACTTAA TCCTTGATCA AATGCCCTAT AAATATTCTA CTACCCCATT   
  
  
+ TGAAAAGGGG TGTTGATTAC AACACATAGC CGTAGGTTTG ACAAAGAGAA TTACCCTTAT TGTTAGAAGC   
  
  
+ ACCATATACT CTTTTCTCTA AGCTCTGATC TTTTTTCATC CTTTTCAGCT TTTTGTCCTT TTAAATCAAA   
  
  
+ TCACTCACTT GAGTATTGGA GGGACGTTCC TCAGGAGTCG AACCACTAAA TTATCTTTTT AAAGAAAAAG   
  
  
+ GGCTTGAAGC CAACCCGATT TAAAAAAACT GCTAATATGT AAGCCTACTC TCTTTCCAGT TCCAACAAAT   
  
  
+ CAAATTTCAG TATGAAACAT CCAGTAAAGG ATTTATCCGT GATAATGATG TAACATATGG TTAAAGGGTC   
  
  
+ ATTTTCAGTT ACATCGGAGC ATGCAATTAA AGTCCCTATA AAATAGGTAG GTGCACGAAC AAGTCACCAT   
  
  
+ CGTCCTTTGC TTTTTGCATG AGTGCATGCT AATTATGAAC TTAATTAATA TCTAAGCAAA ATTAAAAAGT   
  
  
+ AGTTAAAACT TTTTCACCTT TGGAACCTCG TAGCTGTAGC AGTAGCTGCC GCACTCTGGT TCTAGACTTC   
  
  
+ TAGGGACCAA GAGATGCACT CATATGCATC CTCTTCTGTC CTTTTACTGT ATCCCCTTCC CATGTGACCT   
  
  
+ TTTTGCCCGG GTGACTTTTC TGTGAGTGTT GATACAAATT TTGTACGTAA GCTAACTCAT TTACTTCACC   
  
  
+ GTCTCATGAG TTTTACATTA CCGATATTAA TTTAAATGGC AAAGTATTAA ATTTGAGCTA AAATCTCATA   
  
  
+ TATATATTTG TATTAAATGT GTCTGGATTG TCAAAATAAT TTTTGACATA TACAGTCACT ATATACTGAG   
  
  
+ TTATTAAAAT ATATAAAAAT ATGAAAATTT AAAAAATAGT AGTTTAAGGT GACAACACTA AAACAAACAA   
  
  
+ ATAAGATATA AATAGGGAAA AGTTCATCCT ATTTTGTTAT TATTATTTAT TATGTATTTT CAGAATTGTC   
  
  
+ TCTATCACAA GCATAGTATA TGTGTCAACA TCGTGACAAC TCGACCAATC TACAAATAAC TAGTAAATTC   
  
  
+ AAATAAAGTT ACTCTCACCT AGCTTAAATA AGTACACCTT GATTTAATTG TCTTAGCTAT GGAACCTATT   
  
  
+ CCAAACTAGC CAATACTTCC TTTTCAAAAG AATTAGTTTT AAAGTTTTAA CTTTTACGTC CAATAATGCC   
  
  
+ GGCCGTACTA TGAGTTGCTT TCAAGCCAGT AGTAGTACTG TAGTAGGTAA AAATATTAGA ATACACCTAC   
  
  
+ TATATATGAT ATTGCTCTTC ACCGTATCCT TCTGCATACT CATCTTTCCG TCCCGGAGTC CACTATCTCT   
  
  
+ CCTCCATTCA GACACTCTCT CGCTCTAGAG AACTCATAGC TATGGACCGC GTGCCCGACG GTTATCGGGT   
  
  
+ TATGAATTCG GAGCTTCTTC AGCAAGTTAG CCCTGAGTTG CCGGATCCAA CCACCTGGTT CCATGCCCGT   
  
  
+ CTACCCGACC CCATCTCTCA GTCTCCTCTT GGACCCGGTC CATCCTCCCA GCCAACCCAT TTCTACCAGG   
  
  
+ GTTCTGCCTC AGGGAACCAT GTGGTTGGGA TTGCTGACAC GTGGACGGAC CAGATTGTTG CAGGTTCCTC   
  
  
+ GTCCCAGCAG TGAGGTTGCA GCAGAGCCAG CAGGTGTTGA CTGCAGTCGA AGCCATGGAG GAGGACTCTG   
  
  
+ GGATAAGACT GGTCTACGCT CTGCTGACGT GTGCGGAGGC CATCCAACGT GGCGATTTCC GATTGGCTGT   
  
  
+ CTTGTTAGTT AACAAGATGA GCAATGACCT CCTGCCACGC GTCAACCCGT CCTGCGGTAT TGGCAAAGTA   
  
  
+ GCCGGCTACT TCATAGATGC CTTGACCCGA AGACTATACC AGCAGGGCCC AGTGTCGGGC CTAATCGGGT   
  
  
+ CGGTTCTGGC GTACCAGGTG TTGTACGAGC ACTTCTATGA AGCTTGCCCG TTCCTCAAAT TCGCTCACTT   
  
  
+ CACTGCTAAT CAAGCGATTT TAGAGGCATT CGACGGCCAC GATTGTGTCC ACATCATCGA CTTCGGCCTA   
  
  
+ ATCCACGGCT TGCAATGGCC AGCTCTAATC CAAGCCTTGG CTGTTCGGCA CGGTGGCCCG CCTTTCGTAA   
  
  
+ GATTAACCGG AATCGGGCCG CCTTCTGAAT ACGGGTCGTG CTCGCTTCAA CCTATCGGGT CAACACTGGC   
  
  
+ CCAGTTAGCC CTATCCATGA ATGTTGGGTT CGCATTTCGG GCCGTCGCCG TCTCACGGCT CGAGGACATC   
  
  
+ AAACCATGGA TGGTTAAAAC AAGCCCAAAT GAAGTCGTAG CCGTGAACTC TATCTTCCAA CTCCACCGGC   
  
  
+ TAATCGGGTC GGGTATTGAC CCTGTCCTAA ACTGGGTCCG GAGCCTAAAC CCGAAAATTG TGACACTGGC   
  
  
+ GGAGCAAGAG GCGAACCACA ACCAGCCCGA GTTTTTGGCC CGGTTCACGG AGGCATTACA TTACTATTCA   
  
  
+ ACTATGTTCG ACTCGTTAGA GGCTTGTCAA GTCCAGGCCG ACAAGGACCT AGCCGAGCTA TACTTAGAGA   
  
  
+ GAGAGTTATC CAACATCGTC TGCTGTGAGG GGTCGGCTCG GATTGAGAGG CACGAGCCGC TGGCCCAATG   
  
  
+ GAGGGCCCGA ATGGCTCGGG CCGGGTTCAA GAAGGTGGAT ATGGGTAAAA ATGCGTTCAA GCAAGTAAGC   
  
  
+ ATGTTGCTGA GTTTGTCTTC AGCACAAGGG TATTGTGTGG AGGAGAGTGA GGGATGTTTG AAGCTCGGCT   
  
  
+ GGCATGACCG CCCTCTCATT GCGGCTTCGG CTTGGCGAGC GGAGACTCAA GCTGAGAACT CCAGCACTGT   
  
  
+ TGTGCTTGAT GGGTCATCGT CGTGTAGTTC ATCTTCTTA  

- -Up\_Stream \_Len000TTTTTT CTTTCTTTAC CTCTTTGGTT CGTTATACCT ATTTTTTTTT TGTTTCATTA   
  
  
- TTTCCTTCCT ACTCCGAGGT GTAAGGTGAG CGGGCGCGGT GAGTATCCTC TACTTATCAT TTTACACTCC   
  
  
- CCAATTTACT AAACTTTTTT TAGATCATTC CCGGGTGTTG TCATAACCTT GGGTCGAACG GTTCCTTTAC   
  
  
- AGTCTCCCCG ATCGTTACCT GGTCACGTTC TTCCGGTTCG GTGTAAACCA ATCCCTTAAA GTAGGGTAGG   
  
  
- CCGCGATTTT CGTAGTTTTA ATGATGAATT AGGAACTAGT TTACGGGATA TTTATAAGAT GATGGGGTAA   
  
  
- ACTTTTCCCC ACAACTAATG TTGTGTATCG GCATCCAAAC TGTTTCTCTT AATGGGAATA ACAATCTTCG   
  
  
- TGGTATATGA GAAAAGAGAT TCGAGACTAG AAAAAAGTAG GAAAAGTCGA AAAACAGGAA AATTTAGTTT   
  
  
- AGTGAGTGAA CTCATAACCT CCCTGCAAGG AGTCCTCAGC TTGGTGATTT AATAGAAAAA TTTCTTTTTC   
  
  
- CCGAACTTCG GTTGGGCTAA ATTTTTTTGA CGATTATACA TTCGGATGAG AGAAAGGTCA AGGTTGTTTA   
  
  
- GTTTAAAGTC ATACTTTGTA GGTCATTTCC TAAATAGGCA CTATTACTAC ATTGTATACC AATTTCCCAG   
  
  
- TAAAAGTCAA TGTAGCCTCG TACGTTAATT TCAGGGATAT TTTATCCATC CACGTGCTTG TTCAGTGGTA   
  
  
- GCAGGAAACG AAAAACGTAC TCACGTACGA TTAATACTTG AATTAATTAT AGATTCGTTT TAATTTTTCA   
  
  
- TCAATTTTGA AAAAGTGGAA ACCTTGGAGC ATCGACATCG TCATCGACGG CGTGAGACCA AGATCTGAAG   
  
  
- ATCCCTGGTT CTCTACGTGA GTATACGTAG GAGAAGACAG GAAAATGACA TAGGGGAAGG GTACACTGGA   
  
  
- AAAACGGGCC CACTGAAAAG ACACTCACAA CTATGTTTAA AACATGCATT CGATTGAGTA AATGAAGTGG   
  
  
- CAGAGTACTC AAAATGTAAT GGCTATAATT AAATTTACCG TTTCATAATT TAAACTCGAT TTTAGAGTAT   
  
  
- ATATATAAAC ATAATTTACA CAGACCTAAC AGTTTTATTA AAAACTGTAT ATGTCAGTGA TATATGACTC   
  
  
- AATAATTTTA TATATTTTTA TACTTTTAAA TTTTTTATCA TCAAATTCCA CTGTTGTGAT TTTGTTTGTT   
  
  
- TATTCTATAT TTATCCCTTT TCAAGTAGGA TAAAACAATA ATAATAAATA ATACATAAAA GTCTTAACAG   
  
  
- AGATAGTGTT CGTATCATAT ACACAGTTGT AGCACTGTTG AGCTGGTTAG ATGTTTATTG ATCATTTAAG   
  
  
- TTTATTTCAA TGAGAGTGGA TCGAATTTAT TCATGTGGAA CTAAATTAAC AGAATCGATA CCTTGGATAA   
  
  
- GGTTTGATCG GTTATGAAGG AAAAGTTTTC TTAATCAAAA TTTCAAAATT GAAAATGCAG GTTATTACGG   
  
  
- CCGGCATGAT ACTCAACGAA AGTTCGGTCA TCATCATGAC ATCATCCATT TTTATAATCT TATGTGGATG   
  
  
- ATATATACTA TAACGAGAAG TGGCATAGGA AGACGTATGA GTAGAAAGGC AGGGCCTCAG GTGATAGAGA   
  
  
- GGAGGTAAGT CTGTGAGAGA GCGAGATCTC TTGAGTATCG ATACCTGGCG CACGGGCTGC CAATAGCCCA   
  
  
- ATACTTAAGC CTCGAAGAAG TCGTTCAATC GGGACTCAAC GGCCTAGGTT GGTGGACCAA GGTACGGGCA   
  
  
- GATGGGCTGG GGTAGAGAGT CAGAGGAGAA CCTGGGCCAG GTAGGAGGGT CGGTTGGGTA AAGATGGTCC   
  
  
- CAAGACGGAG TCCCTTGGTA CACCAACCCT AACGACTGTG CACCTGCCTG GTCTAACAAC GTCCAAGGAG   
  
  
- CAGGGTCGTC ACTCCAACGT CGTCTCGGTC GTCCACAACT GACGTCAGCT TCGGTACCTC CTCCTGAGAC   
  
  
- CCTATTCTGA CCAGATGCGA GACGACTGCA CACGCCTCCG GTAGGTTGCA CCGCTAAAGG CTAACCGACA   
  
  
- GAACAATCAA TTGTTCTACT CGTTACTGGA GGACGGTGCG CAGTTGGGCA GGACGCCATA ACCGTTTCAT   
  
  
- CGGCCGATGA AGTATCTACG GAACTGGGCT TCTGATATGG TCGTCCCGGG TCACAGCCCG GATTAGCCCA   
  
  
- GCCAAGACCG CATGGTCCAC AACATGCTCG TGAAGATACT TCGAACGGGC AAGGAGTTTA AGCGAGTGAA   
  
  
- GTGACGATTA GTTCGCTAAA ATCTCCGTAA GCTGCCGGTG CTAACACAGG TGTAGTAGCT GAAGCCGGAT   
  
  
- TAGGTGCCGA ACGTTACCGG TCGAGATTAG GTTCGGAACC GACAAGCCGT GCCACCGGGC GGAAAGCATT   
  
  
- CTAATTGGCC TTAGCCCGGC GGAAGACTTA TGCCCAGCAC GAGCGAAGTT GGATAGCCCA GTTGTGACCG   
  
  
- GGTCAATCGG GATAGGTACT TACAACCCAA GCGTAAAGCC CGGCAGCGGC AGAGTGCCGA GCTCCTGTAG   
  
  
- TTTGGTACCT ACCAATTTTG TTCGGGTTTA CTTCAGCATC GGCACTTGAG ATAGAAGGTT GAGGTGGCCG   
  
  
- ATTAGCCCAG CCCATAACTG GGACAGGATT TGACCCAGGC CTCGGATTTG GGCTTTTAAC ACTGTGACCG   
  
  
- CCTCGTTCTC CGCTTGGTGT TGGTCGGGCT CAAAAACCGG GCCAAGTGCC TCCGTAATGT AATGATAAGT   
  
  
- TGATACAAGC TGAGCAATCT CCGAACAGTT CAGGTCCGGC TGTTCCTGGA TCGGCTCGAT ATGAATCTCT   
  
  
- CTCTCAATAG GTTGTAGCAG ACGACACTCC CCAGCCGAGC CTAACTCTCC GTGCTCGGCG ACCGGGTTAC   
  
  
- CTCCCGGGCT TACCGAGCCC GGCCCAAGTT CTTCCACCTA TACCCATTTT TACGCAAGTT CGTTCATTCG   
  
  
- TACAACGACT CAAACAGAAG TCGTGTTCCC ATAACACACC TCCTCTCACT CCCTACAAAC TTCGAGCCGA   
  
  
- CCGTACTGGC GGGAGAGTAA CGCCGAAGCC GAACCGCTCG CCTCTGAGTT CGACTCTTGA GGTCGTGACA   
  
  
- ACACGAACTA CCCAGTAGCA GCACATCAAG TAGAAGAAT

  
  
Motifs Found  

+   

| Site Name | Organism | Position | Strand | Matrix score. | sequence | function |
| --- | --- | --- | --- | --- | --- | --- |
|  | organism | 3185 | + | 4 | motif\_sequence | short\_function |
|  | organism | 3056 | - | 4 | motif\_sequence | short\_function |
|  | organism | 2053 | + | 4 | motif\_sequence | short\_function |
|  | organism | 1985 | - | 4 | motif\_sequence | short\_function |
|  | organism | 2808 | + | 4 | motif\_sequence | short\_function |
|  | organism | 2686 | + | 4 | motif\_sequence | short\_function |
|  | organism | 950 | + | 4 | motif\_sequence | short\_function |
|  | organism | 2646 | + | 4 | motif\_sequence | short\_function |
|  | organism | 945 | + | 4 | motif\_sequence | short\_function |
|  | organism | 2588 | - | 4 | motif\_sequence | short\_function |
|  | organism | 1629 | + | 4 | motif\_sequence | short\_function |
|  | organism | 121 | - | 4 | motif\_sequence | short\_function |
|  | organism | 2425 | + | 4 | motif\_sequence | short\_function |
|  | organism | 1681 | + | 4 | motif\_sequence | short\_function |
|  | organism | 1382 | + | 4 | motif\_sequence | short\_function |

>HU11G00778.1   
+ -Up\_Stream \_Len000AAAAAA GAAAGAAATG GAGAAACCAA GCAATATGGA TAAAAAAAAA ACAAAGTAAT   
  
  
+ AAAGGAAGGA TGAGGCTCCA CATTCCACTC GCCCGCGCCA CTCATAGGAG ATGAATAGTA AAATGTGAGG   
  
  
+ GGTTAAATGA TTTGAAAAAA ATCTAGTAAG GGCCCACAAC AGTATTGGAA CCCAGCTTGC CAAGGAAATG   
  
  
+ TCAGAGGGGC TAGCAATGGA CCAGTGCAAG AAGGCCAAGC CACATTTGGT TAGGGAATTT CATCCCATCC   
  
  
+ GGCGCTAAAA GCATCAAAAT TACTACTTAA TCCTTGATCA AATGCCCTAT AAATATTCTA CTACCCCATT   
  
  
+ TGAAAAGGGG TGTTGATTAC AACACATAGC CGTAGGTTTG ACAAAGAGAA TTACCCTTAT TGTTAGAAGC   
  
  
+ ACCATATACT CTTTTCTCTA AGCTCTGATC TTTTTTCATC CTTTTCAGCT TTTTGTCCTT TTAAATCAAA   
  
  
+ TCACTCACTT GAGTATTGGA GGGACGTTCC TCAGGAGTCG AACCACTAAA TTATCTTTTT AAAGAAAAAG   
  
  
+ GGCTTGAAGC CAACCCGATT TAAAAAAACT GCTAATATGT AAGCCTACTC TCTTTCCAGT TCCAACAAAT   
  
  
+ CAAATTTCAG TATGAAACAT CCAGTAAAGG ATTTATCCGT GATAATGATG TAACATATGG TTAAAGGGTC   
  
  
+ ATTTTCAGTT ACATCGGAGC ATGCAATTAA AGTCCCTATA AAATAGGTAG GTGCACGAAC AAGTCACCAT   
  
  
+ CGTCCTTTGC TTTTTGCATG AGTGCATGCT AATTATGAAC TTAATTAATA TCTAAGCAAA ATTAAAAAGT   
  
  
+ AGTTAAAACT TTTTCACCTT TGGAACCTCG TAGCTGTAGC AGTAGCTGCC GCACTCTGGT TCTAGACTTC   
  
  
+ TAGGGACCAA GAGATGCACT CATATGCATC CTCTTCTGTC CTTTTACTGT ATCCCCTTCC CATGTGACCT   
  
  
+ TTTTGCCCGG GTGACTTTTC TGTGAGTGTT GATACAAATT TTGTACGTAA GCTAACTCAT TTACTTCACC   
  
  
+ GTCTCATGAG TTTTACATTA CCGATATTAA TTTAAATGGC AAAGTATTAA ATTTGAGCTA AAATCTCATA   
  
  
+ TATATATTTG TATTAAATGT GTCTGGATTG TCAAAATAAT TTTTGACATA TACAGTCACT ATATACTGAG   
  
  
+ TTATTAAAAT ATATAAAAAT ATGAAAATTT AAAAAATAGT AGTTTAAGGT GACAACACTA AAACAAACAA   
  
  
+ ATAAGATATA AATAGGGAAA AGTTCATCCT ATTTTGTTAT TATTATTTAT TATGTATTTT CAGAATTGTC   
  
  
+ TCTATCACAA GCATAGTATA TGTGTCAACA TCGTGACAAC TCGACCAATC TACAAATAAC TAGTAAATTC   
  
  
+ AAATAAAGTT ACTCTCACCT AGCTTAAATA AGTACACCTT GATTTAATTG TCTTAGCTAT GGAACCTATT   
  
  
+ CCAAACTAGC CAATACTTCC TTTTCAAAAG AATTAGTTTT AAAGTTTTAA CTTTTACGTC CAATAATGCC   
  
  
+ GGCCGTACTA TGAGTTGCTT TCAAGCCAGT AGTAGTACTG TAGTAGGTAA AAATATTAGA ATACACCTAC   
  
  
+ TATATATGAT ATTGCTCTTC ACCGTATCCT TCTGCATACT CATCTTTCCG TCCCGGAGTC CACTATCTCT   
  
  
+ CCTCCATTCA GACACTCTCT CGCTCTAGAG AACTCATAGC TATGGACCGC GTGCCCGACG GTTATCGGGT   
  
  
+ TATGAATTCG GAGCTTCTTC AGCAAGTTAG CCCTGAGTTG CCGGATCCAA CCACCTGGTT CCATGCCCGT   
  
  
+ CTACCCGACC CCATCTCTCA GTCTCCTCTT GGACCCGGTC CATCCTCCCA GCCAACCCAT TTCTACCAGG   
  
  
+ GTTCTGCCTC AGGGAACCAT GTGGTTGGGA TTGCTGACAC GTGGACGGAC CAGATTGTTG CAGGTTCCTC   
  
  
+ GTCCCAGCAG TGAGGTTGCA GCAGAGCCAG CAGGTGTTGA CTGCAGTCGA AGCCATGGAG GAGGACTCTG   
  
  
+ GGATAAGACT GGTCTACGCT CTGCTGACGT GTGCGGAGGC CATCCAACGT GGCGATTTCC GATTGGCTGT   
  
  
+ CTTGTTAGTT AACAAGATGA GCAATGACCT CCTGCCACGC GTCAACCCGT CCTGCGGTAT TGGCAAAGTA   
  
  
+ GCCGGCTACT TCATAGATGC CTTGACCCGA AGACTATACC AGCAGGGCCC AGTGTCGGGC CTAATCGGGT   
  
  
+ CGGTTCTGGC GTACCAGGTG TTGTACGAGC ACTTCTATGA AGCTTGCCCG TTCCTCAAAT TCGCTCACTT   
  
  
+ CACTGCTAAT CAAGCGATTT TAGAGGCATT CGACGGCCAC GATTGTGTCC ACATCATCGA CTTCGGCCTA   
  
  
+ ATCCACGGCT TGCAATGGCC AGCTCTAATC CAAGCCTTGG CTGTTCGGCA CGGTGGCCCG CCTTTCGTAA   
  
  
+ GATTAACCGG AATCGGGCCG CCTTCTGAAT ACGGGTCGTG CTCGCTTCAA CCTATCGGGT CAACACTGGC   
  
  
+ CCAGTTAGCC CTATCCATGA ATGTTGGGTT CGCATTTCGG GCCGTCGCCG TCTCACGGCT CGAGGACATC   
  
  
+ AAACCATGGA TGGTTAAAAC AAGCCCAAAT GAAGTCGTAG CCGTGAACTC TATCTTCCAA CTCCACCGGC   
  
  
+ TAATCGGGTC GGGTATTGAC CCTGTCCTAA ACTGGGTCCG GAGCCTAAAC CCGAAAATTG TGACACTGGC   
  
  
+ GGAGCAAGAG GCGAACCACA ACCAGCCCGA GTTTTTGGCC CGGTTCACGG AGGCATTACA TTACTATTCA   
  
  
+ ACTATGTTCG ACTCGTTAGA GGCTTGTCAA GTCCAGGCCG ACAAGGACCT AGCCGAGCTA TACTTAGAGA   
  
  
+ GAGAGTTATC CAACATCGTC TGCTGTGAGG GGTCGGCTCG GATTGAGAGG CACGAGCCGC TGGCCCAATG   
  
  
+ GAGGGCCCGA ATGGCTCGGG CCGGGTTCAA GAAGGTGGAT ATGGGTAAAA ATGCGTTCAA GCAAGTAAGC   
  
  
+ ATGTTGCTGA GTTTGTCTTC AGCACAAGGG TATTGTGTGG AGGAGAGTGA GGGATGTTTG AAGCTCGGCT   
  
  
+ GGCATGACCG CCCTCTCATT GCGGCTTCGG CTTGGCGAGC GGAGACTCAA GCTGAGAACT CCAGCACTGT   
  
  
+ TGTGCTTGAT GGGTCATCGT CGTGTAGTTC ATCTTCTTAG   

- -Up\_Stream \_Len

000TTTTTT CTTTCTTTAC CTCTTTGGTT CGTTATACCT ATTTTTTTTT TGTTTCATTA   
  
  
- TTTCCTTCCT ACTCCGAGGT GTAAGGTGAG CGGGCGCGGT GAGTATCCTC TACTTATCAT TTTACACTCC   
  
  
- CCAATTTACT AAACTTTTTT TAGATCATTC CCGGGTGTTG TCATAACCTT GGGTCGAACG GTTCCTTTAC   
  
  
- AGTCTCCCCG ATCGTTACCT GGTCACGTTC TTCCGGTTCG GTGTAAACCA ATCCCTTAAA GTAGGGTAGG   
  
  
- CCGCGATTTT CGTAGTTTTA ATGATGAATT AGGAACTAGT TTACGGGATA TTTATAAGAT GATGGGGTAA   
  
  
- ACTTTTCCCC ACAACTAATG TTGTGTATCG GCATCCAAAC TGTTTCTCTT AATGGGAATA ACAATCTTCG   
  
  
- TGGTATATGA GAAAAGAGAT TCGAGACTAG AAAAAAGTAG GAAAAGTCGA AAAACAGGAA AATTTAGTTT   
  
  
- AGTGAGTGAA CTCATAACCT CCCTGCAAGG AGTCCTCAGC TTGGTGATTT AATAGAAAAA TTTCTTTTTC   
  
  
- CCGAACTTCG GTTGGGCTAA ATTTTTTTGA CGATTATACA TTCGGATGAG AGAAAGGTCA AGGTTGTTTA   
  
  
- GTTTAAAGTC ATACTTTGTA GGTCATTTCC TAAATAGGCA CTATTACTAC ATTGTATACC AATTTCCCAG   
  
  
- TAAAAGTCAA TGTAGCCTCG TACGTTAATT TCAGGGATAT TTTATCCATC CACGTGCTTG TTCAGTGGTA   
  
  
- GCAGGAAACG AAAAACGTAC TCACGTACGA TTAATACTTG AATTAATTAT AGATTCGTTT TAATTTTTCA   
  
  
- TCAATTTTGA AAAAGTGGAA ACCTTGGAGC ATCGACATCG TCATCGACGG CGTGAGACCA AGATCTGAAG   
  
  
- ATCCCTGGTT CTCTACGTGA GTATACGTAG GAGAAGACAG GAAAATGACA TAGGGGAAGG GTACACTGGA   
  
  
- AAAACGGGCC CACTGAAAAG ACACTCACAA CTATGTTTAA AACATGCATT CGATTGAGTA AATGAAGTGG   
  
  
- CAGAGTACTC AAAATGTAAT GGCTATAATT AAATTTACCG TTTCATAATT TAAACTCGAT TTTAGAGTAT   
  
  
- ATATATAAAC ATAATTTACA CAGACCTAAC AGTTTTATTA AAAACTGTAT ATGTCAGTGA TATATGACTC   
  
  
- AATAATTTTA TATATTTTTA TACTTTTAAA TTTTTTATCA TCAAATTCCA CTGTTGTGAT TTTGTTTGTT   
  
  
- TATTCTATAT TTATCCCTTT TCAAGTAGGA TAAAACAATA ATAATAAATA ATACATAAAA GTCTTAACAG   
  
  
- AGATAGTGTT CGTATCATAT ACACAGTTGT AGCACTGTTG AGCTGGTTAG ATGTTTATTG ATCATTTAAG   
  
  
- TTTATTTCAA TGAGAGTGGA TCGAATTTAT TCATGTGGAA CTAAATTAAC AGAATCGATA CCTTGGATAA   
  
  
- GGTTTGATCG GTTATGAAGG AAAAGTTTTC TTAATCAAAA TTTCAAAATT GAAAATGCAG GTTATTACGG   
  
  
- CCGGCATGAT ACTCAACGAA AGTTCGGTCA TCATCATGAC ATCATCCATT TTTATAATCT TATGTGGATG   
  
  
- ATATATACTA TAACGAGAAG TGGCATAGGA AGACGTATGA GTAGAAAGGC AGGGCCTCAG GTGATAGAGA   
  
  
- GGAGGTAAGT CTGTGAGAGA GCGAGATCTC TTGAGTATCG ATACCTGGCG CACGGGCTGC CAATAGCCCA   
  
  
- ATACTTAAGC CTCGAAGAAG TCGTTCAATC GGGACTCAAC GGCCTAGGTT GGTGGACCAA GGTACGGGCA   
  
  
- GATGGGCTGG GGTAGAGAGT CAGAGGAGAA CCTGGGCCAG GTAGGAGGGT CGGTTGGGTA AAGATGGTCC   
  
  
- CAAGACGGAG TCCCTTGGTA CACCAACCCT AACGACTGTG CACCTGCCTG GTCTAACAAC GTCCAAGGAG   
  
  
- CAGGGTCGTC ACTCCAACGT CGTCTCGGTC GTCCACAACT GACGTCAGCT TCGGTACCTC CTCCTGAGAC   
  
  
- CCTATTCTGA CCAGATGCGA GACGACTGCA CACGCCTCCG GTAGGTTGCA CCGCTAAAGG CTAACCGACA   
  
  
- GAACAATCAA TTGTTCTACT CGTTACTGGA GGACGGTGCG CAGTTGGGCA GGACGCCATA ACCGTTTCAT   
  
  
- CGGCCGATGA AGTATCTACG GAACTGGGCT TCTGATATGG TCGTCCCGGG TCACAGCCCG GATTAGCCCA   
  
  
- GCCAAGACCG CATGGTCCAC AACATGCTCG TGAAGATACT TCGAACGGGC AAGGAGTTTA AGCGAGTGAA   
  
  
- GTGACGATTA GTTCGCTAAA ATCTCCGTAA GCTGCCGGTG CTAACACAGG TGTAGTAGCT GAAGCCGGAT   
  
  
- TAGGTGCCGA ACGTTACCGG TCGAGATTAG GTTCGGAACC GACAAGCCGT GCCACCGGGC GGAAAGCATT   
  
  
- CTAATTGGCC TTAGCCCGGC GGAAGACTTA TGCCCAGCAC GAGCGAAGTT GGATAGCCCA GTTGTGACCG   
  
  
- GGTCAATCGG GATAGGTACT TACAACCCAA GCGTAAAGCC CGGCAGCGGC AGAGTGCCGA GCTCCTGTAG   
  
  
- TTTGGTACCT ACCAATTTTG TTCGGGTTTA CTTCAGCATC GGCACTTGAG ATAGAAGGTT GAGGTGGCCG   
  
  
- ATTAGCCCAG CCCATAACTG GGACAGGATT TGACCCAGGC CTCGGATTTG GGCTTTTAAC ACTGTGACCG   
  
  
- CCTCGTTCTC CGCTTGGTGT TGGTCGGGCT CAAAAACCGG GCCAAGTGCC TCCGTAATGT AATGATAAGT   
  
  
- TGATACAAGC TGAGCAATCT CCGAACAGTT CAGGTCCGGC TGTTCCTGGA TCGGCTCGAT ATGAATCTCT   
  
  
- CTCTCAATAG GTTGTAGCAG ACGACACTCC CCAGCCGAGC CTAACTCTCC GTGCTCGGCG ACCGGGTTAC   
  
  
- CTCCCGGGCT TACCGAGCCC GGCCCAAGTT CTTCCACCTA TACCCATTTT TACGCAAGTT CGTTCATTCG   
  
  
- TACAACGACT CAAACAGAAG TCGTGTTCCC ATAACACACC TCCTCTCACT CCCTACAAAC TTCGAGCCGA   
  
  
- CCGTACTGGC GGGAGAGTAA CGCCGAAGCC GAACCGCTCG CCTCTGAGTT CGACTCTTGA GGTCGTGACA   
  
  
- ACACGAACTA CCCAGTAGCA GCACATCAAG TAGAAGAAT

+     A-box

| Site Name | Organism | Position | Strand | Matrix score. | sequence | function |
| --- | --- | --- | --- | --- | --- | --- |
| A-box | Petroselinum crispum | 1662 | + | 6 | CCGTCC | cis-acting regulatory element |
| A-box | Petroselinum crispum | 2151 | + | 6 | CCGTCC | cis-acting regulatory element |
| A-box | Petroselinum crispum | 1937 | - | 6 | CCGTCC | cis-acting regulatory element |

>HU11G00778.1   
+ -Up\_Stream \_Len000AAAAAA GAAAGAAATG GAGAAACCAA GCAATATGGA TAAAAAAAAA ACAAAGTAAT   
  
  
+ AAAGGAAGGA TGAGGCTCCA CATTCCACTC GCCCGCGCCA CTCATAGGAG ATGAATAGTA AAATGTGAGG   
  
  
+ GGTTAAATGA TTTGAAAAAA ATCTAGTAAG GGCCCACAAC AGTATTGGAA CCCAGCTTGC CAAGGAAATG   
  
  
+ TCAGAGGGGC TAGCAATGGA CCAGTGCAAG AAGGCCAAGC CACATTTGGT TAGGGAATTT CATCCCATCC   
  
  
+ GGCGCTAAAA GCATCAAAAT TACTACTTAA TCCTTGATCA AATGCCCTAT AAATATTCTA CTACCCCATT   
  
  
+ TGAAAAGGGG TGTTGATTAC AACACATAGC CGTAGGTTTG ACAAAGAGAA TTACCCTTAT TGTTAGAAGC   
  
  
+ ACCATATACT CTTTTCTCTA AGCTCTGATC TTTTTTCATC CTTTTCAGCT TTTTGTCCTT TTAAATCAAA   
  
  
+ TCACTCACTT GAGTATTGGA GGGACGTTCC TCAGGAGTCG AACCACTAAA TTATCTTTTT AAAGAAAAAG   
  
  
+ GGCTTGAAGC CAACCCGATT TAAAAAAACT GCTAATATGT AAGCCTACTC TCTTTCCAGT TCCAACAAAT   
  
  
+ CAAATTTCAG TATGAAACAT CCAGTAAAGG ATTTATCCGT GATAATGATG TAACATATGG TTAAAGGGTC   
  
  
+ ATTTTCAGTT ACATCGGAGC ATGCAATTAA AGTCCCTATA AAATAGGTAG GTGCACGAAC AAGTCACCAT   
  
  
+ CGTCCTTTGC TTTTTGCATG AGTGCATGCT AATTATGAAC TTAATTAATA TCTAAGCAAA ATTAAAAAGT   
  
  
+ AGTTAAAACT TTTTCACCTT TGGAACCTCG TAGCTGTAGC AGTAGCTGCC GCACTCTGGT TCTAGACTTC   
  
  
+ TAGGGACCAA GAGATGCACT CATATGCATC CTCTTCTGTC CTTTTACTGT ATCCCCTTCC CATGTGACCT   
  
  
+ TTTTGCCCGG GTGACTTTTC TGTGAGTGTT GATACAAATT TTGTACGTAA GCTAACTCAT TTACTTCACC   
  
  
+ GTCTCATGAG TTTTACATTA CCGATATTAA TTTAAATGGC AAAGTATTAA ATTTGAGCTA AAATCTCATA   
  
  
+ TATATATTTG TATTAAATGT GTCTGGATTG TCAAAATAAT TTTTGACATA TACAGTCACT ATATACTGAG   
  
  
+ TTATTAAAAT ATATAAAAAT ATGAAAATTT AAAAAATAGT AGTTTAAGGT GACAACACTA AAACAAACAA   
  
  
+ ATAAGATATA AATAGGGAAA AGTTCATCCT ATTTTGTTAT TATTATTTAT TATGTATTTT CAGAATTGTC   
  
  
+ TCTATCACAA GCATAGTATA TGTGTCAACA TCGTGACAAC TCGACCAATC TACAAATAAC TAGTAAATTC   
  
  
+ AAATAAAGTT ACTCTCACCT AGCTTAAATA AGTACACCTT GATTTAATTG TCTTAGCTAT GGAACCTATT   
  
  
+ CCAAACTAGC CAATACTTCC TTTTCAAAAG AATTAGTTTT AAAGTTTTAA CTTTTACGTC CAATAATGCC   
  
  
+ GGCCGTACTA TGAGTTGCTT TCAAGCCAGT AGTAGTACTG TAGTAGGTAA AAATATTAGA ATACACCTAC   
  
  
+ TATATATGAT ATTGCTCTTC ACCGTATCCT TCTGCATACT CATCTTTCCG TCCCGGAGTC CACTATCTCT   
  
  
+ CCTCCATTCA GACACTCTCT CGCTCTAGAG AACTCATAGC TATGGACCGC GTGCCCGACG GTTATCGGGT   
  
  
+ TATGAATTCG GAGCTTCTTC AGCAAGTTAG CCCTGAGTTG CCGGATCCAA CCACCTGGTT CCATGCCCGT   
  
  
+ CTACCCGACC CCATCTCTCA GTCTCCTCTT GGACCCGGTC CATCCTCCCA GCCAACCCAT TTCTACCAGG   
  
  
+ GTTCTGCCTC AGGGAACCAT GTGGTTGGGA TTGCTGACAC GTGGACGGAC CAGATTGTTG CAGGTTCCTC   
  
  
+ GTCCCAGCAG TGAGGTTGCA GCAGAGCCAG CAGGTGTTGA CTGCAGTCGA AGCCATGGAG GAGGACTCTG   
  
  
+ GGATAAGACT GGTCTACGCT CTGCTGACGT GTGCGGAGGC CATCCAACGT GGCGATTTCC GATTGGCTGT   
  
  
+ CTTGTTAGTT AACAAGATGA GCAATGACCT CCTGCCACGC GTCAACCCGT CCTGCGGTAT TGGCAAAGTA   
  
  
+ GCCGGCTACT TCATAGATGC CTTGACCCGA AGACTATACC AGCAGGGCCC AGTGTCGGGC CTAATCGGGT   
  
  
+ CGGTTCTGGC GTACCAGGTG TTGTACGAGC ACTTCTATGA AGCTTGCCCG TTCCTCAAAT TCGCTCACTT   
  
  
+ CACTGCTAAT CAAGCGATTT TAGAGGCATT CGACGGCCAC GATTGTGTCC ACATCATCGA CTTCGGCCTA   
  
  
+ ATCCACGGCT TGCAATGGCC AGCTCTAATC CAAGCCTTGG CTGTTCGGCA CGGTGGCCCG CCTTTCGTAA   
  
  
+ GATTAACCGG AATCGGGCCG CCTTCTGAAT ACGGGTCGTG CTCGCTTCAA CCTATCGGGT CAACACTGGC   
  
  
+ CCAGTTAGCC CTATCCATGA ATGTTGGGTT CGCATTTCGG GCCGTCGCCG TCTCACGGCT CGAGGACATC   
  
  
+ AAACCATGGA TGGTTAAAAC AAGCCCAAAT GAAGTCGTAG CCGTGAACTC TATCTTCCAA CTCCACCGGC   
  
  
+ TAATCGGGTC GGGTATTGAC CCTGTCCTAA ACTGGGTCCG GAGCCTAAAC CCGAAAATTG TGACACTGGC   
  
  
+ GGAGCAAGAG GCGAACCACA ACCAGCCCGA GTTTTTGGCC CGGTTCACGG AGGCATTACA TTACTATTCA   
  
  
+ ACTATGTTCG ACTCGTTAGA GGCTTGTCAA GTCCAGGCCG ACAAGGACCT AGCCGAGCTA TACTTAGAGA   
  
  
+ GAGAGTTATC CAACATCGTC TGCTGTGAGG GGTCGGCTCG GATTGAGAGG CACGAGCCGC TGGCCCAATG   
  
  
+ GAGGGCCCGA ATGGCTCGGG CCGGGTTCAA GAAGGTGGAT ATGGGTAAAA ATGCGTTCAA GCAAGTAAGC   
  
  
+ ATGTTGCTGA GTTTGTCTTC AGCACAAGGG TATTGTGTGG AGGAGAGTGA GGGATGTTTG AAGCTCGGCT   
  
  
+ GGCATGACCG CCCTCTCATT GCGGCTTCGG CTTGGCGAGC GGAGACTCAA GCTGAGAACT CCAGCACTGT   
  
  
+ TGTGCTTGAT GGGTCATCGT CGTGTAGTTC ATCTTCTTA  

- -Up\_Stream \_Len000TTTTTT CTTTCTTTAC CTCTTTGGTT CGTTATACCT ATTTTTTTTT TGTTTCATTA   
  
  
- TTTCCTTCCT ACTCCGAGGT GTAAGGTGAG CGGGCGCGGT GAGTATCCTC TACTTATCAT TTTACACTCC   
  
  
- CCAATTTACT AAACTTTTTT TAGATCATTC CCGGGTGTTG TCATAACCTT GGGTCGAACG GTTCCTTTAC   
  
  
- AGTCTCCCCG ATCGTTACCT GGTCACGTTC TTCCGGTTCG GTGTAAACCA ATCCCTTAAA GTAGGGTAGG   
  
  
- CCGCGATTTT CGTAGTTTTA ATGATGAATT AGGAACTAGT TTACGGGATA TTTATAAGAT GATGGGGTAA   
  
  
- ACTTTTCCCC ACAACTAATG TTGTGTATCG GCATCCAAAC TGTTTCTCTT AATGGGAATA ACAATCTTCG   
  
  
- TGGTATATGA GAAAAGAGAT TCGAGACTAG AAAAAAGTAG GAAAAGTCGA AAAACAGGAA AATTTAGTTT   
  
  
- AGTGAGTGAA CTCATAACCT CCCTGCAAGG AGTCCTCAGC TTGGTGATTT AATAGAAAAA TTTCTTTTTC   
  
  
- CCGAACTTCG GTTGGGCTAA ATTTTTTTGA CGATTATACA TTCGGATGAG AGAAAGGTCA AGGTTGTTTA   
  
  
- GTTTAAAGTC ATACTTTGTA GGTCATTTCC TAAATAGGCA CTATTACTAC ATTGTATACC AATTTCCCAG   
  
  
- TAAAAGTCAA TGTAGCCTCG TACGTTAATT TCAGGGATAT TTTATCCATC CACGTGCTTG TTCAGTGGTA   
  
  
- GCAGGAAACG AAAAACGTAC TCACGTACGA TTAATACTTG AATTAATTAT AGATTCGTTT TAATTTTTCA   
  
  
- TCAATTTTGA AAAAGTGGAA ACCTTGGAGC ATCGACATCG TCATCGACGG CGTGAGACCA AGATCTGAAG   
  
  
- ATCCCTGGTT CTCTACGTGA GTATACGTAG GAGAAGACAG GAAAATGACA TAGGGGAAGG GTACACTGGA   
  
  
- AAAACGGGCC CACTGAAAAG ACACTCACAA CTATGTTTAA AACATGCATT CGATTGAGTA AATGAAGTGG   
  
  
- CAGAGTACTC AAAATGTAAT GGCTATAATT AAATTTACCG TTTCATAATT TAAACTCGAT TTTAGAGTAT   
  
  
- ATATATAAAC ATAATTTACA CAGACCTAAC AGTTTTATTA AAAACTGTAT ATGTCAGTGA TATATGACTC   
  
  
- AATAATTTTA TATATTTTTA TACTTTTAAA TTTTTTATCA TCAAATTCCA CTGTTGTGAT TTTGTTTGTT   
  
  
- TATTCTATAT TTATCCCTTT TCAAGTAGGA TAAAACAATA ATAATAAATA ATACATAAAA GTCTTAACAG   
  
  
- AGATAGTGTT CGTATCATAT ACACAGTTGT AGCACTGTTG AGCTGGTTAG ATGTTTATTG ATCATTTAAG   
  
  
- TTTATTTCAA TGAGAGTGGA TCGAATTTAT TCATGTGGAA CTAAATTAAC AGAATCGATA CCTTGGATAA   
  
  
- GGTTTGATCG GTTATGAAGG AAAAGTTTTC TTAATCAAAA TTTCAAAATT GAAAATGCAG GTTATTACGG   
  
  
- CCGGCATGAT ACTCAACGAA AGTTCGGTCA TCATCATGAC ATCATCCATT TTTATAATCT TATGTGGATG   
  
  
- ATATATACTA TAACGAGAAG TGGCATAGGA AGACGTATGA GTAGAAAGGC AGGGCCTCAG GTGATAGAGA   
  
  
- GGAGGTAAGT CTGTGAGAGA GCGAGATCTC TTGAGTATCG ATACCTGGCG CACGGGCTGC CAATAGCCCA   
  
  
- ATACTTAAGC CTCGAAGAAG TCGTTCAATC GGGACTCAAC GGCCTAGGTT GGTGGACCAA GGTACGGGCA   
  
  
- GATGGGCTGG GGTAGAGAGT CAGAGGAGAA CCTGGGCCAG GTAGGAGGGT CGGTTGGGTA AAGATGGTCC   
  
  
- CAAGACGGAG TCCCTTGGTA CACCAACCCT AACGACTGTG CACCTGCCTG GTCTAACAAC GTCCAAGGAG   
  
  
- CAGGGTCGTC ACTCCAACGT CGTCTCGGTC GTCCACAACT GACGTCAGCT TCGGTACCTC CTCCTGAGAC   
  
  
- CCTATTCTGA CCAGATGCGA GACGACTGCA CACGCCTCCG GTAGGTTGCA CCGCTAAAGG CTAACCGACA   
  
  
- GAACAATCAA TTGTTCTACT CGTTACTGGA GGACGGTGCG CAGTTGGGCA GGACGCCATA ACCGTTTCAT   
  
  
- CGGCCGATGA AGTATCTACG GAACTGGGCT TCTGATATGG TCGTCCCGGG TCACAGCCCG GATTAGCCCA   
  
  
- GCCAAGACCG CATGGTCCAC AACATGCTCG TGAAGATACT TCGAACGGGC AAGGAGTTTA AGCGAGTGAA   
  
  
- GTGACGATTA GTTCGCTAAA ATCTCCGTAA GCTGCCGGTG CTAACACAGG TGTAGTAGCT GAAGCCGGAT   
  
  
- TAGGTGCCGA ACGTTACCGG TCGAGATTAG GTTCGGAACC GACAAGCCGT GCCACCGGGC GGAAAGCATT   
  
  
- CTAATTGGCC TTAGCCCGGC GGAAGACTTA TGCCCAGCAC GAGCGAAGTT GGATAGCCCA GTTGTGACCG   
  
  
- GGTCAATCGG GATAGGTACT TACAACCCAA GCGTAAAGCC CGGCAGCGGC AGAGTGCCGA GCTCCTGTAG   
  
  
- TTTGGTACCT ACCAATTTTG TTCGGGTTTA CTTCAGCATC GGCACTTGAG ATAGAAGGTT GAGGTGGCCG   
  
  
- ATTAGCCCAG CCCATAACTG GGACAGGATT TGACCCAGGC CTCGGATTTG GGCTTTTAAC ACTGTGACCG   
  
  
- CCTCGTTCTC CGCTTGGTGT TGGTCGGGCT CAAAAACCGG GCCAAGTGCC TCCGTAATGT AATGATAAGT   
  
  
- TGATACAAGC TGAGCAATCT CCGAACAGTT CAGGTCCGGC TGTTCCTGGA TCGGCTCGAT ATGAATCTCT   
  
  
- CTCTCAATAG GTTGTAGCAG ACGACACTCC CCAGCCGAGC CTAACTCTCC GTGCTCGGCG ACCGGGTTAC   
  
  
- CTCCCGGGCT TACCGAGCCC GGCCCAAGTT CTTCCACCTA TACCCATTTT TACGCAAGTT CGTTCATTCG   
  
  
- TACAACGACT CAAACAGAAG TCGTGTTCCC ATAACACACC TCCTCTCACT CCCTACAAAC TTCGAGCCGA   
  
  
- CCGTACTGGC GGGAGAGTAA CGCCGAAGCC GAACCGCTCG CCTCTGAGTT CGACTCTTGA GGTCGTGACA   
  
  
- ACACGAACTA CCCAGTAGCA GCACATCAAG TAGAAGAAT

+     AAGAA-motif

| Site Name | Organism | Position | Strand | Matrix score. | sequence | function |
| --- | --- | --- | --- | --- | --- | --- |
| AAGAA-motif | Avena sativa | 25 | + | 7 | GAAAGAA |  |

>HU11G00778.1   
+ -Up\_Stream \_Len000AAAAAA GAAAGAAATG GAGAAACCAA GCAATATGGA TAAAAAAAAA ACAAAGTAAT   
  
  
+ AAAGGAAGGA TGAGGCTCCA CATTCCACTC GCCCGCGCCA CTCATAGGAG ATGAATAGTA AAATGTGAGG   
  
  
+ GGTTAAATGA TTTGAAAAAA ATCTAGTAAG GGCCCACAAC AGTATTGGAA CCCAGCTTGC CAAGGAAATG   
  
  
+ TCAGAGGGGC TAGCAATGGA CCAGTGCAAG AAGGCCAAGC CACATTTGGT TAGGGAATTT CATCCCATCC   
  
  
+ GGCGCTAAAA GCATCAAAAT TACTACTTAA TCCTTGATCA AATGCCCTAT AAATATTCTA CTACCCCATT   
  
  
+ TGAAAAGGGG TGTTGATTAC AACACATAGC CGTAGGTTTG ACAAAGAGAA TTACCCTTAT TGTTAGAAGC   
  
  
+ ACCATATACT CTTTTCTCTA AGCTCTGATC TTTTTTCATC CTTTTCAGCT TTTTGTCCTT TTAAATCAAA   
  
  
+ TCACTCACTT GAGTATTGGA GGGACGTTCC TCAGGAGTCG AACCACTAAA TTATCTTTTT AAAGAAAAAG   
  
  
+ GGCTTGAAGC CAACCCGATT TAAAAAAACT GCTAATATGT AAGCCTACTC TCTTTCCAGT TCCAACAAAT   
  
  
+ CAAATTTCAG TATGAAACAT CCAGTAAAGG ATTTATCCGT GATAATGATG TAACATATGG TTAAAGGGTC   
  
  
+ ATTTTCAGTT ACATCGGAGC ATGCAATTAA AGTCCCTATA AAATAGGTAG GTGCACGAAC AAGTCACCAT   
  
  
+ CGTCCTTTGC TTTTTGCATG AGTGCATGCT AATTATGAAC TTAATTAATA TCTAAGCAAA ATTAAAAAGT   
  
  
+ AGTTAAAACT TTTTCACCTT TGGAACCTCG TAGCTGTAGC AGTAGCTGCC GCACTCTGGT TCTAGACTTC   
  
  
+ TAGGGACCAA GAGATGCACT CATATGCATC CTCTTCTGTC CTTTTACTGT ATCCCCTTCC CATGTGACCT   
  
  
+ TTTTGCCCGG GTGACTTTTC TGTGAGTGTT GATACAAATT TTGTACGTAA GCTAACTCAT TTACTTCACC   
  
  
+ GTCTCATGAG TTTTACATTA CCGATATTAA TTTAAATGGC AAAGTATTAA ATTTGAGCTA AAATCTCATA   
  
  
+ TATATATTTG TATTAAATGT GTCTGGATTG TCAAAATAAT TTTTGACATA TACAGTCACT ATATACTGAG   
  
  
+ TTATTAAAAT ATATAAAAAT ATGAAAATTT AAAAAATAGT AGTTTAAGGT GACAACACTA AAACAAACAA   
  
  
+ ATAAGATATA AATAGGGAAA AGTTCATCCT ATTTTGTTAT TATTATTTAT TATGTATTTT CAGAATTGTC   
  
  
+ TCTATCACAA GCATAGTATA TGTGTCAACA TCGTGACAAC TCGACCAATC TACAAATAAC TAGTAAATTC   
  
  
+ AAATAAAGTT ACTCTCACCT AGCTTAAATA AGTACACCTT GATTTAATTG TCTTAGCTAT GGAACCTATT   
  
  
+ CCAAACTAGC CAATACTTCC TTTTCAAAAG AATTAGTTTT AAAGTTTTAA CTTTTACGTC CAATAATGCC   
  
  
+ GGCCGTACTA TGAGTTGCTT TCAAGCCAGT AGTAGTACTG TAGTAGGTAA AAATATTAGA ATACACCTAC   
  
  
+ TATATATGAT ATTGCTCTTC ACCGTATCCT TCTGCATACT CATCTTTCCG TCCCGGAGTC CACTATCTCT   
  
  
+ CCTCCATTCA GACACTCTCT CGCTCTAGAG AACTCATAGC TATGGACCGC GTGCCCGACG GTTATCGGGT   
  
  
+ TATGAATTCG GAGCTTCTTC AGCAAGTTAG CCCTGAGTTG CCGGATCCAA CCACCTGGTT CCATGCCCGT   
  
  
+ CTACCCGACC CCATCTCTCA GTCTCCTCTT GGACCCGGTC CATCCTCCCA GCCAACCCAT TTCTACCAGG   
  
  
+ GTTCTGCCTC AGGGAACCAT GTGGTTGGGA TTGCTGACAC GTGGACGGAC CAGATTGTTG CAGGTTCCTC   
  
  
+ GTCCCAGCAG TGAGGTTGCA GCAGAGCCAG CAGGTGTTGA CTGCAGTCGA AGCCATGGAG GAGGACTCTG   
  
  
+ GGATAAGACT GGTCTACGCT CTGCTGACGT GTGCGGAGGC CATCCAACGT GGCGATTTCC GATTGGCTGT   
  
  
+ CTTGTTAGTT AACAAGATGA GCAATGACCT CCTGCCACGC GTCAACCCGT CCTGCGGTAT TGGCAAAGTA   
  
  
+ GCCGGCTACT TCATAGATGC CTTGACCCGA AGACTATACC AGCAGGGCCC AGTGTCGGGC CTAATCGGGT   
  
  
+ CGGTTCTGGC GTACCAGGTG TTGTACGAGC ACTTCTATGA AGCTTGCCCG TTCCTCAAAT TCGCTCACTT   
  
  
+ CACTGCTAAT CAAGCGATTT TAGAGGCATT CGACGGCCAC GATTGTGTCC ACATCATCGA CTTCGGCCTA   
  
  
+ ATCCACGGCT TGCAATGGCC AGCTCTAATC CAAGCCTTGG CTGTTCGGCA CGGTGGCCCG CCTTTCGTAA   
  
  
+ GATTAACCGG AATCGGGCCG CCTTCTGAAT ACGGGTCGTG CTCGCTTCAA CCTATCGGGT CAACACTGGC   
  
  
+ CCAGTTAGCC CTATCCATGA ATGTTGGGTT CGCATTTCGG GCCGTCGCCG TCTCACGGCT CGAGGACATC   
  
  
+ AAACCATGGA TGGTTAAAAC AAGCCCAAAT GAAGTCGTAG CCGTGAACTC TATCTTCCAA CTCCACCGGC   
  
  
+ TAATCGGGTC GGGTATTGAC CCTGTCCTAA ACTGGGTCCG GAGCCTAAAC CCGAAAATTG TGACACTGGC   
  
  
+ GGAGCAAGAG GCGAACCACA ACCAGCCCGA GTTTTTGGCC CGGTTCACGG AGGCATTACA TTACTATTCA   
  
  
+ ACTATGTTCG ACTCGTTAGA GGCTTGTCAA GTCCAGGCCG ACAAGGACCT AGCCGAGCTA TACTTAGAGA   
  
  
+ GAGAGTTATC CAACATCGTC TGCTGTGAGG GGTCGGCTCG GATTGAGAGG CACGAGCCGC TGGCCCAATG   
  
  
+ GAGGGCCCGA ATGGCTCGGG CCGGGTTCAA GAAGGTGGAT ATGGGTAAAA ATGCGTTCAA GCAAGTAAGC   
  
  
+ ATGTTGCTGA GTTTGTCTTC AGCACAAGGG TATTGTGTGG AGGAGAGTGA GGGATGTTTG AAGCTCGGCT   
  
  
+ GGCATGACCG CCCTCTCATT GCGGCTTCGG CTTGGCGAGC GGAGACTCAA GCTGAGAACT CCAGCACTGT   
  
  
+ TGTGCTTGAT GGGTCATCGT CGTGTAGTTC ATCTTCTTA  

- -Up\_Stream \_Len000TTTTTT CTTTCTTTAC CTCTTTGGTT CGTTATACCT ATTTTTTTTT TGTTTCATTA   
  
  
- TTTCCTTCCT ACTCCGAGGT GTAAGGTGAG CGGGCGCGGT GAGTATCCTC TACTTATCAT TTTACACTCC   
  
  
- CCAATTTACT AAACTTTTTT TAGATCATTC CCGGGTGTTG TCATAACCTT GGGTCGAACG GTTCCTTTAC   
  
  
- AGTCTCCCCG ATCGTTACCT GGTCACGTTC TTCCGGTTCG GTGTAAACCA ATCCCTTAAA GTAGGGTAGG   
  
  
- CCGCGATTTT CGTAGTTTTA ATGATGAATT AGGAACTAGT TTACGGGATA TTTATAAGAT GATGGGGTAA   
  
  
- ACTTTTCCCC ACAACTAATG TTGTGTATCG GCATCCAAAC TGTTTCTCTT AATGGGAATA ACAATCTTCG   
  
  
- TGGTATATGA GAAAAGAGAT TCGAGACTAG AAAAAAGTAG GAAAAGTCGA AAAACAGGAA AATTTAGTTT   
  
  
- AGTGAGTGAA CTCATAACCT CCCTGCAAGG AGTCCTCAGC TTGGTGATTT AATAGAAAAA TTTCTTTTTC   
  
  
- CCGAACTTCG GTTGGGCTAA ATTTTTTTGA CGATTATACA TTCGGATGAG AGAAAGGTCA AGGTTGTTTA   
  
  
- GTTTAAAGTC ATACTTTGTA GGTCATTTCC TAAATAGGCA CTATTACTAC ATTGTATACC AATTTCCCAG   
  
  
- TAAAAGTCAA TGTAGCCTCG TACGTTAATT TCAGGGATAT TTTATCCATC CACGTGCTTG TTCAGTGGTA   
  
  
- GCAGGAAACG AAAAACGTAC TCACGTACGA TTAATACTTG AATTAATTAT AGATTCGTTT TAATTTTTCA   
  
  
- TCAATTTTGA AAAAGTGGAA ACCTTGGAGC ATCGACATCG TCATCGACGG CGTGAGACCA AGATCTGAAG   
  
  
- ATCCCTGGTT CTCTACGTGA GTATACGTAG GAGAAGACAG GAAAATGACA TAGGGGAAGG GTACACTGGA   
  
  
- AAAACGGGCC CACTGAAAAG ACACTCACAA CTATGTTTAA AACATGCATT CGATTGAGTA AATGAAGTGG   
  
  
- CAGAGTACTC AAAATGTAAT GGCTATAATT AAATTTACCG TTTCATAATT TAAACTCGAT TTTAGAGTAT   
  
  
- ATATATAAAC ATAATTTACA CAGACCTAAC AGTTTTATTA AAAACTGTAT ATGTCAGTGA TATATGACTC   
  
  
- AATAATTTTA TATATTTTTA TACTTTTAAA TTTTTTATCA TCAAATTCCA CTGTTGTGAT TTTGTTTGTT   
  
  
- TATTCTATAT TTATCCCTTT TCAAGTAGGA TAAAACAATA ATAATAAATA ATACATAAAA GTCTTAACAG   
  
  
- AGATAGTGTT CGTATCATAT ACACAGTTGT AGCACTGTTG AGCTGGTTAG ATGTTTATTG ATCATTTAAG   
  
  
- TTTATTTCAA TGAGAGTGGA TCGAATTTAT TCATGTGGAA CTAAATTAAC AGAATCGATA CCTTGGATAA   
  
  
- GGTTTGATCG GTTATGAAGG AAAAGTTTTC TTAATCAAAA TTTCAAAATT GAAAATGCAG GTTATTACGG   
  
  
- CCGGCATGAT ACTCAACGAA AGTTCGGTCA TCATCATGAC ATCATCCATT TTTATAATCT TATGTGGATG   
  
  
- ATATATACTA TAACGAGAAG TGGCATAGGA AGACGTATGA GTAGAAAGGC AGGGCCTCAG GTGATAGAGA   
  
  
- GGAGGTAAGT CTGTGAGAGA GCGAGATCTC TTGAGTATCG ATACCTGGCG CACGGGCTGC CAATAGCCCA   
  
  
- ATACTTAAGC CTCGAAGAAG TCGTTCAATC GGGACTCAAC GGCCTAGGTT GGTGGACCAA GGTACGGGCA   
  
  
- GATGGGCTGG GGTAGAGAGT CAGAGGAGAA CCTGGGCCAG GTAGGAGGGT CGGTTGGGTA AAGATGGTCC   
  
  
- CAAGACGGAG TCCCTTGGTA CACCAACCCT AACGACTGTG CACCTGCCTG GTCTAACAAC GTCCAAGGAG   
  
  
- CAGGGTCGTC ACTCCAACGT CGTCTCGGTC GTCCACAACT GACGTCAGCT TCGGTACCTC CTCCTGAGAC   
  
  
- CCTATTCTGA CCAGATGCGA GACGACTGCA CACGCCTCCG GTAGGTTGCA CCGCTAAAGG CTAACCGACA   
  
  
- GAACAATCAA TTGTTCTACT CGTTACTGGA GGACGGTGCG CAGTTGGGCA GGACGCCATA ACCGTTTCAT   
  
  
- CGGCCGATGA AGTATCTACG GAACTGGGCT TCTGATATGG TCGTCCCGGG TCACAGCCCG GATTAGCCCA   
  
  
- GCCAAGACCG CATGGTCCAC AACATGCTCG TGAAGATACT TCGAACGGGC AAGGAGTTTA AGCGAGTGAA   
  
  
- GTGACGATTA GTTCGCTAAA ATCTCCGTAA GCTGCCGGTG CTAACACAGG TGTAGTAGCT GAAGCCGGAT   
  
  
- TAGGTGCCGA ACGTTACCGG TCGAGATTAG GTTCGGAACC GACAAGCCGT GCCACCGGGC GGAAAGCATT   
  
  
- CTAATTGGCC TTAGCCCGGC GGAAGACTTA TGCCCAGCAC GAGCGAAGTT GGATAGCCCA GTTGTGACCG   
  
  
- GGTCAATCGG GATAGGTACT TACAACCCAA GCGTAAAGCC CGGCAGCGGC AGAGTGCCGA GCTCCTGTAG   
  
  
- TTTGGTACCT ACCAATTTTG TTCGGGTTTA CTTCAGCATC GGCACTTGAG ATAGAAGGTT GAGGTGGCCG   
  
  
- ATTAGCCCAG CCCATAACTG GGACAGGATT TGACCCAGGC CTCGGATTTG GGCTTTTAAC ACTGTGACCG   
  
  
- CCTCGTTCTC CGCTTGGTGT TGGTCGGGCT CAAAAACCGG GCCAAGTGCC TCCGTAATGT AATGATAAGT   
  
  
- TGATACAAGC TGAGCAATCT CCGAACAGTT CAGGTCCGGC TGTTCCTGGA TCGGCTCGAT ATGAATCTCT   
  
  
- CTCTCAATAG GTTGTAGCAG ACGACACTCC CCAGCCGAGC CTAACTCTCC GTGCTCGGCG ACCGGGTTAC   
  
  
- CTCCCGGGCT TACCGAGCCC GGCCCAAGTT CTTCCACCTA TACCCATTTT TACGCAAGTT CGTTCATTCG   
  
  
- TACAACGACT CAAACAGAAG TCGTGTTCCC ATAACACACC TCCTCTCACT CCCTACAAAC TTCGAGCCGA   
  
  
- CCGTACTGGC GGGAGAGTAA CGCCGAAGCC GAACCGCTCG CCTCTGAGTT CGACTCTTGA GGTCGTGACA   
  
  
- ACACGAACTA CCCAGTAGCA GCACATCAAG TAGAAGAAT

+     ABRE

| Site Name | Organism | Position | Strand | Matrix score. | sequence | function |
| --- | --- | --- | --- | --- | --- | --- |
| ABRE | Arabidopsis thaliana | 1933 | + | 5 | ACGTG | cis-acting element involved in the abscisic acid responsiveness |
| ABRE | Arabidopsis thaliana | 2965 | - | 7 | AACCCGG | cis-acting element involved in the abscisic acid responsiveness |
| ABRE | Oryza sativa | 2138 | - | 9 | GCCGCGTGGC | cis-acting element involved in the abscisic acid responsiveness |
| ABRE | Arabidopsis thaliana | 2081 | + | 5 | ACGTG | cis-acting element involved in the abscisic acid responsiveness |
| ABRE | Arabidopsis thaliana | 2061 | + | 5 | ACGTG | cis-acting element involved in the abscisic acid responsiveness |
| ABRE | Triticum aestivum | 1930 | + | 9 | GACACGTGGC | cis-acting element involved in the abscisic acid responsiveness |
| ABRE | Arabidopsis thaliana | 1932 | - | 6 | CACGTG | cis-acting element involved in the abscisic acid responsiveness |

>HU11G00778.1   
+ -Up\_Stream \_Len000AAAAAA GAAAGAAATG GAGAAACCAA GCAATATGGA TAAAAAAAAA ACAAAGTAAT   
  
  
+ AAAGGAAGGA TGAGGCTCCA CATTCCACTC GCCCGCGCCA CTCATAGGAG ATGAATAGTA AAATGTGAGG   
  
  
+ GGTTAAATGA TTTGAAAAAA ATCTAGTAAG GGCCCACAAC AGTATTGGAA CCCAGCTTGC CAAGGAAATG   
  
  
+ TCAGAGGGGC TAGCAATGGA CCAGTGCAAG AAGGCCAAGC CACATTTGGT TAGGGAATTT CATCCCATCC   
  
  
+ GGCGCTAAAA GCATCAAAAT TACTACTTAA TCCTTGATCA AATGCCCTAT AAATATTCTA CTACCCCATT   
  
  
+ TGAAAAGGGG TGTTGATTAC AACACATAGC CGTAGGTTTG ACAAAGAGAA TTACCCTTAT TGTTAGAAGC   
  
  
+ ACCATATACT CTTTTCTCTA AGCTCTGATC TTTTTTCATC CTTTTCAGCT TTTTGTCCTT TTAAATCAAA   
  
  
+ TCACTCACTT GAGTATTGGA GGGACGTTCC TCAGGAGTCG AACCACTAAA TTATCTTTTT AAAGAAAAAG   
  
  
+ GGCTTGAAGC CAACCCGATT TAAAAAAACT GCTAATATGT AAGCCTACTC TCTTTCCAGT TCCAACAAAT   
  
  
+ CAAATTTCAG TATGAAACAT CCAGTAAAGG ATTTATCCGT GATAATGATG TAACATATGG TTAAAGGGTC   
  
  
+ ATTTTCAGTT ACATCGGAGC ATGCAATTAA AGTCCCTATA AAATAGGTAG GTGCACGAAC AAGTCACCAT   
  
  
+ CGTCCTTTGC TTTTTGCATG AGTGCATGCT AATTATGAAC TTAATTAATA TCTAAGCAAA ATTAAAAAGT   
  
  
+ AGTTAAAACT TTTTCACCTT TGGAACCTCG TAGCTGTAGC AGTAGCTGCC GCACTCTGGT TCTAGACTTC   
  
  
+ TAGGGACCAA GAGATGCACT CATATGCATC CTCTTCTGTC CTTTTACTGT ATCCCCTTCC CATGTGACCT   
  
  
+ TTTTGCCCGG GTGACTTTTC TGTGAGTGTT GATACAAATT TTGTACGTAA GCTAACTCAT TTACTTCACC   
  
  
+ GTCTCATGAG TTTTACATTA CCGATATTAA TTTAAATGGC AAAGTATTAA ATTTGAGCTA AAATCTCATA   
  
  
+ TATATATTTG TATTAAATGT GTCTGGATTG TCAAAATAAT TTTTGACATA TACAGTCACT ATATACTGAG   
  
  
+ TTATTAAAAT ATATAAAAAT ATGAAAATTT AAAAAATAGT AGTTTAAGGT GACAACACTA AAACAAACAA   
  
  
+ ATAAGATATA AATAGGGAAA AGTTCATCCT ATTTTGTTAT TATTATTTAT TATGTATTTT CAGAATTGTC   
  
  
+ TCTATCACAA GCATAGTATA TGTGTCAACA TCGTGACAAC TCGACCAATC TACAAATAAC TAGTAAATTC   
  
  
+ AAATAAAGTT ACTCTCACCT AGCTTAAATA AGTACACCTT GATTTAATTG TCTTAGCTAT GGAACCTATT   
  
  
+ CCAAACTAGC CAATACTTCC TTTTCAAAAG AATTAGTTTT AAAGTTTTAA CTTTTACGTC CAATAATGCC   
  
  
+ GGCCGTACTA TGAGTTGCTT TCAAGCCAGT AGTAGTACTG TAGTAGGTAA AAATATTAGA ATACACCTAC   
  
  
+ TATATATGAT ATTGCTCTTC ACCGTATCCT TCTGCATACT CATCTTTCCG TCCCGGAGTC CACTATCTCT   
  
  
+ CCTCCATTCA GACACTCTCT CGCTCTAGAG AACTCATAGC TATGGACCGC GTGCCCGACG GTTATCGGGT   
  
  
+ TATGAATTCG GAGCTTCTTC AGCAAGTTAG CCCTGAGTTG CCGGATCCAA CCACCTGGTT CCATGCCCGT   
  
  
+ CTACCCGACC CCATCTCTCA GTCTCCTCTT GGACCCGGTC CATCCTCCCA GCCAACCCAT TTCTACCAGG   
  
  
+ GTTCTGCCTC AGGGAACCAT GTGGTTGGGA TTGCTGACAC GTGGACGGAC CAGATTGTTG CAGGTTCCTC   
  
  
+ GTCCCAGCAG TGAGGTTGCA GCAGAGCCAG CAGGTGTTGA CTGCAGTCGA AGCCATGGAG GAGGACTCTG   
  
  
+ GGATAAGACT GGTCTACGCT CTGCTGACGT GTGCGGAGGC CATCCAACGT GGCGATTTCC GATTGGCTGT   
  
  
+ CTTGTTAGTT AACAAGATGA GCAATGACCT CCTGCCACGC GTCAACCCGT CCTGCGGTAT TGGCAAAGTA   
  
  
+ GCCGGCTACT TCATAGATGC CTTGACCCGA AGACTATACC AGCAGGGCCC AGTGTCGGGC CTAATCGGGT   
  
  
+ CGGTTCTGGC GTACCAGGTG TTGTACGAGC ACTTCTATGA AGCTTGCCCG TTCCTCAAAT TCGCTCACTT   
  
  
+ CACTGCTAAT CAAGCGATTT TAGAGGCATT CGACGGCCAC GATTGTGTCC ACATCATCGA CTTCGGCCTA   
  
  
+ ATCCACGGCT TGCAATGGCC AGCTCTAATC CAAGCCTTGG CTGTTCGGCA CGGTGGCCCG CCTTTCGTAA   
  
  
+ GATTAACCGG AATCGGGCCG CCTTCTGAAT ACGGGTCGTG CTCGCTTCAA CCTATCGGGT CAACACTGGC   
  
  
+ CCAGTTAGCC CTATCCATGA ATGTTGGGTT CGCATTTCGG GCCGTCGCCG TCTCACGGCT CGAGGACATC   
  
  
+ AAACCATGGA TGGTTAAAAC AAGCCCAAAT GAAGTCGTAG CCGTGAACTC TATCTTCCAA CTCCACCGGC   
  
  
+ TAATCGGGTC GGGTATTGAC CCTGTCCTAA ACTGGGTCCG GAGCCTAAAC CCGAAAATTG TGACACTGGC   
  
  
+ GGAGCAAGAG GCGAACCACA ACCAGCCCGA GTTTTTGGCC CGGTTCACGG AGGCATTACA TTACTATTCA   
  
  
+ ACTATGTTCG ACTCGTTAGA GGCTTGTCAA GTCCAGGCCG ACAAGGACCT AGCCGAGCTA TACTTAGAGA   
  
  
+ GAGAGTTATC CAACATCGTC TGCTGTGAGG GGTCGGCTCG GATTGAGAGG CACGAGCCGC TGGCCCAATG   
  
  
+ GAGGGCCCGA ATGGCTCGGG CCGGGTTCAA GAAGGTGGAT ATGGGTAAAA ATGCGTTCAA GCAAGTAAGC   
  
  
+ ATGTTGCTGA GTTTGTCTTC AGCACAAGGG TATTGTGTGG AGGAGAGTGA GGGATGTTTG AAGCTCGGCT   
  
  
+ GGCATGACCG CCCTCTCATT GCGGCTTCGG CTTGGCGAGC GGAGACTCAA GCTGAGAACT CCAGCACTGT   
  
  
+ TGTGCTTGAT GGGTCATCGT CGTGTAGTTC ATCTTCTTA  

- -Up\_Stream \_Len000TTTTTT CTTTCTTTAC CTCTTTGGTT CGTTATACCT ATTTTTTTTT TGTTTCATTA   
  
  
- TTTCCTTCCT ACTCCGAGGT GTAAGGTGAG CGGGCGCGGT GAGTATCCTC TACTTATCAT TTTACACTCC   
  
  
- CCAATTTACT AAACTTTTTT TAGATCATTC CCGGGTGTTG TCATAACCTT GGGTCGAACG GTTCCTTTAC   
  
  
- AGTCTCCCCG ATCGTTACCT GGTCACGTTC TTCCGGTTCG GTGTAAACCA ATCCCTTAAA GTAGGGTAGG   
  
  
- CCGCGATTTT CGTAGTTTTA ATGATGAATT AGGAACTAGT TTACGGGATA TTTATAAGAT GATGGGGTAA   
  
  
- ACTTTTCCCC ACAACTAATG TTGTGTATCG GCATCCAAAC TGTTTCTCTT AATGGGAATA ACAATCTTCG   
  
  
- TGGTATATGA GAAAAGAGAT TCGAGACTAG AAAAAAGTAG GAAAAGTCGA AAAACAGGAA AATTTAGTTT   
  
  
- AGTGAGTGAA CTCATAACCT CCCTGCAAGG AGTCCTCAGC TTGGTGATTT AATAGAAAAA TTTCTTTTTC   
  
  
- CCGAACTTCG GTTGGGCTAA ATTTTTTTGA CGATTATACA TTCGGATGAG AGAAAGGTCA AGGTTGTTTA   
  
  
- GTTTAAAGTC ATACTTTGTA GGTCATTTCC TAAATAGGCA CTATTACTAC ATTGTATACC AATTTCCCAG   
  
  
- TAAAAGTCAA TGTAGCCTCG TACGTTAATT TCAGGGATAT TTTATCCATC CACGTGCTTG TTCAGTGGTA   
  
  
- GCAGGAAACG AAAAACGTAC TCACGTACGA TTAATACTTG AATTAATTAT AGATTCGTTT TAATTTTTCA   
  
  
- TCAATTTTGA AAAAGTGGAA ACCTTGGAGC ATCGACATCG TCATCGACGG CGTGAGACCA AGATCTGAAG   
  
  
- ATCCCTGGTT CTCTACGTGA GTATACGTAG GAGAAGACAG GAAAATGACA TAGGGGAAGG GTACACTGGA   
  
  
- AAAACGGGCC CACTGAAAAG ACACTCACAA CTATGTTTAA AACATGCATT CGATTGAGTA AATGAAGTGG   
  
  
- CAGAGTACTC AAAATGTAAT GGCTATAATT AAATTTACCG TTTCATAATT TAAACTCGAT TTTAGAGTAT   
  
  
- ATATATAAAC ATAATTTACA CAGACCTAAC AGTTTTATTA AAAACTGTAT ATGTCAGTGA TATATGACTC   
  
  
- AATAATTTTA TATATTTTTA TACTTTTAAA TTTTTTATCA TCAAATTCCA CTGTTGTGAT TTTGTTTGTT   
  
  
- TATTCTATAT TTATCCCTTT TCAAGTAGGA TAAAACAATA ATAATAAATA ATACATAAAA GTCTTAACAG   
  
  
- AGATAGTGTT CGTATCATAT ACACAGTTGT AGCACTGTTG AGCTGGTTAG ATGTTTATTG ATCATTTAAG   
  
  
- TTTATTTCAA TGAGAGTGGA TCGAATTTAT TCATGTGGAA CTAAATTAAC AGAATCGATA CCTTGGATAA   
  
  
- GGTTTGATCG GTTATGAAGG AAAAGTTTTC TTAATCAAAA TTTCAAAATT GAAAATGCAG GTTATTACGG   
  
  
- CCGGCATGAT ACTCAACGAA AGTTCGGTCA TCATCATGAC ATCATCCATT TTTATAATCT TATGTGGATG   
  
  
- ATATATACTA TAACGAGAAG TGGCATAGGA AGACGTATGA GTAGAAAGGC AGGGCCTCAG GTGATAGAGA   
  
  
- GGAGGTAAGT CTGTGAGAGA GCGAGATCTC TTGAGTATCG ATACCTGGCG CACGGGCTGC CAATAGCCCA   
  
  
- ATACTTAAGC CTCGAAGAAG TCGTTCAATC GGGACTCAAC GGCCTAGGTT GGTGGACCAA GGTACGGGCA   
  
  
- GATGGGCTGG GGTAGAGAGT CAGAGGAGAA CCTGGGCCAG GTAGGAGGGT CGGTTGGGTA AAGATGGTCC   
  
  
- CAAGACGGAG TCCCTTGGTA CACCAACCCT AACGACTGTG CACCTGCCTG GTCTAACAAC GTCCAAGGAG   
  
  
- CAGGGTCGTC ACTCCAACGT CGTCTCGGTC GTCCACAACT GACGTCAGCT TCGGTACCTC CTCCTGAGAC   
  
  
- CCTATTCTGA CCAGATGCGA GACGACTGCA CACGCCTCCG GTAGGTTGCA CCGCTAAAGG CTAACCGACA   
  
  
- GAACAATCAA TTGTTCTACT CGTTACTGGA GGACGGTGCG CAGTTGGGCA GGACGCCATA ACCGTTTCAT   
  
  
- CGGCCGATGA AGTATCTACG GAACTGGGCT TCTGATATGG TCGTCCCGGG TCACAGCCCG GATTAGCCCA   
  
  
- GCCAAGACCG CATGGTCCAC AACATGCTCG TGAAGATACT TCGAACGGGC AAGGAGTTTA AGCGAGTGAA   
  
  
- GTGACGATTA GTTCGCTAAA ATCTCCGTAA GCTGCCGGTG CTAACACAGG TGTAGTAGCT GAAGCCGGAT   
  
  
- TAGGTGCCGA ACGTTACCGG TCGAGATTAG GTTCGGAACC GACAAGCCGT GCCACCGGGC GGAAAGCATT   
  
  
- CTAATTGGCC TTAGCCCGGC GGAAGACTTA TGCCCAGCAC GAGCGAAGTT GGATAGCCCA GTTGTGACCG   
  
  
- GGTCAATCGG GATAGGTACT TACAACCCAA GCGTAAAGCC CGGCAGCGGC AGAGTGCCGA GCTCCTGTAG   
  
  
- TTTGGTACCT ACCAATTTTG TTCGGGTTTA CTTCAGCATC GGCACTTGAG ATAGAAGGTT GAGGTGGCCG   
  
  
- ATTAGCCCAG CCCATAACTG GGACAGGATT TGACCCAGGC CTCGGATTTG GGCTTTTAAC ACTGTGACCG   
  
  
- CCTCGTTCTC CGCTTGGTGT TGGTCGGGCT CAAAAACCGG GCCAAGTGCC TCCGTAATGT AATGATAAGT   
  
  
- TGATACAAGC TGAGCAATCT CCGAACAGTT CAGGTCCGGC TGTTCCTGGA TCGGCTCGAT ATGAATCTCT   
  
  
- CTCTCAATAG GTTGTAGCAG ACGACACTCC CCAGCCGAGC CTAACTCTCC GTGCTCGGCG ACCGGGTTAC   
  
  
- CTCCCGGGCT TACCGAGCCC GGCCCAAGTT CTTCCACCTA TACCCATTTT TACGCAAGTT CGTTCATTCG   
  
  
- TACAACGACT CAAACAGAAG TCGTGTTCCC ATAACACACC TCCTCTCACT CCCTACAAAC TTCGAGCCGA   
  
  
- CCGTACTGGC GGGAGAGTAA CGCCGAAGCC GAACCGCTCG CCTCTGAGTT CGACTCTTGA GGTCGTGACA   
  
  
- ACACGAACTA CCCAGTAGCA GCACATCAAG TAGAAGAAT

+     AP-1

| Site Name | Organism | Position | Strand | Matrix score. | sequence | function |
| --- | --- | --- | --- | --- | --- | --- |
| AP-1 | Arabidopsis thaliana | 1036 | - | 8 | TGAGTTAG |  |

>HU11G00778.1   
+ -Up\_Stream \_Len000AAAAAA GAAAGAAATG GAGAAACCAA GCAATATGGA TAAAAAAAAA ACAAAGTAAT   
  
  
+ AAAGGAAGGA TGAGGCTCCA CATTCCACTC GCCCGCGCCA CTCATAGGAG ATGAATAGTA AAATGTGAGG   
  
  
+ GGTTAAATGA TTTGAAAAAA ATCTAGTAAG GGCCCACAAC AGTATTGGAA CCCAGCTTGC CAAGGAAATG   
  
  
+ TCAGAGGGGC TAGCAATGGA CCAGTGCAAG AAGGCCAAGC CACATTTGGT TAGGGAATTT CATCCCATCC   
  
  
+ GGCGCTAAAA GCATCAAAAT TACTACTTAA TCCTTGATCA AATGCCCTAT AAATATTCTA CTACCCCATT   
  
  
+ TGAAAAGGGG TGTTGATTAC AACACATAGC CGTAGGTTTG ACAAAGAGAA TTACCCTTAT TGTTAGAAGC   
  
  
+ ACCATATACT CTTTTCTCTA AGCTCTGATC TTTTTTCATC CTTTTCAGCT TTTTGTCCTT TTAAATCAAA   
  
  
+ TCACTCACTT GAGTATTGGA GGGACGTTCC TCAGGAGTCG AACCACTAAA TTATCTTTTT AAAGAAAAAG   
  
  
+ GGCTTGAAGC CAACCCGATT TAAAAAAACT GCTAATATGT AAGCCTACTC TCTTTCCAGT TCCAACAAAT   
  
  
+ CAAATTTCAG TATGAAACAT CCAGTAAAGG ATTTATCCGT GATAATGATG TAACATATGG TTAAAGGGTC   
  
  
+ ATTTTCAGTT ACATCGGAGC ATGCAATTAA AGTCCCTATA AAATAGGTAG GTGCACGAAC AAGTCACCAT   
  
  
+ CGTCCTTTGC TTTTTGCATG AGTGCATGCT AATTATGAAC TTAATTAATA TCTAAGCAAA ATTAAAAAGT   
  
  
+ AGTTAAAACT TTTTCACCTT TGGAACCTCG TAGCTGTAGC AGTAGCTGCC GCACTCTGGT TCTAGACTTC   
  
  
+ TAGGGACCAA GAGATGCACT CATATGCATC CTCTTCTGTC CTTTTACTGT ATCCCCTTCC CATGTGACCT   
  
  
+ TTTTGCCCGG GTGACTTTTC TGTGAGTGTT GATACAAATT TTGTACGTAA GCTAACTCAT TTACTTCACC   
  
  
+ GTCTCATGAG TTTTACATTA CCGATATTAA TTTAAATGGC AAAGTATTAA ATTTGAGCTA AAATCTCATA   
  
  
+ TATATATTTG TATTAAATGT GTCTGGATTG TCAAAATAAT TTTTGACATA TACAGTCACT ATATACTGAG   
  
  
+ TTATTAAAAT ATATAAAAAT ATGAAAATTT AAAAAATAGT AGTTTAAGGT GACAACACTA AAACAAACAA   
  
  
+ ATAAGATATA AATAGGGAAA AGTTCATCCT ATTTTGTTAT TATTATTTAT TATGTATTTT CAGAATTGTC   
  
  
+ TCTATCACAA GCATAGTATA TGTGTCAACA TCGTGACAAC TCGACCAATC TACAAATAAC TAGTAAATTC   
  
  
+ AAATAAAGTT ACTCTCACCT AGCTTAAATA AGTACACCTT GATTTAATTG TCTTAGCTAT GGAACCTATT   
  
  
+ CCAAACTAGC CAATACTTCC TTTTCAAAAG AATTAGTTTT AAAGTTTTAA CTTTTACGTC CAATAATGCC   
  
  
+ GGCCGTACTA TGAGTTGCTT TCAAGCCAGT AGTAGTACTG TAGTAGGTAA AAATATTAGA ATACACCTAC   
  
  
+ TATATATGAT ATTGCTCTTC ACCGTATCCT TCTGCATACT CATCTTTCCG TCCCGGAGTC CACTATCTCT   
  
  
+ CCTCCATTCA GACACTCTCT CGCTCTAGAG AACTCATAGC TATGGACCGC GTGCCCGACG GTTATCGGGT   
  
  
+ TATGAATTCG GAGCTTCTTC AGCAAGTTAG CCCTGAGTTG CCGGATCCAA CCACCTGGTT CCATGCCCGT   
  
  
+ CTACCCGACC CCATCTCTCA GTCTCCTCTT GGACCCGGTC CATCCTCCCA GCCAACCCAT TTCTACCAGG   
  
  
+ GTTCTGCCTC AGGGAACCAT GTGGTTGGGA TTGCTGACAC GTGGACGGAC CAGATTGTTG CAGGTTCCTC   
  
  
+ GTCCCAGCAG TGAGGTTGCA GCAGAGCCAG CAGGTGTTGA CTGCAGTCGA AGCCATGGAG GAGGACTCTG   
  
  
+ GGATAAGACT GGTCTACGCT CTGCTGACGT GTGCGGAGGC CATCCAACGT GGCGATTTCC GATTGGCTGT   
  
  
+ CTTGTTAGTT AACAAGATGA GCAATGACCT CCTGCCACGC GTCAACCCGT CCTGCGGTAT TGGCAAAGTA   
  
  
+ GCCGGCTACT TCATAGATGC CTTGACCCGA AGACTATACC AGCAGGGCCC AGTGTCGGGC CTAATCGGGT   
  
  
+ CGGTTCTGGC GTACCAGGTG TTGTACGAGC ACTTCTATGA AGCTTGCCCG TTCCTCAAAT TCGCTCACTT   
  
  
+ CACTGCTAAT CAAGCGATTT TAGAGGCATT CGACGGCCAC GATTGTGTCC ACATCATCGA CTTCGGCCTA   
  
  
+ ATCCACGGCT TGCAATGGCC AGCTCTAATC CAAGCCTTGG CTGTTCGGCA CGGTGGCCCG CCTTTCGTAA   
  
  
+ GATTAACCGG AATCGGGCCG CCTTCTGAAT ACGGGTCGTG CTCGCTTCAA CCTATCGGGT CAACACTGGC   
  
  
+ CCAGTTAGCC CTATCCATGA ATGTTGGGTT CGCATTTCGG GCCGTCGCCG TCTCACGGCT CGAGGACATC   
  
  
+ AAACCATGGA TGGTTAAAAC AAGCCCAAAT GAAGTCGTAG CCGTGAACTC TATCTTCCAA CTCCACCGGC   
  
  
+ TAATCGGGTC GGGTATTGAC CCTGTCCTAA ACTGGGTCCG GAGCCTAAAC CCGAAAATTG TGACACTGGC   
  
  
+ GGAGCAAGAG GCGAACCACA ACCAGCCCGA GTTTTTGGCC CGGTTCACGG AGGCATTACA TTACTATTCA   
  
  
+ ACTATGTTCG ACTCGTTAGA GGCTTGTCAA GTCCAGGCCG ACAAGGACCT AGCCGAGCTA TACTTAGAGA   
  
  
+ GAGAGTTATC CAACATCGTC TGCTGTGAGG GGTCGGCTCG GATTGAGAGG CACGAGCCGC TGGCCCAATG   
  
  
+ GAGGGCCCGA ATGGCTCGGG CCGGGTTCAA GAAGGTGGAT ATGGGTAAAA ATGCGTTCAA GCAAGTAAGC   
  
  
+ ATGTTGCTGA GTTTGTCTTC AGCACAAGGG TATTGTGTGG AGGAGAGTGA GGGATGTTTG AAGCTCGGCT   
  
  
+ GGCATGACCG CCCTCTCATT GCGGCTTCGG CTTGGCGAGC GGAGACTCAA GCTGAGAACT CCAGCACTGT   
  
  
+ TGTGCTTGAT GGGTCATCGT CGTGTAGTTC ATCTTCTTA  

- -Up\_Stream \_Len000TTTTTT CTTTCTTTAC CTCTTTGGTT CGTTATACCT ATTTTTTTTT TGTTTCATTA   
  
  
- TTTCCTTCCT ACTCCGAGGT GTAAGGTGAG CGGGCGCGGT GAGTATCCTC TACTTATCAT TTTACACTCC   
  
  
- CCAATTTACT AAACTTTTTT TAGATCATTC CCGGGTGTTG TCATAACCTT GGGTCGAACG GTTCCTTTAC   
  
  
- AGTCTCCCCG ATCGTTACCT GGTCACGTTC TTCCGGTTCG GTGTAAACCA ATCCCTTAAA GTAGGGTAGG   
  
  
- CCGCGATTTT CGTAGTTTTA ATGATGAATT AGGAACTAGT TTACGGGATA TTTATAAGAT GATGGGGTAA   
  
  
- ACTTTTCCCC ACAACTAATG TTGTGTATCG GCATCCAAAC TGTTTCTCTT AATGGGAATA ACAATCTTCG   
  
  
- TGGTATATGA GAAAAGAGAT TCGAGACTAG AAAAAAGTAG GAAAAGTCGA AAAACAGGAA AATTTAGTTT   
  
  
- AGTGAGTGAA CTCATAACCT CCCTGCAAGG AGTCCTCAGC TTGGTGATTT AATAGAAAAA TTTCTTTTTC   
  
  
- CCGAACTTCG GTTGGGCTAA ATTTTTTTGA CGATTATACA TTCGGATGAG AGAAAGGTCA AGGTTGTTTA   
  
  
- GTTTAAAGTC ATACTTTGTA GGTCATTTCC TAAATAGGCA CTATTACTAC ATTGTATACC AATTTCCCAG   
  
  
- TAAAAGTCAA TGTAGCCTCG TACGTTAATT TCAGGGATAT TTTATCCATC CACGTGCTTG TTCAGTGGTA   
  
  
- GCAGGAAACG AAAAACGTAC TCACGTACGA TTAATACTTG AATTAATTAT AGATTCGTTT TAATTTTTCA   
  
  
- TCAATTTTGA AAAAGTGGAA ACCTTGGAGC ATCGACATCG TCATCGACGG CGTGAGACCA AGATCTGAAG   
  
  
- ATCCCTGGTT CTCTACGTGA GTATACGTAG GAGAAGACAG GAAAATGACA TAGGGGAAGG GTACACTGGA   
  
  
- AAAACGGGCC CACTGAAAAG ACACTCACAA CTATGTTTAA AACATGCATT CGATTGAGTA AATGAAGTGG   
  
  
- CAGAGTACTC AAAATGTAAT GGCTATAATT AAATTTACCG TTTCATAATT TAAACTCGAT TTTAGAGTAT   
  
  
- ATATATAAAC ATAATTTACA CAGACCTAAC AGTTTTATTA AAAACTGTAT ATGTCAGTGA TATATGACTC   
  
  
- AATAATTTTA TATATTTTTA TACTTTTAAA TTTTTTATCA TCAAATTCCA CTGTTGTGAT TTTGTTTGTT   
  
  
- TATTCTATAT TTATCCCTTT TCAAGTAGGA TAAAACAATA ATAATAAATA ATACATAAAA GTCTTAACAG   
  
  
- AGATAGTGTT CGTATCATAT ACACAGTTGT AGCACTGTTG AGCTGGTTAG ATGTTTATTG ATCATTTAAG   
  
  
- TTTATTTCAA TGAGAGTGGA TCGAATTTAT TCATGTGGAA CTAAATTAAC AGAATCGATA CCTTGGATAA   
  
  
- GGTTTGATCG GTTATGAAGG AAAAGTTTTC TTAATCAAAA TTTCAAAATT GAAAATGCAG GTTATTACGG   
  
  
- CCGGCATGAT ACTCAACGAA AGTTCGGTCA TCATCATGAC ATCATCCATT TTTATAATCT TATGTGGATG   
  
  
- ATATATACTA TAACGAGAAG TGGCATAGGA AGACGTATGA GTAGAAAGGC AGGGCCTCAG GTGATAGAGA   
  
  
- GGAGGTAAGT CTGTGAGAGA GCGAGATCTC TTGAGTATCG ATACCTGGCG CACGGGCTGC CAATAGCCCA   
  
  
- ATACTTAAGC CTCGAAGAAG TCGTTCAATC GGGACTCAAC GGCCTAGGTT GGTGGACCAA GGTACGGGCA   
  
  
- GATGGGCTGG GGTAGAGAGT CAGAGGAGAA CCTGGGCCAG GTAGGAGGGT CGGTTGGGTA AAGATGGTCC   
  
  
- CAAGACGGAG TCCCTTGGTA CACCAACCCT AACGACTGTG CACCTGCCTG GTCTAACAAC GTCCAAGGAG   
  
  
- CAGGGTCGTC ACTCCAACGT CGTCTCGGTC GTCCACAACT GACGTCAGCT TCGGTACCTC CTCCTGAGAC   
  
  
- CCTATTCTGA CCAGATGCGA GACGACTGCA CACGCCTCCG GTAGGTTGCA CCGCTAAAGG CTAACCGACA   
  
  
- GAACAATCAA TTGTTCTACT CGTTACTGGA GGACGGTGCG CAGTTGGGCA GGACGCCATA ACCGTTTCAT   
  
  
- CGGCCGATGA AGTATCTACG GAACTGGGCT TCTGATATGG TCGTCCCGGG TCACAGCCCG GATTAGCCCA   
  
  
- GCCAAGACCG CATGGTCCAC AACATGCTCG TGAAGATACT TCGAACGGGC AAGGAGTTTA AGCGAGTGAA   
  
  
- GTGACGATTA GTTCGCTAAA ATCTCCGTAA GCTGCCGGTG CTAACACAGG TGTAGTAGCT GAAGCCGGAT   
  
  
- TAGGTGCCGA ACGTTACCGG TCGAGATTAG GTTCGGAACC GACAAGCCGT GCCACCGGGC GGAAAGCATT   
  
  
- CTAATTGGCC TTAGCCCGGC GGAAGACTTA TGCCCAGCAC GAGCGAAGTT GGATAGCCCA GTTGTGACCG   
  
  
- GGTCAATCGG GATAGGTACT TACAACCCAA GCGTAAAGCC CGGCAGCGGC AGAGTGCCGA GCTCCTGTAG   
  
  
- TTTGGTACCT ACCAATTTTG TTCGGGTTTA CTTCAGCATC GGCACTTGAG ATAGAAGGTT GAGGTGGCCG   
  
  
- ATTAGCCCAG CCCATAACTG GGACAGGATT TGACCCAGGC CTCGGATTTG GGCTTTTAAC ACTGTGACCG   
  
  
- CCTCGTTCTC CGCTTGGTGT TGGTCGGGCT CAAAAACCGG GCCAAGTGCC TCCGTAATGT AATGATAAGT   
  
  
- TGATACAAGC TGAGCAATCT CCGAACAGTT CAGGTCCGGC TGTTCCTGGA TCGGCTCGAT ATGAATCTCT   
  
  
- CTCTCAATAG GTTGTAGCAG ACGACACTCC CCAGCCGAGC CTAACTCTCC GTGCTCGGCG ACCGGGTTAC   
  
  
- CTCCCGGGCT TACCGAGCCC GGCCCAAGTT CTTCCACCTA TACCCATTTT TACGCAAGTT CGTTCATTCG   
  
  
- TACAACGACT CAAACAGAAG TCGTGTTCCC ATAACACACC TCCTCTCACT CCCTACAAAC TTCGAGCCGA   
  
  
- CCGTACTGGC GGGAGAGTAA CGCCGAAGCC GAACCGCTCG CCTCTGAGTT CGACTCTTGA GGTCGTGACA   
  
  
- ACACGAACTA CCCAGTAGCA GCACATCAAG TAGAAGAAT

+     ARE

| Site Name | Organism | Position | Strand | Matrix score. | sequence | function |
| --- | --- | --- | --- | --- | --- | --- |
| ARE | Zea mays | 38 | + | 6 | AAACCA | cis-acting regulatory element essential for the anaerobic induction |
| ARE | Zea mays | 2595 | + | 6 | AAACCA | cis-acting regulatory element essential for the anaerobic induction |

>HU11G00778.1   
+ -Up\_Stream \_Len000AAAAAA GAAAGAAATG GAGAAACCAA GCAATATGGA TAAAAAAAAA ACAAAGTAAT   
  
  
+ AAAGGAAGGA TGAGGCTCCA CATTCCACTC GCCCGCGCCA CTCATAGGAG ATGAATAGTA AAATGTGAGG   
  
  
+ GGTTAAATGA TTTGAAAAAA ATCTAGTAAG GGCCCACAAC AGTATTGGAA CCCAGCTTGC CAAGGAAATG   
  
  
+ TCAGAGGGGC TAGCAATGGA CCAGTGCAAG AAGGCCAAGC CACATTTGGT TAGGGAATTT CATCCCATCC   
  
  
+ GGCGCTAAAA GCATCAAAAT TACTACTTAA TCCTTGATCA AATGCCCTAT AAATATTCTA CTACCCCATT   
  
  
+ TGAAAAGGGG TGTTGATTAC AACACATAGC CGTAGGTTTG ACAAAGAGAA TTACCCTTAT TGTTAGAAGC   
  
  
+ ACCATATACT CTTTTCTCTA AGCTCTGATC TTTTTTCATC CTTTTCAGCT TTTTGTCCTT TTAAATCAAA   
  
  
+ TCACTCACTT GAGTATTGGA GGGACGTTCC TCAGGAGTCG AACCACTAAA TTATCTTTTT AAAGAAAAAG   
  
  
+ GGCTTGAAGC CAACCCGATT TAAAAAAACT GCTAATATGT AAGCCTACTC TCTTTCCAGT TCCAACAAAT   
  
  
+ CAAATTTCAG TATGAAACAT CCAGTAAAGG ATTTATCCGT GATAATGATG TAACATATGG TTAAAGGGTC   
  
  
+ ATTTTCAGTT ACATCGGAGC ATGCAATTAA AGTCCCTATA AAATAGGTAG GTGCACGAAC AAGTCACCAT   
  
  
+ CGTCCTTTGC TTTTTGCATG AGTGCATGCT AATTATGAAC TTAATTAATA TCTAAGCAAA ATTAAAAAGT   
  
  
+ AGTTAAAACT TTTTCACCTT TGGAACCTCG TAGCTGTAGC AGTAGCTGCC GCACTCTGGT TCTAGACTTC   
  
  
+ TAGGGACCAA GAGATGCACT CATATGCATC CTCTTCTGTC CTTTTACTGT ATCCCCTTCC CATGTGACCT   
  
  
+ TTTTGCCCGG GTGACTTTTC TGTGAGTGTT GATACAAATT TTGTACGTAA GCTAACTCAT TTACTTCACC   
  
  
+ GTCTCATGAG TTTTACATTA CCGATATTAA TTTAAATGGC AAAGTATTAA ATTTGAGCTA AAATCTCATA   
  
  
+ TATATATTTG TATTAAATGT GTCTGGATTG TCAAAATAAT TTTTGACATA TACAGTCACT ATATACTGAG   
  
  
+ TTATTAAAAT ATATAAAAAT ATGAAAATTT AAAAAATAGT AGTTTAAGGT GACAACACTA AAACAAACAA   
  
  
+ ATAAGATATA AATAGGGAAA AGTTCATCCT ATTTTGTTAT TATTATTTAT TATGTATTTT CAGAATTGTC   
  
  
+ TCTATCACAA GCATAGTATA TGTGTCAACA TCGTGACAAC TCGACCAATC TACAAATAAC TAGTAAATTC   
  
  
+ AAATAAAGTT ACTCTCACCT AGCTTAAATA AGTACACCTT GATTTAATTG TCTTAGCTAT GGAACCTATT   
  
  
+ CCAAACTAGC CAATACTTCC TTTTCAAAAG AATTAGTTTT AAAGTTTTAA CTTTTACGTC CAATAATGCC   
  
  
+ GGCCGTACTA TGAGTTGCTT TCAAGCCAGT AGTAGTACTG TAGTAGGTAA AAATATTAGA ATACACCTAC   
  
  
+ TATATATGAT ATTGCTCTTC ACCGTATCCT TCTGCATACT CATCTTTCCG TCCCGGAGTC CACTATCTCT   
  
  
+ CCTCCATTCA GACACTCTCT CGCTCTAGAG AACTCATAGC TATGGACCGC GTGCCCGACG GTTATCGGGT   
  
  
+ TATGAATTCG GAGCTTCTTC AGCAAGTTAG CCCTGAGTTG CCGGATCCAA CCACCTGGTT CCATGCCCGT   
  
  
+ CTACCCGACC CCATCTCTCA GTCTCCTCTT GGACCCGGTC CATCCTCCCA GCCAACCCAT TTCTACCAGG   
  
  
+ GTTCTGCCTC AGGGAACCAT GTGGTTGGGA TTGCTGACAC GTGGACGGAC CAGATTGTTG CAGGTTCCTC   
  
  
+ GTCCCAGCAG TGAGGTTGCA GCAGAGCCAG CAGGTGTTGA CTGCAGTCGA AGCCATGGAG GAGGACTCTG   
  
  
+ GGATAAGACT GGTCTACGCT CTGCTGACGT GTGCGGAGGC CATCCAACGT GGCGATTTCC GATTGGCTGT   
  
  
+ CTTGTTAGTT AACAAGATGA GCAATGACCT CCTGCCACGC GTCAACCCGT CCTGCGGTAT TGGCAAAGTA   
  
  
+ GCCGGCTACT TCATAGATGC CTTGACCCGA AGACTATACC AGCAGGGCCC AGTGTCGGGC CTAATCGGGT   
  
  
+ CGGTTCTGGC GTACCAGGTG TTGTACGAGC ACTTCTATGA AGCTTGCCCG TTCCTCAAAT TCGCTCACTT   
  
  
+ CACTGCTAAT CAAGCGATTT TAGAGGCATT CGACGGCCAC GATTGTGTCC ACATCATCGA CTTCGGCCTA   
  
  
+ ATCCACGGCT TGCAATGGCC AGCTCTAATC CAAGCCTTGG CTGTTCGGCA CGGTGGCCCG CCTTTCGTAA   
  
  
+ GATTAACCGG AATCGGGCCG CCTTCTGAAT ACGGGTCGTG CTCGCTTCAA CCTATCGGGT CAACACTGGC   
  
  
+ CCAGTTAGCC CTATCCATGA ATGTTGGGTT CGCATTTCGG GCCGTCGCCG TCTCACGGCT CGAGGACATC   
  
  
+ AAACCATGGA TGGTTAAAAC AAGCCCAAAT GAAGTCGTAG CCGTGAACTC TATCTTCCAA CTCCACCGGC   
  
  
+ TAATCGGGTC GGGTATTGAC CCTGTCCTAA ACTGGGTCCG GAGCCTAAAC CCGAAAATTG TGACACTGGC   
  
  
+ GGAGCAAGAG GCGAACCACA ACCAGCCCGA GTTTTTGGCC CGGTTCACGG AGGCATTACA TTACTATTCA   
  
  
+ ACTATGTTCG ACTCGTTAGA GGCTTGTCAA GTCCAGGCCG ACAAGGACCT AGCCGAGCTA TACTTAGAGA   
  
  
+ GAGAGTTATC CAACATCGTC TGCTGTGAGG GGTCGGCTCG GATTGAGAGG CACGAGCCGC TGGCCCAATG   
  
  
+ GAGGGCCCGA ATGGCTCGGG CCGGGTTCAA GAAGGTGGAT ATGGGTAAAA ATGCGTTCAA GCAAGTAAGC   
  
  
+ ATGTTGCTGA GTTTGTCTTC AGCACAAGGG TATTGTGTGG AGGAGAGTGA GGGATGTTTG AAGCTCGGCT   
  
  
+ GGCATGACCG CCCTCTCATT GCGGCTTCGG CTTGGCGAGC GGAGACTCAA GCTGAGAACT CCAGCACTGT   
  
  
+ TGTGCTTGAT GGGTCATCGT CGTGTAGTTC ATCTTCTTA  

- -Up\_Stream \_Len000TTTTTT CTTTCTTTAC CTCTTTGGTT CGTTATACCT ATTTTTTTTT TGTTTCATTA   
  
  
- TTTCCTTCCT ACTCCGAGGT GTAAGGTGAG CGGGCGCGGT GAGTATCCTC TACTTATCAT TTTACACTCC   
  
  
- CCAATTTACT AAACTTTTTT TAGATCATTC CCGGGTGTTG TCATAACCTT GGGTCGAACG GTTCCTTTAC   
  
  
- AGTCTCCCCG ATCGTTACCT GGTCACGTTC TTCCGGTTCG GTGTAAACCA ATCCCTTAAA GTAGGGTAGG   
  
  
- CCGCGATTTT CGTAGTTTTA ATGATGAATT AGGAACTAGT TTACGGGATA TTTATAAGAT GATGGGGTAA   
  
  
- ACTTTTCCCC ACAACTAATG TTGTGTATCG GCATCCAAAC TGTTTCTCTT AATGGGAATA ACAATCTTCG   
  
  
- TGGTATATGA GAAAAGAGAT TCGAGACTAG AAAAAAGTAG GAAAAGTCGA AAAACAGGAA AATTTAGTTT   
  
  
- AGTGAGTGAA CTCATAACCT CCCTGCAAGG AGTCCTCAGC TTGGTGATTT AATAGAAAAA TTTCTTTTTC   
  
  
- CCGAACTTCG GTTGGGCTAA ATTTTTTTGA CGATTATACA TTCGGATGAG AGAAAGGTCA AGGTTGTTTA   
  
  
- GTTTAAAGTC ATACTTTGTA GGTCATTTCC TAAATAGGCA CTATTACTAC ATTGTATACC AATTTCCCAG   
  
  
- TAAAAGTCAA TGTAGCCTCG TACGTTAATT TCAGGGATAT TTTATCCATC CACGTGCTTG TTCAGTGGTA   
  
  
- GCAGGAAACG AAAAACGTAC TCACGTACGA TTAATACTTG AATTAATTAT AGATTCGTTT TAATTTTTCA   
  
  
- TCAATTTTGA AAAAGTGGAA ACCTTGGAGC ATCGACATCG TCATCGACGG CGTGAGACCA AGATCTGAAG   
  
  
- ATCCCTGGTT CTCTACGTGA GTATACGTAG GAGAAGACAG GAAAATGACA TAGGGGAAGG GTACACTGGA   
  
  
- AAAACGGGCC CACTGAAAAG ACACTCACAA CTATGTTTAA AACATGCATT CGATTGAGTA AATGAAGTGG   
  
  
- CAGAGTACTC AAAATGTAAT GGCTATAATT AAATTTACCG TTTCATAATT TAAACTCGAT TTTAGAGTAT   
  
  
- ATATATAAAC ATAATTTACA CAGACCTAAC AGTTTTATTA AAAACTGTAT ATGTCAGTGA TATATGACTC   
  
  
- AATAATTTTA TATATTTTTA TACTTTTAAA TTTTTTATCA TCAAATTCCA CTGTTGTGAT TTTGTTTGTT   
  
  
- TATTCTATAT TTATCCCTTT TCAAGTAGGA TAAAACAATA ATAATAAATA ATACATAAAA GTCTTAACAG   
  
  
- AGATAGTGTT CGTATCATAT ACACAGTTGT AGCACTGTTG AGCTGGTTAG ATGTTTATTG ATCATTTAAG   
  
  
- TTTATTTCAA TGAGAGTGGA TCGAATTTAT TCATGTGGAA CTAAATTAAC AGAATCGATA CCTTGGATAA   
  
  
- GGTTTGATCG GTTATGAAGG AAAAGTTTTC TTAATCAAAA TTTCAAAATT GAAAATGCAG GTTATTACGG   
  
  
- CCGGCATGAT ACTCAACGAA AGTTCGGTCA TCATCATGAC ATCATCCATT TTTATAATCT TATGTGGATG   
  
  
- ATATATACTA TAACGAGAAG TGGCATAGGA AGACGTATGA GTAGAAAGGC AGGGCCTCAG GTGATAGAGA   
  
  
- GGAGGTAAGT CTGTGAGAGA GCGAGATCTC TTGAGTATCG ATACCTGGCG CACGGGCTGC CAATAGCCCA   
  
  
- ATACTTAAGC CTCGAAGAAG TCGTTCAATC GGGACTCAAC GGCCTAGGTT GGTGGACCAA GGTACGGGCA   
  
  
- GATGGGCTGG GGTAGAGAGT CAGAGGAGAA CCTGGGCCAG GTAGGAGGGT CGGTTGGGTA AAGATGGTCC   
  
  
- CAAGACGGAG TCCCTTGGTA CACCAACCCT AACGACTGTG CACCTGCCTG GTCTAACAAC GTCCAAGGAG   
  
  
- CAGGGTCGTC ACTCCAACGT CGTCTCGGTC GTCCACAACT GACGTCAGCT TCGGTACCTC CTCCTGAGAC   
  
  
- CCTATTCTGA CCAGATGCGA GACGACTGCA CACGCCTCCG GTAGGTTGCA CCGCTAAAGG CTAACCGACA   
  
  
- GAACAATCAA TTGTTCTACT CGTTACTGGA GGACGGTGCG CAGTTGGGCA GGACGCCATA ACCGTTTCAT   
  
  
- CGGCCGATGA AGTATCTACG GAACTGGGCT TCTGATATGG TCGTCCCGGG TCACAGCCCG GATTAGCCCA   
  
  
- GCCAAGACCG CATGGTCCAC AACATGCTCG TGAAGATACT TCGAACGGGC AAGGAGTTTA AGCGAGTGAA   
  
  
- GTGACGATTA GTTCGCTAAA ATCTCCGTAA GCTGCCGGTG CTAACACAGG TGTAGTAGCT GAAGCCGGAT   
  
  
- TAGGTGCCGA ACGTTACCGG TCGAGATTAG GTTCGGAACC GACAAGCCGT GCCACCGGGC GGAAAGCATT   
  
  
- CTAATTGGCC TTAGCCCGGC GGAAGACTTA TGCCCAGCAC GAGCGAAGTT GGATAGCCCA GTTGTGACCG   
  
  
- GGTCAATCGG GATAGGTACT TACAACCCAA GCGTAAAGCC CGGCAGCGGC AGAGTGCCGA GCTCCTGTAG   
  
  
- TTTGGTACCT ACCAATTTTG TTCGGGTTTA CTTCAGCATC GGCACTTGAG ATAGAAGGTT GAGGTGGCCG   
  
  
- ATTAGCCCAG CCCATAACTG GGACAGGATT TGACCCAGGC CTCGGATTTG GGCTTTTAAC ACTGTGACCG   
  
  
- CCTCGTTCTC CGCTTGGTGT TGGTCGGGCT CAAAAACCGG GCCAAGTGCC TCCGTAATGT AATGATAAGT   
  
  
- TGATACAAGC TGAGCAATCT CCGAACAGTT CAGGTCCGGC TGTTCCTGGA TCGGCTCGAT ATGAATCTCT   
  
  
- CTCTCAATAG GTTGTAGCAG ACGACACTCC CCAGCCGAGC CTAACTCTCC GTGCTCGGCG ACCGGGTTAC   
  
  
- CTCCCGGGCT TACCGAGCCC GGCCCAAGTT CTTCCACCTA TACCCATTTT TACGCAAGTT CGTTCATTCG   
  
  
- TACAACGACT CAAACAGAAG TCGTGTTCCC ATAACACACC TCCTCTCACT CCCTACAAAC TTCGAGCCGA   
  
  
- CCGTACTGGC GGGAGAGTAA CGCCGAAGCC GAACCGCTCG CCTCTGAGTT CGACTCTTGA GGTCGTGACA   
  
  
- ACACGAACTA CCCAGTAGCA GCACATCAAG TAGAAGAAT

+     AT~TATA-box

| Site Name | Organism | Position | Strand | Matrix score. | sequence | function |
| --- | --- | --- | --- | --- | --- | --- |
| AT~TATA-box | Arabidopsis thaliana | 1204 | + | 6 | TATATA |  |
| AT~TATA-box | Arabidopsis thaliana | 1125 | + | 6 | TATATA |  |
| AT~TATA-box | Arabidopsis thaliana | 1615 | - | 6 | TATATA |  |
| AT~TATA-box | Arabidopsis thaliana | 1123 | + | 6 | TATATA |  |
| AT~TATA-box | Arabidopsis thaliana | 1184 | + | 6 | TATATA |  |

>HU11G00778.1   
+ -Up\_Stream \_Len000AAAAAA GAAAGAAATG GAGAAACCAA GCAATATGGA TAAAAAAAAA ACAAAGTAAT   
  
  
+ AAAGGAAGGA TGAGGCTCCA CATTCCACTC GCCCGCGCCA CTCATAGGAG ATGAATAGTA AAATGTGAGG   
  
  
+ GGTTAAATGA TTTGAAAAAA ATCTAGTAAG GGCCCACAAC AGTATTGGAA CCCAGCTTGC CAAGGAAATG   
  
  
+ TCAGAGGGGC TAGCAATGGA CCAGTGCAAG AAGGCCAAGC CACATTTGGT TAGGGAATTT CATCCCATCC   
  
  
+ GGCGCTAAAA GCATCAAAAT TACTACTTAA TCCTTGATCA AATGCCCTAT AAATATTCTA CTACCCCATT   
  
  
+ TGAAAAGGGG TGTTGATTAC AACACATAGC CGTAGGTTTG ACAAAGAGAA TTACCCTTAT TGTTAGAAGC   
  
  
+ ACCATATACT CTTTTCTCTA AGCTCTGATC TTTTTTCATC CTTTTCAGCT TTTTGTCCTT TTAAATCAAA   
  
  
+ TCACTCACTT GAGTATTGGA GGGACGTTCC TCAGGAGTCG AACCACTAAA TTATCTTTTT AAAGAAAAAG   
  
  
+ GGCTTGAAGC CAACCCGATT TAAAAAAACT GCTAATATGT AAGCCTACTC TCTTTCCAGT TCCAACAAAT   
  
  
+ CAAATTTCAG TATGAAACAT CCAGTAAAGG ATTTATCCGT GATAATGATG TAACATATGG TTAAAGGGTC   
  
  
+ ATTTTCAGTT ACATCGGAGC ATGCAATTAA AGTCCCTATA AAATAGGTAG GTGCACGAAC AAGTCACCAT   
  
  
+ CGTCCTTTGC TTTTTGCATG AGTGCATGCT AATTATGAAC TTAATTAATA TCTAAGCAAA ATTAAAAAGT   
  
  
+ AGTTAAAACT TTTTCACCTT TGGAACCTCG TAGCTGTAGC AGTAGCTGCC GCACTCTGGT TCTAGACTTC   
  
  
+ TAGGGACCAA GAGATGCACT CATATGCATC CTCTTCTGTC CTTTTACTGT ATCCCCTTCC CATGTGACCT   
  
  
+ TTTTGCCCGG GTGACTTTTC TGTGAGTGTT GATACAAATT TTGTACGTAA GCTAACTCAT TTACTTCACC   
  
  
+ GTCTCATGAG TTTTACATTA CCGATATTAA TTTAAATGGC AAAGTATTAA ATTTGAGCTA AAATCTCATA   
  
  
+ TATATATTTG TATTAAATGT GTCTGGATTG TCAAAATAAT TTTTGACATA TACAGTCACT ATATACTGAG   
  
  
+ TTATTAAAAT ATATAAAAAT ATGAAAATTT AAAAAATAGT AGTTTAAGGT GACAACACTA AAACAAACAA   
  
  
+ ATAAGATATA AATAGGGAAA AGTTCATCCT ATTTTGTTAT TATTATTTAT TATGTATTTT CAGAATTGTC   
  
  
+ TCTATCACAA GCATAGTATA TGTGTCAACA TCGTGACAAC TCGACCAATC TACAAATAAC TAGTAAATTC   
  
  
+ AAATAAAGTT ACTCTCACCT AGCTTAAATA AGTACACCTT GATTTAATTG TCTTAGCTAT GGAACCTATT   
  
  
+ CCAAACTAGC CAATACTTCC TTTTCAAAAG AATTAGTTTT AAAGTTTTAA CTTTTACGTC CAATAATGCC   
  
  
+ GGCCGTACTA TGAGTTGCTT TCAAGCCAGT AGTAGTACTG TAGTAGGTAA AAATATTAGA ATACACCTAC   
  
  
+ TATATATGAT ATTGCTCTTC ACCGTATCCT TCTGCATACT CATCTTTCCG TCCCGGAGTC CACTATCTCT   
  
  
+ CCTCCATTCA GACACTCTCT CGCTCTAGAG AACTCATAGC TATGGACCGC GTGCCCGACG GTTATCGGGT   
  
  
+ TATGAATTCG GAGCTTCTTC AGCAAGTTAG CCCTGAGTTG CCGGATCCAA CCACCTGGTT CCATGCCCGT   
  
  
+ CTACCCGACC CCATCTCTCA GTCTCCTCTT GGACCCGGTC CATCCTCCCA GCCAACCCAT TTCTACCAGG   
  
  
+ GTTCTGCCTC AGGGAACCAT GTGGTTGGGA TTGCTGACAC GTGGACGGAC CAGATTGTTG CAGGTTCCTC   
  
  
+ GTCCCAGCAG TGAGGTTGCA GCAGAGCCAG CAGGTGTTGA CTGCAGTCGA AGCCATGGAG GAGGACTCTG   
  
  
+ GGATAAGACT GGTCTACGCT CTGCTGACGT GTGCGGAGGC CATCCAACGT GGCGATTTCC GATTGGCTGT   
  
  
+ CTTGTTAGTT AACAAGATGA GCAATGACCT CCTGCCACGC GTCAACCCGT CCTGCGGTAT TGGCAAAGTA   
  
  
+ GCCGGCTACT TCATAGATGC CTTGACCCGA AGACTATACC AGCAGGGCCC AGTGTCGGGC CTAATCGGGT   
  
  
+ CGGTTCTGGC GTACCAGGTG TTGTACGAGC ACTTCTATGA AGCTTGCCCG TTCCTCAAAT TCGCTCACTT   
  
  
+ CACTGCTAAT CAAGCGATTT TAGAGGCATT CGACGGCCAC GATTGTGTCC ACATCATCGA CTTCGGCCTA   
  
  
+ ATCCACGGCT TGCAATGGCC AGCTCTAATC CAAGCCTTGG CTGTTCGGCA CGGTGGCCCG CCTTTCGTAA   
  
  
+ GATTAACCGG AATCGGGCCG CCTTCTGAAT ACGGGTCGTG CTCGCTTCAA CCTATCGGGT CAACACTGGC   
  
  
+ CCAGTTAGCC CTATCCATGA ATGTTGGGTT CGCATTTCGG GCCGTCGCCG TCTCACGGCT CGAGGACATC   
  
  
+ AAACCATGGA TGGTTAAAAC AAGCCCAAAT GAAGTCGTAG CCGTGAACTC TATCTTCCAA CTCCACCGGC   
  
  
+ TAATCGGGTC GGGTATTGAC CCTGTCCTAA ACTGGGTCCG GAGCCTAAAC CCGAAAATTG TGACACTGGC   
  
  
+ GGAGCAAGAG GCGAACCACA ACCAGCCCGA GTTTTTGGCC CGGTTCACGG AGGCATTACA TTACTATTCA   
  
  
+ ACTATGTTCG ACTCGTTAGA GGCTTGTCAA GTCCAGGCCG ACAAGGACCT AGCCGAGCTA TACTTAGAGA   
  
  
+ GAGAGTTATC CAACATCGTC TGCTGTGAGG GGTCGGCTCG GATTGAGAGG CACGAGCCGC TGGCCCAATG   
  
  
+ GAGGGCCCGA ATGGCTCGGG CCGGGTTCAA GAAGGTGGAT ATGGGTAAAA ATGCGTTCAA GCAAGTAAGC   
  
  
+ ATGTTGCTGA GTTTGTCTTC AGCACAAGGG TATTGTGTGG AGGAGAGTGA GGGATGTTTG AAGCTCGGCT   
  
  
+ GGCATGACCG CCCTCTCATT GCGGCTTCGG CTTGGCGAGC GGAGACTCAA GCTGAGAACT CCAGCACTGT   
  
  
+ TGTGCTTGAT GGGTCATCGT CGTGTAGTTC ATCTTCTTA  

- -Up\_Stream \_Len000TTTTTT CTTTCTTTAC CTCTTTGGTT CGTTATACCT ATTTTTTTTT TGTTTCATTA   
  
  
- TTTCCTTCCT ACTCCGAGGT GTAAGGTGAG CGGGCGCGGT GAGTATCCTC TACTTATCAT TTTACACTCC   
  
  
- CCAATTTACT AAACTTTTTT TAGATCATTC CCGGGTGTTG TCATAACCTT GGGTCGAACG GTTCCTTTAC   
  
  
- AGTCTCCCCG ATCGTTACCT GGTCACGTTC TTCCGGTTCG GTGTAAACCA ATCCCTTAAA GTAGGGTAGG   
  
  
- CCGCGATTTT CGTAGTTTTA ATGATGAATT AGGAACTAGT TTACGGGATA TTTATAAGAT GATGGGGTAA   
  
  
- ACTTTTCCCC ACAACTAATG TTGTGTATCG GCATCCAAAC TGTTTCTCTT AATGGGAATA ACAATCTTCG   
  
  
- TGGTATATGA GAAAAGAGAT TCGAGACTAG AAAAAAGTAG GAAAAGTCGA AAAACAGGAA AATTTAGTTT   
  
  
- AGTGAGTGAA CTCATAACCT CCCTGCAAGG AGTCCTCAGC TTGGTGATTT AATAGAAAAA TTTCTTTTTC   
  
  
- CCGAACTTCG GTTGGGCTAA ATTTTTTTGA CGATTATACA TTCGGATGAG AGAAAGGTCA AGGTTGTTTA   
  
  
- GTTTAAAGTC ATACTTTGTA GGTCATTTCC TAAATAGGCA CTATTACTAC ATTGTATACC AATTTCCCAG   
  
  
- TAAAAGTCAA TGTAGCCTCG TACGTTAATT TCAGGGATAT TTTATCCATC CACGTGCTTG TTCAGTGGTA   
  
  
- GCAGGAAACG AAAAACGTAC TCACGTACGA TTAATACTTG AATTAATTAT AGATTCGTTT TAATTTTTCA   
  
  
- TCAATTTTGA AAAAGTGGAA ACCTTGGAGC ATCGACATCG TCATCGACGG CGTGAGACCA AGATCTGAAG   
  
  
- ATCCCTGGTT CTCTACGTGA GTATACGTAG GAGAAGACAG GAAAATGACA TAGGGGAAGG GTACACTGGA   
  
  
- AAAACGGGCC CACTGAAAAG ACACTCACAA CTATGTTTAA AACATGCATT CGATTGAGTA AATGAAGTGG   
  
  
- CAGAGTACTC AAAATGTAAT GGCTATAATT AAATTTACCG TTTCATAATT TAAACTCGAT TTTAGAGTAT   
  
  
- ATATATAAAC ATAATTTACA CAGACCTAAC AGTTTTATTA AAAACTGTAT ATGTCAGTGA TATATGACTC   
  
  
- AATAATTTTA TATATTTTTA TACTTTTAAA TTTTTTATCA TCAAATTCCA CTGTTGTGAT TTTGTTTGTT   
  
  
- TATTCTATAT TTATCCCTTT TCAAGTAGGA TAAAACAATA ATAATAAATA ATACATAAAA GTCTTAACAG   
  
  
- AGATAGTGTT CGTATCATAT ACACAGTTGT AGCACTGTTG AGCTGGTTAG ATGTTTATTG ATCATTTAAG   
  
  
- TTTATTTCAA TGAGAGTGGA TCGAATTTAT TCATGTGGAA CTAAATTAAC AGAATCGATA CCTTGGATAA   
  
  
- GGTTTGATCG GTTATGAAGG AAAAGTTTTC TTAATCAAAA TTTCAAAATT GAAAATGCAG GTTATTACGG   
  
  
- CCGGCATGAT ACTCAACGAA AGTTCGGTCA TCATCATGAC ATCATCCATT TTTATAATCT TATGTGGATG   
  
  
- ATATATACTA TAACGAGAAG TGGCATAGGA AGACGTATGA GTAGAAAGGC AGGGCCTCAG GTGATAGAGA   
  
  
- GGAGGTAAGT CTGTGAGAGA GCGAGATCTC TTGAGTATCG ATACCTGGCG CACGGGCTGC CAATAGCCCA   
  
  
- ATACTTAAGC CTCGAAGAAG TCGTTCAATC GGGACTCAAC GGCCTAGGTT GGTGGACCAA GGTACGGGCA   
  
  
- GATGGGCTGG GGTAGAGAGT CAGAGGAGAA CCTGGGCCAG GTAGGAGGGT CGGTTGGGTA AAGATGGTCC   
  
  
- CAAGACGGAG TCCCTTGGTA CACCAACCCT AACGACTGTG CACCTGCCTG GTCTAACAAC GTCCAAGGAG   
  
  
- CAGGGTCGTC ACTCCAACGT CGTCTCGGTC GTCCACAACT GACGTCAGCT TCGGTACCTC CTCCTGAGAC   
  
  
- CCTATTCTGA CCAGATGCGA GACGACTGCA CACGCCTCCG GTAGGTTGCA CCGCTAAAGG CTAACCGACA   
  
  
- GAACAATCAA TTGTTCTACT CGTTACTGGA GGACGGTGCG CAGTTGGGCA GGACGCCATA ACCGTTTCAT   
  
  
- CGGCCGATGA AGTATCTACG GAACTGGGCT TCTGATATGG TCGTCCCGGG TCACAGCCCG GATTAGCCCA   
  
  
- GCCAAGACCG CATGGTCCAC AACATGCTCG TGAAGATACT TCGAACGGGC AAGGAGTTTA AGCGAGTGAA   
  
  
- GTGACGATTA GTTCGCTAAA ATCTCCGTAA GCTGCCGGTG CTAACACAGG TGTAGTAGCT GAAGCCGGAT   
  
  
- TAGGTGCCGA ACGTTACCGG TCGAGATTAG GTTCGGAACC GACAAGCCGT GCCACCGGGC GGAAAGCATT   
  
  
- CTAATTGGCC TTAGCCCGGC GGAAGACTTA TGCCCAGCAC GAGCGAAGTT GGATAGCCCA GTTGTGACCG   
  
  
- GGTCAATCGG GATAGGTACT TACAACCCAA GCGTAAAGCC CGGCAGCGGC AGAGTGCCGA GCTCCTGTAG   
  
  
- TTTGGTACCT ACCAATTTTG TTCGGGTTTA CTTCAGCATC GGCACTTGAG ATAGAAGGTT GAGGTGGCCG   
  
  
- ATTAGCCCAG CCCATAACTG GGACAGGATT TGACCCAGGC CTCGGATTTG GGCTTTTAAC ACTGTGACCG   
  
  
- CCTCGTTCTC CGCTTGGTGT TGGTCGGGCT CAAAAACCGG GCCAAGTGCC TCCGTAATGT AATGATAAGT   
  
  
- TGATACAAGC TGAGCAATCT CCGAACAGTT CAGGTCCGGC TGTTCCTGGA TCGGCTCGAT ATGAATCTCT   
  
  
- CTCTCAATAG GTTGTAGCAG ACGACACTCC CCAGCCGAGC CTAACTCTCC GTGCTCGGCG ACCGGGTTAC   
  
  
- CTCCCGGGCT TACCGAGCCC GGCCCAAGTT CTTCCACCTA TACCCATTTT TACGCAAGTT CGTTCATTCG   
  
  
- TACAACGACT CAAACAGAAG TCGTGTTCCC ATAACACACC TCCTCTCACT CCCTACAAAC TTCGAGCCGA   
  
  
- CCGTACTGGC GGGAGAGTAA CGCCGAAGCC GAACCGCTCG CCTCTGAGTT CGACTCTTGA GGTCGTGACA   
  
  
- ACACGAACTA CCCAGTAGCA GCACATCAAG TAGAAGAAT

+     AuxRR-core

| Site Name | Organism | Position | Strand | Matrix score. | sequence | function |
| --- | --- | --- | --- | --- | --- | --- |
| AuxRR-core | Nicotiana tabacum | 230 | - | 7 | GGTCCAT | cis-acting regulatory element involved in auxin responsiveness |
| AuxRR-core | Nicotiana tabacum | 1726 | - | 7 | GGTCCAT | cis-acting regulatory element involved in auxin responsiveness |
| AuxRR-core | Nicotiana tabacum | 1861 | + | 7 | GGTCCAT | cis-acting regulatory element involved in auxin responsiveness |

>HU11G00778.1   
+ -Up\_Stream \_Len000AAAAAA GAAAGAAATG GAGAAACCAA GCAATATGGA TAAAAAAAAA ACAAAGTAAT   
  
  
+ AAAGGAAGGA TGAGGCTCCA CATTCCACTC GCCCGCGCCA CTCATAGGAG ATGAATAGTA AAATGTGAGG   
  
  
+ GGTTAAATGA TTTGAAAAAA ATCTAGTAAG GGCCCACAAC AGTATTGGAA CCCAGCTTGC CAAGGAAATG   
  
  
+ TCAGAGGGGC TAGCAATGGA CCAGTGCAAG AAGGCCAAGC CACATTTGGT TAGGGAATTT CATCCCATCC   
  
  
+ GGCGCTAAAA GCATCAAAAT TACTACTTAA TCCTTGATCA AATGCCCTAT AAATATTCTA CTACCCCATT   
  
  
+ TGAAAAGGGG TGTTGATTAC AACACATAGC CGTAGGTTTG ACAAAGAGAA TTACCCTTAT TGTTAGAAGC   
  
  
+ ACCATATACT CTTTTCTCTA AGCTCTGATC TTTTTTCATC CTTTTCAGCT TTTTGTCCTT TTAAATCAAA   
  
  
+ TCACTCACTT GAGTATTGGA GGGACGTTCC TCAGGAGTCG AACCACTAAA TTATCTTTTT AAAGAAAAAG   
  
  
+ GGCTTGAAGC CAACCCGATT TAAAAAAACT GCTAATATGT AAGCCTACTC TCTTTCCAGT TCCAACAAAT   
  
  
+ CAAATTTCAG TATGAAACAT CCAGTAAAGG ATTTATCCGT GATAATGATG TAACATATGG TTAAAGGGTC   
  
  
+ ATTTTCAGTT ACATCGGAGC ATGCAATTAA AGTCCCTATA AAATAGGTAG GTGCACGAAC AAGTCACCAT   
  
  
+ CGTCCTTTGC TTTTTGCATG AGTGCATGCT AATTATGAAC TTAATTAATA TCTAAGCAAA ATTAAAAAGT   
  
  
+ AGTTAAAACT TTTTCACCTT TGGAACCTCG TAGCTGTAGC AGTAGCTGCC GCACTCTGGT TCTAGACTTC   
  
  
+ TAGGGACCAA GAGATGCACT CATATGCATC CTCTTCTGTC CTTTTACTGT ATCCCCTTCC CATGTGACCT   
  
  
+ TTTTGCCCGG GTGACTTTTC TGTGAGTGTT GATACAAATT TTGTACGTAA GCTAACTCAT TTACTTCACC   
  
  
+ GTCTCATGAG TTTTACATTA CCGATATTAA TTTAAATGGC AAAGTATTAA ATTTGAGCTA AAATCTCATA   
  
  
+ TATATATTTG TATTAAATGT GTCTGGATTG TCAAAATAAT TTTTGACATA TACAGTCACT ATATACTGAG   
  
  
+ TTATTAAAAT ATATAAAAAT ATGAAAATTT AAAAAATAGT AGTTTAAGGT GACAACACTA AAACAAACAA   
  
  
+ ATAAGATATA AATAGGGAAA AGTTCATCCT ATTTTGTTAT TATTATTTAT TATGTATTTT CAGAATTGTC   
  
  
+ TCTATCACAA GCATAGTATA TGTGTCAACA TCGTGACAAC TCGACCAATC TACAAATAAC TAGTAAATTC   
  
  
+ AAATAAAGTT ACTCTCACCT AGCTTAAATA AGTACACCTT GATTTAATTG TCTTAGCTAT GGAACCTATT   
  
  
+ CCAAACTAGC CAATACTTCC TTTTCAAAAG AATTAGTTTT AAAGTTTTAA CTTTTACGTC CAATAATGCC   
  
  
+ GGCCGTACTA TGAGTTGCTT TCAAGCCAGT AGTAGTACTG TAGTAGGTAA AAATATTAGA ATACACCTAC   
  
  
+ TATATATGAT ATTGCTCTTC ACCGTATCCT TCTGCATACT CATCTTTCCG TCCCGGAGTC CACTATCTCT   
  
  
+ CCTCCATTCA GACACTCTCT CGCTCTAGAG AACTCATAGC TATGGACCGC GTGCCCGACG GTTATCGGGT   
  
  
+ TATGAATTCG GAGCTTCTTC AGCAAGTTAG CCCTGAGTTG CCGGATCCAA CCACCTGGTT CCATGCCCGT   
  
  
+ CTACCCGACC CCATCTCTCA GTCTCCTCTT GGACCCGGTC CATCCTCCCA GCCAACCCAT TTCTACCAGG   
  
  
+ GTTCTGCCTC AGGGAACCAT GTGGTTGGGA TTGCTGACAC GTGGACGGAC CAGATTGTTG CAGGTTCCTC   
  
  
+ GTCCCAGCAG TGAGGTTGCA GCAGAGCCAG CAGGTGTTGA CTGCAGTCGA AGCCATGGAG GAGGACTCTG   
  
  
+ GGATAAGACT GGTCTACGCT CTGCTGACGT GTGCGGAGGC CATCCAACGT GGCGATTTCC GATTGGCTGT   
  
  
+ CTTGTTAGTT AACAAGATGA GCAATGACCT CCTGCCACGC GTCAACCCGT CCTGCGGTAT TGGCAAAGTA   
  
  
+ GCCGGCTACT TCATAGATGC CTTGACCCGA AGACTATACC AGCAGGGCCC AGTGTCGGGC CTAATCGGGT   
  
  
+ CGGTTCTGGC GTACCAGGTG TTGTACGAGC ACTTCTATGA AGCTTGCCCG TTCCTCAAAT TCGCTCACTT   
  
  
+ CACTGCTAAT CAAGCGATTT TAGAGGCATT CGACGGCCAC GATTGTGTCC ACATCATCGA CTTCGGCCTA   
  
  
+ ATCCACGGCT TGCAATGGCC AGCTCTAATC CAAGCCTTGG CTGTTCGGCA CGGTGGCCCG CCTTTCGTAA   
  
  
+ GATTAACCGG AATCGGGCCG CCTTCTGAAT ACGGGTCGTG CTCGCTTCAA CCTATCGGGT CAACACTGGC   
  
  
+ CCAGTTAGCC CTATCCATGA ATGTTGGGTT CGCATTTCGG GCCGTCGCCG TCTCACGGCT CGAGGACATC   
  
  
+ AAACCATGGA TGGTTAAAAC AAGCCCAAAT GAAGTCGTAG CCGTGAACTC TATCTTCCAA CTCCACCGGC   
  
  
+ TAATCGGGTC GGGTATTGAC CCTGTCCTAA ACTGGGTCCG GAGCCTAAAC CCGAAAATTG TGACACTGGC   
  
  
+ GGAGCAAGAG GCGAACCACA ACCAGCCCGA GTTTTTGGCC CGGTTCACGG AGGCATTACA TTACTATTCA   
  
  
+ ACTATGTTCG ACTCGTTAGA GGCTTGTCAA GTCCAGGCCG ACAAGGACCT AGCCGAGCTA TACTTAGAGA   
  
  
+ GAGAGTTATC CAACATCGTC TGCTGTGAGG GGTCGGCTCG GATTGAGAGG CACGAGCCGC TGGCCCAATG   
  
  
+ GAGGGCCCGA ATGGCTCGGG CCGGGTTCAA GAAGGTGGAT ATGGGTAAAA ATGCGTTCAA GCAAGTAAGC   
  
  
+ ATGTTGCTGA GTTTGTCTTC AGCACAAGGG TATTGTGTGG AGGAGAGTGA GGGATGTTTG AAGCTCGGCT   
  
  
+ GGCATGACCG CCCTCTCATT GCGGCTTCGG CTTGGCGAGC GGAGACTCAA GCTGAGAACT CCAGCACTGT   
  
  
+ TGTGCTTGAT GGGTCATCGT CGTGTAGTTC ATCTTCTTA  

- -Up\_Stream \_Len000TTTTTT CTTTCTTTAC CTCTTTGGTT CGTTATACCT ATTTTTTTTT TGTTTCATTA   
  
  
- TTTCCTTCCT ACTCCGAGGT GTAAGGTGAG CGGGCGCGGT GAGTATCCTC TACTTATCAT TTTACACTCC   
  
  
- CCAATTTACT AAACTTTTTT TAGATCATTC CCGGGTGTTG TCATAACCTT GGGTCGAACG GTTCCTTTAC   
  
  
- AGTCTCCCCG ATCGTTACCT GGTCACGTTC TTCCGGTTCG GTGTAAACCA ATCCCTTAAA GTAGGGTAGG   
  
  
- CCGCGATTTT CGTAGTTTTA ATGATGAATT AGGAACTAGT TTACGGGATA TTTATAAGAT GATGGGGTAA   
  
  
- ACTTTTCCCC ACAACTAATG TTGTGTATCG GCATCCAAAC TGTTTCTCTT AATGGGAATA ACAATCTTCG   
  
  
- TGGTATATGA GAAAAGAGAT TCGAGACTAG AAAAAAGTAG GAAAAGTCGA AAAACAGGAA AATTTAGTTT   
  
  
- AGTGAGTGAA CTCATAACCT CCCTGCAAGG AGTCCTCAGC TTGGTGATTT AATAGAAAAA TTTCTTTTTC   
  
  
- CCGAACTTCG GTTGGGCTAA ATTTTTTTGA CGATTATACA TTCGGATGAG AGAAAGGTCA AGGTTGTTTA   
  
  
- GTTTAAAGTC ATACTTTGTA GGTCATTTCC TAAATAGGCA CTATTACTAC ATTGTATACC AATTTCCCAG   
  
  
- TAAAAGTCAA TGTAGCCTCG TACGTTAATT TCAGGGATAT TTTATCCATC CACGTGCTTG TTCAGTGGTA   
  
  
- GCAGGAAACG AAAAACGTAC TCACGTACGA TTAATACTTG AATTAATTAT AGATTCGTTT TAATTTTTCA   
  
  
- TCAATTTTGA AAAAGTGGAA ACCTTGGAGC ATCGACATCG TCATCGACGG CGTGAGACCA AGATCTGAAG   
  
  
- ATCCCTGGTT CTCTACGTGA GTATACGTAG GAGAAGACAG GAAAATGACA TAGGGGAAGG GTACACTGGA   
  
  
- AAAACGGGCC CACTGAAAAG ACACTCACAA CTATGTTTAA AACATGCATT CGATTGAGTA AATGAAGTGG   
  
  
- CAGAGTACTC AAAATGTAAT GGCTATAATT AAATTTACCG TTTCATAATT TAAACTCGAT TTTAGAGTAT   
  
  
- ATATATAAAC ATAATTTACA CAGACCTAAC AGTTTTATTA AAAACTGTAT ATGTCAGTGA TATATGACTC   
  
  
- AATAATTTTA TATATTTTTA TACTTTTAAA TTTTTTATCA TCAAATTCCA CTGTTGTGAT TTTGTTTGTT   
  
  
- TATTCTATAT TTATCCCTTT TCAAGTAGGA TAAAACAATA ATAATAAATA ATACATAAAA GTCTTAACAG   
  
  
- AGATAGTGTT CGTATCATAT ACACAGTTGT AGCACTGTTG AGCTGGTTAG ATGTTTATTG ATCATTTAAG   
  
  
- TTTATTTCAA TGAGAGTGGA TCGAATTTAT TCATGTGGAA CTAAATTAAC AGAATCGATA CCTTGGATAA   
  
  
- GGTTTGATCG GTTATGAAGG AAAAGTTTTC TTAATCAAAA TTTCAAAATT GAAAATGCAG GTTATTACGG   
  
  
- CCGGCATGAT ACTCAACGAA AGTTCGGTCA TCATCATGAC ATCATCCATT TTTATAATCT TATGTGGATG   
  
  
- ATATATACTA TAACGAGAAG TGGCATAGGA AGACGTATGA GTAGAAAGGC AGGGCCTCAG GTGATAGAGA   
  
  
- GGAGGTAAGT CTGTGAGAGA GCGAGATCTC TTGAGTATCG ATACCTGGCG CACGGGCTGC CAATAGCCCA   
  
  
- ATACTTAAGC CTCGAAGAAG TCGTTCAATC GGGACTCAAC GGCCTAGGTT GGTGGACCAA GGTACGGGCA   
  
  
- GATGGGCTGG GGTAGAGAGT CAGAGGAGAA CCTGGGCCAG GTAGGAGGGT CGGTTGGGTA AAGATGGTCC   
  
  
- CAAGACGGAG TCCCTTGGTA CACCAACCCT AACGACTGTG CACCTGCCTG GTCTAACAAC GTCCAAGGAG   
  
  
- CAGGGTCGTC ACTCCAACGT CGTCTCGGTC GTCCACAACT GACGTCAGCT TCGGTACCTC CTCCTGAGAC   
  
  
- CCTATTCTGA CCAGATGCGA GACGACTGCA CACGCCTCCG GTAGGTTGCA CCGCTAAAGG CTAACCGACA   
  
  
- GAACAATCAA TTGTTCTACT CGTTACTGGA GGACGGTGCG CAGTTGGGCA GGACGCCATA ACCGTTTCAT   
  
  
- CGGCCGATGA AGTATCTACG GAACTGGGCT TCTGATATGG TCGTCCCGGG TCACAGCCCG GATTAGCCCA   
  
  
- GCCAAGACCG CATGGTCCAC AACATGCTCG TGAAGATACT TCGAACGGGC AAGGAGTTTA AGCGAGTGAA   
  
  
- GTGACGATTA GTTCGCTAAA ATCTCCGTAA GCTGCCGGTG CTAACACAGG TGTAGTAGCT GAAGCCGGAT   
  
  
- TAGGTGCCGA ACGTTACCGG TCGAGATTAG GTTCGGAACC GACAAGCCGT GCCACCGGGC GGAAAGCATT   
  
  
- CTAATTGGCC TTAGCCCGGC GGAAGACTTA TGCCCAGCAC GAGCGAAGTT GGATAGCCCA GTTGTGACCG   
  
  
- GGTCAATCGG GATAGGTACT TACAACCCAA GCGTAAAGCC CGGCAGCGGC AGAGTGCCGA GCTCCTGTAG   
  
  
- TTTGGTACCT ACCAATTTTG TTCGGGTTTA CTTCAGCATC GGCACTTGAG ATAGAAGGTT GAGGTGGCCG   
  
  
- ATTAGCCCAG CCCATAACTG GGACAGGATT TGACCCAGGC CTCGGATTTG GGCTTTTAAC ACTGTGACCG   
  
  
- CCTCGTTCTC CGCTTGGTGT TGGTCGGGCT CAAAAACCGG GCCAAGTGCC TCCGTAATGT AATGATAAGT   
  
  
- TGATACAAGC TGAGCAATCT CCGAACAGTT CAGGTCCGGC TGTTCCTGGA TCGGCTCGAT ATGAATCTCT   
  
  
- CTCTCAATAG GTTGTAGCAG ACGACACTCC CCAGCCGAGC CTAACTCTCC GTGCTCGGCG ACCGGGTTAC   
  
  
- CTCCCGGGCT TACCGAGCCC GGCCCAAGTT CTTCCACCTA TACCCATTTT TACGCAAGTT CGTTCATTCG   
  
  
- TACAACGACT CAAACAGAAG TCGTGTTCCC ATAACACACC TCCTCTCACT CCCTACAAAC TTCGAGCCGA   
  
  
- CCGTACTGGC GGGAGAGTAA CGCCGAAGCC GAACCGCTCG CCTCTGAGTT CGACTCTTGA GGTCGTGACA   
  
  
- ACACGAACTA CCCAGTAGCA GCACATCAAG TAGAAGAAT

+     Box 4

| Site Name | Organism | Position | Strand | Matrix score. | sequence | function |
| --- | --- | --- | --- | --- | --- | --- |
| Box 4 | Petroselinum crispum | 818 | + | 6 | ATTAAT | part of a conserved DNA module involved in light responsiveness |
| Box 4 | Petroselinum crispum | 1080 | + | 6 | ATTAAT | part of a conserved DNA module involved in light responsiveness |

>HU11G00778.1   
+ -Up\_Stream \_Len000AAAAAA GAAAGAAATG GAGAAACCAA GCAATATGGA TAAAAAAAAA ACAAAGTAAT   
  
  
+ AAAGGAAGGA TGAGGCTCCA CATTCCACTC GCCCGCGCCA CTCATAGGAG ATGAATAGTA AAATGTGAGG   
  
  
+ GGTTAAATGA TTTGAAAAAA ATCTAGTAAG GGCCCACAAC AGTATTGGAA CCCAGCTTGC CAAGGAAATG   
  
  
+ TCAGAGGGGC TAGCAATGGA CCAGTGCAAG AAGGCCAAGC CACATTTGGT TAGGGAATTT CATCCCATCC   
  
  
+ GGCGCTAAAA GCATCAAAAT TACTACTTAA TCCTTGATCA AATGCCCTAT AAATATTCTA CTACCCCATT   
  
  
+ TGAAAAGGGG TGTTGATTAC AACACATAGC CGTAGGTTTG ACAAAGAGAA TTACCCTTAT TGTTAGAAGC   
  
  
+ ACCATATACT CTTTTCTCTA AGCTCTGATC TTTTTTCATC CTTTTCAGCT TTTTGTCCTT TTAAATCAAA   
  
  
+ TCACTCACTT GAGTATTGGA GGGACGTTCC TCAGGAGTCG AACCACTAAA TTATCTTTTT AAAGAAAAAG   
  
  
+ GGCTTGAAGC CAACCCGATT TAAAAAAACT GCTAATATGT AAGCCTACTC TCTTTCCAGT TCCAACAAAT   
  
  
+ CAAATTTCAG TATGAAACAT CCAGTAAAGG ATTTATCCGT GATAATGATG TAACATATGG TTAAAGGGTC   
  
  
+ ATTTTCAGTT ACATCGGAGC ATGCAATTAA AGTCCCTATA AAATAGGTAG GTGCACGAAC AAGTCACCAT   
  
  
+ CGTCCTTTGC TTTTTGCATG AGTGCATGCT AATTATGAAC TTAATTAATA TCTAAGCAAA ATTAAAAAGT   
  
  
+ AGTTAAAACT TTTTCACCTT TGGAACCTCG TAGCTGTAGC AGTAGCTGCC GCACTCTGGT TCTAGACTTC   
  
  
+ TAGGGACCAA GAGATGCACT CATATGCATC CTCTTCTGTC CTTTTACTGT ATCCCCTTCC CATGTGACCT   
  
  
+ TTTTGCCCGG GTGACTTTTC TGTGAGTGTT GATACAAATT TTGTACGTAA GCTAACTCAT TTACTTCACC   
  
  
+ GTCTCATGAG TTTTACATTA CCGATATTAA TTTAAATGGC AAAGTATTAA ATTTGAGCTA AAATCTCATA   
  
  
+ TATATATTTG TATTAAATGT GTCTGGATTG TCAAAATAAT TTTTGACATA TACAGTCACT ATATACTGAG   
  
  
+ TTATTAAAAT ATATAAAAAT ATGAAAATTT AAAAAATAGT AGTTTAAGGT GACAACACTA AAACAAACAA   
  
  
+ ATAAGATATA AATAGGGAAA AGTTCATCCT ATTTTGTTAT TATTATTTAT TATGTATTTT CAGAATTGTC   
  
  
+ TCTATCACAA GCATAGTATA TGTGTCAACA TCGTGACAAC TCGACCAATC TACAAATAAC TAGTAAATTC   
  
  
+ AAATAAAGTT ACTCTCACCT AGCTTAAATA AGTACACCTT GATTTAATTG TCTTAGCTAT GGAACCTATT   
  
  
+ CCAAACTAGC CAATACTTCC TTTTCAAAAG AATTAGTTTT AAAGTTTTAA CTTTTACGTC CAATAATGCC   
  
  
+ GGCCGTACTA TGAGTTGCTT TCAAGCCAGT AGTAGTACTG TAGTAGGTAA AAATATTAGA ATACACCTAC   
  
  
+ TATATATGAT ATTGCTCTTC ACCGTATCCT TCTGCATACT CATCTTTCCG TCCCGGAGTC CACTATCTCT   
  
  
+ CCTCCATTCA GACACTCTCT CGCTCTAGAG AACTCATAGC TATGGACCGC GTGCCCGACG GTTATCGGGT   
  
  
+ TATGAATTCG GAGCTTCTTC AGCAAGTTAG CCCTGAGTTG CCGGATCCAA CCACCTGGTT CCATGCCCGT   
  
  
+ CTACCCGACC CCATCTCTCA GTCTCCTCTT GGACCCGGTC CATCCTCCCA GCCAACCCAT TTCTACCAGG   
  
  
+ GTTCTGCCTC AGGGAACCAT GTGGTTGGGA TTGCTGACAC GTGGACGGAC CAGATTGTTG CAGGTTCCTC   
  
  
+ GTCCCAGCAG TGAGGTTGCA GCAGAGCCAG CAGGTGTTGA CTGCAGTCGA AGCCATGGAG GAGGACTCTG   
  
  
+ GGATAAGACT GGTCTACGCT CTGCTGACGT GTGCGGAGGC CATCCAACGT GGCGATTTCC GATTGGCTGT   
  
  
+ CTTGTTAGTT AACAAGATGA GCAATGACCT CCTGCCACGC GTCAACCCGT CCTGCGGTAT TGGCAAAGTA   
  
  
+ GCCGGCTACT TCATAGATGC CTTGACCCGA AGACTATACC AGCAGGGCCC AGTGTCGGGC CTAATCGGGT   
  
  
+ CGGTTCTGGC GTACCAGGTG TTGTACGAGC ACTTCTATGA AGCTTGCCCG TTCCTCAAAT TCGCTCACTT   
  
  
+ CACTGCTAAT CAAGCGATTT TAGAGGCATT CGACGGCCAC GATTGTGTCC ACATCATCGA CTTCGGCCTA   
  
  
+ ATCCACGGCT TGCAATGGCC AGCTCTAATC CAAGCCTTGG CTGTTCGGCA CGGTGGCCCG CCTTTCGTAA   
  
  
+ GATTAACCGG AATCGGGCCG CCTTCTGAAT ACGGGTCGTG CTCGCTTCAA CCTATCGGGT CAACACTGGC   
  
  
+ CCAGTTAGCC CTATCCATGA ATGTTGGGTT CGCATTTCGG GCCGTCGCCG TCTCACGGCT CGAGGACATC   
  
  
+ AAACCATGGA TGGTTAAAAC AAGCCCAAAT GAAGTCGTAG CCGTGAACTC TATCTTCCAA CTCCACCGGC   
  
  
+ TAATCGGGTC GGGTATTGAC CCTGTCCTAA ACTGGGTCCG GAGCCTAAAC CCGAAAATTG TGACACTGGC   
  
  
+ GGAGCAAGAG GCGAACCACA ACCAGCCCGA GTTTTTGGCC CGGTTCACGG AGGCATTACA TTACTATTCA   
  
  
+ ACTATGTTCG ACTCGTTAGA GGCTTGTCAA GTCCAGGCCG ACAAGGACCT AGCCGAGCTA TACTTAGAGA   
  
  
+ GAGAGTTATC CAACATCGTC TGCTGTGAGG GGTCGGCTCG GATTGAGAGG CACGAGCCGC TGGCCCAATG   
  
  
+ GAGGGCCCGA ATGGCTCGGG CCGGGTTCAA GAAGGTGGAT ATGGGTAAAA ATGCGTTCAA GCAAGTAAGC   
  
  
+ ATGTTGCTGA GTTTGTCTTC AGCACAAGGG TATTGTGTGG AGGAGAGTGA GGGATGTTTG AAGCTCGGCT   
  
  
+ GGCATGACCG CCCTCTCATT GCGGCTTCGG CTTGGCGAGC GGAGACTCAA GCTGAGAACT CCAGCACTGT   
  
  
+ TGTGCTTGAT GGGTCATCGT CGTGTAGTTC ATCTTCTTA  

- -Up\_Stream \_Len000TTTTTT CTTTCTTTAC CTCTTTGGTT CGTTATACCT ATTTTTTTTT TGTTTCATTA   
  
  
- TTTCCTTCCT ACTCCGAGGT GTAAGGTGAG CGGGCGCGGT GAGTATCCTC TACTTATCAT TTTACACTCC   
  
  
- CCAATTTACT AAACTTTTTT TAGATCATTC CCGGGTGTTG TCATAACCTT GGGTCGAACG GTTCCTTTAC   
  
  
- AGTCTCCCCG ATCGTTACCT GGTCACGTTC TTCCGGTTCG GTGTAAACCA ATCCCTTAAA GTAGGGTAGG   
  
  
- CCGCGATTTT CGTAGTTTTA ATGATGAATT AGGAACTAGT TTACGGGATA TTTATAAGAT GATGGGGTAA   
  
  
- ACTTTTCCCC ACAACTAATG TTGTGTATCG GCATCCAAAC TGTTTCTCTT AATGGGAATA ACAATCTTCG   
  
  
- TGGTATATGA GAAAAGAGAT TCGAGACTAG AAAAAAGTAG GAAAAGTCGA AAAACAGGAA AATTTAGTTT   
  
  
- AGTGAGTGAA CTCATAACCT CCCTGCAAGG AGTCCTCAGC TTGGTGATTT AATAGAAAAA TTTCTTTTTC   
  
  
- CCGAACTTCG GTTGGGCTAA ATTTTTTTGA CGATTATACA TTCGGATGAG AGAAAGGTCA AGGTTGTTTA   
  
  
- GTTTAAAGTC ATACTTTGTA GGTCATTTCC TAAATAGGCA CTATTACTAC ATTGTATACC AATTTCCCAG   
  
  
- TAAAAGTCAA TGTAGCCTCG TACGTTAATT TCAGGGATAT TTTATCCATC CACGTGCTTG TTCAGTGGTA   
  
  
- GCAGGAAACG AAAAACGTAC TCACGTACGA TTAATACTTG AATTAATTAT AGATTCGTTT TAATTTTTCA   
  
  
- TCAATTTTGA AAAAGTGGAA ACCTTGGAGC ATCGACATCG TCATCGACGG CGTGAGACCA AGATCTGAAG   
  
  
- ATCCCTGGTT CTCTACGTGA GTATACGTAG GAGAAGACAG GAAAATGACA TAGGGGAAGG GTACACTGGA   
  
  
- AAAACGGGCC CACTGAAAAG ACACTCACAA CTATGTTTAA AACATGCATT CGATTGAGTA AATGAAGTGG   
  
  
- CAGAGTACTC AAAATGTAAT GGCTATAATT AAATTTACCG TTTCATAATT TAAACTCGAT TTTAGAGTAT   
  
  
- ATATATAAAC ATAATTTACA CAGACCTAAC AGTTTTATTA AAAACTGTAT ATGTCAGTGA TATATGACTC   
  
  
- AATAATTTTA TATATTTTTA TACTTTTAAA TTTTTTATCA TCAAATTCCA CTGTTGTGAT TTTGTTTGTT   
  
  
- TATTCTATAT TTATCCCTTT TCAAGTAGGA TAAAACAATA ATAATAAATA ATACATAAAA GTCTTAACAG   
  
  
- AGATAGTGTT CGTATCATAT ACACAGTTGT AGCACTGTTG AGCTGGTTAG ATGTTTATTG ATCATTTAAG   
  
  
- TTTATTTCAA TGAGAGTGGA TCGAATTTAT TCATGTGGAA CTAAATTAAC AGAATCGATA CCTTGGATAA   
  
  
- GGTTTGATCG GTTATGAAGG AAAAGTTTTC TTAATCAAAA TTTCAAAATT GAAAATGCAG GTTATTACGG   
  
  
- CCGGCATGAT ACTCAACGAA AGTTCGGTCA TCATCATGAC ATCATCCATT TTTATAATCT TATGTGGATG   
  
  
- ATATATACTA TAACGAGAAG TGGCATAGGA AGACGTATGA GTAGAAAGGC AGGGCCTCAG GTGATAGAGA   
  
  
- GGAGGTAAGT CTGTGAGAGA GCGAGATCTC TTGAGTATCG ATACCTGGCG CACGGGCTGC CAATAGCCCA   
  
  
- ATACTTAAGC CTCGAAGAAG TCGTTCAATC GGGACTCAAC GGCCTAGGTT GGTGGACCAA GGTACGGGCA   
  
  
- GATGGGCTGG GGTAGAGAGT CAGAGGAGAA CCTGGGCCAG GTAGGAGGGT CGGTTGGGTA AAGATGGTCC   
  
  
- CAAGACGGAG TCCCTTGGTA CACCAACCCT AACGACTGTG CACCTGCCTG GTCTAACAAC GTCCAAGGAG   
  
  
- CAGGGTCGTC ACTCCAACGT CGTCTCGGTC GTCCACAACT GACGTCAGCT TCGGTACCTC CTCCTGAGAC   
  
  
- CCTATTCTGA CCAGATGCGA GACGACTGCA CACGCCTCCG GTAGGTTGCA CCGCTAAAGG CTAACCGACA   
  
  
- GAACAATCAA TTGTTCTACT CGTTACTGGA GGACGGTGCG CAGTTGGGCA GGACGCCATA ACCGTTTCAT   
  
  
- CGGCCGATGA AGTATCTACG GAACTGGGCT TCTGATATGG TCGTCCCGGG TCACAGCCCG GATTAGCCCA   
  
  
- GCCAAGACCG CATGGTCCAC AACATGCTCG TGAAGATACT TCGAACGGGC AAGGAGTTTA AGCGAGTGAA   
  
  
- GTGACGATTA GTTCGCTAAA ATCTCCGTAA GCTGCCGGTG CTAACACAGG TGTAGTAGCT GAAGCCGGAT   
  
  
- TAGGTGCCGA ACGTTACCGG TCGAGATTAG GTTCGGAACC GACAAGCCGT GCCACCGGGC GGAAAGCATT   
  
  
- CTAATTGGCC TTAGCCCGGC GGAAGACTTA TGCCCAGCAC GAGCGAAGTT GGATAGCCCA GTTGTGACCG   
  
  
- GGTCAATCGG GATAGGTACT TACAACCCAA GCGTAAAGCC CGGCAGCGGC AGAGTGCCGA GCTCCTGTAG   
  
  
- TTTGGTACCT ACCAATTTTG TTCGGGTTTA CTTCAGCATC GGCACTTGAG ATAGAAGGTT GAGGTGGCCG   
  
  
- ATTAGCCCAG CCCATAACTG GGACAGGATT TGACCCAGGC CTCGGATTTG GGCTTTTAAC ACTGTGACCG   
  
  
- CCTCGTTCTC CGCTTGGTGT TGGTCGGGCT CAAAAACCGG GCCAAGTGCC TCCGTAATGT AATGATAAGT   
  
  
- TGATACAAGC TGAGCAATCT CCGAACAGTT CAGGTCCGGC TGTTCCTGGA TCGGCTCGAT ATGAATCTCT   
  
  
- CTCTCAATAG GTTGTAGCAG ACGACACTCC CCAGCCGAGC CTAACTCTCC GTGCTCGGCG ACCGGGTTAC   
  
  
- CTCCCGGGCT TACCGAGCCC GGCCCAAGTT CTTCCACCTA TACCCATTTT TACGCAAGTT CGTTCATTCG   
  
  
- TACAACGACT CAAACAGAAG TCGTGTTCCC ATAACACACC TCCTCTCACT CCCTACAAAC TTCGAGCCGA   
  
  
- CCGTACTGGC GGGAGAGTAA CGCCGAAGCC GAACCGCTCG CCTCTGAGTT CGACTCTTGA GGTCGTGACA   
  
  
- ACACGAACTA CCCAGTAGCA GCACATCAAG TAGAAGAAT

+     CAAT-box

| Site Name | Organism | Position | Strand | Matrix score. | sequence | function |
| --- | --- | --- | --- | --- | --- | --- |
| CAAT-box | Nicotiana glutinosa | 1625 | - | 4 | CAAT |  |
| CAAT-box | Arabidopsis thaliana | 1484 | + | 5 | CCAAT | common cis-acting element in promoter and enhancer regions |
| CAAT-box | Nicotiana glutinosa | 2126 | + | 4 | CAAT |  |
| CAAT-box | Nicotiana glutinosa | 3102 | - | 4 | CAAT |  |
| CAAT-box | Nicotiana glutinosa | 2916 | - | 4 | CAAT |  |
| CAAT-box | Nicotiana glutinosa | 2721 | - | 4 | CAAT |  |
| CAAT-box | Nicotiana glutinosa | 2679 | - | 4 | CAAT |  |
| CAAT-box | Pisum sativum | 2620 | + | 5 | CAAAT | common cis-acting element in promoter and enhancer regions |
| CAAT-box | Nicotiana glutinosa | 2397 | + | 4 | CAAT |  |
| CAAT-box | Nicotiana glutinosa | 2940 | + | 4 | CAAT |  |
| CAAT-box | Arabidopsis thaliana | 2939 | + | 5 | CCAAT | common cis-acting element in promoter and enhancer regions |
| CAAT-box | Nicotiana glutinosa | 3046 | - | 4 | CAAT |  |
| CAAT-box | Nicotiana glutinosa | 1485 | + | 4 | CAAT |  |
| CAAT-box | Pisum sativum | 2300 | + | 5 | CAAAT | common cis-acting element in promoter and enhancer regions |
| CAAT-box | Nicotiana glutinosa | 2356 | - | 4 | CAAT |  |
| CAAT-box | Nicotiana glutinosa | 1535 | + | 4 | CAAT |  |
| CAAT-box | Arabidopsis thaliana | 1534 | + | 5 | CCAAT | common cis-acting element in promoter and enhancer regions |
| CAAT-box | Nicotiana glutinosa | 1948 | - | 4 | CAAT |  |
| CAAT-box | Nicotiana glutinosa | 1924 | - | 4 | CAAT |  |
| CAAT-box | Arabidopsis thaliana | 2163 | - | 5 | CCAAT | common cis-acting element in promoter and enhancer regions |
| CAAT-box | Arabidopsis thaliana | 2096 | - | 5 | CCAAT | common cis-acting element in promoter and enhancer regions |
| CAAT-box | Pisum sativum | 1404 | + | 5 | CAAAT | common cis-acting element in promoter and enhancer regions |
| CAAT-box | Pisum sativum | 1387 | + | 5 | CAAAT | common cis-acting element in promoter and enhancer regions |
| CAAT-box | Nicotiana glutinosa | 1380 | + | 4 | CAAT |  |
| CAAT-box | Arabidopsis thaliana | 1379 | + | 5 | CCAAT | common cis-acting element in promoter and enhancer regions |
| CAAT-box | Nicotiana glutinosa | 1151 | - | 4 | CAAT |  |
| CAAT-box | Pisum sativum | 1019 | + | 5 | CAAAT | common cis-acting element in promoter and enhancer regions |
| CAAT-box | Nicotiana glutinosa | 1451 | - | 4 | CAAT |  |
| CAAT-box | Nicotiana glutinosa | 1329 | - | 4 | CAAT |  |
| CAAT-box | Pisum sativum | 1105 | - | 5 | CAAAT | common cis-acting element in promoter and enhancer regions |
| CAAT-box | Pisum sativum | 1262 | + | 5 | CAAAT | common cis-acting element in promoter and enhancer regions |
| CAAT-box | Pisum sativum | 1130 | - | 5 | CAAAT | common cis-acting element in promoter and enhancer regions |
| CAAT-box | Nicotiana glutinosa | 728 | + | 4 | CAAT |  |
| CAAT-box | Pisum sativum | 154 | - | 5 | CAAAT | common cis-acting element in promoter and enhancer regions |
| CAAT-box | Pisum sativum | 630 | + | 5 | CAAAT | common cis-acting element in promoter and enhancer regions |
| CAAT-box | Arabidopsis thaliana | 188 | - | 5 | CCAAT | common cis-acting element in promoter and enhancer regions |
| CAAT-box | Nicotiana glutinosa | 413 | - | 4 | CAAT |  |
| CAAT-box | Pisum sativum | 258 | - | 5 | CAAAT | common cis-acting element in promoter and enhancer regions |
| CAAT-box | Pisum sativum | 491 | + | 5 | CAAAT | common cis-acting element in promoter and enhancer regions |
| CAAT-box | Pisum sativum | 352 | - | 5 | CAAAT | common cis-acting element in promoter and enhancer regions |
| CAAT-box | Arabidopsis thaliana | 509 | - | 5 | CCAAT | common cis-acting element in promoter and enhancer regions |
| CAAT-box | Nicotiana glutinosa | 228 | + | 4 | CAAT |  |
| CAAT-box | Pisum sativum | 323 | + | 5 | CAAAT | common cis-acting element in promoter and enhancer regions |
| CAAT-box | Pisum sativum | 635 | + | 5 | CAAAT | common cis-acting element in promoter and enhancer regions |
| CAAT-box | Nicotiana glutinosa | 46 | + | 4 | CAAT |  |

>HU11G00778.1   
+ -Up\_Stream \_Len000AAAAAA GAAAGAAATG GAGAAACCAA GCAATATGGA TAAAAAAAAA ACAAAGTAAT   
  
  
+ AAAGGAAGGA TGAGGCTCCA CATTCCACTC GCCCGCGCCA CTCATAGGAG ATGAATAGTA AAATGTGAGG   
  
  
+ GGTTAAATGA TTTGAAAAAA ATCTAGTAAG GGCCCACAAC AGTATTGGAA CCCAGCTTGC CAAGGAAATG   
  
  
+ TCAGAGGGGC TAGCAATGGA CCAGTGCAAG AAGGCCAAGC CACATTTGGT TAGGGAATTT CATCCCATCC   
  
  
+ GGCGCTAAAA GCATCAAAAT TACTACTTAA TCCTTGATCA AATGCCCTAT AAATATTCTA CTACCCCATT   
  
  
+ TGAAAAGGGG TGTTGATTAC AACACATAGC CGTAGGTTTG ACAAAGAGAA TTACCCTTAT TGTTAGAAGC   
  
  
+ ACCATATACT CTTTTCTCTA AGCTCTGATC TTTTTTCATC CTTTTCAGCT TTTTGTCCTT TTAAATCAAA   
  
  
+ TCACTCACTT GAGTATTGGA GGGACGTTCC TCAGGAGTCG AACCACTAAA TTATCTTTTT AAAGAAAAAG   
  
  
+ GGCTTGAAGC CAACCCGATT TAAAAAAACT GCTAATATGT AAGCCTACTC TCTTTCCAGT TCCAACAAAT   
  
  
+ CAAATTTCAG TATGAAACAT CCAGTAAAGG ATTTATCCGT GATAATGATG TAACATATGG TTAAAGGGTC   
  
  
+ ATTTTCAGTT ACATCGGAGC ATGCAATTAA AGTCCCTATA AAATAGGTAG GTGCACGAAC AAGTCACCAT   
  
  
+ CGTCCTTTGC TTTTTGCATG AGTGCATGCT AATTATGAAC TTAATTAATA TCTAAGCAAA ATTAAAAAGT   
  
  
+ AGTTAAAACT TTTTCACCTT TGGAACCTCG TAGCTGTAGC AGTAGCTGCC GCACTCTGGT TCTAGACTTC   
  
  
+ TAGGGACCAA GAGATGCACT CATATGCATC CTCTTCTGTC CTTTTACTGT ATCCCCTTCC CATGTGACCT   
  
  
+ TTTTGCCCGG GTGACTTTTC TGTGAGTGTT GATACAAATT TTGTACGTAA GCTAACTCAT TTACTTCACC   
  
  
+ GTCTCATGAG TTTTACATTA CCGATATTAA TTTAAATGGC AAAGTATTAA ATTTGAGCTA AAATCTCATA   
  
  
+ TATATATTTG TATTAAATGT GTCTGGATTG TCAAAATAAT TTTTGACATA TACAGTCACT ATATACTGAG   
  
  
+ TTATTAAAAT ATATAAAAAT ATGAAAATTT AAAAAATAGT AGTTTAAGGT GACAACACTA AAACAAACAA   
  
  
+ ATAAGATATA AATAGGGAAA AGTTCATCCT ATTTTGTTAT TATTATTTAT TATGTATTTT CAGAATTGTC   
  
  
+ TCTATCACAA GCATAGTATA TGTGTCAACA TCGTGACAAC TCGACCAATC TACAAATAAC TAGTAAATTC   
  
  
+ AAATAAAGTT ACTCTCACCT AGCTTAAATA AGTACACCTT GATTTAATTG TCTTAGCTAT GGAACCTATT   
  
  
+ CCAAACTAGC CAATACTTCC TTTTCAAAAG AATTAGTTTT AAAGTTTTAA CTTTTACGTC CAATAATGCC   
  
  
+ GGCCGTACTA TGAGTTGCTT TCAAGCCAGT AGTAGTACTG TAGTAGGTAA AAATATTAGA ATACACCTAC   
  
  
+ TATATATGAT ATTGCTCTTC ACCGTATCCT TCTGCATACT CATCTTTCCG TCCCGGAGTC CACTATCTCT   
  
  
+ CCTCCATTCA GACACTCTCT CGCTCTAGAG AACTCATAGC TATGGACCGC GTGCCCGACG GTTATCGGGT   
  
  
+ TATGAATTCG GAGCTTCTTC AGCAAGTTAG CCCTGAGTTG CCGGATCCAA CCACCTGGTT CCATGCCCGT   
  
  
+ CTACCCGACC CCATCTCTCA GTCTCCTCTT GGACCCGGTC CATCCTCCCA GCCAACCCAT TTCTACCAGG   
  
  
+ GTTCTGCCTC AGGGAACCAT GTGGTTGGGA TTGCTGACAC GTGGACGGAC CAGATTGTTG CAGGTTCCTC   
  
  
+ GTCCCAGCAG TGAGGTTGCA GCAGAGCCAG CAGGTGTTGA CTGCAGTCGA AGCCATGGAG GAGGACTCTG   
  
  
+ GGATAAGACT GGTCTACGCT CTGCTGACGT GTGCGGAGGC CATCCAACGT GGCGATTTCC GATTGGCTGT   
  
  
+ CTTGTTAGTT AACAAGATGA GCAATGACCT CCTGCCACGC GTCAACCCGT CCTGCGGTAT TGGCAAAGTA   
  
  
+ GCCGGCTACT TCATAGATGC CTTGACCCGA AGACTATACC AGCAGGGCCC AGTGTCGGGC CTAATCGGGT   
  
  
+ CGGTTCTGGC GTACCAGGTG TTGTACGAGC ACTTCTATGA AGCTTGCCCG TTCCTCAAAT TCGCTCACTT   
  
  
+ CACTGCTAAT CAAGCGATTT TAGAGGCATT CGACGGCCAC GATTGTGTCC ACATCATCGA CTTCGGCCTA   
  
  
+ ATCCACGGCT TGCAATGGCC AGCTCTAATC CAAGCCTTGG CTGTTCGGCA CGGTGGCCCG CCTTTCGTAA   
  
  
+ GATTAACCGG AATCGGGCCG CCTTCTGAAT ACGGGTCGTG CTCGCTTCAA CCTATCGGGT CAACACTGGC   
  
  
+ CCAGTTAGCC CTATCCATGA ATGTTGGGTT CGCATTTCGG GCCGTCGCCG TCTCACGGCT CGAGGACATC   
  
  
+ AAACCATGGA TGGTTAAAAC AAGCCCAAAT GAAGTCGTAG CCGTGAACTC TATCTTCCAA CTCCACCGGC   
  
  
+ TAATCGGGTC GGGTATTGAC CCTGTCCTAA ACTGGGTCCG GAGCCTAAAC CCGAAAATTG TGACACTGGC   
  
  
+ GGAGCAAGAG GCGAACCACA ACCAGCCCGA GTTTTTGGCC CGGTTCACGG AGGCATTACA TTACTATTCA   
  
  
+ ACTATGTTCG ACTCGTTAGA GGCTTGTCAA GTCCAGGCCG ACAAGGACCT AGCCGAGCTA TACTTAGAGA   
  
  
+ GAGAGTTATC CAACATCGTC TGCTGTGAGG GGTCGGCTCG GATTGAGAGG CACGAGCCGC TGGCCCAATG   
  
  
+ GAGGGCCCGA ATGGCTCGGG CCGGGTTCAA GAAGGTGGAT ATGGGTAAAA ATGCGTTCAA GCAAGTAAGC   
  
  
+ ATGTTGCTGA GTTTGTCTTC AGCACAAGGG TATTGTGTGG AGGAGAGTGA GGGATGTTTG AAGCTCGGCT   
  
  
+ GGCATGACCG CCCTCTCATT GCGGCTTCGG CTTGGCGAGC GGAGACTCAA GCTGAGAACT CCAGCACTGT   
  
  
+ TGTGCTTGAT GGGTCATCGT CGTGTAGTTC ATCTTCTTA  

- -Up\_Stream \_Len000TTTTTT CTTTCTTTAC CTCTTTGGTT CGTTATACCT ATTTTTTTTT TGTTTCATTA   
  
  
- TTTCCTTCCT ACTCCGAGGT GTAAGGTGAG CGGGCGCGGT GAGTATCCTC TACTTATCAT TTTACACTCC   
  
  
- CCAATTTACT AAACTTTTTT TAGATCATTC CCGGGTGTTG TCATAACCTT GGGTCGAACG GTTCCTTTAC   
  
  
- AGTCTCCCCG ATCGTTACCT GGTCACGTTC TTCCGGTTCG GTGTAAACCA ATCCCTTAAA GTAGGGTAGG   
  
  
- CCGCGATTTT CGTAGTTTTA ATGATGAATT AGGAACTAGT TTACGGGATA TTTATAAGAT GATGGGGTAA   
  
  
- ACTTTTCCCC ACAACTAATG TTGTGTATCG GCATCCAAAC TGTTTCTCTT AATGGGAATA ACAATCTTCG   
  
  
- TGGTATATGA GAAAAGAGAT TCGAGACTAG AAAAAAGTAG GAAAAGTCGA AAAACAGGAA AATTTAGTTT   
  
  
- AGTGAGTGAA CTCATAACCT CCCTGCAAGG AGTCCTCAGC TTGGTGATTT AATAGAAAAA TTTCTTTTTC   
  
  
- CCGAACTTCG GTTGGGCTAA ATTTTTTTGA CGATTATACA TTCGGATGAG AGAAAGGTCA AGGTTGTTTA   
  
  
- GTTTAAAGTC ATACTTTGTA GGTCATTTCC TAAATAGGCA CTATTACTAC ATTGTATACC AATTTCCCAG   
  
  
- TAAAAGTCAA TGTAGCCTCG TACGTTAATT TCAGGGATAT TTTATCCATC CACGTGCTTG TTCAGTGGTA   
  
  
- GCAGGAAACG AAAAACGTAC TCACGTACGA TTAATACTTG AATTAATTAT AGATTCGTTT TAATTTTTCA   
  
  
- TCAATTTTGA AAAAGTGGAA ACCTTGGAGC ATCGACATCG TCATCGACGG CGTGAGACCA AGATCTGAAG   
  
  
- ATCCCTGGTT CTCTACGTGA GTATACGTAG GAGAAGACAG GAAAATGACA TAGGGGAAGG GTACACTGGA   
  
  
- AAAACGGGCC CACTGAAAAG ACACTCACAA CTATGTTTAA AACATGCATT CGATTGAGTA AATGAAGTGG   
  
  
- CAGAGTACTC AAAATGTAAT GGCTATAATT AAATTTACCG TTTCATAATT TAAACTCGAT TTTAGAGTAT   
  
  
- ATATATAAAC ATAATTTACA CAGACCTAAC AGTTTTATTA AAAACTGTAT ATGTCAGTGA TATATGACTC   
  
  
- AATAATTTTA TATATTTTTA TACTTTTAAA TTTTTTATCA TCAAATTCCA CTGTTGTGAT TTTGTTTGTT   
  
  
- TATTCTATAT TTATCCCTTT TCAAGTAGGA TAAAACAATA ATAATAAATA ATACATAAAA GTCTTAACAG   
  
  
- AGATAGTGTT CGTATCATAT ACACAGTTGT AGCACTGTTG AGCTGGTTAG ATGTTTATTG ATCATTTAAG   
  
  
- TTTATTTCAA TGAGAGTGGA TCGAATTTAT TCATGTGGAA CTAAATTAAC AGAATCGATA CCTTGGATAA   
  
  
- GGTTTGATCG GTTATGAAGG AAAAGTTTTC TTAATCAAAA TTTCAAAATT GAAAATGCAG GTTATTACGG   
  
  
- CCGGCATGAT ACTCAACGAA AGTTCGGTCA TCATCATGAC ATCATCCATT TTTATAATCT TATGTGGATG   
  
  
- ATATATACTA TAACGAGAAG TGGCATAGGA AGACGTATGA GTAGAAAGGC AGGGCCTCAG GTGATAGAGA   
  
  
- GGAGGTAAGT CTGTGAGAGA GCGAGATCTC TTGAGTATCG ATACCTGGCG CACGGGCTGC CAATAGCCCA   
  
  
- ATACTTAAGC CTCGAAGAAG TCGTTCAATC GGGACTCAAC GGCCTAGGTT GGTGGACCAA GGTACGGGCA   
  
  
- GATGGGCTGG GGTAGAGAGT CAGAGGAGAA CCTGGGCCAG GTAGGAGGGT CGGTTGGGTA AAGATGGTCC   
  
  
- CAAGACGGAG TCCCTTGGTA CACCAACCCT AACGACTGTG CACCTGCCTG GTCTAACAAC GTCCAAGGAG   
  
  
- CAGGGTCGTC ACTCCAACGT CGTCTCGGTC GTCCACAACT GACGTCAGCT TCGGTACCTC CTCCTGAGAC   
  
  
- CCTATTCTGA CCAGATGCGA GACGACTGCA CACGCCTCCG GTAGGTTGCA CCGCTAAAGG CTAACCGACA   
  
  
- GAACAATCAA TTGTTCTACT CGTTACTGGA GGACGGTGCG CAGTTGGGCA GGACGCCATA ACCGTTTCAT   
  
  
- CGGCCGATGA AGTATCTACG GAACTGGGCT TCTGATATGG TCGTCCCGGG TCACAGCCCG GATTAGCCCA   
  
  
- GCCAAGACCG CATGGTCCAC AACATGCTCG TGAAGATACT TCGAACGGGC AAGGAGTTTA AGCGAGTGAA   
  
  
- GTGACGATTA GTTCGCTAAA ATCTCCGTAA GCTGCCGGTG CTAACACAGG TGTAGTAGCT GAAGCCGGAT   
  
  
- TAGGTGCCGA ACGTTACCGG TCGAGATTAG GTTCGGAACC GACAAGCCGT GCCACCGGGC GGAAAGCATT   
  
  
- CTAATTGGCC TTAGCCCGGC GGAAGACTTA TGCCCAGCAC GAGCGAAGTT GGATAGCCCA GTTGTGACCG   
  
  
- GGTCAATCGG GATAGGTACT TACAACCCAA GCGTAAAGCC CGGCAGCGGC AGAGTGCCGA GCTCCTGTAG   
  
  
- TTTGGTACCT ACCAATTTTG TTCGGGTTTA CTTCAGCATC GGCACTTGAG ATAGAAGGTT GAGGTGGCCG   
  
  
- ATTAGCCCAG CCCATAACTG GGACAGGATT TGACCCAGGC CTCGGATTTG GGCTTTTAAC ACTGTGACCG   
  
  
- CCTCGTTCTC CGCTTGGTGT TGGTCGGGCT CAAAAACCGG GCCAAGTGCC TCCGTAATGT AATGATAAGT   
  
  
- TGATACAAGC TGAGCAATCT CCGAACAGTT CAGGTCCGGC TGTTCCTGGA TCGGCTCGAT ATGAATCTCT   
  
  
- CTCTCAATAG GTTGTAGCAG ACGACACTCC CCAGCCGAGC CTAACTCTCC GTGCTCGGCG ACCGGGTTAC   
  
  
- CTCCCGGGCT TACCGAGCCC GGCCCAAGTT CTTCCACCTA TACCCATTTT TACGCAAGTT CGTTCATTCG   
  
  
- TACAACGACT CAAACAGAAG TCGTGTTCCC ATAACACACC TCCTCTCACT CCCTACAAAC TTCGAGCCGA   
  
  
- CCGTACTGGC GGGAGAGTAA CGCCGAAGCC GAACCGCTCG CCTCTGAGTT CGACTCTTGA GGTCGTGACA   
  
  
- ACACGAACTA CCCAGTAGCA GCACATCAAG TAGAAGAAT

+     CAT-box

| Site Name | Organism | Position | Strand | Matrix score. | sequence | function |
| --- | --- | --- | --- | --- | --- | --- |
| CAT-box | Arabidopsis thaliana | 111 | + | 6 | GCCACT | cis-acting regulatory element related to meristem expression |

>HU11G00778.1   
+ -Up\_Stream \_Len000AAAAAA GAAAGAAATG GAGAAACCAA GCAATATGGA TAAAAAAAAA ACAAAGTAAT   
  
  
+ AAAGGAAGGA TGAGGCTCCA CATTCCACTC GCCCGCGCCA CTCATAGGAG ATGAATAGTA AAATGTGAGG   
  
  
+ GGTTAAATGA TTTGAAAAAA ATCTAGTAAG GGCCCACAAC AGTATTGGAA CCCAGCTTGC CAAGGAAATG   
  
  
+ TCAGAGGGGC TAGCAATGGA CCAGTGCAAG AAGGCCAAGC CACATTTGGT TAGGGAATTT CATCCCATCC   
  
  
+ GGCGCTAAAA GCATCAAAAT TACTACTTAA TCCTTGATCA AATGCCCTAT AAATATTCTA CTACCCCATT   
  
  
+ TGAAAAGGGG TGTTGATTAC AACACATAGC CGTAGGTTTG ACAAAGAGAA TTACCCTTAT TGTTAGAAGC   
  
  
+ ACCATATACT CTTTTCTCTA AGCTCTGATC TTTTTTCATC CTTTTCAGCT TTTTGTCCTT TTAAATCAAA   
  
  
+ TCACTCACTT GAGTATTGGA GGGACGTTCC TCAGGAGTCG AACCACTAAA TTATCTTTTT AAAGAAAAAG   
  
  
+ GGCTTGAAGC CAACCCGATT TAAAAAAACT GCTAATATGT AAGCCTACTC TCTTTCCAGT TCCAACAAAT   
  
  
+ CAAATTTCAG TATGAAACAT CCAGTAAAGG ATTTATCCGT GATAATGATG TAACATATGG TTAAAGGGTC   
  
  
+ ATTTTCAGTT ACATCGGAGC ATGCAATTAA AGTCCCTATA AAATAGGTAG GTGCACGAAC AAGTCACCAT   
  
  
+ CGTCCTTTGC TTTTTGCATG AGTGCATGCT AATTATGAAC TTAATTAATA TCTAAGCAAA ATTAAAAAGT   
  
  
+ AGTTAAAACT TTTTCACCTT TGGAACCTCG TAGCTGTAGC AGTAGCTGCC GCACTCTGGT TCTAGACTTC   
  
  
+ TAGGGACCAA GAGATGCACT CATATGCATC CTCTTCTGTC CTTTTACTGT ATCCCCTTCC CATGTGACCT   
  
  
+ TTTTGCCCGG GTGACTTTTC TGTGAGTGTT GATACAAATT TTGTACGTAA GCTAACTCAT TTACTTCACC   
  
  
+ GTCTCATGAG TTTTACATTA CCGATATTAA TTTAAATGGC AAAGTATTAA ATTTGAGCTA AAATCTCATA   
  
  
+ TATATATTTG TATTAAATGT GTCTGGATTG TCAAAATAAT TTTTGACATA TACAGTCACT ATATACTGAG   
  
  
+ TTATTAAAAT ATATAAAAAT ATGAAAATTT AAAAAATAGT AGTTTAAGGT GACAACACTA AAACAAACAA   
  
  
+ ATAAGATATA AATAGGGAAA AGTTCATCCT ATTTTGTTAT TATTATTTAT TATGTATTTT CAGAATTGTC   
  
  
+ TCTATCACAA GCATAGTATA TGTGTCAACA TCGTGACAAC TCGACCAATC TACAAATAAC TAGTAAATTC   
  
  
+ AAATAAAGTT ACTCTCACCT AGCTTAAATA AGTACACCTT GATTTAATTG TCTTAGCTAT GGAACCTATT   
  
  
+ CCAAACTAGC CAATACTTCC TTTTCAAAAG AATTAGTTTT AAAGTTTTAA CTTTTACGTC CAATAATGCC   
  
  
+ GGCCGTACTA TGAGTTGCTT TCAAGCCAGT AGTAGTACTG TAGTAGGTAA AAATATTAGA ATACACCTAC   
  
  
+ TATATATGAT ATTGCTCTTC ACCGTATCCT TCTGCATACT CATCTTTCCG TCCCGGAGTC CACTATCTCT   
  
  
+ CCTCCATTCA GACACTCTCT CGCTCTAGAG AACTCATAGC TATGGACCGC GTGCCCGACG GTTATCGGGT   
  
  
+ TATGAATTCG GAGCTTCTTC AGCAAGTTAG CCCTGAGTTG CCGGATCCAA CCACCTGGTT CCATGCCCGT   
  
  
+ CTACCCGACC CCATCTCTCA GTCTCCTCTT GGACCCGGTC CATCCTCCCA GCCAACCCAT TTCTACCAGG   
  
  
+ GTTCTGCCTC AGGGAACCAT GTGGTTGGGA TTGCTGACAC GTGGACGGAC CAGATTGTTG CAGGTTCCTC   
  
  
+ GTCCCAGCAG TGAGGTTGCA GCAGAGCCAG CAGGTGTTGA CTGCAGTCGA AGCCATGGAG GAGGACTCTG   
  
  
+ GGATAAGACT GGTCTACGCT CTGCTGACGT GTGCGGAGGC CATCCAACGT GGCGATTTCC GATTGGCTGT   
  
  
+ CTTGTTAGTT AACAAGATGA GCAATGACCT CCTGCCACGC GTCAACCCGT CCTGCGGTAT TGGCAAAGTA   
  
  
+ GCCGGCTACT TCATAGATGC CTTGACCCGA AGACTATACC AGCAGGGCCC AGTGTCGGGC CTAATCGGGT   
  
  
+ CGGTTCTGGC GTACCAGGTG TTGTACGAGC ACTTCTATGA AGCTTGCCCG TTCCTCAAAT TCGCTCACTT   
  
  
+ CACTGCTAAT CAAGCGATTT TAGAGGCATT CGACGGCCAC GATTGTGTCC ACATCATCGA CTTCGGCCTA   
  
  
+ ATCCACGGCT TGCAATGGCC AGCTCTAATC CAAGCCTTGG CTGTTCGGCA CGGTGGCCCG CCTTTCGTAA   
  
  
+ GATTAACCGG AATCGGGCCG CCTTCTGAAT ACGGGTCGTG CTCGCTTCAA CCTATCGGGT CAACACTGGC   
  
  
+ CCAGTTAGCC CTATCCATGA ATGTTGGGTT CGCATTTCGG GCCGTCGCCG TCTCACGGCT CGAGGACATC   
  
  
+ AAACCATGGA TGGTTAAAAC AAGCCCAAAT GAAGTCGTAG CCGTGAACTC TATCTTCCAA CTCCACCGGC   
  
  
+ TAATCGGGTC GGGTATTGAC CCTGTCCTAA ACTGGGTCCG GAGCCTAAAC CCGAAAATTG TGACACTGGC   
  
  
+ GGAGCAAGAG GCGAACCACA ACCAGCCCGA GTTTTTGGCC CGGTTCACGG AGGCATTACA TTACTATTCA   
  
  
+ ACTATGTTCG ACTCGTTAGA GGCTTGTCAA GTCCAGGCCG ACAAGGACCT AGCCGAGCTA TACTTAGAGA   
  
  
+ GAGAGTTATC CAACATCGTC TGCTGTGAGG GGTCGGCTCG GATTGAGAGG CACGAGCCGC TGGCCCAATG   
  
  
+ GAGGGCCCGA ATGGCTCGGG CCGGGTTCAA GAAGGTGGAT ATGGGTAAAA ATGCGTTCAA GCAAGTAAGC   
  
  
+ ATGTTGCTGA GTTTGTCTTC AGCACAAGGG TATTGTGTGG AGGAGAGTGA GGGATGTTTG AAGCTCGGCT   
  
  
+ GGCATGACCG CCCTCTCATT GCGGCTTCGG CTTGGCGAGC GGAGACTCAA GCTGAGAACT CCAGCACTGT   
  
  
+ TGTGCTTGAT GGGTCATCGT CGTGTAGTTC ATCTTCTTA  

- -Up\_Stream \_Len000TTTTTT CTTTCTTTAC CTCTTTGGTT CGTTATACCT ATTTTTTTTT TGTTTCATTA   
  
  
- TTTCCTTCCT ACTCCGAGGT GTAAGGTGAG CGGGCGCGGT GAGTATCCTC TACTTATCAT TTTACACTCC   
  
  
- CCAATTTACT AAACTTTTTT TAGATCATTC CCGGGTGTTG TCATAACCTT GGGTCGAACG GTTCCTTTAC   
  
  
- AGTCTCCCCG ATCGTTACCT GGTCACGTTC TTCCGGTTCG GTGTAAACCA ATCCCTTAAA GTAGGGTAGG   
  
  
- CCGCGATTTT CGTAGTTTTA ATGATGAATT AGGAACTAGT TTACGGGATA TTTATAAGAT GATGGGGTAA   
  
  
- ACTTTTCCCC ACAACTAATG TTGTGTATCG GCATCCAAAC TGTTTCTCTT AATGGGAATA ACAATCTTCG   
  
  
- TGGTATATGA GAAAAGAGAT TCGAGACTAG AAAAAAGTAG GAAAAGTCGA AAAACAGGAA AATTTAGTTT   
  
  
- AGTGAGTGAA CTCATAACCT CCCTGCAAGG AGTCCTCAGC TTGGTGATTT AATAGAAAAA TTTCTTTTTC   
  
  
- CCGAACTTCG GTTGGGCTAA ATTTTTTTGA CGATTATACA TTCGGATGAG AGAAAGGTCA AGGTTGTTTA   
  
  
- GTTTAAAGTC ATACTTTGTA GGTCATTTCC TAAATAGGCA CTATTACTAC ATTGTATACC AATTTCCCAG   
  
  
- TAAAAGTCAA TGTAGCCTCG TACGTTAATT TCAGGGATAT TTTATCCATC CACGTGCTTG TTCAGTGGTA   
  
  
- GCAGGAAACG AAAAACGTAC TCACGTACGA TTAATACTTG AATTAATTAT AGATTCGTTT TAATTTTTCA   
  
  
- TCAATTTTGA AAAAGTGGAA ACCTTGGAGC ATCGACATCG TCATCGACGG CGTGAGACCA AGATCTGAAG   
  
  
- ATCCCTGGTT CTCTACGTGA GTATACGTAG GAGAAGACAG GAAAATGACA TAGGGGAAGG GTACACTGGA   
  
  
- AAAACGGGCC CACTGAAAAG ACACTCACAA CTATGTTTAA AACATGCATT CGATTGAGTA AATGAAGTGG   
  
  
- CAGAGTACTC AAAATGTAAT GGCTATAATT AAATTTACCG TTTCATAATT TAAACTCGAT TTTAGAGTAT   
  
  
- ATATATAAAC ATAATTTACA CAGACCTAAC AGTTTTATTA AAAACTGTAT ATGTCAGTGA TATATGACTC   
  
  
- AATAATTTTA TATATTTTTA TACTTTTAAA TTTTTTATCA TCAAATTCCA CTGTTGTGAT TTTGTTTGTT   
  
  
- TATTCTATAT TTATCCCTTT TCAAGTAGGA TAAAACAATA ATAATAAATA ATACATAAAA GTCTTAACAG   
  
  
- AGATAGTGTT CGTATCATAT ACACAGTTGT AGCACTGTTG AGCTGGTTAG ATGTTTATTG ATCATTTAAG   
  
  
- TTTATTTCAA TGAGAGTGGA TCGAATTTAT TCATGTGGAA CTAAATTAAC AGAATCGATA CCTTGGATAA   
  
  
- GGTTTGATCG GTTATGAAGG AAAAGTTTTC TTAATCAAAA TTTCAAAATT GAAAATGCAG GTTATTACGG   
  
  
- CCGGCATGAT ACTCAACGAA AGTTCGGTCA TCATCATGAC ATCATCCATT TTTATAATCT TATGTGGATG   
  
  
- ATATATACTA TAACGAGAAG TGGCATAGGA AGACGTATGA GTAGAAAGGC AGGGCCTCAG GTGATAGAGA   
  
  
- GGAGGTAAGT CTGTGAGAGA GCGAGATCTC TTGAGTATCG ATACCTGGCG CACGGGCTGC CAATAGCCCA   
  
  
- ATACTTAAGC CTCGAAGAAG TCGTTCAATC GGGACTCAAC GGCCTAGGTT GGTGGACCAA GGTACGGGCA   
  
  
- GATGGGCTGG GGTAGAGAGT CAGAGGAGAA CCTGGGCCAG GTAGGAGGGT CGGTTGGGTA AAGATGGTCC   
  
  
- CAAGACGGAG TCCCTTGGTA CACCAACCCT AACGACTGTG CACCTGCCTG GTCTAACAAC GTCCAAGGAG   
  
  
- CAGGGTCGTC ACTCCAACGT CGTCTCGGTC GTCCACAACT GACGTCAGCT TCGGTACCTC CTCCTGAGAC   
  
  
- CCTATTCTGA CCAGATGCGA GACGACTGCA CACGCCTCCG GTAGGTTGCA CCGCTAAAGG CTAACCGACA   
  
  
- GAACAATCAA TTGTTCTACT CGTTACTGGA GGACGGTGCG CAGTTGGGCA GGACGCCATA ACCGTTTCAT   
  
  
- CGGCCGATGA AGTATCTACG GAACTGGGCT TCTGATATGG TCGTCCCGGG TCACAGCCCG GATTAGCCCA   
  
  
- GCCAAGACCG CATGGTCCAC AACATGCTCG TGAAGATACT TCGAACGGGC AAGGAGTTTA AGCGAGTGAA   
  
  
- GTGACGATTA GTTCGCTAAA ATCTCCGTAA GCTGCCGGTG CTAACACAGG TGTAGTAGCT GAAGCCGGAT   
  
  
- TAGGTGCCGA ACGTTACCGG TCGAGATTAG GTTCGGAACC GACAAGCCGT GCCACCGGGC GGAAAGCATT   
  
  
- CTAATTGGCC TTAGCCCGGC GGAAGACTTA TGCCCAGCAC GAGCGAAGTT GGATAGCCCA GTTGTGACCG   
  
  
- GGTCAATCGG GATAGGTACT TACAACCCAA GCGTAAAGCC CGGCAGCGGC AGAGTGCCGA GCTCCTGTAG   
  
  
- TTTGGTACCT ACCAATTTTG TTCGGGTTTA CTTCAGCATC GGCACTTGAG ATAGAAGGTT GAGGTGGCCG   
  
  
- ATTAGCCCAG CCCATAACTG GGACAGGATT TGACCCAGGC CTCGGATTTG GGCTTTTAAC ACTGTGACCG   
  
  
- CCTCGTTCTC CGCTTGGTGT TGGTCGGGCT CAAAAACCGG GCCAAGTGCC TCCGTAATGT AATGATAAGT   
  
  
- TGATACAAGC TGAGCAATCT CCGAACAGTT CAGGTCCGGC TGTTCCTGGA TCGGCTCGAT ATGAATCTCT   
  
  
- CTCTCAATAG GTTGTAGCAG ACGACACTCC CCAGCCGAGC CTAACTCTCC GTGCTCGGCG ACCGGGTTAC   
  
  
- CTCCCGGGCT TACCGAGCCC GGCCCAAGTT CTTCCACCTA TACCCATTTT TACGCAAGTT CGTTCATTCG   
  
  
- TACAACGACT CAAACAGAAG TCGTGTTCCC ATAACACACC TCCTCTCACT CCCTACAAAC TTCGAGCCGA   
  
  
- CCGTACTGGC GGGAGAGTAA CGCCGAAGCC GAACCGCTCG CCTCTGAGTT CGACTCTTGA GGTCGTGACA   
  
  
- ACACGAACTA CCCAGTAGCA GCACATCAAG TAGAAGAAT

+     CCGTCC motif

| Site Name | Organism | Position | Strand | Matrix score. | sequence | function |
| --- | --- | --- | --- | --- | --- | --- |
| CCGTCC motif | Nicotiana tabacum | 2151 | + | 6 | CCGTCC |  |
| CCGTCC motif | Nicotiana tabacum | 1937 | - | 6 | CCGTCC |  |
| CCGTCC motif | Nicotiana tabacum | 1662 | + | 6 | CCGTCC |  |

>HU11G00778.1   
+ -Up\_Stream \_Len000AAAAAA GAAAGAAATG GAGAAACCAA GCAATATGGA TAAAAAAAAA ACAAAGTAAT   
  
  
+ AAAGGAAGGA TGAGGCTCCA CATTCCACTC GCCCGCGCCA CTCATAGGAG ATGAATAGTA AAATGTGAGG   
  
  
+ GGTTAAATGA TTTGAAAAAA ATCTAGTAAG GGCCCACAAC AGTATTGGAA CCCAGCTTGC CAAGGAAATG   
  
  
+ TCAGAGGGGC TAGCAATGGA CCAGTGCAAG AAGGCCAAGC CACATTTGGT TAGGGAATTT CATCCCATCC   
  
  
+ GGCGCTAAAA GCATCAAAAT TACTACTTAA TCCTTGATCA AATGCCCTAT AAATATTCTA CTACCCCATT   
  
  
+ TGAAAAGGGG TGTTGATTAC AACACATAGC CGTAGGTTTG ACAAAGAGAA TTACCCTTAT TGTTAGAAGC   
  
  
+ ACCATATACT CTTTTCTCTA AGCTCTGATC TTTTTTCATC CTTTTCAGCT TTTTGTCCTT TTAAATCAAA   
  
  
+ TCACTCACTT GAGTATTGGA GGGACGTTCC TCAGGAGTCG AACCACTAAA TTATCTTTTT AAAGAAAAAG   
  
  
+ GGCTTGAAGC CAACCCGATT TAAAAAAACT GCTAATATGT AAGCCTACTC TCTTTCCAGT TCCAACAAAT   
  
  
+ CAAATTTCAG TATGAAACAT CCAGTAAAGG ATTTATCCGT GATAATGATG TAACATATGG TTAAAGGGTC   
  
  
+ ATTTTCAGTT ACATCGGAGC ATGCAATTAA AGTCCCTATA AAATAGGTAG GTGCACGAAC AAGTCACCAT   
  
  
+ CGTCCTTTGC TTTTTGCATG AGTGCATGCT AATTATGAAC TTAATTAATA TCTAAGCAAA ATTAAAAAGT   
  
  
+ AGTTAAAACT TTTTCACCTT TGGAACCTCG TAGCTGTAGC AGTAGCTGCC GCACTCTGGT TCTAGACTTC   
  
  
+ TAGGGACCAA GAGATGCACT CATATGCATC CTCTTCTGTC CTTTTACTGT ATCCCCTTCC CATGTGACCT   
  
  
+ TTTTGCCCGG GTGACTTTTC TGTGAGTGTT GATACAAATT TTGTACGTAA GCTAACTCAT TTACTTCACC   
  
  
+ GTCTCATGAG TTTTACATTA CCGATATTAA TTTAAATGGC AAAGTATTAA ATTTGAGCTA AAATCTCATA   
  
  
+ TATATATTTG TATTAAATGT GTCTGGATTG TCAAAATAAT TTTTGACATA TACAGTCACT ATATACTGAG   
  
  
+ TTATTAAAAT ATATAAAAAT ATGAAAATTT AAAAAATAGT AGTTTAAGGT GACAACACTA AAACAAACAA   
  
  
+ ATAAGATATA AATAGGGAAA AGTTCATCCT ATTTTGTTAT TATTATTTAT TATGTATTTT CAGAATTGTC   
  
  
+ TCTATCACAA GCATAGTATA TGTGTCAACA TCGTGACAAC TCGACCAATC TACAAATAAC TAGTAAATTC   
  
  
+ AAATAAAGTT ACTCTCACCT AGCTTAAATA AGTACACCTT GATTTAATTG TCTTAGCTAT GGAACCTATT   
  
  
+ CCAAACTAGC CAATACTTCC TTTTCAAAAG AATTAGTTTT AAAGTTTTAA CTTTTACGTC CAATAATGCC   
  
  
+ GGCCGTACTA TGAGTTGCTT TCAAGCCAGT AGTAGTACTG TAGTAGGTAA AAATATTAGA ATACACCTAC   
  
  
+ TATATATGAT ATTGCTCTTC ACCGTATCCT TCTGCATACT CATCTTTCCG TCCCGGAGTC CACTATCTCT   
  
  
+ CCTCCATTCA GACACTCTCT CGCTCTAGAG AACTCATAGC TATGGACCGC GTGCCCGACG GTTATCGGGT   
  
  
+ TATGAATTCG GAGCTTCTTC AGCAAGTTAG CCCTGAGTTG CCGGATCCAA CCACCTGGTT CCATGCCCGT   
  
  
+ CTACCCGACC CCATCTCTCA GTCTCCTCTT GGACCCGGTC CATCCTCCCA GCCAACCCAT TTCTACCAGG   
  
  
+ GTTCTGCCTC AGGGAACCAT GTGGTTGGGA TTGCTGACAC GTGGACGGAC CAGATTGTTG CAGGTTCCTC   
  
  
+ GTCCCAGCAG TGAGGTTGCA GCAGAGCCAG CAGGTGTTGA CTGCAGTCGA AGCCATGGAG GAGGACTCTG   
  
  
+ GGATAAGACT GGTCTACGCT CTGCTGACGT GTGCGGAGGC CATCCAACGT GGCGATTTCC GATTGGCTGT   
  
  
+ CTTGTTAGTT AACAAGATGA GCAATGACCT CCTGCCACGC GTCAACCCGT CCTGCGGTAT TGGCAAAGTA   
  
  
+ GCCGGCTACT TCATAGATGC CTTGACCCGA AGACTATACC AGCAGGGCCC AGTGTCGGGC CTAATCGGGT   
  
  
+ CGGTTCTGGC GTACCAGGTG TTGTACGAGC ACTTCTATGA AGCTTGCCCG TTCCTCAAAT TCGCTCACTT   
  
  
+ CACTGCTAAT CAAGCGATTT TAGAGGCATT CGACGGCCAC GATTGTGTCC ACATCATCGA CTTCGGCCTA   
  
  
+ ATCCACGGCT TGCAATGGCC AGCTCTAATC CAAGCCTTGG CTGTTCGGCA CGGTGGCCCG CCTTTCGTAA   
  
  
+ GATTAACCGG AATCGGGCCG CCTTCTGAAT ACGGGTCGTG CTCGCTTCAA CCTATCGGGT CAACACTGGC   
  
  
+ CCAGTTAGCC CTATCCATGA ATGTTGGGTT CGCATTTCGG GCCGTCGCCG TCTCACGGCT CGAGGACATC   
  
  
+ AAACCATGGA TGGTTAAAAC AAGCCCAAAT GAAGTCGTAG CCGTGAACTC TATCTTCCAA CTCCACCGGC   
  
  
+ TAATCGGGTC GGGTATTGAC CCTGTCCTAA ACTGGGTCCG GAGCCTAAAC CCGAAAATTG TGACACTGGC   
  
  
+ GGAGCAAGAG GCGAACCACA ACCAGCCCGA GTTTTTGGCC CGGTTCACGG AGGCATTACA TTACTATTCA   
  
  
+ ACTATGTTCG ACTCGTTAGA GGCTTGTCAA GTCCAGGCCG ACAAGGACCT AGCCGAGCTA TACTTAGAGA   
  
  
+ GAGAGTTATC CAACATCGTC TGCTGTGAGG GGTCGGCTCG GATTGAGAGG CACGAGCCGC TGGCCCAATG   
  
  
+ GAGGGCCCGA ATGGCTCGGG CCGGGTTCAA GAAGGTGGAT ATGGGTAAAA ATGCGTTCAA GCAAGTAAGC   
  
  
+ ATGTTGCTGA GTTTGTCTTC AGCACAAGGG TATTGTGTGG AGGAGAGTGA GGGATGTTTG AAGCTCGGCT   
  
  
+ GGCATGACCG CCCTCTCATT GCGGCTTCGG CTTGGCGAGC GGAGACTCAA GCTGAGAACT CCAGCACTGT   
  
  
+ TGTGCTTGAT GGGTCATCGT CGTGTAGTTC ATCTTCTTA  

- -Up\_Stream \_Len000TTTTTT CTTTCTTTAC CTCTTTGGTT CGTTATACCT ATTTTTTTTT TGTTTCATTA   
  
  
- TTTCCTTCCT ACTCCGAGGT GTAAGGTGAG CGGGCGCGGT GAGTATCCTC TACTTATCAT TTTACACTCC   
  
  
- CCAATTTACT AAACTTTTTT TAGATCATTC CCGGGTGTTG TCATAACCTT GGGTCGAACG GTTCCTTTAC   
  
  
- AGTCTCCCCG ATCGTTACCT GGTCACGTTC TTCCGGTTCG GTGTAAACCA ATCCCTTAAA GTAGGGTAGG   
  
  
- CCGCGATTTT CGTAGTTTTA ATGATGAATT AGGAACTAGT TTACGGGATA TTTATAAGAT GATGGGGTAA   
  
  
- ACTTTTCCCC ACAACTAATG TTGTGTATCG GCATCCAAAC TGTTTCTCTT AATGGGAATA ACAATCTTCG   
  
  
- TGGTATATGA GAAAAGAGAT TCGAGACTAG AAAAAAGTAG GAAAAGTCGA AAAACAGGAA AATTTAGTTT   
  
  
- AGTGAGTGAA CTCATAACCT CCCTGCAAGG AGTCCTCAGC TTGGTGATTT AATAGAAAAA TTTCTTTTTC   
  
  
- CCGAACTTCG GTTGGGCTAA ATTTTTTTGA CGATTATACA TTCGGATGAG AGAAAGGTCA AGGTTGTTTA   
  
  
- GTTTAAAGTC ATACTTTGTA GGTCATTTCC TAAATAGGCA CTATTACTAC ATTGTATACC AATTTCCCAG   
  
  
- TAAAAGTCAA TGTAGCCTCG TACGTTAATT TCAGGGATAT TTTATCCATC CACGTGCTTG TTCAGTGGTA   
  
  
- GCAGGAAACG AAAAACGTAC TCACGTACGA TTAATACTTG AATTAATTAT AGATTCGTTT TAATTTTTCA   
  
  
- TCAATTTTGA AAAAGTGGAA ACCTTGGAGC ATCGACATCG TCATCGACGG CGTGAGACCA AGATCTGAAG   
  
  
- ATCCCTGGTT CTCTACGTGA GTATACGTAG GAGAAGACAG GAAAATGACA TAGGGGAAGG GTACACTGGA   
  
  
- AAAACGGGCC CACTGAAAAG ACACTCACAA CTATGTTTAA AACATGCATT CGATTGAGTA AATGAAGTGG   
  
  
- CAGAGTACTC AAAATGTAAT GGCTATAATT AAATTTACCG TTTCATAATT TAAACTCGAT TTTAGAGTAT   
  
  
- ATATATAAAC ATAATTTACA CAGACCTAAC AGTTTTATTA AAAACTGTAT ATGTCAGTGA TATATGACTC   
  
  
- AATAATTTTA TATATTTTTA TACTTTTAAA TTTTTTATCA TCAAATTCCA CTGTTGTGAT TTTGTTTGTT   
  
  
- TATTCTATAT TTATCCCTTT TCAAGTAGGA TAAAACAATA ATAATAAATA ATACATAAAA GTCTTAACAG   
  
  
- AGATAGTGTT CGTATCATAT ACACAGTTGT AGCACTGTTG AGCTGGTTAG ATGTTTATTG ATCATTTAAG   
  
  
- TTTATTTCAA TGAGAGTGGA TCGAATTTAT TCATGTGGAA CTAAATTAAC AGAATCGATA CCTTGGATAA   
  
  
- GGTTTGATCG GTTATGAAGG AAAAGTTTTC TTAATCAAAA TTTCAAAATT GAAAATGCAG GTTATTACGG   
  
  
- CCGGCATGAT ACTCAACGAA AGTTCGGTCA TCATCATGAC ATCATCCATT TTTATAATCT TATGTGGATG   
  
  
- ATATATACTA TAACGAGAAG TGGCATAGGA AGACGTATGA GTAGAAAGGC AGGGCCTCAG GTGATAGAGA   
  
  
- GGAGGTAAGT CTGTGAGAGA GCGAGATCTC TTGAGTATCG ATACCTGGCG CACGGGCTGC CAATAGCCCA   
  
  
- ATACTTAAGC CTCGAAGAAG TCGTTCAATC GGGACTCAAC GGCCTAGGTT GGTGGACCAA GGTACGGGCA   
  
  
- GATGGGCTGG GGTAGAGAGT CAGAGGAGAA CCTGGGCCAG GTAGGAGGGT CGGTTGGGTA AAGATGGTCC   
  
  
- CAAGACGGAG TCCCTTGGTA CACCAACCCT AACGACTGTG CACCTGCCTG GTCTAACAAC GTCCAAGGAG   
  
  
- CAGGGTCGTC ACTCCAACGT CGTCTCGGTC GTCCACAACT GACGTCAGCT TCGGTACCTC CTCCTGAGAC   
  
  
- CCTATTCTGA CCAGATGCGA GACGACTGCA CACGCCTCCG GTAGGTTGCA CCGCTAAAGG CTAACCGACA   
  
  
- GAACAATCAA TTGTTCTACT CGTTACTGGA GGACGGTGCG CAGTTGGGCA GGACGCCATA ACCGTTTCAT   
  
  
- CGGCCGATGA AGTATCTACG GAACTGGGCT TCTGATATGG TCGTCCCGGG TCACAGCCCG GATTAGCCCA   
  
  
- GCCAAGACCG CATGGTCCAC AACATGCTCG TGAAGATACT TCGAACGGGC AAGGAGTTTA AGCGAGTGAA   
  
  
- GTGACGATTA GTTCGCTAAA ATCTCCGTAA GCTGCCGGTG CTAACACAGG TGTAGTAGCT GAAGCCGGAT   
  
  
- TAGGTGCCGA ACGTTACCGG TCGAGATTAG GTTCGGAACC GACAAGCCGT GCCACCGGGC GGAAAGCATT   
  
  
- CTAATTGGCC TTAGCCCGGC GGAAGACTTA TGCCCAGCAC GAGCGAAGTT GGATAGCCCA GTTGTGACCG   
  
  
- GGTCAATCGG GATAGGTACT TACAACCCAA GCGTAAAGCC CGGCAGCGGC AGAGTGCCGA GCTCCTGTAG   
  
  
- TTTGGTACCT ACCAATTTTG TTCGGGTTTA CTTCAGCATC GGCACTTGAG ATAGAAGGTT GAGGTGGCCG   
  
  
- ATTAGCCCAG CCCATAACTG GGACAGGATT TGACCCAGGC CTCGGATTTG GGCTTTTAAC ACTGTGACCG   
  
  
- CCTCGTTCTC CGCTTGGTGT TGGTCGGGCT CAAAAACCGG GCCAAGTGCC TCCGTAATGT AATGATAAGT   
  
  
- TGATACAAGC TGAGCAATCT CCGAACAGTT CAGGTCCGGC TGTTCCTGGA TCGGCTCGAT ATGAATCTCT   
  
  
- CTCTCAATAG GTTGTAGCAG ACGACACTCC CCAGCCGAGC CTAACTCTCC GTGCTCGGCG ACCGGGTTAC   
  
  
- CTCCCGGGCT TACCGAGCCC GGCCCAAGTT CTTCCACCTA TACCCATTTT TACGCAAGTT CGTTCATTCG   
  
  
- TACAACGACT CAAACAGAAG TCGTGTTCCC ATAACACACC TCCTCTCACT CCCTACAAAC TTCGAGCCGA   
  
  
- CCGTACTGGC GGGAGAGTAA CGCCGAAGCC GAACCGCTCG CCTCTGAGTT CGACTCTTGA GGTCGTGACA   
  
  
- ACACGAACTA CCCAGTAGCA GCACATCAAG TAGAAGAAT

+     CCGTCC-box

| Site Name | Organism | Position | Strand | Matrix score. | sequence | function |
| --- | --- | --- | --- | --- | --- | --- |
| CCGTCC-box | Petroselinum hortense | 2151 | + | 6 | CCGTCC |  |
| CCGTCC-box | Petroselinum hortense | 1937 | - | 6 | CCGTCC |  |
| CCGTCC-box | Petroselinum hortense | 1662 | + | 6 | CCGTCC |  |

>HU11G00778.1   
+ -Up\_Stream \_Len000AAAAAA GAAAGAAATG GAGAAACCAA GCAATATGGA TAAAAAAAAA ACAAAGTAAT   
  
  
+ AAAGGAAGGA TGAGGCTCCA CATTCCACTC GCCCGCGCCA CTCATAGGAG ATGAATAGTA AAATGTGAGG   
  
  
+ GGTTAAATGA TTTGAAAAAA ATCTAGTAAG GGCCCACAAC AGTATTGGAA CCCAGCTTGC CAAGGAAATG   
  
  
+ TCAGAGGGGC TAGCAATGGA CCAGTGCAAG AAGGCCAAGC CACATTTGGT TAGGGAATTT CATCCCATCC   
  
  
+ GGCGCTAAAA GCATCAAAAT TACTACTTAA TCCTTGATCA AATGCCCTAT AAATATTCTA CTACCCCATT   
  
  
+ TGAAAAGGGG TGTTGATTAC AACACATAGC CGTAGGTTTG ACAAAGAGAA TTACCCTTAT TGTTAGAAGC   
  
  
+ ACCATATACT CTTTTCTCTA AGCTCTGATC TTTTTTCATC CTTTTCAGCT TTTTGTCCTT TTAAATCAAA   
  
  
+ TCACTCACTT GAGTATTGGA GGGACGTTCC TCAGGAGTCG AACCACTAAA TTATCTTTTT AAAGAAAAAG   
  
  
+ GGCTTGAAGC CAACCCGATT TAAAAAAACT GCTAATATGT AAGCCTACTC TCTTTCCAGT TCCAACAAAT   
  
  
+ CAAATTTCAG TATGAAACAT CCAGTAAAGG ATTTATCCGT GATAATGATG TAACATATGG TTAAAGGGTC   
  
  
+ ATTTTCAGTT ACATCGGAGC ATGCAATTAA AGTCCCTATA AAATAGGTAG GTGCACGAAC AAGTCACCAT   
  
  
+ CGTCCTTTGC TTTTTGCATG AGTGCATGCT AATTATGAAC TTAATTAATA TCTAAGCAAA ATTAAAAAGT   
  
  
+ AGTTAAAACT TTTTCACCTT TGGAACCTCG TAGCTGTAGC AGTAGCTGCC GCACTCTGGT TCTAGACTTC   
  
  
+ TAGGGACCAA GAGATGCACT CATATGCATC CTCTTCTGTC CTTTTACTGT ATCCCCTTCC CATGTGACCT   
  
  
+ TTTTGCCCGG GTGACTTTTC TGTGAGTGTT GATACAAATT TTGTACGTAA GCTAACTCAT TTACTTCACC   
  
  
+ GTCTCATGAG TTTTACATTA CCGATATTAA TTTAAATGGC AAAGTATTAA ATTTGAGCTA AAATCTCATA   
  
  
+ TATATATTTG TATTAAATGT GTCTGGATTG TCAAAATAAT TTTTGACATA TACAGTCACT ATATACTGAG   
  
  
+ TTATTAAAAT ATATAAAAAT ATGAAAATTT AAAAAATAGT AGTTTAAGGT GACAACACTA AAACAAACAA   
  
  
+ ATAAGATATA AATAGGGAAA AGTTCATCCT ATTTTGTTAT TATTATTTAT TATGTATTTT CAGAATTGTC   
  
  
+ TCTATCACAA GCATAGTATA TGTGTCAACA TCGTGACAAC TCGACCAATC TACAAATAAC TAGTAAATTC   
  
  
+ AAATAAAGTT ACTCTCACCT AGCTTAAATA AGTACACCTT GATTTAATTG TCTTAGCTAT GGAACCTATT   
  
  
+ CCAAACTAGC CAATACTTCC TTTTCAAAAG AATTAGTTTT AAAGTTTTAA CTTTTACGTC CAATAATGCC   
  
  
+ GGCCGTACTA TGAGTTGCTT TCAAGCCAGT AGTAGTACTG TAGTAGGTAA AAATATTAGA ATACACCTAC   
  
  
+ TATATATGAT ATTGCTCTTC ACCGTATCCT TCTGCATACT CATCTTTCCG TCCCGGAGTC CACTATCTCT   
  
  
+ CCTCCATTCA GACACTCTCT CGCTCTAGAG AACTCATAGC TATGGACCGC GTGCCCGACG GTTATCGGGT   
  
  
+ TATGAATTCG GAGCTTCTTC AGCAAGTTAG CCCTGAGTTG CCGGATCCAA CCACCTGGTT CCATGCCCGT   
  
  
+ CTACCCGACC CCATCTCTCA GTCTCCTCTT GGACCCGGTC CATCCTCCCA GCCAACCCAT TTCTACCAGG   
  
  
+ GTTCTGCCTC AGGGAACCAT GTGGTTGGGA TTGCTGACAC GTGGACGGAC CAGATTGTTG CAGGTTCCTC   
  
  
+ GTCCCAGCAG TGAGGTTGCA GCAGAGCCAG CAGGTGTTGA CTGCAGTCGA AGCCATGGAG GAGGACTCTG   
  
  
+ GGATAAGACT GGTCTACGCT CTGCTGACGT GTGCGGAGGC CATCCAACGT GGCGATTTCC GATTGGCTGT   
  
  
+ CTTGTTAGTT AACAAGATGA GCAATGACCT CCTGCCACGC GTCAACCCGT CCTGCGGTAT TGGCAAAGTA   
  
  
+ GCCGGCTACT TCATAGATGC CTTGACCCGA AGACTATACC AGCAGGGCCC AGTGTCGGGC CTAATCGGGT   
  
  
+ CGGTTCTGGC GTACCAGGTG TTGTACGAGC ACTTCTATGA AGCTTGCCCG TTCCTCAAAT TCGCTCACTT   
  
  
+ CACTGCTAAT CAAGCGATTT TAGAGGCATT CGACGGCCAC GATTGTGTCC ACATCATCGA CTTCGGCCTA   
  
  
+ ATCCACGGCT TGCAATGGCC AGCTCTAATC CAAGCCTTGG CTGTTCGGCA CGGTGGCCCG CCTTTCGTAA   
  
  
+ GATTAACCGG AATCGGGCCG CCTTCTGAAT ACGGGTCGTG CTCGCTTCAA CCTATCGGGT CAACACTGGC   
  
  
+ CCAGTTAGCC CTATCCATGA ATGTTGGGTT CGCATTTCGG GCCGTCGCCG TCTCACGGCT CGAGGACATC   
  
  
+ AAACCATGGA TGGTTAAAAC AAGCCCAAAT GAAGTCGTAG CCGTGAACTC TATCTTCCAA CTCCACCGGC   
  
  
+ TAATCGGGTC GGGTATTGAC CCTGTCCTAA ACTGGGTCCG GAGCCTAAAC CCGAAAATTG TGACACTGGC   
  
  
+ GGAGCAAGAG GCGAACCACA ACCAGCCCGA GTTTTTGGCC CGGTTCACGG AGGCATTACA TTACTATTCA   
  
  
+ ACTATGTTCG ACTCGTTAGA GGCTTGTCAA GTCCAGGCCG ACAAGGACCT AGCCGAGCTA TACTTAGAGA   
  
  
+ GAGAGTTATC CAACATCGTC TGCTGTGAGG GGTCGGCTCG GATTGAGAGG CACGAGCCGC TGGCCCAATG   
  
  
+ GAGGGCCCGA ATGGCTCGGG CCGGGTTCAA GAAGGTGGAT ATGGGTAAAA ATGCGTTCAA GCAAGTAAGC   
  
  
+ ATGTTGCTGA GTTTGTCTTC AGCACAAGGG TATTGTGTGG AGGAGAGTGA GGGATGTTTG AAGCTCGGCT   
  
  
+ GGCATGACCG CCCTCTCATT GCGGCTTCGG CTTGGCGAGC GGAGACTCAA GCTGAGAACT CCAGCACTGT   
  
  
+ TGTGCTTGAT GGGTCATCGT CGTGTAGTTC ATCTTCTTA  

- -Up\_Stream \_Len000TTTTTT CTTTCTTTAC CTCTTTGGTT CGTTATACCT ATTTTTTTTT TGTTTCATTA   
  
  
- TTTCCTTCCT ACTCCGAGGT GTAAGGTGAG CGGGCGCGGT GAGTATCCTC TACTTATCAT TTTACACTCC   
  
  
- CCAATTTACT AAACTTTTTT TAGATCATTC CCGGGTGTTG TCATAACCTT GGGTCGAACG GTTCCTTTAC   
  
  
- AGTCTCCCCG ATCGTTACCT GGTCACGTTC TTCCGGTTCG GTGTAAACCA ATCCCTTAAA GTAGGGTAGG   
  
  
- CCGCGATTTT CGTAGTTTTA ATGATGAATT AGGAACTAGT TTACGGGATA TTTATAAGAT GATGGGGTAA   
  
  
- ACTTTTCCCC ACAACTAATG TTGTGTATCG GCATCCAAAC TGTTTCTCTT AATGGGAATA ACAATCTTCG   
  
  
- TGGTATATGA GAAAAGAGAT TCGAGACTAG AAAAAAGTAG GAAAAGTCGA AAAACAGGAA AATTTAGTTT   
  
  
- AGTGAGTGAA CTCATAACCT CCCTGCAAGG AGTCCTCAGC TTGGTGATTT AATAGAAAAA TTTCTTTTTC   
  
  
- CCGAACTTCG GTTGGGCTAA ATTTTTTTGA CGATTATACA TTCGGATGAG AGAAAGGTCA AGGTTGTTTA   
  
  
- GTTTAAAGTC ATACTTTGTA GGTCATTTCC TAAATAGGCA CTATTACTAC ATTGTATACC AATTTCCCAG   
  
  
- TAAAAGTCAA TGTAGCCTCG TACGTTAATT TCAGGGATAT TTTATCCATC CACGTGCTTG TTCAGTGGTA   
  
  
- GCAGGAAACG AAAAACGTAC TCACGTACGA TTAATACTTG AATTAATTAT AGATTCGTTT TAATTTTTCA   
  
  
- TCAATTTTGA AAAAGTGGAA ACCTTGGAGC ATCGACATCG TCATCGACGG CGTGAGACCA AGATCTGAAG   
  
  
- ATCCCTGGTT CTCTACGTGA GTATACGTAG GAGAAGACAG GAAAATGACA TAGGGGAAGG GTACACTGGA   
  
  
- AAAACGGGCC CACTGAAAAG ACACTCACAA CTATGTTTAA AACATGCATT CGATTGAGTA AATGAAGTGG   
  
  
- CAGAGTACTC AAAATGTAAT GGCTATAATT AAATTTACCG TTTCATAATT TAAACTCGAT TTTAGAGTAT   
  
  
- ATATATAAAC ATAATTTACA CAGACCTAAC AGTTTTATTA AAAACTGTAT ATGTCAGTGA TATATGACTC   
  
  
- AATAATTTTA TATATTTTTA TACTTTTAAA TTTTTTATCA TCAAATTCCA CTGTTGTGAT TTTGTTTGTT   
  
  
- TATTCTATAT TTATCCCTTT TCAAGTAGGA TAAAACAATA ATAATAAATA ATACATAAAA GTCTTAACAG   
  
  
- AGATAGTGTT CGTATCATAT ACACAGTTGT AGCACTGTTG AGCTGGTTAG ATGTTTATTG ATCATTTAAG   
  
  
- TTTATTTCAA TGAGAGTGGA TCGAATTTAT TCATGTGGAA CTAAATTAAC AGAATCGATA CCTTGGATAA   
  
  
- GGTTTGATCG GTTATGAAGG AAAAGTTTTC TTAATCAAAA TTTCAAAATT GAAAATGCAG GTTATTACGG   
  
  
- CCGGCATGAT ACTCAACGAA AGTTCGGTCA TCATCATGAC ATCATCCATT TTTATAATCT TATGTGGATG   
  
  
- ATATATACTA TAACGAGAAG TGGCATAGGA AGACGTATGA GTAGAAAGGC AGGGCCTCAG GTGATAGAGA   
  
  
- GGAGGTAAGT CTGTGAGAGA GCGAGATCTC TTGAGTATCG ATACCTGGCG CACGGGCTGC CAATAGCCCA   
  
  
- ATACTTAAGC CTCGAAGAAG TCGTTCAATC GGGACTCAAC GGCCTAGGTT GGTGGACCAA GGTACGGGCA   
  
  
- GATGGGCTGG GGTAGAGAGT CAGAGGAGAA CCTGGGCCAG GTAGGAGGGT CGGTTGGGTA AAGATGGTCC   
  
  
- CAAGACGGAG TCCCTTGGTA CACCAACCCT AACGACTGTG CACCTGCCTG GTCTAACAAC GTCCAAGGAG   
  
  
- CAGGGTCGTC ACTCCAACGT CGTCTCGGTC GTCCACAACT GACGTCAGCT TCGGTACCTC CTCCTGAGAC   
  
  
- CCTATTCTGA CCAGATGCGA GACGACTGCA CACGCCTCCG GTAGGTTGCA CCGCTAAAGG CTAACCGACA   
  
  
- GAACAATCAA TTGTTCTACT CGTTACTGGA GGACGGTGCG CAGTTGGGCA GGACGCCATA ACCGTTTCAT   
  
  
- CGGCCGATGA AGTATCTACG GAACTGGGCT TCTGATATGG TCGTCCCGGG TCACAGCCCG GATTAGCCCA   
  
  
- GCCAAGACCG CATGGTCCAC AACATGCTCG TGAAGATACT TCGAACGGGC AAGGAGTTTA AGCGAGTGAA   
  
  
- GTGACGATTA GTTCGCTAAA ATCTCCGTAA GCTGCCGGTG CTAACACAGG TGTAGTAGCT GAAGCCGGAT   
  
  
- TAGGTGCCGA ACGTTACCGG TCGAGATTAG GTTCGGAACC GACAAGCCGT GCCACCGGGC GGAAAGCATT   
  
  
- CTAATTGGCC TTAGCCCGGC GGAAGACTTA TGCCCAGCAC GAGCGAAGTT GGATAGCCCA GTTGTGACCG   
  
  
- GGTCAATCGG GATAGGTACT TACAACCCAA GCGTAAAGCC CGGCAGCGGC AGAGTGCCGA GCTCCTGTAG   
  
  
- TTTGGTACCT ACCAATTTTG TTCGGGTTTA CTTCAGCATC GGCACTTGAG ATAGAAGGTT GAGGTGGCCG   
  
  
- ATTAGCCCAG CCCATAACTG GGACAGGATT TGACCCAGGC CTCGGATTTG GGCTTTTAAC ACTGTGACCG   
  
  
- CCTCGTTCTC CGCTTGGTGT TGGTCGGGCT CAAAAACCGG GCCAAGTGCC TCCGTAATGT AATGATAAGT   
  
  
- TGATACAAGC TGAGCAATCT CCGAACAGTT CAGGTCCGGC TGTTCCTGGA TCGGCTCGAT ATGAATCTCT   
  
  
- CTCTCAATAG GTTGTAGCAG ACGACACTCC CCAGCCGAGC CTAACTCTCC GTGCTCGGCG ACCGGGTTAC   
  
  
- CTCCCGGGCT TACCGAGCCC GGCCCAAGTT CTTCCACCTA TACCCATTTT TACGCAAGTT CGTTCATTCG   
  
  
- TACAACGACT CAAACAGAAG TCGTGTTCCC ATAACACACC TCCTCTCACT CCCTACAAAC TTCGAGCCGA   
  
  
- CCGTACTGGC GGGAGAGTAA CGCCGAAGCC GAACCGCTCG CCTCTGAGTT CGACTCTTGA GGTCGTGACA   
  
  
- ACACGAACTA CCCAGTAGCA GCACATCAAG TAGAAGAAT

+     CGTCA-motif

| Site Name | Organism | Position | Strand | Matrix score. | sequence | function |
| --- | --- | --- | --- | --- | --- | --- |
| CGTCA-motif | Hordeum vulgare | 2144 | + | 5 | CGTCA | cis-acting regulatory element involved in the MeJA-responsiveness |
| CGTCA-motif | Hordeum vulgare | 2059 | - | 5 | CGTCA | cis-acting regulatory element involved in the MeJA-responsiveness |

>HU11G00778.1   
+ -Up\_Stream \_Len000AAAAAA GAAAGAAATG GAGAAACCAA GCAATATGGA TAAAAAAAAA ACAAAGTAAT   
  
  
+ AAAGGAAGGA TGAGGCTCCA CATTCCACTC GCCCGCGCCA CTCATAGGAG ATGAATAGTA AAATGTGAGG   
  
  
+ GGTTAAATGA TTTGAAAAAA ATCTAGTAAG GGCCCACAAC AGTATTGGAA CCCAGCTTGC CAAGGAAATG   
  
  
+ TCAGAGGGGC TAGCAATGGA CCAGTGCAAG AAGGCCAAGC CACATTTGGT TAGGGAATTT CATCCCATCC   
  
  
+ GGCGCTAAAA GCATCAAAAT TACTACTTAA TCCTTGATCA AATGCCCTAT AAATATTCTA CTACCCCATT   
  
  
+ TGAAAAGGGG TGTTGATTAC AACACATAGC CGTAGGTTTG ACAAAGAGAA TTACCCTTAT TGTTAGAAGC   
  
  
+ ACCATATACT CTTTTCTCTA AGCTCTGATC TTTTTTCATC CTTTTCAGCT TTTTGTCCTT TTAAATCAAA   
  
  
+ TCACTCACTT GAGTATTGGA GGGACGTTCC TCAGGAGTCG AACCACTAAA TTATCTTTTT AAAGAAAAAG   
  
  
+ GGCTTGAAGC CAACCCGATT TAAAAAAACT GCTAATATGT AAGCCTACTC TCTTTCCAGT TCCAACAAAT   
  
  
+ CAAATTTCAG TATGAAACAT CCAGTAAAGG ATTTATCCGT GATAATGATG TAACATATGG TTAAAGGGTC   
  
  
+ ATTTTCAGTT ACATCGGAGC ATGCAATTAA AGTCCCTATA AAATAGGTAG GTGCACGAAC AAGTCACCAT   
  
  
+ CGTCCTTTGC TTTTTGCATG AGTGCATGCT AATTATGAAC TTAATTAATA TCTAAGCAAA ATTAAAAAGT   
  
  
+ AGTTAAAACT TTTTCACCTT TGGAACCTCG TAGCTGTAGC AGTAGCTGCC GCACTCTGGT TCTAGACTTC   
  
  
+ TAGGGACCAA GAGATGCACT CATATGCATC CTCTTCTGTC CTTTTACTGT ATCCCCTTCC CATGTGACCT   
  
  
+ TTTTGCCCGG GTGACTTTTC TGTGAGTGTT GATACAAATT TTGTACGTAA GCTAACTCAT TTACTTCACC   
  
  
+ GTCTCATGAG TTTTACATTA CCGATATTAA TTTAAATGGC AAAGTATTAA ATTTGAGCTA AAATCTCATA   
  
  
+ TATATATTTG TATTAAATGT GTCTGGATTG TCAAAATAAT TTTTGACATA TACAGTCACT ATATACTGAG   
  
  
+ TTATTAAAAT ATATAAAAAT ATGAAAATTT AAAAAATAGT AGTTTAAGGT GACAACACTA AAACAAACAA   
  
  
+ ATAAGATATA AATAGGGAAA AGTTCATCCT ATTTTGTTAT TATTATTTAT TATGTATTTT CAGAATTGTC   
  
  
+ TCTATCACAA GCATAGTATA TGTGTCAACA TCGTGACAAC TCGACCAATC TACAAATAAC TAGTAAATTC   
  
  
+ AAATAAAGTT ACTCTCACCT AGCTTAAATA AGTACACCTT GATTTAATTG TCTTAGCTAT GGAACCTATT   
  
  
+ CCAAACTAGC CAATACTTCC TTTTCAAAAG AATTAGTTTT AAAGTTTTAA CTTTTACGTC CAATAATGCC   
  
  
+ GGCCGTACTA TGAGTTGCTT TCAAGCCAGT AGTAGTACTG TAGTAGGTAA AAATATTAGA ATACACCTAC   
  
  
+ TATATATGAT ATTGCTCTTC ACCGTATCCT TCTGCATACT CATCTTTCCG TCCCGGAGTC CACTATCTCT   
  
  
+ CCTCCATTCA GACACTCTCT CGCTCTAGAG AACTCATAGC TATGGACCGC GTGCCCGACG GTTATCGGGT   
  
  
+ TATGAATTCG GAGCTTCTTC AGCAAGTTAG CCCTGAGTTG CCGGATCCAA CCACCTGGTT CCATGCCCGT   
  
  
+ CTACCCGACC CCATCTCTCA GTCTCCTCTT GGACCCGGTC CATCCTCCCA GCCAACCCAT TTCTACCAGG   
  
  
+ GTTCTGCCTC AGGGAACCAT GTGGTTGGGA TTGCTGACAC GTGGACGGAC CAGATTGTTG CAGGTTCCTC   
  
  
+ GTCCCAGCAG TGAGGTTGCA GCAGAGCCAG CAGGTGTTGA CTGCAGTCGA AGCCATGGAG GAGGACTCTG   
  
  
+ GGATAAGACT GGTCTACGCT CTGCTGACGT GTGCGGAGGC CATCCAACGT GGCGATTTCC GATTGGCTGT   
  
  
+ CTTGTTAGTT AACAAGATGA GCAATGACCT CCTGCCACGC GTCAACCCGT CCTGCGGTAT TGGCAAAGTA   
  
  
+ GCCGGCTACT TCATAGATGC CTTGACCCGA AGACTATACC AGCAGGGCCC AGTGTCGGGC CTAATCGGGT   
  
  
+ CGGTTCTGGC GTACCAGGTG TTGTACGAGC ACTTCTATGA AGCTTGCCCG TTCCTCAAAT TCGCTCACTT   
  
  
+ CACTGCTAAT CAAGCGATTT TAGAGGCATT CGACGGCCAC GATTGTGTCC ACATCATCGA CTTCGGCCTA   
  
  
+ ATCCACGGCT TGCAATGGCC AGCTCTAATC CAAGCCTTGG CTGTTCGGCA CGGTGGCCCG CCTTTCGTAA   
  
  
+ GATTAACCGG AATCGGGCCG CCTTCTGAAT ACGGGTCGTG CTCGCTTCAA CCTATCGGGT CAACACTGGC   
  
  
+ CCAGTTAGCC CTATCCATGA ATGTTGGGTT CGCATTTCGG GCCGTCGCCG TCTCACGGCT CGAGGACATC   
  
  
+ AAACCATGGA TGGTTAAAAC AAGCCCAAAT GAAGTCGTAG CCGTGAACTC TATCTTCCAA CTCCACCGGC   
  
  
+ TAATCGGGTC GGGTATTGAC CCTGTCCTAA ACTGGGTCCG GAGCCTAAAC CCGAAAATTG TGACACTGGC   
  
  
+ GGAGCAAGAG GCGAACCACA ACCAGCCCGA GTTTTTGGCC CGGTTCACGG AGGCATTACA TTACTATTCA   
  
  
+ ACTATGTTCG ACTCGTTAGA GGCTTGTCAA GTCCAGGCCG ACAAGGACCT AGCCGAGCTA TACTTAGAGA   
  
  
+ GAGAGTTATC CAACATCGTC TGCTGTGAGG GGTCGGCTCG GATTGAGAGG CACGAGCCGC TGGCCCAATG   
  
  
+ GAGGGCCCGA ATGGCTCGGG CCGGGTTCAA GAAGGTGGAT ATGGGTAAAA ATGCGTTCAA GCAAGTAAGC   
  
  
+ ATGTTGCTGA GTTTGTCTTC AGCACAAGGG TATTGTGTGG AGGAGAGTGA GGGATGTTTG AAGCTCGGCT   
  
  
+ GGCATGACCG CCCTCTCATT GCGGCTTCGG CTTGGCGAGC GGAGACTCAA GCTGAGAACT CCAGCACTGT   
  
  
+ TGTGCTTGAT GGGTCATCGT CGTGTAGTTC ATCTTCTTA  

- -Up\_Stream \_Len000TTTTTT CTTTCTTTAC CTCTTTGGTT CGTTATACCT ATTTTTTTTT TGTTTCATTA   
  
  
- TTTCCTTCCT ACTCCGAGGT GTAAGGTGAG CGGGCGCGGT GAGTATCCTC TACTTATCAT TTTACACTCC   
  
  
- CCAATTTACT AAACTTTTTT TAGATCATTC CCGGGTGTTG TCATAACCTT GGGTCGAACG GTTCCTTTAC   
  
  
- AGTCTCCCCG ATCGTTACCT GGTCACGTTC TTCCGGTTCG GTGTAAACCA ATCCCTTAAA GTAGGGTAGG   
  
  
- CCGCGATTTT CGTAGTTTTA ATGATGAATT AGGAACTAGT TTACGGGATA TTTATAAGAT GATGGGGTAA   
  
  
- ACTTTTCCCC ACAACTAATG TTGTGTATCG GCATCCAAAC TGTTTCTCTT AATGGGAATA ACAATCTTCG   
  
  
- TGGTATATGA GAAAAGAGAT TCGAGACTAG AAAAAAGTAG GAAAAGTCGA AAAACAGGAA AATTTAGTTT   
  
  
- AGTGAGTGAA CTCATAACCT CCCTGCAAGG AGTCCTCAGC TTGGTGATTT AATAGAAAAA TTTCTTTTTC   
  
  
- CCGAACTTCG GTTGGGCTAA ATTTTTTTGA CGATTATACA TTCGGATGAG AGAAAGGTCA AGGTTGTTTA   
  
  
- GTTTAAAGTC ATACTTTGTA GGTCATTTCC TAAATAGGCA CTATTACTAC ATTGTATACC AATTTCCCAG   
  
  
- TAAAAGTCAA TGTAGCCTCG TACGTTAATT TCAGGGATAT TTTATCCATC CACGTGCTTG TTCAGTGGTA   
  
  
- GCAGGAAACG AAAAACGTAC TCACGTACGA TTAATACTTG AATTAATTAT AGATTCGTTT TAATTTTTCA   
  
  
- TCAATTTTGA AAAAGTGGAA ACCTTGGAGC ATCGACATCG TCATCGACGG CGTGAGACCA AGATCTGAAG   
  
  
- ATCCCTGGTT CTCTACGTGA GTATACGTAG GAGAAGACAG GAAAATGACA TAGGGGAAGG GTACACTGGA   
  
  
- AAAACGGGCC CACTGAAAAG ACACTCACAA CTATGTTTAA AACATGCATT CGATTGAGTA AATGAAGTGG   
  
  
- CAGAGTACTC AAAATGTAAT GGCTATAATT AAATTTACCG TTTCATAATT TAAACTCGAT TTTAGAGTAT   
  
  
- ATATATAAAC ATAATTTACA CAGACCTAAC AGTTTTATTA AAAACTGTAT ATGTCAGTGA TATATGACTC   
  
  
- AATAATTTTA TATATTTTTA TACTTTTAAA TTTTTTATCA TCAAATTCCA CTGTTGTGAT TTTGTTTGTT   
  
  
- TATTCTATAT TTATCCCTTT TCAAGTAGGA TAAAACAATA ATAATAAATA ATACATAAAA GTCTTAACAG   
  
  
- AGATAGTGTT CGTATCATAT ACACAGTTGT AGCACTGTTG AGCTGGTTAG ATGTTTATTG ATCATTTAAG   
  
  
- TTTATTTCAA TGAGAGTGGA TCGAATTTAT TCATGTGGAA CTAAATTAAC AGAATCGATA CCTTGGATAA   
  
  
- GGTTTGATCG GTTATGAAGG AAAAGTTTTC TTAATCAAAA TTTCAAAATT GAAAATGCAG GTTATTACGG   
  
  
- CCGGCATGAT ACTCAACGAA AGTTCGGTCA TCATCATGAC ATCATCCATT TTTATAATCT TATGTGGATG   
  
  
- ATATATACTA TAACGAGAAG TGGCATAGGA AGACGTATGA GTAGAAAGGC AGGGCCTCAG GTGATAGAGA   
  
  
- GGAGGTAAGT CTGTGAGAGA GCGAGATCTC TTGAGTATCG ATACCTGGCG CACGGGCTGC CAATAGCCCA   
  
  
- ATACTTAAGC CTCGAAGAAG TCGTTCAATC GGGACTCAAC GGCCTAGGTT GGTGGACCAA GGTACGGGCA   
  
  
- GATGGGCTGG GGTAGAGAGT CAGAGGAGAA CCTGGGCCAG GTAGGAGGGT CGGTTGGGTA AAGATGGTCC   
  
  
- CAAGACGGAG TCCCTTGGTA CACCAACCCT AACGACTGTG CACCTGCCTG GTCTAACAAC GTCCAAGGAG   
  
  
- CAGGGTCGTC ACTCCAACGT CGTCTCGGTC GTCCACAACT GACGTCAGCT TCGGTACCTC CTCCTGAGAC   
  
  
- CCTATTCTGA CCAGATGCGA GACGACTGCA CACGCCTCCG GTAGGTTGCA CCGCTAAAGG CTAACCGACA   
  
  
- GAACAATCAA TTGTTCTACT CGTTACTGGA GGACGGTGCG CAGTTGGGCA GGACGCCATA ACCGTTTCAT   
  
  
- CGGCCGATGA AGTATCTACG GAACTGGGCT TCTGATATGG TCGTCCCGGG TCACAGCCCG GATTAGCCCA   
  
  
- GCCAAGACCG CATGGTCCAC AACATGCTCG TGAAGATACT TCGAACGGGC AAGGAGTTTA AGCGAGTGAA   
  
  
- GTGACGATTA GTTCGCTAAA ATCTCCGTAA GCTGCCGGTG CTAACACAGG TGTAGTAGCT GAAGCCGGAT   
  
  
- TAGGTGCCGA ACGTTACCGG TCGAGATTAG GTTCGGAACC GACAAGCCGT GCCACCGGGC GGAAAGCATT   
  
  
- CTAATTGGCC TTAGCCCGGC GGAAGACTTA TGCCCAGCAC GAGCGAAGTT GGATAGCCCA GTTGTGACCG   
  
  
- GGTCAATCGG GATAGGTACT TACAACCCAA GCGTAAAGCC CGGCAGCGGC AGAGTGCCGA GCTCCTGTAG   
  
  
- TTTGGTACCT ACCAATTTTG TTCGGGTTTA CTTCAGCATC GGCACTTGAG ATAGAAGGTT GAGGTGGCCG   
  
  
- ATTAGCCCAG CCCATAACTG GGACAGGATT TGACCCAGGC CTCGGATTTG GGCTTTTAAC ACTGTGACCG   
  
  
- CCTCGTTCTC CGCTTGGTGT TGGTCGGGCT CAAAAACCGG GCCAAGTGCC TCCGTAATGT AATGATAAGT   
  
  
- TGATACAAGC TGAGCAATCT CCGAACAGTT CAGGTCCGGC TGTTCCTGGA TCGGCTCGAT ATGAATCTCT   
  
  
- CTCTCAATAG GTTGTAGCAG ACGACACTCC CCAGCCGAGC CTAACTCTCC GTGCTCGGCG ACCGGGTTAC   
  
  
- CTCCCGGGCT TACCGAGCCC GGCCCAAGTT CTTCCACCTA TACCCATTTT TACGCAAGTT CGTTCATTCG   
  
  
- TACAACGACT CAAACAGAAG TCGTGTTCCC ATAACACACC TCCTCTCACT CCCTACAAAC TTCGAGCCGA   
  
  
- CCGTACTGGC GGGAGAGTAA CGCCGAAGCC GAACCGCTCG CCTCTGAGTT CGACTCTTGA GGTCGTGACA   
  
  
- ACACGAACTA CCCAGTAGCA GCACATCAAG TAGAAGAAT

+     DRE core

| Site Name | Organism | Position | Strand | Matrix score. | sequence | function |
| --- | --- | --- | --- | --- | --- | --- |
| DRE core | Arabidopsis thaliana | 2906 | - | 6 | GCCGAC |  |
| DRE core | Arabidopsis thaliana | 2841 | + | 6 | GCCGAC |  |

>HU11G00778.1   
+ -Up\_Stream \_Len000AAAAAA GAAAGAAATG GAGAAACCAA GCAATATGGA TAAAAAAAAA ACAAAGTAAT   
  
  
+ AAAGGAAGGA TGAGGCTCCA CATTCCACTC GCCCGCGCCA CTCATAGGAG ATGAATAGTA AAATGTGAGG   
  
  
+ GGTTAAATGA TTTGAAAAAA ATCTAGTAAG GGCCCACAAC AGTATTGGAA CCCAGCTTGC CAAGGAAATG   
  
  
+ TCAGAGGGGC TAGCAATGGA CCAGTGCAAG AAGGCCAAGC CACATTTGGT TAGGGAATTT CATCCCATCC   
  
  
+ GGCGCTAAAA GCATCAAAAT TACTACTTAA TCCTTGATCA AATGCCCTAT AAATATTCTA CTACCCCATT   
  
  
+ TGAAAAGGGG TGTTGATTAC AACACATAGC CGTAGGTTTG ACAAAGAGAA TTACCCTTAT TGTTAGAAGC   
  
  
+ ACCATATACT CTTTTCTCTA AGCTCTGATC TTTTTTCATC CTTTTCAGCT TTTTGTCCTT TTAAATCAAA   
  
  
+ TCACTCACTT GAGTATTGGA GGGACGTTCC TCAGGAGTCG AACCACTAAA TTATCTTTTT AAAGAAAAAG   
  
  
+ GGCTTGAAGC CAACCCGATT TAAAAAAACT GCTAATATGT AAGCCTACTC TCTTTCCAGT TCCAACAAAT   
  
  
+ CAAATTTCAG TATGAAACAT CCAGTAAAGG ATTTATCCGT GATAATGATG TAACATATGG TTAAAGGGTC   
  
  
+ ATTTTCAGTT ACATCGGAGC ATGCAATTAA AGTCCCTATA AAATAGGTAG GTGCACGAAC AAGTCACCAT   
  
  
+ CGTCCTTTGC TTTTTGCATG AGTGCATGCT AATTATGAAC TTAATTAATA TCTAAGCAAA ATTAAAAAGT   
  
  
+ AGTTAAAACT TTTTCACCTT TGGAACCTCG TAGCTGTAGC AGTAGCTGCC GCACTCTGGT TCTAGACTTC   
  
  
+ TAGGGACCAA GAGATGCACT CATATGCATC CTCTTCTGTC CTTTTACTGT ATCCCCTTCC CATGTGACCT   
  
  
+ TTTTGCCCGG GTGACTTTTC TGTGAGTGTT GATACAAATT TTGTACGTAA GCTAACTCAT TTACTTCACC   
  
  
+ GTCTCATGAG TTTTACATTA CCGATATTAA TTTAAATGGC AAAGTATTAA ATTTGAGCTA AAATCTCATA   
  
  
+ TATATATTTG TATTAAATGT GTCTGGATTG TCAAAATAAT TTTTGACATA TACAGTCACT ATATACTGAG   
  
  
+ TTATTAAAAT ATATAAAAAT ATGAAAATTT AAAAAATAGT AGTTTAAGGT GACAACACTA AAACAAACAA   
  
  
+ ATAAGATATA AATAGGGAAA AGTTCATCCT ATTTTGTTAT TATTATTTAT TATGTATTTT CAGAATTGTC   
  
  
+ TCTATCACAA GCATAGTATA TGTGTCAACA TCGTGACAAC TCGACCAATC TACAAATAAC TAGTAAATTC   
  
  
+ AAATAAAGTT ACTCTCACCT AGCTTAAATA AGTACACCTT GATTTAATTG TCTTAGCTAT GGAACCTATT   
  
  
+ CCAAACTAGC CAATACTTCC TTTTCAAAAG AATTAGTTTT AAAGTTTTAA CTTTTACGTC CAATAATGCC   
  
  
+ GGCCGTACTA TGAGTTGCTT TCAAGCCAGT AGTAGTACTG TAGTAGGTAA AAATATTAGA ATACACCTAC   
  
  
+ TATATATGAT ATTGCTCTTC ACCGTATCCT TCTGCATACT CATCTTTCCG TCCCGGAGTC CACTATCTCT   
  
  
+ CCTCCATTCA GACACTCTCT CGCTCTAGAG AACTCATAGC TATGGACCGC GTGCCCGACG GTTATCGGGT   
  
  
+ TATGAATTCG GAGCTTCTTC AGCAAGTTAG CCCTGAGTTG CCGGATCCAA CCACCTGGTT CCATGCCCGT   
  
  
+ CTACCCGACC CCATCTCTCA GTCTCCTCTT GGACCCGGTC CATCCTCCCA GCCAACCCAT TTCTACCAGG   
  
  
+ GTTCTGCCTC AGGGAACCAT GTGGTTGGGA TTGCTGACAC GTGGACGGAC CAGATTGTTG CAGGTTCCTC   
  
  
+ GTCCCAGCAG TGAGGTTGCA GCAGAGCCAG CAGGTGTTGA CTGCAGTCGA AGCCATGGAG GAGGACTCTG   
  
  
+ GGATAAGACT GGTCTACGCT CTGCTGACGT GTGCGGAGGC CATCCAACGT GGCGATTTCC GATTGGCTGT   
  
  
+ CTTGTTAGTT AACAAGATGA GCAATGACCT CCTGCCACGC GTCAACCCGT CCTGCGGTAT TGGCAAAGTA   
  
  
+ GCCGGCTACT TCATAGATGC CTTGACCCGA AGACTATACC AGCAGGGCCC AGTGTCGGGC CTAATCGGGT   
  
  
+ CGGTTCTGGC GTACCAGGTG TTGTACGAGC ACTTCTATGA AGCTTGCCCG TTCCTCAAAT TCGCTCACTT   
  
  
+ CACTGCTAAT CAAGCGATTT TAGAGGCATT CGACGGCCAC GATTGTGTCC ACATCATCGA CTTCGGCCTA   
  
  
+ ATCCACGGCT TGCAATGGCC AGCTCTAATC CAAGCCTTGG CTGTTCGGCA CGGTGGCCCG CCTTTCGTAA   
  
  
+ GATTAACCGG AATCGGGCCG CCTTCTGAAT ACGGGTCGTG CTCGCTTCAA CCTATCGGGT CAACACTGGC   
  
  
+ CCAGTTAGCC CTATCCATGA ATGTTGGGTT CGCATTTCGG GCCGTCGCCG TCTCACGGCT CGAGGACATC   
  
  
+ AAACCATGGA TGGTTAAAAC AAGCCCAAAT GAAGTCGTAG CCGTGAACTC TATCTTCCAA CTCCACCGGC   
  
  
+ TAATCGGGTC GGGTATTGAC CCTGTCCTAA ACTGGGTCCG GAGCCTAAAC CCGAAAATTG TGACACTGGC   
  
  
+ GGAGCAAGAG GCGAACCACA ACCAGCCCGA GTTTTTGGCC CGGTTCACGG AGGCATTACA TTACTATTCA   
  
  
+ ACTATGTTCG ACTCGTTAGA GGCTTGTCAA GTCCAGGCCG ACAAGGACCT AGCCGAGCTA TACTTAGAGA   
  
  
+ GAGAGTTATC CAACATCGTC TGCTGTGAGG GGTCGGCTCG GATTGAGAGG CACGAGCCGC TGGCCCAATG   
  
  
+ GAGGGCCCGA ATGGCTCGGG CCGGGTTCAA GAAGGTGGAT ATGGGTAAAA ATGCGTTCAA GCAAGTAAGC   
  
  
+ ATGTTGCTGA GTTTGTCTTC AGCACAAGGG TATTGTGTGG AGGAGAGTGA GGGATGTTTG AAGCTCGGCT   
  
  
+ GGCATGACCG CCCTCTCATT GCGGCTTCGG CTTGGCGAGC GGAGACTCAA GCTGAGAACT CCAGCACTGT   
  
  
+ TGTGCTTGAT GGGTCATCGT CGTGTAGTTC ATCTTCTTA  

- -Up\_Stream \_Len000TTTTTT CTTTCTTTAC CTCTTTGGTT CGTTATACCT ATTTTTTTTT TGTTTCATTA   
  
  
- TTTCCTTCCT ACTCCGAGGT GTAAGGTGAG CGGGCGCGGT GAGTATCCTC TACTTATCAT TTTACACTCC   
  
  
- CCAATTTACT AAACTTTTTT TAGATCATTC CCGGGTGTTG TCATAACCTT GGGTCGAACG GTTCCTTTAC   
  
  
- AGTCTCCCCG ATCGTTACCT GGTCACGTTC TTCCGGTTCG GTGTAAACCA ATCCCTTAAA GTAGGGTAGG   
  
  
- CCGCGATTTT CGTAGTTTTA ATGATGAATT AGGAACTAGT TTACGGGATA TTTATAAGAT GATGGGGTAA   
  
  
- ACTTTTCCCC ACAACTAATG TTGTGTATCG GCATCCAAAC TGTTTCTCTT AATGGGAATA ACAATCTTCG   
  
  
- TGGTATATGA GAAAAGAGAT TCGAGACTAG AAAAAAGTAG GAAAAGTCGA AAAACAGGAA AATTTAGTTT   
  
  
- AGTGAGTGAA CTCATAACCT CCCTGCAAGG AGTCCTCAGC TTGGTGATTT AATAGAAAAA TTTCTTTTTC   
  
  
- CCGAACTTCG GTTGGGCTAA ATTTTTTTGA CGATTATACA TTCGGATGAG AGAAAGGTCA AGGTTGTTTA   
  
  
- GTTTAAAGTC ATACTTTGTA GGTCATTTCC TAAATAGGCA CTATTACTAC ATTGTATACC AATTTCCCAG   
  
  
- TAAAAGTCAA TGTAGCCTCG TACGTTAATT TCAGGGATAT TTTATCCATC CACGTGCTTG TTCAGTGGTA   
  
  
- GCAGGAAACG AAAAACGTAC TCACGTACGA TTAATACTTG AATTAATTAT AGATTCGTTT TAATTTTTCA   
  
  
- TCAATTTTGA AAAAGTGGAA ACCTTGGAGC ATCGACATCG TCATCGACGG CGTGAGACCA AGATCTGAAG   
  
  
- ATCCCTGGTT CTCTACGTGA GTATACGTAG GAGAAGACAG GAAAATGACA TAGGGGAAGG GTACACTGGA   
  
  
- AAAACGGGCC CACTGAAAAG ACACTCACAA CTATGTTTAA AACATGCATT CGATTGAGTA AATGAAGTGG   
  
  
- CAGAGTACTC AAAATGTAAT GGCTATAATT AAATTTACCG TTTCATAATT TAAACTCGAT TTTAGAGTAT   
  
  
- ATATATAAAC ATAATTTACA CAGACCTAAC AGTTTTATTA AAAACTGTAT ATGTCAGTGA TATATGACTC   
  
  
- AATAATTTTA TATATTTTTA TACTTTTAAA TTTTTTATCA TCAAATTCCA CTGTTGTGAT TTTGTTTGTT   
  
  
- TATTCTATAT TTATCCCTTT TCAAGTAGGA TAAAACAATA ATAATAAATA ATACATAAAA GTCTTAACAG   
  
  
- AGATAGTGTT CGTATCATAT ACACAGTTGT AGCACTGTTG AGCTGGTTAG ATGTTTATTG ATCATTTAAG   
  
  
- TTTATTTCAA TGAGAGTGGA TCGAATTTAT TCATGTGGAA CTAAATTAAC AGAATCGATA CCTTGGATAA   
  
  
- GGTTTGATCG GTTATGAAGG AAAAGTTTTC TTAATCAAAA TTTCAAAATT GAAAATGCAG GTTATTACGG   
  
  
- CCGGCATGAT ACTCAACGAA AGTTCGGTCA TCATCATGAC ATCATCCATT TTTATAATCT TATGTGGATG   
  
  
- ATATATACTA TAACGAGAAG TGGCATAGGA AGACGTATGA GTAGAAAGGC AGGGCCTCAG GTGATAGAGA   
  
  
- GGAGGTAAGT CTGTGAGAGA GCGAGATCTC TTGAGTATCG ATACCTGGCG CACGGGCTGC CAATAGCCCA   
  
  
- ATACTTAAGC CTCGAAGAAG TCGTTCAATC GGGACTCAAC GGCCTAGGTT GGTGGACCAA GGTACGGGCA   
  
  
- GATGGGCTGG GGTAGAGAGT CAGAGGAGAA CCTGGGCCAG GTAGGAGGGT CGGTTGGGTA AAGATGGTCC   
  
  
- CAAGACGGAG TCCCTTGGTA CACCAACCCT AACGACTGTG CACCTGCCTG GTCTAACAAC GTCCAAGGAG   
  
  
- CAGGGTCGTC ACTCCAACGT CGTCTCGGTC GTCCACAACT GACGTCAGCT TCGGTACCTC CTCCTGAGAC   
  
  
- CCTATTCTGA CCAGATGCGA GACGACTGCA CACGCCTCCG GTAGGTTGCA CCGCTAAAGG CTAACCGACA   
  
  
- GAACAATCAA TTGTTCTACT CGTTACTGGA GGACGGTGCG CAGTTGGGCA GGACGCCATA ACCGTTTCAT   
  
  
- CGGCCGATGA AGTATCTACG GAACTGGGCT TCTGATATGG TCGTCCCGGG TCACAGCCCG GATTAGCCCA   
  
  
- GCCAAGACCG CATGGTCCAC AACATGCTCG TGAAGATACT TCGAACGGGC AAGGAGTTTA AGCGAGTGAA   
  
  
- GTGACGATTA GTTCGCTAAA ATCTCCGTAA GCTGCCGGTG CTAACACAGG TGTAGTAGCT GAAGCCGGAT   
  
  
- TAGGTGCCGA ACGTTACCGG TCGAGATTAG GTTCGGAACC GACAAGCCGT GCCACCGGGC GGAAAGCATT   
  
  
- CTAATTGGCC TTAGCCCGGC GGAAGACTTA TGCCCAGCAC GAGCGAAGTT GGATAGCCCA GTTGTGACCG   
  
  
- GGTCAATCGG GATAGGTACT TACAACCCAA GCGTAAAGCC CGGCAGCGGC AGAGTGCCGA GCTCCTGTAG   
  
  
- TTTGGTACCT ACCAATTTTG TTCGGGTTTA CTTCAGCATC GGCACTTGAG ATAGAAGGTT GAGGTGGCCG   
  
  
- ATTAGCCCAG CCCATAACTG GGACAGGATT TGACCCAGGC CTCGGATTTG GGCTTTTAAC ACTGTGACCG   
  
  
- CCTCGTTCTC CGCTTGGTGT TGGTCGGGCT CAAAAACCGG GCCAAGTGCC TCCGTAATGT AATGATAAGT   
  
  
- TGATACAAGC TGAGCAATCT CCGAACAGTT CAGGTCCGGC TGTTCCTGGA TCGGCTCGAT ATGAATCTCT   
  
  
- CTCTCAATAG GTTGTAGCAG ACGACACTCC CCAGCCGAGC CTAACTCTCC GTGCTCGGCG ACCGGGTTAC   
  
  
- CTCCCGGGCT TACCGAGCCC GGCCCAAGTT CTTCCACCTA TACCCATTTT TACGCAAGTT CGTTCATTCG   
  
  
- TACAACGACT CAAACAGAAG TCGTGTTCCC ATAACACACC TCCTCTCACT CCCTACAAAC TTCGAGCCGA   
  
  
- CCGTACTGGC GGGAGAGTAA CGCCGAAGCC GAACCGCTCG CCTCTGAGTT CGACTCTTGA GGTCGTGACA   
  
  
- ACACGAACTA CCCAGTAGCA GCACATCAAG TAGAAGAAT

+     G-Box

| Site Name | Organism | Position | Strand | Matrix score. | sequence | function |
| --- | --- | --- | --- | --- | --- | --- |
| G-Box | Pisum sativum | 2080 | - | 6 | CACGTT | cis-acting regulatory element involved in light responsiveness |
| G-Box | Pisum sativum | 1932 | - | 6 | CACGTG | cis-acting regulatory element involved in light responsiveness |

>HU11G00778.1   
+ -Up\_Stream \_Len000AAAAAA GAAAGAAATG GAGAAACCAA GCAATATGGA TAAAAAAAAA ACAAAGTAAT   
  
  
+ AAAGGAAGGA TGAGGCTCCA CATTCCACTC GCCCGCGCCA CTCATAGGAG ATGAATAGTA AAATGTGAGG   
  
  
+ GGTTAAATGA TTTGAAAAAA ATCTAGTAAG GGCCCACAAC AGTATTGGAA CCCAGCTTGC CAAGGAAATG   
  
  
+ TCAGAGGGGC TAGCAATGGA CCAGTGCAAG AAGGCCAAGC CACATTTGGT TAGGGAATTT CATCCCATCC   
  
  
+ GGCGCTAAAA GCATCAAAAT TACTACTTAA TCCTTGATCA AATGCCCTAT AAATATTCTA CTACCCCATT   
  
  
+ TGAAAAGGGG TGTTGATTAC AACACATAGC CGTAGGTTTG ACAAAGAGAA TTACCCTTAT TGTTAGAAGC   
  
  
+ ACCATATACT CTTTTCTCTA AGCTCTGATC TTTTTTCATC CTTTTCAGCT TTTTGTCCTT TTAAATCAAA   
  
  
+ TCACTCACTT GAGTATTGGA GGGACGTTCC TCAGGAGTCG AACCACTAAA TTATCTTTTT AAAGAAAAAG   
  
  
+ GGCTTGAAGC CAACCCGATT TAAAAAAACT GCTAATATGT AAGCCTACTC TCTTTCCAGT TCCAACAAAT   
  
  
+ CAAATTTCAG TATGAAACAT CCAGTAAAGG ATTTATCCGT GATAATGATG TAACATATGG TTAAAGGGTC   
  
  
+ ATTTTCAGTT ACATCGGAGC ATGCAATTAA AGTCCCTATA AAATAGGTAG GTGCACGAAC AAGTCACCAT   
  
  
+ CGTCCTTTGC TTTTTGCATG AGTGCATGCT AATTATGAAC TTAATTAATA TCTAAGCAAA ATTAAAAAGT   
  
  
+ AGTTAAAACT TTTTCACCTT TGGAACCTCG TAGCTGTAGC AGTAGCTGCC GCACTCTGGT TCTAGACTTC   
  
  
+ TAGGGACCAA GAGATGCACT CATATGCATC CTCTTCTGTC CTTTTACTGT ATCCCCTTCC CATGTGACCT   
  
  
+ TTTTGCCCGG GTGACTTTTC TGTGAGTGTT GATACAAATT TTGTACGTAA GCTAACTCAT TTACTTCACC   
  
  
+ GTCTCATGAG TTTTACATTA CCGATATTAA TTTAAATGGC AAAGTATTAA ATTTGAGCTA AAATCTCATA   
  
  
+ TATATATTTG TATTAAATGT GTCTGGATTG TCAAAATAAT TTTTGACATA TACAGTCACT ATATACTGAG   
  
  
+ TTATTAAAAT ATATAAAAAT ATGAAAATTT AAAAAATAGT AGTTTAAGGT GACAACACTA AAACAAACAA   
  
  
+ ATAAGATATA AATAGGGAAA AGTTCATCCT ATTTTGTTAT TATTATTTAT TATGTATTTT CAGAATTGTC   
  
  
+ TCTATCACAA GCATAGTATA TGTGTCAACA TCGTGACAAC TCGACCAATC TACAAATAAC TAGTAAATTC   
  
  
+ AAATAAAGTT ACTCTCACCT AGCTTAAATA AGTACACCTT GATTTAATTG TCTTAGCTAT GGAACCTATT   
  
  
+ CCAAACTAGC CAATACTTCC TTTTCAAAAG AATTAGTTTT AAAGTTTTAA CTTTTACGTC CAATAATGCC   
  
  
+ GGCCGTACTA TGAGTTGCTT TCAAGCCAGT AGTAGTACTG TAGTAGGTAA AAATATTAGA ATACACCTAC   
  
  
+ TATATATGAT ATTGCTCTTC ACCGTATCCT TCTGCATACT CATCTTTCCG TCCCGGAGTC CACTATCTCT   
  
  
+ CCTCCATTCA GACACTCTCT CGCTCTAGAG AACTCATAGC TATGGACCGC GTGCCCGACG GTTATCGGGT   
  
  
+ TATGAATTCG GAGCTTCTTC AGCAAGTTAG CCCTGAGTTG CCGGATCCAA CCACCTGGTT CCATGCCCGT   
  
  
+ CTACCCGACC CCATCTCTCA GTCTCCTCTT GGACCCGGTC CATCCTCCCA GCCAACCCAT TTCTACCAGG   
  
  
+ GTTCTGCCTC AGGGAACCAT GTGGTTGGGA TTGCTGACAC GTGGACGGAC CAGATTGTTG CAGGTTCCTC   
  
  
+ GTCCCAGCAG TGAGGTTGCA GCAGAGCCAG CAGGTGTTGA CTGCAGTCGA AGCCATGGAG GAGGACTCTG   
  
  
+ GGATAAGACT GGTCTACGCT CTGCTGACGT GTGCGGAGGC CATCCAACGT GGCGATTTCC GATTGGCTGT   
  
  
+ CTTGTTAGTT AACAAGATGA GCAATGACCT CCTGCCACGC GTCAACCCGT CCTGCGGTAT TGGCAAAGTA   
  
  
+ GCCGGCTACT TCATAGATGC CTTGACCCGA AGACTATACC AGCAGGGCCC AGTGTCGGGC CTAATCGGGT   
  
  
+ CGGTTCTGGC GTACCAGGTG TTGTACGAGC ACTTCTATGA AGCTTGCCCG TTCCTCAAAT TCGCTCACTT   
  
  
+ CACTGCTAAT CAAGCGATTT TAGAGGCATT CGACGGCCAC GATTGTGTCC ACATCATCGA CTTCGGCCTA   
  
  
+ ATCCACGGCT TGCAATGGCC AGCTCTAATC CAAGCCTTGG CTGTTCGGCA CGGTGGCCCG CCTTTCGTAA   
  
  
+ GATTAACCGG AATCGGGCCG CCTTCTGAAT ACGGGTCGTG CTCGCTTCAA CCTATCGGGT CAACACTGGC   
  
  
+ CCAGTTAGCC CTATCCATGA ATGTTGGGTT CGCATTTCGG GCCGTCGCCG TCTCACGGCT CGAGGACATC   
  
  
+ AAACCATGGA TGGTTAAAAC AAGCCCAAAT GAAGTCGTAG CCGTGAACTC TATCTTCCAA CTCCACCGGC   
  
  
+ TAATCGGGTC GGGTATTGAC CCTGTCCTAA ACTGGGTCCG GAGCCTAAAC CCGAAAATTG TGACACTGGC   
  
  
+ GGAGCAAGAG GCGAACCACA ACCAGCCCGA GTTTTTGGCC CGGTTCACGG AGGCATTACA TTACTATTCA   
  
  
+ ACTATGTTCG ACTCGTTAGA GGCTTGTCAA GTCCAGGCCG ACAAGGACCT AGCCGAGCTA TACTTAGAGA   
  
  
+ GAGAGTTATC CAACATCGTC TGCTGTGAGG GGTCGGCTCG GATTGAGAGG CACGAGCCGC TGGCCCAATG   
  
  
+ GAGGGCCCGA ATGGCTCGGG CCGGGTTCAA GAAGGTGGAT ATGGGTAAAA ATGCGTTCAA GCAAGTAAGC   
  
  
+ ATGTTGCTGA GTTTGTCTTC AGCACAAGGG TATTGTGTGG AGGAGAGTGA GGGATGTTTG AAGCTCGGCT   
  
  
+ GGCATGACCG CCCTCTCATT GCGGCTTCGG CTTGGCGAGC GGAGACTCAA GCTGAGAACT CCAGCACTGT   
  
  
+ TGTGCTTGAT GGGTCATCGT CGTGTAGTTC ATCTTCTTA  

- -Up\_Stream \_Len000TTTTTT CTTTCTTTAC CTCTTTGGTT CGTTATACCT ATTTTTTTTT TGTTTCATTA   
  
  
- TTTCCTTCCT ACTCCGAGGT GTAAGGTGAG CGGGCGCGGT GAGTATCCTC TACTTATCAT TTTACACTCC   
  
  
- CCAATTTACT AAACTTTTTT TAGATCATTC CCGGGTGTTG TCATAACCTT GGGTCGAACG GTTCCTTTAC   
  
  
- AGTCTCCCCG ATCGTTACCT GGTCACGTTC TTCCGGTTCG GTGTAAACCA ATCCCTTAAA GTAGGGTAGG   
  
  
- CCGCGATTTT CGTAGTTTTA ATGATGAATT AGGAACTAGT TTACGGGATA TTTATAAGAT GATGGGGTAA   
  
  
- ACTTTTCCCC ACAACTAATG TTGTGTATCG GCATCCAAAC TGTTTCTCTT AATGGGAATA ACAATCTTCG   
  
  
- TGGTATATGA GAAAAGAGAT TCGAGACTAG AAAAAAGTAG GAAAAGTCGA AAAACAGGAA AATTTAGTTT   
  
  
- AGTGAGTGAA CTCATAACCT CCCTGCAAGG AGTCCTCAGC TTGGTGATTT AATAGAAAAA TTTCTTTTTC   
  
  
- CCGAACTTCG GTTGGGCTAA ATTTTTTTGA CGATTATACA TTCGGATGAG AGAAAGGTCA AGGTTGTTTA   
  
  
- GTTTAAAGTC ATACTTTGTA GGTCATTTCC TAAATAGGCA CTATTACTAC ATTGTATACC AATTTCCCAG   
  
  
- TAAAAGTCAA TGTAGCCTCG TACGTTAATT TCAGGGATAT TTTATCCATC CACGTGCTTG TTCAGTGGTA   
  
  
- GCAGGAAACG AAAAACGTAC TCACGTACGA TTAATACTTG AATTAATTAT AGATTCGTTT TAATTTTTCA   
  
  
- TCAATTTTGA AAAAGTGGAA ACCTTGGAGC ATCGACATCG TCATCGACGG CGTGAGACCA AGATCTGAAG   
  
  
- ATCCCTGGTT CTCTACGTGA GTATACGTAG GAGAAGACAG GAAAATGACA TAGGGGAAGG GTACACTGGA   
  
  
- AAAACGGGCC CACTGAAAAG ACACTCACAA CTATGTTTAA AACATGCATT CGATTGAGTA AATGAAGTGG   
  
  
- CAGAGTACTC AAAATGTAAT GGCTATAATT AAATTTACCG TTTCATAATT TAAACTCGAT TTTAGAGTAT   
  
  
- ATATATAAAC ATAATTTACA CAGACCTAAC AGTTTTATTA AAAACTGTAT ATGTCAGTGA TATATGACTC   
  
  
- AATAATTTTA TATATTTTTA TACTTTTAAA TTTTTTATCA TCAAATTCCA CTGTTGTGAT TTTGTTTGTT   
  
  
- TATTCTATAT TTATCCCTTT TCAAGTAGGA TAAAACAATA ATAATAAATA ATACATAAAA GTCTTAACAG   
  
  
- AGATAGTGTT CGTATCATAT ACACAGTTGT AGCACTGTTG AGCTGGTTAG ATGTTTATTG ATCATTTAAG   
  
  
- TTTATTTCAA TGAGAGTGGA TCGAATTTAT TCATGTGGAA CTAAATTAAC AGAATCGATA CCTTGGATAA   
  
  
- GGTTTGATCG GTTATGAAGG AAAAGTTTTC TTAATCAAAA TTTCAAAATT GAAAATGCAG GTTATTACGG   
  
  
- CCGGCATGAT ACTCAACGAA AGTTCGGTCA TCATCATGAC ATCATCCATT TTTATAATCT TATGTGGATG   
  
  
- ATATATACTA TAACGAGAAG TGGCATAGGA AGACGTATGA GTAGAAAGGC AGGGCCTCAG GTGATAGAGA   
  
  
- GGAGGTAAGT CTGTGAGAGA GCGAGATCTC TTGAGTATCG ATACCTGGCG CACGGGCTGC CAATAGCCCA   
  
  
- ATACTTAAGC CTCGAAGAAG TCGTTCAATC GGGACTCAAC GGCCTAGGTT GGTGGACCAA GGTACGGGCA   
  
  
- GATGGGCTGG GGTAGAGAGT CAGAGGAGAA CCTGGGCCAG GTAGGAGGGT CGGTTGGGTA AAGATGGTCC   
  
  
- CAAGACGGAG TCCCTTGGTA CACCAACCCT AACGACTGTG CACCTGCCTG GTCTAACAAC GTCCAAGGAG   
  
  
- CAGGGTCGTC ACTCCAACGT CGTCTCGGTC GTCCACAACT GACGTCAGCT TCGGTACCTC CTCCTGAGAC   
  
  
- CCTATTCTGA CCAGATGCGA GACGACTGCA CACGCCTCCG GTAGGTTGCA CCGCTAAAGG CTAACCGACA   
  
  
- GAACAATCAA TTGTTCTACT CGTTACTGGA GGACGGTGCG CAGTTGGGCA GGACGCCATA ACCGTTTCAT   
  
  
- CGGCCGATGA AGTATCTACG GAACTGGGCT TCTGATATGG TCGTCCCGGG TCACAGCCCG GATTAGCCCA   
  
  
- GCCAAGACCG CATGGTCCAC AACATGCTCG TGAAGATACT TCGAACGGGC AAGGAGTTTA AGCGAGTGAA   
  
  
- GTGACGATTA GTTCGCTAAA ATCTCCGTAA GCTGCCGGTG CTAACACAGG TGTAGTAGCT GAAGCCGGAT   
  
  
- TAGGTGCCGA ACGTTACCGG TCGAGATTAG GTTCGGAACC GACAAGCCGT GCCACCGGGC GGAAAGCATT   
  
  
- CTAATTGGCC TTAGCCCGGC GGAAGACTTA TGCCCAGCAC GAGCGAAGTT GGATAGCCCA GTTGTGACCG   
  
  
- GGTCAATCGG GATAGGTACT TACAACCCAA GCGTAAAGCC CGGCAGCGGC AGAGTGCCGA GCTCCTGTAG   
  
  
- TTTGGTACCT ACCAATTTTG TTCGGGTTTA CTTCAGCATC GGCACTTGAG ATAGAAGGTT GAGGTGGCCG   
  
  
- ATTAGCCCAG CCCATAACTG GGACAGGATT TGACCCAGGC CTCGGATTTG GGCTTTTAAC ACTGTGACCG   
  
  
- CCTCGTTCTC CGCTTGGTGT TGGTCGGGCT CAAAAACCGG GCCAAGTGCC TCCGTAATGT AATGATAAGT   
  
  
- TGATACAAGC TGAGCAATCT CCGAACAGTT CAGGTCCGGC TGTTCCTGGA TCGGCTCGAT ATGAATCTCT   
  
  
- CTCTCAATAG GTTGTAGCAG ACGACACTCC CCAGCCGAGC CTAACTCTCC GTGCTCGGCG ACCGGGTTAC   
  
  
- CTCCCGGGCT TACCGAGCCC GGCCCAAGTT CTTCCACCTA TACCCATTTT TACGCAAGTT CGTTCATTCG   
  
  
- TACAACGACT CAAACAGAAG TCGTGTTCCC ATAACACACC TCCTCTCACT CCCTACAAAC TTCGAGCCGA   
  
  
- CCGTACTGGC GGGAGAGTAA CGCCGAAGCC GAACCGCTCG CCTCTGAGTT CGACTCTTGA GGTCGTGACA   
  
  
- ACACGAACTA CCCAGTAGCA GCACATCAAG TAGAAGAAT

+     G-box

| Site Name | Organism | Position | Strand | Matrix score. | sequence | function |
| --- | --- | --- | --- | --- | --- | --- |
| G-box | Zea mays | 3173 | - | 6 | CACGAC | cis-acting regulatory element involved in light responsiveness |
| G-box | Zea mays | 2489 | - | 6 | CACGAC | cis-acting regulatory element involved in light responsiveness |
| G-box | Lycopersicon esculentum | 2137 | - | 11 | tgACACGTGGCA | cis-acting regulatory element involved in light responsiveness |
| G-box | Zea mays | 2060 | - | 6 | CACGTC | cis-acting regulatory element involved in light responsiveness |
| G-box | Arabidopsis thaliana | 1930 | + | 9 | GCCACGTGGA | cis-acting regulatory element involved in light responsiveness |
| G-box | Arabidopsis thaliana | 1932 | - | 6 | CACGTG | cis-acting regulatory element involved in light responsiveness |

>HU11G00778.1   
+ -Up\_Stream \_Len000AAAAAA GAAAGAAATG GAGAAACCAA GCAATATGGA TAAAAAAAAA ACAAAGTAAT   
  
  
+ AAAGGAAGGA TGAGGCTCCA CATTCCACTC GCCCGCGCCA CTCATAGGAG ATGAATAGTA AAATGTGAGG   
  
  
+ GGTTAAATGA TTTGAAAAAA ATCTAGTAAG GGCCCACAAC AGTATTGGAA CCCAGCTTGC CAAGGAAATG   
  
  
+ TCAGAGGGGC TAGCAATGGA CCAGTGCAAG AAGGCCAAGC CACATTTGGT TAGGGAATTT CATCCCATCC   
  
  
+ GGCGCTAAAA GCATCAAAAT TACTACTTAA TCCTTGATCA AATGCCCTAT AAATATTCTA CTACCCCATT   
  
  
+ TGAAAAGGGG TGTTGATTAC AACACATAGC CGTAGGTTTG ACAAAGAGAA TTACCCTTAT TGTTAGAAGC   
  
  
+ ACCATATACT CTTTTCTCTA AGCTCTGATC TTTTTTCATC CTTTTCAGCT TTTTGTCCTT TTAAATCAAA   
  
  
+ TCACTCACTT GAGTATTGGA GGGACGTTCC TCAGGAGTCG AACCACTAAA TTATCTTTTT AAAGAAAAAG   
  
  
+ GGCTTGAAGC CAACCCGATT TAAAAAAACT GCTAATATGT AAGCCTACTC TCTTTCCAGT TCCAACAAAT   
  
  
+ CAAATTTCAG TATGAAACAT CCAGTAAAGG ATTTATCCGT GATAATGATG TAACATATGG TTAAAGGGTC   
  
  
+ ATTTTCAGTT ACATCGGAGC ATGCAATTAA AGTCCCTATA AAATAGGTAG GTGCACGAAC AAGTCACCAT   
  
  
+ CGTCCTTTGC TTTTTGCATG AGTGCATGCT AATTATGAAC TTAATTAATA TCTAAGCAAA ATTAAAAAGT   
  
  
+ AGTTAAAACT TTTTCACCTT TGGAACCTCG TAGCTGTAGC AGTAGCTGCC GCACTCTGGT TCTAGACTTC   
  
  
+ TAGGGACCAA GAGATGCACT CATATGCATC CTCTTCTGTC CTTTTACTGT ATCCCCTTCC CATGTGACCT   
  
  
+ TTTTGCCCGG GTGACTTTTC TGTGAGTGTT GATACAAATT TTGTACGTAA GCTAACTCAT TTACTTCACC   
  
  
+ GTCTCATGAG TTTTACATTA CCGATATTAA TTTAAATGGC AAAGTATTAA ATTTGAGCTA AAATCTCATA   
  
  
+ TATATATTTG TATTAAATGT GTCTGGATTG TCAAAATAAT TTTTGACATA TACAGTCACT ATATACTGAG   
  
  
+ TTATTAAAAT ATATAAAAAT ATGAAAATTT AAAAAATAGT AGTTTAAGGT GACAACACTA AAACAAACAA   
  
  
+ ATAAGATATA AATAGGGAAA AGTTCATCCT ATTTTGTTAT TATTATTTAT TATGTATTTT CAGAATTGTC   
  
  
+ TCTATCACAA GCATAGTATA TGTGTCAACA TCGTGACAAC TCGACCAATC TACAAATAAC TAGTAAATTC   
  
  
+ AAATAAAGTT ACTCTCACCT AGCTTAAATA AGTACACCTT GATTTAATTG TCTTAGCTAT GGAACCTATT   
  
  
+ CCAAACTAGC CAATACTTCC TTTTCAAAAG AATTAGTTTT AAAGTTTTAA CTTTTACGTC CAATAATGCC   
  
  
+ GGCCGTACTA TGAGTTGCTT TCAAGCCAGT AGTAGTACTG TAGTAGGTAA AAATATTAGA ATACACCTAC   
  
  
+ TATATATGAT ATTGCTCTTC ACCGTATCCT TCTGCATACT CATCTTTCCG TCCCGGAGTC CACTATCTCT   
  
  
+ CCTCCATTCA GACACTCTCT CGCTCTAGAG AACTCATAGC TATGGACCGC GTGCCCGACG GTTATCGGGT   
  
  
+ TATGAATTCG GAGCTTCTTC AGCAAGTTAG CCCTGAGTTG CCGGATCCAA CCACCTGGTT CCATGCCCGT   
  
  
+ CTACCCGACC CCATCTCTCA GTCTCCTCTT GGACCCGGTC CATCCTCCCA GCCAACCCAT TTCTACCAGG   
  
  
+ GTTCTGCCTC AGGGAACCAT GTGGTTGGGA TTGCTGACAC GTGGACGGAC CAGATTGTTG CAGGTTCCTC   
  
  
+ GTCCCAGCAG TGAGGTTGCA GCAGAGCCAG CAGGTGTTGA CTGCAGTCGA AGCCATGGAG GAGGACTCTG   
  
  
+ GGATAAGACT GGTCTACGCT CTGCTGACGT GTGCGGAGGC CATCCAACGT GGCGATTTCC GATTGGCTGT   
  
  
+ CTTGTTAGTT AACAAGATGA GCAATGACCT CCTGCCACGC GTCAACCCGT CCTGCGGTAT TGGCAAAGTA   
  
  
+ GCCGGCTACT TCATAGATGC CTTGACCCGA AGACTATACC AGCAGGGCCC AGTGTCGGGC CTAATCGGGT   
  
  
+ CGGTTCTGGC GTACCAGGTG TTGTACGAGC ACTTCTATGA AGCTTGCCCG TTCCTCAAAT TCGCTCACTT   
  
  
+ CACTGCTAAT CAAGCGATTT TAGAGGCATT CGACGGCCAC GATTGTGTCC ACATCATCGA CTTCGGCCTA   
  
  
+ ATCCACGGCT TGCAATGGCC AGCTCTAATC CAAGCCTTGG CTGTTCGGCA CGGTGGCCCG CCTTTCGTAA   
  
  
+ GATTAACCGG AATCGGGCCG CCTTCTGAAT ACGGGTCGTG CTCGCTTCAA CCTATCGGGT CAACACTGGC   
  
  
+ CCAGTTAGCC CTATCCATGA ATGTTGGGTT CGCATTTCGG GCCGTCGCCG TCTCACGGCT CGAGGACATC   
  
  
+ AAACCATGGA TGGTTAAAAC AAGCCCAAAT GAAGTCGTAG CCGTGAACTC TATCTTCCAA CTCCACCGGC   
  
  
+ TAATCGGGTC GGGTATTGAC CCTGTCCTAA ACTGGGTCCG GAGCCTAAAC CCGAAAATTG TGACACTGGC   
  
  
+ GGAGCAAGAG GCGAACCACA ACCAGCCCGA GTTTTTGGCC CGGTTCACGG AGGCATTACA TTACTATTCA   
  
  
+ ACTATGTTCG ACTCGTTAGA GGCTTGTCAA GTCCAGGCCG ACAAGGACCT AGCCGAGCTA TACTTAGAGA   
  
  
+ GAGAGTTATC CAACATCGTC TGCTGTGAGG GGTCGGCTCG GATTGAGAGG CACGAGCCGC TGGCCCAATG   
  
  
+ GAGGGCCCGA ATGGCTCGGG CCGGGTTCAA GAAGGTGGAT ATGGGTAAAA ATGCGTTCAA GCAAGTAAGC   
  
  
+ ATGTTGCTGA GTTTGTCTTC AGCACAAGGG TATTGTGTGG AGGAGAGTGA GGGATGTTTG AAGCTCGGCT   
  
  
+ GGCATGACCG CCCTCTCATT GCGGCTTCGG CTTGGCGAGC GGAGACTCAA GCTGAGAACT CCAGCACTGT   
  
  
+ TGTGCTTGAT GGGTCATCGT CGTGTAGTTC ATCTTCTTA  

- -Up\_Stream \_Len000TTTTTT CTTTCTTTAC CTCTTTGGTT CGTTATACCT ATTTTTTTTT TGTTTCATTA   
  
  
- TTTCCTTCCT ACTCCGAGGT GTAAGGTGAG CGGGCGCGGT GAGTATCCTC TACTTATCAT TTTACACTCC   
  
  
- CCAATTTACT AAACTTTTTT TAGATCATTC CCGGGTGTTG TCATAACCTT GGGTCGAACG GTTCCTTTAC   
  
  
- AGTCTCCCCG ATCGTTACCT GGTCACGTTC TTCCGGTTCG GTGTAAACCA ATCCCTTAAA GTAGGGTAGG   
  
  
- CCGCGATTTT CGTAGTTTTA ATGATGAATT AGGAACTAGT TTACGGGATA TTTATAAGAT GATGGGGTAA   
  
  
- ACTTTTCCCC ACAACTAATG TTGTGTATCG GCATCCAAAC TGTTTCTCTT AATGGGAATA ACAATCTTCG   
  
  
- TGGTATATGA GAAAAGAGAT TCGAGACTAG AAAAAAGTAG GAAAAGTCGA AAAACAGGAA AATTTAGTTT   
  
  
- AGTGAGTGAA CTCATAACCT CCCTGCAAGG AGTCCTCAGC TTGGTGATTT AATAGAAAAA TTTCTTTTTC   
  
  
- CCGAACTTCG GTTGGGCTAA ATTTTTTTGA CGATTATACA TTCGGATGAG AGAAAGGTCA AGGTTGTTTA   
  
  
- GTTTAAAGTC ATACTTTGTA GGTCATTTCC TAAATAGGCA CTATTACTAC ATTGTATACC AATTTCCCAG   
  
  
- TAAAAGTCAA TGTAGCCTCG TACGTTAATT TCAGGGATAT TTTATCCATC CACGTGCTTG TTCAGTGGTA   
  
  
- GCAGGAAACG AAAAACGTAC TCACGTACGA TTAATACTTG AATTAATTAT AGATTCGTTT TAATTTTTCA   
  
  
- TCAATTTTGA AAAAGTGGAA ACCTTGGAGC ATCGACATCG TCATCGACGG CGTGAGACCA AGATCTGAAG   
  
  
- ATCCCTGGTT CTCTACGTGA GTATACGTAG GAGAAGACAG GAAAATGACA TAGGGGAAGG GTACACTGGA   
  
  
- AAAACGGGCC CACTGAAAAG ACACTCACAA CTATGTTTAA AACATGCATT CGATTGAGTA AATGAAGTGG   
  
  
- CAGAGTACTC AAAATGTAAT GGCTATAATT AAATTTACCG TTTCATAATT TAAACTCGAT TTTAGAGTAT   
  
  
- ATATATAAAC ATAATTTACA CAGACCTAAC AGTTTTATTA AAAACTGTAT ATGTCAGTGA TATATGACTC   
  
  
- AATAATTTTA TATATTTTTA TACTTTTAAA TTTTTTATCA TCAAATTCCA CTGTTGTGAT TTTGTTTGTT   
  
  
- TATTCTATAT TTATCCCTTT TCAAGTAGGA TAAAACAATA ATAATAAATA ATACATAAAA GTCTTAACAG   
  
  
- AGATAGTGTT CGTATCATAT ACACAGTTGT AGCACTGTTG AGCTGGTTAG ATGTTTATTG ATCATTTAAG   
  
  
- TTTATTTCAA TGAGAGTGGA TCGAATTTAT TCATGTGGAA CTAAATTAAC AGAATCGATA CCTTGGATAA   
  
  
- GGTTTGATCG GTTATGAAGG AAAAGTTTTC TTAATCAAAA TTTCAAAATT GAAAATGCAG GTTATTACGG   
  
  
- CCGGCATGAT ACTCAACGAA AGTTCGGTCA TCATCATGAC ATCATCCATT TTTATAATCT TATGTGGATG   
  
  
- ATATATACTA TAACGAGAAG TGGCATAGGA AGACGTATGA GTAGAAAGGC AGGGCCTCAG GTGATAGAGA   
  
  
- GGAGGTAAGT CTGTGAGAGA GCGAGATCTC TTGAGTATCG ATACCTGGCG CACGGGCTGC CAATAGCCCA   
  
  
- ATACTTAAGC CTCGAAGAAG TCGTTCAATC GGGACTCAAC GGCCTAGGTT GGTGGACCAA GGTACGGGCA   
  
  
- GATGGGCTGG GGTAGAGAGT CAGAGGAGAA CCTGGGCCAG GTAGGAGGGT CGGTTGGGTA AAGATGGTCC   
  
  
- CAAGACGGAG TCCCTTGGTA CACCAACCCT AACGACTGTG CACCTGCCTG GTCTAACAAC GTCCAAGGAG   
  
  
- CAGGGTCGTC ACTCCAACGT CGTCTCGGTC GTCCACAACT GACGTCAGCT TCGGTACCTC CTCCTGAGAC   
  
  
- CCTATTCTGA CCAGATGCGA GACGACTGCA CACGCCTCCG GTAGGTTGCA CCGCTAAAGG CTAACCGACA   
  
  
- GAACAATCAA TTGTTCTACT CGTTACTGGA GGACGGTGCG CAGTTGGGCA GGACGCCATA ACCGTTTCAT   
  
  
- CGGCCGATGA AGTATCTACG GAACTGGGCT TCTGATATGG TCGTCCCGGG TCACAGCCCG GATTAGCCCA   
  
  
- GCCAAGACCG CATGGTCCAC AACATGCTCG TGAAGATACT TCGAACGGGC AAGGAGTTTA AGCGAGTGAA   
  
  
- GTGACGATTA GTTCGCTAAA ATCTCCGTAA GCTGCCGGTG CTAACACAGG TGTAGTAGCT GAAGCCGGAT   
  
  
- TAGGTGCCGA ACGTTACCGG TCGAGATTAG GTTCGGAACC GACAAGCCGT GCCACCGGGC GGAAAGCATT   
  
  
- CTAATTGGCC TTAGCCCGGC GGAAGACTTA TGCCCAGCAC GAGCGAAGTT GGATAGCCCA GTTGTGACCG   
  
  
- GGTCAATCGG GATAGGTACT TACAACCCAA GCGTAAAGCC CGGCAGCGGC AGAGTGCCGA GCTCCTGTAG   
  
  
- TTTGGTACCT ACCAATTTTG TTCGGGTTTA CTTCAGCATC GGCACTTGAG ATAGAAGGTT GAGGTGGCCG   
  
  
- ATTAGCCCAG CCCATAACTG GGACAGGATT TGACCCAGGC CTCGGATTTG GGCTTTTAAC ACTGTGACCG   
  
  
- CCTCGTTCTC CGCTTGGTGT TGGTCGGGCT CAAAAACCGG GCCAAGTGCC TCCGTAATGT AATGATAAGT   
  
  
- TGATACAAGC TGAGCAATCT CCGAACAGTT CAGGTCCGGC TGTTCCTGGA TCGGCTCGAT ATGAATCTCT   
  
  
- CTCTCAATAG GTTGTAGCAG ACGACACTCC CCAGCCGAGC CTAACTCTCC GTGCTCGGCG ACCGGGTTAC   
  
  
- CTCCCGGGCT TACCGAGCCC GGCCCAAGTT CTTCCACCTA TACCCATTTT TACGCAAGTT CGTTCATTCG   
  
  
- TACAACGACT CAAACAGAAG TCGTGTTCCC ATAACACACC TCCTCTCACT CCCTACAAAC TTCGAGCCGA   
  
  
- CCGTACTGGC GGGAGAGTAA CGCCGAAGCC GAACCGCTCG CCTCTGAGTT CGACTCTTGA GGTCGTGACA   
  
  
- ACACGAACTA CCCAGTAGCA GCACATCAAG TAGAAGAAT

+     GATA-motif

| Site Name | Organism | Position | Strand | Matrix score. | sequence | function |
| --- | --- | --- | --- | --- | --- | --- |
| GATA-motif | Pisum sativum | 2533 | - | 7 | GATAGGG | part of a light responsive element |
| GATA-motif | Solanum tuberosum | 80 | + | 9 | AAGGATAAGG | part of a light responsive element |
| GATA-motif | Solanum tuberosum | 1636 | - | 9 | AAGGATAAGG | part of a light responsive element |

>HU11G00778.1   
+ -Up\_Stream \_Len000AAAAAA GAAAGAAATG GAGAAACCAA GCAATATGGA TAAAAAAAAA ACAAAGTAAT   
  
  
+ AAAGGAAGGA TGAGGCTCCA CATTCCACTC GCCCGCGCCA CTCATAGGAG ATGAATAGTA AAATGTGAGG   
  
  
+ GGTTAAATGA TTTGAAAAAA ATCTAGTAAG GGCCCACAAC AGTATTGGAA CCCAGCTTGC CAAGGAAATG   
  
  
+ TCAGAGGGGC TAGCAATGGA CCAGTGCAAG AAGGCCAAGC CACATTTGGT TAGGGAATTT CATCCCATCC   
  
  
+ GGCGCTAAAA GCATCAAAAT TACTACTTAA TCCTTGATCA AATGCCCTAT AAATATTCTA CTACCCCATT   
  
  
+ TGAAAAGGGG TGTTGATTAC AACACATAGC CGTAGGTTTG ACAAAGAGAA TTACCCTTAT TGTTAGAAGC   
  
  
+ ACCATATACT CTTTTCTCTA AGCTCTGATC TTTTTTCATC CTTTTCAGCT TTTTGTCCTT TTAAATCAAA   
  
  
+ TCACTCACTT GAGTATTGGA GGGACGTTCC TCAGGAGTCG AACCACTAAA TTATCTTTTT AAAGAAAAAG   
  
  
+ GGCTTGAAGC CAACCCGATT TAAAAAAACT GCTAATATGT AAGCCTACTC TCTTTCCAGT TCCAACAAAT   
  
  
+ CAAATTTCAG TATGAAACAT CCAGTAAAGG ATTTATCCGT GATAATGATG TAACATATGG TTAAAGGGTC   
  
  
+ ATTTTCAGTT ACATCGGAGC ATGCAATTAA AGTCCCTATA AAATAGGTAG GTGCACGAAC AAGTCACCAT   
  
  
+ CGTCCTTTGC TTTTTGCATG AGTGCATGCT AATTATGAAC TTAATTAATA TCTAAGCAAA ATTAAAAAGT   
  
  
+ AGTTAAAACT TTTTCACCTT TGGAACCTCG TAGCTGTAGC AGTAGCTGCC GCACTCTGGT TCTAGACTTC   
  
  
+ TAGGGACCAA GAGATGCACT CATATGCATC CTCTTCTGTC CTTTTACTGT ATCCCCTTCC CATGTGACCT   
  
  
+ TTTTGCCCGG GTGACTTTTC TGTGAGTGTT GATACAAATT TTGTACGTAA GCTAACTCAT TTACTTCACC   
  
  
+ GTCTCATGAG TTTTACATTA CCGATATTAA TTTAAATGGC AAAGTATTAA ATTTGAGCTA AAATCTCATA   
  
  
+ TATATATTTG TATTAAATGT GTCTGGATTG TCAAAATAAT TTTTGACATA TACAGTCACT ATATACTGAG   
  
  
+ TTATTAAAAT ATATAAAAAT ATGAAAATTT AAAAAATAGT AGTTTAAGGT GACAACACTA AAACAAACAA   
  
  
+ ATAAGATATA AATAGGGAAA AGTTCATCCT ATTTTGTTAT TATTATTTAT TATGTATTTT CAGAATTGTC   
  
  
+ TCTATCACAA GCATAGTATA TGTGTCAACA TCGTGACAAC TCGACCAATC TACAAATAAC TAGTAAATTC   
  
  
+ AAATAAAGTT ACTCTCACCT AGCTTAAATA AGTACACCTT GATTTAATTG TCTTAGCTAT GGAACCTATT   
  
  
+ CCAAACTAGC CAATACTTCC TTTTCAAAAG AATTAGTTTT AAAGTTTTAA CTTTTACGTC CAATAATGCC   
  
  
+ GGCCGTACTA TGAGTTGCTT TCAAGCCAGT AGTAGTACTG TAGTAGGTAA AAATATTAGA ATACACCTAC   
  
  
+ TATATATGAT ATTGCTCTTC ACCGTATCCT TCTGCATACT CATCTTTCCG TCCCGGAGTC CACTATCTCT   
  
  
+ CCTCCATTCA GACACTCTCT CGCTCTAGAG AACTCATAGC TATGGACCGC GTGCCCGACG GTTATCGGGT   
  
  
+ TATGAATTCG GAGCTTCTTC AGCAAGTTAG CCCTGAGTTG CCGGATCCAA CCACCTGGTT CCATGCCCGT   
  
  
+ CTACCCGACC CCATCTCTCA GTCTCCTCTT GGACCCGGTC CATCCTCCCA GCCAACCCAT TTCTACCAGG   
  
  
+ GTTCTGCCTC AGGGAACCAT GTGGTTGGGA TTGCTGACAC GTGGACGGAC CAGATTGTTG CAGGTTCCTC   
  
  
+ GTCCCAGCAG TGAGGTTGCA GCAGAGCCAG CAGGTGTTGA CTGCAGTCGA AGCCATGGAG GAGGACTCTG   
  
  
+ GGATAAGACT GGTCTACGCT CTGCTGACGT GTGCGGAGGC CATCCAACGT GGCGATTTCC GATTGGCTGT   
  
  
+ CTTGTTAGTT AACAAGATGA GCAATGACCT CCTGCCACGC GTCAACCCGT CCTGCGGTAT TGGCAAAGTA   
  
  
+ GCCGGCTACT TCATAGATGC CTTGACCCGA AGACTATACC AGCAGGGCCC AGTGTCGGGC CTAATCGGGT   
  
  
+ CGGTTCTGGC GTACCAGGTG TTGTACGAGC ACTTCTATGA AGCTTGCCCG TTCCTCAAAT TCGCTCACTT   
  
  
+ CACTGCTAAT CAAGCGATTT TAGAGGCATT CGACGGCCAC GATTGTGTCC ACATCATCGA CTTCGGCCTA   
  
  
+ ATCCACGGCT TGCAATGGCC AGCTCTAATC CAAGCCTTGG CTGTTCGGCA CGGTGGCCCG CCTTTCGTAA   
  
  
+ GATTAACCGG AATCGGGCCG CCTTCTGAAT ACGGGTCGTG CTCGCTTCAA CCTATCGGGT CAACACTGGC   
  
  
+ CCAGTTAGCC CTATCCATGA ATGTTGGGTT CGCATTTCGG GCCGTCGCCG TCTCACGGCT CGAGGACATC   
  
  
+ AAACCATGGA TGGTTAAAAC AAGCCCAAAT GAAGTCGTAG CCGTGAACTC TATCTTCCAA CTCCACCGGC   
  
  
+ TAATCGGGTC GGGTATTGAC CCTGTCCTAA ACTGGGTCCG GAGCCTAAAC CCGAAAATTG TGACACTGGC   
  
  
+ GGAGCAAGAG GCGAACCACA ACCAGCCCGA GTTTTTGGCC CGGTTCACGG AGGCATTACA TTACTATTCA   
  
  
+ ACTATGTTCG ACTCGTTAGA GGCTTGTCAA GTCCAGGCCG ACAAGGACCT AGCCGAGCTA TACTTAGAGA   
  
  
+ GAGAGTTATC CAACATCGTC TGCTGTGAGG GGTCGGCTCG GATTGAGAGG CACGAGCCGC TGGCCCAATG   
  
  
+ GAGGGCCCGA ATGGCTCGGG CCGGGTTCAA GAAGGTGGAT ATGGGTAAAA ATGCGTTCAA GCAAGTAAGC   
  
  
+ ATGTTGCTGA GTTTGTCTTC AGCACAAGGG TATTGTGTGG AGGAGAGTGA GGGATGTTTG AAGCTCGGCT   
  
  
+ GGCATGACCG CCCTCTCATT GCGGCTTCGG CTTGGCGAGC GGAGACTCAA GCTGAGAACT CCAGCACTGT   
  
  
+ TGTGCTTGAT GGGTCATCGT CGTGTAGTTC ATCTTCTTA  

- -Up\_Stream \_Len000TTTTTT CTTTCTTTAC CTCTTTGGTT CGTTATACCT ATTTTTTTTT TGTTTCATTA   
  
  
- TTTCCTTCCT ACTCCGAGGT GTAAGGTGAG CGGGCGCGGT GAGTATCCTC TACTTATCAT TTTACACTCC   
  
  
- CCAATTTACT AAACTTTTTT TAGATCATTC CCGGGTGTTG TCATAACCTT GGGTCGAACG GTTCCTTTAC   
  
  
- AGTCTCCCCG ATCGTTACCT GGTCACGTTC TTCCGGTTCG GTGTAAACCA ATCCCTTAAA GTAGGGTAGG   
  
  
- CCGCGATTTT CGTAGTTTTA ATGATGAATT AGGAACTAGT TTACGGGATA TTTATAAGAT GATGGGGTAA   
  
  
- ACTTTTCCCC ACAACTAATG TTGTGTATCG GCATCCAAAC TGTTTCTCTT AATGGGAATA ACAATCTTCG   
  
  
- TGGTATATGA GAAAAGAGAT TCGAGACTAG AAAAAAGTAG GAAAAGTCGA AAAACAGGAA AATTTAGTTT   
  
  
- AGTGAGTGAA CTCATAACCT CCCTGCAAGG AGTCCTCAGC TTGGTGATTT AATAGAAAAA TTTCTTTTTC   
  
  
- CCGAACTTCG GTTGGGCTAA ATTTTTTTGA CGATTATACA TTCGGATGAG AGAAAGGTCA AGGTTGTTTA   
  
  
- GTTTAAAGTC ATACTTTGTA GGTCATTTCC TAAATAGGCA CTATTACTAC ATTGTATACC AATTTCCCAG   
  
  
- TAAAAGTCAA TGTAGCCTCG TACGTTAATT TCAGGGATAT TTTATCCATC CACGTGCTTG TTCAGTGGTA   
  
  
- GCAGGAAACG AAAAACGTAC TCACGTACGA TTAATACTTG AATTAATTAT AGATTCGTTT TAATTTTTCA   
  
  
- TCAATTTTGA AAAAGTGGAA ACCTTGGAGC ATCGACATCG TCATCGACGG CGTGAGACCA AGATCTGAAG   
  
  
- ATCCCTGGTT CTCTACGTGA GTATACGTAG GAGAAGACAG GAAAATGACA TAGGGGAAGG GTACACTGGA   
  
  
- AAAACGGGCC CACTGAAAAG ACACTCACAA CTATGTTTAA AACATGCATT CGATTGAGTA AATGAAGTGG   
  
  
- CAGAGTACTC AAAATGTAAT GGCTATAATT AAATTTACCG TTTCATAATT TAAACTCGAT TTTAGAGTAT   
  
  
- ATATATAAAC ATAATTTACA CAGACCTAAC AGTTTTATTA AAAACTGTAT ATGTCAGTGA TATATGACTC   
  
  
- AATAATTTTA TATATTTTTA TACTTTTAAA TTTTTTATCA TCAAATTCCA CTGTTGTGAT TTTGTTTGTT   
  
  
- TATTCTATAT TTATCCCTTT TCAAGTAGGA TAAAACAATA ATAATAAATA ATACATAAAA GTCTTAACAG   
  
  
- AGATAGTGTT CGTATCATAT ACACAGTTGT AGCACTGTTG AGCTGGTTAG ATGTTTATTG ATCATTTAAG   
  
  
- TTTATTTCAA TGAGAGTGGA TCGAATTTAT TCATGTGGAA CTAAATTAAC AGAATCGATA CCTTGGATAA   
  
  
- GGTTTGATCG GTTATGAAGG AAAAGTTTTC TTAATCAAAA TTTCAAAATT GAAAATGCAG GTTATTACGG   
  
  
- CCGGCATGAT ACTCAACGAA AGTTCGGTCA TCATCATGAC ATCATCCATT TTTATAATCT TATGTGGATG   
  
  
- ATATATACTA TAACGAGAAG TGGCATAGGA AGACGTATGA GTAGAAAGGC AGGGCCTCAG GTGATAGAGA   
  
  
- GGAGGTAAGT CTGTGAGAGA GCGAGATCTC TTGAGTATCG ATACCTGGCG CACGGGCTGC CAATAGCCCA   
  
  
- ATACTTAAGC CTCGAAGAAG TCGTTCAATC GGGACTCAAC GGCCTAGGTT GGTGGACCAA GGTACGGGCA   
  
  
- GATGGGCTGG GGTAGAGAGT CAGAGGAGAA CCTGGGCCAG GTAGGAGGGT CGGTTGGGTA AAGATGGTCC   
  
  
- CAAGACGGAG TCCCTTGGTA CACCAACCCT AACGACTGTG CACCTGCCTG GTCTAACAAC GTCCAAGGAG   
  
  
- CAGGGTCGTC ACTCCAACGT CGTCTCGGTC GTCCACAACT GACGTCAGCT TCGGTACCTC CTCCTGAGAC   
  
  
- CCTATTCTGA CCAGATGCGA GACGACTGCA CACGCCTCCG GTAGGTTGCA CCGCTAAAGG CTAACCGACA   
  
  
- GAACAATCAA TTGTTCTACT CGTTACTGGA GGACGGTGCG CAGTTGGGCA GGACGCCATA ACCGTTTCAT   
  
  
- CGGCCGATGA AGTATCTACG GAACTGGGCT TCTGATATGG TCGTCCCGGG TCACAGCCCG GATTAGCCCA   
  
  
- GCCAAGACCG CATGGTCCAC AACATGCTCG TGAAGATACT TCGAACGGGC AAGGAGTTTA AGCGAGTGAA   
  
  
- GTGACGATTA GTTCGCTAAA ATCTCCGTAA GCTGCCGGTG CTAACACAGG TGTAGTAGCT GAAGCCGGAT   
  
  
- TAGGTGCCGA ACGTTACCGG TCGAGATTAG GTTCGGAACC GACAAGCCGT GCCACCGGGC GGAAAGCATT   
  
  
- CTAATTGGCC TTAGCCCGGC GGAAGACTTA TGCCCAGCAC GAGCGAAGTT GGATAGCCCA GTTGTGACCG   
  
  
- GGTCAATCGG GATAGGTACT TACAACCCAA GCGTAAAGCC CGGCAGCGGC AGAGTGCCGA GCTCCTGTAG   
  
  
- TTTGGTACCT ACCAATTTTG TTCGGGTTTA CTTCAGCATC GGCACTTGAG ATAGAAGGTT GAGGTGGCCG   
  
  
- ATTAGCCCAG CCCATAACTG GGACAGGATT TGACCCAGGC CTCGGATTTG GGCTTTTAAC ACTGTGACCG   
  
  
- CCTCGTTCTC CGCTTGGTGT TGGTCGGGCT CAAAAACCGG GCCAAGTGCC TCCGTAATGT AATGATAAGT   
  
  
- TGATACAAGC TGAGCAATCT CCGAACAGTT CAGGTCCGGC TGTTCCTGGA TCGGCTCGAT ATGAATCTCT   
  
  
- CTCTCAATAG GTTGTAGCAG ACGACACTCC CCAGCCGAGC CTAACTCTCC GTGCTCGGCG ACCGGGTTAC   
  
  
- CTCCCGGGCT TACCGAGCCC GGCCCAAGTT CTTCCACCTA TACCCATTTT TACGCAAGTT CGTTCATTCG   
  
  
- TACAACGACT CAAACAGAAG TCGTGTTCCC ATAACACACC TCCTCTCACT CCCTACAAAC TTCGAGCCGA   
  
  
- CCGTACTGGC GGGAGAGTAA CGCCGAAGCC GAACCGCTCG CCTCTGAGTT CGACTCTTGA GGTCGTGACA   
  
  
- ACACGAACTA CCCAGTAGCA GCACATCAAG TAGAAGAAT

+     GT1-motif

| Site Name | Organism | Position | Strand | Matrix score. | sequence | function |
| --- | --- | --- | --- | --- | --- | --- |
| GT1-motif | Arabidopsis thaliana | 2606 | + | 6 | GGTTAA | light responsive element |
| GT1-motif | Arabidopsis thaliana | 2457 | - | 6 | GGTTAA | light responsive element |
| GT1-motif | Avena sativa | 2456 | - | 7 | GGTTAAT | light responsive element |
| GT1-motif | Arabidopsis thaliana | 693 | + | 6 | GGTTAA | light responsive element |
| GT1-motif | Arabidopsis thaliana | 145 | + | 6 | GGTTAA | light responsive element |

>HU11G00778.1   
+ -Up\_Stream \_Len000AAAAAA GAAAGAAATG GAGAAACCAA GCAATATGGA TAAAAAAAAA ACAAAGTAAT   
  
  
+ AAAGGAAGGA TGAGGCTCCA CATTCCACTC GCCCGCGCCA CTCATAGGAG ATGAATAGTA AAATGTGAGG   
  
  
+ GGTTAAATGA TTTGAAAAAA ATCTAGTAAG GGCCCACAAC AGTATTGGAA CCCAGCTTGC CAAGGAAATG   
  
  
+ TCAGAGGGGC TAGCAATGGA CCAGTGCAAG AAGGCCAAGC CACATTTGGT TAGGGAATTT CATCCCATCC   
  
  
+ GGCGCTAAAA GCATCAAAAT TACTACTTAA TCCTTGATCA AATGCCCTAT AAATATTCTA CTACCCCATT   
  
  
+ TGAAAAGGGG TGTTGATTAC AACACATAGC CGTAGGTTTG ACAAAGAGAA TTACCCTTAT TGTTAGAAGC   
  
  
+ ACCATATACT CTTTTCTCTA AGCTCTGATC TTTTTTCATC CTTTTCAGCT TTTTGTCCTT TTAAATCAAA   
  
  
+ TCACTCACTT GAGTATTGGA GGGACGTTCC TCAGGAGTCG AACCACTAAA TTATCTTTTT AAAGAAAAAG   
  
  
+ GGCTTGAAGC CAACCCGATT TAAAAAAACT GCTAATATGT AAGCCTACTC TCTTTCCAGT TCCAACAAAT   
  
  
+ CAAATTTCAG TATGAAACAT CCAGTAAAGG ATTTATCCGT GATAATGATG TAACATATGG TTAAAGGGTC   
  
  
+ ATTTTCAGTT ACATCGGAGC ATGCAATTAA AGTCCCTATA AAATAGGTAG GTGCACGAAC AAGTCACCAT   
  
  
+ CGTCCTTTGC TTTTTGCATG AGTGCATGCT AATTATGAAC TTAATTAATA TCTAAGCAAA ATTAAAAAGT   
  
  
+ AGTTAAAACT TTTTCACCTT TGGAACCTCG TAGCTGTAGC AGTAGCTGCC GCACTCTGGT TCTAGACTTC   
  
  
+ TAGGGACCAA GAGATGCACT CATATGCATC CTCTTCTGTC CTTTTACTGT ATCCCCTTCC CATGTGACCT   
  
  
+ TTTTGCCCGG GTGACTTTTC TGTGAGTGTT GATACAAATT TTGTACGTAA GCTAACTCAT TTACTTCACC   
  
  
+ GTCTCATGAG TTTTACATTA CCGATATTAA TTTAAATGGC AAAGTATTAA ATTTGAGCTA AAATCTCATA   
  
  
+ TATATATTTG TATTAAATGT GTCTGGATTG TCAAAATAAT TTTTGACATA TACAGTCACT ATATACTGAG   
  
  
+ TTATTAAAAT ATATAAAAAT ATGAAAATTT AAAAAATAGT AGTTTAAGGT GACAACACTA AAACAAACAA   
  
  
+ ATAAGATATA AATAGGGAAA AGTTCATCCT ATTTTGTTAT TATTATTTAT TATGTATTTT CAGAATTGTC   
  
  
+ TCTATCACAA GCATAGTATA TGTGTCAACA TCGTGACAAC TCGACCAATC TACAAATAAC TAGTAAATTC   
  
  
+ AAATAAAGTT ACTCTCACCT AGCTTAAATA AGTACACCTT GATTTAATTG TCTTAGCTAT GGAACCTATT   
  
  
+ CCAAACTAGC CAATACTTCC TTTTCAAAAG AATTAGTTTT AAAGTTTTAA CTTTTACGTC CAATAATGCC   
  
  
+ GGCCGTACTA TGAGTTGCTT TCAAGCCAGT AGTAGTACTG TAGTAGGTAA AAATATTAGA ATACACCTAC   
  
  
+ TATATATGAT ATTGCTCTTC ACCGTATCCT TCTGCATACT CATCTTTCCG TCCCGGAGTC CACTATCTCT   
  
  
+ CCTCCATTCA GACACTCTCT CGCTCTAGAG AACTCATAGC TATGGACCGC GTGCCCGACG GTTATCGGGT   
  
  
+ TATGAATTCG GAGCTTCTTC AGCAAGTTAG CCCTGAGTTG CCGGATCCAA CCACCTGGTT CCATGCCCGT   
  
  
+ CTACCCGACC CCATCTCTCA GTCTCCTCTT GGACCCGGTC CATCCTCCCA GCCAACCCAT TTCTACCAGG   
  
  
+ GTTCTGCCTC AGGGAACCAT GTGGTTGGGA TTGCTGACAC GTGGACGGAC CAGATTGTTG CAGGTTCCTC   
  
  
+ GTCCCAGCAG TGAGGTTGCA GCAGAGCCAG CAGGTGTTGA CTGCAGTCGA AGCCATGGAG GAGGACTCTG   
  
  
+ GGATAAGACT GGTCTACGCT CTGCTGACGT GTGCGGAGGC CATCCAACGT GGCGATTTCC GATTGGCTGT   
  
  
+ CTTGTTAGTT AACAAGATGA GCAATGACCT CCTGCCACGC GTCAACCCGT CCTGCGGTAT TGGCAAAGTA   
  
  
+ GCCGGCTACT TCATAGATGC CTTGACCCGA AGACTATACC AGCAGGGCCC AGTGTCGGGC CTAATCGGGT   
  
  
+ CGGTTCTGGC GTACCAGGTG TTGTACGAGC ACTTCTATGA AGCTTGCCCG TTCCTCAAAT TCGCTCACTT   
  
  
+ CACTGCTAAT CAAGCGATTT TAGAGGCATT CGACGGCCAC GATTGTGTCC ACATCATCGA CTTCGGCCTA   
  
  
+ ATCCACGGCT TGCAATGGCC AGCTCTAATC CAAGCCTTGG CTGTTCGGCA CGGTGGCCCG CCTTTCGTAA   
  
  
+ GATTAACCGG AATCGGGCCG CCTTCTGAAT ACGGGTCGTG CTCGCTTCAA CCTATCGGGT CAACACTGGC   
  
  
+ CCAGTTAGCC CTATCCATGA ATGTTGGGTT CGCATTTCGG GCCGTCGCCG TCTCACGGCT CGAGGACATC   
  
  
+ AAACCATGGA TGGTTAAAAC AAGCCCAAAT GAAGTCGTAG CCGTGAACTC TATCTTCCAA CTCCACCGGC   
  
  
+ TAATCGGGTC GGGTATTGAC CCTGTCCTAA ACTGGGTCCG GAGCCTAAAC CCGAAAATTG TGACACTGGC   
  
  
+ GGAGCAAGAG GCGAACCACA ACCAGCCCGA GTTTTTGGCC CGGTTCACGG AGGCATTACA TTACTATTCA   
  
  
+ ACTATGTTCG ACTCGTTAGA GGCTTGTCAA GTCCAGGCCG ACAAGGACCT AGCCGAGCTA TACTTAGAGA   
  
  
+ GAGAGTTATC CAACATCGTC TGCTGTGAGG GGTCGGCTCG GATTGAGAGG CACGAGCCGC TGGCCCAATG   
  
  
+ GAGGGCCCGA ATGGCTCGGG CCGGGTTCAA GAAGGTGGAT ATGGGTAAAA ATGCGTTCAA GCAAGTAAGC   
  
  
+ ATGTTGCTGA GTTTGTCTTC AGCACAAGGG TATTGTGTGG AGGAGAGTGA GGGATGTTTG AAGCTCGGCT   
  
  
+ GGCATGACCG CCCTCTCATT GCGGCTTCGG CTTGGCGAGC GGAGACTCAA GCTGAGAACT CCAGCACTGT   
  
  
+ TGTGCTTGAT GGGTCATCGT CGTGTAGTTC ATCTTCTTA  

- -Up\_Stream \_Len000TTTTTT CTTTCTTTAC CTCTTTGGTT CGTTATACCT ATTTTTTTTT TGTTTCATTA   
  
  
- TTTCCTTCCT ACTCCGAGGT GTAAGGTGAG CGGGCGCGGT GAGTATCCTC TACTTATCAT TTTACACTCC   
  
  
- CCAATTTACT AAACTTTTTT TAGATCATTC CCGGGTGTTG TCATAACCTT GGGTCGAACG GTTCCTTTAC   
  
  
- AGTCTCCCCG ATCGTTACCT GGTCACGTTC TTCCGGTTCG GTGTAAACCA ATCCCTTAAA GTAGGGTAGG   
  
  
- CCGCGATTTT CGTAGTTTTA ATGATGAATT AGGAACTAGT TTACGGGATA TTTATAAGAT GATGGGGTAA   
  
  
- ACTTTTCCCC ACAACTAATG TTGTGTATCG GCATCCAAAC TGTTTCTCTT AATGGGAATA ACAATCTTCG   
  
  
- TGGTATATGA GAAAAGAGAT TCGAGACTAG AAAAAAGTAG GAAAAGTCGA AAAACAGGAA AATTTAGTTT   
  
  
- AGTGAGTGAA CTCATAACCT CCCTGCAAGG AGTCCTCAGC TTGGTGATTT AATAGAAAAA TTTCTTTTTC   
  
  
- CCGAACTTCG GTTGGGCTAA ATTTTTTTGA CGATTATACA TTCGGATGAG AGAAAGGTCA AGGTTGTTTA   
  
  
- GTTTAAAGTC ATACTTTGTA GGTCATTTCC TAAATAGGCA CTATTACTAC ATTGTATACC AATTTCCCAG   
  
  
- TAAAAGTCAA TGTAGCCTCG TACGTTAATT TCAGGGATAT TTTATCCATC CACGTGCTTG TTCAGTGGTA   
  
  
- GCAGGAAACG AAAAACGTAC TCACGTACGA TTAATACTTG AATTAATTAT AGATTCGTTT TAATTTTTCA   
  
  
- TCAATTTTGA AAAAGTGGAA ACCTTGGAGC ATCGACATCG TCATCGACGG CGTGAGACCA AGATCTGAAG   
  
  
- ATCCCTGGTT CTCTACGTGA GTATACGTAG GAGAAGACAG GAAAATGACA TAGGGGAAGG GTACACTGGA   
  
  
- AAAACGGGCC CACTGAAAAG ACACTCACAA CTATGTTTAA AACATGCATT CGATTGAGTA AATGAAGTGG   
  
  
- CAGAGTACTC AAAATGTAAT GGCTATAATT AAATTTACCG TTTCATAATT TAAACTCGAT TTTAGAGTAT   
  
  
- ATATATAAAC ATAATTTACA CAGACCTAAC AGTTTTATTA AAAACTGTAT ATGTCAGTGA TATATGACTC   
  
  
- AATAATTTTA TATATTTTTA TACTTTTAAA TTTTTTATCA TCAAATTCCA CTGTTGTGAT TTTGTTTGTT   
  
  
- TATTCTATAT TTATCCCTTT TCAAGTAGGA TAAAACAATA ATAATAAATA ATACATAAAA GTCTTAACAG   
  
  
- AGATAGTGTT CGTATCATAT ACACAGTTGT AGCACTGTTG AGCTGGTTAG ATGTTTATTG ATCATTTAAG   
  
  
- TTTATTTCAA TGAGAGTGGA TCGAATTTAT TCATGTGGAA CTAAATTAAC AGAATCGATA CCTTGGATAA   
  
  
- GGTTTGATCG GTTATGAAGG AAAAGTTTTC TTAATCAAAA TTTCAAAATT GAAAATGCAG GTTATTACGG   
  
  
- CCGGCATGAT ACTCAACGAA AGTTCGGTCA TCATCATGAC ATCATCCATT TTTATAATCT TATGTGGATG   
  
  
- ATATATACTA TAACGAGAAG TGGCATAGGA AGACGTATGA GTAGAAAGGC AGGGCCTCAG GTGATAGAGA   
  
  
- GGAGGTAAGT CTGTGAGAGA GCGAGATCTC TTGAGTATCG ATACCTGGCG CACGGGCTGC CAATAGCCCA   
  
  
- ATACTTAAGC CTCGAAGAAG TCGTTCAATC GGGACTCAAC GGCCTAGGTT GGTGGACCAA GGTACGGGCA   
  
  
- GATGGGCTGG GGTAGAGAGT CAGAGGAGAA CCTGGGCCAG GTAGGAGGGT CGGTTGGGTA AAGATGGTCC   
  
  
- CAAGACGGAG TCCCTTGGTA CACCAACCCT AACGACTGTG CACCTGCCTG GTCTAACAAC GTCCAAGGAG   
  
  
- CAGGGTCGTC ACTCCAACGT CGTCTCGGTC GTCCACAACT GACGTCAGCT TCGGTACCTC CTCCTGAGAC   
  
  
- CCTATTCTGA CCAGATGCGA GACGACTGCA CACGCCTCCG GTAGGTTGCA CCGCTAAAGG CTAACCGACA   
  
  
- GAACAATCAA TTGTTCTACT CGTTACTGGA GGACGGTGCG CAGTTGGGCA GGACGCCATA ACCGTTTCAT   
  
  
- CGGCCGATGA AGTATCTACG GAACTGGGCT TCTGATATGG TCGTCCCGGG TCACAGCCCG GATTAGCCCA   
  
  
- GCCAAGACCG CATGGTCCAC AACATGCTCG TGAAGATACT TCGAACGGGC AAGGAGTTTA AGCGAGTGAA   
  
  
- GTGACGATTA GTTCGCTAAA ATCTCCGTAA GCTGCCGGTG CTAACACAGG TGTAGTAGCT GAAGCCGGAT   
  
  
- TAGGTGCCGA ACGTTACCGG TCGAGATTAG GTTCGGAACC GACAAGCCGT GCCACCGGGC GGAAAGCATT   
  
  
- CTAATTGGCC TTAGCCCGGC GGAAGACTTA TGCCCAGCAC GAGCGAAGTT GGATAGCCCA GTTGTGACCG   
  
  
- GGTCAATCGG GATAGGTACT TACAACCCAA GCGTAAAGCC CGGCAGCGGC AGAGTGCCGA GCTCCTGTAG   
  
  
- TTTGGTACCT ACCAATTTTG TTCGGGTTTA CTTCAGCATC GGCACTTGAG ATAGAAGGTT GAGGTGGCCG   
  
  
- ATTAGCCCAG CCCATAACTG GGACAGGATT TGACCCAGGC CTCGGATTTG GGCTTTTAAC ACTGTGACCG   
  
  
- CCTCGTTCTC CGCTTGGTGT TGGTCGGGCT CAAAAACCGG GCCAAGTGCC TCCGTAATGT AATGATAAGT   
  
  
- TGATACAAGC TGAGCAATCT CCGAACAGTT CAGGTCCGGC TGTTCCTGGA TCGGCTCGAT ATGAATCTCT   
  
  
- CTCTCAATAG GTTGTAGCAG ACGACACTCC CCAGCCGAGC CTAACTCTCC GTGCTCGGCG ACCGGGTTAC   
  
  
- CTCCCGGGCT TACCGAGCCC GGCCCAAGTT CTTCCACCTA TACCCATTTT TACGCAAGTT CGTTCATTCG   
  
  
- TACAACGACT CAAACAGAAG TCGTGTTCCC ATAACACACC TCCTCTCACT CCCTACAAAC TTCGAGCCGA   
  
  
- CCGTACTGGC GGGAGAGTAA CGCCGAAGCC GAACCGCTCG CCTCTGAGTT CGACTCTTGA GGTCGTGACA   
  
  
- ACACGAACTA CCCAGTAGCA GCACATCAAG TAGAAGAAT

+     I-box

| Site Name | Organism | Position | Strand | Matrix score. | sequence | function |
| --- | --- | --- | --- | --- | --- | --- |
| I-box | Zea mays | 1634 | - | 9 | gGATAAGGTG | part of a light responsive element |

>HU11G00778.1   
+ -Up\_Stream \_Len000AAAAAA GAAAGAAATG GAGAAACCAA GCAATATGGA TAAAAAAAAA ACAAAGTAAT   
  
  
+ AAAGGAAGGA TGAGGCTCCA CATTCCACTC GCCCGCGCCA CTCATAGGAG ATGAATAGTA AAATGTGAGG   
  
  
+ GGTTAAATGA TTTGAAAAAA ATCTAGTAAG GGCCCACAAC AGTATTGGAA CCCAGCTTGC CAAGGAAATG   
  
  
+ TCAGAGGGGC TAGCAATGGA CCAGTGCAAG AAGGCCAAGC CACATTTGGT TAGGGAATTT CATCCCATCC   
  
  
+ GGCGCTAAAA GCATCAAAAT TACTACTTAA TCCTTGATCA AATGCCCTAT AAATATTCTA CTACCCCATT   
  
  
+ TGAAAAGGGG TGTTGATTAC AACACATAGC CGTAGGTTTG ACAAAGAGAA TTACCCTTAT TGTTAGAAGC   
  
  
+ ACCATATACT CTTTTCTCTA AGCTCTGATC TTTTTTCATC CTTTTCAGCT TTTTGTCCTT TTAAATCAAA   
  
  
+ TCACTCACTT GAGTATTGGA GGGACGTTCC TCAGGAGTCG AACCACTAAA TTATCTTTTT AAAGAAAAAG   
  
  
+ GGCTTGAAGC CAACCCGATT TAAAAAAACT GCTAATATGT AAGCCTACTC TCTTTCCAGT TCCAACAAAT   
  
  
+ CAAATTTCAG TATGAAACAT CCAGTAAAGG ATTTATCCGT GATAATGATG TAACATATGG TTAAAGGGTC   
  
  
+ ATTTTCAGTT ACATCGGAGC ATGCAATTAA AGTCCCTATA AAATAGGTAG GTGCACGAAC AAGTCACCAT   
  
  
+ CGTCCTTTGC TTTTTGCATG AGTGCATGCT AATTATGAAC TTAATTAATA TCTAAGCAAA ATTAAAAAGT   
  
  
+ AGTTAAAACT TTTTCACCTT TGGAACCTCG TAGCTGTAGC AGTAGCTGCC GCACTCTGGT TCTAGACTTC   
  
  
+ TAGGGACCAA GAGATGCACT CATATGCATC CTCTTCTGTC CTTTTACTGT ATCCCCTTCC CATGTGACCT   
  
  
+ TTTTGCCCGG GTGACTTTTC TGTGAGTGTT GATACAAATT TTGTACGTAA GCTAACTCAT TTACTTCACC   
  
  
+ GTCTCATGAG TTTTACATTA CCGATATTAA TTTAAATGGC AAAGTATTAA ATTTGAGCTA AAATCTCATA   
  
  
+ TATATATTTG TATTAAATGT GTCTGGATTG TCAAAATAAT TTTTGACATA TACAGTCACT ATATACTGAG   
  
  
+ TTATTAAAAT ATATAAAAAT ATGAAAATTT AAAAAATAGT AGTTTAAGGT GACAACACTA AAACAAACAA   
  
  
+ ATAAGATATA AATAGGGAAA AGTTCATCCT ATTTTGTTAT TATTATTTAT TATGTATTTT CAGAATTGTC   
  
  
+ TCTATCACAA GCATAGTATA TGTGTCAACA TCGTGACAAC TCGACCAATC TACAAATAAC TAGTAAATTC   
  
  
+ AAATAAAGTT ACTCTCACCT AGCTTAAATA AGTACACCTT GATTTAATTG TCTTAGCTAT GGAACCTATT   
  
  
+ CCAAACTAGC CAATACTTCC TTTTCAAAAG AATTAGTTTT AAAGTTTTAA CTTTTACGTC CAATAATGCC   
  
  
+ GGCCGTACTA TGAGTTGCTT TCAAGCCAGT AGTAGTACTG TAGTAGGTAA AAATATTAGA ATACACCTAC   
  
  
+ TATATATGAT ATTGCTCTTC ACCGTATCCT TCTGCATACT CATCTTTCCG TCCCGGAGTC CACTATCTCT   
  
  
+ CCTCCATTCA GACACTCTCT CGCTCTAGAG AACTCATAGC TATGGACCGC GTGCCCGACG GTTATCGGGT   
  
  
+ TATGAATTCG GAGCTTCTTC AGCAAGTTAG CCCTGAGTTG CCGGATCCAA CCACCTGGTT CCATGCCCGT   
  
  
+ CTACCCGACC CCATCTCTCA GTCTCCTCTT GGACCCGGTC CATCCTCCCA GCCAACCCAT TTCTACCAGG   
  
  
+ GTTCTGCCTC AGGGAACCAT GTGGTTGGGA TTGCTGACAC GTGGACGGAC CAGATTGTTG CAGGTTCCTC   
  
  
+ GTCCCAGCAG TGAGGTTGCA GCAGAGCCAG CAGGTGTTGA CTGCAGTCGA AGCCATGGAG GAGGACTCTG   
  
  
+ GGATAAGACT GGTCTACGCT CTGCTGACGT GTGCGGAGGC CATCCAACGT GGCGATTTCC GATTGGCTGT   
  
  
+ CTTGTTAGTT AACAAGATGA GCAATGACCT CCTGCCACGC GTCAACCCGT CCTGCGGTAT TGGCAAAGTA   
  
  
+ GCCGGCTACT TCATAGATGC CTTGACCCGA AGACTATACC AGCAGGGCCC AGTGTCGGGC CTAATCGGGT   
  
  
+ CGGTTCTGGC GTACCAGGTG TTGTACGAGC ACTTCTATGA AGCTTGCCCG TTCCTCAAAT TCGCTCACTT   
  
  
+ CACTGCTAAT CAAGCGATTT TAGAGGCATT CGACGGCCAC GATTGTGTCC ACATCATCGA CTTCGGCCTA   
  
  
+ ATCCACGGCT TGCAATGGCC AGCTCTAATC CAAGCCTTGG CTGTTCGGCA CGGTGGCCCG CCTTTCGTAA   
  
  
+ GATTAACCGG AATCGGGCCG CCTTCTGAAT ACGGGTCGTG CTCGCTTCAA CCTATCGGGT CAACACTGGC   
  
  
+ CCAGTTAGCC CTATCCATGA ATGTTGGGTT CGCATTTCGG GCCGTCGCCG TCTCACGGCT CGAGGACATC   
  
  
+ AAACCATGGA TGGTTAAAAC AAGCCCAAAT GAAGTCGTAG CCGTGAACTC TATCTTCCAA CTCCACCGGC   
  
  
+ TAATCGGGTC GGGTATTGAC CCTGTCCTAA ACTGGGTCCG GAGCCTAAAC CCGAAAATTG TGACACTGGC   
  
  
+ GGAGCAAGAG GCGAACCACA ACCAGCCCGA GTTTTTGGCC CGGTTCACGG AGGCATTACA TTACTATTCA   
  
  
+ ACTATGTTCG ACTCGTTAGA GGCTTGTCAA GTCCAGGCCG ACAAGGACCT AGCCGAGCTA TACTTAGAGA   
  
  
+ GAGAGTTATC CAACATCGTC TGCTGTGAGG GGTCGGCTCG GATTGAGAGG CACGAGCCGC TGGCCCAATG   
  
  
+ GAGGGCCCGA ATGGCTCGGG CCGGGTTCAA GAAGGTGGAT ATGGGTAAAA ATGCGTTCAA GCAAGTAAGC   
  
  
+ ATGTTGCTGA GTTTGTCTTC AGCACAAGGG TATTGTGTGG AGGAGAGTGA GGGATGTTTG AAGCTCGGCT   
  
  
+ GGCATGACCG CCCTCTCATT GCGGCTTCGG CTTGGCGAGC GGAGACTCAA GCTGAGAACT CCAGCACTGT   
  
  
+ TGTGCTTGAT GGGTCATCGT CGTGTAGTTC ATCTTCTTA  

- -Up\_Stream \_Len000TTTTTT CTTTCTTTAC CTCTTTGGTT CGTTATACCT ATTTTTTTTT TGTTTCATTA   
  
  
- TTTCCTTCCT ACTCCGAGGT GTAAGGTGAG CGGGCGCGGT GAGTATCCTC TACTTATCAT TTTACACTCC   
  
  
- CCAATTTACT AAACTTTTTT TAGATCATTC CCGGGTGTTG TCATAACCTT GGGTCGAACG GTTCCTTTAC   
  
  
- AGTCTCCCCG ATCGTTACCT GGTCACGTTC TTCCGGTTCG GTGTAAACCA ATCCCTTAAA GTAGGGTAGG   
  
  
- CCGCGATTTT CGTAGTTTTA ATGATGAATT AGGAACTAGT TTACGGGATA TTTATAAGAT GATGGGGTAA   
  
  
- ACTTTTCCCC ACAACTAATG TTGTGTATCG GCATCCAAAC TGTTTCTCTT AATGGGAATA ACAATCTTCG   
  
  
- TGGTATATGA GAAAAGAGAT TCGAGACTAG AAAAAAGTAG GAAAAGTCGA AAAACAGGAA AATTTAGTTT   
  
  
- AGTGAGTGAA CTCATAACCT CCCTGCAAGG AGTCCTCAGC TTGGTGATTT AATAGAAAAA TTTCTTTTTC   
  
  
- CCGAACTTCG GTTGGGCTAA ATTTTTTTGA CGATTATACA TTCGGATGAG AGAAAGGTCA AGGTTGTTTA   
  
  
- GTTTAAAGTC ATACTTTGTA GGTCATTTCC TAAATAGGCA CTATTACTAC ATTGTATACC AATTTCCCAG   
  
  
- TAAAAGTCAA TGTAGCCTCG TACGTTAATT TCAGGGATAT TTTATCCATC CACGTGCTTG TTCAGTGGTA   
  
  
- GCAGGAAACG AAAAACGTAC TCACGTACGA TTAATACTTG AATTAATTAT AGATTCGTTT TAATTTTTCA   
  
  
- TCAATTTTGA AAAAGTGGAA ACCTTGGAGC ATCGACATCG TCATCGACGG CGTGAGACCA AGATCTGAAG   
  
  
- ATCCCTGGTT CTCTACGTGA GTATACGTAG GAGAAGACAG GAAAATGACA TAGGGGAAGG GTACACTGGA   
  
  
- AAAACGGGCC CACTGAAAAG ACACTCACAA CTATGTTTAA AACATGCATT CGATTGAGTA AATGAAGTGG   
  
  
- CAGAGTACTC AAAATGTAAT GGCTATAATT AAATTTACCG TTTCATAATT TAAACTCGAT TTTAGAGTAT   
  
  
- ATATATAAAC ATAATTTACA CAGACCTAAC AGTTTTATTA AAAACTGTAT ATGTCAGTGA TATATGACTC   
  
  
- AATAATTTTA TATATTTTTA TACTTTTAAA TTTTTTATCA TCAAATTCCA CTGTTGTGAT TTTGTTTGTT   
  
  
- TATTCTATAT TTATCCCTTT TCAAGTAGGA TAAAACAATA ATAATAAATA ATACATAAAA GTCTTAACAG   
  
  
- AGATAGTGTT CGTATCATAT ACACAGTTGT AGCACTGTTG AGCTGGTTAG ATGTTTATTG ATCATTTAAG   
  
  
- TTTATTTCAA TGAGAGTGGA TCGAATTTAT TCATGTGGAA CTAAATTAAC AGAATCGATA CCTTGGATAA   
  
  
- GGTTTGATCG GTTATGAAGG AAAAGTTTTC TTAATCAAAA TTTCAAAATT GAAAATGCAG GTTATTACGG   
  
  
- CCGGCATGAT ACTCAACGAA AGTTCGGTCA TCATCATGAC ATCATCCATT TTTATAATCT TATGTGGATG   
  
  
- ATATATACTA TAACGAGAAG TGGCATAGGA AGACGTATGA GTAGAAAGGC AGGGCCTCAG GTGATAGAGA   
  
  
- GGAGGTAAGT CTGTGAGAGA GCGAGATCTC TTGAGTATCG ATACCTGGCG CACGGGCTGC CAATAGCCCA   
  
  
- ATACTTAAGC CTCGAAGAAG TCGTTCAATC GGGACTCAAC GGCCTAGGTT GGTGGACCAA GGTACGGGCA   
  
  
- GATGGGCTGG GGTAGAGAGT CAGAGGAGAA CCTGGGCCAG GTAGGAGGGT CGGTTGGGTA AAGATGGTCC   
  
  
- CAAGACGGAG TCCCTTGGTA CACCAACCCT AACGACTGTG CACCTGCCTG GTCTAACAAC GTCCAAGGAG   
  
  
- CAGGGTCGTC ACTCCAACGT CGTCTCGGTC GTCCACAACT GACGTCAGCT TCGGTACCTC CTCCTGAGAC   
  
  
- CCTATTCTGA CCAGATGCGA GACGACTGCA CACGCCTCCG GTAGGTTGCA CCGCTAAAGG CTAACCGACA   
  
  
- GAACAATCAA TTGTTCTACT CGTTACTGGA GGACGGTGCG CAGTTGGGCA GGACGCCATA ACCGTTTCAT   
  
  
- CGGCCGATGA AGTATCTACG GAACTGGGCT TCTGATATGG TCGTCCCGGG TCACAGCCCG GATTAGCCCA   
  
  
- GCCAAGACCG CATGGTCCAC AACATGCTCG TGAAGATACT TCGAACGGGC AAGGAGTTTA AGCGAGTGAA   
  
  
- GTGACGATTA GTTCGCTAAA ATCTCCGTAA GCTGCCGGTG CTAACACAGG TGTAGTAGCT GAAGCCGGAT   
  
  
- TAGGTGCCGA ACGTTACCGG TCGAGATTAG GTTCGGAACC GACAAGCCGT GCCACCGGGC GGAAAGCATT   
  
  
- CTAATTGGCC TTAGCCCGGC GGAAGACTTA TGCCCAGCAC GAGCGAAGTT GGATAGCCCA GTTGTGACCG   
  
  
- GGTCAATCGG GATAGGTACT TACAACCCAA GCGTAAAGCC CGGCAGCGGC AGAGTGCCGA GCTCCTGTAG   
  
  
- TTTGGTACCT ACCAATTTTG TTCGGGTTTA CTTCAGCATC GGCACTTGAG ATAGAAGGTT GAGGTGGCCG   
  
  
- ATTAGCCCAG CCCATAACTG GGACAGGATT TGACCCAGGC CTCGGATTTG GGCTTTTAAC ACTGTGACCG   
  
  
- CCTCGTTCTC CGCTTGGTGT TGGTCGGGCT CAAAAACCGG GCCAAGTGCC TCCGTAATGT AATGATAAGT   
  
  
- TGATACAAGC TGAGCAATCT CCGAACAGTT CAGGTCCGGC TGTTCCTGGA TCGGCTCGAT ATGAATCTCT   
  
  
- CTCTCAATAG GTTGTAGCAG ACGACACTCC CCAGCCGAGC CTAACTCTCC GTGCTCGGCG ACCGGGTTAC   
  
  
- CTCCCGGGCT TACCGAGCCC GGCCCAAGTT CTTCCACCTA TACCCATTTT TACGCAAGTT CGTTCATTCG   
  
  
- TACAACGACT CAAACAGAAG TCGTGTTCCC ATAACACACC TCCTCTCACT CCCTACAAAC TTCGAGCCGA   
  
  
- CCGTACTGGC GGGAGAGTAA CGCCGAAGCC GAACCGCTCG CCTCTGAGTT CGACTCTTGA GGTCGTGACA   
  
  
- ACACGAACTA CCCAGTAGCA GCACATCAAG TAGAAGAAT

+     LTR

| Site Name | Organism | Position | Strand | Matrix score. | sequence | function |
| --- | --- | --- | --- | --- | --- | --- |
| LTR | Hordeum vulgare | 2715 | + | 6 | CCGAAA | cis-acting element involved in low-temperature responsiveness |
| LTR | Hordeum vulgare | 2559 | - | 6 | CCGAAA | cis-acting element involved in low-temperature responsiveness |

>HU11G00778.1   
+ -Up\_Stream \_Len000AAAAAA GAAAGAAATG GAGAAACCAA GCAATATGGA TAAAAAAAAA ACAAAGTAAT   
  
  
+ AAAGGAAGGA TGAGGCTCCA CATTCCACTC GCCCGCGCCA CTCATAGGAG ATGAATAGTA AAATGTGAGG   
  
  
+ GGTTAAATGA TTTGAAAAAA ATCTAGTAAG GGCCCACAAC AGTATTGGAA CCCAGCTTGC CAAGGAAATG   
  
  
+ TCAGAGGGGC TAGCAATGGA CCAGTGCAAG AAGGCCAAGC CACATTTGGT TAGGGAATTT CATCCCATCC   
  
  
+ GGCGCTAAAA GCATCAAAAT TACTACTTAA TCCTTGATCA AATGCCCTAT AAATATTCTA CTACCCCATT   
  
  
+ TGAAAAGGGG TGTTGATTAC AACACATAGC CGTAGGTTTG ACAAAGAGAA TTACCCTTAT TGTTAGAAGC   
  
  
+ ACCATATACT CTTTTCTCTA AGCTCTGATC TTTTTTCATC CTTTTCAGCT TTTTGTCCTT TTAAATCAAA   
  
  
+ TCACTCACTT GAGTATTGGA GGGACGTTCC TCAGGAGTCG AACCACTAAA TTATCTTTTT AAAGAAAAAG   
  
  
+ GGCTTGAAGC CAACCCGATT TAAAAAAACT GCTAATATGT AAGCCTACTC TCTTTCCAGT TCCAACAAAT   
  
  
+ CAAATTTCAG TATGAAACAT CCAGTAAAGG ATTTATCCGT GATAATGATG TAACATATGG TTAAAGGGTC   
  
  
+ ATTTTCAGTT ACATCGGAGC ATGCAATTAA AGTCCCTATA AAATAGGTAG GTGCACGAAC AAGTCACCAT   
  
  
+ CGTCCTTTGC TTTTTGCATG AGTGCATGCT AATTATGAAC TTAATTAATA TCTAAGCAAA ATTAAAAAGT   
  
  
+ AGTTAAAACT TTTTCACCTT TGGAACCTCG TAGCTGTAGC AGTAGCTGCC GCACTCTGGT TCTAGACTTC   
  
  
+ TAGGGACCAA GAGATGCACT CATATGCATC CTCTTCTGTC CTTTTACTGT ATCCCCTTCC CATGTGACCT   
  
  
+ TTTTGCCCGG GTGACTTTTC TGTGAGTGTT GATACAAATT TTGTACGTAA GCTAACTCAT TTACTTCACC   
  
  
+ GTCTCATGAG TTTTACATTA CCGATATTAA TTTAAATGGC AAAGTATTAA ATTTGAGCTA AAATCTCATA   
  
  
+ TATATATTTG TATTAAATGT GTCTGGATTG TCAAAATAAT TTTTGACATA TACAGTCACT ATATACTGAG   
  
  
+ TTATTAAAAT ATATAAAAAT ATGAAAATTT AAAAAATAGT AGTTTAAGGT GACAACACTA AAACAAACAA   
  
  
+ ATAAGATATA AATAGGGAAA AGTTCATCCT ATTTTGTTAT TATTATTTAT TATGTATTTT CAGAATTGTC   
  
  
+ TCTATCACAA GCATAGTATA TGTGTCAACA TCGTGACAAC TCGACCAATC TACAAATAAC TAGTAAATTC   
  
  
+ AAATAAAGTT ACTCTCACCT AGCTTAAATA AGTACACCTT GATTTAATTG TCTTAGCTAT GGAACCTATT   
  
  
+ CCAAACTAGC CAATACTTCC TTTTCAAAAG AATTAGTTTT AAAGTTTTAA CTTTTACGTC CAATAATGCC   
  
  
+ GGCCGTACTA TGAGTTGCTT TCAAGCCAGT AGTAGTACTG TAGTAGGTAA AAATATTAGA ATACACCTAC   
  
  
+ TATATATGAT ATTGCTCTTC ACCGTATCCT TCTGCATACT CATCTTTCCG TCCCGGAGTC CACTATCTCT   
  
  
+ CCTCCATTCA GACACTCTCT CGCTCTAGAG AACTCATAGC TATGGACCGC GTGCCCGACG GTTATCGGGT   
  
  
+ TATGAATTCG GAGCTTCTTC AGCAAGTTAG CCCTGAGTTG CCGGATCCAA CCACCTGGTT CCATGCCCGT   
  
  
+ CTACCCGACC CCATCTCTCA GTCTCCTCTT GGACCCGGTC CATCCTCCCA GCCAACCCAT TTCTACCAGG   
  
  
+ GTTCTGCCTC AGGGAACCAT GTGGTTGGGA TTGCTGACAC GTGGACGGAC CAGATTGTTG CAGGTTCCTC   
  
  
+ GTCCCAGCAG TGAGGTTGCA GCAGAGCCAG CAGGTGTTGA CTGCAGTCGA AGCCATGGAG GAGGACTCTG   
  
  
+ GGATAAGACT GGTCTACGCT CTGCTGACGT GTGCGGAGGC CATCCAACGT GGCGATTTCC GATTGGCTGT   
  
  
+ CTTGTTAGTT AACAAGATGA GCAATGACCT CCTGCCACGC GTCAACCCGT CCTGCGGTAT TGGCAAAGTA   
  
  
+ GCCGGCTACT TCATAGATGC CTTGACCCGA AGACTATACC AGCAGGGCCC AGTGTCGGGC CTAATCGGGT   
  
  
+ CGGTTCTGGC GTACCAGGTG TTGTACGAGC ACTTCTATGA AGCTTGCCCG TTCCTCAAAT TCGCTCACTT   
  
  
+ CACTGCTAAT CAAGCGATTT TAGAGGCATT CGACGGCCAC GATTGTGTCC ACATCATCGA CTTCGGCCTA   
  
  
+ ATCCACGGCT TGCAATGGCC AGCTCTAATC CAAGCCTTGG CTGTTCGGCA CGGTGGCCCG CCTTTCGTAA   
  
  
+ GATTAACCGG AATCGGGCCG CCTTCTGAAT ACGGGTCGTG CTCGCTTCAA CCTATCGGGT CAACACTGGC   
  
  
+ CCAGTTAGCC CTATCCATGA ATGTTGGGTT CGCATTTCGG GCCGTCGCCG TCTCACGGCT CGAGGACATC   
  
  
+ AAACCATGGA TGGTTAAAAC AAGCCCAAAT GAAGTCGTAG CCGTGAACTC TATCTTCCAA CTCCACCGGC   
  
  
+ TAATCGGGTC GGGTATTGAC CCTGTCCTAA ACTGGGTCCG GAGCCTAAAC CCGAAAATTG TGACACTGGC   
  
  
+ GGAGCAAGAG GCGAACCACA ACCAGCCCGA GTTTTTGGCC CGGTTCACGG AGGCATTACA TTACTATTCA   
  
  
+ ACTATGTTCG ACTCGTTAGA GGCTTGTCAA GTCCAGGCCG ACAAGGACCT AGCCGAGCTA TACTTAGAGA   
  
  
+ GAGAGTTATC CAACATCGTC TGCTGTGAGG GGTCGGCTCG GATTGAGAGG CACGAGCCGC TGGCCCAATG   
  
  
+ GAGGGCCCGA ATGGCTCGGG CCGGGTTCAA GAAGGTGGAT ATGGGTAAAA ATGCGTTCAA GCAAGTAAGC   
  
  
+ ATGTTGCTGA GTTTGTCTTC AGCACAAGGG TATTGTGTGG AGGAGAGTGA GGGATGTTTG AAGCTCGGCT   
  
  
+ GGCATGACCG CCCTCTCATT GCGGCTTCGG CTTGGCGAGC GGAGACTCAA GCTGAGAACT CCAGCACTGT   
  
  
+ TGTGCTTGAT GGGTCATCGT CGTGTAGTTC ATCTTCTTA  

- -Up\_Stream \_Len000TTTTTT CTTTCTTTAC CTCTTTGGTT CGTTATACCT ATTTTTTTTT TGTTTCATTA   
  
  
- TTTCCTTCCT ACTCCGAGGT GTAAGGTGAG CGGGCGCGGT GAGTATCCTC TACTTATCAT TTTACACTCC   
  
  
- CCAATTTACT AAACTTTTTT TAGATCATTC CCGGGTGTTG TCATAACCTT GGGTCGAACG GTTCCTTTAC   
  
  
- AGTCTCCCCG ATCGTTACCT GGTCACGTTC TTCCGGTTCG GTGTAAACCA ATCCCTTAAA GTAGGGTAGG   
  
  
- CCGCGATTTT CGTAGTTTTA ATGATGAATT AGGAACTAGT TTACGGGATA TTTATAAGAT GATGGGGTAA   
  
  
- ACTTTTCCCC ACAACTAATG TTGTGTATCG GCATCCAAAC TGTTTCTCTT AATGGGAATA ACAATCTTCG   
  
  
- TGGTATATGA GAAAAGAGAT TCGAGACTAG AAAAAAGTAG GAAAAGTCGA AAAACAGGAA AATTTAGTTT   
  
  
- AGTGAGTGAA CTCATAACCT CCCTGCAAGG AGTCCTCAGC TTGGTGATTT AATAGAAAAA TTTCTTTTTC   
  
  
- CCGAACTTCG GTTGGGCTAA ATTTTTTTGA CGATTATACA TTCGGATGAG AGAAAGGTCA AGGTTGTTTA   
  
  
- GTTTAAAGTC ATACTTTGTA GGTCATTTCC TAAATAGGCA CTATTACTAC ATTGTATACC AATTTCCCAG   
  
  
- TAAAAGTCAA TGTAGCCTCG TACGTTAATT TCAGGGATAT TTTATCCATC CACGTGCTTG TTCAGTGGTA   
  
  
- GCAGGAAACG AAAAACGTAC TCACGTACGA TTAATACTTG AATTAATTAT AGATTCGTTT TAATTTTTCA   
  
  
- TCAATTTTGA AAAAGTGGAA ACCTTGGAGC ATCGACATCG TCATCGACGG CGTGAGACCA AGATCTGAAG   
  
  
- ATCCCTGGTT CTCTACGTGA GTATACGTAG GAGAAGACAG GAAAATGACA TAGGGGAAGG GTACACTGGA   
  
  
- AAAACGGGCC CACTGAAAAG ACACTCACAA CTATGTTTAA AACATGCATT CGATTGAGTA AATGAAGTGG   
  
  
- CAGAGTACTC AAAATGTAAT GGCTATAATT AAATTTACCG TTTCATAATT TAAACTCGAT TTTAGAGTAT   
  
  
- ATATATAAAC ATAATTTACA CAGACCTAAC AGTTTTATTA AAAACTGTAT ATGTCAGTGA TATATGACTC   
  
  
- AATAATTTTA TATATTTTTA TACTTTTAAA TTTTTTATCA TCAAATTCCA CTGTTGTGAT TTTGTTTGTT   
  
  
- TATTCTATAT TTATCCCTTT TCAAGTAGGA TAAAACAATA ATAATAAATA ATACATAAAA GTCTTAACAG   
  
  
- AGATAGTGTT CGTATCATAT ACACAGTTGT AGCACTGTTG AGCTGGTTAG ATGTTTATTG ATCATTTAAG   
  
  
- TTTATTTCAA TGAGAGTGGA TCGAATTTAT TCATGTGGAA CTAAATTAAC AGAATCGATA CCTTGGATAA   
  
  
- GGTTTGATCG GTTATGAAGG AAAAGTTTTC TTAATCAAAA TTTCAAAATT GAAAATGCAG GTTATTACGG   
  
  
- CCGGCATGAT ACTCAACGAA AGTTCGGTCA TCATCATGAC ATCATCCATT TTTATAATCT TATGTGGATG   
  
  
- ATATATACTA TAACGAGAAG TGGCATAGGA AGACGTATGA GTAGAAAGGC AGGGCCTCAG GTGATAGAGA   
  
  
- GGAGGTAAGT CTGTGAGAGA GCGAGATCTC TTGAGTATCG ATACCTGGCG CACGGGCTGC CAATAGCCCA   
  
  
- ATACTTAAGC CTCGAAGAAG TCGTTCAATC GGGACTCAAC GGCCTAGGTT GGTGGACCAA GGTACGGGCA   
  
  
- GATGGGCTGG GGTAGAGAGT CAGAGGAGAA CCTGGGCCAG GTAGGAGGGT CGGTTGGGTA AAGATGGTCC   
  
  
- CAAGACGGAG TCCCTTGGTA CACCAACCCT AACGACTGTG CACCTGCCTG GTCTAACAAC GTCCAAGGAG   
  
  
- CAGGGTCGTC ACTCCAACGT CGTCTCGGTC GTCCACAACT GACGTCAGCT TCGGTACCTC CTCCTGAGAC   
  
  
- CCTATTCTGA CCAGATGCGA GACGACTGCA CACGCCTCCG GTAGGTTGCA CCGCTAAAGG CTAACCGACA   
  
  
- GAACAATCAA TTGTTCTACT CGTTACTGGA GGACGGTGCG CAGTTGGGCA GGACGCCATA ACCGTTTCAT   
  
  
- CGGCCGATGA AGTATCTACG GAACTGGGCT TCTGATATGG TCGTCCCGGG TCACAGCCCG GATTAGCCCA   
  
  
- GCCAAGACCG CATGGTCCAC AACATGCTCG TGAAGATACT TCGAACGGGC AAGGAGTTTA AGCGAGTGAA   
  
  
- GTGACGATTA GTTCGCTAAA ATCTCCGTAA GCTGCCGGTG CTAACACAGG TGTAGTAGCT GAAGCCGGAT   
  
  
- TAGGTGCCGA ACGTTACCGG TCGAGATTAG GTTCGGAACC GACAAGCCGT GCCACCGGGC GGAAAGCATT   
  
  
- CTAATTGGCC TTAGCCCGGC GGAAGACTTA TGCCCAGCAC GAGCGAAGTT GGATAGCCCA GTTGTGACCG   
  
  
- GGTCAATCGG GATAGGTACT TACAACCCAA GCGTAAAGCC CGGCAGCGGC AGAGTGCCGA GCTCCTGTAG   
  
  
- TTTGGTACCT ACCAATTTTG TTCGGGTTTA CTTCAGCATC GGCACTTGAG ATAGAAGGTT GAGGTGGCCG   
  
  
- ATTAGCCCAG CCCATAACTG GGACAGGATT TGACCCAGGC CTCGGATTTG GGCTTTTAAC ACTGTGACCG   
  
  
- CCTCGTTCTC CGCTTGGTGT TGGTCGGGCT CAAAAACCGG GCCAAGTGCC TCCGTAATGT AATGATAAGT   
  
  
- TGATACAAGC TGAGCAATCT CCGAACAGTT CAGGTCCGGC TGTTCCTGGA TCGGCTCGAT ATGAATCTCT   
  
  
- CTCTCAATAG GTTGTAGCAG ACGACACTCC CCAGCCGAGC CTAACTCTCC GTGCTCGGCG ACCGGGTTAC   
  
  
- CTCCCGGGCT TACCGAGCCC GGCCCAAGTT CTTCCACCTA TACCCATTTT TACGCAAGTT CGTTCATTCG   
  
  
- TACAACGACT CAAACAGAAG TCGTGTTCCC ATAACACACC TCCTCTCACT CCCTACAAAC TTCGAGCCGA   
  
  
- CCGTACTGGC GGGAGAGTAA CGCCGAAGCC GAACCGCTCG CCTCTGAGTT CGACTCTTGA GGTCGTGACA   
  
  
- ACACGAACTA CCCAGTAGCA GCACATCAAG TAGAAGAAT

+     MYB

| Site Name | Organism | Position | Strand | Matrix score. | sequence | function |
| --- | --- | --- | --- | --- | --- | --- |
| MYB | Arabidopsis thaliana | 3151 | - | 6 | CAACAG |  |
| MYB | Arabidopsis thaliana | 2753 | + | 6 | CAACCA |  |
| MYB | Arabidopsis thaliana | 2605 | - | 6 | TAACCA |  |
| MYB | Arabidopsis thaliana | 1916 | - | 6 | CAACCA |  |
| MYB | Arabidopsis thaliana | 692 | - | 6 | TAACCA |  |
| MYB | Arabidopsis thaliana | 1802 | + | 6 | CAACCA |  |
| MYB | Arabidopsis thaliana | 261 | - | 6 | TAACCA |  |
| MYB | Arabidopsis thaliana | 181 | + | 6 | CAACAG |  |

>HU11G00778.1   
+ -Up\_Stream \_Len000AAAAAA GAAAGAAATG GAGAAACCAA GCAATATGGA TAAAAAAAAA ACAAAGTAAT   
  
  
+ AAAGGAAGGA TGAGGCTCCA CATTCCACTC GCCCGCGCCA CTCATAGGAG ATGAATAGTA AAATGTGAGG   
  
  
+ GGTTAAATGA TTTGAAAAAA ATCTAGTAAG GGCCCACAAC AGTATTGGAA CCCAGCTTGC CAAGGAAATG   
  
  
+ TCAGAGGGGC TAGCAATGGA CCAGTGCAAG AAGGCCAAGC CACATTTGGT TAGGGAATTT CATCCCATCC   
  
  
+ GGCGCTAAAA GCATCAAAAT TACTACTTAA TCCTTGATCA AATGCCCTAT AAATATTCTA CTACCCCATT   
  
  
+ TGAAAAGGGG TGTTGATTAC AACACATAGC CGTAGGTTTG ACAAAGAGAA TTACCCTTAT TGTTAGAAGC   
  
  
+ ACCATATACT CTTTTCTCTA AGCTCTGATC TTTTTTCATC CTTTTCAGCT TTTTGTCCTT TTAAATCAAA   
  
  
+ TCACTCACTT GAGTATTGGA GGGACGTTCC TCAGGAGTCG AACCACTAAA TTATCTTTTT AAAGAAAAAG   
  
  
+ GGCTTGAAGC CAACCCGATT TAAAAAAACT GCTAATATGT AAGCCTACTC TCTTTCCAGT TCCAACAAAT   
  
  
+ CAAATTTCAG TATGAAACAT CCAGTAAAGG ATTTATCCGT GATAATGATG TAACATATGG TTAAAGGGTC   
  
  
+ ATTTTCAGTT ACATCGGAGC ATGCAATTAA AGTCCCTATA AAATAGGTAG GTGCACGAAC AAGTCACCAT   
  
  
+ CGTCCTTTGC TTTTTGCATG AGTGCATGCT AATTATGAAC TTAATTAATA TCTAAGCAAA ATTAAAAAGT   
  
  
+ AGTTAAAACT TTTTCACCTT TGGAACCTCG TAGCTGTAGC AGTAGCTGCC GCACTCTGGT TCTAGACTTC   
  
  
+ TAGGGACCAA GAGATGCACT CATATGCATC CTCTTCTGTC CTTTTACTGT ATCCCCTTCC CATGTGACCT   
  
  
+ TTTTGCCCGG GTGACTTTTC TGTGAGTGTT GATACAAATT TTGTACGTAA GCTAACTCAT TTACTTCACC   
  
  
+ GTCTCATGAG TTTTACATTA CCGATATTAA TTTAAATGGC AAAGTATTAA ATTTGAGCTA AAATCTCATA   
  
  
+ TATATATTTG TATTAAATGT GTCTGGATTG TCAAAATAAT TTTTGACATA TACAGTCACT ATATACTGAG   
  
  
+ TTATTAAAAT ATATAAAAAT ATGAAAATTT AAAAAATAGT AGTTTAAGGT GACAACACTA AAACAAACAA   
  
  
+ ATAAGATATA AATAGGGAAA AGTTCATCCT ATTTTGTTAT TATTATTTAT TATGTATTTT CAGAATTGTC   
  
  
+ TCTATCACAA GCATAGTATA TGTGTCAACA TCGTGACAAC TCGACCAATC TACAAATAAC TAGTAAATTC   
  
  
+ AAATAAAGTT ACTCTCACCT AGCTTAAATA AGTACACCTT GATTTAATTG TCTTAGCTAT GGAACCTATT   
  
  
+ CCAAACTAGC CAATACTTCC TTTTCAAAAG AATTAGTTTT AAAGTTTTAA CTTTTACGTC CAATAATGCC   
  
  
+ GGCCGTACTA TGAGTTGCTT TCAAGCCAGT AGTAGTACTG TAGTAGGTAA AAATATTAGA ATACACCTAC   
  
  
+ TATATATGAT ATTGCTCTTC ACCGTATCCT TCTGCATACT CATCTTTCCG TCCCGGAGTC CACTATCTCT   
  
  
+ CCTCCATTCA GACACTCTCT CGCTCTAGAG AACTCATAGC TATGGACCGC GTGCCCGACG GTTATCGGGT   
  
  
+ TATGAATTCG GAGCTTCTTC AGCAAGTTAG CCCTGAGTTG CCGGATCCAA CCACCTGGTT CCATGCCCGT   
  
  
+ CTACCCGACC CCATCTCTCA GTCTCCTCTT GGACCCGGTC CATCCTCCCA GCCAACCCAT TTCTACCAGG   
  
  
+ GTTCTGCCTC AGGGAACCAT GTGGTTGGGA TTGCTGACAC GTGGACGGAC CAGATTGTTG CAGGTTCCTC   
  
  
+ GTCCCAGCAG TGAGGTTGCA GCAGAGCCAG CAGGTGTTGA CTGCAGTCGA AGCCATGGAG GAGGACTCTG   
  
  
+ GGATAAGACT GGTCTACGCT CTGCTGACGT GTGCGGAGGC CATCCAACGT GGCGATTTCC GATTGGCTGT   
  
  
+ CTTGTTAGTT AACAAGATGA GCAATGACCT CCTGCCACGC GTCAACCCGT CCTGCGGTAT TGGCAAAGTA   
  
  
+ GCCGGCTACT TCATAGATGC CTTGACCCGA AGACTATACC AGCAGGGCCC AGTGTCGGGC CTAATCGGGT   
  
  
+ CGGTTCTGGC GTACCAGGTG TTGTACGAGC ACTTCTATGA AGCTTGCCCG TTCCTCAAAT TCGCTCACTT   
  
  
+ CACTGCTAAT CAAGCGATTT TAGAGGCATT CGACGGCCAC GATTGTGTCC ACATCATCGA CTTCGGCCTA   
  
  
+ ATCCACGGCT TGCAATGGCC AGCTCTAATC CAAGCCTTGG CTGTTCGGCA CGGTGGCCCG CCTTTCGTAA   
  
  
+ GATTAACCGG AATCGGGCCG CCTTCTGAAT ACGGGTCGTG CTCGCTTCAA CCTATCGGGT CAACACTGGC   
  
  
+ CCAGTTAGCC CTATCCATGA ATGTTGGGTT CGCATTTCGG GCCGTCGCCG TCTCACGGCT CGAGGACATC   
  
  
+ AAACCATGGA TGGTTAAAAC AAGCCCAAAT GAAGTCGTAG CCGTGAACTC TATCTTCCAA CTCCACCGGC   
  
  
+ TAATCGGGTC GGGTATTGAC CCTGTCCTAA ACTGGGTCCG GAGCCTAAAC CCGAAAATTG TGACACTGGC   
  
  
+ GGAGCAAGAG GCGAACCACA ACCAGCCCGA GTTTTTGGCC CGGTTCACGG AGGCATTACA TTACTATTCA   
  
  
+ ACTATGTTCG ACTCGTTAGA GGCTTGTCAA GTCCAGGCCG ACAAGGACCT AGCCGAGCTA TACTTAGAGA   
  
  
+ GAGAGTTATC CAACATCGTC TGCTGTGAGG GGTCGGCTCG GATTGAGAGG CACGAGCCGC TGGCCCAATG   
  
  
+ GAGGGCCCGA ATGGCTCGGG CCGGGTTCAA GAAGGTGGAT ATGGGTAAAA ATGCGTTCAA GCAAGTAAGC   
  
  
+ ATGTTGCTGA GTTTGTCTTC AGCACAAGGG TATTGTGTGG AGGAGAGTGA GGGATGTTTG AAGCTCGGCT   
  
  
+ GGCATGACCG CCCTCTCATT GCGGCTTCGG CTTGGCGAGC GGAGACTCAA GCTGAGAACT CCAGCACTGT   
  
  
+ TGTGCTTGAT GGGTCATCGT CGTGTAGTTC ATCTTCTTA  

- -Up\_Stream \_Len000TTTTTT CTTTCTTTAC CTCTTTGGTT CGTTATACCT ATTTTTTTTT TGTTTCATTA   
  
  
- TTTCCTTCCT ACTCCGAGGT GTAAGGTGAG CGGGCGCGGT GAGTATCCTC TACTTATCAT TTTACACTCC   
  
  
- CCAATTTACT AAACTTTTTT TAGATCATTC CCGGGTGTTG TCATAACCTT GGGTCGAACG GTTCCTTTAC   
  
  
- AGTCTCCCCG ATCGTTACCT GGTCACGTTC TTCCGGTTCG GTGTAAACCA ATCCCTTAAA GTAGGGTAGG   
  
  
- CCGCGATTTT CGTAGTTTTA ATGATGAATT AGGAACTAGT TTACGGGATA TTTATAAGAT GATGGGGTAA   
  
  
- ACTTTTCCCC ACAACTAATG TTGTGTATCG GCATCCAAAC TGTTTCTCTT AATGGGAATA ACAATCTTCG   
  
  
- TGGTATATGA GAAAAGAGAT TCGAGACTAG AAAAAAGTAG GAAAAGTCGA AAAACAGGAA AATTTAGTTT   
  
  
- AGTGAGTGAA CTCATAACCT CCCTGCAAGG AGTCCTCAGC TTGGTGATTT AATAGAAAAA TTTCTTTTTC   
  
  
- CCGAACTTCG GTTGGGCTAA ATTTTTTTGA CGATTATACA TTCGGATGAG AGAAAGGTCA AGGTTGTTTA   
  
  
- GTTTAAAGTC ATACTTTGTA GGTCATTTCC TAAATAGGCA CTATTACTAC ATTGTATACC AATTTCCCAG   
  
  
- TAAAAGTCAA TGTAGCCTCG TACGTTAATT TCAGGGATAT TTTATCCATC CACGTGCTTG TTCAGTGGTA   
  
  
- GCAGGAAACG AAAAACGTAC TCACGTACGA TTAATACTTG AATTAATTAT AGATTCGTTT TAATTTTTCA   
  
  
- TCAATTTTGA AAAAGTGGAA ACCTTGGAGC ATCGACATCG TCATCGACGG CGTGAGACCA AGATCTGAAG   
  
  
- ATCCCTGGTT CTCTACGTGA GTATACGTAG GAGAAGACAG GAAAATGACA TAGGGGAAGG GTACACTGGA   
  
  
- AAAACGGGCC CACTGAAAAG ACACTCACAA CTATGTTTAA AACATGCATT CGATTGAGTA AATGAAGTGG   
  
  
- CAGAGTACTC AAAATGTAAT GGCTATAATT AAATTTACCG TTTCATAATT TAAACTCGAT TTTAGAGTAT   
  
  
- ATATATAAAC ATAATTTACA CAGACCTAAC AGTTTTATTA AAAACTGTAT ATGTCAGTGA TATATGACTC   
  
  
- AATAATTTTA TATATTTTTA TACTTTTAAA TTTTTTATCA TCAAATTCCA CTGTTGTGAT TTTGTTTGTT   
  
  
- TATTCTATAT TTATCCCTTT TCAAGTAGGA TAAAACAATA ATAATAAATA ATACATAAAA GTCTTAACAG   
  
  
- AGATAGTGTT CGTATCATAT ACACAGTTGT AGCACTGTTG AGCTGGTTAG ATGTTTATTG ATCATTTAAG   
  
  
- TTTATTTCAA TGAGAGTGGA TCGAATTTAT TCATGTGGAA CTAAATTAAC AGAATCGATA CCTTGGATAA   
  
  
- GGTTTGATCG GTTATGAAGG AAAAGTTTTC TTAATCAAAA TTTCAAAATT GAAAATGCAG GTTATTACGG   
  
  
- CCGGCATGAT ACTCAACGAA AGTTCGGTCA TCATCATGAC ATCATCCATT TTTATAATCT TATGTGGATG   
  
  
- ATATATACTA TAACGAGAAG TGGCATAGGA AGACGTATGA GTAGAAAGGC AGGGCCTCAG GTGATAGAGA   
  
  
- GGAGGTAAGT CTGTGAGAGA GCGAGATCTC TTGAGTATCG ATACCTGGCG CACGGGCTGC CAATAGCCCA   
  
  
- ATACTTAAGC CTCGAAGAAG TCGTTCAATC GGGACTCAAC GGCCTAGGTT GGTGGACCAA GGTACGGGCA   
  
  
- GATGGGCTGG GGTAGAGAGT CAGAGGAGAA CCTGGGCCAG GTAGGAGGGT CGGTTGGGTA AAGATGGTCC   
  
  
- CAAGACGGAG TCCCTTGGTA CACCAACCCT AACGACTGTG CACCTGCCTG GTCTAACAAC GTCCAAGGAG   
  
  
- CAGGGTCGTC ACTCCAACGT CGTCTCGGTC GTCCACAACT GACGTCAGCT TCGGTACCTC CTCCTGAGAC   
  
  
- CCTATTCTGA CCAGATGCGA GACGACTGCA CACGCCTCCG GTAGGTTGCA CCGCTAAAGG CTAACCGACA   
  
  
- GAACAATCAA TTGTTCTACT CGTTACTGGA GGACGGTGCG CAGTTGGGCA GGACGCCATA ACCGTTTCAT   
  
  
- CGGCCGATGA AGTATCTACG GAACTGGGCT TCTGATATGG TCGTCCCGGG TCACAGCCCG GATTAGCCCA   
  
  
- GCCAAGACCG CATGGTCCAC AACATGCTCG TGAAGATACT TCGAACGGGC AAGGAGTTTA AGCGAGTGAA   
  
  
- GTGACGATTA GTTCGCTAAA ATCTCCGTAA GCTGCCGGTG CTAACACAGG TGTAGTAGCT GAAGCCGGAT   
  
  
- TAGGTGCCGA ACGTTACCGG TCGAGATTAG GTTCGGAACC GACAAGCCGT GCCACCGGGC GGAAAGCATT   
  
  
- CTAATTGGCC TTAGCCCGGC GGAAGACTTA TGCCCAGCAC GAGCGAAGTT GGATAGCCCA GTTGTGACCG   
  
  
- GGTCAATCGG GATAGGTACT TACAACCCAA GCGTAAAGCC CGGCAGCGGC AGAGTGCCGA GCTCCTGTAG   
  
  
- TTTGGTACCT ACCAATTTTG TTCGGGTTTA CTTCAGCATC GGCACTTGAG ATAGAAGGTT GAGGTGGCCG   
  
  
- ATTAGCCCAG CCCATAACTG GGACAGGATT TGACCCAGGC CTCGGATTTG GGCTTTTAAC ACTGTGACCG   
  
  
- CCTCGTTCTC CGCTTGGTGT TGGTCGGGCT CAAAAACCGG GCCAAGTGCC TCCGTAATGT AATGATAAGT   
  
  
- TGATACAAGC TGAGCAATCT CCGAACAGTT CAGGTCCGGC TGTTCCTGGA TCGGCTCGAT ATGAATCTCT   
  
  
- CTCTCAATAG GTTGTAGCAG ACGACACTCC CCAGCCGAGC CTAACTCTCC GTGCTCGGCG ACCGGGTTAC   
  
  
- CTCCCGGGCT TACCGAGCCC GGCCCAAGTT CTTCCACCTA TACCCATTTT TACGCAAGTT CGTTCATTCG   
  
  
- TACAACGACT CAAACAGAAG TCGTGTTCCC ATAACACACC TCCTCTCACT CCCTACAAAC TTCGAGCCGA   
  
  
- CCGTACTGGC GGGAGAGTAA CGCCGAAGCC GAACCGCTCG CCTCTGAGTT CGACTCTTGA GGTCGTGACA   
  
  
- ACACGAACTA CCCAGTAGCA GCACATCAAG TAGAAGAAT

+     MYB-like sequence

| Site Name | Organism | Position | Strand | Matrix score. | sequence | function |
| --- | --- | --- | --- | --- | --- | --- |
| MYB-like sequence | Arabidopsis thaliana | 261 | - | 6 | TAACCA |  |
| MYB-like sequence | Arabidopsis thaliana | 692 | - | 6 | TAACCA |  |
| MYB-like sequence | Arabidopsis thaliana | 2605 | - | 6 | TAACCA |  |

>HU11G00778.1   
+ -Up\_Stream \_Len000AAAAAA GAAAGAAATG GAGAAACCAA GCAATATGGA TAAAAAAAAA ACAAAGTAAT   
  
  
+ AAAGGAAGGA TGAGGCTCCA CATTCCACTC GCCCGCGCCA CTCATAGGAG ATGAATAGTA AAATGTGAGG   
  
  
+ GGTTAAATGA TTTGAAAAAA ATCTAGTAAG GGCCCACAAC AGTATTGGAA CCCAGCTTGC CAAGGAAATG   
  
  
+ TCAGAGGGGC TAGCAATGGA CCAGTGCAAG AAGGCCAAGC CACATTTGGT TAGGGAATTT CATCCCATCC   
  
  
+ GGCGCTAAAA GCATCAAAAT TACTACTTAA TCCTTGATCA AATGCCCTAT AAATATTCTA CTACCCCATT   
  
  
+ TGAAAAGGGG TGTTGATTAC AACACATAGC CGTAGGTTTG ACAAAGAGAA TTACCCTTAT TGTTAGAAGC   
  
  
+ ACCATATACT CTTTTCTCTA AGCTCTGATC TTTTTTCATC CTTTTCAGCT TTTTGTCCTT TTAAATCAAA   
  
  
+ TCACTCACTT GAGTATTGGA GGGACGTTCC TCAGGAGTCG AACCACTAAA TTATCTTTTT AAAGAAAAAG   
  
  
+ GGCTTGAAGC CAACCCGATT TAAAAAAACT GCTAATATGT AAGCCTACTC TCTTTCCAGT TCCAACAAAT   
  
  
+ CAAATTTCAG TATGAAACAT CCAGTAAAGG ATTTATCCGT GATAATGATG TAACATATGG TTAAAGGGTC   
  
  
+ ATTTTCAGTT ACATCGGAGC ATGCAATTAA AGTCCCTATA AAATAGGTAG GTGCACGAAC AAGTCACCAT   
  
  
+ CGTCCTTTGC TTTTTGCATG AGTGCATGCT AATTATGAAC TTAATTAATA TCTAAGCAAA ATTAAAAAGT   
  
  
+ AGTTAAAACT TTTTCACCTT TGGAACCTCG TAGCTGTAGC AGTAGCTGCC GCACTCTGGT TCTAGACTTC   
  
  
+ TAGGGACCAA GAGATGCACT CATATGCATC CTCTTCTGTC CTTTTACTGT ATCCCCTTCC CATGTGACCT   
  
  
+ TTTTGCCCGG GTGACTTTTC TGTGAGTGTT GATACAAATT TTGTACGTAA GCTAACTCAT TTACTTCACC   
  
  
+ GTCTCATGAG TTTTACATTA CCGATATTAA TTTAAATGGC AAAGTATTAA ATTTGAGCTA AAATCTCATA   
  
  
+ TATATATTTG TATTAAATGT GTCTGGATTG TCAAAATAAT TTTTGACATA TACAGTCACT ATATACTGAG   
  
  
+ TTATTAAAAT ATATAAAAAT ATGAAAATTT AAAAAATAGT AGTTTAAGGT GACAACACTA AAACAAACAA   
  
  
+ ATAAGATATA AATAGGGAAA AGTTCATCCT ATTTTGTTAT TATTATTTAT TATGTATTTT CAGAATTGTC   
  
  
+ TCTATCACAA GCATAGTATA TGTGTCAACA TCGTGACAAC TCGACCAATC TACAAATAAC TAGTAAATTC   
  
  
+ AAATAAAGTT ACTCTCACCT AGCTTAAATA AGTACACCTT GATTTAATTG TCTTAGCTAT GGAACCTATT   
  
  
+ CCAAACTAGC CAATACTTCC TTTTCAAAAG AATTAGTTTT AAAGTTTTAA CTTTTACGTC CAATAATGCC   
  
  
+ GGCCGTACTA TGAGTTGCTT TCAAGCCAGT AGTAGTACTG TAGTAGGTAA AAATATTAGA ATACACCTAC   
  
  
+ TATATATGAT ATTGCTCTTC ACCGTATCCT TCTGCATACT CATCTTTCCG TCCCGGAGTC CACTATCTCT   
  
  
+ CCTCCATTCA GACACTCTCT CGCTCTAGAG AACTCATAGC TATGGACCGC GTGCCCGACG GTTATCGGGT   
  
  
+ TATGAATTCG GAGCTTCTTC AGCAAGTTAG CCCTGAGTTG CCGGATCCAA CCACCTGGTT CCATGCCCGT   
  
  
+ CTACCCGACC CCATCTCTCA GTCTCCTCTT GGACCCGGTC CATCCTCCCA GCCAACCCAT TTCTACCAGG   
  
  
+ GTTCTGCCTC AGGGAACCAT GTGGTTGGGA TTGCTGACAC GTGGACGGAC CAGATTGTTG CAGGTTCCTC   
  
  
+ GTCCCAGCAG TGAGGTTGCA GCAGAGCCAG CAGGTGTTGA CTGCAGTCGA AGCCATGGAG GAGGACTCTG   
  
  
+ GGATAAGACT GGTCTACGCT CTGCTGACGT GTGCGGAGGC CATCCAACGT GGCGATTTCC GATTGGCTGT   
  
  
+ CTTGTTAGTT AACAAGATGA GCAATGACCT CCTGCCACGC GTCAACCCGT CCTGCGGTAT TGGCAAAGTA   
  
  
+ GCCGGCTACT TCATAGATGC CTTGACCCGA AGACTATACC AGCAGGGCCC AGTGTCGGGC CTAATCGGGT   
  
  
+ CGGTTCTGGC GTACCAGGTG TTGTACGAGC ACTTCTATGA AGCTTGCCCG TTCCTCAAAT TCGCTCACTT   
  
  
+ CACTGCTAAT CAAGCGATTT TAGAGGCATT CGACGGCCAC GATTGTGTCC ACATCATCGA CTTCGGCCTA   
  
  
+ ATCCACGGCT TGCAATGGCC AGCTCTAATC CAAGCCTTGG CTGTTCGGCA CGGTGGCCCG CCTTTCGTAA   
  
  
+ GATTAACCGG AATCGGGCCG CCTTCTGAAT ACGGGTCGTG CTCGCTTCAA CCTATCGGGT CAACACTGGC   
  
  
+ CCAGTTAGCC CTATCCATGA ATGTTGGGTT CGCATTTCGG GCCGTCGCCG TCTCACGGCT CGAGGACATC   
  
  
+ AAACCATGGA TGGTTAAAAC AAGCCCAAAT GAAGTCGTAG CCGTGAACTC TATCTTCCAA CTCCACCGGC   
  
  
+ TAATCGGGTC GGGTATTGAC CCTGTCCTAA ACTGGGTCCG GAGCCTAAAC CCGAAAATTG TGACACTGGC   
  
  
+ GGAGCAAGAG GCGAACCACA ACCAGCCCGA GTTTTTGGCC CGGTTCACGG AGGCATTACA TTACTATTCA   
  
  
+ ACTATGTTCG ACTCGTTAGA GGCTTGTCAA GTCCAGGCCG ACAAGGACCT AGCCGAGCTA TACTTAGAGA   
  
  
+ GAGAGTTATC CAACATCGTC TGCTGTGAGG GGTCGGCTCG GATTGAGAGG CACGAGCCGC TGGCCCAATG   
  
  
+ GAGGGCCCGA ATGGCTCGGG CCGGGTTCAA GAAGGTGGAT ATGGGTAAAA ATGCGTTCAA GCAAGTAAGC   
  
  
+ ATGTTGCTGA GTTTGTCTTC AGCACAAGGG TATTGTGTGG AGGAGAGTGA GGGATGTTTG AAGCTCGGCT   
  
  
+ GGCATGACCG CCCTCTCATT GCGGCTTCGG CTTGGCGAGC GGAGACTCAA GCTGAGAACT CCAGCACTGT   
  
  
+ TGTGCTTGAT GGGTCATCGT CGTGTAGTTC ATCTTCTTA  

- -Up\_Stream \_Len000TTTTTT CTTTCTTTAC CTCTTTGGTT CGTTATACCT ATTTTTTTTT TGTTTCATTA   
  
  
- TTTCCTTCCT ACTCCGAGGT GTAAGGTGAG CGGGCGCGGT GAGTATCCTC TACTTATCAT TTTACACTCC   
  
  
- CCAATTTACT AAACTTTTTT TAGATCATTC CCGGGTGTTG TCATAACCTT GGGTCGAACG GTTCCTTTAC   
  
  
- AGTCTCCCCG ATCGTTACCT GGTCACGTTC TTCCGGTTCG GTGTAAACCA ATCCCTTAAA GTAGGGTAGG   
  
  
- CCGCGATTTT CGTAGTTTTA ATGATGAATT AGGAACTAGT TTACGGGATA TTTATAAGAT GATGGGGTAA   
  
  
- ACTTTTCCCC ACAACTAATG TTGTGTATCG GCATCCAAAC TGTTTCTCTT AATGGGAATA ACAATCTTCG   
  
  
- TGGTATATGA GAAAAGAGAT TCGAGACTAG AAAAAAGTAG GAAAAGTCGA AAAACAGGAA AATTTAGTTT   
  
  
- AGTGAGTGAA CTCATAACCT CCCTGCAAGG AGTCCTCAGC TTGGTGATTT AATAGAAAAA TTTCTTTTTC   
  
  
- CCGAACTTCG GTTGGGCTAA ATTTTTTTGA CGATTATACA TTCGGATGAG AGAAAGGTCA AGGTTGTTTA   
  
  
- GTTTAAAGTC ATACTTTGTA GGTCATTTCC TAAATAGGCA CTATTACTAC ATTGTATACC AATTTCCCAG   
  
  
- TAAAAGTCAA TGTAGCCTCG TACGTTAATT TCAGGGATAT TTTATCCATC CACGTGCTTG TTCAGTGGTA   
  
  
- GCAGGAAACG AAAAACGTAC TCACGTACGA TTAATACTTG AATTAATTAT AGATTCGTTT TAATTTTTCA   
  
  
- TCAATTTTGA AAAAGTGGAA ACCTTGGAGC ATCGACATCG TCATCGACGG CGTGAGACCA AGATCTGAAG   
  
  
- ATCCCTGGTT CTCTACGTGA GTATACGTAG GAGAAGACAG GAAAATGACA TAGGGGAAGG GTACACTGGA   
  
  
- AAAACGGGCC CACTGAAAAG ACACTCACAA CTATGTTTAA AACATGCATT CGATTGAGTA AATGAAGTGG   
  
  
- CAGAGTACTC AAAATGTAAT GGCTATAATT AAATTTACCG TTTCATAATT TAAACTCGAT TTTAGAGTAT   
  
  
- ATATATAAAC ATAATTTACA CAGACCTAAC AGTTTTATTA AAAACTGTAT ATGTCAGTGA TATATGACTC   
  
  
- AATAATTTTA TATATTTTTA TACTTTTAAA TTTTTTATCA TCAAATTCCA CTGTTGTGAT TTTGTTTGTT   
  
  
- TATTCTATAT TTATCCCTTT TCAAGTAGGA TAAAACAATA ATAATAAATA ATACATAAAA GTCTTAACAG   
  
  
- AGATAGTGTT CGTATCATAT ACACAGTTGT AGCACTGTTG AGCTGGTTAG ATGTTTATTG ATCATTTAAG   
  
  
- TTTATTTCAA TGAGAGTGGA TCGAATTTAT TCATGTGGAA CTAAATTAAC AGAATCGATA CCTTGGATAA   
  
  
- GGTTTGATCG GTTATGAAGG AAAAGTTTTC TTAATCAAAA TTTCAAAATT GAAAATGCAG GTTATTACGG   
  
  
- CCGGCATGAT ACTCAACGAA AGTTCGGTCA TCATCATGAC ATCATCCATT TTTATAATCT TATGTGGATG   
  
  
- ATATATACTA TAACGAGAAG TGGCATAGGA AGACGTATGA GTAGAAAGGC AGGGCCTCAG GTGATAGAGA   
  
  
- GGAGGTAAGT CTGTGAGAGA GCGAGATCTC TTGAGTATCG ATACCTGGCG CACGGGCTGC CAATAGCCCA   
  
  
- ATACTTAAGC CTCGAAGAAG TCGTTCAATC GGGACTCAAC GGCCTAGGTT GGTGGACCAA GGTACGGGCA   
  
  
- GATGGGCTGG GGTAGAGAGT CAGAGGAGAA CCTGGGCCAG GTAGGAGGGT CGGTTGGGTA AAGATGGTCC   
  
  
- CAAGACGGAG TCCCTTGGTA CACCAACCCT AACGACTGTG CACCTGCCTG GTCTAACAAC GTCCAAGGAG   
  
  
- CAGGGTCGTC ACTCCAACGT CGTCTCGGTC GTCCACAACT GACGTCAGCT TCGGTACCTC CTCCTGAGAC   
  
  
- CCTATTCTGA CCAGATGCGA GACGACTGCA CACGCCTCCG GTAGGTTGCA CCGCTAAAGG CTAACCGACA   
  
  
- GAACAATCAA TTGTTCTACT CGTTACTGGA GGACGGTGCG CAGTTGGGCA GGACGCCATA ACCGTTTCAT   
  
  
- CGGCCGATGA AGTATCTACG GAACTGGGCT TCTGATATGG TCGTCCCGGG TCACAGCCCG GATTAGCCCA   
  
  
- GCCAAGACCG CATGGTCCAC AACATGCTCG TGAAGATACT TCGAACGGGC AAGGAGTTTA AGCGAGTGAA   
  
  
- GTGACGATTA GTTCGCTAAA ATCTCCGTAA GCTGCCGGTG CTAACACAGG TGTAGTAGCT GAAGCCGGAT   
  
  
- TAGGTGCCGA ACGTTACCGG TCGAGATTAG GTTCGGAACC GACAAGCCGT GCCACCGGGC GGAAAGCATT   
  
  
- CTAATTGGCC TTAGCCCGGC GGAAGACTTA TGCCCAGCAC GAGCGAAGTT GGATAGCCCA GTTGTGACCG   
  
  
- GGTCAATCGG GATAGGTACT TACAACCCAA GCGTAAAGCC CGGCAGCGGC AGAGTGCCGA GCTCCTGTAG   
  
  
- TTTGGTACCT ACCAATTTTG TTCGGGTTTA CTTCAGCATC GGCACTTGAG ATAGAAGGTT GAGGTGGCCG   
  
  
- ATTAGCCCAG CCCATAACTG GGACAGGATT TGACCCAGGC CTCGGATTTG GGCTTTTAAC ACTGTGACCG   
  
  
- CCTCGTTCTC CGCTTGGTGT TGGTCGGGCT CAAAAACCGG GCCAAGTGCC TCCGTAATGT AATGATAAGT   
  
  
- TGATACAAGC TGAGCAATCT CCGAACAGTT CAGGTCCGGC TGTTCCTGGA TCGGCTCGAT ATGAATCTCT   
  
  
- CTCTCAATAG GTTGTAGCAG ACGACACTCC CCAGCCGAGC CTAACTCTCC GTGCTCGGCG ACCGGGTTAC   
  
  
- CTCCCGGGCT TACCGAGCCC GGCCCAAGTT CTTCCACCTA TACCCATTTT TACGCAAGTT CGTTCATTCG   
  
  
- TACAACGACT CAAACAGAAG TCGTGTTCCC ATAACACACC TCCTCTCACT CCCTACAAAC TTCGAGCCGA   
  
  
- CCGTACTGGC GGGAGAGTAA CGCCGAAGCC GAACCGCTCG CCTCTGAGTT CGACTCTTGA GGTCGTGACA   
  
  
- ACACGAACTA CCCAGTAGCA GCACATCAAG TAGAAGAAT

+     MYC

| Site Name | Organism | Position | Strand | Matrix score. | sequence | function |
| --- | --- | --- | --- | --- | --- | --- |
| MYC | Arabidopsis thaliana | 2620 | - | 6 | CATTTG |  |
| MYC | Arabidopsis thaliana | 1912 | + | 6 | CATGTG |  |
| MYC | Arabidopsis thaliana | 975 | + | 6 | CATGTG |  |
| MYC | Arabidopsis thaliana | 351 | + | 6 | CATTTG |  |
| MYC | Arabidopsis thaliana | 323 | - | 6 | CATTTG |  |
| MYC | Arabidopsis thaliana | 257 | + | 6 | CATTTG |  |

>HU11G00778.1   
+ -Up\_Stream \_Len000AAAAAA GAAAGAAATG GAGAAACCAA GCAATATGGA TAAAAAAAAA ACAAAGTAAT   
  
  
+ AAAGGAAGGA TGAGGCTCCA CATTCCACTC GCCCGCGCCA CTCATAGGAG ATGAATAGTA AAATGTGAGG   
  
  
+ GGTTAAATGA TTTGAAAAAA ATCTAGTAAG GGCCCACAAC AGTATTGGAA CCCAGCTTGC CAAGGAAATG   
  
  
+ TCAGAGGGGC TAGCAATGGA CCAGTGCAAG AAGGCCAAGC CACATTTGGT TAGGGAATTT CATCCCATCC   
  
  
+ GGCGCTAAAA GCATCAAAAT TACTACTTAA TCCTTGATCA AATGCCCTAT AAATATTCTA CTACCCCATT   
  
  
+ TGAAAAGGGG TGTTGATTAC AACACATAGC CGTAGGTTTG ACAAAGAGAA TTACCCTTAT TGTTAGAAGC   
  
  
+ ACCATATACT CTTTTCTCTA AGCTCTGATC TTTTTTCATC CTTTTCAGCT TTTTGTCCTT TTAAATCAAA   
  
  
+ TCACTCACTT GAGTATTGGA GGGACGTTCC TCAGGAGTCG AACCACTAAA TTATCTTTTT AAAGAAAAAG   
  
  
+ GGCTTGAAGC CAACCCGATT TAAAAAAACT GCTAATATGT AAGCCTACTC TCTTTCCAGT TCCAACAAAT   
  
  
+ CAAATTTCAG TATGAAACAT CCAGTAAAGG ATTTATCCGT GATAATGATG TAACATATGG TTAAAGGGTC   
  
  
+ ATTTTCAGTT ACATCGGAGC ATGCAATTAA AGTCCCTATA AAATAGGTAG GTGCACGAAC AAGTCACCAT   
  
  
+ CGTCCTTTGC TTTTTGCATG AGTGCATGCT AATTATGAAC TTAATTAATA TCTAAGCAAA ATTAAAAAGT   
  
  
+ AGTTAAAACT TTTTCACCTT TGGAACCTCG TAGCTGTAGC AGTAGCTGCC GCACTCTGGT TCTAGACTTC   
  
  
+ TAGGGACCAA GAGATGCACT CATATGCATC CTCTTCTGTC CTTTTACTGT ATCCCCTTCC CATGTGACCT   
  
  
+ TTTTGCCCGG GTGACTTTTC TGTGAGTGTT GATACAAATT TTGTACGTAA GCTAACTCAT TTACTTCACC   
  
  
+ GTCTCATGAG TTTTACATTA CCGATATTAA TTTAAATGGC AAAGTATTAA ATTTGAGCTA AAATCTCATA   
  
  
+ TATATATTTG TATTAAATGT GTCTGGATTG TCAAAATAAT TTTTGACATA TACAGTCACT ATATACTGAG   
  
  
+ TTATTAAAAT ATATAAAAAT ATGAAAATTT AAAAAATAGT AGTTTAAGGT GACAACACTA AAACAAACAA   
  
  
+ ATAAGATATA AATAGGGAAA AGTTCATCCT ATTTTGTTAT TATTATTTAT TATGTATTTT CAGAATTGTC   
  
  
+ TCTATCACAA GCATAGTATA TGTGTCAACA TCGTGACAAC TCGACCAATC TACAAATAAC TAGTAAATTC   
  
  
+ AAATAAAGTT ACTCTCACCT AGCTTAAATA AGTACACCTT GATTTAATTG TCTTAGCTAT GGAACCTATT   
  
  
+ CCAAACTAGC CAATACTTCC TTTTCAAAAG AATTAGTTTT AAAGTTTTAA CTTTTACGTC CAATAATGCC   
  
  
+ GGCCGTACTA TGAGTTGCTT TCAAGCCAGT AGTAGTACTG TAGTAGGTAA AAATATTAGA ATACACCTAC   
  
  
+ TATATATGAT ATTGCTCTTC ACCGTATCCT TCTGCATACT CATCTTTCCG TCCCGGAGTC CACTATCTCT   
  
  
+ CCTCCATTCA GACACTCTCT CGCTCTAGAG AACTCATAGC TATGGACCGC GTGCCCGACG GTTATCGGGT   
  
  
+ TATGAATTCG GAGCTTCTTC AGCAAGTTAG CCCTGAGTTG CCGGATCCAA CCACCTGGTT CCATGCCCGT   
  
  
+ CTACCCGACC CCATCTCTCA GTCTCCTCTT GGACCCGGTC CATCCTCCCA GCCAACCCAT TTCTACCAGG   
  
  
+ GTTCTGCCTC AGGGAACCAT GTGGTTGGGA TTGCTGACAC GTGGACGGAC CAGATTGTTG CAGGTTCCTC   
  
  
+ GTCCCAGCAG TGAGGTTGCA GCAGAGCCAG CAGGTGTTGA CTGCAGTCGA AGCCATGGAG GAGGACTCTG   
  
  
+ GGATAAGACT GGTCTACGCT CTGCTGACGT GTGCGGAGGC CATCCAACGT GGCGATTTCC GATTGGCTGT   
  
  
+ CTTGTTAGTT AACAAGATGA GCAATGACCT CCTGCCACGC GTCAACCCGT CCTGCGGTAT TGGCAAAGTA   
  
  
+ GCCGGCTACT TCATAGATGC CTTGACCCGA AGACTATACC AGCAGGGCCC AGTGTCGGGC CTAATCGGGT   
  
  
+ CGGTTCTGGC GTACCAGGTG TTGTACGAGC ACTTCTATGA AGCTTGCCCG TTCCTCAAAT TCGCTCACTT   
  
  
+ CACTGCTAAT CAAGCGATTT TAGAGGCATT CGACGGCCAC GATTGTGTCC ACATCATCGA CTTCGGCCTA   
  
  
+ ATCCACGGCT TGCAATGGCC AGCTCTAATC CAAGCCTTGG CTGTTCGGCA CGGTGGCCCG CCTTTCGTAA   
  
  
+ GATTAACCGG AATCGGGCCG CCTTCTGAAT ACGGGTCGTG CTCGCTTCAA CCTATCGGGT CAACACTGGC   
  
  
+ CCAGTTAGCC CTATCCATGA ATGTTGGGTT CGCATTTCGG GCCGTCGCCG TCTCACGGCT CGAGGACATC   
  
  
+ AAACCATGGA TGGTTAAAAC AAGCCCAAAT GAAGTCGTAG CCGTGAACTC TATCTTCCAA CTCCACCGGC   
  
  
+ TAATCGGGTC GGGTATTGAC CCTGTCCTAA ACTGGGTCCG GAGCCTAAAC CCGAAAATTG TGACACTGGC   
  
  
+ GGAGCAAGAG GCGAACCACA ACCAGCCCGA GTTTTTGGCC CGGTTCACGG AGGCATTACA TTACTATTCA   
  
  
+ ACTATGTTCG ACTCGTTAGA GGCTTGTCAA GTCCAGGCCG ACAAGGACCT AGCCGAGCTA TACTTAGAGA   
  
  
+ GAGAGTTATC CAACATCGTC TGCTGTGAGG GGTCGGCTCG GATTGAGAGG CACGAGCCGC TGGCCCAATG   
  
  
+ GAGGGCCCGA ATGGCTCGGG CCGGGTTCAA GAAGGTGGAT ATGGGTAAAA ATGCGTTCAA GCAAGTAAGC   
  
  
+ ATGTTGCTGA GTTTGTCTTC AGCACAAGGG TATTGTGTGG AGGAGAGTGA GGGATGTTTG AAGCTCGGCT   
  
  
+ GGCATGACCG CCCTCTCATT GCGGCTTCGG CTTGGCGAGC GGAGACTCAA GCTGAGAACT CCAGCACTGT   
  
  
+ TGTGCTTGAT GGGTCATCGT CGTGTAGTTC ATCTTCTTA  

- -Up\_Stream \_Len000TTTTTT CTTTCTTTAC CTCTTTGGTT CGTTATACCT ATTTTTTTTT TGTTTCATTA   
  
  
- TTTCCTTCCT ACTCCGAGGT GTAAGGTGAG CGGGCGCGGT GAGTATCCTC TACTTATCAT TTTACACTCC   
  
  
- CCAATTTACT AAACTTTTTT TAGATCATTC CCGGGTGTTG TCATAACCTT GGGTCGAACG GTTCCTTTAC   
  
  
- AGTCTCCCCG ATCGTTACCT GGTCACGTTC TTCCGGTTCG GTGTAAACCA ATCCCTTAAA GTAGGGTAGG   
  
  
- CCGCGATTTT CGTAGTTTTA ATGATGAATT AGGAACTAGT TTACGGGATA TTTATAAGAT GATGGGGTAA   
  
  
- ACTTTTCCCC ACAACTAATG TTGTGTATCG GCATCCAAAC TGTTTCTCTT AATGGGAATA ACAATCTTCG   
  
  
- TGGTATATGA GAAAAGAGAT TCGAGACTAG AAAAAAGTAG GAAAAGTCGA AAAACAGGAA AATTTAGTTT   
  
  
- AGTGAGTGAA CTCATAACCT CCCTGCAAGG AGTCCTCAGC TTGGTGATTT AATAGAAAAA TTTCTTTTTC   
  
  
- CCGAACTTCG GTTGGGCTAA ATTTTTTTGA CGATTATACA TTCGGATGAG AGAAAGGTCA AGGTTGTTTA   
  
  
- GTTTAAAGTC ATACTTTGTA GGTCATTTCC TAAATAGGCA CTATTACTAC ATTGTATACC AATTTCCCAG   
  
  
- TAAAAGTCAA TGTAGCCTCG TACGTTAATT TCAGGGATAT TTTATCCATC CACGTGCTTG TTCAGTGGTA   
  
  
- GCAGGAAACG AAAAACGTAC TCACGTACGA TTAATACTTG AATTAATTAT AGATTCGTTT TAATTTTTCA   
  
  
- TCAATTTTGA AAAAGTGGAA ACCTTGGAGC ATCGACATCG TCATCGACGG CGTGAGACCA AGATCTGAAG   
  
  
- ATCCCTGGTT CTCTACGTGA GTATACGTAG GAGAAGACAG GAAAATGACA TAGGGGAAGG GTACACTGGA   
  
  
- AAAACGGGCC CACTGAAAAG ACACTCACAA CTATGTTTAA AACATGCATT CGATTGAGTA AATGAAGTGG   
  
  
- CAGAGTACTC AAAATGTAAT GGCTATAATT AAATTTACCG TTTCATAATT TAAACTCGAT TTTAGAGTAT   
  
  
- ATATATAAAC ATAATTTACA CAGACCTAAC AGTTTTATTA AAAACTGTAT ATGTCAGTGA TATATGACTC   
  
  
- AATAATTTTA TATATTTTTA TACTTTTAAA TTTTTTATCA TCAAATTCCA CTGTTGTGAT TTTGTTTGTT   
  
  
- TATTCTATAT TTATCCCTTT TCAAGTAGGA TAAAACAATA ATAATAAATA ATACATAAAA GTCTTAACAG   
  
  
- AGATAGTGTT CGTATCATAT ACACAGTTGT AGCACTGTTG AGCTGGTTAG ATGTTTATTG ATCATTTAAG   
  
  
- TTTATTTCAA TGAGAGTGGA TCGAATTTAT TCATGTGGAA CTAAATTAAC AGAATCGATA CCTTGGATAA   
  
  
- GGTTTGATCG GTTATGAAGG AAAAGTTTTC TTAATCAAAA TTTCAAAATT GAAAATGCAG GTTATTACGG   
  
  
- CCGGCATGAT ACTCAACGAA AGTTCGGTCA TCATCATGAC ATCATCCATT TTTATAATCT TATGTGGATG   
  
  
- ATATATACTA TAACGAGAAG TGGCATAGGA AGACGTATGA GTAGAAAGGC AGGGCCTCAG GTGATAGAGA   
  
  
- GGAGGTAAGT CTGTGAGAGA GCGAGATCTC TTGAGTATCG ATACCTGGCG CACGGGCTGC CAATAGCCCA   
  
  
- ATACTTAAGC CTCGAAGAAG TCGTTCAATC GGGACTCAAC GGCCTAGGTT GGTGGACCAA GGTACGGGCA   
  
  
- GATGGGCTGG GGTAGAGAGT CAGAGGAGAA CCTGGGCCAG GTAGGAGGGT CGGTTGGGTA AAGATGGTCC   
  
  
- CAAGACGGAG TCCCTTGGTA CACCAACCCT AACGACTGTG CACCTGCCTG GTCTAACAAC GTCCAAGGAG   
  
  
- CAGGGTCGTC ACTCCAACGT CGTCTCGGTC GTCCACAACT GACGTCAGCT TCGGTACCTC CTCCTGAGAC   
  
  
- CCTATTCTGA CCAGATGCGA GACGACTGCA CACGCCTCCG GTAGGTTGCA CCGCTAAAGG CTAACCGACA   
  
  
- GAACAATCAA TTGTTCTACT CGTTACTGGA GGACGGTGCG CAGTTGGGCA GGACGCCATA ACCGTTTCAT   
  
  
- CGGCCGATGA AGTATCTACG GAACTGGGCT TCTGATATGG TCGTCCCGGG TCACAGCCCG GATTAGCCCA   
  
  
- GCCAAGACCG CATGGTCCAC AACATGCTCG TGAAGATACT TCGAACGGGC AAGGAGTTTA AGCGAGTGAA   
  
  
- GTGACGATTA GTTCGCTAAA ATCTCCGTAA GCTGCCGGTG CTAACACAGG TGTAGTAGCT GAAGCCGGAT   
  
  
- TAGGTGCCGA ACGTTACCGG TCGAGATTAG GTTCGGAACC GACAAGCCGT GCCACCGGGC GGAAAGCATT   
  
  
- CTAATTGGCC TTAGCCCGGC GGAAGACTTA TGCCCAGCAC GAGCGAAGTT GGATAGCCCA GTTGTGACCG   
  
  
- GGTCAATCGG GATAGGTACT TACAACCCAA GCGTAAAGCC CGGCAGCGGC AGAGTGCCGA GCTCCTGTAG   
  
  
- TTTGGTACCT ACCAATTTTG TTCGGGTTTA CTTCAGCATC GGCACTTGAG ATAGAAGGTT GAGGTGGCCG   
  
  
- ATTAGCCCAG CCCATAACTG GGACAGGATT TGACCCAGGC CTCGGATTTG GGCTTTTAAC ACTGTGACCG   
  
  
- CCTCGTTCTC CGCTTGGTGT TGGTCGGGCT CAAAAACCGG GCCAAGTGCC TCCGTAATGT AATGATAAGT   
  
  
- TGATACAAGC TGAGCAATCT CCGAACAGTT CAGGTCCGGC TGTTCCTGGA TCGGCTCGAT ATGAATCTCT   
  
  
- CTCTCAATAG GTTGTAGCAG ACGACACTCC CCAGCCGAGC CTAACTCTCC GTGCTCGGCG ACCGGGTTAC   
  
  
- CTCCCGGGCT TACCGAGCCC GGCCCAAGTT CTTCCACCTA TACCCATTTT TACGCAAGTT CGTTCATTCG   
  
  
- TACAACGACT CAAACAGAAG TCGTGTTCCC ATAACACACC TCCTCTCACT CCCTACAAAC TTCGAGCCGA   
  
  
- CCGTACTGGC GGGAGAGTAA CGCCGAAGCC GAACCGCTCG CCTCTGAGTT CGACTCTTGA GGTCGTGACA   
  
  
- ACACGAACTA CCCAGTAGCA GCACATCAAG TAGAAGAAT

+     Myb

| Site Name | Organism | Position | Strand | Matrix score. | sequence | function |
| --- | --- | --- | --- | --- | --- | --- |
| Myb | Arabidopsis thaliana | 2526 | - | 6 | TAACTG |  |
| Myb | Arabidopsis thaliana | 710 | - | 6 | TAACTG |  |

>HU11G00778.1   
+ -Up\_Stream \_Len000AAAAAA GAAAGAAATG GAGAAACCAA GCAATATGGA TAAAAAAAAA ACAAAGTAAT   
  
  
+ AAAGGAAGGA TGAGGCTCCA CATTCCACTC GCCCGCGCCA CTCATAGGAG ATGAATAGTA AAATGTGAGG   
  
  
+ GGTTAAATGA TTTGAAAAAA ATCTAGTAAG GGCCCACAAC AGTATTGGAA CCCAGCTTGC CAAGGAAATG   
  
  
+ TCAGAGGGGC TAGCAATGGA CCAGTGCAAG AAGGCCAAGC CACATTTGGT TAGGGAATTT CATCCCATCC   
  
  
+ GGCGCTAAAA GCATCAAAAT TACTACTTAA TCCTTGATCA AATGCCCTAT AAATATTCTA CTACCCCATT   
  
  
+ TGAAAAGGGG TGTTGATTAC AACACATAGC CGTAGGTTTG ACAAAGAGAA TTACCCTTAT TGTTAGAAGC   
  
  
+ ACCATATACT CTTTTCTCTA AGCTCTGATC TTTTTTCATC CTTTTCAGCT TTTTGTCCTT TTAAATCAAA   
  
  
+ TCACTCACTT GAGTATTGGA GGGACGTTCC TCAGGAGTCG AACCACTAAA TTATCTTTTT AAAGAAAAAG   
  
  
+ GGCTTGAAGC CAACCCGATT TAAAAAAACT GCTAATATGT AAGCCTACTC TCTTTCCAGT TCCAACAAAT   
  
  
+ CAAATTTCAG TATGAAACAT CCAGTAAAGG ATTTATCCGT GATAATGATG TAACATATGG TTAAAGGGTC   
  
  
+ ATTTTCAGTT ACATCGGAGC ATGCAATTAA AGTCCCTATA AAATAGGTAG GTGCACGAAC AAGTCACCAT   
  
  
+ CGTCCTTTGC TTTTTGCATG AGTGCATGCT AATTATGAAC TTAATTAATA TCTAAGCAAA ATTAAAAAGT   
  
  
+ AGTTAAAACT TTTTCACCTT TGGAACCTCG TAGCTGTAGC AGTAGCTGCC GCACTCTGGT TCTAGACTTC   
  
  
+ TAGGGACCAA GAGATGCACT CATATGCATC CTCTTCTGTC CTTTTACTGT ATCCCCTTCC CATGTGACCT   
  
  
+ TTTTGCCCGG GTGACTTTTC TGTGAGTGTT GATACAAATT TTGTACGTAA GCTAACTCAT TTACTTCACC   
  
  
+ GTCTCATGAG TTTTACATTA CCGATATTAA TTTAAATGGC AAAGTATTAA ATTTGAGCTA AAATCTCATA   
  
  
+ TATATATTTG TATTAAATGT GTCTGGATTG TCAAAATAAT TTTTGACATA TACAGTCACT ATATACTGAG   
  
  
+ TTATTAAAAT ATATAAAAAT ATGAAAATTT AAAAAATAGT AGTTTAAGGT GACAACACTA AAACAAACAA   
  
  
+ ATAAGATATA AATAGGGAAA AGTTCATCCT ATTTTGTTAT TATTATTTAT TATGTATTTT CAGAATTGTC   
  
  
+ TCTATCACAA GCATAGTATA TGTGTCAACA TCGTGACAAC TCGACCAATC TACAAATAAC TAGTAAATTC   
  
  
+ AAATAAAGTT ACTCTCACCT AGCTTAAATA AGTACACCTT GATTTAATTG TCTTAGCTAT GGAACCTATT   
  
  
+ CCAAACTAGC CAATACTTCC TTTTCAAAAG AATTAGTTTT AAAGTTTTAA CTTTTACGTC CAATAATGCC   
  
  
+ GGCCGTACTA TGAGTTGCTT TCAAGCCAGT AGTAGTACTG TAGTAGGTAA AAATATTAGA ATACACCTAC   
  
  
+ TATATATGAT ATTGCTCTTC ACCGTATCCT TCTGCATACT CATCTTTCCG TCCCGGAGTC CACTATCTCT   
  
  
+ CCTCCATTCA GACACTCTCT CGCTCTAGAG AACTCATAGC TATGGACCGC GTGCCCGACG GTTATCGGGT   
  
  
+ TATGAATTCG GAGCTTCTTC AGCAAGTTAG CCCTGAGTTG CCGGATCCAA CCACCTGGTT CCATGCCCGT   
  
  
+ CTACCCGACC CCATCTCTCA GTCTCCTCTT GGACCCGGTC CATCCTCCCA GCCAACCCAT TTCTACCAGG   
  
  
+ GTTCTGCCTC AGGGAACCAT GTGGTTGGGA TTGCTGACAC GTGGACGGAC CAGATTGTTG CAGGTTCCTC   
  
  
+ GTCCCAGCAG TGAGGTTGCA GCAGAGCCAG CAGGTGTTGA CTGCAGTCGA AGCCATGGAG GAGGACTCTG   
  
  
+ GGATAAGACT GGTCTACGCT CTGCTGACGT GTGCGGAGGC CATCCAACGT GGCGATTTCC GATTGGCTGT   
  
  
+ CTTGTTAGTT AACAAGATGA GCAATGACCT CCTGCCACGC GTCAACCCGT CCTGCGGTAT TGGCAAAGTA   
  
  
+ GCCGGCTACT TCATAGATGC CTTGACCCGA AGACTATACC AGCAGGGCCC AGTGTCGGGC CTAATCGGGT   
  
  
+ CGGTTCTGGC GTACCAGGTG TTGTACGAGC ACTTCTATGA AGCTTGCCCG TTCCTCAAAT TCGCTCACTT   
  
  
+ CACTGCTAAT CAAGCGATTT TAGAGGCATT CGACGGCCAC GATTGTGTCC ACATCATCGA CTTCGGCCTA   
  
  
+ ATCCACGGCT TGCAATGGCC AGCTCTAATC CAAGCCTTGG CTGTTCGGCA CGGTGGCCCG CCTTTCGTAA   
  
  
+ GATTAACCGG AATCGGGCCG CCTTCTGAAT ACGGGTCGTG CTCGCTTCAA CCTATCGGGT CAACACTGGC   
  
  
+ CCAGTTAGCC CTATCCATGA ATGTTGGGTT CGCATTTCGG GCCGTCGCCG TCTCACGGCT CGAGGACATC   
  
  
+ AAACCATGGA TGGTTAAAAC AAGCCCAAAT GAAGTCGTAG CCGTGAACTC TATCTTCCAA CTCCACCGGC   
  
  
+ TAATCGGGTC GGGTATTGAC CCTGTCCTAA ACTGGGTCCG GAGCCTAAAC CCGAAAATTG TGACACTGGC   
  
  
+ GGAGCAAGAG GCGAACCACA ACCAGCCCGA GTTTTTGGCC CGGTTCACGG AGGCATTACA TTACTATTCA   
  
  
+ ACTATGTTCG ACTCGTTAGA GGCTTGTCAA GTCCAGGCCG ACAAGGACCT AGCCGAGCTA TACTTAGAGA   
  
  
+ GAGAGTTATC CAACATCGTC TGCTGTGAGG GGTCGGCTCG GATTGAGAGG CACGAGCCGC TGGCCCAATG   
  
  
+ GAGGGCCCGA ATGGCTCGGG CCGGGTTCAA GAAGGTGGAT ATGGGTAAAA ATGCGTTCAA GCAAGTAAGC   
  
  
+ ATGTTGCTGA GTTTGTCTTC AGCACAAGGG TATTGTGTGG AGGAGAGTGA GGGATGTTTG AAGCTCGGCT   
  
  
+ GGCATGACCG CCCTCTCATT GCGGCTTCGG CTTGGCGAGC GGAGACTCAA GCTGAGAACT CCAGCACTGT   
  
  
+ TGTGCTTGAT GGGTCATCGT CGTGTAGTTC ATCTTCTTA  

- -Up\_Stream \_Len000TTTTTT CTTTCTTTAC CTCTTTGGTT CGTTATACCT ATTTTTTTTT TGTTTCATTA   
  
  
- TTTCCTTCCT ACTCCGAGGT GTAAGGTGAG CGGGCGCGGT GAGTATCCTC TACTTATCAT TTTACACTCC   
  
  
- CCAATTTACT AAACTTTTTT TAGATCATTC CCGGGTGTTG TCATAACCTT GGGTCGAACG GTTCCTTTAC   
  
  
- AGTCTCCCCG ATCGTTACCT GGTCACGTTC TTCCGGTTCG GTGTAAACCA ATCCCTTAAA GTAGGGTAGG   
  
  
- CCGCGATTTT CGTAGTTTTA ATGATGAATT AGGAACTAGT TTACGGGATA TTTATAAGAT GATGGGGTAA   
  
  
- ACTTTTCCCC ACAACTAATG TTGTGTATCG GCATCCAAAC TGTTTCTCTT AATGGGAATA ACAATCTTCG   
  
  
- TGGTATATGA GAAAAGAGAT TCGAGACTAG AAAAAAGTAG GAAAAGTCGA AAAACAGGAA AATTTAGTTT   
  
  
- AGTGAGTGAA CTCATAACCT CCCTGCAAGG AGTCCTCAGC TTGGTGATTT AATAGAAAAA TTTCTTTTTC   
  
  
- CCGAACTTCG GTTGGGCTAA ATTTTTTTGA CGATTATACA TTCGGATGAG AGAAAGGTCA AGGTTGTTTA   
  
  
- GTTTAAAGTC ATACTTTGTA GGTCATTTCC TAAATAGGCA CTATTACTAC ATTGTATACC AATTTCCCAG   
  
  
- TAAAAGTCAA TGTAGCCTCG TACGTTAATT TCAGGGATAT TTTATCCATC CACGTGCTTG TTCAGTGGTA   
  
  
- GCAGGAAACG AAAAACGTAC TCACGTACGA TTAATACTTG AATTAATTAT AGATTCGTTT TAATTTTTCA   
  
  
- TCAATTTTGA AAAAGTGGAA ACCTTGGAGC ATCGACATCG TCATCGACGG CGTGAGACCA AGATCTGAAG   
  
  
- ATCCCTGGTT CTCTACGTGA GTATACGTAG GAGAAGACAG GAAAATGACA TAGGGGAAGG GTACACTGGA   
  
  
- AAAACGGGCC CACTGAAAAG ACACTCACAA CTATGTTTAA AACATGCATT CGATTGAGTA AATGAAGTGG   
  
  
- CAGAGTACTC AAAATGTAAT GGCTATAATT AAATTTACCG TTTCATAATT TAAACTCGAT TTTAGAGTAT   
  
  
- ATATATAAAC ATAATTTACA CAGACCTAAC AGTTTTATTA AAAACTGTAT ATGTCAGTGA TATATGACTC   
  
  
- AATAATTTTA TATATTTTTA TACTTTTAAA TTTTTTATCA TCAAATTCCA CTGTTGTGAT TTTGTTTGTT   
  
  
- TATTCTATAT TTATCCCTTT TCAAGTAGGA TAAAACAATA ATAATAAATA ATACATAAAA GTCTTAACAG   
  
  
- AGATAGTGTT CGTATCATAT ACACAGTTGT AGCACTGTTG AGCTGGTTAG ATGTTTATTG ATCATTTAAG   
  
  
- TTTATTTCAA TGAGAGTGGA TCGAATTTAT TCATGTGGAA CTAAATTAAC AGAATCGATA CCTTGGATAA   
  
  
- GGTTTGATCG GTTATGAAGG AAAAGTTTTC TTAATCAAAA TTTCAAAATT GAAAATGCAG GTTATTACGG   
  
  
- CCGGCATGAT ACTCAACGAA AGTTCGGTCA TCATCATGAC ATCATCCATT TTTATAATCT TATGTGGATG   
  
  
- ATATATACTA TAACGAGAAG TGGCATAGGA AGACGTATGA GTAGAAAGGC AGGGCCTCAG GTGATAGAGA   
  
  
- GGAGGTAAGT CTGTGAGAGA GCGAGATCTC TTGAGTATCG ATACCTGGCG CACGGGCTGC CAATAGCCCA   
  
  
- ATACTTAAGC CTCGAAGAAG TCGTTCAATC GGGACTCAAC GGCCTAGGTT GGTGGACCAA GGTACGGGCA   
  
  
- GATGGGCTGG GGTAGAGAGT CAGAGGAGAA CCTGGGCCAG GTAGGAGGGT CGGTTGGGTA AAGATGGTCC   
  
  
- CAAGACGGAG TCCCTTGGTA CACCAACCCT AACGACTGTG CACCTGCCTG GTCTAACAAC GTCCAAGGAG   
  
  
- CAGGGTCGTC ACTCCAACGT CGTCTCGGTC GTCCACAACT GACGTCAGCT TCGGTACCTC CTCCTGAGAC   
  
  
- CCTATTCTGA CCAGATGCGA GACGACTGCA CACGCCTCCG GTAGGTTGCA CCGCTAAAGG CTAACCGACA   
  
  
- GAACAATCAA TTGTTCTACT CGTTACTGGA GGACGGTGCG CAGTTGGGCA GGACGCCATA ACCGTTTCAT   
  
  
- CGGCCGATGA AGTATCTACG GAACTGGGCT TCTGATATGG TCGTCCCGGG TCACAGCCCG GATTAGCCCA   
  
  
- GCCAAGACCG CATGGTCCAC AACATGCTCG TGAAGATACT TCGAACGGGC AAGGAGTTTA AGCGAGTGAA   
  
  
- GTGACGATTA GTTCGCTAAA ATCTCCGTAA GCTGCCGGTG CTAACACAGG TGTAGTAGCT GAAGCCGGAT   
  
  
- TAGGTGCCGA ACGTTACCGG TCGAGATTAG GTTCGGAACC GACAAGCCGT GCCACCGGGC GGAAAGCATT   
  
  
- CTAATTGGCC TTAGCCCGGC GGAAGACTTA TGCCCAGCAC GAGCGAAGTT GGATAGCCCA GTTGTGACCG   
  
  
- GGTCAATCGG GATAGGTACT TACAACCCAA GCGTAAAGCC CGGCAGCGGC AGAGTGCCGA GCTCCTGTAG   
  
  
- TTTGGTACCT ACCAATTTTG TTCGGGTTTA CTTCAGCATC GGCACTTGAG ATAGAAGGTT GAGGTGGCCG   
  
  
- ATTAGCCCAG CCCATAACTG GGACAGGATT TGACCCAGGC CTCGGATTTG GGCTTTTAAC ACTGTGACCG   
  
  
- CCTCGTTCTC CGCTTGGTGT TGGTCGGGCT CAAAAACCGG GCCAAGTGCC TCCGTAATGT AATGATAAGT   
  
  
- TGATACAAGC TGAGCAATCT CCGAACAGTT CAGGTCCGGC TGTTCCTGGA TCGGCTCGAT ATGAATCTCT   
  
  
- CTCTCAATAG GTTGTAGCAG ACGACACTCC CCAGCCGAGC CTAACTCTCC GTGCTCGGCG ACCGGGTTAC   
  
  
- CTCCCGGGCT TACCGAGCCC GGCCCAAGTT CTTCCACCTA TACCCATTTT TACGCAAGTT CGTTCATTCG   
  
  
- TACAACGACT CAAACAGAAG TCGTGTTCCC ATAACACACC TCCTCTCACT CCCTACAAAC TTCGAGCCGA   
  
  
- CCGTACTGGC GGGAGAGTAA CGCCGAAGCC GAACCGCTCG CCTCTGAGTT CGACTCTTGA GGTCGTGACA   
  
  
- ACACGAACTA CCCAGTAGCA GCACATCAAG TAGAAGAAT

+     Myb-binding site

| Site Name | Organism | Position | Strand | Matrix score. | sequence | function |
| --- | --- | --- | --- | --- | --- | --- |
| Myb-binding site | Nicotiana tabacum | 181 | + | 6 | CAACAG |  |
| Myb-binding site | Nicotiana tabacum | 3151 | - | 6 | CAACAG |  |

>HU11G00778.1   
+ -Up\_Stream \_Len000AAAAAA GAAAGAAATG GAGAAACCAA GCAATATGGA TAAAAAAAAA ACAAAGTAAT   
  
  
+ AAAGGAAGGA TGAGGCTCCA CATTCCACTC GCCCGCGCCA CTCATAGGAG ATGAATAGTA AAATGTGAGG   
  
  
+ GGTTAAATGA TTTGAAAAAA ATCTAGTAAG GGCCCACAAC AGTATTGGAA CCCAGCTTGC CAAGGAAATG   
  
  
+ TCAGAGGGGC TAGCAATGGA CCAGTGCAAG AAGGCCAAGC CACATTTGGT TAGGGAATTT CATCCCATCC   
  
  
+ GGCGCTAAAA GCATCAAAAT TACTACTTAA TCCTTGATCA AATGCCCTAT AAATATTCTA CTACCCCATT   
  
  
+ TGAAAAGGGG TGTTGATTAC AACACATAGC CGTAGGTTTG ACAAAGAGAA TTACCCTTAT TGTTAGAAGC   
  
  
+ ACCATATACT CTTTTCTCTA AGCTCTGATC TTTTTTCATC CTTTTCAGCT TTTTGTCCTT TTAAATCAAA   
  
  
+ TCACTCACTT GAGTATTGGA GGGACGTTCC TCAGGAGTCG AACCACTAAA TTATCTTTTT AAAGAAAAAG   
  
  
+ GGCTTGAAGC CAACCCGATT TAAAAAAACT GCTAATATGT AAGCCTACTC TCTTTCCAGT TCCAACAAAT   
  
  
+ CAAATTTCAG TATGAAACAT CCAGTAAAGG ATTTATCCGT GATAATGATG TAACATATGG TTAAAGGGTC   
  
  
+ ATTTTCAGTT ACATCGGAGC ATGCAATTAA AGTCCCTATA AAATAGGTAG GTGCACGAAC AAGTCACCAT   
  
  
+ CGTCCTTTGC TTTTTGCATG AGTGCATGCT AATTATGAAC TTAATTAATA TCTAAGCAAA ATTAAAAAGT   
  
  
+ AGTTAAAACT TTTTCACCTT TGGAACCTCG TAGCTGTAGC AGTAGCTGCC GCACTCTGGT TCTAGACTTC   
  
  
+ TAGGGACCAA GAGATGCACT CATATGCATC CTCTTCTGTC CTTTTACTGT ATCCCCTTCC CATGTGACCT   
  
  
+ TTTTGCCCGG GTGACTTTTC TGTGAGTGTT GATACAAATT TTGTACGTAA GCTAACTCAT TTACTTCACC   
  
  
+ GTCTCATGAG TTTTACATTA CCGATATTAA TTTAAATGGC AAAGTATTAA ATTTGAGCTA AAATCTCATA   
  
  
+ TATATATTTG TATTAAATGT GTCTGGATTG TCAAAATAAT TTTTGACATA TACAGTCACT ATATACTGAG   
  
  
+ TTATTAAAAT ATATAAAAAT ATGAAAATTT AAAAAATAGT AGTTTAAGGT GACAACACTA AAACAAACAA   
  
  
+ ATAAGATATA AATAGGGAAA AGTTCATCCT ATTTTGTTAT TATTATTTAT TATGTATTTT CAGAATTGTC   
  
  
+ TCTATCACAA GCATAGTATA TGTGTCAACA TCGTGACAAC TCGACCAATC TACAAATAAC TAGTAAATTC   
  
  
+ AAATAAAGTT ACTCTCACCT AGCTTAAATA AGTACACCTT GATTTAATTG TCTTAGCTAT GGAACCTATT   
  
  
+ CCAAACTAGC CAATACTTCC TTTTCAAAAG AATTAGTTTT AAAGTTTTAA CTTTTACGTC CAATAATGCC   
  
  
+ GGCCGTACTA TGAGTTGCTT TCAAGCCAGT AGTAGTACTG TAGTAGGTAA AAATATTAGA ATACACCTAC   
  
  
+ TATATATGAT ATTGCTCTTC ACCGTATCCT TCTGCATACT CATCTTTCCG TCCCGGAGTC CACTATCTCT   
  
  
+ CCTCCATTCA GACACTCTCT CGCTCTAGAG AACTCATAGC TATGGACCGC GTGCCCGACG GTTATCGGGT   
  
  
+ TATGAATTCG GAGCTTCTTC AGCAAGTTAG CCCTGAGTTG CCGGATCCAA CCACCTGGTT CCATGCCCGT   
  
  
+ CTACCCGACC CCATCTCTCA GTCTCCTCTT GGACCCGGTC CATCCTCCCA GCCAACCCAT TTCTACCAGG   
  
  
+ GTTCTGCCTC AGGGAACCAT GTGGTTGGGA TTGCTGACAC GTGGACGGAC CAGATTGTTG CAGGTTCCTC   
  
  
+ GTCCCAGCAG TGAGGTTGCA GCAGAGCCAG CAGGTGTTGA CTGCAGTCGA AGCCATGGAG GAGGACTCTG   
  
  
+ GGATAAGACT GGTCTACGCT CTGCTGACGT GTGCGGAGGC CATCCAACGT GGCGATTTCC GATTGGCTGT   
  
  
+ CTTGTTAGTT AACAAGATGA GCAATGACCT CCTGCCACGC GTCAACCCGT CCTGCGGTAT TGGCAAAGTA   
  
  
+ GCCGGCTACT TCATAGATGC CTTGACCCGA AGACTATACC AGCAGGGCCC AGTGTCGGGC CTAATCGGGT   
  
  
+ CGGTTCTGGC GTACCAGGTG TTGTACGAGC ACTTCTATGA AGCTTGCCCG TTCCTCAAAT TCGCTCACTT   
  
  
+ CACTGCTAAT CAAGCGATTT TAGAGGCATT CGACGGCCAC GATTGTGTCC ACATCATCGA CTTCGGCCTA   
  
  
+ ATCCACGGCT TGCAATGGCC AGCTCTAATC CAAGCCTTGG CTGTTCGGCA CGGTGGCCCG CCTTTCGTAA   
  
  
+ GATTAACCGG AATCGGGCCG CCTTCTGAAT ACGGGTCGTG CTCGCTTCAA CCTATCGGGT CAACACTGGC   
  
  
+ CCAGTTAGCC CTATCCATGA ATGTTGGGTT CGCATTTCGG GCCGTCGCCG TCTCACGGCT CGAGGACATC   
  
  
+ AAACCATGGA TGGTTAAAAC AAGCCCAAAT GAAGTCGTAG CCGTGAACTC TATCTTCCAA CTCCACCGGC   
  
  
+ TAATCGGGTC GGGTATTGAC CCTGTCCTAA ACTGGGTCCG GAGCCTAAAC CCGAAAATTG TGACACTGGC   
  
  
+ GGAGCAAGAG GCGAACCACA ACCAGCCCGA GTTTTTGGCC CGGTTCACGG AGGCATTACA TTACTATTCA   
  
  
+ ACTATGTTCG ACTCGTTAGA GGCTTGTCAA GTCCAGGCCG ACAAGGACCT AGCCGAGCTA TACTTAGAGA   
  
  
+ GAGAGTTATC CAACATCGTC TGCTGTGAGG GGTCGGCTCG GATTGAGAGG CACGAGCCGC TGGCCCAATG   
  
  
+ GAGGGCCCGA ATGGCTCGGG CCGGGTTCAA GAAGGTGGAT ATGGGTAAAA ATGCGTTCAA GCAAGTAAGC   
  
  
+ ATGTTGCTGA GTTTGTCTTC AGCACAAGGG TATTGTGTGG AGGAGAGTGA GGGATGTTTG AAGCTCGGCT   
  
  
+ GGCATGACCG CCCTCTCATT GCGGCTTCGG CTTGGCGAGC GGAGACTCAA GCTGAGAACT CCAGCACTGT   
  
  
+ TGTGCTTGAT GGGTCATCGT CGTGTAGTTC ATCTTCTTA  

- -Up\_Stream \_Len000TTTTTT CTTTCTTTAC CTCTTTGGTT CGTTATACCT ATTTTTTTTT TGTTTCATTA   
  
  
- TTTCCTTCCT ACTCCGAGGT GTAAGGTGAG CGGGCGCGGT GAGTATCCTC TACTTATCAT TTTACACTCC   
  
  
- CCAATTTACT AAACTTTTTT TAGATCATTC CCGGGTGTTG TCATAACCTT GGGTCGAACG GTTCCTTTAC   
  
  
- AGTCTCCCCG ATCGTTACCT GGTCACGTTC TTCCGGTTCG GTGTAAACCA ATCCCTTAAA GTAGGGTAGG   
  
  
- CCGCGATTTT CGTAGTTTTA ATGATGAATT AGGAACTAGT TTACGGGATA TTTATAAGAT GATGGGGTAA   
  
  
- ACTTTTCCCC ACAACTAATG TTGTGTATCG GCATCCAAAC TGTTTCTCTT AATGGGAATA ACAATCTTCG   
  
  
- TGGTATATGA GAAAAGAGAT TCGAGACTAG AAAAAAGTAG GAAAAGTCGA AAAACAGGAA AATTTAGTTT   
  
  
- AGTGAGTGAA CTCATAACCT CCCTGCAAGG AGTCCTCAGC TTGGTGATTT AATAGAAAAA TTTCTTTTTC   
  
  
- CCGAACTTCG GTTGGGCTAA ATTTTTTTGA CGATTATACA TTCGGATGAG AGAAAGGTCA AGGTTGTTTA   
  
  
- GTTTAAAGTC ATACTTTGTA GGTCATTTCC TAAATAGGCA CTATTACTAC ATTGTATACC AATTTCCCAG   
  
  
- TAAAAGTCAA TGTAGCCTCG TACGTTAATT TCAGGGATAT TTTATCCATC CACGTGCTTG TTCAGTGGTA   
  
  
- GCAGGAAACG AAAAACGTAC TCACGTACGA TTAATACTTG AATTAATTAT AGATTCGTTT TAATTTTTCA   
  
  
- TCAATTTTGA AAAAGTGGAA ACCTTGGAGC ATCGACATCG TCATCGACGG CGTGAGACCA AGATCTGAAG   
  
  
- ATCCCTGGTT CTCTACGTGA GTATACGTAG GAGAAGACAG GAAAATGACA TAGGGGAAGG GTACACTGGA   
  
  
- AAAACGGGCC CACTGAAAAG ACACTCACAA CTATGTTTAA AACATGCATT CGATTGAGTA AATGAAGTGG   
  
  
- CAGAGTACTC AAAATGTAAT GGCTATAATT AAATTTACCG TTTCATAATT TAAACTCGAT TTTAGAGTAT   
  
  
- ATATATAAAC ATAATTTACA CAGACCTAAC AGTTTTATTA AAAACTGTAT ATGTCAGTGA TATATGACTC   
  
  
- AATAATTTTA TATATTTTTA TACTTTTAAA TTTTTTATCA TCAAATTCCA CTGTTGTGAT TTTGTTTGTT   
  
  
- TATTCTATAT TTATCCCTTT TCAAGTAGGA TAAAACAATA ATAATAAATA ATACATAAAA GTCTTAACAG   
  
  
- AGATAGTGTT CGTATCATAT ACACAGTTGT AGCACTGTTG AGCTGGTTAG ATGTTTATTG ATCATTTAAG   
  
  
- TTTATTTCAA TGAGAGTGGA TCGAATTTAT TCATGTGGAA CTAAATTAAC AGAATCGATA CCTTGGATAA   
  
  
- GGTTTGATCG GTTATGAAGG AAAAGTTTTC TTAATCAAAA TTTCAAAATT GAAAATGCAG GTTATTACGG   
  
  
- CCGGCATGAT ACTCAACGAA AGTTCGGTCA TCATCATGAC ATCATCCATT TTTATAATCT TATGTGGATG   
  
  
- ATATATACTA TAACGAGAAG TGGCATAGGA AGACGTATGA GTAGAAAGGC AGGGCCTCAG GTGATAGAGA   
  
  
- GGAGGTAAGT CTGTGAGAGA GCGAGATCTC TTGAGTATCG ATACCTGGCG CACGGGCTGC CAATAGCCCA   
  
  
- ATACTTAAGC CTCGAAGAAG TCGTTCAATC GGGACTCAAC GGCCTAGGTT GGTGGACCAA GGTACGGGCA   
  
  
- GATGGGCTGG GGTAGAGAGT CAGAGGAGAA CCTGGGCCAG GTAGGAGGGT CGGTTGGGTA AAGATGGTCC   
  
  
- CAAGACGGAG TCCCTTGGTA CACCAACCCT AACGACTGTG CACCTGCCTG GTCTAACAAC GTCCAAGGAG   
  
  
- CAGGGTCGTC ACTCCAACGT CGTCTCGGTC GTCCACAACT GACGTCAGCT TCGGTACCTC CTCCTGAGAC   
  
  
- CCTATTCTGA CCAGATGCGA GACGACTGCA CACGCCTCCG GTAGGTTGCA CCGCTAAAGG CTAACCGACA   
  
  
- GAACAATCAA TTGTTCTACT CGTTACTGGA GGACGGTGCG CAGTTGGGCA GGACGCCATA ACCGTTTCAT   
  
  
- CGGCCGATGA AGTATCTACG GAACTGGGCT TCTGATATGG TCGTCCCGGG TCACAGCCCG GATTAGCCCA   
  
  
- GCCAAGACCG CATGGTCCAC AACATGCTCG TGAAGATACT TCGAACGGGC AAGGAGTTTA AGCGAGTGAA   
  
  
- GTGACGATTA GTTCGCTAAA ATCTCCGTAA GCTGCCGGTG CTAACACAGG TGTAGTAGCT GAAGCCGGAT   
  
  
- TAGGTGCCGA ACGTTACCGG TCGAGATTAG GTTCGGAACC GACAAGCCGT GCCACCGGGC GGAAAGCATT   
  
  
- CTAATTGGCC TTAGCCCGGC GGAAGACTTA TGCCCAGCAC GAGCGAAGTT GGATAGCCCA GTTGTGACCG   
  
  
- GGTCAATCGG GATAGGTACT TACAACCCAA GCGTAAAGCC CGGCAGCGGC AGAGTGCCGA GCTCCTGTAG   
  
  
- TTTGGTACCT ACCAATTTTG TTCGGGTTTA CTTCAGCATC GGCACTTGAG ATAGAAGGTT GAGGTGGCCG   
  
  
- ATTAGCCCAG CCCATAACTG GGACAGGATT TGACCCAGGC CTCGGATTTG GGCTTTTAAC ACTGTGACCG   
  
  
- CCTCGTTCTC CGCTTGGTGT TGGTCGGGCT CAAAAACCGG GCCAAGTGCC TCCGTAATGT AATGATAAGT   
  
  
- TGATACAAGC TGAGCAATCT CCGAACAGTT CAGGTCCGGC TGTTCCTGGA TCGGCTCGAT ATGAATCTCT   
  
  
- CTCTCAATAG GTTGTAGCAG ACGACACTCC CCAGCCGAGC CTAACTCTCC GTGCTCGGCG ACCGGGTTAC   
  
  
- CTCCCGGGCT TACCGAGCCC GGCCCAAGTT CTTCCACCTA TACCCATTTT TACGCAAGTT CGTTCATTCG   
  
  
- TACAACGACT CAAACAGAAG TCGTGTTCCC ATAACACACC TCCTCTCACT CCCTACAAAC TTCGAGCCGA   
  
  
- CCGTACTGGC GGGAGAGTAA CGCCGAAGCC GAACCGCTCG CCTCTGAGTT CGACTCTTGA GGTCGTGACA   
  
  
- ACACGAACTA CCCAGTAGCA GCACATCAAG TAGAAGAAT

+     NON

| Site Name | Organism | Position | Strand | Matrix score. | sequence | function |
| --- | --- | --- | --- | --- | --- | --- |
| NON | Nicotiana tabacum | 2345 | + | 10 | CAACGGCCACG |  |

>HU11G00778.1   
+ -Up\_Stream \_Len000AAAAAA GAAAGAAATG GAGAAACCAA GCAATATGGA TAAAAAAAAA ACAAAGTAAT   
  
  
+ AAAGGAAGGA TGAGGCTCCA CATTCCACTC GCCCGCGCCA CTCATAGGAG ATGAATAGTA AAATGTGAGG   
  
  
+ GGTTAAATGA TTTGAAAAAA ATCTAGTAAG GGCCCACAAC AGTATTGGAA CCCAGCTTGC CAAGGAAATG   
  
  
+ TCAGAGGGGC TAGCAATGGA CCAGTGCAAG AAGGCCAAGC CACATTTGGT TAGGGAATTT CATCCCATCC   
  
  
+ GGCGCTAAAA GCATCAAAAT TACTACTTAA TCCTTGATCA AATGCCCTAT AAATATTCTA CTACCCCATT   
  
  
+ TGAAAAGGGG TGTTGATTAC AACACATAGC CGTAGGTTTG ACAAAGAGAA TTACCCTTAT TGTTAGAAGC   
  
  
+ ACCATATACT CTTTTCTCTA AGCTCTGATC TTTTTTCATC CTTTTCAGCT TTTTGTCCTT TTAAATCAAA   
  
  
+ TCACTCACTT GAGTATTGGA GGGACGTTCC TCAGGAGTCG AACCACTAAA TTATCTTTTT AAAGAAAAAG   
  
  
+ GGCTTGAAGC CAACCCGATT TAAAAAAACT GCTAATATGT AAGCCTACTC TCTTTCCAGT TCCAACAAAT   
  
  
+ CAAATTTCAG TATGAAACAT CCAGTAAAGG ATTTATCCGT GATAATGATG TAACATATGG TTAAAGGGTC   
  
  
+ ATTTTCAGTT ACATCGGAGC ATGCAATTAA AGTCCCTATA AAATAGGTAG GTGCACGAAC AAGTCACCAT   
  
  
+ CGTCCTTTGC TTTTTGCATG AGTGCATGCT AATTATGAAC TTAATTAATA TCTAAGCAAA ATTAAAAAGT   
  
  
+ AGTTAAAACT TTTTCACCTT TGGAACCTCG TAGCTGTAGC AGTAGCTGCC GCACTCTGGT TCTAGACTTC   
  
  
+ TAGGGACCAA GAGATGCACT CATATGCATC CTCTTCTGTC CTTTTACTGT ATCCCCTTCC CATGTGACCT   
  
  
+ TTTTGCCCGG GTGACTTTTC TGTGAGTGTT GATACAAATT TTGTACGTAA GCTAACTCAT TTACTTCACC   
  
  
+ GTCTCATGAG TTTTACATTA CCGATATTAA TTTAAATGGC AAAGTATTAA ATTTGAGCTA AAATCTCATA   
  
  
+ TATATATTTG TATTAAATGT GTCTGGATTG TCAAAATAAT TTTTGACATA TACAGTCACT ATATACTGAG   
  
  
+ TTATTAAAAT ATATAAAAAT ATGAAAATTT AAAAAATAGT AGTTTAAGGT GACAACACTA AAACAAACAA   
  
  
+ ATAAGATATA AATAGGGAAA AGTTCATCCT ATTTTGTTAT TATTATTTAT TATGTATTTT CAGAATTGTC   
  
  
+ TCTATCACAA GCATAGTATA TGTGTCAACA TCGTGACAAC TCGACCAATC TACAAATAAC TAGTAAATTC   
  
  
+ AAATAAAGTT ACTCTCACCT AGCTTAAATA AGTACACCTT GATTTAATTG TCTTAGCTAT GGAACCTATT   
  
  
+ CCAAACTAGC CAATACTTCC TTTTCAAAAG AATTAGTTTT AAAGTTTTAA CTTTTACGTC CAATAATGCC   
  
  
+ GGCCGTACTA TGAGTTGCTT TCAAGCCAGT AGTAGTACTG TAGTAGGTAA AAATATTAGA ATACACCTAC   
  
  
+ TATATATGAT ATTGCTCTTC ACCGTATCCT TCTGCATACT CATCTTTCCG TCCCGGAGTC CACTATCTCT   
  
  
+ CCTCCATTCA GACACTCTCT CGCTCTAGAG AACTCATAGC TATGGACCGC GTGCCCGACG GTTATCGGGT   
  
  
+ TATGAATTCG GAGCTTCTTC AGCAAGTTAG CCCTGAGTTG CCGGATCCAA CCACCTGGTT CCATGCCCGT   
  
  
+ CTACCCGACC CCATCTCTCA GTCTCCTCTT GGACCCGGTC CATCCTCCCA GCCAACCCAT TTCTACCAGG   
  
  
+ GTTCTGCCTC AGGGAACCAT GTGGTTGGGA TTGCTGACAC GTGGACGGAC CAGATTGTTG CAGGTTCCTC   
  
  
+ GTCCCAGCAG TGAGGTTGCA GCAGAGCCAG CAGGTGTTGA CTGCAGTCGA AGCCATGGAG GAGGACTCTG   
  
  
+ GGATAAGACT GGTCTACGCT CTGCTGACGT GTGCGGAGGC CATCCAACGT GGCGATTTCC GATTGGCTGT   
  
  
+ CTTGTTAGTT AACAAGATGA GCAATGACCT CCTGCCACGC GTCAACCCGT CCTGCGGTAT TGGCAAAGTA   
  
  
+ GCCGGCTACT TCATAGATGC CTTGACCCGA AGACTATACC AGCAGGGCCC AGTGTCGGGC CTAATCGGGT   
  
  
+ CGGTTCTGGC GTACCAGGTG TTGTACGAGC ACTTCTATGA AGCTTGCCCG TTCCTCAAAT TCGCTCACTT   
  
  
+ CACTGCTAAT CAAGCGATTT TAGAGGCATT CGACGGCCAC GATTGTGTCC ACATCATCGA CTTCGGCCTA   
  
  
+ ATCCACGGCT TGCAATGGCC AGCTCTAATC CAAGCCTTGG CTGTTCGGCA CGGTGGCCCG CCTTTCGTAA   
  
  
+ GATTAACCGG AATCGGGCCG CCTTCTGAAT ACGGGTCGTG CTCGCTTCAA CCTATCGGGT CAACACTGGC   
  
  
+ CCAGTTAGCC CTATCCATGA ATGTTGGGTT CGCATTTCGG GCCGTCGCCG TCTCACGGCT CGAGGACATC   
  
  
+ AAACCATGGA TGGTTAAAAC AAGCCCAAAT GAAGTCGTAG CCGTGAACTC TATCTTCCAA CTCCACCGGC   
  
  
+ TAATCGGGTC GGGTATTGAC CCTGTCCTAA ACTGGGTCCG GAGCCTAAAC CCGAAAATTG TGACACTGGC   
  
  
+ GGAGCAAGAG GCGAACCACA ACCAGCCCGA GTTTTTGGCC CGGTTCACGG AGGCATTACA TTACTATTCA   
  
  
+ ACTATGTTCG ACTCGTTAGA GGCTTGTCAA GTCCAGGCCG ACAAGGACCT AGCCGAGCTA TACTTAGAGA   
  
  
+ GAGAGTTATC CAACATCGTC TGCTGTGAGG GGTCGGCTCG GATTGAGAGG CACGAGCCGC TGGCCCAATG   
  
  
+ GAGGGCCCGA ATGGCTCGGG CCGGGTTCAA GAAGGTGGAT ATGGGTAAAA ATGCGTTCAA GCAAGTAAGC   
  
  
+ ATGTTGCTGA GTTTGTCTTC AGCACAAGGG TATTGTGTGG AGGAGAGTGA GGGATGTTTG AAGCTCGGCT   
  
  
+ GGCATGACCG CCCTCTCATT GCGGCTTCGG CTTGGCGAGC GGAGACTCAA GCTGAGAACT CCAGCACTGT   
  
  
+ TGTGCTTGAT GGGTCATCGT CGTGTAGTTC ATCTTCTTA  

- -Up\_Stream \_Len000TTTTTT CTTTCTTTAC CTCTTTGGTT CGTTATACCT ATTTTTTTTT TGTTTCATTA   
  
  
- TTTCCTTCCT ACTCCGAGGT GTAAGGTGAG CGGGCGCGGT GAGTATCCTC TACTTATCAT TTTACACTCC   
  
  
- CCAATTTACT AAACTTTTTT TAGATCATTC CCGGGTGTTG TCATAACCTT GGGTCGAACG GTTCCTTTAC   
  
  
- AGTCTCCCCG ATCGTTACCT GGTCACGTTC TTCCGGTTCG GTGTAAACCA ATCCCTTAAA GTAGGGTAGG   
  
  
- CCGCGATTTT CGTAGTTTTA ATGATGAATT AGGAACTAGT TTACGGGATA TTTATAAGAT GATGGGGTAA   
  
  
- ACTTTTCCCC ACAACTAATG TTGTGTATCG GCATCCAAAC TGTTTCTCTT AATGGGAATA ACAATCTTCG   
  
  
- TGGTATATGA GAAAAGAGAT TCGAGACTAG AAAAAAGTAG GAAAAGTCGA AAAACAGGAA AATTTAGTTT   
  
  
- AGTGAGTGAA CTCATAACCT CCCTGCAAGG AGTCCTCAGC TTGGTGATTT AATAGAAAAA TTTCTTTTTC   
  
  
- CCGAACTTCG GTTGGGCTAA ATTTTTTTGA CGATTATACA TTCGGATGAG AGAAAGGTCA AGGTTGTTTA   
  
  
- GTTTAAAGTC ATACTTTGTA GGTCATTTCC TAAATAGGCA CTATTACTAC ATTGTATACC AATTTCCCAG   
  
  
- TAAAAGTCAA TGTAGCCTCG TACGTTAATT TCAGGGATAT TTTATCCATC CACGTGCTTG TTCAGTGGTA   
  
  
- GCAGGAAACG AAAAACGTAC TCACGTACGA TTAATACTTG AATTAATTAT AGATTCGTTT TAATTTTTCA   
  
  
- TCAATTTTGA AAAAGTGGAA ACCTTGGAGC ATCGACATCG TCATCGACGG CGTGAGACCA AGATCTGAAG   
  
  
- ATCCCTGGTT CTCTACGTGA GTATACGTAG GAGAAGACAG GAAAATGACA TAGGGGAAGG GTACACTGGA   
  
  
- AAAACGGGCC CACTGAAAAG ACACTCACAA CTATGTTTAA AACATGCATT CGATTGAGTA AATGAAGTGG   
  
  
- CAGAGTACTC AAAATGTAAT GGCTATAATT AAATTTACCG TTTCATAATT TAAACTCGAT TTTAGAGTAT   
  
  
- ATATATAAAC ATAATTTACA CAGACCTAAC AGTTTTATTA AAAACTGTAT ATGTCAGTGA TATATGACTC   
  
  
- AATAATTTTA TATATTTTTA TACTTTTAAA TTTTTTATCA TCAAATTCCA CTGTTGTGAT TTTGTTTGTT   
  
  
- TATTCTATAT TTATCCCTTT TCAAGTAGGA TAAAACAATA ATAATAAATA ATACATAAAA GTCTTAACAG   
  
  
- AGATAGTGTT CGTATCATAT ACACAGTTGT AGCACTGTTG AGCTGGTTAG ATGTTTATTG ATCATTTAAG   
  
  
- TTTATTTCAA TGAGAGTGGA TCGAATTTAT TCATGTGGAA CTAAATTAAC AGAATCGATA CCTTGGATAA   
  
  
- GGTTTGATCG GTTATGAAGG AAAAGTTTTC TTAATCAAAA TTTCAAAATT GAAAATGCAG GTTATTACGG   
  
  
- CCGGCATGAT ACTCAACGAA AGTTCGGTCA TCATCATGAC ATCATCCATT TTTATAATCT TATGTGGATG   
  
  
- ATATATACTA TAACGAGAAG TGGCATAGGA AGACGTATGA GTAGAAAGGC AGGGCCTCAG GTGATAGAGA   
  
  
- GGAGGTAAGT CTGTGAGAGA GCGAGATCTC TTGAGTATCG ATACCTGGCG CACGGGCTGC CAATAGCCCA   
  
  
- ATACTTAAGC CTCGAAGAAG TCGTTCAATC GGGACTCAAC GGCCTAGGTT GGTGGACCAA GGTACGGGCA   
  
  
- GATGGGCTGG GGTAGAGAGT CAGAGGAGAA CCTGGGCCAG GTAGGAGGGT CGGTTGGGTA AAGATGGTCC   
  
  
- CAAGACGGAG TCCCTTGGTA CACCAACCCT AACGACTGTG CACCTGCCTG GTCTAACAAC GTCCAAGGAG   
  
  
- CAGGGTCGTC ACTCCAACGT CGTCTCGGTC GTCCACAACT GACGTCAGCT TCGGTACCTC CTCCTGAGAC   
  
  
- CCTATTCTGA CCAGATGCGA GACGACTGCA CACGCCTCCG GTAGGTTGCA CCGCTAAAGG CTAACCGACA   
  
  
- GAACAATCAA TTGTTCTACT CGTTACTGGA GGACGGTGCG CAGTTGGGCA GGACGCCATA ACCGTTTCAT   
  
  
- CGGCCGATGA AGTATCTACG GAACTGGGCT TCTGATATGG TCGTCCCGGG TCACAGCCCG GATTAGCCCA   
  
  
- GCCAAGACCG CATGGTCCAC AACATGCTCG TGAAGATACT TCGAACGGGC AAGGAGTTTA AGCGAGTGAA   
  
  
- GTGACGATTA GTTCGCTAAA ATCTCCGTAA GCTGCCGGTG CTAACACAGG TGTAGTAGCT GAAGCCGGAT   
  
  
- TAGGTGCCGA ACGTTACCGG TCGAGATTAG GTTCGGAACC GACAAGCCGT GCCACCGGGC GGAAAGCATT   
  
  
- CTAATTGGCC TTAGCCCGGC GGAAGACTTA TGCCCAGCAC GAGCGAAGTT GGATAGCCCA GTTGTGACCG   
  
  
- GGTCAATCGG GATAGGTACT TACAACCCAA GCGTAAAGCC CGGCAGCGGC AGAGTGCCGA GCTCCTGTAG   
  
  
- TTTGGTACCT ACCAATTTTG TTCGGGTTTA CTTCAGCATC GGCACTTGAG ATAGAAGGTT GAGGTGGCCG   
  
  
- ATTAGCCCAG CCCATAACTG GGACAGGATT TGACCCAGGC CTCGGATTTG GGCTTTTAAC ACTGTGACCG   
  
  
- CCTCGTTCTC CGCTTGGTGT TGGTCGGGCT CAAAAACCGG GCCAAGTGCC TCCGTAATGT AATGATAAGT   
  
  
- TGATACAAGC TGAGCAATCT CCGAACAGTT CAGGTCCGGC TGTTCCTGGA TCGGCTCGAT ATGAATCTCT   
  
  
- CTCTCAATAG GTTGTAGCAG ACGACACTCC CCAGCCGAGC CTAACTCTCC GTGCTCGGCG ACCGGGTTAC   
  
  
- CTCCCGGGCT TACCGAGCCC GGCCCAAGTT CTTCCACCTA TACCCATTTT TACGCAAGTT CGTTCATTCG   
  
  
- TACAACGACT CAAACAGAAG TCGTGTTCCC ATAACACACC TCCTCTCACT CCCTACAAAC TTCGAGCCGA   
  
  
- CCGTACTGGC GGGAGAGTAA CGCCGAAGCC GAACCGCTCG CCTCTGAGTT CGACTCTTGA GGTCGTGACA   
  
  
- ACACGAACTA CCCAGTAGCA GCACATCAAG TAGAAGAAT

+     O2-site

| Site Name | Organism | Position | Strand | Matrix score. | sequence | function |
| --- | --- | --- | --- | --- | --- | --- |
| O2-site | Zea mays | 2363 | - | 10 | GATGATGTGG | cis-acting regulatory element involved in zein metabolism regulation |

>HU11G00778.1   
+ -Up\_Stream \_Len000AAAAAA GAAAGAAATG GAGAAACCAA GCAATATGGA TAAAAAAAAA ACAAAGTAAT   
  
  
+ AAAGGAAGGA TGAGGCTCCA CATTCCACTC GCCCGCGCCA CTCATAGGAG ATGAATAGTA AAATGTGAGG   
  
  
+ GGTTAAATGA TTTGAAAAAA ATCTAGTAAG GGCCCACAAC AGTATTGGAA CCCAGCTTGC CAAGGAAATG   
  
  
+ TCAGAGGGGC TAGCAATGGA CCAGTGCAAG AAGGCCAAGC CACATTTGGT TAGGGAATTT CATCCCATCC   
  
  
+ GGCGCTAAAA GCATCAAAAT TACTACTTAA TCCTTGATCA AATGCCCTAT AAATATTCTA CTACCCCATT   
  
  
+ TGAAAAGGGG TGTTGATTAC AACACATAGC CGTAGGTTTG ACAAAGAGAA TTACCCTTAT TGTTAGAAGC   
  
  
+ ACCATATACT CTTTTCTCTA AGCTCTGATC TTTTTTCATC CTTTTCAGCT TTTTGTCCTT TTAAATCAAA   
  
  
+ TCACTCACTT GAGTATTGGA GGGACGTTCC TCAGGAGTCG AACCACTAAA TTATCTTTTT AAAGAAAAAG   
  
  
+ GGCTTGAAGC CAACCCGATT TAAAAAAACT GCTAATATGT AAGCCTACTC TCTTTCCAGT TCCAACAAAT   
  
  
+ CAAATTTCAG TATGAAACAT CCAGTAAAGG ATTTATCCGT GATAATGATG TAACATATGG TTAAAGGGTC   
  
  
+ ATTTTCAGTT ACATCGGAGC ATGCAATTAA AGTCCCTATA AAATAGGTAG GTGCACGAAC AAGTCACCAT   
  
  
+ CGTCCTTTGC TTTTTGCATG AGTGCATGCT AATTATGAAC TTAATTAATA TCTAAGCAAA ATTAAAAAGT   
  
  
+ AGTTAAAACT TTTTCACCTT TGGAACCTCG TAGCTGTAGC AGTAGCTGCC GCACTCTGGT TCTAGACTTC   
  
  
+ TAGGGACCAA GAGATGCACT CATATGCATC CTCTTCTGTC CTTTTACTGT ATCCCCTTCC CATGTGACCT   
  
  
+ TTTTGCCCGG GTGACTTTTC TGTGAGTGTT GATACAAATT TTGTACGTAA GCTAACTCAT TTACTTCACC   
  
  
+ GTCTCATGAG TTTTACATTA CCGATATTAA TTTAAATGGC AAAGTATTAA ATTTGAGCTA AAATCTCATA   
  
  
+ TATATATTTG TATTAAATGT GTCTGGATTG TCAAAATAAT TTTTGACATA TACAGTCACT ATATACTGAG   
  
  
+ TTATTAAAAT ATATAAAAAT ATGAAAATTT AAAAAATAGT AGTTTAAGGT GACAACACTA AAACAAACAA   
  
  
+ ATAAGATATA AATAGGGAAA AGTTCATCCT ATTTTGTTAT TATTATTTAT TATGTATTTT CAGAATTGTC   
  
  
+ TCTATCACAA GCATAGTATA TGTGTCAACA TCGTGACAAC TCGACCAATC TACAAATAAC TAGTAAATTC   
  
  
+ AAATAAAGTT ACTCTCACCT AGCTTAAATA AGTACACCTT GATTTAATTG TCTTAGCTAT GGAACCTATT   
  
  
+ CCAAACTAGC CAATACTTCC TTTTCAAAAG AATTAGTTTT AAAGTTTTAA CTTTTACGTC CAATAATGCC   
  
  
+ GGCCGTACTA TGAGTTGCTT TCAAGCCAGT AGTAGTACTG TAGTAGGTAA AAATATTAGA ATACACCTAC   
  
  
+ TATATATGAT ATTGCTCTTC ACCGTATCCT TCTGCATACT CATCTTTCCG TCCCGGAGTC CACTATCTCT   
  
  
+ CCTCCATTCA GACACTCTCT CGCTCTAGAG AACTCATAGC TATGGACCGC GTGCCCGACG GTTATCGGGT   
  
  
+ TATGAATTCG GAGCTTCTTC AGCAAGTTAG CCCTGAGTTG CCGGATCCAA CCACCTGGTT CCATGCCCGT   
  
  
+ CTACCCGACC CCATCTCTCA GTCTCCTCTT GGACCCGGTC CATCCTCCCA GCCAACCCAT TTCTACCAGG   
  
  
+ GTTCTGCCTC AGGGAACCAT GTGGTTGGGA TTGCTGACAC GTGGACGGAC CAGATTGTTG CAGGTTCCTC   
  
  
+ GTCCCAGCAG TGAGGTTGCA GCAGAGCCAG CAGGTGTTGA CTGCAGTCGA AGCCATGGAG GAGGACTCTG   
  
  
+ GGATAAGACT GGTCTACGCT CTGCTGACGT GTGCGGAGGC CATCCAACGT GGCGATTTCC GATTGGCTGT   
  
  
+ CTTGTTAGTT AACAAGATGA GCAATGACCT CCTGCCACGC GTCAACCCGT CCTGCGGTAT TGGCAAAGTA   
  
  
+ GCCGGCTACT TCATAGATGC CTTGACCCGA AGACTATACC AGCAGGGCCC AGTGTCGGGC CTAATCGGGT   
  
  
+ CGGTTCTGGC GTACCAGGTG TTGTACGAGC ACTTCTATGA AGCTTGCCCG TTCCTCAAAT TCGCTCACTT   
  
  
+ CACTGCTAAT CAAGCGATTT TAGAGGCATT CGACGGCCAC GATTGTGTCC ACATCATCGA CTTCGGCCTA   
  
  
+ ATCCACGGCT TGCAATGGCC AGCTCTAATC CAAGCCTTGG CTGTTCGGCA CGGTGGCCCG CCTTTCGTAA   
  
  
+ GATTAACCGG AATCGGGCCG CCTTCTGAAT ACGGGTCGTG CTCGCTTCAA CCTATCGGGT CAACACTGGC   
  
  
+ CCAGTTAGCC CTATCCATGA ATGTTGGGTT CGCATTTCGG GCCGTCGCCG TCTCACGGCT CGAGGACATC   
  
  
+ AAACCATGGA TGGTTAAAAC AAGCCCAAAT GAAGTCGTAG CCGTGAACTC TATCTTCCAA CTCCACCGGC   
  
  
+ TAATCGGGTC GGGTATTGAC CCTGTCCTAA ACTGGGTCCG GAGCCTAAAC CCGAAAATTG TGACACTGGC   
  
  
+ GGAGCAAGAG GCGAACCACA ACCAGCCCGA GTTTTTGGCC CGGTTCACGG AGGCATTACA TTACTATTCA   
  
  
+ ACTATGTTCG ACTCGTTAGA GGCTTGTCAA GTCCAGGCCG ACAAGGACCT AGCCGAGCTA TACTTAGAGA   
  
  
+ GAGAGTTATC CAACATCGTC TGCTGTGAGG GGTCGGCTCG GATTGAGAGG CACGAGCCGC TGGCCCAATG   
  
  
+ GAGGGCCCGA ATGGCTCGGG CCGGGTTCAA GAAGGTGGAT ATGGGTAAAA ATGCGTTCAA GCAAGTAAGC   
  
  
+ ATGTTGCTGA GTTTGTCTTC AGCACAAGGG TATTGTGTGG AGGAGAGTGA GGGATGTTTG AAGCTCGGCT   
  
  
+ GGCATGACCG CCCTCTCATT GCGGCTTCGG CTTGGCGAGC GGAGACTCAA GCTGAGAACT CCAGCACTGT   
  
  
+ TGTGCTTGAT GGGTCATCGT CGTGTAGTTC ATCTTCTTA  

- -Up\_Stream \_Len000TTTTTT CTTTCTTTAC CTCTTTGGTT CGTTATACCT ATTTTTTTTT TGTTTCATTA   
  
  
- TTTCCTTCCT ACTCCGAGGT GTAAGGTGAG CGGGCGCGGT GAGTATCCTC TACTTATCAT TTTACACTCC   
  
  
- CCAATTTACT AAACTTTTTT TAGATCATTC CCGGGTGTTG TCATAACCTT GGGTCGAACG GTTCCTTTAC   
  
  
- AGTCTCCCCG ATCGTTACCT GGTCACGTTC TTCCGGTTCG GTGTAAACCA ATCCCTTAAA GTAGGGTAGG   
  
  
- CCGCGATTTT CGTAGTTTTA ATGATGAATT AGGAACTAGT TTACGGGATA TTTATAAGAT GATGGGGTAA   
  
  
- ACTTTTCCCC ACAACTAATG TTGTGTATCG GCATCCAAAC TGTTTCTCTT AATGGGAATA ACAATCTTCG   
  
  
- TGGTATATGA GAAAAGAGAT TCGAGACTAG AAAAAAGTAG GAAAAGTCGA AAAACAGGAA AATTTAGTTT   
  
  
- AGTGAGTGAA CTCATAACCT CCCTGCAAGG AGTCCTCAGC TTGGTGATTT AATAGAAAAA TTTCTTTTTC   
  
  
- CCGAACTTCG GTTGGGCTAA ATTTTTTTGA CGATTATACA TTCGGATGAG AGAAAGGTCA AGGTTGTTTA   
  
  
- GTTTAAAGTC ATACTTTGTA GGTCATTTCC TAAATAGGCA CTATTACTAC ATTGTATACC AATTTCCCAG   
  
  
- TAAAAGTCAA TGTAGCCTCG TACGTTAATT TCAGGGATAT TTTATCCATC CACGTGCTTG TTCAGTGGTA   
  
  
- GCAGGAAACG AAAAACGTAC TCACGTACGA TTAATACTTG AATTAATTAT AGATTCGTTT TAATTTTTCA   
  
  
- TCAATTTTGA AAAAGTGGAA ACCTTGGAGC ATCGACATCG TCATCGACGG CGTGAGACCA AGATCTGAAG   
  
  
- ATCCCTGGTT CTCTACGTGA GTATACGTAG GAGAAGACAG GAAAATGACA TAGGGGAAGG GTACACTGGA   
  
  
- AAAACGGGCC CACTGAAAAG ACACTCACAA CTATGTTTAA AACATGCATT CGATTGAGTA AATGAAGTGG   
  
  
- CAGAGTACTC AAAATGTAAT GGCTATAATT AAATTTACCG TTTCATAATT TAAACTCGAT TTTAGAGTAT   
  
  
- ATATATAAAC ATAATTTACA CAGACCTAAC AGTTTTATTA AAAACTGTAT ATGTCAGTGA TATATGACTC   
  
  
- AATAATTTTA TATATTTTTA TACTTTTAAA TTTTTTATCA TCAAATTCCA CTGTTGTGAT TTTGTTTGTT   
  
  
- TATTCTATAT TTATCCCTTT TCAAGTAGGA TAAAACAATA ATAATAAATA ATACATAAAA GTCTTAACAG   
  
  
- AGATAGTGTT CGTATCATAT ACACAGTTGT AGCACTGTTG AGCTGGTTAG ATGTTTATTG ATCATTTAAG   
  
  
- TTTATTTCAA TGAGAGTGGA TCGAATTTAT TCATGTGGAA CTAAATTAAC AGAATCGATA CCTTGGATAA   
  
  
- GGTTTGATCG GTTATGAAGG AAAAGTTTTC TTAATCAAAA TTTCAAAATT GAAAATGCAG GTTATTACGG   
  
  
- CCGGCATGAT ACTCAACGAA AGTTCGGTCA TCATCATGAC ATCATCCATT TTTATAATCT TATGTGGATG   
  
  
- ATATATACTA TAACGAGAAG TGGCATAGGA AGACGTATGA GTAGAAAGGC AGGGCCTCAG GTGATAGAGA   
  
  
- GGAGGTAAGT CTGTGAGAGA GCGAGATCTC TTGAGTATCG ATACCTGGCG CACGGGCTGC CAATAGCCCA   
  
  
- ATACTTAAGC CTCGAAGAAG TCGTTCAATC GGGACTCAAC GGCCTAGGTT GGTGGACCAA GGTACGGGCA   
  
  
- GATGGGCTGG GGTAGAGAGT CAGAGGAGAA CCTGGGCCAG GTAGGAGGGT CGGTTGGGTA AAGATGGTCC   
  
  
- CAAGACGGAG TCCCTTGGTA CACCAACCCT AACGACTGTG CACCTGCCTG GTCTAACAAC GTCCAAGGAG   
  
  
- CAGGGTCGTC ACTCCAACGT CGTCTCGGTC GTCCACAACT GACGTCAGCT TCGGTACCTC CTCCTGAGAC   
  
  
- CCTATTCTGA CCAGATGCGA GACGACTGCA CACGCCTCCG GTAGGTTGCA CCGCTAAAGG CTAACCGACA   
  
  
- GAACAATCAA TTGTTCTACT CGTTACTGGA GGACGGTGCG CAGTTGGGCA GGACGCCATA ACCGTTTCAT   
  
  
- CGGCCGATGA AGTATCTACG GAACTGGGCT TCTGATATGG TCGTCCCGGG TCACAGCCCG GATTAGCCCA   
  
  
- GCCAAGACCG CATGGTCCAC AACATGCTCG TGAAGATACT TCGAACGGGC AAGGAGTTTA AGCGAGTGAA   
  
  
- GTGACGATTA GTTCGCTAAA ATCTCCGTAA GCTGCCGGTG CTAACACAGG TGTAGTAGCT GAAGCCGGAT   
  
  
- TAGGTGCCGA ACGTTACCGG TCGAGATTAG GTTCGGAACC GACAAGCCGT GCCACCGGGC GGAAAGCATT   
  
  
- CTAATTGGCC TTAGCCCGGC GGAAGACTTA TGCCCAGCAC GAGCGAAGTT GGATAGCCCA GTTGTGACCG   
  
  
- GGTCAATCGG GATAGGTACT TACAACCCAA GCGTAAAGCC CGGCAGCGGC AGAGTGCCGA GCTCCTGTAG   
  
  
- TTTGGTACCT ACCAATTTTG TTCGGGTTTA CTTCAGCATC GGCACTTGAG ATAGAAGGTT GAGGTGGCCG   
  
  
- ATTAGCCCAG CCCATAACTG GGACAGGATT TGACCCAGGC CTCGGATTTG GGCTTTTAAC ACTGTGACCG   
  
  
- CCTCGTTCTC CGCTTGGTGT TGGTCGGGCT CAAAAACCGG GCCAAGTGCC TCCGTAATGT AATGATAAGT   
  
  
- TGATACAAGC TGAGCAATCT CCGAACAGTT CAGGTCCGGC TGTTCCTGGA TCGGCTCGAT ATGAATCTCT   
  
  
- CTCTCAATAG GTTGTAGCAG ACGACACTCC CCAGCCGAGC CTAACTCTCC GTGCTCGGCG ACCGGGTTAC   
  
  
- CTCCCGGGCT TACCGAGCCC GGCCCAAGTT CTTCCACCTA TACCCATTTT TACGCAAGTT CGTTCATTCG   
  
  
- TACAACGACT CAAACAGAAG TCGTGTTCCC ATAACACACC TCCTCTCACT CCCTACAAAC TTCGAGCCGA   
  
  
- CCGTACTGGC GGGAGAGTAA CGCCGAAGCC GAACCGCTCG CCTCTGAGTT CGACTCTTGA GGTCGTGACA   
  
  
- ACACGAACTA CCCAGTAGCA GCACATCAAG TAGAAGAAT

+     STRE

| Site Name | Organism | Position | Strand | Matrix score. | sequence | function |
| --- | --- | --- | --- | --- | --- | --- |
| STRE | Arabidopsis thaliana | 2902 | + | 5 | AGGGG |  |
| STRE | Arabidopsis thaliana | 967 | - | 5 | AGGGG |  |
| STRE | Arabidopsis thaliana | 142 | + | 5 | AGGGG |  |
| STRE | Arabidopsis thaliana | 360 | + | 5 | AGGGG |  |
| STRE | Arabidopsis thaliana | 219 | + | 5 | AGGGG |  |

>HU11G00778.1   
+ -Up\_Stream \_Len000AAAAAA GAAAGAAATG GAGAAACCAA GCAATATGGA TAAAAAAAAA ACAAAGTAAT   
  
  
+ AAAGGAAGGA TGAGGCTCCA CATTCCACTC GCCCGCGCCA CTCATAGGAG ATGAATAGTA AAATGTGAGG   
  
  
+ GGTTAAATGA TTTGAAAAAA ATCTAGTAAG GGCCCACAAC AGTATTGGAA CCCAGCTTGC CAAGGAAATG   
  
  
+ TCAGAGGGGC TAGCAATGGA CCAGTGCAAG AAGGCCAAGC CACATTTGGT TAGGGAATTT CATCCCATCC   
  
  
+ GGCGCTAAAA GCATCAAAAT TACTACTTAA TCCTTGATCA AATGCCCTAT AAATATTCTA CTACCCCATT   
  
  
+ TGAAAAGGGG TGTTGATTAC AACACATAGC CGTAGGTTTG ACAAAGAGAA TTACCCTTAT TGTTAGAAGC   
  
  
+ ACCATATACT CTTTTCTCTA AGCTCTGATC TTTTTTCATC CTTTTCAGCT TTTTGTCCTT TTAAATCAAA   
  
  
+ TCACTCACTT GAGTATTGGA GGGACGTTCC TCAGGAGTCG AACCACTAAA TTATCTTTTT AAAGAAAAAG   
  
  
+ GGCTTGAAGC CAACCCGATT TAAAAAAACT GCTAATATGT AAGCCTACTC TCTTTCCAGT TCCAACAAAT   
  
  
+ CAAATTTCAG TATGAAACAT CCAGTAAAGG ATTTATCCGT GATAATGATG TAACATATGG TTAAAGGGTC   
  
  
+ ATTTTCAGTT ACATCGGAGC ATGCAATTAA AGTCCCTATA AAATAGGTAG GTGCACGAAC AAGTCACCAT   
  
  
+ CGTCCTTTGC TTTTTGCATG AGTGCATGCT AATTATGAAC TTAATTAATA TCTAAGCAAA ATTAAAAAGT   
  
  
+ AGTTAAAACT TTTTCACCTT TGGAACCTCG TAGCTGTAGC AGTAGCTGCC GCACTCTGGT TCTAGACTTC   
  
  
+ TAGGGACCAA GAGATGCACT CATATGCATC CTCTTCTGTC CTTTTACTGT ATCCCCTTCC CATGTGACCT   
  
  
+ TTTTGCCCGG GTGACTTTTC TGTGAGTGTT GATACAAATT TTGTACGTAA GCTAACTCAT TTACTTCACC   
  
  
+ GTCTCATGAG TTTTACATTA CCGATATTAA TTTAAATGGC AAAGTATTAA ATTTGAGCTA AAATCTCATA   
  
  
+ TATATATTTG TATTAAATGT GTCTGGATTG TCAAAATAAT TTTTGACATA TACAGTCACT ATATACTGAG   
  
  
+ TTATTAAAAT ATATAAAAAT ATGAAAATTT AAAAAATAGT AGTTTAAGGT GACAACACTA AAACAAACAA   
  
  
+ ATAAGATATA AATAGGGAAA AGTTCATCCT ATTTTGTTAT TATTATTTAT TATGTATTTT CAGAATTGTC   
  
  
+ TCTATCACAA GCATAGTATA TGTGTCAACA TCGTGACAAC TCGACCAATC TACAAATAAC TAGTAAATTC   
  
  
+ AAATAAAGTT ACTCTCACCT AGCTTAAATA AGTACACCTT GATTTAATTG TCTTAGCTAT GGAACCTATT   
  
  
+ CCAAACTAGC CAATACTTCC TTTTCAAAAG AATTAGTTTT AAAGTTTTAA CTTTTACGTC CAATAATGCC   
  
  
+ GGCCGTACTA TGAGTTGCTT TCAAGCCAGT AGTAGTACTG TAGTAGGTAA AAATATTAGA ATACACCTAC   
  
  
+ TATATATGAT ATTGCTCTTC ACCGTATCCT TCTGCATACT CATCTTTCCG TCCCGGAGTC CACTATCTCT   
  
  
+ CCTCCATTCA GACACTCTCT CGCTCTAGAG AACTCATAGC TATGGACCGC GTGCCCGACG GTTATCGGGT   
  
  
+ TATGAATTCG GAGCTTCTTC AGCAAGTTAG CCCTGAGTTG CCGGATCCAA CCACCTGGTT CCATGCCCGT   
  
  
+ CTACCCGACC CCATCTCTCA GTCTCCTCTT GGACCCGGTC CATCCTCCCA GCCAACCCAT TTCTACCAGG   
  
  
+ GTTCTGCCTC AGGGAACCAT GTGGTTGGGA TTGCTGACAC GTGGACGGAC CAGATTGTTG CAGGTTCCTC   
  
  
+ GTCCCAGCAG TGAGGTTGCA GCAGAGCCAG CAGGTGTTGA CTGCAGTCGA AGCCATGGAG GAGGACTCTG   
  
  
+ GGATAAGACT GGTCTACGCT CTGCTGACGT GTGCGGAGGC CATCCAACGT GGCGATTTCC GATTGGCTGT   
  
  
+ CTTGTTAGTT AACAAGATGA GCAATGACCT CCTGCCACGC GTCAACCCGT CCTGCGGTAT TGGCAAAGTA   
  
  
+ GCCGGCTACT TCATAGATGC CTTGACCCGA AGACTATACC AGCAGGGCCC AGTGTCGGGC CTAATCGGGT   
  
  
+ CGGTTCTGGC GTACCAGGTG TTGTACGAGC ACTTCTATGA AGCTTGCCCG TTCCTCAAAT TCGCTCACTT   
  
  
+ CACTGCTAAT CAAGCGATTT TAGAGGCATT CGACGGCCAC GATTGTGTCC ACATCATCGA CTTCGGCCTA   
  
  
+ ATCCACGGCT TGCAATGGCC AGCTCTAATC CAAGCCTTGG CTGTTCGGCA CGGTGGCCCG CCTTTCGTAA   
  
  
+ GATTAACCGG AATCGGGCCG CCTTCTGAAT ACGGGTCGTG CTCGCTTCAA CCTATCGGGT CAACACTGGC   
  
  
+ CCAGTTAGCC CTATCCATGA ATGTTGGGTT CGCATTTCGG GCCGTCGCCG TCTCACGGCT CGAGGACATC   
  
  
+ AAACCATGGA TGGTTAAAAC AAGCCCAAAT GAAGTCGTAG CCGTGAACTC TATCTTCCAA CTCCACCGGC   
  
  
+ TAATCGGGTC GGGTATTGAC CCTGTCCTAA ACTGGGTCCG GAGCCTAAAC CCGAAAATTG TGACACTGGC   
  
  
+ GGAGCAAGAG GCGAACCACA ACCAGCCCGA GTTTTTGGCC CGGTTCACGG AGGCATTACA TTACTATTCA   
  
  
+ ACTATGTTCG ACTCGTTAGA GGCTTGTCAA GTCCAGGCCG ACAAGGACCT AGCCGAGCTA TACTTAGAGA   
  
  
+ GAGAGTTATC CAACATCGTC TGCTGTGAGG GGTCGGCTCG GATTGAGAGG CACGAGCCGC TGGCCCAATG   
  
  
+ GAGGGCCCGA ATGGCTCGGG CCGGGTTCAA GAAGGTGGAT ATGGGTAAAA ATGCGTTCAA GCAAGTAAGC   
  
  
+ ATGTTGCTGA GTTTGTCTTC AGCACAAGGG TATTGTGTGG AGGAGAGTGA GGGATGTTTG AAGCTCGGCT   
  
  
+ GGCATGACCG CCCTCTCATT GCGGCTTCGG CTTGGCGAGC GGAGACTCAA GCTGAGAACT CCAGCACTGT   
  
  
+ TGTGCTTGAT GGGTCATCGT CGTGTAGTTC ATCTTCTTA  

- -Up\_Stream \_Len000TTTTTT CTTTCTTTAC CTCTTTGGTT CGTTATACCT ATTTTTTTTT TGTTTCATTA   
  
  
- TTTCCTTCCT ACTCCGAGGT GTAAGGTGAG CGGGCGCGGT GAGTATCCTC TACTTATCAT TTTACACTCC   
  
  
- CCAATTTACT AAACTTTTTT TAGATCATTC CCGGGTGTTG TCATAACCTT GGGTCGAACG GTTCCTTTAC   
  
  
- AGTCTCCCCG ATCGTTACCT GGTCACGTTC TTCCGGTTCG GTGTAAACCA ATCCCTTAAA GTAGGGTAGG   
  
  
- CCGCGATTTT CGTAGTTTTA ATGATGAATT AGGAACTAGT TTACGGGATA TTTATAAGAT GATGGGGTAA   
  
  
- ACTTTTCCCC ACAACTAATG TTGTGTATCG GCATCCAAAC TGTTTCTCTT AATGGGAATA ACAATCTTCG   
  
  
- TGGTATATGA GAAAAGAGAT TCGAGACTAG AAAAAAGTAG GAAAAGTCGA AAAACAGGAA AATTTAGTTT   
  
  
- AGTGAGTGAA CTCATAACCT CCCTGCAAGG AGTCCTCAGC TTGGTGATTT AATAGAAAAA TTTCTTTTTC   
  
  
- CCGAACTTCG GTTGGGCTAA ATTTTTTTGA CGATTATACA TTCGGATGAG AGAAAGGTCA AGGTTGTTTA   
  
  
- GTTTAAAGTC ATACTTTGTA GGTCATTTCC TAAATAGGCA CTATTACTAC ATTGTATACC AATTTCCCAG   
  
  
- TAAAAGTCAA TGTAGCCTCG TACGTTAATT TCAGGGATAT TTTATCCATC CACGTGCTTG TTCAGTGGTA   
  
  
- GCAGGAAACG AAAAACGTAC TCACGTACGA TTAATACTTG AATTAATTAT AGATTCGTTT TAATTTTTCA   
  
  
- TCAATTTTGA AAAAGTGGAA ACCTTGGAGC ATCGACATCG TCATCGACGG CGTGAGACCA AGATCTGAAG   
  
  
- ATCCCTGGTT CTCTACGTGA GTATACGTAG GAGAAGACAG GAAAATGACA TAGGGGAAGG GTACACTGGA   
  
  
- AAAACGGGCC CACTGAAAAG ACACTCACAA CTATGTTTAA AACATGCATT CGATTGAGTA AATGAAGTGG   
  
  
- CAGAGTACTC AAAATGTAAT GGCTATAATT AAATTTACCG TTTCATAATT TAAACTCGAT TTTAGAGTAT   
  
  
- ATATATAAAC ATAATTTACA CAGACCTAAC AGTTTTATTA AAAACTGTAT ATGTCAGTGA TATATGACTC   
  
  
- AATAATTTTA TATATTTTTA TACTTTTAAA TTTTTTATCA TCAAATTCCA CTGTTGTGAT TTTGTTTGTT   
  
  
- TATTCTATAT TTATCCCTTT TCAAGTAGGA TAAAACAATA ATAATAAATA ATACATAAAA GTCTTAACAG   
  
  
- AGATAGTGTT CGTATCATAT ACACAGTTGT AGCACTGTTG AGCTGGTTAG ATGTTTATTG ATCATTTAAG   
  
  
- TTTATTTCAA TGAGAGTGGA TCGAATTTAT TCATGTGGAA CTAAATTAAC AGAATCGATA CCTTGGATAA   
  
  
- GGTTTGATCG GTTATGAAGG AAAAGTTTTC TTAATCAAAA TTTCAAAATT GAAAATGCAG GTTATTACGG   
  
  
- CCGGCATGAT ACTCAACGAA AGTTCGGTCA TCATCATGAC ATCATCCATT TTTATAATCT TATGTGGATG   
  
  
- ATATATACTA TAACGAGAAG TGGCATAGGA AGACGTATGA GTAGAAAGGC AGGGCCTCAG GTGATAGAGA   
  
  
- GGAGGTAAGT CTGTGAGAGA GCGAGATCTC TTGAGTATCG ATACCTGGCG CACGGGCTGC CAATAGCCCA   
  
  
- ATACTTAAGC CTCGAAGAAG TCGTTCAATC GGGACTCAAC GGCCTAGGTT GGTGGACCAA GGTACGGGCA   
  
  
- GATGGGCTGG GGTAGAGAGT CAGAGGAGAA CCTGGGCCAG GTAGGAGGGT CGGTTGGGTA AAGATGGTCC   
  
  
- CAAGACGGAG TCCCTTGGTA CACCAACCCT AACGACTGTG CACCTGCCTG GTCTAACAAC GTCCAAGGAG   
  
  
- CAGGGTCGTC ACTCCAACGT CGTCTCGGTC GTCCACAACT GACGTCAGCT TCGGTACCTC CTCCTGAGAC   
  
  
- CCTATTCTGA CCAGATGCGA GACGACTGCA CACGCCTCCG GTAGGTTGCA CCGCTAAAGG CTAACCGACA   
  
  
- GAACAATCAA TTGTTCTACT CGTTACTGGA GGACGGTGCG CAGTTGGGCA GGACGCCATA ACCGTTTCAT   
  
  
- CGGCCGATGA AGTATCTACG GAACTGGGCT TCTGATATGG TCGTCCCGGG TCACAGCCCG GATTAGCCCA   
  
  
- GCCAAGACCG CATGGTCCAC AACATGCTCG TGAAGATACT TCGAACGGGC AAGGAGTTTA AGCGAGTGAA   
  
  
- GTGACGATTA GTTCGCTAAA ATCTCCGTAA GCTGCCGGTG CTAACACAGG TGTAGTAGCT GAAGCCGGAT   
  
  
- TAGGTGCCGA ACGTTACCGG TCGAGATTAG GTTCGGAACC GACAAGCCGT GCCACCGGGC GGAAAGCATT   
  
  
- CTAATTGGCC TTAGCCCGGC GGAAGACTTA TGCCCAGCAC GAGCGAAGTT GGATAGCCCA GTTGTGACCG   
  
  
- GGTCAATCGG GATAGGTACT TACAACCCAA GCGTAAAGCC CGGCAGCGGC AGAGTGCCGA GCTCCTGTAG   
  
  
- TTTGGTACCT ACCAATTTTG TTCGGGTTTA CTTCAGCATC GGCACTTGAG ATAGAAGGTT GAGGTGGCCG   
  
  
- ATTAGCCCAG CCCATAACTG GGACAGGATT TGACCCAGGC CTCGGATTTG GGCTTTTAAC ACTGTGACCG   
  
  
- CCTCGTTCTC CGCTTGGTGT TGGTCGGGCT CAAAAACCGG GCCAAGTGCC TCCGTAATGT AATGATAAGT   
  
  
- TGATACAAGC TGAGCAATCT CCGAACAGTT CAGGTCCGGC TGTTCCTGGA TCGGCTCGAT ATGAATCTCT   
  
  
- CTCTCAATAG GTTGTAGCAG ACGACACTCC CCAGCCGAGC CTAACTCTCC GTGCTCGGCG ACCGGGTTAC   
  
  
- CTCCCGGGCT TACCGAGCCC GGCCCAAGTT CTTCCACCTA TACCCATTTT TACGCAAGTT CGTTCATTCG   
  
  
- TACAACGACT CAAACAGAAG TCGTGTTCCC ATAACACACC TCCTCTCACT CCCTACAAAC TTCGAGCCGA   
  
  
- CCGTACTGGC GGGAGAGTAA CGCCGAAGCC GAACCGCTCG CCTCTGAGTT CGACTCTTGA GGTCGTGACA   
  
  
- ACACGAACTA CCCAGTAGCA GCACATCAAG TAGAAGAAT

+     Sp1

| Site Name | Organism | Position | Strand | Matrix score. | sequence | function |
| --- | --- | --- | --- | --- | --- | --- |
| Sp1 | Oryza sativa | 3092 | - | 6 | GGGCGG | light responsive element |

>HU11G00778.1   
+ -Up\_Stream \_Len000AAAAAA GAAAGAAATG GAGAAACCAA GCAATATGGA TAAAAAAAAA ACAAAGTAAT   
  
  
+ AAAGGAAGGA TGAGGCTCCA CATTCCACTC GCCCGCGCCA CTCATAGGAG ATGAATAGTA AAATGTGAGG   
  
  
+ GGTTAAATGA TTTGAAAAAA ATCTAGTAAG GGCCCACAAC AGTATTGGAA CCCAGCTTGC CAAGGAAATG   
  
  
+ TCAGAGGGGC TAGCAATGGA CCAGTGCAAG AAGGCCAAGC CACATTTGGT TAGGGAATTT CATCCCATCC   
  
  
+ GGCGCTAAAA GCATCAAAAT TACTACTTAA TCCTTGATCA AATGCCCTAT AAATATTCTA CTACCCCATT   
  
  
+ TGAAAAGGGG TGTTGATTAC AACACATAGC CGTAGGTTTG ACAAAGAGAA TTACCCTTAT TGTTAGAAGC   
  
  
+ ACCATATACT CTTTTCTCTA AGCTCTGATC TTTTTTCATC CTTTTCAGCT TTTTGTCCTT TTAAATCAAA   
  
  
+ TCACTCACTT GAGTATTGGA GGGACGTTCC TCAGGAGTCG AACCACTAAA TTATCTTTTT AAAGAAAAAG   
  
  
+ GGCTTGAAGC CAACCCGATT TAAAAAAACT GCTAATATGT AAGCCTACTC TCTTTCCAGT TCCAACAAAT   
  
  
+ CAAATTTCAG TATGAAACAT CCAGTAAAGG ATTTATCCGT GATAATGATG TAACATATGG TTAAAGGGTC   
  
  
+ ATTTTCAGTT ACATCGGAGC ATGCAATTAA AGTCCCTATA AAATAGGTAG GTGCACGAAC AAGTCACCAT   
  
  
+ CGTCCTTTGC TTTTTGCATG AGTGCATGCT AATTATGAAC TTAATTAATA TCTAAGCAAA ATTAAAAAGT   
  
  
+ AGTTAAAACT TTTTCACCTT TGGAACCTCG TAGCTGTAGC AGTAGCTGCC GCACTCTGGT TCTAGACTTC   
  
  
+ TAGGGACCAA GAGATGCACT CATATGCATC CTCTTCTGTC CTTTTACTGT ATCCCCTTCC CATGTGACCT   
  
  
+ TTTTGCCCGG GTGACTTTTC TGTGAGTGTT GATACAAATT TTGTACGTAA GCTAACTCAT TTACTTCACC   
  
  
+ GTCTCATGAG TTTTACATTA CCGATATTAA TTTAAATGGC AAAGTATTAA ATTTGAGCTA AAATCTCATA   
  
  
+ TATATATTTG TATTAAATGT GTCTGGATTG TCAAAATAAT TTTTGACATA TACAGTCACT ATATACTGAG   
  
  
+ TTATTAAAAT ATATAAAAAT ATGAAAATTT AAAAAATAGT AGTTTAAGGT GACAACACTA AAACAAACAA   
  
  
+ ATAAGATATA AATAGGGAAA AGTTCATCCT ATTTTGTTAT TATTATTTAT TATGTATTTT CAGAATTGTC   
  
  
+ TCTATCACAA GCATAGTATA TGTGTCAACA TCGTGACAAC TCGACCAATC TACAAATAAC TAGTAAATTC   
  
  
+ AAATAAAGTT ACTCTCACCT AGCTTAAATA AGTACACCTT GATTTAATTG TCTTAGCTAT GGAACCTATT   
  
  
+ CCAAACTAGC CAATACTTCC TTTTCAAAAG AATTAGTTTT AAAGTTTTAA CTTTTACGTC CAATAATGCC   
  
  
+ GGCCGTACTA TGAGTTGCTT TCAAGCCAGT AGTAGTACTG TAGTAGGTAA AAATATTAGA ATACACCTAC   
  
  
+ TATATATGAT ATTGCTCTTC ACCGTATCCT TCTGCATACT CATCTTTCCG TCCCGGAGTC CACTATCTCT   
  
  
+ CCTCCATTCA GACACTCTCT CGCTCTAGAG AACTCATAGC TATGGACCGC GTGCCCGACG GTTATCGGGT   
  
  
+ TATGAATTCG GAGCTTCTTC AGCAAGTTAG CCCTGAGTTG CCGGATCCAA CCACCTGGTT CCATGCCCGT   
  
  
+ CTACCCGACC CCATCTCTCA GTCTCCTCTT GGACCCGGTC CATCCTCCCA GCCAACCCAT TTCTACCAGG   
  
  
+ GTTCTGCCTC AGGGAACCAT GTGGTTGGGA TTGCTGACAC GTGGACGGAC CAGATTGTTG CAGGTTCCTC   
  
  
+ GTCCCAGCAG TGAGGTTGCA GCAGAGCCAG CAGGTGTTGA CTGCAGTCGA AGCCATGGAG GAGGACTCTG   
  
  
+ GGATAAGACT GGTCTACGCT CTGCTGACGT GTGCGGAGGC CATCCAACGT GGCGATTTCC GATTGGCTGT   
  
  
+ CTTGTTAGTT AACAAGATGA GCAATGACCT CCTGCCACGC GTCAACCCGT CCTGCGGTAT TGGCAAAGTA   
  
  
+ GCCGGCTACT TCATAGATGC CTTGACCCGA AGACTATACC AGCAGGGCCC AGTGTCGGGC CTAATCGGGT   
  
  
+ CGGTTCTGGC GTACCAGGTG TTGTACGAGC ACTTCTATGA AGCTTGCCCG TTCCTCAAAT TCGCTCACTT   
  
  
+ CACTGCTAAT CAAGCGATTT TAGAGGCATT CGACGGCCAC GATTGTGTCC ACATCATCGA CTTCGGCCTA   
  
  
+ ATCCACGGCT TGCAATGGCC AGCTCTAATC CAAGCCTTGG CTGTTCGGCA CGGTGGCCCG CCTTTCGTAA   
  
  
+ GATTAACCGG AATCGGGCCG CCTTCTGAAT ACGGGTCGTG CTCGCTTCAA CCTATCGGGT CAACACTGGC   
  
  
+ CCAGTTAGCC CTATCCATGA ATGTTGGGTT CGCATTTCGG GCCGTCGCCG TCTCACGGCT CGAGGACATC   
  
  
+ AAACCATGGA TGGTTAAAAC AAGCCCAAAT GAAGTCGTAG CCGTGAACTC TATCTTCCAA CTCCACCGGC   
  
  
+ TAATCGGGTC GGGTATTGAC CCTGTCCTAA ACTGGGTCCG GAGCCTAAAC CCGAAAATTG TGACACTGGC   
  
  
+ GGAGCAAGAG GCGAACCACA ACCAGCCCGA GTTTTTGGCC CGGTTCACGG AGGCATTACA TTACTATTCA   
  
  
+ ACTATGTTCG ACTCGTTAGA GGCTTGTCAA GTCCAGGCCG ACAAGGACCT AGCCGAGCTA TACTTAGAGA   
  
  
+ GAGAGTTATC CAACATCGTC TGCTGTGAGG GGTCGGCTCG GATTGAGAGG CACGAGCCGC TGGCCCAATG   
  
  
+ GAGGGCCCGA ATGGCTCGGG CCGGGTTCAA GAAGGTGGAT ATGGGTAAAA ATGCGTTCAA GCAAGTAAGC   
  
  
+ ATGTTGCTGA GTTTGTCTTC AGCACAAGGG TATTGTGTGG AGGAGAGTGA GGGATGTTTG AAGCTCGGCT   
  
  
+ GGCATGACCG CCCTCTCATT GCGGCTTCGG CTTGGCGAGC GGAGACTCAA GCTGAGAACT CCAGCACTGT   
  
  
+ TGTGCTTGAT GGGTCATCGT CGTGTAGTTC ATCTTCTTA  

- -Up\_Stream \_Len000TTTTTT CTTTCTTTAC CTCTTTGGTT CGTTATACCT ATTTTTTTTT TGTTTCATTA   
  
  
- TTTCCTTCCT ACTCCGAGGT GTAAGGTGAG CGGGCGCGGT GAGTATCCTC TACTTATCAT TTTACACTCC   
  
  
- CCAATTTACT AAACTTTTTT TAGATCATTC CCGGGTGTTG TCATAACCTT GGGTCGAACG GTTCCTTTAC   
  
  
- AGTCTCCCCG ATCGTTACCT GGTCACGTTC TTCCGGTTCG GTGTAAACCA ATCCCTTAAA GTAGGGTAGG   
  
  
- CCGCGATTTT CGTAGTTTTA ATGATGAATT AGGAACTAGT TTACGGGATA TTTATAAGAT GATGGGGTAA   
  
  
- ACTTTTCCCC ACAACTAATG TTGTGTATCG GCATCCAAAC TGTTTCTCTT AATGGGAATA ACAATCTTCG   
  
  
- TGGTATATGA GAAAAGAGAT TCGAGACTAG AAAAAAGTAG GAAAAGTCGA AAAACAGGAA AATTTAGTTT   
  
  
- AGTGAGTGAA CTCATAACCT CCCTGCAAGG AGTCCTCAGC TTGGTGATTT AATAGAAAAA TTTCTTTTTC   
  
  
- CCGAACTTCG GTTGGGCTAA ATTTTTTTGA CGATTATACA TTCGGATGAG AGAAAGGTCA AGGTTGTTTA   
  
  
- GTTTAAAGTC ATACTTTGTA GGTCATTTCC TAAATAGGCA CTATTACTAC ATTGTATACC AATTTCCCAG   
  
  
- TAAAAGTCAA TGTAGCCTCG TACGTTAATT TCAGGGATAT TTTATCCATC CACGTGCTTG TTCAGTGGTA   
  
  
- GCAGGAAACG AAAAACGTAC TCACGTACGA TTAATACTTG AATTAATTAT AGATTCGTTT TAATTTTTCA   
  
  
- TCAATTTTGA AAAAGTGGAA ACCTTGGAGC ATCGACATCG TCATCGACGG CGTGAGACCA AGATCTGAAG   
  
  
- ATCCCTGGTT CTCTACGTGA GTATACGTAG GAGAAGACAG GAAAATGACA TAGGGGAAGG GTACACTGGA   
  
  
- AAAACGGGCC CACTGAAAAG ACACTCACAA CTATGTTTAA AACATGCATT CGATTGAGTA AATGAAGTGG   
  
  
- CAGAGTACTC AAAATGTAAT GGCTATAATT AAATTTACCG TTTCATAATT TAAACTCGAT TTTAGAGTAT   
  
  
- ATATATAAAC ATAATTTACA CAGACCTAAC AGTTTTATTA AAAACTGTAT ATGTCAGTGA TATATGACTC   
  
  
- AATAATTTTA TATATTTTTA TACTTTTAAA TTTTTTATCA TCAAATTCCA CTGTTGTGAT TTTGTTTGTT   
  
  
- TATTCTATAT TTATCCCTTT TCAAGTAGGA TAAAACAATA ATAATAAATA ATACATAAAA GTCTTAACAG   
  
  
- AGATAGTGTT CGTATCATAT ACACAGTTGT AGCACTGTTG AGCTGGTTAG ATGTTTATTG ATCATTTAAG   
  
  
- TTTATTTCAA TGAGAGTGGA TCGAATTTAT TCATGTGGAA CTAAATTAAC AGAATCGATA CCTTGGATAA   
  
  
- GGTTTGATCG GTTATGAAGG AAAAGTTTTC TTAATCAAAA TTTCAAAATT GAAAATGCAG GTTATTACGG   
  
  
- CCGGCATGAT ACTCAACGAA AGTTCGGTCA TCATCATGAC ATCATCCATT TTTATAATCT TATGTGGATG   
  
  
- ATATATACTA TAACGAGAAG TGGCATAGGA AGACGTATGA GTAGAAAGGC AGGGCCTCAG GTGATAGAGA   
  
  
- GGAGGTAAGT CTGTGAGAGA GCGAGATCTC TTGAGTATCG ATACCTGGCG CACGGGCTGC CAATAGCCCA   
  
  
- ATACTTAAGC CTCGAAGAAG TCGTTCAATC GGGACTCAAC GGCCTAGGTT GGTGGACCAA GGTACGGGCA   
  
  
- GATGGGCTGG GGTAGAGAGT CAGAGGAGAA CCTGGGCCAG GTAGGAGGGT CGGTTGGGTA AAGATGGTCC   
  
  
- CAAGACGGAG TCCCTTGGTA CACCAACCCT AACGACTGTG CACCTGCCTG GTCTAACAAC GTCCAAGGAG   
  
  
- CAGGGTCGTC ACTCCAACGT CGTCTCGGTC GTCCACAACT GACGTCAGCT TCGGTACCTC CTCCTGAGAC   
  
  
- CCTATTCTGA CCAGATGCGA GACGACTGCA CACGCCTCCG GTAGGTTGCA CCGCTAAAGG CTAACCGACA   
  
  
- GAACAATCAA TTGTTCTACT CGTTACTGGA GGACGGTGCG CAGTTGGGCA GGACGCCATA ACCGTTTCAT   
  
  
- CGGCCGATGA AGTATCTACG GAACTGGGCT TCTGATATGG TCGTCCCGGG TCACAGCCCG GATTAGCCCA   
  
  
- GCCAAGACCG CATGGTCCAC AACATGCTCG TGAAGATACT TCGAACGGGC AAGGAGTTTA AGCGAGTGAA   
  
  
- GTGACGATTA GTTCGCTAAA ATCTCCGTAA GCTGCCGGTG CTAACACAGG TGTAGTAGCT GAAGCCGGAT   
  
  
- TAGGTGCCGA ACGTTACCGG TCGAGATTAG GTTCGGAACC GACAAGCCGT GCCACCGGGC GGAAAGCATT   
  
  
- CTAATTGGCC TTAGCCCGGC GGAAGACTTA TGCCCAGCAC GAGCGAAGTT GGATAGCCCA GTTGTGACCG   
  
  
- GGTCAATCGG GATAGGTACT TACAACCCAA GCGTAAAGCC CGGCAGCGGC AGAGTGCCGA GCTCCTGTAG   
  
  
- TTTGGTACCT ACCAATTTTG TTCGGGTTTA CTTCAGCATC GGCACTTGAG ATAGAAGGTT GAGGTGGCCG   
  
  
- ATTAGCCCAG CCCATAACTG GGACAGGATT TGACCCAGGC CTCGGATTTG GGCTTTTAAC ACTGTGACCG   
  
  
- CCTCGTTCTC CGCTTGGTGT TGGTCGGGCT CAAAAACCGG GCCAAGTGCC TCCGTAATGT AATGATAAGT   
  
  
- TGATACAAGC TGAGCAATCT CCGAACAGTT CAGGTCCGGC TGTTCCTGGA TCGGCTCGAT ATGAATCTCT   
  
  
- CTCTCAATAG GTTGTAGCAG ACGACACTCC CCAGCCGAGC CTAACTCTCC GTGCTCGGCG ACCGGGTTAC   
  
  
- CTCCCGGGCT TACCGAGCCC GGCCCAAGTT CTTCCACCTA TACCCATTTT TACGCAAGTT CGTTCATTCG   
  
  
- TACAACGACT CAAACAGAAG TCGTGTTCCC ATAACACACC TCCTCTCACT CCCTACAAAC TTCGAGCCGA   
  
  
- CCGTACTGGC GGGAGAGTAA CGCCGAAGCC GAACCGCTCG CCTCTGAGTT CGACTCTTGA GGTCGTGACA   
  
  
- ACACGAACTA CCCAGTAGCA GCACATCAAG TAGAAGAAT

+     TATA

| Site Name | Organism | Position | Strand | Matrix score. | sequence | function |
| --- | --- | --- | --- | --- | --- | --- |
| TATA | Arabidopsis thaliana | 741 | + | 8 | TATAAAAT |  |

>HU11G00778.1   
+ -Up\_Stream \_Len000AAAAAA GAAAGAAATG GAGAAACCAA GCAATATGGA TAAAAAAAAA ACAAAGTAAT   
  
  
+ AAAGGAAGGA TGAGGCTCCA CATTCCACTC GCCCGCGCCA CTCATAGGAG ATGAATAGTA AAATGTGAGG   
  
  
+ GGTTAAATGA TTTGAAAAAA ATCTAGTAAG GGCCCACAAC AGTATTGGAA CCCAGCTTGC CAAGGAAATG   
  
  
+ TCAGAGGGGC TAGCAATGGA CCAGTGCAAG AAGGCCAAGC CACATTTGGT TAGGGAATTT CATCCCATCC   
  
  
+ GGCGCTAAAA GCATCAAAAT TACTACTTAA TCCTTGATCA AATGCCCTAT AAATATTCTA CTACCCCATT   
  
  
+ TGAAAAGGGG TGTTGATTAC AACACATAGC CGTAGGTTTG ACAAAGAGAA TTACCCTTAT TGTTAGAAGC   
  
  
+ ACCATATACT CTTTTCTCTA AGCTCTGATC TTTTTTCATC CTTTTCAGCT TTTTGTCCTT TTAAATCAAA   
  
  
+ TCACTCACTT GAGTATTGGA GGGACGTTCC TCAGGAGTCG AACCACTAAA TTATCTTTTT AAAGAAAAAG   
  
  
+ GGCTTGAAGC CAACCCGATT TAAAAAAACT GCTAATATGT AAGCCTACTC TCTTTCCAGT TCCAACAAAT   
  
  
+ CAAATTTCAG TATGAAACAT CCAGTAAAGG ATTTATCCGT GATAATGATG TAACATATGG TTAAAGGGTC   
  
  
+ ATTTTCAGTT ACATCGGAGC ATGCAATTAA AGTCCCTATA AAATAGGTAG GTGCACGAAC AAGTCACCAT   
  
  
+ CGTCCTTTGC TTTTTGCATG AGTGCATGCT AATTATGAAC TTAATTAATA TCTAAGCAAA ATTAAAAAGT   
  
  
+ AGTTAAAACT TTTTCACCTT TGGAACCTCG TAGCTGTAGC AGTAGCTGCC GCACTCTGGT TCTAGACTTC   
  
  
+ TAGGGACCAA GAGATGCACT CATATGCATC CTCTTCTGTC CTTTTACTGT ATCCCCTTCC CATGTGACCT   
  
  
+ TTTTGCCCGG GTGACTTTTC TGTGAGTGTT GATACAAATT TTGTACGTAA GCTAACTCAT TTACTTCACC   
  
  
+ GTCTCATGAG TTTTACATTA CCGATATTAA TTTAAATGGC AAAGTATTAA ATTTGAGCTA AAATCTCATA   
  
  
+ TATATATTTG TATTAAATGT GTCTGGATTG TCAAAATAAT TTTTGACATA TACAGTCACT ATATACTGAG   
  
  
+ TTATTAAAAT ATATAAAAAT ATGAAAATTT AAAAAATAGT AGTTTAAGGT GACAACACTA AAACAAACAA   
  
  
+ ATAAGATATA AATAGGGAAA AGTTCATCCT ATTTTGTTAT TATTATTTAT TATGTATTTT CAGAATTGTC   
  
  
+ TCTATCACAA GCATAGTATA TGTGTCAACA TCGTGACAAC TCGACCAATC TACAAATAAC TAGTAAATTC   
  
  
+ AAATAAAGTT ACTCTCACCT AGCTTAAATA AGTACACCTT GATTTAATTG TCTTAGCTAT GGAACCTATT   
  
  
+ CCAAACTAGC CAATACTTCC TTTTCAAAAG AATTAGTTTT AAAGTTTTAA CTTTTACGTC CAATAATGCC   
  
  
+ GGCCGTACTA TGAGTTGCTT TCAAGCCAGT AGTAGTACTG TAGTAGGTAA AAATATTAGA ATACACCTAC   
  
  
+ TATATATGAT ATTGCTCTTC ACCGTATCCT TCTGCATACT CATCTTTCCG TCCCGGAGTC CACTATCTCT   
  
  
+ CCTCCATTCA GACACTCTCT CGCTCTAGAG AACTCATAGC TATGGACCGC GTGCCCGACG GTTATCGGGT   
  
  
+ TATGAATTCG GAGCTTCTTC AGCAAGTTAG CCCTGAGTTG CCGGATCCAA CCACCTGGTT CCATGCCCGT   
  
  
+ CTACCCGACC CCATCTCTCA GTCTCCTCTT GGACCCGGTC CATCCTCCCA GCCAACCCAT TTCTACCAGG   
  
  
+ GTTCTGCCTC AGGGAACCAT GTGGTTGGGA TTGCTGACAC GTGGACGGAC CAGATTGTTG CAGGTTCCTC   
  
  
+ GTCCCAGCAG TGAGGTTGCA GCAGAGCCAG CAGGTGTTGA CTGCAGTCGA AGCCATGGAG GAGGACTCTG   
  
  
+ GGATAAGACT GGTCTACGCT CTGCTGACGT GTGCGGAGGC CATCCAACGT GGCGATTTCC GATTGGCTGT   
  
  
+ CTTGTTAGTT AACAAGATGA GCAATGACCT CCTGCCACGC GTCAACCCGT CCTGCGGTAT TGGCAAAGTA   
  
  
+ GCCGGCTACT TCATAGATGC CTTGACCCGA AGACTATACC AGCAGGGCCC AGTGTCGGGC CTAATCGGGT   
  
  
+ CGGTTCTGGC GTACCAGGTG TTGTACGAGC ACTTCTATGA AGCTTGCCCG TTCCTCAAAT TCGCTCACTT   
  
  
+ CACTGCTAAT CAAGCGATTT TAGAGGCATT CGACGGCCAC GATTGTGTCC ACATCATCGA CTTCGGCCTA   
  
  
+ ATCCACGGCT TGCAATGGCC AGCTCTAATC CAAGCCTTGG CTGTTCGGCA CGGTGGCCCG CCTTTCGTAA   
  
  
+ GATTAACCGG AATCGGGCCG CCTTCTGAAT ACGGGTCGTG CTCGCTTCAA CCTATCGGGT CAACACTGGC   
  
  
+ CCAGTTAGCC CTATCCATGA ATGTTGGGTT CGCATTTCGG GCCGTCGCCG TCTCACGGCT CGAGGACATC   
  
  
+ AAACCATGGA TGGTTAAAAC AAGCCCAAAT GAAGTCGTAG CCGTGAACTC TATCTTCCAA CTCCACCGGC   
  
  
+ TAATCGGGTC GGGTATTGAC CCTGTCCTAA ACTGGGTCCG GAGCCTAAAC CCGAAAATTG TGACACTGGC   
  
  
+ GGAGCAAGAG GCGAACCACA ACCAGCCCGA GTTTTTGGCC CGGTTCACGG AGGCATTACA TTACTATTCA   
  
  
+ ACTATGTTCG ACTCGTTAGA GGCTTGTCAA GTCCAGGCCG ACAAGGACCT AGCCGAGCTA TACTTAGAGA   
  
  
+ GAGAGTTATC CAACATCGTC TGCTGTGAGG GGTCGGCTCG GATTGAGAGG CACGAGCCGC TGGCCCAATG   
  
  
+ GAGGGCCCGA ATGGCTCGGG CCGGGTTCAA GAAGGTGGAT ATGGGTAAAA ATGCGTTCAA GCAAGTAAGC   
  
  
+ ATGTTGCTGA GTTTGTCTTC AGCACAAGGG TATTGTGTGG AGGAGAGTGA GGGATGTTTG AAGCTCGGCT   
  
  
+ GGCATGACCG CCCTCTCATT GCGGCTTCGG CTTGGCGAGC GGAGACTCAA GCTGAGAACT CCAGCACTGT   
  
  
+ TGTGCTTGAT GGGTCATCGT CGTGTAGTTC ATCTTCTTA  

- -Up\_Stream \_Len000TTTTTT CTTTCTTTAC CTCTTTGGTT CGTTATACCT ATTTTTTTTT TGTTTCATTA   
  
  
- TTTCCTTCCT ACTCCGAGGT GTAAGGTGAG CGGGCGCGGT GAGTATCCTC TACTTATCAT TTTACACTCC   
  
  
- CCAATTTACT AAACTTTTTT TAGATCATTC CCGGGTGTTG TCATAACCTT GGGTCGAACG GTTCCTTTAC   
  
  
- AGTCTCCCCG ATCGTTACCT GGTCACGTTC TTCCGGTTCG GTGTAAACCA ATCCCTTAAA GTAGGGTAGG   
  
  
- CCGCGATTTT CGTAGTTTTA ATGATGAATT AGGAACTAGT TTACGGGATA TTTATAAGAT GATGGGGTAA   
  
  
- ACTTTTCCCC ACAACTAATG TTGTGTATCG GCATCCAAAC TGTTTCTCTT AATGGGAATA ACAATCTTCG   
  
  
- TGGTATATGA GAAAAGAGAT TCGAGACTAG AAAAAAGTAG GAAAAGTCGA AAAACAGGAA AATTTAGTTT   
  
  
- AGTGAGTGAA CTCATAACCT CCCTGCAAGG AGTCCTCAGC TTGGTGATTT AATAGAAAAA TTTCTTTTTC   
  
  
- CCGAACTTCG GTTGGGCTAA ATTTTTTTGA CGATTATACA TTCGGATGAG AGAAAGGTCA AGGTTGTTTA   
  
  
- GTTTAAAGTC ATACTTTGTA GGTCATTTCC TAAATAGGCA CTATTACTAC ATTGTATACC AATTTCCCAG   
  
  
- TAAAAGTCAA TGTAGCCTCG TACGTTAATT TCAGGGATAT TTTATCCATC CACGTGCTTG TTCAGTGGTA   
  
  
- GCAGGAAACG AAAAACGTAC TCACGTACGA TTAATACTTG AATTAATTAT AGATTCGTTT TAATTTTTCA   
  
  
- TCAATTTTGA AAAAGTGGAA ACCTTGGAGC ATCGACATCG TCATCGACGG CGTGAGACCA AGATCTGAAG   
  
  
- ATCCCTGGTT CTCTACGTGA GTATACGTAG GAGAAGACAG GAAAATGACA TAGGGGAAGG GTACACTGGA   
  
  
- AAAACGGGCC CACTGAAAAG ACACTCACAA CTATGTTTAA AACATGCATT CGATTGAGTA AATGAAGTGG   
  
  
- CAGAGTACTC AAAATGTAAT GGCTATAATT AAATTTACCG TTTCATAATT TAAACTCGAT TTTAGAGTAT   
  
  
- ATATATAAAC ATAATTTACA CAGACCTAAC AGTTTTATTA AAAACTGTAT ATGTCAGTGA TATATGACTC   
  
  
- AATAATTTTA TATATTTTTA TACTTTTAAA TTTTTTATCA TCAAATTCCA CTGTTGTGAT TTTGTTTGTT   
  
  
- TATTCTATAT TTATCCCTTT TCAAGTAGGA TAAAACAATA ATAATAAATA ATACATAAAA GTCTTAACAG   
  
  
- AGATAGTGTT CGTATCATAT ACACAGTTGT AGCACTGTTG AGCTGGTTAG ATGTTTATTG ATCATTTAAG   
  
  
- TTTATTTCAA TGAGAGTGGA TCGAATTTAT TCATGTGGAA CTAAATTAAC AGAATCGATA CCTTGGATAA   
  
  
- GGTTTGATCG GTTATGAAGG AAAAGTTTTC TTAATCAAAA TTTCAAAATT GAAAATGCAG GTTATTACGG   
  
  
- CCGGCATGAT ACTCAACGAA AGTTCGGTCA TCATCATGAC ATCATCCATT TTTATAATCT TATGTGGATG   
  
  
- ATATATACTA TAACGAGAAG TGGCATAGGA AGACGTATGA GTAGAAAGGC AGGGCCTCAG GTGATAGAGA   
  
  
- GGAGGTAAGT CTGTGAGAGA GCGAGATCTC TTGAGTATCG ATACCTGGCG CACGGGCTGC CAATAGCCCA   
  
  
- ATACTTAAGC CTCGAAGAAG TCGTTCAATC GGGACTCAAC GGCCTAGGTT GGTGGACCAA GGTACGGGCA   
  
  
- GATGGGCTGG GGTAGAGAGT CAGAGGAGAA CCTGGGCCAG GTAGGAGGGT CGGTTGGGTA AAGATGGTCC   
  
  
- CAAGACGGAG TCCCTTGGTA CACCAACCCT AACGACTGTG CACCTGCCTG GTCTAACAAC GTCCAAGGAG   
  
  
- CAGGGTCGTC ACTCCAACGT CGTCTCGGTC GTCCACAACT GACGTCAGCT TCGGTACCTC CTCCTGAGAC   
  
  
- CCTATTCTGA CCAGATGCGA GACGACTGCA CACGCCTCCG GTAGGTTGCA CCGCTAAAGG CTAACCGACA   
  
  
- GAACAATCAA TTGTTCTACT CGTTACTGGA GGACGGTGCG CAGTTGGGCA GGACGCCATA ACCGTTTCAT   
  
  
- CGGCCGATGA AGTATCTACG GAACTGGGCT TCTGATATGG TCGTCCCGGG TCACAGCCCG GATTAGCCCA   
  
  
- GCCAAGACCG CATGGTCCAC AACATGCTCG TGAAGATACT TCGAACGGGC AAGGAGTTTA AGCGAGTGAA   
  
  
- GTGACGATTA GTTCGCTAAA ATCTCCGTAA GCTGCCGGTG CTAACACAGG TGTAGTAGCT GAAGCCGGAT   
  
  
- TAGGTGCCGA ACGTTACCGG TCGAGATTAG GTTCGGAACC GACAAGCCGT GCCACCGGGC GGAAAGCATT   
  
  
- CTAATTGGCC TTAGCCCGGC GGAAGACTTA TGCCCAGCAC GAGCGAAGTT GGATAGCCCA GTTGTGACCG   
  
  
- GGTCAATCGG GATAGGTACT TACAACCCAA GCGTAAAGCC CGGCAGCGGC AGAGTGCCGA GCTCCTGTAG   
  
  
- TTTGGTACCT ACCAATTTTG TTCGGGTTTA CTTCAGCATC GGCACTTGAG ATAGAAGGTT GAGGTGGCCG   
  
  
- ATTAGCCCAG CCCATAACTG GGACAGGATT TGACCCAGGC CTCGGATTTG GGCTTTTAAC ACTGTGACCG   
  
  
- CCTCGTTCTC CGCTTGGTGT TGGTCGGGCT CAAAAACCGG GCCAAGTGCC TCCGTAATGT AATGATAAGT   
  
  
- TGATACAAGC TGAGCAATCT CCGAACAGTT CAGGTCCGGC TGTTCCTGGA TCGGCTCGAT ATGAATCTCT   
  
  
- CTCTCAATAG GTTGTAGCAG ACGACACTCC CCAGCCGAGC CTAACTCTCC GTGCTCGGCG ACCGGGTTAC   
  
  
- CTCCCGGGCT TACCGAGCCC GGCCCAAGTT CTTCCACCTA TACCCATTTT TACGCAAGTT CGTTCATTCG   
  
  
- TACAACGACT CAAACAGAAG TCGTGTTCCC ATAACACACC TCCTCTCACT CCCTACAAAC TTCGAGCCGA   
  
  
- CCGTACTGGC GGGAGAGTAA CGCCGAAGCC GAACCGCTCG CCTCTGAGTT CGACTCTTGA GGTCGTGACA   
  
  
- ACACGAACTA CCCAGTAGCA GCACATCAAG TAGAAGAAT

+     TATA-box

| Site Name | Organism | Position | Strand | Matrix score. | sequence | function |
| --- | --- | --- | --- | --- | --- | --- |
| TATA-box | Arabidopsis thaliana | 1617 | - | 4 | TATA | core promoter element around -30 of transcription start |
| TATA-box | Arabidopsis thaliana | 1615 | - | 6 | TATATA | core promoter element around -30 of transcription start |
| TATA-box | Arabidopsis thaliana | 1127 | + | 4 | TATA | core promoter element around -30 of transcription start |
| TATA-box | Brassica napus | 1126 | + | 6 | ATATAT | core promoter element around -30 of transcription start |
| TATA-box | Arabidopsis thaliana | 1125 | + | 6 | TATATA | core promoter element around -30 of transcription start |
| TATA-box | Brassica napus | 1124 | + | 6 | ATATAT | core promoter element around -30 of transcription start |
| TATA-box | Arabidopsis thaliana | 2863 | - | 4 | TATA | core promoter element around -30 of transcription start |
| TATA-box | Arabidopsis thaliana | 1351 | + | 4 | TATA | core promoter element around -30 of transcription start |
| TATA-box | Arabidopsis thaliana | 1271 | + | 4 | TATA | core promoter element around -30 of transcription start |
| TATA-box | Brassica napus | 1616 | - | 6 | ATATAT | core promoter element around -30 of transcription start |
| TATA-box | Arabidopsis thaliana | 332 | + | 4 | TATA | core promoter element around -30 of transcription start |
| TATA-box | Brassica napus | 1203 | + | 6 | ATATAT | core promoter element around -30 of transcription start |
| TATA-box | Arabidopsis thaliana | 1184 | + | 6 | TATATA | core promoter element around -30 of transcription start |
| TATA-box | Arabidopsis thaliana | 1173 | + | 4 | TATA | core promoter element around -30 of transcription start |
| TATA-box | Arabidopsis thaliana | 2209 | - | 4 | TATA | core promoter element around -30 of transcription start |
| TATA-box | Arabidopsis thaliana | 1186 | + | 4 | TATA | core promoter element around -30 of transcription start |
| TATA-box | Brassica oleracea | 1270 | + | 6 | ATATAA | core promoter element around -30 of transcription start |
| TATA-box | Brassica oleracea | 1205 | + | 6 | ATATAA | core promoter element around -30 of transcription start |
| TATA-box | Arabidopsis thaliana | 1206 | + | 4 | TATA | core promoter element around -30 of transcription start |
| TATA-box | Oryza sativa | 1023 | - | 7 | TACAAAA | core promoter element around -30 of transcription start |
| TATA-box | Arabidopsis thaliana | 739 | + | 9 | ccTATAAAaa | core promoter element around -30 of transcription start |
| TATA-box | Arabidopsis thaliana | 429 | + | 4 | TATA | core promoter element around -30 of transcription start |
| TATA-box | Arabidopsis thaliana | 1123 | + | 6 | TATATA | core promoter element around -30 of transcription start |
| TATA-box | Brassica napus | 1122 | + | 6 | ATATAT | core promoter element around -30 of transcription start |
| TATA-box | Arabidopsis thaliana | 1204 | + | 6 | TATATA | core promoter element around -30 of transcription start |
| TATA-box | Arabidopsis thaliana | 330 | + | 9 | ccTATAAAaa | core promoter element around -30 of transcription start |
| TATA-box | Arabidopsis thaliana | 741 | + | 4 | TATA | core promoter element around -30 of transcription start |

>HU11G00778.1   
+ -Up\_Stream \_Len000AAAAAA GAAAGAAATG GAGAAACCAA GCAATATGGA TAAAAAAAAA ACAAAGTAAT   
  
  
+ AAAGGAAGGA TGAGGCTCCA CATTCCACTC GCCCGCGCCA CTCATAGGAG ATGAATAGTA AAATGTGAGG   
  
  
+ GGTTAAATGA TTTGAAAAAA ATCTAGTAAG GGCCCACAAC AGTATTGGAA CCCAGCTTGC CAAGGAAATG   
  
  
+ TCAGAGGGGC TAGCAATGGA CCAGTGCAAG AAGGCCAAGC CACATTTGGT TAGGGAATTT CATCCCATCC   
  
  
+ GGCGCTAAAA GCATCAAAAT TACTACTTAA TCCTTGATCA AATGCCCTAT AAATATTCTA CTACCCCATT   
  
  
+ TGAAAAGGGG TGTTGATTAC AACACATAGC CGTAGGTTTG ACAAAGAGAA TTACCCTTAT TGTTAGAAGC   
  
  
+ ACCATATACT CTTTTCTCTA AGCTCTGATC TTTTTTCATC CTTTTCAGCT TTTTGTCCTT TTAAATCAAA   
  
  
+ TCACTCACTT GAGTATTGGA GGGACGTTCC TCAGGAGTCG AACCACTAAA TTATCTTTTT AAAGAAAAAG   
  
  
+ GGCTTGAAGC CAACCCGATT TAAAAAAACT GCTAATATGT AAGCCTACTC TCTTTCCAGT TCCAACAAAT   
  
  
+ CAAATTTCAG TATGAAACAT CCAGTAAAGG ATTTATCCGT GATAATGATG TAACATATGG TTAAAGGGTC   
  
  
+ ATTTTCAGTT ACATCGGAGC ATGCAATTAA AGTCCCTATA AAATAGGTAG GTGCACGAAC AAGTCACCAT   
  
  
+ CGTCCTTTGC TTTTTGCATG AGTGCATGCT AATTATGAAC TTAATTAATA TCTAAGCAAA ATTAAAAAGT   
  
  
+ AGTTAAAACT TTTTCACCTT TGGAACCTCG TAGCTGTAGC AGTAGCTGCC GCACTCTGGT TCTAGACTTC   
  
  
+ TAGGGACCAA GAGATGCACT CATATGCATC CTCTTCTGTC CTTTTACTGT ATCCCCTTCC CATGTGACCT   
  
  
+ TTTTGCCCGG GTGACTTTTC TGTGAGTGTT GATACAAATT TTGTACGTAA GCTAACTCAT TTACTTCACC   
  
  
+ GTCTCATGAG TTTTACATTA CCGATATTAA TTTAAATGGC AAAGTATTAA ATTTGAGCTA AAATCTCATA   
  
  
+ TATATATTTG TATTAAATGT GTCTGGATTG TCAAAATAAT TTTTGACATA TACAGTCACT ATATACTGAG   
  
  
+ TTATTAAAAT ATATAAAAAT ATGAAAATTT AAAAAATAGT AGTTTAAGGT GACAACACTA AAACAAACAA   
  
  
+ ATAAGATATA AATAGGGAAA AGTTCATCCT ATTTTGTTAT TATTATTTAT TATGTATTTT CAGAATTGTC   
  
  
+ TCTATCACAA GCATAGTATA TGTGTCAACA TCGTGACAAC TCGACCAATC TACAAATAAC TAGTAAATTC   
  
  
+ AAATAAAGTT ACTCTCACCT AGCTTAAATA AGTACACCTT GATTTAATTG TCTTAGCTAT GGAACCTATT   
  
  
+ CCAAACTAGC CAATACTTCC TTTTCAAAAG AATTAGTTTT AAAGTTTTAA CTTTTACGTC CAATAATGCC   
  
  
+ GGCCGTACTA TGAGTTGCTT TCAAGCCAGT AGTAGTACTG TAGTAGGTAA AAATATTAGA ATACACCTAC   
  
  
+ TATATATGAT ATTGCTCTTC ACCGTATCCT TCTGCATACT CATCTTTCCG TCCCGGAGTC CACTATCTCT   
  
  
+ CCTCCATTCA GACACTCTCT CGCTCTAGAG AACTCATAGC TATGGACCGC GTGCCCGACG GTTATCGGGT   
  
  
+ TATGAATTCG GAGCTTCTTC AGCAAGTTAG CCCTGAGTTG CCGGATCCAA CCACCTGGTT CCATGCCCGT   
  
  
+ CTACCCGACC CCATCTCTCA GTCTCCTCTT GGACCCGGTC CATCCTCCCA GCCAACCCAT TTCTACCAGG   
  
  
+ GTTCTGCCTC AGGGAACCAT GTGGTTGGGA TTGCTGACAC GTGGACGGAC CAGATTGTTG CAGGTTCCTC   
  
  
+ GTCCCAGCAG TGAGGTTGCA GCAGAGCCAG CAGGTGTTGA CTGCAGTCGA AGCCATGGAG GAGGACTCTG   
  
  
+ GGATAAGACT GGTCTACGCT CTGCTGACGT GTGCGGAGGC CATCCAACGT GGCGATTTCC GATTGGCTGT   
  
  
+ CTTGTTAGTT AACAAGATGA GCAATGACCT CCTGCCACGC GTCAACCCGT CCTGCGGTAT TGGCAAAGTA   
  
  
+ GCCGGCTACT TCATAGATGC CTTGACCCGA AGACTATACC AGCAGGGCCC AGTGTCGGGC CTAATCGGGT   
  
  
+ CGGTTCTGGC GTACCAGGTG TTGTACGAGC ACTTCTATGA AGCTTGCCCG TTCCTCAAAT TCGCTCACTT   
  
  
+ CACTGCTAAT CAAGCGATTT TAGAGGCATT CGACGGCCAC GATTGTGTCC ACATCATCGA CTTCGGCCTA   
  
  
+ ATCCACGGCT TGCAATGGCC AGCTCTAATC CAAGCCTTGG CTGTTCGGCA CGGTGGCCCG CCTTTCGTAA   
  
  
+ GATTAACCGG AATCGGGCCG CCTTCTGAAT ACGGGTCGTG CTCGCTTCAA CCTATCGGGT CAACACTGGC   
  
  
+ CCAGTTAGCC CTATCCATGA ATGTTGGGTT CGCATTTCGG GCCGTCGCCG TCTCACGGCT CGAGGACATC   
  
  
+ AAACCATGGA TGGTTAAAAC AAGCCCAAAT GAAGTCGTAG CCGTGAACTC TATCTTCCAA CTCCACCGGC   
  
  
+ TAATCGGGTC GGGTATTGAC CCTGTCCTAA ACTGGGTCCG GAGCCTAAAC CCGAAAATTG TGACACTGGC   
  
  
+ GGAGCAAGAG GCGAACCACA ACCAGCCCGA GTTTTTGGCC CGGTTCACGG AGGCATTACA TTACTATTCA   
  
  
+ ACTATGTTCG ACTCGTTAGA GGCTTGTCAA GTCCAGGCCG ACAAGGACCT AGCCGAGCTA TACTTAGAGA   
  
  
+ GAGAGTTATC CAACATCGTC TGCTGTGAGG GGTCGGCTCG GATTGAGAGG CACGAGCCGC TGGCCCAATG   
  
  
+ GAGGGCCCGA ATGGCTCGGG CCGGGTTCAA GAAGGTGGAT ATGGGTAAAA ATGCGTTCAA GCAAGTAAGC   
  
  
+ ATGTTGCTGA GTTTGTCTTC AGCACAAGGG TATTGTGTGG AGGAGAGTGA GGGATGTTTG AAGCTCGGCT   
  
  
+ GGCATGACCG CCCTCTCATT GCGGCTTCGG CTTGGCGAGC GGAGACTCAA GCTGAGAACT CCAGCACTGT   
  
  
+ TGTGCTTGAT GGGTCATCGT CGTGTAGTTC ATCTTCTTA  

- -Up\_Stream \_Len000TTTTTT CTTTCTTTAC CTCTTTGGTT CGTTATACCT ATTTTTTTTT TGTTTCATTA   
  
  
- TTTCCTTCCT ACTCCGAGGT GTAAGGTGAG CGGGCGCGGT GAGTATCCTC TACTTATCAT TTTACACTCC   
  
  
- CCAATTTACT AAACTTTTTT TAGATCATTC CCGGGTGTTG TCATAACCTT GGGTCGAACG GTTCCTTTAC   
  
  
- AGTCTCCCCG ATCGTTACCT GGTCACGTTC TTCCGGTTCG GTGTAAACCA ATCCCTTAAA GTAGGGTAGG   
  
  
- CCGCGATTTT CGTAGTTTTA ATGATGAATT AGGAACTAGT TTACGGGATA TTTATAAGAT GATGGGGTAA   
  
  
- ACTTTTCCCC ACAACTAATG TTGTGTATCG GCATCCAAAC TGTTTCTCTT AATGGGAATA ACAATCTTCG   
  
  
- TGGTATATGA GAAAAGAGAT TCGAGACTAG AAAAAAGTAG GAAAAGTCGA AAAACAGGAA AATTTAGTTT   
  
  
- AGTGAGTGAA CTCATAACCT CCCTGCAAGG AGTCCTCAGC TTGGTGATTT AATAGAAAAA TTTCTTTTTC   
  
  
- CCGAACTTCG GTTGGGCTAA ATTTTTTTGA CGATTATACA TTCGGATGAG AGAAAGGTCA AGGTTGTTTA   
  
  
- GTTTAAAGTC ATACTTTGTA GGTCATTTCC TAAATAGGCA CTATTACTAC ATTGTATACC AATTTCCCAG   
  
  
- TAAAAGTCAA TGTAGCCTCG TACGTTAATT TCAGGGATAT TTTATCCATC CACGTGCTTG TTCAGTGGTA   
  
  
- GCAGGAAACG AAAAACGTAC TCACGTACGA TTAATACTTG AATTAATTAT AGATTCGTTT TAATTTTTCA   
  
  
- TCAATTTTGA AAAAGTGGAA ACCTTGGAGC ATCGACATCG TCATCGACGG CGTGAGACCA AGATCTGAAG   
  
  
- ATCCCTGGTT CTCTACGTGA GTATACGTAG GAGAAGACAG GAAAATGACA TAGGGGAAGG GTACACTGGA   
  
  
- AAAACGGGCC CACTGAAAAG ACACTCACAA CTATGTTTAA AACATGCATT CGATTGAGTA AATGAAGTGG   
  
  
- CAGAGTACTC AAAATGTAAT GGCTATAATT AAATTTACCG TTTCATAATT TAAACTCGAT TTTAGAGTAT   
  
  
- ATATATAAAC ATAATTTACA CAGACCTAAC AGTTTTATTA AAAACTGTAT ATGTCAGTGA TATATGACTC   
  
  
- AATAATTTTA TATATTTTTA TACTTTTAAA TTTTTTATCA TCAAATTCCA CTGTTGTGAT TTTGTTTGTT   
  
  
- TATTCTATAT TTATCCCTTT TCAAGTAGGA TAAAACAATA ATAATAAATA ATACATAAAA GTCTTAACAG   
  
  
- AGATAGTGTT CGTATCATAT ACACAGTTGT AGCACTGTTG AGCTGGTTAG ATGTTTATTG ATCATTTAAG   
  
  
- TTTATTTCAA TGAGAGTGGA TCGAATTTAT TCATGTGGAA CTAAATTAAC AGAATCGATA CCTTGGATAA   
  
  
- GGTTTGATCG GTTATGAAGG AAAAGTTTTC TTAATCAAAA TTTCAAAATT GAAAATGCAG GTTATTACGG   
  
  
- CCGGCATGAT ACTCAACGAA AGTTCGGTCA TCATCATGAC ATCATCCATT TTTATAATCT TATGTGGATG   
  
  
- ATATATACTA TAACGAGAAG TGGCATAGGA AGACGTATGA GTAGAAAGGC AGGGCCTCAG GTGATAGAGA   
  
  
- GGAGGTAAGT CTGTGAGAGA GCGAGATCTC TTGAGTATCG ATACCTGGCG CACGGGCTGC CAATAGCCCA   
  
  
- ATACTTAAGC CTCGAAGAAG TCGTTCAATC GGGACTCAAC GGCCTAGGTT GGTGGACCAA GGTACGGGCA   
  
  
- GATGGGCTGG GGTAGAGAGT CAGAGGAGAA CCTGGGCCAG GTAGGAGGGT CGGTTGGGTA AAGATGGTCC   
  
  
- CAAGACGGAG TCCCTTGGTA CACCAACCCT AACGACTGTG CACCTGCCTG GTCTAACAAC GTCCAAGGAG   
  
  
- CAGGGTCGTC ACTCCAACGT CGTCTCGGTC GTCCACAACT GACGTCAGCT TCGGTACCTC CTCCTGAGAC   
  
  
- CCTATTCTGA CCAGATGCGA GACGACTGCA CACGCCTCCG GTAGGTTGCA CCGCTAAAGG CTAACCGACA   
  
  
- GAACAATCAA TTGTTCTACT CGTTACTGGA GGACGGTGCG CAGTTGGGCA GGACGCCATA ACCGTTTCAT   
  
  
- CGGCCGATGA AGTATCTACG GAACTGGGCT TCTGATATGG TCGTCCCGGG TCACAGCCCG GATTAGCCCA   
  
  
- GCCAAGACCG CATGGTCCAC AACATGCTCG TGAAGATACT TCGAACGGGC AAGGAGTTTA AGCGAGTGAA   
  
  
- GTGACGATTA GTTCGCTAAA ATCTCCGTAA GCTGCCGGTG CTAACACAGG TGTAGTAGCT GAAGCCGGAT   
  
  
- TAGGTGCCGA ACGTTACCGG TCGAGATTAG GTTCGGAACC GACAAGCCGT GCCACCGGGC GGAAAGCATT   
  
  
- CTAATTGGCC TTAGCCCGGC GGAAGACTTA TGCCCAGCAC GAGCGAAGTT GGATAGCCCA GTTGTGACCG   
  
  
- GGTCAATCGG GATAGGTACT TACAACCCAA GCGTAAAGCC CGGCAGCGGC AGAGTGCCGA GCTCCTGTAG   
  
  
- TTTGGTACCT ACCAATTTTG TTCGGGTTTA CTTCAGCATC GGCACTTGAG ATAGAAGGTT GAGGTGGCCG   
  
  
- ATTAGCCCAG CCCATAACTG GGACAGGATT TGACCCAGGC CTCGGATTTG GGCTTTTAAC ACTGTGACCG   
  
  
- CCTCGTTCTC CGCTTGGTGT TGGTCGGGCT CAAAAACCGG GCCAAGTGCC TCCGTAATGT AATGATAAGT   
  
  
- TGATACAAGC TGAGCAATCT CCGAACAGTT CAGGTCCGGC TGTTCCTGGA TCGGCTCGAT ATGAATCTCT   
  
  
- CTCTCAATAG GTTGTAGCAG ACGACACTCC CCAGCCGAGC CTAACTCTCC GTGCTCGGCG ACCGGGTTAC   
  
  
- CTCCCGGGCT TACCGAGCCC GGCCCAAGTT CTTCCACCTA TACCCATTTT TACGCAAGTT CGTTCATTCG   
  
  
- TACAACGACT CAAACAGAAG TCGTGTTCCC ATAACACACC TCCTCTCACT CCCTACAAAC TTCGAGCCGA   
  
  
- CCGTACTGGC GGGAGAGTAA CGCCGAAGCC GAACCGCTCG CCTCTGAGTT CGACTCTTGA GGTCGTGACA   
  
  
- ACACGAACTA CCCAGTAGCA GCACATCAAG TAGAAGAAT

+     TATC-box

| Site Name | Organism | Position | Strand | Matrix score. | sequence | function |
| --- | --- | --- | --- | --- | --- | --- |
| TATC-box | Oryza sativa | 2033 | - | 7 | TATCCCA | cis-acting element involved in gibberellin-responsiveness |

>HU11G00778.1   
+ -Up\_Stream \_Len000AAAAAA GAAAGAAATG GAGAAACCAA GCAATATGGA TAAAAAAAAA ACAAAGTAAT   
  
  
+ AAAGGAAGGA TGAGGCTCCA CATTCCACTC GCCCGCGCCA CTCATAGGAG ATGAATAGTA AAATGTGAGG   
  
  
+ GGTTAAATGA TTTGAAAAAA ATCTAGTAAG GGCCCACAAC AGTATTGGAA CCCAGCTTGC CAAGGAAATG   
  
  
+ TCAGAGGGGC TAGCAATGGA CCAGTGCAAG AAGGCCAAGC CACATTTGGT TAGGGAATTT CATCCCATCC   
  
  
+ GGCGCTAAAA GCATCAAAAT TACTACTTAA TCCTTGATCA AATGCCCTAT AAATATTCTA CTACCCCATT   
  
  
+ TGAAAAGGGG TGTTGATTAC AACACATAGC CGTAGGTTTG ACAAAGAGAA TTACCCTTAT TGTTAGAAGC   
  
  
+ ACCATATACT CTTTTCTCTA AGCTCTGATC TTTTTTCATC CTTTTCAGCT TTTTGTCCTT TTAAATCAAA   
  
  
+ TCACTCACTT GAGTATTGGA GGGACGTTCC TCAGGAGTCG AACCACTAAA TTATCTTTTT AAAGAAAAAG   
  
  
+ GGCTTGAAGC CAACCCGATT TAAAAAAACT GCTAATATGT AAGCCTACTC TCTTTCCAGT TCCAACAAAT   
  
  
+ CAAATTTCAG TATGAAACAT CCAGTAAAGG ATTTATCCGT GATAATGATG TAACATATGG TTAAAGGGTC   
  
  
+ ATTTTCAGTT ACATCGGAGC ATGCAATTAA AGTCCCTATA AAATAGGTAG GTGCACGAAC AAGTCACCAT   
  
  
+ CGTCCTTTGC TTTTTGCATG AGTGCATGCT AATTATGAAC TTAATTAATA TCTAAGCAAA ATTAAAAAGT   
  
  
+ AGTTAAAACT TTTTCACCTT TGGAACCTCG TAGCTGTAGC AGTAGCTGCC GCACTCTGGT TCTAGACTTC   
  
  
+ TAGGGACCAA GAGATGCACT CATATGCATC CTCTTCTGTC CTTTTACTGT ATCCCCTTCC CATGTGACCT   
  
  
+ TTTTGCCCGG GTGACTTTTC TGTGAGTGTT GATACAAATT TTGTACGTAA GCTAACTCAT TTACTTCACC   
  
  
+ GTCTCATGAG TTTTACATTA CCGATATTAA TTTAAATGGC AAAGTATTAA ATTTGAGCTA AAATCTCATA   
  
  
+ TATATATTTG TATTAAATGT GTCTGGATTG TCAAAATAAT TTTTGACATA TACAGTCACT ATATACTGAG   
  
  
+ TTATTAAAAT ATATAAAAAT ATGAAAATTT AAAAAATAGT AGTTTAAGGT GACAACACTA AAACAAACAA   
  
  
+ ATAAGATATA AATAGGGAAA AGTTCATCCT ATTTTGTTAT TATTATTTAT TATGTATTTT CAGAATTGTC   
  
  
+ TCTATCACAA GCATAGTATA TGTGTCAACA TCGTGACAAC TCGACCAATC TACAAATAAC TAGTAAATTC   
  
  
+ AAATAAAGTT ACTCTCACCT AGCTTAAATA AGTACACCTT GATTTAATTG TCTTAGCTAT GGAACCTATT   
  
  
+ CCAAACTAGC CAATACTTCC TTTTCAAAAG AATTAGTTTT AAAGTTTTAA CTTTTACGTC CAATAATGCC   
  
  
+ GGCCGTACTA TGAGTTGCTT TCAAGCCAGT AGTAGTACTG TAGTAGGTAA AAATATTAGA ATACACCTAC   
  
  
+ TATATATGAT ATTGCTCTTC ACCGTATCCT TCTGCATACT CATCTTTCCG TCCCGGAGTC CACTATCTCT   
  
  
+ CCTCCATTCA GACACTCTCT CGCTCTAGAG AACTCATAGC TATGGACCGC GTGCCCGACG GTTATCGGGT   
  
  
+ TATGAATTCG GAGCTTCTTC AGCAAGTTAG CCCTGAGTTG CCGGATCCAA CCACCTGGTT CCATGCCCGT   
  
  
+ CTACCCGACC CCATCTCTCA GTCTCCTCTT GGACCCGGTC CATCCTCCCA GCCAACCCAT TTCTACCAGG   
  
  
+ GTTCTGCCTC AGGGAACCAT GTGGTTGGGA TTGCTGACAC GTGGACGGAC CAGATTGTTG CAGGTTCCTC   
  
  
+ GTCCCAGCAG TGAGGTTGCA GCAGAGCCAG CAGGTGTTGA CTGCAGTCGA AGCCATGGAG GAGGACTCTG   
  
  
+ GGATAAGACT GGTCTACGCT CTGCTGACGT GTGCGGAGGC CATCCAACGT GGCGATTTCC GATTGGCTGT   
  
  
+ CTTGTTAGTT AACAAGATGA GCAATGACCT CCTGCCACGC GTCAACCCGT CCTGCGGTAT TGGCAAAGTA   
  
  
+ GCCGGCTACT TCATAGATGC CTTGACCCGA AGACTATACC AGCAGGGCCC AGTGTCGGGC CTAATCGGGT   
  
  
+ CGGTTCTGGC GTACCAGGTG TTGTACGAGC ACTTCTATGA AGCTTGCCCG TTCCTCAAAT TCGCTCACTT   
  
  
+ CACTGCTAAT CAAGCGATTT TAGAGGCATT CGACGGCCAC GATTGTGTCC ACATCATCGA CTTCGGCCTA   
  
  
+ ATCCACGGCT TGCAATGGCC AGCTCTAATC CAAGCCTTGG CTGTTCGGCA CGGTGGCCCG CCTTTCGTAA   
  
  
+ GATTAACCGG AATCGGGCCG CCTTCTGAAT ACGGGTCGTG CTCGCTTCAA CCTATCGGGT CAACACTGGC   
  
  
+ CCAGTTAGCC CTATCCATGA ATGTTGGGTT CGCATTTCGG GCCGTCGCCG TCTCACGGCT CGAGGACATC   
  
  
+ AAACCATGGA TGGTTAAAAC AAGCCCAAAT GAAGTCGTAG CCGTGAACTC TATCTTCCAA CTCCACCGGC   
  
  
+ TAATCGGGTC GGGTATTGAC CCTGTCCTAA ACTGGGTCCG GAGCCTAAAC CCGAAAATTG TGACACTGGC   
  
  
+ GGAGCAAGAG GCGAACCACA ACCAGCCCGA GTTTTTGGCC CGGTTCACGG AGGCATTACA TTACTATTCA   
  
  
+ ACTATGTTCG ACTCGTTAGA GGCTTGTCAA GTCCAGGCCG ACAAGGACCT AGCCGAGCTA TACTTAGAGA   
  
  
+ GAGAGTTATC CAACATCGTC TGCTGTGAGG GGTCGGCTCG GATTGAGAGG CACGAGCCGC TGGCCCAATG   
  
  
+ GAGGGCCCGA ATGGCTCGGG CCGGGTTCAA GAAGGTGGAT ATGGGTAAAA ATGCGTTCAA GCAAGTAAGC   
  
  
+ ATGTTGCTGA GTTTGTCTTC AGCACAAGGG TATTGTGTGG AGGAGAGTGA GGGATGTTTG AAGCTCGGCT   
  
  
+ GGCATGACCG CCCTCTCATT GCGGCTTCGG CTTGGCGAGC GGAGACTCAA GCTGAGAACT CCAGCACTGT   
  
  
+ TGTGCTTGAT GGGTCATCGT CGTGTAGTTC ATCTTCTTA  

- -Up\_Stream \_Len000TTTTTT CTTTCTTTAC CTCTTTGGTT CGTTATACCT ATTTTTTTTT TGTTTCATTA   
  
  
- TTTCCTTCCT ACTCCGAGGT GTAAGGTGAG CGGGCGCGGT GAGTATCCTC TACTTATCAT TTTACACTCC   
  
  
- CCAATTTACT AAACTTTTTT TAGATCATTC CCGGGTGTTG TCATAACCTT GGGTCGAACG GTTCCTTTAC   
  
  
- AGTCTCCCCG ATCGTTACCT GGTCACGTTC TTCCGGTTCG GTGTAAACCA ATCCCTTAAA GTAGGGTAGG   
  
  
- CCGCGATTTT CGTAGTTTTA ATGATGAATT AGGAACTAGT TTACGGGATA TTTATAAGAT GATGGGGTAA   
  
  
- ACTTTTCCCC ACAACTAATG TTGTGTATCG GCATCCAAAC TGTTTCTCTT AATGGGAATA ACAATCTTCG   
  
  
- TGGTATATGA GAAAAGAGAT TCGAGACTAG AAAAAAGTAG GAAAAGTCGA AAAACAGGAA AATTTAGTTT   
  
  
- AGTGAGTGAA CTCATAACCT CCCTGCAAGG AGTCCTCAGC TTGGTGATTT AATAGAAAAA TTTCTTTTTC   
  
  
- CCGAACTTCG GTTGGGCTAA ATTTTTTTGA CGATTATACA TTCGGATGAG AGAAAGGTCA AGGTTGTTTA   
  
  
- GTTTAAAGTC ATACTTTGTA GGTCATTTCC TAAATAGGCA CTATTACTAC ATTGTATACC AATTTCCCAG   
  
  
- TAAAAGTCAA TGTAGCCTCG TACGTTAATT TCAGGGATAT TTTATCCATC CACGTGCTTG TTCAGTGGTA   
  
  
- GCAGGAAACG AAAAACGTAC TCACGTACGA TTAATACTTG AATTAATTAT AGATTCGTTT TAATTTTTCA   
  
  
- TCAATTTTGA AAAAGTGGAA ACCTTGGAGC ATCGACATCG TCATCGACGG CGTGAGACCA AGATCTGAAG   
  
  
- ATCCCTGGTT CTCTACGTGA GTATACGTAG GAGAAGACAG GAAAATGACA TAGGGGAAGG GTACACTGGA   
  
  
- AAAACGGGCC CACTGAAAAG ACACTCACAA CTATGTTTAA AACATGCATT CGATTGAGTA AATGAAGTGG   
  
  
- CAGAGTACTC AAAATGTAAT GGCTATAATT AAATTTACCG TTTCATAATT TAAACTCGAT TTTAGAGTAT   
  
  
- ATATATAAAC ATAATTTACA CAGACCTAAC AGTTTTATTA AAAACTGTAT ATGTCAGTGA TATATGACTC   
  
  
- AATAATTTTA TATATTTTTA TACTTTTAAA TTTTTTATCA TCAAATTCCA CTGTTGTGAT TTTGTTTGTT   
  
  
- TATTCTATAT TTATCCCTTT TCAAGTAGGA TAAAACAATA ATAATAAATA ATACATAAAA GTCTTAACAG   
  
  
- AGATAGTGTT CGTATCATAT ACACAGTTGT AGCACTGTTG AGCTGGTTAG ATGTTTATTG ATCATTTAAG   
  
  
- TTTATTTCAA TGAGAGTGGA TCGAATTTAT TCATGTGGAA CTAAATTAAC AGAATCGATA CCTTGGATAA   
  
  
- GGTTTGATCG GTTATGAAGG AAAAGTTTTC TTAATCAAAA TTTCAAAATT GAAAATGCAG GTTATTACGG   
  
  
- CCGGCATGAT ACTCAACGAA AGTTCGGTCA TCATCATGAC ATCATCCATT TTTATAATCT TATGTGGATG   
  
  
- ATATATACTA TAACGAGAAG TGGCATAGGA AGACGTATGA GTAGAAAGGC AGGGCCTCAG GTGATAGAGA   
  
  
- GGAGGTAAGT CTGTGAGAGA GCGAGATCTC TTGAGTATCG ATACCTGGCG CACGGGCTGC CAATAGCCCA   
  
  
- ATACTTAAGC CTCGAAGAAG TCGTTCAATC GGGACTCAAC GGCCTAGGTT GGTGGACCAA GGTACGGGCA   
  
  
- GATGGGCTGG GGTAGAGAGT CAGAGGAGAA CCTGGGCCAG GTAGGAGGGT CGGTTGGGTA AAGATGGTCC   
  
  
- CAAGACGGAG TCCCTTGGTA CACCAACCCT AACGACTGTG CACCTGCCTG GTCTAACAAC GTCCAAGGAG   
  
  
- CAGGGTCGTC ACTCCAACGT CGTCTCGGTC GTCCACAACT GACGTCAGCT TCGGTACCTC CTCCTGAGAC   
  
  
- CCTATTCTGA CCAGATGCGA GACGACTGCA CACGCCTCCG GTAGGTTGCA CCGCTAAAGG CTAACCGACA   
  
  
- GAACAATCAA TTGTTCTACT CGTTACTGGA GGACGGTGCG CAGTTGGGCA GGACGCCATA ACCGTTTCAT   
  
  
- CGGCCGATGA AGTATCTACG GAACTGGGCT TCTGATATGG TCGTCCCGGG TCACAGCCCG GATTAGCCCA   
  
  
- GCCAAGACCG CATGGTCCAC AACATGCTCG TGAAGATACT TCGAACGGGC AAGGAGTTTA AGCGAGTGAA   
  
  
- GTGACGATTA GTTCGCTAAA ATCTCCGTAA GCTGCCGGTG CTAACACAGG TGTAGTAGCT GAAGCCGGAT   
  
  
- TAGGTGCCGA ACGTTACCGG TCGAGATTAG GTTCGGAACC GACAAGCCGT GCCACCGGGC GGAAAGCATT   
  
  
- CTAATTGGCC TTAGCCCGGC GGAAGACTTA TGCCCAGCAC GAGCGAAGTT GGATAGCCCA GTTGTGACCG   
  
  
- GGTCAATCGG GATAGGTACT TACAACCCAA GCGTAAAGCC CGGCAGCGGC AGAGTGCCGA GCTCCTGTAG   
  
  
- TTTGGTACCT ACCAATTTTG TTCGGGTTTA CTTCAGCATC GGCACTTGAG ATAGAAGGTT GAGGTGGCCG   
  
  
- ATTAGCCCAG CCCATAACTG GGACAGGATT TGACCCAGGC CTCGGATTTG GGCTTTTAAC ACTGTGACCG   
  
  
- CCTCGTTCTC CGCTTGGTGT TGGTCGGGCT CAAAAACCGG GCCAAGTGCC TCCGTAATGT AATGATAAGT   
  
  
- TGATACAAGC TGAGCAATCT CCGAACAGTT CAGGTCCGGC TGTTCCTGGA TCGGCTCGAT ATGAATCTCT   
  
  
- CTCTCAATAG GTTGTAGCAG ACGACACTCC CCAGCCGAGC CTAACTCTCC GTGCTCGGCG ACCGGGTTAC   
  
  
- CTCCCGGGCT TACCGAGCCC GGCCCAAGTT CTTCCACCTA TACCCATTTT TACGCAAGTT CGTTCATTCG   
  
  
- TACAACGACT CAAACAGAAG TCGTGTTCCC ATAACACACC TCCTCTCACT CCCTACAAAC TTCGAGCCGA   
  
  
- CCGTACTGGC GGGAGAGTAA CGCCGAAGCC GAACCGCTCG CCTCTGAGTT CGACTCTTGA GGTCGTGACA   
  
  
- ACACGAACTA CCCAGTAGCA GCACATCAAG TAGAAGAAT

+     TC-rich repeats

| Site Name | Organism | Position | Strand | Matrix score. | sequence | function |
| --- | --- | --- | --- | --- | --- | --- |
| TC-rich repeats | Nicotiana tabacum | 263 | - | 9 | ATTCTCTAAC | cis-acting element involved in defense and stress responsiveness |

>HU11G00778.1   
+ -Up\_Stream \_Len000AAAAAA GAAAGAAATG GAGAAACCAA GCAATATGGA TAAAAAAAAA ACAAAGTAAT   
  
  
+ AAAGGAAGGA TGAGGCTCCA CATTCCACTC GCCCGCGCCA CTCATAGGAG ATGAATAGTA AAATGTGAGG   
  
  
+ GGTTAAATGA TTTGAAAAAA ATCTAGTAAG GGCCCACAAC AGTATTGGAA CCCAGCTTGC CAAGGAAATG   
  
  
+ TCAGAGGGGC TAGCAATGGA CCAGTGCAAG AAGGCCAAGC CACATTTGGT TAGGGAATTT CATCCCATCC   
  
  
+ GGCGCTAAAA GCATCAAAAT TACTACTTAA TCCTTGATCA AATGCCCTAT AAATATTCTA CTACCCCATT   
  
  
+ TGAAAAGGGG TGTTGATTAC AACACATAGC CGTAGGTTTG ACAAAGAGAA TTACCCTTAT TGTTAGAAGC   
  
  
+ ACCATATACT CTTTTCTCTA AGCTCTGATC TTTTTTCATC CTTTTCAGCT TTTTGTCCTT TTAAATCAAA   
  
  
+ TCACTCACTT GAGTATTGGA GGGACGTTCC TCAGGAGTCG AACCACTAAA TTATCTTTTT AAAGAAAAAG   
  
  
+ GGCTTGAAGC CAACCCGATT TAAAAAAACT GCTAATATGT AAGCCTACTC TCTTTCCAGT TCCAACAAAT   
  
  
+ CAAATTTCAG TATGAAACAT CCAGTAAAGG ATTTATCCGT GATAATGATG TAACATATGG TTAAAGGGTC   
  
  
+ ATTTTCAGTT ACATCGGAGC ATGCAATTAA AGTCCCTATA AAATAGGTAG GTGCACGAAC AAGTCACCAT   
  
  
+ CGTCCTTTGC TTTTTGCATG AGTGCATGCT AATTATGAAC TTAATTAATA TCTAAGCAAA ATTAAAAAGT   
  
  
+ AGTTAAAACT TTTTCACCTT TGGAACCTCG TAGCTGTAGC AGTAGCTGCC GCACTCTGGT TCTAGACTTC   
  
  
+ TAGGGACCAA GAGATGCACT CATATGCATC CTCTTCTGTC CTTTTACTGT ATCCCCTTCC CATGTGACCT   
  
  
+ TTTTGCCCGG GTGACTTTTC TGTGAGTGTT GATACAAATT TTGTACGTAA GCTAACTCAT TTACTTCACC   
  
  
+ GTCTCATGAG TTTTACATTA CCGATATTAA TTTAAATGGC AAAGTATTAA ATTTGAGCTA AAATCTCATA   
  
  
+ TATATATTTG TATTAAATGT GTCTGGATTG TCAAAATAAT TTTTGACATA TACAGTCACT ATATACTGAG   
  
  
+ TTATTAAAAT ATATAAAAAT ATGAAAATTT AAAAAATAGT AGTTTAAGGT GACAACACTA AAACAAACAA   
  
  
+ ATAAGATATA AATAGGGAAA AGTTCATCCT ATTTTGTTAT TATTATTTAT TATGTATTTT CAGAATTGTC   
  
  
+ TCTATCACAA GCATAGTATA TGTGTCAACA TCGTGACAAC TCGACCAATC TACAAATAAC TAGTAAATTC   
  
  
+ AAATAAAGTT ACTCTCACCT AGCTTAAATA AGTACACCTT GATTTAATTG TCTTAGCTAT GGAACCTATT   
  
  
+ CCAAACTAGC CAATACTTCC TTTTCAAAAG AATTAGTTTT AAAGTTTTAA CTTTTACGTC CAATAATGCC   
  
  
+ GGCCGTACTA TGAGTTGCTT TCAAGCCAGT AGTAGTACTG TAGTAGGTAA AAATATTAGA ATACACCTAC   
  
  
+ TATATATGAT ATTGCTCTTC ACCGTATCCT TCTGCATACT CATCTTTCCG TCCCGGAGTC CACTATCTCT   
  
  
+ CCTCCATTCA GACACTCTCT CGCTCTAGAG AACTCATAGC TATGGACCGC GTGCCCGACG GTTATCGGGT   
  
  
+ TATGAATTCG GAGCTTCTTC AGCAAGTTAG CCCTGAGTTG CCGGATCCAA CCACCTGGTT CCATGCCCGT   
  
  
+ CTACCCGACC CCATCTCTCA GTCTCCTCTT GGACCCGGTC CATCCTCCCA GCCAACCCAT TTCTACCAGG   
  
  
+ GTTCTGCCTC AGGGAACCAT GTGGTTGGGA TTGCTGACAC GTGGACGGAC CAGATTGTTG CAGGTTCCTC   
  
  
+ GTCCCAGCAG TGAGGTTGCA GCAGAGCCAG CAGGTGTTGA CTGCAGTCGA AGCCATGGAG GAGGACTCTG   
  
  
+ GGATAAGACT GGTCTACGCT CTGCTGACGT GTGCGGAGGC CATCCAACGT GGCGATTTCC GATTGGCTGT   
  
  
+ CTTGTTAGTT AACAAGATGA GCAATGACCT CCTGCCACGC GTCAACCCGT CCTGCGGTAT TGGCAAAGTA   
  
  
+ GCCGGCTACT TCATAGATGC CTTGACCCGA AGACTATACC AGCAGGGCCC AGTGTCGGGC CTAATCGGGT   
  
  
+ CGGTTCTGGC GTACCAGGTG TTGTACGAGC ACTTCTATGA AGCTTGCCCG TTCCTCAAAT TCGCTCACTT   
  
  
+ CACTGCTAAT CAAGCGATTT TAGAGGCATT CGACGGCCAC GATTGTGTCC ACATCATCGA CTTCGGCCTA   
  
  
+ ATCCACGGCT TGCAATGGCC AGCTCTAATC CAAGCCTTGG CTGTTCGGCA CGGTGGCCCG CCTTTCGTAA   
  
  
+ GATTAACCGG AATCGGGCCG CCTTCTGAAT ACGGGTCGTG CTCGCTTCAA CCTATCGGGT CAACACTGGC   
  
  
+ CCAGTTAGCC CTATCCATGA ATGTTGGGTT CGCATTTCGG GCCGTCGCCG TCTCACGGCT CGAGGACATC   
  
  
+ AAACCATGGA TGGTTAAAAC AAGCCCAAAT GAAGTCGTAG CCGTGAACTC TATCTTCCAA CTCCACCGGC   
  
  
+ TAATCGGGTC GGGTATTGAC CCTGTCCTAA ACTGGGTCCG GAGCCTAAAC CCGAAAATTG TGACACTGGC   
  
  
+ GGAGCAAGAG GCGAACCACA ACCAGCCCGA GTTTTTGGCC CGGTTCACGG AGGCATTACA TTACTATTCA   
  
  
+ ACTATGTTCG ACTCGTTAGA GGCTTGTCAA GTCCAGGCCG ACAAGGACCT AGCCGAGCTA TACTTAGAGA   
  
  
+ GAGAGTTATC CAACATCGTC TGCTGTGAGG GGTCGGCTCG GATTGAGAGG CACGAGCCGC TGGCCCAATG   
  
  
+ GAGGGCCCGA ATGGCTCGGG CCGGGTTCAA GAAGGTGGAT ATGGGTAAAA ATGCGTTCAA GCAAGTAAGC   
  
  
+ ATGTTGCTGA GTTTGTCTTC AGCACAAGGG TATTGTGTGG AGGAGAGTGA GGGATGTTTG AAGCTCGGCT   
  
  
+ GGCATGACCG CCCTCTCATT GCGGCTTCGG CTTGGCGAGC GGAGACTCAA GCTGAGAACT CCAGCACTGT   
  
  
+ TGTGCTTGAT GGGTCATCGT CGTGTAGTTC ATCTTCTTA  

- -Up\_Stream \_Len000TTTTTT CTTTCTTTAC CTCTTTGGTT CGTTATACCT ATTTTTTTTT TGTTTCATTA   
  
  
- TTTCCTTCCT ACTCCGAGGT GTAAGGTGAG CGGGCGCGGT GAGTATCCTC TACTTATCAT TTTACACTCC   
  
  
- CCAATTTACT AAACTTTTTT TAGATCATTC CCGGGTGTTG TCATAACCTT GGGTCGAACG GTTCCTTTAC   
  
  
- AGTCTCCCCG ATCGTTACCT GGTCACGTTC TTCCGGTTCG GTGTAAACCA ATCCCTTAAA GTAGGGTAGG   
  
  
- CCGCGATTTT CGTAGTTTTA ATGATGAATT AGGAACTAGT TTACGGGATA TTTATAAGAT GATGGGGTAA   
  
  
- ACTTTTCCCC ACAACTAATG TTGTGTATCG GCATCCAAAC TGTTTCTCTT AATGGGAATA ACAATCTTCG   
  
  
- TGGTATATGA GAAAAGAGAT TCGAGACTAG AAAAAAGTAG GAAAAGTCGA AAAACAGGAA AATTTAGTTT   
  
  
- AGTGAGTGAA CTCATAACCT CCCTGCAAGG AGTCCTCAGC TTGGTGATTT AATAGAAAAA TTTCTTTTTC   
  
  
- CCGAACTTCG GTTGGGCTAA ATTTTTTTGA CGATTATACA TTCGGATGAG AGAAAGGTCA AGGTTGTTTA   
  
  
- GTTTAAAGTC ATACTTTGTA GGTCATTTCC TAAATAGGCA CTATTACTAC ATTGTATACC AATTTCCCAG   
  
  
- TAAAAGTCAA TGTAGCCTCG TACGTTAATT TCAGGGATAT TTTATCCATC CACGTGCTTG TTCAGTGGTA   
  
  
- GCAGGAAACG AAAAACGTAC TCACGTACGA TTAATACTTG AATTAATTAT AGATTCGTTT TAATTTTTCA   
  
  
- TCAATTTTGA AAAAGTGGAA ACCTTGGAGC ATCGACATCG TCATCGACGG CGTGAGACCA AGATCTGAAG   
  
  
- ATCCCTGGTT CTCTACGTGA GTATACGTAG GAGAAGACAG GAAAATGACA TAGGGGAAGG GTACACTGGA   
  
  
- AAAACGGGCC CACTGAAAAG ACACTCACAA CTATGTTTAA AACATGCATT CGATTGAGTA AATGAAGTGG   
  
  
- CAGAGTACTC AAAATGTAAT GGCTATAATT AAATTTACCG TTTCATAATT TAAACTCGAT TTTAGAGTAT   
  
  
- ATATATAAAC ATAATTTACA CAGACCTAAC AGTTTTATTA AAAACTGTAT ATGTCAGTGA TATATGACTC   
  
  
- AATAATTTTA TATATTTTTA TACTTTTAAA TTTTTTATCA TCAAATTCCA CTGTTGTGAT TTTGTTTGTT   
  
  
- TATTCTATAT TTATCCCTTT TCAAGTAGGA TAAAACAATA ATAATAAATA ATACATAAAA GTCTTAACAG   
  
  
- AGATAGTGTT CGTATCATAT ACACAGTTGT AGCACTGTTG AGCTGGTTAG ATGTTTATTG ATCATTTAAG   
  
  
- TTTATTTCAA TGAGAGTGGA TCGAATTTAT TCATGTGGAA CTAAATTAAC AGAATCGATA CCTTGGATAA   
  
  
- GGTTTGATCG GTTATGAAGG AAAAGTTTTC TTAATCAAAA TTTCAAAATT GAAAATGCAG GTTATTACGG   
  
  
- CCGGCATGAT ACTCAACGAA AGTTCGGTCA TCATCATGAC ATCATCCATT TTTATAATCT TATGTGGATG   
  
  
- ATATATACTA TAACGAGAAG TGGCATAGGA AGACGTATGA GTAGAAAGGC AGGGCCTCAG GTGATAGAGA   
  
  
- GGAGGTAAGT CTGTGAGAGA GCGAGATCTC TTGAGTATCG ATACCTGGCG CACGGGCTGC CAATAGCCCA   
  
  
- ATACTTAAGC CTCGAAGAAG TCGTTCAATC GGGACTCAAC GGCCTAGGTT GGTGGACCAA GGTACGGGCA   
  
  
- GATGGGCTGG GGTAGAGAGT CAGAGGAGAA CCTGGGCCAG GTAGGAGGGT CGGTTGGGTA AAGATGGTCC   
  
  
- CAAGACGGAG TCCCTTGGTA CACCAACCCT AACGACTGTG CACCTGCCTG GTCTAACAAC GTCCAAGGAG   
  
  
- CAGGGTCGTC ACTCCAACGT CGTCTCGGTC GTCCACAACT GACGTCAGCT TCGGTACCTC CTCCTGAGAC   
  
  
- CCTATTCTGA CCAGATGCGA GACGACTGCA CACGCCTCCG GTAGGTTGCA CCGCTAAAGG CTAACCGACA   
  
  
- GAACAATCAA TTGTTCTACT CGTTACTGGA GGACGGTGCG CAGTTGGGCA GGACGCCATA ACCGTTTCAT   
  
  
- CGGCCGATGA AGTATCTACG GAACTGGGCT TCTGATATGG TCGTCCCGGG TCACAGCCCG GATTAGCCCA   
  
  
- GCCAAGACCG CATGGTCCAC AACATGCTCG TGAAGATACT TCGAACGGGC AAGGAGTTTA AGCGAGTGAA   
  
  
- GTGACGATTA GTTCGCTAAA ATCTCCGTAA GCTGCCGGTG CTAACACAGG TGTAGTAGCT GAAGCCGGAT   
  
  
- TAGGTGCCGA ACGTTACCGG TCGAGATTAG GTTCGGAACC GACAAGCCGT GCCACCGGGC GGAAAGCATT   
  
  
- CTAATTGGCC TTAGCCCGGC GGAAGACTTA TGCCCAGCAC GAGCGAAGTT GGATAGCCCA GTTGTGACCG   
  
  
- GGTCAATCGG GATAGGTACT TACAACCCAA GCGTAAAGCC CGGCAGCGGC AGAGTGCCGA GCTCCTGTAG   
  
  
- TTTGGTACCT ACCAATTTTG TTCGGGTTTA CTTCAGCATC GGCACTTGAG ATAGAAGGTT GAGGTGGCCG   
  
  
- ATTAGCCCAG CCCATAACTG GGACAGGATT TGACCCAGGC CTCGGATTTG GGCTTTTAAC ACTGTGACCG   
  
  
- CCTCGTTCTC CGCTTGGTGT TGGTCGGGCT CAAAAACCGG GCCAAGTGCC TCCGTAATGT AATGATAAGT   
  
  
- TGATACAAGC TGAGCAATCT CCGAACAGTT CAGGTCCGGC TGTTCCTGGA TCGGCTCGAT ATGAATCTCT   
  
  
- CTCTCAATAG GTTGTAGCAG ACGACACTCC CCAGCCGAGC CTAACTCTCC GTGCTCGGCG ACCGGGTTAC   
  
  
- CTCCCGGGCT TACCGAGCCC GGCCCAAGTT CTTCCACCTA TACCCATTTT TACGCAAGTT CGTTCATTCG   
  
  
- TACAACGACT CAAACAGAAG TCGTGTTCCC ATAACACACC TCCTCTCACT CCCTACAAAC TTCGAGCCGA   
  
  
- CCGTACTGGC GGGAGAGTAA CGCCGAAGCC GAACCGCTCG CCTCTGAGTT CGACTCTTGA GGTCGTGACA   
  
  
- ACACGAACTA CCCAGTAGCA GCACATCAAG TAGAAGAAT

+     TCA

| Site Name | Organism | Position | Strand | Matrix score. | sequence | function |
| --- | --- | --- | --- | --- | --- | --- |
| TCA | Pisum sativum | 3183 | + | 9 | TCATCTTCAT |  |

>HU11G00778.1   
+ -Up\_Stream \_Len000AAAAAA GAAAGAAATG GAGAAACCAA GCAATATGGA TAAAAAAAAA ACAAAGTAAT   
  
  
+ AAAGGAAGGA TGAGGCTCCA CATTCCACTC GCCCGCGCCA CTCATAGGAG ATGAATAGTA AAATGTGAGG   
  
  
+ GGTTAAATGA TTTGAAAAAA ATCTAGTAAG GGCCCACAAC AGTATTGGAA CCCAGCTTGC CAAGGAAATG   
  
  
+ TCAGAGGGGC TAGCAATGGA CCAGTGCAAG AAGGCCAAGC CACATTTGGT TAGGGAATTT CATCCCATCC   
  
  
+ GGCGCTAAAA GCATCAAAAT TACTACTTAA TCCTTGATCA AATGCCCTAT AAATATTCTA CTACCCCATT   
  
  
+ TGAAAAGGGG TGTTGATTAC AACACATAGC CGTAGGTTTG ACAAAGAGAA TTACCCTTAT TGTTAGAAGC   
  
  
+ ACCATATACT CTTTTCTCTA AGCTCTGATC TTTTTTCATC CTTTTCAGCT TTTTGTCCTT TTAAATCAAA   
  
  
+ TCACTCACTT GAGTATTGGA GGGACGTTCC TCAGGAGTCG AACCACTAAA TTATCTTTTT AAAGAAAAAG   
  
  
+ GGCTTGAAGC CAACCCGATT TAAAAAAACT GCTAATATGT AAGCCTACTC TCTTTCCAGT TCCAACAAAT   
  
  
+ CAAATTTCAG TATGAAACAT CCAGTAAAGG ATTTATCCGT GATAATGATG TAACATATGG TTAAAGGGTC   
  
  
+ ATTTTCAGTT ACATCGGAGC ATGCAATTAA AGTCCCTATA AAATAGGTAG GTGCACGAAC AAGTCACCAT   
  
  
+ CGTCCTTTGC TTTTTGCATG AGTGCATGCT AATTATGAAC TTAATTAATA TCTAAGCAAA ATTAAAAAGT   
  
  
+ AGTTAAAACT TTTTCACCTT TGGAACCTCG TAGCTGTAGC AGTAGCTGCC GCACTCTGGT TCTAGACTTC   
  
  
+ TAGGGACCAA GAGATGCACT CATATGCATC CTCTTCTGTC CTTTTACTGT ATCCCCTTCC CATGTGACCT   
  
  
+ TTTTGCCCGG GTGACTTTTC TGTGAGTGTT GATACAAATT TTGTACGTAA GCTAACTCAT TTACTTCACC   
  
  
+ GTCTCATGAG TTTTACATTA CCGATATTAA TTTAAATGGC AAAGTATTAA ATTTGAGCTA AAATCTCATA   
  
  
+ TATATATTTG TATTAAATGT GTCTGGATTG TCAAAATAAT TTTTGACATA TACAGTCACT ATATACTGAG   
  
  
+ TTATTAAAAT ATATAAAAAT ATGAAAATTT AAAAAATAGT AGTTTAAGGT GACAACACTA AAACAAACAA   
  
  
+ ATAAGATATA AATAGGGAAA AGTTCATCCT ATTTTGTTAT TATTATTTAT TATGTATTTT CAGAATTGTC   
  
  
+ TCTATCACAA GCATAGTATA TGTGTCAACA TCGTGACAAC TCGACCAATC TACAAATAAC TAGTAAATTC   
  
  
+ AAATAAAGTT ACTCTCACCT AGCTTAAATA AGTACACCTT GATTTAATTG TCTTAGCTAT GGAACCTATT   
  
  
+ CCAAACTAGC CAATACTTCC TTTTCAAAAG AATTAGTTTT AAAGTTTTAA CTTTTACGTC CAATAATGCC   
  
  
+ GGCCGTACTA TGAGTTGCTT TCAAGCCAGT AGTAGTACTG TAGTAGGTAA AAATATTAGA ATACACCTAC   
  
  
+ TATATATGAT ATTGCTCTTC ACCGTATCCT TCTGCATACT CATCTTTCCG TCCCGGAGTC CACTATCTCT   
  
  
+ CCTCCATTCA GACACTCTCT CGCTCTAGAG AACTCATAGC TATGGACCGC GTGCCCGACG GTTATCGGGT   
  
  
+ TATGAATTCG GAGCTTCTTC AGCAAGTTAG CCCTGAGTTG CCGGATCCAA CCACCTGGTT CCATGCCCGT   
  
  
+ CTACCCGACC CCATCTCTCA GTCTCCTCTT GGACCCGGTC CATCCTCCCA GCCAACCCAT TTCTACCAGG   
  
  
+ GTTCTGCCTC AGGGAACCAT GTGGTTGGGA TTGCTGACAC GTGGACGGAC CAGATTGTTG CAGGTTCCTC   
  
  
+ GTCCCAGCAG TGAGGTTGCA GCAGAGCCAG CAGGTGTTGA CTGCAGTCGA AGCCATGGAG GAGGACTCTG   
  
  
+ GGATAAGACT GGTCTACGCT CTGCTGACGT GTGCGGAGGC CATCCAACGT GGCGATTTCC GATTGGCTGT   
  
  
+ CTTGTTAGTT AACAAGATGA GCAATGACCT CCTGCCACGC GTCAACCCGT CCTGCGGTAT TGGCAAAGTA   
  
  
+ GCCGGCTACT TCATAGATGC CTTGACCCGA AGACTATACC AGCAGGGCCC AGTGTCGGGC CTAATCGGGT   
  
  
+ CGGTTCTGGC GTACCAGGTG TTGTACGAGC ACTTCTATGA AGCTTGCCCG TTCCTCAAAT TCGCTCACTT   
  
  
+ CACTGCTAAT CAAGCGATTT TAGAGGCATT CGACGGCCAC GATTGTGTCC ACATCATCGA CTTCGGCCTA   
  
  
+ ATCCACGGCT TGCAATGGCC AGCTCTAATC CAAGCCTTGG CTGTTCGGCA CGGTGGCCCG CCTTTCGTAA   
  
  
+ GATTAACCGG AATCGGGCCG CCTTCTGAAT ACGGGTCGTG CTCGCTTCAA CCTATCGGGT CAACACTGGC   
  
  
+ CCAGTTAGCC CTATCCATGA ATGTTGGGTT CGCATTTCGG GCCGTCGCCG TCTCACGGCT CGAGGACATC   
  
  
+ AAACCATGGA TGGTTAAAAC AAGCCCAAAT GAAGTCGTAG CCGTGAACTC TATCTTCCAA CTCCACCGGC   
  
  
+ TAATCGGGTC GGGTATTGAC CCTGTCCTAA ACTGGGTCCG GAGCCTAAAC CCGAAAATTG TGACACTGGC   
  
  
+ GGAGCAAGAG GCGAACCACA ACCAGCCCGA GTTTTTGGCC CGGTTCACGG AGGCATTACA TTACTATTCA   
  
  
+ ACTATGTTCG ACTCGTTAGA GGCTTGTCAA GTCCAGGCCG ACAAGGACCT AGCCGAGCTA TACTTAGAGA   
  
  
+ GAGAGTTATC CAACATCGTC TGCTGTGAGG GGTCGGCTCG GATTGAGAGG CACGAGCCGC TGGCCCAATG   
  
  
+ GAGGGCCCGA ATGGCTCGGG CCGGGTTCAA GAAGGTGGAT ATGGGTAAAA ATGCGTTCAA GCAAGTAAGC   
  
  
+ ATGTTGCTGA GTTTGTCTTC AGCACAAGGG TATTGTGTGG AGGAGAGTGA GGGATGTTTG AAGCTCGGCT   
  
  
+ GGCATGACCG CCCTCTCATT GCGGCTTCGG CTTGGCGAGC GGAGACTCAA GCTGAGAACT CCAGCACTGT   
  
  
+ TGTGCTTGAT GGGTCATCGT CGTGTAGTTC ATCTTCTTA  

- -Up\_Stream \_Len000TTTTTT CTTTCTTTAC CTCTTTGGTT CGTTATACCT ATTTTTTTTT TGTTTCATTA   
  
  
- TTTCCTTCCT ACTCCGAGGT GTAAGGTGAG CGGGCGCGGT GAGTATCCTC TACTTATCAT TTTACACTCC   
  
  
- CCAATTTACT AAACTTTTTT TAGATCATTC CCGGGTGTTG TCATAACCTT GGGTCGAACG GTTCCTTTAC   
  
  
- AGTCTCCCCG ATCGTTACCT GGTCACGTTC TTCCGGTTCG GTGTAAACCA ATCCCTTAAA GTAGGGTAGG   
  
  
- CCGCGATTTT CGTAGTTTTA ATGATGAATT AGGAACTAGT TTACGGGATA TTTATAAGAT GATGGGGTAA   
  
  
- ACTTTTCCCC ACAACTAATG TTGTGTATCG GCATCCAAAC TGTTTCTCTT AATGGGAATA ACAATCTTCG   
  
  
- TGGTATATGA GAAAAGAGAT TCGAGACTAG AAAAAAGTAG GAAAAGTCGA AAAACAGGAA AATTTAGTTT   
  
  
- AGTGAGTGAA CTCATAACCT CCCTGCAAGG AGTCCTCAGC TTGGTGATTT AATAGAAAAA TTTCTTTTTC   
  
  
- CCGAACTTCG GTTGGGCTAA ATTTTTTTGA CGATTATACA TTCGGATGAG AGAAAGGTCA AGGTTGTTTA   
  
  
- GTTTAAAGTC ATACTTTGTA GGTCATTTCC TAAATAGGCA CTATTACTAC ATTGTATACC AATTTCCCAG   
  
  
- TAAAAGTCAA TGTAGCCTCG TACGTTAATT TCAGGGATAT TTTATCCATC CACGTGCTTG TTCAGTGGTA   
  
  
- GCAGGAAACG AAAAACGTAC TCACGTACGA TTAATACTTG AATTAATTAT AGATTCGTTT TAATTTTTCA   
  
  
- TCAATTTTGA AAAAGTGGAA ACCTTGGAGC ATCGACATCG TCATCGACGG CGTGAGACCA AGATCTGAAG   
  
  
- ATCCCTGGTT CTCTACGTGA GTATACGTAG GAGAAGACAG GAAAATGACA TAGGGGAAGG GTACACTGGA   
  
  
- AAAACGGGCC CACTGAAAAG ACACTCACAA CTATGTTTAA AACATGCATT CGATTGAGTA AATGAAGTGG   
  
  
- CAGAGTACTC AAAATGTAAT GGCTATAATT AAATTTACCG TTTCATAATT TAAACTCGAT TTTAGAGTAT   
  
  
- ATATATAAAC ATAATTTACA CAGACCTAAC AGTTTTATTA AAAACTGTAT ATGTCAGTGA TATATGACTC   
  
  
- AATAATTTTA TATATTTTTA TACTTTTAAA TTTTTTATCA TCAAATTCCA CTGTTGTGAT TTTGTTTGTT   
  
  
- TATTCTATAT TTATCCCTTT TCAAGTAGGA TAAAACAATA ATAATAAATA ATACATAAAA GTCTTAACAG   
  
  
- AGATAGTGTT CGTATCATAT ACACAGTTGT AGCACTGTTG AGCTGGTTAG ATGTTTATTG ATCATTTAAG   
  
  
- TTTATTTCAA TGAGAGTGGA TCGAATTTAT TCATGTGGAA CTAAATTAAC AGAATCGATA CCTTGGATAA   
  
  
- GGTTTGATCG GTTATGAAGG AAAAGTTTTC TTAATCAAAA TTTCAAAATT GAAAATGCAG GTTATTACGG   
  
  
- CCGGCATGAT ACTCAACGAA AGTTCGGTCA TCATCATGAC ATCATCCATT TTTATAATCT TATGTGGATG   
  
  
- ATATATACTA TAACGAGAAG TGGCATAGGA AGACGTATGA GTAGAAAGGC AGGGCCTCAG GTGATAGAGA   
  
  
- GGAGGTAAGT CTGTGAGAGA GCGAGATCTC TTGAGTATCG ATACCTGGCG CACGGGCTGC CAATAGCCCA   
  
  
- ATACTTAAGC CTCGAAGAAG TCGTTCAATC GGGACTCAAC GGCCTAGGTT GGTGGACCAA GGTACGGGCA   
  
  
- GATGGGCTGG GGTAGAGAGT CAGAGGAGAA CCTGGGCCAG GTAGGAGGGT CGGTTGGGTA AAGATGGTCC   
  
  
- CAAGACGGAG TCCCTTGGTA CACCAACCCT AACGACTGTG CACCTGCCTG GTCTAACAAC GTCCAAGGAG   
  
  
- CAGGGTCGTC ACTCCAACGT CGTCTCGGTC GTCCACAACT GACGTCAGCT TCGGTACCTC CTCCTGAGAC   
  
  
- CCTATTCTGA CCAGATGCGA GACGACTGCA CACGCCTCCG GTAGGTTGCA CCGCTAAAGG CTAACCGACA   
  
  
- GAACAATCAA TTGTTCTACT CGTTACTGGA GGACGGTGCG CAGTTGGGCA GGACGCCATA ACCGTTTCAT   
  
  
- CGGCCGATGA AGTATCTACG GAACTGGGCT TCTGATATGG TCGTCCCGGG TCACAGCCCG GATTAGCCCA   
  
  
- GCCAAGACCG CATGGTCCAC AACATGCTCG TGAAGATACT TCGAACGGGC AAGGAGTTTA AGCGAGTGAA   
  
  
- GTGACGATTA GTTCGCTAAA ATCTCCGTAA GCTGCCGGTG CTAACACAGG TGTAGTAGCT GAAGCCGGAT   
  
  
- TAGGTGCCGA ACGTTACCGG TCGAGATTAG GTTCGGAACC GACAAGCCGT GCCACCGGGC GGAAAGCATT   
  
  
- CTAATTGGCC TTAGCCCGGC GGAAGACTTA TGCCCAGCAC GAGCGAAGTT GGATAGCCCA GTTGTGACCG   
  
  
- GGTCAATCGG GATAGGTACT TACAACCCAA GCGTAAAGCC CGGCAGCGGC AGAGTGCCGA GCTCCTGTAG   
  
  
- TTTGGTACCT ACCAATTTTG TTCGGGTTTA CTTCAGCATC GGCACTTGAG ATAGAAGGTT GAGGTGGCCG   
  
  
- ATTAGCCCAG CCCATAACTG GGACAGGATT TGACCCAGGC CTCGGATTTG GGCTTTTAAC ACTGTGACCG   
  
  
- CCTCGTTCTC CGCTTGGTGT TGGTCGGGCT CAAAAACCGG GCCAAGTGCC TCCGTAATGT AATGATAAGT   
  
  
- TGATACAAGC TGAGCAATCT CCGAACAGTT CAGGTCCGGC TGTTCCTGGA TCGGCTCGAT ATGAATCTCT   
  
  
- CTCTCAATAG GTTGTAGCAG ACGACACTCC CCAGCCGAGC CTAACTCTCC GTGCTCGGCG ACCGGGTTAC   
  
  
- CTCCCGGGCT TACCGAGCCC GGCCCAAGTT CTTCCACCTA TACCCATTTT TACGCAAGTT CGTTCATTCG   
  
  
- TACAACGACT CAAACAGAAG TCGTGTTCCC ATAACACACC TCCTCTCACT CCCTACAAAC TTCGAGCCGA   
  
  
- CCGTACTGGC GGGAGAGTAA CGCCGAAGCC GAACCGCTCG CCTCTGAGTT CGACTCTTGA GGTCGTGACA   
  
  
- ACACGAACTA CCCAGTAGCA GCACATCAAG TAGAAGAAT

+     TCA-element

| Site Name | Organism | Position | Strand | Matrix score. | sequence | function |
| --- | --- | --- | --- | --- | --- | --- |
| TCA-element | Brassica oleracea | 944 | - | 9 | TCAGAAGAGG | cis-acting element involved in salicylic acid responsiveness |

>HU11G00778.1   
+ -Up\_Stream \_Len000AAAAAA GAAAGAAATG GAGAAACCAA GCAATATGGA TAAAAAAAAA ACAAAGTAAT   
  
  
+ AAAGGAAGGA TGAGGCTCCA CATTCCACTC GCCCGCGCCA CTCATAGGAG ATGAATAGTA AAATGTGAGG   
  
  
+ GGTTAAATGA TTTGAAAAAA ATCTAGTAAG GGCCCACAAC AGTATTGGAA CCCAGCTTGC CAAGGAAATG   
  
  
+ TCAGAGGGGC TAGCAATGGA CCAGTGCAAG AAGGCCAAGC CACATTTGGT TAGGGAATTT CATCCCATCC   
  
  
+ GGCGCTAAAA GCATCAAAAT TACTACTTAA TCCTTGATCA AATGCCCTAT AAATATTCTA CTACCCCATT   
  
  
+ TGAAAAGGGG TGTTGATTAC AACACATAGC CGTAGGTTTG ACAAAGAGAA TTACCCTTAT TGTTAGAAGC   
  
  
+ ACCATATACT CTTTTCTCTA AGCTCTGATC TTTTTTCATC CTTTTCAGCT TTTTGTCCTT TTAAATCAAA   
  
  
+ TCACTCACTT GAGTATTGGA GGGACGTTCC TCAGGAGTCG AACCACTAAA TTATCTTTTT AAAGAAAAAG   
  
  
+ GGCTTGAAGC CAACCCGATT TAAAAAAACT GCTAATATGT AAGCCTACTC TCTTTCCAGT TCCAACAAAT   
  
  
+ CAAATTTCAG TATGAAACAT CCAGTAAAGG ATTTATCCGT GATAATGATG TAACATATGG TTAAAGGGTC   
  
  
+ ATTTTCAGTT ACATCGGAGC ATGCAATTAA AGTCCCTATA AAATAGGTAG GTGCACGAAC AAGTCACCAT   
  
  
+ CGTCCTTTGC TTTTTGCATG AGTGCATGCT AATTATGAAC TTAATTAATA TCTAAGCAAA ATTAAAAAGT   
  
  
+ AGTTAAAACT TTTTCACCTT TGGAACCTCG TAGCTGTAGC AGTAGCTGCC GCACTCTGGT TCTAGACTTC   
  
  
+ TAGGGACCAA GAGATGCACT CATATGCATC CTCTTCTGTC CTTTTACTGT ATCCCCTTCC CATGTGACCT   
  
  
+ TTTTGCCCGG GTGACTTTTC TGTGAGTGTT GATACAAATT TTGTACGTAA GCTAACTCAT TTACTTCACC   
  
  
+ GTCTCATGAG TTTTACATTA CCGATATTAA TTTAAATGGC AAAGTATTAA ATTTGAGCTA AAATCTCATA   
  
  
+ TATATATTTG TATTAAATGT GTCTGGATTG TCAAAATAAT TTTTGACATA TACAGTCACT ATATACTGAG   
  
  
+ TTATTAAAAT ATATAAAAAT ATGAAAATTT AAAAAATAGT AGTTTAAGGT GACAACACTA AAACAAACAA   
  
  
+ ATAAGATATA AATAGGGAAA AGTTCATCCT ATTTTGTTAT TATTATTTAT TATGTATTTT CAGAATTGTC   
  
  
+ TCTATCACAA GCATAGTATA TGTGTCAACA TCGTGACAAC TCGACCAATC TACAAATAAC TAGTAAATTC   
  
  
+ AAATAAAGTT ACTCTCACCT AGCTTAAATA AGTACACCTT GATTTAATTG TCTTAGCTAT GGAACCTATT   
  
  
+ CCAAACTAGC CAATACTTCC TTTTCAAAAG AATTAGTTTT AAAGTTTTAA CTTTTACGTC CAATAATGCC   
  
  
+ GGCCGTACTA TGAGTTGCTT TCAAGCCAGT AGTAGTACTG TAGTAGGTAA AAATATTAGA ATACACCTAC   
  
  
+ TATATATGAT ATTGCTCTTC ACCGTATCCT TCTGCATACT CATCTTTCCG TCCCGGAGTC CACTATCTCT   
  
  
+ CCTCCATTCA GACACTCTCT CGCTCTAGAG AACTCATAGC TATGGACCGC GTGCCCGACG GTTATCGGGT   
  
  
+ TATGAATTCG GAGCTTCTTC AGCAAGTTAG CCCTGAGTTG CCGGATCCAA CCACCTGGTT CCATGCCCGT   
  
  
+ CTACCCGACC CCATCTCTCA GTCTCCTCTT GGACCCGGTC CATCCTCCCA GCCAACCCAT TTCTACCAGG   
  
  
+ GTTCTGCCTC AGGGAACCAT GTGGTTGGGA TTGCTGACAC GTGGACGGAC CAGATTGTTG CAGGTTCCTC   
  
  
+ GTCCCAGCAG TGAGGTTGCA GCAGAGCCAG CAGGTGTTGA CTGCAGTCGA AGCCATGGAG GAGGACTCTG   
  
  
+ GGATAAGACT GGTCTACGCT CTGCTGACGT GTGCGGAGGC CATCCAACGT GGCGATTTCC GATTGGCTGT   
  
  
+ CTTGTTAGTT AACAAGATGA GCAATGACCT CCTGCCACGC GTCAACCCGT CCTGCGGTAT TGGCAAAGTA   
  
  
+ GCCGGCTACT TCATAGATGC CTTGACCCGA AGACTATACC AGCAGGGCCC AGTGTCGGGC CTAATCGGGT   
  
  
+ CGGTTCTGGC GTACCAGGTG TTGTACGAGC ACTTCTATGA AGCTTGCCCG TTCCTCAAAT TCGCTCACTT   
  
  
+ CACTGCTAAT CAAGCGATTT TAGAGGCATT CGACGGCCAC GATTGTGTCC ACATCATCGA CTTCGGCCTA   
  
  
+ ATCCACGGCT TGCAATGGCC AGCTCTAATC CAAGCCTTGG CTGTTCGGCA CGGTGGCCCG CCTTTCGTAA   
  
  
+ GATTAACCGG AATCGGGCCG CCTTCTGAAT ACGGGTCGTG CTCGCTTCAA CCTATCGGGT CAACACTGGC   
  
  
+ CCAGTTAGCC CTATCCATGA ATGTTGGGTT CGCATTTCGG GCCGTCGCCG TCTCACGGCT CGAGGACATC   
  
  
+ AAACCATGGA TGGTTAAAAC AAGCCCAAAT GAAGTCGTAG CCGTGAACTC TATCTTCCAA CTCCACCGGC   
  
  
+ TAATCGGGTC GGGTATTGAC CCTGTCCTAA ACTGGGTCCG GAGCCTAAAC CCGAAAATTG TGACACTGGC   
  
  
+ GGAGCAAGAG GCGAACCACA ACCAGCCCGA GTTTTTGGCC CGGTTCACGG AGGCATTACA TTACTATTCA   
  
  
+ ACTATGTTCG ACTCGTTAGA GGCTTGTCAA GTCCAGGCCG ACAAGGACCT AGCCGAGCTA TACTTAGAGA   
  
  
+ GAGAGTTATC CAACATCGTC TGCTGTGAGG GGTCGGCTCG GATTGAGAGG CACGAGCCGC TGGCCCAATG   
  
  
+ GAGGGCCCGA ATGGCTCGGG CCGGGTTCAA GAAGGTGGAT ATGGGTAAAA ATGCGTTCAA GCAAGTAAGC   
  
  
+ ATGTTGCTGA GTTTGTCTTC AGCACAAGGG TATTGTGTGG AGGAGAGTGA GGGATGTTTG AAGCTCGGCT   
  
  
+ GGCATGACCG CCCTCTCATT GCGGCTTCGG CTTGGCGAGC GGAGACTCAA GCTGAGAACT CCAGCACTGT   
  
  
+ TGTGCTTGAT GGGTCATCGT CGTGTAGTTC ATCTTCTTA  

- -Up\_Stream \_Len000TTTTTT CTTTCTTTAC CTCTTTGGTT CGTTATACCT ATTTTTTTTT TGTTTCATTA   
  
  
- TTTCCTTCCT ACTCCGAGGT GTAAGGTGAG CGGGCGCGGT GAGTATCCTC TACTTATCAT TTTACACTCC   
  
  
- CCAATTTACT AAACTTTTTT TAGATCATTC CCGGGTGTTG TCATAACCTT GGGTCGAACG GTTCCTTTAC   
  
  
- AGTCTCCCCG ATCGTTACCT GGTCACGTTC TTCCGGTTCG GTGTAAACCA ATCCCTTAAA GTAGGGTAGG   
  
  
- CCGCGATTTT CGTAGTTTTA ATGATGAATT AGGAACTAGT TTACGGGATA TTTATAAGAT GATGGGGTAA   
  
  
- ACTTTTCCCC ACAACTAATG TTGTGTATCG GCATCCAAAC TGTTTCTCTT AATGGGAATA ACAATCTTCG   
  
  
- TGGTATATGA GAAAAGAGAT TCGAGACTAG AAAAAAGTAG GAAAAGTCGA AAAACAGGAA AATTTAGTTT   
  
  
- AGTGAGTGAA CTCATAACCT CCCTGCAAGG AGTCCTCAGC TTGGTGATTT AATAGAAAAA TTTCTTTTTC   
  
  
- CCGAACTTCG GTTGGGCTAA ATTTTTTTGA CGATTATACA TTCGGATGAG AGAAAGGTCA AGGTTGTTTA   
  
  
- GTTTAAAGTC ATACTTTGTA GGTCATTTCC TAAATAGGCA CTATTACTAC ATTGTATACC AATTTCCCAG   
  
  
- TAAAAGTCAA TGTAGCCTCG TACGTTAATT TCAGGGATAT TTTATCCATC CACGTGCTTG TTCAGTGGTA   
  
  
- GCAGGAAACG AAAAACGTAC TCACGTACGA TTAATACTTG AATTAATTAT AGATTCGTTT TAATTTTTCA   
  
  
- TCAATTTTGA AAAAGTGGAA ACCTTGGAGC ATCGACATCG TCATCGACGG CGTGAGACCA AGATCTGAAG   
  
  
- ATCCCTGGTT CTCTACGTGA GTATACGTAG GAGAAGACAG GAAAATGACA TAGGGGAAGG GTACACTGGA   
  
  
- AAAACGGGCC CACTGAAAAG ACACTCACAA CTATGTTTAA AACATGCATT CGATTGAGTA AATGAAGTGG   
  
  
- CAGAGTACTC AAAATGTAAT GGCTATAATT AAATTTACCG TTTCATAATT TAAACTCGAT TTTAGAGTAT   
  
  
- ATATATAAAC ATAATTTACA CAGACCTAAC AGTTTTATTA AAAACTGTAT ATGTCAGTGA TATATGACTC   
  
  
- AATAATTTTA TATATTTTTA TACTTTTAAA TTTTTTATCA TCAAATTCCA CTGTTGTGAT TTTGTTTGTT   
  
  
- TATTCTATAT TTATCCCTTT TCAAGTAGGA TAAAACAATA ATAATAAATA ATACATAAAA GTCTTAACAG   
  
  
- AGATAGTGTT CGTATCATAT ACACAGTTGT AGCACTGTTG AGCTGGTTAG ATGTTTATTG ATCATTTAAG   
  
  
- TTTATTTCAA TGAGAGTGGA TCGAATTTAT TCATGTGGAA CTAAATTAAC AGAATCGATA CCTTGGATAA   
  
  
- GGTTTGATCG GTTATGAAGG AAAAGTTTTC TTAATCAAAA TTTCAAAATT GAAAATGCAG GTTATTACGG   
  
  
- CCGGCATGAT ACTCAACGAA AGTTCGGTCA TCATCATGAC ATCATCCATT TTTATAATCT TATGTGGATG   
  
  
- ATATATACTA TAACGAGAAG TGGCATAGGA AGACGTATGA GTAGAAAGGC AGGGCCTCAG GTGATAGAGA   
  
  
- GGAGGTAAGT CTGTGAGAGA GCGAGATCTC TTGAGTATCG ATACCTGGCG CACGGGCTGC CAATAGCCCA   
  
  
- ATACTTAAGC CTCGAAGAAG TCGTTCAATC GGGACTCAAC GGCCTAGGTT GGTGGACCAA GGTACGGGCA   
  
  
- GATGGGCTGG GGTAGAGAGT CAGAGGAGAA CCTGGGCCAG GTAGGAGGGT CGGTTGGGTA AAGATGGTCC   
  
  
- CAAGACGGAG TCCCTTGGTA CACCAACCCT AACGACTGTG CACCTGCCTG GTCTAACAAC GTCCAAGGAG   
  
  
- CAGGGTCGTC ACTCCAACGT CGTCTCGGTC GTCCACAACT GACGTCAGCT TCGGTACCTC CTCCTGAGAC   
  
  
- CCTATTCTGA CCAGATGCGA GACGACTGCA CACGCCTCCG GTAGGTTGCA CCGCTAAAGG CTAACCGACA   
  
  
- GAACAATCAA TTGTTCTACT CGTTACTGGA GGACGGTGCG CAGTTGGGCA GGACGCCATA ACCGTTTCAT   
  
  
- CGGCCGATGA AGTATCTACG GAACTGGGCT TCTGATATGG TCGTCCCGGG TCACAGCCCG GATTAGCCCA   
  
  
- GCCAAGACCG CATGGTCCAC AACATGCTCG TGAAGATACT TCGAACGGGC AAGGAGTTTA AGCGAGTGAA   
  
  
- GTGACGATTA GTTCGCTAAA ATCTCCGTAA GCTGCCGGTG CTAACACAGG TGTAGTAGCT GAAGCCGGAT   
  
  
- TAGGTGCCGA ACGTTACCGG TCGAGATTAG GTTCGGAACC GACAAGCCGT GCCACCGGGC GGAAAGCATT   
  
  
- CTAATTGGCC TTAGCCCGGC GGAAGACTTA TGCCCAGCAC GAGCGAAGTT GGATAGCCCA GTTGTGACCG   
  
  
- GGTCAATCGG GATAGGTACT TACAACCCAA GCGTAAAGCC CGGCAGCGGC AGAGTGCCGA GCTCCTGTAG   
  
  
- TTTGGTACCT ACCAATTTTG TTCGGGTTTA CTTCAGCATC GGCACTTGAG ATAGAAGGTT GAGGTGGCCG   
  
  
- ATTAGCCCAG CCCATAACTG GGACAGGATT TGACCCAGGC CTCGGATTTG GGCTTTTAAC ACTGTGACCG   
  
  
- CCTCGTTCTC CGCTTGGTGT TGGTCGGGCT CAAAAACCGG GCCAAGTGCC TCCGTAATGT AATGATAAGT   
  
  
- TGATACAAGC TGAGCAATCT CCGAACAGTT CAGGTCCGGC TGTTCCTGGA TCGGCTCGAT ATGAATCTCT   
  
  
- CTCTCAATAG GTTGTAGCAG ACGACACTCC CCAGCCGAGC CTAACTCTCC GTGCTCGGCG ACCGGGTTAC   
  
  
- CTCCCGGGCT TACCGAGCCC GGCCCAAGTT CTTCCACCTA TACCCATTTT TACGCAAGTT CGTTCATTCG   
  
  
- TACAACGACT CAAACAGAAG TCGTGTTCCC ATAACACACC TCCTCTCACT CCCTACAAAC TTCGAGCCGA   
  
  
- CCGTACTGGC GGGAGAGTAA CGCCGAAGCC GAACCGCTCG CCTCTGAGTT CGACTCTTGA GGTCGTGACA   
  
  
- ACACGAACTA CCCAGTAGCA GCACATCAAG TAGAAGAAT

+     TCT-motif

| Site Name | Organism | Position | Strand | Matrix score. | sequence | function |
| --- | --- | --- | --- | --- | --- | --- |
| TCT-motif | Arabidopsis thaliana | 2451 | - | 6 | TCTTAC | part of a light responsive element |

>HU11G00778.1   
+ -Up\_Stream \_Len000AAAAAA GAAAGAAATG GAGAAACCAA GCAATATGGA TAAAAAAAAA ACAAAGTAAT   
  
  
+ AAAGGAAGGA TGAGGCTCCA CATTCCACTC GCCCGCGCCA CTCATAGGAG ATGAATAGTA AAATGTGAGG   
  
  
+ GGTTAAATGA TTTGAAAAAA ATCTAGTAAG GGCCCACAAC AGTATTGGAA CCCAGCTTGC CAAGGAAATG   
  
  
+ TCAGAGGGGC TAGCAATGGA CCAGTGCAAG AAGGCCAAGC CACATTTGGT TAGGGAATTT CATCCCATCC   
  
  
+ GGCGCTAAAA GCATCAAAAT TACTACTTAA TCCTTGATCA AATGCCCTAT AAATATTCTA CTACCCCATT   
  
  
+ TGAAAAGGGG TGTTGATTAC AACACATAGC CGTAGGTTTG ACAAAGAGAA TTACCCTTAT TGTTAGAAGC   
  
  
+ ACCATATACT CTTTTCTCTA AGCTCTGATC TTTTTTCATC CTTTTCAGCT TTTTGTCCTT TTAAATCAAA   
  
  
+ TCACTCACTT GAGTATTGGA GGGACGTTCC TCAGGAGTCG AACCACTAAA TTATCTTTTT AAAGAAAAAG   
  
  
+ GGCTTGAAGC CAACCCGATT TAAAAAAACT GCTAATATGT AAGCCTACTC TCTTTCCAGT TCCAACAAAT   
  
  
+ CAAATTTCAG TATGAAACAT CCAGTAAAGG ATTTATCCGT GATAATGATG TAACATATGG TTAAAGGGTC   
  
  
+ ATTTTCAGTT ACATCGGAGC ATGCAATTAA AGTCCCTATA AAATAGGTAG GTGCACGAAC AAGTCACCAT   
  
  
+ CGTCCTTTGC TTTTTGCATG AGTGCATGCT AATTATGAAC TTAATTAATA TCTAAGCAAA ATTAAAAAGT   
  
  
+ AGTTAAAACT TTTTCACCTT TGGAACCTCG TAGCTGTAGC AGTAGCTGCC GCACTCTGGT TCTAGACTTC   
  
  
+ TAGGGACCAA GAGATGCACT CATATGCATC CTCTTCTGTC CTTTTACTGT ATCCCCTTCC CATGTGACCT   
  
  
+ TTTTGCCCGG GTGACTTTTC TGTGAGTGTT GATACAAATT TTGTACGTAA GCTAACTCAT TTACTTCACC   
  
  
+ GTCTCATGAG TTTTACATTA CCGATATTAA TTTAAATGGC AAAGTATTAA ATTTGAGCTA AAATCTCATA   
  
  
+ TATATATTTG TATTAAATGT GTCTGGATTG TCAAAATAAT TTTTGACATA TACAGTCACT ATATACTGAG   
  
  
+ TTATTAAAAT ATATAAAAAT ATGAAAATTT AAAAAATAGT AGTTTAAGGT GACAACACTA AAACAAACAA   
  
  
+ ATAAGATATA AATAGGGAAA AGTTCATCCT ATTTTGTTAT TATTATTTAT TATGTATTTT CAGAATTGTC   
  
  
+ TCTATCACAA GCATAGTATA TGTGTCAACA TCGTGACAAC TCGACCAATC TACAAATAAC TAGTAAATTC   
  
  
+ AAATAAAGTT ACTCTCACCT AGCTTAAATA AGTACACCTT GATTTAATTG TCTTAGCTAT GGAACCTATT   
  
  
+ CCAAACTAGC CAATACTTCC TTTTCAAAAG AATTAGTTTT AAAGTTTTAA CTTTTACGTC CAATAATGCC   
  
  
+ GGCCGTACTA TGAGTTGCTT TCAAGCCAGT AGTAGTACTG TAGTAGGTAA AAATATTAGA ATACACCTAC   
  
  
+ TATATATGAT ATTGCTCTTC ACCGTATCCT TCTGCATACT CATCTTTCCG TCCCGGAGTC CACTATCTCT   
  
  
+ CCTCCATTCA GACACTCTCT CGCTCTAGAG AACTCATAGC TATGGACCGC GTGCCCGACG GTTATCGGGT   
  
  
+ TATGAATTCG GAGCTTCTTC AGCAAGTTAG CCCTGAGTTG CCGGATCCAA CCACCTGGTT CCATGCCCGT   
  
  
+ CTACCCGACC CCATCTCTCA GTCTCCTCTT GGACCCGGTC CATCCTCCCA GCCAACCCAT TTCTACCAGG   
  
  
+ GTTCTGCCTC AGGGAACCAT GTGGTTGGGA TTGCTGACAC GTGGACGGAC CAGATTGTTG CAGGTTCCTC   
  
  
+ GTCCCAGCAG TGAGGTTGCA GCAGAGCCAG CAGGTGTTGA CTGCAGTCGA AGCCATGGAG GAGGACTCTG   
  
  
+ GGATAAGACT GGTCTACGCT CTGCTGACGT GTGCGGAGGC CATCCAACGT GGCGATTTCC GATTGGCTGT   
  
  
+ CTTGTTAGTT AACAAGATGA GCAATGACCT CCTGCCACGC GTCAACCCGT CCTGCGGTAT TGGCAAAGTA   
  
  
+ GCCGGCTACT TCATAGATGC CTTGACCCGA AGACTATACC AGCAGGGCCC AGTGTCGGGC CTAATCGGGT   
  
  
+ CGGTTCTGGC GTACCAGGTG TTGTACGAGC ACTTCTATGA AGCTTGCCCG TTCCTCAAAT TCGCTCACTT   
  
  
+ CACTGCTAAT CAAGCGATTT TAGAGGCATT CGACGGCCAC GATTGTGTCC ACATCATCGA CTTCGGCCTA   
  
  
+ ATCCACGGCT TGCAATGGCC AGCTCTAATC CAAGCCTTGG CTGTTCGGCA CGGTGGCCCG CCTTTCGTAA   
  
  
+ GATTAACCGG AATCGGGCCG CCTTCTGAAT ACGGGTCGTG CTCGCTTCAA CCTATCGGGT CAACACTGGC   
  
  
+ CCAGTTAGCC CTATCCATGA ATGTTGGGTT CGCATTTCGG GCCGTCGCCG TCTCACGGCT CGAGGACATC   
  
  
+ AAACCATGGA TGGTTAAAAC AAGCCCAAAT GAAGTCGTAG CCGTGAACTC TATCTTCCAA CTCCACCGGC   
  
  
+ TAATCGGGTC GGGTATTGAC CCTGTCCTAA ACTGGGTCCG GAGCCTAAAC CCGAAAATTG TGACACTGGC   
  
  
+ GGAGCAAGAG GCGAACCACA ACCAGCCCGA GTTTTTGGCC CGGTTCACGG AGGCATTACA TTACTATTCA   
  
  
+ ACTATGTTCG ACTCGTTAGA GGCTTGTCAA GTCCAGGCCG ACAAGGACCT AGCCGAGCTA TACTTAGAGA   
  
  
+ GAGAGTTATC CAACATCGTC TGCTGTGAGG GGTCGGCTCG GATTGAGAGG CACGAGCCGC TGGCCCAATG   
  
  
+ GAGGGCCCGA ATGGCTCGGG CCGGGTTCAA GAAGGTGGAT ATGGGTAAAA ATGCGTTCAA GCAAGTAAGC   
  
  
+ ATGTTGCTGA GTTTGTCTTC AGCACAAGGG TATTGTGTGG AGGAGAGTGA GGGATGTTTG AAGCTCGGCT   
  
  
+ GGCATGACCG CCCTCTCATT GCGGCTTCGG CTTGGCGAGC GGAGACTCAA GCTGAGAACT CCAGCACTGT   
  
  
+ TGTGCTTGAT GGGTCATCGT CGTGTAGTTC ATCTTCTTA  

- -Up\_Stream \_Len000TTTTTT CTTTCTTTAC CTCTTTGGTT CGTTATACCT ATTTTTTTTT TGTTTCATTA   
  
  
- TTTCCTTCCT ACTCCGAGGT GTAAGGTGAG CGGGCGCGGT GAGTATCCTC TACTTATCAT TTTACACTCC   
  
  
- CCAATTTACT AAACTTTTTT TAGATCATTC CCGGGTGTTG TCATAACCTT GGGTCGAACG GTTCCTTTAC   
  
  
- AGTCTCCCCG ATCGTTACCT GGTCACGTTC TTCCGGTTCG GTGTAAACCA ATCCCTTAAA GTAGGGTAGG   
  
  
- CCGCGATTTT CGTAGTTTTA ATGATGAATT AGGAACTAGT TTACGGGATA TTTATAAGAT GATGGGGTAA   
  
  
- ACTTTTCCCC ACAACTAATG TTGTGTATCG GCATCCAAAC TGTTTCTCTT AATGGGAATA ACAATCTTCG   
  
  
- TGGTATATGA GAAAAGAGAT TCGAGACTAG AAAAAAGTAG GAAAAGTCGA AAAACAGGAA AATTTAGTTT   
  
  
- AGTGAGTGAA CTCATAACCT CCCTGCAAGG AGTCCTCAGC TTGGTGATTT AATAGAAAAA TTTCTTTTTC   
  
  
- CCGAACTTCG GTTGGGCTAA ATTTTTTTGA CGATTATACA TTCGGATGAG AGAAAGGTCA AGGTTGTTTA   
  
  
- GTTTAAAGTC ATACTTTGTA GGTCATTTCC TAAATAGGCA CTATTACTAC ATTGTATACC AATTTCCCAG   
  
  
- TAAAAGTCAA TGTAGCCTCG TACGTTAATT TCAGGGATAT TTTATCCATC CACGTGCTTG TTCAGTGGTA   
  
  
- GCAGGAAACG AAAAACGTAC TCACGTACGA TTAATACTTG AATTAATTAT AGATTCGTTT TAATTTTTCA   
  
  
- TCAATTTTGA AAAAGTGGAA ACCTTGGAGC ATCGACATCG TCATCGACGG CGTGAGACCA AGATCTGAAG   
  
  
- ATCCCTGGTT CTCTACGTGA GTATACGTAG GAGAAGACAG GAAAATGACA TAGGGGAAGG GTACACTGGA   
  
  
- AAAACGGGCC CACTGAAAAG ACACTCACAA CTATGTTTAA AACATGCATT CGATTGAGTA AATGAAGTGG   
  
  
- CAGAGTACTC AAAATGTAAT GGCTATAATT AAATTTACCG TTTCATAATT TAAACTCGAT TTTAGAGTAT   
  
  
- ATATATAAAC ATAATTTACA CAGACCTAAC AGTTTTATTA AAAACTGTAT ATGTCAGTGA TATATGACTC   
  
  
- AATAATTTTA TATATTTTTA TACTTTTAAA TTTTTTATCA TCAAATTCCA CTGTTGTGAT TTTGTTTGTT   
  
  
- TATTCTATAT TTATCCCTTT TCAAGTAGGA TAAAACAATA ATAATAAATA ATACATAAAA GTCTTAACAG   
  
  
- AGATAGTGTT CGTATCATAT ACACAGTTGT AGCACTGTTG AGCTGGTTAG ATGTTTATTG ATCATTTAAG   
  
  
- TTTATTTCAA TGAGAGTGGA TCGAATTTAT TCATGTGGAA CTAAATTAAC AGAATCGATA CCTTGGATAA   
  
  
- GGTTTGATCG GTTATGAAGG AAAAGTTTTC TTAATCAAAA TTTCAAAATT GAAAATGCAG GTTATTACGG   
  
  
- CCGGCATGAT ACTCAACGAA AGTTCGGTCA TCATCATGAC ATCATCCATT TTTATAATCT TATGTGGATG   
  
  
- ATATATACTA TAACGAGAAG TGGCATAGGA AGACGTATGA GTAGAAAGGC AGGGCCTCAG GTGATAGAGA   
  
  
- GGAGGTAAGT CTGTGAGAGA GCGAGATCTC TTGAGTATCG ATACCTGGCG CACGGGCTGC CAATAGCCCA   
  
  
- ATACTTAAGC CTCGAAGAAG TCGTTCAATC GGGACTCAAC GGCCTAGGTT GGTGGACCAA GGTACGGGCA   
  
  
- GATGGGCTGG GGTAGAGAGT CAGAGGAGAA CCTGGGCCAG GTAGGAGGGT CGGTTGGGTA AAGATGGTCC   
  
  
- CAAGACGGAG TCCCTTGGTA CACCAACCCT AACGACTGTG CACCTGCCTG GTCTAACAAC GTCCAAGGAG   
  
  
- CAGGGTCGTC ACTCCAACGT CGTCTCGGTC GTCCACAACT GACGTCAGCT TCGGTACCTC CTCCTGAGAC   
  
  
- CCTATTCTGA CCAGATGCGA GACGACTGCA CACGCCTCCG GTAGGTTGCA CCGCTAAAGG CTAACCGACA   
  
  
- GAACAATCAA TTGTTCTACT CGTTACTGGA GGACGGTGCG CAGTTGGGCA GGACGCCATA ACCGTTTCAT   
  
  
- CGGCCGATGA AGTATCTACG GAACTGGGCT TCTGATATGG TCGTCCCGGG TCACAGCCCG GATTAGCCCA   
  
  
- GCCAAGACCG CATGGTCCAC AACATGCTCG TGAAGATACT TCGAACGGGC AAGGAGTTTA AGCGAGTGAA   
  
  
- GTGACGATTA GTTCGCTAAA ATCTCCGTAA GCTGCCGGTG CTAACACAGG TGTAGTAGCT GAAGCCGGAT   
  
  
- TAGGTGCCGA ACGTTACCGG TCGAGATTAG GTTCGGAACC GACAAGCCGT GCCACCGGGC GGAAAGCATT   
  
  
- CTAATTGGCC TTAGCCCGGC GGAAGACTTA TGCCCAGCAC GAGCGAAGTT GGATAGCCCA GTTGTGACCG   
  
  
- GGTCAATCGG GATAGGTACT TACAACCCAA GCGTAAAGCC CGGCAGCGGC AGAGTGCCGA GCTCCTGTAG   
  
  
- TTTGGTACCT ACCAATTTTG TTCGGGTTTA CTTCAGCATC GGCACTTGAG ATAGAAGGTT GAGGTGGCCG   
  
  
- ATTAGCCCAG CCCATAACTG GGACAGGATT TGACCCAGGC CTCGGATTTG GGCTTTTAAC ACTGTGACCG   
  
  
- CCTCGTTCTC CGCTTGGTGT TGGTCGGGCT CAAAAACCGG GCCAAGTGCC TCCGTAATGT AATGATAAGT   
  
  
- TGATACAAGC TGAGCAATCT CCGAACAGTT CAGGTCCGGC TGTTCCTGGA TCGGCTCGAT ATGAATCTCT   
  
  
- CTCTCAATAG GTTGTAGCAG ACGACACTCC CCAGCCGAGC CTAACTCTCC GTGCTCGGCG ACCGGGTTAC   
  
  
- CTCCCGGGCT TACCGAGCCC GGCCCAAGTT CTTCCACCTA TACCCATTTT TACGCAAGTT CGTTCATTCG   
  
  
- TACAACGACT CAAACAGAAG TCGTGTTCCC ATAACACACC TCCTCTCACT CCCTACAAAC TTCGAGCCGA   
  
  
- CCGTACTGGC GGGAGAGTAA CGCCGAAGCC GAACCGCTCG CCTCTGAGTT CGACTCTTGA GGTCGTGACA   
  
  
- ACACGAACTA CCCAGTAGCA GCACATCAAG TAGAAGAAT

+     TGACG-motif

| Site Name | Organism | Position | Strand | Matrix score. | sequence | function |
| --- | --- | --- | --- | --- | --- | --- |
| TGACG-motif | Hordeum vulgare | 2144 | - | 5 | TGACG | cis-acting regulatory element involved in the MeJA-responsiveness |
| TGACG-motif | Hordeum vulgare | 2059 | + | 5 | TGACG | cis-acting regulatory element involved in the MeJA-responsiveness |

>HU11G00778.1   
+ -Up\_Stream \_Len000AAAAAA GAAAGAAATG GAGAAACCAA GCAATATGGA TAAAAAAAAA ACAAAGTAAT   
  
  
+ AAAGGAAGGA TGAGGCTCCA CATTCCACTC GCCCGCGCCA CTCATAGGAG ATGAATAGTA AAATGTGAGG   
  
  
+ GGTTAAATGA TTTGAAAAAA ATCTAGTAAG GGCCCACAAC AGTATTGGAA CCCAGCTTGC CAAGGAAATG   
  
  
+ TCAGAGGGGC TAGCAATGGA CCAGTGCAAG AAGGCCAAGC CACATTTGGT TAGGGAATTT CATCCCATCC   
  
  
+ GGCGCTAAAA GCATCAAAAT TACTACTTAA TCCTTGATCA AATGCCCTAT AAATATTCTA CTACCCCATT   
  
  
+ TGAAAAGGGG TGTTGATTAC AACACATAGC CGTAGGTTTG ACAAAGAGAA TTACCCTTAT TGTTAGAAGC   
  
  
+ ACCATATACT CTTTTCTCTA AGCTCTGATC TTTTTTCATC CTTTTCAGCT TTTTGTCCTT TTAAATCAAA   
  
  
+ TCACTCACTT GAGTATTGGA GGGACGTTCC TCAGGAGTCG AACCACTAAA TTATCTTTTT AAAGAAAAAG   
  
  
+ GGCTTGAAGC CAACCCGATT TAAAAAAACT GCTAATATGT AAGCCTACTC TCTTTCCAGT TCCAACAAAT   
  
  
+ CAAATTTCAG TATGAAACAT CCAGTAAAGG ATTTATCCGT GATAATGATG TAACATATGG TTAAAGGGTC   
  
  
+ ATTTTCAGTT ACATCGGAGC ATGCAATTAA AGTCCCTATA AAATAGGTAG GTGCACGAAC AAGTCACCAT   
  
  
+ CGTCCTTTGC TTTTTGCATG AGTGCATGCT AATTATGAAC TTAATTAATA TCTAAGCAAA ATTAAAAAGT   
  
  
+ AGTTAAAACT TTTTCACCTT TGGAACCTCG TAGCTGTAGC AGTAGCTGCC GCACTCTGGT TCTAGACTTC   
  
  
+ TAGGGACCAA GAGATGCACT CATATGCATC CTCTTCTGTC CTTTTACTGT ATCCCCTTCC CATGTGACCT   
  
  
+ TTTTGCCCGG GTGACTTTTC TGTGAGTGTT GATACAAATT TTGTACGTAA GCTAACTCAT TTACTTCACC   
  
  
+ GTCTCATGAG TTTTACATTA CCGATATTAA TTTAAATGGC AAAGTATTAA ATTTGAGCTA AAATCTCATA   
  
  
+ TATATATTTG TATTAAATGT GTCTGGATTG TCAAAATAAT TTTTGACATA TACAGTCACT ATATACTGAG   
  
  
+ TTATTAAAAT ATATAAAAAT ATGAAAATTT AAAAAATAGT AGTTTAAGGT GACAACACTA AAACAAACAA   
  
  
+ ATAAGATATA AATAGGGAAA AGTTCATCCT ATTTTGTTAT TATTATTTAT TATGTATTTT CAGAATTGTC   
  
  
+ TCTATCACAA GCATAGTATA TGTGTCAACA TCGTGACAAC TCGACCAATC TACAAATAAC TAGTAAATTC   
  
  
+ AAATAAAGTT ACTCTCACCT AGCTTAAATA AGTACACCTT GATTTAATTG TCTTAGCTAT GGAACCTATT   
  
  
+ CCAAACTAGC CAATACTTCC TTTTCAAAAG AATTAGTTTT AAAGTTTTAA CTTTTACGTC CAATAATGCC   
  
  
+ GGCCGTACTA TGAGTTGCTT TCAAGCCAGT AGTAGTACTG TAGTAGGTAA AAATATTAGA ATACACCTAC   
  
  
+ TATATATGAT ATTGCTCTTC ACCGTATCCT TCTGCATACT CATCTTTCCG TCCCGGAGTC CACTATCTCT   
  
  
+ CCTCCATTCA GACACTCTCT CGCTCTAGAG AACTCATAGC TATGGACCGC GTGCCCGACG GTTATCGGGT   
  
  
+ TATGAATTCG GAGCTTCTTC AGCAAGTTAG CCCTGAGTTG CCGGATCCAA CCACCTGGTT CCATGCCCGT   
  
  
+ CTACCCGACC CCATCTCTCA GTCTCCTCTT GGACCCGGTC CATCCTCCCA GCCAACCCAT TTCTACCAGG   
  
  
+ GTTCTGCCTC AGGGAACCAT GTGGTTGGGA TTGCTGACAC GTGGACGGAC CAGATTGTTG CAGGTTCCTC   
  
  
+ GTCCCAGCAG TGAGGTTGCA GCAGAGCCAG CAGGTGTTGA CTGCAGTCGA AGCCATGGAG GAGGACTCTG   
  
  
+ GGATAAGACT GGTCTACGCT CTGCTGACGT GTGCGGAGGC CATCCAACGT GGCGATTTCC GATTGGCTGT   
  
  
+ CTTGTTAGTT AACAAGATGA GCAATGACCT CCTGCCACGC GTCAACCCGT CCTGCGGTAT TGGCAAAGTA   
  
  
+ GCCGGCTACT TCATAGATGC CTTGACCCGA AGACTATACC AGCAGGGCCC AGTGTCGGGC CTAATCGGGT   
  
  
+ CGGTTCTGGC GTACCAGGTG TTGTACGAGC ACTTCTATGA AGCTTGCCCG TTCCTCAAAT TCGCTCACTT   
  
  
+ CACTGCTAAT CAAGCGATTT TAGAGGCATT CGACGGCCAC GATTGTGTCC ACATCATCGA CTTCGGCCTA   
  
  
+ ATCCACGGCT TGCAATGGCC AGCTCTAATC CAAGCCTTGG CTGTTCGGCA CGGTGGCCCG CCTTTCGTAA   
  
  
+ GATTAACCGG AATCGGGCCG CCTTCTGAAT ACGGGTCGTG CTCGCTTCAA CCTATCGGGT CAACACTGGC   
  
  
+ CCAGTTAGCC CTATCCATGA ATGTTGGGTT CGCATTTCGG GCCGTCGCCG TCTCACGGCT CGAGGACATC   
  
  
+ AAACCATGGA TGGTTAAAAC AAGCCCAAAT GAAGTCGTAG CCGTGAACTC TATCTTCCAA CTCCACCGGC   
  
  
+ TAATCGGGTC GGGTATTGAC CCTGTCCTAA ACTGGGTCCG GAGCCTAAAC CCGAAAATTG TGACACTGGC   
  
  
+ GGAGCAAGAG GCGAACCACA ACCAGCCCGA GTTTTTGGCC CGGTTCACGG AGGCATTACA TTACTATTCA   
  
  
+ ACTATGTTCG ACTCGTTAGA GGCTTGTCAA GTCCAGGCCG ACAAGGACCT AGCCGAGCTA TACTTAGAGA   
  
  
+ GAGAGTTATC CAACATCGTC TGCTGTGAGG GGTCGGCTCG GATTGAGAGG CACGAGCCGC TGGCCCAATG   
  
  
+ GAGGGCCCGA ATGGCTCGGG CCGGGTTCAA GAAGGTGGAT ATGGGTAAAA ATGCGTTCAA GCAAGTAAGC   
  
  
+ ATGTTGCTGA GTTTGTCTTC AGCACAAGGG TATTGTGTGG AGGAGAGTGA GGGATGTTTG AAGCTCGGCT   
  
  
+ GGCATGACCG CCCTCTCATT GCGGCTTCGG CTTGGCGAGC GGAGACTCAA GCTGAGAACT CCAGCACTGT   
  
  
+ TGTGCTTGAT GGGTCATCGT CGTGTAGTTC ATCTTCTTA  

- -Up\_Stream \_Len000TTTTTT CTTTCTTTAC CTCTTTGGTT CGTTATACCT ATTTTTTTTT TGTTTCATTA   
  
  
- TTTCCTTCCT ACTCCGAGGT GTAAGGTGAG CGGGCGCGGT GAGTATCCTC TACTTATCAT TTTACACTCC   
  
  
- CCAATTTACT AAACTTTTTT TAGATCATTC CCGGGTGTTG TCATAACCTT GGGTCGAACG GTTCCTTTAC   
  
  
- AGTCTCCCCG ATCGTTACCT GGTCACGTTC TTCCGGTTCG GTGTAAACCA ATCCCTTAAA GTAGGGTAGG   
  
  
- CCGCGATTTT CGTAGTTTTA ATGATGAATT AGGAACTAGT TTACGGGATA TTTATAAGAT GATGGGGTAA   
  
  
- ACTTTTCCCC ACAACTAATG TTGTGTATCG GCATCCAAAC TGTTTCTCTT AATGGGAATA ACAATCTTCG   
  
  
- TGGTATATGA GAAAAGAGAT TCGAGACTAG AAAAAAGTAG GAAAAGTCGA AAAACAGGAA AATTTAGTTT   
  
  
- AGTGAGTGAA CTCATAACCT CCCTGCAAGG AGTCCTCAGC TTGGTGATTT AATAGAAAAA TTTCTTTTTC   
  
  
- CCGAACTTCG GTTGGGCTAA ATTTTTTTGA CGATTATACA TTCGGATGAG AGAAAGGTCA AGGTTGTTTA   
  
  
- GTTTAAAGTC ATACTTTGTA GGTCATTTCC TAAATAGGCA CTATTACTAC ATTGTATACC AATTTCCCAG   
  
  
- TAAAAGTCAA TGTAGCCTCG TACGTTAATT TCAGGGATAT TTTATCCATC CACGTGCTTG TTCAGTGGTA   
  
  
- GCAGGAAACG AAAAACGTAC TCACGTACGA TTAATACTTG AATTAATTAT AGATTCGTTT TAATTTTTCA   
  
  
- TCAATTTTGA AAAAGTGGAA ACCTTGGAGC ATCGACATCG TCATCGACGG CGTGAGACCA AGATCTGAAG   
  
  
- ATCCCTGGTT CTCTACGTGA GTATACGTAG GAGAAGACAG GAAAATGACA TAGGGGAAGG GTACACTGGA   
  
  
- AAAACGGGCC CACTGAAAAG ACACTCACAA CTATGTTTAA AACATGCATT CGATTGAGTA AATGAAGTGG   
  
  
- CAGAGTACTC AAAATGTAAT GGCTATAATT AAATTTACCG TTTCATAATT TAAACTCGAT TTTAGAGTAT   
  
  
- ATATATAAAC ATAATTTACA CAGACCTAAC AGTTTTATTA AAAACTGTAT ATGTCAGTGA TATATGACTC   
  
  
- AATAATTTTA TATATTTTTA TACTTTTAAA TTTTTTATCA TCAAATTCCA CTGTTGTGAT TTTGTTTGTT   
  
  
- TATTCTATAT TTATCCCTTT TCAAGTAGGA TAAAACAATA ATAATAAATA ATACATAAAA GTCTTAACAG   
  
  
- AGATAGTGTT CGTATCATAT ACACAGTTGT AGCACTGTTG AGCTGGTTAG ATGTTTATTG ATCATTTAAG   
  
  
- TTTATTTCAA TGAGAGTGGA TCGAATTTAT TCATGTGGAA CTAAATTAAC AGAATCGATA CCTTGGATAA   
  
  
- GGTTTGATCG GTTATGAAGG AAAAGTTTTC TTAATCAAAA TTTCAAAATT GAAAATGCAG GTTATTACGG   
  
  
- CCGGCATGAT ACTCAACGAA AGTTCGGTCA TCATCATGAC ATCATCCATT TTTATAATCT TATGTGGATG   
  
  
- ATATATACTA TAACGAGAAG TGGCATAGGA AGACGTATGA GTAGAAAGGC AGGGCCTCAG GTGATAGAGA   
  
  
- GGAGGTAAGT CTGTGAGAGA GCGAGATCTC TTGAGTATCG ATACCTGGCG CACGGGCTGC CAATAGCCCA   
  
  
- ATACTTAAGC CTCGAAGAAG TCGTTCAATC GGGACTCAAC GGCCTAGGTT GGTGGACCAA GGTACGGGCA   
  
  
- GATGGGCTGG GGTAGAGAGT CAGAGGAGAA CCTGGGCCAG GTAGGAGGGT CGGTTGGGTA AAGATGGTCC   
  
  
- CAAGACGGAG TCCCTTGGTA CACCAACCCT AACGACTGTG CACCTGCCTG GTCTAACAAC GTCCAAGGAG   
  
  
- CAGGGTCGTC ACTCCAACGT CGTCTCGGTC GTCCACAACT GACGTCAGCT TCGGTACCTC CTCCTGAGAC   
  
  
- CCTATTCTGA CCAGATGCGA GACGACTGCA CACGCCTCCG GTAGGTTGCA CCGCTAAAGG CTAACCGACA   
  
  
- GAACAATCAA TTGTTCTACT CGTTACTGGA GGACGGTGCG CAGTTGGGCA GGACGCCATA ACCGTTTCAT   
  
  
- CGGCCGATGA AGTATCTACG GAACTGGGCT TCTGATATGG TCGTCCCGGG TCACAGCCCG GATTAGCCCA   
  
  
- GCCAAGACCG CATGGTCCAC AACATGCTCG TGAAGATACT TCGAACGGGC AAGGAGTTTA AGCGAGTGAA   
  
  
- GTGACGATTA GTTCGCTAAA ATCTCCGTAA GCTGCCGGTG CTAACACAGG TGTAGTAGCT GAAGCCGGAT   
  
  
- TAGGTGCCGA ACGTTACCGG TCGAGATTAG GTTCGGAACC GACAAGCCGT GCCACCGGGC GGAAAGCATT   
  
  
- CTAATTGGCC TTAGCCCGGC GGAAGACTTA TGCCCAGCAC GAGCGAAGTT GGATAGCCCA GTTGTGACCG   
  
  
- GGTCAATCGG GATAGGTACT TACAACCCAA GCGTAAAGCC CGGCAGCGGC AGAGTGCCGA GCTCCTGTAG   
  
  
- TTTGGTACCT ACCAATTTTG TTCGGGTTTA CTTCAGCATC GGCACTTGAG ATAGAAGGTT GAGGTGGCCG   
  
  
- ATTAGCCCAG CCCATAACTG GGACAGGATT TGACCCAGGC CTCGGATTTG GGCTTTTAAC ACTGTGACCG   
  
  
- CCTCGTTCTC CGCTTGGTGT TGGTCGGGCT CAAAAACCGG GCCAAGTGCC TCCGTAATGT AATGATAAGT   
  
  
- TGATACAAGC TGAGCAATCT CCGAACAGTT CAGGTCCGGC TGTTCCTGGA TCGGCTCGAT ATGAATCTCT   
  
  
- CTCTCAATAG GTTGTAGCAG ACGACACTCC CCAGCCGAGC CTAACTCTCC GTGCTCGGCG ACCGGGTTAC   
  
  
- CTCCCGGGCT TACCGAGCCC GGCCCAAGTT CTTCCACCTA TACCCATTTT TACGCAAGTT CGTTCATTCG   
  
  
- TACAACGACT CAAACAGAAG TCGTGTTCCC ATAACACACC TCCTCTCACT CCCTACAAAC TTCGAGCCGA   
  
  
- CCGTACTGGC GGGAGAGTAA CGCCGAAGCC GAACCGCTCG CCTCTGAGTT CGACTCTTGA GGTCGTGACA   
  
  
- ACACGAACTA CCCAGTAGCA GCACATCAAG TAGAAGAAT

+     Unnamed\_\_1

| Site Name | Organism | Position | Strand | Matrix score. | sequence | function |
| --- | --- | --- | --- | --- | --- | --- |
| Unnamed\_\_1 | Zea mays | 2351 | - | 5 | CGTGG |  |
| Unnamed\_\_1 | Zea mays | 2387 | - | 5 | CGTGG |  |
| Unnamed\_\_1 | Zea mays | 2139 | - | 5 | CGTGG |  |
| Unnamed\_\_1 | Zea mays | 2082 | + | 5 | CGTGG |  |
| Unnamed\_\_1 | Glycine max | 811 | + | 11 | GAATTTAATTAA | 60K protein binding site |
| Unnamed\_\_1 | Zea mays | 1934 | + | 5 | CGTGG |  |

>HU11G00778.1   
+ -Up\_Stream \_Len000AAAAAA GAAAGAAATG GAGAAACCAA GCAATATGGA TAAAAAAAAA ACAAAGTAAT   
  
  
+ AAAGGAAGGA TGAGGCTCCA CATTCCACTC GCCCGCGCCA CTCATAGGAG ATGAATAGTA AAATGTGAGG   
  
  
+ GGTTAAATGA TTTGAAAAAA ATCTAGTAAG GGCCCACAAC AGTATTGGAA CCCAGCTTGC CAAGGAAATG   
  
  
+ TCAGAGGGGC TAGCAATGGA CCAGTGCAAG AAGGCCAAGC CACATTTGGT TAGGGAATTT CATCCCATCC   
  
  
+ GGCGCTAAAA GCATCAAAAT TACTACTTAA TCCTTGATCA AATGCCCTAT AAATATTCTA CTACCCCATT   
  
  
+ TGAAAAGGGG TGTTGATTAC AACACATAGC CGTAGGTTTG ACAAAGAGAA TTACCCTTAT TGTTAGAAGC   
  
  
+ ACCATATACT CTTTTCTCTA AGCTCTGATC TTTTTTCATC CTTTTCAGCT TTTTGTCCTT TTAAATCAAA   
  
  
+ TCACTCACTT GAGTATTGGA GGGACGTTCC TCAGGAGTCG AACCACTAAA TTATCTTTTT AAAGAAAAAG   
  
  
+ GGCTTGAAGC CAACCCGATT TAAAAAAACT GCTAATATGT AAGCCTACTC TCTTTCCAGT TCCAACAAAT   
  
  
+ CAAATTTCAG TATGAAACAT CCAGTAAAGG ATTTATCCGT GATAATGATG TAACATATGG TTAAAGGGTC   
  
  
+ ATTTTCAGTT ACATCGGAGC ATGCAATTAA AGTCCCTATA AAATAGGTAG GTGCACGAAC AAGTCACCAT   
  
  
+ CGTCCTTTGC TTTTTGCATG AGTGCATGCT AATTATGAAC TTAATTAATA TCTAAGCAAA ATTAAAAAGT   
  
  
+ AGTTAAAACT TTTTCACCTT TGGAACCTCG TAGCTGTAGC AGTAGCTGCC GCACTCTGGT TCTAGACTTC   
  
  
+ TAGGGACCAA GAGATGCACT CATATGCATC CTCTTCTGTC CTTTTACTGT ATCCCCTTCC CATGTGACCT   
  
  
+ TTTTGCCCGG GTGACTTTTC TGTGAGTGTT GATACAAATT TTGTACGTAA GCTAACTCAT TTACTTCACC   
  
  
+ GTCTCATGAG TTTTACATTA CCGATATTAA TTTAAATGGC AAAGTATTAA ATTTGAGCTA AAATCTCATA   
  
  
+ TATATATTTG TATTAAATGT GTCTGGATTG TCAAAATAAT TTTTGACATA TACAGTCACT ATATACTGAG   
  
  
+ TTATTAAAAT ATATAAAAAT ATGAAAATTT AAAAAATAGT AGTTTAAGGT GACAACACTA AAACAAACAA   
  
  
+ ATAAGATATA AATAGGGAAA AGTTCATCCT ATTTTGTTAT TATTATTTAT TATGTATTTT CAGAATTGTC   
  
  
+ TCTATCACAA GCATAGTATA TGTGTCAACA TCGTGACAAC TCGACCAATC TACAAATAAC TAGTAAATTC   
  
  
+ AAATAAAGTT ACTCTCACCT AGCTTAAATA AGTACACCTT GATTTAATTG TCTTAGCTAT GGAACCTATT   
  
  
+ CCAAACTAGC CAATACTTCC TTTTCAAAAG AATTAGTTTT AAAGTTTTAA CTTTTACGTC CAATAATGCC   
  
  
+ GGCCGTACTA TGAGTTGCTT TCAAGCCAGT AGTAGTACTG TAGTAGGTAA AAATATTAGA ATACACCTAC   
  
  
+ TATATATGAT ATTGCTCTTC ACCGTATCCT TCTGCATACT CATCTTTCCG TCCCGGAGTC CACTATCTCT   
  
  
+ CCTCCATTCA GACACTCTCT CGCTCTAGAG AACTCATAGC TATGGACCGC GTGCCCGACG GTTATCGGGT   
  
  
+ TATGAATTCG GAGCTTCTTC AGCAAGTTAG CCCTGAGTTG CCGGATCCAA CCACCTGGTT CCATGCCCGT   
  
  
+ CTACCCGACC CCATCTCTCA GTCTCCTCTT GGACCCGGTC CATCCTCCCA GCCAACCCAT TTCTACCAGG   
  
  
+ GTTCTGCCTC AGGGAACCAT GTGGTTGGGA TTGCTGACAC GTGGACGGAC CAGATTGTTG CAGGTTCCTC   
  
  
+ GTCCCAGCAG TGAGGTTGCA GCAGAGCCAG CAGGTGTTGA CTGCAGTCGA AGCCATGGAG GAGGACTCTG   
  
  
+ GGATAAGACT GGTCTACGCT CTGCTGACGT GTGCGGAGGC CATCCAACGT GGCGATTTCC GATTGGCTGT   
  
  
+ CTTGTTAGTT AACAAGATGA GCAATGACCT CCTGCCACGC GTCAACCCGT CCTGCGGTAT TGGCAAAGTA   
  
  
+ GCCGGCTACT TCATAGATGC CTTGACCCGA AGACTATACC AGCAGGGCCC AGTGTCGGGC CTAATCGGGT   
  
  
+ CGGTTCTGGC GTACCAGGTG TTGTACGAGC ACTTCTATGA AGCTTGCCCG TTCCTCAAAT TCGCTCACTT   
  
  
+ CACTGCTAAT CAAGCGATTT TAGAGGCATT CGACGGCCAC GATTGTGTCC ACATCATCGA CTTCGGCCTA   
  
  
+ ATCCACGGCT TGCAATGGCC AGCTCTAATC CAAGCCTTGG CTGTTCGGCA CGGTGGCCCG CCTTTCGTAA   
  
  
+ GATTAACCGG AATCGGGCCG CCTTCTGAAT ACGGGTCGTG CTCGCTTCAA CCTATCGGGT CAACACTGGC   
  
  
+ CCAGTTAGCC CTATCCATGA ATGTTGGGTT CGCATTTCGG GCCGTCGCCG TCTCACGGCT CGAGGACATC   
  
  
+ AAACCATGGA TGGTTAAAAC AAGCCCAAAT GAAGTCGTAG CCGTGAACTC TATCTTCCAA CTCCACCGGC   
  
  
+ TAATCGGGTC GGGTATTGAC CCTGTCCTAA ACTGGGTCCG GAGCCTAAAC CCGAAAATTG TGACACTGGC   
  
  
+ GGAGCAAGAG GCGAACCACA ACCAGCCCGA GTTTTTGGCC CGGTTCACGG AGGCATTACA TTACTATTCA   
  
  
+ ACTATGTTCG ACTCGTTAGA GGCTTGTCAA GTCCAGGCCG ACAAGGACCT AGCCGAGCTA TACTTAGAGA   
  
  
+ GAGAGTTATC CAACATCGTC TGCTGTGAGG GGTCGGCTCG GATTGAGAGG CACGAGCCGC TGGCCCAATG   
  
  
+ GAGGGCCCGA ATGGCTCGGG CCGGGTTCAA GAAGGTGGAT ATGGGTAAAA ATGCGTTCAA GCAAGTAAGC   
  
  
+ ATGTTGCTGA GTTTGTCTTC AGCACAAGGG TATTGTGTGG AGGAGAGTGA GGGATGTTTG AAGCTCGGCT   
  
  
+ GGCATGACCG CCCTCTCATT GCGGCTTCGG CTTGGCGAGC GGAGACTCAA GCTGAGAACT CCAGCACTGT   
  
  
+ TGTGCTTGAT GGGTCATCGT CGTGTAGTTC ATCTTCTTA  

- -Up\_Stream \_Len000TTTTTT CTTTCTTTAC CTCTTTGGTT CGTTATACCT ATTTTTTTTT TGTTTCATTA   
  
  
- TTTCCTTCCT ACTCCGAGGT GTAAGGTGAG CGGGCGCGGT GAGTATCCTC TACTTATCAT TTTACACTCC   
  
  
- CCAATTTACT AAACTTTTTT TAGATCATTC CCGGGTGTTG TCATAACCTT GGGTCGAACG GTTCCTTTAC   
  
  
- AGTCTCCCCG ATCGTTACCT GGTCACGTTC TTCCGGTTCG GTGTAAACCA ATCCCTTAAA GTAGGGTAGG   
  
  
- CCGCGATTTT CGTAGTTTTA ATGATGAATT AGGAACTAGT TTACGGGATA TTTATAAGAT GATGGGGTAA   
  
  
- ACTTTTCCCC ACAACTAATG TTGTGTATCG GCATCCAAAC TGTTTCTCTT AATGGGAATA ACAATCTTCG   
  
  
- TGGTATATGA GAAAAGAGAT TCGAGACTAG AAAAAAGTAG GAAAAGTCGA AAAACAGGAA AATTTAGTTT   
  
  
- AGTGAGTGAA CTCATAACCT CCCTGCAAGG AGTCCTCAGC TTGGTGATTT AATAGAAAAA TTTCTTTTTC   
  
  
- CCGAACTTCG GTTGGGCTAA ATTTTTTTGA CGATTATACA TTCGGATGAG AGAAAGGTCA AGGTTGTTTA   
  
  
- GTTTAAAGTC ATACTTTGTA GGTCATTTCC TAAATAGGCA CTATTACTAC ATTGTATACC AATTTCCCAG   
  
  
- TAAAAGTCAA TGTAGCCTCG TACGTTAATT TCAGGGATAT TTTATCCATC CACGTGCTTG TTCAGTGGTA   
  
  
- GCAGGAAACG AAAAACGTAC TCACGTACGA TTAATACTTG AATTAATTAT AGATTCGTTT TAATTTTTCA   
  
  
- TCAATTTTGA AAAAGTGGAA ACCTTGGAGC ATCGACATCG TCATCGACGG CGTGAGACCA AGATCTGAAG   
  
  
- ATCCCTGGTT CTCTACGTGA GTATACGTAG GAGAAGACAG GAAAATGACA TAGGGGAAGG GTACACTGGA   
  
  
- AAAACGGGCC CACTGAAAAG ACACTCACAA CTATGTTTAA AACATGCATT CGATTGAGTA AATGAAGTGG   
  
  
- CAGAGTACTC AAAATGTAAT GGCTATAATT AAATTTACCG TTTCATAATT TAAACTCGAT TTTAGAGTAT   
  
  
- ATATATAAAC ATAATTTACA CAGACCTAAC AGTTTTATTA AAAACTGTAT ATGTCAGTGA TATATGACTC   
  
  
- AATAATTTTA TATATTTTTA TACTTTTAAA TTTTTTATCA TCAAATTCCA CTGTTGTGAT TTTGTTTGTT   
  
  
- TATTCTATAT TTATCCCTTT TCAAGTAGGA TAAAACAATA ATAATAAATA ATACATAAAA GTCTTAACAG   
  
  
- AGATAGTGTT CGTATCATAT ACACAGTTGT AGCACTGTTG AGCTGGTTAG ATGTTTATTG ATCATTTAAG   
  
  
- TTTATTTCAA TGAGAGTGGA TCGAATTTAT TCATGTGGAA CTAAATTAAC AGAATCGATA CCTTGGATAA   
  
  
- GGTTTGATCG GTTATGAAGG AAAAGTTTTC TTAATCAAAA TTTCAAAATT GAAAATGCAG GTTATTACGG   
  
  
- CCGGCATGAT ACTCAACGAA AGTTCGGTCA TCATCATGAC ATCATCCATT TTTATAATCT TATGTGGATG   
  
  
- ATATATACTA TAACGAGAAG TGGCATAGGA AGACGTATGA GTAGAAAGGC AGGGCCTCAG GTGATAGAGA   
  
  
- GGAGGTAAGT CTGTGAGAGA GCGAGATCTC TTGAGTATCG ATACCTGGCG CACGGGCTGC CAATAGCCCA   
  
  
- ATACTTAAGC CTCGAAGAAG TCGTTCAATC GGGACTCAAC GGCCTAGGTT GGTGGACCAA GGTACGGGCA   
  
  
- GATGGGCTGG GGTAGAGAGT CAGAGGAGAA CCTGGGCCAG GTAGGAGGGT CGGTTGGGTA AAGATGGTCC   
  
  
- CAAGACGGAG TCCCTTGGTA CACCAACCCT AACGACTGTG CACCTGCCTG GTCTAACAAC GTCCAAGGAG   
  
  
- CAGGGTCGTC ACTCCAACGT CGTCTCGGTC GTCCACAACT GACGTCAGCT TCGGTACCTC CTCCTGAGAC   
  
  
- CCTATTCTGA CCAGATGCGA GACGACTGCA CACGCCTCCG GTAGGTTGCA CCGCTAAAGG CTAACCGACA   
  
  
- GAACAATCAA TTGTTCTACT CGTTACTGGA GGACGGTGCG CAGTTGGGCA GGACGCCATA ACCGTTTCAT   
  
  
- CGGCCGATGA AGTATCTACG GAACTGGGCT TCTGATATGG TCGTCCCGGG TCACAGCCCG GATTAGCCCA   
  
  
- GCCAAGACCG CATGGTCCAC AACATGCTCG TGAAGATACT TCGAACGGGC AAGGAGTTTA AGCGAGTGAA   
  
  
- GTGACGATTA GTTCGCTAAA ATCTCCGTAA GCTGCCGGTG CTAACACAGG TGTAGTAGCT GAAGCCGGAT   
  
  
- TAGGTGCCGA ACGTTACCGG TCGAGATTAG GTTCGGAACC GACAAGCCGT GCCACCGGGC GGAAAGCATT   
  
  
- CTAATTGGCC TTAGCCCGGC GGAAGACTTA TGCCCAGCAC GAGCGAAGTT GGATAGCCCA GTTGTGACCG   
  
  
- GGTCAATCGG GATAGGTACT TACAACCCAA GCGTAAAGCC CGGCAGCGGC AGAGTGCCGA GCTCCTGTAG   
  
  
- TTTGGTACCT ACCAATTTTG TTCGGGTTTA CTTCAGCATC GGCACTTGAG ATAGAAGGTT GAGGTGGCCG   
  
  
- ATTAGCCCAG CCCATAACTG GGACAGGATT TGACCCAGGC CTCGGATTTG GGCTTTTAAC ACTGTGACCG   
  
  
- CCTCGTTCTC CGCTTGGTGT TGGTCGGGCT CAAAAACCGG GCCAAGTGCC TCCGTAATGT AATGATAAGT   
  
  
- TGATACAAGC TGAGCAATCT CCGAACAGTT CAGGTCCGGC TGTTCCTGGA TCGGCTCGAT ATGAATCTCT   
  
  
- CTCTCAATAG GTTGTAGCAG ACGACACTCC CCAGCCGAGC CTAACTCTCC GTGCTCGGCG ACCGGGTTAC   
  
  
- CTCCCGGGCT TACCGAGCCC GGCCCAAGTT CTTCCACCTA TACCCATTTT TACGCAAGTT CGTTCATTCG   
  
  
- TACAACGACT CAAACAGAAG TCGTGTTCCC ATAACACACC TCCTCTCACT CCCTACAAAC TTCGAGCCGA   
  
  
- CCGTACTGGC GGGAGAGTAA CGCCGAAGCC GAACCGCTCG CCTCTGAGTT CGACTCTTGA GGTCGTGACA   
  
  
- ACACGAACTA CCCAGTAGCA GCACATCAAG TAGAAGAAT

+     Unnamed\_\_4

| Site Name | Organism | Position | Strand | Matrix score. | sequence | function |
| --- | --- | --- | --- | --- | --- | --- |
| Unnamed\_\_4 | Petroselinum hortense | 3143 | + | 4 | CTCC |  |
| Unnamed\_\_4 | Petroselinum hortense | 2783 | - | 4 | CTCC |  |
| Unnamed\_\_4 | Petroselinum hortense | 1683 | + | 4 | CTCC |  |
| Unnamed\_\_4 | Petroselinum hortense | 1669 | - | 4 | CTCC |  |
| Unnamed\_\_4 | Petroselinum hortense | 1847 | + | 4 | CTCC |  |
| Unnamed\_\_4 | Petroselinum hortense | 1686 | + | 4 | CTCC |  |
| Unnamed\_\_4 | Petroselinum hortense | 720 | - | 4 | CTCC |  |
| Unnamed\_\_4 | Petroselinum hortense | 512 | - | 4 | CTCC |  |
| Unnamed\_\_4 | Petroselinum hortense | 2735 | - | 4 | CTCC |  |
| Unnamed\_\_4 | Petroselinum hortense | 528 | - | 4 | CTCC |  |
| Unnamed\_\_4 | Petroselinum hortense | 3125 | - | 4 | CTCC |  |
| Unnamed\_\_4 | Petroselinum hortense | 3056 | - | 4 | CTCC |  |
| Unnamed\_\_4 | Petroselinum hortense | 3053 | - | 4 | CTCC |  |
| Unnamed\_\_4 | Petroselinum hortense | 2704 | - | 4 | CTCC |  |
| Unnamed\_\_4 | Petroselinum hortense | 2133 | + | 4 | CTCC |  |
| Unnamed\_\_4 | Petroselinum hortense | 2655 | + | 4 | CTCC |  |
| Unnamed\_\_4 | Petroselinum hortense | 2944 | - | 4 | CTCC |  |
| Unnamed\_\_4 | Petroselinum hortense | 2024 | - | 4 | CTCC |  |
| Unnamed\_\_4 | Petroselinum hortense | 2021 | - | 4 | CTCC |  |
| Unnamed\_\_4 | Petroselinum hortense | 2069 | - | 4 | CTCC |  |
| Unnamed\_\_4 | Petroselinum hortense | 121 | - | 4 | CTCC |  |
| Unnamed\_\_4 | Petroselinum hortense | 90 | + | 4 | CTCC |  |
| Unnamed\_\_4 | Petroselinum hortense | 34 | - | 4 | CTCC |  |
| Unnamed\_\_4 | Petroselinum hortense | 1764 | - | 4 | CTCC |  |
| Unnamed\_\_4 | Petroselinum hortense | 1869 | + | 4 | CTCC |  |

>HU11G00778.1   
+ -Up\_Stream \_Len000AAAAAA GAAAGAAATG GAGAAACCAA GCAATATGGA TAAAAAAAAA ACAAAGTAAT   
  
  
+ AAAGGAAGGA TGAGGCTCCA CATTCCACTC GCCCGCGCCA CTCATAGGAG ATGAATAGTA AAATGTGAGG   
  
  
+ GGTTAAATGA TTTGAAAAAA ATCTAGTAAG GGCCCACAAC AGTATTGGAA CCCAGCTTGC CAAGGAAATG   
  
  
+ TCAGAGGGGC TAGCAATGGA CCAGTGCAAG AAGGCCAAGC CACATTTGGT TAGGGAATTT CATCCCATCC   
  
  
+ GGCGCTAAAA GCATCAAAAT TACTACTTAA TCCTTGATCA AATGCCCTAT AAATATTCTA CTACCCCATT   
  
  
+ TGAAAAGGGG TGTTGATTAC AACACATAGC CGTAGGTTTG ACAAAGAGAA TTACCCTTAT TGTTAGAAGC   
  
  
+ ACCATATACT CTTTTCTCTA AGCTCTGATC TTTTTTCATC CTTTTCAGCT TTTTGTCCTT TTAAATCAAA   
  
  
+ TCACTCACTT GAGTATTGGA GGGACGTTCC TCAGGAGTCG AACCACTAAA TTATCTTTTT AAAGAAAAAG   
  
  
+ GGCTTGAAGC CAACCCGATT TAAAAAAACT GCTAATATGT AAGCCTACTC TCTTTCCAGT TCCAACAAAT   
  
  
+ CAAATTTCAG TATGAAACAT CCAGTAAAGG ATTTATCCGT GATAATGATG TAACATATGG TTAAAGGGTC   
  
  
+ ATTTTCAGTT ACATCGGAGC ATGCAATTAA AGTCCCTATA AAATAGGTAG GTGCACGAAC AAGTCACCAT   
  
  
+ CGTCCTTTGC TTTTTGCATG AGTGCATGCT AATTATGAAC TTAATTAATA TCTAAGCAAA ATTAAAAAGT   
  
  
+ AGTTAAAACT TTTTCACCTT TGGAACCTCG TAGCTGTAGC AGTAGCTGCC GCACTCTGGT TCTAGACTTC   
  
  
+ TAGGGACCAA GAGATGCACT CATATGCATC CTCTTCTGTC CTTTTACTGT ATCCCCTTCC CATGTGACCT   
  
  
+ TTTTGCCCGG GTGACTTTTC TGTGAGTGTT GATACAAATT TTGTACGTAA GCTAACTCAT TTACTTCACC   
  
  
+ GTCTCATGAG TTTTACATTA CCGATATTAA TTTAAATGGC AAAGTATTAA ATTTGAGCTA AAATCTCATA   
  
  
+ TATATATTTG TATTAAATGT GTCTGGATTG TCAAAATAAT TTTTGACATA TACAGTCACT ATATACTGAG   
  
  
+ TTATTAAAAT ATATAAAAAT ATGAAAATTT AAAAAATAGT AGTTTAAGGT GACAACACTA AAACAAACAA   
  
  
+ ATAAGATATA AATAGGGAAA AGTTCATCCT ATTTTGTTAT TATTATTTAT TATGTATTTT CAGAATTGTC   
  
  
+ TCTATCACAA GCATAGTATA TGTGTCAACA TCGTGACAAC TCGACCAATC TACAAATAAC TAGTAAATTC   
  
  
+ AAATAAAGTT ACTCTCACCT AGCTTAAATA AGTACACCTT GATTTAATTG TCTTAGCTAT GGAACCTATT   
  
  
+ CCAAACTAGC CAATACTTCC TTTTCAAAAG AATTAGTTTT AAAGTTTTAA CTTTTACGTC CAATAATGCC   
  
  
+ GGCCGTACTA TGAGTTGCTT TCAAGCCAGT AGTAGTACTG TAGTAGGTAA AAATATTAGA ATACACCTAC   
  
  
+ TATATATGAT ATTGCTCTTC ACCGTATCCT TCTGCATACT CATCTTTCCG TCCCGGAGTC CACTATCTCT   
  
  
+ CCTCCATTCA GACACTCTCT CGCTCTAGAG AACTCATAGC TATGGACCGC GTGCCCGACG GTTATCGGGT   
  
  
+ TATGAATTCG GAGCTTCTTC AGCAAGTTAG CCCTGAGTTG CCGGATCCAA CCACCTGGTT CCATGCCCGT   
  
  
+ CTACCCGACC CCATCTCTCA GTCTCCTCTT GGACCCGGTC CATCCTCCCA GCCAACCCAT TTCTACCAGG   
  
  
+ GTTCTGCCTC AGGGAACCAT GTGGTTGGGA TTGCTGACAC GTGGACGGAC CAGATTGTTG CAGGTTCCTC   
  
  
+ GTCCCAGCAG TGAGGTTGCA GCAGAGCCAG CAGGTGTTGA CTGCAGTCGA AGCCATGGAG GAGGACTCTG   
  
  
+ GGATAAGACT GGTCTACGCT CTGCTGACGT GTGCGGAGGC CATCCAACGT GGCGATTTCC GATTGGCTGT   
  
  
+ CTTGTTAGTT AACAAGATGA GCAATGACCT CCTGCCACGC GTCAACCCGT CCTGCGGTAT TGGCAAAGTA   
  
  
+ GCCGGCTACT TCATAGATGC CTTGACCCGA AGACTATACC AGCAGGGCCC AGTGTCGGGC CTAATCGGGT   
  
  
+ CGGTTCTGGC GTACCAGGTG TTGTACGAGC ACTTCTATGA AGCTTGCCCG TTCCTCAAAT TCGCTCACTT   
  
  
+ CACTGCTAAT CAAGCGATTT TAGAGGCATT CGACGGCCAC GATTGTGTCC ACATCATCGA CTTCGGCCTA   
  
  
+ ATCCACGGCT TGCAATGGCC AGCTCTAATC CAAGCCTTGG CTGTTCGGCA CGGTGGCCCG CCTTTCGTAA   
  
  
+ GATTAACCGG AATCGGGCCG CCTTCTGAAT ACGGGTCGTG CTCGCTTCAA CCTATCGGGT CAACACTGGC   
  
  
+ CCAGTTAGCC CTATCCATGA ATGTTGGGTT CGCATTTCGG GCCGTCGCCG TCTCACGGCT CGAGGACATC   
  
  
+ AAACCATGGA TGGTTAAAAC AAGCCCAAAT GAAGTCGTAG CCGTGAACTC TATCTTCCAA CTCCACCGGC   
  
  
+ TAATCGGGTC GGGTATTGAC CCTGTCCTAA ACTGGGTCCG GAGCCTAAAC CCGAAAATTG TGACACTGGC   
  
  
+ GGAGCAAGAG GCGAACCACA ACCAGCCCGA GTTTTTGGCC CGGTTCACGG AGGCATTACA TTACTATTCA   
  
  
+ ACTATGTTCG ACTCGTTAGA GGCTTGTCAA GTCCAGGCCG ACAAGGACCT AGCCGAGCTA TACTTAGAGA   
  
  
+ GAGAGTTATC CAACATCGTC TGCTGTGAGG GGTCGGCTCG GATTGAGAGG CACGAGCCGC TGGCCCAATG   
  
  
+ GAGGGCCCGA ATGGCTCGGG CCGGGTTCAA GAAGGTGGAT ATGGGTAAAA ATGCGTTCAA GCAAGTAAGC   
  
  
+ ATGTTGCTGA GTTTGTCTTC AGCACAAGGG TATTGTGTGG AGGAGAGTGA GGGATGTTTG AAGCTCGGCT   
  
  
+ GGCATGACCG CCCTCTCATT GCGGCTTCGG CTTGGCGAGC GGAGACTCAA GCTGAGAACT CCAGCACTGT   
  
  
+ TGTGCTTGAT GGGTCATCGT CGTGTAGTTC ATCTTCTTA  

- -Up\_Stream \_Len000TTTTTT CTTTCTTTAC CTCTTTGGTT CGTTATACCT ATTTTTTTTT TGTTTCATTA   
  
  
- TTTCCTTCCT ACTCCGAGGT GTAAGGTGAG CGGGCGCGGT GAGTATCCTC TACTTATCAT TTTACACTCC   
  
  
- CCAATTTACT AAACTTTTTT TAGATCATTC CCGGGTGTTG TCATAACCTT GGGTCGAACG GTTCCTTTAC   
  
  
- AGTCTCCCCG ATCGTTACCT GGTCACGTTC TTCCGGTTCG GTGTAAACCA ATCCCTTAAA GTAGGGTAGG   
  
  
- CCGCGATTTT CGTAGTTTTA ATGATGAATT AGGAACTAGT TTACGGGATA TTTATAAGAT GATGGGGTAA   
  
  
- ACTTTTCCCC ACAACTAATG TTGTGTATCG GCATCCAAAC TGTTTCTCTT AATGGGAATA ACAATCTTCG   
  
  
- TGGTATATGA GAAAAGAGAT TCGAGACTAG AAAAAAGTAG GAAAAGTCGA AAAACAGGAA AATTTAGTTT   
  
  
- AGTGAGTGAA CTCATAACCT CCCTGCAAGG AGTCCTCAGC TTGGTGATTT AATAGAAAAA TTTCTTTTTC   
  
  
- CCGAACTTCG GTTGGGCTAA ATTTTTTTGA CGATTATACA TTCGGATGAG AGAAAGGTCA AGGTTGTTTA   
  
  
- GTTTAAAGTC ATACTTTGTA GGTCATTTCC TAAATAGGCA CTATTACTAC ATTGTATACC AATTTCCCAG   
  
  
- TAAAAGTCAA TGTAGCCTCG TACGTTAATT TCAGGGATAT TTTATCCATC CACGTGCTTG TTCAGTGGTA   
  
  
- GCAGGAAACG AAAAACGTAC TCACGTACGA TTAATACTTG AATTAATTAT AGATTCGTTT TAATTTTTCA   
  
  
- TCAATTTTGA AAAAGTGGAA ACCTTGGAGC ATCGACATCG TCATCGACGG CGTGAGACCA AGATCTGAAG   
  
  
- ATCCCTGGTT CTCTACGTGA GTATACGTAG GAGAAGACAG GAAAATGACA TAGGGGAAGG GTACACTGGA   
  
  
- AAAACGGGCC CACTGAAAAG ACACTCACAA CTATGTTTAA AACATGCATT CGATTGAGTA AATGAAGTGG   
  
  
- CAGAGTACTC AAAATGTAAT GGCTATAATT AAATTTACCG TTTCATAATT TAAACTCGAT TTTAGAGTAT   
  
  
- ATATATAAAC ATAATTTACA CAGACCTAAC AGTTTTATTA AAAACTGTAT ATGTCAGTGA TATATGACTC   
  
  
- AATAATTTTA TATATTTTTA TACTTTTAAA TTTTTTATCA TCAAATTCCA CTGTTGTGAT TTTGTTTGTT   
  
  
- TATTCTATAT TTATCCCTTT TCAAGTAGGA TAAAACAATA ATAATAAATA ATACATAAAA GTCTTAACAG   
  
  
- AGATAGTGTT CGTATCATAT ACACAGTTGT AGCACTGTTG AGCTGGTTAG ATGTTTATTG ATCATTTAAG   
  
  
- TTTATTTCAA TGAGAGTGGA TCGAATTTAT TCATGTGGAA CTAAATTAAC AGAATCGATA CCTTGGATAA   
  
  
- GGTTTGATCG GTTATGAAGG AAAAGTTTTC TTAATCAAAA TTTCAAAATT GAAAATGCAG GTTATTACGG   
  
  
- CCGGCATGAT ACTCAACGAA AGTTCGGTCA TCATCATGAC ATCATCCATT TTTATAATCT TATGTGGATG   
  
  
- ATATATACTA TAACGAGAAG TGGCATAGGA AGACGTATGA GTAGAAAGGC AGGGCCTCAG GTGATAGAGA   
  
  
- GGAGGTAAGT CTGTGAGAGA GCGAGATCTC TTGAGTATCG ATACCTGGCG CACGGGCTGC CAATAGCCCA   
  
  
- ATACTTAAGC CTCGAAGAAG TCGTTCAATC GGGACTCAAC GGCCTAGGTT GGTGGACCAA GGTACGGGCA   
  
  
- GATGGGCTGG GGTAGAGAGT CAGAGGAGAA CCTGGGCCAG GTAGGAGGGT CGGTTGGGTA AAGATGGTCC   
  
  
- CAAGACGGAG TCCCTTGGTA CACCAACCCT AACGACTGTG CACCTGCCTG GTCTAACAAC GTCCAAGGAG   
  
  
- CAGGGTCGTC ACTCCAACGT CGTCTCGGTC GTCCACAACT GACGTCAGCT TCGGTACCTC CTCCTGAGAC   
  
  
- CCTATTCTGA CCAGATGCGA GACGACTGCA CACGCCTCCG GTAGGTTGCA CCGCTAAAGG CTAACCGACA   
  
  
- GAACAATCAA TTGTTCTACT CGTTACTGGA GGACGGTGCG CAGTTGGGCA GGACGCCATA ACCGTTTCAT   
  
  
- CGGCCGATGA AGTATCTACG GAACTGGGCT TCTGATATGG TCGTCCCGGG TCACAGCCCG GATTAGCCCA   
  
  
- GCCAAGACCG CATGGTCCAC AACATGCTCG TGAAGATACT TCGAACGGGC AAGGAGTTTA AGCGAGTGAA   
  
  
- GTGACGATTA GTTCGCTAAA ATCTCCGTAA GCTGCCGGTG CTAACACAGG TGTAGTAGCT GAAGCCGGAT   
  
  
- TAGGTGCCGA ACGTTACCGG TCGAGATTAG GTTCGGAACC GACAAGCCGT GCCACCGGGC GGAAAGCATT   
  
  
- CTAATTGGCC TTAGCCCGGC GGAAGACTTA TGCCCAGCAC GAGCGAAGTT GGATAGCCCA GTTGTGACCG   
  
  
- GGTCAATCGG GATAGGTACT TACAACCCAA GCGTAAAGCC CGGCAGCGGC AGAGTGCCGA GCTCCTGTAG   
  
  
- TTTGGTACCT ACCAATTTTG TTCGGGTTTA CTTCAGCATC GGCACTTGAG ATAGAAGGTT GAGGTGGCCG   
  
  
- ATTAGCCCAG CCCATAACTG GGACAGGATT TGACCCAGGC CTCGGATTTG GGCTTTTAAC ACTGTGACCG   
  
  
- CCTCGTTCTC CGCTTGGTGT TGGTCGGGCT CAAAAACCGG GCCAAGTGCC TCCGTAATGT AATGATAAGT   
  
  
- TGATACAAGC TGAGCAATCT CCGAACAGTT CAGGTCCGGC TGTTCCTGGA TCGGCTCGAT ATGAATCTCT   
  
  
- CTCTCAATAG GTTGTAGCAG ACGACACTCC CCAGCCGAGC CTAACTCTCC GTGCTCGGCG ACCGGGTTAC   
  
  
- CTCCCGGGCT TACCGAGCCC GGCCCAAGTT CTTCCACCTA TACCCATTTT TACGCAAGTT CGTTCATTCG   
  
  
- TACAACGACT CAAACAGAAG TCGTGTTCCC ATAACACACC TCCTCTCACT CCCTACAAAC TTCGAGCCGA   
  
  
- CCGTACTGGC GGGAGAGTAA CGCCGAAGCC GAACCGCTCG CCTCTGAGTT CGACTCTTGA GGTCGTGACA   
  
  
- ACACGAACTA CCCAGTAGCA GCACATCAAG TAGAAGAAT

+     W box

| Site Name | Organism | Position | Strand | Matrix score. | sequence | function |
| --- | --- | --- | --- | --- | --- | --- |
| W box | Arabidopsis thaliana | 2680 | + | 6 | TTGACC |  |
| W box | Arabidopsis thaliana | 2512 | - | 6 | TTGACC |  |
| W box | Arabidopsis thaliana | 2196 | + | 6 | TTGACC |  |

>HU11G00778.1   
+ -Up\_Stream \_Len000AAAAAA GAAAGAAATG GAGAAACCAA GCAATATGGA TAAAAAAAAA ACAAAGTAAT   
  
  
+ AAAGGAAGGA TGAGGCTCCA CATTCCACTC GCCCGCGCCA CTCATAGGAG ATGAATAGTA AAATGTGAGG   
  
  
+ GGTTAAATGA TTTGAAAAAA ATCTAGTAAG GGCCCACAAC AGTATTGGAA CCCAGCTTGC CAAGGAAATG   
  
  
+ TCAGAGGGGC TAGCAATGGA CCAGTGCAAG AAGGCCAAGC CACATTTGGT TAGGGAATTT CATCCCATCC   
  
  
+ GGCGCTAAAA GCATCAAAAT TACTACTTAA TCCTTGATCA AATGCCCTAT AAATATTCTA CTACCCCATT   
  
  
+ TGAAAAGGGG TGTTGATTAC AACACATAGC CGTAGGTTTG ACAAAGAGAA TTACCCTTAT TGTTAGAAGC   
  
  
+ ACCATATACT CTTTTCTCTA AGCTCTGATC TTTTTTCATC CTTTTCAGCT TTTTGTCCTT TTAAATCAAA   
  
  
+ TCACTCACTT GAGTATTGGA GGGACGTTCC TCAGGAGTCG AACCACTAAA TTATCTTTTT AAAGAAAAAG   
  
  
+ GGCTTGAAGC CAACCCGATT TAAAAAAACT GCTAATATGT AAGCCTACTC TCTTTCCAGT TCCAACAAAT   
  
  
+ CAAATTTCAG TATGAAACAT CCAGTAAAGG ATTTATCCGT GATAATGATG TAACATATGG TTAAAGGGTC   
  
  
+ ATTTTCAGTT ACATCGGAGC ATGCAATTAA AGTCCCTATA AAATAGGTAG GTGCACGAAC AAGTCACCAT   
  
  
+ CGTCCTTTGC TTTTTGCATG AGTGCATGCT AATTATGAAC TTAATTAATA TCTAAGCAAA ATTAAAAAGT   
  
  
+ AGTTAAAACT TTTTCACCTT TGGAACCTCG TAGCTGTAGC AGTAGCTGCC GCACTCTGGT TCTAGACTTC   
  
  
+ TAGGGACCAA GAGATGCACT CATATGCATC CTCTTCTGTC CTTTTACTGT ATCCCCTTCC CATGTGACCT   
  
  
+ TTTTGCCCGG GTGACTTTTC TGTGAGTGTT GATACAAATT TTGTACGTAA GCTAACTCAT TTACTTCACC   
  
  
+ GTCTCATGAG TTTTACATTA CCGATATTAA TTTAAATGGC AAAGTATTAA ATTTGAGCTA AAATCTCATA   
  
  
+ TATATATTTG TATTAAATGT GTCTGGATTG TCAAAATAAT TTTTGACATA TACAGTCACT ATATACTGAG   
  
  
+ TTATTAAAAT ATATAAAAAT ATGAAAATTT AAAAAATAGT AGTTTAAGGT GACAACACTA AAACAAACAA   
  
  
+ ATAAGATATA AATAGGGAAA AGTTCATCCT ATTTTGTTAT TATTATTTAT TATGTATTTT CAGAATTGTC   
  
  
+ TCTATCACAA GCATAGTATA TGTGTCAACA TCGTGACAAC TCGACCAATC TACAAATAAC TAGTAAATTC   
  
  
+ AAATAAAGTT ACTCTCACCT AGCTTAAATA AGTACACCTT GATTTAATTG TCTTAGCTAT GGAACCTATT   
  
  
+ CCAAACTAGC CAATACTTCC TTTTCAAAAG AATTAGTTTT AAAGTTTTAA CTTTTACGTC CAATAATGCC   
  
  
+ GGCCGTACTA TGAGTTGCTT TCAAGCCAGT AGTAGTACTG TAGTAGGTAA AAATATTAGA ATACACCTAC   
  
  
+ TATATATGAT ATTGCTCTTC ACCGTATCCT TCTGCATACT CATCTTTCCG TCCCGGAGTC CACTATCTCT   
  
  
+ CCTCCATTCA GACACTCTCT CGCTCTAGAG AACTCATAGC TATGGACCGC GTGCCCGACG GTTATCGGGT   
  
  
+ TATGAATTCG GAGCTTCTTC AGCAAGTTAG CCCTGAGTTG CCGGATCCAA CCACCTGGTT CCATGCCCGT   
  
  
+ CTACCCGACC CCATCTCTCA GTCTCCTCTT GGACCCGGTC CATCCTCCCA GCCAACCCAT TTCTACCAGG   
  
  
+ GTTCTGCCTC AGGGAACCAT GTGGTTGGGA TTGCTGACAC GTGGACGGAC CAGATTGTTG CAGGTTCCTC   
  
  
+ GTCCCAGCAG TGAGGTTGCA GCAGAGCCAG CAGGTGTTGA CTGCAGTCGA AGCCATGGAG GAGGACTCTG   
  
  
+ GGATAAGACT GGTCTACGCT CTGCTGACGT GTGCGGAGGC CATCCAACGT GGCGATTTCC GATTGGCTGT   
  
  
+ CTTGTTAGTT AACAAGATGA GCAATGACCT CCTGCCACGC GTCAACCCGT CCTGCGGTAT TGGCAAAGTA   
  
  
+ GCCGGCTACT TCATAGATGC CTTGACCCGA AGACTATACC AGCAGGGCCC AGTGTCGGGC CTAATCGGGT   
  
  
+ CGGTTCTGGC GTACCAGGTG TTGTACGAGC ACTTCTATGA AGCTTGCCCG TTCCTCAAAT TCGCTCACTT   
  
  
+ CACTGCTAAT CAAGCGATTT TAGAGGCATT CGACGGCCAC GATTGTGTCC ACATCATCGA CTTCGGCCTA   
  
  
+ ATCCACGGCT TGCAATGGCC AGCTCTAATC CAAGCCTTGG CTGTTCGGCA CGGTGGCCCG CCTTTCGTAA   
  
  
+ GATTAACCGG AATCGGGCCG CCTTCTGAAT ACGGGTCGTG CTCGCTTCAA CCTATCGGGT CAACACTGGC   
  
  
+ CCAGTTAGCC CTATCCATGA ATGTTGGGTT CGCATTTCGG GCCGTCGCCG TCTCACGGCT CGAGGACATC   
  
  
+ AAACCATGGA TGGTTAAAAC AAGCCCAAAT GAAGTCGTAG CCGTGAACTC TATCTTCCAA CTCCACCGGC   
  
  
+ TAATCGGGTC GGGTATTGAC CCTGTCCTAA ACTGGGTCCG GAGCCTAAAC CCGAAAATTG TGACACTGGC   
  
  
+ GGAGCAAGAG GCGAACCACA ACCAGCCCGA GTTTTTGGCC CGGTTCACGG AGGCATTACA TTACTATTCA   
  
  
+ ACTATGTTCG ACTCGTTAGA GGCTTGTCAA GTCCAGGCCG ACAAGGACCT AGCCGAGCTA TACTTAGAGA   
  
  
+ GAGAGTTATC CAACATCGTC TGCTGTGAGG GGTCGGCTCG GATTGAGAGG CACGAGCCGC TGGCCCAATG   
  
  
+ GAGGGCCCGA ATGGCTCGGG CCGGGTTCAA GAAGGTGGAT ATGGGTAAAA ATGCGTTCAA GCAAGTAAGC   
  
  
+ ATGTTGCTGA GTTTGTCTTC AGCACAAGGG TATTGTGTGG AGGAGAGTGA GGGATGTTTG AAGCTCGGCT   
  
  
+ GGCATGACCG CCCTCTCATT GCGGCTTCGG CTTGGCGAGC GGAGACTCAA GCTGAGAACT CCAGCACTGT   
  
  
+ TGTGCTTGAT GGGTCATCGT CGTGTAGTTC ATCTTCTTA  

- -Up\_Stream \_Len000TTTTTT CTTTCTTTAC CTCTTTGGTT CGTTATACCT ATTTTTTTTT TGTTTCATTA   
  
  
- TTTCCTTCCT ACTCCGAGGT GTAAGGTGAG CGGGCGCGGT GAGTATCCTC TACTTATCAT TTTACACTCC   
  
  
- CCAATTTACT AAACTTTTTT TAGATCATTC CCGGGTGTTG TCATAACCTT GGGTCGAACG GTTCCTTTAC   
  
  
- AGTCTCCCCG ATCGTTACCT GGTCACGTTC TTCCGGTTCG GTGTAAACCA ATCCCTTAAA GTAGGGTAGG   
  
  
- CCGCGATTTT CGTAGTTTTA ATGATGAATT AGGAACTAGT TTACGGGATA TTTATAAGAT GATGGGGTAA   
  
  
- ACTTTTCCCC ACAACTAATG TTGTGTATCG GCATCCAAAC TGTTTCTCTT AATGGGAATA ACAATCTTCG   
  
  
- TGGTATATGA GAAAAGAGAT TCGAGACTAG AAAAAAGTAG GAAAAGTCGA AAAACAGGAA AATTTAGTTT   
  
  
- AGTGAGTGAA CTCATAACCT CCCTGCAAGG AGTCCTCAGC TTGGTGATTT AATAGAAAAA TTTCTTTTTC   
  
  
- CCGAACTTCG GTTGGGCTAA ATTTTTTTGA CGATTATACA TTCGGATGAG AGAAAGGTCA AGGTTGTTTA   
  
  
- GTTTAAAGTC ATACTTTGTA GGTCATTTCC TAAATAGGCA CTATTACTAC ATTGTATACC AATTTCCCAG   
  
  
- TAAAAGTCAA TGTAGCCTCG TACGTTAATT TCAGGGATAT TTTATCCATC CACGTGCTTG TTCAGTGGTA   
  
  
- GCAGGAAACG AAAAACGTAC TCACGTACGA TTAATACTTG AATTAATTAT AGATTCGTTT TAATTTTTCA   
  
  
- TCAATTTTGA AAAAGTGGAA ACCTTGGAGC ATCGACATCG TCATCGACGG CGTGAGACCA AGATCTGAAG   
  
  
- ATCCCTGGTT CTCTACGTGA GTATACGTAG GAGAAGACAG GAAAATGACA TAGGGGAAGG GTACACTGGA   
  
  
- AAAACGGGCC CACTGAAAAG ACACTCACAA CTATGTTTAA AACATGCATT CGATTGAGTA AATGAAGTGG   
  
  
- CAGAGTACTC AAAATGTAAT GGCTATAATT AAATTTACCG TTTCATAATT TAAACTCGAT TTTAGAGTAT   
  
  
- ATATATAAAC ATAATTTACA CAGACCTAAC AGTTTTATTA AAAACTGTAT ATGTCAGTGA TATATGACTC   
  
  
- AATAATTTTA TATATTTTTA TACTTTTAAA TTTTTTATCA TCAAATTCCA CTGTTGTGAT TTTGTTTGTT   
  
  
- TATTCTATAT TTATCCCTTT TCAAGTAGGA TAAAACAATA ATAATAAATA ATACATAAAA GTCTTAACAG   
  
  
- AGATAGTGTT CGTATCATAT ACACAGTTGT AGCACTGTTG AGCTGGTTAG ATGTTTATTG ATCATTTAAG   
  
  
- TTTATTTCAA TGAGAGTGGA TCGAATTTAT TCATGTGGAA CTAAATTAAC AGAATCGATA CCTTGGATAA   
  
  
- GGTTTGATCG GTTATGAAGG AAAAGTTTTC TTAATCAAAA TTTCAAAATT GAAAATGCAG GTTATTACGG   
  
  
- CCGGCATGAT ACTCAACGAA AGTTCGGTCA TCATCATGAC ATCATCCATT TTTATAATCT TATGTGGATG   
  
  
- ATATATACTA TAACGAGAAG TGGCATAGGA AGACGTATGA GTAGAAAGGC AGGGCCTCAG GTGATAGAGA   
  
  
- GGAGGTAAGT CTGTGAGAGA GCGAGATCTC TTGAGTATCG ATACCTGGCG CACGGGCTGC CAATAGCCCA   
  
  
- ATACTTAAGC CTCGAAGAAG TCGTTCAATC GGGACTCAAC GGCCTAGGTT GGTGGACCAA GGTACGGGCA   
  
  
- GATGGGCTGG GGTAGAGAGT CAGAGGAGAA CCTGGGCCAG GTAGGAGGGT CGGTTGGGTA AAGATGGTCC   
  
  
- CAAGACGGAG TCCCTTGGTA CACCAACCCT AACGACTGTG CACCTGCCTG GTCTAACAAC GTCCAAGGAG   
  
  
- CAGGGTCGTC ACTCCAACGT CGTCTCGGTC GTCCACAACT GACGTCAGCT TCGGTACCTC CTCCTGAGAC   
  
  
- CCTATTCTGA CCAGATGCGA GACGACTGCA CACGCCTCCG GTAGGTTGCA CCGCTAAAGG CTAACCGACA   
  
  
- GAACAATCAA TTGTTCTACT CGTTACTGGA GGACGGTGCG CAGTTGGGCA GGACGCCATA ACCGTTTCAT   
  
  
- CGGCCGATGA AGTATCTACG GAACTGGGCT TCTGATATGG TCGTCCCGGG TCACAGCCCG GATTAGCCCA   
  
  
- GCCAAGACCG CATGGTCCAC AACATGCTCG TGAAGATACT TCGAACGGGC AAGGAGTTTA AGCGAGTGAA   
  
  
- GTGACGATTA GTTCGCTAAA ATCTCCGTAA GCTGCCGGTG CTAACACAGG TGTAGTAGCT GAAGCCGGAT   
  
  
- TAGGTGCCGA ACGTTACCGG TCGAGATTAG GTTCGGAACC GACAAGCCGT GCCACCGGGC GGAAAGCATT   
  
  
- CTAATTGGCC TTAGCCCGGC GGAAGACTTA TGCCCAGCAC GAGCGAAGTT GGATAGCCCA GTTGTGACCG   
  
  
- GGTCAATCGG GATAGGTACT TACAACCCAA GCGTAAAGCC CGGCAGCGGC AGAGTGCCGA GCTCCTGTAG   
  
  
- TTTGGTACCT ACCAATTTTG TTCGGGTTTA CTTCAGCATC GGCACTTGAG ATAGAAGGTT GAGGTGGCCG   
  
  
- ATTAGCCCAG CCCATAACTG GGACAGGATT TGACCCAGGC CTCGGATTTG GGCTTTTAAC ACTGTGACCG   
  
  
- CCTCGTTCTC CGCTTGGTGT TGGTCGGGCT CAAAAACCGG GCCAAGTGCC TCCGTAATGT AATGATAAGT   
  
  
- TGATACAAGC TGAGCAATCT CCGAACAGTT CAGGTCCGGC TGTTCCTGGA TCGGCTCGAT ATGAATCTCT   
  
  
- CTCTCAATAG GTTGTAGCAG ACGACACTCC CCAGCCGAGC CTAACTCTCC GTGCTCGGCG ACCGGGTTAC   
  
  
- CTCCCGGGCT TACCGAGCCC GGCCCAAGTT CTTCCACCTA TACCCATTTT TACGCAAGTT CGTTCATTCG   
  
  
- TACAACGACT CAAACAGAAG TCGTGTTCCC ATAACACACC TCCTCTCACT CCCTACAAAC TTCGAGCCGA   
  
  
- CCGTACTGGC GGGAGAGTAA CGCCGAAGCC GAACCGCTCG CCTCTGAGTT CGACTCTTGA GGTCGTGACA   
  
  
- ACACGAACTA CCCAGTAGCA GCACATCAAG TAGAAGAAT

+     WRE3

| Site Name | Organism | Position | Strand | Matrix score. | sequence | function |
| --- | --- | --- | --- | --- | --- | --- |
| WRE3 | Pisum sativum | 2977 | - | 6 | CCACCT |  |
| WRE3 | Pisum sativum | 1805 | + | 6 | CCACCT |  |

>HU11G00778.1   
+ -Up\_Stream \_Len000AAAAAA GAAAGAAATG GAGAAACCAA GCAATATGGA TAAAAAAAAA ACAAAGTAAT   
  
  
+ AAAGGAAGGA TGAGGCTCCA CATTCCACTC GCCCGCGCCA CTCATAGGAG ATGAATAGTA AAATGTGAGG   
  
  
+ GGTTAAATGA TTTGAAAAAA ATCTAGTAAG GGCCCACAAC AGTATTGGAA CCCAGCTTGC CAAGGAAATG   
  
  
+ TCAGAGGGGC TAGCAATGGA CCAGTGCAAG AAGGCCAAGC CACATTTGGT TAGGGAATTT CATCCCATCC   
  
  
+ GGCGCTAAAA GCATCAAAAT TACTACTTAA TCCTTGATCA AATGCCCTAT AAATATTCTA CTACCCCATT   
  
  
+ TGAAAAGGGG TGTTGATTAC AACACATAGC CGTAGGTTTG ACAAAGAGAA TTACCCTTAT TGTTAGAAGC   
  
  
+ ACCATATACT CTTTTCTCTA AGCTCTGATC TTTTTTCATC CTTTTCAGCT TTTTGTCCTT TTAAATCAAA   
  
  
+ TCACTCACTT GAGTATTGGA GGGACGTTCC TCAGGAGTCG AACCACTAAA TTATCTTTTT AAAGAAAAAG   
  
  
+ GGCTTGAAGC CAACCCGATT TAAAAAAACT GCTAATATGT AAGCCTACTC TCTTTCCAGT TCCAACAAAT   
  
  
+ CAAATTTCAG TATGAAACAT CCAGTAAAGG ATTTATCCGT GATAATGATG TAACATATGG TTAAAGGGTC   
  
  
+ ATTTTCAGTT ACATCGGAGC ATGCAATTAA AGTCCCTATA AAATAGGTAG GTGCACGAAC AAGTCACCAT   
  
  
+ CGTCCTTTGC TTTTTGCATG AGTGCATGCT AATTATGAAC TTAATTAATA TCTAAGCAAA ATTAAAAAGT   
  
  
+ AGTTAAAACT TTTTCACCTT TGGAACCTCG TAGCTGTAGC AGTAGCTGCC GCACTCTGGT TCTAGACTTC   
  
  
+ TAGGGACCAA GAGATGCACT CATATGCATC CTCTTCTGTC CTTTTACTGT ATCCCCTTCC CATGTGACCT   
  
  
+ TTTTGCCCGG GTGACTTTTC TGTGAGTGTT GATACAAATT TTGTACGTAA GCTAACTCAT TTACTTCACC   
  
  
+ GTCTCATGAG TTTTACATTA CCGATATTAA TTTAAATGGC AAAGTATTAA ATTTGAGCTA AAATCTCATA   
  
  
+ TATATATTTG TATTAAATGT GTCTGGATTG TCAAAATAAT TTTTGACATA TACAGTCACT ATATACTGAG   
  
  
+ TTATTAAAAT ATATAAAAAT ATGAAAATTT AAAAAATAGT AGTTTAAGGT GACAACACTA AAACAAACAA   
  
  
+ ATAAGATATA AATAGGGAAA AGTTCATCCT ATTTTGTTAT TATTATTTAT TATGTATTTT CAGAATTGTC   
  
  
+ TCTATCACAA GCATAGTATA TGTGTCAACA TCGTGACAAC TCGACCAATC TACAAATAAC TAGTAAATTC   
  
  
+ AAATAAAGTT ACTCTCACCT AGCTTAAATA AGTACACCTT GATTTAATTG TCTTAGCTAT GGAACCTATT   
  
  
+ CCAAACTAGC CAATACTTCC TTTTCAAAAG AATTAGTTTT AAAGTTTTAA CTTTTACGTC CAATAATGCC   
  
  
+ GGCCGTACTA TGAGTTGCTT TCAAGCCAGT AGTAGTACTG TAGTAGGTAA AAATATTAGA ATACACCTAC   
  
  
+ TATATATGAT ATTGCTCTTC ACCGTATCCT TCTGCATACT CATCTTTCCG TCCCGGAGTC CACTATCTCT   
  
  
+ CCTCCATTCA GACACTCTCT CGCTCTAGAG AACTCATAGC TATGGACCGC GTGCCCGACG GTTATCGGGT   
  
  
+ TATGAATTCG GAGCTTCTTC AGCAAGTTAG CCCTGAGTTG CCGGATCCAA CCACCTGGTT CCATGCCCGT   
  
  
+ CTACCCGACC CCATCTCTCA GTCTCCTCTT GGACCCGGTC CATCCTCCCA GCCAACCCAT TTCTACCAGG   
  
  
+ GTTCTGCCTC AGGGAACCAT GTGGTTGGGA TTGCTGACAC GTGGACGGAC CAGATTGTTG CAGGTTCCTC   
  
  
+ GTCCCAGCAG TGAGGTTGCA GCAGAGCCAG CAGGTGTTGA CTGCAGTCGA AGCCATGGAG GAGGACTCTG   
  
  
+ GGATAAGACT GGTCTACGCT CTGCTGACGT GTGCGGAGGC CATCCAACGT GGCGATTTCC GATTGGCTGT   
  
  
+ CTTGTTAGTT AACAAGATGA GCAATGACCT CCTGCCACGC GTCAACCCGT CCTGCGGTAT TGGCAAAGTA   
  
  
+ GCCGGCTACT TCATAGATGC CTTGACCCGA AGACTATACC AGCAGGGCCC AGTGTCGGGC CTAATCGGGT   
  
  
+ CGGTTCTGGC GTACCAGGTG TTGTACGAGC ACTTCTATGA AGCTTGCCCG TTCCTCAAAT TCGCTCACTT   
  
  
+ CACTGCTAAT CAAGCGATTT TAGAGGCATT CGACGGCCAC GATTGTGTCC ACATCATCGA CTTCGGCCTA   
  
  
+ ATCCACGGCT TGCAATGGCC AGCTCTAATC CAAGCCTTGG CTGTTCGGCA CGGTGGCCCG CCTTTCGTAA   
  
  
+ GATTAACCGG AATCGGGCCG CCTTCTGAAT ACGGGTCGTG CTCGCTTCAA CCTATCGGGT CAACACTGGC   
  
  
+ CCAGTTAGCC CTATCCATGA ATGTTGGGTT CGCATTTCGG GCCGTCGCCG TCTCACGGCT CGAGGACATC   
  
  
+ AAACCATGGA TGGTTAAAAC AAGCCCAAAT GAAGTCGTAG CCGTGAACTC TATCTTCCAA CTCCACCGGC   
  
  
+ TAATCGGGTC GGGTATTGAC CCTGTCCTAA ACTGGGTCCG GAGCCTAAAC CCGAAAATTG TGACACTGGC   
  
  
+ GGAGCAAGAG GCGAACCACA ACCAGCCCGA GTTTTTGGCC CGGTTCACGG AGGCATTACA TTACTATTCA   
  
  
+ ACTATGTTCG ACTCGTTAGA GGCTTGTCAA GTCCAGGCCG ACAAGGACCT AGCCGAGCTA TACTTAGAGA   
  
  
+ GAGAGTTATC CAACATCGTC TGCTGTGAGG GGTCGGCTCG GATTGAGAGG CACGAGCCGC TGGCCCAATG   
  
  
+ GAGGGCCCGA ATGGCTCGGG CCGGGTTCAA GAAGGTGGAT ATGGGTAAAA ATGCGTTCAA GCAAGTAAGC   
  
  
+ ATGTTGCTGA GTTTGTCTTC AGCACAAGGG TATTGTGTGG AGGAGAGTGA GGGATGTTTG AAGCTCGGCT   
  
  
+ GGCATGACCG CCCTCTCATT GCGGCTTCGG CTTGGCGAGC GGAGACTCAA GCTGAGAACT CCAGCACTGT   
  
  
+ TGTGCTTGAT GGGTCATCGT CGTGTAGTTC ATCTTCTTA  

- -Up\_Stream \_Len000TTTTTT CTTTCTTTAC CTCTTTGGTT CGTTATACCT ATTTTTTTTT TGTTTCATTA   
  
  
- TTTCCTTCCT ACTCCGAGGT GTAAGGTGAG CGGGCGCGGT GAGTATCCTC TACTTATCAT TTTACACTCC   
  
  
- CCAATTTACT AAACTTTTTT TAGATCATTC CCGGGTGTTG TCATAACCTT GGGTCGAACG GTTCCTTTAC   
  
  
- AGTCTCCCCG ATCGTTACCT GGTCACGTTC TTCCGGTTCG GTGTAAACCA ATCCCTTAAA GTAGGGTAGG   
  
  
- CCGCGATTTT CGTAGTTTTA ATGATGAATT AGGAACTAGT TTACGGGATA TTTATAAGAT GATGGGGTAA   
  
  
- ACTTTTCCCC ACAACTAATG TTGTGTATCG GCATCCAAAC TGTTTCTCTT AATGGGAATA ACAATCTTCG   
  
  
- TGGTATATGA GAAAAGAGAT TCGAGACTAG AAAAAAGTAG GAAAAGTCGA AAAACAGGAA AATTTAGTTT   
  
  
- AGTGAGTGAA CTCATAACCT CCCTGCAAGG AGTCCTCAGC TTGGTGATTT AATAGAAAAA TTTCTTTTTC   
  
  
- CCGAACTTCG GTTGGGCTAA ATTTTTTTGA CGATTATACA TTCGGATGAG AGAAAGGTCA AGGTTGTTTA   
  
  
- GTTTAAAGTC ATACTTTGTA GGTCATTTCC TAAATAGGCA CTATTACTAC ATTGTATACC AATTTCCCAG   
  
  
- TAAAAGTCAA TGTAGCCTCG TACGTTAATT TCAGGGATAT TTTATCCATC CACGTGCTTG TTCAGTGGTA   
  
  
- GCAGGAAACG AAAAACGTAC TCACGTACGA TTAATACTTG AATTAATTAT AGATTCGTTT TAATTTTTCA   
  
  
- TCAATTTTGA AAAAGTGGAA ACCTTGGAGC ATCGACATCG TCATCGACGG CGTGAGACCA AGATCTGAAG   
  
  
- ATCCCTGGTT CTCTACGTGA GTATACGTAG GAGAAGACAG GAAAATGACA TAGGGGAAGG GTACACTGGA   
  
  
- AAAACGGGCC CACTGAAAAG ACACTCACAA CTATGTTTAA AACATGCATT CGATTGAGTA AATGAAGTGG   
  
  
- CAGAGTACTC AAAATGTAAT GGCTATAATT AAATTTACCG TTTCATAATT TAAACTCGAT TTTAGAGTAT   
  
  
- ATATATAAAC ATAATTTACA CAGACCTAAC AGTTTTATTA AAAACTGTAT ATGTCAGTGA TATATGACTC   
  
  
- AATAATTTTA TATATTTTTA TACTTTTAAA TTTTTTATCA TCAAATTCCA CTGTTGTGAT TTTGTTTGTT   
  
  
- TATTCTATAT TTATCCCTTT TCAAGTAGGA TAAAACAATA ATAATAAATA ATACATAAAA GTCTTAACAG   
  
  
- AGATAGTGTT CGTATCATAT ACACAGTTGT AGCACTGTTG AGCTGGTTAG ATGTTTATTG ATCATTTAAG   
  
  
- TTTATTTCAA TGAGAGTGGA TCGAATTTAT TCATGTGGAA CTAAATTAAC AGAATCGATA CCTTGGATAA   
  
  
- GGTTTGATCG GTTATGAAGG AAAAGTTTTC TTAATCAAAA TTTCAAAATT GAAAATGCAG GTTATTACGG   
  
  
- CCGGCATGAT ACTCAACGAA AGTTCGGTCA TCATCATGAC ATCATCCATT TTTATAATCT TATGTGGATG   
  
  
- ATATATACTA TAACGAGAAG TGGCATAGGA AGACGTATGA GTAGAAAGGC AGGGCCTCAG GTGATAGAGA   
  
  
- GGAGGTAAGT CTGTGAGAGA GCGAGATCTC TTGAGTATCG ATACCTGGCG CACGGGCTGC CAATAGCCCA   
  
  
- ATACTTAAGC CTCGAAGAAG TCGTTCAATC GGGACTCAAC GGCCTAGGTT GGTGGACCAA GGTACGGGCA   
  
  
- GATGGGCTGG GGTAGAGAGT CAGAGGAGAA CCTGGGCCAG GTAGGAGGGT CGGTTGGGTA AAGATGGTCC   
  
  
- CAAGACGGAG TCCCTTGGTA CACCAACCCT AACGACTGTG CACCTGCCTG GTCTAACAAC GTCCAAGGAG   
  
  
- CAGGGTCGTC ACTCCAACGT CGTCTCGGTC GTCCACAACT GACGTCAGCT TCGGTACCTC CTCCTGAGAC   
  
  
- CCTATTCTGA CCAGATGCGA GACGACTGCA CACGCCTCCG GTAGGTTGCA CCGCTAAAGG CTAACCGACA   
  
  
- GAACAATCAA TTGTTCTACT CGTTACTGGA GGACGGTGCG CAGTTGGGCA GGACGCCATA ACCGTTTCAT   
  
  
- CGGCCGATGA AGTATCTACG GAACTGGGCT TCTGATATGG TCGTCCCGGG TCACAGCCCG GATTAGCCCA   
  
  
- GCCAAGACCG CATGGTCCAC AACATGCTCG TGAAGATACT TCGAACGGGC AAGGAGTTTA AGCGAGTGAA   
  
  
- GTGACGATTA GTTCGCTAAA ATCTCCGTAA GCTGCCGGTG CTAACACAGG TGTAGTAGCT GAAGCCGGAT   
  
  
- TAGGTGCCGA ACGTTACCGG TCGAGATTAG GTTCGGAACC GACAAGCCGT GCCACCGGGC GGAAAGCATT   
  
  
- CTAATTGGCC TTAGCCCGGC GGAAGACTTA TGCCCAGCAC GAGCGAAGTT GGATAGCCCA GTTGTGACCG   
  
  
- GGTCAATCGG GATAGGTACT TACAACCCAA GCGTAAAGCC CGGCAGCGGC AGAGTGCCGA GCTCCTGTAG   
  
  
- TTTGGTACCT ACCAATTTTG TTCGGGTTTA CTTCAGCATC GGCACTTGAG ATAGAAGGTT GAGGTGGCCG   
  
  
- ATTAGCCCAG CCCATAACTG GGACAGGATT TGACCCAGGC CTCGGATTTG GGCTTTTAAC ACTGTGACCG   
  
  
- CCTCGTTCTC CGCTTGGTGT TGGTCGGGCT CAAAAACCGG GCCAAGTGCC TCCGTAATGT AATGATAAGT   
  
  
- TGATACAAGC TGAGCAATCT CCGAACAGTT CAGGTCCGGC TGTTCCTGGA TCGGCTCGAT ATGAATCTCT   
  
  
- CTCTCAATAG GTTGTAGCAG ACGACACTCC CCAGCCGAGC CTAACTCTCC GTGCTCGGCG ACCGGGTTAC   
  
  
- CTCCCGGGCT TACCGAGCCC GGCCCAAGTT CTTCCACCTA TACCCATTTT TACGCAAGTT CGTTCATTCG   
  
  
- TACAACGACT CAAACAGAAG TCGTGTTCCC ATAACACACC TCCTCTCACT CCCTACAAAC TTCGAGCCGA   
  
  
- CCGTACTGGC GGGAGAGTAA CGCCGAAGCC GAACCGCTCG CCTCTGAGTT CGACTCTTGA GGTCGTGACA   
  
  
- ACACGAACTA CCCAGTAGCA GCACATCAAG TAGAAGAAT

+     WUN-motif

| Site Name | Organism | Position | Strand | Matrix score. | sequence | function |
| --- | --- | --- | --- | --- | --- | --- |
| WUN-motif | Nicotiana glutinosa | 301 | + | 9 | AAATTACTA |  |

>HU11G00778.1   
+ -Up\_Stream \_Len000AAAAAA GAAAGAAATG GAGAAACCAA GCAATATGGA TAAAAAAAAA ACAAAGTAAT   
  
  
+ AAAGGAAGGA TGAGGCTCCA CATTCCACTC GCCCGCGCCA CTCATAGGAG ATGAATAGTA AAATGTGAGG   
  
  
+ GGTTAAATGA TTTGAAAAAA ATCTAGTAAG GGCCCACAAC AGTATTGGAA CCCAGCTTGC CAAGGAAATG   
  
  
+ TCAGAGGGGC TAGCAATGGA CCAGTGCAAG AAGGCCAAGC CACATTTGGT TAGGGAATTT CATCCCATCC   
  
  
+ GGCGCTAAAA GCATCAAAAT TACTACTTAA TCCTTGATCA AATGCCCTAT AAATATTCTA CTACCCCATT   
  
  
+ TGAAAAGGGG TGTTGATTAC AACACATAGC CGTAGGTTTG ACAAAGAGAA TTACCCTTAT TGTTAGAAGC   
  
  
+ ACCATATACT CTTTTCTCTA AGCTCTGATC TTTTTTCATC CTTTTCAGCT TTTTGTCCTT TTAAATCAAA   
  
  
+ TCACTCACTT GAGTATTGGA GGGACGTTCC TCAGGAGTCG AACCACTAAA TTATCTTTTT AAAGAAAAAG   
  
  
+ GGCTTGAAGC CAACCCGATT TAAAAAAACT GCTAATATGT AAGCCTACTC TCTTTCCAGT TCCAACAAAT   
  
  
+ CAAATTTCAG TATGAAACAT CCAGTAAAGG ATTTATCCGT GATAATGATG TAACATATGG TTAAAGGGTC   
  
  
+ ATTTTCAGTT ACATCGGAGC ATGCAATTAA AGTCCCTATA AAATAGGTAG GTGCACGAAC AAGTCACCAT   
  
  
+ CGTCCTTTGC TTTTTGCATG AGTGCATGCT AATTATGAAC TTAATTAATA TCTAAGCAAA ATTAAAAAGT   
  
  
+ AGTTAAAACT TTTTCACCTT TGGAACCTCG TAGCTGTAGC AGTAGCTGCC GCACTCTGGT TCTAGACTTC   
  
  
+ TAGGGACCAA GAGATGCACT CATATGCATC CTCTTCTGTC CTTTTACTGT ATCCCCTTCC CATGTGACCT   
  
  
+ TTTTGCCCGG GTGACTTTTC TGTGAGTGTT GATACAAATT TTGTACGTAA GCTAACTCAT TTACTTCACC   
  
  
+ GTCTCATGAG TTTTACATTA CCGATATTAA TTTAAATGGC AAAGTATTAA ATTTGAGCTA AAATCTCATA   
  
  
+ TATATATTTG TATTAAATGT GTCTGGATTG TCAAAATAAT TTTTGACATA TACAGTCACT ATATACTGAG   
  
  
+ TTATTAAAAT ATATAAAAAT ATGAAAATTT AAAAAATAGT AGTTTAAGGT GACAACACTA AAACAAACAA   
  
  
+ ATAAGATATA AATAGGGAAA AGTTCATCCT ATTTTGTTAT TATTATTTAT TATGTATTTT CAGAATTGTC   
  
  
+ TCTATCACAA GCATAGTATA TGTGTCAACA TCGTGACAAC TCGACCAATC TACAAATAAC TAGTAAATTC   
  
  
+ AAATAAAGTT ACTCTCACCT AGCTTAAATA AGTACACCTT GATTTAATTG TCTTAGCTAT GGAACCTATT   
  
  
+ CCAAACTAGC CAATACTTCC TTTTCAAAAG AATTAGTTTT AAAGTTTTAA CTTTTACGTC CAATAATGCC   
  
  
+ GGCCGTACTA TGAGTTGCTT TCAAGCCAGT AGTAGTACTG TAGTAGGTAA AAATATTAGA ATACACCTAC   
  
  
+ TATATATGAT ATTGCTCTTC ACCGTATCCT TCTGCATACT CATCTTTCCG TCCCGGAGTC CACTATCTCT   
  
  
+ CCTCCATTCA GACACTCTCT CGCTCTAGAG AACTCATAGC TATGGACCGC GTGCCCGACG GTTATCGGGT   
  
  
+ TATGAATTCG GAGCTTCTTC AGCAAGTTAG CCCTGAGTTG CCGGATCCAA CCACCTGGTT CCATGCCCGT   
  
  
+ CTACCCGACC CCATCTCTCA GTCTCCTCTT GGACCCGGTC CATCCTCCCA GCCAACCCAT TTCTACCAGG   
  
  
+ GTTCTGCCTC AGGGAACCAT GTGGTTGGGA TTGCTGACAC GTGGACGGAC CAGATTGTTG CAGGTTCCTC   
  
  
+ GTCCCAGCAG TGAGGTTGCA GCAGAGCCAG CAGGTGTTGA CTGCAGTCGA AGCCATGGAG GAGGACTCTG   
  
  
+ GGATAAGACT GGTCTACGCT CTGCTGACGT GTGCGGAGGC CATCCAACGT GGCGATTTCC GATTGGCTGT   
  
  
+ CTTGTTAGTT AACAAGATGA GCAATGACCT CCTGCCACGC GTCAACCCGT CCTGCGGTAT TGGCAAAGTA   
  
  
+ GCCGGCTACT TCATAGATGC CTTGACCCGA AGACTATACC AGCAGGGCCC AGTGTCGGGC CTAATCGGGT   
  
  
+ CGGTTCTGGC GTACCAGGTG TTGTACGAGC ACTTCTATGA AGCTTGCCCG TTCCTCAAAT TCGCTCACTT   
  
  
+ CACTGCTAAT CAAGCGATTT TAGAGGCATT CGACGGCCAC GATTGTGTCC ACATCATCGA CTTCGGCCTA   
  
  
+ ATCCACGGCT TGCAATGGCC AGCTCTAATC CAAGCCTTGG CTGTTCGGCA CGGTGGCCCG CCTTTCGTAA   
  
  
+ GATTAACCGG AATCGGGCCG CCTTCTGAAT ACGGGTCGTG CTCGCTTCAA CCTATCGGGT CAACACTGGC   
  
  
+ CCAGTTAGCC CTATCCATGA ATGTTGGGTT CGCATTTCGG GCCGTCGCCG TCTCACGGCT CGAGGACATC   
  
  
+ AAACCATGGA TGGTTAAAAC AAGCCCAAAT GAAGTCGTAG CCGTGAACTC TATCTTCCAA CTCCACCGGC   
  
  
+ TAATCGGGTC GGGTATTGAC CCTGTCCTAA ACTGGGTCCG GAGCCTAAAC CCGAAAATTG TGACACTGGC   
  
  
+ GGAGCAAGAG GCGAACCACA ACCAGCCCGA GTTTTTGGCC CGGTTCACGG AGGCATTACA TTACTATTCA   
  
  
+ ACTATGTTCG ACTCGTTAGA GGCTTGTCAA GTCCAGGCCG ACAAGGACCT AGCCGAGCTA TACTTAGAGA   
  
  
+ GAGAGTTATC CAACATCGTC TGCTGTGAGG GGTCGGCTCG GATTGAGAGG CACGAGCCGC TGGCCCAATG   
  
  
+ GAGGGCCCGA ATGGCTCGGG CCGGGTTCAA GAAGGTGGAT ATGGGTAAAA ATGCGTTCAA GCAAGTAAGC   
  
  
+ ATGTTGCTGA GTTTGTCTTC AGCACAAGGG TATTGTGTGG AGGAGAGTGA GGGATGTTTG AAGCTCGGCT   
  
  
+ GGCATGACCG CCCTCTCATT GCGGCTTCGG CTTGGCGAGC GGAGACTCAA GCTGAGAACT CCAGCACTGT   
  
  
+ TGTGCTTGAT GGGTCATCGT CGTGTAGTTC ATCTTCTTA  

- -Up\_Stream \_Len000TTTTTT CTTTCTTTAC CTCTTTGGTT CGTTATACCT ATTTTTTTTT TGTTTCATTA   
  
  
- TTTCCTTCCT ACTCCGAGGT GTAAGGTGAG CGGGCGCGGT GAGTATCCTC TACTTATCAT TTTACACTCC   
  
  
- CCAATTTACT AAACTTTTTT TAGATCATTC CCGGGTGTTG TCATAACCTT GGGTCGAACG GTTCCTTTAC   
  
  
- AGTCTCCCCG ATCGTTACCT GGTCACGTTC TTCCGGTTCG GTGTAAACCA ATCCCTTAAA GTAGGGTAGG   
  
  
- CCGCGATTTT CGTAGTTTTA ATGATGAATT AGGAACTAGT TTACGGGATA TTTATAAGAT GATGGGGTAA   
  
  
- ACTTTTCCCC ACAACTAATG TTGTGTATCG GCATCCAAAC TGTTTCTCTT AATGGGAATA ACAATCTTCG   
  
  
- TGGTATATGA GAAAAGAGAT TCGAGACTAG AAAAAAGTAG GAAAAGTCGA AAAACAGGAA AATTTAGTTT   
  
  
- AGTGAGTGAA CTCATAACCT CCCTGCAAGG AGTCCTCAGC TTGGTGATTT AATAGAAAAA TTTCTTTTTC   
  
  
- CCGAACTTCG GTTGGGCTAA ATTTTTTTGA CGATTATACA TTCGGATGAG AGAAAGGTCA AGGTTGTTTA   
  
  
- GTTTAAAGTC ATACTTTGTA GGTCATTTCC TAAATAGGCA CTATTACTAC ATTGTATACC AATTTCCCAG   
  
  
- TAAAAGTCAA TGTAGCCTCG TACGTTAATT TCAGGGATAT TTTATCCATC CACGTGCTTG TTCAGTGGTA   
  
  
- GCAGGAAACG AAAAACGTAC TCACGTACGA TTAATACTTG AATTAATTAT AGATTCGTTT TAATTTTTCA   
  
  
- TCAATTTTGA AAAAGTGGAA ACCTTGGAGC ATCGACATCG TCATCGACGG CGTGAGACCA AGATCTGAAG   
  
  
- ATCCCTGGTT CTCTACGTGA GTATACGTAG GAGAAGACAG GAAAATGACA TAGGGGAAGG GTACACTGGA   
  
  
- AAAACGGGCC CACTGAAAAG ACACTCACAA CTATGTTTAA AACATGCATT CGATTGAGTA AATGAAGTGG   
  
  
- CAGAGTACTC AAAATGTAAT GGCTATAATT AAATTTACCG TTTCATAATT TAAACTCGAT TTTAGAGTAT   
  
  
- ATATATAAAC ATAATTTACA CAGACCTAAC AGTTTTATTA AAAACTGTAT ATGTCAGTGA TATATGACTC   
  
  
- AATAATTTTA TATATTTTTA TACTTTTAAA TTTTTTATCA TCAAATTCCA CTGTTGTGAT TTTGTTTGTT   
  
  
- TATTCTATAT TTATCCCTTT TCAAGTAGGA TAAAACAATA ATAATAAATA ATACATAAAA GTCTTAACAG   
  
  
- AGATAGTGTT CGTATCATAT ACACAGTTGT AGCACTGTTG AGCTGGTTAG ATGTTTATTG ATCATTTAAG   
  
  
- TTTATTTCAA TGAGAGTGGA TCGAATTTAT TCATGTGGAA CTAAATTAAC AGAATCGATA CCTTGGATAA   
  
  
- GGTTTGATCG GTTATGAAGG AAAAGTTTTC TTAATCAAAA TTTCAAAATT GAAAATGCAG GTTATTACGG   
  
  
- CCGGCATGAT ACTCAACGAA AGTTCGGTCA TCATCATGAC ATCATCCATT TTTATAATCT TATGTGGATG   
  
  
- ATATATACTA TAACGAGAAG TGGCATAGGA AGACGTATGA GTAGAAAGGC AGGGCCTCAG GTGATAGAGA   
  
  
- GGAGGTAAGT CTGTGAGAGA GCGAGATCTC TTGAGTATCG ATACCTGGCG CACGGGCTGC CAATAGCCCA   
  
  
- ATACTTAAGC CTCGAAGAAG TCGTTCAATC GGGACTCAAC GGCCTAGGTT GGTGGACCAA GGTACGGGCA   
  
  
- GATGGGCTGG GGTAGAGAGT CAGAGGAGAA CCTGGGCCAG GTAGGAGGGT CGGTTGGGTA AAGATGGTCC   
  
  
- CAAGACGGAG TCCCTTGGTA CACCAACCCT AACGACTGTG CACCTGCCTG GTCTAACAAC GTCCAAGGAG   
  
  
- CAGGGTCGTC ACTCCAACGT CGTCTCGGTC GTCCACAACT GACGTCAGCT TCGGTACCTC CTCCTGAGAC   
  
  
- CCTATTCTGA CCAGATGCGA GACGACTGCA CACGCCTCCG GTAGGTTGCA CCGCTAAAGG CTAACCGACA   
  
  
- GAACAATCAA TTGTTCTACT CGTTACTGGA GGACGGTGCG CAGTTGGGCA GGACGCCATA ACCGTTTCAT   
  
  
- CGGCCGATGA AGTATCTACG GAACTGGGCT TCTGATATGG TCGTCCCGGG TCACAGCCCG GATTAGCCCA   
  
  
- GCCAAGACCG CATGGTCCAC AACATGCTCG TGAAGATACT TCGAACGGGC AAGGAGTTTA AGCGAGTGAA   
  
  
- GTGACGATTA GTTCGCTAAA ATCTCCGTAA GCTGCCGGTG CTAACACAGG TGTAGTAGCT GAAGCCGGAT   
  
  
- TAGGTGCCGA ACGTTACCGG TCGAGATTAG GTTCGGAACC GACAAGCCGT GCCACCGGGC GGAAAGCATT   
  
  
- CTAATTGGCC TTAGCCCGGC GGAAGACTTA TGCCCAGCAC GAGCGAAGTT GGATAGCCCA GTTGTGACCG   
  
  
- GGTCAATCGG GATAGGTACT TACAACCCAA GCGTAAAGCC CGGCAGCGGC AGAGTGCCGA GCTCCTGTAG   
  
  
- TTTGGTACCT ACCAATTTTG TTCGGGTTTA CTTCAGCATC GGCACTTGAG ATAGAAGGTT GAGGTGGCCG   
  
  
- ATTAGCCCAG CCCATAACTG GGACAGGATT TGACCCAGGC CTCGGATTTG GGCTTTTAAC ACTGTGACCG   
  
  
- CCTCGTTCTC CGCTTGGTGT TGGTCGGGCT CAAAAACCGG GCCAAGTGCC TCCGTAATGT AATGATAAGT   
  
  
- TGATACAAGC TGAGCAATCT CCGAACAGTT CAGGTCCGGC TGTTCCTGGA TCGGCTCGAT ATGAATCTCT   
  
  
- CTCTCAATAG GTTGTAGCAG ACGACACTCC CCAGCCGAGC CTAACTCTCC GTGCTCGGCG ACCGGGTTAC   
  
  
- CTCCCGGGCT TACCGAGCCC GGCCCAAGTT CTTCCACCTA TACCCATTTT TACGCAAGTT CGTTCATTCG   
  
  
- TACAACGACT CAAACAGAAG TCGTGTTCCC ATAACACACC TCCTCTCACT CCCTACAAAC TTCGAGCCGA   
  
  
- CCGTACTGGC GGGAGAGTAA CGCCGAAGCC GAACCGCTCG CCTCTGAGTT CGACTCTTGA GGTCGTGACA   
  
  
- ACACGAACTA CCCAGTAGCA GCACATCAAG TAGAAGAAT

+     as-1

| Site Name | Organism | Position | Strand | Matrix score. | sequence | function |
| --- | --- | --- | --- | --- | --- | --- |
| as-1 | Arabidopsis thaliana | 2059 | + | 5 | TGACG |  |
| as-1 | Arabidopsis thaliana | 2144 | - | 5 | TGACG |  |

>HU11G00778.1   
+ -Up\_Stream \_Len000AAAAAA GAAAGAAATG GAGAAACCAA GCAATATGGA TAAAAAAAAA ACAAAGTAAT   
  
  
+ AAAGGAAGGA TGAGGCTCCA CATTCCACTC GCCCGCGCCA CTCATAGGAG ATGAATAGTA AAATGTGAGG   
  
  
+ GGTTAAATGA TTTGAAAAAA ATCTAGTAAG GGCCCACAAC AGTATTGGAA CCCAGCTTGC CAAGGAAATG   
  
  
+ TCAGAGGGGC TAGCAATGGA CCAGTGCAAG AAGGCCAAGC CACATTTGGT TAGGGAATTT CATCCCATCC   
  
  
+ GGCGCTAAAA GCATCAAAAT TACTACTTAA TCCTTGATCA AATGCCCTAT AAATATTCTA CTACCCCATT   
  
  
+ TGAAAAGGGG TGTTGATTAC AACACATAGC CGTAGGTTTG ACAAAGAGAA TTACCCTTAT TGTTAGAAGC   
  
  
+ ACCATATACT CTTTTCTCTA AGCTCTGATC TTTTTTCATC CTTTTCAGCT TTTTGTCCTT TTAAATCAAA   
  
  
+ TCACTCACTT GAGTATTGGA GGGACGTTCC TCAGGAGTCG AACCACTAAA TTATCTTTTT AAAGAAAAAG   
  
  
+ GGCTTGAAGC CAACCCGATT TAAAAAAACT GCTAATATGT AAGCCTACTC TCTTTCCAGT TCCAACAAAT   
  
  
+ CAAATTTCAG TATGAAACAT CCAGTAAAGG ATTTATCCGT GATAATGATG TAACATATGG TTAAAGGGTC   
  
  
+ ATTTTCAGTT ACATCGGAGC ATGCAATTAA AGTCCCTATA AAATAGGTAG GTGCACGAAC AAGTCACCAT   
  
  
+ CGTCCTTTGC TTTTTGCATG AGTGCATGCT AATTATGAAC TTAATTAATA TCTAAGCAAA ATTAAAAAGT   
  
  
+ AGTTAAAACT TTTTCACCTT TGGAACCTCG TAGCTGTAGC AGTAGCTGCC GCACTCTGGT TCTAGACTTC   
  
  
+ TAGGGACCAA GAGATGCACT CATATGCATC CTCTTCTGTC CTTTTACTGT ATCCCCTTCC CATGTGACCT   
  
  
+ TTTTGCCCGG GTGACTTTTC TGTGAGTGTT GATACAAATT TTGTACGTAA GCTAACTCAT TTACTTCACC   
  
  
+ GTCTCATGAG TTTTACATTA CCGATATTAA TTTAAATGGC AAAGTATTAA ATTTGAGCTA AAATCTCATA   
  
  
+ TATATATTTG TATTAAATGT GTCTGGATTG TCAAAATAAT TTTTGACATA TACAGTCACT ATATACTGAG   
  
  
+ TTATTAAAAT ATATAAAAAT ATGAAAATTT AAAAAATAGT AGTTTAAGGT GACAACACTA AAACAAACAA   
  
  
+ ATAAGATATA AATAGGGAAA AGTTCATCCT ATTTTGTTAT TATTATTTAT TATGTATTTT CAGAATTGTC   
  
  
+ TCTATCACAA GCATAGTATA TGTGTCAACA TCGTGACAAC TCGACCAATC TACAAATAAC TAGTAAATTC   
  
  
+ AAATAAAGTT ACTCTCACCT AGCTTAAATA AGTACACCTT GATTTAATTG TCTTAGCTAT GGAACCTATT   
  
  
+ CCAAACTAGC CAATACTTCC TTTTCAAAAG AATTAGTTTT AAAGTTTTAA CTTTTACGTC CAATAATGCC   
  
  
+ GGCCGTACTA TGAGTTGCTT TCAAGCCAGT AGTAGTACTG TAGTAGGTAA AAATATTAGA ATACACCTAC   
  
  
+ TATATATGAT ATTGCTCTTC ACCGTATCCT TCTGCATACT CATCTTTCCG TCCCGGAGTC CACTATCTCT   
  
  
+ CCTCCATTCA GACACTCTCT CGCTCTAGAG AACTCATAGC TATGGACCGC GTGCCCGACG GTTATCGGGT   
  
  
+ TATGAATTCG GAGCTTCTTC AGCAAGTTAG CCCTGAGTTG CCGGATCCAA CCACCTGGTT CCATGCCCGT   
  
  
+ CTACCCGACC CCATCTCTCA GTCTCCTCTT GGACCCGGTC CATCCTCCCA GCCAACCCAT TTCTACCAGG   
  
  
+ GTTCTGCCTC AGGGAACCAT GTGGTTGGGA TTGCTGACAC GTGGACGGAC CAGATTGTTG CAGGTTCCTC   
  
  
+ GTCCCAGCAG TGAGGTTGCA GCAGAGCCAG CAGGTGTTGA CTGCAGTCGA AGCCATGGAG GAGGACTCTG   
  
  
+ GGATAAGACT GGTCTACGCT CTGCTGACGT GTGCGGAGGC CATCCAACGT GGCGATTTCC GATTGGCTGT   
  
  
+ CTTGTTAGTT AACAAGATGA GCAATGACCT CCTGCCACGC GTCAACCCGT CCTGCGGTAT TGGCAAAGTA   
  
  
+ GCCGGCTACT TCATAGATGC CTTGACCCGA AGACTATACC AGCAGGGCCC AGTGTCGGGC CTAATCGGGT   
  
  
+ CGGTTCTGGC GTACCAGGTG TTGTACGAGC ACTTCTATGA AGCTTGCCCG TTCCTCAAAT TCGCTCACTT   
  
  
+ CACTGCTAAT CAAGCGATTT TAGAGGCATT CGACGGCCAC GATTGTGTCC ACATCATCGA CTTCGGCCTA   
  
  
+ ATCCACGGCT TGCAATGGCC AGCTCTAATC CAAGCCTTGG CTGTTCGGCA CGGTGGCCCG CCTTTCGTAA   
  
  
+ GATTAACCGG AATCGGGCCG CCTTCTGAAT ACGGGTCGTG CTCGCTTCAA CCTATCGGGT CAACACTGGC   
  
  
+ CCAGTTAGCC CTATCCATGA ATGTTGGGTT CGCATTTCGG GCCGTCGCCG TCTCACGGCT CGAGGACATC   
  
  
+ AAACCATGGA TGGTTAAAAC AAGCCCAAAT GAAGTCGTAG CCGTGAACTC TATCTTCCAA CTCCACCGGC   
  
  
+ TAATCGGGTC GGGTATTGAC CCTGTCCTAA ACTGGGTCCG GAGCCTAAAC CCGAAAATTG TGACACTGGC   
  
  
+ GGAGCAAGAG GCGAACCACA ACCAGCCCGA GTTTTTGGCC CGGTTCACGG AGGCATTACA TTACTATTCA   
  
  
+ ACTATGTTCG ACTCGTTAGA GGCTTGTCAA GTCCAGGCCG ACAAGGACCT AGCCGAGCTA TACTTAGAGA   
  
  
+ GAGAGTTATC CAACATCGTC TGCTGTGAGG GGTCGGCTCG GATTGAGAGG CACGAGCCGC TGGCCCAATG   
  
  
+ GAGGGCCCGA ATGGCTCGGG CCGGGTTCAA GAAGGTGGAT ATGGGTAAAA ATGCGTTCAA GCAAGTAAGC   
  
  
+ ATGTTGCTGA GTTTGTCTTC AGCACAAGGG TATTGTGTGG AGGAGAGTGA GGGATGTTTG AAGCTCGGCT   
  
  
+ GGCATGACCG CCCTCTCATT GCGGCTTCGG CTTGGCGAGC GGAGACTCAA GCTGAGAACT CCAGCACTGT   
  
  
+ TGTGCTTGAT GGGTCATCGT CGTGTAGTTC ATCTTCTTA  

- -Up\_Stream \_Len000TTTTTT CTTTCTTTAC CTCTTTGGTT CGTTATACCT ATTTTTTTTT TGTTTCATTA   
  
  
- TTTCCTTCCT ACTCCGAGGT GTAAGGTGAG CGGGCGCGGT GAGTATCCTC TACTTATCAT TTTACACTCC   
  
  
- CCAATTTACT AAACTTTTTT TAGATCATTC CCGGGTGTTG TCATAACCTT GGGTCGAACG GTTCCTTTAC   
  
  
- AGTCTCCCCG ATCGTTACCT GGTCACGTTC TTCCGGTTCG GTGTAAACCA ATCCCTTAAA GTAGGGTAGG   
  
  
- CCGCGATTTT CGTAGTTTTA ATGATGAATT AGGAACTAGT TTACGGGATA TTTATAAGAT GATGGGGTAA   
  
  
- ACTTTTCCCC ACAACTAATG TTGTGTATCG GCATCCAAAC TGTTTCTCTT AATGGGAATA ACAATCTTCG   
  
  
- TGGTATATGA GAAAAGAGAT TCGAGACTAG AAAAAAGTAG GAAAAGTCGA AAAACAGGAA AATTTAGTTT   
  
  
- AGTGAGTGAA CTCATAACCT CCCTGCAAGG AGTCCTCAGC TTGGTGATTT AATAGAAAAA TTTCTTTTTC   
  
  
- CCGAACTTCG GTTGGGCTAA ATTTTTTTGA CGATTATACA TTCGGATGAG AGAAAGGTCA AGGTTGTTTA   
  
  
- GTTTAAAGTC ATACTTTGTA GGTCATTTCC TAAATAGGCA CTATTACTAC ATTGTATACC AATTTCCCAG   
  
  
- TAAAAGTCAA TGTAGCCTCG TACGTTAATT TCAGGGATAT TTTATCCATC CACGTGCTTG TTCAGTGGTA   
  
  
- GCAGGAAACG AAAAACGTAC TCACGTACGA TTAATACTTG AATTAATTAT AGATTCGTTT TAATTTTTCA   
  
  
- TCAATTTTGA AAAAGTGGAA ACCTTGGAGC ATCGACATCG TCATCGACGG CGTGAGACCA AGATCTGAAG   
  
  
- ATCCCTGGTT CTCTACGTGA GTATACGTAG GAGAAGACAG GAAAATGACA TAGGGGAAGG GTACACTGGA   
  
  
- AAAACGGGCC CACTGAAAAG ACACTCACAA CTATGTTTAA AACATGCATT CGATTGAGTA AATGAAGTGG   
  
  
- CAGAGTACTC AAAATGTAAT GGCTATAATT AAATTTACCG TTTCATAATT TAAACTCGAT TTTAGAGTAT   
  
  
- ATATATAAAC ATAATTTACA CAGACCTAAC AGTTTTATTA AAAACTGTAT ATGTCAGTGA TATATGACTC   
  
  
- AATAATTTTA TATATTTTTA TACTTTTAAA TTTTTTATCA TCAAATTCCA CTGTTGTGAT TTTGTTTGTT   
  
  
- TATTCTATAT TTATCCCTTT TCAAGTAGGA TAAAACAATA ATAATAAATA ATACATAAAA GTCTTAACAG   
  
  
- AGATAGTGTT CGTATCATAT ACACAGTTGT AGCACTGTTG AGCTGGTTAG ATGTTTATTG ATCATTTAAG   
  
  
- TTTATTTCAA TGAGAGTGGA TCGAATTTAT TCATGTGGAA CTAAATTAAC AGAATCGATA CCTTGGATAA   
  
  
- GGTTTGATCG GTTATGAAGG AAAAGTTTTC TTAATCAAAA TTTCAAAATT GAAAATGCAG GTTATTACGG   
  
  
- CCGGCATGAT ACTCAACGAA AGTTCGGTCA TCATCATGAC ATCATCCATT TTTATAATCT TATGTGGATG   
  
  
- ATATATACTA TAACGAGAAG TGGCATAGGA AGACGTATGA GTAGAAAGGC AGGGCCTCAG GTGATAGAGA   
  
  
- GGAGGTAAGT CTGTGAGAGA GCGAGATCTC TTGAGTATCG ATACCTGGCG CACGGGCTGC CAATAGCCCA   
  
  
- ATACTTAAGC CTCGAAGAAG TCGTTCAATC GGGACTCAAC GGCCTAGGTT GGTGGACCAA GGTACGGGCA   
  
  
- GATGGGCTGG GGTAGAGAGT CAGAGGAGAA CCTGGGCCAG GTAGGAGGGT CGGTTGGGTA AAGATGGTCC   
  
  
- CAAGACGGAG TCCCTTGGTA CACCAACCCT AACGACTGTG CACCTGCCTG GTCTAACAAC GTCCAAGGAG   
  
  
- CAGGGTCGTC ACTCCAACGT CGTCTCGGTC GTCCACAACT GACGTCAGCT TCGGTACCTC CTCCTGAGAC   
  
  
- CCTATTCTGA CCAGATGCGA GACGACTGCA CACGCCTCCG GTAGGTTGCA CCGCTAAAGG CTAACCGACA   
  
  
- GAACAATCAA TTGTTCTACT CGTTACTGGA GGACGGTGCG CAGTTGGGCA GGACGCCATA ACCGTTTCAT   
  
  
- CGGCCGATGA AGTATCTACG GAACTGGGCT TCTGATATGG TCGTCCCGGG TCACAGCCCG GATTAGCCCA   
  
  
- GCCAAGACCG CATGGTCCAC AACATGCTCG TGAAGATACT TCGAACGGGC AAGGAGTTTA AGCGAGTGAA   
  
  
- GTGACGATTA GTTCGCTAAA ATCTCCGTAA GCTGCCGGTG CTAACACAGG TGTAGTAGCT GAAGCCGGAT   
  
  
- TAGGTGCCGA ACGTTACCGG TCGAGATTAG GTTCGGAACC GACAAGCCGT GCCACCGGGC GGAAAGCATT   
  
  
- CTAATTGGCC TTAGCCCGGC GGAAGACTTA TGCCCAGCAC GAGCGAAGTT GGATAGCCCA GTTGTGACCG   
  
  
- GGTCAATCGG GATAGGTACT TACAACCCAA GCGTAAAGCC CGGCAGCGGC AGAGTGCCGA GCTCCTGTAG   
  
  
- TTTGGTACCT ACCAATTTTG TTCGGGTTTA CTTCAGCATC GGCACTTGAG ATAGAAGGTT GAGGTGGCCG   
  
  
- ATTAGCCCAG CCCATAACTG GGACAGGATT TGACCCAGGC CTCGGATTTG GGCTTTTAAC ACTGTGACCG   
  
  
- CCTCGTTCTC CGCTTGGTGT TGGTCGGGCT CAAAAACCGG GCCAAGTGCC TCCGTAATGT AATGATAAGT   
  
  
- TGATACAAGC TGAGCAATCT CCGAACAGTT CAGGTCCGGC TGTTCCTGGA TCGGCTCGAT ATGAATCTCT   
  
  
- CTCTCAATAG GTTGTAGCAG ACGACACTCC CCAGCCGAGC CTAACTCTCC GTGCTCGGCG ACCGGGTTAC   
  
  
- CTCCCGGGCT TACCGAGCCC GGCCCAAGTT CTTCCACCTA TACCCATTTT TACGCAAGTT CGTTCATTCG   
  
  
- TACAACGACT CAAACAGAAG TCGTGTTCCC ATAACACACC TCCTCTCACT CCCTACAAAC TTCGAGCCGA   
  
  
- CCGTACTGGC GGGAGAGTAA CGCCGAAGCC GAACCGCTCG CCTCTGAGTT CGACTCTTGA GGTCGTGACA   
  
  
- ACACGAACTA CCCAGTAGCA GCACATCAAG TAGAAGAAT

+     chs-CMA2a

| Site Name | Organism | Position | Strand | Matrix score. | sequence | function |
| --- | --- | --- | --- | --- | --- | --- |
| chs-CMA2a | Petroselinum crispum | 499 | + | 8 | TCACTTGA | part of a light responsive element |

>HU11G00778.1   
+ -Up\_Stream \_Len000AAAAAA GAAAGAAATG GAGAAACCAA GCAATATGGA TAAAAAAAAA ACAAAGTAAT   
  
  
+ AAAGGAAGGA TGAGGCTCCA CATTCCACTC GCCCGCGCCA CTCATAGGAG ATGAATAGTA AAATGTGAGG   
  
  
+ GGTTAAATGA TTTGAAAAAA ATCTAGTAAG GGCCCACAAC AGTATTGGAA CCCAGCTTGC CAAGGAAATG   
  
  
+ TCAGAGGGGC TAGCAATGGA CCAGTGCAAG AAGGCCAAGC CACATTTGGT TAGGGAATTT CATCCCATCC   
  
  
+ GGCGCTAAAA GCATCAAAAT TACTACTTAA TCCTTGATCA AATGCCCTAT AAATATTCTA CTACCCCATT   
  
  
+ TGAAAAGGGG TGTTGATTAC AACACATAGC CGTAGGTTTG ACAAAGAGAA TTACCCTTAT TGTTAGAAGC   
  
  
+ ACCATATACT CTTTTCTCTA AGCTCTGATC TTTTTTCATC CTTTTCAGCT TTTTGTCCTT TTAAATCAAA   
  
  
+ TCACTCACTT GAGTATTGGA GGGACGTTCC TCAGGAGTCG AACCACTAAA TTATCTTTTT AAAGAAAAAG   
  
  
+ GGCTTGAAGC CAACCCGATT TAAAAAAACT GCTAATATGT AAGCCTACTC TCTTTCCAGT TCCAACAAAT   
  
  
+ CAAATTTCAG TATGAAACAT CCAGTAAAGG ATTTATCCGT GATAATGATG TAACATATGG TTAAAGGGTC   
  
  
+ ATTTTCAGTT ACATCGGAGC ATGCAATTAA AGTCCCTATA AAATAGGTAG GTGCACGAAC AAGTCACCAT   
  
  
+ CGTCCTTTGC TTTTTGCATG AGTGCATGCT AATTATGAAC TTAATTAATA TCTAAGCAAA ATTAAAAAGT   
  
  
+ AGTTAAAACT TTTTCACCTT TGGAACCTCG TAGCTGTAGC AGTAGCTGCC GCACTCTGGT TCTAGACTTC   
  
  
+ TAGGGACCAA GAGATGCACT CATATGCATC CTCTTCTGTC CTTTTACTGT ATCCCCTTCC CATGTGACCT   
  
  
+ TTTTGCCCGG GTGACTTTTC TGTGAGTGTT GATACAAATT TTGTACGTAA GCTAACTCAT TTACTTCACC   
  
  
+ GTCTCATGAG TTTTACATTA CCGATATTAA TTTAAATGGC AAAGTATTAA ATTTGAGCTA AAATCTCATA   
  
  
+ TATATATTTG TATTAAATGT GTCTGGATTG TCAAAATAAT TTTTGACATA TACAGTCACT ATATACTGAG   
  
  
+ TTATTAAAAT ATATAAAAAT ATGAAAATTT AAAAAATAGT AGTTTAAGGT GACAACACTA AAACAAACAA   
  
  
+ ATAAGATATA AATAGGGAAA AGTTCATCCT ATTTTGTTAT TATTATTTAT TATGTATTTT CAGAATTGTC   
  
  
+ TCTATCACAA GCATAGTATA TGTGTCAACA TCGTGACAAC TCGACCAATC TACAAATAAC TAGTAAATTC   
  
  
+ AAATAAAGTT ACTCTCACCT AGCTTAAATA AGTACACCTT GATTTAATTG TCTTAGCTAT GGAACCTATT   
  
  
+ CCAAACTAGC CAATACTTCC TTTTCAAAAG AATTAGTTTT AAAGTTTTAA CTTTTACGTC CAATAATGCC   
  
  
+ GGCCGTACTA TGAGTTGCTT TCAAGCCAGT AGTAGTACTG TAGTAGGTAA AAATATTAGA ATACACCTAC   
  
  
+ TATATATGAT ATTGCTCTTC ACCGTATCCT TCTGCATACT CATCTTTCCG TCCCGGAGTC CACTATCTCT   
  
  
+ CCTCCATTCA GACACTCTCT CGCTCTAGAG AACTCATAGC TATGGACCGC GTGCCCGACG GTTATCGGGT   
  
  
+ TATGAATTCG GAGCTTCTTC AGCAAGTTAG CCCTGAGTTG CCGGATCCAA CCACCTGGTT CCATGCCCGT   
  
  
+ CTACCCGACC CCATCTCTCA GTCTCCTCTT GGACCCGGTC CATCCTCCCA GCCAACCCAT TTCTACCAGG   
  
  
+ GTTCTGCCTC AGGGAACCAT GTGGTTGGGA TTGCTGACAC GTGGACGGAC CAGATTGTTG CAGGTTCCTC   
  
  
+ GTCCCAGCAG TGAGGTTGCA GCAGAGCCAG CAGGTGTTGA CTGCAGTCGA AGCCATGGAG GAGGACTCTG   
  
  
+ GGATAAGACT GGTCTACGCT CTGCTGACGT GTGCGGAGGC CATCCAACGT GGCGATTTCC GATTGGCTGT   
  
  
+ CTTGTTAGTT AACAAGATGA GCAATGACCT CCTGCCACGC GTCAACCCGT CCTGCGGTAT TGGCAAAGTA   
  
  
+ GCCGGCTACT TCATAGATGC CTTGACCCGA AGACTATACC AGCAGGGCCC AGTGTCGGGC CTAATCGGGT   
  
  
+ CGGTTCTGGC GTACCAGGTG TTGTACGAGC ACTTCTATGA AGCTTGCCCG TTCCTCAAAT TCGCTCACTT   
  
  
+ CACTGCTAAT CAAGCGATTT TAGAGGCATT CGACGGCCAC GATTGTGTCC ACATCATCGA CTTCGGCCTA   
  
  
+ ATCCACGGCT TGCAATGGCC AGCTCTAATC CAAGCCTTGG CTGTTCGGCA CGGTGGCCCG CCTTTCGTAA   
  
  
+ GATTAACCGG AATCGGGCCG CCTTCTGAAT ACGGGTCGTG CTCGCTTCAA CCTATCGGGT CAACACTGGC   
  
  
+ CCAGTTAGCC CTATCCATGA ATGTTGGGTT CGCATTTCGG GCCGTCGCCG TCTCACGGCT CGAGGACATC   
  
  
+ AAACCATGGA TGGTTAAAAC AAGCCCAAAT GAAGTCGTAG CCGTGAACTC TATCTTCCAA CTCCACCGGC   
  
  
+ TAATCGGGTC GGGTATTGAC CCTGTCCTAA ACTGGGTCCG GAGCCTAAAC CCGAAAATTG TGACACTGGC   
  
  
+ GGAGCAAGAG GCGAACCACA ACCAGCCCGA GTTTTTGGCC CGGTTCACGG AGGCATTACA TTACTATTCA   
  
  
+ ACTATGTTCG ACTCGTTAGA GGCTTGTCAA GTCCAGGCCG ACAAGGACCT AGCCGAGCTA TACTTAGAGA   
  
  
+ GAGAGTTATC CAACATCGTC TGCTGTGAGG GGTCGGCTCG GATTGAGAGG CACGAGCCGC TGGCCCAATG   
  
  
+ GAGGGCCCGA ATGGCTCGGG CCGGGTTCAA GAAGGTGGAT ATGGGTAAAA ATGCGTTCAA GCAAGTAAGC   
  
  
+ ATGTTGCTGA GTTTGTCTTC AGCACAAGGG TATTGTGTGG AGGAGAGTGA GGGATGTTTG AAGCTCGGCT   
  
  
+ GGCATGACCG CCCTCTCATT GCGGCTTCGG CTTGGCGAGC GGAGACTCAA GCTGAGAACT CCAGCACTGT   
  
  
+ TGTGCTTGAT GGGTCATCGT CGTGTAGTTC ATCTTCTTA  

- -Up\_Stream \_Len000TTTTTT CTTTCTTTAC CTCTTTGGTT CGTTATACCT ATTTTTTTTT TGTTTCATTA   
  
  
- TTTCCTTCCT ACTCCGAGGT GTAAGGTGAG CGGGCGCGGT GAGTATCCTC TACTTATCAT TTTACACTCC   
  
  
- CCAATTTACT AAACTTTTTT TAGATCATTC CCGGGTGTTG TCATAACCTT GGGTCGAACG GTTCCTTTAC   
  
  
- AGTCTCCCCG ATCGTTACCT GGTCACGTTC TTCCGGTTCG GTGTAAACCA ATCCCTTAAA GTAGGGTAGG   
  
  
- CCGCGATTTT CGTAGTTTTA ATGATGAATT AGGAACTAGT TTACGGGATA TTTATAAGAT GATGGGGTAA   
  
  
- ACTTTTCCCC ACAACTAATG TTGTGTATCG GCATCCAAAC TGTTTCTCTT AATGGGAATA ACAATCTTCG   
  
  
- TGGTATATGA GAAAAGAGAT TCGAGACTAG AAAAAAGTAG GAAAAGTCGA AAAACAGGAA AATTTAGTTT   
  
  
- AGTGAGTGAA CTCATAACCT CCCTGCAAGG AGTCCTCAGC TTGGTGATTT AATAGAAAAA TTTCTTTTTC   
  
  
- CCGAACTTCG GTTGGGCTAA ATTTTTTTGA CGATTATACA TTCGGATGAG AGAAAGGTCA AGGTTGTTTA   
  
  
- GTTTAAAGTC ATACTTTGTA GGTCATTTCC TAAATAGGCA CTATTACTAC ATTGTATACC AATTTCCCAG   
  
  
- TAAAAGTCAA TGTAGCCTCG TACGTTAATT TCAGGGATAT TTTATCCATC CACGTGCTTG TTCAGTGGTA   
  
  
- GCAGGAAACG AAAAACGTAC TCACGTACGA TTAATACTTG AATTAATTAT AGATTCGTTT TAATTTTTCA   
  
  
- TCAATTTTGA AAAAGTGGAA ACCTTGGAGC ATCGACATCG TCATCGACGG CGTGAGACCA AGATCTGAAG   
  
  
- ATCCCTGGTT CTCTACGTGA GTATACGTAG GAGAAGACAG GAAAATGACA TAGGGGAAGG GTACACTGGA   
  
  
- AAAACGGGCC CACTGAAAAG ACACTCACAA CTATGTTTAA AACATGCATT CGATTGAGTA AATGAAGTGG   
  
  
- CAGAGTACTC AAAATGTAAT GGCTATAATT AAATTTACCG TTTCATAATT TAAACTCGAT TTTAGAGTAT   
  
  
- ATATATAAAC ATAATTTACA CAGACCTAAC AGTTTTATTA AAAACTGTAT ATGTCAGTGA TATATGACTC   
  
  
- AATAATTTTA TATATTTTTA TACTTTTAAA TTTTTTATCA TCAAATTCCA CTGTTGTGAT TTTGTTTGTT   
  
  
- TATTCTATAT TTATCCCTTT TCAAGTAGGA TAAAACAATA ATAATAAATA ATACATAAAA GTCTTAACAG   
  
  
- AGATAGTGTT CGTATCATAT ACACAGTTGT AGCACTGTTG AGCTGGTTAG ATGTTTATTG ATCATTTAAG   
  
  
- TTTATTTCAA TGAGAGTGGA TCGAATTTAT TCATGTGGAA CTAAATTAAC AGAATCGATA CCTTGGATAA   
  
  
- GGTTTGATCG GTTATGAAGG AAAAGTTTTC TTAATCAAAA TTTCAAAATT GAAAATGCAG GTTATTACGG   
  
  
- CCGGCATGAT ACTCAACGAA AGTTCGGTCA TCATCATGAC ATCATCCATT TTTATAATCT TATGTGGATG   
  
  
- ATATATACTA TAACGAGAAG TGGCATAGGA AGACGTATGA GTAGAAAGGC AGGGCCTCAG GTGATAGAGA   
  
  
- GGAGGTAAGT CTGTGAGAGA GCGAGATCTC TTGAGTATCG ATACCTGGCG CACGGGCTGC CAATAGCCCA   
  
  
- ATACTTAAGC CTCGAAGAAG TCGTTCAATC GGGACTCAAC GGCCTAGGTT GGTGGACCAA GGTACGGGCA   
  
  
- GATGGGCTGG GGTAGAGAGT CAGAGGAGAA CCTGGGCCAG GTAGGAGGGT CGGTTGGGTA AAGATGGTCC   
  
  
- CAAGACGGAG TCCCTTGGTA CACCAACCCT AACGACTGTG CACCTGCCTG GTCTAACAAC GTCCAAGGAG   
  
  
- CAGGGTCGTC ACTCCAACGT CGTCTCGGTC GTCCACAACT GACGTCAGCT TCGGTACCTC CTCCTGAGAC   
  
  
- CCTATTCTGA CCAGATGCGA GACGACTGCA CACGCCTCCG GTAGGTTGCA CCGCTAAAGG CTAACCGACA   
  
  
- GAACAATCAA TTGTTCTACT CGTTACTGGA GGACGGTGCG CAGTTGGGCA GGACGCCATA ACCGTTTCAT   
  
  
- CGGCCGATGA AGTATCTACG GAACTGGGCT TCTGATATGG TCGTCCCGGG TCACAGCCCG GATTAGCCCA   
  
  
- GCCAAGACCG CATGGTCCAC AACATGCTCG TGAAGATACT TCGAACGGGC AAGGAGTTTA AGCGAGTGAA   
  
  
- GTGACGATTA GTTCGCTAAA ATCTCCGTAA GCTGCCGGTG CTAACACAGG TGTAGTAGCT GAAGCCGGAT   
  
  
- TAGGTGCCGA ACGTTACCGG TCGAGATTAG GTTCGGAACC GACAAGCCGT GCCACCGGGC GGAAAGCATT   
  
  
- CTAATTGGCC TTAGCCCGGC GGAAGACTTA TGCCCAGCAC GAGCGAAGTT GGATAGCCCA GTTGTGACCG   
  
  
- GGTCAATCGG GATAGGTACT TACAACCCAA GCGTAAAGCC CGGCAGCGGC AGAGTGCCGA GCTCCTGTAG   
  
  
- TTTGGTACCT ACCAATTTTG TTCGGGTTTA CTTCAGCATC GGCACTTGAG ATAGAAGGTT GAGGTGGCCG   
  
  
- ATTAGCCCAG CCCATAACTG GGACAGGATT TGACCCAGGC CTCGGATTTG GGCTTTTAAC ACTGTGACCG   
  
  
- CCTCGTTCTC CGCTTGGTGT TGGTCGGGCT CAAAAACCGG GCCAAGTGCC TCCGTAATGT AATGATAAGT   
  
  
- TGATACAAGC TGAGCAATCT CCGAACAGTT CAGGTCCGGC TGTTCCTGGA TCGGCTCGAT ATGAATCTCT   
  
  
- CTCTCAATAG GTTGTAGCAG ACGACACTCC CCAGCCGAGC CTAACTCTCC GTGCTCGGCG ACCGGGTTAC   
  
  
- CTCCCGGGCT TACCGAGCCC GGCCCAAGTT CTTCCACCTA TACCCATTTT TACGCAAGTT CGTTCATTCG   
  
  
- TACAACGACT CAAACAGAAG TCGTGTTCCC ATAACACACC TCCTCTCACT CCCTACAAAC TTCGAGCCGA   
  
  
- CCGTACTGGC GGGAGAGTAA CGCCGAAGCC GAACCGCTCG CCTCTGAGTT CGACTCTTGA GGTCGTGACA   
  
  
- ACACGAACTA CCCAGTAGCA GCACATCAAG TAGAAGAAT
