# Supplementary material for: Genome-Wide Identification and Expression Pattern of the GRAS Gene Family in Pitaya (Selenicereus undatus L.)
Source: Biology (Basel). 2022 Dec 21;12(1):11. doi: 10.3390/biology12010011 (PMC9854919; doi:10.3390/biology12010011)
Supplement: Supplementary file 1 [file biology-12-00011-s001.zip › Supplementary file S5/HU11G01134.1_plantcare.html]

Content-Type: text/html; charset=ISO-8859-1


PlantCARE


Webmaster Firefox specific output  
To save the result:
click on the frame with the right mouse button and save the source code as a text file with extension .html  
REFERENCE:PlantCARE: a database of plant cis-acting regulatory elements and a portal to tools for in silico analysis of promoter sequences.  
Lescot, M., Déhais, P., Moreau, Y., De Moor, B., Rouzé ,P.,and Rombauts, S.  
Nucleic Acids Res., Database issue(2002), 30(1):325-327.   


---

>HU11G01134.1   
+ +Up\_Stream \_Len000AGATAG ACATTCAGCC ACTTGCCAGT TCAGAGAGAG ATAGAGAGAG ATTGGGTAAG   
  
  
+ CCCTTTTTGC ACTACTTTTT CTCGCTTCCA TTTTTCTTTC TTAATTTTTA GTGATGATTT TCCAGAAGAT   
  
  
+ TTGATTTATT CATCATGCTT AACCAATTGG GTCTTTGAGT ATTATTGTTT ACTGGGTATT TGATCTTTTC   
  
  
+ ATATTCCTTT GGACTGGGTG GGTGGAACTT TTGAACTTTC TTATTAGTTG GGCATGCCCT TATCTTATTC   
  
  
+ CAATTCAAAA CCCATATTTT TCTTCAATTT TAATTATGGG TATTTTGAAA TTGAATGCTT TCCTTCACAG   
  
  
+ CAATACTATA TTACATATTT TACAGGAAAG TAATATTTTA CAGGAAAGTA TGTTGCTGTT TCAACCCTGT   
  
  
+ TTTGTTTCCT CTCTTTCCTT GGATATGATG TTCATTGCTC TTGTACCAAA CCTAACCTCA ACTTCCTGAA   
  
  
+ TTATTTAAGG CAATATTACC GTGTGCTGGT CAATTTTGGT TGTGGGTTTG TTTCTTAATG TATATGATTT   
  
  
+ ATGGTCAGGA TCCATGCTCC TCTTCCGTCC ACTCTATATC TCTCTCTCTC TCTCTCAATA CAAATTCAGA   
  
  
+ GACTTTCTCT TTCTGGGTTT ACTATTGATT GCAGAAGTTT GTAGTCTTGC GAGGCCTTGA GTCTTCTTCG   
  
  
+ AGGTAGAAAA TTTTGCTTTG CGCTTCCTTC CCCATATTTT AACACCCCTC CCCCCCCAAC GCAAGTTTTC   
  
  
+ TACATTTTTG TGCAATTCTT CAGCTATGAT TCCCCAATTT CACTTGTCAC ATCTCTATTT TCCTCTTTTT   
  
  
+ GGTTTCTTGA TTCTGTAGAA GGTTGTGTAT GAACTTCTTT GTTTCCCTTT TTCCTTTGTT ATTTTTAATG   
  
  
+ TTCCCATAGA TGATATAGAG ATCTGCTTTT CCTTGCTGTG AGAGGTATCT CTGAGAATGC ATGTAGCAAT   
  
  
+ CAAAATTCTG AGTTGTTAAT TCTATTGCAT GTCTATGGGT GATGATTGTG ATGATGTTTT TTCTGTTGCC   
  
  
+ CTCTTTCTCT CTTTCACTGA AATTGCTTTC CAGAATAGTT GTTTGACTCC TTTCTGTATC TTAATTGAGC   
  
  
+ CCTTGCTTTG GTTTGTGCTC TGGGAGTTCT TCTTTAGTAA TCCCTTGCTT GTATTTTCTG AAAGAGCAAG   
  
  
+ CAATAATCCT CATAGCATGT GTATAATCAT ACAATAGCAA CAGCAAACAT GGATCCTGCG CCCAGTTAGG   
  
  
+ TTTGGTTAGG TTAAGATATG CATATTCTTT TCTCTTGAAA AGAGTTGTCT AGGAAGGGTA ATTATGCCCA   
  
  
+ TTTCATGGAT AATAATGGTA CAATTAAGTT TGATTGGCTT AAATACCAAT TATTTAGCGT CTTTTTCTTT   
  
  
+ GGCTGTCCAG GGTTCCATTT CCATAAACAA CTTTGAGTAA GGCATAGAGA ACTAAATTTC TGCCAATTGT   
  
  
+ CAAGTGCAAG TTGCTCATAT GCAACAATAC TTGGAAATTT TGAGCTGAGC TGCTGGCATA TCATGAATAG   
  
  
+ TTCTAATGGT GGCGAGTTAT CACTTATCGT CCCTTGTGTG ACGAATTTCT CTTCCTAGGA AGCCCTCTAG   
  
  
+ TCAATGCCTA TCTGCTGTTG TGGAGTGAAA ATGGATACAT TTAGATCTCT ATAAACCCTA CTTTGGCAAG   
  
  
+ ATCCATGCTG ATTTTAGGAA GCCCTTTGAC CAGAGAATGA CATAACAACC TGATAGGGTG TTACATGCTA   
  
  
+ ACTTCGATTA TTTAATCAAA ATACGGAACT TCTATCAGTC AAAGCCTGCC CCAGGTTCTA CTGAATTAAA   
  
  
+ TTGTATTGAA TTGCTCTCAC TTGCTTATGA CGATTAAATA ACTGGATTTG CTAATTATTC TTTTTATTTT   
  
  
+ ATTCTTGGTG ATGTAGGGAA CTCAGGGAAT TGTAACCATA CCTGTTCCAG CTGAAAAGGG TACGGGAACA   
  
  
+ GTCATAGTTA CAGGGGAATT TCGCAATACA TTTAAGGGAG TAAGAAGCAG GATCATGTCT TTAGTTAGGC   
  
  
+ CGGCAGATAT GTCTGTGACT CCATATGGAA ATCCAACACT GTATTCACTT CAAGGCAATA ATAATAGCTC   
  
  
+ TGGTTTGTCT GGTCAACTGT ATGGCTCTGA AAAGCACAAG ATCAAATATG TGACCGAATC TTTTAATGGT   
  
  
+ CCAAGTTATG GCCTGAAATT CTTTGTTGAT TCTCCATCAG AAGAGCTCAT CCACCCATCA GATGCTACTC   
  
  
+ CTAACCCATT TGACTCTTCT TTCGTGGGCA TAAGACATGA TGAGAGTCCT TACCAAGGAA ATTATGGATC   
  
  
+ AGAATATGGG GTAAGCCCAT CTTCCGATGC AGTTGAATAT GATGAAGGTA ATAAGATAAG GCTAAAACTT   
  
  
+ CAAGAATTGG AGCATGCCCT GCTCGATGAA GATGATGAAG TGGATGAGGT GGCTATAGGG CCCGTGCTAG   
  
  
+ AACACAGCAT GGAGCTTGAT GAAGAGTGGG TCGCCCCATT GAGAAATGTG GTTTTCCATG ATTCACCAAA   
  
  
+ GGAGTCCACC TCCTCAGAGT CATCCAATGT CAGCAGCATT AGCAGCACCA AAGAAGTATC ACCTTGCTCT   
  
  
+ CCGAGTACTC CCAAACAATT GCTCTTTAAT TGTGCAAATA TGATTTCAGA GGGGAATTTT GAGGAAGCCG   
  
  
+ TGACTATGAT CAGCGAGCTT AGACAGATTG TTTCTATCCA AGGGGATCCA GCGCAGAGGA TAGCAGCCTA   
  
  
+ CATGGTAGAA GGCCTTGCTT CCCGTTTGGC TTCATCGGGA AAAGTTCTTT ACAAAGCTTT GAAATGCAAA   
  
  
+ GAGCCACCTT CATTTGACAG GCTAGCGGCT ATGCAAATCC TCTTTGAGGT GTGCCCATGT TTCAAATTCG   
  
  
+ GATTTATGGC CGCAAATGCA GCGATTATGG ATGCAATCAA AAACGAAAAA AGGGTACACA TCATAGATTT   
  
  
+ TGACATAAAC CAAGGGAATC AATACATAAA TCTCATGCAA TCCCTTGCTA AACAAGGTAA CAAGCTGACG   
  
  
+ CACTTGAAGT TGACTGGAGT TGATGACCCT GAGTCAGTTC AACGCCCTAT TGGTGGCCTA AAAAACATCG   
  
  
+ GACAAAGGCT GCAAGCATTA GCTGAATATC TTGGTGTGTC ATTTGAGTTC AAAGCAATAC CTGCTAGAAC   
  
  
+ TCCACTTGTT AACCCGGAAA TGTTAGAATG TCGACCTGCG GAAGCTTTAG TGGTGAACTT TGCCTTCCAG   
  
  
+ CTTCACCACA TGCCTGATGA AAGCGTCTCA ACAATCAACC TCCGAGACCA GCTTCTTCGG ATGGTCAAAA   
  
  
+ GCCTAAACCC AAAGCTAGTA ACCATTGTCG AGCAGGATGT GAACACAAAC ACTACCCCTT TCCTCTCTAG   
  
  
+ GTTTGCTGAG GCATACAGCT ACTATTCTGC TGTTTTTGAG TCTCTTGATG CTACTCTCCC TAGAGATAGC   
  
  
+ CAGGACAGGG TGAATGTGGA GAAGCAGTGC TTGGCACGTG ATGTTGTGAA CATCATTGCA TGTGAAGGGG   
  
  
+ AGGAGAGGAT TGAGCGCTAT GAGGTTGCGG GGAAATGGAG AGCGAGGATG TTGATGGCGG GATTCAGGGC   
  
  
+ TATTCCAATG GGTCAGAAGA CCGGTGATAT GATTCGGAAG CTTATATCAA TGCGGTATTG TGACAGGCTC   
  
  
+ AAGGTAAAAG AGGAGATGGA TGCACTTCAC TTTGGGTGGG AAGATAAAAA CTTAATCGTT ACATCAGCTT   
  
  
+ GGAGGTG  

- +Up\_Stream \_Len000TCTATC TGTAAGTCGG TGAACGGTCA AGTCTCTCTC TATCTCTCTC TAACCCATTC   
  
  
- GGGAAAAACG TGATGAAAAA GAGCGAAGGT AAAAAGAAAG AATTAAAAAT CACTACTAAA AGGTCTTCTA   
  
  
- AACTAAATAA GTAGTACGAA TTGGTTAACC CAGAAACTCA TAATAACAAA TGACCCATAA ACTAGAAAAG   
  
  
- TATAAGGAAA CCTGACCCAC CCACCTTGAA AACTTGAAAG AATAATCAAC CCGTACGGGA ATAGAATAAG   
  
  
- GTTAAGTTTT GGGTATAAAA AGAAGTTAAA ATTAATACCC ATAAAACTTT AACTTACGAA AGGAAGTGTC   
  
  
- GTTATGATAT AATGTATAAA ATGTCCTTTC ATTATAAAAT GTCCTTTCAT ACAACGACAA AGTTGGGACA   
  
  
- AAACAAAGGA GAGAAAGGAA CCTATACTAC AAGTAACGAG AACATGGTTT GGATTGGAGT TGAAGGACTT   
  
  
- AATAAATTCC GTTATAATGG CACACGACCA GTTAAAACCA ACACCCAAAC AAAGAATTAC ATATACTAAA   
  
  
- TACCAGTCCT AGGTACGAGG AGAAGGCAGG TGAGATATAG AGAGAGAGAG AGAGAGTTAT GTTTAAGTCT   
  
  
- CTGAAAGAGA AAGACCCAAA TGATAACTAA CGTCTTCAAA CATCAGAACG CTCCGGAACT CAGAAGAAGC   
  
  
- TCCATCTTTT AAAACGAAAC GCGAAGGAAG GGGTATAAAA TTGTGGGGAG GGGGGGGTTG CGTTCAAAAG   
  
  
- ATGTAAAAAC ACGTTAAGAA GTCGATACTA AGGGGTTAAA GTGAACAGTG TAGAGATAAA AGGAGAAAAA   
  
  
- CCAAAGAACT AAGACATCTT CCAACACATA CTTGAAGAAA CAAAGGGAAA AAGGAAACAA TAAAAATTAC   
  
  
- AAGGGTATCT ACTATATCTC TAGACGAAAA GGAACGACAC TCTCCATAGA GACTCTTACG TACATCGTTA   
  
  
- GTTTTAAGAC TCAACAATTA AGATAACGTA CAGATACCCA CTACTAACAC TACTACAAAA AAGACAACGG   
  
  
- GAGAAAGAGA GAAAGTGACT TTAACGAAAG GTCTTATCAA CAAACTGAGG AAAGACATAG AATTAACTCG   
  
  
- GGAACGAAAC CAAACACGAG ACCCTCAAGA AGAAATCATT AGGGAACGAA CATAAAAGAC TTTCTCGTTC   
  
  
- GTTATTAGGA GTATCGTACA CATATTAGTA TGTTATCGTT GTCGTTTGTA CCTAGGACGC GGGTCAATCC   
  
  
- AAACCAATCC AATTCTATAC GTATAAGAAA AGAGAACTTT TCTCAACAGA TCCTTCCCAT TAATACGGGT   
  
  
- AAAGTACCTA TTATTACCAT GTTAATTCAA ACTAACCGAA TTTATGGTTA ATAAATCGCA GAAAAAGAAA   
  
  
- CCGACAGGTC CCAAGGTAAA GGTATTTGTT GAAACTCATT CCGTATCTCT TGATTTAAAG ACGGTTAACA   
  
  
- GTTCACGTTC AACGAGTATA CGTTGTTATG AACCTTTAAA ACTCGACTCG ACGACCGTAT AGTACTTATC   
  
  
- AAGATTACCA CCGCTCAATA GTGAATAGCA GGGAACACAC TGCTTAAAGA GAAGGATCCT TCGGGAGATC   
  
  
- AGTTACGGAT AGACGACAAC ACCTCACTTT TACCTATGTA AATCTAGAGA TATTTGGGAT GAAACCGTTC   
  
  
- TAGGTACGAC TAAAATCCTT CGGGAAACTG GTCTCTTACT GTATTGTTGG ACTATCCCAC AATGTACGAT   
  
  
- TGAAGCTAAT AAATTAGTTT TATGCCTTGA AGATAGTCAG TTTCGGACGG GGTCCAAGAT GACTTAATTT   
  
  
- AACATAACTT AACGAGAGTG AACGAATACT GCTAATTTAT TGACCTAAAC GATTAATAAG AAAAATAAAA   
  
  
- TAAGAACCAC TACATCCCTT GAGTCCCTTA ACATTGGTAT GGACAAGGTC GACTTTTCCC ATGCCCTTGT   
  
  
- CAGTATCAAT GTCCCCTTAA AGCGTTATGT AAATTCCCTC ATTCTTCGTC CTAGTACAGA AATCAATCCG   
  
  
- GCCGTCTATA CAGACACTGA GGTATACCTT TAGGTTGTGA CATAAGTGAA GTTCCGTTAT TATTATCGAG   
  
  
- ACCAAACAGA CCAGTTGACA TACCGAGACT TTTCGTGTTC TAGTTTATAC ACTGGCTTAG AAAATTACCA   
  
  
- GGTTCAATAC CGGACTTTAA GAAACAACTA AGAGGTAGTC TTCTCGAGTA GGTGGGTAGT CTACGATGAG   
  
  
- GATTGGGTAA ACTGAGAAGA AAGCACCCGT ATTCTGTACT ACTCTCAGGA ATGGTTCCTT TAATACCTAG   
  
  
- TCTTATACCC CATTCGGGTA GAAGGCTACG TCAACTTATA CTACTTCCAT TATTCTATTC CGATTTTGAA   
  
  
- GTTCTTAACC TCGTACGGGA CGAGCTACTT CTACTACTTC ACCTACTCCA CCGATATCCC GGGCACGATC   
  
  
- TTGTGTCGTA CCTCGAACTA CTTCTCACCC AGCGGGGTAA CTCTTTACAC CAAAAGGTAC TAAGTGGTTT   
  
  
- CCTCAGGTGG AGGAGTCTCA GTAGGTTACA GTCGTCGTAA TCGTCGTGGT TTCTTCATAG TGGAACGAGA   
  
  
- GGCTCATGAG GGTTTGTTAA CGAGAAATTA ACACGTTTAT ACTAAAGTCT CCCCTTAAAA CTCCTTCGGC   
  
  
- ACTGATACTA GTCGCTCGAA TCTGTCTAAC AAAGATAGGT TCCCCTAGGT CGCGTCTCCT ATCGTCGGAT   
  
  
- GTACCATCTT CCGGAACGAA GGGCAAACCG AAGTAGCCCT TTTCAAGAAA TGTTTCGAAA CTTTACGTTT   
  
  
- CTCGGTGGAA GTAAACTGTC CGATCGCCGA TACGTTTAGG AGAAACTCCA CACGGGTACA AAGTTTAAGC   
  
  
- CTAAATACCG GCGTTTACGT CGCTAATACC TACGTTAGTT TTTGCTTTTT TCCCATGTGT AGTATCTAAA   
  
  
- ACTGTATTTG GTTCCCTTAG TTATGTATTT AGAGTACGTT AGGGAACGAT TTGTTCCATT GTTCGACTGC   
  
  
- GTGAACTTCA ACTGACCTCA ACTACTGGGA CTCAGTCAAG TTGCGGGATA ACCACCGGAT TTTTTGTAGC   
  
  
- CTGTTTCCGA CGTTCGTAAT CGACTTATAG AACCACACAG TAAACTCAAG TTTCGTTATG GACGATCTTG   
  
  
- AGGTGAACAA TTGGGCCTTT ACAATCTTAC AGCTGGACGC CTTCGAAATC ACCACTTGAA ACGGAAGGTC   
  
  
- GAAGTGGTGT ACGGACTACT TTCGCAGAGT TGTTAGTTGG AGGCTCTGGT CGAAGAAGCC TACCAGTTTT   
  
  
- CGGATTTGGG TTTCGATCAT TGGTAACAGC TCGTCCTACA CTTGTGTTTG TGATGGGGAA AGGAGAGATC   
  
  
- CAAACGACTC CGTATGTCGA TGATAAGACG ACAAAAACTC AGAGAACTAC GATGAGAGGG ATCTCTATCG   
  
  
- GTCCTGTCCC ACTTACACCT CTTCGTCACG AACCGTGCAC TACAACACTT GTAGTAACGT ACACTTCCCC   
  
  
- TCCTCTCCTA ACTCGCGATA CTCCAACGCC CCTTTACCTC TCGCTCCTAC AACTACCGCC CTAAGTCCCG   
  
  
- ATAAGGTTAC CCAGTCTTCT GGCCACTATA CTAAGCCTTC GAATATAGTT ACGCCATAAC ACTGTCCGAG   
  
  
- TTCCATTTTC TCCTCTACCT ACGTGAAGTG AAACCCACCC TTCTATTTTT GAATTAGCAA TGTAGTCGAA   
  
  
- CCTCCAC

  
  
Motifs Found  

+   

| Site Name | Organism | Position | Strand | Matrix score. | sequence | function |
| --- | --- | --- | --- | --- | --- | --- |
|  | organism | 3541 | - | 4 | motif\_sequence | short\_function |
|  | organism | 2591 | + | 4 | motif\_sequence | short\_function |
|  | organism | 2037 | - | 4 | motif\_sequence | short\_function |
|  | organism | 2717 | - | 4 | motif\_sequence | short\_function |
|  | organism | 1936 | + | 4 | motif\_sequence | short\_function |
|  | organism | 912 | + | 4 | motif\_sequence | short\_function |
|  | organism | 3684 | - | 4 | motif\_sequence | short\_function |
|  | organism | 452 | + | 4 | motif\_sequence | short\_function |
|  | organism | 1593 | + | 4 | motif\_sequence | short\_function |
|  | organism | 1407 | + | 4 | motif\_sequence | short\_function |
|  | organism | 3437 | - | 4 | motif\_sequence | short\_function |
|  | organism | 2258 | + | 4 | motif\_sequence | short\_function |
|  | organism | 3656 | - | 4 | motif\_sequence | short\_function |
|  | organism | 1960 | - | 4 | motif\_sequence | short\_function |
|  | organism | 2214 | - | 4 | motif\_sequence | short\_function |
|  | organism | 2475 | - | 4 | motif\_sequence | short\_function |
|  | organism | 1624 | + | 4 | motif\_sequence | short\_function |
|  | organism | 139 | - | 4 | motif\_sequence | short\_function |
|  | organism | 3506 | - | 4 | motif\_sequence | short\_function |
|  | organism | 2333 | + | 4 | motif\_sequence | short\_function |
|  | organism | 584 | + | 4 | motif\_sequence | short\_function |
|  | organism | 935 | + | 4 | motif\_sequence | short\_function |
|  | organism | 3418 | + | 4 | motif\_sequence | short\_function |
|  | organism | 3482 | - | 4 | motif\_sequence | short\_function |
|  | organism | 2412 | - | 4 | motif\_sequence | short\_function |

>HU11G01134.1   
+ +Up\_Stream \_Len000AGATAG ACATTCAGCC ACTTGCCAGT TCAGAGAGAG ATAGAGAGAG ATTGGGTAAG   
  
  
+ CCCTTTTTGC ACTACTTTTT CTCGCTTCCA TTTTTCTTTC TTAATTTTTA GTGATGATTT TCCAGAAGAT   
  
  
+ TTGATTTATT CATCATGCTT AACCAATTGG GTCTTTGAGT ATTATTGTTT ACTGGGTATT TGATCTTTTC   
  
  
+ ATATTCCTTT GGACTGGGTG GGTGGAACTT TTGAACTTTC TTATTAGTTG GGCATGCCCT TATCTTATTC   
  
  
+ CAATTCAAAA CCCATATTTT TCTTCAATTT TAATTATGGG TATTTTGAAA TTGAATGCTT TCCTTCACAG   
  
  
+ CAATACTATA TTACATATTT TACAGGAAAG TAATATTTTA CAGGAAAGTA TGTTGCTGTT TCAACCCTGT   
  
  
+ TTTGTTTCCT CTCTTTCCTT GGATATGATG TTCATTGCTC TTGTACCAAA CCTAACCTCA ACTTCCTGAA   
  
  
+ TTATTTAAGG CAATATTACC GTGTGCTGGT CAATTTTGGT TGTGGGTTTG TTTCTTAATG TATATGATTT   
  
  
+ ATGGTCAGGA TCCATGCTCC TCTTCCGTCC ACTCTATATC TCTCTCTCTC TCTCTCAATA CAAATTCAGA   
  
  
+ GACTTTCTCT TTCTGGGTTT ACTATTGATT GCAGAAGTTT GTAGTCTTGC GAGGCCTTGA GTCTTCTTCG   
  
  
+ AGGTAGAAAA TTTTGCTTTG CGCTTCCTTC CCCATATTTT AACACCCCTC CCCCCCCAAC GCAAGTTTTC   
  
  
+ TACATTTTTG TGCAATTCTT CAGCTATGAT TCCCCAATTT CACTTGTCAC ATCTCTATTT TCCTCTTTTT   
  
  
+ GGTTTCTTGA TTCTGTAGAA GGTTGTGTAT GAACTTCTTT GTTTCCCTTT TTCCTTTGTT ATTTTTAATG   
  
  
+ TTCCCATAGA TGATATAGAG ATCTGCTTTT CCTTGCTGTG AGAGGTATCT CTGAGAATGC ATGTAGCAAT   
  
  
+ CAAAATTCTG AGTTGTTAAT TCTATTGCAT GTCTATGGGT GATGATTGTG ATGATGTTTT TTCTGTTGCC   
  
  
+ CTCTTTCTCT CTTTCACTGA AATTGCTTTC CAGAATAGTT GTTTGACTCC TTTCTGTATC TTAATTGAGC   
  
  
+ CCTTGCTTTG GTTTGTGCTC TGGGAGTTCT TCTTTAGTAA TCCCTTGCTT GTATTTTCTG AAAGAGCAAG   
  
  
+ CAATAATCCT CATAGCATGT GTATAATCAT ACAATAGCAA CAGCAAACAT GGATCCTGCG CCCAGTTAGG   
  
  
+ TTTGGTTAGG TTAAGATATG CATATTCTTT TCTCTTGAAA AGAGTTGTCT AGGAAGGGTA ATTATGCCCA   
  
  
+ TTTCATGGAT AATAATGGTA CAATTAAGTT TGATTGGCTT AAATACCAAT TATTTAGCGT CTTTTTCTTT   
  
  
+ GGCTGTCCAG GGTTCCATTT CCATAAACAA CTTTGAGTAA GGCATAGAGA ACTAAATTTC TGCCAATTGT   
  
  
+ CAAGTGCAAG TTGCTCATAT GCAACAATAC TTGGAAATTT TGAGCTGAGC TGCTGGCATA TCATGAATAG   
  
  
+ TTCTAATGGT GGCGAGTTAT CACTTATCGT CCCTTGTGTG ACGAATTTCT CTTCCTAGGA AGCCCTCTAG   
  
  
+ TCAATGCCTA TCTGCTGTTG TGGAGTGAAA ATGGATACAT TTAGATCTCT ATAAACCCTA CTTTGGCAAG   
  
  
+ ATCCATGCTG ATTTTAGGAA GCCCTTTGAC CAGAGAATGA CATAACAACC TGATAGGGTG TTACATGCTA   
  
  
+ ACTTCGATTA TTTAATCAAA ATACGGAACT TCTATCAGTC AAAGCCTGCC CCAGGTTCTA CTGAATTAAA   
  
  
+ TTGTATTGAA TTGCTCTCAC TTGCTTATGA CGATTAAATA ACTGGATTTG CTAATTATTC TTTTTATTTT   
  
  
+ ATTCTTGGTG ATGTAGGGAA CTCAGGGAAT TGTAACCATA CCTGTTCCAG CTGAAAAGGG TACGGGAACA   
  
  
+ GTCATAGTTA CAGGGGAATT TCGCAATACA TTTAAGGGAG TAAGAAGCAG GATCATGTCT TTAGTTAGGC   
  
  
+ CGGCAGATAT GTCTGTGACT CCATATGGAA ATCCAACACT GTATTCACTT CAAGGCAATA ATAATAGCTC   
  
  
+ TGGTTTGTCT GGTCAACTGT ATGGCTCTGA AAAGCACAAG ATCAAATATG TGACCGAATC TTTTAATGGT   
  
  
+ CCAAGTTATG GCCTGAAATT CTTTGTTGAT TCTCCATCAG AAGAGCTCAT CCACCCATCA GATGCTACTC   
  
  
+ CTAACCCATT TGACTCTTCT TTCGTGGGCA TAAGACATGA TGAGAGTCCT TACCAAGGAA ATTATGGATC   
  
  
+ AGAATATGGG GTAAGCCCAT CTTCCGATGC AGTTGAATAT GATGAAGGTA ATAAGATAAG GCTAAAACTT   
  
  
+ CAAGAATTGG AGCATGCCCT GCTCGATGAA GATGATGAAG TGGATGAGGT GGCTATAGGG CCCGTGCTAG   
  
  
+ AACACAGCAT GGAGCTTGAT GAAGAGTGGG TCGCCCCATT GAGAAATGTG GTTTTCCATG ATTCACCAAA   
  
  
+ GGAGTCCACC TCCTCAGAGT CATCCAATGT CAGCAGCATT AGCAGCACCA AAGAAGTATC ACCTTGCTCT   
  
  
+ CCGAGTACTC CCAAACAATT GCTCTTTAAT TGTGCAAATA TGATTTCAGA GGGGAATTTT GAGGAAGCCG   
  
  
+ TGACTATGAT CAGCGAGCTT AGACAGATTG TTTCTATCCA AGGGGATCCA GCGCAGAGGA TAGCAGCCTA   
  
  
+ CATGGTAGAA GGCCTTGCTT CCCGTTTGGC TTCATCGGGA AAAGTTCTTT ACAAAGCTTT GAAATGCAAA   
  
  
+ GAGCCACCTT CATTTGACAG GCTAGCGGCT ATGCAAATCC TCTTTGAGGT GTGCCCATGT TTCAAATTCG   
  
  
+ GATTTATGGC CGCAAATGCA GCGATTATGG ATGCAATCAA AAACGAAAAA AGGGTACACA TCATAGATTT   
  
  
+ TGACATAAAC CAAGGGAATC AATACATAAA TCTCATGCAA TCCCTTGCTA AACAAGGTAA CAAGCTGACG   
  
  
+ CACTTGAAGT TGACTGGAGT TGATGACCCT GAGTCAGTTC AACGCCCTAT TGGTGGCCTA AAAAACATCG   
  
  
+ GACAAAGGCT GCAAGCATTA GCTGAATATC TTGGTGTGTC ATTTGAGTTC AAAGCAATAC CTGCTAGAAC   
  
  
+ TCCACTTGTT AACCCGGAAA TGTTAGAATG TCGACCTGCG GAAGCTTTAG TGGTGAACTT TGCCTTCCAG   
  
  
+ CTTCACCACA TGCCTGATGA AAGCGTCTCA ACAATCAACC TCCGAGACCA GCTTCTTCGG ATGGTCAAAA   
  
  
+ GCCTAAACCC AAAGCTAGTA ACCATTGTCG AGCAGGATGT GAACACAAAC ACTACCCCTT TCCTCTCTAG   
  
  
+ GTTTGCTGAG GCATACAGCT ACTATTCTGC TGTTTTTGAG TCTCTTGATG CTACTCTCCC TAGAGATAGC   
  
  
+ CAGGACAGGG TGAATGTGGA GAAGCAGTGC TTGGCACGTG ATGTTGTGAA CATCATTGCA TGTGAAGGGG   
  
  
+ AGGAGAGGAT TGAGCGCTAT GAGGTTGCGG GGAAATGGAG AGCGAGGATG TTGATGGCGG GATTCAGGGC   
  
  
+ TATTCCAATG GGTCAGAAGA CCGGTGATAT GATTCGGAAG CTTATATCAA TGCGGTATTG TGACAGGCTC   
  
  
+ AAGGTAAAAG AGGAGATGGA TGCACTTCAC TTTGGGTGGG AAGATAAAAA CTTAATCGTT ACATCAGCTT   
  
  
+ GGAGGTG  

- +Up\_Stream \_Len000TCTATC TGTAAGTCGG TGAACGGTCA AGTCTCTCTC TATCTCTCTC TAACCCATTC   
  
  
- GGGAAAAACG TGATGAAAAA GAGCGAAGGT AAAAAGAAAG AATTAAAAAT CACTACTAAA AGGTCTTCTA   
  
  
- AACTAAATAA GTAGTACGAA TTGGTTAACC CAGAAACTCA TAATAACAAA TGACCCATAA ACTAGAAAAG   
  
  
- TATAAGGAAA CCTGACCCAC CCACCTTGAA AACTTGAAAG AATAATCAAC CCGTACGGGA ATAGAATAAG   
  
  
- GTTAAGTTTT GGGTATAAAA AGAAGTTAAA ATTAATACCC ATAAAACTTT AACTTACGAA AGGAAGTGTC   
  
  
- GTTATGATAT AATGTATAAA ATGTCCTTTC ATTATAAAAT GTCCTTTCAT ACAACGACAA AGTTGGGACA   
  
  
- AAACAAAGGA GAGAAAGGAA CCTATACTAC AAGTAACGAG AACATGGTTT GGATTGGAGT TGAAGGACTT   
  
  
- AATAAATTCC GTTATAATGG CACACGACCA GTTAAAACCA ACACCCAAAC AAAGAATTAC ATATACTAAA   
  
  
- TACCAGTCCT AGGTACGAGG AGAAGGCAGG TGAGATATAG AGAGAGAGAG AGAGAGTTAT GTTTAAGTCT   
  
  
- CTGAAAGAGA AAGACCCAAA TGATAACTAA CGTCTTCAAA CATCAGAACG CTCCGGAACT CAGAAGAAGC   
  
  
- TCCATCTTTT AAAACGAAAC GCGAAGGAAG GGGTATAAAA TTGTGGGGAG GGGGGGGTTG CGTTCAAAAG   
  
  
- ATGTAAAAAC ACGTTAAGAA GTCGATACTA AGGGGTTAAA GTGAACAGTG TAGAGATAAA AGGAGAAAAA   
  
  
- CCAAAGAACT AAGACATCTT CCAACACATA CTTGAAGAAA CAAAGGGAAA AAGGAAACAA TAAAAATTAC   
  
  
- AAGGGTATCT ACTATATCTC TAGACGAAAA GGAACGACAC TCTCCATAGA GACTCTTACG TACATCGTTA   
  
  
- GTTTTAAGAC TCAACAATTA AGATAACGTA CAGATACCCA CTACTAACAC TACTACAAAA AAGACAACGG   
  
  
- GAGAAAGAGA GAAAGTGACT TTAACGAAAG GTCTTATCAA CAAACTGAGG AAAGACATAG AATTAACTCG   
  
  
- GGAACGAAAC CAAACACGAG ACCCTCAAGA AGAAATCATT AGGGAACGAA CATAAAAGAC TTTCTCGTTC   
  
  
- GTTATTAGGA GTATCGTACA CATATTAGTA TGTTATCGTT GTCGTTTGTA CCTAGGACGC GGGTCAATCC   
  
  
- AAACCAATCC AATTCTATAC GTATAAGAAA AGAGAACTTT TCTCAACAGA TCCTTCCCAT TAATACGGGT   
  
  
- AAAGTACCTA TTATTACCAT GTTAATTCAA ACTAACCGAA TTTATGGTTA ATAAATCGCA GAAAAAGAAA   
  
  
- CCGACAGGTC CCAAGGTAAA GGTATTTGTT GAAACTCATT CCGTATCTCT TGATTTAAAG ACGGTTAACA   
  
  
- GTTCACGTTC AACGAGTATA CGTTGTTATG AACCTTTAAA ACTCGACTCG ACGACCGTAT AGTACTTATC   
  
  
- AAGATTACCA CCGCTCAATA GTGAATAGCA GGGAACACAC TGCTTAAAGA GAAGGATCCT TCGGGAGATC   
  
  
- AGTTACGGAT AGACGACAAC ACCTCACTTT TACCTATGTA AATCTAGAGA TATTTGGGAT GAAACCGTTC   
  
  
- TAGGTACGAC TAAAATCCTT CGGGAAACTG GTCTCTTACT GTATTGTTGG ACTATCCCAC AATGTACGAT   
  
  
- TGAAGCTAAT AAATTAGTTT TATGCCTTGA AGATAGTCAG TTTCGGACGG GGTCCAAGAT GACTTAATTT   
  
  
- AACATAACTT AACGAGAGTG AACGAATACT GCTAATTTAT TGACCTAAAC GATTAATAAG AAAAATAAAA   
  
  
- TAAGAACCAC TACATCCCTT GAGTCCCTTA ACATTGGTAT GGACAAGGTC GACTTTTCCC ATGCCCTTGT   
  
  
- CAGTATCAAT GTCCCCTTAA AGCGTTATGT AAATTCCCTC ATTCTTCGTC CTAGTACAGA AATCAATCCG   
  
  
- GCCGTCTATA CAGACACTGA GGTATACCTT TAGGTTGTGA CATAAGTGAA GTTCCGTTAT TATTATCGAG   
  
  
- ACCAAACAGA CCAGTTGACA TACCGAGACT TTTCGTGTTC TAGTTTATAC ACTGGCTTAG AAAATTACCA   
  
  
- GGTTCAATAC CGGACTTTAA GAAACAACTA AGAGGTAGTC TTCTCGAGTA GGTGGGTAGT CTACGATGAG   
  
  
- GATTGGGTAA ACTGAGAAGA AAGCACCCGT ATTCTGTACT ACTCTCAGGA ATGGTTCCTT TAATACCTAG   
  
  
- TCTTATACCC CATTCGGGTA GAAGGCTACG TCAACTTATA CTACTTCCAT TATTCTATTC CGATTTTGAA   
  
  
- GTTCTTAACC TCGTACGGGA CGAGCTACTT CTACTACTTC ACCTACTCCA CCGATATCCC GGGCACGATC   
  
  
- TTGTGTCGTA CCTCGAACTA CTTCTCACCC AGCGGGGTAA CTCTTTACAC CAAAAGGTAC TAAGTGGTTT   
  
  
- CCTCAGGTGG AGGAGTCTCA GTAGGTTACA GTCGTCGTAA TCGTCGTGGT TTCTTCATAG TGGAACGAGA   
  
  
- GGCTCATGAG GGTTTGTTAA CGAGAAATTA ACACGTTTAT ACTAAAGTCT CCCCTTAAAA CTCCTTCGGC   
  
  
- ACTGATACTA GTCGCTCGAA TCTGTCTAAC AAAGATAGGT TCCCCTAGGT CGCGTCTCCT ATCGTCGGAT   
  
  
- GTACCATCTT CCGGAACGAA GGGCAAACCG AAGTAGCCCT TTTCAAGAAA TGTTTCGAAA CTTTACGTTT   
  
  
- CTCGGTGGAA GTAAACTGTC CGATCGCCGA TACGTTTAGG AGAAACTCCA CACGGGTACA AAGTTTAAGC   
  
  
- CTAAATACCG GCGTTTACGT CGCTAATACC TACGTTAGTT TTTGCTTTTT TCCCATGTGT AGTATCTAAA   
  
  
- ACTGTATTTG GTTCCCTTAG TTATGTATTT AGAGTACGTT AGGGAACGAT TTGTTCCATT GTTCGACTGC   
  
  
- GTGAACTTCA ACTGACCTCA ACTACTGGGA CTCAGTCAAG TTGCGGGATA ACCACCGGAT TTTTTGTAGC   
  
  
- CTGTTTCCGA CGTTCGTAAT CGACTTATAG AACCACACAG TAAACTCAAG TTTCGTTATG GACGATCTTG   
  
  
- AGGTGAACAA TTGGGCCTTT ACAATCTTAC AGCTGGACGC CTTCGAAATC ACCACTTGAA ACGGAAGGTC   
  
  
- GAAGTGGTGT ACGGACTACT TTCGCAGAGT TGTTAGTTGG AGGCTCTGGT CGAAGAAGCC TACCAGTTTT   
  
  
- CGGATTTGGG TTTCGATCAT TGGTAACAGC TCGTCCTACA CTTGTGTTTG TGATGGGGAA AGGAGAGATC   
  
  
- CAAACGACTC CGTATGTCGA TGATAAGACG ACAAAAACTC AGAGAACTAC GATGAGAGGG ATCTCTATCG   
  
  
- GTCCTGTCCC ACTTACACCT CTTCGTCACG AACCGTGCAC TACAACACTT GTAGTAACGT ACACTTCCCC   
  
  
- TCCTCTCCTA ACTCGCGATA CTCCAACGCC CCTTTACCTC TCGCTCCTAC AACTACCGCC CTAAGTCCCG   
  
  
- ATAAGGTTAC CCAGTCTTCT GGCCACTATA CTAAGCCTTC GAATATAGTT ACGCCATAAC ACTGTCCGAG   
  
  
- TTCCATTTTC TCCTCTACCT ACGTGAAGTG AAACCCACCC TTCTATTTTT GAATTAGCAA TGTAGTCGAA   
  
  
- CCTCCAC

+     3-AF1 binding site

| Site Name | Organism | Position | Strand | Matrix score. | sequence | function |
| --- | --- | --- | --- | --- | --- | --- |
| 3-AF1 binding site | Solanum tuberosum | 430 | - | 10 | TAAGAGAGGAA | light responsive element |

>HU11G01134.1   
+ +Up\_Stream \_Len000AGATAG ACATTCAGCC ACTTGCCAGT TCAGAGAGAG ATAGAGAGAG ATTGGGTAAG   
  
  
+ CCCTTTTTGC ACTACTTTTT CTCGCTTCCA TTTTTCTTTC TTAATTTTTA GTGATGATTT TCCAGAAGAT   
  
  
+ TTGATTTATT CATCATGCTT AACCAATTGG GTCTTTGAGT ATTATTGTTT ACTGGGTATT TGATCTTTTC   
  
  
+ ATATTCCTTT GGACTGGGTG GGTGGAACTT TTGAACTTTC TTATTAGTTG GGCATGCCCT TATCTTATTC   
  
  
+ CAATTCAAAA CCCATATTTT TCTTCAATTT TAATTATGGG TATTTTGAAA TTGAATGCTT TCCTTCACAG   
  
  
+ CAATACTATA TTACATATTT TACAGGAAAG TAATATTTTA CAGGAAAGTA TGTTGCTGTT TCAACCCTGT   
  
  
+ TTTGTTTCCT CTCTTTCCTT GGATATGATG TTCATTGCTC TTGTACCAAA CCTAACCTCA ACTTCCTGAA   
  
  
+ TTATTTAAGG CAATATTACC GTGTGCTGGT CAATTTTGGT TGTGGGTTTG TTTCTTAATG TATATGATTT   
  
  
+ ATGGTCAGGA TCCATGCTCC TCTTCCGTCC ACTCTATATC TCTCTCTCTC TCTCTCAATA CAAATTCAGA   
  
  
+ GACTTTCTCT TTCTGGGTTT ACTATTGATT GCAGAAGTTT GTAGTCTTGC GAGGCCTTGA GTCTTCTTCG   
  
  
+ AGGTAGAAAA TTTTGCTTTG CGCTTCCTTC CCCATATTTT AACACCCCTC CCCCCCCAAC GCAAGTTTTC   
  
  
+ TACATTTTTG TGCAATTCTT CAGCTATGAT TCCCCAATTT CACTTGTCAC ATCTCTATTT TCCTCTTTTT   
  
  
+ GGTTTCTTGA TTCTGTAGAA GGTTGTGTAT GAACTTCTTT GTTTCCCTTT TTCCTTTGTT ATTTTTAATG   
  
  
+ TTCCCATAGA TGATATAGAG ATCTGCTTTT CCTTGCTGTG AGAGGTATCT CTGAGAATGC ATGTAGCAAT   
  
  
+ CAAAATTCTG AGTTGTTAAT TCTATTGCAT GTCTATGGGT GATGATTGTG ATGATGTTTT TTCTGTTGCC   
  
  
+ CTCTTTCTCT CTTTCACTGA AATTGCTTTC CAGAATAGTT GTTTGACTCC TTTCTGTATC TTAATTGAGC   
  
  
+ CCTTGCTTTG GTTTGTGCTC TGGGAGTTCT TCTTTAGTAA TCCCTTGCTT GTATTTTCTG AAAGAGCAAG   
  
  
+ CAATAATCCT CATAGCATGT GTATAATCAT ACAATAGCAA CAGCAAACAT GGATCCTGCG CCCAGTTAGG   
  
  
+ TTTGGTTAGG TTAAGATATG CATATTCTTT TCTCTTGAAA AGAGTTGTCT AGGAAGGGTA ATTATGCCCA   
  
  
+ TTTCATGGAT AATAATGGTA CAATTAAGTT TGATTGGCTT AAATACCAAT TATTTAGCGT CTTTTTCTTT   
  
  
+ GGCTGTCCAG GGTTCCATTT CCATAAACAA CTTTGAGTAA GGCATAGAGA ACTAAATTTC TGCCAATTGT   
  
  
+ CAAGTGCAAG TTGCTCATAT GCAACAATAC TTGGAAATTT TGAGCTGAGC TGCTGGCATA TCATGAATAG   
  
  
+ TTCTAATGGT GGCGAGTTAT CACTTATCGT CCCTTGTGTG ACGAATTTCT CTTCCTAGGA AGCCCTCTAG   
  
  
+ TCAATGCCTA TCTGCTGTTG TGGAGTGAAA ATGGATACAT TTAGATCTCT ATAAACCCTA CTTTGGCAAG   
  
  
+ ATCCATGCTG ATTTTAGGAA GCCCTTTGAC CAGAGAATGA CATAACAACC TGATAGGGTG TTACATGCTA   
  
  
+ ACTTCGATTA TTTAATCAAA ATACGGAACT TCTATCAGTC AAAGCCTGCC CCAGGTTCTA CTGAATTAAA   
  
  
+ TTGTATTGAA TTGCTCTCAC TTGCTTATGA CGATTAAATA ACTGGATTTG CTAATTATTC TTTTTATTTT   
  
  
+ ATTCTTGGTG ATGTAGGGAA CTCAGGGAAT TGTAACCATA CCTGTTCCAG CTGAAAAGGG TACGGGAACA   
  
  
+ GTCATAGTTA CAGGGGAATT TCGCAATACA TTTAAGGGAG TAAGAAGCAG GATCATGTCT TTAGTTAGGC   
  
  
+ CGGCAGATAT GTCTGTGACT CCATATGGAA ATCCAACACT GTATTCACTT CAAGGCAATA ATAATAGCTC   
  
  
+ TGGTTTGTCT GGTCAACTGT ATGGCTCTGA AAAGCACAAG ATCAAATATG TGACCGAATC TTTTAATGGT   
  
  
+ CCAAGTTATG GCCTGAAATT CTTTGTTGAT TCTCCATCAG AAGAGCTCAT CCACCCATCA GATGCTACTC   
  
  
+ CTAACCCATT TGACTCTTCT TTCGTGGGCA TAAGACATGA TGAGAGTCCT TACCAAGGAA ATTATGGATC   
  
  
+ AGAATATGGG GTAAGCCCAT CTTCCGATGC AGTTGAATAT GATGAAGGTA ATAAGATAAG GCTAAAACTT   
  
  
+ CAAGAATTGG AGCATGCCCT GCTCGATGAA GATGATGAAG TGGATGAGGT GGCTATAGGG CCCGTGCTAG   
  
  
+ AACACAGCAT GGAGCTTGAT GAAGAGTGGG TCGCCCCATT GAGAAATGTG GTTTTCCATG ATTCACCAAA   
  
  
+ GGAGTCCACC TCCTCAGAGT CATCCAATGT CAGCAGCATT AGCAGCACCA AAGAAGTATC ACCTTGCTCT   
  
  
+ CCGAGTACTC CCAAACAATT GCTCTTTAAT TGTGCAAATA TGATTTCAGA GGGGAATTTT GAGGAAGCCG   
  
  
+ TGACTATGAT CAGCGAGCTT AGACAGATTG TTTCTATCCA AGGGGATCCA GCGCAGAGGA TAGCAGCCTA   
  
  
+ CATGGTAGAA GGCCTTGCTT CCCGTTTGGC TTCATCGGGA AAAGTTCTTT ACAAAGCTTT GAAATGCAAA   
  
  
+ GAGCCACCTT CATTTGACAG GCTAGCGGCT ATGCAAATCC TCTTTGAGGT GTGCCCATGT TTCAAATTCG   
  
  
+ GATTTATGGC CGCAAATGCA GCGATTATGG ATGCAATCAA AAACGAAAAA AGGGTACACA TCATAGATTT   
  
  
+ TGACATAAAC CAAGGGAATC AATACATAAA TCTCATGCAA TCCCTTGCTA AACAAGGTAA CAAGCTGACG   
  
  
+ CACTTGAAGT TGACTGGAGT TGATGACCCT GAGTCAGTTC AACGCCCTAT TGGTGGCCTA AAAAACATCG   
  
  
+ GACAAAGGCT GCAAGCATTA GCTGAATATC TTGGTGTGTC ATTTGAGTTC AAAGCAATAC CTGCTAGAAC   
  
  
+ TCCACTTGTT AACCCGGAAA TGTTAGAATG TCGACCTGCG GAAGCTTTAG TGGTGAACTT TGCCTTCCAG   
  
  
+ CTTCACCACA TGCCTGATGA AAGCGTCTCA ACAATCAACC TCCGAGACCA GCTTCTTCGG ATGGTCAAAA   
  
  
+ GCCTAAACCC AAAGCTAGTA ACCATTGTCG AGCAGGATGT GAACACAAAC ACTACCCCTT TCCTCTCTAG   
  
  
+ GTTTGCTGAG GCATACAGCT ACTATTCTGC TGTTTTTGAG TCTCTTGATG CTACTCTCCC TAGAGATAGC   
  
  
+ CAGGACAGGG TGAATGTGGA GAAGCAGTGC TTGGCACGTG ATGTTGTGAA CATCATTGCA TGTGAAGGGG   
  
  
+ AGGAGAGGAT TGAGCGCTAT GAGGTTGCGG GGAAATGGAG AGCGAGGATG TTGATGGCGG GATTCAGGGC   
  
  
+ TATTCCAATG GGTCAGAAGA CCGGTGATAT GATTCGGAAG CTTATATCAA TGCGGTATTG TGACAGGCTC   
  
  
+ AAGGTAAAAG AGGAGATGGA TGCACTTCAC TTTGGGTGGG AAGATAAAAA CTTAATCGTT ACATCAGCTT   
  
  
+ GGAGGTG  

- +Up\_Stream \_Len000TCTATC TGTAAGTCGG TGAACGGTCA AGTCTCTCTC TATCTCTCTC TAACCCATTC   
  
  
- GGGAAAAACG TGATGAAAAA GAGCGAAGGT AAAAAGAAAG AATTAAAAAT CACTACTAAA AGGTCTTCTA   
  
  
- AACTAAATAA GTAGTACGAA TTGGTTAACC CAGAAACTCA TAATAACAAA TGACCCATAA ACTAGAAAAG   
  
  
- TATAAGGAAA CCTGACCCAC CCACCTTGAA AACTTGAAAG AATAATCAAC CCGTACGGGA ATAGAATAAG   
  
  
- GTTAAGTTTT GGGTATAAAA AGAAGTTAAA ATTAATACCC ATAAAACTTT AACTTACGAA AGGAAGTGTC   
  
  
- GTTATGATAT AATGTATAAA ATGTCCTTTC ATTATAAAAT GTCCTTTCAT ACAACGACAA AGTTGGGACA   
  
  
- AAACAAAGGA GAGAAAGGAA CCTATACTAC AAGTAACGAG AACATGGTTT GGATTGGAGT TGAAGGACTT   
  
  
- AATAAATTCC GTTATAATGG CACACGACCA GTTAAAACCA ACACCCAAAC AAAGAATTAC ATATACTAAA   
  
  
- TACCAGTCCT AGGTACGAGG AGAAGGCAGG TGAGATATAG AGAGAGAGAG AGAGAGTTAT GTTTAAGTCT   
  
  
- CTGAAAGAGA AAGACCCAAA TGATAACTAA CGTCTTCAAA CATCAGAACG CTCCGGAACT CAGAAGAAGC   
  
  
- TCCATCTTTT AAAACGAAAC GCGAAGGAAG GGGTATAAAA TTGTGGGGAG GGGGGGGTTG CGTTCAAAAG   
  
  
- ATGTAAAAAC ACGTTAAGAA GTCGATACTA AGGGGTTAAA GTGAACAGTG TAGAGATAAA AGGAGAAAAA   
  
  
- CCAAAGAACT AAGACATCTT CCAACACATA CTTGAAGAAA CAAAGGGAAA AAGGAAACAA TAAAAATTAC   
  
  
- AAGGGTATCT ACTATATCTC TAGACGAAAA GGAACGACAC TCTCCATAGA GACTCTTACG TACATCGTTA   
  
  
- GTTTTAAGAC TCAACAATTA AGATAACGTA CAGATACCCA CTACTAACAC TACTACAAAA AAGACAACGG   
  
  
- GAGAAAGAGA GAAAGTGACT TTAACGAAAG GTCTTATCAA CAAACTGAGG AAAGACATAG AATTAACTCG   
  
  
- GGAACGAAAC CAAACACGAG ACCCTCAAGA AGAAATCATT AGGGAACGAA CATAAAAGAC TTTCTCGTTC   
  
  
- GTTATTAGGA GTATCGTACA CATATTAGTA TGTTATCGTT GTCGTTTGTA CCTAGGACGC GGGTCAATCC   
  
  
- AAACCAATCC AATTCTATAC GTATAAGAAA AGAGAACTTT TCTCAACAGA TCCTTCCCAT TAATACGGGT   
  
  
- AAAGTACCTA TTATTACCAT GTTAATTCAA ACTAACCGAA TTTATGGTTA ATAAATCGCA GAAAAAGAAA   
  
  
- CCGACAGGTC CCAAGGTAAA GGTATTTGTT GAAACTCATT CCGTATCTCT TGATTTAAAG ACGGTTAACA   
  
  
- GTTCACGTTC AACGAGTATA CGTTGTTATG AACCTTTAAA ACTCGACTCG ACGACCGTAT AGTACTTATC   
  
  
- AAGATTACCA CCGCTCAATA GTGAATAGCA GGGAACACAC TGCTTAAAGA GAAGGATCCT TCGGGAGATC   
  
  
- AGTTACGGAT AGACGACAAC ACCTCACTTT TACCTATGTA AATCTAGAGA TATTTGGGAT GAAACCGTTC   
  
  
- TAGGTACGAC TAAAATCCTT CGGGAAACTG GTCTCTTACT GTATTGTTGG ACTATCCCAC AATGTACGAT   
  
  
- TGAAGCTAAT AAATTAGTTT TATGCCTTGA AGATAGTCAG TTTCGGACGG GGTCCAAGAT GACTTAATTT   
  
  
- AACATAACTT AACGAGAGTG AACGAATACT GCTAATTTAT TGACCTAAAC GATTAATAAG AAAAATAAAA   
  
  
- TAAGAACCAC TACATCCCTT GAGTCCCTTA ACATTGGTAT GGACAAGGTC GACTTTTCCC ATGCCCTTGT   
  
  
- CAGTATCAAT GTCCCCTTAA AGCGTTATGT AAATTCCCTC ATTCTTCGTC CTAGTACAGA AATCAATCCG   
  
  
- GCCGTCTATA CAGACACTGA GGTATACCTT TAGGTTGTGA CATAAGTGAA GTTCCGTTAT TATTATCGAG   
  
  
- ACCAAACAGA CCAGTTGACA TACCGAGACT TTTCGTGTTC TAGTTTATAC ACTGGCTTAG AAAATTACCA   
  
  
- GGTTCAATAC CGGACTTTAA GAAACAACTA AGAGGTAGTC TTCTCGAGTA GGTGGGTAGT CTACGATGAG   
  
  
- GATTGGGTAA ACTGAGAAGA AAGCACCCGT ATTCTGTACT ACTCTCAGGA ATGGTTCCTT TAATACCTAG   
  
  
- TCTTATACCC CATTCGGGTA GAAGGCTACG TCAACTTATA CTACTTCCAT TATTCTATTC CGATTTTGAA   
  
  
- GTTCTTAACC TCGTACGGGA CGAGCTACTT CTACTACTTC ACCTACTCCA CCGATATCCC GGGCACGATC   
  
  
- TTGTGTCGTA CCTCGAACTA CTTCTCACCC AGCGGGGTAA CTCTTTACAC CAAAAGGTAC TAAGTGGTTT   
  
  
- CCTCAGGTGG AGGAGTCTCA GTAGGTTACA GTCGTCGTAA TCGTCGTGGT TTCTTCATAG TGGAACGAGA   
  
  
- GGCTCATGAG GGTTTGTTAA CGAGAAATTA ACACGTTTAT ACTAAAGTCT CCCCTTAAAA CTCCTTCGGC   
  
  
- ACTGATACTA GTCGCTCGAA TCTGTCTAAC AAAGATAGGT TCCCCTAGGT CGCGTCTCCT ATCGTCGGAT   
  
  
- GTACCATCTT CCGGAACGAA GGGCAAACCG AAGTAGCCCT TTTCAAGAAA TGTTTCGAAA CTTTACGTTT   
  
  
- CTCGGTGGAA GTAAACTGTC CGATCGCCGA TACGTTTAGG AGAAACTCCA CACGGGTACA AAGTTTAAGC   
  
  
- CTAAATACCG GCGTTTACGT CGCTAATACC TACGTTAGTT TTTGCTTTTT TCCCATGTGT AGTATCTAAA   
  
  
- ACTGTATTTG GTTCCCTTAG TTATGTATTT AGAGTACGTT AGGGAACGAT TTGTTCCATT GTTCGACTGC   
  
  
- GTGAACTTCA ACTGACCTCA ACTACTGGGA CTCAGTCAAG TTGCGGGATA ACCACCGGAT TTTTTGTAGC   
  
  
- CTGTTTCCGA CGTTCGTAAT CGACTTATAG AACCACACAG TAAACTCAAG TTTCGTTATG GACGATCTTG   
  
  
- AGGTGAACAA TTGGGCCTTT ACAATCTTAC AGCTGGACGC CTTCGAAATC ACCACTTGAA ACGGAAGGTC   
  
  
- GAAGTGGTGT ACGGACTACT TTCGCAGAGT TGTTAGTTGG AGGCTCTGGT CGAAGAAGCC TACCAGTTTT   
  
  
- CGGATTTGGG TTTCGATCAT TGGTAACAGC TCGTCCTACA CTTGTGTTTG TGATGGGGAA AGGAGAGATC   
  
  
- CAAACGACTC CGTATGTCGA TGATAAGACG ACAAAAACTC AGAGAACTAC GATGAGAGGG ATCTCTATCG   
  
  
- GTCCTGTCCC ACTTACACCT CTTCGTCACG AACCGTGCAC TACAACACTT GTAGTAACGT ACACTTCCCC   
  
  
- TCCTCTCCTA ACTCGCGATA CTCCAACGCC CCTTTACCTC TCGCTCCTAC AACTACCGCC CTAAGTCCCG   
  
  
- ATAAGGTTAC CCAGTCTTCT GGCCACTATA CTAAGCCTTC GAATATAGTT ACGCCATAAC ACTGTCCGAG   
  
  
- TTCCATTTTC TCCTCTACCT ACGTGAAGTG AAACCCACCC TTCTATTTTT GAATTAGCAA TGTAGTCGAA   
  
  
- CCTCCAC

+     A-box

| Site Name | Organism | Position | Strand | Matrix score. | sequence | function |
| --- | --- | --- | --- | --- | --- | --- |
| A-box | Petroselinum crispum | 589 | + | 6 | CCGTCC | cis-acting regulatory element |

>HU11G01134.1   
+ +Up\_Stream \_Len000AGATAG ACATTCAGCC ACTTGCCAGT TCAGAGAGAG ATAGAGAGAG ATTGGGTAAG   
  
  
+ CCCTTTTTGC ACTACTTTTT CTCGCTTCCA TTTTTCTTTC TTAATTTTTA GTGATGATTT TCCAGAAGAT   
  
  
+ TTGATTTATT CATCATGCTT AACCAATTGG GTCTTTGAGT ATTATTGTTT ACTGGGTATT TGATCTTTTC   
  
  
+ ATATTCCTTT GGACTGGGTG GGTGGAACTT TTGAACTTTC TTATTAGTTG GGCATGCCCT TATCTTATTC   
  
  
+ CAATTCAAAA CCCATATTTT TCTTCAATTT TAATTATGGG TATTTTGAAA TTGAATGCTT TCCTTCACAG   
  
  
+ CAATACTATA TTACATATTT TACAGGAAAG TAATATTTTA CAGGAAAGTA TGTTGCTGTT TCAACCCTGT   
  
  
+ TTTGTTTCCT CTCTTTCCTT GGATATGATG TTCATTGCTC TTGTACCAAA CCTAACCTCA ACTTCCTGAA   
  
  
+ TTATTTAAGG CAATATTACC GTGTGCTGGT CAATTTTGGT TGTGGGTTTG TTTCTTAATG TATATGATTT   
  
  
+ ATGGTCAGGA TCCATGCTCC TCTTCCGTCC ACTCTATATC TCTCTCTCTC TCTCTCAATA CAAATTCAGA   
  
  
+ GACTTTCTCT TTCTGGGTTT ACTATTGATT GCAGAAGTTT GTAGTCTTGC GAGGCCTTGA GTCTTCTTCG   
  
  
+ AGGTAGAAAA TTTTGCTTTG CGCTTCCTTC CCCATATTTT AACACCCCTC CCCCCCCAAC GCAAGTTTTC   
  
  
+ TACATTTTTG TGCAATTCTT CAGCTATGAT TCCCCAATTT CACTTGTCAC ATCTCTATTT TCCTCTTTTT   
  
  
+ GGTTTCTTGA TTCTGTAGAA GGTTGTGTAT GAACTTCTTT GTTTCCCTTT TTCCTTTGTT ATTTTTAATG   
  
  
+ TTCCCATAGA TGATATAGAG ATCTGCTTTT CCTTGCTGTG AGAGGTATCT CTGAGAATGC ATGTAGCAAT   
  
  
+ CAAAATTCTG AGTTGTTAAT TCTATTGCAT GTCTATGGGT GATGATTGTG ATGATGTTTT TTCTGTTGCC   
  
  
+ CTCTTTCTCT CTTTCACTGA AATTGCTTTC CAGAATAGTT GTTTGACTCC TTTCTGTATC TTAATTGAGC   
  
  
+ CCTTGCTTTG GTTTGTGCTC TGGGAGTTCT TCTTTAGTAA TCCCTTGCTT GTATTTTCTG AAAGAGCAAG   
  
  
+ CAATAATCCT CATAGCATGT GTATAATCAT ACAATAGCAA CAGCAAACAT GGATCCTGCG CCCAGTTAGG   
  
  
+ TTTGGTTAGG TTAAGATATG CATATTCTTT TCTCTTGAAA AGAGTTGTCT AGGAAGGGTA ATTATGCCCA   
  
  
+ TTTCATGGAT AATAATGGTA CAATTAAGTT TGATTGGCTT AAATACCAAT TATTTAGCGT CTTTTTCTTT   
  
  
+ GGCTGTCCAG GGTTCCATTT CCATAAACAA CTTTGAGTAA GGCATAGAGA ACTAAATTTC TGCCAATTGT   
  
  
+ CAAGTGCAAG TTGCTCATAT GCAACAATAC TTGGAAATTT TGAGCTGAGC TGCTGGCATA TCATGAATAG   
  
  
+ TTCTAATGGT GGCGAGTTAT CACTTATCGT CCCTTGTGTG ACGAATTTCT CTTCCTAGGA AGCCCTCTAG   
  
  
+ TCAATGCCTA TCTGCTGTTG TGGAGTGAAA ATGGATACAT TTAGATCTCT ATAAACCCTA CTTTGGCAAG   
  
  
+ ATCCATGCTG ATTTTAGGAA GCCCTTTGAC CAGAGAATGA CATAACAACC TGATAGGGTG TTACATGCTA   
  
  
+ ACTTCGATTA TTTAATCAAA ATACGGAACT TCTATCAGTC AAAGCCTGCC CCAGGTTCTA CTGAATTAAA   
  
  
+ TTGTATTGAA TTGCTCTCAC TTGCTTATGA CGATTAAATA ACTGGATTTG CTAATTATTC TTTTTATTTT   
  
  
+ ATTCTTGGTG ATGTAGGGAA CTCAGGGAAT TGTAACCATA CCTGTTCCAG CTGAAAAGGG TACGGGAACA   
  
  
+ GTCATAGTTA CAGGGGAATT TCGCAATACA TTTAAGGGAG TAAGAAGCAG GATCATGTCT TTAGTTAGGC   
  
  
+ CGGCAGATAT GTCTGTGACT CCATATGGAA ATCCAACACT GTATTCACTT CAAGGCAATA ATAATAGCTC   
  
  
+ TGGTTTGTCT GGTCAACTGT ATGGCTCTGA AAAGCACAAG ATCAAATATG TGACCGAATC TTTTAATGGT   
  
  
+ CCAAGTTATG GCCTGAAATT CTTTGTTGAT TCTCCATCAG AAGAGCTCAT CCACCCATCA GATGCTACTC   
  
  
+ CTAACCCATT TGACTCTTCT TTCGTGGGCA TAAGACATGA TGAGAGTCCT TACCAAGGAA ATTATGGATC   
  
  
+ AGAATATGGG GTAAGCCCAT CTTCCGATGC AGTTGAATAT GATGAAGGTA ATAAGATAAG GCTAAAACTT   
  
  
+ CAAGAATTGG AGCATGCCCT GCTCGATGAA GATGATGAAG TGGATGAGGT GGCTATAGGG CCCGTGCTAG   
  
  
+ AACACAGCAT GGAGCTTGAT GAAGAGTGGG TCGCCCCATT GAGAAATGTG GTTTTCCATG ATTCACCAAA   
  
  
+ GGAGTCCACC TCCTCAGAGT CATCCAATGT CAGCAGCATT AGCAGCACCA AAGAAGTATC ACCTTGCTCT   
  
  
+ CCGAGTACTC CCAAACAATT GCTCTTTAAT TGTGCAAATA TGATTTCAGA GGGGAATTTT GAGGAAGCCG   
  
  
+ TGACTATGAT CAGCGAGCTT AGACAGATTG TTTCTATCCA AGGGGATCCA GCGCAGAGGA TAGCAGCCTA   
  
  
+ CATGGTAGAA GGCCTTGCTT CCCGTTTGGC TTCATCGGGA AAAGTTCTTT ACAAAGCTTT GAAATGCAAA   
  
  
+ GAGCCACCTT CATTTGACAG GCTAGCGGCT ATGCAAATCC TCTTTGAGGT GTGCCCATGT TTCAAATTCG   
  
  
+ GATTTATGGC CGCAAATGCA GCGATTATGG ATGCAATCAA AAACGAAAAA AGGGTACACA TCATAGATTT   
  
  
+ TGACATAAAC CAAGGGAATC AATACATAAA TCTCATGCAA TCCCTTGCTA AACAAGGTAA CAAGCTGACG   
  
  
+ CACTTGAAGT TGACTGGAGT TGATGACCCT GAGTCAGTTC AACGCCCTAT TGGTGGCCTA AAAAACATCG   
  
  
+ GACAAAGGCT GCAAGCATTA GCTGAATATC TTGGTGTGTC ATTTGAGTTC AAAGCAATAC CTGCTAGAAC   
  
  
+ TCCACTTGTT AACCCGGAAA TGTTAGAATG TCGACCTGCG GAAGCTTTAG TGGTGAACTT TGCCTTCCAG   
  
  
+ CTTCACCACA TGCCTGATGA AAGCGTCTCA ACAATCAACC TCCGAGACCA GCTTCTTCGG ATGGTCAAAA   
  
  
+ GCCTAAACCC AAAGCTAGTA ACCATTGTCG AGCAGGATGT GAACACAAAC ACTACCCCTT TCCTCTCTAG   
  
  
+ GTTTGCTGAG GCATACAGCT ACTATTCTGC TGTTTTTGAG TCTCTTGATG CTACTCTCCC TAGAGATAGC   
  
  
+ CAGGACAGGG TGAATGTGGA GAAGCAGTGC TTGGCACGTG ATGTTGTGAA CATCATTGCA TGTGAAGGGG   
  
  
+ AGGAGAGGAT TGAGCGCTAT GAGGTTGCGG GGAAATGGAG AGCGAGGATG TTGATGGCGG GATTCAGGGC   
  
  
+ TATTCCAATG GGTCAGAAGA CCGGTGATAT GATTCGGAAG CTTATATCAA TGCGGTATTG TGACAGGCTC   
  
  
+ AAGGTAAAAG AGGAGATGGA TGCACTTCAC TTTGGGTGGG AAGATAAAAA CTTAATCGTT ACATCAGCTT   
  
  
+ GGAGGTG  

- +Up\_Stream \_Len000TCTATC TGTAAGTCGG TGAACGGTCA AGTCTCTCTC TATCTCTCTC TAACCCATTC   
  
  
- GGGAAAAACG TGATGAAAAA GAGCGAAGGT AAAAAGAAAG AATTAAAAAT CACTACTAAA AGGTCTTCTA   
  
  
- AACTAAATAA GTAGTACGAA TTGGTTAACC CAGAAACTCA TAATAACAAA TGACCCATAA ACTAGAAAAG   
  
  
- TATAAGGAAA CCTGACCCAC CCACCTTGAA AACTTGAAAG AATAATCAAC CCGTACGGGA ATAGAATAAG   
  
  
- GTTAAGTTTT GGGTATAAAA AGAAGTTAAA ATTAATACCC ATAAAACTTT AACTTACGAA AGGAAGTGTC   
  
  
- GTTATGATAT AATGTATAAA ATGTCCTTTC ATTATAAAAT GTCCTTTCAT ACAACGACAA AGTTGGGACA   
  
  
- AAACAAAGGA GAGAAAGGAA CCTATACTAC AAGTAACGAG AACATGGTTT GGATTGGAGT TGAAGGACTT   
  
  
- AATAAATTCC GTTATAATGG CACACGACCA GTTAAAACCA ACACCCAAAC AAAGAATTAC ATATACTAAA   
  
  
- TACCAGTCCT AGGTACGAGG AGAAGGCAGG TGAGATATAG AGAGAGAGAG AGAGAGTTAT GTTTAAGTCT   
  
  
- CTGAAAGAGA AAGACCCAAA TGATAACTAA CGTCTTCAAA CATCAGAACG CTCCGGAACT CAGAAGAAGC   
  
  
- TCCATCTTTT AAAACGAAAC GCGAAGGAAG GGGTATAAAA TTGTGGGGAG GGGGGGGTTG CGTTCAAAAG   
  
  
- ATGTAAAAAC ACGTTAAGAA GTCGATACTA AGGGGTTAAA GTGAACAGTG TAGAGATAAA AGGAGAAAAA   
  
  
- CCAAAGAACT AAGACATCTT CCAACACATA CTTGAAGAAA CAAAGGGAAA AAGGAAACAA TAAAAATTAC   
  
  
- AAGGGTATCT ACTATATCTC TAGACGAAAA GGAACGACAC TCTCCATAGA GACTCTTACG TACATCGTTA   
  
  
- GTTTTAAGAC TCAACAATTA AGATAACGTA CAGATACCCA CTACTAACAC TACTACAAAA AAGACAACGG   
  
  
- GAGAAAGAGA GAAAGTGACT TTAACGAAAG GTCTTATCAA CAAACTGAGG AAAGACATAG AATTAACTCG   
  
  
- GGAACGAAAC CAAACACGAG ACCCTCAAGA AGAAATCATT AGGGAACGAA CATAAAAGAC TTTCTCGTTC   
  
  
- GTTATTAGGA GTATCGTACA CATATTAGTA TGTTATCGTT GTCGTTTGTA CCTAGGACGC GGGTCAATCC   
  
  
- AAACCAATCC AATTCTATAC GTATAAGAAA AGAGAACTTT TCTCAACAGA TCCTTCCCAT TAATACGGGT   
  
  
- AAAGTACCTA TTATTACCAT GTTAATTCAA ACTAACCGAA TTTATGGTTA ATAAATCGCA GAAAAAGAAA   
  
  
- CCGACAGGTC CCAAGGTAAA GGTATTTGTT GAAACTCATT CCGTATCTCT TGATTTAAAG ACGGTTAACA   
  
  
- GTTCACGTTC AACGAGTATA CGTTGTTATG AACCTTTAAA ACTCGACTCG ACGACCGTAT AGTACTTATC   
  
  
- AAGATTACCA CCGCTCAATA GTGAATAGCA GGGAACACAC TGCTTAAAGA GAAGGATCCT TCGGGAGATC   
  
  
- AGTTACGGAT AGACGACAAC ACCTCACTTT TACCTATGTA AATCTAGAGA TATTTGGGAT GAAACCGTTC   
  
  
- TAGGTACGAC TAAAATCCTT CGGGAAACTG GTCTCTTACT GTATTGTTGG ACTATCCCAC AATGTACGAT   
  
  
- TGAAGCTAAT AAATTAGTTT TATGCCTTGA AGATAGTCAG TTTCGGACGG GGTCCAAGAT GACTTAATTT   
  
  
- AACATAACTT AACGAGAGTG AACGAATACT GCTAATTTAT TGACCTAAAC GATTAATAAG AAAAATAAAA   
  
  
- TAAGAACCAC TACATCCCTT GAGTCCCTTA ACATTGGTAT GGACAAGGTC GACTTTTCCC ATGCCCTTGT   
  
  
- CAGTATCAAT GTCCCCTTAA AGCGTTATGT AAATTCCCTC ATTCTTCGTC CTAGTACAGA AATCAATCCG   
  
  
- GCCGTCTATA CAGACACTGA GGTATACCTT TAGGTTGTGA CATAAGTGAA GTTCCGTTAT TATTATCGAG   
  
  
- ACCAAACAGA CCAGTTGACA TACCGAGACT TTTCGTGTTC TAGTTTATAC ACTGGCTTAG AAAATTACCA   
  
  
- GGTTCAATAC CGGACTTTAA GAAACAACTA AGAGGTAGTC TTCTCGAGTA GGTGGGTAGT CTACGATGAG   
  
  
- GATTGGGTAA ACTGAGAAGA AAGCACCCGT ATTCTGTACT ACTCTCAGGA ATGGTTCCTT TAATACCTAG   
  
  
- TCTTATACCC CATTCGGGTA GAAGGCTACG TCAACTTATA CTACTTCCAT TATTCTATTC CGATTTTGAA   
  
  
- GTTCTTAACC TCGTACGGGA CGAGCTACTT CTACTACTTC ACCTACTCCA CCGATATCCC GGGCACGATC   
  
  
- TTGTGTCGTA CCTCGAACTA CTTCTCACCC AGCGGGGTAA CTCTTTACAC CAAAAGGTAC TAAGTGGTTT   
  
  
- CCTCAGGTGG AGGAGTCTCA GTAGGTTACA GTCGTCGTAA TCGTCGTGGT TTCTTCATAG TGGAACGAGA   
  
  
- GGCTCATGAG GGTTTGTTAA CGAGAAATTA ACACGTTTAT ACTAAAGTCT CCCCTTAAAA CTCCTTCGGC   
  
  
- ACTGATACTA GTCGCTCGAA TCTGTCTAAC AAAGATAGGT TCCCCTAGGT CGCGTCTCCT ATCGTCGGAT   
  
  
- GTACCATCTT CCGGAACGAA GGGCAAACCG AAGTAGCCCT TTTCAAGAAA TGTTTCGAAA CTTTACGTTT   
  
  
- CTCGGTGGAA GTAAACTGTC CGATCGCCGA TACGTTTAGG AGAAACTCCA CACGGGTACA AAGTTTAAGC   
  
  
- CTAAATACCG GCGTTTACGT CGCTAATACC TACGTTAGTT TTTGCTTTTT TCCCATGTGT AGTATCTAAA   
  
  
- ACTGTATTTG GTTCCCTTAG TTATGTATTT AGAGTACGTT AGGGAACGAT TTGTTCCATT GTTCGACTGC   
  
  
- GTGAACTTCA ACTGACCTCA ACTACTGGGA CTCAGTCAAG TTGCGGGATA ACCACCGGAT TTTTTGTAGC   
  
  
- CTGTTTCCGA CGTTCGTAAT CGACTTATAG AACCACACAG TAAACTCAAG TTTCGTTATG GACGATCTTG   
  
  
- AGGTGAACAA TTGGGCCTTT ACAATCTTAC AGCTGGACGC CTTCGAAATC ACCACTTGAA ACGGAAGGTC   
  
  
- GAAGTGGTGT ACGGACTACT TTCGCAGAGT TGTTAGTTGG AGGCTCTGGT CGAAGAAGCC TACCAGTTTT   
  
  
- CGGATTTGGG TTTCGATCAT TGGTAACAGC TCGTCCTACA CTTGTGTTTG TGATGGGGAA AGGAGAGATC   
  
  
- CAAACGACTC CGTATGTCGA TGATAAGACG ACAAAAACTC AGAGAACTAC GATGAGAGGG ATCTCTATCG   
  
  
- GTCCTGTCCC ACTTACACCT CTTCGTCACG AACCGTGCAC TACAACACTT GTAGTAACGT ACACTTCCCC   
  
  
- TCCTCTCCTA ACTCGCGATA CTCCAACGCC CCTTTACCTC TCGCTCCTAC AACTACCGCC CTAAGTCCCG   
  
  
- ATAAGGTTAC CCAGTCTTCT GGCCACTATA CTAAGCCTTC GAATATAGTT ACGCCATAAC ACTGTCCGAG   
  
  
- TTCCATTTTC TCCTCTACCT ACGTGAAGTG AAACCCACCC TTCTATTTTT GAATTAGCAA TGTAGTCGAA   
  
  
- CCTCCAC

+     AAGAA-motif

| Site Name | Organism | Position | Strand | Matrix score. | sequence | function |
| --- | --- | --- | --- | --- | --- | --- |
| AAGAA-motif | Avena sativa | 2261 | - | 7 | GAAAGAA |  |
| AAGAA-motif | Avena sativa | 108 | - | 7 | GAAAGAA |  |

>HU11G01134.1   
+ +Up\_Stream \_Len000AGATAG ACATTCAGCC ACTTGCCAGT TCAGAGAGAG ATAGAGAGAG ATTGGGTAAG   
  
  
+ CCCTTTTTGC ACTACTTTTT CTCGCTTCCA TTTTTCTTTC TTAATTTTTA GTGATGATTT TCCAGAAGAT   
  
  
+ TTGATTTATT CATCATGCTT AACCAATTGG GTCTTTGAGT ATTATTGTTT ACTGGGTATT TGATCTTTTC   
  
  
+ ATATTCCTTT GGACTGGGTG GGTGGAACTT TTGAACTTTC TTATTAGTTG GGCATGCCCT TATCTTATTC   
  
  
+ CAATTCAAAA CCCATATTTT TCTTCAATTT TAATTATGGG TATTTTGAAA TTGAATGCTT TCCTTCACAG   
  
  
+ CAATACTATA TTACATATTT TACAGGAAAG TAATATTTTA CAGGAAAGTA TGTTGCTGTT TCAACCCTGT   
  
  
+ TTTGTTTCCT CTCTTTCCTT GGATATGATG TTCATTGCTC TTGTACCAAA CCTAACCTCA ACTTCCTGAA   
  
  
+ TTATTTAAGG CAATATTACC GTGTGCTGGT CAATTTTGGT TGTGGGTTTG TTTCTTAATG TATATGATTT   
  
  
+ ATGGTCAGGA TCCATGCTCC TCTTCCGTCC ACTCTATATC TCTCTCTCTC TCTCTCAATA CAAATTCAGA   
  
  
+ GACTTTCTCT TTCTGGGTTT ACTATTGATT GCAGAAGTTT GTAGTCTTGC GAGGCCTTGA GTCTTCTTCG   
  
  
+ AGGTAGAAAA TTTTGCTTTG CGCTTCCTTC CCCATATTTT AACACCCCTC CCCCCCCAAC GCAAGTTTTC   
  
  
+ TACATTTTTG TGCAATTCTT CAGCTATGAT TCCCCAATTT CACTTGTCAC ATCTCTATTT TCCTCTTTTT   
  
  
+ GGTTTCTTGA TTCTGTAGAA GGTTGTGTAT GAACTTCTTT GTTTCCCTTT TTCCTTTGTT ATTTTTAATG   
  
  
+ TTCCCATAGA TGATATAGAG ATCTGCTTTT CCTTGCTGTG AGAGGTATCT CTGAGAATGC ATGTAGCAAT   
  
  
+ CAAAATTCTG AGTTGTTAAT TCTATTGCAT GTCTATGGGT GATGATTGTG ATGATGTTTT TTCTGTTGCC   
  
  
+ CTCTTTCTCT CTTTCACTGA AATTGCTTTC CAGAATAGTT GTTTGACTCC TTTCTGTATC TTAATTGAGC   
  
  
+ CCTTGCTTTG GTTTGTGCTC TGGGAGTTCT TCTTTAGTAA TCCCTTGCTT GTATTTTCTG AAAGAGCAAG   
  
  
+ CAATAATCCT CATAGCATGT GTATAATCAT ACAATAGCAA CAGCAAACAT GGATCCTGCG CCCAGTTAGG   
  
  
+ TTTGGTTAGG TTAAGATATG CATATTCTTT TCTCTTGAAA AGAGTTGTCT AGGAAGGGTA ATTATGCCCA   
  
  
+ TTTCATGGAT AATAATGGTA CAATTAAGTT TGATTGGCTT AAATACCAAT TATTTAGCGT CTTTTTCTTT   
  
  
+ GGCTGTCCAG GGTTCCATTT CCATAAACAA CTTTGAGTAA GGCATAGAGA ACTAAATTTC TGCCAATTGT   
  
  
+ CAAGTGCAAG TTGCTCATAT GCAACAATAC TTGGAAATTT TGAGCTGAGC TGCTGGCATA TCATGAATAG   
  
  
+ TTCTAATGGT GGCGAGTTAT CACTTATCGT CCCTTGTGTG ACGAATTTCT CTTCCTAGGA AGCCCTCTAG   
  
  
+ TCAATGCCTA TCTGCTGTTG TGGAGTGAAA ATGGATACAT TTAGATCTCT ATAAACCCTA CTTTGGCAAG   
  
  
+ ATCCATGCTG ATTTTAGGAA GCCCTTTGAC CAGAGAATGA CATAACAACC TGATAGGGTG TTACATGCTA   
  
  
+ ACTTCGATTA TTTAATCAAA ATACGGAACT TCTATCAGTC AAAGCCTGCC CCAGGTTCTA CTGAATTAAA   
  
  
+ TTGTATTGAA TTGCTCTCAC TTGCTTATGA CGATTAAATA ACTGGATTTG CTAATTATTC TTTTTATTTT   
  
  
+ ATTCTTGGTG ATGTAGGGAA CTCAGGGAAT TGTAACCATA CCTGTTCCAG CTGAAAAGGG TACGGGAACA   
  
  
+ GTCATAGTTA CAGGGGAATT TCGCAATACA TTTAAGGGAG TAAGAAGCAG GATCATGTCT TTAGTTAGGC   
  
  
+ CGGCAGATAT GTCTGTGACT CCATATGGAA ATCCAACACT GTATTCACTT CAAGGCAATA ATAATAGCTC   
  
  
+ TGGTTTGTCT GGTCAACTGT ATGGCTCTGA AAAGCACAAG ATCAAATATG TGACCGAATC TTTTAATGGT   
  
  
+ CCAAGTTATG GCCTGAAATT CTTTGTTGAT TCTCCATCAG AAGAGCTCAT CCACCCATCA GATGCTACTC   
  
  
+ CTAACCCATT TGACTCTTCT TTCGTGGGCA TAAGACATGA TGAGAGTCCT TACCAAGGAA ATTATGGATC   
  
  
+ AGAATATGGG GTAAGCCCAT CTTCCGATGC AGTTGAATAT GATGAAGGTA ATAAGATAAG GCTAAAACTT   
  
  
+ CAAGAATTGG AGCATGCCCT GCTCGATGAA GATGATGAAG TGGATGAGGT GGCTATAGGG CCCGTGCTAG   
  
  
+ AACACAGCAT GGAGCTTGAT GAAGAGTGGG TCGCCCCATT GAGAAATGTG GTTTTCCATG ATTCACCAAA   
  
  
+ GGAGTCCACC TCCTCAGAGT CATCCAATGT CAGCAGCATT AGCAGCACCA AAGAAGTATC ACCTTGCTCT   
  
  
+ CCGAGTACTC CCAAACAATT GCTCTTTAAT TGTGCAAATA TGATTTCAGA GGGGAATTTT GAGGAAGCCG   
  
  
+ TGACTATGAT CAGCGAGCTT AGACAGATTG TTTCTATCCA AGGGGATCCA GCGCAGAGGA TAGCAGCCTA   
  
  
+ CATGGTAGAA GGCCTTGCTT CCCGTTTGGC TTCATCGGGA AAAGTTCTTT ACAAAGCTTT GAAATGCAAA   
  
  
+ GAGCCACCTT CATTTGACAG GCTAGCGGCT ATGCAAATCC TCTTTGAGGT GTGCCCATGT TTCAAATTCG   
  
  
+ GATTTATGGC CGCAAATGCA GCGATTATGG ATGCAATCAA AAACGAAAAA AGGGTACACA TCATAGATTT   
  
  
+ TGACATAAAC CAAGGGAATC AATACATAAA TCTCATGCAA TCCCTTGCTA AACAAGGTAA CAAGCTGACG   
  
  
+ CACTTGAAGT TGACTGGAGT TGATGACCCT GAGTCAGTTC AACGCCCTAT TGGTGGCCTA AAAAACATCG   
  
  
+ GACAAAGGCT GCAAGCATTA GCTGAATATC TTGGTGTGTC ATTTGAGTTC AAAGCAATAC CTGCTAGAAC   
  
  
+ TCCACTTGTT AACCCGGAAA TGTTAGAATG TCGACCTGCG GAAGCTTTAG TGGTGAACTT TGCCTTCCAG   
  
  
+ CTTCACCACA TGCCTGATGA AAGCGTCTCA ACAATCAACC TCCGAGACCA GCTTCTTCGG ATGGTCAAAA   
  
  
+ GCCTAAACCC AAAGCTAGTA ACCATTGTCG AGCAGGATGT GAACACAAAC ACTACCCCTT TCCTCTCTAG   
  
  
+ GTTTGCTGAG GCATACAGCT ACTATTCTGC TGTTTTTGAG TCTCTTGATG CTACTCTCCC TAGAGATAGC   
  
  
+ CAGGACAGGG TGAATGTGGA GAAGCAGTGC TTGGCACGTG ATGTTGTGAA CATCATTGCA TGTGAAGGGG   
  
  
+ AGGAGAGGAT TGAGCGCTAT GAGGTTGCGG GGAAATGGAG AGCGAGGATG TTGATGGCGG GATTCAGGGC   
  
  
+ TATTCCAATG GGTCAGAAGA CCGGTGATAT GATTCGGAAG CTTATATCAA TGCGGTATTG TGACAGGCTC   
  
  
+ AAGGTAAAAG AGGAGATGGA TGCACTTCAC TTTGGGTGGG AAGATAAAAA CTTAATCGTT ACATCAGCTT   
  
  
+ GGAGGTG  

- +Up\_Stream \_Len000TCTATC TGTAAGTCGG TGAACGGTCA AGTCTCTCTC TATCTCTCTC TAACCCATTC   
  
  
- GGGAAAAACG TGATGAAAAA GAGCGAAGGT AAAAAGAAAG AATTAAAAAT CACTACTAAA AGGTCTTCTA   
  
  
- AACTAAATAA GTAGTACGAA TTGGTTAACC CAGAAACTCA TAATAACAAA TGACCCATAA ACTAGAAAAG   
  
  
- TATAAGGAAA CCTGACCCAC CCACCTTGAA AACTTGAAAG AATAATCAAC CCGTACGGGA ATAGAATAAG   
  
  
- GTTAAGTTTT GGGTATAAAA AGAAGTTAAA ATTAATACCC ATAAAACTTT AACTTACGAA AGGAAGTGTC   
  
  
- GTTATGATAT AATGTATAAA ATGTCCTTTC ATTATAAAAT GTCCTTTCAT ACAACGACAA AGTTGGGACA   
  
  
- AAACAAAGGA GAGAAAGGAA CCTATACTAC AAGTAACGAG AACATGGTTT GGATTGGAGT TGAAGGACTT   
  
  
- AATAAATTCC GTTATAATGG CACACGACCA GTTAAAACCA ACACCCAAAC AAAGAATTAC ATATACTAAA   
  
  
- TACCAGTCCT AGGTACGAGG AGAAGGCAGG TGAGATATAG AGAGAGAGAG AGAGAGTTAT GTTTAAGTCT   
  
  
- CTGAAAGAGA AAGACCCAAA TGATAACTAA CGTCTTCAAA CATCAGAACG CTCCGGAACT CAGAAGAAGC   
  
  
- TCCATCTTTT AAAACGAAAC GCGAAGGAAG GGGTATAAAA TTGTGGGGAG GGGGGGGTTG CGTTCAAAAG   
  
  
- ATGTAAAAAC ACGTTAAGAA GTCGATACTA AGGGGTTAAA GTGAACAGTG TAGAGATAAA AGGAGAAAAA   
  
  
- CCAAAGAACT AAGACATCTT CCAACACATA CTTGAAGAAA CAAAGGGAAA AAGGAAACAA TAAAAATTAC   
  
  
- AAGGGTATCT ACTATATCTC TAGACGAAAA GGAACGACAC TCTCCATAGA GACTCTTACG TACATCGTTA   
  
  
- GTTTTAAGAC TCAACAATTA AGATAACGTA CAGATACCCA CTACTAACAC TACTACAAAA AAGACAACGG   
  
  
- GAGAAAGAGA GAAAGTGACT TTAACGAAAG GTCTTATCAA CAAACTGAGG AAAGACATAG AATTAACTCG   
  
  
- GGAACGAAAC CAAACACGAG ACCCTCAAGA AGAAATCATT AGGGAACGAA CATAAAAGAC TTTCTCGTTC   
  
  
- GTTATTAGGA GTATCGTACA CATATTAGTA TGTTATCGTT GTCGTTTGTA CCTAGGACGC GGGTCAATCC   
  
  
- AAACCAATCC AATTCTATAC GTATAAGAAA AGAGAACTTT TCTCAACAGA TCCTTCCCAT TAATACGGGT   
  
  
- AAAGTACCTA TTATTACCAT GTTAATTCAA ACTAACCGAA TTTATGGTTA ATAAATCGCA GAAAAAGAAA   
  
  
- CCGACAGGTC CCAAGGTAAA GGTATTTGTT GAAACTCATT CCGTATCTCT TGATTTAAAG ACGGTTAACA   
  
  
- GTTCACGTTC AACGAGTATA CGTTGTTATG AACCTTTAAA ACTCGACTCG ACGACCGTAT AGTACTTATC   
  
  
- AAGATTACCA CCGCTCAATA GTGAATAGCA GGGAACACAC TGCTTAAAGA GAAGGATCCT TCGGGAGATC   
  
  
- AGTTACGGAT AGACGACAAC ACCTCACTTT TACCTATGTA AATCTAGAGA TATTTGGGAT GAAACCGTTC   
  
  
- TAGGTACGAC TAAAATCCTT CGGGAAACTG GTCTCTTACT GTATTGTTGG ACTATCCCAC AATGTACGAT   
  
  
- TGAAGCTAAT AAATTAGTTT TATGCCTTGA AGATAGTCAG TTTCGGACGG GGTCCAAGAT GACTTAATTT   
  
  
- AACATAACTT AACGAGAGTG AACGAATACT GCTAATTTAT TGACCTAAAC GATTAATAAG AAAAATAAAA   
  
  
- TAAGAACCAC TACATCCCTT GAGTCCCTTA ACATTGGTAT GGACAAGGTC GACTTTTCCC ATGCCCTTGT   
  
  
- CAGTATCAAT GTCCCCTTAA AGCGTTATGT AAATTCCCTC ATTCTTCGTC CTAGTACAGA AATCAATCCG   
  
  
- GCCGTCTATA CAGACACTGA GGTATACCTT TAGGTTGTGA CATAAGTGAA GTTCCGTTAT TATTATCGAG   
  
  
- ACCAAACAGA CCAGTTGACA TACCGAGACT TTTCGTGTTC TAGTTTATAC ACTGGCTTAG AAAATTACCA   
  
  
- GGTTCAATAC CGGACTTTAA GAAACAACTA AGAGGTAGTC TTCTCGAGTA GGTGGGTAGT CTACGATGAG   
  
  
- GATTGGGTAA ACTGAGAAGA AAGCACCCGT ATTCTGTACT ACTCTCAGGA ATGGTTCCTT TAATACCTAG   
  
  
- TCTTATACCC CATTCGGGTA GAAGGCTACG TCAACTTATA CTACTTCCAT TATTCTATTC CGATTTTGAA   
  
  
- GTTCTTAACC TCGTACGGGA CGAGCTACTT CTACTACTTC ACCTACTCCA CCGATATCCC GGGCACGATC   
  
  
- TTGTGTCGTA CCTCGAACTA CTTCTCACCC AGCGGGGTAA CTCTTTACAC CAAAAGGTAC TAAGTGGTTT   
  
  
- CCTCAGGTGG AGGAGTCTCA GTAGGTTACA GTCGTCGTAA TCGTCGTGGT TTCTTCATAG TGGAACGAGA   
  
  
- GGCTCATGAG GGTTTGTTAA CGAGAAATTA ACACGTTTAT ACTAAAGTCT CCCCTTAAAA CTCCTTCGGC   
  
  
- ACTGATACTA GTCGCTCGAA TCTGTCTAAC AAAGATAGGT TCCCCTAGGT CGCGTCTCCT ATCGTCGGAT   
  
  
- GTACCATCTT CCGGAACGAA GGGCAAACCG AAGTAGCCCT TTTCAAGAAA TGTTTCGAAA CTTTACGTTT   
  
  
- CTCGGTGGAA GTAAACTGTC CGATCGCCGA TACGTTTAGG AGAAACTCCA CACGGGTACA AAGTTTAAGC   
  
  
- CTAAATACCG GCGTTTACGT CGCTAATACC TACGTTAGTT TTTGCTTTTT TCCCATGTGT AGTATCTAAA   
  
  
- ACTGTATTTG GTTCCCTTAG TTATGTATTT AGAGTACGTT AGGGAACGAT TTGTTCCATT GTTCGACTGC   
  
  
- GTGAACTTCA ACTGACCTCA ACTACTGGGA CTCAGTCAAG TTGCGGGATA ACCACCGGAT TTTTTGTAGC   
  
  
- CTGTTTCCGA CGTTCGTAAT CGACTTATAG AACCACACAG TAAACTCAAG TTTCGTTATG GACGATCTTG   
  
  
- AGGTGAACAA TTGGGCCTTT ACAATCTTAC AGCTGGACGC CTTCGAAATC ACCACTTGAA ACGGAAGGTC   
  
  
- GAAGTGGTGT ACGGACTACT TTCGCAGAGT TGTTAGTTGG AGGCTCTGGT CGAAGAAGCC TACCAGTTTT   
  
  
- CGGATTTGGG TTTCGATCAT TGGTAACAGC TCGTCCTACA CTTGTGTTTG TGATGGGGAA AGGAGAGATC   
  
  
- CAAACGACTC CGTATGTCGA TGATAAGACG ACAAAAACTC AGAGAACTAC GATGAGAGGG ATCTCTATCG   
  
  
- GTCCTGTCCC ACTTACACCT CTTCGTCACG AACCGTGCAC TACAACACTT GTAGTAACGT ACACTTCCCC   
  
  
- TCCTCTCCTA ACTCGCGATA CTCCAACGCC CCTTTACCTC TCGCTCCTAC AACTACCGCC CTAAGTCCCG   
  
  
- ATAAGGTTAC CCAGTCTTCT GGCCACTATA CTAAGCCTTC GAATATAGTT ACGCCATAAC ACTGTCCGAG   
  
  
- TTCCATTTTC TCCTCTACCT ACGTGAAGTG AAACCCACCC TTCTATTTTT GAATTAGCAA TGTAGTCGAA   
  
  
- CCTCCAC

+     ABRE

| Site Name | Organism | Position | Strand | Matrix score. | sequence | function |
| --- | --- | --- | --- | --- | --- | --- |
| ABRE | Arabidopsis thaliana | 3470 | + | 5 | ACGTG | cis-acting element involved in the abscisic acid responsiveness |
| ABRE | Arabidopsis thaliana | 3165 | + | 7 | AACCCGG | cis-acting element involved in the abscisic acid responsiveness |
| ABRE | Arabidopsis thaliana | 3469 | - | 6 | CACGTG | cis-acting element involved in the abscisic acid responsiveness |

>HU11G01134.1   
+ +Up\_Stream \_Len000AGATAG ACATTCAGCC ACTTGCCAGT TCAGAGAGAG ATAGAGAGAG ATTGGGTAAG   
  
  
+ CCCTTTTTGC ACTACTTTTT CTCGCTTCCA TTTTTCTTTC TTAATTTTTA GTGATGATTT TCCAGAAGAT   
  
  
+ TTGATTTATT CATCATGCTT AACCAATTGG GTCTTTGAGT ATTATTGTTT ACTGGGTATT TGATCTTTTC   
  
  
+ ATATTCCTTT GGACTGGGTG GGTGGAACTT TTGAACTTTC TTATTAGTTG GGCATGCCCT TATCTTATTC   
  
  
+ CAATTCAAAA CCCATATTTT TCTTCAATTT TAATTATGGG TATTTTGAAA TTGAATGCTT TCCTTCACAG   
  
  
+ CAATACTATA TTACATATTT TACAGGAAAG TAATATTTTA CAGGAAAGTA TGTTGCTGTT TCAACCCTGT   
  
  
+ TTTGTTTCCT CTCTTTCCTT GGATATGATG TTCATTGCTC TTGTACCAAA CCTAACCTCA ACTTCCTGAA   
  
  
+ TTATTTAAGG CAATATTACC GTGTGCTGGT CAATTTTGGT TGTGGGTTTG TTTCTTAATG TATATGATTT   
  
  
+ ATGGTCAGGA TCCATGCTCC TCTTCCGTCC ACTCTATATC TCTCTCTCTC TCTCTCAATA CAAATTCAGA   
  
  
+ GACTTTCTCT TTCTGGGTTT ACTATTGATT GCAGAAGTTT GTAGTCTTGC GAGGCCTTGA GTCTTCTTCG   
  
  
+ AGGTAGAAAA TTTTGCTTTG CGCTTCCTTC CCCATATTTT AACACCCCTC CCCCCCCAAC GCAAGTTTTC   
  
  
+ TACATTTTTG TGCAATTCTT CAGCTATGAT TCCCCAATTT CACTTGTCAC ATCTCTATTT TCCTCTTTTT   
  
  
+ GGTTTCTTGA TTCTGTAGAA GGTTGTGTAT GAACTTCTTT GTTTCCCTTT TTCCTTTGTT ATTTTTAATG   
  
  
+ TTCCCATAGA TGATATAGAG ATCTGCTTTT CCTTGCTGTG AGAGGTATCT CTGAGAATGC ATGTAGCAAT   
  
  
+ CAAAATTCTG AGTTGTTAAT TCTATTGCAT GTCTATGGGT GATGATTGTG ATGATGTTTT TTCTGTTGCC   
  
  
+ CTCTTTCTCT CTTTCACTGA AATTGCTTTC CAGAATAGTT GTTTGACTCC TTTCTGTATC TTAATTGAGC   
  
  
+ CCTTGCTTTG GTTTGTGCTC TGGGAGTTCT TCTTTAGTAA TCCCTTGCTT GTATTTTCTG AAAGAGCAAG   
  
  
+ CAATAATCCT CATAGCATGT GTATAATCAT ACAATAGCAA CAGCAAACAT GGATCCTGCG CCCAGTTAGG   
  
  
+ TTTGGTTAGG TTAAGATATG CATATTCTTT TCTCTTGAAA AGAGTTGTCT AGGAAGGGTA ATTATGCCCA   
  
  
+ TTTCATGGAT AATAATGGTA CAATTAAGTT TGATTGGCTT AAATACCAAT TATTTAGCGT CTTTTTCTTT   
  
  
+ GGCTGTCCAG GGTTCCATTT CCATAAACAA CTTTGAGTAA GGCATAGAGA ACTAAATTTC TGCCAATTGT   
  
  
+ CAAGTGCAAG TTGCTCATAT GCAACAATAC TTGGAAATTT TGAGCTGAGC TGCTGGCATA TCATGAATAG   
  
  
+ TTCTAATGGT GGCGAGTTAT CACTTATCGT CCCTTGTGTG ACGAATTTCT CTTCCTAGGA AGCCCTCTAG   
  
  
+ TCAATGCCTA TCTGCTGTTG TGGAGTGAAA ATGGATACAT TTAGATCTCT ATAAACCCTA CTTTGGCAAG   
  
  
+ ATCCATGCTG ATTTTAGGAA GCCCTTTGAC CAGAGAATGA CATAACAACC TGATAGGGTG TTACATGCTA   
  
  
+ ACTTCGATTA TTTAATCAAA ATACGGAACT TCTATCAGTC AAAGCCTGCC CCAGGTTCTA CTGAATTAAA   
  
  
+ TTGTATTGAA TTGCTCTCAC TTGCTTATGA CGATTAAATA ACTGGATTTG CTAATTATTC TTTTTATTTT   
  
  
+ ATTCTTGGTG ATGTAGGGAA CTCAGGGAAT TGTAACCATA CCTGTTCCAG CTGAAAAGGG TACGGGAACA   
  
  
+ GTCATAGTTA CAGGGGAATT TCGCAATACA TTTAAGGGAG TAAGAAGCAG GATCATGTCT TTAGTTAGGC   
  
  
+ CGGCAGATAT GTCTGTGACT CCATATGGAA ATCCAACACT GTATTCACTT CAAGGCAATA ATAATAGCTC   
  
  
+ TGGTTTGTCT GGTCAACTGT ATGGCTCTGA AAAGCACAAG ATCAAATATG TGACCGAATC TTTTAATGGT   
  
  
+ CCAAGTTATG GCCTGAAATT CTTTGTTGAT TCTCCATCAG AAGAGCTCAT CCACCCATCA GATGCTACTC   
  
  
+ CTAACCCATT TGACTCTTCT TTCGTGGGCA TAAGACATGA TGAGAGTCCT TACCAAGGAA ATTATGGATC   
  
  
+ AGAATATGGG GTAAGCCCAT CTTCCGATGC AGTTGAATAT GATGAAGGTA ATAAGATAAG GCTAAAACTT   
  
  
+ CAAGAATTGG AGCATGCCCT GCTCGATGAA GATGATGAAG TGGATGAGGT GGCTATAGGG CCCGTGCTAG   
  
  
+ AACACAGCAT GGAGCTTGAT GAAGAGTGGG TCGCCCCATT GAGAAATGTG GTTTTCCATG ATTCACCAAA   
  
  
+ GGAGTCCACC TCCTCAGAGT CATCCAATGT CAGCAGCATT AGCAGCACCA AAGAAGTATC ACCTTGCTCT   
  
  
+ CCGAGTACTC CCAAACAATT GCTCTTTAAT TGTGCAAATA TGATTTCAGA GGGGAATTTT GAGGAAGCCG   
  
  
+ TGACTATGAT CAGCGAGCTT AGACAGATTG TTTCTATCCA AGGGGATCCA GCGCAGAGGA TAGCAGCCTA   
  
  
+ CATGGTAGAA GGCCTTGCTT CCCGTTTGGC TTCATCGGGA AAAGTTCTTT ACAAAGCTTT GAAATGCAAA   
  
  
+ GAGCCACCTT CATTTGACAG GCTAGCGGCT ATGCAAATCC TCTTTGAGGT GTGCCCATGT TTCAAATTCG   
  
  
+ GATTTATGGC CGCAAATGCA GCGATTATGG ATGCAATCAA AAACGAAAAA AGGGTACACA TCATAGATTT   
  
  
+ TGACATAAAC CAAGGGAATC AATACATAAA TCTCATGCAA TCCCTTGCTA AACAAGGTAA CAAGCTGACG   
  
  
+ CACTTGAAGT TGACTGGAGT TGATGACCCT GAGTCAGTTC AACGCCCTAT TGGTGGCCTA AAAAACATCG   
  
  
+ GACAAAGGCT GCAAGCATTA GCTGAATATC TTGGTGTGTC ATTTGAGTTC AAAGCAATAC CTGCTAGAAC   
  
  
+ TCCACTTGTT AACCCGGAAA TGTTAGAATG TCGACCTGCG GAAGCTTTAG TGGTGAACTT TGCCTTCCAG   
  
  
+ CTTCACCACA TGCCTGATGA AAGCGTCTCA ACAATCAACC TCCGAGACCA GCTTCTTCGG ATGGTCAAAA   
  
  
+ GCCTAAACCC AAAGCTAGTA ACCATTGTCG AGCAGGATGT GAACACAAAC ACTACCCCTT TCCTCTCTAG   
  
  
+ GTTTGCTGAG GCATACAGCT ACTATTCTGC TGTTTTTGAG TCTCTTGATG CTACTCTCCC TAGAGATAGC   
  
  
+ CAGGACAGGG TGAATGTGGA GAAGCAGTGC TTGGCACGTG ATGTTGTGAA CATCATTGCA TGTGAAGGGG   
  
  
+ AGGAGAGGAT TGAGCGCTAT GAGGTTGCGG GGAAATGGAG AGCGAGGATG TTGATGGCGG GATTCAGGGC   
  
  
+ TATTCCAATG GGTCAGAAGA CCGGTGATAT GATTCGGAAG CTTATATCAA TGCGGTATTG TGACAGGCTC   
  
  
+ AAGGTAAAAG AGGAGATGGA TGCACTTCAC TTTGGGTGGG AAGATAAAAA CTTAATCGTT ACATCAGCTT   
  
  
+ GGAGGTG  

- +Up\_Stream \_Len000TCTATC TGTAAGTCGG TGAACGGTCA AGTCTCTCTC TATCTCTCTC TAACCCATTC   
  
  
- GGGAAAAACG TGATGAAAAA GAGCGAAGGT AAAAAGAAAG AATTAAAAAT CACTACTAAA AGGTCTTCTA   
  
  
- AACTAAATAA GTAGTACGAA TTGGTTAACC CAGAAACTCA TAATAACAAA TGACCCATAA ACTAGAAAAG   
  
  
- TATAAGGAAA CCTGACCCAC CCACCTTGAA AACTTGAAAG AATAATCAAC CCGTACGGGA ATAGAATAAG   
  
  
- GTTAAGTTTT GGGTATAAAA AGAAGTTAAA ATTAATACCC ATAAAACTTT AACTTACGAA AGGAAGTGTC   
  
  
- GTTATGATAT AATGTATAAA ATGTCCTTTC ATTATAAAAT GTCCTTTCAT ACAACGACAA AGTTGGGACA   
  
  
- AAACAAAGGA GAGAAAGGAA CCTATACTAC AAGTAACGAG AACATGGTTT GGATTGGAGT TGAAGGACTT   
  
  
- AATAAATTCC GTTATAATGG CACACGACCA GTTAAAACCA ACACCCAAAC AAAGAATTAC ATATACTAAA   
  
  
- TACCAGTCCT AGGTACGAGG AGAAGGCAGG TGAGATATAG AGAGAGAGAG AGAGAGTTAT GTTTAAGTCT   
  
  
- CTGAAAGAGA AAGACCCAAA TGATAACTAA CGTCTTCAAA CATCAGAACG CTCCGGAACT CAGAAGAAGC   
  
  
- TCCATCTTTT AAAACGAAAC GCGAAGGAAG GGGTATAAAA TTGTGGGGAG GGGGGGGTTG CGTTCAAAAG   
  
  
- ATGTAAAAAC ACGTTAAGAA GTCGATACTA AGGGGTTAAA GTGAACAGTG TAGAGATAAA AGGAGAAAAA   
  
  
- CCAAAGAACT AAGACATCTT CCAACACATA CTTGAAGAAA CAAAGGGAAA AAGGAAACAA TAAAAATTAC   
  
  
- AAGGGTATCT ACTATATCTC TAGACGAAAA GGAACGACAC TCTCCATAGA GACTCTTACG TACATCGTTA   
  
  
- GTTTTAAGAC TCAACAATTA AGATAACGTA CAGATACCCA CTACTAACAC TACTACAAAA AAGACAACGG   
  
  
- GAGAAAGAGA GAAAGTGACT TTAACGAAAG GTCTTATCAA CAAACTGAGG AAAGACATAG AATTAACTCG   
  
  
- GGAACGAAAC CAAACACGAG ACCCTCAAGA AGAAATCATT AGGGAACGAA CATAAAAGAC TTTCTCGTTC   
  
  
- GTTATTAGGA GTATCGTACA CATATTAGTA TGTTATCGTT GTCGTTTGTA CCTAGGACGC GGGTCAATCC   
  
  
- AAACCAATCC AATTCTATAC GTATAAGAAA AGAGAACTTT TCTCAACAGA TCCTTCCCAT TAATACGGGT   
  
  
- AAAGTACCTA TTATTACCAT GTTAATTCAA ACTAACCGAA TTTATGGTTA ATAAATCGCA GAAAAAGAAA   
  
  
- CCGACAGGTC CCAAGGTAAA GGTATTTGTT GAAACTCATT CCGTATCTCT TGATTTAAAG ACGGTTAACA   
  
  
- GTTCACGTTC AACGAGTATA CGTTGTTATG AACCTTTAAA ACTCGACTCG ACGACCGTAT AGTACTTATC   
  
  
- AAGATTACCA CCGCTCAATA GTGAATAGCA GGGAACACAC TGCTTAAAGA GAAGGATCCT TCGGGAGATC   
  
  
- AGTTACGGAT AGACGACAAC ACCTCACTTT TACCTATGTA AATCTAGAGA TATTTGGGAT GAAACCGTTC   
  
  
- TAGGTACGAC TAAAATCCTT CGGGAAACTG GTCTCTTACT GTATTGTTGG ACTATCCCAC AATGTACGAT   
  
  
- TGAAGCTAAT AAATTAGTTT TATGCCTTGA AGATAGTCAG TTTCGGACGG GGTCCAAGAT GACTTAATTT   
  
  
- AACATAACTT AACGAGAGTG AACGAATACT GCTAATTTAT TGACCTAAAC GATTAATAAG AAAAATAAAA   
  
  
- TAAGAACCAC TACATCCCTT GAGTCCCTTA ACATTGGTAT GGACAAGGTC GACTTTTCCC ATGCCCTTGT   
  
  
- CAGTATCAAT GTCCCCTTAA AGCGTTATGT AAATTCCCTC ATTCTTCGTC CTAGTACAGA AATCAATCCG   
  
  
- GCCGTCTATA CAGACACTGA GGTATACCTT TAGGTTGTGA CATAAGTGAA GTTCCGTTAT TATTATCGAG   
  
  
- ACCAAACAGA CCAGTTGACA TACCGAGACT TTTCGTGTTC TAGTTTATAC ACTGGCTTAG AAAATTACCA   
  
  
- GGTTCAATAC CGGACTTTAA GAAACAACTA AGAGGTAGTC TTCTCGAGTA GGTGGGTAGT CTACGATGAG   
  
  
- GATTGGGTAA ACTGAGAAGA AAGCACCCGT ATTCTGTACT ACTCTCAGGA ATGGTTCCTT TAATACCTAG   
  
  
- TCTTATACCC CATTCGGGTA GAAGGCTACG TCAACTTATA CTACTTCCAT TATTCTATTC CGATTTTGAA   
  
  
- GTTCTTAACC TCGTACGGGA CGAGCTACTT CTACTACTTC ACCTACTCCA CCGATATCCC GGGCACGATC   
  
  
- TTGTGTCGTA CCTCGAACTA CTTCTCACCC AGCGGGGTAA CTCTTTACAC CAAAAGGTAC TAAGTGGTTT   
  
  
- CCTCAGGTGG AGGAGTCTCA GTAGGTTACA GTCGTCGTAA TCGTCGTGGT TTCTTCATAG TGGAACGAGA   
  
  
- GGCTCATGAG GGTTTGTTAA CGAGAAATTA ACACGTTTAT ACTAAAGTCT CCCCTTAAAA CTCCTTCGGC   
  
  
- ACTGATACTA GTCGCTCGAA TCTGTCTAAC AAAGATAGGT TCCCCTAGGT CGCGTCTCCT ATCGTCGGAT   
  
  
- GTACCATCTT CCGGAACGAA GGGCAAACCG AAGTAGCCCT TTTCAAGAAA TGTTTCGAAA CTTTACGTTT   
  
  
- CTCGGTGGAA GTAAACTGTC CGATCGCCGA TACGTTTAGG AGAAACTCCA CACGGGTACA AAGTTTAAGC   
  
  
- CTAAATACCG GCGTTTACGT CGCTAATACC TACGTTAGTT TTTGCTTTTT TCCCATGTGT AGTATCTAAA   
  
  
- ACTGTATTTG GTTCCCTTAG TTATGTATTT AGAGTACGTT AGGGAACGAT TTGTTCCATT GTTCGACTGC   
  
  
- GTGAACTTCA ACTGACCTCA ACTACTGGGA CTCAGTCAAG TTGCGGGATA ACCACCGGAT TTTTTGTAGC   
  
  
- CTGTTTCCGA CGTTCGTAAT CGACTTATAG AACCACACAG TAAACTCAAG TTTCGTTATG GACGATCTTG   
  
  
- AGGTGAACAA TTGGGCCTTT ACAATCTTAC AGCTGGACGC CTTCGAAATC ACCACTTGAA ACGGAAGGTC   
  
  
- GAAGTGGTGT ACGGACTACT TTCGCAGAGT TGTTAGTTGG AGGCTCTGGT CGAAGAAGCC TACCAGTTTT   
  
  
- CGGATTTGGG TTTCGATCAT TGGTAACAGC TCGTCCTACA CTTGTGTTTG TGATGGGGAA AGGAGAGATC   
  
  
- CAAACGACTC CGTATGTCGA TGATAAGACG ACAAAAACTC AGAGAACTAC GATGAGAGGG ATCTCTATCG   
  
  
- GTCCTGTCCC ACTTACACCT CTTCGTCACG AACCGTGCAC TACAACACTT GTAGTAACGT ACACTTCCCC   
  
  
- TCCTCTCCTA ACTCGCGATA CTCCAACGCC CCTTTACCTC TCGCTCCTAC AACTACCGCC CTAAGTCCCG   
  
  
- ATAAGGTTAC CCAGTCTTCT GGCCACTATA CTAAGCCTTC GAATATAGTT ACGCCATAAC ACTGTCCGAG   
  
  
- TTCCATTTTC TCCTCTACCT ACGTGAAGTG AAACCCACCC TTCTATTTTT GAATTAGCAA TGTAGTCGAA   
  
  
- CCTCCAC

+     AE-box

| Site Name | Organism | Position | Strand | Matrix score. | sequence | function |
| --- | --- | --- | --- | --- | --- | --- |
| AE-box | Arabidopsis thaliana | 542 | - | 8 | AGAAACAA | part of a module for light response |
| AE-box | Arabidopsis thaliana | 2692 | - | 8 | AGAAACAA | part of a module for light response |

>HU11G01134.1   
+ +Up\_Stream \_Len000AGATAG ACATTCAGCC ACTTGCCAGT TCAGAGAGAG ATAGAGAGAG ATTGGGTAAG   
  
  
+ CCCTTTTTGC ACTACTTTTT CTCGCTTCCA TTTTTCTTTC TTAATTTTTA GTGATGATTT TCCAGAAGAT   
  
  
+ TTGATTTATT CATCATGCTT AACCAATTGG GTCTTTGAGT ATTATTGTTT ACTGGGTATT TGATCTTTTC   
  
  
+ ATATTCCTTT GGACTGGGTG GGTGGAACTT TTGAACTTTC TTATTAGTTG GGCATGCCCT TATCTTATTC   
  
  
+ CAATTCAAAA CCCATATTTT TCTTCAATTT TAATTATGGG TATTTTGAAA TTGAATGCTT TCCTTCACAG   
  
  
+ CAATACTATA TTACATATTT TACAGGAAAG TAATATTTTA CAGGAAAGTA TGTTGCTGTT TCAACCCTGT   
  
  
+ TTTGTTTCCT CTCTTTCCTT GGATATGATG TTCATTGCTC TTGTACCAAA CCTAACCTCA ACTTCCTGAA   
  
  
+ TTATTTAAGG CAATATTACC GTGTGCTGGT CAATTTTGGT TGTGGGTTTG TTTCTTAATG TATATGATTT   
  
  
+ ATGGTCAGGA TCCATGCTCC TCTTCCGTCC ACTCTATATC TCTCTCTCTC TCTCTCAATA CAAATTCAGA   
  
  
+ GACTTTCTCT TTCTGGGTTT ACTATTGATT GCAGAAGTTT GTAGTCTTGC GAGGCCTTGA GTCTTCTTCG   
  
  
+ AGGTAGAAAA TTTTGCTTTG CGCTTCCTTC CCCATATTTT AACACCCCTC CCCCCCCAAC GCAAGTTTTC   
  
  
+ TACATTTTTG TGCAATTCTT CAGCTATGAT TCCCCAATTT CACTTGTCAC ATCTCTATTT TCCTCTTTTT   
  
  
+ GGTTTCTTGA TTCTGTAGAA GGTTGTGTAT GAACTTCTTT GTTTCCCTTT TTCCTTTGTT ATTTTTAATG   
  
  
+ TTCCCATAGA TGATATAGAG ATCTGCTTTT CCTTGCTGTG AGAGGTATCT CTGAGAATGC ATGTAGCAAT   
  
  
+ CAAAATTCTG AGTTGTTAAT TCTATTGCAT GTCTATGGGT GATGATTGTG ATGATGTTTT TTCTGTTGCC   
  
  
+ CTCTTTCTCT CTTTCACTGA AATTGCTTTC CAGAATAGTT GTTTGACTCC TTTCTGTATC TTAATTGAGC   
  
  
+ CCTTGCTTTG GTTTGTGCTC TGGGAGTTCT TCTTTAGTAA TCCCTTGCTT GTATTTTCTG AAAGAGCAAG   
  
  
+ CAATAATCCT CATAGCATGT GTATAATCAT ACAATAGCAA CAGCAAACAT GGATCCTGCG CCCAGTTAGG   
  
  
+ TTTGGTTAGG TTAAGATATG CATATTCTTT TCTCTTGAAA AGAGTTGTCT AGGAAGGGTA ATTATGCCCA   
  
  
+ TTTCATGGAT AATAATGGTA CAATTAAGTT TGATTGGCTT AAATACCAAT TATTTAGCGT CTTTTTCTTT   
  
  
+ GGCTGTCCAG GGTTCCATTT CCATAAACAA CTTTGAGTAA GGCATAGAGA ACTAAATTTC TGCCAATTGT   
  
  
+ CAAGTGCAAG TTGCTCATAT GCAACAATAC TTGGAAATTT TGAGCTGAGC TGCTGGCATA TCATGAATAG   
  
  
+ TTCTAATGGT GGCGAGTTAT CACTTATCGT CCCTTGTGTG ACGAATTTCT CTTCCTAGGA AGCCCTCTAG   
  
  
+ TCAATGCCTA TCTGCTGTTG TGGAGTGAAA ATGGATACAT TTAGATCTCT ATAAACCCTA CTTTGGCAAG   
  
  
+ ATCCATGCTG ATTTTAGGAA GCCCTTTGAC CAGAGAATGA CATAACAACC TGATAGGGTG TTACATGCTA   
  
  
+ ACTTCGATTA TTTAATCAAA ATACGGAACT TCTATCAGTC AAAGCCTGCC CCAGGTTCTA CTGAATTAAA   
  
  
+ TTGTATTGAA TTGCTCTCAC TTGCTTATGA CGATTAAATA ACTGGATTTG CTAATTATTC TTTTTATTTT   
  
  
+ ATTCTTGGTG ATGTAGGGAA CTCAGGGAAT TGTAACCATA CCTGTTCCAG CTGAAAAGGG TACGGGAACA   
  
  
+ GTCATAGTTA CAGGGGAATT TCGCAATACA TTTAAGGGAG TAAGAAGCAG GATCATGTCT TTAGTTAGGC   
  
  
+ CGGCAGATAT GTCTGTGACT CCATATGGAA ATCCAACACT GTATTCACTT CAAGGCAATA ATAATAGCTC   
  
  
+ TGGTTTGTCT GGTCAACTGT ATGGCTCTGA AAAGCACAAG ATCAAATATG TGACCGAATC TTTTAATGGT   
  
  
+ CCAAGTTATG GCCTGAAATT CTTTGTTGAT TCTCCATCAG AAGAGCTCAT CCACCCATCA GATGCTACTC   
  
  
+ CTAACCCATT TGACTCTTCT TTCGTGGGCA TAAGACATGA TGAGAGTCCT TACCAAGGAA ATTATGGATC   
  
  
+ AGAATATGGG GTAAGCCCAT CTTCCGATGC AGTTGAATAT GATGAAGGTA ATAAGATAAG GCTAAAACTT   
  
  
+ CAAGAATTGG AGCATGCCCT GCTCGATGAA GATGATGAAG TGGATGAGGT GGCTATAGGG CCCGTGCTAG   
  
  
+ AACACAGCAT GGAGCTTGAT GAAGAGTGGG TCGCCCCATT GAGAAATGTG GTTTTCCATG ATTCACCAAA   
  
  
+ GGAGTCCACC TCCTCAGAGT CATCCAATGT CAGCAGCATT AGCAGCACCA AAGAAGTATC ACCTTGCTCT   
  
  
+ CCGAGTACTC CCAAACAATT GCTCTTTAAT TGTGCAAATA TGATTTCAGA GGGGAATTTT GAGGAAGCCG   
  
  
+ TGACTATGAT CAGCGAGCTT AGACAGATTG TTTCTATCCA AGGGGATCCA GCGCAGAGGA TAGCAGCCTA   
  
  
+ CATGGTAGAA GGCCTTGCTT CCCGTTTGGC TTCATCGGGA AAAGTTCTTT ACAAAGCTTT GAAATGCAAA   
  
  
+ GAGCCACCTT CATTTGACAG GCTAGCGGCT ATGCAAATCC TCTTTGAGGT GTGCCCATGT TTCAAATTCG   
  
  
+ GATTTATGGC CGCAAATGCA GCGATTATGG ATGCAATCAA AAACGAAAAA AGGGTACACA TCATAGATTT   
  
  
+ TGACATAAAC CAAGGGAATC AATACATAAA TCTCATGCAA TCCCTTGCTA AACAAGGTAA CAAGCTGACG   
  
  
+ CACTTGAAGT TGACTGGAGT TGATGACCCT GAGTCAGTTC AACGCCCTAT TGGTGGCCTA AAAAACATCG   
  
  
+ GACAAAGGCT GCAAGCATTA GCTGAATATC TTGGTGTGTC ATTTGAGTTC AAAGCAATAC CTGCTAGAAC   
  
  
+ TCCACTTGTT AACCCGGAAA TGTTAGAATG TCGACCTGCG GAAGCTTTAG TGGTGAACTT TGCCTTCCAG   
  
  
+ CTTCACCACA TGCCTGATGA AAGCGTCTCA ACAATCAACC TCCGAGACCA GCTTCTTCGG ATGGTCAAAA   
  
  
+ GCCTAAACCC AAAGCTAGTA ACCATTGTCG AGCAGGATGT GAACACAAAC ACTACCCCTT TCCTCTCTAG   
  
  
+ GTTTGCTGAG GCATACAGCT ACTATTCTGC TGTTTTTGAG TCTCTTGATG CTACTCTCCC TAGAGATAGC   
  
  
+ CAGGACAGGG TGAATGTGGA GAAGCAGTGC TTGGCACGTG ATGTTGTGAA CATCATTGCA TGTGAAGGGG   
  
  
+ AGGAGAGGAT TGAGCGCTAT GAGGTTGCGG GGAAATGGAG AGCGAGGATG TTGATGGCGG GATTCAGGGC   
  
  
+ TATTCCAATG GGTCAGAAGA CCGGTGATAT GATTCGGAAG CTTATATCAA TGCGGTATTG TGACAGGCTC   
  
  
+ AAGGTAAAAG AGGAGATGGA TGCACTTCAC TTTGGGTGGG AAGATAAAAA CTTAATCGTT ACATCAGCTT   
  
  
+ GGAGGTG  

- +Up\_Stream \_Len000TCTATC TGTAAGTCGG TGAACGGTCA AGTCTCTCTC TATCTCTCTC TAACCCATTC   
  
  
- GGGAAAAACG TGATGAAAAA GAGCGAAGGT AAAAAGAAAG AATTAAAAAT CACTACTAAA AGGTCTTCTA   
  
  
- AACTAAATAA GTAGTACGAA TTGGTTAACC CAGAAACTCA TAATAACAAA TGACCCATAA ACTAGAAAAG   
  
  
- TATAAGGAAA CCTGACCCAC CCACCTTGAA AACTTGAAAG AATAATCAAC CCGTACGGGA ATAGAATAAG   
  
  
- GTTAAGTTTT GGGTATAAAA AGAAGTTAAA ATTAATACCC ATAAAACTTT AACTTACGAA AGGAAGTGTC   
  
  
- GTTATGATAT AATGTATAAA ATGTCCTTTC ATTATAAAAT GTCCTTTCAT ACAACGACAA AGTTGGGACA   
  
  
- AAACAAAGGA GAGAAAGGAA CCTATACTAC AAGTAACGAG AACATGGTTT GGATTGGAGT TGAAGGACTT   
  
  
- AATAAATTCC GTTATAATGG CACACGACCA GTTAAAACCA ACACCCAAAC AAAGAATTAC ATATACTAAA   
  
  
- TACCAGTCCT AGGTACGAGG AGAAGGCAGG TGAGATATAG AGAGAGAGAG AGAGAGTTAT GTTTAAGTCT   
  
  
- CTGAAAGAGA AAGACCCAAA TGATAACTAA CGTCTTCAAA CATCAGAACG CTCCGGAACT CAGAAGAAGC   
  
  
- TCCATCTTTT AAAACGAAAC GCGAAGGAAG GGGTATAAAA TTGTGGGGAG GGGGGGGTTG CGTTCAAAAG   
  
  
- ATGTAAAAAC ACGTTAAGAA GTCGATACTA AGGGGTTAAA GTGAACAGTG TAGAGATAAA AGGAGAAAAA   
  
  
- CCAAAGAACT AAGACATCTT CCAACACATA CTTGAAGAAA CAAAGGGAAA AAGGAAACAA TAAAAATTAC   
  
  
- AAGGGTATCT ACTATATCTC TAGACGAAAA GGAACGACAC TCTCCATAGA GACTCTTACG TACATCGTTA   
  
  
- GTTTTAAGAC TCAACAATTA AGATAACGTA CAGATACCCA CTACTAACAC TACTACAAAA AAGACAACGG   
  
  
- GAGAAAGAGA GAAAGTGACT TTAACGAAAG GTCTTATCAA CAAACTGAGG AAAGACATAG AATTAACTCG   
  
  
- GGAACGAAAC CAAACACGAG ACCCTCAAGA AGAAATCATT AGGGAACGAA CATAAAAGAC TTTCTCGTTC   
  
  
- GTTATTAGGA GTATCGTACA CATATTAGTA TGTTATCGTT GTCGTTTGTA CCTAGGACGC GGGTCAATCC   
  
  
- AAACCAATCC AATTCTATAC GTATAAGAAA AGAGAACTTT TCTCAACAGA TCCTTCCCAT TAATACGGGT   
  
  
- AAAGTACCTA TTATTACCAT GTTAATTCAA ACTAACCGAA TTTATGGTTA ATAAATCGCA GAAAAAGAAA   
  
  
- CCGACAGGTC CCAAGGTAAA GGTATTTGTT GAAACTCATT CCGTATCTCT TGATTTAAAG ACGGTTAACA   
  
  
- GTTCACGTTC AACGAGTATA CGTTGTTATG AACCTTTAAA ACTCGACTCG ACGACCGTAT AGTACTTATC   
  
  
- AAGATTACCA CCGCTCAATA GTGAATAGCA GGGAACACAC TGCTTAAAGA GAAGGATCCT TCGGGAGATC   
  
  
- AGTTACGGAT AGACGACAAC ACCTCACTTT TACCTATGTA AATCTAGAGA TATTTGGGAT GAAACCGTTC   
  
  
- TAGGTACGAC TAAAATCCTT CGGGAAACTG GTCTCTTACT GTATTGTTGG ACTATCCCAC AATGTACGAT   
  
  
- TGAAGCTAAT AAATTAGTTT TATGCCTTGA AGATAGTCAG TTTCGGACGG GGTCCAAGAT GACTTAATTT   
  
  
- AACATAACTT AACGAGAGTG AACGAATACT GCTAATTTAT TGACCTAAAC GATTAATAAG AAAAATAAAA   
  
  
- TAAGAACCAC TACATCCCTT GAGTCCCTTA ACATTGGTAT GGACAAGGTC GACTTTTCCC ATGCCCTTGT   
  
  
- CAGTATCAAT GTCCCCTTAA AGCGTTATGT AAATTCCCTC ATTCTTCGTC CTAGTACAGA AATCAATCCG   
  
  
- GCCGTCTATA CAGACACTGA GGTATACCTT TAGGTTGTGA CATAAGTGAA GTTCCGTTAT TATTATCGAG   
  
  
- ACCAAACAGA CCAGTTGACA TACCGAGACT TTTCGTGTTC TAGTTTATAC ACTGGCTTAG AAAATTACCA   
  
  
- GGTTCAATAC CGGACTTTAA GAAACAACTA AGAGGTAGTC TTCTCGAGTA GGTGGGTAGT CTACGATGAG   
  
  
- GATTGGGTAA ACTGAGAAGA AAGCACCCGT ATTCTGTACT ACTCTCAGGA ATGGTTCCTT TAATACCTAG   
  
  
- TCTTATACCC CATTCGGGTA GAAGGCTACG TCAACTTATA CTACTTCCAT TATTCTATTC CGATTTTGAA   
  
  
- GTTCTTAACC TCGTACGGGA CGAGCTACTT CTACTACTTC ACCTACTCCA CCGATATCCC GGGCACGATC   
  
  
- TTGTGTCGTA CCTCGAACTA CTTCTCACCC AGCGGGGTAA CTCTTTACAC CAAAAGGTAC TAAGTGGTTT   
  
  
- CCTCAGGTGG AGGAGTCTCA GTAGGTTACA GTCGTCGTAA TCGTCGTGGT TTCTTCATAG TGGAACGAGA   
  
  
- GGCTCATGAG GGTTTGTTAA CGAGAAATTA ACACGTTTAT ACTAAAGTCT CCCCTTAAAA CTCCTTCGGC   
  
  
- ACTGATACTA GTCGCTCGAA TCTGTCTAAC AAAGATAGGT TCCCCTAGGT CGCGTCTCCT ATCGTCGGAT   
  
  
- GTACCATCTT CCGGAACGAA GGGCAAACCG AAGTAGCCCT TTTCAAGAAA TGTTTCGAAA CTTTACGTTT   
  
  
- CTCGGTGGAA GTAAACTGTC CGATCGCCGA TACGTTTAGG AGAAACTCCA CACGGGTACA AAGTTTAAGC   
  
  
- CTAAATACCG GCGTTTACGT CGCTAATACC TACGTTAGTT TTTGCTTTTT TCCCATGTGT AGTATCTAAA   
  
  
- ACTGTATTTG GTTCCCTTAG TTATGTATTT AGAGTACGTT AGGGAACGAT TTGTTCCATT GTTCGACTGC   
  
  
- GTGAACTTCA ACTGACCTCA ACTACTGGGA CTCAGTCAAG TTGCGGGATA ACCACCGGAT TTTTTGTAGC   
  
  
- CTGTTTCCGA CGTTCGTAAT CGACTTATAG AACCACACAG TAAACTCAAG TTTCGTTATG GACGATCTTG   
  
  
- AGGTGAACAA TTGGGCCTTT ACAATCTTAC AGCTGGACGC CTTCGAAATC ACCACTTGAA ACGGAAGGTC   
  
  
- GAAGTGGTGT ACGGACTACT TTCGCAGAGT TGTTAGTTGG AGGCTCTGGT CGAAGAAGCC TACCAGTTTT   
  
  
- CGGATTTGGG TTTCGATCAT TGGTAACAGC TCGTCCTACA CTTGTGTTTG TGATGGGGAA AGGAGAGATC   
  
  
- CAAACGACTC CGTATGTCGA TGATAAGACG ACAAAAACTC AGAGAACTAC GATGAGAGGG ATCTCTATCG   
  
  
- GTCCTGTCCC ACTTACACCT CTTCGTCACG AACCGTGCAC TACAACACTT GTAGTAACGT ACACTTCCCC   
  
  
- TCCTCTCCTA ACTCGCGATA CTCCAACGCC CCTTTACCTC TCGCTCCTAC AACTACCGCC CTAAGTCCCG   
  
  
- ATAAGGTTAC CCAGTCTTCT GGCCACTATA CTAAGCCTTC GAATATAGTT ACGCCATAAC ACTGTCCGAG   
  
  
- TTCCATTTTC TCCTCTACCT ACGTGAAGTG AAACCCACCC TTCTATTTTT GAATTAGCAA TGTAGTCGAA   
  
  
- CCTCCAC

+     ARE

| Site Name | Organism | Position | Strand | Matrix score. | sequence | function |
| --- | --- | --- | --- | --- | --- | --- |
| ARE | Zea mays | 1133 | - | 6 | AAACCA | cis-acting regulatory element essential for the anaerobic induction |
| ARE | Zea mays | 2105 | - | 6 | AAACCA | cis-acting regulatory element essential for the anaerobic induction |
| ARE | Zea mays | 2951 | + | 6 | AAACCA | cis-acting regulatory element essential for the anaerobic induction |
| ARE | Zea mays | 844 | - | 6 | AAACCA | cis-acting regulatory element essential for the anaerobic induction |
| ARE | Zea mays | 2503 | - | 6 | AAACCA | cis-acting regulatory element essential for the anaerobic induction |

>HU11G01134.1   
+ +Up\_Stream \_Len000AGATAG ACATTCAGCC ACTTGCCAGT TCAGAGAGAG ATAGAGAGAG ATTGGGTAAG   
  
  
+ CCCTTTTTGC ACTACTTTTT CTCGCTTCCA TTTTTCTTTC TTAATTTTTA GTGATGATTT TCCAGAAGAT   
  
  
+ TTGATTTATT CATCATGCTT AACCAATTGG GTCTTTGAGT ATTATTGTTT ACTGGGTATT TGATCTTTTC   
  
  
+ ATATTCCTTT GGACTGGGTG GGTGGAACTT TTGAACTTTC TTATTAGTTG GGCATGCCCT TATCTTATTC   
  
  
+ CAATTCAAAA CCCATATTTT TCTTCAATTT TAATTATGGG TATTTTGAAA TTGAATGCTT TCCTTCACAG   
  
  
+ CAATACTATA TTACATATTT TACAGGAAAG TAATATTTTA CAGGAAAGTA TGTTGCTGTT TCAACCCTGT   
  
  
+ TTTGTTTCCT CTCTTTCCTT GGATATGATG TTCATTGCTC TTGTACCAAA CCTAACCTCA ACTTCCTGAA   
  
  
+ TTATTTAAGG CAATATTACC GTGTGCTGGT CAATTTTGGT TGTGGGTTTG TTTCTTAATG TATATGATTT   
  
  
+ ATGGTCAGGA TCCATGCTCC TCTTCCGTCC ACTCTATATC TCTCTCTCTC TCTCTCAATA CAAATTCAGA   
  
  
+ GACTTTCTCT TTCTGGGTTT ACTATTGATT GCAGAAGTTT GTAGTCTTGC GAGGCCTTGA GTCTTCTTCG   
  
  
+ AGGTAGAAAA TTTTGCTTTG CGCTTCCTTC CCCATATTTT AACACCCCTC CCCCCCCAAC GCAAGTTTTC   
  
  
+ TACATTTTTG TGCAATTCTT CAGCTATGAT TCCCCAATTT CACTTGTCAC ATCTCTATTT TCCTCTTTTT   
  
  
+ GGTTTCTTGA TTCTGTAGAA GGTTGTGTAT GAACTTCTTT GTTTCCCTTT TTCCTTTGTT ATTTTTAATG   
  
  
+ TTCCCATAGA TGATATAGAG ATCTGCTTTT CCTTGCTGTG AGAGGTATCT CTGAGAATGC ATGTAGCAAT   
  
  
+ CAAAATTCTG AGTTGTTAAT TCTATTGCAT GTCTATGGGT GATGATTGTG ATGATGTTTT TTCTGTTGCC   
  
  
+ CTCTTTCTCT CTTTCACTGA AATTGCTTTC CAGAATAGTT GTTTGACTCC TTTCTGTATC TTAATTGAGC   
  
  
+ CCTTGCTTTG GTTTGTGCTC TGGGAGTTCT TCTTTAGTAA TCCCTTGCTT GTATTTTCTG AAAGAGCAAG   
  
  
+ CAATAATCCT CATAGCATGT GTATAATCAT ACAATAGCAA CAGCAAACAT GGATCCTGCG CCCAGTTAGG   
  
  
+ TTTGGTTAGG TTAAGATATG CATATTCTTT TCTCTTGAAA AGAGTTGTCT AGGAAGGGTA ATTATGCCCA   
  
  
+ TTTCATGGAT AATAATGGTA CAATTAAGTT TGATTGGCTT AAATACCAAT TATTTAGCGT CTTTTTCTTT   
  
  
+ GGCTGTCCAG GGTTCCATTT CCATAAACAA CTTTGAGTAA GGCATAGAGA ACTAAATTTC TGCCAATTGT   
  
  
+ CAAGTGCAAG TTGCTCATAT GCAACAATAC TTGGAAATTT TGAGCTGAGC TGCTGGCATA TCATGAATAG   
  
  
+ TTCTAATGGT GGCGAGTTAT CACTTATCGT CCCTTGTGTG ACGAATTTCT CTTCCTAGGA AGCCCTCTAG   
  
  
+ TCAATGCCTA TCTGCTGTTG TGGAGTGAAA ATGGATACAT TTAGATCTCT ATAAACCCTA CTTTGGCAAG   
  
  
+ ATCCATGCTG ATTTTAGGAA GCCCTTTGAC CAGAGAATGA CATAACAACC TGATAGGGTG TTACATGCTA   
  
  
+ ACTTCGATTA TTTAATCAAA ATACGGAACT TCTATCAGTC AAAGCCTGCC CCAGGTTCTA CTGAATTAAA   
  
  
+ TTGTATTGAA TTGCTCTCAC TTGCTTATGA CGATTAAATA ACTGGATTTG CTAATTATTC TTTTTATTTT   
  
  
+ ATTCTTGGTG ATGTAGGGAA CTCAGGGAAT TGTAACCATA CCTGTTCCAG CTGAAAAGGG TACGGGAACA   
  
  
+ GTCATAGTTA CAGGGGAATT TCGCAATACA TTTAAGGGAG TAAGAAGCAG GATCATGTCT TTAGTTAGGC   
  
  
+ CGGCAGATAT GTCTGTGACT CCATATGGAA ATCCAACACT GTATTCACTT CAAGGCAATA ATAATAGCTC   
  
  
+ TGGTTTGTCT GGTCAACTGT ATGGCTCTGA AAAGCACAAG ATCAAATATG TGACCGAATC TTTTAATGGT   
  
  
+ CCAAGTTATG GCCTGAAATT CTTTGTTGAT TCTCCATCAG AAGAGCTCAT CCACCCATCA GATGCTACTC   
  
  
+ CTAACCCATT TGACTCTTCT TTCGTGGGCA TAAGACATGA TGAGAGTCCT TACCAAGGAA ATTATGGATC   
  
  
+ AGAATATGGG GTAAGCCCAT CTTCCGATGC AGTTGAATAT GATGAAGGTA ATAAGATAAG GCTAAAACTT   
  
  
+ CAAGAATTGG AGCATGCCCT GCTCGATGAA GATGATGAAG TGGATGAGGT GGCTATAGGG CCCGTGCTAG   
  
  
+ AACACAGCAT GGAGCTTGAT GAAGAGTGGG TCGCCCCATT GAGAAATGTG GTTTTCCATG ATTCACCAAA   
  
  
+ GGAGTCCACC TCCTCAGAGT CATCCAATGT CAGCAGCATT AGCAGCACCA AAGAAGTATC ACCTTGCTCT   
  
  
+ CCGAGTACTC CCAAACAATT GCTCTTTAAT TGTGCAAATA TGATTTCAGA GGGGAATTTT GAGGAAGCCG   
  
  
+ TGACTATGAT CAGCGAGCTT AGACAGATTG TTTCTATCCA AGGGGATCCA GCGCAGAGGA TAGCAGCCTA   
  
  
+ CATGGTAGAA GGCCTTGCTT CCCGTTTGGC TTCATCGGGA AAAGTTCTTT ACAAAGCTTT GAAATGCAAA   
  
  
+ GAGCCACCTT CATTTGACAG GCTAGCGGCT ATGCAAATCC TCTTTGAGGT GTGCCCATGT TTCAAATTCG   
  
  
+ GATTTATGGC CGCAAATGCA GCGATTATGG ATGCAATCAA AAACGAAAAA AGGGTACACA TCATAGATTT   
  
  
+ TGACATAAAC CAAGGGAATC AATACATAAA TCTCATGCAA TCCCTTGCTA AACAAGGTAA CAAGCTGACG   
  
  
+ CACTTGAAGT TGACTGGAGT TGATGACCCT GAGTCAGTTC AACGCCCTAT TGGTGGCCTA AAAAACATCG   
  
  
+ GACAAAGGCT GCAAGCATTA GCTGAATATC TTGGTGTGTC ATTTGAGTTC AAAGCAATAC CTGCTAGAAC   
  
  
+ TCCACTTGTT AACCCGGAAA TGTTAGAATG TCGACCTGCG GAAGCTTTAG TGGTGAACTT TGCCTTCCAG   
  
  
+ CTTCACCACA TGCCTGATGA AAGCGTCTCA ACAATCAACC TCCGAGACCA GCTTCTTCGG ATGGTCAAAA   
  
  
+ GCCTAAACCC AAAGCTAGTA ACCATTGTCG AGCAGGATGT GAACACAAAC ACTACCCCTT TCCTCTCTAG   
  
  
+ GTTTGCTGAG GCATACAGCT ACTATTCTGC TGTTTTTGAG TCTCTTGATG CTACTCTCCC TAGAGATAGC   
  
  
+ CAGGACAGGG TGAATGTGGA GAAGCAGTGC TTGGCACGTG ATGTTGTGAA CATCATTGCA TGTGAAGGGG   
  
  
+ AGGAGAGGAT TGAGCGCTAT GAGGTTGCGG GGAAATGGAG AGCGAGGATG TTGATGGCGG GATTCAGGGC   
  
  
+ TATTCCAATG GGTCAGAAGA CCGGTGATAT GATTCGGAAG CTTATATCAA TGCGGTATTG TGACAGGCTC   
  
  
+ AAGGTAAAAG AGGAGATGGA TGCACTTCAC TTTGGGTGGG AAGATAAAAA CTTAATCGTT ACATCAGCTT   
  
  
+ GGAGGTG  

- +Up\_Stream \_Len000TCTATC TGTAAGTCGG TGAACGGTCA AGTCTCTCTC TATCTCTCTC TAACCCATTC   
  
  
- GGGAAAAACG TGATGAAAAA GAGCGAAGGT AAAAAGAAAG AATTAAAAAT CACTACTAAA AGGTCTTCTA   
  
  
- AACTAAATAA GTAGTACGAA TTGGTTAACC CAGAAACTCA TAATAACAAA TGACCCATAA ACTAGAAAAG   
  
  
- TATAAGGAAA CCTGACCCAC CCACCTTGAA AACTTGAAAG AATAATCAAC CCGTACGGGA ATAGAATAAG   
  
  
- GTTAAGTTTT GGGTATAAAA AGAAGTTAAA ATTAATACCC ATAAAACTTT AACTTACGAA AGGAAGTGTC   
  
  
- GTTATGATAT AATGTATAAA ATGTCCTTTC ATTATAAAAT GTCCTTTCAT ACAACGACAA AGTTGGGACA   
  
  
- AAACAAAGGA GAGAAAGGAA CCTATACTAC AAGTAACGAG AACATGGTTT GGATTGGAGT TGAAGGACTT   
  
  
- AATAAATTCC GTTATAATGG CACACGACCA GTTAAAACCA ACACCCAAAC AAAGAATTAC ATATACTAAA   
  
  
- TACCAGTCCT AGGTACGAGG AGAAGGCAGG TGAGATATAG AGAGAGAGAG AGAGAGTTAT GTTTAAGTCT   
  
  
- CTGAAAGAGA AAGACCCAAA TGATAACTAA CGTCTTCAAA CATCAGAACG CTCCGGAACT CAGAAGAAGC   
  
  
- TCCATCTTTT AAAACGAAAC GCGAAGGAAG GGGTATAAAA TTGTGGGGAG GGGGGGGTTG CGTTCAAAAG   
  
  
- ATGTAAAAAC ACGTTAAGAA GTCGATACTA AGGGGTTAAA GTGAACAGTG TAGAGATAAA AGGAGAAAAA   
  
  
- CCAAAGAACT AAGACATCTT CCAACACATA CTTGAAGAAA CAAAGGGAAA AAGGAAACAA TAAAAATTAC   
  
  
- AAGGGTATCT ACTATATCTC TAGACGAAAA GGAACGACAC TCTCCATAGA GACTCTTACG TACATCGTTA   
  
  
- GTTTTAAGAC TCAACAATTA AGATAACGTA CAGATACCCA CTACTAACAC TACTACAAAA AAGACAACGG   
  
  
- GAGAAAGAGA GAAAGTGACT TTAACGAAAG GTCTTATCAA CAAACTGAGG AAAGACATAG AATTAACTCG   
  
  
- GGAACGAAAC CAAACACGAG ACCCTCAAGA AGAAATCATT AGGGAACGAA CATAAAAGAC TTTCTCGTTC   
  
  
- GTTATTAGGA GTATCGTACA CATATTAGTA TGTTATCGTT GTCGTTTGTA CCTAGGACGC GGGTCAATCC   
  
  
- AAACCAATCC AATTCTATAC GTATAAGAAA AGAGAACTTT TCTCAACAGA TCCTTCCCAT TAATACGGGT   
  
  
- AAAGTACCTA TTATTACCAT GTTAATTCAA ACTAACCGAA TTTATGGTTA ATAAATCGCA GAAAAAGAAA   
  
  
- CCGACAGGTC CCAAGGTAAA GGTATTTGTT GAAACTCATT CCGTATCTCT TGATTTAAAG ACGGTTAACA   
  
  
- GTTCACGTTC AACGAGTATA CGTTGTTATG AACCTTTAAA ACTCGACTCG ACGACCGTAT AGTACTTATC   
  
  
- AAGATTACCA CCGCTCAATA GTGAATAGCA GGGAACACAC TGCTTAAAGA GAAGGATCCT TCGGGAGATC   
  
  
- AGTTACGGAT AGACGACAAC ACCTCACTTT TACCTATGTA AATCTAGAGA TATTTGGGAT GAAACCGTTC   
  
  
- TAGGTACGAC TAAAATCCTT CGGGAAACTG GTCTCTTACT GTATTGTTGG ACTATCCCAC AATGTACGAT   
  
  
- TGAAGCTAAT AAATTAGTTT TATGCCTTGA AGATAGTCAG TTTCGGACGG GGTCCAAGAT GACTTAATTT   
  
  
- AACATAACTT AACGAGAGTG AACGAATACT GCTAATTTAT TGACCTAAAC GATTAATAAG AAAAATAAAA   
  
  
- TAAGAACCAC TACATCCCTT GAGTCCCTTA ACATTGGTAT GGACAAGGTC GACTTTTCCC ATGCCCTTGT   
  
  
- CAGTATCAAT GTCCCCTTAA AGCGTTATGT AAATTCCCTC ATTCTTCGTC CTAGTACAGA AATCAATCCG   
  
  
- GCCGTCTATA CAGACACTGA GGTATACCTT TAGGTTGTGA CATAAGTGAA GTTCCGTTAT TATTATCGAG   
  
  
- ACCAAACAGA CCAGTTGACA TACCGAGACT TTTCGTGTTC TAGTTTATAC ACTGGCTTAG AAAATTACCA   
  
  
- GGTTCAATAC CGGACTTTAA GAAACAACTA AGAGGTAGTC TTCTCGAGTA GGTGGGTAGT CTACGATGAG   
  
  
- GATTGGGTAA ACTGAGAAGA AAGCACCCGT ATTCTGTACT ACTCTCAGGA ATGGTTCCTT TAATACCTAG   
  
  
- TCTTATACCC CATTCGGGTA GAAGGCTACG TCAACTTATA CTACTTCCAT TATTCTATTC CGATTTTGAA   
  
  
- GTTCTTAACC TCGTACGGGA CGAGCTACTT CTACTACTTC ACCTACTCCA CCGATATCCC GGGCACGATC   
  
  
- TTGTGTCGTA CCTCGAACTA CTTCTCACCC AGCGGGGTAA CTCTTTACAC CAAAAGGTAC TAAGTGGTTT   
  
  
- CCTCAGGTGG AGGAGTCTCA GTAGGTTACA GTCGTCGTAA TCGTCGTGGT TTCTTCATAG TGGAACGAGA   
  
  
- GGCTCATGAG GGTTTGTTAA CGAGAAATTA ACACGTTTAT ACTAAAGTCT CCCCTTAAAA CTCCTTCGGC   
  
  
- ACTGATACTA GTCGCTCGAA TCTGTCTAAC AAAGATAGGT TCCCCTAGGT CGCGTCTCCT ATCGTCGGAT   
  
  
- GTACCATCTT CCGGAACGAA GGGCAAACCG AAGTAGCCCT TTTCAAGAAA TGTTTCGAAA CTTTACGTTT   
  
  
- CTCGGTGGAA GTAAACTGTC CGATCGCCGA TACGTTTAGG AGAAACTCCA CACGGGTACA AAGTTTAAGC   
  
  
- CTAAATACCG GCGTTTACGT CGCTAATACC TACGTTAGTT TTTGCTTTTT TCCCATGTGT AGTATCTAAA   
  
  
- ACTGTATTTG GTTCCCTTAG TTATGTATTT AGAGTACGTT AGGGAACGAT TTGTTCCATT GTTCGACTGC   
  
  
- GTGAACTTCA ACTGACCTCA ACTACTGGGA CTCAGTCAAG TTGCGGGATA ACCACCGGAT TTTTTGTAGC   
  
  
- CTGTTTCCGA CGTTCGTAAT CGACTTATAG AACCACACAG TAAACTCAAG TTTCGTTATG GACGATCTTG   
  
  
- AGGTGAACAA TTGGGCCTTT ACAATCTTAC AGCTGGACGC CTTCGAAATC ACCACTTGAA ACGGAAGGTC   
  
  
- GAAGTGGTGT ACGGACTACT TTCGCAGAGT TGTTAGTTGG AGGCTCTGGT CGAAGAAGCC TACCAGTTTT   
  
  
- CGGATTTGGG TTTCGATCAT TGGTAACAGC TCGTCCTACA CTTGTGTTTG TGATGGGGAA AGGAGAGATC   
  
  
- CAAACGACTC CGTATGTCGA TGATAAGACG ACAAAAACTC AGAGAACTAC GATGAGAGGG ATCTCTATCG   
  
  
- GTCCTGTCCC ACTTACACCT CTTCGTCACG AACCGTGCAC TACAACACTT GTAGTAACGT ACACTTCCCC   
  
  
- TCCTCTCCTA ACTCGCGATA CTCCAACGCC CCTTTACCTC TCGCTCCTAC AACTACCGCC CTAAGTCCCG   
  
  
- ATAAGGTTAC CCAGTCTTCT GGCCACTATA CTAAGCCTTC GAATATAGTT ACGCCATAAC ACTGTCCGAG   
  
  
- TTCCATTTTC TCCTCTACCT ACGTGAAGTG AAACCCACCC TTCTATTTTT GAATTAGCAA TGTAGTCGAA   
  
  
- CCTCCAC

+     CAAT-box

| Site Name | Organism | Position | Strand | Matrix score. | sequence | function |
| --- | --- | --- | --- | --- | --- | --- |
| CAAT-box | Nicotiana glutinosa | 1616 | + | 4 | CAAT |  |
| CAAT-box | Nicotiana glutinosa | 2691 | - | 4 | CAAT |  |
| CAAT-box | Nicotiana glutinosa | 3622 | + | 4 | CAAT |  |
| CAAT-box | Nicotiana glutinosa | 3631 | - | 4 | CAAT |  |
| CAAT-box | Pisum sativum | 2816 | - | 5 | CAAAT | common cis-acting element in promoter and enhancer regions |
| CAAT-box | Nicotiana glutinosa | 3513 | - | 4 | CAAT |  |
| CAAT-box | Nicotiana glutinosa | 3256 | + | 4 | CAAT |  |
| CAAT-box | Pisum sativum | 2629 | + | 5 | CAAAT | common cis-acting element in promoter and enhancer regions |
| CAAT-box | Nicotiana glutinosa | 2090 | + | 4 | CAAT |  |
| CAAT-box | Nicotiana glutinosa | 2623 | - | 4 | CAAT |  |
| CAAT-box | Nicotiana glutinosa | 2612 | - | 4 | CAAT |  |
| CAAT-box | Nicotiana glutinosa | 2610 | + | 4 | CAAT |  |
| CAAT-box | Nicotiana glutinosa | 2549 | + | 4 | CAAT |  |
| CAAT-box | Arabidopsis thaliana | 2548 | + | 5 | CCAAT | common cis-acting element in promoter and enhancer regions |
| CAAT-box | Nicotiana glutinosa | 458 | - | 4 | CAAT |  |
| CAAT-box | Arabidopsis thaliana | 2390 | - | 5 | CCAAT | common cis-acting element in promoter and enhancer regions |
| CAAT-box | Nicotiana glutinosa | 2492 | - | 4 | CAAT |  |
| CAAT-box | Nicotiana glutinosa | 3489 | - | 4 | CAAT |  |
| CAAT-box | Nicotiana glutinosa | 309 | + | 4 | CAAT |  |
| CAAT-box | Nicotiana glutinosa | 1381 | + | 4 | CAAT |  |
| CAAT-box | Nicotiana glutinosa | 2982 | + | 4 | CAAT |  |
| CAAT-box | Pisum sativum | 625 | + | 5 | CAAAT | common cis-acting element in promoter and enhancer regions |
| CAAT-box | Nicotiana glutinosa | 188 | - | 4 | CAAT |  |
| CAAT-box | Nicotiana glutinosa | 1008 | - | 4 | CAAT |  |
| CAAT-box | Pisum sativum | 2147 | + | 5 | CAAAT | common cis-acting element in promoter and enhancer regions |
| CAAT-box | Arabidopsis thaliana | 1367 | - | 5 | CCAAT | common cis-acting element in promoter and enhancer regions |
| CAAT-box | Nicotiana glutinosa | 1468 | + | 4 | CAAT |  |
| CAAT-box | Nicotiana glutinosa | 809 | + | 4 | CAAT |  |
| CAAT-box | Arabidopsis thaliana | 1380 | + | 5 | CCAAT | common cis-acting element in promoter and enhancer regions |
| CAAT-box | Nicotiana glutinosa | 1118 | - | 4 | CAAT |  |
| CAAT-box | Nicotiana glutinosa | 1076 | - | 4 | CAAT |  |
| CAAT-box | Nicotiana glutinosa | 662 | - | 4 | CAAT |  |
| CAAT-box | Nicotiana glutinosa | 787 | + | 4 | CAAT |  |
| CAAT-box | Nicotiana glutinosa | 1355 | + | 4 | CAAT |  |
| CAAT-box | Arabidopsis thaliana | 65 | - | 5 | CCAAT | common cis-acting element in promoter and enhancer regions |
| CAAT-box | Nicotiana glutinosa | 1470 | - | 4 | CAAT |  |
| CAAT-box | Pisum sativum | 202 | - | 5 | CAAAT | common cis-acting element in promoter and enhancer regions |
| CAAT-box | Nicotiana glutinosa | 1029 | - | 4 | CAAT |  |
| CAAT-box | Nicotiana glutinosa | 168 | + | 4 | CAAT |  |
| CAAT-box | Arabidopsis thaliana | 808 | + | 5 | CCAAT | common cis-acting element in promoter and enhancer regions |
| CAAT-box | Nicotiana glutinosa | 658 | - | 4 | CAAT |  |
| CAAT-box | Arabidopsis thaliana | 1467 | + | 5 | CCAAT | common cis-acting element in promoter and enhancer regions |
| CAAT-box | Arabidopsis thaliana | 284 | + | 5 | CCAAT | common cis-acting element in promoter and enhancer regions |
| CAAT-box | Nicotiana glutinosa | 355 | + | 4 | CAAT |  |
| CAAT-box | Nicotiana glutinosa | 1499 | + | 4 | CAAT |  |
| CAAT-box | Arabidopsis thaliana | 170 | - | 5 | CCAAT | common cis-acting element in promoter and enhancer regions |
| CAAT-box | Nicotiana glutinosa | 525 | + | 4 | CAAT |  |
| CAAT-box | Nicotiana glutinosa | 2908 | + | 4 | CAAT |  |
| CAAT-box | Arabidopsis thaliana | 167 | + | 5 | CCAAT | common cis-acting element in promoter and enhancer regions |
| CAAT-box | Nicotiana glutinosa | 1988 | + | 4 | CAAT |  |
| CAAT-box | Nicotiana glutinosa | 1226 | + | 4 | CAAT |  |
| CAAT-box | Nicotiana glutinosa | 1195 | + | 4 | CAAT |  |
| CAAT-box | Nicotiana glutinosa | 620 | + | 4 | CAAT |  |
| CAAT-box | Nicotiana glutinosa | 1923 | - | 4 | CAAT |  |
| CAAT-box | Nicotiana glutinosa | 285 | + | 4 | CAAT |  |
| CAAT-box | Nicotiana glutinosa | 1834 | - | 4 | CAAT |  |
| CAAT-box | Pisum sativum | 2252 | - | 5 | CAAAT | common cis-acting element in promoter and enhancer regions |
| CAAT-box | Arabidopsis thaliana | 807 | + | 8 | CCCAATTT | common cis-acting element in promoter and enhancer regions |
| CAAT-box | Pisum sativum | 143 | - | 5 | CAAAT | common cis-acting element in promoter and enhancer regions |
| CAAT-box | Nicotiana glutinosa | 3580 | + | 4 | CAAT |  |
| CAAT-box | Nicotiana glutinosa | 981 | + | 4 | CAAT |  |
| CAAT-box | Nicotiana glutinosa | 1824 | - | 4 | CAAT |  |
| CAAT-box | Nicotiana glutinosa | 334 | - | 4 | CAAT |  |
| CAAT-box | Nicotiana glutinosa | 2964 | + | 4 | CAAT |  |
| CAAT-box | Pisum sativum | 1870 | - | 5 | CAAAT | common cis-acting element in promoter and enhancer regions |
| CAAT-box | Nicotiana glutinosa | 505 | + | 4 | CAAT |  |
| CAAT-box | Nicotiana glutinosa | 3318 | - | 4 | CAAT |  |
| CAAT-box | Pisum sativum | 2887 | + | 5 | CAAAT | common cis-acting element in promoter and enhancer regions |
| CAAT-box | Nicotiana glutinosa | 1829 | - | 4 | CAAT |  |
| CAAT-box | Arabidopsis thaliana | 3579 | + | 5 | CCAAT | common cis-acting element in promoter and enhancer regions |
| CAAT-box | Pisum sativum | 2867 | + | 5 | CAAAT | common cis-acting element in promoter and enhancer regions |
| CAAT-box | Nicotiana glutinosa | 3139 | + | 4 | CAAT |  |
| CAAT-box | Arabidopsis thaliana | 3063 | - | 5 | CCAAT | common cis-acting element in promoter and enhancer regions |
| CAAT-box | Pisum sativum | 2838 | + | 5 | CAAAT | common cis-acting element in promoter and enhancer regions |
| CAAT-box | Pisum sativum | 3125 | - | 5 | CAAAT | common cis-acting element in promoter and enhancer regions |

>HU11G01134.1   
+ +Up\_Stream \_Len000AGATAG ACATTCAGCC ACTTGCCAGT TCAGAGAGAG ATAGAGAGAG ATTGGGTAAG   
  
  
+ CCCTTTTTGC ACTACTTTTT CTCGCTTCCA TTTTTCTTTC TTAATTTTTA GTGATGATTT TCCAGAAGAT   
  
  
+ TTGATTTATT CATCATGCTT AACCAATTGG GTCTTTGAGT ATTATTGTTT ACTGGGTATT TGATCTTTTC   
  
  
+ ATATTCCTTT GGACTGGGTG GGTGGAACTT TTGAACTTTC TTATTAGTTG GGCATGCCCT TATCTTATTC   
  
  
+ CAATTCAAAA CCCATATTTT TCTTCAATTT TAATTATGGG TATTTTGAAA TTGAATGCTT TCCTTCACAG   
  
  
+ CAATACTATA TTACATATTT TACAGGAAAG TAATATTTTA CAGGAAAGTA TGTTGCTGTT TCAACCCTGT   
  
  
+ TTTGTTTCCT CTCTTTCCTT GGATATGATG TTCATTGCTC TTGTACCAAA CCTAACCTCA ACTTCCTGAA   
  
  
+ TTATTTAAGG CAATATTACC GTGTGCTGGT CAATTTTGGT TGTGGGTTTG TTTCTTAATG TATATGATTT   
  
  
+ ATGGTCAGGA TCCATGCTCC TCTTCCGTCC ACTCTATATC TCTCTCTCTC TCTCTCAATA CAAATTCAGA   
  
  
+ GACTTTCTCT TTCTGGGTTT ACTATTGATT GCAGAAGTTT GTAGTCTTGC GAGGCCTTGA GTCTTCTTCG   
  
  
+ AGGTAGAAAA TTTTGCTTTG CGCTTCCTTC CCCATATTTT AACACCCCTC CCCCCCCAAC GCAAGTTTTC   
  
  
+ TACATTTTTG TGCAATTCTT CAGCTATGAT TCCCCAATTT CACTTGTCAC ATCTCTATTT TCCTCTTTTT   
  
  
+ GGTTTCTTGA TTCTGTAGAA GGTTGTGTAT GAACTTCTTT GTTTCCCTTT TTCCTTTGTT ATTTTTAATG   
  
  
+ TTCCCATAGA TGATATAGAG ATCTGCTTTT CCTTGCTGTG AGAGGTATCT CTGAGAATGC ATGTAGCAAT   
  
  
+ CAAAATTCTG AGTTGTTAAT TCTATTGCAT GTCTATGGGT GATGATTGTG ATGATGTTTT TTCTGTTGCC   
  
  
+ CTCTTTCTCT CTTTCACTGA AATTGCTTTC CAGAATAGTT GTTTGACTCC TTTCTGTATC TTAATTGAGC   
  
  
+ CCTTGCTTTG GTTTGTGCTC TGGGAGTTCT TCTTTAGTAA TCCCTTGCTT GTATTTTCTG AAAGAGCAAG   
  
  
+ CAATAATCCT CATAGCATGT GTATAATCAT ACAATAGCAA CAGCAAACAT GGATCCTGCG CCCAGTTAGG   
  
  
+ TTTGGTTAGG TTAAGATATG CATATTCTTT TCTCTTGAAA AGAGTTGTCT AGGAAGGGTA ATTATGCCCA   
  
  
+ TTTCATGGAT AATAATGGTA CAATTAAGTT TGATTGGCTT AAATACCAAT TATTTAGCGT CTTTTTCTTT   
  
  
+ GGCTGTCCAG GGTTCCATTT CCATAAACAA CTTTGAGTAA GGCATAGAGA ACTAAATTTC TGCCAATTGT   
  
  
+ CAAGTGCAAG TTGCTCATAT GCAACAATAC TTGGAAATTT TGAGCTGAGC TGCTGGCATA TCATGAATAG   
  
  
+ TTCTAATGGT GGCGAGTTAT CACTTATCGT CCCTTGTGTG ACGAATTTCT CTTCCTAGGA AGCCCTCTAG   
  
  
+ TCAATGCCTA TCTGCTGTTG TGGAGTGAAA ATGGATACAT TTAGATCTCT ATAAACCCTA CTTTGGCAAG   
  
  
+ ATCCATGCTG ATTTTAGGAA GCCCTTTGAC CAGAGAATGA CATAACAACC TGATAGGGTG TTACATGCTA   
  
  
+ ACTTCGATTA TTTAATCAAA ATACGGAACT TCTATCAGTC AAAGCCTGCC CCAGGTTCTA CTGAATTAAA   
  
  
+ TTGTATTGAA TTGCTCTCAC TTGCTTATGA CGATTAAATA ACTGGATTTG CTAATTATTC TTTTTATTTT   
  
  
+ ATTCTTGGTG ATGTAGGGAA CTCAGGGAAT TGTAACCATA CCTGTTCCAG CTGAAAAGGG TACGGGAACA   
  
  
+ GTCATAGTTA CAGGGGAATT TCGCAATACA TTTAAGGGAG TAAGAAGCAG GATCATGTCT TTAGTTAGGC   
  
  
+ CGGCAGATAT GTCTGTGACT CCATATGGAA ATCCAACACT GTATTCACTT CAAGGCAATA ATAATAGCTC   
  
  
+ TGGTTTGTCT GGTCAACTGT ATGGCTCTGA AAAGCACAAG ATCAAATATG TGACCGAATC TTTTAATGGT   
  
  
+ CCAAGTTATG GCCTGAAATT CTTTGTTGAT TCTCCATCAG AAGAGCTCAT CCACCCATCA GATGCTACTC   
  
  
+ CTAACCCATT TGACTCTTCT TTCGTGGGCA TAAGACATGA TGAGAGTCCT TACCAAGGAA ATTATGGATC   
  
  
+ AGAATATGGG GTAAGCCCAT CTTCCGATGC AGTTGAATAT GATGAAGGTA ATAAGATAAG GCTAAAACTT   
  
  
+ CAAGAATTGG AGCATGCCCT GCTCGATGAA GATGATGAAG TGGATGAGGT GGCTATAGGG CCCGTGCTAG   
  
  
+ AACACAGCAT GGAGCTTGAT GAAGAGTGGG TCGCCCCATT GAGAAATGTG GTTTTCCATG ATTCACCAAA   
  
  
+ GGAGTCCACC TCCTCAGAGT CATCCAATGT CAGCAGCATT AGCAGCACCA AAGAAGTATC ACCTTGCTCT   
  
  
+ CCGAGTACTC CCAAACAATT GCTCTTTAAT TGTGCAAATA TGATTTCAGA GGGGAATTTT GAGGAAGCCG   
  
  
+ TGACTATGAT CAGCGAGCTT AGACAGATTG TTTCTATCCA AGGGGATCCA GCGCAGAGGA TAGCAGCCTA   
  
  
+ CATGGTAGAA GGCCTTGCTT CCCGTTTGGC TTCATCGGGA AAAGTTCTTT ACAAAGCTTT GAAATGCAAA   
  
  
+ GAGCCACCTT CATTTGACAG GCTAGCGGCT ATGCAAATCC TCTTTGAGGT GTGCCCATGT TTCAAATTCG   
  
  
+ GATTTATGGC CGCAAATGCA GCGATTATGG ATGCAATCAA AAACGAAAAA AGGGTACACA TCATAGATTT   
  
  
+ TGACATAAAC CAAGGGAATC AATACATAAA TCTCATGCAA TCCCTTGCTA AACAAGGTAA CAAGCTGACG   
  
  
+ CACTTGAAGT TGACTGGAGT TGATGACCCT GAGTCAGTTC AACGCCCTAT TGGTGGCCTA AAAAACATCG   
  
  
+ GACAAAGGCT GCAAGCATTA GCTGAATATC TTGGTGTGTC ATTTGAGTTC AAAGCAATAC CTGCTAGAAC   
  
  
+ TCCACTTGTT AACCCGGAAA TGTTAGAATG TCGACCTGCG GAAGCTTTAG TGGTGAACTT TGCCTTCCAG   
  
  
+ CTTCACCACA TGCCTGATGA AAGCGTCTCA ACAATCAACC TCCGAGACCA GCTTCTTCGG ATGGTCAAAA   
  
  
+ GCCTAAACCC AAAGCTAGTA ACCATTGTCG AGCAGGATGT GAACACAAAC ACTACCCCTT TCCTCTCTAG   
  
  
+ GTTTGCTGAG GCATACAGCT ACTATTCTGC TGTTTTTGAG TCTCTTGATG CTACTCTCCC TAGAGATAGC   
  
  
+ CAGGACAGGG TGAATGTGGA GAAGCAGTGC TTGGCACGTG ATGTTGTGAA CATCATTGCA TGTGAAGGGG   
  
  
+ AGGAGAGGAT TGAGCGCTAT GAGGTTGCGG GGAAATGGAG AGCGAGGATG TTGATGGCGG GATTCAGGGC   
  
  
+ TATTCCAATG GGTCAGAAGA CCGGTGATAT GATTCGGAAG CTTATATCAA TGCGGTATTG TGACAGGCTC   
  
  
+ AAGGTAAAAG AGGAGATGGA TGCACTTCAC TTTGGGTGGG AAGATAAAAA CTTAATCGTT ACATCAGCTT   
  
  
+ GGAGGTG  

- +Up\_Stream \_Len000TCTATC TGTAAGTCGG TGAACGGTCA AGTCTCTCTC TATCTCTCTC TAACCCATTC   
  
  
- GGGAAAAACG TGATGAAAAA GAGCGAAGGT AAAAAGAAAG AATTAAAAAT CACTACTAAA AGGTCTTCTA   
  
  
- AACTAAATAA GTAGTACGAA TTGGTTAACC CAGAAACTCA TAATAACAAA TGACCCATAA ACTAGAAAAG   
  
  
- TATAAGGAAA CCTGACCCAC CCACCTTGAA AACTTGAAAG AATAATCAAC CCGTACGGGA ATAGAATAAG   
  
  
- GTTAAGTTTT GGGTATAAAA AGAAGTTAAA ATTAATACCC ATAAAACTTT AACTTACGAA AGGAAGTGTC   
  
  
- GTTATGATAT AATGTATAAA ATGTCCTTTC ATTATAAAAT GTCCTTTCAT ACAACGACAA AGTTGGGACA   
  
  
- AAACAAAGGA GAGAAAGGAA CCTATACTAC AAGTAACGAG AACATGGTTT GGATTGGAGT TGAAGGACTT   
  
  
- AATAAATTCC GTTATAATGG CACACGACCA GTTAAAACCA ACACCCAAAC AAAGAATTAC ATATACTAAA   
  
  
- TACCAGTCCT AGGTACGAGG AGAAGGCAGG TGAGATATAG AGAGAGAGAG AGAGAGTTAT GTTTAAGTCT   
  
  
- CTGAAAGAGA AAGACCCAAA TGATAACTAA CGTCTTCAAA CATCAGAACG CTCCGGAACT CAGAAGAAGC   
  
  
- TCCATCTTTT AAAACGAAAC GCGAAGGAAG GGGTATAAAA TTGTGGGGAG GGGGGGGTTG CGTTCAAAAG   
  
  
- ATGTAAAAAC ACGTTAAGAA GTCGATACTA AGGGGTTAAA GTGAACAGTG TAGAGATAAA AGGAGAAAAA   
  
  
- CCAAAGAACT AAGACATCTT CCAACACATA CTTGAAGAAA CAAAGGGAAA AAGGAAACAA TAAAAATTAC   
  
  
- AAGGGTATCT ACTATATCTC TAGACGAAAA GGAACGACAC TCTCCATAGA GACTCTTACG TACATCGTTA   
  
  
- GTTTTAAGAC TCAACAATTA AGATAACGTA CAGATACCCA CTACTAACAC TACTACAAAA AAGACAACGG   
  
  
- GAGAAAGAGA GAAAGTGACT TTAACGAAAG GTCTTATCAA CAAACTGAGG AAAGACATAG AATTAACTCG   
  
  
- GGAACGAAAC CAAACACGAG ACCCTCAAGA AGAAATCATT AGGGAACGAA CATAAAAGAC TTTCTCGTTC   
  
  
- GTTATTAGGA GTATCGTACA CATATTAGTA TGTTATCGTT GTCGTTTGTA CCTAGGACGC GGGTCAATCC   
  
  
- AAACCAATCC AATTCTATAC GTATAAGAAA AGAGAACTTT TCTCAACAGA TCCTTCCCAT TAATACGGGT   
  
  
- AAAGTACCTA TTATTACCAT GTTAATTCAA ACTAACCGAA TTTATGGTTA ATAAATCGCA GAAAAAGAAA   
  
  
- CCGACAGGTC CCAAGGTAAA GGTATTTGTT GAAACTCATT CCGTATCTCT TGATTTAAAG ACGGTTAACA   
  
  
- GTTCACGTTC AACGAGTATA CGTTGTTATG AACCTTTAAA ACTCGACTCG ACGACCGTAT AGTACTTATC   
  
  
- AAGATTACCA CCGCTCAATA GTGAATAGCA GGGAACACAC TGCTTAAAGA GAAGGATCCT TCGGGAGATC   
  
  
- AGTTACGGAT AGACGACAAC ACCTCACTTT TACCTATGTA AATCTAGAGA TATTTGGGAT GAAACCGTTC   
  
  
- TAGGTACGAC TAAAATCCTT CGGGAAACTG GTCTCTTACT GTATTGTTGG ACTATCCCAC AATGTACGAT   
  
  
- TGAAGCTAAT AAATTAGTTT TATGCCTTGA AGATAGTCAG TTTCGGACGG GGTCCAAGAT GACTTAATTT   
  
  
- AACATAACTT AACGAGAGTG AACGAATACT GCTAATTTAT TGACCTAAAC GATTAATAAG AAAAATAAAA   
  
  
- TAAGAACCAC TACATCCCTT GAGTCCCTTA ACATTGGTAT GGACAAGGTC GACTTTTCCC ATGCCCTTGT   
  
  
- CAGTATCAAT GTCCCCTTAA AGCGTTATGT AAATTCCCTC ATTCTTCGTC CTAGTACAGA AATCAATCCG   
  
  
- GCCGTCTATA CAGACACTGA GGTATACCTT TAGGTTGTGA CATAAGTGAA GTTCCGTTAT TATTATCGAG   
  
  
- ACCAAACAGA CCAGTTGACA TACCGAGACT TTTCGTGTTC TAGTTTATAC ACTGGCTTAG AAAATTACCA   
  
  
- GGTTCAATAC CGGACTTTAA GAAACAACTA AGAGGTAGTC TTCTCGAGTA GGTGGGTAGT CTACGATGAG   
  
  
- GATTGGGTAA ACTGAGAAGA AAGCACCCGT ATTCTGTACT ACTCTCAGGA ATGGTTCCTT TAATACCTAG   
  
  
- TCTTATACCC CATTCGGGTA GAAGGCTACG TCAACTTATA CTACTTCCAT TATTCTATTC CGATTTTGAA   
  
  
- GTTCTTAACC TCGTACGGGA CGAGCTACTT CTACTACTTC ACCTACTCCA CCGATATCCC GGGCACGATC   
  
  
- TTGTGTCGTA CCTCGAACTA CTTCTCACCC AGCGGGGTAA CTCTTTACAC CAAAAGGTAC TAAGTGGTTT   
  
  
- CCTCAGGTGG AGGAGTCTCA GTAGGTTACA GTCGTCGTAA TCGTCGTGGT TTCTTCATAG TGGAACGAGA   
  
  
- GGCTCATGAG GGTTTGTTAA CGAGAAATTA ACACGTTTAT ACTAAAGTCT CCCCTTAAAA CTCCTTCGGC   
  
  
- ACTGATACTA GTCGCTCGAA TCTGTCTAAC AAAGATAGGT TCCCCTAGGT CGCGTCTCCT ATCGTCGGAT   
  
  
- GTACCATCTT CCGGAACGAA GGGCAAACCG AAGTAGCCCT TTTCAAGAAA TGTTTCGAAA CTTTACGTTT   
  
  
- CTCGGTGGAA GTAAACTGTC CGATCGCCGA TACGTTTAGG AGAAACTCCA CACGGGTACA AAGTTTAAGC   
  
  
- CTAAATACCG GCGTTTACGT CGCTAATACC TACGTTAGTT TTTGCTTTTT TCCCATGTGT AGTATCTAAA   
  
  
- ACTGTATTTG GTTCCCTTAG TTATGTATTT AGAGTACGTT AGGGAACGAT TTGTTCCATT GTTCGACTGC   
  
  
- GTGAACTTCA ACTGACCTCA ACTACTGGGA CTCAGTCAAG TTGCGGGATA ACCACCGGAT TTTTTGTAGC   
  
  
- CTGTTTCCGA CGTTCGTAAT CGACTTATAG AACCACACAG TAAACTCAAG TTTCGTTATG GACGATCTTG   
  
  
- AGGTGAACAA TTGGGCCTTT ACAATCTTAC AGCTGGACGC CTTCGAAATC ACCACTTGAA ACGGAAGGTC   
  
  
- GAAGTGGTGT ACGGACTACT TTCGCAGAGT TGTTAGTTGG AGGCTCTGGT CGAAGAAGCC TACCAGTTTT   
  
  
- CGGATTTGGG TTTCGATCAT TGGTAACAGC TCGTCCTACA CTTGTGTTTG TGATGGGGAA AGGAGAGATC   
  
  
- CAAACGACTC CGTATGTCGA TGATAAGACG ACAAAAACTC AGAGAACTAC GATGAGAGGG ATCTCTATCG   
  
  
- GTCCTGTCCC ACTTACACCT CTTCGTCACG AACCGTGCAC TACAACACTT GTAGTAACGT ACACTTCCCC   
  
  
- TCCTCTCCTA ACTCGCGATA CTCCAACGCC CCTTTACCTC TCGCTCCTAC AACTACCGCC CTAAGTCCCG   
  
  
- ATAAGGTTAC CCAGTCTTCT GGCCACTATA CTAAGCCTTC GAATATAGTT ACGCCATAAC ACTGTCCGAG   
  
  
- TTCCATTTTC TCCTCTACCT ACGTGAAGTG AAACCCACCC TTCTATTTTT GAATTAGCAA TGTAGTCGAA   
  
  
- CCTCCAC

+     CAT-box

| Site Name | Organism | Position | Strand | Matrix score. | sequence | function |
| --- | --- | --- | --- | --- | --- | --- |
| CAT-box | Arabidopsis thaliana | 32 | + | 6 | GCCACT | cis-acting regulatory element related to meristem expression |

>HU11G01134.1   
+ +Up\_Stream \_Len000AGATAG ACATTCAGCC ACTTGCCAGT TCAGAGAGAG ATAGAGAGAG ATTGGGTAAG   
  
  
+ CCCTTTTTGC ACTACTTTTT CTCGCTTCCA TTTTTCTTTC TTAATTTTTA GTGATGATTT TCCAGAAGAT   
  
  
+ TTGATTTATT CATCATGCTT AACCAATTGG GTCTTTGAGT ATTATTGTTT ACTGGGTATT TGATCTTTTC   
  
  
+ ATATTCCTTT GGACTGGGTG GGTGGAACTT TTGAACTTTC TTATTAGTTG GGCATGCCCT TATCTTATTC   
  
  
+ CAATTCAAAA CCCATATTTT TCTTCAATTT TAATTATGGG TATTTTGAAA TTGAATGCTT TCCTTCACAG   
  
  
+ CAATACTATA TTACATATTT TACAGGAAAG TAATATTTTA CAGGAAAGTA TGTTGCTGTT TCAACCCTGT   
  
  
+ TTTGTTTCCT CTCTTTCCTT GGATATGATG TTCATTGCTC TTGTACCAAA CCTAACCTCA ACTTCCTGAA   
  
  
+ TTATTTAAGG CAATATTACC GTGTGCTGGT CAATTTTGGT TGTGGGTTTG TTTCTTAATG TATATGATTT   
  
  
+ ATGGTCAGGA TCCATGCTCC TCTTCCGTCC ACTCTATATC TCTCTCTCTC TCTCTCAATA CAAATTCAGA   
  
  
+ GACTTTCTCT TTCTGGGTTT ACTATTGATT GCAGAAGTTT GTAGTCTTGC GAGGCCTTGA GTCTTCTTCG   
  
  
+ AGGTAGAAAA TTTTGCTTTG CGCTTCCTTC CCCATATTTT AACACCCCTC CCCCCCCAAC GCAAGTTTTC   
  
  
+ TACATTTTTG TGCAATTCTT CAGCTATGAT TCCCCAATTT CACTTGTCAC ATCTCTATTT TCCTCTTTTT   
  
  
+ GGTTTCTTGA TTCTGTAGAA GGTTGTGTAT GAACTTCTTT GTTTCCCTTT TTCCTTTGTT ATTTTTAATG   
  
  
+ TTCCCATAGA TGATATAGAG ATCTGCTTTT CCTTGCTGTG AGAGGTATCT CTGAGAATGC ATGTAGCAAT   
  
  
+ CAAAATTCTG AGTTGTTAAT TCTATTGCAT GTCTATGGGT GATGATTGTG ATGATGTTTT TTCTGTTGCC   
  
  
+ CTCTTTCTCT CTTTCACTGA AATTGCTTTC CAGAATAGTT GTTTGACTCC TTTCTGTATC TTAATTGAGC   
  
  
+ CCTTGCTTTG GTTTGTGCTC TGGGAGTTCT TCTTTAGTAA TCCCTTGCTT GTATTTTCTG AAAGAGCAAG   
  
  
+ CAATAATCCT CATAGCATGT GTATAATCAT ACAATAGCAA CAGCAAACAT GGATCCTGCG CCCAGTTAGG   
  
  
+ TTTGGTTAGG TTAAGATATG CATATTCTTT TCTCTTGAAA AGAGTTGTCT AGGAAGGGTA ATTATGCCCA   
  
  
+ TTTCATGGAT AATAATGGTA CAATTAAGTT TGATTGGCTT AAATACCAAT TATTTAGCGT CTTTTTCTTT   
  
  
+ GGCTGTCCAG GGTTCCATTT CCATAAACAA CTTTGAGTAA GGCATAGAGA ACTAAATTTC TGCCAATTGT   
  
  
+ CAAGTGCAAG TTGCTCATAT GCAACAATAC TTGGAAATTT TGAGCTGAGC TGCTGGCATA TCATGAATAG   
  
  
+ TTCTAATGGT GGCGAGTTAT CACTTATCGT CCCTTGTGTG ACGAATTTCT CTTCCTAGGA AGCCCTCTAG   
  
  
+ TCAATGCCTA TCTGCTGTTG TGGAGTGAAA ATGGATACAT TTAGATCTCT ATAAACCCTA CTTTGGCAAG   
  
  
+ ATCCATGCTG ATTTTAGGAA GCCCTTTGAC CAGAGAATGA CATAACAACC TGATAGGGTG TTACATGCTA   
  
  
+ ACTTCGATTA TTTAATCAAA ATACGGAACT TCTATCAGTC AAAGCCTGCC CCAGGTTCTA CTGAATTAAA   
  
  
+ TTGTATTGAA TTGCTCTCAC TTGCTTATGA CGATTAAATA ACTGGATTTG CTAATTATTC TTTTTATTTT   
  
  
+ ATTCTTGGTG ATGTAGGGAA CTCAGGGAAT TGTAACCATA CCTGTTCCAG CTGAAAAGGG TACGGGAACA   
  
  
+ GTCATAGTTA CAGGGGAATT TCGCAATACA TTTAAGGGAG TAAGAAGCAG GATCATGTCT TTAGTTAGGC   
  
  
+ CGGCAGATAT GTCTGTGACT CCATATGGAA ATCCAACACT GTATTCACTT CAAGGCAATA ATAATAGCTC   
  
  
+ TGGTTTGTCT GGTCAACTGT ATGGCTCTGA AAAGCACAAG ATCAAATATG TGACCGAATC TTTTAATGGT   
  
  
+ CCAAGTTATG GCCTGAAATT CTTTGTTGAT TCTCCATCAG AAGAGCTCAT CCACCCATCA GATGCTACTC   
  
  
+ CTAACCCATT TGACTCTTCT TTCGTGGGCA TAAGACATGA TGAGAGTCCT TACCAAGGAA ATTATGGATC   
  
  
+ AGAATATGGG GTAAGCCCAT CTTCCGATGC AGTTGAATAT GATGAAGGTA ATAAGATAAG GCTAAAACTT   
  
  
+ CAAGAATTGG AGCATGCCCT GCTCGATGAA GATGATGAAG TGGATGAGGT GGCTATAGGG CCCGTGCTAG   
  
  
+ AACACAGCAT GGAGCTTGAT GAAGAGTGGG TCGCCCCATT GAGAAATGTG GTTTTCCATG ATTCACCAAA   
  
  
+ GGAGTCCACC TCCTCAGAGT CATCCAATGT CAGCAGCATT AGCAGCACCA AAGAAGTATC ACCTTGCTCT   
  
  
+ CCGAGTACTC CCAAACAATT GCTCTTTAAT TGTGCAAATA TGATTTCAGA GGGGAATTTT GAGGAAGCCG   
  
  
+ TGACTATGAT CAGCGAGCTT AGACAGATTG TTTCTATCCA AGGGGATCCA GCGCAGAGGA TAGCAGCCTA   
  
  
+ CATGGTAGAA GGCCTTGCTT CCCGTTTGGC TTCATCGGGA AAAGTTCTTT ACAAAGCTTT GAAATGCAAA   
  
  
+ GAGCCACCTT CATTTGACAG GCTAGCGGCT ATGCAAATCC TCTTTGAGGT GTGCCCATGT TTCAAATTCG   
  
  
+ GATTTATGGC CGCAAATGCA GCGATTATGG ATGCAATCAA AAACGAAAAA AGGGTACACA TCATAGATTT   
  
  
+ TGACATAAAC CAAGGGAATC AATACATAAA TCTCATGCAA TCCCTTGCTA AACAAGGTAA CAAGCTGACG   
  
  
+ CACTTGAAGT TGACTGGAGT TGATGACCCT GAGTCAGTTC AACGCCCTAT TGGTGGCCTA AAAAACATCG   
  
  
+ GACAAAGGCT GCAAGCATTA GCTGAATATC TTGGTGTGTC ATTTGAGTTC AAAGCAATAC CTGCTAGAAC   
  
  
+ TCCACTTGTT AACCCGGAAA TGTTAGAATG TCGACCTGCG GAAGCTTTAG TGGTGAACTT TGCCTTCCAG   
  
  
+ CTTCACCACA TGCCTGATGA AAGCGTCTCA ACAATCAACC TCCGAGACCA GCTTCTTCGG ATGGTCAAAA   
  
  
+ GCCTAAACCC AAAGCTAGTA ACCATTGTCG AGCAGGATGT GAACACAAAC ACTACCCCTT TCCTCTCTAG   
  
  
+ GTTTGCTGAG GCATACAGCT ACTATTCTGC TGTTTTTGAG TCTCTTGATG CTACTCTCCC TAGAGATAGC   
  
  
+ CAGGACAGGG TGAATGTGGA GAAGCAGTGC TTGGCACGTG ATGTTGTGAA CATCATTGCA TGTGAAGGGG   
  
  
+ AGGAGAGGAT TGAGCGCTAT GAGGTTGCGG GGAAATGGAG AGCGAGGATG TTGATGGCGG GATTCAGGGC   
  
  
+ TATTCCAATG GGTCAGAAGA CCGGTGATAT GATTCGGAAG CTTATATCAA TGCGGTATTG TGACAGGCTC   
  
  
+ AAGGTAAAAG AGGAGATGGA TGCACTTCAC TTTGGGTGGG AAGATAAAAA CTTAATCGTT ACATCAGCTT   
  
  
+ GGAGGTG  

- +Up\_Stream \_Len000TCTATC TGTAAGTCGG TGAACGGTCA AGTCTCTCTC TATCTCTCTC TAACCCATTC   
  
  
- GGGAAAAACG TGATGAAAAA GAGCGAAGGT AAAAAGAAAG AATTAAAAAT CACTACTAAA AGGTCTTCTA   
  
  
- AACTAAATAA GTAGTACGAA TTGGTTAACC CAGAAACTCA TAATAACAAA TGACCCATAA ACTAGAAAAG   
  
  
- TATAAGGAAA CCTGACCCAC CCACCTTGAA AACTTGAAAG AATAATCAAC CCGTACGGGA ATAGAATAAG   
  
  
- GTTAAGTTTT GGGTATAAAA AGAAGTTAAA ATTAATACCC ATAAAACTTT AACTTACGAA AGGAAGTGTC   
  
  
- GTTATGATAT AATGTATAAA ATGTCCTTTC ATTATAAAAT GTCCTTTCAT ACAACGACAA AGTTGGGACA   
  
  
- AAACAAAGGA GAGAAAGGAA CCTATACTAC AAGTAACGAG AACATGGTTT GGATTGGAGT TGAAGGACTT   
  
  
- AATAAATTCC GTTATAATGG CACACGACCA GTTAAAACCA ACACCCAAAC AAAGAATTAC ATATACTAAA   
  
  
- TACCAGTCCT AGGTACGAGG AGAAGGCAGG TGAGATATAG AGAGAGAGAG AGAGAGTTAT GTTTAAGTCT   
  
  
- CTGAAAGAGA AAGACCCAAA TGATAACTAA CGTCTTCAAA CATCAGAACG CTCCGGAACT CAGAAGAAGC   
  
  
- TCCATCTTTT AAAACGAAAC GCGAAGGAAG GGGTATAAAA TTGTGGGGAG GGGGGGGTTG CGTTCAAAAG   
  
  
- ATGTAAAAAC ACGTTAAGAA GTCGATACTA AGGGGTTAAA GTGAACAGTG TAGAGATAAA AGGAGAAAAA   
  
  
- CCAAAGAACT AAGACATCTT CCAACACATA CTTGAAGAAA CAAAGGGAAA AAGGAAACAA TAAAAATTAC   
  
  
- AAGGGTATCT ACTATATCTC TAGACGAAAA GGAACGACAC TCTCCATAGA GACTCTTACG TACATCGTTA   
  
  
- GTTTTAAGAC TCAACAATTA AGATAACGTA CAGATACCCA CTACTAACAC TACTACAAAA AAGACAACGG   
  
  
- GAGAAAGAGA GAAAGTGACT TTAACGAAAG GTCTTATCAA CAAACTGAGG AAAGACATAG AATTAACTCG   
  
  
- GGAACGAAAC CAAACACGAG ACCCTCAAGA AGAAATCATT AGGGAACGAA CATAAAAGAC TTTCTCGTTC   
  
  
- GTTATTAGGA GTATCGTACA CATATTAGTA TGTTATCGTT GTCGTTTGTA CCTAGGACGC GGGTCAATCC   
  
  
- AAACCAATCC AATTCTATAC GTATAAGAAA AGAGAACTTT TCTCAACAGA TCCTTCCCAT TAATACGGGT   
  
  
- AAAGTACCTA TTATTACCAT GTTAATTCAA ACTAACCGAA TTTATGGTTA ATAAATCGCA GAAAAAGAAA   
  
  
- CCGACAGGTC CCAAGGTAAA GGTATTTGTT GAAACTCATT CCGTATCTCT TGATTTAAAG ACGGTTAACA   
  
  
- GTTCACGTTC AACGAGTATA CGTTGTTATG AACCTTTAAA ACTCGACTCG ACGACCGTAT AGTACTTATC   
  
  
- AAGATTACCA CCGCTCAATA GTGAATAGCA GGGAACACAC TGCTTAAAGA GAAGGATCCT TCGGGAGATC   
  
  
- AGTTACGGAT AGACGACAAC ACCTCACTTT TACCTATGTA AATCTAGAGA TATTTGGGAT GAAACCGTTC   
  
  
- TAGGTACGAC TAAAATCCTT CGGGAAACTG GTCTCTTACT GTATTGTTGG ACTATCCCAC AATGTACGAT   
  
  
- TGAAGCTAAT AAATTAGTTT TATGCCTTGA AGATAGTCAG TTTCGGACGG GGTCCAAGAT GACTTAATTT   
  
  
- AACATAACTT AACGAGAGTG AACGAATACT GCTAATTTAT TGACCTAAAC GATTAATAAG AAAAATAAAA   
  
  
- TAAGAACCAC TACATCCCTT GAGTCCCTTA ACATTGGTAT GGACAAGGTC GACTTTTCCC ATGCCCTTGT   
  
  
- CAGTATCAAT GTCCCCTTAA AGCGTTATGT AAATTCCCTC ATTCTTCGTC CTAGTACAGA AATCAATCCG   
  
  
- GCCGTCTATA CAGACACTGA GGTATACCTT TAGGTTGTGA CATAAGTGAA GTTCCGTTAT TATTATCGAG   
  
  
- ACCAAACAGA CCAGTTGACA TACCGAGACT TTTCGTGTTC TAGTTTATAC ACTGGCTTAG AAAATTACCA   
  
  
- GGTTCAATAC CGGACTTTAA GAAACAACTA AGAGGTAGTC TTCTCGAGTA GGTGGGTAGT CTACGATGAG   
  
  
- GATTGGGTAA ACTGAGAAGA AAGCACCCGT ATTCTGTACT ACTCTCAGGA ATGGTTCCTT TAATACCTAG   
  
  
- TCTTATACCC CATTCGGGTA GAAGGCTACG TCAACTTATA CTACTTCCAT TATTCTATTC CGATTTTGAA   
  
  
- GTTCTTAACC TCGTACGGGA CGAGCTACTT CTACTACTTC ACCTACTCCA CCGATATCCC GGGCACGATC   
  
  
- TTGTGTCGTA CCTCGAACTA CTTCTCACCC AGCGGGGTAA CTCTTTACAC CAAAAGGTAC TAAGTGGTTT   
  
  
- CCTCAGGTGG AGGAGTCTCA GTAGGTTACA GTCGTCGTAA TCGTCGTGGT TTCTTCATAG TGGAACGAGA   
  
  
- GGCTCATGAG GGTTTGTTAA CGAGAAATTA ACACGTTTAT ACTAAAGTCT CCCCTTAAAA CTCCTTCGGC   
  
  
- ACTGATACTA GTCGCTCGAA TCTGTCTAAC AAAGATAGGT TCCCCTAGGT CGCGTCTCCT ATCGTCGGAT   
  
  
- GTACCATCTT CCGGAACGAA GGGCAAACCG AAGTAGCCCT TTTCAAGAAA TGTTTCGAAA CTTTACGTTT   
  
  
- CTCGGTGGAA GTAAACTGTC CGATCGCCGA TACGTTTAGG AGAAACTCCA CACGGGTACA AAGTTTAAGC   
  
  
- CTAAATACCG GCGTTTACGT CGCTAATACC TACGTTAGTT TTTGCTTTTT TCCCATGTGT AGTATCTAAA   
  
  
- ACTGTATTTG GTTCCCTTAG TTATGTATTT AGAGTACGTT AGGGAACGAT TTGTTCCATT GTTCGACTGC   
  
  
- GTGAACTTCA ACTGACCTCA ACTACTGGGA CTCAGTCAAG TTGCGGGATA ACCACCGGAT TTTTTGTAGC   
  
  
- CTGTTTCCGA CGTTCGTAAT CGACTTATAG AACCACACAG TAAACTCAAG TTTCGTTATG GACGATCTTG   
  
  
- AGGTGAACAA TTGGGCCTTT ACAATCTTAC AGCTGGACGC CTTCGAAATC ACCACTTGAA ACGGAAGGTC   
  
  
- GAAGTGGTGT ACGGACTACT TTCGCAGAGT TGTTAGTTGG AGGCTCTGGT CGAAGAAGCC TACCAGTTTT   
  
  
- CGGATTTGGG TTTCGATCAT TGGTAACAGC TCGTCCTACA CTTGTGTTTG TGATGGGGAA AGGAGAGATC   
  
  
- CAAACGACTC CGTATGTCGA TGATAAGACG ACAAAAACTC AGAGAACTAC GATGAGAGGG ATCTCTATCG   
  
  
- GTCCTGTCCC ACTTACACCT CTTCGTCACG AACCGTGCAC TACAACACTT GTAGTAACGT ACACTTCCCC   
  
  
- TCCTCTCCTA ACTCGCGATA CTCCAACGCC CCTTTACCTC TCGCTCCTAC AACTACCGCC CTAAGTCCCG   
  
  
- ATAAGGTTAC CCAGTCTTCT GGCCACTATA CTAAGCCTTC GAATATAGTT ACGCCATAAC ACTGTCCGAG   
  
  
- TTCCATTTTC TCCTCTACCT ACGTGAAGTG AAACCCACCC TTCTATTTTT GAATTAGCAA TGTAGTCGAA   
  
  
- CCTCCAC

+     CCGTCC motif

| Site Name | Organism | Position | Strand | Matrix score. | sequence | function |
| --- | --- | --- | --- | --- | --- | --- |
| CCGTCC motif | Nicotiana tabacum | 589 | + | 6 | CCGTCC |  |

>HU11G01134.1   
+ +Up\_Stream \_Len000AGATAG ACATTCAGCC ACTTGCCAGT TCAGAGAGAG ATAGAGAGAG ATTGGGTAAG   
  
  
+ CCCTTTTTGC ACTACTTTTT CTCGCTTCCA TTTTTCTTTC TTAATTTTTA GTGATGATTT TCCAGAAGAT   
  
  
+ TTGATTTATT CATCATGCTT AACCAATTGG GTCTTTGAGT ATTATTGTTT ACTGGGTATT TGATCTTTTC   
  
  
+ ATATTCCTTT GGACTGGGTG GGTGGAACTT TTGAACTTTC TTATTAGTTG GGCATGCCCT TATCTTATTC   
  
  
+ CAATTCAAAA CCCATATTTT TCTTCAATTT TAATTATGGG TATTTTGAAA TTGAATGCTT TCCTTCACAG   
  
  
+ CAATACTATA TTACATATTT TACAGGAAAG TAATATTTTA CAGGAAAGTA TGTTGCTGTT TCAACCCTGT   
  
  
+ TTTGTTTCCT CTCTTTCCTT GGATATGATG TTCATTGCTC TTGTACCAAA CCTAACCTCA ACTTCCTGAA   
  
  
+ TTATTTAAGG CAATATTACC GTGTGCTGGT CAATTTTGGT TGTGGGTTTG TTTCTTAATG TATATGATTT   
  
  
+ ATGGTCAGGA TCCATGCTCC TCTTCCGTCC ACTCTATATC TCTCTCTCTC TCTCTCAATA CAAATTCAGA   
  
  
+ GACTTTCTCT TTCTGGGTTT ACTATTGATT GCAGAAGTTT GTAGTCTTGC GAGGCCTTGA GTCTTCTTCG   
  
  
+ AGGTAGAAAA TTTTGCTTTG CGCTTCCTTC CCCATATTTT AACACCCCTC CCCCCCCAAC GCAAGTTTTC   
  
  
+ TACATTTTTG TGCAATTCTT CAGCTATGAT TCCCCAATTT CACTTGTCAC ATCTCTATTT TCCTCTTTTT   
  
  
+ GGTTTCTTGA TTCTGTAGAA GGTTGTGTAT GAACTTCTTT GTTTCCCTTT TTCCTTTGTT ATTTTTAATG   
  
  
+ TTCCCATAGA TGATATAGAG ATCTGCTTTT CCTTGCTGTG AGAGGTATCT CTGAGAATGC ATGTAGCAAT   
  
  
+ CAAAATTCTG AGTTGTTAAT TCTATTGCAT GTCTATGGGT GATGATTGTG ATGATGTTTT TTCTGTTGCC   
  
  
+ CTCTTTCTCT CTTTCACTGA AATTGCTTTC CAGAATAGTT GTTTGACTCC TTTCTGTATC TTAATTGAGC   
  
  
+ CCTTGCTTTG GTTTGTGCTC TGGGAGTTCT TCTTTAGTAA TCCCTTGCTT GTATTTTCTG AAAGAGCAAG   
  
  
+ CAATAATCCT CATAGCATGT GTATAATCAT ACAATAGCAA CAGCAAACAT GGATCCTGCG CCCAGTTAGG   
  
  
+ TTTGGTTAGG TTAAGATATG CATATTCTTT TCTCTTGAAA AGAGTTGTCT AGGAAGGGTA ATTATGCCCA   
  
  
+ TTTCATGGAT AATAATGGTA CAATTAAGTT TGATTGGCTT AAATACCAAT TATTTAGCGT CTTTTTCTTT   
  
  
+ GGCTGTCCAG GGTTCCATTT CCATAAACAA CTTTGAGTAA GGCATAGAGA ACTAAATTTC TGCCAATTGT   
  
  
+ CAAGTGCAAG TTGCTCATAT GCAACAATAC TTGGAAATTT TGAGCTGAGC TGCTGGCATA TCATGAATAG   
  
  
+ TTCTAATGGT GGCGAGTTAT CACTTATCGT CCCTTGTGTG ACGAATTTCT CTTCCTAGGA AGCCCTCTAG   
  
  
+ TCAATGCCTA TCTGCTGTTG TGGAGTGAAA ATGGATACAT TTAGATCTCT ATAAACCCTA CTTTGGCAAG   
  
  
+ ATCCATGCTG ATTTTAGGAA GCCCTTTGAC CAGAGAATGA CATAACAACC TGATAGGGTG TTACATGCTA   
  
  
+ ACTTCGATTA TTTAATCAAA ATACGGAACT TCTATCAGTC AAAGCCTGCC CCAGGTTCTA CTGAATTAAA   
  
  
+ TTGTATTGAA TTGCTCTCAC TTGCTTATGA CGATTAAATA ACTGGATTTG CTAATTATTC TTTTTATTTT   
  
  
+ ATTCTTGGTG ATGTAGGGAA CTCAGGGAAT TGTAACCATA CCTGTTCCAG CTGAAAAGGG TACGGGAACA   
  
  
+ GTCATAGTTA CAGGGGAATT TCGCAATACA TTTAAGGGAG TAAGAAGCAG GATCATGTCT TTAGTTAGGC   
  
  
+ CGGCAGATAT GTCTGTGACT CCATATGGAA ATCCAACACT GTATTCACTT CAAGGCAATA ATAATAGCTC   
  
  
+ TGGTTTGTCT GGTCAACTGT ATGGCTCTGA AAAGCACAAG ATCAAATATG TGACCGAATC TTTTAATGGT   
  
  
+ CCAAGTTATG GCCTGAAATT CTTTGTTGAT TCTCCATCAG AAGAGCTCAT CCACCCATCA GATGCTACTC   
  
  
+ CTAACCCATT TGACTCTTCT TTCGTGGGCA TAAGACATGA TGAGAGTCCT TACCAAGGAA ATTATGGATC   
  
  
+ AGAATATGGG GTAAGCCCAT CTTCCGATGC AGTTGAATAT GATGAAGGTA ATAAGATAAG GCTAAAACTT   
  
  
+ CAAGAATTGG AGCATGCCCT GCTCGATGAA GATGATGAAG TGGATGAGGT GGCTATAGGG CCCGTGCTAG   
  
  
+ AACACAGCAT GGAGCTTGAT GAAGAGTGGG TCGCCCCATT GAGAAATGTG GTTTTCCATG ATTCACCAAA   
  
  
+ GGAGTCCACC TCCTCAGAGT CATCCAATGT CAGCAGCATT AGCAGCACCA AAGAAGTATC ACCTTGCTCT   
  
  
+ CCGAGTACTC CCAAACAATT GCTCTTTAAT TGTGCAAATA TGATTTCAGA GGGGAATTTT GAGGAAGCCG   
  
  
+ TGACTATGAT CAGCGAGCTT AGACAGATTG TTTCTATCCA AGGGGATCCA GCGCAGAGGA TAGCAGCCTA   
  
  
+ CATGGTAGAA GGCCTTGCTT CCCGTTTGGC TTCATCGGGA AAAGTTCTTT ACAAAGCTTT GAAATGCAAA   
  
  
+ GAGCCACCTT CATTTGACAG GCTAGCGGCT ATGCAAATCC TCTTTGAGGT GTGCCCATGT TTCAAATTCG   
  
  
+ GATTTATGGC CGCAAATGCA GCGATTATGG ATGCAATCAA AAACGAAAAA AGGGTACACA TCATAGATTT   
  
  
+ TGACATAAAC CAAGGGAATC AATACATAAA TCTCATGCAA TCCCTTGCTA AACAAGGTAA CAAGCTGACG   
  
  
+ CACTTGAAGT TGACTGGAGT TGATGACCCT GAGTCAGTTC AACGCCCTAT TGGTGGCCTA AAAAACATCG   
  
  
+ GACAAAGGCT GCAAGCATTA GCTGAATATC TTGGTGTGTC ATTTGAGTTC AAAGCAATAC CTGCTAGAAC   
  
  
+ TCCACTTGTT AACCCGGAAA TGTTAGAATG TCGACCTGCG GAAGCTTTAG TGGTGAACTT TGCCTTCCAG   
  
  
+ CTTCACCACA TGCCTGATGA AAGCGTCTCA ACAATCAACC TCCGAGACCA GCTTCTTCGG ATGGTCAAAA   
  
  
+ GCCTAAACCC AAAGCTAGTA ACCATTGTCG AGCAGGATGT GAACACAAAC ACTACCCCTT TCCTCTCTAG   
  
  
+ GTTTGCTGAG GCATACAGCT ACTATTCTGC TGTTTTTGAG TCTCTTGATG CTACTCTCCC TAGAGATAGC   
  
  
+ CAGGACAGGG TGAATGTGGA GAAGCAGTGC TTGGCACGTG ATGTTGTGAA CATCATTGCA TGTGAAGGGG   
  
  
+ AGGAGAGGAT TGAGCGCTAT GAGGTTGCGG GGAAATGGAG AGCGAGGATG TTGATGGCGG GATTCAGGGC   
  
  
+ TATTCCAATG GGTCAGAAGA CCGGTGATAT GATTCGGAAG CTTATATCAA TGCGGTATTG TGACAGGCTC   
  
  
+ AAGGTAAAAG AGGAGATGGA TGCACTTCAC TTTGGGTGGG AAGATAAAAA CTTAATCGTT ACATCAGCTT   
  
  
+ GGAGGTG  

- +Up\_Stream \_Len000TCTATC TGTAAGTCGG TGAACGGTCA AGTCTCTCTC TATCTCTCTC TAACCCATTC   
  
  
- GGGAAAAACG TGATGAAAAA GAGCGAAGGT AAAAAGAAAG AATTAAAAAT CACTACTAAA AGGTCTTCTA   
  
  
- AACTAAATAA GTAGTACGAA TTGGTTAACC CAGAAACTCA TAATAACAAA TGACCCATAA ACTAGAAAAG   
  
  
- TATAAGGAAA CCTGACCCAC CCACCTTGAA AACTTGAAAG AATAATCAAC CCGTACGGGA ATAGAATAAG   
  
  
- GTTAAGTTTT GGGTATAAAA AGAAGTTAAA ATTAATACCC ATAAAACTTT AACTTACGAA AGGAAGTGTC   
  
  
- GTTATGATAT AATGTATAAA ATGTCCTTTC ATTATAAAAT GTCCTTTCAT ACAACGACAA AGTTGGGACA   
  
  
- AAACAAAGGA GAGAAAGGAA CCTATACTAC AAGTAACGAG AACATGGTTT GGATTGGAGT TGAAGGACTT   
  
  
- AATAAATTCC GTTATAATGG CACACGACCA GTTAAAACCA ACACCCAAAC AAAGAATTAC ATATACTAAA   
  
  
- TACCAGTCCT AGGTACGAGG AGAAGGCAGG TGAGATATAG AGAGAGAGAG AGAGAGTTAT GTTTAAGTCT   
  
  
- CTGAAAGAGA AAGACCCAAA TGATAACTAA CGTCTTCAAA CATCAGAACG CTCCGGAACT CAGAAGAAGC   
  
  
- TCCATCTTTT AAAACGAAAC GCGAAGGAAG GGGTATAAAA TTGTGGGGAG GGGGGGGTTG CGTTCAAAAG   
  
  
- ATGTAAAAAC ACGTTAAGAA GTCGATACTA AGGGGTTAAA GTGAACAGTG TAGAGATAAA AGGAGAAAAA   
  
  
- CCAAAGAACT AAGACATCTT CCAACACATA CTTGAAGAAA CAAAGGGAAA AAGGAAACAA TAAAAATTAC   
  
  
- AAGGGTATCT ACTATATCTC TAGACGAAAA GGAACGACAC TCTCCATAGA GACTCTTACG TACATCGTTA   
  
  
- GTTTTAAGAC TCAACAATTA AGATAACGTA CAGATACCCA CTACTAACAC TACTACAAAA AAGACAACGG   
  
  
- GAGAAAGAGA GAAAGTGACT TTAACGAAAG GTCTTATCAA CAAACTGAGG AAAGACATAG AATTAACTCG   
  
  
- GGAACGAAAC CAAACACGAG ACCCTCAAGA AGAAATCATT AGGGAACGAA CATAAAAGAC TTTCTCGTTC   
  
  
- GTTATTAGGA GTATCGTACA CATATTAGTA TGTTATCGTT GTCGTTTGTA CCTAGGACGC GGGTCAATCC   
  
  
- AAACCAATCC AATTCTATAC GTATAAGAAA AGAGAACTTT TCTCAACAGA TCCTTCCCAT TAATACGGGT   
  
  
- AAAGTACCTA TTATTACCAT GTTAATTCAA ACTAACCGAA TTTATGGTTA ATAAATCGCA GAAAAAGAAA   
  
  
- CCGACAGGTC CCAAGGTAAA GGTATTTGTT GAAACTCATT CCGTATCTCT TGATTTAAAG ACGGTTAACA   
  
  
- GTTCACGTTC AACGAGTATA CGTTGTTATG AACCTTTAAA ACTCGACTCG ACGACCGTAT AGTACTTATC   
  
  
- AAGATTACCA CCGCTCAATA GTGAATAGCA GGGAACACAC TGCTTAAAGA GAAGGATCCT TCGGGAGATC   
  
  
- AGTTACGGAT AGACGACAAC ACCTCACTTT TACCTATGTA AATCTAGAGA TATTTGGGAT GAAACCGTTC   
  
  
- TAGGTACGAC TAAAATCCTT CGGGAAACTG GTCTCTTACT GTATTGTTGG ACTATCCCAC AATGTACGAT   
  
  
- TGAAGCTAAT AAATTAGTTT TATGCCTTGA AGATAGTCAG TTTCGGACGG GGTCCAAGAT GACTTAATTT   
  
  
- AACATAACTT AACGAGAGTG AACGAATACT GCTAATTTAT TGACCTAAAC GATTAATAAG AAAAATAAAA   
  
  
- TAAGAACCAC TACATCCCTT GAGTCCCTTA ACATTGGTAT GGACAAGGTC GACTTTTCCC ATGCCCTTGT   
  
  
- CAGTATCAAT GTCCCCTTAA AGCGTTATGT AAATTCCCTC ATTCTTCGTC CTAGTACAGA AATCAATCCG   
  
  
- GCCGTCTATA CAGACACTGA GGTATACCTT TAGGTTGTGA CATAAGTGAA GTTCCGTTAT TATTATCGAG   
  
  
- ACCAAACAGA CCAGTTGACA TACCGAGACT TTTCGTGTTC TAGTTTATAC ACTGGCTTAG AAAATTACCA   
  
  
- GGTTCAATAC CGGACTTTAA GAAACAACTA AGAGGTAGTC TTCTCGAGTA GGTGGGTAGT CTACGATGAG   
  
  
- GATTGGGTAA ACTGAGAAGA AAGCACCCGT ATTCTGTACT ACTCTCAGGA ATGGTTCCTT TAATACCTAG   
  
  
- TCTTATACCC CATTCGGGTA GAAGGCTACG TCAACTTATA CTACTTCCAT TATTCTATTC CGATTTTGAA   
  
  
- GTTCTTAACC TCGTACGGGA CGAGCTACTT CTACTACTTC ACCTACTCCA CCGATATCCC GGGCACGATC   
  
  
- TTGTGTCGTA CCTCGAACTA CTTCTCACCC AGCGGGGTAA CTCTTTACAC CAAAAGGTAC TAAGTGGTTT   
  
  
- CCTCAGGTGG AGGAGTCTCA GTAGGTTACA GTCGTCGTAA TCGTCGTGGT TTCTTCATAG TGGAACGAGA   
  
  
- GGCTCATGAG GGTTTGTTAA CGAGAAATTA ACACGTTTAT ACTAAAGTCT CCCCTTAAAA CTCCTTCGGC   
  
  
- ACTGATACTA GTCGCTCGAA TCTGTCTAAC AAAGATAGGT TCCCCTAGGT CGCGTCTCCT ATCGTCGGAT   
  
  
- GTACCATCTT CCGGAACGAA GGGCAAACCG AAGTAGCCCT TTTCAAGAAA TGTTTCGAAA CTTTACGTTT   
  
  
- CTCGGTGGAA GTAAACTGTC CGATCGCCGA TACGTTTAGG AGAAACTCCA CACGGGTACA AAGTTTAAGC   
  
  
- CTAAATACCG GCGTTTACGT CGCTAATACC TACGTTAGTT TTTGCTTTTT TCCCATGTGT AGTATCTAAA   
  
  
- ACTGTATTTG GTTCCCTTAG TTATGTATTT AGAGTACGTT AGGGAACGAT TTGTTCCATT GTTCGACTGC   
  
  
- GTGAACTTCA ACTGACCTCA ACTACTGGGA CTCAGTCAAG TTGCGGGATA ACCACCGGAT TTTTTGTAGC   
  
  
- CTGTTTCCGA CGTTCGTAAT CGACTTATAG AACCACACAG TAAACTCAAG TTTCGTTATG GACGATCTTG   
  
  
- AGGTGAACAA TTGGGCCTTT ACAATCTTAC AGCTGGACGC CTTCGAAATC ACCACTTGAA ACGGAAGGTC   
  
  
- GAAGTGGTGT ACGGACTACT TTCGCAGAGT TGTTAGTTGG AGGCTCTGGT CGAAGAAGCC TACCAGTTTT   
  
  
- CGGATTTGGG TTTCGATCAT TGGTAACAGC TCGTCCTACA CTTGTGTTTG TGATGGGGAA AGGAGAGATC   
  
  
- CAAACGACTC CGTATGTCGA TGATAAGACG ACAAAAACTC AGAGAACTAC GATGAGAGGG ATCTCTATCG   
  
  
- GTCCTGTCCC ACTTACACCT CTTCGTCACG AACCGTGCAC TACAACACTT GTAGTAACGT ACACTTCCCC   
  
  
- TCCTCTCCTA ACTCGCGATA CTCCAACGCC CCTTTACCTC TCGCTCCTAC AACTACCGCC CTAAGTCCCG   
  
  
- ATAAGGTTAC CCAGTCTTCT GGCCACTATA CTAAGCCTTC GAATATAGTT ACGCCATAAC ACTGTCCGAG   
  
  
- TTCCATTTTC TCCTCTACCT ACGTGAAGTG AAACCCACCC TTCTATTTTT GAATTAGCAA TGTAGTCGAA   
  
  
- CCTCCAC

+     CCGTCC-box

| Site Name | Organism | Position | Strand | Matrix score. | sequence | function |
| --- | --- | --- | --- | --- | --- | --- |
| CCGTCC-box | Petroselinum hortense | 589 | + | 6 | CCGTCC |  |

>HU11G01134.1   
+ +Up\_Stream \_Len000AGATAG ACATTCAGCC ACTTGCCAGT TCAGAGAGAG ATAGAGAGAG ATTGGGTAAG   
  
  
+ CCCTTTTTGC ACTACTTTTT CTCGCTTCCA TTTTTCTTTC TTAATTTTTA GTGATGATTT TCCAGAAGAT   
  
  
+ TTGATTTATT CATCATGCTT AACCAATTGG GTCTTTGAGT ATTATTGTTT ACTGGGTATT TGATCTTTTC   
  
  
+ ATATTCCTTT GGACTGGGTG GGTGGAACTT TTGAACTTTC TTATTAGTTG GGCATGCCCT TATCTTATTC   
  
  
+ CAATTCAAAA CCCATATTTT TCTTCAATTT TAATTATGGG TATTTTGAAA TTGAATGCTT TCCTTCACAG   
  
  
+ CAATACTATA TTACATATTT TACAGGAAAG TAATATTTTA CAGGAAAGTA TGTTGCTGTT TCAACCCTGT   
  
  
+ TTTGTTTCCT CTCTTTCCTT GGATATGATG TTCATTGCTC TTGTACCAAA CCTAACCTCA ACTTCCTGAA   
  
  
+ TTATTTAAGG CAATATTACC GTGTGCTGGT CAATTTTGGT TGTGGGTTTG TTTCTTAATG TATATGATTT   
  
  
+ ATGGTCAGGA TCCATGCTCC TCTTCCGTCC ACTCTATATC TCTCTCTCTC TCTCTCAATA CAAATTCAGA   
  
  
+ GACTTTCTCT TTCTGGGTTT ACTATTGATT GCAGAAGTTT GTAGTCTTGC GAGGCCTTGA GTCTTCTTCG   
  
  
+ AGGTAGAAAA TTTTGCTTTG CGCTTCCTTC CCCATATTTT AACACCCCTC CCCCCCCAAC GCAAGTTTTC   
  
  
+ TACATTTTTG TGCAATTCTT CAGCTATGAT TCCCCAATTT CACTTGTCAC ATCTCTATTT TCCTCTTTTT   
  
  
+ GGTTTCTTGA TTCTGTAGAA GGTTGTGTAT GAACTTCTTT GTTTCCCTTT TTCCTTTGTT ATTTTTAATG   
  
  
+ TTCCCATAGA TGATATAGAG ATCTGCTTTT CCTTGCTGTG AGAGGTATCT CTGAGAATGC ATGTAGCAAT   
  
  
+ CAAAATTCTG AGTTGTTAAT TCTATTGCAT GTCTATGGGT GATGATTGTG ATGATGTTTT TTCTGTTGCC   
  
  
+ CTCTTTCTCT CTTTCACTGA AATTGCTTTC CAGAATAGTT GTTTGACTCC TTTCTGTATC TTAATTGAGC   
  
  
+ CCTTGCTTTG GTTTGTGCTC TGGGAGTTCT TCTTTAGTAA TCCCTTGCTT GTATTTTCTG AAAGAGCAAG   
  
  
+ CAATAATCCT CATAGCATGT GTATAATCAT ACAATAGCAA CAGCAAACAT GGATCCTGCG CCCAGTTAGG   
  
  
+ TTTGGTTAGG TTAAGATATG CATATTCTTT TCTCTTGAAA AGAGTTGTCT AGGAAGGGTA ATTATGCCCA   
  
  
+ TTTCATGGAT AATAATGGTA CAATTAAGTT TGATTGGCTT AAATACCAAT TATTTAGCGT CTTTTTCTTT   
  
  
+ GGCTGTCCAG GGTTCCATTT CCATAAACAA CTTTGAGTAA GGCATAGAGA ACTAAATTTC TGCCAATTGT   
  
  
+ CAAGTGCAAG TTGCTCATAT GCAACAATAC TTGGAAATTT TGAGCTGAGC TGCTGGCATA TCATGAATAG   
  
  
+ TTCTAATGGT GGCGAGTTAT CACTTATCGT CCCTTGTGTG ACGAATTTCT CTTCCTAGGA AGCCCTCTAG   
  
  
+ TCAATGCCTA TCTGCTGTTG TGGAGTGAAA ATGGATACAT TTAGATCTCT ATAAACCCTA CTTTGGCAAG   
  
  
+ ATCCATGCTG ATTTTAGGAA GCCCTTTGAC CAGAGAATGA CATAACAACC TGATAGGGTG TTACATGCTA   
  
  
+ ACTTCGATTA TTTAATCAAA ATACGGAACT TCTATCAGTC AAAGCCTGCC CCAGGTTCTA CTGAATTAAA   
  
  
+ TTGTATTGAA TTGCTCTCAC TTGCTTATGA CGATTAAATA ACTGGATTTG CTAATTATTC TTTTTATTTT   
  
  
+ ATTCTTGGTG ATGTAGGGAA CTCAGGGAAT TGTAACCATA CCTGTTCCAG CTGAAAAGGG TACGGGAACA   
  
  
+ GTCATAGTTA CAGGGGAATT TCGCAATACA TTTAAGGGAG TAAGAAGCAG GATCATGTCT TTAGTTAGGC   
  
  
+ CGGCAGATAT GTCTGTGACT CCATATGGAA ATCCAACACT GTATTCACTT CAAGGCAATA ATAATAGCTC   
  
  
+ TGGTTTGTCT GGTCAACTGT ATGGCTCTGA AAAGCACAAG ATCAAATATG TGACCGAATC TTTTAATGGT   
  
  
+ CCAAGTTATG GCCTGAAATT CTTTGTTGAT TCTCCATCAG AAGAGCTCAT CCACCCATCA GATGCTACTC   
  
  
+ CTAACCCATT TGACTCTTCT TTCGTGGGCA TAAGACATGA TGAGAGTCCT TACCAAGGAA ATTATGGATC   
  
  
+ AGAATATGGG GTAAGCCCAT CTTCCGATGC AGTTGAATAT GATGAAGGTA ATAAGATAAG GCTAAAACTT   
  
  
+ CAAGAATTGG AGCATGCCCT GCTCGATGAA GATGATGAAG TGGATGAGGT GGCTATAGGG CCCGTGCTAG   
  
  
+ AACACAGCAT GGAGCTTGAT GAAGAGTGGG TCGCCCCATT GAGAAATGTG GTTTTCCATG ATTCACCAAA   
  
  
+ GGAGTCCACC TCCTCAGAGT CATCCAATGT CAGCAGCATT AGCAGCACCA AAGAAGTATC ACCTTGCTCT   
  
  
+ CCGAGTACTC CCAAACAATT GCTCTTTAAT TGTGCAAATA TGATTTCAGA GGGGAATTTT GAGGAAGCCG   
  
  
+ TGACTATGAT CAGCGAGCTT AGACAGATTG TTTCTATCCA AGGGGATCCA GCGCAGAGGA TAGCAGCCTA   
  
  
+ CATGGTAGAA GGCCTTGCTT CCCGTTTGGC TTCATCGGGA AAAGTTCTTT ACAAAGCTTT GAAATGCAAA   
  
  
+ GAGCCACCTT CATTTGACAG GCTAGCGGCT ATGCAAATCC TCTTTGAGGT GTGCCCATGT TTCAAATTCG   
  
  
+ GATTTATGGC CGCAAATGCA GCGATTATGG ATGCAATCAA AAACGAAAAA AGGGTACACA TCATAGATTT   
  
  
+ TGACATAAAC CAAGGGAATC AATACATAAA TCTCATGCAA TCCCTTGCTA AACAAGGTAA CAAGCTGACG   
  
  
+ CACTTGAAGT TGACTGGAGT TGATGACCCT GAGTCAGTTC AACGCCCTAT TGGTGGCCTA AAAAACATCG   
  
  
+ GACAAAGGCT GCAAGCATTA GCTGAATATC TTGGTGTGTC ATTTGAGTTC AAAGCAATAC CTGCTAGAAC   
  
  
+ TCCACTTGTT AACCCGGAAA TGTTAGAATG TCGACCTGCG GAAGCTTTAG TGGTGAACTT TGCCTTCCAG   
  
  
+ CTTCACCACA TGCCTGATGA AAGCGTCTCA ACAATCAACC TCCGAGACCA GCTTCTTCGG ATGGTCAAAA   
  
  
+ GCCTAAACCC AAAGCTAGTA ACCATTGTCG AGCAGGATGT GAACACAAAC ACTACCCCTT TCCTCTCTAG   
  
  
+ GTTTGCTGAG GCATACAGCT ACTATTCTGC TGTTTTTGAG TCTCTTGATG CTACTCTCCC TAGAGATAGC   
  
  
+ CAGGACAGGG TGAATGTGGA GAAGCAGTGC TTGGCACGTG ATGTTGTGAA CATCATTGCA TGTGAAGGGG   
  
  
+ AGGAGAGGAT TGAGCGCTAT GAGGTTGCGG GGAAATGGAG AGCGAGGATG TTGATGGCGG GATTCAGGGC   
  
  
+ TATTCCAATG GGTCAGAAGA CCGGTGATAT GATTCGGAAG CTTATATCAA TGCGGTATTG TGACAGGCTC   
  
  
+ AAGGTAAAAG AGGAGATGGA TGCACTTCAC TTTGGGTGGG AAGATAAAAA CTTAATCGTT ACATCAGCTT   
  
  
+ GGAGGTG  

- +Up\_Stream \_Len000TCTATC TGTAAGTCGG TGAACGGTCA AGTCTCTCTC TATCTCTCTC TAACCCATTC   
  
  
- GGGAAAAACG TGATGAAAAA GAGCGAAGGT AAAAAGAAAG AATTAAAAAT CACTACTAAA AGGTCTTCTA   
  
  
- AACTAAATAA GTAGTACGAA TTGGTTAACC CAGAAACTCA TAATAACAAA TGACCCATAA ACTAGAAAAG   
  
  
- TATAAGGAAA CCTGACCCAC CCACCTTGAA AACTTGAAAG AATAATCAAC CCGTACGGGA ATAGAATAAG   
  
  
- GTTAAGTTTT GGGTATAAAA AGAAGTTAAA ATTAATACCC ATAAAACTTT AACTTACGAA AGGAAGTGTC   
  
  
- GTTATGATAT AATGTATAAA ATGTCCTTTC ATTATAAAAT GTCCTTTCAT ACAACGACAA AGTTGGGACA   
  
  
- AAACAAAGGA GAGAAAGGAA CCTATACTAC AAGTAACGAG AACATGGTTT GGATTGGAGT TGAAGGACTT   
  
  
- AATAAATTCC GTTATAATGG CACACGACCA GTTAAAACCA ACACCCAAAC AAAGAATTAC ATATACTAAA   
  
  
- TACCAGTCCT AGGTACGAGG AGAAGGCAGG TGAGATATAG AGAGAGAGAG AGAGAGTTAT GTTTAAGTCT   
  
  
- CTGAAAGAGA AAGACCCAAA TGATAACTAA CGTCTTCAAA CATCAGAACG CTCCGGAACT CAGAAGAAGC   
  
  
- TCCATCTTTT AAAACGAAAC GCGAAGGAAG GGGTATAAAA TTGTGGGGAG GGGGGGGTTG CGTTCAAAAG   
  
  
- ATGTAAAAAC ACGTTAAGAA GTCGATACTA AGGGGTTAAA GTGAACAGTG TAGAGATAAA AGGAGAAAAA   
  
  
- CCAAAGAACT AAGACATCTT CCAACACATA CTTGAAGAAA CAAAGGGAAA AAGGAAACAA TAAAAATTAC   
  
  
- AAGGGTATCT ACTATATCTC TAGACGAAAA GGAACGACAC TCTCCATAGA GACTCTTACG TACATCGTTA   
  
  
- GTTTTAAGAC TCAACAATTA AGATAACGTA CAGATACCCA CTACTAACAC TACTACAAAA AAGACAACGG   
  
  
- GAGAAAGAGA GAAAGTGACT TTAACGAAAG GTCTTATCAA CAAACTGAGG AAAGACATAG AATTAACTCG   
  
  
- GGAACGAAAC CAAACACGAG ACCCTCAAGA AGAAATCATT AGGGAACGAA CATAAAAGAC TTTCTCGTTC   
  
  
- GTTATTAGGA GTATCGTACA CATATTAGTA TGTTATCGTT GTCGTTTGTA CCTAGGACGC GGGTCAATCC   
  
  
- AAACCAATCC AATTCTATAC GTATAAGAAA AGAGAACTTT TCTCAACAGA TCCTTCCCAT TAATACGGGT   
  
  
- AAAGTACCTA TTATTACCAT GTTAATTCAA ACTAACCGAA TTTATGGTTA ATAAATCGCA GAAAAAGAAA   
  
  
- CCGACAGGTC CCAAGGTAAA GGTATTTGTT GAAACTCATT CCGTATCTCT TGATTTAAAG ACGGTTAACA   
  
  
- GTTCACGTTC AACGAGTATA CGTTGTTATG AACCTTTAAA ACTCGACTCG ACGACCGTAT AGTACTTATC   
  
  
- AAGATTACCA CCGCTCAATA GTGAATAGCA GGGAACACAC TGCTTAAAGA GAAGGATCCT TCGGGAGATC   
  
  
- AGTTACGGAT AGACGACAAC ACCTCACTTT TACCTATGTA AATCTAGAGA TATTTGGGAT GAAACCGTTC   
  
  
- TAGGTACGAC TAAAATCCTT CGGGAAACTG GTCTCTTACT GTATTGTTGG ACTATCCCAC AATGTACGAT   
  
  
- TGAAGCTAAT AAATTAGTTT TATGCCTTGA AGATAGTCAG TTTCGGACGG GGTCCAAGAT GACTTAATTT   
  
  
- AACATAACTT AACGAGAGTG AACGAATACT GCTAATTTAT TGACCTAAAC GATTAATAAG AAAAATAAAA   
  
  
- TAAGAACCAC TACATCCCTT GAGTCCCTTA ACATTGGTAT GGACAAGGTC GACTTTTCCC ATGCCCTTGT   
  
  
- CAGTATCAAT GTCCCCTTAA AGCGTTATGT AAATTCCCTC ATTCTTCGTC CTAGTACAGA AATCAATCCG   
  
  
- GCCGTCTATA CAGACACTGA GGTATACCTT TAGGTTGTGA CATAAGTGAA GTTCCGTTAT TATTATCGAG   
  
  
- ACCAAACAGA CCAGTTGACA TACCGAGACT TTTCGTGTTC TAGTTTATAC ACTGGCTTAG AAAATTACCA   
  
  
- GGTTCAATAC CGGACTTTAA GAAACAACTA AGAGGTAGTC TTCTCGAGTA GGTGGGTAGT CTACGATGAG   
  
  
- GATTGGGTAA ACTGAGAAGA AAGCACCCGT ATTCTGTACT ACTCTCAGGA ATGGTTCCTT TAATACCTAG   
  
  
- TCTTATACCC CATTCGGGTA GAAGGCTACG TCAACTTATA CTACTTCCAT TATTCTATTC CGATTTTGAA   
  
  
- GTTCTTAACC TCGTACGGGA CGAGCTACTT CTACTACTTC ACCTACTCCA CCGATATCCC GGGCACGATC   
  
  
- TTGTGTCGTA CCTCGAACTA CTTCTCACCC AGCGGGGTAA CTCTTTACAC CAAAAGGTAC TAAGTGGTTT   
  
  
- CCTCAGGTGG AGGAGTCTCA GTAGGTTACA GTCGTCGTAA TCGTCGTGGT TTCTTCATAG TGGAACGAGA   
  
  
- GGCTCATGAG GGTTTGTTAA CGAGAAATTA ACACGTTTAT ACTAAAGTCT CCCCTTAAAA CTCCTTCGGC   
  
  
- ACTGATACTA GTCGCTCGAA TCTGTCTAAC AAAGATAGGT TCCCCTAGGT CGCGTCTCCT ATCGTCGGAT   
  
  
- GTACCATCTT CCGGAACGAA GGGCAAACCG AAGTAGCCCT TTTCAAGAAA TGTTTCGAAA CTTTACGTTT   
  
  
- CTCGGTGGAA GTAAACTGTC CGATCGCCGA TACGTTTAGG AGAAACTCCA CACGGGTACA AAGTTTAAGC   
  
  
- CTAAATACCG GCGTTTACGT CGCTAATACC TACGTTAGTT TTTGCTTTTT TCCCATGTGT AGTATCTAAA   
  
  
- ACTGTATTTG GTTCCCTTAG TTATGTATTT AGAGTACGTT AGGGAACGAT TTGTTCCATT GTTCGACTGC   
  
  
- GTGAACTTCA ACTGACCTCA ACTACTGGGA CTCAGTCAAG TTGCGGGATA ACCACCGGAT TTTTTGTAGC   
  
  
- CTGTTTCCGA CGTTCGTAAT CGACTTATAG AACCACACAG TAAACTCAAG TTTCGTTATG GACGATCTTG   
  
  
- AGGTGAACAA TTGGGCCTTT ACAATCTTAC AGCTGGACGC CTTCGAAATC ACCACTTGAA ACGGAAGGTC   
  
  
- GAAGTGGTGT ACGGACTACT TTCGCAGAGT TGTTAGTTGG AGGCTCTGGT CGAAGAAGCC TACCAGTTTT   
  
  
- CGGATTTGGG TTTCGATCAT TGGTAACAGC TCGTCCTACA CTTGTGTTTG TGATGGGGAA AGGAGAGATC   
  
  
- CAAACGACTC CGTATGTCGA TGATAAGACG ACAAAAACTC AGAGAACTAC GATGAGAGGG ATCTCTATCG   
  
  
- GTCCTGTCCC ACTTACACCT CTTCGTCACG AACCGTGCAC TACAACACTT GTAGTAACGT ACACTTCCCC   
  
  
- TCCTCTCCTA ACTCGCGATA CTCCAACGCC CCTTTACCTC TCGCTCCTAC AACTACCGCC CTAAGTCCCG   
  
  
- ATAAGGTTAC CCAGTCTTCT GGCCACTATA CTAAGCCTTC GAATATAGTT ACGCCATAAC ACTGTCCGAG   
  
  
- TTCCATTTTC TCCTCTACCT ACGTGAAGTG AAACCCACCC TTCTATTTTT GAATTAGCAA TGTAGTCGAA   
  
  
- CCTCCAC

+     CGTCA-motif

| Site Name | Organism | Position | Strand | Matrix score. | sequence | function |
| --- | --- | --- | --- | --- | --- | --- |
| CGTCA-motif | Hordeum vulgare | 1583 | - | 5 | CGTCA | cis-acting regulatory element involved in the MeJA-responsiveness |
| CGTCA-motif | Hordeum vulgare | 3010 | - | 5 | CGTCA | cis-acting regulatory element involved in the MeJA-responsiveness |
| CGTCA-motif | Hordeum vulgare | 1852 | - | 5 | CGTCA | cis-acting regulatory element involved in the MeJA-responsiveness |

>HU11G01134.1   
+ +Up\_Stream \_Len000AGATAG ACATTCAGCC ACTTGCCAGT TCAGAGAGAG ATAGAGAGAG ATTGGGTAAG   
  
  
+ CCCTTTTTGC ACTACTTTTT CTCGCTTCCA TTTTTCTTTC TTAATTTTTA GTGATGATTT TCCAGAAGAT   
  
  
+ TTGATTTATT CATCATGCTT AACCAATTGG GTCTTTGAGT ATTATTGTTT ACTGGGTATT TGATCTTTTC   
  
  
+ ATATTCCTTT GGACTGGGTG GGTGGAACTT TTGAACTTTC TTATTAGTTG GGCATGCCCT TATCTTATTC   
  
  
+ CAATTCAAAA CCCATATTTT TCTTCAATTT TAATTATGGG TATTTTGAAA TTGAATGCTT TCCTTCACAG   
  
  
+ CAATACTATA TTACATATTT TACAGGAAAG TAATATTTTA CAGGAAAGTA TGTTGCTGTT TCAACCCTGT   
  
  
+ TTTGTTTCCT CTCTTTCCTT GGATATGATG TTCATTGCTC TTGTACCAAA CCTAACCTCA ACTTCCTGAA   
  
  
+ TTATTTAAGG CAATATTACC GTGTGCTGGT CAATTTTGGT TGTGGGTTTG TTTCTTAATG TATATGATTT   
  
  
+ ATGGTCAGGA TCCATGCTCC TCTTCCGTCC ACTCTATATC TCTCTCTCTC TCTCTCAATA CAAATTCAGA   
  
  
+ GACTTTCTCT TTCTGGGTTT ACTATTGATT GCAGAAGTTT GTAGTCTTGC GAGGCCTTGA GTCTTCTTCG   
  
  
+ AGGTAGAAAA TTTTGCTTTG CGCTTCCTTC CCCATATTTT AACACCCCTC CCCCCCCAAC GCAAGTTTTC   
  
  
+ TACATTTTTG TGCAATTCTT CAGCTATGAT TCCCCAATTT CACTTGTCAC ATCTCTATTT TCCTCTTTTT   
  
  
+ GGTTTCTTGA TTCTGTAGAA GGTTGTGTAT GAACTTCTTT GTTTCCCTTT TTCCTTTGTT ATTTTTAATG   
  
  
+ TTCCCATAGA TGATATAGAG ATCTGCTTTT CCTTGCTGTG AGAGGTATCT CTGAGAATGC ATGTAGCAAT   
  
  
+ CAAAATTCTG AGTTGTTAAT TCTATTGCAT GTCTATGGGT GATGATTGTG ATGATGTTTT TTCTGTTGCC   
  
  
+ CTCTTTCTCT CTTTCACTGA AATTGCTTTC CAGAATAGTT GTTTGACTCC TTTCTGTATC TTAATTGAGC   
  
  
+ CCTTGCTTTG GTTTGTGCTC TGGGAGTTCT TCTTTAGTAA TCCCTTGCTT GTATTTTCTG AAAGAGCAAG   
  
  
+ CAATAATCCT CATAGCATGT GTATAATCAT ACAATAGCAA CAGCAAACAT GGATCCTGCG CCCAGTTAGG   
  
  
+ TTTGGTTAGG TTAAGATATG CATATTCTTT TCTCTTGAAA AGAGTTGTCT AGGAAGGGTA ATTATGCCCA   
  
  
+ TTTCATGGAT AATAATGGTA CAATTAAGTT TGATTGGCTT AAATACCAAT TATTTAGCGT CTTTTTCTTT   
  
  
+ GGCTGTCCAG GGTTCCATTT CCATAAACAA CTTTGAGTAA GGCATAGAGA ACTAAATTTC TGCCAATTGT   
  
  
+ CAAGTGCAAG TTGCTCATAT GCAACAATAC TTGGAAATTT TGAGCTGAGC TGCTGGCATA TCATGAATAG   
  
  
+ TTCTAATGGT GGCGAGTTAT CACTTATCGT CCCTTGTGTG ACGAATTTCT CTTCCTAGGA AGCCCTCTAG   
  
  
+ TCAATGCCTA TCTGCTGTTG TGGAGTGAAA ATGGATACAT TTAGATCTCT ATAAACCCTA CTTTGGCAAG   
  
  
+ ATCCATGCTG ATTTTAGGAA GCCCTTTGAC CAGAGAATGA CATAACAACC TGATAGGGTG TTACATGCTA   
  
  
+ ACTTCGATTA TTTAATCAAA ATACGGAACT TCTATCAGTC AAAGCCTGCC CCAGGTTCTA CTGAATTAAA   
  
  
+ TTGTATTGAA TTGCTCTCAC TTGCTTATGA CGATTAAATA ACTGGATTTG CTAATTATTC TTTTTATTTT   
  
  
+ ATTCTTGGTG ATGTAGGGAA CTCAGGGAAT TGTAACCATA CCTGTTCCAG CTGAAAAGGG TACGGGAACA   
  
  
+ GTCATAGTTA CAGGGGAATT TCGCAATACA TTTAAGGGAG TAAGAAGCAG GATCATGTCT TTAGTTAGGC   
  
  
+ CGGCAGATAT GTCTGTGACT CCATATGGAA ATCCAACACT GTATTCACTT CAAGGCAATA ATAATAGCTC   
  
  
+ TGGTTTGTCT GGTCAACTGT ATGGCTCTGA AAAGCACAAG ATCAAATATG TGACCGAATC TTTTAATGGT   
  
  
+ CCAAGTTATG GCCTGAAATT CTTTGTTGAT TCTCCATCAG AAGAGCTCAT CCACCCATCA GATGCTACTC   
  
  
+ CTAACCCATT TGACTCTTCT TTCGTGGGCA TAAGACATGA TGAGAGTCCT TACCAAGGAA ATTATGGATC   
  
  
+ AGAATATGGG GTAAGCCCAT CTTCCGATGC AGTTGAATAT GATGAAGGTA ATAAGATAAG GCTAAAACTT   
  
  
+ CAAGAATTGG AGCATGCCCT GCTCGATGAA GATGATGAAG TGGATGAGGT GGCTATAGGG CCCGTGCTAG   
  
  
+ AACACAGCAT GGAGCTTGAT GAAGAGTGGG TCGCCCCATT GAGAAATGTG GTTTTCCATG ATTCACCAAA   
  
  
+ GGAGTCCACC TCCTCAGAGT CATCCAATGT CAGCAGCATT AGCAGCACCA AAGAAGTATC ACCTTGCTCT   
  
  
+ CCGAGTACTC CCAAACAATT GCTCTTTAAT TGTGCAAATA TGATTTCAGA GGGGAATTTT GAGGAAGCCG   
  
  
+ TGACTATGAT CAGCGAGCTT AGACAGATTG TTTCTATCCA AGGGGATCCA GCGCAGAGGA TAGCAGCCTA   
  
  
+ CATGGTAGAA GGCCTTGCTT CCCGTTTGGC TTCATCGGGA AAAGTTCTTT ACAAAGCTTT GAAATGCAAA   
  
  
+ GAGCCACCTT CATTTGACAG GCTAGCGGCT ATGCAAATCC TCTTTGAGGT GTGCCCATGT TTCAAATTCG   
  
  
+ GATTTATGGC CGCAAATGCA GCGATTATGG ATGCAATCAA AAACGAAAAA AGGGTACACA TCATAGATTT   
  
  
+ TGACATAAAC CAAGGGAATC AATACATAAA TCTCATGCAA TCCCTTGCTA AACAAGGTAA CAAGCTGACG   
  
  
+ CACTTGAAGT TGACTGGAGT TGATGACCCT GAGTCAGTTC AACGCCCTAT TGGTGGCCTA AAAAACATCG   
  
  
+ GACAAAGGCT GCAAGCATTA GCTGAATATC TTGGTGTGTC ATTTGAGTTC AAAGCAATAC CTGCTAGAAC   
  
  
+ TCCACTTGTT AACCCGGAAA TGTTAGAATG TCGACCTGCG GAAGCTTTAG TGGTGAACTT TGCCTTCCAG   
  
  
+ CTTCACCACA TGCCTGATGA AAGCGTCTCA ACAATCAACC TCCGAGACCA GCTTCTTCGG ATGGTCAAAA   
  
  
+ GCCTAAACCC AAAGCTAGTA ACCATTGTCG AGCAGGATGT GAACACAAAC ACTACCCCTT TCCTCTCTAG   
  
  
+ GTTTGCTGAG GCATACAGCT ACTATTCTGC TGTTTTTGAG TCTCTTGATG CTACTCTCCC TAGAGATAGC   
  
  
+ CAGGACAGGG TGAATGTGGA GAAGCAGTGC TTGGCACGTG ATGTTGTGAA CATCATTGCA TGTGAAGGGG   
  
  
+ AGGAGAGGAT TGAGCGCTAT GAGGTTGCGG GGAAATGGAG AGCGAGGATG TTGATGGCGG GATTCAGGGC   
  
  
+ TATTCCAATG GGTCAGAAGA CCGGTGATAT GATTCGGAAG CTTATATCAA TGCGGTATTG TGACAGGCTC   
  
  
+ AAGGTAAAAG AGGAGATGGA TGCACTTCAC TTTGGGTGGG AAGATAAAAA CTTAATCGTT ACATCAGCTT   
  
  
+ GGAGGTG  

- +Up\_Stream \_Len000TCTATC TGTAAGTCGG TGAACGGTCA AGTCTCTCTC TATCTCTCTC TAACCCATTC   
  
  
- GGGAAAAACG TGATGAAAAA GAGCGAAGGT AAAAAGAAAG AATTAAAAAT CACTACTAAA AGGTCTTCTA   
  
  
- AACTAAATAA GTAGTACGAA TTGGTTAACC CAGAAACTCA TAATAACAAA TGACCCATAA ACTAGAAAAG   
  
  
- TATAAGGAAA CCTGACCCAC CCACCTTGAA AACTTGAAAG AATAATCAAC CCGTACGGGA ATAGAATAAG   
  
  
- GTTAAGTTTT GGGTATAAAA AGAAGTTAAA ATTAATACCC ATAAAACTTT AACTTACGAA AGGAAGTGTC   
  
  
- GTTATGATAT AATGTATAAA ATGTCCTTTC ATTATAAAAT GTCCTTTCAT ACAACGACAA AGTTGGGACA   
  
  
- AAACAAAGGA GAGAAAGGAA CCTATACTAC AAGTAACGAG AACATGGTTT GGATTGGAGT TGAAGGACTT   
  
  
- AATAAATTCC GTTATAATGG CACACGACCA GTTAAAACCA ACACCCAAAC AAAGAATTAC ATATACTAAA   
  
  
- TACCAGTCCT AGGTACGAGG AGAAGGCAGG TGAGATATAG AGAGAGAGAG AGAGAGTTAT GTTTAAGTCT   
  
  
- CTGAAAGAGA AAGACCCAAA TGATAACTAA CGTCTTCAAA CATCAGAACG CTCCGGAACT CAGAAGAAGC   
  
  
- TCCATCTTTT AAAACGAAAC GCGAAGGAAG GGGTATAAAA TTGTGGGGAG GGGGGGGTTG CGTTCAAAAG   
  
  
- ATGTAAAAAC ACGTTAAGAA GTCGATACTA AGGGGTTAAA GTGAACAGTG TAGAGATAAA AGGAGAAAAA   
  
  
- CCAAAGAACT AAGACATCTT CCAACACATA CTTGAAGAAA CAAAGGGAAA AAGGAAACAA TAAAAATTAC   
  
  
- AAGGGTATCT ACTATATCTC TAGACGAAAA GGAACGACAC TCTCCATAGA GACTCTTACG TACATCGTTA   
  
  
- GTTTTAAGAC TCAACAATTA AGATAACGTA CAGATACCCA CTACTAACAC TACTACAAAA AAGACAACGG   
  
  
- GAGAAAGAGA GAAAGTGACT TTAACGAAAG GTCTTATCAA CAAACTGAGG AAAGACATAG AATTAACTCG   
  
  
- GGAACGAAAC CAAACACGAG ACCCTCAAGA AGAAATCATT AGGGAACGAA CATAAAAGAC TTTCTCGTTC   
  
  
- GTTATTAGGA GTATCGTACA CATATTAGTA TGTTATCGTT GTCGTTTGTA CCTAGGACGC GGGTCAATCC   
  
  
- AAACCAATCC AATTCTATAC GTATAAGAAA AGAGAACTTT TCTCAACAGA TCCTTCCCAT TAATACGGGT   
  
  
- AAAGTACCTA TTATTACCAT GTTAATTCAA ACTAACCGAA TTTATGGTTA ATAAATCGCA GAAAAAGAAA   
  
  
- CCGACAGGTC CCAAGGTAAA GGTATTTGTT GAAACTCATT CCGTATCTCT TGATTTAAAG ACGGTTAACA   
  
  
- GTTCACGTTC AACGAGTATA CGTTGTTATG AACCTTTAAA ACTCGACTCG ACGACCGTAT AGTACTTATC   
  
  
- AAGATTACCA CCGCTCAATA GTGAATAGCA GGGAACACAC TGCTTAAAGA GAAGGATCCT TCGGGAGATC   
  
  
- AGTTACGGAT AGACGACAAC ACCTCACTTT TACCTATGTA AATCTAGAGA TATTTGGGAT GAAACCGTTC   
  
  
- TAGGTACGAC TAAAATCCTT CGGGAAACTG GTCTCTTACT GTATTGTTGG ACTATCCCAC AATGTACGAT   
  
  
- TGAAGCTAAT AAATTAGTTT TATGCCTTGA AGATAGTCAG TTTCGGACGG GGTCCAAGAT GACTTAATTT   
  
  
- AACATAACTT AACGAGAGTG AACGAATACT GCTAATTTAT TGACCTAAAC GATTAATAAG AAAAATAAAA   
  
  
- TAAGAACCAC TACATCCCTT GAGTCCCTTA ACATTGGTAT GGACAAGGTC GACTTTTCCC ATGCCCTTGT   
  
  
- CAGTATCAAT GTCCCCTTAA AGCGTTATGT AAATTCCCTC ATTCTTCGTC CTAGTACAGA AATCAATCCG   
  
  
- GCCGTCTATA CAGACACTGA GGTATACCTT TAGGTTGTGA CATAAGTGAA GTTCCGTTAT TATTATCGAG   
  
  
- ACCAAACAGA CCAGTTGACA TACCGAGACT TTTCGTGTTC TAGTTTATAC ACTGGCTTAG AAAATTACCA   
  
  
- GGTTCAATAC CGGACTTTAA GAAACAACTA AGAGGTAGTC TTCTCGAGTA GGTGGGTAGT CTACGATGAG   
  
  
- GATTGGGTAA ACTGAGAAGA AAGCACCCGT ATTCTGTACT ACTCTCAGGA ATGGTTCCTT TAATACCTAG   
  
  
- TCTTATACCC CATTCGGGTA GAAGGCTACG TCAACTTATA CTACTTCCAT TATTCTATTC CGATTTTGAA   
  
  
- GTTCTTAACC TCGTACGGGA CGAGCTACTT CTACTACTTC ACCTACTCCA CCGATATCCC GGGCACGATC   
  
  
- TTGTGTCGTA CCTCGAACTA CTTCTCACCC AGCGGGGTAA CTCTTTACAC CAAAAGGTAC TAAGTGGTTT   
  
  
- CCTCAGGTGG AGGAGTCTCA GTAGGTTACA GTCGTCGTAA TCGTCGTGGT TTCTTCATAG TGGAACGAGA   
  
  
- GGCTCATGAG GGTTTGTTAA CGAGAAATTA ACACGTTTAT ACTAAAGTCT CCCCTTAAAA CTCCTTCGGC   
  
  
- ACTGATACTA GTCGCTCGAA TCTGTCTAAC AAAGATAGGT TCCCCTAGGT CGCGTCTCCT ATCGTCGGAT   
  
  
- GTACCATCTT CCGGAACGAA GGGCAAACCG AAGTAGCCCT TTTCAAGAAA TGTTTCGAAA CTTTACGTTT   
  
  
- CTCGGTGGAA GTAAACTGTC CGATCGCCGA TACGTTTAGG AGAAACTCCA CACGGGTACA AAGTTTAAGC   
  
  
- CTAAATACCG GCGTTTACGT CGCTAATACC TACGTTAGTT TTTGCTTTTT TCCCATGTGT AGTATCTAAA   
  
  
- ACTGTATTTG GTTCCCTTAG TTATGTATTT AGAGTACGTT AGGGAACGAT TTGTTCCATT GTTCGACTGC   
  
  
- GTGAACTTCA ACTGACCTCA ACTACTGGGA CTCAGTCAAG TTGCGGGATA ACCACCGGAT TTTTTGTAGC   
  
  
- CTGTTTCCGA CGTTCGTAAT CGACTTATAG AACCACACAG TAAACTCAAG TTTCGTTATG GACGATCTTG   
  
  
- AGGTGAACAA TTGGGCCTTT ACAATCTTAC AGCTGGACGC CTTCGAAATC ACCACTTGAA ACGGAAGGTC   
  
  
- GAAGTGGTGT ACGGACTACT TTCGCAGAGT TGTTAGTTGG AGGCTCTGGT CGAAGAAGCC TACCAGTTTT   
  
  
- CGGATTTGGG TTTCGATCAT TGGTAACAGC TCGTCCTACA CTTGTGTTTG TGATGGGGAA AGGAGAGATC   
  
  
- CAAACGACTC CGTATGTCGA TGATAAGACG ACAAAAACTC AGAGAACTAC GATGAGAGGG ATCTCTATCG   
  
  
- GTCCTGTCCC ACTTACACCT CTTCGTCACG AACCGTGCAC TACAACACTT GTAGTAACGT ACACTTCCCC   
  
  
- TCCTCTCCTA ACTCGCGATA CTCCAACGCC CCTTTACCTC TCGCTCCTAC AACTACCGCC CTAAGTCCCG   
  
  
- ATAAGGTTAC CCAGTCTTCT GGCCACTATA CTAAGCCTTC GAATATAGTT ACGCCATAAC ACTGTCCGAG   
  
  
- TTCCATTTTC TCCTCTACCT ACGTGAAGTG AAACCCACCC TTCTATTTTT GAATTAGCAA TGTAGTCGAA   
  
  
- CCTCCAC

+     G-Box

| Site Name | Organism | Position | Strand | Matrix score. | sequence | function |
| --- | --- | --- | --- | --- | --- | --- |
| G-Box | Pisum sativum | 3469 | - | 6 | CACGTG | cis-acting regulatory element involved in light responsiveness |

>HU11G01134.1   
+ +Up\_Stream \_Len000AGATAG ACATTCAGCC ACTTGCCAGT TCAGAGAGAG ATAGAGAGAG ATTGGGTAAG   
  
  
+ CCCTTTTTGC ACTACTTTTT CTCGCTTCCA TTTTTCTTTC TTAATTTTTA GTGATGATTT TCCAGAAGAT   
  
  
+ TTGATTTATT CATCATGCTT AACCAATTGG GTCTTTGAGT ATTATTGTTT ACTGGGTATT TGATCTTTTC   
  
  
+ ATATTCCTTT GGACTGGGTG GGTGGAACTT TTGAACTTTC TTATTAGTTG GGCATGCCCT TATCTTATTC   
  
  
+ CAATTCAAAA CCCATATTTT TCTTCAATTT TAATTATGGG TATTTTGAAA TTGAATGCTT TCCTTCACAG   
  
  
+ CAATACTATA TTACATATTT TACAGGAAAG TAATATTTTA CAGGAAAGTA TGTTGCTGTT TCAACCCTGT   
  
  
+ TTTGTTTCCT CTCTTTCCTT GGATATGATG TTCATTGCTC TTGTACCAAA CCTAACCTCA ACTTCCTGAA   
  
  
+ TTATTTAAGG CAATATTACC GTGTGCTGGT CAATTTTGGT TGTGGGTTTG TTTCTTAATG TATATGATTT   
  
  
+ ATGGTCAGGA TCCATGCTCC TCTTCCGTCC ACTCTATATC TCTCTCTCTC TCTCTCAATA CAAATTCAGA   
  
  
+ GACTTTCTCT TTCTGGGTTT ACTATTGATT GCAGAAGTTT GTAGTCTTGC GAGGCCTTGA GTCTTCTTCG   
  
  
+ AGGTAGAAAA TTTTGCTTTG CGCTTCCTTC CCCATATTTT AACACCCCTC CCCCCCCAAC GCAAGTTTTC   
  
  
+ TACATTTTTG TGCAATTCTT CAGCTATGAT TCCCCAATTT CACTTGTCAC ATCTCTATTT TCCTCTTTTT   
  
  
+ GGTTTCTTGA TTCTGTAGAA GGTTGTGTAT GAACTTCTTT GTTTCCCTTT TTCCTTTGTT ATTTTTAATG   
  
  
+ TTCCCATAGA TGATATAGAG ATCTGCTTTT CCTTGCTGTG AGAGGTATCT CTGAGAATGC ATGTAGCAAT   
  
  
+ CAAAATTCTG AGTTGTTAAT TCTATTGCAT GTCTATGGGT GATGATTGTG ATGATGTTTT TTCTGTTGCC   
  
  
+ CTCTTTCTCT CTTTCACTGA AATTGCTTTC CAGAATAGTT GTTTGACTCC TTTCTGTATC TTAATTGAGC   
  
  
+ CCTTGCTTTG GTTTGTGCTC TGGGAGTTCT TCTTTAGTAA TCCCTTGCTT GTATTTTCTG AAAGAGCAAG   
  
  
+ CAATAATCCT CATAGCATGT GTATAATCAT ACAATAGCAA CAGCAAACAT GGATCCTGCG CCCAGTTAGG   
  
  
+ TTTGGTTAGG TTAAGATATG CATATTCTTT TCTCTTGAAA AGAGTTGTCT AGGAAGGGTA ATTATGCCCA   
  
  
+ TTTCATGGAT AATAATGGTA CAATTAAGTT TGATTGGCTT AAATACCAAT TATTTAGCGT CTTTTTCTTT   
  
  
+ GGCTGTCCAG GGTTCCATTT CCATAAACAA CTTTGAGTAA GGCATAGAGA ACTAAATTTC TGCCAATTGT   
  
  
+ CAAGTGCAAG TTGCTCATAT GCAACAATAC TTGGAAATTT TGAGCTGAGC TGCTGGCATA TCATGAATAG   
  
  
+ TTCTAATGGT GGCGAGTTAT CACTTATCGT CCCTTGTGTG ACGAATTTCT CTTCCTAGGA AGCCCTCTAG   
  
  
+ TCAATGCCTA TCTGCTGTTG TGGAGTGAAA ATGGATACAT TTAGATCTCT ATAAACCCTA CTTTGGCAAG   
  
  
+ ATCCATGCTG ATTTTAGGAA GCCCTTTGAC CAGAGAATGA CATAACAACC TGATAGGGTG TTACATGCTA   
  
  
+ ACTTCGATTA TTTAATCAAA ATACGGAACT TCTATCAGTC AAAGCCTGCC CCAGGTTCTA CTGAATTAAA   
  
  
+ TTGTATTGAA TTGCTCTCAC TTGCTTATGA CGATTAAATA ACTGGATTTG CTAATTATTC TTTTTATTTT   
  
  
+ ATTCTTGGTG ATGTAGGGAA CTCAGGGAAT TGTAACCATA CCTGTTCCAG CTGAAAAGGG TACGGGAACA   
  
  
+ GTCATAGTTA CAGGGGAATT TCGCAATACA TTTAAGGGAG TAAGAAGCAG GATCATGTCT TTAGTTAGGC   
  
  
+ CGGCAGATAT GTCTGTGACT CCATATGGAA ATCCAACACT GTATTCACTT CAAGGCAATA ATAATAGCTC   
  
  
+ TGGTTTGTCT GGTCAACTGT ATGGCTCTGA AAAGCACAAG ATCAAATATG TGACCGAATC TTTTAATGGT   
  
  
+ CCAAGTTATG GCCTGAAATT CTTTGTTGAT TCTCCATCAG AAGAGCTCAT CCACCCATCA GATGCTACTC   
  
  
+ CTAACCCATT TGACTCTTCT TTCGTGGGCA TAAGACATGA TGAGAGTCCT TACCAAGGAA ATTATGGATC   
  
  
+ AGAATATGGG GTAAGCCCAT CTTCCGATGC AGTTGAATAT GATGAAGGTA ATAAGATAAG GCTAAAACTT   
  
  
+ CAAGAATTGG AGCATGCCCT GCTCGATGAA GATGATGAAG TGGATGAGGT GGCTATAGGG CCCGTGCTAG   
  
  
+ AACACAGCAT GGAGCTTGAT GAAGAGTGGG TCGCCCCATT GAGAAATGTG GTTTTCCATG ATTCACCAAA   
  
  
+ GGAGTCCACC TCCTCAGAGT CATCCAATGT CAGCAGCATT AGCAGCACCA AAGAAGTATC ACCTTGCTCT   
  
  
+ CCGAGTACTC CCAAACAATT GCTCTTTAAT TGTGCAAATA TGATTTCAGA GGGGAATTTT GAGGAAGCCG   
  
  
+ TGACTATGAT CAGCGAGCTT AGACAGATTG TTTCTATCCA AGGGGATCCA GCGCAGAGGA TAGCAGCCTA   
  
  
+ CATGGTAGAA GGCCTTGCTT CCCGTTTGGC TTCATCGGGA AAAGTTCTTT ACAAAGCTTT GAAATGCAAA   
  
  
+ GAGCCACCTT CATTTGACAG GCTAGCGGCT ATGCAAATCC TCTTTGAGGT GTGCCCATGT TTCAAATTCG   
  
  
+ GATTTATGGC CGCAAATGCA GCGATTATGG ATGCAATCAA AAACGAAAAA AGGGTACACA TCATAGATTT   
  
  
+ TGACATAAAC CAAGGGAATC AATACATAAA TCTCATGCAA TCCCTTGCTA AACAAGGTAA CAAGCTGACG   
  
  
+ CACTTGAAGT TGACTGGAGT TGATGACCCT GAGTCAGTTC AACGCCCTAT TGGTGGCCTA AAAAACATCG   
  
  
+ GACAAAGGCT GCAAGCATTA GCTGAATATC TTGGTGTGTC ATTTGAGTTC AAAGCAATAC CTGCTAGAAC   
  
  
+ TCCACTTGTT AACCCGGAAA TGTTAGAATG TCGACCTGCG GAAGCTTTAG TGGTGAACTT TGCCTTCCAG   
  
  
+ CTTCACCACA TGCCTGATGA AAGCGTCTCA ACAATCAACC TCCGAGACCA GCTTCTTCGG ATGGTCAAAA   
  
  
+ GCCTAAACCC AAAGCTAGTA ACCATTGTCG AGCAGGATGT GAACACAAAC ACTACCCCTT TCCTCTCTAG   
  
  
+ GTTTGCTGAG GCATACAGCT ACTATTCTGC TGTTTTTGAG TCTCTTGATG CTACTCTCCC TAGAGATAGC   
  
  
+ CAGGACAGGG TGAATGTGGA GAAGCAGTGC TTGGCACGTG ATGTTGTGAA CATCATTGCA TGTGAAGGGG   
  
  
+ AGGAGAGGAT TGAGCGCTAT GAGGTTGCGG GGAAATGGAG AGCGAGGATG TTGATGGCGG GATTCAGGGC   
  
  
+ TATTCCAATG GGTCAGAAGA CCGGTGATAT GATTCGGAAG CTTATATCAA TGCGGTATTG TGACAGGCTC   
  
  
+ AAGGTAAAAG AGGAGATGGA TGCACTTCAC TTTGGGTGGG AAGATAAAAA CTTAATCGTT ACATCAGCTT   
  
  
+ GGAGGTG  

- +Up\_Stream \_Len000TCTATC TGTAAGTCGG TGAACGGTCA AGTCTCTCTC TATCTCTCTC TAACCCATTC   
  
  
- GGGAAAAACG TGATGAAAAA GAGCGAAGGT AAAAAGAAAG AATTAAAAAT CACTACTAAA AGGTCTTCTA   
  
  
- AACTAAATAA GTAGTACGAA TTGGTTAACC CAGAAACTCA TAATAACAAA TGACCCATAA ACTAGAAAAG   
  
  
- TATAAGGAAA CCTGACCCAC CCACCTTGAA AACTTGAAAG AATAATCAAC CCGTACGGGA ATAGAATAAG   
  
  
- GTTAAGTTTT GGGTATAAAA AGAAGTTAAA ATTAATACCC ATAAAACTTT AACTTACGAA AGGAAGTGTC   
  
  
- GTTATGATAT AATGTATAAA ATGTCCTTTC ATTATAAAAT GTCCTTTCAT ACAACGACAA AGTTGGGACA   
  
  
- AAACAAAGGA GAGAAAGGAA CCTATACTAC AAGTAACGAG AACATGGTTT GGATTGGAGT TGAAGGACTT   
  
  
- AATAAATTCC GTTATAATGG CACACGACCA GTTAAAACCA ACACCCAAAC AAAGAATTAC ATATACTAAA   
  
  
- TACCAGTCCT AGGTACGAGG AGAAGGCAGG TGAGATATAG AGAGAGAGAG AGAGAGTTAT GTTTAAGTCT   
  
  
- CTGAAAGAGA AAGACCCAAA TGATAACTAA CGTCTTCAAA CATCAGAACG CTCCGGAACT CAGAAGAAGC   
  
  
- TCCATCTTTT AAAACGAAAC GCGAAGGAAG GGGTATAAAA TTGTGGGGAG GGGGGGGTTG CGTTCAAAAG   
  
  
- ATGTAAAAAC ACGTTAAGAA GTCGATACTA AGGGGTTAAA GTGAACAGTG TAGAGATAAA AGGAGAAAAA   
  
  
- CCAAAGAACT AAGACATCTT CCAACACATA CTTGAAGAAA CAAAGGGAAA AAGGAAACAA TAAAAATTAC   
  
  
- AAGGGTATCT ACTATATCTC TAGACGAAAA GGAACGACAC TCTCCATAGA GACTCTTACG TACATCGTTA   
  
  
- GTTTTAAGAC TCAACAATTA AGATAACGTA CAGATACCCA CTACTAACAC TACTACAAAA AAGACAACGG   
  
  
- GAGAAAGAGA GAAAGTGACT TTAACGAAAG GTCTTATCAA CAAACTGAGG AAAGACATAG AATTAACTCG   
  
  
- GGAACGAAAC CAAACACGAG ACCCTCAAGA AGAAATCATT AGGGAACGAA CATAAAAGAC TTTCTCGTTC   
  
  
- GTTATTAGGA GTATCGTACA CATATTAGTA TGTTATCGTT GTCGTTTGTA CCTAGGACGC GGGTCAATCC   
  
  
- AAACCAATCC AATTCTATAC GTATAAGAAA AGAGAACTTT TCTCAACAGA TCCTTCCCAT TAATACGGGT   
  
  
- AAAGTACCTA TTATTACCAT GTTAATTCAA ACTAACCGAA TTTATGGTTA ATAAATCGCA GAAAAAGAAA   
  
  
- CCGACAGGTC CCAAGGTAAA GGTATTTGTT GAAACTCATT CCGTATCTCT TGATTTAAAG ACGGTTAACA   
  
  
- GTTCACGTTC AACGAGTATA CGTTGTTATG AACCTTTAAA ACTCGACTCG ACGACCGTAT AGTACTTATC   
  
  
- AAGATTACCA CCGCTCAATA GTGAATAGCA GGGAACACAC TGCTTAAAGA GAAGGATCCT TCGGGAGATC   
  
  
- AGTTACGGAT AGACGACAAC ACCTCACTTT TACCTATGTA AATCTAGAGA TATTTGGGAT GAAACCGTTC   
  
  
- TAGGTACGAC TAAAATCCTT CGGGAAACTG GTCTCTTACT GTATTGTTGG ACTATCCCAC AATGTACGAT   
  
  
- TGAAGCTAAT AAATTAGTTT TATGCCTTGA AGATAGTCAG TTTCGGACGG GGTCCAAGAT GACTTAATTT   
  
  
- AACATAACTT AACGAGAGTG AACGAATACT GCTAATTTAT TGACCTAAAC GATTAATAAG AAAAATAAAA   
  
  
- TAAGAACCAC TACATCCCTT GAGTCCCTTA ACATTGGTAT GGACAAGGTC GACTTTTCCC ATGCCCTTGT   
  
  
- CAGTATCAAT GTCCCCTTAA AGCGTTATGT AAATTCCCTC ATTCTTCGTC CTAGTACAGA AATCAATCCG   
  
  
- GCCGTCTATA CAGACACTGA GGTATACCTT TAGGTTGTGA CATAAGTGAA GTTCCGTTAT TATTATCGAG   
  
  
- ACCAAACAGA CCAGTTGACA TACCGAGACT TTTCGTGTTC TAGTTTATAC ACTGGCTTAG AAAATTACCA   
  
  
- GGTTCAATAC CGGACTTTAA GAAACAACTA AGAGGTAGTC TTCTCGAGTA GGTGGGTAGT CTACGATGAG   
  
  
- GATTGGGTAA ACTGAGAAGA AAGCACCCGT ATTCTGTACT ACTCTCAGGA ATGGTTCCTT TAATACCTAG   
  
  
- TCTTATACCC CATTCGGGTA GAAGGCTACG TCAACTTATA CTACTTCCAT TATTCTATTC CGATTTTGAA   
  
  
- GTTCTTAACC TCGTACGGGA CGAGCTACTT CTACTACTTC ACCTACTCCA CCGATATCCC GGGCACGATC   
  
  
- TTGTGTCGTA CCTCGAACTA CTTCTCACCC AGCGGGGTAA CTCTTTACAC CAAAAGGTAC TAAGTGGTTT   
  
  
- CCTCAGGTGG AGGAGTCTCA GTAGGTTACA GTCGTCGTAA TCGTCGTGGT TTCTTCATAG TGGAACGAGA   
  
  
- GGCTCATGAG GGTTTGTTAA CGAGAAATTA ACACGTTTAT ACTAAAGTCT CCCCTTAAAA CTCCTTCGGC   
  
  
- ACTGATACTA GTCGCTCGAA TCTGTCTAAC AAAGATAGGT TCCCCTAGGT CGCGTCTCCT ATCGTCGGAT   
  
  
- GTACCATCTT CCGGAACGAA GGGCAAACCG AAGTAGCCCT TTTCAAGAAA TGTTTCGAAA CTTTACGTTT   
  
  
- CTCGGTGGAA GTAAACTGTC CGATCGCCGA TACGTTTAGG AGAAACTCCA CACGGGTACA AAGTTTAAGC   
  
  
- CTAAATACCG GCGTTTACGT CGCTAATACC TACGTTAGTT TTTGCTTTTT TCCCATGTGT AGTATCTAAA   
  
  
- ACTGTATTTG GTTCCCTTAG TTATGTATTT AGAGTACGTT AGGGAACGAT TTGTTCCATT GTTCGACTGC   
  
  
- GTGAACTTCA ACTGACCTCA ACTACTGGGA CTCAGTCAAG TTGCGGGATA ACCACCGGAT TTTTTGTAGC   
  
  
- CTGTTTCCGA CGTTCGTAAT CGACTTATAG AACCACACAG TAAACTCAAG TTTCGTTATG GACGATCTTG   
  
  
- AGGTGAACAA TTGGGCCTTT ACAATCTTAC AGCTGGACGC CTTCGAAATC ACCACTTGAA ACGGAAGGTC   
  
  
- GAAGTGGTGT ACGGACTACT TTCGCAGAGT TGTTAGTTGG AGGCTCTGGT CGAAGAAGCC TACCAGTTTT   
  
  
- CGGATTTGGG TTTCGATCAT TGGTAACAGC TCGTCCTACA CTTGTGTTTG TGATGGGGAA AGGAGAGATC   
  
  
- CAAACGACTC CGTATGTCGA TGATAAGACG ACAAAAACTC AGAGAACTAC GATGAGAGGG ATCTCTATCG   
  
  
- GTCCTGTCCC ACTTACACCT CTTCGTCACG AACCGTGCAC TACAACACTT GTAGTAACGT ACACTTCCCC   
  
  
- TCCTCTCCTA ACTCGCGATA CTCCAACGCC CCTTTACCTC TCGCTCCTAC AACTACCGCC CTAAGTCCCG   
  
  
- ATAAGGTTAC CCAGTCTTCT GGCCACTATA CTAAGCCTTC GAATATAGTT ACGCCATAAC ACTGTCCGAG   
  
  
- TTCCATTTTC TCCTCTACCT ACGTGAAGTG AAACCCACCC TTCTATTTTT GAATTAGCAA TGTAGTCGAA   
  
  
- CCTCCAC

+     G-box

| Site Name | Organism | Position | Strand | Matrix score. | sequence | function |
| --- | --- | --- | --- | --- | --- | --- |
| G-box | Arabidopsis thaliana | 3469 | - | 6 | CACGTG | cis-acting regulatory element involved in light responsiveness |

>HU11G01134.1   
+ +Up\_Stream \_Len000AGATAG ACATTCAGCC ACTTGCCAGT TCAGAGAGAG ATAGAGAGAG ATTGGGTAAG   
  
  
+ CCCTTTTTGC ACTACTTTTT CTCGCTTCCA TTTTTCTTTC TTAATTTTTA GTGATGATTT TCCAGAAGAT   
  
  
+ TTGATTTATT CATCATGCTT AACCAATTGG GTCTTTGAGT ATTATTGTTT ACTGGGTATT TGATCTTTTC   
  
  
+ ATATTCCTTT GGACTGGGTG GGTGGAACTT TTGAACTTTC TTATTAGTTG GGCATGCCCT TATCTTATTC   
  
  
+ CAATTCAAAA CCCATATTTT TCTTCAATTT TAATTATGGG TATTTTGAAA TTGAATGCTT TCCTTCACAG   
  
  
+ CAATACTATA TTACATATTT TACAGGAAAG TAATATTTTA CAGGAAAGTA TGTTGCTGTT TCAACCCTGT   
  
  
+ TTTGTTTCCT CTCTTTCCTT GGATATGATG TTCATTGCTC TTGTACCAAA CCTAACCTCA ACTTCCTGAA   
  
  
+ TTATTTAAGG CAATATTACC GTGTGCTGGT CAATTTTGGT TGTGGGTTTG TTTCTTAATG TATATGATTT   
  
  
+ ATGGTCAGGA TCCATGCTCC TCTTCCGTCC ACTCTATATC TCTCTCTCTC TCTCTCAATA CAAATTCAGA   
  
  
+ GACTTTCTCT TTCTGGGTTT ACTATTGATT GCAGAAGTTT GTAGTCTTGC GAGGCCTTGA GTCTTCTTCG   
  
  
+ AGGTAGAAAA TTTTGCTTTG CGCTTCCTTC CCCATATTTT AACACCCCTC CCCCCCCAAC GCAAGTTTTC   
  
  
+ TACATTTTTG TGCAATTCTT CAGCTATGAT TCCCCAATTT CACTTGTCAC ATCTCTATTT TCCTCTTTTT   
  
  
+ GGTTTCTTGA TTCTGTAGAA GGTTGTGTAT GAACTTCTTT GTTTCCCTTT TTCCTTTGTT ATTTTTAATG   
  
  
+ TTCCCATAGA TGATATAGAG ATCTGCTTTT CCTTGCTGTG AGAGGTATCT CTGAGAATGC ATGTAGCAAT   
  
  
+ CAAAATTCTG AGTTGTTAAT TCTATTGCAT GTCTATGGGT GATGATTGTG ATGATGTTTT TTCTGTTGCC   
  
  
+ CTCTTTCTCT CTTTCACTGA AATTGCTTTC CAGAATAGTT GTTTGACTCC TTTCTGTATC TTAATTGAGC   
  
  
+ CCTTGCTTTG GTTTGTGCTC TGGGAGTTCT TCTTTAGTAA TCCCTTGCTT GTATTTTCTG AAAGAGCAAG   
  
  
+ CAATAATCCT CATAGCATGT GTATAATCAT ACAATAGCAA CAGCAAACAT GGATCCTGCG CCCAGTTAGG   
  
  
+ TTTGGTTAGG TTAAGATATG CATATTCTTT TCTCTTGAAA AGAGTTGTCT AGGAAGGGTA ATTATGCCCA   
  
  
+ TTTCATGGAT AATAATGGTA CAATTAAGTT TGATTGGCTT AAATACCAAT TATTTAGCGT CTTTTTCTTT   
  
  
+ GGCTGTCCAG GGTTCCATTT CCATAAACAA CTTTGAGTAA GGCATAGAGA ACTAAATTTC TGCCAATTGT   
  
  
+ CAAGTGCAAG TTGCTCATAT GCAACAATAC TTGGAAATTT TGAGCTGAGC TGCTGGCATA TCATGAATAG   
  
  
+ TTCTAATGGT GGCGAGTTAT CACTTATCGT CCCTTGTGTG ACGAATTTCT CTTCCTAGGA AGCCCTCTAG   
  
  
+ TCAATGCCTA TCTGCTGTTG TGGAGTGAAA ATGGATACAT TTAGATCTCT ATAAACCCTA CTTTGGCAAG   
  
  
+ ATCCATGCTG ATTTTAGGAA GCCCTTTGAC CAGAGAATGA CATAACAACC TGATAGGGTG TTACATGCTA   
  
  
+ ACTTCGATTA TTTAATCAAA ATACGGAACT TCTATCAGTC AAAGCCTGCC CCAGGTTCTA CTGAATTAAA   
  
  
+ TTGTATTGAA TTGCTCTCAC TTGCTTATGA CGATTAAATA ACTGGATTTG CTAATTATTC TTTTTATTTT   
  
  
+ ATTCTTGGTG ATGTAGGGAA CTCAGGGAAT TGTAACCATA CCTGTTCCAG CTGAAAAGGG TACGGGAACA   
  
  
+ GTCATAGTTA CAGGGGAATT TCGCAATACA TTTAAGGGAG TAAGAAGCAG GATCATGTCT TTAGTTAGGC   
  
  
+ CGGCAGATAT GTCTGTGACT CCATATGGAA ATCCAACACT GTATTCACTT CAAGGCAATA ATAATAGCTC   
  
  
+ TGGTTTGTCT GGTCAACTGT ATGGCTCTGA AAAGCACAAG ATCAAATATG TGACCGAATC TTTTAATGGT   
  
  
+ CCAAGTTATG GCCTGAAATT CTTTGTTGAT TCTCCATCAG AAGAGCTCAT CCACCCATCA GATGCTACTC   
  
  
+ CTAACCCATT TGACTCTTCT TTCGTGGGCA TAAGACATGA TGAGAGTCCT TACCAAGGAA ATTATGGATC   
  
  
+ AGAATATGGG GTAAGCCCAT CTTCCGATGC AGTTGAATAT GATGAAGGTA ATAAGATAAG GCTAAAACTT   
  
  
+ CAAGAATTGG AGCATGCCCT GCTCGATGAA GATGATGAAG TGGATGAGGT GGCTATAGGG CCCGTGCTAG   
  
  
+ AACACAGCAT GGAGCTTGAT GAAGAGTGGG TCGCCCCATT GAGAAATGTG GTTTTCCATG ATTCACCAAA   
  
  
+ GGAGTCCACC TCCTCAGAGT CATCCAATGT CAGCAGCATT AGCAGCACCA AAGAAGTATC ACCTTGCTCT   
  
  
+ CCGAGTACTC CCAAACAATT GCTCTTTAAT TGTGCAAATA TGATTTCAGA GGGGAATTTT GAGGAAGCCG   
  
  
+ TGACTATGAT CAGCGAGCTT AGACAGATTG TTTCTATCCA AGGGGATCCA GCGCAGAGGA TAGCAGCCTA   
  
  
+ CATGGTAGAA GGCCTTGCTT CCCGTTTGGC TTCATCGGGA AAAGTTCTTT ACAAAGCTTT GAAATGCAAA   
  
  
+ GAGCCACCTT CATTTGACAG GCTAGCGGCT ATGCAAATCC TCTTTGAGGT GTGCCCATGT TTCAAATTCG   
  
  
+ GATTTATGGC CGCAAATGCA GCGATTATGG ATGCAATCAA AAACGAAAAA AGGGTACACA TCATAGATTT   
  
  
+ TGACATAAAC CAAGGGAATC AATACATAAA TCTCATGCAA TCCCTTGCTA AACAAGGTAA CAAGCTGACG   
  
  
+ CACTTGAAGT TGACTGGAGT TGATGACCCT GAGTCAGTTC AACGCCCTAT TGGTGGCCTA AAAAACATCG   
  
  
+ GACAAAGGCT GCAAGCATTA GCTGAATATC TTGGTGTGTC ATTTGAGTTC AAAGCAATAC CTGCTAGAAC   
  
  
+ TCCACTTGTT AACCCGGAAA TGTTAGAATG TCGACCTGCG GAAGCTTTAG TGGTGAACTT TGCCTTCCAG   
  
  
+ CTTCACCACA TGCCTGATGA AAGCGTCTCA ACAATCAACC TCCGAGACCA GCTTCTTCGG ATGGTCAAAA   
  
  
+ GCCTAAACCC AAAGCTAGTA ACCATTGTCG AGCAGGATGT GAACACAAAC ACTACCCCTT TCCTCTCTAG   
  
  
+ GTTTGCTGAG GCATACAGCT ACTATTCTGC TGTTTTTGAG TCTCTTGATG CTACTCTCCC TAGAGATAGC   
  
  
+ CAGGACAGGG TGAATGTGGA GAAGCAGTGC TTGGCACGTG ATGTTGTGAA CATCATTGCA TGTGAAGGGG   
  
  
+ AGGAGAGGAT TGAGCGCTAT GAGGTTGCGG GGAAATGGAG AGCGAGGATG TTGATGGCGG GATTCAGGGC   
  
  
+ TATTCCAATG GGTCAGAAGA CCGGTGATAT GATTCGGAAG CTTATATCAA TGCGGTATTG TGACAGGCTC   
  
  
+ AAGGTAAAAG AGGAGATGGA TGCACTTCAC TTTGGGTGGG AAGATAAAAA CTTAATCGTT ACATCAGCTT   
  
  
+ GGAGGTG  

- +Up\_Stream \_Len000TCTATC TGTAAGTCGG TGAACGGTCA AGTCTCTCTC TATCTCTCTC TAACCCATTC   
  
  
- GGGAAAAACG TGATGAAAAA GAGCGAAGGT AAAAAGAAAG AATTAAAAAT CACTACTAAA AGGTCTTCTA   
  
  
- AACTAAATAA GTAGTACGAA TTGGTTAACC CAGAAACTCA TAATAACAAA TGACCCATAA ACTAGAAAAG   
  
  
- TATAAGGAAA CCTGACCCAC CCACCTTGAA AACTTGAAAG AATAATCAAC CCGTACGGGA ATAGAATAAG   
  
  
- GTTAAGTTTT GGGTATAAAA AGAAGTTAAA ATTAATACCC ATAAAACTTT AACTTACGAA AGGAAGTGTC   
  
  
- GTTATGATAT AATGTATAAA ATGTCCTTTC ATTATAAAAT GTCCTTTCAT ACAACGACAA AGTTGGGACA   
  
  
- AAACAAAGGA GAGAAAGGAA CCTATACTAC AAGTAACGAG AACATGGTTT GGATTGGAGT TGAAGGACTT   
  
  
- AATAAATTCC GTTATAATGG CACACGACCA GTTAAAACCA ACACCCAAAC AAAGAATTAC ATATACTAAA   
  
  
- TACCAGTCCT AGGTACGAGG AGAAGGCAGG TGAGATATAG AGAGAGAGAG AGAGAGTTAT GTTTAAGTCT   
  
  
- CTGAAAGAGA AAGACCCAAA TGATAACTAA CGTCTTCAAA CATCAGAACG CTCCGGAACT CAGAAGAAGC   
  
  
- TCCATCTTTT AAAACGAAAC GCGAAGGAAG GGGTATAAAA TTGTGGGGAG GGGGGGGTTG CGTTCAAAAG   
  
  
- ATGTAAAAAC ACGTTAAGAA GTCGATACTA AGGGGTTAAA GTGAACAGTG TAGAGATAAA AGGAGAAAAA   
  
  
- CCAAAGAACT AAGACATCTT CCAACACATA CTTGAAGAAA CAAAGGGAAA AAGGAAACAA TAAAAATTAC   
  
  
- AAGGGTATCT ACTATATCTC TAGACGAAAA GGAACGACAC TCTCCATAGA GACTCTTACG TACATCGTTA   
  
  
- GTTTTAAGAC TCAACAATTA AGATAACGTA CAGATACCCA CTACTAACAC TACTACAAAA AAGACAACGG   
  
  
- GAGAAAGAGA GAAAGTGACT TTAACGAAAG GTCTTATCAA CAAACTGAGG AAAGACATAG AATTAACTCG   
  
  
- GGAACGAAAC CAAACACGAG ACCCTCAAGA AGAAATCATT AGGGAACGAA CATAAAAGAC TTTCTCGTTC   
  
  
- GTTATTAGGA GTATCGTACA CATATTAGTA TGTTATCGTT GTCGTTTGTA CCTAGGACGC GGGTCAATCC   
  
  
- AAACCAATCC AATTCTATAC GTATAAGAAA AGAGAACTTT TCTCAACAGA TCCTTCCCAT TAATACGGGT   
  
  
- AAAGTACCTA TTATTACCAT GTTAATTCAA ACTAACCGAA TTTATGGTTA ATAAATCGCA GAAAAAGAAA   
  
  
- CCGACAGGTC CCAAGGTAAA GGTATTTGTT GAAACTCATT CCGTATCTCT TGATTTAAAG ACGGTTAACA   
  
  
- GTTCACGTTC AACGAGTATA CGTTGTTATG AACCTTTAAA ACTCGACTCG ACGACCGTAT AGTACTTATC   
  
  
- AAGATTACCA CCGCTCAATA GTGAATAGCA GGGAACACAC TGCTTAAAGA GAAGGATCCT TCGGGAGATC   
  
  
- AGTTACGGAT AGACGACAAC ACCTCACTTT TACCTATGTA AATCTAGAGA TATTTGGGAT GAAACCGTTC   
  
  
- TAGGTACGAC TAAAATCCTT CGGGAAACTG GTCTCTTACT GTATTGTTGG ACTATCCCAC AATGTACGAT   
  
  
- TGAAGCTAAT AAATTAGTTT TATGCCTTGA AGATAGTCAG TTTCGGACGG GGTCCAAGAT GACTTAATTT   
  
  
- AACATAACTT AACGAGAGTG AACGAATACT GCTAATTTAT TGACCTAAAC GATTAATAAG AAAAATAAAA   
  
  
- TAAGAACCAC TACATCCCTT GAGTCCCTTA ACATTGGTAT GGACAAGGTC GACTTTTCCC ATGCCCTTGT   
  
  
- CAGTATCAAT GTCCCCTTAA AGCGTTATGT AAATTCCCTC ATTCTTCGTC CTAGTACAGA AATCAATCCG   
  
  
- GCCGTCTATA CAGACACTGA GGTATACCTT TAGGTTGTGA CATAAGTGAA GTTCCGTTAT TATTATCGAG   
  
  
- ACCAAACAGA CCAGTTGACA TACCGAGACT TTTCGTGTTC TAGTTTATAC ACTGGCTTAG AAAATTACCA   
  
  
- GGTTCAATAC CGGACTTTAA GAAACAACTA AGAGGTAGTC TTCTCGAGTA GGTGGGTAGT CTACGATGAG   
  
  
- GATTGGGTAA ACTGAGAAGA AAGCACCCGT ATTCTGTACT ACTCTCAGGA ATGGTTCCTT TAATACCTAG   
  
  
- TCTTATACCC CATTCGGGTA GAAGGCTACG TCAACTTATA CTACTTCCAT TATTCTATTC CGATTTTGAA   
  
  
- GTTCTTAACC TCGTACGGGA CGAGCTACTT CTACTACTTC ACCTACTCCA CCGATATCCC GGGCACGATC   
  
  
- TTGTGTCGTA CCTCGAACTA CTTCTCACCC AGCGGGGTAA CTCTTTACAC CAAAAGGTAC TAAGTGGTTT   
  
  
- CCTCAGGTGG AGGAGTCTCA GTAGGTTACA GTCGTCGTAA TCGTCGTGGT TTCTTCATAG TGGAACGAGA   
  
  
- GGCTCATGAG GGTTTGTTAA CGAGAAATTA ACACGTTTAT ACTAAAGTCT CCCCTTAAAA CTCCTTCGGC   
  
  
- ACTGATACTA GTCGCTCGAA TCTGTCTAAC AAAGATAGGT TCCCCTAGGT CGCGTCTCCT ATCGTCGGAT   
  
  
- GTACCATCTT CCGGAACGAA GGGCAAACCG AAGTAGCCCT TTTCAAGAAA TGTTTCGAAA CTTTACGTTT   
  
  
- CTCGGTGGAA GTAAACTGTC CGATCGCCGA TACGTTTAGG AGAAACTCCA CACGGGTACA AAGTTTAAGC   
  
  
- CTAAATACCG GCGTTTACGT CGCTAATACC TACGTTAGTT TTTGCTTTTT TCCCATGTGT AGTATCTAAA   
  
  
- ACTGTATTTG GTTCCCTTAG TTATGTATTT AGAGTACGTT AGGGAACGAT TTGTTCCATT GTTCGACTGC   
  
  
- GTGAACTTCA ACTGACCTCA ACTACTGGGA CTCAGTCAAG TTGCGGGATA ACCACCGGAT TTTTTGTAGC   
  
  
- CTGTTTCCGA CGTTCGTAAT CGACTTATAG AACCACACAG TAAACTCAAG TTTCGTTATG GACGATCTTG   
  
  
- AGGTGAACAA TTGGGCCTTT ACAATCTTAC AGCTGGACGC CTTCGAAATC ACCACTTGAA ACGGAAGGTC   
  
  
- GAAGTGGTGT ACGGACTACT TTCGCAGAGT TGTTAGTTGG AGGCTCTGGT CGAAGAAGCC TACCAGTTTT   
  
  
- CGGATTTGGG TTTCGATCAT TGGTAACAGC TCGTCCTACA CTTGTGTTTG TGATGGGGAA AGGAGAGATC   
  
  
- CAAACGACTC CGTATGTCGA TGATAAGACG ACAAAAACTC AGAGAACTAC GATGAGAGGG ATCTCTATCG   
  
  
- GTCCTGTCCC ACTTACACCT CTTCGTCACG AACCGTGCAC TACAACACTT GTAGTAACGT ACACTTCCCC   
  
  
- TCCTCTCCTA ACTCGCGATA CTCCAACGCC CCTTTACCTC TCGCTCCTAC AACTACCGCC CTAAGTCCCG   
  
  
- ATAAGGTTAC CCAGTCTTCT GGCCACTATA CTAAGCCTTC GAATATAGTT ACGCCATAAC ACTGTCCGAG   
  
  
- TTCCATTTTC TCCTCTACCT ACGTGAAGTG AAACCCACCC TTCTATTTTT GAATTAGCAA TGTAGTCGAA   
  
  
- CCTCCAC

+     GARE-motif

| Site Name | Organism | Position | Strand | Matrix score. | sequence | function |
| --- | --- | --- | --- | --- | --- | --- |
| GARE-motif | Brassica oleracea | 1046 | + | 7 | TCTGTTG | gibberellin-responsive element |

>HU11G01134.1   
+ +Up\_Stream \_Len000AGATAG ACATTCAGCC ACTTGCCAGT TCAGAGAGAG ATAGAGAGAG ATTGGGTAAG   
  
  
+ CCCTTTTTGC ACTACTTTTT CTCGCTTCCA TTTTTCTTTC TTAATTTTTA GTGATGATTT TCCAGAAGAT   
  
  
+ TTGATTTATT CATCATGCTT AACCAATTGG GTCTTTGAGT ATTATTGTTT ACTGGGTATT TGATCTTTTC   
  
  
+ ATATTCCTTT GGACTGGGTG GGTGGAACTT TTGAACTTTC TTATTAGTTG GGCATGCCCT TATCTTATTC   
  
  
+ CAATTCAAAA CCCATATTTT TCTTCAATTT TAATTATGGG TATTTTGAAA TTGAATGCTT TCCTTCACAG   
  
  
+ CAATACTATA TTACATATTT TACAGGAAAG TAATATTTTA CAGGAAAGTA TGTTGCTGTT TCAACCCTGT   
  
  
+ TTTGTTTCCT CTCTTTCCTT GGATATGATG TTCATTGCTC TTGTACCAAA CCTAACCTCA ACTTCCTGAA   
  
  
+ TTATTTAAGG CAATATTACC GTGTGCTGGT CAATTTTGGT TGTGGGTTTG TTTCTTAATG TATATGATTT   
  
  
+ ATGGTCAGGA TCCATGCTCC TCTTCCGTCC ACTCTATATC TCTCTCTCTC TCTCTCAATA CAAATTCAGA   
  
  
+ GACTTTCTCT TTCTGGGTTT ACTATTGATT GCAGAAGTTT GTAGTCTTGC GAGGCCTTGA GTCTTCTTCG   
  
  
+ AGGTAGAAAA TTTTGCTTTG CGCTTCCTTC CCCATATTTT AACACCCCTC CCCCCCCAAC GCAAGTTTTC   
  
  
+ TACATTTTTG TGCAATTCTT CAGCTATGAT TCCCCAATTT CACTTGTCAC ATCTCTATTT TCCTCTTTTT   
  
  
+ GGTTTCTTGA TTCTGTAGAA GGTTGTGTAT GAACTTCTTT GTTTCCCTTT TTCCTTTGTT ATTTTTAATG   
  
  
+ TTCCCATAGA TGATATAGAG ATCTGCTTTT CCTTGCTGTG AGAGGTATCT CTGAGAATGC ATGTAGCAAT   
  
  
+ CAAAATTCTG AGTTGTTAAT TCTATTGCAT GTCTATGGGT GATGATTGTG ATGATGTTTT TTCTGTTGCC   
  
  
+ CTCTTTCTCT CTTTCACTGA AATTGCTTTC CAGAATAGTT GTTTGACTCC TTTCTGTATC TTAATTGAGC   
  
  
+ CCTTGCTTTG GTTTGTGCTC TGGGAGTTCT TCTTTAGTAA TCCCTTGCTT GTATTTTCTG AAAGAGCAAG   
  
  
+ CAATAATCCT CATAGCATGT GTATAATCAT ACAATAGCAA CAGCAAACAT GGATCCTGCG CCCAGTTAGG   
  
  
+ TTTGGTTAGG TTAAGATATG CATATTCTTT TCTCTTGAAA AGAGTTGTCT AGGAAGGGTA ATTATGCCCA   
  
  
+ TTTCATGGAT AATAATGGTA CAATTAAGTT TGATTGGCTT AAATACCAAT TATTTAGCGT CTTTTTCTTT   
  
  
+ GGCTGTCCAG GGTTCCATTT CCATAAACAA CTTTGAGTAA GGCATAGAGA ACTAAATTTC TGCCAATTGT   
  
  
+ CAAGTGCAAG TTGCTCATAT GCAACAATAC TTGGAAATTT TGAGCTGAGC TGCTGGCATA TCATGAATAG   
  
  
+ TTCTAATGGT GGCGAGTTAT CACTTATCGT CCCTTGTGTG ACGAATTTCT CTTCCTAGGA AGCCCTCTAG   
  
  
+ TCAATGCCTA TCTGCTGTTG TGGAGTGAAA ATGGATACAT TTAGATCTCT ATAAACCCTA CTTTGGCAAG   
  
  
+ ATCCATGCTG ATTTTAGGAA GCCCTTTGAC CAGAGAATGA CATAACAACC TGATAGGGTG TTACATGCTA   
  
  
+ ACTTCGATTA TTTAATCAAA ATACGGAACT TCTATCAGTC AAAGCCTGCC CCAGGTTCTA CTGAATTAAA   
  
  
+ TTGTATTGAA TTGCTCTCAC TTGCTTATGA CGATTAAATA ACTGGATTTG CTAATTATTC TTTTTATTTT   
  
  
+ ATTCTTGGTG ATGTAGGGAA CTCAGGGAAT TGTAACCATA CCTGTTCCAG CTGAAAAGGG TACGGGAACA   
  
  
+ GTCATAGTTA CAGGGGAATT TCGCAATACA TTTAAGGGAG TAAGAAGCAG GATCATGTCT TTAGTTAGGC   
  
  
+ CGGCAGATAT GTCTGTGACT CCATATGGAA ATCCAACACT GTATTCACTT CAAGGCAATA ATAATAGCTC   
  
  
+ TGGTTTGTCT GGTCAACTGT ATGGCTCTGA AAAGCACAAG ATCAAATATG TGACCGAATC TTTTAATGGT   
  
  
+ CCAAGTTATG GCCTGAAATT CTTTGTTGAT TCTCCATCAG AAGAGCTCAT CCACCCATCA GATGCTACTC   
  
  
+ CTAACCCATT TGACTCTTCT TTCGTGGGCA TAAGACATGA TGAGAGTCCT TACCAAGGAA ATTATGGATC   
  
  
+ AGAATATGGG GTAAGCCCAT CTTCCGATGC AGTTGAATAT GATGAAGGTA ATAAGATAAG GCTAAAACTT   
  
  
+ CAAGAATTGG AGCATGCCCT GCTCGATGAA GATGATGAAG TGGATGAGGT GGCTATAGGG CCCGTGCTAG   
  
  
+ AACACAGCAT GGAGCTTGAT GAAGAGTGGG TCGCCCCATT GAGAAATGTG GTTTTCCATG ATTCACCAAA   
  
  
+ GGAGTCCACC TCCTCAGAGT CATCCAATGT CAGCAGCATT AGCAGCACCA AAGAAGTATC ACCTTGCTCT   
  
  
+ CCGAGTACTC CCAAACAATT GCTCTTTAAT TGTGCAAATA TGATTTCAGA GGGGAATTTT GAGGAAGCCG   
  
  
+ TGACTATGAT CAGCGAGCTT AGACAGATTG TTTCTATCCA AGGGGATCCA GCGCAGAGGA TAGCAGCCTA   
  
  
+ CATGGTAGAA GGCCTTGCTT CCCGTTTGGC TTCATCGGGA AAAGTTCTTT ACAAAGCTTT GAAATGCAAA   
  
  
+ GAGCCACCTT CATTTGACAG GCTAGCGGCT ATGCAAATCC TCTTTGAGGT GTGCCCATGT TTCAAATTCG   
  
  
+ GATTTATGGC CGCAAATGCA GCGATTATGG ATGCAATCAA AAACGAAAAA AGGGTACACA TCATAGATTT   
  
  
+ TGACATAAAC CAAGGGAATC AATACATAAA TCTCATGCAA TCCCTTGCTA AACAAGGTAA CAAGCTGACG   
  
  
+ CACTTGAAGT TGACTGGAGT TGATGACCCT GAGTCAGTTC AACGCCCTAT TGGTGGCCTA AAAAACATCG   
  
  
+ GACAAAGGCT GCAAGCATTA GCTGAATATC TTGGTGTGTC ATTTGAGTTC AAAGCAATAC CTGCTAGAAC   
  
  
+ TCCACTTGTT AACCCGGAAA TGTTAGAATG TCGACCTGCG GAAGCTTTAG TGGTGAACTT TGCCTTCCAG   
  
  
+ CTTCACCACA TGCCTGATGA AAGCGTCTCA ACAATCAACC TCCGAGACCA GCTTCTTCGG ATGGTCAAAA   
  
  
+ GCCTAAACCC AAAGCTAGTA ACCATTGTCG AGCAGGATGT GAACACAAAC ACTACCCCTT TCCTCTCTAG   
  
  
+ GTTTGCTGAG GCATACAGCT ACTATTCTGC TGTTTTTGAG TCTCTTGATG CTACTCTCCC TAGAGATAGC   
  
  
+ CAGGACAGGG TGAATGTGGA GAAGCAGTGC TTGGCACGTG ATGTTGTGAA CATCATTGCA TGTGAAGGGG   
  
  
+ AGGAGAGGAT TGAGCGCTAT GAGGTTGCGG GGAAATGGAG AGCGAGGATG TTGATGGCGG GATTCAGGGC   
  
  
+ TATTCCAATG GGTCAGAAGA CCGGTGATAT GATTCGGAAG CTTATATCAA TGCGGTATTG TGACAGGCTC   
  
  
+ AAGGTAAAAG AGGAGATGGA TGCACTTCAC TTTGGGTGGG AAGATAAAAA CTTAATCGTT ACATCAGCTT   
  
  
+ GGAGGTG  

- +Up\_Stream \_Len000TCTATC TGTAAGTCGG TGAACGGTCA AGTCTCTCTC TATCTCTCTC TAACCCATTC   
  
  
- GGGAAAAACG TGATGAAAAA GAGCGAAGGT AAAAAGAAAG AATTAAAAAT CACTACTAAA AGGTCTTCTA   
  
  
- AACTAAATAA GTAGTACGAA TTGGTTAACC CAGAAACTCA TAATAACAAA TGACCCATAA ACTAGAAAAG   
  
  
- TATAAGGAAA CCTGACCCAC CCACCTTGAA AACTTGAAAG AATAATCAAC CCGTACGGGA ATAGAATAAG   
  
  
- GTTAAGTTTT GGGTATAAAA AGAAGTTAAA ATTAATACCC ATAAAACTTT AACTTACGAA AGGAAGTGTC   
  
  
- GTTATGATAT AATGTATAAA ATGTCCTTTC ATTATAAAAT GTCCTTTCAT ACAACGACAA AGTTGGGACA   
  
  
- AAACAAAGGA GAGAAAGGAA CCTATACTAC AAGTAACGAG AACATGGTTT GGATTGGAGT TGAAGGACTT   
  
  
- AATAAATTCC GTTATAATGG CACACGACCA GTTAAAACCA ACACCCAAAC AAAGAATTAC ATATACTAAA   
  
  
- TACCAGTCCT AGGTACGAGG AGAAGGCAGG TGAGATATAG AGAGAGAGAG AGAGAGTTAT GTTTAAGTCT   
  
  
- CTGAAAGAGA AAGACCCAAA TGATAACTAA CGTCTTCAAA CATCAGAACG CTCCGGAACT CAGAAGAAGC   
  
  
- TCCATCTTTT AAAACGAAAC GCGAAGGAAG GGGTATAAAA TTGTGGGGAG GGGGGGGTTG CGTTCAAAAG   
  
  
- ATGTAAAAAC ACGTTAAGAA GTCGATACTA AGGGGTTAAA GTGAACAGTG TAGAGATAAA AGGAGAAAAA   
  
  
- CCAAAGAACT AAGACATCTT CCAACACATA CTTGAAGAAA CAAAGGGAAA AAGGAAACAA TAAAAATTAC   
  
  
- AAGGGTATCT ACTATATCTC TAGACGAAAA GGAACGACAC TCTCCATAGA GACTCTTACG TACATCGTTA   
  
  
- GTTTTAAGAC TCAACAATTA AGATAACGTA CAGATACCCA CTACTAACAC TACTACAAAA AAGACAACGG   
  
  
- GAGAAAGAGA GAAAGTGACT TTAACGAAAG GTCTTATCAA CAAACTGAGG AAAGACATAG AATTAACTCG   
  
  
- GGAACGAAAC CAAACACGAG ACCCTCAAGA AGAAATCATT AGGGAACGAA CATAAAAGAC TTTCTCGTTC   
  
  
- GTTATTAGGA GTATCGTACA CATATTAGTA TGTTATCGTT GTCGTTTGTA CCTAGGACGC GGGTCAATCC   
  
  
- AAACCAATCC AATTCTATAC GTATAAGAAA AGAGAACTTT TCTCAACAGA TCCTTCCCAT TAATACGGGT   
  
  
- AAAGTACCTA TTATTACCAT GTTAATTCAA ACTAACCGAA TTTATGGTTA ATAAATCGCA GAAAAAGAAA   
  
  
- CCGACAGGTC CCAAGGTAAA GGTATTTGTT GAAACTCATT CCGTATCTCT TGATTTAAAG ACGGTTAACA   
  
  
- GTTCACGTTC AACGAGTATA CGTTGTTATG AACCTTTAAA ACTCGACTCG ACGACCGTAT AGTACTTATC   
  
  
- AAGATTACCA CCGCTCAATA GTGAATAGCA GGGAACACAC TGCTTAAAGA GAAGGATCCT TCGGGAGATC   
  
  
- AGTTACGGAT AGACGACAAC ACCTCACTTT TACCTATGTA AATCTAGAGA TATTTGGGAT GAAACCGTTC   
  
  
- TAGGTACGAC TAAAATCCTT CGGGAAACTG GTCTCTTACT GTATTGTTGG ACTATCCCAC AATGTACGAT   
  
  
- TGAAGCTAAT AAATTAGTTT TATGCCTTGA AGATAGTCAG TTTCGGACGG GGTCCAAGAT GACTTAATTT   
  
  
- AACATAACTT AACGAGAGTG AACGAATACT GCTAATTTAT TGACCTAAAC GATTAATAAG AAAAATAAAA   
  
  
- TAAGAACCAC TACATCCCTT GAGTCCCTTA ACATTGGTAT GGACAAGGTC GACTTTTCCC ATGCCCTTGT   
  
  
- CAGTATCAAT GTCCCCTTAA AGCGTTATGT AAATTCCCTC ATTCTTCGTC CTAGTACAGA AATCAATCCG   
  
  
- GCCGTCTATA CAGACACTGA GGTATACCTT TAGGTTGTGA CATAAGTGAA GTTCCGTTAT TATTATCGAG   
  
  
- ACCAAACAGA CCAGTTGACA TACCGAGACT TTTCGTGTTC TAGTTTATAC ACTGGCTTAG AAAATTACCA   
  
  
- GGTTCAATAC CGGACTTTAA GAAACAACTA AGAGGTAGTC TTCTCGAGTA GGTGGGTAGT CTACGATGAG   
  
  
- GATTGGGTAA ACTGAGAAGA AAGCACCCGT ATTCTGTACT ACTCTCAGGA ATGGTTCCTT TAATACCTAG   
  
  
- TCTTATACCC CATTCGGGTA GAAGGCTACG TCAACTTATA CTACTTCCAT TATTCTATTC CGATTTTGAA   
  
  
- GTTCTTAACC TCGTACGGGA CGAGCTACTT CTACTACTTC ACCTACTCCA CCGATATCCC GGGCACGATC   
  
  
- TTGTGTCGTA CCTCGAACTA CTTCTCACCC AGCGGGGTAA CTCTTTACAC CAAAAGGTAC TAAGTGGTTT   
  
  
- CCTCAGGTGG AGGAGTCTCA GTAGGTTACA GTCGTCGTAA TCGTCGTGGT TTCTTCATAG TGGAACGAGA   
  
  
- GGCTCATGAG GGTTTGTTAA CGAGAAATTA ACACGTTTAT ACTAAAGTCT CCCCTTAAAA CTCCTTCGGC   
  
  
- ACTGATACTA GTCGCTCGAA TCTGTCTAAC AAAGATAGGT TCCCCTAGGT CGCGTCTCCT ATCGTCGGAT   
  
  
- GTACCATCTT CCGGAACGAA GGGCAAACCG AAGTAGCCCT TTTCAAGAAA TGTTTCGAAA CTTTACGTTT   
  
  
- CTCGGTGGAA GTAAACTGTC CGATCGCCGA TACGTTTAGG AGAAACTCCA CACGGGTACA AAGTTTAAGC   
  
  
- CTAAATACCG GCGTTTACGT CGCTAATACC TACGTTAGTT TTTGCTTTTT TCCCATGTGT AGTATCTAAA   
  
  
- ACTGTATTTG GTTCCCTTAG TTATGTATTT AGAGTACGTT AGGGAACGAT TTGTTCCATT GTTCGACTGC   
  
  
- GTGAACTTCA ACTGACCTCA ACTACTGGGA CTCAGTCAAG TTGCGGGATA ACCACCGGAT TTTTTGTAGC   
  
  
- CTGTTTCCGA CGTTCGTAAT CGACTTATAG AACCACACAG TAAACTCAAG TTTCGTTATG GACGATCTTG   
  
  
- AGGTGAACAA TTGGGCCTTT ACAATCTTAC AGCTGGACGC CTTCGAAATC ACCACTTGAA ACGGAAGGTC   
  
  
- GAAGTGGTGT ACGGACTACT TTCGCAGAGT TGTTAGTTGG AGGCTCTGGT CGAAGAAGCC TACCAGTTTT   
  
  
- CGGATTTGGG TTTCGATCAT TGGTAACAGC TCGTCCTACA CTTGTGTTTG TGATGGGGAA AGGAGAGATC   
  
  
- CAAACGACTC CGTATGTCGA TGATAAGACG ACAAAAACTC AGAGAACTAC GATGAGAGGG ATCTCTATCG   
  
  
- GTCCTGTCCC ACTTACACCT CTTCGTCACG AACCGTGCAC TACAACACTT GTAGTAACGT ACACTTCCCC   
  
  
- TCCTCTCCTA ACTCGCGATA CTCCAACGCC CCTTTACCTC TCGCTCCTAC AACTACCGCC CTAAGTCCCG   
  
  
- ATAAGGTTAC CCAGTCTTCT GGCCACTATA CTAAGCCTTC GAATATAGTT ACGCCATAAC ACTGTCCGAG   
  
  
- TTCCATTTTC TCCTCTACCT ACGTGAAGTG AAACCCACCC TTCTATTTTT GAATTAGCAA TGTAGTCGAA   
  
  
- CCTCCAC

+     GATA-motif

| Site Name | Organism | Position | Strand | Matrix score. | sequence | function |
| --- | --- | --- | --- | --- | --- | --- |
| GATA-motif | Pisum sativum | 1736 | + | 7 | GATAGGG | part of a light responsive element |

>HU11G01134.1   
+ +Up\_Stream \_Len000AGATAG ACATTCAGCC ACTTGCCAGT TCAGAGAGAG ATAGAGAGAG ATTGGGTAAG   
  
  
+ CCCTTTTTGC ACTACTTTTT CTCGCTTCCA TTTTTCTTTC TTAATTTTTA GTGATGATTT TCCAGAAGAT   
  
  
+ TTGATTTATT CATCATGCTT AACCAATTGG GTCTTTGAGT ATTATTGTTT ACTGGGTATT TGATCTTTTC   
  
  
+ ATATTCCTTT GGACTGGGTG GGTGGAACTT TTGAACTTTC TTATTAGTTG GGCATGCCCT TATCTTATTC   
  
  
+ CAATTCAAAA CCCATATTTT TCTTCAATTT TAATTATGGG TATTTTGAAA TTGAATGCTT TCCTTCACAG   
  
  
+ CAATACTATA TTACATATTT TACAGGAAAG TAATATTTTA CAGGAAAGTA TGTTGCTGTT TCAACCCTGT   
  
  
+ TTTGTTTCCT CTCTTTCCTT GGATATGATG TTCATTGCTC TTGTACCAAA CCTAACCTCA ACTTCCTGAA   
  
  
+ TTATTTAAGG CAATATTACC GTGTGCTGGT CAATTTTGGT TGTGGGTTTG TTTCTTAATG TATATGATTT   
  
  
+ ATGGTCAGGA TCCATGCTCC TCTTCCGTCC ACTCTATATC TCTCTCTCTC TCTCTCAATA CAAATTCAGA   
  
  
+ GACTTTCTCT TTCTGGGTTT ACTATTGATT GCAGAAGTTT GTAGTCTTGC GAGGCCTTGA GTCTTCTTCG   
  
  
+ AGGTAGAAAA TTTTGCTTTG CGCTTCCTTC CCCATATTTT AACACCCCTC CCCCCCCAAC GCAAGTTTTC   
  
  
+ TACATTTTTG TGCAATTCTT CAGCTATGAT TCCCCAATTT CACTTGTCAC ATCTCTATTT TCCTCTTTTT   
  
  
+ GGTTTCTTGA TTCTGTAGAA GGTTGTGTAT GAACTTCTTT GTTTCCCTTT TTCCTTTGTT ATTTTTAATG   
  
  
+ TTCCCATAGA TGATATAGAG ATCTGCTTTT CCTTGCTGTG AGAGGTATCT CTGAGAATGC ATGTAGCAAT   
  
  
+ CAAAATTCTG AGTTGTTAAT TCTATTGCAT GTCTATGGGT GATGATTGTG ATGATGTTTT TTCTGTTGCC   
  
  
+ CTCTTTCTCT CTTTCACTGA AATTGCTTTC CAGAATAGTT GTTTGACTCC TTTCTGTATC TTAATTGAGC   
  
  
+ CCTTGCTTTG GTTTGTGCTC TGGGAGTTCT TCTTTAGTAA TCCCTTGCTT GTATTTTCTG AAAGAGCAAG   
  
  
+ CAATAATCCT CATAGCATGT GTATAATCAT ACAATAGCAA CAGCAAACAT GGATCCTGCG CCCAGTTAGG   
  
  
+ TTTGGTTAGG TTAAGATATG CATATTCTTT TCTCTTGAAA AGAGTTGTCT AGGAAGGGTA ATTATGCCCA   
  
  
+ TTTCATGGAT AATAATGGTA CAATTAAGTT TGATTGGCTT AAATACCAAT TATTTAGCGT CTTTTTCTTT   
  
  
+ GGCTGTCCAG GGTTCCATTT CCATAAACAA CTTTGAGTAA GGCATAGAGA ACTAAATTTC TGCCAATTGT   
  
  
+ CAAGTGCAAG TTGCTCATAT GCAACAATAC TTGGAAATTT TGAGCTGAGC TGCTGGCATA TCATGAATAG   
  
  
+ TTCTAATGGT GGCGAGTTAT CACTTATCGT CCCTTGTGTG ACGAATTTCT CTTCCTAGGA AGCCCTCTAG   
  
  
+ TCAATGCCTA TCTGCTGTTG TGGAGTGAAA ATGGATACAT TTAGATCTCT ATAAACCCTA CTTTGGCAAG   
  
  
+ ATCCATGCTG ATTTTAGGAA GCCCTTTGAC CAGAGAATGA CATAACAACC TGATAGGGTG TTACATGCTA   
  
  
+ ACTTCGATTA TTTAATCAAA ATACGGAACT TCTATCAGTC AAAGCCTGCC CCAGGTTCTA CTGAATTAAA   
  
  
+ TTGTATTGAA TTGCTCTCAC TTGCTTATGA CGATTAAATA ACTGGATTTG CTAATTATTC TTTTTATTTT   
  
  
+ ATTCTTGGTG ATGTAGGGAA CTCAGGGAAT TGTAACCATA CCTGTTCCAG CTGAAAAGGG TACGGGAACA   
  
  
+ GTCATAGTTA CAGGGGAATT TCGCAATACA TTTAAGGGAG TAAGAAGCAG GATCATGTCT TTAGTTAGGC   
  
  
+ CGGCAGATAT GTCTGTGACT CCATATGGAA ATCCAACACT GTATTCACTT CAAGGCAATA ATAATAGCTC   
  
  
+ TGGTTTGTCT GGTCAACTGT ATGGCTCTGA AAAGCACAAG ATCAAATATG TGACCGAATC TTTTAATGGT   
  
  
+ CCAAGTTATG GCCTGAAATT CTTTGTTGAT TCTCCATCAG AAGAGCTCAT CCACCCATCA GATGCTACTC   
  
  
+ CTAACCCATT TGACTCTTCT TTCGTGGGCA TAAGACATGA TGAGAGTCCT TACCAAGGAA ATTATGGATC   
  
  
+ AGAATATGGG GTAAGCCCAT CTTCCGATGC AGTTGAATAT GATGAAGGTA ATAAGATAAG GCTAAAACTT   
  
  
+ CAAGAATTGG AGCATGCCCT GCTCGATGAA GATGATGAAG TGGATGAGGT GGCTATAGGG CCCGTGCTAG   
  
  
+ AACACAGCAT GGAGCTTGAT GAAGAGTGGG TCGCCCCATT GAGAAATGTG GTTTTCCATG ATTCACCAAA   
  
  
+ GGAGTCCACC TCCTCAGAGT CATCCAATGT CAGCAGCATT AGCAGCACCA AAGAAGTATC ACCTTGCTCT   
  
  
+ CCGAGTACTC CCAAACAATT GCTCTTTAAT TGTGCAAATA TGATTTCAGA GGGGAATTTT GAGGAAGCCG   
  
  
+ TGACTATGAT CAGCGAGCTT AGACAGATTG TTTCTATCCA AGGGGATCCA GCGCAGAGGA TAGCAGCCTA   
  
  
+ CATGGTAGAA GGCCTTGCTT CCCGTTTGGC TTCATCGGGA AAAGTTCTTT ACAAAGCTTT GAAATGCAAA   
  
  
+ GAGCCACCTT CATTTGACAG GCTAGCGGCT ATGCAAATCC TCTTTGAGGT GTGCCCATGT TTCAAATTCG   
  
  
+ GATTTATGGC CGCAAATGCA GCGATTATGG ATGCAATCAA AAACGAAAAA AGGGTACACA TCATAGATTT   
  
  
+ TGACATAAAC CAAGGGAATC AATACATAAA TCTCATGCAA TCCCTTGCTA AACAAGGTAA CAAGCTGACG   
  
  
+ CACTTGAAGT TGACTGGAGT TGATGACCCT GAGTCAGTTC AACGCCCTAT TGGTGGCCTA AAAAACATCG   
  
  
+ GACAAAGGCT GCAAGCATTA GCTGAATATC TTGGTGTGTC ATTTGAGTTC AAAGCAATAC CTGCTAGAAC   
  
  
+ TCCACTTGTT AACCCGGAAA TGTTAGAATG TCGACCTGCG GAAGCTTTAG TGGTGAACTT TGCCTTCCAG   
  
  
+ CTTCACCACA TGCCTGATGA AAGCGTCTCA ACAATCAACC TCCGAGACCA GCTTCTTCGG ATGGTCAAAA   
  
  
+ GCCTAAACCC AAAGCTAGTA ACCATTGTCG AGCAGGATGT GAACACAAAC ACTACCCCTT TCCTCTCTAG   
  
  
+ GTTTGCTGAG GCATACAGCT ACTATTCTGC TGTTTTTGAG TCTCTTGATG CTACTCTCCC TAGAGATAGC   
  
  
+ CAGGACAGGG TGAATGTGGA GAAGCAGTGC TTGGCACGTG ATGTTGTGAA CATCATTGCA TGTGAAGGGG   
  
  
+ AGGAGAGGAT TGAGCGCTAT GAGGTTGCGG GGAAATGGAG AGCGAGGATG TTGATGGCGG GATTCAGGGC   
  
  
+ TATTCCAATG GGTCAGAAGA CCGGTGATAT GATTCGGAAG CTTATATCAA TGCGGTATTG TGACAGGCTC   
  
  
+ AAGGTAAAAG AGGAGATGGA TGCACTTCAC TTTGGGTGGG AAGATAAAAA CTTAATCGTT ACATCAGCTT   
  
  
+ GGAGGTG  

- +Up\_Stream \_Len000TCTATC TGTAAGTCGG TGAACGGTCA AGTCTCTCTC TATCTCTCTC TAACCCATTC   
  
  
- GGGAAAAACG TGATGAAAAA GAGCGAAGGT AAAAAGAAAG AATTAAAAAT CACTACTAAA AGGTCTTCTA   
  
  
- AACTAAATAA GTAGTACGAA TTGGTTAACC CAGAAACTCA TAATAACAAA TGACCCATAA ACTAGAAAAG   
  
  
- TATAAGGAAA CCTGACCCAC CCACCTTGAA AACTTGAAAG AATAATCAAC CCGTACGGGA ATAGAATAAG   
  
  
- GTTAAGTTTT GGGTATAAAA AGAAGTTAAA ATTAATACCC ATAAAACTTT AACTTACGAA AGGAAGTGTC   
  
  
- GTTATGATAT AATGTATAAA ATGTCCTTTC ATTATAAAAT GTCCTTTCAT ACAACGACAA AGTTGGGACA   
  
  
- AAACAAAGGA GAGAAAGGAA CCTATACTAC AAGTAACGAG AACATGGTTT GGATTGGAGT TGAAGGACTT   
  
  
- AATAAATTCC GTTATAATGG CACACGACCA GTTAAAACCA ACACCCAAAC AAAGAATTAC ATATACTAAA   
  
  
- TACCAGTCCT AGGTACGAGG AGAAGGCAGG TGAGATATAG AGAGAGAGAG AGAGAGTTAT GTTTAAGTCT   
  
  
- CTGAAAGAGA AAGACCCAAA TGATAACTAA CGTCTTCAAA CATCAGAACG CTCCGGAACT CAGAAGAAGC   
  
  
- TCCATCTTTT AAAACGAAAC GCGAAGGAAG GGGTATAAAA TTGTGGGGAG GGGGGGGTTG CGTTCAAAAG   
  
  
- ATGTAAAAAC ACGTTAAGAA GTCGATACTA AGGGGTTAAA GTGAACAGTG TAGAGATAAA AGGAGAAAAA   
  
  
- CCAAAGAACT AAGACATCTT CCAACACATA CTTGAAGAAA CAAAGGGAAA AAGGAAACAA TAAAAATTAC   
  
  
- AAGGGTATCT ACTATATCTC TAGACGAAAA GGAACGACAC TCTCCATAGA GACTCTTACG TACATCGTTA   
  
  
- GTTTTAAGAC TCAACAATTA AGATAACGTA CAGATACCCA CTACTAACAC TACTACAAAA AAGACAACGG   
  
  
- GAGAAAGAGA GAAAGTGACT TTAACGAAAG GTCTTATCAA CAAACTGAGG AAAGACATAG AATTAACTCG   
  
  
- GGAACGAAAC CAAACACGAG ACCCTCAAGA AGAAATCATT AGGGAACGAA CATAAAAGAC TTTCTCGTTC   
  
  
- GTTATTAGGA GTATCGTACA CATATTAGTA TGTTATCGTT GTCGTTTGTA CCTAGGACGC GGGTCAATCC   
  
  
- AAACCAATCC AATTCTATAC GTATAAGAAA AGAGAACTTT TCTCAACAGA TCCTTCCCAT TAATACGGGT   
  
  
- AAAGTACCTA TTATTACCAT GTTAATTCAA ACTAACCGAA TTTATGGTTA ATAAATCGCA GAAAAAGAAA   
  
  
- CCGACAGGTC CCAAGGTAAA GGTATTTGTT GAAACTCATT CCGTATCTCT TGATTTAAAG ACGGTTAACA   
  
  
- GTTCACGTTC AACGAGTATA CGTTGTTATG AACCTTTAAA ACTCGACTCG ACGACCGTAT AGTACTTATC   
  
  
- AAGATTACCA CCGCTCAATA GTGAATAGCA GGGAACACAC TGCTTAAAGA GAAGGATCCT TCGGGAGATC   
  
  
- AGTTACGGAT AGACGACAAC ACCTCACTTT TACCTATGTA AATCTAGAGA TATTTGGGAT GAAACCGTTC   
  
  
- TAGGTACGAC TAAAATCCTT CGGGAAACTG GTCTCTTACT GTATTGTTGG ACTATCCCAC AATGTACGAT   
  
  
- TGAAGCTAAT AAATTAGTTT TATGCCTTGA AGATAGTCAG TTTCGGACGG GGTCCAAGAT GACTTAATTT   
  
  
- AACATAACTT AACGAGAGTG AACGAATACT GCTAATTTAT TGACCTAAAC GATTAATAAG AAAAATAAAA   
  
  
- TAAGAACCAC TACATCCCTT GAGTCCCTTA ACATTGGTAT GGACAAGGTC GACTTTTCCC ATGCCCTTGT   
  
  
- CAGTATCAAT GTCCCCTTAA AGCGTTATGT AAATTCCCTC ATTCTTCGTC CTAGTACAGA AATCAATCCG   
  
  
- GCCGTCTATA CAGACACTGA GGTATACCTT TAGGTTGTGA CATAAGTGAA GTTCCGTTAT TATTATCGAG   
  
  
- ACCAAACAGA CCAGTTGACA TACCGAGACT TTTCGTGTTC TAGTTTATAC ACTGGCTTAG AAAATTACCA   
  
  
- GGTTCAATAC CGGACTTTAA GAAACAACTA AGAGGTAGTC TTCTCGAGTA GGTGGGTAGT CTACGATGAG   
  
  
- GATTGGGTAA ACTGAGAAGA AAGCACCCGT ATTCTGTACT ACTCTCAGGA ATGGTTCCTT TAATACCTAG   
  
  
- TCTTATACCC CATTCGGGTA GAAGGCTACG TCAACTTATA CTACTTCCAT TATTCTATTC CGATTTTGAA   
  
  
- GTTCTTAACC TCGTACGGGA CGAGCTACTT CTACTACTTC ACCTACTCCA CCGATATCCC GGGCACGATC   
  
  
- TTGTGTCGTA CCTCGAACTA CTTCTCACCC AGCGGGGTAA CTCTTTACAC CAAAAGGTAC TAAGTGGTTT   
  
  
- CCTCAGGTGG AGGAGTCTCA GTAGGTTACA GTCGTCGTAA TCGTCGTGGT TTCTTCATAG TGGAACGAGA   
  
  
- GGCTCATGAG GGTTTGTTAA CGAGAAATTA ACACGTTTAT ACTAAAGTCT CCCCTTAAAA CTCCTTCGGC   
  
  
- ACTGATACTA GTCGCTCGAA TCTGTCTAAC AAAGATAGGT TCCCCTAGGT CGCGTCTCCT ATCGTCGGAT   
  
  
- GTACCATCTT CCGGAACGAA GGGCAAACCG AAGTAGCCCT TTTCAAGAAA TGTTTCGAAA CTTTACGTTT   
  
  
- CTCGGTGGAA GTAAACTGTC CGATCGCCGA TACGTTTAGG AGAAACTCCA CACGGGTACA AAGTTTAAGC   
  
  
- CTAAATACCG GCGTTTACGT CGCTAATACC TACGTTAGTT TTTGCTTTTT TCCCATGTGT AGTATCTAAA   
  
  
- ACTGTATTTG GTTCCCTTAG TTATGTATTT AGAGTACGTT AGGGAACGAT TTGTTCCATT GTTCGACTGC   
  
  
- GTGAACTTCA ACTGACCTCA ACTACTGGGA CTCAGTCAAG TTGCGGGATA ACCACCGGAT TTTTTGTAGC   
  
  
- CTGTTTCCGA CGTTCGTAAT CGACTTATAG AACCACACAG TAAACTCAAG TTTCGTTATG GACGATCTTG   
  
  
- AGGTGAACAA TTGGGCCTTT ACAATCTTAC AGCTGGACGC CTTCGAAATC ACCACTTGAA ACGGAAGGTC   
  
  
- GAAGTGGTGT ACGGACTACT TTCGCAGAGT TGTTAGTTGG AGGCTCTGGT CGAAGAAGCC TACCAGTTTT   
  
  
- CGGATTTGGG TTTCGATCAT TGGTAACAGC TCGTCCTACA CTTGTGTTTG TGATGGGGAA AGGAGAGATC   
  
  
- CAAACGACTC CGTATGTCGA TGATAAGACG ACAAAAACTC AGAGAACTAC GATGAGAGGG ATCTCTATCG   
  
  
- GTCCTGTCCC ACTTACACCT CTTCGTCACG AACCGTGCAC TACAACACTT GTAGTAACGT ACACTTCCCC   
  
  
- TCCTCTCCTA ACTCGCGATA CTCCAACGCC CCTTTACCTC TCGCTCCTAC AACTACCGCC CTAAGTCCCG   
  
  
- ATAAGGTTAC CCAGTCTTCT GGCCACTATA CTAAGCCTTC GAATATAGTT ACGCCATAAC ACTGTCCGAG   
  
  
- TTCCATTTTC TCCTCTACCT ACGTGAAGTG AAACCCACCC TTCTATTTTT GAATTAGCAA TGTAGTCGAA   
  
  
- CCTCCAC

+     GCN4\_motif

| Site Name | Organism | Position | Strand | Matrix score. | sequence | function |
| --- | --- | --- | --- | --- | --- | --- |
| GCN4\_motif | Oryza sativa | 3044 | + | 7 | TGAGTCA | cis-regulatory element involved in endosperm expression |

>HU11G01134.1   
+ +Up\_Stream \_Len000AGATAG ACATTCAGCC ACTTGCCAGT TCAGAGAGAG ATAGAGAGAG ATTGGGTAAG   
  
  
+ CCCTTTTTGC ACTACTTTTT CTCGCTTCCA TTTTTCTTTC TTAATTTTTA GTGATGATTT TCCAGAAGAT   
  
  
+ TTGATTTATT CATCATGCTT AACCAATTGG GTCTTTGAGT ATTATTGTTT ACTGGGTATT TGATCTTTTC   
  
  
+ ATATTCCTTT GGACTGGGTG GGTGGAACTT TTGAACTTTC TTATTAGTTG GGCATGCCCT TATCTTATTC   
  
  
+ CAATTCAAAA CCCATATTTT TCTTCAATTT TAATTATGGG TATTTTGAAA TTGAATGCTT TCCTTCACAG   
  
  
+ CAATACTATA TTACATATTT TACAGGAAAG TAATATTTTA CAGGAAAGTA TGTTGCTGTT TCAACCCTGT   
  
  
+ TTTGTTTCCT CTCTTTCCTT GGATATGATG TTCATTGCTC TTGTACCAAA CCTAACCTCA ACTTCCTGAA   
  
  
+ TTATTTAAGG CAATATTACC GTGTGCTGGT CAATTTTGGT TGTGGGTTTG TTTCTTAATG TATATGATTT   
  
  
+ ATGGTCAGGA TCCATGCTCC TCTTCCGTCC ACTCTATATC TCTCTCTCTC TCTCTCAATA CAAATTCAGA   
  
  
+ GACTTTCTCT TTCTGGGTTT ACTATTGATT GCAGAAGTTT GTAGTCTTGC GAGGCCTTGA GTCTTCTTCG   
  
  
+ AGGTAGAAAA TTTTGCTTTG CGCTTCCTTC CCCATATTTT AACACCCCTC CCCCCCCAAC GCAAGTTTTC   
  
  
+ TACATTTTTG TGCAATTCTT CAGCTATGAT TCCCCAATTT CACTTGTCAC ATCTCTATTT TCCTCTTTTT   
  
  
+ GGTTTCTTGA TTCTGTAGAA GGTTGTGTAT GAACTTCTTT GTTTCCCTTT TTCCTTTGTT ATTTTTAATG   
  
  
+ TTCCCATAGA TGATATAGAG ATCTGCTTTT CCTTGCTGTG AGAGGTATCT CTGAGAATGC ATGTAGCAAT   
  
  
+ CAAAATTCTG AGTTGTTAAT TCTATTGCAT GTCTATGGGT GATGATTGTG ATGATGTTTT TTCTGTTGCC   
  
  
+ CTCTTTCTCT CTTTCACTGA AATTGCTTTC CAGAATAGTT GTTTGACTCC TTTCTGTATC TTAATTGAGC   
  
  
+ CCTTGCTTTG GTTTGTGCTC TGGGAGTTCT TCTTTAGTAA TCCCTTGCTT GTATTTTCTG AAAGAGCAAG   
  
  
+ CAATAATCCT CATAGCATGT GTATAATCAT ACAATAGCAA CAGCAAACAT GGATCCTGCG CCCAGTTAGG   
  
  
+ TTTGGTTAGG TTAAGATATG CATATTCTTT TCTCTTGAAA AGAGTTGTCT AGGAAGGGTA ATTATGCCCA   
  
  
+ TTTCATGGAT AATAATGGTA CAATTAAGTT TGATTGGCTT AAATACCAAT TATTTAGCGT CTTTTTCTTT   
  
  
+ GGCTGTCCAG GGTTCCATTT CCATAAACAA CTTTGAGTAA GGCATAGAGA ACTAAATTTC TGCCAATTGT   
  
  
+ CAAGTGCAAG TTGCTCATAT GCAACAATAC TTGGAAATTT TGAGCTGAGC TGCTGGCATA TCATGAATAG   
  
  
+ TTCTAATGGT GGCGAGTTAT CACTTATCGT CCCTTGTGTG ACGAATTTCT CTTCCTAGGA AGCCCTCTAG   
  
  
+ TCAATGCCTA TCTGCTGTTG TGGAGTGAAA ATGGATACAT TTAGATCTCT ATAAACCCTA CTTTGGCAAG   
  
  
+ ATCCATGCTG ATTTTAGGAA GCCCTTTGAC CAGAGAATGA CATAACAACC TGATAGGGTG TTACATGCTA   
  
  
+ ACTTCGATTA TTTAATCAAA ATACGGAACT TCTATCAGTC AAAGCCTGCC CCAGGTTCTA CTGAATTAAA   
  
  
+ TTGTATTGAA TTGCTCTCAC TTGCTTATGA CGATTAAATA ACTGGATTTG CTAATTATTC TTTTTATTTT   
  
  
+ ATTCTTGGTG ATGTAGGGAA CTCAGGGAAT TGTAACCATA CCTGTTCCAG CTGAAAAGGG TACGGGAACA   
  
  
+ GTCATAGTTA CAGGGGAATT TCGCAATACA TTTAAGGGAG TAAGAAGCAG GATCATGTCT TTAGTTAGGC   
  
  
+ CGGCAGATAT GTCTGTGACT CCATATGGAA ATCCAACACT GTATTCACTT CAAGGCAATA ATAATAGCTC   
  
  
+ TGGTTTGTCT GGTCAACTGT ATGGCTCTGA AAAGCACAAG ATCAAATATG TGACCGAATC TTTTAATGGT   
  
  
+ CCAAGTTATG GCCTGAAATT CTTTGTTGAT TCTCCATCAG AAGAGCTCAT CCACCCATCA GATGCTACTC   
  
  
+ CTAACCCATT TGACTCTTCT TTCGTGGGCA TAAGACATGA TGAGAGTCCT TACCAAGGAA ATTATGGATC   
  
  
+ AGAATATGGG GTAAGCCCAT CTTCCGATGC AGTTGAATAT GATGAAGGTA ATAAGATAAG GCTAAAACTT   
  
  
+ CAAGAATTGG AGCATGCCCT GCTCGATGAA GATGATGAAG TGGATGAGGT GGCTATAGGG CCCGTGCTAG   
  
  
+ AACACAGCAT GGAGCTTGAT GAAGAGTGGG TCGCCCCATT GAGAAATGTG GTTTTCCATG ATTCACCAAA   
  
  
+ GGAGTCCACC TCCTCAGAGT CATCCAATGT CAGCAGCATT AGCAGCACCA AAGAAGTATC ACCTTGCTCT   
  
  
+ CCGAGTACTC CCAAACAATT GCTCTTTAAT TGTGCAAATA TGATTTCAGA GGGGAATTTT GAGGAAGCCG   
  
  
+ TGACTATGAT CAGCGAGCTT AGACAGATTG TTTCTATCCA AGGGGATCCA GCGCAGAGGA TAGCAGCCTA   
  
  
+ CATGGTAGAA GGCCTTGCTT CCCGTTTGGC TTCATCGGGA AAAGTTCTTT ACAAAGCTTT GAAATGCAAA   
  
  
+ GAGCCACCTT CATTTGACAG GCTAGCGGCT ATGCAAATCC TCTTTGAGGT GTGCCCATGT TTCAAATTCG   
  
  
+ GATTTATGGC CGCAAATGCA GCGATTATGG ATGCAATCAA AAACGAAAAA AGGGTACACA TCATAGATTT   
  
  
+ TGACATAAAC CAAGGGAATC AATACATAAA TCTCATGCAA TCCCTTGCTA AACAAGGTAA CAAGCTGACG   
  
  
+ CACTTGAAGT TGACTGGAGT TGATGACCCT GAGTCAGTTC AACGCCCTAT TGGTGGCCTA AAAAACATCG   
  
  
+ GACAAAGGCT GCAAGCATTA GCTGAATATC TTGGTGTGTC ATTTGAGTTC AAAGCAATAC CTGCTAGAAC   
  
  
+ TCCACTTGTT AACCCGGAAA TGTTAGAATG TCGACCTGCG GAAGCTTTAG TGGTGAACTT TGCCTTCCAG   
  
  
+ CTTCACCACA TGCCTGATGA AAGCGTCTCA ACAATCAACC TCCGAGACCA GCTTCTTCGG ATGGTCAAAA   
  
  
+ GCCTAAACCC AAAGCTAGTA ACCATTGTCG AGCAGGATGT GAACACAAAC ACTACCCCTT TCCTCTCTAG   
  
  
+ GTTTGCTGAG GCATACAGCT ACTATTCTGC TGTTTTTGAG TCTCTTGATG CTACTCTCCC TAGAGATAGC   
  
  
+ CAGGACAGGG TGAATGTGGA GAAGCAGTGC TTGGCACGTG ATGTTGTGAA CATCATTGCA TGTGAAGGGG   
  
  
+ AGGAGAGGAT TGAGCGCTAT GAGGTTGCGG GGAAATGGAG AGCGAGGATG TTGATGGCGG GATTCAGGGC   
  
  
+ TATTCCAATG GGTCAGAAGA CCGGTGATAT GATTCGGAAG CTTATATCAA TGCGGTATTG TGACAGGCTC   
  
  
+ AAGGTAAAAG AGGAGATGGA TGCACTTCAC TTTGGGTGGG AAGATAAAAA CTTAATCGTT ACATCAGCTT   
  
  
+ GGAGGTG  

- +Up\_Stream \_Len000TCTATC TGTAAGTCGG TGAACGGTCA AGTCTCTCTC TATCTCTCTC TAACCCATTC   
  
  
- GGGAAAAACG TGATGAAAAA GAGCGAAGGT AAAAAGAAAG AATTAAAAAT CACTACTAAA AGGTCTTCTA   
  
  
- AACTAAATAA GTAGTACGAA TTGGTTAACC CAGAAACTCA TAATAACAAA TGACCCATAA ACTAGAAAAG   
  
  
- TATAAGGAAA CCTGACCCAC CCACCTTGAA AACTTGAAAG AATAATCAAC CCGTACGGGA ATAGAATAAG   
  
  
- GTTAAGTTTT GGGTATAAAA AGAAGTTAAA ATTAATACCC ATAAAACTTT AACTTACGAA AGGAAGTGTC   
  
  
- GTTATGATAT AATGTATAAA ATGTCCTTTC ATTATAAAAT GTCCTTTCAT ACAACGACAA AGTTGGGACA   
  
  
- AAACAAAGGA GAGAAAGGAA CCTATACTAC AAGTAACGAG AACATGGTTT GGATTGGAGT TGAAGGACTT   
  
  
- AATAAATTCC GTTATAATGG CACACGACCA GTTAAAACCA ACACCCAAAC AAAGAATTAC ATATACTAAA   
  
  
- TACCAGTCCT AGGTACGAGG AGAAGGCAGG TGAGATATAG AGAGAGAGAG AGAGAGTTAT GTTTAAGTCT   
  
  
- CTGAAAGAGA AAGACCCAAA TGATAACTAA CGTCTTCAAA CATCAGAACG CTCCGGAACT CAGAAGAAGC   
  
  
- TCCATCTTTT AAAACGAAAC GCGAAGGAAG GGGTATAAAA TTGTGGGGAG GGGGGGGTTG CGTTCAAAAG   
  
  
- ATGTAAAAAC ACGTTAAGAA GTCGATACTA AGGGGTTAAA GTGAACAGTG TAGAGATAAA AGGAGAAAAA   
  
  
- CCAAAGAACT AAGACATCTT CCAACACATA CTTGAAGAAA CAAAGGGAAA AAGGAAACAA TAAAAATTAC   
  
  
- AAGGGTATCT ACTATATCTC TAGACGAAAA GGAACGACAC TCTCCATAGA GACTCTTACG TACATCGTTA   
  
  
- GTTTTAAGAC TCAACAATTA AGATAACGTA CAGATACCCA CTACTAACAC TACTACAAAA AAGACAACGG   
  
  
- GAGAAAGAGA GAAAGTGACT TTAACGAAAG GTCTTATCAA CAAACTGAGG AAAGACATAG AATTAACTCG   
  
  
- GGAACGAAAC CAAACACGAG ACCCTCAAGA AGAAATCATT AGGGAACGAA CATAAAAGAC TTTCTCGTTC   
  
  
- GTTATTAGGA GTATCGTACA CATATTAGTA TGTTATCGTT GTCGTTTGTA CCTAGGACGC GGGTCAATCC   
  
  
- AAACCAATCC AATTCTATAC GTATAAGAAA AGAGAACTTT TCTCAACAGA TCCTTCCCAT TAATACGGGT   
  
  
- AAAGTACCTA TTATTACCAT GTTAATTCAA ACTAACCGAA TTTATGGTTA ATAAATCGCA GAAAAAGAAA   
  
  
- CCGACAGGTC CCAAGGTAAA GGTATTTGTT GAAACTCATT CCGTATCTCT TGATTTAAAG ACGGTTAACA   
  
  
- GTTCACGTTC AACGAGTATA CGTTGTTATG AACCTTTAAA ACTCGACTCG ACGACCGTAT AGTACTTATC   
  
  
- AAGATTACCA CCGCTCAATA GTGAATAGCA GGGAACACAC TGCTTAAAGA GAAGGATCCT TCGGGAGATC   
  
  
- AGTTACGGAT AGACGACAAC ACCTCACTTT TACCTATGTA AATCTAGAGA TATTTGGGAT GAAACCGTTC   
  
  
- TAGGTACGAC TAAAATCCTT CGGGAAACTG GTCTCTTACT GTATTGTTGG ACTATCCCAC AATGTACGAT   
  
  
- TGAAGCTAAT AAATTAGTTT TATGCCTTGA AGATAGTCAG TTTCGGACGG GGTCCAAGAT GACTTAATTT   
  
  
- AACATAACTT AACGAGAGTG AACGAATACT GCTAATTTAT TGACCTAAAC GATTAATAAG AAAAATAAAA   
  
  
- TAAGAACCAC TACATCCCTT GAGTCCCTTA ACATTGGTAT GGACAAGGTC GACTTTTCCC ATGCCCTTGT   
  
  
- CAGTATCAAT GTCCCCTTAA AGCGTTATGT AAATTCCCTC ATTCTTCGTC CTAGTACAGA AATCAATCCG   
  
  
- GCCGTCTATA CAGACACTGA GGTATACCTT TAGGTTGTGA CATAAGTGAA GTTCCGTTAT TATTATCGAG   
  
  
- ACCAAACAGA CCAGTTGACA TACCGAGACT TTTCGTGTTC TAGTTTATAC ACTGGCTTAG AAAATTACCA   
  
  
- GGTTCAATAC CGGACTTTAA GAAACAACTA AGAGGTAGTC TTCTCGAGTA GGTGGGTAGT CTACGATGAG   
  
  
- GATTGGGTAA ACTGAGAAGA AAGCACCCGT ATTCTGTACT ACTCTCAGGA ATGGTTCCTT TAATACCTAG   
  
  
- TCTTATACCC CATTCGGGTA GAAGGCTACG TCAACTTATA CTACTTCCAT TATTCTATTC CGATTTTGAA   
  
  
- GTTCTTAACC TCGTACGGGA CGAGCTACTT CTACTACTTC ACCTACTCCA CCGATATCCC GGGCACGATC   
  
  
- TTGTGTCGTA CCTCGAACTA CTTCTCACCC AGCGGGGTAA CTCTTTACAC CAAAAGGTAC TAAGTGGTTT   
  
  
- CCTCAGGTGG AGGAGTCTCA GTAGGTTACA GTCGTCGTAA TCGTCGTGGT TTCTTCATAG TGGAACGAGA   
  
  
- GGCTCATGAG GGTTTGTTAA CGAGAAATTA ACACGTTTAT ACTAAAGTCT CCCCTTAAAA CTCCTTCGGC   
  
  
- ACTGATACTA GTCGCTCGAA TCTGTCTAAC AAAGATAGGT TCCCCTAGGT CGCGTCTCCT ATCGTCGGAT   
  
  
- GTACCATCTT CCGGAACGAA GGGCAAACCG AAGTAGCCCT TTTCAAGAAA TGTTTCGAAA CTTTACGTTT   
  
  
- CTCGGTGGAA GTAAACTGTC CGATCGCCGA TACGTTTAGG AGAAACTCCA CACGGGTACA AAGTTTAAGC   
  
  
- CTAAATACCG GCGTTTACGT CGCTAATACC TACGTTAGTT TTTGCTTTTT TCCCATGTGT AGTATCTAAA   
  
  
- ACTGTATTTG GTTCCCTTAG TTATGTATTT AGAGTACGTT AGGGAACGAT TTGTTCCATT GTTCGACTGC   
  
  
- GTGAACTTCA ACTGACCTCA ACTACTGGGA CTCAGTCAAG TTGCGGGATA ACCACCGGAT TTTTTGTAGC   
  
  
- CTGTTTCCGA CGTTCGTAAT CGACTTATAG AACCACACAG TAAACTCAAG TTTCGTTATG GACGATCTTG   
  
  
- AGGTGAACAA TTGGGCCTTT ACAATCTTAC AGCTGGACGC CTTCGAAATC ACCACTTGAA ACGGAAGGTC   
  
  
- GAAGTGGTGT ACGGACTACT TTCGCAGAGT TGTTAGTTGG AGGCTCTGGT CGAAGAAGCC TACCAGTTTT   
  
  
- CGGATTTGGG TTTCGATCAT TGGTAACAGC TCGTCCTACA CTTGTGTTTG TGATGGGGAA AGGAGAGATC   
  
  
- CAAACGACTC CGTATGTCGA TGATAAGACG ACAAAAACTC AGAGAACTAC GATGAGAGGG ATCTCTATCG   
  
  
- GTCCTGTCCC ACTTACACCT CTTCGTCACG AACCGTGCAC TACAACACTT GTAGTAACGT ACACTTCCCC   
  
  
- TCCTCTCCTA ACTCGCGATA CTCCAACGCC CCTTTACCTC TCGCTCCTAC AACTACCGCC CTAAGTCCCG   
  
  
- ATAAGGTTAC CCAGTCTTCT GGCCACTATA CTAAGCCTTC GAATATAGTT ACGCCATAAC ACTGTCCGAG   
  
  
- TTCCATTTTC TCCTCTACCT ACGTGAAGTG AAACCCACCC TTCTATTTTT GAATTAGCAA TGTAGTCGAA   
  
  
- CCTCCAC

+     GT1-motif

| Site Name | Organism | Position | Strand | Matrix score. | sequence | function |
| --- | --- | --- | --- | --- | --- | --- |
| GT1-motif | Arabidopsis thaliana | 1273 | + | 6 | GGTTAA | light responsive element |
| GT1-motif | Arabidopsis thaliana | 163 | - | 6 | GGTTAA | light responsive element |
| GT1-motif | Arabidopsis thaliana | 3163 | - | 6 | GGTTAA | light responsive element |

>HU11G01134.1   
+ +Up\_Stream \_Len000AGATAG ACATTCAGCC ACTTGCCAGT TCAGAGAGAG ATAGAGAGAG ATTGGGTAAG   
  
  
+ CCCTTTTTGC ACTACTTTTT CTCGCTTCCA TTTTTCTTTC TTAATTTTTA GTGATGATTT TCCAGAAGAT   
  
  
+ TTGATTTATT CATCATGCTT AACCAATTGG GTCTTTGAGT ATTATTGTTT ACTGGGTATT TGATCTTTTC   
  
  
+ ATATTCCTTT GGACTGGGTG GGTGGAACTT TTGAACTTTC TTATTAGTTG GGCATGCCCT TATCTTATTC   
  
  
+ CAATTCAAAA CCCATATTTT TCTTCAATTT TAATTATGGG TATTTTGAAA TTGAATGCTT TCCTTCACAG   
  
  
+ CAATACTATA TTACATATTT TACAGGAAAG TAATATTTTA CAGGAAAGTA TGTTGCTGTT TCAACCCTGT   
  
  
+ TTTGTTTCCT CTCTTTCCTT GGATATGATG TTCATTGCTC TTGTACCAAA CCTAACCTCA ACTTCCTGAA   
  
  
+ TTATTTAAGG CAATATTACC GTGTGCTGGT CAATTTTGGT TGTGGGTTTG TTTCTTAATG TATATGATTT   
  
  
+ ATGGTCAGGA TCCATGCTCC TCTTCCGTCC ACTCTATATC TCTCTCTCTC TCTCTCAATA CAAATTCAGA   
  
  
+ GACTTTCTCT TTCTGGGTTT ACTATTGATT GCAGAAGTTT GTAGTCTTGC GAGGCCTTGA GTCTTCTTCG   
  
  
+ AGGTAGAAAA TTTTGCTTTG CGCTTCCTTC CCCATATTTT AACACCCCTC CCCCCCCAAC GCAAGTTTTC   
  
  
+ TACATTTTTG TGCAATTCTT CAGCTATGAT TCCCCAATTT CACTTGTCAC ATCTCTATTT TCCTCTTTTT   
  
  
+ GGTTTCTTGA TTCTGTAGAA GGTTGTGTAT GAACTTCTTT GTTTCCCTTT TTCCTTTGTT ATTTTTAATG   
  
  
+ TTCCCATAGA TGATATAGAG ATCTGCTTTT CCTTGCTGTG AGAGGTATCT CTGAGAATGC ATGTAGCAAT   
  
  
+ CAAAATTCTG AGTTGTTAAT TCTATTGCAT GTCTATGGGT GATGATTGTG ATGATGTTTT TTCTGTTGCC   
  
  
+ CTCTTTCTCT CTTTCACTGA AATTGCTTTC CAGAATAGTT GTTTGACTCC TTTCTGTATC TTAATTGAGC   
  
  
+ CCTTGCTTTG GTTTGTGCTC TGGGAGTTCT TCTTTAGTAA TCCCTTGCTT GTATTTTCTG AAAGAGCAAG   
  
  
+ CAATAATCCT CATAGCATGT GTATAATCAT ACAATAGCAA CAGCAAACAT GGATCCTGCG CCCAGTTAGG   
  
  
+ TTTGGTTAGG TTAAGATATG CATATTCTTT TCTCTTGAAA AGAGTTGTCT AGGAAGGGTA ATTATGCCCA   
  
  
+ TTTCATGGAT AATAATGGTA CAATTAAGTT TGATTGGCTT AAATACCAAT TATTTAGCGT CTTTTTCTTT   
  
  
+ GGCTGTCCAG GGTTCCATTT CCATAAACAA CTTTGAGTAA GGCATAGAGA ACTAAATTTC TGCCAATTGT   
  
  
+ CAAGTGCAAG TTGCTCATAT GCAACAATAC TTGGAAATTT TGAGCTGAGC TGCTGGCATA TCATGAATAG   
  
  
+ TTCTAATGGT GGCGAGTTAT CACTTATCGT CCCTTGTGTG ACGAATTTCT CTTCCTAGGA AGCCCTCTAG   
  
  
+ TCAATGCCTA TCTGCTGTTG TGGAGTGAAA ATGGATACAT TTAGATCTCT ATAAACCCTA CTTTGGCAAG   
  
  
+ ATCCATGCTG ATTTTAGGAA GCCCTTTGAC CAGAGAATGA CATAACAACC TGATAGGGTG TTACATGCTA   
  
  
+ ACTTCGATTA TTTAATCAAA ATACGGAACT TCTATCAGTC AAAGCCTGCC CCAGGTTCTA CTGAATTAAA   
  
  
+ TTGTATTGAA TTGCTCTCAC TTGCTTATGA CGATTAAATA ACTGGATTTG CTAATTATTC TTTTTATTTT   
  
  
+ ATTCTTGGTG ATGTAGGGAA CTCAGGGAAT TGTAACCATA CCTGTTCCAG CTGAAAAGGG TACGGGAACA   
  
  
+ GTCATAGTTA CAGGGGAATT TCGCAATACA TTTAAGGGAG TAAGAAGCAG GATCATGTCT TTAGTTAGGC   
  
  
+ CGGCAGATAT GTCTGTGACT CCATATGGAA ATCCAACACT GTATTCACTT CAAGGCAATA ATAATAGCTC   
  
  
+ TGGTTTGTCT GGTCAACTGT ATGGCTCTGA AAAGCACAAG ATCAAATATG TGACCGAATC TTTTAATGGT   
  
  
+ CCAAGTTATG GCCTGAAATT CTTTGTTGAT TCTCCATCAG AAGAGCTCAT CCACCCATCA GATGCTACTC   
  
  
+ CTAACCCATT TGACTCTTCT TTCGTGGGCA TAAGACATGA TGAGAGTCCT TACCAAGGAA ATTATGGATC   
  
  
+ AGAATATGGG GTAAGCCCAT CTTCCGATGC AGTTGAATAT GATGAAGGTA ATAAGATAAG GCTAAAACTT   
  
  
+ CAAGAATTGG AGCATGCCCT GCTCGATGAA GATGATGAAG TGGATGAGGT GGCTATAGGG CCCGTGCTAG   
  
  
+ AACACAGCAT GGAGCTTGAT GAAGAGTGGG TCGCCCCATT GAGAAATGTG GTTTTCCATG ATTCACCAAA   
  
  
+ GGAGTCCACC TCCTCAGAGT CATCCAATGT CAGCAGCATT AGCAGCACCA AAGAAGTATC ACCTTGCTCT   
  
  
+ CCGAGTACTC CCAAACAATT GCTCTTTAAT TGTGCAAATA TGATTTCAGA GGGGAATTTT GAGGAAGCCG   
  
  
+ TGACTATGAT CAGCGAGCTT AGACAGATTG TTTCTATCCA AGGGGATCCA GCGCAGAGGA TAGCAGCCTA   
  
  
+ CATGGTAGAA GGCCTTGCTT CCCGTTTGGC TTCATCGGGA AAAGTTCTTT ACAAAGCTTT GAAATGCAAA   
  
  
+ GAGCCACCTT CATTTGACAG GCTAGCGGCT ATGCAAATCC TCTTTGAGGT GTGCCCATGT TTCAAATTCG   
  
  
+ GATTTATGGC CGCAAATGCA GCGATTATGG ATGCAATCAA AAACGAAAAA AGGGTACACA TCATAGATTT   
  
  
+ TGACATAAAC CAAGGGAATC AATACATAAA TCTCATGCAA TCCCTTGCTA AACAAGGTAA CAAGCTGACG   
  
  
+ CACTTGAAGT TGACTGGAGT TGATGACCCT GAGTCAGTTC AACGCCCTAT TGGTGGCCTA AAAAACATCG   
  
  
+ GACAAAGGCT GCAAGCATTA GCTGAATATC TTGGTGTGTC ATTTGAGTTC AAAGCAATAC CTGCTAGAAC   
  
  
+ TCCACTTGTT AACCCGGAAA TGTTAGAATG TCGACCTGCG GAAGCTTTAG TGGTGAACTT TGCCTTCCAG   
  
  
+ CTTCACCACA TGCCTGATGA AAGCGTCTCA ACAATCAACC TCCGAGACCA GCTTCTTCGG ATGGTCAAAA   
  
  
+ GCCTAAACCC AAAGCTAGTA ACCATTGTCG AGCAGGATGT GAACACAAAC ACTACCCCTT TCCTCTCTAG   
  
  
+ GTTTGCTGAG GCATACAGCT ACTATTCTGC TGTTTTTGAG TCTCTTGATG CTACTCTCCC TAGAGATAGC   
  
  
+ CAGGACAGGG TGAATGTGGA GAAGCAGTGC TTGGCACGTG ATGTTGTGAA CATCATTGCA TGTGAAGGGG   
  
  
+ AGGAGAGGAT TGAGCGCTAT GAGGTTGCGG GGAAATGGAG AGCGAGGATG TTGATGGCGG GATTCAGGGC   
  
  
+ TATTCCAATG GGTCAGAAGA CCGGTGATAT GATTCGGAAG CTTATATCAA TGCGGTATTG TGACAGGCTC   
  
  
+ AAGGTAAAAG AGGAGATGGA TGCACTTCAC TTTGGGTGGG AAGATAAAAA CTTAATCGTT ACATCAGCTT   
  
  
+ GGAGGTG  

- +Up\_Stream \_Len000TCTATC TGTAAGTCGG TGAACGGTCA AGTCTCTCTC TATCTCTCTC TAACCCATTC   
  
  
- GGGAAAAACG TGATGAAAAA GAGCGAAGGT AAAAAGAAAG AATTAAAAAT CACTACTAAA AGGTCTTCTA   
  
  
- AACTAAATAA GTAGTACGAA TTGGTTAACC CAGAAACTCA TAATAACAAA TGACCCATAA ACTAGAAAAG   
  
  
- TATAAGGAAA CCTGACCCAC CCACCTTGAA AACTTGAAAG AATAATCAAC CCGTACGGGA ATAGAATAAG   
  
  
- GTTAAGTTTT GGGTATAAAA AGAAGTTAAA ATTAATACCC ATAAAACTTT AACTTACGAA AGGAAGTGTC   
  
  
- GTTATGATAT AATGTATAAA ATGTCCTTTC ATTATAAAAT GTCCTTTCAT ACAACGACAA AGTTGGGACA   
  
  
- AAACAAAGGA GAGAAAGGAA CCTATACTAC AAGTAACGAG AACATGGTTT GGATTGGAGT TGAAGGACTT   
  
  
- AATAAATTCC GTTATAATGG CACACGACCA GTTAAAACCA ACACCCAAAC AAAGAATTAC ATATACTAAA   
  
  
- TACCAGTCCT AGGTACGAGG AGAAGGCAGG TGAGATATAG AGAGAGAGAG AGAGAGTTAT GTTTAAGTCT   
  
  
- CTGAAAGAGA AAGACCCAAA TGATAACTAA CGTCTTCAAA CATCAGAACG CTCCGGAACT CAGAAGAAGC   
  
  
- TCCATCTTTT AAAACGAAAC GCGAAGGAAG GGGTATAAAA TTGTGGGGAG GGGGGGGTTG CGTTCAAAAG   
  
  
- ATGTAAAAAC ACGTTAAGAA GTCGATACTA AGGGGTTAAA GTGAACAGTG TAGAGATAAA AGGAGAAAAA   
  
  
- CCAAAGAACT AAGACATCTT CCAACACATA CTTGAAGAAA CAAAGGGAAA AAGGAAACAA TAAAAATTAC   
  
  
- AAGGGTATCT ACTATATCTC TAGACGAAAA GGAACGACAC TCTCCATAGA GACTCTTACG TACATCGTTA   
  
  
- GTTTTAAGAC TCAACAATTA AGATAACGTA CAGATACCCA CTACTAACAC TACTACAAAA AAGACAACGG   
  
  
- GAGAAAGAGA GAAAGTGACT TTAACGAAAG GTCTTATCAA CAAACTGAGG AAAGACATAG AATTAACTCG   
  
  
- GGAACGAAAC CAAACACGAG ACCCTCAAGA AGAAATCATT AGGGAACGAA CATAAAAGAC TTTCTCGTTC   
  
  
- GTTATTAGGA GTATCGTACA CATATTAGTA TGTTATCGTT GTCGTTTGTA CCTAGGACGC GGGTCAATCC   
  
  
- AAACCAATCC AATTCTATAC GTATAAGAAA AGAGAACTTT TCTCAACAGA TCCTTCCCAT TAATACGGGT   
  
  
- AAAGTACCTA TTATTACCAT GTTAATTCAA ACTAACCGAA TTTATGGTTA ATAAATCGCA GAAAAAGAAA   
  
  
- CCGACAGGTC CCAAGGTAAA GGTATTTGTT GAAACTCATT CCGTATCTCT TGATTTAAAG ACGGTTAACA   
  
  
- GTTCACGTTC AACGAGTATA CGTTGTTATG AACCTTTAAA ACTCGACTCG ACGACCGTAT AGTACTTATC   
  
  
- AAGATTACCA CCGCTCAATA GTGAATAGCA GGGAACACAC TGCTTAAAGA GAAGGATCCT TCGGGAGATC   
  
  
- AGTTACGGAT AGACGACAAC ACCTCACTTT TACCTATGTA AATCTAGAGA TATTTGGGAT GAAACCGTTC   
  
  
- TAGGTACGAC TAAAATCCTT CGGGAAACTG GTCTCTTACT GTATTGTTGG ACTATCCCAC AATGTACGAT   
  
  
- TGAAGCTAAT AAATTAGTTT TATGCCTTGA AGATAGTCAG TTTCGGACGG GGTCCAAGAT GACTTAATTT   
  
  
- AACATAACTT AACGAGAGTG AACGAATACT GCTAATTTAT TGACCTAAAC GATTAATAAG AAAAATAAAA   
  
  
- TAAGAACCAC TACATCCCTT GAGTCCCTTA ACATTGGTAT GGACAAGGTC GACTTTTCCC ATGCCCTTGT   
  
  
- CAGTATCAAT GTCCCCTTAA AGCGTTATGT AAATTCCCTC ATTCTTCGTC CTAGTACAGA AATCAATCCG   
  
  
- GCCGTCTATA CAGACACTGA GGTATACCTT TAGGTTGTGA CATAAGTGAA GTTCCGTTAT TATTATCGAG   
  
  
- ACCAAACAGA CCAGTTGACA TACCGAGACT TTTCGTGTTC TAGTTTATAC ACTGGCTTAG AAAATTACCA   
  
  
- GGTTCAATAC CGGACTTTAA GAAACAACTA AGAGGTAGTC TTCTCGAGTA GGTGGGTAGT CTACGATGAG   
  
  
- GATTGGGTAA ACTGAGAAGA AAGCACCCGT ATTCTGTACT ACTCTCAGGA ATGGTTCCTT TAATACCTAG   
  
  
- TCTTATACCC CATTCGGGTA GAAGGCTACG TCAACTTATA CTACTTCCAT TATTCTATTC CGATTTTGAA   
  
  
- GTTCTTAACC TCGTACGGGA CGAGCTACTT CTACTACTTC ACCTACTCCA CCGATATCCC GGGCACGATC   
  
  
- TTGTGTCGTA CCTCGAACTA CTTCTCACCC AGCGGGGTAA CTCTTTACAC CAAAAGGTAC TAAGTGGTTT   
  
  
- CCTCAGGTGG AGGAGTCTCA GTAGGTTACA GTCGTCGTAA TCGTCGTGGT TTCTTCATAG TGGAACGAGA   
  
  
- GGCTCATGAG GGTTTGTTAA CGAGAAATTA ACACGTTTAT ACTAAAGTCT CCCCTTAAAA CTCCTTCGGC   
  
  
- ACTGATACTA GTCGCTCGAA TCTGTCTAAC AAAGATAGGT TCCCCTAGGT CGCGTCTCCT ATCGTCGGAT   
  
  
- GTACCATCTT CCGGAACGAA GGGCAAACCG AAGTAGCCCT TTTCAAGAAA TGTTTCGAAA CTTTACGTTT   
  
  
- CTCGGTGGAA GTAAACTGTC CGATCGCCGA TACGTTTAGG AGAAACTCCA CACGGGTACA AAGTTTAAGC   
  
  
- CTAAATACCG GCGTTTACGT CGCTAATACC TACGTTAGTT TTTGCTTTTT TCCCATGTGT AGTATCTAAA   
  
  
- ACTGTATTTG GTTCCCTTAG TTATGTATTT AGAGTACGTT AGGGAACGAT TTGTTCCATT GTTCGACTGC   
  
  
- GTGAACTTCA ACTGACCTCA ACTACTGGGA CTCAGTCAAG TTGCGGGATA ACCACCGGAT TTTTTGTAGC   
  
  
- CTGTTTCCGA CGTTCGTAAT CGACTTATAG AACCACACAG TAAACTCAAG TTTCGTTATG GACGATCTTG   
  
  
- AGGTGAACAA TTGGGCCTTT ACAATCTTAC AGCTGGACGC CTTCGAAATC ACCACTTGAA ACGGAAGGTC   
  
  
- GAAGTGGTGT ACGGACTACT TTCGCAGAGT TGTTAGTTGG AGGCTCTGGT CGAAGAAGCC TACCAGTTTT   
  
  
- CGGATTTGGG TTTCGATCAT TGGTAACAGC TCGTCCTACA CTTGTGTTTG TGATGGGGAA AGGAGAGATC   
  
  
- CAAACGACTC CGTATGTCGA TGATAAGACG ACAAAAACTC AGAGAACTAC GATGAGAGGG ATCTCTATCG   
  
  
- GTCCTGTCCC ACTTACACCT CTTCGTCACG AACCGTGCAC TACAACACTT GTAGTAACGT ACACTTCCCC   
  
  
- TCCTCTCCTA ACTCGCGATA CTCCAACGCC CCTTTACCTC TCGCTCCTAC AACTACCGCC CTAAGTCCCG   
  
  
- ATAAGGTTAC CCAGTCTTCT GGCCACTATA CTAAGCCTTC GAATATAGTT ACGCCATAAC ACTGTCCGAG   
  
  
- TTCCATTTTC TCCTCTACCT ACGTGAAGTG AAACCCACCC TTCTATTTTT GAATTAGCAA TGTAGTCGAA   
  
  
- CCTCCAC

+     I-box

| Site Name | Organism | Position | Strand | Matrix score. | sequence | function |
| --- | --- | --- | --- | --- | --- | --- |
| I-box | Larix laricina | 2739 | + | 9 | GTATAAGGCC | part of a light responsive element |
| I-box | Zea mays | 2426 | + | 9 | gGATAAGGTG | part of a light responsive element |
| I-box | Gossypium hirsutum | 2367 | + | 11 | AAGATAAGGCT | part of a light responsive element |
| I-box | Triticum aestivum | 2368 | + | 8 | AGATAAGG | part of a light responsive element |
| I-box | Triticum aestivum | 272 | - | 8 | AGATAAGG | part of a light responsive element |

>HU11G01134.1   
+ +Up\_Stream \_Len000AGATAG ACATTCAGCC ACTTGCCAGT TCAGAGAGAG ATAGAGAGAG ATTGGGTAAG   
  
  
+ CCCTTTTTGC ACTACTTTTT CTCGCTTCCA TTTTTCTTTC TTAATTTTTA GTGATGATTT TCCAGAAGAT   
  
  
+ TTGATTTATT CATCATGCTT AACCAATTGG GTCTTTGAGT ATTATTGTTT ACTGGGTATT TGATCTTTTC   
  
  
+ ATATTCCTTT GGACTGGGTG GGTGGAACTT TTGAACTTTC TTATTAGTTG GGCATGCCCT TATCTTATTC   
  
  
+ CAATTCAAAA CCCATATTTT TCTTCAATTT TAATTATGGG TATTTTGAAA TTGAATGCTT TCCTTCACAG   
  
  
+ CAATACTATA TTACATATTT TACAGGAAAG TAATATTTTA CAGGAAAGTA TGTTGCTGTT TCAACCCTGT   
  
  
+ TTTGTTTCCT CTCTTTCCTT GGATATGATG TTCATTGCTC TTGTACCAAA CCTAACCTCA ACTTCCTGAA   
  
  
+ TTATTTAAGG CAATATTACC GTGTGCTGGT CAATTTTGGT TGTGGGTTTG TTTCTTAATG TATATGATTT   
  
  
+ ATGGTCAGGA TCCATGCTCC TCTTCCGTCC ACTCTATATC TCTCTCTCTC TCTCTCAATA CAAATTCAGA   
  
  
+ GACTTTCTCT TTCTGGGTTT ACTATTGATT GCAGAAGTTT GTAGTCTTGC GAGGCCTTGA GTCTTCTTCG   
  
  
+ AGGTAGAAAA TTTTGCTTTG CGCTTCCTTC CCCATATTTT AACACCCCTC CCCCCCCAAC GCAAGTTTTC   
  
  
+ TACATTTTTG TGCAATTCTT CAGCTATGAT TCCCCAATTT CACTTGTCAC ATCTCTATTT TCCTCTTTTT   
  
  
+ GGTTTCTTGA TTCTGTAGAA GGTTGTGTAT GAACTTCTTT GTTTCCCTTT TTCCTTTGTT ATTTTTAATG   
  
  
+ TTCCCATAGA TGATATAGAG ATCTGCTTTT CCTTGCTGTG AGAGGTATCT CTGAGAATGC ATGTAGCAAT   
  
  
+ CAAAATTCTG AGTTGTTAAT TCTATTGCAT GTCTATGGGT GATGATTGTG ATGATGTTTT TTCTGTTGCC   
  
  
+ CTCTTTCTCT CTTTCACTGA AATTGCTTTC CAGAATAGTT GTTTGACTCC TTTCTGTATC TTAATTGAGC   
  
  
+ CCTTGCTTTG GTTTGTGCTC TGGGAGTTCT TCTTTAGTAA TCCCTTGCTT GTATTTTCTG AAAGAGCAAG   
  
  
+ CAATAATCCT CATAGCATGT GTATAATCAT ACAATAGCAA CAGCAAACAT GGATCCTGCG CCCAGTTAGG   
  
  
+ TTTGGTTAGG TTAAGATATG CATATTCTTT TCTCTTGAAA AGAGTTGTCT AGGAAGGGTA ATTATGCCCA   
  
  
+ TTTCATGGAT AATAATGGTA CAATTAAGTT TGATTGGCTT AAATACCAAT TATTTAGCGT CTTTTTCTTT   
  
  
+ GGCTGTCCAG GGTTCCATTT CCATAAACAA CTTTGAGTAA GGCATAGAGA ACTAAATTTC TGCCAATTGT   
  
  
+ CAAGTGCAAG TTGCTCATAT GCAACAATAC TTGGAAATTT TGAGCTGAGC TGCTGGCATA TCATGAATAG   
  
  
+ TTCTAATGGT GGCGAGTTAT CACTTATCGT CCCTTGTGTG ACGAATTTCT CTTCCTAGGA AGCCCTCTAG   
  
  
+ TCAATGCCTA TCTGCTGTTG TGGAGTGAAA ATGGATACAT TTAGATCTCT ATAAACCCTA CTTTGGCAAG   
  
  
+ ATCCATGCTG ATTTTAGGAA GCCCTTTGAC CAGAGAATGA CATAACAACC TGATAGGGTG TTACATGCTA   
  
  
+ ACTTCGATTA TTTAATCAAA ATACGGAACT TCTATCAGTC AAAGCCTGCC CCAGGTTCTA CTGAATTAAA   
  
  
+ TTGTATTGAA TTGCTCTCAC TTGCTTATGA CGATTAAATA ACTGGATTTG CTAATTATTC TTTTTATTTT   
  
  
+ ATTCTTGGTG ATGTAGGGAA CTCAGGGAAT TGTAACCATA CCTGTTCCAG CTGAAAAGGG TACGGGAACA   
  
  
+ GTCATAGTTA CAGGGGAATT TCGCAATACA TTTAAGGGAG TAAGAAGCAG GATCATGTCT TTAGTTAGGC   
  
  
+ CGGCAGATAT GTCTGTGACT CCATATGGAA ATCCAACACT GTATTCACTT CAAGGCAATA ATAATAGCTC   
  
  
+ TGGTTTGTCT GGTCAACTGT ATGGCTCTGA AAAGCACAAG ATCAAATATG TGACCGAATC TTTTAATGGT   
  
  
+ CCAAGTTATG GCCTGAAATT CTTTGTTGAT TCTCCATCAG AAGAGCTCAT CCACCCATCA GATGCTACTC   
  
  
+ CTAACCCATT TGACTCTTCT TTCGTGGGCA TAAGACATGA TGAGAGTCCT TACCAAGGAA ATTATGGATC   
  
  
+ AGAATATGGG GTAAGCCCAT CTTCCGATGC AGTTGAATAT GATGAAGGTA ATAAGATAAG GCTAAAACTT   
  
  
+ CAAGAATTGG AGCATGCCCT GCTCGATGAA GATGATGAAG TGGATGAGGT GGCTATAGGG CCCGTGCTAG   
  
  
+ AACACAGCAT GGAGCTTGAT GAAGAGTGGG TCGCCCCATT GAGAAATGTG GTTTTCCATG ATTCACCAAA   
  
  
+ GGAGTCCACC TCCTCAGAGT CATCCAATGT CAGCAGCATT AGCAGCACCA AAGAAGTATC ACCTTGCTCT   
  
  
+ CCGAGTACTC CCAAACAATT GCTCTTTAAT TGTGCAAATA TGATTTCAGA GGGGAATTTT GAGGAAGCCG   
  
  
+ TGACTATGAT CAGCGAGCTT AGACAGATTG TTTCTATCCA AGGGGATCCA GCGCAGAGGA TAGCAGCCTA   
  
  
+ CATGGTAGAA GGCCTTGCTT CCCGTTTGGC TTCATCGGGA AAAGTTCTTT ACAAAGCTTT GAAATGCAAA   
  
  
+ GAGCCACCTT CATTTGACAG GCTAGCGGCT ATGCAAATCC TCTTTGAGGT GTGCCCATGT TTCAAATTCG   
  
  
+ GATTTATGGC CGCAAATGCA GCGATTATGG ATGCAATCAA AAACGAAAAA AGGGTACACA TCATAGATTT   
  
  
+ TGACATAAAC CAAGGGAATC AATACATAAA TCTCATGCAA TCCCTTGCTA AACAAGGTAA CAAGCTGACG   
  
  
+ CACTTGAAGT TGACTGGAGT TGATGACCCT GAGTCAGTTC AACGCCCTAT TGGTGGCCTA AAAAACATCG   
  
  
+ GACAAAGGCT GCAAGCATTA GCTGAATATC TTGGTGTGTC ATTTGAGTTC AAAGCAATAC CTGCTAGAAC   
  
  
+ TCCACTTGTT AACCCGGAAA TGTTAGAATG TCGACCTGCG GAAGCTTTAG TGGTGAACTT TGCCTTCCAG   
  
  
+ CTTCACCACA TGCCTGATGA AAGCGTCTCA ACAATCAACC TCCGAGACCA GCTTCTTCGG ATGGTCAAAA   
  
  
+ GCCTAAACCC AAAGCTAGTA ACCATTGTCG AGCAGGATGT GAACACAAAC ACTACCCCTT TCCTCTCTAG   
  
  
+ GTTTGCTGAG GCATACAGCT ACTATTCTGC TGTTTTTGAG TCTCTTGATG CTACTCTCCC TAGAGATAGC   
  
  
+ CAGGACAGGG TGAATGTGGA GAAGCAGTGC TTGGCACGTG ATGTTGTGAA CATCATTGCA TGTGAAGGGG   
  
  
+ AGGAGAGGAT TGAGCGCTAT GAGGTTGCGG GGAAATGGAG AGCGAGGATG TTGATGGCGG GATTCAGGGC   
  
  
+ TATTCCAATG GGTCAGAAGA CCGGTGATAT GATTCGGAAG CTTATATCAA TGCGGTATTG TGACAGGCTC   
  
  
+ AAGGTAAAAG AGGAGATGGA TGCACTTCAC TTTGGGTGGG AAGATAAAAA CTTAATCGTT ACATCAGCTT   
  
  
+ GGAGGTG  

- +Up\_Stream \_Len000TCTATC TGTAAGTCGG TGAACGGTCA AGTCTCTCTC TATCTCTCTC TAACCCATTC   
  
  
- GGGAAAAACG TGATGAAAAA GAGCGAAGGT AAAAAGAAAG AATTAAAAAT CACTACTAAA AGGTCTTCTA   
  
  
- AACTAAATAA GTAGTACGAA TTGGTTAACC CAGAAACTCA TAATAACAAA TGACCCATAA ACTAGAAAAG   
  
  
- TATAAGGAAA CCTGACCCAC CCACCTTGAA AACTTGAAAG AATAATCAAC CCGTACGGGA ATAGAATAAG   
  
  
- GTTAAGTTTT GGGTATAAAA AGAAGTTAAA ATTAATACCC ATAAAACTTT AACTTACGAA AGGAAGTGTC   
  
  
- GTTATGATAT AATGTATAAA ATGTCCTTTC ATTATAAAAT GTCCTTTCAT ACAACGACAA AGTTGGGACA   
  
  
- AAACAAAGGA GAGAAAGGAA CCTATACTAC AAGTAACGAG AACATGGTTT GGATTGGAGT TGAAGGACTT   
  
  
- AATAAATTCC GTTATAATGG CACACGACCA GTTAAAACCA ACACCCAAAC AAAGAATTAC ATATACTAAA   
  
  
- TACCAGTCCT AGGTACGAGG AGAAGGCAGG TGAGATATAG AGAGAGAGAG AGAGAGTTAT GTTTAAGTCT   
  
  
- CTGAAAGAGA AAGACCCAAA TGATAACTAA CGTCTTCAAA CATCAGAACG CTCCGGAACT CAGAAGAAGC   
  
  
- TCCATCTTTT AAAACGAAAC GCGAAGGAAG GGGTATAAAA TTGTGGGGAG GGGGGGGTTG CGTTCAAAAG   
  
  
- ATGTAAAAAC ACGTTAAGAA GTCGATACTA AGGGGTTAAA GTGAACAGTG TAGAGATAAA AGGAGAAAAA   
  
  
- CCAAAGAACT AAGACATCTT CCAACACATA CTTGAAGAAA CAAAGGGAAA AAGGAAACAA TAAAAATTAC   
  
  
- AAGGGTATCT ACTATATCTC TAGACGAAAA GGAACGACAC TCTCCATAGA GACTCTTACG TACATCGTTA   
  
  
- GTTTTAAGAC TCAACAATTA AGATAACGTA CAGATACCCA CTACTAACAC TACTACAAAA AAGACAACGG   
  
  
- GAGAAAGAGA GAAAGTGACT TTAACGAAAG GTCTTATCAA CAAACTGAGG AAAGACATAG AATTAACTCG   
  
  
- GGAACGAAAC CAAACACGAG ACCCTCAAGA AGAAATCATT AGGGAACGAA CATAAAAGAC TTTCTCGTTC   
  
  
- GTTATTAGGA GTATCGTACA CATATTAGTA TGTTATCGTT GTCGTTTGTA CCTAGGACGC GGGTCAATCC   
  
  
- AAACCAATCC AATTCTATAC GTATAAGAAA AGAGAACTTT TCTCAACAGA TCCTTCCCAT TAATACGGGT   
  
  
- AAAGTACCTA TTATTACCAT GTTAATTCAA ACTAACCGAA TTTATGGTTA ATAAATCGCA GAAAAAGAAA   
  
  
- CCGACAGGTC CCAAGGTAAA GGTATTTGTT GAAACTCATT CCGTATCTCT TGATTTAAAG ACGGTTAACA   
  
  
- GTTCACGTTC AACGAGTATA CGTTGTTATG AACCTTTAAA ACTCGACTCG ACGACCGTAT AGTACTTATC   
  
  
- AAGATTACCA CCGCTCAATA GTGAATAGCA GGGAACACAC TGCTTAAAGA GAAGGATCCT TCGGGAGATC   
  
  
- AGTTACGGAT AGACGACAAC ACCTCACTTT TACCTATGTA AATCTAGAGA TATTTGGGAT GAAACCGTTC   
  
  
- TAGGTACGAC TAAAATCCTT CGGGAAACTG GTCTCTTACT GTATTGTTGG ACTATCCCAC AATGTACGAT   
  
  
- TGAAGCTAAT AAATTAGTTT TATGCCTTGA AGATAGTCAG TTTCGGACGG GGTCCAAGAT GACTTAATTT   
  
  
- AACATAACTT AACGAGAGTG AACGAATACT GCTAATTTAT TGACCTAAAC GATTAATAAG AAAAATAAAA   
  
  
- TAAGAACCAC TACATCCCTT GAGTCCCTTA ACATTGGTAT GGACAAGGTC GACTTTTCCC ATGCCCTTGT   
  
  
- CAGTATCAAT GTCCCCTTAA AGCGTTATGT AAATTCCCTC ATTCTTCGTC CTAGTACAGA AATCAATCCG   
  
  
- GCCGTCTATA CAGACACTGA GGTATACCTT TAGGTTGTGA CATAAGTGAA GTTCCGTTAT TATTATCGAG   
  
  
- ACCAAACAGA CCAGTTGACA TACCGAGACT TTTCGTGTTC TAGTTTATAC ACTGGCTTAG AAAATTACCA   
  
  
- GGTTCAATAC CGGACTTTAA GAAACAACTA AGAGGTAGTC TTCTCGAGTA GGTGGGTAGT CTACGATGAG   
  
  
- GATTGGGTAA ACTGAGAAGA AAGCACCCGT ATTCTGTACT ACTCTCAGGA ATGGTTCCTT TAATACCTAG   
  
  
- TCTTATACCC CATTCGGGTA GAAGGCTACG TCAACTTATA CTACTTCCAT TATTCTATTC CGATTTTGAA   
  
  
- GTTCTTAACC TCGTACGGGA CGAGCTACTT CTACTACTTC ACCTACTCCA CCGATATCCC GGGCACGATC   
  
  
- TTGTGTCGTA CCTCGAACTA CTTCTCACCC AGCGGGGTAA CTCTTTACAC CAAAAGGTAC TAAGTGGTTT   
  
  
- CCTCAGGTGG AGGAGTCTCA GTAGGTTACA GTCGTCGTAA TCGTCGTGGT TTCTTCATAG TGGAACGAGA   
  
  
- GGCTCATGAG GGTTTGTTAA CGAGAAATTA ACACGTTTAT ACTAAAGTCT CCCCTTAAAA CTCCTTCGGC   
  
  
- ACTGATACTA GTCGCTCGAA TCTGTCTAAC AAAGATAGGT TCCCCTAGGT CGCGTCTCCT ATCGTCGGAT   
  
  
- GTACCATCTT CCGGAACGAA GGGCAAACCG AAGTAGCCCT TTTCAAGAAA TGTTTCGAAA CTTTACGTTT   
  
  
- CTCGGTGGAA GTAAACTGTC CGATCGCCGA TACGTTTAGG AGAAACTCCA CACGGGTACA AAGTTTAAGC   
  
  
- CTAAATACCG GCGTTTACGT CGCTAATACC TACGTTAGTT TTTGCTTTTT TCCCATGTGT AGTATCTAAA   
  
  
- ACTGTATTTG GTTCCCTTAG TTATGTATTT AGAGTACGTT AGGGAACGAT TTGTTCCATT GTTCGACTGC   
  
  
- GTGAACTTCA ACTGACCTCA ACTACTGGGA CTCAGTCAAG TTGCGGGATA ACCACCGGAT TTTTTGTAGC   
  
  
- CTGTTTCCGA CGTTCGTAAT CGACTTATAG AACCACACAG TAAACTCAAG TTTCGTTATG GACGATCTTG   
  
  
- AGGTGAACAA TTGGGCCTTT ACAATCTTAC AGCTGGACGC CTTCGAAATC ACCACTTGAA ACGGAAGGTC   
  
  
- GAAGTGGTGT ACGGACTACT TTCGCAGAGT TGTTAGTTGG AGGCTCTGGT CGAAGAAGCC TACCAGTTTT   
  
  
- CGGATTTGGG TTTCGATCAT TGGTAACAGC TCGTCCTACA CTTGTGTTTG TGATGGGGAA AGGAGAGATC   
  
  
- CAAACGACTC CGTATGTCGA TGATAAGACG ACAAAAACTC AGAGAACTAC GATGAGAGGG ATCTCTATCG   
  
  
- GTCCTGTCCC ACTTACACCT CTTCGTCACG AACCGTGCAC TACAACACTT GTAGTAACGT ACACTTCCCC   
  
  
- TCCTCTCCTA ACTCGCGATA CTCCAACGCC CCTTTACCTC TCGCTCCTAC AACTACCGCC CTAAGTCCCG   
  
  
- ATAAGGTTAC CCAGTCTTCT GGCCACTATA CTAAGCCTTC GAATATAGTT ACGCCATAAC ACTGTCCGAG   
  
  
- TTCCATTTTC TCCTCTACCT ACGTGAAGTG AAACCCACCC TTCTATTTTT GAATTAGCAA TGTAGTCGAA   
  
  
- CCTCCAC

+     MBS

| Site Name | Organism | Position | Strand | Matrix score. | sequence | function |
| --- | --- | --- | --- | --- | --- | --- |
| MBS | Arabidopsis thaliana | 2344 | - | 6 | CAACTG | MYB binding site involved in drought-inducibility |
| MBS | Arabidopsis thaliana | 2118 | + | 6 | CAACTG | MYB binding site involved in drought-inducibility |

>HU11G01134.1   
+ +Up\_Stream \_Len000AGATAG ACATTCAGCC ACTTGCCAGT TCAGAGAGAG ATAGAGAGAG ATTGGGTAAG   
  
  
+ CCCTTTTTGC ACTACTTTTT CTCGCTTCCA TTTTTCTTTC TTAATTTTTA GTGATGATTT TCCAGAAGAT   
  
  
+ TTGATTTATT CATCATGCTT AACCAATTGG GTCTTTGAGT ATTATTGTTT ACTGGGTATT TGATCTTTTC   
  
  
+ ATATTCCTTT GGACTGGGTG GGTGGAACTT TTGAACTTTC TTATTAGTTG GGCATGCCCT TATCTTATTC   
  
  
+ CAATTCAAAA CCCATATTTT TCTTCAATTT TAATTATGGG TATTTTGAAA TTGAATGCTT TCCTTCACAG   
  
  
+ CAATACTATA TTACATATTT TACAGGAAAG TAATATTTTA CAGGAAAGTA TGTTGCTGTT TCAACCCTGT   
  
  
+ TTTGTTTCCT CTCTTTCCTT GGATATGATG TTCATTGCTC TTGTACCAAA CCTAACCTCA ACTTCCTGAA   
  
  
+ TTATTTAAGG CAATATTACC GTGTGCTGGT CAATTTTGGT TGTGGGTTTG TTTCTTAATG TATATGATTT   
  
  
+ ATGGTCAGGA TCCATGCTCC TCTTCCGTCC ACTCTATATC TCTCTCTCTC TCTCTCAATA CAAATTCAGA   
  
  
+ GACTTTCTCT TTCTGGGTTT ACTATTGATT GCAGAAGTTT GTAGTCTTGC GAGGCCTTGA GTCTTCTTCG   
  
  
+ AGGTAGAAAA TTTTGCTTTG CGCTTCCTTC CCCATATTTT AACACCCCTC CCCCCCCAAC GCAAGTTTTC   
  
  
+ TACATTTTTG TGCAATTCTT CAGCTATGAT TCCCCAATTT CACTTGTCAC ATCTCTATTT TCCTCTTTTT   
  
  
+ GGTTTCTTGA TTCTGTAGAA GGTTGTGTAT GAACTTCTTT GTTTCCCTTT TTCCTTTGTT ATTTTTAATG   
  
  
+ TTCCCATAGA TGATATAGAG ATCTGCTTTT CCTTGCTGTG AGAGGTATCT CTGAGAATGC ATGTAGCAAT   
  
  
+ CAAAATTCTG AGTTGTTAAT TCTATTGCAT GTCTATGGGT GATGATTGTG ATGATGTTTT TTCTGTTGCC   
  
  
+ CTCTTTCTCT CTTTCACTGA AATTGCTTTC CAGAATAGTT GTTTGACTCC TTTCTGTATC TTAATTGAGC   
  
  
+ CCTTGCTTTG GTTTGTGCTC TGGGAGTTCT TCTTTAGTAA TCCCTTGCTT GTATTTTCTG AAAGAGCAAG   
  
  
+ CAATAATCCT CATAGCATGT GTATAATCAT ACAATAGCAA CAGCAAACAT GGATCCTGCG CCCAGTTAGG   
  
  
+ TTTGGTTAGG TTAAGATATG CATATTCTTT TCTCTTGAAA AGAGTTGTCT AGGAAGGGTA ATTATGCCCA   
  
  
+ TTTCATGGAT AATAATGGTA CAATTAAGTT TGATTGGCTT AAATACCAAT TATTTAGCGT CTTTTTCTTT   
  
  
+ GGCTGTCCAG GGTTCCATTT CCATAAACAA CTTTGAGTAA GGCATAGAGA ACTAAATTTC TGCCAATTGT   
  
  
+ CAAGTGCAAG TTGCTCATAT GCAACAATAC TTGGAAATTT TGAGCTGAGC TGCTGGCATA TCATGAATAG   
  
  
+ TTCTAATGGT GGCGAGTTAT CACTTATCGT CCCTTGTGTG ACGAATTTCT CTTCCTAGGA AGCCCTCTAG   
  
  
+ TCAATGCCTA TCTGCTGTTG TGGAGTGAAA ATGGATACAT TTAGATCTCT ATAAACCCTA CTTTGGCAAG   
  
  
+ ATCCATGCTG ATTTTAGGAA GCCCTTTGAC CAGAGAATGA CATAACAACC TGATAGGGTG TTACATGCTA   
  
  
+ ACTTCGATTA TTTAATCAAA ATACGGAACT TCTATCAGTC AAAGCCTGCC CCAGGTTCTA CTGAATTAAA   
  
  
+ TTGTATTGAA TTGCTCTCAC TTGCTTATGA CGATTAAATA ACTGGATTTG CTAATTATTC TTTTTATTTT   
  
  
+ ATTCTTGGTG ATGTAGGGAA CTCAGGGAAT TGTAACCATA CCTGTTCCAG CTGAAAAGGG TACGGGAACA   
  
  
+ GTCATAGTTA CAGGGGAATT TCGCAATACA TTTAAGGGAG TAAGAAGCAG GATCATGTCT TTAGTTAGGC   
  
  
+ CGGCAGATAT GTCTGTGACT CCATATGGAA ATCCAACACT GTATTCACTT CAAGGCAATA ATAATAGCTC   
  
  
+ TGGTTTGTCT GGTCAACTGT ATGGCTCTGA AAAGCACAAG ATCAAATATG TGACCGAATC TTTTAATGGT   
  
  
+ CCAAGTTATG GCCTGAAATT CTTTGTTGAT TCTCCATCAG AAGAGCTCAT CCACCCATCA GATGCTACTC   
  
  
+ CTAACCCATT TGACTCTTCT TTCGTGGGCA TAAGACATGA TGAGAGTCCT TACCAAGGAA ATTATGGATC   
  
  
+ AGAATATGGG GTAAGCCCAT CTTCCGATGC AGTTGAATAT GATGAAGGTA ATAAGATAAG GCTAAAACTT   
  
  
+ CAAGAATTGG AGCATGCCCT GCTCGATGAA GATGATGAAG TGGATGAGGT GGCTATAGGG CCCGTGCTAG   
  
  
+ AACACAGCAT GGAGCTTGAT GAAGAGTGGG TCGCCCCATT GAGAAATGTG GTTTTCCATG ATTCACCAAA   
  
  
+ GGAGTCCACC TCCTCAGAGT CATCCAATGT CAGCAGCATT AGCAGCACCA AAGAAGTATC ACCTTGCTCT   
  
  
+ CCGAGTACTC CCAAACAATT GCTCTTTAAT TGTGCAAATA TGATTTCAGA GGGGAATTTT GAGGAAGCCG   
  
  
+ TGACTATGAT CAGCGAGCTT AGACAGATTG TTTCTATCCA AGGGGATCCA GCGCAGAGGA TAGCAGCCTA   
  
  
+ CATGGTAGAA GGCCTTGCTT CCCGTTTGGC TTCATCGGGA AAAGTTCTTT ACAAAGCTTT GAAATGCAAA   
  
  
+ GAGCCACCTT CATTTGACAG GCTAGCGGCT ATGCAAATCC TCTTTGAGGT GTGCCCATGT TTCAAATTCG   
  
  
+ GATTTATGGC CGCAAATGCA GCGATTATGG ATGCAATCAA AAACGAAAAA AGGGTACACA TCATAGATTT   
  
  
+ TGACATAAAC CAAGGGAATC AATACATAAA TCTCATGCAA TCCCTTGCTA AACAAGGTAA CAAGCTGACG   
  
  
+ CACTTGAAGT TGACTGGAGT TGATGACCCT GAGTCAGTTC AACGCCCTAT TGGTGGCCTA AAAAACATCG   
  
  
+ GACAAAGGCT GCAAGCATTA GCTGAATATC TTGGTGTGTC ATTTGAGTTC AAAGCAATAC CTGCTAGAAC   
  
  
+ TCCACTTGTT AACCCGGAAA TGTTAGAATG TCGACCTGCG GAAGCTTTAG TGGTGAACTT TGCCTTCCAG   
  
  
+ CTTCACCACA TGCCTGATGA AAGCGTCTCA ACAATCAACC TCCGAGACCA GCTTCTTCGG ATGGTCAAAA   
  
  
+ GCCTAAACCC AAAGCTAGTA ACCATTGTCG AGCAGGATGT GAACACAAAC ACTACCCCTT TCCTCTCTAG   
  
  
+ GTTTGCTGAG GCATACAGCT ACTATTCTGC TGTTTTTGAG TCTCTTGATG CTACTCTCCC TAGAGATAGC   
  
  
+ CAGGACAGGG TGAATGTGGA GAAGCAGTGC TTGGCACGTG ATGTTGTGAA CATCATTGCA TGTGAAGGGG   
  
  
+ AGGAGAGGAT TGAGCGCTAT GAGGTTGCGG GGAAATGGAG AGCGAGGATG TTGATGGCGG GATTCAGGGC   
  
  
+ TATTCCAATG GGTCAGAAGA CCGGTGATAT GATTCGGAAG CTTATATCAA TGCGGTATTG TGACAGGCTC   
  
  
+ AAGGTAAAAG AGGAGATGGA TGCACTTCAC TTTGGGTGGG AAGATAAAAA CTTAATCGTT ACATCAGCTT   
  
  
+ GGAGGTG  

- +Up\_Stream \_Len000TCTATC TGTAAGTCGG TGAACGGTCA AGTCTCTCTC TATCTCTCTC TAACCCATTC   
  
  
- GGGAAAAACG TGATGAAAAA GAGCGAAGGT AAAAAGAAAG AATTAAAAAT CACTACTAAA AGGTCTTCTA   
  
  
- AACTAAATAA GTAGTACGAA TTGGTTAACC CAGAAACTCA TAATAACAAA TGACCCATAA ACTAGAAAAG   
  
  
- TATAAGGAAA CCTGACCCAC CCACCTTGAA AACTTGAAAG AATAATCAAC CCGTACGGGA ATAGAATAAG   
  
  
- GTTAAGTTTT GGGTATAAAA AGAAGTTAAA ATTAATACCC ATAAAACTTT AACTTACGAA AGGAAGTGTC   
  
  
- GTTATGATAT AATGTATAAA ATGTCCTTTC ATTATAAAAT GTCCTTTCAT ACAACGACAA AGTTGGGACA   
  
  
- AAACAAAGGA GAGAAAGGAA CCTATACTAC AAGTAACGAG AACATGGTTT GGATTGGAGT TGAAGGACTT   
  
  
- AATAAATTCC GTTATAATGG CACACGACCA GTTAAAACCA ACACCCAAAC AAAGAATTAC ATATACTAAA   
  
  
- TACCAGTCCT AGGTACGAGG AGAAGGCAGG TGAGATATAG AGAGAGAGAG AGAGAGTTAT GTTTAAGTCT   
  
  
- CTGAAAGAGA AAGACCCAAA TGATAACTAA CGTCTTCAAA CATCAGAACG CTCCGGAACT CAGAAGAAGC   
  
  
- TCCATCTTTT AAAACGAAAC GCGAAGGAAG GGGTATAAAA TTGTGGGGAG GGGGGGGTTG CGTTCAAAAG   
  
  
- ATGTAAAAAC ACGTTAAGAA GTCGATACTA AGGGGTTAAA GTGAACAGTG TAGAGATAAA AGGAGAAAAA   
  
  
- CCAAAGAACT AAGACATCTT CCAACACATA CTTGAAGAAA CAAAGGGAAA AAGGAAACAA TAAAAATTAC   
  
  
- AAGGGTATCT ACTATATCTC TAGACGAAAA GGAACGACAC TCTCCATAGA GACTCTTACG TACATCGTTA   
  
  
- GTTTTAAGAC TCAACAATTA AGATAACGTA CAGATACCCA CTACTAACAC TACTACAAAA AAGACAACGG   
  
  
- GAGAAAGAGA GAAAGTGACT TTAACGAAAG GTCTTATCAA CAAACTGAGG AAAGACATAG AATTAACTCG   
  
  
- GGAACGAAAC CAAACACGAG ACCCTCAAGA AGAAATCATT AGGGAACGAA CATAAAAGAC TTTCTCGTTC   
  
  
- GTTATTAGGA GTATCGTACA CATATTAGTA TGTTATCGTT GTCGTTTGTA CCTAGGACGC GGGTCAATCC   
  
  
- AAACCAATCC AATTCTATAC GTATAAGAAA AGAGAACTTT TCTCAACAGA TCCTTCCCAT TAATACGGGT   
  
  
- AAAGTACCTA TTATTACCAT GTTAATTCAA ACTAACCGAA TTTATGGTTA ATAAATCGCA GAAAAAGAAA   
  
  
- CCGACAGGTC CCAAGGTAAA GGTATTTGTT GAAACTCATT CCGTATCTCT TGATTTAAAG ACGGTTAACA   
  
  
- GTTCACGTTC AACGAGTATA CGTTGTTATG AACCTTTAAA ACTCGACTCG ACGACCGTAT AGTACTTATC   
  
  
- AAGATTACCA CCGCTCAATA GTGAATAGCA GGGAACACAC TGCTTAAAGA GAAGGATCCT TCGGGAGATC   
  
  
- AGTTACGGAT AGACGACAAC ACCTCACTTT TACCTATGTA AATCTAGAGA TATTTGGGAT GAAACCGTTC   
  
  
- TAGGTACGAC TAAAATCCTT CGGGAAACTG GTCTCTTACT GTATTGTTGG ACTATCCCAC AATGTACGAT   
  
  
- TGAAGCTAAT AAATTAGTTT TATGCCTTGA AGATAGTCAG TTTCGGACGG GGTCCAAGAT GACTTAATTT   
  
  
- AACATAACTT AACGAGAGTG AACGAATACT GCTAATTTAT TGACCTAAAC GATTAATAAG AAAAATAAAA   
  
  
- TAAGAACCAC TACATCCCTT GAGTCCCTTA ACATTGGTAT GGACAAGGTC GACTTTTCCC ATGCCCTTGT   
  
  
- CAGTATCAAT GTCCCCTTAA AGCGTTATGT AAATTCCCTC ATTCTTCGTC CTAGTACAGA AATCAATCCG   
  
  
- GCCGTCTATA CAGACACTGA GGTATACCTT TAGGTTGTGA CATAAGTGAA GTTCCGTTAT TATTATCGAG   
  
  
- ACCAAACAGA CCAGTTGACA TACCGAGACT TTTCGTGTTC TAGTTTATAC ACTGGCTTAG AAAATTACCA   
  
  
- GGTTCAATAC CGGACTTTAA GAAACAACTA AGAGGTAGTC TTCTCGAGTA GGTGGGTAGT CTACGATGAG   
  
  
- GATTGGGTAA ACTGAGAAGA AAGCACCCGT ATTCTGTACT ACTCTCAGGA ATGGTTCCTT TAATACCTAG   
  
  
- TCTTATACCC CATTCGGGTA GAAGGCTACG TCAACTTATA CTACTTCCAT TATTCTATTC CGATTTTGAA   
  
  
- GTTCTTAACC TCGTACGGGA CGAGCTACTT CTACTACTTC ACCTACTCCA CCGATATCCC GGGCACGATC   
  
  
- TTGTGTCGTA CCTCGAACTA CTTCTCACCC AGCGGGGTAA CTCTTTACAC CAAAAGGTAC TAAGTGGTTT   
  
  
- CCTCAGGTGG AGGAGTCTCA GTAGGTTACA GTCGTCGTAA TCGTCGTGGT TTCTTCATAG TGGAACGAGA   
  
  
- GGCTCATGAG GGTTTGTTAA CGAGAAATTA ACACGTTTAT ACTAAAGTCT CCCCTTAAAA CTCCTTCGGC   
  
  
- ACTGATACTA GTCGCTCGAA TCTGTCTAAC AAAGATAGGT TCCCCTAGGT CGCGTCTCCT ATCGTCGGAT   
  
  
- GTACCATCTT CCGGAACGAA GGGCAAACCG AAGTAGCCCT TTTCAAGAAA TGTTTCGAAA CTTTACGTTT   
  
  
- CTCGGTGGAA GTAAACTGTC CGATCGCCGA TACGTTTAGG AGAAACTCCA CACGGGTACA AAGTTTAAGC   
  
  
- CTAAATACCG GCGTTTACGT CGCTAATACC TACGTTAGTT TTTGCTTTTT TCCCATGTGT AGTATCTAAA   
  
  
- ACTGTATTTG GTTCCCTTAG TTATGTATTT AGAGTACGTT AGGGAACGAT TTGTTCCATT GTTCGACTGC   
  
  
- GTGAACTTCA ACTGACCTCA ACTACTGGGA CTCAGTCAAG TTGCGGGATA ACCACCGGAT TTTTTGTAGC   
  
  
- CTGTTTCCGA CGTTCGTAAT CGACTTATAG AACCACACAG TAAACTCAAG TTTCGTTATG GACGATCTTG   
  
  
- AGGTGAACAA TTGGGCCTTT ACAATCTTAC AGCTGGACGC CTTCGAAATC ACCACTTGAA ACGGAAGGTC   
  
  
- GAAGTGGTGT ACGGACTACT TTCGCAGAGT TGTTAGTTGG AGGCTCTGGT CGAAGAAGCC TACCAGTTTT   
  
  
- CGGATTTGGG TTTCGATCAT TGGTAACAGC TCGTCCTACA CTTGTGTTTG TGATGGGGAA AGGAGAGATC   
  
  
- CAAACGACTC CGTATGTCGA TGATAAGACG ACAAAAACTC AGAGAACTAC GATGAGAGGG ATCTCTATCG   
  
  
- GTCCTGTCCC ACTTACACCT CTTCGTCACG AACCGTGCAC TACAACACTT GTAGTAACGT ACACTTCCCC   
  
  
- TCCTCTCCTA ACTCGCGATA CTCCAACGCC CCTTTACCTC TCGCTCCTAC AACTACCGCC CTAAGTCCCG   
  
  
- ATAAGGTTAC CCAGTCTTCT GGCCACTATA CTAAGCCTTC GAATATAGTT ACGCCATAAC ACTGTCCGAG   
  
  
- TTCCATTTTC TCCTCTACCT ACGTGAAGTG AAACCCACCC TTCTATTTTT GAATTAGCAA TGTAGTCGAA   
  
  
- CCTCCAC

+     MRE

| Site Name | Organism | Position | Strand | Matrix score. | sequence | function |
| --- | --- | --- | --- | --- | --- | --- |
| MRE | Petroselinum crispum | 1270 | - | 7 | AACCTAA | MYB binding site involved in light responsiveness |
| MRE | Petroselinum crispum | 473 | + | 7 | AACCTAA | MYB binding site involved in light responsiveness |
| MRE | Petroselinum crispum | 1260 | - | 7 | AACCTAA | MYB binding site involved in light responsiveness |

>HU11G01134.1   
+ +Up\_Stream \_Len000AGATAG ACATTCAGCC ACTTGCCAGT TCAGAGAGAG ATAGAGAGAG ATTGGGTAAG   
  
  
+ CCCTTTTTGC ACTACTTTTT CTCGCTTCCA TTTTTCTTTC TTAATTTTTA GTGATGATTT TCCAGAAGAT   
  
  
+ TTGATTTATT CATCATGCTT AACCAATTGG GTCTTTGAGT ATTATTGTTT ACTGGGTATT TGATCTTTTC   
  
  
+ ATATTCCTTT GGACTGGGTG GGTGGAACTT TTGAACTTTC TTATTAGTTG GGCATGCCCT TATCTTATTC   
  
  
+ CAATTCAAAA CCCATATTTT TCTTCAATTT TAATTATGGG TATTTTGAAA TTGAATGCTT TCCTTCACAG   
  
  
+ CAATACTATA TTACATATTT TACAGGAAAG TAATATTTTA CAGGAAAGTA TGTTGCTGTT TCAACCCTGT   
  
  
+ TTTGTTTCCT CTCTTTCCTT GGATATGATG TTCATTGCTC TTGTACCAAA CCTAACCTCA ACTTCCTGAA   
  
  
+ TTATTTAAGG CAATATTACC GTGTGCTGGT CAATTTTGGT TGTGGGTTTG TTTCTTAATG TATATGATTT   
  
  
+ ATGGTCAGGA TCCATGCTCC TCTTCCGTCC ACTCTATATC TCTCTCTCTC TCTCTCAATA CAAATTCAGA   
  
  
+ GACTTTCTCT TTCTGGGTTT ACTATTGATT GCAGAAGTTT GTAGTCTTGC GAGGCCTTGA GTCTTCTTCG   
  
  
+ AGGTAGAAAA TTTTGCTTTG CGCTTCCTTC CCCATATTTT AACACCCCTC CCCCCCCAAC GCAAGTTTTC   
  
  
+ TACATTTTTG TGCAATTCTT CAGCTATGAT TCCCCAATTT CACTTGTCAC ATCTCTATTT TCCTCTTTTT   
  
  
+ GGTTTCTTGA TTCTGTAGAA GGTTGTGTAT GAACTTCTTT GTTTCCCTTT TTCCTTTGTT ATTTTTAATG   
  
  
+ TTCCCATAGA TGATATAGAG ATCTGCTTTT CCTTGCTGTG AGAGGTATCT CTGAGAATGC ATGTAGCAAT   
  
  
+ CAAAATTCTG AGTTGTTAAT TCTATTGCAT GTCTATGGGT GATGATTGTG ATGATGTTTT TTCTGTTGCC   
  
  
+ CTCTTTCTCT CTTTCACTGA AATTGCTTTC CAGAATAGTT GTTTGACTCC TTTCTGTATC TTAATTGAGC   
  
  
+ CCTTGCTTTG GTTTGTGCTC TGGGAGTTCT TCTTTAGTAA TCCCTTGCTT GTATTTTCTG AAAGAGCAAG   
  
  
+ CAATAATCCT CATAGCATGT GTATAATCAT ACAATAGCAA CAGCAAACAT GGATCCTGCG CCCAGTTAGG   
  
  
+ TTTGGTTAGG TTAAGATATG CATATTCTTT TCTCTTGAAA AGAGTTGTCT AGGAAGGGTA ATTATGCCCA   
  
  
+ TTTCATGGAT AATAATGGTA CAATTAAGTT TGATTGGCTT AAATACCAAT TATTTAGCGT CTTTTTCTTT   
  
  
+ GGCTGTCCAG GGTTCCATTT CCATAAACAA CTTTGAGTAA GGCATAGAGA ACTAAATTTC TGCCAATTGT   
  
  
+ CAAGTGCAAG TTGCTCATAT GCAACAATAC TTGGAAATTT TGAGCTGAGC TGCTGGCATA TCATGAATAG   
  
  
+ TTCTAATGGT GGCGAGTTAT CACTTATCGT CCCTTGTGTG ACGAATTTCT CTTCCTAGGA AGCCCTCTAG   
  
  
+ TCAATGCCTA TCTGCTGTTG TGGAGTGAAA ATGGATACAT TTAGATCTCT ATAAACCCTA CTTTGGCAAG   
  
  
+ ATCCATGCTG ATTTTAGGAA GCCCTTTGAC CAGAGAATGA CATAACAACC TGATAGGGTG TTACATGCTA   
  
  
+ ACTTCGATTA TTTAATCAAA ATACGGAACT TCTATCAGTC AAAGCCTGCC CCAGGTTCTA CTGAATTAAA   
  
  
+ TTGTATTGAA TTGCTCTCAC TTGCTTATGA CGATTAAATA ACTGGATTTG CTAATTATTC TTTTTATTTT   
  
  
+ ATTCTTGGTG ATGTAGGGAA CTCAGGGAAT TGTAACCATA CCTGTTCCAG CTGAAAAGGG TACGGGAACA   
  
  
+ GTCATAGTTA CAGGGGAATT TCGCAATACA TTTAAGGGAG TAAGAAGCAG GATCATGTCT TTAGTTAGGC   
  
  
+ CGGCAGATAT GTCTGTGACT CCATATGGAA ATCCAACACT GTATTCACTT CAAGGCAATA ATAATAGCTC   
  
  
+ TGGTTTGTCT GGTCAACTGT ATGGCTCTGA AAAGCACAAG ATCAAATATG TGACCGAATC TTTTAATGGT   
  
  
+ CCAAGTTATG GCCTGAAATT CTTTGTTGAT TCTCCATCAG AAGAGCTCAT CCACCCATCA GATGCTACTC   
  
  
+ CTAACCCATT TGACTCTTCT TTCGTGGGCA TAAGACATGA TGAGAGTCCT TACCAAGGAA ATTATGGATC   
  
  
+ AGAATATGGG GTAAGCCCAT CTTCCGATGC AGTTGAATAT GATGAAGGTA ATAAGATAAG GCTAAAACTT   
  
  
+ CAAGAATTGG AGCATGCCCT GCTCGATGAA GATGATGAAG TGGATGAGGT GGCTATAGGG CCCGTGCTAG   
  
  
+ AACACAGCAT GGAGCTTGAT GAAGAGTGGG TCGCCCCATT GAGAAATGTG GTTTTCCATG ATTCACCAAA   
  
  
+ GGAGTCCACC TCCTCAGAGT CATCCAATGT CAGCAGCATT AGCAGCACCA AAGAAGTATC ACCTTGCTCT   
  
  
+ CCGAGTACTC CCAAACAATT GCTCTTTAAT TGTGCAAATA TGATTTCAGA GGGGAATTTT GAGGAAGCCG   
  
  
+ TGACTATGAT CAGCGAGCTT AGACAGATTG TTTCTATCCA AGGGGATCCA GCGCAGAGGA TAGCAGCCTA   
  
  
+ CATGGTAGAA GGCCTTGCTT CCCGTTTGGC TTCATCGGGA AAAGTTCTTT ACAAAGCTTT GAAATGCAAA   
  
  
+ GAGCCACCTT CATTTGACAG GCTAGCGGCT ATGCAAATCC TCTTTGAGGT GTGCCCATGT TTCAAATTCG   
  
  
+ GATTTATGGC CGCAAATGCA GCGATTATGG ATGCAATCAA AAACGAAAAA AGGGTACACA TCATAGATTT   
  
  
+ TGACATAAAC CAAGGGAATC AATACATAAA TCTCATGCAA TCCCTTGCTA AACAAGGTAA CAAGCTGACG   
  
  
+ CACTTGAAGT TGACTGGAGT TGATGACCCT GAGTCAGTTC AACGCCCTAT TGGTGGCCTA AAAAACATCG   
  
  
+ GACAAAGGCT GCAAGCATTA GCTGAATATC TTGGTGTGTC ATTTGAGTTC AAAGCAATAC CTGCTAGAAC   
  
  
+ TCCACTTGTT AACCCGGAAA TGTTAGAATG TCGACCTGCG GAAGCTTTAG TGGTGAACTT TGCCTTCCAG   
  
  
+ CTTCACCACA TGCCTGATGA AAGCGTCTCA ACAATCAACC TCCGAGACCA GCTTCTTCGG ATGGTCAAAA   
  
  
+ GCCTAAACCC AAAGCTAGTA ACCATTGTCG AGCAGGATGT GAACACAAAC ACTACCCCTT TCCTCTCTAG   
  
  
+ GTTTGCTGAG GCATACAGCT ACTATTCTGC TGTTTTTGAG TCTCTTGATG CTACTCTCCC TAGAGATAGC   
  
  
+ CAGGACAGGG TGAATGTGGA GAAGCAGTGC TTGGCACGTG ATGTTGTGAA CATCATTGCA TGTGAAGGGG   
  
  
+ AGGAGAGGAT TGAGCGCTAT GAGGTTGCGG GGAAATGGAG AGCGAGGATG TTGATGGCGG GATTCAGGGC   
  
  
+ TATTCCAATG GGTCAGAAGA CCGGTGATAT GATTCGGAAG CTTATATCAA TGCGGTATTG TGACAGGCTC   
  
  
+ AAGGTAAAAG AGGAGATGGA TGCACTTCAC TTTGGGTGGG AAGATAAAAA CTTAATCGTT ACATCAGCTT   
  
  
+ GGAGGTG  

- +Up\_Stream \_Len000TCTATC TGTAAGTCGG TGAACGGTCA AGTCTCTCTC TATCTCTCTC TAACCCATTC   
  
  
- GGGAAAAACG TGATGAAAAA GAGCGAAGGT AAAAAGAAAG AATTAAAAAT CACTACTAAA AGGTCTTCTA   
  
  
- AACTAAATAA GTAGTACGAA TTGGTTAACC CAGAAACTCA TAATAACAAA TGACCCATAA ACTAGAAAAG   
  
  
- TATAAGGAAA CCTGACCCAC CCACCTTGAA AACTTGAAAG AATAATCAAC CCGTACGGGA ATAGAATAAG   
  
  
- GTTAAGTTTT GGGTATAAAA AGAAGTTAAA ATTAATACCC ATAAAACTTT AACTTACGAA AGGAAGTGTC   
  
  
- GTTATGATAT AATGTATAAA ATGTCCTTTC ATTATAAAAT GTCCTTTCAT ACAACGACAA AGTTGGGACA   
  
  
- AAACAAAGGA GAGAAAGGAA CCTATACTAC AAGTAACGAG AACATGGTTT GGATTGGAGT TGAAGGACTT   
  
  
- AATAAATTCC GTTATAATGG CACACGACCA GTTAAAACCA ACACCCAAAC AAAGAATTAC ATATACTAAA   
  
  
- TACCAGTCCT AGGTACGAGG AGAAGGCAGG TGAGATATAG AGAGAGAGAG AGAGAGTTAT GTTTAAGTCT   
  
  
- CTGAAAGAGA AAGACCCAAA TGATAACTAA CGTCTTCAAA CATCAGAACG CTCCGGAACT CAGAAGAAGC   
  
  
- TCCATCTTTT AAAACGAAAC GCGAAGGAAG GGGTATAAAA TTGTGGGGAG GGGGGGGTTG CGTTCAAAAG   
  
  
- ATGTAAAAAC ACGTTAAGAA GTCGATACTA AGGGGTTAAA GTGAACAGTG TAGAGATAAA AGGAGAAAAA   
  
  
- CCAAAGAACT AAGACATCTT CCAACACATA CTTGAAGAAA CAAAGGGAAA AAGGAAACAA TAAAAATTAC   
  
  
- AAGGGTATCT ACTATATCTC TAGACGAAAA GGAACGACAC TCTCCATAGA GACTCTTACG TACATCGTTA   
  
  
- GTTTTAAGAC TCAACAATTA AGATAACGTA CAGATACCCA CTACTAACAC TACTACAAAA AAGACAACGG   
  
  
- GAGAAAGAGA GAAAGTGACT TTAACGAAAG GTCTTATCAA CAAACTGAGG AAAGACATAG AATTAACTCG   
  
  
- GGAACGAAAC CAAACACGAG ACCCTCAAGA AGAAATCATT AGGGAACGAA CATAAAAGAC TTTCTCGTTC   
  
  
- GTTATTAGGA GTATCGTACA CATATTAGTA TGTTATCGTT GTCGTTTGTA CCTAGGACGC GGGTCAATCC   
  
  
- AAACCAATCC AATTCTATAC GTATAAGAAA AGAGAACTTT TCTCAACAGA TCCTTCCCAT TAATACGGGT   
  
  
- AAAGTACCTA TTATTACCAT GTTAATTCAA ACTAACCGAA TTTATGGTTA ATAAATCGCA GAAAAAGAAA   
  
  
- CCGACAGGTC CCAAGGTAAA GGTATTTGTT GAAACTCATT CCGTATCTCT TGATTTAAAG ACGGTTAACA   
  
  
- GTTCACGTTC AACGAGTATA CGTTGTTATG AACCTTTAAA ACTCGACTCG ACGACCGTAT AGTACTTATC   
  
  
- AAGATTACCA CCGCTCAATA GTGAATAGCA GGGAACACAC TGCTTAAAGA GAAGGATCCT TCGGGAGATC   
  
  
- AGTTACGGAT AGACGACAAC ACCTCACTTT TACCTATGTA AATCTAGAGA TATTTGGGAT GAAACCGTTC   
  
  
- TAGGTACGAC TAAAATCCTT CGGGAAACTG GTCTCTTACT GTATTGTTGG ACTATCCCAC AATGTACGAT   
  
  
- TGAAGCTAAT AAATTAGTTT TATGCCTTGA AGATAGTCAG TTTCGGACGG GGTCCAAGAT GACTTAATTT   
  
  
- AACATAACTT AACGAGAGTG AACGAATACT GCTAATTTAT TGACCTAAAC GATTAATAAG AAAAATAAAA   
  
  
- TAAGAACCAC TACATCCCTT GAGTCCCTTA ACATTGGTAT GGACAAGGTC GACTTTTCCC ATGCCCTTGT   
  
  
- CAGTATCAAT GTCCCCTTAA AGCGTTATGT AAATTCCCTC ATTCTTCGTC CTAGTACAGA AATCAATCCG   
  
  
- GCCGTCTATA CAGACACTGA GGTATACCTT TAGGTTGTGA CATAAGTGAA GTTCCGTTAT TATTATCGAG   
  
  
- ACCAAACAGA CCAGTTGACA TACCGAGACT TTTCGTGTTC TAGTTTATAC ACTGGCTTAG AAAATTACCA   
  
  
- GGTTCAATAC CGGACTTTAA GAAACAACTA AGAGGTAGTC TTCTCGAGTA GGTGGGTAGT CTACGATGAG   
  
  
- GATTGGGTAA ACTGAGAAGA AAGCACCCGT ATTCTGTACT ACTCTCAGGA ATGGTTCCTT TAATACCTAG   
  
  
- TCTTATACCC CATTCGGGTA GAAGGCTACG TCAACTTATA CTACTTCCAT TATTCTATTC CGATTTTGAA   
  
  
- GTTCTTAACC TCGTACGGGA CGAGCTACTT CTACTACTTC ACCTACTCCA CCGATATCCC GGGCACGATC   
  
  
- TTGTGTCGTA CCTCGAACTA CTTCTCACCC AGCGGGGTAA CTCTTTACAC CAAAAGGTAC TAAGTGGTTT   
  
  
- CCTCAGGTGG AGGAGTCTCA GTAGGTTACA GTCGTCGTAA TCGTCGTGGT TTCTTCATAG TGGAACGAGA   
  
  
- GGCTCATGAG GGTTTGTTAA CGAGAAATTA ACACGTTTAT ACTAAAGTCT CCCCTTAAAA CTCCTTCGGC   
  
  
- ACTGATACTA GTCGCTCGAA TCTGTCTAAC AAAGATAGGT TCCCCTAGGT CGCGTCTCCT ATCGTCGGAT   
  
  
- GTACCATCTT CCGGAACGAA GGGCAAACCG AAGTAGCCCT TTTCAAGAAA TGTTTCGAAA CTTTACGTTT   
  
  
- CTCGGTGGAA GTAAACTGTC CGATCGCCGA TACGTTTAGG AGAAACTCCA CACGGGTACA AAGTTTAAGC   
  
  
- CTAAATACCG GCGTTTACGT CGCTAATACC TACGTTAGTT TTTGCTTTTT TCCCATGTGT AGTATCTAAA   
  
  
- ACTGTATTTG GTTCCCTTAG TTATGTATTT AGAGTACGTT AGGGAACGAT TTGTTCCATT GTTCGACTGC   
  
  
- GTGAACTTCA ACTGACCTCA ACTACTGGGA CTCAGTCAAG TTGCGGGATA ACCACCGGAT TTTTTGTAGC   
  
  
- CTGTTTCCGA CGTTCGTAAT CGACTTATAG AACCACACAG TAAACTCAAG TTTCGTTATG GACGATCTTG   
  
  
- AGGTGAACAA TTGGGCCTTT ACAATCTTAC AGCTGGACGC CTTCGAAATC ACCACTTGAA ACGGAAGGTC   
  
  
- GAAGTGGTGT ACGGACTACT TTCGCAGAGT TGTTAGTTGG AGGCTCTGGT CGAAGAAGCC TACCAGTTTT   
  
  
- CGGATTTGGG TTTCGATCAT TGGTAACAGC TCGTCCTACA CTTGTGTTTG TGATGGGGAA AGGAGAGATC   
  
  
- CAAACGACTC CGTATGTCGA TGATAAGACG ACAAAAACTC AGAGAACTAC GATGAGAGGG ATCTCTATCG   
  
  
- GTCCTGTCCC ACTTACACCT CTTCGTCACG AACCGTGCAC TACAACACTT GTAGTAACGT ACACTTCCCC   
  
  
- TCCTCTCCTA ACTCGCGATA CTCCAACGCC CCTTTACCTC TCGCTCCTAC AACTACCGCC CTAAGTCCCG   
  
  
- ATAAGGTTAC CCAGTCTTCT GGCCACTATA CTAAGCCTTC GAATATAGTT ACGCCATAAC ACTGTCCGAG   
  
  
- TTCCATTTTC TCCTCTACCT ACGTGAAGTG AAACCCACCC TTCTATTTTT GAATTAGCAA TGTAGTCGAA   
  
  
- CCTCCAC

+     MYB

| Site Name | Organism | Position | Strand | Matrix score. | sequence | function |
| --- | --- | --- | --- | --- | --- | --- |
| MYB | Arabidopsis thaliana | 1927 | + | 6 | TAACCA |  |
| MYB | Arabidopsis thaliana | 531 | - | 6 | CAACCA |  |
| MYB | Arabidopsis thaliana | 164 | + | 6 | TAACCA |  |
| MYB | Arabidopsis thaliana | 1267 | - | 6 | TAACCA |  |
| MYB | Arabidopsis thaliana | 1047 | - | 6 | CAACAG |  |
| MYB | Arabidopsis thaliana | 1232 | + | 6 | CAACAG |  |
| MYB | Arabidopsis thaliana | 1629 | - | 6 | CAACAG |  |
| MYB | Arabidopsis thaliana | 3313 | + | 6 | TAACCA |  |

>HU11G01134.1   
+ +Up\_Stream \_Len000AGATAG ACATTCAGCC ACTTGCCAGT TCAGAGAGAG ATAGAGAGAG ATTGGGTAAG   
  
  
+ CCCTTTTTGC ACTACTTTTT CTCGCTTCCA TTTTTCTTTC TTAATTTTTA GTGATGATTT TCCAGAAGAT   
  
  
+ TTGATTTATT CATCATGCTT AACCAATTGG GTCTTTGAGT ATTATTGTTT ACTGGGTATT TGATCTTTTC   
  
  
+ ATATTCCTTT GGACTGGGTG GGTGGAACTT TTGAACTTTC TTATTAGTTG GGCATGCCCT TATCTTATTC   
  
  
+ CAATTCAAAA CCCATATTTT TCTTCAATTT TAATTATGGG TATTTTGAAA TTGAATGCTT TCCTTCACAG   
  
  
+ CAATACTATA TTACATATTT TACAGGAAAG TAATATTTTA CAGGAAAGTA TGTTGCTGTT TCAACCCTGT   
  
  
+ TTTGTTTCCT CTCTTTCCTT GGATATGATG TTCATTGCTC TTGTACCAAA CCTAACCTCA ACTTCCTGAA   
  
  
+ TTATTTAAGG CAATATTACC GTGTGCTGGT CAATTTTGGT TGTGGGTTTG TTTCTTAATG TATATGATTT   
  
  
+ ATGGTCAGGA TCCATGCTCC TCTTCCGTCC ACTCTATATC TCTCTCTCTC TCTCTCAATA CAAATTCAGA   
  
  
+ GACTTTCTCT TTCTGGGTTT ACTATTGATT GCAGAAGTTT GTAGTCTTGC GAGGCCTTGA GTCTTCTTCG   
  
  
+ AGGTAGAAAA TTTTGCTTTG CGCTTCCTTC CCCATATTTT AACACCCCTC CCCCCCCAAC GCAAGTTTTC   
  
  
+ TACATTTTTG TGCAATTCTT CAGCTATGAT TCCCCAATTT CACTTGTCAC ATCTCTATTT TCCTCTTTTT   
  
  
+ GGTTTCTTGA TTCTGTAGAA GGTTGTGTAT GAACTTCTTT GTTTCCCTTT TTCCTTTGTT ATTTTTAATG   
  
  
+ TTCCCATAGA TGATATAGAG ATCTGCTTTT CCTTGCTGTG AGAGGTATCT CTGAGAATGC ATGTAGCAAT   
  
  
+ CAAAATTCTG AGTTGTTAAT TCTATTGCAT GTCTATGGGT GATGATTGTG ATGATGTTTT TTCTGTTGCC   
  
  
+ CTCTTTCTCT CTTTCACTGA AATTGCTTTC CAGAATAGTT GTTTGACTCC TTTCTGTATC TTAATTGAGC   
  
  
+ CCTTGCTTTG GTTTGTGCTC TGGGAGTTCT TCTTTAGTAA TCCCTTGCTT GTATTTTCTG AAAGAGCAAG   
  
  
+ CAATAATCCT CATAGCATGT GTATAATCAT ACAATAGCAA CAGCAAACAT GGATCCTGCG CCCAGTTAGG   
  
  
+ TTTGGTTAGG TTAAGATATG CATATTCTTT TCTCTTGAAA AGAGTTGTCT AGGAAGGGTA ATTATGCCCA   
  
  
+ TTTCATGGAT AATAATGGTA CAATTAAGTT TGATTGGCTT AAATACCAAT TATTTAGCGT CTTTTTCTTT   
  
  
+ GGCTGTCCAG GGTTCCATTT CCATAAACAA CTTTGAGTAA GGCATAGAGA ACTAAATTTC TGCCAATTGT   
  
  
+ CAAGTGCAAG TTGCTCATAT GCAACAATAC TTGGAAATTT TGAGCTGAGC TGCTGGCATA TCATGAATAG   
  
  
+ TTCTAATGGT GGCGAGTTAT CACTTATCGT CCCTTGTGTG ACGAATTTCT CTTCCTAGGA AGCCCTCTAG   
  
  
+ TCAATGCCTA TCTGCTGTTG TGGAGTGAAA ATGGATACAT TTAGATCTCT ATAAACCCTA CTTTGGCAAG   
  
  
+ ATCCATGCTG ATTTTAGGAA GCCCTTTGAC CAGAGAATGA CATAACAACC TGATAGGGTG TTACATGCTA   
  
  
+ ACTTCGATTA TTTAATCAAA ATACGGAACT TCTATCAGTC AAAGCCTGCC CCAGGTTCTA CTGAATTAAA   
  
  
+ TTGTATTGAA TTGCTCTCAC TTGCTTATGA CGATTAAATA ACTGGATTTG CTAATTATTC TTTTTATTTT   
  
  
+ ATTCTTGGTG ATGTAGGGAA CTCAGGGAAT TGTAACCATA CCTGTTCCAG CTGAAAAGGG TACGGGAACA   
  
  
+ GTCATAGTTA CAGGGGAATT TCGCAATACA TTTAAGGGAG TAAGAAGCAG GATCATGTCT TTAGTTAGGC   
  
  
+ CGGCAGATAT GTCTGTGACT CCATATGGAA ATCCAACACT GTATTCACTT CAAGGCAATA ATAATAGCTC   
  
  
+ TGGTTTGTCT GGTCAACTGT ATGGCTCTGA AAAGCACAAG ATCAAATATG TGACCGAATC TTTTAATGGT   
  
  
+ CCAAGTTATG GCCTGAAATT CTTTGTTGAT TCTCCATCAG AAGAGCTCAT CCACCCATCA GATGCTACTC   
  
  
+ CTAACCCATT TGACTCTTCT TTCGTGGGCA TAAGACATGA TGAGAGTCCT TACCAAGGAA ATTATGGATC   
  
  
+ AGAATATGGG GTAAGCCCAT CTTCCGATGC AGTTGAATAT GATGAAGGTA ATAAGATAAG GCTAAAACTT   
  
  
+ CAAGAATTGG AGCATGCCCT GCTCGATGAA GATGATGAAG TGGATGAGGT GGCTATAGGG CCCGTGCTAG   
  
  
+ AACACAGCAT GGAGCTTGAT GAAGAGTGGG TCGCCCCATT GAGAAATGTG GTTTTCCATG ATTCACCAAA   
  
  
+ GGAGTCCACC TCCTCAGAGT CATCCAATGT CAGCAGCATT AGCAGCACCA AAGAAGTATC ACCTTGCTCT   
  
  
+ CCGAGTACTC CCAAACAATT GCTCTTTAAT TGTGCAAATA TGATTTCAGA GGGGAATTTT GAGGAAGCCG   
  
  
+ TGACTATGAT CAGCGAGCTT AGACAGATTG TTTCTATCCA AGGGGATCCA GCGCAGAGGA TAGCAGCCTA   
  
  
+ CATGGTAGAA GGCCTTGCTT CCCGTTTGGC TTCATCGGGA AAAGTTCTTT ACAAAGCTTT GAAATGCAAA   
  
  
+ GAGCCACCTT CATTTGACAG GCTAGCGGCT ATGCAAATCC TCTTTGAGGT GTGCCCATGT TTCAAATTCG   
  
  
+ GATTTATGGC CGCAAATGCA GCGATTATGG ATGCAATCAA AAACGAAAAA AGGGTACACA TCATAGATTT   
  
  
+ TGACATAAAC CAAGGGAATC AATACATAAA TCTCATGCAA TCCCTTGCTA AACAAGGTAA CAAGCTGACG   
  
  
+ CACTTGAAGT TGACTGGAGT TGATGACCCT GAGTCAGTTC AACGCCCTAT TGGTGGCCTA AAAAACATCG   
  
  
+ GACAAAGGCT GCAAGCATTA GCTGAATATC TTGGTGTGTC ATTTGAGTTC AAAGCAATAC CTGCTAGAAC   
  
  
+ TCCACTTGTT AACCCGGAAA TGTTAGAATG TCGACCTGCG GAAGCTTTAG TGGTGAACTT TGCCTTCCAG   
  
  
+ CTTCACCACA TGCCTGATGA AAGCGTCTCA ACAATCAACC TCCGAGACCA GCTTCTTCGG ATGGTCAAAA   
  
  
+ GCCTAAACCC AAAGCTAGTA ACCATTGTCG AGCAGGATGT GAACACAAAC ACTACCCCTT TCCTCTCTAG   
  
  
+ GTTTGCTGAG GCATACAGCT ACTATTCTGC TGTTTTTGAG TCTCTTGATG CTACTCTCCC TAGAGATAGC   
  
  
+ CAGGACAGGG TGAATGTGGA GAAGCAGTGC TTGGCACGTG ATGTTGTGAA CATCATTGCA TGTGAAGGGG   
  
  
+ AGGAGAGGAT TGAGCGCTAT GAGGTTGCGG GGAAATGGAG AGCGAGGATG TTGATGGCGG GATTCAGGGC   
  
  
+ TATTCCAATG GGTCAGAAGA CCGGTGATAT GATTCGGAAG CTTATATCAA TGCGGTATTG TGACAGGCTC   
  
  
+ AAGGTAAAAG AGGAGATGGA TGCACTTCAC TTTGGGTGGG AAGATAAAAA CTTAATCGTT ACATCAGCTT   
  
  
+ GGAGGTG  

- +Up\_Stream \_Len000TCTATC TGTAAGTCGG TGAACGGTCA AGTCTCTCTC TATCTCTCTC TAACCCATTC   
  
  
- GGGAAAAACG TGATGAAAAA GAGCGAAGGT AAAAAGAAAG AATTAAAAAT CACTACTAAA AGGTCTTCTA   
  
  
- AACTAAATAA GTAGTACGAA TTGGTTAACC CAGAAACTCA TAATAACAAA TGACCCATAA ACTAGAAAAG   
  
  
- TATAAGGAAA CCTGACCCAC CCACCTTGAA AACTTGAAAG AATAATCAAC CCGTACGGGA ATAGAATAAG   
  
  
- GTTAAGTTTT GGGTATAAAA AGAAGTTAAA ATTAATACCC ATAAAACTTT AACTTACGAA AGGAAGTGTC   
  
  
- GTTATGATAT AATGTATAAA ATGTCCTTTC ATTATAAAAT GTCCTTTCAT ACAACGACAA AGTTGGGACA   
  
  
- AAACAAAGGA GAGAAAGGAA CCTATACTAC AAGTAACGAG AACATGGTTT GGATTGGAGT TGAAGGACTT   
  
  
- AATAAATTCC GTTATAATGG CACACGACCA GTTAAAACCA ACACCCAAAC AAAGAATTAC ATATACTAAA   
  
  
- TACCAGTCCT AGGTACGAGG AGAAGGCAGG TGAGATATAG AGAGAGAGAG AGAGAGTTAT GTTTAAGTCT   
  
  
- CTGAAAGAGA AAGACCCAAA TGATAACTAA CGTCTTCAAA CATCAGAACG CTCCGGAACT CAGAAGAAGC   
  
  
- TCCATCTTTT AAAACGAAAC GCGAAGGAAG GGGTATAAAA TTGTGGGGAG GGGGGGGTTG CGTTCAAAAG   
  
  
- ATGTAAAAAC ACGTTAAGAA GTCGATACTA AGGGGTTAAA GTGAACAGTG TAGAGATAAA AGGAGAAAAA   
  
  
- CCAAAGAACT AAGACATCTT CCAACACATA CTTGAAGAAA CAAAGGGAAA AAGGAAACAA TAAAAATTAC   
  
  
- AAGGGTATCT ACTATATCTC TAGACGAAAA GGAACGACAC TCTCCATAGA GACTCTTACG TACATCGTTA   
  
  
- GTTTTAAGAC TCAACAATTA AGATAACGTA CAGATACCCA CTACTAACAC TACTACAAAA AAGACAACGG   
  
  
- GAGAAAGAGA GAAAGTGACT TTAACGAAAG GTCTTATCAA CAAACTGAGG AAAGACATAG AATTAACTCG   
  
  
- GGAACGAAAC CAAACACGAG ACCCTCAAGA AGAAATCATT AGGGAACGAA CATAAAAGAC TTTCTCGTTC   
  
  
- GTTATTAGGA GTATCGTACA CATATTAGTA TGTTATCGTT GTCGTTTGTA CCTAGGACGC GGGTCAATCC   
  
  
- AAACCAATCC AATTCTATAC GTATAAGAAA AGAGAACTTT TCTCAACAGA TCCTTCCCAT TAATACGGGT   
  
  
- AAAGTACCTA TTATTACCAT GTTAATTCAA ACTAACCGAA TTTATGGTTA ATAAATCGCA GAAAAAGAAA   
  
  
- CCGACAGGTC CCAAGGTAAA GGTATTTGTT GAAACTCATT CCGTATCTCT TGATTTAAAG ACGGTTAACA   
  
  
- GTTCACGTTC AACGAGTATA CGTTGTTATG AACCTTTAAA ACTCGACTCG ACGACCGTAT AGTACTTATC   
  
  
- AAGATTACCA CCGCTCAATA GTGAATAGCA GGGAACACAC TGCTTAAAGA GAAGGATCCT TCGGGAGATC   
  
  
- AGTTACGGAT AGACGACAAC ACCTCACTTT TACCTATGTA AATCTAGAGA TATTTGGGAT GAAACCGTTC   
  
  
- TAGGTACGAC TAAAATCCTT CGGGAAACTG GTCTCTTACT GTATTGTTGG ACTATCCCAC AATGTACGAT   
  
  
- TGAAGCTAAT AAATTAGTTT TATGCCTTGA AGATAGTCAG TTTCGGACGG GGTCCAAGAT GACTTAATTT   
  
  
- AACATAACTT AACGAGAGTG AACGAATACT GCTAATTTAT TGACCTAAAC GATTAATAAG AAAAATAAAA   
  
  
- TAAGAACCAC TACATCCCTT GAGTCCCTTA ACATTGGTAT GGACAAGGTC GACTTTTCCC ATGCCCTTGT   
  
  
- CAGTATCAAT GTCCCCTTAA AGCGTTATGT AAATTCCCTC ATTCTTCGTC CTAGTACAGA AATCAATCCG   
  
  
- GCCGTCTATA CAGACACTGA GGTATACCTT TAGGTTGTGA CATAAGTGAA GTTCCGTTAT TATTATCGAG   
  
  
- ACCAAACAGA CCAGTTGACA TACCGAGACT TTTCGTGTTC TAGTTTATAC ACTGGCTTAG AAAATTACCA   
  
  
- GGTTCAATAC CGGACTTTAA GAAACAACTA AGAGGTAGTC TTCTCGAGTA GGTGGGTAGT CTACGATGAG   
  
  
- GATTGGGTAA ACTGAGAAGA AAGCACCCGT ATTCTGTACT ACTCTCAGGA ATGGTTCCTT TAATACCTAG   
  
  
- TCTTATACCC CATTCGGGTA GAAGGCTACG TCAACTTATA CTACTTCCAT TATTCTATTC CGATTTTGAA   
  
  
- GTTCTTAACC TCGTACGGGA CGAGCTACTT CTACTACTTC ACCTACTCCA CCGATATCCC GGGCACGATC   
  
  
- TTGTGTCGTA CCTCGAACTA CTTCTCACCC AGCGGGGTAA CTCTTTACAC CAAAAGGTAC TAAGTGGTTT   
  
  
- CCTCAGGTGG AGGAGTCTCA GTAGGTTACA GTCGTCGTAA TCGTCGTGGT TTCTTCATAG TGGAACGAGA   
  
  
- GGCTCATGAG GGTTTGTTAA CGAGAAATTA ACACGTTTAT ACTAAAGTCT CCCCTTAAAA CTCCTTCGGC   
  
  
- ACTGATACTA GTCGCTCGAA TCTGTCTAAC AAAGATAGGT TCCCCTAGGT CGCGTCTCCT ATCGTCGGAT   
  
  
- GTACCATCTT CCGGAACGAA GGGCAAACCG AAGTAGCCCT TTTCAAGAAA TGTTTCGAAA CTTTACGTTT   
  
  
- CTCGGTGGAA GTAAACTGTC CGATCGCCGA TACGTTTAGG AGAAACTCCA CACGGGTACA AAGTTTAAGC   
  
  
- CTAAATACCG GCGTTTACGT CGCTAATACC TACGTTAGTT TTTGCTTTTT TCCCATGTGT AGTATCTAAA   
  
  
- ACTGTATTTG GTTCCCTTAG TTATGTATTT AGAGTACGTT AGGGAACGAT TTGTTCCATT GTTCGACTGC   
  
  
- GTGAACTTCA ACTGACCTCA ACTACTGGGA CTCAGTCAAG TTGCGGGATA ACCACCGGAT TTTTTGTAGC   
  
  
- CTGTTTCCGA CGTTCGTAAT CGACTTATAG AACCACACAG TAAACTCAAG TTTCGTTATG GACGATCTTG   
  
  
- AGGTGAACAA TTGGGCCTTT ACAATCTTAC AGCTGGACGC CTTCGAAATC ACCACTTGAA ACGGAAGGTC   
  
  
- GAAGTGGTGT ACGGACTACT TTCGCAGAGT TGTTAGTTGG AGGCTCTGGT CGAAGAAGCC TACCAGTTTT   
  
  
- CGGATTTGGG TTTCGATCAT TGGTAACAGC TCGTCCTACA CTTGTGTTTG TGATGGGGAA AGGAGAGATC   
  
  
- CAAACGACTC CGTATGTCGA TGATAAGACG ACAAAAACTC AGAGAACTAC GATGAGAGGG ATCTCTATCG   
  
  
- GTCCTGTCCC ACTTACACCT CTTCGTCACG AACCGTGCAC TACAACACTT GTAGTAACGT ACACTTCCCC   
  
  
- TCCTCTCCTA ACTCGCGATA CTCCAACGCC CCTTTACCTC TCGCTCCTAC AACTACCGCC CTAAGTCCCG   
  
  
- ATAAGGTTAC CCAGTCTTCT GGCCACTATA CTAAGCCTTC GAATATAGTT ACGCCATAAC ACTGTCCGAG   
  
  
- TTCCATTTTC TCCTCTACCT ACGTGAAGTG AAACCCACCC TTCTATTTTT GAATTAGCAA TGTAGTCGAA   
  
  
- CCTCCAC

+     MYB-like sequence

| Site Name | Organism | Position | Strand | Matrix score. | sequence | function |
| --- | --- | --- | --- | --- | --- | --- |
| MYB-like sequence | Arabidopsis thaliana | 3313 | + | 6 | TAACCA |  |
| MYB-like sequence | Arabidopsis thaliana | 164 | + | 6 | TAACCA |  |
| MYB-like sequence | Arabidopsis thaliana | 1267 | - | 6 | TAACCA |  |
| MYB-like sequence | Arabidopsis thaliana | 1927 | + | 6 | TAACCA |  |

>HU11G01134.1   
+ +Up\_Stream \_Len000AGATAG ACATTCAGCC ACTTGCCAGT TCAGAGAGAG ATAGAGAGAG ATTGGGTAAG   
  
  
+ CCCTTTTTGC ACTACTTTTT CTCGCTTCCA TTTTTCTTTC TTAATTTTTA GTGATGATTT TCCAGAAGAT   
  
  
+ TTGATTTATT CATCATGCTT AACCAATTGG GTCTTTGAGT ATTATTGTTT ACTGGGTATT TGATCTTTTC   
  
  
+ ATATTCCTTT GGACTGGGTG GGTGGAACTT TTGAACTTTC TTATTAGTTG GGCATGCCCT TATCTTATTC   
  
  
+ CAATTCAAAA CCCATATTTT TCTTCAATTT TAATTATGGG TATTTTGAAA TTGAATGCTT TCCTTCACAG   
  
  
+ CAATACTATA TTACATATTT TACAGGAAAG TAATATTTTA CAGGAAAGTA TGTTGCTGTT TCAACCCTGT   
  
  
+ TTTGTTTCCT CTCTTTCCTT GGATATGATG TTCATTGCTC TTGTACCAAA CCTAACCTCA ACTTCCTGAA   
  
  
+ TTATTTAAGG CAATATTACC GTGTGCTGGT CAATTTTGGT TGTGGGTTTG TTTCTTAATG TATATGATTT   
  
  
+ ATGGTCAGGA TCCATGCTCC TCTTCCGTCC ACTCTATATC TCTCTCTCTC TCTCTCAATA CAAATTCAGA   
  
  
+ GACTTTCTCT TTCTGGGTTT ACTATTGATT GCAGAAGTTT GTAGTCTTGC GAGGCCTTGA GTCTTCTTCG   
  
  
+ AGGTAGAAAA TTTTGCTTTG CGCTTCCTTC CCCATATTTT AACACCCCTC CCCCCCCAAC GCAAGTTTTC   
  
  
+ TACATTTTTG TGCAATTCTT CAGCTATGAT TCCCCAATTT CACTTGTCAC ATCTCTATTT TCCTCTTTTT   
  
  
+ GGTTTCTTGA TTCTGTAGAA GGTTGTGTAT GAACTTCTTT GTTTCCCTTT TTCCTTTGTT ATTTTTAATG   
  
  
+ TTCCCATAGA TGATATAGAG ATCTGCTTTT CCTTGCTGTG AGAGGTATCT CTGAGAATGC ATGTAGCAAT   
  
  
+ CAAAATTCTG AGTTGTTAAT TCTATTGCAT GTCTATGGGT GATGATTGTG ATGATGTTTT TTCTGTTGCC   
  
  
+ CTCTTTCTCT CTTTCACTGA AATTGCTTTC CAGAATAGTT GTTTGACTCC TTTCTGTATC TTAATTGAGC   
  
  
+ CCTTGCTTTG GTTTGTGCTC TGGGAGTTCT TCTTTAGTAA TCCCTTGCTT GTATTTTCTG AAAGAGCAAG   
  
  
+ CAATAATCCT CATAGCATGT GTATAATCAT ACAATAGCAA CAGCAAACAT GGATCCTGCG CCCAGTTAGG   
  
  
+ TTTGGTTAGG TTAAGATATG CATATTCTTT TCTCTTGAAA AGAGTTGTCT AGGAAGGGTA ATTATGCCCA   
  
  
+ TTTCATGGAT AATAATGGTA CAATTAAGTT TGATTGGCTT AAATACCAAT TATTTAGCGT CTTTTTCTTT   
  
  
+ GGCTGTCCAG GGTTCCATTT CCATAAACAA CTTTGAGTAA GGCATAGAGA ACTAAATTTC TGCCAATTGT   
  
  
+ CAAGTGCAAG TTGCTCATAT GCAACAATAC TTGGAAATTT TGAGCTGAGC TGCTGGCATA TCATGAATAG   
  
  
+ TTCTAATGGT GGCGAGTTAT CACTTATCGT CCCTTGTGTG ACGAATTTCT CTTCCTAGGA AGCCCTCTAG   
  
  
+ TCAATGCCTA TCTGCTGTTG TGGAGTGAAA ATGGATACAT TTAGATCTCT ATAAACCCTA CTTTGGCAAG   
  
  
+ ATCCATGCTG ATTTTAGGAA GCCCTTTGAC CAGAGAATGA CATAACAACC TGATAGGGTG TTACATGCTA   
  
  
+ ACTTCGATTA TTTAATCAAA ATACGGAACT TCTATCAGTC AAAGCCTGCC CCAGGTTCTA CTGAATTAAA   
  
  
+ TTGTATTGAA TTGCTCTCAC TTGCTTATGA CGATTAAATA ACTGGATTTG CTAATTATTC TTTTTATTTT   
  
  
+ ATTCTTGGTG ATGTAGGGAA CTCAGGGAAT TGTAACCATA CCTGTTCCAG CTGAAAAGGG TACGGGAACA   
  
  
+ GTCATAGTTA CAGGGGAATT TCGCAATACA TTTAAGGGAG TAAGAAGCAG GATCATGTCT TTAGTTAGGC   
  
  
+ CGGCAGATAT GTCTGTGACT CCATATGGAA ATCCAACACT GTATTCACTT CAAGGCAATA ATAATAGCTC   
  
  
+ TGGTTTGTCT GGTCAACTGT ATGGCTCTGA AAAGCACAAG ATCAAATATG TGACCGAATC TTTTAATGGT   
  
  
+ CCAAGTTATG GCCTGAAATT CTTTGTTGAT TCTCCATCAG AAGAGCTCAT CCACCCATCA GATGCTACTC   
  
  
+ CTAACCCATT TGACTCTTCT TTCGTGGGCA TAAGACATGA TGAGAGTCCT TACCAAGGAA ATTATGGATC   
  
  
+ AGAATATGGG GTAAGCCCAT CTTCCGATGC AGTTGAATAT GATGAAGGTA ATAAGATAAG GCTAAAACTT   
  
  
+ CAAGAATTGG AGCATGCCCT GCTCGATGAA GATGATGAAG TGGATGAGGT GGCTATAGGG CCCGTGCTAG   
  
  
+ AACACAGCAT GGAGCTTGAT GAAGAGTGGG TCGCCCCATT GAGAAATGTG GTTTTCCATG ATTCACCAAA   
  
  
+ GGAGTCCACC TCCTCAGAGT CATCCAATGT CAGCAGCATT AGCAGCACCA AAGAAGTATC ACCTTGCTCT   
  
  
+ CCGAGTACTC CCAAACAATT GCTCTTTAAT TGTGCAAATA TGATTTCAGA GGGGAATTTT GAGGAAGCCG   
  
  
+ TGACTATGAT CAGCGAGCTT AGACAGATTG TTTCTATCCA AGGGGATCCA GCGCAGAGGA TAGCAGCCTA   
  
  
+ CATGGTAGAA GGCCTTGCTT CCCGTTTGGC TTCATCGGGA AAAGTTCTTT ACAAAGCTTT GAAATGCAAA   
  
  
+ GAGCCACCTT CATTTGACAG GCTAGCGGCT ATGCAAATCC TCTTTGAGGT GTGCCCATGT TTCAAATTCG   
  
  
+ GATTTATGGC CGCAAATGCA GCGATTATGG ATGCAATCAA AAACGAAAAA AGGGTACACA TCATAGATTT   
  
  
+ TGACATAAAC CAAGGGAATC AATACATAAA TCTCATGCAA TCCCTTGCTA AACAAGGTAA CAAGCTGACG   
  
  
+ CACTTGAAGT TGACTGGAGT TGATGACCCT GAGTCAGTTC AACGCCCTAT TGGTGGCCTA AAAAACATCG   
  
  
+ GACAAAGGCT GCAAGCATTA GCTGAATATC TTGGTGTGTC ATTTGAGTTC AAAGCAATAC CTGCTAGAAC   
  
  
+ TCCACTTGTT AACCCGGAAA TGTTAGAATG TCGACCTGCG GAAGCTTTAG TGGTGAACTT TGCCTTCCAG   
  
  
+ CTTCACCACA TGCCTGATGA AAGCGTCTCA ACAATCAACC TCCGAGACCA GCTTCTTCGG ATGGTCAAAA   
  
  
+ GCCTAAACCC AAAGCTAGTA ACCATTGTCG AGCAGGATGT GAACACAAAC ACTACCCCTT TCCTCTCTAG   
  
  
+ GTTTGCTGAG GCATACAGCT ACTATTCTGC TGTTTTTGAG TCTCTTGATG CTACTCTCCC TAGAGATAGC   
  
  
+ CAGGACAGGG TGAATGTGGA GAAGCAGTGC TTGGCACGTG ATGTTGTGAA CATCATTGCA TGTGAAGGGG   
  
  
+ AGGAGAGGAT TGAGCGCTAT GAGGTTGCGG GGAAATGGAG AGCGAGGATG TTGATGGCGG GATTCAGGGC   
  
  
+ TATTCCAATG GGTCAGAAGA CCGGTGATAT GATTCGGAAG CTTATATCAA TGCGGTATTG TGACAGGCTC   
  
  
+ AAGGTAAAAG AGGAGATGGA TGCACTTCAC TTTGGGTGGG AAGATAAAAA CTTAATCGTT ACATCAGCTT   
  
  
+ GGAGGTG  

- +Up\_Stream \_Len000TCTATC TGTAAGTCGG TGAACGGTCA AGTCTCTCTC TATCTCTCTC TAACCCATTC   
  
  
- GGGAAAAACG TGATGAAAAA GAGCGAAGGT AAAAAGAAAG AATTAAAAAT CACTACTAAA AGGTCTTCTA   
  
  
- AACTAAATAA GTAGTACGAA TTGGTTAACC CAGAAACTCA TAATAACAAA TGACCCATAA ACTAGAAAAG   
  
  
- TATAAGGAAA CCTGACCCAC CCACCTTGAA AACTTGAAAG AATAATCAAC CCGTACGGGA ATAGAATAAG   
  
  
- GTTAAGTTTT GGGTATAAAA AGAAGTTAAA ATTAATACCC ATAAAACTTT AACTTACGAA AGGAAGTGTC   
  
  
- GTTATGATAT AATGTATAAA ATGTCCTTTC ATTATAAAAT GTCCTTTCAT ACAACGACAA AGTTGGGACA   
  
  
- AAACAAAGGA GAGAAAGGAA CCTATACTAC AAGTAACGAG AACATGGTTT GGATTGGAGT TGAAGGACTT   
  
  
- AATAAATTCC GTTATAATGG CACACGACCA GTTAAAACCA ACACCCAAAC AAAGAATTAC ATATACTAAA   
  
  
- TACCAGTCCT AGGTACGAGG AGAAGGCAGG TGAGATATAG AGAGAGAGAG AGAGAGTTAT GTTTAAGTCT   
  
  
- CTGAAAGAGA AAGACCCAAA TGATAACTAA CGTCTTCAAA CATCAGAACG CTCCGGAACT CAGAAGAAGC   
  
  
- TCCATCTTTT AAAACGAAAC GCGAAGGAAG GGGTATAAAA TTGTGGGGAG GGGGGGGTTG CGTTCAAAAG   
  
  
- ATGTAAAAAC ACGTTAAGAA GTCGATACTA AGGGGTTAAA GTGAACAGTG TAGAGATAAA AGGAGAAAAA   
  
  
- CCAAAGAACT AAGACATCTT CCAACACATA CTTGAAGAAA CAAAGGGAAA AAGGAAACAA TAAAAATTAC   
  
  
- AAGGGTATCT ACTATATCTC TAGACGAAAA GGAACGACAC TCTCCATAGA GACTCTTACG TACATCGTTA   
  
  
- GTTTTAAGAC TCAACAATTA AGATAACGTA CAGATACCCA CTACTAACAC TACTACAAAA AAGACAACGG   
  
  
- GAGAAAGAGA GAAAGTGACT TTAACGAAAG GTCTTATCAA CAAACTGAGG AAAGACATAG AATTAACTCG   
  
  
- GGAACGAAAC CAAACACGAG ACCCTCAAGA AGAAATCATT AGGGAACGAA CATAAAAGAC TTTCTCGTTC   
  
  
- GTTATTAGGA GTATCGTACA CATATTAGTA TGTTATCGTT GTCGTTTGTA CCTAGGACGC GGGTCAATCC   
  
  
- AAACCAATCC AATTCTATAC GTATAAGAAA AGAGAACTTT TCTCAACAGA TCCTTCCCAT TAATACGGGT   
  
  
- AAAGTACCTA TTATTACCAT GTTAATTCAA ACTAACCGAA TTTATGGTTA ATAAATCGCA GAAAAAGAAA   
  
  
- CCGACAGGTC CCAAGGTAAA GGTATTTGTT GAAACTCATT CCGTATCTCT TGATTTAAAG ACGGTTAACA   
  
  
- GTTCACGTTC AACGAGTATA CGTTGTTATG AACCTTTAAA ACTCGACTCG ACGACCGTAT AGTACTTATC   
  
  
- AAGATTACCA CCGCTCAATA GTGAATAGCA GGGAACACAC TGCTTAAAGA GAAGGATCCT TCGGGAGATC   
  
  
- AGTTACGGAT AGACGACAAC ACCTCACTTT TACCTATGTA AATCTAGAGA TATTTGGGAT GAAACCGTTC   
  
  
- TAGGTACGAC TAAAATCCTT CGGGAAACTG GTCTCTTACT GTATTGTTGG ACTATCCCAC AATGTACGAT   
  
  
- TGAAGCTAAT AAATTAGTTT TATGCCTTGA AGATAGTCAG TTTCGGACGG GGTCCAAGAT GACTTAATTT   
  
  
- AACATAACTT AACGAGAGTG AACGAATACT GCTAATTTAT TGACCTAAAC GATTAATAAG AAAAATAAAA   
  
  
- TAAGAACCAC TACATCCCTT GAGTCCCTTA ACATTGGTAT GGACAAGGTC GACTTTTCCC ATGCCCTTGT   
  
  
- CAGTATCAAT GTCCCCTTAA AGCGTTATGT AAATTCCCTC ATTCTTCGTC CTAGTACAGA AATCAATCCG   
  
  
- GCCGTCTATA CAGACACTGA GGTATACCTT TAGGTTGTGA CATAAGTGAA GTTCCGTTAT TATTATCGAG   
  
  
- ACCAAACAGA CCAGTTGACA TACCGAGACT TTTCGTGTTC TAGTTTATAC ACTGGCTTAG AAAATTACCA   
  
  
- GGTTCAATAC CGGACTTTAA GAAACAACTA AGAGGTAGTC TTCTCGAGTA GGTGGGTAGT CTACGATGAG   
  
  
- GATTGGGTAA ACTGAGAAGA AAGCACCCGT ATTCTGTACT ACTCTCAGGA ATGGTTCCTT TAATACCTAG   
  
  
- TCTTATACCC CATTCGGGTA GAAGGCTACG TCAACTTATA CTACTTCCAT TATTCTATTC CGATTTTGAA   
  
  
- GTTCTTAACC TCGTACGGGA CGAGCTACTT CTACTACTTC ACCTACTCCA CCGATATCCC GGGCACGATC   
  
  
- TTGTGTCGTA CCTCGAACTA CTTCTCACCC AGCGGGGTAA CTCTTTACAC CAAAAGGTAC TAAGTGGTTT   
  
  
- CCTCAGGTGG AGGAGTCTCA GTAGGTTACA GTCGTCGTAA TCGTCGTGGT TTCTTCATAG TGGAACGAGA   
  
  
- GGCTCATGAG GGTTTGTTAA CGAGAAATTA ACACGTTTAT ACTAAAGTCT CCCCTTAAAA CTCCTTCGGC   
  
  
- ACTGATACTA GTCGCTCGAA TCTGTCTAAC AAAGATAGGT TCCCCTAGGT CGCGTCTCCT ATCGTCGGAT   
  
  
- GTACCATCTT CCGGAACGAA GGGCAAACCG AAGTAGCCCT TTTCAAGAAA TGTTTCGAAA CTTTACGTTT   
  
  
- CTCGGTGGAA GTAAACTGTC CGATCGCCGA TACGTTTAGG AGAAACTCCA CACGGGTACA AAGTTTAAGC   
  
  
- CTAAATACCG GCGTTTACGT CGCTAATACC TACGTTAGTT TTTGCTTTTT TCCCATGTGT AGTATCTAAA   
  
  
- ACTGTATTTG GTTCCCTTAG TTATGTATTT AGAGTACGTT AGGGAACGAT TTGTTCCATT GTTCGACTGC   
  
  
- GTGAACTTCA ACTGACCTCA ACTACTGGGA CTCAGTCAAG TTGCGGGATA ACCACCGGAT TTTTTGTAGC   
  
  
- CTGTTTCCGA CGTTCGTAAT CGACTTATAG AACCACACAG TAAACTCAAG TTTCGTTATG GACGATCTTG   
  
  
- AGGTGAACAA TTGGGCCTTT ACAATCTTAC AGCTGGACGC CTTCGAAATC ACCACTTGAA ACGGAAGGTC   
  
  
- GAAGTGGTGT ACGGACTACT TTCGCAGAGT TGTTAGTTGG AGGCTCTGGT CGAAGAAGCC TACCAGTTTT   
  
  
- CGGATTTGGG TTTCGATCAT TGGTAACAGC TCGTCCTACA CTTGTGTTTG TGATGGGGAA AGGAGAGATC   
  
  
- CAAACGACTC CGTATGTCGA TGATAAGACG ACAAAAACTC AGAGAACTAC GATGAGAGGG ATCTCTATCG   
  
  
- GTCCTGTCCC ACTTACACCT CTTCGTCACG AACCGTGCAC TACAACACTT GTAGTAACGT ACACTTCCCC   
  
  
- TCCTCTCCTA ACTCGCGATA CTCCAACGCC CCTTTACCTC TCGCTCCTAC AACTACCGCC CTAAGTCCCG   
  
  
- ATAAGGTTAC CCAGTCTTCT GGCCACTATA CTAAGCCTTC GAATATAGTT ACGCCATAAC ACTGTCCGAG   
  
  
- TTCCATTTTC TCCTCTACCT ACGTGAAGTG AAACCCACCC TTCTATTTTT GAATTAGCAA TGTAGTCGAA   
  
  
- CCTCCAC

+     MYC

| Site Name | Organism | Position | Strand | Matrix score. | sequence | function |
| --- | --- | --- | --- | --- | --- | --- |
| MYC | Arabidopsis thaliana | 3493 | + | 6 | CATGTG |  |
| MYC | Arabidopsis thaliana | 2610 | - | 6 | CAATTG |  |
| MYC | Arabidopsis thaliana | 2815 | + | 6 | CATTTG |  |
| MYC | Arabidopsis thaliana | 2251 | + | 6 | CATTTG |  |
| MYC | Arabidopsis thaliana | 3231 | - | 6 | CATGTG |  |
| MYC | Arabidopsis thaliana | 2887 | - | 6 | CATTTG |  |
| MYC | Arabidopsis thaliana | 3124 | + | 6 | CATTTG |  |
| MYC | Arabidopsis thaliana | 168 | + | 6 | CAATTG |  |
| MYC | Arabidopsis thaliana | 1468 | + | 6 | CAATTG |  |
| MYC | Arabidopsis thaliana | 1210 | + | 6 | CATGTG |  |

>HU11G01134.1   
+ +Up\_Stream \_Len000AGATAG ACATTCAGCC ACTTGCCAGT TCAGAGAGAG ATAGAGAGAG ATTGGGTAAG   
  
  
+ CCCTTTTTGC ACTACTTTTT CTCGCTTCCA TTTTTCTTTC TTAATTTTTA GTGATGATTT TCCAGAAGAT   
  
  
+ TTGATTTATT CATCATGCTT AACCAATTGG GTCTTTGAGT ATTATTGTTT ACTGGGTATT TGATCTTTTC   
  
  
+ ATATTCCTTT GGACTGGGTG GGTGGAACTT TTGAACTTTC TTATTAGTTG GGCATGCCCT TATCTTATTC   
  
  
+ CAATTCAAAA CCCATATTTT TCTTCAATTT TAATTATGGG TATTTTGAAA TTGAATGCTT TCCTTCACAG   
  
  
+ CAATACTATA TTACATATTT TACAGGAAAG TAATATTTTA CAGGAAAGTA TGTTGCTGTT TCAACCCTGT   
  
  
+ TTTGTTTCCT CTCTTTCCTT GGATATGATG TTCATTGCTC TTGTACCAAA CCTAACCTCA ACTTCCTGAA   
  
  
+ TTATTTAAGG CAATATTACC GTGTGCTGGT CAATTTTGGT TGTGGGTTTG TTTCTTAATG TATATGATTT   
  
  
+ ATGGTCAGGA TCCATGCTCC TCTTCCGTCC ACTCTATATC TCTCTCTCTC TCTCTCAATA CAAATTCAGA   
  
  
+ GACTTTCTCT TTCTGGGTTT ACTATTGATT GCAGAAGTTT GTAGTCTTGC GAGGCCTTGA GTCTTCTTCG   
  
  
+ AGGTAGAAAA TTTTGCTTTG CGCTTCCTTC CCCATATTTT AACACCCCTC CCCCCCCAAC GCAAGTTTTC   
  
  
+ TACATTTTTG TGCAATTCTT CAGCTATGAT TCCCCAATTT CACTTGTCAC ATCTCTATTT TCCTCTTTTT   
  
  
+ GGTTTCTTGA TTCTGTAGAA GGTTGTGTAT GAACTTCTTT GTTTCCCTTT TTCCTTTGTT ATTTTTAATG   
  
  
+ TTCCCATAGA TGATATAGAG ATCTGCTTTT CCTTGCTGTG AGAGGTATCT CTGAGAATGC ATGTAGCAAT   
  
  
+ CAAAATTCTG AGTTGTTAAT TCTATTGCAT GTCTATGGGT GATGATTGTG ATGATGTTTT TTCTGTTGCC   
  
  
+ CTCTTTCTCT CTTTCACTGA AATTGCTTTC CAGAATAGTT GTTTGACTCC TTTCTGTATC TTAATTGAGC   
  
  
+ CCTTGCTTTG GTTTGTGCTC TGGGAGTTCT TCTTTAGTAA TCCCTTGCTT GTATTTTCTG AAAGAGCAAG   
  
  
+ CAATAATCCT CATAGCATGT GTATAATCAT ACAATAGCAA CAGCAAACAT GGATCCTGCG CCCAGTTAGG   
  
  
+ TTTGGTTAGG TTAAGATATG CATATTCTTT TCTCTTGAAA AGAGTTGTCT AGGAAGGGTA ATTATGCCCA   
  
  
+ TTTCATGGAT AATAATGGTA CAATTAAGTT TGATTGGCTT AAATACCAAT TATTTAGCGT CTTTTTCTTT   
  
  
+ GGCTGTCCAG GGTTCCATTT CCATAAACAA CTTTGAGTAA GGCATAGAGA ACTAAATTTC TGCCAATTGT   
  
  
+ CAAGTGCAAG TTGCTCATAT GCAACAATAC TTGGAAATTT TGAGCTGAGC TGCTGGCATA TCATGAATAG   
  
  
+ TTCTAATGGT GGCGAGTTAT CACTTATCGT CCCTTGTGTG ACGAATTTCT CTTCCTAGGA AGCCCTCTAG   
  
  
+ TCAATGCCTA TCTGCTGTTG TGGAGTGAAA ATGGATACAT TTAGATCTCT ATAAACCCTA CTTTGGCAAG   
  
  
+ ATCCATGCTG ATTTTAGGAA GCCCTTTGAC CAGAGAATGA CATAACAACC TGATAGGGTG TTACATGCTA   
  
  
+ ACTTCGATTA TTTAATCAAA ATACGGAACT TCTATCAGTC AAAGCCTGCC CCAGGTTCTA CTGAATTAAA   
  
  
+ TTGTATTGAA TTGCTCTCAC TTGCTTATGA CGATTAAATA ACTGGATTTG CTAATTATTC TTTTTATTTT   
  
  
+ ATTCTTGGTG ATGTAGGGAA CTCAGGGAAT TGTAACCATA CCTGTTCCAG CTGAAAAGGG TACGGGAACA   
  
  
+ GTCATAGTTA CAGGGGAATT TCGCAATACA TTTAAGGGAG TAAGAAGCAG GATCATGTCT TTAGTTAGGC   
  
  
+ CGGCAGATAT GTCTGTGACT CCATATGGAA ATCCAACACT GTATTCACTT CAAGGCAATA ATAATAGCTC   
  
  
+ TGGTTTGTCT GGTCAACTGT ATGGCTCTGA AAAGCACAAG ATCAAATATG TGACCGAATC TTTTAATGGT   
  
  
+ CCAAGTTATG GCCTGAAATT CTTTGTTGAT TCTCCATCAG AAGAGCTCAT CCACCCATCA GATGCTACTC   
  
  
+ CTAACCCATT TGACTCTTCT TTCGTGGGCA TAAGACATGA TGAGAGTCCT TACCAAGGAA ATTATGGATC   
  
  
+ AGAATATGGG GTAAGCCCAT CTTCCGATGC AGTTGAATAT GATGAAGGTA ATAAGATAAG GCTAAAACTT   
  
  
+ CAAGAATTGG AGCATGCCCT GCTCGATGAA GATGATGAAG TGGATGAGGT GGCTATAGGG CCCGTGCTAG   
  
  
+ AACACAGCAT GGAGCTTGAT GAAGAGTGGG TCGCCCCATT GAGAAATGTG GTTTTCCATG ATTCACCAAA   
  
  
+ GGAGTCCACC TCCTCAGAGT CATCCAATGT CAGCAGCATT AGCAGCACCA AAGAAGTATC ACCTTGCTCT   
  
  
+ CCGAGTACTC CCAAACAATT GCTCTTTAAT TGTGCAAATA TGATTTCAGA GGGGAATTTT GAGGAAGCCG   
  
  
+ TGACTATGAT CAGCGAGCTT AGACAGATTG TTTCTATCCA AGGGGATCCA GCGCAGAGGA TAGCAGCCTA   
  
  
+ CATGGTAGAA GGCCTTGCTT CCCGTTTGGC TTCATCGGGA AAAGTTCTTT ACAAAGCTTT GAAATGCAAA   
  
  
+ GAGCCACCTT CATTTGACAG GCTAGCGGCT ATGCAAATCC TCTTTGAGGT GTGCCCATGT TTCAAATTCG   
  
  
+ GATTTATGGC CGCAAATGCA GCGATTATGG ATGCAATCAA AAACGAAAAA AGGGTACACA TCATAGATTT   
  
  
+ TGACATAAAC CAAGGGAATC AATACATAAA TCTCATGCAA TCCCTTGCTA AACAAGGTAA CAAGCTGACG   
  
  
+ CACTTGAAGT TGACTGGAGT TGATGACCCT GAGTCAGTTC AACGCCCTAT TGGTGGCCTA AAAAACATCG   
  
  
+ GACAAAGGCT GCAAGCATTA GCTGAATATC TTGGTGTGTC ATTTGAGTTC AAAGCAATAC CTGCTAGAAC   
  
  
+ TCCACTTGTT AACCCGGAAA TGTTAGAATG TCGACCTGCG GAAGCTTTAG TGGTGAACTT TGCCTTCCAG   
  
  
+ CTTCACCACA TGCCTGATGA AAGCGTCTCA ACAATCAACC TCCGAGACCA GCTTCTTCGG ATGGTCAAAA   
  
  
+ GCCTAAACCC AAAGCTAGTA ACCATTGTCG AGCAGGATGT GAACACAAAC ACTACCCCTT TCCTCTCTAG   
  
  
+ GTTTGCTGAG GCATACAGCT ACTATTCTGC TGTTTTTGAG TCTCTTGATG CTACTCTCCC TAGAGATAGC   
  
  
+ CAGGACAGGG TGAATGTGGA GAAGCAGTGC TTGGCACGTG ATGTTGTGAA CATCATTGCA TGTGAAGGGG   
  
  
+ AGGAGAGGAT TGAGCGCTAT GAGGTTGCGG GGAAATGGAG AGCGAGGATG TTGATGGCGG GATTCAGGGC   
  
  
+ TATTCCAATG GGTCAGAAGA CCGGTGATAT GATTCGGAAG CTTATATCAA TGCGGTATTG TGACAGGCTC   
  
  
+ AAGGTAAAAG AGGAGATGGA TGCACTTCAC TTTGGGTGGG AAGATAAAAA CTTAATCGTT ACATCAGCTT   
  
  
+ GGAGGTG  

- +Up\_Stream \_Len000TCTATC TGTAAGTCGG TGAACGGTCA AGTCTCTCTC TATCTCTCTC TAACCCATTC   
  
  
- GGGAAAAACG TGATGAAAAA GAGCGAAGGT AAAAAGAAAG AATTAAAAAT CACTACTAAA AGGTCTTCTA   
  
  
- AACTAAATAA GTAGTACGAA TTGGTTAACC CAGAAACTCA TAATAACAAA TGACCCATAA ACTAGAAAAG   
  
  
- TATAAGGAAA CCTGACCCAC CCACCTTGAA AACTTGAAAG AATAATCAAC CCGTACGGGA ATAGAATAAG   
  
  
- GTTAAGTTTT GGGTATAAAA AGAAGTTAAA ATTAATACCC ATAAAACTTT AACTTACGAA AGGAAGTGTC   
  
  
- GTTATGATAT AATGTATAAA ATGTCCTTTC ATTATAAAAT GTCCTTTCAT ACAACGACAA AGTTGGGACA   
  
  
- AAACAAAGGA GAGAAAGGAA CCTATACTAC AAGTAACGAG AACATGGTTT GGATTGGAGT TGAAGGACTT   
  
  
- AATAAATTCC GTTATAATGG CACACGACCA GTTAAAACCA ACACCCAAAC AAAGAATTAC ATATACTAAA   
  
  
- TACCAGTCCT AGGTACGAGG AGAAGGCAGG TGAGATATAG AGAGAGAGAG AGAGAGTTAT GTTTAAGTCT   
  
  
- CTGAAAGAGA AAGACCCAAA TGATAACTAA CGTCTTCAAA CATCAGAACG CTCCGGAACT CAGAAGAAGC   
  
  
- TCCATCTTTT AAAACGAAAC GCGAAGGAAG GGGTATAAAA TTGTGGGGAG GGGGGGGTTG CGTTCAAAAG   
  
  
- ATGTAAAAAC ACGTTAAGAA GTCGATACTA AGGGGTTAAA GTGAACAGTG TAGAGATAAA AGGAGAAAAA   
  
  
- CCAAAGAACT AAGACATCTT CCAACACATA CTTGAAGAAA CAAAGGGAAA AAGGAAACAA TAAAAATTAC   
  
  
- AAGGGTATCT ACTATATCTC TAGACGAAAA GGAACGACAC TCTCCATAGA GACTCTTACG TACATCGTTA   
  
  
- GTTTTAAGAC TCAACAATTA AGATAACGTA CAGATACCCA CTACTAACAC TACTACAAAA AAGACAACGG   
  
  
- GAGAAAGAGA GAAAGTGACT TTAACGAAAG GTCTTATCAA CAAACTGAGG AAAGACATAG AATTAACTCG   
  
  
- GGAACGAAAC CAAACACGAG ACCCTCAAGA AGAAATCATT AGGGAACGAA CATAAAAGAC TTTCTCGTTC   
  
  
- GTTATTAGGA GTATCGTACA CATATTAGTA TGTTATCGTT GTCGTTTGTA CCTAGGACGC GGGTCAATCC   
  
  
- AAACCAATCC AATTCTATAC GTATAAGAAA AGAGAACTTT TCTCAACAGA TCCTTCCCAT TAATACGGGT   
  
  
- AAAGTACCTA TTATTACCAT GTTAATTCAA ACTAACCGAA TTTATGGTTA ATAAATCGCA GAAAAAGAAA   
  
  
- CCGACAGGTC CCAAGGTAAA GGTATTTGTT GAAACTCATT CCGTATCTCT TGATTTAAAG ACGGTTAACA   
  
  
- GTTCACGTTC AACGAGTATA CGTTGTTATG AACCTTTAAA ACTCGACTCG ACGACCGTAT AGTACTTATC   
  
  
- AAGATTACCA CCGCTCAATA GTGAATAGCA GGGAACACAC TGCTTAAAGA GAAGGATCCT TCGGGAGATC   
  
  
- AGTTACGGAT AGACGACAAC ACCTCACTTT TACCTATGTA AATCTAGAGA TATTTGGGAT GAAACCGTTC   
  
  
- TAGGTACGAC TAAAATCCTT CGGGAAACTG GTCTCTTACT GTATTGTTGG ACTATCCCAC AATGTACGAT   
  
  
- TGAAGCTAAT AAATTAGTTT TATGCCTTGA AGATAGTCAG TTTCGGACGG GGTCCAAGAT GACTTAATTT   
  
  
- AACATAACTT AACGAGAGTG AACGAATACT GCTAATTTAT TGACCTAAAC GATTAATAAG AAAAATAAAA   
  
  
- TAAGAACCAC TACATCCCTT GAGTCCCTTA ACATTGGTAT GGACAAGGTC GACTTTTCCC ATGCCCTTGT   
  
  
- CAGTATCAAT GTCCCCTTAA AGCGTTATGT AAATTCCCTC ATTCTTCGTC CTAGTACAGA AATCAATCCG   
  
  
- GCCGTCTATA CAGACACTGA GGTATACCTT TAGGTTGTGA CATAAGTGAA GTTCCGTTAT TATTATCGAG   
  
  
- ACCAAACAGA CCAGTTGACA TACCGAGACT TTTCGTGTTC TAGTTTATAC ACTGGCTTAG AAAATTACCA   
  
  
- GGTTCAATAC CGGACTTTAA GAAACAACTA AGAGGTAGTC TTCTCGAGTA GGTGGGTAGT CTACGATGAG   
  
  
- GATTGGGTAA ACTGAGAAGA AAGCACCCGT ATTCTGTACT ACTCTCAGGA ATGGTTCCTT TAATACCTAG   
  
  
- TCTTATACCC CATTCGGGTA GAAGGCTACG TCAACTTATA CTACTTCCAT TATTCTATTC CGATTTTGAA   
  
  
- GTTCTTAACC TCGTACGGGA CGAGCTACTT CTACTACTTC ACCTACTCCA CCGATATCCC GGGCACGATC   
  
  
- TTGTGTCGTA CCTCGAACTA CTTCTCACCC AGCGGGGTAA CTCTTTACAC CAAAAGGTAC TAAGTGGTTT   
  
  
- CCTCAGGTGG AGGAGTCTCA GTAGGTTACA GTCGTCGTAA TCGTCGTGGT TTCTTCATAG TGGAACGAGA   
  
  
- GGCTCATGAG GGTTTGTTAA CGAGAAATTA ACACGTTTAT ACTAAAGTCT CCCCTTAAAA CTCCTTCGGC   
  
  
- ACTGATACTA GTCGCTCGAA TCTGTCTAAC AAAGATAGGT TCCCCTAGGT CGCGTCTCCT ATCGTCGGAT   
  
  
- GTACCATCTT CCGGAACGAA GGGCAAACCG AAGTAGCCCT TTTCAAGAAA TGTTTCGAAA CTTTACGTTT   
  
  
- CTCGGTGGAA GTAAACTGTC CGATCGCCGA TACGTTTAGG AGAAACTCCA CACGGGTACA AAGTTTAAGC   
  
  
- CTAAATACCG GCGTTTACGT CGCTAATACC TACGTTAGTT TTTGCTTTTT TCCCATGTGT AGTATCTAAA   
  
  
- ACTGTATTTG GTTCCCTTAG TTATGTATTT AGAGTACGTT AGGGAACGAT TTGTTCCATT GTTCGACTGC   
  
  
- GTGAACTTCA ACTGACCTCA ACTACTGGGA CTCAGTCAAG TTGCGGGATA ACCACCGGAT TTTTTGTAGC   
  
  
- CTGTTTCCGA CGTTCGTAAT CGACTTATAG AACCACACAG TAAACTCAAG TTTCGTTATG GACGATCTTG   
  
  
- AGGTGAACAA TTGGGCCTTT ACAATCTTAC AGCTGGACGC CTTCGAAATC ACCACTTGAA ACGGAAGGTC   
  
  
- GAAGTGGTGT ACGGACTACT TTCGCAGAGT TGTTAGTTGG AGGCTCTGGT CGAAGAAGCC TACCAGTTTT   
  
  
- CGGATTTGGG TTTCGATCAT TGGTAACAGC TCGTCCTACA CTTGTGTTTG TGATGGGGAA AGGAGAGATC   
  
  
- CAAACGACTC CGTATGTCGA TGATAAGACG ACAAAAACTC AGAGAACTAC GATGAGAGGG ATCTCTATCG   
  
  
- GTCCTGTCCC ACTTACACCT CTTCGTCACG AACCGTGCAC TACAACACTT GTAGTAACGT ACACTTCCCC   
  
  
- TCCTCTCCTA ACTCGCGATA CTCCAACGCC CCTTTACCTC TCGCTCCTAC AACTACCGCC CTAAGTCCCG   
  
  
- ATAAGGTTAC CCAGTCTTCT GGCCACTATA CTAAGCCTTC GAATATAGTT ACGCCATAAC ACTGTCCGAG   
  
  
- TTCCATTTTC TCCTCTACCT ACGTGAAGTG AAACCCACCC TTCTATTTTT GAATTAGCAA TGTAGTCGAA   
  
  
- CCTCCAC

+     Myb

| Site Name | Organism | Position | Strand | Matrix score. | sequence | function |
| --- | --- | --- | --- | --- | --- | --- |
| Myb | Arabidopsis thaliana | 2118 | + | 6 | CAACTG |  |
| Myb | Arabidopsis thaliana | 1863 | + | 6 | TAACTG |  |
| Myb | Arabidopsis thaliana | 1257 | - | 6 | TAACTG |  |
| Myb | Arabidopsis thaliana | 2344 | - | 6 | CAACTG |  |

>HU11G01134.1   
+ +Up\_Stream \_Len000AGATAG ACATTCAGCC ACTTGCCAGT TCAGAGAGAG ATAGAGAGAG ATTGGGTAAG   
  
  
+ CCCTTTTTGC ACTACTTTTT CTCGCTTCCA TTTTTCTTTC TTAATTTTTA GTGATGATTT TCCAGAAGAT   
  
  
+ TTGATTTATT CATCATGCTT AACCAATTGG GTCTTTGAGT ATTATTGTTT ACTGGGTATT TGATCTTTTC   
  
  
+ ATATTCCTTT GGACTGGGTG GGTGGAACTT TTGAACTTTC TTATTAGTTG GGCATGCCCT TATCTTATTC   
  
  
+ CAATTCAAAA CCCATATTTT TCTTCAATTT TAATTATGGG TATTTTGAAA TTGAATGCTT TCCTTCACAG   
  
  
+ CAATACTATA TTACATATTT TACAGGAAAG TAATATTTTA CAGGAAAGTA TGTTGCTGTT TCAACCCTGT   
  
  
+ TTTGTTTCCT CTCTTTCCTT GGATATGATG TTCATTGCTC TTGTACCAAA CCTAACCTCA ACTTCCTGAA   
  
  
+ TTATTTAAGG CAATATTACC GTGTGCTGGT CAATTTTGGT TGTGGGTTTG TTTCTTAATG TATATGATTT   
  
  
+ ATGGTCAGGA TCCATGCTCC TCTTCCGTCC ACTCTATATC TCTCTCTCTC TCTCTCAATA CAAATTCAGA   
  
  
+ GACTTTCTCT TTCTGGGTTT ACTATTGATT GCAGAAGTTT GTAGTCTTGC GAGGCCTTGA GTCTTCTTCG   
  
  
+ AGGTAGAAAA TTTTGCTTTG CGCTTCCTTC CCCATATTTT AACACCCCTC CCCCCCCAAC GCAAGTTTTC   
  
  
+ TACATTTTTG TGCAATTCTT CAGCTATGAT TCCCCAATTT CACTTGTCAC ATCTCTATTT TCCTCTTTTT   
  
  
+ GGTTTCTTGA TTCTGTAGAA GGTTGTGTAT GAACTTCTTT GTTTCCCTTT TTCCTTTGTT ATTTTTAATG   
  
  
+ TTCCCATAGA TGATATAGAG ATCTGCTTTT CCTTGCTGTG AGAGGTATCT CTGAGAATGC ATGTAGCAAT   
  
  
+ CAAAATTCTG AGTTGTTAAT TCTATTGCAT GTCTATGGGT GATGATTGTG ATGATGTTTT TTCTGTTGCC   
  
  
+ CTCTTTCTCT CTTTCACTGA AATTGCTTTC CAGAATAGTT GTTTGACTCC TTTCTGTATC TTAATTGAGC   
  
  
+ CCTTGCTTTG GTTTGTGCTC TGGGAGTTCT TCTTTAGTAA TCCCTTGCTT GTATTTTCTG AAAGAGCAAG   
  
  
+ CAATAATCCT CATAGCATGT GTATAATCAT ACAATAGCAA CAGCAAACAT GGATCCTGCG CCCAGTTAGG   
  
  
+ TTTGGTTAGG TTAAGATATG CATATTCTTT TCTCTTGAAA AGAGTTGTCT AGGAAGGGTA ATTATGCCCA   
  
  
+ TTTCATGGAT AATAATGGTA CAATTAAGTT TGATTGGCTT AAATACCAAT TATTTAGCGT CTTTTTCTTT   
  
  
+ GGCTGTCCAG GGTTCCATTT CCATAAACAA CTTTGAGTAA GGCATAGAGA ACTAAATTTC TGCCAATTGT   
  
  
+ CAAGTGCAAG TTGCTCATAT GCAACAATAC TTGGAAATTT TGAGCTGAGC TGCTGGCATA TCATGAATAG   
  
  
+ TTCTAATGGT GGCGAGTTAT CACTTATCGT CCCTTGTGTG ACGAATTTCT CTTCCTAGGA AGCCCTCTAG   
  
  
+ TCAATGCCTA TCTGCTGTTG TGGAGTGAAA ATGGATACAT TTAGATCTCT ATAAACCCTA CTTTGGCAAG   
  
  
+ ATCCATGCTG ATTTTAGGAA GCCCTTTGAC CAGAGAATGA CATAACAACC TGATAGGGTG TTACATGCTA   
  
  
+ ACTTCGATTA TTTAATCAAA ATACGGAACT TCTATCAGTC AAAGCCTGCC CCAGGTTCTA CTGAATTAAA   
  
  
+ TTGTATTGAA TTGCTCTCAC TTGCTTATGA CGATTAAATA ACTGGATTTG CTAATTATTC TTTTTATTTT   
  
  
+ ATTCTTGGTG ATGTAGGGAA CTCAGGGAAT TGTAACCATA CCTGTTCCAG CTGAAAAGGG TACGGGAACA   
  
  
+ GTCATAGTTA CAGGGGAATT TCGCAATACA TTTAAGGGAG TAAGAAGCAG GATCATGTCT TTAGTTAGGC   
  
  
+ CGGCAGATAT GTCTGTGACT CCATATGGAA ATCCAACACT GTATTCACTT CAAGGCAATA ATAATAGCTC   
  
  
+ TGGTTTGTCT GGTCAACTGT ATGGCTCTGA AAAGCACAAG ATCAAATATG TGACCGAATC TTTTAATGGT   
  
  
+ CCAAGTTATG GCCTGAAATT CTTTGTTGAT TCTCCATCAG AAGAGCTCAT CCACCCATCA GATGCTACTC   
  
  
+ CTAACCCATT TGACTCTTCT TTCGTGGGCA TAAGACATGA TGAGAGTCCT TACCAAGGAA ATTATGGATC   
  
  
+ AGAATATGGG GTAAGCCCAT CTTCCGATGC AGTTGAATAT GATGAAGGTA ATAAGATAAG GCTAAAACTT   
  
  
+ CAAGAATTGG AGCATGCCCT GCTCGATGAA GATGATGAAG TGGATGAGGT GGCTATAGGG CCCGTGCTAG   
  
  
+ AACACAGCAT GGAGCTTGAT GAAGAGTGGG TCGCCCCATT GAGAAATGTG GTTTTCCATG ATTCACCAAA   
  
  
+ GGAGTCCACC TCCTCAGAGT CATCCAATGT CAGCAGCATT AGCAGCACCA AAGAAGTATC ACCTTGCTCT   
  
  
+ CCGAGTACTC CCAAACAATT GCTCTTTAAT TGTGCAAATA TGATTTCAGA GGGGAATTTT GAGGAAGCCG   
  
  
+ TGACTATGAT CAGCGAGCTT AGACAGATTG TTTCTATCCA AGGGGATCCA GCGCAGAGGA TAGCAGCCTA   
  
  
+ CATGGTAGAA GGCCTTGCTT CCCGTTTGGC TTCATCGGGA AAAGTTCTTT ACAAAGCTTT GAAATGCAAA   
  
  
+ GAGCCACCTT CATTTGACAG GCTAGCGGCT ATGCAAATCC TCTTTGAGGT GTGCCCATGT TTCAAATTCG   
  
  
+ GATTTATGGC CGCAAATGCA GCGATTATGG ATGCAATCAA AAACGAAAAA AGGGTACACA TCATAGATTT   
  
  
+ TGACATAAAC CAAGGGAATC AATACATAAA TCTCATGCAA TCCCTTGCTA AACAAGGTAA CAAGCTGACG   
  
  
+ CACTTGAAGT TGACTGGAGT TGATGACCCT GAGTCAGTTC AACGCCCTAT TGGTGGCCTA AAAAACATCG   
  
  
+ GACAAAGGCT GCAAGCATTA GCTGAATATC TTGGTGTGTC ATTTGAGTTC AAAGCAATAC CTGCTAGAAC   
  
  
+ TCCACTTGTT AACCCGGAAA TGTTAGAATG TCGACCTGCG GAAGCTTTAG TGGTGAACTT TGCCTTCCAG   
  
  
+ CTTCACCACA TGCCTGATGA AAGCGTCTCA ACAATCAACC TCCGAGACCA GCTTCTTCGG ATGGTCAAAA   
  
  
+ GCCTAAACCC AAAGCTAGTA ACCATTGTCG AGCAGGATGT GAACACAAAC ACTACCCCTT TCCTCTCTAG   
  
  
+ GTTTGCTGAG GCATACAGCT ACTATTCTGC TGTTTTTGAG TCTCTTGATG CTACTCTCCC TAGAGATAGC   
  
  
+ CAGGACAGGG TGAATGTGGA GAAGCAGTGC TTGGCACGTG ATGTTGTGAA CATCATTGCA TGTGAAGGGG   
  
  
+ AGGAGAGGAT TGAGCGCTAT GAGGTTGCGG GGAAATGGAG AGCGAGGATG TTGATGGCGG GATTCAGGGC   
  
  
+ TATTCCAATG GGTCAGAAGA CCGGTGATAT GATTCGGAAG CTTATATCAA TGCGGTATTG TGACAGGCTC   
  
  
+ AAGGTAAAAG AGGAGATGGA TGCACTTCAC TTTGGGTGGG AAGATAAAAA CTTAATCGTT ACATCAGCTT   
  
  
+ GGAGGTG  

- +Up\_Stream \_Len000TCTATC TGTAAGTCGG TGAACGGTCA AGTCTCTCTC TATCTCTCTC TAACCCATTC   
  
  
- GGGAAAAACG TGATGAAAAA GAGCGAAGGT AAAAAGAAAG AATTAAAAAT CACTACTAAA AGGTCTTCTA   
  
  
- AACTAAATAA GTAGTACGAA TTGGTTAACC CAGAAACTCA TAATAACAAA TGACCCATAA ACTAGAAAAG   
  
  
- TATAAGGAAA CCTGACCCAC CCACCTTGAA AACTTGAAAG AATAATCAAC CCGTACGGGA ATAGAATAAG   
  
  
- GTTAAGTTTT GGGTATAAAA AGAAGTTAAA ATTAATACCC ATAAAACTTT AACTTACGAA AGGAAGTGTC   
  
  
- GTTATGATAT AATGTATAAA ATGTCCTTTC ATTATAAAAT GTCCTTTCAT ACAACGACAA AGTTGGGACA   
  
  
- AAACAAAGGA GAGAAAGGAA CCTATACTAC AAGTAACGAG AACATGGTTT GGATTGGAGT TGAAGGACTT   
  
  
- AATAAATTCC GTTATAATGG CACACGACCA GTTAAAACCA ACACCCAAAC AAAGAATTAC ATATACTAAA   
  
  
- TACCAGTCCT AGGTACGAGG AGAAGGCAGG TGAGATATAG AGAGAGAGAG AGAGAGTTAT GTTTAAGTCT   
  
  
- CTGAAAGAGA AAGACCCAAA TGATAACTAA CGTCTTCAAA CATCAGAACG CTCCGGAACT CAGAAGAAGC   
  
  
- TCCATCTTTT AAAACGAAAC GCGAAGGAAG GGGTATAAAA TTGTGGGGAG GGGGGGGTTG CGTTCAAAAG   
  
  
- ATGTAAAAAC ACGTTAAGAA GTCGATACTA AGGGGTTAAA GTGAACAGTG TAGAGATAAA AGGAGAAAAA   
  
  
- CCAAAGAACT AAGACATCTT CCAACACATA CTTGAAGAAA CAAAGGGAAA AAGGAAACAA TAAAAATTAC   
  
  
- AAGGGTATCT ACTATATCTC TAGACGAAAA GGAACGACAC TCTCCATAGA GACTCTTACG TACATCGTTA   
  
  
- GTTTTAAGAC TCAACAATTA AGATAACGTA CAGATACCCA CTACTAACAC TACTACAAAA AAGACAACGG   
  
  
- GAGAAAGAGA GAAAGTGACT TTAACGAAAG GTCTTATCAA CAAACTGAGG AAAGACATAG AATTAACTCG   
  
  
- GGAACGAAAC CAAACACGAG ACCCTCAAGA AGAAATCATT AGGGAACGAA CATAAAAGAC TTTCTCGTTC   
  
  
- GTTATTAGGA GTATCGTACA CATATTAGTA TGTTATCGTT GTCGTTTGTA CCTAGGACGC GGGTCAATCC   
  
  
- AAACCAATCC AATTCTATAC GTATAAGAAA AGAGAACTTT TCTCAACAGA TCCTTCCCAT TAATACGGGT   
  
  
- AAAGTACCTA TTATTACCAT GTTAATTCAA ACTAACCGAA TTTATGGTTA ATAAATCGCA GAAAAAGAAA   
  
  
- CCGACAGGTC CCAAGGTAAA GGTATTTGTT GAAACTCATT CCGTATCTCT TGATTTAAAG ACGGTTAACA   
  
  
- GTTCACGTTC AACGAGTATA CGTTGTTATG AACCTTTAAA ACTCGACTCG ACGACCGTAT AGTACTTATC   
  
  
- AAGATTACCA CCGCTCAATA GTGAATAGCA GGGAACACAC TGCTTAAAGA GAAGGATCCT TCGGGAGATC   
  
  
- AGTTACGGAT AGACGACAAC ACCTCACTTT TACCTATGTA AATCTAGAGA TATTTGGGAT GAAACCGTTC   
  
  
- TAGGTACGAC TAAAATCCTT CGGGAAACTG GTCTCTTACT GTATTGTTGG ACTATCCCAC AATGTACGAT   
  
  
- TGAAGCTAAT AAATTAGTTT TATGCCTTGA AGATAGTCAG TTTCGGACGG GGTCCAAGAT GACTTAATTT   
  
  
- AACATAACTT AACGAGAGTG AACGAATACT GCTAATTTAT TGACCTAAAC GATTAATAAG AAAAATAAAA   
  
  
- TAAGAACCAC TACATCCCTT GAGTCCCTTA ACATTGGTAT GGACAAGGTC GACTTTTCCC ATGCCCTTGT   
  
  
- CAGTATCAAT GTCCCCTTAA AGCGTTATGT AAATTCCCTC ATTCTTCGTC CTAGTACAGA AATCAATCCG   
  
  
- GCCGTCTATA CAGACACTGA GGTATACCTT TAGGTTGTGA CATAAGTGAA GTTCCGTTAT TATTATCGAG   
  
  
- ACCAAACAGA CCAGTTGACA TACCGAGACT TTTCGTGTTC TAGTTTATAC ACTGGCTTAG AAAATTACCA   
  
  
- GGTTCAATAC CGGACTTTAA GAAACAACTA AGAGGTAGTC TTCTCGAGTA GGTGGGTAGT CTACGATGAG   
  
  
- GATTGGGTAA ACTGAGAAGA AAGCACCCGT ATTCTGTACT ACTCTCAGGA ATGGTTCCTT TAATACCTAG   
  
  
- TCTTATACCC CATTCGGGTA GAAGGCTACG TCAACTTATA CTACTTCCAT TATTCTATTC CGATTTTGAA   
  
  
- GTTCTTAACC TCGTACGGGA CGAGCTACTT CTACTACTTC ACCTACTCCA CCGATATCCC GGGCACGATC   
  
  
- TTGTGTCGTA CCTCGAACTA CTTCTCACCC AGCGGGGTAA CTCTTTACAC CAAAAGGTAC TAAGTGGTTT   
  
  
- CCTCAGGTGG AGGAGTCTCA GTAGGTTACA GTCGTCGTAA TCGTCGTGGT TTCTTCATAG TGGAACGAGA   
  
  
- GGCTCATGAG GGTTTGTTAA CGAGAAATTA ACACGTTTAT ACTAAAGTCT CCCCTTAAAA CTCCTTCGGC   
  
  
- ACTGATACTA GTCGCTCGAA TCTGTCTAAC AAAGATAGGT TCCCCTAGGT CGCGTCTCCT ATCGTCGGAT   
  
  
- GTACCATCTT CCGGAACGAA GGGCAAACCG AAGTAGCCCT TTTCAAGAAA TGTTTCGAAA CTTTACGTTT   
  
  
- CTCGGTGGAA GTAAACTGTC CGATCGCCGA TACGTTTAGG AGAAACTCCA CACGGGTACA AAGTTTAAGC   
  
  
- CTAAATACCG GCGTTTACGT CGCTAATACC TACGTTAGTT TTTGCTTTTT TCCCATGTGT AGTATCTAAA   
  
  
- ACTGTATTTG GTTCCCTTAG TTATGTATTT AGAGTACGTT AGGGAACGAT TTGTTCCATT GTTCGACTGC   
  
  
- GTGAACTTCA ACTGACCTCA ACTACTGGGA CTCAGTCAAG TTGCGGGATA ACCACCGGAT TTTTTGTAGC   
  
  
- CTGTTTCCGA CGTTCGTAAT CGACTTATAG AACCACACAG TAAACTCAAG TTTCGTTATG GACGATCTTG   
  
  
- AGGTGAACAA TTGGGCCTTT ACAATCTTAC AGCTGGACGC CTTCGAAATC ACCACTTGAA ACGGAAGGTC   
  
  
- GAAGTGGTGT ACGGACTACT TTCGCAGAGT TGTTAGTTGG AGGCTCTGGT CGAAGAAGCC TACCAGTTTT   
  
  
- CGGATTTGGG TTTCGATCAT TGGTAACAGC TCGTCCTACA CTTGTGTTTG TGATGGGGAA AGGAGAGATC   
  
  
- CAAACGACTC CGTATGTCGA TGATAAGACG ACAAAAACTC AGAGAACTAC GATGAGAGGG ATCTCTATCG   
  
  
- GTCCTGTCCC ACTTACACCT CTTCGTCACG AACCGTGCAC TACAACACTT GTAGTAACGT ACACTTCCCC   
  
  
- TCCTCTCCTA ACTCGCGATA CTCCAACGCC CCTTTACCTC TCGCTCCTAC AACTACCGCC CTAAGTCCCG   
  
  
- ATAAGGTTAC CCAGTCTTCT GGCCACTATA CTAAGCCTTC GAATATAGTT ACGCCATAAC ACTGTCCGAG   
  
  
- TTCCATTTTC TCCTCTACCT ACGTGAAGTG AAACCCACCC TTCTATTTTT GAATTAGCAA TGTAGTCGAA   
  
  
- CCTCCAC

+     Myb-binding site

| Site Name | Organism | Position | Strand | Matrix score. | sequence | function |
| --- | --- | --- | --- | --- | --- | --- |
| Myb-binding site | Nicotiana tabacum | 1629 | - | 6 | CAACAG |  |
| Myb-binding site | Nicotiana tabacum | 1047 | - | 6 | CAACAG |  |
| Myb-binding site | Nicotiana tabacum | 1232 | + | 6 | CAACAG |  |

>HU11G01134.1   
+ +Up\_Stream \_Len000AGATAG ACATTCAGCC ACTTGCCAGT TCAGAGAGAG ATAGAGAGAG ATTGGGTAAG   
  
  
+ CCCTTTTTGC ACTACTTTTT CTCGCTTCCA TTTTTCTTTC TTAATTTTTA GTGATGATTT TCCAGAAGAT   
  
  
+ TTGATTTATT CATCATGCTT AACCAATTGG GTCTTTGAGT ATTATTGTTT ACTGGGTATT TGATCTTTTC   
  
  
+ ATATTCCTTT GGACTGGGTG GGTGGAACTT TTGAACTTTC TTATTAGTTG GGCATGCCCT TATCTTATTC   
  
  
+ CAATTCAAAA CCCATATTTT TCTTCAATTT TAATTATGGG TATTTTGAAA TTGAATGCTT TCCTTCACAG   
  
  
+ CAATACTATA TTACATATTT TACAGGAAAG TAATATTTTA CAGGAAAGTA TGTTGCTGTT TCAACCCTGT   
  
  
+ TTTGTTTCCT CTCTTTCCTT GGATATGATG TTCATTGCTC TTGTACCAAA CCTAACCTCA ACTTCCTGAA   
  
  
+ TTATTTAAGG CAATATTACC GTGTGCTGGT CAATTTTGGT TGTGGGTTTG TTTCTTAATG TATATGATTT   
  
  
+ ATGGTCAGGA TCCATGCTCC TCTTCCGTCC ACTCTATATC TCTCTCTCTC TCTCTCAATA CAAATTCAGA   
  
  
+ GACTTTCTCT TTCTGGGTTT ACTATTGATT GCAGAAGTTT GTAGTCTTGC GAGGCCTTGA GTCTTCTTCG   
  
  
+ AGGTAGAAAA TTTTGCTTTG CGCTTCCTTC CCCATATTTT AACACCCCTC CCCCCCCAAC GCAAGTTTTC   
  
  
+ TACATTTTTG TGCAATTCTT CAGCTATGAT TCCCCAATTT CACTTGTCAC ATCTCTATTT TCCTCTTTTT   
  
  
+ GGTTTCTTGA TTCTGTAGAA GGTTGTGTAT GAACTTCTTT GTTTCCCTTT TTCCTTTGTT ATTTTTAATG   
  
  
+ TTCCCATAGA TGATATAGAG ATCTGCTTTT CCTTGCTGTG AGAGGTATCT CTGAGAATGC ATGTAGCAAT   
  
  
+ CAAAATTCTG AGTTGTTAAT TCTATTGCAT GTCTATGGGT GATGATTGTG ATGATGTTTT TTCTGTTGCC   
  
  
+ CTCTTTCTCT CTTTCACTGA AATTGCTTTC CAGAATAGTT GTTTGACTCC TTTCTGTATC TTAATTGAGC   
  
  
+ CCTTGCTTTG GTTTGTGCTC TGGGAGTTCT TCTTTAGTAA TCCCTTGCTT GTATTTTCTG AAAGAGCAAG   
  
  
+ CAATAATCCT CATAGCATGT GTATAATCAT ACAATAGCAA CAGCAAACAT GGATCCTGCG CCCAGTTAGG   
  
  
+ TTTGGTTAGG TTAAGATATG CATATTCTTT TCTCTTGAAA AGAGTTGTCT AGGAAGGGTA ATTATGCCCA   
  
  
+ TTTCATGGAT AATAATGGTA CAATTAAGTT TGATTGGCTT AAATACCAAT TATTTAGCGT CTTTTTCTTT   
  
  
+ GGCTGTCCAG GGTTCCATTT CCATAAACAA CTTTGAGTAA GGCATAGAGA ACTAAATTTC TGCCAATTGT   
  
  
+ CAAGTGCAAG TTGCTCATAT GCAACAATAC TTGGAAATTT TGAGCTGAGC TGCTGGCATA TCATGAATAG   
  
  
+ TTCTAATGGT GGCGAGTTAT CACTTATCGT CCCTTGTGTG ACGAATTTCT CTTCCTAGGA AGCCCTCTAG   
  
  
+ TCAATGCCTA TCTGCTGTTG TGGAGTGAAA ATGGATACAT TTAGATCTCT ATAAACCCTA CTTTGGCAAG   
  
  
+ ATCCATGCTG ATTTTAGGAA GCCCTTTGAC CAGAGAATGA CATAACAACC TGATAGGGTG TTACATGCTA   
  
  
+ ACTTCGATTA TTTAATCAAA ATACGGAACT TCTATCAGTC AAAGCCTGCC CCAGGTTCTA CTGAATTAAA   
  
  
+ TTGTATTGAA TTGCTCTCAC TTGCTTATGA CGATTAAATA ACTGGATTTG CTAATTATTC TTTTTATTTT   
  
  
+ ATTCTTGGTG ATGTAGGGAA CTCAGGGAAT TGTAACCATA CCTGTTCCAG CTGAAAAGGG TACGGGAACA   
  
  
+ GTCATAGTTA CAGGGGAATT TCGCAATACA TTTAAGGGAG TAAGAAGCAG GATCATGTCT TTAGTTAGGC   
  
  
+ CGGCAGATAT GTCTGTGACT CCATATGGAA ATCCAACACT GTATTCACTT CAAGGCAATA ATAATAGCTC   
  
  
+ TGGTTTGTCT GGTCAACTGT ATGGCTCTGA AAAGCACAAG ATCAAATATG TGACCGAATC TTTTAATGGT   
  
  
+ CCAAGTTATG GCCTGAAATT CTTTGTTGAT TCTCCATCAG AAGAGCTCAT CCACCCATCA GATGCTACTC   
  
  
+ CTAACCCATT TGACTCTTCT TTCGTGGGCA TAAGACATGA TGAGAGTCCT TACCAAGGAA ATTATGGATC   
  
  
+ AGAATATGGG GTAAGCCCAT CTTCCGATGC AGTTGAATAT GATGAAGGTA ATAAGATAAG GCTAAAACTT   
  
  
+ CAAGAATTGG AGCATGCCCT GCTCGATGAA GATGATGAAG TGGATGAGGT GGCTATAGGG CCCGTGCTAG   
  
  
+ AACACAGCAT GGAGCTTGAT GAAGAGTGGG TCGCCCCATT GAGAAATGTG GTTTTCCATG ATTCACCAAA   
  
  
+ GGAGTCCACC TCCTCAGAGT CATCCAATGT CAGCAGCATT AGCAGCACCA AAGAAGTATC ACCTTGCTCT   
  
  
+ CCGAGTACTC CCAAACAATT GCTCTTTAAT TGTGCAAATA TGATTTCAGA GGGGAATTTT GAGGAAGCCG   
  
  
+ TGACTATGAT CAGCGAGCTT AGACAGATTG TTTCTATCCA AGGGGATCCA GCGCAGAGGA TAGCAGCCTA   
  
  
+ CATGGTAGAA GGCCTTGCTT CCCGTTTGGC TTCATCGGGA AAAGTTCTTT ACAAAGCTTT GAAATGCAAA   
  
  
+ GAGCCACCTT CATTTGACAG GCTAGCGGCT ATGCAAATCC TCTTTGAGGT GTGCCCATGT TTCAAATTCG   
  
  
+ GATTTATGGC CGCAAATGCA GCGATTATGG ATGCAATCAA AAACGAAAAA AGGGTACACA TCATAGATTT   
  
  
+ TGACATAAAC CAAGGGAATC AATACATAAA TCTCATGCAA TCCCTTGCTA AACAAGGTAA CAAGCTGACG   
  
  
+ CACTTGAAGT TGACTGGAGT TGATGACCCT GAGTCAGTTC AACGCCCTAT TGGTGGCCTA AAAAACATCG   
  
  
+ GACAAAGGCT GCAAGCATTA GCTGAATATC TTGGTGTGTC ATTTGAGTTC AAAGCAATAC CTGCTAGAAC   
  
  
+ TCCACTTGTT AACCCGGAAA TGTTAGAATG TCGACCTGCG GAAGCTTTAG TGGTGAACTT TGCCTTCCAG   
  
  
+ CTTCACCACA TGCCTGATGA AAGCGTCTCA ACAATCAACC TCCGAGACCA GCTTCTTCGG ATGGTCAAAA   
  
  
+ GCCTAAACCC AAAGCTAGTA ACCATTGTCG AGCAGGATGT GAACACAAAC ACTACCCCTT TCCTCTCTAG   
  
  
+ GTTTGCTGAG GCATACAGCT ACTATTCTGC TGTTTTTGAG TCTCTTGATG CTACTCTCCC TAGAGATAGC   
  
  
+ CAGGACAGGG TGAATGTGGA GAAGCAGTGC TTGGCACGTG ATGTTGTGAA CATCATTGCA TGTGAAGGGG   
  
  
+ AGGAGAGGAT TGAGCGCTAT GAGGTTGCGG GGAAATGGAG AGCGAGGATG TTGATGGCGG GATTCAGGGC   
  
  
+ TATTCCAATG GGTCAGAAGA CCGGTGATAT GATTCGGAAG CTTATATCAA TGCGGTATTG TGACAGGCTC   
  
  
+ AAGGTAAAAG AGGAGATGGA TGCACTTCAC TTTGGGTGGG AAGATAAAAA CTTAATCGTT ACATCAGCTT   
  
  
+ GGAGGTG  

- +Up\_Stream \_Len000TCTATC TGTAAGTCGG TGAACGGTCA AGTCTCTCTC TATCTCTCTC TAACCCATTC   
  
  
- GGGAAAAACG TGATGAAAAA GAGCGAAGGT AAAAAGAAAG AATTAAAAAT CACTACTAAA AGGTCTTCTA   
  
  
- AACTAAATAA GTAGTACGAA TTGGTTAACC CAGAAACTCA TAATAACAAA TGACCCATAA ACTAGAAAAG   
  
  
- TATAAGGAAA CCTGACCCAC CCACCTTGAA AACTTGAAAG AATAATCAAC CCGTACGGGA ATAGAATAAG   
  
  
- GTTAAGTTTT GGGTATAAAA AGAAGTTAAA ATTAATACCC ATAAAACTTT AACTTACGAA AGGAAGTGTC   
  
  
- GTTATGATAT AATGTATAAA ATGTCCTTTC ATTATAAAAT GTCCTTTCAT ACAACGACAA AGTTGGGACA   
  
  
- AAACAAAGGA GAGAAAGGAA CCTATACTAC AAGTAACGAG AACATGGTTT GGATTGGAGT TGAAGGACTT   
  
  
- AATAAATTCC GTTATAATGG CACACGACCA GTTAAAACCA ACACCCAAAC AAAGAATTAC ATATACTAAA   
  
  
- TACCAGTCCT AGGTACGAGG AGAAGGCAGG TGAGATATAG AGAGAGAGAG AGAGAGTTAT GTTTAAGTCT   
  
  
- CTGAAAGAGA AAGACCCAAA TGATAACTAA CGTCTTCAAA CATCAGAACG CTCCGGAACT CAGAAGAAGC   
  
  
- TCCATCTTTT AAAACGAAAC GCGAAGGAAG GGGTATAAAA TTGTGGGGAG GGGGGGGTTG CGTTCAAAAG   
  
  
- ATGTAAAAAC ACGTTAAGAA GTCGATACTA AGGGGTTAAA GTGAACAGTG TAGAGATAAA AGGAGAAAAA   
  
  
- CCAAAGAACT AAGACATCTT CCAACACATA CTTGAAGAAA CAAAGGGAAA AAGGAAACAA TAAAAATTAC   
  
  
- AAGGGTATCT ACTATATCTC TAGACGAAAA GGAACGACAC TCTCCATAGA GACTCTTACG TACATCGTTA   
  
  
- GTTTTAAGAC TCAACAATTA AGATAACGTA CAGATACCCA CTACTAACAC TACTACAAAA AAGACAACGG   
  
  
- GAGAAAGAGA GAAAGTGACT TTAACGAAAG GTCTTATCAA CAAACTGAGG AAAGACATAG AATTAACTCG   
  
  
- GGAACGAAAC CAAACACGAG ACCCTCAAGA AGAAATCATT AGGGAACGAA CATAAAAGAC TTTCTCGTTC   
  
  
- GTTATTAGGA GTATCGTACA CATATTAGTA TGTTATCGTT GTCGTTTGTA CCTAGGACGC GGGTCAATCC   
  
  
- AAACCAATCC AATTCTATAC GTATAAGAAA AGAGAACTTT TCTCAACAGA TCCTTCCCAT TAATACGGGT   
  
  
- AAAGTACCTA TTATTACCAT GTTAATTCAA ACTAACCGAA TTTATGGTTA ATAAATCGCA GAAAAAGAAA   
  
  
- CCGACAGGTC CCAAGGTAAA GGTATTTGTT GAAACTCATT CCGTATCTCT TGATTTAAAG ACGGTTAACA   
  
  
- GTTCACGTTC AACGAGTATA CGTTGTTATG AACCTTTAAA ACTCGACTCG ACGACCGTAT AGTACTTATC   
  
  
- AAGATTACCA CCGCTCAATA GTGAATAGCA GGGAACACAC TGCTTAAAGA GAAGGATCCT TCGGGAGATC   
  
  
- AGTTACGGAT AGACGACAAC ACCTCACTTT TACCTATGTA AATCTAGAGA TATTTGGGAT GAAACCGTTC   
  
  
- TAGGTACGAC TAAAATCCTT CGGGAAACTG GTCTCTTACT GTATTGTTGG ACTATCCCAC AATGTACGAT   
  
  
- TGAAGCTAAT AAATTAGTTT TATGCCTTGA AGATAGTCAG TTTCGGACGG GGTCCAAGAT GACTTAATTT   
  
  
- AACATAACTT AACGAGAGTG AACGAATACT GCTAATTTAT TGACCTAAAC GATTAATAAG AAAAATAAAA   
  
  
- TAAGAACCAC TACATCCCTT GAGTCCCTTA ACATTGGTAT GGACAAGGTC GACTTTTCCC ATGCCCTTGT   
  
  
- CAGTATCAAT GTCCCCTTAA AGCGTTATGT AAATTCCCTC ATTCTTCGTC CTAGTACAGA AATCAATCCG   
  
  
- GCCGTCTATA CAGACACTGA GGTATACCTT TAGGTTGTGA CATAAGTGAA GTTCCGTTAT TATTATCGAG   
  
  
- ACCAAACAGA CCAGTTGACA TACCGAGACT TTTCGTGTTC TAGTTTATAC ACTGGCTTAG AAAATTACCA   
  
  
- GGTTCAATAC CGGACTTTAA GAAACAACTA AGAGGTAGTC TTCTCGAGTA GGTGGGTAGT CTACGATGAG   
  
  
- GATTGGGTAA ACTGAGAAGA AAGCACCCGT ATTCTGTACT ACTCTCAGGA ATGGTTCCTT TAATACCTAG   
  
  
- TCTTATACCC CATTCGGGTA GAAGGCTACG TCAACTTATA CTACTTCCAT TATTCTATTC CGATTTTGAA   
  
  
- GTTCTTAACC TCGTACGGGA CGAGCTACTT CTACTACTTC ACCTACTCCA CCGATATCCC GGGCACGATC   
  
  
- TTGTGTCGTA CCTCGAACTA CTTCTCACCC AGCGGGGTAA CTCTTTACAC CAAAAGGTAC TAAGTGGTTT   
  
  
- CCTCAGGTGG AGGAGTCTCA GTAGGTTACA GTCGTCGTAA TCGTCGTGGT TTCTTCATAG TGGAACGAGA   
  
  
- GGCTCATGAG GGTTTGTTAA CGAGAAATTA ACACGTTTAT ACTAAAGTCT CCCCTTAAAA CTCCTTCGGC   
  
  
- ACTGATACTA GTCGCTCGAA TCTGTCTAAC AAAGATAGGT TCCCCTAGGT CGCGTCTCCT ATCGTCGGAT   
  
  
- GTACCATCTT CCGGAACGAA GGGCAAACCG AAGTAGCCCT TTTCAAGAAA TGTTTCGAAA CTTTACGTTT   
  
  
- CTCGGTGGAA GTAAACTGTC CGATCGCCGA TACGTTTAGG AGAAACTCCA CACGGGTACA AAGTTTAAGC   
  
  
- CTAAATACCG GCGTTTACGT CGCTAATACC TACGTTAGTT TTTGCTTTTT TCCCATGTGT AGTATCTAAA   
  
  
- ACTGTATTTG GTTCCCTTAG TTATGTATTT AGAGTACGTT AGGGAACGAT TTGTTCCATT GTTCGACTGC   
  
  
- GTGAACTTCA ACTGACCTCA ACTACTGGGA CTCAGTCAAG TTGCGGGATA ACCACCGGAT TTTTTGTAGC   
  
  
- CTGTTTCCGA CGTTCGTAAT CGACTTATAG AACCACACAG TAAACTCAAG TTTCGTTATG GACGATCTTG   
  
  
- AGGTGAACAA TTGGGCCTTT ACAATCTTAC AGCTGGACGC CTTCGAAATC ACCACTTGAA ACGGAAGGTC   
  
  
- GAAGTGGTGT ACGGACTACT TTCGCAGAGT TGTTAGTTGG AGGCTCTGGT CGAAGAAGCC TACCAGTTTT   
  
  
- CGGATTTGGG TTTCGATCAT TGGTAACAGC TCGTCCTACA CTTGTGTTTG TGATGGGGAA AGGAGAGATC   
  
  
- CAAACGACTC CGTATGTCGA TGATAAGACG ACAAAAACTC AGAGAACTAC GATGAGAGGG ATCTCTATCG   
  
  
- GTCCTGTCCC ACTTACACCT CTTCGTCACG AACCGTGCAC TACAACACTT GTAGTAACGT ACACTTCCCC   
  
  
- TCCTCTCCTA ACTCGCGATA CTCCAACGCC CCTTTACCTC TCGCTCCTAC AACTACCGCC CTAAGTCCCG   
  
  
- ATAAGGTTAC CCAGTCTTCT GGCCACTATA CTAAGCCTTC GAATATAGTT ACGCCATAAC ACTGTCCGAG   
  
  
- TTCCATTTTC TCCTCTACCT ACGTGAAGTG AAACCCACCC TTCTATTTTT GAATTAGCAA TGTAGTCGAA   
  
  
- CCTCCAC

+     O2-site

| Site Name | Organism | Position | Strand | Matrix score. | sequence | function |
| --- | --- | --- | --- | --- | --- | --- |
| O2-site | Zea mays | 2418 | + | 9 | GATGATGTGG | cis-acting regulatory element involved in zein metabolism regulation |
| O2-site | Zea mays | 2427 | + | 9 | GATGATGTGG | cis-acting regulatory element involved in zein metabolism regulation |

>HU11G01134.1   
+ +Up\_Stream \_Len000AGATAG ACATTCAGCC ACTTGCCAGT TCAGAGAGAG ATAGAGAGAG ATTGGGTAAG   
  
  
+ CCCTTTTTGC ACTACTTTTT CTCGCTTCCA TTTTTCTTTC TTAATTTTTA GTGATGATTT TCCAGAAGAT   
  
  
+ TTGATTTATT CATCATGCTT AACCAATTGG GTCTTTGAGT ATTATTGTTT ACTGGGTATT TGATCTTTTC   
  
  
+ ATATTCCTTT GGACTGGGTG GGTGGAACTT TTGAACTTTC TTATTAGTTG GGCATGCCCT TATCTTATTC   
  
  
+ CAATTCAAAA CCCATATTTT TCTTCAATTT TAATTATGGG TATTTTGAAA TTGAATGCTT TCCTTCACAG   
  
  
+ CAATACTATA TTACATATTT TACAGGAAAG TAATATTTTA CAGGAAAGTA TGTTGCTGTT TCAACCCTGT   
  
  
+ TTTGTTTCCT CTCTTTCCTT GGATATGATG TTCATTGCTC TTGTACCAAA CCTAACCTCA ACTTCCTGAA   
  
  
+ TTATTTAAGG CAATATTACC GTGTGCTGGT CAATTTTGGT TGTGGGTTTG TTTCTTAATG TATATGATTT   
  
  
+ ATGGTCAGGA TCCATGCTCC TCTTCCGTCC ACTCTATATC TCTCTCTCTC TCTCTCAATA CAAATTCAGA   
  
  
+ GACTTTCTCT TTCTGGGTTT ACTATTGATT GCAGAAGTTT GTAGTCTTGC GAGGCCTTGA GTCTTCTTCG   
  
  
+ AGGTAGAAAA TTTTGCTTTG CGCTTCCTTC CCCATATTTT AACACCCCTC CCCCCCCAAC GCAAGTTTTC   
  
  
+ TACATTTTTG TGCAATTCTT CAGCTATGAT TCCCCAATTT CACTTGTCAC ATCTCTATTT TCCTCTTTTT   
  
  
+ GGTTTCTTGA TTCTGTAGAA GGTTGTGTAT GAACTTCTTT GTTTCCCTTT TTCCTTTGTT ATTTTTAATG   
  
  
+ TTCCCATAGA TGATATAGAG ATCTGCTTTT CCTTGCTGTG AGAGGTATCT CTGAGAATGC ATGTAGCAAT   
  
  
+ CAAAATTCTG AGTTGTTAAT TCTATTGCAT GTCTATGGGT GATGATTGTG ATGATGTTTT TTCTGTTGCC   
  
  
+ CTCTTTCTCT CTTTCACTGA AATTGCTTTC CAGAATAGTT GTTTGACTCC TTTCTGTATC TTAATTGAGC   
  
  
+ CCTTGCTTTG GTTTGTGCTC TGGGAGTTCT TCTTTAGTAA TCCCTTGCTT GTATTTTCTG AAAGAGCAAG   
  
  
+ CAATAATCCT CATAGCATGT GTATAATCAT ACAATAGCAA CAGCAAACAT GGATCCTGCG CCCAGTTAGG   
  
  
+ TTTGGTTAGG TTAAGATATG CATATTCTTT TCTCTTGAAA AGAGTTGTCT AGGAAGGGTA ATTATGCCCA   
  
  
+ TTTCATGGAT AATAATGGTA CAATTAAGTT TGATTGGCTT AAATACCAAT TATTTAGCGT CTTTTTCTTT   
  
  
+ GGCTGTCCAG GGTTCCATTT CCATAAACAA CTTTGAGTAA GGCATAGAGA ACTAAATTTC TGCCAATTGT   
  
  
+ CAAGTGCAAG TTGCTCATAT GCAACAATAC TTGGAAATTT TGAGCTGAGC TGCTGGCATA TCATGAATAG   
  
  
+ TTCTAATGGT GGCGAGTTAT CACTTATCGT CCCTTGTGTG ACGAATTTCT CTTCCTAGGA AGCCCTCTAG   
  
  
+ TCAATGCCTA TCTGCTGTTG TGGAGTGAAA ATGGATACAT TTAGATCTCT ATAAACCCTA CTTTGGCAAG   
  
  
+ ATCCATGCTG ATTTTAGGAA GCCCTTTGAC CAGAGAATGA CATAACAACC TGATAGGGTG TTACATGCTA   
  
  
+ ACTTCGATTA TTTAATCAAA ATACGGAACT TCTATCAGTC AAAGCCTGCC CCAGGTTCTA CTGAATTAAA   
  
  
+ TTGTATTGAA TTGCTCTCAC TTGCTTATGA CGATTAAATA ACTGGATTTG CTAATTATTC TTTTTATTTT   
  
  
+ ATTCTTGGTG ATGTAGGGAA CTCAGGGAAT TGTAACCATA CCTGTTCCAG CTGAAAAGGG TACGGGAACA   
  
  
+ GTCATAGTTA CAGGGGAATT TCGCAATACA TTTAAGGGAG TAAGAAGCAG GATCATGTCT TTAGTTAGGC   
  
  
+ CGGCAGATAT GTCTGTGACT CCATATGGAA ATCCAACACT GTATTCACTT CAAGGCAATA ATAATAGCTC   
  
  
+ TGGTTTGTCT GGTCAACTGT ATGGCTCTGA AAAGCACAAG ATCAAATATG TGACCGAATC TTTTAATGGT   
  
  
+ CCAAGTTATG GCCTGAAATT CTTTGTTGAT TCTCCATCAG AAGAGCTCAT CCACCCATCA GATGCTACTC   
  
  
+ CTAACCCATT TGACTCTTCT TTCGTGGGCA TAAGACATGA TGAGAGTCCT TACCAAGGAA ATTATGGATC   
  
  
+ AGAATATGGG GTAAGCCCAT CTTCCGATGC AGTTGAATAT GATGAAGGTA ATAAGATAAG GCTAAAACTT   
  
  
+ CAAGAATTGG AGCATGCCCT GCTCGATGAA GATGATGAAG TGGATGAGGT GGCTATAGGG CCCGTGCTAG   
  
  
+ AACACAGCAT GGAGCTTGAT GAAGAGTGGG TCGCCCCATT GAGAAATGTG GTTTTCCATG ATTCACCAAA   
  
  
+ GGAGTCCACC TCCTCAGAGT CATCCAATGT CAGCAGCATT AGCAGCACCA AAGAAGTATC ACCTTGCTCT   
  
  
+ CCGAGTACTC CCAAACAATT GCTCTTTAAT TGTGCAAATA TGATTTCAGA GGGGAATTTT GAGGAAGCCG   
  
  
+ TGACTATGAT CAGCGAGCTT AGACAGATTG TTTCTATCCA AGGGGATCCA GCGCAGAGGA TAGCAGCCTA   
  
  
+ CATGGTAGAA GGCCTTGCTT CCCGTTTGGC TTCATCGGGA AAAGTTCTTT ACAAAGCTTT GAAATGCAAA   
  
  
+ GAGCCACCTT CATTTGACAG GCTAGCGGCT ATGCAAATCC TCTTTGAGGT GTGCCCATGT TTCAAATTCG   
  
  
+ GATTTATGGC CGCAAATGCA GCGATTATGG ATGCAATCAA AAACGAAAAA AGGGTACACA TCATAGATTT   
  
  
+ TGACATAAAC CAAGGGAATC AATACATAAA TCTCATGCAA TCCCTTGCTA AACAAGGTAA CAAGCTGACG   
  
  
+ CACTTGAAGT TGACTGGAGT TGATGACCCT GAGTCAGTTC AACGCCCTAT TGGTGGCCTA AAAAACATCG   
  
  
+ GACAAAGGCT GCAAGCATTA GCTGAATATC TTGGTGTGTC ATTTGAGTTC AAAGCAATAC CTGCTAGAAC   
  
  
+ TCCACTTGTT AACCCGGAAA TGTTAGAATG TCGACCTGCG GAAGCTTTAG TGGTGAACTT TGCCTTCCAG   
  
  
+ CTTCACCACA TGCCTGATGA AAGCGTCTCA ACAATCAACC TCCGAGACCA GCTTCTTCGG ATGGTCAAAA   
  
  
+ GCCTAAACCC AAAGCTAGTA ACCATTGTCG AGCAGGATGT GAACACAAAC ACTACCCCTT TCCTCTCTAG   
  
  
+ GTTTGCTGAG GCATACAGCT ACTATTCTGC TGTTTTTGAG TCTCTTGATG CTACTCTCCC TAGAGATAGC   
  
  
+ CAGGACAGGG TGAATGTGGA GAAGCAGTGC TTGGCACGTG ATGTTGTGAA CATCATTGCA TGTGAAGGGG   
  
  
+ AGGAGAGGAT TGAGCGCTAT GAGGTTGCGG GGAAATGGAG AGCGAGGATG TTGATGGCGG GATTCAGGGC   
  
  
+ TATTCCAATG GGTCAGAAGA CCGGTGATAT GATTCGGAAG CTTATATCAA TGCGGTATTG TGACAGGCTC   
  
  
+ AAGGTAAAAG AGGAGATGGA TGCACTTCAC TTTGGGTGGG AAGATAAAAA CTTAATCGTT ACATCAGCTT   
  
  
+ GGAGGTG  

- +Up\_Stream \_Len000TCTATC TGTAAGTCGG TGAACGGTCA AGTCTCTCTC TATCTCTCTC TAACCCATTC   
  
  
- GGGAAAAACG TGATGAAAAA GAGCGAAGGT AAAAAGAAAG AATTAAAAAT CACTACTAAA AGGTCTTCTA   
  
  
- AACTAAATAA GTAGTACGAA TTGGTTAACC CAGAAACTCA TAATAACAAA TGACCCATAA ACTAGAAAAG   
  
  
- TATAAGGAAA CCTGACCCAC CCACCTTGAA AACTTGAAAG AATAATCAAC CCGTACGGGA ATAGAATAAG   
  
  
- GTTAAGTTTT GGGTATAAAA AGAAGTTAAA ATTAATACCC ATAAAACTTT AACTTACGAA AGGAAGTGTC   
  
  
- GTTATGATAT AATGTATAAA ATGTCCTTTC ATTATAAAAT GTCCTTTCAT ACAACGACAA AGTTGGGACA   
  
  
- AAACAAAGGA GAGAAAGGAA CCTATACTAC AAGTAACGAG AACATGGTTT GGATTGGAGT TGAAGGACTT   
  
  
- AATAAATTCC GTTATAATGG CACACGACCA GTTAAAACCA ACACCCAAAC AAAGAATTAC ATATACTAAA   
  
  
- TACCAGTCCT AGGTACGAGG AGAAGGCAGG TGAGATATAG AGAGAGAGAG AGAGAGTTAT GTTTAAGTCT   
  
  
- CTGAAAGAGA AAGACCCAAA TGATAACTAA CGTCTTCAAA CATCAGAACG CTCCGGAACT CAGAAGAAGC   
  
  
- TCCATCTTTT AAAACGAAAC GCGAAGGAAG GGGTATAAAA TTGTGGGGAG GGGGGGGTTG CGTTCAAAAG   
  
  
- ATGTAAAAAC ACGTTAAGAA GTCGATACTA AGGGGTTAAA GTGAACAGTG TAGAGATAAA AGGAGAAAAA   
  
  
- CCAAAGAACT AAGACATCTT CCAACACATA CTTGAAGAAA CAAAGGGAAA AAGGAAACAA TAAAAATTAC   
  
  
- AAGGGTATCT ACTATATCTC TAGACGAAAA GGAACGACAC TCTCCATAGA GACTCTTACG TACATCGTTA   
  
  
- GTTTTAAGAC TCAACAATTA AGATAACGTA CAGATACCCA CTACTAACAC TACTACAAAA AAGACAACGG   
  
  
- GAGAAAGAGA GAAAGTGACT TTAACGAAAG GTCTTATCAA CAAACTGAGG AAAGACATAG AATTAACTCG   
  
  
- GGAACGAAAC CAAACACGAG ACCCTCAAGA AGAAATCATT AGGGAACGAA CATAAAAGAC TTTCTCGTTC   
  
  
- GTTATTAGGA GTATCGTACA CATATTAGTA TGTTATCGTT GTCGTTTGTA CCTAGGACGC GGGTCAATCC   
  
  
- AAACCAATCC AATTCTATAC GTATAAGAAA AGAGAACTTT TCTCAACAGA TCCTTCCCAT TAATACGGGT   
  
  
- AAAGTACCTA TTATTACCAT GTTAATTCAA ACTAACCGAA TTTATGGTTA ATAAATCGCA GAAAAAGAAA   
  
  
- CCGACAGGTC CCAAGGTAAA GGTATTTGTT GAAACTCATT CCGTATCTCT TGATTTAAAG ACGGTTAACA   
  
  
- GTTCACGTTC AACGAGTATA CGTTGTTATG AACCTTTAAA ACTCGACTCG ACGACCGTAT AGTACTTATC   
  
  
- AAGATTACCA CCGCTCAATA GTGAATAGCA GGGAACACAC TGCTTAAAGA GAAGGATCCT TCGGGAGATC   
  
  
- AGTTACGGAT AGACGACAAC ACCTCACTTT TACCTATGTA AATCTAGAGA TATTTGGGAT GAAACCGTTC   
  
  
- TAGGTACGAC TAAAATCCTT CGGGAAACTG GTCTCTTACT GTATTGTTGG ACTATCCCAC AATGTACGAT   
  
  
- TGAAGCTAAT AAATTAGTTT TATGCCTTGA AGATAGTCAG TTTCGGACGG GGTCCAAGAT GACTTAATTT   
  
  
- AACATAACTT AACGAGAGTG AACGAATACT GCTAATTTAT TGACCTAAAC GATTAATAAG AAAAATAAAA   
  
  
- TAAGAACCAC TACATCCCTT GAGTCCCTTA ACATTGGTAT GGACAAGGTC GACTTTTCCC ATGCCCTTGT   
  
  
- CAGTATCAAT GTCCCCTTAA AGCGTTATGT AAATTCCCTC ATTCTTCGTC CTAGTACAGA AATCAATCCG   
  
  
- GCCGTCTATA CAGACACTGA GGTATACCTT TAGGTTGTGA CATAAGTGAA GTTCCGTTAT TATTATCGAG   
  
  
- ACCAAACAGA CCAGTTGACA TACCGAGACT TTTCGTGTTC TAGTTTATAC ACTGGCTTAG AAAATTACCA   
  
  
- GGTTCAATAC CGGACTTTAA GAAACAACTA AGAGGTAGTC TTCTCGAGTA GGTGGGTAGT CTACGATGAG   
  
  
- GATTGGGTAA ACTGAGAAGA AAGCACCCGT ATTCTGTACT ACTCTCAGGA ATGGTTCCTT TAATACCTAG   
  
  
- TCTTATACCC CATTCGGGTA GAAGGCTACG TCAACTTATA CTACTTCCAT TATTCTATTC CGATTTTGAA   
  
  
- GTTCTTAACC TCGTACGGGA CGAGCTACTT CTACTACTTC ACCTACTCCA CCGATATCCC GGGCACGATC   
  
  
- TTGTGTCGTA CCTCGAACTA CTTCTCACCC AGCGGGGTAA CTCTTTACAC CAAAAGGTAC TAAGTGGTTT   
  
  
- CCTCAGGTGG AGGAGTCTCA GTAGGTTACA GTCGTCGTAA TCGTCGTGGT TTCTTCATAG TGGAACGAGA   
  
  
- GGCTCATGAG GGTTTGTTAA CGAGAAATTA ACACGTTTAT ACTAAAGTCT CCCCTTAAAA CTCCTTCGGC   
  
  
- ACTGATACTA GTCGCTCGAA TCTGTCTAAC AAAGATAGGT TCCCCTAGGT CGCGTCTCCT ATCGTCGGAT   
  
  
- GTACCATCTT CCGGAACGAA GGGCAAACCG AAGTAGCCCT TTTCAAGAAA TGTTTCGAAA CTTTACGTTT   
  
  
- CTCGGTGGAA GTAAACTGTC CGATCGCCGA TACGTTTAGG AGAAACTCCA CACGGGTACA AAGTTTAAGC   
  
  
- CTAAATACCG GCGTTTACGT CGCTAATACC TACGTTAGTT TTTGCTTTTT TCCCATGTGT AGTATCTAAA   
  
  
- ACTGTATTTG GTTCCCTTAG TTATGTATTT AGAGTACGTT AGGGAACGAT TTGTTCCATT GTTCGACTGC   
  
  
- GTGAACTTCA ACTGACCTCA ACTACTGGGA CTCAGTCAAG TTGCGGGATA ACCACCGGAT TTTTTGTAGC   
  
  
- CTGTTTCCGA CGTTCGTAAT CGACTTATAG AACCACACAG TAAACTCAAG TTTCGTTATG GACGATCTTG   
  
  
- AGGTGAACAA TTGGGCCTTT ACAATCTTAC AGCTGGACGC CTTCGAAATC ACCACTTGAA ACGGAAGGTC   
  
  
- GAAGTGGTGT ACGGACTACT TTCGCAGAGT TGTTAGTTGG AGGCTCTGGT CGAAGAAGCC TACCAGTTTT   
  
  
- CGGATTTGGG TTTCGATCAT TGGTAACAGC TCGTCCTACA CTTGTGTTTG TGATGGGGAA AGGAGAGATC   
  
  
- CAAACGACTC CGTATGTCGA TGATAAGACG ACAAAAACTC AGAGAACTAC GATGAGAGGG ATCTCTATCG   
  
  
- GTCCTGTCCC ACTTACACCT CTTCGTCACG AACCGTGCAC TACAACACTT GTAGTAACGT ACACTTCCCC   
  
  
- TCCTCTCCTA ACTCGCGATA CTCCAACGCC CCTTTACCTC TCGCTCCTAC AACTACCGCC CTAAGTCCCG   
  
  
- ATAAGGTTAC CCAGTCTTCT GGCCACTATA CTAAGCCTTC GAATATAGTT ACGCCATAAC ACTGTCCGAG   
  
  
- TTCCATTTTC TCCTCTACCT ACGTGAAGTG AAACCCACCC TTCTATTTTT GAATTAGCAA TGTAGTCGAA   
  
  
- CCTCCAC

+     STRE

| Site Name | Organism | Position | Strand | Matrix score. | sequence | function |
| --- | --- | --- | --- | --- | --- | --- |
| STRE | Arabidopsis thaliana | 3500 | + | 5 | AGGGG |  |
| STRE | Arabidopsis thaliana | 3349 | - | 5 | AGGGG |  |
| STRE | Arabidopsis thaliana | 1976 | + | 5 | AGGGG |  |
| STRE | Arabidopsis thaliana | 2705 | + | 5 | AGGGG |  |
| STRE | Arabidopsis thaliana | 749 | - | 5 | AGGGG |  |
| STRE | Arabidopsis thaliana | 2644 | + | 5 | AGGGG |  |

>HU11G01134.1   
+ +Up\_Stream \_Len000AGATAG ACATTCAGCC ACTTGCCAGT TCAGAGAGAG ATAGAGAGAG ATTGGGTAAG   
  
  
+ CCCTTTTTGC ACTACTTTTT CTCGCTTCCA TTTTTCTTTC TTAATTTTTA GTGATGATTT TCCAGAAGAT   
  
  
+ TTGATTTATT CATCATGCTT AACCAATTGG GTCTTTGAGT ATTATTGTTT ACTGGGTATT TGATCTTTTC   
  
  
+ ATATTCCTTT GGACTGGGTG GGTGGAACTT TTGAACTTTC TTATTAGTTG GGCATGCCCT TATCTTATTC   
  
  
+ CAATTCAAAA CCCATATTTT TCTTCAATTT TAATTATGGG TATTTTGAAA TTGAATGCTT TCCTTCACAG   
  
  
+ CAATACTATA TTACATATTT TACAGGAAAG TAATATTTTA CAGGAAAGTA TGTTGCTGTT TCAACCCTGT   
  
  
+ TTTGTTTCCT CTCTTTCCTT GGATATGATG TTCATTGCTC TTGTACCAAA CCTAACCTCA ACTTCCTGAA   
  
  
+ TTATTTAAGG CAATATTACC GTGTGCTGGT CAATTTTGGT TGTGGGTTTG TTTCTTAATG TATATGATTT   
  
  
+ ATGGTCAGGA TCCATGCTCC TCTTCCGTCC ACTCTATATC TCTCTCTCTC TCTCTCAATA CAAATTCAGA   
  
  
+ GACTTTCTCT TTCTGGGTTT ACTATTGATT GCAGAAGTTT GTAGTCTTGC GAGGCCTTGA GTCTTCTTCG   
  
  
+ AGGTAGAAAA TTTTGCTTTG CGCTTCCTTC CCCATATTTT AACACCCCTC CCCCCCCAAC GCAAGTTTTC   
  
  
+ TACATTTTTG TGCAATTCTT CAGCTATGAT TCCCCAATTT CACTTGTCAC ATCTCTATTT TCCTCTTTTT   
  
  
+ GGTTTCTTGA TTCTGTAGAA GGTTGTGTAT GAACTTCTTT GTTTCCCTTT TTCCTTTGTT ATTTTTAATG   
  
  
+ TTCCCATAGA TGATATAGAG ATCTGCTTTT CCTTGCTGTG AGAGGTATCT CTGAGAATGC ATGTAGCAAT   
  
  
+ CAAAATTCTG AGTTGTTAAT TCTATTGCAT GTCTATGGGT GATGATTGTG ATGATGTTTT TTCTGTTGCC   
  
  
+ CTCTTTCTCT CTTTCACTGA AATTGCTTTC CAGAATAGTT GTTTGACTCC TTTCTGTATC TTAATTGAGC   
  
  
+ CCTTGCTTTG GTTTGTGCTC TGGGAGTTCT TCTTTAGTAA TCCCTTGCTT GTATTTTCTG AAAGAGCAAG   
  
  
+ CAATAATCCT CATAGCATGT GTATAATCAT ACAATAGCAA CAGCAAACAT GGATCCTGCG CCCAGTTAGG   
  
  
+ TTTGGTTAGG TTAAGATATG CATATTCTTT TCTCTTGAAA AGAGTTGTCT AGGAAGGGTA ATTATGCCCA   
  
  
+ TTTCATGGAT AATAATGGTA CAATTAAGTT TGATTGGCTT AAATACCAAT TATTTAGCGT CTTTTTCTTT   
  
  
+ GGCTGTCCAG GGTTCCATTT CCATAAACAA CTTTGAGTAA GGCATAGAGA ACTAAATTTC TGCCAATTGT   
  
  
+ CAAGTGCAAG TTGCTCATAT GCAACAATAC TTGGAAATTT TGAGCTGAGC TGCTGGCATA TCATGAATAG   
  
  
+ TTCTAATGGT GGCGAGTTAT CACTTATCGT CCCTTGTGTG ACGAATTTCT CTTCCTAGGA AGCCCTCTAG   
  
  
+ TCAATGCCTA TCTGCTGTTG TGGAGTGAAA ATGGATACAT TTAGATCTCT ATAAACCCTA CTTTGGCAAG   
  
  
+ ATCCATGCTG ATTTTAGGAA GCCCTTTGAC CAGAGAATGA CATAACAACC TGATAGGGTG TTACATGCTA   
  
  
+ ACTTCGATTA TTTAATCAAA ATACGGAACT TCTATCAGTC AAAGCCTGCC CCAGGTTCTA CTGAATTAAA   
  
  
+ TTGTATTGAA TTGCTCTCAC TTGCTTATGA CGATTAAATA ACTGGATTTG CTAATTATTC TTTTTATTTT   
  
  
+ ATTCTTGGTG ATGTAGGGAA CTCAGGGAAT TGTAACCATA CCTGTTCCAG CTGAAAAGGG TACGGGAACA   
  
  
+ GTCATAGTTA CAGGGGAATT TCGCAATACA TTTAAGGGAG TAAGAAGCAG GATCATGTCT TTAGTTAGGC   
  
  
+ CGGCAGATAT GTCTGTGACT CCATATGGAA ATCCAACACT GTATTCACTT CAAGGCAATA ATAATAGCTC   
  
  
+ TGGTTTGTCT GGTCAACTGT ATGGCTCTGA AAAGCACAAG ATCAAATATG TGACCGAATC TTTTAATGGT   
  
  
+ CCAAGTTATG GCCTGAAATT CTTTGTTGAT TCTCCATCAG AAGAGCTCAT CCACCCATCA GATGCTACTC   
  
  
+ CTAACCCATT TGACTCTTCT TTCGTGGGCA TAAGACATGA TGAGAGTCCT TACCAAGGAA ATTATGGATC   
  
  
+ AGAATATGGG GTAAGCCCAT CTTCCGATGC AGTTGAATAT GATGAAGGTA ATAAGATAAG GCTAAAACTT   
  
  
+ CAAGAATTGG AGCATGCCCT GCTCGATGAA GATGATGAAG TGGATGAGGT GGCTATAGGG CCCGTGCTAG   
  
  
+ AACACAGCAT GGAGCTTGAT GAAGAGTGGG TCGCCCCATT GAGAAATGTG GTTTTCCATG ATTCACCAAA   
  
  
+ GGAGTCCACC TCCTCAGAGT CATCCAATGT CAGCAGCATT AGCAGCACCA AAGAAGTATC ACCTTGCTCT   
  
  
+ CCGAGTACTC CCAAACAATT GCTCTTTAAT TGTGCAAATA TGATTTCAGA GGGGAATTTT GAGGAAGCCG   
  
  
+ TGACTATGAT CAGCGAGCTT AGACAGATTG TTTCTATCCA AGGGGATCCA GCGCAGAGGA TAGCAGCCTA   
  
  
+ CATGGTAGAA GGCCTTGCTT CCCGTTTGGC TTCATCGGGA AAAGTTCTTT ACAAAGCTTT GAAATGCAAA   
  
  
+ GAGCCACCTT CATTTGACAG GCTAGCGGCT ATGCAAATCC TCTTTGAGGT GTGCCCATGT TTCAAATTCG   
  
  
+ GATTTATGGC CGCAAATGCA GCGATTATGG ATGCAATCAA AAACGAAAAA AGGGTACACA TCATAGATTT   
  
  
+ TGACATAAAC CAAGGGAATC AATACATAAA TCTCATGCAA TCCCTTGCTA AACAAGGTAA CAAGCTGACG   
  
  
+ CACTTGAAGT TGACTGGAGT TGATGACCCT GAGTCAGTTC AACGCCCTAT TGGTGGCCTA AAAAACATCG   
  
  
+ GACAAAGGCT GCAAGCATTA GCTGAATATC TTGGTGTGTC ATTTGAGTTC AAAGCAATAC CTGCTAGAAC   
  
  
+ TCCACTTGTT AACCCGGAAA TGTTAGAATG TCGACCTGCG GAAGCTTTAG TGGTGAACTT TGCCTTCCAG   
  
  
+ CTTCACCACA TGCCTGATGA AAGCGTCTCA ACAATCAACC TCCGAGACCA GCTTCTTCGG ATGGTCAAAA   
  
  
+ GCCTAAACCC AAAGCTAGTA ACCATTGTCG AGCAGGATGT GAACACAAAC ACTACCCCTT TCCTCTCTAG   
  
  
+ GTTTGCTGAG GCATACAGCT ACTATTCTGC TGTTTTTGAG TCTCTTGATG CTACTCTCCC TAGAGATAGC   
  
  
+ CAGGACAGGG TGAATGTGGA GAAGCAGTGC TTGGCACGTG ATGTTGTGAA CATCATTGCA TGTGAAGGGG   
  
  
+ AGGAGAGGAT TGAGCGCTAT GAGGTTGCGG GGAAATGGAG AGCGAGGATG TTGATGGCGG GATTCAGGGC   
  
  
+ TATTCCAATG GGTCAGAAGA CCGGTGATAT GATTCGGAAG CTTATATCAA TGCGGTATTG TGACAGGCTC   
  
  
+ AAGGTAAAAG AGGAGATGGA TGCACTTCAC TTTGGGTGGG AAGATAAAAA CTTAATCGTT ACATCAGCTT   
  
  
+ GGAGGTG  

- +Up\_Stream \_Len000TCTATC TGTAAGTCGG TGAACGGTCA AGTCTCTCTC TATCTCTCTC TAACCCATTC   
  
  
- GGGAAAAACG TGATGAAAAA GAGCGAAGGT AAAAAGAAAG AATTAAAAAT CACTACTAAA AGGTCTTCTA   
  
  
- AACTAAATAA GTAGTACGAA TTGGTTAACC CAGAAACTCA TAATAACAAA TGACCCATAA ACTAGAAAAG   
  
  
- TATAAGGAAA CCTGACCCAC CCACCTTGAA AACTTGAAAG AATAATCAAC CCGTACGGGA ATAGAATAAG   
  
  
- GTTAAGTTTT GGGTATAAAA AGAAGTTAAA ATTAATACCC ATAAAACTTT AACTTACGAA AGGAAGTGTC   
  
  
- GTTATGATAT AATGTATAAA ATGTCCTTTC ATTATAAAAT GTCCTTTCAT ACAACGACAA AGTTGGGACA   
  
  
- AAACAAAGGA GAGAAAGGAA CCTATACTAC AAGTAACGAG AACATGGTTT GGATTGGAGT TGAAGGACTT   
  
  
- AATAAATTCC GTTATAATGG CACACGACCA GTTAAAACCA ACACCCAAAC AAAGAATTAC ATATACTAAA   
  
  
- TACCAGTCCT AGGTACGAGG AGAAGGCAGG TGAGATATAG AGAGAGAGAG AGAGAGTTAT GTTTAAGTCT   
  
  
- CTGAAAGAGA AAGACCCAAA TGATAACTAA CGTCTTCAAA CATCAGAACG CTCCGGAACT CAGAAGAAGC   
  
  
- TCCATCTTTT AAAACGAAAC GCGAAGGAAG GGGTATAAAA TTGTGGGGAG GGGGGGGTTG CGTTCAAAAG   
  
  
- ATGTAAAAAC ACGTTAAGAA GTCGATACTA AGGGGTTAAA GTGAACAGTG TAGAGATAAA AGGAGAAAAA   
  
  
- CCAAAGAACT AAGACATCTT CCAACACATA CTTGAAGAAA CAAAGGGAAA AAGGAAACAA TAAAAATTAC   
  
  
- AAGGGTATCT ACTATATCTC TAGACGAAAA GGAACGACAC TCTCCATAGA GACTCTTACG TACATCGTTA   
  
  
- GTTTTAAGAC TCAACAATTA AGATAACGTA CAGATACCCA CTACTAACAC TACTACAAAA AAGACAACGG   
  
  
- GAGAAAGAGA GAAAGTGACT TTAACGAAAG GTCTTATCAA CAAACTGAGG AAAGACATAG AATTAACTCG   
  
  
- GGAACGAAAC CAAACACGAG ACCCTCAAGA AGAAATCATT AGGGAACGAA CATAAAAGAC TTTCTCGTTC   
  
  
- GTTATTAGGA GTATCGTACA CATATTAGTA TGTTATCGTT GTCGTTTGTA CCTAGGACGC GGGTCAATCC   
  
  
- AAACCAATCC AATTCTATAC GTATAAGAAA AGAGAACTTT TCTCAACAGA TCCTTCCCAT TAATACGGGT   
  
  
- AAAGTACCTA TTATTACCAT GTTAATTCAA ACTAACCGAA TTTATGGTTA ATAAATCGCA GAAAAAGAAA   
  
  
- CCGACAGGTC CCAAGGTAAA GGTATTTGTT GAAACTCATT CCGTATCTCT TGATTTAAAG ACGGTTAACA   
  
  
- GTTCACGTTC AACGAGTATA CGTTGTTATG AACCTTTAAA ACTCGACTCG ACGACCGTAT AGTACTTATC   
  
  
- AAGATTACCA CCGCTCAATA GTGAATAGCA GGGAACACAC TGCTTAAAGA GAAGGATCCT TCGGGAGATC   
  
  
- AGTTACGGAT AGACGACAAC ACCTCACTTT TACCTATGTA AATCTAGAGA TATTTGGGAT GAAACCGTTC   
  
  
- TAGGTACGAC TAAAATCCTT CGGGAAACTG GTCTCTTACT GTATTGTTGG ACTATCCCAC AATGTACGAT   
  
  
- TGAAGCTAAT AAATTAGTTT TATGCCTTGA AGATAGTCAG TTTCGGACGG GGTCCAAGAT GACTTAATTT   
  
  
- AACATAACTT AACGAGAGTG AACGAATACT GCTAATTTAT TGACCTAAAC GATTAATAAG AAAAATAAAA   
  
  
- TAAGAACCAC TACATCCCTT GAGTCCCTTA ACATTGGTAT GGACAAGGTC GACTTTTCCC ATGCCCTTGT   
  
  
- CAGTATCAAT GTCCCCTTAA AGCGTTATGT AAATTCCCTC ATTCTTCGTC CTAGTACAGA AATCAATCCG   
  
  
- GCCGTCTATA CAGACACTGA GGTATACCTT TAGGTTGTGA CATAAGTGAA GTTCCGTTAT TATTATCGAG   
  
  
- ACCAAACAGA CCAGTTGACA TACCGAGACT TTTCGTGTTC TAGTTTATAC ACTGGCTTAG AAAATTACCA   
  
  
- GGTTCAATAC CGGACTTTAA GAAACAACTA AGAGGTAGTC TTCTCGAGTA GGTGGGTAGT CTACGATGAG   
  
  
- GATTGGGTAA ACTGAGAAGA AAGCACCCGT ATTCTGTACT ACTCTCAGGA ATGGTTCCTT TAATACCTAG   
  
  
- TCTTATACCC CATTCGGGTA GAAGGCTACG TCAACTTATA CTACTTCCAT TATTCTATTC CGATTTTGAA   
  
  
- GTTCTTAACC TCGTACGGGA CGAGCTACTT CTACTACTTC ACCTACTCCA CCGATATCCC GGGCACGATC   
  
  
- TTGTGTCGTA CCTCGAACTA CTTCTCACCC AGCGGGGTAA CTCTTTACAC CAAAAGGTAC TAAGTGGTTT   
  
  
- CCTCAGGTGG AGGAGTCTCA GTAGGTTACA GTCGTCGTAA TCGTCGTGGT TTCTTCATAG TGGAACGAGA   
  
  
- GGCTCATGAG GGTTTGTTAA CGAGAAATTA ACACGTTTAT ACTAAAGTCT CCCCTTAAAA CTCCTTCGGC   
  
  
- ACTGATACTA GTCGCTCGAA TCTGTCTAAC AAAGATAGGT TCCCCTAGGT CGCGTCTCCT ATCGTCGGAT   
  
  
- GTACCATCTT CCGGAACGAA GGGCAAACCG AAGTAGCCCT TTTCAAGAAA TGTTTCGAAA CTTTACGTTT   
  
  
- CTCGGTGGAA GTAAACTGTC CGATCGCCGA TACGTTTAGG AGAAACTCCA CACGGGTACA AAGTTTAAGC   
  
  
- CTAAATACCG GCGTTTACGT CGCTAATACC TACGTTAGTT TTTGCTTTTT TCCCATGTGT AGTATCTAAA   
  
  
- ACTGTATTTG GTTCCCTTAG TTATGTATTT AGAGTACGTT AGGGAACGAT TTGTTCCATT GTTCGACTGC   
  
  
- GTGAACTTCA ACTGACCTCA ACTACTGGGA CTCAGTCAAG TTGCGGGATA ACCACCGGAT TTTTTGTAGC   
  
  
- CTGTTTCCGA CGTTCGTAAT CGACTTATAG AACCACACAG TAAACTCAAG TTTCGTTATG GACGATCTTG   
  
  
- AGGTGAACAA TTGGGCCTTT ACAATCTTAC AGCTGGACGC CTTCGAAATC ACCACTTGAA ACGGAAGGTC   
  
  
- GAAGTGGTGT ACGGACTACT TTCGCAGAGT TGTTAGTTGG AGGCTCTGGT CGAAGAAGCC TACCAGTTTT   
  
  
- CGGATTTGGG TTTCGATCAT TGGTAACAGC TCGTCCTACA CTTGTGTTTG TGATGGGGAA AGGAGAGATC   
  
  
- CAAACGACTC CGTATGTCGA TGATAAGACG ACAAAAACTC AGAGAACTAC GATGAGAGGG ATCTCTATCG   
  
  
- GTCCTGTCCC ACTTACACCT CTTCGTCACG AACCGTGCAC TACAACACTT GTAGTAACGT ACACTTCCCC   
  
  
- TCCTCTCCTA ACTCGCGATA CTCCAACGCC CCTTTACCTC TCGCTCCTAC AACTACCGCC CTAAGTCCCG   
  
  
- ATAAGGTTAC CCAGTCTTCT GGCCACTATA CTAAGCCTTC GAATATAGTT ACGCCATAAC ACTGTCCGAG   
  
  
- TTCCATTTTC TCCTCTACCT ACGTGAAGTG AAACCCACCC TTCTATTTTT GAATTAGCAA TGTAGTCGAA   
  
  
- CCTCCAC

+     TATA-box

| Site Name | Organism | Position | Strand | Matrix score. | sequence | function |
| --- | --- | --- | --- | --- | --- | --- |
| TATA-box | Arabidopsis thaliana | 599 | + | 4 | TATA | core promoter element around -30 of transcription start |
| TATA-box | Arabidopsis thaliana | 3617 | - | 4 | TATA | core promoter element around -30 of transcription start |
| TATA-box | Helianthus annuus | 1214 | - | 6 | TATACA | core promoter element around -30 of transcription start |
| TATA-box | Arabidopsis thaliana | 555 | + | 4 | TATA | core promoter element around -30 of transcription start |
| TATA-box | Arabidopsis thaliana | 3616 | - | 5 | TATAA | core promoter element around -30 of transcription start |
| TATA-box | Arabidopsis thaliana | 1216 | + | 4 | TATA | core promoter element around -30 of transcription start |
| TATA-box | Arabidopsis thaliana | 928 | + | 4 | TATA | core promoter element around -30 of transcription start |
| TATA-box | Helianthus annuus | 553 | - | 6 | TATACA | core promoter element around -30 of transcription start |
| TATA-box | Arabidopsis thaliana | 361 | + | 4 | TATA | core promoter element around -30 of transcription start |
| TATA-box | Arabidopsis thaliana | 2438 | - | 4 | TATA | core promoter element around -30 of transcription start |
| TATA-box | Arabidopsis thaliana | 1664 | + | 4 | TATA | core promoter element around -30 of transcription start |
| TATA-box | Arabidopsis thaliana | 2967 | + | 9 | taTATAAAtc | core promoter element around -30 of transcription start |

>HU11G01134.1   
+ +Up\_Stream \_Len000AGATAG ACATTCAGCC ACTTGCCAGT TCAGAGAGAG ATAGAGAGAG ATTGGGTAAG   
  
  
+ CCCTTTTTGC ACTACTTTTT CTCGCTTCCA TTTTTCTTTC TTAATTTTTA GTGATGATTT TCCAGAAGAT   
  
  
+ TTGATTTATT CATCATGCTT AACCAATTGG GTCTTTGAGT ATTATTGTTT ACTGGGTATT TGATCTTTTC   
  
  
+ ATATTCCTTT GGACTGGGTG GGTGGAACTT TTGAACTTTC TTATTAGTTG GGCATGCCCT TATCTTATTC   
  
  
+ CAATTCAAAA CCCATATTTT TCTTCAATTT TAATTATGGG TATTTTGAAA TTGAATGCTT TCCTTCACAG   
  
  
+ CAATACTATA TTACATATTT TACAGGAAAG TAATATTTTA CAGGAAAGTA TGTTGCTGTT TCAACCCTGT   
  
  
+ TTTGTTTCCT CTCTTTCCTT GGATATGATG TTCATTGCTC TTGTACCAAA CCTAACCTCA ACTTCCTGAA   
  
  
+ TTATTTAAGG CAATATTACC GTGTGCTGGT CAATTTTGGT TGTGGGTTTG TTTCTTAATG TATATGATTT   
  
  
+ ATGGTCAGGA TCCATGCTCC TCTTCCGTCC ACTCTATATC TCTCTCTCTC TCTCTCAATA CAAATTCAGA   
  
  
+ GACTTTCTCT TTCTGGGTTT ACTATTGATT GCAGAAGTTT GTAGTCTTGC GAGGCCTTGA GTCTTCTTCG   
  
  
+ AGGTAGAAAA TTTTGCTTTG CGCTTCCTTC CCCATATTTT AACACCCCTC CCCCCCCAAC GCAAGTTTTC   
  
  
+ TACATTTTTG TGCAATTCTT CAGCTATGAT TCCCCAATTT CACTTGTCAC ATCTCTATTT TCCTCTTTTT   
  
  
+ GGTTTCTTGA TTCTGTAGAA GGTTGTGTAT GAACTTCTTT GTTTCCCTTT TTCCTTTGTT ATTTTTAATG   
  
  
+ TTCCCATAGA TGATATAGAG ATCTGCTTTT CCTTGCTGTG AGAGGTATCT CTGAGAATGC ATGTAGCAAT   
  
  
+ CAAAATTCTG AGTTGTTAAT TCTATTGCAT GTCTATGGGT GATGATTGTG ATGATGTTTT TTCTGTTGCC   
  
  
+ CTCTTTCTCT CTTTCACTGA AATTGCTTTC CAGAATAGTT GTTTGACTCC TTTCTGTATC TTAATTGAGC   
  
  
+ CCTTGCTTTG GTTTGTGCTC TGGGAGTTCT TCTTTAGTAA TCCCTTGCTT GTATTTTCTG AAAGAGCAAG   
  
  
+ CAATAATCCT CATAGCATGT GTATAATCAT ACAATAGCAA CAGCAAACAT GGATCCTGCG CCCAGTTAGG   
  
  
+ TTTGGTTAGG TTAAGATATG CATATTCTTT TCTCTTGAAA AGAGTTGTCT AGGAAGGGTA ATTATGCCCA   
  
  
+ TTTCATGGAT AATAATGGTA CAATTAAGTT TGATTGGCTT AAATACCAAT TATTTAGCGT CTTTTTCTTT   
  
  
+ GGCTGTCCAG GGTTCCATTT CCATAAACAA CTTTGAGTAA GGCATAGAGA ACTAAATTTC TGCCAATTGT   
  
  
+ CAAGTGCAAG TTGCTCATAT GCAACAATAC TTGGAAATTT TGAGCTGAGC TGCTGGCATA TCATGAATAG   
  
  
+ TTCTAATGGT GGCGAGTTAT CACTTATCGT CCCTTGTGTG ACGAATTTCT CTTCCTAGGA AGCCCTCTAG   
  
  
+ TCAATGCCTA TCTGCTGTTG TGGAGTGAAA ATGGATACAT TTAGATCTCT ATAAACCCTA CTTTGGCAAG   
  
  
+ ATCCATGCTG ATTTTAGGAA GCCCTTTGAC CAGAGAATGA CATAACAACC TGATAGGGTG TTACATGCTA   
  
  
+ ACTTCGATTA TTTAATCAAA ATACGGAACT TCTATCAGTC AAAGCCTGCC CCAGGTTCTA CTGAATTAAA   
  
  
+ TTGTATTGAA TTGCTCTCAC TTGCTTATGA CGATTAAATA ACTGGATTTG CTAATTATTC TTTTTATTTT   
  
  
+ ATTCTTGGTG ATGTAGGGAA CTCAGGGAAT TGTAACCATA CCTGTTCCAG CTGAAAAGGG TACGGGAACA   
  
  
+ GTCATAGTTA CAGGGGAATT TCGCAATACA TTTAAGGGAG TAAGAAGCAG GATCATGTCT TTAGTTAGGC   
  
  
+ CGGCAGATAT GTCTGTGACT CCATATGGAA ATCCAACACT GTATTCACTT CAAGGCAATA ATAATAGCTC   
  
  
+ TGGTTTGTCT GGTCAACTGT ATGGCTCTGA AAAGCACAAG ATCAAATATG TGACCGAATC TTTTAATGGT   
  
  
+ CCAAGTTATG GCCTGAAATT CTTTGTTGAT TCTCCATCAG AAGAGCTCAT CCACCCATCA GATGCTACTC   
  
  
+ CTAACCCATT TGACTCTTCT TTCGTGGGCA TAAGACATGA TGAGAGTCCT TACCAAGGAA ATTATGGATC   
  
  
+ AGAATATGGG GTAAGCCCAT CTTCCGATGC AGTTGAATAT GATGAAGGTA ATAAGATAAG GCTAAAACTT   
  
  
+ CAAGAATTGG AGCATGCCCT GCTCGATGAA GATGATGAAG TGGATGAGGT GGCTATAGGG CCCGTGCTAG   
  
  
+ AACACAGCAT GGAGCTTGAT GAAGAGTGGG TCGCCCCATT GAGAAATGTG GTTTTCCATG ATTCACCAAA   
  
  
+ GGAGTCCACC TCCTCAGAGT CATCCAATGT CAGCAGCATT AGCAGCACCA AAGAAGTATC ACCTTGCTCT   
  
  
+ CCGAGTACTC CCAAACAATT GCTCTTTAAT TGTGCAAATA TGATTTCAGA GGGGAATTTT GAGGAAGCCG   
  
  
+ TGACTATGAT CAGCGAGCTT AGACAGATTG TTTCTATCCA AGGGGATCCA GCGCAGAGGA TAGCAGCCTA   
  
  
+ CATGGTAGAA GGCCTTGCTT CCCGTTTGGC TTCATCGGGA AAAGTTCTTT ACAAAGCTTT GAAATGCAAA   
  
  
+ GAGCCACCTT CATTTGACAG GCTAGCGGCT ATGCAAATCC TCTTTGAGGT GTGCCCATGT TTCAAATTCG   
  
  
+ GATTTATGGC CGCAAATGCA GCGATTATGG ATGCAATCAA AAACGAAAAA AGGGTACACA TCATAGATTT   
  
  
+ TGACATAAAC CAAGGGAATC AATACATAAA TCTCATGCAA TCCCTTGCTA AACAAGGTAA CAAGCTGACG   
  
  
+ CACTTGAAGT TGACTGGAGT TGATGACCCT GAGTCAGTTC AACGCCCTAT TGGTGGCCTA AAAAACATCG   
  
  
+ GACAAAGGCT GCAAGCATTA GCTGAATATC TTGGTGTGTC ATTTGAGTTC AAAGCAATAC CTGCTAGAAC   
  
  
+ TCCACTTGTT AACCCGGAAA TGTTAGAATG TCGACCTGCG GAAGCTTTAG TGGTGAACTT TGCCTTCCAG   
  
  
+ CTTCACCACA TGCCTGATGA AAGCGTCTCA ACAATCAACC TCCGAGACCA GCTTCTTCGG ATGGTCAAAA   
  
  
+ GCCTAAACCC AAAGCTAGTA ACCATTGTCG AGCAGGATGT GAACACAAAC ACTACCCCTT TCCTCTCTAG   
  
  
+ GTTTGCTGAG GCATACAGCT ACTATTCTGC TGTTTTTGAG TCTCTTGATG CTACTCTCCC TAGAGATAGC   
  
  
+ CAGGACAGGG TGAATGTGGA GAAGCAGTGC TTGGCACGTG ATGTTGTGAA CATCATTGCA TGTGAAGGGG   
  
  
+ AGGAGAGGAT TGAGCGCTAT GAGGTTGCGG GGAAATGGAG AGCGAGGATG TTGATGGCGG GATTCAGGGC   
  
  
+ TATTCCAATG GGTCAGAAGA CCGGTGATAT GATTCGGAAG CTTATATCAA TGCGGTATTG TGACAGGCTC   
  
  
+ AAGGTAAAAG AGGAGATGGA TGCACTTCAC TTTGGGTGGG AAGATAAAAA CTTAATCGTT ACATCAGCTT   
  
  
+ GGAGGTG  

- +Up\_Stream \_Len000TCTATC TGTAAGTCGG TGAACGGTCA AGTCTCTCTC TATCTCTCTC TAACCCATTC   
  
  
- GGGAAAAACG TGATGAAAAA GAGCGAAGGT AAAAAGAAAG AATTAAAAAT CACTACTAAA AGGTCTTCTA   
  
  
- AACTAAATAA GTAGTACGAA TTGGTTAACC CAGAAACTCA TAATAACAAA TGACCCATAA ACTAGAAAAG   
  
  
- TATAAGGAAA CCTGACCCAC CCACCTTGAA AACTTGAAAG AATAATCAAC CCGTACGGGA ATAGAATAAG   
  
  
- GTTAAGTTTT GGGTATAAAA AGAAGTTAAA ATTAATACCC ATAAAACTTT AACTTACGAA AGGAAGTGTC   
  
  
- GTTATGATAT AATGTATAAA ATGTCCTTTC ATTATAAAAT GTCCTTTCAT ACAACGACAA AGTTGGGACA   
  
  
- AAACAAAGGA GAGAAAGGAA CCTATACTAC AAGTAACGAG AACATGGTTT GGATTGGAGT TGAAGGACTT   
  
  
- AATAAATTCC GTTATAATGG CACACGACCA GTTAAAACCA ACACCCAAAC AAAGAATTAC ATATACTAAA   
  
  
- TACCAGTCCT AGGTACGAGG AGAAGGCAGG TGAGATATAG AGAGAGAGAG AGAGAGTTAT GTTTAAGTCT   
  
  
- CTGAAAGAGA AAGACCCAAA TGATAACTAA CGTCTTCAAA CATCAGAACG CTCCGGAACT CAGAAGAAGC   
  
  
- TCCATCTTTT AAAACGAAAC GCGAAGGAAG GGGTATAAAA TTGTGGGGAG GGGGGGGTTG CGTTCAAAAG   
  
  
- ATGTAAAAAC ACGTTAAGAA GTCGATACTA AGGGGTTAAA GTGAACAGTG TAGAGATAAA AGGAGAAAAA   
  
  
- CCAAAGAACT AAGACATCTT CCAACACATA CTTGAAGAAA CAAAGGGAAA AAGGAAACAA TAAAAATTAC   
  
  
- AAGGGTATCT ACTATATCTC TAGACGAAAA GGAACGACAC TCTCCATAGA GACTCTTACG TACATCGTTA   
  
  
- GTTTTAAGAC TCAACAATTA AGATAACGTA CAGATACCCA CTACTAACAC TACTACAAAA AAGACAACGG   
  
  
- GAGAAAGAGA GAAAGTGACT TTAACGAAAG GTCTTATCAA CAAACTGAGG AAAGACATAG AATTAACTCG   
  
  
- GGAACGAAAC CAAACACGAG ACCCTCAAGA AGAAATCATT AGGGAACGAA CATAAAAGAC TTTCTCGTTC   
  
  
- GTTATTAGGA GTATCGTACA CATATTAGTA TGTTATCGTT GTCGTTTGTA CCTAGGACGC GGGTCAATCC   
  
  
- AAACCAATCC AATTCTATAC GTATAAGAAA AGAGAACTTT TCTCAACAGA TCCTTCCCAT TAATACGGGT   
  
  
- AAAGTACCTA TTATTACCAT GTTAATTCAA ACTAACCGAA TTTATGGTTA ATAAATCGCA GAAAAAGAAA   
  
  
- CCGACAGGTC CCAAGGTAAA GGTATTTGTT GAAACTCATT CCGTATCTCT TGATTTAAAG ACGGTTAACA   
  
  
- GTTCACGTTC AACGAGTATA CGTTGTTATG AACCTTTAAA ACTCGACTCG ACGACCGTAT AGTACTTATC   
  
  
- AAGATTACCA CCGCTCAATA GTGAATAGCA GGGAACACAC TGCTTAAAGA GAAGGATCCT TCGGGAGATC   
  
  
- AGTTACGGAT AGACGACAAC ACCTCACTTT TACCTATGTA AATCTAGAGA TATTTGGGAT GAAACCGTTC   
  
  
- TAGGTACGAC TAAAATCCTT CGGGAAACTG GTCTCTTACT GTATTGTTGG ACTATCCCAC AATGTACGAT   
  
  
- TGAAGCTAAT AAATTAGTTT TATGCCTTGA AGATAGTCAG TTTCGGACGG GGTCCAAGAT GACTTAATTT   
  
  
- AACATAACTT AACGAGAGTG AACGAATACT GCTAATTTAT TGACCTAAAC GATTAATAAG AAAAATAAAA   
  
  
- TAAGAACCAC TACATCCCTT GAGTCCCTTA ACATTGGTAT GGACAAGGTC GACTTTTCCC ATGCCCTTGT   
  
  
- CAGTATCAAT GTCCCCTTAA AGCGTTATGT AAATTCCCTC ATTCTTCGTC CTAGTACAGA AATCAATCCG   
  
  
- GCCGTCTATA CAGACACTGA GGTATACCTT TAGGTTGTGA CATAAGTGAA GTTCCGTTAT TATTATCGAG   
  
  
- ACCAAACAGA CCAGTTGACA TACCGAGACT TTTCGTGTTC TAGTTTATAC ACTGGCTTAG AAAATTACCA   
  
  
- GGTTCAATAC CGGACTTTAA GAAACAACTA AGAGGTAGTC TTCTCGAGTA GGTGGGTAGT CTACGATGAG   
  
  
- GATTGGGTAA ACTGAGAAGA AAGCACCCGT ATTCTGTACT ACTCTCAGGA ATGGTTCCTT TAATACCTAG   
  
  
- TCTTATACCC CATTCGGGTA GAAGGCTACG TCAACTTATA CTACTTCCAT TATTCTATTC CGATTTTGAA   
  
  
- GTTCTTAACC TCGTACGGGA CGAGCTACTT CTACTACTTC ACCTACTCCA CCGATATCCC GGGCACGATC   
  
  
- TTGTGTCGTA CCTCGAACTA CTTCTCACCC AGCGGGGTAA CTCTTTACAC CAAAAGGTAC TAAGTGGTTT   
  
  
- CCTCAGGTGG AGGAGTCTCA GTAGGTTACA GTCGTCGTAA TCGTCGTGGT TTCTTCATAG TGGAACGAGA   
  
  
- GGCTCATGAG GGTTTGTTAA CGAGAAATTA ACACGTTTAT ACTAAAGTCT CCCCTTAAAA CTCCTTCGGC   
  
  
- ACTGATACTA GTCGCTCGAA TCTGTCTAAC AAAGATAGGT TCCCCTAGGT CGCGTCTCCT ATCGTCGGAT   
  
  
- GTACCATCTT CCGGAACGAA GGGCAAACCG AAGTAGCCCT TTTCAAGAAA TGTTTCGAAA CTTTACGTTT   
  
  
- CTCGGTGGAA GTAAACTGTC CGATCGCCGA TACGTTTAGG AGAAACTCCA CACGGGTACA AAGTTTAAGC   
  
  
- CTAAATACCG GCGTTTACGT CGCTAATACC TACGTTAGTT TTTGCTTTTT TCCCATGTGT AGTATCTAAA   
  
  
- ACTGTATTTG GTTCCCTTAG TTATGTATTT AGAGTACGTT AGGGAACGAT TTGTTCCATT GTTCGACTGC   
  
  
- GTGAACTTCA ACTGACCTCA ACTACTGGGA CTCAGTCAAG TTGCGGGATA ACCACCGGAT TTTTTGTAGC   
  
  
- CTGTTTCCGA CGTTCGTAAT CGACTTATAG AACCACACAG TAAACTCAAG TTTCGTTATG GACGATCTTG   
  
  
- AGGTGAACAA TTGGGCCTTT ACAATCTTAC AGCTGGACGC CTTCGAAATC ACCACTTGAA ACGGAAGGTC   
  
  
- GAAGTGGTGT ACGGACTACT TTCGCAGAGT TGTTAGTTGG AGGCTCTGGT CGAAGAAGCC TACCAGTTTT   
  
  
- CGGATTTGGG TTTCGATCAT TGGTAACAGC TCGTCCTACA CTTGTGTTTG TGATGGGGAA AGGAGAGATC   
  
  
- CAAACGACTC CGTATGTCGA TGATAAGACG ACAAAAACTC AGAGAACTAC GATGAGAGGG ATCTCTATCG   
  
  
- GTCCTGTCCC ACTTACACCT CTTCGTCACG AACCGTGCAC TACAACACTT GTAGTAACGT ACACTTCCCC   
  
  
- TCCTCTCCTA ACTCGCGATA CTCCAACGCC CCTTTACCTC TCGCTCCTAC AACTACCGCC CTAAGTCCCG   
  
  
- ATAAGGTTAC CCAGTCTTCT GGCCACTATA CTAAGCCTTC GAATATAGTT ACGCCATAAC ACTGTCCGAG   
  
  
- TTCCATTTTC TCCTCTACCT ACGTGAAGTG AAACCCACCC TTCTATTTTT GAATTAGCAA TGTAGTCGAA   
  
  
- CCTCCAC

+     TCA

| Site Name | Organism | Position | Strand | Matrix score. | sequence | function |
| --- | --- | --- | --- | --- | --- | --- |
| TCA | Pisum sativum | 2410 | - | 10 | TCATCTTCAT |  |

>HU11G01134.1   
+ +Up\_Stream \_Len000AGATAG ACATTCAGCC ACTTGCCAGT TCAGAGAGAG ATAGAGAGAG ATTGGGTAAG   
  
  
+ CCCTTTTTGC ACTACTTTTT CTCGCTTCCA TTTTTCTTTC TTAATTTTTA GTGATGATTT TCCAGAAGAT   
  
  
+ TTGATTTATT CATCATGCTT AACCAATTGG GTCTTTGAGT ATTATTGTTT ACTGGGTATT TGATCTTTTC   
  
  
+ ATATTCCTTT GGACTGGGTG GGTGGAACTT TTGAACTTTC TTATTAGTTG GGCATGCCCT TATCTTATTC   
  
  
+ CAATTCAAAA CCCATATTTT TCTTCAATTT TAATTATGGG TATTTTGAAA TTGAATGCTT TCCTTCACAG   
  
  
+ CAATACTATA TTACATATTT TACAGGAAAG TAATATTTTA CAGGAAAGTA TGTTGCTGTT TCAACCCTGT   
  
  
+ TTTGTTTCCT CTCTTTCCTT GGATATGATG TTCATTGCTC TTGTACCAAA CCTAACCTCA ACTTCCTGAA   
  
  
+ TTATTTAAGG CAATATTACC GTGTGCTGGT CAATTTTGGT TGTGGGTTTG TTTCTTAATG TATATGATTT   
  
  
+ ATGGTCAGGA TCCATGCTCC TCTTCCGTCC ACTCTATATC TCTCTCTCTC TCTCTCAATA CAAATTCAGA   
  
  
+ GACTTTCTCT TTCTGGGTTT ACTATTGATT GCAGAAGTTT GTAGTCTTGC GAGGCCTTGA GTCTTCTTCG   
  
  
+ AGGTAGAAAA TTTTGCTTTG CGCTTCCTTC CCCATATTTT AACACCCCTC CCCCCCCAAC GCAAGTTTTC   
  
  
+ TACATTTTTG TGCAATTCTT CAGCTATGAT TCCCCAATTT CACTTGTCAC ATCTCTATTT TCCTCTTTTT   
  
  
+ GGTTTCTTGA TTCTGTAGAA GGTTGTGTAT GAACTTCTTT GTTTCCCTTT TTCCTTTGTT ATTTTTAATG   
  
  
+ TTCCCATAGA TGATATAGAG ATCTGCTTTT CCTTGCTGTG AGAGGTATCT CTGAGAATGC ATGTAGCAAT   
  
  
+ CAAAATTCTG AGTTGTTAAT TCTATTGCAT GTCTATGGGT GATGATTGTG ATGATGTTTT TTCTGTTGCC   
  
  
+ CTCTTTCTCT CTTTCACTGA AATTGCTTTC CAGAATAGTT GTTTGACTCC TTTCTGTATC TTAATTGAGC   
  
  
+ CCTTGCTTTG GTTTGTGCTC TGGGAGTTCT TCTTTAGTAA TCCCTTGCTT GTATTTTCTG AAAGAGCAAG   
  
  
+ CAATAATCCT CATAGCATGT GTATAATCAT ACAATAGCAA CAGCAAACAT GGATCCTGCG CCCAGTTAGG   
  
  
+ TTTGGTTAGG TTAAGATATG CATATTCTTT TCTCTTGAAA AGAGTTGTCT AGGAAGGGTA ATTATGCCCA   
  
  
+ TTTCATGGAT AATAATGGTA CAATTAAGTT TGATTGGCTT AAATACCAAT TATTTAGCGT CTTTTTCTTT   
  
  
+ GGCTGTCCAG GGTTCCATTT CCATAAACAA CTTTGAGTAA GGCATAGAGA ACTAAATTTC TGCCAATTGT   
  
  
+ CAAGTGCAAG TTGCTCATAT GCAACAATAC TTGGAAATTT TGAGCTGAGC TGCTGGCATA TCATGAATAG   
  
  
+ TTCTAATGGT GGCGAGTTAT CACTTATCGT CCCTTGTGTG ACGAATTTCT CTTCCTAGGA AGCCCTCTAG   
  
  
+ TCAATGCCTA TCTGCTGTTG TGGAGTGAAA ATGGATACAT TTAGATCTCT ATAAACCCTA CTTTGGCAAG   
  
  
+ ATCCATGCTG ATTTTAGGAA GCCCTTTGAC CAGAGAATGA CATAACAACC TGATAGGGTG TTACATGCTA   
  
  
+ ACTTCGATTA TTTAATCAAA ATACGGAACT TCTATCAGTC AAAGCCTGCC CCAGGTTCTA CTGAATTAAA   
  
  
+ TTGTATTGAA TTGCTCTCAC TTGCTTATGA CGATTAAATA ACTGGATTTG CTAATTATTC TTTTTATTTT   
  
  
+ ATTCTTGGTG ATGTAGGGAA CTCAGGGAAT TGTAACCATA CCTGTTCCAG CTGAAAAGGG TACGGGAACA   
  
  
+ GTCATAGTTA CAGGGGAATT TCGCAATACA TTTAAGGGAG TAAGAAGCAG GATCATGTCT TTAGTTAGGC   
  
  
+ CGGCAGATAT GTCTGTGACT CCATATGGAA ATCCAACACT GTATTCACTT CAAGGCAATA ATAATAGCTC   
  
  
+ TGGTTTGTCT GGTCAACTGT ATGGCTCTGA AAAGCACAAG ATCAAATATG TGACCGAATC TTTTAATGGT   
  
  
+ CCAAGTTATG GCCTGAAATT CTTTGTTGAT TCTCCATCAG AAGAGCTCAT CCACCCATCA GATGCTACTC   
  
  
+ CTAACCCATT TGACTCTTCT TTCGTGGGCA TAAGACATGA TGAGAGTCCT TACCAAGGAA ATTATGGATC   
  
  
+ AGAATATGGG GTAAGCCCAT CTTCCGATGC AGTTGAATAT GATGAAGGTA ATAAGATAAG GCTAAAACTT   
  
  
+ CAAGAATTGG AGCATGCCCT GCTCGATGAA GATGATGAAG TGGATGAGGT GGCTATAGGG CCCGTGCTAG   
  
  
+ AACACAGCAT GGAGCTTGAT GAAGAGTGGG TCGCCCCATT GAGAAATGTG GTTTTCCATG ATTCACCAAA   
  
  
+ GGAGTCCACC TCCTCAGAGT CATCCAATGT CAGCAGCATT AGCAGCACCA AAGAAGTATC ACCTTGCTCT   
  
  
+ CCGAGTACTC CCAAACAATT GCTCTTTAAT TGTGCAAATA TGATTTCAGA GGGGAATTTT GAGGAAGCCG   
  
  
+ TGACTATGAT CAGCGAGCTT AGACAGATTG TTTCTATCCA AGGGGATCCA GCGCAGAGGA TAGCAGCCTA   
  
  
+ CATGGTAGAA GGCCTTGCTT CCCGTTTGGC TTCATCGGGA AAAGTTCTTT ACAAAGCTTT GAAATGCAAA   
  
  
+ GAGCCACCTT CATTTGACAG GCTAGCGGCT ATGCAAATCC TCTTTGAGGT GTGCCCATGT TTCAAATTCG   
  
  
+ GATTTATGGC CGCAAATGCA GCGATTATGG ATGCAATCAA AAACGAAAAA AGGGTACACA TCATAGATTT   
  
  
+ TGACATAAAC CAAGGGAATC AATACATAAA TCTCATGCAA TCCCTTGCTA AACAAGGTAA CAAGCTGACG   
  
  
+ CACTTGAAGT TGACTGGAGT TGATGACCCT GAGTCAGTTC AACGCCCTAT TGGTGGCCTA AAAAACATCG   
  
  
+ GACAAAGGCT GCAAGCATTA GCTGAATATC TTGGTGTGTC ATTTGAGTTC AAAGCAATAC CTGCTAGAAC   
  
  
+ TCCACTTGTT AACCCGGAAA TGTTAGAATG TCGACCTGCG GAAGCTTTAG TGGTGAACTT TGCCTTCCAG   
  
  
+ CTTCACCACA TGCCTGATGA AAGCGTCTCA ACAATCAACC TCCGAGACCA GCTTCTTCGG ATGGTCAAAA   
  
  
+ GCCTAAACCC AAAGCTAGTA ACCATTGTCG AGCAGGATGT GAACACAAAC ACTACCCCTT TCCTCTCTAG   
  
  
+ GTTTGCTGAG GCATACAGCT ACTATTCTGC TGTTTTTGAG TCTCTTGATG CTACTCTCCC TAGAGATAGC   
  
  
+ CAGGACAGGG TGAATGTGGA GAAGCAGTGC TTGGCACGTG ATGTTGTGAA CATCATTGCA TGTGAAGGGG   
  
  
+ AGGAGAGGAT TGAGCGCTAT GAGGTTGCGG GGAAATGGAG AGCGAGGATG TTGATGGCGG GATTCAGGGC   
  
  
+ TATTCCAATG GGTCAGAAGA CCGGTGATAT GATTCGGAAG CTTATATCAA TGCGGTATTG TGACAGGCTC   
  
  
+ AAGGTAAAAG AGGAGATGGA TGCACTTCAC TTTGGGTGGG AAGATAAAAA CTTAATCGTT ACATCAGCTT   
  
  
+ GGAGGTG  

- +Up\_Stream \_Len000TCTATC TGTAAGTCGG TGAACGGTCA AGTCTCTCTC TATCTCTCTC TAACCCATTC   
  
  
- GGGAAAAACG TGATGAAAAA GAGCGAAGGT AAAAAGAAAG AATTAAAAAT CACTACTAAA AGGTCTTCTA   
  
  
- AACTAAATAA GTAGTACGAA TTGGTTAACC CAGAAACTCA TAATAACAAA TGACCCATAA ACTAGAAAAG   
  
  
- TATAAGGAAA CCTGACCCAC CCACCTTGAA AACTTGAAAG AATAATCAAC CCGTACGGGA ATAGAATAAG   
  
  
- GTTAAGTTTT GGGTATAAAA AGAAGTTAAA ATTAATACCC ATAAAACTTT AACTTACGAA AGGAAGTGTC   
  
  
- GTTATGATAT AATGTATAAA ATGTCCTTTC ATTATAAAAT GTCCTTTCAT ACAACGACAA AGTTGGGACA   
  
  
- AAACAAAGGA GAGAAAGGAA CCTATACTAC AAGTAACGAG AACATGGTTT GGATTGGAGT TGAAGGACTT   
  
  
- AATAAATTCC GTTATAATGG CACACGACCA GTTAAAACCA ACACCCAAAC AAAGAATTAC ATATACTAAA   
  
  
- TACCAGTCCT AGGTACGAGG AGAAGGCAGG TGAGATATAG AGAGAGAGAG AGAGAGTTAT GTTTAAGTCT   
  
  
- CTGAAAGAGA AAGACCCAAA TGATAACTAA CGTCTTCAAA CATCAGAACG CTCCGGAACT CAGAAGAAGC   
  
  
- TCCATCTTTT AAAACGAAAC GCGAAGGAAG GGGTATAAAA TTGTGGGGAG GGGGGGGTTG CGTTCAAAAG   
  
  
- ATGTAAAAAC ACGTTAAGAA GTCGATACTA AGGGGTTAAA GTGAACAGTG TAGAGATAAA AGGAGAAAAA   
  
  
- CCAAAGAACT AAGACATCTT CCAACACATA CTTGAAGAAA CAAAGGGAAA AAGGAAACAA TAAAAATTAC   
  
  
- AAGGGTATCT ACTATATCTC TAGACGAAAA GGAACGACAC TCTCCATAGA GACTCTTACG TACATCGTTA   
  
  
- GTTTTAAGAC TCAACAATTA AGATAACGTA CAGATACCCA CTACTAACAC TACTACAAAA AAGACAACGG   
  
  
- GAGAAAGAGA GAAAGTGACT TTAACGAAAG GTCTTATCAA CAAACTGAGG AAAGACATAG AATTAACTCG   
  
  
- GGAACGAAAC CAAACACGAG ACCCTCAAGA AGAAATCATT AGGGAACGAA CATAAAAGAC TTTCTCGTTC   
  
  
- GTTATTAGGA GTATCGTACA CATATTAGTA TGTTATCGTT GTCGTTTGTA CCTAGGACGC GGGTCAATCC   
  
  
- AAACCAATCC AATTCTATAC GTATAAGAAA AGAGAACTTT TCTCAACAGA TCCTTCCCAT TAATACGGGT   
  
  
- AAAGTACCTA TTATTACCAT GTTAATTCAA ACTAACCGAA TTTATGGTTA ATAAATCGCA GAAAAAGAAA   
  
  
- CCGACAGGTC CCAAGGTAAA GGTATTTGTT GAAACTCATT CCGTATCTCT TGATTTAAAG ACGGTTAACA   
  
  
- GTTCACGTTC AACGAGTATA CGTTGTTATG AACCTTTAAA ACTCGACTCG ACGACCGTAT AGTACTTATC   
  
  
- AAGATTACCA CCGCTCAATA GTGAATAGCA GGGAACACAC TGCTTAAAGA GAAGGATCCT TCGGGAGATC   
  
  
- AGTTACGGAT AGACGACAAC ACCTCACTTT TACCTATGTA AATCTAGAGA TATTTGGGAT GAAACCGTTC   
  
  
- TAGGTACGAC TAAAATCCTT CGGGAAACTG GTCTCTTACT GTATTGTTGG ACTATCCCAC AATGTACGAT   
  
  
- TGAAGCTAAT AAATTAGTTT TATGCCTTGA AGATAGTCAG TTTCGGACGG GGTCCAAGAT GACTTAATTT   
  
  
- AACATAACTT AACGAGAGTG AACGAATACT GCTAATTTAT TGACCTAAAC GATTAATAAG AAAAATAAAA   
  
  
- TAAGAACCAC TACATCCCTT GAGTCCCTTA ACATTGGTAT GGACAAGGTC GACTTTTCCC ATGCCCTTGT   
  
  
- CAGTATCAAT GTCCCCTTAA AGCGTTATGT AAATTCCCTC ATTCTTCGTC CTAGTACAGA AATCAATCCG   
  
  
- GCCGTCTATA CAGACACTGA GGTATACCTT TAGGTTGTGA CATAAGTGAA GTTCCGTTAT TATTATCGAG   
  
  
- ACCAAACAGA CCAGTTGACA TACCGAGACT TTTCGTGTTC TAGTTTATAC ACTGGCTTAG AAAATTACCA   
  
  
- GGTTCAATAC CGGACTTTAA GAAACAACTA AGAGGTAGTC TTCTCGAGTA GGTGGGTAGT CTACGATGAG   
  
  
- GATTGGGTAA ACTGAGAAGA AAGCACCCGT ATTCTGTACT ACTCTCAGGA ATGGTTCCTT TAATACCTAG   
  
  
- TCTTATACCC CATTCGGGTA GAAGGCTACG TCAACTTATA CTACTTCCAT TATTCTATTC CGATTTTGAA   
  
  
- GTTCTTAACC TCGTACGGGA CGAGCTACTT CTACTACTTC ACCTACTCCA CCGATATCCC GGGCACGATC   
  
  
- TTGTGTCGTA CCTCGAACTA CTTCTCACCC AGCGGGGTAA CTCTTTACAC CAAAAGGTAC TAAGTGGTTT   
  
  
- CCTCAGGTGG AGGAGTCTCA GTAGGTTACA GTCGTCGTAA TCGTCGTGGT TTCTTCATAG TGGAACGAGA   
  
  
- GGCTCATGAG GGTTTGTTAA CGAGAAATTA ACACGTTTAT ACTAAAGTCT CCCCTTAAAA CTCCTTCGGC   
  
  
- ACTGATACTA GTCGCTCGAA TCTGTCTAAC AAAGATAGGT TCCCCTAGGT CGCGTCTCCT ATCGTCGGAT   
  
  
- GTACCATCTT CCGGAACGAA GGGCAAACCG AAGTAGCCCT TTTCAAGAAA TGTTTCGAAA CTTTACGTTT   
  
  
- CTCGGTGGAA GTAAACTGTC CGATCGCCGA TACGTTTAGG AGAAACTCCA CACGGGTACA AAGTTTAAGC   
  
  
- CTAAATACCG GCGTTTACGT CGCTAATACC TACGTTAGTT TTTGCTTTTT TCCCATGTGT AGTATCTAAA   
  
  
- ACTGTATTTG GTTCCCTTAG TTATGTATTT AGAGTACGTT AGGGAACGAT TTGTTCCATT GTTCGACTGC   
  
  
- GTGAACTTCA ACTGACCTCA ACTACTGGGA CTCAGTCAAG TTGCGGGATA ACCACCGGAT TTTTTGTAGC   
  
  
- CTGTTTCCGA CGTTCGTAAT CGACTTATAG AACCACACAG TAAACTCAAG TTTCGTTATG GACGATCTTG   
  
  
- AGGTGAACAA TTGGGCCTTT ACAATCTTAC AGCTGGACGC CTTCGAAATC ACCACTTGAA ACGGAAGGTC   
  
  
- GAAGTGGTGT ACGGACTACT TTCGCAGAGT TGTTAGTTGG AGGCTCTGGT CGAAGAAGCC TACCAGTTTT   
  
  
- CGGATTTGGG TTTCGATCAT TGGTAACAGC TCGTCCTACA CTTGTGTTTG TGATGGGGAA AGGAGAGATC   
  
  
- CAAACGACTC CGTATGTCGA TGATAAGACG ACAAAAACTC AGAGAACTAC GATGAGAGGG ATCTCTATCG   
  
  
- GTCCTGTCCC ACTTACACCT CTTCGTCACG AACCGTGCAC TACAACACTT GTAGTAACGT ACACTTCCCC   
  
  
- TCCTCTCCTA ACTCGCGATA CTCCAACGCC CCTTTACCTC TCGCTCCTAC AACTACCGCC CTAAGTCCCG   
  
  
- ATAAGGTTAC CCAGTCTTCT GGCCACTATA CTAAGCCTTC GAATATAGTT ACGCCATAAC ACTGTCCGAG   
  
  
- TTCCATTTTC TCCTCTACCT ACGTGAAGTG AAACCCACCC TTCTATTTTT GAATTAGCAA TGTAGTCGAA   
  
  
- CCTCCAC

+     TCA-element

| Site Name | Organism | Position | Strand | Matrix score. | sequence | function |
| --- | --- | --- | --- | --- | --- | --- |
| TCA-element | Brassica oleracea | 2211 | + | 9 | TCAGAAGAGG | cis-acting element involved in salicylic acid responsiveness |
| TCA-element | Nicotiana tabacum | 296 | + | 9 | CCATCTTTTT | cis-acting element involved in salicylic acid responsiveness |

>HU11G01134.1   
+ +Up\_Stream \_Len000AGATAG ACATTCAGCC ACTTGCCAGT TCAGAGAGAG ATAGAGAGAG ATTGGGTAAG   
  
  
+ CCCTTTTTGC ACTACTTTTT CTCGCTTCCA TTTTTCTTTC TTAATTTTTA GTGATGATTT TCCAGAAGAT   
  
  
+ TTGATTTATT CATCATGCTT AACCAATTGG GTCTTTGAGT ATTATTGTTT ACTGGGTATT TGATCTTTTC   
  
  
+ ATATTCCTTT GGACTGGGTG GGTGGAACTT TTGAACTTTC TTATTAGTTG GGCATGCCCT TATCTTATTC   
  
  
+ CAATTCAAAA CCCATATTTT TCTTCAATTT TAATTATGGG TATTTTGAAA TTGAATGCTT TCCTTCACAG   
  
  
+ CAATACTATA TTACATATTT TACAGGAAAG TAATATTTTA CAGGAAAGTA TGTTGCTGTT TCAACCCTGT   
  
  
+ TTTGTTTCCT CTCTTTCCTT GGATATGATG TTCATTGCTC TTGTACCAAA CCTAACCTCA ACTTCCTGAA   
  
  
+ TTATTTAAGG CAATATTACC GTGTGCTGGT CAATTTTGGT TGTGGGTTTG TTTCTTAATG TATATGATTT   
  
  
+ ATGGTCAGGA TCCATGCTCC TCTTCCGTCC ACTCTATATC TCTCTCTCTC TCTCTCAATA CAAATTCAGA   
  
  
+ GACTTTCTCT TTCTGGGTTT ACTATTGATT GCAGAAGTTT GTAGTCTTGC GAGGCCTTGA GTCTTCTTCG   
  
  
+ AGGTAGAAAA TTTTGCTTTG CGCTTCCTTC CCCATATTTT AACACCCCTC CCCCCCCAAC GCAAGTTTTC   
  
  
+ TACATTTTTG TGCAATTCTT CAGCTATGAT TCCCCAATTT CACTTGTCAC ATCTCTATTT TCCTCTTTTT   
  
  
+ GGTTTCTTGA TTCTGTAGAA GGTTGTGTAT GAACTTCTTT GTTTCCCTTT TTCCTTTGTT ATTTTTAATG   
  
  
+ TTCCCATAGA TGATATAGAG ATCTGCTTTT CCTTGCTGTG AGAGGTATCT CTGAGAATGC ATGTAGCAAT   
  
  
+ CAAAATTCTG AGTTGTTAAT TCTATTGCAT GTCTATGGGT GATGATTGTG ATGATGTTTT TTCTGTTGCC   
  
  
+ CTCTTTCTCT CTTTCACTGA AATTGCTTTC CAGAATAGTT GTTTGACTCC TTTCTGTATC TTAATTGAGC   
  
  
+ CCTTGCTTTG GTTTGTGCTC TGGGAGTTCT TCTTTAGTAA TCCCTTGCTT GTATTTTCTG AAAGAGCAAG   
  
  
+ CAATAATCCT CATAGCATGT GTATAATCAT ACAATAGCAA CAGCAAACAT GGATCCTGCG CCCAGTTAGG   
  
  
+ TTTGGTTAGG TTAAGATATG CATATTCTTT TCTCTTGAAA AGAGTTGTCT AGGAAGGGTA ATTATGCCCA   
  
  
+ TTTCATGGAT AATAATGGTA CAATTAAGTT TGATTGGCTT AAATACCAAT TATTTAGCGT CTTTTTCTTT   
  
  
+ GGCTGTCCAG GGTTCCATTT CCATAAACAA CTTTGAGTAA GGCATAGAGA ACTAAATTTC TGCCAATTGT   
  
  
+ CAAGTGCAAG TTGCTCATAT GCAACAATAC TTGGAAATTT TGAGCTGAGC TGCTGGCATA TCATGAATAG   
  
  
+ TTCTAATGGT GGCGAGTTAT CACTTATCGT CCCTTGTGTG ACGAATTTCT CTTCCTAGGA AGCCCTCTAG   
  
  
+ TCAATGCCTA TCTGCTGTTG TGGAGTGAAA ATGGATACAT TTAGATCTCT ATAAACCCTA CTTTGGCAAG   
  
  
+ ATCCATGCTG ATTTTAGGAA GCCCTTTGAC CAGAGAATGA CATAACAACC TGATAGGGTG TTACATGCTA   
  
  
+ ACTTCGATTA TTTAATCAAA ATACGGAACT TCTATCAGTC AAAGCCTGCC CCAGGTTCTA CTGAATTAAA   
  
  
+ TTGTATTGAA TTGCTCTCAC TTGCTTATGA CGATTAAATA ACTGGATTTG CTAATTATTC TTTTTATTTT   
  
  
+ ATTCTTGGTG ATGTAGGGAA CTCAGGGAAT TGTAACCATA CCTGTTCCAG CTGAAAAGGG TACGGGAACA   
  
  
+ GTCATAGTTA CAGGGGAATT TCGCAATACA TTTAAGGGAG TAAGAAGCAG GATCATGTCT TTAGTTAGGC   
  
  
+ CGGCAGATAT GTCTGTGACT CCATATGGAA ATCCAACACT GTATTCACTT CAAGGCAATA ATAATAGCTC   
  
  
+ TGGTTTGTCT GGTCAACTGT ATGGCTCTGA AAAGCACAAG ATCAAATATG TGACCGAATC TTTTAATGGT   
  
  
+ CCAAGTTATG GCCTGAAATT CTTTGTTGAT TCTCCATCAG AAGAGCTCAT CCACCCATCA GATGCTACTC   
  
  
+ CTAACCCATT TGACTCTTCT TTCGTGGGCA TAAGACATGA TGAGAGTCCT TACCAAGGAA ATTATGGATC   
  
  
+ AGAATATGGG GTAAGCCCAT CTTCCGATGC AGTTGAATAT GATGAAGGTA ATAAGATAAG GCTAAAACTT   
  
  
+ CAAGAATTGG AGCATGCCCT GCTCGATGAA GATGATGAAG TGGATGAGGT GGCTATAGGG CCCGTGCTAG   
  
  
+ AACACAGCAT GGAGCTTGAT GAAGAGTGGG TCGCCCCATT GAGAAATGTG GTTTTCCATG ATTCACCAAA   
  
  
+ GGAGTCCACC TCCTCAGAGT CATCCAATGT CAGCAGCATT AGCAGCACCA AAGAAGTATC ACCTTGCTCT   
  
  
+ CCGAGTACTC CCAAACAATT GCTCTTTAAT TGTGCAAATA TGATTTCAGA GGGGAATTTT GAGGAAGCCG   
  
  
+ TGACTATGAT CAGCGAGCTT AGACAGATTG TTTCTATCCA AGGGGATCCA GCGCAGAGGA TAGCAGCCTA   
  
  
+ CATGGTAGAA GGCCTTGCTT CCCGTTTGGC TTCATCGGGA AAAGTTCTTT ACAAAGCTTT GAAATGCAAA   
  
  
+ GAGCCACCTT CATTTGACAG GCTAGCGGCT ATGCAAATCC TCTTTGAGGT GTGCCCATGT TTCAAATTCG   
  
  
+ GATTTATGGC CGCAAATGCA GCGATTATGG ATGCAATCAA AAACGAAAAA AGGGTACACA TCATAGATTT   
  
  
+ TGACATAAAC CAAGGGAATC AATACATAAA TCTCATGCAA TCCCTTGCTA AACAAGGTAA CAAGCTGACG   
  
  
+ CACTTGAAGT TGACTGGAGT TGATGACCCT GAGTCAGTTC AACGCCCTAT TGGTGGCCTA AAAAACATCG   
  
  
+ GACAAAGGCT GCAAGCATTA GCTGAATATC TTGGTGTGTC ATTTGAGTTC AAAGCAATAC CTGCTAGAAC   
  
  
+ TCCACTTGTT AACCCGGAAA TGTTAGAATG TCGACCTGCG GAAGCTTTAG TGGTGAACTT TGCCTTCCAG   
  
  
+ CTTCACCACA TGCCTGATGA AAGCGTCTCA ACAATCAACC TCCGAGACCA GCTTCTTCGG ATGGTCAAAA   
  
  
+ GCCTAAACCC AAAGCTAGTA ACCATTGTCG AGCAGGATGT GAACACAAAC ACTACCCCTT TCCTCTCTAG   
  
  
+ GTTTGCTGAG GCATACAGCT ACTATTCTGC TGTTTTTGAG TCTCTTGATG CTACTCTCCC TAGAGATAGC   
  
  
+ CAGGACAGGG TGAATGTGGA GAAGCAGTGC TTGGCACGTG ATGTTGTGAA CATCATTGCA TGTGAAGGGG   
  
  
+ AGGAGAGGAT TGAGCGCTAT GAGGTTGCGG GGAAATGGAG AGCGAGGATG TTGATGGCGG GATTCAGGGC   
  
  
+ TATTCCAATG GGTCAGAAGA CCGGTGATAT GATTCGGAAG CTTATATCAA TGCGGTATTG TGACAGGCTC   
  
  
+ AAGGTAAAAG AGGAGATGGA TGCACTTCAC TTTGGGTGGG AAGATAAAAA CTTAATCGTT ACATCAGCTT   
  
  
+ GGAGGTG  

- +Up\_Stream \_Len000TCTATC TGTAAGTCGG TGAACGGTCA AGTCTCTCTC TATCTCTCTC TAACCCATTC   
  
  
- GGGAAAAACG TGATGAAAAA GAGCGAAGGT AAAAAGAAAG AATTAAAAAT CACTACTAAA AGGTCTTCTA   
  
  
- AACTAAATAA GTAGTACGAA TTGGTTAACC CAGAAACTCA TAATAACAAA TGACCCATAA ACTAGAAAAG   
  
  
- TATAAGGAAA CCTGACCCAC CCACCTTGAA AACTTGAAAG AATAATCAAC CCGTACGGGA ATAGAATAAG   
  
  
- GTTAAGTTTT GGGTATAAAA AGAAGTTAAA ATTAATACCC ATAAAACTTT AACTTACGAA AGGAAGTGTC   
  
  
- GTTATGATAT AATGTATAAA ATGTCCTTTC ATTATAAAAT GTCCTTTCAT ACAACGACAA AGTTGGGACA   
  
  
- AAACAAAGGA GAGAAAGGAA CCTATACTAC AAGTAACGAG AACATGGTTT GGATTGGAGT TGAAGGACTT   
  
  
- AATAAATTCC GTTATAATGG CACACGACCA GTTAAAACCA ACACCCAAAC AAAGAATTAC ATATACTAAA   
  
  
- TACCAGTCCT AGGTACGAGG AGAAGGCAGG TGAGATATAG AGAGAGAGAG AGAGAGTTAT GTTTAAGTCT   
  
  
- CTGAAAGAGA AAGACCCAAA TGATAACTAA CGTCTTCAAA CATCAGAACG CTCCGGAACT CAGAAGAAGC   
  
  
- TCCATCTTTT AAAACGAAAC GCGAAGGAAG GGGTATAAAA TTGTGGGGAG GGGGGGGTTG CGTTCAAAAG   
  
  
- ATGTAAAAAC ACGTTAAGAA GTCGATACTA AGGGGTTAAA GTGAACAGTG TAGAGATAAA AGGAGAAAAA   
  
  
- CCAAAGAACT AAGACATCTT CCAACACATA CTTGAAGAAA CAAAGGGAAA AAGGAAACAA TAAAAATTAC   
  
  
- AAGGGTATCT ACTATATCTC TAGACGAAAA GGAACGACAC TCTCCATAGA GACTCTTACG TACATCGTTA   
  
  
- GTTTTAAGAC TCAACAATTA AGATAACGTA CAGATACCCA CTACTAACAC TACTACAAAA AAGACAACGG   
  
  
- GAGAAAGAGA GAAAGTGACT TTAACGAAAG GTCTTATCAA CAAACTGAGG AAAGACATAG AATTAACTCG   
  
  
- GGAACGAAAC CAAACACGAG ACCCTCAAGA AGAAATCATT AGGGAACGAA CATAAAAGAC TTTCTCGTTC   
  
  
- GTTATTAGGA GTATCGTACA CATATTAGTA TGTTATCGTT GTCGTTTGTA CCTAGGACGC GGGTCAATCC   
  
  
- AAACCAATCC AATTCTATAC GTATAAGAAA AGAGAACTTT TCTCAACAGA TCCTTCCCAT TAATACGGGT   
  
  
- AAAGTACCTA TTATTACCAT GTTAATTCAA ACTAACCGAA TTTATGGTTA ATAAATCGCA GAAAAAGAAA   
  
  
- CCGACAGGTC CCAAGGTAAA GGTATTTGTT GAAACTCATT CCGTATCTCT TGATTTAAAG ACGGTTAACA   
  
  
- GTTCACGTTC AACGAGTATA CGTTGTTATG AACCTTTAAA ACTCGACTCG ACGACCGTAT AGTACTTATC   
  
  
- AAGATTACCA CCGCTCAATA GTGAATAGCA GGGAACACAC TGCTTAAAGA GAAGGATCCT TCGGGAGATC   
  
  
- AGTTACGGAT AGACGACAAC ACCTCACTTT TACCTATGTA AATCTAGAGA TATTTGGGAT GAAACCGTTC   
  
  
- TAGGTACGAC TAAAATCCTT CGGGAAACTG GTCTCTTACT GTATTGTTGG ACTATCCCAC AATGTACGAT   
  
  
- TGAAGCTAAT AAATTAGTTT TATGCCTTGA AGATAGTCAG TTTCGGACGG GGTCCAAGAT GACTTAATTT   
  
  
- AACATAACTT AACGAGAGTG AACGAATACT GCTAATTTAT TGACCTAAAC GATTAATAAG AAAAATAAAA   
  
  
- TAAGAACCAC TACATCCCTT GAGTCCCTTA ACATTGGTAT GGACAAGGTC GACTTTTCCC ATGCCCTTGT   
  
  
- CAGTATCAAT GTCCCCTTAA AGCGTTATGT AAATTCCCTC ATTCTTCGTC CTAGTACAGA AATCAATCCG   
  
  
- GCCGTCTATA CAGACACTGA GGTATACCTT TAGGTTGTGA CATAAGTGAA GTTCCGTTAT TATTATCGAG   
  
  
- ACCAAACAGA CCAGTTGACA TACCGAGACT TTTCGTGTTC TAGTTTATAC ACTGGCTTAG AAAATTACCA   
  
  
- GGTTCAATAC CGGACTTTAA GAAACAACTA AGAGGTAGTC TTCTCGAGTA GGTGGGTAGT CTACGATGAG   
  
  
- GATTGGGTAA ACTGAGAAGA AAGCACCCGT ATTCTGTACT ACTCTCAGGA ATGGTTCCTT TAATACCTAG   
  
  
- TCTTATACCC CATTCGGGTA GAAGGCTACG TCAACTTATA CTACTTCCAT TATTCTATTC CGATTTTGAA   
  
  
- GTTCTTAACC TCGTACGGGA CGAGCTACTT CTACTACTTC ACCTACTCCA CCGATATCCC GGGCACGATC   
  
  
- TTGTGTCGTA CCTCGAACTA CTTCTCACCC AGCGGGGTAA CTCTTTACAC CAAAAGGTAC TAAGTGGTTT   
  
  
- CCTCAGGTGG AGGAGTCTCA GTAGGTTACA GTCGTCGTAA TCGTCGTGGT TTCTTCATAG TGGAACGAGA   
  
  
- GGCTCATGAG GGTTTGTTAA CGAGAAATTA ACACGTTTAT ACTAAAGTCT CCCCTTAAAA CTCCTTCGGC   
  
  
- ACTGATACTA GTCGCTCGAA TCTGTCTAAC AAAGATAGGT TCCCCTAGGT CGCGTCTCCT ATCGTCGGAT   
  
  
- GTACCATCTT CCGGAACGAA GGGCAAACCG AAGTAGCCCT TTTCAAGAAA TGTTTCGAAA CTTTACGTTT   
  
  
- CTCGGTGGAA GTAAACTGTC CGATCGCCGA TACGTTTAGG AGAAACTCCA CACGGGTACA AAGTTTAAGC   
  
  
- CTAAATACCG GCGTTTACGT CGCTAATACC TACGTTAGTT TTTGCTTTTT TCCCATGTGT AGTATCTAAA   
  
  
- ACTGTATTTG GTTCCCTTAG TTATGTATTT AGAGTACGTT AGGGAACGAT TTGTTCCATT GTTCGACTGC   
  
  
- GTGAACTTCA ACTGACCTCA ACTACTGGGA CTCAGTCAAG TTGCGGGATA ACCACCGGAT TTTTTGTAGC   
  
  
- CTGTTTCCGA CGTTCGTAAT CGACTTATAG AACCACACAG TAAACTCAAG TTTCGTTATG GACGATCTTG   
  
  
- AGGTGAACAA TTGGGCCTTT ACAATCTTAC AGCTGGACGC CTTCGAAATC ACCACTTGAA ACGGAAGGTC   
  
  
- GAAGTGGTGT ACGGACTACT TTCGCAGAGT TGTTAGTTGG AGGCTCTGGT CGAAGAAGCC TACCAGTTTT   
  
  
- CGGATTTGGG TTTCGATCAT TGGTAACAGC TCGTCCTACA CTTGTGTTTG TGATGGGGAA AGGAGAGATC   
  
  
- CAAACGACTC CGTATGTCGA TGATAAGACG ACAAAAACTC AGAGAACTAC GATGAGAGGG ATCTCTATCG   
  
  
- GTCCTGTCCC ACTTACACCT CTTCGTCACG AACCGTGCAC TACAACACTT GTAGTAACGT ACACTTCCCC   
  
  
- TCCTCTCCTA ACTCGCGATA CTCCAACGCC CCTTTACCTC TCGCTCCTAC AACTACCGCC CTAAGTCCCG   
  
  
- ATAAGGTTAC CCAGTCTTCT GGCCACTATA CTAAGCCTTC GAATATAGTT ACGCCATAAC ACTGTCCGAG   
  
  
- TTCCATTTTC TCCTCTACCT ACGTGAAGTG AAACCCACCC TTCTATTTTT GAATTAGCAA TGTAGTCGAA   
  
  
- CCTCCAC

+     TCCC-motif

| Site Name | Organism | Position | Strand | Matrix score. | sequence | function |
| --- | --- | --- | --- | --- | --- | --- |
| TCCC-motif | Spinacia oleracea | 3419 | + | 7 | TCTCCCT | part of a light responsive element |

>HU11G01134.1   
+ +Up\_Stream \_Len000AGATAG ACATTCAGCC ACTTGCCAGT TCAGAGAGAG ATAGAGAGAG ATTGGGTAAG   
  
  
+ CCCTTTTTGC ACTACTTTTT CTCGCTTCCA TTTTTCTTTC TTAATTTTTA GTGATGATTT TCCAGAAGAT   
  
  
+ TTGATTTATT CATCATGCTT AACCAATTGG GTCTTTGAGT ATTATTGTTT ACTGGGTATT TGATCTTTTC   
  
  
+ ATATTCCTTT GGACTGGGTG GGTGGAACTT TTGAACTTTC TTATTAGTTG GGCATGCCCT TATCTTATTC   
  
  
+ CAATTCAAAA CCCATATTTT TCTTCAATTT TAATTATGGG TATTTTGAAA TTGAATGCTT TCCTTCACAG   
  
  
+ CAATACTATA TTACATATTT TACAGGAAAG TAATATTTTA CAGGAAAGTA TGTTGCTGTT TCAACCCTGT   
  
  
+ TTTGTTTCCT CTCTTTCCTT GGATATGATG TTCATTGCTC TTGTACCAAA CCTAACCTCA ACTTCCTGAA   
  
  
+ TTATTTAAGG CAATATTACC GTGTGCTGGT CAATTTTGGT TGTGGGTTTG TTTCTTAATG TATATGATTT   
  
  
+ ATGGTCAGGA TCCATGCTCC TCTTCCGTCC ACTCTATATC TCTCTCTCTC TCTCTCAATA CAAATTCAGA   
  
  
+ GACTTTCTCT TTCTGGGTTT ACTATTGATT GCAGAAGTTT GTAGTCTTGC GAGGCCTTGA GTCTTCTTCG   
  
  
+ AGGTAGAAAA TTTTGCTTTG CGCTTCCTTC CCCATATTTT AACACCCCTC CCCCCCCAAC GCAAGTTTTC   
  
  
+ TACATTTTTG TGCAATTCTT CAGCTATGAT TCCCCAATTT CACTTGTCAC ATCTCTATTT TCCTCTTTTT   
  
  
+ GGTTTCTTGA TTCTGTAGAA GGTTGTGTAT GAACTTCTTT GTTTCCCTTT TTCCTTTGTT ATTTTTAATG   
  
  
+ TTCCCATAGA TGATATAGAG ATCTGCTTTT CCTTGCTGTG AGAGGTATCT CTGAGAATGC ATGTAGCAAT   
  
  
+ CAAAATTCTG AGTTGTTAAT TCTATTGCAT GTCTATGGGT GATGATTGTG ATGATGTTTT TTCTGTTGCC   
  
  
+ CTCTTTCTCT CTTTCACTGA AATTGCTTTC CAGAATAGTT GTTTGACTCC TTTCTGTATC TTAATTGAGC   
  
  
+ CCTTGCTTTG GTTTGTGCTC TGGGAGTTCT TCTTTAGTAA TCCCTTGCTT GTATTTTCTG AAAGAGCAAG   
  
  
+ CAATAATCCT CATAGCATGT GTATAATCAT ACAATAGCAA CAGCAAACAT GGATCCTGCG CCCAGTTAGG   
  
  
+ TTTGGTTAGG TTAAGATATG CATATTCTTT TCTCTTGAAA AGAGTTGTCT AGGAAGGGTA ATTATGCCCA   
  
  
+ TTTCATGGAT AATAATGGTA CAATTAAGTT TGATTGGCTT AAATACCAAT TATTTAGCGT CTTTTTCTTT   
  
  
+ GGCTGTCCAG GGTTCCATTT CCATAAACAA CTTTGAGTAA GGCATAGAGA ACTAAATTTC TGCCAATTGT   
  
  
+ CAAGTGCAAG TTGCTCATAT GCAACAATAC TTGGAAATTT TGAGCTGAGC TGCTGGCATA TCATGAATAG   
  
  
+ TTCTAATGGT GGCGAGTTAT CACTTATCGT CCCTTGTGTG ACGAATTTCT CTTCCTAGGA AGCCCTCTAG   
  
  
+ TCAATGCCTA TCTGCTGTTG TGGAGTGAAA ATGGATACAT TTAGATCTCT ATAAACCCTA CTTTGGCAAG   
  
  
+ ATCCATGCTG ATTTTAGGAA GCCCTTTGAC CAGAGAATGA CATAACAACC TGATAGGGTG TTACATGCTA   
  
  
+ ACTTCGATTA TTTAATCAAA ATACGGAACT TCTATCAGTC AAAGCCTGCC CCAGGTTCTA CTGAATTAAA   
  
  
+ TTGTATTGAA TTGCTCTCAC TTGCTTATGA CGATTAAATA ACTGGATTTG CTAATTATTC TTTTTATTTT   
  
  
+ ATTCTTGGTG ATGTAGGGAA CTCAGGGAAT TGTAACCATA CCTGTTCCAG CTGAAAAGGG TACGGGAACA   
  
  
+ GTCATAGTTA CAGGGGAATT TCGCAATACA TTTAAGGGAG TAAGAAGCAG GATCATGTCT TTAGTTAGGC   
  
  
+ CGGCAGATAT GTCTGTGACT CCATATGGAA ATCCAACACT GTATTCACTT CAAGGCAATA ATAATAGCTC   
  
  
+ TGGTTTGTCT GGTCAACTGT ATGGCTCTGA AAAGCACAAG ATCAAATATG TGACCGAATC TTTTAATGGT   
  
  
+ CCAAGTTATG GCCTGAAATT CTTTGTTGAT TCTCCATCAG AAGAGCTCAT CCACCCATCA GATGCTACTC   
  
  
+ CTAACCCATT TGACTCTTCT TTCGTGGGCA TAAGACATGA TGAGAGTCCT TACCAAGGAA ATTATGGATC   
  
  
+ AGAATATGGG GTAAGCCCAT CTTCCGATGC AGTTGAATAT GATGAAGGTA ATAAGATAAG GCTAAAACTT   
  
  
+ CAAGAATTGG AGCATGCCCT GCTCGATGAA GATGATGAAG TGGATGAGGT GGCTATAGGG CCCGTGCTAG   
  
  
+ AACACAGCAT GGAGCTTGAT GAAGAGTGGG TCGCCCCATT GAGAAATGTG GTTTTCCATG ATTCACCAAA   
  
  
+ GGAGTCCACC TCCTCAGAGT CATCCAATGT CAGCAGCATT AGCAGCACCA AAGAAGTATC ACCTTGCTCT   
  
  
+ CCGAGTACTC CCAAACAATT GCTCTTTAAT TGTGCAAATA TGATTTCAGA GGGGAATTTT GAGGAAGCCG   
  
  
+ TGACTATGAT CAGCGAGCTT AGACAGATTG TTTCTATCCA AGGGGATCCA GCGCAGAGGA TAGCAGCCTA   
  
  
+ CATGGTAGAA GGCCTTGCTT CCCGTTTGGC TTCATCGGGA AAAGTTCTTT ACAAAGCTTT GAAATGCAAA   
  
  
+ GAGCCACCTT CATTTGACAG GCTAGCGGCT ATGCAAATCC TCTTTGAGGT GTGCCCATGT TTCAAATTCG   
  
  
+ GATTTATGGC CGCAAATGCA GCGATTATGG ATGCAATCAA AAACGAAAAA AGGGTACACA TCATAGATTT   
  
  
+ TGACATAAAC CAAGGGAATC AATACATAAA TCTCATGCAA TCCCTTGCTA AACAAGGTAA CAAGCTGACG   
  
  
+ CACTTGAAGT TGACTGGAGT TGATGACCCT GAGTCAGTTC AACGCCCTAT TGGTGGCCTA AAAAACATCG   
  
  
+ GACAAAGGCT GCAAGCATTA GCTGAATATC TTGGTGTGTC ATTTGAGTTC AAAGCAATAC CTGCTAGAAC   
  
  
+ TCCACTTGTT AACCCGGAAA TGTTAGAATG TCGACCTGCG GAAGCTTTAG TGGTGAACTT TGCCTTCCAG   
  
  
+ CTTCACCACA TGCCTGATGA AAGCGTCTCA ACAATCAACC TCCGAGACCA GCTTCTTCGG ATGGTCAAAA   
  
  
+ GCCTAAACCC AAAGCTAGTA ACCATTGTCG AGCAGGATGT GAACACAAAC ACTACCCCTT TCCTCTCTAG   
  
  
+ GTTTGCTGAG GCATACAGCT ACTATTCTGC TGTTTTTGAG TCTCTTGATG CTACTCTCCC TAGAGATAGC   
  
  
+ CAGGACAGGG TGAATGTGGA GAAGCAGTGC TTGGCACGTG ATGTTGTGAA CATCATTGCA TGTGAAGGGG   
  
  
+ AGGAGAGGAT TGAGCGCTAT GAGGTTGCGG GGAAATGGAG AGCGAGGATG TTGATGGCGG GATTCAGGGC   
  
  
+ TATTCCAATG GGTCAGAAGA CCGGTGATAT GATTCGGAAG CTTATATCAA TGCGGTATTG TGACAGGCTC   
  
  
+ AAGGTAAAAG AGGAGATGGA TGCACTTCAC TTTGGGTGGG AAGATAAAAA CTTAATCGTT ACATCAGCTT   
  
  
+ GGAGGTG  

- +Up\_Stream \_Len000TCTATC TGTAAGTCGG TGAACGGTCA AGTCTCTCTC TATCTCTCTC TAACCCATTC   
  
  
- GGGAAAAACG TGATGAAAAA GAGCGAAGGT AAAAAGAAAG AATTAAAAAT CACTACTAAA AGGTCTTCTA   
  
  
- AACTAAATAA GTAGTACGAA TTGGTTAACC CAGAAACTCA TAATAACAAA TGACCCATAA ACTAGAAAAG   
  
  
- TATAAGGAAA CCTGACCCAC CCACCTTGAA AACTTGAAAG AATAATCAAC CCGTACGGGA ATAGAATAAG   
  
  
- GTTAAGTTTT GGGTATAAAA AGAAGTTAAA ATTAATACCC ATAAAACTTT AACTTACGAA AGGAAGTGTC   
  
  
- GTTATGATAT AATGTATAAA ATGTCCTTTC ATTATAAAAT GTCCTTTCAT ACAACGACAA AGTTGGGACA   
  
  
- AAACAAAGGA GAGAAAGGAA CCTATACTAC AAGTAACGAG AACATGGTTT GGATTGGAGT TGAAGGACTT   
  
  
- AATAAATTCC GTTATAATGG CACACGACCA GTTAAAACCA ACACCCAAAC AAAGAATTAC ATATACTAAA   
  
  
- TACCAGTCCT AGGTACGAGG AGAAGGCAGG TGAGATATAG AGAGAGAGAG AGAGAGTTAT GTTTAAGTCT   
  
  
- CTGAAAGAGA AAGACCCAAA TGATAACTAA CGTCTTCAAA CATCAGAACG CTCCGGAACT CAGAAGAAGC   
  
  
- TCCATCTTTT AAAACGAAAC GCGAAGGAAG GGGTATAAAA TTGTGGGGAG GGGGGGGTTG CGTTCAAAAG   
  
  
- ATGTAAAAAC ACGTTAAGAA GTCGATACTA AGGGGTTAAA GTGAACAGTG TAGAGATAAA AGGAGAAAAA   
  
  
- CCAAAGAACT AAGACATCTT CCAACACATA CTTGAAGAAA CAAAGGGAAA AAGGAAACAA TAAAAATTAC   
  
  
- AAGGGTATCT ACTATATCTC TAGACGAAAA GGAACGACAC TCTCCATAGA GACTCTTACG TACATCGTTA   
  
  
- GTTTTAAGAC TCAACAATTA AGATAACGTA CAGATACCCA CTACTAACAC TACTACAAAA AAGACAACGG   
  
  
- GAGAAAGAGA GAAAGTGACT TTAACGAAAG GTCTTATCAA CAAACTGAGG AAAGACATAG AATTAACTCG   
  
  
- GGAACGAAAC CAAACACGAG ACCCTCAAGA AGAAATCATT AGGGAACGAA CATAAAAGAC TTTCTCGTTC   
  
  
- GTTATTAGGA GTATCGTACA CATATTAGTA TGTTATCGTT GTCGTTTGTA CCTAGGACGC GGGTCAATCC   
  
  
- AAACCAATCC AATTCTATAC GTATAAGAAA AGAGAACTTT TCTCAACAGA TCCTTCCCAT TAATACGGGT   
  
  
- AAAGTACCTA TTATTACCAT GTTAATTCAA ACTAACCGAA TTTATGGTTA ATAAATCGCA GAAAAAGAAA   
  
  
- CCGACAGGTC CCAAGGTAAA GGTATTTGTT GAAACTCATT CCGTATCTCT TGATTTAAAG ACGGTTAACA   
  
  
- GTTCACGTTC AACGAGTATA CGTTGTTATG AACCTTTAAA ACTCGACTCG ACGACCGTAT AGTACTTATC   
  
  
- AAGATTACCA CCGCTCAATA GTGAATAGCA GGGAACACAC TGCTTAAAGA GAAGGATCCT TCGGGAGATC   
  
  
- AGTTACGGAT AGACGACAAC ACCTCACTTT TACCTATGTA AATCTAGAGA TATTTGGGAT GAAACCGTTC   
  
  
- TAGGTACGAC TAAAATCCTT CGGGAAACTG GTCTCTTACT GTATTGTTGG ACTATCCCAC AATGTACGAT   
  
  
- TGAAGCTAAT AAATTAGTTT TATGCCTTGA AGATAGTCAG TTTCGGACGG GGTCCAAGAT GACTTAATTT   
  
  
- AACATAACTT AACGAGAGTG AACGAATACT GCTAATTTAT TGACCTAAAC GATTAATAAG AAAAATAAAA   
  
  
- TAAGAACCAC TACATCCCTT GAGTCCCTTA ACATTGGTAT GGACAAGGTC GACTTTTCCC ATGCCCTTGT   
  
  
- CAGTATCAAT GTCCCCTTAA AGCGTTATGT AAATTCCCTC ATTCTTCGTC CTAGTACAGA AATCAATCCG   
  
  
- GCCGTCTATA CAGACACTGA GGTATACCTT TAGGTTGTGA CATAAGTGAA GTTCCGTTAT TATTATCGAG   
  
  
- ACCAAACAGA CCAGTTGACA TACCGAGACT TTTCGTGTTC TAGTTTATAC ACTGGCTTAG AAAATTACCA   
  
  
- GGTTCAATAC CGGACTTTAA GAAACAACTA AGAGGTAGTC TTCTCGAGTA GGTGGGTAGT CTACGATGAG   
  
  
- GATTGGGTAA ACTGAGAAGA AAGCACCCGT ATTCTGTACT ACTCTCAGGA ATGGTTCCTT TAATACCTAG   
  
  
- TCTTATACCC CATTCGGGTA GAAGGCTACG TCAACTTATA CTACTTCCAT TATTCTATTC CGATTTTGAA   
  
  
- GTTCTTAACC TCGTACGGGA CGAGCTACTT CTACTACTTC ACCTACTCCA CCGATATCCC GGGCACGATC   
  
  
- TTGTGTCGTA CCTCGAACTA CTTCTCACCC AGCGGGGTAA CTCTTTACAC CAAAAGGTAC TAAGTGGTTT   
  
  
- CCTCAGGTGG AGGAGTCTCA GTAGGTTACA GTCGTCGTAA TCGTCGTGGT TTCTTCATAG TGGAACGAGA   
  
  
- GGCTCATGAG GGTTTGTTAA CGAGAAATTA ACACGTTTAT ACTAAAGTCT CCCCTTAAAA CTCCTTCGGC   
  
  
- ACTGATACTA GTCGCTCGAA TCTGTCTAAC AAAGATAGGT TCCCCTAGGT CGCGTCTCCT ATCGTCGGAT   
  
  
- GTACCATCTT CCGGAACGAA GGGCAAACCG AAGTAGCCCT TTTCAAGAAA TGTTTCGAAA CTTTACGTTT   
  
  
- CTCGGTGGAA GTAAACTGTC CGATCGCCGA TACGTTTAGG AGAAACTCCA CACGGGTACA AAGTTTAAGC   
  
  
- CTAAATACCG GCGTTTACGT CGCTAATACC TACGTTAGTT TTTGCTTTTT TCCCATGTGT AGTATCTAAA   
  
  
- ACTGTATTTG GTTCCCTTAG TTATGTATTT AGAGTACGTT AGGGAACGAT TTGTTCCATT GTTCGACTGC   
  
  
- GTGAACTTCA ACTGACCTCA ACTACTGGGA CTCAGTCAAG TTGCGGGATA ACCACCGGAT TTTTTGTAGC   
  
  
- CTGTTTCCGA CGTTCGTAAT CGACTTATAG AACCACACAG TAAACTCAAG TTTCGTTATG GACGATCTTG   
  
  
- AGGTGAACAA TTGGGCCTTT ACAATCTTAC AGCTGGACGC CTTCGAAATC ACCACTTGAA ACGGAAGGTC   
  
  
- GAAGTGGTGT ACGGACTACT TTCGCAGAGT TGTTAGTTGG AGGCTCTGGT CGAAGAAGCC TACCAGTTTT   
  
  
- CGGATTTGGG TTTCGATCAT TGGTAACAGC TCGTCCTACA CTTGTGTTTG TGATGGGGAA AGGAGAGATC   
  
  
- CAAACGACTC CGTATGTCGA TGATAAGACG ACAAAAACTC AGAGAACTAC GATGAGAGGG ATCTCTATCG   
  
  
- GTCCTGTCCC ACTTACACCT CTTCGTCACG AACCGTGCAC TACAACACTT GTAGTAACGT ACACTTCCCC   
  
  
- TCCTCTCCTA ACTCGCGATA CTCCAACGCC CCTTTACCTC TCGCTCCTAC AACTACCGCC CTAAGTCCCG   
  
  
- ATAAGGTTAC CCAGTCTTCT GGCCACTATA CTAAGCCTTC GAATATAGTT ACGCCATAAC ACTGTCCGAG   
  
  
- TTCCATTTTC TCCTCTACCT ACGTGAAGTG AAACCCACCC TTCTATTTTT GAATTAGCAA TGTAGTCGAA   
  
  
- CCTCCAC

+     TCT-motif

| Site Name | Organism | Position | Strand | Matrix score. | sequence | function |
| --- | --- | --- | --- | --- | --- | --- |
| TCT-motif | Arabidopsis thaliana | 2004 | - | 6 | TCTTAC | part of a light responsive element |

>HU11G01134.1   
+ +Up\_Stream \_Len000AGATAG ACATTCAGCC ACTTGCCAGT TCAGAGAGAG ATAGAGAGAG ATTGGGTAAG   
  
  
+ CCCTTTTTGC ACTACTTTTT CTCGCTTCCA TTTTTCTTTC TTAATTTTTA GTGATGATTT TCCAGAAGAT   
  
  
+ TTGATTTATT CATCATGCTT AACCAATTGG GTCTTTGAGT ATTATTGTTT ACTGGGTATT TGATCTTTTC   
  
  
+ ATATTCCTTT GGACTGGGTG GGTGGAACTT TTGAACTTTC TTATTAGTTG GGCATGCCCT TATCTTATTC   
  
  
+ CAATTCAAAA CCCATATTTT TCTTCAATTT TAATTATGGG TATTTTGAAA TTGAATGCTT TCCTTCACAG   
  
  
+ CAATACTATA TTACATATTT TACAGGAAAG TAATATTTTA CAGGAAAGTA TGTTGCTGTT TCAACCCTGT   
  
  
+ TTTGTTTCCT CTCTTTCCTT GGATATGATG TTCATTGCTC TTGTACCAAA CCTAACCTCA ACTTCCTGAA   
  
  
+ TTATTTAAGG CAATATTACC GTGTGCTGGT CAATTTTGGT TGTGGGTTTG TTTCTTAATG TATATGATTT   
  
  
+ ATGGTCAGGA TCCATGCTCC TCTTCCGTCC ACTCTATATC TCTCTCTCTC TCTCTCAATA CAAATTCAGA   
  
  
+ GACTTTCTCT TTCTGGGTTT ACTATTGATT GCAGAAGTTT GTAGTCTTGC GAGGCCTTGA GTCTTCTTCG   
  
  
+ AGGTAGAAAA TTTTGCTTTG CGCTTCCTTC CCCATATTTT AACACCCCTC CCCCCCCAAC GCAAGTTTTC   
  
  
+ TACATTTTTG TGCAATTCTT CAGCTATGAT TCCCCAATTT CACTTGTCAC ATCTCTATTT TCCTCTTTTT   
  
  
+ GGTTTCTTGA TTCTGTAGAA GGTTGTGTAT GAACTTCTTT GTTTCCCTTT TTCCTTTGTT ATTTTTAATG   
  
  
+ TTCCCATAGA TGATATAGAG ATCTGCTTTT CCTTGCTGTG AGAGGTATCT CTGAGAATGC ATGTAGCAAT   
  
  
+ CAAAATTCTG AGTTGTTAAT TCTATTGCAT GTCTATGGGT GATGATTGTG ATGATGTTTT TTCTGTTGCC   
  
  
+ CTCTTTCTCT CTTTCACTGA AATTGCTTTC CAGAATAGTT GTTTGACTCC TTTCTGTATC TTAATTGAGC   
  
  
+ CCTTGCTTTG GTTTGTGCTC TGGGAGTTCT TCTTTAGTAA TCCCTTGCTT GTATTTTCTG AAAGAGCAAG   
  
  
+ CAATAATCCT CATAGCATGT GTATAATCAT ACAATAGCAA CAGCAAACAT GGATCCTGCG CCCAGTTAGG   
  
  
+ TTTGGTTAGG TTAAGATATG CATATTCTTT TCTCTTGAAA AGAGTTGTCT AGGAAGGGTA ATTATGCCCA   
  
  
+ TTTCATGGAT AATAATGGTA CAATTAAGTT TGATTGGCTT AAATACCAAT TATTTAGCGT CTTTTTCTTT   
  
  
+ GGCTGTCCAG GGTTCCATTT CCATAAACAA CTTTGAGTAA GGCATAGAGA ACTAAATTTC TGCCAATTGT   
  
  
+ CAAGTGCAAG TTGCTCATAT GCAACAATAC TTGGAAATTT TGAGCTGAGC TGCTGGCATA TCATGAATAG   
  
  
+ TTCTAATGGT GGCGAGTTAT CACTTATCGT CCCTTGTGTG ACGAATTTCT CTTCCTAGGA AGCCCTCTAG   
  
  
+ TCAATGCCTA TCTGCTGTTG TGGAGTGAAA ATGGATACAT TTAGATCTCT ATAAACCCTA CTTTGGCAAG   
  
  
+ ATCCATGCTG ATTTTAGGAA GCCCTTTGAC CAGAGAATGA CATAACAACC TGATAGGGTG TTACATGCTA   
  
  
+ ACTTCGATTA TTTAATCAAA ATACGGAACT TCTATCAGTC AAAGCCTGCC CCAGGTTCTA CTGAATTAAA   
  
  
+ TTGTATTGAA TTGCTCTCAC TTGCTTATGA CGATTAAATA ACTGGATTTG CTAATTATTC TTTTTATTTT   
  
  
+ ATTCTTGGTG ATGTAGGGAA CTCAGGGAAT TGTAACCATA CCTGTTCCAG CTGAAAAGGG TACGGGAACA   
  
  
+ GTCATAGTTA CAGGGGAATT TCGCAATACA TTTAAGGGAG TAAGAAGCAG GATCATGTCT TTAGTTAGGC   
  
  
+ CGGCAGATAT GTCTGTGACT CCATATGGAA ATCCAACACT GTATTCACTT CAAGGCAATA ATAATAGCTC   
  
  
+ TGGTTTGTCT GGTCAACTGT ATGGCTCTGA AAAGCACAAG ATCAAATATG TGACCGAATC TTTTAATGGT   
  
  
+ CCAAGTTATG GCCTGAAATT CTTTGTTGAT TCTCCATCAG AAGAGCTCAT CCACCCATCA GATGCTACTC   
  
  
+ CTAACCCATT TGACTCTTCT TTCGTGGGCA TAAGACATGA TGAGAGTCCT TACCAAGGAA ATTATGGATC   
  
  
+ AGAATATGGG GTAAGCCCAT CTTCCGATGC AGTTGAATAT GATGAAGGTA ATAAGATAAG GCTAAAACTT   
  
  
+ CAAGAATTGG AGCATGCCCT GCTCGATGAA GATGATGAAG TGGATGAGGT GGCTATAGGG CCCGTGCTAG   
  
  
+ AACACAGCAT GGAGCTTGAT GAAGAGTGGG TCGCCCCATT GAGAAATGTG GTTTTCCATG ATTCACCAAA   
  
  
+ GGAGTCCACC TCCTCAGAGT CATCCAATGT CAGCAGCATT AGCAGCACCA AAGAAGTATC ACCTTGCTCT   
  
  
+ CCGAGTACTC CCAAACAATT GCTCTTTAAT TGTGCAAATA TGATTTCAGA GGGGAATTTT GAGGAAGCCG   
  
  
+ TGACTATGAT CAGCGAGCTT AGACAGATTG TTTCTATCCA AGGGGATCCA GCGCAGAGGA TAGCAGCCTA   
  
  
+ CATGGTAGAA GGCCTTGCTT CCCGTTTGGC TTCATCGGGA AAAGTTCTTT ACAAAGCTTT GAAATGCAAA   
  
  
+ GAGCCACCTT CATTTGACAG GCTAGCGGCT ATGCAAATCC TCTTTGAGGT GTGCCCATGT TTCAAATTCG   
  
  
+ GATTTATGGC CGCAAATGCA GCGATTATGG ATGCAATCAA AAACGAAAAA AGGGTACACA TCATAGATTT   
  
  
+ TGACATAAAC CAAGGGAATC AATACATAAA TCTCATGCAA TCCCTTGCTA AACAAGGTAA CAAGCTGACG   
  
  
+ CACTTGAAGT TGACTGGAGT TGATGACCCT GAGTCAGTTC AACGCCCTAT TGGTGGCCTA AAAAACATCG   
  
  
+ GACAAAGGCT GCAAGCATTA GCTGAATATC TTGGTGTGTC ATTTGAGTTC AAAGCAATAC CTGCTAGAAC   
  
  
+ TCCACTTGTT AACCCGGAAA TGTTAGAATG TCGACCTGCG GAAGCTTTAG TGGTGAACTT TGCCTTCCAG   
  
  
+ CTTCACCACA TGCCTGATGA AAGCGTCTCA ACAATCAACC TCCGAGACCA GCTTCTTCGG ATGGTCAAAA   
  
  
+ GCCTAAACCC AAAGCTAGTA ACCATTGTCG AGCAGGATGT GAACACAAAC ACTACCCCTT TCCTCTCTAG   
  
  
+ GTTTGCTGAG GCATACAGCT ACTATTCTGC TGTTTTTGAG TCTCTTGATG CTACTCTCCC TAGAGATAGC   
  
  
+ CAGGACAGGG TGAATGTGGA GAAGCAGTGC TTGGCACGTG ATGTTGTGAA CATCATTGCA TGTGAAGGGG   
  
  
+ AGGAGAGGAT TGAGCGCTAT GAGGTTGCGG GGAAATGGAG AGCGAGGATG TTGATGGCGG GATTCAGGGC   
  
  
+ TATTCCAATG GGTCAGAAGA CCGGTGATAT GATTCGGAAG CTTATATCAA TGCGGTATTG TGACAGGCTC   
  
  
+ AAGGTAAAAG AGGAGATGGA TGCACTTCAC TTTGGGTGGG AAGATAAAAA CTTAATCGTT ACATCAGCTT   
  
  
+ GGAGGTG  

- +Up\_Stream \_Len000TCTATC TGTAAGTCGG TGAACGGTCA AGTCTCTCTC TATCTCTCTC TAACCCATTC   
  
  
- GGGAAAAACG TGATGAAAAA GAGCGAAGGT AAAAAGAAAG AATTAAAAAT CACTACTAAA AGGTCTTCTA   
  
  
- AACTAAATAA GTAGTACGAA TTGGTTAACC CAGAAACTCA TAATAACAAA TGACCCATAA ACTAGAAAAG   
  
  
- TATAAGGAAA CCTGACCCAC CCACCTTGAA AACTTGAAAG AATAATCAAC CCGTACGGGA ATAGAATAAG   
  
  
- GTTAAGTTTT GGGTATAAAA AGAAGTTAAA ATTAATACCC ATAAAACTTT AACTTACGAA AGGAAGTGTC   
  
  
- GTTATGATAT AATGTATAAA ATGTCCTTTC ATTATAAAAT GTCCTTTCAT ACAACGACAA AGTTGGGACA   
  
  
- AAACAAAGGA GAGAAAGGAA CCTATACTAC AAGTAACGAG AACATGGTTT GGATTGGAGT TGAAGGACTT   
  
  
- AATAAATTCC GTTATAATGG CACACGACCA GTTAAAACCA ACACCCAAAC AAAGAATTAC ATATACTAAA   
  
  
- TACCAGTCCT AGGTACGAGG AGAAGGCAGG TGAGATATAG AGAGAGAGAG AGAGAGTTAT GTTTAAGTCT   
  
  
- CTGAAAGAGA AAGACCCAAA TGATAACTAA CGTCTTCAAA CATCAGAACG CTCCGGAACT CAGAAGAAGC   
  
  
- TCCATCTTTT AAAACGAAAC GCGAAGGAAG GGGTATAAAA TTGTGGGGAG GGGGGGGTTG CGTTCAAAAG   
  
  
- ATGTAAAAAC ACGTTAAGAA GTCGATACTA AGGGGTTAAA GTGAACAGTG TAGAGATAAA AGGAGAAAAA   
  
  
- CCAAAGAACT AAGACATCTT CCAACACATA CTTGAAGAAA CAAAGGGAAA AAGGAAACAA TAAAAATTAC   
  
  
- AAGGGTATCT ACTATATCTC TAGACGAAAA GGAACGACAC TCTCCATAGA GACTCTTACG TACATCGTTA   
  
  
- GTTTTAAGAC TCAACAATTA AGATAACGTA CAGATACCCA CTACTAACAC TACTACAAAA AAGACAACGG   
  
  
- GAGAAAGAGA GAAAGTGACT TTAACGAAAG GTCTTATCAA CAAACTGAGG AAAGACATAG AATTAACTCG   
  
  
- GGAACGAAAC CAAACACGAG ACCCTCAAGA AGAAATCATT AGGGAACGAA CATAAAAGAC TTTCTCGTTC   
  
  
- GTTATTAGGA GTATCGTACA CATATTAGTA TGTTATCGTT GTCGTTTGTA CCTAGGACGC GGGTCAATCC   
  
  
- AAACCAATCC AATTCTATAC GTATAAGAAA AGAGAACTTT TCTCAACAGA TCCTTCCCAT TAATACGGGT   
  
  
- AAAGTACCTA TTATTACCAT GTTAATTCAA ACTAACCGAA TTTATGGTTA ATAAATCGCA GAAAAAGAAA   
  
  
- CCGACAGGTC CCAAGGTAAA GGTATTTGTT GAAACTCATT CCGTATCTCT TGATTTAAAG ACGGTTAACA   
  
  
- GTTCACGTTC AACGAGTATA CGTTGTTATG AACCTTTAAA ACTCGACTCG ACGACCGTAT AGTACTTATC   
  
  
- AAGATTACCA CCGCTCAATA GTGAATAGCA GGGAACACAC TGCTTAAAGA GAAGGATCCT TCGGGAGATC   
  
  
- AGTTACGGAT AGACGACAAC ACCTCACTTT TACCTATGTA AATCTAGAGA TATTTGGGAT GAAACCGTTC   
  
  
- TAGGTACGAC TAAAATCCTT CGGGAAACTG GTCTCTTACT GTATTGTTGG ACTATCCCAC AATGTACGAT   
  
  
- TGAAGCTAAT AAATTAGTTT TATGCCTTGA AGATAGTCAG TTTCGGACGG GGTCCAAGAT GACTTAATTT   
  
  
- AACATAACTT AACGAGAGTG AACGAATACT GCTAATTTAT TGACCTAAAC GATTAATAAG AAAAATAAAA   
  
  
- TAAGAACCAC TACATCCCTT GAGTCCCTTA ACATTGGTAT GGACAAGGTC GACTTTTCCC ATGCCCTTGT   
  
  
- CAGTATCAAT GTCCCCTTAA AGCGTTATGT AAATTCCCTC ATTCTTCGTC CTAGTACAGA AATCAATCCG   
  
  
- GCCGTCTATA CAGACACTGA GGTATACCTT TAGGTTGTGA CATAAGTGAA GTTCCGTTAT TATTATCGAG   
  
  
- ACCAAACAGA CCAGTTGACA TACCGAGACT TTTCGTGTTC TAGTTTATAC ACTGGCTTAG AAAATTACCA   
  
  
- GGTTCAATAC CGGACTTTAA GAAACAACTA AGAGGTAGTC TTCTCGAGTA GGTGGGTAGT CTACGATGAG   
  
  
- GATTGGGTAA ACTGAGAAGA AAGCACCCGT ATTCTGTACT ACTCTCAGGA ATGGTTCCTT TAATACCTAG   
  
  
- TCTTATACCC CATTCGGGTA GAAGGCTACG TCAACTTATA CTACTTCCAT TATTCTATTC CGATTTTGAA   
  
  
- GTTCTTAACC TCGTACGGGA CGAGCTACTT CTACTACTTC ACCTACTCCA CCGATATCCC GGGCACGATC   
  
  
- TTGTGTCGTA CCTCGAACTA CTTCTCACCC AGCGGGGTAA CTCTTTACAC CAAAAGGTAC TAAGTGGTTT   
  
  
- CCTCAGGTGG AGGAGTCTCA GTAGGTTACA GTCGTCGTAA TCGTCGTGGT TTCTTCATAG TGGAACGAGA   
  
  
- GGCTCATGAG GGTTTGTTAA CGAGAAATTA ACACGTTTAT ACTAAAGTCT CCCCTTAAAA CTCCTTCGGC   
  
  
- ACTGATACTA GTCGCTCGAA TCTGTCTAAC AAAGATAGGT TCCCCTAGGT CGCGTCTCCT ATCGTCGGAT   
  
  
- GTACCATCTT CCGGAACGAA GGGCAAACCG AAGTAGCCCT TTTCAAGAAA TGTTTCGAAA CTTTACGTTT   
  
  
- CTCGGTGGAA GTAAACTGTC CGATCGCCGA TACGTTTAGG AGAAACTCCA CACGGGTACA AAGTTTAAGC   
  
  
- CTAAATACCG GCGTTTACGT CGCTAATACC TACGTTAGTT TTTGCTTTTT TCCCATGTGT AGTATCTAAA   
  
  
- ACTGTATTTG GTTCCCTTAG TTATGTATTT AGAGTACGTT AGGGAACGAT TTGTTCCATT GTTCGACTGC   
  
  
- GTGAACTTCA ACTGACCTCA ACTACTGGGA CTCAGTCAAG TTGCGGGATA ACCACCGGAT TTTTTGTAGC   
  
  
- CTGTTTCCGA CGTTCGTAAT CGACTTATAG AACCACACAG TAAACTCAAG TTTCGTTATG GACGATCTTG   
  
  
- AGGTGAACAA TTGGGCCTTT ACAATCTTAC AGCTGGACGC CTTCGAAATC ACCACTTGAA ACGGAAGGTC   
  
  
- GAAGTGGTGT ACGGACTACT TTCGCAGAGT TGTTAGTTGG AGGCTCTGGT CGAAGAAGCC TACCAGTTTT   
  
  
- CGGATTTGGG TTTCGATCAT TGGTAACAGC TCGTCCTACA CTTGTGTTTG TGATGGGGAA AGGAGAGATC   
  
  
- CAAACGACTC CGTATGTCGA TGATAAGACG ACAAAAACTC AGAGAACTAC GATGAGAGGG ATCTCTATCG   
  
  
- GTCCTGTCCC ACTTACACCT CTTCGTCACG AACCGTGCAC TACAACACTT GTAGTAACGT ACACTTCCCC   
  
  
- TCCTCTCCTA ACTCGCGATA CTCCAACGCC CCTTTACCTC TCGCTCCTAC AACTACCGCC CTAAGTCCCG   
  
  
- ATAAGGTTAC CCAGTCTTCT GGCCACTATA CTAAGCCTTC GAATATAGTT ACGCCATAAC ACTGTCCGAG   
  
  
- TTCCATTTTC TCCTCTACCT ACGTGAAGTG AAACCCACCC TTCTATTTTT GAATTAGCAA TGTAGTCGAA   
  
  
- CCTCCAC

+     TGACG-motif

| Site Name | Organism | Position | Strand | Matrix score. | sequence | function |
| --- | --- | --- | --- | --- | --- | --- |
| TGACG-motif | Hordeum vulgare | 1852 | + | 5 | TGACG | cis-acting regulatory element involved in the MeJA-responsiveness |
| TGACG-motif | Hordeum vulgare | 1583 | + | 5 | TGACG | cis-acting regulatory element involved in the MeJA-responsiveness |
| TGACG-motif | Hordeum vulgare | 3010 | + | 5 | TGACG | cis-acting regulatory element involved in the MeJA-responsiveness |

>HU11G01134.1   
+ +Up\_Stream \_Len000AGATAG ACATTCAGCC ACTTGCCAGT TCAGAGAGAG ATAGAGAGAG ATTGGGTAAG   
  
  
+ CCCTTTTTGC ACTACTTTTT CTCGCTTCCA TTTTTCTTTC TTAATTTTTA GTGATGATTT TCCAGAAGAT   
  
  
+ TTGATTTATT CATCATGCTT AACCAATTGG GTCTTTGAGT ATTATTGTTT ACTGGGTATT TGATCTTTTC   
  
  
+ ATATTCCTTT GGACTGGGTG GGTGGAACTT TTGAACTTTC TTATTAGTTG GGCATGCCCT TATCTTATTC   
  
  
+ CAATTCAAAA CCCATATTTT TCTTCAATTT TAATTATGGG TATTTTGAAA TTGAATGCTT TCCTTCACAG   
  
  
+ CAATACTATA TTACATATTT TACAGGAAAG TAATATTTTA CAGGAAAGTA TGTTGCTGTT TCAACCCTGT   
  
  
+ TTTGTTTCCT CTCTTTCCTT GGATATGATG TTCATTGCTC TTGTACCAAA CCTAACCTCA ACTTCCTGAA   
  
  
+ TTATTTAAGG CAATATTACC GTGTGCTGGT CAATTTTGGT TGTGGGTTTG TTTCTTAATG TATATGATTT   
  
  
+ ATGGTCAGGA TCCATGCTCC TCTTCCGTCC ACTCTATATC TCTCTCTCTC TCTCTCAATA CAAATTCAGA   
  
  
+ GACTTTCTCT TTCTGGGTTT ACTATTGATT GCAGAAGTTT GTAGTCTTGC GAGGCCTTGA GTCTTCTTCG   
  
  
+ AGGTAGAAAA TTTTGCTTTG CGCTTCCTTC CCCATATTTT AACACCCCTC CCCCCCCAAC GCAAGTTTTC   
  
  
+ TACATTTTTG TGCAATTCTT CAGCTATGAT TCCCCAATTT CACTTGTCAC ATCTCTATTT TCCTCTTTTT   
  
  
+ GGTTTCTTGA TTCTGTAGAA GGTTGTGTAT GAACTTCTTT GTTTCCCTTT TTCCTTTGTT ATTTTTAATG   
  
  
+ TTCCCATAGA TGATATAGAG ATCTGCTTTT CCTTGCTGTG AGAGGTATCT CTGAGAATGC ATGTAGCAAT   
  
  
+ CAAAATTCTG AGTTGTTAAT TCTATTGCAT GTCTATGGGT GATGATTGTG ATGATGTTTT TTCTGTTGCC   
  
  
+ CTCTTTCTCT CTTTCACTGA AATTGCTTTC CAGAATAGTT GTTTGACTCC TTTCTGTATC TTAATTGAGC   
  
  
+ CCTTGCTTTG GTTTGTGCTC TGGGAGTTCT TCTTTAGTAA TCCCTTGCTT GTATTTTCTG AAAGAGCAAG   
  
  
+ CAATAATCCT CATAGCATGT GTATAATCAT ACAATAGCAA CAGCAAACAT GGATCCTGCG CCCAGTTAGG   
  
  
+ TTTGGTTAGG TTAAGATATG CATATTCTTT TCTCTTGAAA AGAGTTGTCT AGGAAGGGTA ATTATGCCCA   
  
  
+ TTTCATGGAT AATAATGGTA CAATTAAGTT TGATTGGCTT AAATACCAAT TATTTAGCGT CTTTTTCTTT   
  
  
+ GGCTGTCCAG GGTTCCATTT CCATAAACAA CTTTGAGTAA GGCATAGAGA ACTAAATTTC TGCCAATTGT   
  
  
+ CAAGTGCAAG TTGCTCATAT GCAACAATAC TTGGAAATTT TGAGCTGAGC TGCTGGCATA TCATGAATAG   
  
  
+ TTCTAATGGT GGCGAGTTAT CACTTATCGT CCCTTGTGTG ACGAATTTCT CTTCCTAGGA AGCCCTCTAG   
  
  
+ TCAATGCCTA TCTGCTGTTG TGGAGTGAAA ATGGATACAT TTAGATCTCT ATAAACCCTA CTTTGGCAAG   
  
  
+ ATCCATGCTG ATTTTAGGAA GCCCTTTGAC CAGAGAATGA CATAACAACC TGATAGGGTG TTACATGCTA   
  
  
+ ACTTCGATTA TTTAATCAAA ATACGGAACT TCTATCAGTC AAAGCCTGCC CCAGGTTCTA CTGAATTAAA   
  
  
+ TTGTATTGAA TTGCTCTCAC TTGCTTATGA CGATTAAATA ACTGGATTTG CTAATTATTC TTTTTATTTT   
  
  
+ ATTCTTGGTG ATGTAGGGAA CTCAGGGAAT TGTAACCATA CCTGTTCCAG CTGAAAAGGG TACGGGAACA   
  
  
+ GTCATAGTTA CAGGGGAATT TCGCAATACA TTTAAGGGAG TAAGAAGCAG GATCATGTCT TTAGTTAGGC   
  
  
+ CGGCAGATAT GTCTGTGACT CCATATGGAA ATCCAACACT GTATTCACTT CAAGGCAATA ATAATAGCTC   
  
  
+ TGGTTTGTCT GGTCAACTGT ATGGCTCTGA AAAGCACAAG ATCAAATATG TGACCGAATC TTTTAATGGT   
  
  
+ CCAAGTTATG GCCTGAAATT CTTTGTTGAT TCTCCATCAG AAGAGCTCAT CCACCCATCA GATGCTACTC   
  
  
+ CTAACCCATT TGACTCTTCT TTCGTGGGCA TAAGACATGA TGAGAGTCCT TACCAAGGAA ATTATGGATC   
  
  
+ AGAATATGGG GTAAGCCCAT CTTCCGATGC AGTTGAATAT GATGAAGGTA ATAAGATAAG GCTAAAACTT   
  
  
+ CAAGAATTGG AGCATGCCCT GCTCGATGAA GATGATGAAG TGGATGAGGT GGCTATAGGG CCCGTGCTAG   
  
  
+ AACACAGCAT GGAGCTTGAT GAAGAGTGGG TCGCCCCATT GAGAAATGTG GTTTTCCATG ATTCACCAAA   
  
  
+ GGAGTCCACC TCCTCAGAGT CATCCAATGT CAGCAGCATT AGCAGCACCA AAGAAGTATC ACCTTGCTCT   
  
  
+ CCGAGTACTC CCAAACAATT GCTCTTTAAT TGTGCAAATA TGATTTCAGA GGGGAATTTT GAGGAAGCCG   
  
  
+ TGACTATGAT CAGCGAGCTT AGACAGATTG TTTCTATCCA AGGGGATCCA GCGCAGAGGA TAGCAGCCTA   
  
  
+ CATGGTAGAA GGCCTTGCTT CCCGTTTGGC TTCATCGGGA AAAGTTCTTT ACAAAGCTTT GAAATGCAAA   
  
  
+ GAGCCACCTT CATTTGACAG GCTAGCGGCT ATGCAAATCC TCTTTGAGGT GTGCCCATGT TTCAAATTCG   
  
  
+ GATTTATGGC CGCAAATGCA GCGATTATGG ATGCAATCAA AAACGAAAAA AGGGTACACA TCATAGATTT   
  
  
+ TGACATAAAC CAAGGGAATC AATACATAAA TCTCATGCAA TCCCTTGCTA AACAAGGTAA CAAGCTGACG   
  
  
+ CACTTGAAGT TGACTGGAGT TGATGACCCT GAGTCAGTTC AACGCCCTAT TGGTGGCCTA AAAAACATCG   
  
  
+ GACAAAGGCT GCAAGCATTA GCTGAATATC TTGGTGTGTC ATTTGAGTTC AAAGCAATAC CTGCTAGAAC   
  
  
+ TCCACTTGTT AACCCGGAAA TGTTAGAATG TCGACCTGCG GAAGCTTTAG TGGTGAACTT TGCCTTCCAG   
  
  
+ CTTCACCACA TGCCTGATGA AAGCGTCTCA ACAATCAACC TCCGAGACCA GCTTCTTCGG ATGGTCAAAA   
  
  
+ GCCTAAACCC AAAGCTAGTA ACCATTGTCG AGCAGGATGT GAACACAAAC ACTACCCCTT TCCTCTCTAG   
  
  
+ GTTTGCTGAG GCATACAGCT ACTATTCTGC TGTTTTTGAG TCTCTTGATG CTACTCTCCC TAGAGATAGC   
  
  
+ CAGGACAGGG TGAATGTGGA GAAGCAGTGC TTGGCACGTG ATGTTGTGAA CATCATTGCA TGTGAAGGGG   
  
  
+ AGGAGAGGAT TGAGCGCTAT GAGGTTGCGG GGAAATGGAG AGCGAGGATG TTGATGGCGG GATTCAGGGC   
  
  
+ TATTCCAATG GGTCAGAAGA CCGGTGATAT GATTCGGAAG CTTATATCAA TGCGGTATTG TGACAGGCTC   
  
  
+ AAGGTAAAAG AGGAGATGGA TGCACTTCAC TTTGGGTGGG AAGATAAAAA CTTAATCGTT ACATCAGCTT   
  
  
+ GGAGGTG  

- +Up\_Stream \_Len000TCTATC TGTAAGTCGG TGAACGGTCA AGTCTCTCTC TATCTCTCTC TAACCCATTC   
  
  
- GGGAAAAACG TGATGAAAAA GAGCGAAGGT AAAAAGAAAG AATTAAAAAT CACTACTAAA AGGTCTTCTA   
  
  
- AACTAAATAA GTAGTACGAA TTGGTTAACC CAGAAACTCA TAATAACAAA TGACCCATAA ACTAGAAAAG   
  
  
- TATAAGGAAA CCTGACCCAC CCACCTTGAA AACTTGAAAG AATAATCAAC CCGTACGGGA ATAGAATAAG   
  
  
- GTTAAGTTTT GGGTATAAAA AGAAGTTAAA ATTAATACCC ATAAAACTTT AACTTACGAA AGGAAGTGTC   
  
  
- GTTATGATAT AATGTATAAA ATGTCCTTTC ATTATAAAAT GTCCTTTCAT ACAACGACAA AGTTGGGACA   
  
  
- AAACAAAGGA GAGAAAGGAA CCTATACTAC AAGTAACGAG AACATGGTTT GGATTGGAGT TGAAGGACTT   
  
  
- AATAAATTCC GTTATAATGG CACACGACCA GTTAAAACCA ACACCCAAAC AAAGAATTAC ATATACTAAA   
  
  
- TACCAGTCCT AGGTACGAGG AGAAGGCAGG TGAGATATAG AGAGAGAGAG AGAGAGTTAT GTTTAAGTCT   
  
  
- CTGAAAGAGA AAGACCCAAA TGATAACTAA CGTCTTCAAA CATCAGAACG CTCCGGAACT CAGAAGAAGC   
  
  
- TCCATCTTTT AAAACGAAAC GCGAAGGAAG GGGTATAAAA TTGTGGGGAG GGGGGGGTTG CGTTCAAAAG   
  
  
- ATGTAAAAAC ACGTTAAGAA GTCGATACTA AGGGGTTAAA GTGAACAGTG TAGAGATAAA AGGAGAAAAA   
  
  
- CCAAAGAACT AAGACATCTT CCAACACATA CTTGAAGAAA CAAAGGGAAA AAGGAAACAA TAAAAATTAC   
  
  
- AAGGGTATCT ACTATATCTC TAGACGAAAA GGAACGACAC TCTCCATAGA GACTCTTACG TACATCGTTA   
  
  
- GTTTTAAGAC TCAACAATTA AGATAACGTA CAGATACCCA CTACTAACAC TACTACAAAA AAGACAACGG   
  
  
- GAGAAAGAGA GAAAGTGACT TTAACGAAAG GTCTTATCAA CAAACTGAGG AAAGACATAG AATTAACTCG   
  
  
- GGAACGAAAC CAAACACGAG ACCCTCAAGA AGAAATCATT AGGGAACGAA CATAAAAGAC TTTCTCGTTC   
  
  
- GTTATTAGGA GTATCGTACA CATATTAGTA TGTTATCGTT GTCGTTTGTA CCTAGGACGC GGGTCAATCC   
  
  
- AAACCAATCC AATTCTATAC GTATAAGAAA AGAGAACTTT TCTCAACAGA TCCTTCCCAT TAATACGGGT   
  
  
- AAAGTACCTA TTATTACCAT GTTAATTCAA ACTAACCGAA TTTATGGTTA ATAAATCGCA GAAAAAGAAA   
  
  
- CCGACAGGTC CCAAGGTAAA GGTATTTGTT GAAACTCATT CCGTATCTCT TGATTTAAAG ACGGTTAACA   
  
  
- GTTCACGTTC AACGAGTATA CGTTGTTATG AACCTTTAAA ACTCGACTCG ACGACCGTAT AGTACTTATC   
  
  
- AAGATTACCA CCGCTCAATA GTGAATAGCA GGGAACACAC TGCTTAAAGA GAAGGATCCT TCGGGAGATC   
  
  
- AGTTACGGAT AGACGACAAC ACCTCACTTT TACCTATGTA AATCTAGAGA TATTTGGGAT GAAACCGTTC   
  
  
- TAGGTACGAC TAAAATCCTT CGGGAAACTG GTCTCTTACT GTATTGTTGG ACTATCCCAC AATGTACGAT   
  
  
- TGAAGCTAAT AAATTAGTTT TATGCCTTGA AGATAGTCAG TTTCGGACGG GGTCCAAGAT GACTTAATTT   
  
  
- AACATAACTT AACGAGAGTG AACGAATACT GCTAATTTAT TGACCTAAAC GATTAATAAG AAAAATAAAA   
  
  
- TAAGAACCAC TACATCCCTT GAGTCCCTTA ACATTGGTAT GGACAAGGTC GACTTTTCCC ATGCCCTTGT   
  
  
- CAGTATCAAT GTCCCCTTAA AGCGTTATGT AAATTCCCTC ATTCTTCGTC CTAGTACAGA AATCAATCCG   
  
  
- GCCGTCTATA CAGACACTGA GGTATACCTT TAGGTTGTGA CATAAGTGAA GTTCCGTTAT TATTATCGAG   
  
  
- ACCAAACAGA CCAGTTGACA TACCGAGACT TTTCGTGTTC TAGTTTATAC ACTGGCTTAG AAAATTACCA   
  
  
- GGTTCAATAC CGGACTTTAA GAAACAACTA AGAGGTAGTC TTCTCGAGTA GGTGGGTAGT CTACGATGAG   
  
  
- GATTGGGTAA ACTGAGAAGA AAGCACCCGT ATTCTGTACT ACTCTCAGGA ATGGTTCCTT TAATACCTAG   
  
  
- TCTTATACCC CATTCGGGTA GAAGGCTACG TCAACTTATA CTACTTCCAT TATTCTATTC CGATTTTGAA   
  
  
- GTTCTTAACC TCGTACGGGA CGAGCTACTT CTACTACTTC ACCTACTCCA CCGATATCCC GGGCACGATC   
  
  
- TTGTGTCGTA CCTCGAACTA CTTCTCACCC AGCGGGGTAA CTCTTTACAC CAAAAGGTAC TAAGTGGTTT   
  
  
- CCTCAGGTGG AGGAGTCTCA GTAGGTTACA GTCGTCGTAA TCGTCGTGGT TTCTTCATAG TGGAACGAGA   
  
  
- GGCTCATGAG GGTTTGTTAA CGAGAAATTA ACACGTTTAT ACTAAAGTCT CCCCTTAAAA CTCCTTCGGC   
  
  
- ACTGATACTA GTCGCTCGAA TCTGTCTAAC AAAGATAGGT TCCCCTAGGT CGCGTCTCCT ATCGTCGGAT   
  
  
- GTACCATCTT CCGGAACGAA GGGCAAACCG AAGTAGCCCT TTTCAAGAAA TGTTTCGAAA CTTTACGTTT   
  
  
- CTCGGTGGAA GTAAACTGTC CGATCGCCGA TACGTTTAGG AGAAACTCCA CACGGGTACA AAGTTTAAGC   
  
  
- CTAAATACCG GCGTTTACGT CGCTAATACC TACGTTAGTT TTTGCTTTTT TCCCATGTGT AGTATCTAAA   
  
  
- ACTGTATTTG GTTCCCTTAG TTATGTATTT AGAGTACGTT AGGGAACGAT TTGTTCCATT GTTCGACTGC   
  
  
- GTGAACTTCA ACTGACCTCA ACTACTGGGA CTCAGTCAAG TTGCGGGATA ACCACCGGAT TTTTTGTAGC   
  
  
- CTGTTTCCGA CGTTCGTAAT CGACTTATAG AACCACACAG TAAACTCAAG TTTCGTTATG GACGATCTTG   
  
  
- AGGTGAACAA TTGGGCCTTT ACAATCTTAC AGCTGGACGC CTTCGAAATC ACCACTTGAA ACGGAAGGTC   
  
  
- GAAGTGGTGT ACGGACTACT TTCGCAGAGT TGTTAGTTGG AGGCTCTGGT CGAAGAAGCC TACCAGTTTT   
  
  
- CGGATTTGGG TTTCGATCAT TGGTAACAGC TCGTCCTACA CTTGTGTTTG TGATGGGGAA AGGAGAGATC   
  
  
- CAAACGACTC CGTATGTCGA TGATAAGACG ACAAAAACTC AGAGAACTAC GATGAGAGGG ATCTCTATCG   
  
  
- GTCCTGTCCC ACTTACACCT CTTCGTCACG AACCGTGCAC TACAACACTT GTAGTAACGT ACACTTCCCC   
  
  
- TCCTCTCCTA ACTCGCGATA CTCCAACGCC CCTTTACCTC TCGCTCCTAC AACTACCGCC CTAAGTCCCG   
  
  
- ATAAGGTTAC CCAGTCTTCT GGCCACTATA CTAAGCCTTC GAATATAGTT ACGCCATAAC ACTGTCCGAG   
  
  
- TTCCATTTTC TCCTCTACCT ACGTGAAGTG AAACCCACCC TTCTATTTTT GAATTAGCAA TGTAGTCGAA   
  
  
- CCTCCAC

+     Unnamed\_\_1

| Site Name | Organism | Position | Strand | Matrix score. | sequence | function |
| --- | --- | --- | --- | --- | --- | --- |
| Unnamed\_\_1 | Zea mays | 2267 | + | 5 | CGTGG |  |

>HU11G01134.1   
+ +Up\_Stream \_Len000AGATAG ACATTCAGCC ACTTGCCAGT TCAGAGAGAG ATAGAGAGAG ATTGGGTAAG   
  
  
+ CCCTTTTTGC ACTACTTTTT CTCGCTTCCA TTTTTCTTTC TTAATTTTTA GTGATGATTT TCCAGAAGAT   
  
  
+ TTGATTTATT CATCATGCTT AACCAATTGG GTCTTTGAGT ATTATTGTTT ACTGGGTATT TGATCTTTTC   
  
  
+ ATATTCCTTT GGACTGGGTG GGTGGAACTT TTGAACTTTC TTATTAGTTG GGCATGCCCT TATCTTATTC   
  
  
+ CAATTCAAAA CCCATATTTT TCTTCAATTT TAATTATGGG TATTTTGAAA TTGAATGCTT TCCTTCACAG   
  
  
+ CAATACTATA TTACATATTT TACAGGAAAG TAATATTTTA CAGGAAAGTA TGTTGCTGTT TCAACCCTGT   
  
  
+ TTTGTTTCCT CTCTTTCCTT GGATATGATG TTCATTGCTC TTGTACCAAA CCTAACCTCA ACTTCCTGAA   
  
  
+ TTATTTAAGG CAATATTACC GTGTGCTGGT CAATTTTGGT TGTGGGTTTG TTTCTTAATG TATATGATTT   
  
  
+ ATGGTCAGGA TCCATGCTCC TCTTCCGTCC ACTCTATATC TCTCTCTCTC TCTCTCAATA CAAATTCAGA   
  
  
+ GACTTTCTCT TTCTGGGTTT ACTATTGATT GCAGAAGTTT GTAGTCTTGC GAGGCCTTGA GTCTTCTTCG   
  
  
+ AGGTAGAAAA TTTTGCTTTG CGCTTCCTTC CCCATATTTT AACACCCCTC CCCCCCCAAC GCAAGTTTTC   
  
  
+ TACATTTTTG TGCAATTCTT CAGCTATGAT TCCCCAATTT CACTTGTCAC ATCTCTATTT TCCTCTTTTT   
  
  
+ GGTTTCTTGA TTCTGTAGAA GGTTGTGTAT GAACTTCTTT GTTTCCCTTT TTCCTTTGTT ATTTTTAATG   
  
  
+ TTCCCATAGA TGATATAGAG ATCTGCTTTT CCTTGCTGTG AGAGGTATCT CTGAGAATGC ATGTAGCAAT   
  
  
+ CAAAATTCTG AGTTGTTAAT TCTATTGCAT GTCTATGGGT GATGATTGTG ATGATGTTTT TTCTGTTGCC   
  
  
+ CTCTTTCTCT CTTTCACTGA AATTGCTTTC CAGAATAGTT GTTTGACTCC TTTCTGTATC TTAATTGAGC   
  
  
+ CCTTGCTTTG GTTTGTGCTC TGGGAGTTCT TCTTTAGTAA TCCCTTGCTT GTATTTTCTG AAAGAGCAAG   
  
  
+ CAATAATCCT CATAGCATGT GTATAATCAT ACAATAGCAA CAGCAAACAT GGATCCTGCG CCCAGTTAGG   
  
  
+ TTTGGTTAGG TTAAGATATG CATATTCTTT TCTCTTGAAA AGAGTTGTCT AGGAAGGGTA ATTATGCCCA   
  
  
+ TTTCATGGAT AATAATGGTA CAATTAAGTT TGATTGGCTT AAATACCAAT TATTTAGCGT CTTTTTCTTT   
  
  
+ GGCTGTCCAG GGTTCCATTT CCATAAACAA CTTTGAGTAA GGCATAGAGA ACTAAATTTC TGCCAATTGT   
  
  
+ CAAGTGCAAG TTGCTCATAT GCAACAATAC TTGGAAATTT TGAGCTGAGC TGCTGGCATA TCATGAATAG   
  
  
+ TTCTAATGGT GGCGAGTTAT CACTTATCGT CCCTTGTGTG ACGAATTTCT CTTCCTAGGA AGCCCTCTAG   
  
  
+ TCAATGCCTA TCTGCTGTTG TGGAGTGAAA ATGGATACAT TTAGATCTCT ATAAACCCTA CTTTGGCAAG   
  
  
+ ATCCATGCTG ATTTTAGGAA GCCCTTTGAC CAGAGAATGA CATAACAACC TGATAGGGTG TTACATGCTA   
  
  
+ ACTTCGATTA TTTAATCAAA ATACGGAACT TCTATCAGTC AAAGCCTGCC CCAGGTTCTA CTGAATTAAA   
  
  
+ TTGTATTGAA TTGCTCTCAC TTGCTTATGA CGATTAAATA ACTGGATTTG CTAATTATTC TTTTTATTTT   
  
  
+ ATTCTTGGTG ATGTAGGGAA CTCAGGGAAT TGTAACCATA CCTGTTCCAG CTGAAAAGGG TACGGGAACA   
  
  
+ GTCATAGTTA CAGGGGAATT TCGCAATACA TTTAAGGGAG TAAGAAGCAG GATCATGTCT TTAGTTAGGC   
  
  
+ CGGCAGATAT GTCTGTGACT CCATATGGAA ATCCAACACT GTATTCACTT CAAGGCAATA ATAATAGCTC   
  
  
+ TGGTTTGTCT GGTCAACTGT ATGGCTCTGA AAAGCACAAG ATCAAATATG TGACCGAATC TTTTAATGGT   
  
  
+ CCAAGTTATG GCCTGAAATT CTTTGTTGAT TCTCCATCAG AAGAGCTCAT CCACCCATCA GATGCTACTC   
  
  
+ CTAACCCATT TGACTCTTCT TTCGTGGGCA TAAGACATGA TGAGAGTCCT TACCAAGGAA ATTATGGATC   
  
  
+ AGAATATGGG GTAAGCCCAT CTTCCGATGC AGTTGAATAT GATGAAGGTA ATAAGATAAG GCTAAAACTT   
  
  
+ CAAGAATTGG AGCATGCCCT GCTCGATGAA GATGATGAAG TGGATGAGGT GGCTATAGGG CCCGTGCTAG   
  
  
+ AACACAGCAT GGAGCTTGAT GAAGAGTGGG TCGCCCCATT GAGAAATGTG GTTTTCCATG ATTCACCAAA   
  
  
+ GGAGTCCACC TCCTCAGAGT CATCCAATGT CAGCAGCATT AGCAGCACCA AAGAAGTATC ACCTTGCTCT   
  
  
+ CCGAGTACTC CCAAACAATT GCTCTTTAAT TGTGCAAATA TGATTTCAGA GGGGAATTTT GAGGAAGCCG   
  
  
+ TGACTATGAT CAGCGAGCTT AGACAGATTG TTTCTATCCA AGGGGATCCA GCGCAGAGGA TAGCAGCCTA   
  
  
+ CATGGTAGAA GGCCTTGCTT CCCGTTTGGC TTCATCGGGA AAAGTTCTTT ACAAAGCTTT GAAATGCAAA   
  
  
+ GAGCCACCTT CATTTGACAG GCTAGCGGCT ATGCAAATCC TCTTTGAGGT GTGCCCATGT TTCAAATTCG   
  
  
+ GATTTATGGC CGCAAATGCA GCGATTATGG ATGCAATCAA AAACGAAAAA AGGGTACACA TCATAGATTT   
  
  
+ TGACATAAAC CAAGGGAATC AATACATAAA TCTCATGCAA TCCCTTGCTA AACAAGGTAA CAAGCTGACG   
  
  
+ CACTTGAAGT TGACTGGAGT TGATGACCCT GAGTCAGTTC AACGCCCTAT TGGTGGCCTA AAAAACATCG   
  
  
+ GACAAAGGCT GCAAGCATTA GCTGAATATC TTGGTGTGTC ATTTGAGTTC AAAGCAATAC CTGCTAGAAC   
  
  
+ TCCACTTGTT AACCCGGAAA TGTTAGAATG TCGACCTGCG GAAGCTTTAG TGGTGAACTT TGCCTTCCAG   
  
  
+ CTTCACCACA TGCCTGATGA AAGCGTCTCA ACAATCAACC TCCGAGACCA GCTTCTTCGG ATGGTCAAAA   
  
  
+ GCCTAAACCC AAAGCTAGTA ACCATTGTCG AGCAGGATGT GAACACAAAC ACTACCCCTT TCCTCTCTAG   
  
  
+ GTTTGCTGAG GCATACAGCT ACTATTCTGC TGTTTTTGAG TCTCTTGATG CTACTCTCCC TAGAGATAGC   
  
  
+ CAGGACAGGG TGAATGTGGA GAAGCAGTGC TTGGCACGTG ATGTTGTGAA CATCATTGCA TGTGAAGGGG   
  
  
+ AGGAGAGGAT TGAGCGCTAT GAGGTTGCGG GGAAATGGAG AGCGAGGATG TTGATGGCGG GATTCAGGGC   
  
  
+ TATTCCAATG GGTCAGAAGA CCGGTGATAT GATTCGGAAG CTTATATCAA TGCGGTATTG TGACAGGCTC   
  
  
+ AAGGTAAAAG AGGAGATGGA TGCACTTCAC TTTGGGTGGG AAGATAAAAA CTTAATCGTT ACATCAGCTT   
  
  
+ GGAGGTG  

- +Up\_Stream \_Len000TCTATC TGTAAGTCGG TGAACGGTCA AGTCTCTCTC TATCTCTCTC TAACCCATTC   
  
  
- GGGAAAAACG TGATGAAAAA GAGCGAAGGT AAAAAGAAAG AATTAAAAAT CACTACTAAA AGGTCTTCTA   
  
  
- AACTAAATAA GTAGTACGAA TTGGTTAACC CAGAAACTCA TAATAACAAA TGACCCATAA ACTAGAAAAG   
  
  
- TATAAGGAAA CCTGACCCAC CCACCTTGAA AACTTGAAAG AATAATCAAC CCGTACGGGA ATAGAATAAG   
  
  
- GTTAAGTTTT GGGTATAAAA AGAAGTTAAA ATTAATACCC ATAAAACTTT AACTTACGAA AGGAAGTGTC   
  
  
- GTTATGATAT AATGTATAAA ATGTCCTTTC ATTATAAAAT GTCCTTTCAT ACAACGACAA AGTTGGGACA   
  
  
- AAACAAAGGA GAGAAAGGAA CCTATACTAC AAGTAACGAG AACATGGTTT GGATTGGAGT TGAAGGACTT   
  
  
- AATAAATTCC GTTATAATGG CACACGACCA GTTAAAACCA ACACCCAAAC AAAGAATTAC ATATACTAAA   
  
  
- TACCAGTCCT AGGTACGAGG AGAAGGCAGG TGAGATATAG AGAGAGAGAG AGAGAGTTAT GTTTAAGTCT   
  
  
- CTGAAAGAGA AAGACCCAAA TGATAACTAA CGTCTTCAAA CATCAGAACG CTCCGGAACT CAGAAGAAGC   
  
  
- TCCATCTTTT AAAACGAAAC GCGAAGGAAG GGGTATAAAA TTGTGGGGAG GGGGGGGTTG CGTTCAAAAG   
  
  
- ATGTAAAAAC ACGTTAAGAA GTCGATACTA AGGGGTTAAA GTGAACAGTG TAGAGATAAA AGGAGAAAAA   
  
  
- CCAAAGAACT AAGACATCTT CCAACACATA CTTGAAGAAA CAAAGGGAAA AAGGAAACAA TAAAAATTAC   
  
  
- AAGGGTATCT ACTATATCTC TAGACGAAAA GGAACGACAC TCTCCATAGA GACTCTTACG TACATCGTTA   
  
  
- GTTTTAAGAC TCAACAATTA AGATAACGTA CAGATACCCA CTACTAACAC TACTACAAAA AAGACAACGG   
  
  
- GAGAAAGAGA GAAAGTGACT TTAACGAAAG GTCTTATCAA CAAACTGAGG AAAGACATAG AATTAACTCG   
  
  
- GGAACGAAAC CAAACACGAG ACCCTCAAGA AGAAATCATT AGGGAACGAA CATAAAAGAC TTTCTCGTTC   
  
  
- GTTATTAGGA GTATCGTACA CATATTAGTA TGTTATCGTT GTCGTTTGTA CCTAGGACGC GGGTCAATCC   
  
  
- AAACCAATCC AATTCTATAC GTATAAGAAA AGAGAACTTT TCTCAACAGA TCCTTCCCAT TAATACGGGT   
  
  
- AAAGTACCTA TTATTACCAT GTTAATTCAA ACTAACCGAA TTTATGGTTA ATAAATCGCA GAAAAAGAAA   
  
  
- CCGACAGGTC CCAAGGTAAA GGTATTTGTT GAAACTCATT CCGTATCTCT TGATTTAAAG ACGGTTAACA   
  
  
- GTTCACGTTC AACGAGTATA CGTTGTTATG AACCTTTAAA ACTCGACTCG ACGACCGTAT AGTACTTATC   
  
  
- AAGATTACCA CCGCTCAATA GTGAATAGCA GGGAACACAC TGCTTAAAGA GAAGGATCCT TCGGGAGATC   
  
  
- AGTTACGGAT AGACGACAAC ACCTCACTTT TACCTATGTA AATCTAGAGA TATTTGGGAT GAAACCGTTC   
  
  
- TAGGTACGAC TAAAATCCTT CGGGAAACTG GTCTCTTACT GTATTGTTGG ACTATCCCAC AATGTACGAT   
  
  
- TGAAGCTAAT AAATTAGTTT TATGCCTTGA AGATAGTCAG TTTCGGACGG GGTCCAAGAT GACTTAATTT   
  
  
- AACATAACTT AACGAGAGTG AACGAATACT GCTAATTTAT TGACCTAAAC GATTAATAAG AAAAATAAAA   
  
  
- TAAGAACCAC TACATCCCTT GAGTCCCTTA ACATTGGTAT GGACAAGGTC GACTTTTCCC ATGCCCTTGT   
  
  
- CAGTATCAAT GTCCCCTTAA AGCGTTATGT AAATTCCCTC ATTCTTCGTC CTAGTACAGA AATCAATCCG   
  
  
- GCCGTCTATA CAGACACTGA GGTATACCTT TAGGTTGTGA CATAAGTGAA GTTCCGTTAT TATTATCGAG   
  
  
- ACCAAACAGA CCAGTTGACA TACCGAGACT TTTCGTGTTC TAGTTTATAC ACTGGCTTAG AAAATTACCA   
  
  
- GGTTCAATAC CGGACTTTAA GAAACAACTA AGAGGTAGTC TTCTCGAGTA GGTGGGTAGT CTACGATGAG   
  
  
- GATTGGGTAA ACTGAGAAGA AAGCACCCGT ATTCTGTACT ACTCTCAGGA ATGGTTCCTT TAATACCTAG   
  
  
- TCTTATACCC CATTCGGGTA GAAGGCTACG TCAACTTATA CTACTTCCAT TATTCTATTC CGATTTTGAA   
  
  
- GTTCTTAACC TCGTACGGGA CGAGCTACTT CTACTACTTC ACCTACTCCA CCGATATCCC GGGCACGATC   
  
  
- TTGTGTCGTA CCTCGAACTA CTTCTCACCC AGCGGGGTAA CTCTTTACAC CAAAAGGTAC TAAGTGGTTT   
  
  
- CCTCAGGTGG AGGAGTCTCA GTAGGTTACA GTCGTCGTAA TCGTCGTGGT TTCTTCATAG TGGAACGAGA   
  
  
- GGCTCATGAG GGTTTGTTAA CGAGAAATTA ACACGTTTAT ACTAAAGTCT CCCCTTAAAA CTCCTTCGGC   
  
  
- ACTGATACTA GTCGCTCGAA TCTGTCTAAC AAAGATAGGT TCCCCTAGGT CGCGTCTCCT ATCGTCGGAT   
  
  
- GTACCATCTT CCGGAACGAA GGGCAAACCG AAGTAGCCCT TTTCAAGAAA TGTTTCGAAA CTTTACGTTT   
  
  
- CTCGGTGGAA GTAAACTGTC CGATCGCCGA TACGTTTAGG AGAAACTCCA CACGGGTACA AAGTTTAAGC   
  
  
- CTAAATACCG GCGTTTACGT CGCTAATACC TACGTTAGTT TTTGCTTTTT TCCCATGTGT AGTATCTAAA   
  
  
- ACTGTATTTG GTTCCCTTAG TTATGTATTT AGAGTACGTT AGGGAACGAT TTGTTCCATT GTTCGACTGC   
  
  
- GTGAACTTCA ACTGACCTCA ACTACTGGGA CTCAGTCAAG TTGCGGGATA ACCACCGGAT TTTTTGTAGC   
  
  
- CTGTTTCCGA CGTTCGTAAT CGACTTATAG AACCACACAG TAAACTCAAG TTTCGTTATG GACGATCTTG   
  
  
- AGGTGAACAA TTGGGCCTTT ACAATCTTAC AGCTGGACGC CTTCGAAATC ACCACTTGAA ACGGAAGGTC   
  
  
- GAAGTGGTGT ACGGACTACT TTCGCAGAGT TGTTAGTTGG AGGCTCTGGT CGAAGAAGCC TACCAGTTTT   
  
  
- CGGATTTGGG TTTCGATCAT TGGTAACAGC TCGTCCTACA CTTGTGTTTG TGATGGGGAA AGGAGAGATC   
  
  
- CAAACGACTC CGTATGTCGA TGATAAGACG ACAAAAACTC AGAGAACTAC GATGAGAGGG ATCTCTATCG   
  
  
- GTCCTGTCCC ACTTACACCT CTTCGTCACG AACCGTGCAC TACAACACTT GTAGTAACGT ACACTTCCCC   
  
  
- TCCTCTCCTA ACTCGCGATA CTCCAACGCC CCTTTACCTC TCGCTCCTAC AACTACCGCC CTAAGTCCCG   
  
  
- ATAAGGTTAC CCAGTCTTCT GGCCACTATA CTAAGCCTTC GAATATAGTT ACGCCATAAC ACTGTCCGAG   
  
  
- TTCCATTTTC TCCTCTACCT ACGTGAAGTG AAACCCACCC TTCTATTTTT GAATTAGCAA TGTAGTCGAA   
  
  
- CCTCCAC

+     Unnamed\_\_2

| Site Name | Organism | Position | Strand | Matrix score. | sequence | function |
| --- | --- | --- | --- | --- | --- | --- |
| Unnamed\_\_2 | Petroselinum hortense | 1262 | - | 9 | AACCTAACCT |  |
| Unnamed\_\_2 | Petroselinum hortense | 1267 | - | 9 | AACCTAACCT |  |
| Unnamed\_\_2 | Petroselinum hortense | 473 | + | 10 | AACCTAACCT |  |

>HU11G01134.1   
+ +Up\_Stream \_Len000AGATAG ACATTCAGCC ACTTGCCAGT TCAGAGAGAG ATAGAGAGAG ATTGGGTAAG   
  
  
+ CCCTTTTTGC ACTACTTTTT CTCGCTTCCA TTTTTCTTTC TTAATTTTTA GTGATGATTT TCCAGAAGAT   
  
  
+ TTGATTTATT CATCATGCTT AACCAATTGG GTCTTTGAGT ATTATTGTTT ACTGGGTATT TGATCTTTTC   
  
  
+ ATATTCCTTT GGACTGGGTG GGTGGAACTT TTGAACTTTC TTATTAGTTG GGCATGCCCT TATCTTATTC   
  
  
+ CAATTCAAAA CCCATATTTT TCTTCAATTT TAATTATGGG TATTTTGAAA TTGAATGCTT TCCTTCACAG   
  
  
+ CAATACTATA TTACATATTT TACAGGAAAG TAATATTTTA CAGGAAAGTA TGTTGCTGTT TCAACCCTGT   
  
  
+ TTTGTTTCCT CTCTTTCCTT GGATATGATG TTCATTGCTC TTGTACCAAA CCTAACCTCA ACTTCCTGAA   
  
  
+ TTATTTAAGG CAATATTACC GTGTGCTGGT CAATTTTGGT TGTGGGTTTG TTTCTTAATG TATATGATTT   
  
  
+ ATGGTCAGGA TCCATGCTCC TCTTCCGTCC ACTCTATATC TCTCTCTCTC TCTCTCAATA CAAATTCAGA   
  
  
+ GACTTTCTCT TTCTGGGTTT ACTATTGATT GCAGAAGTTT GTAGTCTTGC GAGGCCTTGA GTCTTCTTCG   
  
  
+ AGGTAGAAAA TTTTGCTTTG CGCTTCCTTC CCCATATTTT AACACCCCTC CCCCCCCAAC GCAAGTTTTC   
  
  
+ TACATTTTTG TGCAATTCTT CAGCTATGAT TCCCCAATTT CACTTGTCAC ATCTCTATTT TCCTCTTTTT   
  
  
+ GGTTTCTTGA TTCTGTAGAA GGTTGTGTAT GAACTTCTTT GTTTCCCTTT TTCCTTTGTT ATTTTTAATG   
  
  
+ TTCCCATAGA TGATATAGAG ATCTGCTTTT CCTTGCTGTG AGAGGTATCT CTGAGAATGC ATGTAGCAAT   
  
  
+ CAAAATTCTG AGTTGTTAAT TCTATTGCAT GTCTATGGGT GATGATTGTG ATGATGTTTT TTCTGTTGCC   
  
  
+ CTCTTTCTCT CTTTCACTGA AATTGCTTTC CAGAATAGTT GTTTGACTCC TTTCTGTATC TTAATTGAGC   
  
  
+ CCTTGCTTTG GTTTGTGCTC TGGGAGTTCT TCTTTAGTAA TCCCTTGCTT GTATTTTCTG AAAGAGCAAG   
  
  
+ CAATAATCCT CATAGCATGT GTATAATCAT ACAATAGCAA CAGCAAACAT GGATCCTGCG CCCAGTTAGG   
  
  
+ TTTGGTTAGG TTAAGATATG CATATTCTTT TCTCTTGAAA AGAGTTGTCT AGGAAGGGTA ATTATGCCCA   
  
  
+ TTTCATGGAT AATAATGGTA CAATTAAGTT TGATTGGCTT AAATACCAAT TATTTAGCGT CTTTTTCTTT   
  
  
+ GGCTGTCCAG GGTTCCATTT CCATAAACAA CTTTGAGTAA GGCATAGAGA ACTAAATTTC TGCCAATTGT   
  
  
+ CAAGTGCAAG TTGCTCATAT GCAACAATAC TTGGAAATTT TGAGCTGAGC TGCTGGCATA TCATGAATAG   
  
  
+ TTCTAATGGT GGCGAGTTAT CACTTATCGT CCCTTGTGTG ACGAATTTCT CTTCCTAGGA AGCCCTCTAG   
  
  
+ TCAATGCCTA TCTGCTGTTG TGGAGTGAAA ATGGATACAT TTAGATCTCT ATAAACCCTA CTTTGGCAAG   
  
  
+ ATCCATGCTG ATTTTAGGAA GCCCTTTGAC CAGAGAATGA CATAACAACC TGATAGGGTG TTACATGCTA   
  
  
+ ACTTCGATTA TTTAATCAAA ATACGGAACT TCTATCAGTC AAAGCCTGCC CCAGGTTCTA CTGAATTAAA   
  
  
+ TTGTATTGAA TTGCTCTCAC TTGCTTATGA CGATTAAATA ACTGGATTTG CTAATTATTC TTTTTATTTT   
  
  
+ ATTCTTGGTG ATGTAGGGAA CTCAGGGAAT TGTAACCATA CCTGTTCCAG CTGAAAAGGG TACGGGAACA   
  
  
+ GTCATAGTTA CAGGGGAATT TCGCAATACA TTTAAGGGAG TAAGAAGCAG GATCATGTCT TTAGTTAGGC   
  
  
+ CGGCAGATAT GTCTGTGACT CCATATGGAA ATCCAACACT GTATTCACTT CAAGGCAATA ATAATAGCTC   
  
  
+ TGGTTTGTCT GGTCAACTGT ATGGCTCTGA AAAGCACAAG ATCAAATATG TGACCGAATC TTTTAATGGT   
  
  
+ CCAAGTTATG GCCTGAAATT CTTTGTTGAT TCTCCATCAG AAGAGCTCAT CCACCCATCA GATGCTACTC   
  
  
+ CTAACCCATT TGACTCTTCT TTCGTGGGCA TAAGACATGA TGAGAGTCCT TACCAAGGAA ATTATGGATC   
  
  
+ AGAATATGGG GTAAGCCCAT CTTCCGATGC AGTTGAATAT GATGAAGGTA ATAAGATAAG GCTAAAACTT   
  
  
+ CAAGAATTGG AGCATGCCCT GCTCGATGAA GATGATGAAG TGGATGAGGT GGCTATAGGG CCCGTGCTAG   
  
  
+ AACACAGCAT GGAGCTTGAT GAAGAGTGGG TCGCCCCATT GAGAAATGTG GTTTTCCATG ATTCACCAAA   
  
  
+ GGAGTCCACC TCCTCAGAGT CATCCAATGT CAGCAGCATT AGCAGCACCA AAGAAGTATC ACCTTGCTCT   
  
  
+ CCGAGTACTC CCAAACAATT GCTCTTTAAT TGTGCAAATA TGATTTCAGA GGGGAATTTT GAGGAAGCCG   
  
  
+ TGACTATGAT CAGCGAGCTT AGACAGATTG TTTCTATCCA AGGGGATCCA GCGCAGAGGA TAGCAGCCTA   
  
  
+ CATGGTAGAA GGCCTTGCTT CCCGTTTGGC TTCATCGGGA AAAGTTCTTT ACAAAGCTTT GAAATGCAAA   
  
  
+ GAGCCACCTT CATTTGACAG GCTAGCGGCT ATGCAAATCC TCTTTGAGGT GTGCCCATGT TTCAAATTCG   
  
  
+ GATTTATGGC CGCAAATGCA GCGATTATGG ATGCAATCAA AAACGAAAAA AGGGTACACA TCATAGATTT   
  
  
+ TGACATAAAC CAAGGGAATC AATACATAAA TCTCATGCAA TCCCTTGCTA AACAAGGTAA CAAGCTGACG   
  
  
+ CACTTGAAGT TGACTGGAGT TGATGACCCT GAGTCAGTTC AACGCCCTAT TGGTGGCCTA AAAAACATCG   
  
  
+ GACAAAGGCT GCAAGCATTA GCTGAATATC TTGGTGTGTC ATTTGAGTTC AAAGCAATAC CTGCTAGAAC   
  
  
+ TCCACTTGTT AACCCGGAAA TGTTAGAATG TCGACCTGCG GAAGCTTTAG TGGTGAACTT TGCCTTCCAG   
  
  
+ CTTCACCACA TGCCTGATGA AAGCGTCTCA ACAATCAACC TCCGAGACCA GCTTCTTCGG ATGGTCAAAA   
  
  
+ GCCTAAACCC AAAGCTAGTA ACCATTGTCG AGCAGGATGT GAACACAAAC ACTACCCCTT TCCTCTCTAG   
  
  
+ GTTTGCTGAG GCATACAGCT ACTATTCTGC TGTTTTTGAG TCTCTTGATG CTACTCTCCC TAGAGATAGC   
  
  
+ CAGGACAGGG TGAATGTGGA GAAGCAGTGC TTGGCACGTG ATGTTGTGAA CATCATTGCA TGTGAAGGGG   
  
  
+ AGGAGAGGAT TGAGCGCTAT GAGGTTGCGG GGAAATGGAG AGCGAGGATG TTGATGGCGG GATTCAGGGC   
  
  
+ TATTCCAATG GGTCAGAAGA CCGGTGATAT GATTCGGAAG CTTATATCAA TGCGGTATTG TGACAGGCTC   
  
  
+ AAGGTAAAAG AGGAGATGGA TGCACTTCAC TTTGGGTGGG AAGATAAAAA CTTAATCGTT ACATCAGCTT   
  
  
+ GGAGGTG  

- +Up\_Stream \_Len000TCTATC TGTAAGTCGG TGAACGGTCA AGTCTCTCTC TATCTCTCTC TAACCCATTC   
  
  
- GGGAAAAACG TGATGAAAAA GAGCGAAGGT AAAAAGAAAG AATTAAAAAT CACTACTAAA AGGTCTTCTA   
  
  
- AACTAAATAA GTAGTACGAA TTGGTTAACC CAGAAACTCA TAATAACAAA TGACCCATAA ACTAGAAAAG   
  
  
- TATAAGGAAA CCTGACCCAC CCACCTTGAA AACTTGAAAG AATAATCAAC CCGTACGGGA ATAGAATAAG   
  
  
- GTTAAGTTTT GGGTATAAAA AGAAGTTAAA ATTAATACCC ATAAAACTTT AACTTACGAA AGGAAGTGTC   
  
  
- GTTATGATAT AATGTATAAA ATGTCCTTTC ATTATAAAAT GTCCTTTCAT ACAACGACAA AGTTGGGACA   
  
  
- AAACAAAGGA GAGAAAGGAA CCTATACTAC AAGTAACGAG AACATGGTTT GGATTGGAGT TGAAGGACTT   
  
  
- AATAAATTCC GTTATAATGG CACACGACCA GTTAAAACCA ACACCCAAAC AAAGAATTAC ATATACTAAA   
  
  
- TACCAGTCCT AGGTACGAGG AGAAGGCAGG TGAGATATAG AGAGAGAGAG AGAGAGTTAT GTTTAAGTCT   
  
  
- CTGAAAGAGA AAGACCCAAA TGATAACTAA CGTCTTCAAA CATCAGAACG CTCCGGAACT CAGAAGAAGC   
  
  
- TCCATCTTTT AAAACGAAAC GCGAAGGAAG GGGTATAAAA TTGTGGGGAG GGGGGGGTTG CGTTCAAAAG   
  
  
- ATGTAAAAAC ACGTTAAGAA GTCGATACTA AGGGGTTAAA GTGAACAGTG TAGAGATAAA AGGAGAAAAA   
  
  
- CCAAAGAACT AAGACATCTT CCAACACATA CTTGAAGAAA CAAAGGGAAA AAGGAAACAA TAAAAATTAC   
  
  
- AAGGGTATCT ACTATATCTC TAGACGAAAA GGAACGACAC TCTCCATAGA GACTCTTACG TACATCGTTA   
  
  
- GTTTTAAGAC TCAACAATTA AGATAACGTA CAGATACCCA CTACTAACAC TACTACAAAA AAGACAACGG   
  
  
- GAGAAAGAGA GAAAGTGACT TTAACGAAAG GTCTTATCAA CAAACTGAGG AAAGACATAG AATTAACTCG   
  
  
- GGAACGAAAC CAAACACGAG ACCCTCAAGA AGAAATCATT AGGGAACGAA CATAAAAGAC TTTCTCGTTC   
  
  
- GTTATTAGGA GTATCGTACA CATATTAGTA TGTTATCGTT GTCGTTTGTA CCTAGGACGC GGGTCAATCC   
  
  
- AAACCAATCC AATTCTATAC GTATAAGAAA AGAGAACTTT TCTCAACAGA TCCTTCCCAT TAATACGGGT   
  
  
- AAAGTACCTA TTATTACCAT GTTAATTCAA ACTAACCGAA TTTATGGTTA ATAAATCGCA GAAAAAGAAA   
  
  
- CCGACAGGTC CCAAGGTAAA GGTATTTGTT GAAACTCATT CCGTATCTCT TGATTTAAAG ACGGTTAACA   
  
  
- GTTCACGTTC AACGAGTATA CGTTGTTATG AACCTTTAAA ACTCGACTCG ACGACCGTAT AGTACTTATC   
  
  
- AAGATTACCA CCGCTCAATA GTGAATAGCA GGGAACACAC TGCTTAAAGA GAAGGATCCT TCGGGAGATC   
  
  
- AGTTACGGAT AGACGACAAC ACCTCACTTT TACCTATGTA AATCTAGAGA TATTTGGGAT GAAACCGTTC   
  
  
- TAGGTACGAC TAAAATCCTT CGGGAAACTG GTCTCTTACT GTATTGTTGG ACTATCCCAC AATGTACGAT   
  
  
- TGAAGCTAAT AAATTAGTTT TATGCCTTGA AGATAGTCAG TTTCGGACGG GGTCCAAGAT GACTTAATTT   
  
  
- AACATAACTT AACGAGAGTG AACGAATACT GCTAATTTAT TGACCTAAAC GATTAATAAG AAAAATAAAA   
  
  
- TAAGAACCAC TACATCCCTT GAGTCCCTTA ACATTGGTAT GGACAAGGTC GACTTTTCCC ATGCCCTTGT   
  
  
- CAGTATCAAT GTCCCCTTAA AGCGTTATGT AAATTCCCTC ATTCTTCGTC CTAGTACAGA AATCAATCCG   
  
  
- GCCGTCTATA CAGACACTGA GGTATACCTT TAGGTTGTGA CATAAGTGAA GTTCCGTTAT TATTATCGAG   
  
  
- ACCAAACAGA CCAGTTGACA TACCGAGACT TTTCGTGTTC TAGTTTATAC ACTGGCTTAG AAAATTACCA   
  
  
- GGTTCAATAC CGGACTTTAA GAAACAACTA AGAGGTAGTC TTCTCGAGTA GGTGGGTAGT CTACGATGAG   
  
  
- GATTGGGTAA ACTGAGAAGA AAGCACCCGT ATTCTGTACT ACTCTCAGGA ATGGTTCCTT TAATACCTAG   
  
  
- TCTTATACCC CATTCGGGTA GAAGGCTACG TCAACTTATA CTACTTCCAT TATTCTATTC CGATTTTGAA   
  
  
- GTTCTTAACC TCGTACGGGA CGAGCTACTT CTACTACTTC ACCTACTCCA CCGATATCCC GGGCACGATC   
  
  
- TTGTGTCGTA CCTCGAACTA CTTCTCACCC AGCGGGGTAA CTCTTTACAC CAAAAGGTAC TAAGTGGTTT   
  
  
- CCTCAGGTGG AGGAGTCTCA GTAGGTTACA GTCGTCGTAA TCGTCGTGGT TTCTTCATAG TGGAACGAGA   
  
  
- GGCTCATGAG GGTTTGTTAA CGAGAAATTA ACACGTTTAT ACTAAAGTCT CCCCTTAAAA CTCCTTCGGC   
  
  
- ACTGATACTA GTCGCTCGAA TCTGTCTAAC AAAGATAGGT TCCCCTAGGT CGCGTCTCCT ATCGTCGGAT   
  
  
- GTACCATCTT CCGGAACGAA GGGCAAACCG AAGTAGCCCT TTTCAAGAAA TGTTTCGAAA CTTTACGTTT   
  
  
- CTCGGTGGAA GTAAACTGTC CGATCGCCGA TACGTTTAGG AGAAACTCCA CACGGGTACA AAGTTTAAGC   
  
  
- CTAAATACCG GCGTTTACGT CGCTAATACC TACGTTAGTT TTTGCTTTTT TCCCATGTGT AGTATCTAAA   
  
  
- ACTGTATTTG GTTCCCTTAG TTATGTATTT AGAGTACGTT AGGGAACGAT TTGTTCCATT GTTCGACTGC   
  
  
- GTGAACTTCA ACTGACCTCA ACTACTGGGA CTCAGTCAAG TTGCGGGATA ACCACCGGAT TTTTTGTAGC   
  
  
- CTGTTTCCGA CGTTCGTAAT CGACTTATAG AACCACACAG TAAACTCAAG TTTCGTTATG GACGATCTTG   
  
  
- AGGTGAACAA TTGGGCCTTT ACAATCTTAC AGCTGGACGC CTTCGAAATC ACCACTTGAA ACGGAAGGTC   
  
  
- GAAGTGGTGT ACGGACTACT TTCGCAGAGT TGTTAGTTGG AGGCTCTGGT CGAAGAAGCC TACCAGTTTT   
  
  
- CGGATTTGGG TTTCGATCAT TGGTAACAGC TCGTCCTACA CTTGTGTTTG TGATGGGGAA AGGAGAGATC   
  
  
- CAAACGACTC CGTATGTCGA TGATAAGACG ACAAAAACTC AGAGAACTAC GATGAGAGGG ATCTCTATCG   
  
  
- GTCCTGTCCC ACTTACACCT CTTCGTCACG AACCGTGCAC TACAACACTT GTAGTAACGT ACACTTCCCC   
  
  
- TCCTCTCCTA ACTCGCGATA CTCCAACGCC CCTTTACCTC TCGCTCCTAC AACTACCGCC CTAAGTCCCG   
  
  
- ATAAGGTTAC CCAGTCTTCT GGCCACTATA CTAAGCCTTC GAATATAGTT ACGCCATAAC ACTGTCCGAG   
  
  
- TTCCATTTTC TCCTCTACCT ACGTGAAGTG AAACCCACCC TTCTATTTTT GAATTAGCAA TGTAGTCGAA   
  
  
- CCTCCAC

+     Unnamed\_\_4

| Site Name | Organism | Position | Strand | Matrix score. | sequence | function |
| --- | --- | --- | --- | --- | --- | --- |
| Unnamed\_\_4 | Petroselinum hortense | 3715 | - | 4 | CTCC |  |
| Unnamed\_\_4 | Petroselinum hortense | 3656 | - | 4 | CTCC |  |
| Unnamed\_\_4 | Petroselinum hortense | 3506 | - | 4 | CTCC |  |
| Unnamed\_\_4 | Petroselinum hortense | 3452 | - | 4 | CTCC |  |
| Unnamed\_\_4 | Petroselinum hortense | 2053 | + | 4 | CTCC |  |
| Unnamed\_\_4 | Petroselinum hortense | 2593 | + | 4 | CTCC |  |
| Unnamed\_\_4 | Petroselinum hortense | 2534 | + | 4 | CTCC |  |
| Unnamed\_\_4 | Petroselinum hortense | 1636 | - | 4 | CTCC |  |
| Unnamed\_\_4 | Petroselinum hortense | 1101 | + | 4 | CTCC |  |
| Unnamed\_\_4 | Petroselinum hortense | 3420 | + | 4 | CTCC |  |
| Unnamed\_\_4 | Petroselinum hortense | 2465 | - | 4 | CTCC |  |
| Unnamed\_\_4 | Petroselinum hortense | 2602 | + | 4 | CTCC |  |
| Unnamed\_\_4 | Petroselinum hortense | 752 | + | 4 | CTCC |  |
| Unnamed\_\_4 | Petroselinum hortense | 581 | + | 4 | CTCC |  |
| Unnamed\_\_4 | Petroselinum hortense | 2242 | + | 4 | CTCC |  |
| Unnamed\_\_4 | Petroselinum hortense | 3503 | - | 4 | CTCC |  |
| Unnamed\_\_4 | Petroselinum hortense | 2001 | - | 4 | CTCC |  |
| Unnamed\_\_4 | Petroselinum hortense | 2393 | - | 4 | CTCC |  |
| Unnamed\_\_4 | Petroselinum hortense | 3030 | - | 4 | CTCC |  |
| Unnamed\_\_4 | Petroselinum hortense | 1147 | - | 4 | CTCC |  |
| Unnamed\_\_4 | Petroselinum hortense | 3541 | - | 4 | CTCC |  |
| Unnamed\_\_4 | Petroselinum hortense | 2525 | - | 4 | CTCC |  |
| Unnamed\_\_4 | Petroselinum hortense | 3264 | + | 4 | CTCC |  |
| Unnamed\_\_4 | Petroselinum hortense | 2206 | + | 4 | CTCC |  |
| Unnamed\_\_4 | Petroselinum hortense | 3154 | + | 4 | CTCC |  |

>HU11G01134.1   
+ +Up\_Stream \_Len000AGATAG ACATTCAGCC ACTTGCCAGT TCAGAGAGAG ATAGAGAGAG ATTGGGTAAG   
  
  
+ CCCTTTTTGC ACTACTTTTT CTCGCTTCCA TTTTTCTTTC TTAATTTTTA GTGATGATTT TCCAGAAGAT   
  
  
+ TTGATTTATT CATCATGCTT AACCAATTGG GTCTTTGAGT ATTATTGTTT ACTGGGTATT TGATCTTTTC   
  
  
+ ATATTCCTTT GGACTGGGTG GGTGGAACTT TTGAACTTTC TTATTAGTTG GGCATGCCCT TATCTTATTC   
  
  
+ CAATTCAAAA CCCATATTTT TCTTCAATTT TAATTATGGG TATTTTGAAA TTGAATGCTT TCCTTCACAG   
  
  
+ CAATACTATA TTACATATTT TACAGGAAAG TAATATTTTA CAGGAAAGTA TGTTGCTGTT TCAACCCTGT   
  
  
+ TTTGTTTCCT CTCTTTCCTT GGATATGATG TTCATTGCTC TTGTACCAAA CCTAACCTCA ACTTCCTGAA   
  
  
+ TTATTTAAGG CAATATTACC GTGTGCTGGT CAATTTTGGT TGTGGGTTTG TTTCTTAATG TATATGATTT   
  
  
+ ATGGTCAGGA TCCATGCTCC TCTTCCGTCC ACTCTATATC TCTCTCTCTC TCTCTCAATA CAAATTCAGA   
  
  
+ GACTTTCTCT TTCTGGGTTT ACTATTGATT GCAGAAGTTT GTAGTCTTGC GAGGCCTTGA GTCTTCTTCG   
  
  
+ AGGTAGAAAA TTTTGCTTTG CGCTTCCTTC CCCATATTTT AACACCCCTC CCCCCCCAAC GCAAGTTTTC   
  
  
+ TACATTTTTG TGCAATTCTT CAGCTATGAT TCCCCAATTT CACTTGTCAC ATCTCTATTT TCCTCTTTTT   
  
  
+ GGTTTCTTGA TTCTGTAGAA GGTTGTGTAT GAACTTCTTT GTTTCCCTTT TTCCTTTGTT ATTTTTAATG   
  
  
+ TTCCCATAGA TGATATAGAG ATCTGCTTTT CCTTGCTGTG AGAGGTATCT CTGAGAATGC ATGTAGCAAT   
  
  
+ CAAAATTCTG AGTTGTTAAT TCTATTGCAT GTCTATGGGT GATGATTGTG ATGATGTTTT TTCTGTTGCC   
  
  
+ CTCTTTCTCT CTTTCACTGA AATTGCTTTC CAGAATAGTT GTTTGACTCC TTTCTGTATC TTAATTGAGC   
  
  
+ CCTTGCTTTG GTTTGTGCTC TGGGAGTTCT TCTTTAGTAA TCCCTTGCTT GTATTTTCTG AAAGAGCAAG   
  
  
+ CAATAATCCT CATAGCATGT GTATAATCAT ACAATAGCAA CAGCAAACAT GGATCCTGCG CCCAGTTAGG   
  
  
+ TTTGGTTAGG TTAAGATATG CATATTCTTT TCTCTTGAAA AGAGTTGTCT AGGAAGGGTA ATTATGCCCA   
  
  
+ TTTCATGGAT AATAATGGTA CAATTAAGTT TGATTGGCTT AAATACCAAT TATTTAGCGT CTTTTTCTTT   
  
  
+ GGCTGTCCAG GGTTCCATTT CCATAAACAA CTTTGAGTAA GGCATAGAGA ACTAAATTTC TGCCAATTGT   
  
  
+ CAAGTGCAAG TTGCTCATAT GCAACAATAC TTGGAAATTT TGAGCTGAGC TGCTGGCATA TCATGAATAG   
  
  
+ TTCTAATGGT GGCGAGTTAT CACTTATCGT CCCTTGTGTG ACGAATTTCT CTTCCTAGGA AGCCCTCTAG   
  
  
+ TCAATGCCTA TCTGCTGTTG TGGAGTGAAA ATGGATACAT TTAGATCTCT ATAAACCCTA CTTTGGCAAG   
  
  
+ ATCCATGCTG ATTTTAGGAA GCCCTTTGAC CAGAGAATGA CATAACAACC TGATAGGGTG TTACATGCTA   
  
  
+ ACTTCGATTA TTTAATCAAA ATACGGAACT TCTATCAGTC AAAGCCTGCC CCAGGTTCTA CTGAATTAAA   
  
  
+ TTGTATTGAA TTGCTCTCAC TTGCTTATGA CGATTAAATA ACTGGATTTG CTAATTATTC TTTTTATTTT   
  
  
+ ATTCTTGGTG ATGTAGGGAA CTCAGGGAAT TGTAACCATA CCTGTTCCAG CTGAAAAGGG TACGGGAACA   
  
  
+ GTCATAGTTA CAGGGGAATT TCGCAATACA TTTAAGGGAG TAAGAAGCAG GATCATGTCT TTAGTTAGGC   
  
  
+ CGGCAGATAT GTCTGTGACT CCATATGGAA ATCCAACACT GTATTCACTT CAAGGCAATA ATAATAGCTC   
  
  
+ TGGTTTGTCT GGTCAACTGT ATGGCTCTGA AAAGCACAAG ATCAAATATG TGACCGAATC TTTTAATGGT   
  
  
+ CCAAGTTATG GCCTGAAATT CTTTGTTGAT TCTCCATCAG AAGAGCTCAT CCACCCATCA GATGCTACTC   
  
  
+ CTAACCCATT TGACTCTTCT TTCGTGGGCA TAAGACATGA TGAGAGTCCT TACCAAGGAA ATTATGGATC   
  
  
+ AGAATATGGG GTAAGCCCAT CTTCCGATGC AGTTGAATAT GATGAAGGTA ATAAGATAAG GCTAAAACTT   
  
  
+ CAAGAATTGG AGCATGCCCT GCTCGATGAA GATGATGAAG TGGATGAGGT GGCTATAGGG CCCGTGCTAG   
  
  
+ AACACAGCAT GGAGCTTGAT GAAGAGTGGG TCGCCCCATT GAGAAATGTG GTTTTCCATG ATTCACCAAA   
  
  
+ GGAGTCCACC TCCTCAGAGT CATCCAATGT CAGCAGCATT AGCAGCACCA AAGAAGTATC ACCTTGCTCT   
  
  
+ CCGAGTACTC CCAAACAATT GCTCTTTAAT TGTGCAAATA TGATTTCAGA GGGGAATTTT GAGGAAGCCG   
  
  
+ TGACTATGAT CAGCGAGCTT AGACAGATTG TTTCTATCCA AGGGGATCCA GCGCAGAGGA TAGCAGCCTA   
  
  
+ CATGGTAGAA GGCCTTGCTT CCCGTTTGGC TTCATCGGGA AAAGTTCTTT ACAAAGCTTT GAAATGCAAA   
  
  
+ GAGCCACCTT CATTTGACAG GCTAGCGGCT ATGCAAATCC TCTTTGAGGT GTGCCCATGT TTCAAATTCG   
  
  
+ GATTTATGGC CGCAAATGCA GCGATTATGG ATGCAATCAA AAACGAAAAA AGGGTACACA TCATAGATTT   
  
  
+ TGACATAAAC CAAGGGAATC AATACATAAA TCTCATGCAA TCCCTTGCTA AACAAGGTAA CAAGCTGACG   
  
  
+ CACTTGAAGT TGACTGGAGT TGATGACCCT GAGTCAGTTC AACGCCCTAT TGGTGGCCTA AAAAACATCG   
  
  
+ GACAAAGGCT GCAAGCATTA GCTGAATATC TTGGTGTGTC ATTTGAGTTC AAAGCAATAC CTGCTAGAAC   
  
  
+ TCCACTTGTT AACCCGGAAA TGTTAGAATG TCGACCTGCG GAAGCTTTAG TGGTGAACTT TGCCTTCCAG   
  
  
+ CTTCACCACA TGCCTGATGA AAGCGTCTCA ACAATCAACC TCCGAGACCA GCTTCTTCGG ATGGTCAAAA   
  
  
+ GCCTAAACCC AAAGCTAGTA ACCATTGTCG AGCAGGATGT GAACACAAAC ACTACCCCTT TCCTCTCTAG   
  
  
+ GTTTGCTGAG GCATACAGCT ACTATTCTGC TGTTTTTGAG TCTCTTGATG CTACTCTCCC TAGAGATAGC   
  
  
+ CAGGACAGGG TGAATGTGGA GAAGCAGTGC TTGGCACGTG ATGTTGTGAA CATCATTGCA TGTGAAGGGG   
  
  
+ AGGAGAGGAT TGAGCGCTAT GAGGTTGCGG GGAAATGGAG AGCGAGGATG TTGATGGCGG GATTCAGGGC   
  
  
+ TATTCCAATG GGTCAGAAGA CCGGTGATAT GATTCGGAAG CTTATATCAA TGCGGTATTG TGACAGGCTC   
  
  
+ AAGGTAAAAG AGGAGATGGA TGCACTTCAC TTTGGGTGGG AAGATAAAAA CTTAATCGTT ACATCAGCTT   
  
  
+ GGAGGTG  

- +Up\_Stream \_Len000TCTATC TGTAAGTCGG TGAACGGTCA AGTCTCTCTC TATCTCTCTC TAACCCATTC   
  
  
- GGGAAAAACG TGATGAAAAA GAGCGAAGGT AAAAAGAAAG AATTAAAAAT CACTACTAAA AGGTCTTCTA   
  
  
- AACTAAATAA GTAGTACGAA TTGGTTAACC CAGAAACTCA TAATAACAAA TGACCCATAA ACTAGAAAAG   
  
  
- TATAAGGAAA CCTGACCCAC CCACCTTGAA AACTTGAAAG AATAATCAAC CCGTACGGGA ATAGAATAAG   
  
  
- GTTAAGTTTT GGGTATAAAA AGAAGTTAAA ATTAATACCC ATAAAACTTT AACTTACGAA AGGAAGTGTC   
  
  
- GTTATGATAT AATGTATAAA ATGTCCTTTC ATTATAAAAT GTCCTTTCAT ACAACGACAA AGTTGGGACA   
  
  
- AAACAAAGGA GAGAAAGGAA CCTATACTAC AAGTAACGAG AACATGGTTT GGATTGGAGT TGAAGGACTT   
  
  
- AATAAATTCC GTTATAATGG CACACGACCA GTTAAAACCA ACACCCAAAC AAAGAATTAC ATATACTAAA   
  
  
- TACCAGTCCT AGGTACGAGG AGAAGGCAGG TGAGATATAG AGAGAGAGAG AGAGAGTTAT GTTTAAGTCT   
  
  
- CTGAAAGAGA AAGACCCAAA TGATAACTAA CGTCTTCAAA CATCAGAACG CTCCGGAACT CAGAAGAAGC   
  
  
- TCCATCTTTT AAAACGAAAC GCGAAGGAAG GGGTATAAAA TTGTGGGGAG GGGGGGGTTG CGTTCAAAAG   
  
  
- ATGTAAAAAC ACGTTAAGAA GTCGATACTA AGGGGTTAAA GTGAACAGTG TAGAGATAAA AGGAGAAAAA   
  
  
- CCAAAGAACT AAGACATCTT CCAACACATA CTTGAAGAAA CAAAGGGAAA AAGGAAACAA TAAAAATTAC   
  
  
- AAGGGTATCT ACTATATCTC TAGACGAAAA GGAACGACAC TCTCCATAGA GACTCTTACG TACATCGTTA   
  
  
- GTTTTAAGAC TCAACAATTA AGATAACGTA CAGATACCCA CTACTAACAC TACTACAAAA AAGACAACGG   
  
  
- GAGAAAGAGA GAAAGTGACT TTAACGAAAG GTCTTATCAA CAAACTGAGG AAAGACATAG AATTAACTCG   
  
  
- GGAACGAAAC CAAACACGAG ACCCTCAAGA AGAAATCATT AGGGAACGAA CATAAAAGAC TTTCTCGTTC   
  
  
- GTTATTAGGA GTATCGTACA CATATTAGTA TGTTATCGTT GTCGTTTGTA CCTAGGACGC GGGTCAATCC   
  
  
- AAACCAATCC AATTCTATAC GTATAAGAAA AGAGAACTTT TCTCAACAGA TCCTTCCCAT TAATACGGGT   
  
  
- AAAGTACCTA TTATTACCAT GTTAATTCAA ACTAACCGAA TTTATGGTTA ATAAATCGCA GAAAAAGAAA   
  
  
- CCGACAGGTC CCAAGGTAAA GGTATTTGTT GAAACTCATT CCGTATCTCT TGATTTAAAG ACGGTTAACA   
  
  
- GTTCACGTTC AACGAGTATA CGTTGTTATG AACCTTTAAA ACTCGACTCG ACGACCGTAT AGTACTTATC   
  
  
- AAGATTACCA CCGCTCAATA GTGAATAGCA GGGAACACAC TGCTTAAAGA GAAGGATCCT TCGGGAGATC   
  
  
- AGTTACGGAT AGACGACAAC ACCTCACTTT TACCTATGTA AATCTAGAGA TATTTGGGAT GAAACCGTTC   
  
  
- TAGGTACGAC TAAAATCCTT CGGGAAACTG GTCTCTTACT GTATTGTTGG ACTATCCCAC AATGTACGAT   
  
  
- TGAAGCTAAT AAATTAGTTT TATGCCTTGA AGATAGTCAG TTTCGGACGG GGTCCAAGAT GACTTAATTT   
  
  
- AACATAACTT AACGAGAGTG AACGAATACT GCTAATTTAT TGACCTAAAC GATTAATAAG AAAAATAAAA   
  
  
- TAAGAACCAC TACATCCCTT GAGTCCCTTA ACATTGGTAT GGACAAGGTC GACTTTTCCC ATGCCCTTGT   
  
  
- CAGTATCAAT GTCCCCTTAA AGCGTTATGT AAATTCCCTC ATTCTTCGTC CTAGTACAGA AATCAATCCG   
  
  
- GCCGTCTATA CAGACACTGA GGTATACCTT TAGGTTGTGA CATAAGTGAA GTTCCGTTAT TATTATCGAG   
  
  
- ACCAAACAGA CCAGTTGACA TACCGAGACT TTTCGTGTTC TAGTTTATAC ACTGGCTTAG AAAATTACCA   
  
  
- GGTTCAATAC CGGACTTTAA GAAACAACTA AGAGGTAGTC TTCTCGAGTA GGTGGGTAGT CTACGATGAG   
  
  
- GATTGGGTAA ACTGAGAAGA AAGCACCCGT ATTCTGTACT ACTCTCAGGA ATGGTTCCTT TAATACCTAG   
  
  
- TCTTATACCC CATTCGGGTA GAAGGCTACG TCAACTTATA CTACTTCCAT TATTCTATTC CGATTTTGAA   
  
  
- GTTCTTAACC TCGTACGGGA CGAGCTACTT CTACTACTTC ACCTACTCCA CCGATATCCC GGGCACGATC   
  
  
- TTGTGTCGTA CCTCGAACTA CTTCTCACCC AGCGGGGTAA CTCTTTACAC CAAAAGGTAC TAAGTGGTTT   
  
  
- CCTCAGGTGG AGGAGTCTCA GTAGGTTACA GTCGTCGTAA TCGTCGTGGT TTCTTCATAG TGGAACGAGA   
  
  
- GGCTCATGAG GGTTTGTTAA CGAGAAATTA ACACGTTTAT ACTAAAGTCT CCCCTTAAAA CTCCTTCGGC   
  
  
- ACTGATACTA GTCGCTCGAA TCTGTCTAAC AAAGATAGGT TCCCCTAGGT CGCGTCTCCT ATCGTCGGAT   
  
  
- GTACCATCTT CCGGAACGAA GGGCAAACCG AAGTAGCCCT TTTCAAGAAA TGTTTCGAAA CTTTACGTTT   
  
  
- CTCGGTGGAA GTAAACTGTC CGATCGCCGA TACGTTTAGG AGAAACTCCA CACGGGTACA AAGTTTAAGC   
  
  
- CTAAATACCG GCGTTTACGT CGCTAATACC TACGTTAGTT TTTGCTTTTT TCCCATGTGT AGTATCTAAA   
  
  
- ACTGTATTTG GTTCCCTTAG TTATGTATTT AGAGTACGTT AGGGAACGAT TTGTTCCATT GTTCGACTGC   
  
  
- GTGAACTTCA ACTGACCTCA ACTACTGGGA CTCAGTCAAG TTGCGGGATA ACCACCGGAT TTTTTGTAGC   
  
  
- CTGTTTCCGA CGTTCGTAAT CGACTTATAG AACCACACAG TAAACTCAAG TTTCGTTATG GACGATCTTG   
  
  
- AGGTGAACAA TTGGGCCTTT ACAATCTTAC AGCTGGACGC CTTCGAAATC ACCACTTGAA ACGGAAGGTC   
  
  
- GAAGTGGTGT ACGGACTACT TTCGCAGAGT TGTTAGTTGG AGGCTCTGGT CGAAGAAGCC TACCAGTTTT   
  
  
- CGGATTTGGG TTTCGATCAT TGGTAACAGC TCGTCCTACA CTTGTGTTTG TGATGGGGAA AGGAGAGATC   
  
  
- CAAACGACTC CGTATGTCGA TGATAAGACG ACAAAAACTC AGAGAACTAC GATGAGAGGG ATCTCTATCG   
  
  
- GTCCTGTCCC ACTTACACCT CTTCGTCACG AACCGTGCAC TACAACACTT GTAGTAACGT ACACTTCCCC   
  
  
- TCCTCTCCTA ACTCGCGATA CTCCAACGCC CCTTTACCTC TCGCTCCTAC AACTACCGCC CTAAGTCCCG   
  
  
- ATAAGGTTAC CCAGTCTTCT GGCCACTATA CTAAGCCTTC GAATATAGTT ACGCCATAAC ACTGTCCGAG   
  
  
- TTCCATTTTC TCCTCTACCT ACGTGAAGTG AAACCCACCC TTCTATTTTT GAATTAGCAA TGTAGTCGAA   
  
  
- CCTCCAC

+     W box

| Site Name | Organism | Position | Strand | Matrix score. | sequence | function |
| --- | --- | --- | --- | --- | --- | --- |
| W box | Arabidopsis thaliana | 522 | - | 6 | TTGACC |  |
| W box | Arabidopsis thaliana | 2115 | - | 6 | TTGACC |  |
| W box | Arabidopsis thaliana | 3287 | - | 6 | TTGACC |  |
| W box | Arabidopsis thaliana | 1710 | + | 6 | TTGACC |  |

>HU11G01134.1   
+ +Up\_Stream \_Len000AGATAG ACATTCAGCC ACTTGCCAGT TCAGAGAGAG ATAGAGAGAG ATTGGGTAAG   
  
  
+ CCCTTTTTGC ACTACTTTTT CTCGCTTCCA TTTTTCTTTC TTAATTTTTA GTGATGATTT TCCAGAAGAT   
  
  
+ TTGATTTATT CATCATGCTT AACCAATTGG GTCTTTGAGT ATTATTGTTT ACTGGGTATT TGATCTTTTC   
  
  
+ ATATTCCTTT GGACTGGGTG GGTGGAACTT TTGAACTTTC TTATTAGTTG GGCATGCCCT TATCTTATTC   
  
  
+ CAATTCAAAA CCCATATTTT TCTTCAATTT TAATTATGGG TATTTTGAAA TTGAATGCTT TCCTTCACAG   
  
  
+ CAATACTATA TTACATATTT TACAGGAAAG TAATATTTTA CAGGAAAGTA TGTTGCTGTT TCAACCCTGT   
  
  
+ TTTGTTTCCT CTCTTTCCTT GGATATGATG TTCATTGCTC TTGTACCAAA CCTAACCTCA ACTTCCTGAA   
  
  
+ TTATTTAAGG CAATATTACC GTGTGCTGGT CAATTTTGGT TGTGGGTTTG TTTCTTAATG TATATGATTT   
  
  
+ ATGGTCAGGA TCCATGCTCC TCTTCCGTCC ACTCTATATC TCTCTCTCTC TCTCTCAATA CAAATTCAGA   
  
  
+ GACTTTCTCT TTCTGGGTTT ACTATTGATT GCAGAAGTTT GTAGTCTTGC GAGGCCTTGA GTCTTCTTCG   
  
  
+ AGGTAGAAAA TTTTGCTTTG CGCTTCCTTC CCCATATTTT AACACCCCTC CCCCCCCAAC GCAAGTTTTC   
  
  
+ TACATTTTTG TGCAATTCTT CAGCTATGAT TCCCCAATTT CACTTGTCAC ATCTCTATTT TCCTCTTTTT   
  
  
+ GGTTTCTTGA TTCTGTAGAA GGTTGTGTAT GAACTTCTTT GTTTCCCTTT TTCCTTTGTT ATTTTTAATG   
  
  
+ TTCCCATAGA TGATATAGAG ATCTGCTTTT CCTTGCTGTG AGAGGTATCT CTGAGAATGC ATGTAGCAAT   
  
  
+ CAAAATTCTG AGTTGTTAAT TCTATTGCAT GTCTATGGGT GATGATTGTG ATGATGTTTT TTCTGTTGCC   
  
  
+ CTCTTTCTCT CTTTCACTGA AATTGCTTTC CAGAATAGTT GTTTGACTCC TTTCTGTATC TTAATTGAGC   
  
  
+ CCTTGCTTTG GTTTGTGCTC TGGGAGTTCT TCTTTAGTAA TCCCTTGCTT GTATTTTCTG AAAGAGCAAG   
  
  
+ CAATAATCCT CATAGCATGT GTATAATCAT ACAATAGCAA CAGCAAACAT GGATCCTGCG CCCAGTTAGG   
  
  
+ TTTGGTTAGG TTAAGATATG CATATTCTTT TCTCTTGAAA AGAGTTGTCT AGGAAGGGTA ATTATGCCCA   
  
  
+ TTTCATGGAT AATAATGGTA CAATTAAGTT TGATTGGCTT AAATACCAAT TATTTAGCGT CTTTTTCTTT   
  
  
+ GGCTGTCCAG GGTTCCATTT CCATAAACAA CTTTGAGTAA GGCATAGAGA ACTAAATTTC TGCCAATTGT   
  
  
+ CAAGTGCAAG TTGCTCATAT GCAACAATAC TTGGAAATTT TGAGCTGAGC TGCTGGCATA TCATGAATAG   
  
  
+ TTCTAATGGT GGCGAGTTAT CACTTATCGT CCCTTGTGTG ACGAATTTCT CTTCCTAGGA AGCCCTCTAG   
  
  
+ TCAATGCCTA TCTGCTGTTG TGGAGTGAAA ATGGATACAT TTAGATCTCT ATAAACCCTA CTTTGGCAAG   
  
  
+ ATCCATGCTG ATTTTAGGAA GCCCTTTGAC CAGAGAATGA CATAACAACC TGATAGGGTG TTACATGCTA   
  
  
+ ACTTCGATTA TTTAATCAAA ATACGGAACT TCTATCAGTC AAAGCCTGCC CCAGGTTCTA CTGAATTAAA   
  
  
+ TTGTATTGAA TTGCTCTCAC TTGCTTATGA CGATTAAATA ACTGGATTTG CTAATTATTC TTTTTATTTT   
  
  
+ ATTCTTGGTG ATGTAGGGAA CTCAGGGAAT TGTAACCATA CCTGTTCCAG CTGAAAAGGG TACGGGAACA   
  
  
+ GTCATAGTTA CAGGGGAATT TCGCAATACA TTTAAGGGAG TAAGAAGCAG GATCATGTCT TTAGTTAGGC   
  
  
+ CGGCAGATAT GTCTGTGACT CCATATGGAA ATCCAACACT GTATTCACTT CAAGGCAATA ATAATAGCTC   
  
  
+ TGGTTTGTCT GGTCAACTGT ATGGCTCTGA AAAGCACAAG ATCAAATATG TGACCGAATC TTTTAATGGT   
  
  
+ CCAAGTTATG GCCTGAAATT CTTTGTTGAT TCTCCATCAG AAGAGCTCAT CCACCCATCA GATGCTACTC   
  
  
+ CTAACCCATT TGACTCTTCT TTCGTGGGCA TAAGACATGA TGAGAGTCCT TACCAAGGAA ATTATGGATC   
  
  
+ AGAATATGGG GTAAGCCCAT CTTCCGATGC AGTTGAATAT GATGAAGGTA ATAAGATAAG GCTAAAACTT   
  
  
+ CAAGAATTGG AGCATGCCCT GCTCGATGAA GATGATGAAG TGGATGAGGT GGCTATAGGG CCCGTGCTAG   
  
  
+ AACACAGCAT GGAGCTTGAT GAAGAGTGGG TCGCCCCATT GAGAAATGTG GTTTTCCATG ATTCACCAAA   
  
  
+ GGAGTCCACC TCCTCAGAGT CATCCAATGT CAGCAGCATT AGCAGCACCA AAGAAGTATC ACCTTGCTCT   
  
  
+ CCGAGTACTC CCAAACAATT GCTCTTTAAT TGTGCAAATA TGATTTCAGA GGGGAATTTT GAGGAAGCCG   
  
  
+ TGACTATGAT CAGCGAGCTT AGACAGATTG TTTCTATCCA AGGGGATCCA GCGCAGAGGA TAGCAGCCTA   
  
  
+ CATGGTAGAA GGCCTTGCTT CCCGTTTGGC TTCATCGGGA AAAGTTCTTT ACAAAGCTTT GAAATGCAAA   
  
  
+ GAGCCACCTT CATTTGACAG GCTAGCGGCT ATGCAAATCC TCTTTGAGGT GTGCCCATGT TTCAAATTCG   
  
  
+ GATTTATGGC CGCAAATGCA GCGATTATGG ATGCAATCAA AAACGAAAAA AGGGTACACA TCATAGATTT   
  
  
+ TGACATAAAC CAAGGGAATC AATACATAAA TCTCATGCAA TCCCTTGCTA AACAAGGTAA CAAGCTGACG   
  
  
+ CACTTGAAGT TGACTGGAGT TGATGACCCT GAGTCAGTTC AACGCCCTAT TGGTGGCCTA AAAAACATCG   
  
  
+ GACAAAGGCT GCAAGCATTA GCTGAATATC TTGGTGTGTC ATTTGAGTTC AAAGCAATAC CTGCTAGAAC   
  
  
+ TCCACTTGTT AACCCGGAAA TGTTAGAATG TCGACCTGCG GAAGCTTTAG TGGTGAACTT TGCCTTCCAG   
  
  
+ CTTCACCACA TGCCTGATGA AAGCGTCTCA ACAATCAACC TCCGAGACCA GCTTCTTCGG ATGGTCAAAA   
  
  
+ GCCTAAACCC AAAGCTAGTA ACCATTGTCG AGCAGGATGT GAACACAAAC ACTACCCCTT TCCTCTCTAG   
  
  
+ GTTTGCTGAG GCATACAGCT ACTATTCTGC TGTTTTTGAG TCTCTTGATG CTACTCTCCC TAGAGATAGC   
  
  
+ CAGGACAGGG TGAATGTGGA GAAGCAGTGC TTGGCACGTG ATGTTGTGAA CATCATTGCA TGTGAAGGGG   
  
  
+ AGGAGAGGAT TGAGCGCTAT GAGGTTGCGG GGAAATGGAG AGCGAGGATG TTGATGGCGG GATTCAGGGC   
  
  
+ TATTCCAATG GGTCAGAAGA CCGGTGATAT GATTCGGAAG CTTATATCAA TGCGGTATTG TGACAGGCTC   
  
  
+ AAGGTAAAAG AGGAGATGGA TGCACTTCAC TTTGGGTGGG AAGATAAAAA CTTAATCGTT ACATCAGCTT   
  
  
+ GGAGGTG  

- +Up\_Stream \_Len000TCTATC TGTAAGTCGG TGAACGGTCA AGTCTCTCTC TATCTCTCTC TAACCCATTC   
  
  
- GGGAAAAACG TGATGAAAAA GAGCGAAGGT AAAAAGAAAG AATTAAAAAT CACTACTAAA AGGTCTTCTA   
  
  
- AACTAAATAA GTAGTACGAA TTGGTTAACC CAGAAACTCA TAATAACAAA TGACCCATAA ACTAGAAAAG   
  
  
- TATAAGGAAA CCTGACCCAC CCACCTTGAA AACTTGAAAG AATAATCAAC CCGTACGGGA ATAGAATAAG   
  
  
- GTTAAGTTTT GGGTATAAAA AGAAGTTAAA ATTAATACCC ATAAAACTTT AACTTACGAA AGGAAGTGTC   
  
  
- GTTATGATAT AATGTATAAA ATGTCCTTTC ATTATAAAAT GTCCTTTCAT ACAACGACAA AGTTGGGACA   
  
  
- AAACAAAGGA GAGAAAGGAA CCTATACTAC AAGTAACGAG AACATGGTTT GGATTGGAGT TGAAGGACTT   
  
  
- AATAAATTCC GTTATAATGG CACACGACCA GTTAAAACCA ACACCCAAAC AAAGAATTAC ATATACTAAA   
  
  
- TACCAGTCCT AGGTACGAGG AGAAGGCAGG TGAGATATAG AGAGAGAGAG AGAGAGTTAT GTTTAAGTCT   
  
  
- CTGAAAGAGA AAGACCCAAA TGATAACTAA CGTCTTCAAA CATCAGAACG CTCCGGAACT CAGAAGAAGC   
  
  
- TCCATCTTTT AAAACGAAAC GCGAAGGAAG GGGTATAAAA TTGTGGGGAG GGGGGGGTTG CGTTCAAAAG   
  
  
- ATGTAAAAAC ACGTTAAGAA GTCGATACTA AGGGGTTAAA GTGAACAGTG TAGAGATAAA AGGAGAAAAA   
  
  
- CCAAAGAACT AAGACATCTT CCAACACATA CTTGAAGAAA CAAAGGGAAA AAGGAAACAA TAAAAATTAC   
  
  
- AAGGGTATCT ACTATATCTC TAGACGAAAA GGAACGACAC TCTCCATAGA GACTCTTACG TACATCGTTA   
  
  
- GTTTTAAGAC TCAACAATTA AGATAACGTA CAGATACCCA CTACTAACAC TACTACAAAA AAGACAACGG   
  
  
- GAGAAAGAGA GAAAGTGACT TTAACGAAAG GTCTTATCAA CAAACTGAGG AAAGACATAG AATTAACTCG   
  
  
- GGAACGAAAC CAAACACGAG ACCCTCAAGA AGAAATCATT AGGGAACGAA CATAAAAGAC TTTCTCGTTC   
  
  
- GTTATTAGGA GTATCGTACA CATATTAGTA TGTTATCGTT GTCGTTTGTA CCTAGGACGC GGGTCAATCC   
  
  
- AAACCAATCC AATTCTATAC GTATAAGAAA AGAGAACTTT TCTCAACAGA TCCTTCCCAT TAATACGGGT   
  
  
- AAAGTACCTA TTATTACCAT GTTAATTCAA ACTAACCGAA TTTATGGTTA ATAAATCGCA GAAAAAGAAA   
  
  
- CCGACAGGTC CCAAGGTAAA GGTATTTGTT GAAACTCATT CCGTATCTCT TGATTTAAAG ACGGTTAACA   
  
  
- GTTCACGTTC AACGAGTATA CGTTGTTATG AACCTTTAAA ACTCGACTCG ACGACCGTAT AGTACTTATC   
  
  
- AAGATTACCA CCGCTCAATA GTGAATAGCA GGGAACACAC TGCTTAAAGA GAAGGATCCT TCGGGAGATC   
  
  
- AGTTACGGAT AGACGACAAC ACCTCACTTT TACCTATGTA AATCTAGAGA TATTTGGGAT GAAACCGTTC   
  
  
- TAGGTACGAC TAAAATCCTT CGGGAAACTG GTCTCTTACT GTATTGTTGG ACTATCCCAC AATGTACGAT   
  
  
- TGAAGCTAAT AAATTAGTTT TATGCCTTGA AGATAGTCAG TTTCGGACGG GGTCCAAGAT GACTTAATTT   
  
  
- AACATAACTT AACGAGAGTG AACGAATACT GCTAATTTAT TGACCTAAAC GATTAATAAG AAAAATAAAA   
  
  
- TAAGAACCAC TACATCCCTT GAGTCCCTTA ACATTGGTAT GGACAAGGTC GACTTTTCCC ATGCCCTTGT   
  
  
- CAGTATCAAT GTCCCCTTAA AGCGTTATGT AAATTCCCTC ATTCTTCGTC CTAGTACAGA AATCAATCCG   
  
  
- GCCGTCTATA CAGACACTGA GGTATACCTT TAGGTTGTGA CATAAGTGAA GTTCCGTTAT TATTATCGAG   
  
  
- ACCAAACAGA CCAGTTGACA TACCGAGACT TTTCGTGTTC TAGTTTATAC ACTGGCTTAG AAAATTACCA   
  
  
- GGTTCAATAC CGGACTTTAA GAAACAACTA AGAGGTAGTC TTCTCGAGTA GGTGGGTAGT CTACGATGAG   
  
  
- GATTGGGTAA ACTGAGAAGA AAGCACCCGT ATTCTGTACT ACTCTCAGGA ATGGTTCCTT TAATACCTAG   
  
  
- TCTTATACCC CATTCGGGTA GAAGGCTACG TCAACTTATA CTACTTCCAT TATTCTATTC CGATTTTGAA   
  
  
- GTTCTTAACC TCGTACGGGA CGAGCTACTT CTACTACTTC ACCTACTCCA CCGATATCCC GGGCACGATC   
  
  
- TTGTGTCGTA CCTCGAACTA CTTCTCACCC AGCGGGGTAA CTCTTTACAC CAAAAGGTAC TAAGTGGTTT   
  
  
- CCTCAGGTGG AGGAGTCTCA GTAGGTTACA GTCGTCGTAA TCGTCGTGGT TTCTTCATAG TGGAACGAGA   
  
  
- GGCTCATGAG GGTTTGTTAA CGAGAAATTA ACACGTTTAT ACTAAAGTCT CCCCTTAAAA CTCCTTCGGC   
  
  
- ACTGATACTA GTCGCTCGAA TCTGTCTAAC AAAGATAGGT TCCCCTAGGT CGCGTCTCCT ATCGTCGGAT   
  
  
- GTACCATCTT CCGGAACGAA GGGCAAACCG AAGTAGCCCT TTTCAAGAAA TGTTTCGAAA CTTTACGTTT   
  
  
- CTCGGTGGAA GTAAACTGTC CGATCGCCGA TACGTTTAGG AGAAACTCCA CACGGGTACA AAGTTTAAGC   
  
  
- CTAAATACCG GCGTTTACGT CGCTAATACC TACGTTAGTT TTTGCTTTTT TCCCATGTGT AGTATCTAAA   
  
  
- ACTGTATTTG GTTCCCTTAG TTATGTATTT AGAGTACGTT AGGGAACGAT TTGTTCCATT GTTCGACTGC   
  
  
- GTGAACTTCA ACTGACCTCA ACTACTGGGA CTCAGTCAAG TTGCGGGATA ACCACCGGAT TTTTTGTAGC   
  
  
- CTGTTTCCGA CGTTCGTAAT CGACTTATAG AACCACACAG TAAACTCAAG TTTCGTTATG GACGATCTTG   
  
  
- AGGTGAACAA TTGGGCCTTT ACAATCTTAC AGCTGGACGC CTTCGAAATC ACCACTTGAA ACGGAAGGTC   
  
  
- GAAGTGGTGT ACGGACTACT TTCGCAGAGT TGTTAGTTGG AGGCTCTGGT CGAAGAAGCC TACCAGTTTT   
  
  
- CGGATTTGGG TTTCGATCAT TGGTAACAGC TCGTCCTACA CTTGTGTTTG TGATGGGGAA AGGAGAGATC   
  
  
- CAAACGACTC CGTATGTCGA TGATAAGACG ACAAAAACTC AGAGAACTAC GATGAGAGGG ATCTCTATCG   
  
  
- GTCCTGTCCC ACTTACACCT CTTCGTCACG AACCGTGCAC TACAACACTT GTAGTAACGT ACACTTCCCC   
  
  
- TCCTCTCCTA ACTCGCGATA CTCCAACGCC CCTTTACCTC TCGCTCCTAC AACTACCGCC CTAAGTCCCG   
  
  
- ATAAGGTTAC CCAGTCTTCT GGCCACTATA CTAAGCCTTC GAATATAGTT ACGCCATAAC ACTGTCCGAG   
  
  
- TTCCATTTTC TCCTCTACCT ACGTGAAGTG AAACCCACCC TTCTATTTTT GAATTAGCAA TGTAGTCGAA   
  
  
- CCTCCAC

+     WRE3

| Site Name | Organism | Position | Strand | Matrix score. | sequence | function |
| --- | --- | --- | --- | --- | --- | --- |
| WRE3 | Pisum sativum | 2808 | + | 6 | CCACCT |  |
| WRE3 | Pisum sativum | 2530 | + | 6 | CCACCT |  |
| WRE3 | Pisum sativum | 2431 | - | 6 | CCACCT |  |

>HU11G01134.1   
+ +Up\_Stream \_Len000AGATAG ACATTCAGCC ACTTGCCAGT TCAGAGAGAG ATAGAGAGAG ATTGGGTAAG   
  
  
+ CCCTTTTTGC ACTACTTTTT CTCGCTTCCA TTTTTCTTTC TTAATTTTTA GTGATGATTT TCCAGAAGAT   
  
  
+ TTGATTTATT CATCATGCTT AACCAATTGG GTCTTTGAGT ATTATTGTTT ACTGGGTATT TGATCTTTTC   
  
  
+ ATATTCCTTT GGACTGGGTG GGTGGAACTT TTGAACTTTC TTATTAGTTG GGCATGCCCT TATCTTATTC   
  
  
+ CAATTCAAAA CCCATATTTT TCTTCAATTT TAATTATGGG TATTTTGAAA TTGAATGCTT TCCTTCACAG   
  
  
+ CAATACTATA TTACATATTT TACAGGAAAG TAATATTTTA CAGGAAAGTA TGTTGCTGTT TCAACCCTGT   
  
  
+ TTTGTTTCCT CTCTTTCCTT GGATATGATG TTCATTGCTC TTGTACCAAA CCTAACCTCA ACTTCCTGAA   
  
  
+ TTATTTAAGG CAATATTACC GTGTGCTGGT CAATTTTGGT TGTGGGTTTG TTTCTTAATG TATATGATTT   
  
  
+ ATGGTCAGGA TCCATGCTCC TCTTCCGTCC ACTCTATATC TCTCTCTCTC TCTCTCAATA CAAATTCAGA   
  
  
+ GACTTTCTCT TTCTGGGTTT ACTATTGATT GCAGAAGTTT GTAGTCTTGC GAGGCCTTGA GTCTTCTTCG   
  
  
+ AGGTAGAAAA TTTTGCTTTG CGCTTCCTTC CCCATATTTT AACACCCCTC CCCCCCCAAC GCAAGTTTTC   
  
  
+ TACATTTTTG TGCAATTCTT CAGCTATGAT TCCCCAATTT CACTTGTCAC ATCTCTATTT TCCTCTTTTT   
  
  
+ GGTTTCTTGA TTCTGTAGAA GGTTGTGTAT GAACTTCTTT GTTTCCCTTT TTCCTTTGTT ATTTTTAATG   
  
  
+ TTCCCATAGA TGATATAGAG ATCTGCTTTT CCTTGCTGTG AGAGGTATCT CTGAGAATGC ATGTAGCAAT   
  
  
+ CAAAATTCTG AGTTGTTAAT TCTATTGCAT GTCTATGGGT GATGATTGTG ATGATGTTTT TTCTGTTGCC   
  
  
+ CTCTTTCTCT CTTTCACTGA AATTGCTTTC CAGAATAGTT GTTTGACTCC TTTCTGTATC TTAATTGAGC   
  
  
+ CCTTGCTTTG GTTTGTGCTC TGGGAGTTCT TCTTTAGTAA TCCCTTGCTT GTATTTTCTG AAAGAGCAAG   
  
  
+ CAATAATCCT CATAGCATGT GTATAATCAT ACAATAGCAA CAGCAAACAT GGATCCTGCG CCCAGTTAGG   
  
  
+ TTTGGTTAGG TTAAGATATG CATATTCTTT TCTCTTGAAA AGAGTTGTCT AGGAAGGGTA ATTATGCCCA   
  
  
+ TTTCATGGAT AATAATGGTA CAATTAAGTT TGATTGGCTT AAATACCAAT TATTTAGCGT CTTTTTCTTT   
  
  
+ GGCTGTCCAG GGTTCCATTT CCATAAACAA CTTTGAGTAA GGCATAGAGA ACTAAATTTC TGCCAATTGT   
  
  
+ CAAGTGCAAG TTGCTCATAT GCAACAATAC TTGGAAATTT TGAGCTGAGC TGCTGGCATA TCATGAATAG   
  
  
+ TTCTAATGGT GGCGAGTTAT CACTTATCGT CCCTTGTGTG ACGAATTTCT CTTCCTAGGA AGCCCTCTAG   
  
  
+ TCAATGCCTA TCTGCTGTTG TGGAGTGAAA ATGGATACAT TTAGATCTCT ATAAACCCTA CTTTGGCAAG   
  
  
+ ATCCATGCTG ATTTTAGGAA GCCCTTTGAC CAGAGAATGA CATAACAACC TGATAGGGTG TTACATGCTA   
  
  
+ ACTTCGATTA TTTAATCAAA ATACGGAACT TCTATCAGTC AAAGCCTGCC CCAGGTTCTA CTGAATTAAA   
  
  
+ TTGTATTGAA TTGCTCTCAC TTGCTTATGA CGATTAAATA ACTGGATTTG CTAATTATTC TTTTTATTTT   
  
  
+ ATTCTTGGTG ATGTAGGGAA CTCAGGGAAT TGTAACCATA CCTGTTCCAG CTGAAAAGGG TACGGGAACA   
  
  
+ GTCATAGTTA CAGGGGAATT TCGCAATACA TTTAAGGGAG TAAGAAGCAG GATCATGTCT TTAGTTAGGC   
  
  
+ CGGCAGATAT GTCTGTGACT CCATATGGAA ATCCAACACT GTATTCACTT CAAGGCAATA ATAATAGCTC   
  
  
+ TGGTTTGTCT GGTCAACTGT ATGGCTCTGA AAAGCACAAG ATCAAATATG TGACCGAATC TTTTAATGGT   
  
  
+ CCAAGTTATG GCCTGAAATT CTTTGTTGAT TCTCCATCAG AAGAGCTCAT CCACCCATCA GATGCTACTC   
  
  
+ CTAACCCATT TGACTCTTCT TTCGTGGGCA TAAGACATGA TGAGAGTCCT TACCAAGGAA ATTATGGATC   
  
  
+ AGAATATGGG GTAAGCCCAT CTTCCGATGC AGTTGAATAT GATGAAGGTA ATAAGATAAG GCTAAAACTT   
  
  
+ CAAGAATTGG AGCATGCCCT GCTCGATGAA GATGATGAAG TGGATGAGGT GGCTATAGGG CCCGTGCTAG   
  
  
+ AACACAGCAT GGAGCTTGAT GAAGAGTGGG TCGCCCCATT GAGAAATGTG GTTTTCCATG ATTCACCAAA   
  
  
+ GGAGTCCACC TCCTCAGAGT CATCCAATGT CAGCAGCATT AGCAGCACCA AAGAAGTATC ACCTTGCTCT   
  
  
+ CCGAGTACTC CCAAACAATT GCTCTTTAAT TGTGCAAATA TGATTTCAGA GGGGAATTTT GAGGAAGCCG   
  
  
+ TGACTATGAT CAGCGAGCTT AGACAGATTG TTTCTATCCA AGGGGATCCA GCGCAGAGGA TAGCAGCCTA   
  
  
+ CATGGTAGAA GGCCTTGCTT CCCGTTTGGC TTCATCGGGA AAAGTTCTTT ACAAAGCTTT GAAATGCAAA   
  
  
+ GAGCCACCTT CATTTGACAG GCTAGCGGCT ATGCAAATCC TCTTTGAGGT GTGCCCATGT TTCAAATTCG   
  
  
+ GATTTATGGC CGCAAATGCA GCGATTATGG ATGCAATCAA AAACGAAAAA AGGGTACACA TCATAGATTT   
  
  
+ TGACATAAAC CAAGGGAATC AATACATAAA TCTCATGCAA TCCCTTGCTA AACAAGGTAA CAAGCTGACG   
  
  
+ CACTTGAAGT TGACTGGAGT TGATGACCCT GAGTCAGTTC AACGCCCTAT TGGTGGCCTA AAAAACATCG   
  
  
+ GACAAAGGCT GCAAGCATTA GCTGAATATC TTGGTGTGTC ATTTGAGTTC AAAGCAATAC CTGCTAGAAC   
  
  
+ TCCACTTGTT AACCCGGAAA TGTTAGAATG TCGACCTGCG GAAGCTTTAG TGGTGAACTT TGCCTTCCAG   
  
  
+ CTTCACCACA TGCCTGATGA AAGCGTCTCA ACAATCAACC TCCGAGACCA GCTTCTTCGG ATGGTCAAAA   
  
  
+ GCCTAAACCC AAAGCTAGTA ACCATTGTCG AGCAGGATGT GAACACAAAC ACTACCCCTT TCCTCTCTAG   
  
  
+ GTTTGCTGAG GCATACAGCT ACTATTCTGC TGTTTTTGAG TCTCTTGATG CTACTCTCCC TAGAGATAGC   
  
  
+ CAGGACAGGG TGAATGTGGA GAAGCAGTGC TTGGCACGTG ATGTTGTGAA CATCATTGCA TGTGAAGGGG   
  
  
+ AGGAGAGGAT TGAGCGCTAT GAGGTTGCGG GGAAATGGAG AGCGAGGATG TTGATGGCGG GATTCAGGGC   
  
  
+ TATTCCAATG GGTCAGAAGA CCGGTGATAT GATTCGGAAG CTTATATCAA TGCGGTATTG TGACAGGCTC   
  
  
+ AAGGTAAAAG AGGAGATGGA TGCACTTCAC TTTGGGTGGG AAGATAAAAA CTTAATCGTT ACATCAGCTT   
  
  
+ GGAGGTG  

- +Up\_Stream \_Len000TCTATC TGTAAGTCGG TGAACGGTCA AGTCTCTCTC TATCTCTCTC TAACCCATTC   
  
  
- GGGAAAAACG TGATGAAAAA GAGCGAAGGT AAAAAGAAAG AATTAAAAAT CACTACTAAA AGGTCTTCTA   
  
  
- AACTAAATAA GTAGTACGAA TTGGTTAACC CAGAAACTCA TAATAACAAA TGACCCATAA ACTAGAAAAG   
  
  
- TATAAGGAAA CCTGACCCAC CCACCTTGAA AACTTGAAAG AATAATCAAC CCGTACGGGA ATAGAATAAG   
  
  
- GTTAAGTTTT GGGTATAAAA AGAAGTTAAA ATTAATACCC ATAAAACTTT AACTTACGAA AGGAAGTGTC   
  
  
- GTTATGATAT AATGTATAAA ATGTCCTTTC ATTATAAAAT GTCCTTTCAT ACAACGACAA AGTTGGGACA   
  
  
- AAACAAAGGA GAGAAAGGAA CCTATACTAC AAGTAACGAG AACATGGTTT GGATTGGAGT TGAAGGACTT   
  
  
- AATAAATTCC GTTATAATGG CACACGACCA GTTAAAACCA ACACCCAAAC AAAGAATTAC ATATACTAAA   
  
  
- TACCAGTCCT AGGTACGAGG AGAAGGCAGG TGAGATATAG AGAGAGAGAG AGAGAGTTAT GTTTAAGTCT   
  
  
- CTGAAAGAGA AAGACCCAAA TGATAACTAA CGTCTTCAAA CATCAGAACG CTCCGGAACT CAGAAGAAGC   
  
  
- TCCATCTTTT AAAACGAAAC GCGAAGGAAG GGGTATAAAA TTGTGGGGAG GGGGGGGTTG CGTTCAAAAG   
  
  
- ATGTAAAAAC ACGTTAAGAA GTCGATACTA AGGGGTTAAA GTGAACAGTG TAGAGATAAA AGGAGAAAAA   
  
  
- CCAAAGAACT AAGACATCTT CCAACACATA CTTGAAGAAA CAAAGGGAAA AAGGAAACAA TAAAAATTAC   
  
  
- AAGGGTATCT ACTATATCTC TAGACGAAAA GGAACGACAC TCTCCATAGA GACTCTTACG TACATCGTTA   
  
  
- GTTTTAAGAC TCAACAATTA AGATAACGTA CAGATACCCA CTACTAACAC TACTACAAAA AAGACAACGG   
  
  
- GAGAAAGAGA GAAAGTGACT TTAACGAAAG GTCTTATCAA CAAACTGAGG AAAGACATAG AATTAACTCG   
  
  
- GGAACGAAAC CAAACACGAG ACCCTCAAGA AGAAATCATT AGGGAACGAA CATAAAAGAC TTTCTCGTTC   
  
  
- GTTATTAGGA GTATCGTACA CATATTAGTA TGTTATCGTT GTCGTTTGTA CCTAGGACGC GGGTCAATCC   
  
  
- AAACCAATCC AATTCTATAC GTATAAGAAA AGAGAACTTT TCTCAACAGA TCCTTCCCAT TAATACGGGT   
  
  
- AAAGTACCTA TTATTACCAT GTTAATTCAA ACTAACCGAA TTTATGGTTA ATAAATCGCA GAAAAAGAAA   
  
  
- CCGACAGGTC CCAAGGTAAA GGTATTTGTT GAAACTCATT CCGTATCTCT TGATTTAAAG ACGGTTAACA   
  
  
- GTTCACGTTC AACGAGTATA CGTTGTTATG AACCTTTAAA ACTCGACTCG ACGACCGTAT AGTACTTATC   
  
  
- AAGATTACCA CCGCTCAATA GTGAATAGCA GGGAACACAC TGCTTAAAGA GAAGGATCCT TCGGGAGATC   
  
  
- AGTTACGGAT AGACGACAAC ACCTCACTTT TACCTATGTA AATCTAGAGA TATTTGGGAT GAAACCGTTC   
  
  
- TAGGTACGAC TAAAATCCTT CGGGAAACTG GTCTCTTACT GTATTGTTGG ACTATCCCAC AATGTACGAT   
  
  
- TGAAGCTAAT AAATTAGTTT TATGCCTTGA AGATAGTCAG TTTCGGACGG GGTCCAAGAT GACTTAATTT   
  
  
- AACATAACTT AACGAGAGTG AACGAATACT GCTAATTTAT TGACCTAAAC GATTAATAAG AAAAATAAAA   
  
  
- TAAGAACCAC TACATCCCTT GAGTCCCTTA ACATTGGTAT GGACAAGGTC GACTTTTCCC ATGCCCTTGT   
  
  
- CAGTATCAAT GTCCCCTTAA AGCGTTATGT AAATTCCCTC ATTCTTCGTC CTAGTACAGA AATCAATCCG   
  
  
- GCCGTCTATA CAGACACTGA GGTATACCTT TAGGTTGTGA CATAAGTGAA GTTCCGTTAT TATTATCGAG   
  
  
- ACCAAACAGA CCAGTTGACA TACCGAGACT TTTCGTGTTC TAGTTTATAC ACTGGCTTAG AAAATTACCA   
  
  
- GGTTCAATAC CGGACTTTAA GAAACAACTA AGAGGTAGTC TTCTCGAGTA GGTGGGTAGT CTACGATGAG   
  
  
- GATTGGGTAA ACTGAGAAGA AAGCACCCGT ATTCTGTACT ACTCTCAGGA ATGGTTCCTT TAATACCTAG   
  
  
- TCTTATACCC CATTCGGGTA GAAGGCTACG TCAACTTATA CTACTTCCAT TATTCTATTC CGATTTTGAA   
  
  
- GTTCTTAACC TCGTACGGGA CGAGCTACTT CTACTACTTC ACCTACTCCA CCGATATCCC GGGCACGATC   
  
  
- TTGTGTCGTA CCTCGAACTA CTTCTCACCC AGCGGGGTAA CTCTTTACAC CAAAAGGTAC TAAGTGGTTT   
  
  
- CCTCAGGTGG AGGAGTCTCA GTAGGTTACA GTCGTCGTAA TCGTCGTGGT TTCTTCATAG TGGAACGAGA   
  
  
- GGCTCATGAG GGTTTGTTAA CGAGAAATTA ACACGTTTAT ACTAAAGTCT CCCCTTAAAA CTCCTTCGGC   
  
  
- ACTGATACTA GTCGCTCGAA TCTGTCTAAC AAAGATAGGT TCCCCTAGGT CGCGTCTCCT ATCGTCGGAT   
  
  
- GTACCATCTT CCGGAACGAA GGGCAAACCG AAGTAGCCCT TTTCAAGAAA TGTTTCGAAA CTTTACGTTT   
  
  
- CTCGGTGGAA GTAAACTGTC CGATCGCCGA TACGTTTAGG AGAAACTCCA CACGGGTACA AAGTTTAAGC   
  
  
- CTAAATACCG GCGTTTACGT CGCTAATACC TACGTTAGTT TTTGCTTTTT TCCCATGTGT AGTATCTAAA   
  
  
- ACTGTATTTG GTTCCCTTAG TTATGTATTT AGAGTACGTT AGGGAACGAT TTGTTCCATT GTTCGACTGC   
  
  
- GTGAACTTCA ACTGACCTCA ACTACTGGGA CTCAGTCAAG TTGCGGGATA ACCACCGGAT TTTTTGTAGC   
  
  
- CTGTTTCCGA CGTTCGTAAT CGACTTATAG AACCACACAG TAAACTCAAG TTTCGTTATG GACGATCTTG   
  
  
- AGGTGAACAA TTGGGCCTTT ACAATCTTAC AGCTGGACGC CTTCGAAATC ACCACTTGAA ACGGAAGGTC   
  
  
- GAAGTGGTGT ACGGACTACT TTCGCAGAGT TGTTAGTTGG AGGCTCTGGT CGAAGAAGCC TACCAGTTTT   
  
  
- CGGATTTGGG TTTCGATCAT TGGTAACAGC TCGTCCTACA CTTGTGTTTG TGATGGGGAA AGGAGAGATC   
  
  
- CAAACGACTC CGTATGTCGA TGATAAGACG ACAAAAACTC AGAGAACTAC GATGAGAGGG ATCTCTATCG   
  
  
- GTCCTGTCCC ACTTACACCT CTTCGTCACG AACCGTGCAC TACAACACTT GTAGTAACGT ACACTTCCCC   
  
  
- TCCTCTCCTA ACTCGCGATA CTCCAACGCC CCTTTACCTC TCGCTCCTAC AACTACCGCC CTAAGTCCCG   
  
  
- ATAAGGTTAC CCAGTCTTCT GGCCACTATA CTAAGCCTTC GAATATAGTT ACGCCATAAC ACTGTCCGAG   
  
  
- TTCCATTTTC TCCTCTACCT ACGTGAAGTG AAACCCACCC TTCTATTTTT GAATTAGCAA TGTAGTCGAA   
  
  
- CCTCCAC

+     Y-box

| Site Name | Organism | Position | Strand | Matrix score. | sequence | function |
| --- | --- | --- | --- | --- | --- | --- |
| Y-box | Lemna gibba | 3449 | + | 11 | TGTGGAGGAGCA | ? |

>HU11G01134.1   
+ +Up\_Stream \_Len000AGATAG ACATTCAGCC ACTTGCCAGT TCAGAGAGAG ATAGAGAGAG ATTGGGTAAG
[truncated: 28,603 more chars]
