# Supplementary material for: Genome-Wide Identification and Expression Pattern of the GRAS Gene Family in Pitaya (Selenicereus undatus L.)
Source: Biology (Basel). 2022 Dec 21;12(1):11. doi: 10.3390/biology12010011 (PMC9854919; doi:10.3390/biology12010011)
Supplement: Supplementary file 1 [file biology-12-00011-s001.zip › Supplementary file S5/HU11G01529.1_plantcare.html]

Content-Type: text/html; charset=ISO-8859-1


PlantCARE


Webmaster Firefox specific output  
To save the result:
click on the frame with the right mouse button and save the source code as a text file with extension .html  
REFERENCE:PlantCARE: a database of plant cis-acting regulatory elements and a portal to tools for in silico analysis of promoter sequences.  
Lescot, M., Déhais, P., Moreau, Y., De Moor, B., Rouzé ,P.,and Rombauts, S.  
Nucleic Acids Res., Database issue(2002), 30(1):325-327.   


---

>HU11G01529.1   
+ +Up\_Stream \_Len000GAAAGA AGATTTAAGG AAATCGATGG TACACGGCAA AGCTAGAAAC AGGAACTGTA   
  
  
+ TTTTTATGTT TTTTTTTTGT TGGAAGGGGG GGGGGGGGAG CGGGCGAGAT GTAACCCGTA TCTAGATGGA   
  
  
+ TAATCTACGT GACAAGACCT TCATTTGATC AAGGTACATC ATGAATATTT AACCCTTAAC TTCTTTGAGC   
  
  
+ CTTACAAGAG ATTGTCTGAG GTTTTTAGGG TGCACGATAT CATGCCTGAC TAGACCTGTG TAGACACAAT   
  
  
+ CTTCAGCCCT TATTGATAGC TGTTCGATAT GCATGCTGCC TAAAAATTGG AATAGTTGGC ACCTAACATT   
  
  
+ CTTTTACATC TTGCTCCTCC TTGGTGTAGA GTTAGATGTG CTAGCACAAT ACAATGGGTC CCGATATTTT   
  
  
+ GCATCTGGTT TCTGAAAGAT AATATTTGAT GATCCTGTCT TGTGCTGATG GGCAAAAGCT AAATTGTAAG   
  
  
+ AATATGTAGT TTATTTACAA GACATATGCT TAAGCTAGAG GTCATGTACT TGCATATTTT TGTGCAGCTT   
  
  
+ TTGAGGTTGT ATGCCCCTTT GAGACAAATT CATAGTTGAA TTCATGACAA GATTATGTTT GGCAATGACT   
  
  
+ TTGATTATTA AATTGGCCTT TATGCTATCC TATCCTGTGT GCTCAAATGT TATTTATCAG ATAATATTAG   
  
  
+ TTTACTCTTT CTTCCCTGTC TTGCAAAGAT TTTAAATCCT AAATTATGCA AATGTTTATC TTTAAAATGA   
  
  
+ CCTGCTACTC TCTGTGCCTG ATTTTATTTG TTTCCCCGTT GAAATTTTTT TTATGATTCC ACTTTTCAAG   
  
  
+ CAGCTTAGAC AGGCGTTATT AGGTAATTAT TTTCCGCTTT GGATTGTAGT AAATTGAAAC AGAAATGCCT   
  
  
+ CACTAAACCG CTCTCTGCTC TTTGTGTTGA AGAACCAATC CCATTCAAAC TCTTCTTTCT TTTTTGAAAT   
  
  
+ TCAATCCCGC TTCAGTCAAA TGTCTAACAG AAACAGCCTC GTGTGCAATT CTACTATATA CGGTTAATGC   
  
  
+ TGTGCATGTA TTACTTCCCA ACATCTTACC CAGGACTAGG AGGGACCTTG GTAGCCTGTT CTGCTTGAAA   
  
  
+ AATTTGTCAT TATCACCTGA ATTTGTATTA AATACATAAA TCTTTGTCTT TCAATTTGTG GCTCCACATT   
  
  
+ TCAATTTGTG GCTCCACACG CTTTTGAAAA CTTTATGAGA CCTAATATAT TAGAGAACAT CTGATCGTGA   
  
  
+ TTGTTAATTC CAGTTTTATA ATCATACTAA TTAGTAAATC ACTGAAGGTC ATATTGCATA TTGTTTTTGC   
  
  
+ CTAATGATTG GCTTATTCAA CTATTCCTGA ATAGTTTAGT TTCCAGAATC TTTAGAAGAA ACCTGGTCTG   
  
  
+ GGTATTATCG AGTATTTTTC TCAGTTCATG AAAGGCTTTG ATTCAGTGTG TGTGCTGTCA TGTCTAGGCA   
  
  
+ TGATTGAGTA TTTTTATTTG CATATCCAGG CATTGTGCCT AGACAAATGT CCTGAATCTT AGTGGCTGTT   
  
  
+ AGGTCTGAAC GGACACTTTT CTTGCTGTTT TTCCTTTCCG AGTGGCATCT TTAGCTTTTT CAAACTACCA   
  
  
+ AAGGACTATT AAACGAGGCC CTGCATATGC TGGAAAACTG CCTCTTAAGC TATGTTTATG CAAAAATGGA   
  
  
+ GGACCTTGAA CTGTTCAATG TACCTACAGT CTGAGGCACA GAGCCAATAG ACATAATAGA TAGTTGGATA   
  
  
+ TTCTTTTTAT ATTTAATTCA CAGTAGCGAC AACCCCAGCA TGAAACGTAG CCACGTAGGG GGGGAAAAAG   
  
  
+ GGTCTCCATC TGTTTATATA TTCTTGTCGC TTATGGTGCT TCTGTTATGT AATGACAAAG GAATTTGTGT   
  
  
+ TGGGATTATT CCGCTTCACT CCGTCTCACT AACTTTACGG TTTCAGTCCT AATTCTCTCT TTTTTTCTTT   
  
  
+ TGTTTTTTTT CCTGAGCTTG TTTAGTCTGT TTTCTTCTGT GTGTCTTTCT GATTATGGAA GCCATACAGG   
  
  
+ AGAAGAATGA CGATCTTTTG AGTCTTAGCT TGGCCATTGT TAGTCATTCT AACCATGCAG AAACGAAGGT   
  
  
+ GAAGAGGAAG AGGCATGATG TCTCTAATTC TCTGATCGCA ACAAGAGAAG AGGGTCGGGA TAAGAAAATA   
  
  
+ TTTCAGCTGC TTCAAGAACG AGAGAGAATG CTAAGCATCG GGCAAAGAAG AAAAGGCGTT ATTCAAGAAG   
  
  
+ GGAAGGGTCT TCATCTGATC CATTTGCTTC TCATGTCTGC TACCCTAATC AGCGAAAACA ATATCAGCTC   
  
  
+ AGCTGTTAAT CATTTGCTTG AGTTGTACAA GAATGTTTCT GTAAATGGTG ATTCTGGGCA GCGCGTTGCT   
  
  
+ GCTTATTTTG CTGATGGGTT AACTGCGAGG TTCTTAACCC AGAACTCTCC CTTTTATTGC ATGGTAATGA   
  
  
+ GTAAACCGAC ACCTGCAGAG GAGTTTATCG CCTTCACTCA TTTGTACAGG GTGTCTCCAT TCTATCAGTT   
  
  
+ TGCCCATTTT ACTGCTAATC AGGCCATCTT TGAGGCATTC GAGAGGGAAG AAGAGAACAA CAGCTGGGCA   
  
  
+ CTCCATGTCA TTGATTTTGA CATCTCTCAT GGCTTCCAGT GGCCGTCTCT CATTCAGTCT CTTTCTGAGA   
  
  
+ AGGCTACTGC TTCAAGCCAT TCACTCTCTC TTCGAATCAC AGGTTTTGCT AGAACTCTGG AAGAACTGAT   
  
  
+ TGAGACTGAG AACAGATTAG TAAGTTTTGC TGAAAGCTTC CGGAACATAC TATTTGAATT CCACGGGTGT   
  
  
+ TTGAGGGGTT CAGAGCTGGT GAACTTGAAG AAAAGGGAGA ATGAAACAGT TGCCGTAAAT CTGGTGTTTC   
  
  
+ ACCTCAGCAG TTTCGATGAC ACCCTGAAGG TCTCAGACAC CTTAACCGCT GTACATTCAC TGAAACCTTC   
  
  
+ TATAATGGTA CTAGTTGAAA GAGAAGGAAC CCGAAATGGA TCAGGATTCA TATCAGGTTT CATGGATTCT   
  
  
+ TTGCATTATT ATGCTGCAAT GTTCGATTCA TTAAATGATT GCCTCCCACG GGAGAGTCCC GAGAGACTGA   
  
  
+ GCATAGAGAA GAACCATCTT GGGAGAGAGA TCAAGGAGGC GGTGACTTGG GAGAAGGACG AGATGAGCTA   
  
  
+ CGGGAAGTTC GAGATAATGG AGACTTGGAA AAAGAGGATG GAAATTCATG GGTTTTCGGG GATCAGGCTG   
  
  
+ AGTTCCAAGG CGACAATTCA AGCCAAGCTC CTGCTCAAAA TGGGCAGCCA TTACTGTCCC CAGTTTGCAG   
  
  
+ GGGATTCTGA AGGAGGAGTT GGCGGGTTCA GAGTGTATGA GAGAGATGAG GGGGAAGCCA TTTCTCTGGT   
  
  
+ TTGGCAAGGC AGGTTCCTCG CAACAGCCTC TGCATGGCGC TGTGTATG  

- +Up\_Stream \_Len000CTTTCT TCTAAATTCC TTTAGCTACC ATGTGCCGTT TCGATCTTTG TCCTTGACAT   
  
  
- AAAAATACAA AAAAAAAACA ACCTTCCCCC CCCCCCCCTC GCCCGCTCTA CATTGGGCAT AGATCTACCT   
  
  
- ATTAGATGCA CTGTTCTGGA AGTAAACTAG TTCCATGTAG TACTTATAAA TTGGGAATTG AAGAAACTCG   
  
  
- GAATGTTCTC TAACAGACTC CAAAAATCCC ACGTGCTATA GTACGGACTG ATCTGGACAC ATCTGTGTTA   
  
  
- GAAGTCGGGA ATAACTATCG ACAAGCTATA CGTACGACGG ATTTTTAACC TTATCAACCG TGGATTGTAA   
  
  
- GAAAATGTAG AACGAGGAGG AACCACATCT CAATCTACAC GATCGTGTTA TGTTACCCAG GGCTATAAAA   
  
  
- CGTAGACCAA AGACTTTCTA TTATAAACTA CTAGGACAGA ACACGACTAC CCGTTTTCGA TTTAACATTC   
  
  
- TTATACATCA AATAAATGTT CTGTATACGA ATTCGATCTC CAGTACATGA ACGTATAAAA ACACGTCGAA   
  
  
- AACTCCAACA TACGGGGAAA CTCTGTTTAA GTATCAACTT AAGTACTGTT CTAATACAAA CCGTTACTGA   
  
  
- AACTAATAAT TTAACCGGAA ATACGATAGG ATAGGACACA CGAGTTTACA ATAAATAGTC TATTATAATC   
  
  
- AAATGAGAAA GAAGGGACAG AACGTTTCTA AAATTTAGGA TTTAATACGT TTACAAATAG AAATTTTACT   
  
  
- GGACGATGAG AGACACGGAC TAAAATAAAC AAAGGGGCAA CTTTAAAAAA AATACTAAGG TGAAAAGTTC   
  
  
- GTCGAATCTG TCCGCAATAA TCCATTAATA AAAGGCGAAA CCTAACATCA TTTAACTTTG TCTTTACGGA   
  
  
- GTGATTTGGC GAGAGACGAG AAACACAACT TCTTGGTTAG GGTAAGTTTG AGAAGAAAGA AAAAACTTTA   
  
  
- AGTTAGGGCG AAGTCAGTTT ACAGATTGTC TTTGTCGGAG CACACGTTAA GATGATATAT GCCAATTACG   
  
  
- ACACGTACAT AATGAAGGGT TGTAGAATGG GTCCTGATCC TCCCTGGAAC CATCGGACAA GACGAACTTT   
  
  
- TTAAACAGTA ATAGTGGACT TAAACATAAT TTATGTATTT AGAAACAGAA AGTTAAACAC CGAGGTGTAA   
  
  
- AGTTAAACAC CGAGGTGTGC GAAAACTTTT GAAATACTCT GGATTATATA ATCTCTTGTA GACTAGCACT   
  
  
- AACAATTAAG GTCAAAATAT TAGTATGATT AATCATTTAG TGACTTCCAG TATAACGTAT AACAAAAACG   
  
  
- GATTACTAAC CGAATAAGTT GATAAGGACT TATCAAATCA AAGGTCTTAG AAATCTTCTT TGGACCAGAC   
  
  
- CCATAATAGC TCATAAAAAG AGTCAAGTAC TTTCCGAAAC TAAGTCACAC ACACGACAGT ACAGATCCGT   
  
  
- ACTAACTCAT AAAAATAAAC GTATAGGTCC GTAACACGGA TCTGTTTACA GGACTTAGAA TCACCGACAA   
  
  
- TCCAGACTTG CCTGTGAAAA GAACGACAAA AAGGAAAGGC TCACCGTAGA AATCGAAAAA GTTTGATGGT   
  
  
- TTCCTGATAA TTTGCTCCGG GACGTATACG ACCTTTTGAC GGAGAATTCG ATACAAATAC GTTTTTACCT   
  
  
- CCTGGAACTT GACAAGTTAC ATGGATGTCA GACTCCGTGT CTCGGTTATC TGTATTATCT ATCAACCTAT   
  
  
- AAGAAAAATA TAAATTAAGT GTCATCGCTG TTGGGGTCGT ACTTTGCATC GGTGCATCCC CCCCTTTTTC   
  
  
- CCAGAGGTAG ACAAATATAT AAGAACAGCG AATACCACGA AGACAATACA TTACTGTTTC CTTAAACACA   
  
  
- ACCCTAATAA GGCGAAGTGA GGCAGAGTGA TTGAAATGCC AAAGTCAGGA TTAAGAGAGA AAAAAAGAAA   
  
  
- ACAAAAAAAA GGACTCGAAC AAATCAGACA AAAGAAGACA CACAGAAAGA CTAATACCTT CGGTATGTCC   
  
  
- TCTTCTTACT GCTAGAAAAC TCAGAATCGA ACCGGTAACA ATCAGTAAGA TTGGTACGTC TTTGCTTCCA   
  
  
- CTTCTCCTTC TCCGTACTAC AGAGATTAAG AGACTAGCGT TGTTCTCTTC TCCCAGCCCT ATTCTTTTAT   
  
  
- AAAGTCGACG AAGTTCTTGC TCTCTCTTAC GATTCGTAGC CCGTTTCTTC TTTTCCGCAA TAAGTTCTTC   
  
  
- CCTTCCCAGA AGTAGACTAG GTAAACGAAG AGTACAGACG ATGGGATTAG TCGCTTTTGT TATAGTCGAG   
  
  
- TCGACAATTA GTAAACGAAC TCAACATGTT CTTACAAAGA CATTTACCAC TAAGACCCGT CGCGCAACGA   
  
  
- CGAATAAAAC GACTACCCAA TTGACGCTCC AAGAATTGGG TCTTGAGAGG GAAAATAACG TACCATTACT   
  
  
- CATTTGGCTG TGGACGTCTC CTCAAATAGC GGAAGTGAGT AAACATGTCC CACAGAGGTA AGATAGTCAA   
  
  
- ACGGGTAAAA TGACGATTAG TCCGGTAGAA ACTCCGTAAG CTCTCCCTTC TTCTCTTGTT GTCGACCCGT   
  
  
- GAGGTACAGT AACTAAAACT GTAGAGAGTA CCGAAGGTCA CCGGCAGAGA GTAAGTCAGA GAAAGACTCT   
  
  
- TCCGATGACG AAGTTCGGTA AGTGAGAGAG AAGCTTAGTG TCCAAAACGA TCTTGAGACC TTCTTGACTA   
  
  
- ACTCTGACTC TTGTCTAATC ATTCAAAACG ACTTTCGAAG GCCTTGTATG ATAAACTTAA GGTGCCCACA   
  
  
- AACTCCCCAA GTCTCGACCA CTTGAACTTC TTTTCCCTCT TACTTTGTCA ACGGCATTTA GACCACAAAG   
  
  
- TGGAGTCGTC AAAGCTACTG TGGGACTTCC AGAGTCTGTG GAATTGGCGA CATGTAAGTG ACTTTGGAAG   
  
  
- ATATTACCAT GATCAACTTT CTCTTCCTTG GGCTTTACCT AGTCCTAAGT ATAGTCCAAA GTACCTAAGA   
  
  
- AACGTAATAA TACGACGTTA CAAGCTAAGT AATTTACTAA CGGAGGGTGC CCTCTCAGGG CTCTCTGACT   
  
  
- CGTATCTCTT CTTGGTAGAA CCCTCTCTCT AGTTCCTCCG CCACTGAACC CTCTTCCTGC TCTACTCGAT   
  
  
- GCCCTTCAAG CTCTATTACC TCTGAACCTT TTTCTCCTAC CTTTAAGTAC CCAAAAGCCC CTAGTCCGAC   
  
  
- TCAAGGTTCC GCTGTTAAGT TCGGTTCGAG GACGAGTTTT ACCCGTCGGT AATGACAGGG GTCAAACGTC   
  
  
- CCCTAAGACT TCCTCCTCAA CCGCCCAAGT CTCACATACT CTCTCTACTC CCCCTTCGGT AAAGAGACCA   
  
  
- AACCGTTCCG TCCAAGGAGC GTTGTCGGAG ACGTACCGCG ACACATAC

  
  
Motifs Found  

+   

| Site Name | Organism | Position | Strand | Matrix score. | sequence | function |
| --- | --- | --- | --- | --- | --- | --- |
|  | organism | 3392 | + | 4 | motif\_sequence | short\_function |
|  | organism | 3278 | + | 4 | motif\_sequence | short\_function |
|  | organism | 2469 | - | 4 | motif\_sequence | short\_function |
|  | organism | 147 | + | 4 | motif\_sequence | short\_function |
|  | organism | 2105 | - | 4 | motif\_sequence | short\_function |
|  | organism | 1702 | + | 4 | motif\_sequence | short\_function |
|  | organism | 390 | + | 4 | motif\_sequence | short\_function |
|  | organism | 2923 | + | 4 | motif\_sequence | short\_function |
|  | organism | 1521 | + | 4 | motif\_sequence | short\_function |
|  | organism | 964 | + | 4 | motif\_sequence | short\_function |
|  | organism | 2574 | - | 4 | motif\_sequence | short\_function |
|  | organism | 304 | + | 4 | motif\_sequence | short\_function |
|  | organism | 3033 | + | 4 | motif\_sequence | short\_function |
|  | organism | 2111 | - | 4 | motif\_sequence | short\_function |
|  | organism | 380 | - | 4 | motif\_sequence | short\_function |
|  | organism | 538 | + | 4 | motif\_sequence | short\_function |
|  | organism | 3106 | - | 4 | motif\_sequence | short\_function |
|  | organism | 2925 | - | 4 | motif\_sequence | short\_function |
|  | organism | 1110 | + | 4 | motif\_sequence | short\_function |
|  | organism | 1695 | + | 4 | motif\_sequence | short\_function |
|  | organism | 2744 | - | 4 | motif\_sequence | short\_function |
|  | organism | 283 | + | 4 | motif\_sequence | short\_function |
|  | organism | 2498 | - | 4 | motif\_sequence | short\_function |
|  | organism | 1054 | + | 4 | motif\_sequence | short\_function |
|  | organism | 3065 | - | 4 | motif\_sequence | short\_function |
|  | organism | 786 | + | 4 | motif\_sequence | short\_function |
|  | organism | 2692 | + | 4 | motif\_sequence | short\_function |
|  | organism | 23 | - | 4 | motif\_sequence | short\_function |
|  | organism | 2777 | - | 4 | motif\_sequence | short\_function |
|  | organism | 927 | + | 4 | motif\_sequence | short\_function |
|  | organism | 2151 | - | 4 | motif\_sequence | short\_function |
|  | organism | 2429 | + | 4 | motif\_sequence | short\_function |
|  | organism | 1720 | - | 4 | motif\_sequence | short\_function |
|  | organism | 178 | - | 4 | motif\_sequence | short\_function |
|  | organism | 1249 | - | 4 | motif\_sequence | short\_function |

>HU11G01529.1   
+ +Up\_Stream \_Len000GAAAGA AGATTTAAGG AAATCGATGG TACACGGCAA AGCTAGAAAC AGGAACTGTA   
  
  
+ TTTTTATGTT TTTTTTTTGT TGGAAGGGGG GGGGGGGGAG CGGGCGAGAT GTAACCCGTA TCTAGATGGA   
  
  
+ TAATCTACGT GACAAGACCT TCATTTGATC AAGGTACATC ATGAATATTT AACCCTTAAC TTCTTTGAGC   
  
  
+ CTTACAAGAG ATTGTCTGAG GTTTTTAGGG TGCACGATAT CATGCCTGAC TAGACCTGTG TAGACACAAT   
  
  
+ CTTCAGCCCT TATTGATAGC TGTTCGATAT GCATGCTGCC TAAAAATTGG AATAGTTGGC ACCTAACATT   
  
  
+ CTTTTACATC TTGCTCCTCC TTGGTGTAGA GTTAGATGTG CTAGCACAAT ACAATGGGTC CCGATATTTT   
  
  
+ GCATCTGGTT TCTGAAAGAT AATATTTGAT GATCCTGTCT TGTGCTGATG GGCAAAAGCT AAATTGTAAG   
  
  
+ AATATGTAGT TTATTTACAA GACATATGCT TAAGCTAGAG GTCATGTACT TGCATATTTT TGTGCAGCTT   
  
  
+ TTGAGGTTGT ATGCCCCTTT GAGACAAATT CATAGTTGAA TTCATGACAA GATTATGTTT GGCAATGACT   
  
  
+ TTGATTATTA AATTGGCCTT TATGCTATCC TATCCTGTGT GCTCAAATGT TATTTATCAG ATAATATTAG   
  
  
+ TTTACTCTTT CTTCCCTGTC TTGCAAAGAT TTTAAATCCT AAATTATGCA AATGTTTATC TTTAAAATGA   
  
  
+ CCTGCTACTC TCTGTGCCTG ATTTTATTTG TTTCCCCGTT GAAATTTTTT TTATGATTCC ACTTTTCAAG   
  
  
+ CAGCTTAGAC AGGCGTTATT AGGTAATTAT TTTCCGCTTT GGATTGTAGT AAATTGAAAC AGAAATGCCT   
  
  
+ CACTAAACCG CTCTCTGCTC TTTGTGTTGA AGAACCAATC CCATTCAAAC TCTTCTTTCT TTTTTGAAAT   
  
  
+ TCAATCCCGC TTCAGTCAAA TGTCTAACAG AAACAGCCTC GTGTGCAATT CTACTATATA CGGTTAATGC   
  
  
+ TGTGCATGTA TTACTTCCCA ACATCTTACC CAGGACTAGG AGGGACCTTG GTAGCCTGTT CTGCTTGAAA   
  
  
+ AATTTGTCAT TATCACCTGA ATTTGTATTA AATACATAAA TCTTTGTCTT TCAATTTGTG GCTCCACATT   
  
  
+ TCAATTTGTG GCTCCACACG CTTTTGAAAA CTTTATGAGA CCTAATATAT TAGAGAACAT CTGATCGTGA   
  
  
+ TTGTTAATTC CAGTTTTATA ATCATACTAA TTAGTAAATC ACTGAAGGTC ATATTGCATA TTGTTTTTGC   
  
  
+ CTAATGATTG GCTTATTCAA CTATTCCTGA ATAGTTTAGT TTCCAGAATC TTTAGAAGAA ACCTGGTCTG   
  
  
+ GGTATTATCG AGTATTTTTC TCAGTTCATG AAAGGCTTTG ATTCAGTGTG TGTGCTGTCA TGTCTAGGCA   
  
  
+ TGATTGAGTA TTTTTATTTG CATATCCAGG CATTGTGCCT AGACAAATGT CCTGAATCTT AGTGGCTGTT   
  
  
+ AGGTCTGAAC GGACACTTTT CTTGCTGTTT TTCCTTTCCG AGTGGCATCT TTAGCTTTTT CAAACTACCA   
  
  
+ AAGGACTATT AAACGAGGCC CTGCATATGC TGGAAAACTG CCTCTTAAGC TATGTTTATG CAAAAATGGA   
  
  
+ GGACCTTGAA CTGTTCAATG TACCTACAGT CTGAGGCACA GAGCCAATAG ACATAATAGA TAGTTGGATA   
  
  
+ TTCTTTTTAT ATTTAATTCA CAGTAGCGAC AACCCCAGCA TGAAACGTAG CCACGTAGGG GGGGAAAAAG   
  
  
+ GGTCTCCATC TGTTTATATA TTCTTGTCGC TTATGGTGCT TCTGTTATGT AATGACAAAG GAATTTGTGT   
  
  
+ TGGGATTATT CCGCTTCACT CCGTCTCACT AACTTTACGG TTTCAGTCCT AATTCTCTCT TTTTTTCTTT   
  
  
+ TGTTTTTTTT CCTGAGCTTG TTTAGTCTGT TTTCTTCTGT GTGTCTTTCT GATTATGGAA GCCATACAGG   
  
  
+ AGAAGAATGA CGATCTTTTG AGTCTTAGCT TGGCCATTGT TAGTCATTCT AACCATGCAG AAACGAAGGT   
  
  
+ GAAGAGGAAG AGGCATGATG TCTCTAATTC TCTGATCGCA ACAAGAGAAG AGGGTCGGGA TAAGAAAATA   
  
  
+ TTTCAGCTGC TTCAAGAACG AGAGAGAATG CTAAGCATCG GGCAAAGAAG AAAAGGCGTT ATTCAAGAAG   
  
  
+ GGAAGGGTCT TCATCTGATC CATTTGCTTC TCATGTCTGC TACCCTAATC AGCGAAAACA ATATCAGCTC   
  
  
+ AGCTGTTAAT CATTTGCTTG AGTTGTACAA GAATGTTTCT GTAAATGGTG ATTCTGGGCA GCGCGTTGCT   
  
  
+ GCTTATTTTG CTGATGGGTT AACTGCGAGG TTCTTAACCC AGAACTCTCC CTTTTATTGC ATGGTAATGA   
  
  
+ GTAAACCGAC ACCTGCAGAG GAGTTTATCG CCTTCACTCA TTTGTACAGG GTGTCTCCAT TCTATCAGTT   
  
  
+ TGCCCATTTT ACTGCTAATC AGGCCATCTT TGAGGCATTC GAGAGGGAAG AAGAGAACAA CAGCTGGGCA   
  
  
+ CTCCATGTCA TTGATTTTGA CATCTCTCAT GGCTTCCAGT GGCCGTCTCT CATTCAGTCT CTTTCTGAGA   
  
  
+ AGGCTACTGC TTCAAGCCAT TCACTCTCTC TTCGAATCAC AGGTTTTGCT AGAACTCTGG AAGAACTGAT   
  
  
+ TGAGACTGAG AACAGATTAG TAAGTTTTGC TGAAAGCTTC CGGAACATAC TATTTGAATT CCACGGGTGT   
  
  
+ TTGAGGGGTT CAGAGCTGGT GAACTTGAAG AAAAGGGAGA ATGAAACAGT TGCCGTAAAT CTGGTGTTTC   
  
  
+ ACCTCAGCAG TTTCGATGAC ACCCTGAAGG TCTCAGACAC CTTAACCGCT GTACATTCAC TGAAACCTTC   
  
  
+ TATAATGGTA CTAGTTGAAA GAGAAGGAAC CCGAAATGGA TCAGGATTCA TATCAGGTTT CATGGATTCT   
  
  
+ TTGCATTATT ATGCTGCAAT GTTCGATTCA TTAAATGATT GCCTCCCACG GGAGAGTCCC GAGAGACTGA   
  
  
+ GCATAGAGAA GAACCATCTT GGGAGAGAGA TCAAGGAGGC GGTGACTTGG GAGAAGGACG AGATGAGCTA   
  
  
+ CGGGAAGTTC GAGATAATGG AGACTTGGAA AAAGAGGATG GAAATTCATG GGTTTTCGGG GATCAGGCTG   
  
  
+ AGTTCCAAGG CGACAATTCA AGCCAAGCTC CTGCTCAAAA TGGGCAGCCA TTACTGTCCC CAGTTTGCAG   
  
  
+ GGGATTCTGA AGGAGGAGTT GGCGGGTTCA GAGTGTATGA GAGAGATGAG GGGGAAGCCA TTTCTCTGGT   
  
  
+ TTGGCAAGGC AGGTTCCTCG CAACAGCCTC TGCATGGCGC TGTGTATG  

- +Up\_Stream \_Len000CTTTCT TCTAAATTCC TTTAGCTACC ATGTGCCGTT TCGATCTTTG TCCTTGACAT   
  
  
- AAAAATACAA AAAAAAAACA ACCTTCCCCC CCCCCCCCTC GCCCGCTCTA CATTGGGCAT AGATCTACCT   
  
  
- ATTAGATGCA CTGTTCTGGA AGTAAACTAG TTCCATGTAG TACTTATAAA TTGGGAATTG AAGAAACTCG   
  
  
- GAATGTTCTC TAACAGACTC CAAAAATCCC ACGTGCTATA GTACGGACTG ATCTGGACAC ATCTGTGTTA   
  
  
- GAAGTCGGGA ATAACTATCG ACAAGCTATA CGTACGACGG ATTTTTAACC TTATCAACCG TGGATTGTAA   
  
  
- GAAAATGTAG AACGAGGAGG AACCACATCT CAATCTACAC GATCGTGTTA TGTTACCCAG GGCTATAAAA   
  
  
- CGTAGACCAA AGACTTTCTA TTATAAACTA CTAGGACAGA ACACGACTAC CCGTTTTCGA TTTAACATTC   
  
  
- TTATACATCA AATAAATGTT CTGTATACGA ATTCGATCTC CAGTACATGA ACGTATAAAA ACACGTCGAA   
  
  
- AACTCCAACA TACGGGGAAA CTCTGTTTAA GTATCAACTT AAGTACTGTT CTAATACAAA CCGTTACTGA   
  
  
- AACTAATAAT TTAACCGGAA ATACGATAGG ATAGGACACA CGAGTTTACA ATAAATAGTC TATTATAATC   
  
  
- AAATGAGAAA GAAGGGACAG AACGTTTCTA AAATTTAGGA TTTAATACGT TTACAAATAG AAATTTTACT   
  
  
- GGACGATGAG AGACACGGAC TAAAATAAAC AAAGGGGCAA CTTTAAAAAA AATACTAAGG TGAAAAGTTC   
  
  
- GTCGAATCTG TCCGCAATAA TCCATTAATA AAAGGCGAAA CCTAACATCA TTTAACTTTG TCTTTACGGA   
  
  
- GTGATTTGGC GAGAGACGAG AAACACAACT TCTTGGTTAG GGTAAGTTTG AGAAGAAAGA AAAAACTTTA   
  
  
- AGTTAGGGCG AAGTCAGTTT ACAGATTGTC TTTGTCGGAG CACACGTTAA GATGATATAT GCCAATTACG   
  
  
- ACACGTACAT AATGAAGGGT TGTAGAATGG GTCCTGATCC TCCCTGGAAC CATCGGACAA GACGAACTTT   
  
  
- TTAAACAGTA ATAGTGGACT TAAACATAAT TTATGTATTT AGAAACAGAA AGTTAAACAC CGAGGTGTAA   
  
  
- AGTTAAACAC CGAGGTGTGC GAAAACTTTT GAAATACTCT GGATTATATA ATCTCTTGTA GACTAGCACT   
  
  
- AACAATTAAG GTCAAAATAT TAGTATGATT AATCATTTAG TGACTTCCAG TATAACGTAT AACAAAAACG   
  
  
- GATTACTAAC CGAATAAGTT GATAAGGACT TATCAAATCA AAGGTCTTAG AAATCTTCTT TGGACCAGAC   
  
  
- CCATAATAGC TCATAAAAAG AGTCAAGTAC TTTCCGAAAC TAAGTCACAC ACACGACAGT ACAGATCCGT   
  
  
- ACTAACTCAT AAAAATAAAC GTATAGGTCC GTAACACGGA TCTGTTTACA GGACTTAGAA TCACCGACAA   
  
  
- TCCAGACTTG CCTGTGAAAA GAACGACAAA AAGGAAAGGC TCACCGTAGA AATCGAAAAA GTTTGATGGT   
  
  
- TTCCTGATAA TTTGCTCCGG GACGTATACG ACCTTTTGAC GGAGAATTCG ATACAAATAC GTTTTTACCT   
  
  
- CCTGGAACTT GACAAGTTAC ATGGATGTCA GACTCCGTGT CTCGGTTATC TGTATTATCT ATCAACCTAT   
  
  
- AAGAAAAATA TAAATTAAGT GTCATCGCTG TTGGGGTCGT ACTTTGCATC GGTGCATCCC CCCCTTTTTC   
  
  
- CCAGAGGTAG ACAAATATAT AAGAACAGCG AATACCACGA AGACAATACA TTACTGTTTC CTTAAACACA   
  
  
- ACCCTAATAA GGCGAAGTGA GGCAGAGTGA TTGAAATGCC AAAGTCAGGA TTAAGAGAGA AAAAAAGAAA   
  
  
- ACAAAAAAAA GGACTCGAAC AAATCAGACA AAAGAAGACA CACAGAAAGA CTAATACCTT CGGTATGTCC   
  
  
- TCTTCTTACT GCTAGAAAAC TCAGAATCGA ACCGGTAACA ATCAGTAAGA TTGGTACGTC TTTGCTTCCA   
  
  
- CTTCTCCTTC TCCGTACTAC AGAGATTAAG AGACTAGCGT TGTTCTCTTC TCCCAGCCCT ATTCTTTTAT   
  
  
- AAAGTCGACG AAGTTCTTGC TCTCTCTTAC GATTCGTAGC CCGTTTCTTC TTTTCCGCAA TAAGTTCTTC   
  
  
- CCTTCCCAGA AGTAGACTAG GTAAACGAAG AGTACAGACG ATGGGATTAG TCGCTTTTGT TATAGTCGAG   
  
  
- TCGACAATTA GTAAACGAAC TCAACATGTT CTTACAAAGA CATTTACCAC TAAGACCCGT CGCGCAACGA   
  
  
- CGAATAAAAC GACTACCCAA TTGACGCTCC AAGAATTGGG TCTTGAGAGG GAAAATAACG TACCATTACT   
  
  
- CATTTGGCTG TGGACGTCTC CTCAAATAGC GGAAGTGAGT AAACATGTCC CACAGAGGTA AGATAGTCAA   
  
  
- ACGGGTAAAA TGACGATTAG TCCGGTAGAA ACTCCGTAAG CTCTCCCTTC TTCTCTTGTT GTCGACCCGT   
  
  
- GAGGTACAGT AACTAAAACT GTAGAGAGTA CCGAAGGTCA CCGGCAGAGA GTAAGTCAGA GAAAGACTCT   
  
  
- TCCGATGACG AAGTTCGGTA AGTGAGAGAG AAGCTTAGTG TCCAAAACGA TCTTGAGACC TTCTTGACTA   
  
  
- ACTCTGACTC TTGTCTAATC ATTCAAAACG ACTTTCGAAG GCCTTGTATG ATAAACTTAA GGTGCCCACA   
  
  
- AACTCCCCAA GTCTCGACCA CTTGAACTTC TTTTCCCTCT TACTTTGTCA ACGGCATTTA GACCACAAAG   
  
  
- TGGAGTCGTC AAAGCTACTG TGGGACTTCC AGAGTCTGTG GAATTGGCGA CATGTAAGTG ACTTTGGAAG   
  
  
- ATATTACCAT GATCAACTTT CTCTTCCTTG GGCTTTACCT AGTCCTAAGT ATAGTCCAAA GTACCTAAGA   
  
  
- AACGTAATAA TACGACGTTA CAAGCTAAGT AATTTACTAA CGGAGGGTGC CCTCTCAGGG CTCTCTGACT   
  
  
- CGTATCTCTT CTTGGTAGAA CCCTCTCTCT AGTTCCTCCG CCACTGAACC CTCTTCCTGC TCTACTCGAT   
  
  
- GCCCTTCAAG CTCTATTACC TCTGAACCTT TTTCTCCTAC CTTTAAGTAC CCAAAAGCCC CTAGTCCGAC   
  
  
- TCAAGGTTCC GCTGTTAAGT TCGGTTCGAG GACGAGTTTT ACCCGTCGGT AATGACAGGG GTCAAACGTC   
  
  
- CCCTAAGACT TCCTCCTCAA CCGCCCAAGT CTCACATACT CTCTCTACTC CCCCTTCGGT AAAGAGACCA   
  
  
- AACCGTTCCG TCCAAGGAGC GTTGTCGGAG ACGTACCGCG ACACATAC

+     AAGAA-motif

| Site Name | Organism | Position | Strand | Matrix score. | sequence | function |
| --- | --- | --- | --- | --- | --- | --- |
| AAGAA-motif | Avena sativa | 19 | + | 7 | GAAAGAA |  |
| AAGAA-motif | Avena sativa | 967 | - | 7 | GAAAGAA |  |

>HU11G01529.1   
+ +Up\_Stream \_Len000GAAAGA AGATTTAAGG AAATCGATGG TACACGGCAA AGCTAGAAAC AGGAACTGTA   
  
  
+ TTTTTATGTT TTTTTTTTGT TGGAAGGGGG GGGGGGGGAG CGGGCGAGAT GTAACCCGTA TCTAGATGGA   
  
  
+ TAATCTACGT GACAAGACCT TCATTTGATC AAGGTACATC ATGAATATTT AACCCTTAAC TTCTTTGAGC   
  
  
+ CTTACAAGAG ATTGTCTGAG GTTTTTAGGG TGCACGATAT CATGCCTGAC TAGACCTGTG TAGACACAAT   
  
  
+ CTTCAGCCCT TATTGATAGC TGTTCGATAT GCATGCTGCC TAAAAATTGG AATAGTTGGC ACCTAACATT   
  
  
+ CTTTTACATC TTGCTCCTCC TTGGTGTAGA GTTAGATGTG CTAGCACAAT ACAATGGGTC CCGATATTTT   
  
  
+ GCATCTGGTT TCTGAAAGAT AATATTTGAT GATCCTGTCT TGTGCTGATG GGCAAAAGCT AAATTGTAAG   
  
  
+ AATATGTAGT TTATTTACAA GACATATGCT TAAGCTAGAG GTCATGTACT TGCATATTTT TGTGCAGCTT   
  
  
+ TTGAGGTTGT ATGCCCCTTT GAGACAAATT CATAGTTGAA TTCATGACAA GATTATGTTT GGCAATGACT   
  
  
+ TTGATTATTA AATTGGCCTT TATGCTATCC TATCCTGTGT GCTCAAATGT TATTTATCAG ATAATATTAG   
  
  
+ TTTACTCTTT CTTCCCTGTC TTGCAAAGAT TTTAAATCCT AAATTATGCA AATGTTTATC TTTAAAATGA   
  
  
+ CCTGCTACTC TCTGTGCCTG ATTTTATTTG TTTCCCCGTT GAAATTTTTT TTATGATTCC ACTTTTCAAG   
  
  
+ CAGCTTAGAC AGGCGTTATT AGGTAATTAT TTTCCGCTTT GGATTGTAGT AAATTGAAAC AGAAATGCCT   
  
  
+ CACTAAACCG CTCTCTGCTC TTTGTGTTGA AGAACCAATC CCATTCAAAC TCTTCTTTCT TTTTTGAAAT   
  
  
+ TCAATCCCGC TTCAGTCAAA TGTCTAACAG AAACAGCCTC GTGTGCAATT CTACTATATA CGGTTAATGC   
  
  
+ TGTGCATGTA TTACTTCCCA ACATCTTACC CAGGACTAGG AGGGACCTTG GTAGCCTGTT CTGCTTGAAA   
  
  
+ AATTTGTCAT TATCACCTGA ATTTGTATTA AATACATAAA TCTTTGTCTT TCAATTTGTG GCTCCACATT   
  
  
+ TCAATTTGTG GCTCCACACG CTTTTGAAAA CTTTATGAGA CCTAATATAT TAGAGAACAT CTGATCGTGA   
  
  
+ TTGTTAATTC CAGTTTTATA ATCATACTAA TTAGTAAATC ACTGAAGGTC ATATTGCATA TTGTTTTTGC   
  
  
+ CTAATGATTG GCTTATTCAA CTATTCCTGA ATAGTTTAGT TTCCAGAATC TTTAGAAGAA ACCTGGTCTG   
  
  
+ GGTATTATCG AGTATTTTTC TCAGTTCATG AAAGGCTTTG ATTCAGTGTG TGTGCTGTCA TGTCTAGGCA   
  
  
+ TGATTGAGTA TTTTTATTTG CATATCCAGG CATTGTGCCT AGACAAATGT CCTGAATCTT AGTGGCTGTT   
  
  
+ AGGTCTGAAC GGACACTTTT CTTGCTGTTT TTCCTTTCCG AGTGGCATCT TTAGCTTTTT CAAACTACCA   
  
  
+ AAGGACTATT AAACGAGGCC CTGCATATGC TGGAAAACTG CCTCTTAAGC TATGTTTATG CAAAAATGGA   
  
  
+ GGACCTTGAA CTGTTCAATG TACCTACAGT CTGAGGCACA GAGCCAATAG ACATAATAGA TAGTTGGATA   
  
  
+ TTCTTTTTAT ATTTAATTCA CAGTAGCGAC AACCCCAGCA TGAAACGTAG CCACGTAGGG GGGGAAAAAG   
  
  
+ GGTCTCCATC TGTTTATATA TTCTTGTCGC TTATGGTGCT TCTGTTATGT AATGACAAAG GAATTTGTGT   
  
  
+ TGGGATTATT CCGCTTCACT CCGTCTCACT AACTTTACGG TTTCAGTCCT AATTCTCTCT TTTTTTCTTT   
  
  
+ TGTTTTTTTT CCTGAGCTTG TTTAGTCTGT TTTCTTCTGT GTGTCTTTCT GATTATGGAA GCCATACAGG   
  
  
+ AGAAGAATGA CGATCTTTTG AGTCTTAGCT TGGCCATTGT TAGTCATTCT AACCATGCAG AAACGAAGGT   
  
  
+ GAAGAGGAAG AGGCATGATG TCTCTAATTC TCTGATCGCA ACAAGAGAAG AGGGTCGGGA TAAGAAAATA   
  
  
+ TTTCAGCTGC TTCAAGAACG AGAGAGAATG CTAAGCATCG GGCAAAGAAG AAAAGGCGTT ATTCAAGAAG   
  
  
+ GGAAGGGTCT TCATCTGATC CATTTGCTTC TCATGTCTGC TACCCTAATC AGCGAAAACA ATATCAGCTC   
  
  
+ AGCTGTTAAT CATTTGCTTG AGTTGTACAA GAATGTTTCT GTAAATGGTG ATTCTGGGCA GCGCGTTGCT   
  
  
+ GCTTATTTTG CTGATGGGTT AACTGCGAGG TTCTTAACCC AGAACTCTCC CTTTTATTGC ATGGTAATGA   
  
  
+ GTAAACCGAC ACCTGCAGAG GAGTTTATCG CCTTCACTCA TTTGTACAGG GTGTCTCCAT TCTATCAGTT   
  
  
+ TGCCCATTTT ACTGCTAATC AGGCCATCTT TGAGGCATTC GAGAGGGAAG AAGAGAACAA CAGCTGGGCA   
  
  
+ CTCCATGTCA TTGATTTTGA CATCTCTCAT GGCTTCCAGT GGCCGTCTCT CATTCAGTCT CTTTCTGAGA   
  
  
+ AGGCTACTGC TTCAAGCCAT TCACTCTCTC TTCGAATCAC AGGTTTTGCT AGAACTCTGG AAGAACTGAT   
  
  
+ TGAGACTGAG AACAGATTAG TAAGTTTTGC TGAAAGCTTC CGGAACATAC TATTTGAATT CCACGGGTGT   
  
  
+ TTGAGGGGTT CAGAGCTGGT GAACTTGAAG AAAAGGGAGA ATGAAACAGT TGCCGTAAAT CTGGTGTTTC   
  
  
+ ACCTCAGCAG TTTCGATGAC ACCCTGAAGG TCTCAGACAC CTTAACCGCT GTACATTCAC TGAAACCTTC   
  
  
+ TATAATGGTA CTAGTTGAAA GAGAAGGAAC CCGAAATGGA TCAGGATTCA TATCAGGTTT CATGGATTCT   
  
  
+ TTGCATTATT ATGCTGCAAT GTTCGATTCA TTAAATGATT GCCTCCCACG GGAGAGTCCC GAGAGACTGA   
  
  
+ GCATAGAGAA GAACCATCTT GGGAGAGAGA TCAAGGAGGC GGTGACTTGG GAGAAGGACG AGATGAGCTA   
  
  
+ CGGGAAGTTC GAGATAATGG AGACTTGGAA AAAGAGGATG GAAATTCATG GGTTTTCGGG GATCAGGCTG   
  
  
+ AGTTCCAAGG CGACAATTCA AGCCAAGCTC CTGCTCAAAA TGGGCAGCCA TTACTGTCCC CAGTTTGCAG   
  
  
+ GGGATTCTGA AGGAGGAGTT GGCGGGTTCA GAGTGTATGA GAGAGATGAG GGGGAAGCCA TTTCTCTGGT   
  
  
+ TTGGCAAGGC AGGTTCCTCG CAACAGCCTC TGCATGGCGC TGTGTATG  

- +Up\_Stream \_Len000CTTTCT TCTAAATTCC TTTAGCTACC ATGTGCCGTT TCGATCTTTG TCCTTGACAT   
  
  
- AAAAATACAA AAAAAAAACA ACCTTCCCCC CCCCCCCCTC GCCCGCTCTA CATTGGGCAT AGATCTACCT   
  
  
- ATTAGATGCA CTGTTCTGGA AGTAAACTAG TTCCATGTAG TACTTATAAA TTGGGAATTG AAGAAACTCG   
  
  
- GAATGTTCTC TAACAGACTC CAAAAATCCC ACGTGCTATA GTACGGACTG ATCTGGACAC ATCTGTGTTA   
  
  
- GAAGTCGGGA ATAACTATCG ACAAGCTATA CGTACGACGG ATTTTTAACC TTATCAACCG TGGATTGTAA   
  
  
- GAAAATGTAG AACGAGGAGG AACCACATCT CAATCTACAC GATCGTGTTA TGTTACCCAG GGCTATAAAA   
  
  
- CGTAGACCAA AGACTTTCTA TTATAAACTA CTAGGACAGA ACACGACTAC CCGTTTTCGA TTTAACATTC   
  
  
- TTATACATCA AATAAATGTT CTGTATACGA ATTCGATCTC CAGTACATGA ACGTATAAAA ACACGTCGAA   
  
  
- AACTCCAACA TACGGGGAAA CTCTGTTTAA GTATCAACTT AAGTACTGTT CTAATACAAA CCGTTACTGA   
  
  
- AACTAATAAT TTAACCGGAA ATACGATAGG ATAGGACACA CGAGTTTACA ATAAATAGTC TATTATAATC   
  
  
- AAATGAGAAA GAAGGGACAG AACGTTTCTA AAATTTAGGA TTTAATACGT TTACAAATAG AAATTTTACT   
  
  
- GGACGATGAG AGACACGGAC TAAAATAAAC AAAGGGGCAA CTTTAAAAAA AATACTAAGG TGAAAAGTTC   
  
  
- GTCGAATCTG TCCGCAATAA TCCATTAATA AAAGGCGAAA CCTAACATCA TTTAACTTTG TCTTTACGGA   
  
  
- GTGATTTGGC GAGAGACGAG AAACACAACT TCTTGGTTAG GGTAAGTTTG AGAAGAAAGA AAAAACTTTA   
  
  
- AGTTAGGGCG AAGTCAGTTT ACAGATTGTC TTTGTCGGAG CACACGTTAA GATGATATAT GCCAATTACG   
  
  
- ACACGTACAT AATGAAGGGT TGTAGAATGG GTCCTGATCC TCCCTGGAAC CATCGGACAA GACGAACTTT   
  
  
- TTAAACAGTA ATAGTGGACT TAAACATAAT TTATGTATTT AGAAACAGAA AGTTAAACAC CGAGGTGTAA   
  
  
- AGTTAAACAC CGAGGTGTGC GAAAACTTTT GAAATACTCT GGATTATATA ATCTCTTGTA GACTAGCACT   
  
  
- AACAATTAAG GTCAAAATAT TAGTATGATT AATCATTTAG TGACTTCCAG TATAACGTAT AACAAAAACG   
  
  
- GATTACTAAC CGAATAAGTT GATAAGGACT TATCAAATCA AAGGTCTTAG AAATCTTCTT TGGACCAGAC   
  
  
- CCATAATAGC TCATAAAAAG AGTCAAGTAC TTTCCGAAAC TAAGTCACAC ACACGACAGT ACAGATCCGT   
  
  
- ACTAACTCAT AAAAATAAAC GTATAGGTCC GTAACACGGA TCTGTTTACA GGACTTAGAA TCACCGACAA   
  
  
- TCCAGACTTG CCTGTGAAAA GAACGACAAA AAGGAAAGGC TCACCGTAGA AATCGAAAAA GTTTGATGGT   
  
  
- TTCCTGATAA TTTGCTCCGG GACGTATACG ACCTTTTGAC GGAGAATTCG ATACAAATAC GTTTTTACCT   
  
  
- CCTGGAACTT GACAAGTTAC ATGGATGTCA GACTCCGTGT CTCGGTTATC TGTATTATCT ATCAACCTAT   
  
  
- AAGAAAAATA TAAATTAAGT GTCATCGCTG TTGGGGTCGT ACTTTGCATC GGTGCATCCC CCCCTTTTTC   
  
  
- CCAGAGGTAG ACAAATATAT AAGAACAGCG AATACCACGA AGACAATACA TTACTGTTTC CTTAAACACA   
  
  
- ACCCTAATAA GGCGAAGTGA GGCAGAGTGA TTGAAATGCC AAAGTCAGGA TTAAGAGAGA AAAAAAGAAA   
  
  
- ACAAAAAAAA GGACTCGAAC AAATCAGACA AAAGAAGACA CACAGAAAGA CTAATACCTT CGGTATGTCC   
  
  
- TCTTCTTACT GCTAGAAAAC TCAGAATCGA ACCGGTAACA ATCAGTAAGA TTGGTACGTC TTTGCTTCCA   
  
  
- CTTCTCCTTC TCCGTACTAC AGAGATTAAG AGACTAGCGT TGTTCTCTTC TCCCAGCCCT ATTCTTTTAT   
  
  
- AAAGTCGACG AAGTTCTTGC TCTCTCTTAC GATTCGTAGC CCGTTTCTTC TTTTCCGCAA TAAGTTCTTC   
  
  
- CCTTCCCAGA AGTAGACTAG GTAAACGAAG AGTACAGACG ATGGGATTAG TCGCTTTTGT TATAGTCGAG   
  
  
- TCGACAATTA GTAAACGAAC TCAACATGTT CTTACAAAGA CATTTACCAC TAAGACCCGT CGCGCAACGA   
  
  
- CGAATAAAAC GACTACCCAA TTGACGCTCC AAGAATTGGG TCTTGAGAGG GAAAATAACG TACCATTACT   
  
  
- CATTTGGCTG TGGACGTCTC CTCAAATAGC GGAAGTGAGT AAACATGTCC CACAGAGGTA AGATAGTCAA   
  
  
- ACGGGTAAAA TGACGATTAG TCCGGTAGAA ACTCCGTAAG CTCTCCCTTC TTCTCTTGTT GTCGACCCGT   
  
  
- GAGGTACAGT AACTAAAACT GTAGAGAGTA CCGAAGGTCA CCGGCAGAGA GTAAGTCAGA GAAAGACTCT   
  
  
- TCCGATGACG AAGTTCGGTA AGTGAGAGAG AAGCTTAGTG TCCAAAACGA TCTTGAGACC TTCTTGACTA   
  
  
- ACTCTGACTC TTGTCTAATC ATTCAAAACG ACTTTCGAAG GCCTTGTATG ATAAACTTAA GGTGCCCACA   
  
  
- AACTCCCCAA GTCTCGACCA CTTGAACTTC TTTTCCCTCT TACTTTGTCA ACGGCATTTA GACCACAAAG   
  
  
- TGGAGTCGTC AAAGCTACTG TGGGACTTCC AGAGTCTGTG GAATTGGCGA CATGTAAGTG ACTTTGGAAG   
  
  
- ATATTACCAT GATCAACTTT CTCTTCCTTG GGCTTTACCT AGTCCTAAGT ATAGTCCAAA GTACCTAAGA   
  
  
- AACGTAATAA TACGACGTTA CAAGCTAAGT AATTTACTAA CGGAGGGTGC CCTCTCAGGG CTCTCTGACT   
  
  
- CGTATCTCTT CTTGGTAGAA CCCTCTCTCT AGTTCCTCCG CCACTGAACC CTCTTCCTGC TCTACTCGAT   
  
  
- GCCCTTCAAG CTCTATTACC TCTGAACCTT TTTCTCCTAC CTTTAAGTAC CCAAAAGCCC CTAGTCCGAC   
  
  
- TCAAGGTTCC GCTGTTAAGT TCGGTTCGAG GACGAGTTTT ACCCGTCGGT AATGACAGGG GTCAAACGTC   
  
  
- CCCTAAGACT TCCTCCTCAA CCGCCCAAGT CTCACATACT CTCTCTACTC CCCCTTCGGT AAAGAGACCA   
  
  
- AACCGTTCCG TCCAAGGAGC GTTGTCGGAG ACGTACCGCG ACACATAC

+     ABRE

| Site Name | Organism | Position | Strand | Matrix score. | sequence | function |
| --- | --- | --- | --- | --- | --- | --- |
| ABRE | Arabidopsis thaliana | 1806 | - | 5 | ACGTG | cis-acting element involved in the abscisic acid responsiveness |
| ABRE | Arabidopsis thaliana | 151 | + | 5 | ACGTG | cis-acting element involved in the abscisic acid responsiveness |

>HU11G01529.1   
+ +Up\_Stream \_Len000GAAAGA AGATTTAAGG AAATCGATGG TACACGGCAA AGCTAGAAAC AGGAACTGTA   
  
  
+ TTTTTATGTT TTTTTTTTGT TGGAAGGGGG GGGGGGGGAG CGGGCGAGAT GTAACCCGTA TCTAGATGGA   
  
  
+ TAATCTACGT GACAAGACCT TCATTTGATC AAGGTACATC ATGAATATTT AACCCTTAAC TTCTTTGAGC   
  
  
+ CTTACAAGAG ATTGTCTGAG GTTTTTAGGG TGCACGATAT CATGCCTGAC TAGACCTGTG TAGACACAAT   
  
  
+ CTTCAGCCCT TATTGATAGC TGTTCGATAT GCATGCTGCC TAAAAATTGG AATAGTTGGC ACCTAACATT   
  
  
+ CTTTTACATC TTGCTCCTCC TTGGTGTAGA GTTAGATGTG CTAGCACAAT ACAATGGGTC CCGATATTTT   
  
  
+ GCATCTGGTT TCTGAAAGAT AATATTTGAT GATCCTGTCT TGTGCTGATG GGCAAAAGCT AAATTGTAAG   
  
  
+ AATATGTAGT TTATTTACAA GACATATGCT TAAGCTAGAG GTCATGTACT TGCATATTTT TGTGCAGCTT   
  
  
+ TTGAGGTTGT ATGCCCCTTT GAGACAAATT CATAGTTGAA TTCATGACAA GATTATGTTT GGCAATGACT   
  
  
+ TTGATTATTA AATTGGCCTT TATGCTATCC TATCCTGTGT GCTCAAATGT TATTTATCAG ATAATATTAG   
  
  
+ TTTACTCTTT CTTCCCTGTC TTGCAAAGAT TTTAAATCCT AAATTATGCA AATGTTTATC TTTAAAATGA   
  
  
+ CCTGCTACTC TCTGTGCCTG ATTTTATTTG TTTCCCCGTT GAAATTTTTT TTATGATTCC ACTTTTCAAG   
  
  
+ CAGCTTAGAC AGGCGTTATT AGGTAATTAT TTTCCGCTTT GGATTGTAGT AAATTGAAAC AGAAATGCCT   
  
  
+ CACTAAACCG CTCTCTGCTC TTTGTGTTGA AGAACCAATC CCATTCAAAC TCTTCTTTCT TTTTTGAAAT   
  
  
+ TCAATCCCGC TTCAGTCAAA TGTCTAACAG AAACAGCCTC GTGTGCAATT CTACTATATA CGGTTAATGC   
  
  
+ TGTGCATGTA TTACTTCCCA ACATCTTACC CAGGACTAGG AGGGACCTTG GTAGCCTGTT CTGCTTGAAA   
  
  
+ AATTTGTCAT TATCACCTGA ATTTGTATTA AATACATAAA TCTTTGTCTT TCAATTTGTG GCTCCACATT   
  
  
+ TCAATTTGTG GCTCCACACG CTTTTGAAAA CTTTATGAGA CCTAATATAT TAGAGAACAT CTGATCGTGA   
  
  
+ TTGTTAATTC CAGTTTTATA ATCATACTAA TTAGTAAATC ACTGAAGGTC ATATTGCATA TTGTTTTTGC   
  
  
+ CTAATGATTG GCTTATTCAA CTATTCCTGA ATAGTTTAGT TTCCAGAATC TTTAGAAGAA ACCTGGTCTG   
  
  
+ GGTATTATCG AGTATTTTTC TCAGTTCATG AAAGGCTTTG ATTCAGTGTG TGTGCTGTCA TGTCTAGGCA   
  
  
+ TGATTGAGTA TTTTTATTTG CATATCCAGG CATTGTGCCT AGACAAATGT CCTGAATCTT AGTGGCTGTT   
  
  
+ AGGTCTGAAC GGACACTTTT CTTGCTGTTT TTCCTTTCCG AGTGGCATCT TTAGCTTTTT CAAACTACCA   
  
  
+ AAGGACTATT AAACGAGGCC CTGCATATGC TGGAAAACTG CCTCTTAAGC TATGTTTATG CAAAAATGGA   
  
  
+ GGACCTTGAA CTGTTCAATG TACCTACAGT CTGAGGCACA GAGCCAATAG ACATAATAGA TAGTTGGATA   
  
  
+ TTCTTTTTAT ATTTAATTCA CAGTAGCGAC AACCCCAGCA TGAAACGTAG CCACGTAGGG GGGGAAAAAG   
  
  
+ GGTCTCCATC TGTTTATATA TTCTTGTCGC TTATGGTGCT TCTGTTATGT AATGACAAAG GAATTTGTGT   
  
  
+ TGGGATTATT CCGCTTCACT CCGTCTCACT AACTTTACGG TTTCAGTCCT AATTCTCTCT TTTTTTCTTT   
  
  
+ TGTTTTTTTT CCTGAGCTTG TTTAGTCTGT TTTCTTCTGT GTGTCTTTCT GATTATGGAA GCCATACAGG   
  
  
+ AGAAGAATGA CGATCTTTTG AGTCTTAGCT TGGCCATTGT TAGTCATTCT AACCATGCAG AAACGAAGGT   
  
  
+ GAAGAGGAAG AGGCATGATG TCTCTAATTC TCTGATCGCA ACAAGAGAAG AGGGTCGGGA TAAGAAAATA   
  
  
+ TTTCAGCTGC TTCAAGAACG AGAGAGAATG CTAAGCATCG GGCAAAGAAG AAAAGGCGTT ATTCAAGAAG   
  
  
+ GGAAGGGTCT TCATCTGATC CATTTGCTTC TCATGTCTGC TACCCTAATC AGCGAAAACA ATATCAGCTC   
  
  
+ AGCTGTTAAT CATTTGCTTG AGTTGTACAA GAATGTTTCT GTAAATGGTG ATTCTGGGCA GCGCGTTGCT   
  
  
+ GCTTATTTTG CTGATGGGTT AACTGCGAGG TTCTTAACCC AGAACTCTCC CTTTTATTGC ATGGTAATGA   
  
  
+ GTAAACCGAC ACCTGCAGAG GAGTTTATCG CCTTCACTCA TTTGTACAGG GTGTCTCCAT TCTATCAGTT   
  
  
+ TGCCCATTTT ACTGCTAATC AGGCCATCTT TGAGGCATTC GAGAGGGAAG AAGAGAACAA CAGCTGGGCA   
  
  
+ CTCCATGTCA TTGATTTTGA CATCTCTCAT GGCTTCCAGT GGCCGTCTCT CATTCAGTCT CTTTCTGAGA   
  
  
+ AGGCTACTGC TTCAAGCCAT TCACTCTCTC TTCGAATCAC AGGTTTTGCT AGAACTCTGG AAGAACTGAT   
  
  
+ TGAGACTGAG AACAGATTAG TAAGTTTTGC TGAAAGCTTC CGGAACATAC TATTTGAATT CCACGGGTGT   
  
  
+ TTGAGGGGTT CAGAGCTGGT GAACTTGAAG AAAAGGGAGA ATGAAACAGT TGCCGTAAAT CTGGTGTTTC   
  
  
+ ACCTCAGCAG TTTCGATGAC ACCCTGAAGG TCTCAGACAC CTTAACCGCT GTACATTCAC TGAAACCTTC   
  
  
+ TATAATGGTA CTAGTTGAAA GAGAAGGAAC CCGAAATGGA TCAGGATTCA TATCAGGTTT CATGGATTCT   
  
  
+ TTGCATTATT ATGCTGCAAT GTTCGATTCA TTAAATGATT GCCTCCCACG GGAGAGTCCC GAGAGACTGA   
  
  
+ GCATAGAGAA GAACCATCTT GGGAGAGAGA TCAAGGAGGC GGTGACTTGG GAGAAGGACG AGATGAGCTA   
  
  
+ CGGGAAGTTC GAGATAATGG AGACTTGGAA AAAGAGGATG GAAATTCATG GGTTTTCGGG GATCAGGCTG   
  
  
+ AGTTCCAAGG CGACAATTCA AGCCAAGCTC CTGCTCAAAA TGGGCAGCCA TTACTGTCCC CAGTTTGCAG   
  
  
+ GGGATTCTGA AGGAGGAGTT GGCGGGTTCA GAGTGTATGA GAGAGATGAG GGGGAAGCCA TTTCTCTGGT   
  
  
+ TTGGCAAGGC AGGTTCCTCG CAACAGCCTC TGCATGGCGC TGTGTATG  

- +Up\_Stream \_Len000CTTTCT TCTAAATTCC TTTAGCTACC ATGTGCCGTT TCGATCTTTG TCCTTGACAT   
  
  
- AAAAATACAA AAAAAAAACA ACCTTCCCCC CCCCCCCCTC GCCCGCTCTA CATTGGGCAT AGATCTACCT   
  
  
- ATTAGATGCA CTGTTCTGGA AGTAAACTAG TTCCATGTAG TACTTATAAA TTGGGAATTG AAGAAACTCG   
  
  
- GAATGTTCTC TAACAGACTC CAAAAATCCC ACGTGCTATA GTACGGACTG ATCTGGACAC ATCTGTGTTA   
  
  
- GAAGTCGGGA ATAACTATCG ACAAGCTATA CGTACGACGG ATTTTTAACC TTATCAACCG TGGATTGTAA   
  
  
- GAAAATGTAG AACGAGGAGG AACCACATCT CAATCTACAC GATCGTGTTA TGTTACCCAG GGCTATAAAA   
  
  
- CGTAGACCAA AGACTTTCTA TTATAAACTA CTAGGACAGA ACACGACTAC CCGTTTTCGA TTTAACATTC   
  
  
- TTATACATCA AATAAATGTT CTGTATACGA ATTCGATCTC CAGTACATGA ACGTATAAAA ACACGTCGAA   
  
  
- AACTCCAACA TACGGGGAAA CTCTGTTTAA GTATCAACTT AAGTACTGTT CTAATACAAA CCGTTACTGA   
  
  
- AACTAATAAT TTAACCGGAA ATACGATAGG ATAGGACACA CGAGTTTACA ATAAATAGTC TATTATAATC   
  
  
- AAATGAGAAA GAAGGGACAG AACGTTTCTA AAATTTAGGA TTTAATACGT TTACAAATAG AAATTTTACT   
  
  
- GGACGATGAG AGACACGGAC TAAAATAAAC AAAGGGGCAA CTTTAAAAAA AATACTAAGG TGAAAAGTTC   
  
  
- GTCGAATCTG TCCGCAATAA TCCATTAATA AAAGGCGAAA CCTAACATCA TTTAACTTTG TCTTTACGGA   
  
  
- GTGATTTGGC GAGAGACGAG AAACACAACT TCTTGGTTAG GGTAAGTTTG AGAAGAAAGA AAAAACTTTA   
  
  
- AGTTAGGGCG AAGTCAGTTT ACAGATTGTC TTTGTCGGAG CACACGTTAA GATGATATAT GCCAATTACG   
  
  
- ACACGTACAT AATGAAGGGT TGTAGAATGG GTCCTGATCC TCCCTGGAAC CATCGGACAA GACGAACTTT   
  
  
- TTAAACAGTA ATAGTGGACT TAAACATAAT TTATGTATTT AGAAACAGAA AGTTAAACAC CGAGGTGTAA   
  
  
- AGTTAAACAC CGAGGTGTGC GAAAACTTTT GAAATACTCT GGATTATATA ATCTCTTGTA GACTAGCACT   
  
  
- AACAATTAAG GTCAAAATAT TAGTATGATT AATCATTTAG TGACTTCCAG TATAACGTAT AACAAAAACG   
  
  
- GATTACTAAC CGAATAAGTT GATAAGGACT TATCAAATCA AAGGTCTTAG AAATCTTCTT TGGACCAGAC   
  
  
- CCATAATAGC TCATAAAAAG AGTCAAGTAC TTTCCGAAAC TAAGTCACAC ACACGACAGT ACAGATCCGT   
  
  
- ACTAACTCAT AAAAATAAAC GTATAGGTCC GTAACACGGA TCTGTTTACA GGACTTAGAA TCACCGACAA   
  
  
- TCCAGACTTG CCTGTGAAAA GAACGACAAA AAGGAAAGGC TCACCGTAGA AATCGAAAAA GTTTGATGGT   
  
  
- TTCCTGATAA TTTGCTCCGG GACGTATACG ACCTTTTGAC GGAGAATTCG ATACAAATAC GTTTTTACCT   
  
  
- CCTGGAACTT GACAAGTTAC ATGGATGTCA GACTCCGTGT CTCGGTTATC TGTATTATCT ATCAACCTAT   
  
  
- AAGAAAAATA TAAATTAAGT GTCATCGCTG TTGGGGTCGT ACTTTGCATC GGTGCATCCC CCCCTTTTTC   
  
  
- CCAGAGGTAG ACAAATATAT AAGAACAGCG AATACCACGA AGACAATACA TTACTGTTTC CTTAAACACA   
  
  
- ACCCTAATAA GGCGAAGTGA GGCAGAGTGA TTGAAATGCC AAAGTCAGGA TTAAGAGAGA AAAAAAGAAA   
  
  
- ACAAAAAAAA GGACTCGAAC AAATCAGACA AAAGAAGACA CACAGAAAGA CTAATACCTT CGGTATGTCC   
  
  
- TCTTCTTACT GCTAGAAAAC TCAGAATCGA ACCGGTAACA ATCAGTAAGA TTGGTACGTC TTTGCTTCCA   
  
  
- CTTCTCCTTC TCCGTACTAC AGAGATTAAG AGACTAGCGT TGTTCTCTTC TCCCAGCCCT ATTCTTTTAT   
  
  
- AAAGTCGACG AAGTTCTTGC TCTCTCTTAC GATTCGTAGC CCGTTTCTTC TTTTCCGCAA TAAGTTCTTC   
  
  
- CCTTCCCAGA AGTAGACTAG GTAAACGAAG AGTACAGACG ATGGGATTAG TCGCTTTTGT TATAGTCGAG   
  
  
- TCGACAATTA GTAAACGAAC TCAACATGTT CTTACAAAGA CATTTACCAC TAAGACCCGT CGCGCAACGA   
  
  
- CGAATAAAAC GACTACCCAA TTGACGCTCC AAGAATTGGG TCTTGAGAGG GAAAATAACG TACCATTACT   
  
  
- CATTTGGCTG TGGACGTCTC CTCAAATAGC GGAAGTGAGT AAACATGTCC CACAGAGGTA AGATAGTCAA   
  
  
- ACGGGTAAAA TGACGATTAG TCCGGTAGAA ACTCCGTAAG CTCTCCCTTC TTCTCTTGTT GTCGACCCGT   
  
  
- GAGGTACAGT AACTAAAACT GTAGAGAGTA CCGAAGGTCA CCGGCAGAGA GTAAGTCAGA GAAAGACTCT   
  
  
- TCCGATGACG AAGTTCGGTA AGTGAGAGAG AAGCTTAGTG TCCAAAACGA TCTTGAGACC TTCTTGACTA   
  
  
- ACTCTGACTC TTGTCTAATC ATTCAAAACG ACTTTCGAAG GCCTTGTATG ATAAACTTAA GGTGCCCACA   
  
  
- AACTCCCCAA GTCTCGACCA CTTGAACTTC TTTTCCCTCT TACTTTGTCA ACGGCATTTA GACCACAAAG   
  
  
- TGGAGTCGTC AAAGCTACTG TGGGACTTCC AGAGTCTGTG GAATTGGCGA CATGTAAGTG ACTTTGGAAG   
  
  
- ATATTACCAT GATCAACTTT CTCTTCCTTG GGCTTTACCT AGTCCTAAGT ATAGTCCAAA GTACCTAAGA   
  
  
- AACGTAATAA TACGACGTTA CAAGCTAAGT AATTTACTAA CGGAGGGTGC CCTCTCAGGG CTCTCTGACT   
  
  
- CGTATCTCTT CTTGGTAGAA CCCTCTCTCT AGTTCCTCCG CCACTGAACC CTCTTCCTGC TCTACTCGAT   
  
  
- GCCCTTCAAG CTCTATTACC TCTGAACCTT TTTCTCCTAC CTTTAAGTAC CCAAAAGCCC CTAGTCCGAC   
  
  
- TCAAGGTTCC GCTGTTAAGT TCGGTTCGAG GACGAGTTTT ACCCGTCGGT AATGACAGGG GTCAAACGTC   
  
  
- CCCTAAGACT TCCTCCTCAA CCGCCCAAGT CTCACATACT CTCTCTACTC CCCCTTCGGT AAAGAGACCA   
  
  
- AACCGTTCCG TCCAAGGAGC GTTGTCGGAG ACGTACCGCG ACACATAC

+     ABRE3a

| Site Name | Organism | Position | Strand | Matrix score. | sequence | function |
| --- | --- | --- | --- | --- | --- | --- |
| ABRE3a | Zea mays | 1806 | - | 6 | TACGTG |  |
| ABRE3a | Zea mays | 150 | + | 6 | TACGTG |  |

>HU11G01529.1   
+ +Up\_Stream \_Len000GAAAGA AGATTTAAGG AAATCGATGG TACACGGCAA AGCTAGAAAC AGGAACTGTA   
  
  
+ TTTTTATGTT TTTTTTTTGT TGGAAGGGGG GGGGGGGGAG CGGGCGAGAT GTAACCCGTA TCTAGATGGA   
  
  
+ TAATCTACGT GACAAGACCT TCATTTGATC AAGGTACATC ATGAATATTT AACCCTTAAC TTCTTTGAGC   
  
  
+ CTTACAAGAG ATTGTCTGAG GTTTTTAGGG TGCACGATAT CATGCCTGAC TAGACCTGTG TAGACACAAT   
  
  
+ CTTCAGCCCT TATTGATAGC TGTTCGATAT GCATGCTGCC TAAAAATTGG AATAGTTGGC ACCTAACATT   
  
  
+ CTTTTACATC TTGCTCCTCC TTGGTGTAGA GTTAGATGTG CTAGCACAAT ACAATGGGTC CCGATATTTT   
  
  
+ GCATCTGGTT TCTGAAAGAT AATATTTGAT GATCCTGTCT TGTGCTGATG GGCAAAAGCT AAATTGTAAG   
  
  
+ AATATGTAGT TTATTTACAA GACATATGCT TAAGCTAGAG GTCATGTACT TGCATATTTT TGTGCAGCTT   
  
  
+ TTGAGGTTGT ATGCCCCTTT GAGACAAATT CATAGTTGAA TTCATGACAA GATTATGTTT GGCAATGACT   
  
  
+ TTGATTATTA AATTGGCCTT TATGCTATCC TATCCTGTGT GCTCAAATGT TATTTATCAG ATAATATTAG   
  
  
+ TTTACTCTTT CTTCCCTGTC TTGCAAAGAT TTTAAATCCT AAATTATGCA AATGTTTATC TTTAAAATGA   
  
  
+ CCTGCTACTC TCTGTGCCTG ATTTTATTTG TTTCCCCGTT GAAATTTTTT TTATGATTCC ACTTTTCAAG   
  
  
+ CAGCTTAGAC AGGCGTTATT AGGTAATTAT TTTCCGCTTT GGATTGTAGT AAATTGAAAC AGAAATGCCT   
  
  
+ CACTAAACCG CTCTCTGCTC TTTGTGTTGA AGAACCAATC CCATTCAAAC TCTTCTTTCT TTTTTGAAAT   
  
  
+ TCAATCCCGC TTCAGTCAAA TGTCTAACAG AAACAGCCTC GTGTGCAATT CTACTATATA CGGTTAATGC   
  
  
+ TGTGCATGTA TTACTTCCCA ACATCTTACC CAGGACTAGG AGGGACCTTG GTAGCCTGTT CTGCTTGAAA   
  
  
+ AATTTGTCAT TATCACCTGA ATTTGTATTA AATACATAAA TCTTTGTCTT TCAATTTGTG GCTCCACATT   
  
  
+ TCAATTTGTG GCTCCACACG CTTTTGAAAA CTTTATGAGA CCTAATATAT TAGAGAACAT CTGATCGTGA   
  
  
+ TTGTTAATTC CAGTTTTATA ATCATACTAA TTAGTAAATC ACTGAAGGTC ATATTGCATA TTGTTTTTGC   
  
  
+ CTAATGATTG GCTTATTCAA CTATTCCTGA ATAGTTTAGT TTCCAGAATC TTTAGAAGAA ACCTGGTCTG   
  
  
+ GGTATTATCG AGTATTTTTC TCAGTTCATG AAAGGCTTTG ATTCAGTGTG TGTGCTGTCA TGTCTAGGCA   
  
  
+ TGATTGAGTA TTTTTATTTG CATATCCAGG CATTGTGCCT AGACAAATGT CCTGAATCTT AGTGGCTGTT   
  
  
+ AGGTCTGAAC GGACACTTTT CTTGCTGTTT TTCCTTTCCG AGTGGCATCT TTAGCTTTTT CAAACTACCA   
  
  
+ AAGGACTATT AAACGAGGCC CTGCATATGC TGGAAAACTG CCTCTTAAGC TATGTTTATG CAAAAATGGA   
  
  
+ GGACCTTGAA CTGTTCAATG TACCTACAGT CTGAGGCACA GAGCCAATAG ACATAATAGA TAGTTGGATA   
  
  
+ TTCTTTTTAT ATTTAATTCA CAGTAGCGAC AACCCCAGCA TGAAACGTAG CCACGTAGGG GGGGAAAAAG   
  
  
+ GGTCTCCATC TGTTTATATA TTCTTGTCGC TTATGGTGCT TCTGTTATGT AATGACAAAG GAATTTGTGT   
  
  
+ TGGGATTATT CCGCTTCACT CCGTCTCACT AACTTTACGG TTTCAGTCCT AATTCTCTCT TTTTTTCTTT   
  
  
+ TGTTTTTTTT CCTGAGCTTG TTTAGTCTGT TTTCTTCTGT GTGTCTTTCT GATTATGGAA GCCATACAGG   
  
  
+ AGAAGAATGA CGATCTTTTG AGTCTTAGCT TGGCCATTGT TAGTCATTCT AACCATGCAG AAACGAAGGT   
  
  
+ GAAGAGGAAG AGGCATGATG TCTCTAATTC TCTGATCGCA ACAAGAGAAG AGGGTCGGGA TAAGAAAATA   
  
  
+ TTTCAGCTGC TTCAAGAACG AGAGAGAATG CTAAGCATCG GGCAAAGAAG AAAAGGCGTT ATTCAAGAAG   
  
  
+ GGAAGGGTCT TCATCTGATC CATTTGCTTC TCATGTCTGC TACCCTAATC AGCGAAAACA ATATCAGCTC   
  
  
+ AGCTGTTAAT CATTTGCTTG AGTTGTACAA GAATGTTTCT GTAAATGGTG ATTCTGGGCA GCGCGTTGCT   
  
  
+ GCTTATTTTG CTGATGGGTT AACTGCGAGG TTCTTAACCC AGAACTCTCC CTTTTATTGC ATGGTAATGA   
  
  
+ GTAAACCGAC ACCTGCAGAG GAGTTTATCG CCTTCACTCA TTTGTACAGG GTGTCTCCAT TCTATCAGTT   
  
  
+ TGCCCATTTT ACTGCTAATC AGGCCATCTT TGAGGCATTC GAGAGGGAAG AAGAGAACAA CAGCTGGGCA   
  
  
+ CTCCATGTCA TTGATTTTGA CATCTCTCAT GGCTTCCAGT GGCCGTCTCT CATTCAGTCT CTTTCTGAGA   
  
  
+ AGGCTACTGC TTCAAGCCAT TCACTCTCTC TTCGAATCAC AGGTTTTGCT AGAACTCTGG AAGAACTGAT   
  
  
+ TGAGACTGAG AACAGATTAG TAAGTTTTGC TGAAAGCTTC CGGAACATAC TATTTGAATT CCACGGGTGT   
  
  
+ TTGAGGGGTT CAGAGCTGGT GAACTTGAAG AAAAGGGAGA ATGAAACAGT TGCCGTAAAT CTGGTGTTTC   
  
  
+ ACCTCAGCAG TTTCGATGAC ACCCTGAAGG TCTCAGACAC CTTAACCGCT GTACATTCAC TGAAACCTTC   
  
  
+ TATAATGGTA CTAGTTGAAA GAGAAGGAAC CCGAAATGGA TCAGGATTCA TATCAGGTTT CATGGATTCT   
  
  
+ TTGCATTATT ATGCTGCAAT GTTCGATTCA TTAAATGATT GCCTCCCACG GGAGAGTCCC GAGAGACTGA   
  
  
+ GCATAGAGAA GAACCATCTT GGGAGAGAGA TCAAGGAGGC GGTGACTTGG GAGAAGGACG AGATGAGCTA   
  
  
+ CGGGAAGTTC GAGATAATGG AGACTTGGAA AAAGAGGATG GAAATTCATG GGTTTTCGGG GATCAGGCTG   
  
  
+ AGTTCCAAGG CGACAATTCA AGCCAAGCTC CTGCTCAAAA TGGGCAGCCA TTACTGTCCC CAGTTTGCAG   
  
  
+ GGGATTCTGA AGGAGGAGTT GGCGGGTTCA GAGTGTATGA GAGAGATGAG GGGGAAGCCA TTTCTCTGGT   
  
  
+ TTGGCAAGGC AGGTTCCTCG CAACAGCCTC TGCATGGCGC TGTGTATG  

- +Up\_Stream \_Len000CTTTCT TCTAAATTCC TTTAGCTACC ATGTGCCGTT TCGATCTTTG TCCTTGACAT   
  
  
- AAAAATACAA AAAAAAAACA ACCTTCCCCC CCCCCCCCTC GCCCGCTCTA CATTGGGCAT AGATCTACCT   
  
  
- ATTAGATGCA CTGTTCTGGA AGTAAACTAG TTCCATGTAG TACTTATAAA TTGGGAATTG AAGAAACTCG   
  
  
- GAATGTTCTC TAACAGACTC CAAAAATCCC ACGTGCTATA GTACGGACTG ATCTGGACAC ATCTGTGTTA   
  
  
- GAAGTCGGGA ATAACTATCG ACAAGCTATA CGTACGACGG ATTTTTAACC TTATCAACCG TGGATTGTAA   
  
  
- GAAAATGTAG AACGAGGAGG AACCACATCT CAATCTACAC GATCGTGTTA TGTTACCCAG GGCTATAAAA   
  
  
- CGTAGACCAA AGACTTTCTA TTATAAACTA CTAGGACAGA ACACGACTAC CCGTTTTCGA TTTAACATTC   
  
  
- TTATACATCA AATAAATGTT CTGTATACGA ATTCGATCTC CAGTACATGA ACGTATAAAA ACACGTCGAA   
  
  
- AACTCCAACA TACGGGGAAA CTCTGTTTAA GTATCAACTT AAGTACTGTT CTAATACAAA CCGTTACTGA   
  
  
- AACTAATAAT TTAACCGGAA ATACGATAGG ATAGGACACA CGAGTTTACA ATAAATAGTC TATTATAATC   
  
  
- AAATGAGAAA GAAGGGACAG AACGTTTCTA AAATTTAGGA TTTAATACGT TTACAAATAG AAATTTTACT   
  
  
- GGACGATGAG AGACACGGAC TAAAATAAAC AAAGGGGCAA CTTTAAAAAA AATACTAAGG TGAAAAGTTC   
  
  
- GTCGAATCTG TCCGCAATAA TCCATTAATA AAAGGCGAAA CCTAACATCA TTTAACTTTG TCTTTACGGA   
  
  
- GTGATTTGGC GAGAGACGAG AAACACAACT TCTTGGTTAG GGTAAGTTTG AGAAGAAAGA AAAAACTTTA   
  
  
- AGTTAGGGCG AAGTCAGTTT ACAGATTGTC TTTGTCGGAG CACACGTTAA GATGATATAT GCCAATTACG   
  
  
- ACACGTACAT AATGAAGGGT TGTAGAATGG GTCCTGATCC TCCCTGGAAC CATCGGACAA GACGAACTTT   
  
  
- TTAAACAGTA ATAGTGGACT TAAACATAAT TTATGTATTT AGAAACAGAA AGTTAAACAC CGAGGTGTAA   
  
  
- AGTTAAACAC CGAGGTGTGC GAAAACTTTT GAAATACTCT GGATTATATA ATCTCTTGTA GACTAGCACT   
  
  
- AACAATTAAG GTCAAAATAT TAGTATGATT AATCATTTAG TGACTTCCAG TATAACGTAT AACAAAAACG   
  
  
- GATTACTAAC CGAATAAGTT GATAAGGACT TATCAAATCA AAGGTCTTAG AAATCTTCTT TGGACCAGAC   
  
  
- CCATAATAGC TCATAAAAAG AGTCAAGTAC TTTCCGAAAC TAAGTCACAC ACACGACAGT ACAGATCCGT   
  
  
- ACTAACTCAT AAAAATAAAC GTATAGGTCC GTAACACGGA TCTGTTTACA GGACTTAGAA TCACCGACAA   
  
  
- TCCAGACTTG CCTGTGAAAA GAACGACAAA AAGGAAAGGC TCACCGTAGA AATCGAAAAA GTTTGATGGT   
  
  
- TTCCTGATAA TTTGCTCCGG GACGTATACG ACCTTTTGAC GGAGAATTCG ATACAAATAC GTTTTTACCT   
  
  
- CCTGGAACTT GACAAGTTAC ATGGATGTCA GACTCCGTGT CTCGGTTATC TGTATTATCT ATCAACCTAT   
  
  
- AAGAAAAATA TAAATTAAGT GTCATCGCTG TTGGGGTCGT ACTTTGCATC GGTGCATCCC CCCCTTTTTC   
  
  
- CCAGAGGTAG ACAAATATAT AAGAACAGCG AATACCACGA AGACAATACA TTACTGTTTC CTTAAACACA   
  
  
- ACCCTAATAA GGCGAAGTGA GGCAGAGTGA TTGAAATGCC AAAGTCAGGA TTAAGAGAGA AAAAAAGAAA   
  
  
- ACAAAAAAAA GGACTCGAAC AAATCAGACA AAAGAAGACA CACAGAAAGA CTAATACCTT CGGTATGTCC   
  
  
- TCTTCTTACT GCTAGAAAAC TCAGAATCGA ACCGGTAACA ATCAGTAAGA TTGGTACGTC TTTGCTTCCA   
  
  
- CTTCTCCTTC TCCGTACTAC AGAGATTAAG AGACTAGCGT TGTTCTCTTC TCCCAGCCCT ATTCTTTTAT   
  
  
- AAAGTCGACG AAGTTCTTGC TCTCTCTTAC GATTCGTAGC CCGTTTCTTC TTTTCCGCAA TAAGTTCTTC   
  
  
- CCTTCCCAGA AGTAGACTAG GTAAACGAAG AGTACAGACG ATGGGATTAG TCGCTTTTGT TATAGTCGAG   
  
  
- TCGACAATTA GTAAACGAAC TCAACATGTT CTTACAAAGA CATTTACCAC TAAGACCCGT CGCGCAACGA   
  
  
- CGAATAAAAC GACTACCCAA TTGACGCTCC AAGAATTGGG TCTTGAGAGG GAAAATAACG TACCATTACT   
  
  
- CATTTGGCTG TGGACGTCTC CTCAAATAGC GGAAGTGAGT AAACATGTCC CACAGAGGTA AGATAGTCAA   
  
  
- ACGGGTAAAA TGACGATTAG TCCGGTAGAA ACTCCGTAAG CTCTCCCTTC TTCTCTTGTT GTCGACCCGT   
  
  
- GAGGTACAGT AACTAAAACT GTAGAGAGTA CCGAAGGTCA CCGGCAGAGA GTAAGTCAGA GAAAGACTCT   
  
  
- TCCGATGACG AAGTTCGGTA AGTGAGAGAG AAGCTTAGTG TCCAAAACGA TCTTGAGACC TTCTTGACTA   
  
  
- ACTCTGACTC TTGTCTAATC ATTCAAAACG ACTTTCGAAG GCCTTGTATG ATAAACTTAA GGTGCCCACA   
  
  
- AACTCCCCAA GTCTCGACCA CTTGAACTTC TTTTCCCTCT TACTTTGTCA ACGGCATTTA GACCACAAAG   
  
  
- TGGAGTCGTC AAAGCTACTG TGGGACTTCC AGAGTCTGTG GAATTGGCGA CATGTAAGTG ACTTTGGAAG   
  
  
- ATATTACCAT GATCAACTTT CTCTTCCTTG GGCTTTACCT AGTCCTAAGT ATAGTCCAAA GTACCTAAGA   
  
  
- AACGTAATAA TACGACGTTA CAAGCTAAGT AATTTACTAA CGGAGGGTGC CCTCTCAGGG CTCTCTGACT   
  
  
- CGTATCTCTT CTTGGTAGAA CCCTCTCTCT AGTTCCTCCG CCACTGAACC CTCTTCCTGC TCTACTCGAT   
  
  
- GCCCTTCAAG CTCTATTACC TCTGAACCTT TTTCTCCTAC CTTTAAGTAC CCAAAAGCCC CTAGTCCGAC   
  
  
- TCAAGGTTCC GCTGTTAAGT TCGGTTCGAG GACGAGTTTT ACCCGTCGGT AATGACAGGG GTCAAACGTC   
  
  
- CCCTAAGACT TCCTCCTCAA CCGCCCAAGT CTCACATACT CTCTCTACTC CCCCTTCGGT AAAGAGACCA   
  
  
- AACCGTTCCG TCCAAGGAGC GTTGTCGGAG ACGTACCGCG ACACATAC

+     ABRE4

| Site Name | Organism | Position | Strand | Matrix score. | sequence | function |
| --- | --- | --- | --- | --- | --- | --- |
| ABRE4 | Zea mays | 150 | - | 6 | CACGTA |  |
| ABRE4 | Zea mays | 1806 | + | 6 | CACGTA |  |

>HU11G01529.1   
+ +Up\_Stream \_Len000GAAAGA AGATTTAAGG AAATCGATGG TACACGGCAA AGCTAGAAAC AGGAACTGTA   
  
  
+ TTTTTATGTT TTTTTTTTGT TGGAAGGGGG GGGGGGGGAG CGGGCGAGAT GTAACCCGTA TCTAGATGGA   
  
  
+ TAATCTACGT GACAAGACCT TCATTTGATC AAGGTACATC ATGAATATTT AACCCTTAAC TTCTTTGAGC   
  
  
+ CTTACAAGAG ATTGTCTGAG GTTTTTAGGG TGCACGATAT CATGCCTGAC TAGACCTGTG TAGACACAAT   
  
  
+ CTTCAGCCCT TATTGATAGC TGTTCGATAT GCATGCTGCC TAAAAATTGG AATAGTTGGC ACCTAACATT   
  
  
+ CTTTTACATC TTGCTCCTCC TTGGTGTAGA GTTAGATGTG CTAGCACAAT ACAATGGGTC CCGATATTTT   
  
  
+ GCATCTGGTT TCTGAAAGAT AATATTTGAT GATCCTGTCT TGTGCTGATG GGCAAAAGCT AAATTGTAAG   
  
  
+ AATATGTAGT TTATTTACAA GACATATGCT TAAGCTAGAG GTCATGTACT TGCATATTTT TGTGCAGCTT   
  
  
+ TTGAGGTTGT ATGCCCCTTT GAGACAAATT CATAGTTGAA TTCATGACAA GATTATGTTT GGCAATGACT   
  
  
+ TTGATTATTA AATTGGCCTT TATGCTATCC TATCCTGTGT GCTCAAATGT TATTTATCAG ATAATATTAG   
  
  
+ TTTACTCTTT CTTCCCTGTC TTGCAAAGAT TTTAAATCCT AAATTATGCA AATGTTTATC TTTAAAATGA   
  
  
+ CCTGCTACTC TCTGTGCCTG ATTTTATTTG TTTCCCCGTT GAAATTTTTT TTATGATTCC ACTTTTCAAG   
  
  
+ CAGCTTAGAC AGGCGTTATT AGGTAATTAT TTTCCGCTTT GGATTGTAGT AAATTGAAAC AGAAATGCCT   
  
  
+ CACTAAACCG CTCTCTGCTC TTTGTGTTGA AGAACCAATC CCATTCAAAC TCTTCTTTCT TTTTTGAAAT   
  
  
+ TCAATCCCGC TTCAGTCAAA TGTCTAACAG AAACAGCCTC GTGTGCAATT CTACTATATA CGGTTAATGC   
  
  
+ TGTGCATGTA TTACTTCCCA ACATCTTACC CAGGACTAGG AGGGACCTTG GTAGCCTGTT CTGCTTGAAA   
  
  
+ AATTTGTCAT TATCACCTGA ATTTGTATTA AATACATAAA TCTTTGTCTT TCAATTTGTG GCTCCACATT   
  
  
+ TCAATTTGTG GCTCCACACG CTTTTGAAAA CTTTATGAGA CCTAATATAT TAGAGAACAT CTGATCGTGA   
  
  
+ TTGTTAATTC CAGTTTTATA ATCATACTAA TTAGTAAATC ACTGAAGGTC ATATTGCATA TTGTTTTTGC   
  
  
+ CTAATGATTG GCTTATTCAA CTATTCCTGA ATAGTTTAGT TTCCAGAATC TTTAGAAGAA ACCTGGTCTG   
  
  
+ GGTATTATCG AGTATTTTTC TCAGTTCATG AAAGGCTTTG ATTCAGTGTG TGTGCTGTCA TGTCTAGGCA   
  
  
+ TGATTGAGTA TTTTTATTTG CATATCCAGG CATTGTGCCT AGACAAATGT CCTGAATCTT AGTGGCTGTT   
  
  
+ AGGTCTGAAC GGACACTTTT CTTGCTGTTT TTCCTTTCCG AGTGGCATCT TTAGCTTTTT CAAACTACCA   
  
  
+ AAGGACTATT AAACGAGGCC CTGCATATGC TGGAAAACTG CCTCTTAAGC TATGTTTATG CAAAAATGGA   
  
  
+ GGACCTTGAA CTGTTCAATG TACCTACAGT CTGAGGCACA GAGCCAATAG ACATAATAGA TAGTTGGATA   
  
  
+ TTCTTTTTAT ATTTAATTCA CAGTAGCGAC AACCCCAGCA TGAAACGTAG CCACGTAGGG GGGGAAAAAG   
  
  
+ GGTCTCCATC TGTTTATATA TTCTTGTCGC TTATGGTGCT TCTGTTATGT AATGACAAAG GAATTTGTGT   
  
  
+ TGGGATTATT CCGCTTCACT CCGTCTCACT AACTTTACGG TTTCAGTCCT AATTCTCTCT TTTTTTCTTT   
  
  
+ TGTTTTTTTT CCTGAGCTTG TTTAGTCTGT TTTCTTCTGT GTGTCTTTCT GATTATGGAA GCCATACAGG   
  
  
+ AGAAGAATGA CGATCTTTTG AGTCTTAGCT TGGCCATTGT TAGTCATTCT AACCATGCAG AAACGAAGGT   
  
  
+ GAAGAGGAAG AGGCATGATG TCTCTAATTC TCTGATCGCA ACAAGAGAAG AGGGTCGGGA TAAGAAAATA   
  
  
+ TTTCAGCTGC TTCAAGAACG AGAGAGAATG CTAAGCATCG GGCAAAGAAG AAAAGGCGTT ATTCAAGAAG   
  
  
+ GGAAGGGTCT TCATCTGATC CATTTGCTTC TCATGTCTGC TACCCTAATC AGCGAAAACA ATATCAGCTC   
  
  
+ AGCTGTTAAT CATTTGCTTG AGTTGTACAA GAATGTTTCT GTAAATGGTG ATTCTGGGCA GCGCGTTGCT   
  
  
+ GCTTATTTTG CTGATGGGTT AACTGCGAGG TTCTTAACCC AGAACTCTCC CTTTTATTGC ATGGTAATGA   
  
  
+ GTAAACCGAC ACCTGCAGAG GAGTTTATCG CCTTCACTCA TTTGTACAGG GTGTCTCCAT TCTATCAGTT   
  
  
+ TGCCCATTTT ACTGCTAATC AGGCCATCTT TGAGGCATTC GAGAGGGAAG AAGAGAACAA CAGCTGGGCA   
  
  
+ CTCCATGTCA TTGATTTTGA CATCTCTCAT GGCTTCCAGT GGCCGTCTCT CATTCAGTCT CTTTCTGAGA   
  
  
+ AGGCTACTGC TTCAAGCCAT TCACTCTCTC TTCGAATCAC AGGTTTTGCT AGAACTCTGG AAGAACTGAT   
  
  
+ TGAGACTGAG AACAGATTAG TAAGTTTTGC TGAAAGCTTC CGGAACATAC TATTTGAATT CCACGGGTGT   
  
  
+ TTGAGGGGTT CAGAGCTGGT GAACTTGAAG AAAAGGGAGA ATGAAACAGT TGCCGTAAAT CTGGTGTTTC   
  
  
+ ACCTCAGCAG TTTCGATGAC ACCCTGAAGG TCTCAGACAC CTTAACCGCT GTACATTCAC TGAAACCTTC   
  
  
+ TATAATGGTA CTAGTTGAAA GAGAAGGAAC CCGAAATGGA TCAGGATTCA TATCAGGTTT CATGGATTCT   
  
  
+ TTGCATTATT ATGCTGCAAT GTTCGATTCA TTAAATGATT GCCTCCCACG GGAGAGTCCC GAGAGACTGA   
  
  
+ GCATAGAGAA GAACCATCTT GGGAGAGAGA TCAAGGAGGC GGTGACTTGG GAGAAGGACG AGATGAGCTA   
  
  
+ CGGGAAGTTC GAGATAATGG AGACTTGGAA AAAGAGGATG GAAATTCATG GGTTTTCGGG GATCAGGCTG   
  
  
+ AGTTCCAAGG CGACAATTCA AGCCAAGCTC CTGCTCAAAA TGGGCAGCCA TTACTGTCCC CAGTTTGCAG   
  
  
+ GGGATTCTGA AGGAGGAGTT GGCGGGTTCA GAGTGTATGA GAGAGATGAG GGGGAAGCCA TTTCTCTGGT   
  
  
+ TTGGCAAGGC AGGTTCCTCG CAACAGCCTC TGCATGGCGC TGTGTATG  

- +Up\_Stream \_Len000CTTTCT TCTAAATTCC TTTAGCTACC ATGTGCCGTT TCGATCTTTG TCCTTGACAT   
  
  
- AAAAATACAA AAAAAAAACA ACCTTCCCCC CCCCCCCCTC GCCCGCTCTA CATTGGGCAT AGATCTACCT   
  
  
- ATTAGATGCA CTGTTCTGGA AGTAAACTAG TTCCATGTAG TACTTATAAA TTGGGAATTG AAGAAACTCG   
  
  
- GAATGTTCTC TAACAGACTC CAAAAATCCC ACGTGCTATA GTACGGACTG ATCTGGACAC ATCTGTGTTA   
  
  
- GAAGTCGGGA ATAACTATCG ACAAGCTATA CGTACGACGG ATTTTTAACC TTATCAACCG TGGATTGTAA   
  
  
- GAAAATGTAG AACGAGGAGG AACCACATCT CAATCTACAC GATCGTGTTA TGTTACCCAG GGCTATAAAA   
  
  
- CGTAGACCAA AGACTTTCTA TTATAAACTA CTAGGACAGA ACACGACTAC CCGTTTTCGA TTTAACATTC   
  
  
- TTATACATCA AATAAATGTT CTGTATACGA ATTCGATCTC CAGTACATGA ACGTATAAAA ACACGTCGAA   
  
  
- AACTCCAACA TACGGGGAAA CTCTGTTTAA GTATCAACTT AAGTACTGTT CTAATACAAA CCGTTACTGA   
  
  
- AACTAATAAT TTAACCGGAA ATACGATAGG ATAGGACACA CGAGTTTACA ATAAATAGTC TATTATAATC   
  
  
- AAATGAGAAA GAAGGGACAG AACGTTTCTA AAATTTAGGA TTTAATACGT TTACAAATAG AAATTTTACT   
  
  
- GGACGATGAG AGACACGGAC TAAAATAAAC AAAGGGGCAA CTTTAAAAAA AATACTAAGG TGAAAAGTTC   
  
  
- GTCGAATCTG TCCGCAATAA TCCATTAATA AAAGGCGAAA CCTAACATCA TTTAACTTTG TCTTTACGGA   
  
  
- GTGATTTGGC GAGAGACGAG AAACACAACT TCTTGGTTAG GGTAAGTTTG AGAAGAAAGA AAAAACTTTA   
  
  
- AGTTAGGGCG AAGTCAGTTT ACAGATTGTC TTTGTCGGAG CACACGTTAA GATGATATAT GCCAATTACG   
  
  
- ACACGTACAT AATGAAGGGT TGTAGAATGG GTCCTGATCC TCCCTGGAAC CATCGGACAA GACGAACTTT   
  
  
- TTAAACAGTA ATAGTGGACT TAAACATAAT TTATGTATTT AGAAACAGAA AGTTAAACAC CGAGGTGTAA   
  
  
- AGTTAAACAC CGAGGTGTGC GAAAACTTTT GAAATACTCT GGATTATATA ATCTCTTGTA GACTAGCACT   
  
  
- AACAATTAAG GTCAAAATAT TAGTATGATT AATCATTTAG TGACTTCCAG TATAACGTAT AACAAAAACG   
  
  
- GATTACTAAC CGAATAAGTT GATAAGGACT TATCAAATCA AAGGTCTTAG AAATCTTCTT TGGACCAGAC   
  
  
- CCATAATAGC TCATAAAAAG AGTCAAGTAC TTTCCGAAAC TAAGTCACAC ACACGACAGT ACAGATCCGT   
  
  
- ACTAACTCAT AAAAATAAAC GTATAGGTCC GTAACACGGA TCTGTTTACA GGACTTAGAA TCACCGACAA   
  
  
- TCCAGACTTG CCTGTGAAAA GAACGACAAA AAGGAAAGGC TCACCGTAGA AATCGAAAAA GTTTGATGGT   
  
  
- TTCCTGATAA TTTGCTCCGG GACGTATACG ACCTTTTGAC GGAGAATTCG ATACAAATAC GTTTTTACCT   
  
  
- CCTGGAACTT GACAAGTTAC ATGGATGTCA GACTCCGTGT CTCGGTTATC TGTATTATCT ATCAACCTAT   
  
  
- AAGAAAAATA TAAATTAAGT GTCATCGCTG TTGGGGTCGT ACTTTGCATC GGTGCATCCC CCCCTTTTTC   
  
  
- CCAGAGGTAG ACAAATATAT AAGAACAGCG AATACCACGA AGACAATACA TTACTGTTTC CTTAAACACA   
  
  
- ACCCTAATAA GGCGAAGTGA GGCAGAGTGA TTGAAATGCC AAAGTCAGGA TTAAGAGAGA AAAAAAGAAA   
  
  
- ACAAAAAAAA GGACTCGAAC AAATCAGACA AAAGAAGACA CACAGAAAGA CTAATACCTT CGGTATGTCC   
  
  
- TCTTCTTACT GCTAGAAAAC TCAGAATCGA ACCGGTAACA ATCAGTAAGA TTGGTACGTC TTTGCTTCCA   
  
  
- CTTCTCCTTC TCCGTACTAC AGAGATTAAG AGACTAGCGT TGTTCTCTTC TCCCAGCCCT ATTCTTTTAT   
  
  
- AAAGTCGACG AAGTTCTTGC TCTCTCTTAC GATTCGTAGC CCGTTTCTTC TTTTCCGCAA TAAGTTCTTC   
  
  
- CCTTCCCAGA AGTAGACTAG GTAAACGAAG AGTACAGACG ATGGGATTAG TCGCTTTTGT TATAGTCGAG   
  
  
- TCGACAATTA GTAAACGAAC TCAACATGTT CTTACAAAGA CATTTACCAC TAAGACCCGT CGCGCAACGA   
  
  
- CGAATAAAAC GACTACCCAA TTGACGCTCC AAGAATTGGG TCTTGAGAGG GAAAATAACG TACCATTACT   
  
  
- CATTTGGCTG TGGACGTCTC CTCAAATAGC GGAAGTGAGT AAACATGTCC CACAGAGGTA AGATAGTCAA   
  
  
- ACGGGTAAAA TGACGATTAG TCCGGTAGAA ACTCCGTAAG CTCTCCCTTC TTCTCTTGTT GTCGACCCGT   
  
  
- GAGGTACAGT AACTAAAACT GTAGAGAGTA CCGAAGGTCA CCGGCAGAGA GTAAGTCAGA GAAAGACTCT   
  
  
- TCCGATGACG AAGTTCGGTA AGTGAGAGAG AAGCTTAGTG TCCAAAACGA TCTTGAGACC TTCTTGACTA   
  
  
- ACTCTGACTC TTGTCTAATC ATTCAAAACG ACTTTCGAAG GCCTTGTATG ATAAACTTAA GGTGCCCACA   
  
  
- AACTCCCCAA GTCTCGACCA CTTGAACTTC TTTTCCCTCT TACTTTGTCA ACGGCATTTA GACCACAAAG   
  
  
- TGGAGTCGTC AAAGCTACTG TGGGACTTCC AGAGTCTGTG GAATTGGCGA CATGTAAGTG ACTTTGGAAG   
  
  
- ATATTACCAT GATCAACTTT CTCTTCCTTG GGCTTTACCT AGTCCTAAGT ATAGTCCAAA GTACCTAAGA   
  
  
- AACGTAATAA TACGACGTTA CAAGCTAAGT AATTTACTAA CGGAGGGTGC CCTCTCAGGG CTCTCTGACT   
  
  
- CGTATCTCTT CTTGGTAGAA CCCTCTCTCT AGTTCCTCCG CCACTGAACC CTCTTCCTGC TCTACTCGAT   
  
  
- GCCCTTCAAG CTCTATTACC TCTGAACCTT TTTCTCCTAC CTTTAAGTAC CCAAAAGCCC CTAGTCCGAC   
  
  
- TCAAGGTTCC GCTGTTAAGT TCGGTTCGAG GACGAGTTTT ACCCGTCGGT AATGACAGGG GTCAAACGTC   
  
  
- CCCTAAGACT TCCTCCTCAA CCGCCCAAGT CTCACATACT CTCTCTACTC CCCCTTCGGT AAAGAGACCA   
  
  
- AACCGTTCCG TCCAAGGAGC GTTGTCGGAG ACGTACCGCG ACACATAC

+     ARE

| Site Name | Organism | Position | Strand | Matrix score. | sequence | function |
| --- | --- | --- | --- | --- | --- | --- |
| ARE | Zea mays | 3361 | - | 6 | AAACCA | cis-acting regulatory element essential for the anaerobic induction |
| ARE | Zea mays | 430 | - | 6 | AAACCA | cis-acting regulatory element essential for the anaerobic induction |

>HU11G01529.1   
+ +Up\_Stream \_Len000GAAAGA AGATTTAAGG AAATCGATGG TACACGGCAA AGCTAGAAAC AGGAACTGTA   
  
  
+ TTTTTATGTT TTTTTTTTGT TGGAAGGGGG GGGGGGGGAG CGGGCGAGAT GTAACCCGTA TCTAGATGGA   
  
  
+ TAATCTACGT GACAAGACCT TCATTTGATC AAGGTACATC ATGAATATTT AACCCTTAAC TTCTTTGAGC   
  
  
+ CTTACAAGAG ATTGTCTGAG GTTTTTAGGG TGCACGATAT CATGCCTGAC TAGACCTGTG TAGACACAAT   
  
  
+ CTTCAGCCCT TATTGATAGC TGTTCGATAT GCATGCTGCC TAAAAATTGG AATAGTTGGC ACCTAACATT   
  
  
+ CTTTTACATC TTGCTCCTCC TTGGTGTAGA GTTAGATGTG CTAGCACAAT ACAATGGGTC CCGATATTTT   
  
  
+ GCATCTGGTT TCTGAAAGAT AATATTTGAT GATCCTGTCT TGTGCTGATG GGCAAAAGCT AAATTGTAAG   
  
  
+ AATATGTAGT TTATTTACAA GACATATGCT TAAGCTAGAG GTCATGTACT TGCATATTTT TGTGCAGCTT   
  
  
+ TTGAGGTTGT ATGCCCCTTT GAGACAAATT CATAGTTGAA TTCATGACAA GATTATGTTT GGCAATGACT   
  
  
+ TTGATTATTA AATTGGCCTT TATGCTATCC TATCCTGTGT GCTCAAATGT TATTTATCAG ATAATATTAG   
  
  
+ TTTACTCTTT CTTCCCTGTC TTGCAAAGAT TTTAAATCCT AAATTATGCA AATGTTTATC TTTAAAATGA   
  
  
+ CCTGCTACTC TCTGTGCCTG ATTTTATTTG TTTCCCCGTT GAAATTTTTT TTATGATTCC ACTTTTCAAG   
  
  
+ CAGCTTAGAC AGGCGTTATT AGGTAATTAT TTTCCGCTTT GGATTGTAGT AAATTGAAAC AGAAATGCCT   
  
  
+ CACTAAACCG CTCTCTGCTC TTTGTGTTGA AGAACCAATC CCATTCAAAC TCTTCTTTCT TTTTTGAAAT   
  
  
+ TCAATCCCGC TTCAGTCAAA TGTCTAACAG AAACAGCCTC GTGTGCAATT CTACTATATA CGGTTAATGC   
  
  
+ TGTGCATGTA TTACTTCCCA ACATCTTACC CAGGACTAGG AGGGACCTTG GTAGCCTGTT CTGCTTGAAA   
  
  
+ AATTTGTCAT TATCACCTGA ATTTGTATTA AATACATAAA TCTTTGTCTT TCAATTTGTG GCTCCACATT   
  
  
+ TCAATTTGTG GCTCCACACG CTTTTGAAAA CTTTATGAGA CCTAATATAT TAGAGAACAT CTGATCGTGA   
  
  
+ TTGTTAATTC CAGTTTTATA ATCATACTAA TTAGTAAATC ACTGAAGGTC ATATTGCATA TTGTTTTTGC   
  
  
+ CTAATGATTG GCTTATTCAA CTATTCCTGA ATAGTTTAGT TTCCAGAATC TTTAGAAGAA ACCTGGTCTG   
  
  
+ GGTATTATCG AGTATTTTTC TCAGTTCATG AAAGGCTTTG ATTCAGTGTG TGTGCTGTCA TGTCTAGGCA   
  
  
+ TGATTGAGTA TTTTTATTTG CATATCCAGG CATTGTGCCT AGACAAATGT CCTGAATCTT AGTGGCTGTT   
  
  
+ AGGTCTGAAC GGACACTTTT CTTGCTGTTT TTCCTTTCCG AGTGGCATCT TTAGCTTTTT CAAACTACCA   
  
  
+ AAGGACTATT AAACGAGGCC CTGCATATGC TGGAAAACTG CCTCTTAAGC TATGTTTATG CAAAAATGGA   
  
  
+ GGACCTTGAA CTGTTCAATG TACCTACAGT CTGAGGCACA GAGCCAATAG ACATAATAGA TAGTTGGATA   
  
  
+ TTCTTTTTAT ATTTAATTCA CAGTAGCGAC AACCCCAGCA TGAAACGTAG CCACGTAGGG GGGGAAAAAG   
  
  
+ GGTCTCCATC TGTTTATATA TTCTTGTCGC TTATGGTGCT TCTGTTATGT AATGACAAAG GAATTTGTGT   
  
  
+ TGGGATTATT CCGCTTCACT CCGTCTCACT AACTTTACGG TTTCAGTCCT AATTCTCTCT TTTTTTCTTT   
  
  
+ TGTTTTTTTT CCTGAGCTTG TTTAGTCTGT TTTCTTCTGT GTGTCTTTCT GATTATGGAA GCCATACAGG   
  
  
+ AGAAGAATGA CGATCTTTTG AGTCTTAGCT TGGCCATTGT TAGTCATTCT AACCATGCAG AAACGAAGGT   
  
  
+ GAAGAGGAAG AGGCATGATG TCTCTAATTC TCTGATCGCA ACAAGAGAAG AGGGTCGGGA TAAGAAAATA   
  
  
+ TTTCAGCTGC TTCAAGAACG AGAGAGAATG CTAAGCATCG GGCAAAGAAG AAAAGGCGTT ATTCAAGAAG   
  
  
+ GGAAGGGTCT TCATCTGATC CATTTGCTTC TCATGTCTGC TACCCTAATC AGCGAAAACA ATATCAGCTC   
  
  
+ AGCTGTTAAT CATTTGCTTG AGTTGTACAA GAATGTTTCT GTAAATGGTG ATTCTGGGCA GCGCGTTGCT   
  
  
+ GCTTATTTTG CTGATGGGTT AACTGCGAGG TTCTTAACCC AGAACTCTCC CTTTTATTGC ATGGTAATGA   
  
  
+ GTAAACCGAC ACCTGCAGAG GAGTTTATCG CCTTCACTCA TTTGTACAGG GTGTCTCCAT TCTATCAGTT   
  
  
+ TGCCCATTTT ACTGCTAATC AGGCCATCTT TGAGGCATTC GAGAGGGAAG AAGAGAACAA CAGCTGGGCA   
  
  
+ CTCCATGTCA TTGATTTTGA CATCTCTCAT GGCTTCCAGT GGCCGTCTCT CATTCAGTCT CTTTCTGAGA   
  
  
+ AGGCTACTGC TTCAAGCCAT TCACTCTCTC TTCGAATCAC AGGTTTTGCT AGAACTCTGG AAGAACTGAT   
  
  
+ TGAGACTGAG AACAGATTAG TAAGTTTTGC TGAAAGCTTC CGGAACATAC TATTTGAATT CCACGGGTGT   
  
  
+ TTGAGGGGTT CAGAGCTGGT GAACTTGAAG AAAAGGGAGA ATGAAACAGT TGCCGTAAAT CTGGTGTTTC   
  
  
+ ACCTCAGCAG TTTCGATGAC ACCCTGAAGG TCTCAGACAC CTTAACCGCT GTACATTCAC TGAAACCTTC   
  
  
+ TATAATGGTA CTAGTTGAAA GAGAAGGAAC CCGAAATGGA TCAGGATTCA TATCAGGTTT CATGGATTCT   
  
  
+ TTGCATTATT ATGCTGCAAT GTTCGATTCA TTAAATGATT GCCTCCCACG GGAGAGTCCC GAGAGACTGA   
  
  
+ GCATAGAGAA GAACCATCTT GGGAGAGAGA TCAAGGAGGC GGTGACTTGG GAGAAGGACG AGATGAGCTA   
  
  
+ CGGGAAGTTC GAGATAATGG AGACTTGGAA AAAGAGGATG GAAATTCATG GGTTTTCGGG GATCAGGCTG   
  
  
+ AGTTCCAAGG CGACAATTCA AGCCAAGCTC CTGCTCAAAA TGGGCAGCCA TTACTGTCCC CAGTTTGCAG   
  
  
+ GGGATTCTGA AGGAGGAGTT GGCGGGTTCA GAGTGTATGA GAGAGATGAG GGGGAAGCCA TTTCTCTGGT   
  
  
+ TTGGCAAGGC AGGTTCCTCG CAACAGCCTC TGCATGGCGC TGTGTATG  

- +Up\_Stream \_Len000CTTTCT TCTAAATTCC TTTAGCTACC ATGTGCCGTT TCGATCTTTG TCCTTGACAT   
  
  
- AAAAATACAA AAAAAAAACA ACCTTCCCCC CCCCCCCCTC GCCCGCTCTA CATTGGGCAT AGATCTACCT   
  
  
- ATTAGATGCA CTGTTCTGGA AGTAAACTAG TTCCATGTAG TACTTATAAA TTGGGAATTG AAGAAACTCG   
  
  
- GAATGTTCTC TAACAGACTC CAAAAATCCC ACGTGCTATA GTACGGACTG ATCTGGACAC ATCTGTGTTA   
  
  
- GAAGTCGGGA ATAACTATCG ACAAGCTATA CGTACGACGG ATTTTTAACC TTATCAACCG TGGATTGTAA   
  
  
- GAAAATGTAG AACGAGGAGG AACCACATCT CAATCTACAC GATCGTGTTA TGTTACCCAG GGCTATAAAA   
  
  
- CGTAGACCAA AGACTTTCTA TTATAAACTA CTAGGACAGA ACACGACTAC CCGTTTTCGA TTTAACATTC   
  
  
- TTATACATCA AATAAATGTT CTGTATACGA ATTCGATCTC CAGTACATGA ACGTATAAAA ACACGTCGAA   
  
  
- AACTCCAACA TACGGGGAAA CTCTGTTTAA GTATCAACTT AAGTACTGTT CTAATACAAA CCGTTACTGA   
  
  
- AACTAATAAT TTAACCGGAA ATACGATAGG ATAGGACACA CGAGTTTACA ATAAATAGTC TATTATAATC   
  
  
- AAATGAGAAA GAAGGGACAG AACGTTTCTA AAATTTAGGA TTTAATACGT TTACAAATAG AAATTTTACT   
  
  
- GGACGATGAG AGACACGGAC TAAAATAAAC AAAGGGGCAA CTTTAAAAAA AATACTAAGG TGAAAAGTTC   
  
  
- GTCGAATCTG TCCGCAATAA TCCATTAATA AAAGGCGAAA CCTAACATCA TTTAACTTTG TCTTTACGGA   
  
  
- GTGATTTGGC GAGAGACGAG AAACACAACT TCTTGGTTAG GGTAAGTTTG AGAAGAAAGA AAAAACTTTA   
  
  
- AGTTAGGGCG AAGTCAGTTT ACAGATTGTC TTTGTCGGAG CACACGTTAA GATGATATAT GCCAATTACG   
  
  
- ACACGTACAT AATGAAGGGT TGTAGAATGG GTCCTGATCC TCCCTGGAAC CATCGGACAA GACGAACTTT   
  
  
- TTAAACAGTA ATAGTGGACT TAAACATAAT TTATGTATTT AGAAACAGAA AGTTAAACAC CGAGGTGTAA   
  
  
- AGTTAAACAC CGAGGTGTGC GAAAACTTTT GAAATACTCT GGATTATATA ATCTCTTGTA GACTAGCACT   
  
  
- AACAATTAAG GTCAAAATAT TAGTATGATT AATCATTTAG TGACTTCCAG TATAACGTAT AACAAAAACG   
  
  
- GATTACTAAC CGAATAAGTT GATAAGGACT TATCAAATCA AAGGTCTTAG AAATCTTCTT TGGACCAGAC   
  
  
- CCATAATAGC TCATAAAAAG AGTCAAGTAC TTTCCGAAAC TAAGTCACAC ACACGACAGT ACAGATCCGT   
  
  
- ACTAACTCAT AAAAATAAAC GTATAGGTCC GTAACACGGA TCTGTTTACA GGACTTAGAA TCACCGACAA   
  
  
- TCCAGACTTG CCTGTGAAAA GAACGACAAA AAGGAAAGGC TCACCGTAGA AATCGAAAAA GTTTGATGGT   
  
  
- TTCCTGATAA TTTGCTCCGG GACGTATACG ACCTTTTGAC GGAGAATTCG ATACAAATAC GTTTTTACCT   
  
  
- CCTGGAACTT GACAAGTTAC ATGGATGTCA GACTCCGTGT CTCGGTTATC TGTATTATCT ATCAACCTAT   
  
  
- AAGAAAAATA TAAATTAAGT GTCATCGCTG TTGGGGTCGT ACTTTGCATC GGTGCATCCC CCCCTTTTTC   
  
  
- CCAGAGGTAG ACAAATATAT AAGAACAGCG AATACCACGA AGACAATACA TTACTGTTTC CTTAAACACA   
  
  
- ACCCTAATAA GGCGAAGTGA GGCAGAGTGA TTGAAATGCC AAAGTCAGGA TTAAGAGAGA AAAAAAGAAA   
  
  
- ACAAAAAAAA GGACTCGAAC AAATCAGACA AAAGAAGACA CACAGAAAGA CTAATACCTT CGGTATGTCC   
  
  
- TCTTCTTACT GCTAGAAAAC TCAGAATCGA ACCGGTAACA ATCAGTAAGA TTGGTACGTC TTTGCTTCCA   
  
  
- CTTCTCCTTC TCCGTACTAC AGAGATTAAG AGACTAGCGT TGTTCTCTTC TCCCAGCCCT ATTCTTTTAT   
  
  
- AAAGTCGACG AAGTTCTTGC TCTCTCTTAC GATTCGTAGC CCGTTTCTTC TTTTCCGCAA TAAGTTCTTC   
  
  
- CCTTCCCAGA AGTAGACTAG GTAAACGAAG AGTACAGACG ATGGGATTAG TCGCTTTTGT TATAGTCGAG   
  
  
- TCGACAATTA GTAAACGAAC TCAACATGTT CTTACAAAGA CATTTACCAC TAAGACCCGT CGCGCAACGA   
  
  
- CGAATAAAAC GACTACCCAA TTGACGCTCC AAGAATTGGG TCTTGAGAGG GAAAATAACG TACCATTACT   
  
  
- CATTTGGCTG TGGACGTCTC CTCAAATAGC GGAAGTGAGT AAACATGTCC CACAGAGGTA AGATAGTCAA   
  
  
- ACGGGTAAAA TGACGATTAG TCCGGTAGAA ACTCCGTAAG CTCTCCCTTC TTCTCTTGTT GTCGACCCGT   
  
  
- GAGGTACAGT AACTAAAACT GTAGAGAGTA CCGAAGGTCA CCGGCAGAGA GTAAGTCAGA GAAAGACTCT   
  
  
- TCCGATGACG AAGTTCGGTA AGTGAGAGAG AAGCTTAGTG TCCAAAACGA TCTTGAGACC TTCTTGACTA   
  
  
- ACTCTGACTC TTGTCTAATC ATTCAAAACG ACTTTCGAAG GCCTTGTATG ATAAACTTAA GGTGCCCACA   
  
  
- AACTCCCCAA GTCTCGACCA CTTGAACTTC TTTTCCCTCT TACTTTGTCA ACGGCATTTA GACCACAAAG   
  
  
- TGGAGTCGTC AAAGCTACTG TGGGACTTCC AGAGTCTGTG GAATTGGCGA CATGTAAGTG ACTTTGGAAG   
  
  
- ATATTACCAT GATCAACTTT CTCTTCCTTG GGCTTTACCT AGTCCTAAGT ATAGTCCAAA GTACCTAAGA   
  
  
- AACGTAATAA TACGACGTTA CAAGCTAAGT AATTTACTAA CGGAGGGTGC CCTCTCAGGG CTCTCTGACT   
  
  
- CGTATCTCTT CTTGGTAGAA CCCTCTCTCT AGTTCCTCCG CCACTGAACC CTCTTCCTGC TCTACTCGAT   
  
  
- GCCCTTCAAG CTCTATTACC TCTGAACCTT TTTCTCCTAC CTTTAAGTAC CCAAAAGCCC CTAGTCCGAC   
  
  
- TCAAGGTTCC GCTGTTAAGT TCGGTTCGAG GACGAGTTTT ACCCGTCGGT AATGACAGGG GTCAAACGTC   
  
  
- CCCTAAGACT TCCTCCTCAA CCGCCCAAGT CTCACATACT CTCTCTACTC CCCCTTCGGT AAAGAGACCA   
  
  
- AACCGTTCCG TCCAAGGAGC GTTGTCGGAG ACGTACCGCG ACACATAC

+     AT~TATA-box

| Site Name | Organism | Position | Strand | Matrix score. | sequence | function |
| --- | --- | --- | --- | --- | --- | --- |
| AT~TATA-box | Arabidopsis thaliana | 1837 | - | 8 | TATATAAA |  |
| AT~TATA-box | Arabidopsis thaliana | 1839 | - | 6 | TATATA |  |
| AT~TATA-box | Arabidopsis thaliana | 1039 | + | 6 | TATATA |  |

>HU11G01529.1   
+ +Up\_Stream \_Len000GAAAGA AGATTTAAGG AAATCGATGG TACACGGCAA AGCTAGAAAC AGGAACTGTA   
  
  
+ TTTTTATGTT TTTTTTTTGT TGGAAGGGGG GGGGGGGGAG CGGGCGAGAT GTAACCCGTA TCTAGATGGA   
  
  
+ TAATCTACGT GACAAGACCT TCATTTGATC AAGGTACATC ATGAATATTT AACCCTTAAC TTCTTTGAGC   
  
  
+ CTTACAAGAG ATTGTCTGAG GTTTTTAGGG TGCACGATAT CATGCCTGAC TAGACCTGTG TAGACACAAT   
  
  
+ CTTCAGCCCT TATTGATAGC TGTTCGATAT GCATGCTGCC TAAAAATTGG AATAGTTGGC ACCTAACATT   
  
  
+ CTTTTACATC TTGCTCCTCC TTGGTGTAGA GTTAGATGTG CTAGCACAAT ACAATGGGTC CCGATATTTT   
  
  
+ GCATCTGGTT TCTGAAAGAT AATATTTGAT GATCCTGTCT TGTGCTGATG GGCAAAAGCT AAATTGTAAG   
  
  
+ AATATGTAGT TTATTTACAA GACATATGCT TAAGCTAGAG GTCATGTACT TGCATATTTT TGTGCAGCTT   
  
  
+ TTGAGGTTGT ATGCCCCTTT GAGACAAATT CATAGTTGAA TTCATGACAA GATTATGTTT GGCAATGACT   
  
  
+ TTGATTATTA AATTGGCCTT TATGCTATCC TATCCTGTGT GCTCAAATGT TATTTATCAG ATAATATTAG   
  
  
+ TTTACTCTTT CTTCCCTGTC TTGCAAAGAT TTTAAATCCT AAATTATGCA AATGTTTATC TTTAAAATGA   
  
  
+ CCTGCTACTC TCTGTGCCTG ATTTTATTTG TTTCCCCGTT GAAATTTTTT TTATGATTCC ACTTTTCAAG   
  
  
+ CAGCTTAGAC AGGCGTTATT AGGTAATTAT TTTCCGCTTT GGATTGTAGT AAATTGAAAC AGAAATGCCT   
  
  
+ CACTAAACCG CTCTCTGCTC TTTGTGTTGA AGAACCAATC CCATTCAAAC TCTTCTTTCT TTTTTGAAAT   
  
  
+ TCAATCCCGC TTCAGTCAAA TGTCTAACAG AAACAGCCTC GTGTGCAATT CTACTATATA CGGTTAATGC   
  
  
+ TGTGCATGTA TTACTTCCCA ACATCTTACC CAGGACTAGG AGGGACCTTG GTAGCCTGTT CTGCTTGAAA   
  
  
+ AATTTGTCAT TATCACCTGA ATTTGTATTA AATACATAAA TCTTTGTCTT TCAATTTGTG GCTCCACATT   
  
  
+ TCAATTTGTG GCTCCACACG CTTTTGAAAA CTTTATGAGA CCTAATATAT TAGAGAACAT CTGATCGTGA   
  
  
+ TTGTTAATTC CAGTTTTATA ATCATACTAA TTAGTAAATC ACTGAAGGTC ATATTGCATA TTGTTTTTGC   
  
  
+ CTAATGATTG GCTTATTCAA CTATTCCTGA ATAGTTTAGT TTCCAGAATC TTTAGAAGAA ACCTGGTCTG   
  
  
+ GGTATTATCG AGTATTTTTC TCAGTTCATG AAAGGCTTTG ATTCAGTGTG TGTGCTGTCA TGTCTAGGCA   
  
  
+ TGATTGAGTA TTTTTATTTG CATATCCAGG CATTGTGCCT AGACAAATGT CCTGAATCTT AGTGGCTGTT   
  
  
+ AGGTCTGAAC GGACACTTTT CTTGCTGTTT TTCCTTTCCG AGTGGCATCT TTAGCTTTTT CAAACTACCA   
  
  
+ AAGGACTATT AAACGAGGCC CTGCATATGC TGGAAAACTG CCTCTTAAGC TATGTTTATG CAAAAATGGA   
  
  
+ GGACCTTGAA CTGTTCAATG TACCTACAGT CTGAGGCACA GAGCCAATAG ACATAATAGA TAGTTGGATA   
  
  
+ TTCTTTTTAT ATTTAATTCA CAGTAGCGAC AACCCCAGCA TGAAACGTAG CCACGTAGGG GGGGAAAAAG   
  
  
+ GGTCTCCATC TGTTTATATA TTCTTGTCGC TTATGGTGCT TCTGTTATGT AATGACAAAG GAATTTGTGT   
  
  
+ TGGGATTATT CCGCTTCACT CCGTCTCACT AACTTTACGG TTTCAGTCCT AATTCTCTCT TTTTTTCTTT   
  
  
+ TGTTTTTTTT CCTGAGCTTG TTTAGTCTGT TTTCTTCTGT GTGTCTTTCT GATTATGGAA GCCATACAGG   
  
  
+ AGAAGAATGA CGATCTTTTG AGTCTTAGCT TGGCCATTGT TAGTCATTCT AACCATGCAG AAACGAAGGT   
  
  
+ GAAGAGGAAG AGGCATGATG TCTCTAATTC TCTGATCGCA ACAAGAGAAG AGGGTCGGGA TAAGAAAATA   
  
  
+ TTTCAGCTGC TTCAAGAACG AGAGAGAATG CTAAGCATCG GGCAAAGAAG AAAAGGCGTT ATTCAAGAAG   
  
  
+ GGAAGGGTCT TCATCTGATC CATTTGCTTC TCATGTCTGC TACCCTAATC AGCGAAAACA ATATCAGCTC   
  
  
+ AGCTGTTAAT CATTTGCTTG AGTTGTACAA GAATGTTTCT GTAAATGGTG ATTCTGGGCA GCGCGTTGCT   
  
  
+ GCTTATTTTG CTGATGGGTT AACTGCGAGG TTCTTAACCC AGAACTCTCC CTTTTATTGC ATGGTAATGA   
  
  
+ GTAAACCGAC ACCTGCAGAG GAGTTTATCG CCTTCACTCA TTTGTACAGG GTGTCTCCAT TCTATCAGTT   
  
  
+ TGCCCATTTT ACTGCTAATC AGGCCATCTT TGAGGCATTC GAGAGGGAAG AAGAGAACAA CAGCTGGGCA   
  
  
+ CTCCATGTCA TTGATTTTGA CATCTCTCAT GGCTTCCAGT GGCCGTCTCT CATTCAGTCT CTTTCTGAGA   
  
  
+ AGGCTACTGC TTCAAGCCAT TCACTCTCTC TTCGAATCAC AGGTTTTGCT AGAACTCTGG AAGAACTGAT   
  
  
+ TGAGACTGAG AACAGATTAG TAAGTTTTGC TGAAAGCTTC CGGAACATAC TATTTGAATT CCACGGGTGT   
  
  
+ TTGAGGGGTT CAGAGCTGGT GAACTTGAAG AAAAGGGAGA ATGAAACAGT TGCCGTAAAT CTGGTGTTTC   
  
  
+ ACCTCAGCAG TTTCGATGAC ACCCTGAAGG TCTCAGACAC CTTAACCGCT GTACATTCAC TGAAACCTTC   
  
  
+ TATAATGGTA CTAGTTGAAA GAGAAGGAAC CCGAAATGGA TCAGGATTCA TATCAGGTTT CATGGATTCT   
  
  
+ TTGCATTATT ATGCTGCAAT GTTCGATTCA TTAAATGATT GCCTCCCACG GGAGAGTCCC GAGAGACTGA   
  
  
+ GCATAGAGAA GAACCATCTT GGGAGAGAGA TCAAGGAGGC GGTGACTTGG GAGAAGGACG AGATGAGCTA   
  
  
+ CGGGAAGTTC GAGATAATGG AGACTTGGAA AAAGAGGATG GAAATTCATG GGTTTTCGGG GATCAGGCTG   
  
  
+ AGTTCCAAGG CGACAATTCA AGCCAAGCTC CTGCTCAAAA TGGGCAGCCA TTACTGTCCC CAGTTTGCAG   
  
  
+ GGGATTCTGA AGGAGGAGTT GGCGGGTTCA GAGTGTATGA GAGAGATGAG GGGGAAGCCA TTTCTCTGGT   
  
  
+ TTGGCAAGGC AGGTTCCTCG CAACAGCCTC TGCATGGCGC TGTGTATG  

- +Up\_Stream \_Len000CTTTCT TCTAAATTCC TTTAGCTACC ATGTGCCGTT TCGATCTTTG TCCTTGACAT   
  
  
- AAAAATACAA AAAAAAAACA ACCTTCCCCC CCCCCCCCTC GCCCGCTCTA CATTGGGCAT AGATCTACCT   
  
  
- ATTAGATGCA CTGTTCTGGA AGTAAACTAG TTCCATGTAG TACTTATAAA TTGGGAATTG AAGAAACTCG   
  
  
- GAATGTTCTC TAACAGACTC CAAAAATCCC ACGTGCTATA GTACGGACTG ATCTGGACAC ATCTGTGTTA   
  
  
- GAAGTCGGGA ATAACTATCG ACAAGCTATA CGTACGACGG ATTTTTAACC TTATCAACCG TGGATTGTAA   
  
  
- GAAAATGTAG AACGAGGAGG AACCACATCT CAATCTACAC GATCGTGTTA TGTTACCCAG GGCTATAAAA   
  
  
- CGTAGACCAA AGACTTTCTA TTATAAACTA CTAGGACAGA ACACGACTAC CCGTTTTCGA TTTAACATTC   
  
  
- TTATACATCA AATAAATGTT CTGTATACGA ATTCGATCTC CAGTACATGA ACGTATAAAA ACACGTCGAA   
  
  
- AACTCCAACA TACGGGGAAA CTCTGTTTAA GTATCAACTT AAGTACTGTT CTAATACAAA CCGTTACTGA   
  
  
- AACTAATAAT TTAACCGGAA ATACGATAGG ATAGGACACA CGAGTTTACA ATAAATAGTC TATTATAATC   
  
  
- AAATGAGAAA GAAGGGACAG AACGTTTCTA AAATTTAGGA TTTAATACGT TTACAAATAG AAATTTTACT   
  
  
- GGACGATGAG AGACACGGAC TAAAATAAAC AAAGGGGCAA CTTTAAAAAA AATACTAAGG TGAAAAGTTC   
  
  
- GTCGAATCTG TCCGCAATAA TCCATTAATA AAAGGCGAAA CCTAACATCA TTTAACTTTG TCTTTACGGA   
  
  
- GTGATTTGGC GAGAGACGAG AAACACAACT TCTTGGTTAG GGTAAGTTTG AGAAGAAAGA AAAAACTTTA   
  
  
- AGTTAGGGCG AAGTCAGTTT ACAGATTGTC TTTGTCGGAG CACACGTTAA GATGATATAT GCCAATTACG   
  
  
- ACACGTACAT AATGAAGGGT TGTAGAATGG GTCCTGATCC TCCCTGGAAC CATCGGACAA GACGAACTTT   
  
  
- TTAAACAGTA ATAGTGGACT TAAACATAAT TTATGTATTT AGAAACAGAA AGTTAAACAC CGAGGTGTAA   
  
  
- AGTTAAACAC CGAGGTGTGC GAAAACTTTT GAAATACTCT GGATTATATA ATCTCTTGTA GACTAGCACT   
  
  
- AACAATTAAG GTCAAAATAT TAGTATGATT AATCATTTAG TGACTTCCAG TATAACGTAT AACAAAAACG   
  
  
- GATTACTAAC CGAATAAGTT GATAAGGACT TATCAAATCA AAGGTCTTAG AAATCTTCTT TGGACCAGAC   
  
  
- CCATAATAGC TCATAAAAAG AGTCAAGTAC TTTCCGAAAC TAAGTCACAC ACACGACAGT ACAGATCCGT   
  
  
- ACTAACTCAT AAAAATAAAC GTATAGGTCC GTAACACGGA TCTGTTTACA GGACTTAGAA TCACCGACAA   
  
  
- TCCAGACTTG CCTGTGAAAA GAACGACAAA AAGGAAAGGC TCACCGTAGA AATCGAAAAA GTTTGATGGT   
  
  
- TTCCTGATAA TTTGCTCCGG GACGTATACG ACCTTTTGAC GGAGAATTCG ATACAAATAC GTTTTTACCT   
  
  
- CCTGGAACTT GACAAGTTAC ATGGATGTCA GACTCCGTGT CTCGGTTATC TGTATTATCT ATCAACCTAT   
  
  
- AAGAAAAATA TAAATTAAGT GTCATCGCTG TTGGGGTCGT ACTTTGCATC GGTGCATCCC CCCCTTTTTC   
  
  
- CCAGAGGTAG ACAAATATAT AAGAACAGCG AATACCACGA AGACAATACA TTACTGTTTC CTTAAACACA   
  
  
- ACCCTAATAA GGCGAAGTGA GGCAGAGTGA TTGAAATGCC AAAGTCAGGA TTAAGAGAGA AAAAAAGAAA   
  
  
- ACAAAAAAAA GGACTCGAAC AAATCAGACA AAAGAAGACA CACAGAAAGA CTAATACCTT CGGTATGTCC   
  
  
- TCTTCTTACT GCTAGAAAAC TCAGAATCGA ACCGGTAACA ATCAGTAAGA TTGGTACGTC TTTGCTTCCA   
  
  
- CTTCTCCTTC TCCGTACTAC AGAGATTAAG AGACTAGCGT TGTTCTCTTC TCCCAGCCCT ATTCTTTTAT   
  
  
- AAAGTCGACG AAGTTCTTGC TCTCTCTTAC GATTCGTAGC CCGTTTCTTC TTTTCCGCAA TAAGTTCTTC   
  
  
- CCTTCCCAGA AGTAGACTAG GTAAACGAAG AGTACAGACG ATGGGATTAG TCGCTTTTGT TATAGTCGAG   
  
  
- TCGACAATTA GTAAACGAAC TCAACATGTT CTTACAAAGA CATTTACCAC TAAGACCCGT CGCGCAACGA   
  
  
- CGAATAAAAC GACTACCCAA TTGACGCTCC AAGAATTGGG TCTTGAGAGG GAAAATAACG TACCATTACT   
  
  
- CATTTGGCTG TGGACGTCTC CTCAAATAGC GGAAGTGAGT AAACATGTCC CACAGAGGTA AGATAGTCAA   
  
  
- ACGGGTAAAA TGACGATTAG TCCGGTAGAA ACTCCGTAAG CTCTCCCTTC TTCTCTTGTT GTCGACCCGT   
  
  
- GAGGTACAGT AACTAAAACT GTAGAGAGTA CCGAAGGTCA CCGGCAGAGA GTAAGTCAGA GAAAGACTCT   
  
  
- TCCGATGACG AAGTTCGGTA AGTGAGAGAG AAGCTTAGTG TCCAAAACGA TCTTGAGACC TTCTTGACTA   
  
  
- ACTCTGACTC TTGTCTAATC ATTCAAAACG ACTTTCGAAG GCCTTGTATG ATAAACTTAA GGTGCCCACA   
  
  
- AACTCCCCAA GTCTCGACCA CTTGAACTTC TTTTCCCTCT TACTTTGTCA ACGGCATTTA GACCACAAAG   
  
  
- TGGAGTCGTC AAAGCTACTG TGGGACTTCC AGAGTCTGTG GAATTGGCGA CATGTAAGTG ACTTTGGAAG   
  
  
- ATATTACCAT GATCAACTTT CTCTTCCTTG GGCTTTACCT AGTCCTAAGT ATAGTCCAAA GTACCTAAGA   
  
  
- AACGTAATAA TACGACGTTA CAAGCTAAGT AATTTACTAA CGGAGGGTGC CCTCTCAGGG CTCTCTGACT   
  
  
- CGTATCTCTT CTTGGTAGAA CCCTCTCTCT AGTTCCTCCG CCACTGAACC CTCTTCCTGC TCTACTCGAT   
  
  
- GCCCTTCAAG CTCTATTACC TCTGAACCTT TTTCTCCTAC CTTTAAGTAC CCAAAAGCCC CTAGTCCGAC   
  
  
- TCAAGGTTCC GCTGTTAAGT TCGGTTCGAG GACGAGTTTT ACCCGTCGGT AATGACAGGG GTCAAACGTC   
  
  
- CCCTAAGACT TCCTCCTCAA CCGCCCAAGT CTCACATACT CTCTCTACTC CCCCTTCGGT AAAGAGACCA   
  
  
- AACCGTTCCG TCCAAGGAGC GTTGTCGGAG ACGTACCGCG ACACATAC

+     Box II -like sequence

| Site Name | Organism | Position | Strand | Matrix score. | sequence | function |
| --- | --- | --- | --- | --- | --- | --- |
| Box II -like sequence | Oryza sativa | 42 | - | 10 | TCCGTGTACCA | cis-acting regulatory element |

>HU11G01529.1   
+ +Up\_Stream \_Len000GAAAGA AGATTTAAGG AAATCGATGG TACACGGCAA AGCTAGAAAC AGGAACTGTA   
  
  
+ TTTTTATGTT TTTTTTTTGT TGGAAGGGGG GGGGGGGGAG CGGGCGAGAT GTAACCCGTA TCTAGATGGA   
  
  
+ TAATCTACGT GACAAGACCT TCATTTGATC AAGGTACATC ATGAATATTT AACCCTTAAC TTCTTTGAGC   
  
  
+ CTTACAAGAG ATTGTCTGAG GTTTTTAGGG TGCACGATAT CATGCCTGAC TAGACCTGTG TAGACACAAT   
  
  
+ CTTCAGCCCT TATTGATAGC TGTTCGATAT GCATGCTGCC TAAAAATTGG AATAGTTGGC ACCTAACATT   
  
  
+ CTTTTACATC TTGCTCCTCC TTGGTGTAGA GTTAGATGTG CTAGCACAAT ACAATGGGTC CCGATATTTT   
  
  
+ GCATCTGGTT TCTGAAAGAT AATATTTGAT GATCCTGTCT TGTGCTGATG GGCAAAAGCT AAATTGTAAG   
  
  
+ AATATGTAGT TTATTTACAA GACATATGCT TAAGCTAGAG GTCATGTACT TGCATATTTT TGTGCAGCTT   
  
  
+ TTGAGGTTGT ATGCCCCTTT GAGACAAATT CATAGTTGAA TTCATGACAA GATTATGTTT GGCAATGACT   
  
  
+ TTGATTATTA AATTGGCCTT TATGCTATCC TATCCTGTGT GCTCAAATGT TATTTATCAG ATAATATTAG   
  
  
+ TTTACTCTTT CTTCCCTGTC TTGCAAAGAT TTTAAATCCT AAATTATGCA AATGTTTATC TTTAAAATGA   
  
  
+ CCTGCTACTC TCTGTGCCTG ATTTTATTTG TTTCCCCGTT GAAATTTTTT TTATGATTCC ACTTTTCAAG   
  
  
+ CAGCTTAGAC AGGCGTTATT AGGTAATTAT TTTCCGCTTT GGATTGTAGT AAATTGAAAC AGAAATGCCT   
  
  
+ CACTAAACCG CTCTCTGCTC TTTGTGTTGA AGAACCAATC CCATTCAAAC TCTTCTTTCT TTTTTGAAAT   
  
  
+ TCAATCCCGC TTCAGTCAAA TGTCTAACAG AAACAGCCTC GTGTGCAATT CTACTATATA CGGTTAATGC   
  
  
+ TGTGCATGTA TTACTTCCCA ACATCTTACC CAGGACTAGG AGGGACCTTG GTAGCCTGTT CTGCTTGAAA   
  
  
+ AATTTGTCAT TATCACCTGA ATTTGTATTA AATACATAAA TCTTTGTCTT TCAATTTGTG GCTCCACATT   
  
  
+ TCAATTTGTG GCTCCACACG CTTTTGAAAA CTTTATGAGA CCTAATATAT TAGAGAACAT CTGATCGTGA   
  
  
+ TTGTTAATTC CAGTTTTATA ATCATACTAA TTAGTAAATC ACTGAAGGTC ATATTGCATA TTGTTTTTGC   
  
  
+ CTAATGATTG GCTTATTCAA CTATTCCTGA ATAGTTTAGT TTCCAGAATC TTTAGAAGAA ACCTGGTCTG   
  
  
+ GGTATTATCG AGTATTTTTC TCAGTTCATG AAAGGCTTTG ATTCAGTGTG TGTGCTGTCA TGTCTAGGCA   
  
  
+ TGATTGAGTA TTTTTATTTG CATATCCAGG CATTGTGCCT AGACAAATGT CCTGAATCTT AGTGGCTGTT   
  
  
+ AGGTCTGAAC GGACACTTTT CTTGCTGTTT TTCCTTTCCG AGTGGCATCT TTAGCTTTTT CAAACTACCA   
  
  
+ AAGGACTATT AAACGAGGCC CTGCATATGC TGGAAAACTG CCTCTTAAGC TATGTTTATG CAAAAATGGA   
  
  
+ GGACCTTGAA CTGTTCAATG TACCTACAGT CTGAGGCACA GAGCCAATAG ACATAATAGA TAGTTGGATA   
  
  
+ TTCTTTTTAT ATTTAATTCA CAGTAGCGAC AACCCCAGCA TGAAACGTAG CCACGTAGGG GGGGAAAAAG   
  
  
+ GGTCTCCATC TGTTTATATA TTCTTGTCGC TTATGGTGCT TCTGTTATGT AATGACAAAG GAATTTGTGT   
  
  
+ TGGGATTATT CCGCTTCACT CCGTCTCACT AACTTTACGG TTTCAGTCCT AATTCTCTCT TTTTTTCTTT   
  
  
+ TGTTTTTTTT CCTGAGCTTG TTTAGTCTGT TTTCTTCTGT GTGTCTTTCT GATTATGGAA GCCATACAGG   
  
  
+ AGAAGAATGA CGATCTTTTG AGTCTTAGCT TGGCCATTGT TAGTCATTCT AACCATGCAG AAACGAAGGT   
  
  
+ GAAGAGGAAG AGGCATGATG TCTCTAATTC TCTGATCGCA ACAAGAGAAG AGGGTCGGGA TAAGAAAATA   
  
  
+ TTTCAGCTGC TTCAAGAACG AGAGAGAATG CTAAGCATCG GGCAAAGAAG AAAAGGCGTT ATTCAAGAAG   
  
  
+ GGAAGGGTCT TCATCTGATC CATTTGCTTC TCATGTCTGC TACCCTAATC AGCGAAAACA ATATCAGCTC   
  
  
+ AGCTGTTAAT CATTTGCTTG AGTTGTACAA GAATGTTTCT GTAAATGGTG ATTCTGGGCA GCGCGTTGCT   
  
  
+ GCTTATTTTG CTGATGGGTT AACTGCGAGG TTCTTAACCC AGAACTCTCC CTTTTATTGC ATGGTAATGA   
  
  
+ GTAAACCGAC ACCTGCAGAG GAGTTTATCG CCTTCACTCA TTTGTACAGG GTGTCTCCAT TCTATCAGTT   
  
  
+ TGCCCATTTT ACTGCTAATC AGGCCATCTT TGAGGCATTC GAGAGGGAAG AAGAGAACAA CAGCTGGGCA   
  
  
+ CTCCATGTCA TTGATTTTGA CATCTCTCAT GGCTTCCAGT GGCCGTCTCT CATTCAGTCT CTTTCTGAGA   
  
  
+ AGGCTACTGC TTCAAGCCAT TCACTCTCTC TTCGAATCAC AGGTTTTGCT AGAACTCTGG AAGAACTGAT   
  
  
+ TGAGACTGAG AACAGATTAG TAAGTTTTGC TGAAAGCTTC CGGAACATAC TATTTGAATT CCACGGGTGT   
  
  
+ TTGAGGGGTT CAGAGCTGGT GAACTTGAAG AAAAGGGAGA ATGAAACAGT TGCCGTAAAT CTGGTGTTTC   
  
  
+ ACCTCAGCAG TTTCGATGAC ACCCTGAAGG TCTCAGACAC CTTAACCGCT GTACATTCAC TGAAACCTTC   
  
  
+ TATAATGGTA CTAGTTGAAA GAGAAGGAAC CCGAAATGGA TCAGGATTCA TATCAGGTTT CATGGATTCT   
  
  
+ TTGCATTATT ATGCTGCAAT GTTCGATTCA TTAAATGATT GCCTCCCACG GGAGAGTCCC GAGAGACTGA   
  
  
+ GCATAGAGAA GAACCATCTT GGGAGAGAGA TCAAGGAGGC GGTGACTTGG GAGAAGGACG AGATGAGCTA   
  
  
+ CGGGAAGTTC GAGATAATGG AGACTTGGAA AAAGAGGATG GAAATTCATG GGTTTTCGGG GATCAGGCTG   
  
  
+ AGTTCCAAGG CGACAATTCA AGCCAAGCTC CTGCTCAAAA TGGGCAGCCA TTACTGTCCC CAGTTTGCAG   
  
  
+ GGGATTCTGA AGGAGGAGTT GGCGGGTTCA GAGTGTATGA GAGAGATGAG GGGGAAGCCA TTTCTCTGGT   
  
  
+ TTGGCAAGGC AGGTTCCTCG CAACAGCCTC TGCATGGCGC TGTGTATG  

- +Up\_Stream \_Len000CTTTCT TCTAAATTCC TTTAGCTACC ATGTGCCGTT TCGATCTTTG TCCTTGACAT   
  
  
- AAAAATACAA AAAAAAAACA ACCTTCCCCC CCCCCCCCTC GCCCGCTCTA CATTGGGCAT AGATCTACCT   
  
  
- ATTAGATGCA CTGTTCTGGA AGTAAACTAG TTCCATGTAG TACTTATAAA TTGGGAATTG AAGAAACTCG   
  
  
- GAATGTTCTC TAACAGACTC CAAAAATCCC ACGTGCTATA GTACGGACTG ATCTGGACAC ATCTGTGTTA   
  
  
- GAAGTCGGGA ATAACTATCG ACAAGCTATA CGTACGACGG ATTTTTAACC TTATCAACCG TGGATTGTAA   
  
  
- GAAAATGTAG AACGAGGAGG AACCACATCT CAATCTACAC GATCGTGTTA TGTTACCCAG GGCTATAAAA   
  
  
- CGTAGACCAA AGACTTTCTA TTATAAACTA CTAGGACAGA ACACGACTAC CCGTTTTCGA TTTAACATTC   
  
  
- TTATACATCA AATAAATGTT CTGTATACGA ATTCGATCTC CAGTACATGA ACGTATAAAA ACACGTCGAA   
  
  
- AACTCCAACA TACGGGGAAA CTCTGTTTAA GTATCAACTT AAGTACTGTT CTAATACAAA CCGTTACTGA   
  
  
- AACTAATAAT TTAACCGGAA ATACGATAGG ATAGGACACA CGAGTTTACA ATAAATAGTC TATTATAATC   
  
  
- AAATGAGAAA GAAGGGACAG AACGTTTCTA AAATTTAGGA TTTAATACGT TTACAAATAG AAATTTTACT   
  
  
- GGACGATGAG AGACACGGAC TAAAATAAAC AAAGGGGCAA CTTTAAAAAA AATACTAAGG TGAAAAGTTC   
  
  
- GTCGAATCTG TCCGCAATAA TCCATTAATA AAAGGCGAAA CCTAACATCA TTTAACTTTG TCTTTACGGA   
  
  
- GTGATTTGGC GAGAGACGAG AAACACAACT TCTTGGTTAG GGTAAGTTTG AGAAGAAAGA AAAAACTTTA   
  
  
- AGTTAGGGCG AAGTCAGTTT ACAGATTGTC TTTGTCGGAG CACACGTTAA GATGATATAT GCCAATTACG   
  
  
- ACACGTACAT AATGAAGGGT TGTAGAATGG GTCCTGATCC TCCCTGGAAC CATCGGACAA GACGAACTTT   
  
  
- TTAAACAGTA ATAGTGGACT TAAACATAAT TTATGTATTT AGAAACAGAA AGTTAAACAC CGAGGTGTAA   
  
  
- AGTTAAACAC CGAGGTGTGC GAAAACTTTT GAAATACTCT GGATTATATA ATCTCTTGTA GACTAGCACT   
  
  
- AACAATTAAG GTCAAAATAT TAGTATGATT AATCATTTAG TGACTTCCAG TATAACGTAT AACAAAAACG   
  
  
- GATTACTAAC CGAATAAGTT GATAAGGACT TATCAAATCA AAGGTCTTAG AAATCTTCTT TGGACCAGAC   
  
  
- CCATAATAGC TCATAAAAAG AGTCAAGTAC TTTCCGAAAC TAAGTCACAC ACACGACAGT ACAGATCCGT   
  
  
- ACTAACTCAT AAAAATAAAC GTATAGGTCC GTAACACGGA TCTGTTTACA GGACTTAGAA TCACCGACAA   
  
  
- TCCAGACTTG CCTGTGAAAA GAACGACAAA AAGGAAAGGC TCACCGTAGA AATCGAAAAA GTTTGATGGT   
  
  
- TTCCTGATAA TTTGCTCCGG GACGTATACG ACCTTTTGAC GGAGAATTCG ATACAAATAC GTTTTTACCT   
  
  
- CCTGGAACTT GACAAGTTAC ATGGATGTCA GACTCCGTGT CTCGGTTATC TGTATTATCT ATCAACCTAT   
  
  
- AAGAAAAATA TAAATTAAGT GTCATCGCTG TTGGGGTCGT ACTTTGCATC GGTGCATCCC CCCCTTTTTC   
  
  
- CCAGAGGTAG ACAAATATAT AAGAACAGCG AATACCACGA AGACAATACA TTACTGTTTC CTTAAACACA   
  
  
- ACCCTAATAA GGCGAAGTGA GGCAGAGTGA TTGAAATGCC AAAGTCAGGA TTAAGAGAGA AAAAAAGAAA   
  
  
- ACAAAAAAAA GGACTCGAAC AAATCAGACA AAAGAAGACA CACAGAAAGA CTAATACCTT CGGTATGTCC   
  
  
- TCTTCTTACT GCTAGAAAAC TCAGAATCGA ACCGGTAACA ATCAGTAAGA TTGGTACGTC TTTGCTTCCA   
  
  
- CTTCTCCTTC TCCGTACTAC AGAGATTAAG AGACTAGCGT TGTTCTCTTC TCCCAGCCCT ATTCTTTTAT   
  
  
- AAAGTCGACG AAGTTCTTGC TCTCTCTTAC GATTCGTAGC CCGTTTCTTC TTTTCCGCAA TAAGTTCTTC   
  
  
- CCTTCCCAGA AGTAGACTAG GTAAACGAAG AGTACAGACG ATGGGATTAG TCGCTTTTGT TATAGTCGAG   
  
  
- TCGACAATTA GTAAACGAAC TCAACATGTT CTTACAAAGA CATTTACCAC TAAGACCCGT CGCGCAACGA   
  
  
- CGAATAAAAC GACTACCCAA TTGACGCTCC AAGAATTGGG TCTTGAGAGG GAAAATAACG TACCATTACT   
  
  
- CATTTGGCTG TGGACGTCTC CTCAAATAGC GGAAGTGAGT AAACATGTCC CACAGAGGTA AGATAGTCAA   
  
  
- ACGGGTAAAA TGACGATTAG TCCGGTAGAA ACTCCGTAAG CTCTCCCTTC TTCTCTTGTT GTCGACCCGT   
  
  
- GAGGTACAGT AACTAAAACT GTAGAGAGTA CCGAAGGTCA CCGGCAGAGA GTAAGTCAGA GAAAGACTCT   
  
  
- TCCGATGACG AAGTTCGGTA AGTGAGAGAG AAGCTTAGTG TCCAAAACGA TCTTGAGACC TTCTTGACTA   
  
  
- ACTCTGACTC TTGTCTAATC ATTCAAAACG ACTTTCGAAG GCCTTGTATG ATAAACTTAA GGTGCCCACA   
  
  
- AACTCCCCAA GTCTCGACCA CTTGAACTTC TTTTCCCTCT TACTTTGTCA ACGGCATTTA GACCACAAAG   
  
  
- TGGAGTCGTC AAAGCTACTG TGGGACTTCC AGAGTCTGTG GAATTGGCGA CATGTAAGTG ACTTTGGAAG   
  
  
- ATATTACCAT GATCAACTTT CTCTTCCTTG GGCTTTACCT AGTCCTAAGT ATAGTCCAAA GTACCTAAGA   
  
  
- AACGTAATAA TACGACGTTA CAAGCTAAGT AATTTACTAA CGGAGGGTGC CCTCTCAGGG CTCTCTGACT   
  
  
- CGTATCTCTT CTTGGTAGAA CCCTCTCTCT AGTTCCTCCG CCACTGAACC CTCTTCCTGC TCTACTCGAT   
  
  
- GCCCTTCAAG CTCTATTACC TCTGAACCTT TTTCTCCTAC CTTTAAGTAC CCAAAAGCCC CTAGTCCGAC   
  
  
- TCAAGGTTCC GCTGTTAAGT TCGGTTCGAG GACGAGTTTT ACCCGTCGGT AATGACAGGG GTCAAACGTC   
  
  
- CCCTAAGACT TCCTCCTCAA CCGCCCAAGT CTCACATACT CTCTCTACTC CCCCTTCGGT AAAGAGACCA   
  
  
- AACCGTTCCG TCCAAGGAGC GTTGTCGGAG ACGTACCGCG ACACATAC

+     CAAT-box

| Site Name | Organism | Position | Strand | Matrix score. | sequence | function |
| --- | --- | --- | --- | --- | --- | --- |
| CAAT-box | Nicotiana glutinosa | 2440 | - | 4 | CAAT |  |
| CAAT-box | Arabidopsis thaliana | 1728 | + | 5 | CCAAT | common cis-acting element in promoter and enhancer regions |
| CAAT-box | Pisum sativum | 1178 | - | 5 | CAAAT | common cis-acting element in promoter and enhancer regions |
| CAAT-box | Nicotiana glutinosa | 225 | - | 4 | CAAT |  |
| CAAT-box | Pisum sativum | 589 | + | 5 | CAAAT | common cis-acting element in promoter and enhancer regions |
| CAAT-box | Nicotiana glutinosa | 887 | - | 4 | CAAT |  |
| CAAT-box | Pisum sativum | 2266 | - | 5 | CAAAT | common cis-acting element in promoter and enhancer regions |
| CAAT-box | Nicotiana glutinosa | 3238 | + | 4 | CAAT |  |
| CAAT-box | Nicotiana glutinosa | 296 | - | 4 | CAAT |  |
| CAAT-box | Nicotiana glutinosa | 1729 | + | 4 | CAAT |  |
| CAAT-box | Arabidopsis thaliana | 330 | - | 5 | CCAAT | common cis-acting element in promoter and enhancer regions |
| CAAT-box | Pisum sativum | 753 | + | 5 | CAAAT | common cis-acting element in promoter and enhancer regions |
| CAAT-box | Petunia hybrida | 339 | - | 7 | TGCCAAC | common cis-acting element in promoter and enhancer regions |
| CAAT-box | Nicotiana glutinosa | 3031 | + | 4 | CAAT |  |
| CAAT-box | Pisum sativum | 2786 | - | 5 | CAAAT | common cis-acting element in promoter and enhancer regions |
| CAAT-box | Nicotiana glutinosa | 1030 | + | 4 | CAAT |  |
| CAAT-box | Nicotiana glutinosa | 1506 | - | 4 | CAAT |  |
| CAAT-box | Pisum sativum | 1490 | - | 5 | CAAAT | common cis-acting element in promoter and enhancer regions |
| CAAT-box | Pisum sativum | 800 | - | 5 | CAAAT | common cis-acting element in promoter and enhancer regions |
| CAAT-box | Pisum sativum | 2494 | - | 5 | CAAAT | common cis-acting element in promoter and enhancer regions |
| CAAT-box | Pisum sativum | 678 | + | 5 | CAAAT | common cis-acting element in promoter and enhancer regions |
| CAAT-box | Nicotiana glutinosa | 627 | + | 4 | CAAT |  |
| CAAT-box | Pisum sativum | 167 | - | 5 | CAAAT | common cis-acting element in promoter and enhancer regions |
| CAAT-box | Pisum sativum | 1887 | - | 5 | CAAAT | common cis-acting element in promoter and enhancer regions |
| CAAT-box | Nicotiana glutinosa | 406 | + | 4 | CAAT |  |
| CAAT-box | Nicotiana glutinosa | 281 | + | 4 | CAAT |  |
| CAAT-box | Nicotiana glutinosa | 2604 | - | 4 | CAAT |  |
| CAAT-box | Nicotiana glutinosa | 2733 | - | 4 | CAAT |  |
| CAAT-box | Nicotiana glutinosa | 1176 | + | 4 | CAAT |  |
| CAAT-box | Arabidopsis thaliana | 1341 | - | 5 | CCAAT | common cis-acting element in promoter and enhancer regions |
| CAAT-box | Nicotiana glutinosa | 1324 | - | 4 | CAAT |  |
| CAAT-box | Nicotiana glutinosa | 1264 | - | 4 | CAAT |  |
| CAAT-box | Nicotiana glutinosa | 2303 | + | 4 | CAAT |  |
| CAAT-box | Nicotiana glutinosa | 1700 | + | 4 | CAAT |  |
| CAAT-box | Pisum sativum | 1518 | + | 5 | CAAAT | common cis-acting element in promoter and enhancer regions |
| CAAT-box | Pisum sativum | 2326 | - | 5 | CAAAT | common cis-acting element in promoter and enhancer regions |
| CAAT-box | Pisum sativum | 1198 | - | 5 | CAAAT | common cis-acting element in promoter and enhancer regions |
| CAAT-box | Arabidopsis thaliana | 646 | - | 5 | CCAAT | common cis-acting element in promoter and enhancer regions |
| CAAT-box | Nicotiana glutinosa | 401 | + | 4 | CAAT |  |
| CAAT-box | Nicotiana glutinosa | 1477 | - | 4 | CAAT |  |
| CAAT-box | Pisum sativum | 1001 | + | 5 | CAAAT | common cis-acting element in promoter and enhancer regions |
| CAAT-box | Nicotiana glutinosa | 950 | + | 4 | CAAT |  |
| CAAT-box | Nicotiana glutinosa | 487 | - | 4 | CAAT |  |
| CAAT-box | Nicotiana glutinosa | 3052 | - | 4 | CAAT |  |
| CAAT-box | Pisum sativum | 448 | - | 5 | CAAAT | common cis-acting element in promoter and enhancer regions |
| CAAT-box | Nicotiana glutinosa | 1317 | - | 4 | CAAT |  |
| CAAT-box | Nicotiana glutinosa | 1196 | + | 4 | CAAT |  |
| CAAT-box | Nicotiana glutinosa | 2070 | - | 4 | CAAT |  |
| CAAT-box | Nicotiana glutinosa | 897 | - | 4 | CAAT |  |
| CAAT-box | Arabidopsis thaliana | 949 | + | 5 | CCAAT | common cis-acting element in promoter and enhancer regions |
| CAAT-box | Nicotiana glutinosa | 986 | + | 4 | CAAT |  |
| CAAT-box | Pisum sativum | 1145 | - | 5 | CAAAT | common cis-acting element in promoter and enhancer regions |
| CAAT-box | Pisum sativum | 1126 | - | 5 | CAAAT | common cis-acting element in promoter and enhancer regions |

>HU11G01529.1   
+ +Up\_Stream \_Len000GAAAGA AGATTTAAGG AAATCGATGG TACACGGCAA AGCTAGAAAC AGGAACTGTA   
  
  
+ TTTTTATGTT TTTTTTTTGT TGGAAGGGGG GGGGGGGGAG CGGGCGAGAT GTAACCCGTA TCTAGATGGA   
  
  
+ TAATCTACGT GACAAGACCT TCATTTGATC AAGGTACATC ATGAATATTT AACCCTTAAC TTCTTTGAGC   
  
  
+ CTTACAAGAG ATTGTCTGAG GTTTTTAGGG TGCACGATAT CATGCCTGAC TAGACCTGTG TAGACACAAT   
  
  
+ CTTCAGCCCT TATTGATAGC TGTTCGATAT GCATGCTGCC TAAAAATTGG AATAGTTGGC ACCTAACATT   
  
  
+ CTTTTACATC TTGCTCCTCC TTGGTGTAGA GTTAGATGTG CTAGCACAAT ACAATGGGTC CCGATATTTT   
  
  
+ GCATCTGGTT TCTGAAAGAT AATATTTGAT GATCCTGTCT TGTGCTGATG GGCAAAAGCT AAATTGTAAG   
  
  
+ AATATGTAGT TTATTTACAA GACATATGCT TAAGCTAGAG GTCATGTACT TGCATATTTT TGTGCAGCTT   
  
  
+ TTGAGGTTGT ATGCCCCTTT GAGACAAATT CATAGTTGAA TTCATGACAA GATTATGTTT GGCAATGACT   
  
  
+ TTGATTATTA AATTGGCCTT TATGCTATCC TATCCTGTGT GCTCAAATGT TATTTATCAG ATAATATTAG   
  
  
+ TTTACTCTTT CTTCCCTGTC TTGCAAAGAT TTTAAATCCT AAATTATGCA AATGTTTATC TTTAAAATGA   
  
  
+ CCTGCTACTC TCTGTGCCTG ATTTTATTTG TTTCCCCGTT GAAATTTTTT TTATGATTCC ACTTTTCAAG   
  
  
+ CAGCTTAGAC AGGCGTTATT AGGTAATTAT TTTCCGCTTT GGATTGTAGT AAATTGAAAC AGAAATGCCT   
  
  
+ CACTAAACCG CTCTCTGCTC TTTGTGTTGA AGAACCAATC CCATTCAAAC TCTTCTTTCT TTTTTGAAAT   
  
  
+ TCAATCCCGC TTCAGTCAAA TGTCTAACAG AAACAGCCTC GTGTGCAATT CTACTATATA CGGTTAATGC   
  
  
+ TGTGCATGTA TTACTTCCCA ACATCTTACC CAGGACTAGG AGGGACCTTG GTAGCCTGTT CTGCTTGAAA   
  
  
+ AATTTGTCAT TATCACCTGA ATTTGTATTA AATACATAAA TCTTTGTCTT TCAATTTGTG GCTCCACATT   
  
  
+ TCAATTTGTG GCTCCACACG CTTTTGAAAA CTTTATGAGA CCTAATATAT TAGAGAACAT CTGATCGTGA   
  
  
+ TTGTTAATTC CAGTTTTATA ATCATACTAA TTAGTAAATC ACTGAAGGTC ATATTGCATA TTGTTTTTGC   
  
  
+ CTAATGATTG GCTTATTCAA CTATTCCTGA ATAGTTTAGT TTCCAGAATC TTTAGAAGAA ACCTGGTCTG   
  
  
+ GGTATTATCG AGTATTTTTC TCAGTTCATG AAAGGCTTTG ATTCAGTGTG TGTGCTGTCA TGTCTAGGCA   
  
  
+ TGATTGAGTA TTTTTATTTG CATATCCAGG CATTGTGCCT AGACAAATGT CCTGAATCTT AGTGGCTGTT   
  
  
+ AGGTCTGAAC GGACACTTTT CTTGCTGTTT TTCCTTTCCG AGTGGCATCT TTAGCTTTTT CAAACTACCA   
  
  
+ AAGGACTATT AAACGAGGCC CTGCATATGC TGGAAAACTG CCTCTTAAGC TATGTTTATG CAAAAATGGA   
  
  
+ GGACCTTGAA CTGTTCAATG TACCTACAGT CTGAGGCACA GAGCCAATAG ACATAATAGA TAGTTGGATA   
  
  
+ TTCTTTTTAT ATTTAATTCA CAGTAGCGAC AACCCCAGCA TGAAACGTAG CCACGTAGGG GGGGAAAAAG   
  
  
+ GGTCTCCATC TGTTTATATA TTCTTGTCGC TTATGGTGCT TCTGTTATGT AATGACAAAG GAATTTGTGT   
  
  
+ TGGGATTATT CCGCTTCACT CCGTCTCACT AACTTTACGG TTTCAGTCCT AATTCTCTCT TTTTTTCTTT   
  
  
+ TGTTTTTTTT CCTGAGCTTG TTTAGTCTGT TTTCTTCTGT GTGTCTTTCT GATTATGGAA GCCATACAGG   
  
  
+ AGAAGAATGA CGATCTTTTG AGTCTTAGCT TGGCCATTGT TAGTCATTCT AACCATGCAG AAACGAAGGT   
  
  
+ GAAGAGGAAG AGGCATGATG TCTCTAATTC TCTGATCGCA ACAAGAGAAG AGGGTCGGGA TAAGAAAATA   
  
  
+ TTTCAGCTGC TTCAAGAACG AGAGAGAATG CTAAGCATCG GGCAAAGAAG AAAAGGCGTT ATTCAAGAAG   
  
  
+ GGAAGGGTCT TCATCTGATC CATTTGCTTC TCATGTCTGC TACCCTAATC AGCGAAAACA ATATCAGCTC   
  
  
+ AGCTGTTAAT CATTTGCTTG AGTTGTACAA GAATGTTTCT GTAAATGGTG ATTCTGGGCA GCGCGTTGCT   
  
  
+ GCTTATTTTG CTGATGGGTT AACTGCGAGG TTCTTAACCC AGAACTCTCC CTTTTATTGC ATGGTAATGA   
  
  
+ GTAAACCGAC ACCTGCAGAG GAGTTTATCG CCTTCACTCA TTTGTACAGG GTGTCTCCAT TCTATCAGTT   
  
  
+ TGCCCATTTT ACTGCTAATC AGGCCATCTT TGAGGCATTC GAGAGGGAAG AAGAGAACAA CAGCTGGGCA   
  
  
+ CTCCATGTCA TTGATTTTGA CATCTCTCAT GGCTTCCAGT GGCCGTCTCT CATTCAGTCT CTTTCTGAGA   
  
  
+ AGGCTACTGC TTCAAGCCAT TCACTCTCTC TTCGAATCAC AGGTTTTGCT AGAACTCTGG AAGAACTGAT   
  
  
+ TGAGACTGAG AACAGATTAG TAAGTTTTGC TGAAAGCTTC CGGAACATAC TATTTGAATT CCACGGGTGT   
  
  
+ TTGAGGGGTT CAGAGCTGGT GAACTTGAAG AAAAGGGAGA ATGAAACAGT TGCCGTAAAT CTGGTGTTTC   
  
  
+ ACCTCAGCAG TTTCGATGAC ACCCTGAAGG TCTCAGACAC CTTAACCGCT GTACATTCAC TGAAACCTTC   
  
  
+ TATAATGGTA CTAGTTGAAA GAGAAGGAAC CCGAAATGGA TCAGGATTCA TATCAGGTTT CATGGATTCT   
  
  
+ TTGCATTATT ATGCTGCAAT GTTCGATTCA TTAAATGATT GCCTCCCACG GGAGAGTCCC GAGAGACTGA   
  
  
+ GCATAGAGAA GAACCATCTT GGGAGAGAGA TCAAGGAGGC GGTGACTTGG GAGAAGGACG AGATGAGCTA   
  
  
+ CGGGAAGTTC GAGATAATGG AGACTTGGAA AAAGAGGATG GAAATTCATG GGTTTTCGGG GATCAGGCTG   
  
  
+ AGTTCCAAGG CGACAATTCA AGCCAAGCTC CTGCTCAAAA TGGGCAGCCA TTACTGTCCC CAGTTTGCAG   
  
  
+ GGGATTCTGA AGGAGGAGTT GGCGGGTTCA GAGTGTATGA GAGAGATGAG GGGGAAGCCA TTTCTCTGGT   
  
  
+ TTGGCAAGGC AGGTTCCTCG CAACAGCCTC TGCATGGCGC TGTGTATG  

- +Up\_Stream \_Len000CTTTCT TCTAAATTCC TTTAGCTACC ATGTGCCGTT TCGATCTTTG TCCTTGACAT   
  
  
- AAAAATACAA AAAAAAAACA ACCTTCCCCC CCCCCCCCTC GCCCGCTCTA CATTGGGCAT AGATCTACCT   
  
  
- ATTAGATGCA CTGTTCTGGA AGTAAACTAG TTCCATGTAG TACTTATAAA TTGGGAATTG AAGAAACTCG   
  
  
- GAATGTTCTC TAACAGACTC CAAAAATCCC ACGTGCTATA GTACGGACTG ATCTGGACAC ATCTGTGTTA   
  
  
- GAAGTCGGGA ATAACTATCG ACAAGCTATA CGTACGACGG ATTTTTAACC TTATCAACCG TGGATTGTAA   
  
  
- GAAAATGTAG AACGAGGAGG AACCACATCT CAATCTACAC GATCGTGTTA TGTTACCCAG GGCTATAAAA   
  
  
- CGTAGACCAA AGACTTTCTA TTATAAACTA CTAGGACAGA ACACGACTAC CCGTTTTCGA TTTAACATTC   
  
  
- TTATACATCA AATAAATGTT CTGTATACGA ATTCGATCTC CAGTACATGA ACGTATAAAA ACACGTCGAA   
  
  
- AACTCCAACA TACGGGGAAA CTCTGTTTAA GTATCAACTT AAGTACTGTT CTAATACAAA CCGTTACTGA   
  
  
- AACTAATAAT TTAACCGGAA ATACGATAGG ATAGGACACA CGAGTTTACA ATAAATAGTC TATTATAATC   
  
  
- AAATGAGAAA GAAGGGACAG AACGTTTCTA AAATTTAGGA TTTAATACGT TTACAAATAG AAATTTTACT   
  
  
- GGACGATGAG AGACACGGAC TAAAATAAAC AAAGGGGCAA CTTTAAAAAA AATACTAAGG TGAAAAGTTC   
  
  
- GTCGAATCTG TCCGCAATAA TCCATTAATA AAAGGCGAAA CCTAACATCA TTTAACTTTG TCTTTACGGA   
  
  
- GTGATTTGGC GAGAGACGAG AAACACAACT TCTTGGTTAG GGTAAGTTTG AGAAGAAAGA AAAAACTTTA   
  
  
- AGTTAGGGCG AAGTCAGTTT ACAGATTGTC TTTGTCGGAG CACACGTTAA GATGATATAT GCCAATTACG   
  
  
- ACACGTACAT AATGAAGGGT TGTAGAATGG GTCCTGATCC TCCCTGGAAC CATCGGACAA GACGAACTTT   
  
  
- TTAAACAGTA ATAGTGGACT TAAACATAAT TTATGTATTT AGAAACAGAA AGTTAAACAC CGAGGTGTAA   
  
  
- AGTTAAACAC CGAGGTGTGC GAAAACTTTT GAAATACTCT GGATTATATA ATCTCTTGTA GACTAGCACT   
  
  
- AACAATTAAG GTCAAAATAT TAGTATGATT AATCATTTAG TGACTTCCAG TATAACGTAT AACAAAAACG   
  
  
- GATTACTAAC CGAATAAGTT GATAAGGACT TATCAAATCA AAGGTCTTAG AAATCTTCTT TGGACCAGAC   
  
  
- CCATAATAGC TCATAAAAAG AGTCAAGTAC TTTCCGAAAC TAAGTCACAC ACACGACAGT ACAGATCCGT   
  
  
- ACTAACTCAT AAAAATAAAC GTATAGGTCC GTAACACGGA TCTGTTTACA GGACTTAGAA TCACCGACAA   
  
  
- TCCAGACTTG CCTGTGAAAA GAACGACAAA AAGGAAAGGC TCACCGTAGA AATCGAAAAA GTTTGATGGT   
  
  
- TTCCTGATAA TTTGCTCCGG GACGTATACG ACCTTTTGAC GGAGAATTCG ATACAAATAC GTTTTTACCT   
  
  
- CCTGGAACTT GACAAGTTAC ATGGATGTCA GACTCCGTGT CTCGGTTATC TGTATTATCT ATCAACCTAT   
  
  
- AAGAAAAATA TAAATTAAGT GTCATCGCTG TTGGGGTCGT ACTTTGCATC GGTGCATCCC CCCCTTTTTC   
  
  
- CCAGAGGTAG ACAAATATAT AAGAACAGCG AATACCACGA AGACAATACA TTACTGTTTC CTTAAACACA   
  
  
- ACCCTAATAA GGCGAAGTGA GGCAGAGTGA TTGAAATGCC AAAGTCAGGA TTAAGAGAGA AAAAAAGAAA   
  
  
- ACAAAAAAAA GGACTCGAAC AAATCAGACA AAAGAAGACA CACAGAAAGA CTAATACCTT CGGTATGTCC   
  
  
- TCTTCTTACT GCTAGAAAAC TCAGAATCGA ACCGGTAACA ATCAGTAAGA TTGGTACGTC TTTGCTTCCA   
  
  
- CTTCTCCTTC TCCGTACTAC AGAGATTAAG AGACTAGCGT TGTTCTCTTC TCCCAGCCCT ATTCTTTTAT   
  
  
- AAAGTCGACG AAGTTCTTGC TCTCTCTTAC GATTCGTAGC CCGTTTCTTC TTTTCCGCAA TAAGTTCTTC   
  
  
- CCTTCCCAGA AGTAGACTAG GTAAACGAAG AGTACAGACG ATGGGATTAG TCGCTTTTGT TATAGTCGAG   
  
  
- TCGACAATTA GTAAACGAAC TCAACATGTT CTTACAAAGA CATTTACCAC TAAGACCCGT CGCGCAACGA   
  
  
- CGAATAAAAC GACTACCCAA TTGACGCTCC AAGAATTGGG TCTTGAGAGG GAAAATAACG TACCATTACT   
  
  
- CATTTGGCTG TGGACGTCTC CTCAAATAGC GGAAGTGAGT AAACATGTCC CACAGAGGTA AGATAGTCAA   
  
  
- ACGGGTAAAA TGACGATTAG TCCGGTAGAA ACTCCGTAAG CTCTCCCTTC TTCTCTTGTT GTCGACCCGT   
  
  
- GAGGTACAGT AACTAAAACT GTAGAGAGTA CCGAAGGTCA CCGGCAGAGA GTAAGTCAGA GAAAGACTCT   
  
  
- TCCGATGACG AAGTTCGGTA AGTGAGAGAG AAGCTTAGTG TCCAAAACGA TCTTGAGACC TTCTTGACTA   
  
  
- ACTCTGACTC TTGTCTAATC ATTCAAAACG ACTTTCGAAG GCCTTGTATG ATAAACTTAA GGTGCCCACA   
  
  
- AACTCCCCAA GTCTCGACCA CTTGAACTTC TTTTCCCTCT TACTTTGTCA ACGGCATTTA GACCACAAAG   
  
  
- TGGAGTCGTC AAAGCTACTG TGGGACTTCC AGAGTCTGTG GAATTGGCGA CATGTAAGTG ACTTTGGAAG   
  
  
- ATATTACCAT GATCAACTTT CTCTTCCTTG GGCTTTACCT AGTCCTAAGT ATAGTCCAAA GTACCTAAGA   
  
  
- AACGTAATAA TACGACGTTA CAAGCTAAGT AATTTACTAA CGGAGGGTGC CCTCTCAGGG CTCTCTGACT   
  
  
- CGTATCTCTT CTTGGTAGAA CCCTCTCTCT AGTTCCTCCG CCACTGAACC CTCTTCCTGC TCTACTCGAT   
  
  
- GCCCTTCAAG CTCTATTACC TCTGAACCTT TTTCTCCTAC CTTTAAGTAC CCAAAAGCCC CTAGTCCGAC   
  
  
- TCAAGGTTCC GCTGTTAAGT TCGGTTCGAG GACGAGTTTT ACCCGTCGGT AATGACAGGG GTCAAACGTC   
  
  
- CCCTAAGACT TCCTCCTCAA CCGCCCAAGT CTCACATACT CTCTCTACTC CCCCTTCGGT AAAGAGACCA   
  
  
- AACCGTTCCG TCCAAGGAGC GTTGTCGGAG ACGTACCGCG ACACATAC

+     CAT-box

| Site Name | Organism | Position | Strand | Matrix score. | sequence | function |
| --- | --- | --- | --- | --- | --- | --- |
| CAT-box | Arabidopsis thaliana | 2632 | - | 6 | GCCACT | cis-acting regulatory element related to meristem expression |
| CAT-box | Arabidopsis thaliana | 1585 | - | 6 | GCCACT | cis-acting regulatory element related to meristem expression |
| CAT-box | Arabidopsis thaliana | 1535 | - | 6 | GCCACT | cis-acting regulatory element related to meristem expression |

>HU11G01529.1   
+ +Up\_Stream \_Len000GAAAGA AGATTTAAGG AAATCGATGG TACACGGCAA AGCTAGAAAC AGGAACTGTA   
  
  
+ TTTTTATGTT TTTTTTTTGT TGGAAGGGGG GGGGGGGGAG CGGGCGAGAT GTAACCCGTA TCTAGATGGA   
  
  
+ TAATCTACGT GACAAGACCT TCATTTGATC AAGGTACATC ATGAATATTT AACCCTTAAC TTCTTTGAGC   
  
  
+ CTTACAAGAG ATTGTCTGAG GTTTTTAGGG TGCACGATAT CATGCCTGAC TAGACCTGTG TAGACACAAT   
  
  
+ CTTCAGCCCT TATTGATAGC TGTTCGATAT GCATGCTGCC TAAAAATTGG AATAGTTGGC ACCTAACATT   
  
  
+ CTTTTACATC TTGCTCCTCC TTGGTGTAGA GTTAGATGTG CTAGCACAAT ACAATGGGTC CCGATATTTT   
  
  
+ GCATCTGGTT TCTGAAAGAT AATATTTGAT GATCCTGTCT TGTGCTGATG GGCAAAAGCT AAATTGTAAG   
  
  
+ AATATGTAGT TTATTTACAA GACATATGCT TAAGCTAGAG GTCATGTACT TGCATATTTT TGTGCAGCTT   
  
  
+ TTGAGGTTGT ATGCCCCTTT GAGACAAATT CATAGTTGAA TTCATGACAA GATTATGTTT GGCAATGACT   
  
  
+ TTGATTATTA AATTGGCCTT TATGCTATCC TATCCTGTGT GCTCAAATGT TATTTATCAG ATAATATTAG   
  
  
+ TTTACTCTTT CTTCCCTGTC TTGCAAAGAT TTTAAATCCT AAATTATGCA AATGTTTATC TTTAAAATGA   
  
  
+ CCTGCTACTC TCTGTGCCTG ATTTTATTTG TTTCCCCGTT GAAATTTTTT TTATGATTCC ACTTTTCAAG   
  
  
+ CAGCTTAGAC AGGCGTTATT AGGTAATTAT TTTCCGCTTT GGATTGTAGT AAATTGAAAC AGAAATGCCT   
  
  
+ CACTAAACCG CTCTCTGCTC TTTGTGTTGA AGAACCAATC CCATTCAAAC TCTTCTTTCT TTTTTGAAAT   
  
  
+ TCAATCCCGC TTCAGTCAAA TGTCTAACAG AAACAGCCTC GTGTGCAATT CTACTATATA CGGTTAATGC   
  
  
+ TGTGCATGTA TTACTTCCCA ACATCTTACC CAGGACTAGG AGGGACCTTG GTAGCCTGTT CTGCTTGAAA   
  
  
+ AATTTGTCAT TATCACCTGA ATTTGTATTA AATACATAAA TCTTTGTCTT TCAATTTGTG GCTCCACATT   
  
  
+ TCAATTTGTG GCTCCACACG CTTTTGAAAA CTTTATGAGA CCTAATATAT TAGAGAACAT CTGATCGTGA   
  
  
+ TTGTTAATTC CAGTTTTATA ATCATACTAA TTAGTAAATC ACTGAAGGTC ATATTGCATA TTGTTTTTGC   
  
  
+ CTAATGATTG GCTTATTCAA CTATTCCTGA ATAGTTTAGT TTCCAGAATC TTTAGAAGAA ACCTGGTCTG   
  
  
+ GGTATTATCG AGTATTTTTC TCAGTTCATG AAAGGCTTTG ATTCAGTGTG TGTGCTGTCA TGTCTAGGCA   
  
  
+ TGATTGAGTA TTTTTATTTG CATATCCAGG CATTGTGCCT AGACAAATGT CCTGAATCTT AGTGGCTGTT   
  
  
+ AGGTCTGAAC GGACACTTTT CTTGCTGTTT TTCCTTTCCG AGTGGCATCT TTAGCTTTTT CAAACTACCA   
  
  
+ AAGGACTATT AAACGAGGCC CTGCATATGC TGGAAAACTG CCTCTTAAGC TATGTTTATG CAAAAATGGA   
  
  
+ GGACCTTGAA CTGTTCAATG TACCTACAGT CTGAGGCACA GAGCCAATAG ACATAATAGA TAGTTGGATA   
  
  
+ TTCTTTTTAT ATTTAATTCA CAGTAGCGAC AACCCCAGCA TGAAACGTAG CCACGTAGGG GGGGAAAAAG   
  
  
+ GGTCTCCATC TGTTTATATA TTCTTGTCGC TTATGGTGCT TCTGTTATGT AATGACAAAG GAATTTGTGT   
  
  
+ TGGGATTATT CCGCTTCACT CCGTCTCACT AACTTTACGG TTTCAGTCCT AATTCTCTCT TTTTTTCTTT   
  
  
+ TGTTTTTTTT CCTGAGCTTG TTTAGTCTGT TTTCTTCTGT GTGTCTTTCT GATTATGGAA GCCATACAGG   
  
  
+ AGAAGAATGA CGATCTTTTG AGTCTTAGCT TGGCCATTGT TAGTCATTCT AACCATGCAG AAACGAAGGT   
  
  
+ GAAGAGGAAG AGGCATGATG TCTCTAATTC TCTGATCGCA ACAAGAGAAG AGGGTCGGGA TAAGAAAATA   
  
  
+ TTTCAGCTGC TTCAAGAACG AGAGAGAATG CTAAGCATCG GGCAAAGAAG AAAAGGCGTT ATTCAAGAAG   
  
  
+ GGAAGGGTCT TCATCTGATC CATTTGCTTC TCATGTCTGC TACCCTAATC AGCGAAAACA ATATCAGCTC   
  
  
+ AGCTGTTAAT CATTTGCTTG AGTTGTACAA GAATGTTTCT GTAAATGGTG ATTCTGGGCA GCGCGTTGCT   
  
  
+ GCTTATTTTG CTGATGGGTT AACTGCGAGG TTCTTAACCC AGAACTCTCC CTTTTATTGC ATGGTAATGA   
  
  
+ GTAAACCGAC ACCTGCAGAG GAGTTTATCG CCTTCACTCA TTTGTACAGG GTGTCTCCAT TCTATCAGTT   
  
  
+ TGCCCATTTT ACTGCTAATC AGGCCATCTT TGAGGCATTC GAGAGGGAAG AAGAGAACAA CAGCTGGGCA   
  
  
+ CTCCATGTCA TTGATTTTGA CATCTCTCAT GGCTTCCAGT GGCCGTCTCT CATTCAGTCT CTTTCTGAGA   
  
  
+ AGGCTACTGC TTCAAGCCAT TCACTCTCTC TTCGAATCAC AGGTTTTGCT AGAACTCTGG AAGAACTGAT   
  
  
+ TGAGACTGAG AACAGATTAG TAAGTTTTGC TGAAAGCTTC CGGAACATAC TATTTGAATT CCACGGGTGT   
  
  
+ TTGAGGGGTT CAGAGCTGGT GAACTTGAAG AAAAGGGAGA ATGAAACAGT TGCCGTAAAT CTGGTGTTTC   
  
  
+ ACCTCAGCAG TTTCGATGAC ACCCTGAAGG TCTCAGACAC CTTAACCGCT GTACATTCAC TGAAACCTTC   
  
  
+ TATAATGGTA CTAGTTGAAA GAGAAGGAAC CCGAAATGGA TCAGGATTCA TATCAGGTTT CATGGATTCT   
  
  
+ TTGCATTATT ATGCTGCAAT GTTCGATTCA TTAAATGATT GCCTCCCACG GGAGAGTCCC GAGAGACTGA   
  
  
+ GCATAGAGAA GAACCATCTT GGGAGAGAGA TCAAGGAGGC GGTGACTTGG GAGAAGGACG AGATGAGCTA   
  
  
+ CGGGAAGTTC GAGATAATGG AGACTTGGAA AAAGAGGATG GAAATTCATG GGTTTTCGGG GATCAGGCTG   
  
  
+ AGTTCCAAGG CGACAATTCA AGCCAAGCTC CTGCTCAAAA TGGGCAGCCA TTACTGTCCC CAGTTTGCAG   
  
  
+ GGGATTCTGA AGGAGGAGTT GGCGGGTTCA GAGTGTATGA GAGAGATGAG GGGGAAGCCA TTTCTCTGGT   
  
  
+ TTGGCAAGGC AGGTTCCTCG CAACAGCCTC TGCATGGCGC TGTGTATG  

- +Up\_Stream \_Len000CTTTCT TCTAAATTCC TTTAGCTACC ATGTGCCGTT TCGATCTTTG TCCTTGACAT   
  
  
- AAAAATACAA AAAAAAAACA ACCTTCCCCC CCCCCCCCTC GCCCGCTCTA CATTGGGCAT AGATCTACCT   
  
  
- ATTAGATGCA CTGTTCTGGA AGTAAACTAG TTCCATGTAG TACTTATAAA TTGGGAATTG AAGAAACTCG   
  
  
- GAATGTTCTC TAACAGACTC CAAAAATCCC ACGTGCTATA GTACGGACTG ATCTGGACAC ATCTGTGTTA   
  
  
- GAAGTCGGGA ATAACTATCG ACAAGCTATA CGTACGACGG ATTTTTAACC TTATCAACCG TGGATTGTAA   
  
  
- GAAAATGTAG AACGAGGAGG AACCACATCT CAATCTACAC GATCGTGTTA TGTTACCCAG GGCTATAAAA   
  
  
- CGTAGACCAA AGACTTTCTA TTATAAACTA CTAGGACAGA ACACGACTAC CCGTTTTCGA TTTAACATTC   
  
  
- TTATACATCA AATAAATGTT CTGTATACGA ATTCGATCTC CAGTACATGA ACGTATAAAA ACACGTCGAA   
  
  
- AACTCCAACA TACGGGGAAA CTCTGTTTAA GTATCAACTT AAGTACTGTT CTAATACAAA CCGTTACTGA   
  
  
- AACTAATAAT TTAACCGGAA ATACGATAGG ATAGGACACA CGAGTTTACA ATAAATAGTC TATTATAATC   
  
  
- AAATGAGAAA GAAGGGACAG AACGTTTCTA AAATTTAGGA TTTAATACGT TTACAAATAG AAATTTTACT   
  
  
- GGACGATGAG AGACACGGAC TAAAATAAAC AAAGGGGCAA CTTTAAAAAA AATACTAAGG TGAAAAGTTC   
  
  
- GTCGAATCTG TCCGCAATAA TCCATTAATA AAAGGCGAAA CCTAACATCA TTTAACTTTG TCTTTACGGA   
  
  
- GTGATTTGGC GAGAGACGAG AAACACAACT TCTTGGTTAG GGTAAGTTTG AGAAGAAAGA AAAAACTTTA   
  
  
- AGTTAGGGCG AAGTCAGTTT ACAGATTGTC TTTGTCGGAG CACACGTTAA GATGATATAT GCCAATTACG   
  
  
- ACACGTACAT AATGAAGGGT TGTAGAATGG GTCCTGATCC TCCCTGGAAC CATCGGACAA GACGAACTTT   
  
  
- TTAAACAGTA ATAGTGGACT TAAACATAAT TTATGTATTT AGAAACAGAA AGTTAAACAC CGAGGTGTAA   
  
  
- AGTTAAACAC CGAGGTGTGC GAAAACTTTT GAAATACTCT GGATTATATA ATCTCTTGTA GACTAGCACT   
  
  
- AACAATTAAG GTCAAAATAT TAGTATGATT AATCATTTAG TGACTTCCAG TATAACGTAT AACAAAAACG   
  
  
- GATTACTAAC CGAATAAGTT GATAAGGACT TATCAAATCA AAGGTCTTAG AAATCTTCTT TGGACCAGAC   
  
  
- CCATAATAGC TCATAAAAAG AGTCAAGTAC TTTCCGAAAC TAAGTCACAC ACACGACAGT ACAGATCCGT   
  
  
- ACTAACTCAT AAAAATAAAC GTATAGGTCC GTAACACGGA TCTGTTTACA GGACTTAGAA TCACCGACAA   
  
  
- TCCAGACTTG CCTGTGAAAA GAACGACAAA AAGGAAAGGC TCACCGTAGA AATCGAAAAA GTTTGATGGT   
  
  
- TTCCTGATAA TTTGCTCCGG GACGTATACG ACCTTTTGAC GGAGAATTCG ATACAAATAC GTTTTTACCT   
  
  
- CCTGGAACTT GACAAGTTAC ATGGATGTCA GACTCCGTGT CTCGGTTATC TGTATTATCT ATCAACCTAT   
  
  
- AAGAAAAATA TAAATTAAGT GTCATCGCTG TTGGGGTCGT ACTTTGCATC GGTGCATCCC CCCCTTTTTC   
  
  
- CCAGAGGTAG ACAAATATAT AAGAACAGCG AATACCACGA AGACAATACA TTACTGTTTC CTTAAACACA   
  
  
- ACCCTAATAA GGCGAAGTGA GGCAGAGTGA TTGAAATGCC AAAGTCAGGA TTAAGAGAGA AAAAAAGAAA   
  
  
- ACAAAAAAAA GGACTCGAAC AAATCAGACA AAAGAAGACA CACAGAAAGA CTAATACCTT CGGTATGTCC   
  
  
- TCTTCTTACT GCTAGAAAAC TCAGAATCGA ACCGGTAACA ATCAGTAAGA TTGGTACGTC TTTGCTTCCA   
  
  
- CTTCTCCTTC TCCGTACTAC AGAGATTAAG AGACTAGCGT TGTTCTCTTC TCCCAGCCCT ATTCTTTTAT   
  
  
- AAAGTCGACG AAGTTCTTGC TCTCTCTTAC GATTCGTAGC CCGTTTCTTC TTTTCCGCAA TAAGTTCTTC   
  
  
- CCTTCCCAGA AGTAGACTAG GTAAACGAAG AGTACAGACG ATGGGATTAG TCGCTTTTGT TATAGTCGAG   
  
  
- TCGACAATTA GTAAACGAAC TCAACATGTT CTTACAAAGA CATTTACCAC TAAGACCCGT CGCGCAACGA   
  
  
- CGAATAAAAC GACTACCCAA TTGACGCTCC AAGAATTGGG TCTTGAGAGG GAAAATAACG TACCATTACT   
  
  
- CATTTGGCTG TGGACGTCTC CTCAAATAGC GGAAGTGAGT AAACATGTCC CACAGAGGTA AGATAGTCAA   
  
  
- ACGGGTAAAA TGACGATTAG TCCGGTAGAA ACTCCGTAAG CTCTCCCTTC TTCTCTTGTT GTCGACCCGT   
  
  
- GAGGTACAGT AACTAAAACT GTAGAGAGTA CCGAAGGTCA CCGGCAGAGA GTAAGTCAGA GAAAGACTCT   
  
  
- TCCGATGACG AAGTTCGGTA AGTGAGAGAG AAGCTTAGTG TCCAAAACGA TCTTGAGACC TTCTTGACTA   
  
  
- ACTCTGACTC TTGTCTAATC ATTCAAAACG ACTTTCGAAG GCCTTGTATG ATAAACTTAA GGTGCCCACA   
  
  
- AACTCCCCAA GTCTCGACCA CTTGAACTTC TTTTCCCTCT TACTTTGTCA ACGGCATTTA GACCACAAAG   
  
  
- TGGAGTCGTC AAAGCTACTG TGGGACTTCC AGAGTCTGTG GAATTGGCGA CATGTAAGTG ACTTTGGAAG   
  
  
- ATATTACCAT GATCAACTTT CTCTTCCTTG GGCTTTACCT AGTCCTAAGT ATAGTCCAAA GTACCTAAGA   
  
  
- AACGTAATAA TACGACGTTA CAAGCTAAGT AATTTACTAA CGGAGGGTGC CCTCTCAGGG CTCTCTGACT   
  
  
- CGTATCTCTT CTTGGTAGAA CCCTCTCTCT AGTTCCTCCG CCACTGAACC CTCTTCCTGC TCTACTCGAT   
  
  
- GCCCTTCAAG CTCTATTACC TCTGAACCTT TTTCTCCTAC CTTTAAGTAC CCAAAAGCCC CTAGTCCGAC   
  
  
- TCAAGGTTCC GCTGTTAAGT TCGGTTCGAG GACGAGTTTT ACCCGTCGGT AATGACAGGG GTCAAACGTC   
  
  
- CCCTAAGACT TCCTCCTCAA CCGCCCAAGT CTCACATACT CTCTCTACTC CCCCTTCGGT AAAGAGACCA   
  
  
- AACCGTTCCG TCCAAGGAGC GTTGTCGGAG ACGTACCGCG ACACATAC

+     CCAAT-box

| Site Name | Organism | Position | Strand | Matrix score. | sequence | function |
| --- | --- | --- | --- | --- | --- | --- |
| CCAAT-box | Hordeum vulgare | 810 | - | 6 | CAACGG | MYBHv1 binding site |

>HU11G01529.1   
+ +Up\_Stream \_Len000GAAAGA AGATTTAAGG AAATCGATGG TACACGGCAA AGCTAGAAAC AGGAACTGTA   
  
  
+ TTTTTATGTT TTTTTTTTGT TGGAAGGGGG GGGGGGGGAG CGGGCGAGAT GTAACCCGTA TCTAGATGGA   
  
  
+ TAATCTACGT GACAAGACCT TCATTTGATC AAGGTACATC ATGAATATTT AACCCTTAAC TTCTTTGAGC   
  
  
+ CTTACAAGAG ATTGTCTGAG GTTTTTAGGG TGCACGATAT CATGCCTGAC TAGACCTGTG TAGACACAAT   
  
  
+ CTTCAGCCCT TATTGATAGC TGTTCGATAT GCATGCTGCC TAAAAATTGG AATAGTTGGC ACCTAACATT   
  
  
+ CTTTTACATC TTGCTCCTCC TTGGTGTAGA GTTAGATGTG CTAGCACAAT ACAATGGGTC CCGATATTTT   
  
  
+ GCATCTGGTT TCTGAAAGAT AATATTTGAT GATCCTGTCT TGTGCTGATG GGCAAAAGCT AAATTGTAAG   
  
  
+ AATATGTAGT TTATTTACAA GACATATGCT TAAGCTAGAG GTCATGTACT TGCATATTTT TGTGCAGCTT   
  
  
+ TTGAGGTTGT ATGCCCCTTT GAGACAAATT CATAGTTGAA TTCATGACAA GATTATGTTT GGCAATGACT   
  
  
+ TTGATTATTA AATTGGCCTT TATGCTATCC TATCCTGTGT GCTCAAATGT TATTTATCAG ATAATATTAG   
  
  
+ TTTACTCTTT CTTCCCTGTC TTGCAAAGAT TTTAAATCCT AAATTATGCA AATGTTTATC TTTAAAATGA   
  
  
+ CCTGCTACTC TCTGTGCCTG ATTTTATTTG TTTCCCCGTT GAAATTTTTT TTATGATTCC ACTTTTCAAG   
  
  
+ CAGCTTAGAC AGGCGTTATT AGGTAATTAT TTTCCGCTTT GGATTGTAGT AAATTGAAAC AGAAATGCCT   
  
  
+ CACTAAACCG CTCTCTGCTC TTTGTGTTGA AGAACCAATC CCATTCAAAC TCTTCTTTCT TTTTTGAAAT   
  
  
+ TCAATCCCGC TTCAGTCAAA TGTCTAACAG AAACAGCCTC GTGTGCAATT CTACTATATA CGGTTAATGC   
  
  
+ TGTGCATGTA TTACTTCCCA ACATCTTACC CAGGACTAGG AGGGACCTTG GTAGCCTGTT CTGCTTGAAA   
  
  
+ AATTTGTCAT TATCACCTGA ATTTGTATTA AATACATAAA TCTTTGTCTT TCAATTTGTG GCTCCACATT   
  
  
+ TCAATTTGTG GCTCCACACG CTTTTGAAAA CTTTATGAGA CCTAATATAT TAGAGAACAT CTGATCGTGA   
  
  
+ TTGTTAATTC CAGTTTTATA ATCATACTAA TTAGTAAATC ACTGAAGGTC ATATTGCATA TTGTTTTTGC   
  
  
+ CTAATGATTG GCTTATTCAA CTATTCCTGA ATAGTTTAGT TTCCAGAATC TTTAGAAGAA ACCTGGTCTG   
  
  
+ GGTATTATCG AGTATTTTTC TCAGTTCATG AAAGGCTTTG ATTCAGTGTG TGTGCTGTCA TGTCTAGGCA   
  
  
+ TGATTGAGTA TTTTTATTTG CATATCCAGG CATTGTGCCT AGACAAATGT CCTGAATCTT AGTGGCTGTT   
  
  
+ AGGTCTGAAC GGACACTTTT CTTGCTGTTT TTCCTTTCCG AGTGGCATCT TTAGCTTTTT CAAACTACCA   
  
  
+ AAGGACTATT AAACGAGGCC CTGCATATGC TGGAAAACTG CCTCTTAAGC TATGTTTATG CAAAAATGGA   
  
  
+ GGACCTTGAA CTGTTCAATG TACCTACAGT CTGAGGCACA GAGCCAATAG ACATAATAGA TAGTTGGATA   
  
  
+ TTCTTTTTAT ATTTAATTCA CAGTAGCGAC AACCCCAGCA TGAAACGTAG CCACGTAGGG GGGGAAAAAG   
  
  
+ GGTCTCCATC TGTTTATATA TTCTTGTCGC TTATGGTGCT TCTGTTATGT AATGACAAAG GAATTTGTGT   
  
  
+ TGGGATTATT CCGCTTCACT CCGTCTCACT AACTTTACGG TTTCAGTCCT AATTCTCTCT TTTTTTCTTT   
  
  
+ TGTTTTTTTT CCTGAGCTTG TTTAGTCTGT TTTCTTCTGT GTGTCTTTCT GATTATGGAA GCCATACAGG   
  
  
+ AGAAGAATGA CGATCTTTTG AGTCTTAGCT TGGCCATTGT TAGTCATTCT AACCATGCAG AAACGAAGGT   
  
  
+ GAAGAGGAAG AGGCATGATG TCTCTAATTC TCTGATCGCA ACAAGAGAAG AGGGTCGGGA TAAGAAAATA   
  
  
+ TTTCAGCTGC TTCAAGAACG AGAGAGAATG CTAAGCATCG GGCAAAGAAG AAAAGGCGTT ATTCAAGAAG   
  
  
+ GGAAGGGTCT TCATCTGATC CATTTGCTTC TCATGTCTGC TACCCTAATC AGCGAAAACA ATATCAGCTC   
  
  
+ AGCTGTTAAT CATTTGCTTG AGTTGTACAA GAATGTTTCT GTAAATGGTG ATTCTGGGCA GCGCGTTGCT   
  
  
+ GCTTATTTTG CTGATGGGTT AACTGCGAGG TTCTTAACCC AGAACTCTCC CTTTTATTGC ATGGTAATGA   
  
  
+ GTAAACCGAC ACCTGCAGAG GAGTTTATCG CCTTCACTCA TTTGTACAGG GTGTCTCCAT TCTATCAGTT   
  
  
+ TGCCCATTTT ACTGCTAATC AGGCCATCTT TGAGGCATTC GAGAGGGAAG AAGAGAACAA CAGCTGGGCA   
  
  
+ CTCCATGTCA TTGATTTTGA CATCTCTCAT GGCTTCCAGT GGCCGTCTCT CATTCAGTCT CTTTCTGAGA   
  
  
+ AGGCTACTGC TTCAAGCCAT TCACTCTCTC TTCGAATCAC AGGTTTTGCT AGAACTCTGG AAGAACTGAT   
  
  
+ TGAGACTGAG AACAGATTAG TAAGTTTTGC TGAAAGCTTC CGGAACATAC TATTTGAATT CCACGGGTGT   
  
  
+ TTGAGGGGTT CAGAGCTGGT GAACTTGAAG AAAAGGGAGA ATGAAACAGT TGCCGTAAAT CTGGTGTTTC   
  
  
+ ACCTCAGCAG TTTCGATGAC ACCCTGAAGG TCTCAGACAC CTTAACCGCT GTACATTCAC TGAAACCTTC   
  
  
+ TATAATGGTA CTAGTTGAAA GAGAAGGAAC CCGAAATGGA TCAGGATTCA TATCAGGTTT CATGGATTCT   
  
  
+ TTGCATTATT ATGCTGCAAT GTTCGATTCA TTAAATGATT GCCTCCCACG GGAGAGTCCC GAGAGACTGA   
  
  
+ GCATAGAGAA GAACCATCTT GGGAGAGAGA TCAAGGAGGC GGTGACTTGG GAGAAGGACG AGATGAGCTA   
  
  
+ CGGGAAGTTC GAGATAATGG AGACTTGGAA AAAGAGGATG GAAATTCATG GGTTTTCGGG GATCAGGCTG   
  
  
+ AGTTCCAAGG CGACAATTCA AGCCAAGCTC CTGCTCAAAA TGGGCAGCCA TTACTGTCCC CAGTTTGCAG   
  
  
+ GGGATTCTGA AGGAGGAGTT GGCGGGTTCA GAGTGTATGA GAGAGATGAG GGGGAAGCCA TTTCTCTGGT   
  
  
+ TTGGCAAGGC AGGTTCCTCG CAACAGCCTC TGCATGGCGC TGTGTATG  

- +Up\_Stream \_Len000CTTTCT TCTAAATTCC TTTAGCTACC ATGTGCCGTT TCGATCTTTG TCCTTGACAT   
  
  
- AAAAATACAA AAAAAAAACA ACCTTCCCCC CCCCCCCCTC GCCCGCTCTA CATTGGGCAT AGATCTACCT   
  
  
- ATTAGATGCA CTGTTCTGGA AGTAAACTAG TTCCATGTAG TACTTATAAA TTGGGAATTG AAGAAACTCG   
  
  
- GAATGTTCTC TAACAGACTC CAAAAATCCC ACGTGCTATA GTACGGACTG ATCTGGACAC ATCTGTGTTA   
  
  
- GAAGTCGGGA ATAACTATCG ACAAGCTATA CGTACGACGG ATTTTTAACC TTATCAACCG TGGATTGTAA   
  
  
- GAAAATGTAG AACGAGGAGG AACCACATCT CAATCTACAC GATCGTGTTA TGTTACCCAG GGCTATAAAA   
  
  
- CGTAGACCAA AGACTTTCTA TTATAAACTA CTAGGACAGA ACACGACTAC CCGTTTTCGA TTTAACATTC   
  
  
- TTATACATCA AATAAATGTT CTGTATACGA ATTCGATCTC CAGTACATGA ACGTATAAAA ACACGTCGAA   
  
  
- AACTCCAACA TACGGGGAAA CTCTGTTTAA GTATCAACTT AAGTACTGTT CTAATACAAA CCGTTACTGA   
  
  
- AACTAATAAT TTAACCGGAA ATACGATAGG ATAGGACACA CGAGTTTACA ATAAATAGTC TATTATAATC   
  
  
- AAATGAGAAA GAAGGGACAG AACGTTTCTA AAATTTAGGA TTTAATACGT TTACAAATAG AAATTTTACT   
  
  
- GGACGATGAG AGACACGGAC TAAAATAAAC AAAGGGGCAA CTTTAAAAAA AATACTAAGG TGAAAAGTTC   
  
  
- GTCGAATCTG TCCGCAATAA TCCATTAATA AAAGGCGAAA CCTAACATCA TTTAACTTTG TCTTTACGGA   
  
  
- GTGATTTGGC GAGAGACGAG AAACACAACT TCTTGGTTAG GGTAAGTTTG AGAAGAAAGA AAAAACTTTA   
  
  
- AGTTAGGGCG AAGTCAGTTT ACAGATTGTC TTTGTCGGAG CACACGTTAA GATGATATAT GCCAATTACG   
  
  
- ACACGTACAT AATGAAGGGT TGTAGAATGG GTCCTGATCC TCCCTGGAAC CATCGGACAA GACGAACTTT   
  
  
- TTAAACAGTA ATAGTGGACT TAAACATAAT TTATGTATTT AGAAACAGAA AGTTAAACAC CGAGGTGTAA   
  
  
- AGTTAAACAC CGAGGTGTGC GAAAACTTTT GAAATACTCT GGATTATATA ATCTCTTGTA GACTAGCACT   
  
  
- AACAATTAAG GTCAAAATAT TAGTATGATT AATCATTTAG TGACTTCCAG TATAACGTAT AACAAAAACG   
  
  
- GATTACTAAC CGAATAAGTT GATAAGGACT TATCAAATCA AAGGTCTTAG AAATCTTCTT TGGACCAGAC   
  
  
- CCATAATAGC TCATAAAAAG AGTCAAGTAC TTTCCGAAAC TAAGTCACAC ACACGACAGT ACAGATCCGT   
  
  
- ACTAACTCAT AAAAATAAAC GTATAGGTCC GTAACACGGA TCTGTTTACA GGACTTAGAA TCACCGACAA   
  
  
- TCCAGACTTG CCTGTGAAAA GAACGACAAA AAGGAAAGGC TCACCGTAGA AATCGAAAAA GTTTGATGGT   
  
  
- TTCCTGATAA TTTGCTCCGG GACGTATACG ACCTTTTGAC GGAGAATTCG ATACAAATAC GTTTTTACCT   
  
  
- CCTGGAACTT GACAAGTTAC ATGGATGTCA GACTCCGTGT CTCGGTTATC TGTATTATCT ATCAACCTAT   
  
  
- AAGAAAAATA TAAATTAAGT GTCATCGCTG TTGGGGTCGT ACTTTGCATC GGTGCATCCC CCCCTTTTTC   
  
  
- CCAGAGGTAG ACAAATATAT AAGAACAGCG AATACCACGA AGACAATACA TTACTGTTTC CTTAAACACA   
  
  
- ACCCTAATAA GGCGAAGTGA GGCAGAGTGA TTGAAATGCC AAAGTCAGGA TTAAGAGAGA AAAAAAGAAA   
  
  
- ACAAAAAAAA GGACTCGAAC AAATCAGACA AAAGAAGACA CACAGAAAGA CTAATACCTT CGGTATGTCC   
  
  
- TCTTCTTACT GCTAGAAAAC TCAGAATCGA ACCGGTAACA ATCAGTAAGA TTGGTACGTC TTTGCTTCCA   
  
  
- CTTCTCCTTC TCCGTACTAC AGAGATTAAG AGACTAGCGT TGTTCTCTTC TCCCAGCCCT ATTCTTTTAT   
  
  
- AAAGTCGACG AAGTTCTTGC TCTCTCTTAC GATTCGTAGC CCGTTTCTTC TTTTCCGCAA TAAGTTCTTC   
  
  
- CCTTCCCAGA AGTAGACTAG GTAAACGAAG AGTACAGACG ATGGGATTAG TCGCTTTTGT TATAGTCGAG   
  
  
- TCGACAATTA GTAAACGAAC TCAACATGTT CTTACAAAGA CATTTACCAC TAAGACCCGT CGCGCAACGA   
  
  
- CGAATAAAAC GACTACCCAA TTGACGCTCC AAGAATTGGG TCTTGAGAGG GAAAATAACG TACCATTACT   
  
  
- CATTTGGCTG TGGACGTCTC CTCAAATAGC GGAAGTGAGT AAACATGTCC CACAGAGGTA AGATAGTCAA   
  
  
- ACGGGTAAAA TGACGATTAG TCCGGTAGAA ACTCCGTAAG CTCTCCCTTC TTCTCTTGTT GTCGACCCGT   
  
  
- GAGGTACAGT AACTAAAACT GTAGAGAGTA CCGAAGGTCA CCGGCAGAGA GTAAGTCAGA GAAAGACTCT   
  
  
- TCCGATGACG AAGTTCGGTA AGTGAGAGAG AAGCTTAGTG TCCAAAACGA TCTTGAGACC TTCTTGACTA   
  
  
- ACTCTGACTC TTGTCTAATC ATTCAAAACG ACTTTCGAAG GCCTTGTATG ATAAACTTAA GGTGCCCACA   
  
  
- AACTCCCCAA GTCTCGACCA CTTGAACTTC TTTTCCCTCT TACTTTGTCA ACGGCATTTA GACCACAAAG   
  
  
- TGGAGTCGTC AAAGCTACTG TGGGACTTCC AGAGTCTGTG GAATTGGCGA CATGTAAGTG ACTTTGGAAG   
  
  
- ATATTACCAT GATCAACTTT CTCTTCCTTG GGCTTTACCT AGTCCTAAGT ATAGTCCAAA GTACCTAAGA   
  
  
- AACGTAATAA TACGACGTTA CAAGCTAAGT AATTTACTAA CGGAGGGTGC CCTCTCAGGG CTCTCTGACT   
  
  
- CGTATCTCTT CTTGGTAGAA CCCTCTCTCT AGTTCCTCCG CCACTGAACC CTCTTCCTGC TCTACTCGAT   
  
  
- GCCCTTCAAG CTCTATTACC TCTGAACCTT TTTCTCCTAC CTTTAAGTAC CCAAAAGCCC CTAGTCCGAC   
  
  
- TCAAGGTTCC GCTGTTAAGT TCGGTTCGAG GACGAGTTTT ACCCGTCGGT AATGACAGGG GTCAAACGTC   
  
  
- CCCTAAGACT TCCTCCTCAA CCGCCCAAGT CTCACATACT CTCTCTACTC CCCCTTCGGT AAAGAGACCA   
  
  
- AACCGTTCCG TCCAAGGAGC GTTGTCGGAG ACGTACCGCG ACACATAC

+     CGTCA-motif

| Site Name | Organism | Position | Strand | Matrix score. | sequence | function |
| --- | --- | --- | --- | --- | --- | --- |
| CGTCA-motif | Hordeum vulgare | 2042 | - | 5 | CGTCA | cis-acting regulatory element involved in the MeJA-responsiveness |

>HU11G01529.1   
+ +Up\_Stream \_Len000GAAAGA AGATTTAAGG AAATCGATGG TACACGGCAA AGCTAGAAAC AGGAACTGTA   
  
  
+ TTTTTATGTT TTTTTTTTGT TGGAAGGGGG GGGGGGGGAG CGGGCGAGAT GTAACCCGTA TCTAGATGGA   
  
  
+ TAATCTACGT GACAAGACCT TCATTTGATC AAGGTACATC ATGAATATTT AACCCTTAAC TTCTTTGAGC   
  
  
+ CTTACAAGAG ATTGTCTGAG GTTTTTAGGG TGCACGATAT CATGCCTGAC TAGACCTGTG TAGACACAAT   
  
  
+ CTTCAGCCCT TATTGATAGC TGTTCGATAT GCATGCTGCC TAAAAATTGG AATAGTTGGC ACCTAACATT   
  
  
+ CTTTTACATC TTGCTCCTCC TTGGTGTAGA GTTAGATGTG CTAGCACAAT ACAATGGGTC CCGATATTTT   
  
  
+ GCATCTGGTT TCTGAAAGAT AATATTTGAT GATCCTGTCT TGTGCTGATG GGCAAAAGCT AAATTGTAAG   
  
  
+ AATATGTAGT TTATTTACAA GACATATGCT TAAGCTAGAG GTCATGTACT TGCATATTTT TGTGCAGCTT   
  
  
+ TTGAGGTTGT ATGCCCCTTT GAGACAAATT CATAGTTGAA TTCATGACAA GATTATGTTT GGCAATGACT   
  
  
+ TTGATTATTA AATTGGCCTT TATGCTATCC TATCCTGTGT GCTCAAATGT TATTTATCAG ATAATATTAG   
  
  
+ TTTACTCTTT CTTCCCTGTC TTGCAAAGAT TTTAAATCCT AAATTATGCA AATGTTTATC TTTAAAATGA   
  
  
+ CCTGCTACTC TCTGTGCCTG ATTTTATTTG TTTCCCCGTT GAAATTTTTT TTATGATTCC ACTTTTCAAG   
  
  
+ CAGCTTAGAC AGGCGTTATT AGGTAATTAT TTTCCGCTTT GGATTGTAGT AAATTGAAAC AGAAATGCCT   
  
  
+ CACTAAACCG CTCTCTGCTC TTTGTGTTGA AGAACCAATC CCATTCAAAC TCTTCTTTCT TTTTTGAAAT   
  
  
+ TCAATCCCGC TTCAGTCAAA TGTCTAACAG AAACAGCCTC GTGTGCAATT CTACTATATA CGGTTAATGC   
  
  
+ TGTGCATGTA TTACTTCCCA ACATCTTACC CAGGACTAGG AGGGACCTTG GTAGCCTGTT CTGCTTGAAA   
  
  
+ AATTTGTCAT TATCACCTGA ATTTGTATTA AATACATAAA TCTTTGTCTT TCAATTTGTG GCTCCACATT   
  
  
+ TCAATTTGTG GCTCCACACG CTTTTGAAAA CTTTATGAGA CCTAATATAT TAGAGAACAT CTGATCGTGA   
  
  
+ TTGTTAATTC CAGTTTTATA ATCATACTAA TTAGTAAATC ACTGAAGGTC ATATTGCATA TTGTTTTTGC   
  
  
+ CTAATGATTG GCTTATTCAA CTATTCCTGA ATAGTTTAGT TTCCAGAATC TTTAGAAGAA ACCTGGTCTG   
  
  
+ GGTATTATCG AGTATTTTTC TCAGTTCATG AAAGGCTTTG ATTCAGTGTG TGTGCTGTCA TGTCTAGGCA   
  
  
+ TGATTGAGTA TTTTTATTTG CATATCCAGG CATTGTGCCT AGACAAATGT CCTGAATCTT AGTGGCTGTT   
  
  
+ AGGTCTGAAC GGACACTTTT CTTGCTGTTT TTCCTTTCCG AGTGGCATCT TTAGCTTTTT CAAACTACCA   
  
  
+ AAGGACTATT AAACGAGGCC CTGCATATGC TGGAAAACTG CCTCTTAAGC TATGTTTATG CAAAAATGGA   
  
  
+ GGACCTTGAA CTGTTCAATG TACCTACAGT CTGAGGCACA GAGCCAATAG ACATAATAGA TAGTTGGATA   
  
  
+ TTCTTTTTAT ATTTAATTCA CAGTAGCGAC AACCCCAGCA TGAAACGTAG CCACGTAGGG GGGGAAAAAG   
  
  
+ GGTCTCCATC TGTTTATATA TTCTTGTCGC TTATGGTGCT TCTGTTATGT AATGACAAAG GAATTTGTGT   
  
  
+ TGGGATTATT CCGCTTCACT CCGTCTCACT AACTTTACGG TTTCAGTCCT AATTCTCTCT TTTTTTCTTT   
  
  
+ TGTTTTTTTT CCTGAGCTTG TTTAGTCTGT TTTCTTCTGT GTGTCTTTCT GATTATGGAA GCCATACAGG   
  
  
+ AGAAGAATGA CGATCTTTTG AGTCTTAGCT TGGCCATTGT TAGTCATTCT AACCATGCAG AAACGAAGGT   
  
  
+ GAAGAGGAAG AGGCATGATG TCTCTAATTC TCTGATCGCA ACAAGAGAAG AGGGTCGGGA TAAGAAAATA   
  
  
+ TTTCAGCTGC TTCAAGAACG AGAGAGAATG CTAAGCATCG GGCAAAGAAG AAAAGGCGTT ATTCAAGAAG   
  
  
+ GGAAGGGTCT TCATCTGATC CATTTGCTTC TCATGTCTGC TACCCTAATC AGCGAAAACA ATATCAGCTC   
  
  
+ AGCTGTTAAT CATTTGCTTG AGTTGTACAA GAATGTTTCT GTAAATGGTG ATTCTGGGCA GCGCGTTGCT   
  
  
+ GCTTATTTTG CTGATGGGTT AACTGCGAGG TTCTTAACCC AGAACTCTCC CTTTTATTGC ATGGTAATGA   
  
  
+ GTAAACCGAC ACCTGCAGAG GAGTTTATCG CCTTCACTCA TTTGTACAGG GTGTCTCCAT TCTATCAGTT   
  
  
+ TGCCCATTTT ACTGCTAATC AGGCCATCTT TGAGGCATTC GAGAGGGAAG AAGAGAACAA CAGCTGGGCA   
  
  
+ CTCCATGTCA TTGATTTTGA CATCTCTCAT GGCTTCCAGT GGCCGTCTCT CATTCAGTCT CTTTCTGAGA   
  
  
+ AGGCTACTGC TTCAAGCCAT TCACTCTCTC TTCGAATCAC AGGTTTTGCT AGAACTCTGG AAGAACTGAT   
  
  
+ TGAGACTGAG AACAGATTAG TAAGTTTTGC TGAAAGCTTC CGGAACATAC TATTTGAATT CCACGGGTGT   
  
  
+ TTGAGGGGTT CAGAGCTGGT GAACTTGAAG AAAAGGGAGA ATGAAACAGT TGCCGTAAAT CTGGTGTTTC   
  
  
+ ACCTCAGCAG TTTCGATGAC ACCCTGAAGG TCTCAGACAC CTTAACCGCT GTACATTCAC TGAAACCTTC   
  
  
+ TATAATGGTA CTAGTTGAAA GAGAAGGAAC CCGAAATGGA TCAGGATTCA TATCAGGTTT CATGGATTCT   
  
  
+ TTGCATTATT ATGCTGCAAT GTTCGATTCA TTAAATGATT GCCTCCCACG GGAGAGTCCC GAGAGACTGA   
  
  
+ GCATAGAGAA GAACCATCTT GGGAGAGAGA TCAAGGAGGC GGTGACTTGG GAGAAGGACG AGATGAGCTA   
  
  
+ CGGGAAGTTC GAGATAATGG AGACTTGGAA AAAGAGGATG GAAATTCATG GGTTTTCGGG GATCAGGCTG   
  
  
+ AGTTCCAAGG CGACAATTCA AGCCAAGCTC CTGCTCAAAA TGGGCAGCCA TTACTGTCCC CAGTTTGCAG   
  
  
+ GGGATTCTGA AGGAGGAGTT GGCGGGTTCA GAGTGTATGA GAGAGATGAG GGGGAAGCCA TTTCTCTGGT   
  
  
+ TTGGCAAGGC AGGTTCCTCG CAACAGCCTC TGCATGGCGC TGTGTATG  

- +Up\_Stream \_Len000CTTTCT TCTAAATTCC TTTAGCTACC ATGTGCCGTT TCGATCTTTG TCCTTGACAT   
  
  
- AAAAATACAA AAAAAAAACA ACCTTCCCCC CCCCCCCCTC GCCCGCTCTA CATTGGGCAT AGATCTACCT   
  
  
- ATTAGATGCA CTGTTCTGGA AGTAAACTAG TTCCATGTAG TACTTATAAA TTGGGAATTG AAGAAACTCG   
  
  
- GAATGTTCTC TAACAGACTC CAAAAATCCC ACGTGCTATA GTACGGACTG ATCTGGACAC ATCTGTGTTA   
  
  
- GAAGTCGGGA ATAACTATCG ACAAGCTATA CGTACGACGG ATTTTTAACC TTATCAACCG TGGATTGTAA   
  
  
- GAAAATGTAG AACGAGGAGG AACCACATCT CAATCTACAC GATCGTGTTA TGTTACCCAG GGCTATAAAA   
  
  
- CGTAGACCAA AGACTTTCTA TTATAAACTA CTAGGACAGA ACACGACTAC CCGTTTTCGA TTTAACATTC   
  
  
- TTATACATCA AATAAATGTT CTGTATACGA ATTCGATCTC CAGTACATGA ACGTATAAAA ACACGTCGAA   
  
  
- AACTCCAACA TACGGGGAAA CTCTGTTTAA GTATCAACTT AAGTACTGTT CTAATACAAA CCGTTACTGA   
  
  
- AACTAATAAT TTAACCGGAA ATACGATAGG ATAGGACACA CGAGTTTACA ATAAATAGTC TATTATAATC   
  
  
- AAATGAGAAA GAAGGGACAG AACGTTTCTA AAATTTAGGA TTTAATACGT TTACAAATAG AAATTTTACT   
  
  
- GGACGATGAG AGACACGGAC TAAAATAAAC AAAGGGGCAA CTTTAAAAAA AATACTAAGG TGAAAAGTTC   
  
  
- GTCGAATCTG TCCGCAATAA TCCATTAATA AAAGGCGAAA CCTAACATCA TTTAACTTTG TCTTTACGGA   
  
  
- GTGATTTGGC GAGAGACGAG AAACACAACT TCTTGGTTAG GGTAAGTTTG AGAAGAAAGA AAAAACTTTA   
  
  
- AGTTAGGGCG AAGTCAGTTT ACAGATTGTC TTTGTCGGAG CACACGTTAA GATGATATAT GCCAATTACG   
  
  
- ACACGTACAT AATGAAGGGT TGTAGAATGG GTCCTGATCC TCCCTGGAAC CATCGGACAA GACGAACTTT   
  
  
- TTAAACAGTA ATAGTGGACT TAAACATAAT TTATGTATTT AGAAACAGAA AGTTAAACAC CGAGGTGTAA   
  
  
- AGTTAAACAC CGAGGTGTGC GAAAACTTTT GAAATACTCT GGATTATATA ATCTCTTGTA GACTAGCACT   
  
  
- AACAATTAAG GTCAAAATAT TAGTATGATT AATCATTTAG TGACTTCCAG TATAACGTAT AACAAAAACG   
  
  
- GATTACTAAC CGAATAAGTT GATAAGGACT TATCAAATCA AAGGTCTTAG AAATCTTCTT TGGACCAGAC   
  
  
- CCATAATAGC TCATAAAAAG AGTCAAGTAC TTTCCGAAAC TAAGTCACAC ACACGACAGT ACAGATCCGT   
  
  
- ACTAACTCAT AAAAATAAAC GTATAGGTCC GTAACACGGA TCTGTTTACA GGACTTAGAA TCACCGACAA   
  
  
- TCCAGACTTG CCTGTGAAAA GAACGACAAA AAGGAAAGGC TCACCGTAGA AATCGAAAAA GTTTGATGGT   
  
  
- TTCCTGATAA TTTGCTCCGG GACGTATACG ACCTTTTGAC GGAGAATTCG ATACAAATAC GTTTTTACCT   
  
  
- CCTGGAACTT GACAAGTTAC ATGGATGTCA GACTCCGTGT CTCGGTTATC TGTATTATCT ATCAACCTAT   
  
  
- AAGAAAAATA TAAATTAAGT GTCATCGCTG TTGGGGTCGT ACTTTGCATC GGTGCATCCC CCCCTTTTTC   
  
  
- CCAGAGGTAG ACAAATATAT AAGAACAGCG AATACCACGA AGACAATACA TTACTGTTTC CTTAAACACA   
  
  
- ACCCTAATAA GGCGAAGTGA GGCAGAGTGA TTGAAATGCC AAAGTCAGGA TTAAGAGAGA AAAAAAGAAA   
  
  
- ACAAAAAAAA GGACTCGAAC AAATCAGACA AAAGAAGACA CACAGAAAGA CTAATACCTT CGGTATGTCC   
  
  
- TCTTCTTACT GCTAGAAAAC TCAGAATCGA ACCGGTAACA ATCAGTAAGA TTGGTACGTC TTTGCTTCCA   
  
  
- CTTCTCCTTC TCCGTACTAC AGAGATTAAG AGACTAGCGT TGTTCTCTTC TCCCAGCCCT ATTCTTTTAT   
  
  
- AAAGTCGACG AAGTTCTTGC TCTCTCTTAC GATTCGTAGC CCGTTTCTTC TTTTCCGCAA TAAGTTCTTC   
  
  
- CCTTCCCAGA AGTAGACTAG GTAAACGAAG AGTACAGACG ATGGGATTAG TCGCTTTTGT TATAGTCGAG   
  
  
- TCGACAATTA GTAAACGAAC TCAACATGTT CTTACAAAGA CATTTACCAC TAAGACCCGT CGCGCAACGA   
  
  
- CGAATAAAAC GACTACCCAA TTGACGCTCC AAGAATTGGG TCTTGAGAGG GAAAATAACG TACCATTACT   
  
  
- CATTTGGCTG TGGACGTCTC CTCAAATAGC GGAAGTGAGT AAACATGTCC CACAGAGGTA AGATAGTCAA   
  
  
- ACGGGTAAAA TGACGATTAG TCCGGTAGAA ACTCCGTAAG CTCTCCCTTC TTCTCTTGTT GTCGACCCGT   
  
  
- GAGGTACAGT AACTAAAACT GTAGAGAGTA CCGAAGGTCA CCGGCAGAGA GTAAGTCAGA GAAAGACTCT   
  
  
- TCCGATGACG AAGTTCGGTA AGTGAGAGAG AAGCTTAGTG TCCAAAACGA TCTTGAGACC TTCTTGACTA   
  
  
- ACTCTGACTC TTGTCTAATC ATTCAAAACG ACTTTCGAAG GCCTTGTATG ATAAACTTAA GGTGCCCACA   
  
  
- AACTCCCCAA GTCTCGACCA CTTGAACTTC TTTTCCCTCT TACTTTGTCA ACGGCATTTA GACCACAAAG   
  
  
- TGGAGTCGTC AAAGCTACTG TGGGACTTCC AGAGTCTGTG GAATTGGCGA CATGTAAGTG ACTTTGGAAG   
  
  
- ATATTACCAT GATCAACTTT CTCTTCCTTG GGCTTTACCT AGTCCTAAGT ATAGTCCAAA GTACCTAAGA   
  
  
- AACGTAATAA TACGACGTTA CAAGCTAAGT AATTTACTAA CGGAGGGTGC CCTCTCAGGG CTCTCTGACT   
  
  
- CGTATCTCTT CTTGGTAGAA CCCTCTCTCT AGTTCCTCCG CCACTGAACC CTCTTCCTGC TCTACTCGAT   
  
  
- GCCCTTCAAG CTCTATTACC TCTGAACCTT TTTCTCCTAC CTTTAAGTAC CCAAAAGCCC CTAGTCCGAC   
  
  
- TCAAGGTTCC GCTGTTAAGT TCGGTTCGAG GACGAGTTTT ACCCGTCGGT AATGACAGGG GTCAAACGTC   
  
  
- CCCTAAGACT TCCTCCTCAA CCGCCCAAGT CTCACATACT CTCTCTACTC CCCCTTCGGT AAAGAGACCA   
  
  
- AACCGTTCCG TCCAAGGAGC GTTGTCGGAG ACGTACCGCG ACACATAC

+     ERE

| Site Name | Organism | Position | Strand | Matrix score. | sequence | function |
| --- | --- | --- | --- | --- | --- | --- |
| ERE | Nicotiana glutinos | 765 | - | 8 | ATTTTAAA |  |
| ERE | Nicotiana glutinos | 733 | + | 8 | ATTTTAAA |  |

>HU11G01529.1   
+ +Up\_Stream \_Len000GAAAGA AGATTTAAGG AAATCGATGG TACACGGCAA AGCTAGAAAC AGGAACTGTA   
  
  
+ TTTTTATGTT TTTTTTTTGT TGGAAGGGGG GGGGGGGGAG CGGGCGAGAT GTAACCCGTA TCTAGATGGA   
  
  
+ TAATCTACGT GACAAGACCT TCATTTGATC AAGGTACATC ATGAATATTT AACCCTTAAC TTCTTTGAGC   
  
  
+ CTTACAAGAG ATTGTCTGAG GTTTTTAGGG TGCACGATAT CATGCCTGAC TAGACCTGTG TAGACACAAT   
  
  
+ CTTCAGCCCT TATTGATAGC TGTTCGATAT GCATGCTGCC TAAAAATTGG AATAGTTGGC ACCTAACATT   
  
  
+ CTTTTACATC TTGCTCCTCC TTGGTGTAGA GTTAGATGTG CTAGCACAAT ACAATGGGTC CCGATATTTT   
  
  
+ GCATCTGGTT TCTGAAAGAT AATATTTGAT GATCCTGTCT TGTGCTGATG GGCAAAAGCT AAATTGTAAG   
  
  
+ AATATGTAGT TTATTTACAA GACATATGCT TAAGCTAGAG GTCATGTACT TGCATATTTT TGTGCAGCTT   
  
  
+ TTGAGGTTGT ATGCCCCTTT GAGACAAATT CATAGTTGAA TTCATGACAA GATTATGTTT GGCAATGACT   
  
  
+ TTGATTATTA AATTGGCCTT TATGCTATCC TATCCTGTGT GCTCAAATGT TATTTATCAG ATAATATTAG   
  
  
+ TTTACTCTTT CTTCCCTGTC TTGCAAAGAT TTTAAATCCT AAATTATGCA AATGTTTATC TTTAAAATGA   
  
  
+ CCTGCTACTC TCTGTGCCTG ATTTTATTTG TTTCCCCGTT GAAATTTTTT TTATGATTCC ACTTTTCAAG   
  
  
+ CAGCTTAGAC AGGCGTTATT AGGTAATTAT TTTCCGCTTT GGATTGTAGT AAATTGAAAC AGAAATGCCT   
  
  
+ CACTAAACCG CTCTCTGCTC TTTGTGTTGA AGAACCAATC CCATTCAAAC TCTTCTTTCT TTTTTGAAAT   
  
  
+ TCAATCCCGC TTCAGTCAAA TGTCTAACAG AAACAGCCTC GTGTGCAATT CTACTATATA CGGTTAATGC   
  
  
+ TGTGCATGTA TTACTTCCCA ACATCTTACC CAGGACTAGG AGGGACCTTG GTAGCCTGTT CTGCTTGAAA   
  
  
+ AATTTGTCAT TATCACCTGA ATTTGTATTA AATACATAAA TCTTTGTCTT TCAATTTGTG GCTCCACATT   
  
  
+ TCAATTTGTG GCTCCACACG CTTTTGAAAA CTTTATGAGA CCTAATATAT TAGAGAACAT CTGATCGTGA   
  
  
+ TTGTTAATTC CAGTTTTATA ATCATACTAA TTAGTAAATC ACTGAAGGTC ATATTGCATA TTGTTTTTGC   
  
  
+ CTAATGATTG GCTTATTCAA CTATTCCTGA ATAGTTTAGT TTCCAGAATC TTTAGAAGAA ACCTGGTCTG   
  
  
+ GGTATTATCG AGTATTTTTC TCAGTTCATG AAAGGCTTTG ATTCAGTGTG TGTGCTGTCA TGTCTAGGCA   
  
  
+ TGATTGAGTA TTTTTATTTG CATATCCAGG CATTGTGCCT AGACAAATGT CCTGAATCTT AGTGGCTGTT   
  
  
+ AGGTCTGAAC GGACACTTTT CTTGCTGTTT TTCCTTTCCG AGTGGCATCT TTAGCTTTTT CAAACTACCA   
  
  
+ AAGGACTATT AAACGAGGCC CTGCATATGC TGGAAAACTG CCTCTTAAGC TATGTTTATG CAAAAATGGA   
  
  
+ GGACCTTGAA CTGTTCAATG TACCTACAGT CTGAGGCACA GAGCCAATAG ACATAATAGA TAGTTGGATA   
  
  
+ TTCTTTTTAT ATTTAATTCA CAGTAGCGAC AACCCCAGCA TGAAACGTAG CCACGTAGGG GGGGAAAAAG   
  
  
+ GGTCTCCATC TGTTTATATA TTCTTGTCGC TTATGGTGCT TCTGTTATGT AATGACAAAG GAATTTGTGT   
  
  
+ TGGGATTATT CCGCTTCACT CCGTCTCACT AACTTTACGG TTTCAGTCCT AATTCTCTCT TTTTTTCTTT   
  
  
+ TGTTTTTTTT CCTGAGCTTG TTTAGTCTGT TTTCTTCTGT GTGTCTTTCT GATTATGGAA GCCATACAGG   
  
  
+ AGAAGAATGA CGATCTTTTG AGTCTTAGCT TGGCCATTGT TAGTCATTCT AACCATGCAG AAACGAAGGT   
  
  
+ GAAGAGGAAG AGGCATGATG TCTCTAATTC TCTGATCGCA ACAAGAGAAG AGGGTCGGGA TAAGAAAATA   
  
  
+ TTTCAGCTGC TTCAAGAACG AGAGAGAATG CTAAGCATCG GGCAAAGAAG AAAAGGCGTT ATTCAAGAAG   
  
  
+ GGAAGGGTCT TCATCTGATC CATTTGCTTC TCATGTCTGC TACCCTAATC AGCGAAAACA ATATCAGCTC   
  
  
+ AGCTGTTAAT CATTTGCTTG AGTTGTACAA GAATGTTTCT GTAAATGGTG ATTCTGGGCA GCGCGTTGCT   
  
  
+ GCTTATTTTG CTGATGGGTT AACTGCGAGG TTCTTAACCC AGAACTCTCC CTTTTATTGC ATGGTAATGA   
  
  
+ GTAAACCGAC ACCTGCAGAG GAGTTTATCG CCTTCACTCA TTTGTACAGG GTGTCTCCAT TCTATCAGTT   
  
  
+ TGCCCATTTT ACTGCTAATC AGGCCATCTT TGAGGCATTC GAGAGGGAAG AAGAGAACAA CAGCTGGGCA   
  
  
+ CTCCATGTCA TTGATTTTGA CATCTCTCAT GGCTTCCAGT GGCCGTCTCT CATTCAGTCT CTTTCTGAGA   
  
  
+ AGGCTACTGC TTCAAGCCAT TCACTCTCTC TTCGAATCAC AGGTTTTGCT AGAACTCTGG AAGAACTGAT   
  
  
+ TGAGACTGAG AACAGATTAG TAAGTTTTGC TGAAAGCTTC CGGAACATAC TATTTGAATT CCACGGGTGT   
  
  
+ TTGAGGGGTT CAGAGCTGGT GAACTTGAAG AAAAGGGAGA ATGAAACAGT TGCCGTAAAT CTGGTGTTTC   
  
  
+ ACCTCAGCAG TTTCGATGAC ACCCTGAAGG TCTCAGACAC CTTAACCGCT GTACATTCAC TGAAACCTTC   
  
  
+ TATAATGGTA CTAGTTGAAA GAGAAGGAAC CCGAAATGGA TCAGGATTCA TATCAGGTTT CATGGATTCT   
  
  
+ TTGCATTATT ATGCTGCAAT GTTCGATTCA TTAAATGATT GCCTCCCACG GGAGAGTCCC GAGAGACTGA   
  
  
+ GCATAGAGAA GAACCATCTT GGGAGAGAGA TCAAGGAGGC GGTGACTTGG GAGAAGGACG AGATGAGCTA   
  
  
+ CGGGAAGTTC GAGATAATGG AGACTTGGAA AAAGAGGATG GAAATTCATG GGTTTTCGGG GATCAGGCTG   
  
  
+ AGTTCCAAGG CGACAATTCA AGCCAAGCTC CTGCTCAAAA TGGGCAGCCA TTACTGTCCC CAGTTTGCAG   
  
  
+ GGGATTCTGA AGGAGGAGTT GGCGGGTTCA GAGTGTATGA GAGAGATGAG GGGGAAGCCA TTTCTCTGGT   
  
  
+ TTGGCAAGGC AGGTTCCTCG CAACAGCCTC TGCATGGCGC TGTGTATG  

- +Up\_Stream \_Len000CTTTCT TCTAAATTCC TTTAGCTACC ATGTGCCGTT TCGATCTTTG TCCTTGACAT   
  
  
- AAAAATACAA AAAAAAAACA ACCTTCCCCC CCCCCCCCTC GCCCGCTCTA CATTGGGCAT AGATCTACCT   
  
  
- ATTAGATGCA CTGTTCTGGA AGTAAACTAG TTCCATGTAG TACTTATAAA TTGGGAATTG AAGAAACTCG   
  
  
- GAATGTTCTC TAACAGACTC CAAAAATCCC ACGTGCTATA GTACGGACTG ATCTGGACAC ATCTGTGTTA   
  
  
- GAAGTCGGGA ATAACTATCG ACAAGCTATA CGTACGACGG ATTTTTAACC TTATCAACCG TGGATTGTAA   
  
  
- GAAAATGTAG AACGAGGAGG AACCACATCT CAATCTACAC GATCGTGTTA TGTTACCCAG GGCTATAAAA   
  
  
- CGTAGACCAA AGACTTTCTA TTATAAACTA CTAGGACAGA ACACGACTAC CCGTTTTCGA TTTAACATTC   
  
  
- TTATACATCA AATAAATGTT CTGTATACGA ATTCGATCTC CAGTACATGA ACGTATAAAA ACACGTCGAA   
  
  
- AACTCCAACA TACGGGGAAA CTCTGTTTAA GTATCAACTT AAGTACTGTT CTAATACAAA CCGTTACTGA   
  
  
- AACTAATAAT TTAACCGGAA ATACGATAGG ATAGGACACA CGAGTTTACA ATAAATAGTC TATTATAATC   
  
  
- AAATGAGAAA GAAGGGACAG AACGTTTCTA AAATTTAGGA TTTAATACGT TTACAAATAG AAATTTTACT   
  
  
- GGACGATGAG AGACACGGAC TAAAATAAAC AAAGGGGCAA CTTTAAAAAA AATACTAAGG TGAAAAGTTC   
  
  
- GTCGAATCTG TCCGCAATAA TCCATTAATA AAAGGCGAAA CCTAACATCA TTTAACTTTG TCTTTACGGA   
  
  
- GTGATTTGGC GAGAGACGAG AAACACAACT TCTTGGTTAG GGTAAGTTTG AGAAGAAAGA AAAAACTTTA   
  
  
- AGTTAGGGCG AAGTCAGTTT ACAGATTGTC TTTGTCGGAG CACACGTTAA GATGATATAT GCCAATTACG   
  
  
- ACACGTACAT AATGAAGGGT TGTAGAATGG GTCCTGATCC TCCCTGGAAC CATCGGACAA GACGAACTTT   
  
  
- TTAAACAGTA ATAGTGGACT TAAACATAAT TTATGTATTT AGAAACAGAA AGTTAAACAC CGAGGTGTAA   
  
  
- AGTTAAACAC CGAGGTGTGC GAAAACTTTT GAAATACTCT GGATTATATA ATCTCTTGTA GACTAGCACT   
  
  
- AACAATTAAG GTCAAAATAT TAGTATGATT AATCATTTAG TGACTTCCAG TATAACGTAT AACAAAAACG   
  
  
- GATTACTAAC CGAATAAGTT GATAAGGACT TATCAAATCA AAGGTCTTAG AAATCTTCTT TGGACCAGAC   
  
  
- CCATAATAGC TCATAAAAAG AGTCAAGTAC TTTCCGAAAC TAAGTCACAC ACACGACAGT ACAGATCCGT   
  
  
- ACTAACTCAT AAAAATAAAC GTATAGGTCC GTAACACGGA TCTGTTTACA GGACTTAGAA TCACCGACAA   
  
  
- TCCAGACTTG CCTGTGAAAA GAACGACAAA AAGGAAAGGC TCACCGTAGA AATCGAAAAA GTTTGATGGT   
  
  
- TTCCTGATAA TTTGCTCCGG GACGTATACG ACCTTTTGAC GGAGAATTCG ATACAAATAC GTTTTTACCT   
  
  
- CCTGGAACTT GACAAGTTAC ATGGATGTCA GACTCCGTGT CTCGGTTATC TGTATTATCT ATCAACCTAT   
  
  
- AAGAAAAATA TAAATTAAGT GTCATCGCTG TTGGGGTCGT ACTTTGCATC GGTGCATCCC CCCCTTTTTC   
  
  
- CCAGAGGTAG ACAAATATAT AAGAACAGCG AATACCACGA AGACAATACA TTACTGTTTC CTTAAACACA   
  
  
- ACCCTAATAA GGCGAAGTGA GGCAGAGTGA TTGAAATGCC AAAGTCAGGA TTAAGAGAGA AAAAAAGAAA   
  
  
- ACAAAAAAAA GGACTCGAAC AAATCAGACA AAAGAAGACA CACAGAAAGA CTAATACCTT CGGTATGTCC   
  
  
- TCTTCTTACT GCTAGAAAAC TCAGAATCGA ACCGGTAACA ATCAGTAAGA TTGGTACGTC TTTGCTTCCA   
  
  
- CTTCTCCTTC TCCGTACTAC AGAGATTAAG AGACTAGCGT TGTTCTCTTC TCCCAGCCCT ATTCTTTTAT   
  
  
- AAAGTCGACG AAGTTCTTGC TCTCTCTTAC GATTCGTAGC CCGTTTCTTC TTTTCCGCAA TAAGTTCTTC   
  
  
- CCTTCCCAGA AGTAGACTAG GTAAACGAAG AGTACAGACG ATGGGATTAG TCGCTTTTGT TATAGTCGAG   
  
  
- TCGACAATTA GTAAACGAAC TCAACATGTT CTTACAAAGA CATTTACCAC TAAGACCCGT CGCGCAACGA   
  
  
- CGAATAAAAC GACTACCCAA TTGACGCTCC AAGAATTGGG TCTTGAGAGG GAAAATAACG TACCATTACT   
  
  
- CATTTGGCTG TGGACGTCTC CTCAAATAGC GGAAGTGAGT AAACATGTCC CACAGAGGTA AGATAGTCAA   
  
  
- ACGGGTAAAA TGACGATTAG TCCGGTAGAA ACTCCGTAAG CTCTCCCTTC TTCTCTTGTT GTCGACCCGT   
  
  
- GAGGTACAGT AACTAAAACT GTAGAGAGTA CCGAAGGTCA CCGGCAGAGA GTAAGTCAGA GAAAGACTCT   
  
  
- TCCGATGACG AAGTTCGGTA AGTGAGAGAG AAGCTTAGTG TCCAAAACGA TCTTGAGACC TTCTTGACTA   
  
  
- ACTCTGACTC TTGTCTAATC ATTCAAAACG ACTTTCGAAG GCCTTGTATG ATAAACTTAA GGTGCCCACA   
  
  
- AACTCCCCAA GTCTCGACCA CTTGAACTTC TTTTCCCTCT TACTTTGTCA ACGGCATTTA GACCACAAAG   
  
  
- TGGAGTCGTC AAAGCTACTG TGGGACTTCC AGAGTCTGTG GAATTGGCGA CATGTAAGTG ACTTTGGAAG   
  
  
- ATATTACCAT GATCAACTTT CTCTTCCTTG GGCTTTACCT AGTCCTAAGT ATAGTCCAAA GTACCTAAGA   
  
  
- AACGTAATAA TACGACGTTA CAAGCTAAGT AATTTACTAA CGGAGGGTGC CCTCTCAGGG CTCTCTGACT   
  
  
- CGTATCTCTT CTTGGTAGAA CCCTCTCTCT AGTTCCTCCG CCACTGAACC CTCTTCCTGC TCTACTCGAT   
  
  
- GCCCTTCAAG CTCTATTACC TCTGAACCTT TTTCTCCTAC CTTTAAGTAC CCAAAAGCCC CTAGTCCGAC   
  
  
- TCAAGGTTCC GCTGTTAAGT TCGGTTCGAG GACGAGTTTT ACCCGTCGGT AATGACAGGG GTCAAACGTC   
  
  
- CCCTAAGACT TCCTCCTCAA CCGCCCAAGT CTCACATACT CTCTCTACTC CCCCTTCGGT AAAGAGACCA   
  
  
- AACCGTTCCG TCCAAGGAGC GTTGTCGGAG ACGTACCGCG ACACATAC

+     G-box

| Site Name | Organism | Position | Strand | Matrix score. | sequence | function |
| --- | --- | --- | --- | --- | --- | --- |
| G-box | Arabidopsis thaliana | 1806 | - | 6 | TACGTG | cis-acting regulatory element involved in light responsiveness |
| G-box | Arabidopsis thaliana | 150 | + | 6 | TACGTG | cis-acting regulatory element involved in light responsiveness |

>HU11G01529.1   
+ +Up\_Stream \_Len000GAAAGA AGATTTAAGG AAATCGATGG TACACGGCAA AGCTAGAAAC AGGAACTGTA   
  
  
+ TTTTTATGTT TTTTTTTTGT TGGAAGGGGG GGGGGGGGAG CGGGCGAGAT GTAACCCGTA TCTAGATGGA   
  
  
+ TAATCTACGT GACAAGACCT TCATTTGATC AAGGTACATC ATGAATATTT AACCCTTAAC TTCTTTGAGC   
  
  
+ CTTACAAGAG ATTGTCTGAG GTTTTTAGGG TGCACGATAT CATGCCTGAC TAGACCTGTG TAGACACAAT   
  
  
+ CTTCAGCCCT TATTGATAGC TGTTCGATAT GCATGCTGCC TAAAAATTGG AATAGTTGGC ACCTAACATT   
  
  
+ CTTTTACATC TTGCTCCTCC TTGGTGTAGA GTTAGATGTG CTAGCACAAT ACAATGGGTC CCGATATTTT   
  
  
+ GCATCTGGTT TCTGAAAGAT AATATTTGAT GATCCTGTCT TGTGCTGATG GGCAAAAGCT AAATTGTAAG   
  
  
+ AATATGTAGT TTATTTACAA GACATATGCT TAAGCTAGAG GTCATGTACT TGCATATTTT TGTGCAGCTT   
  
  
+ TTGAGGTTGT ATGCCCCTTT GAGACAAATT CATAGTTGAA TTCATGACAA GATTATGTTT GGCAATGACT   
  
  
+ TTGATTATTA AATTGGCCTT TATGCTATCC TATCCTGTGT GCTCAAATGT TATTTATCAG ATAATATTAG   
  
  
+ TTTACTCTTT CTTCCCTGTC TTGCAAAGAT TTTAAATCCT AAATTATGCA AATGTTTATC TTTAAAATGA   
  
  
+ CCTGCTACTC TCTGTGCCTG ATTTTATTTG TTTCCCCGTT GAAATTTTTT TTATGATTCC ACTTTTCAAG   
  
  
+ CAGCTTAGAC AGGCGTTATT AGGTAATTAT TTTCCGCTTT GGATTGTAGT AAATTGAAAC AGAAATGCCT   
  
  
+ CACTAAACCG CTCTCTGCTC TTTGTGTTGA AGAACCAATC CCATTCAAAC TCTTCTTTCT TTTTTGAAAT   
  
  
+ TCAATCCCGC TTCAGTCAAA TGTCTAACAG AAACAGCCTC GTGTGCAATT CTACTATATA CGGTTAATGC   
  
  
+ TGTGCATGTA TTACTTCCCA ACATCTTACC CAGGACTAGG AGGGACCTTG GTAGCCTGTT CTGCTTGAAA   
  
  
+ AATTTGTCAT TATCACCTGA ATTTGTATTA AATACATAAA TCTTTGTCTT TCAATTTGTG GCTCCACATT   
  
  
+ TCAATTTGTG GCTCCACACG CTTTTGAAAA CTTTATGAGA CCTAATATAT TAGAGAACAT CTGATCGTGA   
  
  
+ TTGTTAATTC CAGTTTTATA ATCATACTAA TTAGTAAATC ACTGAAGGTC ATATTGCATA TTGTTTTTGC   
  
  
+ CTAATGATTG GCTTATTCAA CTATTCCTGA ATAGTTTAGT TTCCAGAATC TTTAGAAGAA ACCTGGTCTG   
  
  
+ GGTATTATCG AGTATTTTTC TCAGTTCATG AAAGGCTTTG ATTCAGTGTG TGTGCTGTCA TGTCTAGGCA   
  
  
+ TGATTGAGTA TTTTTATTTG CATATCCAGG CATTGTGCCT AGACAAATGT CCTGAATCTT AGTGGCTGTT   
  
  
+ AGGTCTGAAC GGACACTTTT CTTGCTGTTT TTCCTTTCCG AGTGGCATCT TTAGCTTTTT CAAACTACCA   
  
  
+ AAGGACTATT AAACGAGGCC CTGCATATGC TGGAAAACTG CCTCTTAAGC TATGTTTATG CAAAAATGGA   
  
  
+ GGACCTTGAA CTGTTCAATG TACCTACAGT CTGAGGCACA GAGCCAATAG ACATAATAGA TAGTTGGATA   
  
  
+ TTCTTTTTAT ATTTAATTCA CAGTAGCGAC AACCCCAGCA TGAAACGTAG CCACGTAGGG GGGGAAAAAG   
  
  
+ GGTCTCCATC TGTTTATATA TTCTTGTCGC TTATGGTGCT TCTGTTATGT AATGACAAAG GAATTTGTGT   
  
  
+ TGGGATTATT CCGCTTCACT CCGTCTCACT AACTTTACGG TTTCAGTCCT AATTCTCTCT TTTTTTCTTT   
  
  
+ TGTTTTTTTT CCTGAGCTTG TTTAGTCTGT TTTCTTCTGT GTGTCTTTCT GATTATGGAA GCCATACAGG   
  
  
+ AGAAGAATGA CGATCTTTTG AGTCTTAGCT TGGCCATTGT TAGTCATTCT AACCATGCAG AAACGAAGGT   
  
  
+ GAAGAGGAAG AGGCATGATG TCTCTAATTC TCTGATCGCA ACAAGAGAAG AGGGTCGGGA TAAGAAAATA   
  
  
+ TTTCAGCTGC TTCAAGAACG AGAGAGAATG CTAAGCATCG GGCAAAGAAG AAAAGGCGTT ATTCAAGAAG   
  
  
+ GGAAGGGTCT TCATCTGATC CATTTGCTTC TCATGTCTGC TACCCTAATC AGCGAAAACA ATATCAGCTC   
  
  
+ AGCTGTTAAT CATTTGCTTG AGTTGTACAA GAATGTTTCT GTAAATGGTG ATTCTGGGCA GCGCGTTGCT   
  
  
+ GCTTATTTTG CTGATGGGTT AACTGCGAGG TTCTTAACCC AGAACTCTCC CTTTTATTGC ATGGTAATGA   
  
  
+ GTAAACCGAC ACCTGCAGAG GAGTTTATCG CCTTCACTCA TTTGTACAGG GTGTCTCCAT TCTATCAGTT   
  
  
+ TGCCCATTTT ACTGCTAATC AGGCCATCTT TGAGGCATTC GAGAGGGAAG AAGAGAACAA CAGCTGGGCA   
  
  
+ CTCCATGTCA TTGATTTTGA CATCTCTCAT GGCTTCCAGT GGCCGTCTCT CATTCAGTCT CTTTCTGAGA   
  
  
+ AGGCTACTGC TTCAAGCCAT TCACTCTCTC TTCGAATCAC AGGTTTTGCT AGAACTCTGG AAGAACTGAT   
  
  
+ TGAGACTGAG AACAGATTAG TAAGTTTTGC TGAAAGCTTC CGGAACATAC TATTTGAATT CCACGGGTGT   
  
  
+ TTGAGGGGTT CAGAGCTGGT GAACTTGAAG AAAAGGGAGA ATGAAACAGT TGCCGTAAAT CTGGTGTTTC   
  
  
+ ACCTCAGCAG TTTCGATGAC ACCCTGAAGG TCTCAGACAC CTTAACCGCT GTACATTCAC TGAAACCTTC   
  
  
+ TATAATGGTA CTAGTTGAAA GAGAAGGAAC CCGAAATGGA TCAGGATTCA TATCAGGTTT CATGGATTCT   
  
  
+ TTGCATTATT ATGCTGCAAT GTTCGATTCA TTAAATGATT GCCTCCCACG GGAGAGTCCC GAGAGACTGA   
  
  
+ GCATAGAGAA GAACCATCTT GGGAGAGAGA TCAAGGAGGC GGTGACTTGG GAGAAGGACG AGATGAGCTA   
  
  
+ CGGGAAGTTC GAGATAATGG AGACTTGGAA AAAGAGGATG GAAATTCATG GGTTTTCGGG GATCAGGCTG   
  
  
+ AGTTCCAAGG CGACAATTCA AGCCAAGCTC CTGCTCAAAA TGGGCAGCCA TTACTGTCCC CAGTTTGCAG   
  
  
+ GGGATTCTGA AGGAGGAGTT GGCGGGTTCA GAGTGTATGA GAGAGATGAG GGGGAAGCCA TTTCTCTGGT   
  
  
+ TTGGCAAGGC AGGTTCCTCG CAACAGCCTC TGCATGGCGC TGTGTATG  

- +Up\_Stream \_Len000CTTTCT TCTAAATTCC TTTAGCTACC ATGTGCCGTT TCGATCTTTG TCCTTGACAT   
  
  
- AAAAATACAA AAAAAAAACA ACCTTCCCCC CCCCCCCCTC GCCCGCTCTA CATTGGGCAT AGATCTACCT   
  
  
- ATTAGATGCA CTGTTCTGGA AGTAAACTAG TTCCATGTAG TACTTATAAA TTGGGAATTG AAGAAACTCG   
  
  
- GAATGTTCTC TAACAGACTC CAAAAATCCC ACGTGCTATA GTACGGACTG ATCTGGACAC ATCTGTGTTA   
  
  
- GAAGTCGGGA ATAACTATCG ACAAGCTATA CGTACGACGG ATTTTTAACC TTATCAACCG TGGATTGTAA   
  
  
- GAAAATGTAG AACGAGGAGG AACCACATCT CAATCTACAC GATCGTGTTA TGTTACCCAG GGCTATAAAA   
  
  
- CGTAGACCAA AGACTTTCTA TTATAAACTA CTAGGACAGA ACACGACTAC CCGTTTTCGA TTTAACATTC   
  
  
- TTATACATCA AATAAATGTT CTGTATACGA ATTCGATCTC CAGTACATGA ACGTATAAAA ACACGTCGAA   
  
  
- AACTCCAACA TACGGGGAAA CTCTGTTTAA GTATCAACTT AAGTACTGTT CTAATACAAA CCGTTACTGA   
  
  
- AACTAATAAT TTAACCGGAA ATACGATAGG ATAGGACACA CGAGTTTACA ATAAATAGTC TATTATAATC   
  
  
- AAATGAGAAA GAAGGGACAG AACGTTTCTA AAATTTAGGA TTTAATACGT TTACAAATAG AAATTTTACT   
  
  
- GGACGATGAG AGACACGGAC TAAAATAAAC AAAGGGGCAA CTTTAAAAAA AATACTAAGG TGAAAAGTTC   
  
  
- GTCGAATCTG TCCGCAATAA TCCATTAATA AAAGGCGAAA CCTAACATCA TTTAACTTTG TCTTTACGGA   
  
  
- GTGATTTGGC GAGAGACGAG AAACACAACT TCTTGGTTAG GGTAAGTTTG AGAAGAAAGA AAAAACTTTA   
  
  
- AGTTAGGGCG AAGTCAGTTT ACAGATTGTC TTTGTCGGAG CACACGTTAA GATGATATAT GCCAATTACG   
  
  
- ACACGTACAT AATGAAGGGT TGTAGAATGG GTCCTGATCC TCCCTGGAAC CATCGGACAA GACGAACTTT   
  
  
- TTAAACAGTA ATAGTGGACT TAAACATAAT TTATGTATTT AGAAACAGAA AGTTAAACAC CGAGGTGTAA   
  
  
- AGTTAAACAC CGAGGTGTGC GAAAACTTTT GAAATACTCT GGATTATATA ATCTCTTGTA GACTAGCACT   
  
  
- AACAATTAAG GTCAAAATAT TAGTATGATT AATCATTTAG TGACTTCCAG TATAACGTAT AACAAAAACG   
  
  
- GATTACTAAC CGAATAAGTT GATAAGGACT TATCAAATCA AAGGTCTTAG AAATCTTCTT TGGACCAGAC   
  
  
- CCATAATAGC TCATAAAAAG AGTCAAGTAC TTTCCGAAAC TAAGTCACAC ACACGACAGT ACAGATCCGT   
  
  
- ACTAACTCAT AAAAATAAAC GTATAGGTCC GTAACACGGA TCTGTTTACA GGACTTAGAA TCACCGACAA   
  
  
- TCCAGACTTG CCTGTGAAAA GAACGACAAA AAGGAAAGGC TCACCGTAGA AATCGAAAAA GTTTGATGGT   
  
  
- TTCCTGATAA TTTGCTCCGG GACGTATACG ACCTTTTGAC GGAGAATTCG ATACAAATAC GTTTTTACCT   
  
  
- CCTGGAACTT GACAAGTTAC ATGGATGTCA GACTCCGTGT CTCGGTTATC TGTATTATCT ATCAACCTAT   
  
  
- AAGAAAAATA TAAATTAAGT GTCATCGCTG TTGGGGTCGT ACTTTGCATC GGTGCATCCC CCCCTTTTTC   
  
  
- CCAGAGGTAG ACAAATATAT AAGAACAGCG AATACCACGA AGACAATACA TTACTGTTTC CTTAAACACA   
  
  
- ACCCTAATAA GGCGAAGTGA GGCAGAGTGA TTGAAATGCC AAAGTCAGGA TTAAGAGAGA AAAAAAGAAA   
  
  
- ACAAAAAAAA GGACTCGAAC AAATCAGACA AAAGAAGACA CACAGAAAGA CTAATACCTT CGGTATGTCC   
  
  
- TCTTCTTACT GCTAGAAAAC TCAGAATCGA ACCGGTAACA ATCAGTAAGA TTGGTACGTC TTTGCTTCCA   
  
  
- CTTCTCCTTC TCCGTACTAC AGAGATTAAG AGACTAGCGT TGTTCTCTTC TCCCAGCCCT ATTCTTTTAT   
  
  
- AAAGTCGACG AAGTTCTTGC TCTCTCTTAC GATTCGTAGC CCGTTTCTTC TTTTCCGCAA TAAGTTCTTC   
  
  
- CCTTCCCAGA AGTAGACTAG GTAAACGAAG AGTACAGACG ATGGGATTAG TCGCTTTTGT TATAGTCGAG   
  
  
- TCGACAATTA GTAAACGAAC TCAACATGTT CTTACAAAGA CATTTACCAC TAAGACCCGT CGCGCAACGA   
  
  
- CGAATAAAAC GACTACCCAA TTGACGCTCC AAGAATTGGG TCTTGAGAGG GAAAATAACG TACCATTACT   
  
  
- CATTTGGCTG TGGACGTCTC CTCAAATAGC GGAAGTGAGT AAACATGTCC CACAGAGGTA AGATAGTCAA   
  
  
- ACGGGTAAAA TGACGATTAG TCCGGTAGAA ACTCCGTAAG CTCTCCCTTC TTCTCTTGTT GTCGACCCGT   
  
  
- GAGGTACAGT AACTAAAACT GTAGAGAGTA CCGAAGGTCA CCGGCAGAGA GTAAGTCAGA GAAAGACTCT   
  
  
- TCCGATGACG AAGTTCGGTA AGTGAGAGAG AAGCTTAGTG TCCAAAACGA TCTTGAGACC TTCTTGACTA   
  
  
- ACTCTGACTC TTGTCTAATC ATTCAAAACG ACTTTCGAAG GCCTTGTATG ATAAACTTAA GGTGCCCACA   
  
  
- AACTCCCCAA GTCTCGACCA CTTGAACTTC TTTTCCCTCT TACTTTGTCA ACGGCATTTA GACCACAAAG   
  
  
- TGGAGTCGTC AAAGCTACTG TGGGACTTCC AGAGTCTGTG GAATTGGCGA CATGTAAGTG ACTTTGGAAG   
  
  
- ATATTACCAT GATCAACTTT CTCTTCCTTG GGCTTTACCT AGTCCTAAGT ATAGTCCAAA GTACCTAAGA   
  
  
- AACGTAATAA TACGACGTTA CAAGCTAAGT AATTTACTAA CGGAGGGTGC CCTCTCAGGG CTCTCTGACT   
  
  
- CGTATCTCTT CTTGGTAGAA CCCTCTCTCT AGTTCCTCCG CCACTGAACC CTCTTCCTGC TCTACTCGAT   
  
  
- GCCCTTCAAG CTCTATTACC TCTGAACCTT TTTCTCCTAC CTTTAAGTAC CCAAAAGCCC CTAGTCCGAC   
  
  
- TCAAGGTTCC GCTGTTAAGT TCGGTTCGAG GACGAGTTTT ACCCGTCGGT AATGACAGGG GTCAAACGTC   
  
  
- CCCTAAGACT TCCTCCTCAA CCGCCCAAGT CTCACATACT CTCTCTACTC CCCCTTCGGT AAAGAGACCA   
  
  
- AACCGTTCCG TCCAAGGAGC GTTGTCGGAG ACGTACCGCG ACACATAC

+     GATA-motif

| Site Name | Organism | Position | Strand | Matrix score. | sequence | function |
| --- | --- | --- | --- | --- | --- | --- |
| GATA-motif | Arabidopsis thaliana | 662 | - | 7 | GATAGGA | part of a light responsive element |
| GATA-motif | Arabidopsis thaliana | 440 | + | 10 | AAGATAAGATT | part of a light responsive element |

>HU11G01529.1   
+ +Up\_Stream \_Len000GAAAGA AGATTTAAGG AAATCGATGG TACACGGCAA AGCTAGAAAC AGGAACTGTA   
  
  
+ TTTTTATGTT TTTTTTTTGT TGGAAGGGGG GGGGGGGGAG CGGGCGAGAT GTAACCCGTA TCTAGATGGA   
  
  
+ TAATCTACGT GACAAGACCT TCATTTGATC AAGGTACATC ATGAATATTT AACCCTTAAC TTCTTTGAGC   
  
  
+ CTTACAAGAG ATTGTCTGAG GTTTTTAGGG TGCACGATAT CATGCCTGAC TAGACCTGTG TAGACACAAT   
  
  
+ CTTCAGCCCT TATTGATAGC TGTTCGATAT GCATGCTGCC TAAAAATTGG AATAGTTGGC ACCTAACATT   
  
  
+ CTTTTACATC TTGCTCCTCC TTGGTGTAGA GTTAGATGTG CTAGCACAAT ACAATGGGTC CCGATATTTT   
  
  
+ GCATCTGGTT TCTGAAAGAT AATATTTGAT GATCCTGTCT TGTGCTGATG GGCAAAAGCT AAATTGTAAG   
  
  
+ AATATGTAGT TTATTTACAA GACATATGCT TAAGCTAGAG GTCATGTACT TGCATATTTT TGTGCAGCTT   
  
  
+ TTGAGGTTGT ATGCCCCTTT GAGACAAATT CATAGTTGAA TTCATGACAA GATTATGTTT GGCAATGACT   
  
  
+ TTGATTATTA AATTGGCCTT TATGCTATCC TATCCTGTGT GCTCAAATGT TATTTATCAG ATAATATTAG   
  
  
+ TTTACTCTTT CTTCCCTGTC TTGCAAAGAT TTTAAATCCT AAATTATGCA AATGTTTATC TTTAAAATGA   
  
  
+ CCTGCTACTC TCTGTGCCTG ATTTTATTTG TTTCCCCGTT GAAATTTTTT TTATGATTCC ACTTTTCAAG   
  
  
+ CAGCTTAGAC AGGCGTTATT AGGTAATTAT TTTCCGCTTT GGATTGTAGT AAATTGAAAC AGAAATGCCT   
  
  
+ CACTAAACCG CTCTCTGCTC TTTGTGTTGA AGAACCAATC CCATTCAAAC TCTTCTTTCT TTTTTGAAAT   
  
  
+ TCAATCCCGC TTCAGTCAAA TGTCTAACAG AAACAGCCTC GTGTGCAATT CTACTATATA CGGTTAATGC   
  
  
+ TGTGCATGTA TTACTTCCCA ACATCTTACC CAGGACTAGG AGGGACCTTG GTAGCCTGTT CTGCTTGAAA   
  
  
+ AATTTGTCAT TATCACCTGA ATTTGTATTA AATACATAAA TCTTTGTCTT TCAATTTGTG GCTCCACATT   
  
  
+ TCAATTTGTG GCTCCACACG CTTTTGAAAA CTTTATGAGA CCTAATATAT TAGAGAACAT CTGATCGTGA   
  
  
+ TTGTTAATTC CAGTTTTATA ATCATACTAA TTAGTAAATC ACTGAAGGTC ATATTGCATA TTGTTTTTGC   
  
  
+ CTAATGATTG GCTTATTCAA CTATTCCTGA ATAGTTTAGT TTCCAGAATC TTTAGAAGAA ACCTGGTCTG   
  
  
+ GGTATTATCG AGTATTTTTC TCAGTTCATG AAAGGCTTTG ATTCAGTGTG TGTGCTGTCA TGTCTAGGCA   
  
  
+ TGATTGAGTA TTTTTATTTG CATATCCAGG CATTGTGCCT AGACAAATGT CCTGAATCTT AGTGGCTGTT   
  
  
+ AGGTCTGAAC GGACACTTTT CTTGCTGTTT TTCCTTTCCG AGTGGCATCT TTAGCTTTTT CAAACTACCA   
  
  
+ AAGGACTATT AAACGAGGCC CTGCATATGC TGGAAAACTG CCTCTTAAGC TATGTTTATG CAAAAATGGA   
  
  
+ GGACCTTGAA CTGTTCAATG TACCTACAGT CTGAGGCACA GAGCCAATAG ACATAATAGA TAGTTGGATA   
  
  
+ TTCTTTTTAT ATTTAATTCA CAGTAGCGAC AACCCCAGCA TGAAACGTAG CCACGTAGGG GGGGAAAAAG   
  
  
+ GGTCTCCATC TGTTTATATA TTCTTGTCGC TTATGGTGCT TCTGTTATGT AATGACAAAG GAATTTGTGT   
  
  
+ TGGGATTATT CCGCTTCACT CCGTCTCACT AACTTTACGG TTTCAGTCCT AATTCTCTCT TTTTTTCTTT   
  
  
+ TGTTTTTTTT CCTGAGCTTG TTTAGTCTGT TTTCTTCTGT GTGTCTTTCT GATTATGGAA GCCATACAGG   
  
  
+ AGAAGAATGA CGATCTTTTG AGTCTTAGCT TGGCCATTGT TAGTCATTCT AACCATGCAG AAACGAAGGT   
  
  
+ GAAGAGGAAG AGGCATGATG TCTCTAATTC TCTGATCGCA ACAAGAGAAG AGGGTCGGGA TAAGAAAATA   
  
  
+ TTTCAGCTGC TTCAAGAACG AGAGAGAATG CTAAGCATCG GGCAAAGAAG AAAAGGCGTT ATTCAAGAAG   
  
  
+ GGAAGGGTCT TCATCTGATC CATTTGCTTC TCATGTCTGC TACCCTAATC AGCGAAAACA ATATCAGCTC   
  
  
+ AGCTGTTAAT CATTTGCTTG AGTTGTACAA GAATGTTTCT GTAAATGGTG ATTCTGGGCA GCGCGTTGCT   
  
  
+ GCTTATTTTG CTGATGGGTT AACTGCGAGG TTCTTAACCC AGAACTCTCC CTTTTATTGC ATGGTAATGA   
  
  
+ GTAAACCGAC ACCTGCAGAG GAGTTTATCG CCTTCACTCA TTTGTACAGG GTGTCTCCAT TCTATCAGTT   
  
  
+ TGCCCATTTT ACTGCTAATC AGGCCATCTT TGAGGCATTC GAGAGGGAAG AAGAGAACAA CAGCTGGGCA   
  
  
+ CTCCATGTCA TTGATTTTGA CATCTCTCAT GGCTTCCAGT GGCCGTCTCT CATTCAGTCT CTTTCTGAGA   
  
  
+ AGGCTACTGC TTCAAGCCAT TCACTCTCTC TTCGAATCAC AGGTTTTGCT AGAACTCTGG AAGAACTGAT   
  
  
+ TGAGACTGAG AACAGATTAG TAAGTTTTGC TGAAAGCTTC CGGAACATAC TATTTGAATT CCACGGGTGT   
  
  
+ TTGAGGGGTT CAGAGCTGGT GAACTTGAAG AAAAGGGAGA ATGAAACAGT TGCCGTAAAT CTGGTGTTTC   
  
  
+ ACCTCAGCAG TTTCGATGAC ACCCTGAAGG TCTCAGACAC CTTAACCGCT GTACATTCAC TGAAACCTTC   
  
  
+ TATAATGGTA CTAGTTGAAA GAGAAGGAAC CCGAAATGGA TCAGGATTCA TATCAGGTTT CATGGATTCT   
  
  
+ TTGCATTATT ATGCTGCAAT GTTCGATTCA TTAAATGATT GCCTCCCACG GGAGAGTCCC GAGAGACTGA   
  
  
+ GCATAGAGAA GAACCATCTT GGGAGAGAGA TCAAGGAGGC GGTGACTTGG GAGAAGGACG AGATGAGCTA   
  
  
+ CGGGAAGTTC GAGATAATGG AGACTTGGAA AAAGAGGATG GAAATTCATG GGTTTTCGGG GATCAGGCTG   
  
  
+ AGTTCCAAGG CGACAATTCA AGCCAAGCTC CTGCTCAAAA TGGGCAGCCA TTACTGTCCC CAGTTTGCAG   
  
  
+ GGGATTCTGA AGGAGGAGTT GGCGGGTTCA GAGTGTATGA GAGAGATGAG GGGGAAGCCA TTTCTCTGGT   
  
  
+ TTGGCAAGGC AGGTTCCTCG CAACAGCCTC TGCATGGCGC TGTGTATG  

- +Up\_Stream \_Len000CTTTCT TCTAAATTCC TTTAGCTACC ATGTGCCGTT TCGATCTTTG TCCTTGACAT   
  
  
- AAAAATACAA AAAAAAAACA ACCTTCCCCC CCCCCCCCTC GCCCGCTCTA CATTGGGCAT AGATCTACCT   
  
  
- ATTAGATGCA CTGTTCTGGA AGTAAACTAG TTCCATGTAG TACTTATAAA TTGGGAATTG AAGAAACTCG   
  
  
- GAATGTTCTC TAACAGACTC CAAAAATCCC ACGTGCTATA GTACGGACTG ATCTGGACAC ATCTGTGTTA   
  
  
- GAAGTCGGGA ATAACTATCG ACAAGCTATA CGTACGACGG ATTTTTAACC TTATCAACCG TGGATTGTAA   
  
  
- GAAAATGTAG AACGAGGAGG AACCACATCT CAATCTACAC GATCGTGTTA TGTTACCCAG GGCTATAAAA   
  
  
- CGTAGACCAA AGACTTTCTA TTATAAACTA CTAGGACAGA ACACGACTAC CCGTTTTCGA TTTAACATTC   
  
  
- TTATACATCA AATAAATGTT CTGTATACGA ATTCGATCTC CAGTACATGA ACGTATAAAA ACACGTCGAA   
  
  
- AACTCCAACA TACGGGGAAA CTCTGTTTAA GTATCAACTT AAGTACTGTT CTAATACAAA CCGTTACTGA   
  
  
- AACTAATAAT TTAACCGGAA ATACGATAGG ATAGGACACA CGAGTTTACA ATAAATAGTC TATTATAATC   
  
  
- AAATGAGAAA GAAGGGACAG AACGTTTCTA AAATTTAGGA TTTAATACGT TTACAAATAG AAATTTTACT   
  
  
- GGACGATGAG AGACACGGAC TAAAATAAAC AAAGGGGCAA CTTTAAAAAA AATACTAAGG TGAAAAGTTC   
  
  
- GTCGAATCTG TCCGCAATAA TCCATTAATA AAAGGCGAAA CCTAACATCA TTTAACTTTG TCTTTACGGA   
  
  
- GTGATTTGGC GAGAGACGAG AAACACAACT TCTTGGTTAG GGTAAGTTTG AGAAGAAAGA AAAAACTTTA   
  
  
- AGTTAGGGCG AAGTCAGTTT ACAGATTGTC TTTGTCGGAG CACACGTTAA GATGATATAT GCCAATTACG   
  
  
- ACACGTACAT AATGAAGGGT TGTAGAATGG GTCCTGATCC TCCCTGGAAC CATCGGACAA GACGAACTTT   
  
  
- TTAAACAGTA ATAGTGGACT TAAACATAAT TTATGTATTT AGAAACAGAA AGTTAAACAC CGAGGTGTAA   
  
  
- AGTTAAACAC CGAGGTGTGC GAAAACTTTT GAAATACTCT GGATTATATA ATCTCTTGTA GACTAGCACT   
  
  
- AACAATTAAG GTCAAAATAT TAGTATGATT AATCATTTAG TGACTTCCAG TATAACGTAT AACAAAAACG   
  
  
- GATTACTAAC CGAATAAGTT GATAAGGACT TATCAAATCA AAGGTCTTAG AAATCTTCTT TGGACCAGAC   
  
  
- CCATAATAGC TCATAAAAAG AGTCAAGTAC TTTCCGAAAC TAAGTCACAC ACACGACAGT ACAGATCCGT   
  
  
- ACTAACTCAT AAAAATAAAC GTATAGGTCC GTAACACGGA TCTGTTTACA GGACTTAGAA TCACCGACAA   
  
  
- TCCAGACTTG CCTGTGAAAA GAACGACAAA AAGGAAAGGC TCACCGTAGA AATCGAAAAA GTTTGATGGT   
  
  
- TTCCTGATAA TTTGCTCCGG GACGTATACG ACCTTTTGAC GGAGAATTCG ATACAAATAC GTTTTTACCT   
  
  
- CCTGGAACTT GACAAGTTAC ATGGATGTCA GACTCCGTGT CTCGGTTATC TGTATTATCT ATCAACCTAT   
  
  
- AAGAAAAATA TAAATTAAGT GTCATCGCTG TTGGGGTCGT ACTTTGCATC GGTGCATCCC CCCCTTTTTC   
  
  
- CCAGAGGTAG ACAAATATAT AAGAACAGCG AATACCACGA AGACAATACA TTACTGTTTC CTTAAACACA   
  
  
- ACCCTAATAA GGCGAAGTGA GGCAGAGTGA TTGAAATGCC AAAGTCAGGA TTAAGAGAGA AAAAAAGAAA   
  
  
- ACAAAAAAAA GGACTCGAAC AAATCAGACA AAAGAAGACA CACAGAAAGA CTAATACCTT CGGTATGTCC   
  
  
- TCTTCTTACT GCTAGAAAAC TCAGAATCGA ACCGGTAACA ATCAGTAAGA TTGGTACGTC TTTGCTTCCA   
  
  
- CTTCTCCTTC TCCGTACTAC AGAGATTAAG AGACTAGCGT TGTTCTCTTC TCCCAGCCCT ATTCTTTTAT   
  
  
- AAAGTCGACG AAGTTCTTGC TCTCTCTTAC GATTCGTAGC CCGTTTCTTC TTTTCCGCAA TAAGTTCTTC   
  
  
- CCTTCCCAGA AGTAGACTAG GTAAACGAAG AGTACAGACG ATGGGATTAG TCGCTTTTGT TATAGTCGAG   
  
  
- TCGACAATTA GTAAACGAAC TCAACATGTT CTTACAAAGA CATTTACCAC TAAGACCCGT CGCGCAACGA   
  
  
- CGAATAAAAC GACTACCCAA TTGACGCTCC AAGAATTGGG TCTTGAGAGG GAAAATAACG TACCATTACT   
  
  
- CATTTGGCTG TGGACGTCTC CTCAAATAGC GGAAGTGAGT AAACATGTCC CACAGAGGTA AGATAGTCAA   
  
  
- ACGGGTAAAA TGACGATTAG TCCGGTAGAA ACTCCGTAAG CTCTCCCTTC TTCTCTTGTT GTCGACCCGT   
  
  
- GAGGTACAGT AACTAAAACT GTAGAGAGTA CCGAAGGTCA CCGGCAGAGA GTAAGTCAGA GAAAGACTCT   
  
  
- TCCGATGACG AAGTTCGGTA AGTGAGAGAG AAGCTTAGTG TCCAAAACGA TCTTGAGACC TTCTTGACTA   
  
  
- ACTCTGACTC TTGTCTAATC ATTCAAAACG ACTTTCGAAG GCCTTGTATG ATAAACTTAA GGTGCCCACA   
  
  
- AACTCCCCAA GTCTCGACCA CTTGAACTTC TTTTCCCTCT TACTTTGTCA ACGGCATTTA GACCACAAAG   
  
  
- TGGAGTCGTC AAAGCTACTG TGGGACTTCC AGAGTCTGTG GAATTGGCGA CATGTAAGTG ACTTTGGAAG   
  
  
- ATATTACCAT GATCAACTTT CTCTTCCTTG GGCTTTACCT AGTCCTAAGT ATAGTCCAAA GTACCTAAGA   
  
  
- AACGTAATAA TACGACGTTA CAAGCTAAGT AATTTACTAA CGGAGGGTGC CCTCTCAGGG CTCTCTGACT   
  
  
- CGTATCTCTT CTTGGTAGAA CCCTCTCTCT AGTTCCTCCG CCACTGAACC CTCTTCCTGC TCTACTCGAT   
  
  
- GCCCTTCAAG CTCTATTACC TCTGAACCTT TTTCTCCTAC CTTTAAGTAC CCAAAAGCCC CTAGTCCGAC   
  
  
- TCAAGGTTCC GCTGTTAAGT TCGGTTCGAG GACGAGTTTT ACCCGTCGGT AATGACAGGG GTCAAACGTC   
  
  
- CCCTAAGACT TCCTCCTCAA CCGCCCAAGT CTCACATACT CTCTCTACTC CCCCTTCGGT AAAGAGACCA   
  
  
- AACCGTTCCG TCCAAGGAGC GTTGTCGGAG ACGTACCGCG ACACATAC

+     GT1-motif

| Site Name | Organism | Position | Strand | Matrix score. | sequence | function |
| --- | --- | --- | --- | --- | --- | --- |
| GT1-motif | Arabidopsis thaliana | 2418 | - | 6 | GGTTAA | light responsive element |
| GT1-motif | Arabidopsis thaliana | 2401 | + | 6 | GGTTAA | light responsive element |
| GT1-motif | Arabidopsis thaliana | 2916 | - | 6 | GGTTAA | light responsive element |
| GT1-motif | Avena sativa | 1046 | + | 7 | GGTTAAT | light responsive element |
| GT1-motif | Arabidopsis thaliana | 193 | - | 6 | GGTTAA | light responsive element |

>HU11G01529.1   
+ +Up\_Stream \_Len000GAAAGA AGATTTAAGG AAATCGATGG TACACGGCAA AGCTAGAAAC AGGAACTGTA   
  
  
+ TTTTTATGTT TTTTTTTTGT TGGAAGGGGG GGGGGGGGAG CGGGCGAGAT GTAACCCGTA TCTAGATGGA   
  
  
+ TAATCTACGT GACAAGACCT TCATTTGATC AAGGTACATC ATGAATATTT AACCCTTAAC TTCTTTGAGC   
  
  
+ CTTACAAGAG ATTGTCTGAG GTTTTTAGGG TGCACGATAT CATGCCTGAC TAGACCTGTG TAGACACAAT   
  
  
+ CTTCAGCCCT TATTGATAGC TGTTCGATAT GCATGCTGCC TAAAAATTGG AATAGTTGGC ACCTAACATT   
  
  
+ CTTTTACATC TTGCTCCTCC TTGGTGTAGA GTTAGATGTG CTAGCACAAT ACAATGGGTC CCGATATTTT   
  
  
+ GCATCTGGTT TCTGAAAGAT AATATTTGAT GATCCTGTCT TGTGCTGATG GGCAAAAGCT AAATTGTAAG   
  
  
+ AATATGTAGT TTATTTACAA GACATATGCT TAAGCTAGAG GTCATGTACT TGCATATTTT TGTGCAGCTT   
  
  
+ TTGAGGTTGT ATGCCCCTTT GAGACAAATT CATAGTTGAA TTCATGACAA GATTATGTTT GGCAATGACT   
  
  
+ TTGATTATTA AATTGGCCTT TATGCTATCC TATCCTGTGT GCTCAAATGT TATTTATCAG ATAATATTAG   
  
  
+ TTTACTCTTT CTTCCCTGTC TTGCAAAGAT TTTAAATCCT AAATTATGCA AATGTTTATC TTTAAAATGA   
  
  
+ CCTGCTACTC TCTGTGCCTG ATTTTATTTG TTTCCCCGTT GAAATTTTTT TTATGATTCC ACTTTTCAAG   
  
  
+ CAGCTTAGAC AGGCGTTATT AGGTAATTAT TTTCCGCTTT GGATTGTAGT AAATTGAAAC AGAAATGCCT   
  
  
+ CACTAAACCG CTCTCTGCTC TTTGTGTTGA AGAACCAATC CCATTCAAAC TCTTCTTTCT TTTTTGAAAT   
  
  
+ TCAATCCCGC TTCAGTCAAA TGTCTAACAG AAACAGCCTC GTGTGCAATT CTACTATATA CGGTTAATGC   
  
  
+ TGTGCATGTA TTACTTCCCA ACATCTTACC CAGGACTAGG AGGGACCTTG GTAGCCTGTT CTGCTTGAAA   
  
  
+ AATTTGTCAT TATCACCTGA ATTTGTATTA AATACATAAA TCTTTGTCTT TCAATTTGTG GCTCCACATT   
  
  
+ TCAATTTGTG GCTCCACACG CTTTTGAAAA CTTTATGAGA CCTAATATAT TAGAGAACAT CTGATCGTGA   
  
  
+ TTGTTAATTC CAGTTTTATA ATCATACTAA TTAGTAAATC ACTGAAGGTC ATATTGCATA TTGTTTTTGC   
  
  
+ CTAATGATTG GCTTATTCAA CTATTCCTGA ATAGTTTAGT TTCCAGAATC TTTAGAAGAA ACCTGGTCTG   
  
  
+ GGTATTATCG AGTATTTTTC TCAGTTCATG AAAGGCTTTG ATTCAGTGTG TGTGCTGTCA TGTCTAGGCA   
  
  
+ TGATTGAGTA TTTTTATTTG CATATCCAGG CATTGTGCCT AGACAAATGT CCTGAATCTT AGTGGCTGTT   
  
  
+ AGGTCTGAAC GGACACTTTT CTTGCTGTTT TTCCTTTCCG AGTGGCATCT TTAGCTTTTT CAAACTACCA   
  
  
+ AAGGACTATT AAACGAGGCC CTGCATATGC TGGAAAACTG CCTCTTAAGC TATGTTTATG CAAAAATGGA   
  
  
+ GGACCTTGAA CTGTTCAATG TACCTACAGT CTGAGGCACA GAGCCAATAG ACATAATAGA TAGTTGGATA   
  
  
+ TTCTTTTTAT ATTTAATTCA CAGTAGCGAC AACCCCAGCA TGAAACGTAG CCACGTAGGG GGGGAAAAAG   
  
  
+ GGTCTCCATC TGTTTATATA TTCTTGTCGC TTATGGTGCT TCTGTTATGT AATGACAAAG GAATTTGTGT   
  
  
+ TGGGATTATT CCGCTTCACT CCGTCTCACT AACTTTACGG TTTCAGTCCT AATTCTCTCT TTTTTTCTTT   
  
  
+ TGTTTTTTTT CCTGAGCTTG TTTAGTCTGT TTTCTTCTGT GTGTCTTTCT GATTATGGAA GCCATACAGG   
  
  
+ AGAAGAATGA CGATCTTTTG AGTCTTAGCT TGGCCATTGT TAGTCATTCT AACCATGCAG AAACGAAGGT   
  
  
+ GAAGAGGAAG AGGCATGATG TCTCTAATTC TCTGATCGCA ACAAGAGAAG AGGGTCGGGA TAAGAAAATA   
  
  
+ TTTCAGCTGC TTCAAGAACG AGAGAGAATG CTAAGCATCG GGCAAAGAAG AAAAGGCGTT ATTCAAGAAG   
  
  
+ GGAAGGGTCT TCATCTGATC CATTTGCTTC TCATGTCTGC TACCCTAATC AGCGAAAACA ATATCAGCTC   
  
  
+ AGCTGTTAAT CATTTGCTTG AGTTGTACAA GAATGTTTCT GTAAATGGTG ATTCTGGGCA GCGCGTTGCT   
  
  
+ GCTTATTTTG CTGATGGGTT AACTGCGAGG TTCTTAACCC AGAACTCTCC CTTTTATTGC ATGGTAATGA   
  
  
+ GTAAACCGAC ACCTGCAGAG GAGTTTATCG CCTTCACTCA TTTGTACAGG GTGTCTCCAT TCTATCAGTT   
  
  
+ TGCCCATTTT ACTGCTAATC AGGCCATCTT TGAGGCATTC GAGAGGGAAG AAGAGAACAA CAGCTGGGCA   
  
  
+ CTCCATGTCA TTGATTTTGA CATCTCTCAT GGCTTCCAGT GGCCGTCTCT CATTCAGTCT CTTTCTGAGA   
  
  
+ AGGCTACTGC TTCAAGCCAT TCACTCTCTC TTCGAATCAC AGGTTTTGCT AGAACTCTGG AAGAACTGAT   
  
  
+ TGAGACTGAG AACAGATTAG TAAGTTTTGC TGAAAGCTTC CGGAACATAC TATTTGAATT CCACGGGTGT   
  
  
+ TTGAGGGGTT CAGAGCTGGT GAACTTGAAG AAAAGGGAGA ATGAAACAGT TGCCGTAAAT CTGGTGTTTC   
  
  
+ ACCTCAGCAG TTTCGATGAC ACCCTGAAGG TCTCAGACAC CTTAACCGCT GTACATTCAC TGAAACCTTC   
  
  
+ TATAATGGTA CTAGTTGAAA GAGAAGGAAC CCGAAATGGA TCAGGATTCA TATCAGGTTT CATGGATTCT   
  
  
+ TTGCATTATT ATGCTGCAAT GTTCGATTCA TTAAATGATT GCCTCCCACG GGAGAGTCCC GAGAGACTGA   
  
  
+ GCATAGAGAA GAACCATCTT GGGAGAGAGA TCAAGGAGGC GGTGACTTGG GAGAAGGACG AGATGAGCTA   
  
  
+ CGGGAAGTTC GAGATAATGG AGACTTGGAA AAAGAGGATG GAAATTCATG GGTTTTCGGG GATCAGGCTG   
  
  
+ AGTTCCAAGG CGACAATTCA AGCCAAGCTC CTGCTCAAAA TGGGCAGCCA TTACTGTCCC CAGTTTGCAG   
  
  
+ GGGATTCTGA AGGAGGAGTT GGCGGGTTCA GAGTGTATGA GAGAGATGAG GGGGAAGCCA TTTCTCTGGT   
  
  
+ TTGGCAAGGC AGGTTCCTCG CAACAGCCTC TGCATGGCGC TGTGTATG  

- +Up\_Stream \_Len000CTTTCT TCTAAATTCC TTTAGCTACC ATGTGCCGTT TCGATCTTTG TCCTTGACAT   
  
  
- AAAAATACAA AAAAAAAACA ACCTTCCCCC CCCCCCCCTC GCCCGCTCTA CATTGGGCAT AGATCTACCT   
  
  
- ATTAGATGCA CTGTTCTGGA AGTAAACTAG TTCCATGTAG TACTTATAAA TTGGGAATTG AAGAAACTCG   
  
  
- GAATGTTCTC TAACAGACTC CAAAAATCCC ACGTGCTATA GTACGGACTG ATCTGGACAC ATCTGTGTTA   
  
  
- GAAGTCGGGA ATAACTATCG ACAAGCTATA CGTACGACGG ATTTTTAACC TTATCAACCG TGGATTGTAA   
  
  
- GAAAATGTAG AACGAGGAGG AACCACATCT CAATCTACAC GATCGTGTTA TGTTACCCAG GGCTATAAAA   
  
  
- CGTAGACCAA AGACTTTCTA TTATAAACTA CTAGGACAGA ACACGACTAC CCGTTTTCGA TTTAACATTC   
  
  
- TTATACATCA AATAAATGTT CTGTATACGA ATTCGATCTC CAGTACATGA ACGTATAAAA ACACGTCGAA   
  
  
- AACTCCAACA TACGGGGAAA CTCTGTTTAA GTATCAACTT AAGTACTGTT CTAATACAAA CCGTTACTGA   
  
  
- AACTAATAAT TTAACCGGAA ATACGATAGG ATAGGACACA CGAGTTTACA ATAAATAGTC TATTATAATC   
  
  
- AAATGAGAAA GAAGGGACAG AACGTTTCTA AAATTTAGGA TTTAATACGT TTACAAATAG AAATTTTACT   
  
  
- GGACGATGAG AGACACGGAC TAAAATAAAC AAAGGGGCAA CTTTAAAAAA AATACTAAGG TGAAAAGTTC   
  
  
- GTCGAATCTG TCCGCAATAA TCCATTAATA AAAGGCGAAA CCTAACATCA TTTAACTTTG TCTTTACGGA   
  
  
- GTGATTTGGC GAGAGACGAG AAACACAACT TCTTGGTTAG GGTAAGTTTG AGAAGAAAGA AAAAACTTTA   
  
  
- AGTTAGGGCG AAGTCAGTTT ACAGATTGTC TTTGTCGGAG CACACGTTAA GATGATATAT GCCAATTACG   
  
  
- ACACGTACAT AATGAAGGGT TGTAGAATGG GTCCTGATCC TCCCTGGAAC CATCGGACAA GACGAACTTT   
  
  
- TTAAACAGTA ATAGTGGACT TAAACATAAT TTATGTATTT AGAAACAGAA AGTTAAACAC CGAGGTGTAA   
  
  
- AGTTAAACAC CGAGGTGTGC GAAAACTTTT GAAATACTCT GGATTATATA ATCTCTTGTA GACTAGCACT   
  
  
- AACAATTAAG GTCAAAATAT TAGTATGATT AATCATTTAG TGACTTCCAG TATAACGTAT AACAAAAACG   
  
  
- GATTACTAAC CGAATAAGTT GATAAGGACT TATCAAATCA AAGGTCTTAG AAATCTTCTT TGGACCAGAC   
  
  
- CCATAATAGC TCATAAAAAG AGTCAAGTAC TTTCCGAAAC TAAGTCACAC ACACGACAGT ACAGATCCGT   
  
  
- ACTAACTCAT AAAAATAAAC GTATAGGTCC GTAACACGGA TCTGTTTACA GGACTTAGAA TCACCGACAA   
  
  
- TCCAGACTTG CCTGTGAAAA GAACGACAAA AAGGAAAGGC TCACCGTAGA AATCGAAAAA GTTTGATGGT   
  
  
- TTCCTGATAA TTTGCTCCGG GACGTATACG ACCTTTTGAC GGAGAATTCG ATACAAATAC GTTTTTACCT   
  
  
- CCTGGAACTT GACAAGTTAC ATGGATGTCA GACTCCGTGT CTCGGTTATC TGTATTATCT ATCAACCTAT   
  
  
- AAGAAAAATA TAAATTAAGT GTCATCGCTG TTGGGGTCGT ACTTTGCATC GGTGCATCCC CCCCTTTTTC   
  
  
- CCAGAGGTAG ACAAATATAT AAGAACAGCG AATACCACGA AGACAATACA TTACTGTTTC CTTAAACACA   
  
  
- ACCCTAATAA GGCGAAGTGA GGCAGAGTGA TTGAAATGCC AAAGTCAGGA TTAAGAGAGA AAAAAAGAAA   
  
  
- ACAAAAAAAA GGACTCGAAC AAATCAGACA AAAGAAGACA CACAGAAAGA CTAATACCTT CGGTATGTCC   
  
  
- TCTTCTTACT GCTAGAAAAC TCAGAATCGA ACCGGTAACA ATCAGTAAGA TTGGTACGTC TTTGCTTCCA   
  
  
- CTTCTCCTTC TCCGTACTAC AGAGATTAAG AGACTAGCGT TGTTCTCTTC TCCCAGCCCT ATTCTTTTAT   
  
  
- AAAGTCGACG AAGTTCTTGC TCTCTCTTAC GATTCGTAGC CCGTTTCTTC TTTTCCGCAA TAAGTTCTTC   
  
  
- CCTTCCCAGA AGTAGACTAG GTAAACGAAG AGTACAGACG ATGGGATTAG TCGCTTTTGT TATAGTCGAG   
  
  
- TCGACAATTA GTAAACGAAC TCAACATGTT CTTACAAAGA CATTTACCAC TAAGACCCGT CGCGCAACGA   
  
  
- CGAATAAAAC GACTACCCAA TTGACGCTCC AAGAATTGGG TCTTGAGAGG GAAAATAACG TACCATTACT   
  
  
- CATTTGGCTG TGGACGTCTC CTCAAATAGC GGAAGTGAGT AAACATGTCC CACAGAGGTA AGATAGTCAA   
  
  
- ACGGGTAAAA TGACGATTAG TCCGGTAGAA ACTCCGTAAG CTCTCCCTTC TTCTCTTGTT GTCGACCCGT   
  
  
- GAGGTACAGT AACTAAAACT GTAGAGAGTA CCGAAGGTCA CCGGCAGAGA GTAAGTCAGA GAAAGACTCT   
  
  
- TCCGATGACG AAGTTCGGTA AGTGAGAGAG AAGCTTAGTG TCCAAAACGA TCTTGAGACC TTCTTGACTA   
  
  
- ACTCTGACTC TTGTCTAATC ATTCAAAACG ACTTTCGAAG GCCTTGTATG ATAAACTTAA GGTGCCCACA   
  
  
- AACTCCCCAA GTCTCGACCA CTTGAACTTC TTTTCCCTCT TACTTTGTCA ACGGCATTTA GACCACAAAG   
  
  
- TGGAGTCGTC AAAGCTACTG TGGGACTTCC AGAGTCTGTG GAATTGGCGA CATGTAAGTG ACTTTGGAAG   
  
  
- ATATTACCAT GATCAACTTT CTCTTCCTTG GGCTTTACCT AGTCCTAAGT ATAGTCCAAA GTACCTAAGA   
  
  
- AACGTAATAA TACGACGTTA CAAGCTAAGT AATTTACTAA CGGAGGGTGC CCTCTCAGGG CTCTCTGACT   
  
  
- CGTATCTCTT CTTGGTAGAA CCCTCTCTCT AGTTCCTCCG CCACTGAACC CTCTTCCTGC TCTACTCGAT   
  
  
- GCCCTTCAAG CTCTATTACC TCTGAACCTT TTTCTCCTAC CTTTAAGTAC CCAAAAGCCC CTAGTCCGAC   
  
  
- TCAAGGTTCC GCTGTTAAGT TCGGTTCGAG GACGAGTTTT ACCCGTCGGT AATGACAGGG GTCAAACGTC   
  
  
- CCCTAAGACT TCCTCCTCAA CCGCCCAAGT CTCACATACT CTCTCTACTC CCCCTTCGGT AAAGAGACCA   
  
  
- AACCGTTCCG TCCAAGGAGC GTTGTCGGAG ACGTACCGCG ACACATAC

+     I-box

| Site Name | Organism | Position | Strand | Matrix score. | sequence | function |
| --- | --- | --- | --- | --- | --- | --- |
| I-box | Zea mays | 2912 | - | 9 | gGATAAGGTG | part of a light responsive element |

>HU11G01529.1   
+ +Up\_Stream \_Len000GAAAGA AGATTTAAGG AAATCGATGG TACACGGCAA AGCTAGAAAC AGGAACTGTA   
  
  
+ TTTTTATGTT TTTTTTTTGT TGGAAGGGGG GGGGGGGGAG CGGGCGAGAT GTAACCCGTA TCTAGATGGA   
  
  
+ TAATCTACGT GACAAGACCT TCATTTGATC AAGGTACATC ATGAATATTT AACCCTTAAC TTCTTTGAGC   
  
  
+ CTTACAAGAG ATTGTCTGAG GTTTTTAGGG TGCACGATAT CATGCCTGAC TAGACCTGTG TAGACACAAT   
  
  
+ CTTCAGCCCT TATTGATAGC TGTTCGATAT GCATGCTGCC TAAAAATTGG AATAGTTGGC ACCTAACATT   
  
  
+ CTTTTACATC TTGCTCCTCC TTGGTGTAGA GTTAGATGTG CTAGCACAAT ACAATGGGTC CCGATATTTT   
  
  
+ GCATCTGGTT TCTGAAAGAT AATATTTGAT GATCCTGTCT TGTGCTGATG GGCAAAAGCT AAATTGTAAG   
  
  
+ AATATGTAGT TTATTTACAA GACATATGCT TAAGCTAGAG GTCATGTACT TGCATATTTT TGTGCAGCTT   
  
  
+ TTGAGGTTGT ATGCCCCTTT GAGACAAATT CATAGTTGAA TTCATGACAA GATTATGTTT GGCAATGACT   
  
  
+ TTGATTATTA AATTGGCCTT TATGCTATCC TATCCTGTGT GCTCAAATGT TATTTATCAG ATAATATTAG   
  
  
+ TTTACTCTTT CTTCCCTGTC TTGCAAAGAT TTTAAATCCT AAATTATGCA AATGTTTATC TTTAAAATGA   
  
  
+ CCTGCTACTC TCTGTGCCTG ATTTTATTTG TTTCCCCGTT GAAATTTTTT TTATGATTCC ACTTTTCAAG   
  
  
+ CAGCTTAGAC AGGCGTTATT AGGTAATTAT TTTCCGCTTT GGATTGTAGT AAATTGAAAC AGAAATGCCT   
  
  
+ CACTAAACCG CTCTCTGCTC TTTGTGTTGA AGAACCAATC CCATTCAAAC TCTTCTTTCT TTTTTGAAAT   
  
  
+ TCAATCCCGC TTCAGTCAAA TGTCTAACAG AAACAGCCTC GTGTGCAATT CTACTATATA CGGTTAATGC   
  
  
+ TGTGCATGTA TTACTTCCCA ACATCTTACC CAGGACTAGG AGGGACCTTG GTAGCCTGTT CTGCTTGAAA   
  
  
+ AATTTGTCAT TATCACCTGA ATTTGTATTA AATACATAAA TCTTTGTCTT TCAATTTGTG GCTCCACATT   
  
  
+ TCAATTTGTG GCTCCACACG CTTTTGAAAA CTTTATGAGA CCTAATATAT TAGAGAACAT CTGATCGTGA   
  
  
+ TTGTTAATTC CAGTTTTATA ATCATACTAA TTAGTAAATC ACTGAAGGTC ATATTGCATA TTGTTTTTGC   
  
  
+ CTAATGATTG GCTTATTCAA CTATTCCTGA ATAGTTTAGT TTCCAGAATC TTTAGAAGAA ACCTGGTCTG   
  
  
+ GGTATTATCG AGTATTTTTC TCAGTTCATG AAAGGCTTTG ATTCAGTGTG TGTGCTGTCA TGTCTAGGCA   
  
  
+ TGATTGAGTA TTTTTATTTG CATATCCAGG CATTGTGCCT AGACAAATGT CCTGAATCTT AGTGGCTGTT   
  
  
+ AGGTCTGAAC GGACACTTTT CTTGCTGTTT TTCCTTTCCG AGTGGCATCT TTAGCTTTTT CAAACTACCA   
  
  
+ AAGGACTATT AAACGAGGCC CTGCATATGC TGGAAAACTG CCTCTTAAGC TATGTTTATG CAAAAATGGA   
  
  
+ GGACCTTGAA CTGTTCAATG TACCTACAGT CTGAGGCACA GAGCCAATAG ACATAATAGA TAGTTGGATA   
  
  
+ TTCTTTTTAT ATTTAATTCA CAGTAGCGAC AACCCCAGCA TGAAACGTAG CCACGTAGGG GGGGAAAAAG   
  
  
+ GGTCTCCATC TGTTTATATA TTCTTGTCGC TTATGGTGCT TCTGTTATGT AATGACAAAG GAATTTGTGT   
  
  
+ TGGGATTATT CCGCTTCACT CCGTCTCACT AACTTTACGG TTTCAGTCCT AATTCTCTCT TTTTTTCTTT   
  
  
+ TGTTTTTTTT CCTGAGCTTG TTTAGTCTGT TTTCTTCTGT GTGTCTTTCT GATTATGGAA GCCATACAGG   
  
  
+ AGAAGAATGA CGATCTTTTG AGTCTTAGCT TGGCCATTGT TAGTCATTCT AACCATGCAG AAACGAAGGT   
  
  
+ GAAGAGGAAG AGGCATGATG TCTCTAATTC TCTGATCGCA ACAAGAGAAG AGGGTCGGGA TAAGAAAATA   
  
  
+ TTTCAGCTGC TTCAAGAACG AGAGAGAATG CTAAGCATCG GGCAAAGAAG AAAAGGCGTT ATTCAAGAAG   
  
  
+ GGAAGGGTCT TCATCTGATC CATTTGCTTC TCATGTCTGC TACCCTAATC AGCGAAAACA ATATCAGCTC   
  
  
+ AGCTGTTAAT CATTTGCTTG AGTTGTACAA GAATGTTTCT GTAAATGGTG ATTCTGGGCA GCGCGTTGCT   
  
  
+ GCTTATTTTG CTGATGGGTT AACTGCGAGG TTCTTAACCC AGAACTCTCC CTTTTATTGC ATGGTAATGA   
  
  
+ GTAAACCGAC ACCTGCAGAG GAGTTTATCG CCTTCACTCA TTTGTACAGG GTGTCTCCAT TCTATCAGTT   
  
  
+ TGCCCATTTT ACTGCTAATC AGGCCATCTT TGAGGCATTC GAGAGGGAAG AAGAGAACAA CAGCTGGGCA   
  
  
+ CTCCATGTCA TTGATTTTGA CATCTCTCAT GGCTTCCAGT GGCCGTCTCT CATTCAGTCT CTTTCTGAGA   
  
  
+ AGGCTACTGC TTCAAGCCAT TCACTCTCTC TTCGAATCAC AGGTTTTGCT AGAACTCTGG AAGAACTGAT   
  
  
+ TGAGACTGAG AACAGATTAG TAAGTTTTGC TGAAAGCTTC CGGAACATAC TATTTGAATT CCACGGGTGT   
  
  
+ TTGAGGGGTT CAGAGCTGGT GAACTTGAAG AAAAGGGAGA ATGAAACAGT TGCCGTAAAT CTGGTGTTTC   
  
  
+ ACCTCAGCAG TTTCGATGAC ACCCTGAAGG TCTCAGACAC CTTAACCGCT GTACATTCAC TGAAACCTTC   
  
  
+ TATAATGGTA CTAGTTGAAA GAGAAGGAAC CCGAAATGGA TCAGGATTCA TATCAGGTTT CATGGATTCT   
  
  
+ TTGCATTATT ATGCTGCAAT GTTCGATTCA TTAAATGATT GCCTCCCACG GGAGAGTCCC GAGAGACTGA   
  
  
+ GCATAGAGAA GAACCATCTT GGGAGAGAGA TCAAGGAGGC GGTGACTTGG GAGAAGGACG AGATGAGCTA   
  
  
+ CGGGAAGTTC GAGATAATGG AGACTTGGAA AAAGAGGATG GAAATTCATG GGTTTTCGGG GATCAGGCTG   
  
  
+ AGTTCCAAGG CGACAATTCA AGCCAAGCTC CTGCTCAAAA TGGGCAGCCA TTACTGTCCC CAGTTTGCAG   
  
  
+ GGGATTCTGA AGGAGGAGTT GGCGGGTTCA GAGTGTATGA GAGAGATGAG GGGGAAGCCA TTTCTCTGGT   
  
  
+ TTGGCAAGGC AGGTTCCTCG CAACAGCCTC TGCATGGCGC TGTGTATG  

- +Up\_Stream \_Len000CTTTCT TCTAAATTCC TTTAGCTACC ATGTGCCGTT TCGATCTTTG TCCTTGACAT   
  
  
- AAAAATACAA AAAAAAAACA ACCTTCCCCC CCCCCCCCTC GCCCGCTCTA CATTGGGCAT AGATCTACCT   
  
  
- ATTAGATGCA CTGTTCTGGA AGTAAACTAG TTCCATGTAG TACTTATAAA TTGGGAATTG AAGAAACTCG   
  
  
- GAATGTTCTC TAACAGACTC CAAAAATCCC ACGTGCTATA GTACGGACTG ATCTGGACAC ATCTGTGTTA   
  
  
- GAAGTCGGGA ATAACTATCG ACAAGCTATA CGTACGACGG ATTTTTAACC TTATCAACCG TGGATTGTAA   
  
  
- GAAAATGTAG AACGAGGAGG AACCACATCT CAATCTACAC GATCGTGTTA TGTTACCCAG GGCTATAAAA   
  
  
- CGTAGACCAA AGACTTTCTA TTATAAACTA CTAGGACAGA ACACGACTAC CCGTTTTCGA TTTAACATTC   
  
  
- TTATACATCA AATAAATGTT CTGTATACGA ATTCGATCTC CAGTACATGA ACGTATAAAA ACACGTCGAA   
  
  
- AACTCCAACA TACGGGGAAA CTCTGTTTAA GTATCAACTT AAGTACTGTT CTAATACAAA CCGTTACTGA   
  
  
- AACTAATAAT TTAACCGGAA ATACGATAGG ATAGGACACA CGAGTTTACA ATAAATAGTC TATTATAATC   
  
  
- AAATGAGAAA GAAGGGACAG AACGTTTCTA AAATTTAGGA TTTAATACGT TTACAAATAG AAATTTTACT   
  
  
- GGACGATGAG AGACACGGAC TAAAATAAAC AAAGGGGCAA CTTTAAAAAA AATACTAAGG TGAAAAGTTC   
  
  
- GTCGAATCTG TCCGCAATAA TCCATTAATA AAAGGCGAAA CCTAACATCA TTTAACTTTG TCTTTACGGA   
  
  
- GTGATTTGGC GAGAGACGAG AAACACAACT TCTTGGTTAG GGTAAGTTTG AGAAGAAAGA AAAAACTTTA   
  
  
- AGTTAGGGCG AAGTCAGTTT ACAGATTGTC TTTGTCGGAG CACACGTTAA GATGATATAT GCCAATTACG   
  
  
- ACACGTACAT AATGAAGGGT TGTAGAATGG GTCCTGATCC TCCCTGGAAC CATCGGACAA GACGAACTTT   
  
  
- TTAAACAGTA ATAGTGGACT TAAACATAAT TTATGTATTT AGAAACAGAA AGTTAAACAC CGAGGTGTAA   
  
  
- AGTTAAACAC CGAGGTGTGC GAAAACTTTT GAAATACTCT GGATTATATA ATCTCTTGTA GACTAGCACT   
  
  
- AACAATTAAG GTCAAAATAT TAGTATGATT AATCATTTAG TGACTTCCAG TATAACGTAT AACAAAAACG   
  
  
- GATTACTAAC CGAATAAGTT GATAAGGACT TATCAAATCA AAGGTCTTAG AAATCTTCTT TGGACCAGAC   
  
  
- CCATAATAGC TCATAAAAAG AGTCAAGTAC TTTCCGAAAC TAAGTCACAC ACACGACAGT ACAGATCCGT   
  
  
- ACTAACTCAT AAAAATAAAC GTATAGGTCC GTAACACGGA TCTGTTTACA GGACTTAGAA TCACCGACAA   
  
  
- TCCAGACTTG CCTGTGAAAA GAACGACAAA AAGGAAAGGC TCACCGTAGA AATCGAAAAA GTTTGATGGT   
  
  
- TTCCTGATAA TTTGCTCCGG GACGTATACG ACCTTTTGAC GGAGAATTCG ATACAAATAC GTTTTTACCT   
  
  
- CCTGGAACTT GACAAGTTAC ATGGATGTCA GACTCCGTGT CTCGGTTATC TGTATTATCT ATCAACCTAT   
  
  
- AAGAAAAATA TAAATTAAGT GTCATCGCTG TTGGGGTCGT ACTTTGCATC GGTGCATCCC CCCCTTTTTC   
  
  
- CCAGAGGTAG ACAAATATAT AAGAACAGCG AATACCACGA AGACAATACA TTACTGTTTC CTTAAACACA   
  
  
- ACCCTAATAA GGCGAAGTGA GGCAGAGTGA TTGAAATGCC AAAGTCAGGA TTAAGAGAGA AAAAAAGAAA   
  
  
- ACAAAAAAAA GGACTCGAAC AAATCAGACA AAAGAAGACA CACAGAAAGA CTAATACCTT CGGTATGTCC   
  
  
- TCTTCTTACT GCTAGAAAAC TCAGAATCGA ACCGGTAACA ATCAGTAAGA TTGGTACGTC TTTGCTTCCA   
  
  
- CTTCTCCTTC TCCGTACTAC AGAGATTAAG AGACTAGCGT TGTTCTCTTC TCCCAGCCCT ATTCTTTTAT   
  
  
- AAAGTCGACG AAGTTCTTGC TCTCTCTTAC GATTCGTAGC CCGTTTCTTC TTTTCCGCAA TAAGTTCTTC   
  
  
- CCTTCCCAGA AGTAGACTAG GTAAACGAAG AGTACAGACG ATGGGATTAG TCGCTTTTGT TATAGTCGAG   
  
  
- TCGACAATTA GTAAACGAAC TCAACATGTT CTTACAAAGA CATTTACCAC TAAGACCCGT CGCGCAACGA   
  
  
- CGAATAAAAC GACTACCCAA TTGACGCTCC AAGAATTGGG TCTTGAGAGG GAAAATAACG TACCATTACT   
  
  
- CATTTGGCTG TGGACGTCTC CTCAAATAGC GGAAGTGAGT AAACATGTCC CACAGAGGTA AGATAGTCAA   
  
  
- ACGGGTAAAA TGACGATTAG TCCGGTAGAA ACTCCGTAAG CTCTCCCTTC TTCTCTTGTT GTCGACCCGT   
  
  
- GAGGTACAGT AACTAAAACT GTAGAGAGTA CCGAAGGTCA CCGGCAGAGA GTAAGTCAGA GAAAGACTCT   
  
  
- TCCGATGACG AAGTTCGGTA AGTGAGAGAG AAGCTTAGTG TCCAAAACGA TCTTGAGACC TTCTTGACTA   
  
  
- ACTCTGACTC TTGTCTAATC ATTCAAAACG ACTTTCGAAG GCCTTGTATG ATAAACTTAA GGTGCCCACA   
  
  
- AACTCCCCAA GTCTCGACCA CTTGAACTTC TTTTCCCTCT TACTTTGTCA ACGGCATTTA GACCACAAAG   
  
  
- TGGAGTCGTC AAAGCTACTG TGGGACTTCC AGAGTCTGTG GAATTGGCGA CATGTAAGTG ACTTTGGAAG   
  
  
- ATATTACCAT GATCAACTTT CTCTTCCTTG GGCTTTACCT AGTCCTAAGT ATAGTCCAAA GTACCTAAGA   
  
  
- AACGTAATAA TACGACGTTA CAAGCTAAGT AATTTACTAA CGGAGGGTGC CCTCTCAGGG CTCTCTGACT   
  
  
- CGTATCTCTT CTTGGTAGAA CCCTCTCTCT AGTTCCTCCG CCACTGAACC CTCTTCCTGC TCTACTCGAT   
  
  
- GCCCTTCAAG CTCTATTACC TCTGAACCTT TTTCTCCTAC CTTTAAGTAC CCAAAAGCCC CTAGTCCGAC   
  
  
- TCAAGGTTCC GCTGTTAAGT TCGGTTCGAG GACGAGTTTT ACCCGTCGGT AATGACAGGG GTCAAACGTC   
  
  
- CCCTAAGACT TCCTCCTCAA CCGCCCAAGT CTCACATACT CTCTCTACTC CCCCTTCGGT AAAGAGACCA   
  
  
- AACCGTTCCG TCCAAGGAGC GTTGTCGGAG ACGTACCGCG ACACATAC

+     LTR

| Site Name | Organism | Position | Strand | Matrix score. | sequence | function |
| --- | --- | --- | --- | --- | --- | --- |
| LTR | Hordeum vulgare | 3208 | - | 6 | CCGAAA | cis-acting element involved in low-temperature responsiveness |
| LTR | Hordeum vulgare | 2975 | + | 6 | CCGAAA | cis-acting element involved in low-temperature responsiveness |

>HU11G01529.1   
+ +Up\_Stream \_Len000GAAAGA AGATTTAAGG AAATCGATGG TACACGGCAA AGCTAGAAAC AGGAACTGTA   
  
  
+ TTTTTATGTT TTTTTTTTGT TGGAAGGGGG GGGGGGGGAG CGGGCGAGAT GTAACCCGTA TCTAGATGGA   
  
  
+ TAATCTACGT GACAAGACCT TCATTTGATC AAGGTACATC ATGAATATTT AACCCTTAAC TTCTTTGAGC   
  
  
+ CTTACAAGAG ATTGTCTGAG GTTTTTAGGG TGCACGATAT CATGCCTGAC TAGACCTGTG TAGACACAAT   
  
  
+ CTTCAGCCCT TATTGATAGC TGTTCGATAT GCATGCTGCC TAAAAATTGG AATAGTTGGC ACCTAACATT   
  
  
+ CTTTTACATC TTGCTCCTCC TTGGTGTAGA GTTAGATGTG CTAGCACAAT ACAATGGGTC CCGATATTTT   
  
  
+ GCATCTGGTT TCTGAAAGAT AATATTTGAT GATCCTGTCT TGTGCTGATG GGCAAAAGCT AAATTGTAAG   
  
  
+ AATATGTAGT TTATTTACAA GACATATGCT TAAGCTAGAG GTCATGTACT TGCATATTTT TGTGCAGCTT   
  
  
+ TTGAGGTTGT ATGCCCCTTT GAGACAAATT CATAGTTGAA TTCATGACAA GATTATGTTT GGCAATGACT   
  
  
+ TTGATTATTA AATTGGCCTT TATGCTATCC TATCCTGTGT GCTCAAATGT TATTTATCAG ATAATATTAG   
  
  
+ TTTACTCTTT CTTCCCTGTC TTGCAAAGAT TTTAAATCCT AAATTATGCA AATGTTTATC TTTAAAATGA   
  
  
+ CCTGCTACTC TCTGTGCCTG ATTTTATTTG TTTCCCCGTT GAAATTTTTT TTATGATTCC ACTTTTCAAG   
  
  
+ CAGCTTAGAC AGGCGTTATT AGGTAATTAT TTTCCGCTTT GGATTGTAGT AAATTGAAAC AGAAATGCCT   
  
  
+ CACTAAACCG CTCTCTGCTC TTTGTGTTGA AGAACCAATC CCATTCAAAC TCTTCTTTCT TTTTTGAAAT   
  
  
+ TCAATCCCGC TTCAGTCAAA TGTCTAACAG AAACAGCCTC GTGTGCAATT CTACTATATA CGGTTAATGC   
  
  
+ TGTGCATGTA TTACTTCCCA ACATCTTACC CAGGACTAGG AGGGACCTTG GTAGCCTGTT CTGCTTGAAA   
  
  
+ AATTTGTCAT TATCACCTGA ATTTGTATTA AATACATAAA TCTTTGTCTT TCAATTTGTG GCTCCACATT   
  
  
+ TCAATTTGTG GCTCCACACG CTTTTGAAAA CTTTATGAGA CCTAATATAT TAGAGAACAT CTGATCGTGA   
  
  
+ TTGTTAATTC CAGTTTTATA ATCATACTAA TTAGTAAATC ACTGAAGGTC ATATTGCATA TTGTTTTTGC   
  
  
+ CTAATGATTG GCTTATTCAA CTATTCCTGA ATAGTTTAGT TTCCAGAATC TTTAGAAGAA ACCTGGTCTG   
  
  
+ GGTATTATCG AGTATTTTTC TCAGTTCATG AAAGGCTTTG ATTCAGTGTG TGTGCTGTCA TGTCTAGGCA   
  
  
+ TGATTGAGTA TTTTTATTTG CATATCCAGG CATTGTGCCT AGACAAATGT CCTGAATCTT AGTGGCTGTT   
  
  
+ AGGTCTGAAC GGACACTTTT CTTGCTGTTT TTCCTTTCCG AGTGGCATCT TTAGCTTTTT CAAACTACCA   
  
  
+ AAGGACTATT AAACGAGGCC CTGCATATGC TGGAAAACTG CCTCTTAAGC TATGTTTATG CAAAAATGGA   
  
  
+ GGACCTTGAA CTGTTCAATG TACCTACAGT CTGAGGCACA GAGCCAATAG ACATAATAGA TAGTTGGATA   
  
  
+ TTCTTTTTAT ATTTAATTCA CAGTAGCGAC AACCCCAGCA TGAAACGTAG CCACGTAGGG GGGGAAAAAG   
  
  
+ GGTCTCCATC TGTTTATATA TTCTTGTCGC TTATGGTGCT TCTGTTATGT AATGACAAAG GAATTTGTGT   
  
  
+ TGGGATTATT CCGCTTCACT CCGTCTCACT AACTTTACGG TTTCAGTCCT AATTCTCTCT TTTTTTCTTT   
  
  
+ TGTTTTTTTT CCTGAGCTTG TTTAGTCTGT TTTCTTCTGT GTGTCTTTCT GATTATGGAA GCCATACAGG   
  
  
+ AGAAGAATGA CGATCTTTTG AGTCTTAGCT TGGCCATTGT TAGTCATTCT AACCATGCAG AAACGAAGGT   
  
  
+ GAAGAGGAAG AGGCATGATG TCTCTAATTC TCTGATCGCA ACAAGAGAAG AGGGTCGGGA TAAGAAAATA   
  
  
+ TTTCAGCTGC TTCAAGAACG AGAGAGAATG CTAAGCATCG GGCAAAGAAG AAAAGGCGTT ATTCAAGAAG   
  
  
+ GGAAGGGTCT TCATCTGATC CATTTGCTTC TCATGTCTGC TACCCTAATC AGCGAAAACA ATATCAGCTC   
  
  
+ AGCTGTTAAT CATTTGCTTG AGTTGTACAA GAATGTTTCT GTAAATGGTG ATTCTGGGCA GCGCGTTGCT   
  
  
+ GCTTATTTTG CTGATGGGTT AACTGCGAGG TTCTTAACCC AGAACTCTCC CTTTTATTGC ATGGTAATGA   
  
  
+ GTAAACCGAC ACCTGCAGAG GAGTTTATCG CCTTCACTCA TTTGTACAGG GTGTCTCCAT TCTATCAGTT   
  
  
+ TGCCCATTTT ACTGCTAATC AGGCCATCTT TGAGGCATTC GAGAGGGAAG AAGAGAACAA CAGCTGGGCA   
  
  
+ CTCCATGTCA TTGATTTTGA CATCTCTCAT GGCTTCCAGT GGCCGTCTCT CATTCAGTCT CTTTCTGAGA   
  
  
+ AGGCTACTGC TTCAAGCCAT TCACTCTCTC TTCGAATCAC AGGTTTTGCT AGAACTCTGG AAGAACTGAT   
  
  
+ TGAGACTGAG AACAGATTAG TAAGTTTTGC TGAAAGCTTC CGGAACATAC TATTTGAATT CCACGGGTGT   
  
  
+ TTGAGGGGTT CAGAGCTGGT GAACTTGAAG AAAAGGGAGA ATGAAACAGT TGCCGTAAAT CTGGTGTTTC   
  
  
+ ACCTCAGCAG TTTCGATGAC ACCCTGAAGG TCTCAGACAC CTTAACCGCT GTACATTCAC TGAAACCTTC   
  
  
+ TATAATGGTA CTAGTTGAAA GAGAAGGAAC CCGAAATGGA TCAGGATTCA TATCAGGTTT CATGGATTCT   
  
  
+ TTGCATTATT ATGCTGCAAT GTTCGATTCA TTAAATGATT GCCTCCCACG GGAGAGTCCC GAGAGACTGA   
  
  
+ GCATAGAGAA GAACCATCTT GGGAGAGAGA TCAAGGAGGC GGTGACTTGG GAGAAGGACG AGATGAGCTA   
  
  
+ CGGGAAGTTC GAGATAATGG AGACTTGGAA AAAGAGGATG GAAATTCATG GGTTTTCGGG GATCAGGCTG   
  
  
+ AGTTCCAAGG CGACAATTCA AGCCAAGCTC CTGCTCAAAA TGGGCAGCCA TTACTGTCCC CAGTTTGCAG   
  
  
+ GGGATTCTGA AGGAGGAGTT GGCGGGTTCA GAGTGTATGA GAGAGATGAG GGGGAAGCCA TTTCTCTGGT   
  
  
+ TTGGCAAGGC AGGTTCCTCG CAACAGCCTC TGCATGGCGC TGTGTATG  

- +Up\_Stream \_Len000CTTTCT TCTAAATTCC TTTAGCTACC ATGTGCCGTT TCGATCTTTG TCCTTGACAT   
  
  
- AAAAATACAA AAAAAAAACA ACCTTCCCCC CCCCCCCCTC GCCCGCTCTA CATTGGGCAT AGATCTACCT   
  
  
- ATTAGATGCA CTGTTCTGGA AGTAAACTAG TTCCATGTAG TACTTATAAA TTGGGAATTG AAGAAACTCG   
  
  
- GAATGTTCTC TAACAGACTC CAAAAATCCC ACGTGCTATA GTACGGACTG ATCTGGACAC ATCTGTGTTA   
  
  
- GAAGTCGGGA ATAACTATCG ACAAGCTATA CGTACGACGG ATTTTTAACC TTATCAACCG TGGATTGTAA   
  
  
- GAAAATGTAG AACGAGGAGG AACCACATCT CAATCTACAC GATCGTGTTA TGTTACCCAG GGCTATAAAA   
  
  
- CGTAGACCAA AGACTTTCTA TTATAAACTA CTAGGACAGA ACACGACTAC CCGTTTTCGA TTTAACATTC   
  
  
- TTATACATCA AATAAATGTT CTGTATACGA ATTCGATCTC CAGTACATGA ACGTATAAAA ACACGTCGAA   
  
  
- AACTCCAACA TACGGGGAAA CTCTGTTTAA GTATCAACTT AAGTACTGTT CTAATACAAA CCGTTACTGA   
  
  
- AACTAATAAT TTAACCGGAA ATACGATAGG ATAGGACACA CGAGTTTACA ATAAATAGTC TATTATAATC   
  
  
- AAATGAGAAA GAAGGGACAG AACGTTTCTA AAATTTAGGA TTTAATACGT TTACAAATAG AAATTTTACT   
  
  
- GGACGATGAG AGACACGGAC TAAAATAAAC AAAGGGGCAA CTTTAAAAAA AATACTAAGG TGAAAAGTTC   
  
  
- GTCGAATCTG TCCGCAATAA TCCATTAATA AAAGGCGAAA CCTAACATCA TTTAACTTTG TCTTTACGGA   
  
  
- GTGATTTGGC GAGAGACGAG AAACACAACT TCTTGGTTAG GGTAAGTTTG AGAAGAAAGA AAAAACTTTA   
  
  
- AGTTAGGGCG AAGTCAGTTT ACAGATTGTC TTTGTCGGAG CACACGTTAA GATGATATAT GCCAATTACG   
  
  
- ACACGTACAT AATGAAGGGT TGTAGAATGG GTCCTGATCC TCCCTGGAAC CATCGGACAA GACGAACTTT   
  
  
- TTAAACAGTA ATAGTGGACT TAAACATAAT TTATGTATTT AGAAACAGAA AGTTAAACAC CGAGGTGTAA   
  
  
- AGTTAAACAC CGAGGTGTGC GAAAACTTTT GAAATACTCT GGATTATATA ATCTCTTGTA GACTAGCACT   
  
  
- AACAATTAAG GTCAAAATAT TAGTATGATT AATCATTTAG TGACTTCCAG TATAACGTAT AACAAAAACG   
  
  
- GATTACTAAC CGAATAAGTT GATAAGGACT TATCAAATCA AAGGTCTTAG AAATCTTCTT TGGACCAGAC   
  
  
- CCATAATAGC TCATAAAAAG AGTCAAGTAC TTTCCGAAAC TAAGTCACAC ACACGACAGT ACAGATCCGT   
  
  
- ACTAACTCAT AAAAATAAAC GTATAGGTCC GTAACACGGA TCTGTTTACA GGACTTAGAA TCACCGACAA   
  
  
- TCCAGACTTG CCTGTGAAAA GAACGACAAA AAGGAAAGGC TCACCGTAGA AATCGAAAAA GTTTGATGGT   
  
  
- TTCCTGATAA TTTGCTCCGG GACGTATACG ACCTTTTGAC GGAGAATTCG ATACAAATAC GTTTTTACCT   
  
  
- CCTGGAACTT GACAAGTTAC ATGGATGTCA GACTCCGTGT CTCGGTTATC TGTATTATCT ATCAACCTAT   
  
  
- AAGAAAAATA TAAATTAAGT GTCATCGCTG TTGGGGTCGT ACTTTGCATC GGTGCATCCC CCCCTTTTTC   
  
  
- CCAGAGGTAG ACAAATATAT AAGAACAGCG AATACCACGA AGACAATACA TTACTGTTTC CTTAAACACA   
  
  
- ACCCTAATAA GGCGAAGTGA GGCAGAGTGA TTGAAATGCC AAAGTCAGGA TTAAGAGAGA AAAAAAGAAA   
  
  
- ACAAAAAAAA GGACTCGAAC AAATCAGACA AAAGAAGACA CACAGAAAGA CTAATACCTT CGGTATGTCC   
  
  
- TCTTCTTACT GCTAGAAAAC TCAGAATCGA ACCGGTAACA ATCAGTAAGA TTGGTACGTC TTTGCTTCCA   
  
  
- CTTCTCCTTC TCCGTACTAC AGAGATTAAG AGACTAGCGT TGTTCTCTTC TCCCAGCCCT ATTCTTTTAT   
  
  
- AAAGTCGACG AAGTTCTTGC TCTCTCTTAC GATTCGTAGC CCGTTTCTTC TTTTCCGCAA TAAGTTCTTC   
  
  
- CCTTCCCAGA AGTAGACTAG GTAAACGAAG AGTACAGACG ATGGGATTAG TCGCTTTTGT TATAGTCGAG   
  
  
- TCGACAATTA GTAAACGAAC TCAACATGTT CTTACAAAGA CATTTACCAC TAAGACCCGT CGCGCAACGA   
  
  
- CGAATAAAAC GACTACCCAA TTGACGCTCC AAGAATTGGG TCTTGAGAGG GAAAATAACG TACCATTACT   
  
  
- CATTTGGCTG TGGACGTCTC CTCAAATAGC GGAAGTGAGT AAACATGTCC CACAGAGGTA AGATAGTCAA   
  
  
- ACGGGTAAAA TGACGATTAG TCCGGTAGAA ACTCCGTAAG CTCTCCCTTC TTCTCTTGTT GTCGACCCGT   
  
  
- GAGGTACAGT AACTAAAACT GTAGAGAGTA CCGAAGGTCA CCGGCAGAGA GTAAGTCAGA GAAAGACTCT   
  
  
- TCCGATGACG AAGTTCGGTA AGTGAGAGAG AAGCTTAGTG TCCAAAACGA TCTTGAGACC TTCTTGACTA   
  
  
- ACTCTGACTC TTGTCTAATC ATTCAAAACG ACTTTCGAAG GCCTTGTATG ATAAACTTAA GGTGCCCACA   
  
  
- AACTCCCCAA GTCTCGACCA CTTGAACTTC TTTTCCCTCT TACTTTGTCA ACGGCATTTA GACCACAAAG   
  
  
- TGGAGTCGTC AAAGCTACTG TGGGACTTCC AGAGTCTGTG GAATTGGCGA CATGTAAGTG ACTTTGGAAG   
  
  
- ATATTACCAT GATCAACTTT CTCTTCCTTG GGCTTTACCT AGTCCTAAGT ATAGTCCAAA GTACCTAAGA   
  
  
- AACGTAATAA TACGACGTTA CAAGCTAAGT AATTTACTAA CGGAGGGTGC CCTCTCAGGG CTCTCTGACT   
  
  
- CGTATCTCTT CTTGGTAGAA CCCTCTCTCT AGTTCCTCCG CCACTGAACC CTCTTCCTGC TCTACTCGAT   
  
  
- GCCCTTCAAG CTCTATTACC TCTGAACCTT TTTCTCCTAC CTTTAAGTAC CCAAAAGCCC CTAGTCCGAC   
  
  
- TCAAGGTTCC GCTGTTAAGT TCGGTTCGAG GACGAGTTTT ACCCGTCGGT AATGACAGGG GTCAAACGTC   
  
  
- CCCTAAGACT TCCTCCTCAA CCGCCCAAGT CTCACATACT CTCTCTACTC CCCCTTCGGT AAAGAGACCA   
  
  
- AACCGTTCCG TCCAAGGAGC GTTGTCGGAG ACGTACCGCG ACACATAC

+     MBS

| Site Name | Organism | Position | Strand | Matrix score. | sequence | function |
| --- | --- | --- | --- | --- | --- | --- |
| MBS | Arabidopsis thaliana | 2851 | - | 6 | CAACTG | MYB binding site involved in drought-inducibility |

>HU11G01529.1   
+ +Up\_Stream \_Len000GAAAGA AGATTTAAGG AAATCGATGG TACACGGCAA AGCTAGAAAC AGGAACTGTA   
  
  
+ TTTTTATGTT TTTTTTTTGT TGGAAGGGGG GGGGGGGGAG CGGGCGAGAT GTAACCCGTA TCTAGATGGA   
  
  
+ TAATCTACGT GACAAGACCT TCATTTGATC AAGGTACATC ATGAATATTT AACCCTTAAC TTCTTTGAGC   
  
  
+ CTTACAAGAG ATTGTCTGAG GTTTTTAGGG TGCACGATAT CATGCCTGAC TAGACCTGTG TAGACACAAT   
  
  
+ CTTCAGCCCT TATTGATAGC TGTTCGATAT GCATGCTGCC TAAAAATTGG AATAGTTGGC ACCTAACATT   
  
  
+ CTTTTACATC TTGCTCCTCC TTGGTGTAGA GTTAGATGTG CTAGCACAAT ACAATGGGTC CCGATATTTT   
  
  
+ GCATCTGGTT TCTGAAAGAT AATATTTGAT GATCCTGTCT TGTGCTGATG GGCAAAAGCT AAATTGTAAG   
  
  
+ AATATGTAGT TTATTTACAA GACATATGCT TAAGCTAGAG GTCATGTACT TGCATATTTT TGTGCAGCTT   
  
  
+ TTGAGGTTGT ATGCCCCTTT GAGACAAATT CATAGTTGAA TTCATGACAA GATTATGTTT GGCAATGACT   
  
  
+ TTGATTATTA AATTGGCCTT TATGCTATCC TATCCTGTGT GCTCAAATGT TATTTATCAG ATAATATTAG   
  
  
+ TTTACTCTTT CTTCCCTGTC TTGCAAAGAT TTTAAATCCT AAATTATGCA AATGTTTATC TTTAAAATGA   
  
  
+ CCTGCTACTC TCTGTGCCTG ATTTTATTTG TTTCCCCGTT GAAATTTTTT TTATGATTCC ACTTTTCAAG   
  
  
+ CAGCTTAGAC AGGCGTTATT AGGTAATTAT TTTCCGCTTT GGATTGTAGT AAATTGAAAC AGAAATGCCT   
  
  
+ CACTAAACCG CTCTCTGCTC TTTGTGTTGA AGAACCAATC CCATTCAAAC TCTTCTTTCT TTTTTGAAAT   
  
  
+ TCAATCCCGC TTCAGTCAAA TGTCTAACAG AAACAGCCTC GTGTGCAATT CTACTATATA CGGTTAATGC   
  
  
+ TGTGCATGTA TTACTTCCCA ACATCTTACC CAGGACTAGG AGGGACCTTG GTAGCCTGTT CTGCTTGAAA   
  
  
+ AATTTGTCAT TATCACCTGA ATTTGTATTA AATACATAAA TCTTTGTCTT TCAATTTGTG GCTCCACATT   
  
  
+ TCAATTTGTG GCTCCACACG CTTTTGAAAA CTTTATGAGA CCTAATATAT TAGAGAACAT CTGATCGTGA   
  
  
+ TTGTTAATTC CAGTTTTATA ATCATACTAA TTAGTAAATC ACTGAAGGTC ATATTGCATA TTGTTTTTGC   
  
  
+ CTAATGATTG GCTTATTCAA CTATTCCTGA ATAGTTTAGT TTCCAGAATC TTTAGAAGAA ACCTGGTCTG   
  
  
+ GGTATTATCG AGTATTTTTC TCAGTTCATG AAAGGCTTTG ATTCAGTGTG TGTGCTGTCA TGTCTAGGCA   
  
  
+ TGATTGAGTA TTTTTATTTG CATATCCAGG CATTGTGCCT AGACAAATGT CCTGAATCTT AGTGGCTGTT   
  
  
+ AGGTCTGAAC GGACACTTTT CTTGCTGTTT TTCCTTTCCG AGTGGCATCT TTAGCTTTTT CAAACTACCA   
  
  
+ AAGGACTATT AAACGAGGCC CTGCATATGC TGGAAAACTG CCTCTTAAGC TATGTTTATG CAAAAATGGA   
  
  
+ GGACCTTGAA CTGTTCAATG TACCTACAGT CTGAGGCACA GAGCCAATAG ACATAATAGA TAGTTGGATA   
  
  
+ TTCTTTTTAT ATTTAATTCA CAGTAGCGAC AACCCCAGCA TGAAACGTAG CCACGTAGGG GGGGAAAAAG   
  
  
+ GGTCTCCATC TGTTTATATA TTCTTGTCGC TTATGGTGCT TCTGTTATGT AATGACAAAG GAATTTGTGT   
  
  
+ TGGGATTATT CCGCTTCACT CCGTCTCACT AACTTTACGG TTTCAGTCCT AATTCTCTCT TTTTTTCTTT   
  
  
+ TGTTTTTTTT CCTGAGCTTG TTTAGTCTGT TTTCTTCTGT GTGTCTTTCT GATTATGGAA GCCATACAGG   
  
  
+ AGAAGAATGA CGATCTTTTG AGTCTTAGCT TGGCCATTGT TAGTCATTCT AACCATGCAG AAACGAAGGT   
  
  
+ GAAGAGGAAG AGGCATGATG TCTCTAATTC TCTGATCGCA ACAAGAGAAG AGGGTCGGGA TAAGAAAATA   
  
  
+ TTTCAGCTGC TTCAAGAACG AGAGAGAATG CTAAGCATCG GGCAAAGAAG AAAAGGCGTT ATTCAAGAAG   
  
  
+ GGAAGGGTCT TCATCTGATC CATTTGCTTC TCATGTCTGC TACCCTAATC AGCGAAAACA ATATCAGCTC   
  
  
+ AGCTGTTAAT CATTTGCTTG AGTTGTACAA GAATGTTTCT GTAAATGGTG ATTCTGGGCA GCGCGTTGCT   
  
  
+ GCTTATTTTG CTGATGGGTT AACTGCGAGG TTCTTAACCC AGAACTCTCC CTTTTATTGC ATGGTAATGA   
  
  
+ GTAAACCGAC ACCTGCAGAG GAGTTTATCG CCTTCACTCA TTTGTACAGG GTGTCTCCAT TCTATCAGTT   
  
  
+ TGCCCATTTT ACTGCTAATC AGGCCATCTT TGAGGCATTC GAGAGGGAAG AAGAGAACAA CAGCTGGGCA   
  
  
+ CTCCATGTCA TTGATTTTGA CATCTCTCAT GGCTTCCAGT GGCCGTCTCT CATTCAGTCT CTTTCTGAGA   
  
  
+ AGGCTACTGC TTCAAGCCAT TCACTCTCTC TTCGAATCAC AGGTTTTGCT AGAACTCTGG AAGAACTGAT   
  
  
+ TGAGACTGAG AACAGATTAG TAAGTTTTGC TGAAAGCTTC CGGAACATAC TATTTGAATT CCACGGGTGT   
  
  
+ TTGAGGGGTT CAGAGCTGGT GAACTTGAAG AAAAGGGAGA ATGAAACAGT TGCCGTAAAT CTGGTGTTTC   
  
  
+ ACCTCAGCAG TTTCGATGAC ACCCTGAAGG TCTCAGACAC CTTAACCGCT GTACATTCAC TGAAACCTTC   
  
  
+ TATAATGGTA CTAGTTGAAA GAGAAGGAAC CCGAAATGGA TCAGGATTCA TATCAGGTTT CATGGATTCT   
  
  
+ TTGCATTATT ATGCTGCAAT GTTCGATTCA TTAAATGATT GCCTCCCACG GGAGAGTCCC GAGAGACTGA   
  
  
+ GCATAGAGAA GAACCATCTT GGGAGAGAGA TCAAGGAGGC GGTGACTTGG GAGAAGGACG AGATGAGCTA   
  
  
+ CGGGAAGTTC GAGATAATGG AGACTTGGAA AAAGAGGATG GAAATTCATG GGTTTTCGGG GATCAGGCTG   
  
  
+ AGTTCCAAGG CGACAATTCA AGCCAAGCTC CTGCTCAAAA TGGGCAGCCA TTACTGTCCC CAGTTTGCAG   
  
  
+ GGGATTCTGA AGGAGGAGTT GGCGGGTTCA GAGTGTATGA GAGAGATGAG GGGGAAGCCA TTTCTCTGGT   
  
  
+ TTGGCAAGGC AGGTTCCTCG CAACAGCCTC TGCATGGCGC TGTGTATG  

- +Up\_Stream \_Len000CTTTCT TCTAAATTCC TTTAGCTACC ATGTGCCGTT TCGATCTTTG TCCTTGACAT   
  
  
- AAAAATACAA AAAAAAAACA ACCTTCCCCC CCCCCCCCTC GCCCGCTCTA CATTGGGCAT AGATCTACCT   
  
  
- ATTAGATGCA CTGTTCTGGA AGTAAACTAG TTCCATGTAG TACTTATAAA TTGGGAATTG AAGAAACTCG   
  
  
- GAATGTTCTC TAACAGACTC CAAAAATCCC ACGTGCTATA GTACGGACTG ATCTGGACAC ATCTGTGTTA   
  
  
- GAAGTCGGGA ATAACTATCG ACAAGCTATA CGTACGACGG ATTTTTAACC TTATCAACCG TGGATTGTAA   
  
  
- GAAAATGTAG AACGAGGAGG AACCACATCT CAATCTACAC GATCGTGTTA TGTTACCCAG GGCTATAAAA   
  
  
- CGTAGACCAA AGACTTTCTA TTATAAACTA CTAGGACAGA ACACGACTAC CCGTTTTCGA TTTAACATTC   
  
  
- TTATACATCA AATAAATGTT CTGTATACGA ATTCGATCTC CAGTACATGA ACGTATAAAA ACACGTCGAA   
  
  
- AACTCCAACA TACGGGGAAA CTCTGTTTAA GTATCAACTT AAGTACTGTT CTAATACAAA CCGTTACTGA   
  
  
- AACTAATAAT TTAACCGGAA ATACGATAGG ATAGGACACA CGAGTTTACA ATAAATAGTC TATTATAATC   
  
  
- AAATGAGAAA GAAGGGACAG AACGTTTCTA AAATTTAGGA TTTAATACGT TTACAAATAG AAATTTTACT   
  
  
- GGACGATGAG AGACACGGAC TAAAATAAAC AAAGGGGCAA CTTTAAAAAA AATACTAAGG TGAAAAGTTC   
  
  
- GTCGAATCTG TCCGCAATAA TCCATTAATA AAAGGCGAAA CCTAACATCA TTTAACTTTG TCTTTACGGA   
  
  
- GTGATTTGGC GAGAGACGAG AAACACAACT TCTTGGTTAG GGTAAGTTTG AGAAGAAAGA AAAAACTTTA   
  
  
- AGTTAGGGCG AAGTCAGTTT ACAGATTGTC TTTGTCGGAG CACACGTTAA GATGATATAT GCCAATTACG   
  
  
- ACACGTACAT AATGAAGGGT TGTAGAATGG GTCCTGATCC TCCCTGGAAC CATCGGACAA GACGAACTTT   
  
  
- TTAAACAGTA ATAGTGGACT TAAACATAAT TTATGTATTT AGAAACAGAA AGTTAAACAC CGAGGTGTAA   
  
  
- AGTTAAACAC CGAGGTGTGC GAAAACTTTT GAAATACTCT GGATTATATA ATCTCTTGTA GACTAGCACT   
  
  
- AACAATTAAG GTCAAAATAT TAGTATGATT AATCATTTAG TGACTTCCAG TATAACGTAT AACAAAAACG   
  
  
- GATTACTAAC CGAATAAGTT GATAAGGACT TATCAAATCA AAGGTCTTAG AAATCTTCTT TGGACCAGAC   
  
  
- CCATAATAGC TCATAAAAAG AGTCAAGTAC TTTCCGAAAC TAAGTCACAC ACACGACAGT ACAGATCCGT   
  
  
- ACTAACTCAT AAAAATAAAC GTATAGGTCC GTAACACGGA TCTGTTTACA GGACTTAGAA TCACCGACAA   
  
  
- TCCAGACTTG CCTGTGAAAA GAACGACAAA AAGGAAAGGC TCACCGTAGA AATCGAAAAA GTTTGATGGT   
  
  
- TTCCTGATAA TTTGCTCCGG GACGTATACG ACCTTTTGAC GGAGAATTCG ATACAAATAC GTTTTTACCT   
  
  
- CCTGGAACTT GACAAGTTAC ATGGATGTCA GACTCCGTGT CTCGGTTATC TGTATTATCT ATCAACCTAT   
  
  
- AAGAAAAATA TAAATTAAGT GTCATCGCTG TTGGGGTCGT ACTTTGCATC GGTGCATCCC CCCCTTTTTC   
  
  
- CCAGAGGTAG ACAAATATAT AAGAACAGCG AATACCACGA AGACAATACA TTACTGTTTC CTTAAACACA   
  
  
- ACCCTAATAA GGCGAAGTGA GGCAGAGTGA TTGAAATGCC AAAGTCAGGA TTAAGAGAGA AAAAAAGAAA   
  
  
- ACAAAAAAAA GGACTCGAAC AAATCAGACA AAAGAAGACA CACAGAAAGA CTAATACCTT CGGTATGTCC   
  
  
- TCTTCTTACT GCTAGAAAAC TCAGAATCGA ACCGGTAACA ATCAGTAAGA TTGGTACGTC TTTGCTTCCA   
  
  
- CTTCTCCTTC TCCGTACTAC AGAGATTAAG AGACTAGCGT TGTTCTCTTC TCCCAGCCCT ATTCTTTTAT   
  
  
- AAAGTCGACG AAGTTCTTGC TCTCTCTTAC GATTCGTAGC CCGTTTCTTC TTTTCCGCAA TAAGTTCTTC   
  
  
- CCTTCCCAGA AGTAGACTAG GTAAACGAAG AGTACAGACG ATGGGATTAG TCGCTTTTGT TATAGTCGAG   
  
  
- TCGACAATTA GTAAACGAAC TCAACATGTT CTTACAAAGA CATTTACCAC TAAGACCCGT CGCGCAACGA   
  
  
- CGAATAAAAC GACTACCCAA TTGACGCTCC AAGAATTGGG TCTTGAGAGG GAAAATAACG TACCATTACT   
  
  
- CATTTGGCTG TGGACGTCTC CTCAAATAGC GGAAGTGAGT AAACATGTCC CACAGAGGTA AGATAGTCAA   
  
  
- ACGGGTAAAA TGACGATTAG TCCGGTAGAA ACTCCGTAAG CTCTCCCTTC TTCTCTTGTT GTCGACCCGT   
  
  
- GAGGTACAGT AACTAAAACT GTAGAGAGTA CCGAAGGTCA CCGGCAGAGA GTAAGTCAGA GAAAGACTCT   
  
  
- TCCGATGACG AAGTTCGGTA AGTGAGAGAG AAGCTTAGTG TCCAAAACGA TCTTGAGACC TTCTTGACTA   
  
  
- ACTCTGACTC TTGTCTAATC ATTCAAAACG ACTTTCGAAG GCCTTGTATG ATAAACTTAA GGTGCCCACA   
  
  
- AACTCCCCAA GTCTCGACCA CTTGAACTTC TTTTCCCTCT TACTTTGTCA ACGGCATTTA GACCACAAAG   
  
  
- TGGAGTCGTC AAAGCTACTG TGGGACTTCC AGAGTCTGTG GAATTGGCGA CATGTAAGTG ACTTTGGAAG   
  
  
- ATATTACCAT GATCAACTTT CTCTTCCTTG GGCTTTACCT AGTCCTAAGT ATAGTCCAAA GTACCTAAGA   
  
  
- AACGTAATAA TACGACGTTA CAAGCTAAGT AATTTACTAA CGGAGGGTGC CCTCTCAGGG CTCTCTGACT   
  
  
- CGTATCTCTT CTTGGTAGAA CCCTCTCTCT AGTTCCTCCG CCACTGAACC CTCTTCCTGC TCTACTCGAT   
  
  
- GCCCTTCAAG CTCTATTACC TCTGAACCTT TTTCTCCTAC CTTTAAGTAC CCAAAAGCCC CTAGTCCGAC   
  
  
- TCAAGGTTCC GCTGTTAAGT TCGGTTCGAG GACGAGTTTT ACCCGTCGGT AATGACAGGG GTCAAACGTC   
  
  
- CCCTAAGACT TCCTCCTCAA CCGCCCAAGT CTCACATACT CTCTCTACTC CCCCTTCGGT AAAGAGACCA   
  
  
- AACCGTTCCG TCCAAGGAGC GTTGTCGGAG ACGTACCGCG ACACATAC

+     MYB

| Site Name | Organism | Position | Strand | Matrix score. | sequence | function |
| --- | --- | --- | --- | --- | --- | --- |
| MYB | Arabidopsis thaliana | 3385 | + | 6 | CAACAG |  |
| MYB | Arabidopsis thaliana | 2582 | + | 6 | CAACAG |  |
| MYB | Arabidopsis thaliana | 2084 | + | 6 | TAACCA |  |

>HU11G01529.1   
+ +Up\_Stream \_Len000GAAAGA AGATTTAAGG AAATCGATGG TACACGGCAA AGCTAGAAAC AGGAACTGTA   
  
  
+ TTTTTATGTT TTTTTTTTGT TGGAAGGGGG GGGGGGGGAG CGGGCGAGAT GTAACCCGTA TCTAGATGGA   
  
  
+ TAATCTACGT GACAAGACCT TCATTTGATC AAGGTACATC ATGAATATTT AACCCTTAAC TTCTTTGAGC   
  
  
+ CTTACAAGAG ATTGTCTGAG GTTTTTAGGG TGCACGATAT CATGCCTGAC TAGACCTGTG TAGACACAAT   
  
  
+ CTTCAGCCCT TATTGATAGC TGTTCGATAT GCATGCTGCC TAAAAATTGG AATAGTTGGC ACCTAACATT   
  
  
+ CTTTTACATC TTGCTCCTCC TTGGTGTAGA GTTAGATGTG CTAGCACAAT ACAATGGGTC CCGATATTTT   
  
  
+ GCATCTGGTT TCTGAAAGAT AATATTTGAT GATCCTGTCT TGTGCTGATG GGCAAAAGCT AAATTGTAAG   
  
  
+ AATATGTAGT TTATTTACAA GACATATGCT TAAGCTAGAG GTCATGTACT TGCATATTTT TGTGCAGCTT   
  
  
+ TTGAGGTTGT ATGCCCCTTT GAGACAAATT CATAGTTGAA TTCATGACAA GATTATGTTT GGCAATGACT   
  
  
+ TTGATTATTA AATTGGCCTT TATGCTATCC TATCCTGTGT GCTCAAATGT TATTTATCAG ATAATATTAG   
  
  
+ TTTACTCTTT CTTCCCTGTC TTGCAAAGAT TTTAAATCCT AAATTATGCA AATGTTTATC TTTAAAATGA   
  
  
+ CCTGCTACTC TCTGTGCCTG ATTTTATTTG TTTCCCCGTT GAAATTTTTT TTATGATTCC ACTTTTCAAG   
  
  
+ CAGCTTAGAC AGGCGTTATT AGGTAATTAT TTTCCGCTTT GGATTGTAGT AAATTGAAAC AGAAATGCCT   
  
  
+ CACTAAACCG CTCTCTGCTC TTTGTGTTGA AGAACCAATC CCATTCAAAC TCTTCTTTCT TTTTTGAAAT   
  
  
+ TCAATCCCGC TTCAGTCAAA TGTCTAACAG AAACAGCCTC GTGTGCAATT CTACTATATA CGGTTAATGC   
  
  
+ TGTGCATGTA TTACTTCCCA ACATCTTACC CAGGACTAGG AGGGACCTTG GTAGCCTGTT CTGCTTGAAA   
  
  
+ AATTTGTCAT TATCACCTGA ATTTGTATTA AATACATAAA TCTTTGTCTT TCAATTTGTG GCTCCACATT   
  
  
+ TCAATTTGTG GCTCCACACG CTTTTGAAAA CTTTATGAGA CCTAATATAT TAGAGAACAT CTGATCGTGA   
  
  
+ TTGTTAATTC CAGTTTTATA ATCATACTAA TTAGTAAATC ACTGAAGGTC ATATTGCATA TTGTTTTTGC   
  
  
+ CTAATGATTG GCTTATTCAA CTATTCCTGA ATAGTTTAGT TTCCAGAATC TTTAGAAGAA ACCTGGTCTG   
  
  
+ GGTATTATCG AGTATTTTTC TCAGTTCATG AAAGGCTTTG ATTCAGTGTG TGTGCTGTCA TGTCTAGGCA   
  
  
+ TGATTGAGTA TTTTTATTTG CATATCCAGG CATTGTGCCT AGACAAATGT CCTGAATCTT AGTGGCTGTT   
  
  
+ AGGTCTGAAC GGACACTTTT CTTGCTGTTT TTCCTTTCCG AGTGGCATCT TTAGCTTTTT CAAACTACCA   
  
  
+ AAGGACTATT AAACGAGGCC CTGCATATGC TGGAAAACTG CCTCTTAAGC TATGTTTATG CAAAAATGGA   
  
  
+ GGACCTTGAA CTGTTCAATG TACCTACAGT CTGAGGCACA GAGCCAATAG ACATAATAGA TAGTTGGATA   
  
  
+ TTCTTTTTAT ATTTAATTCA CAGTAGCGAC AACCCCAGCA TGAAACGTAG CCACGTAGGG GGGGAAAAAG   
  
  
+ GGTCTCCATC TGTTTATATA TTCTTGTCGC TTATGGTGCT TCTGTTATGT AATGACAAAG GAATTTGTGT   
  
  
+ TGGGATTATT CCGCTTCACT CCGTCTCACT AACTTTACGG TTTCAGTCCT AATTCTCTCT TTTTTTCTTT   
  
  
+ TGTTTTTTTT CCTGAGCTTG TTTAGTCTGT TTTCTTCTGT GTGTCTTTCT GATTATGGAA GCCATACAGG   
  
  
+ AGAAGAATGA CGATCTTTTG AGTCTTAGCT TGGCCATTGT TAGTCATTCT AACCATGCAG AAACGAAGGT   
  
  
+ GAAGAGGAAG AGGCATGATG TCTCTAATTC TCTGATCGCA ACAAGAGAAG AGGGTCGGGA TAAGAAAATA   
  
  
+ TTTCAGCTGC TTCAAGAACG AGAGAGAATG CTAAGCATCG GGCAAAGAAG AAAAGGCGTT ATTCAAGAAG   
  
  
+ GGAAGGGTCT TCATCTGATC CATTTGCTTC TCATGTCTGC TACCCTAATC AGCGAAAACA ATATCAGCTC   
  
  
+ AGCTGTTAAT CATTTGCTTG AGTTGTACAA GAATGTTTCT GTAAATGGTG ATTCTGGGCA GCGCGTTGCT   
  
  
+ GCTTATTTTG CTGATGGGTT AACTGCGAGG TTCTTAACCC AGAACTCTCC CTTTTATTGC ATGGTAATGA   
  
  
+ GTAAACCGAC ACCTGCAGAG GAGTTTATCG CCTTCACTCA TTTGTACAGG GTGTCTCCAT TCTATCAGTT   
  
  
+ TGCCCATTTT ACTGCTAATC AGGCCATCTT TGAGGCATTC GAGAGGGAAG AAGAGAACAA CAGCTGGGCA   
  
  
+ CTCCATGTCA TTGATTTTGA CATCTCTCAT GGCTTCCAGT GGCCGTCTCT CATTCAGTCT CTTTCTGAGA   
  
  
+ AGGCTACTGC TTCAAGCCAT TCACTCTCTC TTCGAATCAC AGGTTTTGCT AGAACTCTGG AAGAACTGAT   
  
  
+ TGAGACTGAG AACAGATTAG TAAGTTTTGC TGAAAGCTTC CGGAACATAC TATTTGAATT CCACGGGTGT   
  
  
+ TTGAGGGGTT CAGAGCTGGT GAACTTGAAG AAAAGGGAGA ATGAAACAGT TGCCGTAAAT CTGGTGTTTC   
  
  
+ ACCTCAGCAG TTTCGATGAC ACCCTGAAGG TCTCAGACAC CTTAACCGCT GTACATTCAC TGAAACCTTC   
  
  
+ TATAATGGTA CTAGTTGAAA GAGAAGGAAC CCGAAATGGA TCAGGATTCA TATCAGGTTT CATGGATTCT   
  
  
+ TTGCATTATT ATGCTGCAAT GTTCGATTCA TTAAATGATT GCCTCCCACG GGAGAGTCCC GAGAGACTGA   
  
  
+ GCATAGAGAA GAACCATCTT GGGAGAGAGA TCAAGGAGGC GGTGACTTGG GAGAAGGACG AGATGAGCTA   
  
  
+ CGGGAAGTTC GAGATAATGG AGACTTGGAA AAAGAGGATG GAAATTCATG GGTTTTCGGG GATCAGGCTG   
  
  
+ AGTTCCAAGG CGACAATTCA AGCCAAGCTC CTGCTCAAAA TGGGCAGCCA TTACTGTCCC CAGTTTGCAG   
  
  
+ GGGATTCTGA AGGAGGAGTT GGCGGGTTCA GAGTGTATGA GAGAGATGAG GGGGAAGCCA TTTCTCTGGT   
  
  
+ TTGGCAAGGC AGGTTCCTCG CAACAGCCTC TGCATGGCGC TGTGTATG  

- +Up\_Stream \_Len000CTTTCT TCTAAATTCC TTTAGCTACC ATGTGCCGTT TCGATCTTTG TCCTTGACAT   
  
  
- AAAAATACAA AAAAAAAACA ACCTTCCCCC CCCCCCCCTC GCCCGCTCTA CATTGGGCAT AGATCTACCT   
  
  
- ATTAGATGCA CTGTTCTGGA AGTAAACTAG TTCCATGTAG TACTTATAAA TTGGGAATTG AAGAAACTCG   
  
  
- GAATGTTCTC TAACAGACTC CAAAAATCCC ACGTGCTATA GTACGGACTG ATCTGGACAC ATCTGTGTTA   
  
  
- GAAGTCGGGA ATAACTATCG ACAAGCTATA CGTACGACGG ATTTTTAACC TTATCAACCG TGGATTGTAA   
  
  
- GAAAATGTAG AACGAGGAGG AACCACATCT CAATCTACAC GATCGTGTTA TGTTACCCAG GGCTATAAAA   
  
  
- CGTAGACCAA AGACTTTCTA TTATAAACTA CTAGGACAGA ACACGACTAC CCGTTTTCGA TTTAACATTC   
  
  
- TTATACATCA AATAAATGTT CTGTATACGA ATTCGATCTC CAGTACATGA ACGTATAAAA ACACGTCGAA   
  
  
- AACTCCAACA TACGGGGAAA CTCTGTTTAA GTATCAACTT AAGTACTGTT CTAATACAAA CCGTTACTGA   
  
  
- AACTAATAAT TTAACCGGAA ATACGATAGG ATAGGACACA CGAGTTTACA ATAAATAGTC TATTATAATC   
  
  
- AAATGAGAAA GAAGGGACAG AACGTTTCTA AAATTTAGGA TTTAATACGT TTACAAATAG AAATTTTACT   
  
  
- GGACGATGAG AGACACGGAC TAAAATAAAC AAAGGGGCAA CTTTAAAAAA AATACTAAGG TGAAAAGTTC   
  
  
- GTCGAATCTG TCCGCAATAA TCCATTAATA AAAGGCGAAA CCTAACATCA TTTAACTTTG TCTTTACGGA   
  
  
- GTGATTTGGC GAGAGACGAG AAACACAACT TCTTGGTTAG GGTAAGTTTG AGAAGAAAGA AAAAACTTTA   
  
  
- AGTTAGGGCG AAGTCAGTTT ACAGATTGTC TTTGTCGGAG CACACGTTAA GATGATATAT GCCAATTACG   
  
  
- ACACGTACAT AATGAAGGGT TGTAGAATGG GTCCTGATCC TCCCTGGAAC CATCGGACAA GACGAACTTT   
  
  
- TTAAACAGTA ATAGTGGACT TAAACATAAT TTATGTATTT AGAAACAGAA AGTTAAACAC CGAGGTGTAA   
  
  
- AGTTAAACAC CGAGGTGTGC GAAAACTTTT GAAATACTCT GGATTATATA ATCTCTTGTA GACTAGCACT   
  
  
- AACAATTAAG GTCAAAATAT TAGTATGATT AATCATTTAG TGACTTCCAG TATAACGTAT AACAAAAACG   
  
  
- GATTACTAAC CGAATAAGTT GATAAGGACT TATCAAATCA AAGGTCTTAG AAATCTTCTT TGGACCAGAC   
  
  
- CCATAATAGC TCATAAAAAG AGTCAAGTAC TTTCCGAAAC TAAGTCACAC ACACGACAGT ACAGATCCGT   
  
  
- ACTAACTCAT AAAAATAAAC GTATAGGTCC GTAACACGGA TCTGTTTACA GGACTTAGAA TCACCGACAA   
  
  
- TCCAGACTTG CCTGTGAAAA GAACGACAAA AAGGAAAGGC TCACCGTAGA AATCGAAAAA GTTTGATGGT   
  
  
- TTCCTGATAA TTTGCTCCGG GACGTATACG ACCTTTTGAC GGAGAATTCG ATACAAATAC GTTTTTACCT   
  
  
- CCTGGAACTT GACAAGTTAC ATGGATGTCA GACTCCGTGT CTCGGTTATC TGTATTATCT ATCAACCTAT   
  
  
- AAGAAAAATA TAAATTAAGT GTCATCGCTG TTGGGGTCGT ACTTTGCATC GGTGCATCCC CCCCTTTTTC   
  
  
- CCAGAGGTAG ACAAATATAT AAGAACAGCG AATACCACGA AGACAATACA TTACTGTTTC CTTAAACACA   
  
  
- ACCCTAATAA GGCGAAGTGA GGCAGAGTGA TTGAAATGCC AAAGTCAGGA TTAAGAGAGA AAAAAAGAAA   
  
  
- ACAAAAAAAA GGACTCGAAC AAATCAGACA AAAGAAGACA CACAGAAAGA CTAATACCTT CGGTATGTCC   
  
  
- TCTTCTTACT GCTAGAAAAC TCAGAATCGA ACCGGTAACA ATCAGTAAGA TTGGTACGTC TTTGCTTCCA   
  
  
- CTTCTCCTTC TCCGTACTAC AGAGATTAAG AGACTAGCGT TGTTCTCTTC TCCCAGCCCT ATTCTTTTAT   
  
  
- AAAGTCGACG AAGTTCTTGC TCTCTCTTAC GATTCGTAGC CCGTTTCTTC TTTTCCGCAA TAAGTTCTTC   
  
  
- CCTTCCCAGA AGTAGACTAG GTAAACGAAG AGTACAGACG ATGGGATTAG TCGCTTTTGT TATAGTCGAG   
  
  
- TCGACAATTA GTAAACGAAC TCAACATGTT CTTACAAAGA CATTTACCAC TAAGACCCGT CGCGCAACGA   
  
  
- CGAATAAAAC GACTACCCAA TTGACGCTCC AAGAATTGGG TCTTGAGAGG GAAAATAACG TACCATTACT   
  
  
- CATTTGGCTG TGGACGTCTC CTCAAATAGC GGAAGTGAGT AAACATGTCC CACAGAGGTA AGATAGTCAA   
  
  
- ACGGGTAAAA TGACGATTAG TCCGGTAGAA ACTCCGTAAG CTCTCCCTTC TTCTCTTGTT GTCGACCCGT   
  
  
- GAGGTACAGT AACTAAAACT GTAGAGAGTA CCGAAGGTCA CCGGCAGAGA GTAAGTCAGA GAAAGACTCT   
  
  
- TCCGATGACG AAGTTCGGTA AGTGAGAGAG AAGCTTAGTG TCCAAAACGA TCTTGAGACC TTCTTGACTA   
  
  
- ACTCTGACTC TTGTCTAATC ATTCAAAACG ACTTTCGAAG GCCTTGTATG ATAAACTTAA GGTGCCCACA   
  
  
- AACTCCCCAA GTCTCGACCA CTTGAACTTC TTTTCCCTCT TACTTTGTCA ACGGCATTTA GACCACAAAG   
  
  
- TGGAGTCGTC AAAGCTACTG TGGGACTTCC AGAGTCTGTG GAATTGGCGA CATGTAAGTG ACTTTGGAAG   
  
  
- ATATTACCAT GATCAACTTT CTCTTCCTTG GGCTTTACCT AGTCCTAAGT ATAGTCCAAA GTACCTAAGA   
  
  
- AACGTAATAA TACGACGTTA CAAGCTAAGT AATTTACTAA CGGAGGGTGC CCTCTCAGGG CTCTCTGACT   
  
  
- CGTATCTCTT CTTGGTAGAA CCCTCTCTCT AGTTCCTCCG CCACTGAACC CTCTTCCTGC TCTACTCGAT   
  
  
- GCCCTTCAAG CTCTATTACC TCTGAACCTT TTTCTCCTAC CTTTAAGTAC CCAAAAGCCC CTAGTCCGAC   
  
  
- TCAAGGTTCC GCTGTTAAGT TCGGTTCGAG GACGAGTTTT ACCCGTCGGT AATGACAGGG GTCAAACGTC   
  
  
- CCCTAAGACT TCCTCCTCAA CCGCCCAAGT CTCACATACT CTCTCTACTC CCCCTTCGGT AAAGAGACCA   
  
  
- AACCGTTCCG TCCAAGGAGC GTTGTCGGAG ACGTACCGCG ACACATAC

+     MYB recognition site

| Site Name | Organism | Position | Strand | Matrix score. | sequence | function |
| --- | --- | --- | --- | --- | --- | --- |
| MYB recognition site | Arabidopsis thaliana | 810 | + | 6 | CCGTTG |  |

>HU11G01529.1   
+ +Up\_Stream \_Len000GAAAGA AGATTTAAGG AAATCGATGG TACACGGCAA AGCTAGAAAC AGGAACTGTA   
  
  
+ TTTTTATGTT TTTTTTTTGT TGGAAGGGGG GGGGGGGGAG CGGGCGAGAT GTAACCCGTA TCTAGATGGA   
  
  
+ TAATCTACGT GACAAGACCT TCATTTGATC AAGGTACATC ATGAATATTT AACCCTTAAC TTCTTTGAGC   
  
  
+ CTTACAAGAG ATTGTCTGAG GTTTTTAGGG TGCACGATAT CATGCCTGAC TAGACCTGTG TAGACACAAT   
  
  
+ CTTCAGCCCT TATTGATAGC TGTTCGATAT GCATGCTGCC TAAAAATTGG AATAGTTGGC ACCTAACATT   
  
  
+ CTTTTACATC TTGCTCCTCC TTGGTGTAGA GTTAGATGTG CTAGCACAAT ACAATGGGTC CCGATATTTT   
  
  
+ GCATCTGGTT TCTGAAAGAT AATATTTGAT GATCCTGTCT TGTGCTGATG GGCAAAAGCT AAATTGTAAG   
  
  
+ AATATGTAGT TTATTTACAA GACATATGCT TAAGCTAGAG GTCATGTACT TGCATATTTT TGTGCAGCTT   
  
  
+ TTGAGGTTGT ATGCCCCTTT GAGACAAATT CATAGTTGAA TTCATGACAA GATTATGTTT GGCAATGACT   
  
  
+ TTGATTATTA AATTGGCCTT TATGCTATCC TATCCTGTGT GCTCAAATGT TATTTATCAG ATAATATTAG   
  
  
+ TTTACTCTTT CTTCCCTGTC TTGCAAAGAT TTTAAATCCT AAATTATGCA AATGTTTATC TTTAAAATGA   
  
  
+ CCTGCTACTC TCTGTGCCTG ATTTTATTTG TTTCCCCGTT GAAATTTTTT TTATGATTCC ACTTTTCAAG   
  
  
+ CAGCTTAGAC AGGCGTTATT AGGTAATTAT TTTCCGCTTT GGATTGTAGT AAATTGAAAC AGAAATGCCT   
  
  
+ CACTAAACCG CTCTCTGCTC TTTGTGTTGA AGAACCAATC CCATTCAAAC TCTTCTTTCT TTTTTGAAAT   
  
  
+ TCAATCCCGC TTCAGTCAAA TGTCTAACAG AAACAGCCTC GTGTGCAATT CTACTATATA CGGTTAATGC   
  
  
+ TGTGCATGTA TTACTTCCCA ACATCTTACC CAGGACTAGG AGGGACCTTG GTAGCCTGTT CTGCTTGAAA   
  
  
+ AATTTGTCAT TATCACCTGA ATTTGTATTA AATACATAAA TCTTTGTCTT TCAATTTGTG GCTCCACATT   
  
  
+ TCAATTTGTG GCTCCACACG CTTTTGAAAA CTTTATGAGA CCTAATATAT TAGAGAACAT CTGATCGTGA   
  
  
+ TTGTTAATTC CAGTTTTATA ATCATACTAA TTAGTAAATC ACTGAAGGTC ATATTGCATA TTGTTTTTGC   
  
  
+ CTAATGATTG GCTTATTCAA CTATTCCTGA ATAGTTTAGT TTCCAGAATC TTTAGAAGAA ACCTGGTCTG   
  
  
+ GGTATTATCG AGTATTTTTC TCAGTTCATG AAAGGCTTTG ATTCAGTGTG TGTGCTGTCA TGTCTAGGCA   
  
  
+ TGATTGAGTA TTTTTATTTG CATATCCAGG CATTGTGCCT AGACAAATGT CCTGAATCTT AGTGGCTGTT   
  
  
+ AGGTCTGAAC GGACACTTTT CTTGCTGTTT TTCCTTTCCG AGTGGCATCT TTAGCTTTTT CAAACTACCA   
  
  
+ AAGGACTATT AAACGAGGCC CTGCATATGC TGGAAAACTG CCTCTTAAGC TATGTTTATG CAAAAATGGA   
  
  
+ GGACCTTGAA CTGTTCAATG TACCTACAGT CTGAGGCACA GAGCCAATAG ACATAATAGA TAGTTGGATA   
  
  
+ TTCTTTTTAT ATTTAATTCA CAGTAGCGAC AACCCCAGCA TGAAACGTAG CCACGTAGGG GGGGAAAAAG   
  
  
+ GGTCTCCATC TGTTTATATA TTCTTGTCGC TTATGGTGCT TCTGTTATGT AATGACAAAG GAATTTGTGT   
  
  
+ TGGGATTATT CCGCTTCACT CCGTCTCACT AACTTTACGG TTTCAGTCCT AATTCTCTCT TTTTTTCTTT   
  
  
+ TGTTTTTTTT CCTGAGCTTG TTTAGTCTGT TTTCTTCTGT GTGTCTTTCT GATTATGGAA GCCATACAGG   
  
  
+ AGAAGAATGA CGATCTTTTG AGTCTTAGCT TGGCCATTGT TAGTCATTCT AACCATGCAG AAACGAAGGT   
  
  
+ GAAGAGGAAG AGGCATGATG TCTCTAATTC TCTGATCGCA ACAAGAGAAG AGGGTCGGGA TAAGAAAATA   
  
  
+ TTTCAGCTGC TTCAAGAACG AGAGAGAATG CTAAGCATCG GGCAAAGAAG AAAAGGCGTT ATTCAAGAAG   
  
  
+ GGAAGGGTCT TCATCTGATC CATTTGCTTC TCATGTCTGC TACCCTAATC AGCGAAAACA ATATCAGCTC   
  
  
+ AGCTGTTAAT CATTTGCTTG AGTTGTACAA GAATGTTTCT GTAAATGGTG ATTCTGGGCA GCGCGTTGCT   
  
  
+ GCTTATTTTG CTGATGGGTT AACTGCGAGG TTCTTAACCC AGAACTCTCC CTTTTATTGC ATGGTAATGA   
  
  
+ GTAAACCGAC ACCTGCAGAG GAGTTTATCG CCTTCACTCA TTTGTACAGG GTGTCTCCAT TCTATCAGTT   
  
  
+ TGCCCATTTT ACTGCTAATC AGGCCATCTT TGAGGCATTC GAGAGGGAAG AAGAGAACAA CAGCTGGGCA   
  
  
+ CTCCATGTCA TTGATTTTGA CATCTCTCAT GGCTTCCAGT GGCCGTCTCT CATTCAGTCT CTTTCTGAGA   
  
  
+ AGGCTACTGC TTCAAGCCAT TCACTCTCTC TTCGAATCAC AGGTTTTGCT AGAACTCTGG AAGAACTGAT   
  
  
+ TGAGACTGAG AACAGATTAG TAAGTTTTGC TGAAAGCTTC CGGAACATAC TATTTGAATT CCACGGGTGT   
  
  
+ TTGAGGGGTT CAGAGCTGGT GAACTTGAAG AAAAGGGAGA ATGAAACAGT TGCCGTAAAT CTGGTGTTTC   
  
  
+ ACCTCAGCAG TTTCGATGAC ACCCTGAAGG TCTCAGACAC CTTAACCGCT GTACATTCAC TGAAACCTTC   
  
  
+ TATAATGGTA CTAGTTGAAA GAGAAGGAAC CCGAAATGGA TCAGGATTCA TATCAGGTTT CATGGATTCT   
  
  
+ TTGCATTATT ATGCTGCAAT GTTCGATTCA TTAAATGATT GCCTCCCACG GGAGAGTCCC GAGAGACTGA   
  
  
+ GCATAGAGAA GAACCATCTT GGGAGAGAGA TCAAGGAGGC GGTGACTTGG GAGAAGGACG AGATGAGCTA   
  
  
+ CGGGAAGTTC GAGATAATGG AGACTTGGAA AAAGAGGATG GAAATTCATG GGTTTTCGGG GATCAGGCTG   
  
  
+ AGTTCCAAGG CGACAATTCA AGCCAAGCTC CTGCTCAAAA TGGGCAGCCA TTACTGTCCC CAGTTTGCAG   
  
  
+ GGGATTCTGA AGGAGGAGTT GGCGGGTTCA GAGTGTATGA GAGAGATGAG GGGGAAGCCA TTTCTCTGGT   
  
  
+ TTGGCAAGGC AGGTTCCTCG CAACAGCCTC TGCATGGCGC TGTGTATG  

- +Up\_Stream \_Len000CTTTCT TCTAAATTCC TTTAGCTACC ATGTGCCGTT TCGATCTTTG TCCTTGACAT   
  
  
- AAAAATACAA AAAAAAAACA ACCTTCCCCC CCCCCCCCTC GCCCGCTCTA CATTGGGCAT AGATCTACCT   
  
  
- ATTAGATGCA CTGTTCTGGA AGTAAACTAG TTCCATGTAG TACTTATAAA TTGGGAATTG AAGAAACTCG   
  
  
- GAATGTTCTC TAACAGACTC CAAAAATCCC ACGTGCTATA GTACGGACTG ATCTGGACAC ATCTGTGTTA   
  
  
- GAAGTCGGGA ATAACTATCG ACAAGCTATA CGTACGACGG ATTTTTAACC TTATCAACCG TGGATTGTAA   
  
  
- GAAAATGTAG AACGAGGAGG AACCACATCT CAATCTACAC GATCGTGTTA TGTTACCCAG GGCTATAAAA   
  
  
- CGTAGACCAA AGACTTTCTA TTATAAACTA CTAGGACAGA ACACGACTAC CCGTTTTCGA TTTAACATTC   
  
  
- TTATACATCA AATAAATGTT CTGTATACGA ATTCGATCTC CAGTACATGA ACGTATAAAA ACACGTCGAA   
  
  
- AACTCCAACA TACGGGGAAA CTCTGTTTAA GTATCAACTT AAGTACTGTT CTAATACAAA CCGTTACTGA   
  
  
- AACTAATAAT TTAACCGGAA ATACGATAGG ATAGGACACA CGAGTTTACA ATAAATAGTC TATTATAATC   
  
  
- AAATGAGAAA GAAGGGACAG AACGTTTCTA AAATTTAGGA TTTAATACGT TTACAAATAG AAATTTTACT   
  
  
- GGACGATGAG AGACACGGAC TAAAATAAAC AAAGGGGCAA CTTTAAAAAA AATACTAAGG TGAAAAGTTC   
  
  
- GTCGAATCTG TCCGCAATAA TCCATTAATA AAAGGCGAAA CCTAACATCA TTTAACTTTG TCTTTACGGA   
  
  
- GTGATTTGGC GAGAGACGAG AAACACAACT TCTTGGTTAG GGTAAGTTTG AGAAGAAAGA AAAAACTTTA   
  
  
- AGTTAGGGCG AAGTCAGTTT ACAGATTGTC TTTGTCGGAG CACACGTTAA GATGATATAT GCCAATTACG   
  
  
- ACACGTACAT AATGAAGGGT TGTAGAATGG GTCCTGATCC TCCCTGGAAC CATCGGACAA GACGAACTTT   
  
  
- TTAAACAGTA ATAGTGGACT TAAACATAAT TTATGTATTT AGAAACAGAA AGTTAAACAC CGAGGTGTAA   
  
  
- AGTTAAACAC CGAGGTGTGC GAAAACTTTT GAAATACTCT GGATTATATA ATCTCTTGTA GACTAGCACT   
  
  
- AACAATTAAG GTCAAAATAT TAGTATGATT AATCATTTAG TGACTTCCAG TATAACGTAT AACAAAAACG   
  
  
- GATTACTAAC CGAATAAGTT GATAAGGACT TATCAAATCA AAGGTCTTAG AAATCTTCTT TGGACCAGAC   
  
  
- CCATAATAGC TCATAAAAAG AGTCAAGTAC TTTCCGAAAC TAAGTCACAC ACACGACAGT ACAGATCCGT   
  
  
- ACTAACTCAT AAAAATAAAC GTATAGGTCC GTAACACGGA TCTGTTTACA GGACTTAGAA TCACCGACAA   
  
  
- TCCAGACTTG CCTGTGAAAA GAACGACAAA AAGGAAAGGC TCACCGTAGA AATCGAAAAA GTTTGATGGT   
  
  
- TTCCTGATAA TTTGCTCCGG GACGTATACG ACCTTTTGAC GGAGAATTCG ATACAAATAC GTTTTTACCT   
  
  
- CCTGGAACTT GACAAGTTAC ATGGATGTCA GACTCCGTGT CTCGGTTATC TGTATTATCT ATCAACCTAT   
  
  
- AAGAAAAATA TAAATTAAGT GTCATCGCTG TTGGGGTCGT ACTTTGCATC GGTGCATCCC CCCCTTTTTC   
  
  
- CCAGAGGTAG ACAAATATAT AAGAACAGCG AATACCACGA AGACAATACA TTACTGTTTC CTTAAACACA   
  
  
- ACCCTAATAA GGCGAAGTGA GGCAGAGTGA TTGAAATGCC AAAGTCAGGA TTAAGAGAGA AAAAAAGAAA   
  
  
- ACAAAAAAAA GGACTCGAAC AAATCAGACA AAAGAAGACA CACAGAAAGA CTAATACCTT CGGTATGTCC   
  
  
- TCTTCTTACT GCTAGAAAAC TCAGAATCGA ACCGGTAACA ATCAGTAAGA TTGGTACGTC TTTGCTTCCA   
  
  
- CTTCTCCTTC TCCGTACTAC AGAGATTAAG AGACTAGCGT TGTTCTCTTC TCCCAGCCCT ATTCTTTTAT   
  
  
- AAAGTCGACG AAGTTCTTGC TCTCTCTTAC GATTCGTAGC CCGTTTCTTC TTTTCCGCAA TAAGTTCTTC   
  
  
- CCTTCCCAGA AGTAGACTAG GTAAACGAAG AGTACAGACG ATGGGATTAG TCGCTTTTGT TATAGTCGAG   
  
  
- TCGACAATTA GTAAACGAAC TCAACATGTT CTTACAAAGA CATTTACCAC TAAGACCCGT CGCGCAACGA   
  
  
- CGAATAAAAC GACTACCCAA TTGACGCTCC AAGAATTGGG TCTTGAGAGG GAAAATAACG TACCATTACT   
  
  
- CATTTGGCTG TGGACGTCTC CTCAAATAGC GGAAGTGAGT AAACATGTCC CACAGAGGTA AGATAGTCAA   
  
  
- ACGGGTAAAA TGACGATTAG TCCGGTAGAA ACTCCGTAAG CTCTCCCTTC TTCTCTTGTT GTCGACCCGT   
  
  
- GAGGTACAGT AACTAAAACT GTAGAGAGTA CCGAAGGTCA CCGGCAGAGA GTAAGTCAGA GAAAGACTCT   
  
  
- TCCGATGACG AAGTTCGGTA AGTGAGAGAG AAGCTTAGTG TCCAAAACGA TCTTGAGACC TTCTTGACTA   
  
  
- ACTCTGACTC TTGTCTAATC ATTCAAAACG ACTTTCGAAG GCCTTGTATG ATAAACTTAA GGTGCCCACA   
  
  
- AACTCCCCAA GTCTCGACCA CTTGAACTTC TTTTCCCTCT TACTTTGTCA ACGGCATTTA GACCACAAAG   
  
  
- TGGAGTCGTC AAAGCTACTG TGGGACTTCC AGAGTCTGTG GAATTGGCGA CATGTAAGTG ACTTTGGAAG   
  
  
- ATATTACCAT GATCAACTTT CTCTTCCTTG GGCTTTACCT AGTCCTAAGT ATAGTCCAAA GTACCTAAGA   
  
  
- AACGTAATAA TACGACGTTA CAAGCTAAGT AATTTACTAA CGGAGGGTGC CCTCTCAGGG CTCTCTGACT   
  
  
- CGTATCTCTT CTTGGTAGAA CCCTCTCTCT AGTTCCTCCG CCACTGAACC CTCTTCCTGC TCTACTCGAT   
  
  
- GCCCTTCAAG CTCTATTACC TCTGAACCTT TTTCTCCTAC CTTTAAGTAC CCAAAAGCCC CTAGTCCGAC   
  
  
- TCAAGGTTCC GCTGTTAAGT TCGGTTCGAG GACGAGTTTT ACCCGTCGGT AATGACAGGG GTCAAACGTC   
  
  
- CCCTAAGACT TCCTCCTCAA CCGCCCAAGT CTCACATACT CTCTCTACTC CCCCTTCGGT AAAGAGACCA   
  
  
- AACCGTTCCG TCCAAGGAGC GTTGTCGGAG ACGTACCGCG ACACATAC

+     MYB-like sequence

| Site Name | Organism | Position | Strand | Matrix score. | sequence | function |
| --- | --- | --- | --- | --- | --- | --- |
| MYB-like sequence | Arabidopsis thaliana | 2084 | + | 6 | TAACCA |  |

>HU11G01529.1   
+ +Up\_Stream \_Len000GAAAGA AGATTTAAGG AAATCGATGG TACACGGCAA AGCTAGAAAC AGGAACTGTA   
  
  
+ TTTTTATGTT TTTTTTTTGT TGGAAGGGGG GGGGGGGGAG CGGGCGAGAT GTAACCCGTA TCTAGATGGA   
  
  
+ TAATCTACGT GACAAGACCT TCATTTGATC AAGGTACATC ATGAATATTT AACCCTTAAC TTCTTTGAGC   
  
  
+ CTTACAAGAG ATTGTCTGAG GTTTTTAGGG TGCACGATAT CATGCCTGAC TAGACCTGTG TAGACACAAT   
  
  
+ CTTCAGCCCT TATTGATAGC TGTTCGATAT GCATGCTGCC TAAAAATTGG AATAGTTGGC ACCTAACATT   
  
  
+ CTTTTACATC TTGCTCCTCC TTGGTGTAGA GTTAGATGTG CTAGCACAAT ACAATGGGTC CCGATATTTT   
  
  
+ GCATCTGGTT TCTGAAAGAT AATATTTGAT GATCCTGTCT TGTGCTGATG GGCAAAAGCT AAATTGTAAG   
  
  
+ AATATGTAGT TTATTTACAA GACATATGCT TAAGCTAGAG GTCATGTACT TGCATATTTT TGTGCAGCTT   
  
  
+ TTGAGGTTGT ATGCCCCTTT GAGACAAATT CATAGTTGAA TTCATGACAA GATTATGTTT GGCAATGACT   
  
  
+ TTGATTATTA AATTGGCCTT TATGCTATCC TATCCTGTGT GCTCAAATGT TATTTATCAG ATAATATTAG   
  
  
+ TTTACTCTTT CTTCCCTGTC TTGCAAAGAT TTTAAATCCT AAATTATGCA AATGTTTATC TTTAAAATGA   
  
  
+ CCTGCTACTC TCTGTGCCTG ATTTTATTTG TTTCCCCGTT GAAATTTTTT TTATGATTCC ACTTTTCAAG   
  
  
+ CAGCTTAGAC AGGCGTTATT AGGTAATTAT TTTCCGCTTT GGATTGTAGT AAATTGAAAC AGAAATGCCT   
  
  
+ CACTAAACCG CTCTCTGCTC TTTGTGTTGA AGAACCAATC CCATTCAAAC TCTTCTTTCT TTTTTGAAAT   
  
  
+ TCAATCCCGC TTCAGTCAAA TGTCTAACAG AAACAGCCTC GTGTGCAATT CTACTATATA CGGTTAATGC   
  
  
+ TGTGCATGTA TTACTTCCCA ACATCTTACC CAGGACTAGG AGGGACCTTG GTAGCCTGTT CTGCTTGAAA   
  
  
+ AATTTGTCAT TATCACCTGA ATTTGTATTA AATACATAAA TCTTTGTCTT TCAATTTGTG GCTCCACATT   
  
  
+ TCAATTTGTG GCTCCACACG CTTTTGAAAA CTTTATGAGA CCTAATATAT TAGAGAACAT CTGATCGTGA   
  
  
+ TTGTTAATTC CAGTTTTATA ATCATACTAA TTAGTAAATC ACTGAAGGTC ATATTGCATA TTGTTTTTGC   
  
  
+ CTAATGATTG GCTTATTCAA CTATTCCTGA ATAGTTTAGT TTCCAGAATC TTTAGAAGAA ACCTGGTCTG   
  
  
+ GGTATTATCG AGTATTTTTC TCAGTTCATG AAAGGCTTTG ATTCAGTGTG TGTGCTGTCA TGTCTAGGCA   
  
  
+ TGATTGAGTA TTTTTATTTG CATATCCAGG CATTGTGCCT AGACAAATGT CCTGAATCTT AGTGGCTGTT   
  
  
+ AGGTCTGAAC GGACACTTTT CTTGCTGTTT TTCCTTTCCG AGTGGCATCT TTAGCTTTTT CAAACTACCA   
  
  
+ AAGGACTATT AAACGAGGCC CTGCATATGC TGGAAAACTG CCTCTTAAGC TATGTTTATG CAAAAATGGA   
  
  
+ GGACCTTGAA CTGTTCAATG TACCTACAGT CTGAGGCACA GAGCCAATAG ACATAATAGA TAGTTGGATA   
  
  
+ TTCTTTTTAT ATTTAATTCA CAGTAGCGAC AACCCCAGCA TGAAACGTAG CCACGTAGGG GGGGAAAAAG   
  
  
+ GGTCTCCATC TGTTTATATA TTCTTGTCGC TTATGGTGCT TCTGTTATGT AATGACAAAG GAATTTGTGT   
  
  
+ TGGGATTATT CCGCTTCACT CCGTCTCACT AACTTTACGG TTTCAGTCCT AATTCTCTCT TTTTTTCTTT   
  
  
+ TGTTTTTTTT CCTGAGCTTG TTTAGTCTGT TTTCTTCTGT GTGTCTTTCT GATTATGGAA GCCATACAGG   
  
  
+ AGAAGAATGA CGATCTTTTG AGTCTTAGCT TGGCCATTGT TAGTCATTCT AACCATGCAG AAACGAAGGT   
  
  
+ GAAGAGGAAG AGGCATGATG TCTCTAATTC TCTGATCGCA ACAAGAGAAG AGGGTCGGGA TAAGAAAATA   
  
  
+ TTTCAGCTGC TTCAAGAACG AGAGAGAATG CTAAGCATCG GGCAAAGAAG AAAAGGCGTT ATTCAAGAAG   
  
  
+ GGAAGGGTCT TCATCTGATC CATTTGCTTC TCATGTCTGC TACCCTAATC AGCGAAAACA ATATCAGCTC   
  
  
+ AGCTGTTAAT CATTTGCTTG AGTTGTACAA GAATGTTTCT GTAAATGGTG ATTCTGGGCA GCGCGTTGCT   
  
  
+ GCTTATTTTG CTGATGGGTT AACTGCGAGG TTCTTAACCC AGAACTCTCC CTTTTATTGC ATGGTAATGA   
  
  
+ GTAAACCGAC ACCTGCAGAG GAGTTTATCG CCTTCACTCA TTTGTACAGG GTGTCTCCAT TCTATCAGTT   
  
  
+ TGCCCATTTT ACTGCTAATC AGGCCATCTT TGAGGCATTC GAGAGGGAAG AAGAGAACAA CAGCTGGGCA   
  
  
+ CTCCATGTCA TTGATTTTGA CATCTCTCAT GGCTTCCAGT GGCCGTCTCT CATTCAGTCT CTTTCTGAGA   
  
  
+ AGGCTACTGC TTCAAGCCAT TCACTCTCTC TTCGAATCAC AGGTTTTGCT AGAACTCTGG AAGAACTGAT   
  
  
+ TGAGACTGAG AACAGATTAG TAAGTTTTGC TGAAAGCTTC CGGAACATAC TATTTGAATT CCACGGGTGT   
  
  
+ TTGAGGGGTT CAGAGCTGGT GAACTTGAAG AAAAGGGAGA ATGAAACAGT TGCCGTAAAT CTGGTGTTTC   
  
  
+ ACCTCAGCAG TTTCGATGAC ACCCTGAAGG TCTCAGACAC CTTAACCGCT GTACATTCAC TGAAACCTTC   
  
  
+ TATAATGGTA CTAGTTGAAA GAGAAGGAAC CCGAAATGGA TCAGGATTCA TATCAGGTTT CATGGATTCT   
  
  
+ TTGCATTATT ATGCTGCAAT GTTCGATTCA TTAAATGATT GCCTCCCACG GGAGAGTCCC GAGAGACTGA   
  
  
+ GCATAGAGAA GAACCATCTT GGGAGAGAGA TCAAGGAGGC GGTGACTTGG GAGAAGGACG AGATGAGCTA   
  
  
+ CGGGAAGTTC GAGATAATGG AGACTTGGAA AAAGAGGATG GAAATTCATG GGTTTTCGGG GATCAGGCTG   
  
  
+ AGTTCCAAGG CGACAATTCA AGCCAAGCTC CTGCTCAAAA TGGGCAGCCA TTACTGTCCC CAGTTTGCAG   
  
  
+ GGGATTCTGA AGGAGGAGTT GGCGGGTTCA GAGTGTATGA GAGAGATGAG GGGGAAGCCA TTTCTCTGGT   
  
  
+ TTGGCAAGGC AGGTTCCTCG CAACAGCCTC TGCATGGCGC TGTGTATG  

- +Up\_Stream \_Len000CTTTCT TCTAAATTCC TTTAGCTACC ATGTGCCGTT TCGATCTTTG TCCTTGACAT   
  
  
- AAAAATACAA AAAAAAAACA ACCTTCCCCC CCCCCCCCTC GCCCGCTCTA CATTGGGCAT AGATCTACCT   
  
  
- ATTAGATGCA CTGTTCTGGA AGTAAACTAG TTCCATGTAG TACTTATAAA TTGGGAATTG AAGAAACTCG   
  
  
- GAATGTTCTC TAACAGACTC CAAAAATCCC ACGTGCTATA GTACGGACTG ATCTGGACAC ATCTGTGTTA   
  
  
- GAAGTCGGGA ATAACTATCG ACAAGCTATA CGTACGACGG ATTTTTAACC TTATCAACCG TGGATTGTAA   
  
  
- GAAAATGTAG AACGAGGAGG AACCACATCT CAATCTACAC GATCGTGTTA TGTTACCCAG GGCTATAAAA   
  
  
- CGTAGACCAA AGACTTTCTA TTATAAACTA CTAGGACAGA ACACGACTAC CCGTTTTCGA TTTAACATTC   
  
  
- TTATACATCA AATAAATGTT CTGTATACGA ATTCGATCTC CAGTACATGA ACGTATAAAA ACACGTCGAA   
  
  
- AACTCCAACA TACGGGGAAA CTCTGTTTAA GTATCAACTT AAGTACTGTT CTAATACAAA CCGTTACTGA   
  
  
- AACTAATAAT TTAACCGGAA ATACGATAGG ATAGGACACA CGAGTTTACA ATAAATAGTC TATTATAATC   
  
  
- AAATGAGAAA GAAGGGACAG AACGTTTCTA AAATTTAGGA TTTAATACGT TTACAAATAG AAATTTTACT   
  
  
- GGACGATGAG AGACACGGAC TAAAATAAAC AAAGGGGCAA CTTTAAAAAA AATACTAAGG TGAAAAGTTC   
  
  
- GTCGAATCTG TCCGCAATAA TCCATTAATA AAAGGCGAAA CCTAACATCA TTTAACTTTG TCTTTACGGA   
  
  
- GTGATTTGGC GAGAGACGAG AAACACAACT TCTTGGTTAG GGTAAGTTTG AGAAGAAAGA AAAAACTTTA   
  
  
- AGTTAGGGCG AAGTCAGTTT ACAGATTGTC TTTGTCGGAG CACACGTTAA GATGATATAT GCCAATTACG   
  
  
- ACACGTACAT AATGAAGGGT TGTAGAATGG GTCCTGATCC TCCCTGGAAC CATCGGACAA GACGAACTTT   
  
  
- TTAAACAGTA ATAGTGGACT TAAACATAAT TTATGTATTT AGAAACAGAA AGTTAAACAC CGAGGTGTAA   
  
  
- AGTTAAACAC CGAGGTGTGC GAAAACTTTT GAAATACTCT GGATTATATA ATCTCTTGTA GACTAGCACT   
  
  
- AACAATTAAG GTCAAAATAT TAGTATGATT AATCATTTAG TGACTTCCAG TATAACGTAT AACAAAAACG   
  
  
- GATTACTAAC CGAATAAGTT GATAAGGACT TATCAAATCA AAGGTCTTAG AAATCTTCTT TGGACCAGAC   
  
  
- CCATAATAGC TCATAAAAAG AGTCAAGTAC TTTCCGAAAC TAAGTCACAC ACACGACAGT ACAGATCCGT   
  
  
- ACTAACTCAT AAAAATAAAC GTATAGGTCC GTAACACGGA TCTGTTTACA GGACTTAGAA TCACCGACAA   
  
  
- TCCAGACTTG CCTGTGAAAA GAACGACAAA AAGGAAAGGC TCACCGTAGA AATCGAAAAA GTTTGATGGT   
  
  
- TTCCTGATAA TTTGCTCCGG GACGTATACG ACCTTTTGAC GGAGAATTCG ATACAAATAC GTTTTTACCT   
  
  
- CCTGGAACTT GACAAGTTAC ATGGATGTCA GACTCCGTGT CTCGGTTATC TGTATTATCT ATCAACCTAT   
  
  
- AAGAAAAATA TAAATTAAGT GTCATCGCTG TTGGGGTCGT ACTTTGCATC GGTGCATCCC CCCCTTTTTC   
  
  
- CCAGAGGTAG ACAAATATAT AAGAACAGCG AATACCACGA AGACAATACA TTACTGTTTC CTTAAACACA   
  
  
- ACCCTAATAA GGCGAAGTGA GGCAGAGTGA TTGAAATGCC AAAGTCAGGA TTAAGAGAGA AAAAAAGAAA   
  
  
- ACAAAAAAAA GGACTCGAAC AAATCAGACA AAAGAAGACA CACAGAAAGA CTAATACCTT CGGTATGTCC   
  
  
- TCTTCTTACT GCTAGAAAAC TCAGAATCGA ACCGGTAACA ATCAGTAAGA TTGGTACGTC TTTGCTTCCA   
  
  
- CTTCTCCTTC TCCGTACTAC AGAGATTAAG AGACTAGCGT TGTTCTCTTC TCCCAGCCCT ATTCTTTTAT   
  
  
- AAAGTCGACG AAGTTCTTGC TCTCTCTTAC GATTCGTAGC CCGTTTCTTC TTTTCCGCAA TAAGTTCTTC   
  
  
- CCTTCCCAGA AGTAGACTAG GTAAACGAAG AGTACAGACG ATGGGATTAG TCGCTTTTGT TATAGTCGAG   
  
  
- TCGACAATTA GTAAACGAAC TCAACATGTT CTTACAAAGA CATTTACCAC TAAGACCCGT CGCGCAACGA   
  
  
- CGAATAAAAC GACTACCCAA TTGACGCTCC AAGAATTGGG TCTTGAGAGG GAAAATAACG TACCATTACT   
  
  
- CATTTGGCTG TGGACGTCTC CTCAAATAGC GGAAGTGAGT AAACATGTCC CACAGAGGTA AGATAGTCAA   
  
  
- ACGGGTAAAA TGACGATTAG TCCGGTAGAA ACTCCGTAAG CTCTCCCTTC TTCTCTTGTT GTCGACCCGT   
  
  
- GAGGTACAGT AACTAAAACT GTAGAGAGTA CCGAAGGTCA CCGGCAGAGA GTAAGTCAGA GAAAGACTCT   
  
  
- TCCGATGACG AAGTTCGGTA AGTGAGAGAG AAGCTTAGTG TCCAAAACGA TCTTGAGACC TTCTTGACTA   
  
  
- ACTCTGACTC TTGTCTAATC ATTCAAAACG ACTTTCGAAG GCCTTGTATG ATAAACTTAA GGTGCCCACA   
  
  
- AACTCCCCAA GTCTCGACCA CTTGAACTTC TTTTCCCTCT TACTTTGTCA ACGGCATTTA GACCACAAAG   
  
  
- TGGAGTCGTC AAAGCTACTG TGGGACTTCC AGAGTCTGTG GAATTGGCGA CATGTAAGTG ACTTTGGAAG   
  
  
- ATATTACCAT GATCAACTTT CTCTTCCTTG GGCTTTACCT AGTCCTAAGT ATAGTCCAAA GTACCTAAGA   
  
  
- AACGTAATAA TACGACGTTA CAAGCTAAGT AATTTACTAA CGGAGGGTGC CCTCTCAGGG CTCTCTGACT   
  
  
- CGTATCTCTT CTTGGTAGAA CCCTCTCTCT AGTTCCTCCG CCACTGAACC CTCTTCCTGC TCTACTCGAT   
  
  
- GCCCTTCAAG CTCTATTACC TCTGAACCTT TTTCTCCTAC CTTTAAGTAC CCAAAAGCCC CTAGTCCGAC   
  
  
- TCAAGGTTCC GCTGTTAAGT TCGGTTCGAG GACGAGTTTT ACCCGTCGGT AATGACAGGG GTCAAACGTC   
  
  
- CCCTAAGACT TCCTCCTCAA CCGCCCAAGT CTCACATACT CTCTCTACTC CCCCTTCGGT AAAGAGACCA   
  
  
- AACCGTTCCG TCCAAGGAGC GTTGTCGGAG ACGTACCGCG ACACATAC

+     MYC

| Site Name | Organism | Position | Strand | Matrix score. | sequence | function |
| --- | --- | --- | --- | --- | --- | --- |
| MYC | Arabidopsis thaliana | 753 | - | 6 | CATTTG |  |
| MYC | Arabidopsis thaliana | 1518 | - | 6 | CATTTG |  |
| MYC | Arabidopsis thaliana | 2265 | + | 6 | CATTTG |  |
| MYC | Arabidopsis thaliana | 1001 | - | 6 | CATTTG |  |
| MYC | Arabidopsis thaliana | 166 | + | 6 | CATTTG |  |
| MYC | Arabidopsis thaliana | 2325 | + | 6 | CATTTG |  |
| MYC | Arabidopsis thaliana | 678 | - | 6 | CATTTG |  |
| MYC | Arabidopsis thaliana | 2493 | + | 6 | CATTTG |  |

>HU11G01529.1   
+ +Up\_Stream \_Len000GAAAGA AGATTTAAGG AAATCGATGG TACACGGCAA AGCTAGAAAC AGGAACTGTA   
  
  
+ TTTTTATGTT TTTTTTTTGT TGGAAGGGGG GGGGGGGGAG CGGGCGAGAT GTAACCCGTA TCTAGATGGA   
  
  
+ TAATCTACGT GACAAGACCT TCATTTGATC AAGGTACATC ATGAATATTT AACCCTTAAC TTCTTTGAGC   
  
  
+ CTTACAAGAG ATTGTCTGAG GTTTTTAGGG TGCACGATAT CATGCCTGAC TAGACCTGTG TAGACACAAT   
  
  
+ CTTCAGCCCT TATTGATAGC TGTTCGATAT GCATGCTGCC TAAAAATTGG AATAGTTGGC ACCTAACATT   
  
  
+ CTTTTACATC TTGCTCCTCC TTGGTGTAGA GTTAGATGTG CTAGCACAAT ACAATGGGTC CCGATATTTT   
  
  
+ GCATCTGGTT TCTGAAAGAT AATATTTGAT GATCCTGTCT TGTGCTGATG GGCAAAAGCT AAATTGTAAG   
  
  
+ AATATGTAGT TTATTTACAA GACATATGCT TAAGCTAGAG GTCATGTACT TGCATATTTT TGTGCAGCTT   
  
  
+ TTGAGGTTGT ATGCCCCTTT GAGACAAATT CATAGTTGAA TTCATGACAA GATTATGTTT GGCAATGACT   
  
  
+ TTGATTATTA AATTGGCCTT TATGCTATCC TATCCTGTGT GCTCAAATGT TATTTATCAG ATAATATTAG   
  
  
+ TTTACTCTTT CTTCCCTGTC TTGCAAAGAT TTTAAATCCT AAATTATGCA AATGTTTATC TTTAAAATGA   
  
  
+ CCTGCTACTC TCTGTGCCTG ATTTTATTTG TTTCCCCGTT GAAATTTTTT TTATGATTCC ACTTTTCAAG   
  
  
+ CAGCTTAGAC AGGCGTTATT AGGTAATTAT TTTCCGCTTT GGATTGTAGT AAATTGAAAC AGAAATGCCT   
  
  
+ CACTAAACCG CTCTCTGCTC TTTGTGTTGA AGAACCAATC CCATTCAAAC TCTTCTTTCT TTTTTGAAAT   
  
  
+ TCAATCCCGC TTCAGTCAAA TGTCTAACAG AAACAGCCTC GTGTGCAATT CTACTATATA CGGTTAATGC   
  
  
+ TGTGCATGTA TTACTTCCCA ACATCTTACC CAGGACTAGG AGGGACCTTG GTAGCCTGTT CTGCTTGAAA   
  
  
+ AATTTGTCAT TATCACCTGA ATTTGTATTA AATACATAAA TCTTTGTCTT TCAATTTGTG GCTCCACATT   
  
  
+ TCAATTTGTG GCTCCACACG CTTTTGAAAA CTTTATGAGA CCTAATATAT TAGAGAACAT CTGATCGTGA   
  
  
+ TTGTTAATTC CAGTTTTATA ATCATACTAA TTAGTAAATC ACTGAAGGTC ATATTGCATA TTGTTTTTGC   
  
  
+ CTAATGATTG GCTTATTCAA CTATTCCTGA ATAGTTTAGT TTCCAGAATC TTTAGAAGAA ACCTGGTCTG   
  
  
+ GGTATTATCG AGTATTTTTC TCAGTTCATG AAAGGCTTTG ATTCAGTGTG TGTGCTGTCA TGTCTAGGCA   
  
  
+ TGATTGAGTA TTTTTATTTG CATATCCAGG CATTGTGCCT AGACAAATGT CCTGAATCTT AGTGGCTGTT   
  
  
+ AGGTCTGAAC GGACACTTTT CTTGCTGTTT TTCCTTTCCG AGTGGCATCT TTAGCTTTTT CAAACTACCA   
  
  
+ AAGGACTATT AAACGAGGCC CTGCATATGC TGGAAAACTG CCTCTTAAGC TATGTTTATG CAAAAATGGA   
  
  
+ GGACCTTGAA CTGTTCAATG TACCTACAGT CTGAGGCACA GAGCCAATAG ACATAATAGA TAGTTGGATA   
  
  
+ TTCTTTTTAT ATTTAATTCA CAGTAGCGAC AACCCCAGCA TGAAACGTAG CCACGTAGGG GGGGAAAAAG   
  
  
+ GGTCTCCATC TGTTTATATA TTCTTGTCGC TTATGGTGCT TCTGTTATGT AATGACAAAG GAATTTGTGT   
  
  
+ TGGGATTATT CCGCTTCACT CCGTCTCACT AACTTTACGG TTTCAGTCCT AATTCTCTCT TTTTTTCTTT   
  
  
+ TGTTTTTTTT CCTGAGCTTG TTTAGTCTGT TTTCTTCTGT GTGTCTTTCT GATTATGGAA GCCATACAGG   
  
  
+ AGAAGAATGA CGATCTTTTG AGTCTTAGCT TGGCCATTGT TAGTCATTCT AACCATGCAG AAACGAAGGT   
  
  
+ GAAGAGGAAG AGGCATGATG TCTCTAATTC TCTGATCGCA ACAAGAGAAG AGGGTCGGGA TAAGAAAATA   
  
  
+ TTTCAGCTGC TTCAAGAACG AGAGAGAATG CTAAGCATCG GGCAAAGAAG AAAAGGCGTT ATTCAAGAAG   
  
  
+ GGAAGGGTCT TCATCTGATC CATTTGCTTC TCATGTCTGC TACCCTAATC AGCGAAAACA ATATCAGCTC   
  
  
+ AGCTGTTAAT CATTTGCTTG AGTTGTACAA GAATGTTTCT GTAAATGGTG ATTCTGGGCA GCGCGTTGCT   
  
  
+ GCTTATTTTG CTGATGGGTT AACTGCGAGG TTCTTAACCC AGAACTCTCC CTTTTATTGC ATGGTAATGA   
  
  
+ GTAAACCGAC ACCTGCAGAG GAGTTTATCG CCTTCACTCA TTTGTACAGG GTGTCTCCAT TCTATCAGTT   
  
  
+ TGCCCATTTT ACTGCTAATC AGGCCATCTT TGAGGCATTC GAGAGGGAAG AAGAGAACAA CAGCTGGGCA   
  
  
+ CTCCATGTCA TTGATTTTGA CATCTCTCAT GGCTTCCAGT GGCCGTCTCT CATTCAGTCT CTTTCTGAGA   
  
  
+ AGGCTACTGC TTCAAGCCAT TCACTCTCTC TTCGAATCAC AGGTTTTGCT AGAACTCTGG AAGAACTGAT   
  
  
+ TGAGACTGAG AACAGATTAG TAAGTTTTGC TGAAAGCTTC CGGAACATAC TATTTGAATT CCACGGGTGT   
  
  
+ TTGAGGGGTT CAGAGCTGGT GAACTTGAAG AAAAGGGAGA ATGAAACAGT TGCCGTAAAT CTGGTGTTTC   
  
  
+ ACCTCAGCAG TTTCGATGAC ACCCTGAAGG TCTCAGACAC CTTAACCGCT GTACATTCAC TGAAACCTTC   
  
  
+ TATAATGGTA CTAGTTGAAA GAGAAGGAAC CCGAAATGGA TCAGGATTCA TATCAGGTTT CATGGATTCT   
  
  
+ TTGCATTATT ATGCTGCAAT GTTCGATTCA TTAAATGATT GCCTCCCACG GGAGAGTCCC GAGAGACTGA   
  
  
+ GCATAGAGAA GAACCATCTT GGGAGAGAGA TCAAGGAGGC GGTGACTTGG GAGAAGGACG AGATGAGCTA   
  
  
+ CGGGAAGTTC GAGATAATGG AGACTTGGAA AAAGAGGATG GAAATTCATG GGTTTTCGGG GATCAGGCTG   
  
  
+ AGTTCCAAGG CGACAATTCA AGCCAAGCTC CTGCTCAAAA TGGGCAGCCA TTACTGTCCC CAGTTTGCAG   
  
  
+ GGGATTCTGA AGGAGGAGTT GGCGGGTTCA GAGTGTATGA GAGAGATGAG GGGGAAGCCA TTTCTCTGGT   
  
  
+ TTGGCAAGGC AGGTTCCTCG CAACAGCCTC TGCATGGCGC TGTGTATG  

- +Up\_Stream \_Len000CTTTCT TCTAAATTCC TTTAGCTACC ATGTGCCGTT TCGATCTTTG TCCTTGACAT   
  
  
- AAAAATACAA AAAAAAAACA ACCTTCCCCC CCCCCCCCTC GCCCGCTCTA CATTGGGCAT AGATCTACCT   
  
  
- ATTAGATGCA CTGTTCTGGA AGTAAACTAG TTCCATGTAG TACTTATAAA TTGGGAATTG AAGAAACTCG   
  
  
- GAATGTTCTC TAACAGACTC CAAAAATCCC ACGTGCTATA GTACGGACTG ATCTGGACAC ATCTGTGTTA   
  
  
- GAAGTCGGGA ATAACTATCG ACAAGCTATA CGTACGACGG ATTTTTAACC TTATCAACCG TGGATTGTAA   
  
  
- GAAAATGTAG AACGAGGAGG AACCACATCT CAATCTACAC GATCGTGTTA TGTTACCCAG GGCTATAAAA   
  
  
- CGTAGACCAA AGACTTTCTA TTATAAACTA CTAGGACAGA ACACGACTAC CCGTTTTCGA TTTAACATTC   
  
  
- TTATACATCA AATAAATGTT CTGTATACGA ATTCGATCTC CAGTACATGA ACGTATAAAA ACACGTCGAA   
  
  
- AACTCCAACA TACGGGGAAA CTCTGTTTAA GTATCAACTT AAGTACTGTT CTAATACAAA CCGTTACTGA   
  
  
- AACTAATAAT TTAACCGGAA ATACGATAGG ATAGGACACA CGAGTTTACA ATAAATAGTC TATTATAATC   
  
  
- AAATGAGAAA GAAGGGACAG AACGTTTCTA AAATTTAGGA TTTAATACGT TTACAAATAG AAATTTTACT   
  
  
- GGACGATGAG AGACACGGAC TAAAATAAAC AAAGGGGCAA CTTTAAAAAA AATACTAAGG TGAAAAGTTC   
  
  
- GTCGAATCTG TCCGCAATAA TCCATTAATA AAAGGCGAAA CCTAACATCA TTTAACTTTG TCTTTACGGA   
  
  
- GTGATTTGGC GAGAGACGAG AAACACAACT TCTTGGTTAG GGTAAGTTTG AGAAGAAAGA AAAAACTTTA   
  
  
- AGTTAGGGCG AAGTCAGTTT ACAGATTGTC TTTGTCGGAG CACACGTTAA GATGATATAT GCCAATTACG   
  
  
- ACACGTACAT AATGAAGGGT TGTAGAATGG GTCCTGATCC TCCCTGGAAC CATCGGACAA GACGAACTTT   
  
  
- TTAAACAGTA ATAGTGGACT TAAACATAAT TTATGTATTT AGAAACAGAA AGTTAAACAC CGAGGTGTAA   
  
  
- AGTTAAACAC CGAGGTGTGC GAAAACTTTT GAAATACTCT GGATTATATA ATCTCTTGTA GACTAGCACT   
  
  
- AACAATTAAG GTCAAAATAT TAGTATGATT AATCATTTAG TGACTTCCAG TATAACGTAT AACAAAAACG   
  
  
- GATTACTAAC CGAATAAGTT GATAAGGACT TATCAAATCA AAGGTCTTAG AAATCTTCTT TGGACCAGAC   
  
  
- CCATAATAGC TCATAAAAAG AGTCAAGTAC TTTCCGAAAC TAAGTCACAC ACACGACAGT ACAGATCCGT   
  
  
- ACTAACTCAT AAAAATAAAC GTATAGGTCC GTAACACGGA TCTGTTTACA GGACTTAGAA TCACCGACAA   
  
  
- TCCAGACTTG CCTGTGAAAA GAACGACAAA AAGGAAAGGC TCACCGTAGA AATCGAAAAA GTTTGATGGT   
  
  
- TTCCTGATAA TTTGCTCCGG GACGTATACG ACCTTTTGAC GGAGAATTCG ATACAAATAC GTTTTTACCT   
  
  
- CCTGGAACTT GACAAGTTAC ATGGATGTCA GACTCCGTGT CTCGGTTATC TGTATTATCT ATCAACCTAT   
  
  
- AAGAAAAATA TAAATTAAGT GTCATCGCTG TTGGGGTCGT ACTTTGCATC GGTGCATCCC CCCCTTTTTC   
  
  
- CCAGAGGTAG ACAAATATAT AAGAACAGCG AATACCACGA AGACAATACA TTACTGTTTC CTTAAACACA   
  
  
- ACCCTAATAA GGCGAAGTGA GGCAGAGTGA TTGAAATGCC AAAGTCAGGA TTAAGAGAGA AAAAAAGAAA   
  
  
- ACAAAAAAAA GGACTCGAAC AAATCAGACA AAAGAAGACA CACAGAAAGA CTAATACCTT CGGTATGTCC   
  
  
- TCTTCTTACT GCTAGAAAAC TCAGAATCGA ACCGGTAACA ATCAGTAAGA TTGGTACGTC TTTGCTTCCA   
  
  
- CTTCTCCTTC TCCGTACTAC AGAGATTAAG AGACTAGCGT TGTTCTCTTC TCCCAGCCCT ATTCTTTTAT   
  
  
- AAAGTCGACG AAGTTCTTGC TCTCTCTTAC GATTCGTAGC CCGTTTCTTC TTTTCCGCAA TAAGTTCTTC   
  
  
- CCTTCCCAGA AGTAGACTAG GTAAACGAAG AGTACAGACG ATGGGATTAG TCGCTTTTGT TATAGTCGAG   
  
  
- TCGACAATTA GTAAACGAAC TCAACATGTT CTTACAAAGA CATTTACCAC TAAGACCCGT CGCGCAACGA   
  
  
- CGAATAAAAC GACTACCCAA TTGACGCTCC AAGAATTGGG TCTTGAGAGG GAAAATAACG TACCATTACT   
  
  
- CATTTGGCTG TGGACGTCTC CTCAAATAGC GGAAGTGAGT AAACATGTCC CACAGAGGTA AGATAGTCAA   
  
  
- ACGGGTAAAA TGACGATTAG TCCGGTAGAA ACTCCGTAAG CTCTCCCTTC TTCTCTTGTT GTCGACCCGT   
  
  
- GAGGTACAGT AACTAAAACT GTAGAGAGTA CCGAAGGTCA CCGGCAGAGA GTAAGTCAGA GAAAGACTCT   
  
  
- TCCGATGACG AAGTTCGGTA AGTGAGAGAG AAGCTTAGTG TCCAAAACGA TCTTGAGACC TTCTTGACTA   
  
  
- ACTCTGACTC TTGTCTAATC ATTCAAAACG ACTTTCGAAG GCCTTGTATG ATAAACTTAA GGTGCCCACA   
  
  
- AACTCCCCAA GTCTCGACCA CTTGAACTTC TTTTCCCTCT TACTTTGTCA ACGGCATTTA GACCACAAAG   
  
  
- TGGAGTCGTC AAAGCTACTG TGGGACTTCC AGAGTCTGTG GAATTGGCGA CATGTAAGTG ACTTTGGAAG   
  
  
- ATATTACCAT GATCAACTTT CTCTTCCTTG GGCTTTACCT AGTCCTAAGT ATAGTCCAAA GTACCTAAGA   
  
  
- AACGTAATAA TACGACGTTA CAAGCTAAGT AATTTACTAA CGGAGGGTGC CCTCTCAGGG CTCTCTGACT   
  
  
- CGTATCTCTT CTTGGTAGAA CCCTCTCTCT AGTTCCTCCG CCACTGAACC CTCTTCCTGC TCTACTCGAT   
  
  
- GCCCTTCAAG CTCTATTACC TCTGAACCTT TTTCTCCTAC CTTTAAGTAC CCAAAAGCCC CTAGTCCGAC   
  
  
- TCAAGGTTCC GCTGTTAAGT TCGGTTCGAG GACGAGTTTT ACCCGTCGGT AATGACAGGG GTCAAACGTC   
  
  
- CCCTAAGACT TCCTCCTCAA CCGCCCAAGT CTCACATACT CTCTCTACTC CCCCTTCGGT AAAGAGACCA   
  
  
- AACCGTTCCG TCCAAGGAGC GTTGTCGGAG ACGTACCGCG ACACATAC

+     Myb

| Site Name | Organism | Position | Strand | Matrix score. | sequence | function |
| --- | --- | --- | --- | --- | --- | --- |
| Myb | Arabidopsis thaliana | 2851 | - | 6 | CAACTG |  |
| Myb | Arabidopsis thaliana | 2404 | + | 6 | TAACTG |  |

>HU11G01529.1   
+ +Up\_Stream \_Len000GAAAGA AGATTTAAGG AAATCGATGG TACACGGCAA AGCTAGAAAC AGGAACTGTA   
  
  
+ TTTTTATGTT TTTTTTTTGT TGGAAGGGGG GGGGGGGGAG CGGGCGAGAT GTAACCCGTA TCTAGATGGA   
  
  
+ TAATCTACGT GACAAGACCT TCATTTGATC AAGGTACATC ATGAATATTT AACCCTTAAC TTCTTTGAGC   
  
  
+ CTTACAAGAG ATTGTCTGAG GTTTTTAGGG TGCACGATAT CATGCCTGAC TAGACCTGTG TAGACACAAT   
  
  
+ CTTCAGCCCT TATTGATAGC TGTTCGATAT GCATGCTGCC TAAAAATTGG AATAGTTGGC ACCTAACATT   
  
  
+ CTTTTACATC TTGCTCCTCC TTGGTGTAGA GTTAGATGTG CTAGCACAAT ACAATGGGTC CCGATATTTT   
  
  
+ GCATCTGGTT TCTGAAAGAT AATATTTGAT GATCCTGTCT TGTGCTGATG GGCAAAAGCT AAATTGTAAG   
  
  
+ AATATGTAGT TTATTTACAA GACATATGCT TAAGCTAGAG GTCATGTACT TGCATATTTT TGTGCAGCTT   
  
  
+ TTGAGGTTGT ATGCCCCTTT GAGACAAATT CATAGTTGAA TTCATGACAA GATTATGTTT GGCAATGACT   
  
  
+ TTGATTATTA AATTGGCCTT TATGCTATCC TATCCTGTGT GCTCAAATGT TATTTATCAG ATAATATTAG   
  
  
+ TTTACTCTTT CTTCCCTGTC TTGCAAAGAT TTTAAATCCT AAATTATGCA AATGTTTATC TTTAAAATGA   
  
  
+ CCTGCTACTC TCTGTGCCTG ATTTTATTTG TTTCCCCGTT GAAATTTTTT TTATGATTCC ACTTTTCAAG   
  
  
+ CAGCTTAGAC AGGCGTTATT AGGTAATTAT TTTCCGCTTT GGATTGTAGT AAATTGAAAC AGAAATGCCT   
  
  
+ CACTAAACCG CTCTCTGCTC TTTGTGTTGA AGAACCAATC CCATTCAAAC TCTTCTTTCT TTTTTGAAAT   
  
  
+ TCAATCCCGC TTCAGTCAAA TGTCTAACAG AAACAGCCTC GTGTGCAATT CTACTATATA CGGTTAATGC   
  
  
+ TGTGCATGTA TTACTTCCCA ACATCTTACC CAGGACTAGG AGGGACCTTG GTAGCCTGTT CTGCTTGAAA   
  
  
+ AATTTGTCAT TATCACCTGA ATTTGTATTA AATACATAAA TCTTTGTCTT TCAATTTGTG GCTCCACATT   
  
  
+ TCAATTTGTG GCTCCACACG CTTTTGAAAA CTTTATGAGA CCTAATATAT TAGAGAACAT CTGATCGTGA   
  
  
+ TTGTTAATTC CAGTTTTATA ATCATACTAA TTAGTAAATC ACTGAAGGTC ATATTGCATA TTGTTTTTGC   
  
  
+ CTAATGATTG GCTTATTCAA CTATTCCTGA ATAGTTTAGT TTCCAGAATC TTTAGAAGAA ACCTGGTCTG   
  
  
+ GGTATTATCG AGTATTTTTC TCAGTTCATG AAAGGCTTTG ATTCAGTGTG TGTGCTGTCA TGTCTAGGCA   
  
  
+ TGATTGAGTA TTTTTATTTG CATATCCAGG CATTGTGCCT AGACAAATGT CCTGAATCTT AGTGGCTGTT   
  
  
+ AGGTCTGAAC GGACACTTTT CTTGCTGTTT TTCCTTTCCG AGTGGCATCT TTAGCTTTTT CAAACTACCA   
  
  
+ AAGGACTATT AAACGAGGCC CTGCATATGC TGGAAAACTG CCTCTTAAGC TATGTTTATG CAAAAATGGA   
  
  
+ GGACCTTGAA CTGTTCAATG TACCTACAGT CTGAGGCACA GAGCCAATAG ACATAATAGA TAGTTGGATA   
  
  
+ TTCTTTTTAT ATTTAATTCA CAGTAGCGAC AACCCCAGCA TGAAACGTAG CCACGTAGGG GGGGAAAAAG   
  
  
+ GGTCTCCATC TGTTTATATA TTCTTGTCGC TTATGGTGCT TCTGTTATGT AATGACAAAG GAATTTGTGT   
  
  
+ TGGGATTATT CCGCTTCACT CCGTCTCACT AACTTTACGG TTTCAGTCCT AATTCTCTCT TTTTTTCTTT   
  
  
+ TGTTTTTTTT CCTGAGCTTG TTTAGTCTGT TTTCTTCTGT GTGTCTTTCT GATTATGGAA GCCATACAGG   
  
  
+ AGAAGAATGA CGATCTTTTG AGTCTTAGCT TGGCCATTGT TAGTCATTCT AACCATGCAG AAACGAAGGT   
  
  
+ GAAGAGGAAG AGGCATGATG TCTCTAATTC TCTGATCGCA ACAAGAGAAG AGGGTCGGGA TAAGAAAATA   
  
  
+ TTTCAGCTGC TTCAAGAACG AGAGAGAATG CTAAGCATCG GGCAAAGAAG AAAAGGCGTT ATTCAAGAAG   
  
  
+ GGAAGGGTCT TCATCTGATC CATTTGCTTC TCATGTCTGC TACCCTAATC AGCGAAAACA ATATCAGCTC   
  
  
+ AGCTGTTAAT CATTTGCTTG AGTTGTACAA GAATGTTTCT GTAAATGGTG ATTCTGGGCA GCGCGTTGCT   
  
  
+ GCTTATTTTG CTGATGGGTT AACTGCGAGG TTCTTAACCC AGAACTCTCC CTTTTATTGC ATGGTAATGA   
  
  
+ GTAAACCGAC ACCTGCAGAG GAGTTTATCG CCTTCACTCA TTTGTACAGG GTGTCTCCAT TCTATCAGTT   
  
  
+ TGCCCATTTT ACTGCTAATC AGGCCATCTT TGAGGCATTC GAGAGGGAAG AAGAGAACAA CAGCTGGGCA   
  
  
+ CTCCATGTCA TTGATTTTGA CATCTCTCAT GGCTTCCAGT GGCCGTCTCT CATTCAGTCT CTTTCTGAGA   
  
  
+ AGGCTACTGC TTCAAGCCAT TCACTCTCTC TTCGAATCAC AGGTTTTGCT AGAACTCTGG AAGAACTGAT   
  
  
+ TGAGACTGAG AACAGATTAG TAAGTTTTGC TGAAAGCTTC CGGAACATAC TATTTGAATT CCACGGGTGT   
  
  
+ TTGAGGGGTT CAGAGCTGGT GAACTTGAAG AAAAGGGAGA ATGAAACAGT TGCCGTAAAT CTGGTGTTTC   
  
  
+ ACCTCAGCAG TTTCGATGAC ACCCTGAAGG TCTCAGACAC CTTAACCGCT GTACATTCAC TGAAACCTTC   
  
  
+ TATAATGGTA CTAGTTGAAA GAGAAGGAAC CCGAAATGGA TCAGGATTCA TATCAGGTTT CATGGATTCT   
  
  
+ TTGCATTATT ATGCTGCAAT GTTCGATTCA TTAAATGATT GCCTCCCACG GGAGAGTCCC GAGAGACTGA   
  
  
+ GCATAGAGAA GAACCATCTT GGGAGAGAGA TCAAGGAGGC GGTGACTTGG GAGAAGGACG AGATGAGCTA   
  
  
+ CGGGAAGTTC GAGATAATGG AGACTTGGAA AAAGAGGATG GAAATTCATG GGTTTTCGGG GATCAGGCTG   
  
  
+ AGTTCCAAGG CGACAATTCA AGCCAAGCTC CTGCTCAAAA TGGGCAGCCA TTACTGTCCC CAGTTTGCAG   
  
  
+ GGGATTCTGA AGGAGGAGTT GGCGGGTTCA GAGTGTATGA GAGAGATGAG GGGGAAGCCA TTTCTCTGGT   
  
  
+ TTGGCAAGGC AGGTTCCTCG CAACAGCCTC TGCATGGCGC TGTGTATG  

- +Up\_Stream \_Len000CTTTCT TCTAAATTCC TTTAGCTACC ATGTGCCGTT TCGATCTTTG TCCTTGACAT   
  
  
- AAAAATACAA AAAAAAAACA ACCTTCCCCC CCCCCCCCTC GCCCGCTCTA CATTGGGCAT AGATCTACCT   
  
  
- ATTAGATGCA CTGTTCTGGA AGTAAACTAG TTCCATGTAG TACTTATAAA TTGGGAATTG AAGAAACTCG   
  
  
- GAATGTTCTC TAACAGACTC CAAAAATCCC ACGTGCTATA GTACGGACTG ATCTGGACAC ATCTGTGTTA   
  
  
- GAAGTCGGGA ATAACTATCG ACAAGCTATA CGTACGACGG ATTTTTAACC TTATCAACCG TGGATTGTAA   
  
  
- GAAAATGTAG AACGAGGAGG AACCACATCT CAATCTACAC GATCGTGTTA TGTTACCCAG GGCTATAAAA   
  
  
- CGTAGACCAA AGACTTTCTA TTATAAACTA CTAGGACAGA ACACGACTAC CCGTTTTCGA TTTAACATTC   
  
  
- TTATACATCA AATAAATGTT CTGTATACGA ATTCGATCTC CAGTACATGA ACGTATAAAA ACACGTCGAA   
  
  
- AACTCCAACA TACGGGGAAA CTCTGTTTAA GTATCAACTT AAGTACTGTT CTAATACAAA CCGTTACTGA   
  
  
- AACTAATAAT TTAACCGGAA ATACGATAGG ATAGGACACA CGAGTTTACA ATAAATAGTC TATTATAATC   
  
  
- AAATGAGAAA GAAGGGACAG AACGTTTCTA AAATTTAGGA TTTAATACGT TTACAAATAG AAATTTTACT   
  
  
- GGACGATGAG AGACACGGAC TAAAATAAAC AAAGGGGCAA CTTTAAAAAA AATACTAAGG TGAAAAGTTC   
  
  
- GTCGAATCTG TCCGCAATAA TCCATTAATA AAAGGCGAAA CCTAACATCA TTTAACTTTG TCTTTACGGA   
  
  
- GTGATTTGGC GAGAGACGAG AAACACAACT TCTTGGTTAG GGTAAGTTTG AGAAGAAAGA AAAAACTTTA   
  
  
- AGTTAGGGCG AAGTCAGTTT ACAGATTGTC TTTGTCGGAG CACACGTTAA GATGATATAT GCCAATTACG   
  
  
- ACACGTACAT AATGAAGGGT TGTAGAATGG GTCCTGATCC TCCCTGGAAC CATCGGACAA GACGAACTTT   
  
  
- TTAAACAGTA ATAGTGGACT TAAACATAAT TTATGTATTT AGAAACAGAA AGTTAAACAC CGAGGTGTAA   
  
  
- AGTTAAACAC CGAGGTGTGC GAAAACTTTT GAAATACTCT GGATTATATA ATCTCTTGTA GACTAGCACT   
  
  
- AACAATTAAG GTCAAAATAT TAGTATGATT AATCATTTAG TGACTTCCAG TATAACGTAT AACAAAAACG   
  
  
- GATTACTAAC CGAATAAGTT GATAAGGACT TATCAAATCA AAGGTCTTAG AAATCTTCTT TGGACCAGAC   
  
  
- CCATAATAGC TCATAAAAAG AGTCAAGTAC TTTCCGAAAC TAAGTCACAC ACACGACAGT ACAGATCCGT   
  
  
- ACTAACTCAT AAAAATAAAC GTATAGGTCC GTAACACGGA TCTGTTTACA GGACTTAGAA TCACCGACAA   
  
  
- TCCAGACTTG CCTGTGAAAA GAACGACAAA AAGGAAAGGC TCACCGTAGA AATCGAAAAA GTTTGATGGT   
  
  
- TTCCTGATAA TTTGCTCCGG GACGTATACG ACCTTTTGAC GGAGAATTCG ATACAAATAC GTTTTTACCT   
  
  
- CCTGGAACTT GACAAGTTAC ATGGATGTCA GACTCCGTGT CTCGGTTATC TGTATTATCT ATCAACCTAT   
  
  
- AAGAAAAATA TAAATTAAGT GTCATCGCTG TTGGGGTCGT ACTTTGCATC GGTGCATCCC CCCCTTTTTC   
  
  
- CCAGAGGTAG ACAAATATAT AAGAACAGCG AATACCACGA AGACAATACA TTACTGTTTC CTTAAACACA   
  
  
- ACCCTAATAA GGCGAAGTGA GGCAGAGTGA TTGAAATGCC AAAGTCAGGA TTAAGAGAGA AAAAAAGAAA   
  
  
- ACAAAAAAAA GGACTCGAAC AAATCAGACA AAAGAAGACA CACAGAAAGA CTAATACCTT CGGTATGTCC   
  
  
- TCTTCTTACT GCTAGAAAAC TCAGAATCGA ACCGGTAACA ATCAGTAAGA TTGGTACGTC TTTGCTTCCA   
  
  
- CTTCTCCTTC TCCGTACTAC AGAGATTAAG AGACTAGCGT TGTTCTCTTC TCCCAGCCCT ATTCTTTTAT   
  
  
- AAAGTCGACG AAGTTCTTGC TCTCTCTTAC GATTCGTAGC CCGTTTCTTC TTTTCCGCAA TAAGTTCTTC   
  
  
- CCTTCCCAGA AGTAGACTAG GTAAACGAAG AGTACAGACG ATGGGATTAG TCGCTTTTGT TATAGTCGAG   
  
  
- TCGACAATTA GTAAACGAAC TCAACATGTT CTTACAAAGA CATTTACCAC TAAGACCCGT CGCGCAACGA   
  
  
- CGAATAAAAC GACTACCCAA TTGACGCTCC AAGAATTGGG TCTTGAGAGG GAAAATAACG TACCATTACT   
  
  
- CATTTGGCTG TGGACGTCTC CTCAAATAGC GGAAGTGAGT AAACATGTCC CACAGAGGTA AGATAGTCAA   
  
  
- ACGGGTAAAA TGACGATTAG TCCGGTAGAA ACTCCGTAAG CTCTCCCTTC TTCTCTTGTT GTCGACCCGT   
  
  
- GAGGTACAGT AACTAAAACT GTAGAGAGTA CCGAAGGTCA CCGGCAGAGA GTAAGTCAGA GAAAGACTCT   
  
  
- TCCGATGACG AAGTTCGGTA AGTGAGAGAG AAGCTTAGTG TCCAAAACGA TCTTGAGACC TTCTTGACTA   
  
  
- ACTCTGACTC TTGTCTAATC ATTCAAAACG ACTTTCGAAG GCCTTGTATG ATAAACTTAA GGTGCCCACA   
  
  
- AACTCCCCAA GTCTCGACCA CTTGAACTTC TTTTCCCTCT TACTTTGTCA ACGGCATTTA GACCACAAAG   
  
  
- TGGAGTCGTC AAAGCTACTG TGGGACTTCC AGAGTCTGTG GAATTGGCGA CATGTAAGTG ACTTTGGAAG   
  
  
- ATATTACCAT GATCAACTTT CTCTTCCTTG GGCTTTACCT AGTCCTAAGT ATAGTCCAAA GTACCTAAGA   
  
  
- AACGTAATAA TACGACGTTA CAAGCTAAGT AATTTACTAA CGGAGGGTGC CCTCTCAGGG CTCTCTGACT   
  
  
- CGTATCTCTT CTTGGTAGAA CCCTCTCTCT AGTTCCTCCG CCACTGAACC CTCTTCCTGC TCTACTCGAT   
  
  
- GCCCTTCAAG CTCTATTACC TCTGAACCTT TTTCTCCTAC CTTTAAGTAC CCAAAAGCCC CTAGTCCGAC   
  
  
- TCAAGGTTCC GCTGTTAAGT TCGGTTCGAG GACGAGTTTT ACCCGTCGGT AATGACAGGG GTCAAACGTC   
  
  
- CCCTAAGACT TCCTCCTCAA CCGCCCAAGT CTCACATACT CTCTCTACTC CCCCTTCGGT AAAGAGACCA   
  
  
- AACCGTTCCG TCCAAGGAGC GTTGTCGGAG ACGTACCGCG ACACATAC

+     Myb-binding site

| Site Name | Organism | Position | Strand | Matrix score. | sequence | function |
| --- | --- | --- | --- | --- | --- | --- |
| Myb-binding site | Nicotiana tabacum | 3385 | + | 6 | CAACAG |  |
| Myb-binding site | Nicotiana tabacum | 2582 | + | 6 | CAACAG |  |

>HU11G01529.1   
+ +Up\_Stream \_Len000GAAAGA AGATTTAAGG AAATCGATGG TACACGGCAA AGCTAGAAAC AGGAACTGTA   
  
  
+ TTTTTATGTT TTTTTTTTGT TGGAAGGGGG GGGGGGGGAG CGGGCGAGAT GTAACCCGTA TCTAGATGGA   
  
  
+ TAATCTACGT GACAAGACCT TCATTTGATC AAGGTACATC ATGAATATTT AACCCTTAAC TTCTTTGAGC   
  
  
+ CTTACAAGAG ATTGTCTGAG GTTTTTAGGG TGCACGATAT CATGCCTGAC TAGACCTGTG TAGACACAAT   
  
  
+ CTTCAGCCCT TATTGATAGC TGTTCGATAT GCATGCTGCC TAAAAATTGG AATAGTTGGC ACCTAACATT   
  
  
+ CTTTTACATC TTGCTCCTCC TTGGTGTAGA GTTAGATGTG CTAGCACAAT ACAATGGGTC CCGATATTTT   
  
  
+ GCATCTGGTT TCTGAAAGAT AATATTTGAT GATCCTGTCT TGTGCTGATG GGCAAAAGCT AAATTGTAAG   
  
  
+ AATATGTAGT TTATTTACAA GACATATGCT TAAGCTAGAG GTCATGTACT TGCATATTTT TGTGCAGCTT   
  
  
+ TTGAGGTTGT ATGCCCCTTT GAGACAAATT CATAGTTGAA TTCATGACAA GATTATGTTT GGCAATGACT   
  
  
+ TTGATTATTA AATTGGCCTT TATGCTATCC TATCCTGTGT GCTCAAATGT TATTTATCAG ATAATATTAG   
  
  
+ TTTACTCTTT CTTCCCTGTC TTGCAAAGAT TTTAAATCCT AAATTATGCA AATGTTTATC TTTAAAATGA   
  
  
+ CCTGCTACTC TCTGTGCCTG ATTTTATTTG TTTCCCCGTT GAAATTTTTT TTATGATTCC ACTTTTCAAG   
  
  
+ CAGCTTAGAC AGGCGTTATT AGGTAATTAT TTTCCGCTTT GGATTGTAGT AAATTGAAAC AGAAATGCCT   
  
  
+ CACTAAACCG CTCTCTGCTC TTTGTGTTGA AGAACCAATC CCATTCAAAC TCTTCTTTCT TTTTTGAAAT   
  
  
+ TCAATCCCGC TTCAGTCAAA TGTCTAACAG AAACAGCCTC GTGTGCAATT CTACTATATA CGGTTAATGC   
  
  
+ TGTGCATGTA TTACTTCCCA ACATCTTACC CAGGACTAGG AGGGACCTTG GTAGCCTGTT CTGCTTGAAA   
  
  
+ AATTTGTCAT TATCACCTGA ATTTGTATTA AATACATAAA TCTTTGTCTT TCAATTTGTG GCTCCACATT   
  
  
+ TCAATTTGTG GCTCCACACG CTTTTGAAAA CTTTATGAGA CCTAATATAT TAGAGAACAT CTGATCGTGA   
  
  
+ TTGTTAATTC CAGTTTTATA ATCATACTAA TTAGTAAATC ACTGAAGGTC ATATTGCATA TTGTTTTTGC   
  
  
+ CTAATGATTG GCTTATTCAA CTATTCCTGA ATAGTTTAGT TTCCAGAATC TTTAGAAGAA ACCTGGTCTG   
  
  
+ GGTATTATCG AGTATTTTTC TCAGTTCATG AAAGGCTTTG ATTCAGTGTG TGTGCTGTCA TGTCTAGGCA   
  
  
+ TGATTGAGTA TTTTTATTTG CATATCCAGG CATTGTGCCT AGACAAATGT CCTGAATCTT AGTGGCTGTT   
  
  
+ AGGTCTGAAC GGACACTTTT CTTGCTGTTT TTCCTTTCCG AGTGGCATCT TTAGCTTTTT CAAACTACCA   
  
  
+ AAGGACTATT AAACGAGGCC CTGCATATGC TGGAAAACTG CCTCTTAAGC TATGTTTATG CAAAAATGGA   
  
  
+ GGACCTTGAA CTGTTCAATG TACCTACAGT CTGAGGCACA GAGCCAATAG ACATAATAGA TAGTTGGATA   
  
  
+ TTCTTTTTAT ATTTAATTCA CAGTAGCGAC AACCCCAGCA TGAAACGTAG CCACGTAGGG GGGGAAAAAG   
  
  
+ GGTCTCCATC TGTTTATATA TTCTTGTCGC TTATGGTGCT TCTGTTATGT AATGACAAAG GAATTTGTGT   
  
  
+ TGGGATTATT CCGCTTCACT CCGTCTCACT AACTTTACGG TTTCAGTCCT AATTCTCTCT TTTTTTCTTT   
  
  
+ TGTTTTTTTT CCTGAGCTTG TTTAGTCTGT TTTCTTCTGT GTGTCTTTCT GATTATGGAA GCCATACAGG   
  
  
+ AGAAGAATGA CGATCTTTTG AGTCTTAGCT TGGCCATTGT TAGTCATTCT AACCATGCAG AAACGAAGGT   
  
  
+ GAAGAGGAAG AGGCATGATG TCTCTAATTC TCTGATCGCA ACAAGAGAAG AGGGTCGGGA TAAGAAAATA   
  
  
+ TTTCAGCTGC TTCAAGAACG AGAGAGAATG CTAAGCATCG GGCAAAGAAG AAAAGGCGTT ATTCAAGAAG   
  
  
+ GGAAGGGTCT TCATCTGATC CATTTGCTTC TCATGTCTGC TACCCTAATC AGCGAAAACA ATATCAGCTC   
  
  
+ AGCTGTTAAT CATTTGCTTG AGTTGTACAA GAATGTTTCT GTAAATGGTG ATTCTGGGCA GCGCGTTGCT   
  
  
+ GCTTATTTTG CTGATGGGTT AACTGCGAGG TTCTTAACCC AGAACTCTCC CTTTTATTGC ATGGTAATGA   
  
  
+ GTAAACCGAC ACCTGCAGAG GAGTTTATCG CCTTCACTCA TTTGTACAGG GTGTCTCCAT TCTATCAGTT   
  
  
+ TGCCCATTTT ACTGCTAATC AGGCCATCTT TGAGGCATTC GAGAGGGAAG AAGAGAACAA CAGCTGGGCA   
  
  
+ CTCCATGTCA TTGATTTTGA CATCTCTCAT GGCTTCCAGT GGCCGTCTCT CATTCAGTCT CTTTCTGAGA   
  
  
+ AGGCTACTGC TTCAAGCCAT TCACTCTCTC TTCGAATCAC AGGTTTTGCT AGAACTCTGG AAGAACTGAT   
  
  
+ TGAGACTGAG AACAGATTAG TAAGTTTTGC TGAAAGCTTC CGGAACATAC TATTTGAATT CCACGGGTGT   
  
  
+ TTGAGGGGTT CAGAGCTGGT GAACTTGAAG AAAAGGGAGA ATGAAACAGT TGCCGTAAAT CTGGTGTTTC   
  
  
+ ACCTCAGCAG TTTCGATGAC ACCCTGAAGG TCTCAGACAC CTTAACCGCT GTACATTCAC TGAAACCTTC   
  
  
+ TATAATGGTA CTAGTTGAAA GAGAAGGAAC CCGAAATGGA TCAGGATTCA TATCAGGTTT CATGGATTCT   
  
  
+ TTGCATTATT ATGCTGCAAT GTTCGATTCA TTAAATGATT GCCTCCCACG GGAGAGTCCC GAGAGACTGA   
  
  
+ GCATAGAGAA GAACCATCTT GGGAGAGAGA TCAAGGAGGC GGTGACTTGG GAGAAGGACG AGATGAGCTA   
  
  
+ CGGGAAGTTC GAGATAATGG AGACTTGGAA AAAGAGGATG GAAATTCATG GGTTTTCGGG GATCAGGCTG   
  
  
+ AGTTCCAAGG CGACAATTCA AGCCAAGCTC CTGCTCAAAA TGGGCAGCCA TTACTGTCCC CAGTTTGCAG   
  
  
+ GGGATTCTGA AGGAGGAGTT GGCGGGTTCA GAGTGTATGA GAGAGATGAG GGGGAAGCCA TTTCTCTGGT   
  
  
+ TTGGCAAGGC AGGTTCCTCG CAACAGCCTC TGCATGGCGC TGTGTATG  

- +Up\_Stream \_Len000CTTTCT TCTAAATTCC TTTAGCTACC ATGTGCCGTT TCGATCTTTG TCCTTGACAT   
  
  
- AAAAATACAA AAAAAAAACA ACCTTCCCCC CCCCCCCCTC GCCCGCTCTA CATTGGGCAT AGATCTACCT   
  
  
- ATTAGATGCA CTGTTCTGGA AGTAAACTAG TTCCATGTAG TACTTATAAA TTGGGAATTG AAGAAACTCG   
  
  
- GAATGTTCTC TAACAGACTC CAAAAATCCC ACGTGCTATA GTACGGACTG ATCTGGACAC ATCTGTGTTA   
  
  
- GAAGTCGGGA ATAACTATCG ACAAGCTATA CGTACGACGG ATTTTTAACC TTATCAACCG TGGATTGTAA   
  
  
- GAAAATGTAG AACGAGGAGG AACCACATCT CAATCTACAC GATCGTGTTA TGTTACCCAG GGCTATAAAA   
  
  
- CGTAGACCAA AGACTTTCTA TTATAAACTA CTAGGACAGA ACACGACTAC CCGTTTTCGA TTTAACATTC   
  
  
- TTATACATCA AATAAATGTT CTGTATACGA ATTCGATCTC CAGTACATGA ACGTATAAAA ACACGTCGAA   
  
  
- AACTCCAACA TACGGGGAAA CTCTGTTTAA GTATCAACTT AAGTACTGTT CTAATACAAA CCGTTACTGA   
  
  
- AACTAATAAT TTAACCGGAA ATACGATAGG ATAGGACACA CGAGTTTACA ATAAATAGTC TATTATAATC   
  
  
- AAATGAGAAA GAAGGGACAG AACGTTTCTA AAATTTAGGA TTTAATACGT TTACAAATAG AAATTTTACT   
  
  
- GGACGATGAG AGACACGGAC TAAAATAAAC AAAGGGGCAA CTTTAAAAAA AATACTAAGG TGAAAAGTTC   
  
  
- GTCGAATCTG TCCGCAATAA TCCATTAATA AAAGGCGAAA CCTAACATCA TTTAACTTTG TCTTTACGGA   
  
  
- GTGATTTGGC GAGAGACGAG AAACACAACT TCTTGGTTAG GGTAAGTTTG AGAAGAAAGA AAAAACTTTA   
  
  
- AGTTAGGGCG AAGTCAGTTT ACAGATTGTC TTTGTCGGAG CACACGTTAA GATGATATAT GCCAATTACG   
  
  
- ACACGTACAT AATGAAGGGT TGTAGAATGG GTCCTGATCC TCCCTGGAAC CATCGGACAA GACGAACTTT   
  
  
- TTAAACAGTA ATAGTGGACT TAAACATAAT TTATGTATTT AGAAACAGAA AGTTAAACAC CGAGGTGTAA   
  
  
- AGTTAAACAC CGAGGTGTGC GAAAACTTTT GAAATACTCT GGATTATATA ATCTCTTGTA GACTAGCACT   
  
  
- AACAATTAAG GTCAAAATAT TAGTATGATT AATCATTTAG TGACTTCCAG TATAACGTAT AACAAAAACG   
  
  
- GATTACTAAC CGAATAAGTT GATAAGGACT TATCAAATCA AAGGTCTTAG AAATCTTCTT TGGACCAGAC   
  
  
- CCATAATAGC TCATAAAAAG AGTCAAGTAC TTTCCGAAAC TAAGTCACAC ACACGACAGT ACAGATCCGT   
  
  
- ACTAACTCAT AAAAATAAAC GTATAGGTCC GTAACACGGA TCTGTTTACA GGACTTAGAA TCACCGACAA   
  
  
- TCCAGACTTG CCTGTGAAAA GAACGACAAA AAGGAAAGGC TCACCGTAGA AATCGAAAAA GTTTGATGGT   
  
  
- TTCCTGATAA TTTGCTCCGG GACGTATACG ACCTTTTGAC GGAGAATTCG ATACAAATAC GTTTTTACCT   
  
  
- CCTGGAACTT GACAAGTTAC ATGGATGTCA GACTCCGTGT CTCGGTTATC TGTATTATCT ATCAACCTAT   
  
  
- AAGAAAAATA TAAATTAAGT GTCATCGCTG TTGGGGTCGT ACTTTGCATC GGTGCATCCC CCCCTTTTTC   
  
  
- CCAGAGGTAG ACAAATATAT AAGAACAGCG AATACCACGA AGACAATACA TTACTGTTTC CTTAAACACA   
  
  
- ACCCTAATAA GGCGAAGTGA GGCAGAGTGA TTGAAATGCC AAAGTCAGGA TTAAGAGAGA AAAAAAGAAA   
  
  
- ACAAAAAAAA GGACTCGAAC AAATCAGACA AAAGAAGACA CACAGAAAGA CTAATACCTT CGGTATGTCC   
  
  
- TCTTCTTACT GCTAGAAAAC TCAGAATCGA ACCGGTAACA ATCAGTAAGA TTGGTACGTC TTTGCTTCCA   
  
  
- CTTCTCCTTC TCCGTACTAC AGAGATTAAG AGACTAGCGT TGTTCTCTTC TCCCAGCCCT ATTCTTTTAT   
  
  
- AAAGTCGACG AAGTTCTTGC TCTCTCTTAC GATTCGTAGC CCGTTTCTTC TTTTCCGCAA TAAGTTCTTC   
  
  
- CCTTCCCAGA AGTAGACTAG GTAAACGAAG AGTACAGACG ATGGGATTAG TCGCTTTTGT TATAGTCGAG   
  
  
- TCGACAATTA GTAAACGAAC TCAACATGTT CTTACAAAGA CATTTACCAC TAAGACCCGT CGCGCAACGA   
  
  
- CGAATAAAAC GACTACCCAA TTGACGCTCC AAGAATTGGG TCTTGAGAGG GAAAATAACG TACCATTACT   
  
  
- CATTTGGCTG TGGACGTCTC CTCAAATAGC GGAAGTGAGT AAACATGTCC CACAGAGGTA AGATAGTCAA   
  
  
- ACGGGTAAAA TGACGATTAG TCCGGTAGAA ACTCCGTAAG CTCTCCCTTC TTCTCTTGTT GTCGACCCGT   
  
  
- GAGGTACAGT AACTAAAACT GTAGAGAGTA CCGAAGGTCA CCGGCAGAGA GTAAGTCAGA GAAAGACTCT   
  
  
- TCCGATGACG AAGTTCGGTA AGTGAGAGAG AAGCTTAGTG TCCAAAACGA TCTTGAGACC TTCTTGACTA   
  
  
- ACTCTGACTC TTGTCTAATC ATTCAAAACG ACTTTCGAAG GCCTTGTATG ATAAACTTAA GGTGCCCACA   
  
  
- AACTCCCCAA GTCTCGACCA CTTGAACTTC TTTTCCCTCT TACTTTGTCA ACGGCATTTA GACCACAAAG   
  
  
- TGGAGTCGTC AAAGCTACTG TGGGACTTCC AGAGTCTGTG GAATTGGCGA CATGTAAGTG ACTTTGGAAG   
  
  
- ATATTACCAT GATCAACTTT CTCTTCCTTG GGCTTTACCT AGTCCTAAGT ATAGTCCAAA GTACCTAAGA   
  
  
- AACGTAATAA TACGACGTTA CAAGCTAAGT AATTTACTAA CGGAGGGTGC CCTCTCAGGG CTCTCTGACT   
  
  
- CGTATCTCTT CTTGGTAGAA CCCTCTCTCT AGTTCCTCCG CCACTGAACC CTCTTCCTGC TCTACTCGAT   
  
  
- GCCCTTCAAG CTCTATTACC TCTGAACCTT TTTCTCCTAC CTTTAAGTAC CCAAAAGCCC CTAGTCCGAC   
  
  
- TCAAGGTTCC GCTGTTAAGT TCGGTTCGAG GACGAGTTTT ACCCGTCGGT AATGACAGGG GTCAAACGTC   
  
  
- CCCTAAGACT TCCTCCTCAA CCGCCCAAGT CTCACATACT CTCTCTACTC CCCCTTCGGT AAAGAGACCA   
  
  
- AACCGTTCCG TCCAAGGAGC GTTGTCGGAG ACGTACCGCG ACACATAC

+     O2-site

| Site Name | Organism | Position | Strand | Matrix score. | sequence | function |
| --- | --- | --- | --- | --- | --- | --- |
| O2-site | Zea mays | 2597 | - | 9 | GATGACATGG | cis-acting regulatory element involved in zein metabolism regulation |

>HU11G01529.1   
+ +Up\_Stream \_Len000GAAAGA AGATTTAAGG AAATCGATGG TACACGGCAA AGCTAGAAAC AGGAACTGTA   
  
  
+ TTTTTATGTT TTTTTTTTGT TGGAAGGGGG GGGGGGGGAG CGGGCGAGAT GTAACCCGTA TCTAGATGGA   
  
  
+ TAATCTACGT GACAAGACCT TCATTTGATC AAGGTACATC ATGAATATTT AACCCTTAAC TTCTTTGAGC   
  
  
+ CTTACAAGAG ATTGTCTGAG GTTTTTAGGG TGCACGATAT CATGCCTGAC TAGACCTGTG TAGACACAAT   
  
  
+ CTTCAGCCCT TATTGATAGC TGTTCGATAT GCATGCTGCC TAAAAATTGG AATAGTTGGC ACCTAACATT   
  
  
+ CTTTTACATC TTGCTCCTCC TTGGTGTAGA GTTAGATGTG CTAGCACAAT ACAATGGGTC CCGATATTTT   
  
  
+ GCATCTGGTT TCTGAAAGAT AATATTTGAT GATCCTGTCT TGTGCTGATG GGCAAAAGCT AAATTGTAAG   
  
  
+ AATATGTAGT TTATTTACAA GACATATGCT TAAGCTAGAG GTCATGTACT TGCATATTTT TGTGCAGCTT   
  
  
+ TTGAGGTTGT ATGCCCCTTT GAGACAAATT CATAGTTGAA TTCATGACAA GATTATGTTT GGCAATGACT   
  
  
+ TTGATTATTA AATTGGCCTT TATGCTATCC TATCCTGTGT GCTCAAATGT TATTTATCAG ATAATATTAG   
  
  
+ TTTACTCTTT CTTCCCTGTC TTGCAAAGAT TTTAAATCCT AAATTATGCA AATGTTTATC TTTAAAATGA   
  
  
+ CCTGCTACTC TCTGTGCCTG ATTTTATTTG TTTCCCCGTT GAAATTTTTT TTATGATTCC ACTTTTCAAG   
  
  
+ CAGCTTAGAC AGGCGTTATT AGGTAATTAT TTTCCGCTTT GGATTGTAGT AAATTGAAAC AGAAATGCCT   
  
  
+ CACTAAACCG CTCTCTGCTC TTTGTGTTGA AGAACCAATC CCATTCAAAC TCTTCTTTCT TTTTTGAAAT   
  
  
+ TCAATCCCGC TTCAGTCAAA TGTCTAACAG AAACAGCCTC GTGTGCAATT CTACTATATA CGGTTAATGC   
  
  
+ TGTGCATGTA TTACTTCCCA ACATCTTACC CAGGACTAGG AGGGACCTTG GTAGCCTGTT CTGCTTGAAA   
  
  
+ AATTTGTCAT TATCACCTGA ATTTGTATTA AATACATAAA TCTTTGTCTT TCAATTTGTG GCTCCACATT   
  
  
+ TCAATTTGTG GCTCCACACG CTTTTGAAAA CTTTATGAGA CCTAATATAT TAGAGAACAT CTGATCGTGA   
  
  
+ TTGTTAATTC CAGTTTTATA ATCATACTAA TTAGTAAATC ACTGAAGGTC ATATTGCATA TTGTTTTTGC   
  
  
+ CTAATGATTG GCTTATTCAA CTATTCCTGA ATAGTTTAGT TTCCAGAATC TTTAGAAGAA ACCTGGTCTG   
  
  
+ GGTATTATCG AGTATTTTTC TCAGTTCATG AAAGGCTTTG ATTCAGTGTG TGTGCTGTCA TGTCTAGGCA   
  
  
+ TGATTGAGTA TTTTTATTTG CATATCCAGG CATTGTGCCT AGACAAATGT CCTGAATCTT AGTGGCTGTT   
  
  
+ AGGTCTGAAC GGACACTTTT CTTGCTGTTT TTCCTTTCCG AGTGGCATCT TTAGCTTTTT CAAACTACCA   
  
  
+ AAGGACTATT AAACGAGGCC CTGCATATGC TGGAAAACTG CCTCTTAAGC TATGTTTATG CAAAAATGGA   
  
  
+ GGACCTTGAA CTGTTCAATG TACCTACAGT CTGAGGCACA GAGCCAATAG ACATAATAGA TAGTTGGATA   
  
  
+ TTCTTTTTAT ATTTAATTCA CAGTAGCGAC AACCCCAGCA TGAAACGTAG CCACGTAGGG GGGGAAAAAG   
  
  
+ GGTCTCCATC TGTTTATATA TTCTTGTCGC TTATGGTGCT TCTGTTATGT AATGACAAAG GAATTTGTGT   
  
  
+ TGGGATTATT CCGCTTCACT CCGTCTCACT AACTTTACGG TTTCAGTCCT AATTCTCTCT TTTTTTCTTT   
  
  
+ TGTTTTTTTT CCTGAGCTTG TTTAGTCTGT TTTCTTCTGT GTGTCTTTCT GATTATGGAA GCCATACAGG   
  
  
+ AGAAGAATGA CGATCTTTTG AGTCTTAGCT TGGCCATTGT TAGTCATTCT AACCATGCAG AAACGAAGGT   
  
  
+ GAAGAGGAAG AGGCATGATG TCTCTAATTC TCTGATCGCA ACAAGAGAAG AGGGTCGGGA TAAGAAAATA   
  
  
+ TTTCAGCTGC TTCAAGAACG AGAGAGAATG CTAAGCATCG GGCAAAGAAG AAAAGGCGTT ATTCAAGAAG   
  
  
+ GGAAGGGTCT TCATCTGATC CATTTGCTTC TCATGTCTGC TACCCTAATC AGCGAAAACA ATATCAGCTC   
  
  
+ AGCTGTTAAT CATTTGCTTG AGTTGTACAA GAATGTTTCT GTAAATGGTG ATTCTGGGCA GCGCGTTGCT   
  
  
+ GCTTATTTTG CTGATGGGTT AACTGCGAGG TTCTTAACCC AGAACTCTCC CTTTTATTGC ATGGTAATGA   
  
  
+ GTAAACCGAC ACCTGCAGAG GAGTTTATCG CCTTCACTCA TTTGTACAGG GTGTCTCCAT TCTATCAGTT   
  
  
+ TGCCCATTTT ACTGCTAATC AGGCCATCTT TGAGGCATTC GAGAGGGAAG AAGAGAACAA CAGCTGGGCA   
  
  
+ CTCCATGTCA TTGATTTTGA CATCTCTCAT GGCTTCCAGT GGCCGTCTCT CATTCAGTCT CTTTCTGAGA   
  
  
+ AGGCTACTGC TTCAAGCCAT TCACTCTCTC TTCGAATCAC AGGTTTTGCT AGAACTCTGG AAGAACTGAT   
  
  
+ TGAGACTGAG AACAGATTAG TAAGTTTTGC TGAAAGCTTC CGGAACATAC TATTTGAATT CCACGGGTGT   
  
  
+ TTGAGGGGTT CAGAGCTGGT GAACTTGAAG AAAAGGGAGA ATGAAACAGT TGCCGTAAAT CTGGTGTTTC   
  
  
+ ACCTCAGCAG TTTCGATGAC ACCCTGAAGG TCTCAGACAC CTTAACCGCT GTACATTCAC TGAAACCTTC   
  
  
+ TATAATGGTA CTAGTTGAAA GAGAAGGAAC CCGAAATGGA TCAGGATTCA TATCAGGTTT CATGGATTCT   
  
  
+ TTGCATTATT ATGCTGCAAT GTTCGATTCA TTAAATGATT GCCTCCCACG GGAGAGTCCC GAGAGACTGA   
  
  
+ GCATAGAGAA GAACCATCTT GGGAGAGAGA TCAAGGAGGC GGTGACTTGG GAGAAGGACG AGATGAGCTA   
  
  
+ CGGGAAGTTC GAGATAATGG AGACTTGGAA AAAGAGGATG GAAATTCATG GGTTTTCGGG GATCAGGCTG   
  
  
+ AGTTCCAAGG CGACAATTCA AGCCAAGCTC CTGCTCAAAA TGGGCAGCCA TTACTGTCCC CAGTTTGCAG   
  
  
+ GGGATTCTGA AGGAGGAGTT GGCGGGTTCA GAGTGTATGA GAGAGATGAG GGGGAAGCCA TTTCTCTGGT   
  
  
+ TTGGCAAGGC AGGTTCCTCG CAACAGCCTC TGCATGGCGC TGTGTATG  

- +Up\_Stream \_Len000CTTTCT TCTAAATTCC TTTAGCTACC ATGTGCCGTT TCGATCTTTG TCCTTGACAT   
  
  
- AAAAATACAA AAAAAAAACA ACCTTCCCCC CCCCCCCCTC GCCCGCTCTA CATTGGGCAT AGATCTACCT   
  
  
- ATTAGATGCA CTGTTCTGGA AGTAAACTAG TTCCATGTAG TACTTATAAA TTGGGAATTG AAGAAACTCG   
  
  
- GAATGTTCTC TAACAGACTC CAAAAATCCC ACGTGCTATA GTACGGACTG ATCTGGACAC ATCTGTGTTA   
  
  
- GAAGTCGGGA ATAACTATCG ACAAGCTATA CGTACGACGG ATTTTTAACC TTATCAACCG TGGATTGTAA   
  
  
- GAAAATGTAG AACGAGGAGG AACCACATCT CAATCTACAC GATCGTGTTA TGTTACCCAG GGCTATAAAA   
  
  
- CGTAGACCAA AGACTTTCTA TTATAAACTA CTAGGACAGA ACACGACTAC CCGTTTTCGA TTTAACATTC   
  
  
- TTATACATCA AATAAATGTT CTGTATACGA ATTCGATCTC CAGTACATGA ACGTATAAAA ACACGTCGAA   
  
  
- AACTCCAACA TACGGGGAAA CTCTGTTTAA GTATCAACTT AAGTACTGTT CTAATACAAA CCGTTACTGA   
  
  
- AACTAATAAT TTAACCGGAA ATACGATAGG ATAGGACACA CGAGTTTACA ATAAATAGTC TATTATAATC   
  
  
- AAATGAGAAA GAAGGGACAG AACGTTTCTA AAATTTAGGA TTTAATACGT TTACAAATAG AAATTTTACT   
  
  
- GGACGATGAG AGACACGGAC TAAAATAAAC AAAGGGGCAA CTTTAAAAAA AATACTAAGG TGAAAAGTTC   
  
  
- GTCGAATCTG TCCGCAATAA TCCATTAATA AAAGGCGAAA CCTAACATCA TTTAACTTTG TCTTTACGGA   
  
  
- GTGATTTGGC GAGAGACGAG AAACACAACT TCTTGGTTAG GGTAAGTTTG AGAAGAAAGA AAAAACTTTA   
  
  
- AGTTAGGGCG AAGTCAGTTT ACAGATTGTC TTTGTCGGAG CACACGTTAA GATGATATAT GCCAATTACG   
  
  
- ACACGTACAT AATGAAGGGT TGTAGAATGG GTCCTGATCC TCCCTGGAAC CATCGGACAA GACGAACTTT   
  
  
- TTAAACAGTA ATAGTGGACT TAAACATAAT TTATGTATTT AGAAACAGAA AGTTAAACAC CGAGGTGTAA   
  
  
- AGTTAAACAC CGAGGTGTGC GAAAACTTTT GAAATACTCT GGATTATATA ATCTCTTGTA GACTAGCACT   
  
  
- AACAATTAAG GTCAAAATAT TAGTATGATT AATCATTTAG TGACTTCCAG TATAACGTAT AACAAAAACG   
  
  
- GATTACTAAC CGAATAAGTT GATAAGGACT TATCAAATCA AAGGTCTTAG AAATCTTCTT TGGACCAGAC   
  
  
- CCATAATAGC TCATAAAAAG AGTCAAGTAC TTTCCGAAAC TAAGTCACAC ACACGACAGT ACAGATCCGT   
  
  
- ACTAACTCAT AAAAATAAAC GTATAGGTCC GTAACACGGA TCTGTTTACA GGACTTAGAA TCACCGACAA   
  
  
- TCCAGACTTG CCTGTGAAAA GAACGACAAA AAGGAAAGGC TCACCGTAGA AATCGAAAAA GTTTGATGGT   
  
  
- TTCCTGATAA TTTGCTCCGG GACGTATACG ACCTTTTGAC GGAGAATTCG ATACAAATAC GTTTTTACCT   
  
  
- CCTGGAACTT GACAAGTTAC ATGGATGTCA GACTCCGTGT CTCGGTTATC TGTATTATCT ATCAACCTAT   
  
  
- AAGAAAAATA TAAATTAAGT GTCATCGCTG TTGGGGTCGT ACTTTGCATC GGTGCATCCC CCCCTTTTTC   
  
  
- CCAGAGGTAG ACAAATATAT AAGAACAGCG AATACCACGA AGACAATACA TTACTGTTTC CTTAAACACA   
  
  
- ACCCTAATAA GGCGAAGTGA GGCAGAGTGA TTGAAATGCC AAAGTCAGGA TTAAGAGAGA AAAAAAGAAA   
  
  
- ACAAAAAAAA GGACTCGAAC AAATCAGACA AAAGAAGACA CACAGAAAGA CTAATACCTT CGGTATGTCC   
  
  
- TCTTCTTACT GCTAGAAAAC TCAGAATCGA ACCGGTAACA ATCAGTAAGA TTGGTACGTC TTTGCTTCCA   
  
  
- CTTCTCCTTC TCCGTACTAC AGAGATTAAG AGACTAGCGT TGTTCTCTTC TCCCAGCCCT ATTCTTTTAT   
  
  
- AAAGTCGACG AAGTTCTTGC TCTCTCTTAC GATTCGTAGC CCGTTTCTTC TTTTCCGCAA TAAGTTCTTC   
  
  
- CCTTCCCAGA AGTAGACTAG GTAAACGAAG AGTACAGACG ATGGGATTAG TCGCTTTTGT TATAGTCGAG   
  
  
- TCGACAATTA GTAAACGAAC TCAACATGTT CTTACAAAGA CATTTACCAC TAAGACCCGT CGCGCAACGA   
  
  
- CGAATAAAAC GACTACCCAA TTGACGCTCC AAGAATTGGG TCTTGAGAGG GAAAATAACG TACCATTACT   
  
  
- CATTTGGCTG TGGACGTCTC CTCAAATAGC GGAAGTGAGT AAACATGTCC CACAGAGGTA AGATAGTCAA   
  
  
- ACGGGTAAAA TGACGATTAG TCCGGTAGAA ACTCCGTAAG CTCTCCCTTC TTCTCTTGTT GTCGACCCGT   
  
  
- GAGGTACAGT AACTAAAACT GTAGAGAGTA CCGAAGGTCA CCGGCAGAGA GTAAGTCAGA GAAAGACTCT   
  
  
- TCCGATGACG AAGTTCGGTA AGTGAGAGAG AAGCTTAGTG TCCAAAACGA TCTTGAGACC TTCTTGACTA   
  
  
- ACTCTGACTC TTGTCTAATC ATTCAAAACG ACTTTCGAAG GCCTTGTATG ATAAACTTAA GGTGCCCACA   
  
  
- AACTCCCCAA GTCTCGACCA CTTGAACTTC TTTTCCCTCT TACTTTGTCA ACGGCATTTA GACCACAAAG   
  
  
- TGGAGTCGTC AAAGCTACTG TGGGACTTCC AGAGTCTGTG GAATTGGCGA CATGTAAGTG ACTTTGGAAG   
  
  
- ATATTACCAT GATCAACTTT CTCTTCCTTG GGCTTTACCT AGTCCTAAGT ATAGTCCAAA GTACCTAAGA   
  
  
- AACGTAATAA TACGACGTTA CAAGCTAAGT AATTTACTAA CGGAGGGTGC CCTCTCAGGG CTCTCTGACT   
  
  
- CGTATCTCTT CTTGGTAGAA CCCTCTCTCT AGTTCCTCCG CCACTGAACC CTCTTCCTGC TCTACTCGAT   
  
  
- GCCCTTCAAG CTCTATTACC TCTGAACCTT TTTCTCCTAC CTTTAAGTAC CCAAAAGCCC CTAGTCCGAC   
  
  
- TCAAGGTTCC GCTGTTAAGT TCGGTTCGAG GACGAGTTTT ACCCGTCGGT AATGACAGGG GTCAAACGTC   
  
  
- CCCTAAGACT TCCTCCTCAA CCGCCCAAGT CTCACATACT CTCTCTACTC CCCCTTCGGT AAAGAGACCA   
  
  
- AACCGTTCCG TCCAAGGAGC GTTGTCGGAG ACGTACCGCG ACACATAC

+     STRE

| Site Name | Organism | Position | Strand | Matrix score. | sequence | function |
| --- | --- | --- | --- | --- | --- | --- |
| STRE | Arabidopsis thaliana | 3343 | + | 5 | AGGGG |  |
| STRE | Arabidopsis thaliana | 3293 | + | 5 | AGGGG |  |
| STRE | Arabidopsis thaliana | 578 | - | 5 | AGGGG |  |
| STRE | Arabidopsis thaliana | 99 | + | 5 | AGGGG |  |
| STRE | Arabidopsis thaliana | 2808 | + | 5 | AGGGG |  |
| STRE | Arabidopsis thaliana | 1811 | + | 5 | AGGGG |  |

>HU11G01529.1   
+ +Up\_Stream \_Len000GAAAGA AGATTTAAGG AAATCGATGG TACACGGCAA AGCTAGAAAC AGGAACTGTA   
  
  
+ TTTTTATGTT TTTTTTTTGT TGGAAGGGGG GGGGGGGGAG CGGGCGAGAT GTAACCCGTA TCTAGATGGA   
  
  
+ TAATCTACGT GACAAGACCT TCATTTGATC AAGGTACATC ATGAATATTT AACCCTTAAC TTCTTTGAGC   
  
  
+ CTTACAAGAG ATTGTCTGAG GTTTTTAGGG TGCACGATAT CATGCCTGAC TAGACCTGTG TAGACACAAT   
  
  
+ CTTCAGCCCT TATTGATAGC TGTTCGATAT GCATGCTGCC TAAAAATTGG AATAGTTGGC ACCTAACATT   
  
  
+ CTTTTACATC TTGCTCCTCC TTGGTGTAGA GTTAGATGTG CTAGCACAAT ACAATGGGTC CCGATATTTT   
  
  
+ GCATCTGGTT TCTGAAAGAT AATATTTGAT GATCCTGTCT TGTGCTGATG GGCAAAAGCT AAATTGTAAG   
  
  
+ AATATGTAGT TTATTTACAA GACATATGCT TAAGCTAGAG GTCATGTACT TGCATATTTT TGTGCAGCTT   
  
  
+ TTGAGGTTGT ATGCCCCTTT GAGACAAATT CATAGTTGAA TTCATGACAA GATTATGTTT GGCAATGACT   
  
  
+ TTGATTATTA AATTGGCCTT TATGCTATCC TATCCTGTGT GCTCAAATGT TATTTATCAG ATAATATTAG   
  
  
+ TTTACTCTTT CTTCCCTGTC TTGCAAAGAT TTTAAATCCT AAATTATGCA AATGTTTATC TTTAAAATGA   
  
  
+ CCTGCTACTC TCTGTGCCTG ATTTTATTTG TTTCCCCGTT GAAATTTTTT TTATGATTCC ACTTTTCAAG   
  
  
+ CAGCTTAGAC AGGCGTTATT AGGTAATTAT TTTCCGCTTT GGATTGTAGT AAATTGAAAC AGAAATGCCT   
  
  
+ CACTAAACCG CTCTCTGCTC TTTGTGTTGA AGAACCAATC CCATTCAAAC TCTTCTTTCT TTTTTGAAAT   
  
  
+ TCAATCCCGC TTCAGTCAAA TGTCTAACAG AAACAGCCTC GTGTGCAATT CTACTATATA CGGTTAATGC   
  
  
+ TGTGCATGTA TTACTTCCCA ACATCTTACC CAGGACTAGG AGGGACCTTG GTAGCCTGTT CTGCTTGAAA   
  
  
+ AATTTGTCAT TATCACCTGA ATTTGTATTA AATACATAAA TCTTTGTCTT TCAATTTGTG GCTCCACATT   
  
  
+ TCAATTTGTG GCTCCACACG CTTTTGAAAA CTTTATGAGA CCTAATATAT TAGAGAACAT CTGATCGTGA   
  
  
+ TTGTTAATTC CAGTTTTATA ATCATACTAA TTAGTAAATC ACTGAAGGTC ATATTGCATA TTGTTTTTGC   
  
  
+ CTAATGATTG GCTTATTCAA CTATTCCTGA ATAGTTTAGT TTCCAGAATC TTTAGAAGAA ACCTGGTCTG   
  
  
+ GGTATTATCG AGTATTTTTC TCAGTTCATG AAAGGCTTTG ATTCAGTGTG TGTGCTGTCA TGTCTAGGCA   
  
  
+ TGATTGAGTA TTTTTATTTG CATATCCAGG CATTGTGCCT AGACAAATGT CCTGAATCTT AGTGGCTGTT   
  
  
+ AGGTCTGAAC GGACACTTTT CTTGCTGTTT TTCCTTTCCG AGTGGCATCT TTAGCTTTTT CAAACTACCA   
  
  
+ AAGGACTATT AAACGAGGCC CTGCATATGC TGGAAAACTG CCTCTTAAGC TATGTTTATG CAAAAATGGA   
  
  
+ GGACCTTGAA CTGTTCAATG TACCTACAGT CTGAGGCACA GAGCCAATAG ACATAATAGA TAGTTGGATA   
  
  
+ TTCTTTTTAT ATTTAATTCA CAGTAGCGAC AACCCCAGCA TGAAACGTAG CCACGTAGGG GGGGAAAAAG   
  
  
+ GGTCTCCATC TGTTTATATA TTCTTGTCGC TTATGGTGCT TCTGTTATGT AATGACAAAG GAATTTGTGT   
  
  
+ TGGGATTATT CCGCTTCACT CCGTCTCACT AACTTTACGG TTTCAGTCCT AATTCTCTCT TTTTTTCTTT   
  
  
+ TGTTTTTTTT CCTGAGCTTG TTTAGTCTGT TTTCTTCTGT GTGTCTTTCT GATTATGGAA GCCATACAGG   
  
  
+ AGAAGAATGA CGATCTTTTG AGTCTTAGCT TGGCCATTGT TAGTCATTCT AACCATGCAG AAACGAAGGT   
  
  
+ GAAGAGGAAG AGGCATGATG TCTCTAATTC TCTGATCGCA ACAAGAGAAG AGGGTCGGGA TAAGAAAATA   
  
  
+ TTTCAGCTGC TTCAAGAACG AGAGAGAATG CTAAGCATCG GGCAAAGAAG AAAAGGCGTT ATTCAAGAAG   
  
  
+ GGAAGGGTCT TCATCTGATC CATTTGCTTC TCATGTCTGC TACCCTAATC AGCGAAAACA ATATCAGCTC   
  
  
+ AGCTGTTAAT CATTTGCTTG AGTTGTACAA GAATGTTTCT GTAAATGGTG ATTCTGGGCA GCGCGTTGCT   
  
  
+ GCTTATTTTG CTGATGGGTT AACTGCGAGG TTCTTAACCC AGAACTCTCC CTTTTATTGC ATGGTAATGA   
  
  
+ GTAAACCGAC ACCTGCAGAG GAGTTTATCG CCTTCACTCA TTTGTACAGG GTGTCTCCAT TCTATCAGTT   
  
  
+ TGCCCATTTT ACTGCTAATC AGGCCATCTT TGAGGCATTC GAGAGGGAAG AAGAGAACAA CAGCTGGGCA   
  
  
+ CTCCATGTCA TTGATTTTGA CATCTCTCAT GGCTTCCAGT GGCCGTCTCT CATTCAGTCT CTTTCTGAGA   
  
  
+ AGGCTACTGC TTCAAGCCAT TCACTCTCTC TTCGAATCAC AGGTTTTGCT AGAACTCTGG AAGAACTGAT   
  
  
+ TGAGACTGAG AACAGATTAG TAAGTTTTGC TGAAAGCTTC CGGAACATAC TATTTGAATT CCACGGGTGT   
  
  
+ TTGAGGGGTT CAGAGCTGGT GAACTTGAAG AAAAGGGAGA ATGAAACAGT TGCCGTAAAT CTGGTGTTTC   
  
  
+ ACCTCAGCAG TTTCGATGAC ACCCTGAAGG TCTCAGACAC CTTAACCGCT GTACATTCAC TGAAACCTTC   
  
  
+ TATAATGGTA CTAGTTGAAA GAGAAGGAAC CCGAAATGGA TCAGGATTCA TATCAGGTTT CATGGATTCT   
  
  
+ TTGCATTATT ATGCTGCAAT GTTCGATTCA TTAAATGATT GCCTCCCACG GGAGAGTCCC GAGAGACTGA   
  
  
+ GCATAGAGAA GAACCATCTT GGGAGAGAGA TCAAGGAGGC GGTGACTTGG GAGAAGGACG AGATGAGCTA   
  
  
+ CGGGAAGTTC GAGATAATGG AGACTTGGAA AAAGAGGATG GAAATTCATG GGTTTTCGGG GATCAGGCTG   
  
  
+ AGTTCCAAGG CGACAATTCA AGCCAAGCTC CTGCTCAAAA TGGGCAGCCA TTACTGTCCC CAGTTTGCAG   
  
  
+ GGGATTCTGA AGGAGGAGTT GGCGGGTTCA GAGTGTATGA GAGAGATGAG GGGGAAGCCA TTTCTCTGGT   
  
  
+ TTGGCAAGGC AGGTTCCTCG CAACAGCCTC TGCATGGCGC TGTGTATG  

- +Up\_Stream \_Len000CTTTCT TCTAAATTCC TTTAGCTACC ATGTGCCGTT TCGATCTTTG TCCTTGACAT   
  
  
- AAAAATACAA AAAAAAAACA ACCTTCCCCC CCCCCCCCTC GCCCGCTCTA CATTGGGCAT AGATCTACCT   
  
  
- ATTAGATGCA CTGTTCTGGA AGTAAACTAG TTCCATGTAG TACTTATAAA TTGGGAATTG AAGAAACTCG   
  
  
- GAATGTTCTC TAACAGACTC CAAAAATCCC ACGTGCTATA GTACGGACTG ATCTGGACAC ATCTGTGTTA   
  
  
- GAAGTCGGGA ATAACTATCG ACAAGCTATA CGTACGACGG ATTTTTAACC TTATCAACCG TGGATTGTAA   
  
  
- GAAAATGTAG AACGAGGAGG AACCACATCT CAATCTACAC GATCGTGTTA TGTTACCCAG GGCTATAAAA   
  
  
- CGTAGACCAA AGACTTTCTA TTATAAACTA CTAGGACAGA ACACGACTAC CCGTTTTCGA TTTAACATTC   
  
  
- TTATACATCA AATAAATGTT CTGTATACGA ATTCGATCTC CAGTACATGA ACGTATAAAA ACACGTCGAA   
  
  
- AACTCCAACA TACGGGGAAA CTCTGTTTAA GTATCAACTT AAGTACTGTT CTAATACAAA CCGTTACTGA   
  
  
- AACTAATAAT TTAACCGGAA ATACGATAGG ATAGGACACA CGAGTTTACA ATAAATAGTC TATTATAATC   
  
  
- AAATGAGAAA GAAGGGACAG AACGTTTCTA AAATTTAGGA TTTAATACGT TTACAAATAG AAATTTTACT   
  
  
- GGACGATGAG AGACACGGAC TAAAATAAAC AAAGGGGCAA CTTTAAAAAA AATACTAAGG TGAAAAGTTC   
  
  
- GTCGAATCTG TCCGCAATAA TCCATTAATA AAAGGCGAAA CCTAACATCA TTTAACTTTG TCTTTACGGA   
  
  
- GTGATTTGGC GAGAGACGAG AAACACAACT TCTTGGTTAG GGTAAGTTTG AGAAGAAAGA AAAAACTTTA   
  
  
- AGTTAGGGCG AAGTCAGTTT ACAGATTGTC TTTGTCGGAG CACACGTTAA GATGATATAT GCCAATTACG   
  
  
- ACACGTACAT AATGAAGGGT TGTAGAATGG GTCCTGATCC TCCCTGGAAC CATCGGACAA GACGAACTTT   
  
  
- TTAAACAGTA ATAGTGGACT TAAACATAAT TTATGTATTT AGAAACAGAA AGTTAAACAC CGAGGTGTAA   
  
  
- AGTTAAACAC CGAGGTGTGC GAAAACTTTT GAAATACTCT GGATTATATA ATCTCTTGTA GACTAGCACT   
  
  
- AACAATTAAG GTCAAAATAT TAGTATGATT AATCATTTAG TGACTTCCAG TATAACGTAT AACAAAAACG   
  
  
- GATTACTAAC CGAATAAGTT GATAAGGACT TATCAAATCA AAGGTCTTAG AAATCTTCTT TGGACCAGAC   
  
  
- CCATAATAGC TCATAAAAAG AGTCAAGTAC TTTCCGAAAC TAAGTCACAC ACACGACAGT ACAGATCCGT   
  
  
- ACTAACTCAT AAAAATAAAC GTATAGGTCC GTAACACGGA TCTGTTTACA GGACTTAGAA TCACCGACAA   
  
  
- TCCAGACTTG CCTGTGAAAA GAACGACAAA AAGGAAAGGC TCACCGTAGA AATCGAAAAA GTTTGATGGT   
  
  
- TTCCTGATAA TTTGCTCCGG GACGTATACG ACCTTTTGAC GGAGAATTCG ATACAAATAC GTTTTTACCT   
  
  
- CCTGGAACTT GACAAGTTAC ATGGATGTCA GACTCCGTGT CTCGGTTATC TGTATTATCT ATCAACCTAT   
  
  
- AAGAAAAATA TAAATTAAGT GTCATCGCTG TTGGGGTCGT ACTTTGCATC GGTGCATCCC CCCCTTTTTC   
  
  
- CCAGAGGTAG ACAAATATAT AAGAACAGCG AATACCACGA AGACAATACA TTACTGTTTC CTTAAACACA   
  
  
- ACCCTAATAA GGCGAAGTGA GGCAGAGTGA TTGAAATGCC AAAGTCAGGA TTAAGAGAGA AAAAAAGAAA   
  
  
- ACAAAAAAAA GGACTCGAAC AAATCAGACA AAAGAAGACA CACAGAAAGA CTAATACCTT CGGTATGTCC   
  
  
- TCTTCTTACT GCTAGAAAAC TCAGAATCGA ACCGGTAACA ATCAGTAAGA TTGGTACGTC TTTGCTTCCA   
  
  
- CTTCTCCTTC TCCGTACTAC AGAGATTAAG AGACTAGCGT TGTTCTCTTC TCCCAGCCCT ATTCTTTTAT   
  
  
- AAAGTCGACG AAGTTCTTGC TCTCTCTTAC GATTCGTAGC CCGTTTCTTC TTTTCCGCAA TAAGTTCTTC   
  
  
- CCTTCCCAGA AGTAGACTAG GTAAACGAAG AGTACAGACG ATGGGATTAG TCGCTTTTGT TATAGTCGAG   
  
  
- TCGACAATTA GTAAACGAAC TCAACATGTT CTTACAAAGA CATTTACCAC TAAGACCCGT CGCGCAACGA   
  
  
- CGAATAAAAC GACTACCCAA TTGACGCTCC AAGAATTGGG TCTTGAGAGG GAAAATAACG TACCATTACT   
  
  
- CATTTGGCTG TGGACGTCTC CTCAAATAGC GGAAGTGAGT AAACATGTCC CACAGAGGTA AGATAGTCAA   
  
  
- ACGGGTAAAA TGACGATTAG TCCGGTAGAA ACTCCGTAAG CTCTCCCTTC TTCTCTTGTT GTCGACCCGT   
  
  
- GAGGTACAGT AACTAAAACT GTAGAGAGTA CCGAAGGTCA CCGGCAGAGA GTAAGTCAGA GAAAGACTCT   
  
  
- TCCGATGACG AAGTTCGGTA AGTGAGAGAG AAGCTTAGTG TCCAAAACGA TCTTGAGACC TTCTTGACTA   
  
  
- ACTCTGACTC TTGTCTAATC ATTCAAAACG ACTTTCGAAG GCCTTGTATG ATAAACTTAA GGTGCCCACA   
  
  
- AACTCCCCAA GTCTCGACCA CTTGAACTTC TTTTCCCTCT TACTTTGTCA ACGGCATTTA GACCACAAAG   
  
  
- TGGAGTCGTC AAAGCTACTG TGGGACTTCC AGAGTCTGTG GAATTGGCGA CATGTAAGTG ACTTTGGAAG   
  
  
- ATATTACCAT GATCAACTTT CTCTTCCTTG GGCTTTACCT AGTCCTAAGT ATAGTCCAAA GTACCTAAGA   
  
  
- AACGTAATAA TACGACGTTA CAAGCTAAGT AATTTACTAA CGGAGGGTGC CCTCTCAGGG CTCTCTGACT   
  
  
- CGTATCTCTT CTTGGTAGAA CCCTCTCTCT AGTTCCTCCG CCACTGAACC CTCTTCCTGC TCTACTCGAT   
  
  
- GCCCTTCAAG CTCTATTACC TCTGAACCTT TTTCTCCTAC CTTTAAGTAC CCAAAAGCCC CTAGTCCGAC   
  
  
- TCAAGGTTCC GCTGTTAAGT TCGGTTCGAG GACGAGTTTT ACCCGTCGGT AATGACAGGG GTCAAACGTC   
  
  
- CCCTAAGACT TCCTCCTCAA CCGCCCAAGT CTCACATACT CTCTCTACTC CCCCTTCGGT AAAGAGACCA   
  
  
- AACCGTTCCG TCCAAGGAGC GTTGTCGGAG ACGTACCGCG ACACATAC

+     TATA-box

| Site Name | Organism | Position | Strand | Matrix score. | sequence | function |
| --- | --- | --- | --- | --- | --- | --- |
| TATA-box | Arabidopsis thaliana | 1761 | - | 5 | TATAA | core promoter element around -30 of transcription start |
| TATA-box | Arabidopsis thaliana | 1841 | - | 4 | TATA | core promoter element around -30 of transcription start |
| TATA-box | Arabidopsis thaliana | 1838 | - | 7 | TATATAA | core promoter element around -30 of transcription start |
| TATA-box | Arabidopsis thaliana | 1381 | - | 8 | TAAAGATT | core promoter element around -30 of transcription start |
| TATA-box | Arabidopsis thaliana | 1762 | - | 4 | TATA | core promoter element around -30 of transcription start |
| TATA-box | Pisum sativum | 1759 | - | 7 | TATAAAA | core promoter element around -30 of transcription start |
| TATA-box | Helianthus annuus | 1760 | - | 6 | TATAAA | core promoter element around -30 of transcription start |
| TATA-box | Arabidopsis thaliana | 2945 | - | 4 | TATA | core promoter element around -30 of transcription start |
| TATA-box | Arabidopsis thaliana | 1839 | - | 6 | TATATA | core promoter element around -30 of transcription start |
| TATA-box | Arabidopsis thaliana | 1041 | + | 4 | TATA | core promoter element around -30 of transcription start |
| TATA-box | Pisum sativum | 1278 | - | 7 | TATAAAA | core promoter element around -30 of transcription start |
| TATA-box | Helianthus annuus | 1837 | - | 6 | TATAAA | core promoter element around -30 of transcription start |
| TATA-box | Helianthus annuus | 1279 | - | 6 | TATAAA | core promoter element around -30 of transcription start |
| TATA-box | Arabidopsis thaliana | 1157 | + | 9 | taTATAAAtc | core promoter element around -30 of transcription start |
| TATA-box | Arabidopsis thaliana | 1280 | - | 5 | TATAA | core promoter element around -30 of transcription start |
| TATA-box | Brassica napus | 1840 | - | 6 | ATATAT | core promoter element around -30 of transcription start |
| TATA-box | Arabidopsis thaliana | 1039 | + | 6 | TATATA | core promoter element around -30 of transcription start |
| TATA-box | Arabidopsis thaliana | 1281 | + | 4 | TATA | core promoter element around -30 of transcription start |
| TATA-box | Brassica napus | 1239 | + | 6 | ATATAT | core promoter element around -30 of transcription start |
| TATA-box | Arabidopsis thaliana | 1240 | + | 4 | TATA | core promoter element around -30 of transcription start |

>HU11G01529.1   
+ +Up\_Stream \_Len000GAAAGA AGATTTAAGG AAATCGATGG TACACGGCAA AGCTAGAAAC AGGAACTGTA   
  
  
+ TTTTTATGTT TTTTTTTTGT TGGAAGGGGG GGGGGGGGAG CGGGCGAGAT GTAACCCGTA TCTAGATGGA   
  
  
+ TAATCTACGT GACAAGACCT TCATTTGATC AAGGTACATC ATGAATATTT AACCCTTAAC TTCTTTGAGC   
  
  
+ CTTACAAGAG ATTGTCTGAG GTTTTTAGGG TGCACGATAT CATGCCTGAC TAGACCTGTG TAGACACAAT   
  
  
+ CTTCAGCCCT TATTGATAGC TGTTCGATAT GCATGCTGCC TAAAAATTGG AATAGTTGGC ACCTAACATT   
  
  
+ CTTTTACATC TTGCTCCTCC TTGGTGTAGA GTTAGATGTG CTAGCACAAT ACAATGGGTC CCGATATTTT   
  
  
+ GCATCTGGTT TCTGAAAGAT AATATTTGAT GATCCTGTCT TGTGCTGATG GGCAAAAGCT AAATTGTAAG   
  
  
+ AATATGTAGT TTATTTACAA GACATATGCT TAAGCTAGAG GTCATGTACT TGCATATTTT TGTGCAGCTT   
  
  
+ TTGAGGTTGT ATGCCCCTTT GAGACAAATT CATAGTTGAA TTCATGACAA GATTATGTTT GGCAATGACT   
  
  
+ TTGATTATTA AATTGGCCTT TATGCTATCC TATCCTGTGT GCTCAAATGT TATTTATCAG ATAATATTAG   
  
  
+ TTTACTCTTT CTTCCCTGTC TTGCAAAGAT TTTAAATCCT AAATTATGCA AATGTTTATC TTTAAAATGA   
  
  
+ CCTGCTACTC TCTGTGCCTG ATTTTATTTG TTTCCCCGTT GAAATTTTTT TTATGATTCC ACTTTTCAAG   
  
  
+ CAGCTTAGAC AGGCGTTATT AGGTAATTAT TTTCCGCTTT GGATTGTAGT AAATTGAAAC AGAAATGCCT   
  
  
+ CACTAAACCG CTCTCTGCTC TTTGTGTTGA AGAACCAATC CCATTCAAAC TCTTCTTTCT TTTTTGAAAT   
  
  
+ TCAATCCCGC TTCAGTCAAA TGTCTAACAG AAACAGCCTC GTGTGCAATT CTACTATATA CGGTTAATGC   
  
  
+ TGTGCATGTA TTACTTCCCA ACATCTTACC CAGGACTAGG AGGGACCTTG GTAGCCTGTT CTGCTTGAAA   
  
  
+ AATTTGTCAT TATCACCTGA ATTTGTATTA AATACATAAA TCTTTGTCTT TCAATTTGTG GCTCCACATT   
  
  
+ TCAATTTGTG GCTCCACACG CTTTTGAAAA CTTTATGAGA CCTAATATAT TAGAGAACAT CTGATCGTGA   
  
  
+ TTGTTAATTC CAGTTTTATA ATCATACTAA TTAGTAAATC ACTGAAGGTC ATATTGCATA TTGTTTTTGC   
  
  
+ CTAATGATTG GCTTATTCAA CTATTCCTGA ATAGTTTAGT TTCCAGAATC TTTAGAAGAA ACCTGGTCTG   
  
  
+ GGTATTATCG AGTATTTTTC TCAGTTCATG AAAGGCTTTG ATTCAGTGTG TGTGCTGTCA TGTCTAGGCA   
  
  
+ TGATTGAGTA TTTTTATTTG CATATCCAGG CATTGTGCCT AGACAAATGT CCTGAATCTT AGTGGCTGTT   
  
  
+ AGGTCTGAAC GGACACTTTT CTTGCTGTTT TTCCTTTCCG AGTGGCATCT TTAGCTTTTT CAAACTACCA   
  
  
+ AAGGACTATT AAACGAGGCC CTGCATATGC TGGAAAACTG CCTCTTAAGC TATGTTTATG CAAAAATGGA   
  
  
+ GGACCTTGAA CTGTTCAATG TACCTACAGT CTGAGGCACA GAGCCAATAG ACATAATAGA TAGTTGGATA   
  
  
+ TTCTTTTTAT ATTTAATTCA CAGTAGCGAC AACCCCAGCA TGAAACGTAG CCACGTAGGG GGGGAAAAAG   
  
  
+ GGTCTCCATC TGTTTATATA TTCTTGTCGC TTATGGTGCT TCTGTTATGT AATGACAAAG GAATTTGTGT   
  
  
+ TGGGATTATT CCGCTTCACT CCGTCTCACT AACTTTACGG TTTCAGTCCT AATTCTCTCT TTTTTTCTTT   
  
  
+ TGTTTTTTTT CCTGAGCTTG TTTAGTCTGT TTTCTTCTGT GTGTCTTTCT GATTATGGAA GCCATACAGG   
  
  
+ AGAAGAATGA CGATCTTTTG AGTCTTAGCT TGGCCATTGT TAGTCATTCT AACCATGCAG AAACGAAGGT   
  
  
+ GAAGAGGAAG AGGCATGATG TCTCTAATTC TCTGATCGCA ACAAGAGAAG AGGGTCGGGA TAAGAAAATA   
  
  
+ TTTCAGCTGC TTCAAGAACG AGAGAGAATG CTAAGCATCG GGCAAAGAAG AAAAGGCGTT ATTCAAGAAG   
  
  
+ GGAAGGGTCT TCATCTGATC CATTTGCTTC TCATGTCTGC TACCCTAATC AGCGAAAACA ATATCAGCTC   
  
  
+ AGCTGTTAAT CATTTGCTTG AGTTGTACAA GAATGTTTCT GTAAATGGTG ATTCTGGGCA GCGCGTTGCT   
  
  
+ GCTTATTTTG CTGATGGGTT AACTGCGAGG TTCTTAACCC AGAACTCTCC CTTTTATTGC ATGGTAATGA   
  
  
+ GTAAACCGAC ACCTGCAGAG GAGTTTATCG CCTTCACTCA TTTGTACAGG GTGTCTCCAT TCTATCAGTT   
  
  
+ TGCCCATTTT ACTGCTAATC AGGCCATCTT TGAGGCATTC GAGAGGGAAG AAGAGAACAA CAGCTGGGCA   
  
  
+ CTCCATGTCA TTGATTTTGA CATCTCTCAT GGCTTCCAGT GGCCGTCTCT CATTCAGTCT CTTTCTGAGA   
  
  
+ AGGCTACTGC TTCAAGCCAT TCACTCTCTC TTCGAATCAC AGGTTTTGCT AGAACTCTGG AAGAACTGAT   
  
  
+ TGAGACTGAG AACAGATTAG TAAGTTTTGC TGAAAGCTTC CGGAACATAC TATTTGAATT CCACGGGTGT   
  
  
+ TTGAGGGGTT CAGAGCTGGT GAACTTGAAG AAAAGGGAGA ATGAAACAGT TGCCGTAAAT CTGGTGTTTC   
  
  
+ ACCTCAGCAG TTTCGATGAC ACCCTGAAGG TCTCAGACAC CTTAACCGCT GTACATTCAC TGAAACCTTC   
  
  
+ TATAATGGTA CTAGTTGAAA GAGAAGGAAC CCGAAATGGA TCAGGATTCA TATCAGGTTT CATGGATTCT   
  
  
+ TTGCATTATT ATGCTGCAAT GTTCGATTCA TTAAATGATT GCCTCCCACG GGAGAGTCCC GAGAGACTGA   
  
  
+ GCATAGAGAA GAACCATCTT GGGAGAGAGA TCAAGGAGGC GGTGACTTGG GAGAAGGACG AGATGAGCTA   
  
  
+ CGGGAAGTTC GAGATAATGG AGACTTGGAA AAAGAGGATG GAAATTCATG GGTTTTCGGG GATCAGGCTG   
  
  
+ AGTTCCAAGG CGACAATTCA AGCCAAGCTC CTGCTCAAAA TGGGCAGCCA TTACTGTCCC CAGTTTGCAG   
  
  
+ GGGATTCTGA AGGAGGAGTT GGCGGGTTCA GAGTGTATGA GAGAGATGAG GGGGAAGCCA TTTCTCTGGT   
  
  
+ TTGGCAAGGC AGGTTCCTCG CAACAGCCTC TGCATGGCGC TGTGTATG  

- +Up\_Stream \_Len000CTTTCT TCTAAATTCC TTTAGCTACC ATGTGCCGTT TCGATCTTTG TCCTTGACAT   
  
  
- AAAAATACAA AAAAAAAACA ACCTTCCCCC CCCCCCCCTC GCCCGCTCTA CATTGGGCAT AGATCTACCT   
  
  
- ATTAGATGCA CTGTTCTGGA AGTAAACTAG TTCCATGTAG TACTTATAAA TTGGGAATTG AAGAAACTCG   
  
  
- GAATGTTCTC TAACAGACTC CAAAAATCCC ACGTGCTATA GTACGGACTG ATCTGGACAC ATCTGTGTTA   
  
  
- GAAGTCGGGA ATAACTATCG ACAAGCTATA CGTACGACGG ATTTTTAACC TTATCAACCG TGGATTGTAA   
  
  
- GAAAATGTAG AACGAGGAGG AACCACATCT CAATCTACAC GATCGTGTTA TGTTACCCAG GGCTATAAAA   
  
  
- CGTAGACCAA AGACTTTCTA TTATAAACTA CTAGGACAGA ACACGACTAC CCGTTTTCGA TTTAACATTC   
  
  
- TTATACATCA AATAAATGTT CTGTATACGA ATTCGATCTC CAGTACATGA ACGTATAAAA ACACGTCGAA   
  
  
- AACTCCAACA TACGGGGAAA CTCTGTTTAA GTATCAACTT AAGTACTGTT CTAATACAAA CCGTTACTGA   
  
  
- AACTAATAAT TTAACCGGAA ATACGATAGG ATAGGACACA CGAGTTTACA ATAAATAGTC TATTATAATC   
  
  
- AAATGAGAAA GAAGGGACAG AACGTTTCTA AAATTTAGGA TTTAATACGT TTACAAATAG AAATTTTACT   
  
  
- GGACGATGAG AGACACGGAC TAAAATAAAC AAAGGGGCAA CTTTAAAAAA AATACTAAGG TGAAAAGTTC   
  
  
- GTCGAATCTG TCCGCAATAA TCCATTAATA AAAGGCGAAA CCTAACATCA TTTAACTTTG TCTTTACGGA   
  
  
- GTGATTTGGC GAGAGACGAG AAACACAACT TCTTGGTTAG GGTAAGTTTG AGAAGAAAGA AAAAACTTTA   
  
  
- AGTTAGGGCG AAGTCAGTTT ACAGATTGTC TTTGTCGGAG CACACGTTAA GATGATATAT GCCAATTACG   
  
  
- ACACGTACAT AATGAAGGGT TGTAGAATGG GTCCTGATCC TCCCTGGAAC CATCGGACAA GACGAACTTT   
  
  
- TTAAACAGTA ATAGTGGACT TAAACATAAT TTATGTATTT AGAAACAGAA AGTTAAACAC CGAGGTGTAA   
  
  
- AGTTAAACAC CGAGGTGTGC GAAAACTTTT GAAATACTCT GGATTATATA ATCTCTTGTA GACTAGCACT   
  
  
- AACAATTAAG GTCAAAATAT TAGTATGATT AATCATTTAG TGACTTCCAG TATAACGTAT AACAAAAACG   
  
  
- GATTACTAAC CGAATAAGTT GATAAGGACT TATCAAATCA AAGGTCTTAG AAATCTTCTT TGGACCAGAC   
  
  
- CCATAATAGC TCATAAAAAG AGTCAAGTAC TTTCCGAAAC TAAGTCACAC ACACGACAGT ACAGATCCGT   
  
  
- ACTAACTCAT AAAAATAAAC GTATAGGTCC GTAACACGGA TCTGTTTACA GGACTTAGAA TCACCGACAA   
  
  
- TCCAGACTTG CCTGTGAAAA GAACGACAAA AAGGAAAGGC TCACCGTAGA AATCGAAAAA GTTTGATGGT   
  
  
- TTCCTGATAA TTTGCTCCGG GACGTATACG ACCTTTTGAC GGAGAATTCG ATACAAATAC GTTTTTACCT   
  
  
- CCTGGAACTT GACAAGTTAC ATGGATGTCA GACTCCGTGT CTCGGTTATC TGTATTATCT ATCAACCTAT   
  
  
- AAGAAAAATA TAAATTAAGT GTCATCGCTG TTGGGGTCGT ACTTTGCATC GGTGCATCCC CCCCTTTTTC   
  
  
- CCAGAGGTAG ACAAATATAT AAGAACAGCG AATACCACGA AGACAATACA TTACTGTTTC CTTAAACACA   
  
  
- ACCCTAATAA GGCGAAGTGA GGCAGAGTGA TTGAAATGCC AAAGTCAGGA TTAAGAGAGA AAAAAAGAAA   
  
  
- ACAAAAAAAA GGACTCGAAC AAATCAGACA AAAGAAGACA CACAGAAAGA CTAATACCTT CGGTATGTCC   
  
  
- TCTTCTTACT GCTAGAAAAC TCAGAATCGA ACCGGTAACA ATCAGTAAGA TTGGTACGTC TTTGCTTCCA   
  
  
- CTTCTCCTTC TCCGTACTAC AGAGATTAAG AGACTAGCGT TGTTCTCTTC TCCCAGCCCT ATTCTTTTAT   
  
  
- AAAGTCGACG AAGTTCTTGC TCTCTCTTAC GATTCGTAGC CCGTTTCTTC TTTTCCGCAA TAAGTTCTTC   
  
  
- CCTTCCCAGA AGTAGACTAG GTAAACGAAG AGTACAGACG ATGGGATTAG TCGCTTTTGT TATAGTCGAG   
  
  
- TCGACAATTA GTAAACGAAC TCAACATGTT CTTACAAAGA CATTTACCAC TAAGACCCGT CGCGCAACGA   
  
  
- CGAATAAAAC GACTACCCAA TTGACGCTCC AAGAATTGGG TCTTGAGAGG GAAAATAACG TACCATTACT   
  
  
- CATTTGGCTG TGGACGTCTC CTCAAATAGC GGAAGTGAGT AAACATGTCC CACAGAGGTA AGATAGTCAA   
  
  
- ACGGGTAAAA TGACGATTAG TCCGGTAGAA ACTCCGTAAG CTCTCCCTTC TTCTCTTGTT GTCGACCCGT   
  
  
- GAGGTACAGT AACTAAAACT GTAGAGAGTA CCGAAGGTCA CCGGCAGAGA GTAAGTCAGA GAAAGACTCT   
  
  
- TCCGATGACG AAGTTCGGTA AGTGAGAGAG AAGCTTAGTG TCCAAAACGA TCTTGAGACC TTCTTGACTA   
  
  
- ACTCTGACTC TTGTCTAATC ATTCAAAACG ACTTTCGAAG GCCTTGTATG ATAAACTTAA GGTGCCCACA   
  
  
- AACTCCCCAA GTCTCGACCA CTTGAACTTC TTTTCCCTCT TACTTTGTCA ACGGCATTTA GACCACAAAG   
  
  
- TGGAGTCGTC AAAGCTACTG TGGGACTTCC AGAGTCTGTG GAATTGGCGA CATGTAAGTG ACTTTGGAAG   
  
  
- ATATTACCAT GATCAACTTT CTCTTCCTTG GGCTTTACCT AGTCCTAAGT ATAGTCCAAA GTACCTAAGA   
  
  
- AACGTAATAA TACGACGTTA CAAGCTAAGT AATTTACTAA CGGAGGGTGC CCTCTCAGGG CTCTCTGACT   
  
  
- CGTATCTCTT CTTGGTAGAA CCCTCTCTCT AGTTCCTCCG CCACTGAACC CTCTTCCTGC TCTACTCGAT   
  
  
- GCCCTTCAAG CTCTATTACC TCTGAACCTT TTTCTCCTAC CTTTAAGTAC CCAAAAGCCC CTAGTCCGAC   
  
  
- TCAAGGTTCC GCTGTTAAGT TCGGTTCGAG GACGAGTTTT ACCCGTCGGT AATGACAGGG GTCAAACGTC   
  
  
- CCCTAAGACT TCCTCCTCAA CCGCCCAAGT CTCACATACT CTCTCTACTC CCCCTTCGGT AAAGAGACCA   
  
  
- AACCGTTCCG TCCAAGGAGC GTTGTCGGAG ACGTACCGCG ACACATAC

+     TCA-element

| Site Name | Organism | Position | Strand | Matrix score. | sequence | function |
| --- | --- | --- | --- | --- | --- | --- |
| TCA-element | Nicotiana tabacum | 1830 | + | 9 | CCATCTTTTT | cis-acting element involved in salicylic acid responsiveness |

>HU11G01529.1   
+ +Up\_Stream \_Len000GAAAGA AGATTTAAGG AAATCGATGG TACACGGCAA AGCTAGAAAC AGGAACTGTA   
  
  
+ TTTTTATGTT TTTTTTTTGT TGGAAGGGGG GGGGGGGGAG CGGGCGAGAT GTAACCCGTA TCTAGATGGA   
  
  
+ TAATCTACGT GACAAGACCT TCATTTGATC AAGGTACATC ATGAATATTT AACCCTTAAC TTCTTTGAGC   
  
  
+ CTTACAAGAG ATTGTCTGAG GTTTTTAGGG TGCACGATAT CATGCCTGAC TAGACCTGTG TAGACACAAT   
  
  
+ CTTCAGCCCT TATTGATAGC TGTTCGATAT GCATGCTGCC TAAAAATTGG AATAGTTGGC ACCTAACATT   
  
  
+ CTTTTACATC TTGCTCCTCC TTGGTGTAGA GTTAGATGTG CTAGCACAAT ACAATGGGTC CCGATATTTT   
  
  
+ GCATCTGGTT TCTGAAAGAT AATATTTGAT GATCCTGTCT TGTGCTGATG GGCAAAAGCT AAATTGTAAG   
  
  
+ AATATGTAGT TTATTTACAA GACATATGCT TAAGCTAGAG GTCATGTACT TGCATATTTT TGTGCAGCTT   
  
  
+ TTGAGGTTGT ATGCCCCTTT GAGACAAATT CATAGTTGAA TTCATGACAA GATTATGTTT GGCAATGACT   
  
  
+ TTGATTATTA AATTGGCCTT TATGCTATCC TATCCTGTGT GCTCAAATGT TATTTATCAG ATAATATTAG   
  
  
+ TTTACTCTTT CTTCCCTGTC TTGCAAAGAT TTTAAATCCT AAATTATGCA AATGTTTATC TTTAAAATGA   
  
  
+ CCTGCTACTC TCTGTGCCTG ATTTTATTTG TTTCCCCGTT GAAATTTTTT TTATGATTCC ACTTTTCAAG   
  
  
+ CAGCTTAGAC AGGCGTTATT AGGTAATTAT TTTCCGCTTT GGATTGTAGT AAATTGAAAC AGAAATGCCT   
  
  
+ CACTAAACCG CTCTCTGCTC TTTGTGTTGA AGAACCAATC CCATTCAAAC TCTTCTTTCT TTTTTGAAAT   
  
  
+ TCAATCCCGC TTCAGTCAAA TGTCTAACAG AAACAGCCTC GTGTGCAATT CTACTATATA CGGTTAATGC   
  
  
+ TGTGCATGTA TTACTTCCCA ACATCTTACC CAGGACTAGG AGGGACCTTG GTAGCCTGTT CTGCTTGAAA   
  
  
+ AATTTGTCAT TATCACCTGA ATTTGTATTA AATACATAAA TCTTTGTCTT TCAATTTGTG GCTCCACATT   
  
  
+ TCAATTTGTG GCTCCACACG CTTTTGAAAA CTTTATGAGA CCTAATATAT TAGAGAACAT CTGATCGTGA   
  
  
+ TTGTTAATTC CAGTTTTATA ATCATACTAA TTAGTAAATC ACTGAAGGTC ATATTGCATA TTGTTTTTGC   
  
  
+ CTAATGATTG GCTTATTCAA CTATTCCTGA ATAGTTTAGT TTCCAGAATC TTTAGAAGAA ACCTGGTCTG   
  
  
+ GGTATTATCG AGTATTTTTC TCAGTTCATG AAAGGCTTTG ATTCAGTGTG TGTGCTGTCA TGTCTAGGCA   
  
  
+ TGATTGAGTA TTTTTATTTG CATATCCAGG CATTGTGCCT AGACAAATGT CCTGAATCTT AGTGGCTGTT   
  
  
+ AGGTCTGAAC GGACACTTTT CTTGCTGTTT TTCCTTTCCG AGTGGCATCT TTAGCTTTTT CAAACTACCA   
  
  
+ AAGGACTATT AAACGAGGCC CTGCATATGC TGGAAAACTG CCTCTTAAGC TATGTTTATG CAAAAATGGA   
  
  
+ GGACCTTGAA CTGTTCAATG TACCTACAGT CTGAGGCACA GAGCCAATAG ACATAATAGA TAGTTGGATA   
  
  
+ TTCTTTTTAT ATTTAATTCA CAGTAGCGAC AACCCCAGCA TGAAACGTAG CCACGTAGGG GGGGAAAAAG   
  
  
+ GGTCTCCATC TGTTTATATA TTCTTGTCGC TTATGGTGCT TCTGTTATGT AATGACAAAG GAATTTGTGT   
  
  
+ TGGGATTATT CCGCTTCACT CCGTCTCACT AACTTTACGG TTTCAGTCCT AATTCTCTCT TTTTTTCTTT   
  
  
+ TGTTTTTTTT CCTGAGCTTG TTTAGTCTGT TTTCTTCTGT GTGTCTTTCT GATTATGGAA GCCATACAGG   
  
  
+ AGAAGAATGA CGATCTTTTG AGTCTTAGCT TGGCCATTGT TAGTCATTCT AACCATGCAG AAACGAAGGT   
  
  
+ GAAGAGGAAG AGGCATGATG TCTCTAATTC TCTGATCGCA ACAAGAGAAG AGGGTCGGGA TAAGAAAATA   
  
  
+ TTTCAGCTGC TTCAAGAACG AGAGAGAATG CTAAGCATCG GGCAAAGAAG AAAAGGCGTT ATTCAAGAAG   
  
  
+ GGAAGGGTCT TCATCTGATC CATTTGCTTC TCATGTCTGC TACCCTAATC AGCGAAAACA ATATCAGCTC   
  
  
+ AGCTGTTAAT CATTTGCTTG AGTTGTACAA GAATGTTTCT GTAAATGGTG ATTCTGGGCA GCGCGTTGCT   
  
  
+ GCTTATTTTG CTGATGGGTT AACTGCGAGG TTCTTAACCC AGAACTCTCC CTTTTATTGC ATGGTAATGA   
  
  
+ GTAAACCGAC ACCTGCAGAG GAGTTTATCG CCTTCACTCA TTTGTACAGG GTGTCTCCAT TCTATCAGTT   
  
  
+ TGCCCATTTT ACTGCTAATC AGGCCATCTT TGAGGCATTC GAGAGGGAAG AAGAGAACAA CAGCTGGGCA   
  
  
+ CTCCATGTCA TTGATTTTGA CATCTCTCAT GGCTTCCAGT GGCCGTCTCT CATTCAGTCT CTTTCTGAGA   
  
  
+ AGGCTACTGC TTCAAGCCAT TCACTCTCTC TTCGAATCAC AGGTTTTGCT AGAACTCTGG AAGAACTGAT   
  
  
+ TGAGACTGAG AACAGATTAG TAAGTTTTGC TGAAAGCTTC CGGAACATAC TATTTGAATT CCACGGGTGT   
  
  
+ TTGAGGGGTT CAGAGCTGGT GAACTTGAAG AAAAGGGAGA ATGAAACAGT TGCCGTAAAT CTGGTGTTTC   
  
  
+ ACCTCAGCAG TTTCGATGAC ACCCTGAAGG TCTCAGACAC CTTAACCGCT GTACATTCAC TGAAACCTTC   
  
  
+ TATAATGGTA CTAGTTGAAA GAGAAGGAAC CCGAAATGGA TCAGGATTCA TATCAGGTTT CATGGATTCT   
  
  
+ TTGCATTATT ATGCTGCAAT GTTCGATTCA TTAAATGATT GCCTCCCACG GGAGAGTCCC GAGAGACTGA   
  
  
+ GCATAGAGAA GAACCATCTT GGGAGAGAGA TCAAGGAGGC GGTGACTTGG GAGAAGGACG AGATGAGCTA   
  
  
+ CGGGAAGTTC GAGATAATGG AGACTTGGAA AAAGAGGATG GAAATTCATG GGTTTTCGGG GATCAGGCTG   
  
  
+ AGTTCCAAGG CGACAATTCA AGCCAAGCTC CTGCTCAAAA TGGGCAGCCA TTACTGTCCC CAGTTTGCAG   
  
  
+ GGGATTCTGA AGGAGGAGTT GGCGGGTTCA GAGTGTATGA GAGAGATGAG GGGGAAGCCA TTTCTCTGGT   
  
  
+ TTGGCAAGGC AGGTTCCTCG CAACAGCCTC TGCATGGCGC TGTGTATG  

- +Up\_Stream \_Len000CTTTCT TCTAAATTCC TTTAGCTACC ATGTGCCGTT TCGATCTTTG TCCTTGACAT   
  
  
- AAAAATACAA AAAAAAAACA ACCTTCCCCC CCCCCCCCTC GCCCGCTCTA CATTGGGCAT AGATCTACCT   
  
  
- ATTAGATGCA CTGTTCTGGA AGTAAACTAG TTCCATGTAG TACTTATAAA TTGGGAATTG AAGAAACTCG   
  
  
- GAATGTTCTC TAACAGACTC CAAAAATCCC ACGTGCTATA GTACGGACTG ATCTGGACAC ATCTGTGTTA   
  
  
- GAAGTCGGGA ATAACTATCG ACAAGCTATA CGTACGACGG ATTTTTAACC TTATCAACCG TGGATTGTAA   
  
  
- GAAAATGTAG AACGAGGAGG AACCACATCT CAATCTACAC GATCGTGTTA TGTTACCCAG GGCTATAAAA   
  
  
- CGTAGACCAA AGACTTTCTA TTATAAACTA CTAGGACAGA ACACGACTAC CCGTTTTCGA TTTAACATTC   
  
  
- TTATACATCA AATAAATGTT CTGTATACGA ATTCGATCTC CAGTACATGA ACGTATAAAA ACACGTCGAA   
  
  
- AACTCCAACA TACGGGGAAA CTCTGTTTAA GTATCAACTT AAGTACTGTT CTAATACAAA CCGTTACTGA   
  
  
- AACTAATAAT TTAACCGGAA ATACGATAGG ATAGGACACA CGAGTTTACA ATAAATAGTC TATTATAATC   
  
  
- AAATGAGAAA GAAGGGACAG AACGTTTCTA AAATTTAGGA TTTAATACGT TTACAAATAG AAATTTTACT   
  
  
- GGACGATGAG AGACACGGAC TAAAATAAAC AAAGGGGCAA CTTTAAAAAA AATACTAAGG TGAAAAGTTC   
  
  
- GTCGAATCTG TCCGCAATAA TCCATTAATA AAAGGCGAAA CCTAACATCA TTTAACTTTG TCTTTACGGA   
  
  
- GTGATTTGGC GAGAGACGAG AAACACAACT TCTTGGTTAG GGTAAGTTTG AGAAGAAAGA AAAAACTTTA   
  
  
- AGTTAGGGCG AAGTCAGTTT ACAGATTGTC TTTGTCGGAG CACACGTTAA GATGATATAT GCCAATTACG   
  
  
- ACACGTACAT AATGAAGGGT TGTAGAATGG GTCCTGATCC TCCCTGGAAC CATCGGACAA GACGAACTTT   
  
  
- TTAAACAGTA ATAGTGGACT TAAACATAAT TTATGTATTT AGAAACAGAA AGTTAAACAC CGAGGTGTAA   
  
  
- AGTTAAACAC CGAGGTGTGC GAAAACTTTT GAAATACTCT GGATTATATA ATCTCTTGTA GACTAGCACT   
  
  
- AACAATTAAG GTCAAAATAT TAGTATGATT AATCATTTAG TGACTTCCAG TATAACGTAT AACAAAAACG   
  
  
- GATTACTAAC CGAATAAGTT GATAAGGACT TATCAAATCA AAGGTCTTAG AAATCTTCTT TGGACCAGAC   
  
  
- CCATAATAGC TCATAAAAAG AGTCAAGTAC TTTCCGAAAC TAAGTCACAC ACACGACAGT ACAGATCCGT   
  
  
- ACTAACTCAT AAAAATAAAC GTATAGGTCC GTAACACGGA TCTGTTTACA GGACTTAGAA TCACCGACAA   
  
  
- TCCAGACTTG CCTGTGAAAA GAACGACAAA AAGGAAAGGC TCACCGTAGA AATCGAAAAA GTTTGATGGT   
  
  
- TTCCTGATAA TTTGCTCCGG GACGTATACG ACCTTTTGAC GGAGAATTCG ATACAAATAC GTTTTTACCT   
  
  
- CCTGGAACTT GACAAGTTAC ATGGATGTCA GACTCCGTGT CTCGGTTATC TGTATTATCT ATCAACCTAT   
  
  
- AAGAAAAATA TAAATTAAGT GTCATCGCTG TTGGGGTCGT ACTTTGCATC GGTGCATCCC CCCCTTTTTC   
  
  
- CCAGAGGTAG ACAAATATAT AAGAACAGCG AATACCACGA AGACAATACA TTACTGTTTC CTTAAACACA   
  
  
- ACCCTAATAA GGCGAAGTGA GGCAGAGTGA TTGAAATGCC AAAGTCAGGA TTAAGAGAGA AAAAAAGAAA   
  
  
- ACAAAAAAAA GGACTCGAAC AAATCAGACA AAAGAAGACA CACAGAAAGA CTAATACCTT CGGTATGTCC   
  
  
- TCTTCTTACT GCTAGAAAAC TCAGAATCGA ACCGGTAACA ATCAGTAAGA TTGGTACGTC TTTGCTTCCA   
  
  
- CTTCTCCTTC TCCGTACTAC AGAGATTAAG AGACTAGCGT TGTTCTCTTC TCCCAGCCCT ATTCTTTTAT   
  
  
- AAAGTCGACG AAGTTCTTGC TCTCTCTTAC GATTCGTAGC CCGTTTCTTC TTTTCCGCAA TAAGTTCTTC   
  
  
- CCTTCCCAGA AGTAGACTAG GTAAACGAAG AGTACAGACG ATGGGATTAG TCGCTTTTGT TATAGTCGAG   
  
  
- TCGACAATTA GTAAACGAAC TCAACATGTT CTTACAAAGA CATTTACCAC TAAGACCCGT CGCGCAACGA   
  
  
- CGAATAAAAC GACTACCCAA TTGACGCTCC AAGAATTGGG TCTTGAGAGG GAAAATAACG TACCATTACT   
  
  
- CATTTGGCTG TGGACGTCTC CTCAAATAGC GGAAGTGAGT AAACATGTCC CACAGAGGTA AGATAGTCAA   
  
  
- ACGGGTAAAA TGACGATTAG TCCGGTAGAA ACTCCGTAAG CTCTCCCTTC TTCTCTTGTT GTCGACCCGT   
  
  
- GAGGTACAGT AACTAAAACT GTAGAGAGTA CCGAAGGTCA CCGGCAGAGA GTAAGTCAGA GAAAGACTCT   
  
  
- TCCGATGACG AAGTTCGGTA AGTGAGAGAG AAGCTTAGTG TCCAAAACGA TCTTGAGACC TTCTTGACTA   
  
  
- ACTCTGACTC TTGTCTAATC ATTCAAAACG ACTTTCGAAG GCCTTGTATG ATAAACTTAA GGTGCCCACA   
  
  
- AACTCCCCAA GTCTCGACCA CTTGAACTTC TTTTCCCTCT TACTTTGTCA ACGGCATTTA GACCACAAAG   
  
  
- TGGAGTCGTC AAAGCTACTG TGGGACTTCC AGAGTCTGTG GAATTGGCGA CATGTAAGTG ACTTTGGAAG   
  
  
- ATATTACCAT GATCAACTTT CTCTTCCTTG GGCTTTACCT AGTCCTAAGT ATAGTCCAAA GTACCTAAGA   
  
  
- AACGTAATAA TACGACGTTA CAAGCTAAGT AATTTACTAA CGGAGGGTGC CCTCTCAGGG CTCTCTGACT   
  
  
- CGTATCTCTT CTTGGTAGAA CCCTCTCTCT AGTTCCTCCG CCACTGAACC CTCTTCCTGC TCTACTCGAT   
  
  
- GCCCTTCAAG CTCTATTACC TCTGAACCTT TTTCTCCTAC CTTTAAGTAC CCAAAAGCCC CTAGTCCGAC   
  
  
- TCAAGGTTCC GCTGTTAAGT TCGGTTCGAG GACGAGTTTT ACCCGTCGGT AATGACAGGG GTCAAACGTC   
  
  
- CCCTAAGACT TCCTCCTCAA CCGCCCAAGT CTCACATACT CTCTCTACTC CCCCTTCGGT AAAGAGACCA   
  
  
- AACCGTTCCG TCCAAGGAGC GTTGTCGGAG ACGTACCGCG ACACATAC

+     TCCC-motif

| Site Name | Organism | Position | Strand | Matrix score. | sequence | function |
| --- | --- | --- | --- | --- | --- | --- |
| TCCC-motif | Spinacia oleracea | 2838 | - | 7 | TCTCCCT | part of a light responsive element |
| TCCC-motif | Spinacia oleracea | 2430 | + | 7 | TCTCCCT | part of a light responsive element |

>HU11G01529.1   
+ +Up\_Stream \_Len000GAAAGA AGATTTAAGG AAATCGATGG TACACGGCAA AGCTAGAAAC AGGAACTGTA   
  
  
+ TTTTTATGTT TTTTTTTTGT TGGAAGGGGG GGGGGGGGAG CGGGCGAGAT GTAACCCGTA TCTAGATGGA   
  
  
+ TAATCTACGT GACAAGACCT TCATTTGATC AAGGTACATC ATGAATATTT AACCCTTAAC TTCTTTGAGC   
  
  
+ CTTACAAGAG ATTGTCTGAG GTTTTTAGGG TGCACGATAT CATGCCTGAC TAGACCTGTG TAGACACAAT   
  
  
+ CTTCAGCCCT TATTGATAGC TGTTCGATAT GCATGCTGCC TAAAAATTGG AATAGTTGGC ACCTAACATT   
  
  
+ CTTTTACATC TTGCTCCTCC TTGGTGTAGA GTTAGATGTG CTAGCACAAT ACAATGGGTC CCGATATTTT   
  
  
+ GCATCTGGTT TCTGAAAGAT AATATTTGAT GATCCTGTCT TGTGCTGATG GGCAAAAGCT AAATTGTAAG   
  
  
+ AATATGTAGT TTATTTACAA GACATATGCT TAAGCTAGAG GTCATGTACT TGCATATTTT TGTGCAGCTT   
  
  
+ TTGAGGTTGT ATGCCCCTTT GAGACAAATT CATAGTTGAA TTCATGACAA GATTATGTTT GGCAATGACT   
  
  
+ TTGATTATTA AATTGGCCTT TATGCTATCC TATCCTGTGT GCTCAAATGT TATTTATCAG ATAATATTAG   
  
  
+ TTTACTCTTT CTTCCCTGTC TTGCAAAGAT TTTAAATCCT AAATTATGCA AATGTTTATC TTTAAAATGA   
  
  
+ CCTGCTACTC TCTGTGCCTG ATTTTATTTG TTTCCCCGTT GAAATTTTTT TTATGATTCC ACTTTTCAAG   
  
  
+ CAGCTTAGAC AGGCGTTATT AGGTAATTAT TTTCCGCTTT GGATTGTAGT AAATTGAAAC AGAAATGCCT   
  
  
+ CACTAAACCG CTCTCTGCTC TTTGTGTTGA AGAACCAATC CCATTCAAAC TCTTCTTTCT TTTTTGAAAT   
  
  
+ TCAATCCCGC TTCAGTCAAA TGTCTAACAG AAACAGCCTC GTGTGCAATT CTACTATATA CGGTTAATGC   
  
  
+ TGTGCATGTA TTACTTCCCA ACATCTTACC CAGGACTAGG AGGGACCTTG GTAGCCTGTT CTGCTTGAAA   
  
  
+ AATTTGTCAT TATCACCTGA ATTTGTATTA AATACATAAA TCTTTGTCTT TCAATTTGTG GCTCCACATT   
  
  
+ TCAATTTGTG GCTCCACACG CTTTTGAAAA CTTTATGAGA CCTAATATAT TAGAGAACAT CTGATCGTGA   
  
  
+ TTGTTAATTC CAGTTTTATA ATCATACTAA TTAGTAAATC ACTGAAGGTC ATATTGCATA TTGTTTTTGC   
  
  
+ CTAATGATTG GCTTATTCAA CTATTCCTGA ATAGTTTAGT TTCCAGAATC TTTAGAAGAA ACCTGGTCTG   
  
  
+ GGTATTATCG AGTATTTTTC TCAGTTCATG AAAGGCTTTG ATTCAGTGTG TGTGCTGTCA TGTCTAGGCA   
  
  
+ TGATTGAGTA TTTTTATTTG CATATCCAGG CATTGTGCCT AGACAAATGT CCTGAATCTT AGTGGCTGTT   
  
  
+ AGGTCTGAAC GGACACTTTT CTTGCTGTTT TTCCTTTCCG AGTGGCATCT TTAGCTTTTT CAAACTACCA   
  
  
+ AAGGACTATT AAACGAGGCC CTGCATATGC TGGAAAACTG CCTCTTAAGC TATGTTTATG CAAAAATGGA   
  
  
+ GGACCTTGAA CTGTTCAATG TACCTACAGT CTGAGGCACA GAGCCAATAG ACATAATAGA TAGTTGGATA   
  
  
+ TTCTTTTTAT ATTTAATTCA CAGTAGCGAC AACCCCAGCA TGAAACGTAG CCACGTAGGG GGGGAAAAAG   
  
  
+ GGTCTCCATC TGTTTATATA TTCTTGTCGC TTATGGTGCT TCTGTTATGT AATGACAAAG GAATTTGTGT   
  
  
+ TGGGATTATT CCGCTTCACT CCGTCTCACT AACTTTACGG TTTCAGTCCT AATTCTCTCT TTTTTTCTTT   
  
  
+ TGTTTTTTTT CCTGAGCTTG TTTAGTCTGT TTTCTTCTGT GTGTCTTTCT GATTATGGAA GCCATACAGG   
  
  
+ AGAAGAATGA CGATCTTTTG AGTCTTAGCT TGGCCATTGT TAGTCATTCT AACCATGCAG AAACGAAGGT   
  
  
+ GAAGAGGAAG AGGCATGATG TCTCTAATTC TCTGATCGCA ACAAGAGAAG AGGGTCGGGA TAAGAAAATA   
  
  
+ TTTCAGCTGC TTCAAGAACG AGAGAGAATG CTAAGCATCG GGCAAAGAAG AAAAGGCGTT ATTCAAGAAG   
  
  
+ GGAAGGGTCT TCATCTGATC CATTTGCTTC TCATGTCTGC TACCCTAATC AGCGAAAACA ATATCAGCTC   
  
  
+ AGCTGTTAAT CATTTGCTTG AGTTGTACAA GAATGTTTCT GTAAATGGTG ATTCTGGGCA GCGCGTTGCT   
  
  
+ GCTTATTTTG CTGATGGGTT AACTGCGAGG TTCTTAACCC AGAACTCTCC CTTTTATTGC ATGGTAATGA   
  
  
+ GTAAACCGAC ACCTGCAGAG GAGTTTATCG CCTTCACTCA TTTGTACAGG GTGTCTCCAT TCTATCAGTT   
  
  
+ TGCCCATTTT ACTGCTAATC AGGCCATCTT TGAGGCATTC GAGAGGGAAG AAGAGAACAA CAGCTGGGCA   
  
  
+ CTCCATGTCA TTGATTTTGA CATCTCTCAT GGCTTCCAGT GGCCGTCTCT CATTCAGTCT CTTTCTGAGA   
  
  
+ AGGCTACTGC TTCAAGCCAT TCACTCTCTC TTCGAATCAC AGGTTTTGCT AGAACTCTGG AAGAACTGAT   
  
  
+ TGAGACTGAG AACAGATTAG TAAGTTTTGC TGAAAGCTTC CGGAACATAC TATTTGAATT CCACGGGTGT   
  
  
+ TTGAGGGGTT CAGAGCTGGT GAACTTGAAG AAAAGGGAGA ATGAAACAGT TGCCGTAAAT CTGGTGTTTC   
  
  
+ ACCTCAGCAG TTTCGATGAC ACCCTGAAGG TCTCAGACAC CTTAACCGCT GTACATTCAC TGAAACCTTC   
  
  
+ TATAATGGTA CTAGTTGAAA GAGAAGGAAC CCGAAATGGA TCAGGATTCA TATCAGGTTT CATGGATTCT   
  
  
+ TTGCATTATT ATGCTGCAAT GTTCGATTCA TTAAATGATT GCCTCCCACG GGAGAGTCCC GAGAGACTGA   
  
  
+ GCATAGAGAA GAACCATCTT GGGAGAGAGA TCAAGGAGGC GGTGACTTGG GAGAAGGACG AGATGAGCTA   
  
  
+ CGGGAAGTTC GAGATAATGG AGACTTGGAA AAAGAGGATG GAAATTCATG GGTTTTCGGG GATCAGGCTG   
  
  
+ AGTTCCAAGG CGACAATTCA AGCCAAGCTC CTGCTCAAAA TGGGCAGCCA TTACTGTCCC CAGTTTGCAG   
  
  
+ GGGATTCTGA AGGAGGAGTT GGCGGGTTCA GAGTGTATGA GAGAGATGAG GGGGAAGCCA TTTCTCTGGT   
  
  
+ TTGGCAAGGC AGGTTCCTCG CAACAGCCTC TGCATGGCGC TGTGTATG  

- +Up\_Stream \_Len000CTTTCT TCTAAATTCC TTTAGCTACC ATGTGCCGTT TCGATCTTTG TCCTTGACAT   
  
  
- AAAAATACAA AAAAAAAACA ACCTTCCCCC CCCCCCCCTC GCCCGCTCTA CATTGGGCAT AGATCTACCT   
  
  
- ATTAGATGCA CTGTTCTGGA AGTAAACTAG TTCCATGTAG TACTTATAAA TTGGGAATTG AAGAAACTCG   
  
  
- GAATGTTCTC TAACAGACTC CAAAAATCCC ACGTGCTATA GTACGGACTG ATCTGGACAC ATCTGTGTTA   
  
  
- GAAGTCGGGA ATAACTATCG ACAAGCTATA CGTACGACGG ATTTTTAACC TTATCAACCG TGGATTGTAA   
  
  
- GAAAATGTAG AACGAGGAGG AACCACATCT CAATCTACAC GATCGTGTTA TGTTACCCAG GGCTATAAAA   
  
  
- CGTAGACCAA AGACTTTCTA TTATAAACTA CTAGGACAGA ACACGACTAC CCGTTTTCGA TTTAACATTC   
  
  
- TTATACATCA AATAAATGTT CTGTATACGA ATTCGATCTC CAGTACATGA ACGTATAAAA ACACGTCGAA   
  
  
- AACTCCAACA TACGGGGAAA CTCTGTTTAA GTATCAACTT AAGTACTGTT CTAATACAAA CCGTTACTGA   
  
  
- AACTAATAAT TTAACCGGAA ATACGATAGG ATAGGACACA CGAGTTTACA ATAAATAGTC TATTATAATC   
  
  
- AAATGAGAAA GAAGGGACAG AACGTTTCTA AAATTTAGGA TTTAATACGT TTACAAATAG AAATTTTACT   
  
  
- GGACGATGAG AGACACGGAC TAAAATAAAC AAAGGGGCAA CTTTAAAAAA AATACTAAGG TGAAAAGTTC   
  
  
- GTCGAATCTG TCCGCAATAA TCCATTAATA AAAGGCGAAA CCTAACATCA TTTAACTTTG TCTTTACGGA   
  
  
- GTGATTTGGC GAGAGACGAG AAACACAACT TCTTGGTTAG GGTAAGTTTG AGAAGAAAGA AAAAACTTTA   
  
  
- AGTTAGGGCG AAGTCAGTTT ACAGATTGTC TTTGTCGGAG CACACGTTAA GATGATATAT GCCAATTACG   
  
  
- ACACGTACAT AATGAAGGGT TGTAGAATGG GTCCTGATCC TCCCTGGAAC CATCGGACAA GACGAACTTT   
  
  
- TTAAACAGTA ATAGTGGACT TAAACATAAT TTATGTATTT AGAAACAGAA AGTTAAACAC CGAGGTGTAA   
  
  
- AGTTAAACAC CGAGGTGTGC GAAAACTTTT GAAATACTCT GGATTATATA ATCTCTTGTA GACTAGCACT   
  
  
- AACAATTAAG GTCAAAATAT TAGTATGATT AATCATTTAG TGACTTCCAG TATAACGTAT AACAAAAACG   
  
  
- GATTACTAAC CGAATAAGTT GATAAGGACT TATCAAATCA AAGGTCTTAG AAATCTTCTT TGGACCAGAC   
  
  
- CCATAATAGC TCATAAAAAG AGTCAAGTAC TTTCCGAAAC TAAGTCACAC ACACGACAGT ACAGATCCGT   
  
  
- ACTAACTCAT AAAAATAAAC GTATAGGTCC GTAACACGGA TCTGTTTACA GGACTTAGAA TCACCGACAA   
  
  
- TCCAGACTTG CCTGTGAAAA GAACGACAAA AAGGAAAGGC TCACCGTAGA AATCGAAAAA GTTTGATGGT   
  
  
- TTCCTGATAA TTTGCTCCGG GACGTATACG ACCTTTTGAC GGAGAATTCG ATACAAATAC GTTTTTACCT   
  
  
- CCTGGAACTT GACAAGTTAC ATGGATGTCA GACTCCGTGT CTCGGTTATC TGTATTATCT ATCAACCTAT   
  
  
- AAGAAAAATA TAAATTAAGT GTCATCGCTG TTGGGGTCGT ACTTTGCATC GGTGCATCCC CCCCTTTTTC   
  
  
- CCAGAGGTAG ACAAATATAT AAGAACAGCG AATACCACGA AGACAATACA TTACTGTTTC CTTAAACACA   
  
  
- ACCCTAATAA GGCGAAGTGA GGCAGAGTGA TTGAAATGCC AAAGTCAGGA TTAAGAGAGA AAAAAAGAAA   
  
  
- ACAAAAAAAA GGACTCGAAC AAATCAGACA AAAGAAGACA CACAGAAAGA CTAATACCTT CGGTATGTCC   
  
  
- TCTTCTTACT GCTAGAAAAC TCAGAATCGA ACCGGTAACA ATCAGTAAGA TTGGTACGTC TTTGCTTCCA   
  
  
- CTTCTCCTTC TCCGTACTAC AGAGATTAAG AGACTAGCGT TGTTCTCTTC TCCCAGCCCT ATTCTTTTAT   
  
  
- AAAGTCGACG AAGTTCTTGC TCTCTCTTAC GATTCGTAGC CCGTTTCTTC TTTTCCGCAA TAAGTTCTTC   
  
  
- CCTTCCCAGA AGTAGACTAG GTAAACGAAG AGTACAGACG ATGGGATTAG TCGCTTTTGT TATAGTCGAG   
  
  
- TCGACAATTA GTAAACGAAC TCAACATGTT CTTACAAAGA CATTTACCAC TAAGACCCGT CGCGCAACGA   
  
  
- CGAATAAAAC GACTACCCAA TTGACGCTCC AAGAATTGGG TCTTGAGAGG GAAAATAACG TACCATTACT   
  
  
- CATTTGGCTG TGGACGTCTC CTCAAATAGC GGAAGTGAGT AAACATGTCC CACAGAGGTA AGATAGTCAA   
  
  
- ACGGGTAAAA TGACGATTAG TCCGGTAGAA ACTCCGTAAG CTCTCCCTTC TTCTCTTGTT GTCGACCCGT   
  
  
- GAGGTACAGT AACTAAAACT GTAGAGAGTA CCGAAGGTCA CCGGCAGAGA GTAAGTCAGA GAAAGACTCT   
  
  
- TCCGATGACG AAGTTCGGTA AGTGAGAGAG AAGCTTAGTG TCCAAAACGA TCTTGAGACC TTCTTGACTA   
  
  
- ACTCTGACTC TTGTCTAATC ATTCAAAACG ACTTTCGAAG GCCTTGTATG ATAAACTTAA GGTGCCCACA   
  
  
- AACTCCCCAA GTCTCGACCA CTTGAACTTC TTTTCCCTCT TACTTTGTCA ACGGCATTTA GACCACAAAG   
  
  
- TGGAGTCGTC AAAGCTACTG TGGGACTTCC AGAGTCTGTG GAATTGGCGA CATGTAAGTG ACTTTGGAAG   
  
  
- ATATTACCAT GATCAACTTT CTCTTCCTTG GGCTTTACCT AGTCCTAAGT ATAGTCCAAA GTACCTAAGA   
  
  
- AACGTAATAA TACGACGTTA CAAGCTAAGT AATTTACTAA CGGAGGGTGC CCTCTCAGGG CTCTCTGACT   
  
  
- CGTATCTCTT CTTGGTAGAA CCCTCTCTCT AGTTCCTCCG CCACTGAACC CTCTTCCTGC TCTACTCGAT   
  
  
- GCCCTTCAAG CTCTATTACC TCTGAACCTT TTTCTCCTAC CTTTAAGTAC CCAAAAGCCC CTAGTCCGAC   
  
  
- TCAAGGTTCC GCTGTTAAGT TCGGTTCGAG GACGAGTTTT ACCCGTCGGT AATGACAGGG GTCAAACGTC   
  
  
- CCCTAAGACT TCCTCCTCAA CCGCCCAAGT CTCACATACT CTCTCTACTC CCCCTTCGGT AAAGAGACCA   
  
  
- AACCGTTCCG TCCAAGGAGC GTTGTCGGAG ACGTACCGCG ACACATAC

+     TCT-motif

| Site Name | Organism | Position | Strand | Matrix score. | sequence | function |
| --- | --- | --- | --- | --- | --- | --- |
| TCT-motif | Arabidopsis thaliana | 490 | - | 6 | TCTTAC | part of a light responsive element |
| TCT-motif | Arabidopsis thaliana | 1078 | + | 6 | TCTTAC | part of a light responsive element |

>HU11G01529.1   
+ +Up\_Stream \_Len000GAAAGA AGATTTAAGG AAATCGATGG TACACGGCAA AGCTAGAAAC AGGAACTGTA   
  
  
+ TTTTTATGTT TTTTTTTTGT TGGAAGGGGG GGGGGGGGAG CGGGCGAGAT GTAACCCGTA TCTAGATGGA   
  
  
+ TAATCTACGT GACAAGACCT TCATTTGATC AAGGTACATC ATGAATATTT AACCCTTAAC TTCTTTGAGC   
  
  
+ CTTACAAGAG ATTGTCTGAG GTTTTTAGGG TGCACGATAT CATGCCTGAC TAGACCTGTG TAGACACAAT   
  
  
+ CTTCAGCCCT TATTGATAGC TGTTCGATAT GCATGCTGCC TAAAAATTGG AATAGTTGGC ACCTAACATT   
  
  
+ CTTTTACATC TTGCTCCTCC TTGGTGTAGA GTTAGATGTG CTAGCACAAT ACAATGGGTC CCGATATTTT   
  
  
+ GCATCTGGTT TCTGAAAGAT AATATTTGAT GATCCTGTCT TGTGCTGATG GGCAAAAGCT AAATTGTAAG   
  
  
+ AATATGTAGT TTATTTACAA GACATATGCT TAAGCTAGAG GTCATGTACT TGCATATTTT TGTGCAGCTT   
  
  
+ TTGAGGTTGT ATGCCCCTTT GAGACAAATT CATAGTTGAA TTCATGACAA GATTATGTTT GGCAATGACT   
  
  
+ TTGATTATTA AATTGGCCTT TATGCTATCC TATCCTGTGT GCTCAAATGT TATTTATCAG ATAATATTAG   
  
  
+ TTTACTCTTT CTTCCCTGTC TTGCAAAGAT TTTAAATCCT AAATTATGCA AATGTTTATC TTTAAAATGA   
  
  
+ CCTGCTACTC TCTGTGCCTG ATTTTATTTG TTTCCCCGTT GAAATTTTTT TTATGATTCC ACTTTTCAAG   
  
  
+ CAGCTTAGAC AGGCGTTATT AGGTAATTAT TTTCCGCTTT GGATTGTAGT AAATTGAAAC AGAAATGCCT   
  
  
+ CACTAAACCG CTCTCTGCTC TTTGTGTTGA AGAACCAATC CCATTCAAAC TCTTCTTTCT TTTTTGAAAT   
  
  
+ TCAATCCCGC TTCAGTCAAA TGTCTAACAG AAACAGCCTC GTGTGCAATT CTACTATATA CGGTTAATGC   
  
  
+ TGTGCATGTA TTACTTCCCA ACATCTTACC CAGGACTAGG AGGGACCTTG GTAGCCTGTT CTGCTTGAAA   
  
  
+ AATTTGTCAT TATCACCTGA ATTTGTATTA AATACATAAA TCTTTGTCTT TCAATTTGTG GCTCCACATT   
  
  
+ TCAATTTGTG GCTCCACACG CTTTTGAAAA CTTTATGAGA CCTAATATAT TAGAGAACAT CTGATCGTGA   
  
  
+ TTGTTAATTC CAGTTTTATA ATCATACTAA TTAGTAAATC ACTGAAGGTC ATATTGCATA TTGTTTTTGC   
  
  
+ CTAATGATTG GCTTATTCAA CTATTCCTGA ATAGTTTAGT TTCCAGAATC TTTAGAAGAA ACCTGGTCTG   
  
  
+ GGTATTATCG AGTATTTTTC TCAGTTCATG AAAGGCTTTG ATTCAGTGTG TGTGCTGTCA TGTCTAGGCA   
  
  
+ TGATTGAGTA TTTTTATTTG CATATCCAGG CATTGTGCCT AGACAAATGT CCTGAATCTT AGTGGCTGTT   
  
  
+ AGGTCTGAAC GGACACTTTT CTTGCTGTTT TTCCTTTCCG AGTGGCATCT TTAGCTTTTT CAAACTACCA   
  
  
+ AAGGACTATT AAACGAGGCC CTGCATATGC TGGAAAACTG CCTCTTAAGC TATGTTTATG CAAAAATGGA   
  
  
+ GGACCTTGAA CTGTTCAATG TACCTACAGT CTGAGGCACA GAGCCAATAG ACATAATAGA TAGTTGGATA   
  
  
+ TTCTTTTTAT ATTTAATTCA CAGTAGCGAC AACCCCAGCA TGAAACGTAG CCACGTAGGG GGGGAAAAAG   
  
  
+ GGTCTCCATC TGTTTATATA TTCTTGTCGC TTATGGTGCT TCTGTTATGT AATGACAAAG GAATTTGTGT   
  
  
+ TGGGATTATT CCGCTTCACT CCGTCTCACT AACTTTACGG TTTCAGTCCT AATTCTCTCT TTTTTTCTTT   
  
  
+ TGTTTTTTTT CCTGAGCTTG TTTAGTCTGT TTTCTTCTGT GTGTCTTTCT GATTATGGAA GCCATACAGG   
  
  
+ AGAAGAATGA CGATCTTTTG AGTCTTAGCT TGGCCATTGT TAGTCATTCT AACCATGCAG AAACGAAGGT   
  
  
+ GAAGAGGAAG AGGCATGATG TCTCTAATTC TCTGATCGCA ACAAGAGAAG AGGGTCGGGA TAAGAAAATA   
  
  
+ TTTCAGCTGC TTCAAGAACG AGAGAGAATG CTAAGCATCG GGCAAAGAAG AAAAGGCGTT ATTCAAGAAG   
  
  
+ GGAAGGGTCT TCATCTGATC CATTTGCTTC TCATGTCTGC TACCCTAATC AGCGAAAACA ATATCAGCTC   
  
  
+ AGCTGTTAAT CATTTGCTTG AGTTGTACAA GAATGTTTCT GTAAATGGTG ATTCTGGGCA GCGCGTTGCT   
  
  
+ GCTTATTTTG CTGATGGGTT AACTGCGAGG TTCTTAACCC AGAACTCTCC CTTTTATTGC ATGGTAATGA   
  
  
+ GTAAACCGAC ACCTGCAGAG GAGTTTATCG CCTTCACTCA TTTGTACAGG GTGTCTCCAT TCTATCAGTT   
  
  
+ TGCCCATTTT ACTGCTAATC AGGCCATCTT TGAGGCATTC GAGAGGGAAG AAGAGAACAA CAGCTGGGCA   
  
  
+ CTCCATGTCA TTGATTTTGA CATCTCTCAT GGCTTCCAGT GGCCGTCTCT CATTCAGTCT CTTTCTGAGA   
  
  
+ AGGCTACTGC TTCAAGCCAT TCACTCTCTC TTCGAATCAC AGGTTTTGCT AGAACTCTGG AAGAACTGAT   
  
  
+ TGAGACTGAG AACAGATTAG TAAGTTTTGC TGAAAGCTTC CGGAACATAC TATTTGAATT CCACGGGTGT   
  
  
+ TTGAGGGGTT CAGAGCTGGT GAACTTGAAG AAAAGGGAGA ATGAAACAGT TGCCGTAAAT CTGGTGTTTC   
  
  
+ ACCTCAGCAG TTTCGATGAC ACCCTGAAGG TCTCAGACAC CTTAACCGCT GTACATTCAC TGAAACCTTC   
  
  
+ TATAATGGTA CTAGTTGAAA GAGAAGGAAC CCGAAATGGA TCAGGATTCA TATCAGGTTT CATGGATTCT   
  
  
+ TTGCATTATT ATGCTGCAAT GTTCGATTCA TTAAATGATT GCCTCCCACG GGAGAGTCCC GAGAGACTGA   
  
  
+ GCATAGAGAA GAACCATCTT GGGAGAGAGA TCAAGGAGGC GGTGACTTGG GAGAAGGACG AGATGAGCTA   
  
  
+ CGGGAAGTTC GAGATAATGG AGACTTGGAA AAAGAGGATG GAAATTCATG GGTTTTCGGG GATCAGGCTG   
  
  
+ AGTTCCAAGG CGACAATTCA AGCCAAGCTC CTGCTCAAAA TGGGCAGCCA TTACTGTCCC CAGTTTGCAG   
  
  
+ GGGATTCTGA AGGAGGAGTT GGCGGGTTCA GAGTGTATGA GAGAGATGAG GGGGAAGCCA TTTCTCTGGT   
  
  
+ TTGGCAAGGC AGGTTCCTCG CAACAGCCTC TGCATGGCGC TGTGTATG  

- +Up\_Stream \_Len000CTTTCT TCTAAATTCC TTTAGCTACC ATGTGCCGTT TCGATCTTTG TCCTTGACAT   
  
  
- AAAAATACAA AAAAAAAACA ACCTTCCCCC CCCCCCCCTC GCCCGCTCTA CATTGGGCAT AGATCTACCT   
  
  
- ATTAGATGCA CTGTTCTGGA AGTAAACTAG TTCCATGTAG TACTTATAAA TTGGGAATTG AAGAAACTCG   
  
  
- GAATGTTCTC TAACAGACTC CAAAAATCCC ACGTGCTATA GTACGGACTG ATCTGGACAC ATCTGTGTTA   
  
  
- GAAGTCGGGA ATAACTATCG ACAAGCTATA CGTACGACGG ATTTTTAACC TTATCAACCG TGGATTGTAA   
  
  
- GAAAATGTAG AACGAGGAGG AACCACATCT CAATCTACAC GATCGTGTTA TGTTACCCAG GGCTATAAAA   
  
  
- CGTAGACCAA AGACTTTCTA TTATAAACTA CTAGGACAGA ACACGACTAC CCGTTTTCGA TTTAACATTC   
  
  
- TTATACATCA AATAAATGTT CTGTATACGA ATTCGATCTC CAGTACATGA ACGTATAAAA ACACGTCGAA   
  
  
- AACTCCAACA TACGGGGAAA CTCTGTTTAA GTATCAACTT AAGTACTGTT CTAATACAAA CCGTTACTGA   
  
  
- AACTAATAAT TTAACCGGAA ATACGATAGG ATAGGACACA CGAGTTTACA ATAAATAGTC TATTATAATC   
  
  
- AAATGAGAAA GAAGGGACAG AACGTTTCTA AAATTTAGGA TTTAATACGT TTACAAATAG AAATTTTACT   
  
  
- GGACGATGAG AGACACGGAC TAAAATAAAC AAAGGGGCAA CTTTAAAAAA AATACTAAGG TGAAAAGTTC   
  
  
- GTCGAATCTG TCCGCAATAA TCCATTAATA AAAGGCGAAA CCTAACATCA TTTAACTTTG TCTTTACGGA   
  
  
- GTGATTTGGC GAGAGACGAG AAACACAACT TCTTGGTTAG GGTAAGTTTG AGAAGAAAGA AAAAACTTTA   
  
  
- AGTTAGGGCG AAGTCAGTTT ACAGATTGTC TTTGTCGGAG CACACGTTAA GATGATATAT GCCAATTACG   
  
  
- ACACGTACAT AATGAAGGGT TGTAGAATGG GTCCTGATCC TCCCTGGAAC CATCGGACAA GACGAACTTT   
  
  
- TTAAACAGTA ATAGTGGACT TAAACATAAT TTATGTATTT AGAAACAGAA AGTTAAACAC CGAGGTGTAA   
  
  
- AGTTAAACAC CGAGGTGTGC GAAAACTTTT GAAATACTCT GGATTATATA ATCTCTTGTA GACTAGCACT   
  
  
- AACAATTAAG GTCAAAATAT TAGTATGATT AATCATTTAG TGACTTCCAG TATAACGTAT AACAAAAACG   
  
  
- GATTACTAAC CGAATAAGTT GATAAGGACT TATCAAATCA AAGGTCTTAG AAATCTTCTT TGGACCAGAC   
  
  
- CCATAATAGC TCATAAAAAG AGTCAAGTAC TTTCCGAAAC TAAGTCACAC ACACGACAGT ACAGATCCGT   
  
  
- ACTAACTCAT AAAAATAAAC GTATAGGTCC GTAACACGGA TCTGTTTACA GGACTTAGAA TCACCGACAA   
  
  
- TCCAGACTTG CCTGTGAAAA GAACGACAAA AAGGAAAGGC TCACCGTAGA AATCGAAAAA GTTTGATGGT   
  
  
- TTCCTGATAA TTTGCTCCGG GACGTATACG ACCTTTTGAC GGAGAATTCG ATACAAATAC GTTTTTACCT   
  
  
- CCTGGAACTT GACAAGTTAC ATGGATGTCA GACTCCGTGT CTCGGTTATC TGTATTATCT ATCAACCTAT   
  
  
- AAGAAAAATA TAAATTAAGT GTCATCGCTG TTGGGGTCGT ACTTTGCATC GGTGCATCCC CCCCTTTTTC   
  
  
- CCAGAGGTAG ACAAATATAT AAGAACAGCG AATACCACGA AGACAATACA TTACTGTTTC CTTAAACACA   
  
  
- ACCCTAATAA GGCGAAGTGA GGCAGAGTGA TTGAAATGCC AAAGTCAGGA TTAAGAGAGA AAAAAAGAAA   
  
  
- ACAAAAAAAA GGACTCGAAC AAATCAGACA AAAGAAGACA CACAGAAAGA CTAATACCTT CGGTATGTCC   
  
  
- TCTTCTTACT GCTAGAAAAC TCAGAATCGA ACCGGTAACA ATCAGTAAGA TTGGTACGTC TTTGCTTCCA   
  
  
- CTTCTCCTTC TCCGTACTAC AGAGATTAAG AGACTAGCGT TGTTCTCTTC TCCCAGCCCT ATTCTTTTAT   
  
  
- AAAGTCGACG AAGTTCTTGC TCTCTCTTAC GATTCGTAGC CCGTTTCTTC TTTTCCGCAA TAAGTTCTTC   
  
  
- CCTTCCCAGA AGTAGACTAG GTAAACGAAG AGTACAGACG ATGGGATTAG TCGCTTTTGT TATAGTCGAG   
  
  
- TCGACAATTA GTAAACGAAC TCAACATGTT CTTACAAAGA CATTTACCAC TAAGACCCGT CGCGCAACGA   
  
  
- CGAATAAAAC GACTACCCAA TTGACGCTCC AAGAATTGGG TCTTGAGAGG GAAAATAACG TACCATTACT   
  
  
- CATTTGGCTG TGGACGTCTC CTCAAATAGC GGAAGTGAGT AAACATGTCC CACAGAGGTA AGATAGTCAA   
  
  
- ACGGGTAAAA TGACGATTAG TCCGGTAGAA ACTCCGTAAG CTCTCCCTTC TTCTCTTGTT GTCGACCCGT   
  
  
- GAGGTACAGT AACTAAAACT GTAGAGAGTA CCGAAGGTCA CCGGCAGAGA GTAAGTCAGA GAAAGACTCT   
  
  
- TCCGATGACG AAGTTCGGTA AGTGAGAGAG AAGCTTAGTG TCCAAAACGA TCTTGAGACC TTCTTGACTA   
  
  
- ACTCTGACTC TTGTCTAATC ATTCAAAACG ACTTTCGAAG GCCTTGTATG ATAAACTTAA GGTGCCCACA   
  
  
- AACTCCCCAA GTCTCGACCA CTTGAACTTC TTTTCCCTCT TACTTTGTCA ACGGCATTTA GACCACAAAG   
  
  
- TGGAGTCGTC AAAGCTACTG TGGGACTTCC AGAGTCTGTG GAATTGGCGA CATGTAAGTG ACTTTGGAAG   
  
  
- ATATTACCAT GATCAACTTT CTCTTCCTTG GGCTTTACCT AGTCCTAAGT ATAGTCCAAA GTACCTAAGA   
  
  
- AACGTAATAA TACGACGTTA CAAGCTAAGT AATTTACTAA CGGAGGGTGC CCTCTCAGGG CTCTCTGACT   
  
  
- CGTATCTCTT CTTGGTAGAA CCCTCTCTCT AGTTCCTCCG CCACTGAACC CTCTTCCTGC TCTACTCGAT   
  
  
- GCCCTTCAAG CTCTATTACC TCTGAACCTT TTTCTCCTAC CTTTAAGTAC CCAAAAGCCC CTAGTCCGAC   
  
  
- TCAAGGTTCC GCTGTTAAGT TCGGTTCGAG GACGAGTTTT ACCCGTCGGT AATGACAGGG GTCAAACGTC   
  
  
- CCCTAAGACT TCCTCCTCAA CCGCCCAAGT CTCACATACT CTCTCTACTC CCCCTTCGGT AAAGAGACCA   
  
  
- AACCGTTCCG TCCAAGGAGC GTTGTCGGAG ACGTACCGCG ACACATAC

+     TGACG-motif

| Site Name | Organism | Position | Strand | Matrix score. | sequence | function |
| --- | --- | --- | --- | --- | --- | --- |
| TGACG-motif | Hordeum vulgare | 2042 | + | 5 | TGACG | cis-acting regulatory element involved in the MeJA-responsiveness |

>HU11G01529.1   
+ +Up\_Stream \_Len000GAAAGA AGATTTAAGG AAATCGATGG TACACGGCAA AGCTAGAAAC AGGAACTGTA   
  
  
+ TTTTTATGTT TTTTTTTTGT TGGAAGGGGG GGGGGGGGAG CGGGCGAGAT GTAACCCGTA TCTAGATGGA   
  
  
+ TAATCTACGT GACAAGACCT TCATTTGATC AAGGTACATC ATGAATATTT AACCCTTAAC TTCTTTGAGC   
  
  
+ CTTACAAGAG ATTGTCTGAG GTTTTTAGGG TGCACGATAT CATGCCTGAC TAGACCTGTG TAGACACAAT   
  
  
+ CTTCAGCCCT TATTGATAGC TGTTCGATAT GCATGCTGCC TAAAAATTGG AATAGTTGGC ACCTAACATT   
  
  
+ CTTTTACATC TTGCTCCTCC TTGGTGTAGA GTTAGATGTG CTAGCACAAT ACAATGGGTC CCGATATTTT   
  
  
+ GCATCTGGTT TCTGAAAGAT AATATTTGAT GATCCTGTCT TGTGCTGATG GGCAAAAGCT AAATTGTAAG   
  
  
+ AATATGTAGT TTATTTACAA GACATATGCT TAAGCTAGAG GTCATGTACT TGCATATTTT TGTGCAGCTT   
  
  
+ TTGAGGTTGT ATGCCCCTTT GAGACAAATT CATAGTTGAA TTCATGACAA GATTATGTTT GGCAATGACT   
  
  
+ TTGATTATTA AATTGGCCTT TATGCTATCC TATCCTGTGT GCTCAAATGT TATTTATCAG ATAATATTAG   
  
  
+ TTTACTCTTT CTTCCCTGTC TTGCAAAGAT TTTAAATCCT AAATTATGCA AATGTTTATC TTTAAAATGA   
  
  
+ CCTGCTACTC TCTGTGCCTG ATTTTATTTG TTTCCCCGTT GAAATTTTTT TTATGATTCC ACTTTTCAAG   
  
  
+ CAGCTTAGAC AGGCGTTATT AGGTAATTAT TTTCCGCTTT GGATTGTAGT AAATTGAAAC AGAAATGCCT   
  
  
+ CACTAAACCG CTCTCTGCTC TTTGTGTTGA AGAACCAATC CCATTCAAAC TCTTCTTTCT TTTTTGAAAT   
  
  
+ TCAATCCCGC TTCAGTCAAA TGTCTAACAG AAACAGCCTC GTGTGCAATT CTACTATATA CGGTTAATGC   
  
  
+ TGTGCATGTA TTACTTCCCA ACATCTTACC CAGGACTAGG AGGGACCTTG GTAGCCTGTT CTGCTTGAAA   
  
  
+ AATTTGTCAT TATCACCTGA ATTTGTATTA AATACATAAA TCTTTGTCTT TCAATTTGTG GCTCCACATT   
  
  
+ TCAATTTGTG GCTCCACACG CTTTTGAAAA CTTTATGAGA CCTAATATAT TAGAGAACAT CTGATCGTGA   
  
  
+ TTGTTAATTC CAGTTTTATA ATCATACTAA TTAGTAAATC ACTGAAGGTC ATATTGCATA TTGTTTTTGC   
  
  
+ CTAATGATTG GCTTATTCAA CTATTCCTGA ATAGTTTAGT TTCCAGAATC TTTAGAAGAA ACCTGGTCTG   
  
  
+ GGTATTATCG AGTATTTTTC TCAGTTCATG AAAGGCTTTG ATTCAGTGTG TGTGCTGTCA TGTCTAGGCA   
  
  
+ TGATTGAGTA TTTTTATTTG CATATCCAGG CATTGTGCCT AGACAAATGT CCTGAATCTT AGTGGCTGTT   
  
  
+ AGGTCTGAAC GGACACTTTT CTTGCTGTTT TTCCTTTCCG AGTGGCATCT TTAGCTTTTT CAAACTACCA   
  
  
+ AAGGACTATT AAACGAGGCC CTGCATATGC TGGAAAACTG CCTCTTAAGC TATGTTTATG CAAAAATGGA   
  
  
+ GGACCTTGAA CTGTTCAATG TACCTACAGT CTGAGGCACA GAGCCAATAG ACATAATAGA TAGTTGGATA   
  
  
+ TTCTTTTTAT ATTTAATTCA CAGTAGCGAC AACCCCAGCA TGAAACGTAG CCACGTAGGG GGGGAAAAAG   
  
  
+ GGTCTCCATC TGTTTATATA TTCTTGTCGC TTATGGTGCT TCTGTTATGT AATGACAAAG GAATTTGTGT   
  
  
+ TGGGATTATT CCGCTTCACT CCGTCTCACT AACTTTACGG TTTCAGTCCT AATTCTCTCT TTTTTTCTTT   
  
  
+ TGTTTTTTTT CCTGAGCTTG TTTAGTCTGT TTTCTTCTGT GTGTCTTTCT GATTATGGAA GCCATACAGG   
  
  
+ AGAAGAATGA CGATCTTTTG AGTCTTAGCT TGGCCATTGT TAGTCATTCT AACCATGCAG AAACGAAGGT   
  
  
+ GAAGAGGAAG AGGCATGATG TCTCTAATTC TCTGATCGCA ACAAGAGAAG AGGGTCGGGA TAAGAAAATA   
  
  
+ TTTCAGCTGC TTCAAGAACG AGAGAGAATG CTAAGCATCG GGCAAAGAAG AAAAGGCGTT ATTCAAGAAG   
  
  
+ GGAAGGGTCT TCATCTGATC CATTTGCTTC TCATGTCTGC TACCCTAATC AGCGAAAACA ATATCAGCTC   
  
  
+ AGCTGTTAAT CATTTGCTTG AGTTGTACAA GAATGTTTCT GTAAATGGTG ATTCTGGGCA GCGCGTTGCT   
  
  
+ GCTTATTTTG CTGATGGGTT AACTGCGAGG TTCTTAACCC AGAACTCTCC CTTTTATTGC ATGGTAATGA   
  
  
+ GTAAACCGAC ACCTGCAGAG GAGTTTATCG CCTTCACTCA TTTGTACAGG GTGTCTCCAT TCTATCAGTT   
  
  
+ TGCCCATTTT ACTGCTAATC AGGCCATCTT TGAGGCATTC GAGAGGGAAG AAGAGAACAA CAGCTGGGCA   
  
  
+ CTCCATGTCA TTGATTTTGA CATCTCTCAT GGCTTCCAGT GGCCGTCTCT CATTCAGTCT CTTTCTGAGA   
  
  
+ AGGCTACTGC TTCAAGCCAT TCACTCTCTC TTCGAATCAC AGGTTTTGCT AGAACTCTGG AAGAACTGAT   
  
  
+ TGAGACTGAG AACAGATTAG TAAGTTTTGC TGAAAGCTTC CGGAACATAC TATTTGAATT CCACGGGTGT   
  
  
+ TTGAGGGGTT CAGAGCTGGT GAACTTGAAG AAAAGGGAGA ATGAAACAGT TGCCGTAAAT CTGGTGTTTC   
  
  
+ ACCTCAGCAG TTTCGATGAC ACCCTGAAGG TCTCAGACAC CTTAACCGCT GTACATTCAC TGAAACCTTC   
  
  
+ TATAATGGTA CTAGTTGAAA GAGAAGGAAC CCGAAATGGA TCAGGATTCA TATCAGGTTT CATGGATTCT   
  
  
+ TTGCATTATT ATGCTGCAAT GTTCGATTCA TTAAATGATT GCCTCCCACG GGAGAGTCCC GAGAGACTGA   
  
  
+ GCATAGAGAA GAACCATCTT GGGAGAGAGA TCAAGGAGGC GGTGACTTGG GAGAAGGACG AGATGAGCTA   
  
  
+ CGGGAAGTTC GAGATAATGG AGACTTGGAA AAAGAGGATG GAAATTCATG GGTTTTCGGG GATCAGGCTG   
  
  
+ AGTTCCAAGG CGACAATTCA AGCCAAGCTC CTGCTCAAAA TGGGCAGCCA TTACTGTCCC CAGTTTGCAG   
  
  
+ GGGATTCTGA AGGAGGAGTT GGCGGGTTCA GAGTGTATGA GAGAGATGAG GGGGAAGCCA TTTCTCTGGT   
  
  
+ TTGGCAAGGC AGGTTCCTCG CAACAGCCTC TGCATGGCGC TGTGTATG  

- +Up\_Stream \_Len000CTTTCT TCTAAATTCC TTTAGCTACC ATGTGCCGTT TCGATCTTTG TCCTTGACAT   
  
  
- AAAAATACAA AAAAAAAACA ACCTTCCCCC CCCCCCCCTC GCCCGCTCTA CATTGGGCAT AGATCTACCT   
  
  
- ATTAGATGCA CTGTTCTGGA AGTAAACTAG TTCCATGTAG TACTTATAAA TTGGGAATTG AAGAAACTCG   
  
  
- GAATGTTCTC TAACAGACTC CAAAAATCCC ACGTGCTATA GTACGGACTG ATCTGGACAC ATCTGTGTTA   
  
  
- GAAGTCGGGA ATAACTATCG ACAAGCTATA CGTACGACGG ATTTTTAACC TTATCAACCG TGGATTGTAA   
  
  
- GAAAATGTAG AACGAGGAGG AACCACATCT CAATCTACAC GATCGTGTTA TGTTACCCAG GGCTATAAAA   
  
  
- CGTAGACCAA AGACTTTCTA TTATAAACTA CTAGGACAGA ACACGACTAC CCGTTTTCGA TTTAACATTC   
  
  
- TTATACATCA AATAAATGTT CTGTATACGA ATTCGATCTC CAGTACATGA ACGTATAAAA ACACGTCGAA   
  
  
- AACTCCAACA TACGGGGAAA CTCTGTTTAA GTATCAACTT AAGTACTGTT CTAATACAAA CCGTTACTGA   
  
  
- AACTAATAAT TTAACCGGAA ATACGATAGG ATAGGACACA CGAGTTTACA ATAAATAGTC TATTATAATC   
  
  
- AAATGAGAAA GAAGGGACAG AACGTTTCTA AAATTTAGGA TTTAATACGT TTACAAATAG AAATTTTACT   
  
  
- GGACGATGAG AGACACGGAC TAAAATAAAC AAAGGGGCAA CTTTAAAAAA AATACTAAGG TGAAAAGTTC   
  
  
- GTCGAATCTG TCCGCAATAA TCCATTAATA AAAGGCGAAA CCTAACATCA TTTAACTTTG TCTTTACGGA   
  
  
- GTGATTTGGC GAGAGACGAG AAACACAACT TCTTGGTTAG GGTAAGTTTG AGAAGAAAGA AAAAACTTTA   
  
  
- AGTTAGGGCG AAGTCAGTTT ACAGATTGTC TTTGTCGGAG CACACGTTAA GATGATATAT GCCAATTACG   
  
  
- ACACGTACAT AATGAAGGGT TGTAGAATGG GTCCTGATCC TCCCTGGAAC CATCGGACAA GACGAACTTT   
  
  
- TTAAACAGTA ATAGTGGACT TAAACATAAT TTATGTATTT AGAAACAGAA AGTTAAACAC CGAGGTGTAA   
  
  
- AGTTAAACAC CGAGGTGTGC GAAAACTTTT GAAATACTCT GGATTATATA ATCTCTTGTA GACTAGCACT   
  
  
- AACAATTAAG GTCAAAATAT TAGTATGATT AATCATTTAG TGACTTCCAG TATAACGTAT AACAAAAACG   
  
  
- GATTACTAAC CGAATAAGTT GATAAGGACT TATCAAATCA AAGGTCTTAG AAATCTTCTT TGGACCAGAC   
  
  
- CCATAATAGC TCATAAAAAG AGTCAAGTAC TTTCCGAAAC TAAGTCACAC ACACGACAGT ACAGATCCGT   
  
  
- ACTAACTCAT AAAAATAAAC GTATAGGTCC GTAACACGGA TCTGTTTACA GGACTTAGAA TCACCGACAA   
  
  
- TCCAGACTTG CCTGTGAAAA GAACGACAAA AAGGAAAGGC TCACCGTAGA AATCGAAAAA GTTTGATGGT   
  
  
- TTCCTGATAA TTTGCTCCGG GACGTATACG ACCTTTTGAC GGAGAATTCG ATACAAATAC GTTTTTACCT   
  
  
- CCTGGAACTT GACAAGTTAC ATGGATGTCA GACTCCGTGT CTCGGTTATC TGTATTATCT ATCAACCTAT   
  
  
- AAGAAAAATA TAAATTAAGT GTCATCGCTG TTGGGGTCGT ACTTTGCATC GGTGCATCCC CCCCTTTTTC   
  
  
- CCAGAGGTAG ACAAATATAT AAGAACAGCG AATACCACGA AGACAATACA TTACTGTTTC CTTAAACACA   
  
  
- ACCCTAATAA GGCGAAGTGA GGCAGAGTGA TTGAAATGCC AAAGTCAGGA TTAAGAGAGA AAAAAAGAAA   
  
  
- ACAAAAAAAA GGACTCGAAC AAATCAGACA AAAGAAGACA CACAGAAAGA CTAATACCTT CGGTATGTCC   
  
  
- TCTTCTTACT GCTAGAAAAC TCAGAATCGA ACCGGTAACA ATCAGTAAGA TTGGTACGTC TTTGCTTCCA   
  
  
- CTTCTCCTTC TCCGTACTAC AGAGATTAAG AGACTAGCGT TGTTCTCTTC TCCCAGCCCT ATTCTTTTAT   
  
  
- AAAGTCGACG AAGTTCTTGC TCTCTCTTAC GATTCGTAGC CCGTTTCTTC TTTTCCGCAA TAAGTTCTTC   
  
  
- CCTTCCCAGA AGTAGACTAG GTAAACGAAG AGTACAGACG ATGGGATTAG TCGCTTTTGT TATAGTCGAG   
  
  
- TCGACAATTA GTAAACGAAC TCAACATGTT CTTACAAAGA CATTTACCAC TAAGACCCGT CGCGCAACGA   
  
  
- CGAATAAAAC GACTACCCAA TTGACGCTCC AAGAATTGGG TCTTGAGAGG GAAAATAACG TACCATTACT   
  
  
- CATTTGGCTG TGGACGTCTC CTCAAATAGC GGAAGTGAGT AAACATGTCC CACAGAGGTA AGATAGTCAA   
  
  
- ACGGGTAAAA TGACGATTAG TCCGGTAGAA ACTCCGTAAG CTCTCCCTTC TTCTCTTGTT GTCGACCCGT   
  
  
- GAGGTACAGT AACTAAAACT GTAGAGAGTA CCGAAGGTCA CCGGCAGAGA GTAAGTCAGA GAAAGACTCT   
  
  
- TCCGATGACG AAGTTCGGTA AGTGAGAGAG AAGCTTAGTG TCCAAAACGA TCTTGAGACC TTCTTGACTA   
  
  
- ACTCTGACTC TTGTCTAATC ATTCAAAACG ACTTTCGAAG GCCTTGTATG ATAAACTTAA GGTGCCCACA   
  
  
- AACTCCCCAA GTCTCGACCA CTTGAACTTC TTTTCCCTCT TACTTTGTCA ACGGCATTTA GACCACAAAG   
  
  
- TGGAGTCGTC AAAGCTACTG TGGGACTTCC AGAGTCTGTG GAATTGGCGA CATGTAAGTG ACTTTGGAAG   
  
  
- ATATTACCAT GATCAACTTT CTCTTCCTTG GGCTTTACCT AGTCCTAAGT ATAGTCCAAA GTACCTAAGA   
  
  
- AACGTAATAA TACGACGTTA CAAGCTAAGT AATTTACTAA CGGAGGGTGC CCTCTCAGGG CTCTCTGACT   
  
  
- CGTATCTCTT CTTGGTAGAA CCCTCTCTCT AGTTCCTCCG CCACTGAACC CTCTTCCTGC TCTACTCGAT   
  
  
- GCCCTTCAAG CTCTATTACC TCTGAACCTT TTTCTCCTAC CTTTAAGTAC CCAAAAGCCC CTAGTCCGAC   
  
  
- TCAAGGTTCC GCTGTTAAGT TCGGTTCGAG GACGAGTTTT ACCCGTCGGT AATGACAGGG GTCAAACGTC   
  
  
- CCCTAAGACT TCCTCCTCAA CCGCCCAAGT CTCACATACT CTCTCTACTC CCCCTTCGGT AAAGAGACCA   
  
  
- AACCGTTCCG TCCAAGGAGC GTTGTCGGAG ACGTACCGCG ACACATAC

+     Unnamed\_\_1

| Site Name | Organism | Position | Strand | Matrix score. | sequence | function |
| --- | --- | --- | --- | --- | --- | --- |
| Unnamed\_\_1 | Zea mays | 3060 | - | 5 | CGTGG |  |
| Unnamed\_\_1 | Zea mays | 2795 | - | 5 | CGTGG |  |
| Unnamed\_\_1 | Zea mays | 1805 | - | 5 | CGTGG |  |

>HU11G01529.1   
+ +Up\_Stream \_Len000GAAAGA AGATTTAAGG AAATCGATGG TACACGGCAA AGCTAGAAAC AGGAACTGTA   
  
  
+ TTTTTATGTT TTTTTTTTGT TGGAAGGGGG GGGGGGGGAG CGGGCGAGAT GTAACCCGTA TCTAGATGGA   
  
  
+ TAATCTACGT GACAAGACCT TCATTTGATC AAGGTACATC ATGAATATTT AACCCTTAAC TTCTTTGAGC   
  
  
+ CTTACAAGAG ATTGTCTGAG GTTTTTAGGG TGCACGATAT CATGCCTGAC TAGACCTGTG TAGACACAAT   
  
  
+ CTTCAGCCCT TATTGATAGC TGTTCGATAT GCATGCTGCC TAAAAATTGG AATAGTTGGC ACCTAACATT   
  
  
+ CTTTTACATC TTGCTCCTCC TTGGTGTAGA GTTAGATGTG CTAGCACAAT ACAATGGGTC CCGATATTTT   
  
  
+ GCATCTGGTT TCTGAAAGAT AATATTTGAT GATCCTGTCT TGTGCTGATG GGCAAAAGCT AAATTGTAAG   
  
  
+ AATATGTAGT TTATTTACAA GACATATGCT TAAGCTAGAG GTCATGTACT TGCATATTTT TGTGCAGCTT   
  
  
+ TTGAGGTTGT ATGCCCCTTT GAGACAAATT CATAGTTGAA TTCATGACAA GATTATGTTT GGCAATGACT   
  
  
+ TTGATTATTA AATTGGCCTT TATGCTATCC TATCCTGTGT GCTCAAATGT TATTTATCAG ATAATATTAG   
  
  
+ TTTACTCTTT CTTCCCTGTC TTGCAAAGAT TTTAAATCCT AAATTATGCA AATGTTTATC TTTAAAATGA   
  
  
+ CCTGCTACTC TCTGTGCCTG ATTTTATTTG TTTCCCCGTT GAAATTTTTT TTATGATTCC ACTTTTCAAG   
  
  
+ CAGCTTAGAC AGGCGTTATT AGGTAATTAT TTTCCGCTTT GGATTGTAGT AAATTGAAAC AGAAATGCCT   
  
  
+ CACTAAACCG CTCTCTGCTC TTTGTGTTGA AGAACCAATC CCATTCAAAC TCTTCTTTCT TTTTTGAAAT   
  
  
+ TCAATCCCGC TTCAGTCAAA TGTCTAACAG AAACAGCCTC GTGTGCAATT CTACTATATA CGGTTAATGC   
  
  
+ TGTGCATGTA TTACTTCCCA ACATCTTACC CAGGACTAGG AGGGACCTTG GTAGCCTGTT CTGCTTGAAA   
  
  
+ AATTTGTCAT TATCACCTGA ATTTGTATTA AATACATAAA TCTTTGTCTT TCAATTTGTG GCTCCACATT   
  
  
+ TCAATTTGTG GCTCCACACG CTTTTGAAAA CTTTATGAGA CCTAATATAT TAGAGAACAT CTGATCGTGA   
  
  
+ TTGTTAATTC CAGTTTTATA ATCATACTAA TTAGTAAATC ACTGAAGGTC ATATTGCATA TTGTTTTTGC   
  
  
+ CTAATGATTG GCTTATTCAA CTATTCCTGA ATAGTTTAGT TTCCAGAATC TTTAGAAGAA ACCTGGTCTG   
  
  
+ GGTATTATCG AGTATTTTTC TCAGTTCATG AAAGGCTTTG ATTCAGTGTG TGTGCTGTCA TGTCTAGGCA   
  
  
+ TGATTGAGTA TTTTTATTTG CATATCCAGG CATTGTGCCT AGACAAATGT CCTGAATCTT AGTGGCTGTT   
  
  
+ AGGTCTGAAC GGACACTTTT CTTGCTGTTT TTCCTTTCCG AGTGGCATCT TTAGCTTTTT CAAACTACCA   
  
  
+ AAGGACTATT AAACGAGGCC CTGCATATGC TGGAAAACTG CCTCTTAAGC TATGTTTATG CAAAAATGGA   
  
  
+ GGACCTTGAA CTGTTCAATG TACCTACAGT CTGAGGCACA GAGCCAATAG ACATAATAGA TAGTTGGATA   
  
  
+ TTCTTTTTAT ATTTAATTCA CAGTAGCGAC AACCCCAGCA TGAAACGTAG CCACGTAGGG GGGGAAAAAG   
  
  
+ GGTCTCCATC TGTTTATATA TTCTTGTCGC TTATGGTGCT TCTGTTATGT AATGACAAAG GAATTTGTGT   
  
  
+ TGGGATTATT CCGCTTCACT CCGTCTCACT AACTTTACGG TTTCAGTCCT AATTCTCTCT TTTTTTCTTT   
  
  
+ TGTTTTTTTT CCTGAGCTTG TTTAGTCTGT TTTCTTCTGT GTGTCTTTCT GATTATGGAA GCCATACAGG   
  
  
+ AGAAGAATGA CGATCTTTTG AGTCTTAGCT TGGCCATTGT TAGTCATTCT AACCATGCAG AAACGAAGGT   
  
  
+ GAAGAGGAAG AGGCATGATG TCTCTAATTC TCTGATCGCA ACAAGAGAAG AGGGTCGGGA TAAGAAAATA   
  
  
+ TTTCAGCTGC TTCAAGAACG AGAGAGAATG CTAAGCATCG GGCAAAGAAG AAAAGGCGTT ATTCAAGAAG   
  
  
+ GGAAGGGTCT TCATCTGATC CATTTGCTTC TCATGTCTGC TACCCTAATC AGCGAAAACA ATATCAGCTC   
  
  
+ AGCTGTTAAT CATTTGCTTG AGTTGTACAA GAATGTTTCT GTAAATGGTG ATTCTGGGCA GCGCGTTGCT   
  
  
+ GCTTATTTTG CTGATGGGTT AACTGCGAGG TTCTTAACCC AGAACTCTCC CTTTTATTGC ATGGTAATGA   
  
  
+ GTAAACCGAC ACCTGCAGAG GAGTTTATCG CCTTCACTCA TTTGTACAGG GTGTCTCCAT TCTATCAGTT   
  
  
+ TGCCCATTTT ACTGCTAATC AGGCCATCTT TGAGGCATTC GAGAGGGAAG AAGAGAACAA CAGCTGGGCA   
  
  
+ CTCCATGTCA TTGATTTTGA CATCTCTCAT GGCTTCCAGT GGCCGTCTCT CATTCAGTCT CTTTCTGAGA   
  
  
+ AGGCTACTGC TTCAAGCCAT TCACTCTCTC TTCGAATCAC AGGTTTTGCT AGAACTCTGG AAGAACTGAT   
  
  
+ TGAGACTGAG AACAGATTAG TAAGTTTTGC TGAAAGCTTC CGGAACATAC TATTTGAATT CCACGGGTGT   
  
  
+ TTGAGGGGTT CAGAGCTGGT GAACTTGAAG AAAAGGGAGA ATGAAACAGT TGCCGTAAAT CTGGTGTTTC   
  
  
+ ACCTCAGCAG TTTCGATGAC ACCCTGAAGG TCTCAGACAC CTTAACCGCT GTACATTCAC TGAAACCTTC   
  
  
+ TATAATGGTA CTAGTTGAAA GAGAAGGAAC CCGAAATGGA TCAGGATTCA TATCAGGTTT CATGGATTCT   
  
  
+ TTGCATTATT ATGCTGCAAT GTTCGATTCA TTAAATGATT GCCTCCCACG GGAGAGTCCC GAGAGACTGA   
  
  
+ GCATAGAGAA GAACCATCTT GGGAGAGAGA TCAAGGAGGC GGTGACTTGG GAGAAGGACG AGATGAGCTA   
  
  
+ CGGGAAGTTC GAGATAATGG AGACTTGGAA AAAGAGGATG GAAATTCATG GGTTTTCGGG GATCAGGCTG   
  
  
+ AGTTCCAAGG CGACAATTCA AGCCAAGCTC CTGCTCAAAA TGGGCAGCCA TTACTGTCCC CAGTTTGCAG   
  
  
+ GGGATTCTGA AGGAGGAGTT GGCGGGTTCA GAGTGTATGA GAGAGATGAG GGGGAAGCCA TTTCTCTGGT   
  
  
+ TTGGCAAGGC AGGTTCCTCG CAACAGCCTC TGCATGGCGC TGTGTATG  

- +Up\_Stream \_Len000CTTTCT TCTAAATTCC TTTAGCTACC ATGTGCCGTT TCGATCTTTG TCCTTGACAT   
  
  
- AAAAATACAA AAAAAAAACA ACCTTCCCCC CCCCCCCCTC GCCCGCTCTA CATTGGGCAT AGATCTACCT   
  
  
- ATTAGATGCA CTGTTCTGGA AGTAAACTAG TTCCATGTAG TACTTATAAA TTGGGAATTG AAGAAACTCG   
  
  
- GAATGTTCTC TAACAGACTC CAAAAATCCC ACGTGCTATA GTACGGACTG ATCTGGACAC ATCTGTGTTA   
  
  
- GAAGTCGGGA ATAACTATCG ACAAGCTATA CGTACGACGG ATTTTTAACC TTATCAACCG TGGATTGTAA   
  
  
- GAAAATGTAG AACGAGGAGG AACCACATCT CAATCTACAC GATCGTGTTA TGTTACCCAG GGCTATAAAA   
  
  
- CGTAGACCAA AGACTTTCTA TTATAAACTA CTAGGACAGA ACACGACTAC CCGTTTTCGA TTTAACATTC   
  
  
- TTATACATCA AATAAATGTT CTGTATACGA ATTCGATCTC CAGTACATGA ACGTATAAAA ACACGTCGAA   
  
  
- AACTCCAACA TACGGGGAAA CTCTGTTTAA GTATCAACTT AAGTACTGTT CTAATACAAA CCGTTACTGA   
  
  
- AACTAATAAT TTAACCGGAA ATACGATAGG ATAGGACACA CGAGTTTACA ATAAATAGTC TATTATAATC   
  
  
- AAATGAGAAA GAAGGGACAG AACGTTTCTA AAATTTAGGA TTTAATACGT TTACAAATAG AAATTTTACT   
  
  
- GGACGATGAG AGACACGGAC TAAAATAAAC AAAGGGGCAA CTTTAAAAAA AATACTAAGG TGAAAAGTTC   
  
  
- GTCGAATCTG TCCGCAATAA TCCATTAATA AAAGGCGAAA CCTAACATCA TTTAACTTTG TCTTTACGGA   
  
  
- GTGATTTGGC GAGAGACGAG AAACACAACT TCTTGGTTAG GGTAAGTTTG AGAAGAAAGA AAAAACTTTA   
  
  
- AGTTAGGGCG AAGTCAGTTT ACAGATTGTC TTTGTCGGAG CACACGTTAA GATGATATAT GCCAATTACG   
  
  
- ACACGTACAT AATGAAGGGT TGTAGAATGG GTCCTGATCC TCCCTGGAAC CATCGGACAA GACGAACTTT   
  
  
- TTAAACAGTA ATAGTGGACT TAAACATAAT TTATGTATTT AGAAACAGAA AGTTAAACAC CGAGGTGTAA   
  
  
- AGTTAAACAC CGAGGTGTGC GAAAACTTTT GAAATACTCT GGATTATATA ATCTCTTGTA GACTAGCACT   
  
  
- AACAATTAAG GTCAAAATAT TAGTATGATT AATCATTTAG TGACTTCCAG TATAACGTAT AACAAAAACG   
  
  
- GATTACTAAC CGAATAAGTT GATAAGGACT TATCAAATCA AAGGTCTTAG AAATCTTCTT TGGACCAGAC   
  
  
- CCATAATAGC TCATAAAAAG AGTCAAGTAC TTTCCGAAAC TAAGTCACAC ACACGACAGT ACAGATCCGT   
  
  
- ACTAACTCAT AAAAATAAAC GTATAGGTCC GTAACACGGA TCTGTTTACA GGACTTAGAA TCACCGACAA   
  
  
- TCCAGACTTG CCTGTGAAAA GAACGACAAA AAGGAAAGGC TCACCGTAGA AATCGAAAAA GTTTGATGGT   
  
  
- TTCCTGATAA TTTGCTCCGG GACGTATACG ACCTTTTGAC GGAGAATTCG ATACAAATAC GTTTTTACCT   
  
  
- CCTGGAACTT GACAAGTTAC ATGGATGTCA GACTCCGTGT CTCGGTTATC TGTATTATCT ATCAACCTAT   
  
  
- AAGAAAAATA TAAATTAAGT GTCATCGCTG TTGGGGTCGT ACTTTGCATC GGTGCATCCC CCCCTTTTTC   
  
  
- CCAGAGGTAG ACAAATATAT AAGAACAGCG AATACCACGA AGACAATACA TTACTGTTTC CTTAAACACA   
  
  
- ACCCTAATAA GGCGAAGTGA GGCAGAGTGA TTGAAATGCC AAAGTCAGGA TTAAGAGAGA AAAAAAGAAA   
  
  
- ACAAAAAAAA GGACTCGAAC AAATCAGACA AAAGAAGACA CACAGAAAGA CTAATACCTT CGGTATGTCC   
  
  
- TCTTCTTACT GCTAGAAAAC TCAGAATCGA ACCGGTAACA ATCAGTAAGA TTGGTACGTC TTTGCTTCCA   
  
  
- CTTCTCCTTC TCCGTACTAC AGAGATTAAG AGACTAGCGT TGTTCTCTTC TCCCAGCCCT ATTCTTTTAT   
  
  
- AAAGTCGACG AAGTTCTTGC TCTCTCTTAC GATTCGTAGC CCGTTTCTTC TTTTCCGCAA TAAGTTCTTC   
  
  
- CCTTCCCAGA AGTAGACTAG GTAAACGAAG AGTACAGACG ATGGGATTAG TCGCTTTTGT TATAGTCGAG   
  
  
- TCGACAATTA GTAAACGAAC TCAACATGTT CTTACAAAGA CATTTACCAC TAAGACCCGT CGCGCAACGA   
  
  
- CGAATAAAAC GACTACCCAA TTGACGCTCC AAGAATTGGG TCTTGAGAGG GAAAATAACG TACCATTACT   
  
  
- CATTTGGCTG TGGACGTCTC CTCAAATAGC GGAAGTGAGT AAACATGTCC CACAGAGGTA AGATAGTCAA   
  
  
- ACGGGTAAAA TGACGATTAG TCCGGTAGAA ACTCCGTAAG CTCTCCCTTC TTCTCTTGTT GTCGACCCGT   
  
  
- GAGGTACAGT AACTAAAACT GTAGAGAGTA CCGAAGGTCA CCGGCAGAGA GTAAGTCAGA GAAAGACTCT   
  
  
- TCCGATGACG AAGTTCGGTA AGTGAGAGAG AAGCTTAGTG TCCAAAACGA TCTTGAGACC TTCTTGACTA   
  
  
- ACTCTGACTC TTGTCTAATC ATTCAAAACG ACTTTCGAAG GCCTTGTATG ATAAACTTAA GGTGCCCACA   
  
  
- AACTCCCCAA GTCTCGACCA CTTGAACTTC TTTTCCCTCT TACTTTGTCA ACGGCATTTA GACCACAAAG   
  
  
- TGGAGTCGTC AAAGCTACTG TGGGACTTCC AGAGTCTGTG GAATTGGCGA CATGTAAGTG ACTTTGGAAG   
  
  
- ATATTACCAT GATCAACTTT CTCTTCCTTG GGCTTTACCT AGTCCTAAGT ATAGTCCAAA GTACCTAAGA   
  
  
- AACGTAATAA TACGACGTTA CAAGCTAAGT AATTTACTAA CGGAGGGTGC CCTCTCAGGG CTCTCTGACT   
  
  
- CGTATCTCTT CTTGGTAGAA CCCTCTCTCT AGTTCCTCCG CCACTGAACC CTCTTCCTGC TCTACTCGAT   
  
  
- GCCCTTCAAG CTCTATTACC TCTGAACCTT TTTCTCCTAC CTTTAAGTAC CCAAAAGCCC CTAGTCCGAC   
  
  
- TCAAGGTTCC GCTGTTAAGT TCGGTTCGAG GACGAGTTTT ACCCGTCGGT AATGACAGGG GTCAAACGTC   
  
  
- CCCTAAGACT TCCTCCTCAA CCGCCCAAGT CTCACATACT CTCTCTACTC CCCCTTCGGT AAAGAGACCA   
  
  
- AACCGTTCCG TCCAAGGAGC GTTGTCGGAG ACGTACCGCG ACACATAC

+     Unnamed\_\_4

| Site Name | Organism | Position | Strand | Matrix score. | sequence | function |
| --- | --- | --- | --- | --- | --- | --- |
| Unnamed\_\_4 | Petroselinum hortense | 3306 | - | 4 | CTCC |  |
| Unnamed\_\_4 | Petroselinum hortense | 3134 | - | 4 | CTCC |  |
| Unnamed\_\_4 | Petroselinum hortense | 1186 | + | 4 | CTCC |  |
| Unnamed\_\_4 | Petroselinum hortense | 368 | + | 4 | CTCC |  |
| Unnamed\_\_4 | Petroselinum hortense | 3252 | + | 4 | CTCC |  |
| Unnamed\_\_4 | Petroselinum hortense | 2840 | - | 4 | CTCC |  |
| Unnamed\_\_4 | Petroselinum hortense | 1682 | - | 4 | CTCC |  |
| Unnamed\_\_4 | Petroselinum hortense | 3309 | - | 4 | CTCC |  |
| Unnamed\_\_4 | Petroselinum hortense | 3057 | + | 4 | CTCC |  |
| Unnamed\_\_4 | Petroselinum hortense | 111 | - | 4 | CTCC |  |
| Unnamed\_\_4 | Petroselinum hortense | 2431 | + | 4 | CTCC |  |
| Unnamed\_\_4 | Petroselinum hortense | 1828 | + | 4 | CTCC |  |
| Unnamed\_\_4 | Petroselinum hortense | 1206 | + | 4 | CTCC |  |
| Unnamed\_\_4 | Petroselinum hortense | 3065 | - | 4 | CTCC |  |
| Unnamed\_\_4 | Petroselinum hortense | 3106 | - | 4 | CTCC |  |
| Unnamed\_\_4 | Petroselinum hortense | 371 | + | 4 | CTCC |  |
| Unnamed\_\_4 | Petroselinum hortense | 2595 | + | 4 | CTCC |  |
| Unnamed\_\_4 | Petroselinum hortense | 2474 | - | 4 | CTCC |  |
| Unnamed\_\_4 | Petroselinum hortense | 1093 | - | 4 | CTCC |  |
| Unnamed\_\_4 | Petroselinum hortense | 3173 | - | 4 | CTCC |  |
| Unnamed\_\_4 | Petroselinum hortense | 2509 | + | 4 | CTCC |  |
| Unnamed\_\_4 | Petroselinum hortense | 2033 | - | 4 | CTCC |  |
| Unnamed\_\_4 | Petroselinum hortense | 1913 | + | 4 | CTCC |  |
| Unnamed\_\_4 | Petroselinum hortense | 3119 | - | 4 | CTCC |  |

>HU11G01529.1   
+ +Up\_Stream \_Len000GAAAGA AGATTTAAGG AAATCGATGG TACACGGCAA AGCTAGAAAC AGGAACTGTA   
  
  
+ TTTTTATGTT TTTTTTTTGT TGGAAGGGGG GGGGGGGGAG CGGGCGAGAT GTAACCCGTA TCTAGATGGA   
  
  
+ TAATCTACGT GACAAGACCT TCATTTGATC AAGGTACATC ATGAATATTT AACCCTTAAC TTCTTTGAGC   
  
  
+ CTTACAAGAG ATTGTCTGAG GTTTTTAGGG TGCACGATAT CATGCCTGAC TAGACCTGTG TAGACACAAT   
  
  
+ CTTCAGCCCT TATTGATAGC TGTTCGATAT GCATGCTGCC TAAAAATTGG AATAGTTGGC ACCTAACATT   
  
  
+ CTTTTACATC TTGCTCCTCC TTGGTGTAGA GTTAGATGTG CTAGCACAAT ACAATGGGTC CCGATATTTT   
  
  
+ GCATCTGGTT TCTGAAAGAT AATATTTGAT GATCCTGTCT TGTGCTGATG GGCAAAAGCT AAATTGTAAG   
  
  
+ AATATGTAGT TTATTTACAA GACATATGCT TAAGCTAGAG GTCATGTACT TGCATATTTT TGTGCAGCTT   
  
  
+ TTGAGGTTGT ATGCCCCTTT GAGACAAATT CATAGTTGAA TTCATGACAA GATTATGTTT GGCAATGACT   
  
  
+ TTGATTATTA AATTGGCCTT TATGCTATCC TATCCTGTGT GCTCAAATGT TATTTATCAG ATAATATTAG   
  
  
+ TTTACTCTTT CTTCCCTGTC TTGCAAAGAT TTTAAATCCT AAATTATGCA AATGTTTATC TTTAAAATGA   
  
  
+ CCTGCTACTC TCTGTGCCTG ATTTTATTTG TTTCCCCGTT GAAATTTTTT TTATGATTCC ACTTTTCAAG   
  
  
+ CAGCTTAGAC AGGCGTTATT AGGTAATTAT TTTCCGCTTT GGATTGTAGT AAATTGAAAC AGAAATGCCT   
  
  
+ CACTAAACCG CTCTCTGCTC TTTGTGTTGA AGAACCAATC CCATTCAAAC TCTTCTTTCT TTTTTGAAAT   
  
  
+ TCAATCCCGC TTCAGTCAAA TGTCTAACAG AAACAGCCTC GTGTGCAATT CTACTATATA CGGTTAATGC   
  
  
+ TGTGCATGTA TTACTTCCCA ACATCTTACC CAGGACTAGG AGGGACCTTG GTAGCCTGTT CTGCTTGAAA   
  
  
+ AATTTGTCAT TATCACCTGA ATTTGTATTA AATACATAAA TCTTTGTCTT TCAATTTGTG GCTCCACATT   
  
  
+ TCAATTTGTG GCTCCACACG CTTTTGAAAA CTTTATGAGA CCTAATATAT TAGAGAACAT CTGATCGTGA   
  
  
+ TTGTTAATTC CAGTTTTATA ATCATACTAA TTAGTAAATC ACTGAAGGTC ATATTGCATA TTGTTTTTGC   
  
  
+ CTAATGATTG GCTTATTCAA CTATTCCTGA ATAGTTTAGT TTCCAGAATC TTTAGAAGAA ACCTGGTCTG   
  
  
+ GGTATTATCG AGTATTTTTC TCAGTTCATG AAAGGCTTTG ATTCAGTGTG TGTGCTGTCA TGTCTAGGCA   
  
  
+ TGATTGAGTA TTTTTATTTG CATATCCAGG CATTGTGCCT AGACAAATGT CCTGAATCTT AGTGGCTGTT   
  
  
+ AGGTCTGAAC GGACACTTTT CTTGCTGTTT TTCCTTTCCG AGTGGCATCT TTAGCTTTTT CAAACTACCA   
  
  
+ AAGGACTATT AAACGAGGCC CTGCATATGC TGGAAAACTG CCTCTTAAGC TATGTTTATG CAAAAATGGA   
  
  
+ GGACCTTGAA CTGTTCAATG TACCTACAGT CTGAGGCACA GAGCCAATAG ACATAATAGA TAGTTGGATA   
  
  
+ TTCTTTTTAT ATTTAATTCA CAGTAGCGAC AACCCCAGCA TGAAACGTAG CCACGTAGGG GGGGAAAAAG   
  
  
+ GGTCTCCATC TGTTTATATA TTCTTGTCGC TTATGGTGCT TCTGTTATGT AATGACAAAG GAATTTGTGT   
  
  
+ TGGGATTATT CCGCTTCACT CCGTCTCACT AACTTTACGG TTTCAGTCCT AATTCTCTCT TTTTTTCTTT   
  
  
+ TGTTTTTTTT CCTGAGCTTG TTTAGTCTGT TTTCTTCTGT GTGTCTTTCT GATTATGGAA GCCATACAGG   
  
  
+ AGAAGAATGA CGATCTTTTG AGTCTTAGCT TGGCCATTGT TAGTCATTCT AACCATGCAG AAACGAAGGT   
  
  
+ GAAGAGGAAG AGGCATGATG TCTCTAATTC TCTGATCGCA ACAAGAGAAG AGGGTCGGGA TAAGAAAATA   
  
  
+ TTTCAGCTGC TTCAAGAACG AGAGAGAATG CTAAGCATCG GGCAAAGAAG AAAAGGCGTT ATTCAAGAAG   
  
  
+ GGAAGGGTCT TCATCTGATC CATTTGCTTC TCATGTCTGC TACCCTAATC AGCGAAAACA ATATCAGCTC   
  
  
+ AGCTGTTAAT CATTTGCTTG AGTTGTACAA GAATGTTTCT GTAAATGGTG ATTCTGGGCA GCGCGTTGCT   
  
  
+ GCTTATTTTG CTGATGGGTT AACTGCGAGG TTCTTAACCC AGAACTCTCC CTTTTATTGC ATGGTAATGA   
  
  
+ GTAAACCGAC ACCTGCAGAG GAGTTTATCG CCTTCACTCA TTTGTACAGG GTGTCTCCAT TCTATCAGTT   
  
  
+ TGCCCATTTT ACTGCTAATC AGGCCATCTT TGAGGCATTC GAGAGGGAAG AAGAGAACAA CAGCTGGGCA   
  
  
+ CTCCATGTCA TTGATTTTGA CATCTCTCAT GGCTTCCAGT GGCCGTCTCT CATTCAGTCT CTTTCTGAGA   
  
  
+ AGGCTACTGC TTCAAGCCAT TCACTCTCTC TTCGAATCAC AGGTTTTGCT AGAACTCTGG AAGAACTGAT   
  
  
+ TGAGACTGAG AACAGATTAG TAAGTTTTGC TGAAAGCTTC CGGAACATAC TATTTGAATT CCACGGGTGT   
  
  
+ TTGAGGGGTT CAGAGCTGGT GAACTTGAAG AAAAGGGAGA ATGAAACAGT TGCCGTAAAT CTGGTGTTTC   
  
  
+ ACCTCAGCAG TTTCGATGAC ACCCTGAAGG TCTCAGACAC CTTAACCGCT GTACATTCAC TGAAACCTTC   
  
  
+ TATAATGGTA CTAGTTGAAA GAGAAGGAAC CCGAAATGGA TCAGGATTCA TATCAGGTTT CATGGATTCT   
  
  
+ TTGCATTATT ATGCTGCAAT GTTCGATTCA TTAAATGATT GCCTCCCACG GGAGAGTCCC GAGAGACTGA   
  
  
+ GCATAGAGAA GAACCATCTT GGGAGAGAGA TCAAGGAGGC GGTGACTTGG GAGAAGGACG AGATGAGCTA   
  
  
+ CGGGAAGTTC GAGATAATGG AGACTTGGAA AAAGAGGATG GAAATTCATG GGTTTTCGGG GATCAGGCTG   
  
  
+ AGTTCCAAGG CGACAATTCA AGCCAAGCTC CTGCTCAAAA TGGGCAGCCA TTACTGTCCC CAGTTTGCAG   
  
  
+ GGGATTCTGA AGGAGGAGTT GGCGGGTTCA GAGTGTATGA GAGAGATGAG GGGGAAGCCA TTTCTCTGGT   
  
  
+ TTGGCAAGGC AGGTTCCTCG CAACAGCCTC TGCATGGCGC TGTGTATG  

- +Up\_Stream \_Len000CTTTCT TCTAAATTCC TTTAGCTACC ATGTGCCGTT TCGATCTTTG TCCTTGACAT   
  
  
- AAAAATACAA AAAAAAAACA ACCTTCCCCC CCCCCCCCTC GCCCGCTCTA CATTGGGCAT AGATCTACCT   
  
  
- ATTAGATGCA CTGTTCTGGA AGTAAACTAG TTCCATGTAG TACTTATAAA TTGGGAATTG AAGAAACTCG   
  
  
- GAATGTTCTC TAACAGACTC CAAAAATCCC ACGTGCTATA GTACGGACTG ATCTGGACAC ATCTGTGTTA   
  
  
- GAAGTCGGGA ATAACTATCG ACAAGCTATA CGTACGACGG ATTTTTAACC TTATCAACCG TGGATTGTAA   
  
  
- GAAAATGTAG AACGAGGAGG AACCACATCT CAATCTACAC GATCGTGTTA TGTTACCCAG GGCTATAAAA   
  
  
- CGTAGACCAA AGACTTTCTA TTATAAACTA CTAGGACAGA ACACGACTAC CCGTTTTCGA TTTAACATTC   
  
  
- TTATACATCA AATAAATGTT CTGTATACGA ATTCGATCTC CAGTACATGA ACGTATAAAA ACACGTCGAA   
  
  
- AACTCCAACA TACGGGGAAA CTCTGTTTAA GTATCAACTT AAGTACTGTT CTAATACAAA CCGTTACTGA   
  
  
- AACTAATAAT TTAACCGGAA ATACGATAGG ATAGGACACA CGAGTTTACA ATAAATAGTC TATTATAATC   
  
  
- AAATGAGAAA GAAGGGACAG AACGTTTCTA AAATTTAGGA TTTAATACGT TTACAAATAG AAATTTTACT   
  
  
- GGACGATGAG AGACACGGAC TAAAATAAAC AAAGGGGCAA CTTTAAAAAA AATACTAAGG TGAAAAGTTC   
  
  
- GTCGAATCTG TCCGCAATAA TCCATTAATA AAAGGCGAAA CCTAACATCA TTTAACTTTG TCTTTACGGA   
  
  
- GTGATTTGGC GAGAGACGAG AAACACAACT TCTTGGTTAG GGTAAGTTTG AGAAGAAAGA AAAAACTTTA   
  
  
- AGTTAGGGCG AAGTCAGTTT ACAGATTGTC TTTGTCGGAG CACACGTTAA GATGATATAT GCCAATTACG   
  
  
- ACACGTACAT AATGAAGGGT TGTAGAATGG GTCCTGATCC TCCCTGGAAC CATCGGACAA GACGAACTTT   
  
  
- TTAAACAGTA ATAGTGGACT TAAACATAAT TTATGTATTT AGAAACAGAA AGTTAAACAC CGAGGTGTAA   
  
  
- AGTTAAACAC CGAGGTGTGC GAAAACTTTT GAAATACTCT GGATTATATA ATCTCTTGTA GACTAGCACT   
  
  
- AACAATTAAG GTCAAAATAT TAGTATGATT AATCATTTAG TGACTTCCAG TATAACGTAT AACAAAAACG   
  
  
- GATTACTAAC CGAATAAGTT GATAAGGACT TATCAAATCA AAGGTCTTAG AAATCTTCTT TGGACCAGAC   
  
  
- CCATAATAGC TCATAAAAAG AGTCAAGTAC TTTCCGAAAC TAAGTCACAC ACACGACAGT ACAGATCCGT   
  
  
- ACTAACTCAT AAAAATAAAC GTATAGGTCC GTAACACGGA TCTGTTTACA GGACTTAGAA TCACCGACAA   
  
  
- TCCAGACTTG CCTGTGAAAA GAACGACAAA AAGGAAAGGC TCACCGTAGA AATCGAAAAA GTTTGATGGT   
  
  
- TTCCTGATAA TTTGCTCCGG GACGTATACG ACCTTTTGAC GGAGAATTCG ATACAAATAC GTTTTTACCT   
  
  
- CCTGGAACTT GACAAGTTAC ATGGATGTCA GACTCCGTGT CTCGGTTATC TGTATTATCT ATCAACCTAT   
  
  
- AAGAAAAATA TAAATTAAGT GTCATCGCTG TTGGGGTCGT ACTTTGCATC GGTGCATCCC CCCCTTTTTC   
  
  
- CCAGAGGTAG ACAAATATAT AAGAACAGCG AATACCACGA AGACAATACA TTACTGTTTC CTTAAACACA   
  
  
- ACCCTAATAA GGCGAAGTGA GGCAGAGTGA TTGAAATGCC AAAGTCAGGA TTAAGAGAGA AAAAAAGAAA   
  
  
- ACAAAAAAAA GGACTCGAAC AAATCAGACA AAAGAAGACA CACAGAAAGA CTAATACCTT CGGTATGTCC   
  
  
- TCTTCTTACT GCTAGAAAAC TCAGAATCGA ACCGGTAACA ATCAGTAAGA TTGGTACGTC TTTGCTTCCA   
  
  
- CTTCTCCTTC TCCGTACTAC AGAGATTAAG AGACTAGCGT TGTTCTCTTC TCCCAGCCCT ATTCTTTTAT   
  
  
- AAAGTCGACG AAGTTCTTGC TCTCTCTTAC GATTCGTAGC CCGTTTCTTC TTTTCCGCAA TAAGTTCTTC   
  
  
- CCTTCCCAGA AGTAGACTAG GTAAACGAAG AGTACAGACG ATGGGATTAG TCGCTTTTGT TATAGTCGAG   
  
  
- TCGACAATTA GTAAACGAAC TCAACATGTT CTTACAAAGA CATTTACCAC TAAGACCCGT CGCGCAACGA   
  
  
- CGAATAAAAC GACTACCCAA TTGACGCTCC AAGAATTGGG TCTTGAGAGG GAAAATAACG TACCATTACT   
  
  
- CATTTGGCTG TGGACGTCTC CTCAAATAGC GGAAGTGAGT AAACATGTCC CACAGAGGTA AGATAGTCAA   
  
  
- ACGGGTAAAA TGACGATTAG TCCGGTAGAA ACTCCGTAAG CTCTCCCTTC TTCTCTTGTT GTCGACCCGT   
  
  
- GAGGTACAGT AACTAAAACT GTAGAGAGTA CCGAAGGTCA CCGGCAGAGA GTAAGTCAGA GAAAGACTCT   
  
  
- TCCGATGACG AAGTTCGGTA AGTGAGAGAG AAGCTTAGTG TCCAAAACGA TCTTGAGACC TTCTTGACTA   
  
  
- ACTCTGACTC TTGTCTAATC ATTCAAAACG ACTTTCGAAG GCCTTGTATG ATAAACTTAA GGTGCCCACA   
  
  
- AACTCCCCAA GTCTCGACCA CTTGAACTTC TTTTCCCTCT TACTTTGTCA ACGGCATTTA GACCACAAAG   
  
  
- TGGAGTCGTC AAAGCTACTG TGGGACTTCC AGAGTCTGTG GAATTGGCGA CATGTAAGTG ACTTTGGAAG   
  
  
- ATATTACCAT GATCAACTTT CTCTTCCTTG GGCTTTACCT AGTCCTAAGT ATAGTCCAAA GTACCTAAGA   
  
  
- AACGTAATAA TACGACGTTA CAAGCTAAGT AATTTACTAA CGGAGGGTGC CCTCTCAGGG CTCTCTGACT   
  
  
- CGTATCTCTT CTTGGTAGAA CCCTCTCTCT AGTTCCTCCG CCACTGAACC CTCTTCCTGC TCTACTCGAT   
  
  
- GCCCTTCAAG CTCTATTACC TCTGAACCTT TTTCTCCTAC CTTTAAGTAC CCAAAAGCCC CTAGTCCGAC   
  
  
- TCAAGGTTCC GCTGTTAAGT TCGGTTCGAG GACGAGTTTT ACCCGTCGGT AATGACAGGG GTCAAACGTC   
  
  
- CCCTAAGACT TCCTCCTCAA CCGCCCAAGT CTCACATACT CTCTCTACTC CCCCTTCGGT AAAGAGACCA   
  
  
- AACCGTTCCG TCCAAGGAGC GTTGTCGGAG ACGTACCGCG ACACATAC

+     as-1

| Site Name | Organism | Position | Strand | Matrix score. | sequence | function |
| --- | --- | --- | --- | --- | --- | --- |
| as-1 | Arabidopsis thaliana | 2042 | + | 5 | TGACG |  |

>HU11G01529.1   
+ +Up\_Stream \_Len000GAAAGA AGATTTAAGG AAATCGATGG TACACGGCAA AGCTAGAAAC AGGAACTGTA   
  
  
+ TTTTTATGTT TTTTTTTTGT TGGAAGGGGG GGGGGGGGAG CGGGCGAGAT GTAACCCGTA TCTAGATGGA   
  
  
+ TAATCTACGT GACAAGACCT TCATTTGATC AAGGTACATC ATGAATATTT AACCCTTAAC TTCTTTGAGC   
  
  
+ CTTACAAGAG ATTGTCTGAG GTTTTTAGGG TGCACGATAT CATGCCTGAC TAGACCTGTG TAGACACAAT   
  
  
+ CTTCAGCCCT TATTGATAGC TGTTCGATAT GCATGCTGCC TAAAAATTGG AATAGTTGGC ACCTAACATT   
  
  
+ CTTTTACATC TTGCTCCTCC TTGGTGTAGA GTTAGATGTG CTAGCACAAT ACAATGGGTC CCGATATTTT   
  
  
+ GCATCTGGTT TCTGAAAGAT AATATTTGAT GATCCTGTCT TGTGCTGATG GGCAAAAGCT AAATTGTAAG   
  
  
+ AATATGTAGT TTATTTACAA GACATATGCT TAAGCTAGAG GTCATGTACT TGCATATTTT TGTGCAGCTT   
  
  
+ TTGAGGTTGT ATGCCCCTTT GAGACAAATT CATAGTTGAA TTCATGACAA GATTATGTTT GGCAATGACT   
  
  
+ TTGATTATTA AATTGGCCTT TATGCTATCC TATCCTGTGT GCTCAAATGT TATTTATCAG ATAATATTAG   
  
  
+ TTTACTCTTT CTTCCCTGTC TTGCAAAGAT TTTAAATCCT AAATTATGCA AATGTTTATC TTTAAAATGA   
  
  
+ CCTGCTACTC TCTGTGCCTG ATTTTATTTG TTTCCCCGTT GAAATTTTTT TTATGATTCC ACTTTTCAAG   
  
  
+ CAGCTTAGAC AGGCGTTATT AGGTAATTAT TTTCCGCTTT GGATTGTAGT AAATTGAAAC AGAAATGCCT   
  
  
+ CACTAAACCG CTCTCTGCTC TTTGTGTTGA AGAACCAATC CCATTCAAAC TCTTCTTTCT TTTTTGAAAT   
  
  
+ TCAATCCCGC TTCAGTCAAA TGTCTAACAG AAACAGCCTC GTGTGCAATT CTACTATATA CGGTTAATGC   
  
  
+ TGTGCATGTA TTACTTCCCA ACATCTTACC CAGGACTAGG AGGGACCTTG GTAGCCTGTT CTGCTTGAAA   
  
  
+ AATTTGTCAT TATCACCTGA ATTTGTATTA AATACATAAA TCTTTGTCTT TCAATTTGTG GCTCCACATT   
  
  
+ TCAATTTGTG GCTCCACACG CTTTTGAAAA CTTTATGAGA CCTAATATAT TAGAGAACAT CTGATCGTGA   
  
  
+ TTGTTAATTC CAGTTTTATA ATCATACTAA TTAGTAAATC ACTGAAGGTC ATATTGCATA TTGTTTTTGC   
  
  
+ CTAATGATTG GCTTATTCAA CTATTCCTGA ATAGTTTAGT TTCCAGAATC TTTAGAAGAA ACCTGGTCTG   
  
  
+ GGTATTATCG AGTATTTTTC TCAGTTCATG AAAGGCTTTG ATTCAGTGTG TGTGCTGTCA TGTCTAGGCA   
  
  
+ TGATTGAGTA TTTTTATTTG CATATCCAGG CATTGTGCCT AGACAAATGT CCTGAATCTT AGTGGCTGTT   
  
  
+ AGGTCTGAAC GGACACTTTT CTTGCTGTTT TTCCTTTCCG AGTGGCATCT TTAGCTTTTT CAAACTACCA   
  
  
+ AAGGACTATT AAACGAGGCC CTGCATATGC TGGAAAACTG CCTCTTAAGC TATGTTTATG CAAAAATGGA   
  
  
+ GGACCTTGAA CTGTTCAATG TACCTACAGT CTGAGGCACA GAGCCAATAG ACATAATAGA TAGTTGGATA   
  
  
+ TTCTTTTTAT ATTTAATTCA CAGTAGCGAC AACCCCAGCA TGAAACGTAG CCACGTAGGG GGGGAAAAAG   
  
  
+ GGTCTCCATC TGTTTATATA TTCTTGTCGC TTATGGTGCT TCTGTTATGT AATGACAAAG GAATTTGTGT   
  
  
+ TGGGATTATT CCGCTTCACT CCGTCTCACT AACTTTACGG TTTCAGTCCT AATTCTCTCT TTTTTTCTTT   
  
  
+ TGTTTTTTTT CCTGAGCTTG TTTAGTCTGT TTTCTTCTGT GTGTCTTTCT GATTATGGAA GCCATACAGG   
  
  
+ AGAAGAATGA CGATCTTTTG AGTCTTAGCT TGGCCATTGT TAGTCATTCT AACCATGCAG AAACGAAGGT   
  
  
+ GAAGAGGAAG AGGCATGATG TCTCTAATTC TCTGATCGCA ACAAGAGAAG AGGGTCGGGA TAAGAAAATA   
  
  
+ TTTCAGCTGC TTCAAGAACG AGAGAGAATG CTAAGCATCG GGCAAAGAAG AAAAGGCGTT ATTCAAGAAG   
  
  
+ GGAAGGGTCT TCATCTGATC CATTTGCTTC TCATGTCTGC TACCCTAATC AGCGAAAACA ATATCAGCTC   
  
  
+ AGCTGTTAAT CATTTGCTTG AGTTGTACAA GAATGTTTCT GTAAATGGTG ATTCTGGGCA GCGCGTTGCT   
  
  
+ GCTTATTTTG CTGATGGGTT AACTGCGAGG TTCTTAACCC AGAACTCTCC CTTTTATTGC ATGGTAATGA   
  
  
+ GTAAACCGAC ACCTGCAGAG GAGTTTATCG CCTTCACTCA TTTGTACAGG GTGTCTCCAT TCTATCAGTT   
  
  
+ TGCCCATTTT ACTGCTAATC AGGCCATCTT TGAGGCATTC GAGAGGGAAG AAGAGAACAA CAGCTGGGCA   
  
  
+ CTCCATGTCA TTGATTTTGA CATCTCTCAT GGCTTCCAGT GGCCGTCTCT CATTCAGTCT CTTTCTGAGA   
  
  
+ AGGCTACTGC TTCAAGCCAT TCACTCTCTC TTCGAATCAC AGGTTTTGCT AGAACTCTGG AAGAACTGAT   
  
  
+ TGAGACTGAG AACAGATTAG TAAGTTTTGC TGAAAGCTTC CGGAACATAC TATTTGAATT CCACGGGTGT   
  
  
+ TTGAGGGGTT CAGAGCTGGT GAACTTGAAG AAAAGGGAGA ATGAAACAGT TGCCGTAAAT CTGGTGTTTC   
  
  
+ ACCTCAGCAG TTTCGATGAC ACCCTGAAGG TCTCAGACAC CTTAACCGCT GTACATTCAC TGAAACCTTC   
  
  
+ TATAATGGTA CTAGTTGAAA GAGAAGGAAC CCGAAATGGA TCAGGATTCA TATCAGGTTT CATGGATTCT   
  
  
+ TTGCATTATT ATGCTGCAAT GTTCGATTCA TTAAATGATT GCCTCCCACG GGAGAGTCCC GAGAGACTGA   
  
  
+ GCATAGAGAA GAACCATCTT GGGAGAGAGA TCAAGGAGGC GGTGACTTGG GAGAAGGACG AGATGAGCTA   
  
  
+ CGGGAAGTTC GAGATAATGG AGACTTGGAA AAAGAGGATG GAAATTCATG GGTTTTCGGG GATCAGGCTG   
  
  
+ AGTTCCAAGG CGACAATTCA AGCCAAGCTC CTGCTCAAAA TGGGCAGCCA TTACTGTCCC CAGTTTGCAG   
  
  
+ GGGATTCTGA AGGAGGAGTT GGCGGGTTCA GAGTGTATGA GAGAGATGAG GGGGAAGCCA TTTCTCTGGT   
  
  
+ TTGGCAAGGC AGGTTCCTCG CAACAGCCTC TGCATGGCGC TGTGTATG  

- +Up\_Stream \_Len000CTTTCT TCTAAATTCC TTTAGCTACC ATGTGCCGTT TCGATCTTTG TCCTTGACAT   
  
  
- AAAAATACAA AAAAAAAACA ACCTTCCCCC CCCCCCCCTC GCCCGCTCTA CATTGGGCAT AGATCTACCT   
  
  
- ATTAGATGCA CTGTTCTGGA AGTAAACTAG TTCCATGTAG TACTTATAAA TTGGGAATTG AAGAAACTCG   
  
  
- GAATGTTCTC TAACAGACTC CAAAAATCCC ACGTGCTATA GTACGGACTG ATCTGGACAC ATCTGTGTTA   
  
  
- GAAGTCGGGA ATAACTATCG ACAAGCTATA CGTACGACGG ATTTTTAACC TTATCAACCG TGGATTGTAA   
  
  
- GAAAATGTAG AACGAGGAGG AACCACATCT CAATCTACAC GATCGTGTTA TGTTACCCAG GGCTATAAAA   
  
  
- CGTAGACCAA AGACTTTCTA TTATAAACTA CTAGGACAGA ACACGACTAC CCGTTTTCGA TTTAACATTC   
  
  
- TTATACATCA AATAAATGTT CTGTATACGA ATTCGATCTC CAGTACATGA ACGTATAAAA ACACGTCGAA   
  
  
- AACTCCAACA TACGGGGAAA CTCTGTTTAA GTATCAACTT AAGTACTGTT CTAATACAAA CCGTTACTGA   
  
  
- AACTAATAAT TTAACCGGAA ATACGATAGG ATAGGACACA CGAGTTTACA ATAAATAGTC TATTATAATC   
  
  
- AAATGAGAAA GAAGGGACAG AACGTTTCTA AAATTTAGGA TTTAATACGT TTACAAATAG AAATTTTACT   
  
  
- GGACGATGAG AGACACGGAC TAAAATAAAC AAAGGGGCAA CTTTAAAAAA AATACTAAGG TGAAAAGTTC   
  
  
- GTCGAATCTG TCCGCAATAA TCCATTAATA AAAGGCGAAA CCTAACATCA TTTAACTTTG TCTTTACGGA   
  
  
- GTGATTTGGC GAGAGACGAG AAACACAACT TCTTGGTTAG GGTAAGTTTG AGAAGAAAGA AAAAACTTTA   
  
  
- AGTTAGGGCG AAGTCAGTTT ACAGATTGTC TTTGTCGGAG CACACGTTAA GATGATATAT GCCAATTACG   
  
  
- ACACGTACAT AATGAAGGGT TGTAGAATGG GTCCTGATCC TCCCTGGAAC CATCGGACAA GACGAACTTT   
  
  
- TTAAACAGTA ATAGTGGACT TAAACATAAT TTATGTATTT AGAAACAGAA AGTTAAACAC CGAGGTGTAA   
  
  
- AGTTAAACAC CGAGGTGTGC GAAAACTTTT GAAATACTCT GGATTATATA ATCTCTTGTA GACTAGCACT   
  
  
- AACAATTAAG GTCAAAATAT TAGTATGATT AATCATTTAG TGACTTCCAG TATAACGTAT AACAAAAACG   
  
  
- GATTACTAAC CGAATAAGTT GATAAGGACT TATCAAATCA AAGGTCTTAG AAATCTTCTT TGGACCAGAC   
  
  
- CCATAATAGC TCATAAAAAG AGTCAAGTAC TTTCCGAAAC TAAGTCACAC ACACGACAGT ACAGATCCGT   
  
  
- ACTAACTCAT AAAAATAAAC GTATAGGTCC GTAACACGGA TCTGTTTACA GGACTTAGAA TCACCGACAA   
  
  
- TCCAGACTTG CCTGTGAAAA GAACGACAAA AAGGAAAGGC TCACCGTAGA AATCGAAAAA GTTTGATGGT   
  
  
- TTCCTGATAA TTTGCTCCGG GACGTATACG ACCTTTTGAC GGAGAATTCG ATACAAATAC GTTTTTACCT   
  
  
- CCTGGAACTT GACAAGTTAC ATGGATGTCA GACTCCGTGT CTCGGTTATC TGTATTATCT ATCAACCTAT   
  
  
- AAGAAAAATA TAAATTAAGT GTCATCGCTG TTGGGGTCGT ACTTTGCATC GGTGCATCCC CCCCTTTTTC   
  
  
- CCAGAGGTAG ACAAATATAT AAGAACAGCG AATACCACGA AGACAATACA TTACTGTTTC CTTAAACACA   
  
  
- ACCCTAATAA GGCGAAGTGA GGCAGAGTGA TTGAAATGCC AAAGTCAGGA TTAAGAGAGA AAAAAAGAAA   
  
  
- ACAAAAAAAA GGACTCGAAC AAATCAGACA AAAGAAGACA CACAGAAAGA CTAATACCTT CGGTATGTCC   
  
  
- TCTTCTTACT GCTAGAAAAC TCAGAATCGA ACCGGTAACA ATCAGTAAGA TTGGTACGTC TTTGCTTCCA   
  
  
- CTTCTCCTTC TCCGTACTAC AGAGATTAAG AGACTAGCGT TGTTCTCTTC TCCCAGCCCT ATTCTTTTAT   
  
  
- AAAGTCGACG AAGTTCTTGC TCTCTCTTAC GATTCGTAGC CCGTTTCTTC TTTTCCGCAA TAAGTTCTTC   
  
  
- CCTTCCCAGA AGTAGACTAG GTAAACGAAG AGTACAGACG ATGGGATTAG TCGCTTTTGT TATAGTCGAG   
  
  
- TCGACAATTA GTAAACGAAC TCAACATGTT CTTACAAAGA CATTTACCAC TAAGACCCGT CGCGCAACGA   
  
  
- CGAATAAAAC GACTACCCAA TTGACGCTCC AAGAATTGGG TCTTGAGAGG GAAAATAACG TACCATTACT   
  
  
- CATTTGGCTG TGGACGTCTC CTCAAATAGC GGAAGTGAGT AAACATGTCC CACAGAGGTA AGATAGTCAA   
  
  
- ACGGGTAAAA TGACGATTAG TCCGGTAGAA ACTCCGTAAG CTCTCCCTTC TTCTCTTGTT GTCGACCCGT   
  
  
- GAGGTACAGT AACTAAAACT GTAGAGAGTA CCGAAGGTCA CCGGCAGAGA GTAAGTCAGA GAAAGACTCT   
  
  
- TCCGATGACG AAGTTCGGTA AGTGAGAGAG AAGCTTAGTG TCCAAAACGA TCTTGAGACC TTCTTGACTA   
  
  
- ACTCTGACTC TTGTCTAATC ATTCAAAACG ACTTTCGAAG GCCTTGTATG ATAAACTTAA GGTGCCCACA   
  
  
- AACTCCCCAA GTCTCGACCA CTTGAACTTC TTTTCCCTCT TACTTTGTCA ACGGCATTTA GACCACAAAG   
  
  
- TGGAGTCGTC AAAGCTACTG TGGGACTTCC AGAGTCTGTG GAATTGGCGA CATGTAAGTG ACTTTGGAAG   
  
  
- ATATTACCAT GATCAACTTT CTCTTCCTTG GGCTTTACCT AGTCCTAAGT ATAGTCCAAA GTACCTAAGA   
  
  
- AACGTAATAA TACGACGTTA CAAGCTAAGT AATTTACTAA CGGAGGGTGC CCTCTCAGGG CTCTCTGACT   
  
  
- CGTATCTCTT CTTGGTAGAA CCCTCTCTCT AGTTCCTCCG CCACTGAACC CTCTTCCTGC TCTACTCGAT   
  
  
- GCCCTTCAAG CTCTATTACC TCTGAACCTT TTTCTCCTAC CTTTAAGTAC CCAAAAGCCC CTAGTCCGAC   
  
  
- TCAAGGTTCC GCTGTTAAGT TCGGTTCGAG GACGAGTTTT ACCCGTCGGT AATGACAGGG GTCAAACGTC   
  
  
- CCCTAAGACT TCCTCCTCAA CCGCCCAAGT CTCACATACT CTCTCTACTC CCCCTTCGGT AAAGAGACCA   
  
  
- AACCGTTCCG TCCAAGGAGC GTTGTCGGAG ACGTACCGCG ACACATAC
