# Supplementary figures and images for: Genome-Wide Identification and Expression Pattern of the GRAS Gene Family in Pitaya (Selenicereus undatus L.)
Source: Biology (Basel). 2022 Dec 21;12(1):11. doi: 10.3390/biology12010011 (PMC9854919; doi:10.3390/biology12010011)

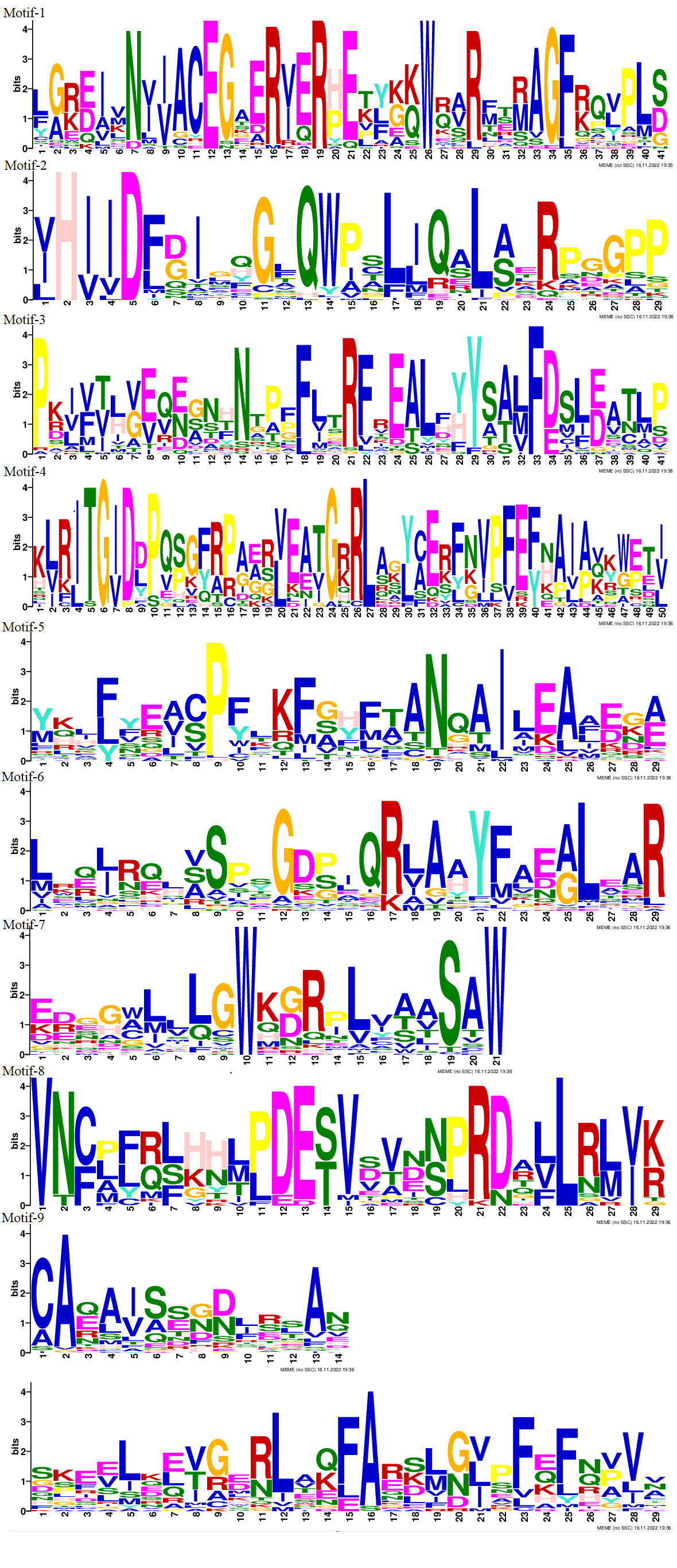

Supplement: Supplementary file 1 [file biology-12-00011-s001.zip › Figure S1.png]

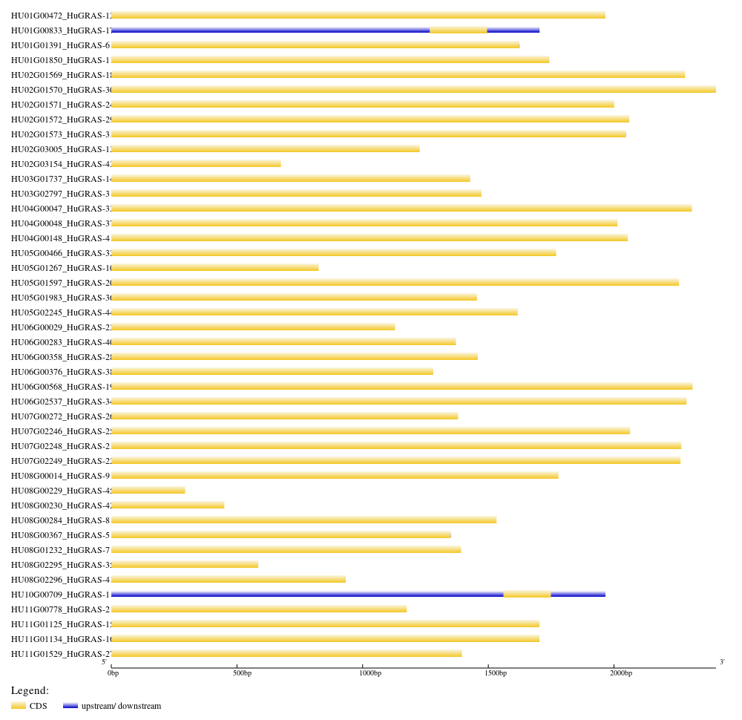

Supplement: Supplementary file 1 [file biology-12-00011-s001.zip › Figure S2.png]

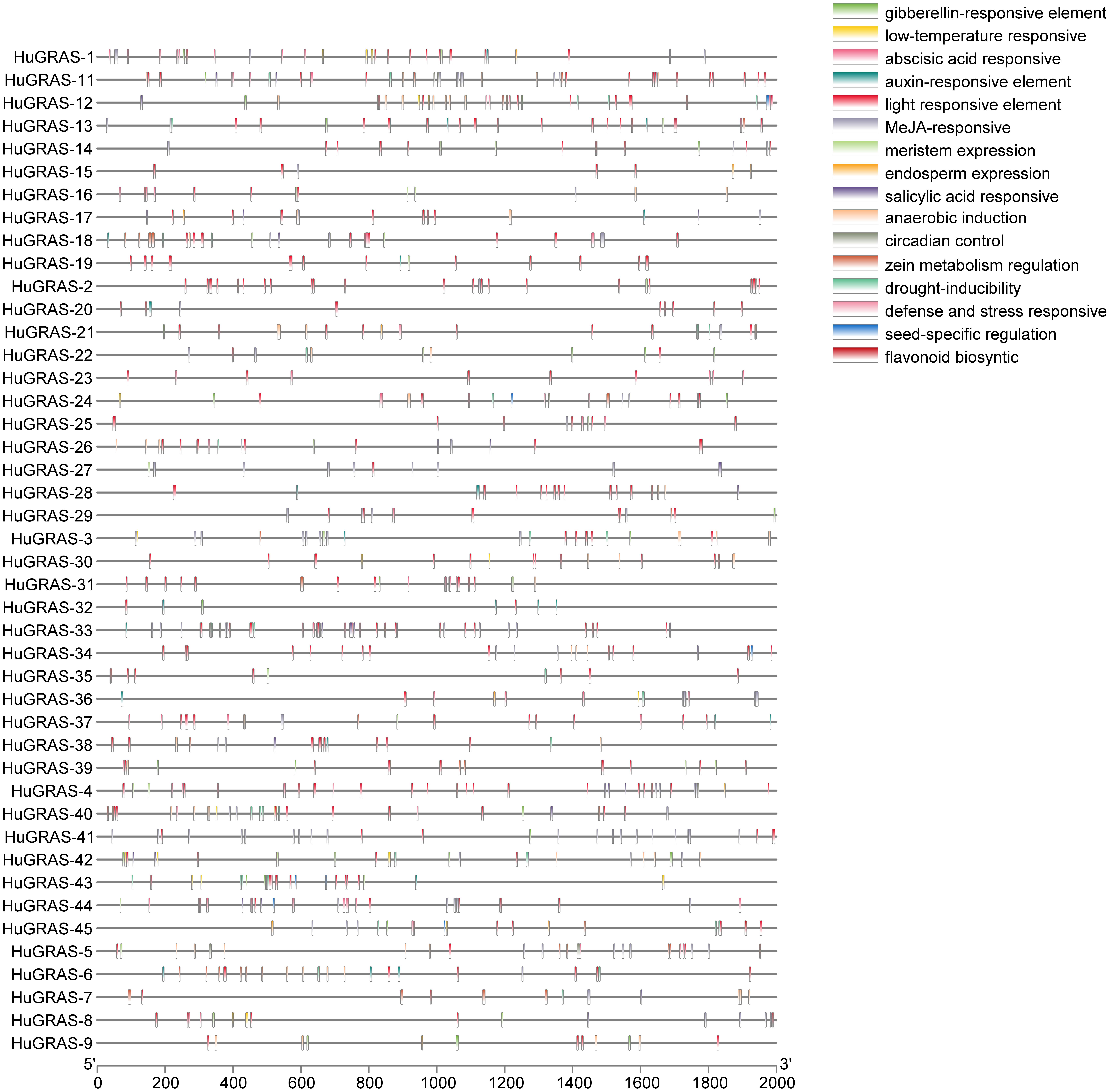

Supplement: Supplementary file 1 [file biology-12-00011-s001.zip › Figure S3.jpg]
